# Unraveling the mystery: a Mendelian randomized exploration of gut microbiota and different types of obesity

**DOI:** 10.3389/fcimb.2024.1352109

**Published:** 2024-02-05

**Authors:** Siyuan Liu, Fan Li, Yunjia Cai, Linan Ren, Lin Sun, Xiaokun Gang, Guixia Wang

**Affiliations:** ^1^ Department of Endocrinology and Metabolism, The First Hospital of Jilin University, Changchun, Jilin, China; ^2^ Department of Gastroenterology, The First Hospital of Jilin University, Changchun, Jilin, China

**Keywords:** gut microbiota, obesity, Mendelian randomization, causal relationship, stratification

## Abstract

**Background:**

Numerous studies have demonstrated the influence of gut microbiota on the development of obesity. In this study, we utilized Mendelian randomization (MR) analysis to investigate the gut microbiota characteristics among different types of obese patients, aiming to elucidate the underlying mechanisms and provide novel insights for obesity treatment.

**Methods:**

Two-sample multivariable Mendelian randomization (MR) analysis was employed to assess causal relationships between gut microbiota and various obesity subtypes. Gut microbiota data were obtained from the international consortium MiBioGen, and data on obese individuals were sourced from the Finnish National Biobank FinnGen. Eligible single-nucleotide polymorphisms (SNPs) were selected as instrumental variables. Various analytical methods, including inverse variance weighted (IVW), MR-Egger regression, weighted median, MR-RAPS, and Lasso regression, were applied. Sensitivity analyses for quality control included MR-Egger intercept tests, Cochran’s Q tests, and leave-one-out analyses and others.

**Results:**

Mendelian randomization studies revealed distinct gut microbiota profiles among European populations with different obesity subtypes. Following multivariable MR analysis, we found that *Ruminococcaceae UCG010* [*Odds Ratio (OR)*: 0.842, *95% confidence interval (CI)*: 0.766-0.926, Adjusted *P* value: 0.028] independently reduced the risk of obesity induced by excessive calorie intake, while *Butyricimonas* [*OR*: 4.252, *95% CI*: 2.177-8.307, Adjusted *P* value: 0.002] independently increased the risk of medication-induced obesity. For localized adiposity, *Pasteurellaceae* [*OR*: 0.213, *95% CI*: 0.115-0.395, Adjusted *P* value: <0.001] acted as a protective factor. In the case of extreme obesity with alveolar hypoventilation, *lactobacillus* [*OR*: 0.724, *95% CI*: 0.609-0.860, Adjusted *P* value: 0.035] reduced the risk of its occurrence. Additionally, six gut microbiota may have potential roles in the onset of different types of obesity. Specifically, the *Ruminococcus* torques group may increase the risk of its occurrence. *Desulfovibrio* and *Catenabacterium* may serve as protective factors in the onset of Drug-induced obesity. *Oxalobacteraceae*, *Actinomycetaceae*, and *Ruminiclostridium 9*, on the other hand, could potentially increase the risk of Drug-induced obesity. No evidence of heterogeneity or horizontal pleiotropy among SNPs was found in the above studies (all *P* values for Q test and MR-Egger intercept > 0.05).

**Conclusion:**

Gut microbiota abundance is causally related to obesity, with distinct gut microbiota profiles observed among different obesity subtypes. Four bacterial species, including *Ruminococcaceae UCG010*, *Butyricimonas*, *Pasteurellaceae* and *lactobacillus* independently influence the development of various types of obesity. Probiotic and prebiotic supplementation may represent a novel approach in future obesity management.

## 1 Introduction

Obesity is a chronic metabolic disorder, and with its global incidence steadily rising, it has become a significant economic and health concern worldwide (Vlassopoulos and Lean, 2016). Obesity is often associated with cardiovascular diseases, diabetes, musculoskeletal disorders (especially osteoarthritis), and cancers (including ovarian, liver, and colon cancers, *etc*). Consequently, obesity and its related issues have gained increasing attention (Haslam and James, 2005). Obesity is recognized as a multifactorial disorder, with its etiology involving a multitude of factors including genetics, environment, behavior, and psychology. The gut microbiome stands out as a significant environmental determinant contributing to its onset. The human gut microbiota comprises approximately 100 trillion species and carries genes numbering about 150 times that of the human genome (Marchesi et al., 2016). The gut microbiota plays a pivotal role in maintaining normal intestinal function and host health, participating in metabolic homeostasis. Consequently, alterations in the gut microbiota contribute significantly to the development of metabolic disorders. The gut microbiota is primarily influenced by environmental factors, particularly diet, and exhibits variation based on ethnicity, possibly owing to disparities in dietary customs across regions (Deschasaux et al., 2018; Rothschild et al., 2018). While the host genome plays a central role in shaping the composition of the gut microbiota, numerous geographic and environmental factors, including diet, lifestyle, sanitation, and medication use, can lead to variations in the gut microbiota. Research suggests that the impact of genes on the gut microbiota appears to be less pronounced, whereas environmental factors (especially diet) hold greater influence (Rothschild et al., 2018).

In recent years, an increasing body of evidence has substantiated a causal relationship between the gut microbiota and obesity. Bäckhed et al. transplanted the gut microbiota of normal mice into germ-free mice, resulting in a significant increase in body fat percentage even without altering food intake (Backhed et al., 2004). Li et al. observed that the genus *Akkermansia* exhibited a protective effect against childhood obesity and BMI (Li et al., 2023). There are several potential mechanisms underlying the association between the gut microbiota and obesity: The gut microbial community enhances energy intake from food by producing efficient enzymes for the degradation of dietary nutrients (Blaut, 2015). Dietary fibers, through metabolites such as short-chain fatty acids (SCFAs) produced by the gut microbiota, not only serve as an energy source but also interact with G protein-coupled receptor (GPR) 41 and GPR43 (Lin et al., 2012) to modulate lipid and glucose metabolism. Additionally, *AMPK* is involved in mediating the effects of SCFAs, contributing not only to the aforementioned metabolic regulations but also to cholesterol metabolism (den Besten et al., 2013); In addition to the aforementioned effects, SCFA can also influence the production of hormones such as leptin, peptide YY (PYY), ghrelin, insulin, and glucagon-like peptide-1 (GLP-1). These hormones all play crucial roles in the development and occurrence of obesity (Fukumoto et al., 2003; Neuman et al., 2015). Furthermore, the gut microbiota can also impact the integrity of the intestinal barrier, modulate plasma lipopolysaccharide (LPS) levels, and further influence the occurrence and progression of metabolic endotoxemia and low-grade inflammation in the body, thereby either promoting or reducing the development of obesity (Tsukumo et al., 2015).

While the fundamental cause of obesity is an excessive calorie intake compared to expenditure, variations in the human gut microbiota ecosystem may be a pivotal factor influencing energy homeostasis. Specifically, individuals prone to obesity might exhibit distinctive alterations in gut microbiota, as opposed to normal or lean populations (Ley et al., 2005). According to the ICD-10 coding (The, 2019), we included localized adiposity, obesity (including calorie-induced obesity, drug-induced obesity, extreme obesity with alveolar hypoventilation) in our study, and analyzed the gut microbiota characteristics of patients with different types of obesity. This study explores the gut microbiota characteristics in various obesity types, aiming to enhance comprehension of obesity pathogenesis and provide insights into novel therapeutic avenues for obese individuals.

## 2 Methods

### 2.1 Source of datasets

In this study, we obtained whole-genome association study (GWAS) data on gut microbiota from the International MiBioGen Consortium. The database coordinated 16S rRNA gene sequencing profiles and genetic typing data for 18,340 participants from 24 cohorts across the United States, Canada, Israel, South Korea, Germany, Denmark, the Netherlands, Belgium, Sweden, Finland, and the United Kingdom. This comprehensive meta-analysis of autosomal human genetic variations and their associations with the gut microbiota involved a large-scale, multi-ethnic, whole-genome approach (Kurilshikov et al., 2021). Details of recruitment criteria, sample size, and ethical approval can be found in the **Supplementary Note 1, 2, Supplementary Table 9**. GWAS datasets on obesity were obtained from the FinnGen research project, which originates from the Finnish National Biobank Network. Participants in FinnGen were recruited from 2017 to 2023. This database encompasses data from 473,681 participants, including 15,045 individuals with obesity due to excessive caloric intake, 240 individuals with drug-induced obesity, 1,064 individuals with extreme obesity associated with alveolar hypoventilation, and 132 individuals with localized adiposity. The control group consisted of varying numbers of individuals, ranging from 355,786 to 355,902 (Kurki et al., 2023). The inclusion and exclusion criteria for participants were determined based on the ICD-10 codes of the primary diagnosis during hospitalization. Pharmaceuticals inducing obesity include Corticosteroids, antihypertensive drugs (β-adrenergic blockers), neuropsychiatric medications (Clozapine, olanzapine, quetiapine), antidepressants (amitriptyline, nortriptyline, and mirtazapine), and antiepileptic drugs (valproate and carbamazepine). Specific disease codes are provided in **Table 1**. Participants in FinnGen provided informed consent for biobank research on basis of the Finnish Biobank Act. The Coordinating Ethics Committee of the Hospital District of Helsinki and Uusimaa (HUS) approved the FinnGen study protocol (number HUS/990/2017). Due to variations in race and population stratification, the exposure and outcome databases were derived from European populations. The study population includes both males and females, mitigating potential biases arising from population stratification (Emdin et al., 2017).

**Table 1:** Dataset information, disease ICD-10 codes, and confounders identification.

Datasets	NCase	Sample Size	Year	Author	Gender	Population	NSNP	ICD10 Codes	Confounders
Gut microbiota abundance	14306	14306	2021	Kurilshikov	MF	European	5547067	–	–
Localized adiposity	132	355918	2023	FINNGEN	MF	European	20167370	E65	E, M, D
Obesity due to excess calories	15045	370947	2023	FINNGEN	MF	European	20167370	E66.0	M, D
Drug-induced obesity	240	356142	2023	FINNGEN	MF	European	20167370	E66.1	E, D
Extreme obesity with alveolar hypoventilation	1064	356966	2023	FINNGEN	MF	European	20167370	E66.2	E, M, D

NCase, Number of Cases; NSNP, Number of Available SNPs; MF, Males and Females; E, Excessive intake of calories; M, Medications (e.g., hormones.); D, Endocrine Disorders (e.g., hypothalamic disorder.).

### 2.2 Study approach

**Figure 1:**
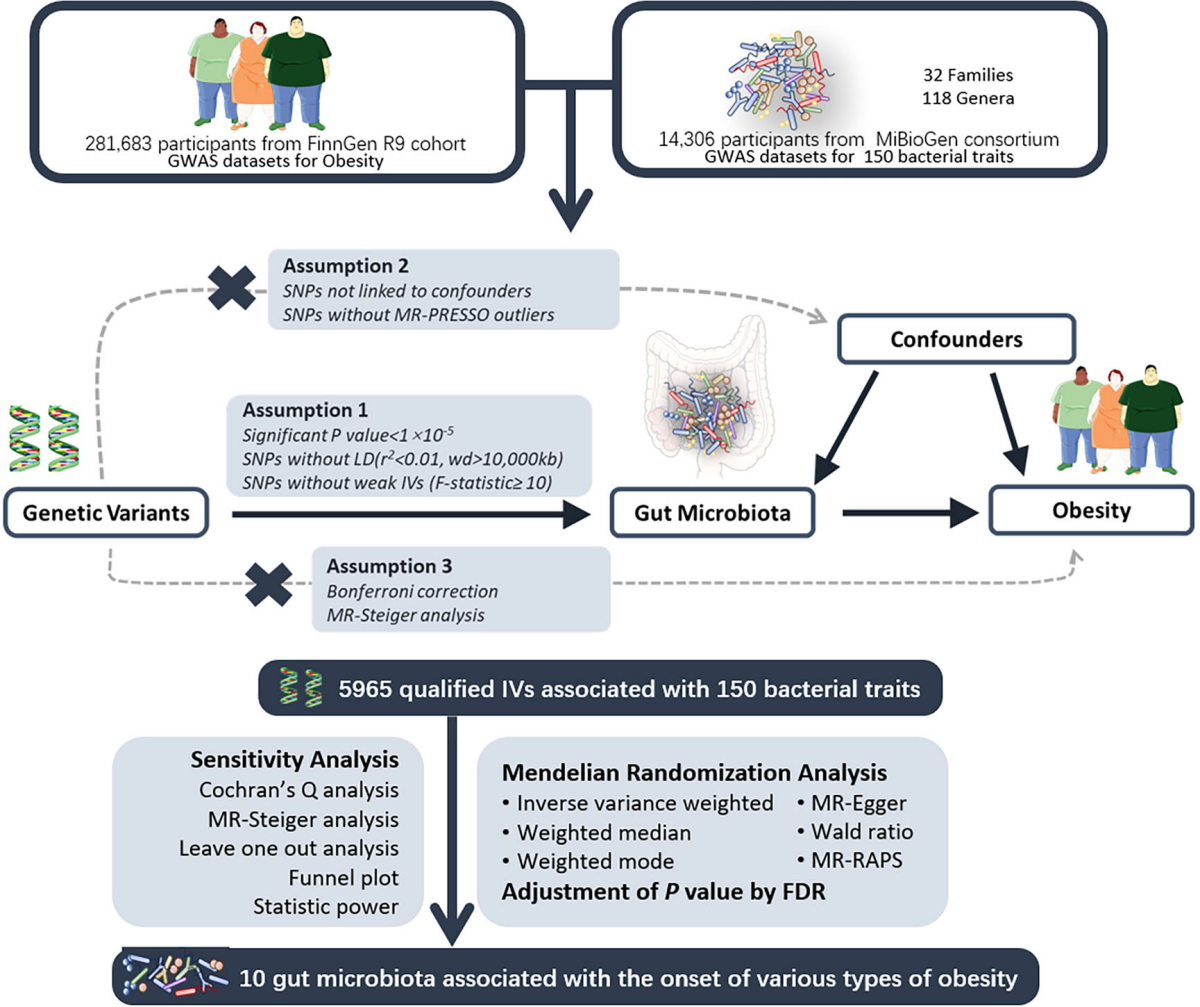
The study design of the MR study of the associations of gut microbiota on obesity. UV, univariable; MV, multivariable; MR, Mendelian Randomization; GWAS, Genome-Wide Association Study; SNP, Single Nucleotide Polymorphism, used as instrumental variables for exposure and outcome; LD, Linkage Disequilibrium; MR-PRESSO, Mendelian Randomization Pleiotropy RESidual Sum and Outlier; FDR, False Discovery Rate. Parts of the figure were drawn by using pictures from Servier Medical Art.

In this study, a two-sample Mendelian randomization (MR) design was employed to investigate the causal effects of gut microbiota on various types of obesity (**Figure 1**). Genetic variants associated with gut microbiota were selected as instrumental variables (IVs). Multiple analytical methods, including inverse variance-weighted (IVW), MR-Egger regression, weighted median, and MR-RAPS, were utilized. Additionally, sensitivity analyses were conducted using MR-Egger intercept test, Cochran’s Q test, and leave-one-out analysis for quality control purposes. A MR analysis must adhere to the following three assumptions: 1) the relevance assumption: there is a strong association between instrumental variables and exposure factors; 2) the independence assumption: instrumental variables must be independent of confounding factors influencing both exposure and outcomes; 3) the exclusion assumption: instrumental variables should not directly impact the outcome variable.

### 2.3 Instrumental variable selection

In line with the study design and assumptions described above, our initial step involved extracting SNPs significantly correlated with both exposure and outcomes using a *p*-value threshold of *p*<1×10^-5^. A linkage disequilibrium (LD) threshold (*r^2^* <0.01, *kb*=0000) was applied to ensure SNP independence by eliminating linkage disequilibrium. Subsequently, we calculated the *F*-value for each SNP as an indicator of strength, and SNPs with *F*<10 were deemed weak instrumental variables and removed (Burgess et al., 2017). During the process of outcome data extraction, any missing SNPs in the outcome database were replaced with highly correlated proxy SNPs. Additionally, during dataset matching, this study excluded ambiguous and palindromic SNPs. Each SNP was examined using the PhenoScanner database (Staley et al., 2016), and SNPs associated with distinct confounding factors were excluded based on different outcomes (as shown in **Table 1**). MR-PRESSO was utilized to detect and eliminate SNPs with levels of horizontal pleiotropy outliers. MR-Steiger analysis was conducted on all SNPs to verify the direction of causal estimates, and SNPs with erroneous directions were removed according to the method described by (Li et al., 2022). Subsequently, a Bonferroni correction (*P*<0.05/n, where n represents the number of remaining SNPs) was applied to remove SNPs directly correlated with outcomes. Following this stringent selection process, the SNPs that remained were deemed as qualified instrumental variables.

### 2.4 Univariable MR analysis

We employed a variety of MR methods to assess the causal relationship between gut microbiota and obesity, including Inverse Variance Weighting (IVW), MR-Egger regression, weighted median, weighted mode, and MR-PAPS method.

The IVW method is a weighted linear regression without an intercept term that uses the reciprocal of the variance of the genetic associations’ product with the outcome as weights (Burgess et al., 2013). Although some have suggested weighted methods with second-order weights, in practice, utilizing first-order standard errors is often reasonable (Burgess et al., 2016). The IVW method is divided into fixed-effects (FE) and multiplicative random-effects (MRE) models based on the consistency of SNP effect differences. In contrast to the IVW method, MR-Egger includes an intercept term that allows for detection of SNP-level pleiotropy (Burgess and Thompson, 2017).

Although the aforementioned methods are commonly used in MR analysis, the weighted average of ratio estimates will differ from zero if the ratio estimate of a single variant is non-zero, leading to a “0% breakdown point” inferences issue where a single incorrect data point can cause estimates to exhibit arbitrary large bias. To address the “0% breakdown point” issue, we also employed two consensus methods. We considered the weighted median method with a 50% breakdown point (based on the majority valid assumption) and the weighted mode method with a higher breakdown point (based on the plurality valid assumption) (Bowden et al., 2016a; Burgess et al., 2019).

Finally, we also employed a common modeling approach, the MR-Robust Adjusted Profile Score (RAPS) method. This method directly models pleiotropic effects of genetic variants using a random-effects distribution, which is likely to perform well when pleiotropic effects are truly normally distributed about zero. To mitigate the increased statistical Type I error rate due to multiple testing, we applied the False Discovery Rate (FDR) method to correct the results, and an adjusted p-value <0.05 was considered a significant causal effect.

### 2.5 Multivariable MR analysis

Building upon the results of univariable MR analysis, we conducted multivariable MR (MVMR) analysis using the same parameters with various methods.Multivariable MR is an extension of the standard univariable MR, incorporating instrumental variables related to one or more gut microbiota and enabling the estimation of direct causal effects of each microbiota in a single analysis (Burgess and Thompson, 2015; Sanderson et al., 2019). Additionally, all included SNPs must be independent of each other. In this study, multivariable analyses were performed using the IVW method, MR-Egger method, Weighted median method, and Lasso method to elucidate gut microbiota with independent causal effects.

### 2.6 Sensitivity analysis

Cochran’s Q test was employed to assess heterogeneity among IVs, considering SNPs with Q test *P*-value < 0.05 as heterogeneous; MR-Egger intercept was computed to determine the presence of pleiotropy among SNPs, and an intercept significance *P*-value < 0.05 indicates horizontal pleiotropy. Given the adoption of various MR analysis methods, in the absence of heterogeneity and pleiotropy among SNPs, we considered the results from the IVW-random effects model as the primary results; in the presence of heterogeneity, we integrated results from the IVW-random effects model and the weighted median method; in the presence of pleiotropy, we regarded results from the MR-Egger method as the primary results (Burgess et al., 2019). Additionally, we employed the MR-Steiger model to validate the estimated overall direction for result robustness; to ascertain the presence of influential SNPs, we conducted a leave-one-out sensitivity test. The MR-Egger regression requires fulfillment of the Instrument Strength Independent of Direct Effect (InSIDE) assumption (Burgess and Thompson, 2017) and the No Measurement Error (NoME) assumption (Bowden et al., 2016b). We generated a funnel plot and calculated the *I^2^* statistic to confirm the validity of these assumptions. Correction for causal estimates is necessary when *I^2^* < 90%, and MR-Egger is the primary analytical method (Bowden et al., 2016b).

### 2.7 Visualization of results

We created a heatmap for the overall MR results and depicted significant MR findings using forest plots. Additionally, for each set of MR analyses, scatter plots and regression curve plots were generated, along with forest plots illustrating the effects of individual SNPs. These visualizations will be presented in the **Supplementary Materials**.

### 2.8 Statistical analysis software

All statistical analyses and visualizations in this study were conducted using R software (version 4.1.2) with the “TwoSampleMR,” “MR-PRESSO,” “mr.raps,” and “forestploter” packages, as well as several foundational R packages.

## 3 Results

### 3.1 Instrument variable selection

Initially, we screened a total of 7671 SNPs associated with gut microbiota. No weak instruments with *F*<10 were identified. Among them, 388 SNPs were excluded due to missing data in the outcome database, 1716 SNPs were removed as ambiguous or palindromic SNPs during dataset integration, and 99 SNPs were deleted after PhenoScanner retrieval revealed associations with confounding factors. MR-PRESSO testing identified 25 SNPs with horizontal pleiotropy. MR-Steiger analysis did not reveal any SNPs with incorrect causal directions. After Bonferroni correction, 18 SNPs directly related to the outcome were removed. In the end, 5965 qualified SNPs were included in the study.

### 3.2 Mendelian randomization analysis

**Figure 2:**
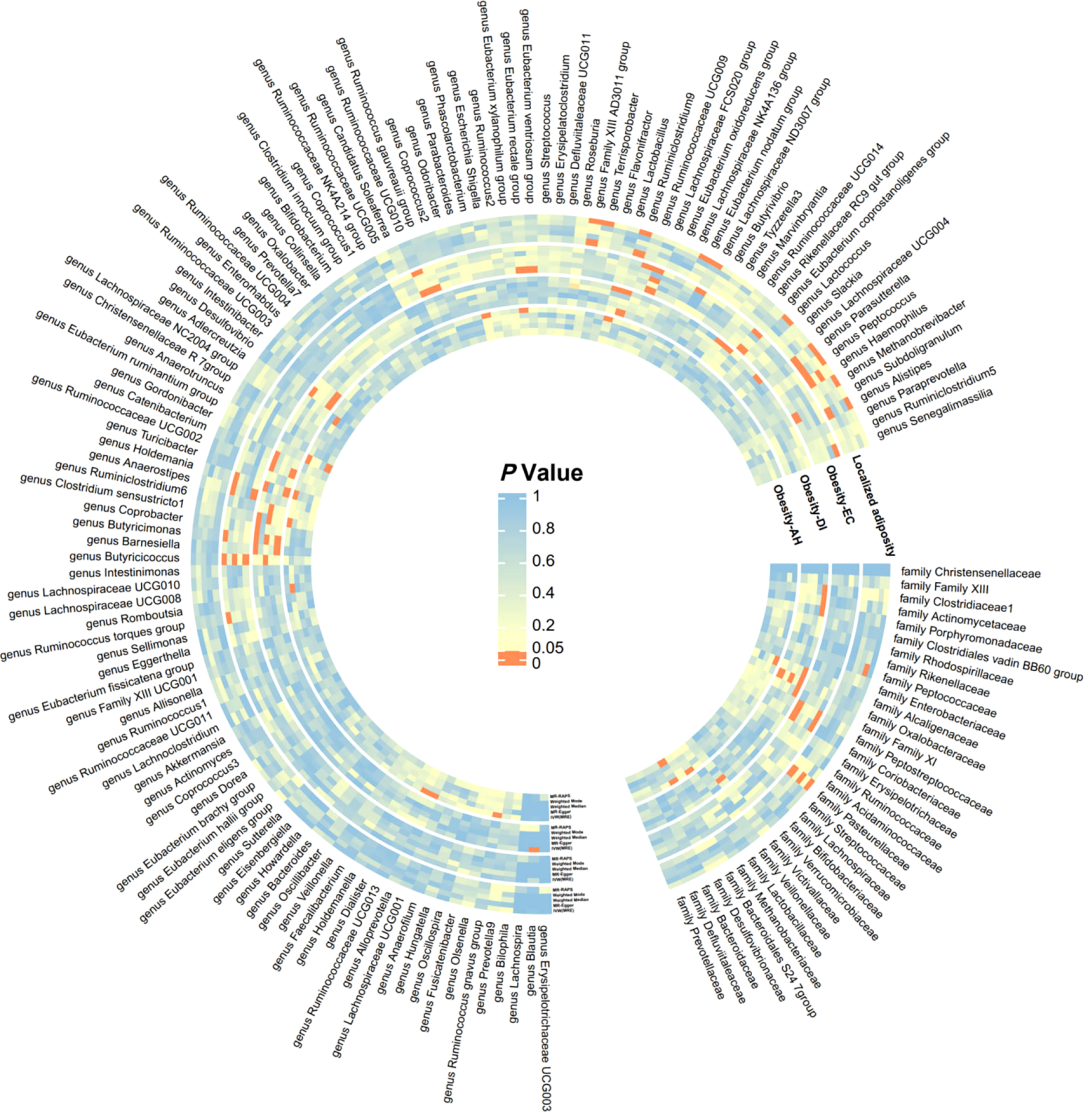
Significance heatmap of MR analysis. IVW, inverse variance weighted; MR, Mendelian randomization; RAPS, Robust Adjusted Profile Score.

**Figure 3:**
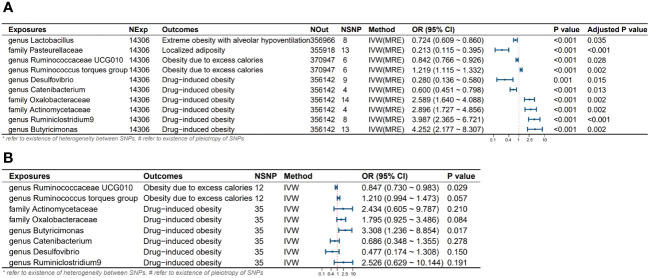
Results and forest plot of the MR analysis. **(A)** Results and forest plot of the significant UVMR analysis; **(B)** Results and forest plot of the MVMR analysis; IVW(MRE), inverse variance weighted (multiplicative random effects model); CI, confidence interval; NExp, sample size of exposure dataset; NOut, sample size of outcome dataset; NSNP, number of SNP included in MR analysis; *refer to existence of heterogeneity of SNPs, #refer to existence of pleiotropy between SNPs.

In this study, MR analysis was conducted for a total of 750 batches, and detailed results for all methods can be found in **Supplementary Table 2**. A total of 60 batches exhibited positive results in the MR analysis. However, after FDR correction, only 10 batches showed significant causal relationships (adjusted *P*-value < 0.05). We created a heatmap with red indicating significant findings (**Figure 2**) and a forest plot based on the main methods in the significant MR results (**Figure 3**). We conducted a comprehensive sensitivity analysis on all MR results. The results of the Q-test are provided in **Supplementary Table 6**, indicating heterogeneity in 13 batches. After comparing the results of IVW (MRE) and Weighted median methods for these batches, we found consistent conclusions between the two methods. The MR-Egger intercept test results can be found in **Supplementary Table 7**, indicating horizontal pleiotropy in the analysis of 7 batches. Therefore, we employed the MR-Egger method as the primary analytical approach for these 7 batches without the need for causal correction (*I^2^* > 90%). The remaining batches were analyzed using the IVW (MRE) method as the primary approach. This study did not identify SNPs that strongly influenced the significant results. Leave-one-out analysis results are available in **Supplementary Figure 3**. The MR-Steiger test did not identify overall directional errors in the analysis results.

### 3.3 Impact of gut microbiota abundance changes on obesity onset

Through univariate and multivariate MR analyses, we identified four intestinal microbial communities that influence the occurrence of obesity. Additionally, we discovered six potential influential microbial communities, which were no longer significant after the multivariate analysis. *Ruminococcaceae UCG010* independently reduces the risk of obesity due to excess calorie intake (*Odds Ratio (OR)*: 0.842, *95% confidence interval (CI)*: 0.766-0.926, Adjusted *P* value: 0.028). *Butyricimonas* independently increases the risk of obesity due to excess calorie intake (*OR*: 4.252, *95% CI*: 2.177-8.307, Adjusted *P* value: 0.002). *Pasteurellaceae* is a protective factor for localized adiposity population (*OR*: 0.213, *95% CI*: 0.115-0.395, Adjusted *P* value: <0.001). *Lactobacillus* reduces the risk of extreme obesity with alveolar hypoventilation (*OR*: 0.724, *95% CI*: 0.609-0.860, Adjusted *P* value: 0.035). Although the following bacteria are no longer significant after multivariable MR, we still consider them potential factors influencing the onset of obesity. Specifically, the *Ruminococcus* torques group may potentially increase the risk of its onset. *Desulfovibrio*, and *Catenabacterium* may act as protective factors in the development of Drug-induced obesity in the population. *Oxalobacteraceae*, *Actinomycetaceae*, and *Ruminiclostridium* 9 may potentially increase the risk of Drug-induced obesity. No heterogeneity and horizontal pleiotropy were observed among the SNPs in the above-mentioned study (all *P* values for Q test and MR-Egger intercept > 0.05) (**Table 2**).

**Table 2:** Sensitivity analysis of significant MR results.

Exposures	Outcomes	Pval for MR-Egger intercept	Q of IVW	Pval for Q test of IVW	Q of MR-Egger	Pval for Q test of MR-Egger	MR-Steiger
*genus Lactobacillus*	Extreme obesity with alveolar hypoventilation	0.762	1.934	0.963	1.833	0.934	TRUE
*family Pasteurellaceae*	Localized adiposity	0.586	6.389	0.895	6.075	0.868	TRUE
*genus Ruminococcaceae UCG010*	Obesity due to excess calories	0.482	2.022	0.846	1.424	0.840	TRUE
*genus Ruminococcus torques group*	Obesity due to excess calories	0.599	0.906	0.970	0.581	0.965	TRUE
*genus Desulfovibrio*	Drug-induced obesity	0.432	5.655	0.686	4.961	0.665	TRUE
*genus Catenibacterium*	Drug-induced obesity	0.602	0.385	0.943	0.009	0.996	TRUE
*family Oxalobacteraceae*	Drug-induced obesity	0.769	10.855	0.623	10.765	0.549	TRUE
*family Actinomycetaceae*	Drug-induced obesity	0.730	0.676	0.879	0.518	0.772	TRUE
*genus Ruminiclostridium9*	Drug-induced obesity	0.882	1.199	0.991	1.175	0.978	TRUE
*genus Butyricimonas*	Drug-induced obesity	0.540	7.901	0.793	7.501	0.757	TRUE

MR analysis with less than 3 SNPs are not available for Cochran’s Q test. IVW, inverse variance weighted method; MVMR, multivariable mendelian randomization.

## 4 Discussion

In this study, we found significant differences in the gut microbiota characteristics among different types of obese populations, revealing a close association between gut microbiota and obesity. Through univariate and multivariate Mendelian randomization (MR) analyses, we identified significant alterations in the abundance of *Ruminococcaceae UCG010*, *Butyricimonas*, *Lactobacillus*, and *Pasteurellaceae* in populations with Obesity due to excess calories, drug-induced obesity, extreme obesity with alveolar hypoventilation, and localized adiposity, respectively, which confirms the independent association of these four-gut microbiota with obesity. Additionally, we observed potential correlations between obesity and six other gut microbiota.

Previous studies, both in rodents and human clinical research, consistently demonstrated a common pattern in the gut microbiota of obese individuals, characterized by a relative decrease in the abundance of *Bacteroidetes* and an increase in *Firmicutes*. Reiner and Mary, among others, observed an increase in *Firmicutes* and a decrease in *Bacteroidetes* abundance in mice exposed to a high-fat diet (Hildebrandt et al., 2009; Jumpertz et al., 2011). A study conducted on the Ukrainian population found a significant increase in *Firmicutes* and a decrease in *Bacteroidetes* levels in adult obese individuals in the country. Furthermore, the *Firmicutes/Bacteroidetes* ratio was positively correlated with BMI (Koliada et al., 2017). Ley et al. also reported a higher abundance of *Firmicutes* and a lower abundance of *Bacteroidetes* in obese populations, with the proportion of *Bacteroidetes* gradually increasing with the frequency of low-calorie (that is, low-fat and low-carbohydrate) diets (Ley et al., 2006). At the genus level, the gut microbiota of overweight or obese individuals exhibited differences compared to the healthy group. Million et al. identified a positive association between *Lactobacillus reuteri* and obesity, while *Lactobacillus reuteri*, *Escherichia coli*, and *Methanobrevibacter smithii* showed a negative correlation with obesity (Million et al., 2013). Yun et al. conducted 16S rRNA gene sequencing on healthy, overweight, and obese populations and identified a positive association of *Paraprevotellaceae* with overweight individuals, while *Acidaminococcus* and *Adlercreutzia* showed a positive correlation with obesity; *Eggerthella* exhibited a negative correlation with both overweight and obese patients (Yun et al., 2017).

In contrast to the aforementioned studies, our investigation of obese patients in a European population, where both the exposure and outcome were European individuals, revealed a decrease in the abundance of *Firmicutes* and *Bacteroidetes* in their gut microbiota, accompanied by an increase in *Bacteroidetes* abundance. We believe that the primary reason for the observed differences lies in the variation in the study populations. Previous related studies primarily focused on individuals with obesity primarily attributed to excessive calorie intake. In contrast, our study conducted a more detailed stratification of obese patients, incorporating multiple outcome variables for Mendelian randomization analysis. Consequently, we observed a decrease in the abundance of *Ruminococcaceae UCG010* (a family within the *Ruminococcaceae* family) in individuals with obesity resulting from excessive calorie intake. This bacterium belongs to the *Firmicutes* phylum, and its production of short-chain fatty acids (SCFAs), primarily butyrate, serves as a major energy source for colonocytes (Kang et al., 2017). Butyrate not only contributes to extra fat deposition in the body but also binds to G protein-coupled receptors (GPR) 41 and GPR43 (Lin et al., 2012), promoting the expression of satiety hormones such as peptide YY (PYY) and leptin in adipocytes (Louis and Flint, 2017; Cuevas-Sierra et al., 2019). Additionally, it stimulates hepatic fat synthesis and intestinal motility (Chakraborti, 2015). Furthermore, butyrate can induce L cells to secrete glucagon-like peptide-1 (GLP-1), attenuating obesity (Yadav et al., 2013). Therefore, we proposed that *Ruminococcaceae UCG010* serves as a protective factor against Obesity due to excess calories.

It is noteworthy that this study represents the first analysis of the gut microbiota characteristics in individuals with drug-induced obesity, extreme obesity with alveolar hypoventilation, and localized adiposity. The findings have revealed some intriguing results. We observed an increased abundance of *Butyricimonas* in the gut microbiota of individuals with drug-induced obesity. *Butyricimonas*, the Gram-negative anaerobic bacterium within the *Bacteroidota* phylum, *Odoribacteraceae* family, is considered to significantly ameliorate obesity induced by a high-fat diet and reduce hepatic fat deposition (Rodriguez et al., 2020; Lee et al., 2022). However, in this study, *Butyricimonas* not only had no discernible impact on the population with obesity due to excess calorie intake but rather increased the risk of developing drug-induced obesity. Several potential reasons may underlie this paradox: Gram-negative bacteria contain lipopolysaccharides (LPS) as an intrinsic component of their cell wall, which is considered an endotoxin. In healthy individuals, blood levels of LPS are typically low. LPS is a potent activator of toll-like receptor 4 (TLR4) (Lim and Staudt, 2013). The binding of LPS to TLR4 can activate a wide array of cellular signaling pathways, inducing inflammation responses and the expression and secretion of cytokines within adipocytes and macrophages, leading to insulin resistance development and increased obesity (Medzhitov and Horng, 2009; Abdallah Ismail et al., 2011). Furthermore, the increase in LPS can directly enhance intestinal permeability, further exacerbating metabolic endotoxemia and inflammatory responses (Saad et al., 2016). Additionally, it is possible that *Butyricimonas* amplifies the effects of drugs in the process of inducing obesity, thereby further promoting or exacerbating obesity. Our hypothesis gains additional support from a report detailing the isolation of a new anaerobic bacterial species, *Butyricimonas phoceensis* sp. nov., from a severely obese 57-year-old French woman with a BMI of 55.8 kg/m² (Togo et al., 2016). Further research is required to delve into the specific mechanistic correlations between *Butyricimonas* and drug-induced obesity.

In individuals with extreme obesity accompanied by alveolar hypoventilation, we observed a decreased abundance of *Lactobacillus*, a genus within the *Lactobacillaceae* family, *Firmicutes* phylum. Previous research has consistently demonstrated the beneficial effects of many *Lactobacillus* strains in mitigating obesity in calorie-induced obesity populations. Wei et al. observed that *Loigolactobacillus coryniformis* and *Lacticasebacillus paracase*i reduced the body weight, Lee’s index, and fat index in high-fat diet-fed mice, significantly alleviating obesity (Song et al., 2021). Kang et al. also confirmed the significant role of *Lactobacillus acidophilus* in combating obesity, suppressing inflammation, increasing energy metabolism, and regulating lipid metabolism (Kang et al., 2022). *Lactobacillus* can restore the intestinal barrier, reduce intestinal permeability, inhibit bacteria and LPS entry into the liver, further suppressing the *TLR4/NF-κB* signaling pathway, and reducing inflammation. Additionally, this bacterium can increase the production of short-chain fatty acids (SCFAs) and regulate bile acid metabolism (Carvalho and Saad, 2013). In this study, we identified *Lactobacillus* as a protective factor in individuals with extreme obesity accompanied by alveolar hypoventilation. This represents the first discovery of this bacterium’s newfound prominence in this context. However, the specific mechanisms remain incompletely understood, necessitating further in-depth research.

Lastly, we observed a decreased abundance of *Pasteurellaceae* in individuals with localized adiposity. There is limited research regarding the association between *Pasteurellaceae* and obesity. This bacterium belongs to the Gram-negative anaerobic bacteria within the phylum *Proteobacteria*, comprising 13 genera and 65 species (Bonaventura et al., 2010). Most of its members live in symbiosis on the mucosal surfaces of birds and mammals, particularly in the upper respiratory tract (Kokotovic et al., 2007). Some of the human pathogens within this family include *Haemophilus influenzae* (St Geme, 2002) and *Aggregatibacter* species (Peng et al., 2019), which can cause various diseases such as meningitis, otitis media, chancroid, pneumonia, periodontitis, among others (Mullen et al., 2007). Maria et al. conducted a comparative genomic analysis of *Pasteurellaceae* and identified numerous unique conserved signature indels (CSIs). These CSIs were found to be uniquely shared among all sequenced *Pasteurella* species/strains and were not present in other bacteria. Hence, it is not out of the realm of possibility that Pasteurellaceae’s potential role in ameliorating localized adiposity may be linked to these unique CSIs. However, due to the limited research in this area, further validation is required (Bonaventura et al., 2010).

With the increasing annual incidence of obesity, obesity, as one of the key global economic and health concerns, is receiving growing attention. Obesity not only affects the quality of people’s life but also increases the risk of various short-term and long-term complications, thereby reducing life expectancy. Therefore, the treatment of obesity is currently a focal point of interest. To date, apart from lifestyle interventions, there are some anti-obesity medications available that are effective. However, they still have limitations, such as the potential for adverse events and higher costs (Aron-Wisnewsky et al., 2021)​​. Therefore, in order to reverse the global obesity trend and its associated management costs, there is a need to develop and implement safe and cost-effective public health interventions.

It is known that probiotics can alter the composition and population of the gastrointestinal microbiota and can be used to prevent or ameliorate associated gastrointestinal diseases. However, both have demonstrated promising preventative or therapeutic effects on non-gastrointestinal diseases, such as obesity, type 2 diabetes, and cardiovascular diseases, in animal models. Although human data are equivocal, it offers a novel perspective for their potential clinical use in managing diseases (Forgie et al., 2019) ​.

In this study, based on databases comprising European populations for both exposure and outcomes, we conducted univariable and multivariable Mendelian randomization (MR) analyses. This study is the first to comprehensively stratify obesity and unveil distinctive gut microbiota features among various obesity types. It confirms the causal relationship between gut microbiota and obesity. It is our hope that this work will provide novel diagnostic and therapeutic avenues for different types of obesity in future clinical practice. Furthermore, our study rigorously controlled for confounding factors related to obesity, rendering the conclusions more reliable compared to previous observational studies. Nevertheless, this study has certain limitations. Firstly, our study was confined to European populations. Given the substantial differences in dietary habits and genetic traits among diverse populations, the findings of this study may not be directly applicable to populations outside of Europe. Secondly, this study did not provide a molecular mechanism explaining how gut microbiota precisely influences the occurrence of thrombotic diseases. Thirdly, due to the limitations of the gut microbiota genome-wide association study (GWAS) databases used in this study, some gut microbiota beyond the scope of these databases may have been inadvertently overlooked.

## 5 Conclusion

There is a causal relationship between gut microbiota abundance and obesity, with distinct gut microbiota profiles observed in different obese populations. The bacteria, including *Ruminococcaceae UCG010*, *Butyricimonas*, *Pasteurellaceae* and *lactobacillus* independently influence the development of various types of obesity. Additionally, there are six gut microbiota species with potential associations with the development of obesity. The adjunctive therapy of probiotics and prebiotics may emerge as a novel approach in the future treatment of obesity.

## Data availability statement

Publicly available datasets were analyzed in this study. This data can be found here: https://mibiogen.gcc.rug.nl/menu/main/home; https://www.finngen.fi/en/access_results.

## Author contributions

SL: Writing – original draft. FL: Writing – original draft. YC: Investigation, Writing – review & editing. LR: Writing – review & editing. LS: Supervision, Writing – review & editing. XG: Supervision, Writing – review & editing. GW: Funding acquisition, Supervision, Writing – review & editing.
