# Supplementary material for: Unraveling the mystery: a Mendelian randomized exploration of gut microbiota and different types of obesity
Source: Front Cell Infect Microbiol. 2024 Feb 5;14:1352109. doi: 10.3389/fcimb.2024.1352109 (PMC10875079; doi:10.3389/fcimb.2024.1352109)
Supplement: Supplementary file 1 [file DataSheet_1.pdf]

Batch 1 : Gut microbiota abundance (family Acidaminococcaceae id.2166) on Localized adiposity

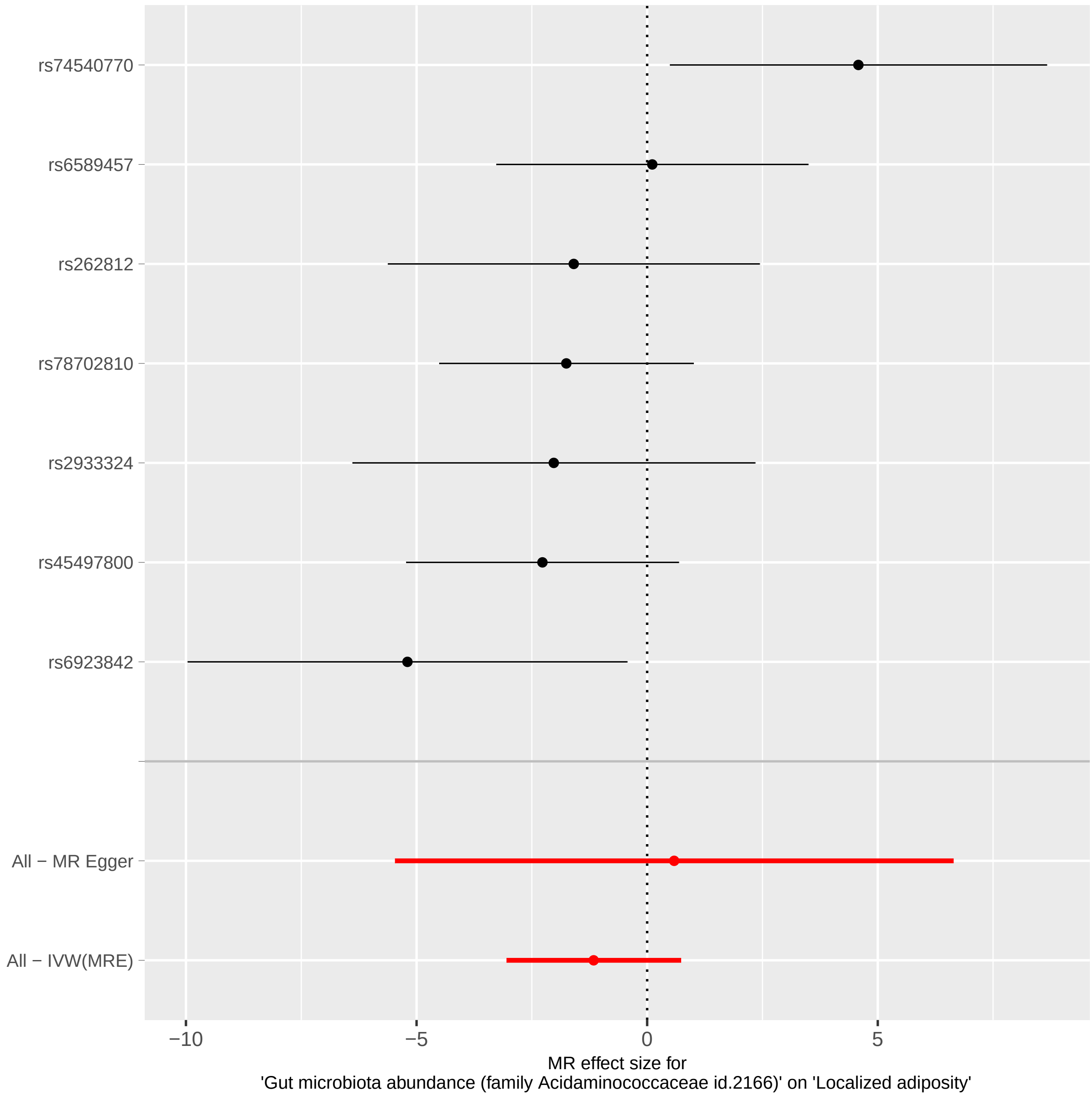

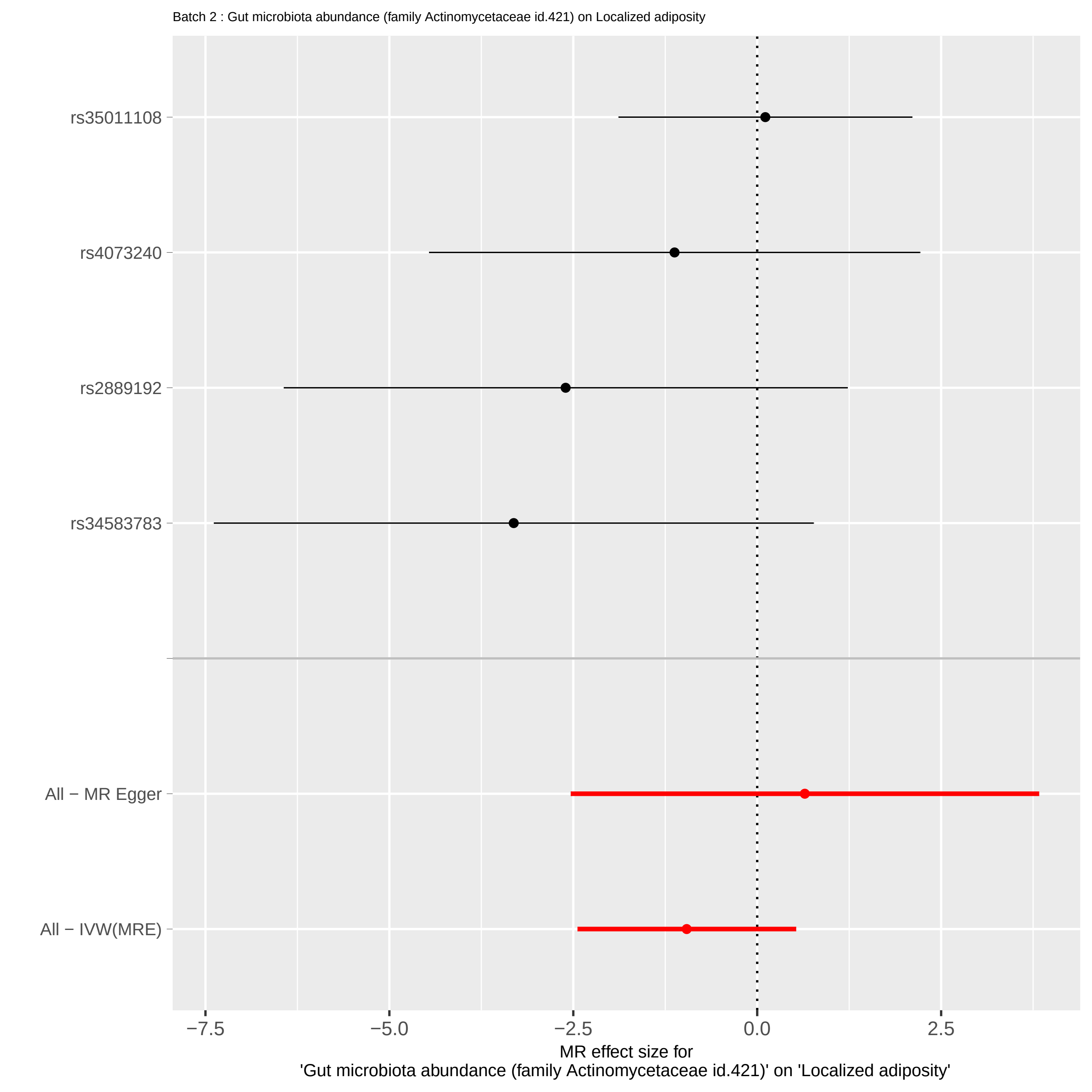

Batch 2 : Gut microbiota abundance (family Actinomycetaceae id.421) on Localized adiposity
rs35011108
rs4073240
rs2889192
rs34583783
All − MR Egger
All − IVW(MRE)
−7.5
−5.0
−2.5
0.0
2.5
MR effect size for
'Gut microbiota abundance (family Actinomycetaceae id.421)' on 'Localized adiposity'

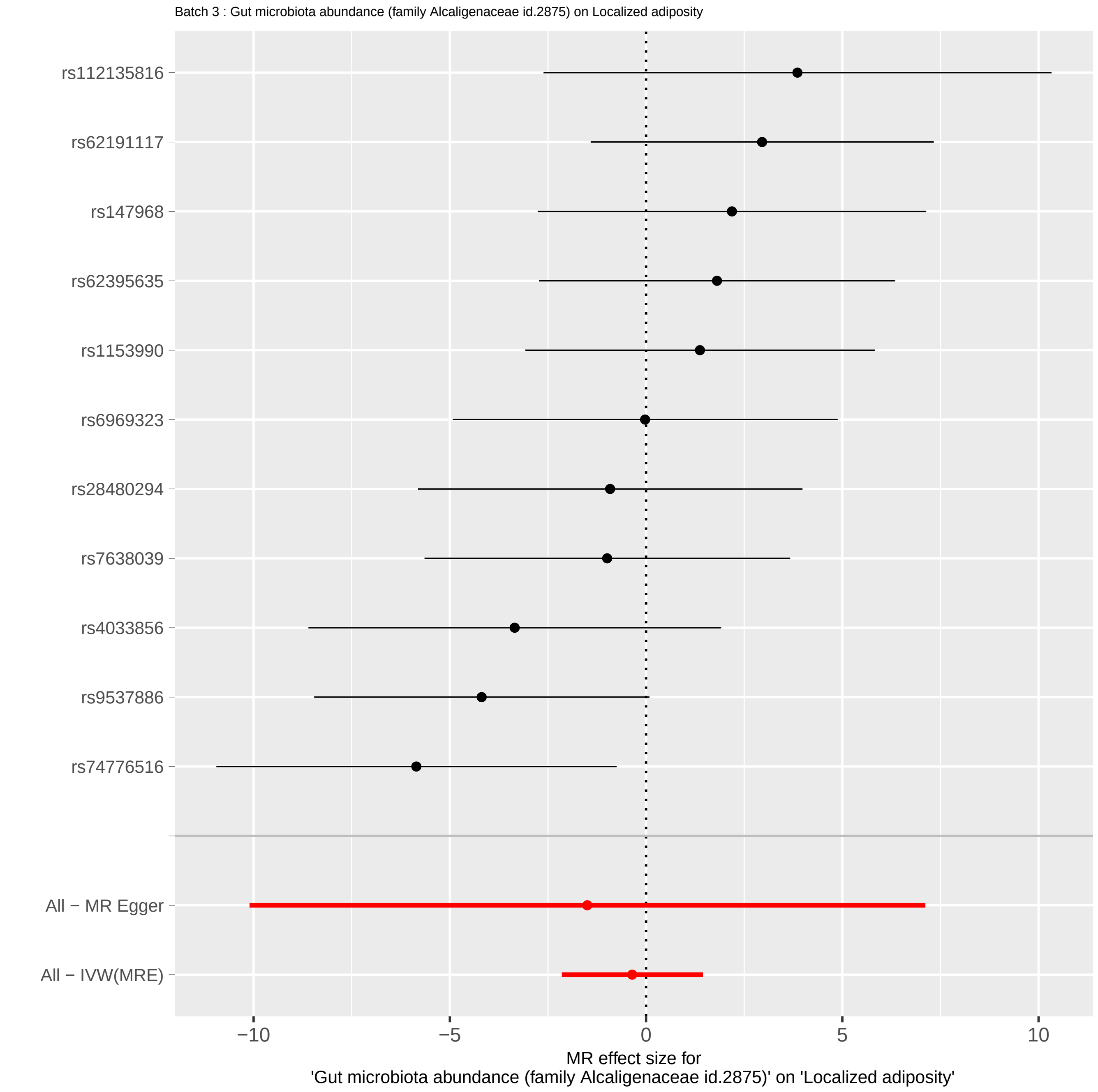
Batch 3 : Gut microbiota abundance (family Alcaligenaceae id.2875) on Localized adiposity
rs112135816
rs62191117
rs147968
rs62395635
rs1153990
rs6969323
rs28480294
rs7638039
rs4033856
rs9537886
rs74776516
All − MR Egger
All − IVW(MRE)
−10
−5
0
5
10
MR effect size for
'Gut microbiota abundance (family Alcaligenaceae id.2875)' on 'Localized adiposity'

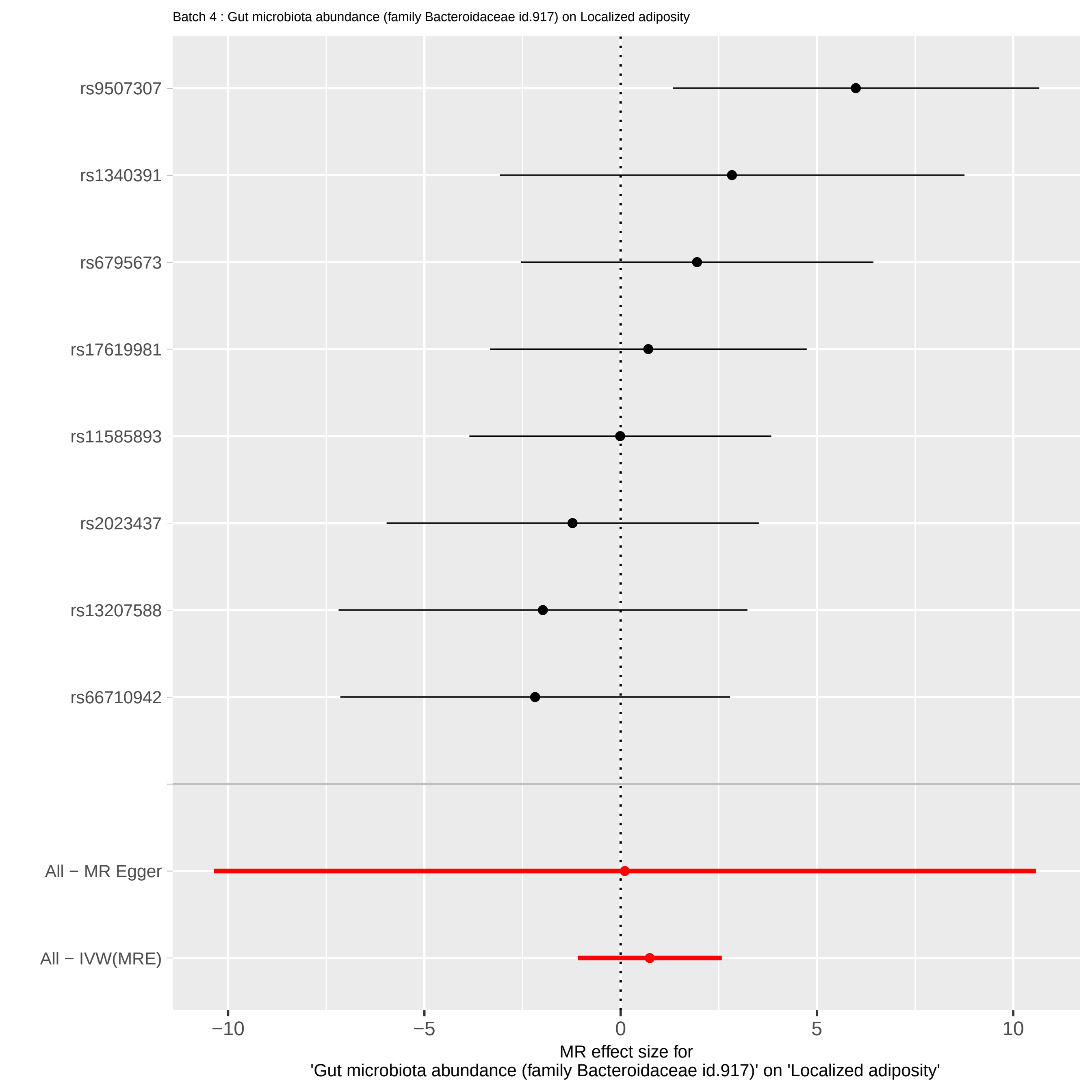
Batch 4 : Gut microbiota abundance (family Bacteroidaceae id.917) on Localized adiposity
rs9507307
rs1340391
rs6795673
rs17619981
rs11585893
rs2023437
rs13207588
rs66710942
All − MR Egger
All − IVW(MRE)
−10
−5
0
5
10
MR effect size for
'Gut microbiota abundance (family Bacteroidaceae id.917)' on 'Localized adiposity'

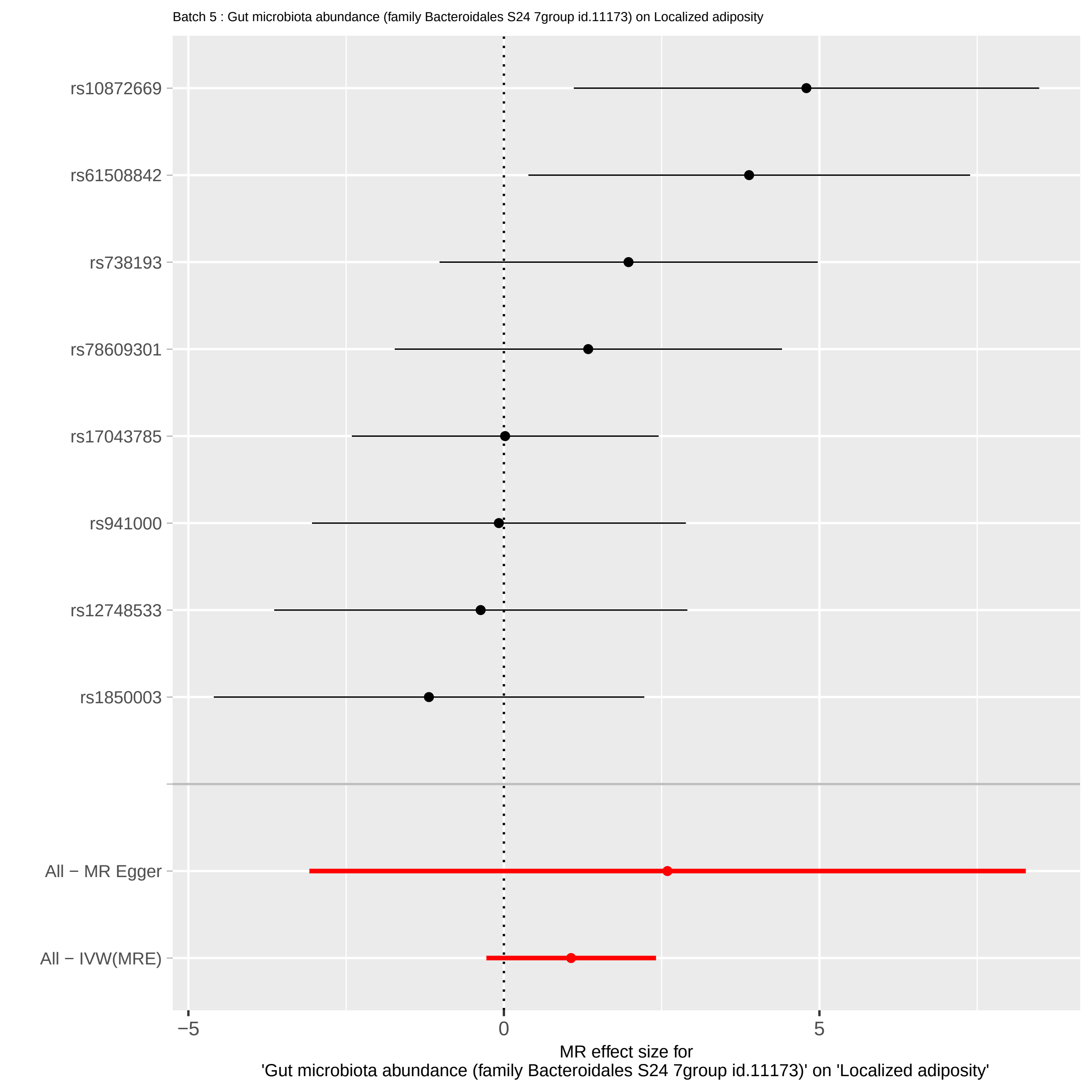

Batch 5 : Gut microbiota abundance (family Bacteroidales S24 7group id.11173) on Localized adiposity
rs10872669
rs61508842
rs738193
rs78609301
rs17043785
rs941000
rs12748533
rs1850003
All − MR Egger
All − IVW(MRE)
−5
0
5
MR effect size for
'Gut microbiota abundance (family Bacteroidales S24 7group id.11173)' on 'Localized adiposity'

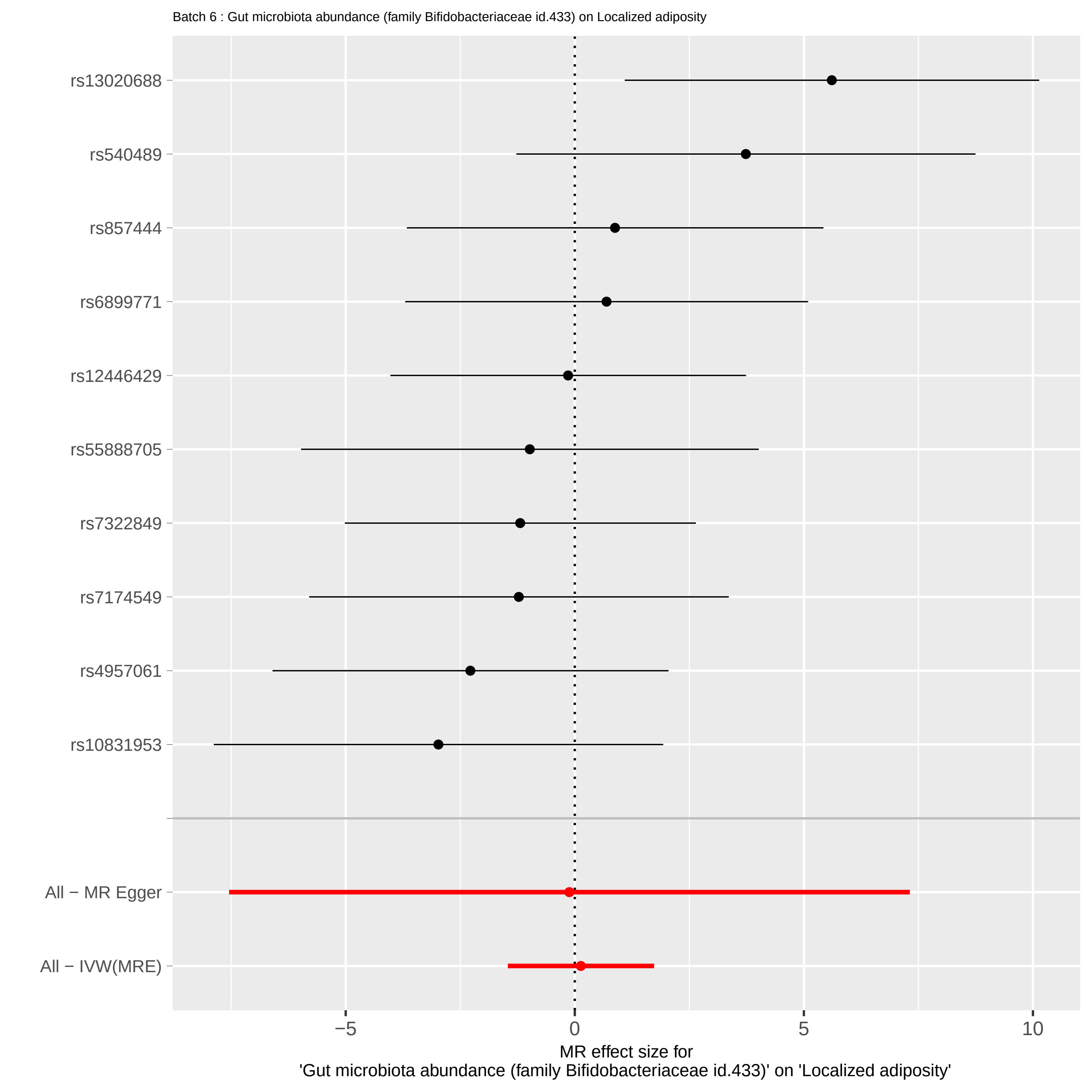
Batch 6 : Gut microbiota abundance (family Bifidobacteriaceae id.433) on Localized adiposity
rs13020688
rs540489
rs857444
rs6899771
rs12446429
rs55888705
rs7322849
rs7174549
rs4957061
rs10831953
All − MR Egger
All − IVW(MRE)
−5
0
5
10
MR effect size for
'Gut microbiota abundance (family Bifidobacteriaceae id.433)' on 'Localized adiposity'

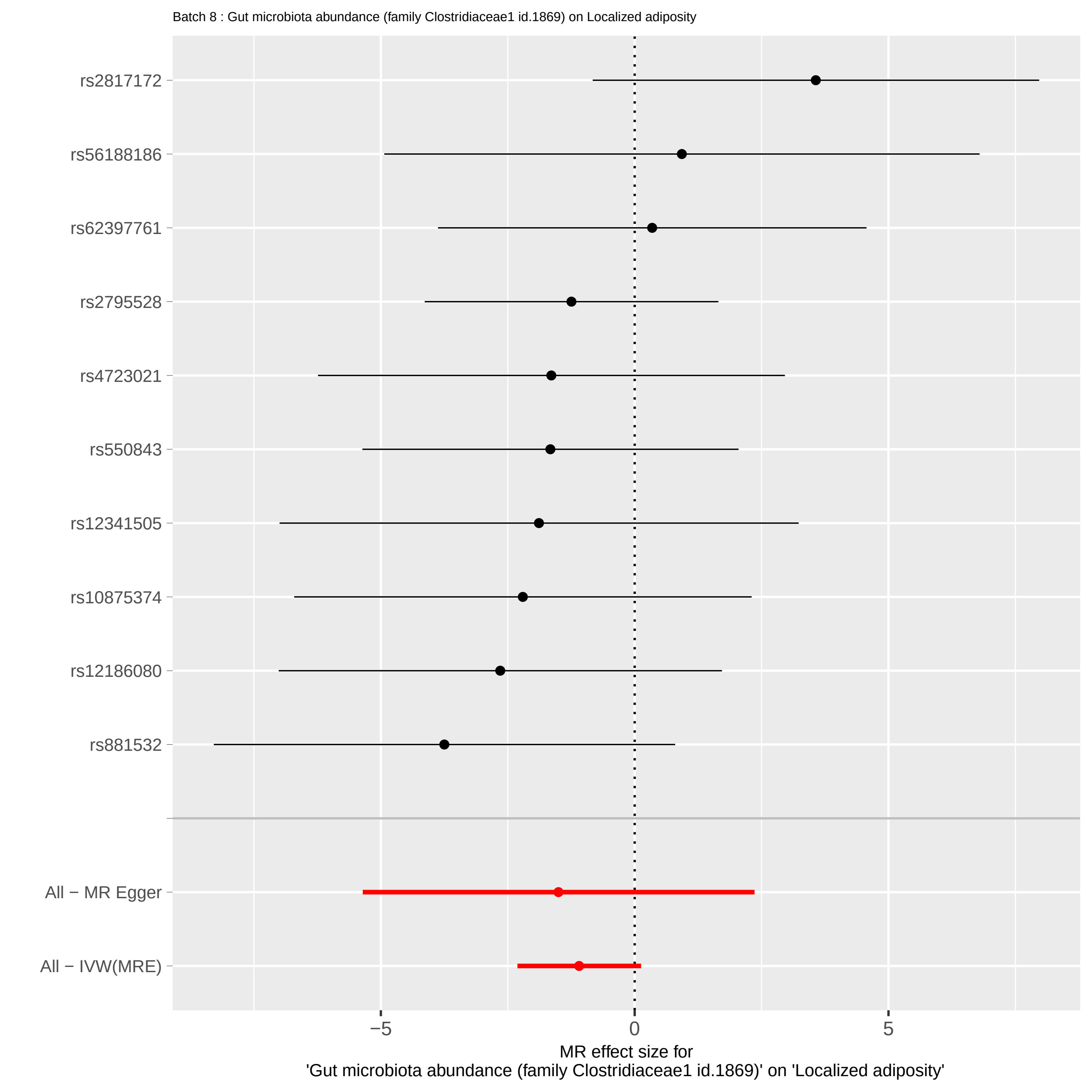

Batch 8 : Gut microbiota abundance (family Clostridiaceae1 id.1869) on Localized adiposity
rs2817172
rs56188186
rs62397761
rs2795528
rs4723021
rs550843
rs12341505
rs10875374
rs12186080
rs881532
All − MR Egger
All − IVW(MRE)
−5
0
5
MR effect size for
'Gut microbiota abundance (family Clostridiaceae1 id.1869)' on 'Localized adiposity'

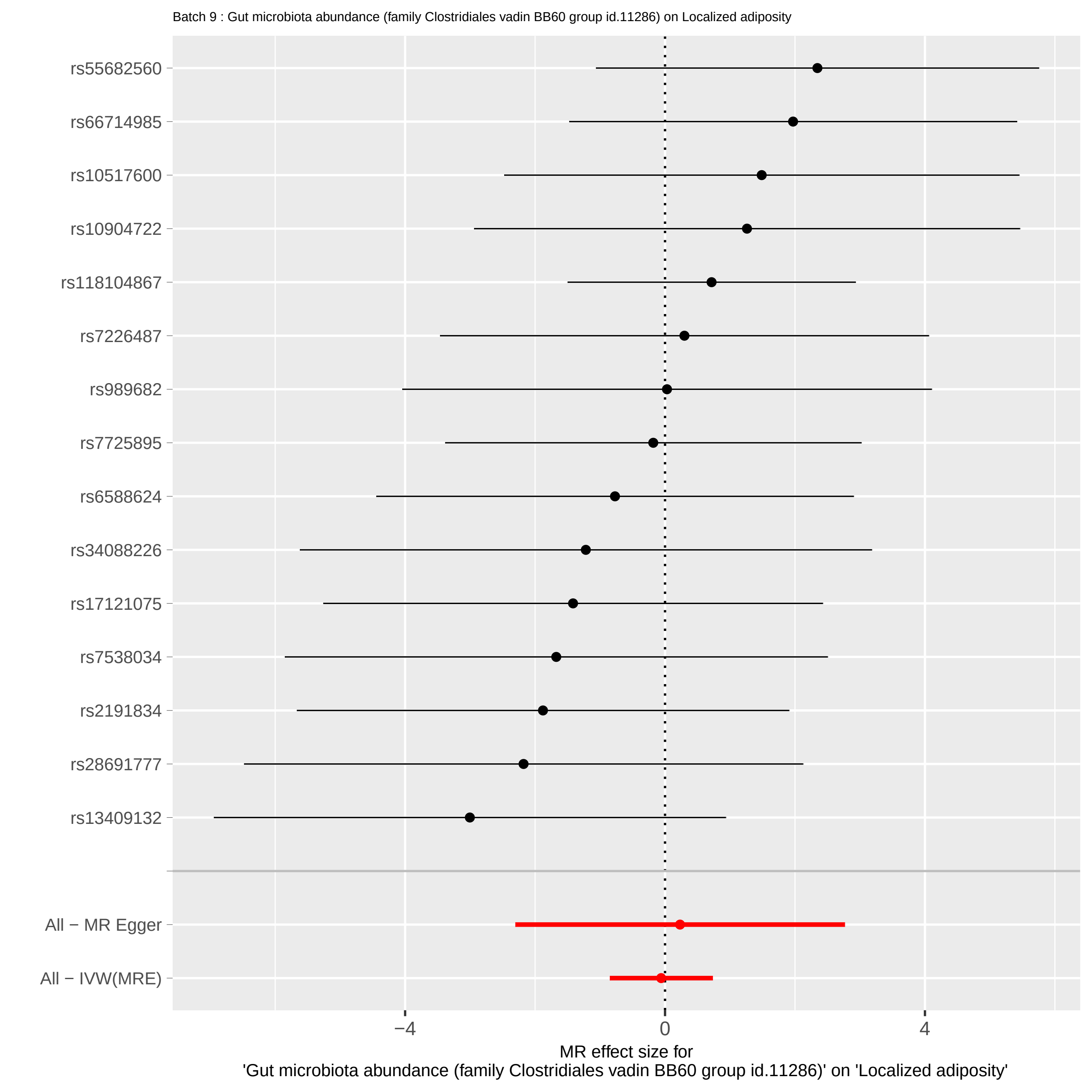
Batch 9 : Gut microbiota abundance (family Clostridiales vadin BB60 group id.11286) on Localized adiposity
rs55682560
rs66714985
rs10517600
rs10904722
rs118104867
rs7226487
rs989682
rs7725895
rs6588624
rs34088226
rs17121075
rs7538034
rs2191834
rs28691777
rs13409132
All − MR Egger
All − IVW(MRE)
−4
0
4
MR effect size for
'Gut microbiota abundance (family Clostridiales vadin BB60 group id.11286)' on 'Localized adiposity'

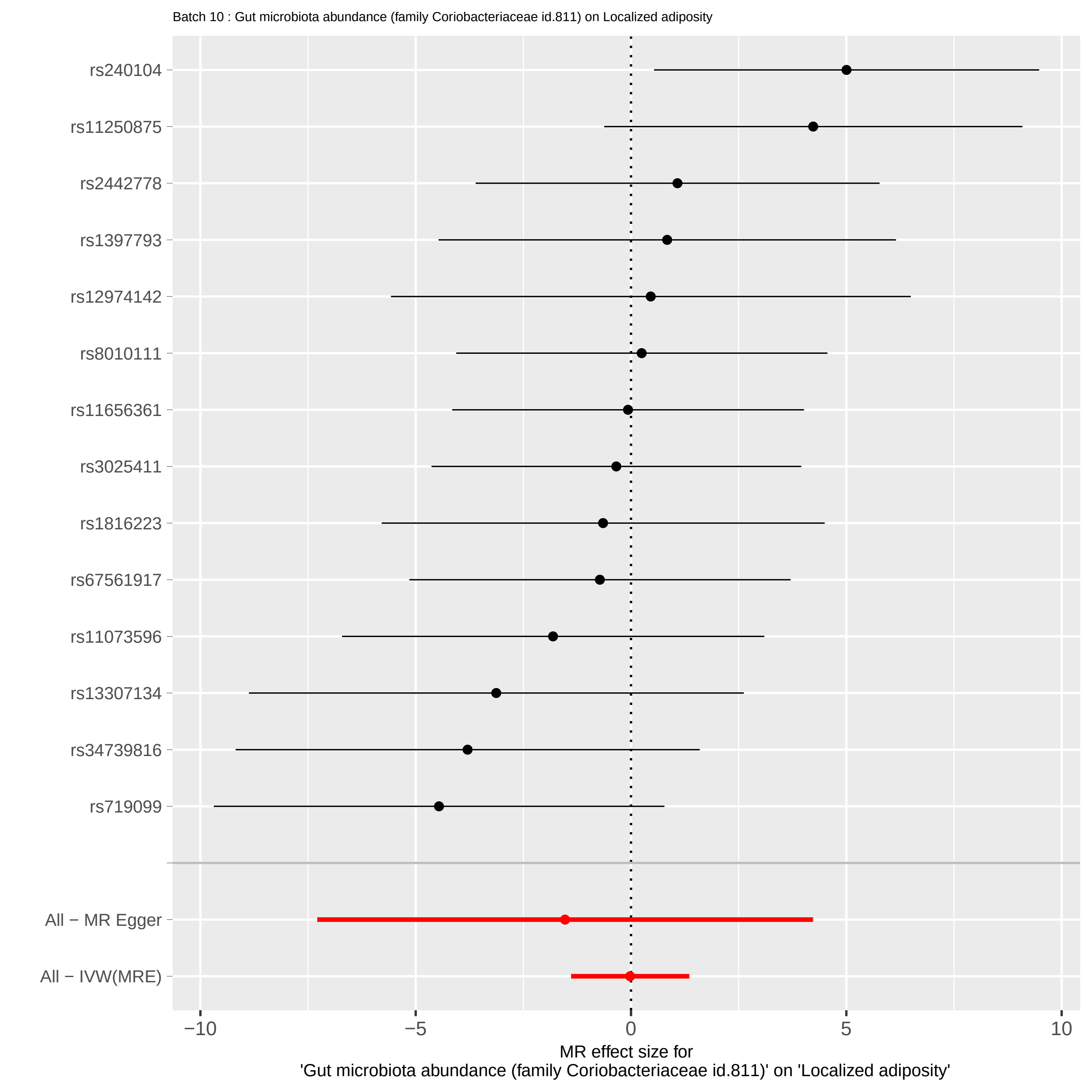
Batch 10 : Gut microbiota abundance (family Coriobacteriaceae id.811) on Localized adiposity
rs240104
rs11250875
rs2442778
rs1397793
rs12974142
rs8010111
rs11656361
rs3025411
rs1816223
rs67561917
rs11073596
rs13307134
rs34739816
rs719099
All − MR Egger
All − IVW(MRE)
−10
−5
0
5
10
MR effect size for
'Gut microbiota abundance (family Coriobacteriaceae id.811)' on 'Localized adiposity'

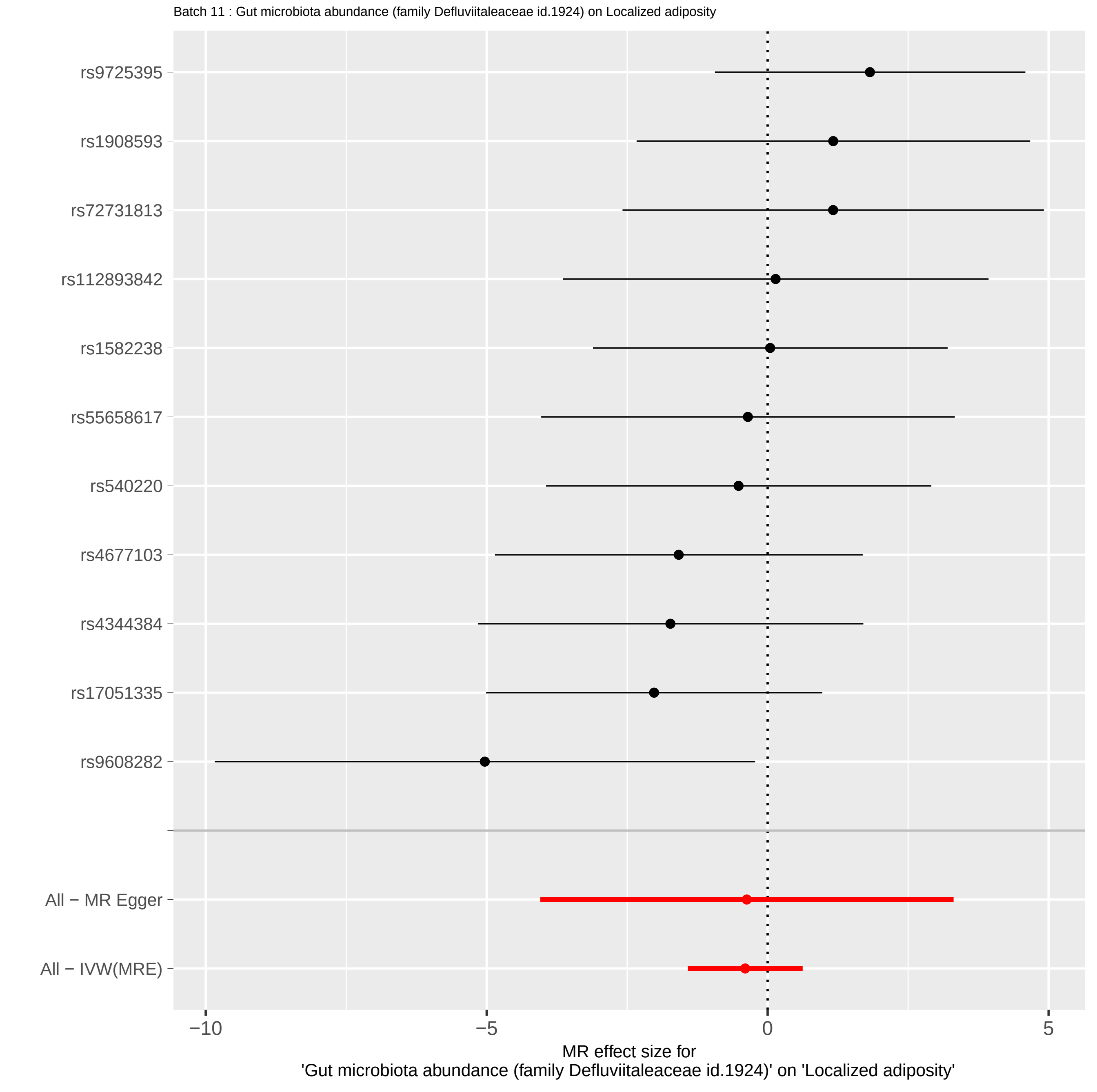

Batch 11 : Gut microbiota abundance (family Defluviitaleaceae id.1924) on Localized adiposity
rs9725395
rs1908593
rs72731813
rs112893842
rs1582238
rs55658617
rs540220
rs4677103
rs4344384
rs17051335
rs9608282
All − MR Egger
All − IVW(MRE)
−10
−5
0
5
MR effect size for
'Gut microbiota abundance (family Defluviitaleaceae id.1924)' on 'Localized adiposity'

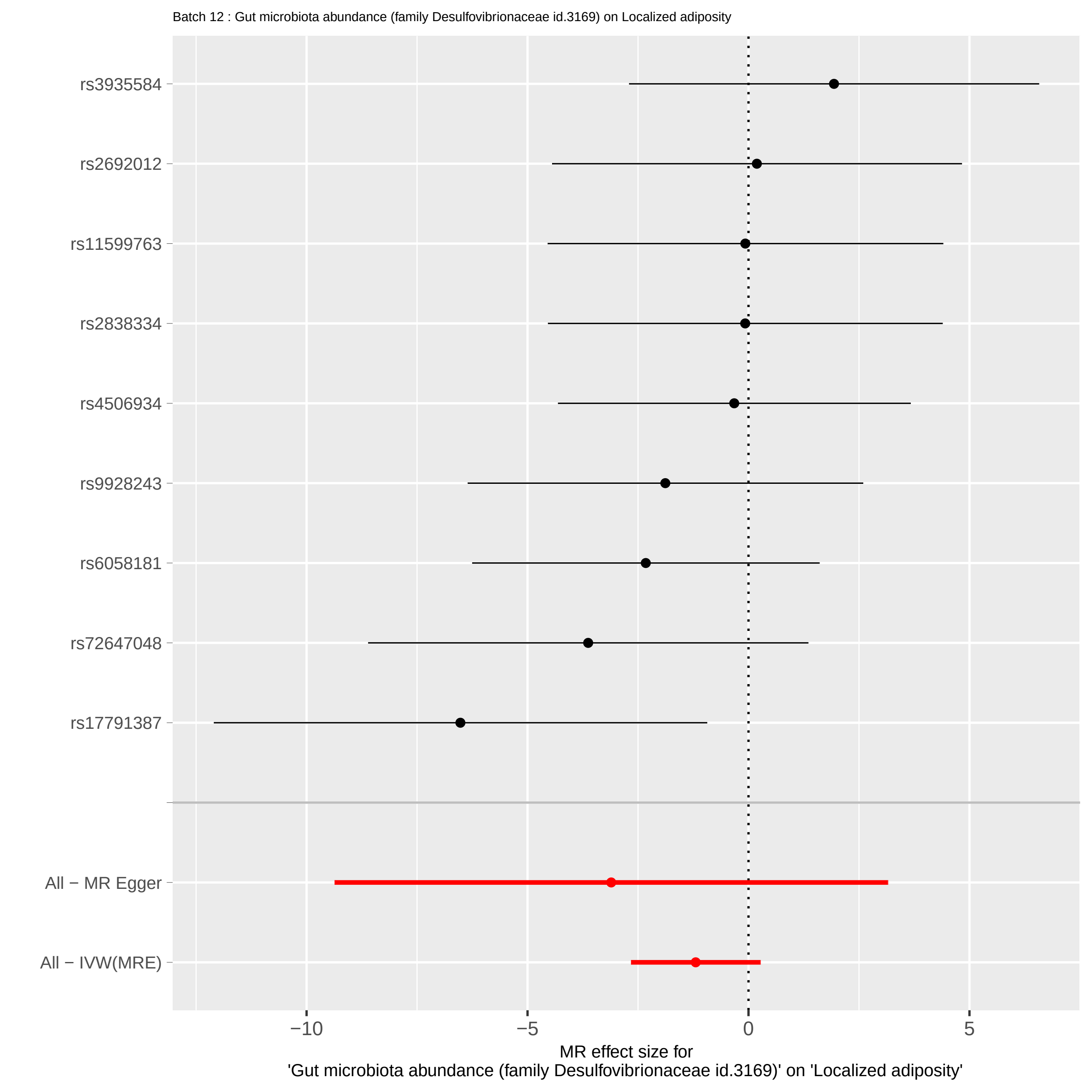

Batch 12 : Gut microbiota abundance (family Desulfovibrionaceae id.3169) on Localized adiposity
rs3935584
rs2692012
rs11599763
rs2838334
rs4506934
rs9928243
rs6058181
rs72647048
rs17791387
All − MR Egger
All − IVW(MRE)
−10
−5
0
5
MR effect size for
'Gut microbiota abundance (family Desulfovibrionaceae id.3169)' on 'Localized adiposity'

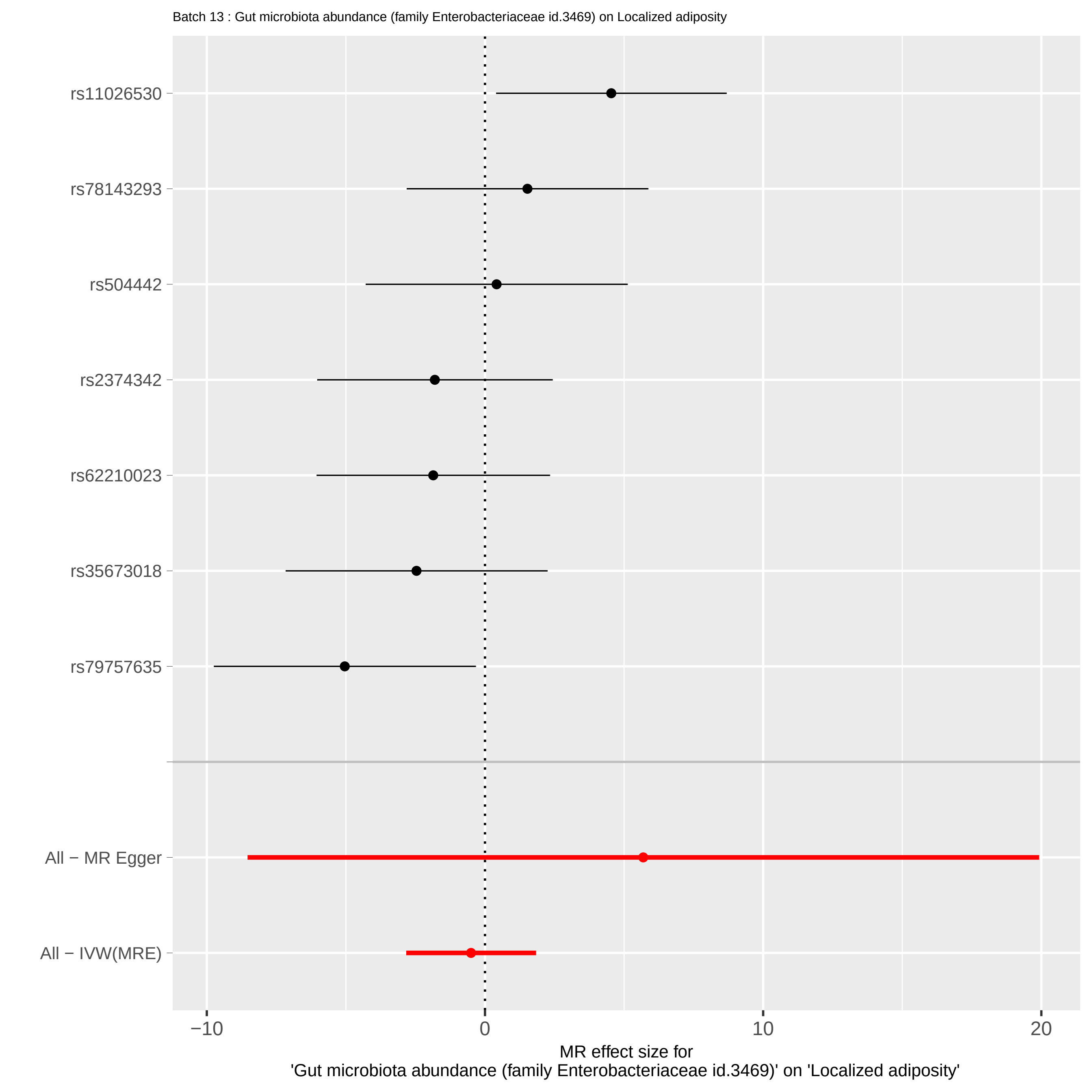
Batch 13 : Gut microbiota abundance (family Enterobacteriaceae id.3469) on Localized adiposity
rs11026530
rs78143293
rs504442
rs2374342
rs62210023
rs35673018
rs79757635
All − MR Egger
All − IVW(MRE)
−10
0
10
20
MR effect size for
'Gut microbiota abundance (family Enterobacteriaceae id.3469)' on 'Localized adiposity'

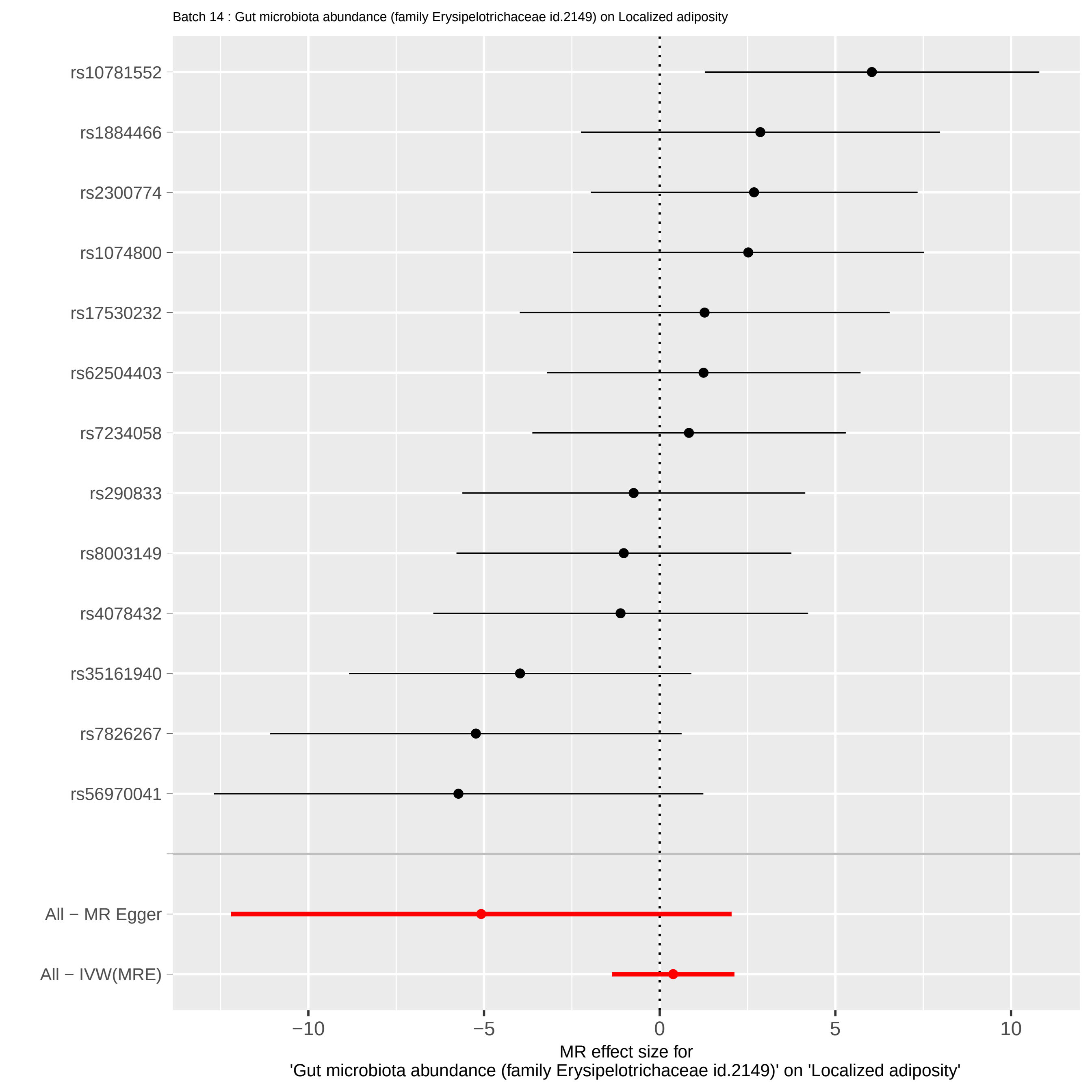
Batch 14 : Gut microbiota abundance (family Erysipelotrichaceae id.2149) on Localized adiposity
rs10781552
rs1884466
rs2300774
rs1074800
rs17530232
rs62504403
rs7234058
rs290833
rs8003149
rs4078432
rs35161940
rs7826267
rs56970041
All − MR Egger
All − IVW(MRE)
−10
−5
0
5
10
MR effect size for
'Gut microbiota abundance (family Erysipelotrichaceae id.2149)' on 'Localized adiposity'

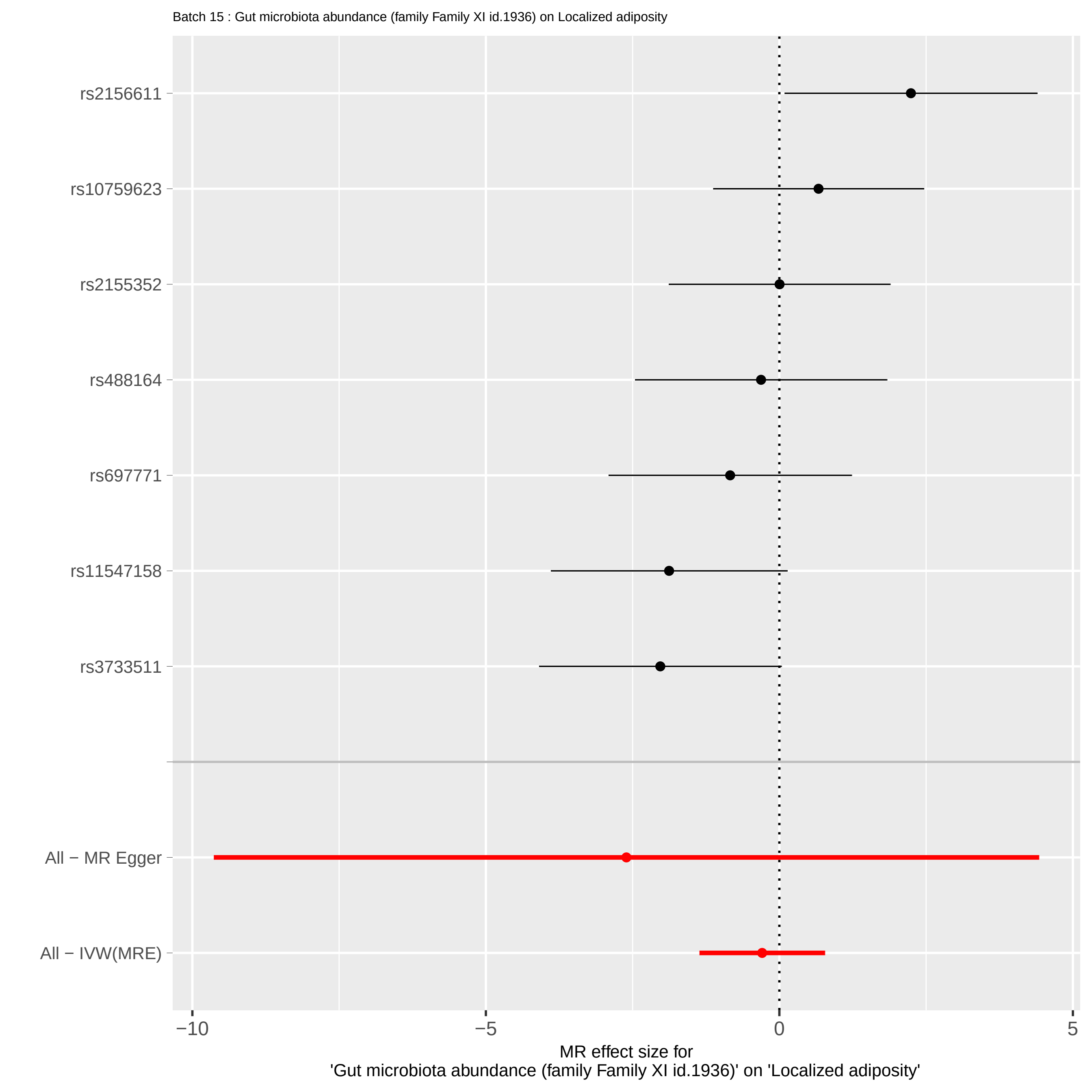
Batch 15 : Gut microbiota abundance (family Family XI id.1936) on Localized adiposity
rs2156611
rs10759623
rs2155352
rs488164
rs697771
rs11547158
rs3733511
All − MR Egger
All − IVW(MRE)
−10
−5
0
5
MR effect size for
'Gut microbiota abundance (family Family XI id.1936)' on 'Localized adiposity'

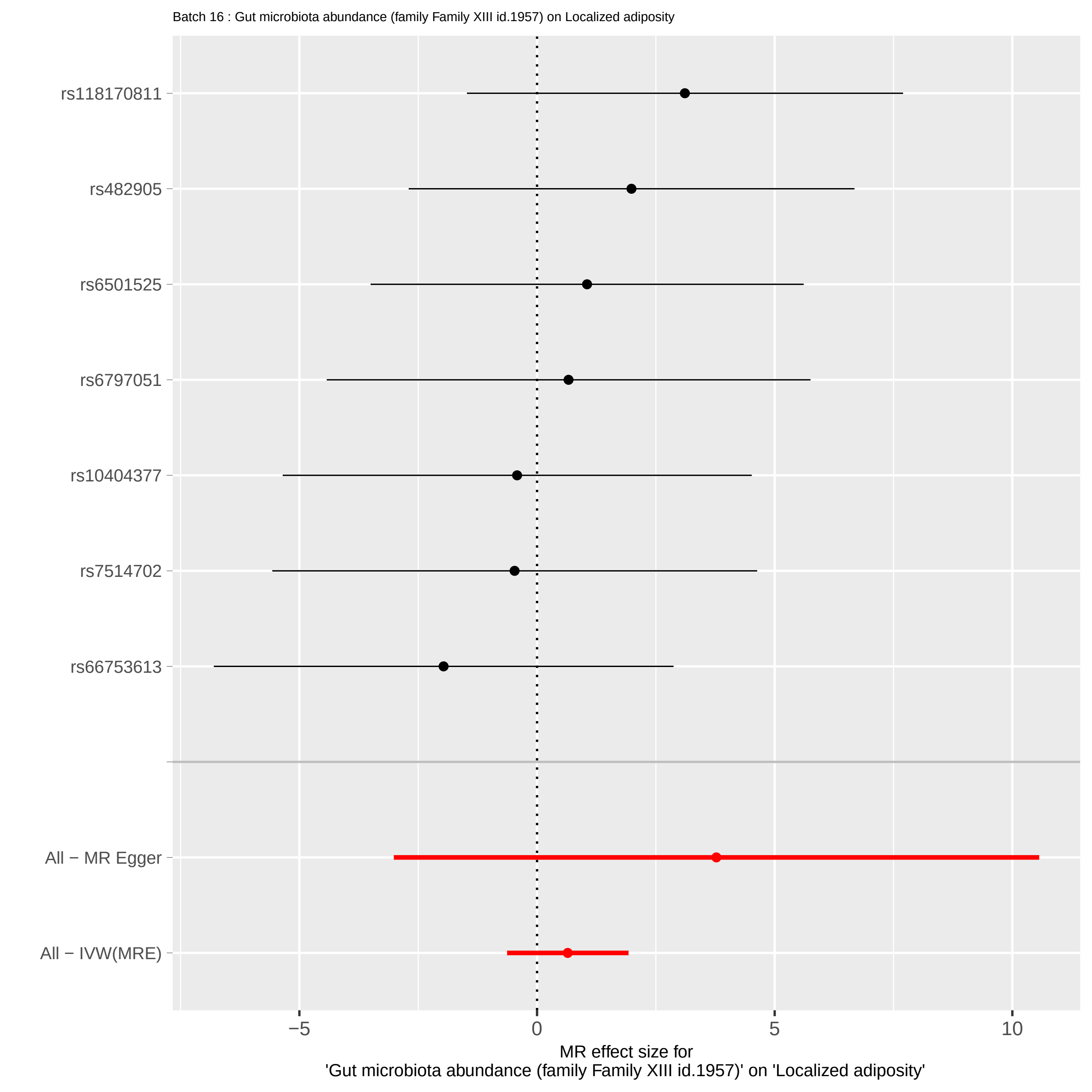

Batch 16 : Gut microbiota abundance (family Family XIII id.1957) on Localized adiposity
rs118170811
rs482905
rs6501525
rs6797051
rs10404377
rs7514702
rs66753613
All − MR Egger
All − IVW(MRE)
−5
0
5
10
MR effect size for
'Gut microbiota abundance (family Family XIII id.1957)' on 'Localized adiposity'

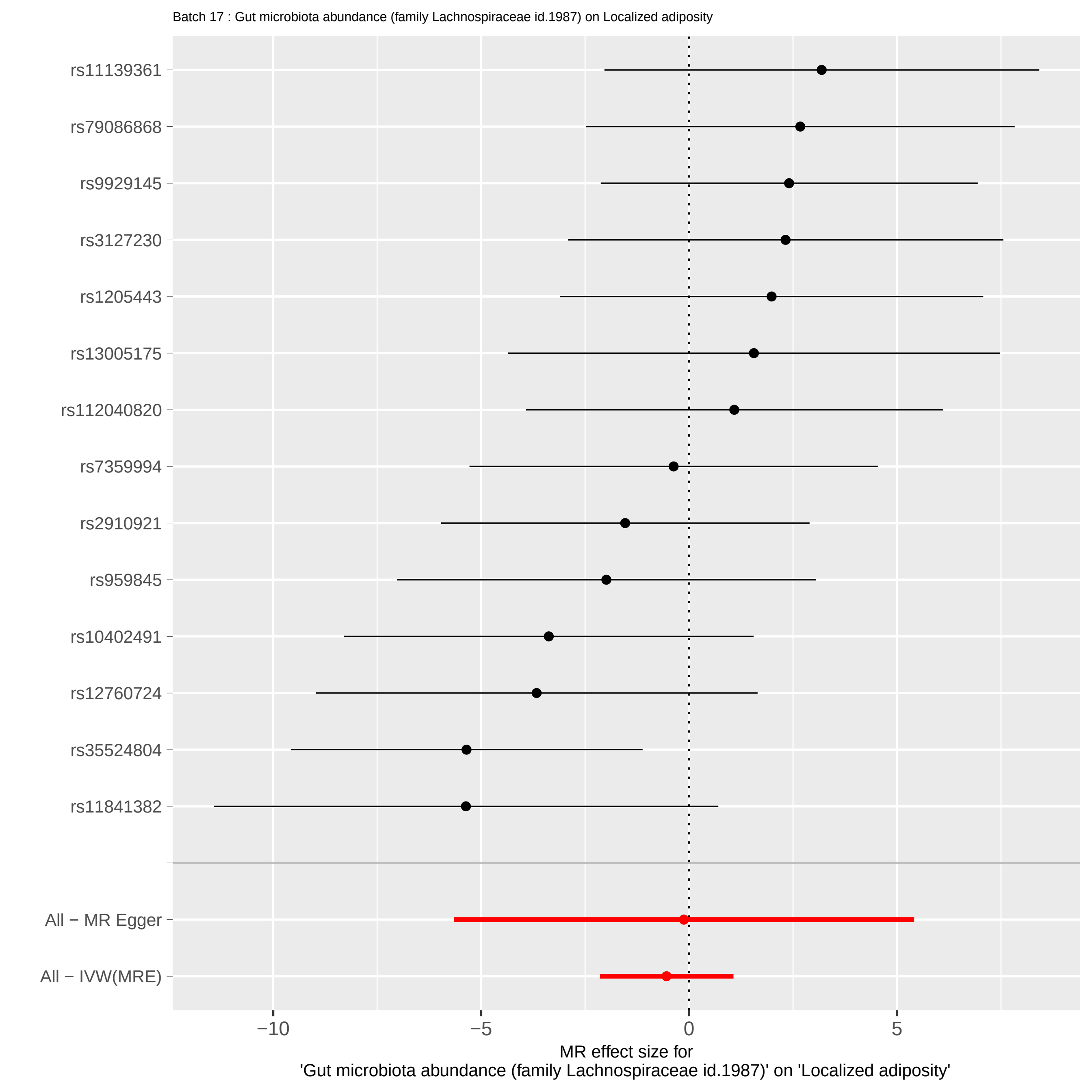
Batch 17 : Gut microbiota abundance (family Lachnospiraceae id.1987) on Localized adiposity
rs11139361
rs79086868
rs9929145
rs3127230
rs1205443
rs13005175
rs112040820
rs7359994
rs2910921
rs959845
rs10402491
rs12760724
rs35524804
rs11841382
All − MR Egger
All − IVW(MRE)
−10
−5
0
5
MR effect size for
'Gut microbiota abundance (family Lachnospiraceae id.1987)' on 'Localized adiposity'

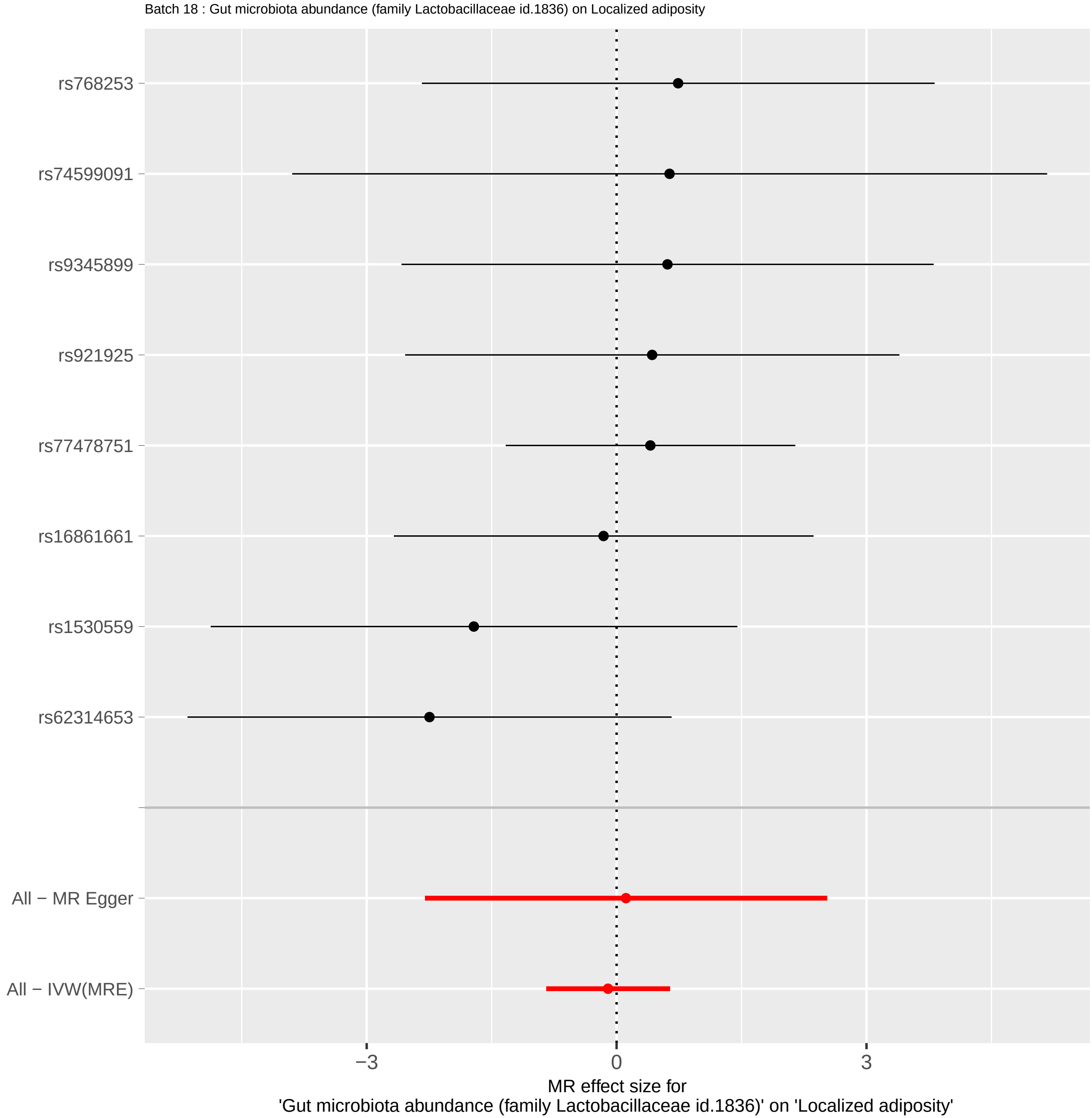

Batch 18 : Gut microbiota abundance (family Lactobacillaceae id.1836) on Localized adiposity
rs768253
rs74599091
rs9345899
rs921925
rs77478751
rs16861661
rs1530559
rs62314653
All − MR Egger
All − IVW(MRE)
−3
0
3
MR effect size for
'Gut microbiota abundance (family Lactobacillaceae id.1836)' on 'Localized adiposity'

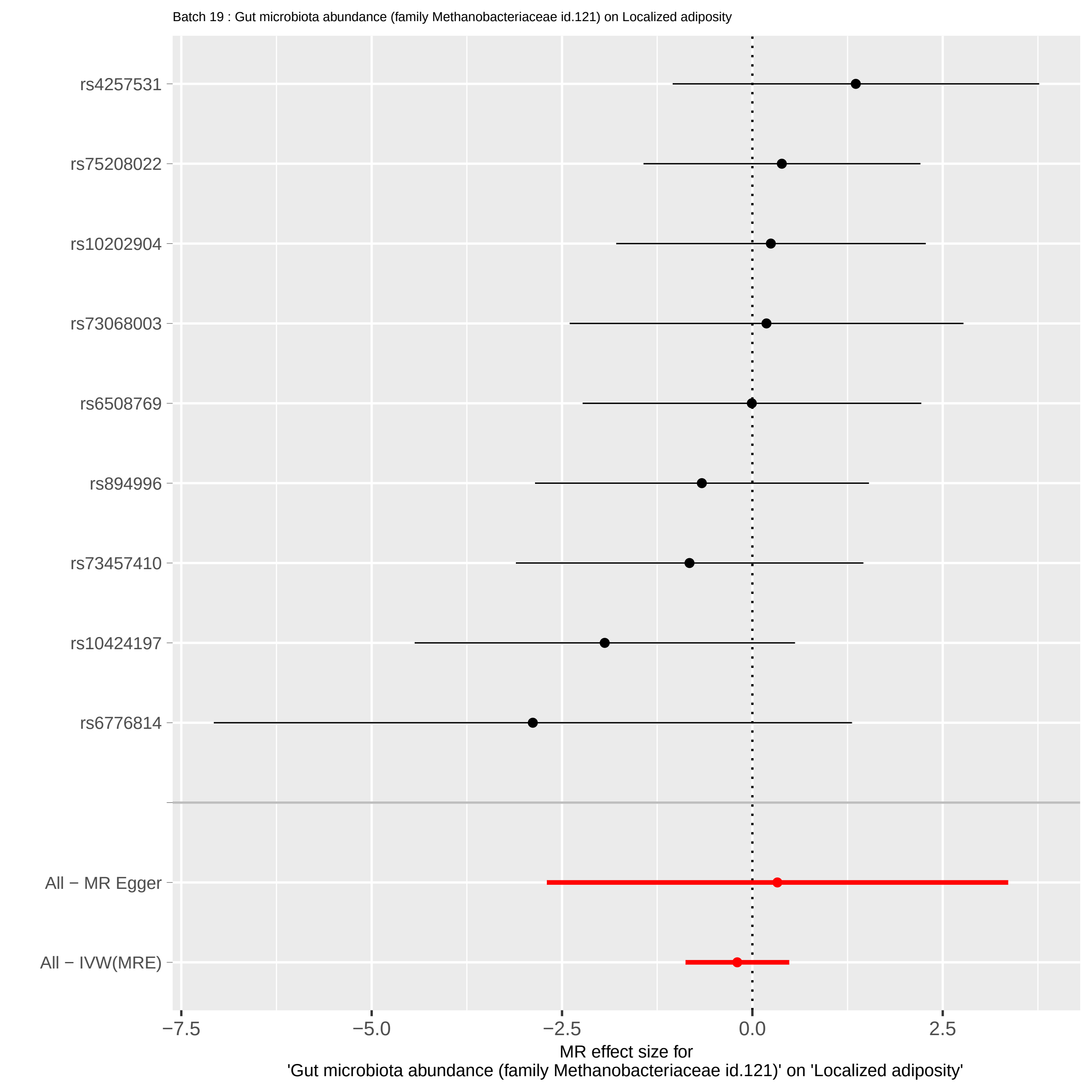
Batch 19 : Gut microbiota abundance (family Methanobacteriaceae id.121) on Localized adiposity
rs4257531
rs75208022
rs10202904
rs73068003
rs6508769
rs894996
rs73457410
rs10424197
rs6776814
All − MR Egger
All − IVW(MRE)
−7.5
−5.0
−2.5
0.0
2.5
MR effect size for
'Gut microbiota abundance (family Methanobacteriaceae id.121)' on 'Localized adiposity'

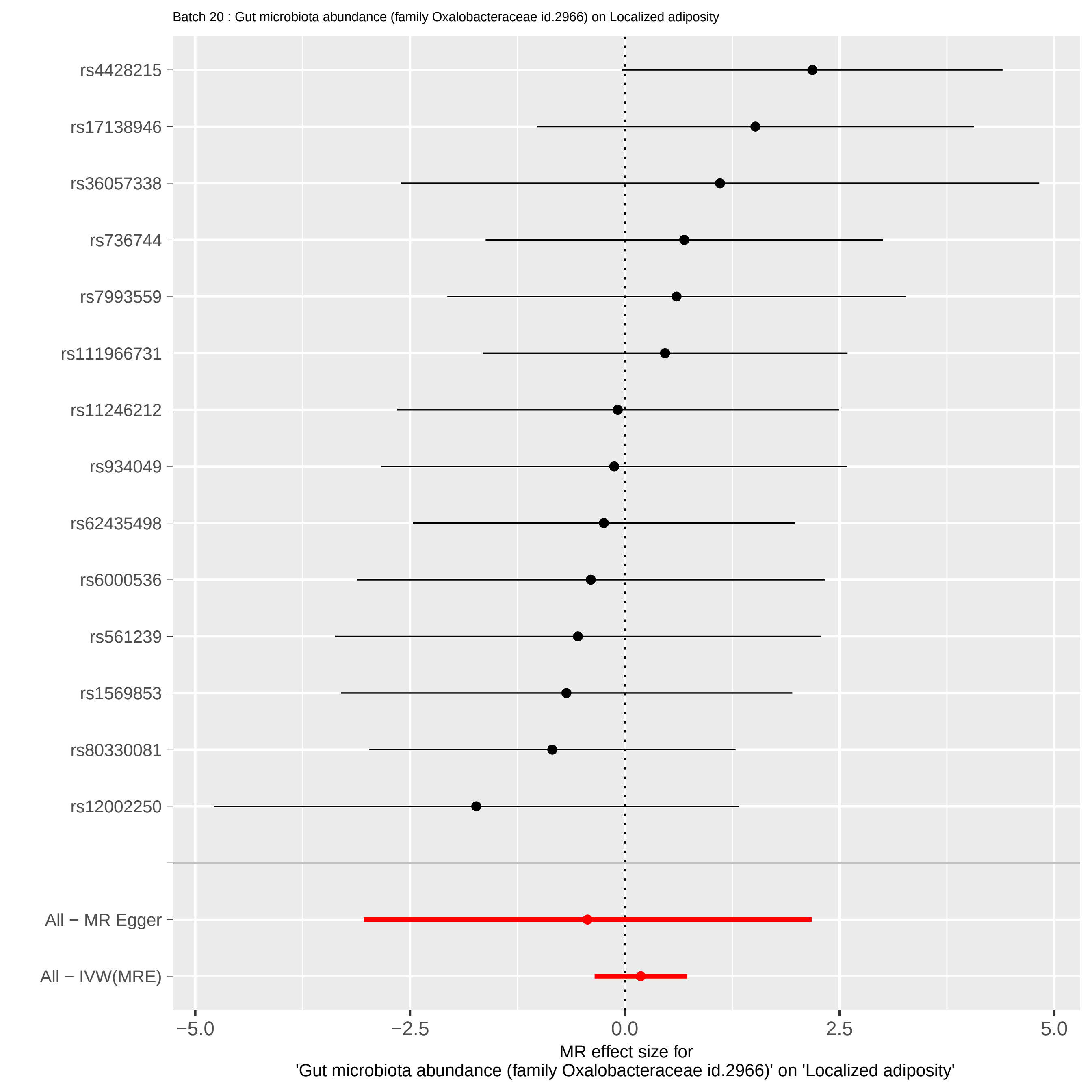
Batch 20 : Gut microbiota abundance (family Oxalobacteraceae id.2966) on Localized adiposity
rs4428215
rs17138946
rs36057338
rs736744
rs7993559
rs111966731
rs11246212
rs934049
rs62435498
rs6000536
rs561239
rs1569853
rs80330081
rs12002250
All − MR Egger
All − IVW(MRE)
−5.0
−2.5
0.0
2.5
5.0
MR effect size for
'Gut microbiota abundance (family Oxalobacteraceae id.2966)' on 'Localized adiposity'

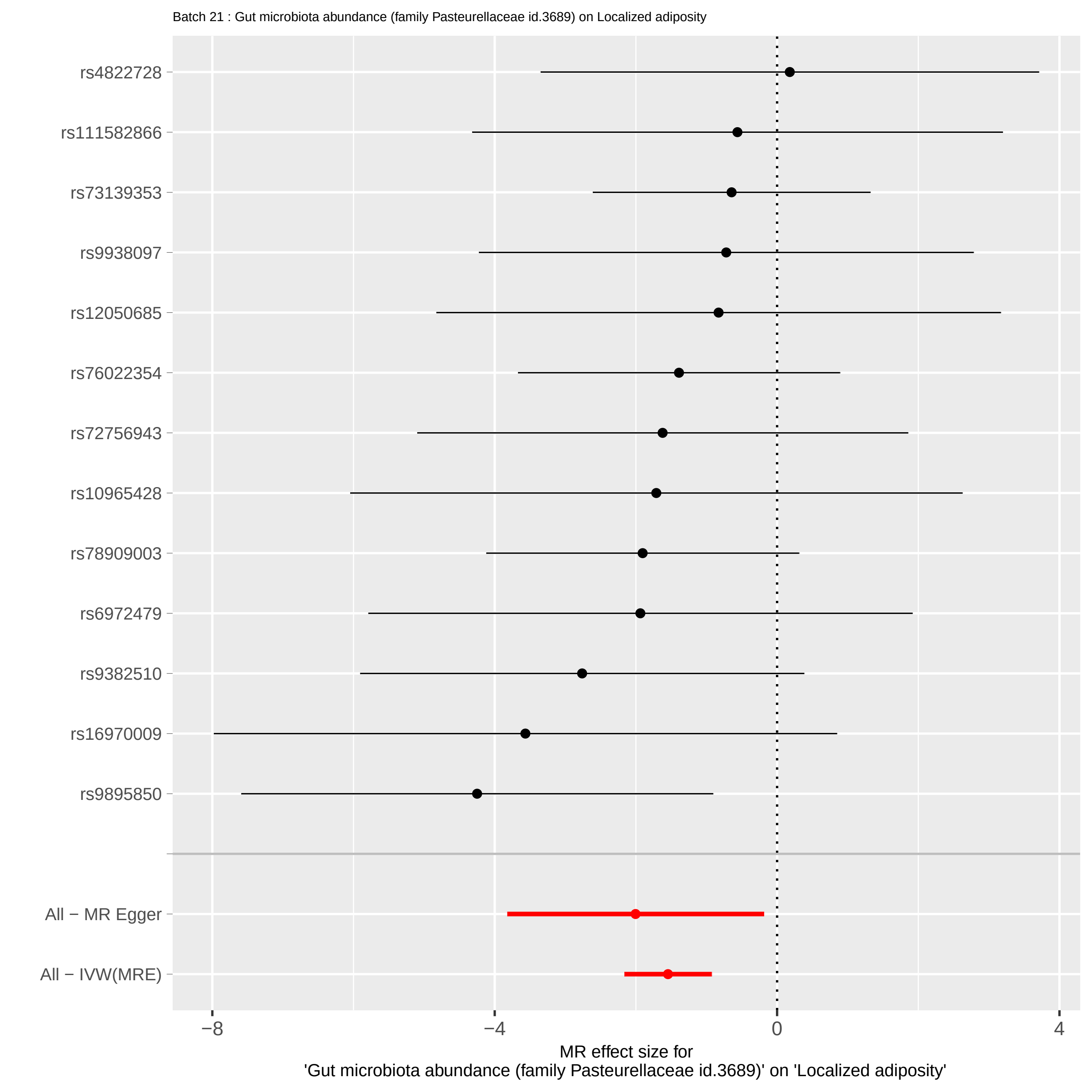

Batch 21 : Gut microbiota abundance (family Pasteurellaceae id.3689) on Localized adiposity
rs4822728
rs111582866
rs73139353
rs9938097
rs12050685
rs76022354
rs72756943
rs10965428
rs78909003
rs6972479
rs9382510
rs16970009
rs9895850
All − MR Egger
All − IVW(MRE)
−8
−4
0
4
MR effect size for
'Gut microbiota abundance (family Pasteurellaceae id.3689)' on 'Localized adiposity'

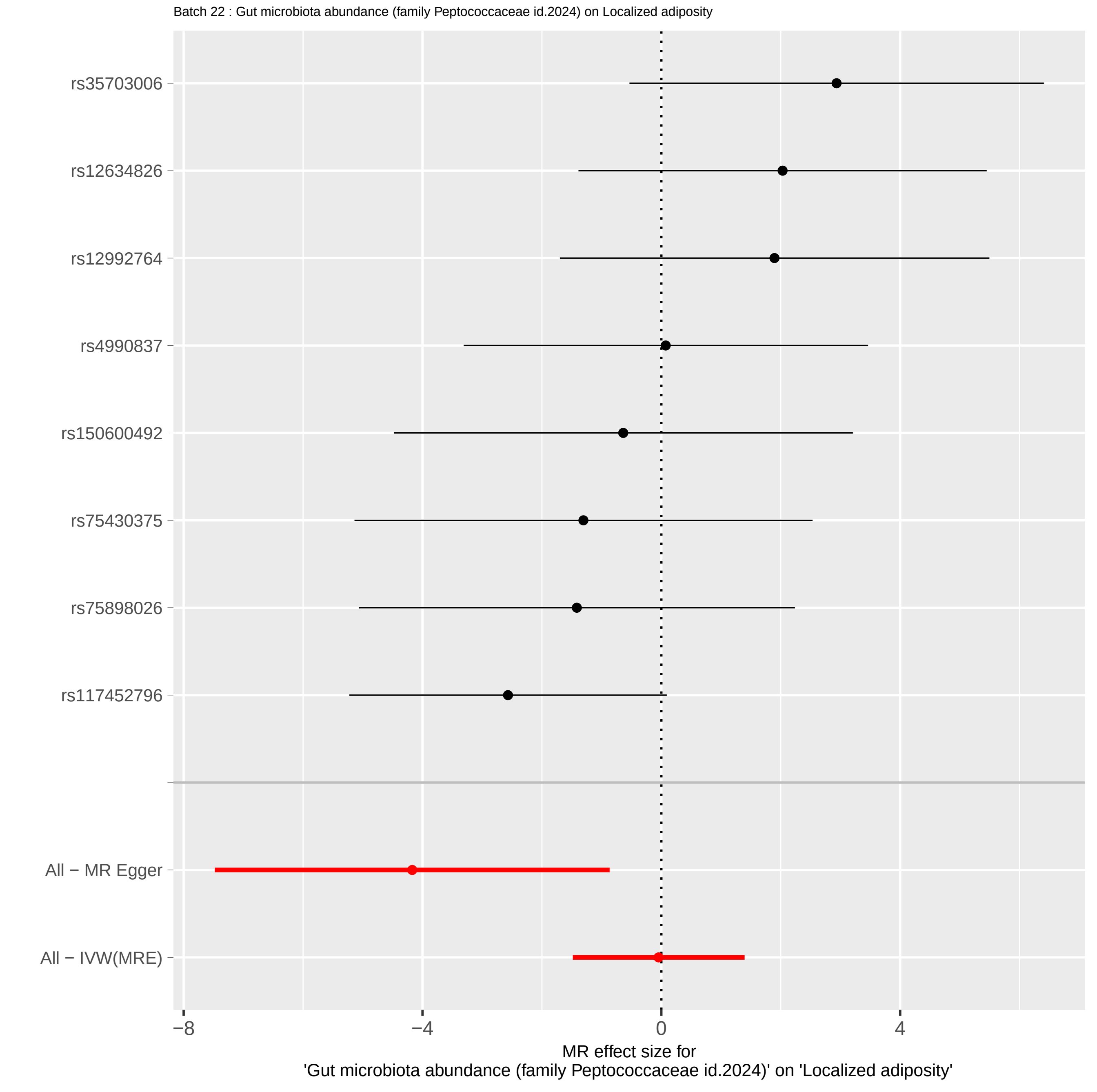
Batch 22 : Gut microbiota abundance (family Peptococcaceae id.2024) on Localized adiposity
rs35703006
rs12634826
rs12992764
rs4990837
rs150600492
rs75430375
rs75898026
rs117452796
All − MR Egger
All − IVW(MRE)
−8
−4
0
4
MR effect size for
'Gut microbiota abundance (family Peptococcaceae id.2024)' on 'Localized adiposity'

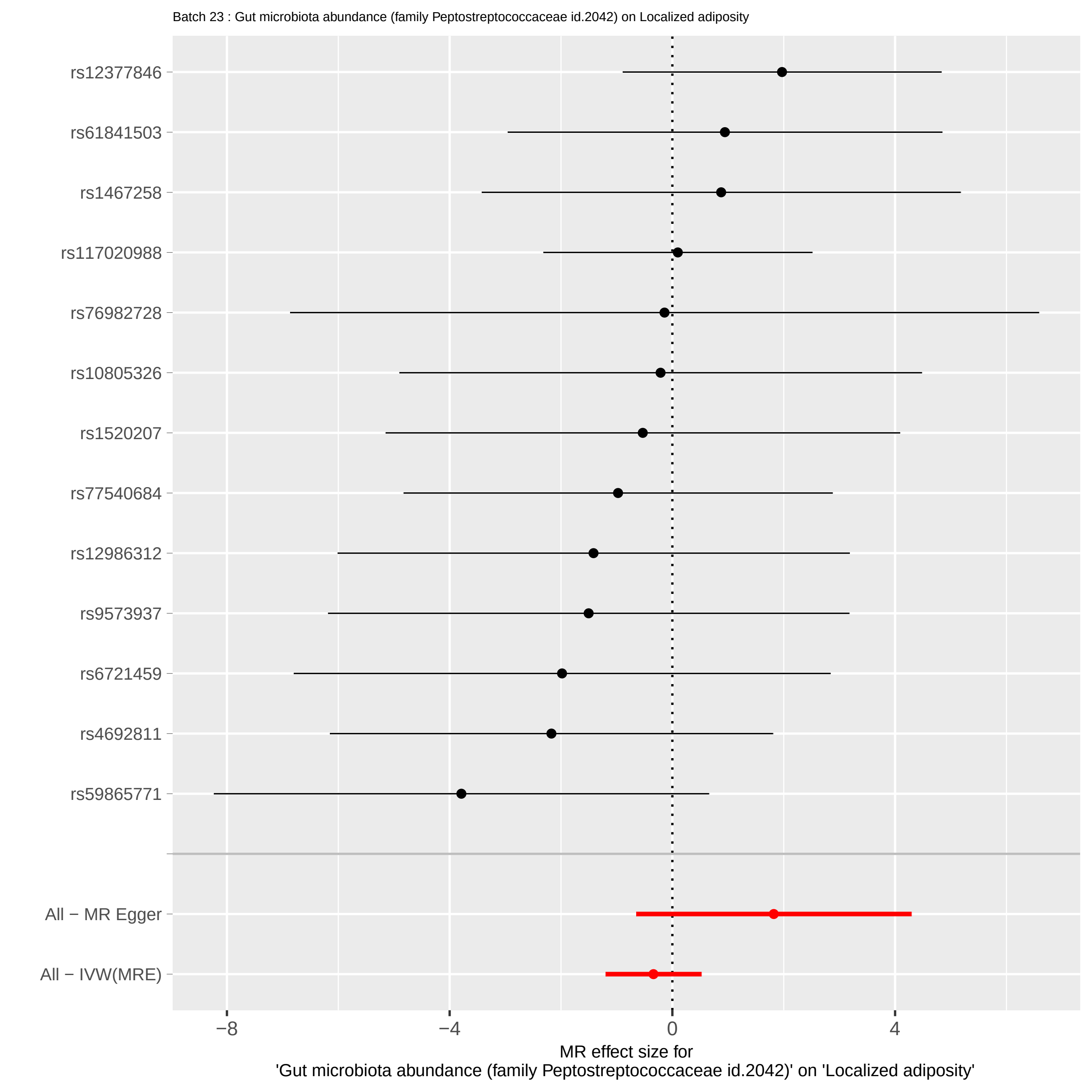
Batch 23 : Gut microbiota abundance (family Peptostreptococcaceae id.2042) on Localized adiposity
rs12377846
rs61841503
rs1467258
rs117020988
rs76982728
rs10805326
rs1520207
rs77540684
rs12986312
rs9573937
rs6721459
rs4692811
rs59865771
All − MR Egger
All − IVW(MRE)
−8
−4
0
4
MR effect size for
'Gut microbiota abundance (family Peptostreptococcaceae id.2042)' on 'Localized adiposity'

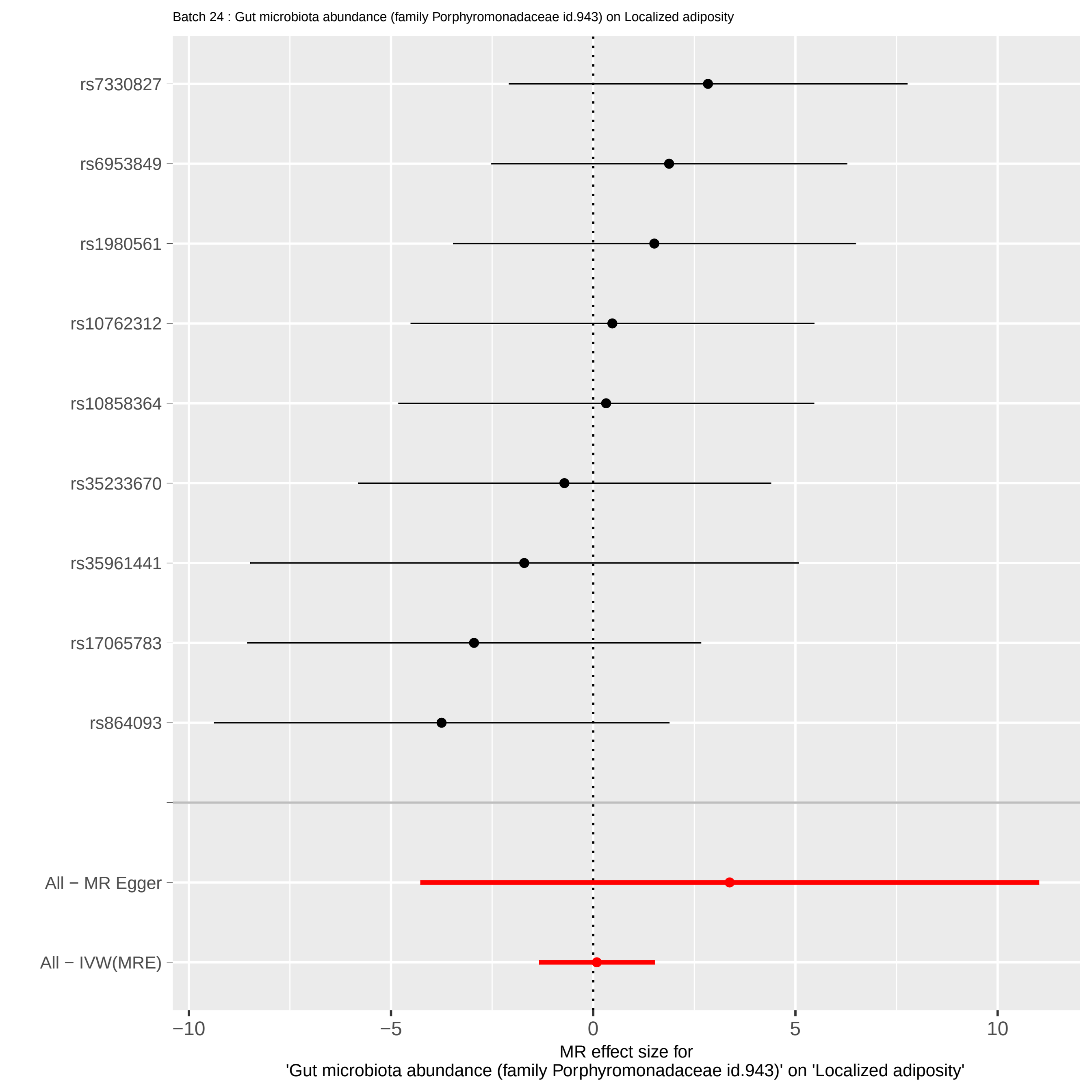

Batch 24 : Gut microbiota abundance (family Porphyromonadaceae id.943) on Localized adiposity
rs7330827
rs6953849
rs1980561
rs10762312
rs10858364
rs35233670
rs35961441
rs17065783
rs864093
All − MR Egger
All − IVW(MRE)
−10
−5
0
5
10
MR effect size for
'Gut microbiota abundance (family Porphyromonadaceae id.943)' on 'Localized adiposity'

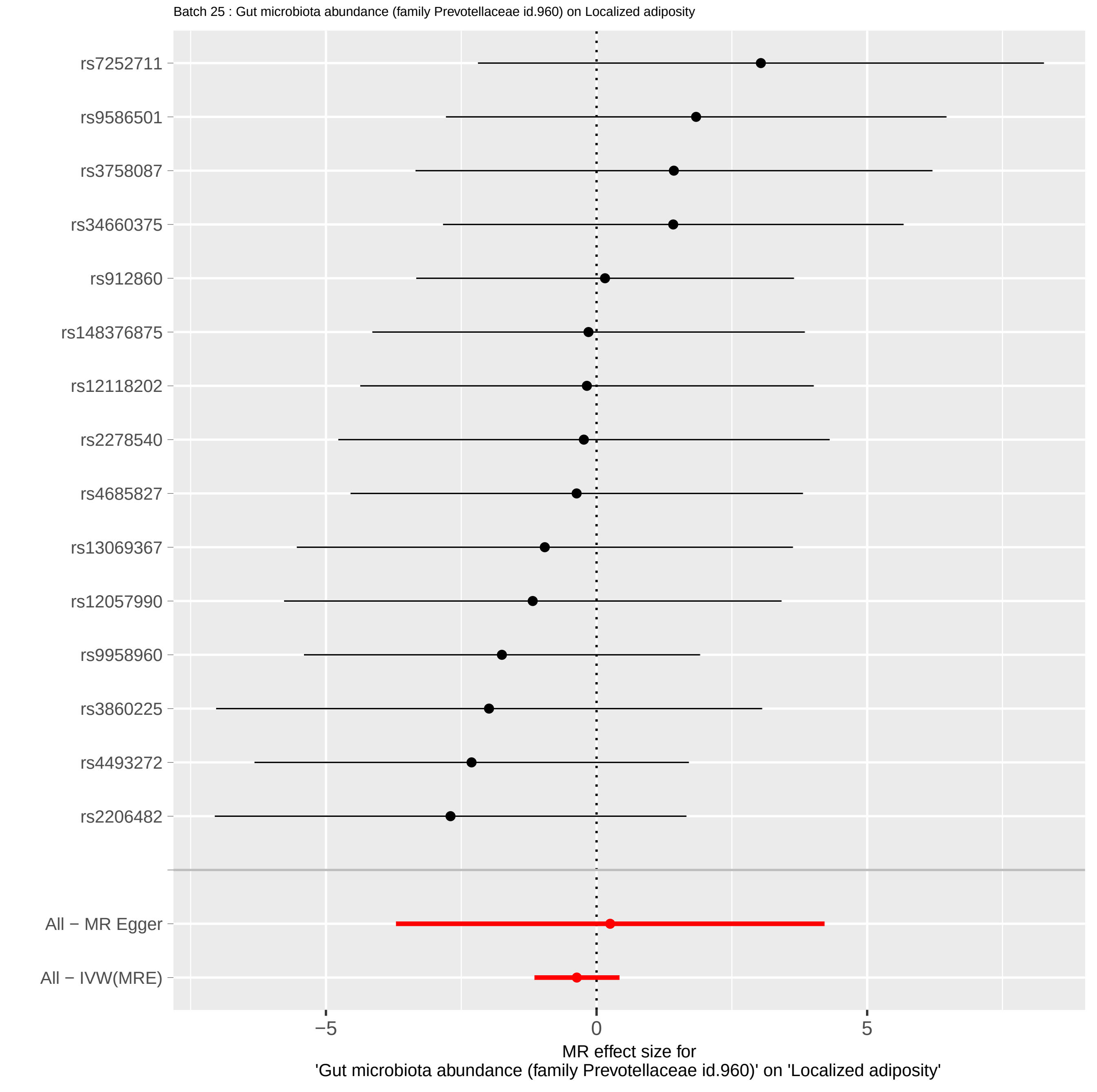
Batch 25 : Gut microbiota abundance (family Prevotellaceae id.960) on Localized adiposity
rs7252711
rs9586501
rs3758087
rs34660375
rs912860
rs148376875
rs12118202
rs2278540
rs4685827
rs13069367
rs12057990
rs9958960
rs3860225
rs4493272
rs2206482
All − MR Egger
All − IVW(MRE)
−5
0
5
MR effect size for
'Gut microbiota abundance (family Prevotellaceae id.960)' on 'Localized adiposity'

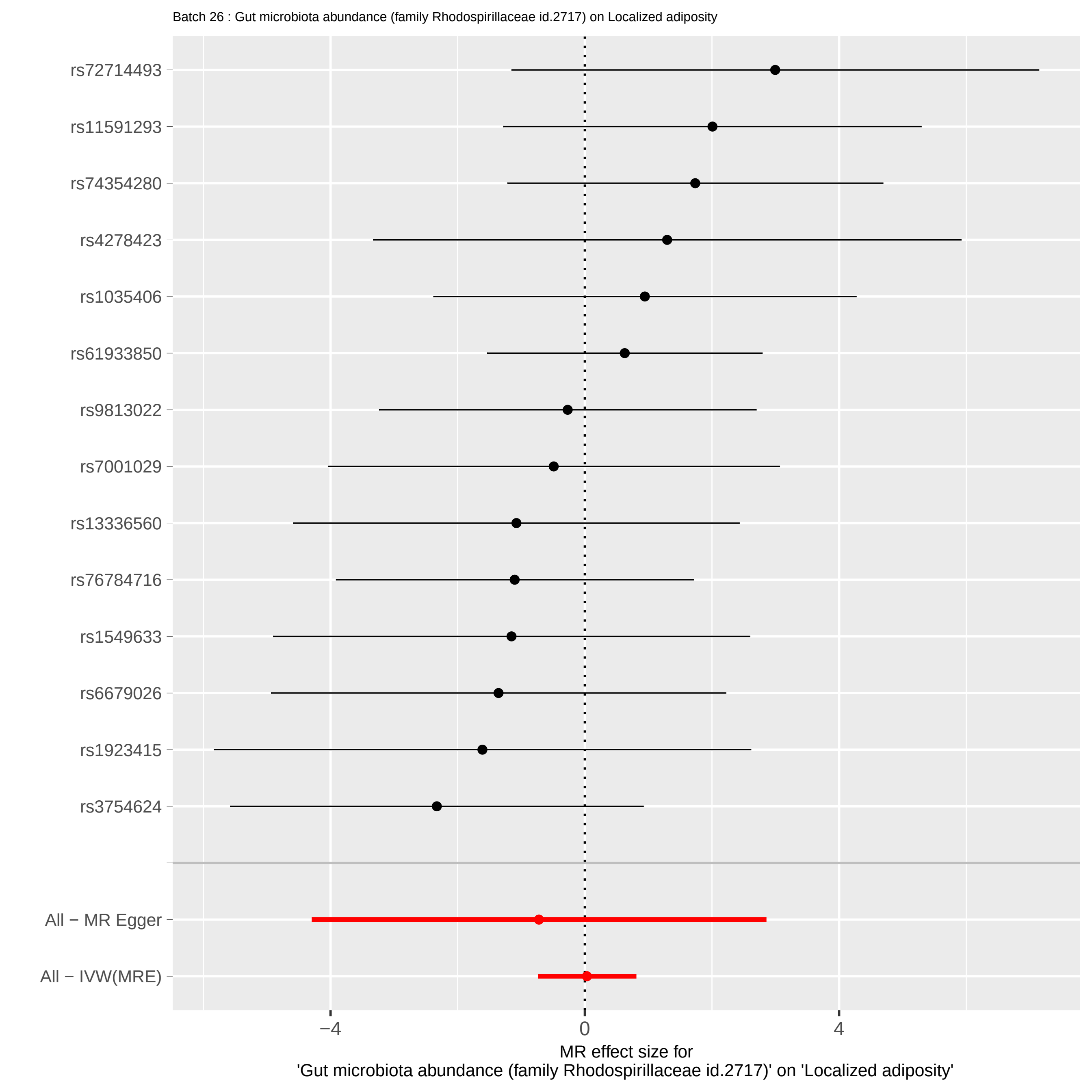

Batch 26 : Gut microbiota abundance (family Rhodospirillaceae id.2717) on Localized adiposity
rs72714493
rs11591293
rs74354280
rs4278423
rs1035406
rs61933850
rs9813022
rs7001029
rs13336560
rs76784716
rs1549633
rs6679026
rs1923415
rs3754624
All − MR Egger
All − IVW(MRE)
−4
0
4
MR effect size for
'Gut microbiota abundance (family Rhodospirillaceae id.2717)' on 'Localized adiposity'

Batch 27 : Gut microbiota abundance (family Rikenellaceae id.967) on Localized adiposity

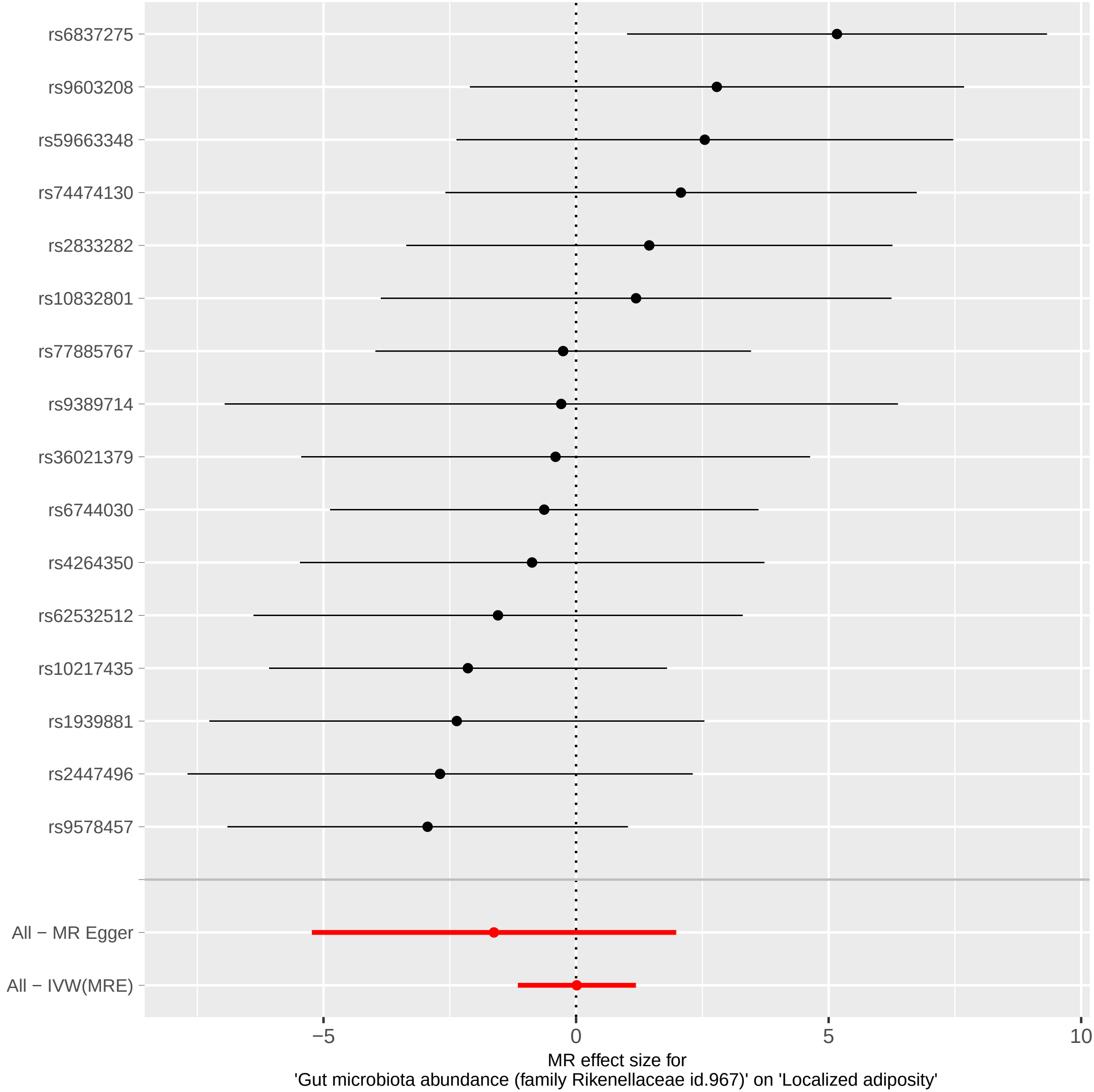

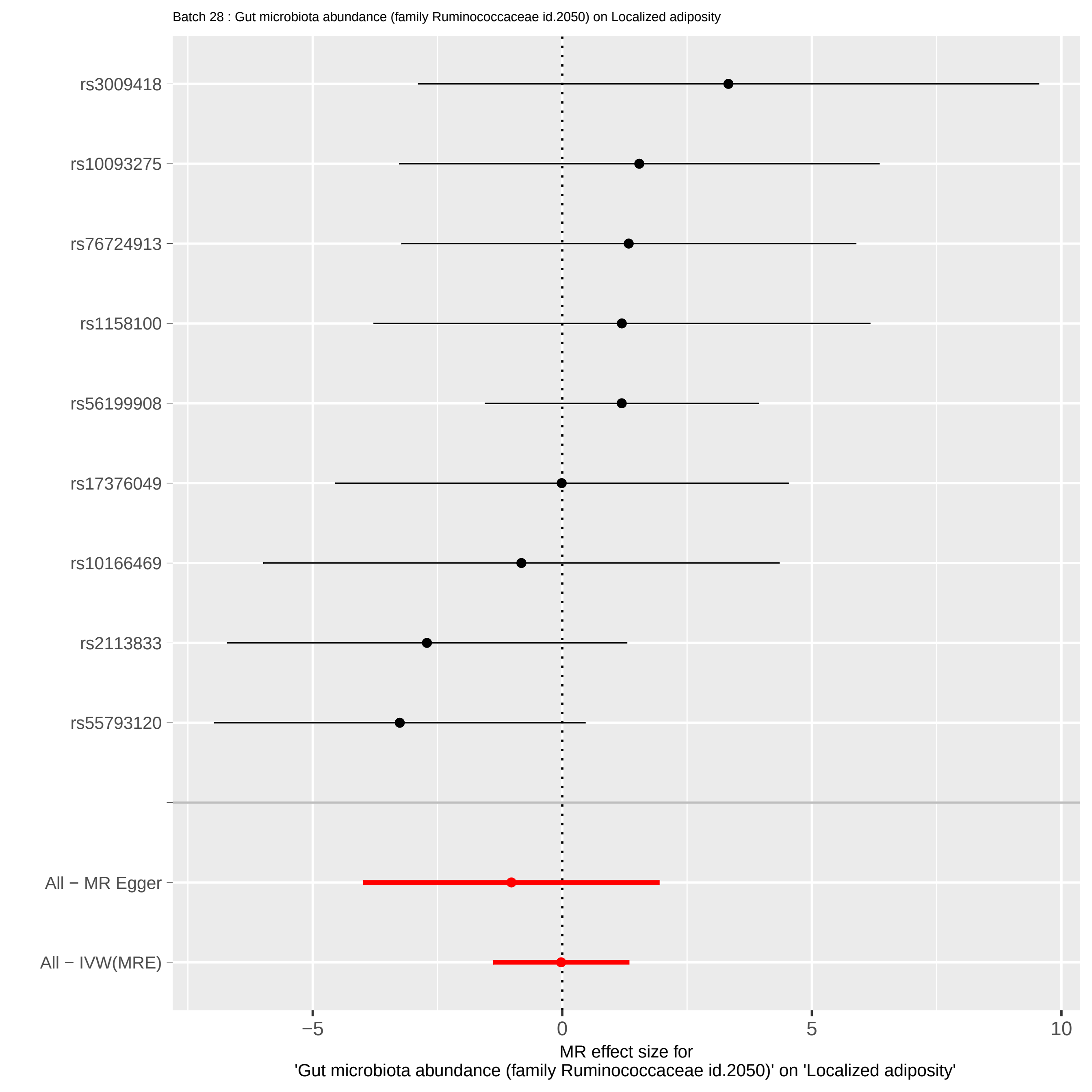
Batch 28 : Gut microbiota abundance (family Ruminococcaceae id.2050) on Localized adiposity
rs3009418
rs10093275
rs76724913
rs1158100
rs56199908
rs17376049
rs10166469
rs2113833
rs55793120
All − MR Egger
All − IVW(MRE)
−5
0
5
10
MR effect size for
'Gut microbiota abundance (family Ruminococcaceae id.2050)' on 'Localized adiposity'

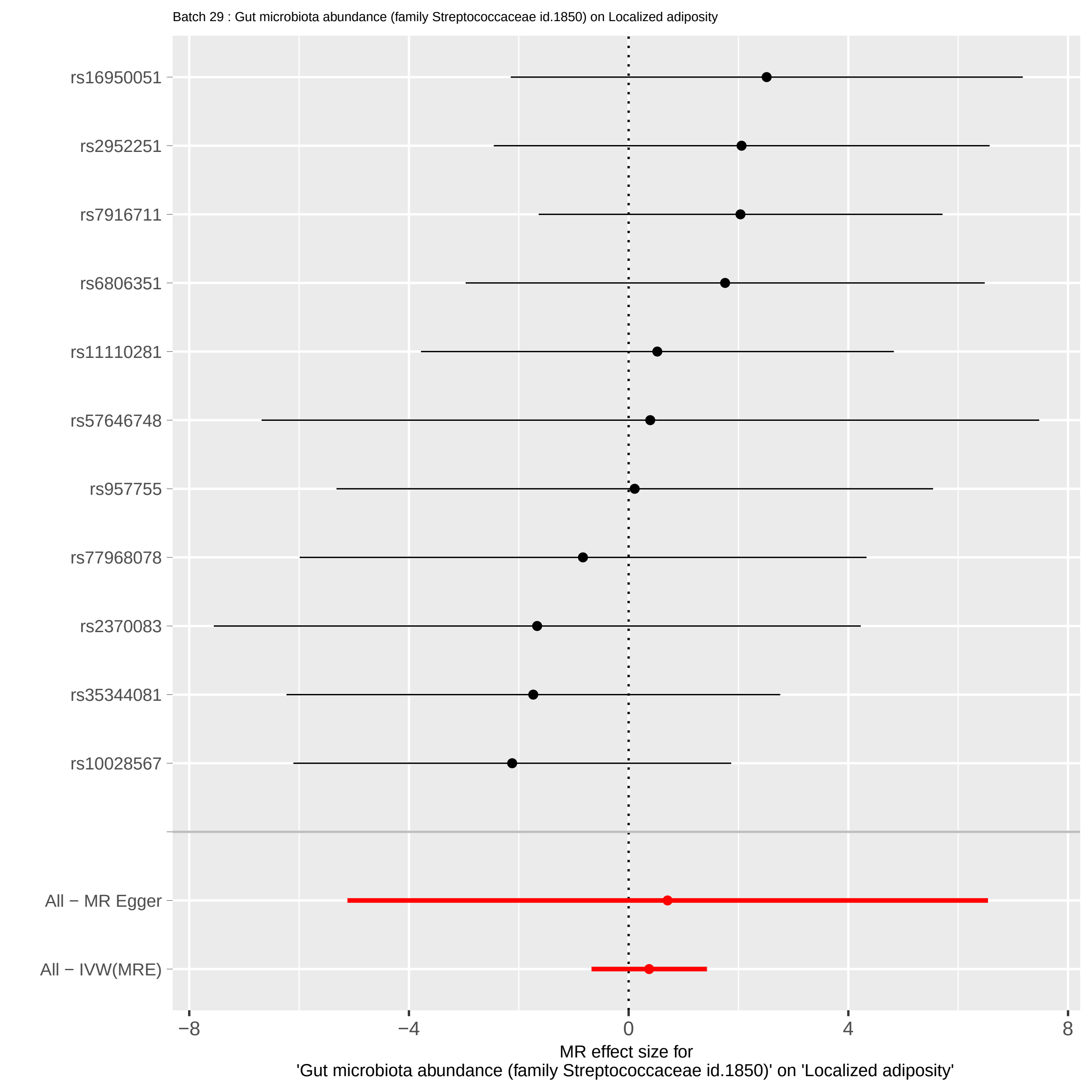

Batch 29 : Gut microbiota abundance (family Streptococcaceae id.1850) on Localized adiposity
rs16950051
rs2952251
rs7916711
rs6806351
rs11110281
rs57646748
rs957755
rs77968078
rs2370083
rs35344081
rs10028567
All − MR Egger
All − IVW(MRE)
−8
−4
0
4
8
MR effect size for
'Gut microbiota abundance (family Streptococcaceae id.1850)' on 'Localized adiposity'

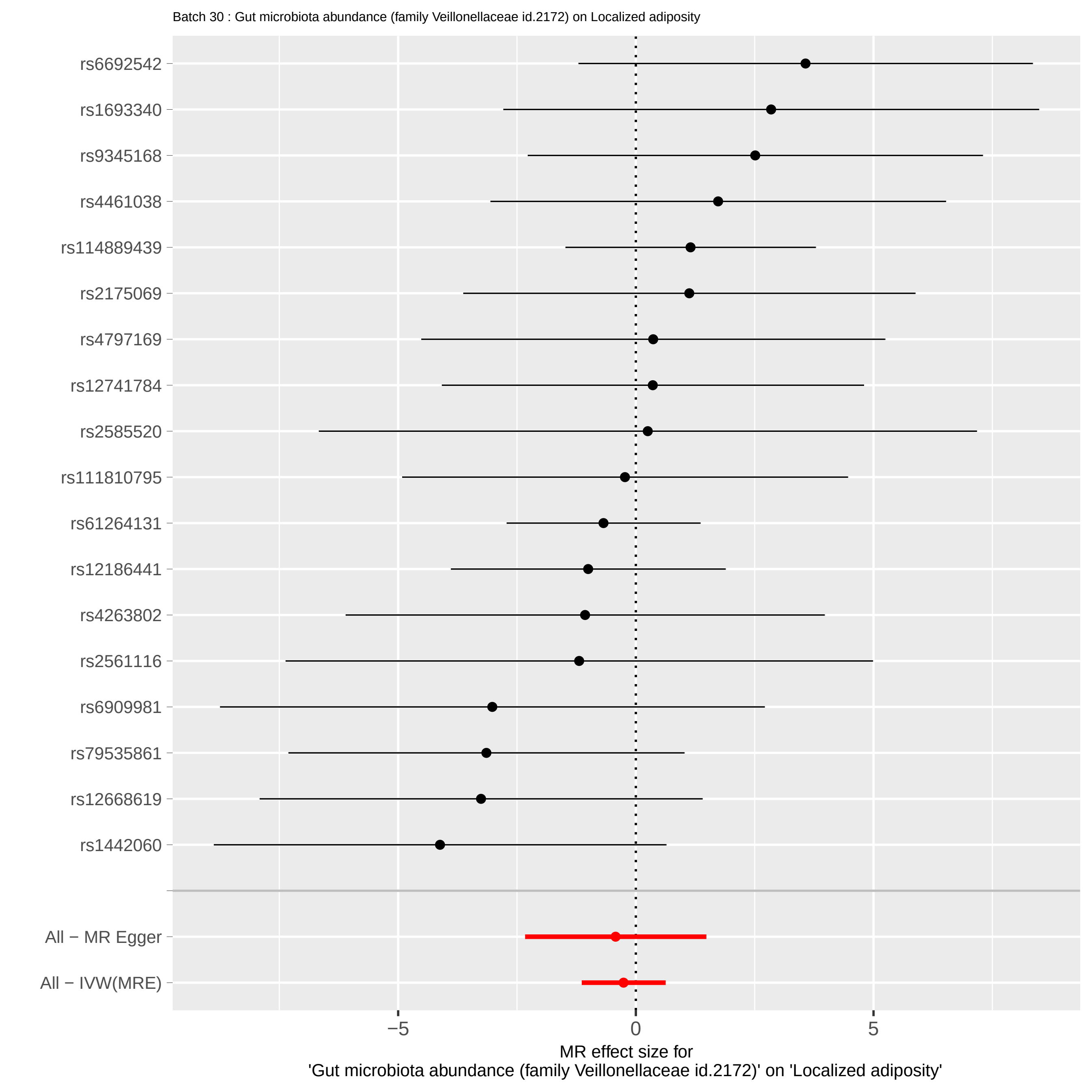

Batch 30 : Gut microbiota abundance (family Veillonellaceae id.2172) on Localized adiposity
rs6692542
rs1693340
rs9345168
rs4461038
rs114889439
rs2175069
rs4797169
rs12741784
rs2585520
rs111810795
rs61264131
rs12186441
rs4263802
rs2561116
rs6909981
rs79535861
rs12668619
rs1442060
All − MR Egger
All − IVW(MRE)
−5
0
5
MR effect size for
'Gut microbiota abundance (family Veillonellaceae id.2172)' on 'Localized adiposity'

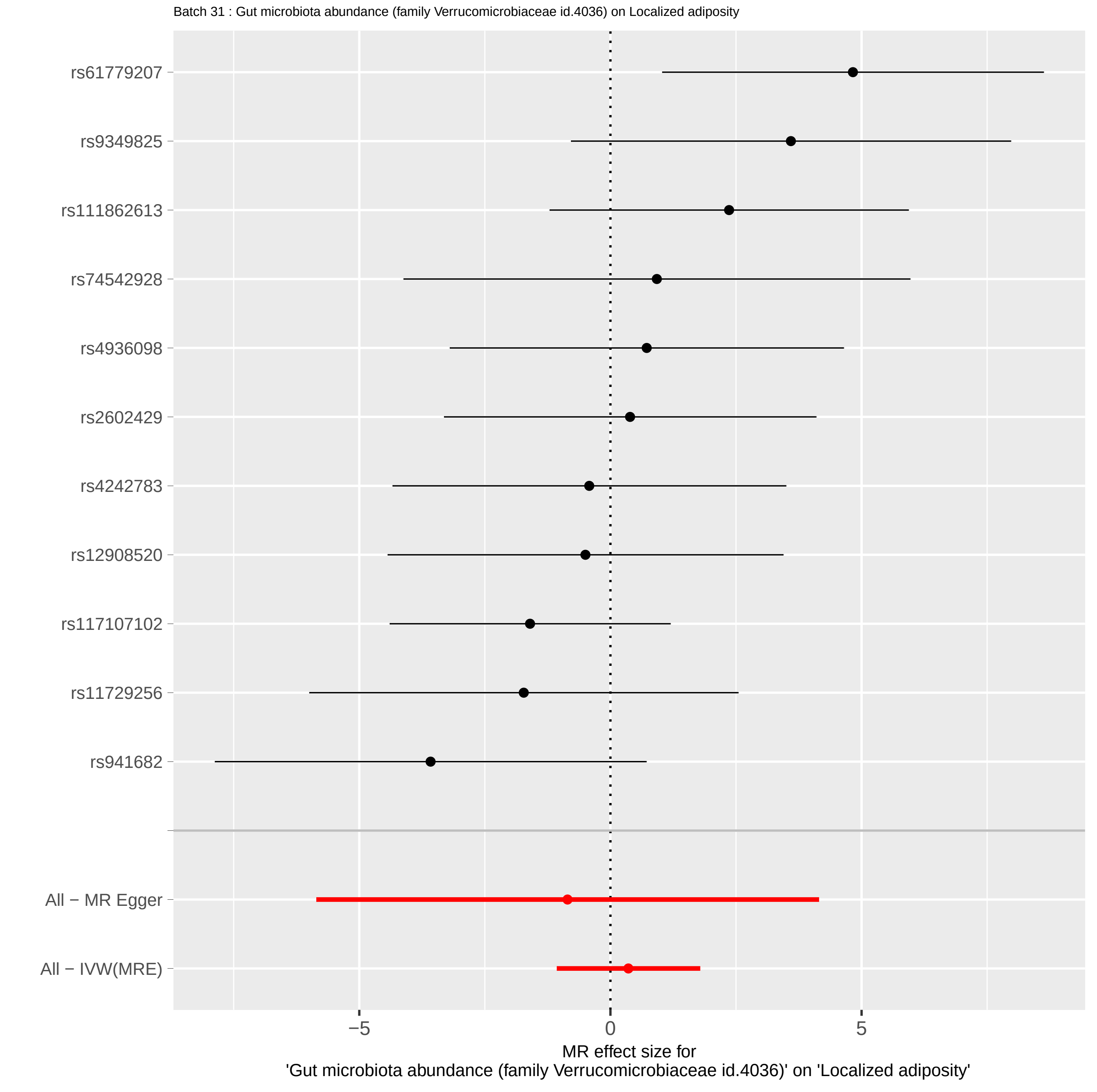

Batch 31 : Gut microbiota abundance (family Verrucomicrobiaceae id.4036) on Localized adiposity
rs61779207
rs9349825
rs111862613
rs74542928
rs4936098
rs2602429
rs4242783
rs12908520
rs117107102
rs11729256
rs941682
All − MR Egger
All − IVW(MRE)
−5
0
5
MR effect size for
'Gut microbiota abundance (family Verrucomicrobiaceae id.4036)' on 'Localized adiposity'

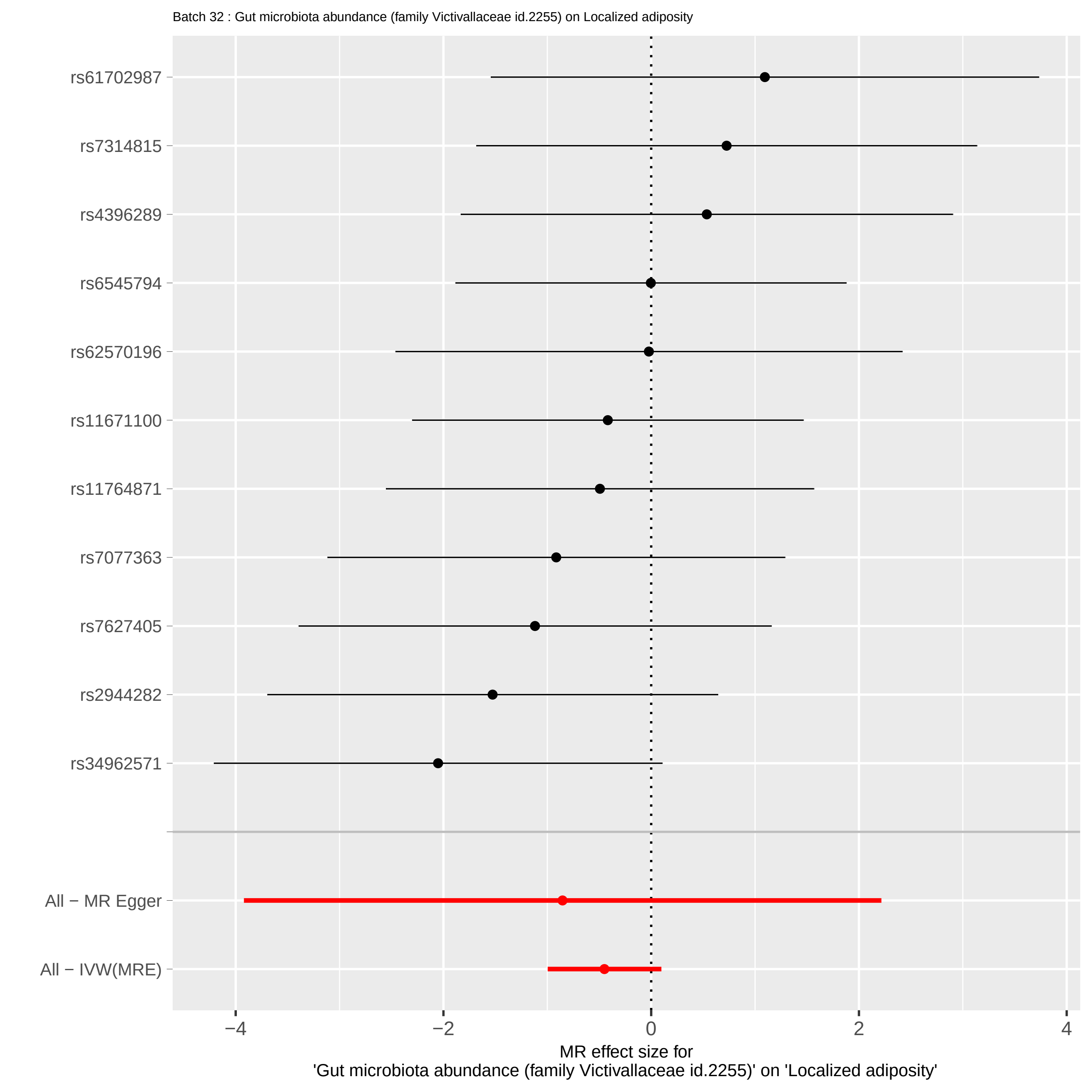

Batch 32 : Gut microbiota abundance (family Victivallaceae id.2255) on Localized adiposity
rs61702987
rs7314815
rs4396289
rs6545794
rs62570196
rs11671100
rs11764871
rs7077363
rs7627405
rs2944282
rs34962571
All − MR Egger
All − IVW(MRE)
−4
−2
0
2
4
MR effect size for
'Gut microbiota abundance (family Victivallaceae id.2255)' on 'Localized adiposity'

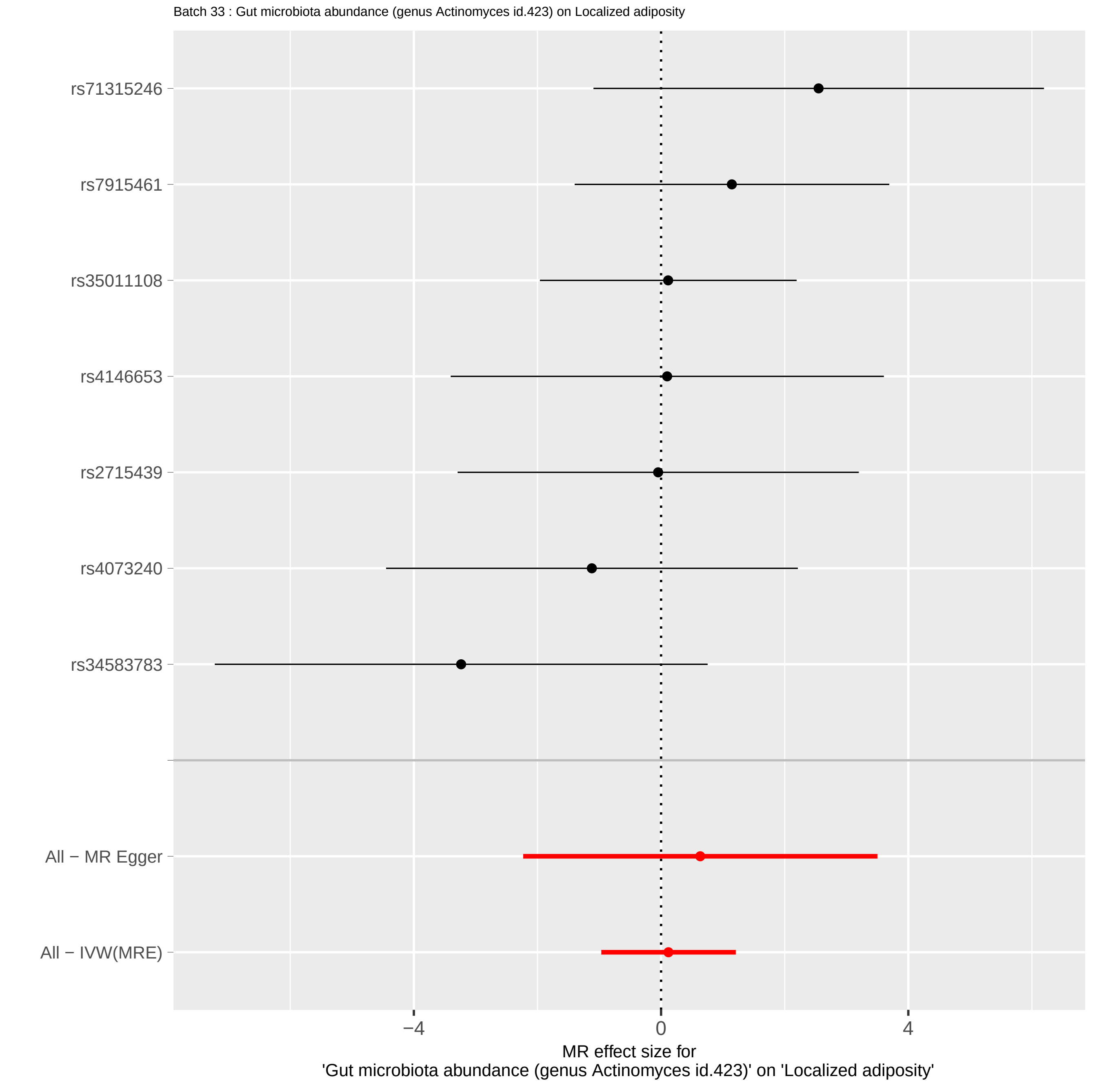
Batch 33 : Gut microbiota abundance (genus Actinomyces id.423) on Localized adiposity
rs71315246
rs7915461
rs35011108
rs4146653
rs2715439
rs4073240
rs34583783
All − MR Egger
All − IVW(MRE)
−4
0
4
MR effect size for
'Gut microbiota abundance (genus Actinomyces id.423)' on 'Localized adiposity'

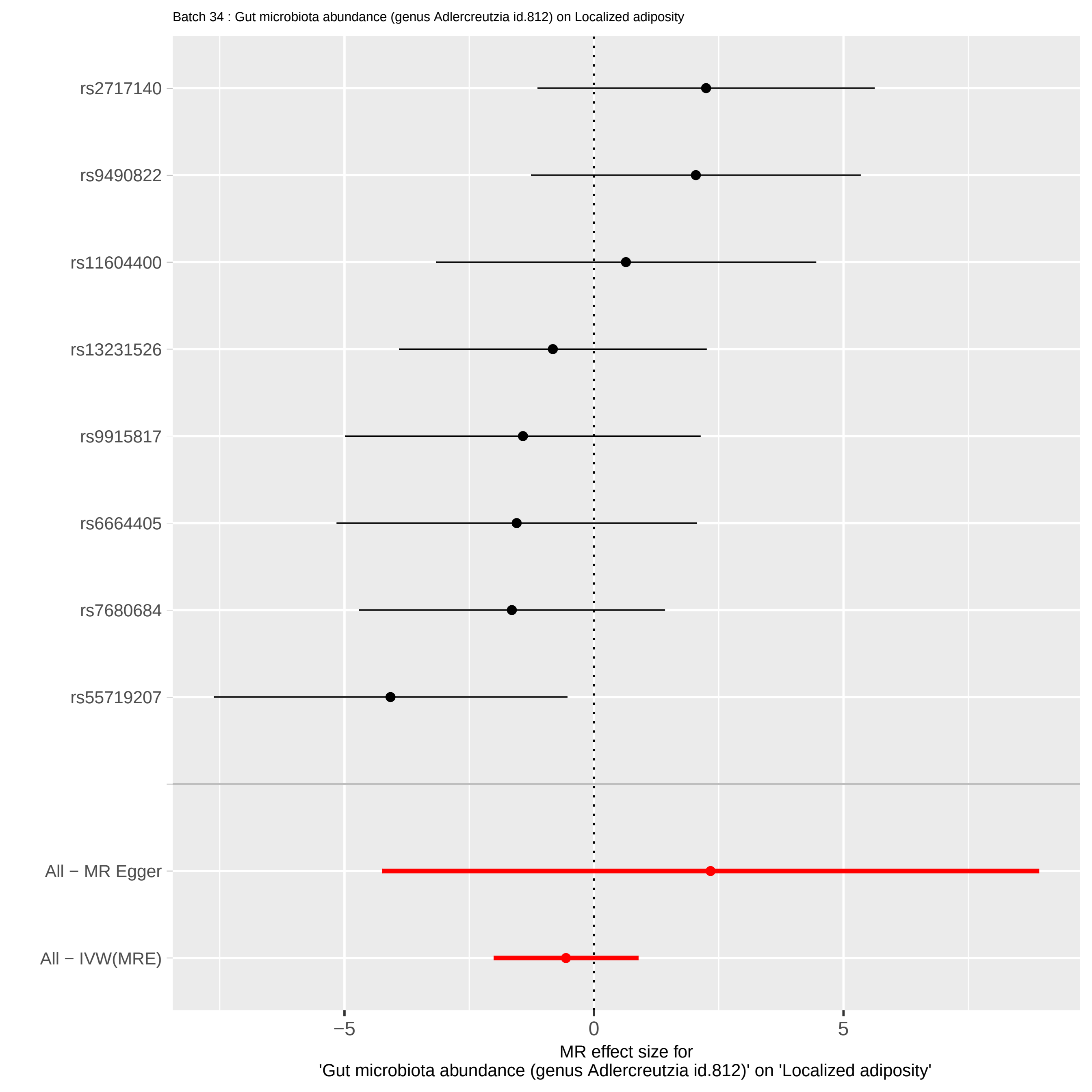
Batch 34 : Gut microbiota abundance (genus Adlercreutzia id.812) on Localized adiposity
rs2717140
rs9490822
rs11604400
rs13231526
rs9915817
rs6664405
rs7680684
rs55719207
All − MR Egger
All − IVW(MRE)
−5
0
5
MR effect size for
'Gut microbiota abundance (genus Adlercreutzia id.812)' on 'Localized adiposity'

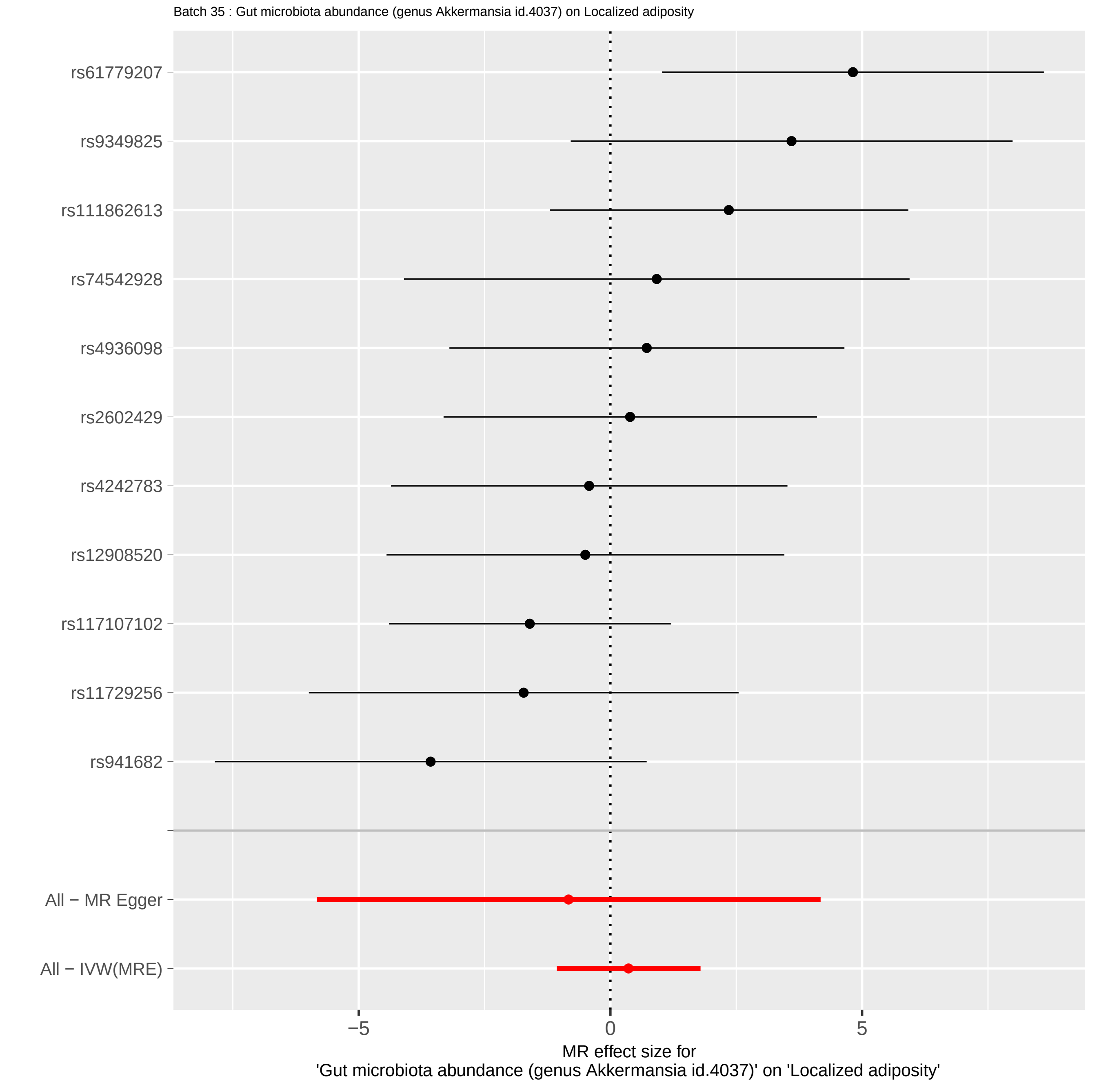

Batch 35 : Gut microbiota abundance (genus Akkermansia id.4037) on Localized adiposity
rs61779207
rs9349825
rs111862613
rs74542928
rs4936098
rs2602429
rs4242783
rs12908520
rs117107102
rs11729256
rs941682
All − MR Egger
All − IVW(MRE)
−5
0
5
MR effect size for
'Gut microbiota abundance (genus Akkermansia id.4037)' on 'Localized adiposity'

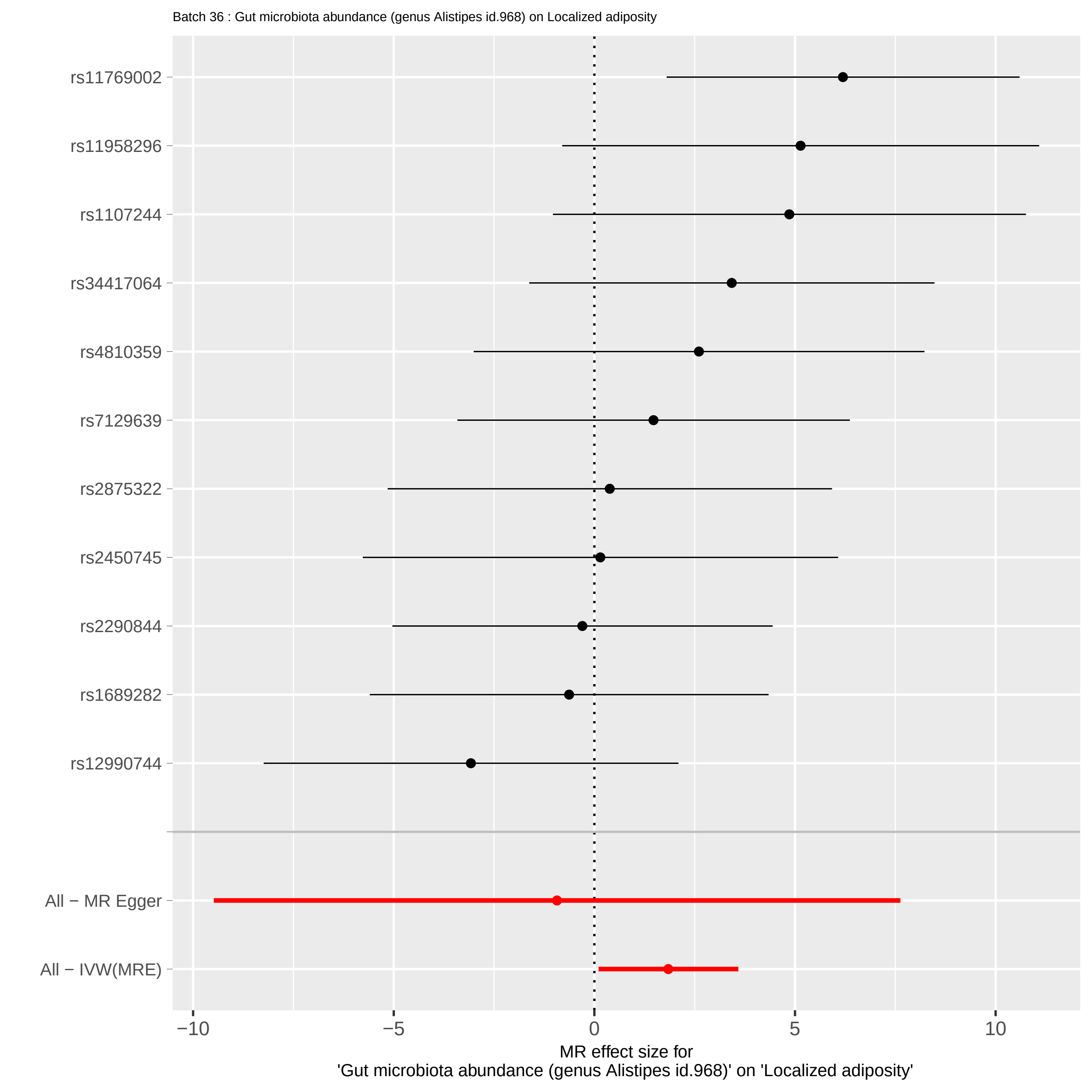

Batch 36 : Gut microbiota abundance (genus Alistipes id.968) on Localized adiposity
rs11769002
rs11958296
rs1107244
rs34417064
rs4810359
rs7129639
rs2875322
rs2450745
rs2290844
rs1689282
rs12990744
All − MR Egger
All − IVW(MRE)
−10
−5
0
5
10
MR effect size for
'Gut microbiota abundance (genus Alistipes id.968)' on 'Localized adiposity'

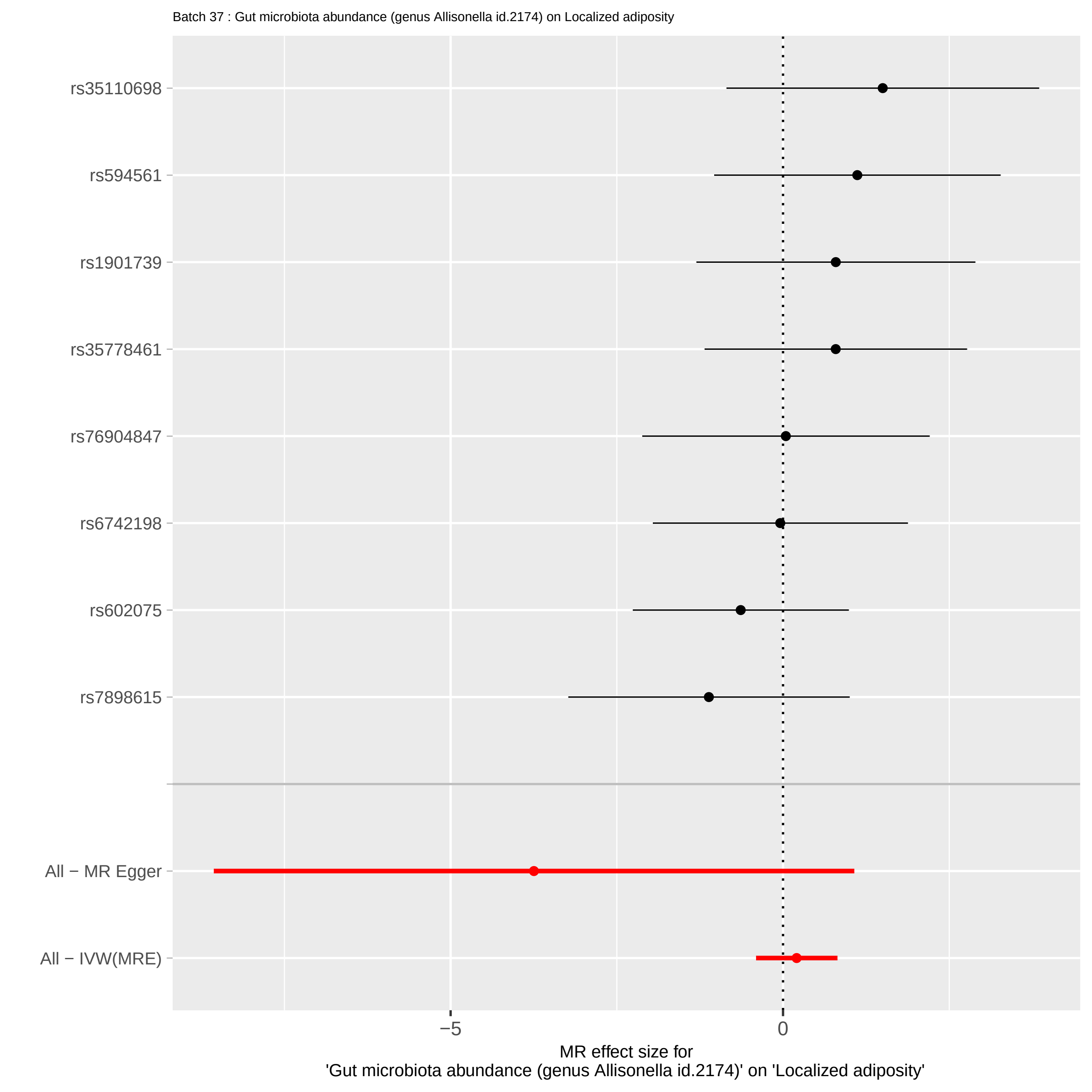

Batch 37 : Gut microbiota abundance (genus Allisonella id.2174) on Localized adiposity
rs35110698
rs594561
rs1901739
rs35778461
rs76904847
rs6742198
rs602075
rs7898615
All − MR Egger
All − IVW(MRE)
−5
0
MR effect size for
'Gut microbiota abundance (genus Allisonella id.2174)' on 'Localized adiposity'

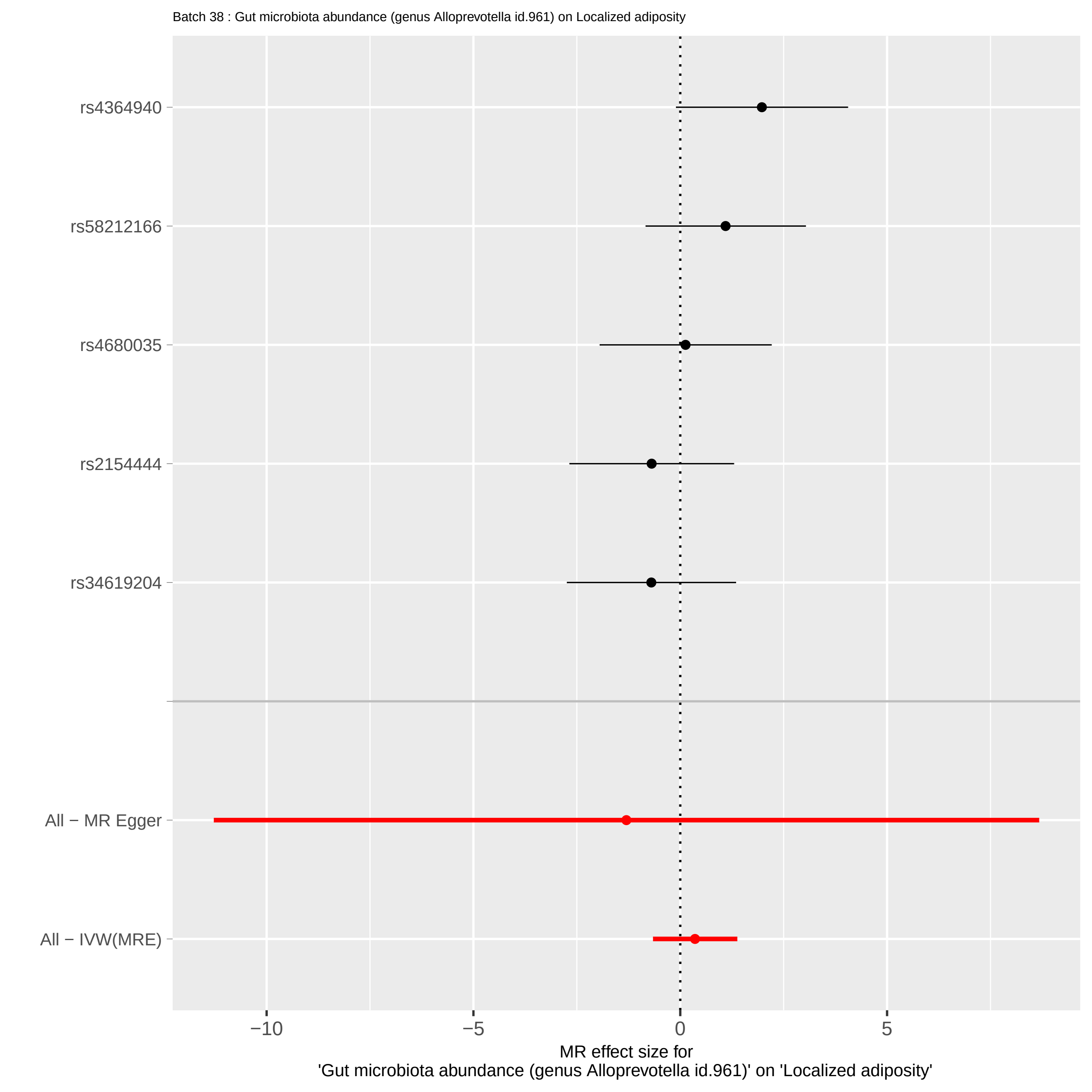
Batch 38 : Gut microbiota abundance (genus Alloprevotella id.961) on Localized adiposity
rs4364940
rs58212166
rs4680035
rs2154444
rs34619204
All − MR Egger
All − IVW(MRE)
−10
−5
0
5
MR effect size for
'Gut microbiota abundance (genus Alloprevotella id.961)' on 'Localized adiposity'

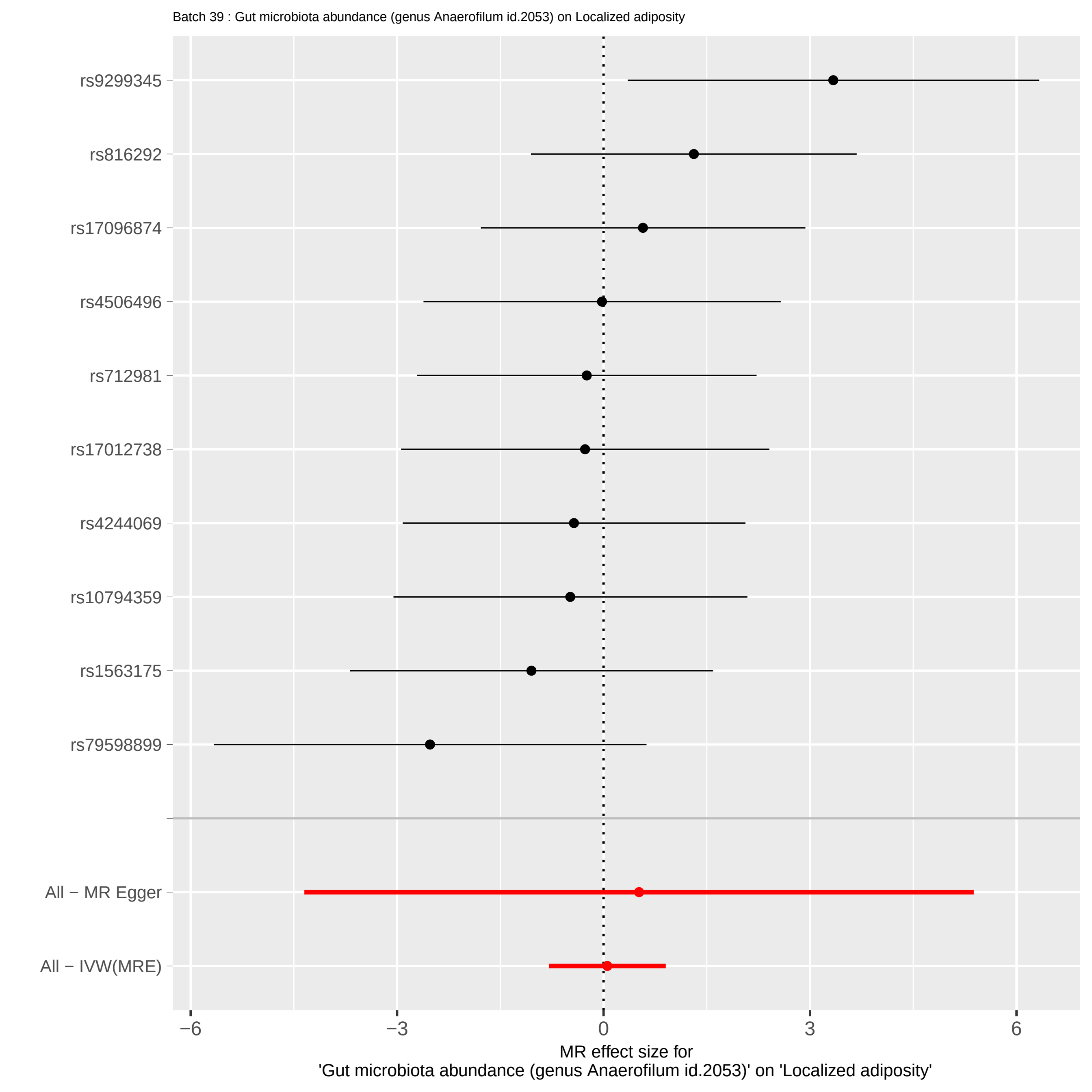

Batch 39 : Gut microbiota abundance (genus Anaerofilum id.2053) on Localized adiposity
rs9299345
rs816292
rs17096874
rs4506496
rs712981
rs17012738
rs4244069
rs10794359
rs1563175
rs79598899
All − MR Egger
All − IVW(MRE)
−6
−3
0
3
6
MR effect size for
'Gut microbiota abundance (genus Anaerofilum id.2053)' on 'Localized adiposity'

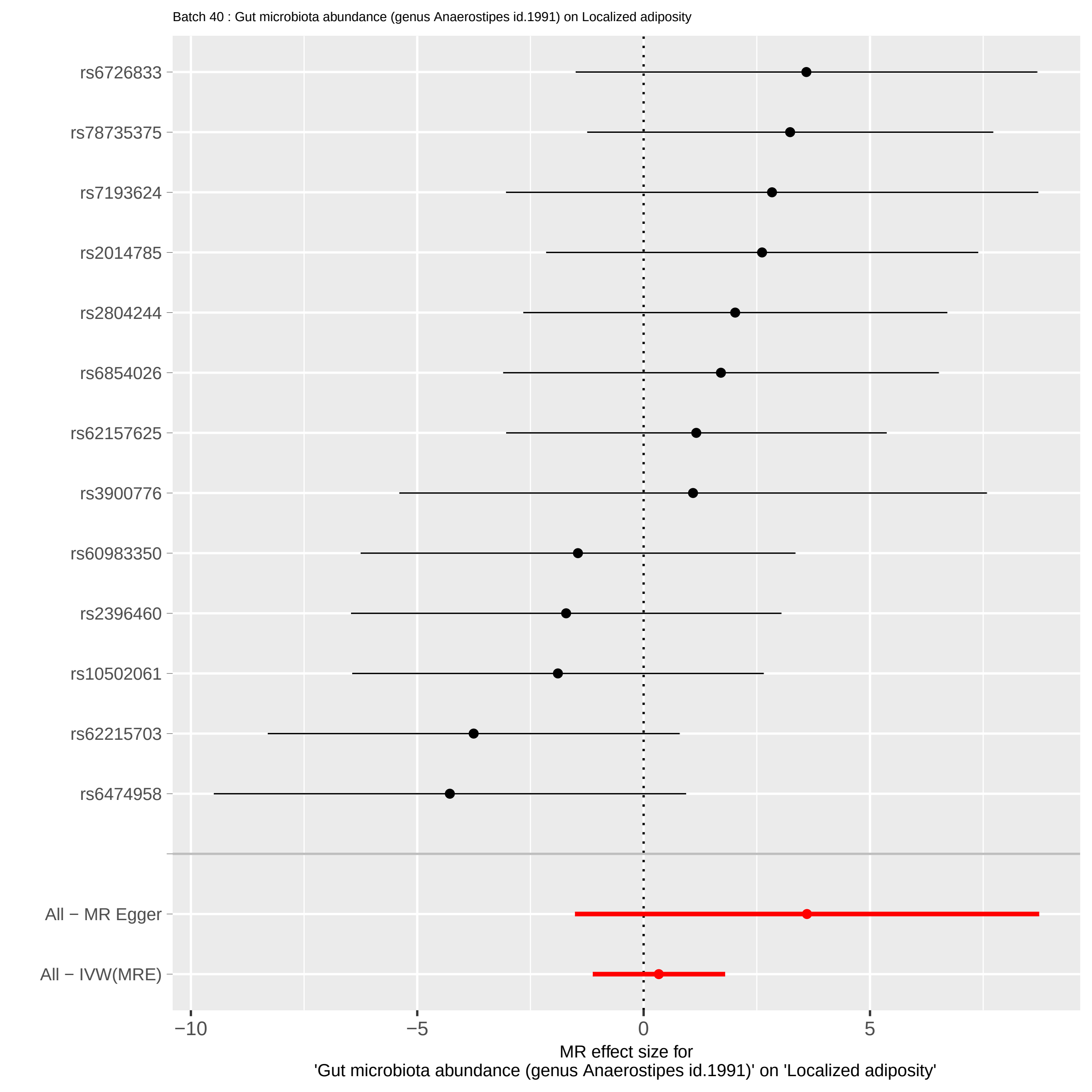

Batch 40 : Gut microbiota abundance (genus Anaerostipes id.1991) on Localized adiposity
rs6726833
rs78735375
rs7193624
rs2014785
rs2804244
rs6854026
rs62157625
rs3900776
rs60983350
rs2396460
rs10502061
rs62215703
rs6474958
All − MR Egger
All − IVW(MRE)
−10
−5
0
5
MR effect size for
'Gut microbiota abundance (genus Anaerostipes id.1991)' on 'Localized adiposity'

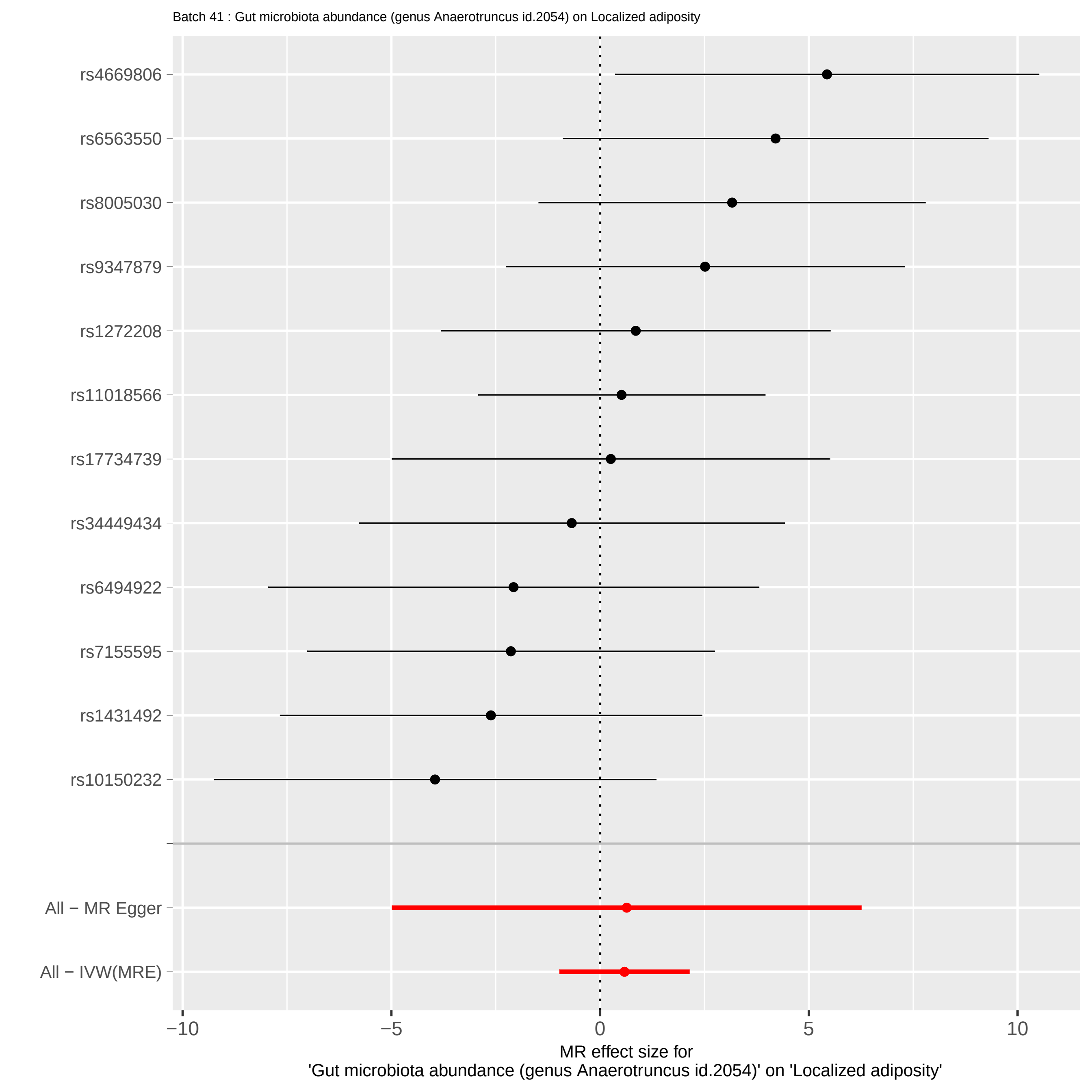
Batch 41 : Gut microbiota abundance (genus Anaerotruncus id.2054) on Localized adiposity
rs4669806
rs6563550
rs8005030
rs9347879
rs1272208
rs11018566
rs17734739
rs34449434
rs6494922
rs7155595
rs1431492
rs10150232
All − MR Egger
All − IVW(MRE)
−10
−5
0
5
10
MR effect size for
'Gut microbiota abundance (genus Anaerotruncus id.2054)' on 'Localized adiposity'

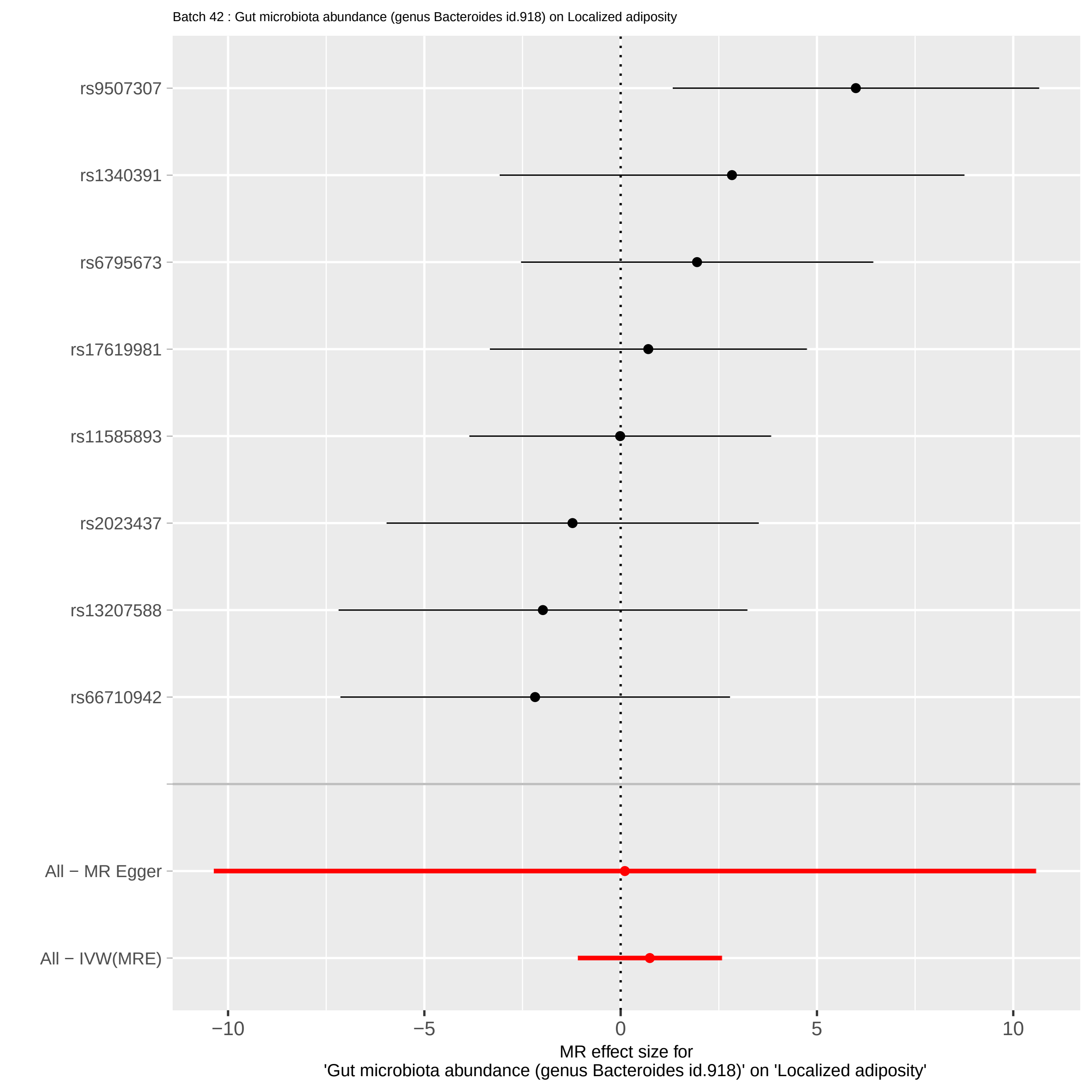

Batch 42 : Gut microbiota abundance (genus Bacteroides id.918) on Localized adiposity
rs9507307
rs1340391
rs6795673
rs17619981
rs11585893
rs2023437
rs13207588
rs66710942
All − MR Egger
All − IVW(MRE)
−10
−5
0
5
10
MR effect size for
'Gut microbiota abundance (genus Bacteroides id.918)' on 'Localized adiposity'

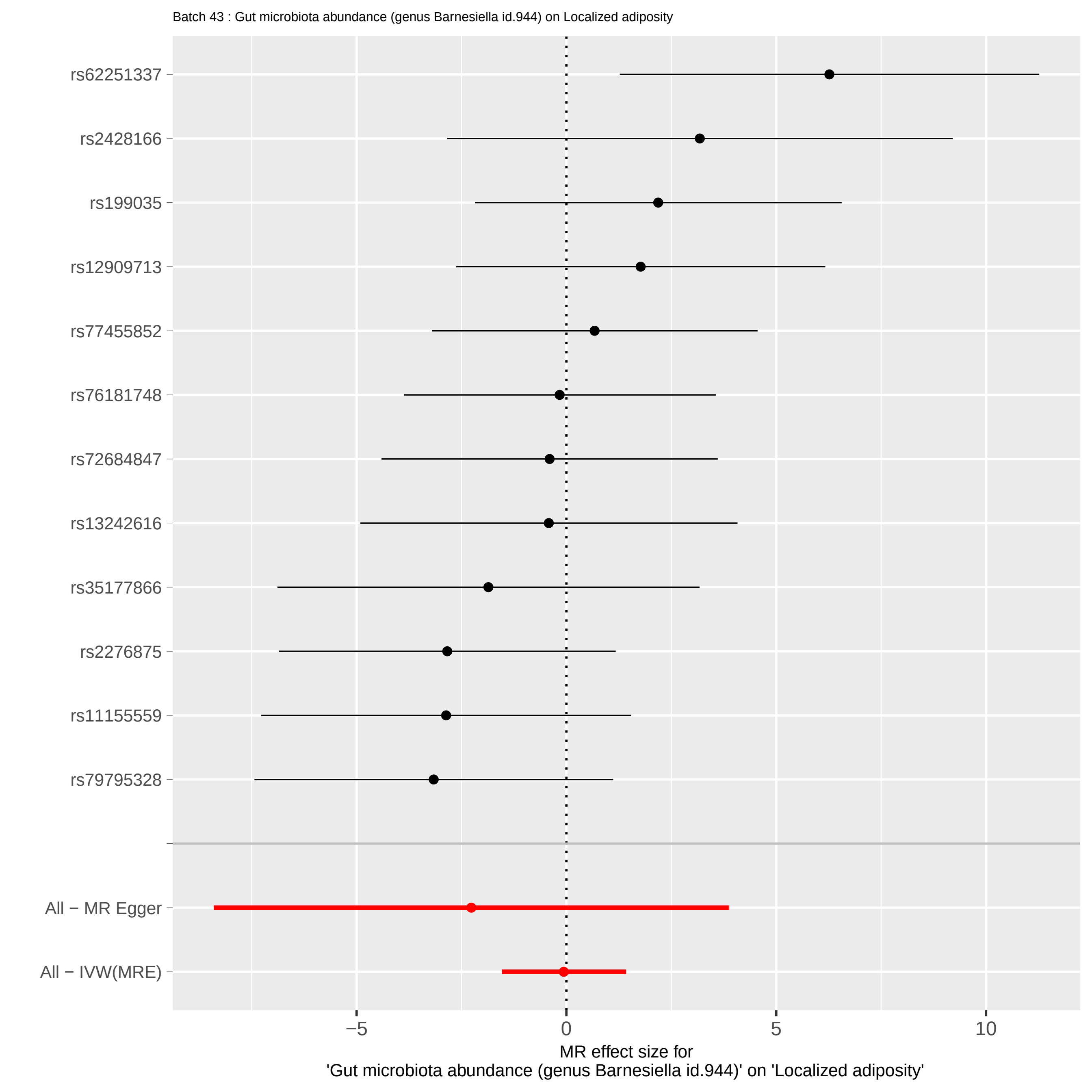

Batch 43 : Gut microbiota abundance (genus Barnesiella id.944) on Localized adiposity
rs62251337
rs2428166
rs199035
rs12909713
rs77455852
rs76181748
rs72684847
rs13242616
rs35177866
rs2276875
rs11155559
rs79795328
All − MR Egger
All − IVW(MRE)
−5
0
5
10
MR effect size for
'Gut microbiota abundance (genus Barnesiella id.944)' on 'Localized adiposity'

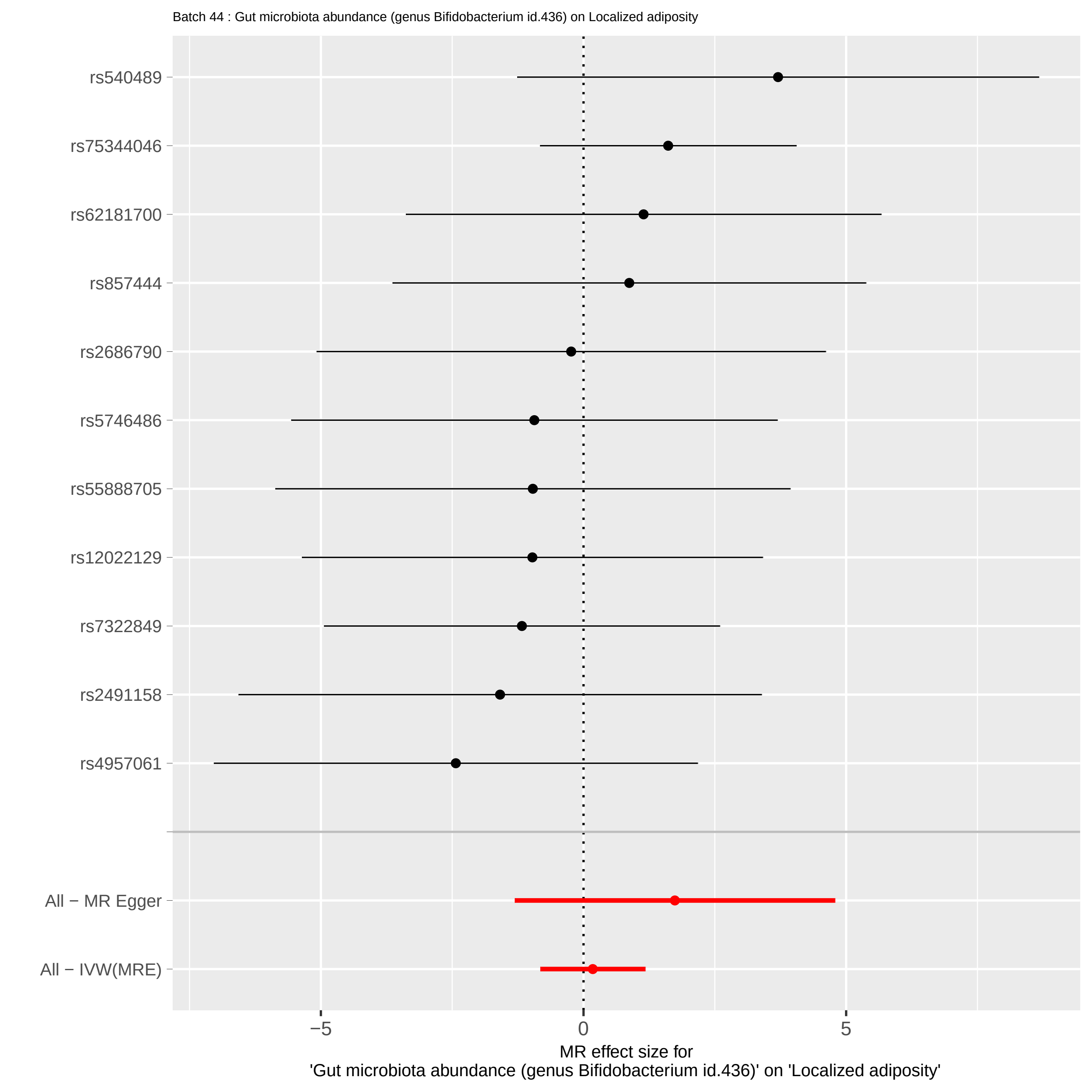

Batch 44 : Gut microbiota abundance (genus Bifidobacterium id.436) on Localized adiposity
rs540489
rs75344046
rs62181700
rs857444
rs2686790
rs5746486
rs55888705
rs12022129
rs7322849
rs2491158
rs4957061
All − MR Egger
All − IVW(MRE)
−5
0
5
MR effect size for
'Gut microbiota abundance (genus Bifidobacterium id.436)' on 'Localized adiposity'

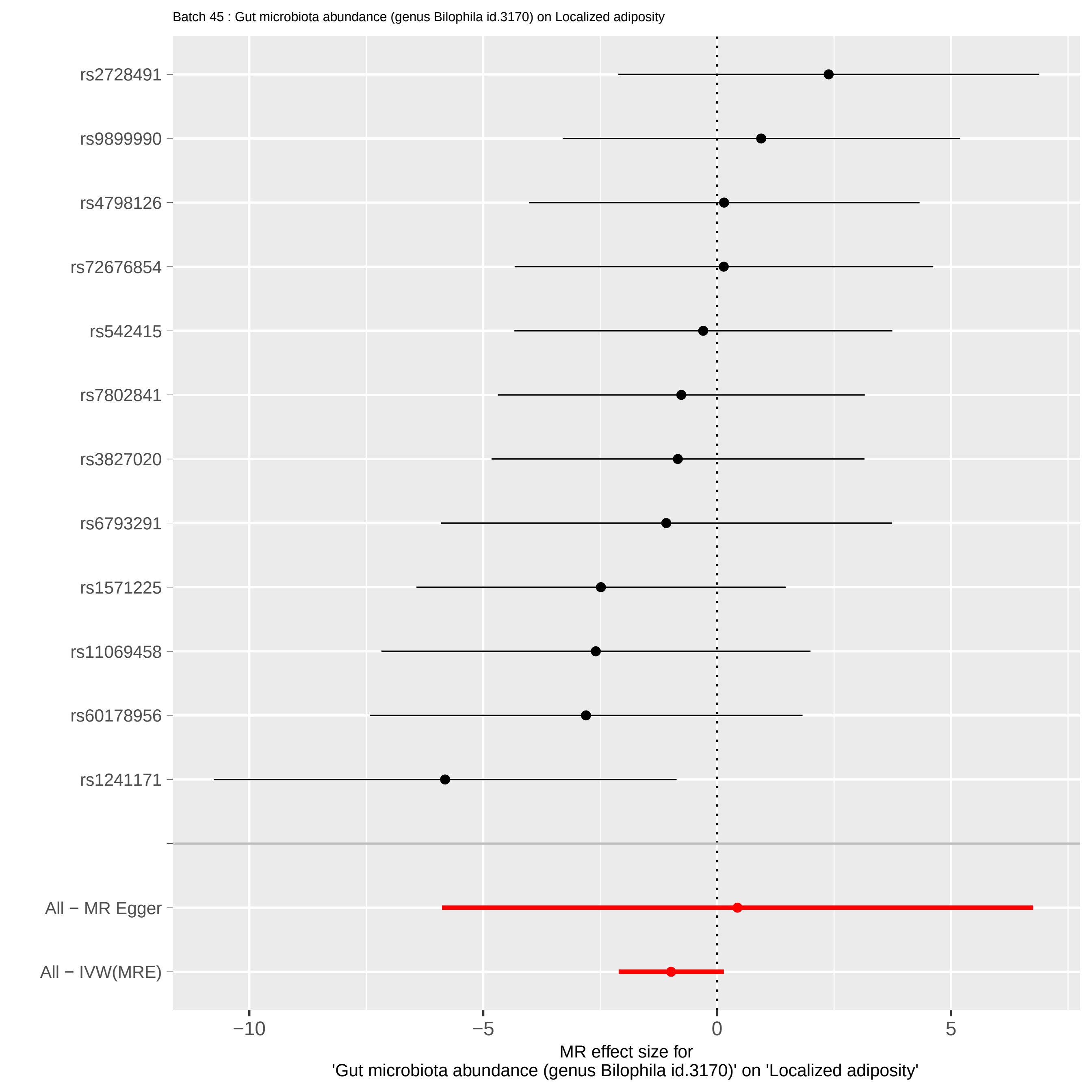
Batch 45 : Gut microbiota abundance (genus Bilophila id.3170) on Localized adiposity
rs2728491
rs9899990
rs4798126
rs72676854
rs542415
rs7802841
rs3827020
rs6793291
rs1571225
rs11069458
rs60178956
rs1241171
All − MR Egger
All − IVW(MRE)
−10
−5
0
5
MR effect size for
'Gut microbiota abundance (genus Bilophila id.3170)' on 'Localized adiposity'

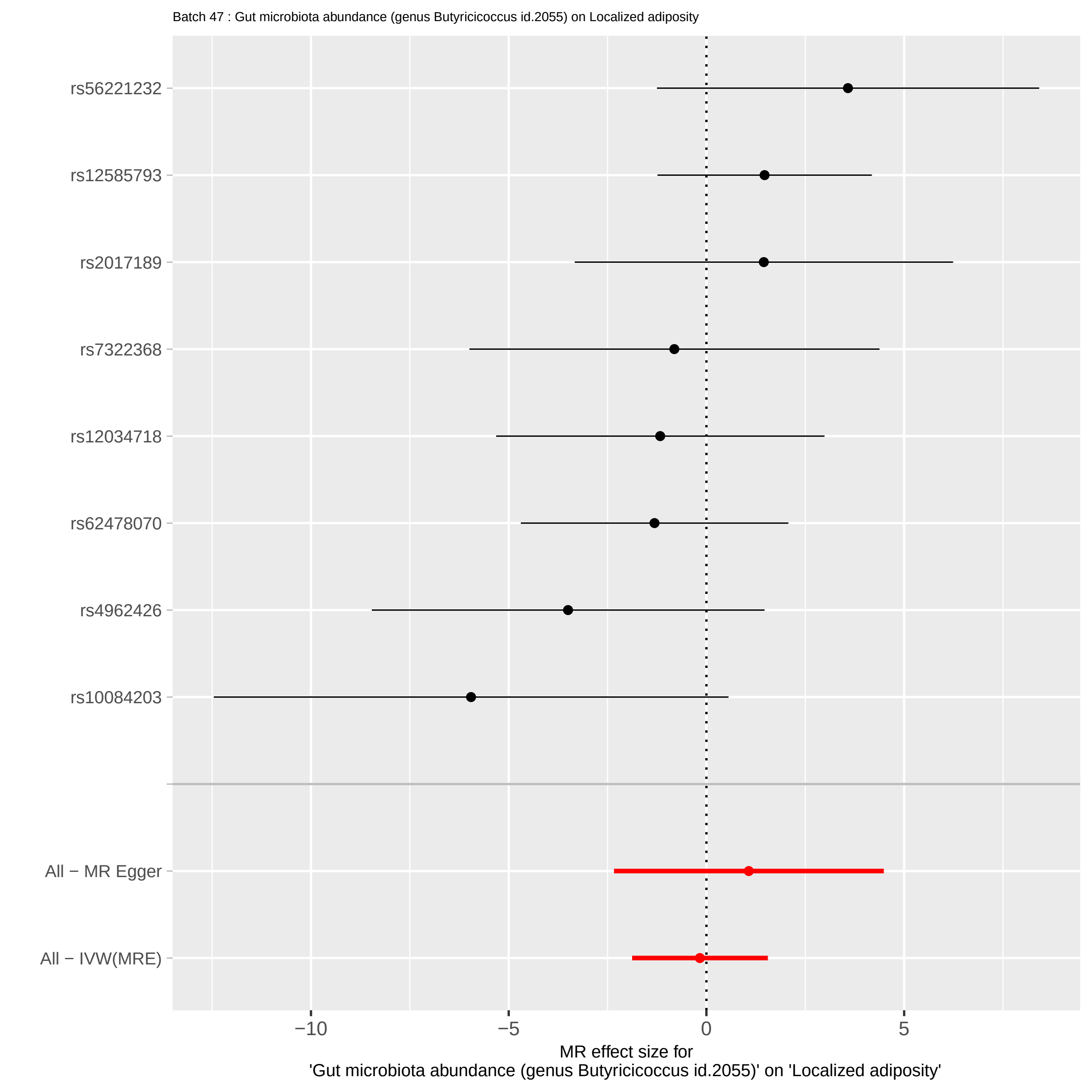
Batch 47 : Gut microbiota abundance (genus Butyricicoccus id.2055) on Localized adiposity
rs56221232
rs12585793
rs2017189
rs7322368
rs12034718
rs62478070
rs4962426
rs10084203
All − MR Egger
All − IVW(MRE)
−10
−5
0
5
MR effect size for
'Gut microbiota abundance (genus Butyricicoccus id.2055)' on 'Localized adiposity'

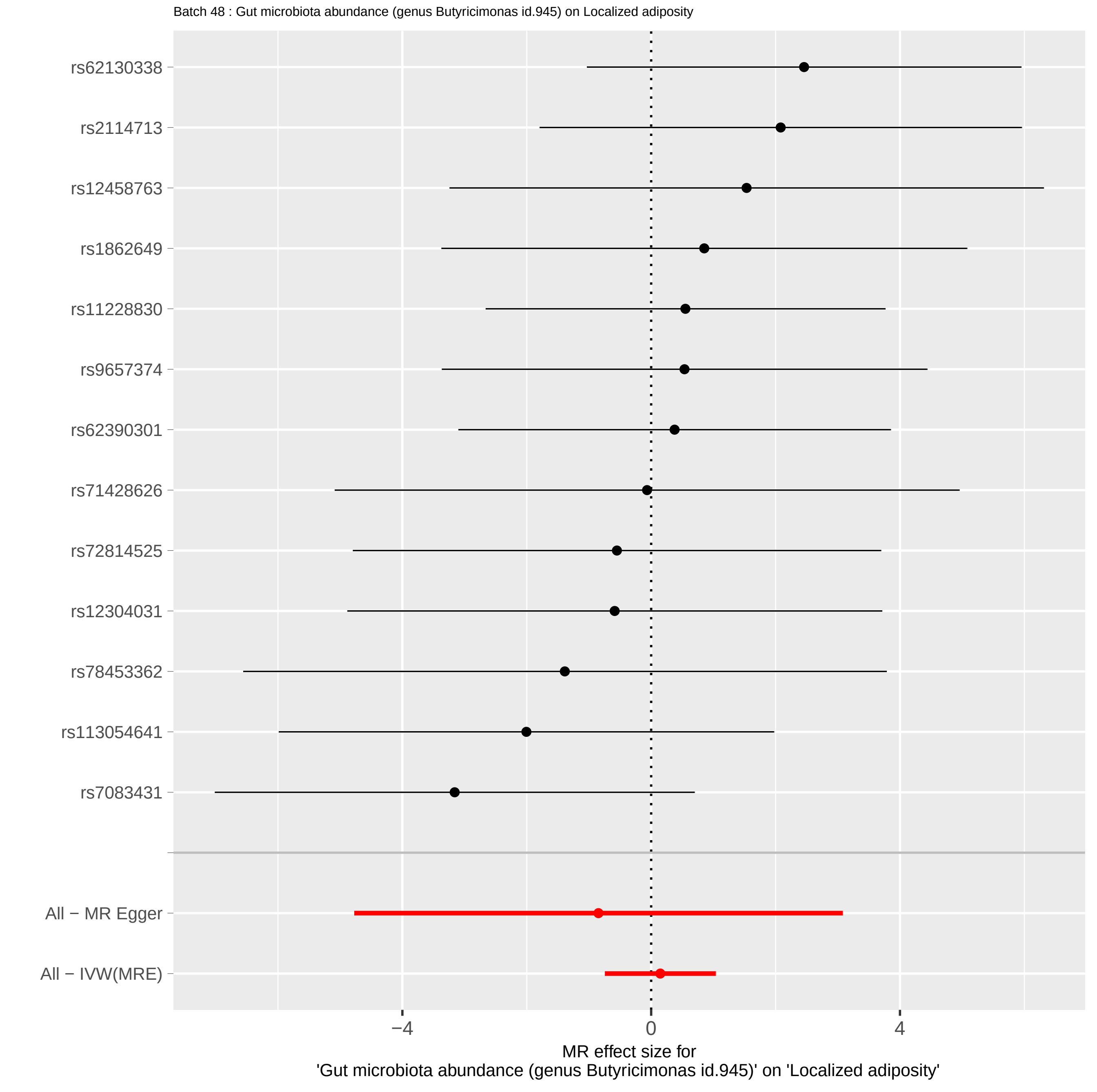

Batch 48 : Gut microbiota abundance (genus Butyricimonas id.945) on Localized adiposity
rs62130338
rs2114713
rs12458763
rs1862649
rs11228830
rs9657374
rs62390301
rs71428626
rs72814525
rs12304031
rs78453362
rs113054641
rs7083431
All − MR Egger
All − IVW(MRE)
−4
0
4
MR effect size for
'Gut microbiota abundance (genus Butyricimonas id.945)' on 'Localized adiposity'

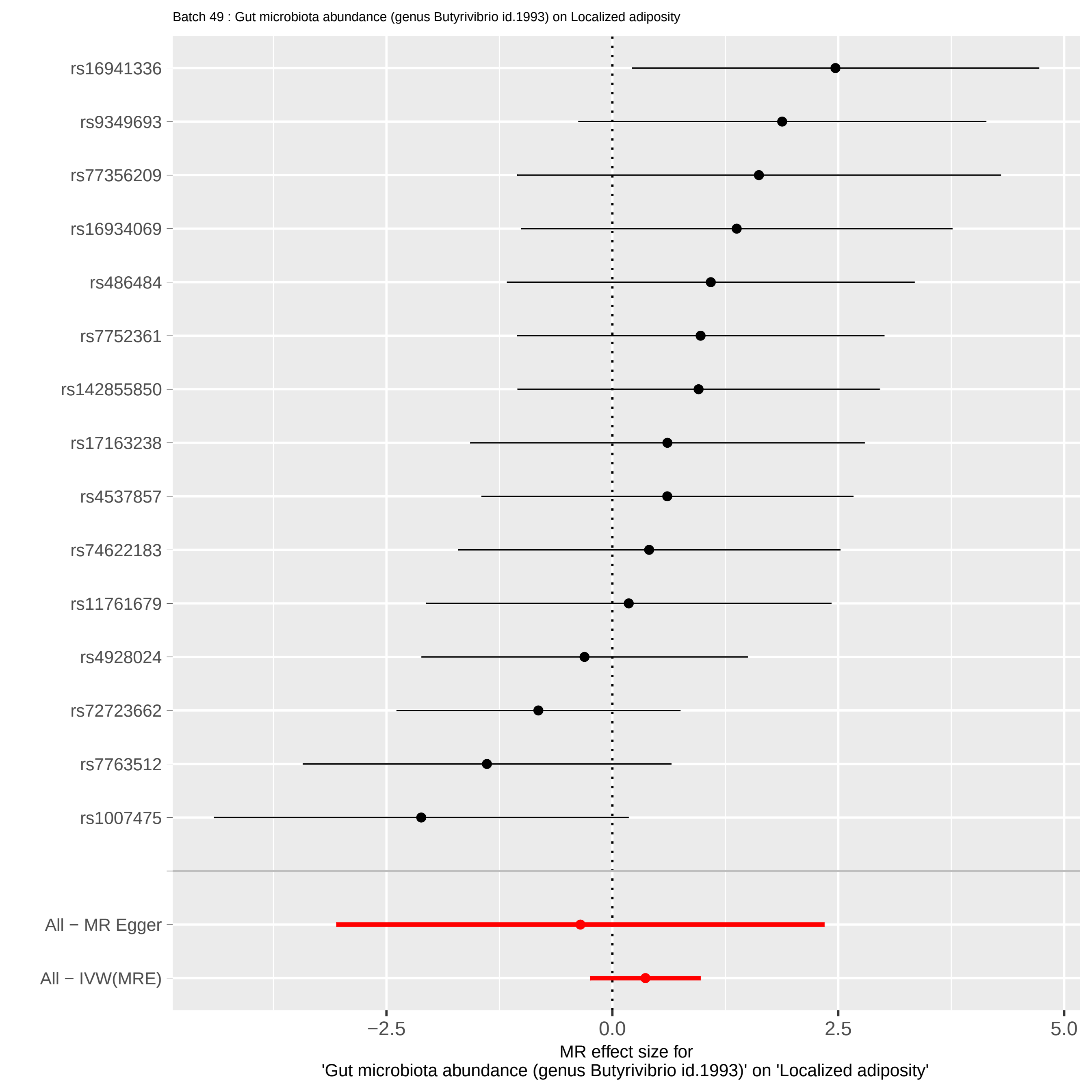
Batch 49 : Gut microbiota abundance (genus Butyrivibrio id.1993) on Localized adiposity
rs16941336
rs9349693
rs77356209
rs16934069
rs486484
rs7752361
rs142855850
rs17163238
rs4537857
rs74622183
rs11761679
rs4928024
rs72723662
rs7763512
rs1007475
All − MR Egger
All − IVW(MRE)
−2.5
0.0
2.5
5.0
MR effect size for
'Gut microbiota abundance (genus Butyrivibrio id.1993)' on 'Localized adiposity'

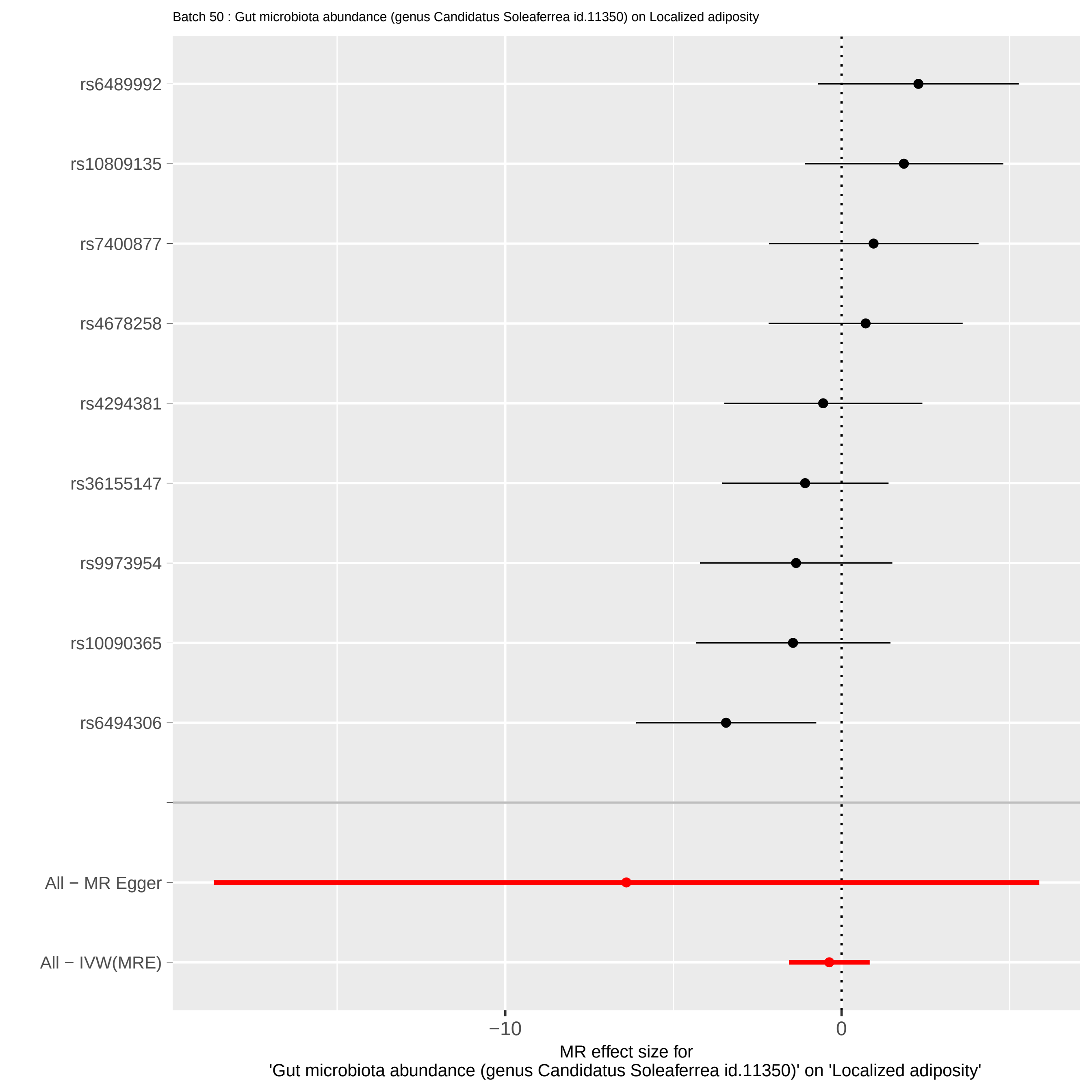

Batch 50 : Gut microbiota abundance (genus Candidatus Soleaferrea id.11350) on Localized adiposity
rs6489992
rs10809135
rs7400877
rs4678258
rs4294381
rs36155147
rs9973954
rs10090365
rs6494306
All − MR Egger
All − IVW(MRE)
−10
0
MR effect size for
'Gut microbiota abundance (genus Candidatus Soleaferrea id.11350)' on 'Localized adiposity'

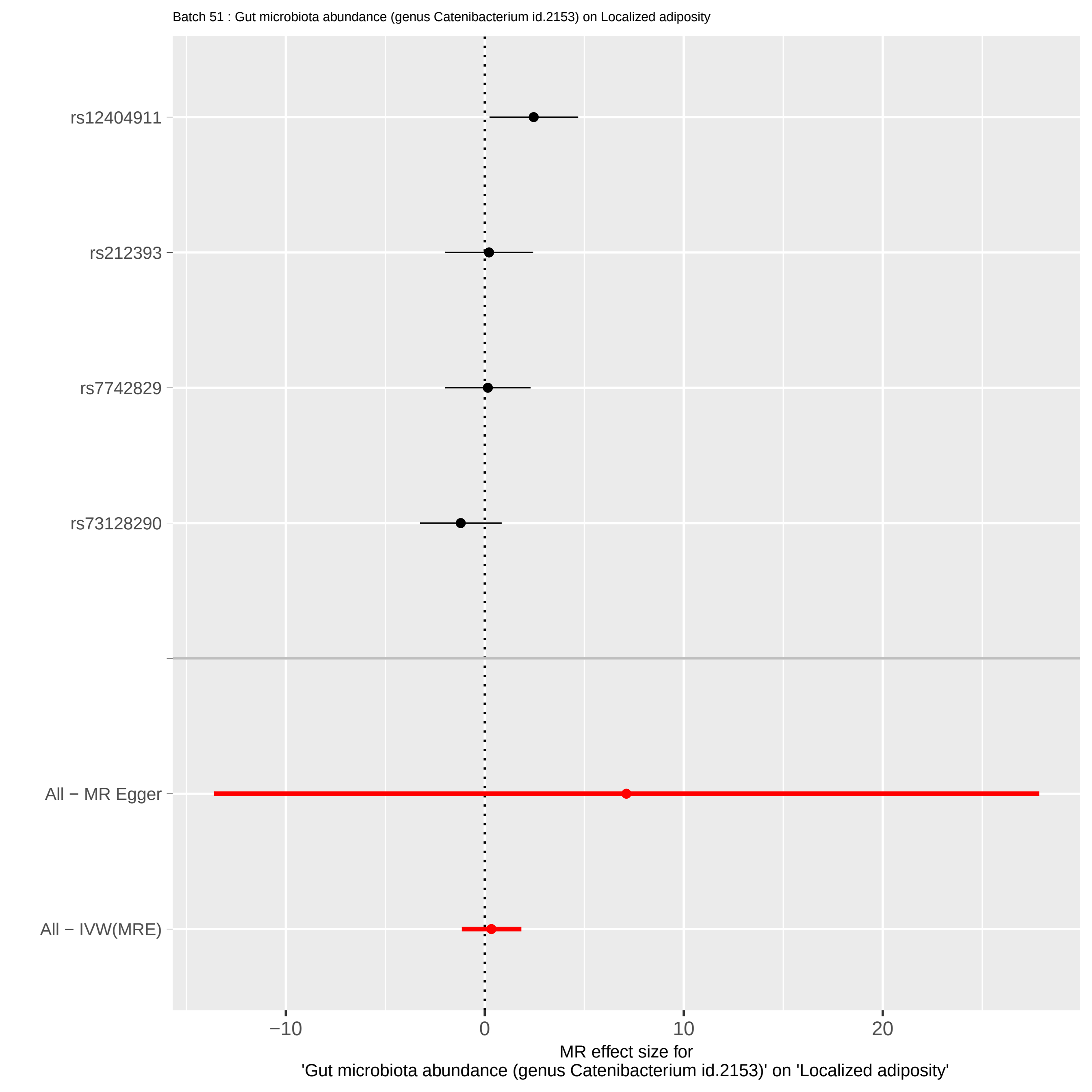

Batch 51 : Gut microbiota abundance (genus Catenibacterium id.2153) on Localized adiposity
rs12404911
rs212393
rs7742829
rs73128290
All − MR Egger
All − IVW(MRE)
−10
0
10
20
MR effect size for
'Gut microbiota abundance (genus Catenibacterium id.2153)' on 'Localized adiposity'

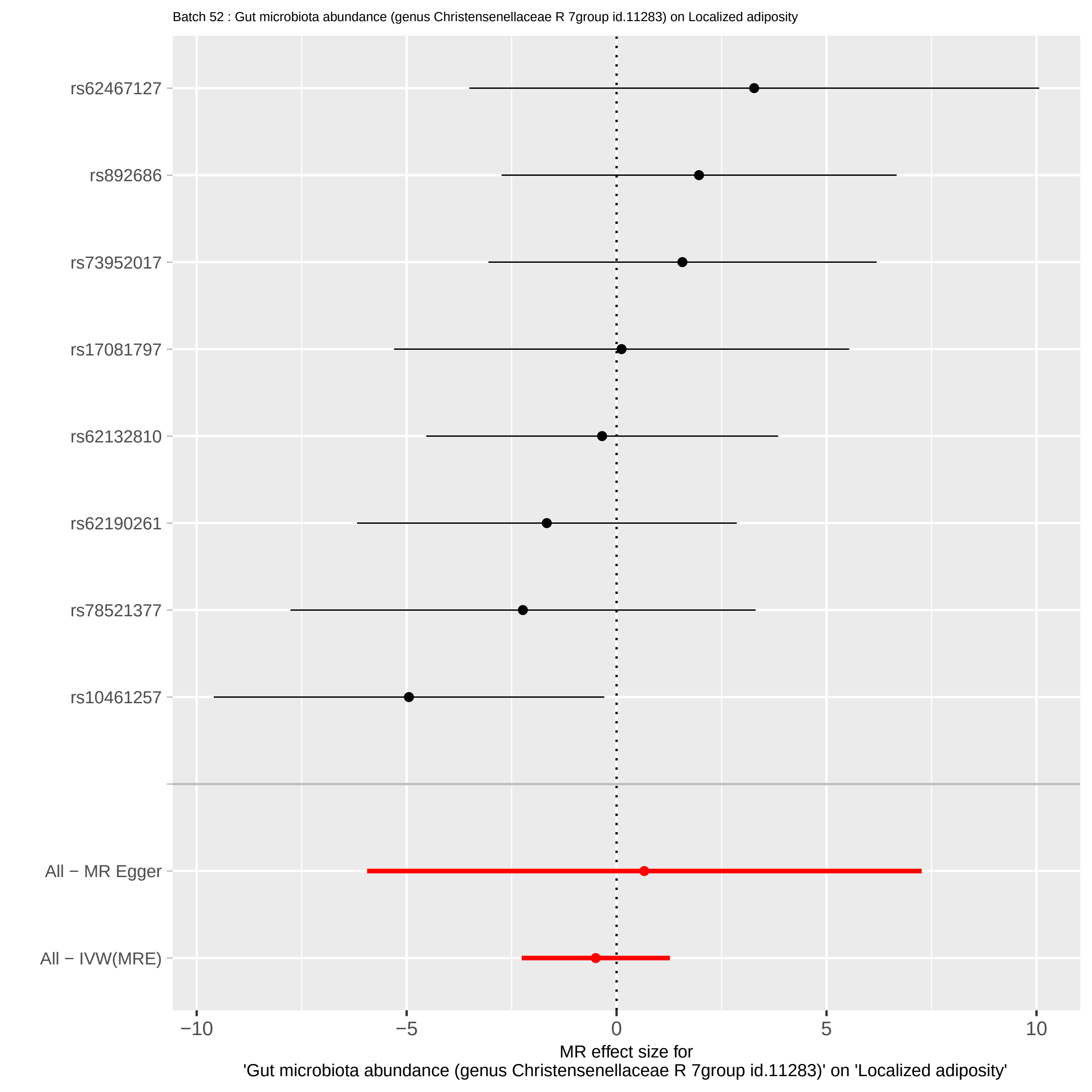

Batch 52 : Gut microbiota abundance (genus Christensenellaceae R 7group id.11283) on Localized adiposity
rs62467127
rs892686
rs73952017
rs17081797
rs62132810
rs62190261
rs78521377
rs10461257
All − MR Egger
All − IVW(MRE)
−10
−5
0
5
10
MR effect size for
'Gut microbiota abundance (genus Christensenellaceae R 7group id.11283)' on 'Localized adiposity'

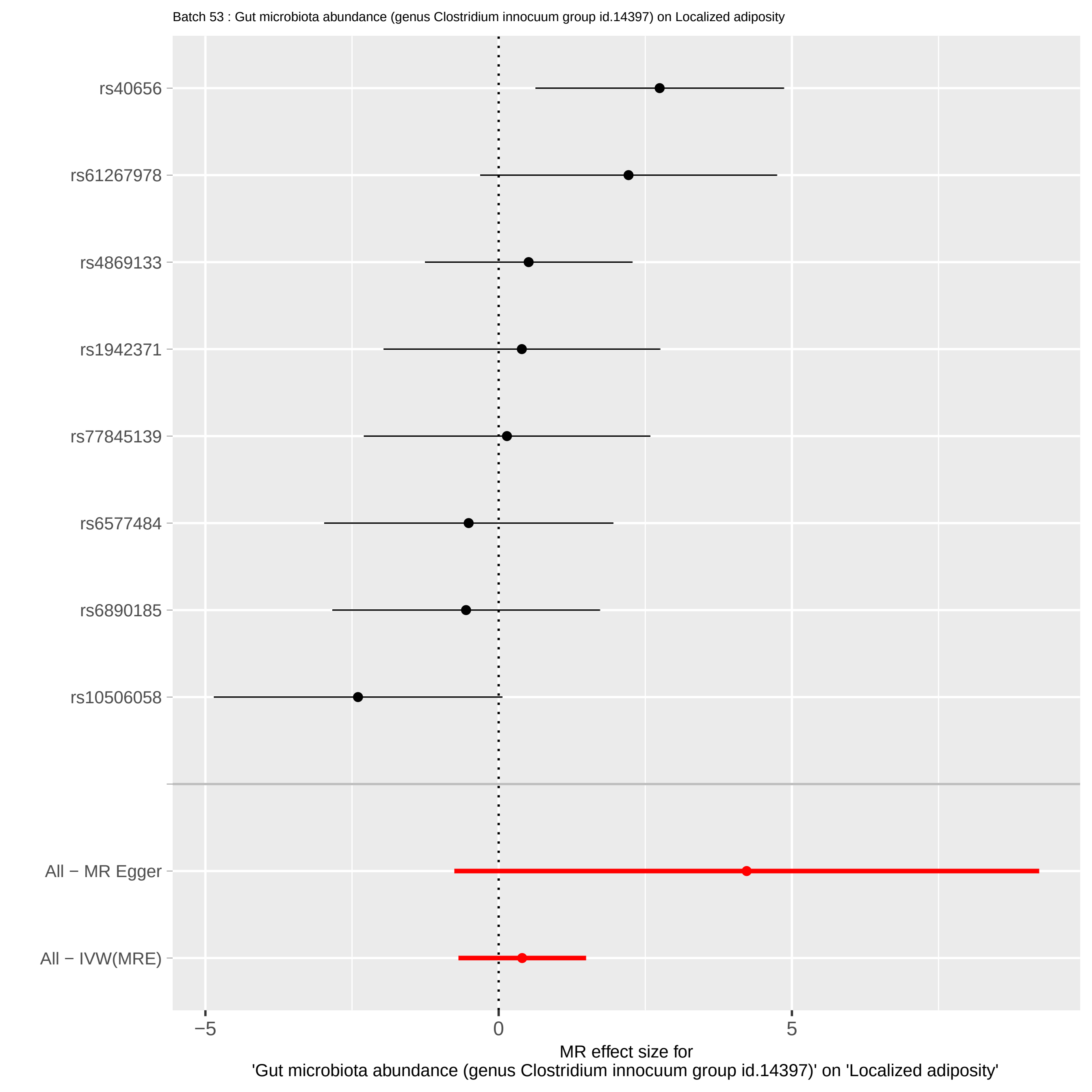
Batch 53 : Gut microbiota abundance (genus Clostridium innocuum group id.14397) on Localized adiposity
rs40656
rs61267978
rs4869133
rs1942371
rs77845139
rs6577484
rs6890185
rs10506058
All − MR Egger
All − IVW(MRE)
−5
0
5
MR effect size for
'Gut microbiota abundance (genus Clostridium innocuum group id.14397)' on 'Localized adiposity'

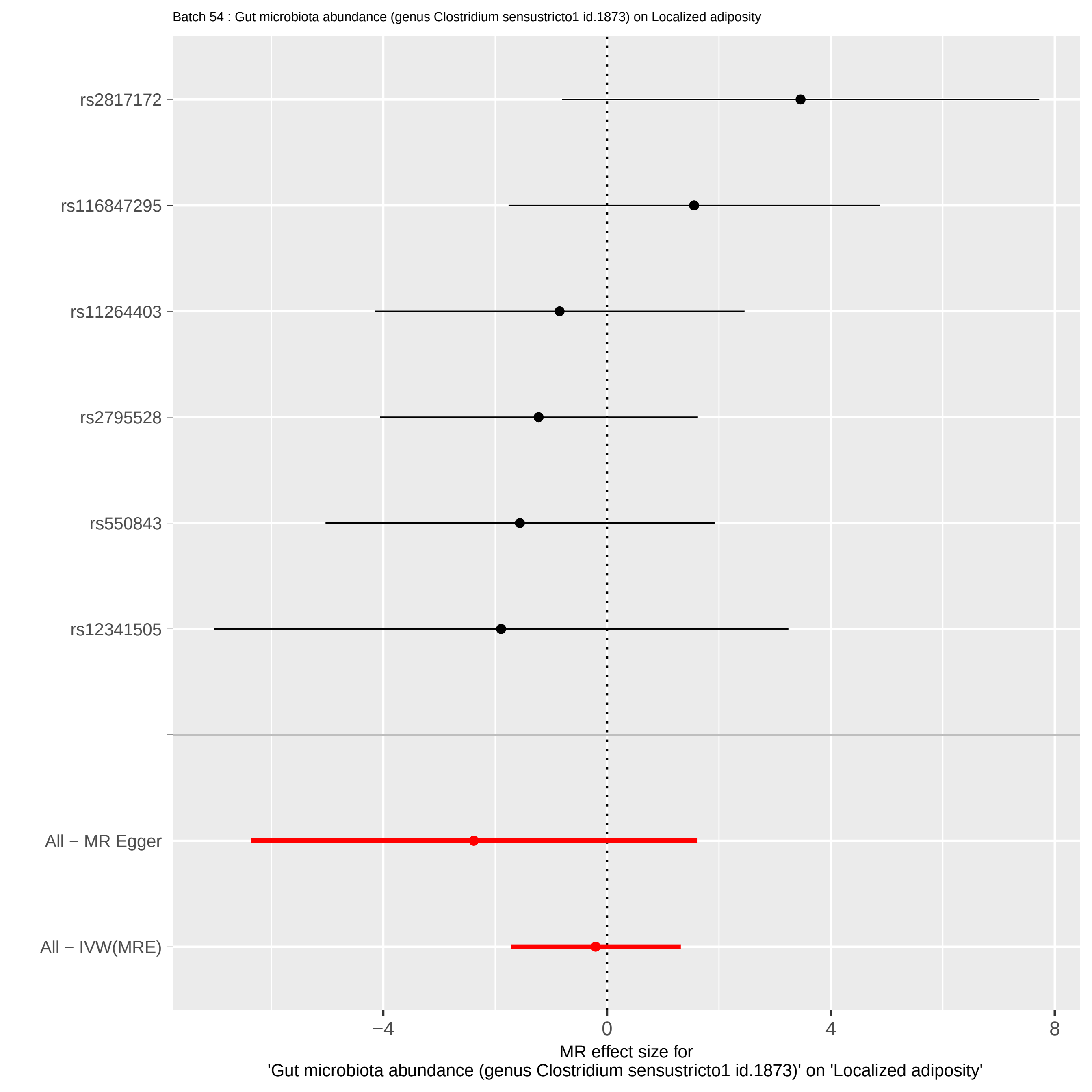

Batch 54 : Gut microbiota abundance (genus Clostridium sensustricto1 id.1873) on Localized adiposity
rs2817172
rs116847295
rs11264403
rs2795528
rs550843
rs12341505
All − MR Egger
All − IVW(MRE)
−4
0
4
8
MR effect size for
'Gut microbiota abundance (genus Clostridium sensustricto1 id.1873)' on 'Localized adiposity'

Batch 55 : Gut microbiota abundance (genus Collinsella id.815) on Localized adiposity

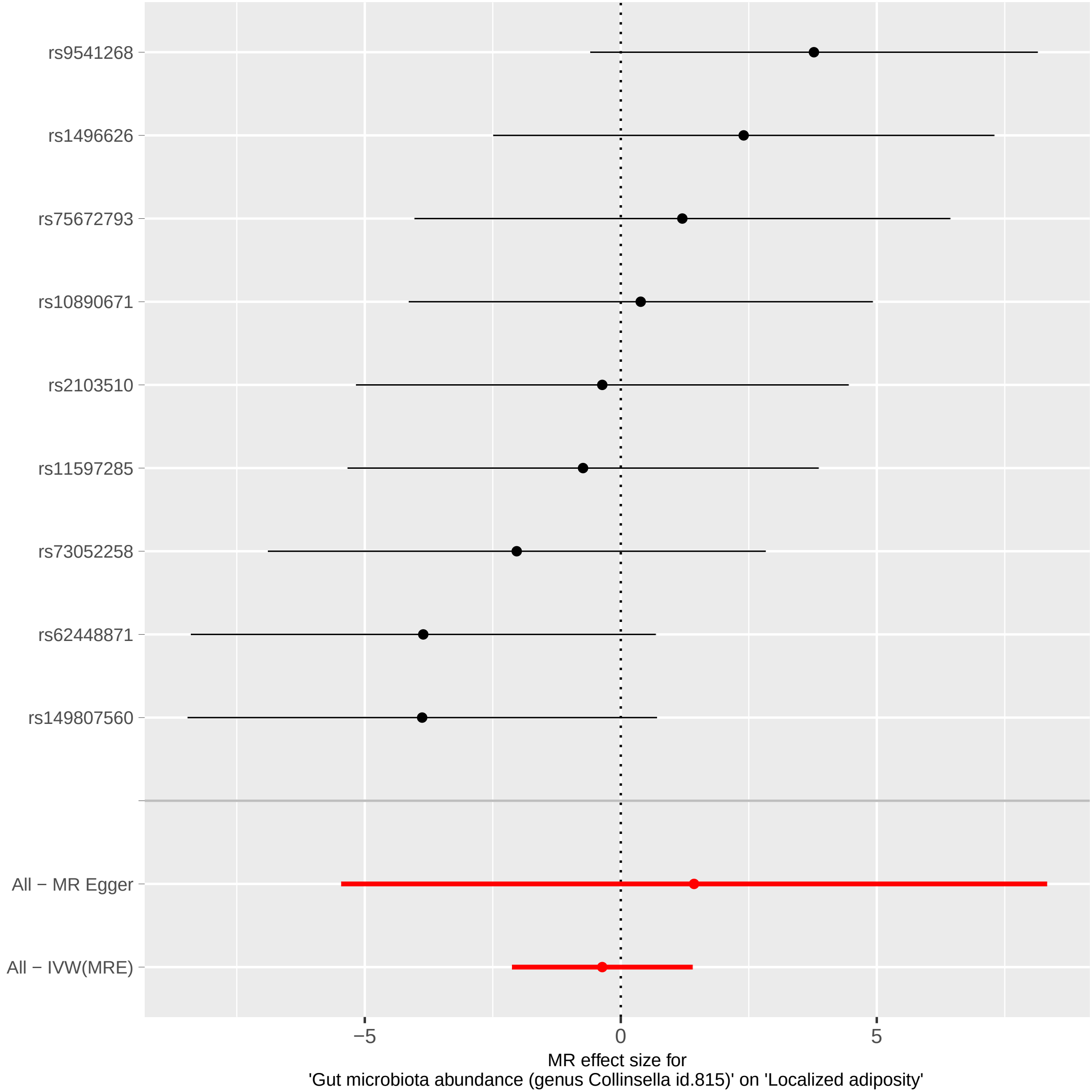

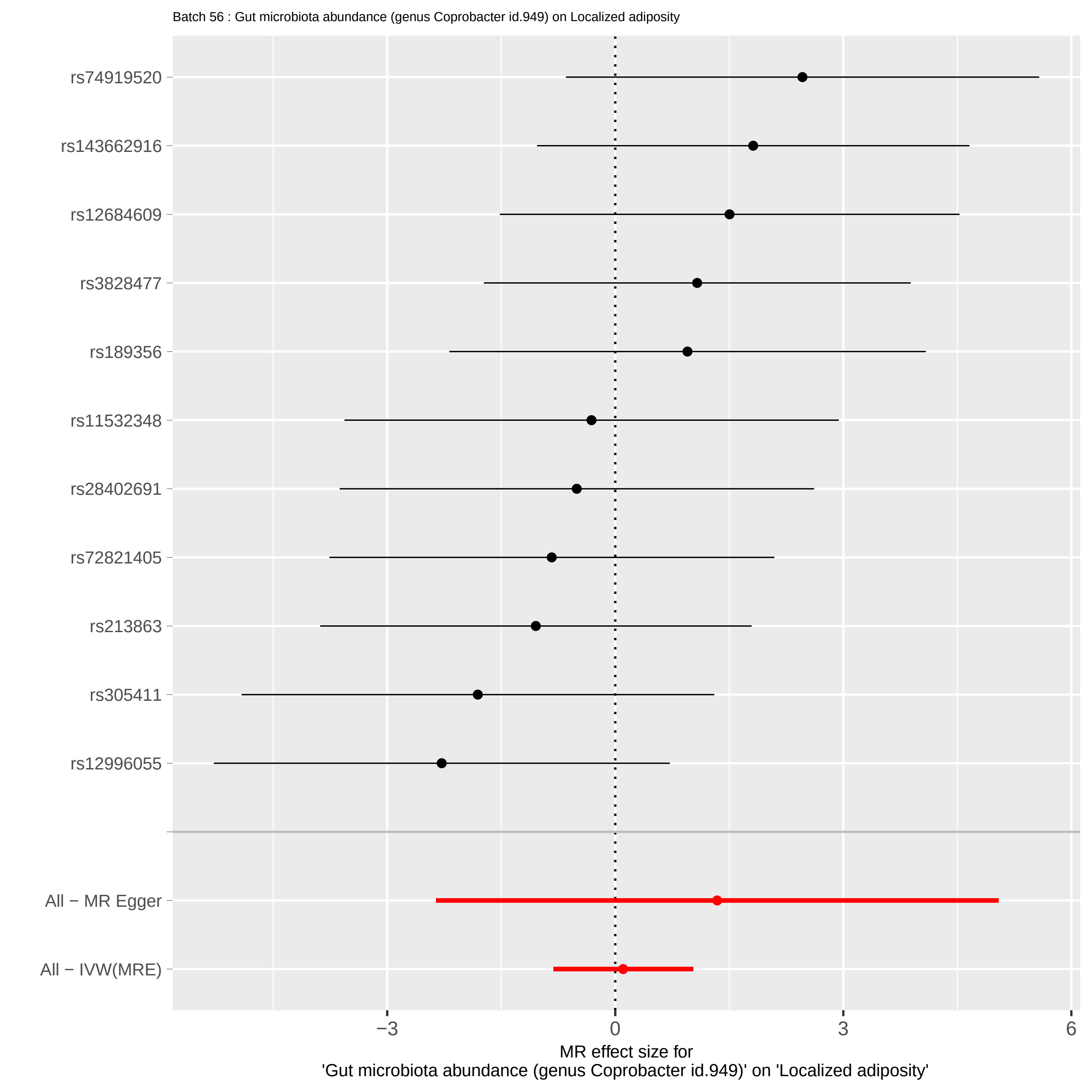
Batch 56 : Gut microbiota abundance (genus Coprobacter id.949) on Localized adiposity
rs74919520
rs143662916
rs12684609
rs3828477
rs189356
rs11532348
rs28402691
rs72821405
rs213863
rs305411
rs12996055
All − MR Egger
All − IVW(MRE)
−3
0
3
6
MR effect size for
'Gut microbiota abundance (genus Coprobacter id.949)' on 'Localized adiposity'

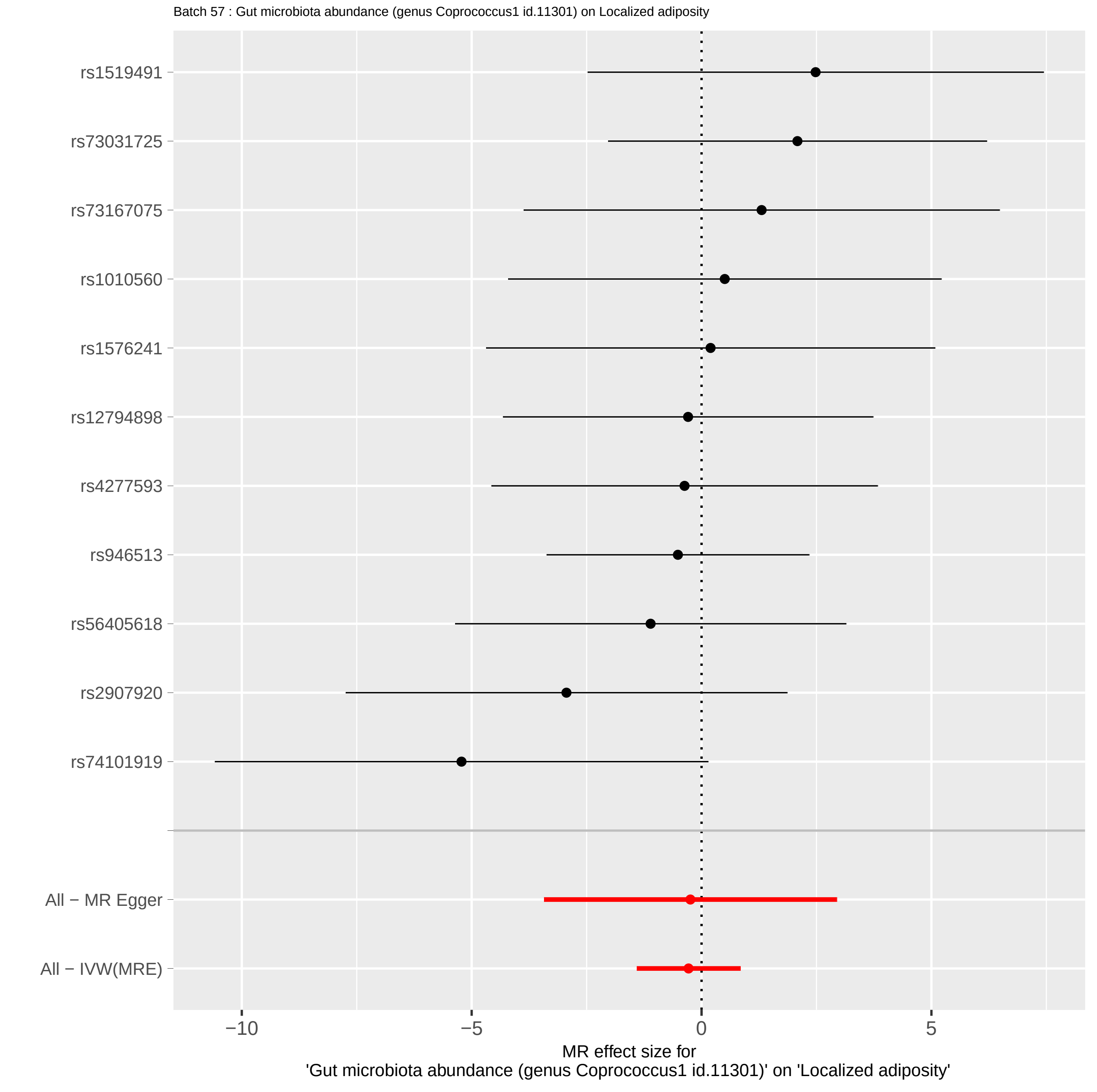

Batch 57 : Gut microbiota abundance (genus Coprococcus1 id.11301) on Localized adiposity
rs1519491
rs73031725
rs73167075
rs1010560
rs1576241
rs12794898
rs4277593
rs946513
rs56405618
rs2907920
rs74101919
All − MR Egger
All − IVW(MRE)
−10
−5
0
5
MR effect size for
'Gut microbiota abundance (genus Coprococcus1 id.11301)' on 'Localized adiposity'

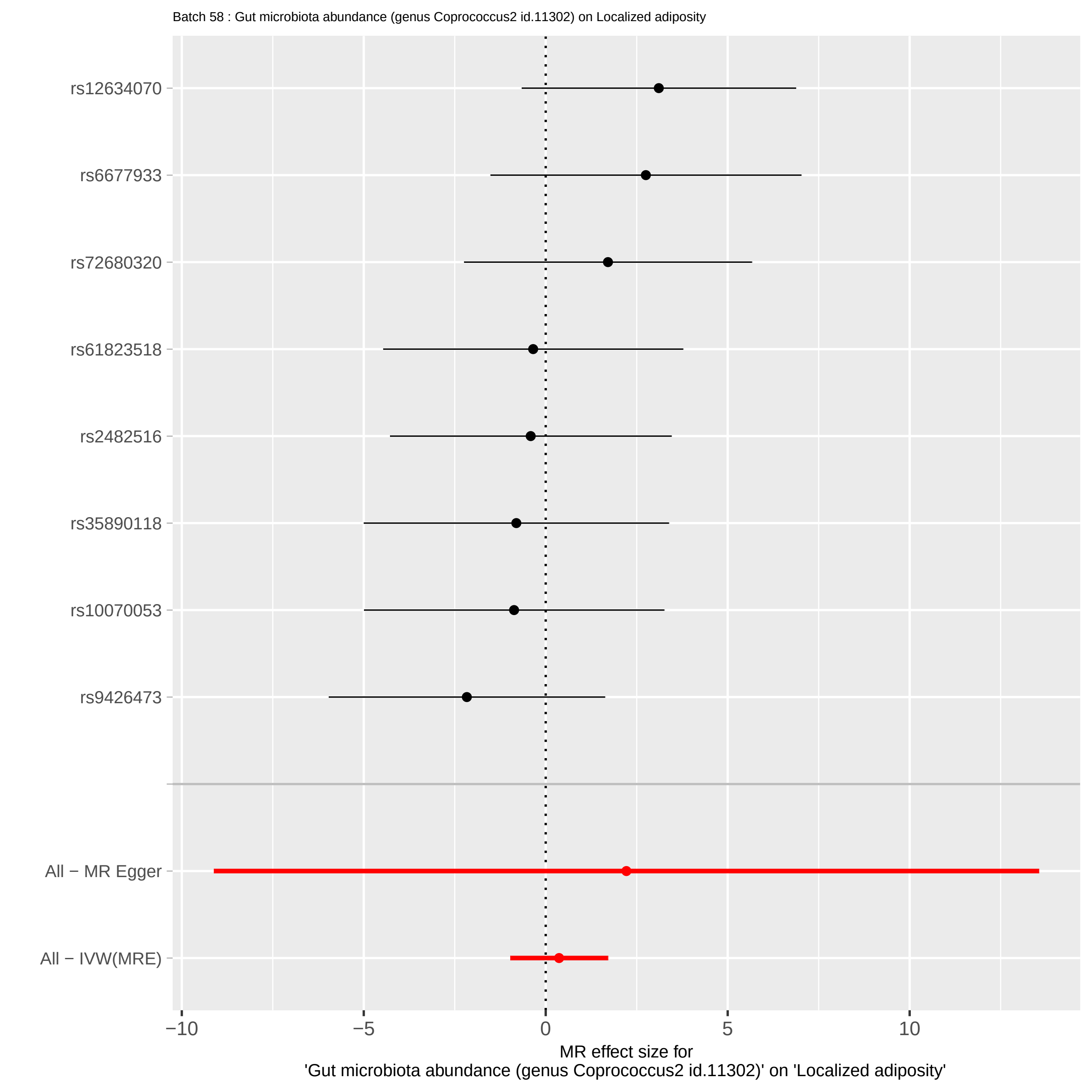

Batch 58 : Gut microbiota abundance (genus Coprococcus2 id.11302) on Localized adiposity
rs12634070
rs6677933
rs72680320
rs61823518
rs2482516
rs35890118
rs10070053
rs9426473
All − MR Egger
All − IVW(MRE)
−10
−5
0
5
10
MR effect size for
'Gut microbiota abundance (genus Coprococcus2 id.11302)' on 'Localized adiposity'

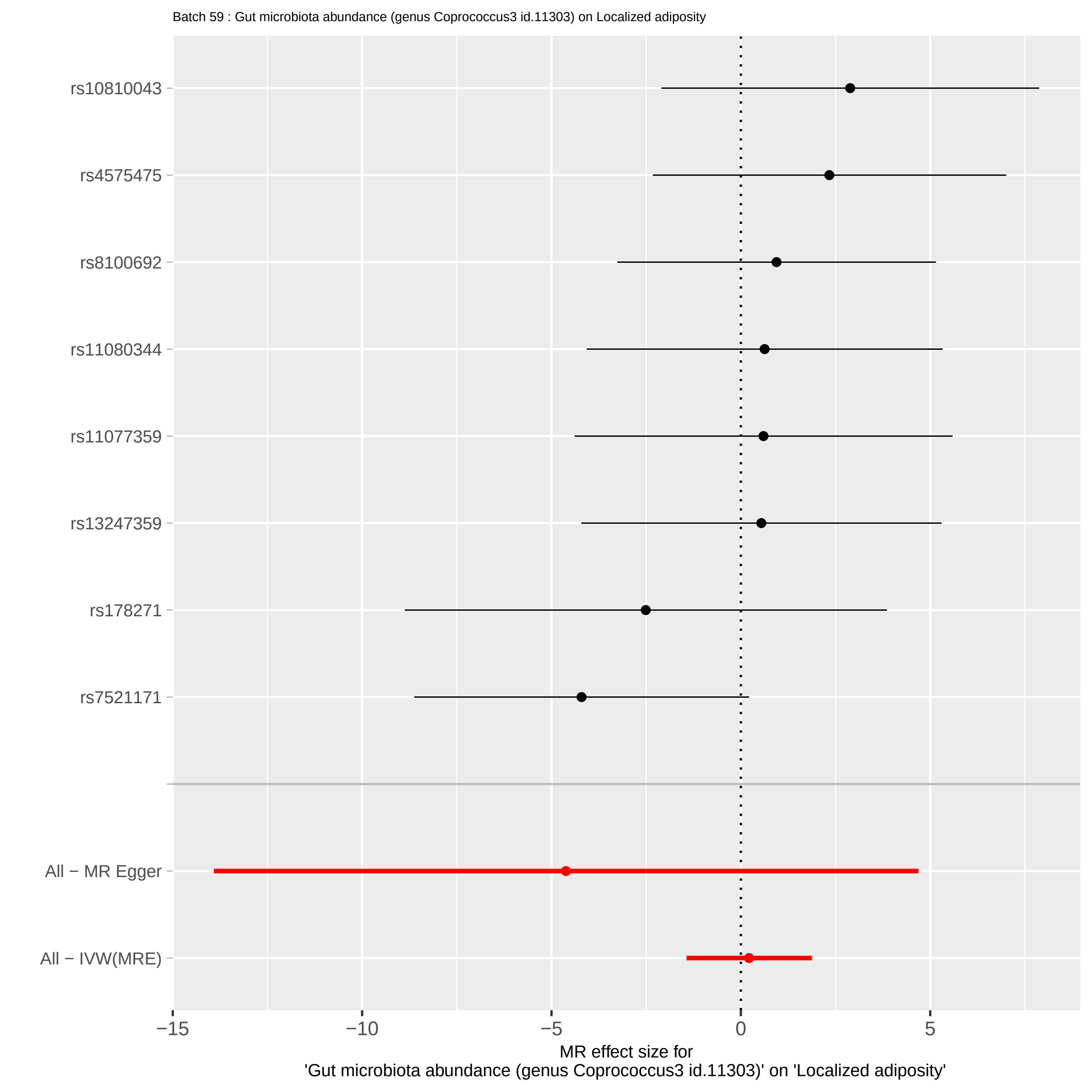

Batch 59 : Gut microbiota abundance (genus Coprococcus3 id.11303) on Localized adiposity
rs10810043
rs4575475
rs8100692
rs11080344
rs11077359
rs13247359
rs178271
rs7521171
All − MR Egger
All − IVW(MRE)
−15
−10
−5
0
5
MR effect size for
'Gut microbiota abundance (genus Coprococcus3 id.11303)' on 'Localized adiposity'

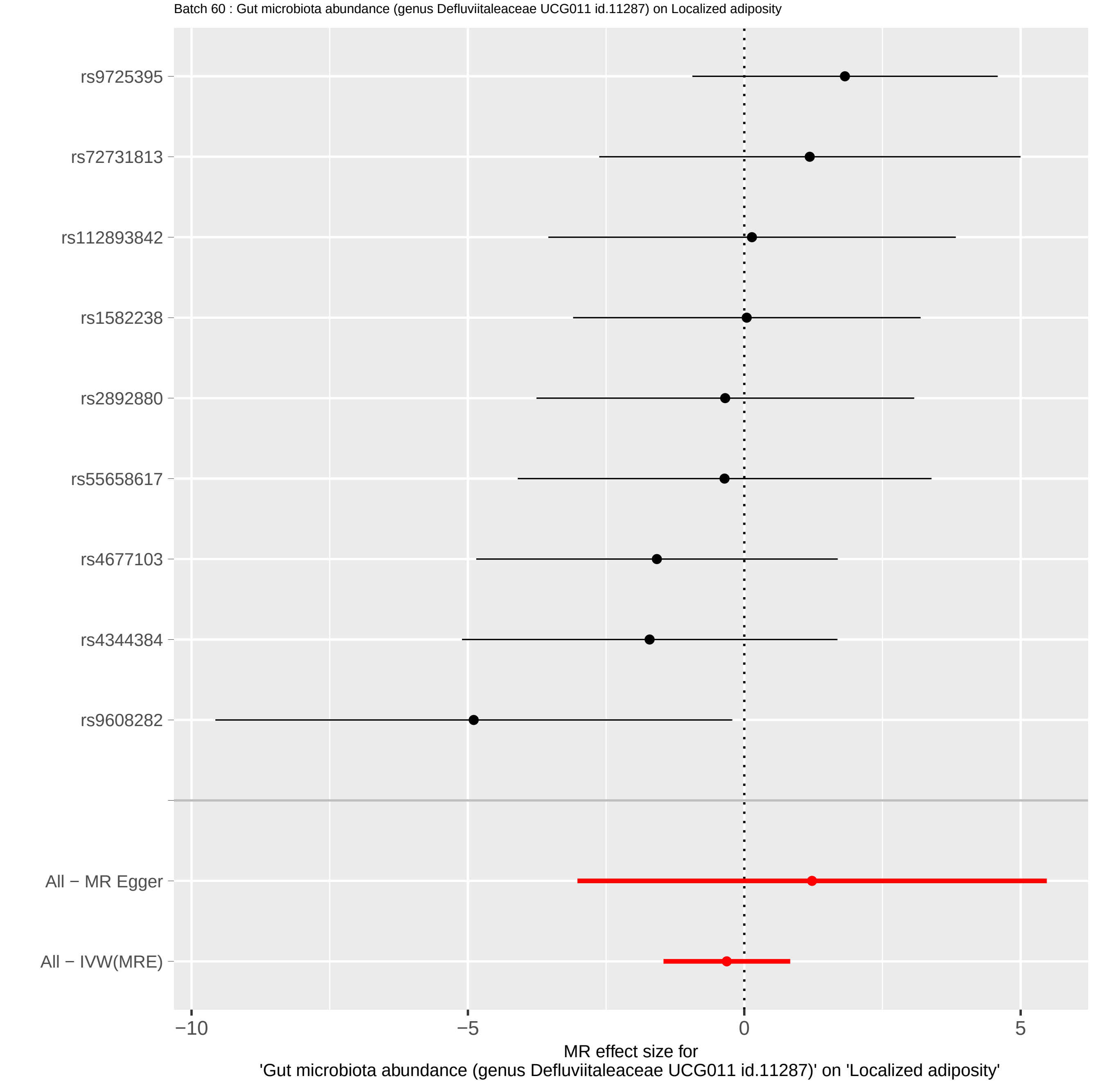

Batch 60 : Gut microbiota abundance (genus Defluviitaleaceae UCG011 id.11287) on Localized adiposity
rs9725395
rs72731813
rs112893842
rs1582238
rs2892880
rs55658617
rs4677103
rs4344384
rs9608282
All − MR Egger
All − IVW(MRE)
−10
−5
0
5
MR effect size for
'Gut microbiota abundance (genus Defluviitaleaceae UCG011 id.11287)' on 'Localized adiposity'

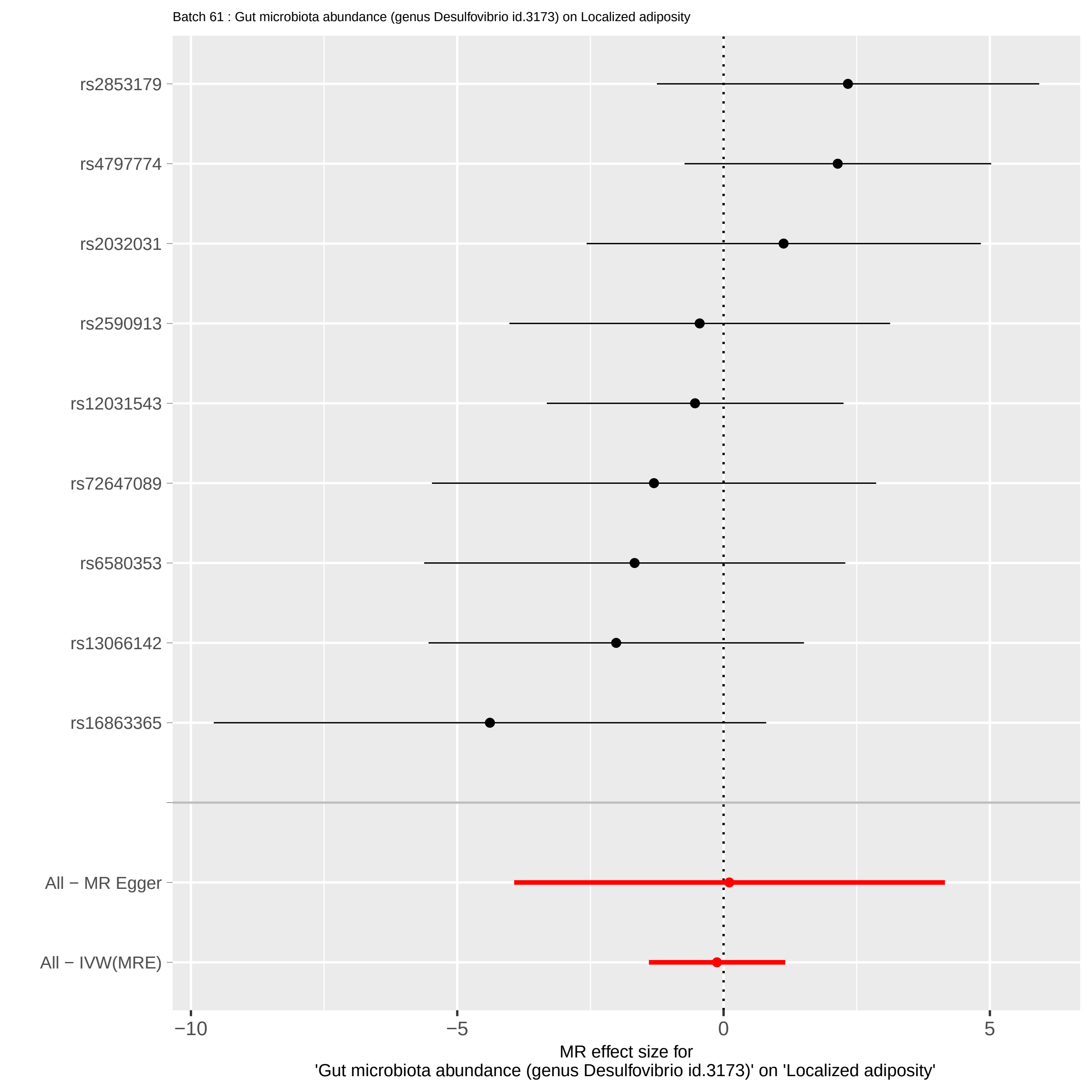
Batch 61 : Gut microbiota abundance (genus Desulfovibrio id.3173) on Localized adiposity
rs2853179
rs4797774
rs2032031
rs2590913
rs12031543
rs72647089
rs6580353
rs13066142
rs16863365
All − MR Egger
All − IVW(MRE)
−10
−5
0
5
MR effect size for
'Gut microbiota abundance (genus Desulfovibrio id.3173)' on 'Localized adiposity'

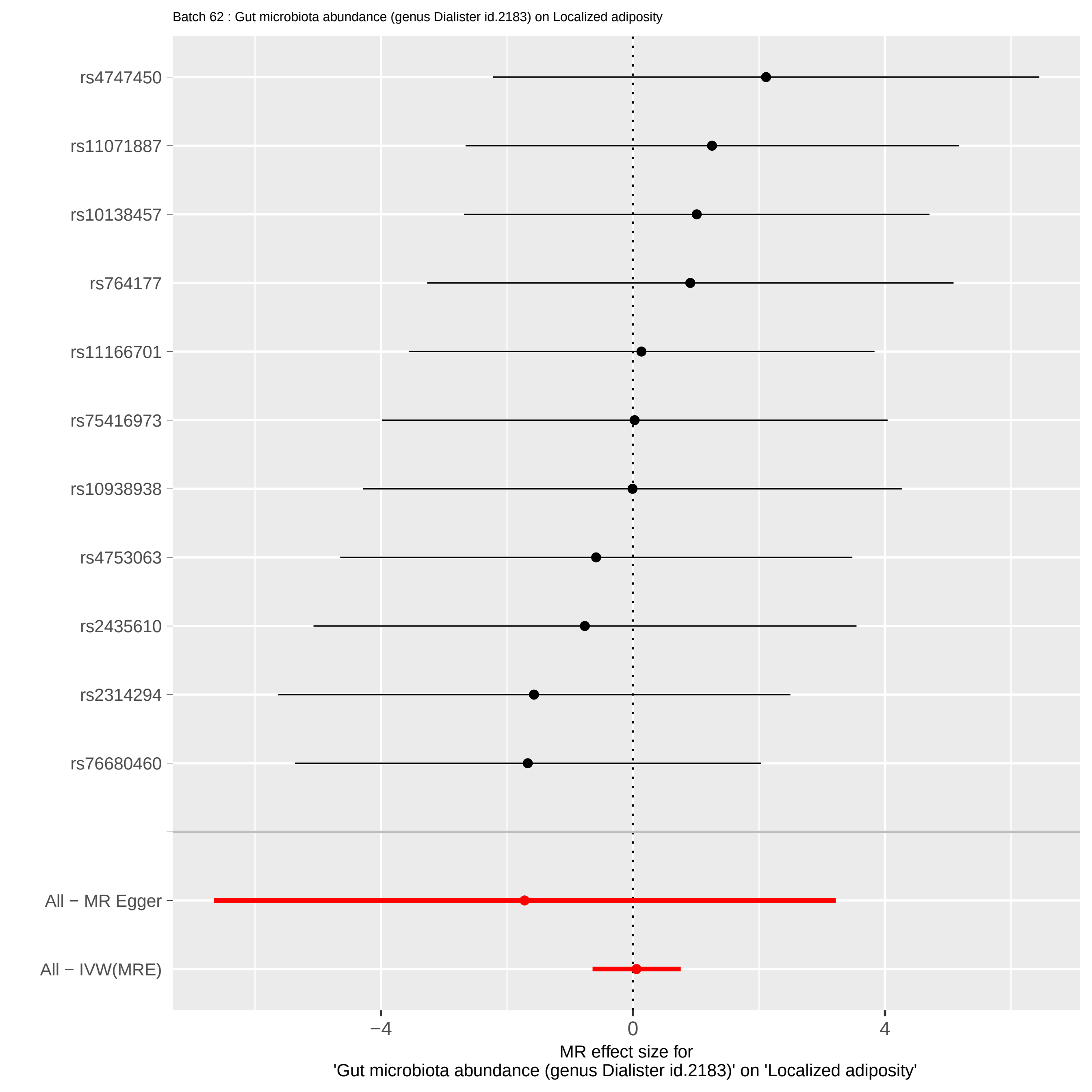

Batch 62 : Gut microbiota abundance (genus Dialister id.2183) on Localized adiposity
rs4747450
rs11071887
rs10138457
rs764177
rs11166701
rs75416973
rs10938938
rs4753063
rs2435610
rs2314294
rs76680460
All − MR Egger
All − IVW(MRE)
−4
0
4
MR effect size for
'Gut microbiota abundance (genus Dialister id.2183)' on 'Localized adiposity'

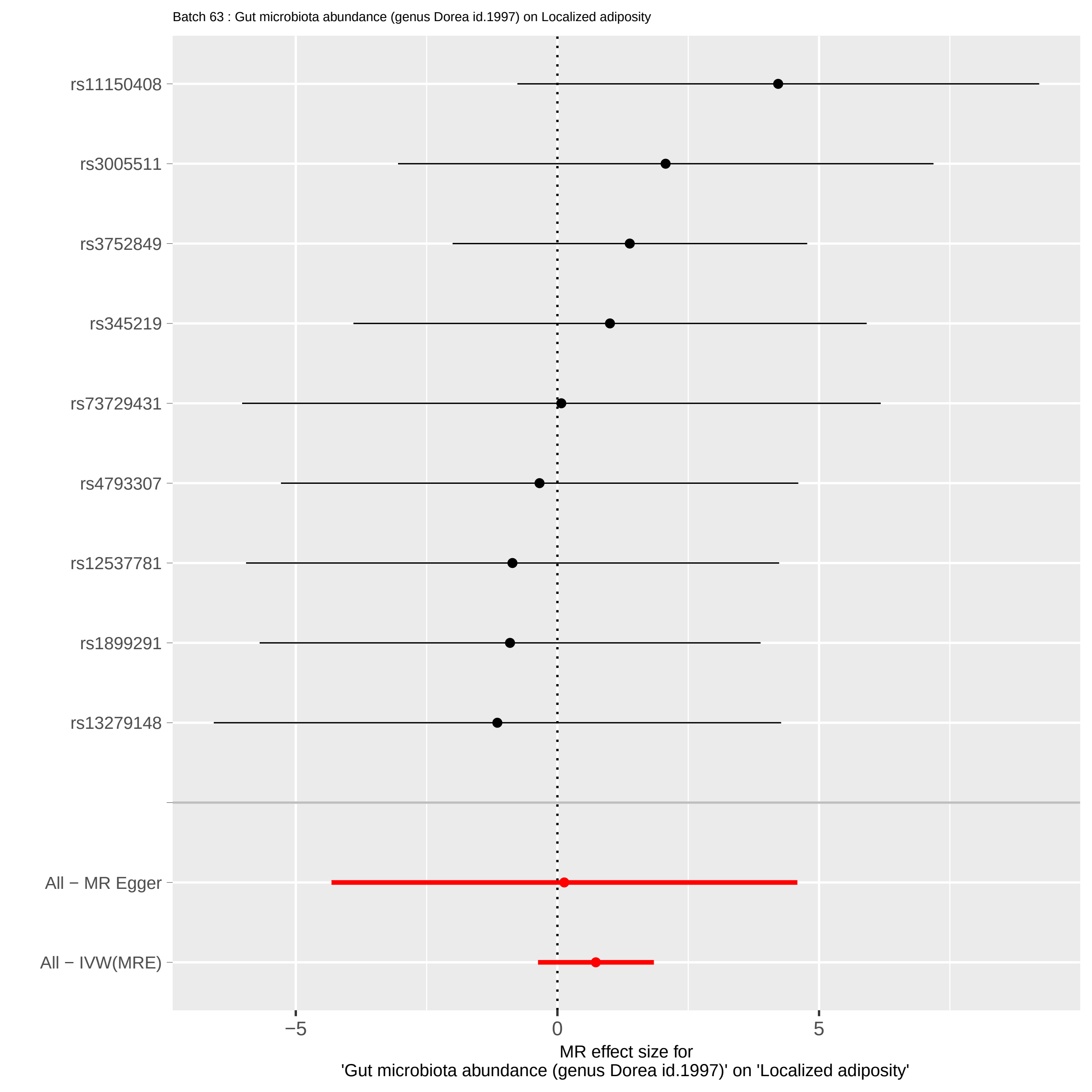

Batch 63 : Gut microbiota abundance (genus Dorea id.1997) on Localized adiposity
rs11150408
rs3005511
rs3752849
rs345219
rs73729431
rs4793307
rs12537781
rs1899291
rs13279148
All − MR Egger
All − IVW(MRE)
−5
0
5
MR effect size for
'Gut microbiota abundance (genus Dorea id.1997)' on 'Localized adiposity'

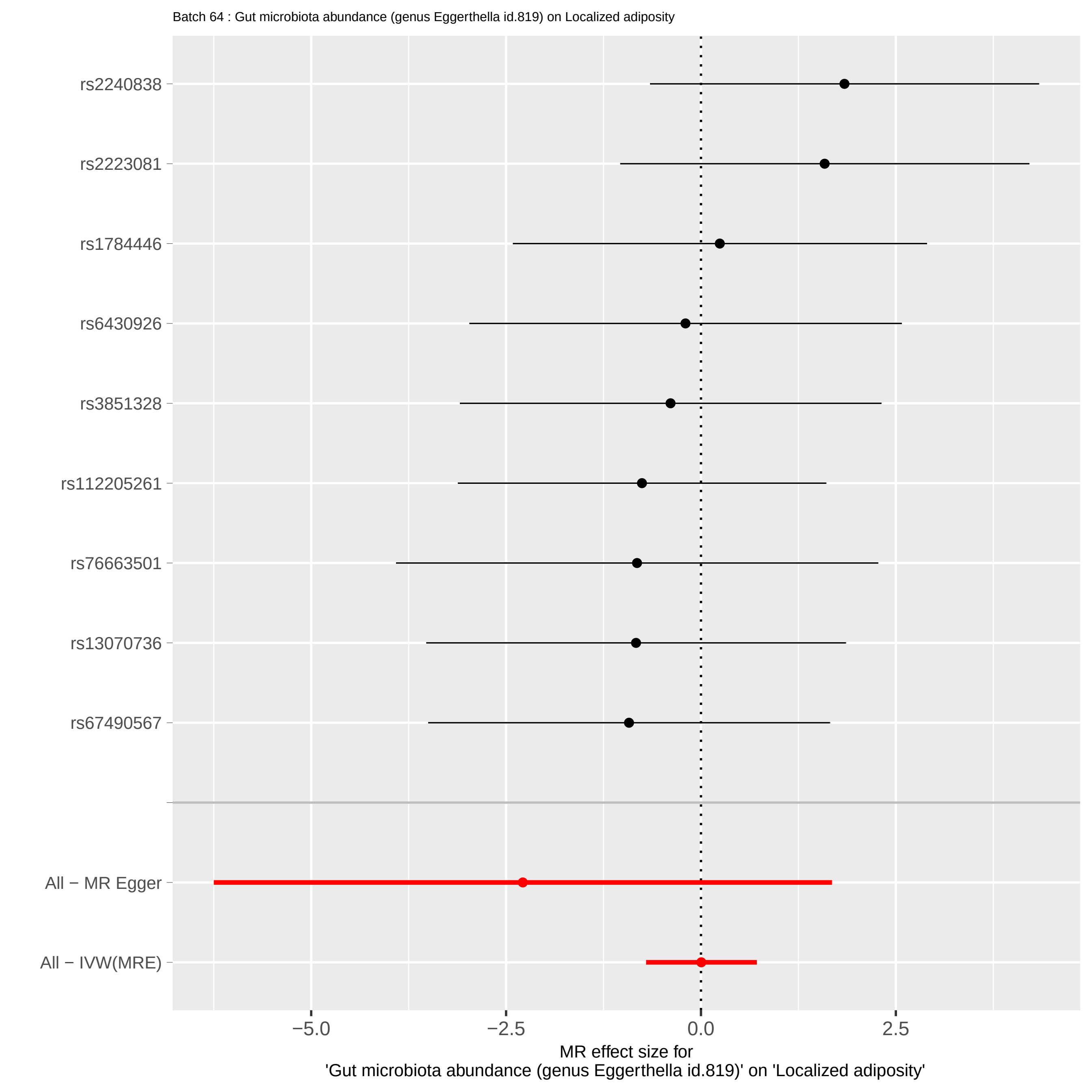

Batch 64 : Gut microbiota abundance (genus Eggerthella id.819) on Localized adiposity
rs2240838
rs2223081
rs1784446
rs6430926
rs3851328
rs112205261
rs76663501
rs13070736
rs67490567
All − MR Egger
All − IVW(MRE)
−5.0
−2.5
0.0
2.5
MR effect size for
'Gut microbiota abundance (genus Eggerthella id.819)' on 'Localized adiposity'

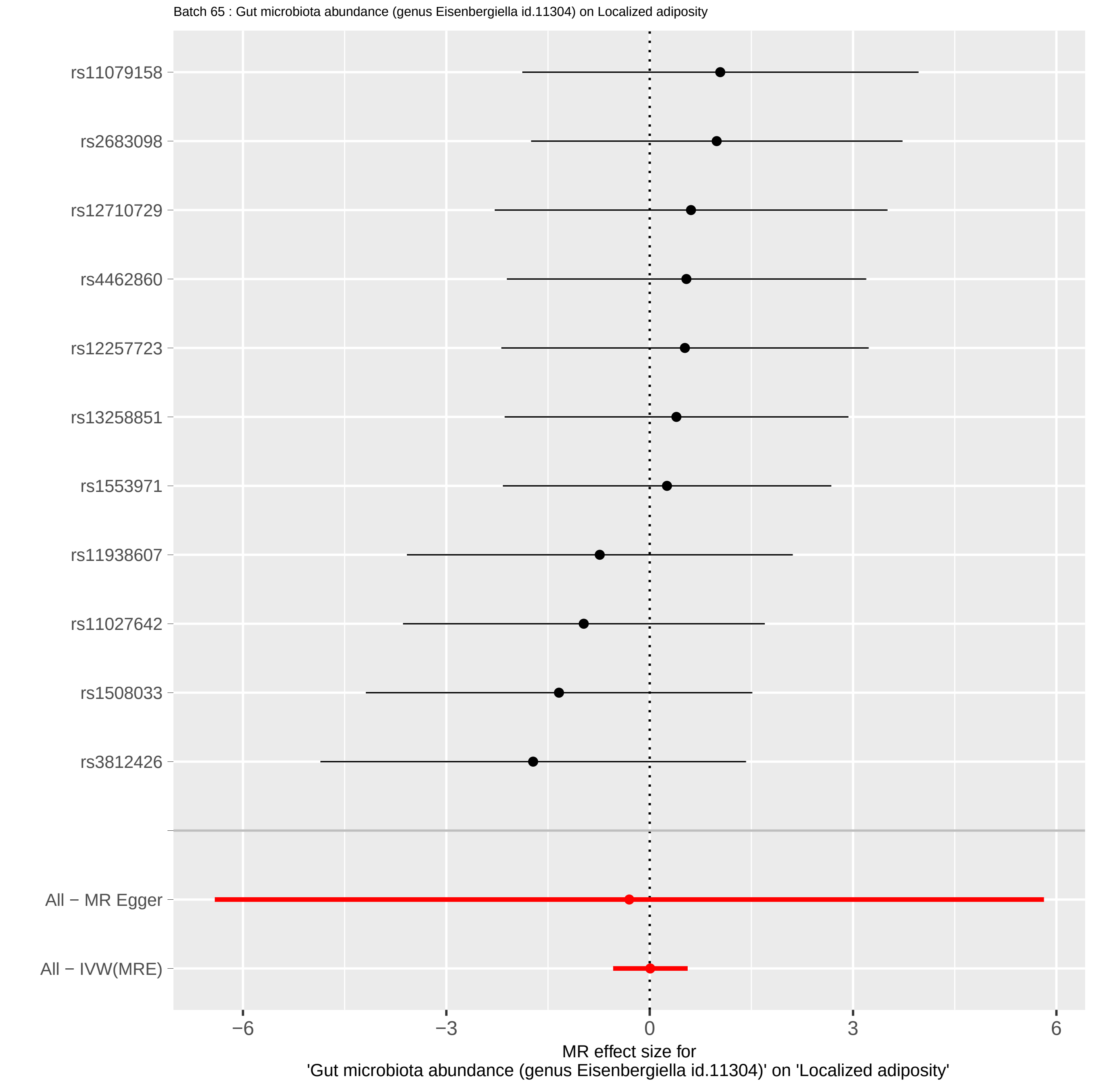
Batch 65 : Gut microbiota abundance (genus Eisenbergiella id.11304) on Localized adiposity
rs11079158
rs2683098
rs12710729
rs4462860
rs12257723
rs13258851
rs1553971
rs11938607
rs11027642
rs1508033
rs3812426
All − MR Egger
All − IVW(MRE)
−6
−3
0
3
6
MR effect size for
'Gut microbiota abundance (genus Eisenbergiella id.11304)' on 'Localized adiposity'

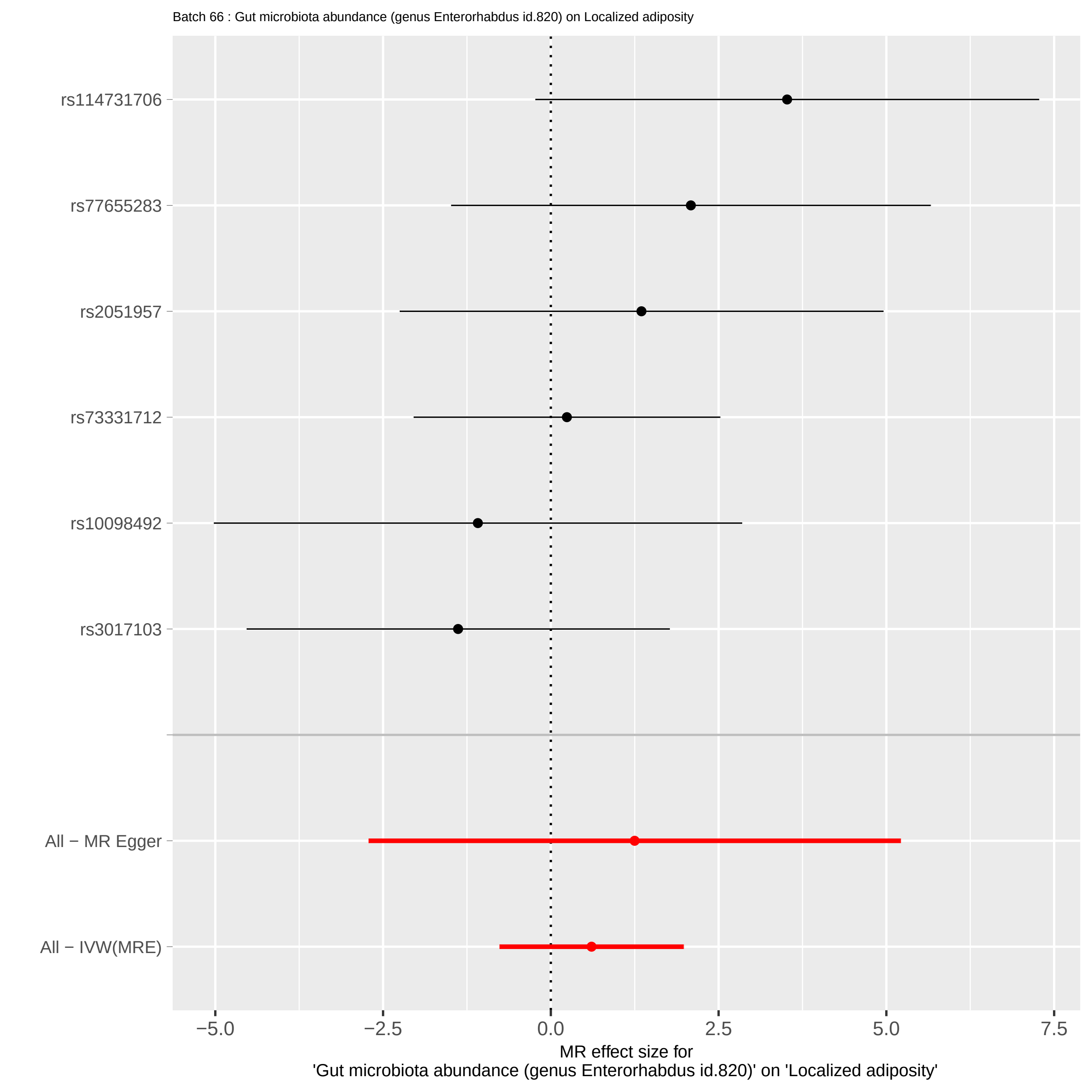

Batch 66 : Gut microbiota abundance (genus Enterorhabdus id.820) on Localized adiposity
rs114731706
rs77655283
rs2051957
rs73331712
rs10098492
rs3017103
All − MR Egger
All − IVW(MRE)
−5.0
−2.5
0.0
2.5
5.0
7.5
MR effect size for
'Gut microbiota abundance (genus Enterorhabdus id.820)' on 'Localized adiposity'

Batch 67 : Gut microbiota abundance (genus Erysipelatoclostridium id.11381) on Localized adiposity

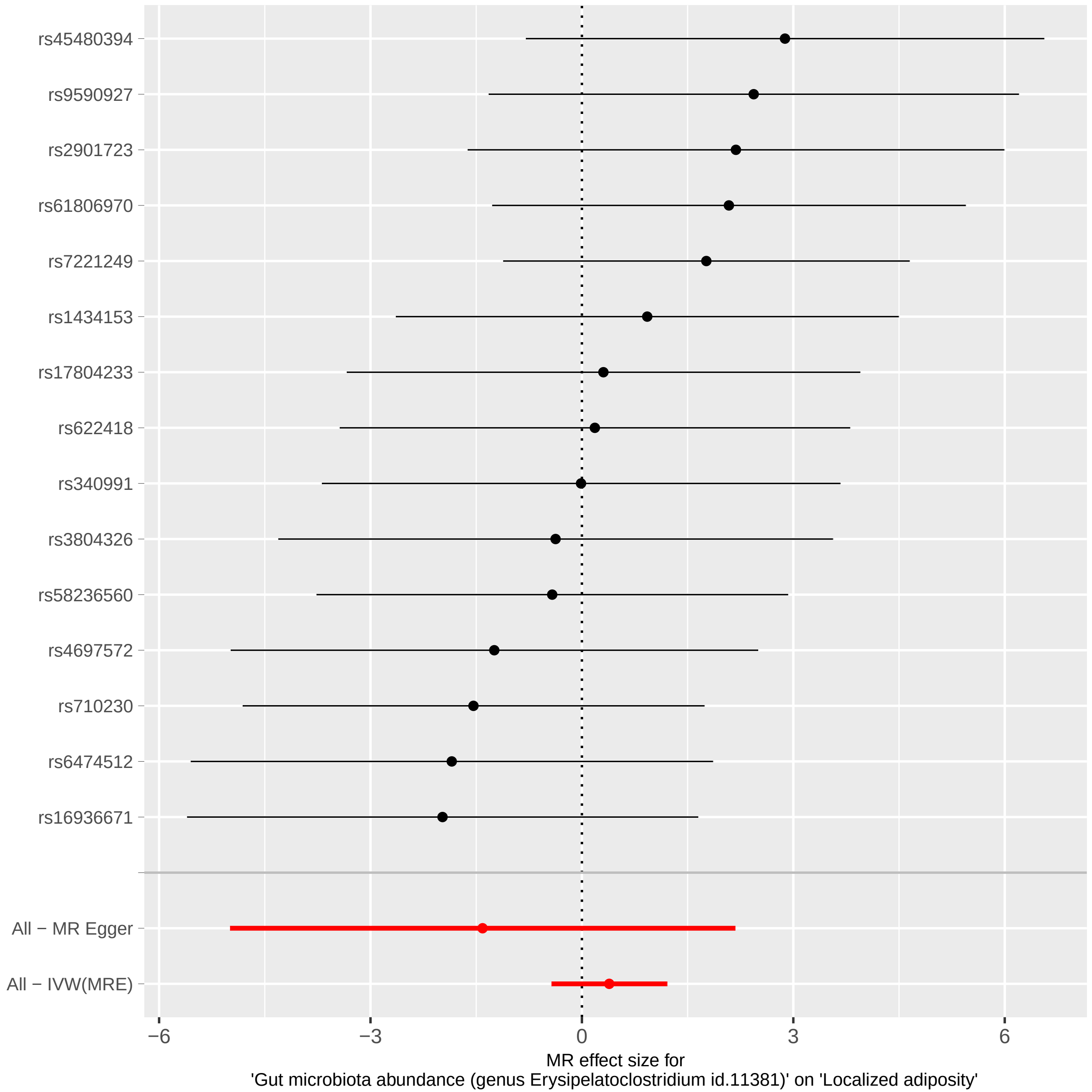

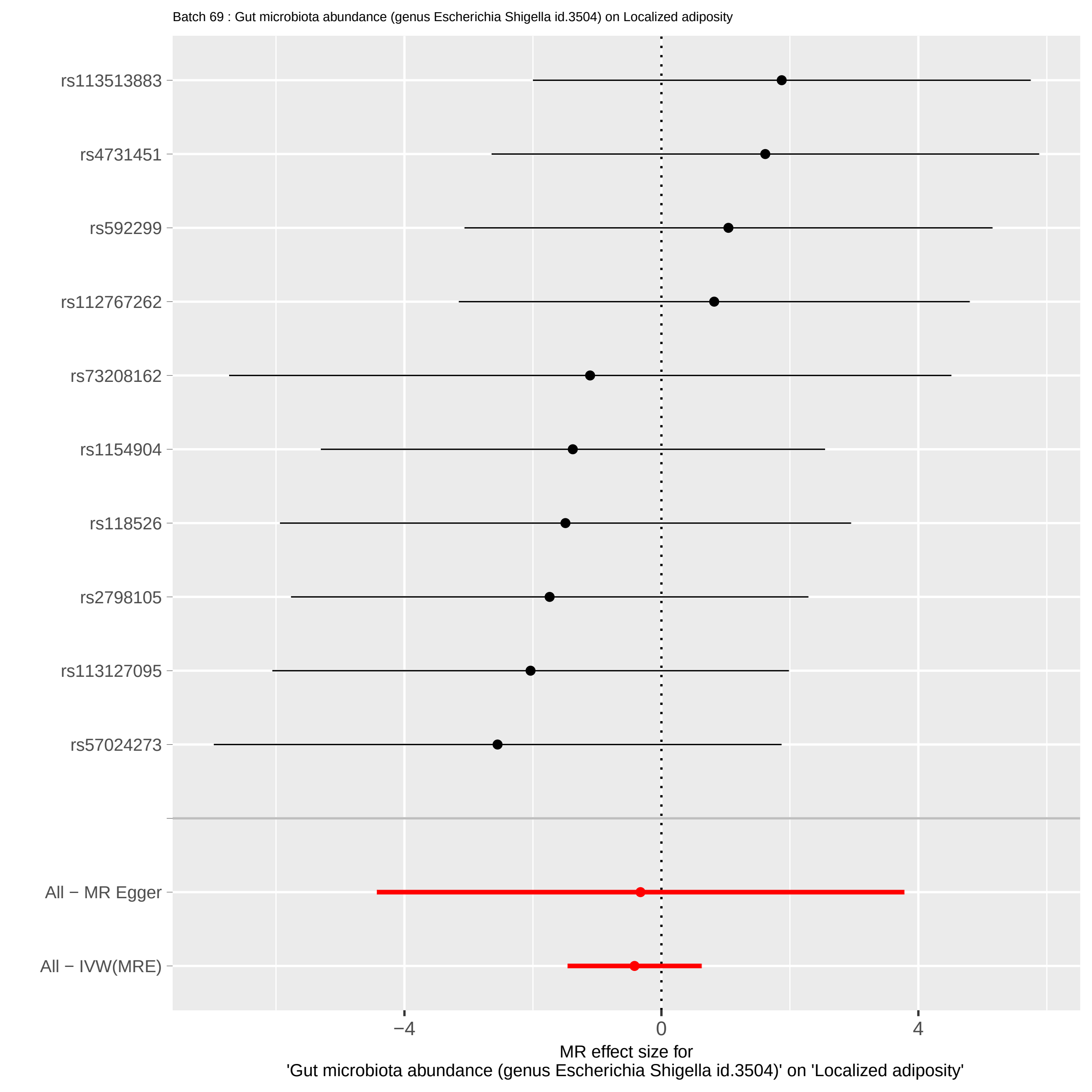

Batch 69 : Gut microbiota abundance (genus Escherichia Shigella id.3504) on Localized adiposity
rs113513883
rs4731451
rs592299
rs112767262
rs73208162
rs1154904
rs118526
rs2798105
rs113127095
rs57024273
All − MR Egger
All − IVW(MRE)
−4
0
4
MR effect size for
'Gut microbiota abundance (genus Escherichia Shigella id.3504)' on 'Localized adiposity'

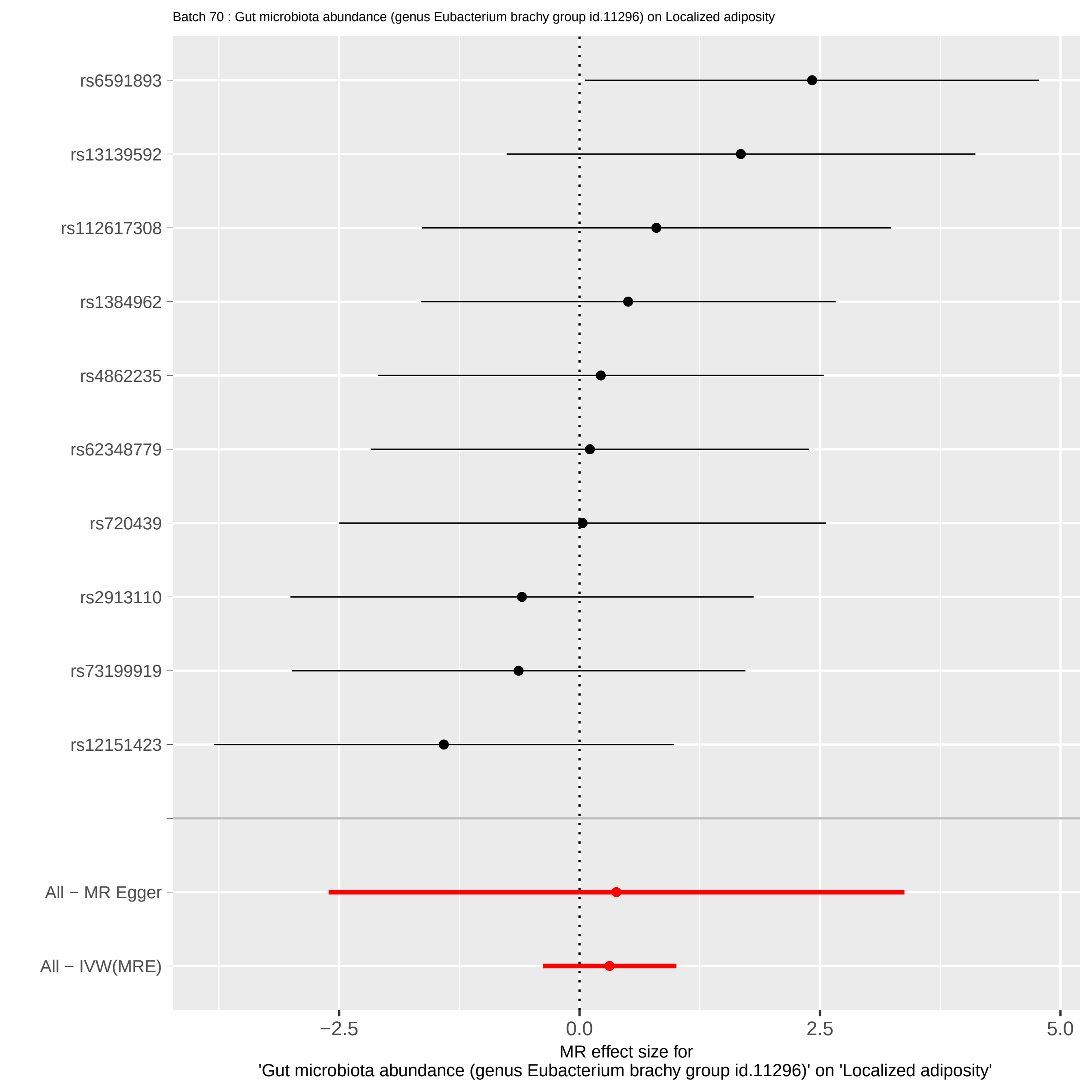

Batch 70 : Gut microbiota abundance (genus Eubacterium brachy group id.11296) on Localized adiposity
rs6591893
rs13139592
rs112617308
rs1384962
rs4862235
rs62348779
rs720439
rs2913110
rs73199919
rs12151423
All − MR Egger
All − IVW(MRE)
−2.5
0.0
2.5
5.0
MR effect size for
'Gut microbiota abundance (genus Eubacterium brachy group id.11296)' on 'Localized adiposity'

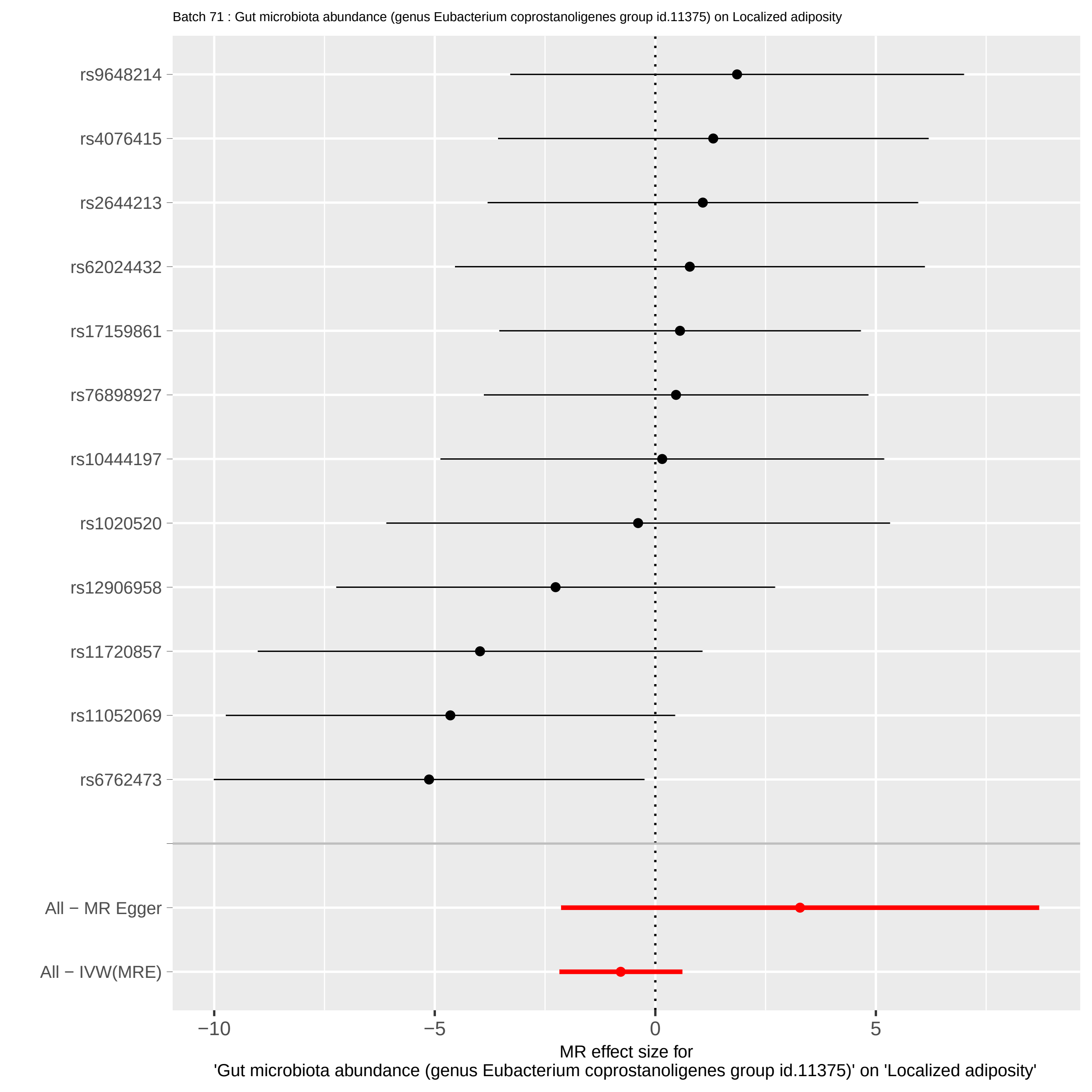
Batch 71 : Gut microbiota abundance (genus Eubacterium coprostanoligenes group id.11375) on Localized adiposity
rs9648214
rs4076415
rs2644213
rs62024432
rs17159861
rs76898927
rs10444197
rs1020520
rs12906958
rs11720857
rs11052069
rs6762473
All − MR Egger
All − IVW(MRE)
−10
−5
0
5
MR effect size for
'Gut microbiota abundance (genus Eubacterium coprostanoligenes group id.11375)' on 'Localized adiposity'

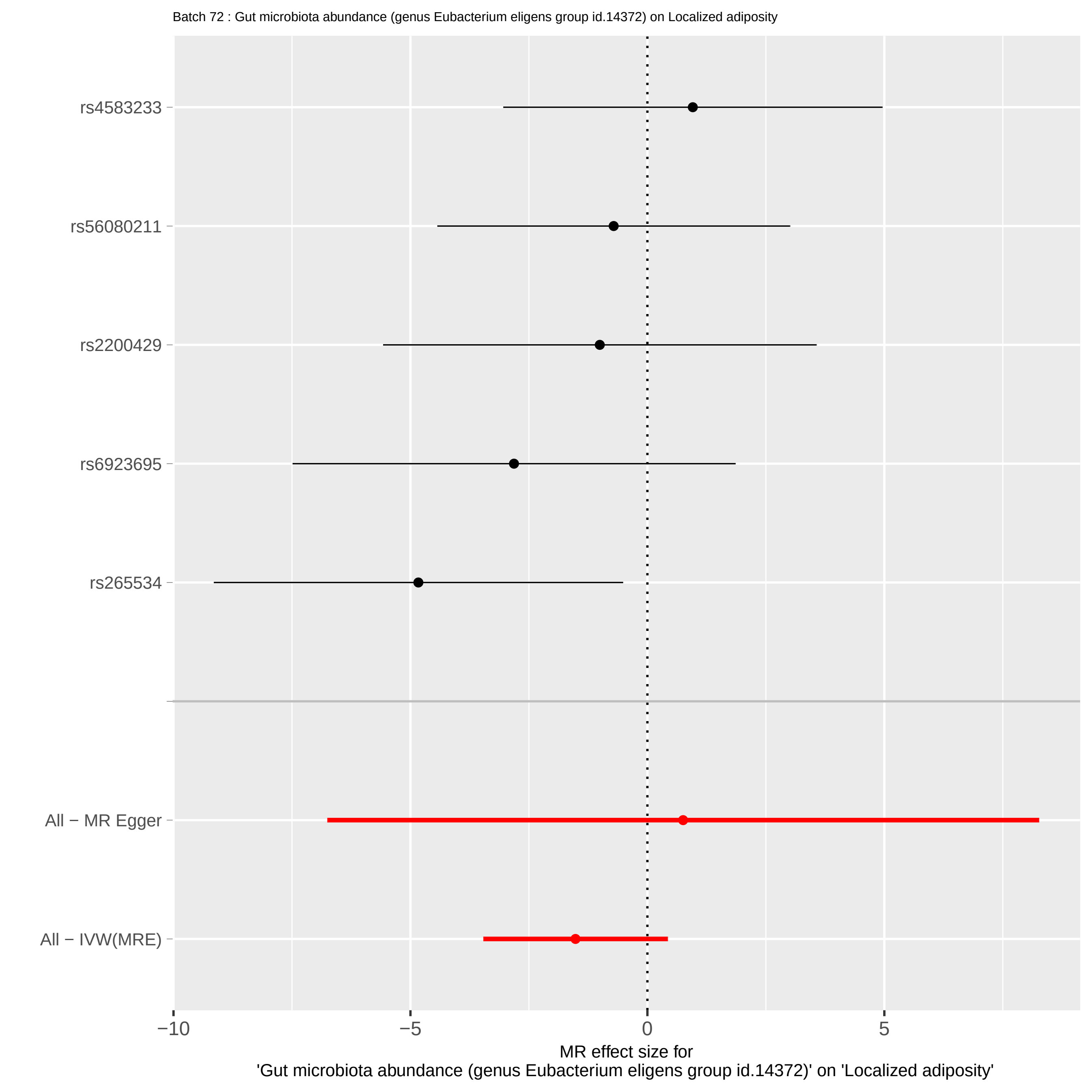
Batch 72 : Gut microbiota abundance (genus Eubacterium eligens group id.14372) on Localized adiposity
rs4583233
rs56080211
rs2200429
rs6923695
rs265534
All − MR Egger
All − IVW(MRE)
−10
−5
0
5
MR effect size for
'Gut microbiota abundance (genus Eubacterium eligens group id.14372)' on 'Localized adiposity'

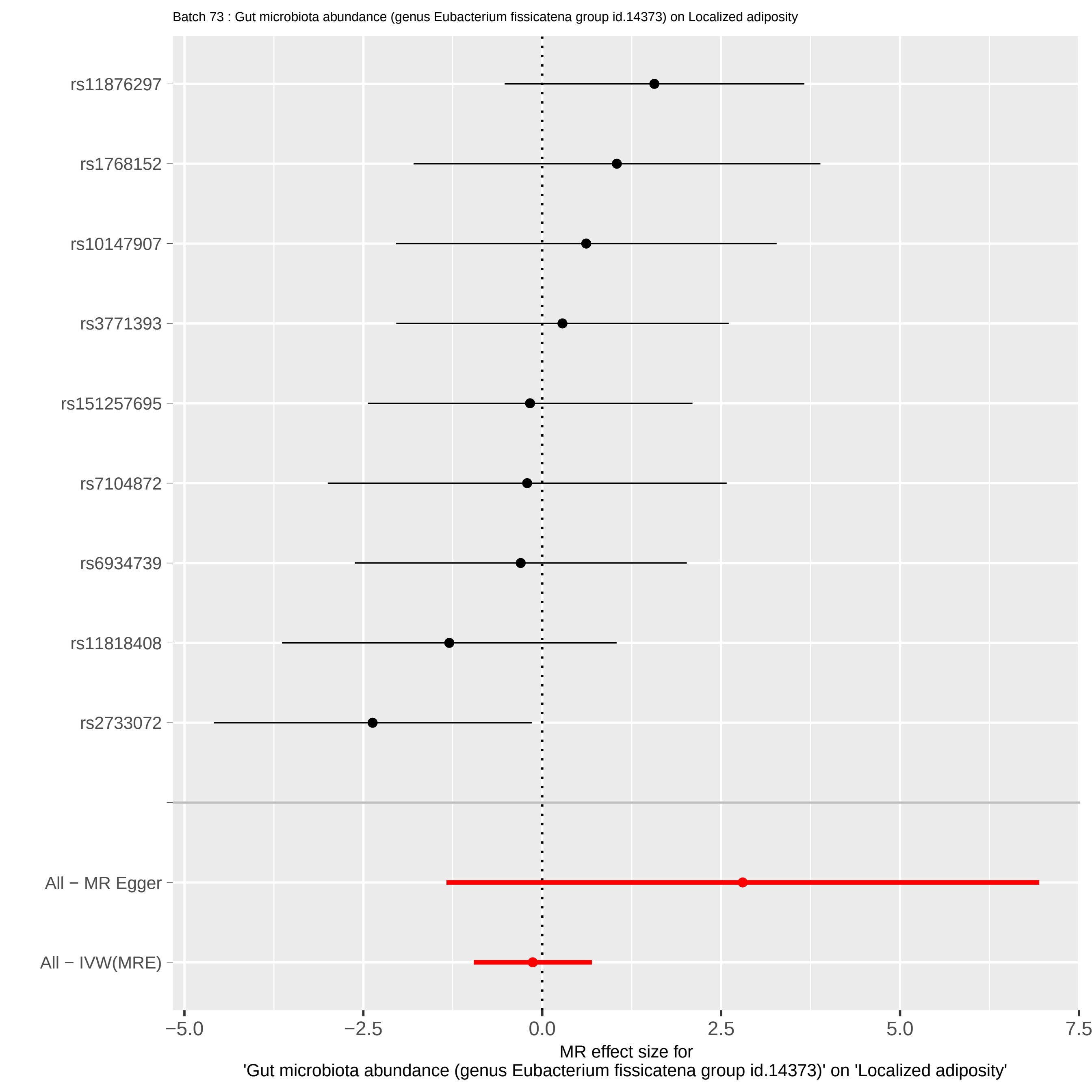

Batch 73 : Gut microbiota abundance (genus Eubacterium fissicatena group id.14373) on Localized adiposity
rs11876297
rs1768152
rs10147907
rs3771393
rs151257695
rs7104872
rs6934739
rs11818408
rs2733072
All − MR Egger
All − IVW(MRE)
−5.0
−2.5
0.0
2.5
5.0
7.5
MR effect size for
'Gut microbiota abundance (genus Eubacterium fissicatena group id.14373)' on 'Localized adiposity'

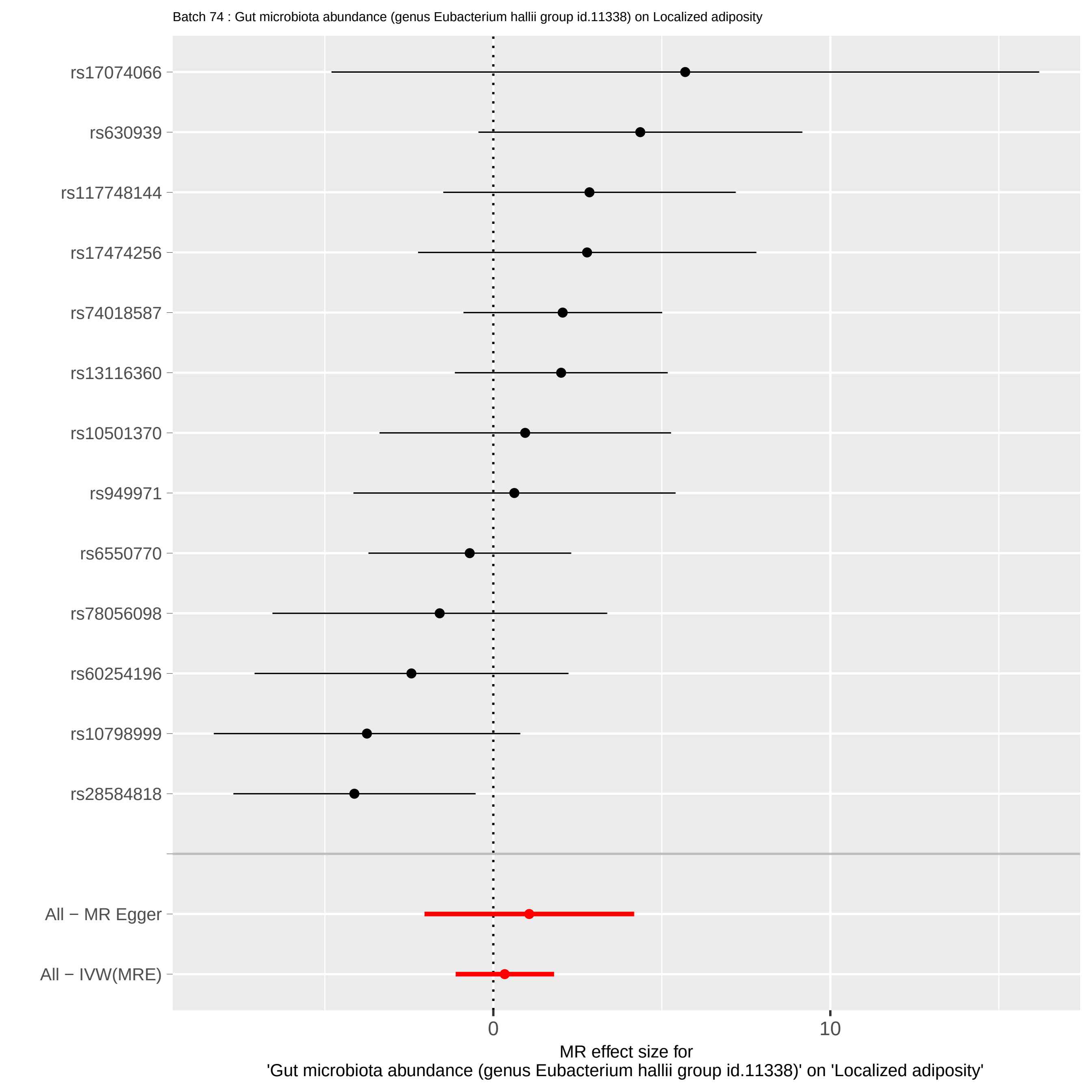

Batch 74 : Gut microbiota abundance (genus Eubacterium hallii group id.11338) on Localized adiposity
rs17074066
rs630939
rs117748144
rs17474256
rs74018587
rs13116360
rs10501370
rs949971
rs6550770
rs78056098
rs60254196
rs10798999
rs28584818
All − MR Egger
All − IVW(MRE)
0
10
MR effect size for
'Gut microbiota abundance (genus Eubacterium hallii group id.11338)' on 'Localized adiposity'

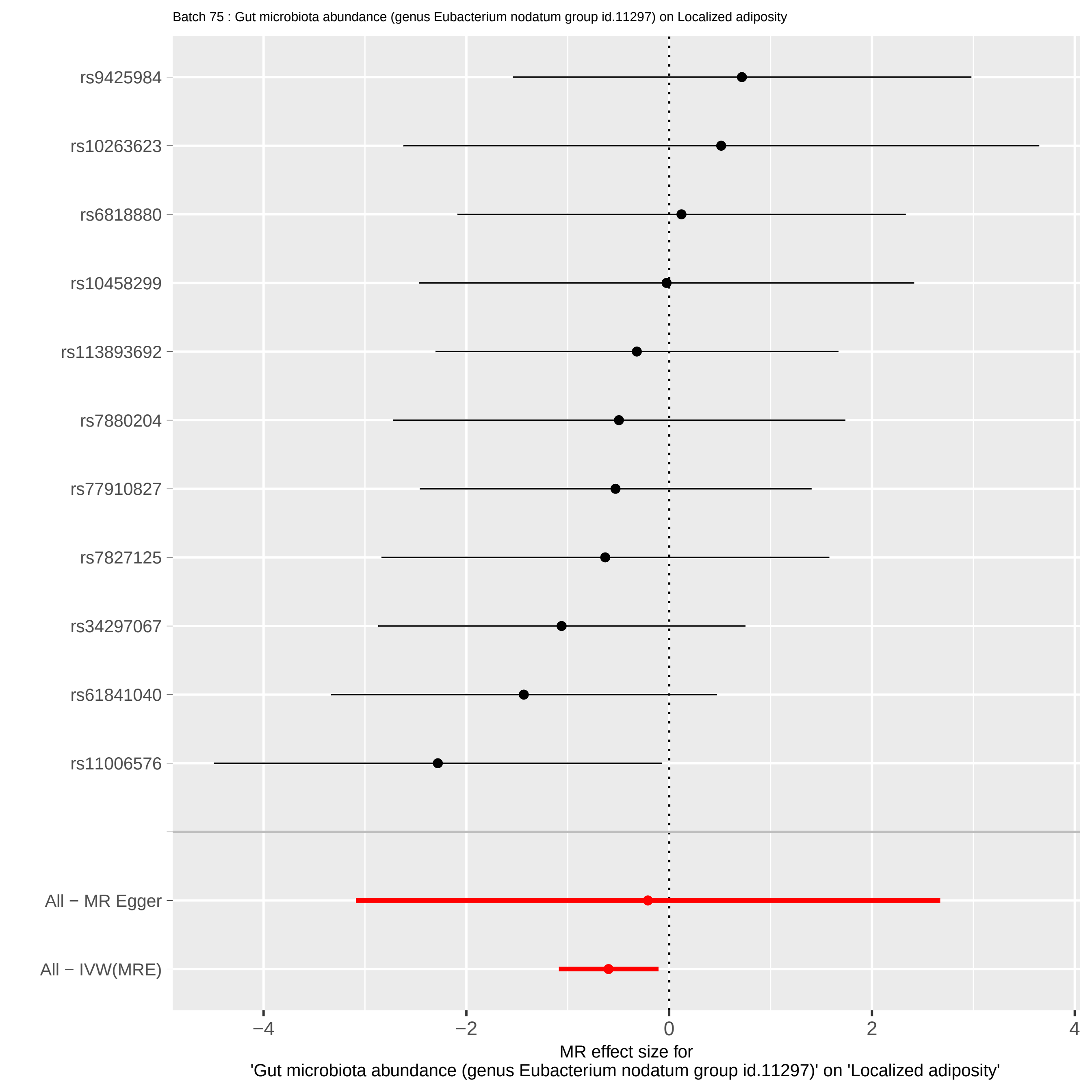

Batch 75 : Gut microbiota abundance (genus Eubacterium nodatum group id.11297) on Localized adiposity
rs9425984
rs10263623
rs6818880
rs10458299
rs113893692
rs7880204
rs77910827
rs7827125
rs34297067
rs61841040
rs11006576
All − MR Egger
All − IVW(MRE)
−4
−2
0
2
4
MR effect size for
'Gut microbiota abundance (genus Eubacterium nodatum group id.11297)' on 'Localized adiposity'

Batch 76 : Gut microbiota abundance (genus Eubacterium oxidoreducens group id.11339) on Localized adiposity

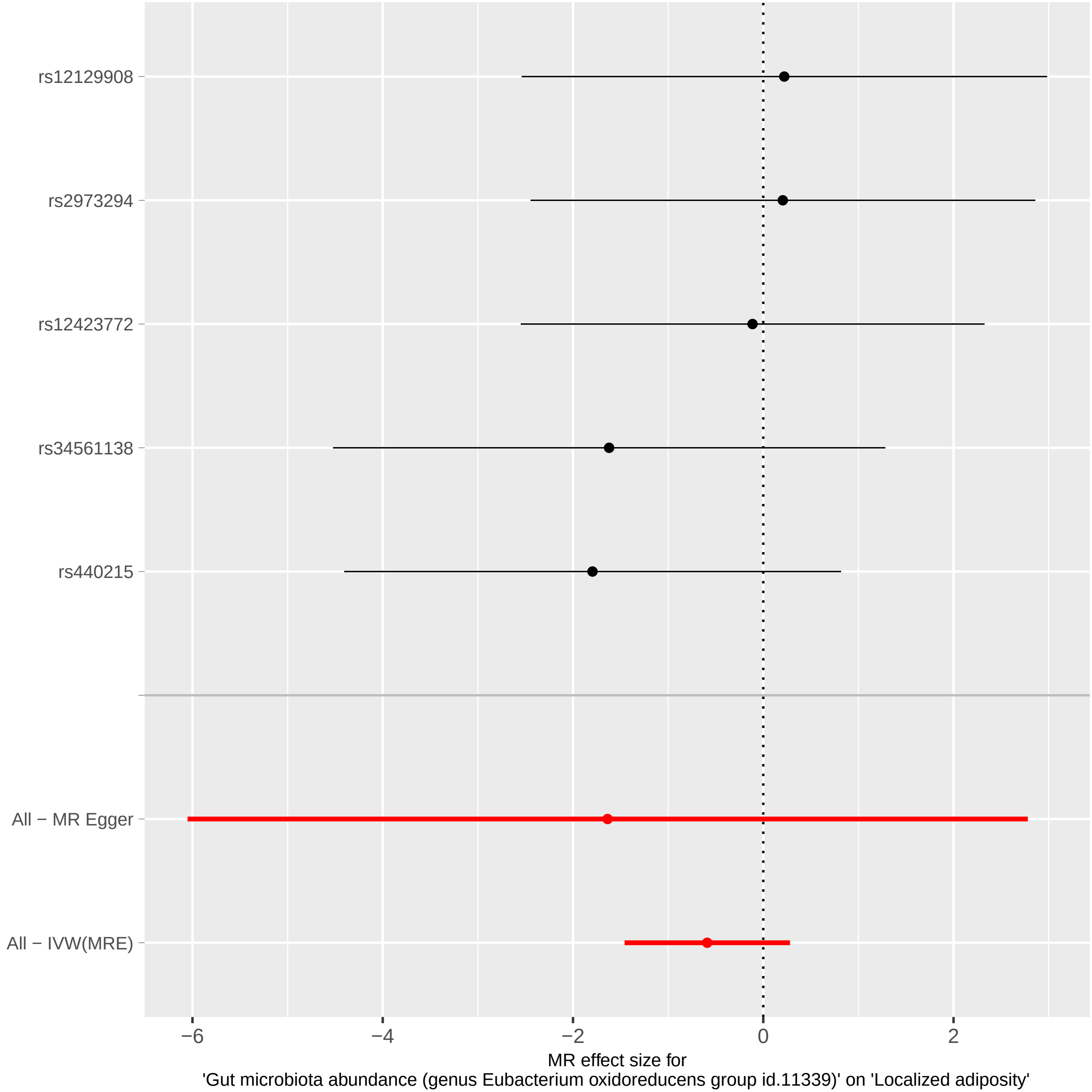

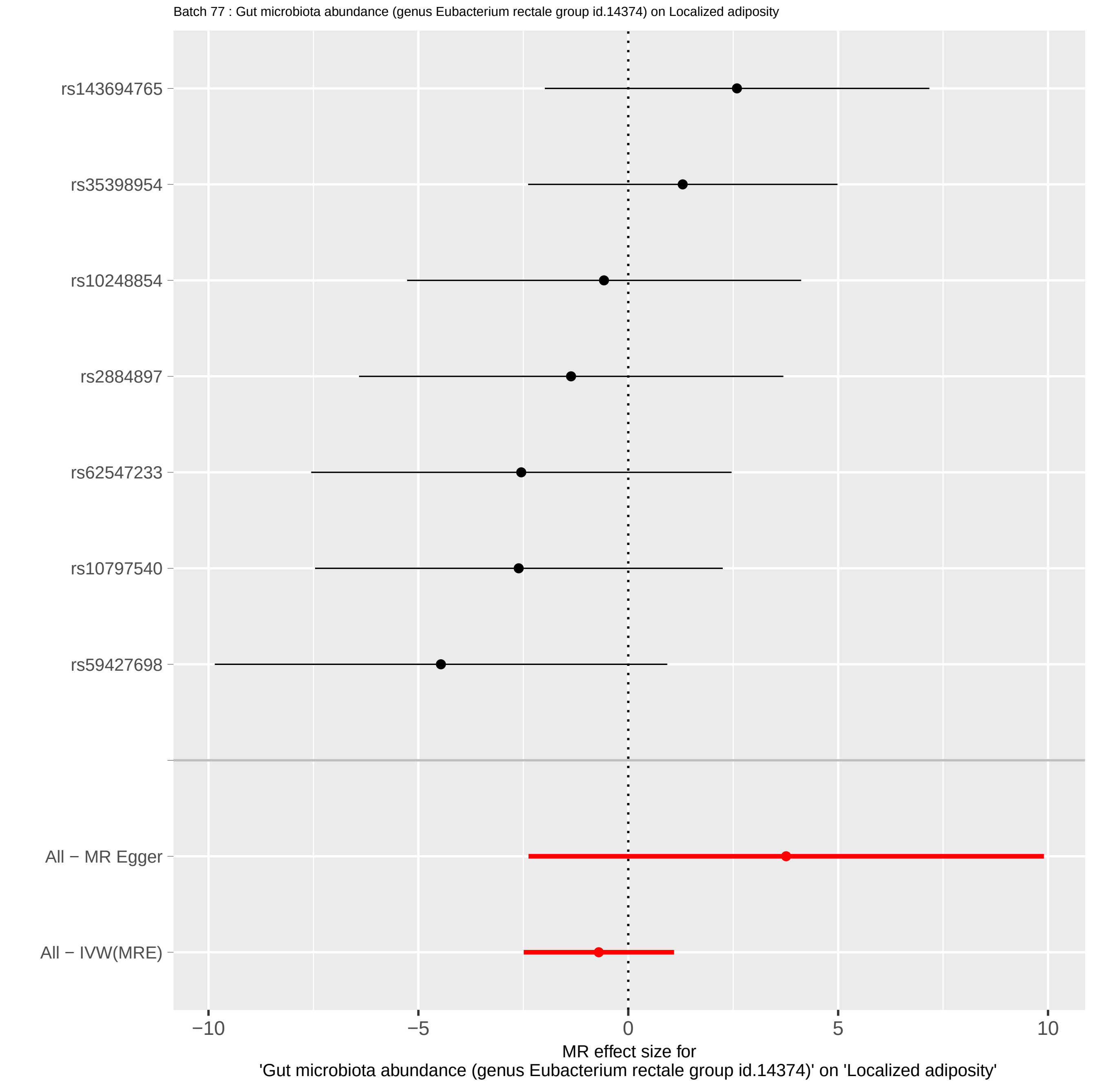

Batch 77 : Gut microbiota abundance (genus Eubacterium rectale group id.14374) on Localized adiposity
rs143694765
rs35398954
rs10248854
rs2884897
rs62547233
rs10797540
rs59427698
All − MR Egger
All − IVW(MRE)
−10
−5
0
5
10
MR effect size for
'Gut microbiota abundance (genus Eubacterium rectale group id.14374)' on 'Localized adiposity'

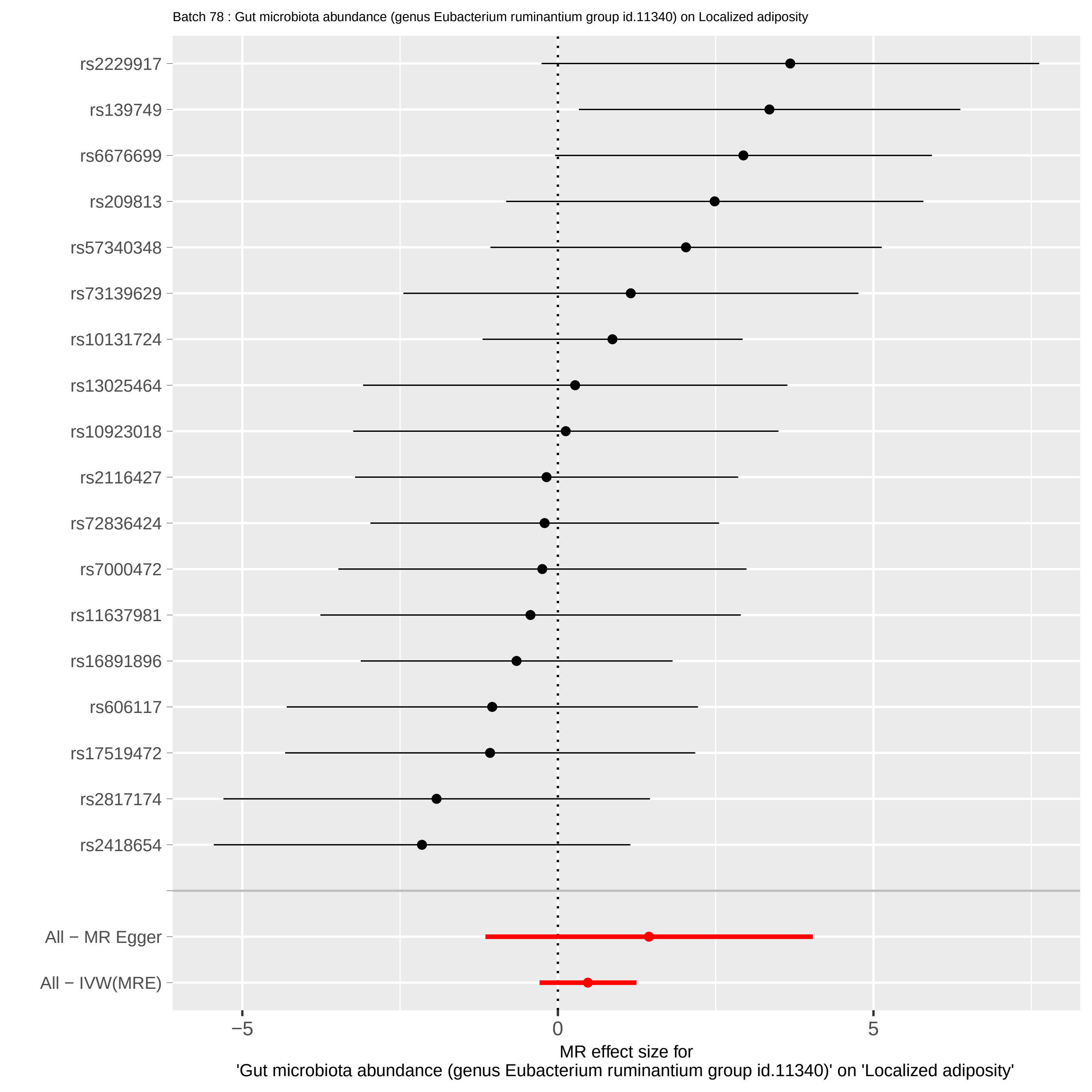

Batch 78 : Gut microbiota abundance (genus Eubacterium ruminantium group id.11340) on Localized adiposity
rs2229917
rs139749
rs6676699
rs209813
rs57340348
rs73139629
rs10131724
rs13025464
rs10923018
rs2116427
rs72836424
rs7000472
rs11637981
rs16891896
rs606117
rs17519472
rs2817174
rs2418654
All − MR Egger
All − IVW(MRE)
−5
0
5
MR effect size for
'Gut microbiota abundance (genus Eubacterium ruminantium group id.11340)' on 'Localized adiposity'

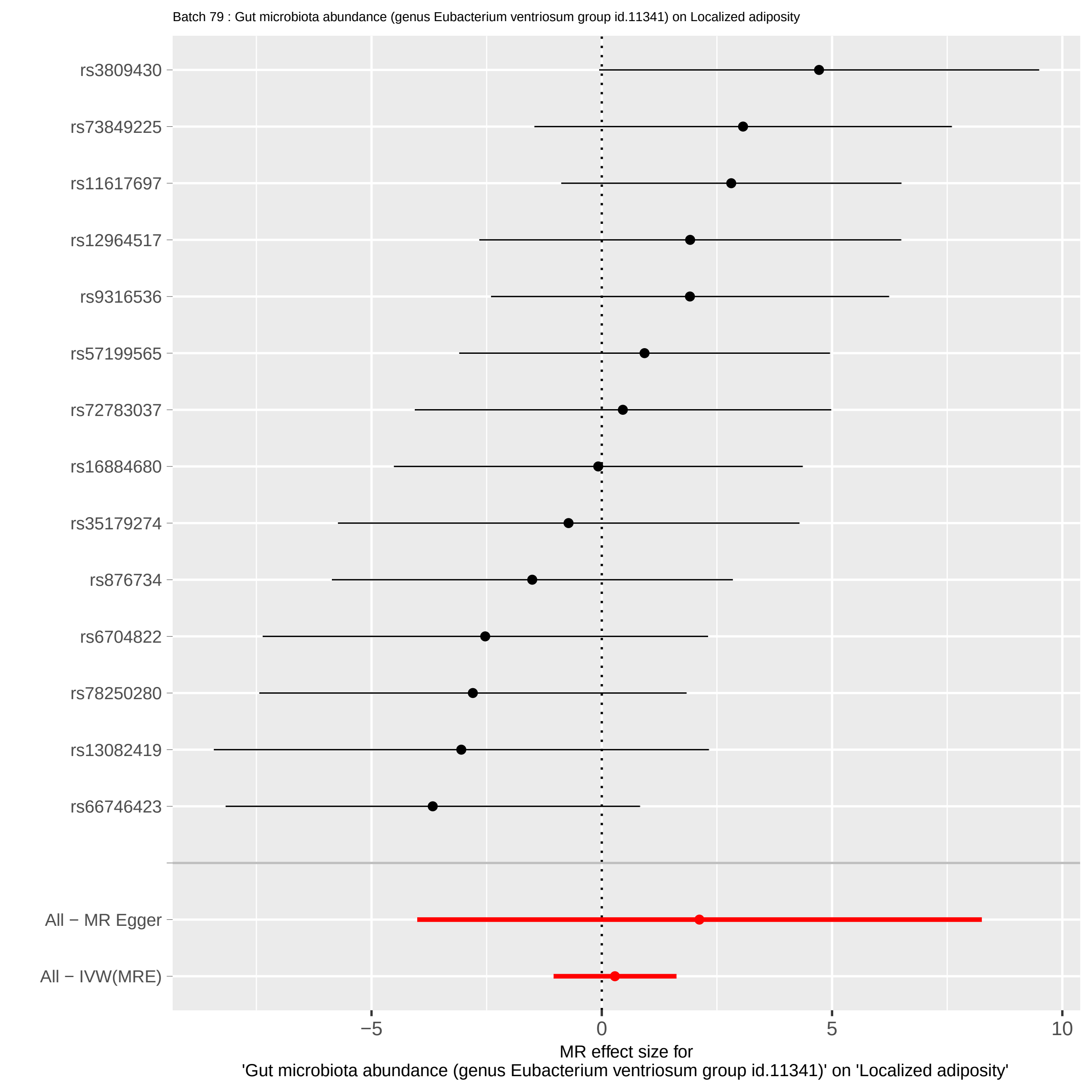
Batch 79 : Gut microbiota abundance (genus Eubacterium ventriosum group id.11341) on Localized adiposity
rs3809430
rs73849225
rs11617697
rs12964517
rs9316536
rs57199565
rs72783037
rs16884680
rs35179274
rs876734
rs6704822
rs78250280
rs13082419
rs66746423
All − MR Egger
All − IVW(MRE)
−5
0
5
10
MR effect size for
'Gut microbiota abundance (genus Eubacterium ventriosum group id.11341)' on 'Localized adiposity'

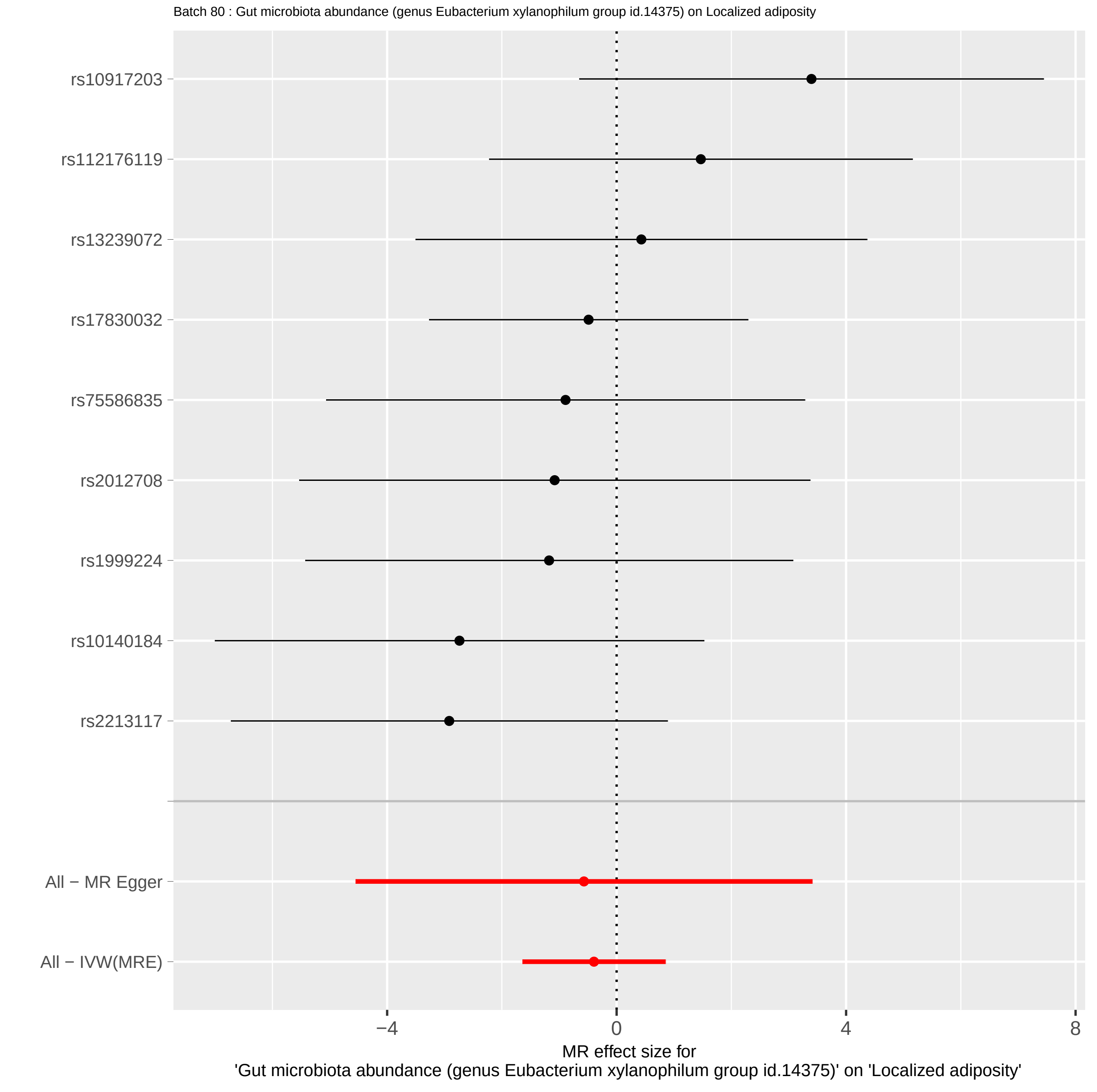

Batch 80 : Gut microbiota abundance (genus Eubacterium xylanophilum group id.14375) on Localized adiposity
rs10917203
rs112176119
rs13239072
rs17830032
rs75586835
rs2012708
rs1999224
rs10140184
rs2213117
All − MR Egger
All − IVW(MRE)
−4
0
4
8
MR effect size for
'Gut microbiota abundance (genus Eubacterium xylanophilum group id.14375)' on 'Localized adiposity'

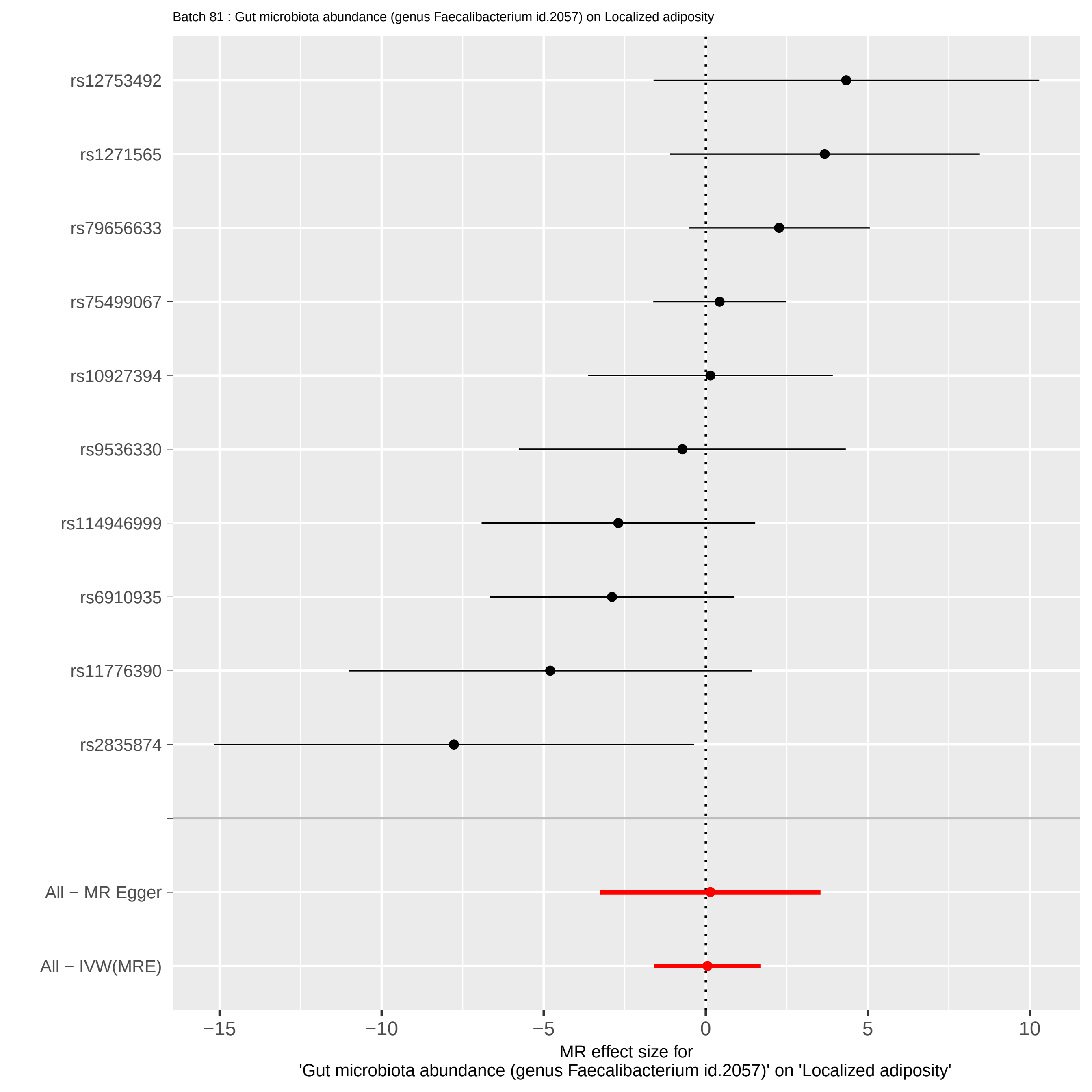

Batch 81 : Gut microbiota abundance (genus Faecalibacterium id.2057) on Localized adiposity
rs12753492
rs1271565
rs79656633
rs75499067
rs10927394
rs9536330
rs114946999
rs6910935
rs11776390
rs2835874
All − MR Egger
All − IVW(MRE)
−15
−10
−5
0
5
10
MR effect size for
'Gut microbiota abundance (genus Faecalibacterium id.2057)' on 'Localized adiposity'

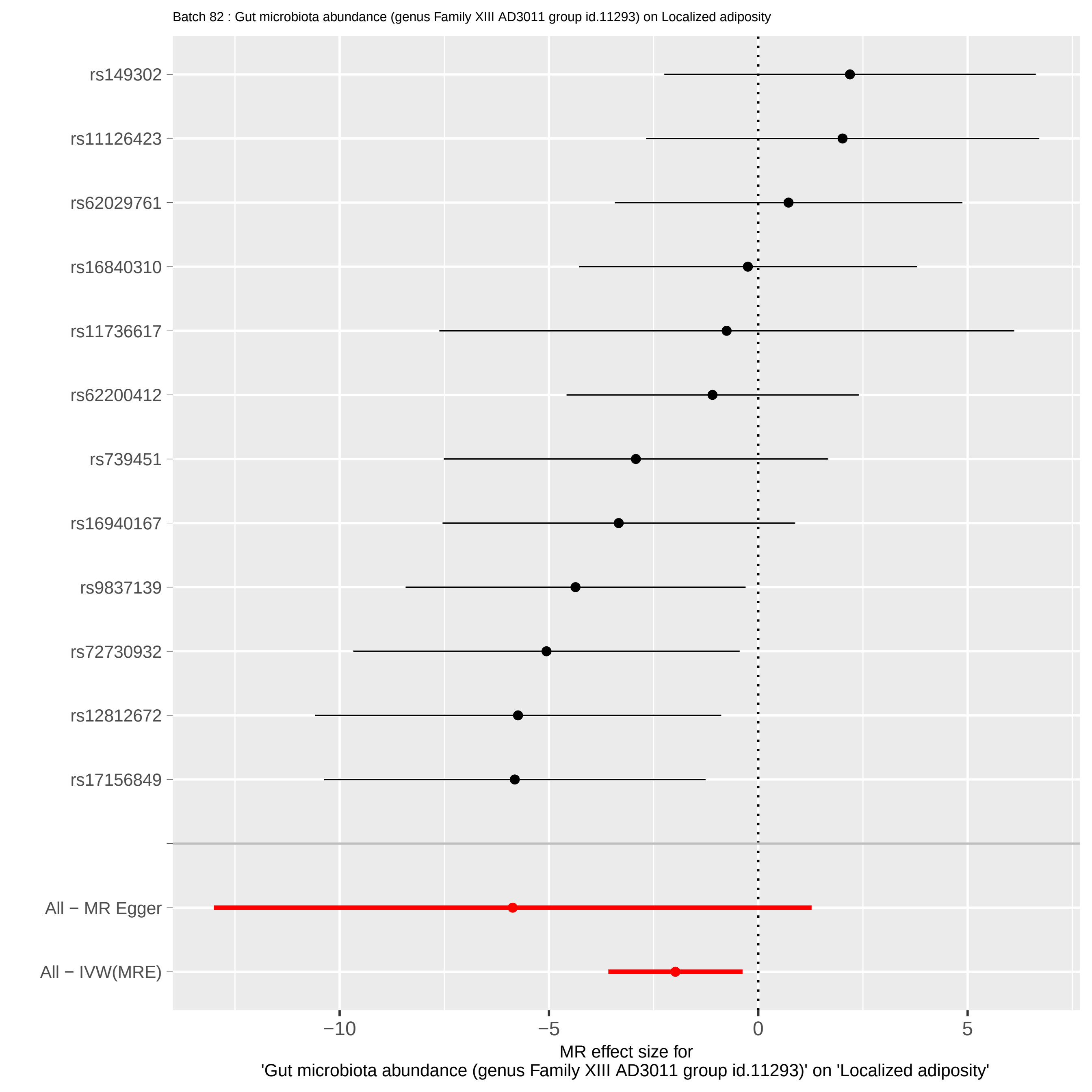

Batch 82 : Gut microbiota abundance (genus Family XIII AD3011 group id.11293) on Localized adiposity
rs149302
rs11126423
rs62029761
rs16840310
rs11736617
rs62200412
rs739451
rs16940167
rs9837139
rs72730932
rs12812672
rs17156849
All − MR Egger
All − IVW(MRE)
−10
−5
0
5
MR effect size for
'Gut microbiota abundance (genus Family XIII AD3011 group id.11293)' on 'Localized adiposity'

Batch 83 : Gut microbiota abundance (genus Family XIII UCG001 id.11294) on Localized adiposity

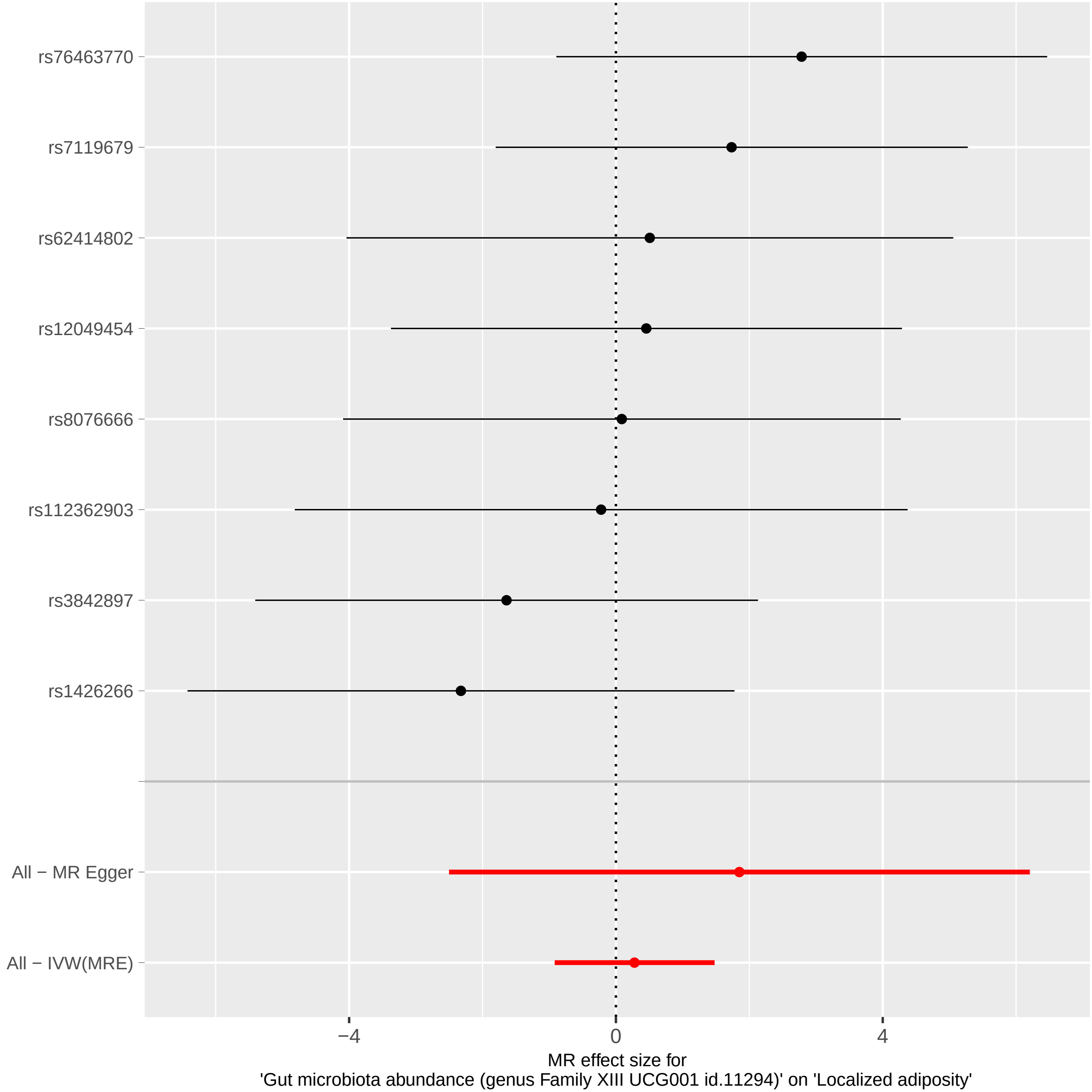

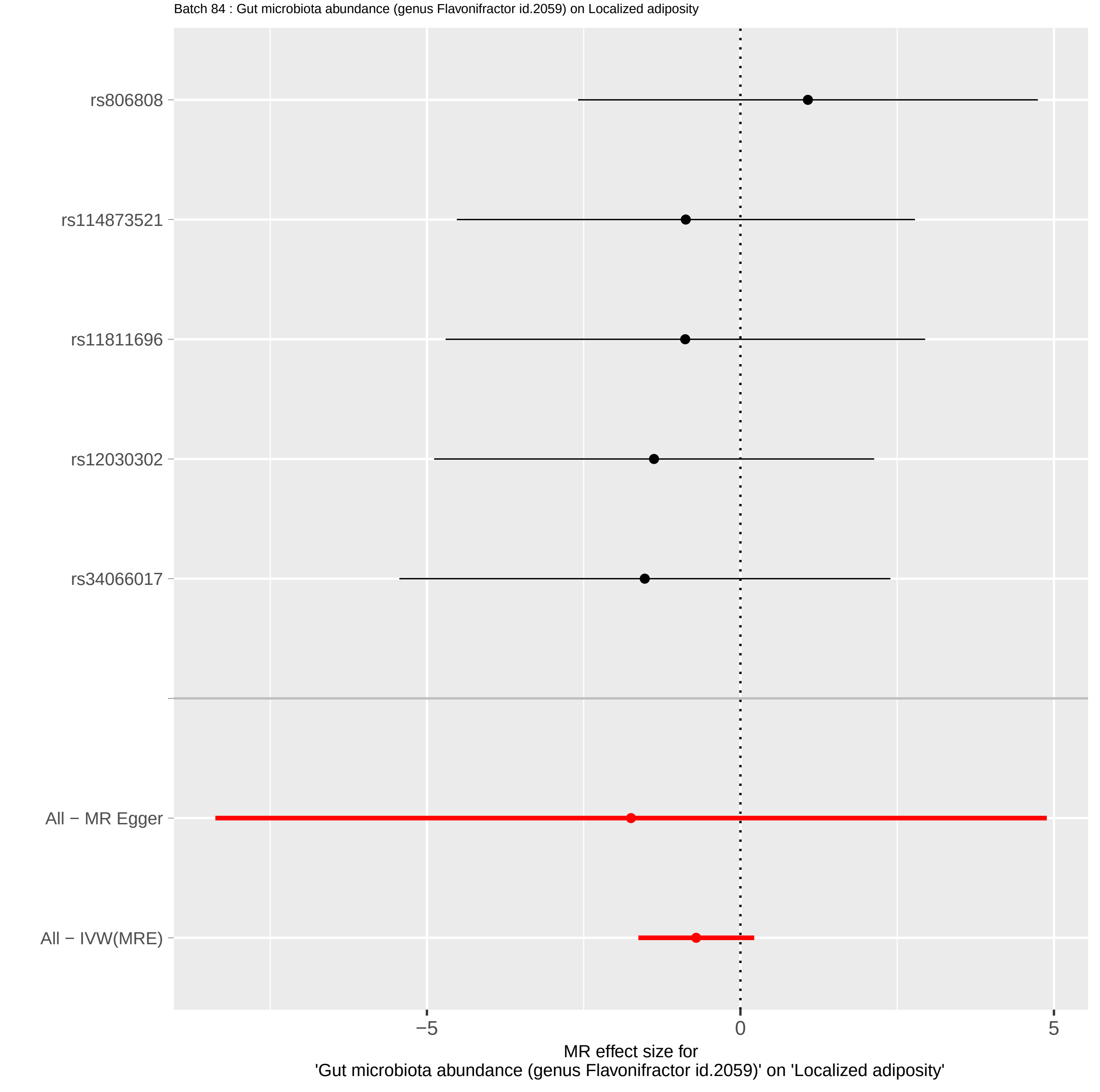

Batch 84 : Gut microbiota abundance (genus Flavonifractor id.2059) on Localized adiposity
rs806808
rs114873521
rs11811696
rs12030302
rs34066017
All − MR Egger
All − IVW(MRE)
−5
0
5
MR effect size for
'Gut microbiota abundance (genus Flavonifractor id.2059)' on 'Localized adiposity'

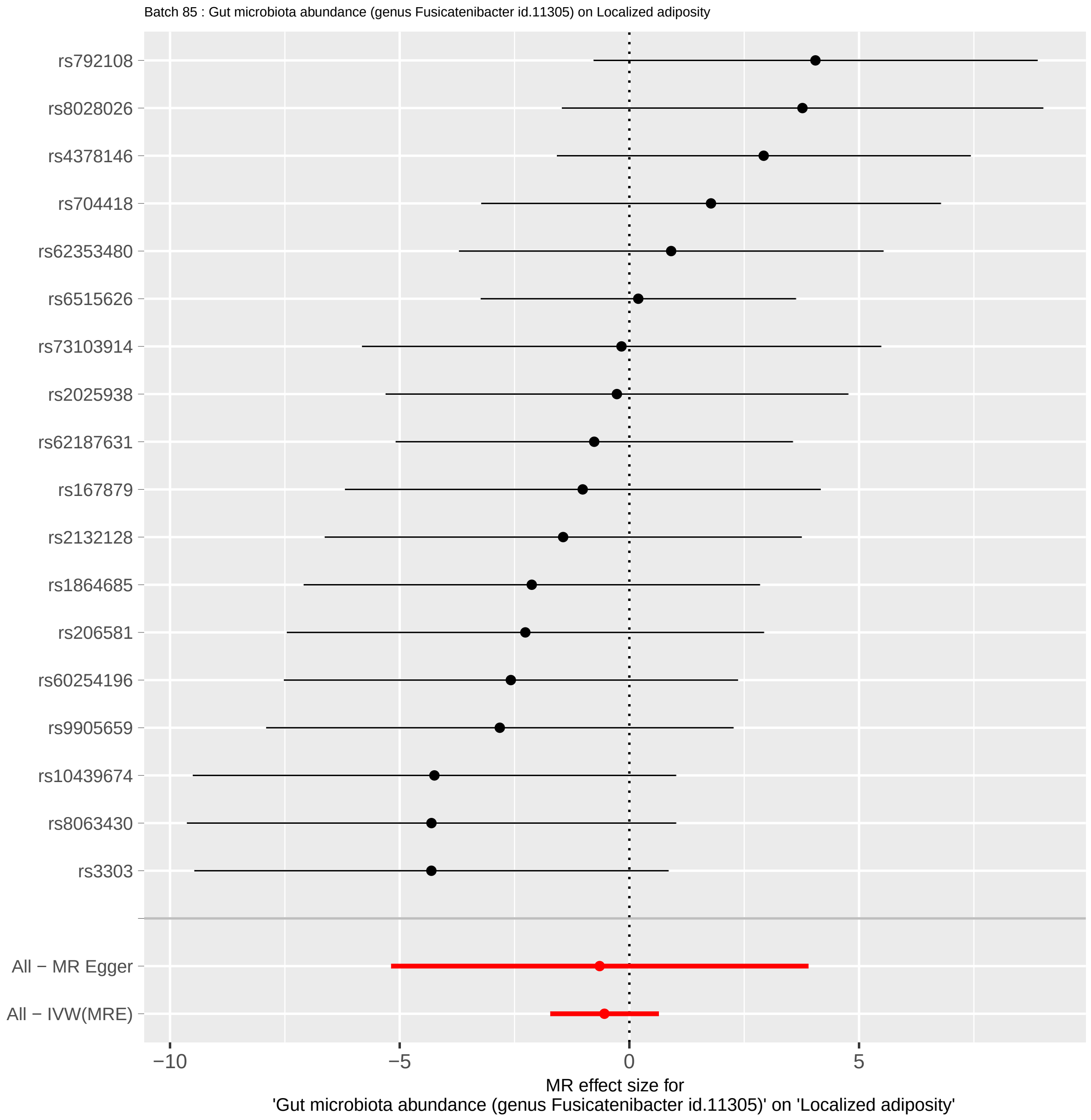

Batch 85 : Gut microbiota abundance (genus Fusicatenibacter id.11305) on Localized adiposity
rs792108
rs8028026
rs4378146
rs704418
rs62353480
rs6515626
rs73103914
rs2025938
rs62187631
rs167879
rs2132128
rs1864685
rs206581
rs60254196
rs9905659
rs10439674
rs8063430
rs3303
All − MR Egger
All − IVW(MRE)
−10
−5
0
5
MR effect size for
'Gut microbiota abundance (genus Fusicatenibacter id.11305)' on 'Localized adiposity'

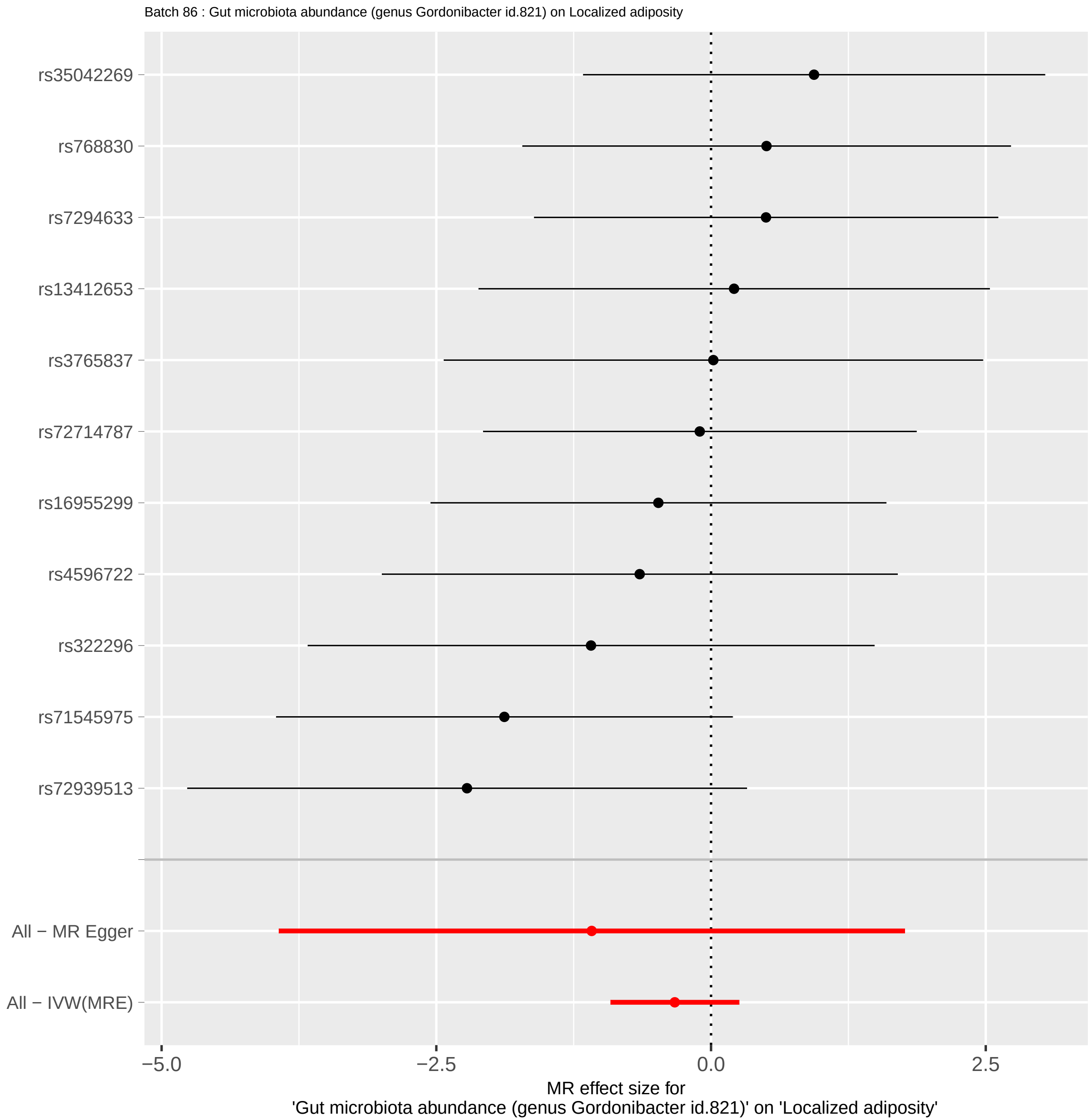
Batch 86 : Gut microbiota abundance (genus Gordonibacter id.821) on Localized adiposity
rs35042269
rs768830
rs7294633
rs13412653
rs3765837
rs72714787
rs16955299
rs4596722
rs322296
rs71545975
rs72939513
All − MR Egger
All − IVW(MRE)
−5.0
−2.5
0.0
2.5
MR effect size for
'Gut microbiota abundance (genus Gordonibacter id.821)' on 'Localized adiposity'

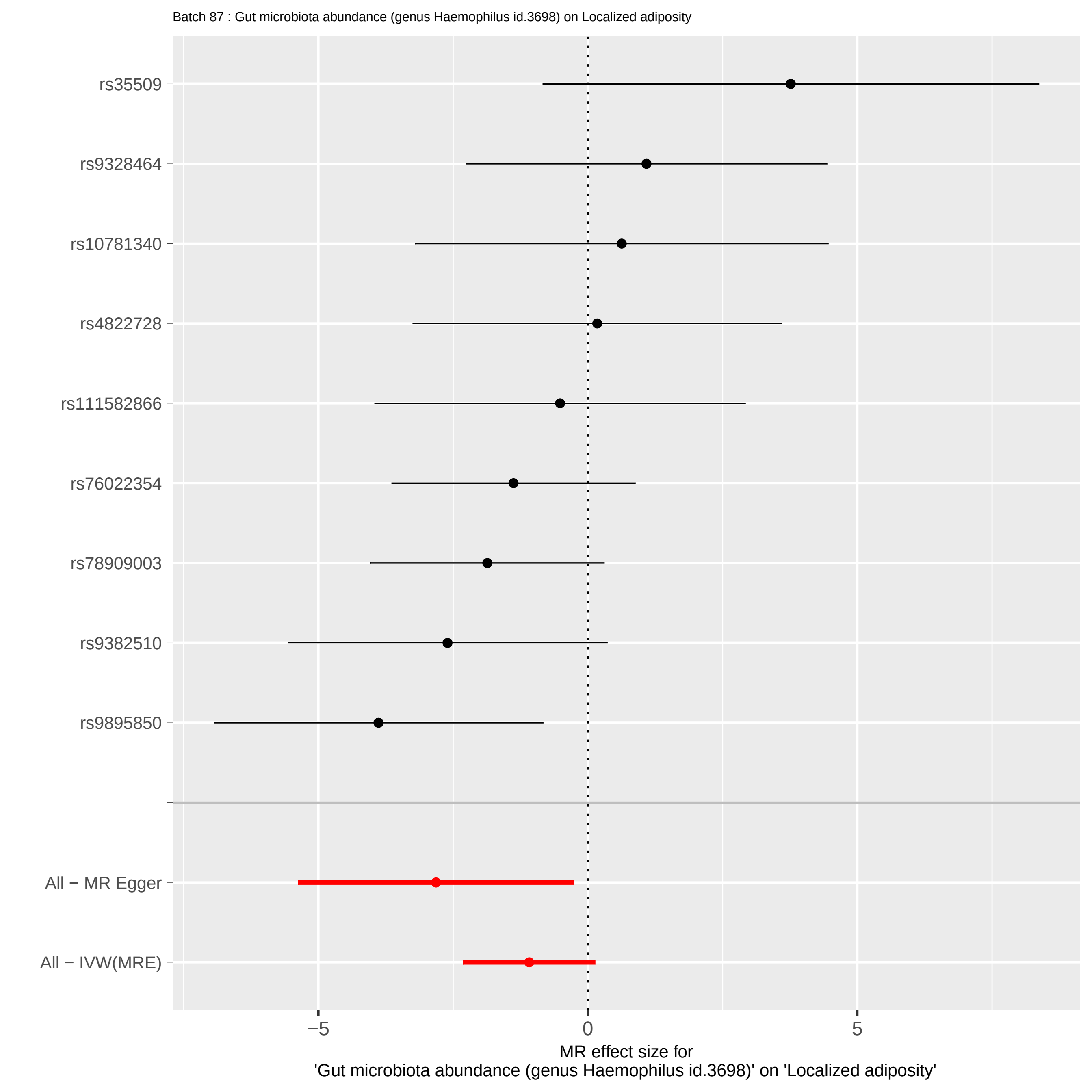
Batch 87 : Gut microbiota abundance (genus Haemophilus id.3698) on Localized adiposity
rs35509
rs9328464
rs10781340
rs4822728
rs111582866
rs76022354
rs78909003
rs9382510
rs9895850
All − MR Egger
All − IVW(MRE)
−5
0
5
MR effect size for
'Gut microbiota abundance (genus Haemophilus id.3698)' on 'Localized adiposity'

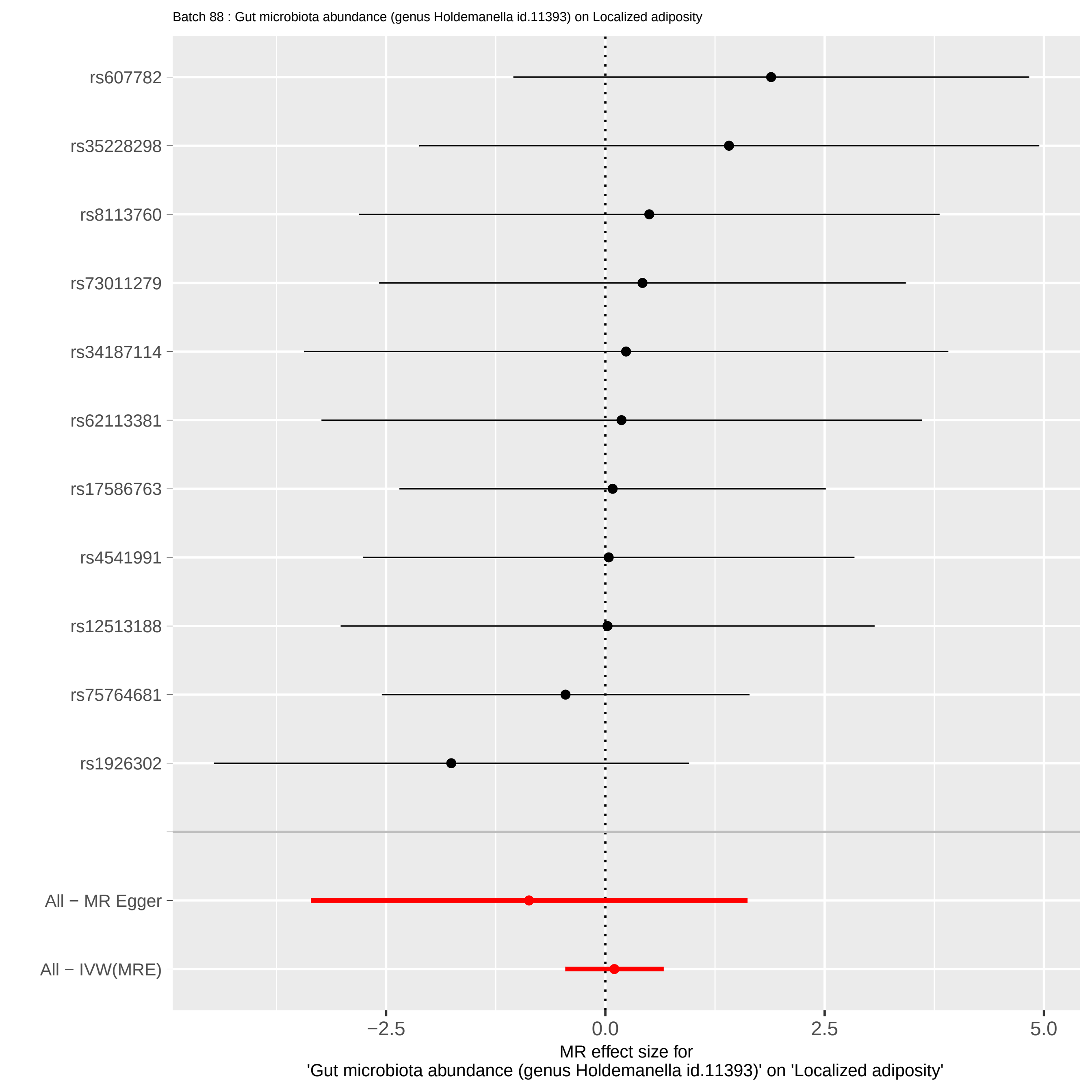
Batch 88 : Gut microbiota abundance (genus Holdemanella id.11393) on Localized adiposity
rs607782
rs35228298
rs8113760
rs73011279
rs34187114
rs62113381
rs17586763
rs4541991
rs12513188
rs75764681
rs1926302
All − MR Egger
All − IVW(MRE)
−2.5
0.0
2.5
5.0
MR effect size for
'Gut microbiota abundance (genus Holdemanella id.11393)' on 'Localized adiposity'

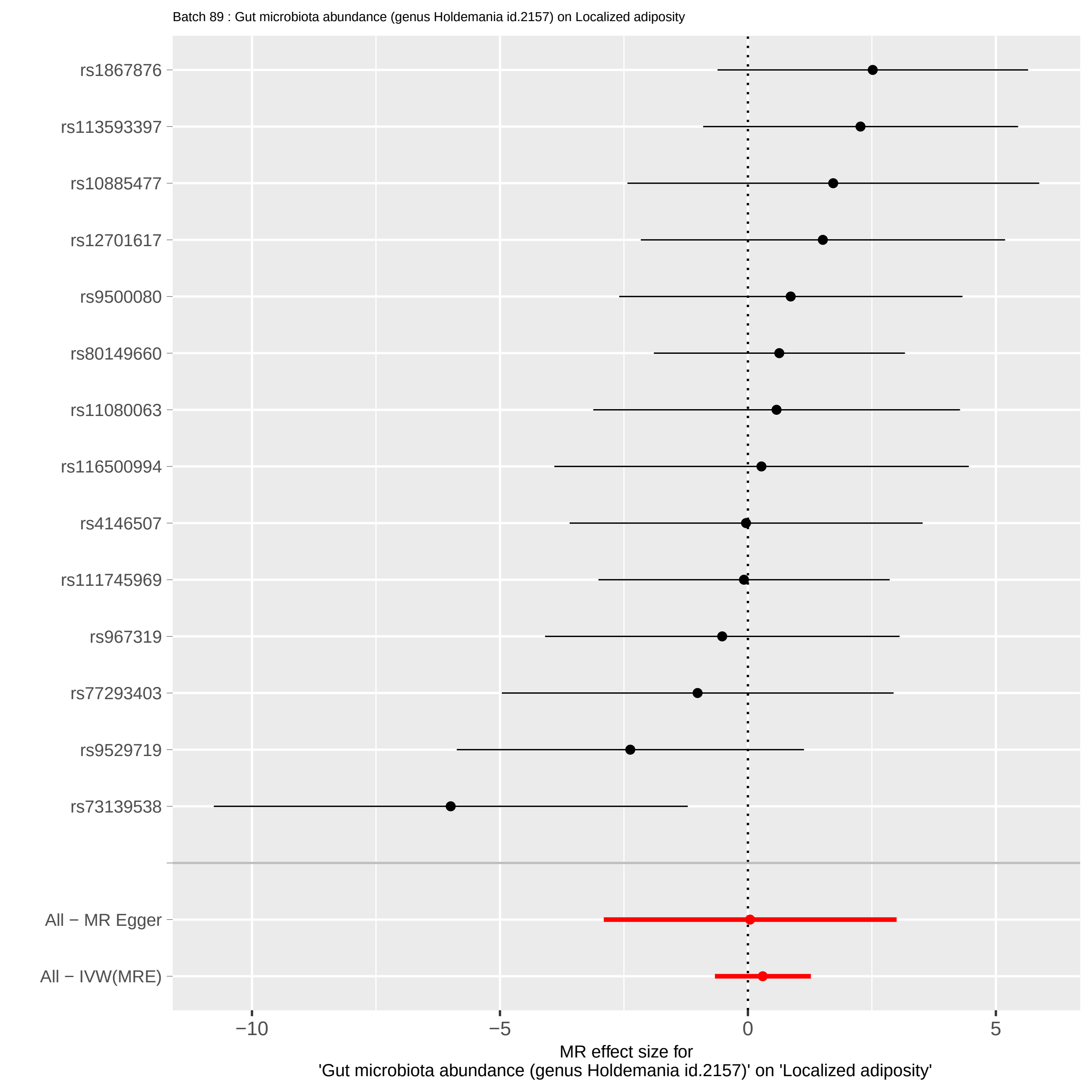

Batch 89 : Gut microbiota abundance (genus Holdemania id.2157) on Localized adiposity
rs1867876
rs113593397
rs10885477
rs12701617
rs9500080
rs80149660
rs11080063
rs116500994
rs4146507
rs111745969
rs967319
rs77293403
rs9529719
rs73139538
All − MR Egger
All − IVW(MRE)
−10
−5
0
5
MR effect size for
'Gut microbiota abundance (genus Holdemania id.2157)' on 'Localized adiposity'

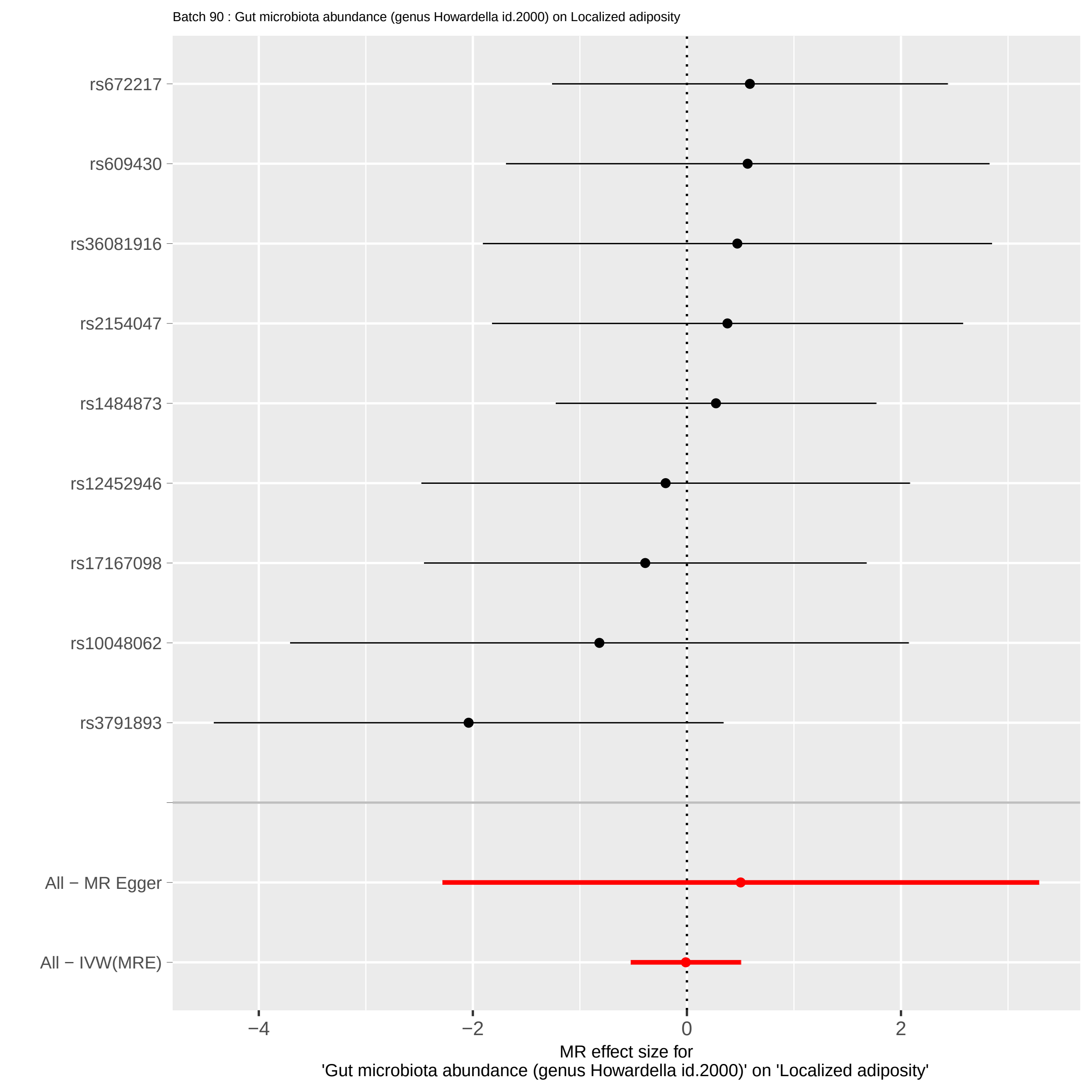

Batch 90 : Gut microbiota abundance (genus Howardella id.2000) on Localized adiposity
rs672217
rs609430
rs36081916
rs2154047
rs1484873
rs12452946
rs17167098
rs10048062
rs3791893
All − MR Egger
All − IVW(MRE)
−4
−2
0
2
MR effect size for
'Gut microbiota abundance (genus Howardella id.2000)' on 'Localized adiposity'

Batch 91 : Gut microbiota abundance (genus Hungatella id.11306) on Localized adiposity

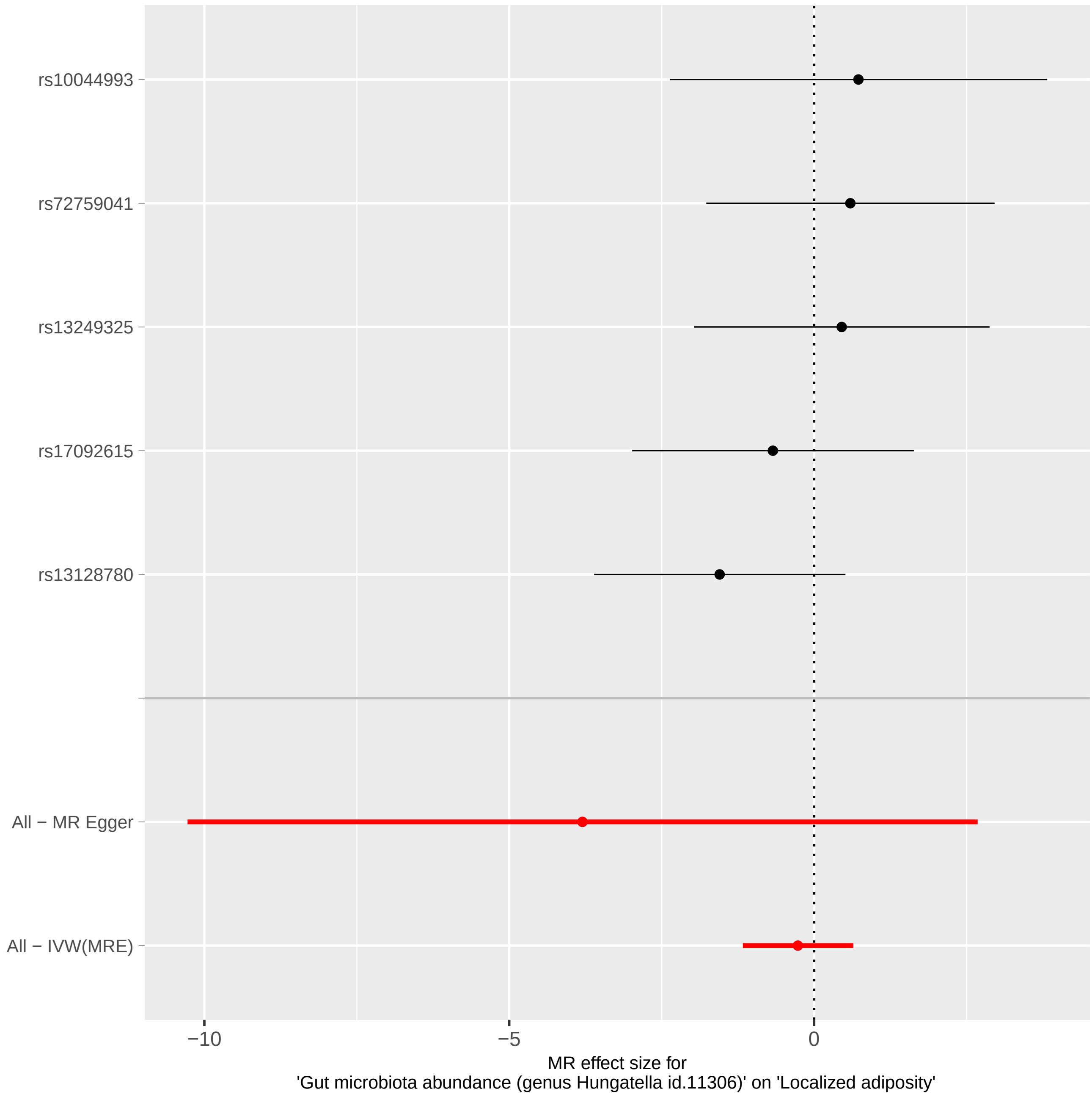

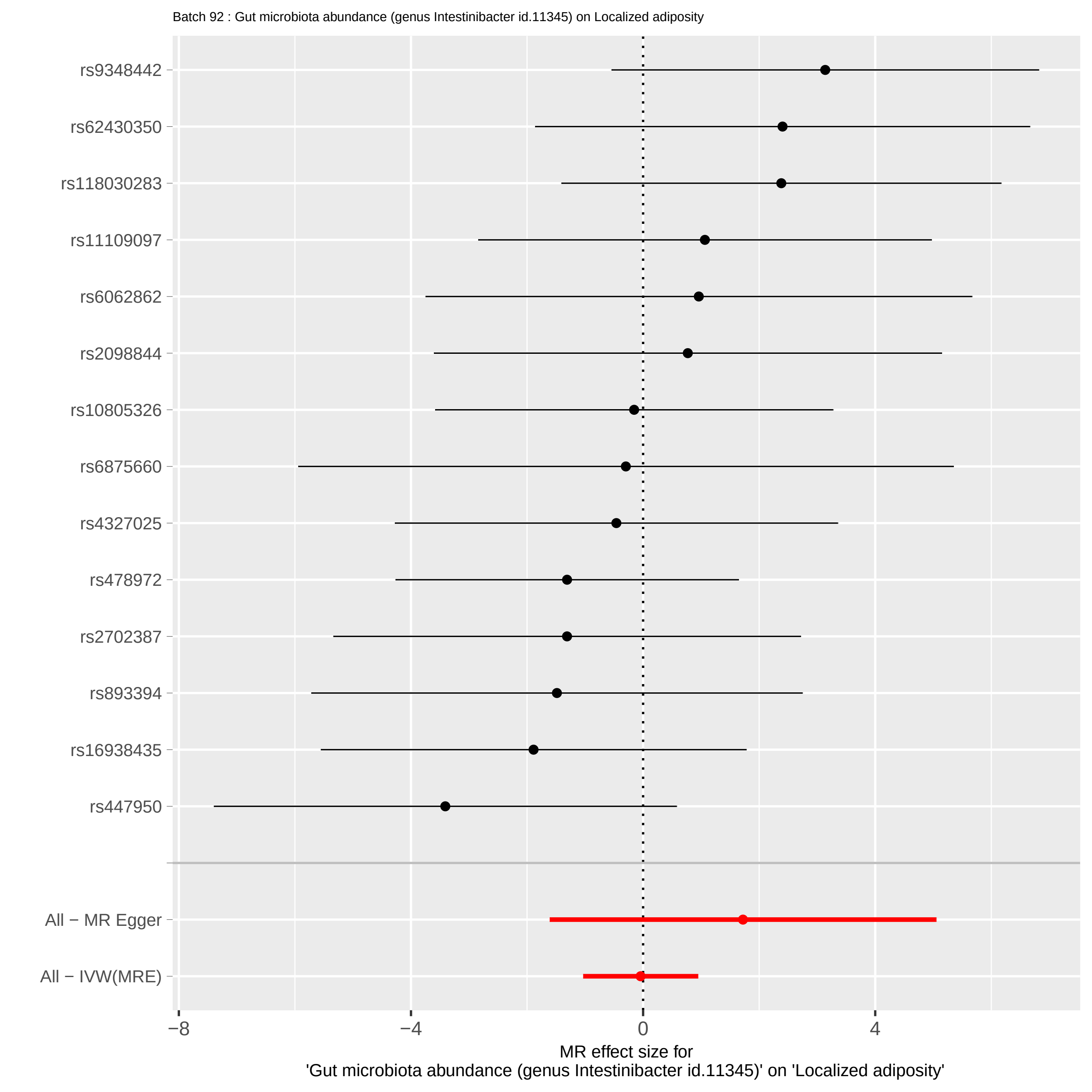
Batch 92 : Gut microbiota abundance (genus Intestinibacter id.11345) on Localized adiposity
rs9348442
rs62430350
rs118030283
rs11109097
rs6062862
rs2098844
rs10805326
rs6875660
rs4327025
rs478972
rs2702387
rs893394
rs16938435
rs447950
All − MR Egger
All − IVW(MRE)
−8
−4
0
4
MR effect size for
'Gut microbiota abundance (genus Intestinibacter id.11345)' on 'Localized adiposity'

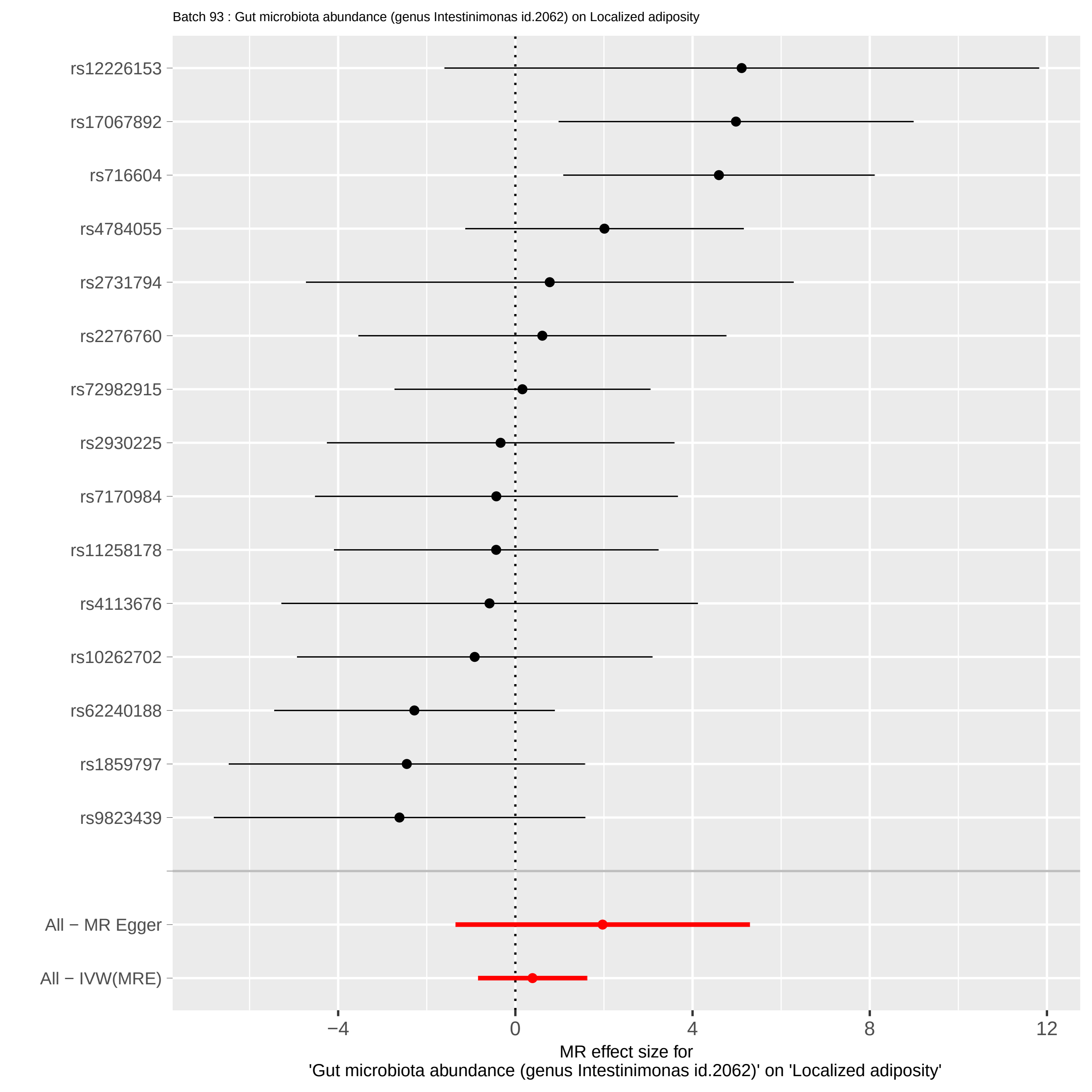

Batch 93 : Gut microbiota abundance (genus Intestinimonas id.2062) on Localized adiposity
rs12226153
rs17067892
rs716604
rs4784055
rs2731794
rs2276760
rs72982915
rs2930225
rs7170984
rs11258178
rs4113676
rs10262702
rs62240188
rs1859797
rs9823439
All − MR Egger
All − IVW(MRE)
−4
0
4
8
12
MR effect size for
'Gut microbiota abundance (genus Intestinimonas id.2062)' on 'Localized adiposity'

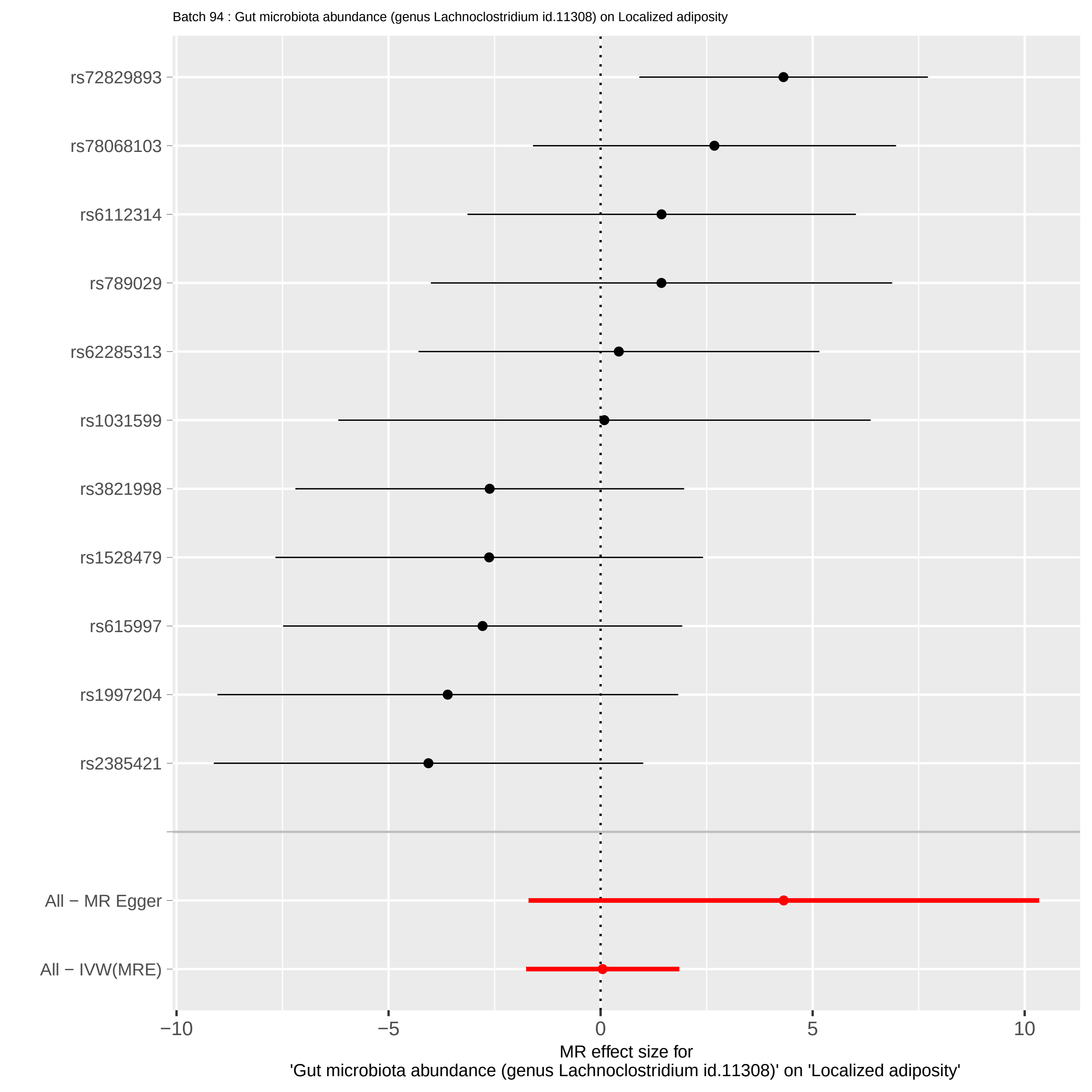

Batch 94 : Gut microbiota abundance (genus Lachnoclostridium id.11308) on Localized adiposity
rs72829893
rs78068103
rs6112314
rs789029
rs62285313
rs1031599
rs3821998
rs1528479
rs615997
rs1997204
rs2385421
All − MR Egger
All − IVW(MRE)
−10
−5
0
5
10
MR effect size for
'Gut microbiota abundance (genus Lachnoclostridium id.11308)' on 'Localized adiposity'

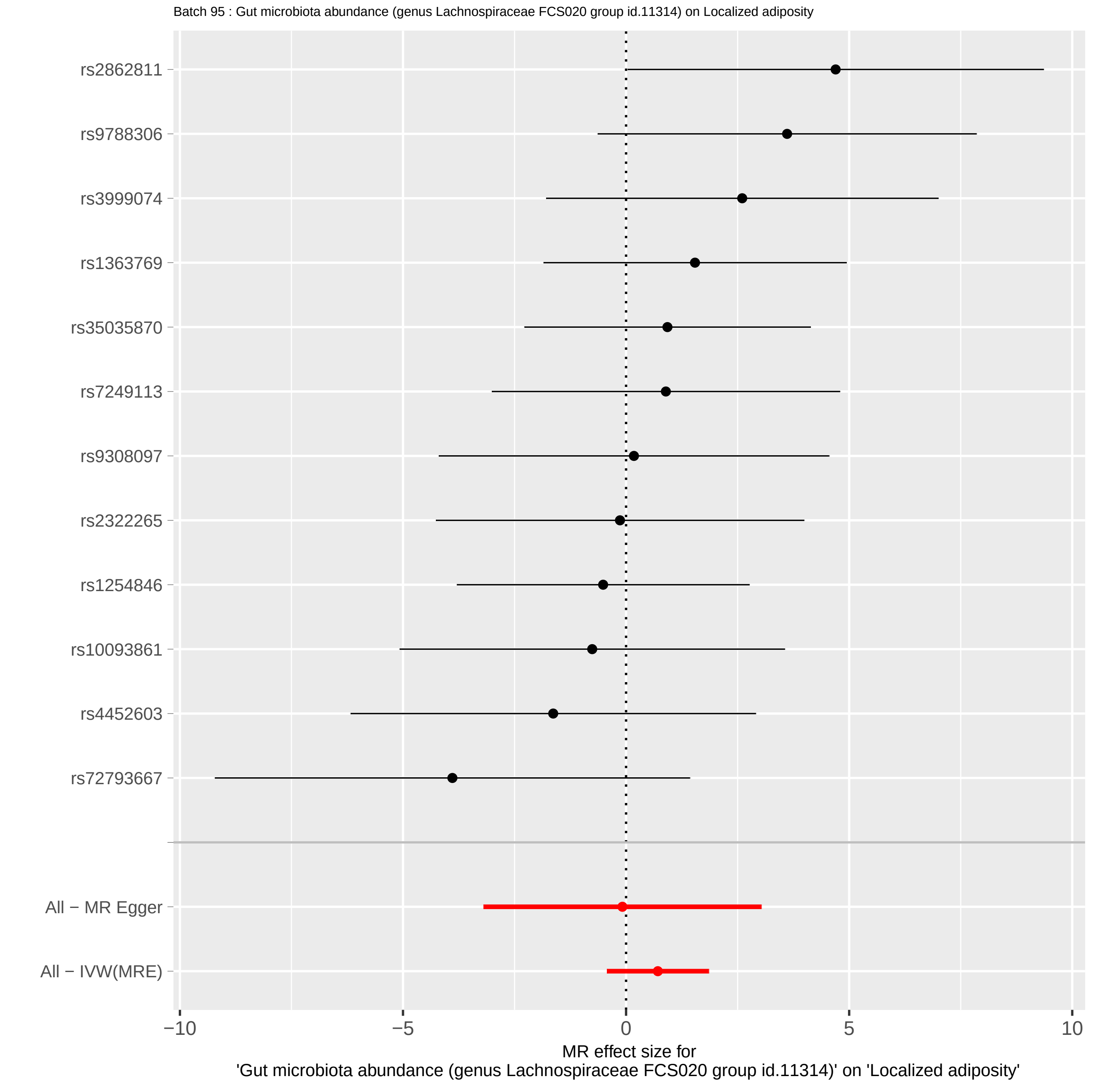

Batch 95 : Gut microbiota abundance (genus Lachnospiraceae FCS020 group id.11314) on Localized adiposity
rs2862811
rs9788306
rs3999074
rs1363769
rs35035870
rs7249113
rs9308097
rs2322265
rs1254846
rs10093861
rs4452603
rs72793667
All − MR Egger
All − IVW(MRE)
−10
−5
0
5
10
MR effect size for
'Gut microbiota abundance (genus Lachnospiraceae FCS020 group id.11314)' on 'Localized adiposity'

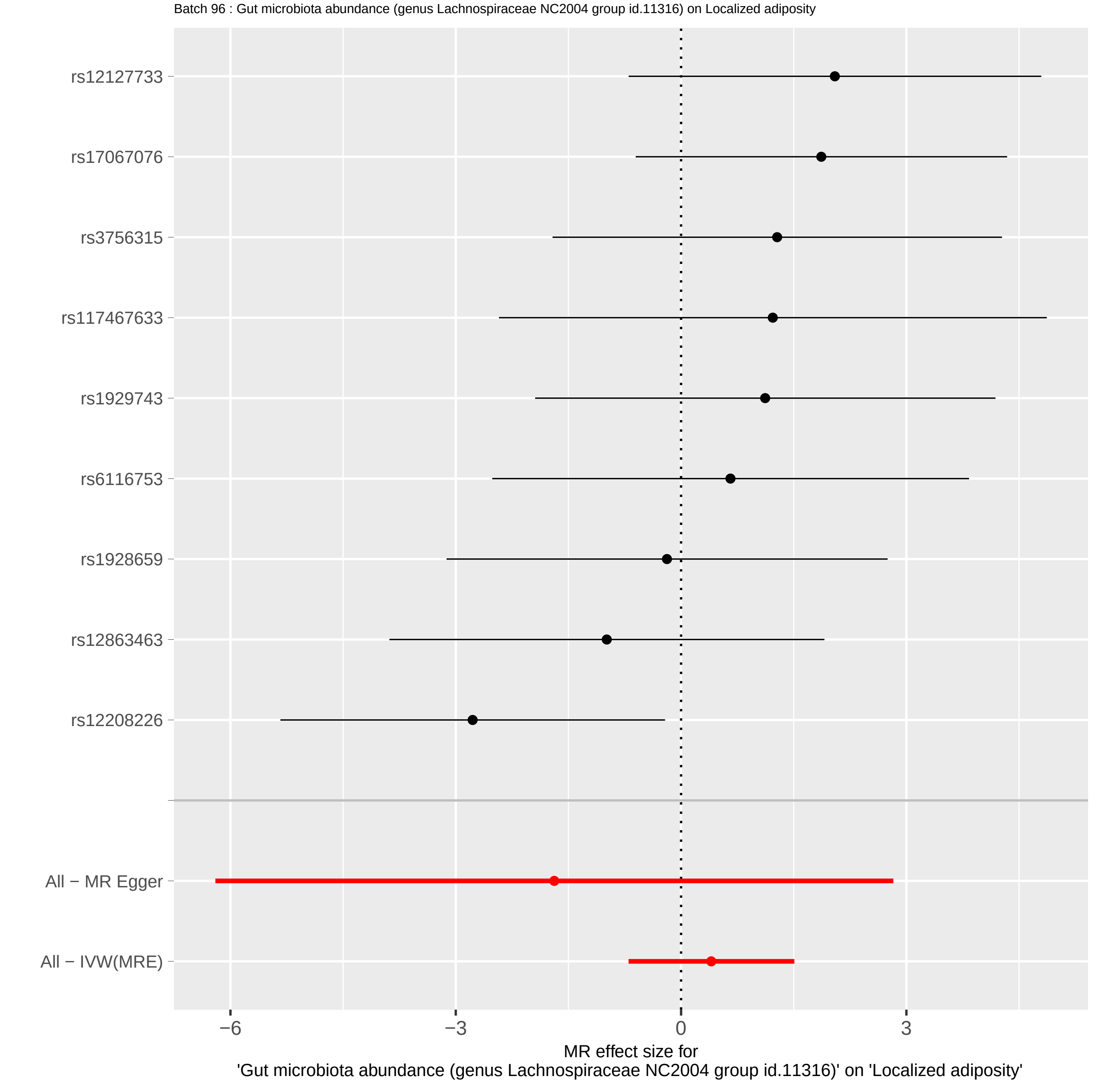

Batch 96 : Gut microbiota abundance (genus Lachnospiraceae NC2004 group id.11316) on Localized adiposity
rs12127733
rs17067076
rs3756315
rs117467633
rs1929743
rs6116753
rs1928659
rs12863463
rs12208226
All − MR Egger
All − IVW(MRE)
−6
−3
0
3
MR effect size for
'Gut microbiota abundance (genus Lachnospiraceae NC2004 group id.11316)' on 'Localized adiposity'

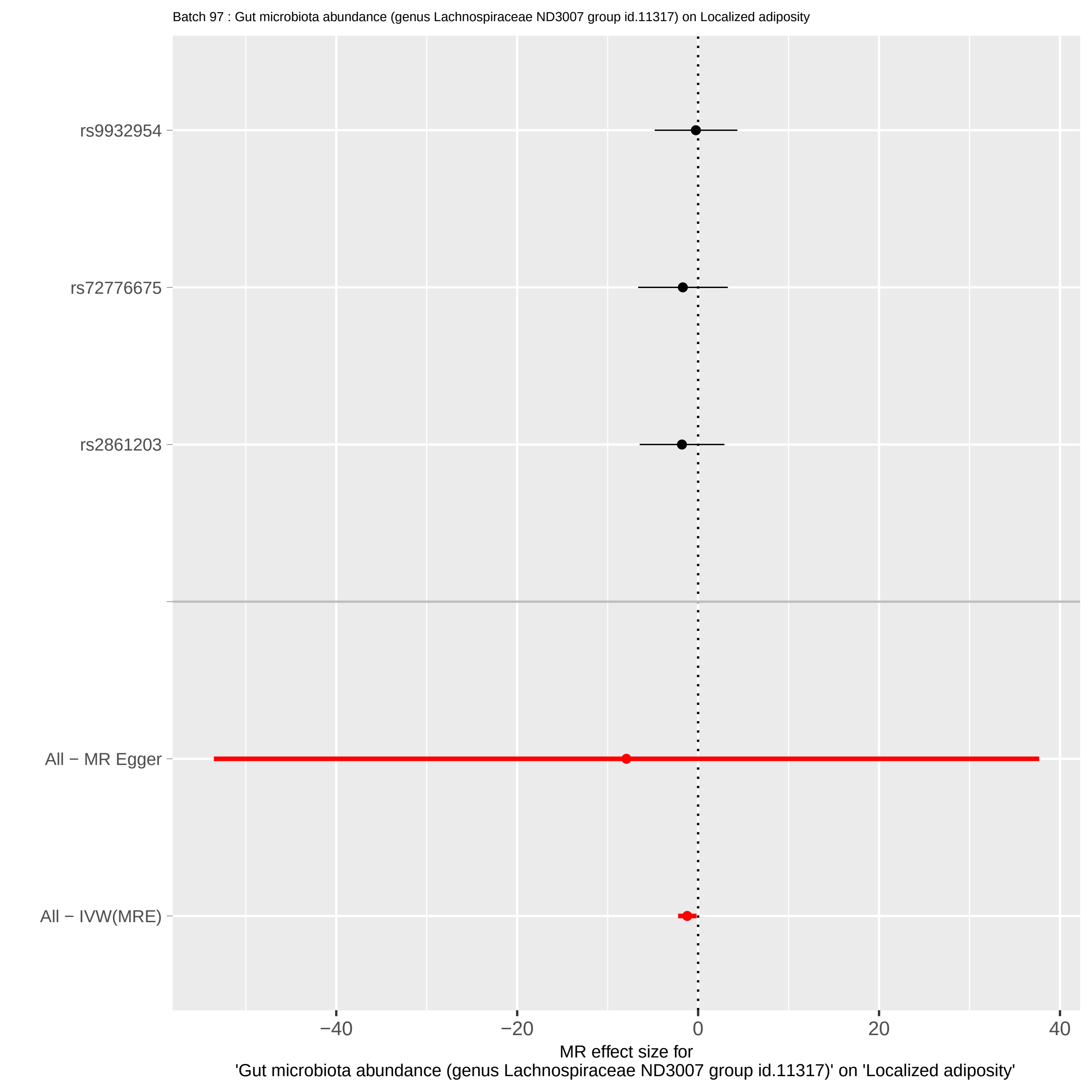

Batch 97 : Gut microbiota abundance (genus Lachnospiraceae ND3007 group id.11317) on Localized adiposity
rs9932954
rs72776675
rs2861203
All − MR Egger
All − IVW(MRE)
−40
−20
0
20
40
MR effect size for
'Gut microbiota abundance (genus Lachnospiraceae ND3007 group id.11317)' on 'Localized adiposity'

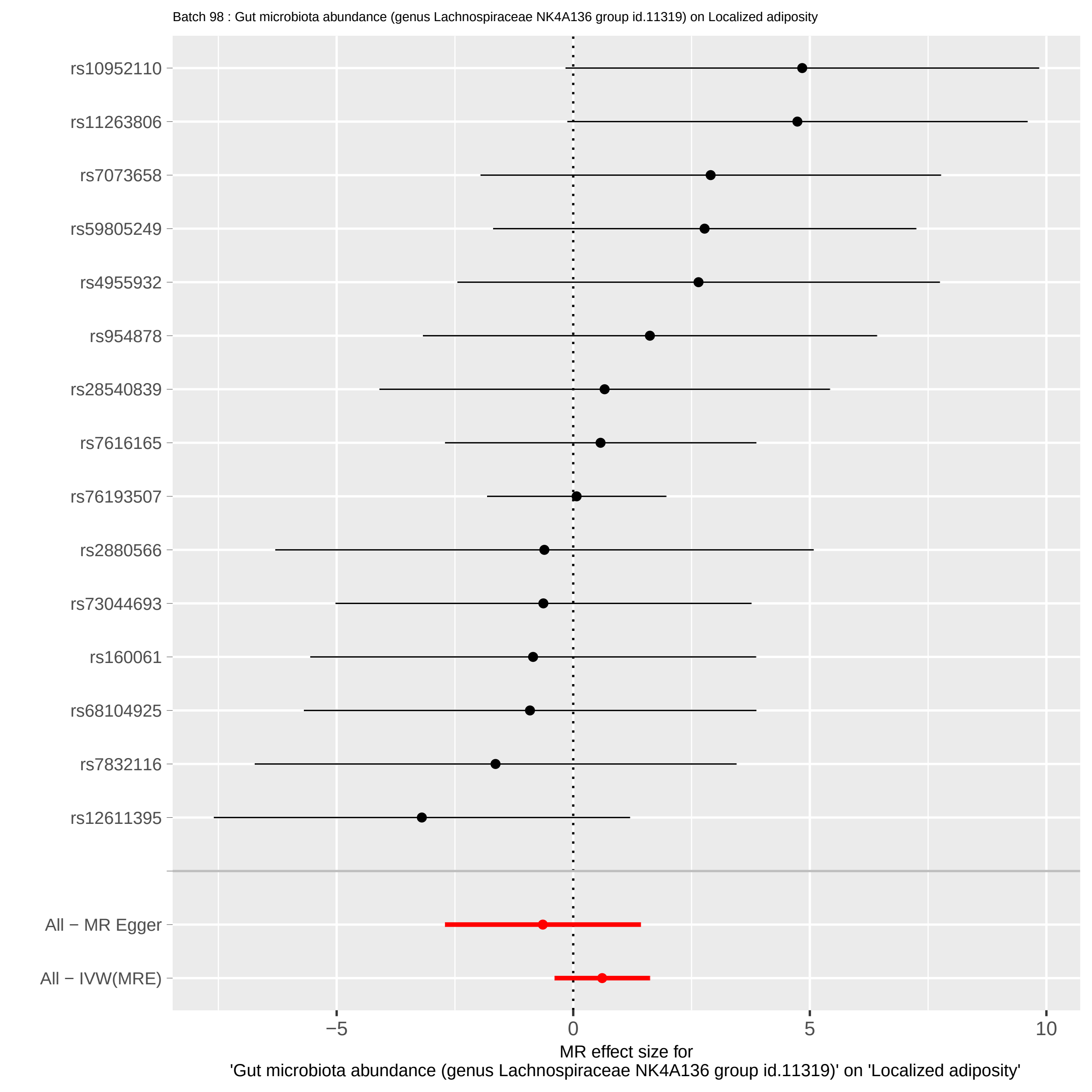
Batch 98 : Gut microbiota abundance (genus Lachnospiraceae NK4A136 group id.11319) on Localized adiposity
rs10952110
rs11263806
rs7073658
rs59805249
rs4955932
rs954878
rs28540839
rs7616165
rs76193507
rs2880566
rs73044693
rs160061
rs68104925
rs7832116
rs12611395
All − MR Egger
All − IVW(MRE)
−5
0
5
10
MR effect size for
'Gut microbiota abundance (genus Lachnospiraceae NK4A136 group id.11319)' on 'Localized adiposity'

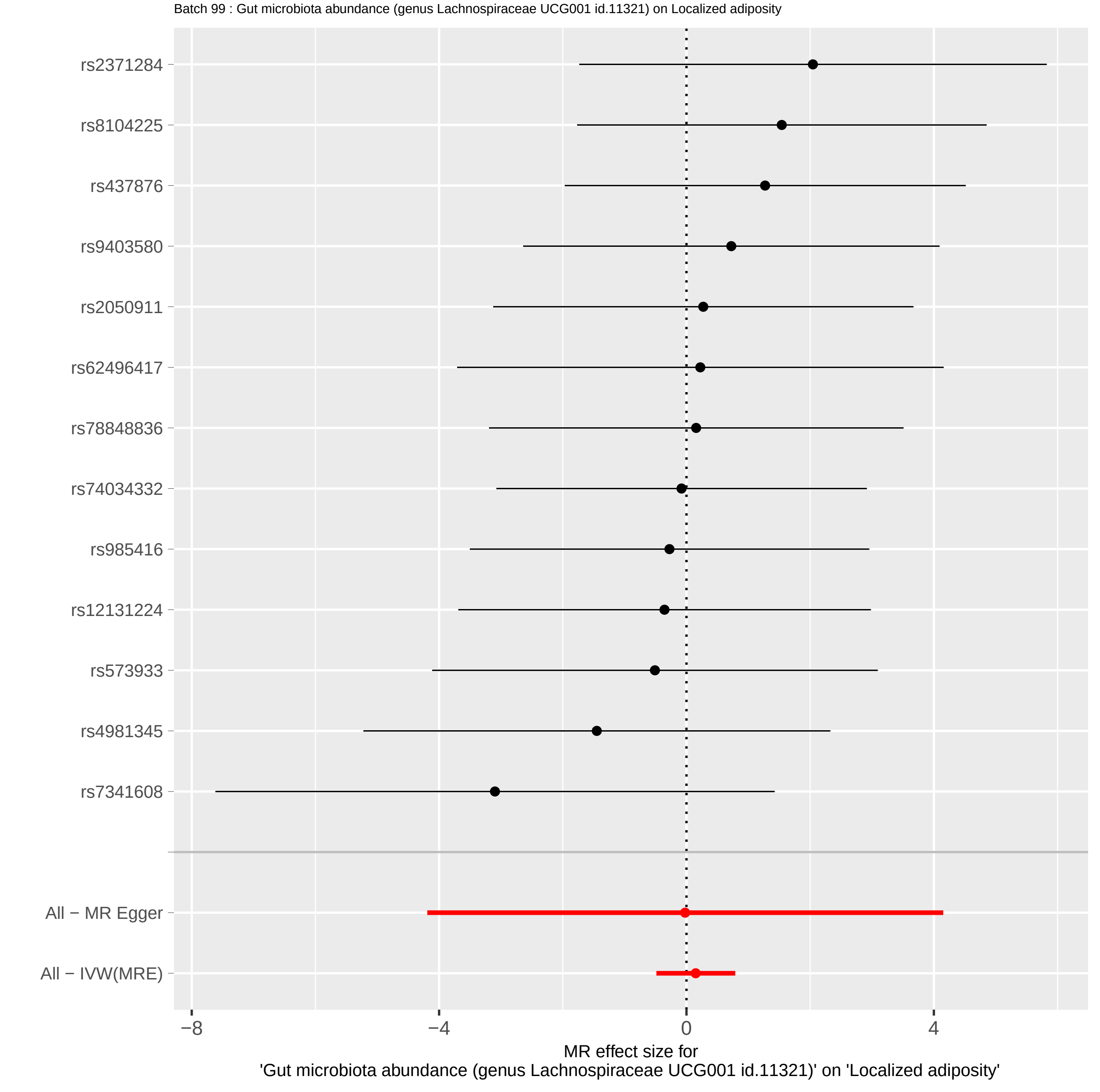

Batch 99 : Gut microbiota abundance (genus Lachnospiraceae UCG001 id.11321) on Localized adiposity
rs2371284
rs8104225
rs437876
rs9403580
rs2050911
rs62496417
rs78848836
rs74034332
rs985416
rs12131224
rs573933
rs4981345
rs7341608
All − MR Egger
All − IVW(MRE)
−8
−4
0
4
MR effect size for
'Gut microbiota abundance (genus Lachnospiraceae UCG001 id.11321)' on 'Localized adiposity'

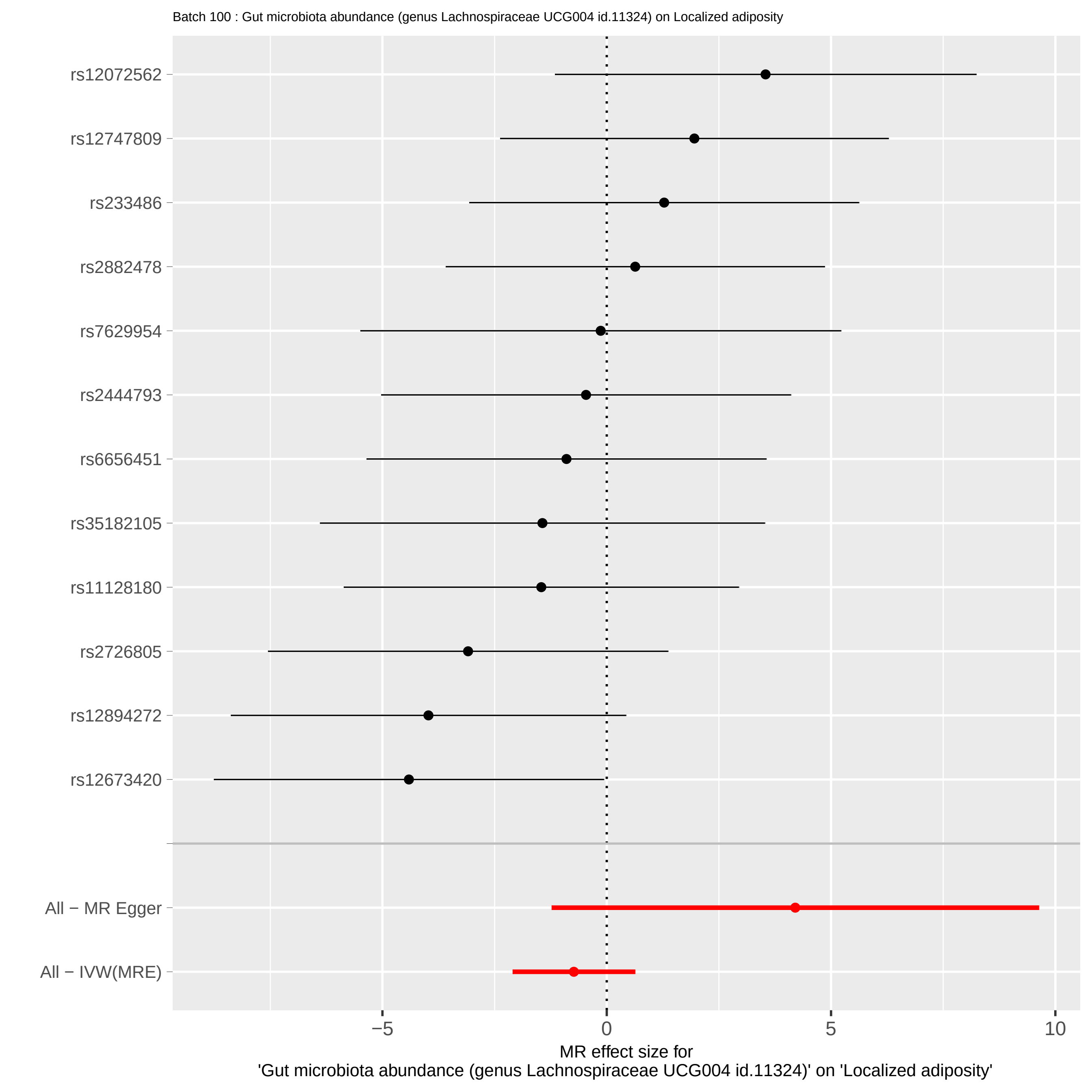
Batch 100 : Gut microbiota abundance (genus Lachnospiraceae UCG004 id.11324) on Localized adiposity
rs12072562
rs12747809
rs233486
rs2882478
rs7629954
rs2444793
rs6656451
rs35182105
rs11128180
rs2726805
rs12894272
rs12673420
All − MR Egger
All − IVW(MRE)
−5
0
5
10
MR effect size for
'Gut microbiota abundance (genus Lachnospiraceae UCG004 id.11324)' on 'Localized adiposity'

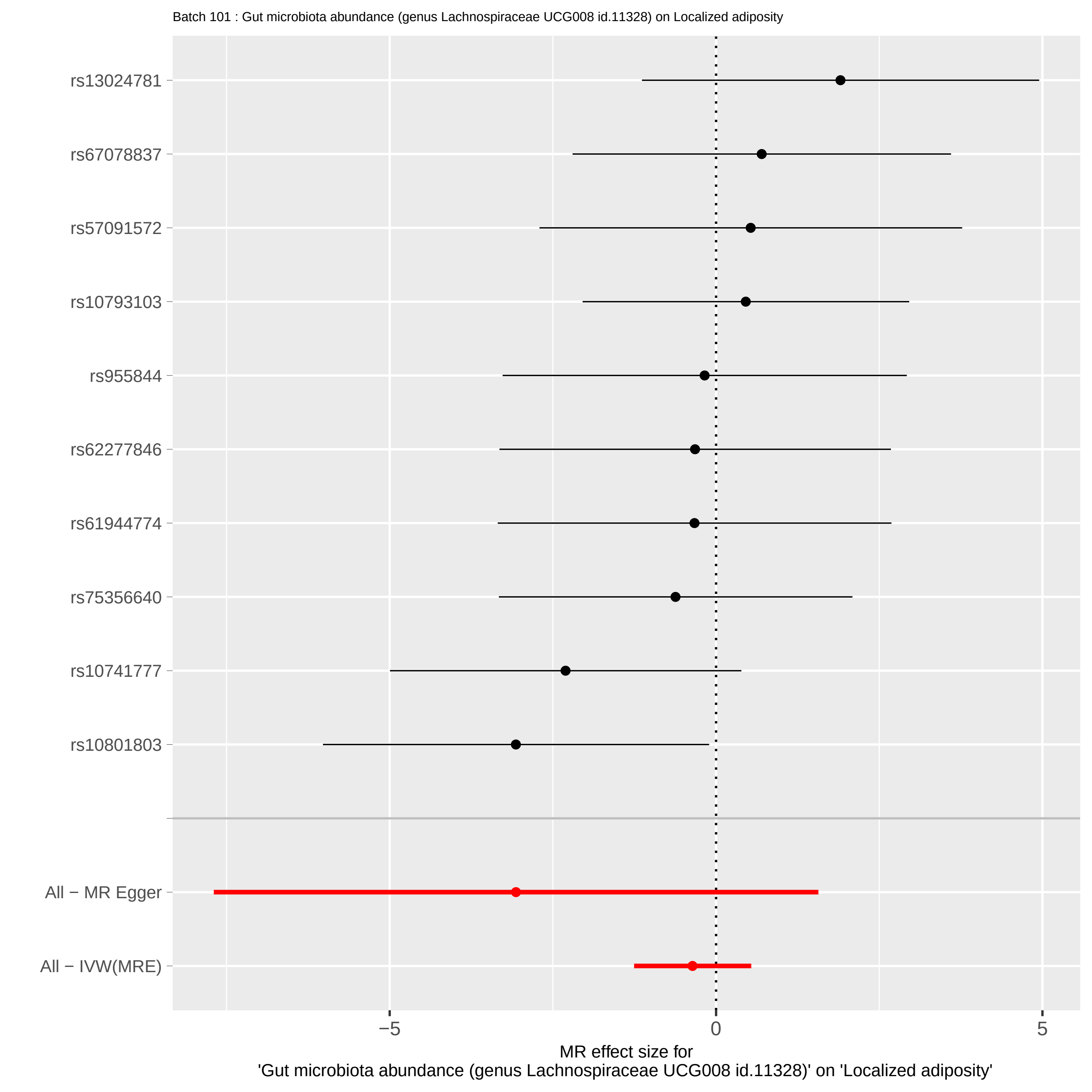

Batch 101 : Gut microbiota abundance (genus Lachnospiraceae UCG008 id.11328) on Localized adiposity
rs13024781
rs67078837
rs57091572
rs10793103
rs955844
rs62277846
rs61944774
rs75356640
rs10741777
rs10801803
All − MR Egger
All − IVW(MRE)
−5
0
5
MR effect size for
'Gut microbiota abundance (genus Lachnospiraceae UCG008 id.11328)' on 'Localized adiposity'

Batch 102 : Gut microbiota abundance (genus Lachnospiraceae UCG010 id.11330) on Localized adiposity

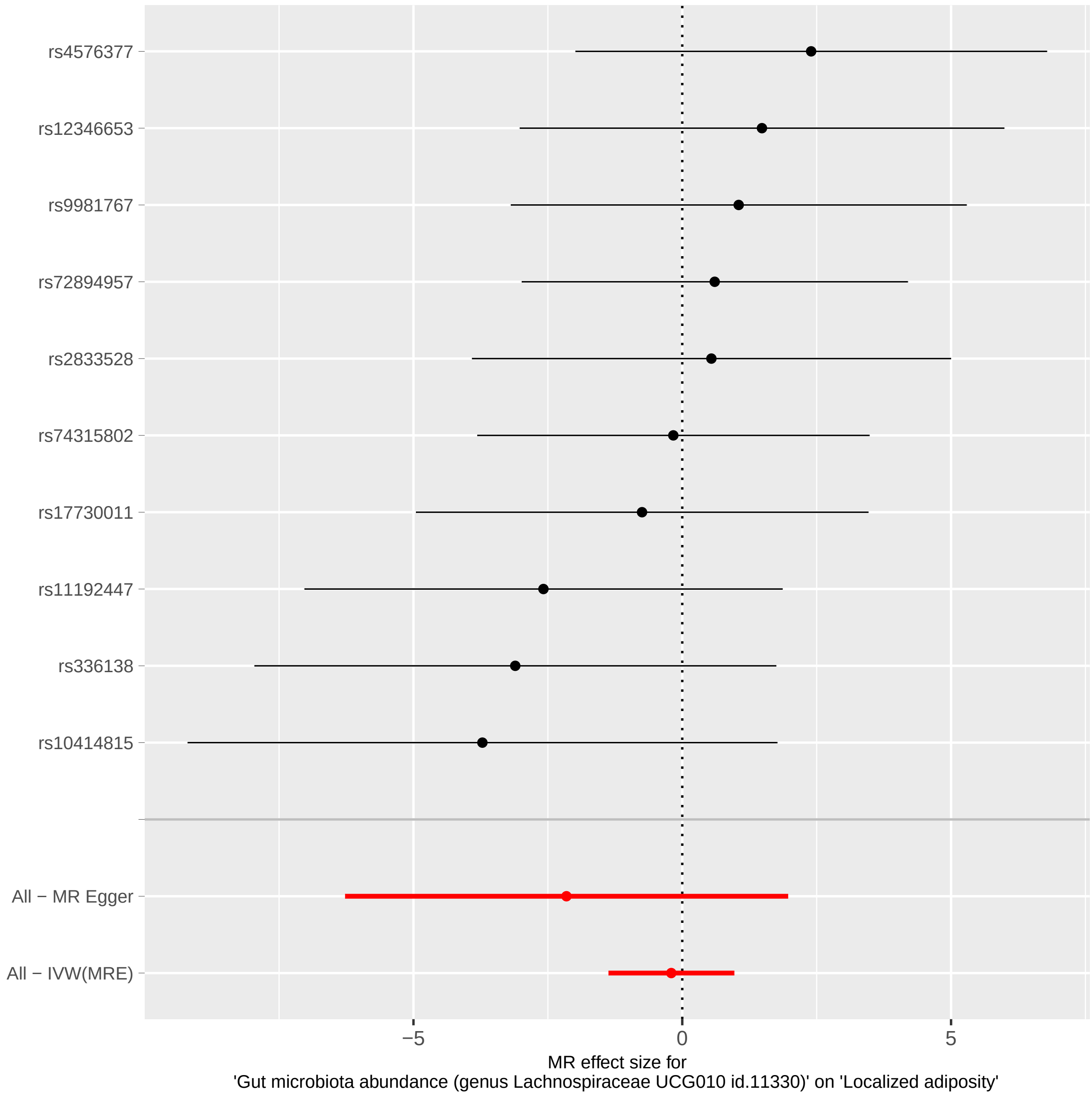

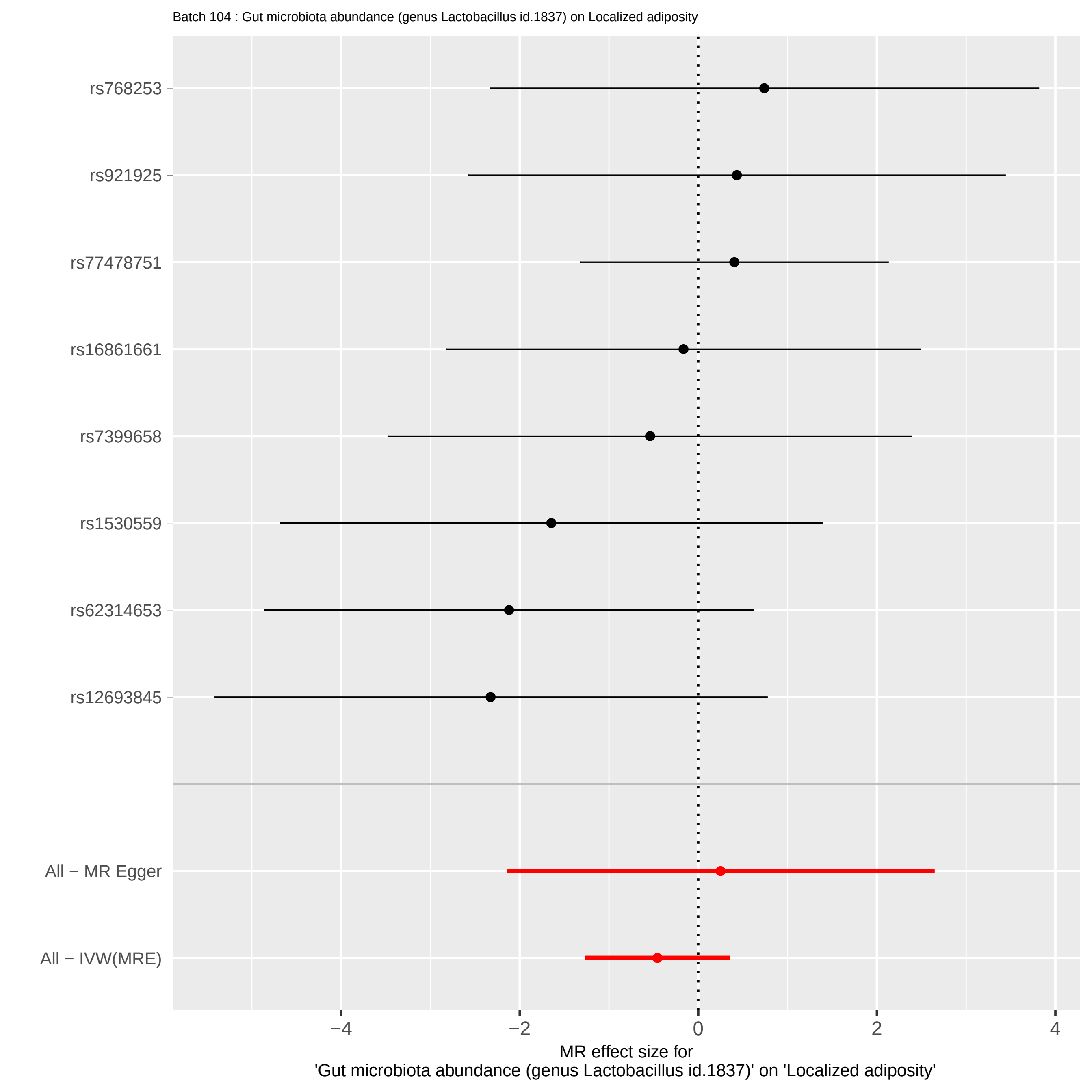
Batch 104 : Gut microbiota abundance (genus Lactobacillus id.1837) on Localized adiposity
rs768253
rs921925
rs77478751
rs16861661
rs7399658
rs1530559
rs62314653
rs12693845
All − MR Egger
All − IVW(MRE)
−4
−2
0
2
4
MR effect size for
'Gut microbiota abundance (genus Lactobacillus id.1837)' on 'Localized adiposity'

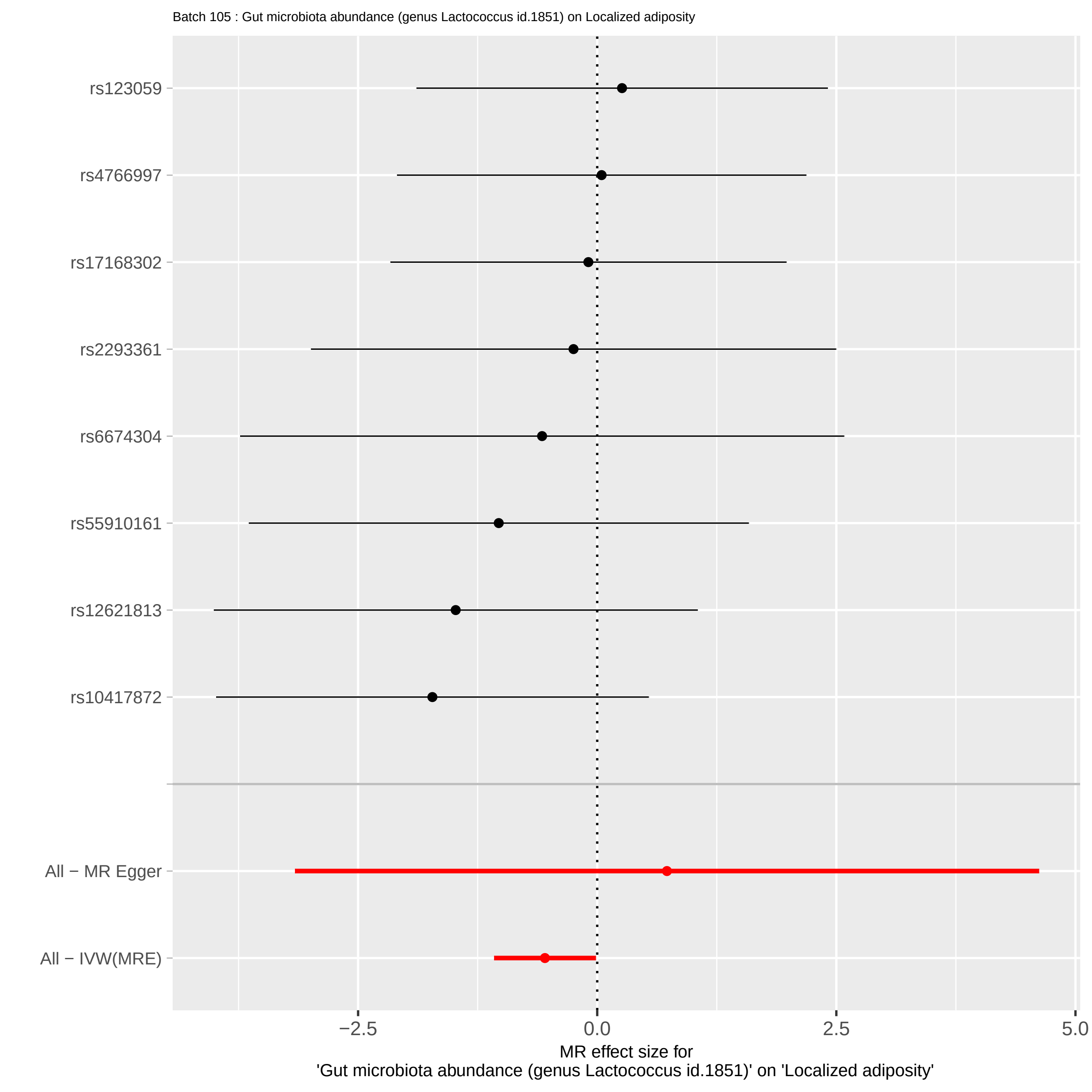

Batch 105 : Gut microbiota abundance (genus Lactococcus id.1851) on Localized adiposity
rs123059
rs4766997
rs17168302
rs2293361
rs6674304
rs55910161
rs12621813
rs10417872
All − MR Egger
All − IVW(MRE)
−2.5
0.0
2.5
5.0
MR effect size for
'Gut microbiota abundance (genus Lactococcus id.1851)' on 'Localized adiposity'

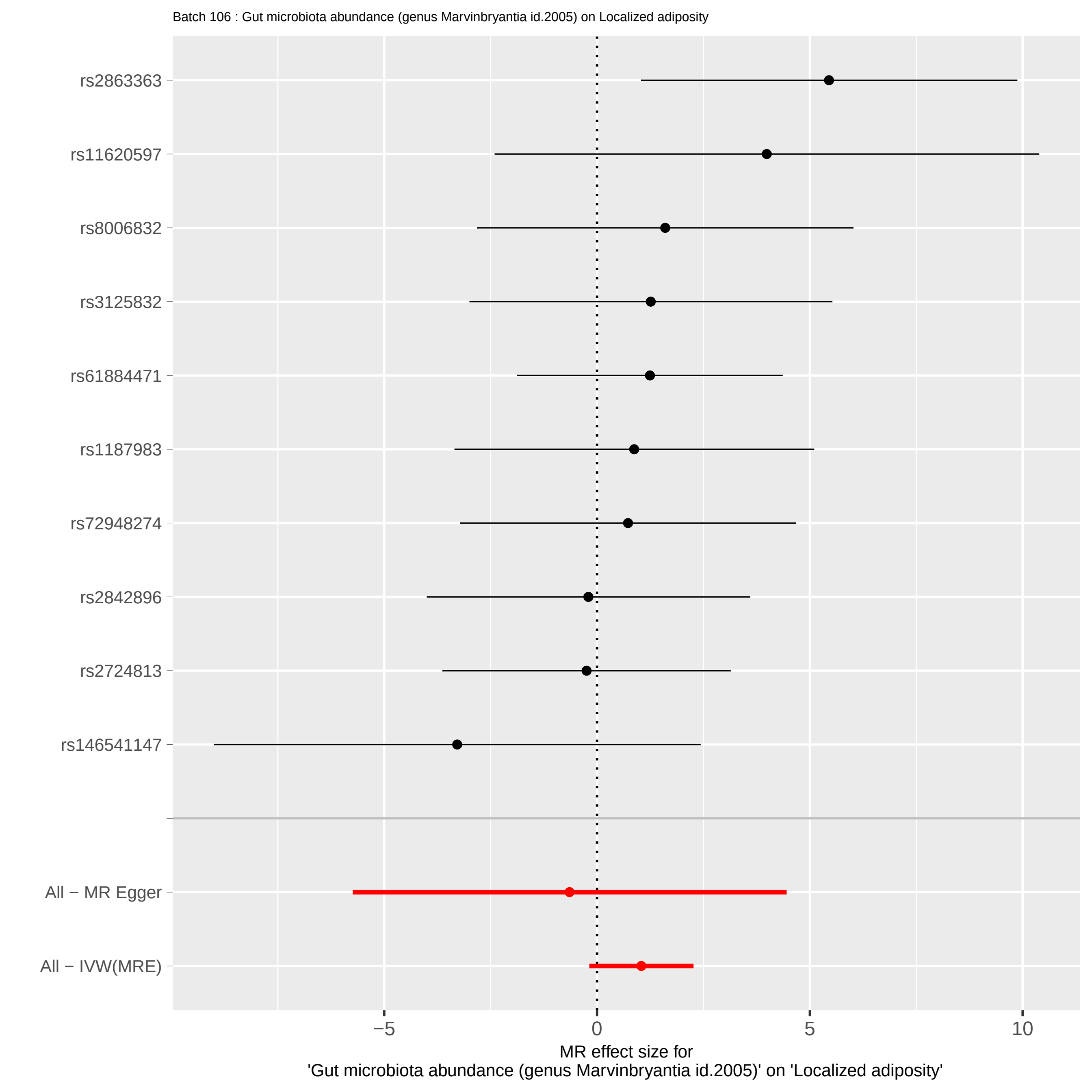

Batch 106 : Gut microbiota abundance (genus Marvinbryantia id.2005) on Localized adiposity
rs2863363
rs11620597
rs8006832
rs3125832
rs61884471
rs1187983
rs72948274
rs2842896
rs2724813
rs146541147
All − MR Egger
All − IVW(MRE)
−5
0
5
10
MR effect size for
'Gut microbiota abundance (genus Marvinbryantia id.2005)' on 'Localized adiposity'

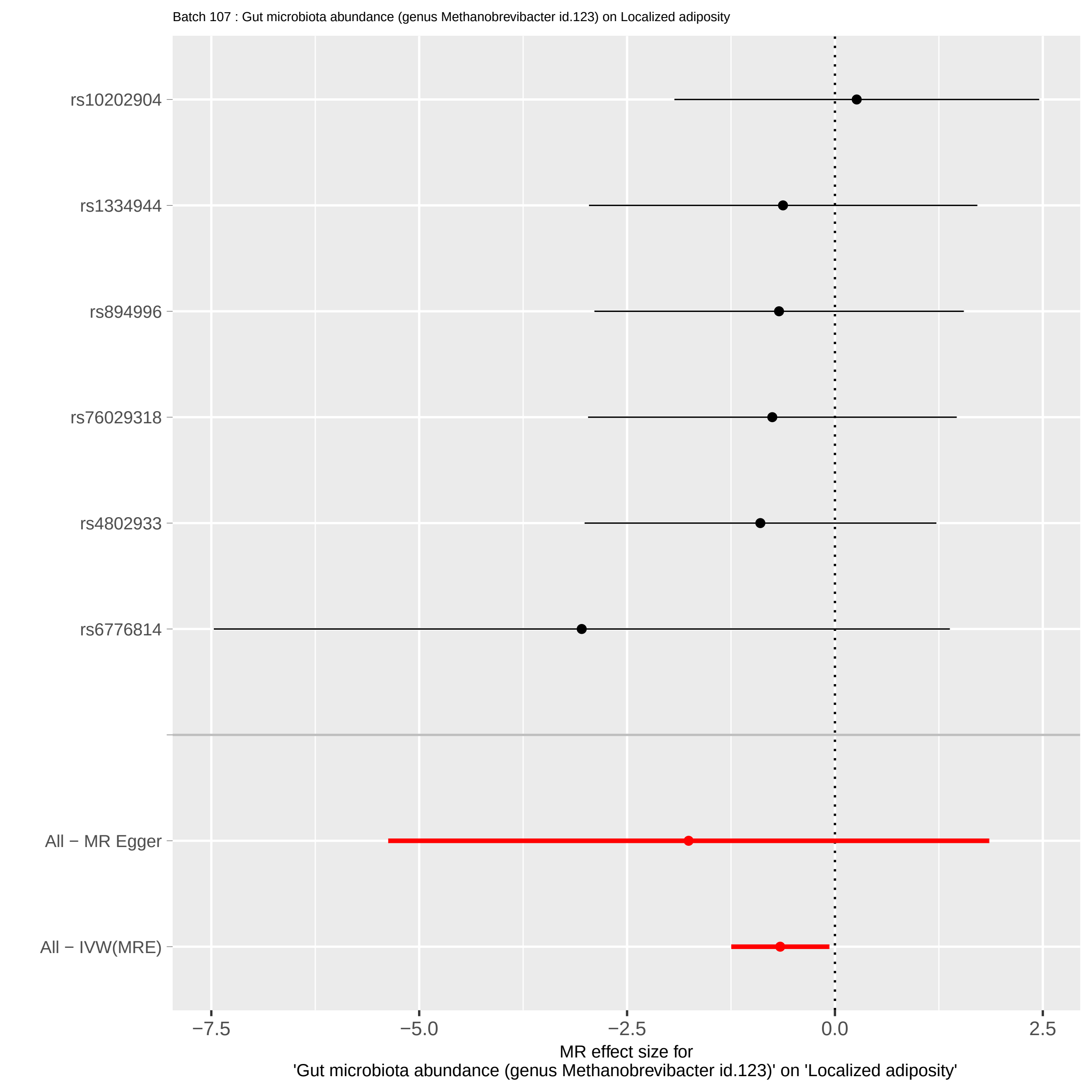

Batch 107 : Gut microbiota abundance (genus Methanobrevibacter id.123) on Localized adiposity
rs10202904
rs1334944
rs894996
rs76029318
rs4802933
rs6776814
All − MR Egger
All − IVW(MRE)
−7.5
−5.0
−2.5
0.0
2.5
MR effect size for
'Gut microbiota abundance (genus Methanobrevibacter id.123)' on 'Localized adiposity'

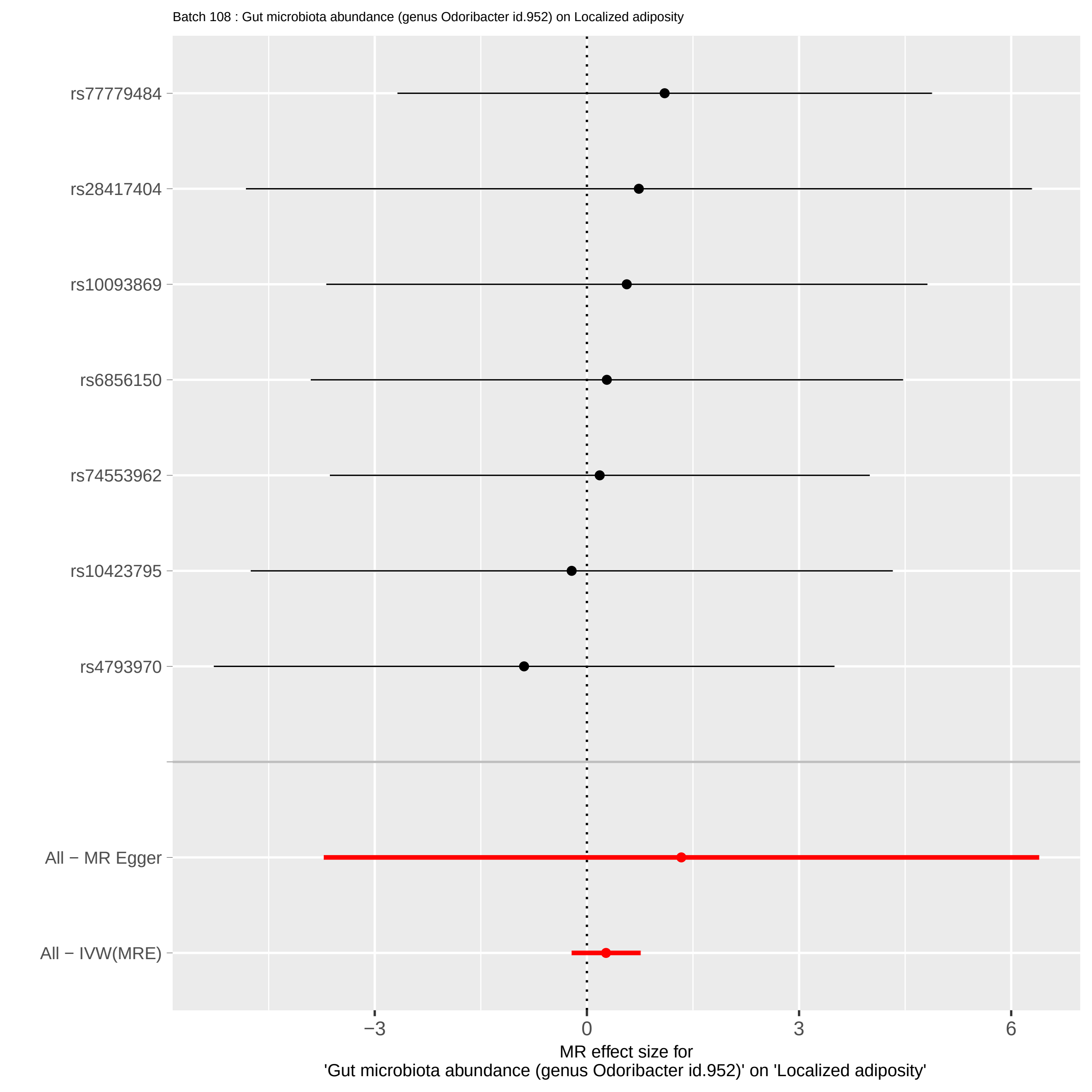
Batch 108 : Gut microbiota abundance (genus Odoribacter id.952) on Localized adiposity
rs77779484
rs28417404
rs10093869
rs6856150
rs74553962
rs10423795
rs4793970
All − MR Egger
All − IVW(MRE)
−3
0
3
6
MR effect size for
'Gut microbiota abundance (genus Odoribacter id.952)' on 'Localized adiposity'

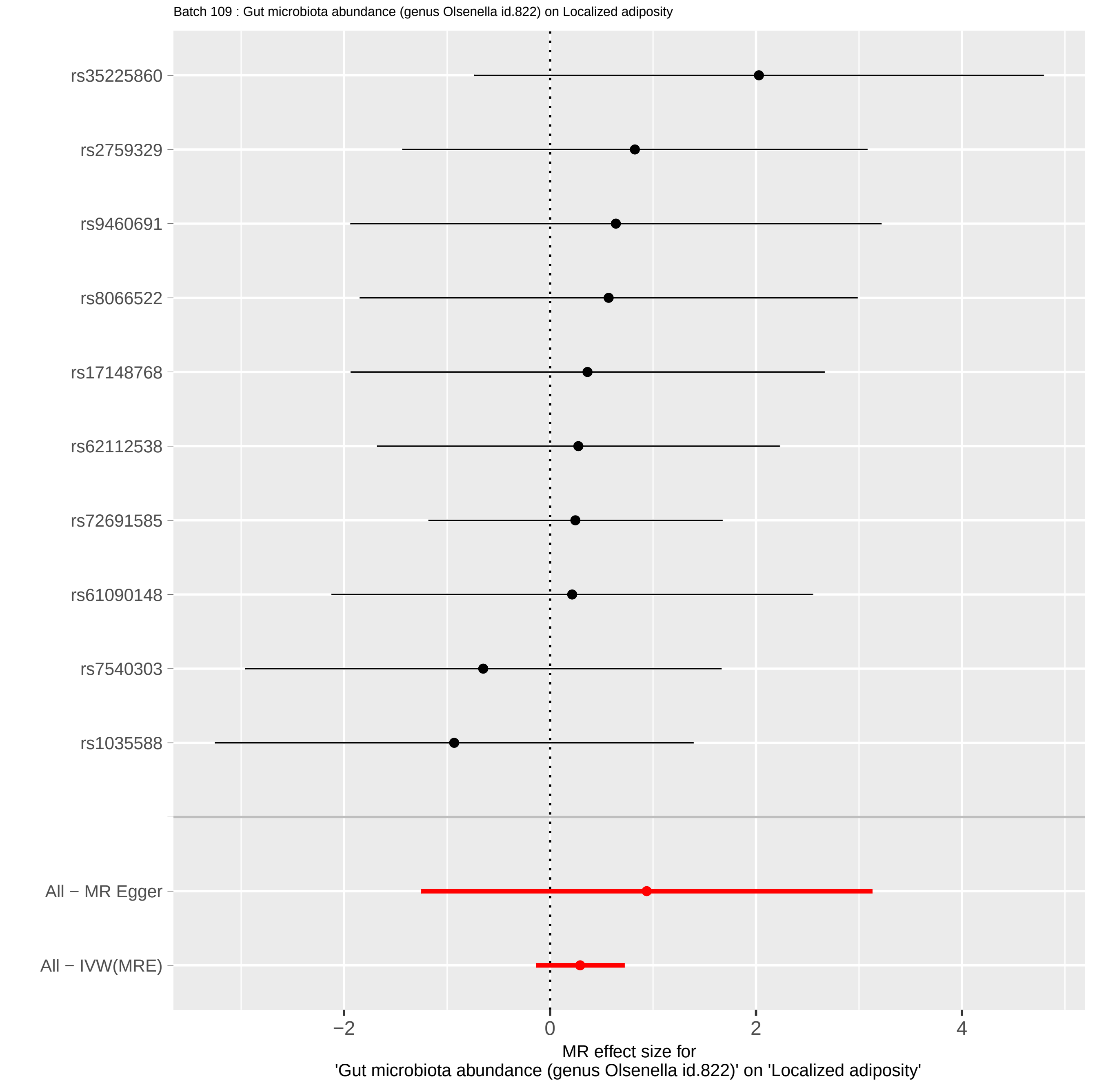
Batch 109 : Gut microbiota abundance (genus Olsenella id.822) on Localized adiposity
rs35225860
rs2759329
rs9460691
rs8066522
rs17148768
rs62112538
rs72691585
rs61090148
rs7540303
rs1035588
All − MR Egger
All − IVW(MRE)
−2
0
2
4
MR effect size for
'Gut microbiota abundance (genus Olsenella id.822)' on 'Localized adiposity'

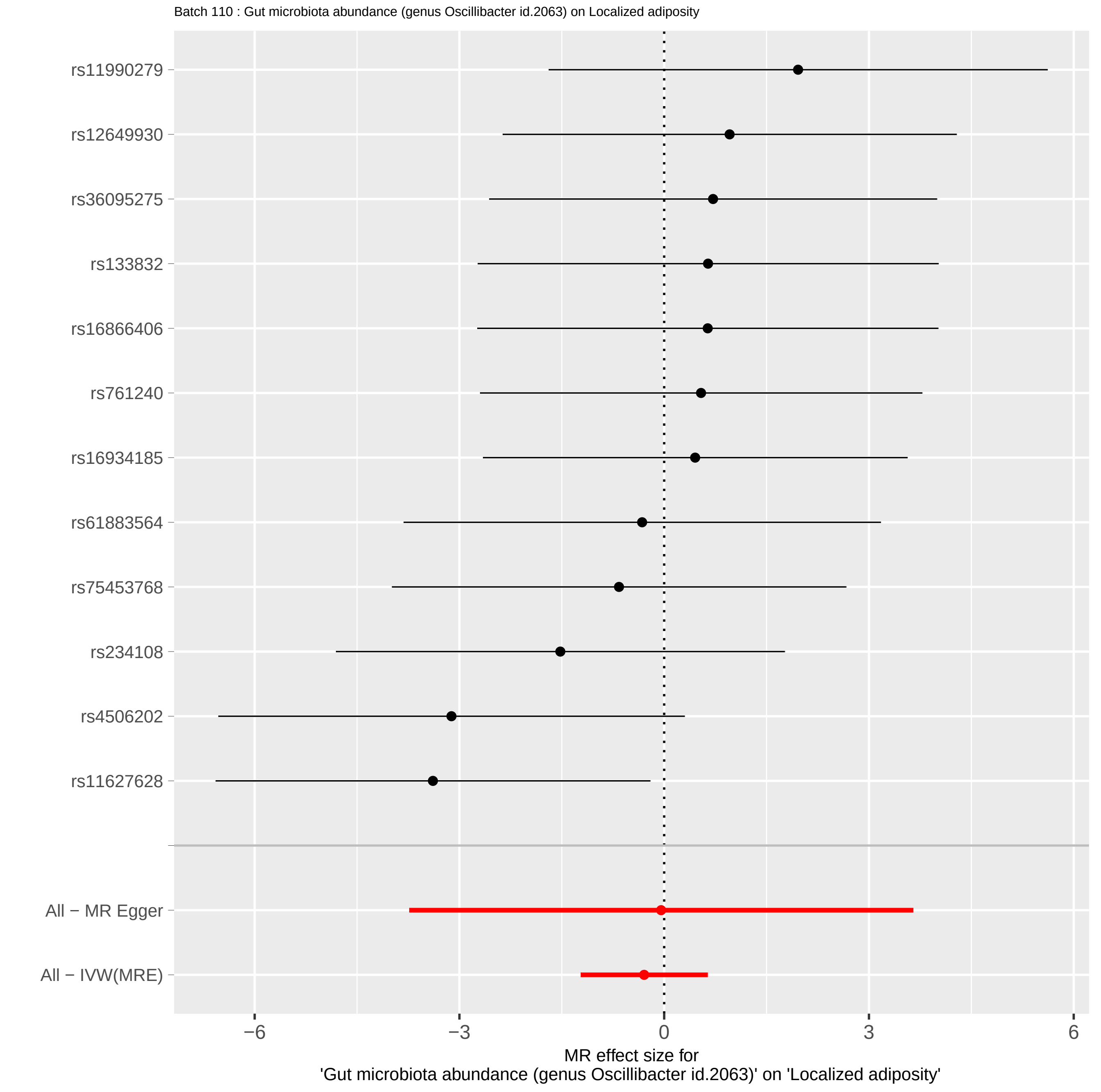
Batch 110 : Gut microbiota abundance (genus Oscillibacter id.2063) on Localized adiposity
rs11990279
rs12649930
rs36095275
rs133832
rs16866406
rs761240
rs16934185
rs61883564
rs75453768
rs234108
rs4506202
rs11627628
All − MR Egger
All − IVW(MRE)
−6
−3
0
3
6
MR effect size for
'Gut microbiota abundance (genus Oscillibacter id.2063)' on 'Localized adiposity'

Batch 111 : Gut microbiota abundance (genus Oscillospira id.2064) on Localized adiposity

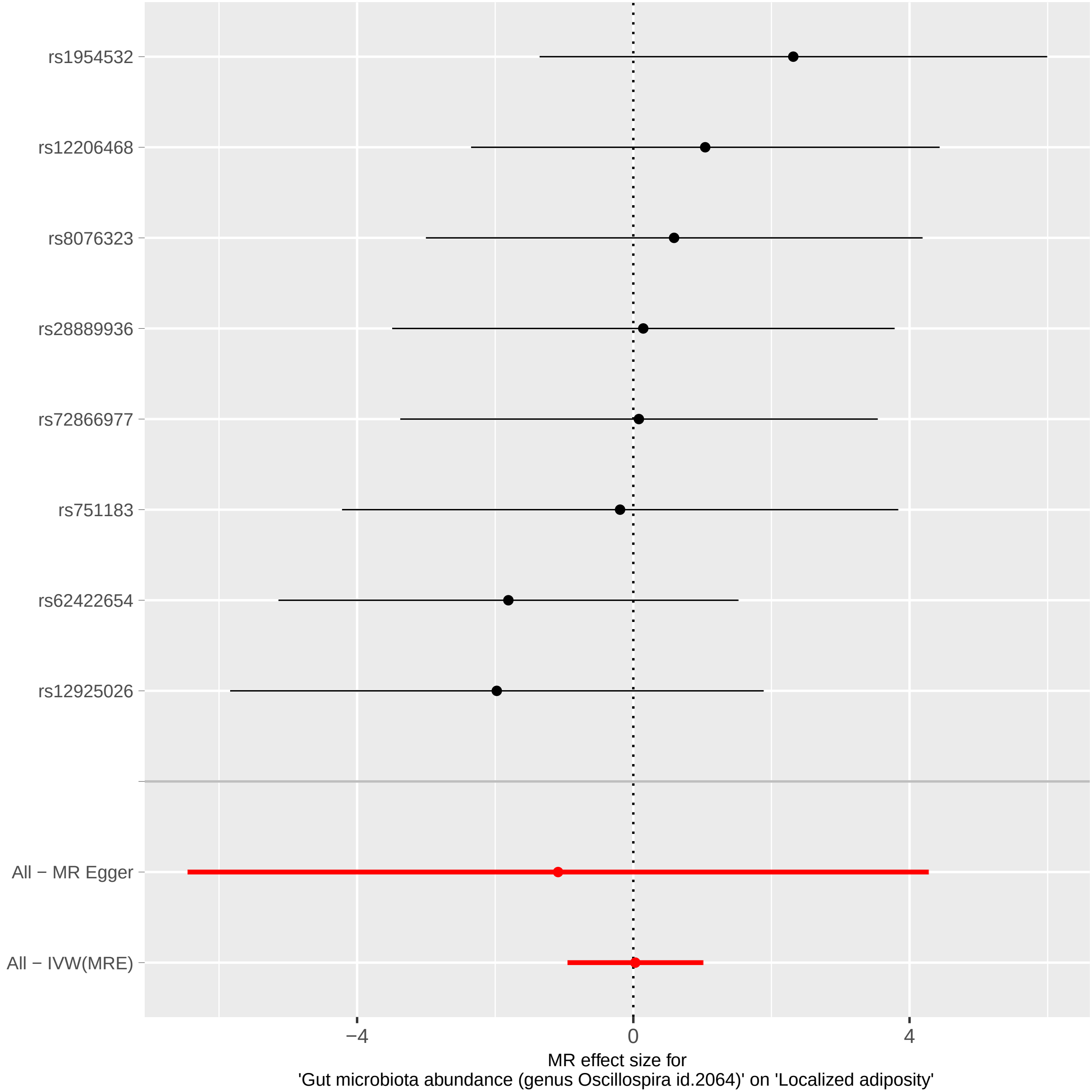

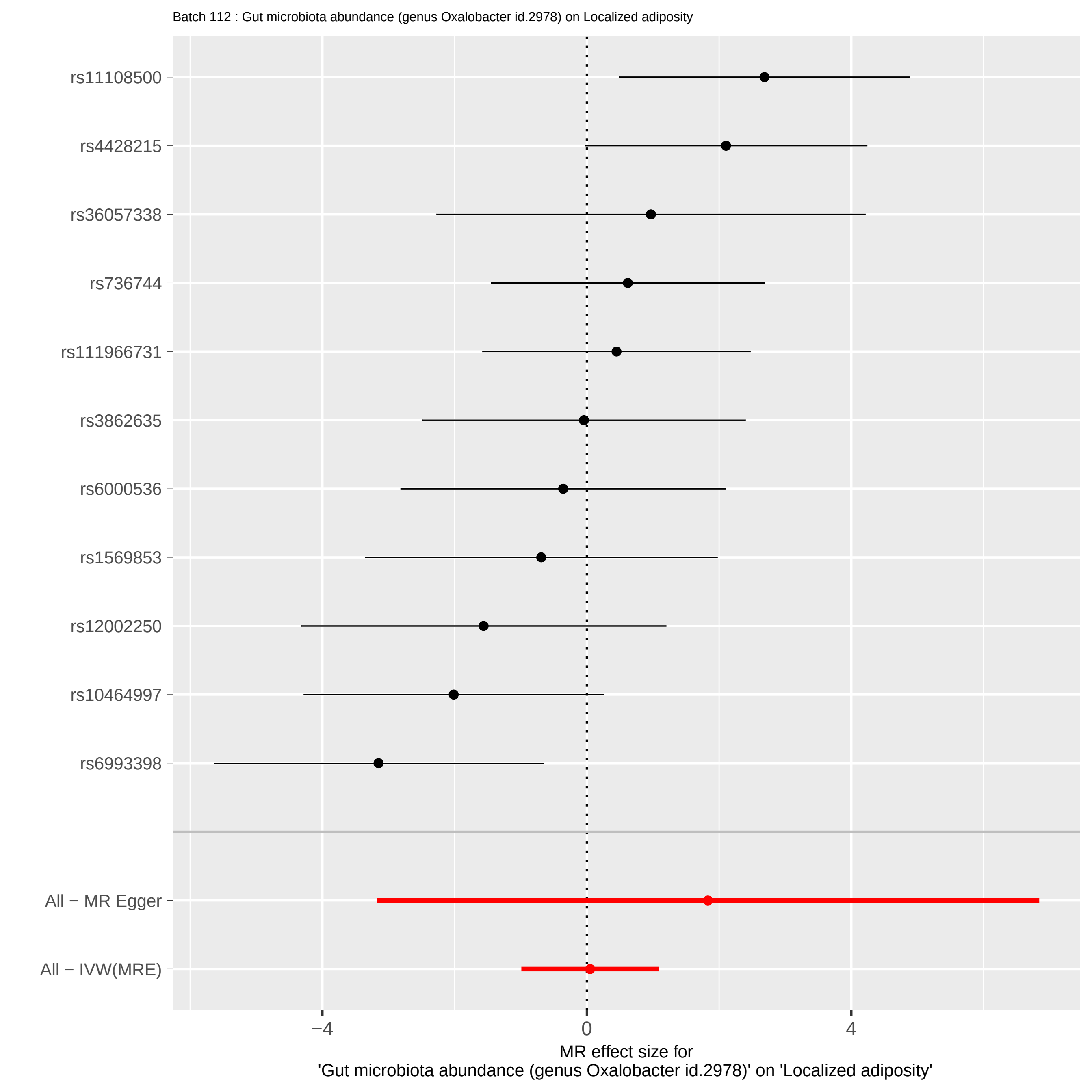
Batch 112 : Gut microbiota abundance (genus Oxalobacter id.2978) on Localized adiposity
rs11108500
rs4428215
rs36057338
rs736744
rs111966731
rs3862635
rs6000536
rs1569853
rs12002250
rs10464997
rs6993398
All − MR Egger
All − IVW(MRE)
−4
0
4
MR effect size for
'Gut microbiota abundance (genus Oxalobacter id.2978)' on 'Localized adiposity'

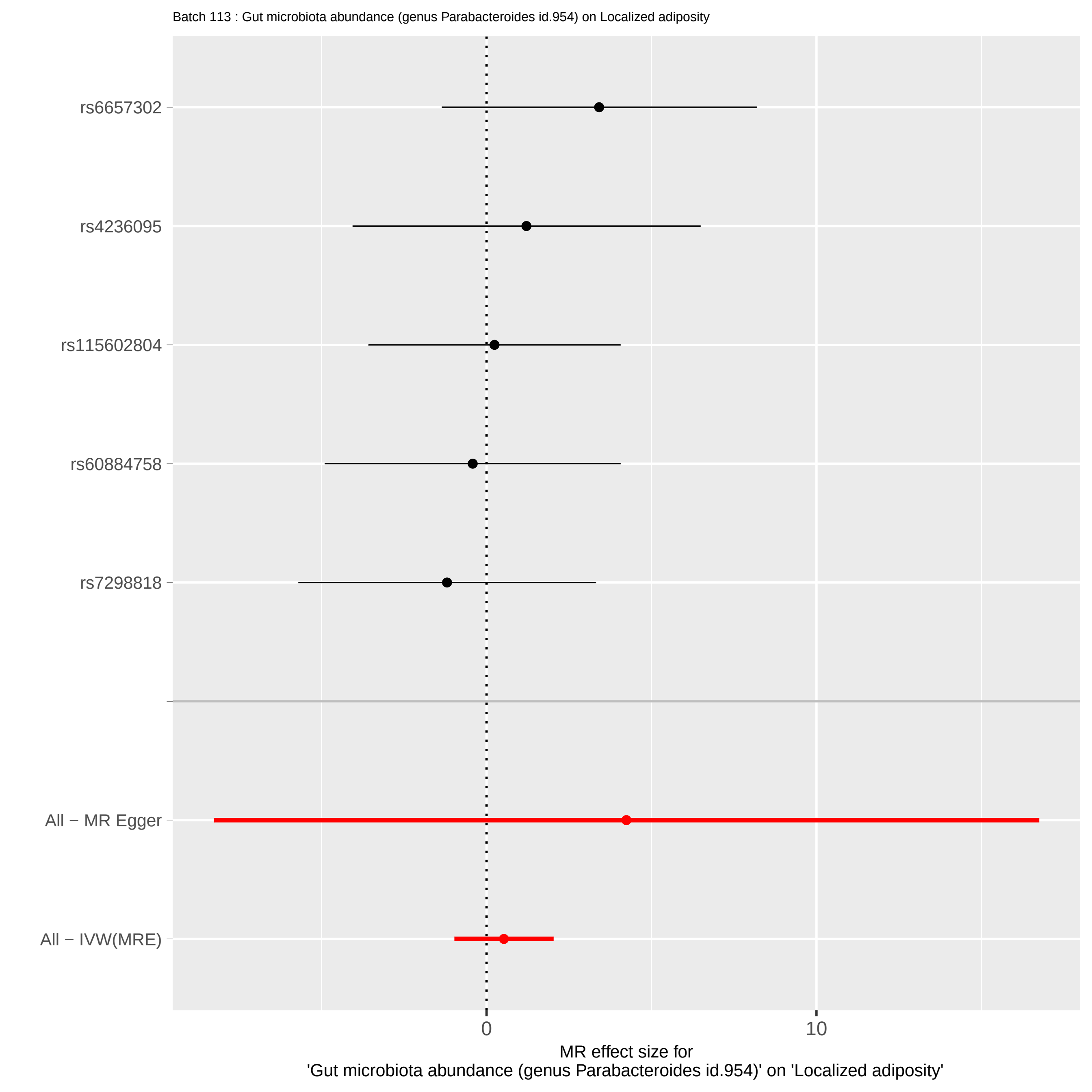

Batch 113 : Gut microbiota abundance (genus Parabacteroides id.954) on Localized adiposity
rs6657302
rs4236095
rs115602804
rs60884758
rs7298818
All − MR Egger
All − IVW(MRE)
0
10
MR effect size for
'Gut microbiota abundance (genus Parabacteroides id.954)' on 'Localized adiposity'

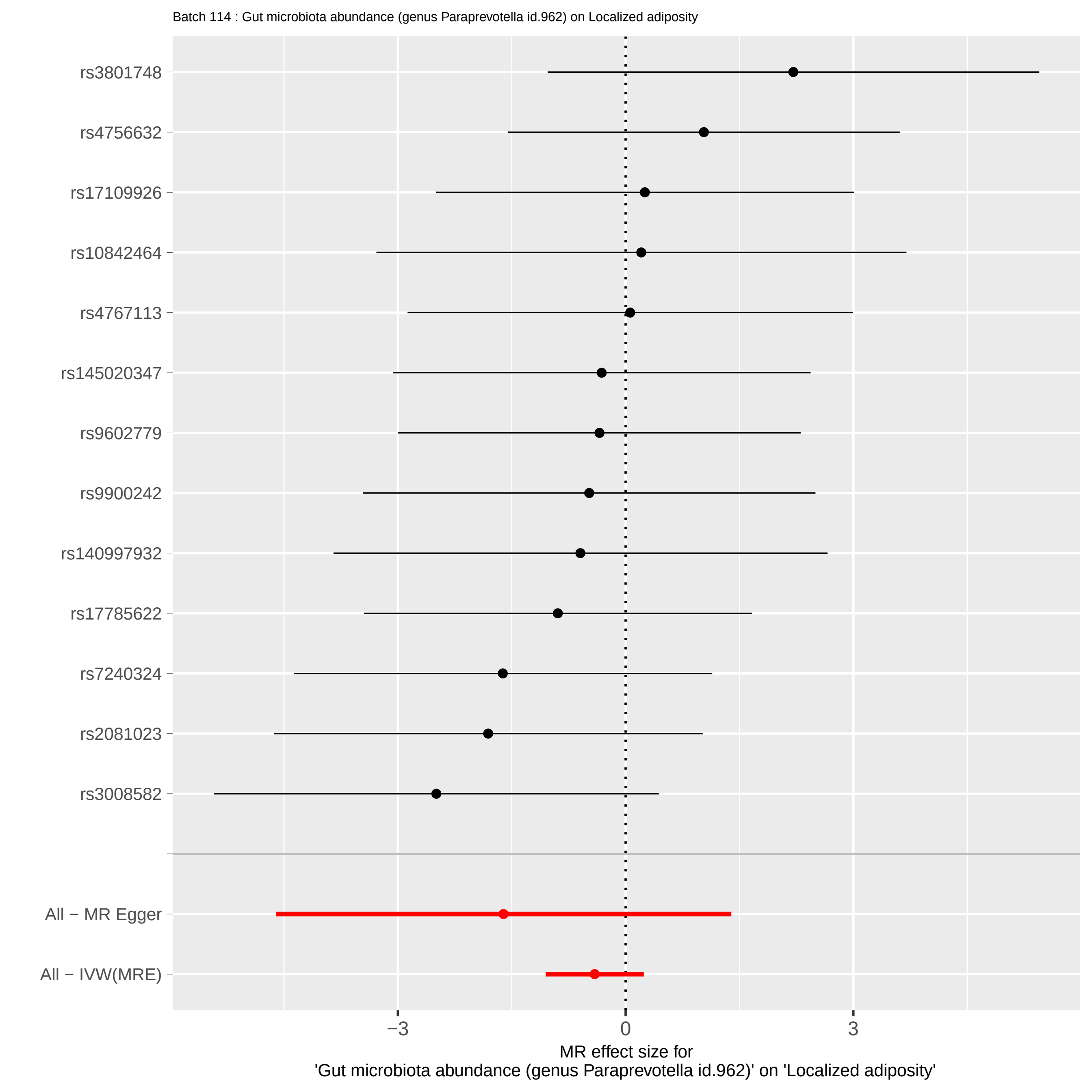
Batch 114 : Gut microbiota abundance (genus Paraprevotella id.962) on Localized adiposity
rs3801748
rs4756632
rs17109926
rs10842464
rs4767113
rs145020347
rs9602779
rs9900242
rs140997932
rs17785622
rs7240324
rs2081023
rs3008582
All − MR Egger
All − IVW(MRE)
−3
0
3
MR effect size for
'Gut microbiota abundance (genus Paraprevotella id.962)' on 'Localized adiposity'

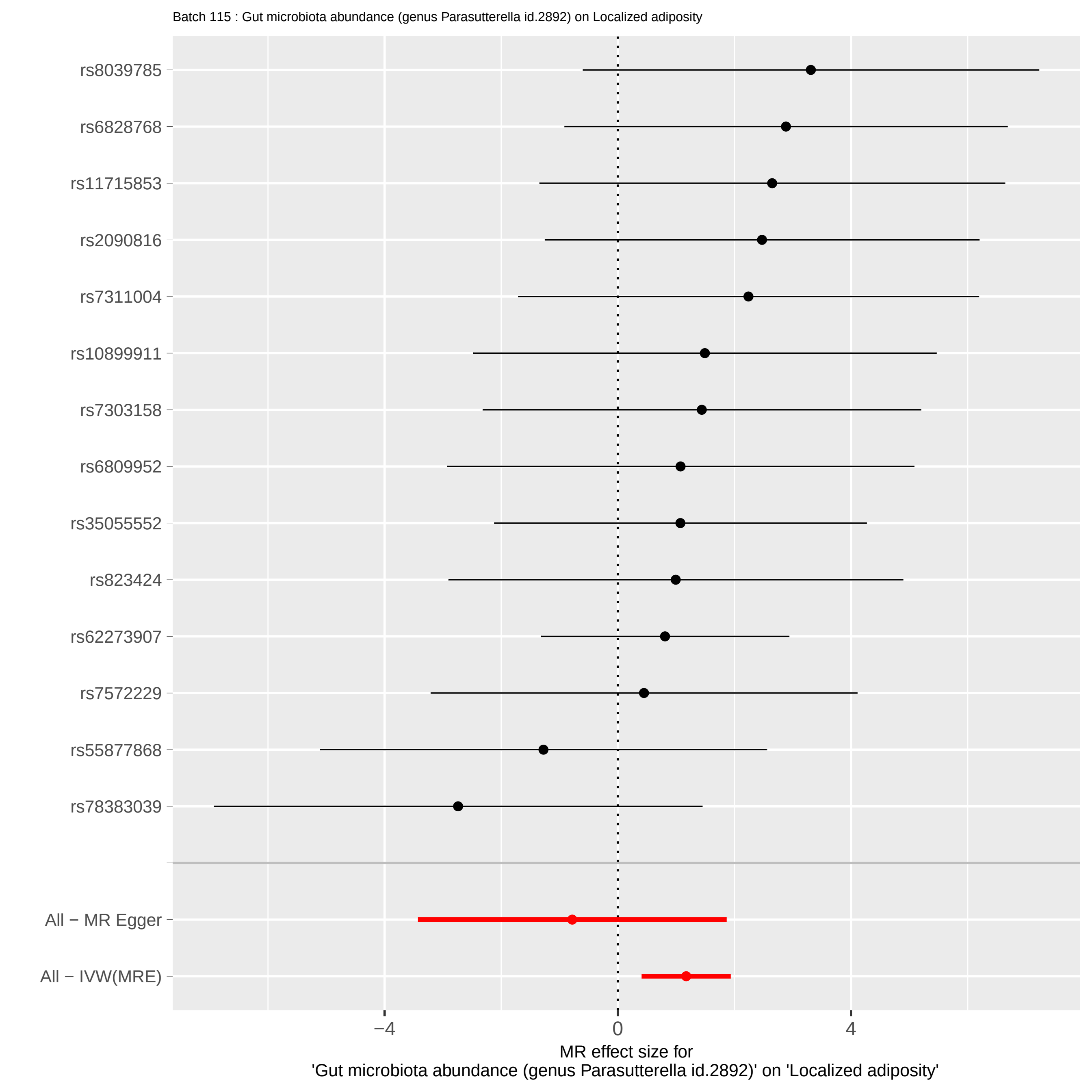
Batch 115 : Gut microbiota abundance (genus Parasutterella id.2892) on Localized adiposity
rs8039785
rs6828768
rs11715853
rs2090816
rs7311004
rs10899911
rs7303158
rs6809952
rs35055552
rs823424
rs62273907
rs7572229
rs55877868
rs78383039
All − MR Egger
All − IVW(MRE)
−4
0
4
MR effect size for
'Gut microbiota abundance (genus Parasutterella id.2892)' on 'Localized adiposity'

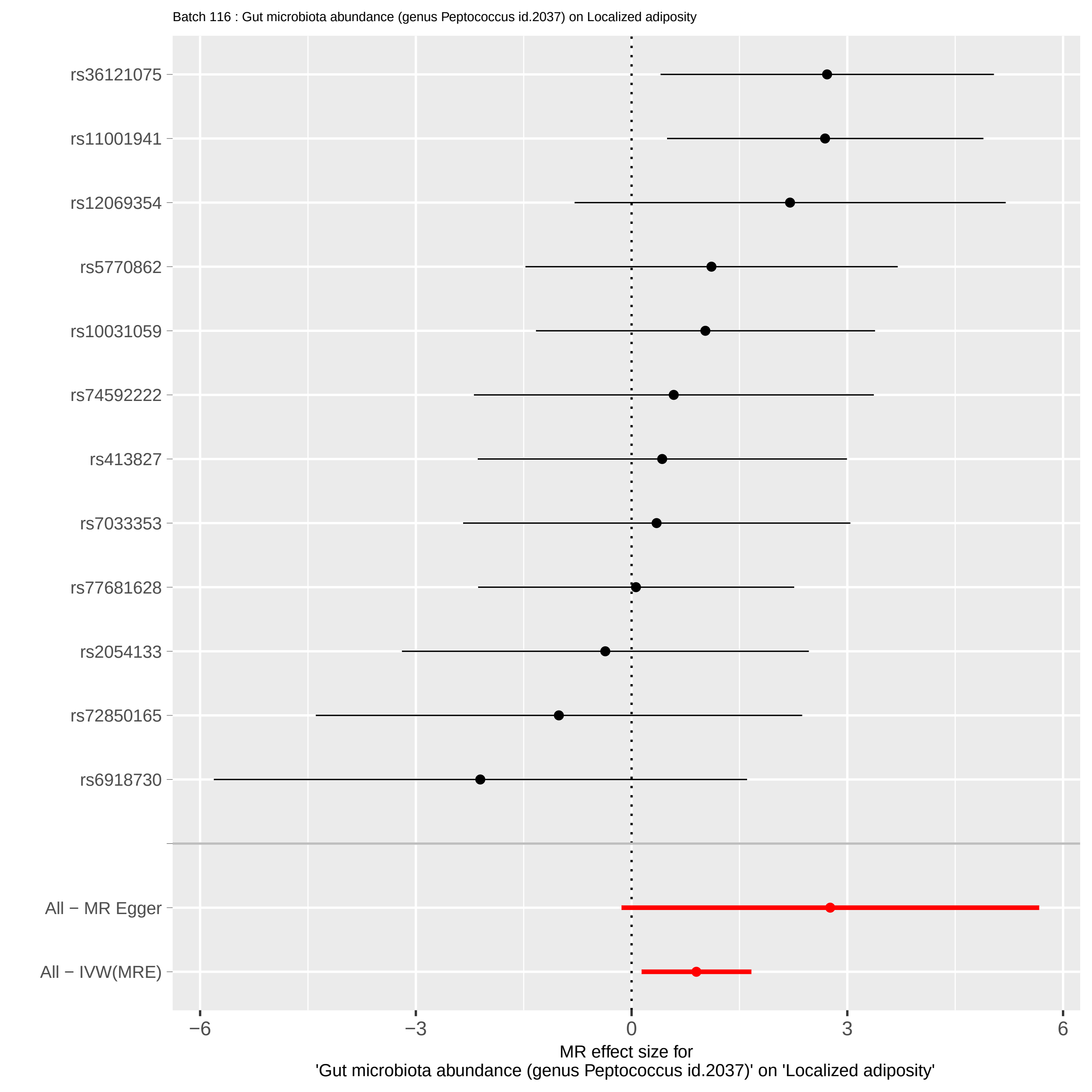

Batch 116 : Gut microbiota abundance (genus Peptococcus id.2037) on Localized adiposity
rs36121075
rs11001941
rs12069354
rs5770862
rs10031059
rs74592222
rs413827
rs7033353
rs77681628
rs2054133
rs72850165
rs6918730
All − MR Egger
All − IVW(MRE)
−6
−3
0
3
6
MR effect size for
'Gut microbiota abundance (genus Peptococcus id.2037)' on 'Localized adiposity'

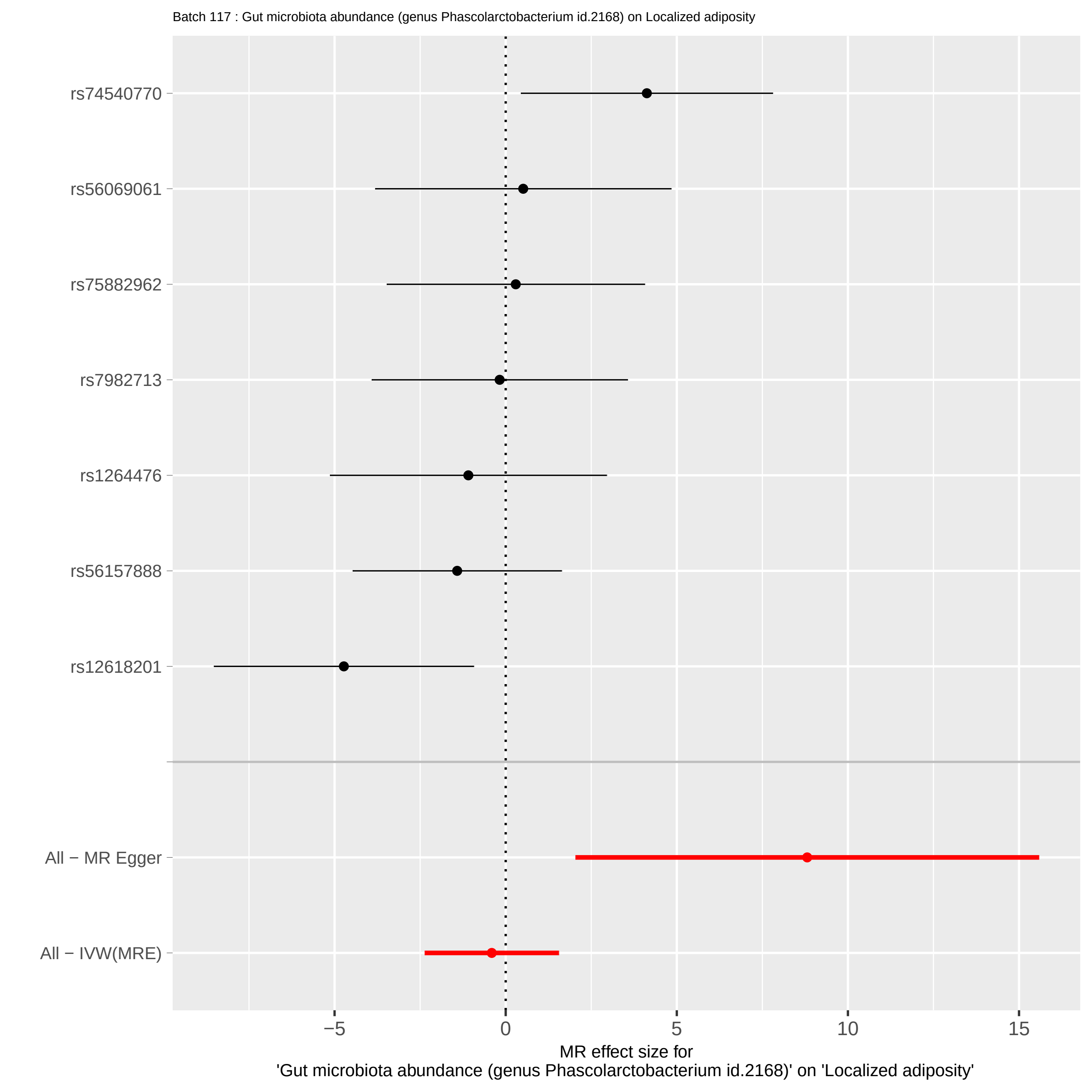
Batch 117 : Gut microbiota abundance (genus Phascolarctobacterium id.2168) on Localized adiposity
rs74540770
rs56069061
rs75882962
rs7982713
rs1264476
rs56157888
rs12618201
All − MR Egger
All − IVW(MRE)
−5
0
5
10
15
MR effect size for
'Gut microbiota abundance (genus Phascolarctobacterium id.2168)' on 'Localized adiposity'

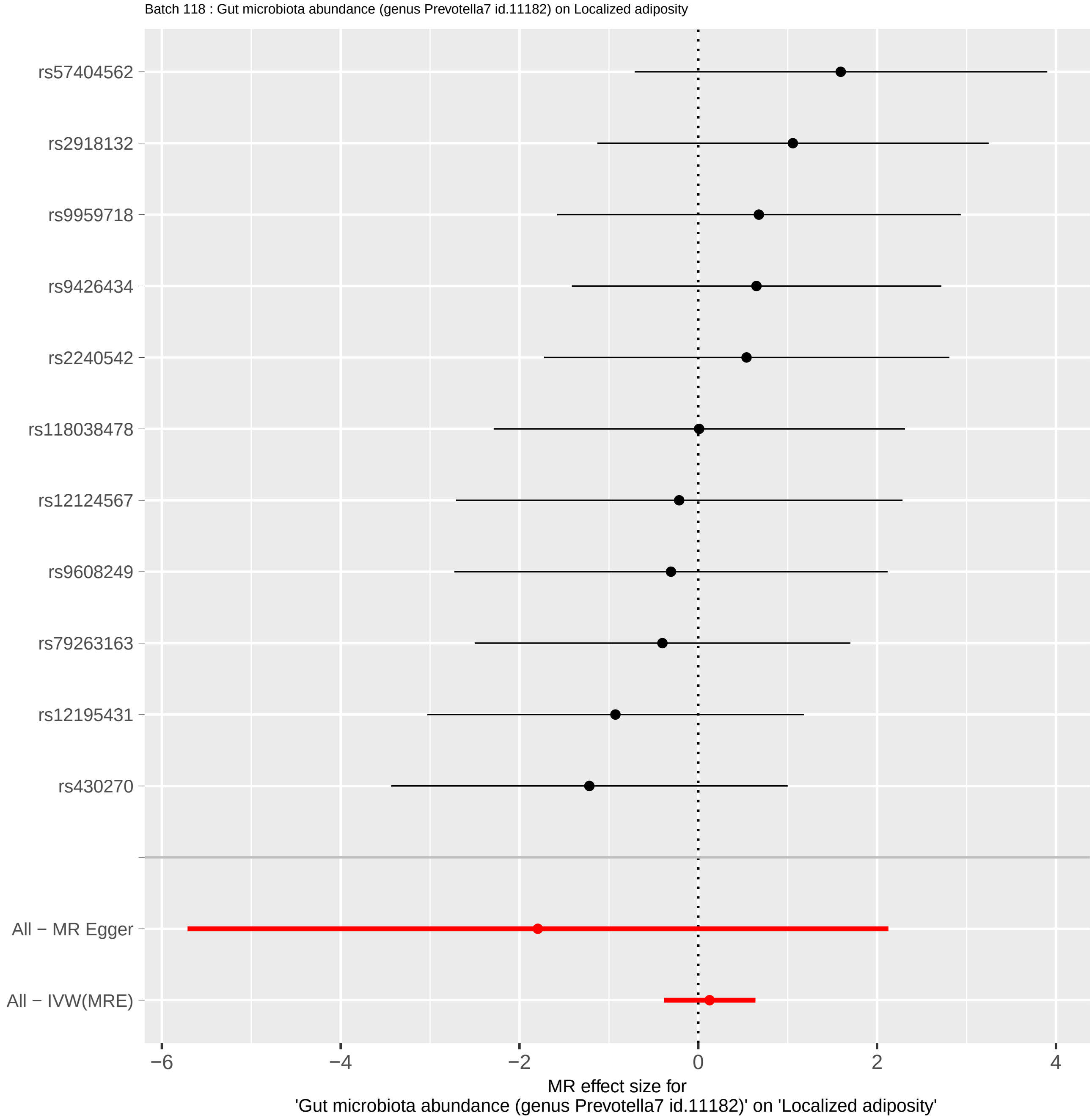

Batch 118 : Gut microbiota abundance (genus Prevotella7 id.11182) on Localized adiposity
rs57404562
rs2918132
rs9959718
rs9426434
rs2240542
rs118038478
rs12124567
rs9608249
rs79263163
rs12195431
rs430270
All − MR Egger
All − IVW(MRE)
−6
−4
−2
0
2
4
MR effect size for
'Gut microbiota abundance (genus Prevotella7 id.11182)' on 'Localized adiposity'

Batch 119 : Gut microbiota abundance (genus Prevotella9 id.11183) on Localized adiposity

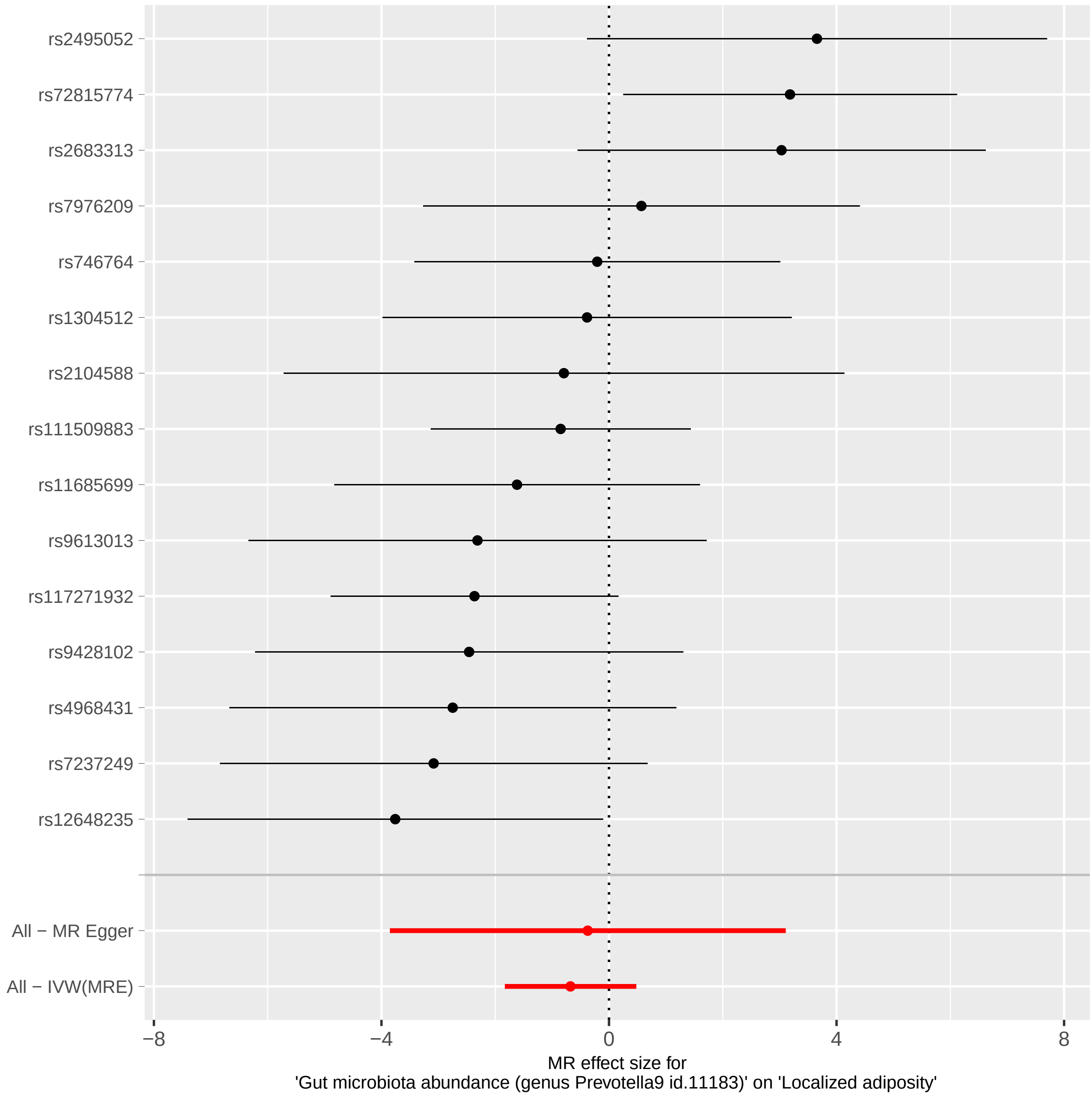

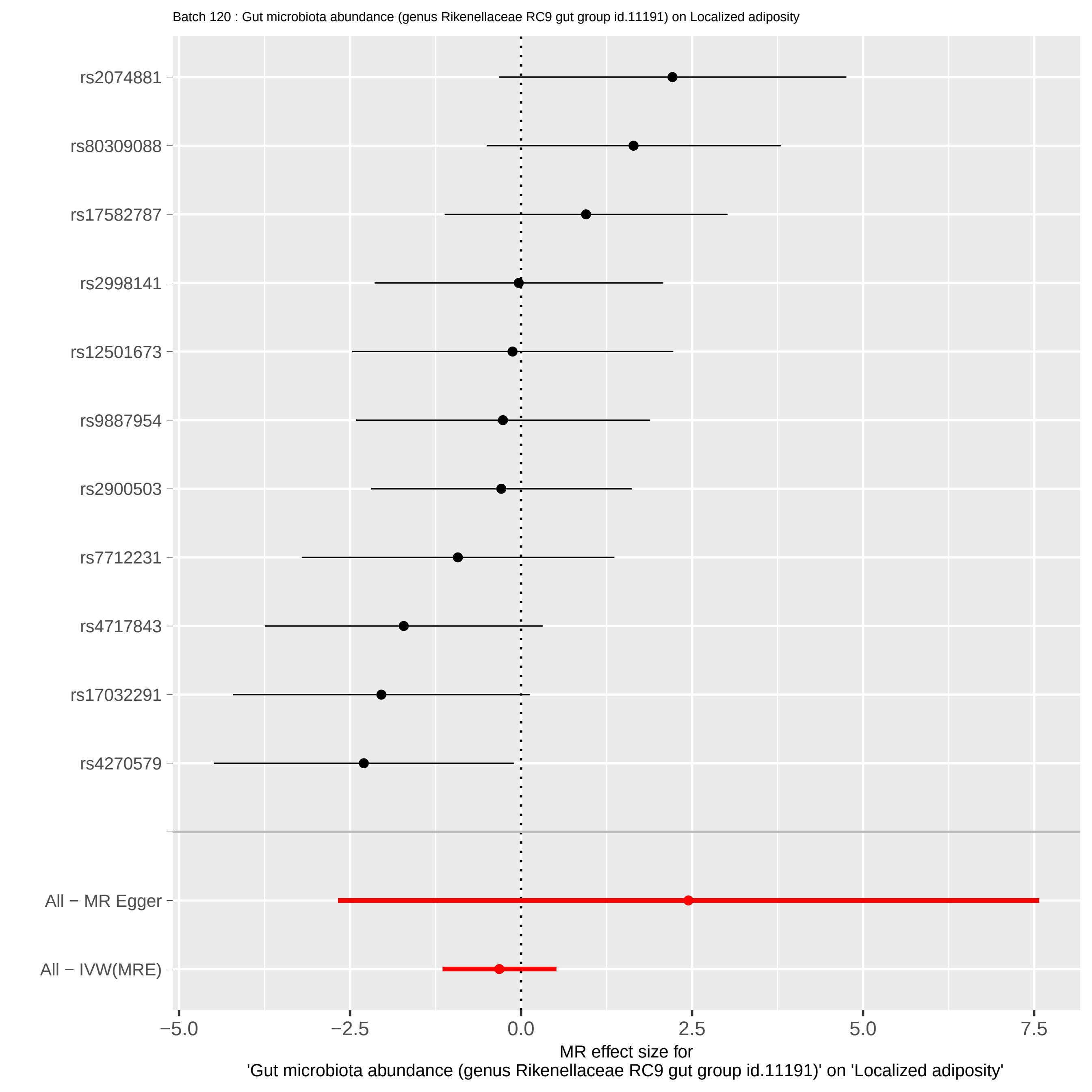
Batch 120 : Gut microbiota abundance (genus Rikenellaceae RC9 gut group id.11191) on Localized adiposity
rs2074881
rs80309088
rs17582787
rs2998141
rs12501673
rs9887954
rs2900503
rs7712231
rs4717843
rs17032291
rs4270579
All − MR Egger
All − IVW(MRE)
−5.0
−2.5
0.0
2.5
5.0
7.5
MR effect size for
'Gut microbiota abundance (genus Rikenellaceae RC9 gut group id.11191)' on 'Localized adiposity'

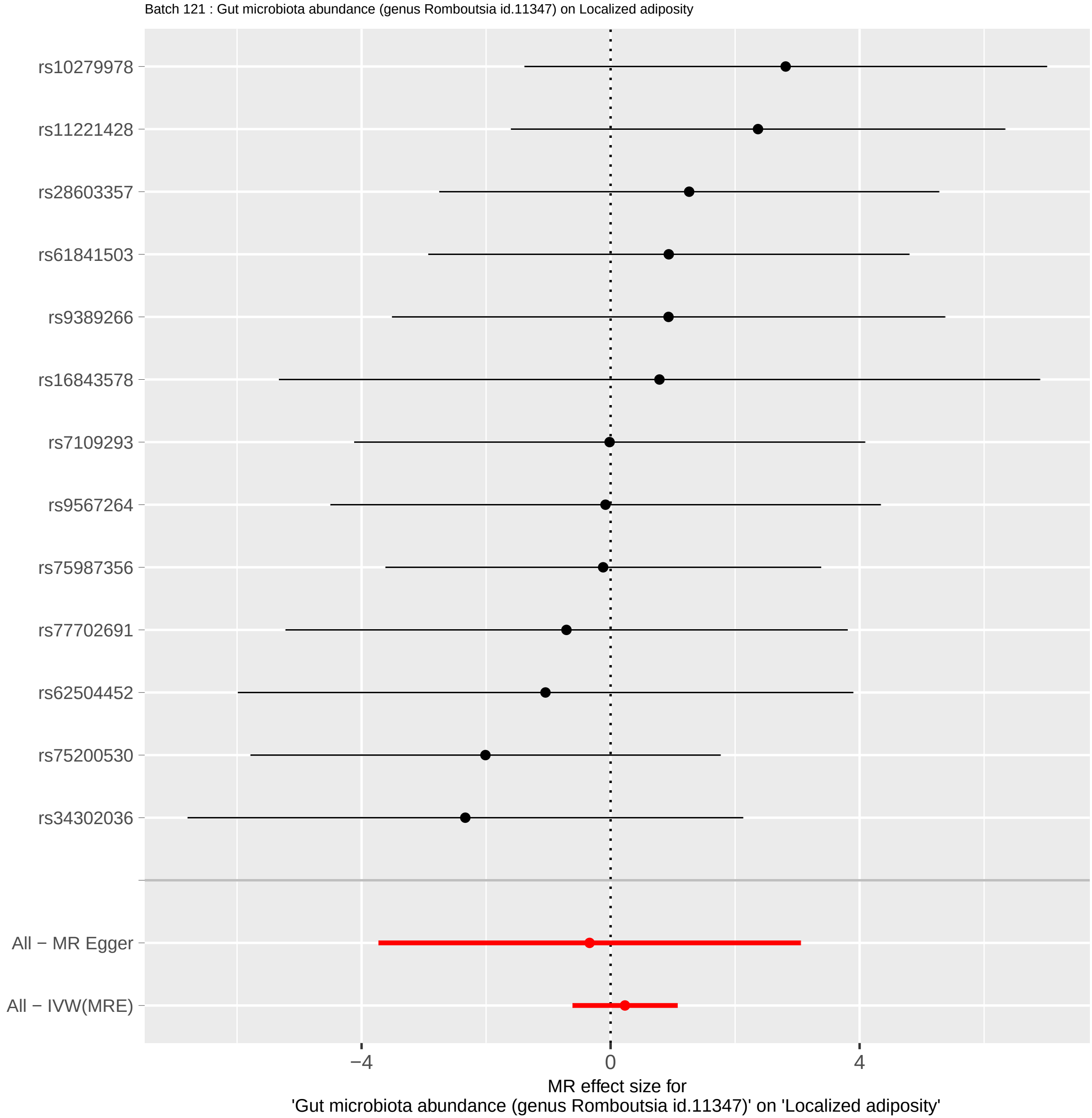

Batch 121 : Gut microbiota abundance (genus Romboutsia id.11347) on Localized adiposity
rs10279978
rs11221428
rs28603357
rs61841503
rs9389266
rs16843578
rs7109293
rs9567264
rs75987356
rs77702691
rs62504452
rs75200530
rs34302036
All − MR Egger
All − IVW(MRE)
−4
0
4
MR effect size for
'Gut microbiota abundance (genus Romboutsia id.11347)' on 'Localized adiposity'

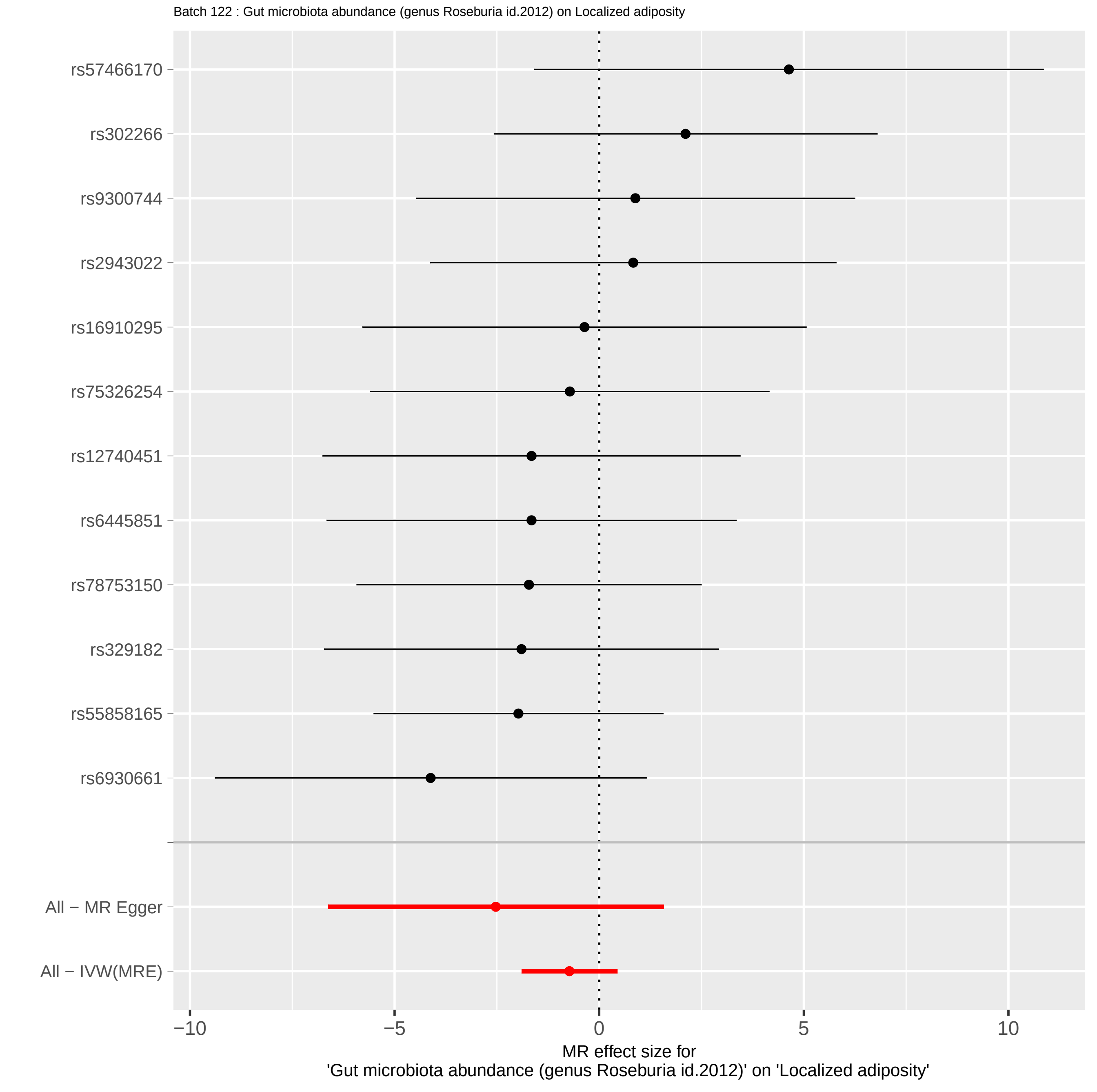

Batch 122 : Gut microbiota abundance (genus Roseburia id.2012) on Localized adiposity
rs57466170
rs302266
rs9300744
rs2943022
rs16910295
rs75326254
rs12740451
rs6445851
rs78753150
rs329182
rs55858165
rs6930661
All − MR Egger
All − IVW(MRE)
−10
−5
0
5
10
MR effect size for
'Gut microbiota abundance (genus Roseburia id.2012)' on 'Localized adiposity'

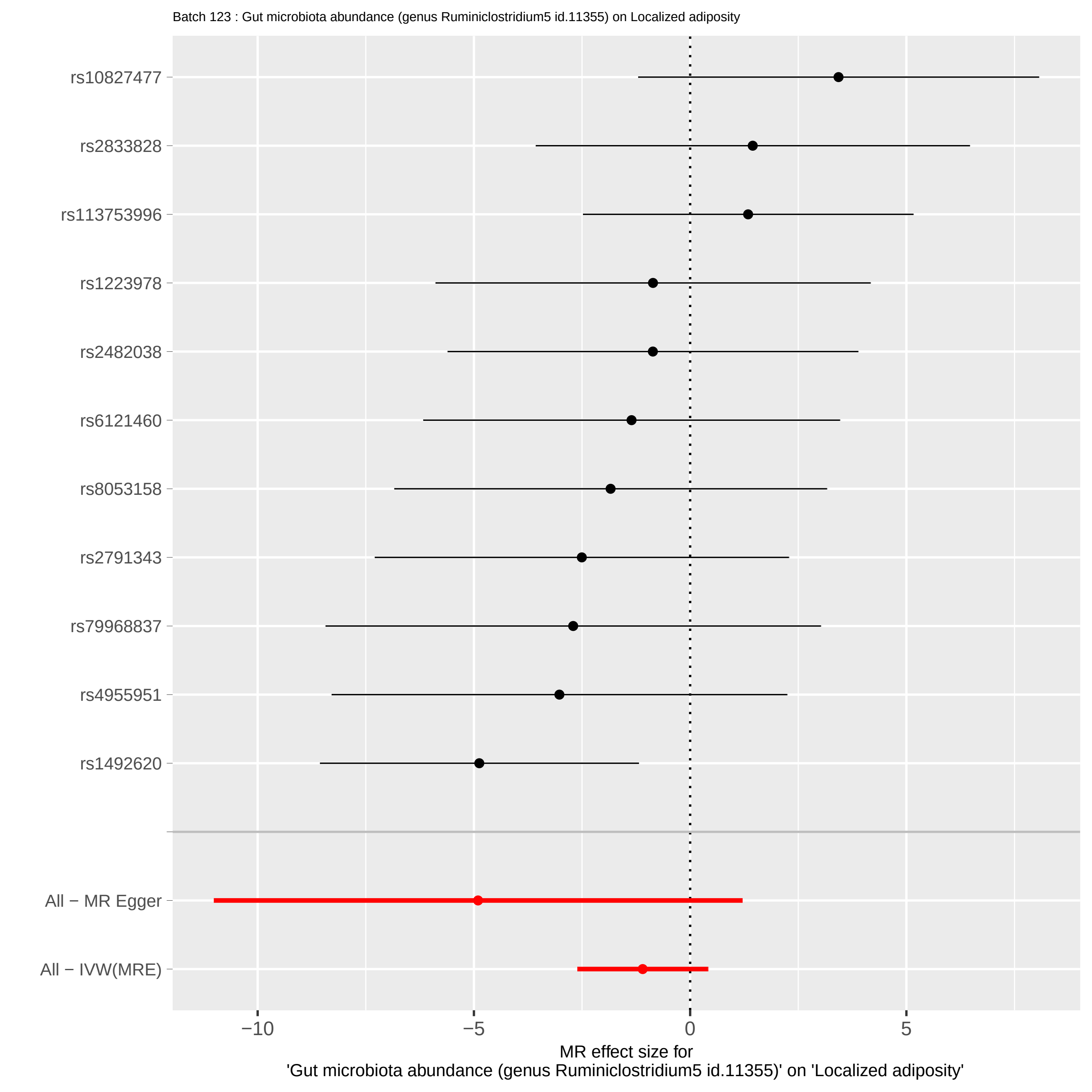
Batch 123 : Gut microbiota abundance (genus Ruminiclostridium5 id.11355) on Localized adiposity
rs10827477
rs2833828
rs113753996
rs1223978
rs2482038
rs6121460
rs8053158
rs2791343
rs79968837
rs4955951
rs1492620
All − MR Egger
All − IVW(MRE)
−10
−5
0
5
MR effect size for
'Gut microbiota abundance (genus Ruminiclostridium5 id.11355)' on 'Localized adiposity'

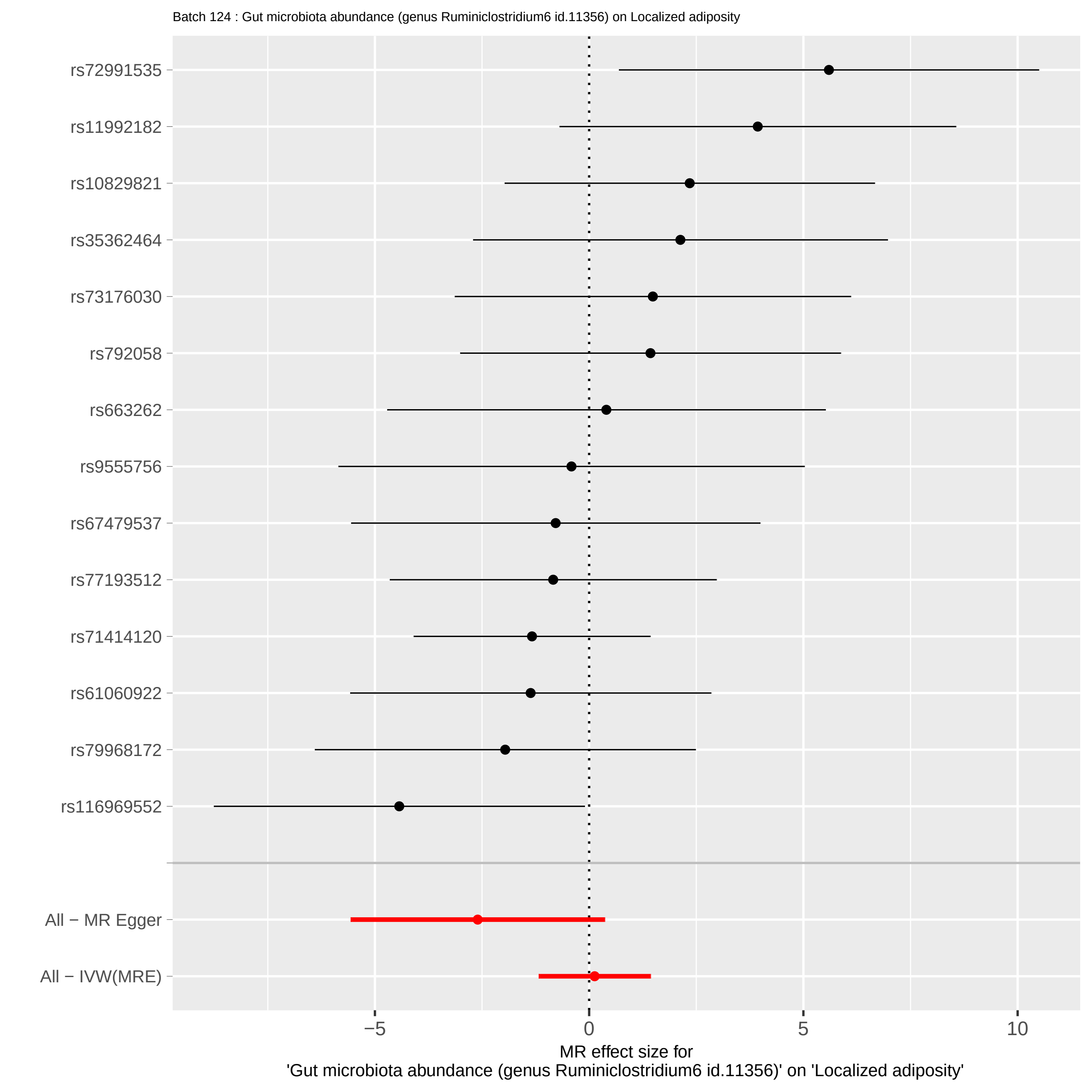
Batch 124 : Gut microbiota abundance (genus Ruminiclostridium6 id.11356) on Localized adiposity
rs72991535
rs11992182
rs10829821
rs35362464
rs73176030
rs792058
rs663262
rs9555756
rs67479537
rs77193512
rs71414120
rs61060922
rs79968172
rs116969552
All − MR Egger
All − IVW(MRE)
−5
0
5
10
MR effect size for
'Gut microbiota abundance (genus Ruminiclostridium6 id.11356)' on 'Localized adiposity'

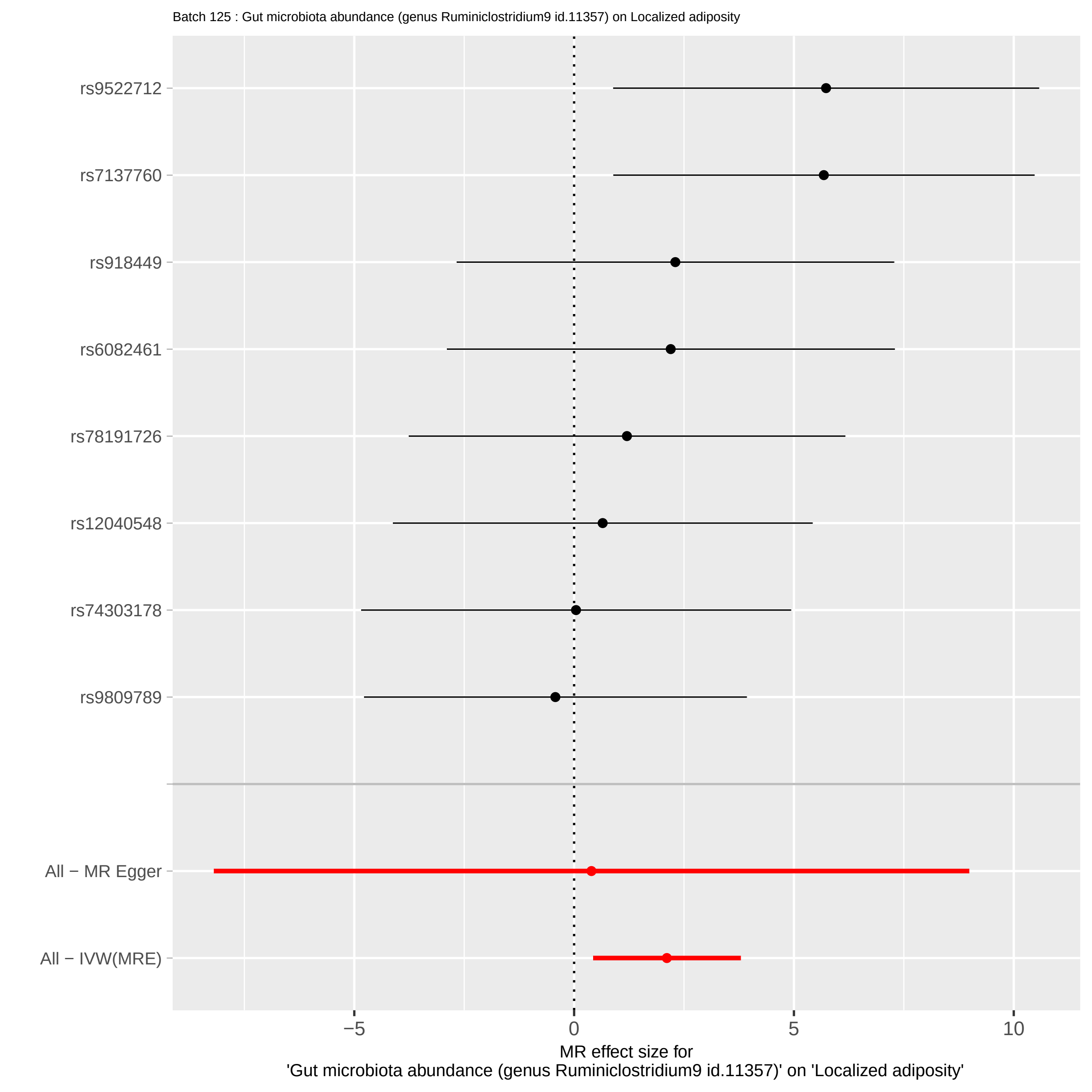

Batch 125 : Gut microbiota abundance (genus Ruminiclostridium9 id.11357) on Localized adiposity
rs9522712
rs7137760
rs918449
rs6082461
rs78191726
rs12040548
rs74303178
rs9809789
All − MR Egger
All − IVW(MRE)
−5
0
5
10
MR effect size for
'Gut microbiota abundance (genus Ruminiclostridium9 id.11357)' on 'Localized adiposity'

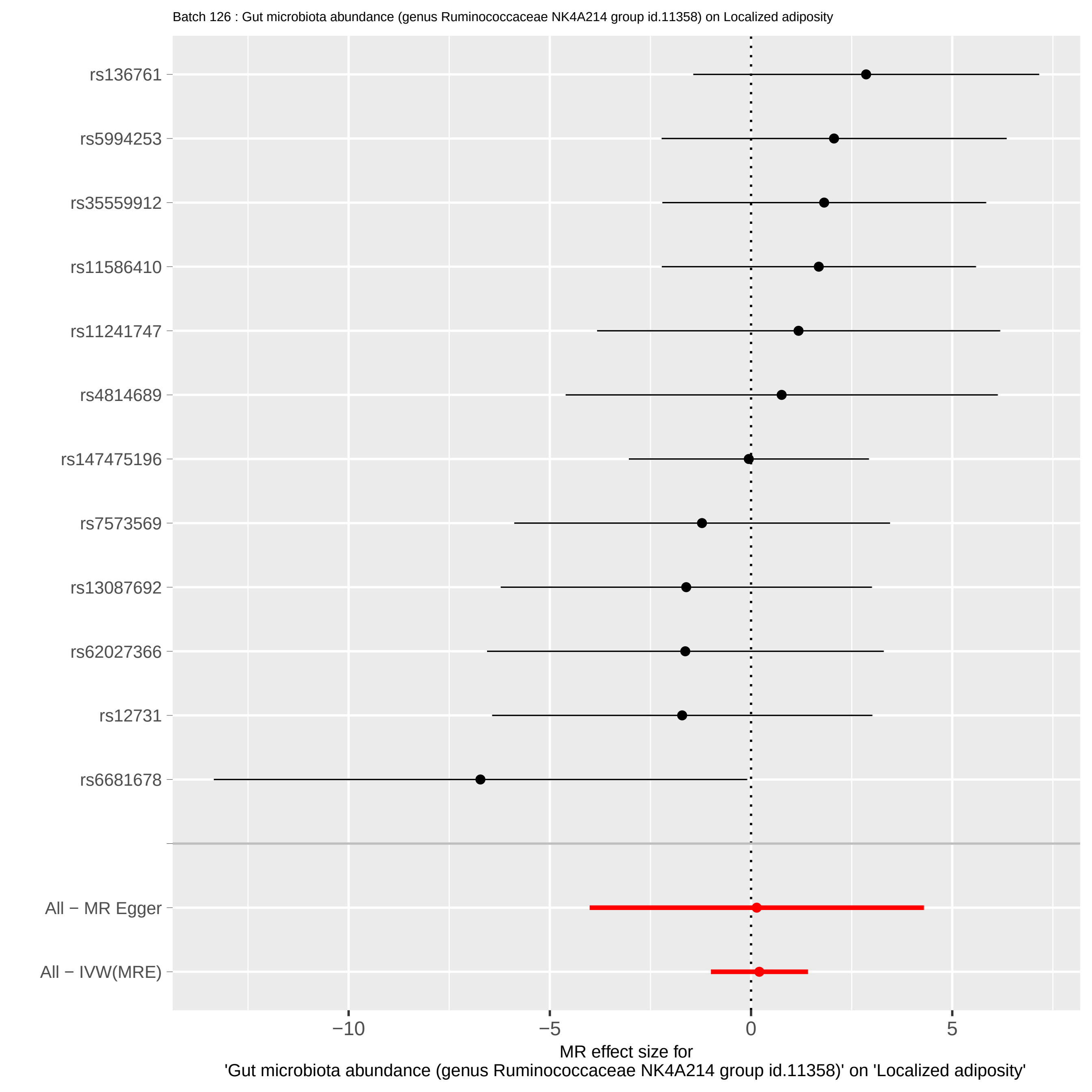
Batch 126 : Gut microbiota abundance (genus Ruminococcaceae NK4A214 group id.11358) on Localized adiposity
rs136761
rs5994253
rs35559912
rs11586410
rs11241747
rs4814689
rs147475196
rs7573569
rs13087692
rs62027366
rs12731
rs6681678
All − MR Egger
All − IVW(MRE)
−10
−5
0
5
MR effect size for
'Gut microbiota abundance (genus Ruminococcaceae NK4A214 group id.11358)' on 'Localized adiposity'

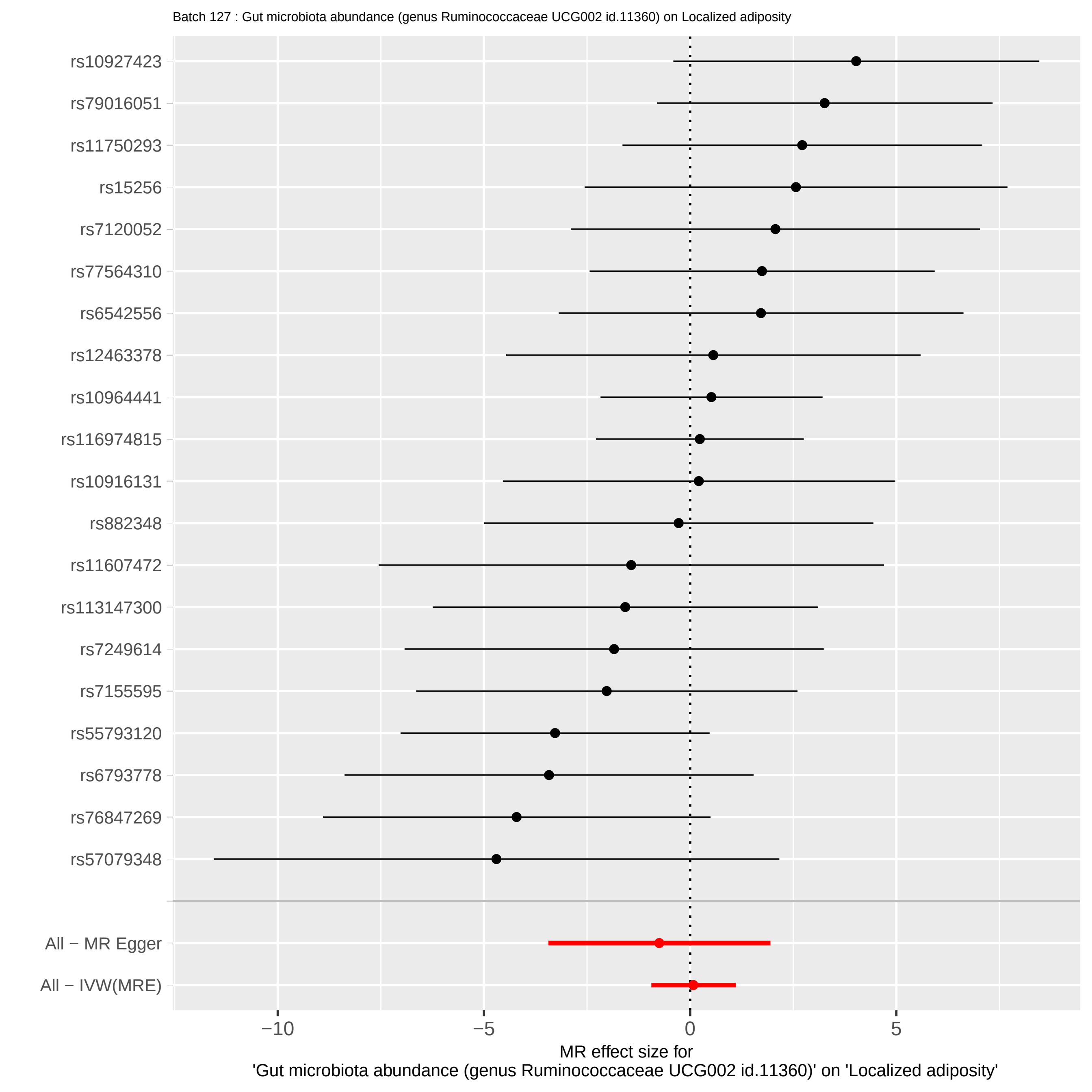

Batch 127 : Gut microbiota abundance (genus Ruminococcaceae UCG002 id.11360) on Localized adiposity
rs10927423
rs79016051
rs11750293
rs15256
rs7120052
rs77564310
rs6542556
rs12463378
rs10964441
rs116974815
rs10916131
rs882348
rs11607472
rs113147300
rs7249614
rs7155595
rs55793120
rs6793778
rs76847269
rs57079348
All − MR Egger
All − IVW(MRE)
−10
−5
0
5
MR effect size for
'Gut microbiota abundance (genus Ruminococcaceae UCG002 id.11360)' on 'Localized adiposity'

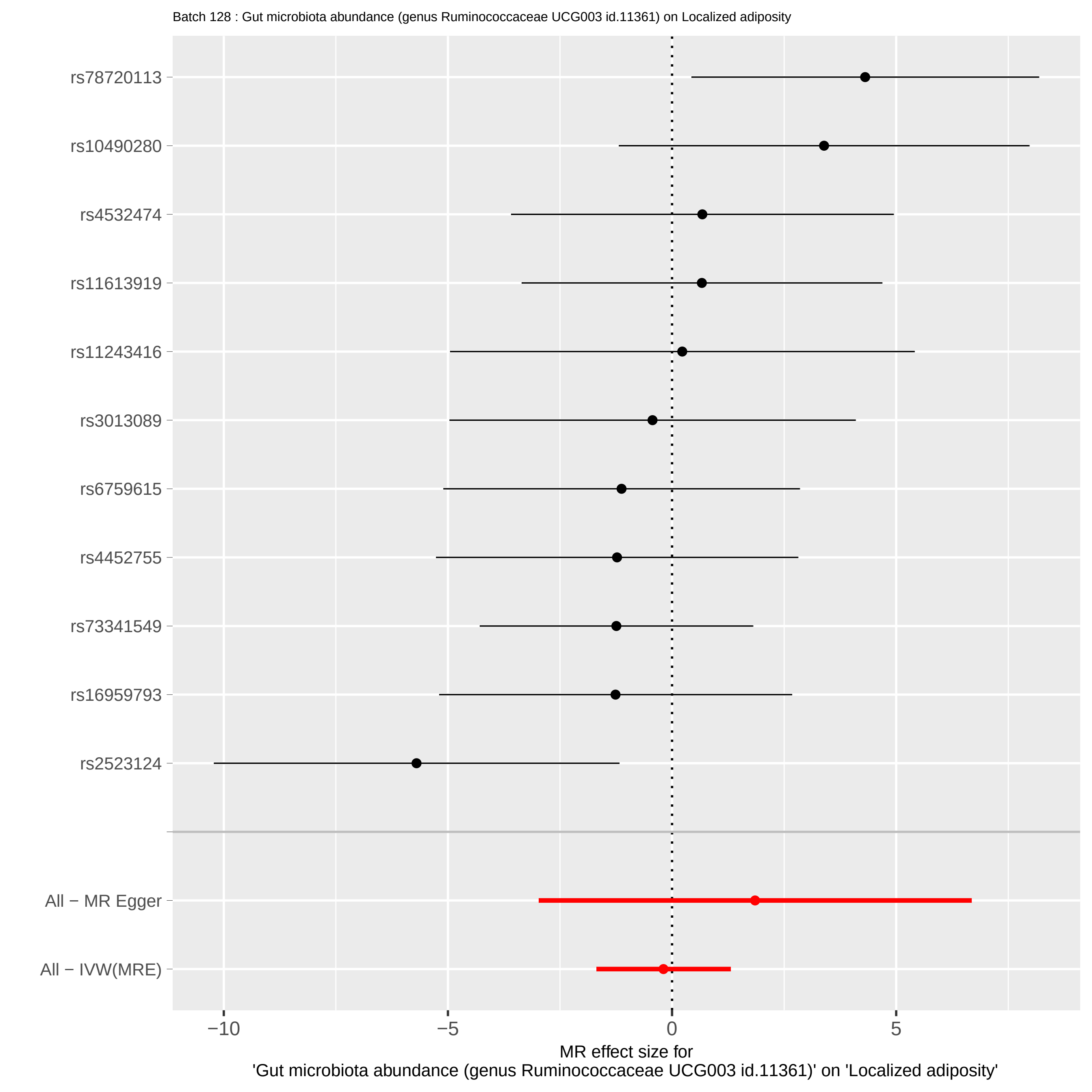

Batch 128 : Gut microbiota abundance (genus Ruminococcaceae UCG003 id.11361) on Localized adiposity
rs78720113
rs10490280
rs4532474
rs11613919
rs11243416
rs3013089
rs6759615
rs4452755
rs73341549
rs16959793
rs2523124
All − MR Egger
All − IVW(MRE)
−10
−5
0
5
MR effect size for
'Gut microbiota abundance (genus Ruminococcaceae UCG003 id.11361)' on 'Localized adiposity'

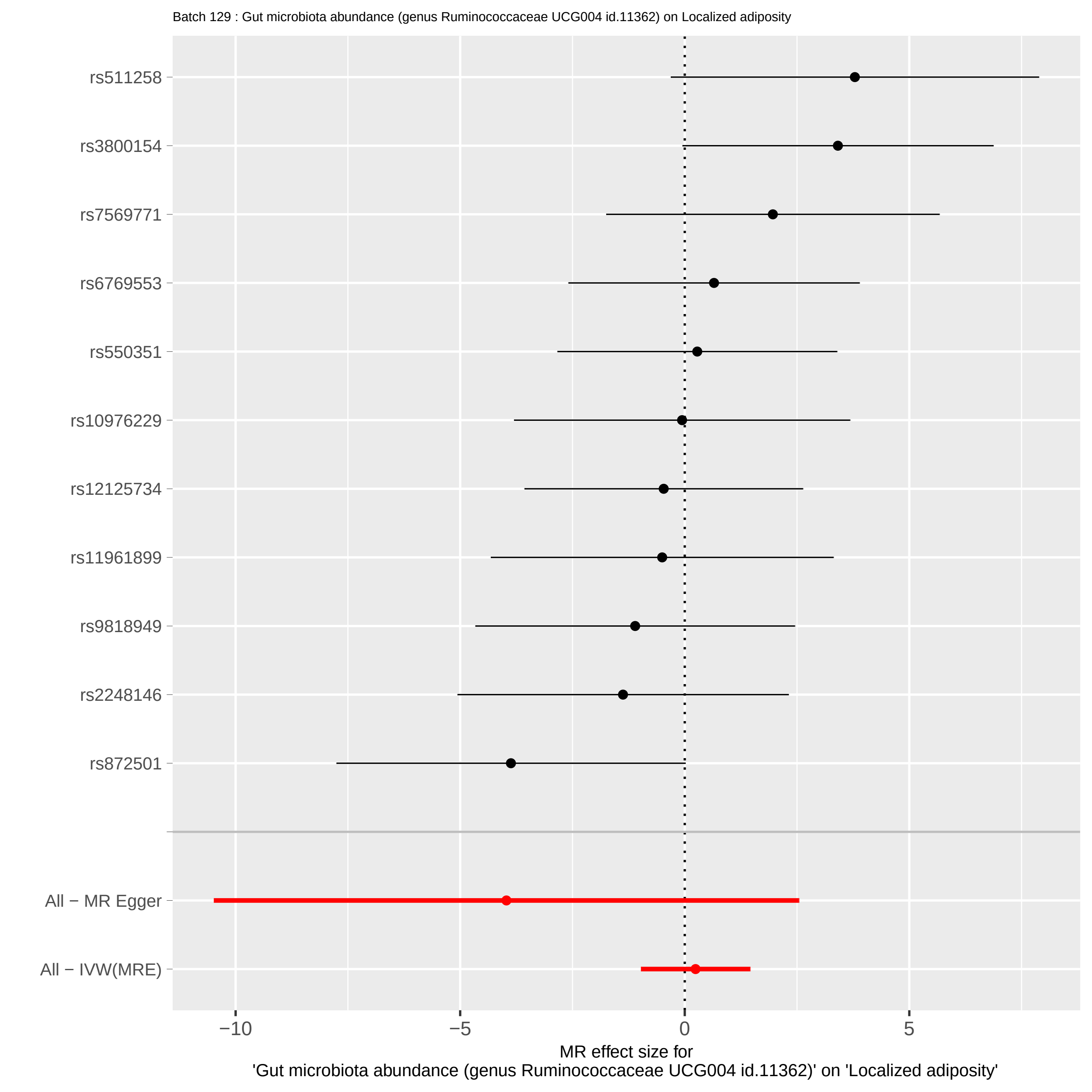
Batch 129 : Gut microbiota abundance (genus Ruminococcaceae UCG004 id.11362) on Localized adiposity
rs511258
rs3800154
rs7569771
rs6769553
rs550351
rs10976229
rs12125734
rs11961899
rs9818949
rs2248146
rs872501
All − MR Egger
All − IVW(MRE)
−10
−5
0
5
MR effect size for
'Gut microbiota abundance (genus Ruminococcaceae UCG004 id.11362)' on 'Localized adiposity'

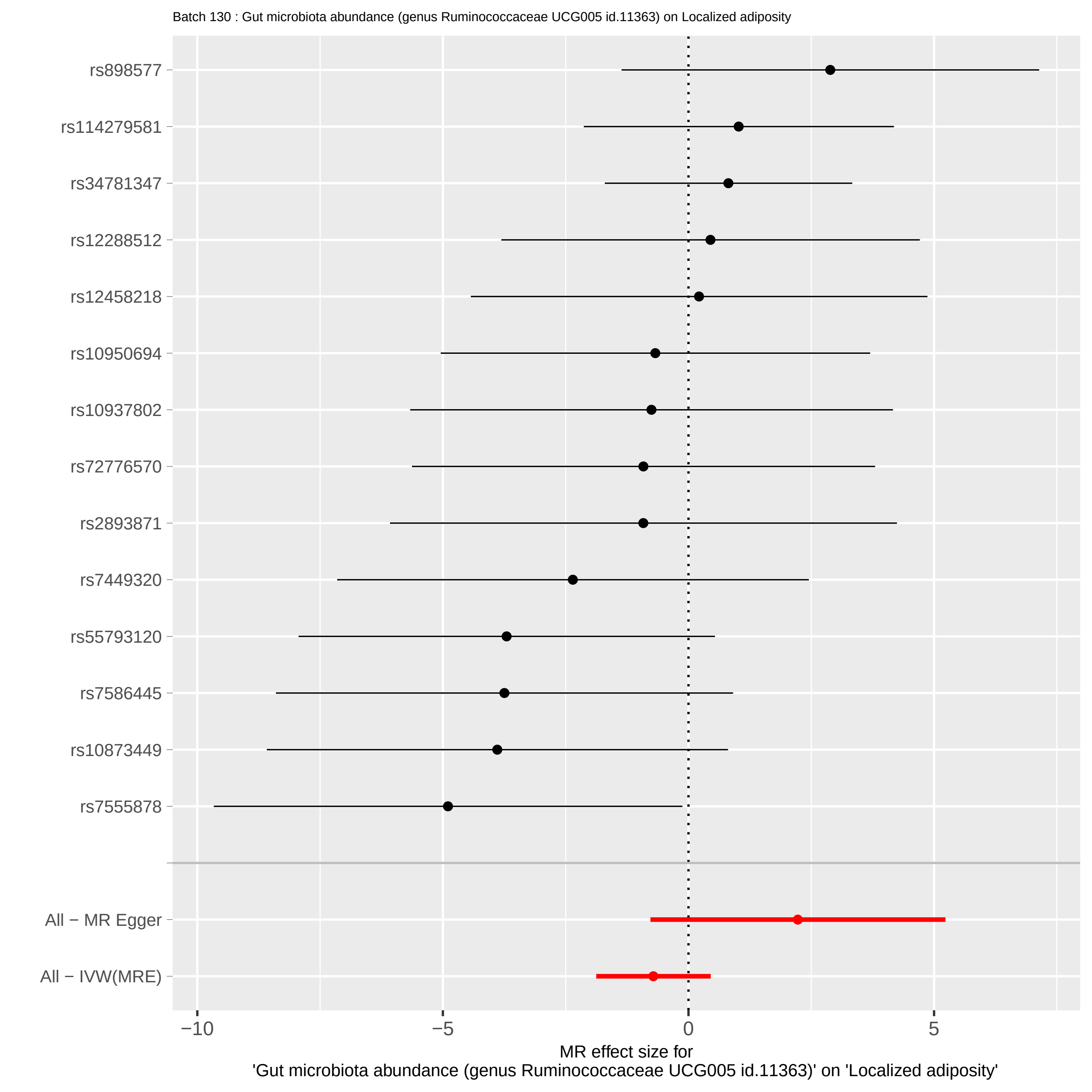

Batch 130 : Gut microbiota abundance (genus Ruminococcaceae UCG005 id.11363) on Localized adiposity
rs898577
rs114279581
rs34781347
rs12288512
rs12458218
rs10950694
rs10937802
rs72776570
rs2893871
rs7449320
rs55793120
rs7586445
rs10873449
rs7555878
All − MR Egger
All − IVW(MRE)
−10
−5
0
5
MR effect size for
'Gut microbiota abundance (genus Ruminococcaceae UCG005 id.11363)' on 'Localized adiposity'

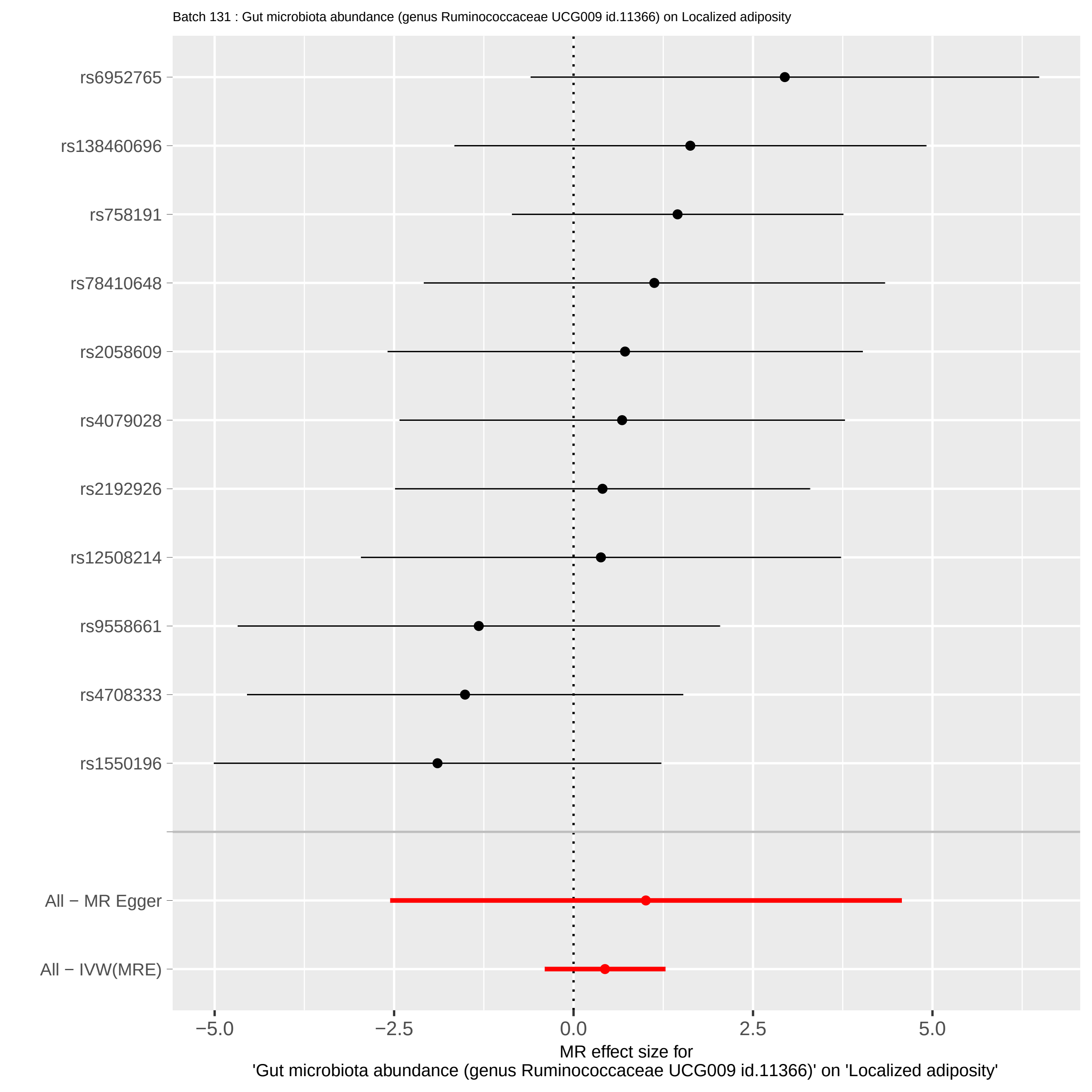

Batch 131 : Gut microbiota abundance (genus Ruminococcaceae UCG009 id.11366) on Localized adiposity
rs6952765
rs138460696
rs758191
rs78410648
rs2058609
rs4079028
rs2192926
rs12508214
rs9558661
rs4708333
rs1550196
All − MR Egger
All − IVW(MRE)
−5.0
−2.5
0.0
2.5
5.0
MR effect size for
'Gut microbiota abundance (genus Ruminococcaceae UCG009 id.11366)' on 'Localized adiposity'

Batch 132 : Gut microbiota abundance (genus Ruminococcaceae UCG010 id.11367) on Localized adiposity

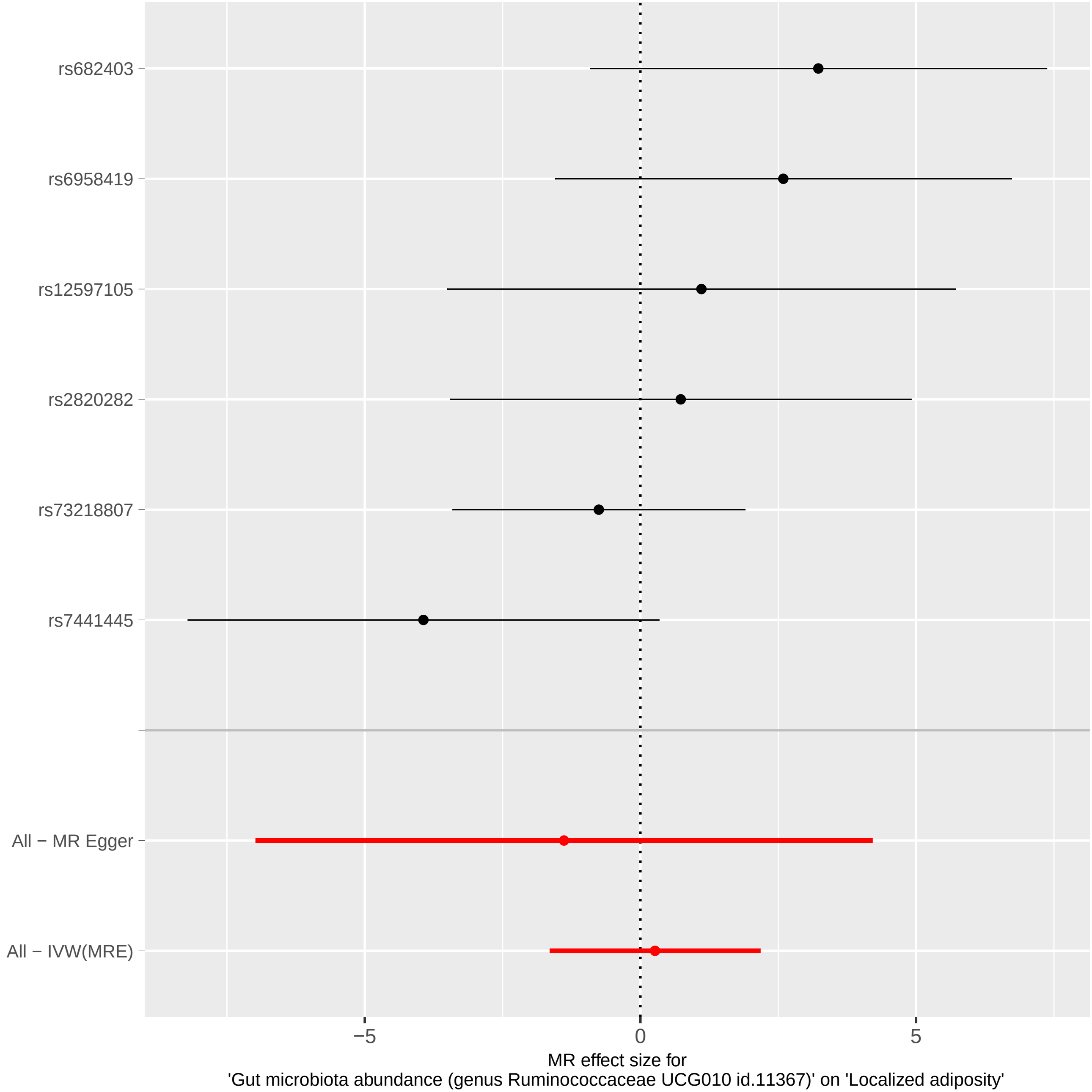

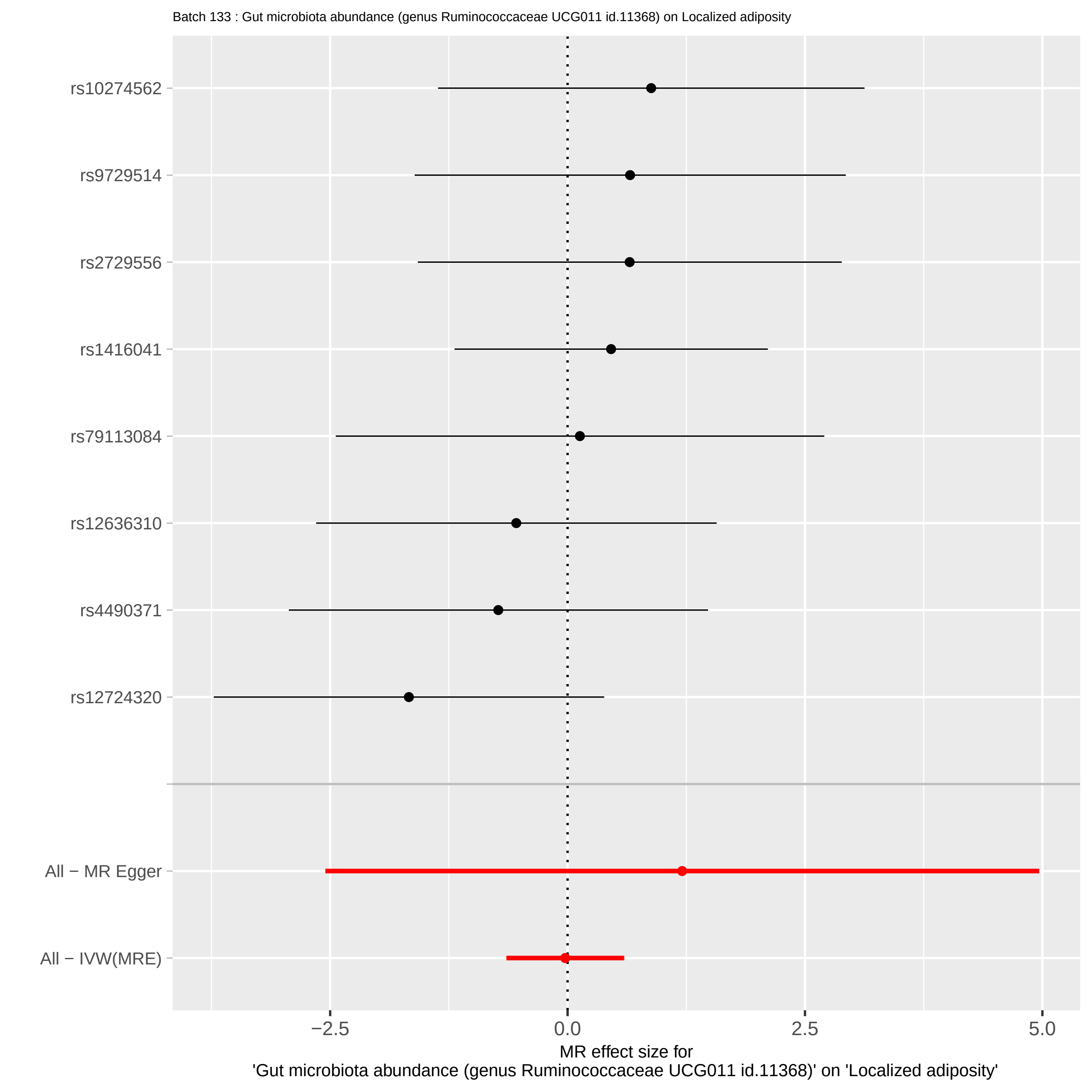

Batch 133 : Gut microbiota abundance (genus Ruminococcaceae UCG011 id.11368) on Localized adiposity
rs10274562
rs9729514
rs2729556
rs1416041
rs79113084
rs12636310
rs4490371
rs12724320
All − MR Egger
All − IVW(MRE)
−2.5
0.0
2.5
5.0
MR effect size for
'Gut microbiota abundance (genus Ruminococcaceae UCG011 id.11368)' on 'Localized adiposity'

Batch 134 : Gut microbiota abundance (genus Ruminococcaceae UCG013 id.11370) on Localized adiposity

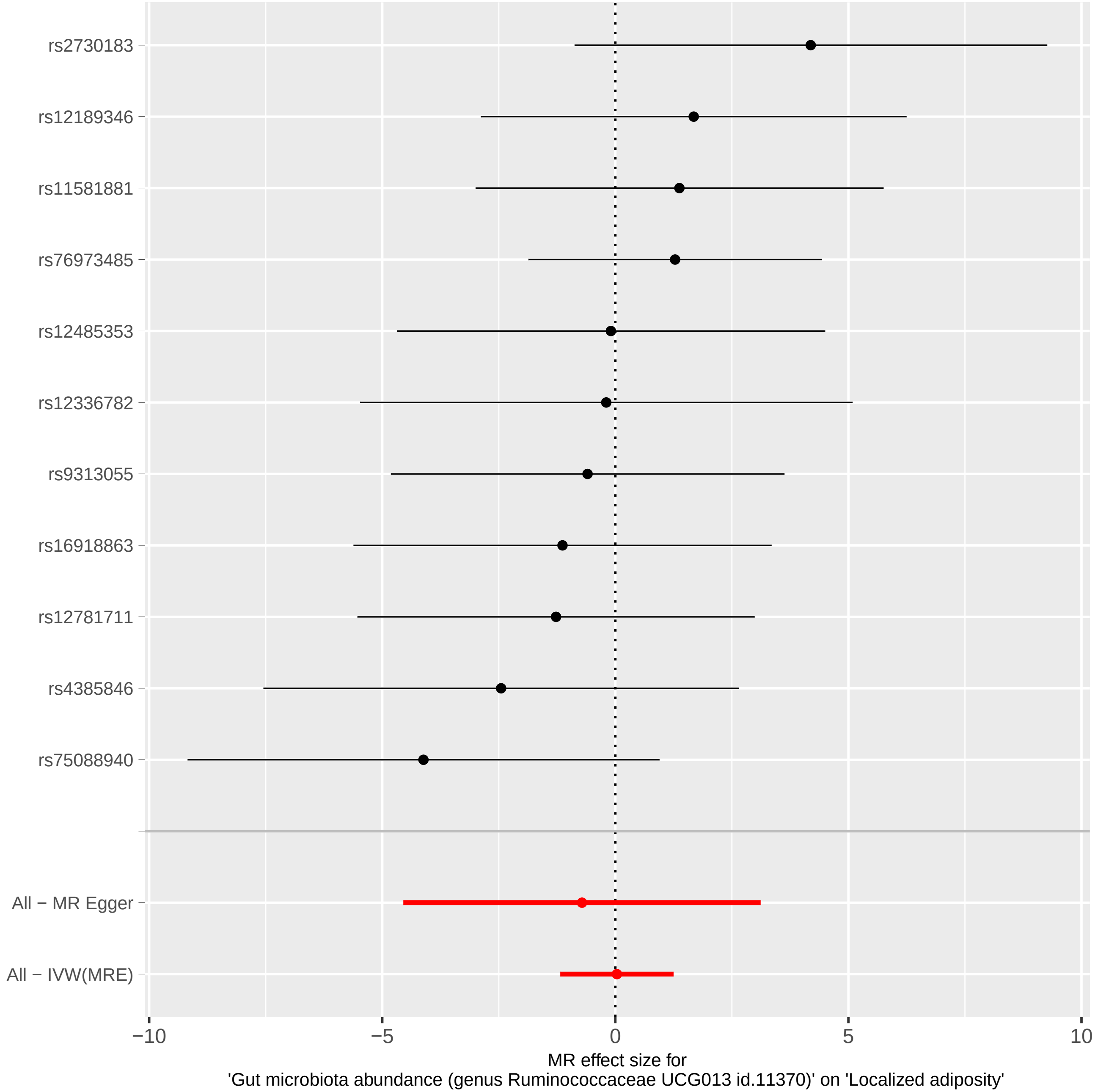

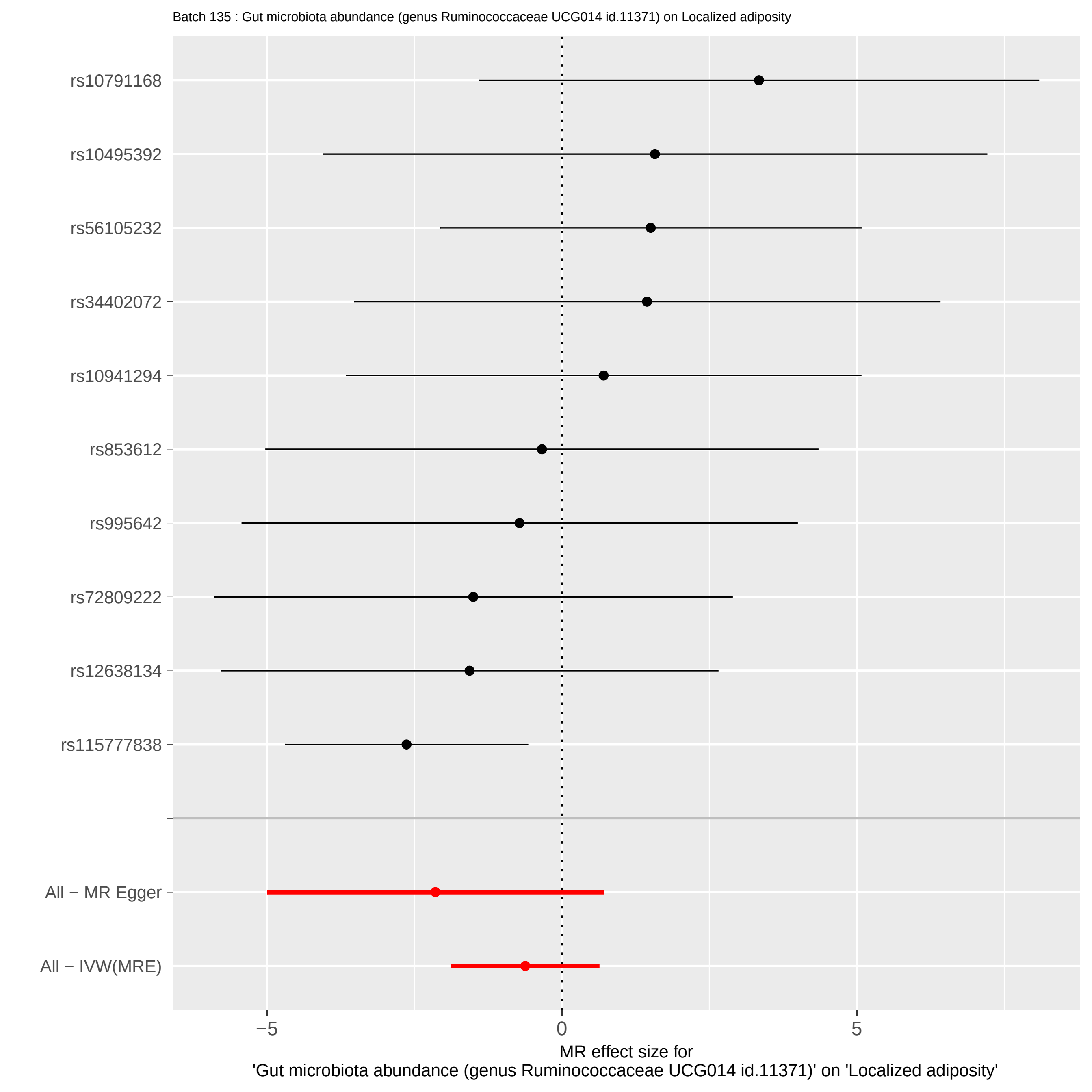

Batch 135 : Gut microbiota abundance (genus Ruminococcaceae UCG014 id.11371) on Localized adiposity
rs10791168
rs10495392
rs56105232
rs34402072
rs10941294
rs853612
rs995642
rs72809222
rs12638134
rs115777838
All − MR Egger
All − IVW(MRE)
−5
0
5
MR effect size for
'Gut microbiota abundance (genus Ruminococcaceae UCG014 id.11371)' on 'Localized adiposity'

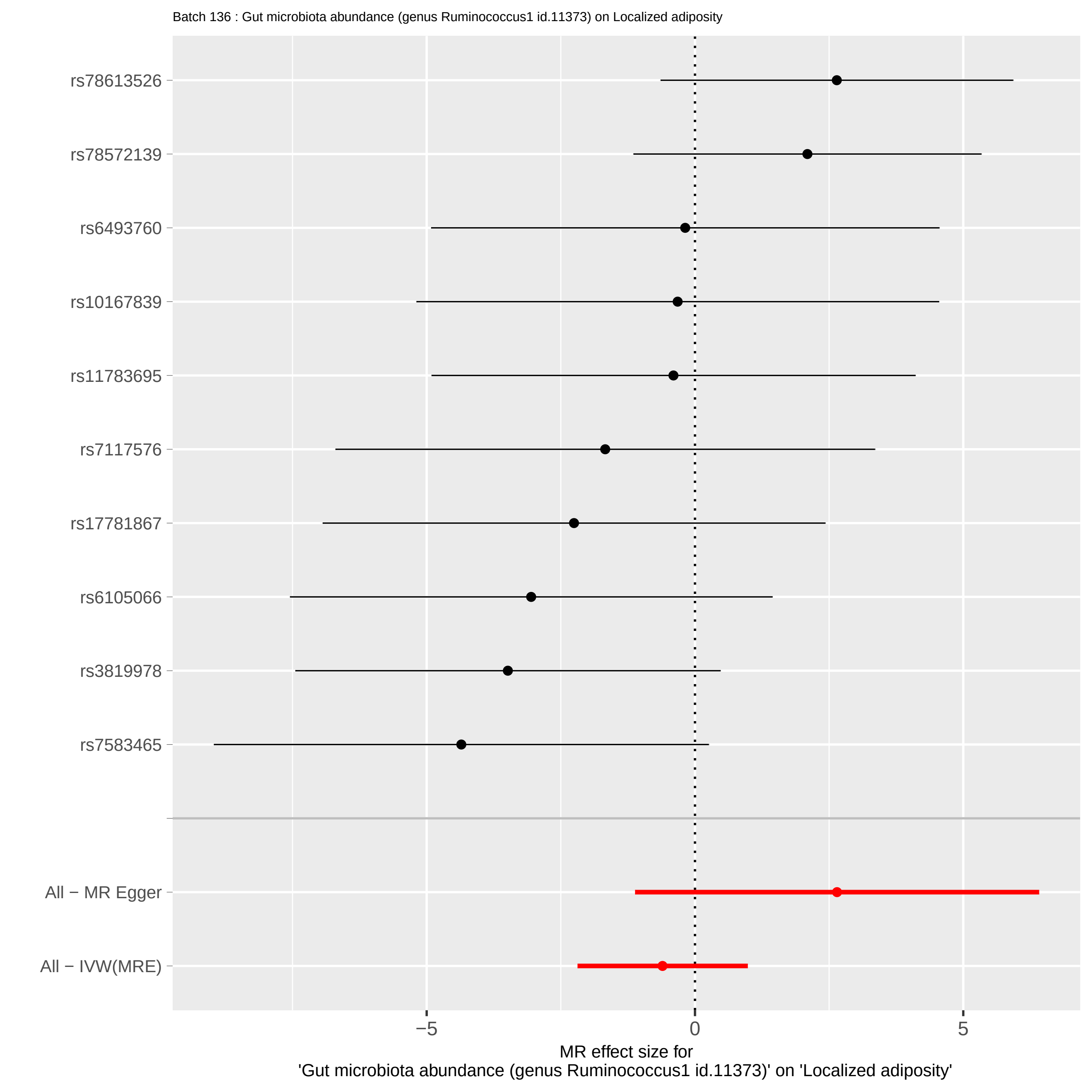

Batch 136 : Gut microbiota abundance (genus Ruminococcus1 id.11373) on Localized adiposity
rs78613526
rs78572139
rs6493760
rs10167839
rs11783695
rs7117576
rs17781867
rs6105066
rs3819978
rs7583465
All − MR Egger
All − IVW(MRE)
−5
0
5
MR effect size for
'Gut microbiota abundance (genus Ruminococcus1 id.11373)' on 'Localized adiposity'

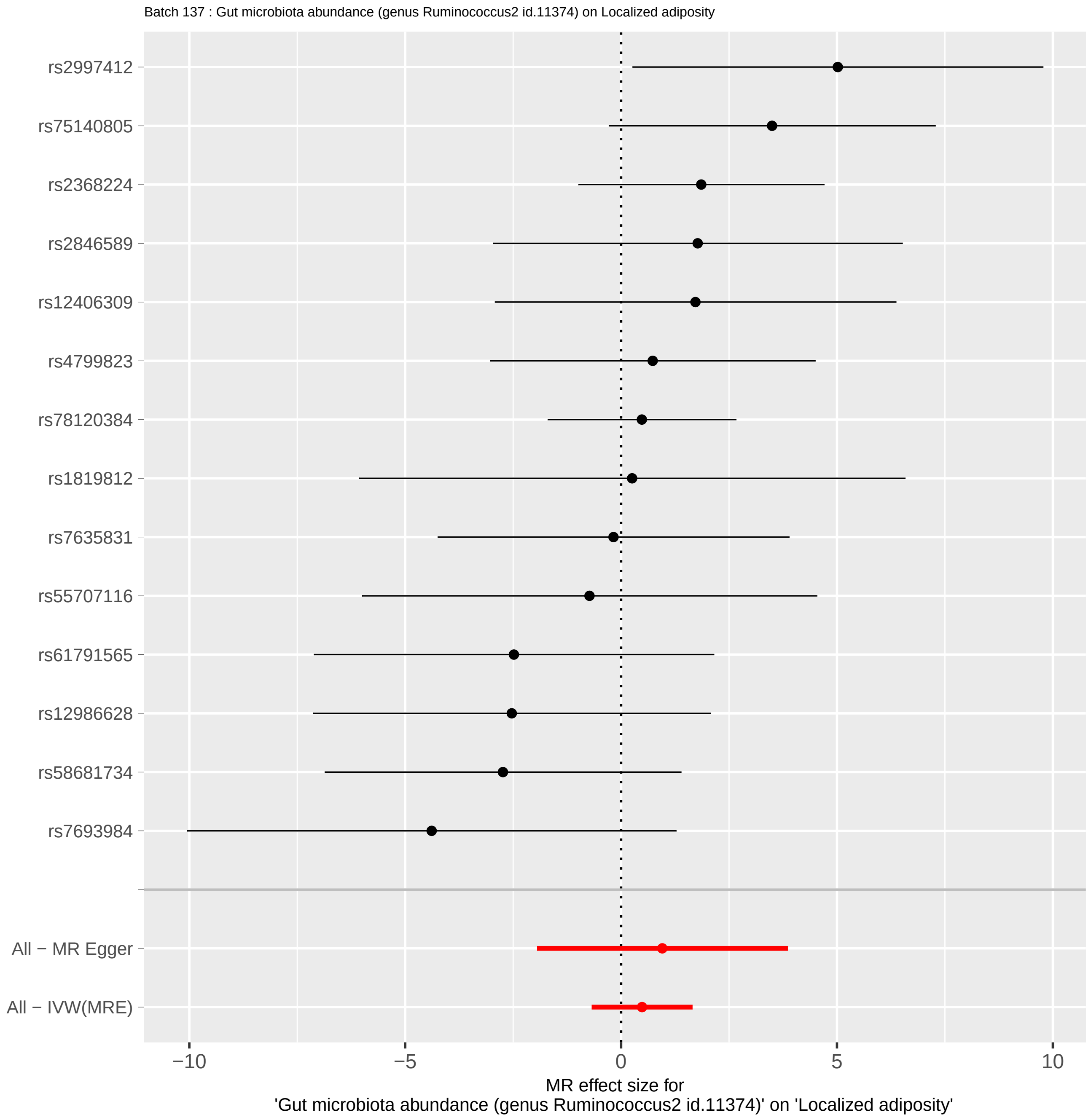

Batch 137 : Gut microbiota abundance (genus Ruminococcus2 id.11374) on Localized adiposity
rs2997412
rs75140805
rs2368224
rs2846589
rs12406309
rs4799823
rs78120384
rs1819812
rs7635831
rs55707116
rs61791565
rs12986628
rs58681734
rs7693984
All − MR Egger
All − IVW(MRE)
−10
−5
0
5
10
MR effect size for
'Gut microbiota abundance (genus Ruminococcus2 id.11374)' on 'Localized adiposity'

Batch 138 : Gut microbiota abundance (genus Ruminococcus gauvreauii group id.11342) on Localized adiposity

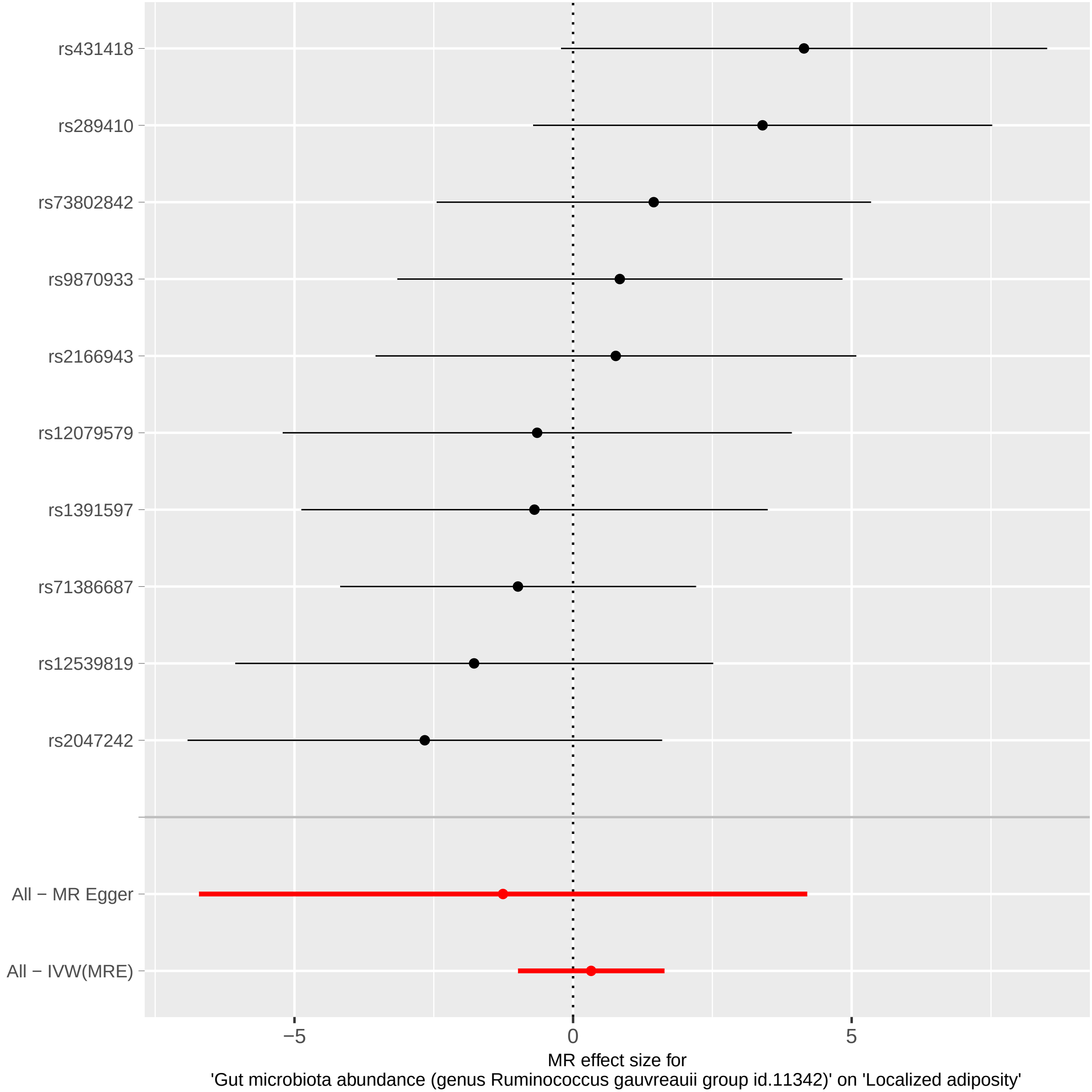

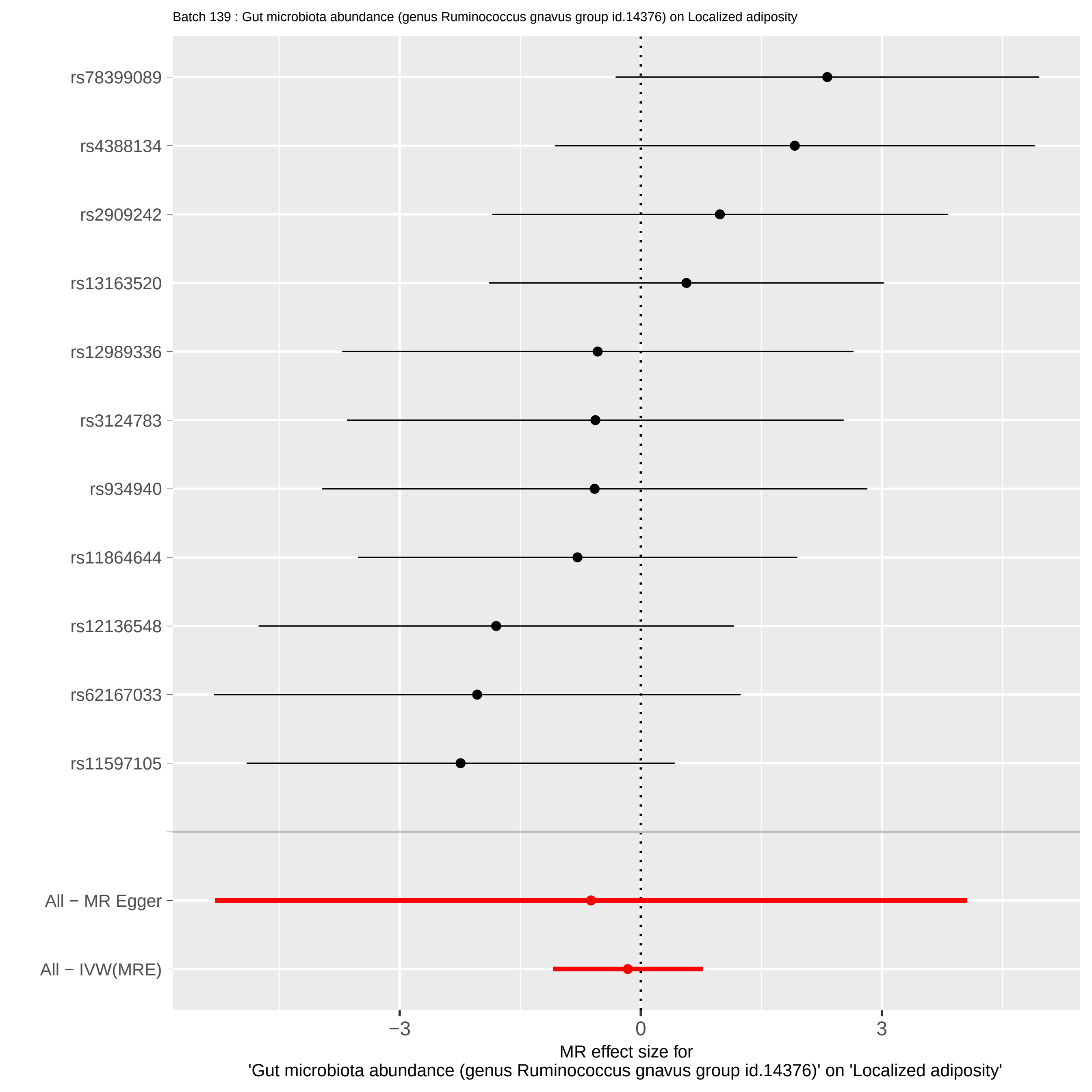
Batch 139 : Gut microbiota abundance (genus Ruminococcus gnavus group id.14376) on Localized adiposity
rs78399089
rs4388134
rs2909242
rs13163520
rs12989336
rs3124783
rs934940
rs11864644
rs12136548
rs62167033
rs11597105
All − MR Egger
All − IVW(MRE)
−3
0
3
MR effect size for
'Gut microbiota abundance (genus Ruminococcus gnavus group id.14376)' on 'Localized adiposity'

Batch 140 : Gut microbiota abundance (genus Ruminococcus torques group id.14377) on Localized adiposity

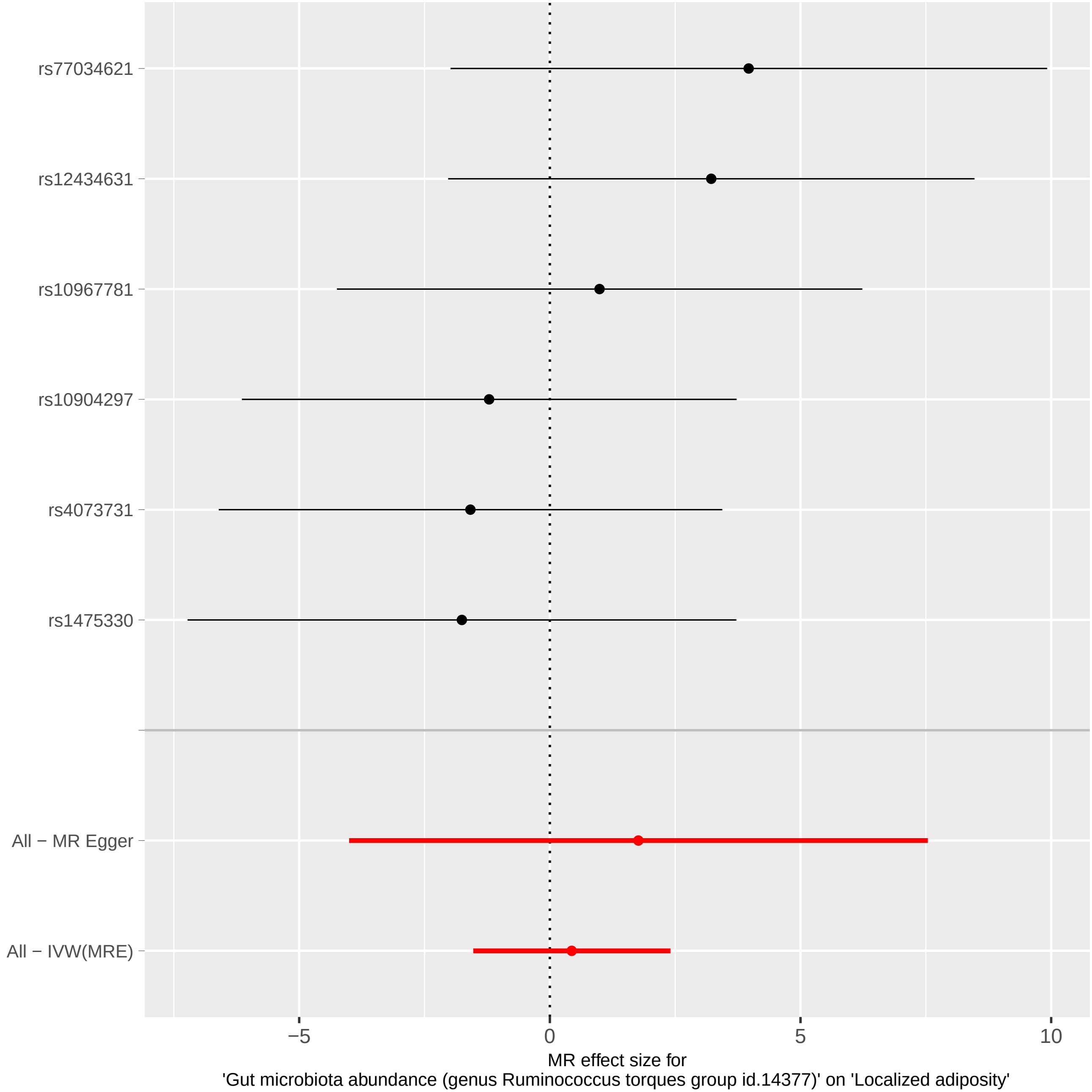

Batch 141 : Gut microbiota abundance (genus Sellimonas id.14369) on Localized adiposity

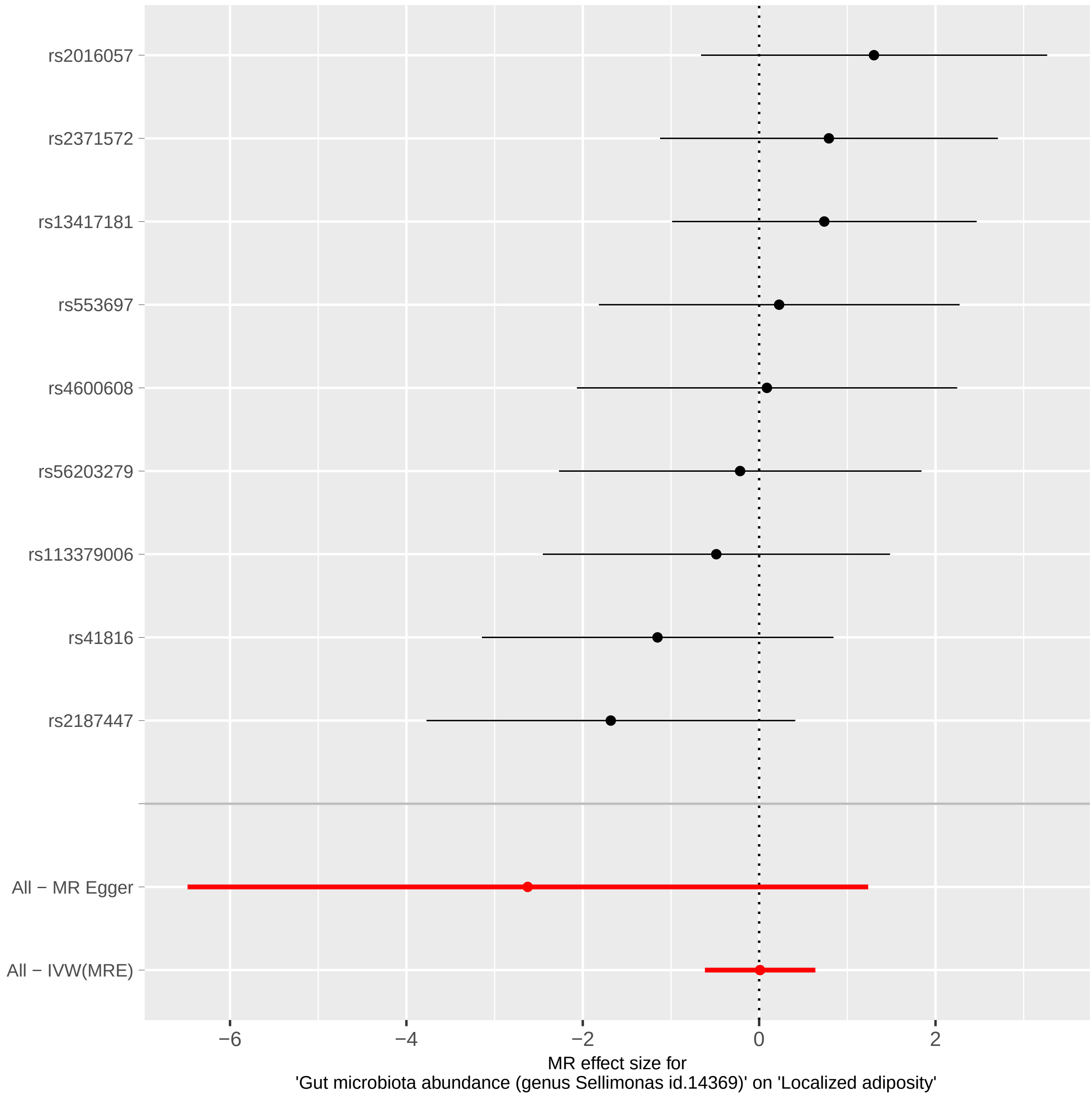

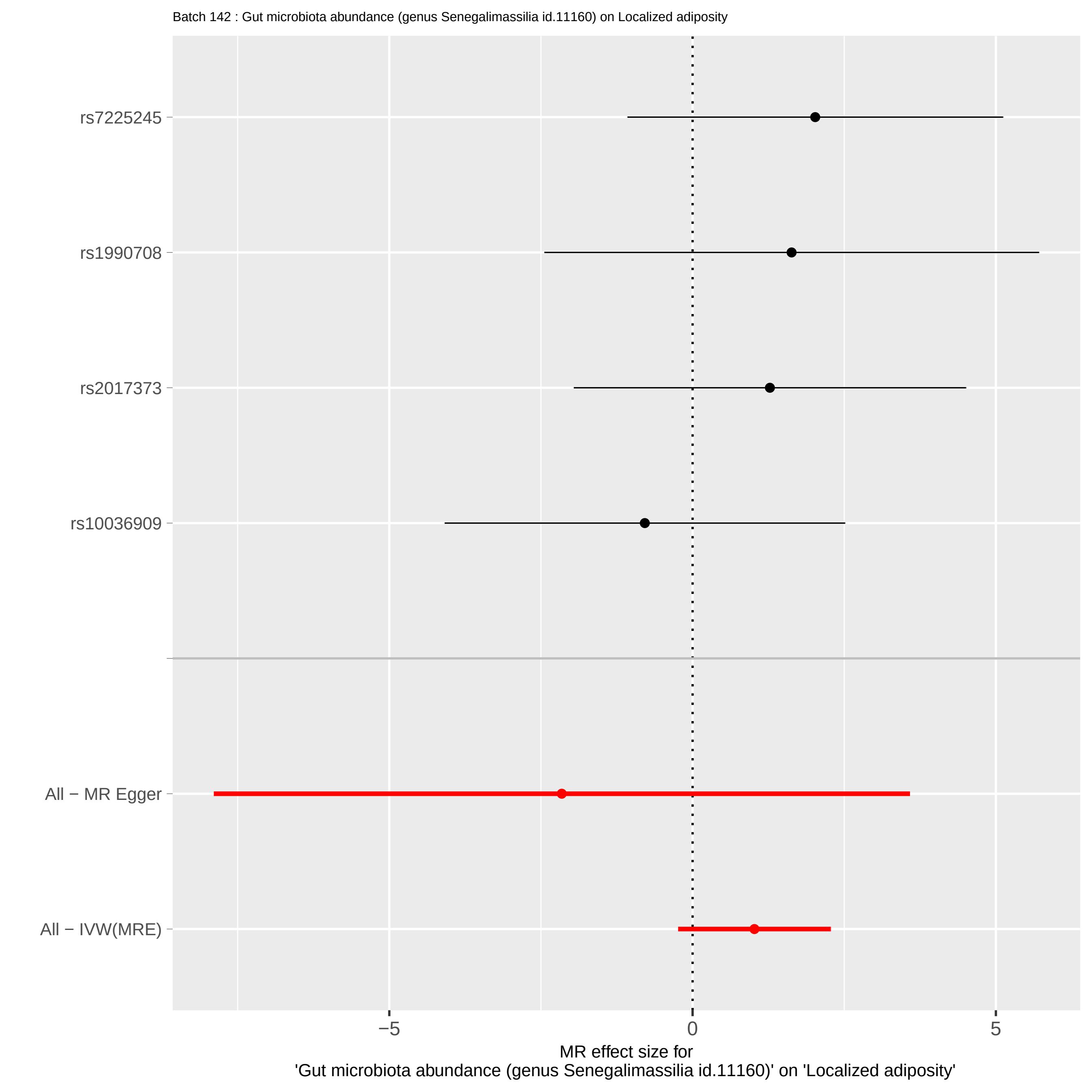

Batch 142 : Gut microbiota abundance (genus Senegalimassilia id.11160) on Localized adiposity
rs7225245
rs1990708
rs2017373
rs10036909
All − MR Egger
All − IVW(MRE)
−5
0
5
MR effect size for
'Gut microbiota abundance (genus Senegalimassilia id.11160)' on 'Localized adiposity'

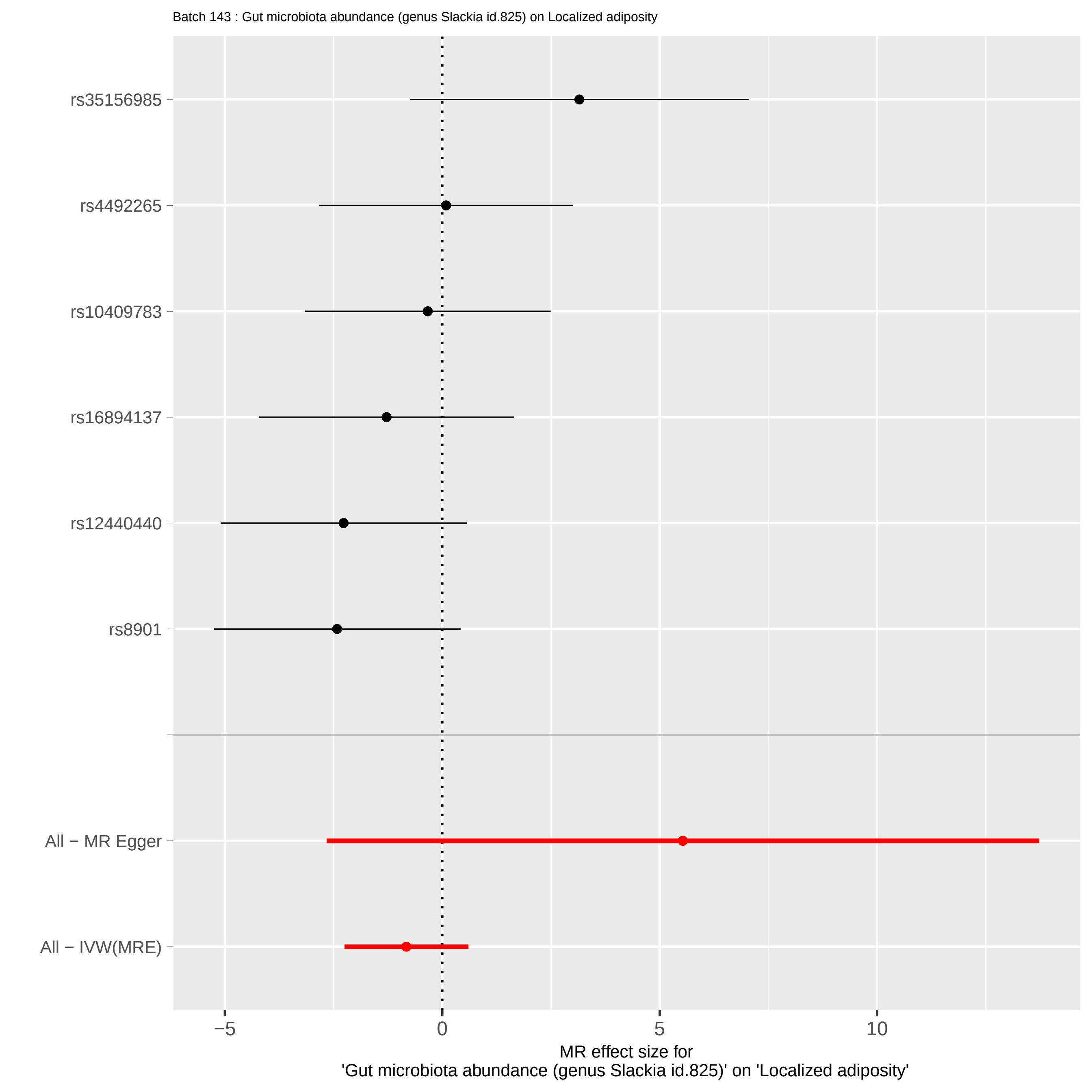
Batch 143 : Gut microbiota abundance (genus Slackia id.825) on Localized adiposity
rs35156985
rs4492265
rs10409783
rs16894137
rs12440440
rs8901
All − MR Egger
All − IVW(MRE)
−5
0
5
10
MR effect size for
'Gut microbiota abundance (genus Slackia id.825)' on 'Localized adiposity'

Batch 144 : Gut microbiota abundance (genus Streptococcus id.1853) on Localized adiposity

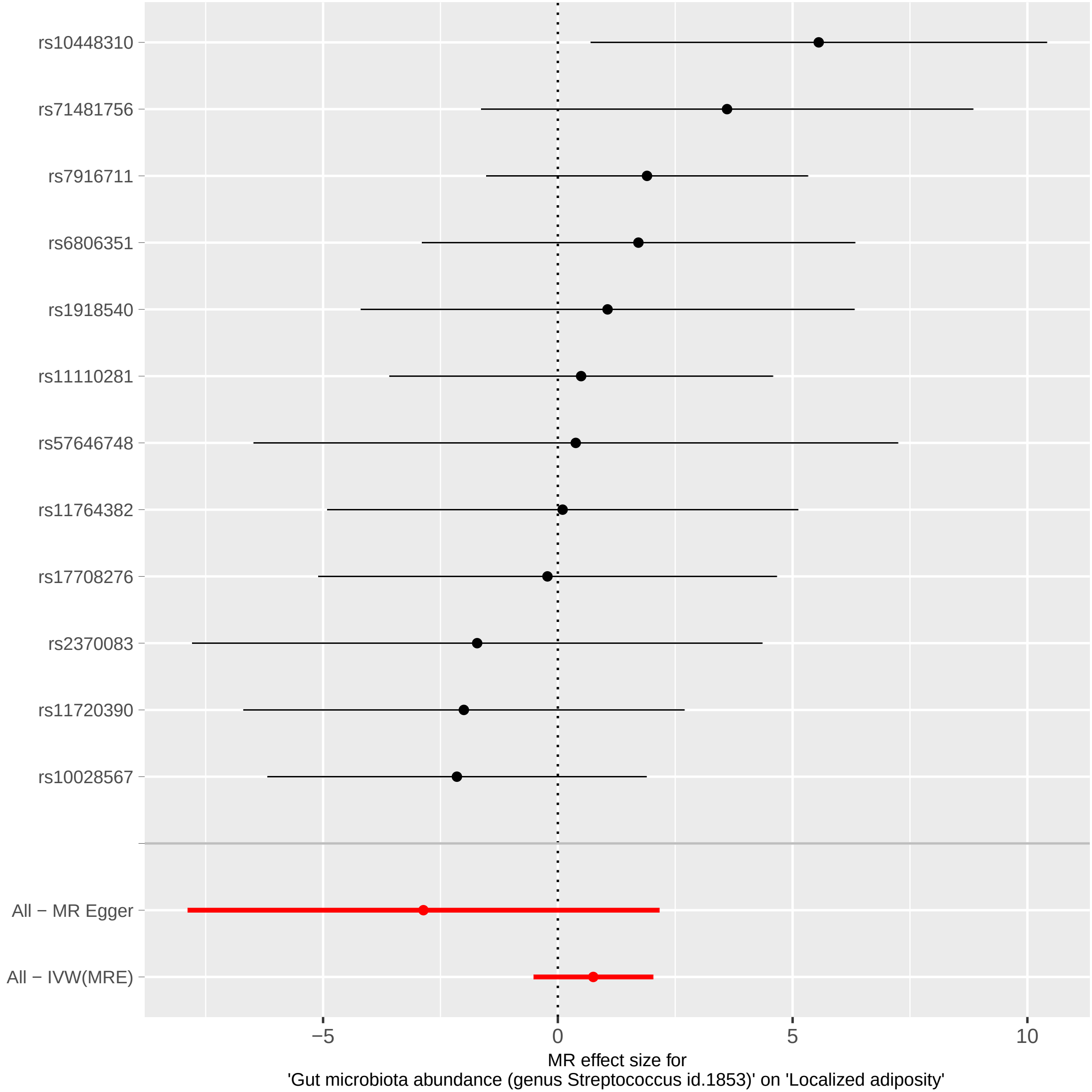

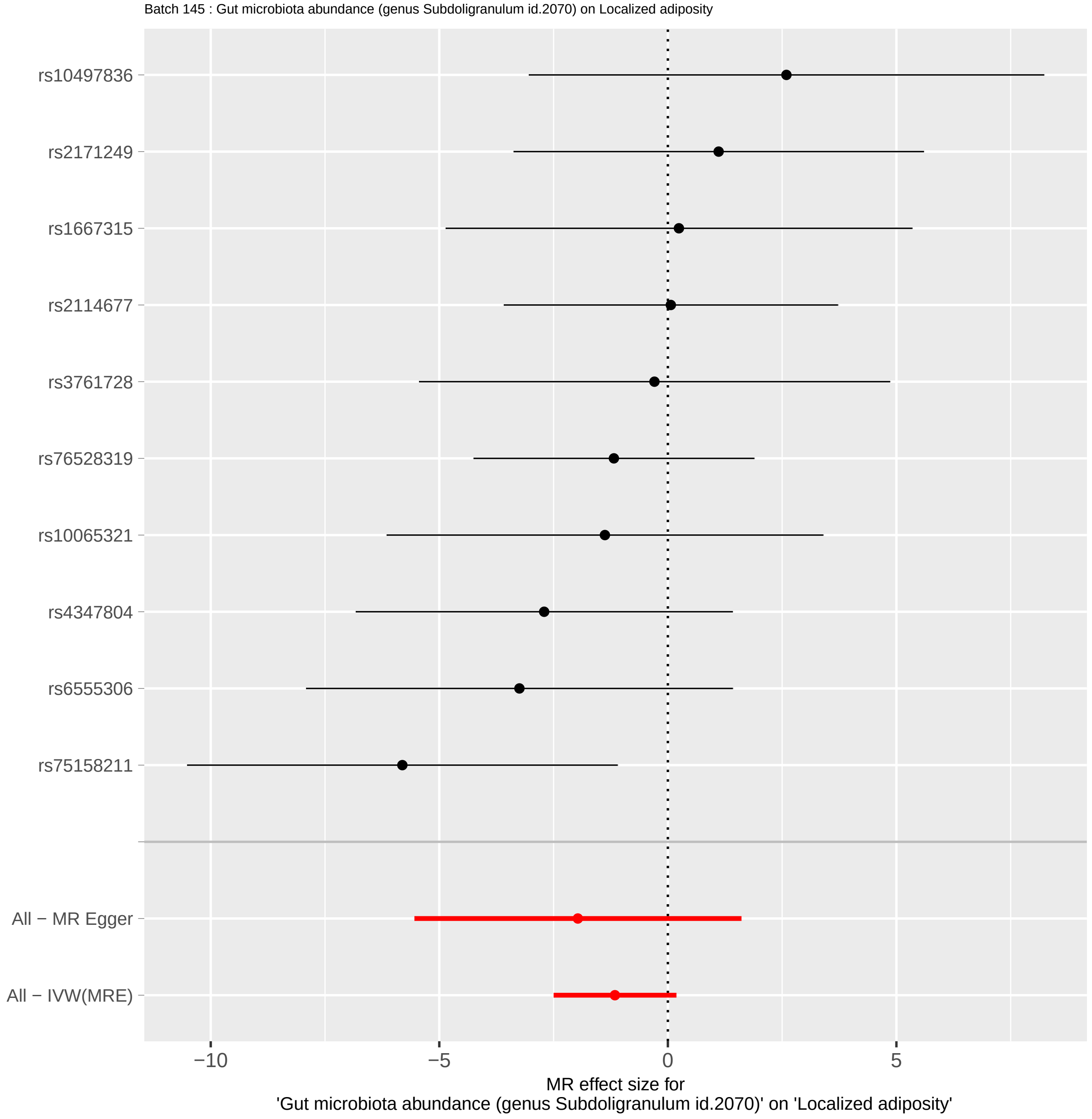

Batch 145 : Gut microbiota abundance (genus Subdoligranulum id.2070) on Localized adiposity
rs10497836
rs2171249
rs1667315
rs2114677
rs3761728
rs76528319
rs10065321
rs4347804
rs6555306
rs75158211
All − MR Egger
All − IVW(MRE)
−10
−5
0
5
MR effect size for
'Gut microbiota abundance (genus Subdoligranulum id.2070)' on 'Localized adiposity'

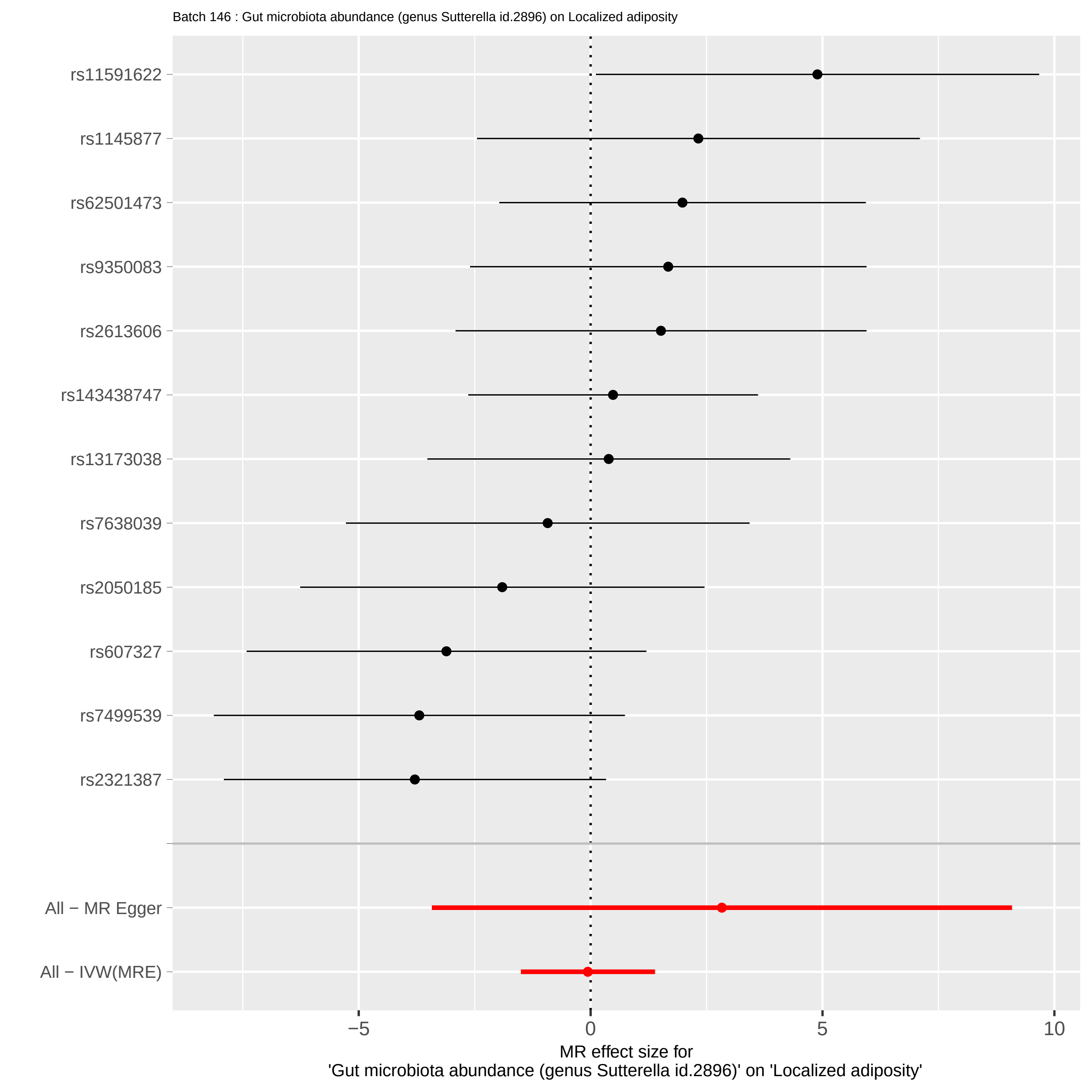

Batch 146 : Gut microbiota abundance (genus Sutterella id.2896) on Localized adiposity
rs11591622
rs1145877
rs62501473
rs9350083
rs2613606
rs143438747
rs13173038
rs7638039
rs2050185
rs607327
rs7499539
rs2321387
All − MR Egger
All − IVW(MRE)
−5
0
5
10
MR effect size for
'Gut microbiota abundance (genus Sutterella id.2896)' on 'Localized adiposity'

Batch 147 : Gut microbiota abundance (genus Terrisporobacter id.11348) on Localized adiposity

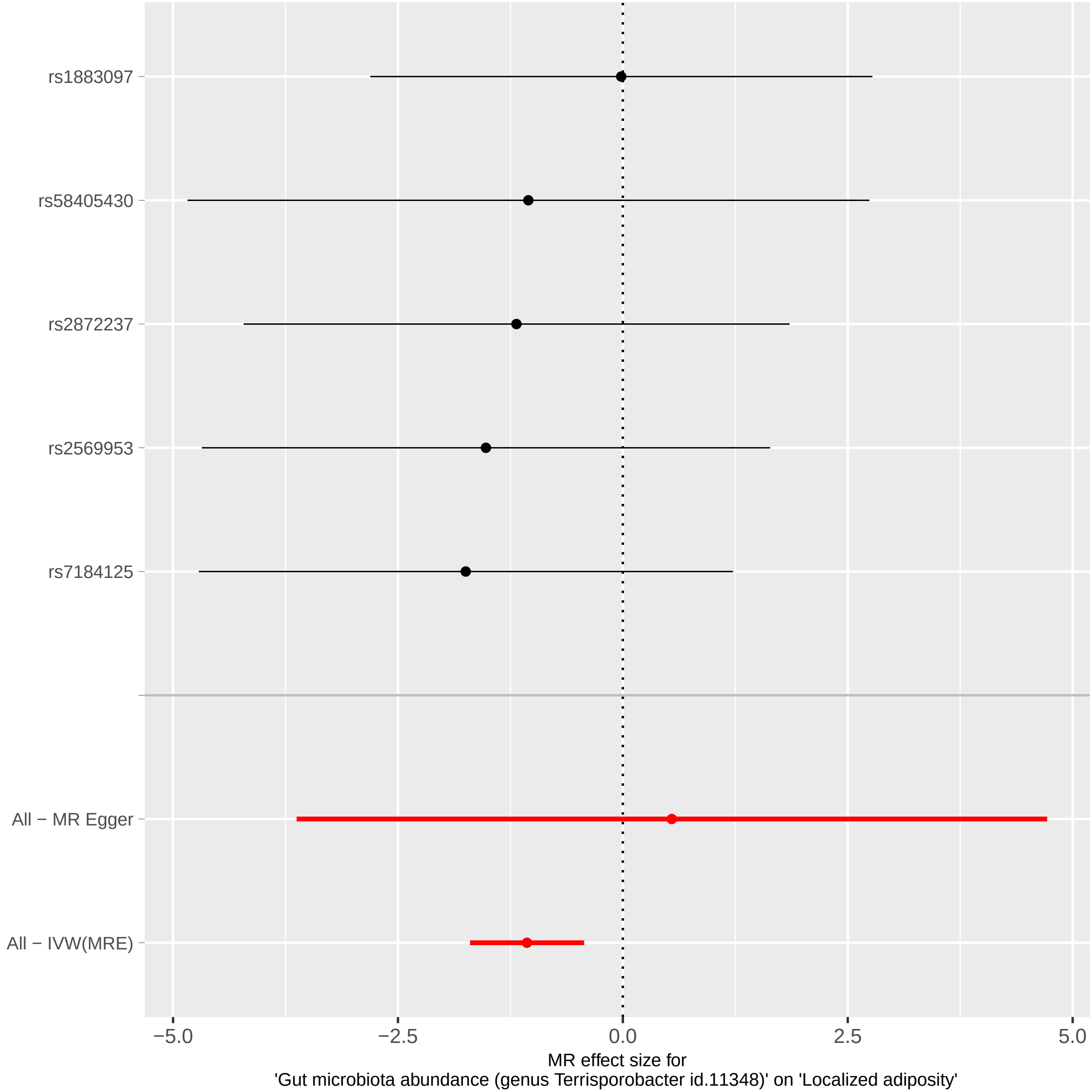

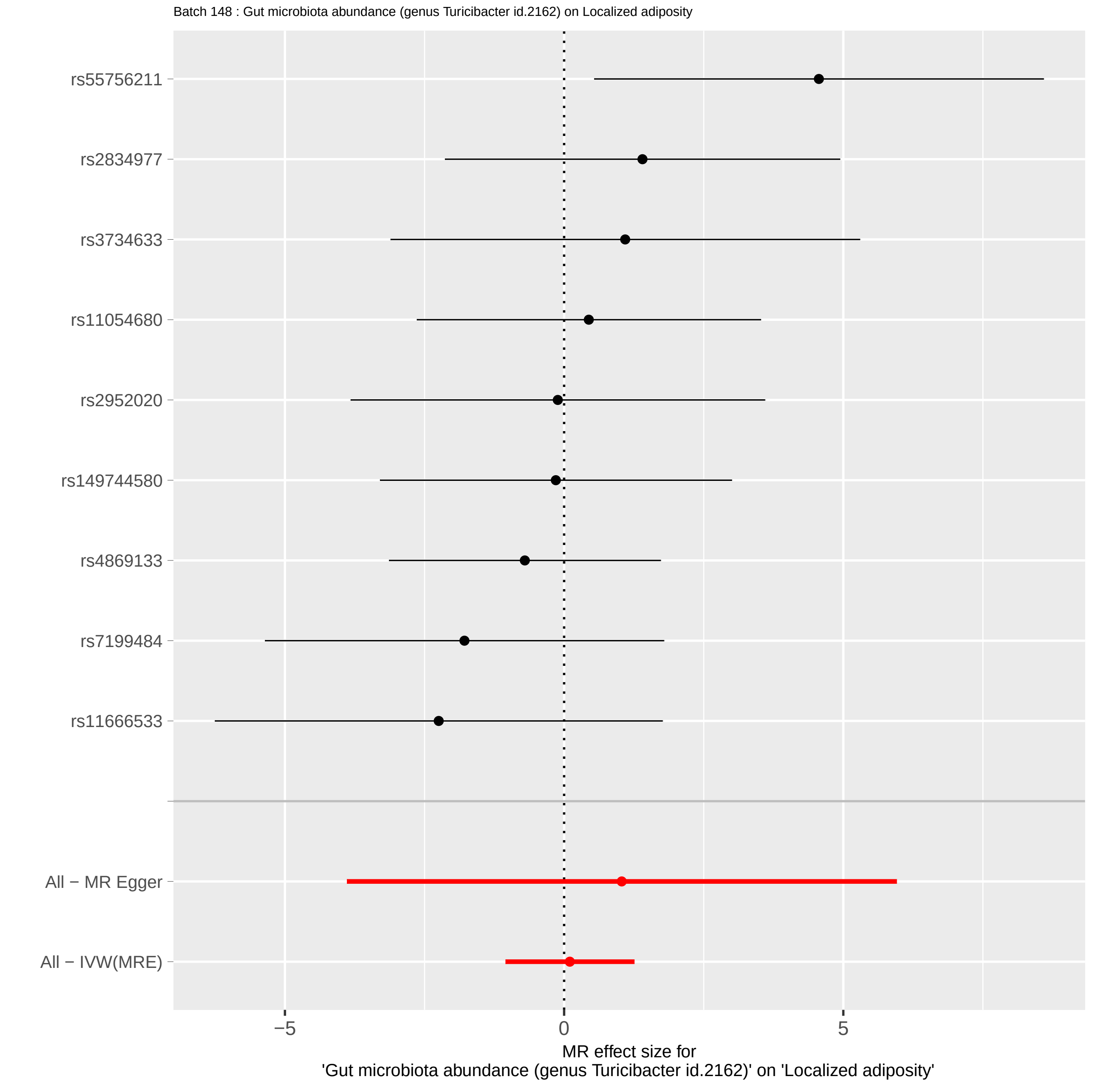

Batch 148 : Gut microbiota abundance (genus Turicibacter id.2162) on Localized adiposity
rs55756211
rs2834977
rs3734633
rs11054680
rs2952020
rs149744580
rs4869133
rs7199484
rs11666533
All − MR Egger
All − IVW(MRE)
−5
0
5
MR effect size for
'Gut microbiota abundance (genus Turicibacter id.2162)' on 'Localized adiposity'

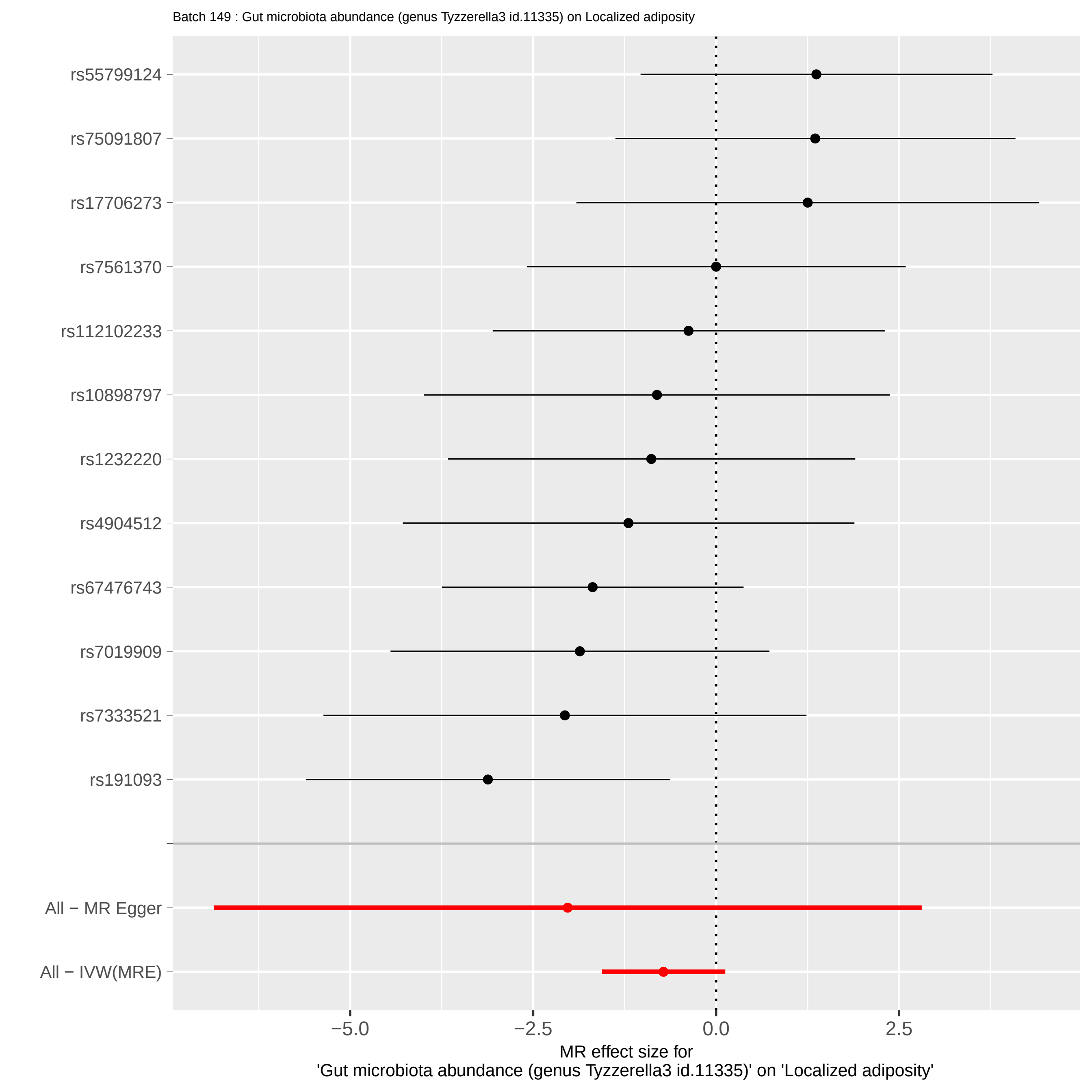
Batch 149 : Gut microbiota abundance (genus Tyzzerella3 id.11335) on Localized adiposity
rs55799124
rs75091807
rs17706273
rs7561370
rs112102233
rs10898797
rs1232220
rs4904512
rs67476743
rs7019909
rs7333521
rs191093
All − MR Egger
All − IVW(MRE)
−5.0
−2.5
0.0
2.5
MR effect size for
'Gut microbiota abundance (genus Tyzzerella3 id.11335)' on 'Localized adiposity'

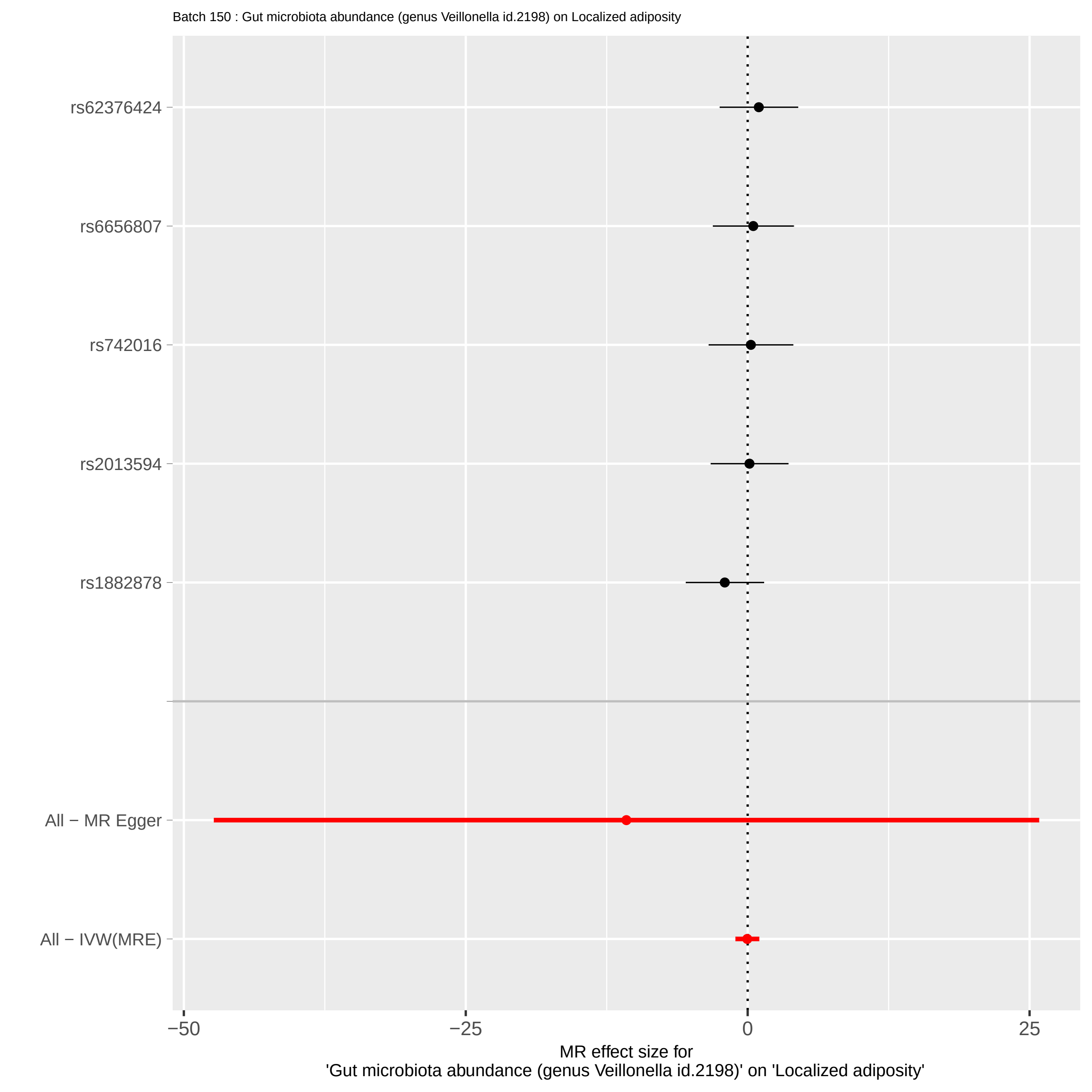

Batch 150 : Gut microbiota abundance (genus Veillonella id.2198) on Localized adiposity
rs62376424
rs6656807
rs742016
rs2013594
rs1882878
All − MR Egger
All − IVW(MRE)
−50
−25
0
25
MR effect size for
'Gut microbiota abundance (genus Veillonella id.2198)' on 'Localized adiposity'

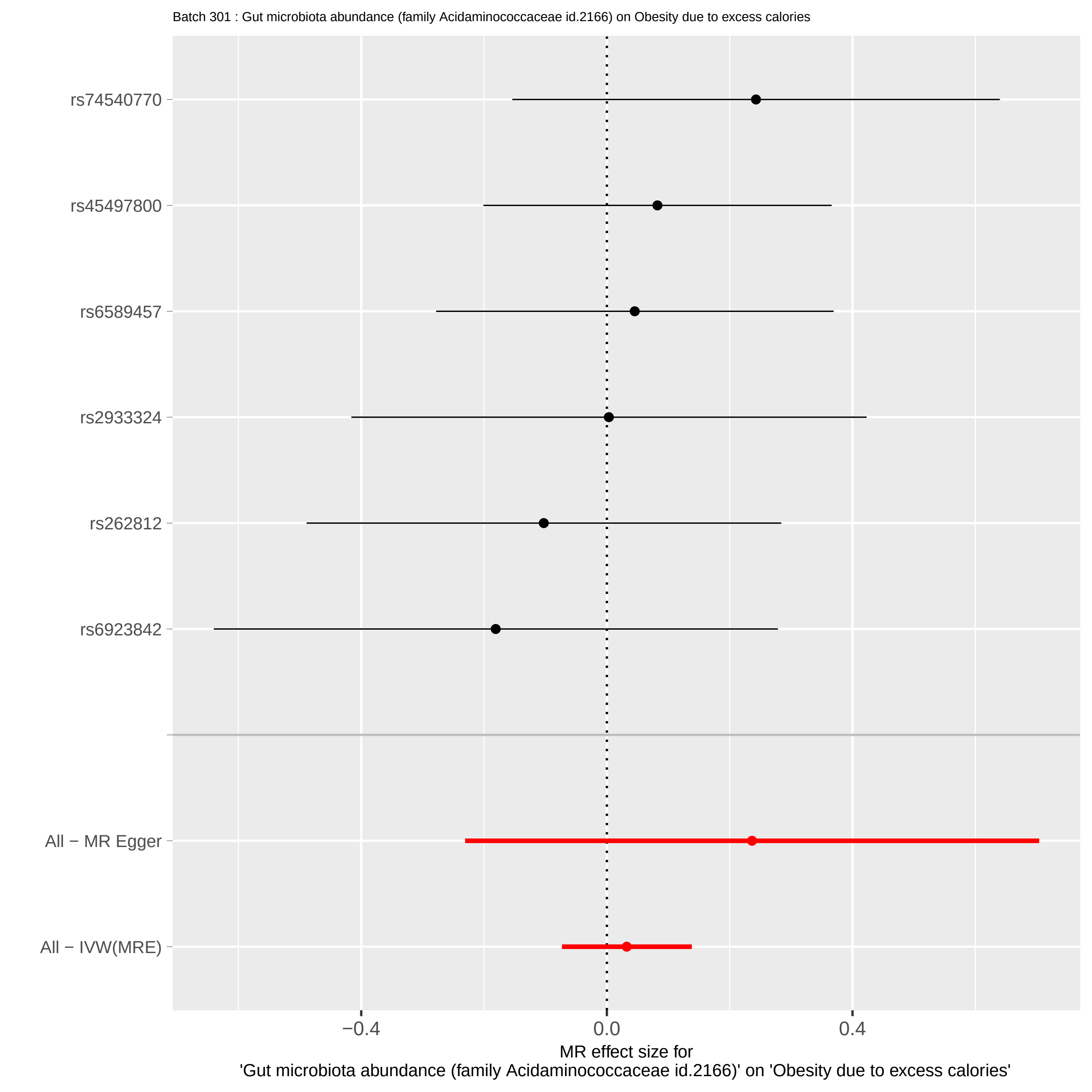
Batch 301 : Gut microbiota abundance (family Acidaminococcaceae id.2166) on Obesity due to excess calories
rs74540770
rs45497800
rs6589457
rs2933324
rs262812
rs6923842
All − MR Egger
All − IVW(MRE)
−0.4
0.0
0.4
MR effect size for
'Gut microbiota abundance (family Acidaminococcaceae id.2166)' on 'Obesity due to excess calories'

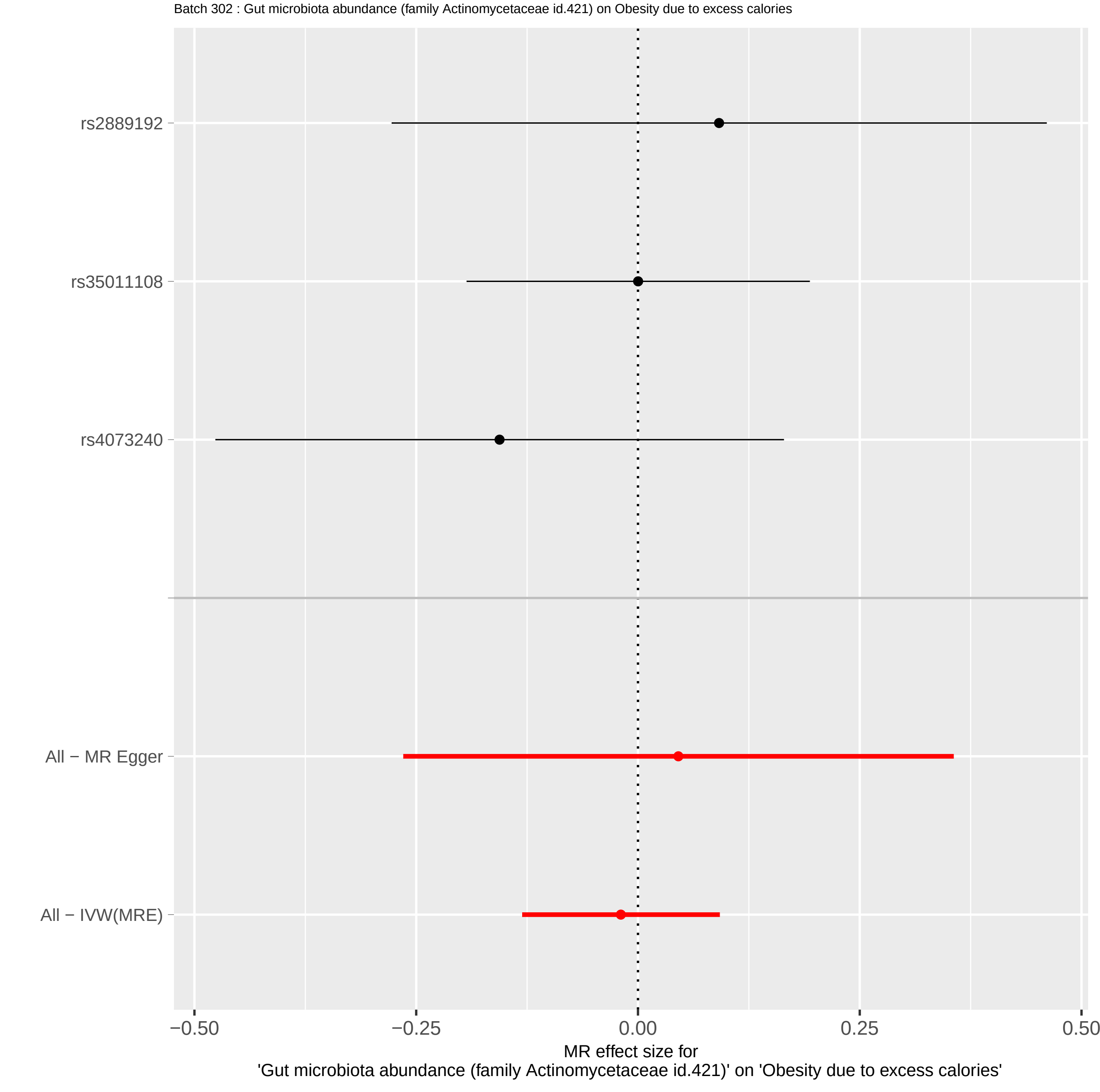
Batch 302 : Gut microbiota abundance (family Actinomycetaceae id.421) on Obesity due to excess calories
rs2889192
rs35011108
rs4073240
All − MR Egger
All − IVW(MRE)
−0.50
−0.25
0.00
0.25
0.50
MR effect size for
'Gut microbiota abundance (family Actinomycetaceae id.421)' on 'Obesity due to excess calories'

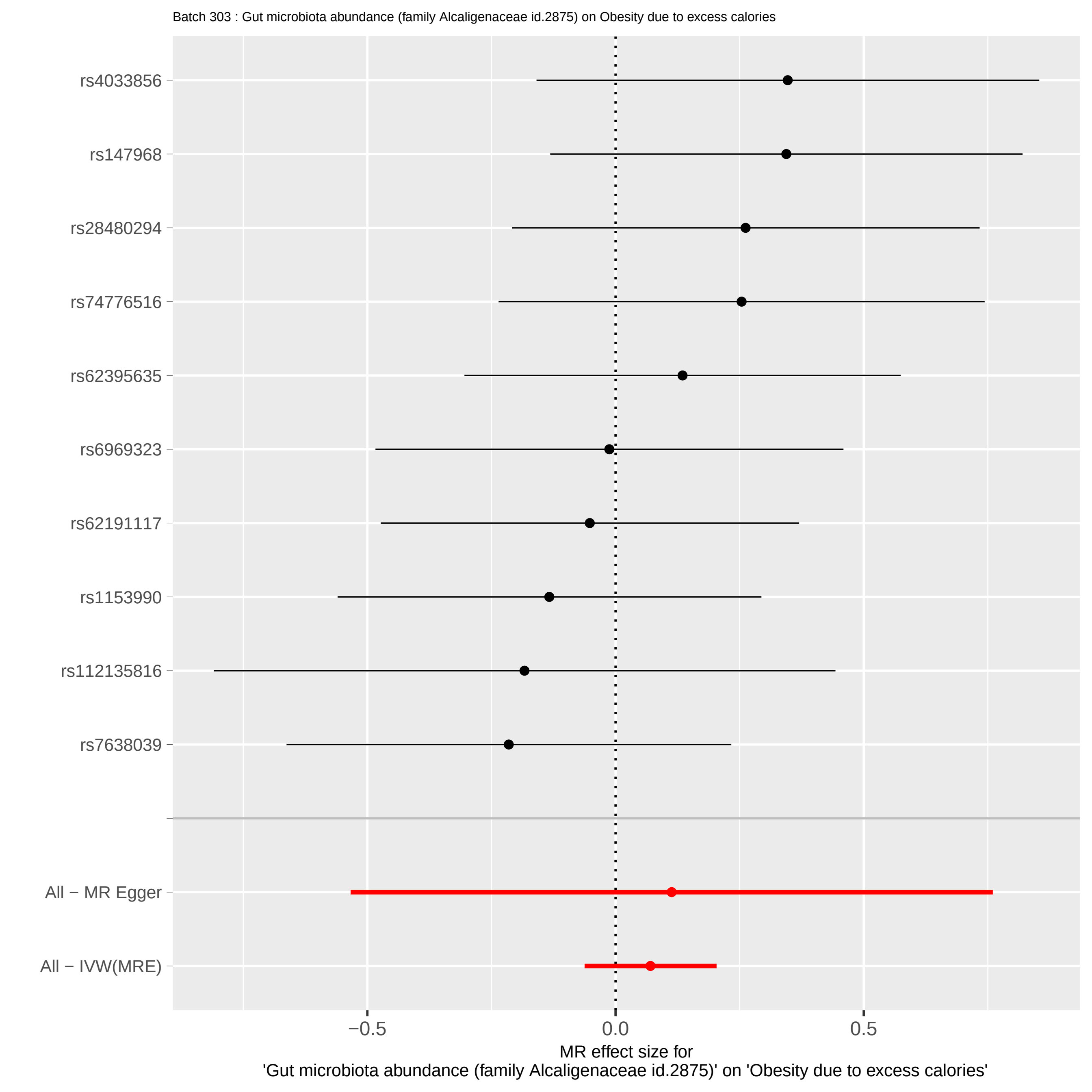

Batch 303 : Gut microbiota abundance (family Alcaligenaceae id.2875) on Obesity due to excess calories
rs4033856
rs147968
rs28480294
rs74776516
rs62395635
rs6969323
rs62191117
rs1153990
rs112135816
rs7638039
All − MR Egger
All − IVW(MRE)
−0.5
0.0
0.5
MR effect size for
'Gut microbiota abundance (family Alcaligenaceae id.2875)' on 'Obesity due to excess calories'

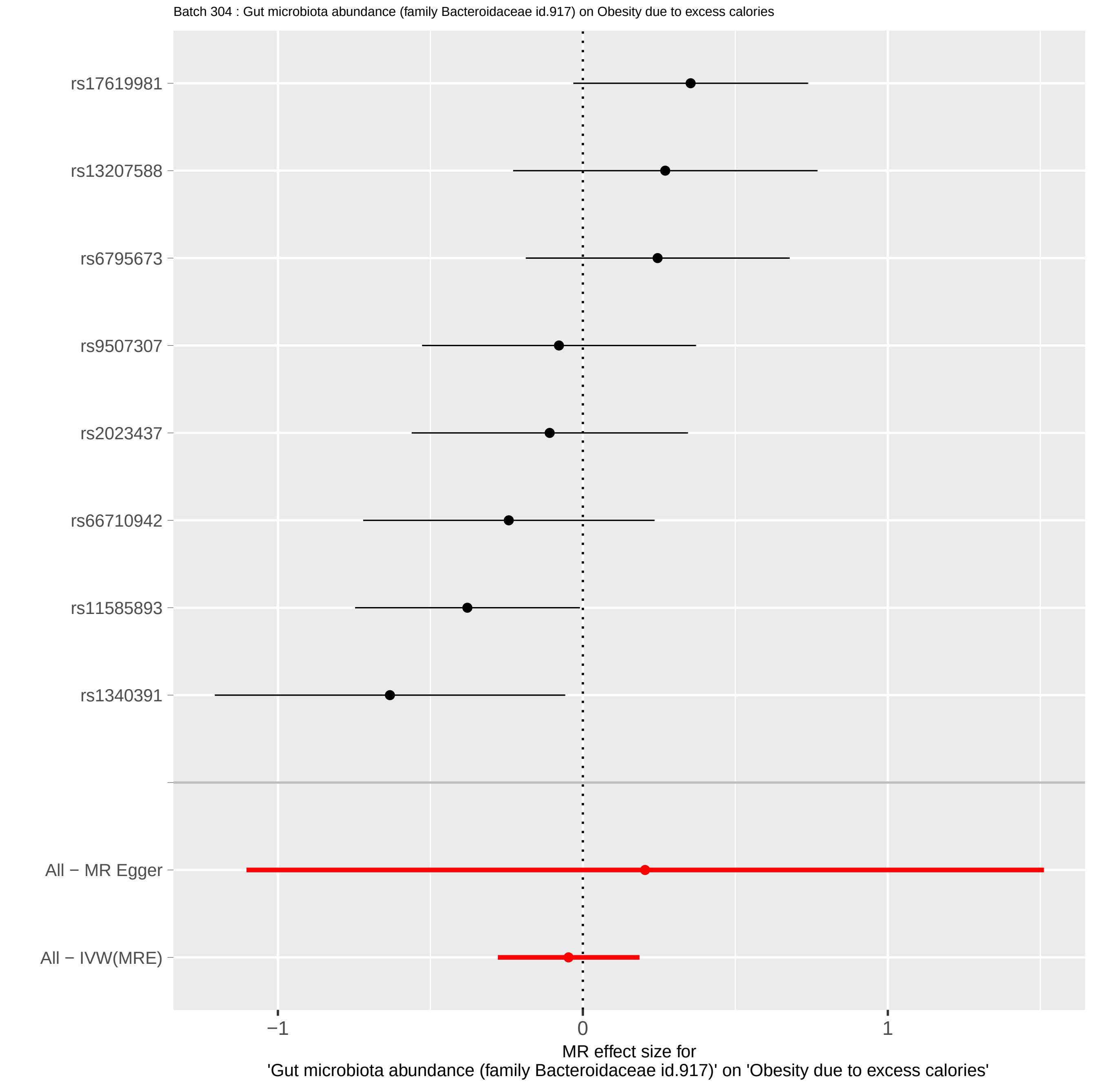
Batch 304 : Gut microbiota abundance (family Bacteroidaceae id.917) on Obesity due to excess calories
rs17619981
rs13207588
rs6795673
rs9507307
rs2023437
rs66710942
rs11585893
rs1340391
All − MR Egger
All − IVW(MRE)
−1
0
1
MR effect size for
'Gut microbiota abundance (family Bacteroidaceae id.917)' on 'Obesity due to excess calories'

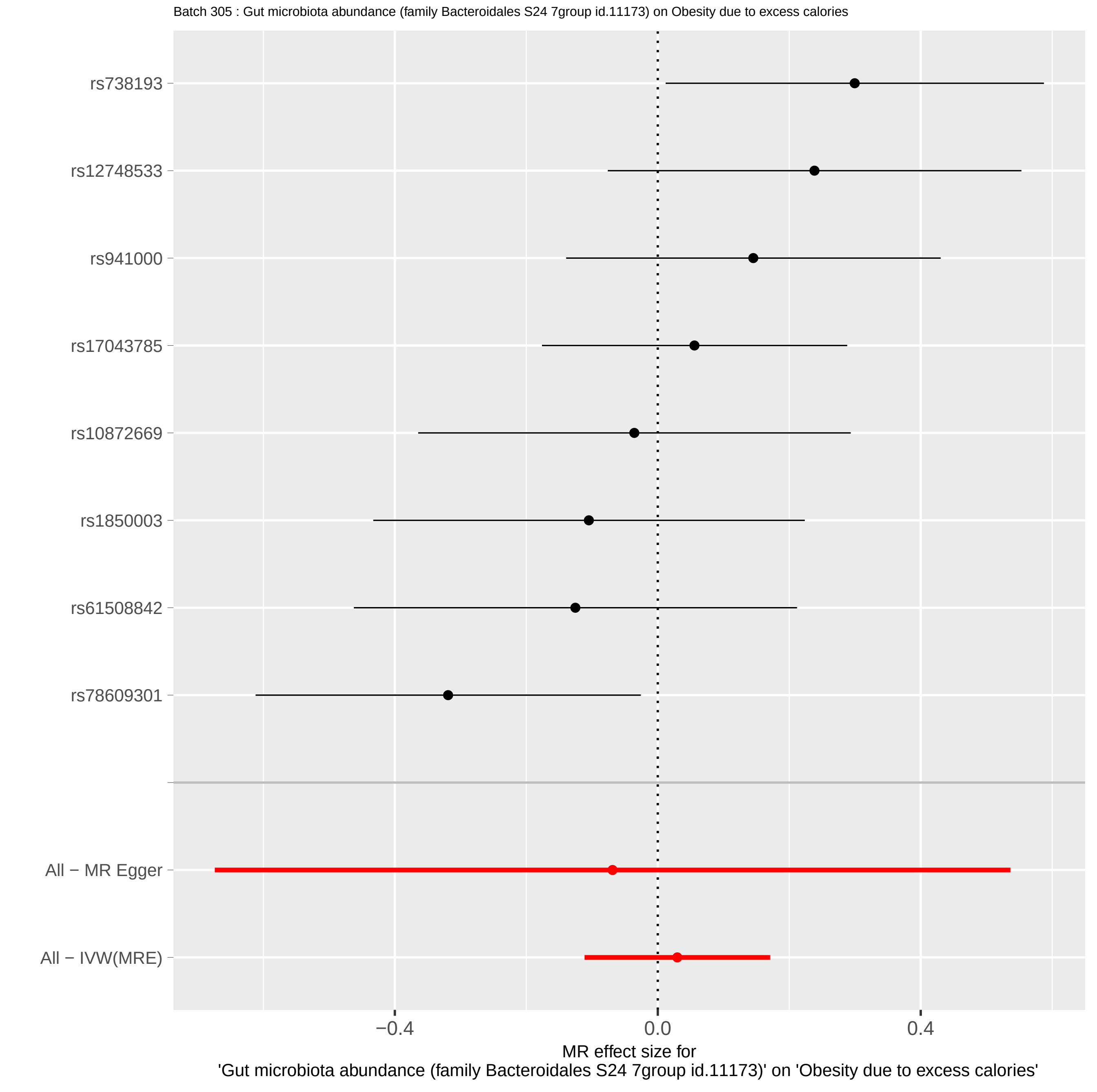
Batch 305 : Gut microbiota abundance (family Bacteroidales S24 7group id.11173) on Obesity due to excess calories
rs738193
rs12748533
rs941000
rs17043785
rs10872669
rs1850003
rs61508842
rs78609301
All − MR Egger
All − IVW(MRE)
−0.4
0.0
0.4
MR effect size for
'Gut microbiota abundance (family Bacteroidales S24 7group id.11173)' on 'Obesity due to excess calories'

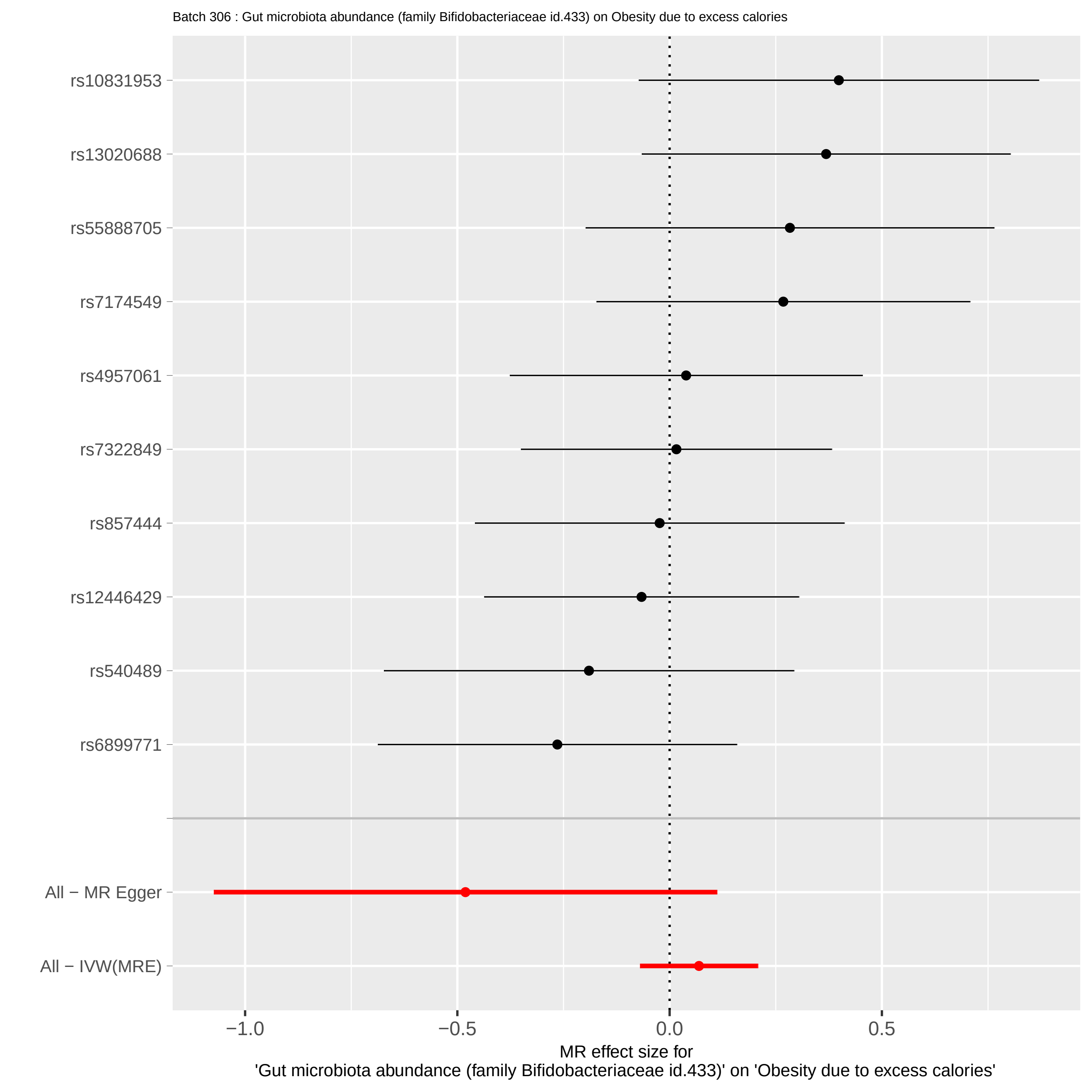

Batch 306 : Gut microbiota abundance (family Bifidobacteriaceae id.433) on Obesity due to excess calories
rs10831953
rs13020688
rs55888705
rs7174549
rs4957061
rs7322849
rs857444
rs12446429
rs540489
rs6899771
All − MR Egger
All − IVW(MRE)
−1.0
−0.5
0.0
0.5
MR effect size for
'Gut microbiota abundance (family Bifidobacteriaceae id.433)' on 'Obesity due to excess calories'

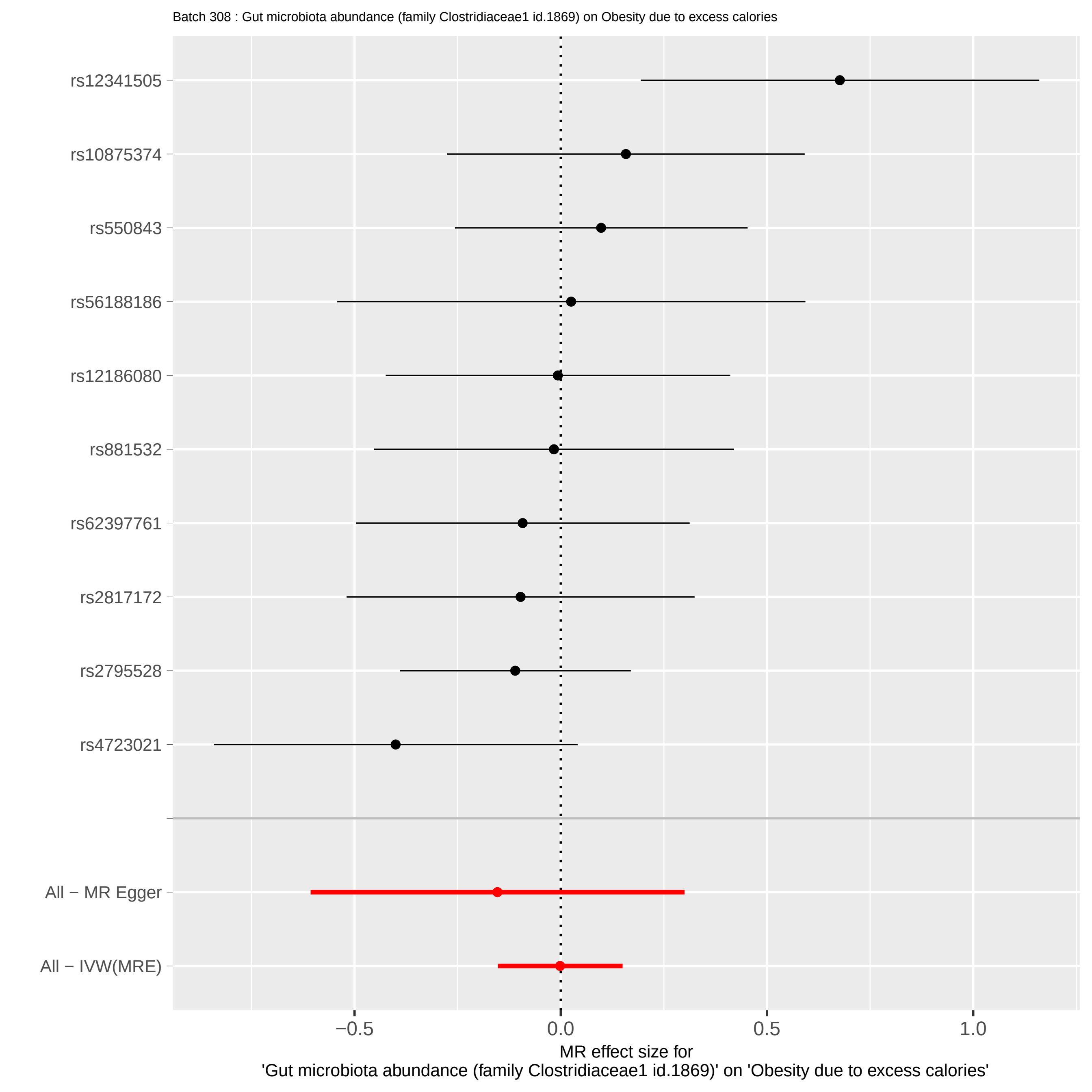
Batch 308 : Gut microbiota abundance (family Clostridiaceae1 id.1869) on Obesity due to excess calories
rs12341505
rs10875374
rs550843
rs56188186
rs12186080
rs881532
rs62397761
rs2817172
rs2795528
rs4723021
All − MR Egger
All − IVW(MRE)
−0.5
0.0
0.5
1.0
MR effect size for
'Gut microbiota abundance (family Clostridiaceae1 id.1869)' on 'Obesity due to excess calories'

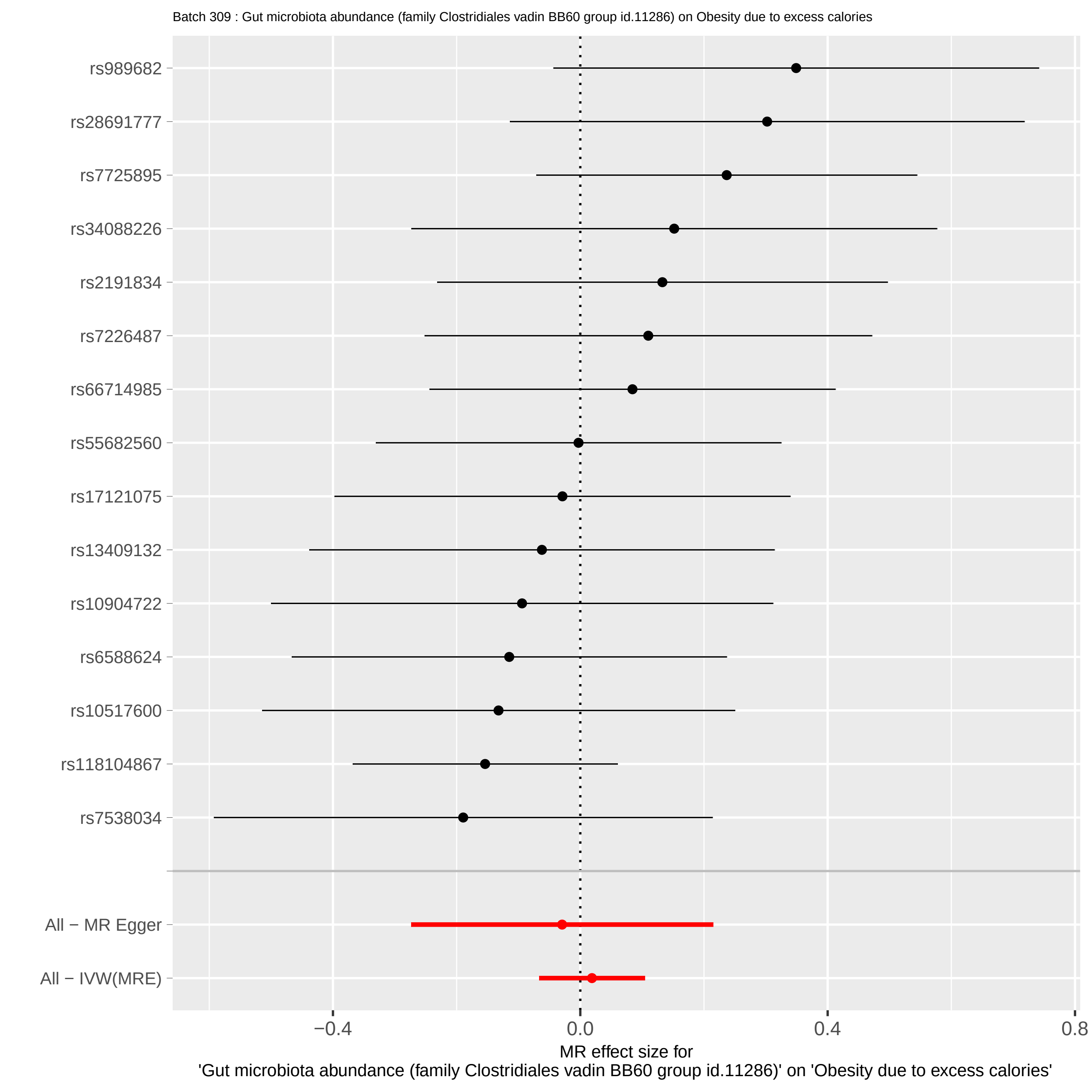

Batch 309 : Gut microbiota abundance (family Clostridiales vadin BB60 group id.11286) on Obesity due to excess calories
rs989682
rs28691777
rs7725895
rs34088226
rs2191834
rs7226487
rs66714985
rs55682560
rs17121075
rs13409132
rs10904722
rs6588624
rs10517600
rs118104867
rs7538034
All − MR Egger
All − IVW(MRE)
−0.4
0.0
0.4
0.8
MR effect size for
'Gut microbiota abundance (family Clostridiales vadin BB60 group id.11286)' on 'Obesity due to excess calories'

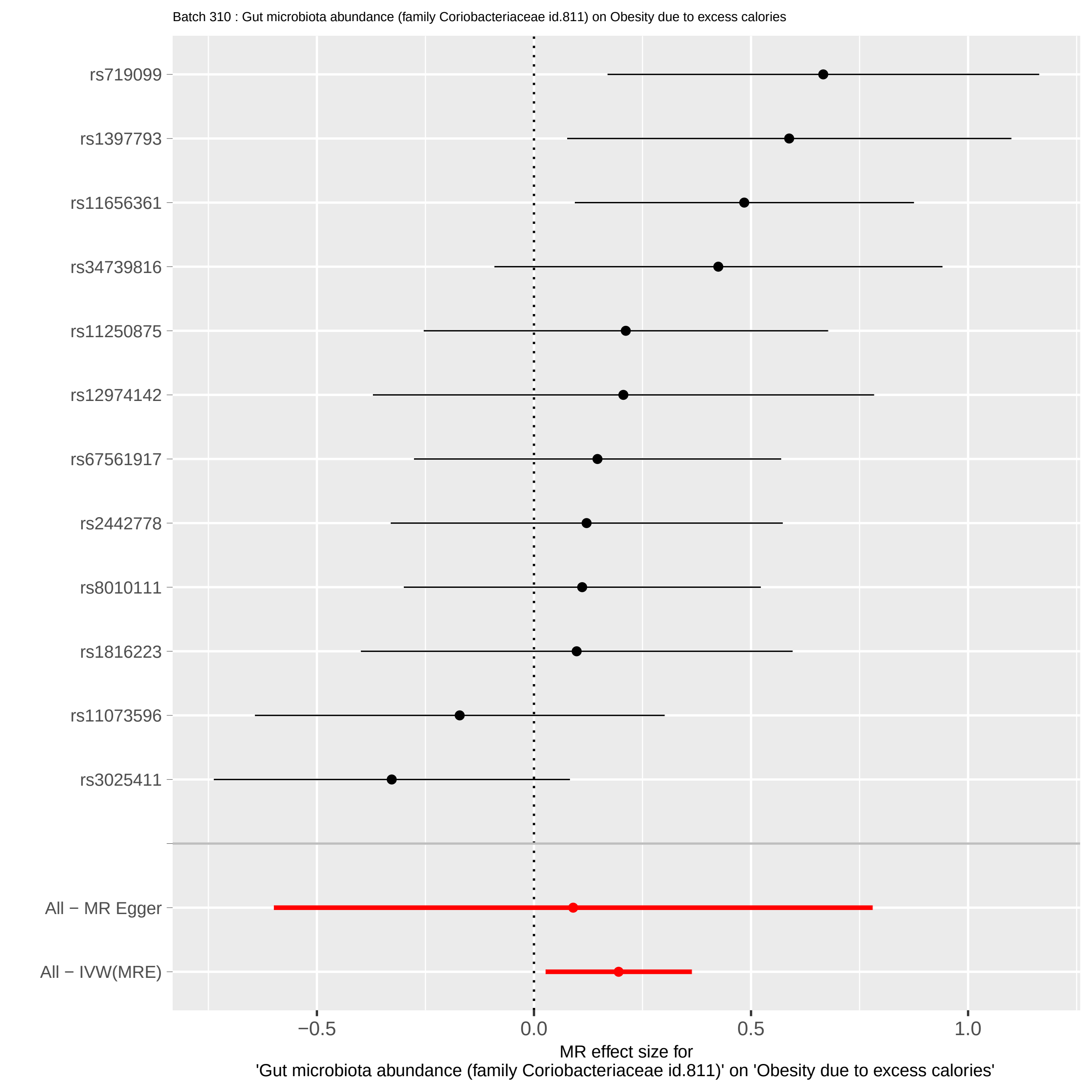

Batch 310 : Gut microbiota abundance (family Coriobacteriaceae id.811) on Obesity due to excess calories
rs719099
rs1397793
rs11656361
rs34739816
rs11250875
rs12974142
rs67561917
rs2442778
rs8010111
rs1816223
rs11073596
rs3025411
All − MR Egger
All − IVW(MRE)
−0.5
0.0
0.5
1.0
MR effect size for
'Gut microbiota abundance (family Coriobacteriaceae id.811)' on 'Obesity due to excess calories'

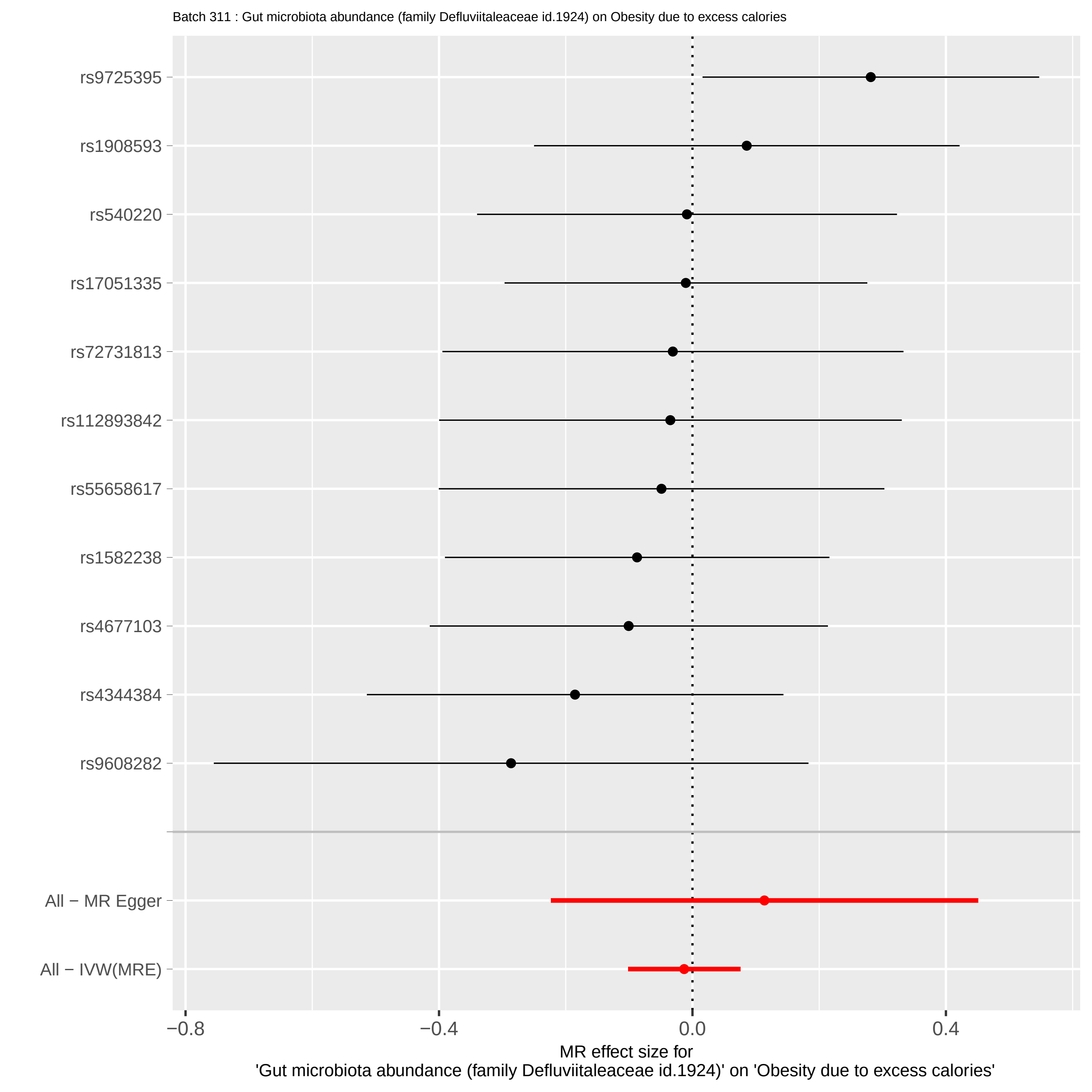

Batch 311 : Gut microbiota abundance (family Defluviitaleaceae id.1924) on Obesity due to excess calories
rs9725395
rs1908593
rs540220
rs17051335
rs72731813
rs112893842
rs55658617
rs1582238
rs4677103
rs4344384
rs9608282
All − MR Egger
All − IVW(MRE)
−0.8
−0.4
0.0
0.4
MR effect size for
'Gut microbiota abundance (family Defluviitaleaceae id.1924)' on 'Obesity due to excess calories'

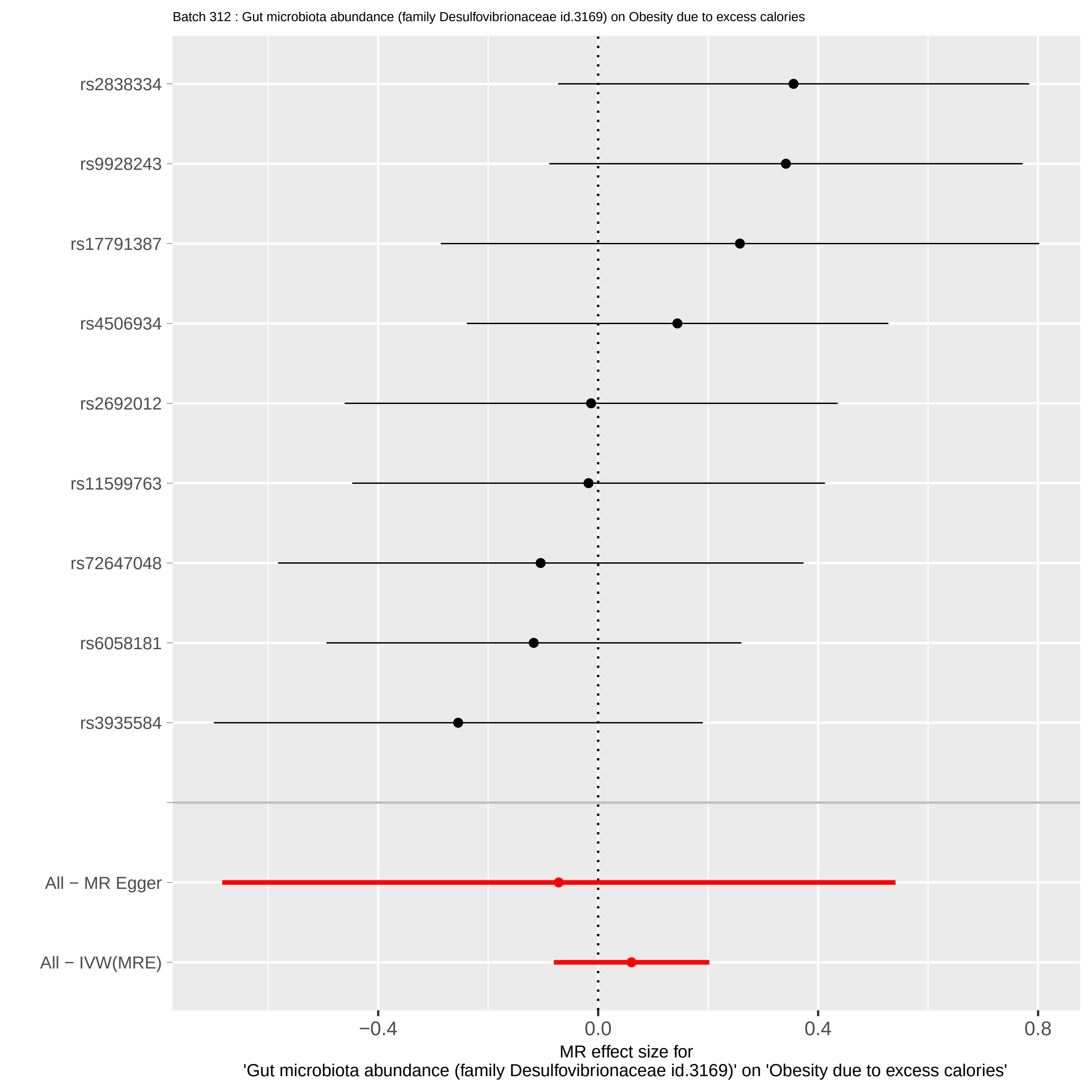
Batch 312 : Gut microbiota abundance (family Desulfovibrionaceae id.3169) on Obesity due to excess calories
rs2838334
rs9928243
rs17791387
rs4506934
rs2692012
rs11599763
rs72647048
rs6058181
rs3935584
All − MR Egger
All − IVW(MRE)
−0.4
0.0
0.4
0.8
MR effect size for
'Gut microbiota abundance (family Desulfovibrionaceae id.3169)' on 'Obesity due to excess calories'

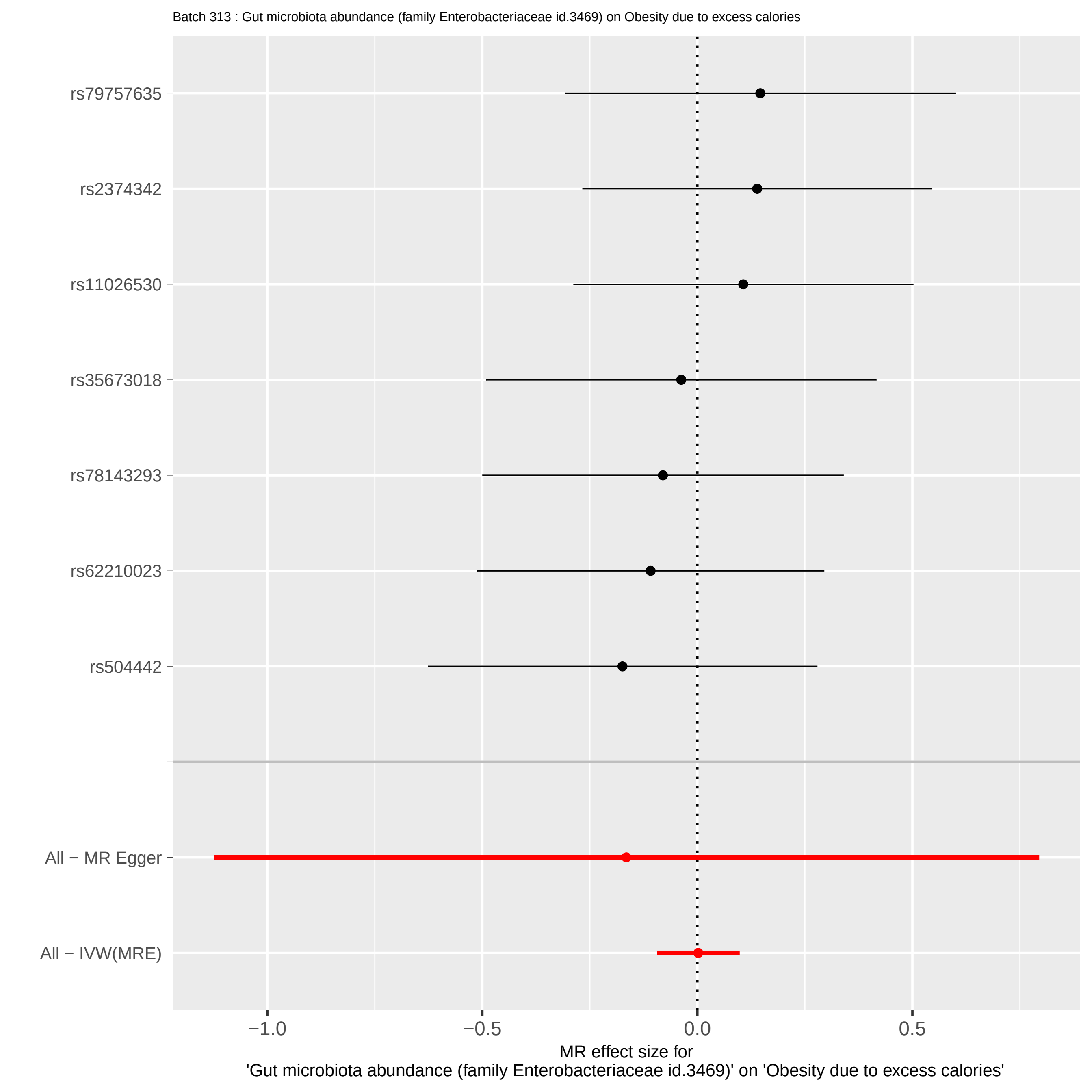

Batch 313 : Gut microbiota abundance (family Enterobacteriaceae id.3469) on Obesity due to excess calories
rs79757635
rs2374342
rs11026530
rs35673018
rs78143293
rs62210023
rs504442
All − MR Egger
All − IVW(MRE)
−1.0
−0.5
0.0
0.5
MR effect size for
'Gut microbiota abundance (family Enterobacteriaceae id.3469)' on 'Obesity due to excess calories'

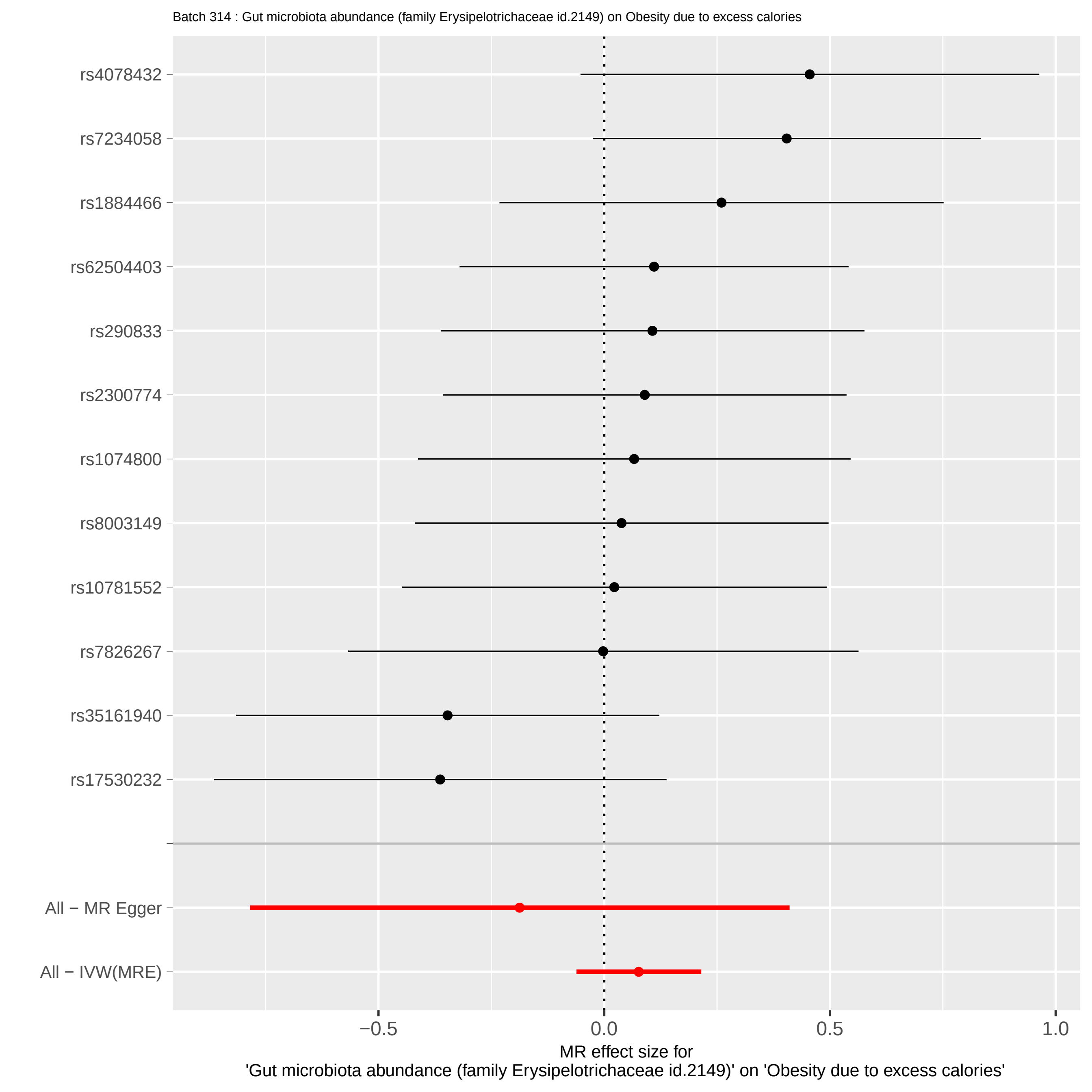

Batch 314 : Gut microbiota abundance (family Erysipelotrichaceae id.2149) on Obesity due to excess calories
rs4078432
rs7234058
rs1884466
rs62504403
rs290833
rs2300774
rs1074800
rs8003149
rs10781552
rs7826267
rs35161940
rs17530232
All − MR Egger
All − IVW(MRE)
−0.5
0.0
0.5
1.0
MR effect size for
'Gut microbiota abundance (family Erysipelotrichaceae id.2149)' on 'Obesity due to excess calories'

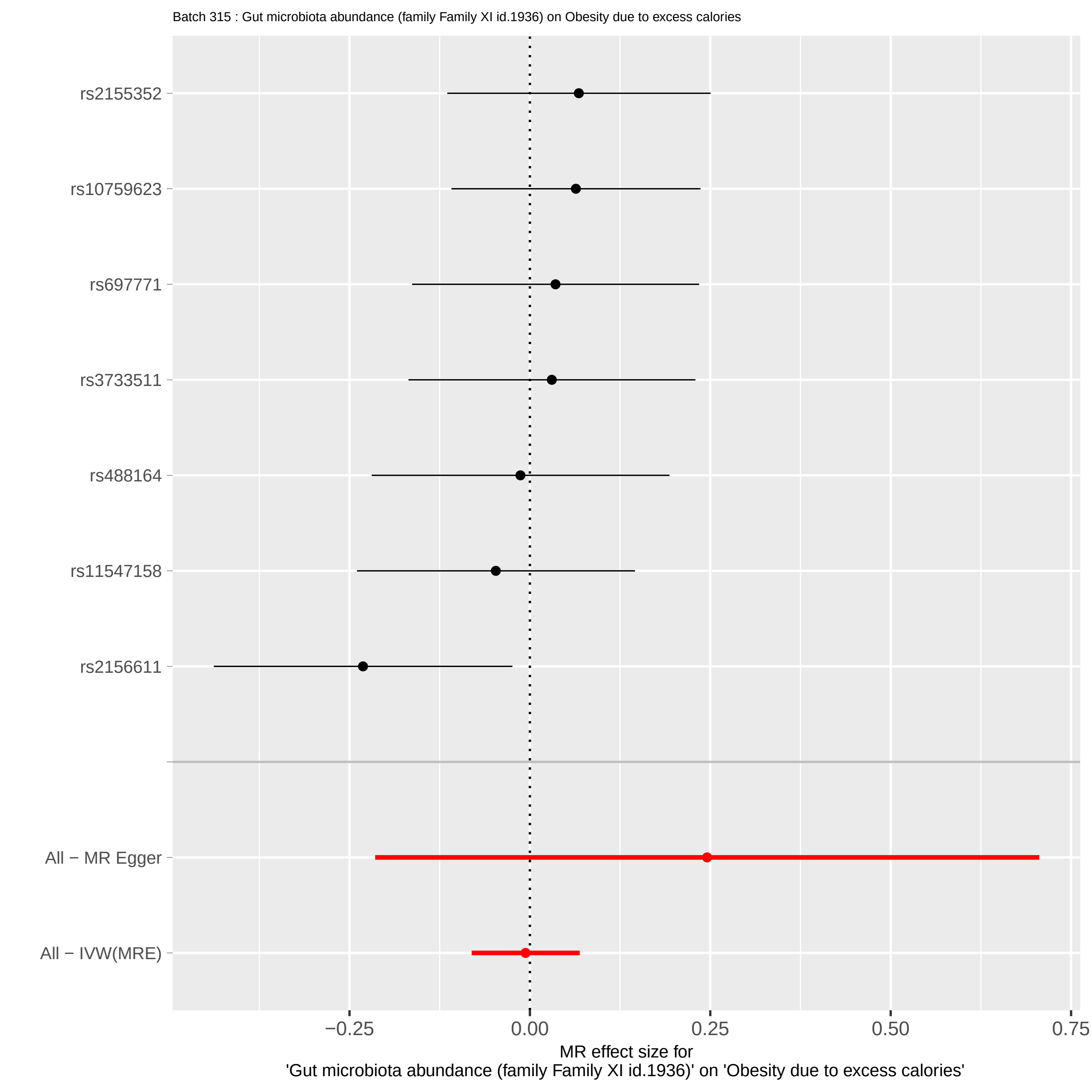
Batch 315 : Gut microbiota abundance (family Family XI id.1936) on Obesity due to excess calories
rs2155352
rs10759623
rs697771
rs3733511
rs488164
rs11547158
rs2156611
All − MR Egger
All − IVW(MRE)
−0.25
0.00
0.25
0.50
0.75
MR effect size for
'Gut microbiota abundance (family Family XI id.1936)' on 'Obesity due to excess calories'

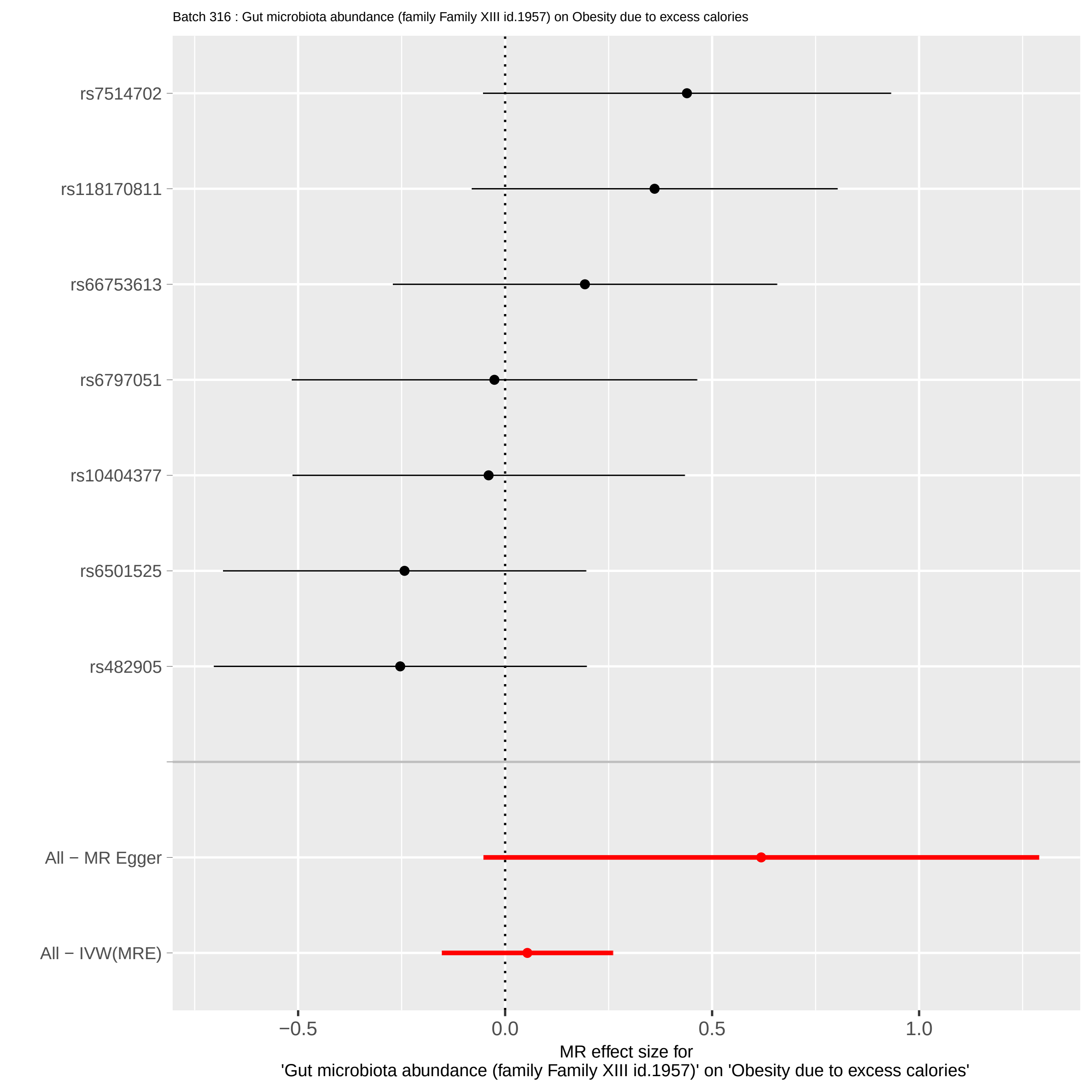
Batch 316 : Gut microbiota abundance (family Family XIII id.1957) on Obesity due to excess calories
rs7514702
rs118170811
rs66753613
rs6797051
rs10404377
rs6501525
rs482905
All − MR Egger
All − IVW(MRE)
−0.5
0.0
0.5
1.0
MR effect size for
'Gut microbiota abundance (family Family XIII id.1957)' on 'Obesity due to excess calories'

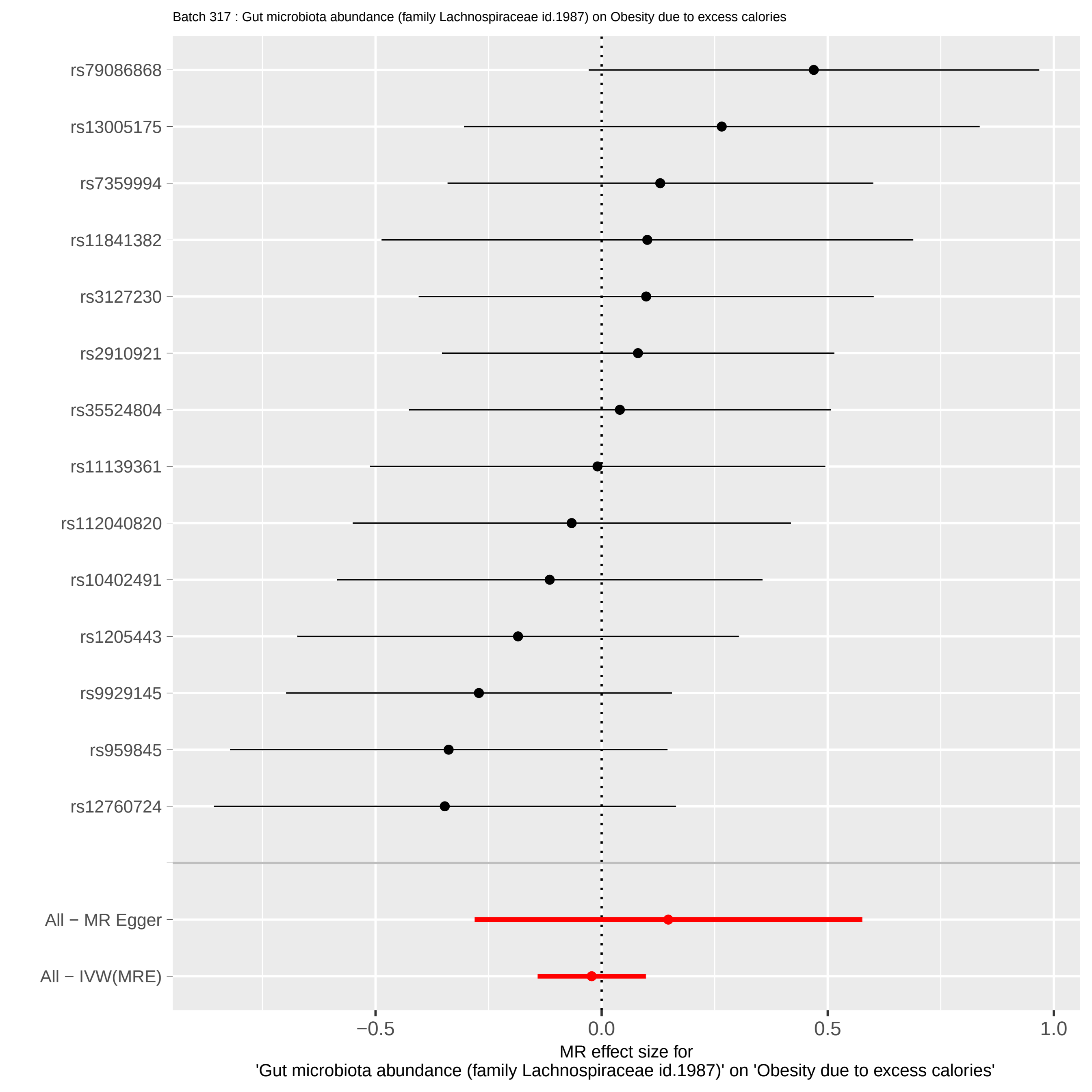

Batch 317 : Gut microbiota abundance (family Lachnospiraceae id.1987) on Obesity due to excess calories
rs79086868
rs13005175
rs7359994
rs11841382
rs3127230
rs2910921
rs35524804
rs11139361
rs112040820
rs10402491
rs1205443
rs9929145
rs959845
rs12760724
All − MR Egger
All − IVW(MRE)
−0.5
0.0
0.5
1.0
MR effect size for
'Gut microbiota abundance (family Lachnospiraceae id.1987)' on 'Obesity due to excess calories'

Batch 318 : Gut microbiota abundance (family Lactobacillaceae id.1836) on Obesity due to excess calories

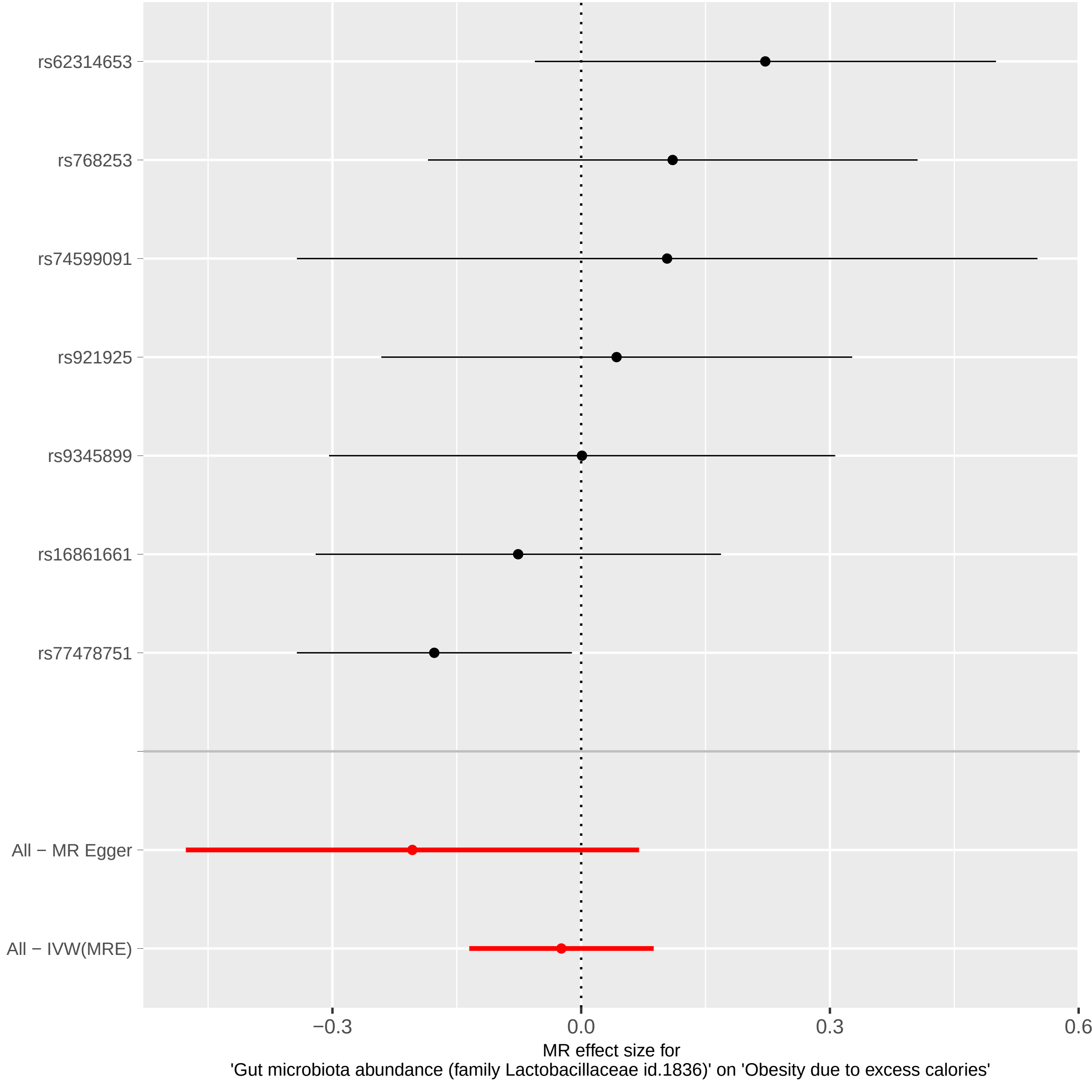

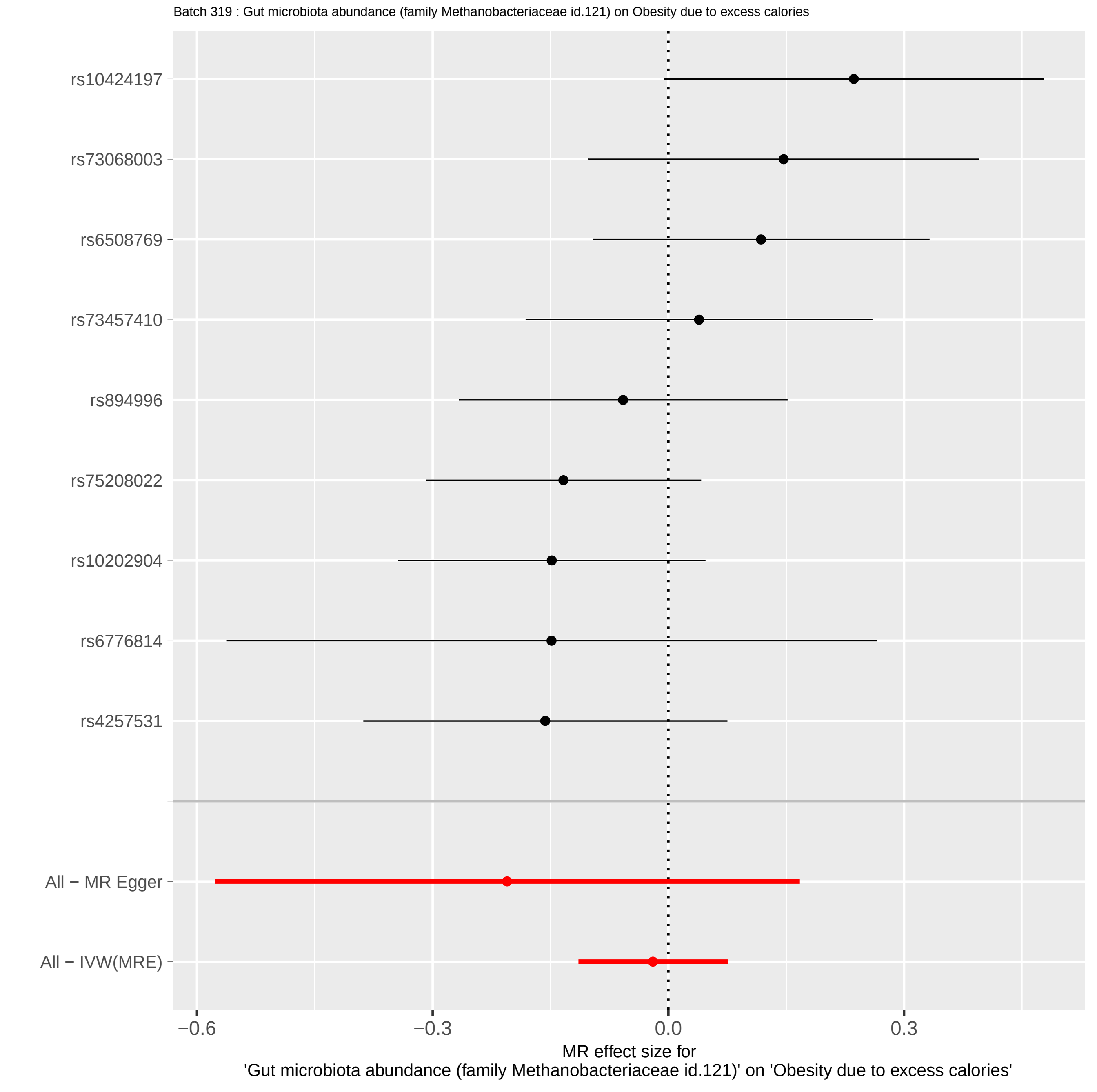

Batch 319 : Gut microbiota abundance (family Methanobacteriaceae id.121) on Obesity due to excess calories
rs10424197
rs73068003
rs6508769
rs73457410
rs894996
rs75208022
rs10202904
rs6776814
rs4257531
All − MR Egger
All − IVW(MRE)
−0.6
−0.3
0.0
0.3
MR effect size for
'Gut microbiota abundance (family Methanobacteriaceae id.121)' on 'Obesity due to excess calories'

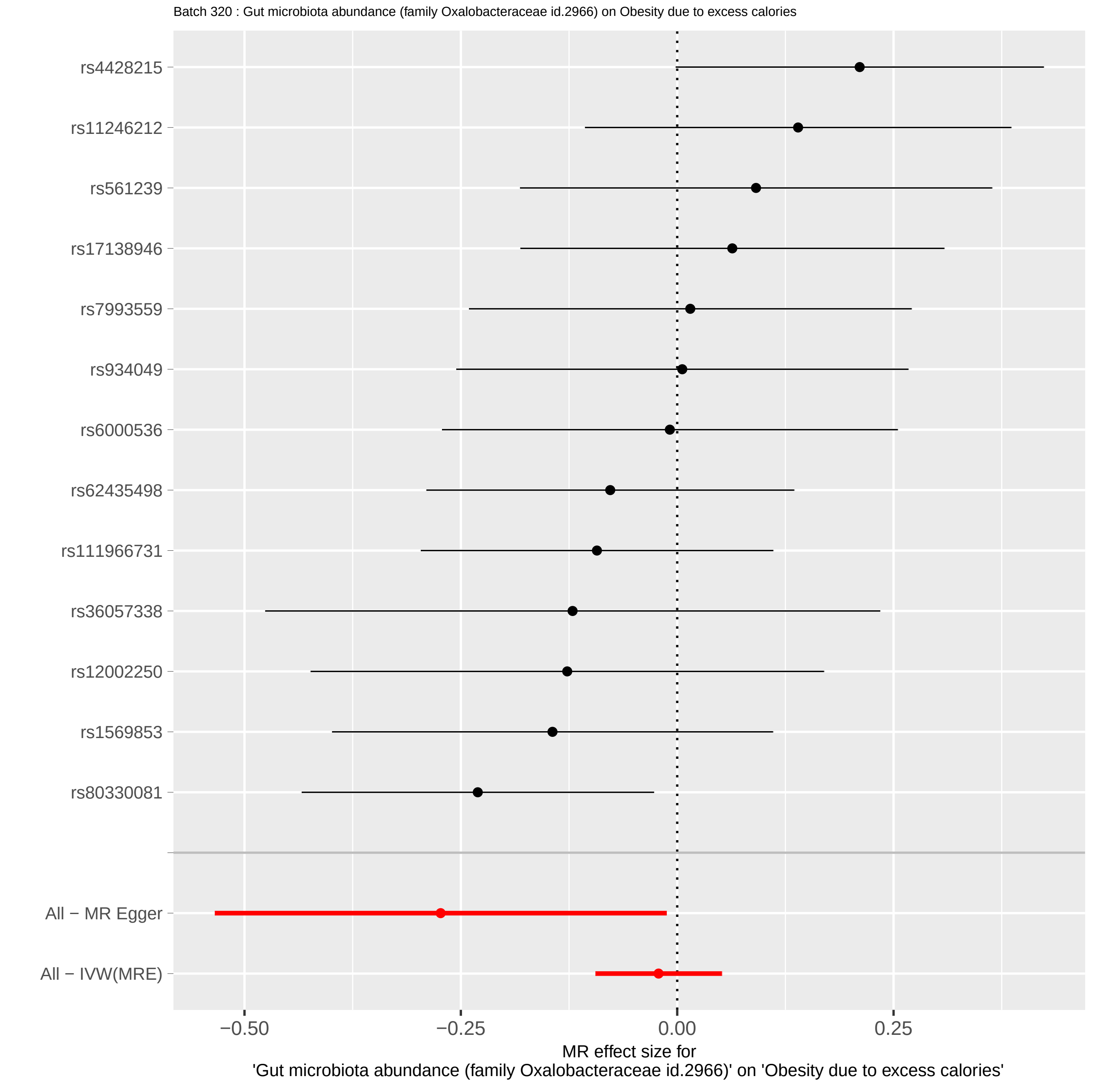

Batch 320 : Gut microbiota abundance (family Oxalobacteraceae id.2966) on Obesity due to excess calories
rs4428215
rs11246212
rs561239
rs17138946
rs7993559
rs934049
rs6000536
rs62435498
rs111966731
rs36057338
rs12002250
rs1569853
rs80330081
All − MR Egger
All − IVW(MRE)
−0.50
−0.25
0.00
0.25
MR effect size for
'Gut microbiota abundance (family Oxalobacteraceae id.2966)' on 'Obesity due to excess calories'

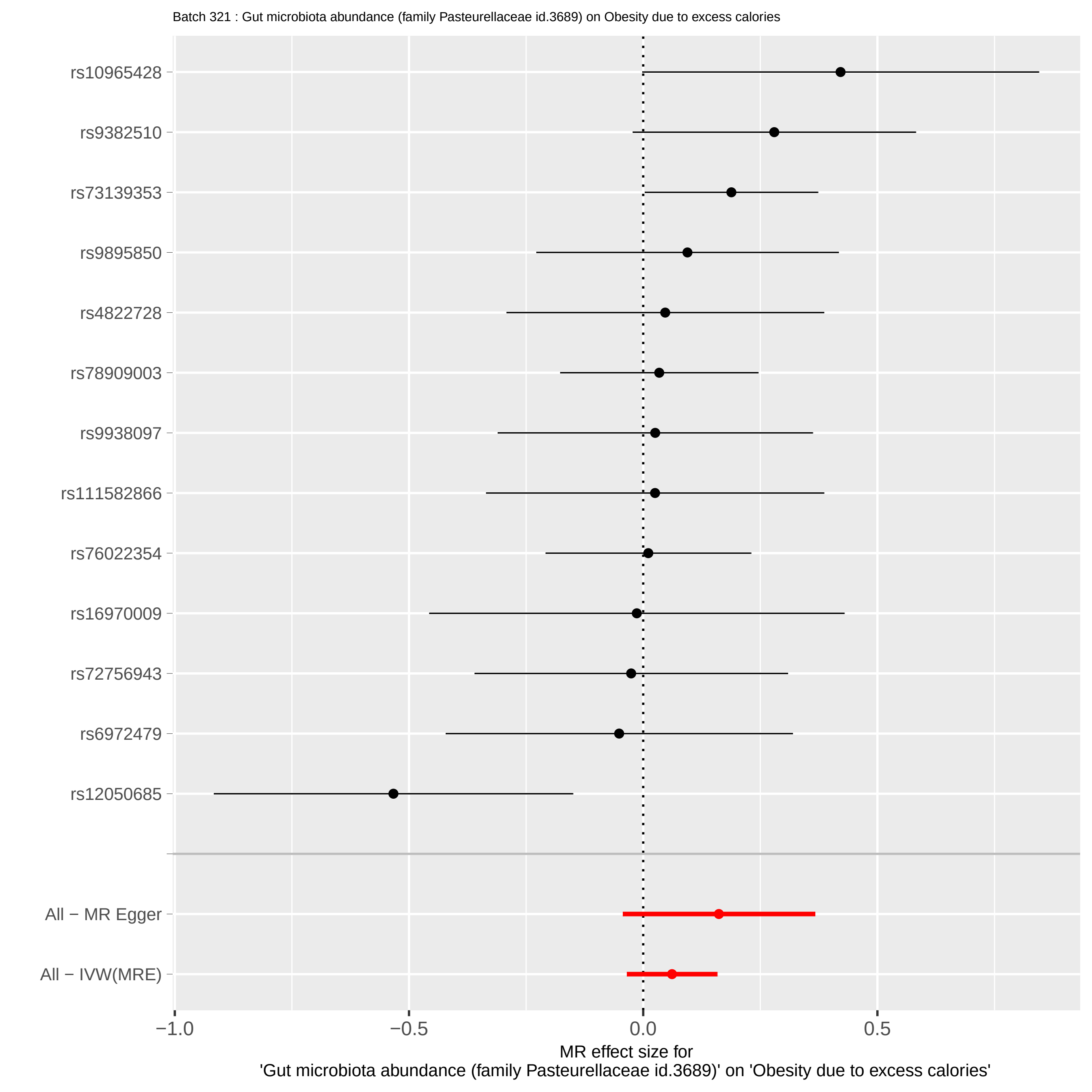

Batch 321 : Gut microbiota abundance (family Pasteurellaceae id.3689) on Obesity due to excess calories
rs10965428
rs9382510
rs73139353
rs9895850
rs4822728
rs78909003
rs9938097
rs111582866
rs76022354
rs16970009
rs72756943
rs6972479
rs12050685
All − MR Egger
All − IVW(MRE)
−1.0
−0.5
0.0
0.5
MR effect size for
'Gut microbiota abundance (family Pasteurellaceae id.3689)' on 'Obesity due to excess calories'

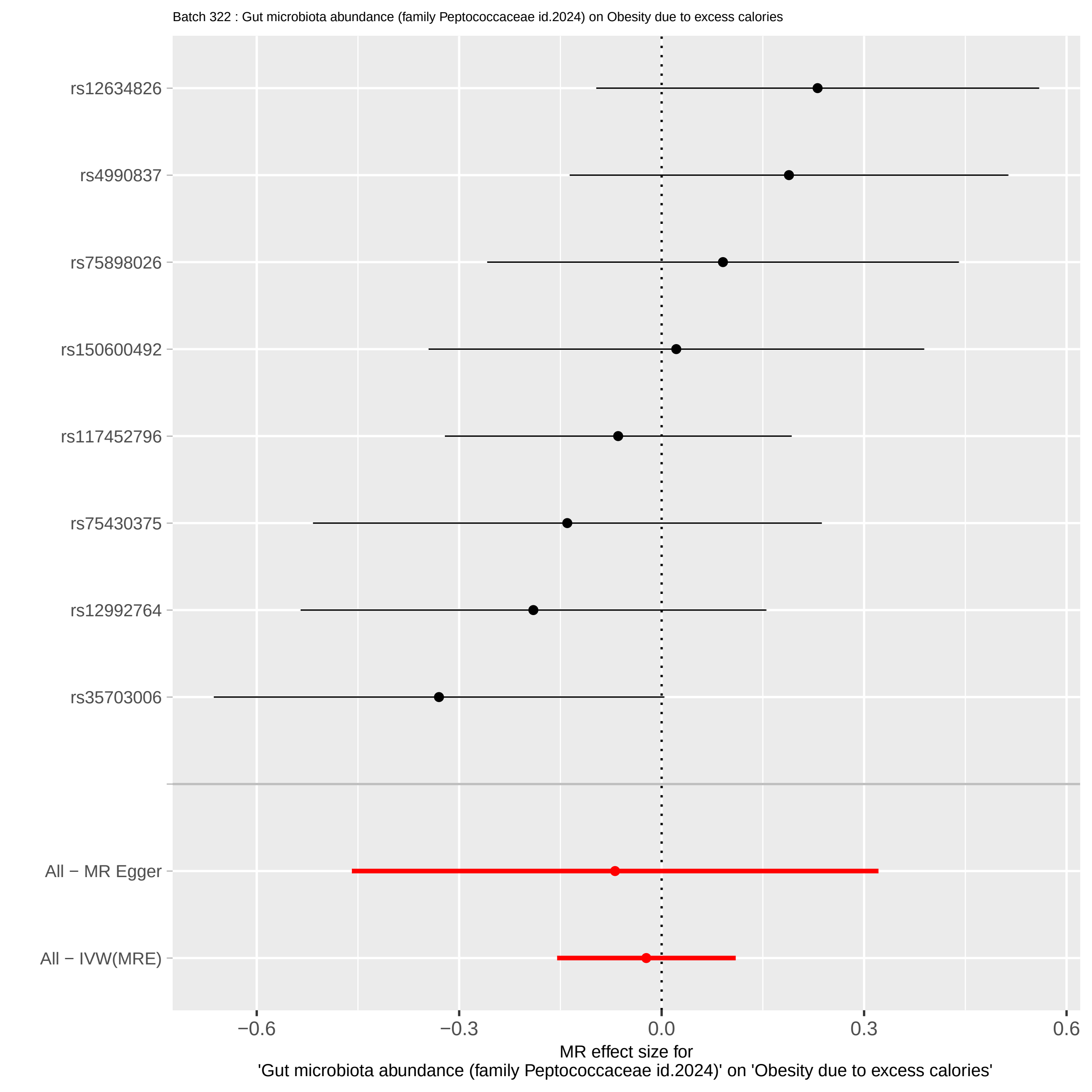

Batch 322 : Gut microbiota abundance (family Peptococcaceae id.2024) on Obesity due to excess calories
rs12634826
rs4990837
rs75898026
rs150600492
rs117452796
rs75430375
rs12992764
rs35703006
All − MR Egger
All − IVW(MRE)
−0.6
−0.3
0.0
0.3
0.6
MR effect size for
'Gut microbiota abundance (family Peptococcaceae id.2024)' on 'Obesity due to excess calories'

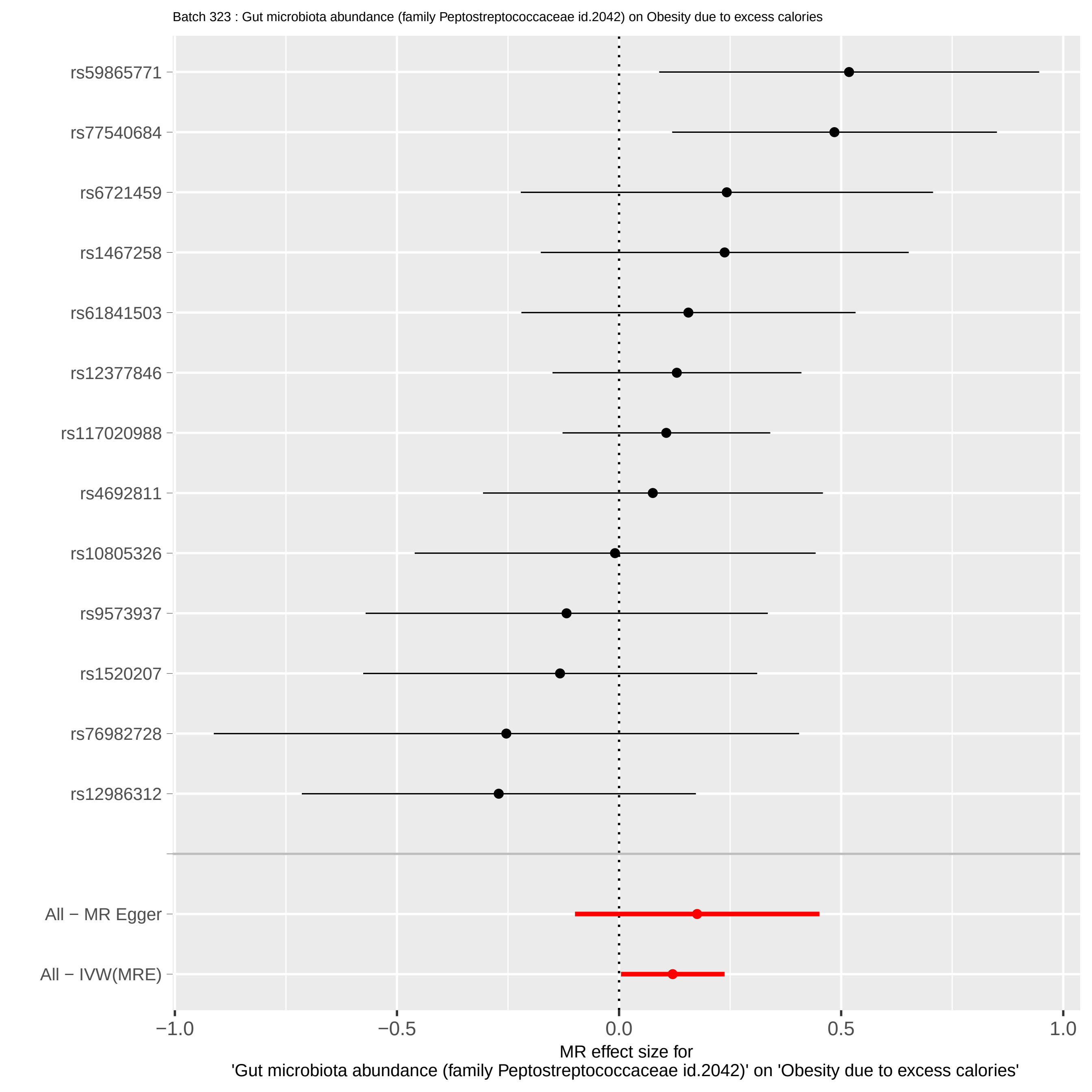

Batch 323 : Gut microbiota abundance (family Peptostreptococcaceae id.2042) on Obesity due to excess calories
rs59865771
rs77540684
rs6721459
rs1467258
rs61841503
rs12377846
rs117020988
rs4692811
rs10805326
rs9573937
rs1520207
rs76982728
rs12986312
All − MR Egger
All − IVW(MRE)
−1.0
−0.5
0.0
0.5
1.0
MR effect size for
'Gut microbiota abundance (family Peptostreptococcaceae id.2042)' on 'Obesity due to excess calories'

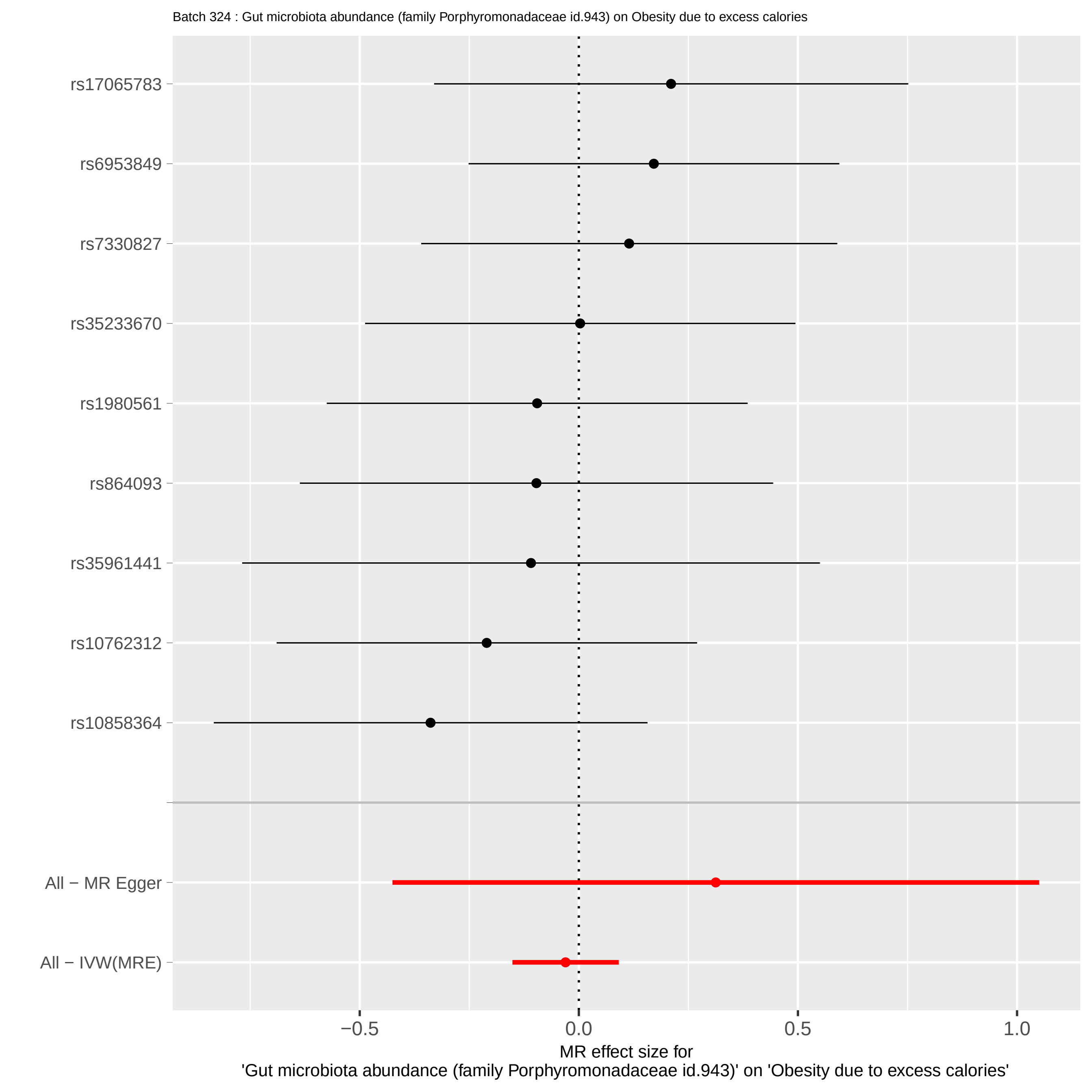

Batch 324 : Gut microbiota abundance (family Porphyromonadaceae id.943) on Obesity due to excess calories
rs17065783
rs6953849
rs7330827
rs35233670
rs1980561
rs864093
rs35961441
rs10762312
rs10858364
All − MR Egger
All − IVW(MRE)
−0.5
0.0
0.5
1.0
MR effect size for
'Gut microbiota abundance (family Porphyromonadaceae id.943)' on 'Obesity due to excess calories'

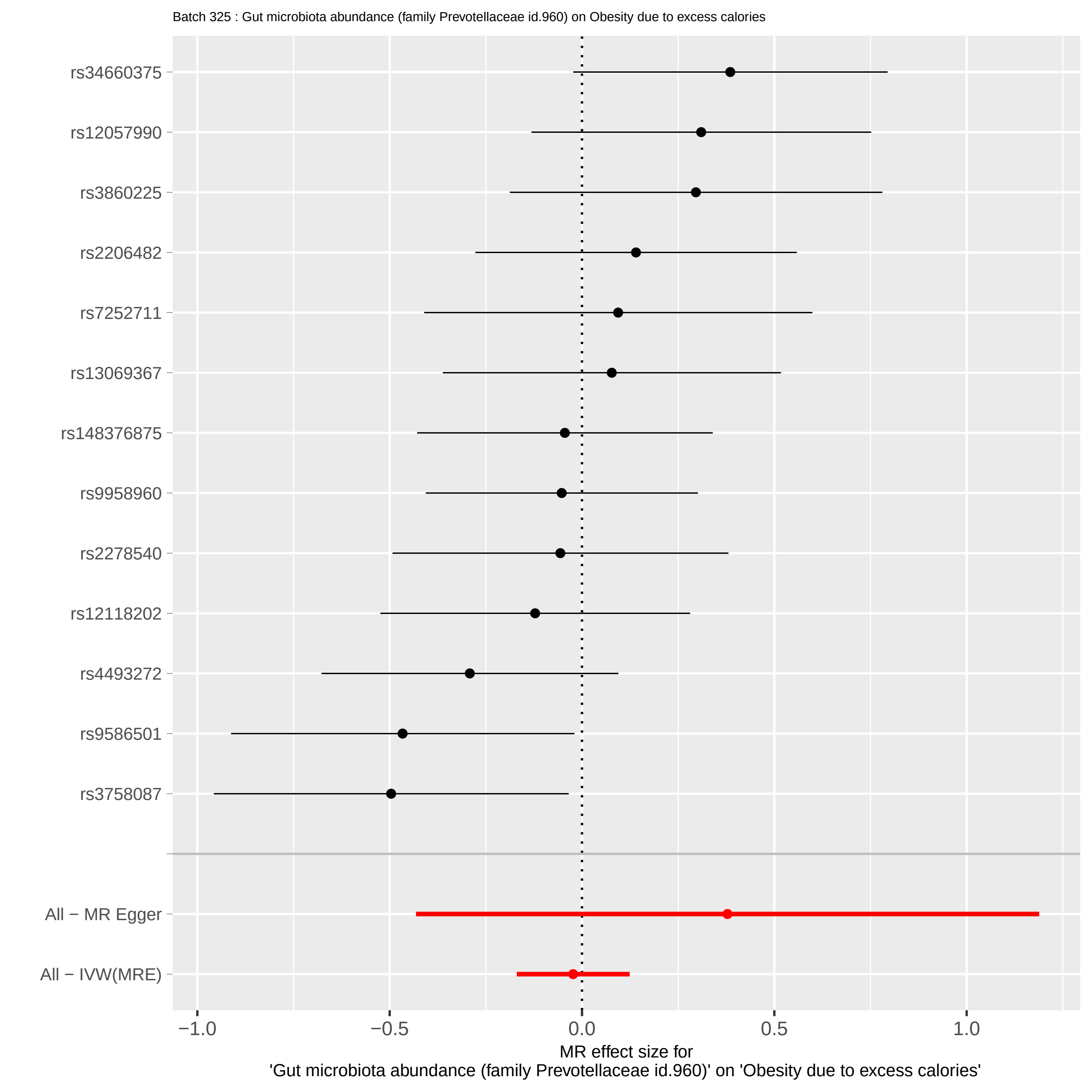
Batch 325 : Gut microbiota abundance (family Prevotellaceae id.960) on Obesity due to excess calories
rs34660375
rs12057990
rs3860225
rs2206482
rs7252711
rs13069367
rs148376875
rs9958960
rs2278540
rs12118202
rs4493272
rs9586501
rs3758087
All − MR Egger
All − IVW(MRE)
−1.0
−0.5
0.0
0.5
1.0
MR effect size for
'Gut microbiota abundance (family Prevotellaceae id.960)' on 'Obesity due to excess calories'

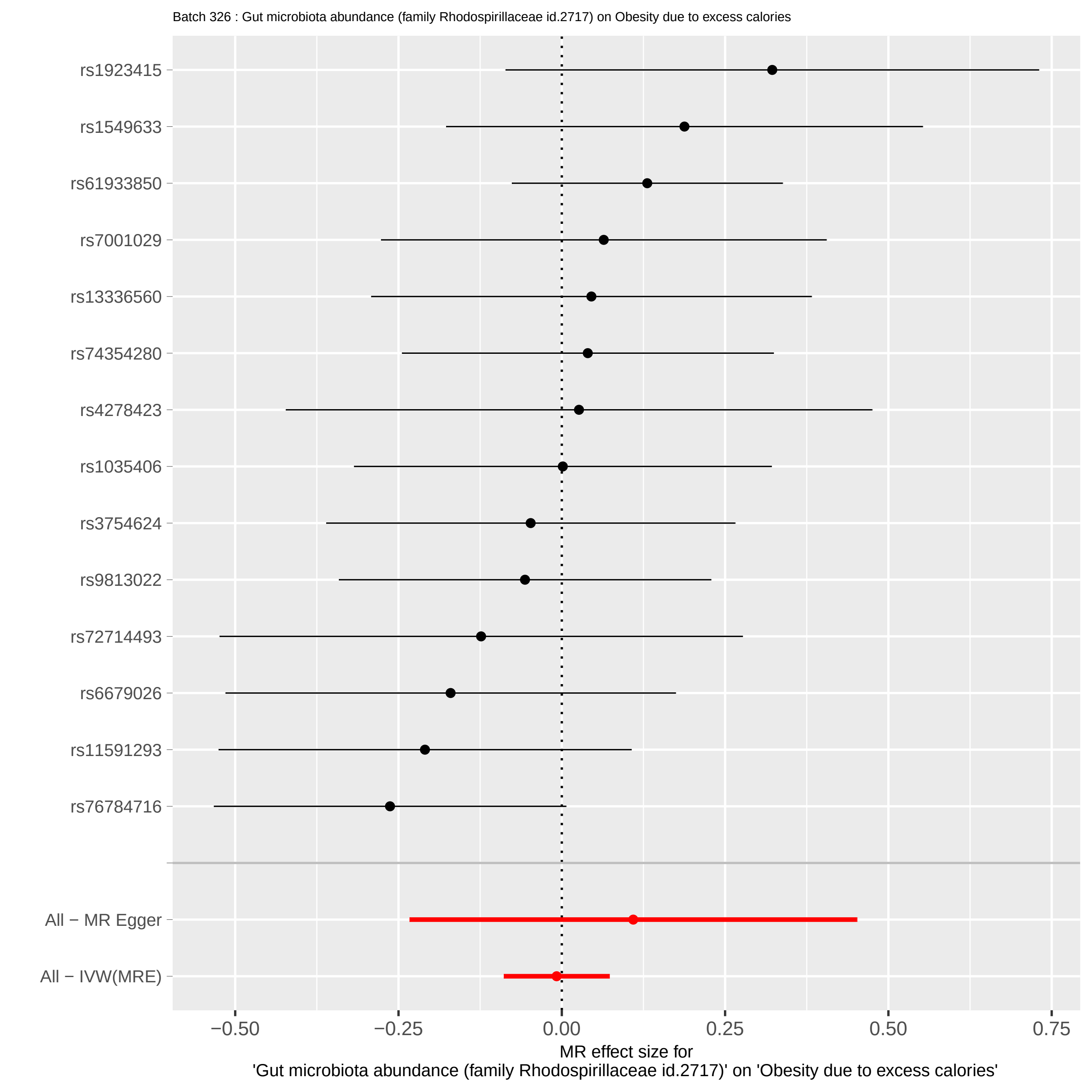

Batch 326 : Gut microbiota abundance (family Rhodospirillaceae id.2717) on Obesity due to excess calories
rs1923415
rs1549633
rs61933850
rs7001029
rs13336560
rs74354280
rs4278423
rs1035406
rs3754624
rs9813022
rs72714493
rs6679026
rs11591293
rs76784716
All − MR Egger
All − IVW(MRE)
−0.50
−0.25
0.00
0.25
0.50
0.75
MR effect size for
'Gut microbiota abundance (family Rhodospirillaceae id.2717)' on 'Obesity due to excess calories'

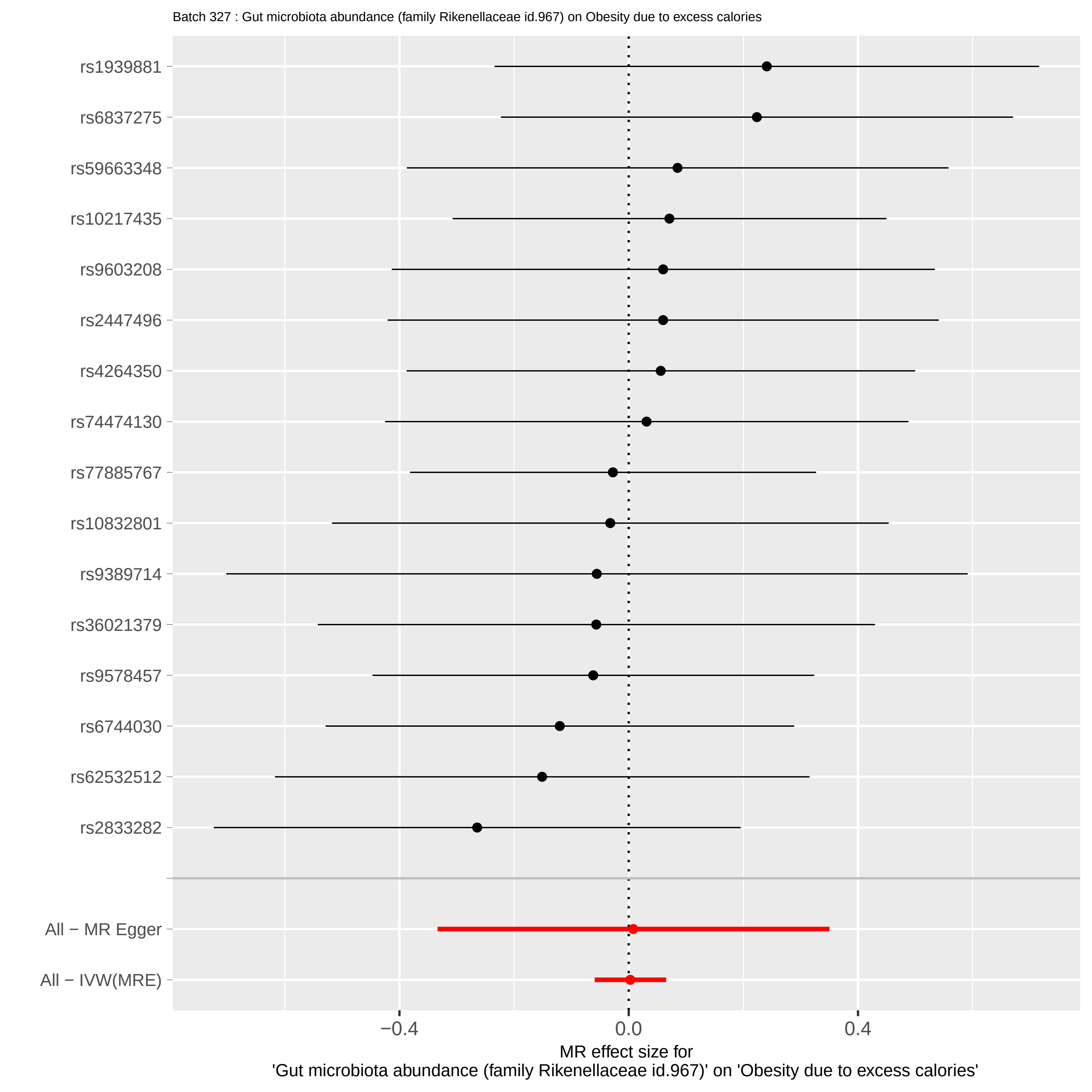
Batch 327 : Gut microbiota abundance (family Rikenellaceae id.967) on Obesity due to excess calories
rs1939881
rs6837275
rs59663348
rs10217435
rs9603208
rs2447496
rs4264350
rs74474130
rs77885767
rs10832801
rs9389714
rs36021379
rs9578457
rs6744030
rs62532512
rs2833282
All − MR Egger
All − IVW(MRE)
−0.4
0.0
0.4
MR effect size for
'Gut microbiota abundance (family Rikenellaceae id.967)' on 'Obesity due to excess calories'

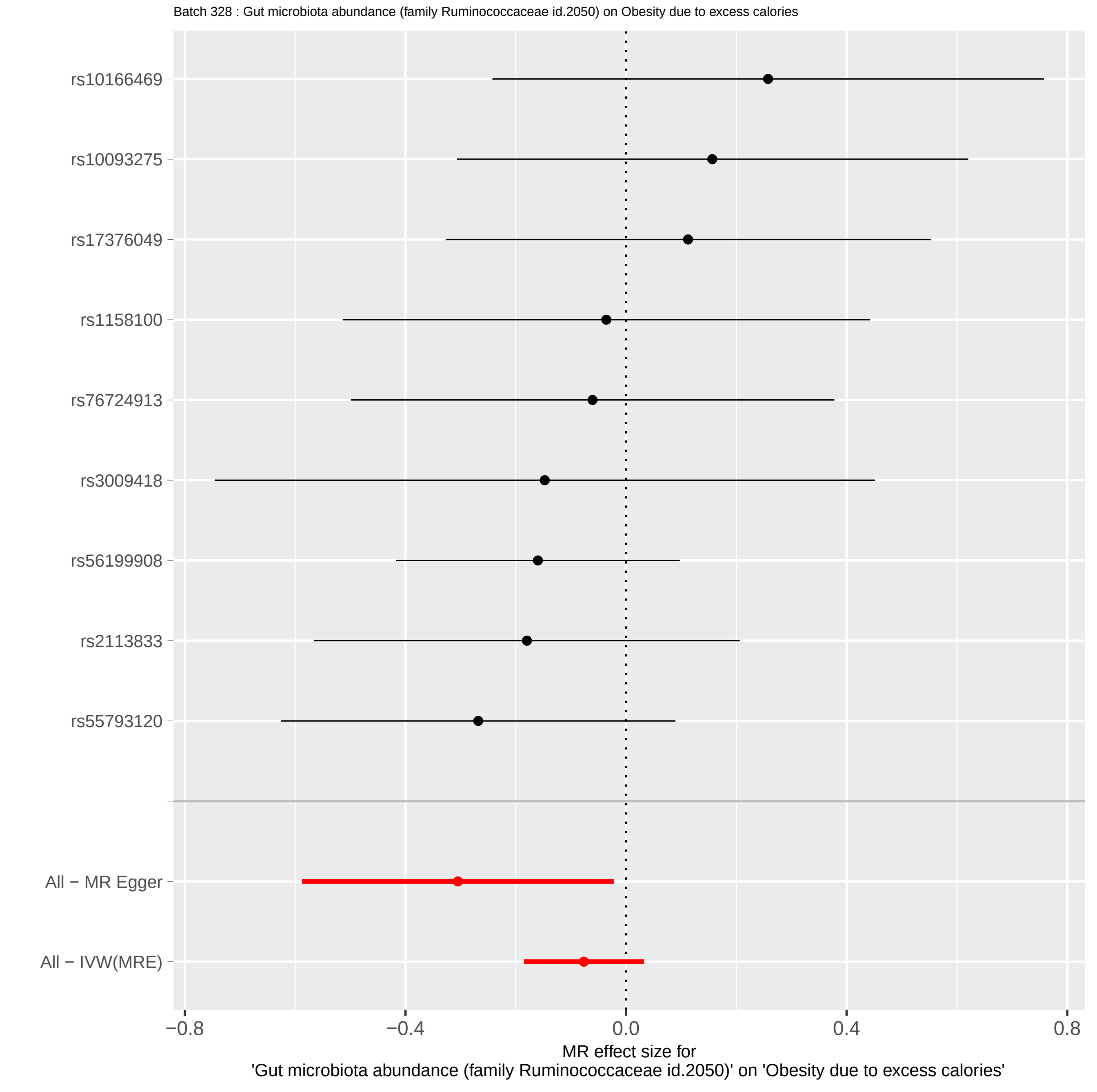

Batch 328 : Gut microbiota abundance (family Ruminococcaceae id.2050) on Obesity due to excess calories
rs10166469
rs10093275
rs17376049
rs1158100
rs76724913
rs3009418
rs56199908
rs2113833
rs55793120
All − MR Egger
All − IVW(MRE)
−0.8
−0.4
0.0
0.4
0.8
MR effect size for
'Gut microbiota abundance (family Ruminococcaceae id.2050)' on 'Obesity due to excess calories'

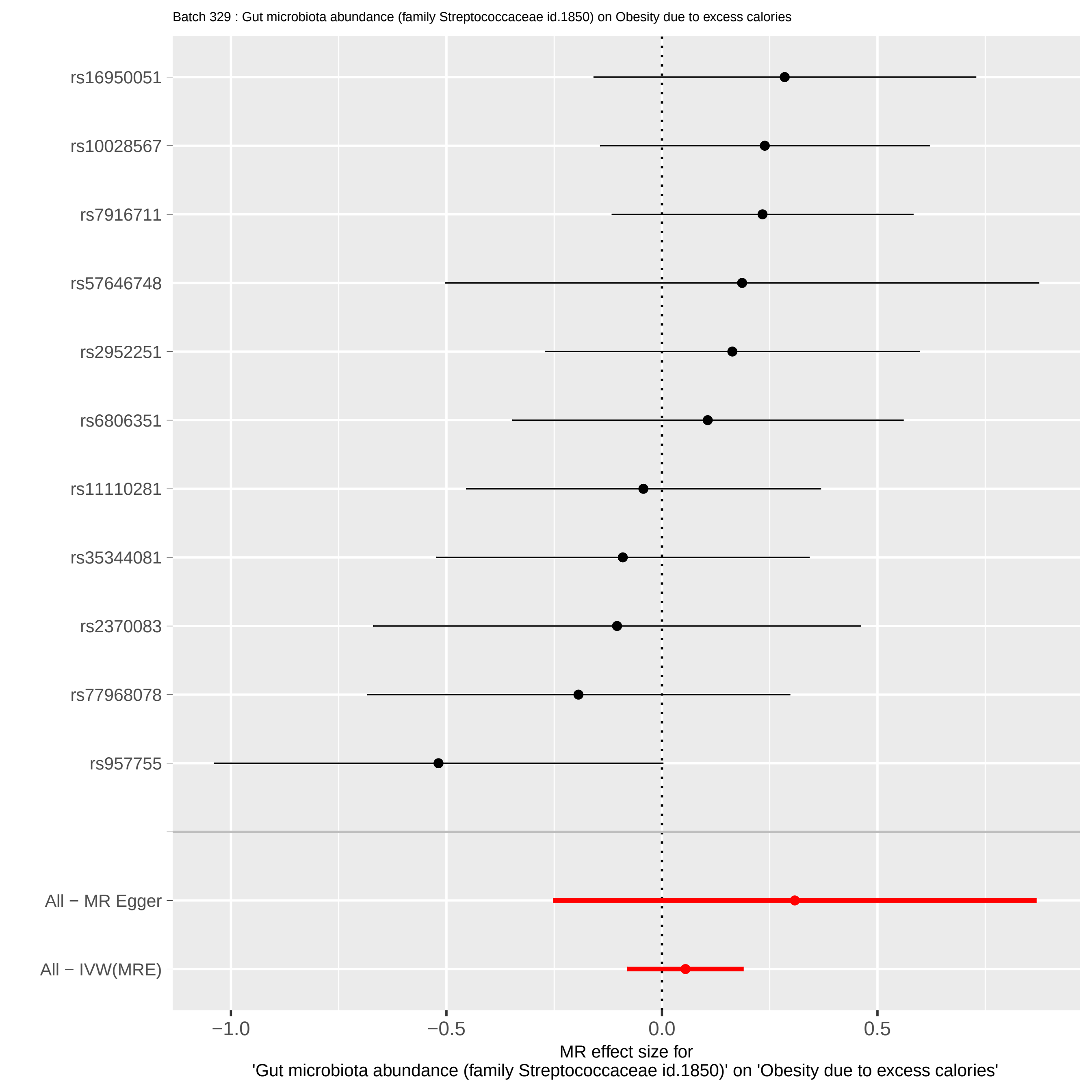

Batch 329 : Gut microbiota abundance (family Streptococcaceae id.1850) on Obesity due to excess calories
rs16950051
rs10028567
rs7916711
rs57646748
rs2952251
rs6806351
rs11110281
rs35344081
rs2370083
rs77968078
rs957755
All − MR Egger
All − IVW(MRE)
−1.0
−0.5
0.0
0.5
MR effect size for
'Gut microbiota abundance (family Streptococcaceae id.1850)' on 'Obesity due to excess calories'

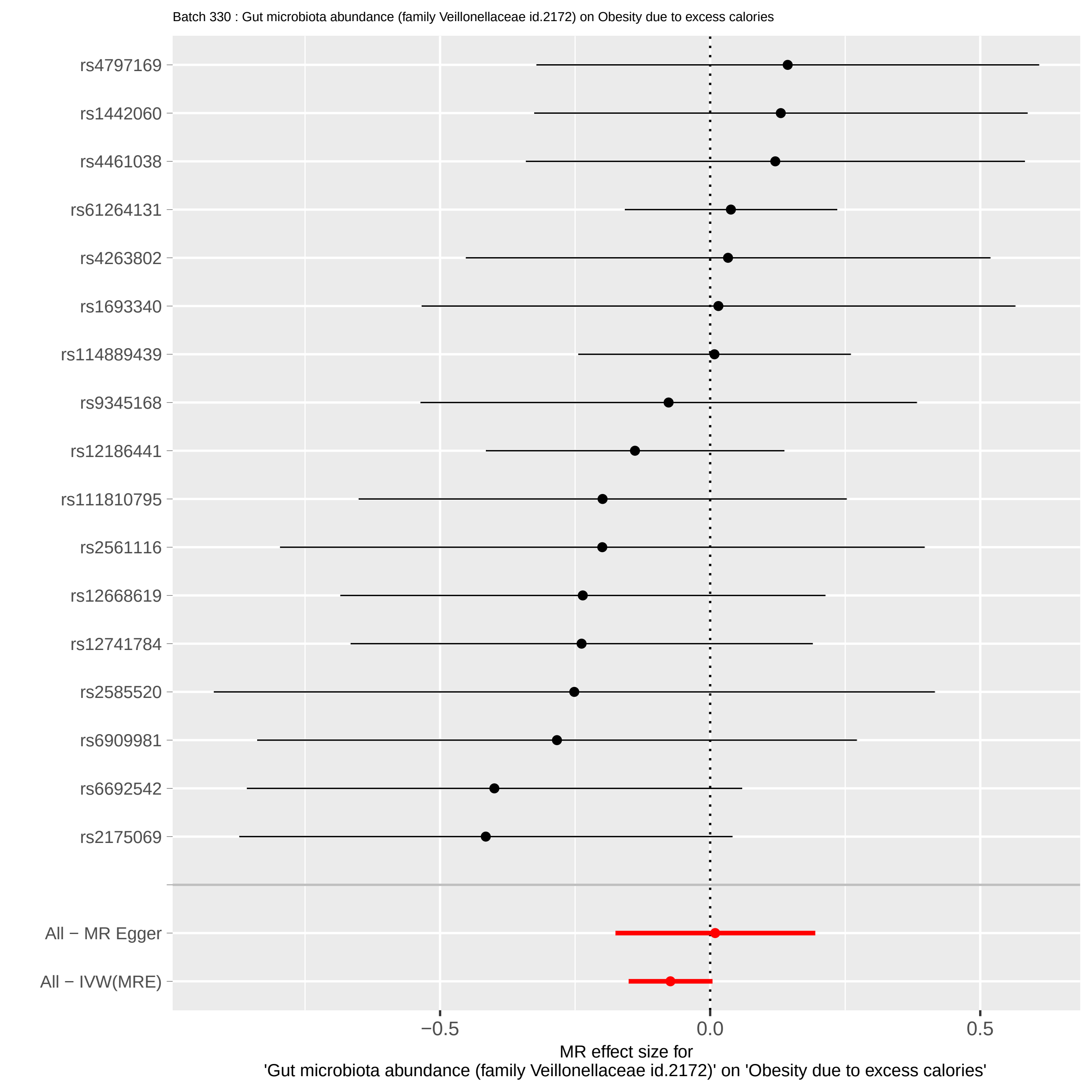

Batch 330 : Gut microbiota abundance (family Veillonellaceae id.2172) on Obesity due to excess calories
rs4797169
rs1442060
rs4461038
rs61264131
rs4263802
rs1693340
rs114889439
rs9345168
rs12186441
rs111810795
rs2561116
rs12668619
rs12741784
rs2585520
rs6909981
rs6692542
rs2175069
All − MR Egger
All − IVW(MRE)
−0.5
0.0
0.5
MR effect size for
'Gut microbiota abundance (family Veillonellaceae id.2172)' on 'Obesity due to excess calories'

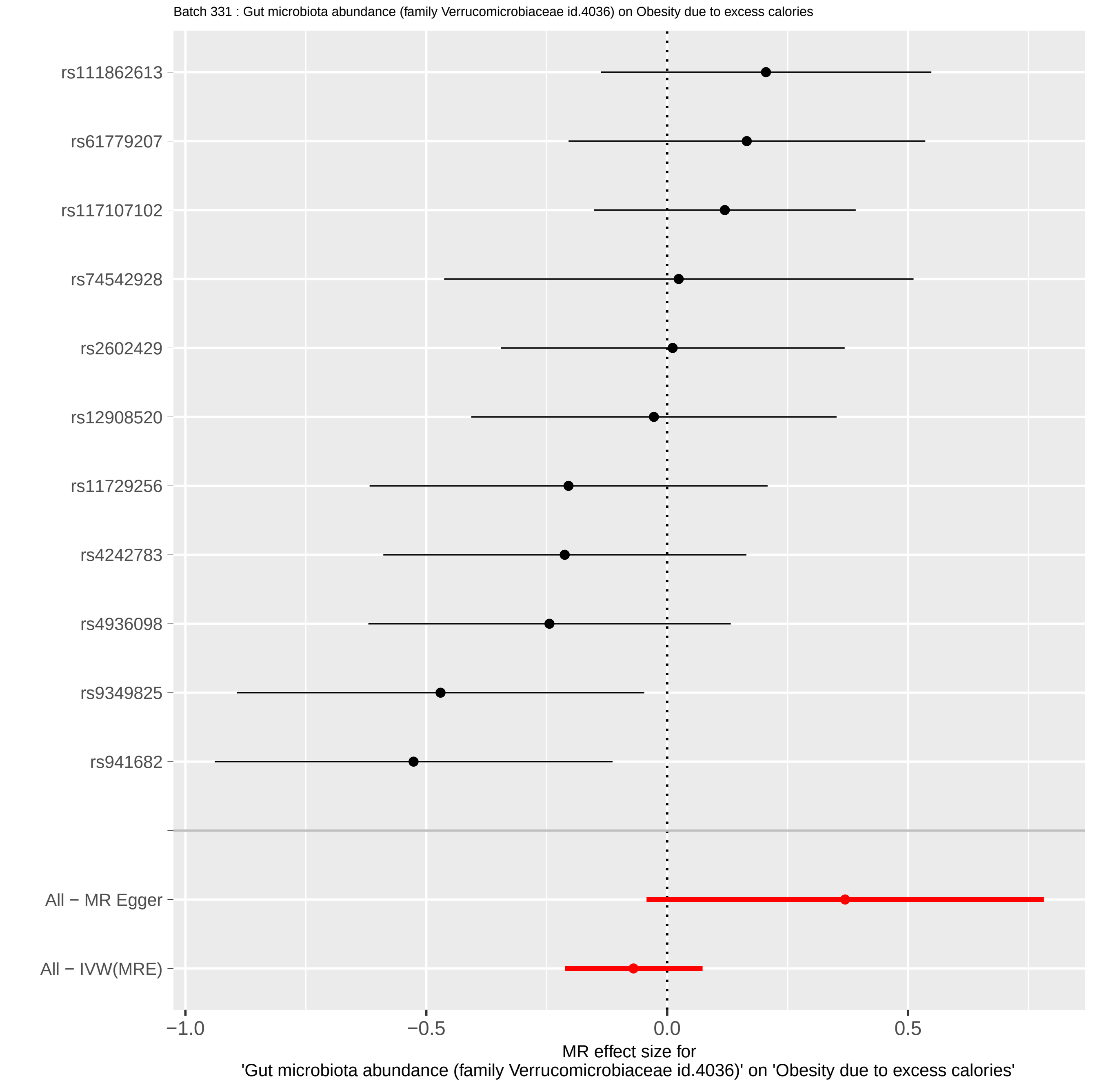
Batch 331 : Gut microbiota abundance (family Verrucomicrobiaceae id.4036) on Obesity due to excess calories
rs111862613
rs61779207
rs117107102
rs74542928
rs2602429
rs12908520
rs11729256
rs4242783
rs4936098
rs9349825
rs941682
All − MR Egger
All − IVW(MRE)
−1.0
−0.5
0.0
0.5
MR effect size for
'Gut microbiota abundance (family Verrucomicrobiaceae id.4036)' on 'Obesity due to excess calories'

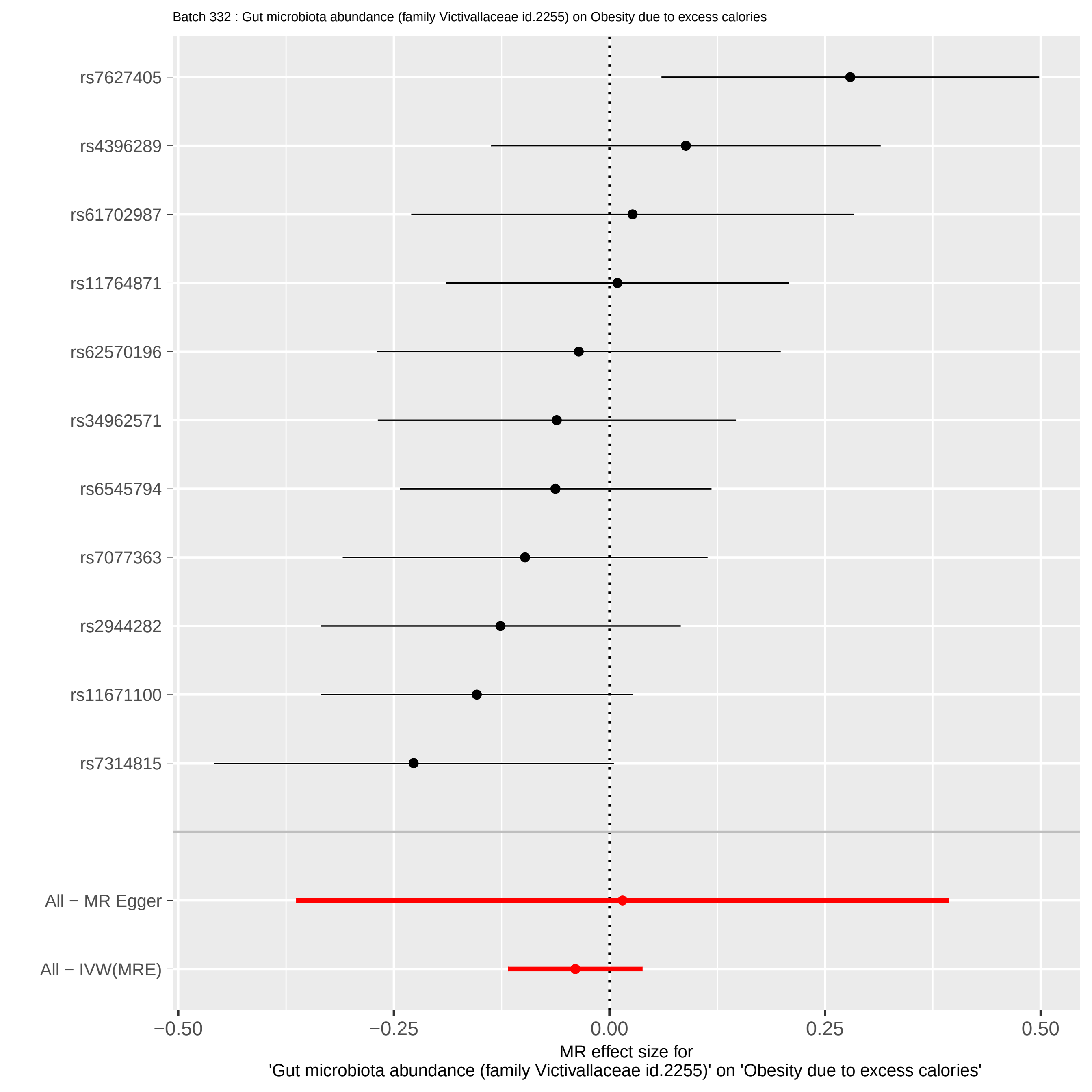
Batch 332 : Gut microbiota abundance (family Victivallaceae id.2255) on Obesity due to excess calories
rs7627405
rs4396289
rs61702987
rs11764871
rs62570196
rs34962571
rs6545794
rs7077363
rs2944282
rs11671100
rs7314815
All − MR Egger
All − IVW(MRE)
−0.50
−0.25
0.00
0.25
0.50
MR effect size for
'Gut microbiota abundance (family Victivallaceae id.2255)' on 'Obesity due to excess calories'

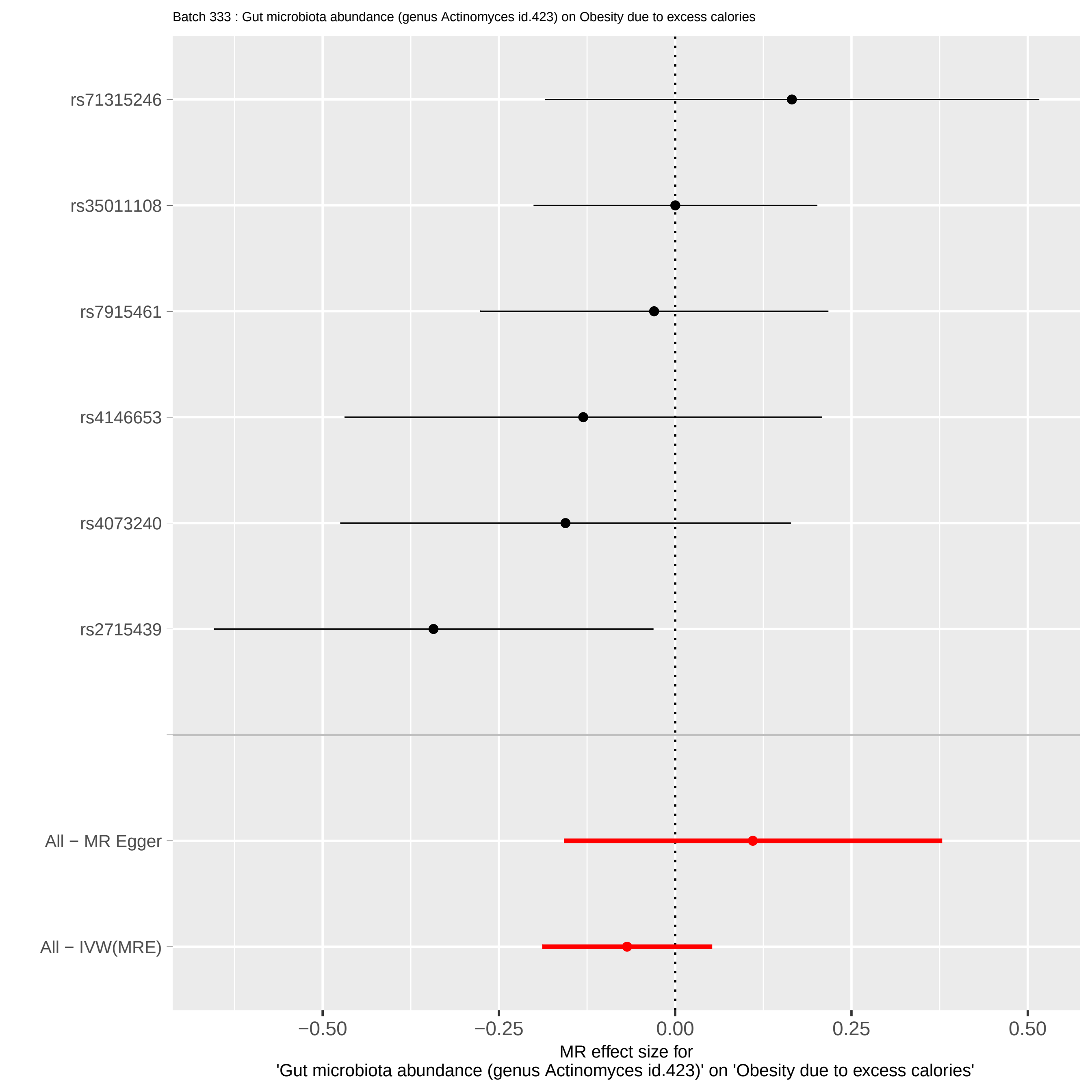
Batch 333 : Gut microbiota abundance (genus Actinomyces id.423) on Obesity due to excess calories
rs71315246
rs35011108
rs7915461
rs4146653
rs4073240
rs2715439
All − MR Egger
All − IVW(MRE)
−0.50
−0.25
0.00
0.25
0.50
MR effect size for
'Gut microbiota abundance (genus Actinomyces id.423)' on 'Obesity due to excess calories'

Batch 334 : Gut microbiota abundance (genus Adlercreutzia id.812) on Obesity due to excess calories

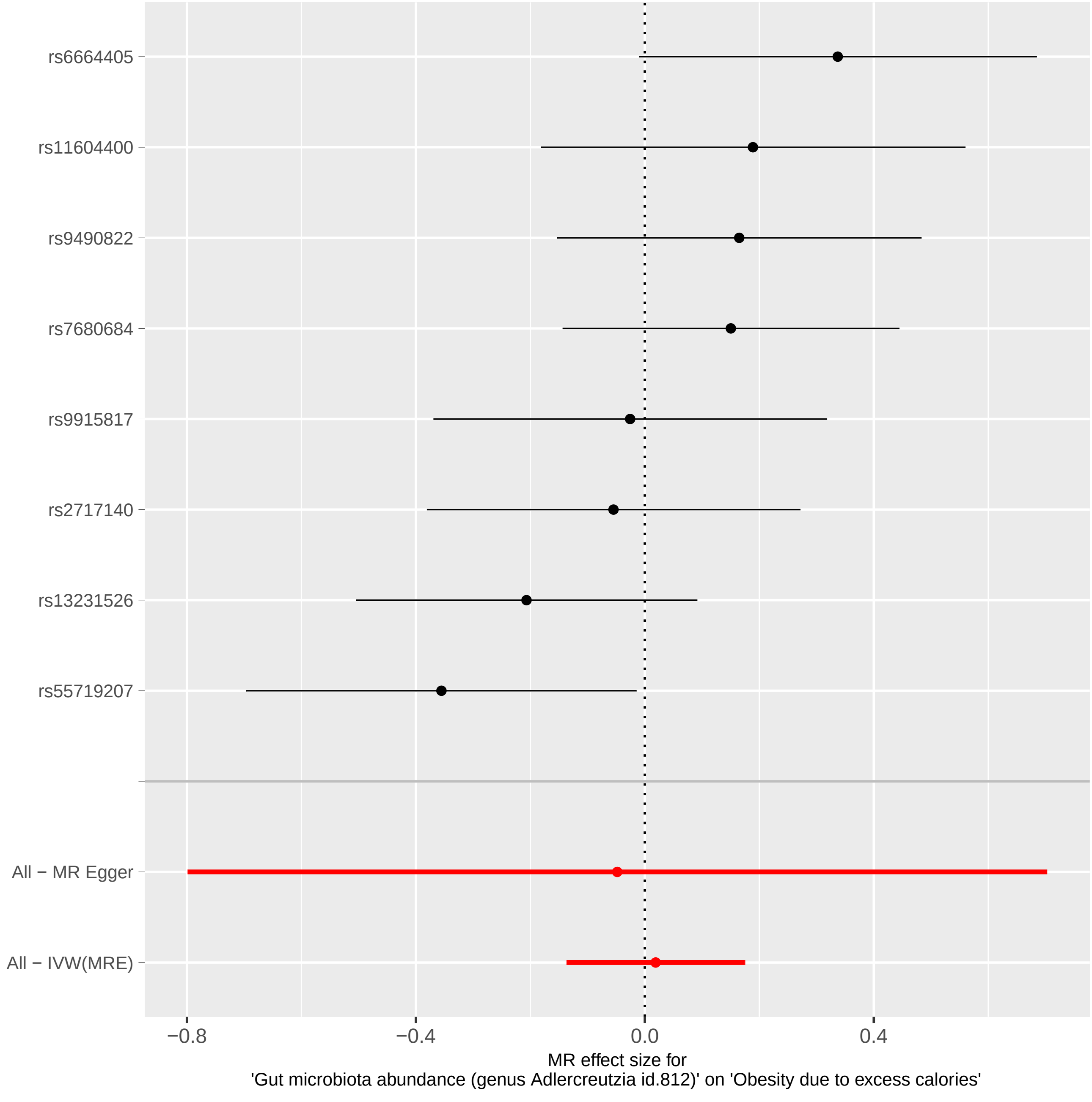

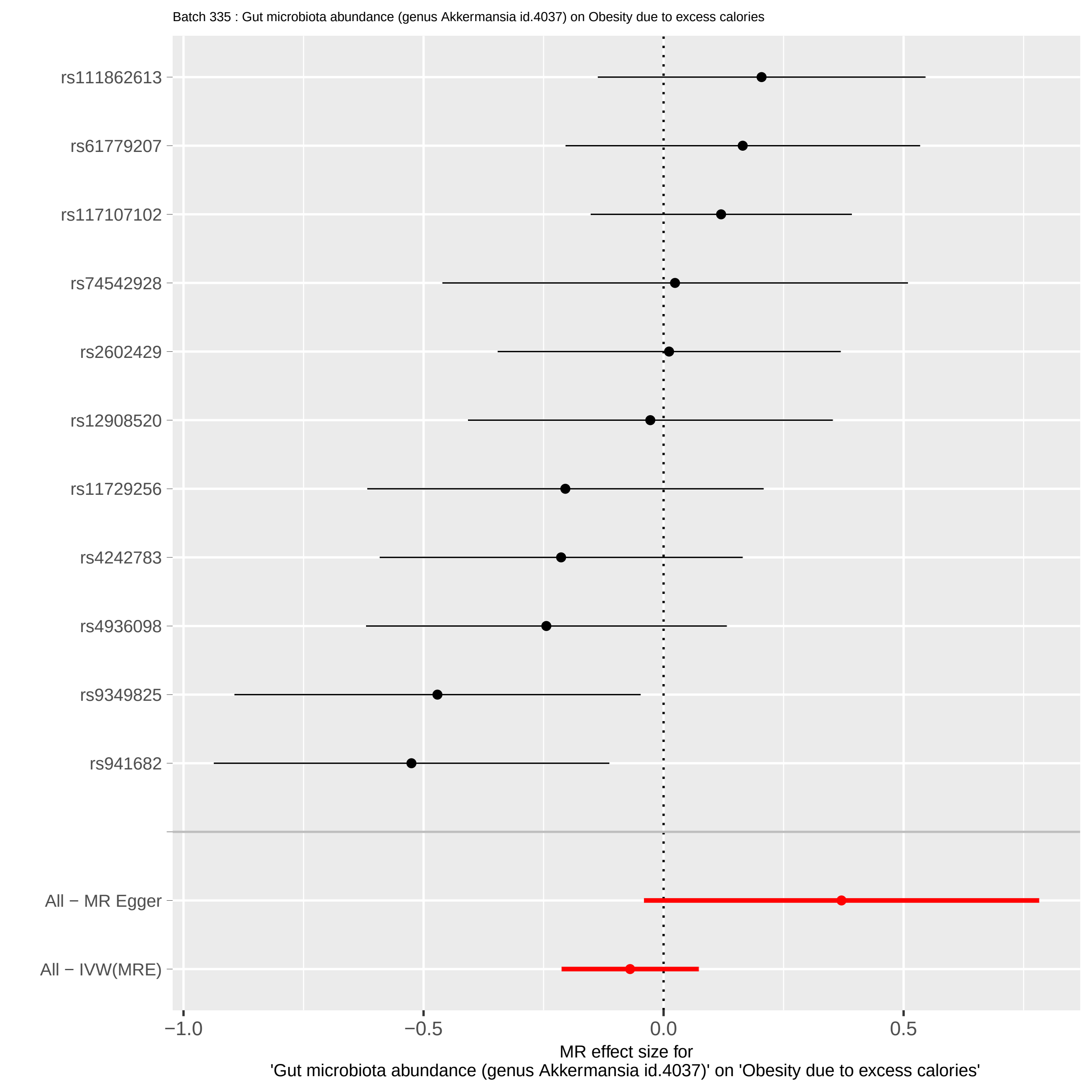

Batch 335 : Gut microbiota abundance (genus Akkermansia id.4037) on Obesity due to excess calories
rs111862613
rs61779207
rs117107102
rs74542928
rs2602429
rs12908520
rs11729256
rs4242783
rs4936098
rs9349825
rs941682
All − MR Egger
All − IVW(MRE)
−1.0
−0.5
0.0
0.5
MR effect size for
'Gut microbiota abundance (genus Akkermansia id.4037)' on 'Obesity due to excess calories'

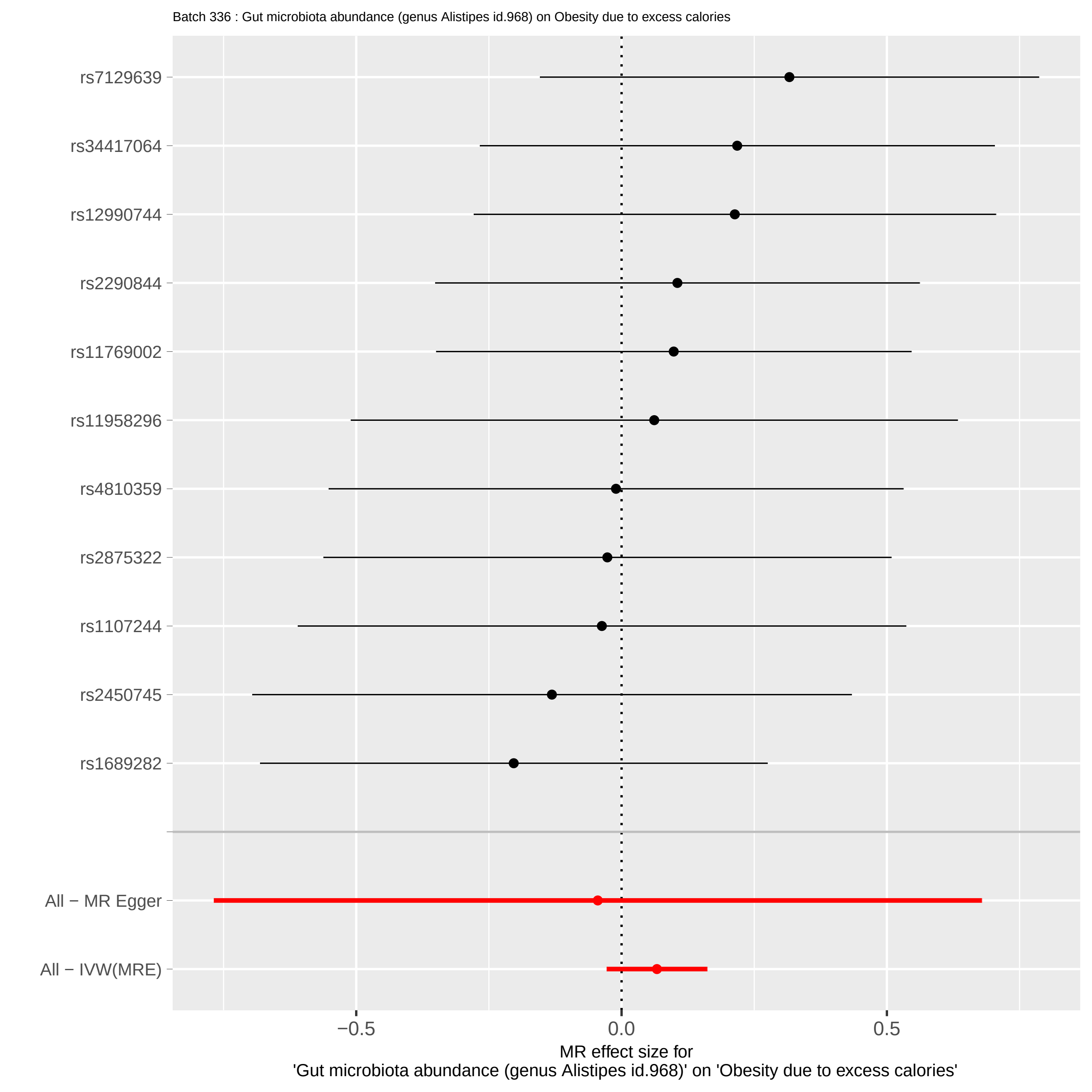

Batch 336 : Gut microbiota abundance (genus Alistipes id.968) on Obesity due to excess calories
rs7129639
rs34417064
rs12990744
rs2290844
rs11769002
rs11958296
rs4810359
rs2875322
rs1107244
rs2450745
rs1689282
All − MR Egger
All − IVW(MRE)
−0.5
0.0
0.5
MR effect size for
'Gut microbiota abundance (genus Alistipes id.968)' on 'Obesity due to excess calories'

Batch 337 : Gut microbiota abundance (genus Allisonella id.2174) on Obesity due to excess calories

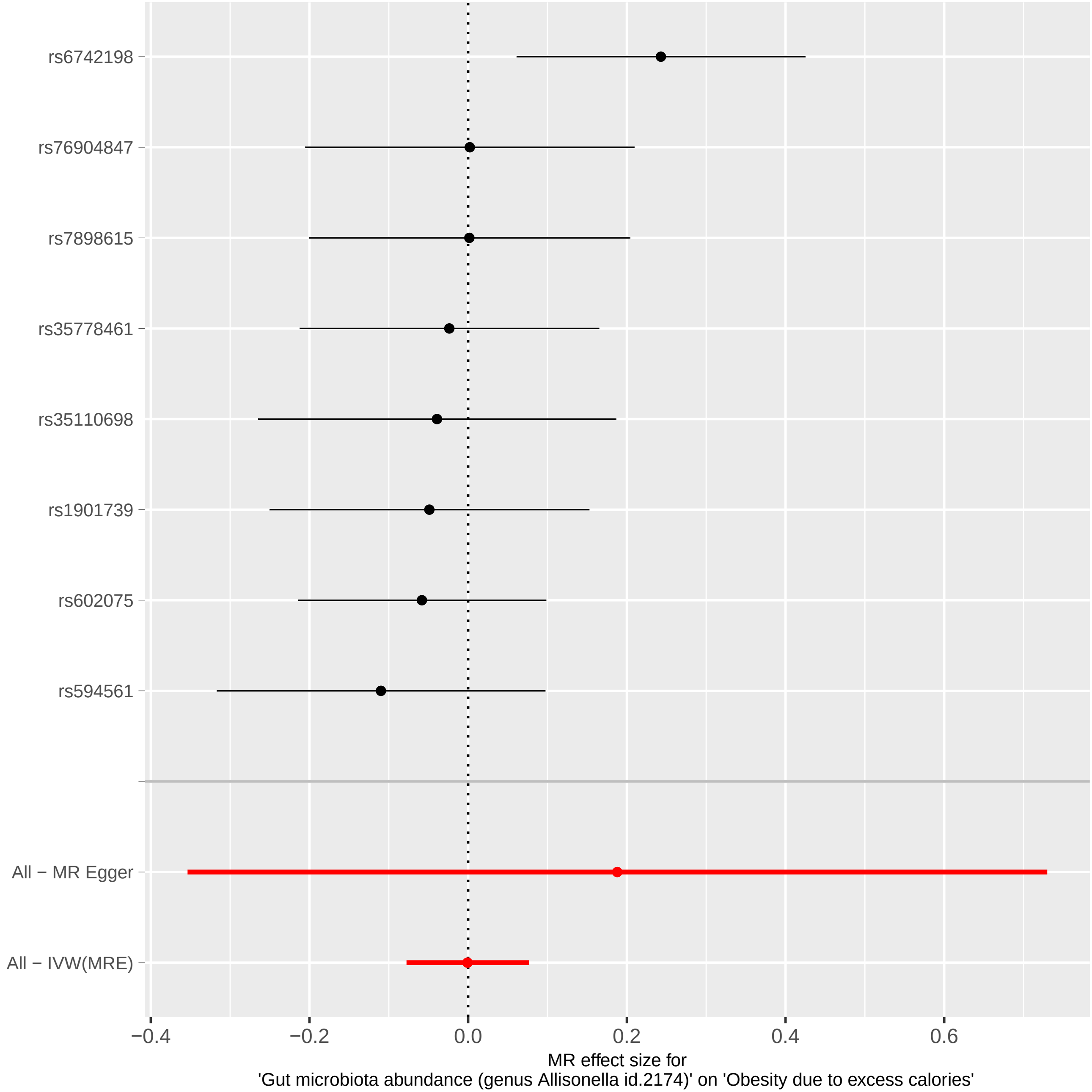

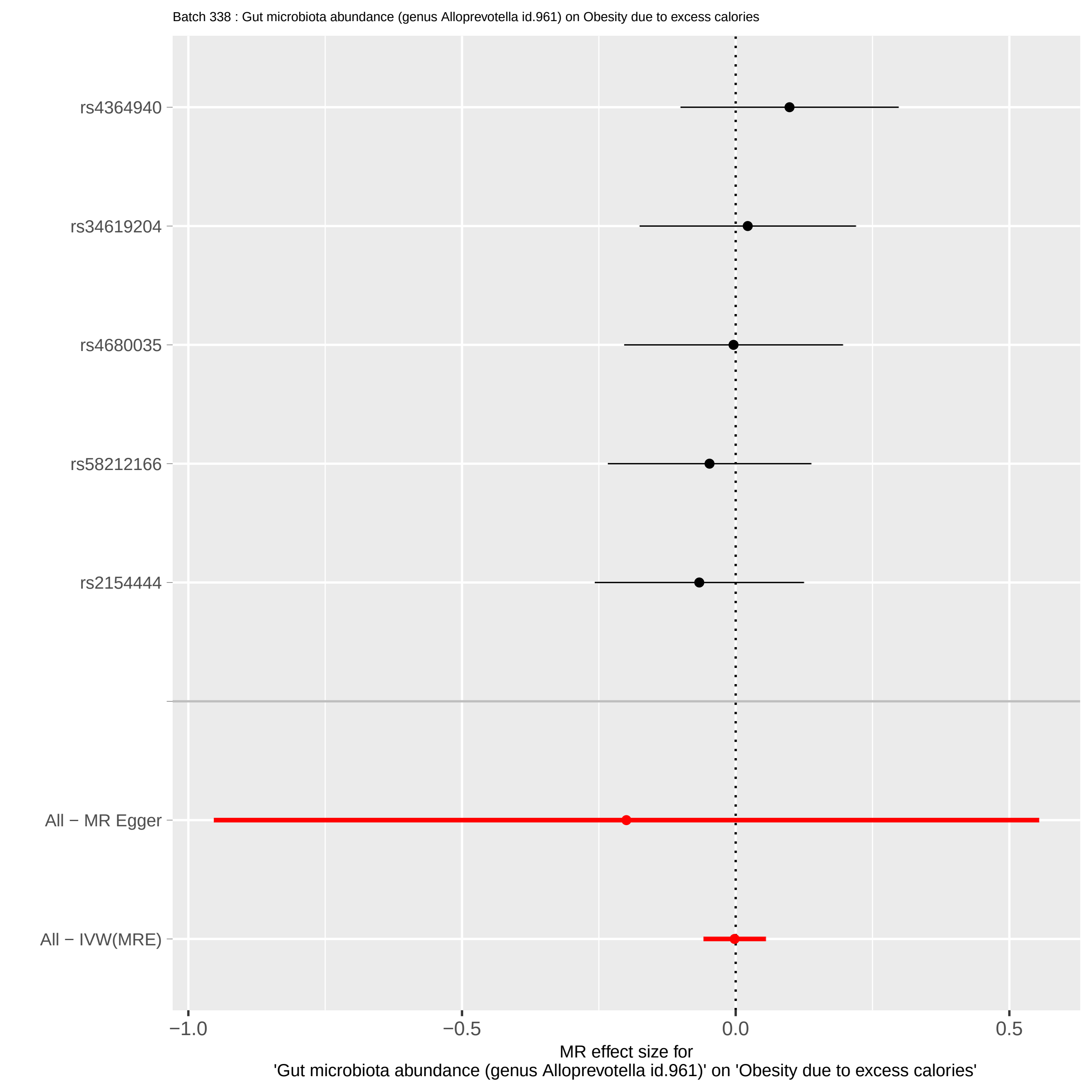

Batch 338 : Gut microbiota abundance (genus Alloprevotella id.961) on Obesity due to excess calories
rs4364940
rs34619204
rs4680035
rs58212166
rs2154444
All − MR Egger
All − IVW(MRE)
−1.0
−0.5
0.0
0.5
MR effect size for
'Gut microbiota abundance (genus Alloprevotella id.961)' on 'Obesity due to excess calories'

Batch 339 : Gut microbiota abundance (genus Anaerofilum id.2053) on Obesity due to excess calories

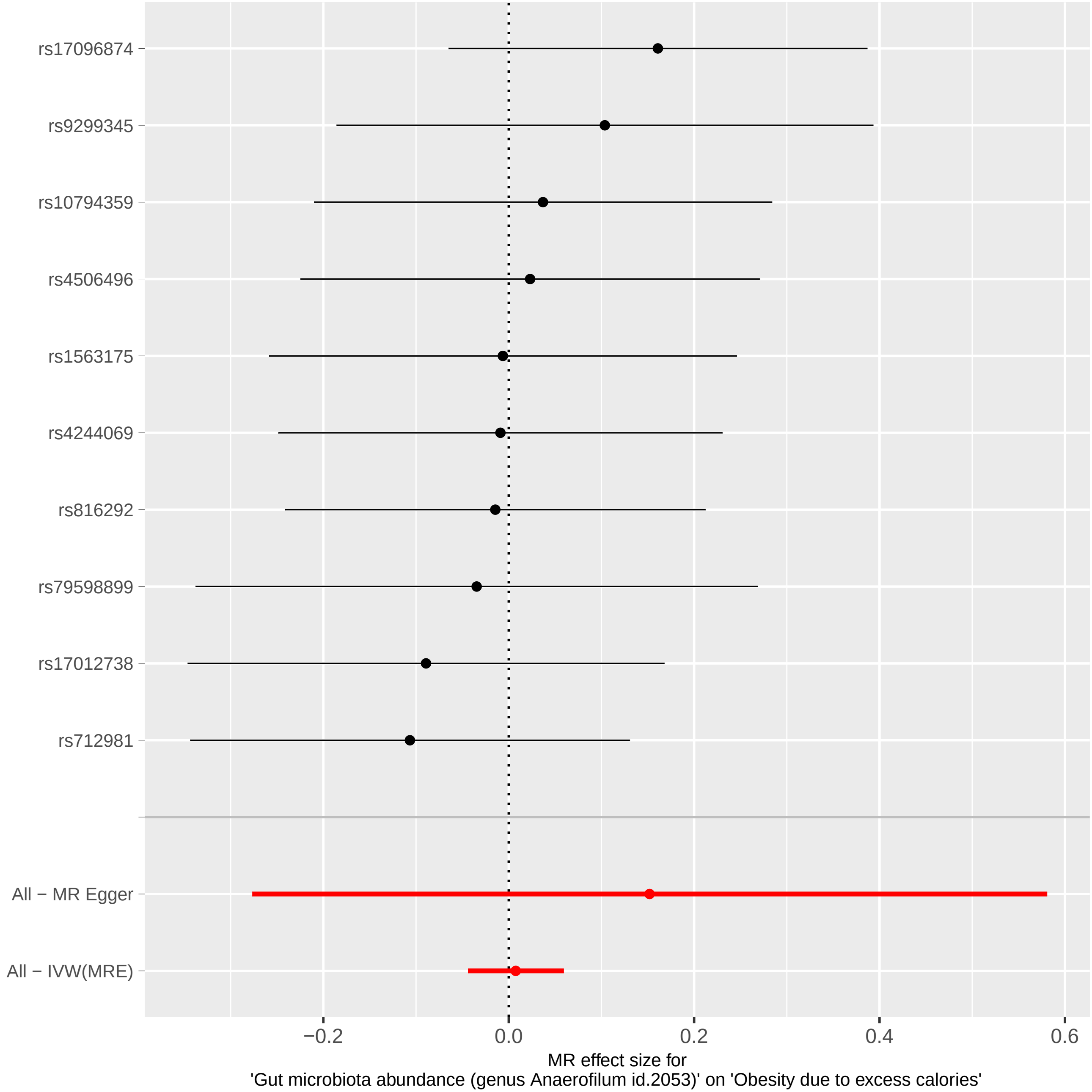

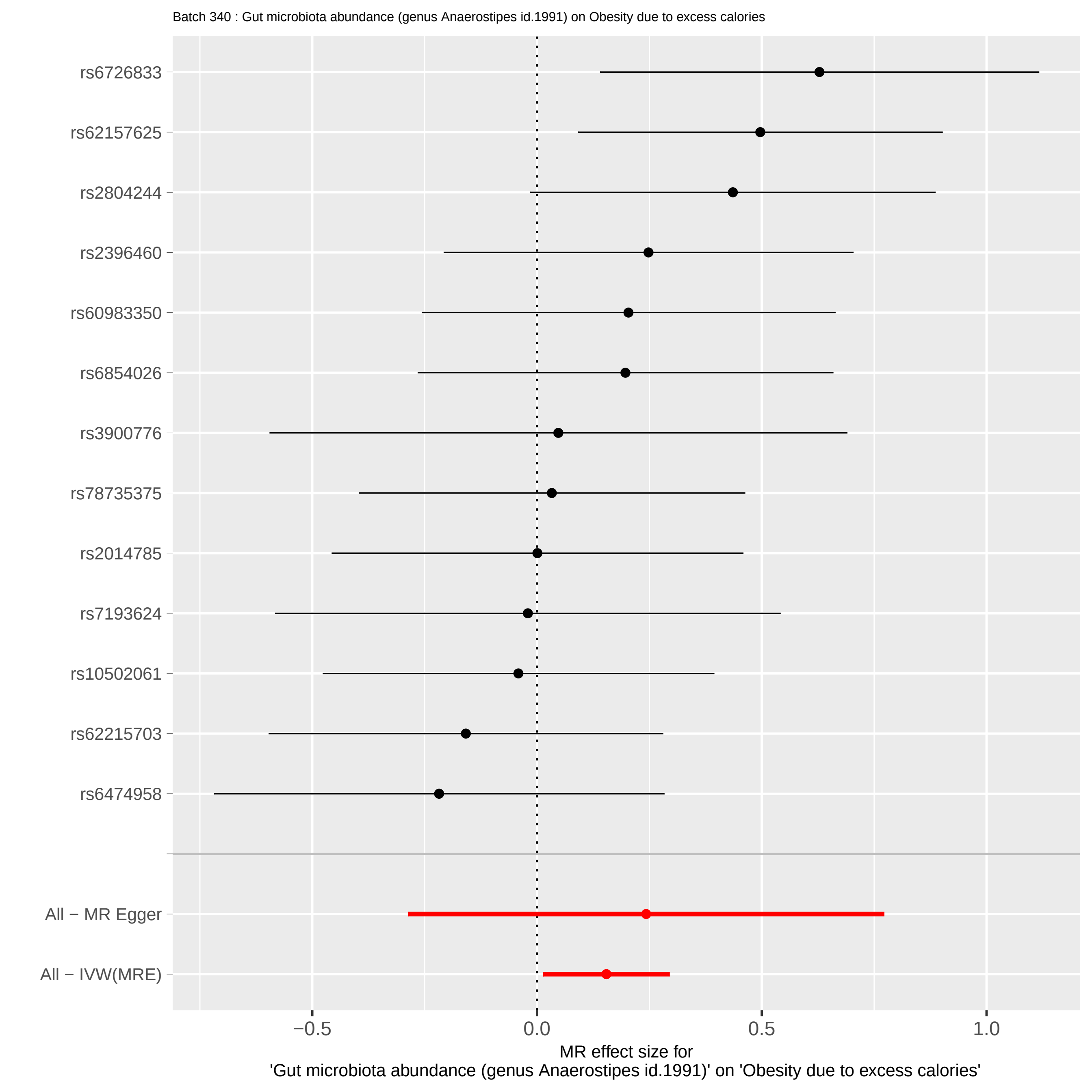

Batch 340 : Gut microbiota abundance (genus Anaerostipes id.1991) on Obesity due to excess calories
rs6726833
rs62157625
rs2804244
rs2396460
rs60983350
rs6854026
rs3900776
rs78735375
rs2014785
rs7193624
rs10502061
rs62215703
rs6474958
All − MR Egger
All − IVW(MRE)
−0.5
0.0
0.5
1.0
MR effect size for
'Gut microbiota abundance (genus Anaerostipes id.1991)' on 'Obesity due to excess calories'

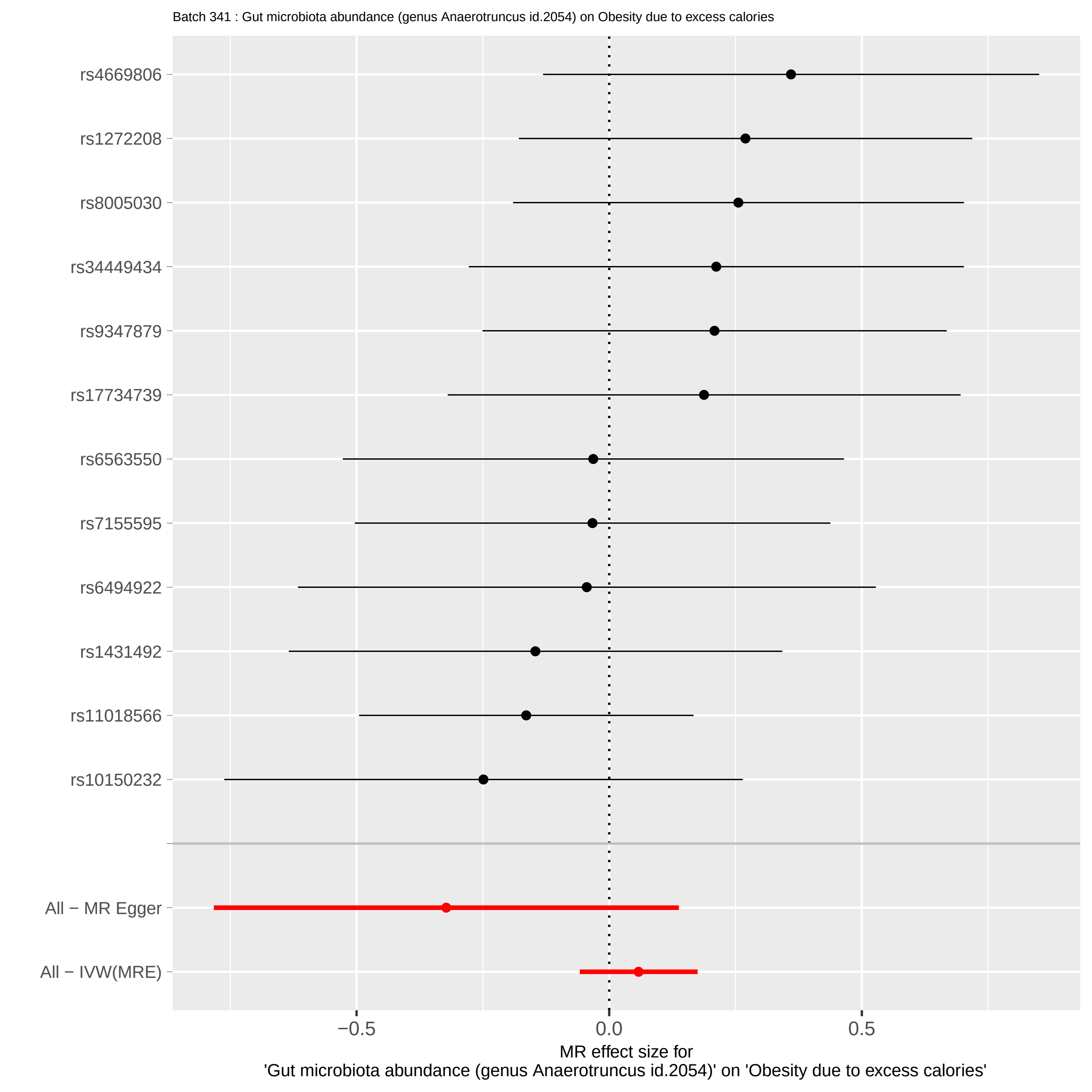

Batch 341 : Gut microbiota abundance (genus Anaerotruncus id.2054) on Obesity due to excess calories
rs4669806
rs1272208
rs8005030
rs34449434
rs9347879
rs17734739
rs6563550
rs7155595
rs6494922
rs1431492
rs11018566
rs10150232
All − MR Egger
All − IVW(MRE)
−0.5
0.0
0.5
MR effect size for
'Gut microbiota abundance (genus Anaerotruncus id.2054)' on 'Obesity due to excess calories'

Batch 342 : Gut microbiota abundance (genus Bacteroides id.918) on Obesity due to excess calories

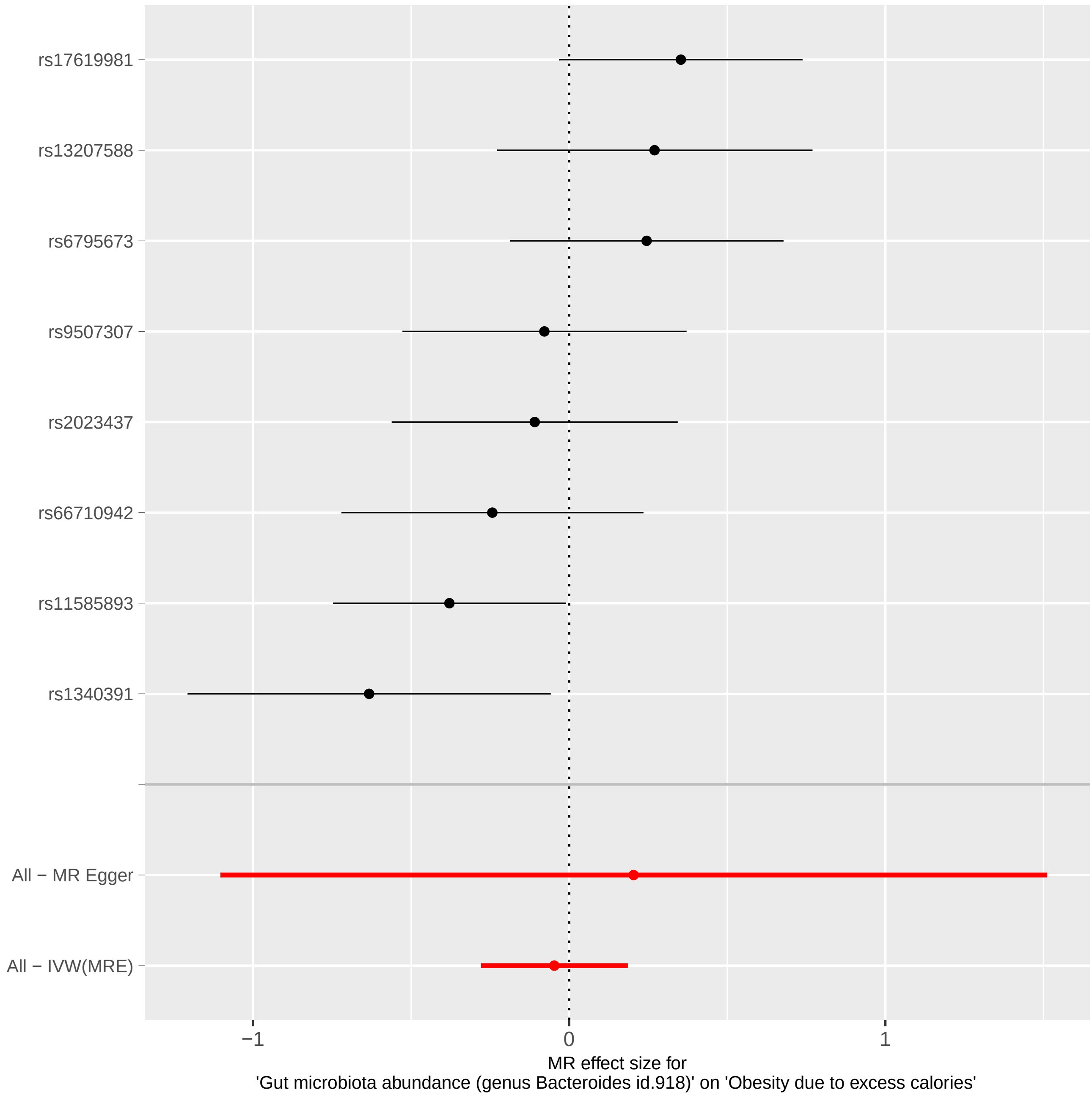

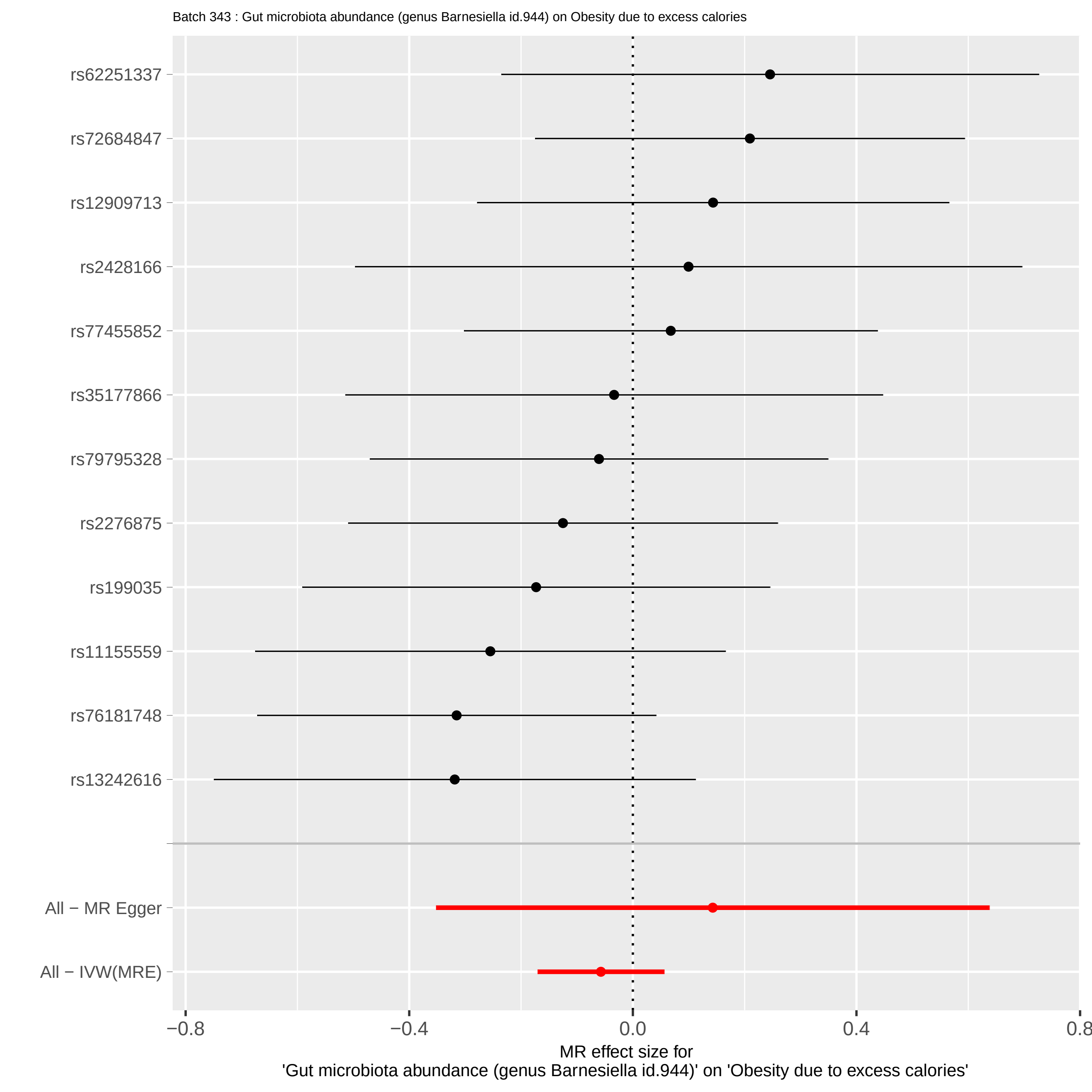
Batch 343 : Gut microbiota abundance (genus Barnesiella id.944) on Obesity due to excess calories
rs62251337
rs72684847
rs12909713
rs2428166
rs77455852
rs35177866
rs79795328
rs2276875
rs199035
rs11155559
rs76181748
rs13242616
All − MR Egger
All − IVW(MRE)
−0.8
−0.4
0.0
0.4
0.8
MR effect size for
'Gut microbiota abundance (genus Barnesiella id.944)' on 'Obesity due to excess calories'

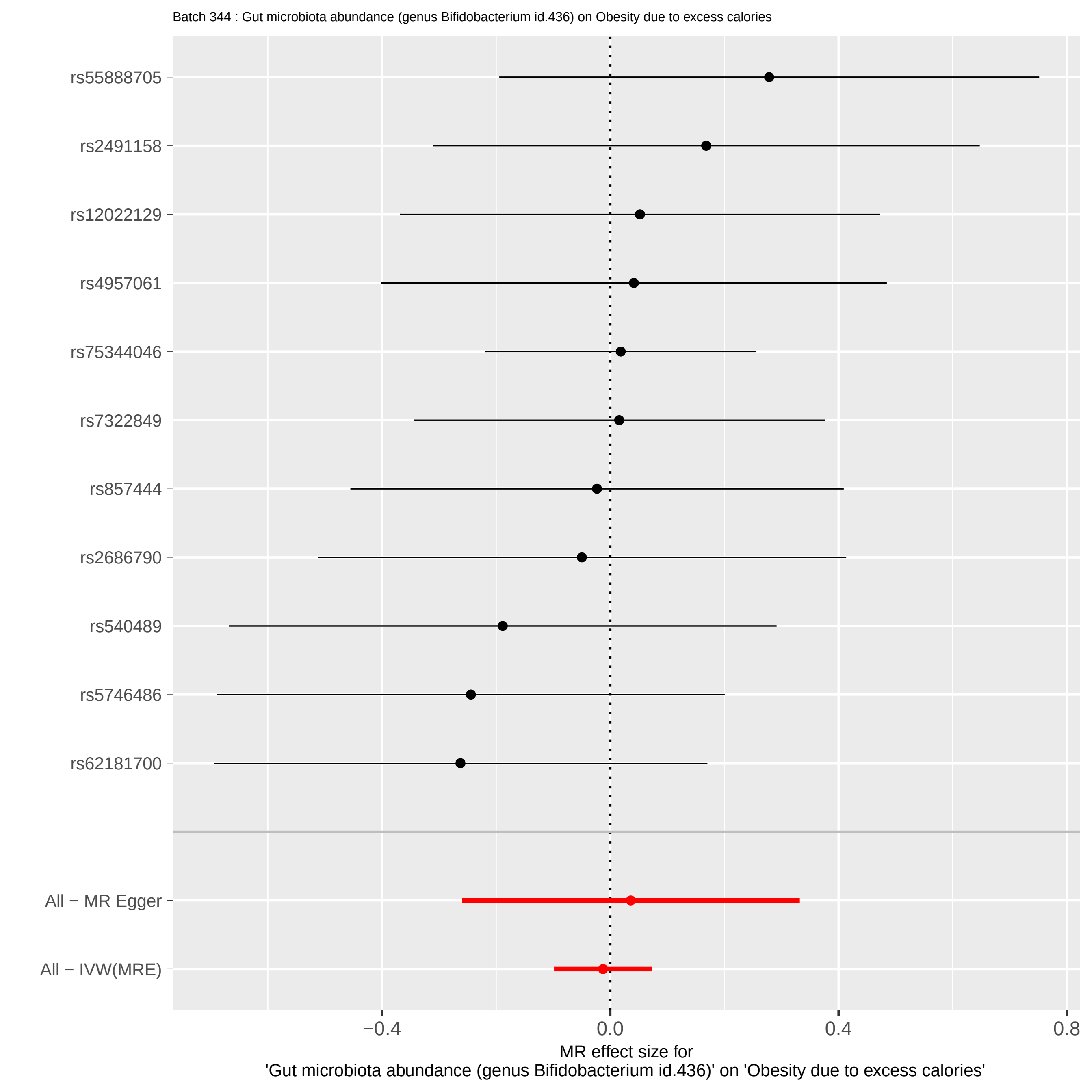

Batch 344 : Gut microbiota abundance (genus Bifidobacterium id.436) on Obesity due to excess calories
rs55888705
rs2491158
rs12022129
rs4957061
rs75344046
rs7322849
rs857444
rs2686790
rs540489
rs5746486
rs62181700
All − MR Egger
All − IVW(MRE)
−0.4
0.0
0.4
0.8
MR effect size for
'Gut microbiota abundance (genus Bifidobacterium id.436)' on 'Obesity due to excess calories'

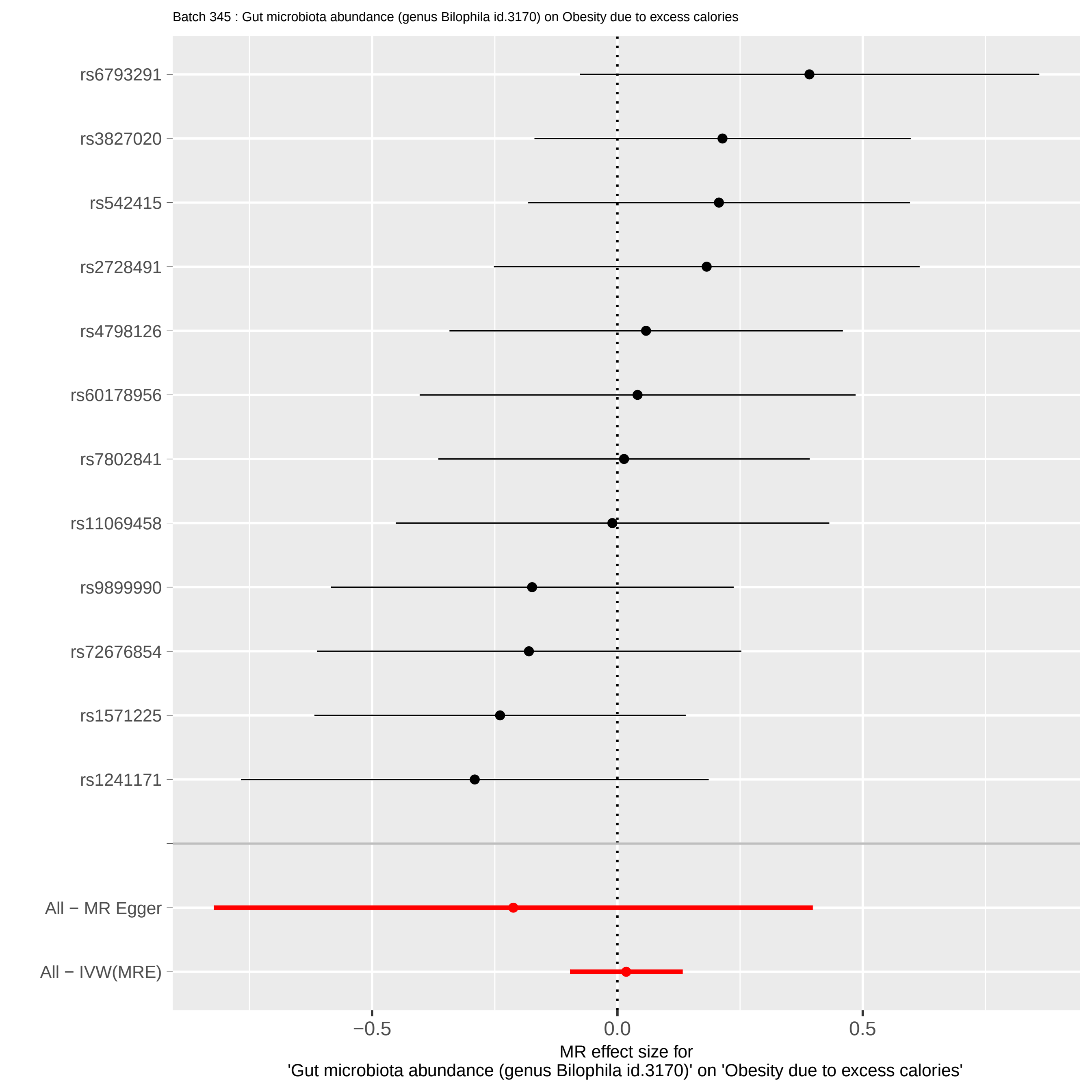

Batch 345 : Gut microbiota abundance (genus Bilophila id.3170) on Obesity due to excess calories
rs6793291
rs3827020
rs542415
rs2728491
rs4798126
rs60178956
rs7802841
rs11069458
rs9899990
rs72676854
rs1571225
rs1241171
All − MR Egger
All − IVW(MRE)
−0.5
0.0
0.5
MR effect size for
'Gut microbiota abundance (genus Bilophila id.3170)' on 'Obesity due to excess calories'

Batch 347 : Gut microbiota abundance (genus Butyricicoccus id.2055) on Obesity due to excess calories

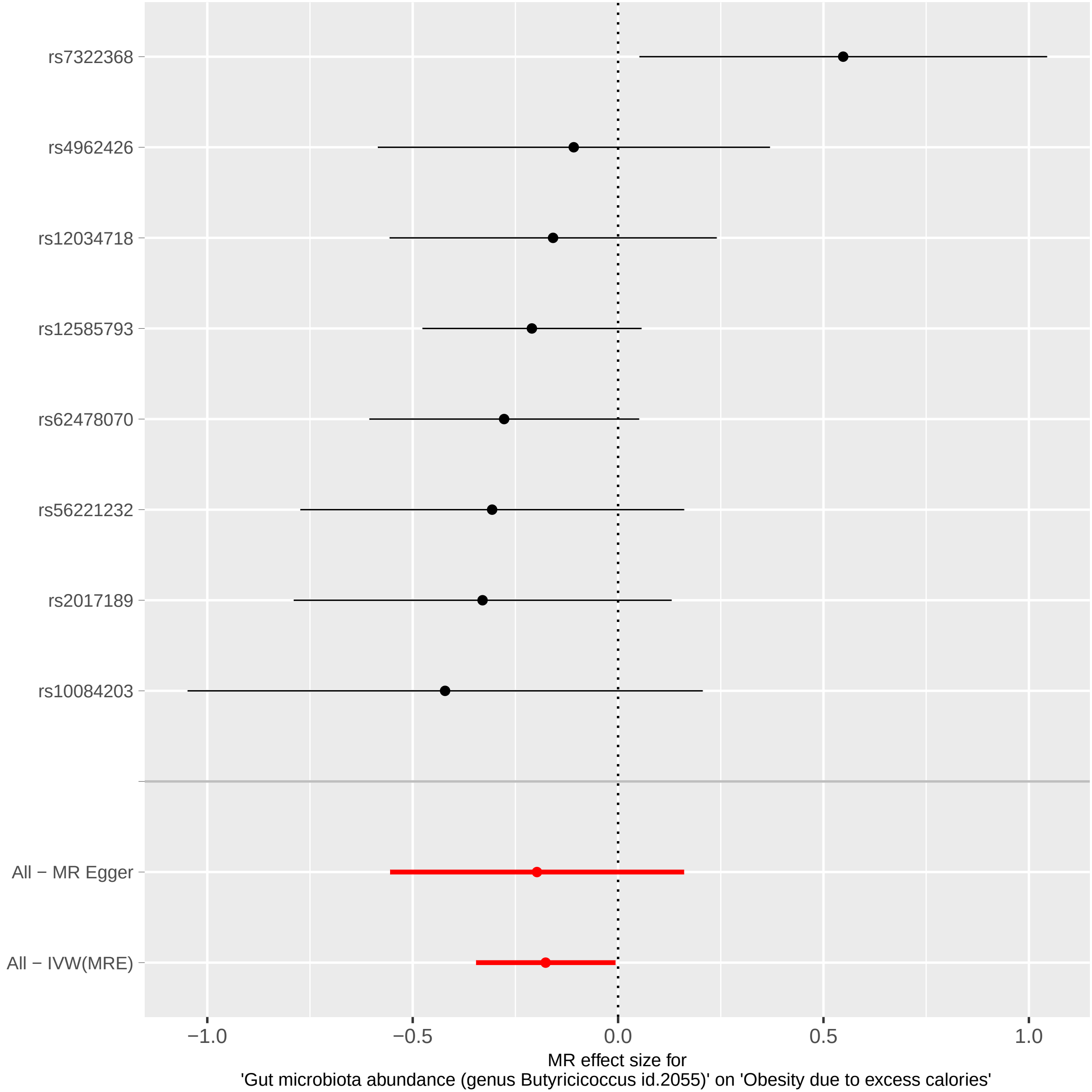

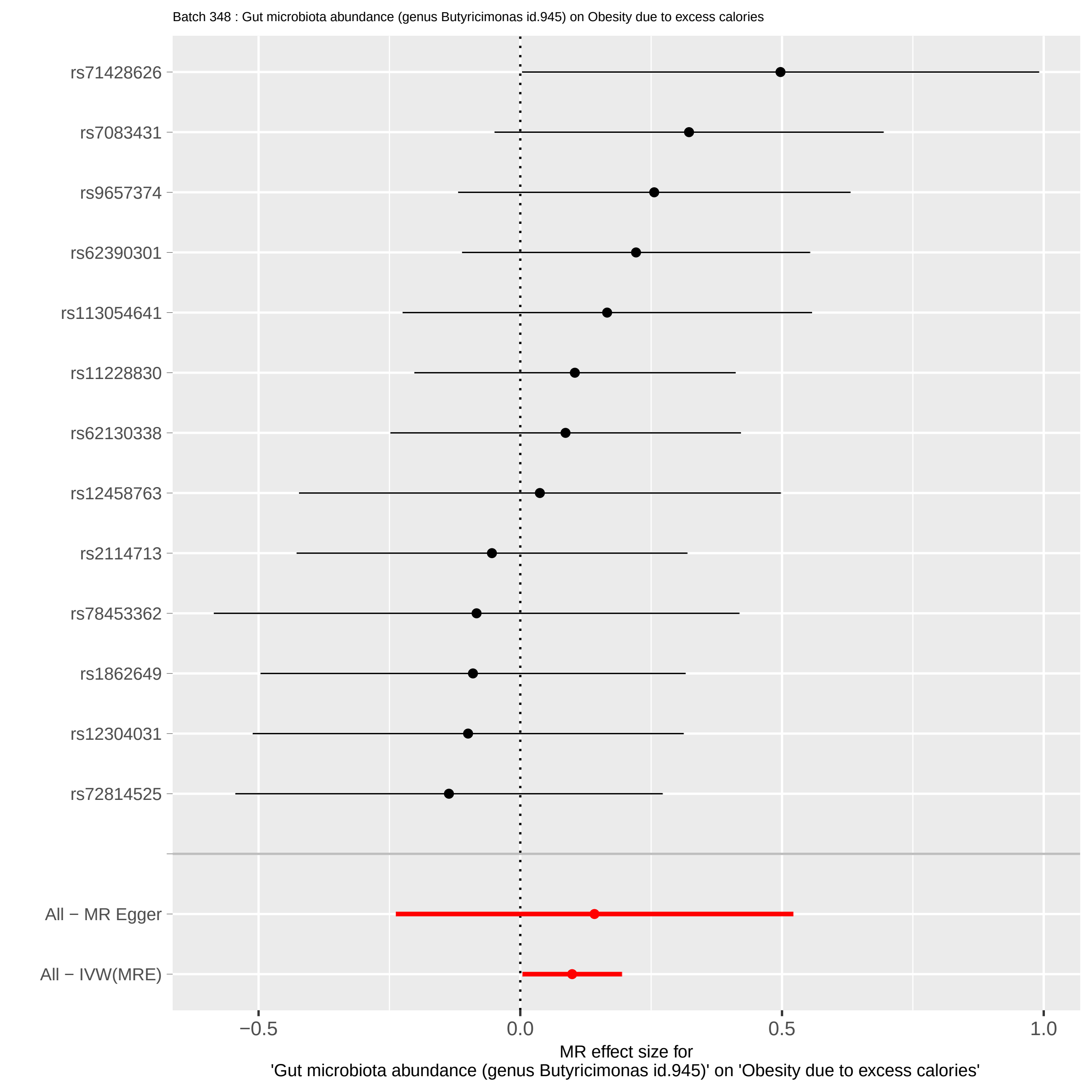
Batch 348 : Gut microbiota abundance (genus Butyricimonas id.945) on Obesity due to excess calories
rs71428626
rs7083431
rs9657374
rs62390301
rs113054641
rs11228830
rs62130338
rs12458763
rs2114713
rs78453362
rs1862649
rs12304031
rs72814525
All − MR Egger
All − IVW(MRE)
−0.5
0.0
0.5
1.0
MR effect size for
'Gut microbiota abundance (genus Butyricimonas id.945)' on 'Obesity due to excess calories'

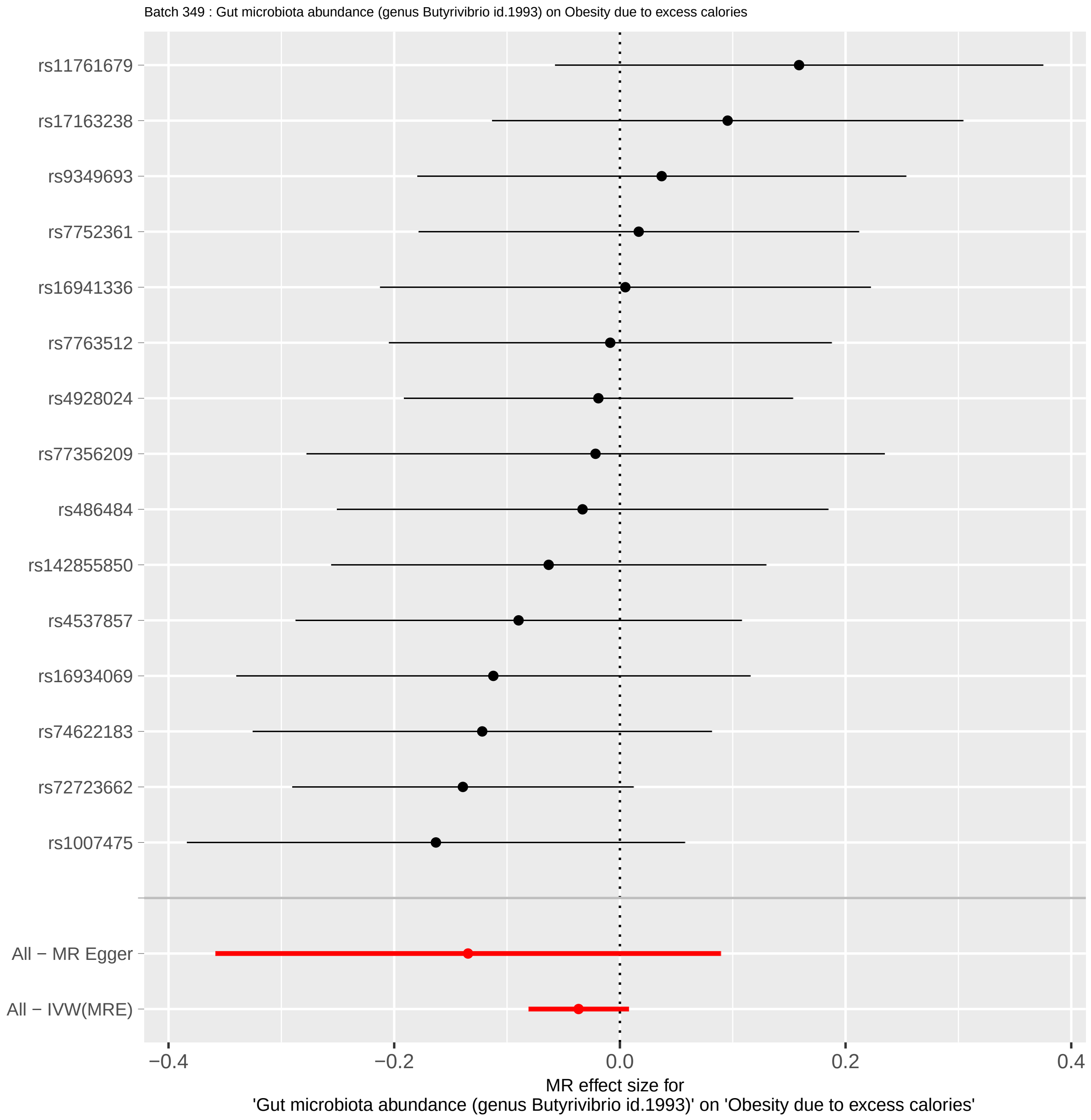
Batch 349 : Gut microbiota abundance (genus Butyrivibrio id.1993) on Obesity due to excess calories
rs11761679
rs17163238
rs9349693
rs7752361
rs16941336
rs7763512
rs4928024
rs77356209
rs486484
rs142855850
rs4537857
rs16934069
rs74622183
rs72723662
rs1007475
All − MR Egger
All − IVW(MRE)
−0.4
−0.2
0.0
0.2
0.4
MR effect size for
'Gut microbiota abundance (genus Butyrivibrio id.1993)' on 'Obesity due to excess calories'

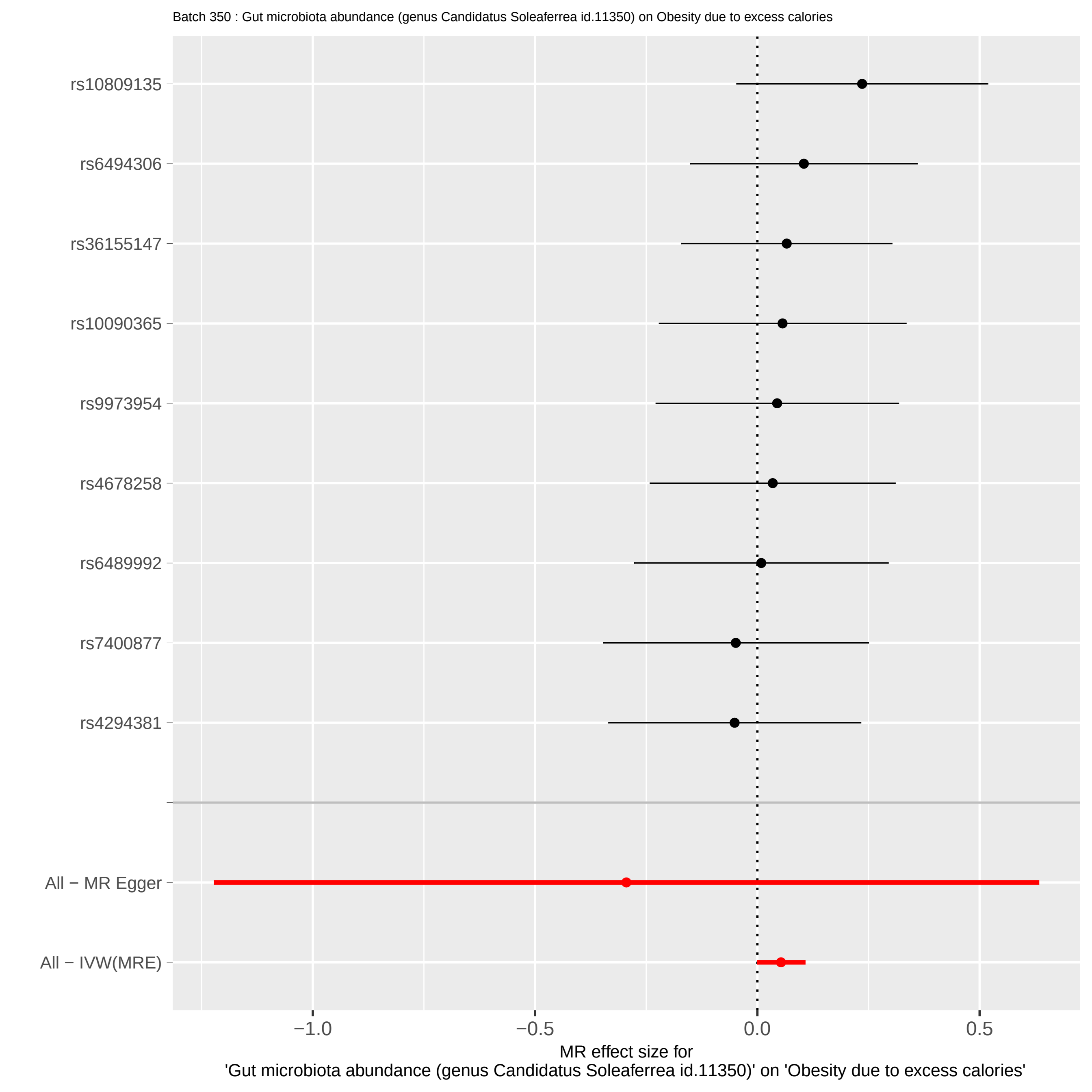

Batch 350 : Gut microbiota abundance (genus Candidatus Soleaferrea id.11350) on Obesity due to excess calories
rs10809135
rs6494306
rs36155147
rs10090365
rs9973954
rs4678258
rs6489992
rs7400877
rs4294381
All − MR Egger
All − IVW(MRE)
−1.0
−0.5
0.0
0.5
MR effect size for
'Gut microbiota abundance (genus Candidatus Soleaferrea id.11350)' on 'Obesity due to excess calories'

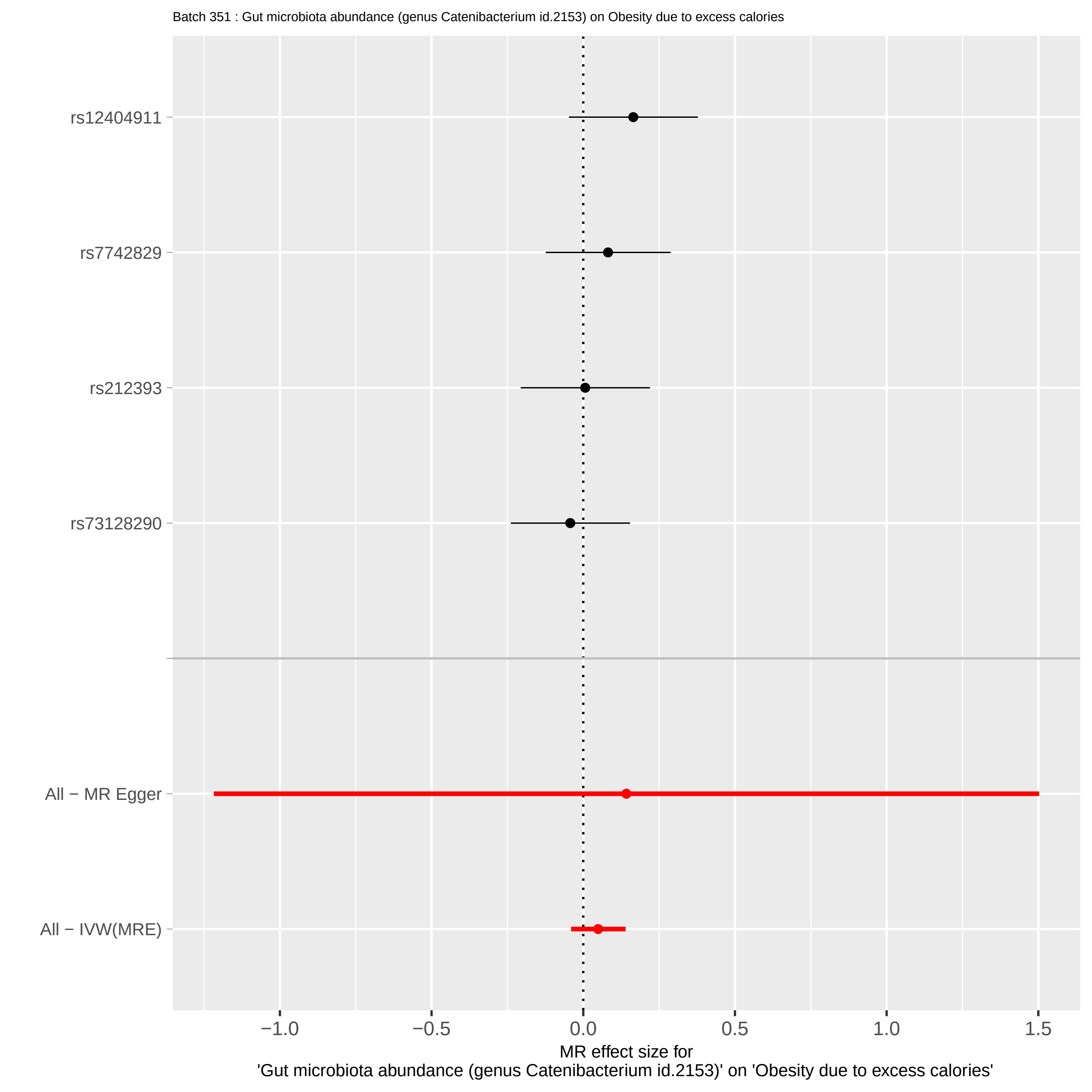
Batch 351 : Gut microbiota abundance (genus Catenibacterium id.2153) on Obesity due to excess calories
rs12404911
rs7742829
rs212393
rs73128290
All − MR Egger
All − IVW(MRE)
−1.0
−0.5
0.0
0.5
1.0
1.5
MR effect size for
'Gut microbiota abundance (genus Catenibacterium id.2153)' on 'Obesity due to excess calories'

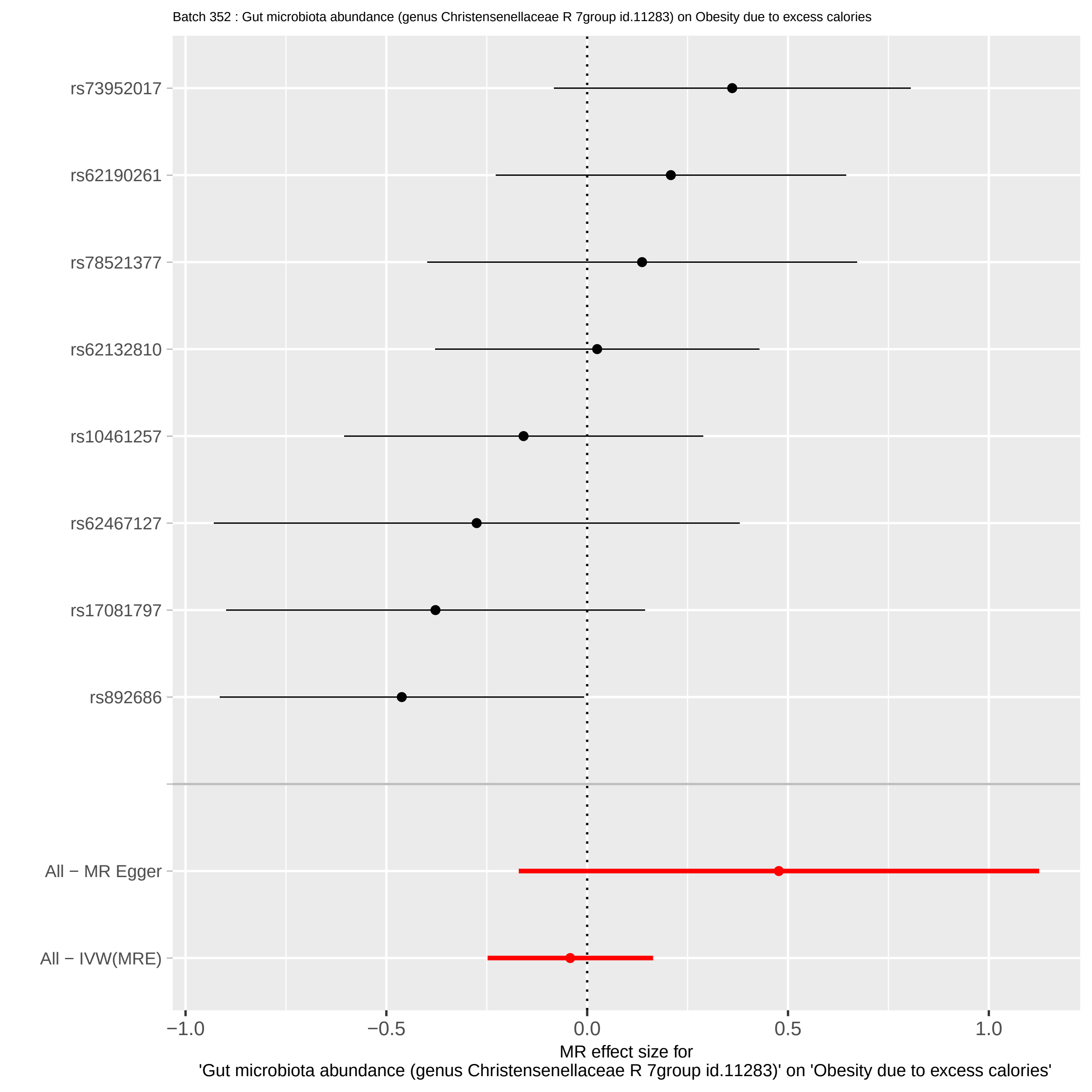

Batch 352 : Gut microbiota abundance (genus Christensenellaceae R 7group id.11283) on Obesity due to excess calories
rs73952017
rs62190261
rs78521377
rs62132810
rs10461257
rs62467127
rs17081797
rs892686
All − MR Egger
All − IVW(MRE)
−1.0
−0.5
0.0
0.5
1.0
MR effect size for
'Gut microbiota abundance (genus Christensenellaceae R 7group id.11283)' on 'Obesity due to excess calories'

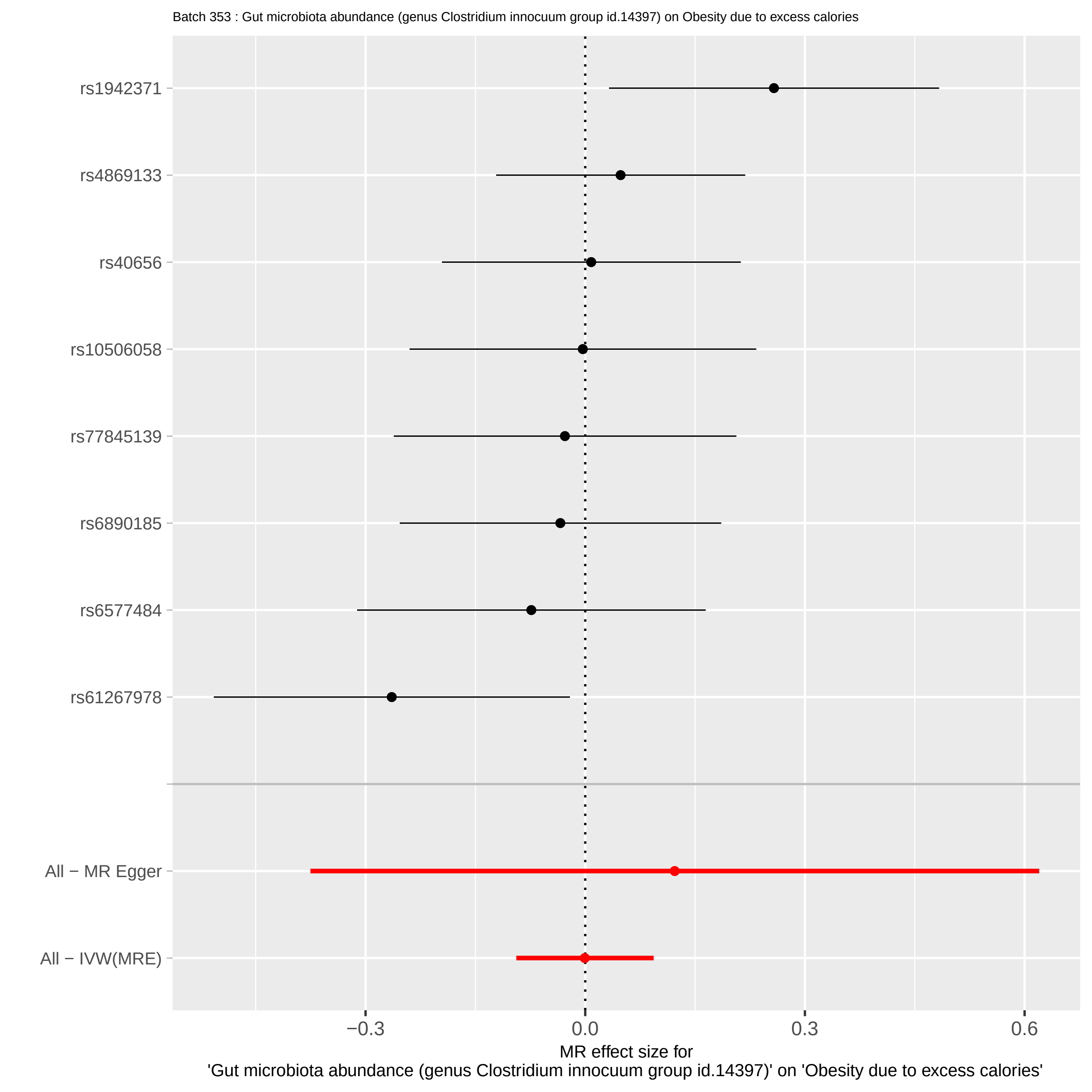

Batch 353 : Gut microbiota abundance (genus Clostridium innocuum group id.14397) on Obesity due to excess calories
rs1942371
rs4869133
rs40656
rs10506058
rs77845139
rs6890185
rs6577484
rs61267978
All − MR Egger
All − IVW(MRE)
−0.3
0.0
0.3
0.6
MR effect size for
'Gut microbiota abundance (genus Clostridium innocuum group id.14397)' on 'Obesity due to excess calories'

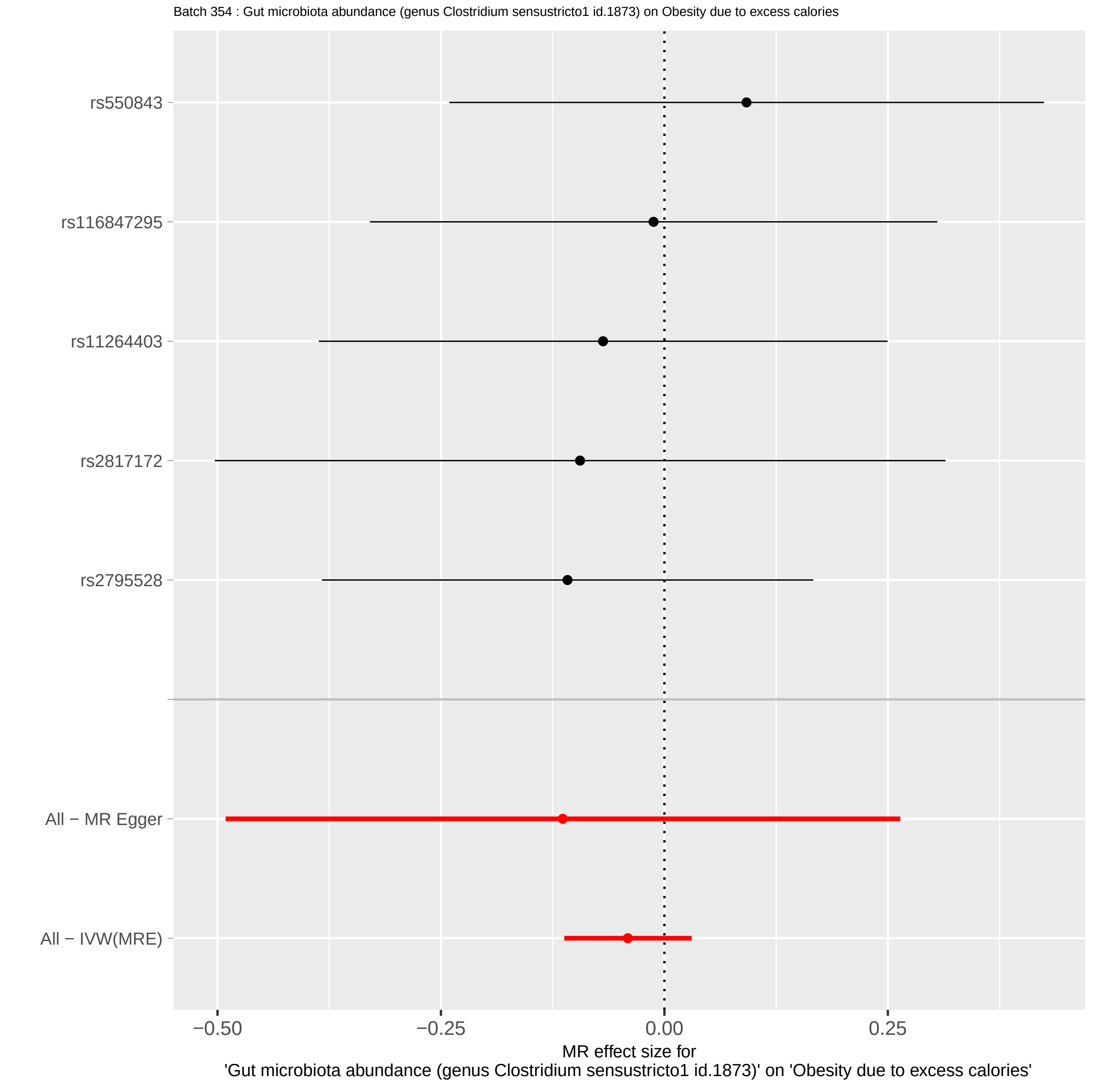
Batch 354 : Gut microbiota abundance (genus Clostridium sensustricto1 id.1873) on Obesity due to excess calories
rs550843
rs116847295
rs11264403
rs2817172
rs2795528
All − MR Egger
All − IVW(MRE)
−0.50
−0.25
0.00
0.25
MR effect size for
'Gut microbiota abundance (genus Clostridium sensustricto1 id.1873)' on 'Obesity due to excess calories'

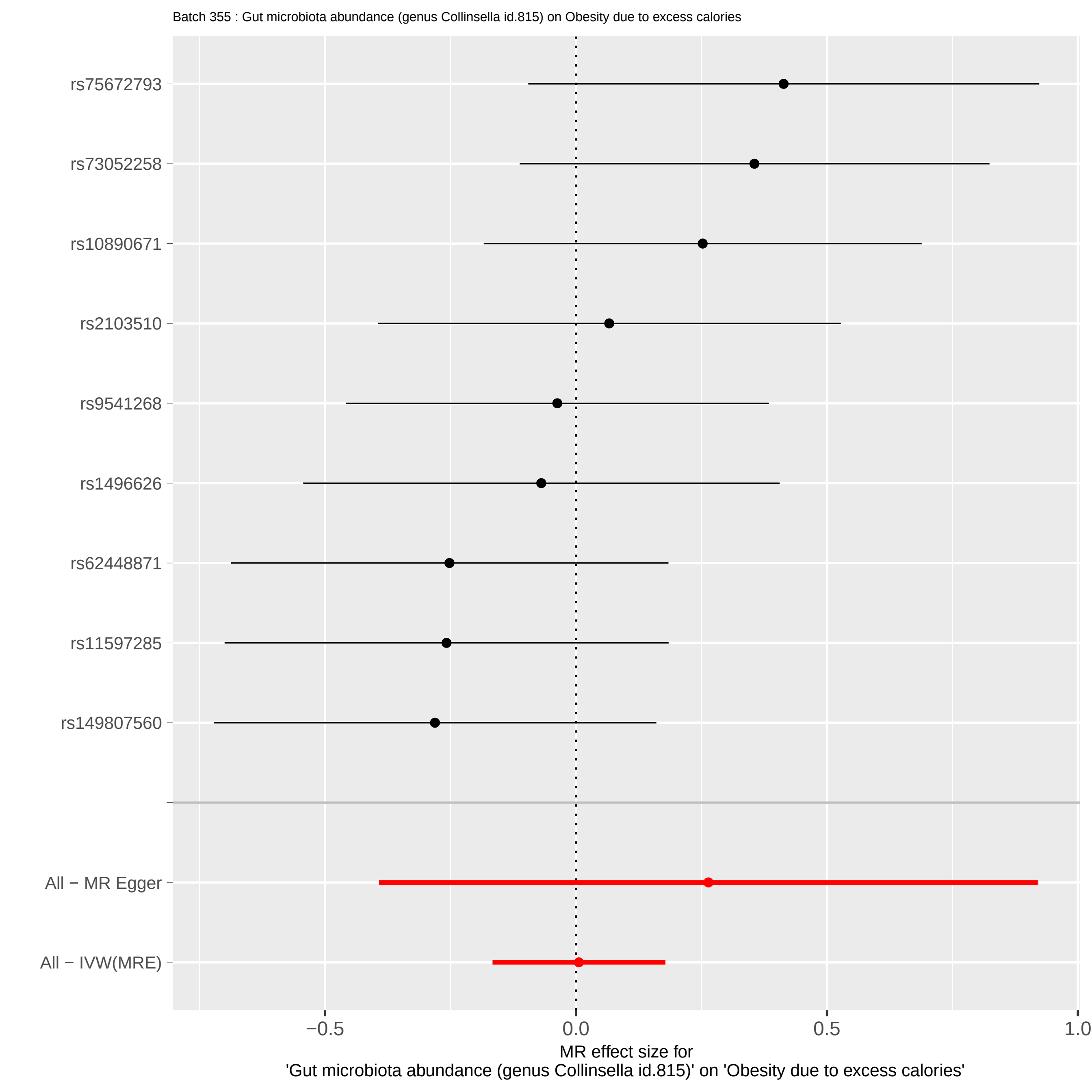
Batch 355 : Gut microbiota abundance (genus Collinsella id.815) on Obesity due to excess calories
rs75672793
rs73052258
rs10890671
rs2103510
rs9541268
rs1496626
rs62448871
rs11597285
rs149807560
All − MR Egger
All − IVW(MRE)
−0.5
0.0
0.5
1.0
MR effect size for
'Gut microbiota abundance (genus Collinsella id.815)' on 'Obesity due to excess calories'

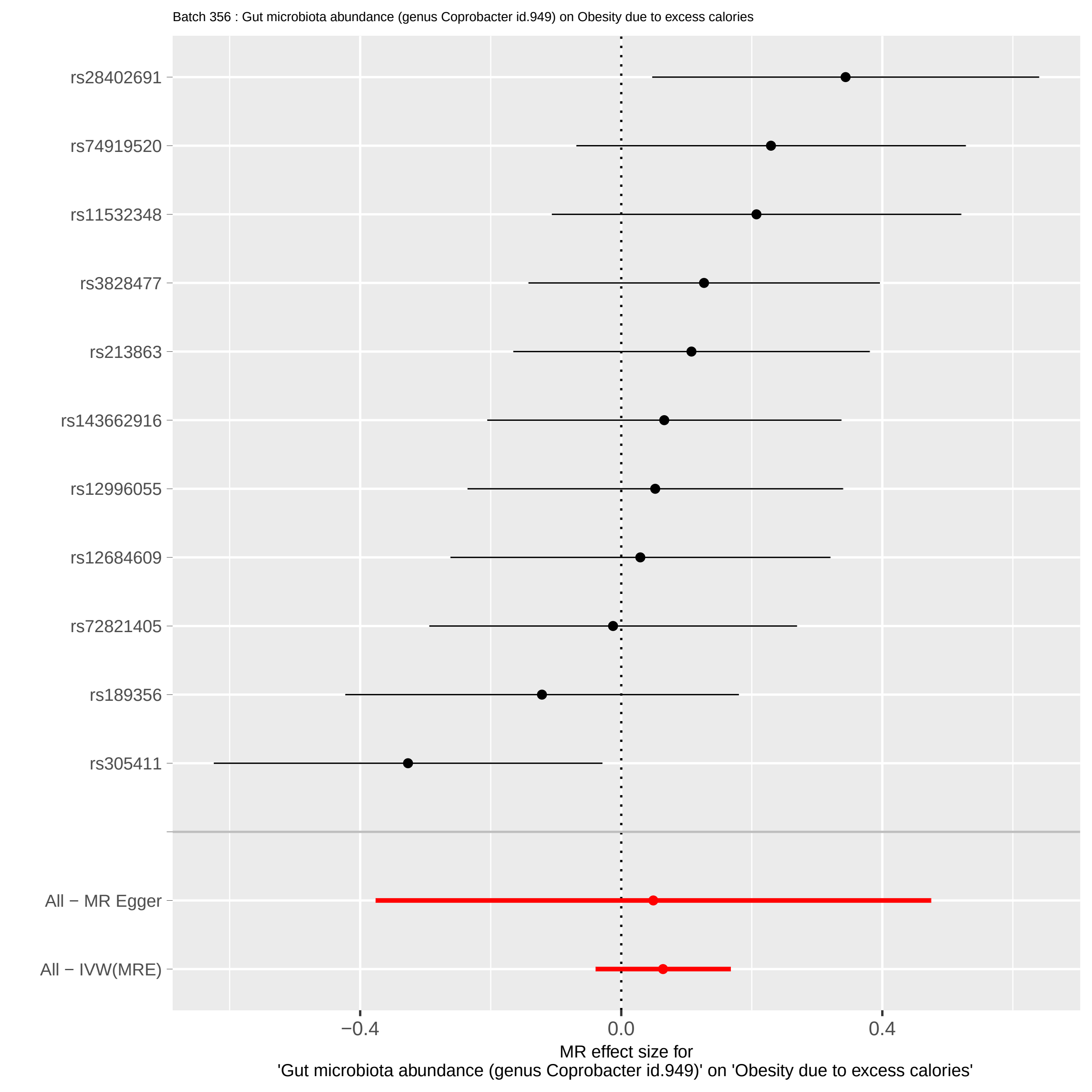
Batch 356 : Gut microbiota abundance (genus Coprobacter id.949) on Obesity due to excess calories
rs28402691
rs74919520
rs11532348
rs3828477
rs213863
rs143662916
rs12996055
rs12684609
rs72821405
rs189356
rs305411
All − MR Egger
All − IVW(MRE)
−0.4
0.0
0.4
MR effect size for
'Gut microbiota abundance (genus Coprobacter id.949)' on 'Obesity due to excess calories'

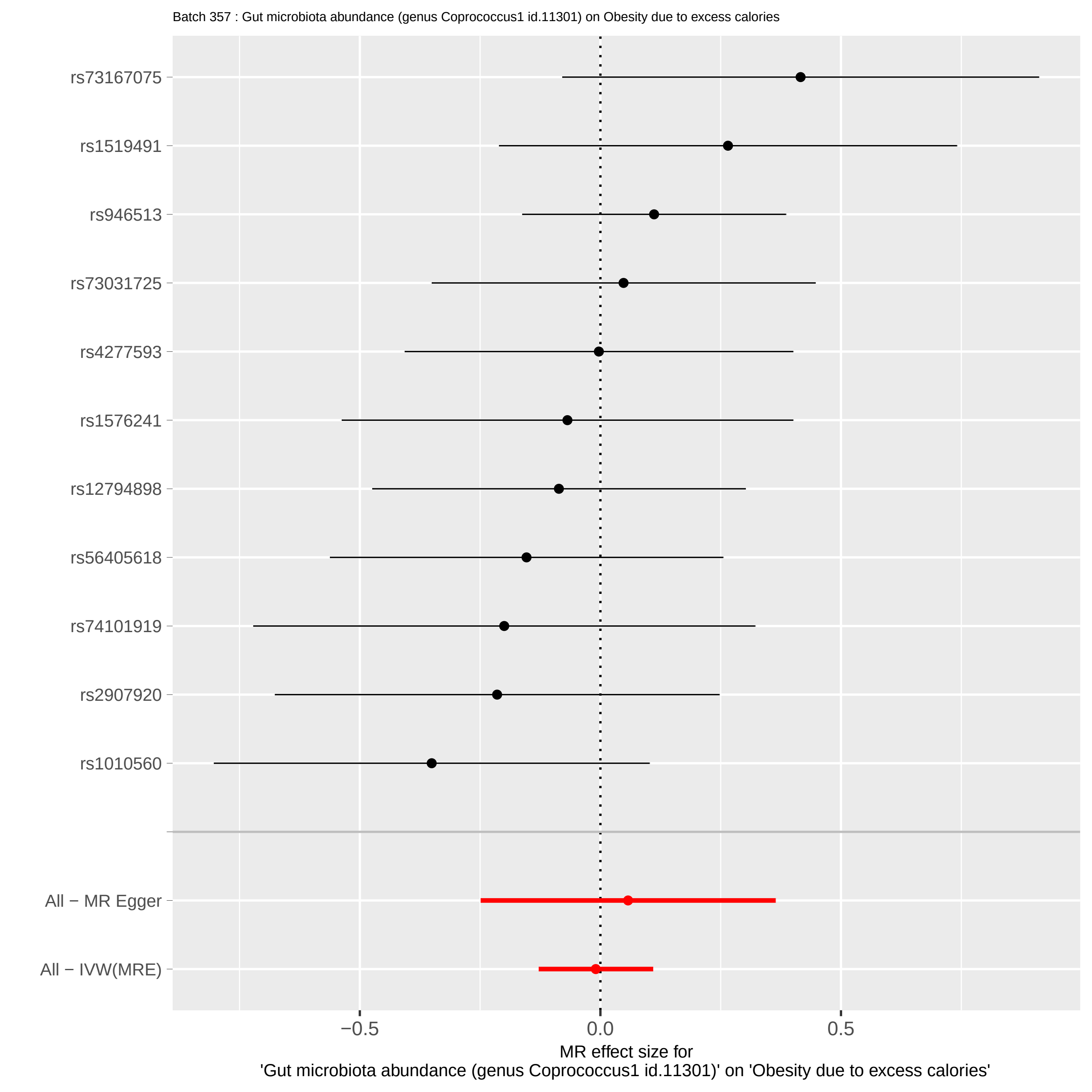

Batch 357 : Gut microbiota abundance (genus Coprococcus1 id.11301) on Obesity due to excess calories
rs73167075
rs1519491
rs946513
rs73031725
rs4277593
rs1576241
rs12794898
rs56405618
rs74101919
rs2907920
rs1010560
All − MR Egger
All − IVW(MRE)
−0.5
0.0
0.5
MR effect size for
'Gut microbiota abundance (genus Coprococcus1 id.11301)' on 'Obesity due to excess calories'

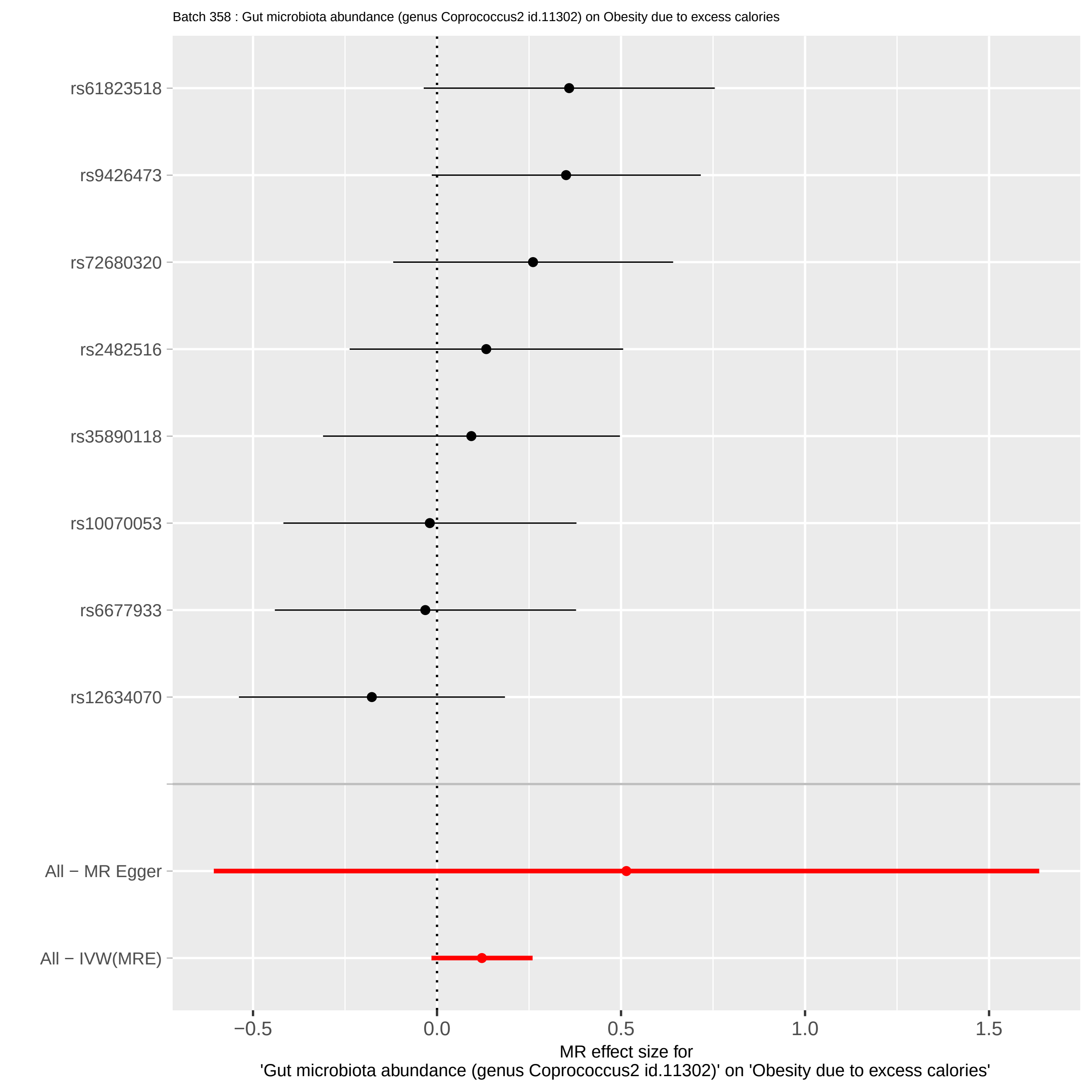
Batch 358 : Gut microbiota abundance (genus Coprococcus2 id.11302) on Obesity due to excess calories
rs61823518
rs9426473
rs72680320
rs2482516
rs35890118
rs10070053
rs6677933
rs12634070
All − MR Egger
All − IVW(MRE)
−0.5
0.0
0.5
1.0
1.5
MR effect size for
'Gut microbiota abundance (genus Coprococcus2 id.11302)' on 'Obesity due to excess calories'

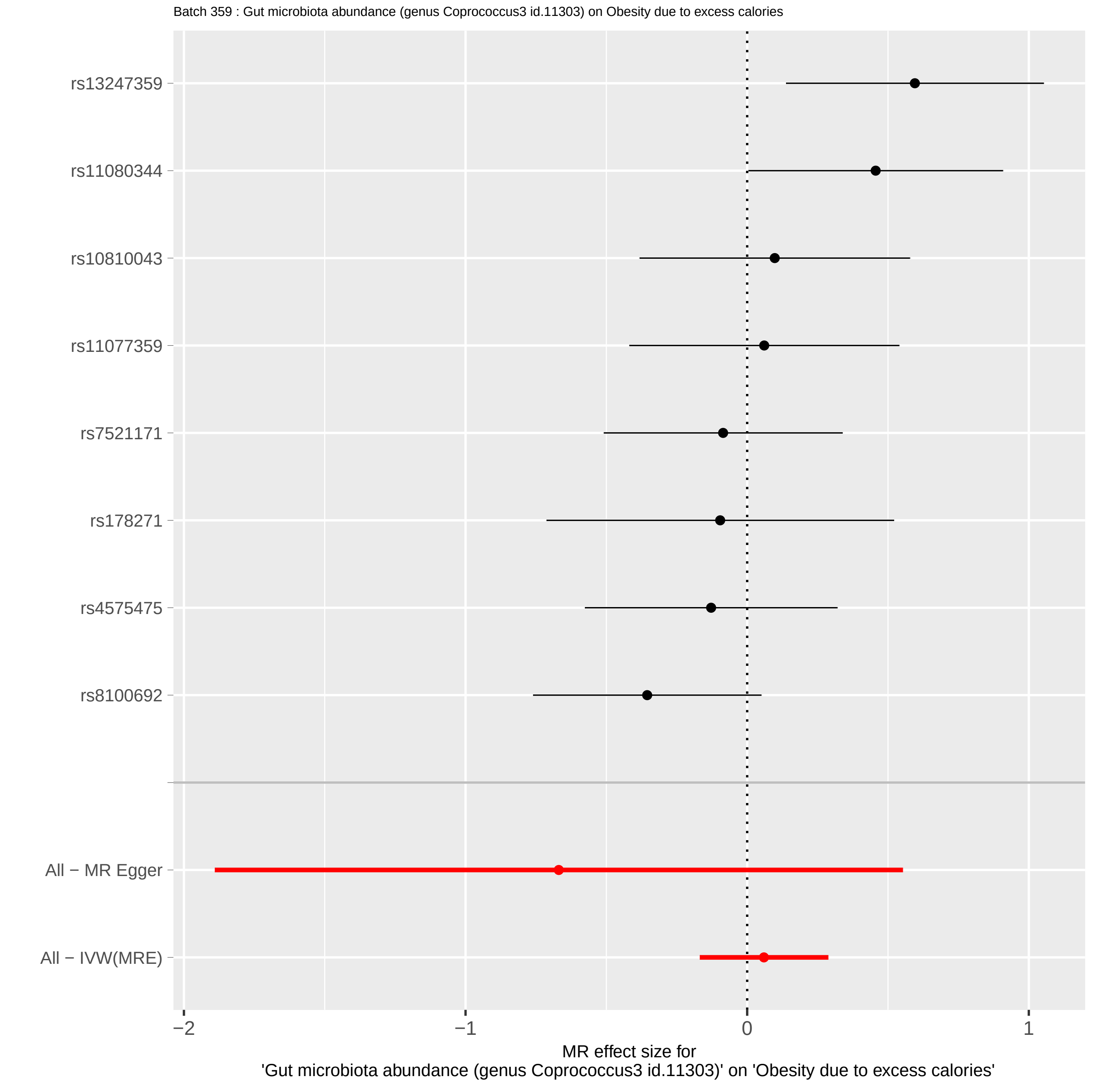
Batch 359 : Gut microbiota abundance (genus Coprococcus3 id.11303) on Obesity due to excess calories
rs13247359
rs11080344
rs10810043
rs11077359
rs7521171
rs178271
rs4575475
rs8100692
All − MR Egger
All − IVW(MRE)
−2
−1
0
1
MR effect size for
'Gut microbiota abundance (genus Coprococcus3 id.11303)' on 'Obesity due to excess calories'

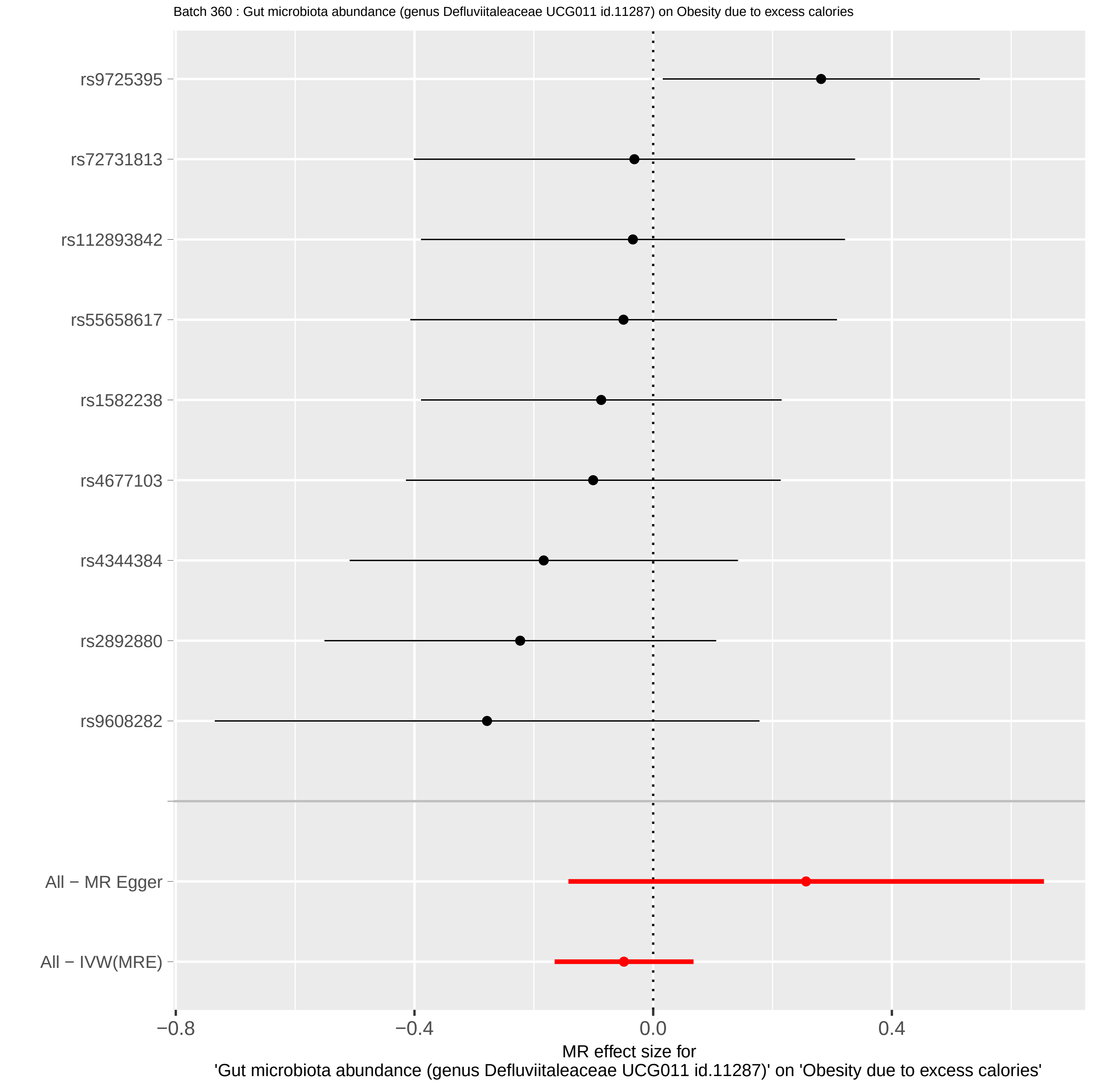

Batch 360 : Gut microbiota abundance (genus Defluviitaleaceae UCG011 id.11287) on Obesity due to excess calories
rs9725395
rs72731813
rs112893842
rs55658617
rs1582238
rs4677103
rs4344384
rs2892880
rs9608282
All − MR Egger
All − IVW(MRE)
−0.8
−0.4
0.0
0.4
MR effect size for
'Gut microbiota abundance (genus Defluviitaleaceae UCG011 id.11287)' on 'Obesity due to excess calories'

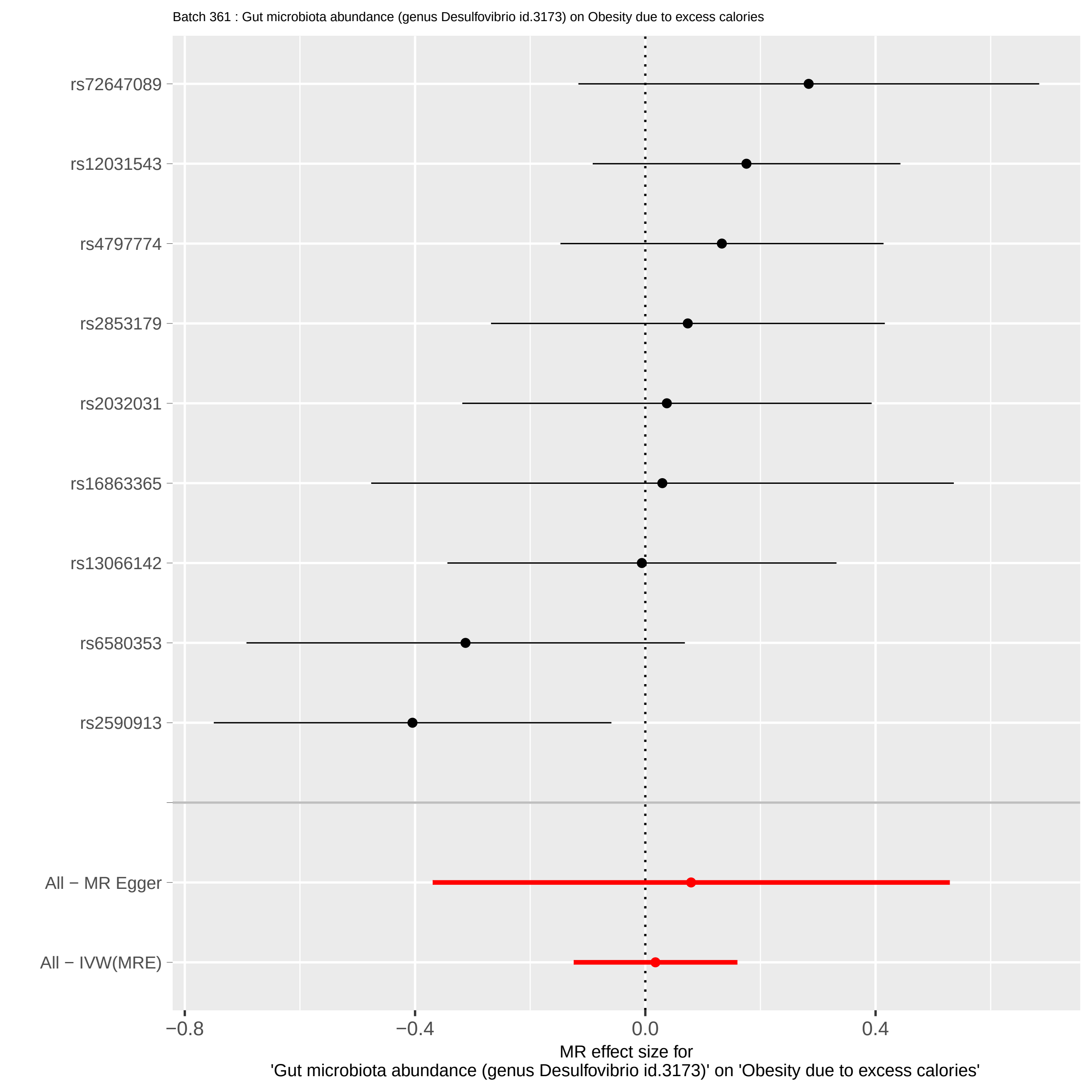
Batch 361 : Gut microbiota abundance (genus Desulfovibrio id.3173) on Obesity due to excess calories
rs72647089
rs12031543
rs4797774
rs2853179
rs2032031
rs16863365
rs13066142
rs6580353
rs2590913
All − MR Egger
All − IVW(MRE)
−0.8
−0.4
0.0
0.4
MR effect size for
'Gut microbiota abundance (genus Desulfovibrio id.3173)' on 'Obesity due to excess calories'

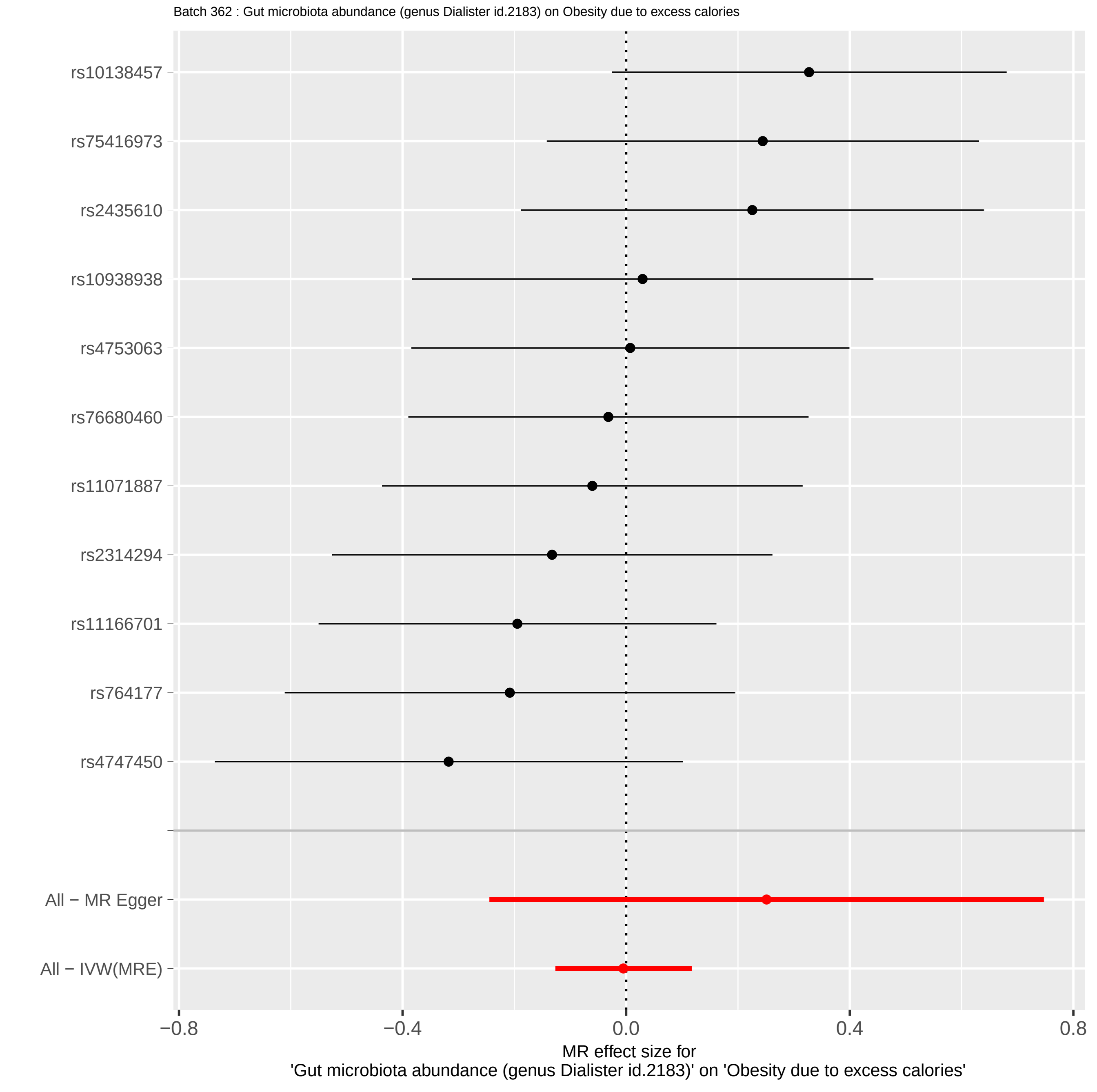

Batch 362 : Gut microbiota abundance (genus Dialister id.2183) on Obesity due to excess calories
rs10138457
rs75416973
rs2435610
rs10938938
rs4753063
rs76680460
rs11071887
rs2314294
rs11166701
rs764177
rs4747450
All − MR Egger
All − IVW(MRE)
−0.8
−0.4
0.0
0.4
0.8
MR effect size for
'Gut microbiota abundance (genus Dialister id.2183)' on 'Obesity due to excess calories'

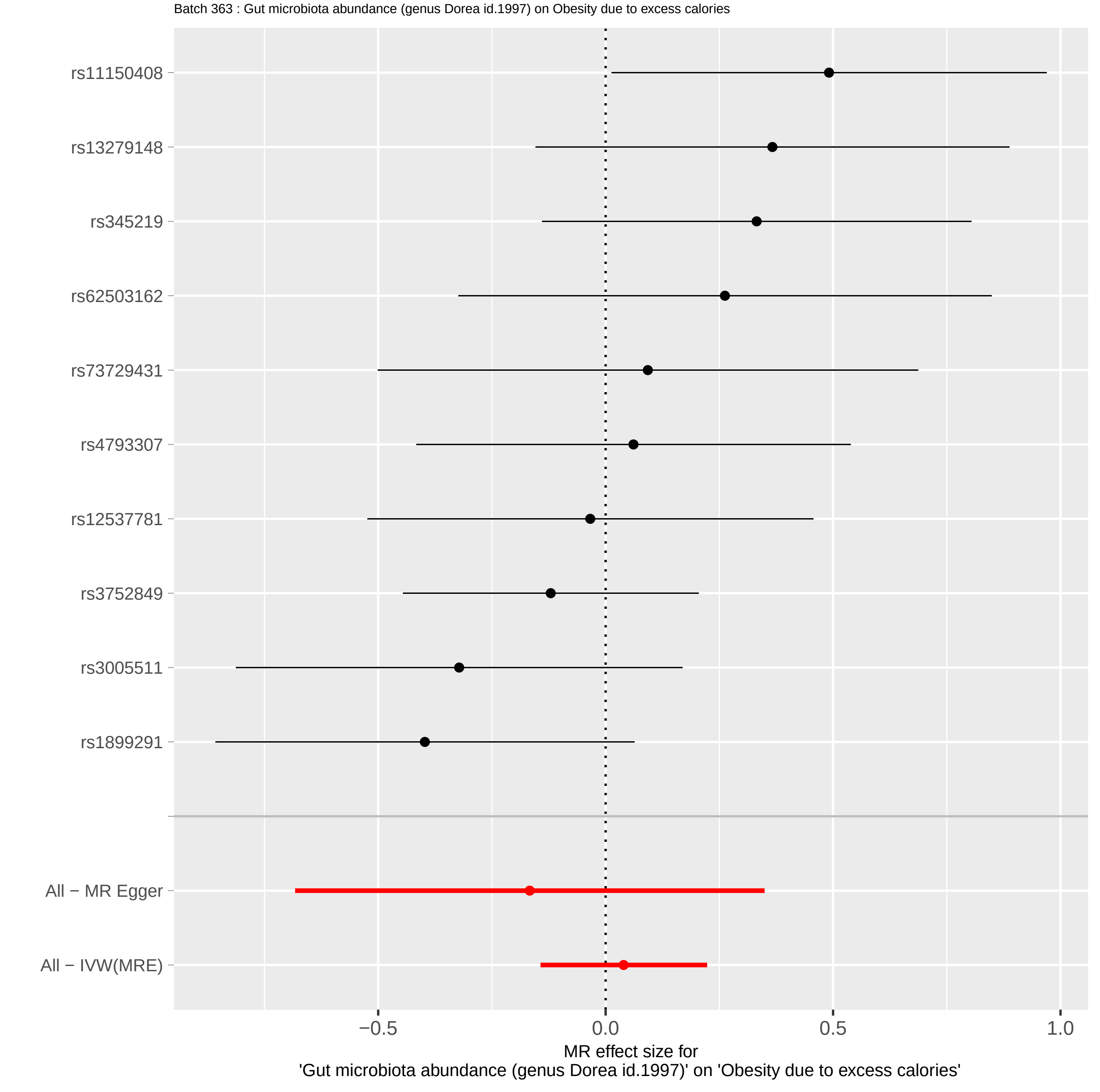

Batch 363 : Gut microbiota abundance (genus Dorea id.1997) on Obesity due to excess calories
rs11150408
rs13279148
rs345219
rs62503162
rs73729431
rs4793307
rs12537781
rs3752849
rs3005511
rs1899291
All − MR Egger
All − IVW(MRE)
−0.5
0.0
0.5
1.0
MR effect size for
'Gut microbiota abundance (genus Dorea id.1997)' on 'Obesity due to excess calories'

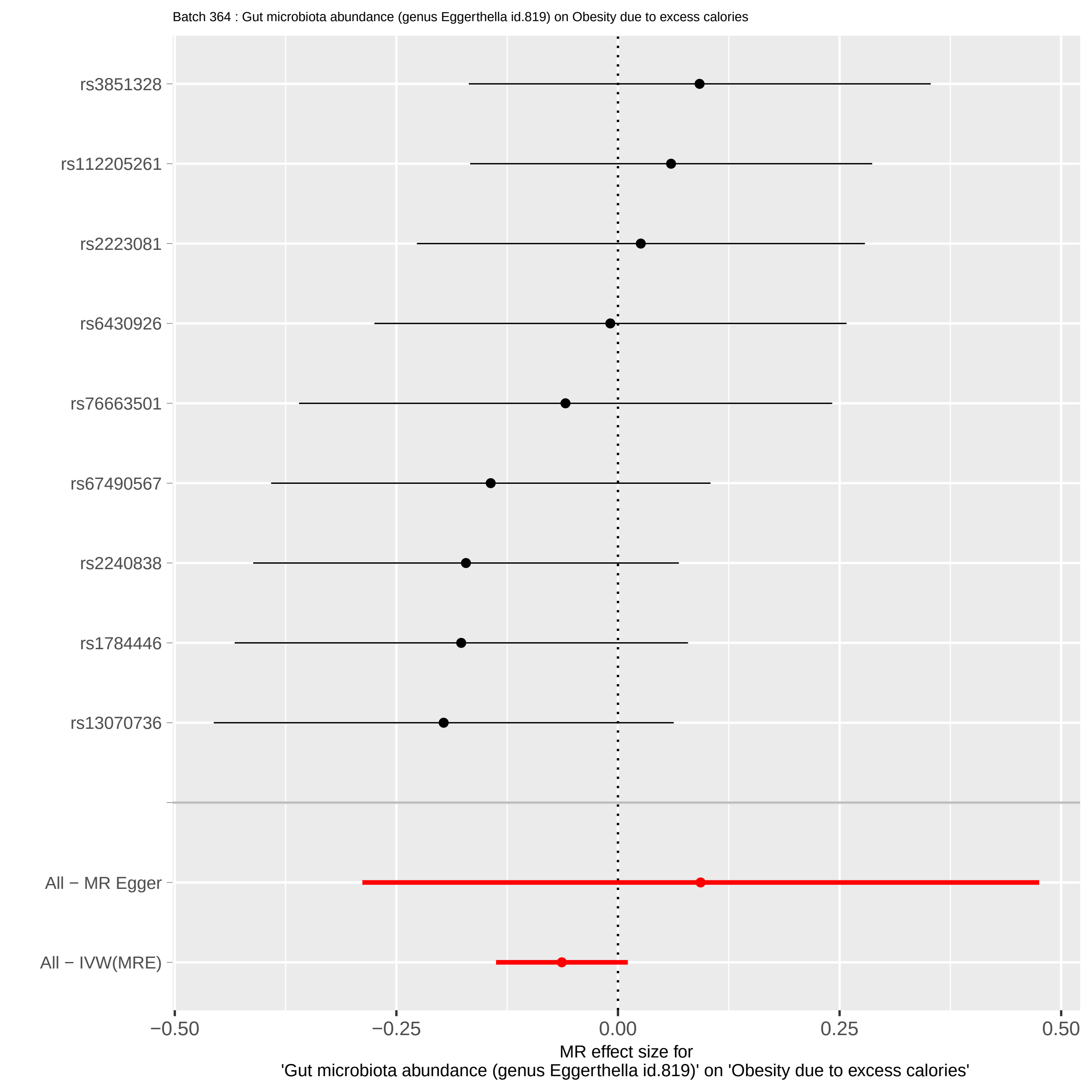
Batch 364 : Gut microbiota abundance (genus Eggerthella id.819) on Obesity due to excess calories
rs3851328
rs112205261
rs2223081
rs6430926
rs76663501
rs67490567
rs2240838
rs1784446
rs13070736
All − MR Egger
All − IVW(MRE)
−0.50
−0.25
0.00
0.25
0.50
MR effect size for
'Gut microbiota abundance (genus Eggerthella id.819)' on 'Obesity due to excess calories'

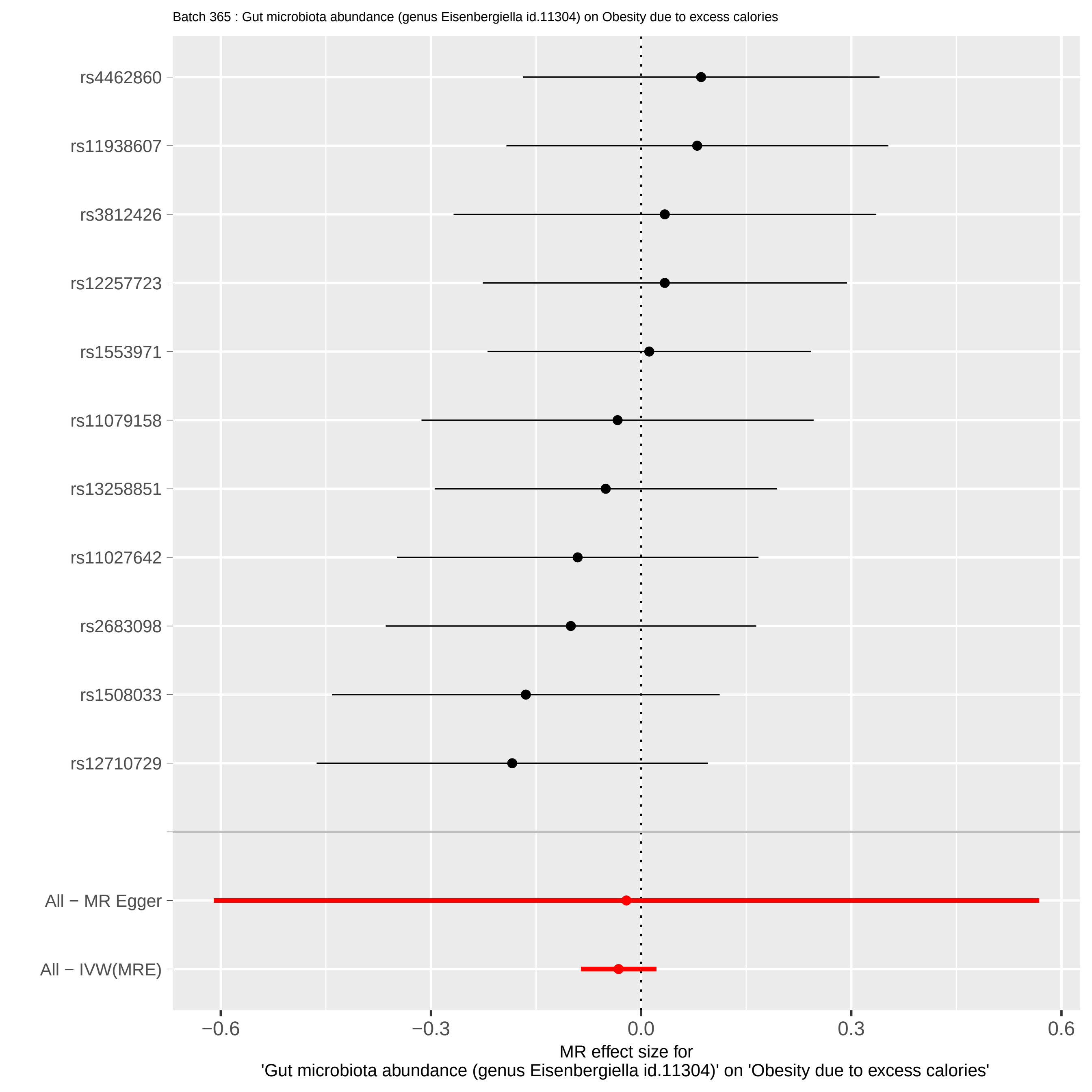

Batch 365 : Gut microbiota abundance (genus Eisenbergiella id.11304) on Obesity due to excess calories
rs4462860
rs11938607
rs3812426
rs12257723
rs1553971
rs11079158
rs13258851
rs11027642
rs2683098
rs1508033
rs12710729
All − MR Egger
All − IVW(MRE)
−0.6
−0.3
0.0
0.3
0.6
MR effect size for
'Gut microbiota abundance (genus Eisenbergiella id.11304)' on 'Obesity due to excess calories'

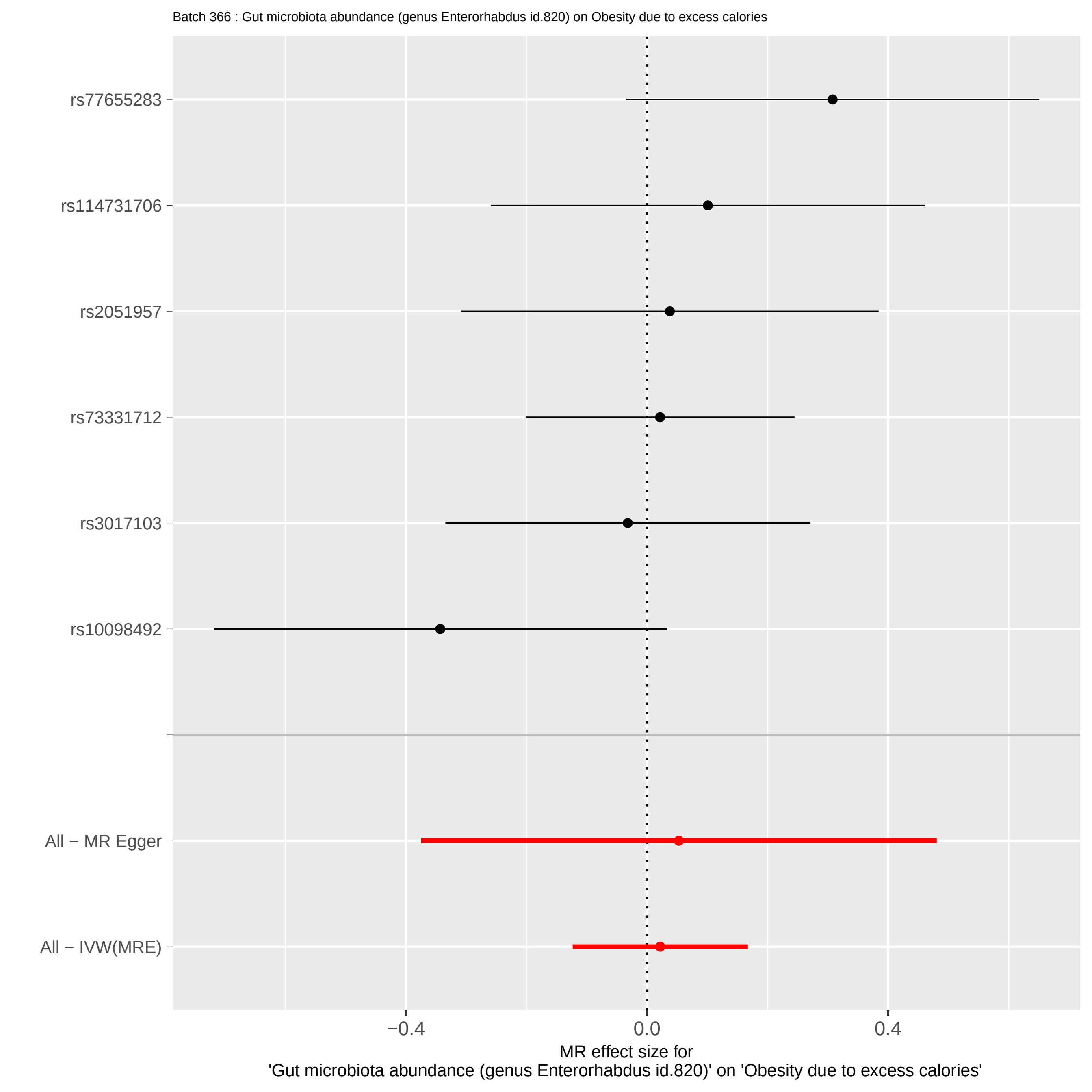

Batch 366 : Gut microbiota abundance (genus Enterorhabdus id.820) on Obesity due to excess calories
rs77655283
rs114731706
rs2051957
rs73331712
rs3017103
rs10098492
All − MR Egger
All − IVW(MRE)
−0.4
0.0
0.4
MR effect size for
'Gut microbiota abundance (genus Enterorhabdus id.820)' on 'Obesity due to excess calories'

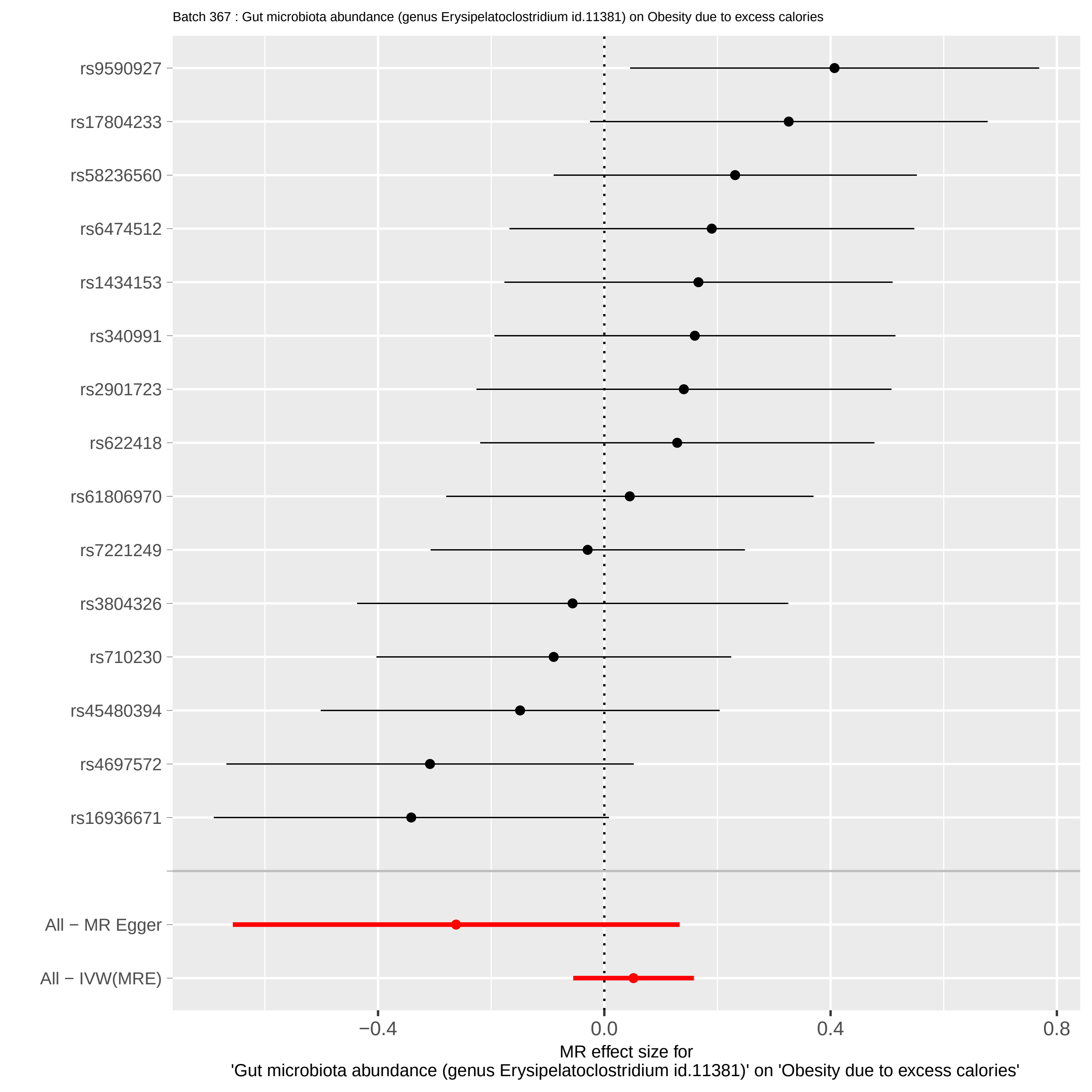

Batch 367 : Gut microbiota abundance (genus Erysipelatoclostridium id.11381) on Obesity due to excess calories
rs9590927
rs17804233
rs58236560
rs6474512
rs1434153
rs340991
rs2901723
rs622418
rs61806970
rs7221249
rs3804326
rs710230
rs45480394
rs4697572
rs16936671
All − MR Egger
All − IVW(MRE)
−0.4
0.0
0.4
0.8
MR effect size for
'Gut microbiota abundance (genus Erysipelatoclostridium id.11381)' on 'Obesity due to excess calories'

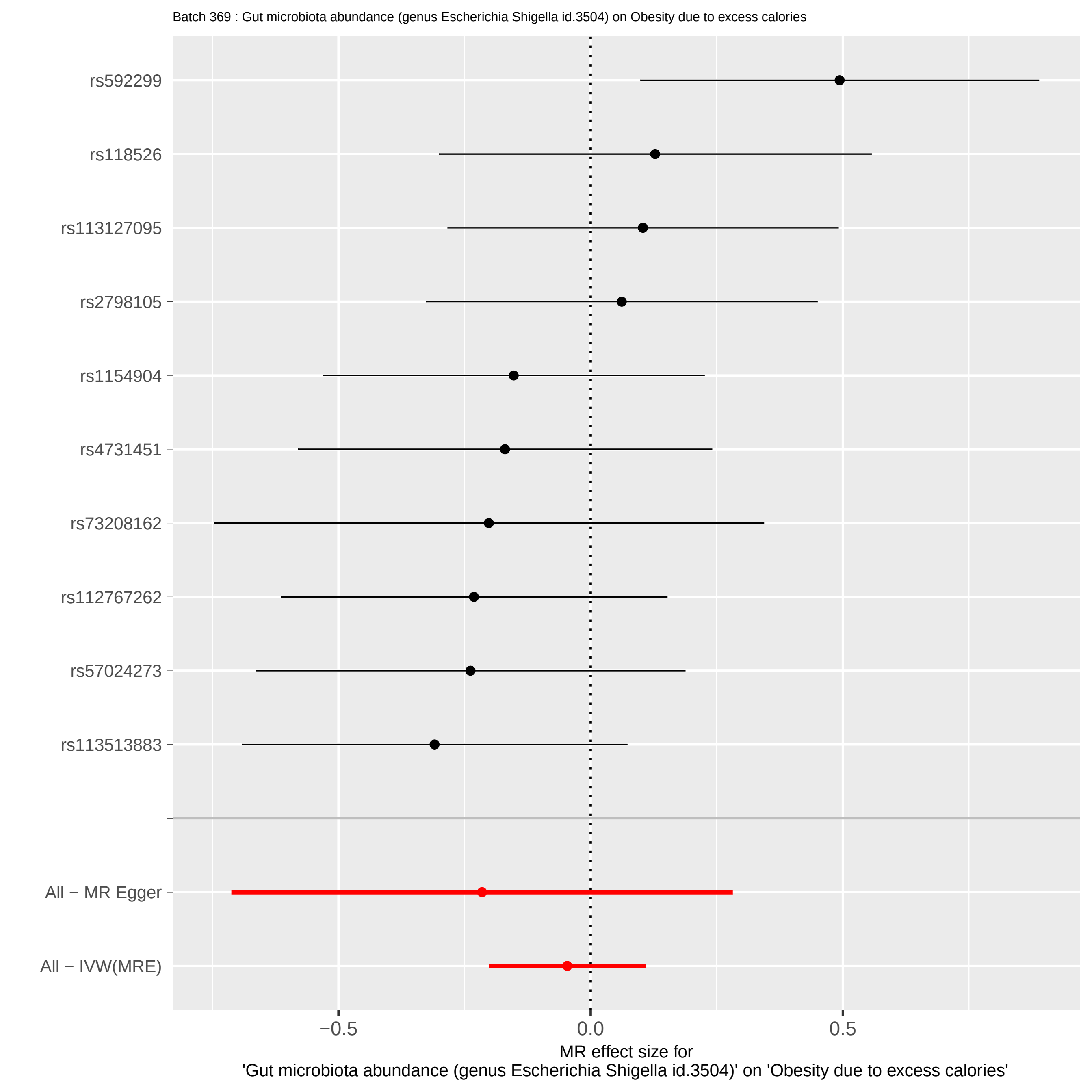

Batch 369 : Gut microbiota abundance (genus Escherichia Shigella id.3504) on Obesity due to excess calories
rs592299
rs118526
rs113127095
rs2798105
rs1154904
rs4731451
rs73208162
rs112767262
rs57024273
rs113513883
All − MR Egger
All − IVW(MRE)
−0.5
0.0
0.5
MR effect size for
'Gut microbiota abundance (genus Escherichia Shigella id.3504)' on 'Obesity due to excess calories'

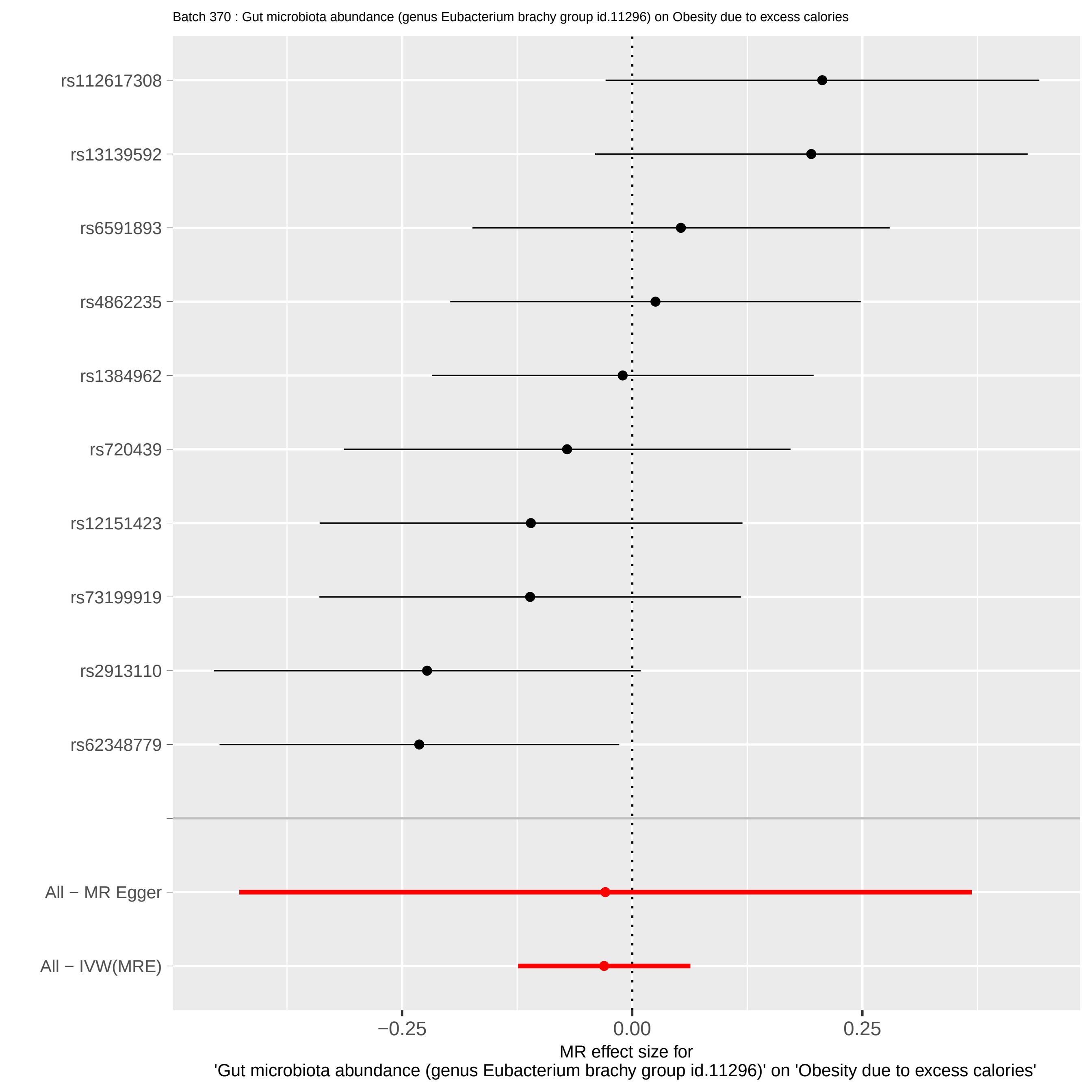

Batch 370 : Gut microbiota abundance (genus Eubacterium brachy group id.11296) on Obesity due to excess calories
rs112617308
rs13139592
rs6591893
rs4862235
rs1384962
rs720439
rs12151423
rs73199919
rs2913110
rs62348779
All − MR Egger
All − IVW(MRE)
−0.25
0.00
0.25
MR effect size for
'Gut microbiota abundance (genus Eubacterium brachy group id.11296)' on 'Obesity due to excess calories'

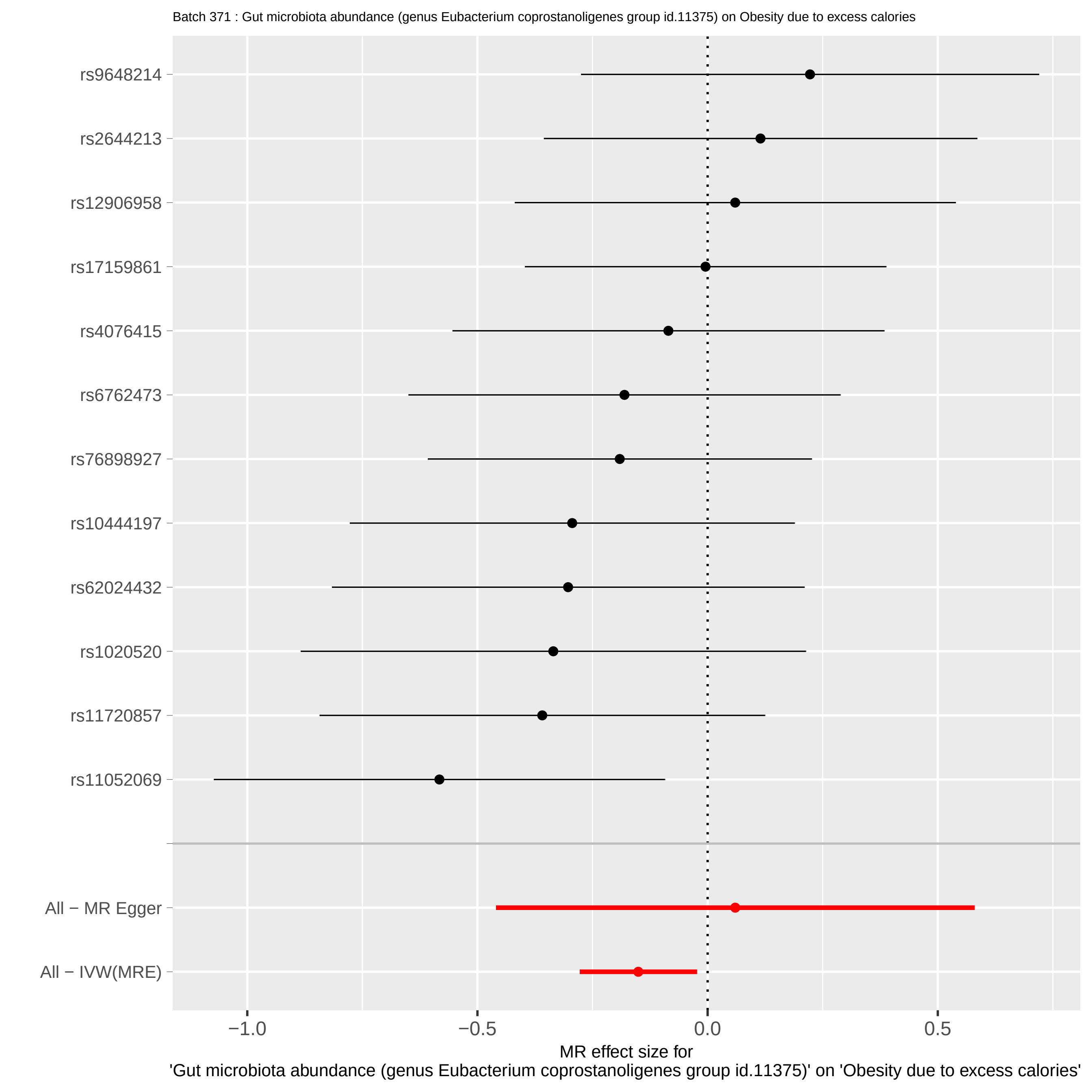
Batch 371 : Gut microbiota abundance (genus Eubacterium coprostanoligenes group id.11375) on Obesity due to excess calories
rs9648214
rs2644213
rs12906958
rs17159861
rs4076415
rs6762473
rs76898927
rs10444197
rs62024432
rs1020520
rs11720857
rs11052069
All − MR Egger
All − IVW(MRE)
−1.0
−0.5
0.0
0.5
MR effect size for
'Gut microbiota abundance (genus Eubacterium coprostanoligenes group id.11375)' on 'Obesity due to excess calories'

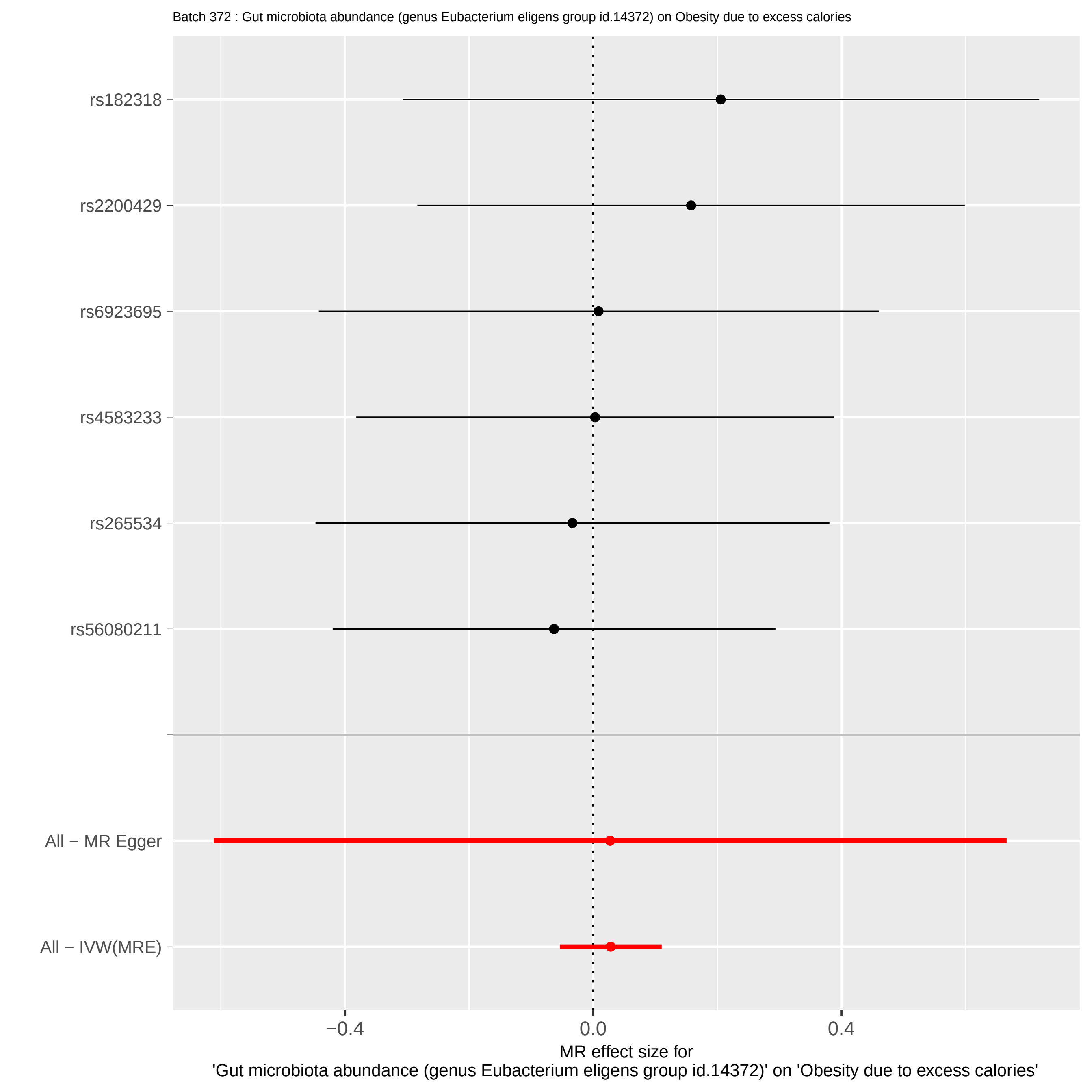

Batch 372 : Gut microbiota abundance (genus Eubacterium eligens group id.14372) on Obesity due to excess calories
rs182318
rs2200429
rs6923695
rs4583233
rs265534
rs56080211
All − MR Egger
All − IVW(MRE)
−0.4
0.0
0.4
MR effect size for
'Gut microbiota abundance (genus Eubacterium eligens group id.14372)' on 'Obesity due to excess calories'

Batch 373 : Gut microbiota abundance (genus Eubacterium fissicatena group id.14373) on Obesity due to excess calories

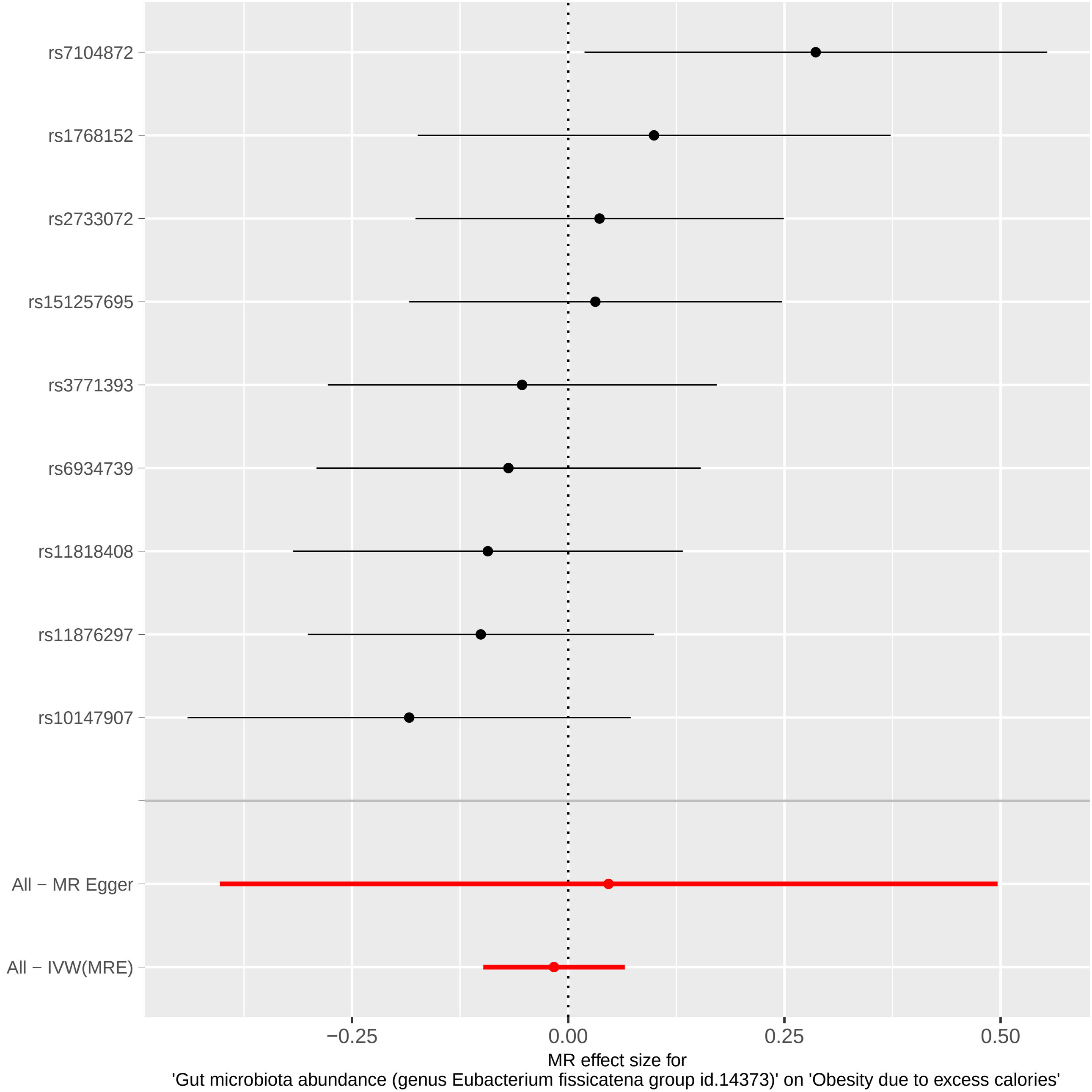

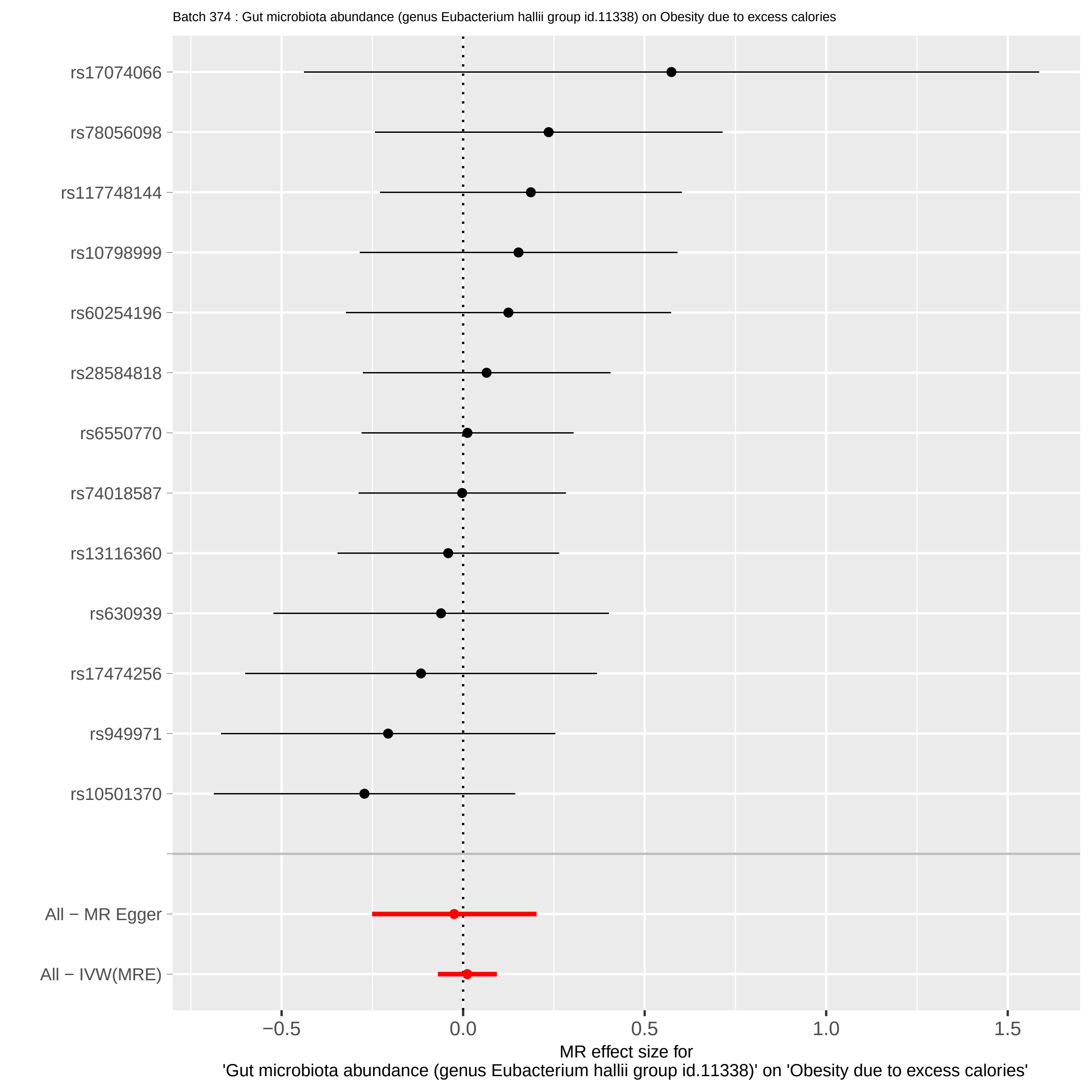
Batch 374 : Gut microbiota abundance (genus Eubacterium hallii group id.11338) on Obesity due to excess calories
rs17074066
rs78056098
rs117748144
rs10798999
rs60254196
rs28584818
rs6550770
rs74018587
rs13116360
rs630939
rs17474256
rs949971
rs10501370
All − MR Egger
All − IVW(MRE)
−0.5
0.0
0.5
1.0
1.5
MR effect size for
'Gut microbiota abundance (genus Eubacterium hallii group id.11338)' on 'Obesity due to excess calories'

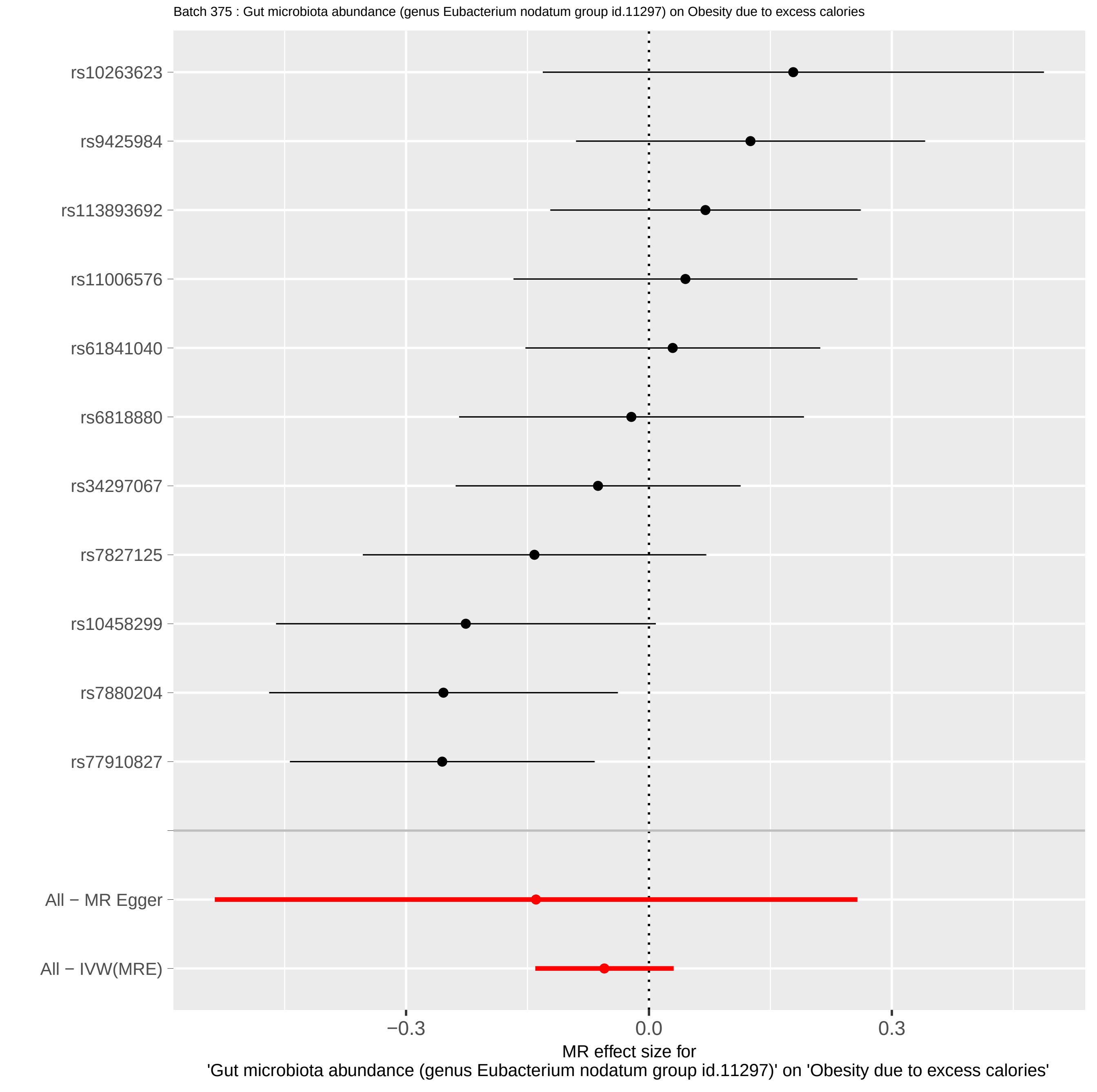
Batch 375 : Gut microbiota abundance (genus Eubacterium nodatum group id.11297) on Obesity due to excess calories
rs10263623
rs9425984
rs113893692
rs11006576
rs61841040
rs6818880
rs34297067
rs7827125
rs10458299
rs7880204
rs77910827
All − MR Egger
All − IVW(MRE)
−0.3
0.0
0.3
MR effect size for
'Gut microbiota abundance (genus Eubacterium nodatum group id.11297)' on 'Obesity due to excess calories'

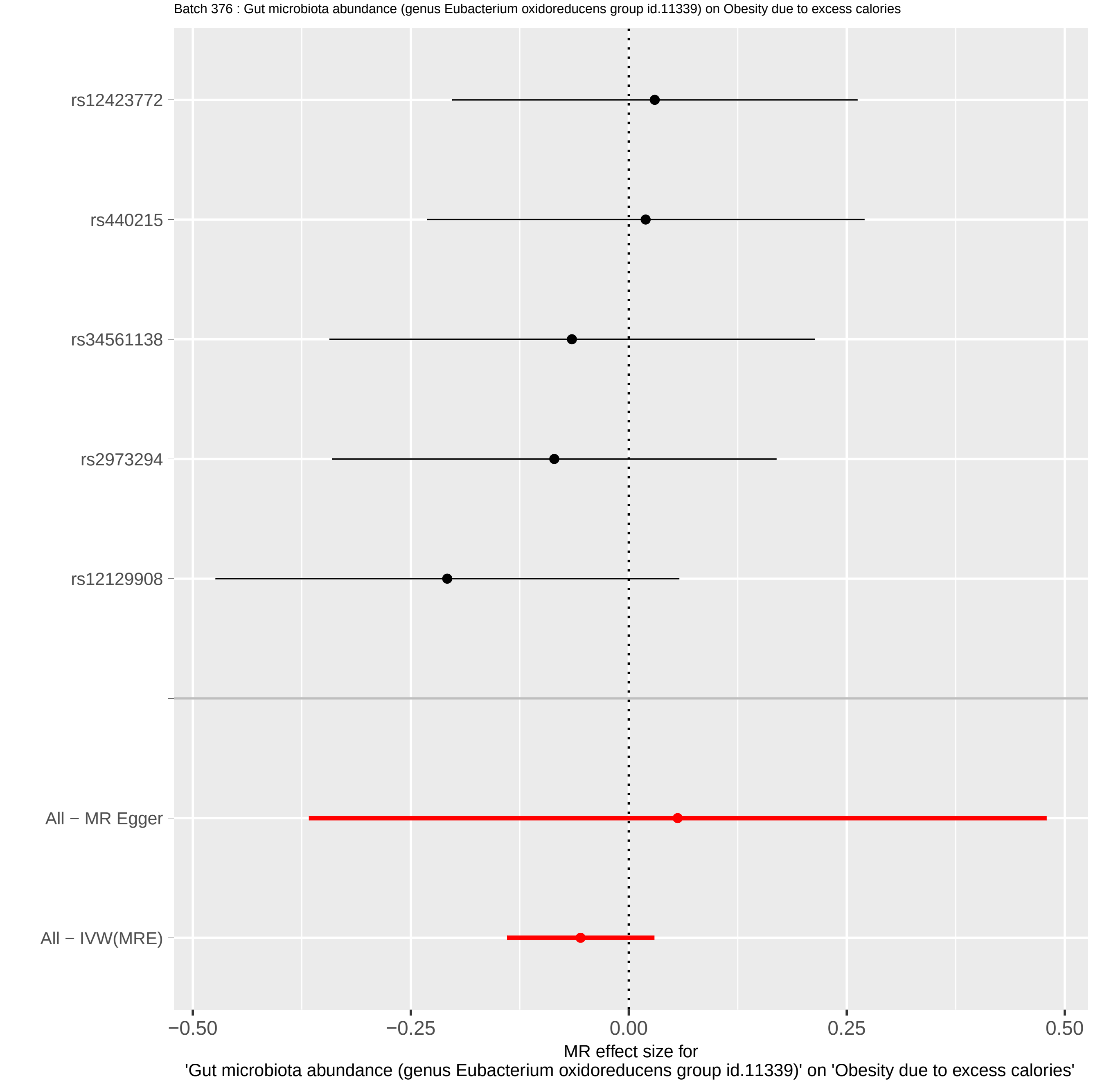
Batch 376 : Gut microbiota abundance (genus Eubacterium oxidoreducens group id.11339) on Obesity due to excess calories
rs12423772
rs440215
rs34561138
rs2973294
rs12129908
All − MR Egger
All − IVW(MRE)
−0.50
−0.25
0.00
0.25
0.50
MR effect size for
'Gut microbiota abundance (genus Eubacterium oxidoreducens group id.11339)' on 'Obesity due to excess calories'

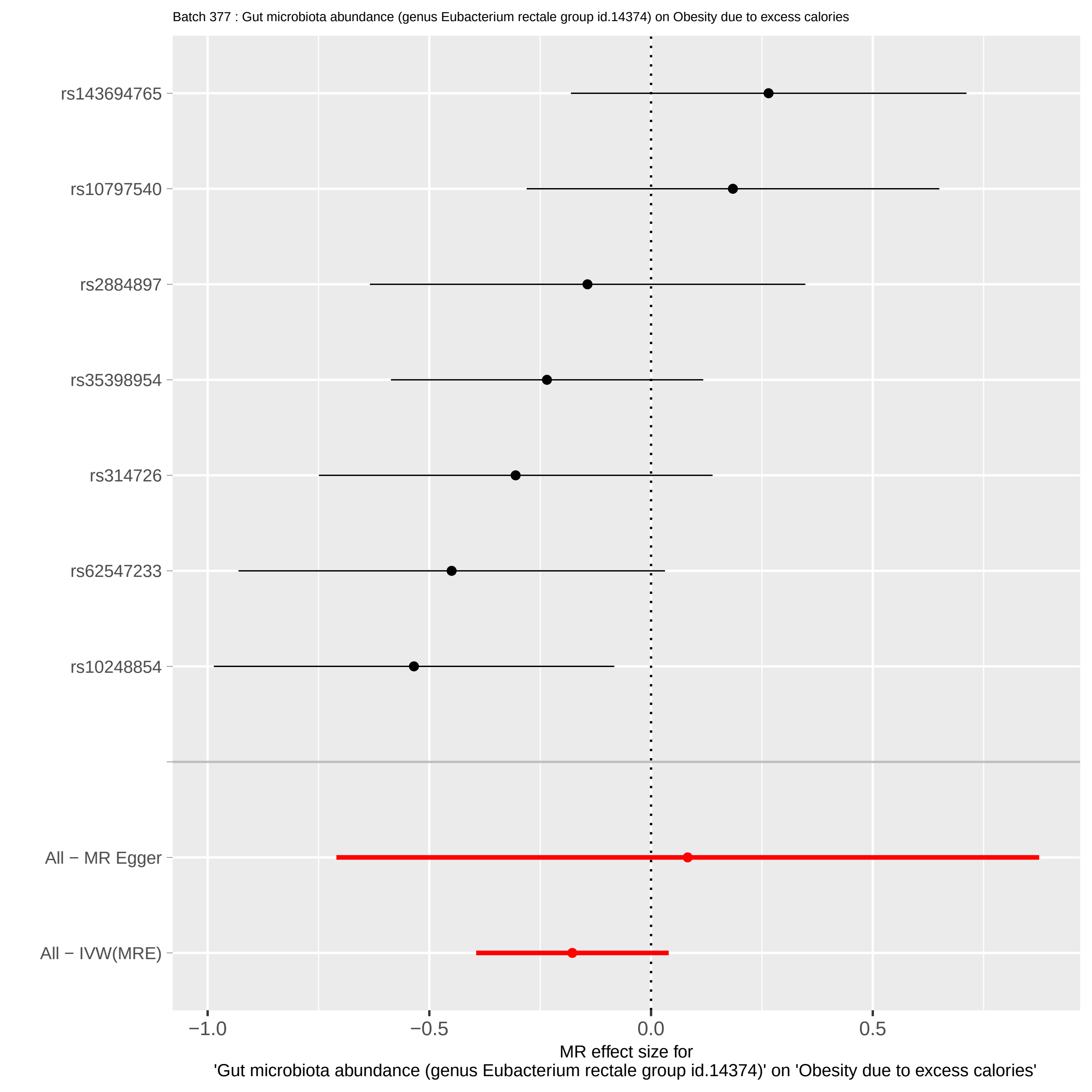
Batch 377 : Gut microbiota abundance (genus Eubacterium rectale group id.14374) on Obesity due to excess calories
rs143694765
rs10797540
rs2884897
rs35398954
rs314726
rs62547233
rs10248854
All − MR Egger
All − IVW(MRE)
−1.0
−0.5
0.0
0.5
MR effect size for
'Gut microbiota abundance (genus Eubacterium rectale group id.14374)' on 'Obesity due to excess calories'

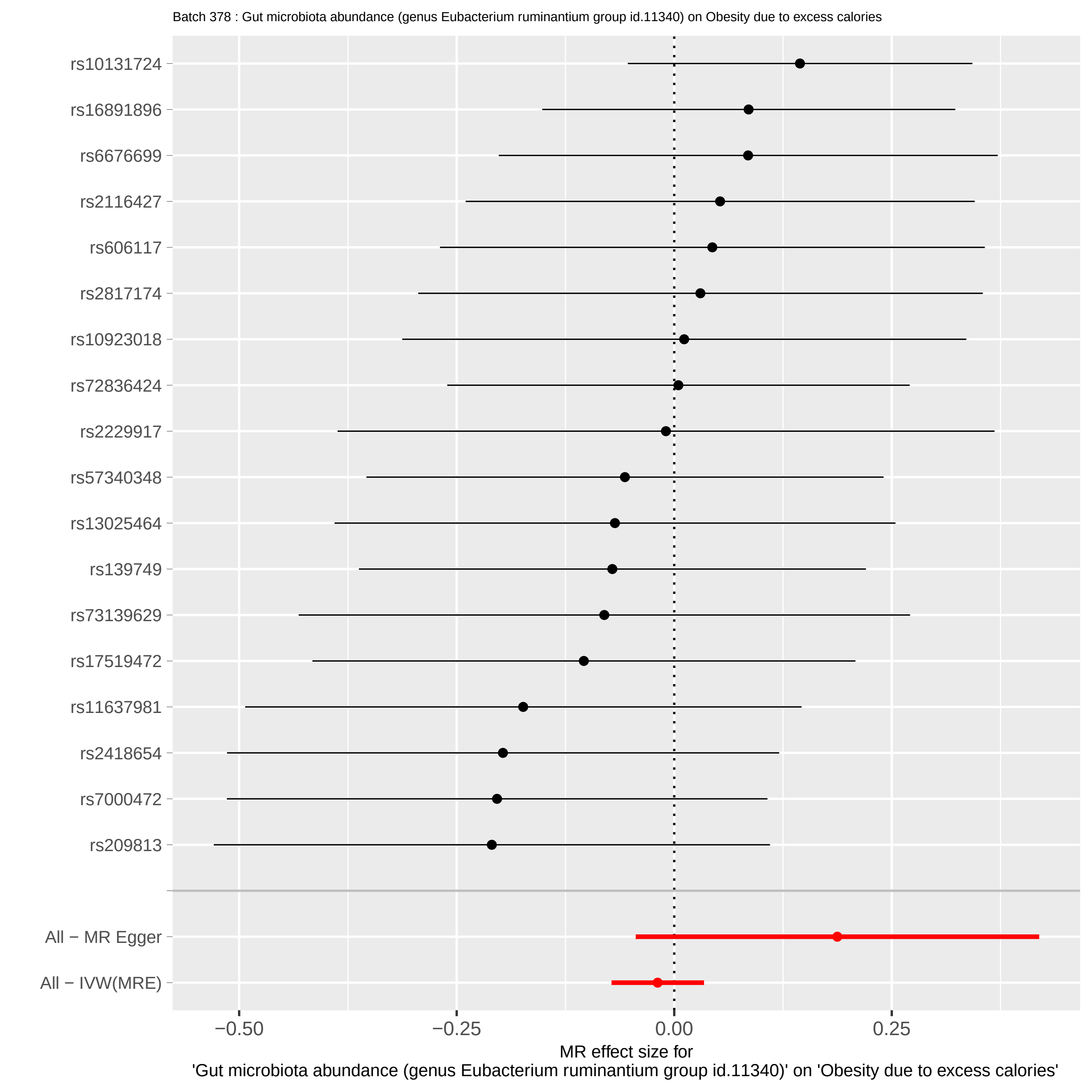

Batch 378 : Gut microbiota abundance (genus Eubacterium ruminantium group id.11340) on Obesity due to excess calories
rs10131724
rs16891896
rs6676699
rs2116427
rs606117
rs2817174
rs10923018
rs72836424
rs2229917
rs57340348
rs13025464
rs139749
rs73139629
rs17519472
rs11637981
rs2418654
rs7000472
rs209813
All − MR Egger
All − IVW(MRE)
−0.50
−0.25
0.00
0.25
MR effect size for
'Gut microbiota abundance (genus Eubacterium ruminantium group id.11340)' on 'Obesity due to excess calories'

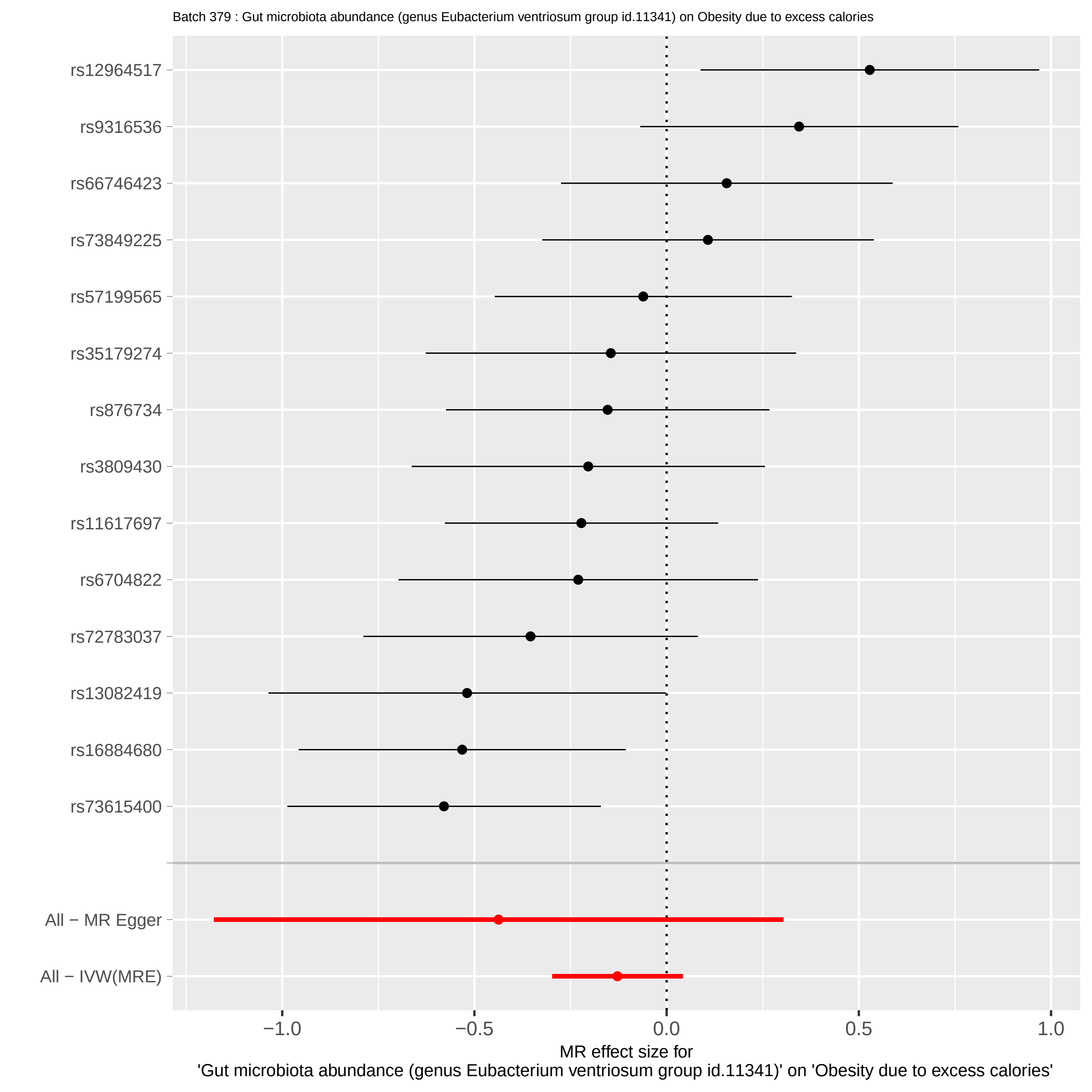

Batch 379 : Gut microbiota abundance (genus Eubacterium ventriosum group id.11341) on Obesity due to excess calories
rs12964517
rs9316536
rs66746423
rs73849225
rs57199565
rs35179274
rs876734
rs3809430
rs11617697
rs6704822
rs72783037
rs13082419
rs16884680
rs73615400
All − MR Egger
All − IVW(MRE)
−1.0
−0.5
0.0
0.5
1.0
MR effect size for
'Gut microbiota abundance (genus Eubacterium ventriosum group id.11341)' on 'Obesity due to excess calories'

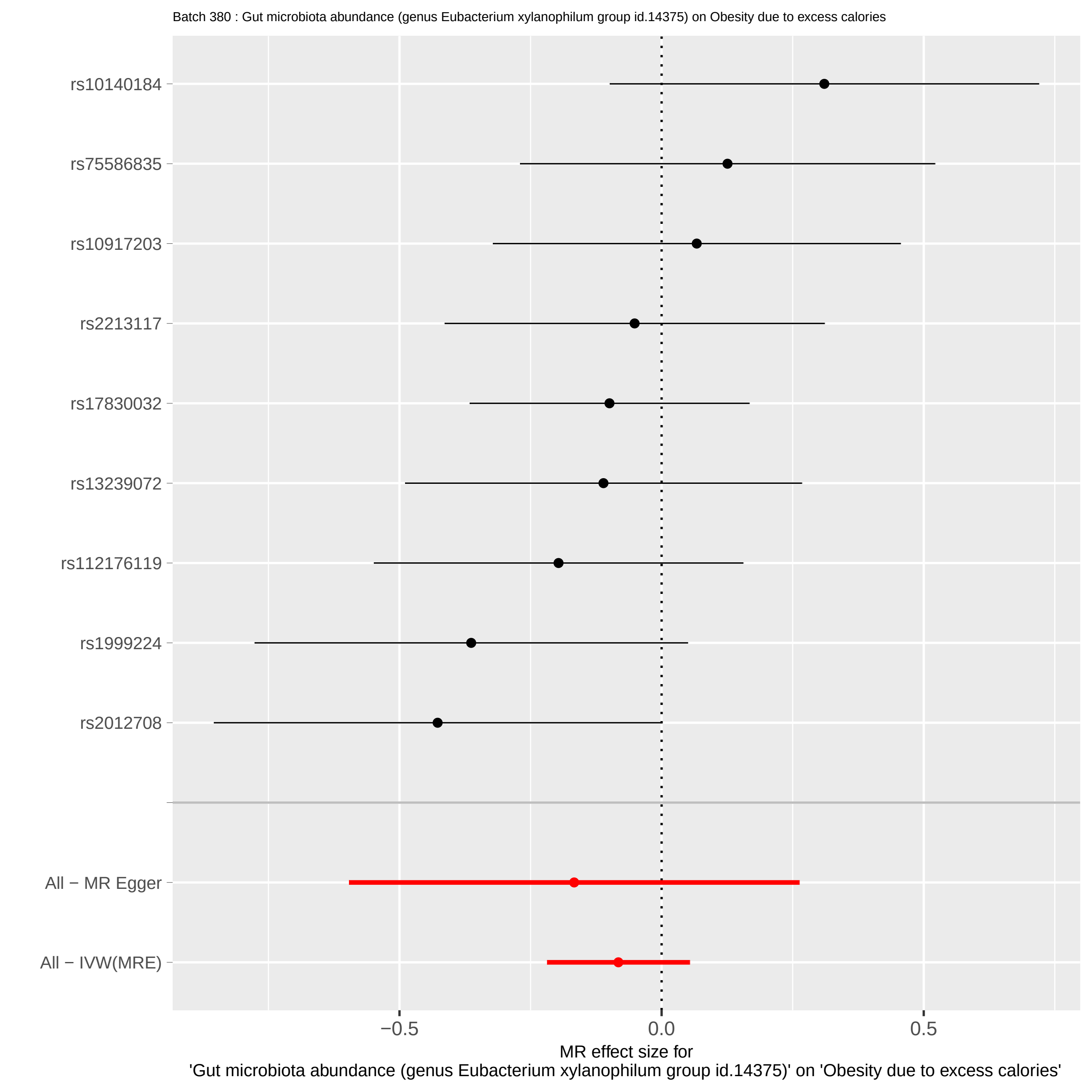
Batch 380 : Gut microbiota abundance (genus Eubacterium xylanophilum group id.14375) on Obesity due to excess calories
rs10140184
rs75586835
rs10917203
rs2213117
rs17830032
rs13239072
rs112176119
rs1999224
rs2012708
All − MR Egger
All − IVW(MRE)
−0.5
0.0
0.5
MR effect size for
'Gut microbiota abundance (genus Eubacterium xylanophilum group id.14375)' on 'Obesity due to excess calories'

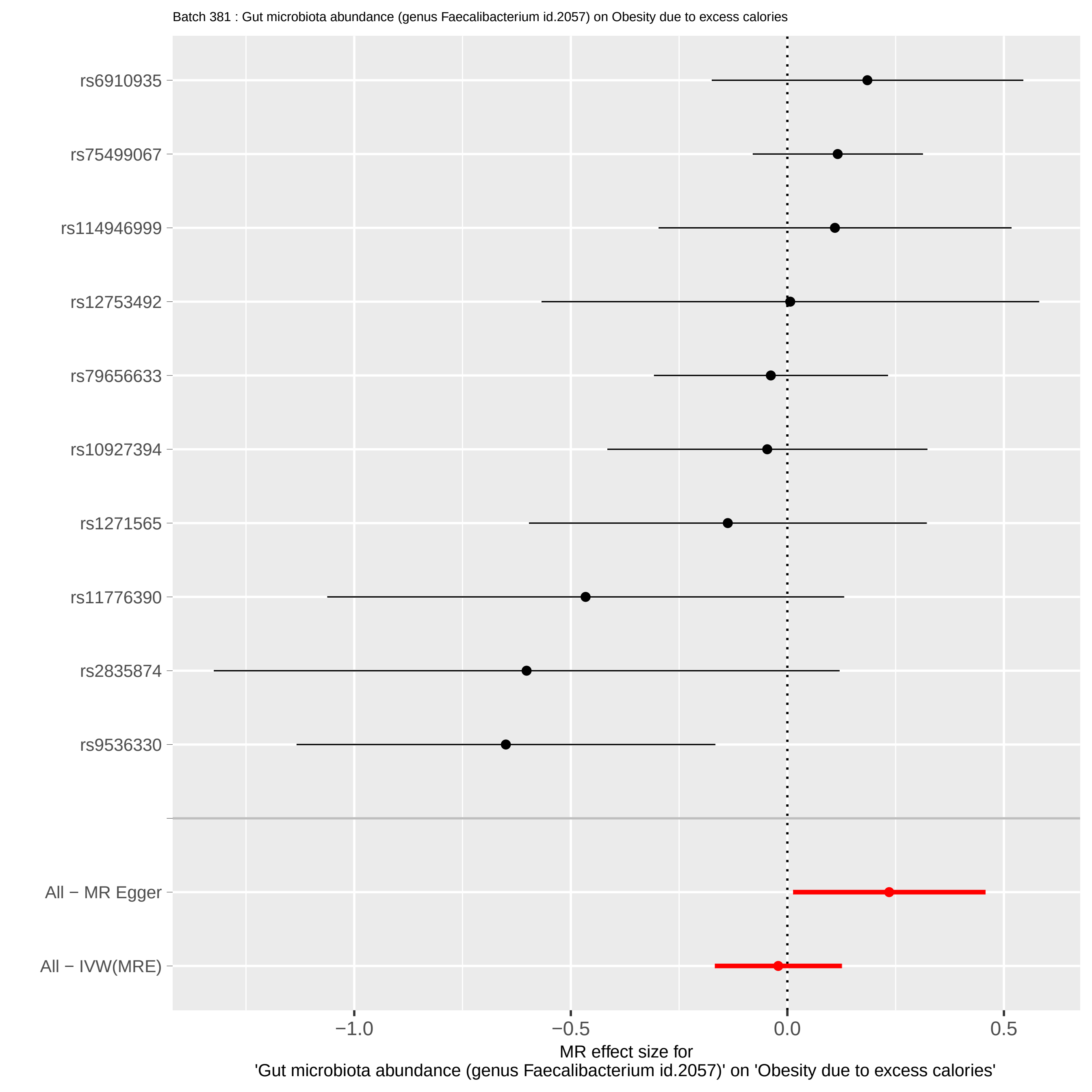
Batch 381 : Gut microbiota abundance (genus Faecalibacterium id.2057) on Obesity due to excess calories
rs6910935
rs75499067
rs114946999
rs12753492
rs79656633
rs10927394
rs1271565
rs11776390
rs2835874
rs9536330
All − MR Egger
All − IVW(MRE)
−1.0
−0.5
0.0
0.5
MR effect size for
'Gut microbiota abundance (genus Faecalibacterium id.2057)' on 'Obesity due to excess calories'

Batch 382 : Gut microbiota abundance (genus Family XIII AD3011 group id.11293) on Obesity due to excess calories

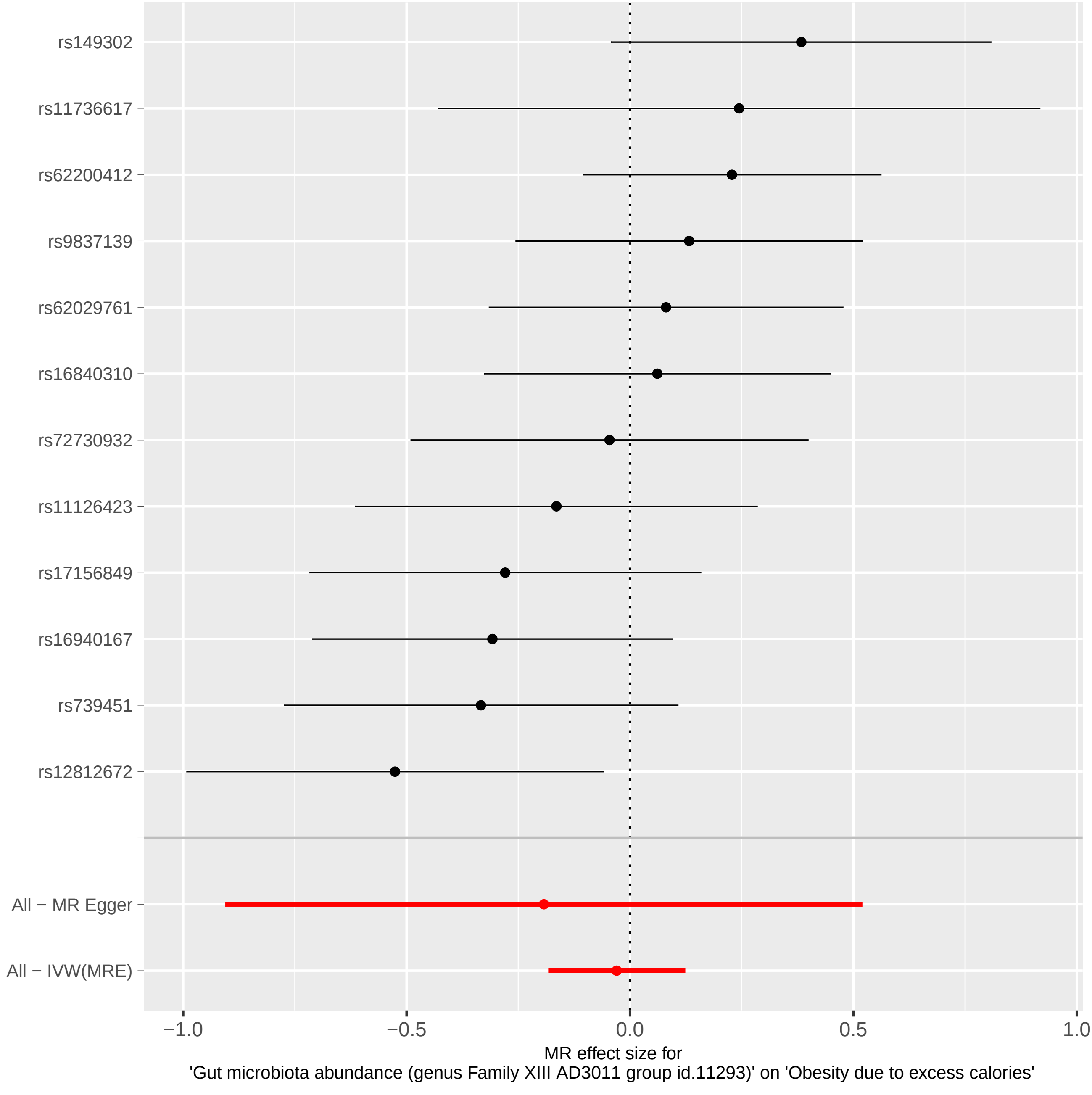

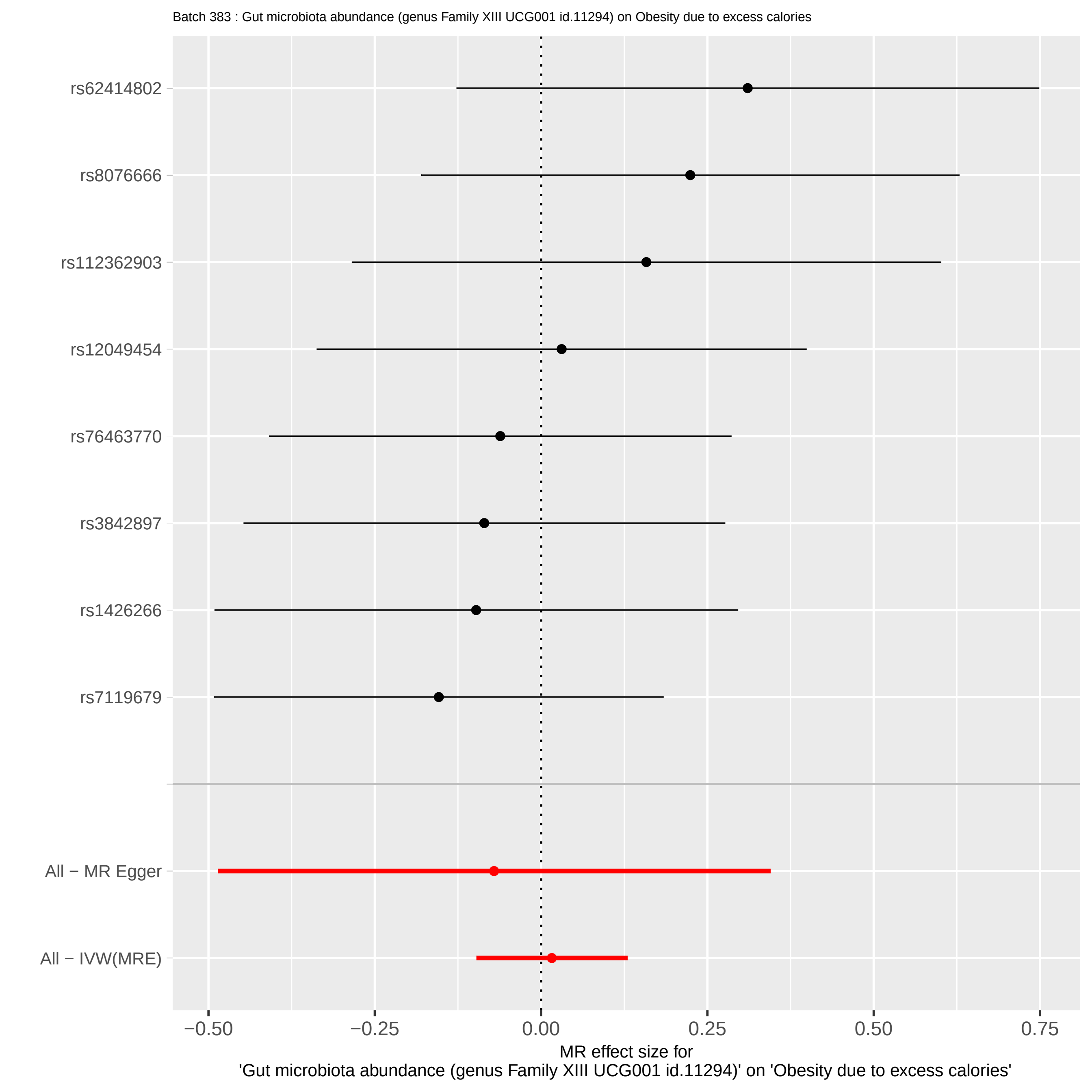

Batch 383 : Gut microbiota abundance (genus Family XIII UCG001 id.11294) on Obesity due to excess calories
rs62414802
rs8076666
rs112362903
rs12049454
rs76463770
rs3842897
rs1426266
rs7119679
All − MR Egger
All − IVW(MRE)
−0.50
−0.25
0.00
0.25
0.50
0.75
MR effect size for
'Gut microbiota abundance (genus Family XIII UCG001 id.11294)' on 'Obesity due to excess calories'

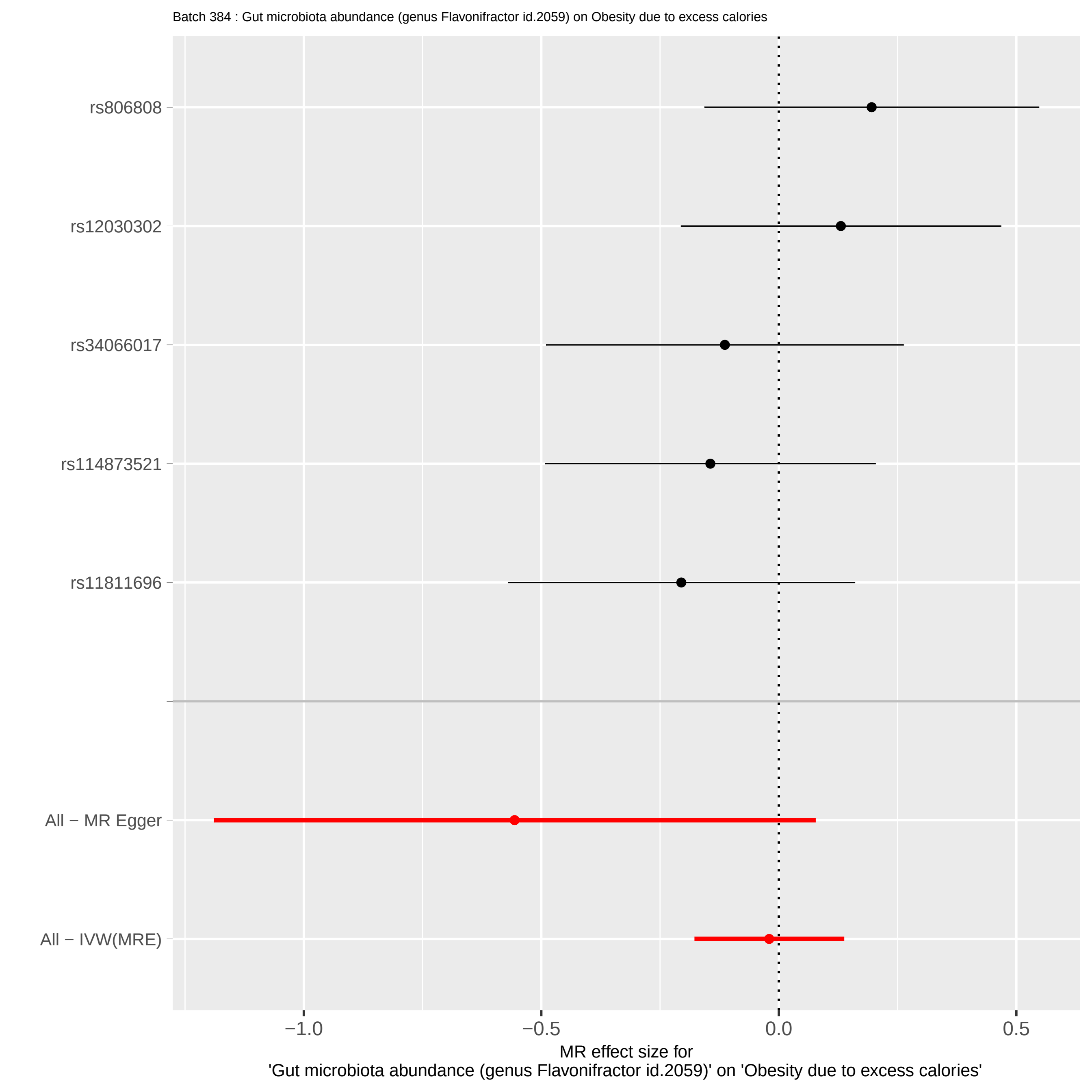

Batch 384 : Gut microbiota abundance (genus Flavonifractor id.2059) on Obesity due to excess calories
rs806808
rs12030302
rs34066017
rs114873521
rs11811696
All − MR Egger
All − IVW(MRE)
−1.0
−0.5
0.0
0.5
MR effect size for
'Gut microbiota abundance (genus Flavonifractor id.2059)' on 'Obesity due to excess calories'

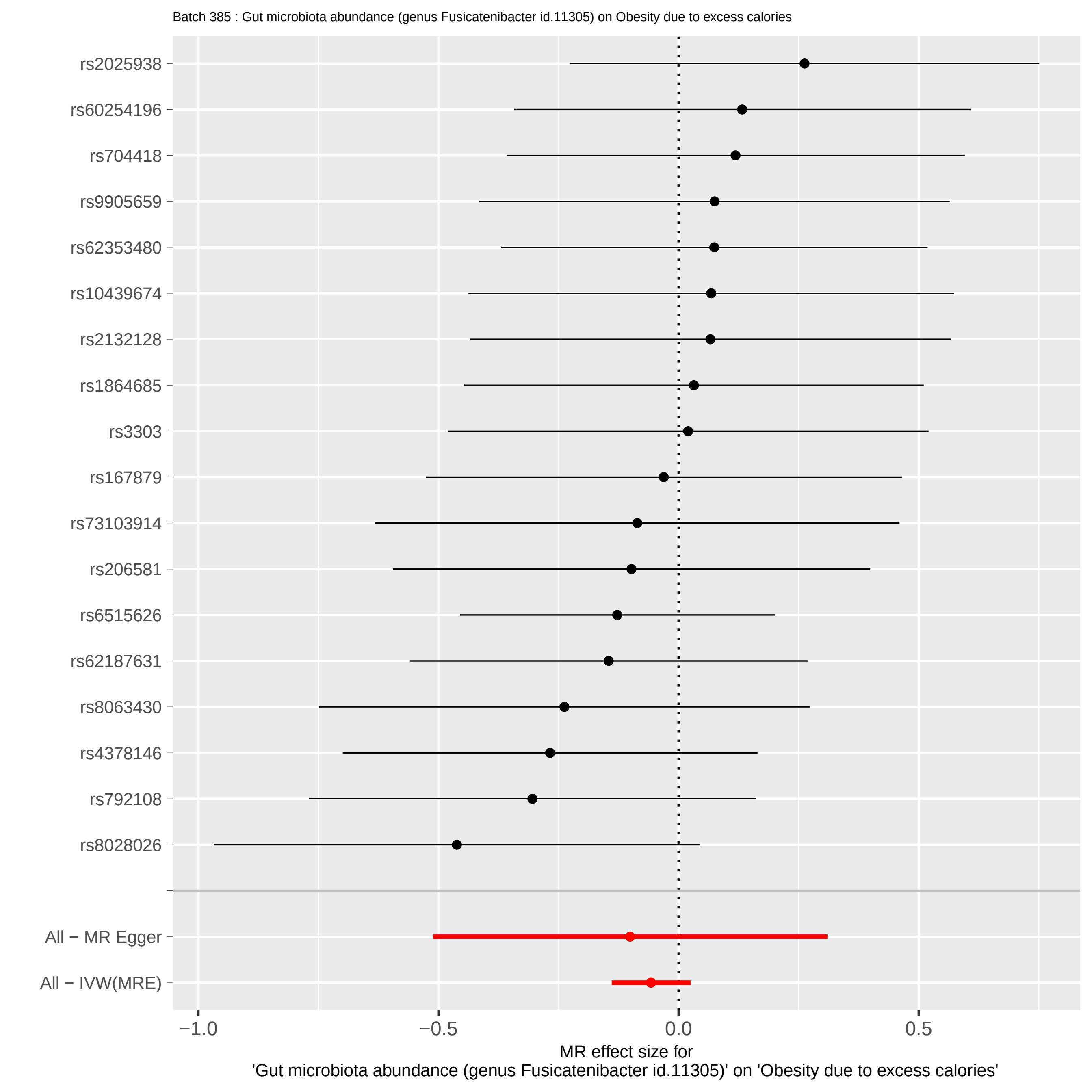

Batch 385 : Gut microbiota abundance (genus Fusicatenibacter id.11305) on Obesity due to excess calories
rs2025938
rs60254196
rs704418
rs9905659
rs62353480
rs10439674
rs2132128
rs1864685
rs3303
rs167879
rs73103914
rs206581
rs6515626
rs62187631
rs8063430
rs4378146
rs792108
rs8028026
All − MR Egger
All − IVW(MRE)
−1.0
−0.5
0.0
0.5
MR effect size for
'Gut microbiota abundance (genus Fusicatenibacter id.11305)' on 'Obesity due to excess calories'

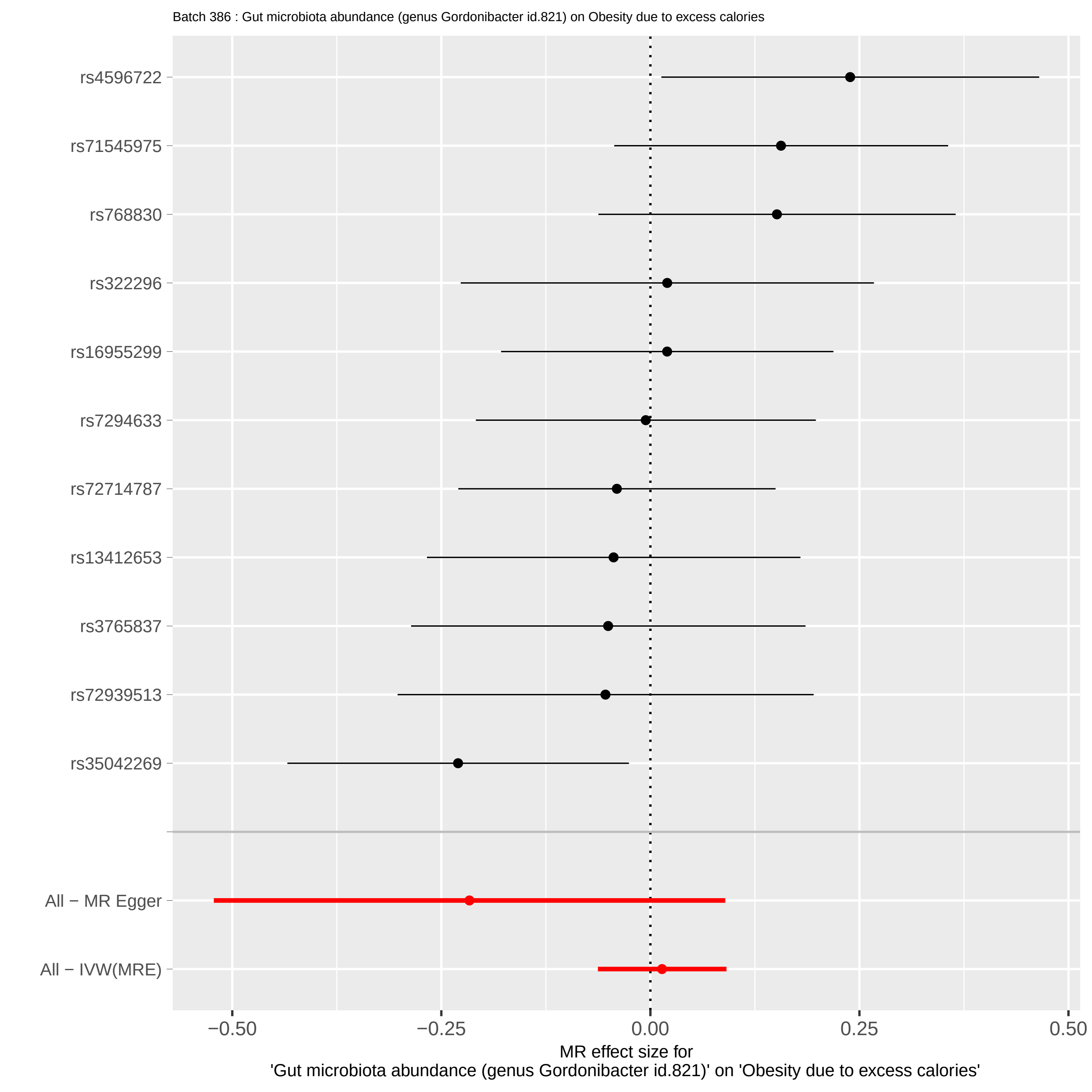

Batch 386 : Gut microbiota abundance (genus Gordonibacter id.821) on Obesity due to excess calories
rs4596722
rs71545975
rs768830
rs322296
rs16955299
rs7294633
rs72714787
rs13412653
rs3765837
rs72939513
rs35042269
All − MR Egger
All − IVW(MRE)
−0.50
−0.25
0.00
0.25
0.50
MR effect size for
'Gut microbiota abundance (genus Gordonibacter id.821)' on 'Obesity due to excess calories'

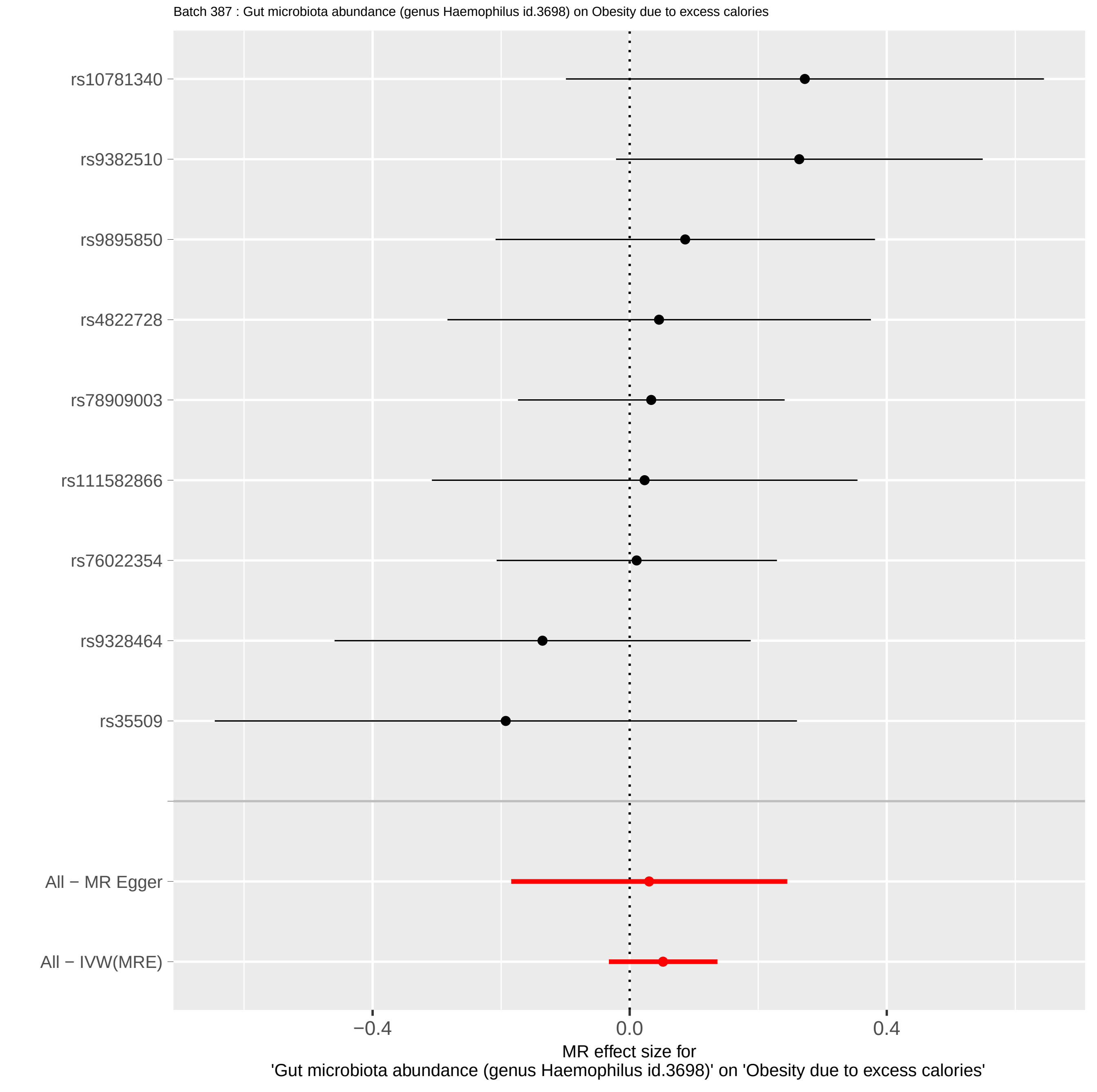

Batch 387 : Gut microbiota abundance (genus Haemophilus id.3698) on Obesity due to excess calories
rs10781340
rs9382510
rs9895850
rs4822728
rs78909003
rs111582866
rs76022354
rs9328464
rs35509
All − MR Egger
All − IVW(MRE)
−0.4
0.0
0.4
MR effect size for
'Gut microbiota abundance (genus Haemophilus id.3698)' on 'Obesity due to excess calories'

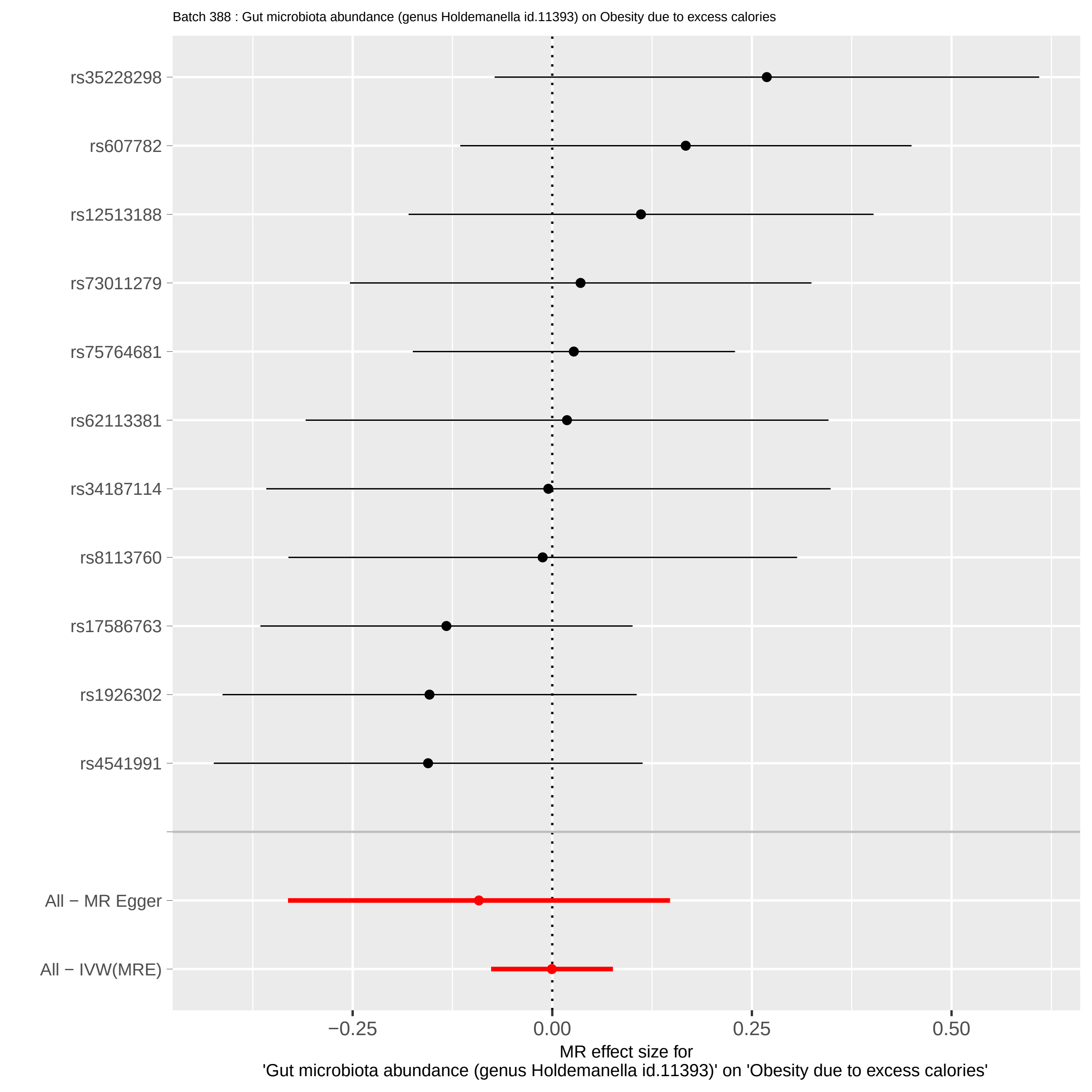

Batch 388 : Gut microbiota abundance (genus Holdemanella id.11393) on Obesity due to excess calories
rs35228298
rs607782
rs12513188
rs73011279
rs75764681
rs62113381
rs34187114
rs8113760
rs17586763
rs1926302
rs4541991
All − MR Egger
All − IVW(MRE)
−0.25
0.00
0.25
0.50
MR effect size for
'Gut microbiota abundance (genus Holdemanella id.11393)' on 'Obesity due to excess calories'

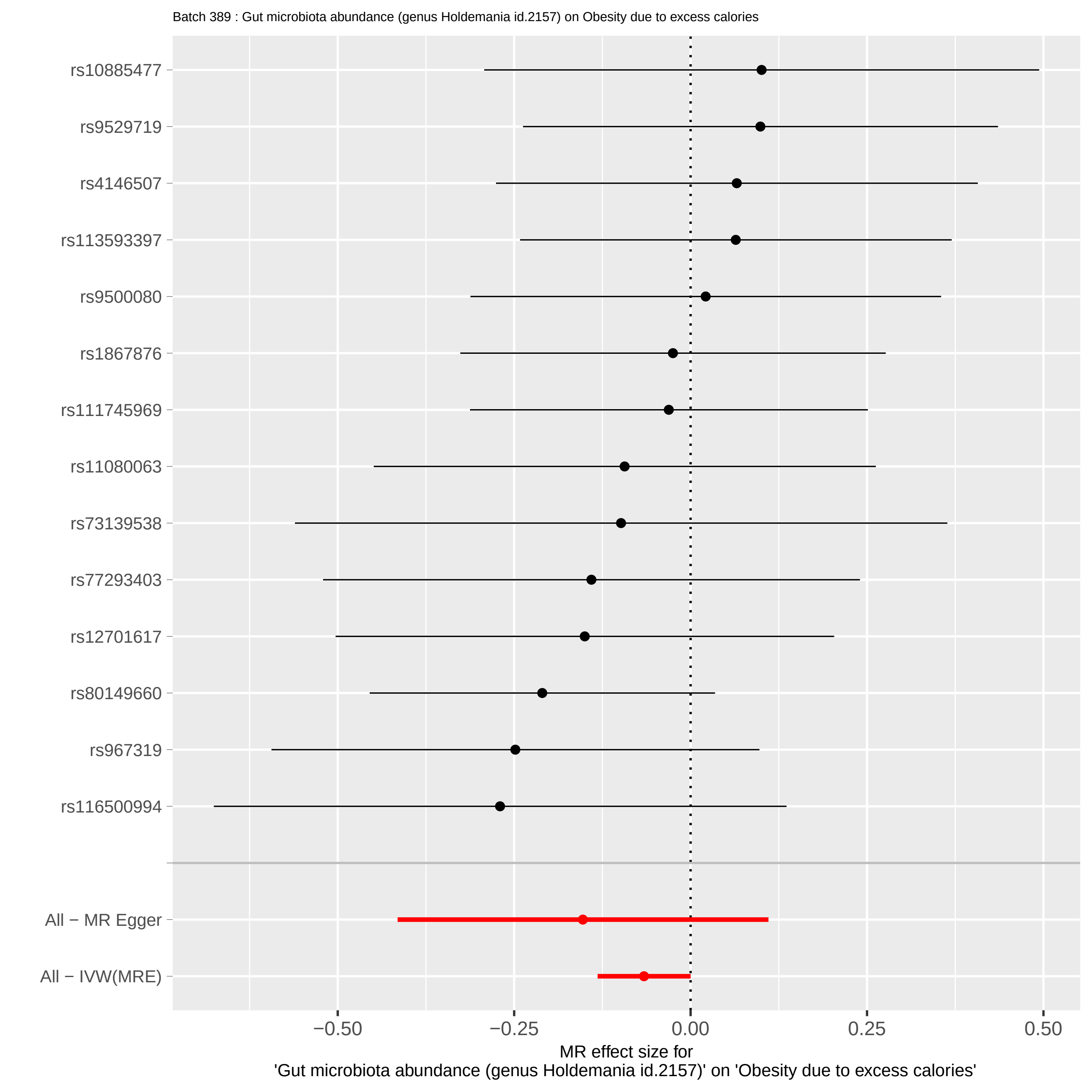
Batch 389 : Gut microbiota abundance (genus Holdemania id.2157) on Obesity due to excess calories
rs10885477
rs9529719
rs4146507
rs113593397
rs9500080
rs1867876
rs111745969
rs11080063
rs73139538
rs77293403
rs12701617
rs80149660
rs967319
rs116500994
All − MR Egger
All − IVW(MRE)
−0.50
−0.25
0.00
0.25
0.50
MR effect size for
'Gut microbiota abundance (genus Holdemania id.2157)' on 'Obesity due to excess calories'

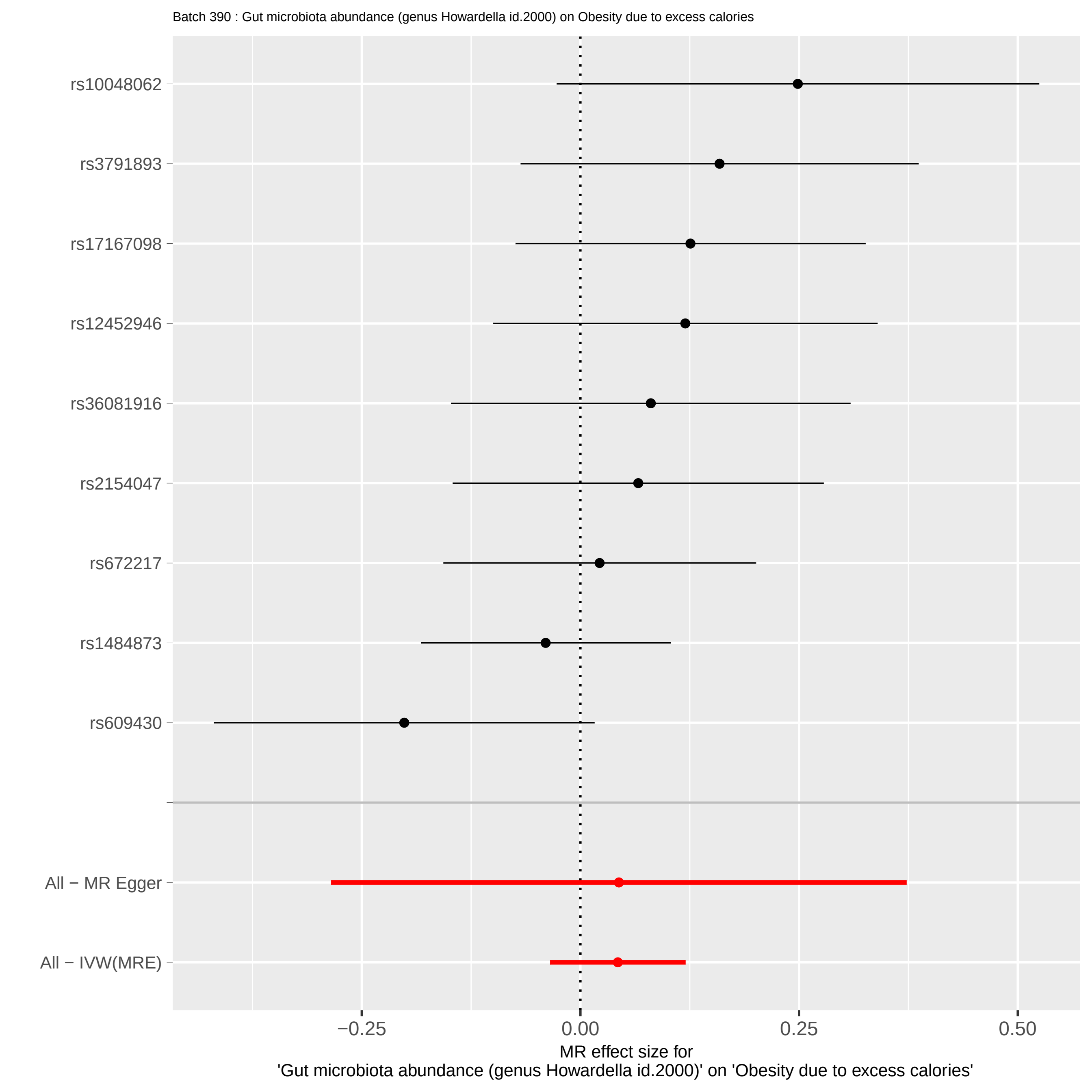
Batch 390 : Gut microbiota abundance (genus Howardella id.2000) on Obesity due to excess calories
rs10048062
rs3791893
rs17167098
rs12452946
rs36081916
rs2154047
rs672217
rs1484873
rs609430
All − MR Egger
All − IVW(MRE)
−0.25
0.00
0.25
0.50
MR effect size for
'Gut microbiota abundance (genus Howardella id.2000)' on 'Obesity due to excess calories'

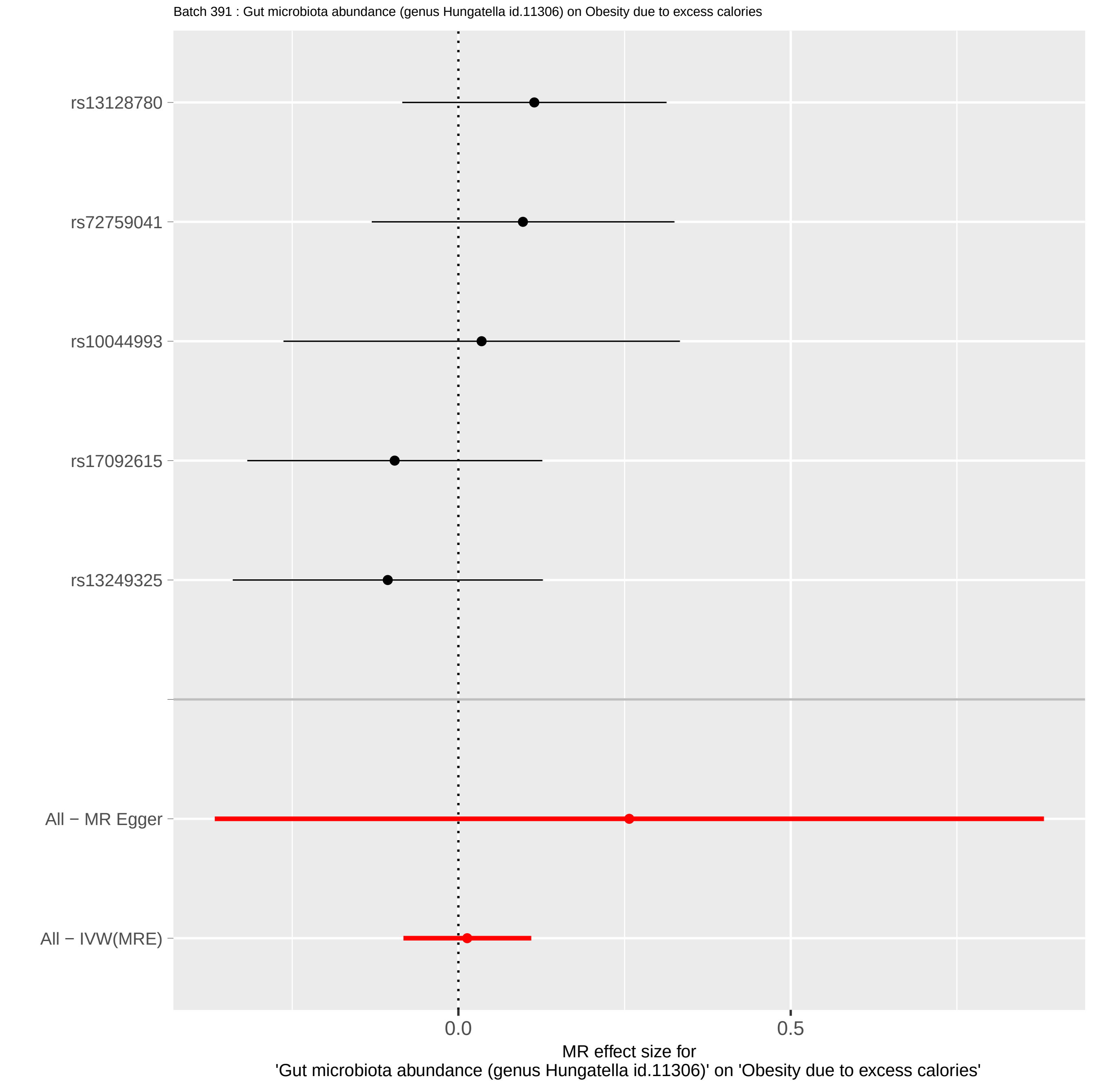
Batch 391 : Gut microbiota abundance (genus Hungatella id.11306) on Obesity due to excess calories
rs13128780
rs72759041
rs10044993
rs17092615
rs13249325
All − MR Egger
All − IVW(MRE)
0.0
0.5
MR effect size for
'Gut microbiota abundance (genus Hungatella id.11306)' on 'Obesity due to excess calories'

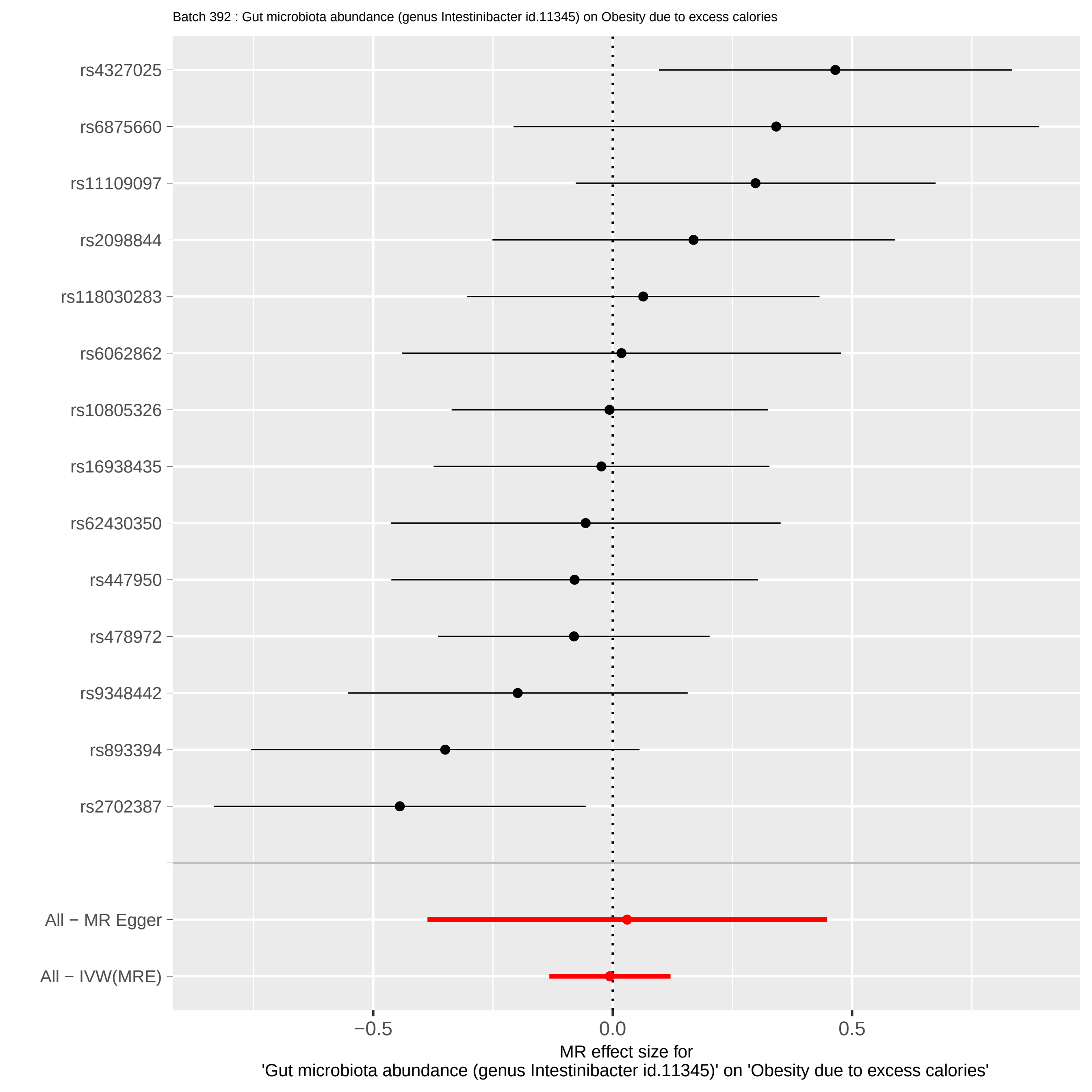

Batch 392 : Gut microbiota abundance (genus Intestinibacter id.11345) on Obesity due to excess calories
rs4327025
rs6875660
rs11109097
rs2098844
rs118030283
rs6062862
rs10805326
rs16938435
rs62430350
rs447950
rs478972
rs9348442
rs893394
rs2702387
All − MR Egger
All − IVW(MRE)
−0.5
0.0
0.5
MR effect size for
'Gut microbiota abundance (genus Intestinibacter id.11345)' on 'Obesity due to excess calories'

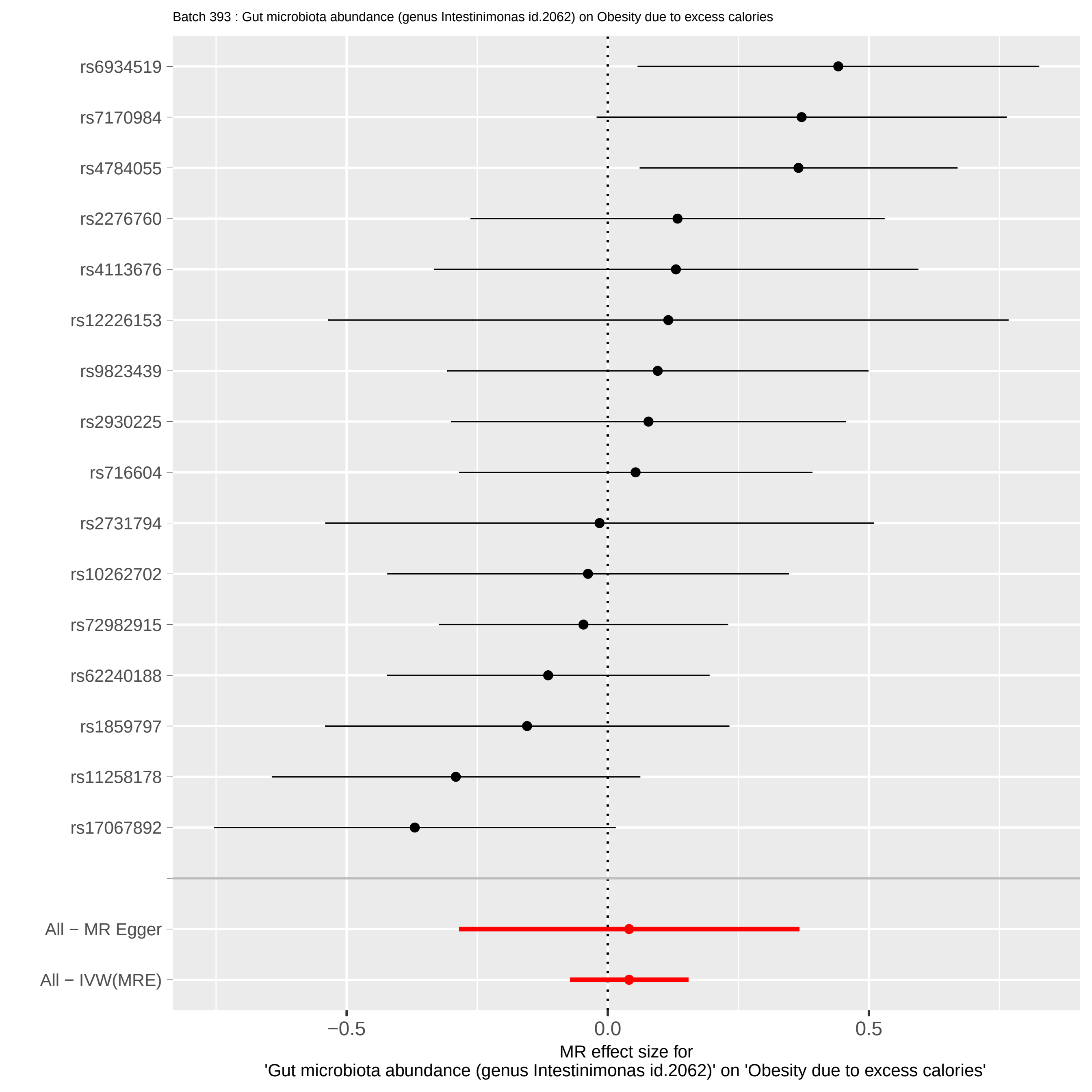

Batch 393 : Gut microbiota abundance (genus Intestinimonas id.2062) on Obesity due to excess calories
rs6934519
rs7170984
rs4784055
rs2276760
rs4113676
rs12226153
rs9823439
rs2930225
rs716604
rs2731794
rs10262702
rs72982915
rs62240188
rs1859797
rs11258178
rs17067892
All − MR Egger
All − IVW(MRE)
−0.5
0.0
0.5
MR effect size for
'Gut microbiota abundance (genus Intestinimonas id.2062)' on 'Obesity due to excess calories'

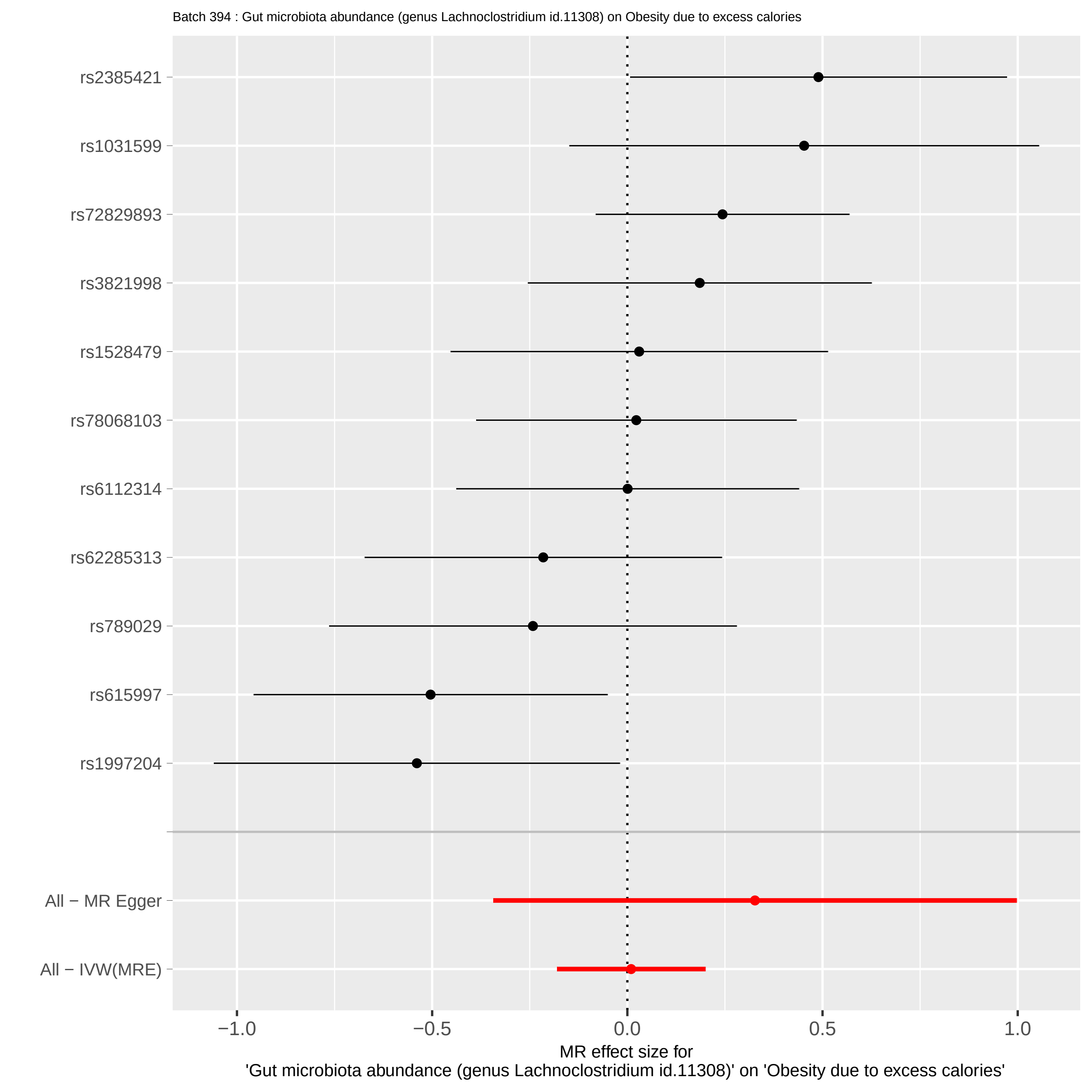

Batch 394 : Gut microbiota abundance (genus Lachnoclostridium id.11308) on Obesity due to excess calories
rs2385421
rs1031599
rs72829893
rs3821998
rs1528479
rs78068103
rs6112314
rs62285313
rs789029
rs615997
rs1997204
All − MR Egger
All − IVW(MRE)
−1.0
−0.5
0.0
0.5
1.0
MR effect size for
'Gut microbiota abundance (genus Lachnoclostridium id.11308)' on 'Obesity due to excess calories'

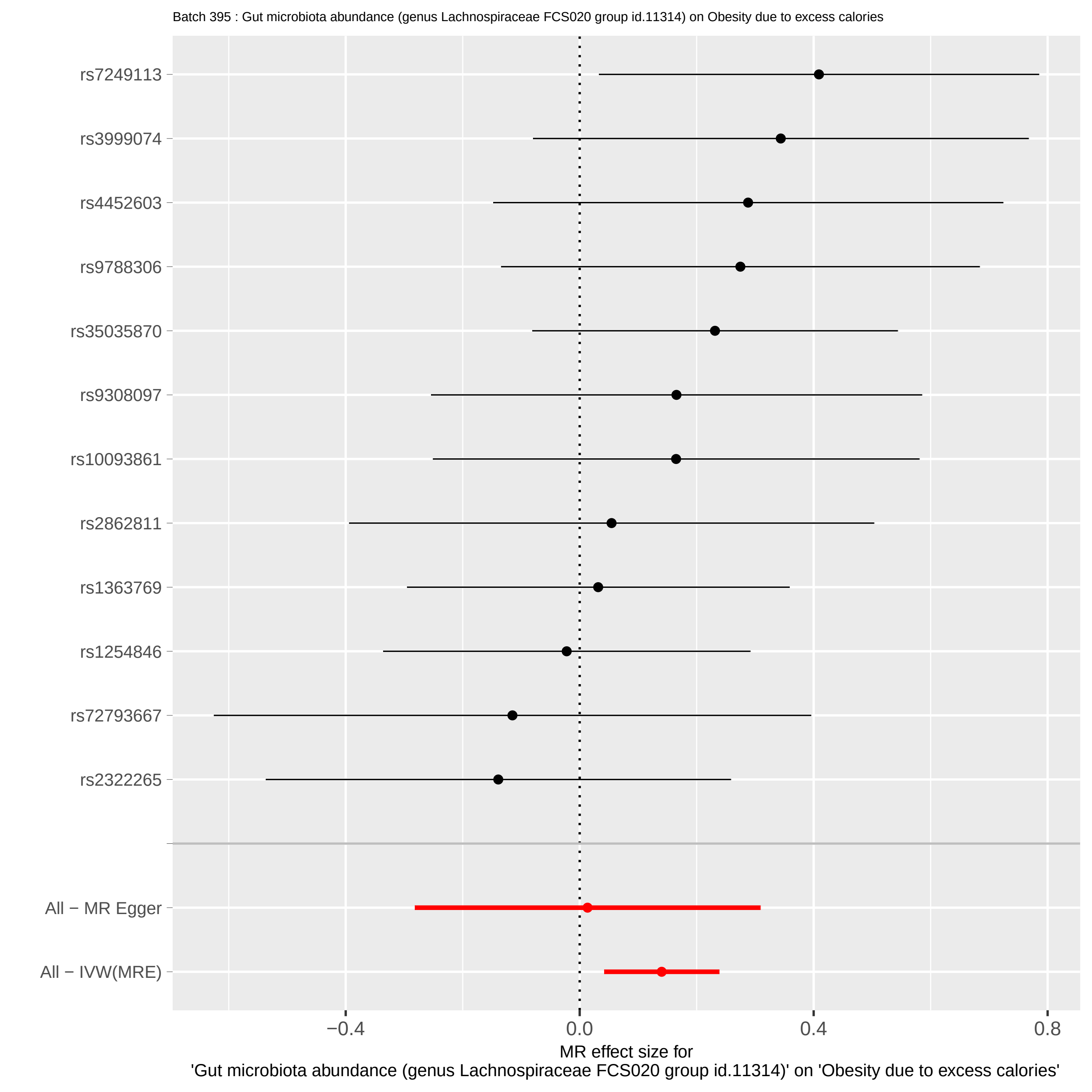

Batch 395 : Gut microbiota abundance (genus Lachnospiraceae FCS020 group id.11314) on Obesity due to excess calories
rs7249113
rs3999074
rs4452603
rs9788306
rs35035870
rs9308097
rs10093861
rs2862811
rs1363769
rs1254846
rs72793667
rs2322265
All − MR Egger
All − IVW(MRE)
−0.4
0.0
0.4
0.8
MR effect size for
'Gut microbiota abundance (genus Lachnospiraceae FCS020 group id.11314)' on 'Obesity due to excess calories'

Batch 396 : Gut microbiota abundance (genus Lachnospiraceae NC2004 group id.11316) on Obesity due to excess calories

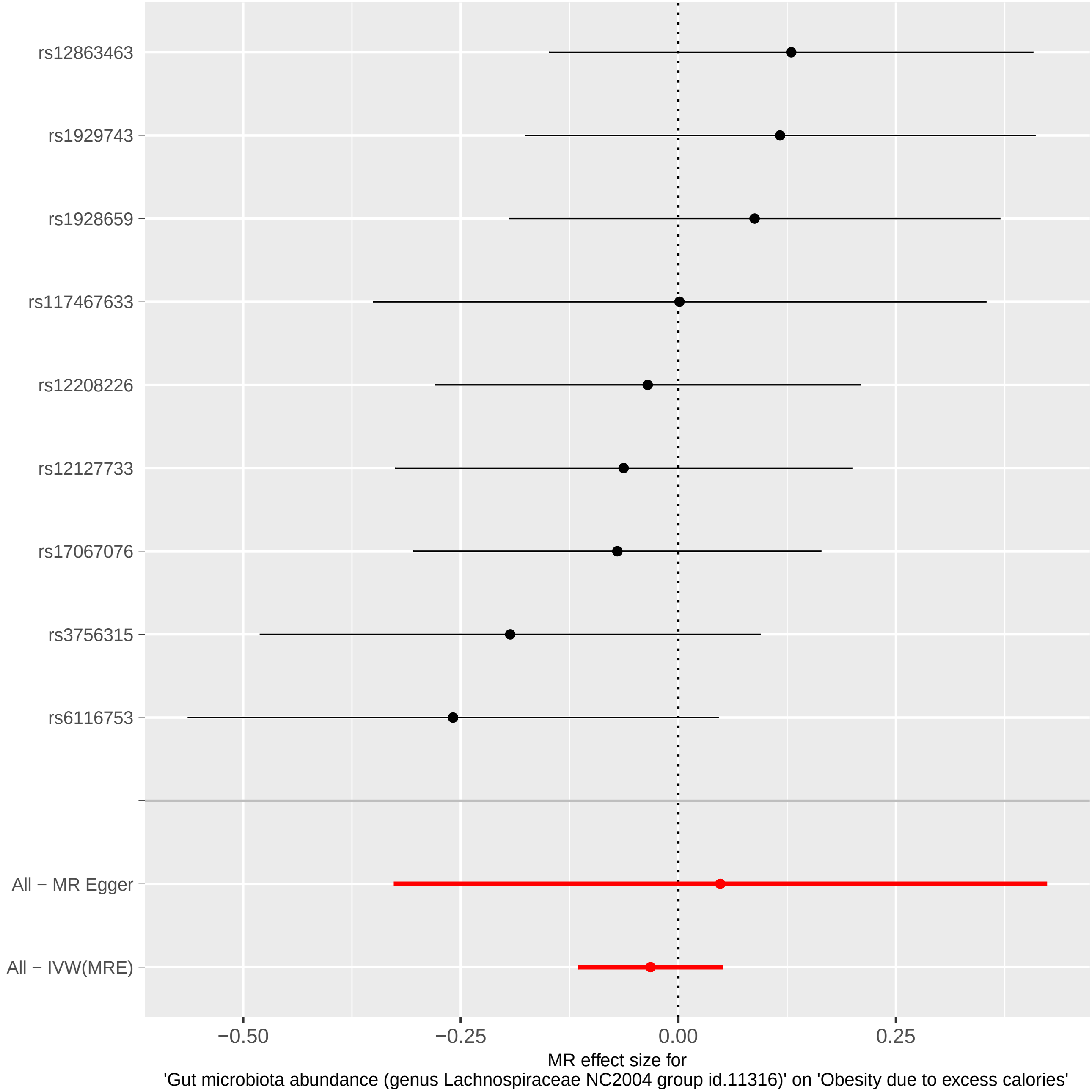

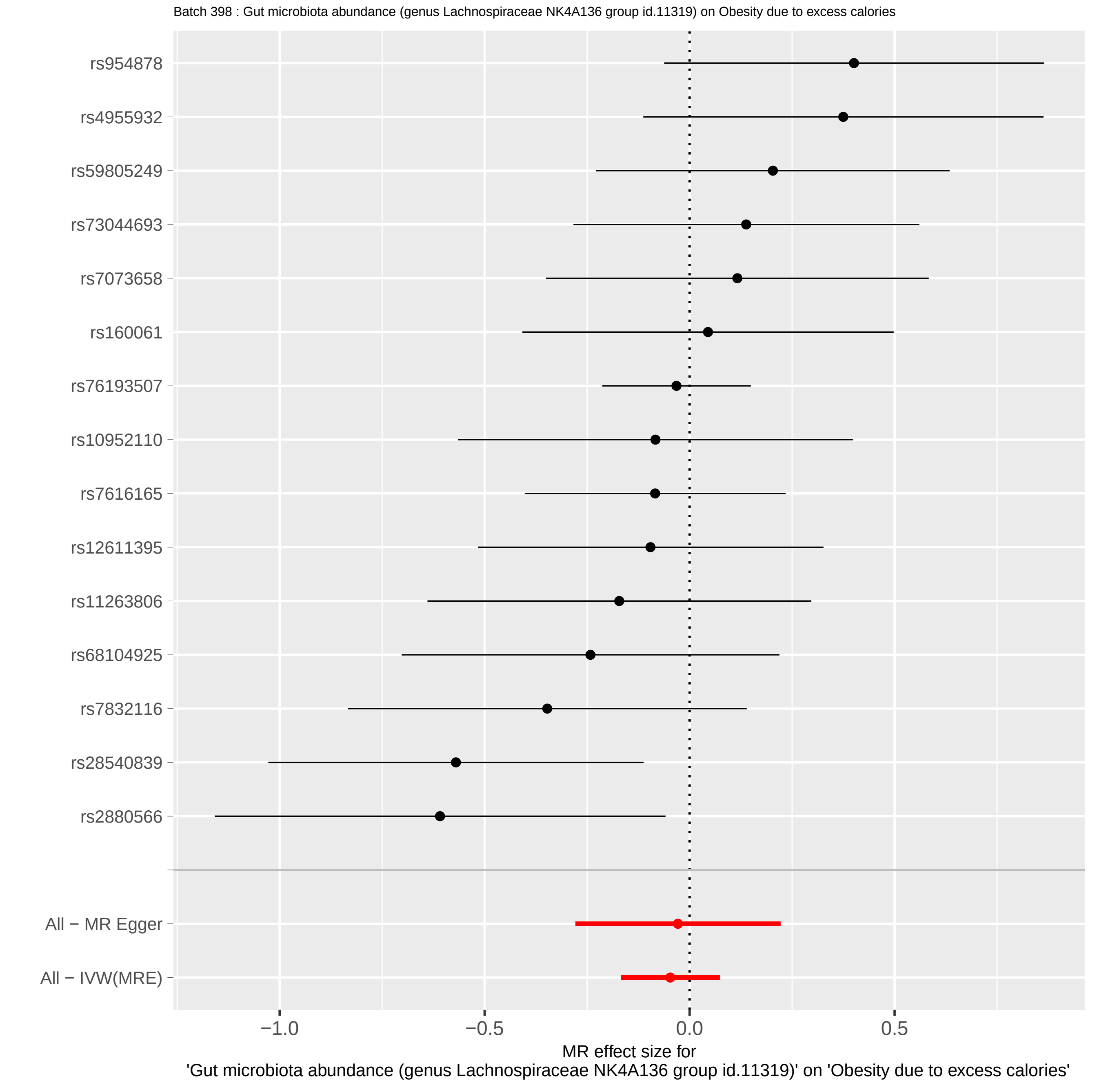
Batch 398 : Gut microbiota abundance (genus Lachnospiraceae NK4A136 group id.11319) on Obesity due to excess calories
rs954878
rs4955932
rs59805249
rs73044693
rs7073658
rs160061
rs76193507
rs10952110
rs7616165
rs12611395
rs11263806
rs68104925
rs7832116
rs28540839
rs2880566
All − MR Egger
All − IVW(MRE)
−1.0
−0.5
0.0
0.5
MR effect size for
'Gut microbiota abundance (genus Lachnospiraceae NK4A136 group id.11319)' on 'Obesity due to excess calories'

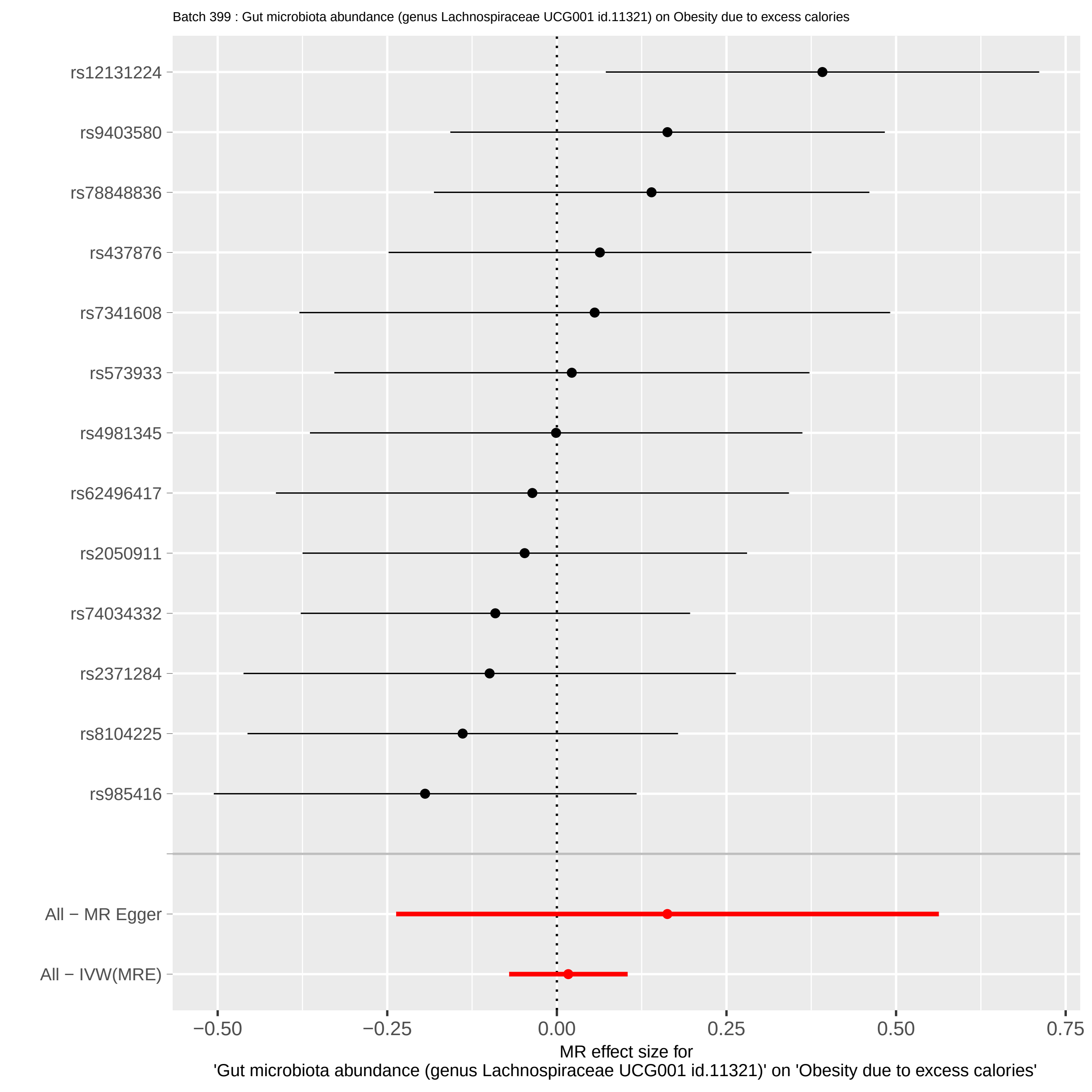
Batch 399 : Gut microbiota abundance (genus Lachnospiraceae UCG001 id.11321) on Obesity due to excess calories
rs12131224
rs9403580
rs78848836
rs437876
rs7341608
rs573933
rs4981345
rs62496417
rs2050911
rs74034332
rs2371284
rs8104225
rs985416
All − MR Egger
All − IVW(MRE)
−0.50
−0.25
0.00
0.25
0.50
0.75
MR effect size for
'Gut microbiota abundance (genus Lachnospiraceae UCG001 id.11321)' on 'Obesity due to excess calories'

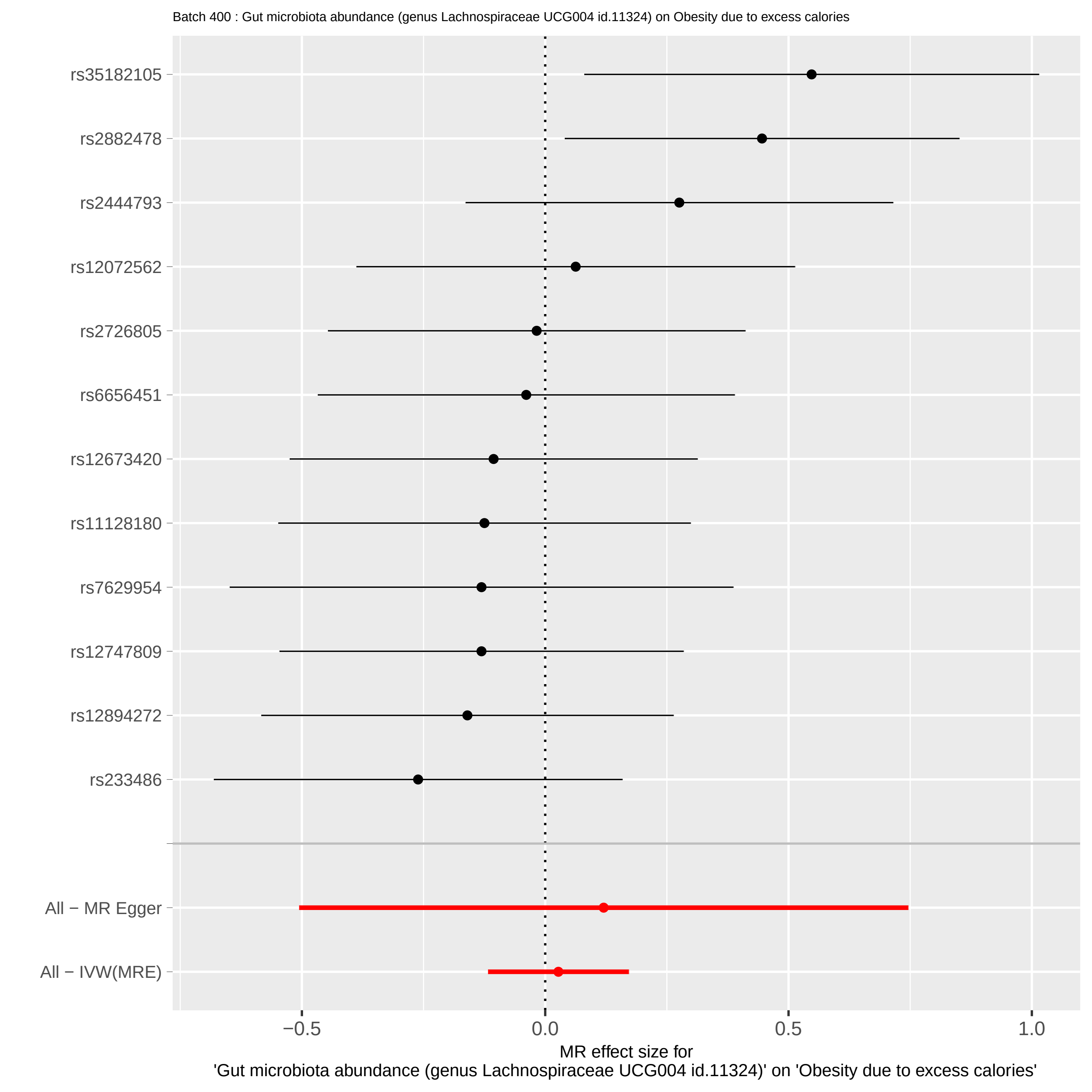

Batch 400 : Gut microbiota abundance (genus Lachnospiraceae UCG004 id.11324) on Obesity due to excess calories
rs35182105
rs2882478
rs2444793
rs12072562
rs2726805
rs6656451
rs12673420
rs11128180
rs7629954
rs12747809
rs12894272
rs233486
All − MR Egger
All − IVW(MRE)
−0.5
0.0
0.5
1.0
MR effect size for
'Gut microbiota abundance (genus Lachnospiraceae UCG004 id.11324)' on 'Obesity due to excess calories'

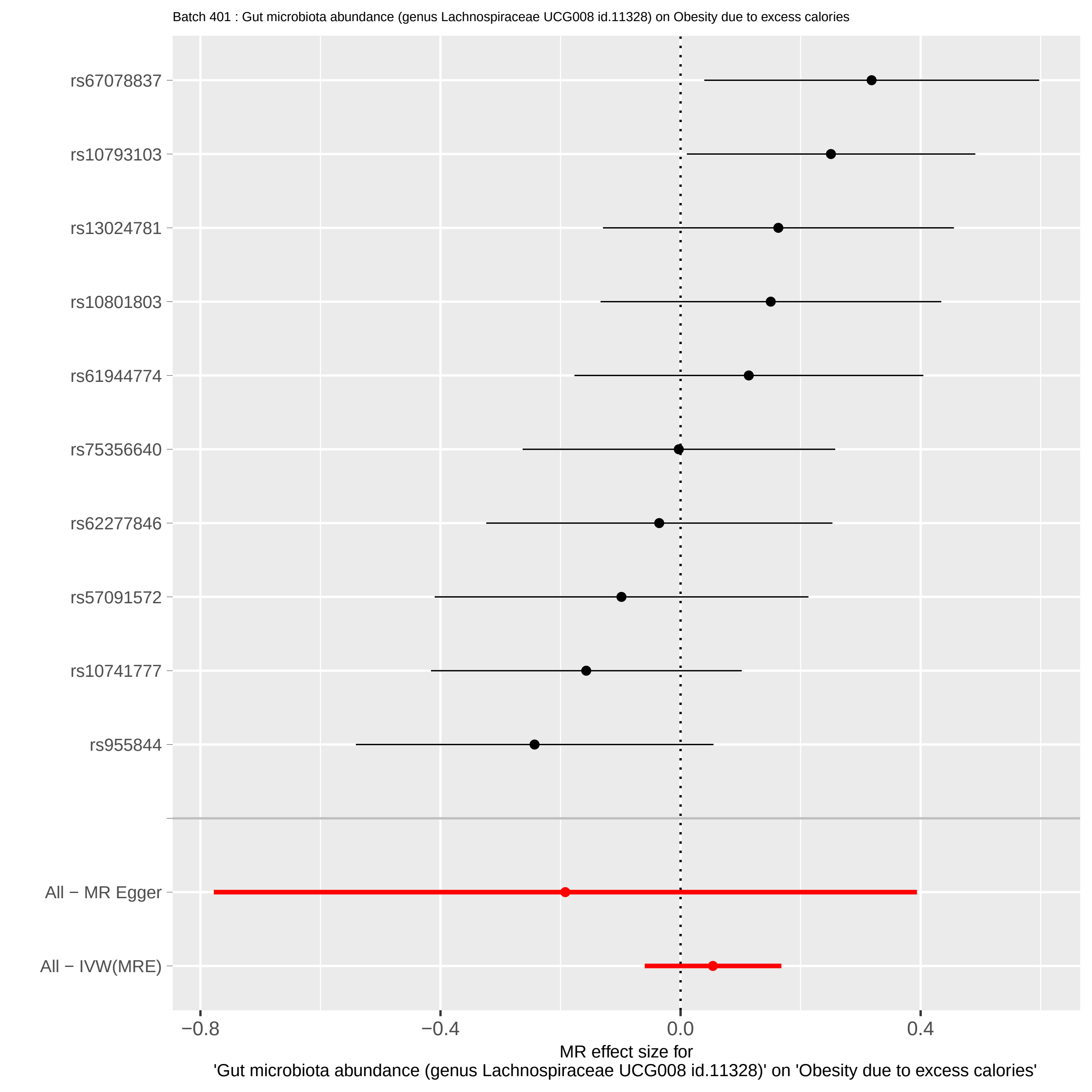

Batch 401 : Gut microbiota abundance (genus Lachnospiraceae UCG008 id.11328) on Obesity due to excess calories
rs67078837
rs10793103
rs13024781
rs10801803
rs61944774
rs75356640
rs62277846
rs57091572
rs10741777
rs955844
All − MR Egger
All − IVW(MRE)
−0.8
−0.4
0.0
0.4
MR effect size for
'Gut microbiota abundance (genus Lachnospiraceae UCG008 id.11328)' on 'Obesity due to excess calories'

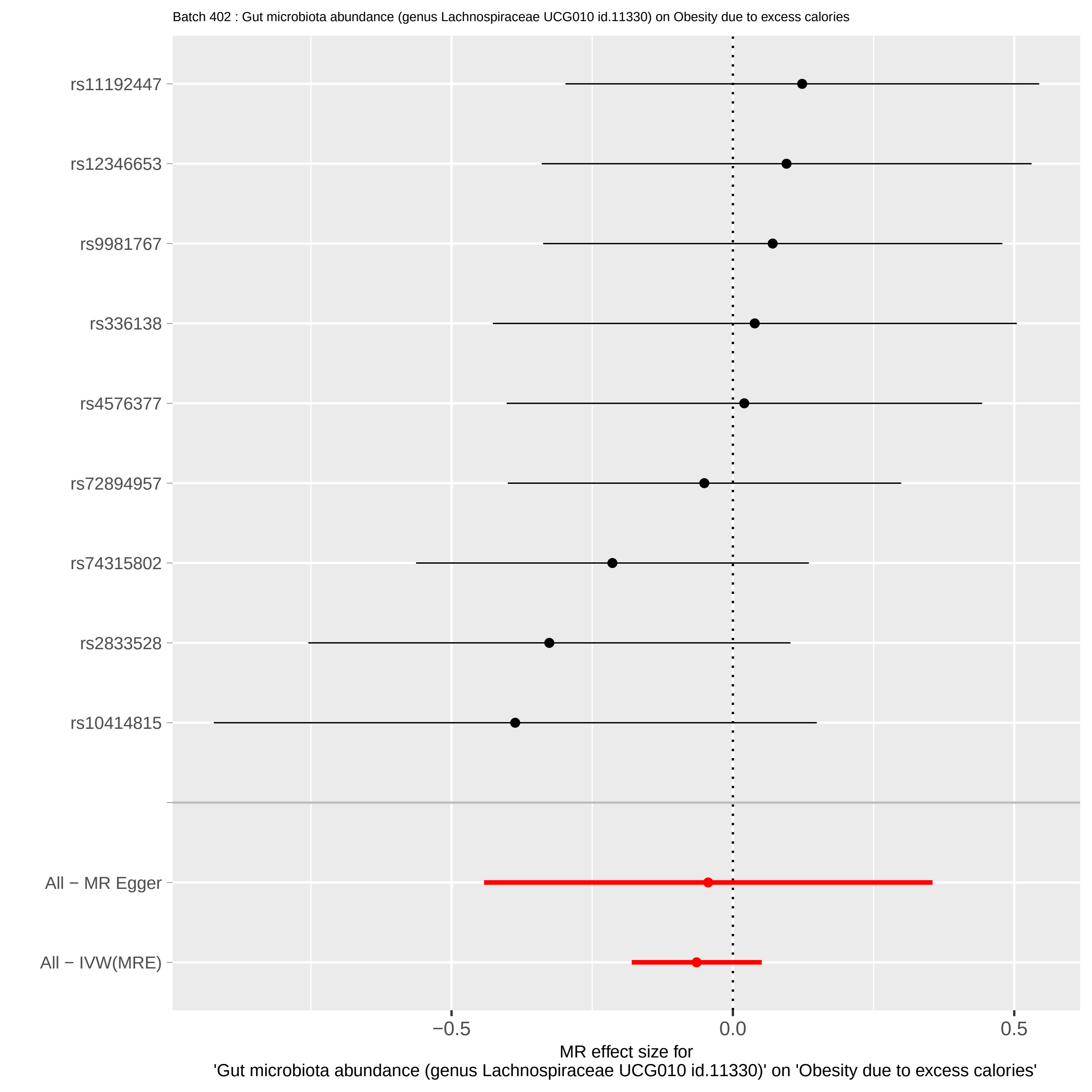
Batch 402 : Gut microbiota abundance (genus Lachnospiraceae UCG010 id.11330) on Obesity due to excess calories
rs11192447
rs12346653
rs9981767
rs336138
rs4576377
rs72894957
rs74315802
rs2833528
rs10414815
All − MR Egger
All − IVW(MRE)
−0.5
0.0
0.5
MR effect size for
'Gut microbiota abundance (genus Lachnospiraceae UCG010 id.11330)' on 'Obesity due to excess calories'

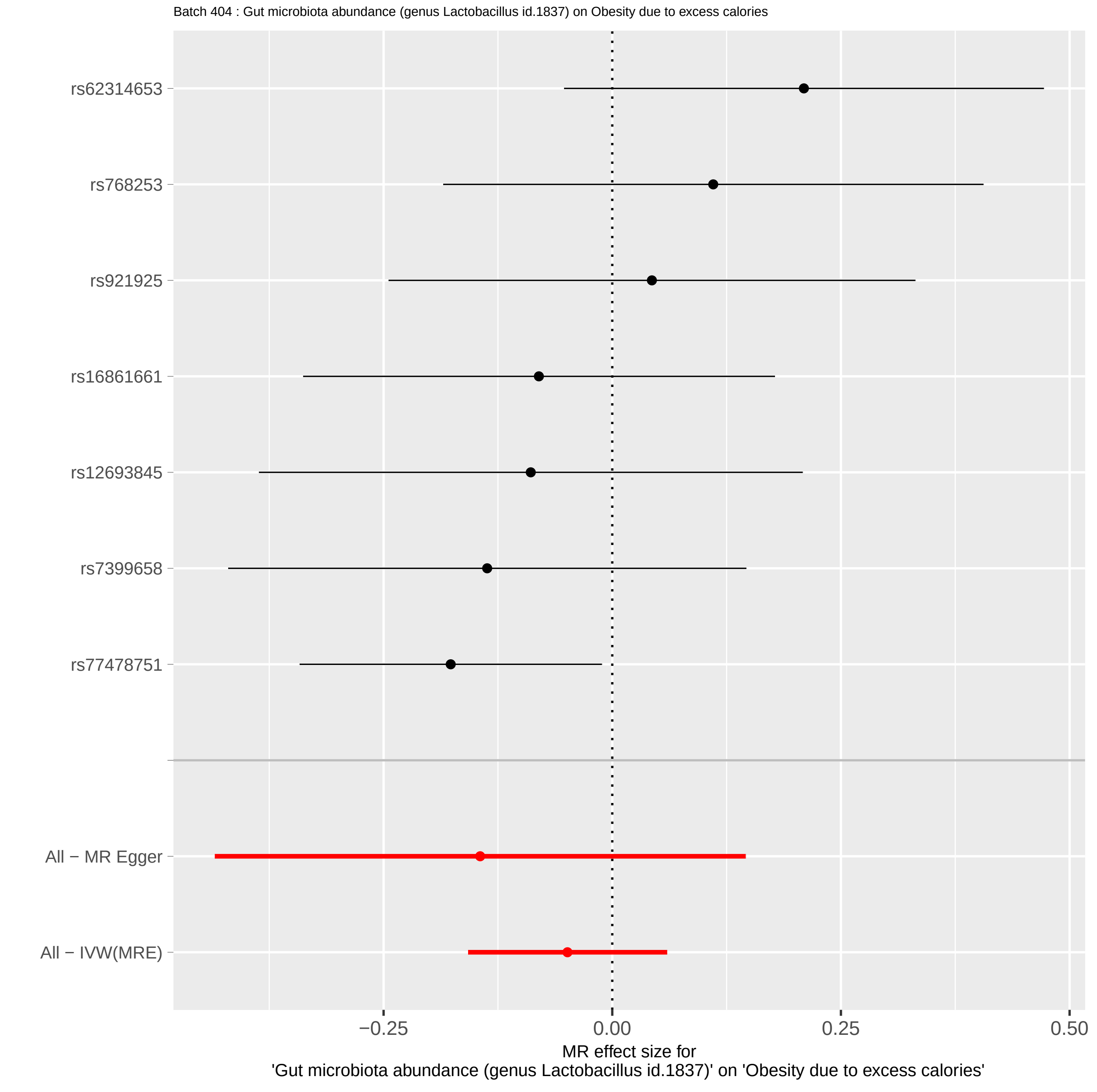

Batch 404 : Gut microbiota abundance (genus Lactobacillus id.1837) on Obesity due to excess calories
rs62314653
rs768253
rs921925
rs16861661
rs12693845
rs7399658
rs77478751
All − MR Egger
All − IVW(MRE)
−0.25
0.00
0.25
0.50
MR effect size for
'Gut microbiota abundance (genus Lactobacillus id.1837)' on 'Obesity due to excess calories'

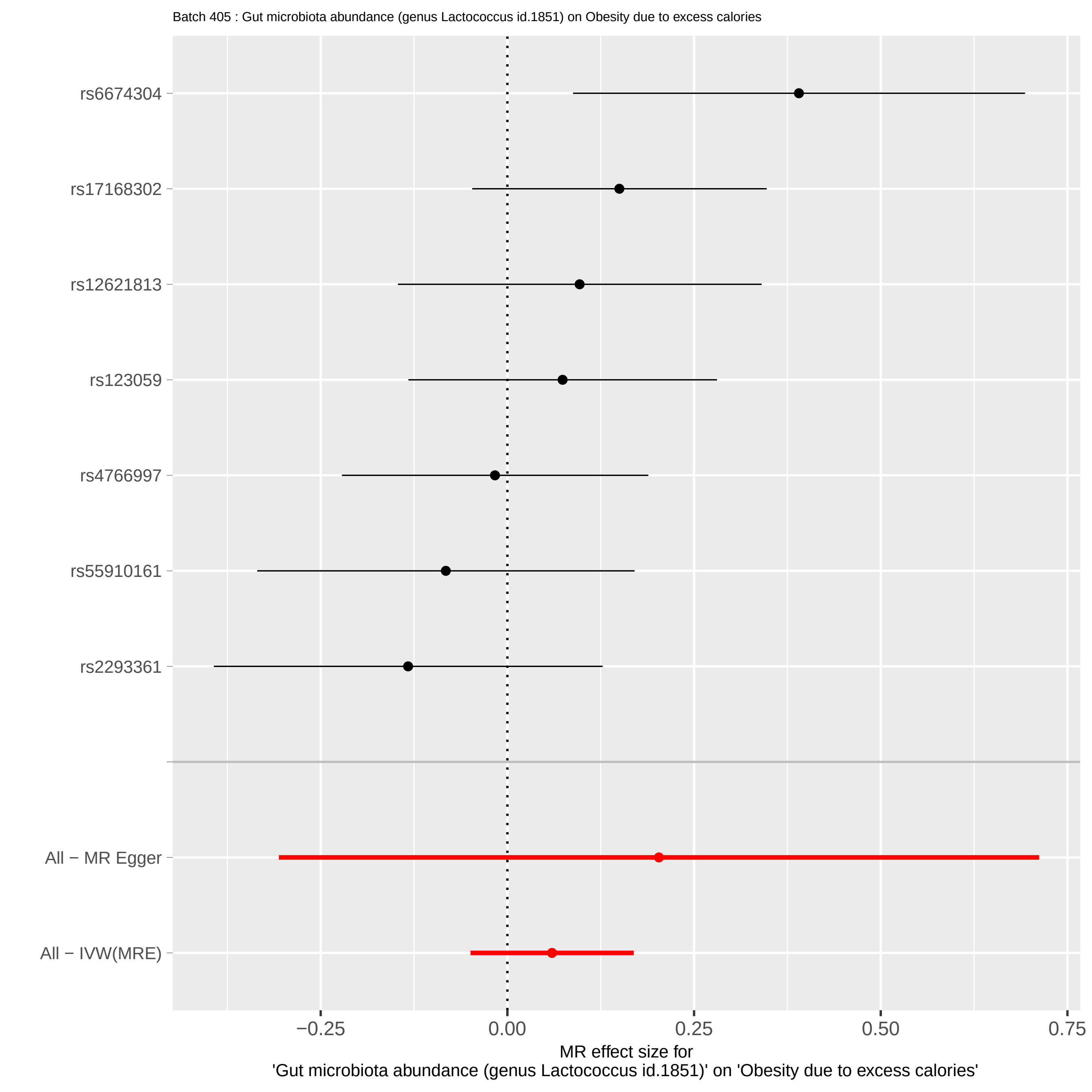

Batch 405 : Gut microbiota abundance (genus Lactococcus id.1851) on Obesity due to excess calories
rs6674304
rs17168302
rs12621813
rs123059
rs4766997
rs55910161
rs2293361
All − MR Egger
All − IVW(MRE)
−0.25
0.00
0.25
0.50
0.75
MR effect size for
'Gut microbiota abundance (genus Lactococcus id.1851)' on 'Obesity due to excess calories'

Batch 406 : Gut microbiota abundance (genus Marvinbryantia id.2005) on Obesity due to excess calories

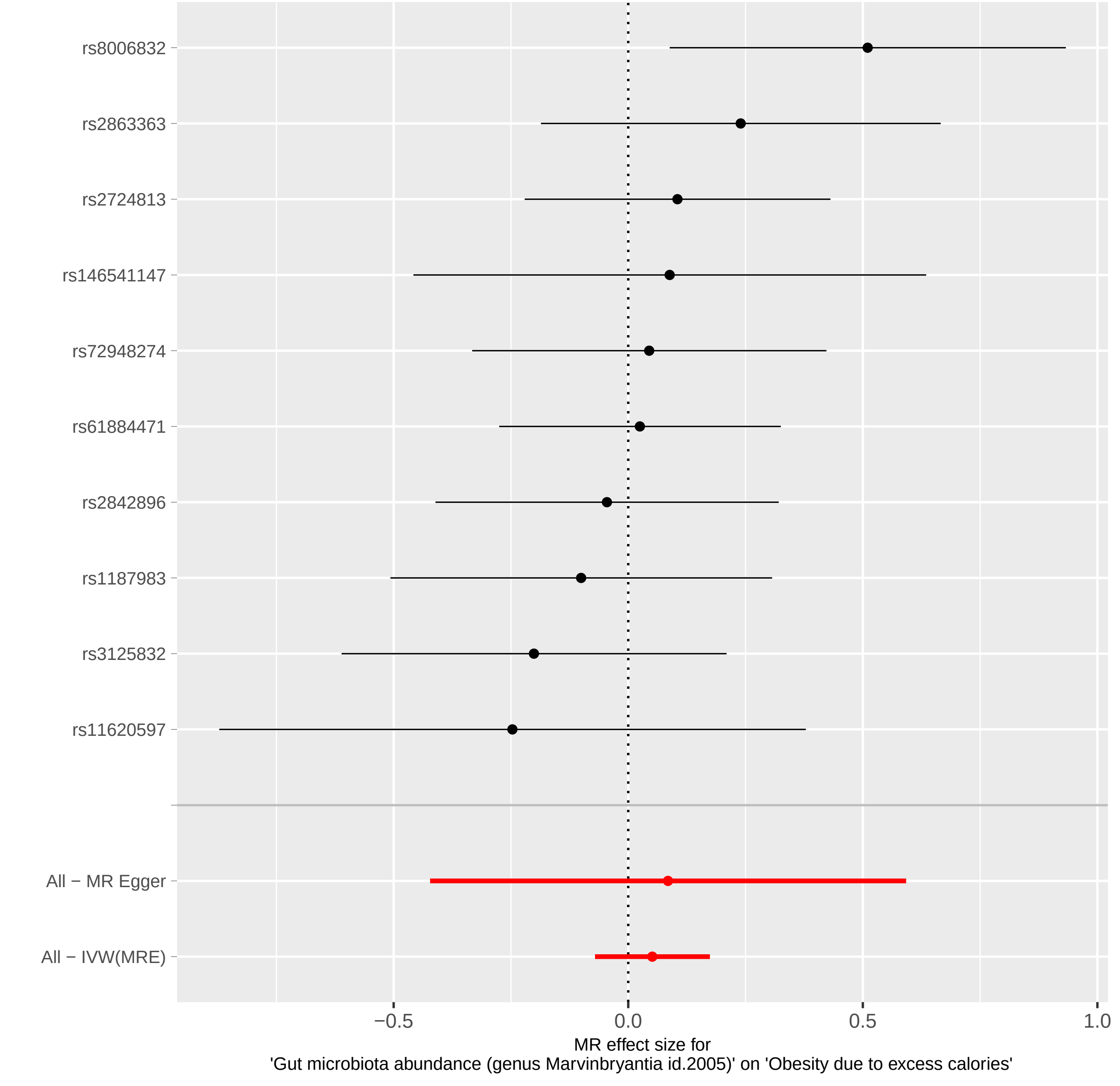

Batch 407 : Gut microbiota abundance (genus Methanobrevibacter id.123) on Obesity due to excess calories

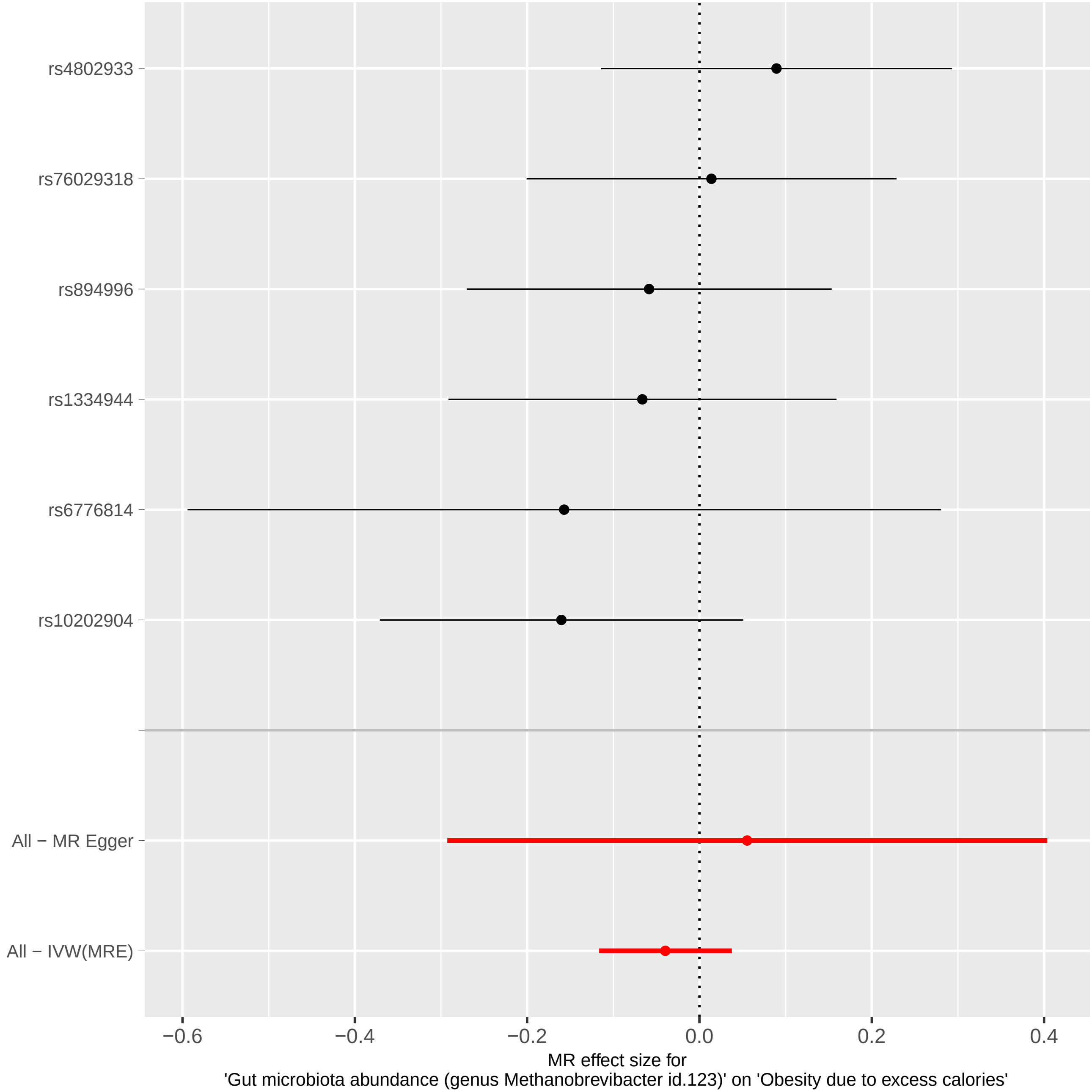

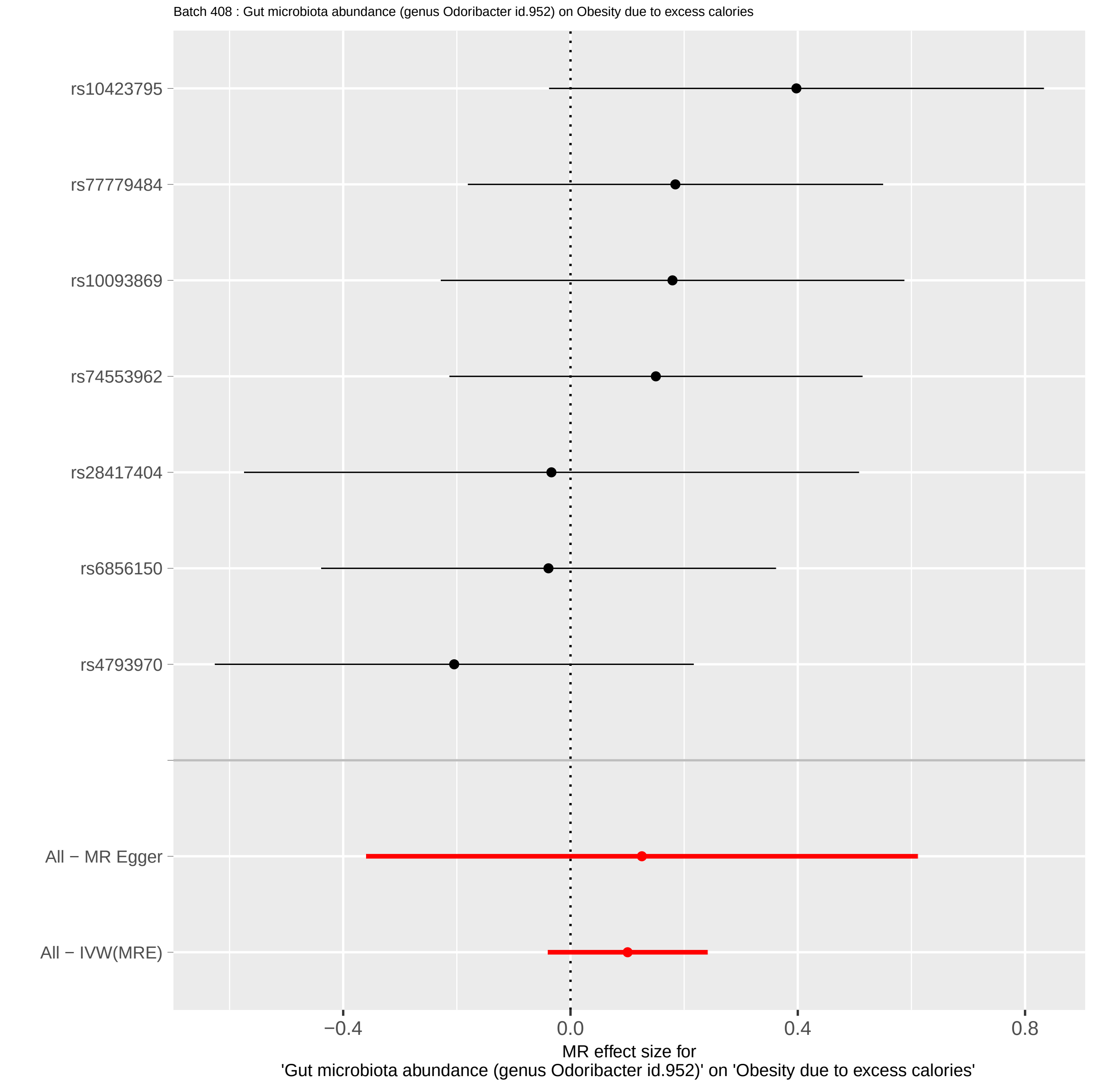

Batch 408 : Gut microbiota abundance (genus Odoribacter id.952) on Obesity due to excess calories
rs10423795
rs77779484
rs10093869
rs74553962
rs28417404
rs6856150
rs4793970
All − MR Egger
All − IVW(MRE)
−0.4
0.0
0.4
0.8
MR effect size for
'Gut microbiota abundance (genus Odoribacter id.952)' on 'Obesity due to excess calories'

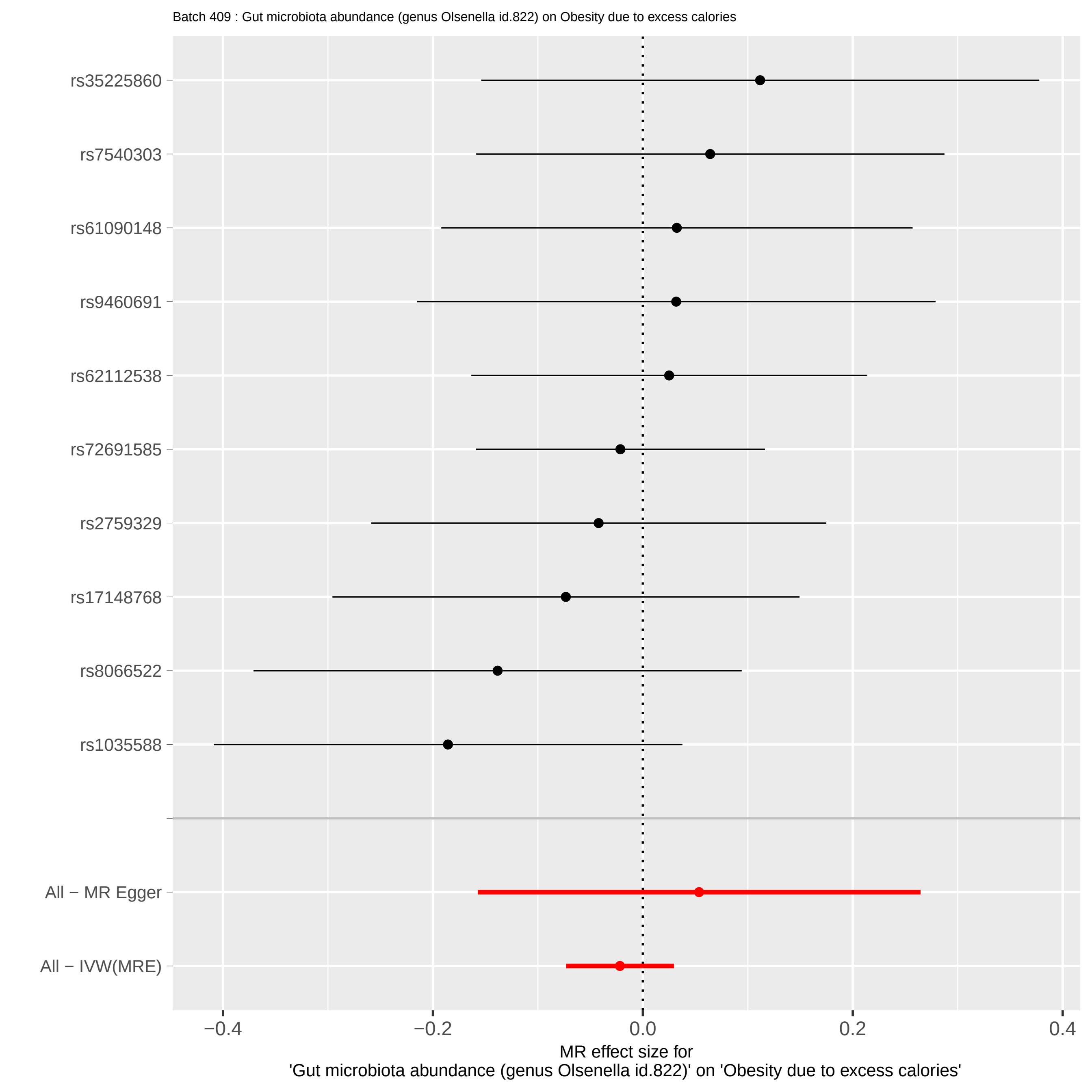

Batch 409 : Gut microbiota abundance (genus Olsenella id.822) on Obesity due to excess calories
rs35225860
rs7540303
rs61090148
rs9460691
rs62112538
rs72691585
rs2759329
rs17148768
rs8066522
rs1035588
All − MR Egger
All − IVW(MRE)
−0.4
−0.2
0.0
0.2
0.4
MR effect size for
'Gut microbiota abundance (genus Olsenella id.822)' on 'Obesity due to excess calories'

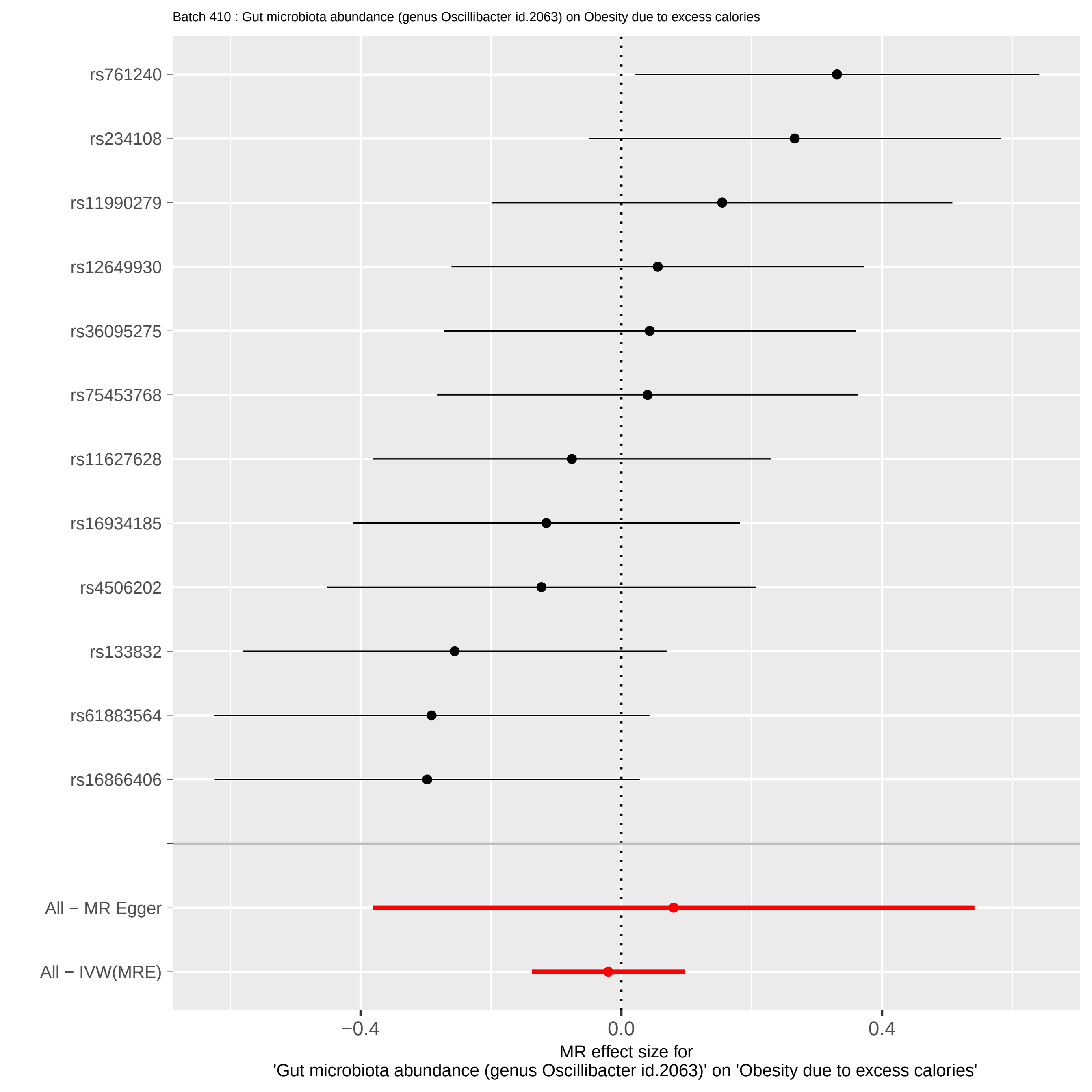

Batch 410 : Gut microbiota abundance (genus Oscillibacter id.2063) on Obesity due to excess calories
rs761240
rs234108
rs11990279
rs12649930
rs36095275
rs75453768
rs11627628
rs16934185
rs4506202
rs133832
rs61883564
rs16866406
All − MR Egger
All − IVW(MRE)
−0.4
0.0
0.4
MR effect size for
'Gut microbiota abundance (genus Oscillibacter id.2063)' on 'Obesity due to excess calories'

Batch 411 : Gut microbiota abundance (genus Oscillospira id.2064) on Obesity due to excess calories

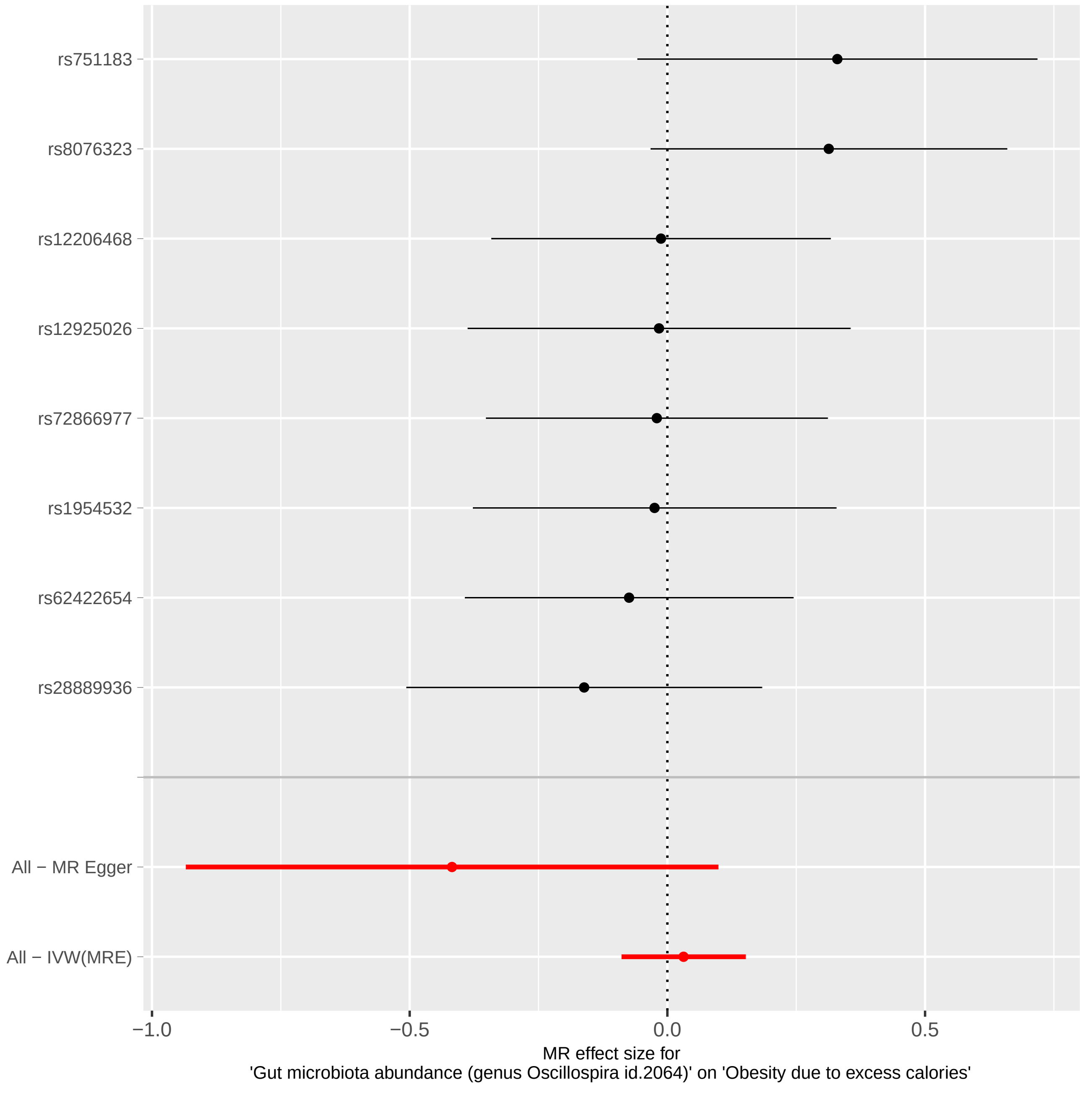

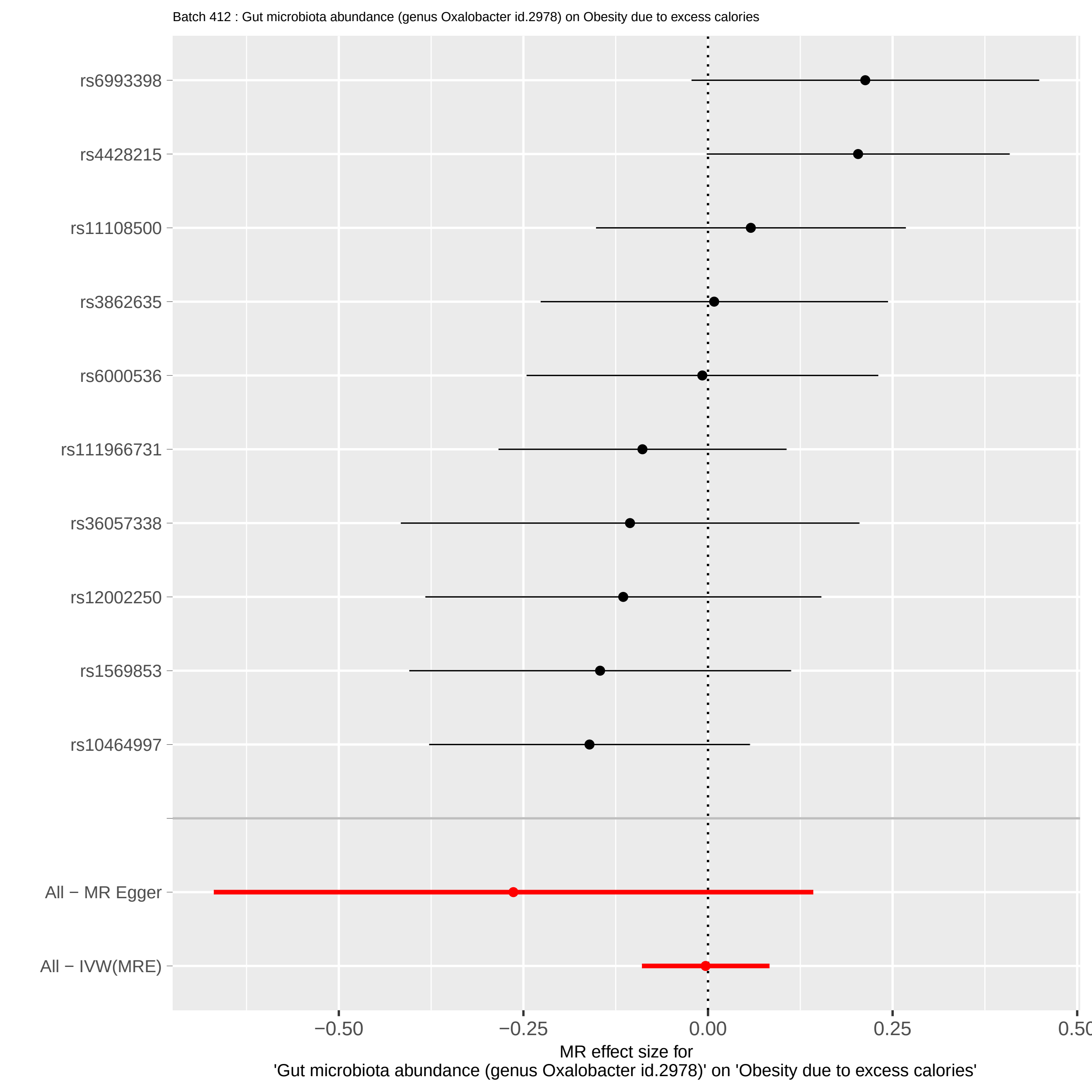

Batch 412 : Gut microbiota abundance (genus Oxalobacter id.2978) on Obesity due to excess calories
rs6993398
rs4428215
rs11108500
rs3862635
rs6000536
rs111966731
rs36057338
rs12002250
rs1569853
rs10464997
All − MR Egger
All − IVW(MRE)
−0.50
−0.25
0.00
0.25
0.50
MR effect size for
'Gut microbiota abundance (genus Oxalobacter id.2978)' on 'Obesity due to excess calories'

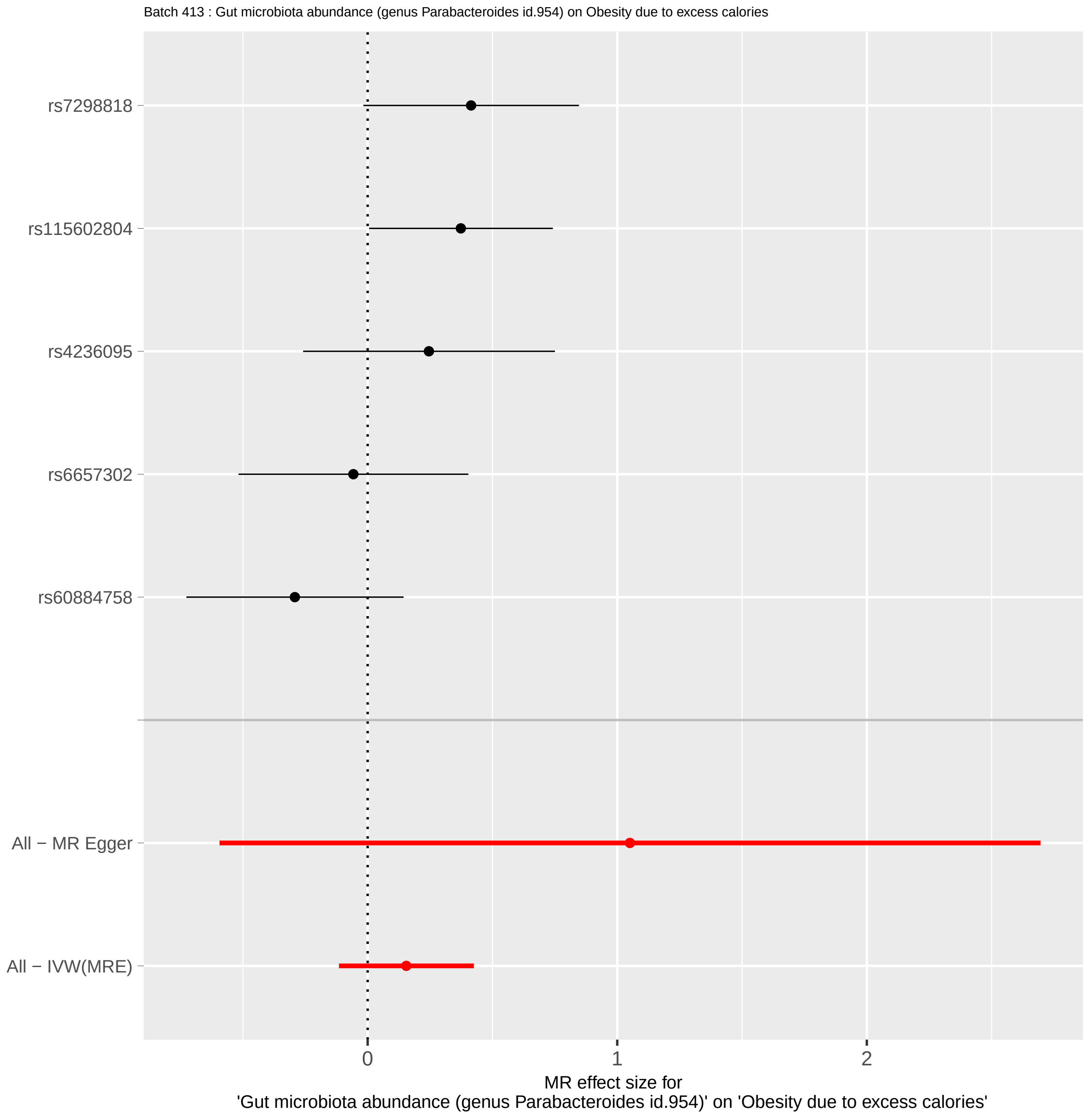
Batch 413 : Gut microbiota abundance (genus Parabacteroides id.954) on Obesity due to excess calories
rs7298818
rs115602804
rs4236095
rs6657302
rs60884758
All − MR Egger
All − IVW(MRE)
0
1
2
MR effect size for
'Gut microbiota abundance (genus Parabacteroides id.954)' on 'Obesity due to excess calories'

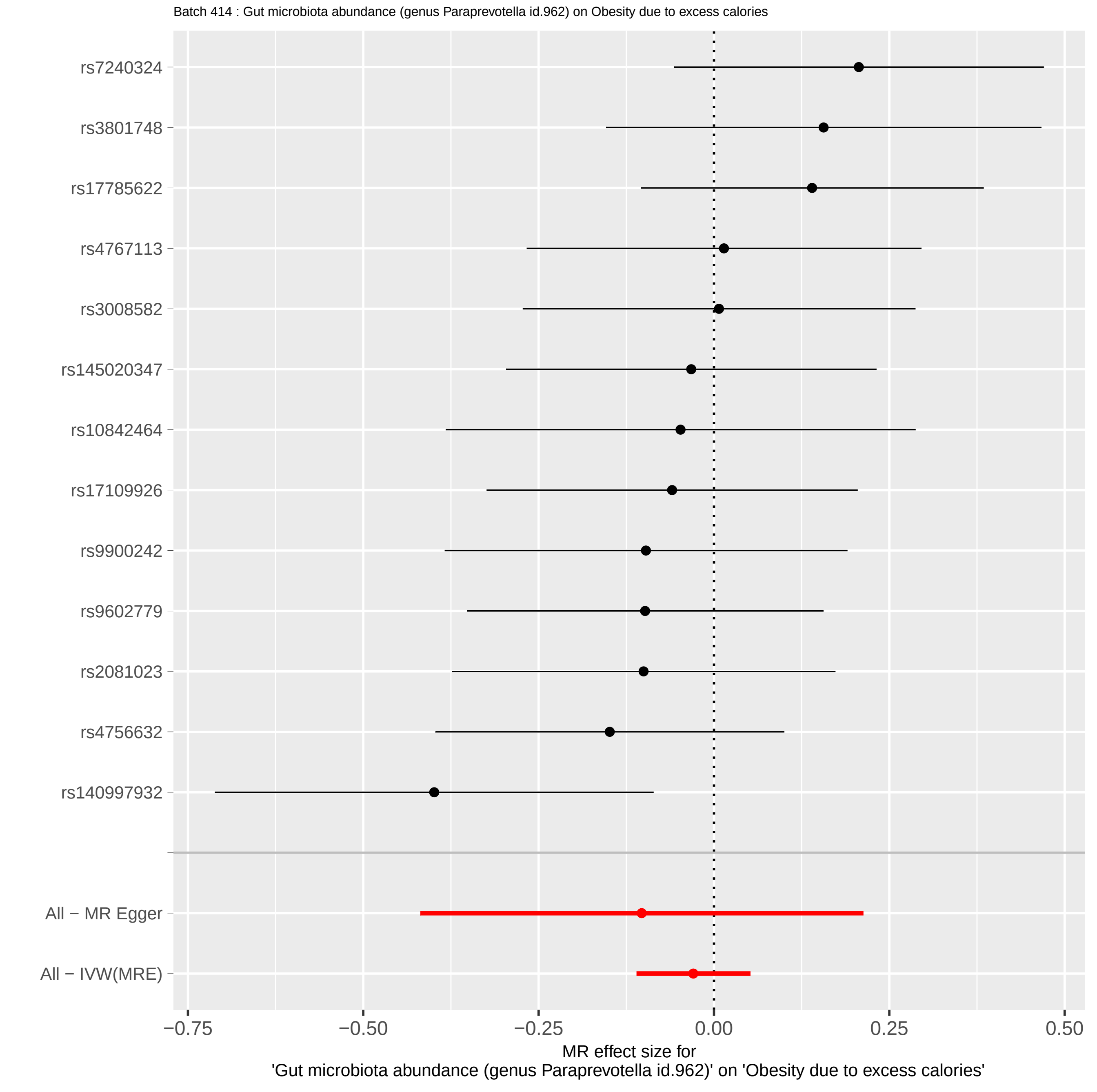

Batch 414 : Gut microbiota abundance (genus Paraprevotella id.962) on Obesity due to excess calories
rs7240324
rs3801748
rs17785622
rs4767113
rs3008582
rs145020347
rs10842464
rs17109926
rs9900242
rs9602779
rs2081023
rs4756632
rs140997932
All − MR Egger
All − IVW(MRE)
−0.75
−0.50
−0.25
0.00
0.25
0.50
MR effect size for
'Gut microbiota abundance (genus Paraprevotella id.962)' on 'Obesity due to excess calories'

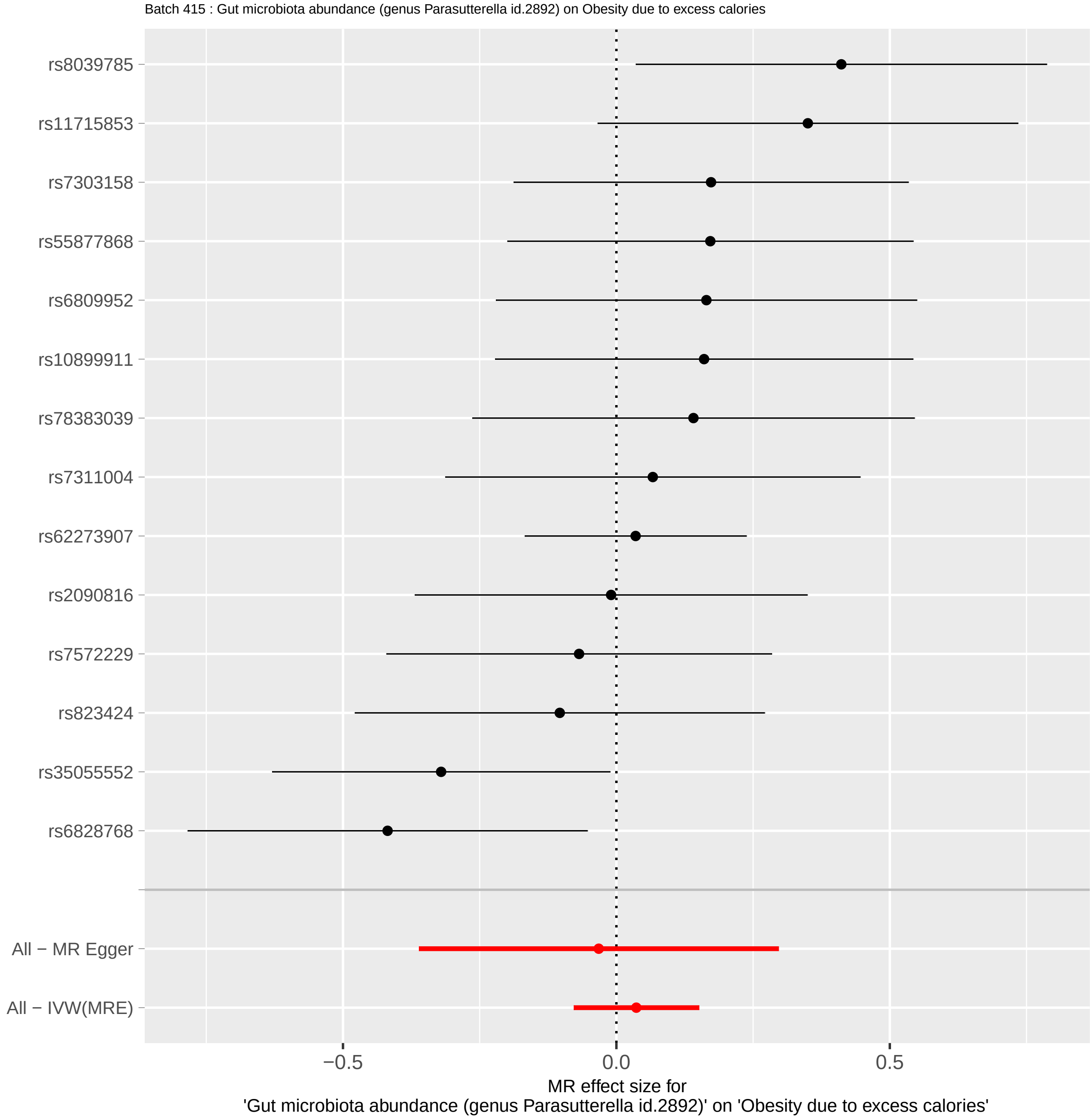

Batch 415 : Gut microbiota abundance (genus Parasutterella id.2892) on Obesity due to excess calories
rs8039785
rs11715853
rs7303158
rs55877868
rs6809952
rs10899911
rs78383039
rs7311004
rs62273907
rs2090816
rs7572229
rs823424
rs35055552
rs6828768
All − MR Egger
All − IVW(MRE)
−0.5
0.0
0.5
MR effect size for
'Gut microbiota abundance (genus Parasutterella id.2892)' on 'Obesity due to excess calories'

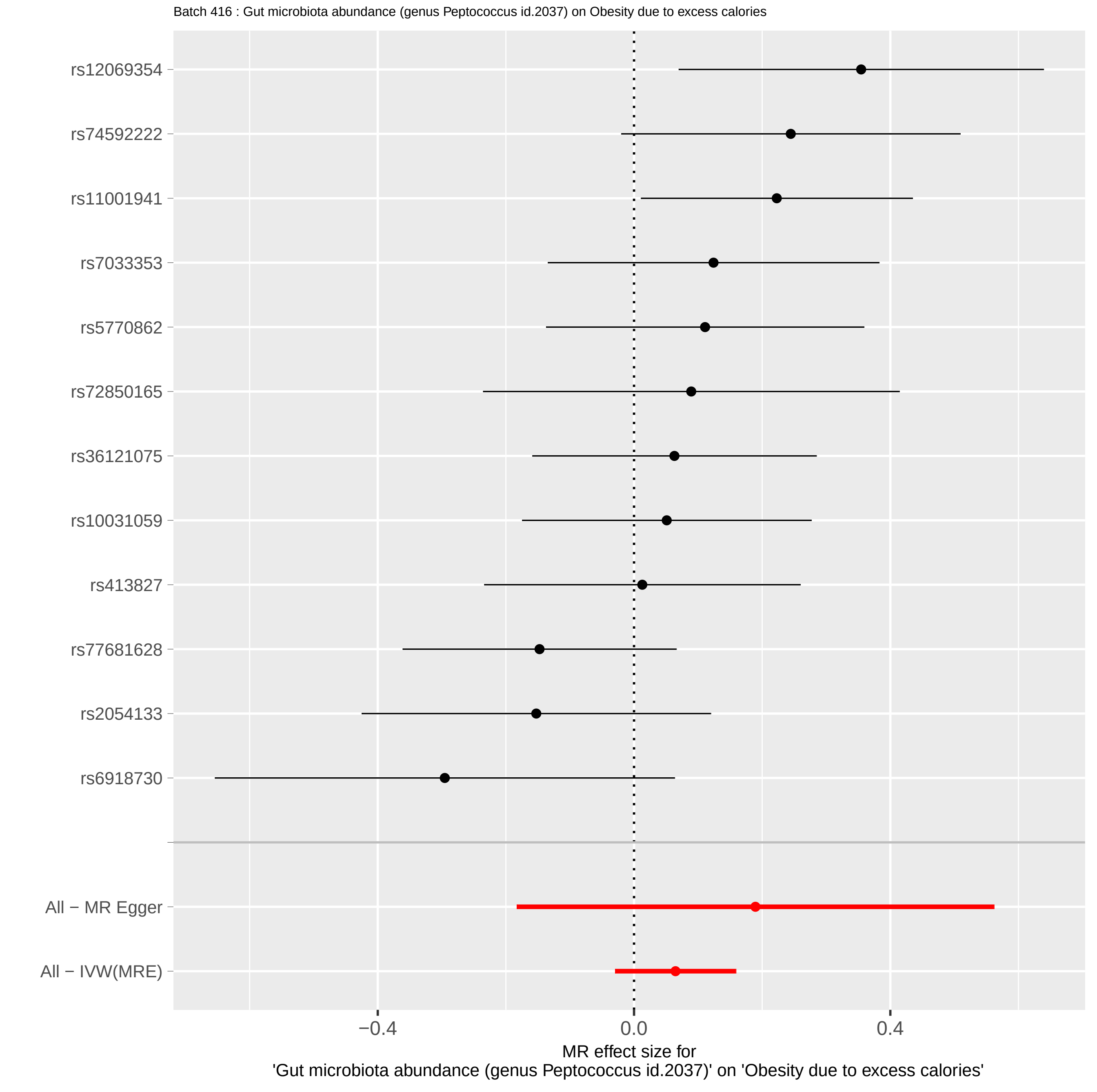

Batch 416 : Gut microbiota abundance (genus Peptococcus id.2037) on Obesity due to excess calories
rs12069354
rs74592222
rs11001941
rs7033353
rs5770862
rs72850165
rs36121075
rs10031059
rs413827
rs77681628
rs2054133
rs6918730
All − MR Egger
All − IVW(MRE)
−0.4
0.0
0.4
MR effect size for
'Gut microbiota abundance (genus Peptococcus id.2037)' on 'Obesity due to excess calories'

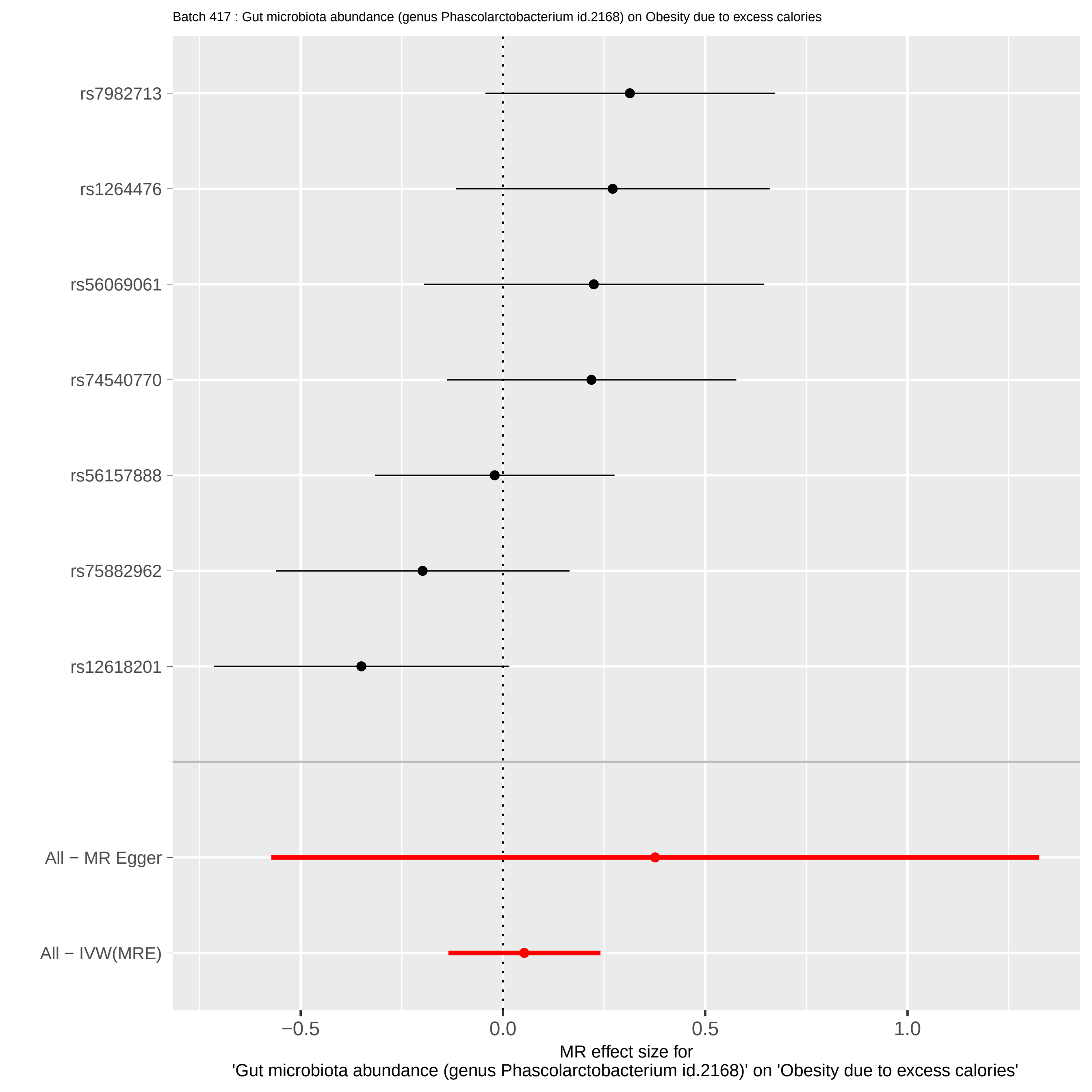
Batch 417 : Gut microbiota abundance (genus Phascolarctobacterium id.2168) on Obesity due to excess calories
rs7982713
rs1264476
rs56069061
rs74540770
rs56157888
rs75882962
rs12618201
All − MR Egger
All − IVW(MRE)
−0.5
0.0
0.5
1.0
MR effect size for
'Gut microbiota abundance (genus Phascolarctobacterium id.2168)' on 'Obesity due to excess calories'

Batch 418 : Gut microbiota abundance (genus Prevotella7 id.11182) on Obesity due to excess calories

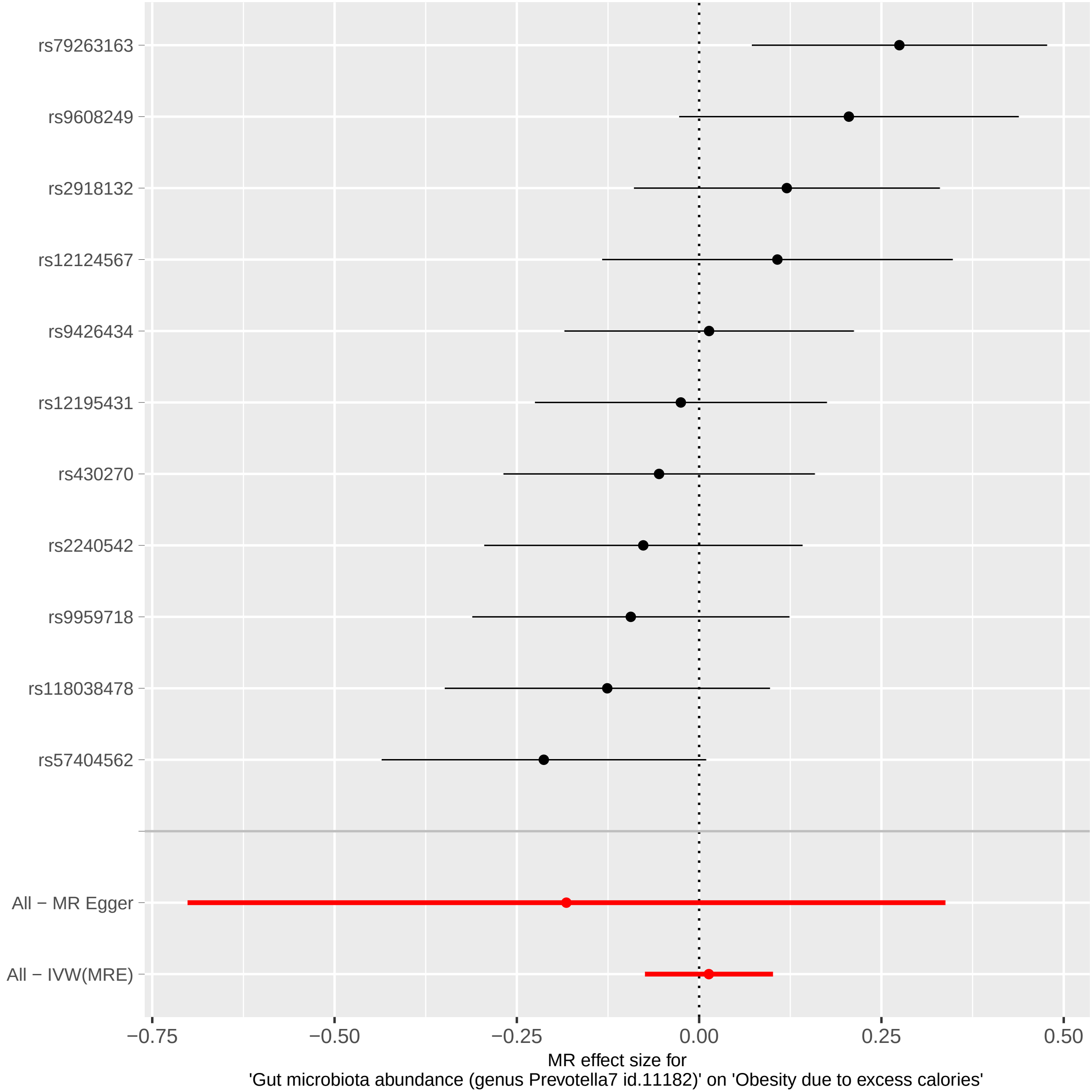

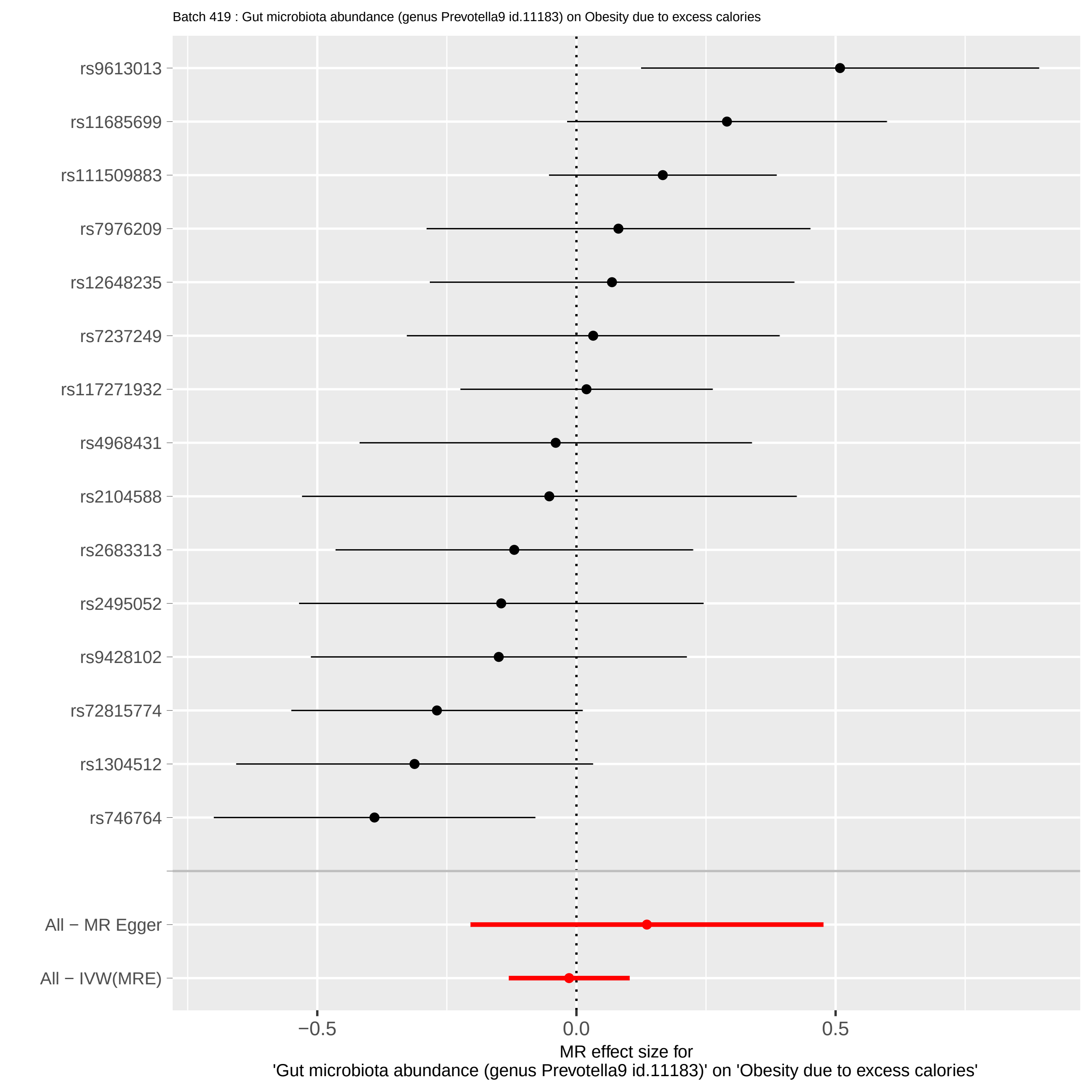
Batch 419 : Gut microbiota abundance (genus Prevotella9 id.11183) on Obesity due to excess calories
rs9613013
rs11685699
rs111509883
rs7976209
rs12648235
rs7237249
rs117271932
rs4968431
rs2104588
rs2683313
rs2495052
rs9428102
rs72815774
rs1304512
rs746764
All − MR Egger
All − IVW(MRE)
−0.5
0.0
0.5
MR effect size for
'Gut microbiota abundance (genus Prevotella9 id.11183)' on 'Obesity due to excess calories'

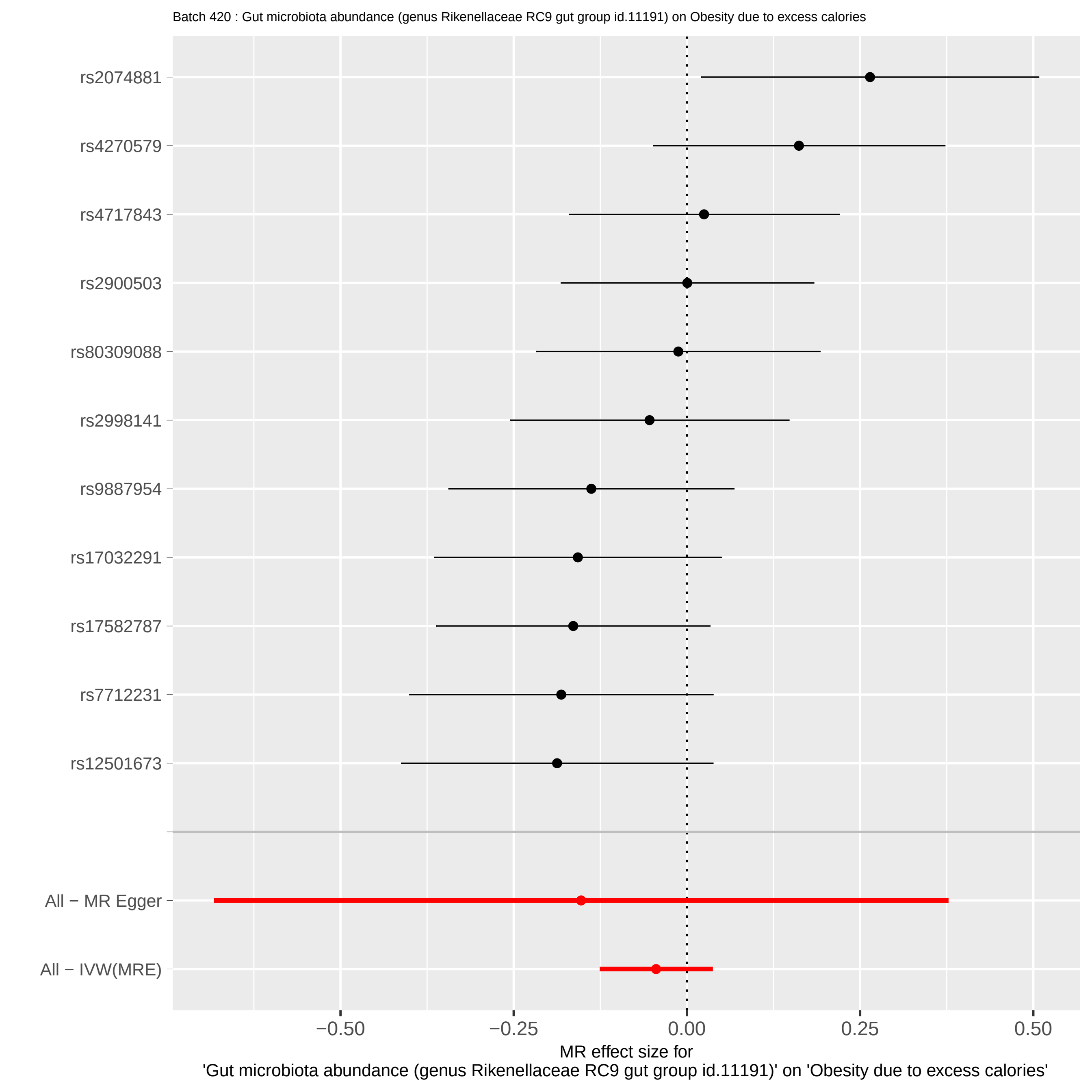

Batch 420 : Gut microbiota abundance (genus Rikenellaceae RC9 gut group id.11191) on Obesity due to excess calories
rs2074881
rs4270579
rs4717843
rs2900503
rs80309088
rs2998141
rs9887954
rs17032291
rs17582787
rs7712231
rs12501673
All − MR Egger
All − IVW(MRE)
−0.50
−0.25
0.00
0.25
0.50
MR effect size for
'Gut microbiota abundance (genus Rikenellaceae RC9 gut group id.11191)' on 'Obesity due to excess calories'

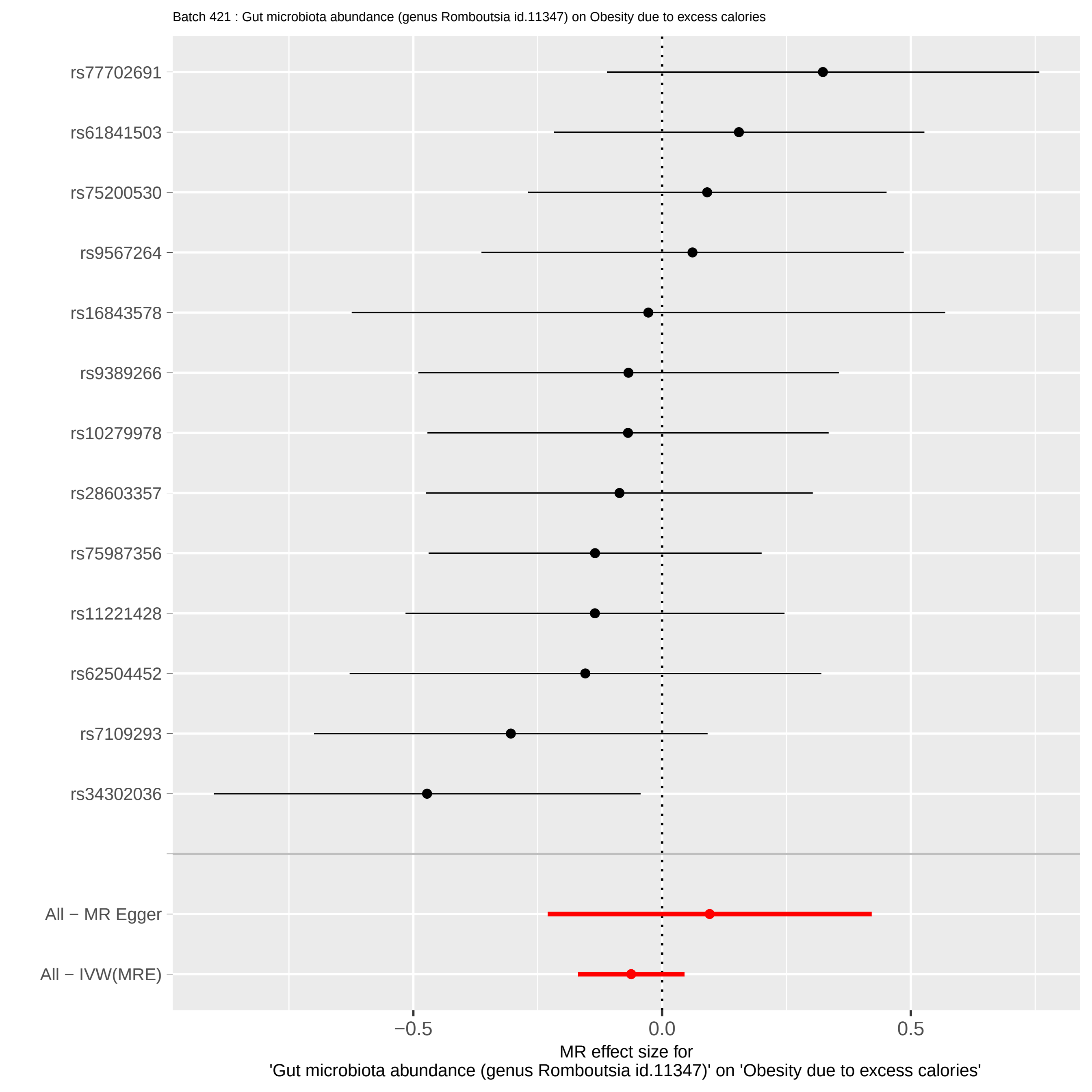

Batch 421 : Gut microbiota abundance (genus Romboutsia id.11347) on Obesity due to excess calories
rs77702691
rs61841503
rs75200530
rs9567264
rs16843578
rs9389266
rs10279978
rs28603357
rs75987356
rs11221428
rs62504452
rs7109293
rs34302036
All − MR Egger
All − IVW(MRE)
−0.5
0.0
0.5
MR effect size for
'Gut microbiota abundance (genus Romboutsia id.11347)' on 'Obesity due to excess calories'

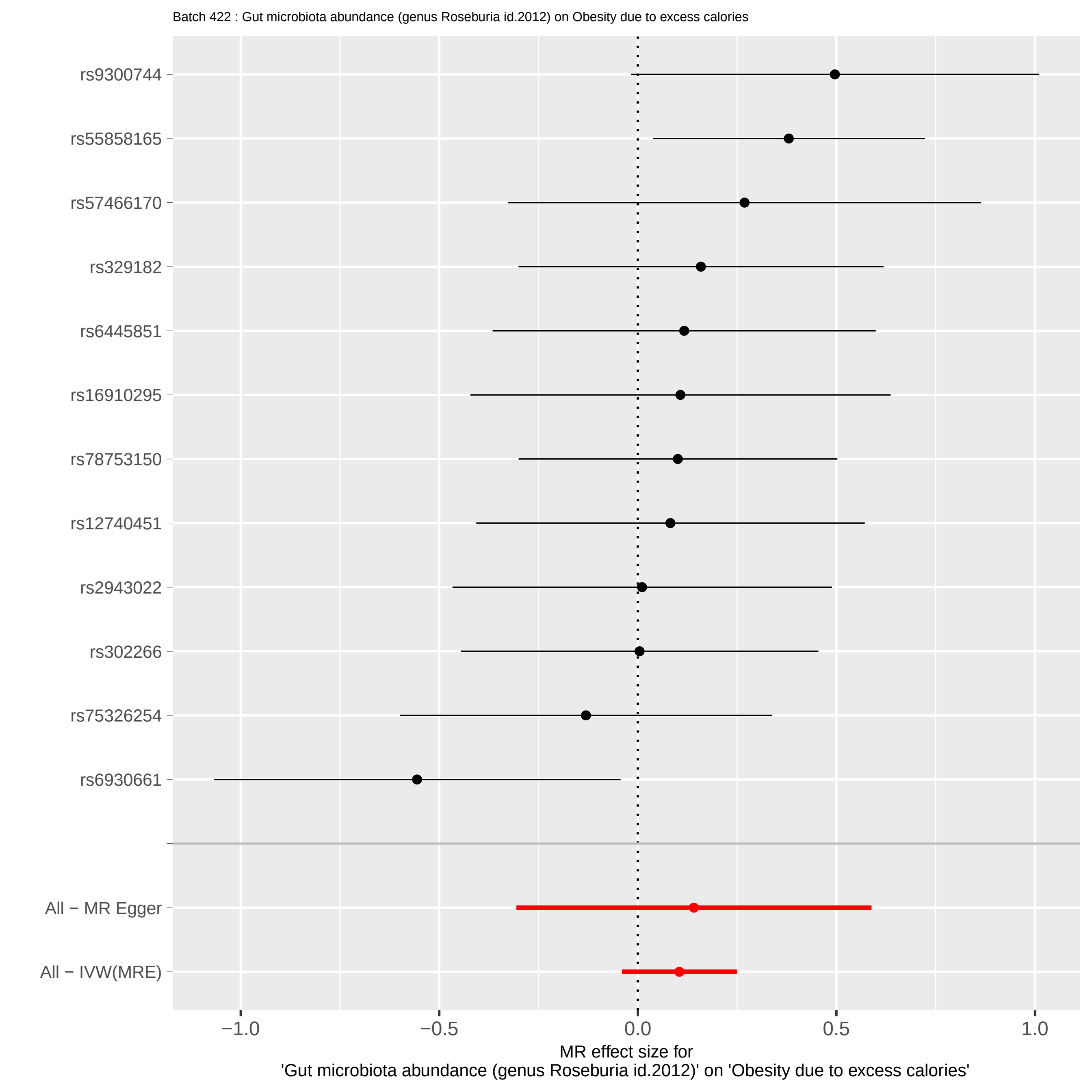

Batch 422 : Gut microbiota abundance (genus Roseburia id.2012) on Obesity due to excess calories
rs9300744
rs55858165
rs57466170
rs329182
rs6445851
rs16910295
rs78753150
rs12740451
rs2943022
rs302266
rs75326254
rs6930661
All − MR Egger
All − IVW(MRE)
−1.0
−0.5
0.0
0.5
1.0
MR effect size for
'Gut microbiota abundance (genus Roseburia id.2012)' on 'Obesity due to excess calories'

Batch 423 : Gut microbiota abundance (genus Ruminiclostridium5 id.11355) on Obesity due to excess calories

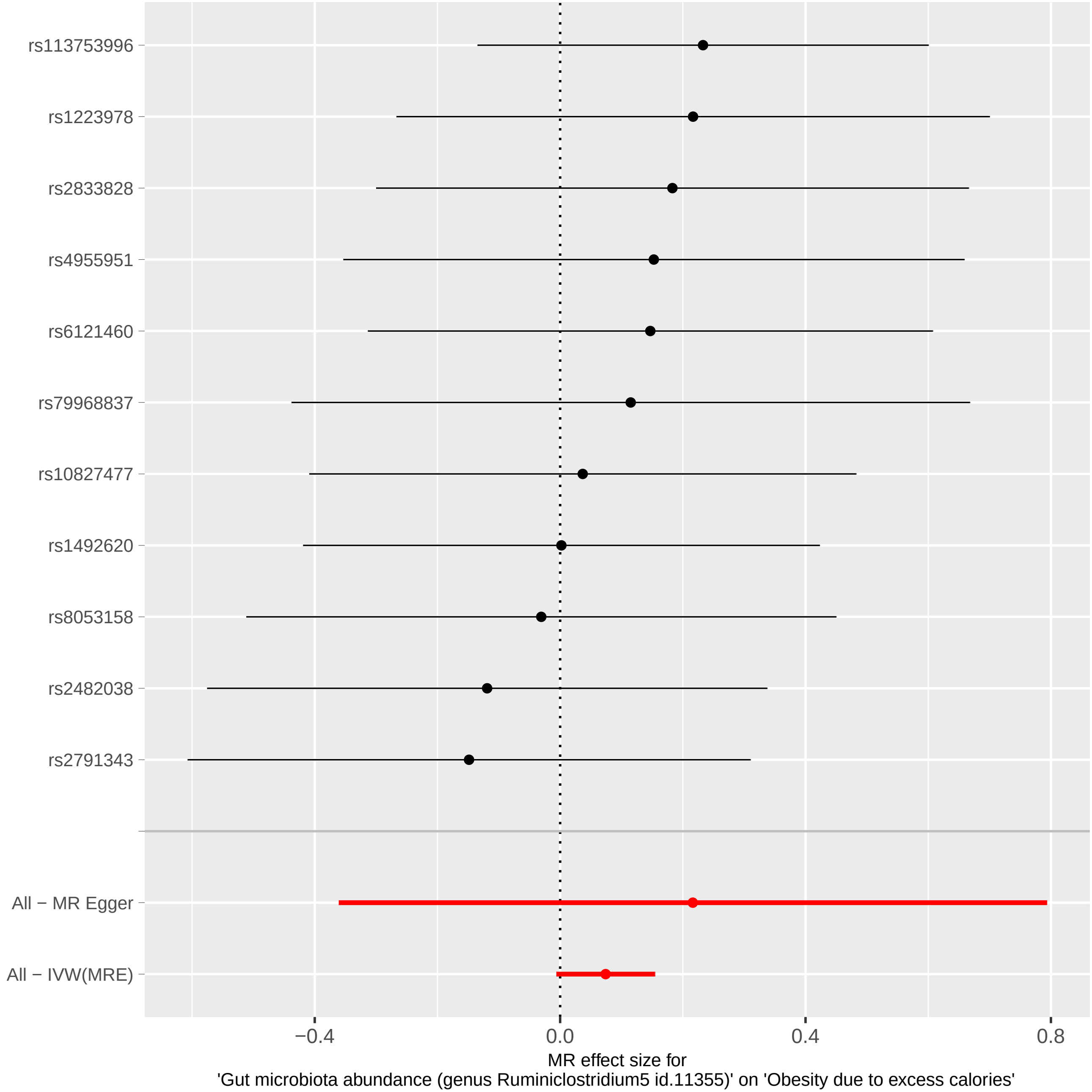

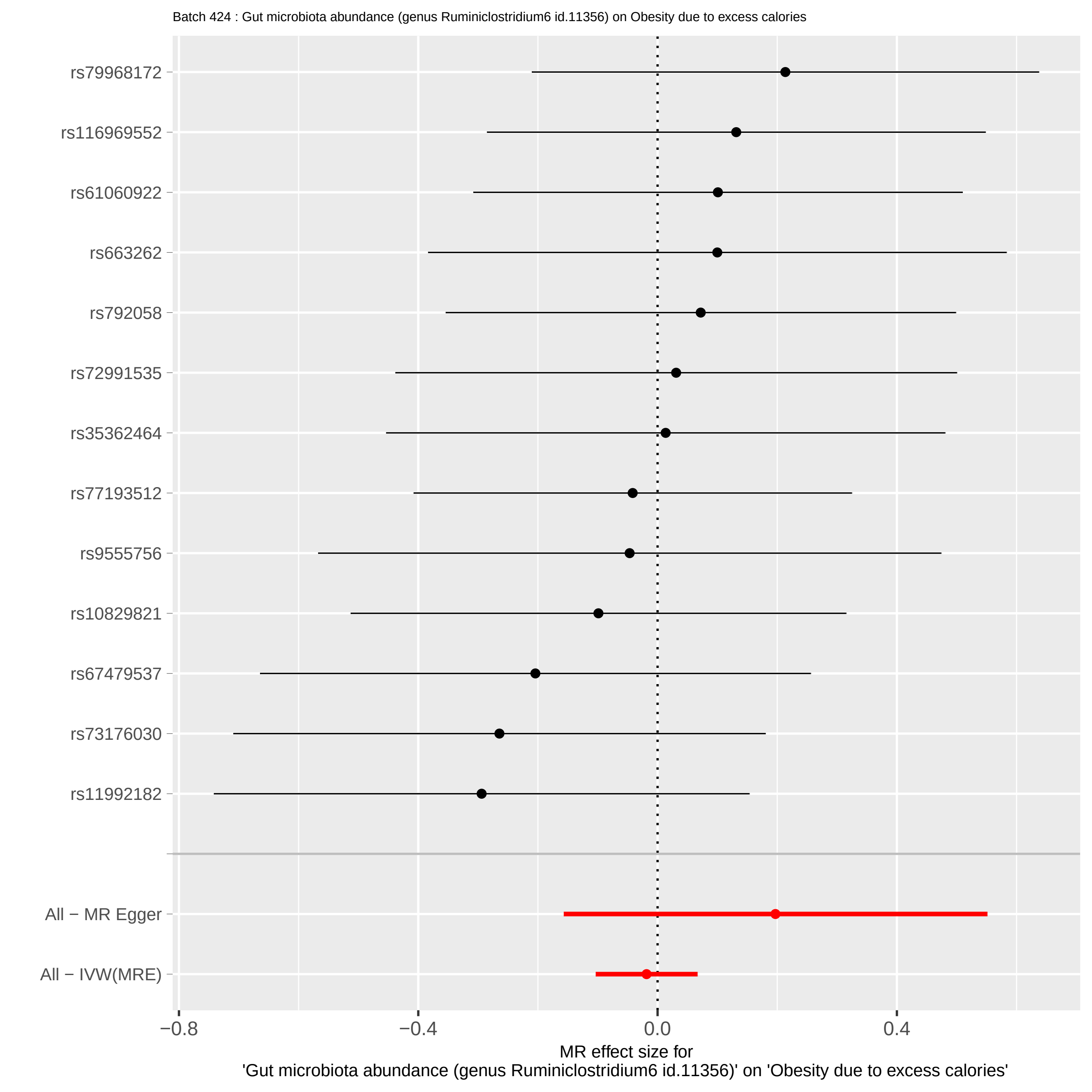

Batch 424 : Gut microbiota abundance (genus Ruminiclostridium6 id.11356) on Obesity due to excess calories
rs79968172
rs116969552
rs61060922
rs663262
rs792058
rs72991535
rs35362464
rs77193512
rs9555756
rs10829821
rs67479537
rs73176030
rs11992182
All − MR Egger
All − IVW(MRE)
−0.8
−0.4
0.0
0.4
MR effect size for
'Gut microbiota abundance (genus Ruminiclostridium6 id.11356)' on 'Obesity due to excess calories'

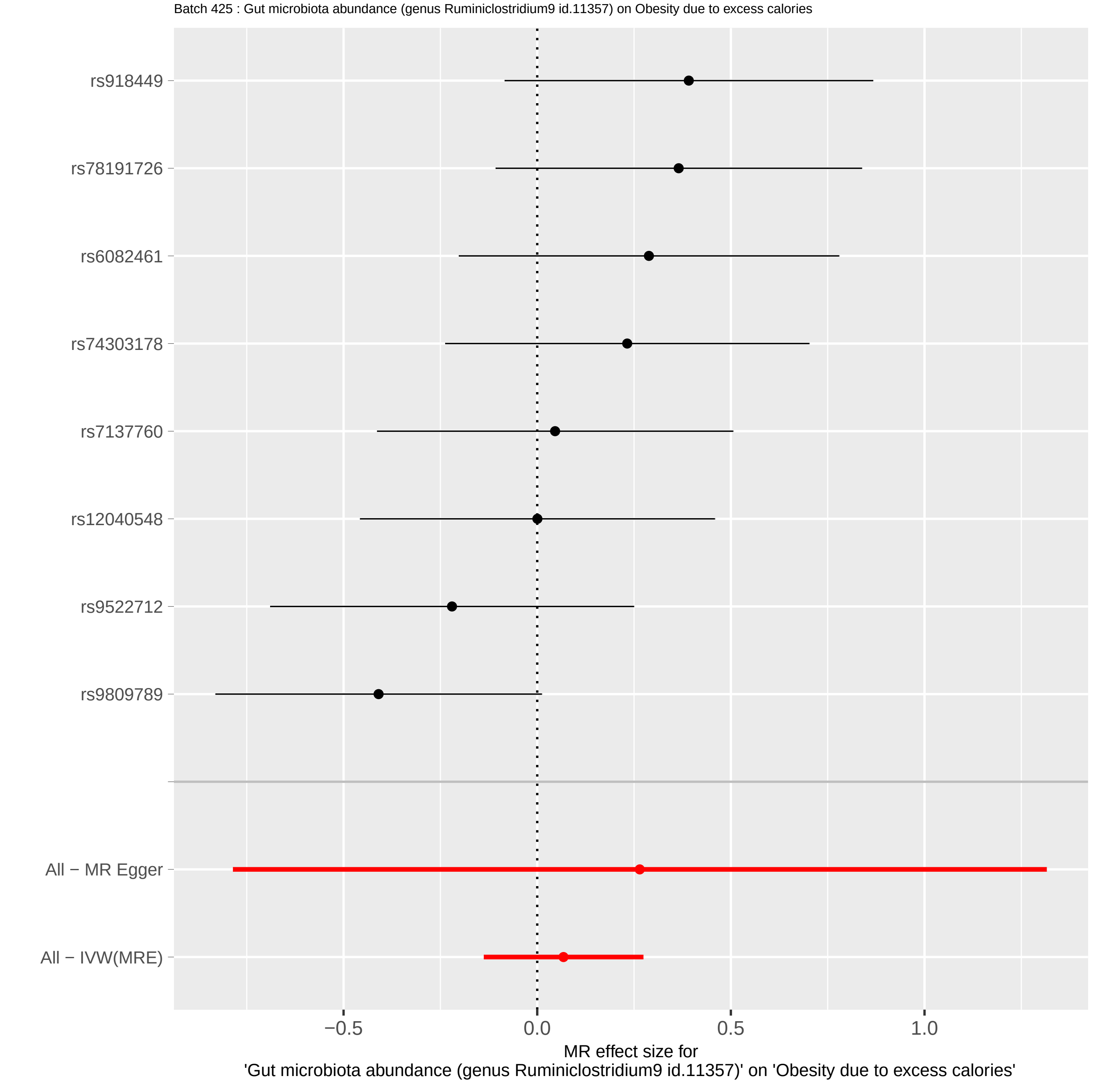

Batch 425 : Gut microbiota abundance (genus Ruminiclostridium9 id.11357) on Obesity due to excess calories
rs918449
rs78191726
rs6082461
rs74303178
rs7137760
rs12040548
rs9522712
rs9809789
All − MR Egger
All − IVW(MRE)
−0.5
0.0
0.5
1.0
MR effect size for
'Gut microbiota abundance (genus Ruminiclostridium9 id.11357)' on 'Obesity due to excess calories'

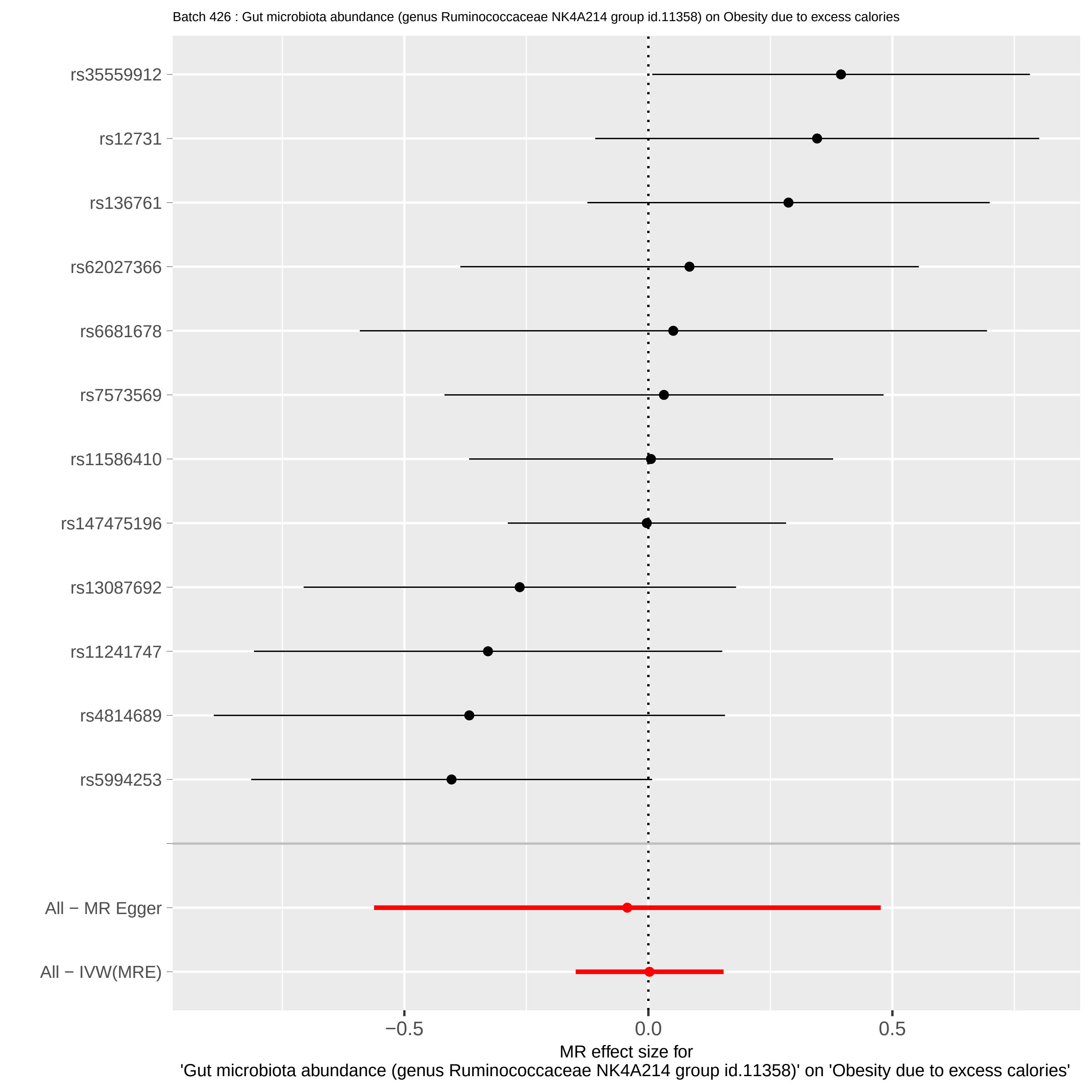
Batch 426 : Gut microbiota abundance (genus Ruminococcaceae NK4A214 group id.11358) on Obesity due to excess calories
rs35559912
rs12731
rs136761
rs62027366
rs6681678
rs7573569
rs11586410
rs147475196
rs13087692
rs11241747
rs4814689
rs5994253
All − MR Egger
All − IVW(MRE)
−0.5
0.0
0.5
MR effect size for
'Gut microbiota abundance (genus Ruminococcaceae NK4A214 group id.11358)' on 'Obesity due to excess calories'

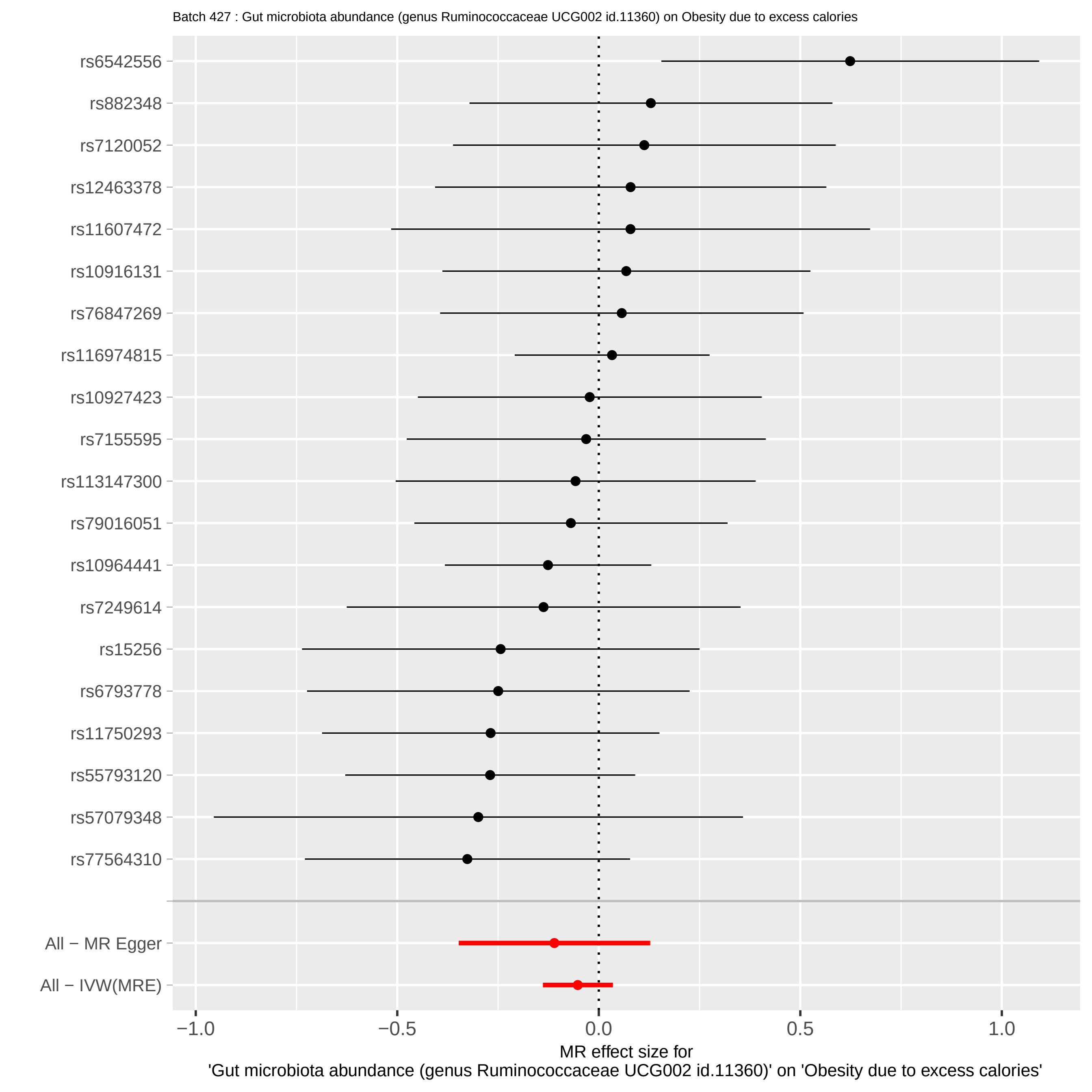

Batch 427 : Gut microbiota abundance (genus Ruminococcaceae UCG002 id.11360) on Obesity due to excess calories
rs6542556
rs882348
rs7120052
rs12463378
rs11607472
rs10916131
rs76847269
rs116974815
rs10927423
rs7155595
rs113147300
rs79016051
rs10964441
rs7249614
rs15256
rs6793778
rs11750293
rs55793120
rs57079348
rs77564310
All − MR Egger
All − IVW(MRE)
−1.0
−0.5
0.0
0.5
1.0
MR effect size for
'Gut microbiota abundance (genus Ruminococcaceae UCG002 id.11360)' on 'Obesity due to excess calories'

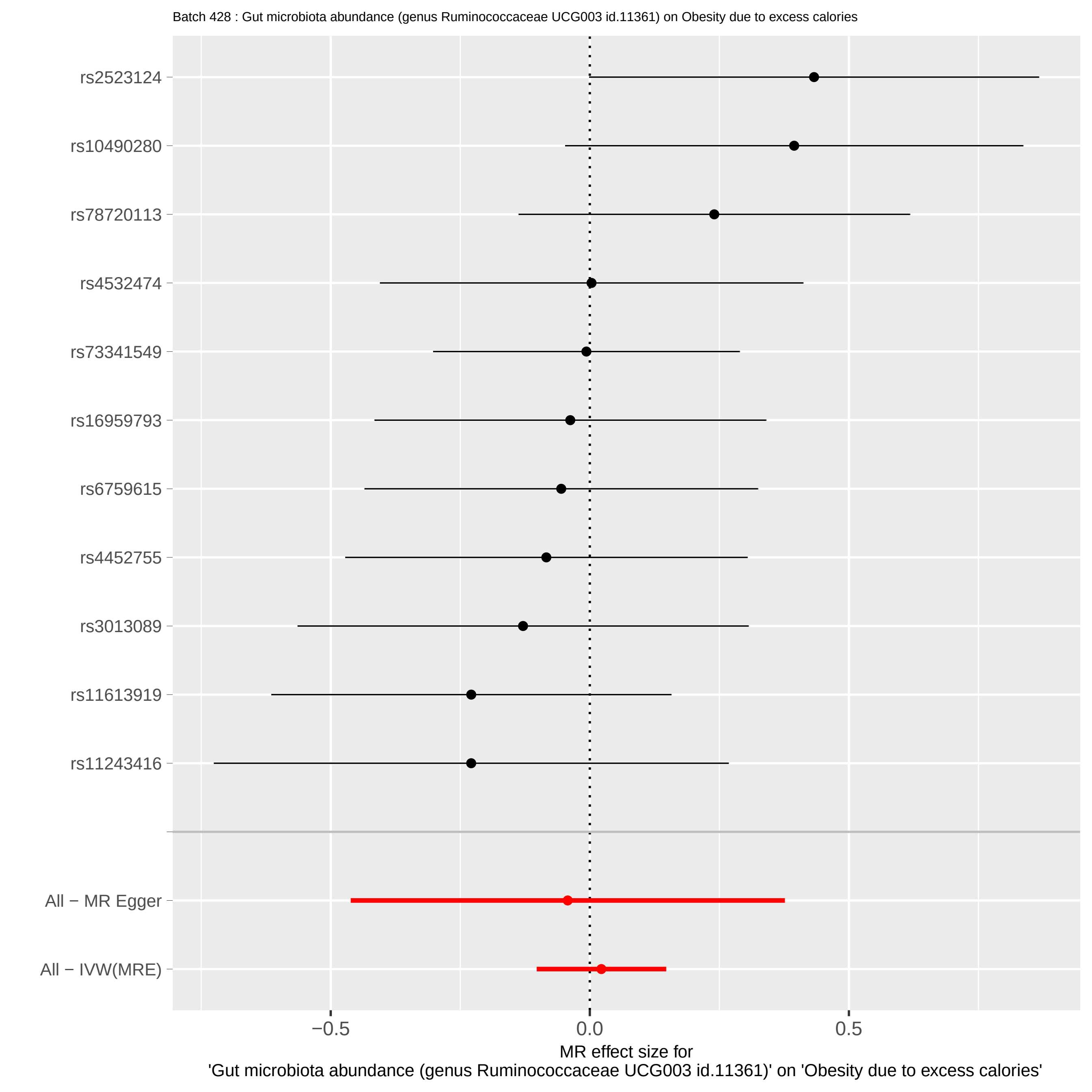

Batch 428 : Gut microbiota abundance (genus Ruminococcaceae UCG003 id.11361) on Obesity due to excess calories
rs2523124
rs10490280
rs78720113
rs4532474
rs73341549
rs16959793
rs6759615
rs4452755
rs3013089
rs11613919
rs11243416
All − MR Egger
All − IVW(MRE)
−0.5
0.0
0.5
MR effect size for
'Gut microbiota abundance (genus Ruminococcaceae UCG003 id.11361)' on 'Obesity due to excess calories'

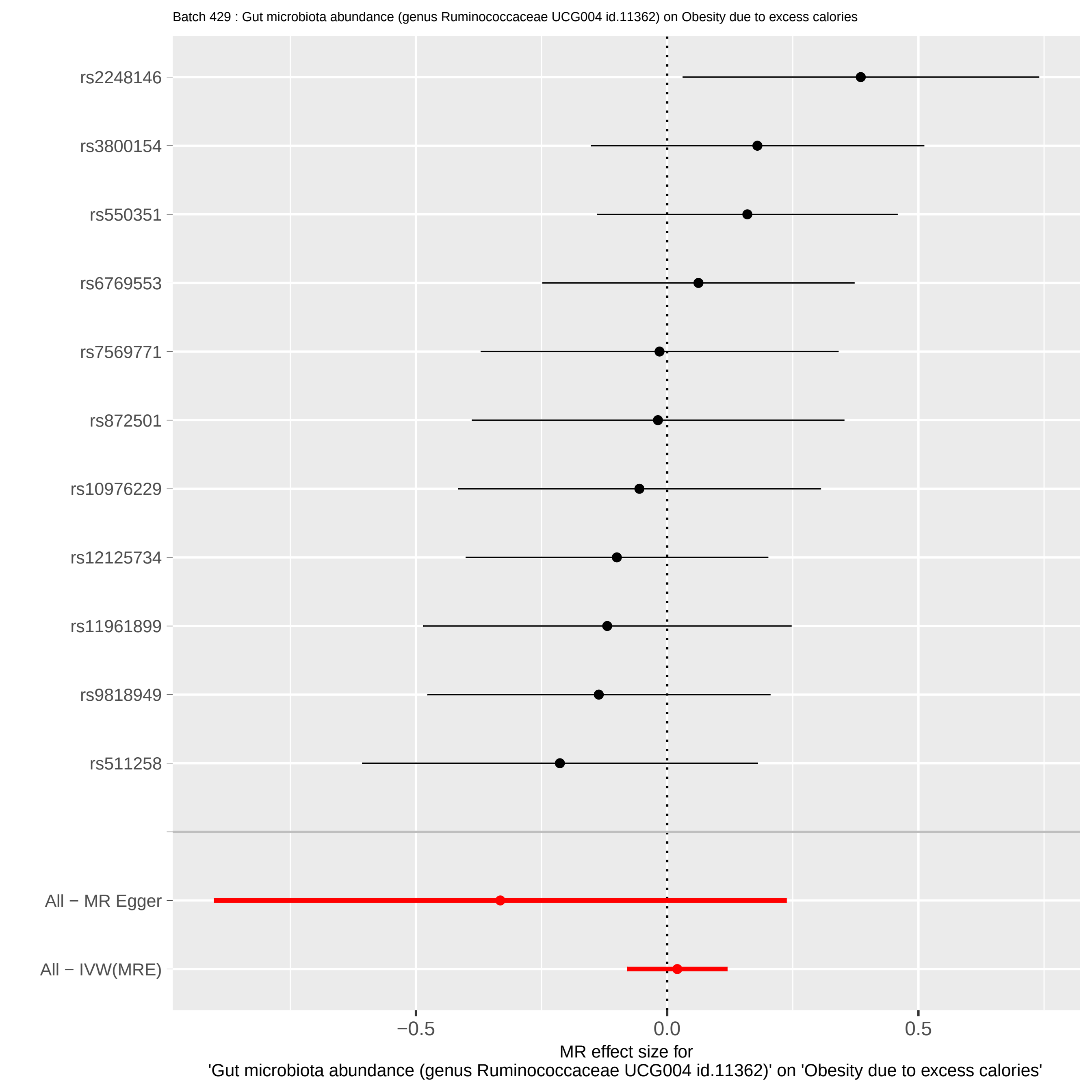

Batch 429 : Gut microbiota abundance (genus Ruminococcaceae UCG004 id.11362) on Obesity due to excess calories
rs2248146
rs3800154
rs550351
rs6769553
rs7569771
rs872501
rs10976229
rs12125734
rs11961899
rs9818949
rs511258
All − MR Egger
All − IVW(MRE)
−0.5
0.0
0.5
MR effect size for
'Gut microbiota abundance (genus Ruminococcaceae UCG004 id.11362)' on 'Obesity due to excess calories'

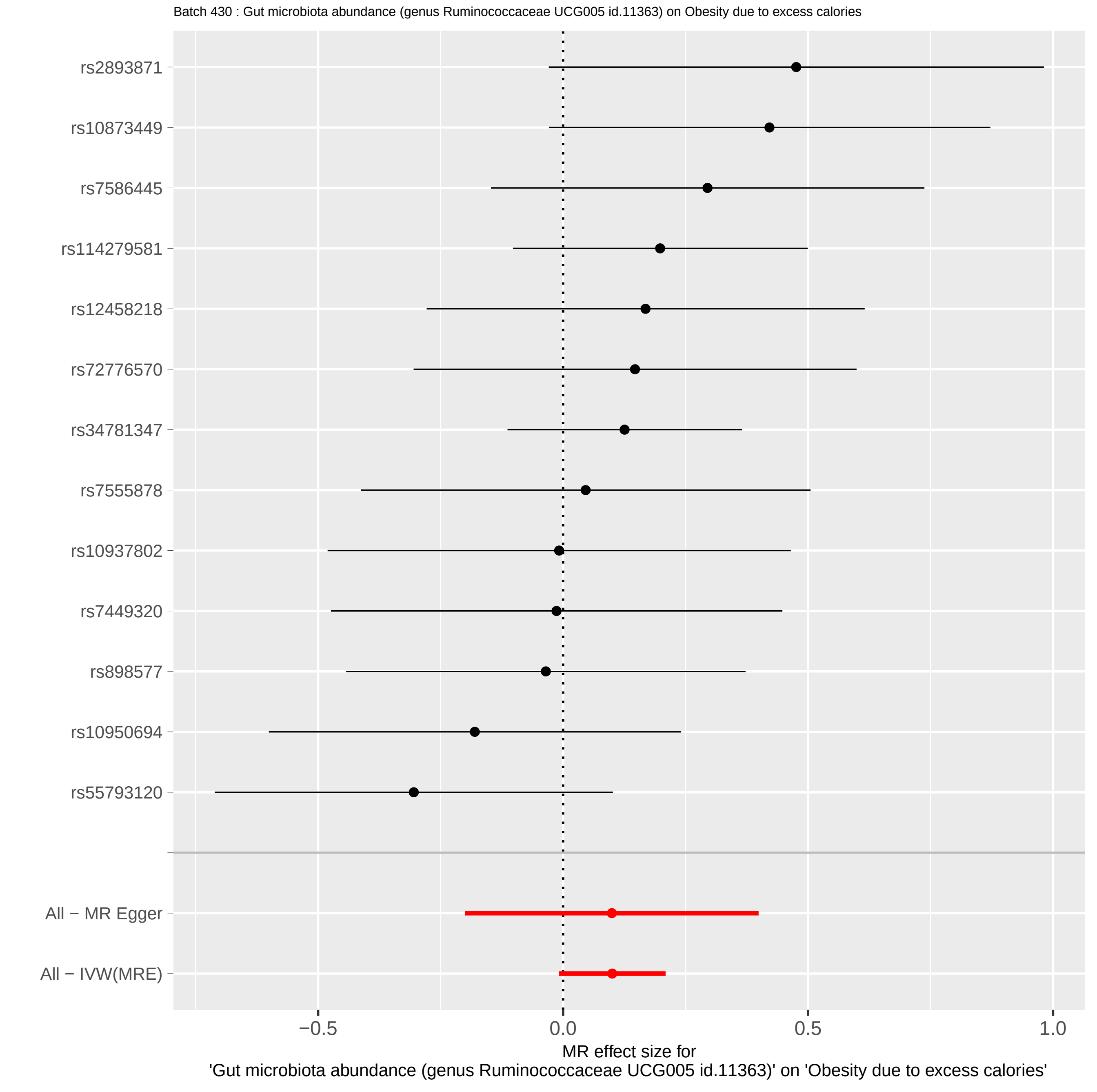

Batch 430 : Gut microbiota abundance (genus Ruminococcaceae UCG005 id.11363) on Obesity due to excess calories
rs2893871
rs10873449
rs7586445
rs114279581
rs12458218
rs72776570
rs34781347
rs7555878
rs10937802
rs7449320
rs898577
rs10950694
rs55793120
All − MR Egger
All − IVW(MRE)
−0.5
0.0
0.5
1.0
MR effect size for
'Gut microbiota abundance (genus Ruminococcaceae UCG005 id.11363)' on 'Obesity due to excess calories'

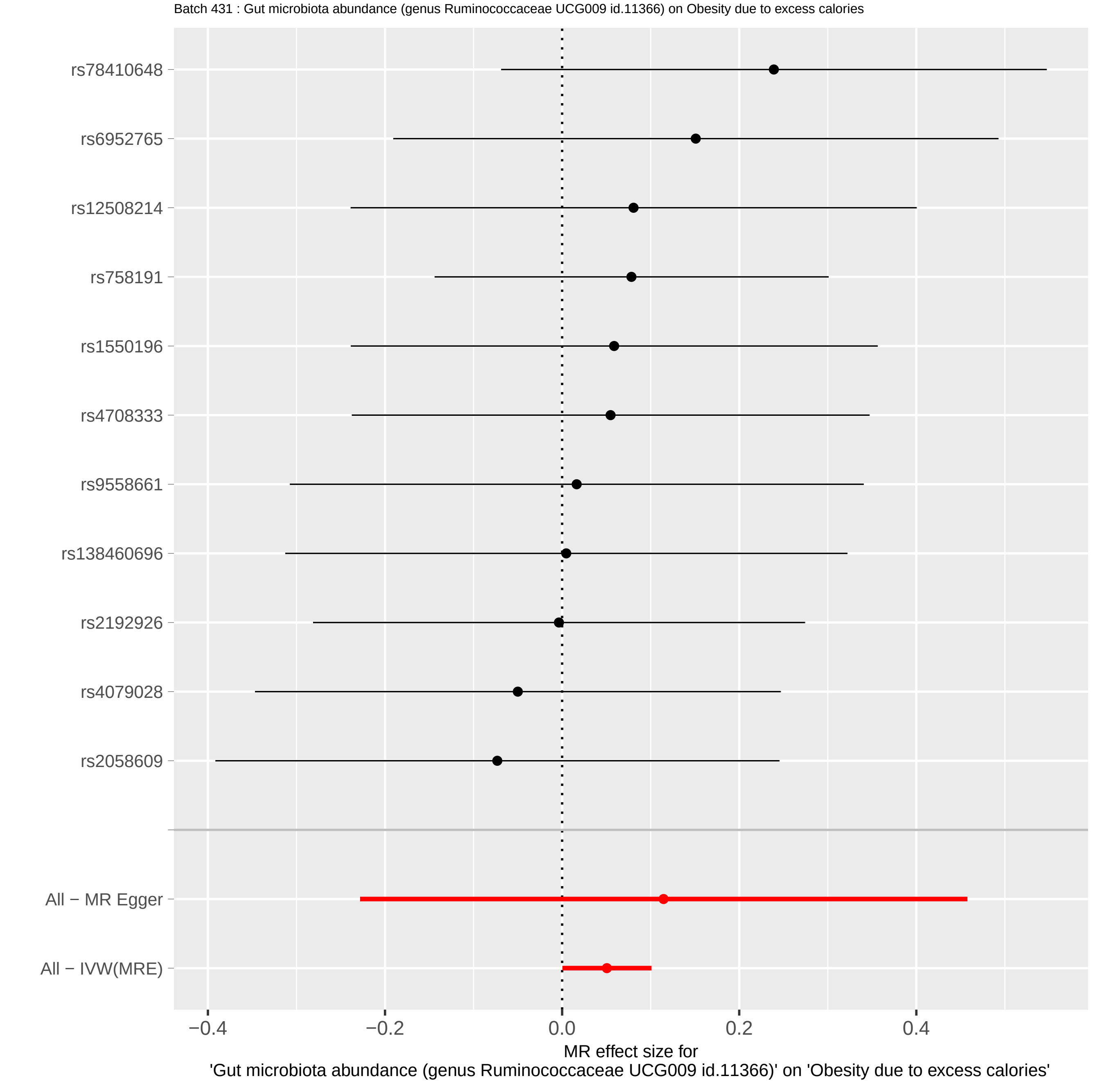
Batch 431 : Gut microbiota abundance (genus Ruminococcaceae UCG009 id.11366) on Obesity due to excess calories
rs78410648
rs6952765
rs12508214
rs758191
rs1550196
rs4708333
rs9558661
rs138460696
rs2192926
rs4079028
rs2058609
All − MR Egger
All − IVW(MRE)
−0.4
−0.2
0.0
0.2
0.4
MR effect size for
'Gut microbiota abundance (genus Ruminococcaceae UCG009 id.11366)' on 'Obesity due to excess calories'

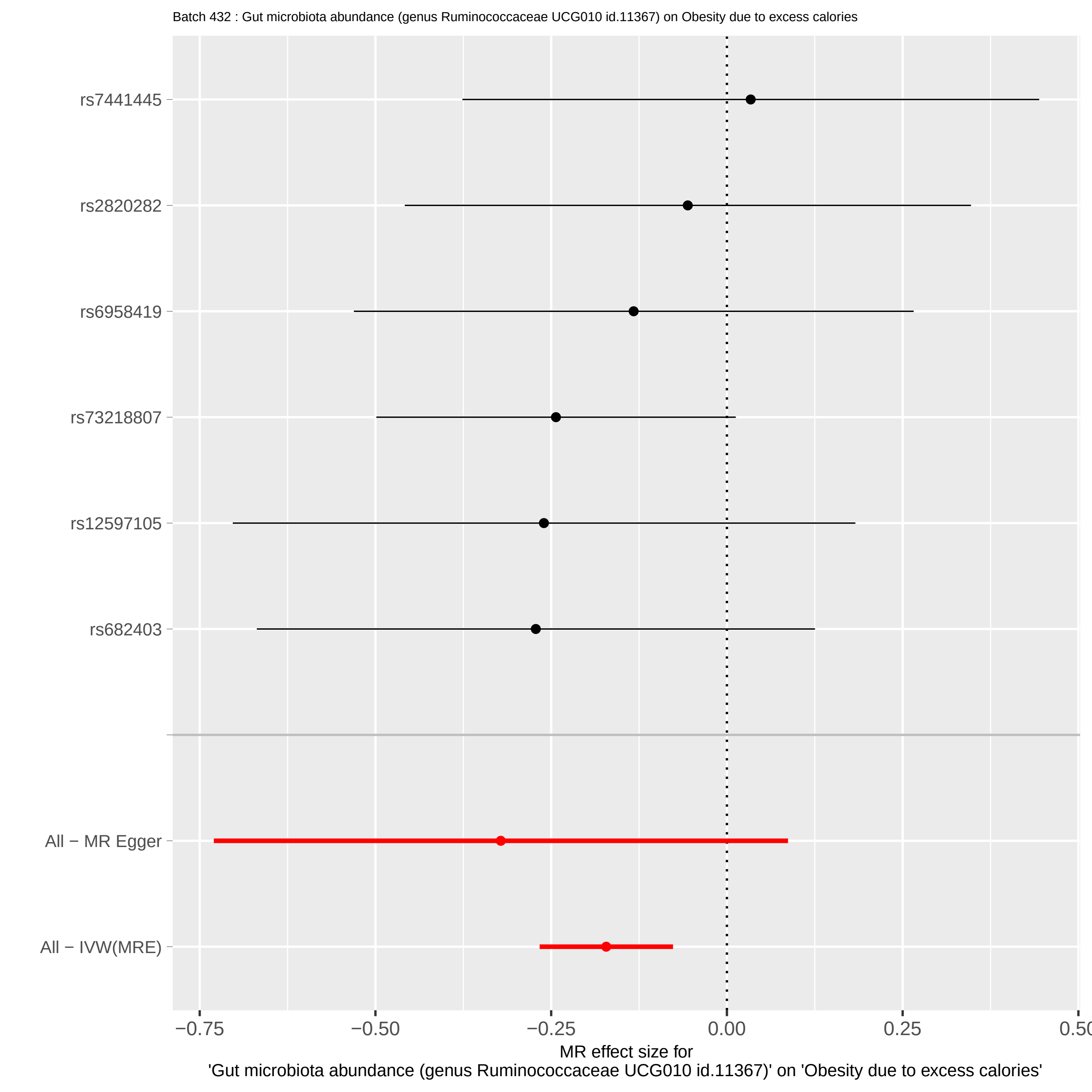

Batch 432 : Gut microbiota abundance (genus Ruminococcaceae UCG010 id.11367) on Obesity due to excess calories
rs7441445
rs2820282
rs6958419
rs73218807
rs12597105
rs682403
All − MR Egger
All − IVW(MRE)
−0.75
−0.50
−0.25
0.00
0.25
0.50
MR effect size for
'Gut microbiota abundance (genus Ruminococcaceae UCG010 id.11367)' on 'Obesity due to excess calories'

Batch 433 : Gut microbiota abundance (genus Ruminococcaceae UCG011 id.11368) on Obesity due to excess calories

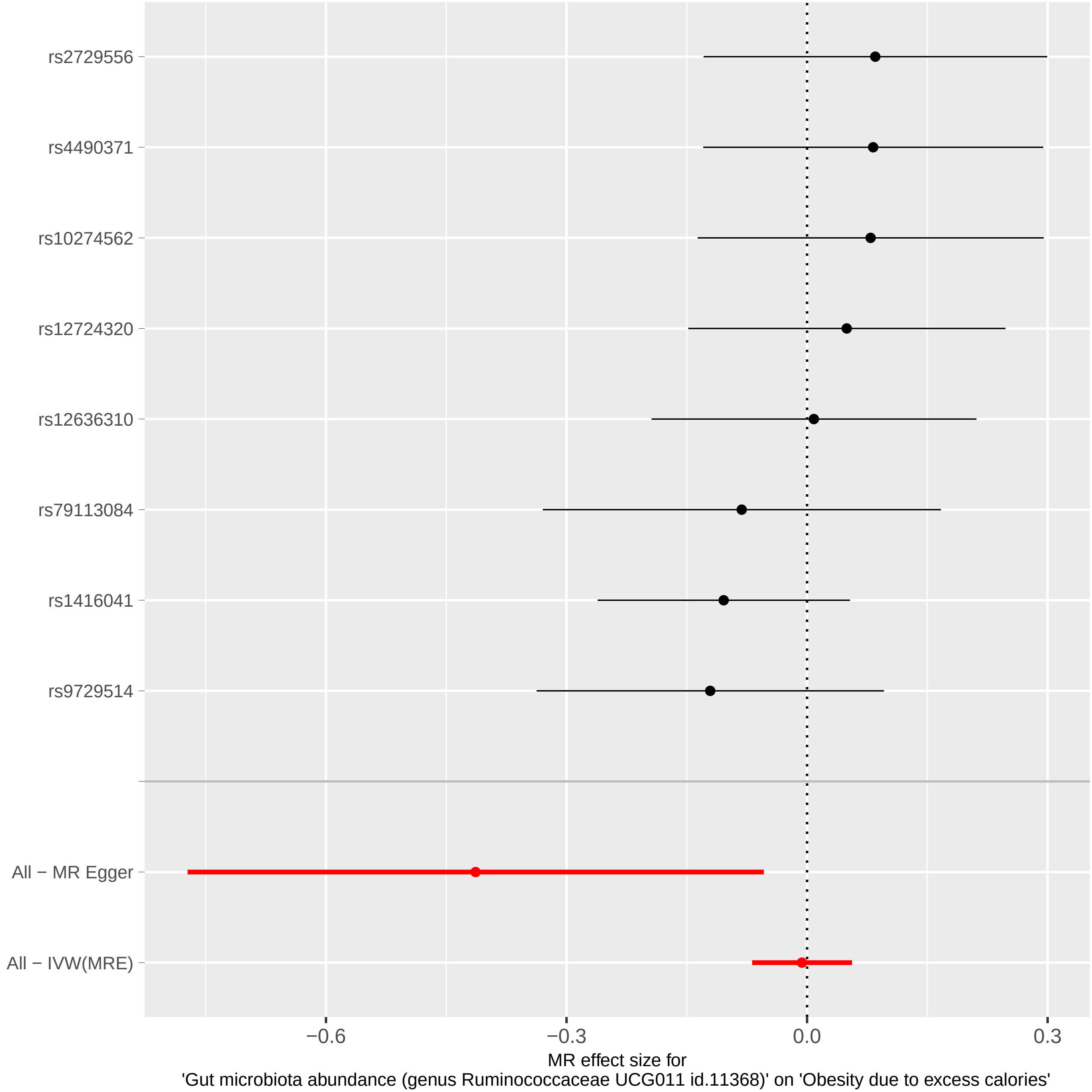

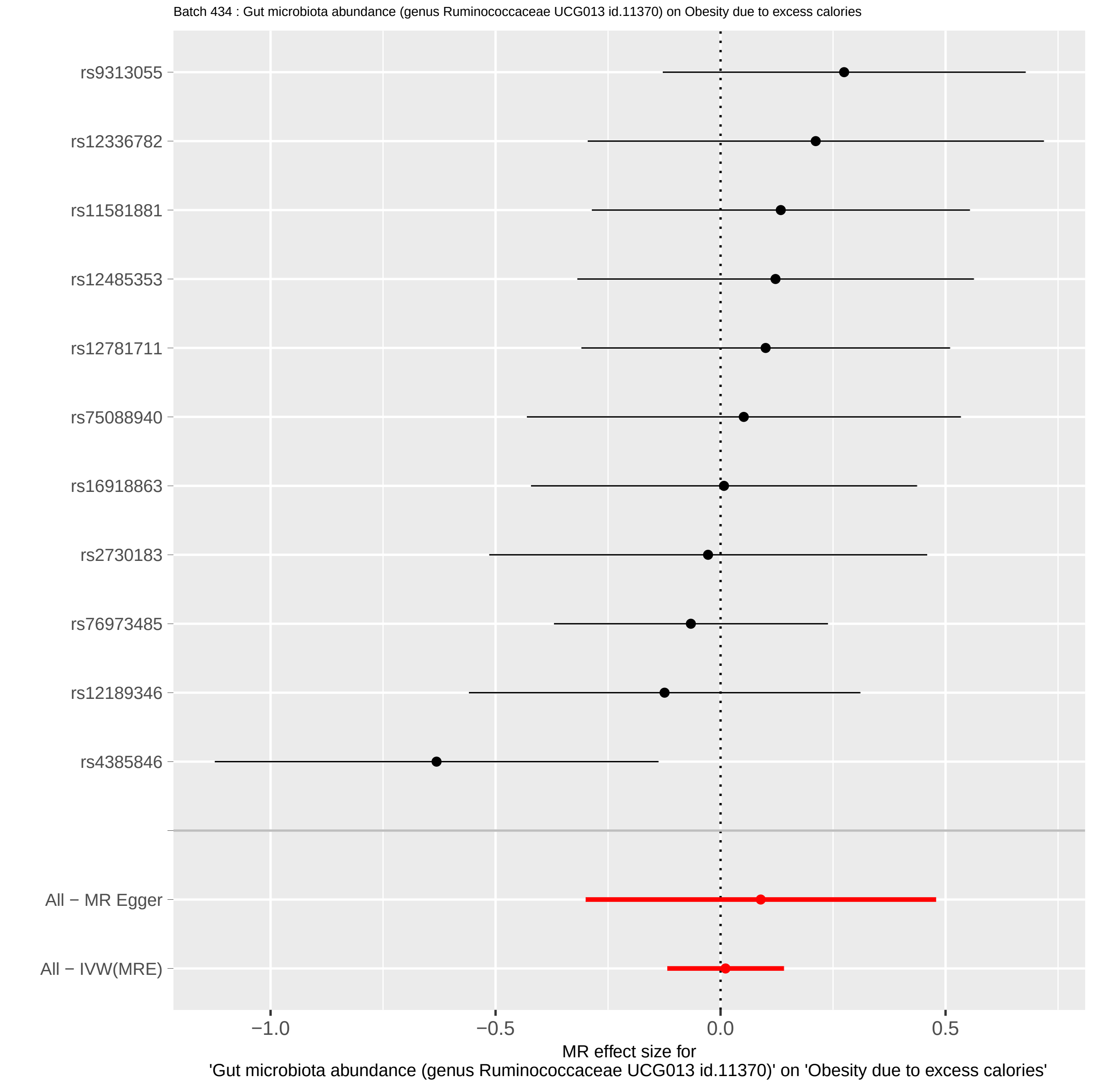
Batch 434 : Gut microbiota abundance (genus Ruminococcaceae UCG013 id.11370) on Obesity due to excess calories
rs9313055
rs12336782
rs11581881
rs12485353
rs12781711
rs75088940
rs16918863
rs2730183
rs76973485
rs12189346
rs4385846
All − MR Egger
All − IVW(MRE)
−1.0
−0.5
0.0
0.5
MR effect size for
'Gut microbiota abundance (genus Ruminococcaceae UCG013 id.11370)' on 'Obesity due to excess calories'

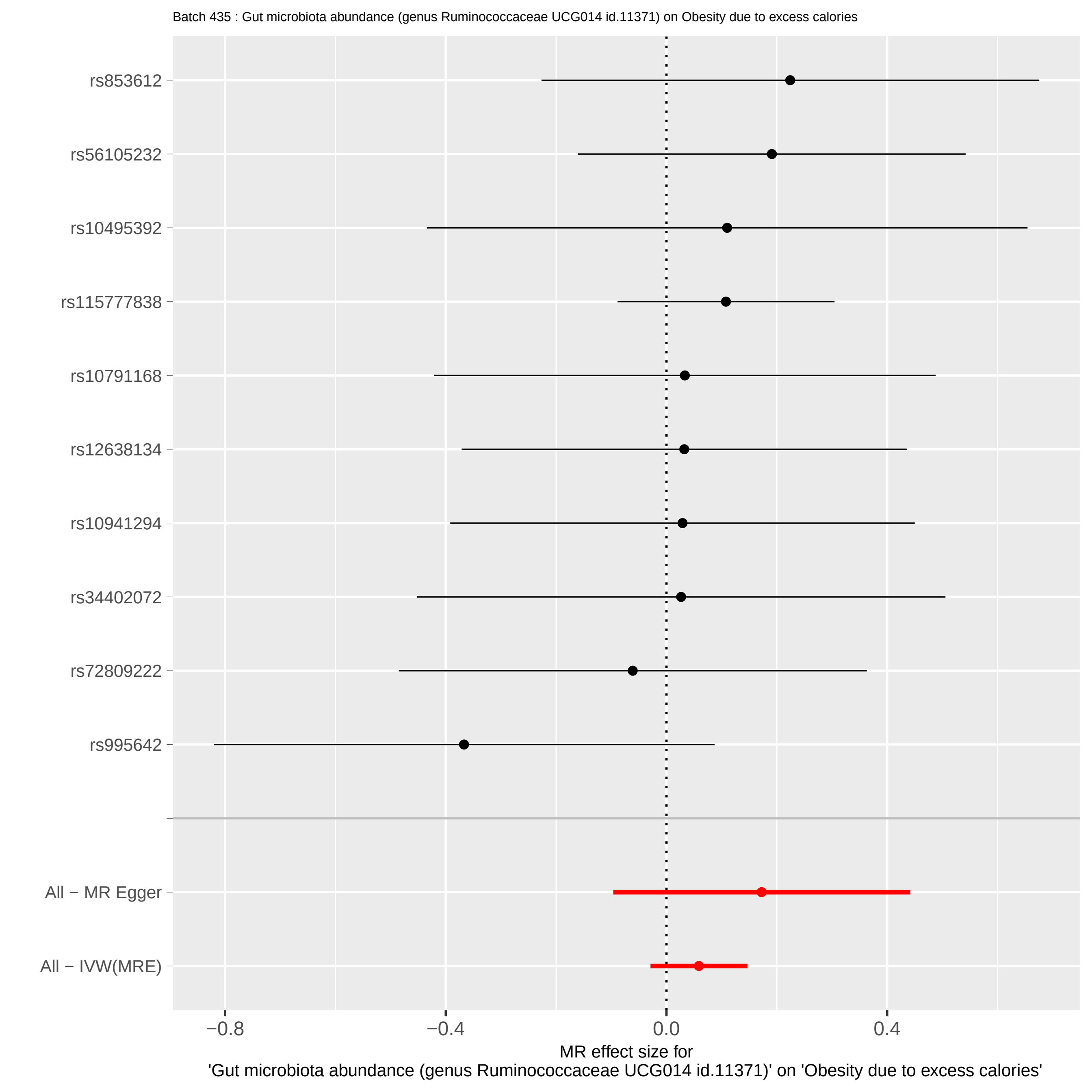
Batch 435 : Gut microbiota abundance (genus Ruminococcaceae UCG014 id.11371) on Obesity due to excess calories
rs853612
rs56105232
rs10495392
rs115777838
rs10791168
rs12638134
rs10941294
rs34402072
rs72809222
rs995642
All − MR Egger
All − IVW(MRE)
−0.8
−0.4
0.0
0.4
MR effect size for
'Gut microbiota abundance (genus Ruminococcaceae UCG014 id.11371)' on 'Obesity due to excess calories'

Batch 436 : Gut microbiota abundance (genus Ruminococcus1 id.11373) on Obesity due to excess calories

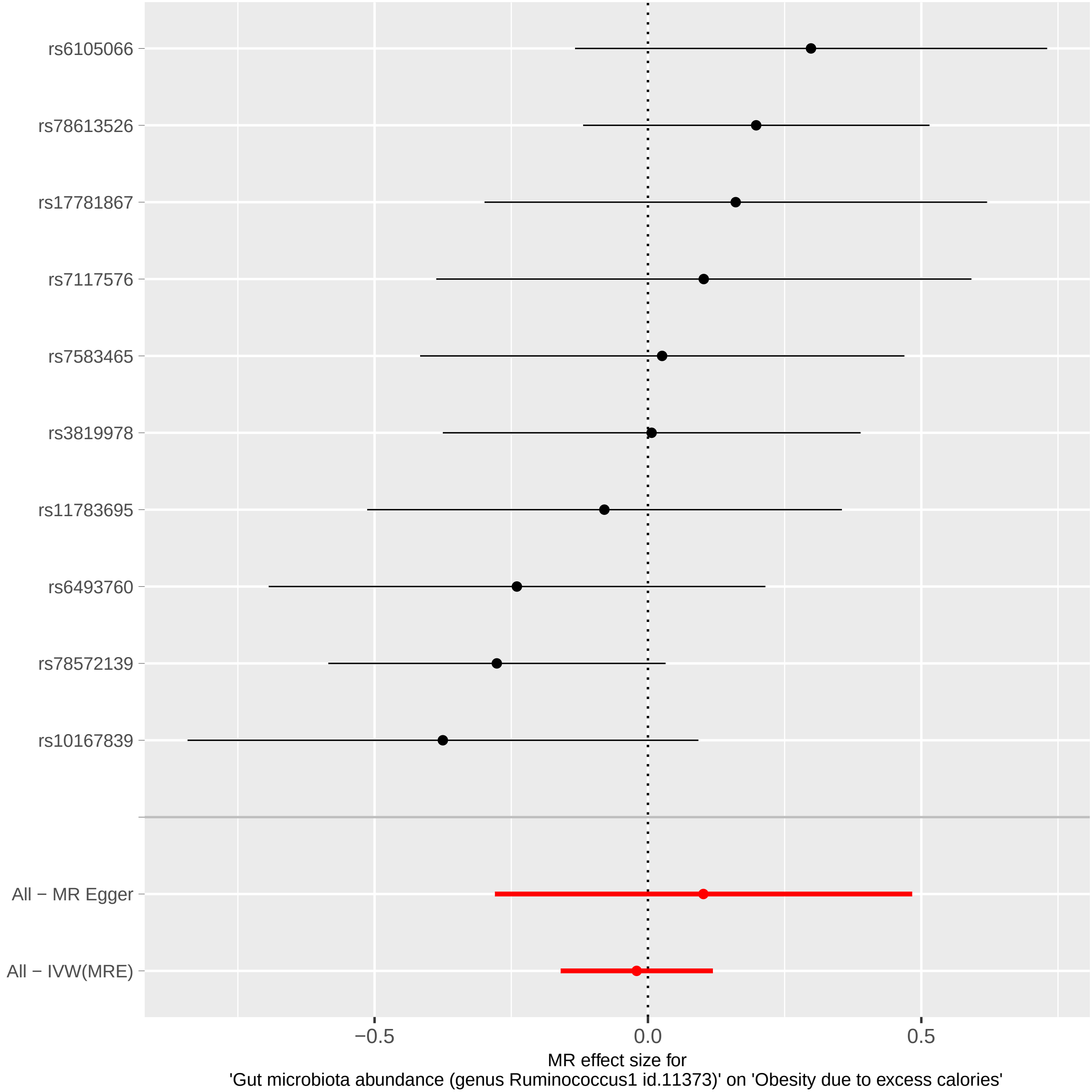

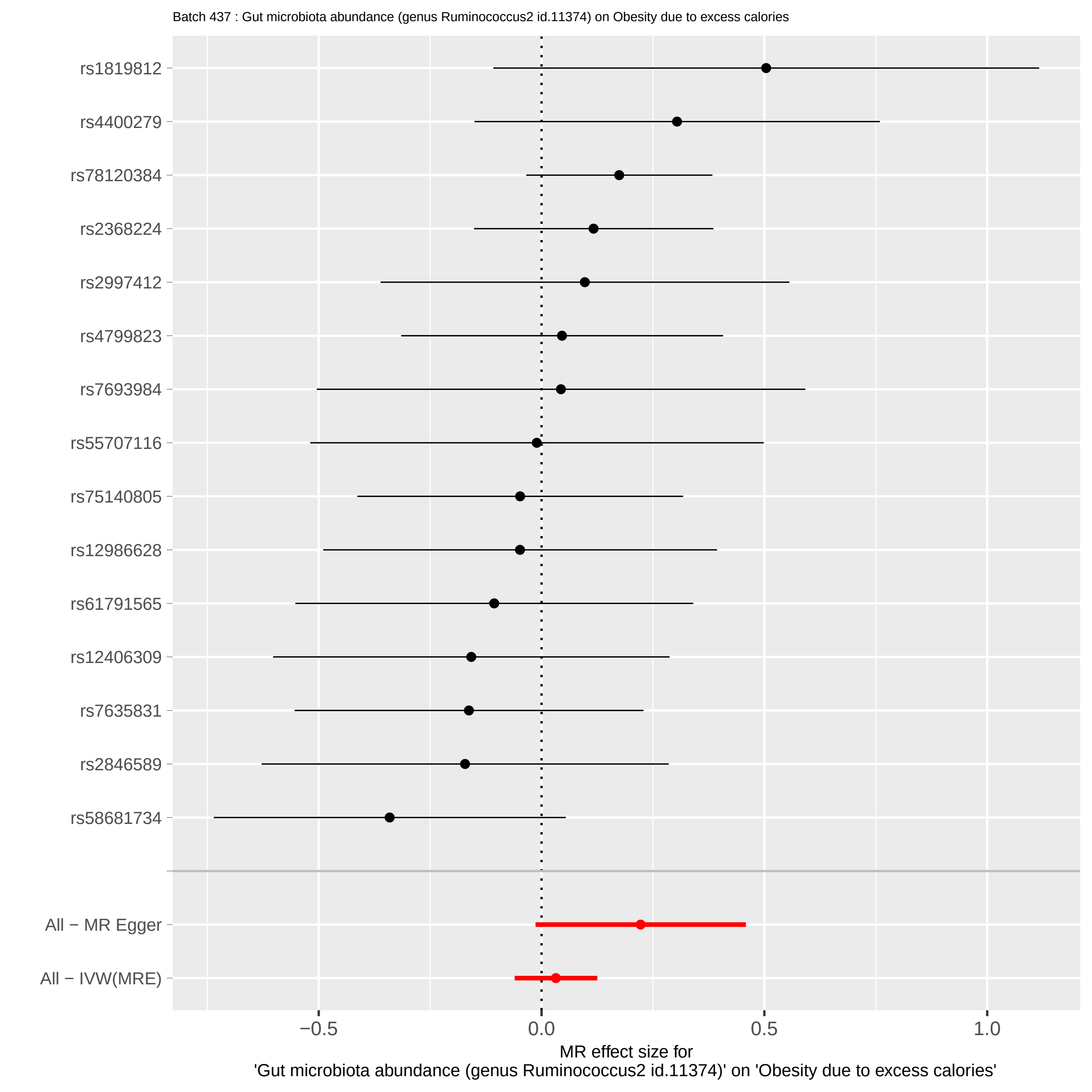

Batch 437 : Gut microbiota abundance (genus Ruminococcus2 id.11374) on Obesity due to excess calories
rs1819812
rs4400279
rs78120384
rs2368224
rs2997412
rs4799823
rs7693984
rs55707116
rs75140805
rs12986628
rs61791565
rs12406309
rs7635831
rs2846589
rs58681734
All − MR Egger
All − IVW(MRE)
−0.5
0.0
0.5
1.0
MR effect size for
'Gut microbiota abundance (genus Ruminococcus2 id.11374)' on 'Obesity due to excess calories'

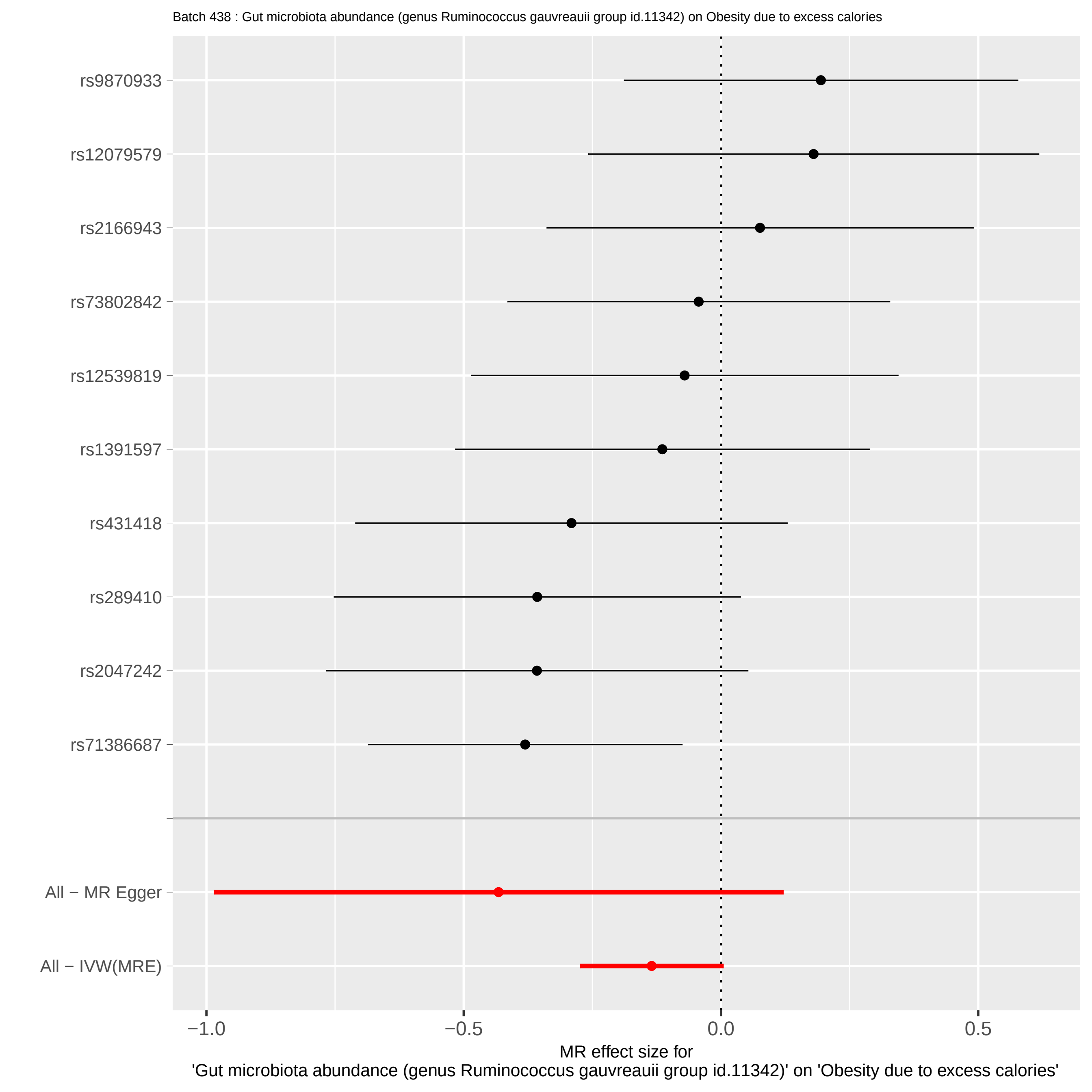

Batch 438 : Gut microbiota abundance (genus Ruminococcus gauvreauii group id.11342) on Obesity due to excess calories
rs9870933
rs12079579
rs2166943
rs73802842
rs12539819
rs1391597
rs431418
rs289410
rs2047242
rs71386687
All − MR Egger
All − IVW(MRE)
−1.0
−0.5
0.0
0.5
MR effect size for
'Gut microbiota abundance (genus Ruminococcus gauvreauii group id.11342)' on 'Obesity due to excess calories'

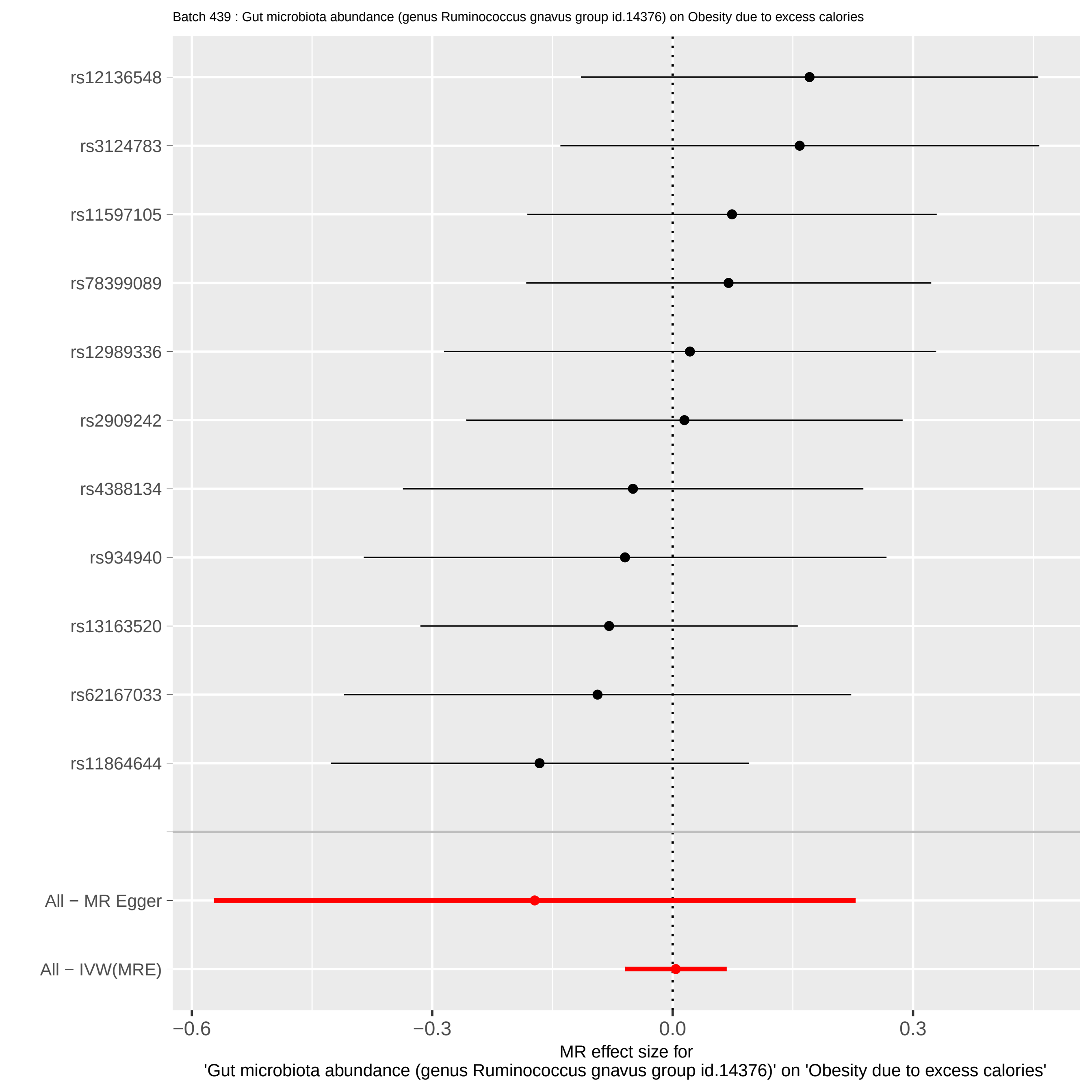
Batch 439 : Gut microbiota abundance (genus Ruminococcus gnavus group id.14376) on Obesity due to excess calories
rs12136548
rs3124783
rs11597105
rs78399089
rs12989336
rs2909242
rs4388134
rs934940
rs13163520
rs62167033
rs11864644
All − MR Egger
All − IVW(MRE)
−0.6
−0.3
0.0
0.3
MR effect size for
'Gut microbiota abundance (genus Ruminococcus gnavus group id.14376)' on 'Obesity due to excess calories'

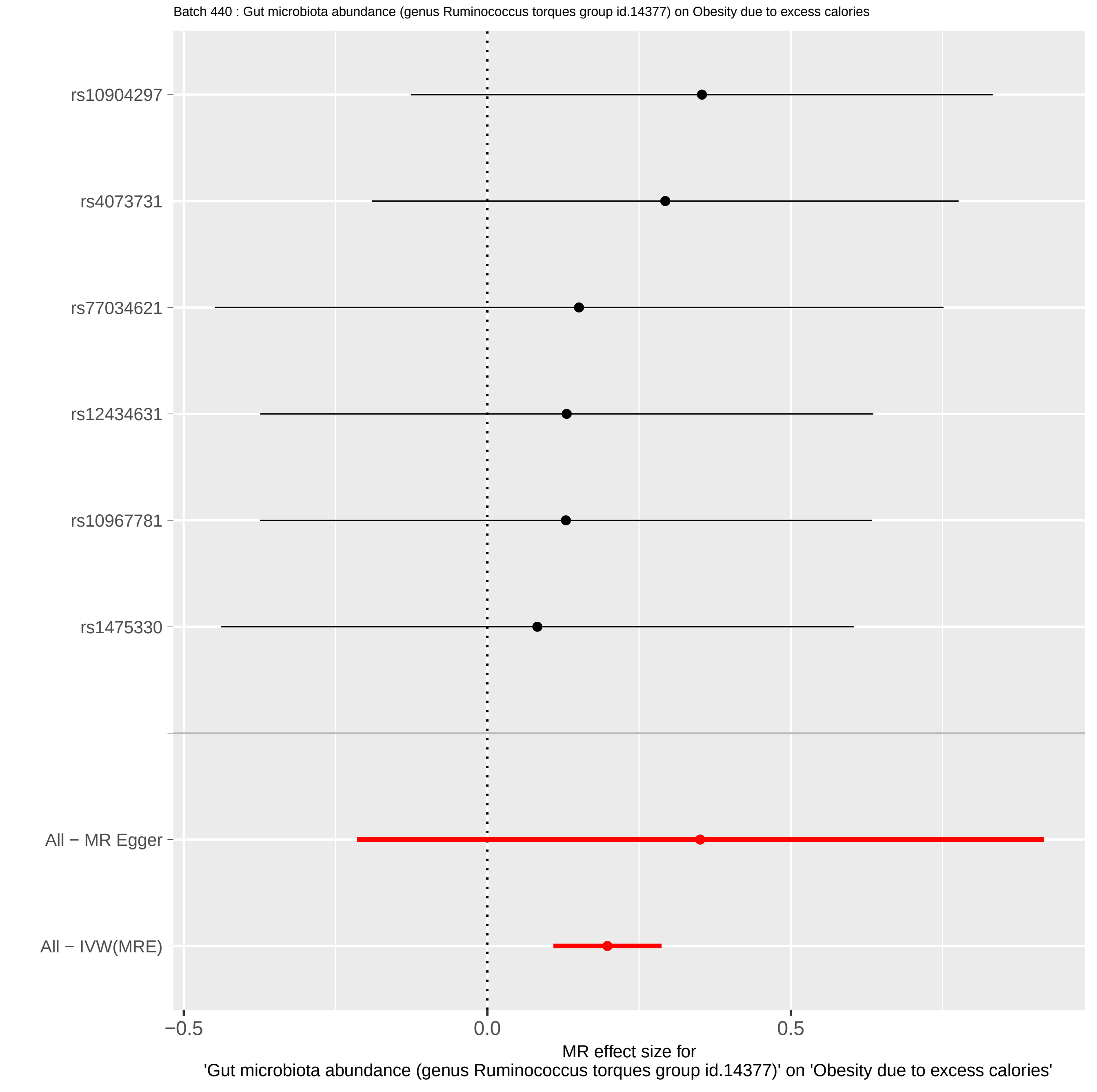

Batch 440 : Gut microbiota abundance (genus Ruminococcus torques group id.14377) on Obesity due to excess calories
rs10904297
rs4073731
rs77034621
rs12434631
rs10967781
rs1475330
All − MR Egger
All − IVW(MRE)
−0.5
0.0
0.5
MR effect size for
'Gut microbiota abundance (genus Ruminococcus torques group id.14377)' on 'Obesity due to excess calories'

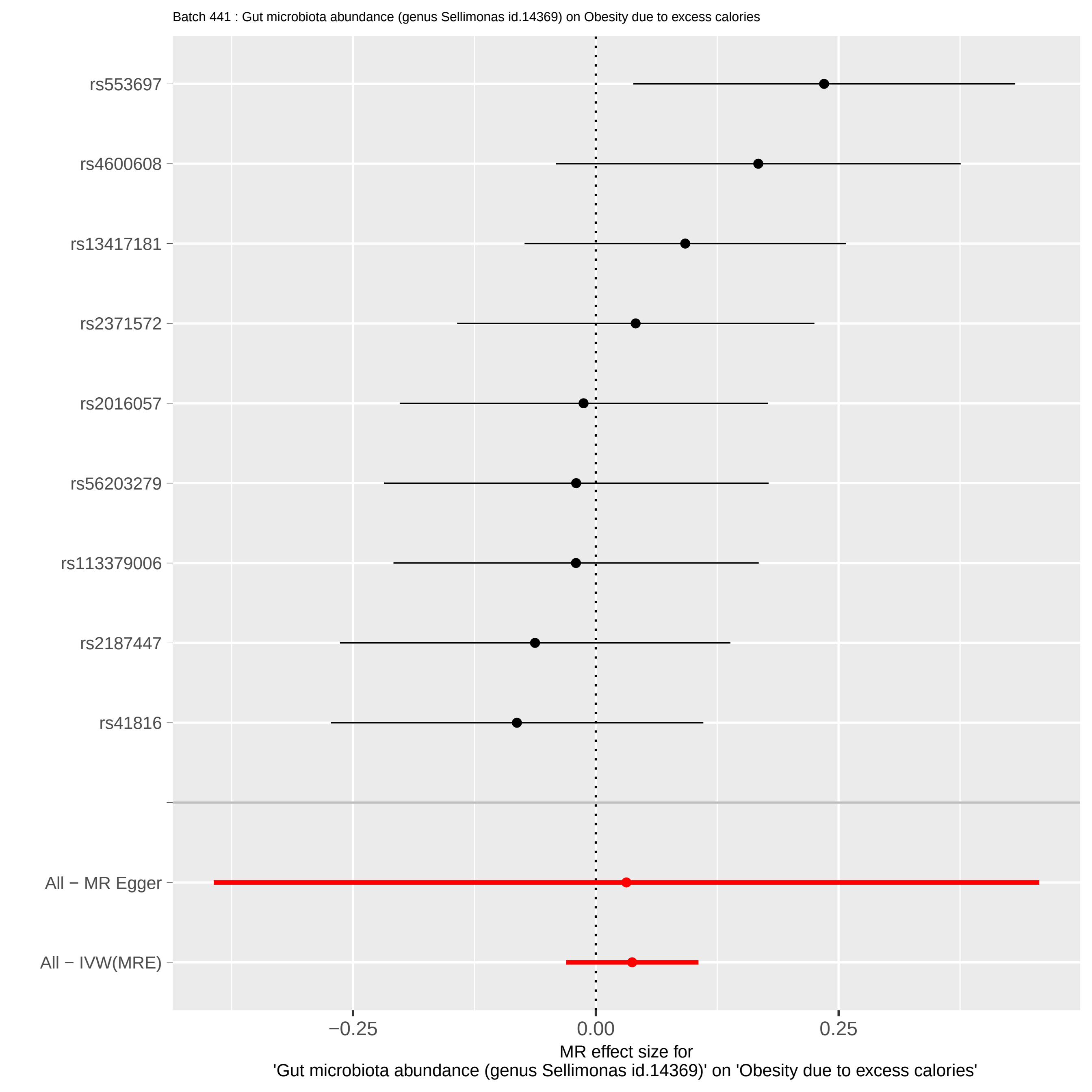
Batch 441 : Gut microbiota abundance (genus Sellimonas id.14369) on Obesity due to excess calories
rs553697
rs4600608
rs13417181
rs2371572
rs2016057
rs56203279
rs113379006
rs2187447
rs41816
All − MR Egger
All − IVW(MRE)
−0.25
0.00
0.25
MR effect size for
'Gut microbiota abundance (genus Sellimonas id.14369)' on 'Obesity due to excess calories'

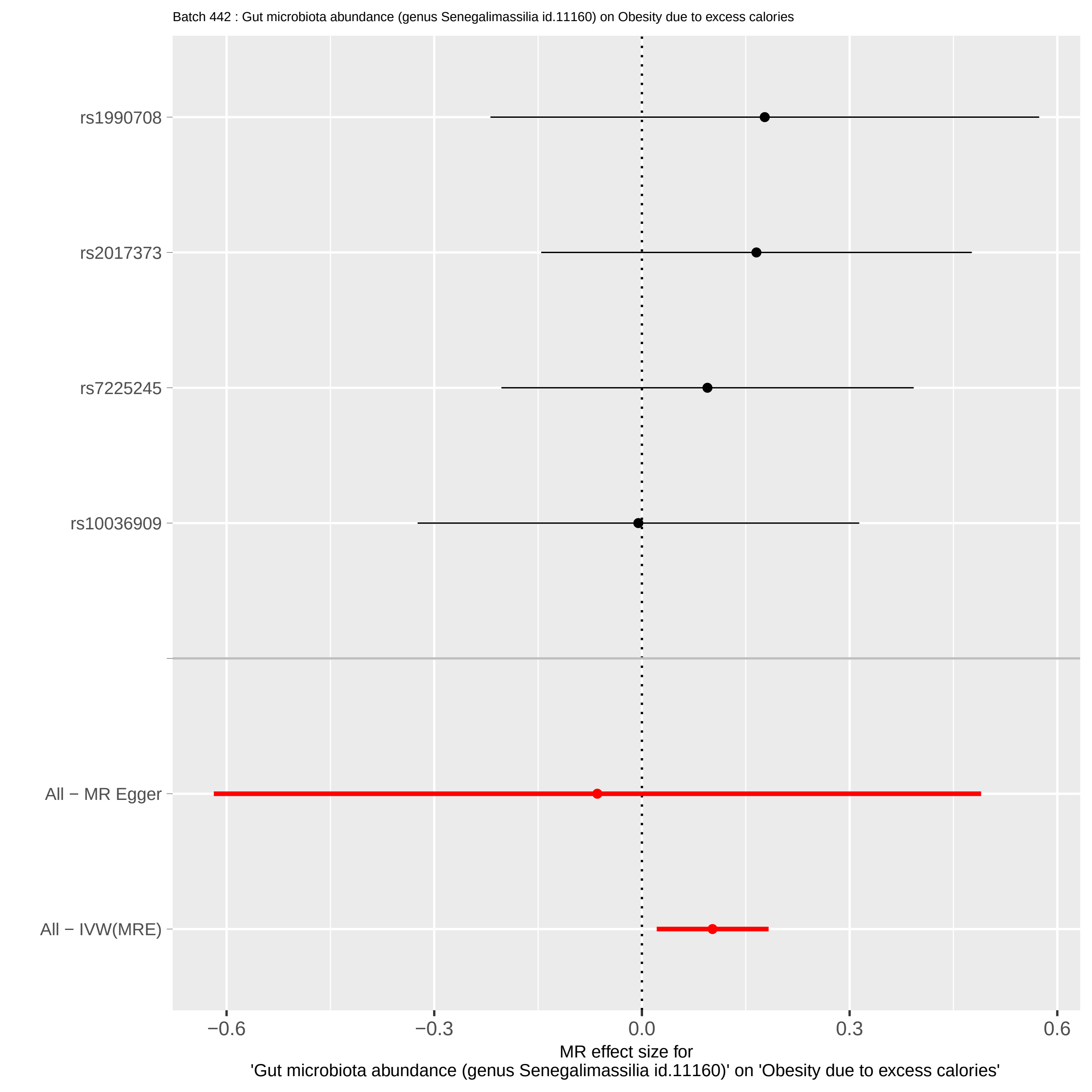

Batch 442 : Gut microbiota abundance (genus Senegalimassilia id.11160) on Obesity due to excess calories
rs1990708
rs2017373
rs7225245
rs10036909
All − MR Egger
All − IVW(MRE)
−0.6
−0.3
0.0
0.3
0.6
MR effect size for
'Gut microbiota abundance (genus Senegalimassilia id.11160)' on 'Obesity due to excess calories'

Batch 443 : Gut microbiota abundance (genus Slackia id.825) on Obesity due to excess calories

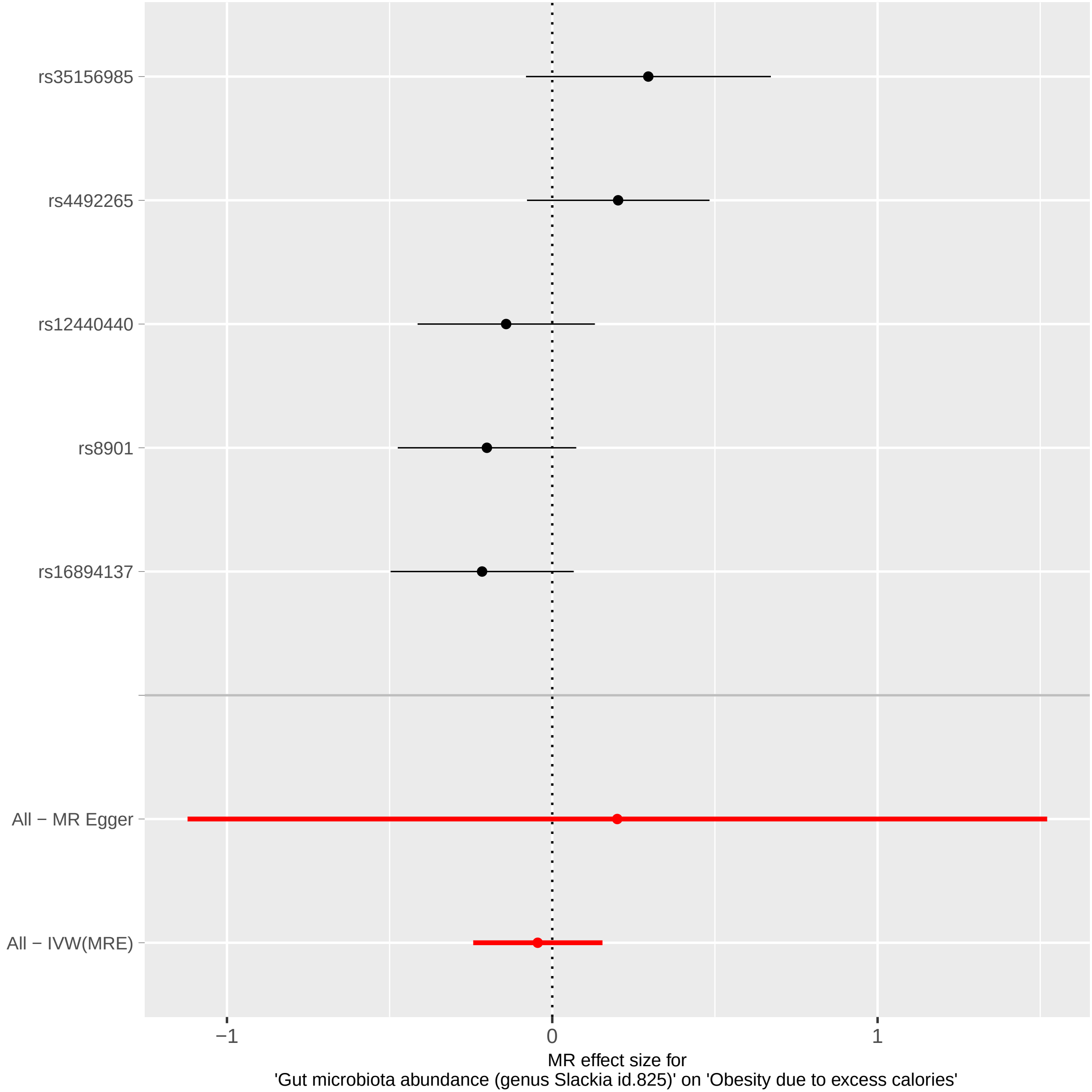

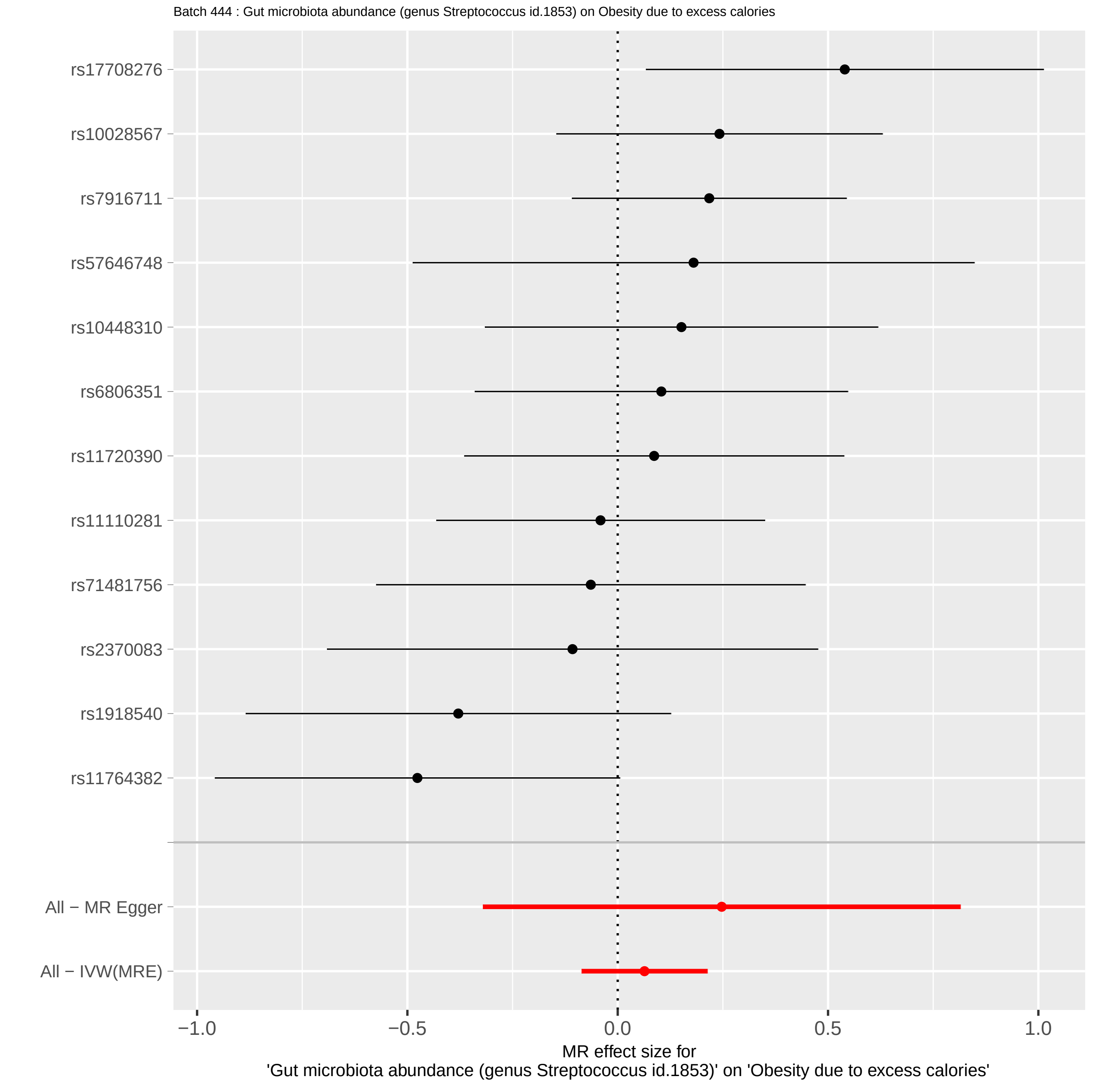

Batch 444 : Gut microbiota abundance (genus Streptococcus id.1853) on Obesity due to excess calories
rs17708276
rs10028567
rs7916711
rs57646748
rs10448310
rs6806351
rs11720390
rs11110281
rs71481756
rs2370083
rs1918540
rs11764382
All − MR Egger
All − IVW(MRE)
−1.0
−0.5
0.0
0.5
1.0
MR effect size for
'Gut microbiota abundance (genus Streptococcus id.1853)' on 'Obesity due to excess calories'

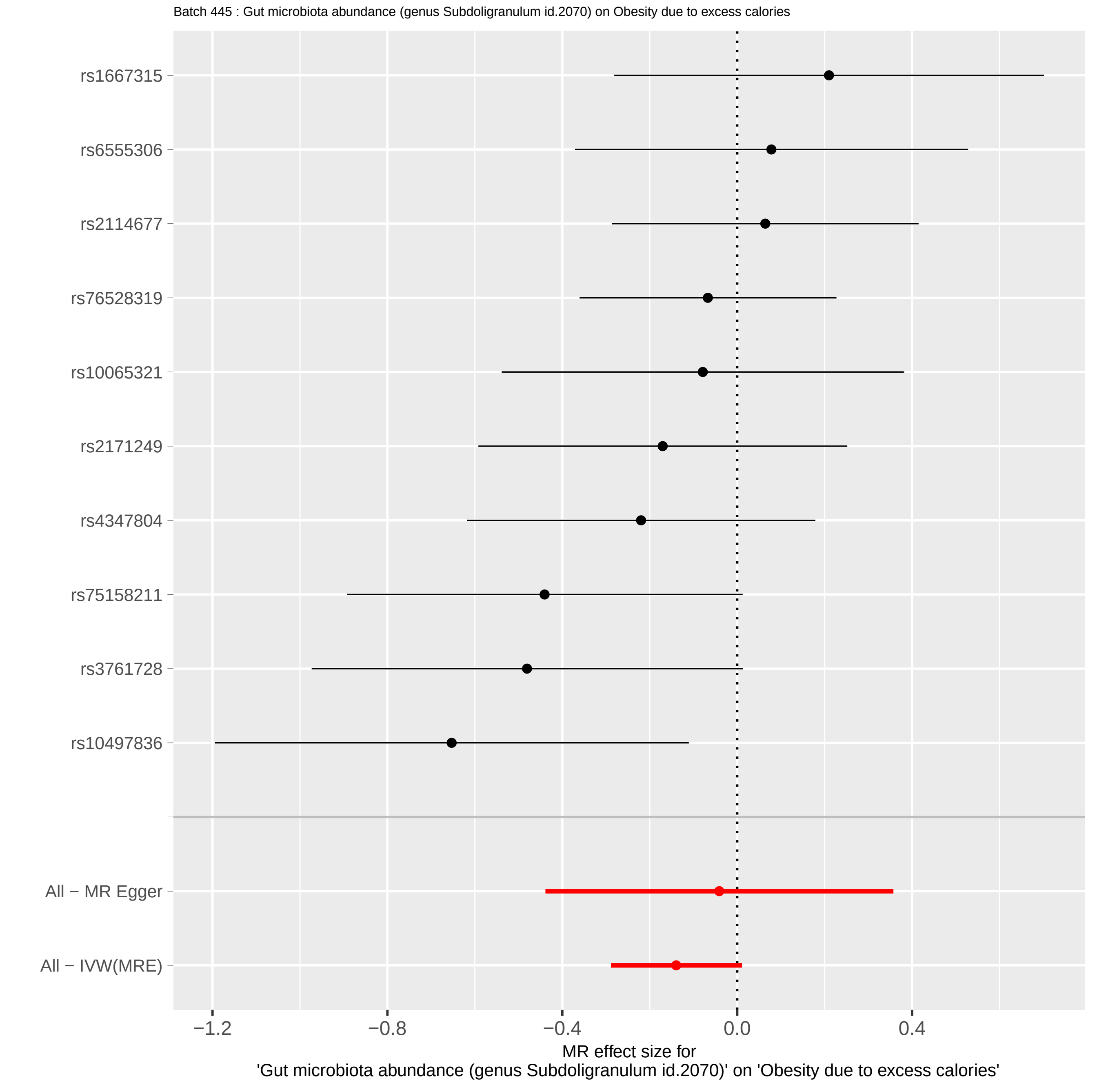

Batch 445 : Gut microbiota abundance (genus Subdoligranulum id.2070) on Obesity due to excess calories
rs1667315
rs6555306
rs2114677
rs76528319
rs10065321
rs2171249
rs4347804
rs75158211
rs3761728
rs10497836
All − MR Egger
All − IVW(MRE)
−1.2
−0.8
−0.4
0.0
0.4
MR effect size for
'Gut microbiota abundance (genus Subdoligranulum id.2070)' on 'Obesity due to excess calories'

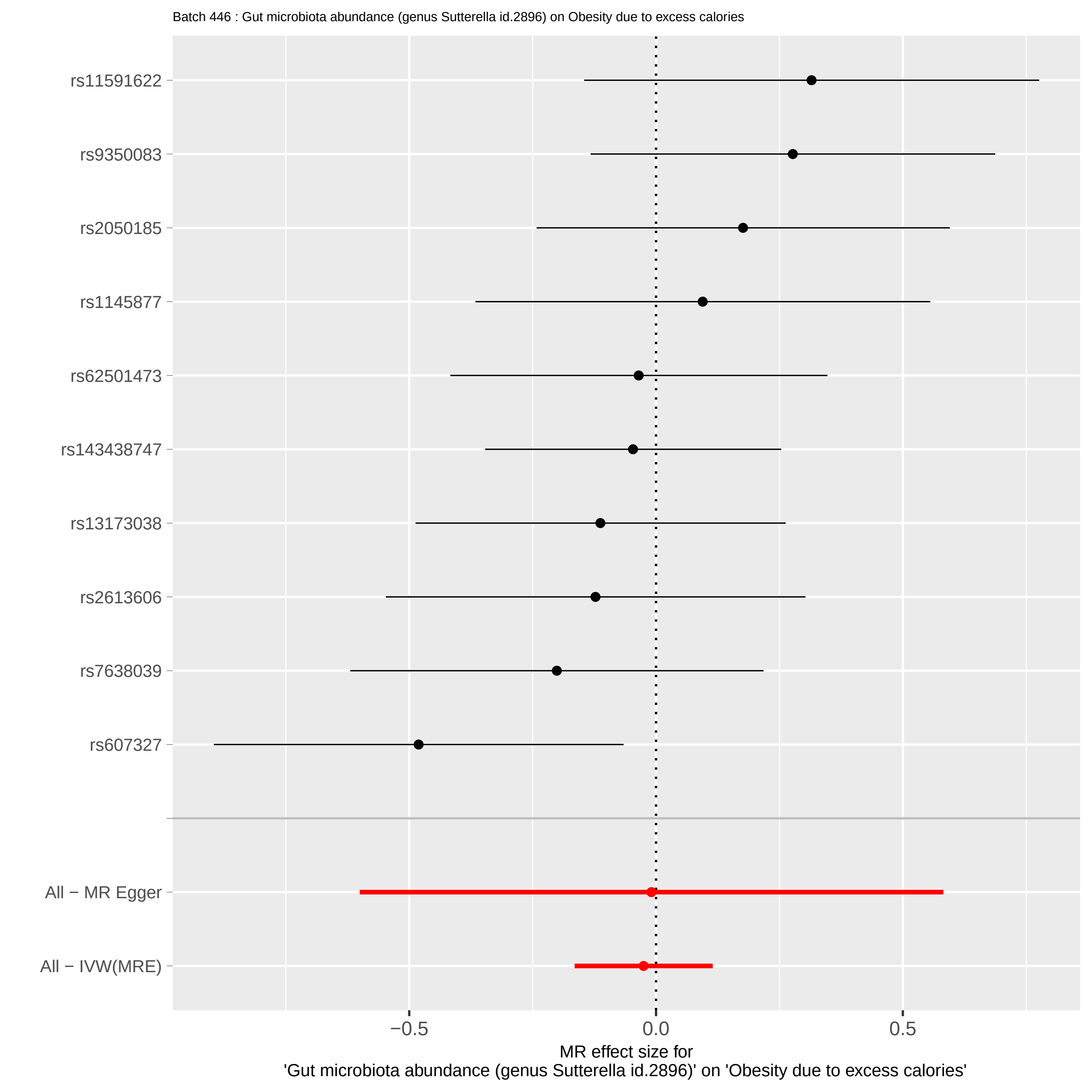

Batch 446 : Gut microbiota abundance (genus Sutterella id.2896) on Obesity due to excess calories
rs11591622
rs9350083
rs2050185
rs1145877
rs62501473
rs143438747
rs13173038
rs2613606
rs7638039
rs607327
All − MR Egger
All − IVW(MRE)
−0.5
0.0
0.5
MR effect size for
'Gut microbiota abundance (genus Sutterella id.2896)' on 'Obesity due to excess calories'

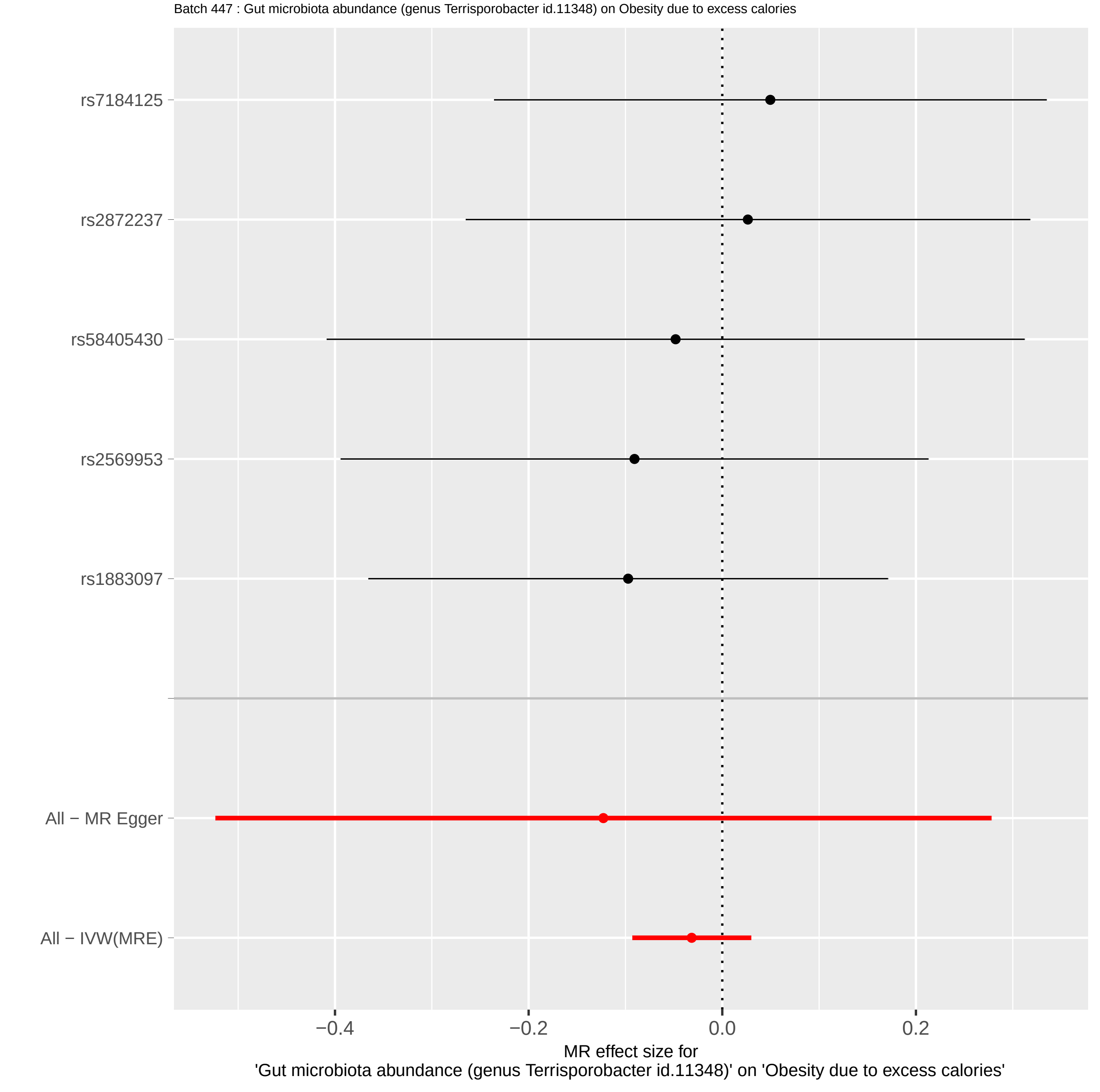
Batch 447 : Gut microbiota abundance (genus Terrisporobacter id.11348) on Obesity due to excess calories
rs7184125
rs2872237
rs58405430
rs2569953
rs1883097
All − MR Egger
All − IVW(MRE)
−0.4
−0.2
0.0
0.2
MR effect size for
'Gut microbiota abundance (genus Terrisporobacter id.11348)' on 'Obesity due to excess calories'

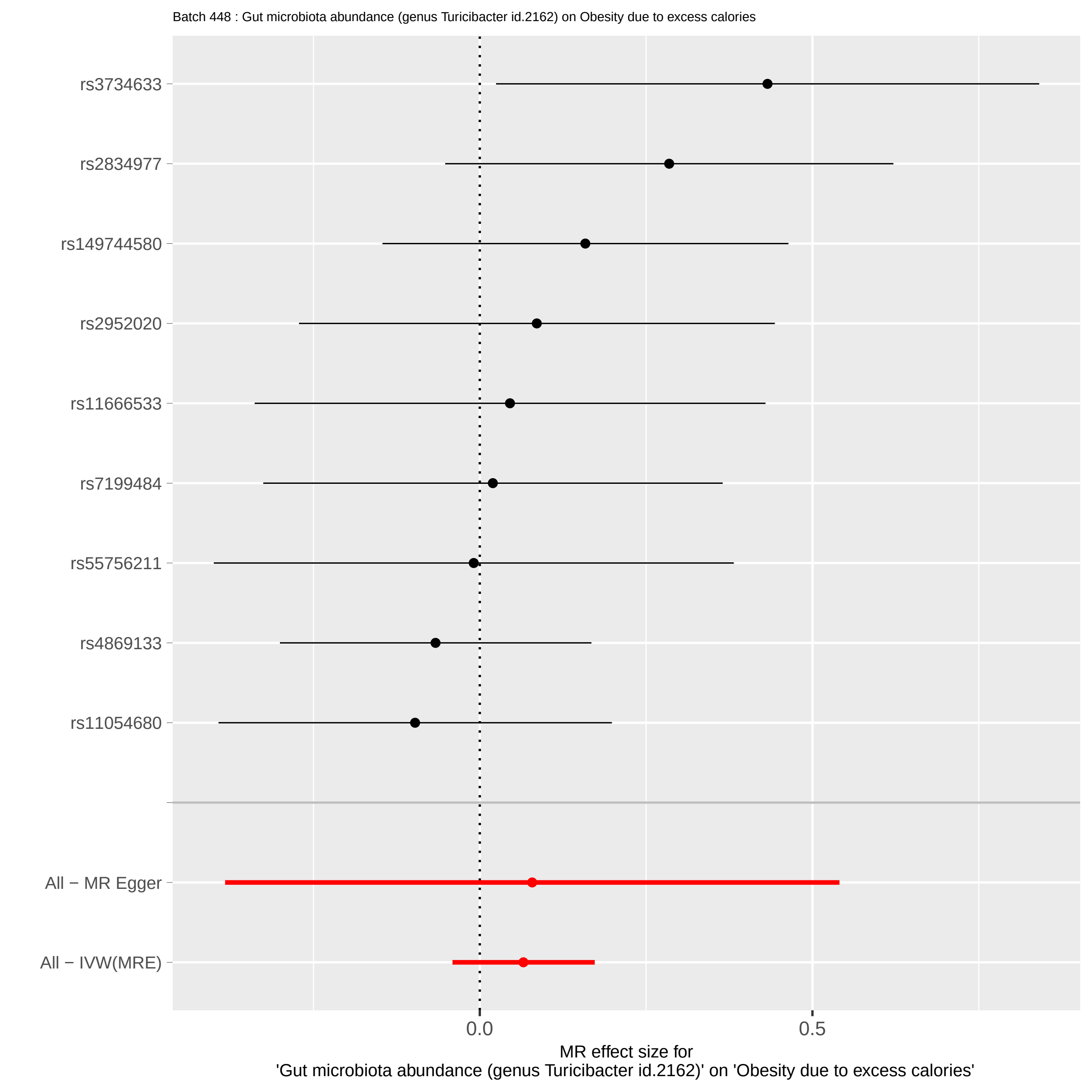
Batch 448 : Gut microbiota abundance (genus Turicibacter id.2162) on Obesity due to excess calories
rs3734633
rs2834977
rs149744580
rs2952020
rs11666533
rs7199484
rs55756211
rs4869133
rs11054680
All − MR Egger
All − IVW(MRE)
0.0
0.5
MR effect size for
'Gut microbiota abundance (genus Turicibacter id.2162)' on 'Obesity due to excess calories'

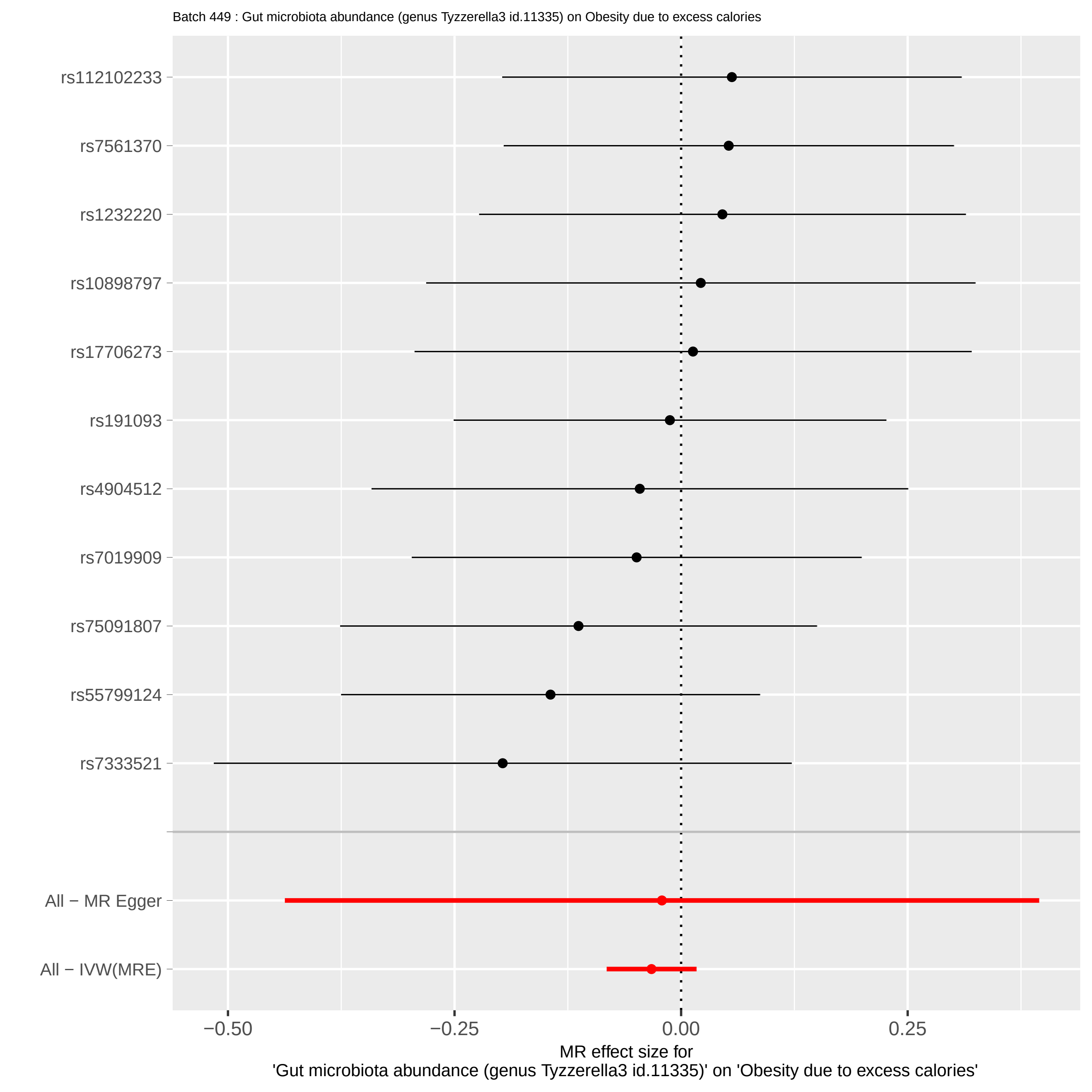
Batch 449 : Gut microbiota abundance (genus Tyzzerella3 id.11335) on Obesity due to excess calories
rs112102233
rs7561370
rs1232220
rs10898797
rs17706273
rs191093
rs4904512
rs7019909
rs75091807
rs55799124
rs7333521
All − MR Egger
All − IVW(MRE)
−0.50
−0.25
0.00
0.25
MR effect size for
'Gut microbiota abundance (genus Tyzzerella3 id.11335)' on 'Obesity due to excess calories'

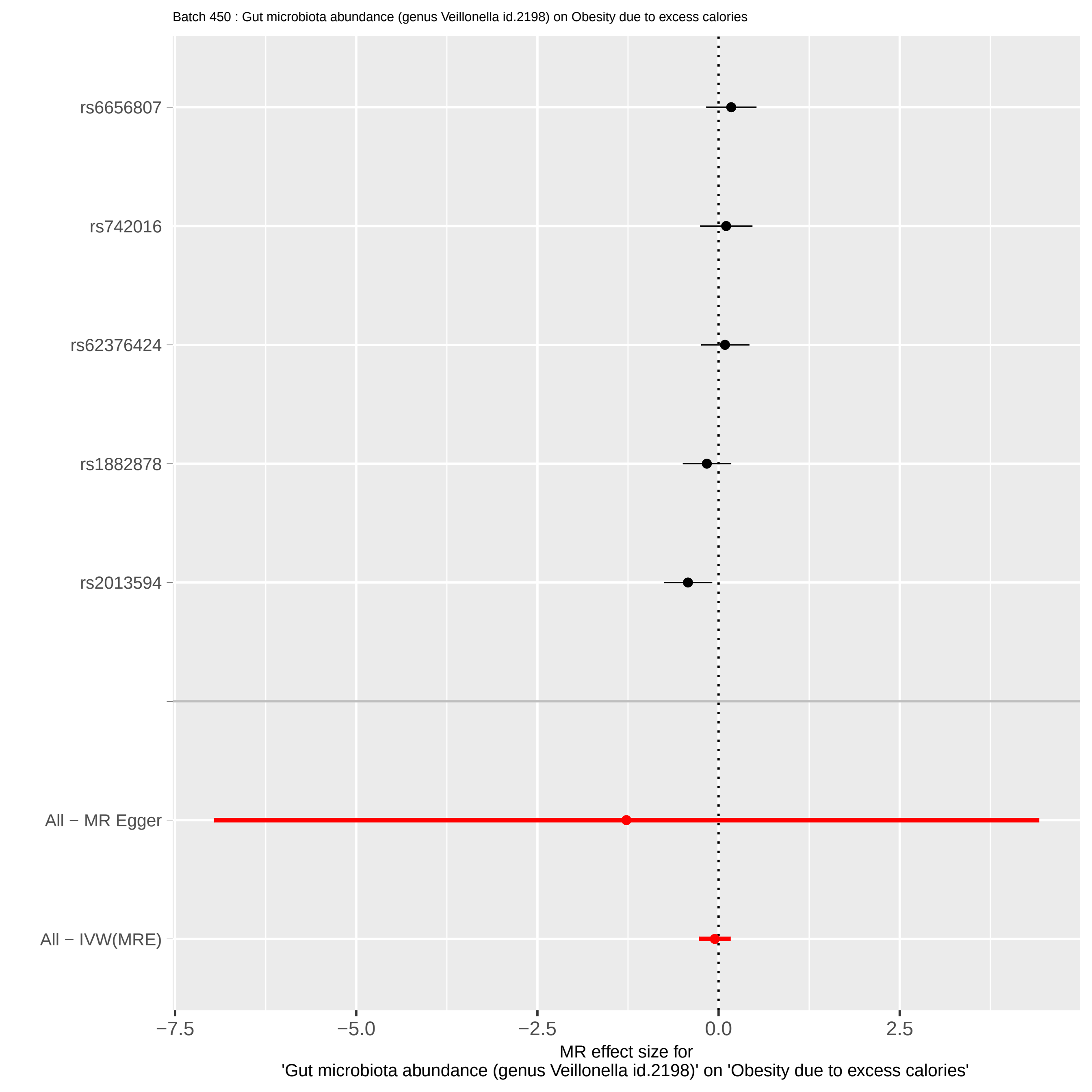

Batch 450 : Gut microbiota abundance (genus Veillonella id.2198) on Obesity due to excess calories
rs6656807
rs742016
rs62376424
rs1882878
rs2013594
All − MR Egger
All − IVW(MRE)
−7.5
−5.0
−2.5
0.0
2.5
MR effect size for
'Gut microbiota abundance (genus Veillonella id.2198)' on 'Obesity due to excess calories'

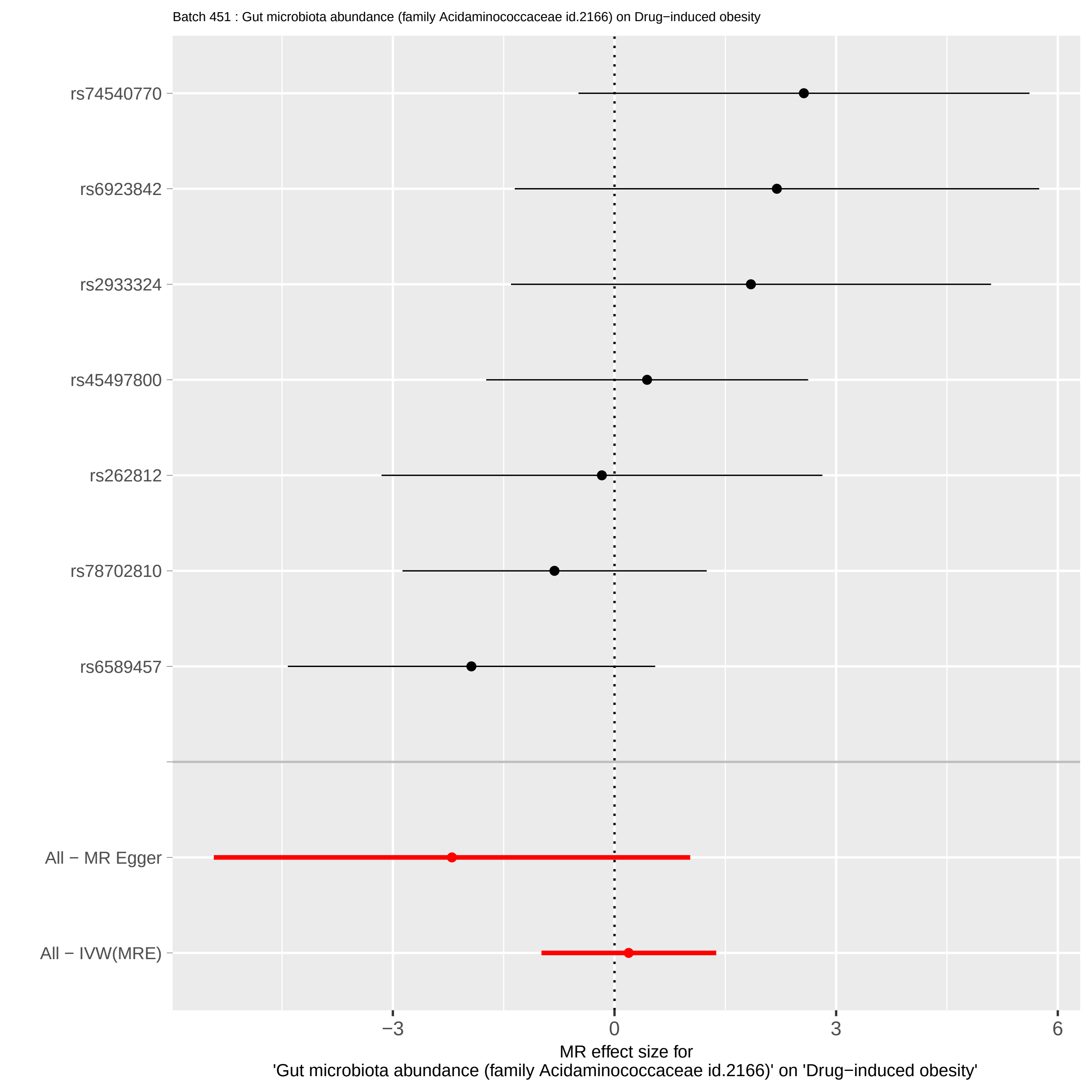
Batch 451 : Gut microbiota abundance (family Acidaminococcaceae id.2166) on Drug−induced obesity
rs74540770
rs6923842
rs2933324
rs45497800
rs262812
rs78702810
rs6589457
All − MR Egger
All − IVW(MRE)
−3
0
3
6
MR effect size for
'Gut microbiota abundance (family Acidaminococcaceae id.2166)' on 'Drug−induced obesity'

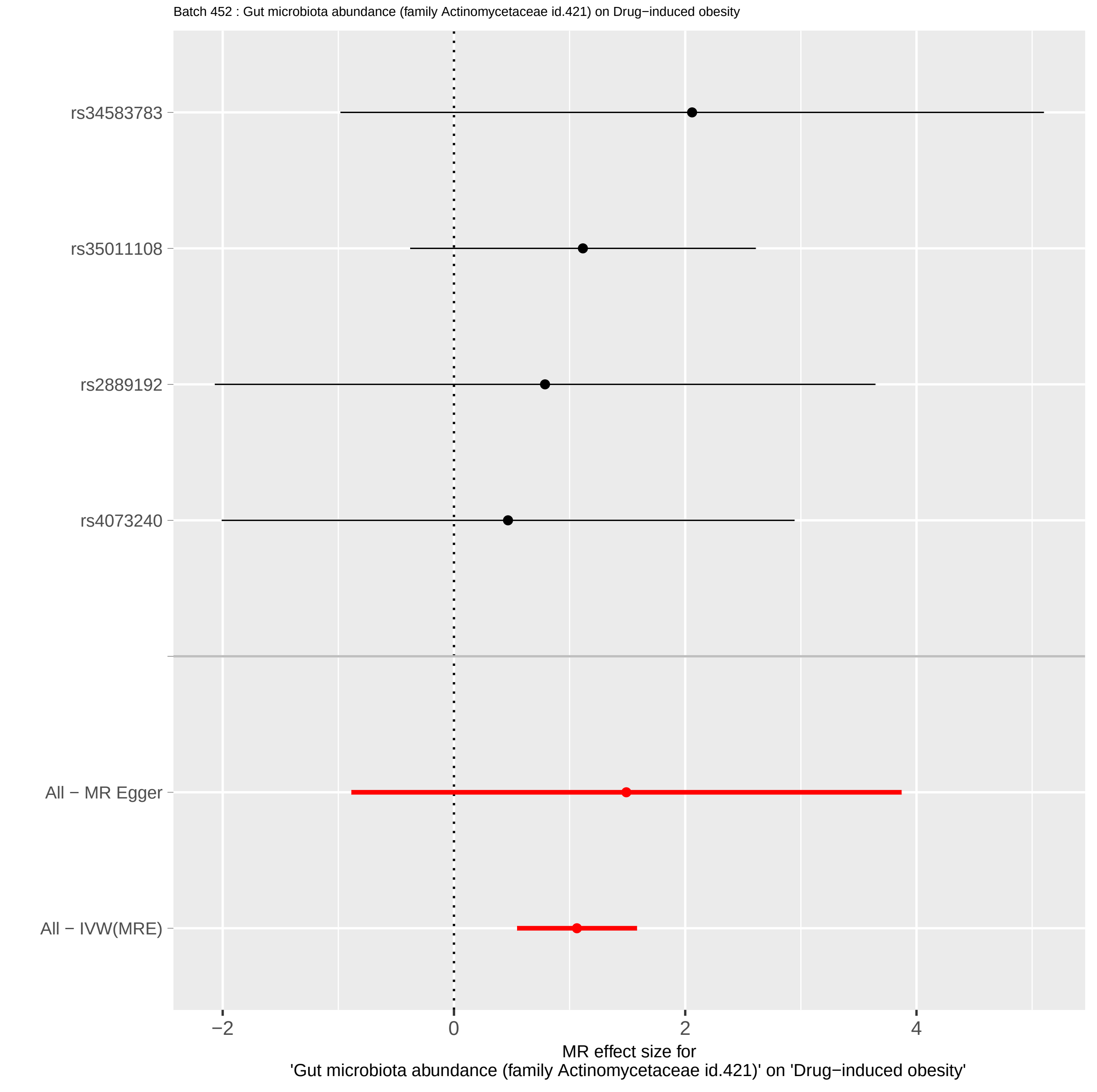

Batch 452 : Gut microbiota abundance (family Actinomycetaceae id.421) on Drug−induced obesity
rs34583783
rs35011108
rs2889192
rs4073240
All − MR Egger
All − IVW(MRE)
−2
0
2
4
MR effect size for
'Gut microbiota abundance (family Actinomycetaceae id.421)' on 'Drug−induced obesity'

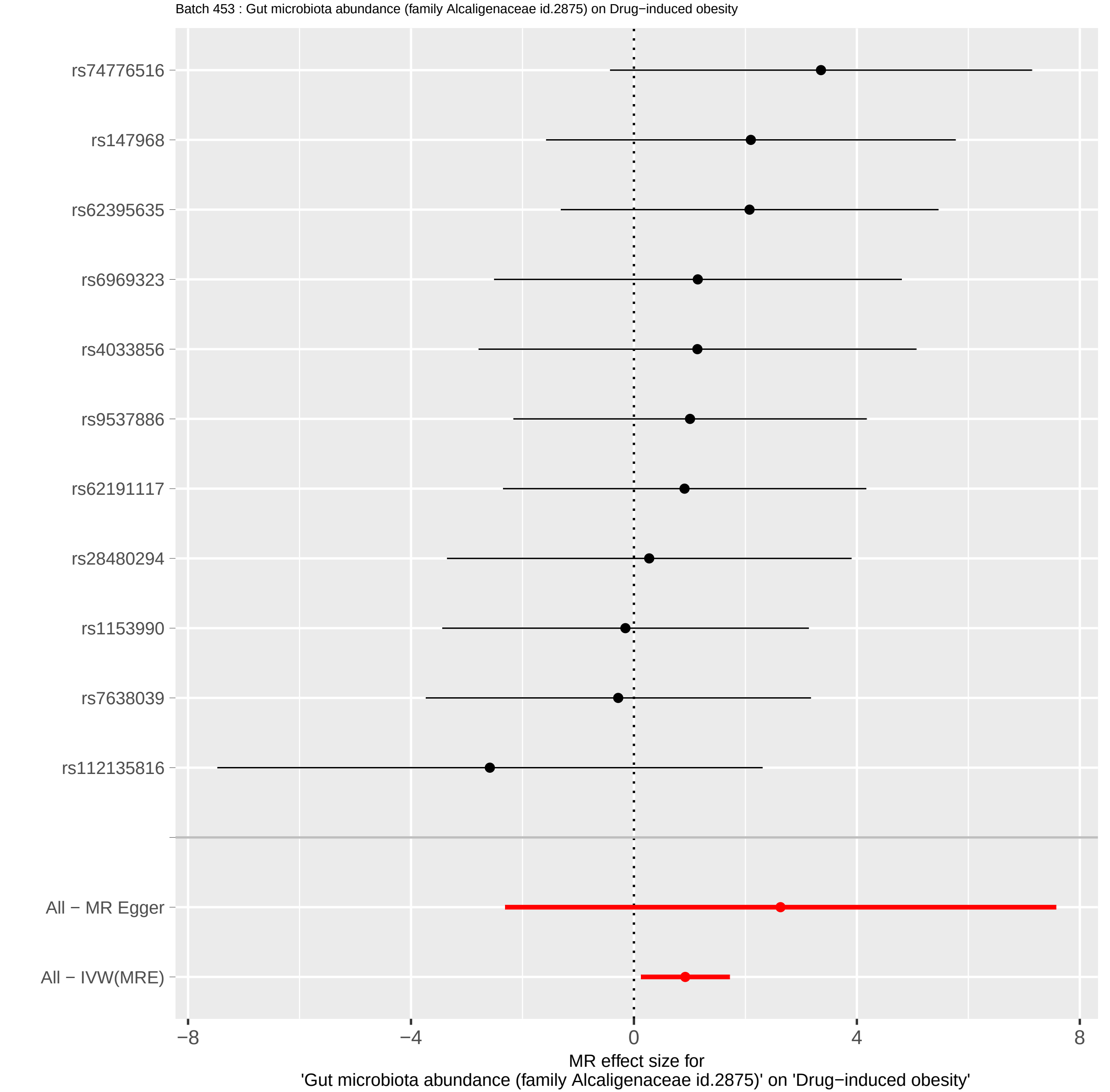

Batch 453 : Gut microbiota abundance (family Alcaligenaceae id.2875) on Drug−induced obesity
rs74776516
rs147968
rs62395635
rs6969323
rs4033856
rs9537886
rs62191117
rs28480294
rs1153990
rs7638039
rs112135816
All − MR Egger
All − IVW(MRE)
−8
−4
0
4
8
MR effect size for
'Gut microbiota abundance (family Alcaligenaceae id.2875)' on 'Drug−induced obesity'

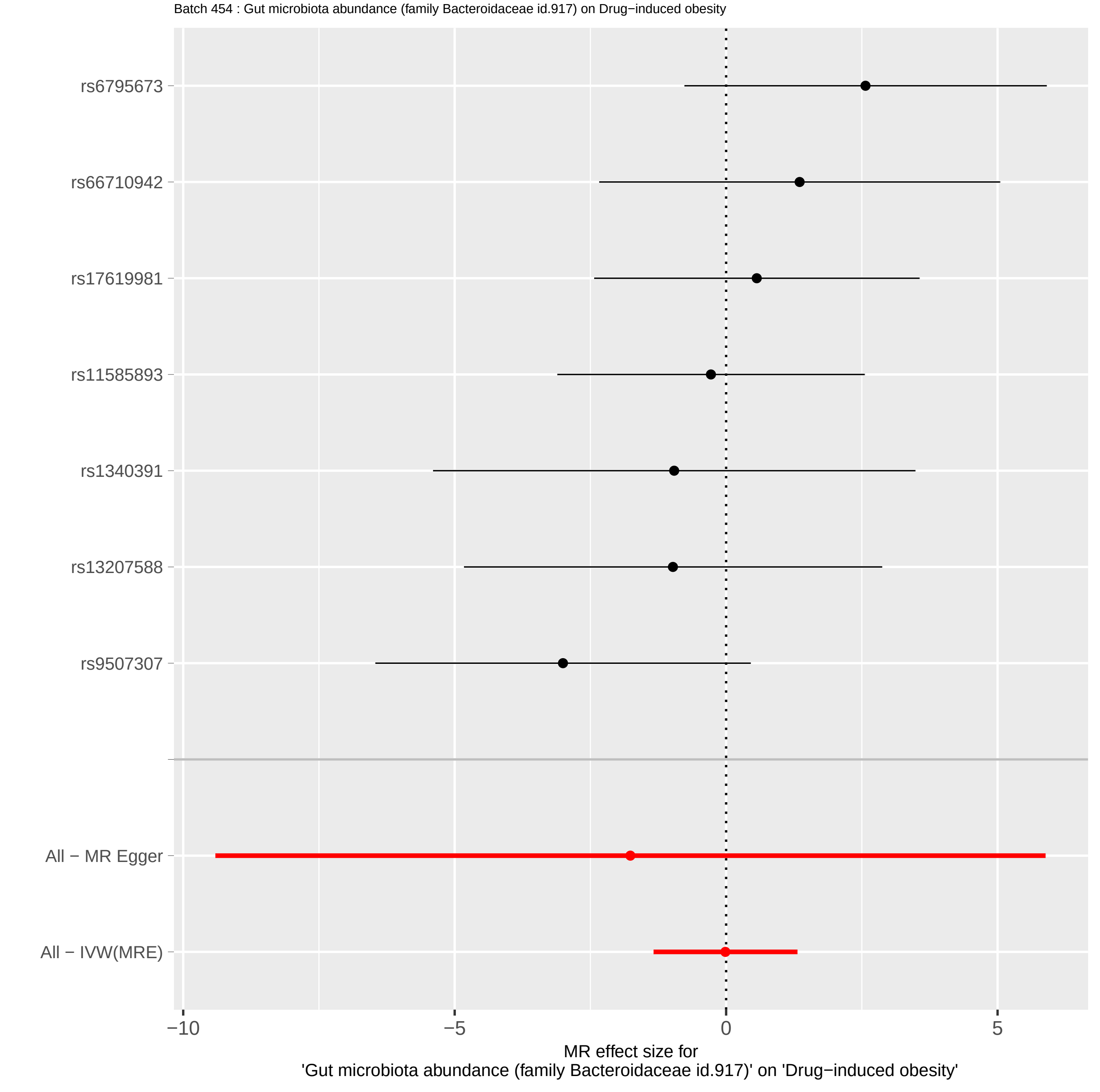

Batch 454 : Gut microbiota abundance (family Bacteroidaceae id.917) on Drug−induced obesity
rs6795673
rs66710942
rs17619981
rs11585893
rs1340391
rs13207588
rs9507307
All − MR Egger
All − IVW(MRE)
−10
−5
0
5
MR effect size for
'Gut microbiota abundance (family Bacteroidaceae id.917)' on 'Drug−induced obesity'

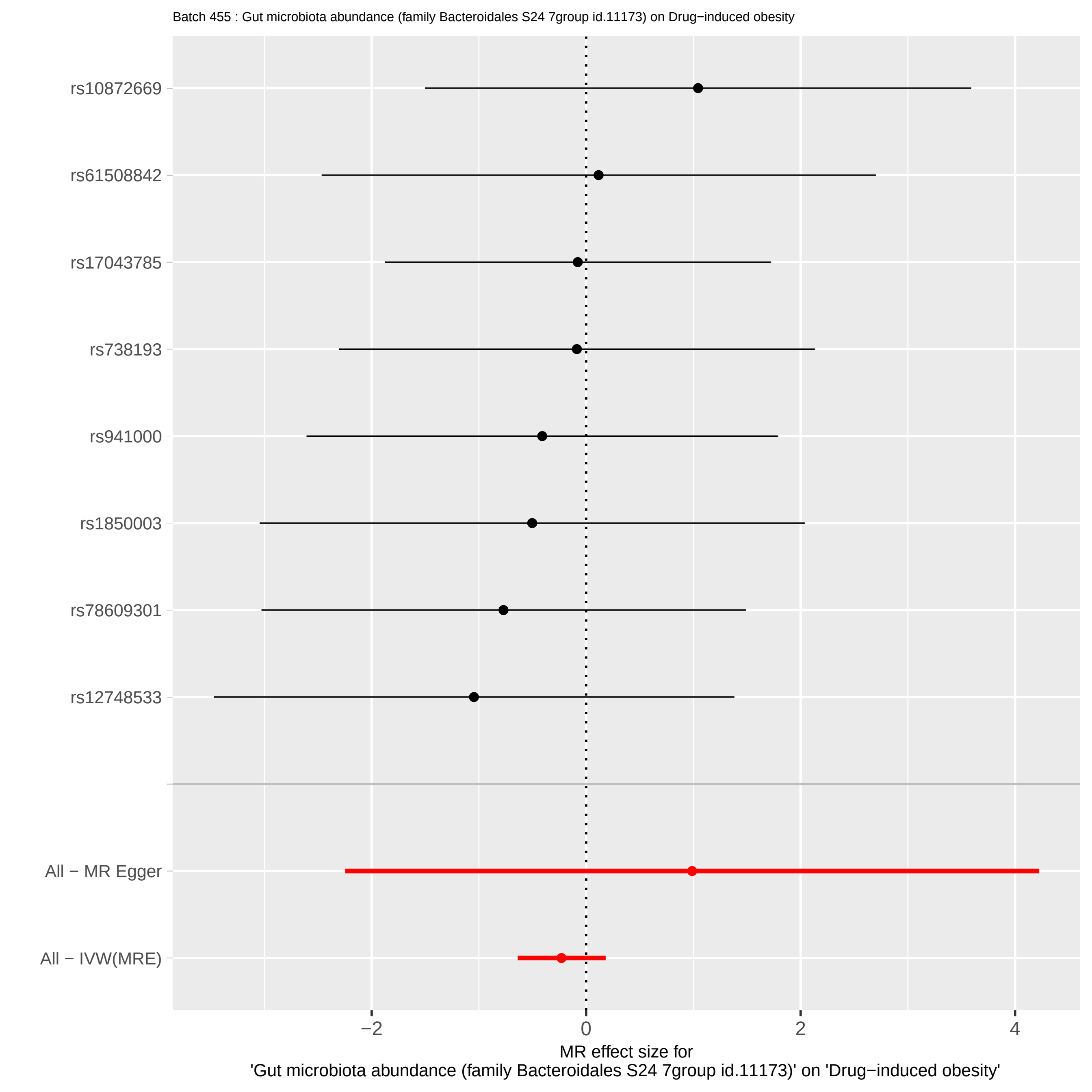

Batch 455 : Gut microbiota abundance (family Bacteroidales S24 7group id.11173) on Drug−induced obesity
rs10872669
rs61508842
rs17043785
rs738193
rs941000
rs1850003
rs78609301
rs12748533
All − MR Egger
All − IVW(MRE)
−2
0
2
4
MR effect size for
'Gut microbiota abundance (family Bacteroidales S24 7group id.11173)' on 'Drug−induced obesity'

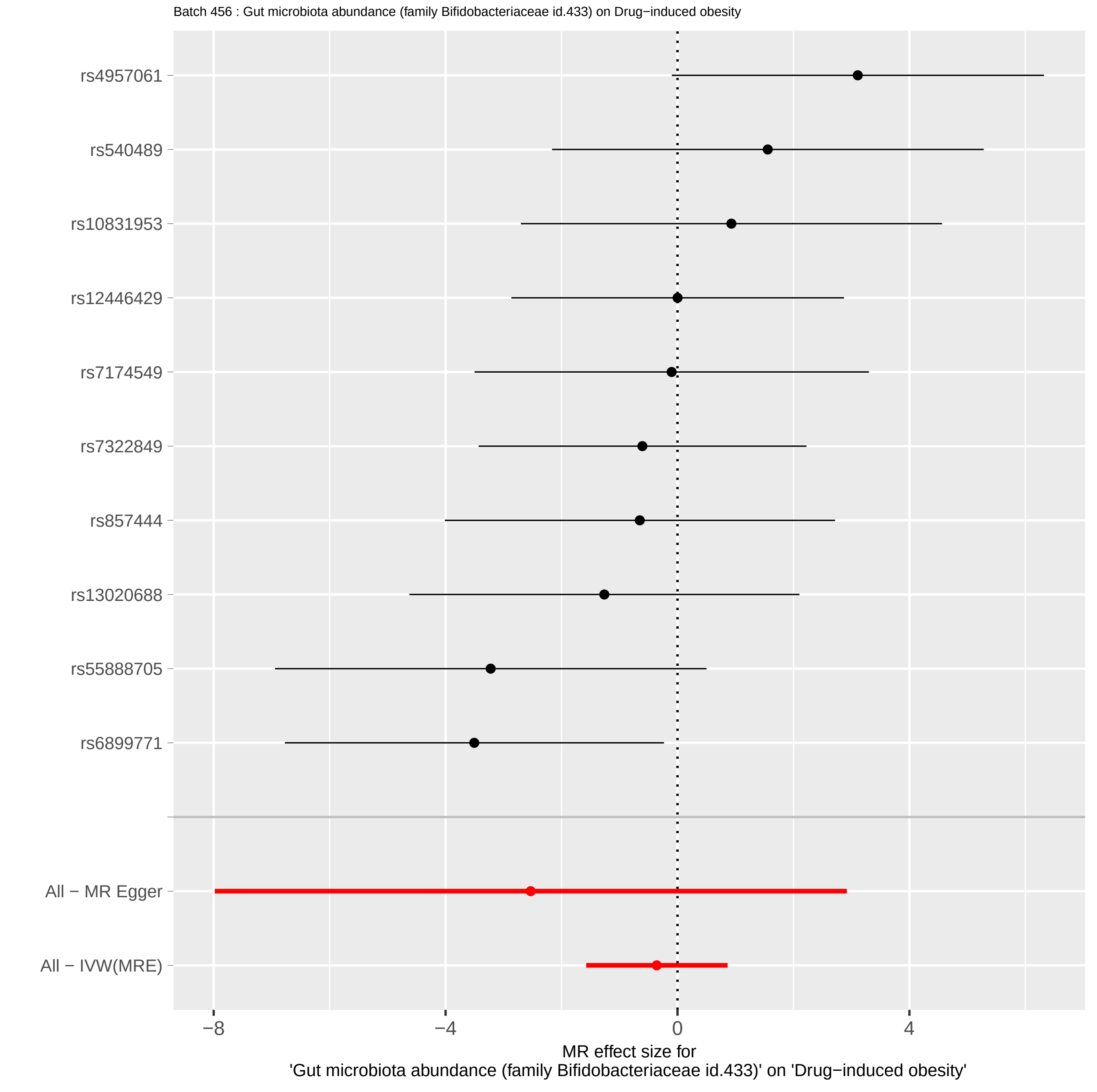

Batch 456 : Gut microbiota abundance (family Bifidobacteriaceae id.433) on Drug−induced obesity
rs4957061
rs540489
rs10831953
rs12446429
rs7174549
rs7322849
rs857444
rs13020688
rs55888705
rs6899771
All − MR Egger
All − IVW(MRE)
−8
−4
0
4
MR effect size for
'Gut microbiota abundance (family Bifidobacteriaceae id.433)' on 'Drug−induced obesity'

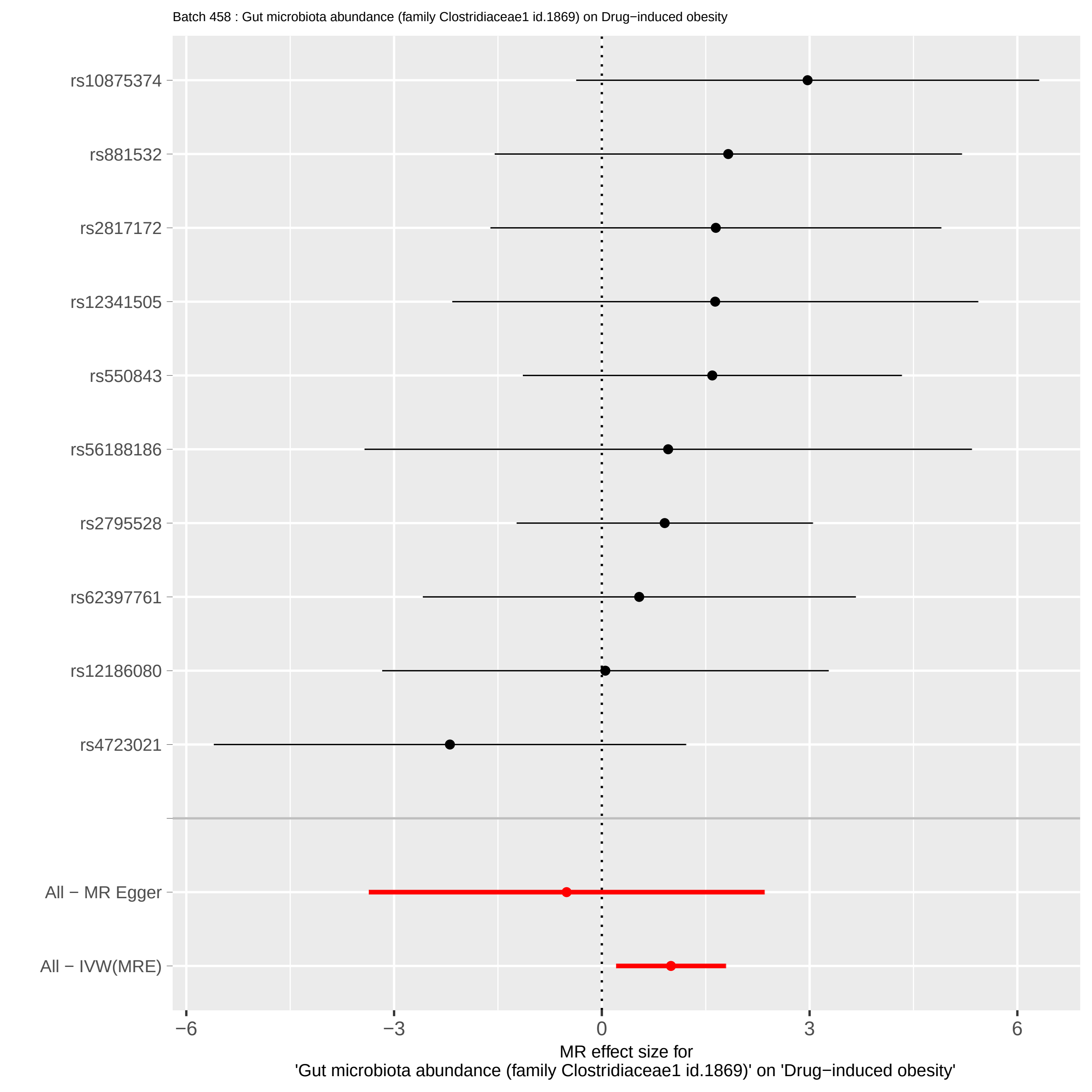

Batch 458 : Gut microbiota abundance (family Clostridiaceae1 id.1869) on Drug−induced obesity
rs10875374
rs881532
rs2817172
rs12341505
rs550843
rs56188186
rs2795528
rs62397761
rs12186080
rs4723021
All − MR Egger
All − IVW(MRE)
−6
−3
0
3
6
MR effect size for
'Gut microbiota abundance (family Clostridiaceae1 id.1869)' on 'Drug−induced obesity'

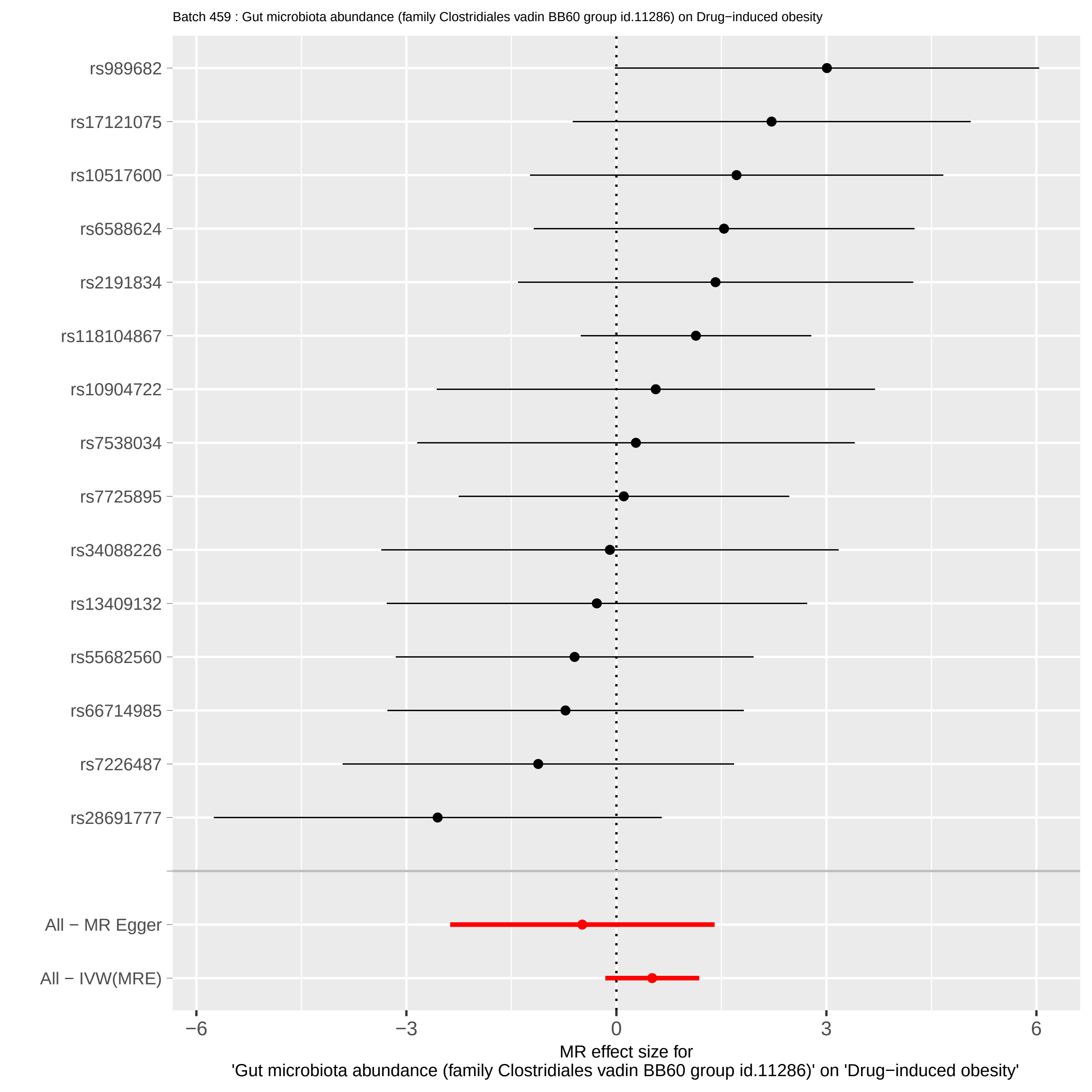

Batch 459 : Gut microbiota abundance (family Clostridiales vadin BB60 group id.11286) on Drug−induced obesity
rs989682
rs17121075
rs10517600
rs6588624
rs2191834
rs118104867
rs10904722
rs7538034
rs7725895
rs34088226
rs13409132
rs55682560
rs66714985
rs7226487
rs28691777
All − MR Egger
All − IVW(MRE)
−6
−3
0
3
6
MR effect size for
'Gut microbiota abundance (family Clostridiales vadin BB60 group id.11286)' on 'Drug−induced obesity'

Batch 460 : Gut microbiota abundance (family Coriobacteriaceae id.811) on Drug−induced obesity

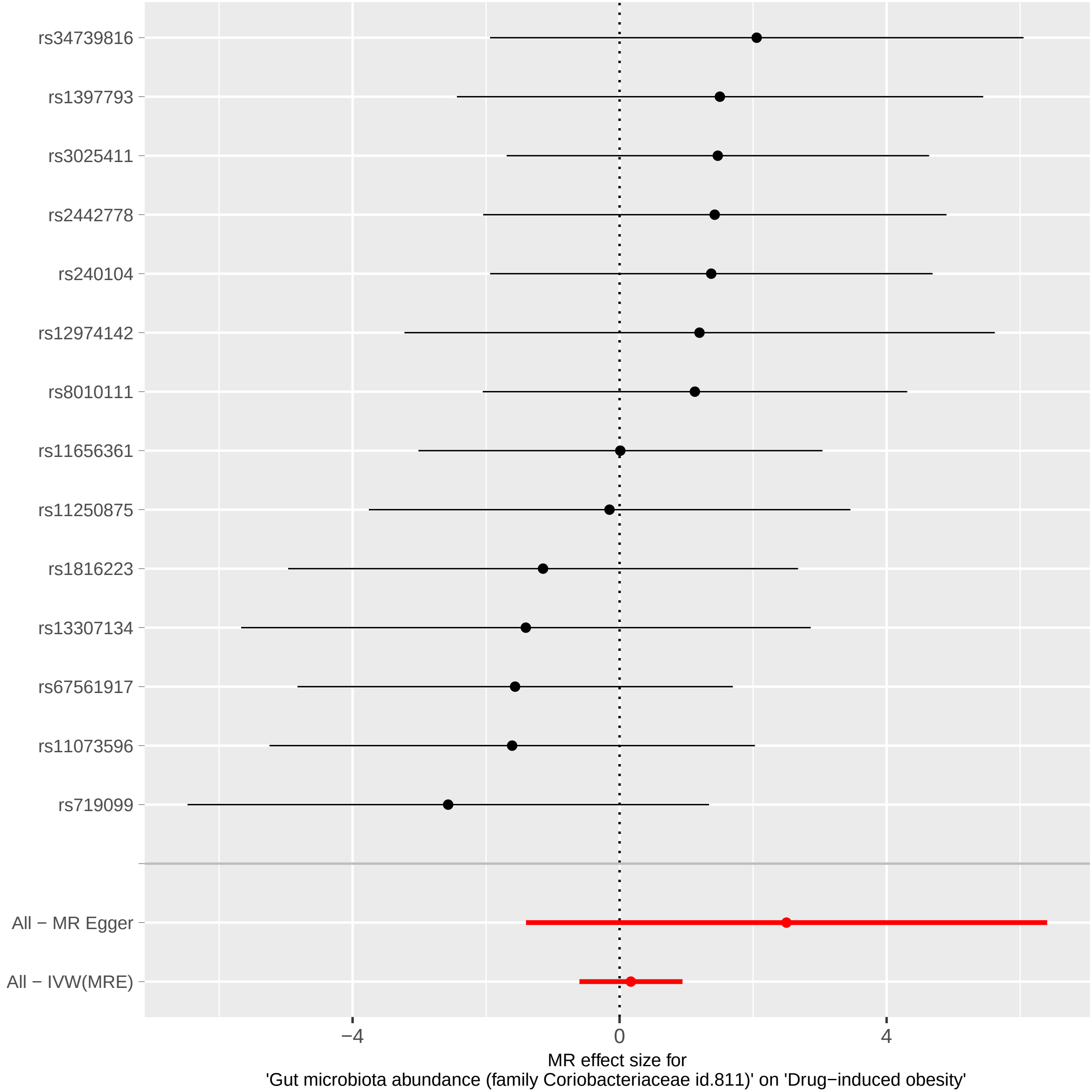

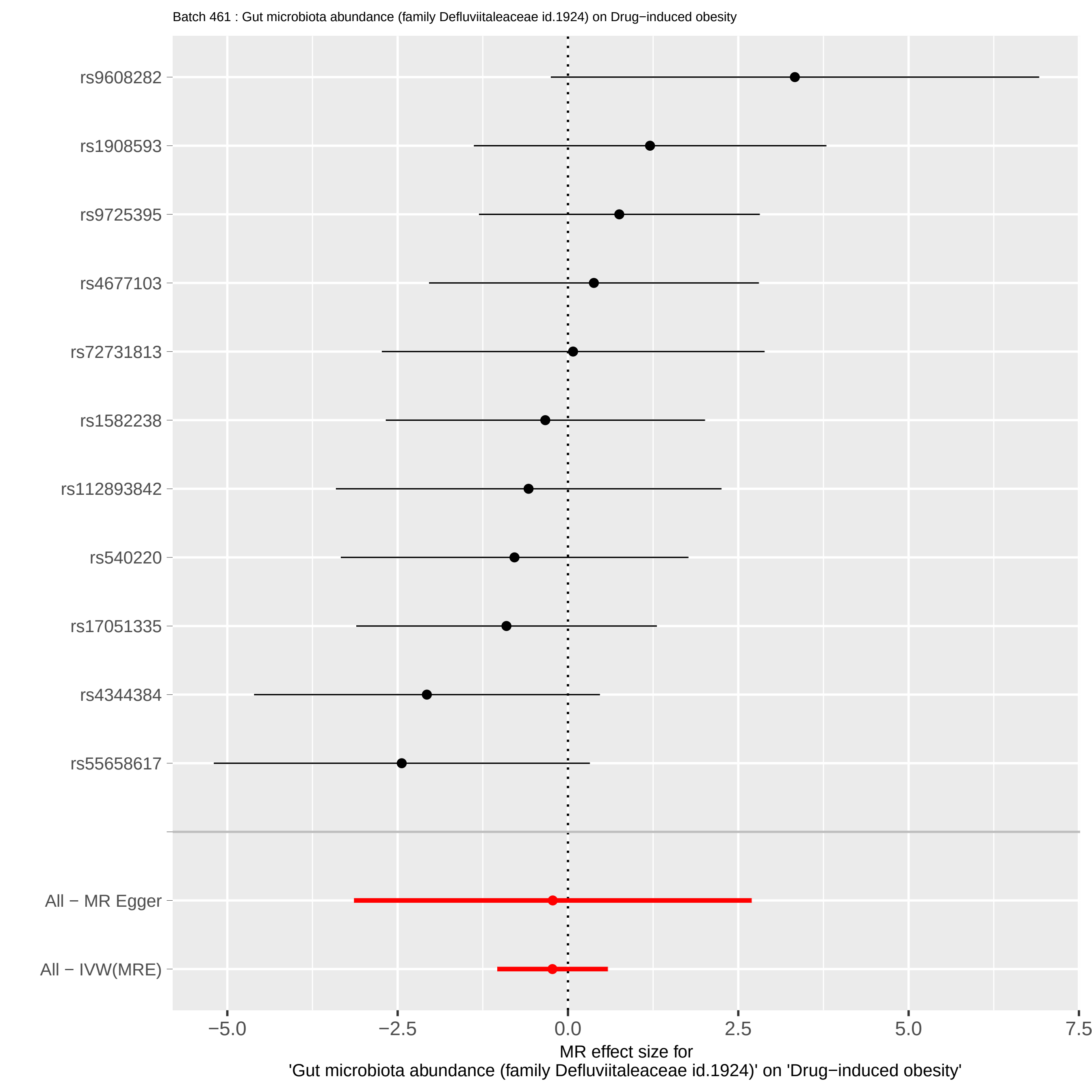

Batch 461 : Gut microbiota abundance (family Defluviitaleaceae id.1924) on Drug−induced obesity
rs9608282
rs1908593
rs9725395
rs4677103
rs72731813
rs1582238
rs112893842
rs540220
rs17051335
rs4344384
rs55658617
All − MR Egger
All − IVW(MRE)
−5.0
−2.5
0.0
2.5
5.0
7.5
MR effect size for
'Gut microbiota abundance (family Defluviitaleaceae id.1924)' on 'Drug−induced obesity'

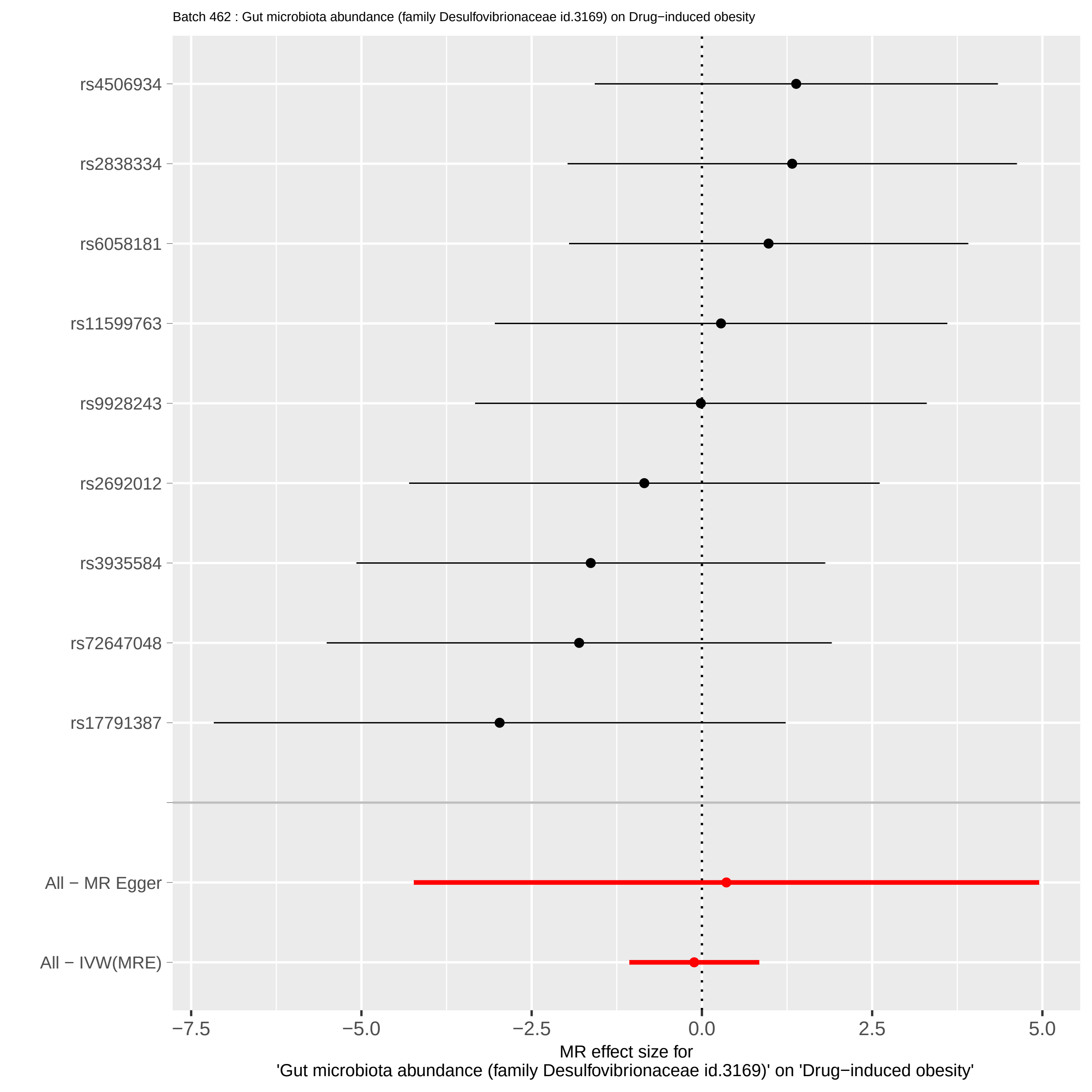

Batch 462 : Gut microbiota abundance (family Desulfovibrionaceae id.3169) on Drug−induced obesity
rs4506934
rs2838334
rs6058181
rs11599763
rs9928243
rs2692012
rs3935584
rs72647048
rs17791387
All − MR Egger
All − IVW(MRE)
−7.5
−5.0
−2.5
0.0
2.5
5.0
MR effect size for
'Gut microbiota abundance (family Desulfovibrionaceae id.3169)' on 'Drug−induced obesity'

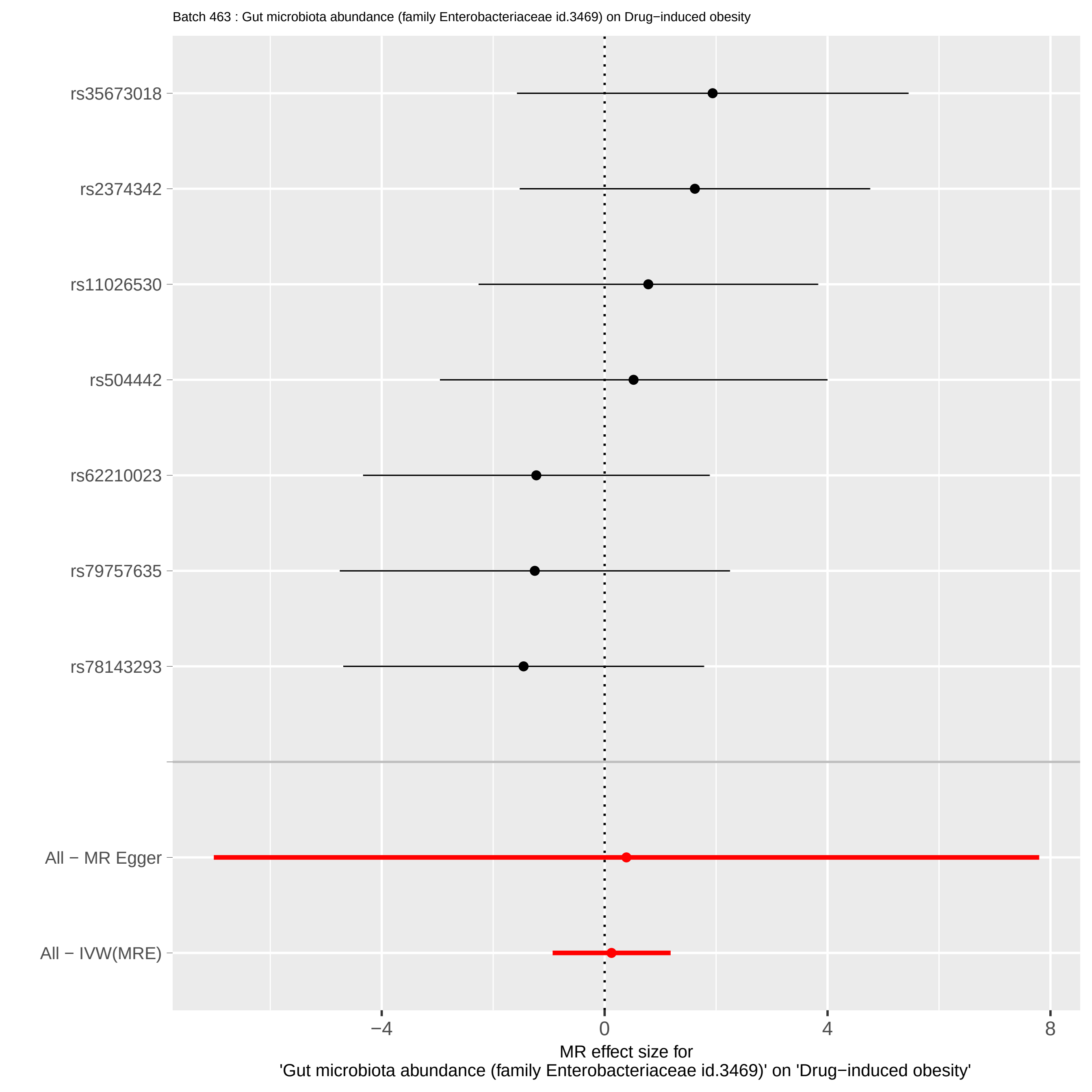

Batch 463 : Gut microbiota abundance (family Enterobacteriaceae id.3469) on Drug−induced obesity
rs35673018
rs2374342
rs11026530
rs504442
rs62210023
rs79757635
rs78143293
All − MR Egger
All − IVW(MRE)
−4
0
4
8
MR effect size for
'Gut microbiota abundance (family Enterobacteriaceae id.3469)' on 'Drug−induced obesity'

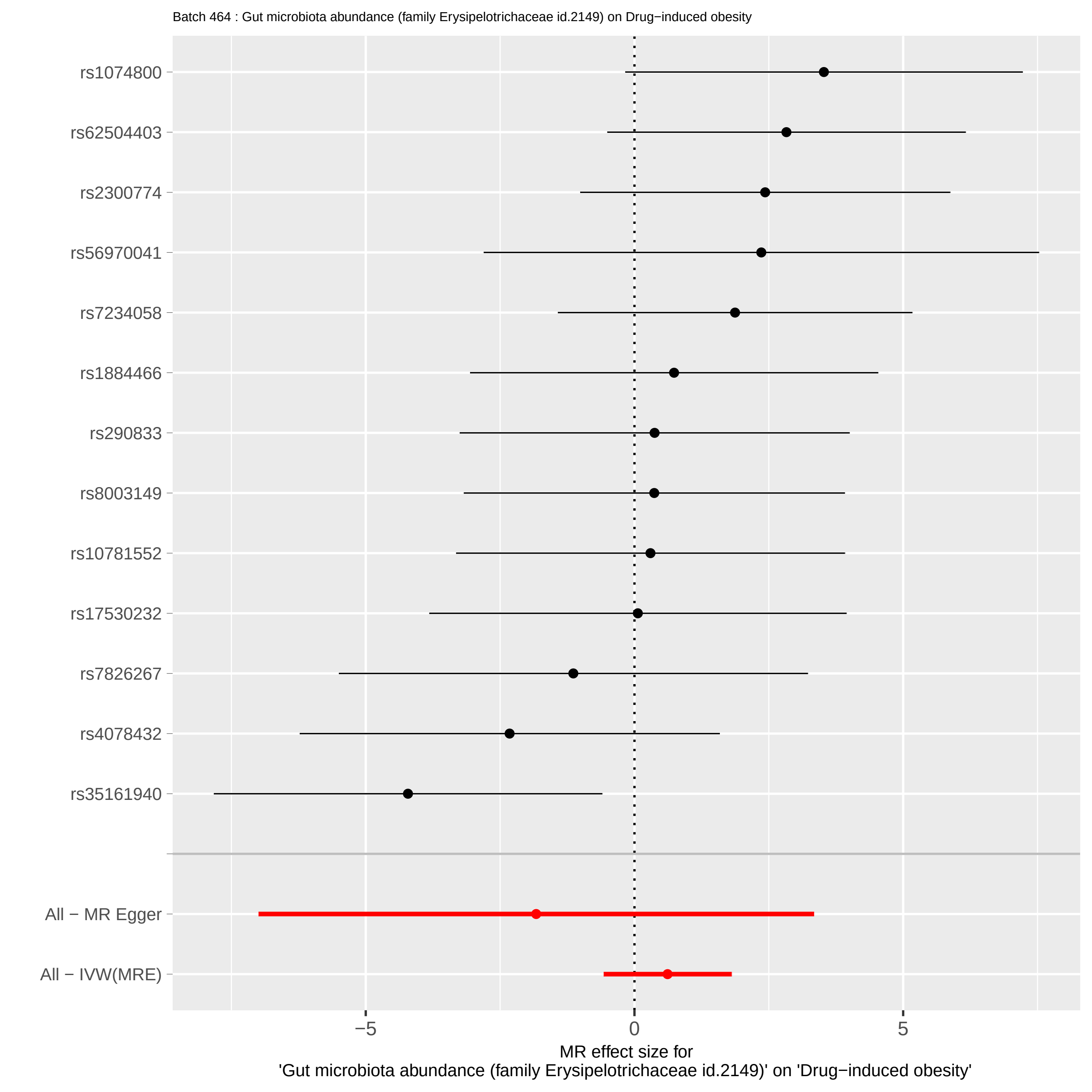

Batch 464 : Gut microbiota abundance (family Erysipelotrichaceae id.2149) on Drug−induced obesity
rs1074800
rs62504403
rs2300774
rs56970041
rs7234058
rs1884466
rs290833
rs8003149
rs10781552
rs17530232
rs7826267
rs4078432
rs35161940
All − MR Egger
All − IVW(MRE)
−5
0
5
MR effect size for
'Gut microbiota abundance (family Erysipelotrichaceae id.2149)' on 'Drug−induced obesity'

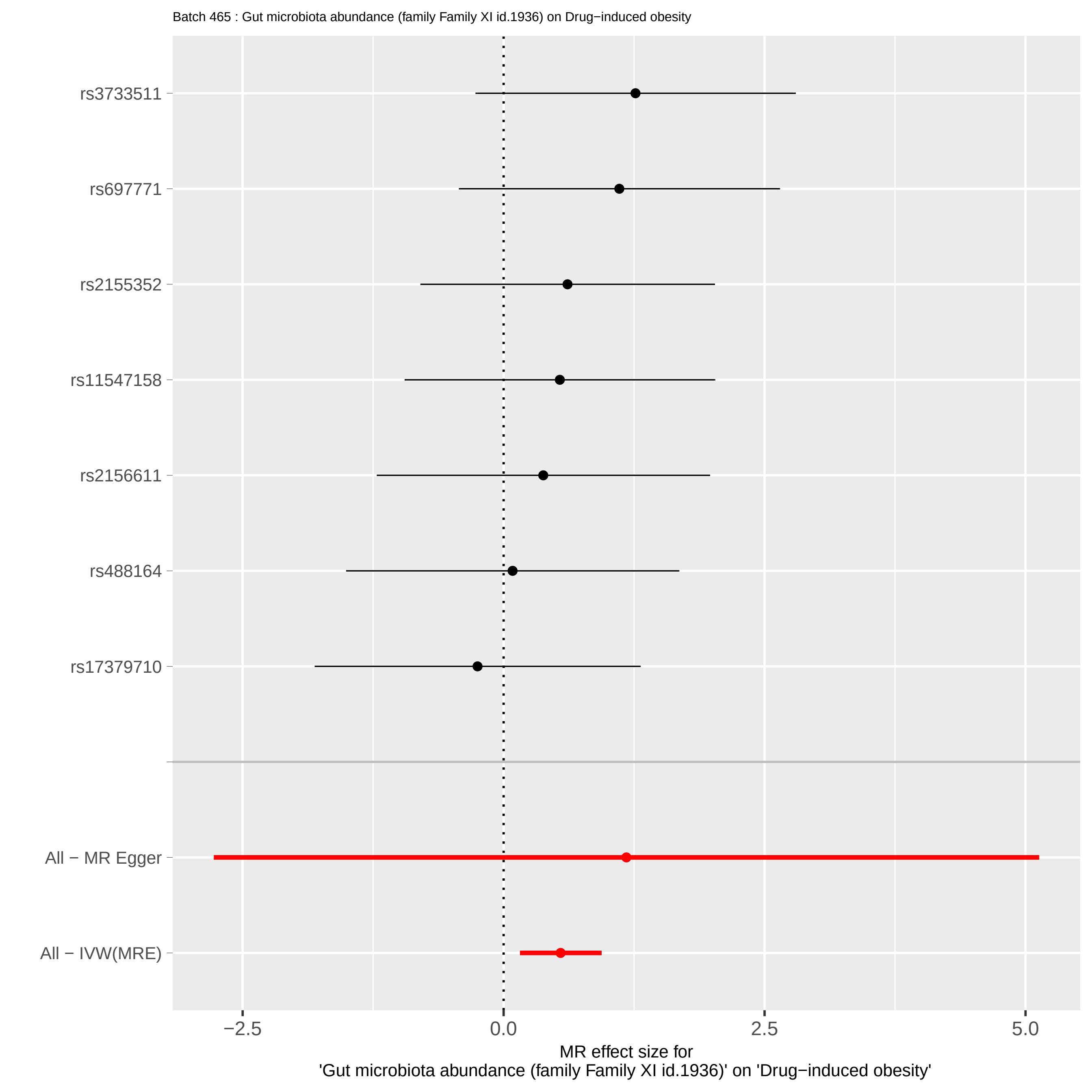

Batch 465 : Gut microbiota abundance (family Family XI id.1936) on Drug−induced obesity
rs3733511
rs697771
rs2155352
rs11547158
rs2156611
rs488164
rs17379710
All − MR Egger
All − IVW(MRE)
−2.5
0.0
2.5
5.0
MR effect size for
'Gut microbiota abundance (family Family XI id.1936)' on 'Drug−induced obesity'

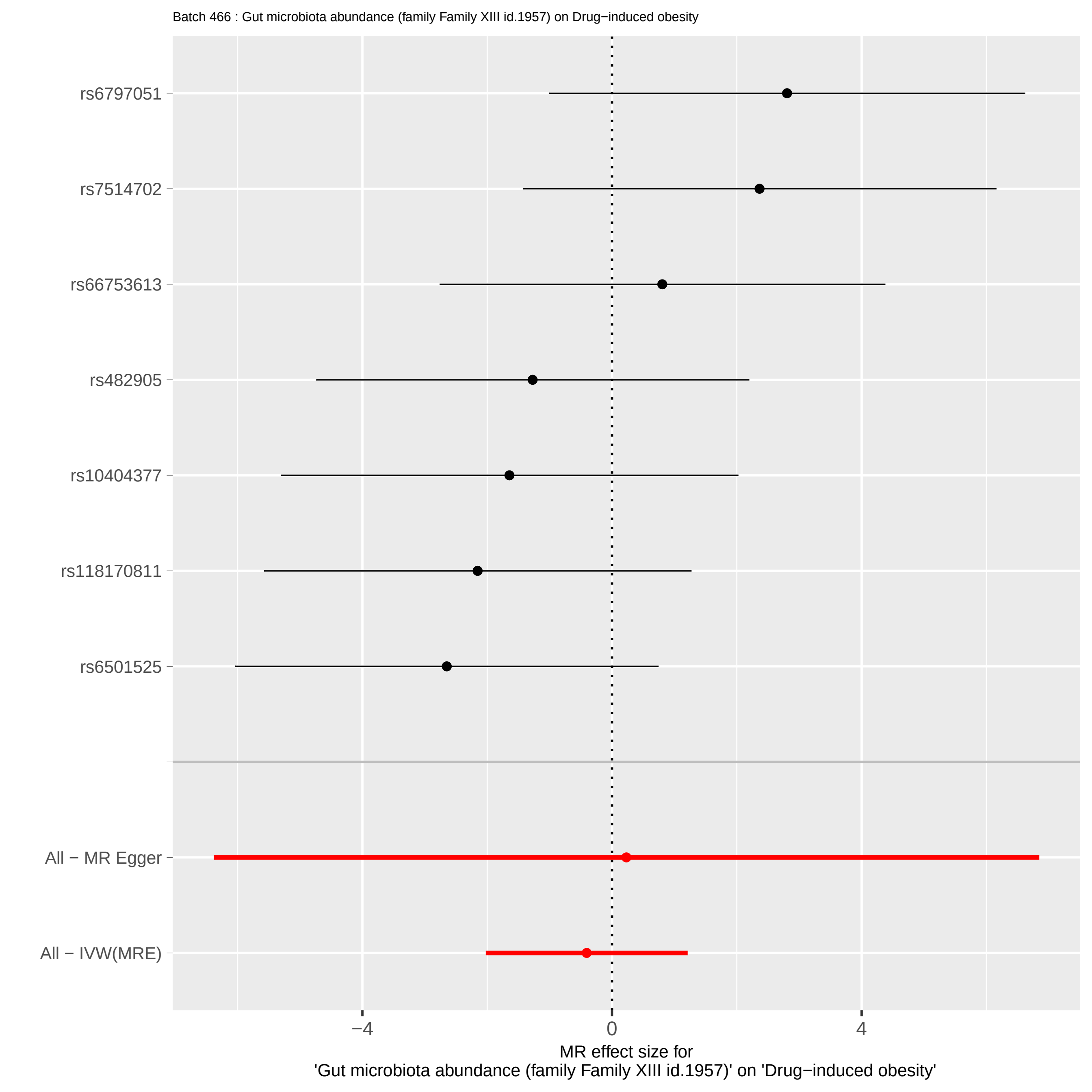
Batch 466 : Gut microbiota abundance (family Family XIII id.1957) on Drug−induced obesity
rs6797051
rs7514702
rs66753613
rs482905
rs10404377
rs118170811
rs6501525
All − MR Egger
All − IVW(MRE)
−4
0
4
MR effect size for
'Gut microbiota abundance (family Family XIII id.1957)' on 'Drug−induced obesity'

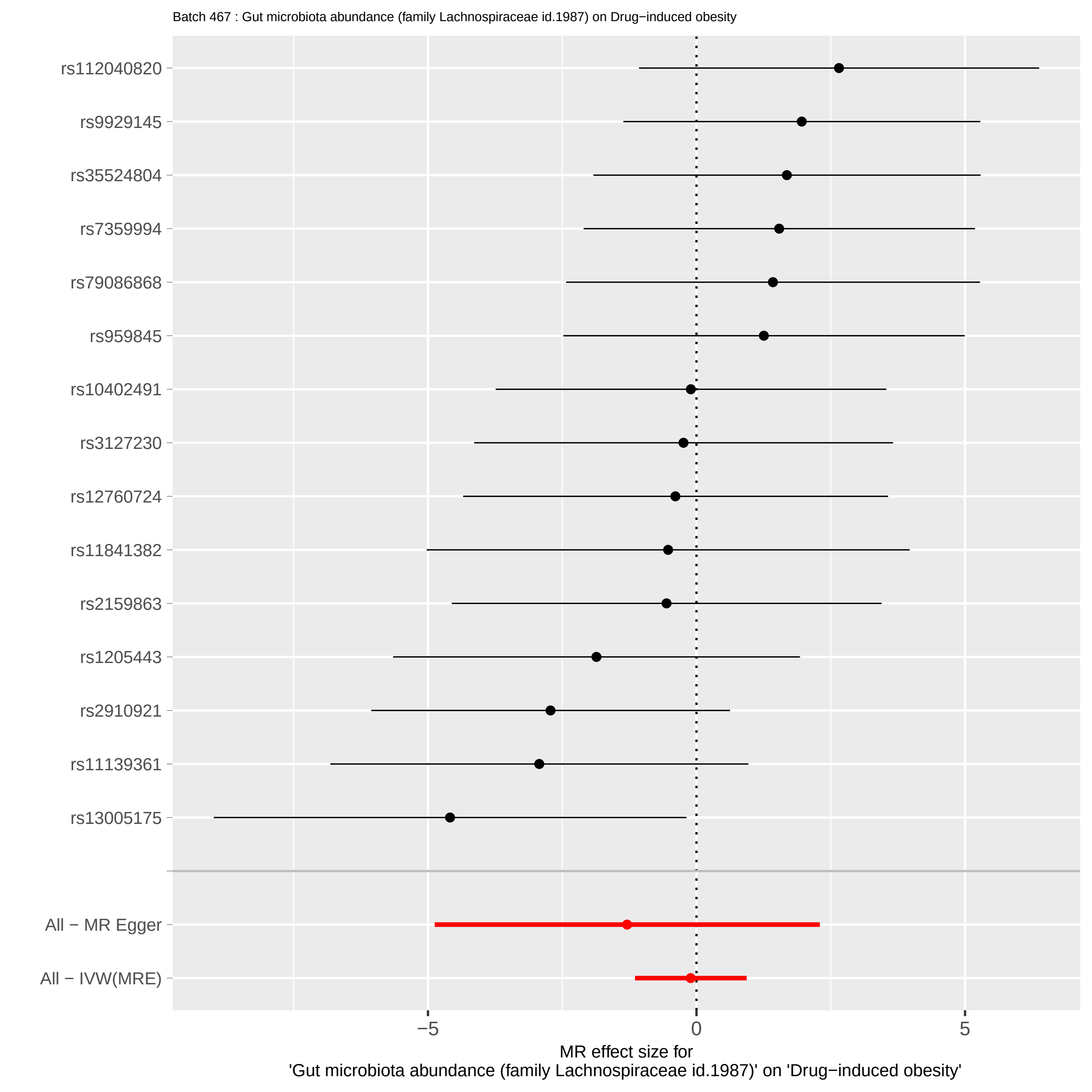
Batch 467 : Gut microbiota abundance (family Lachnospiraceae id.1987) on Drug−induced obesity
rs112040820
rs9929145
rs35524804
rs7359994
rs79086868
rs959845
rs10402491
rs3127230
rs12760724
rs11841382
rs2159863
rs1205443
rs2910921
rs11139361
rs13005175
All − MR Egger
All − IVW(MRE)
−5
0
5
MR effect size for
'Gut microbiota abundance (family Lachnospiraceae id.1987)' on 'Drug−induced obesity'

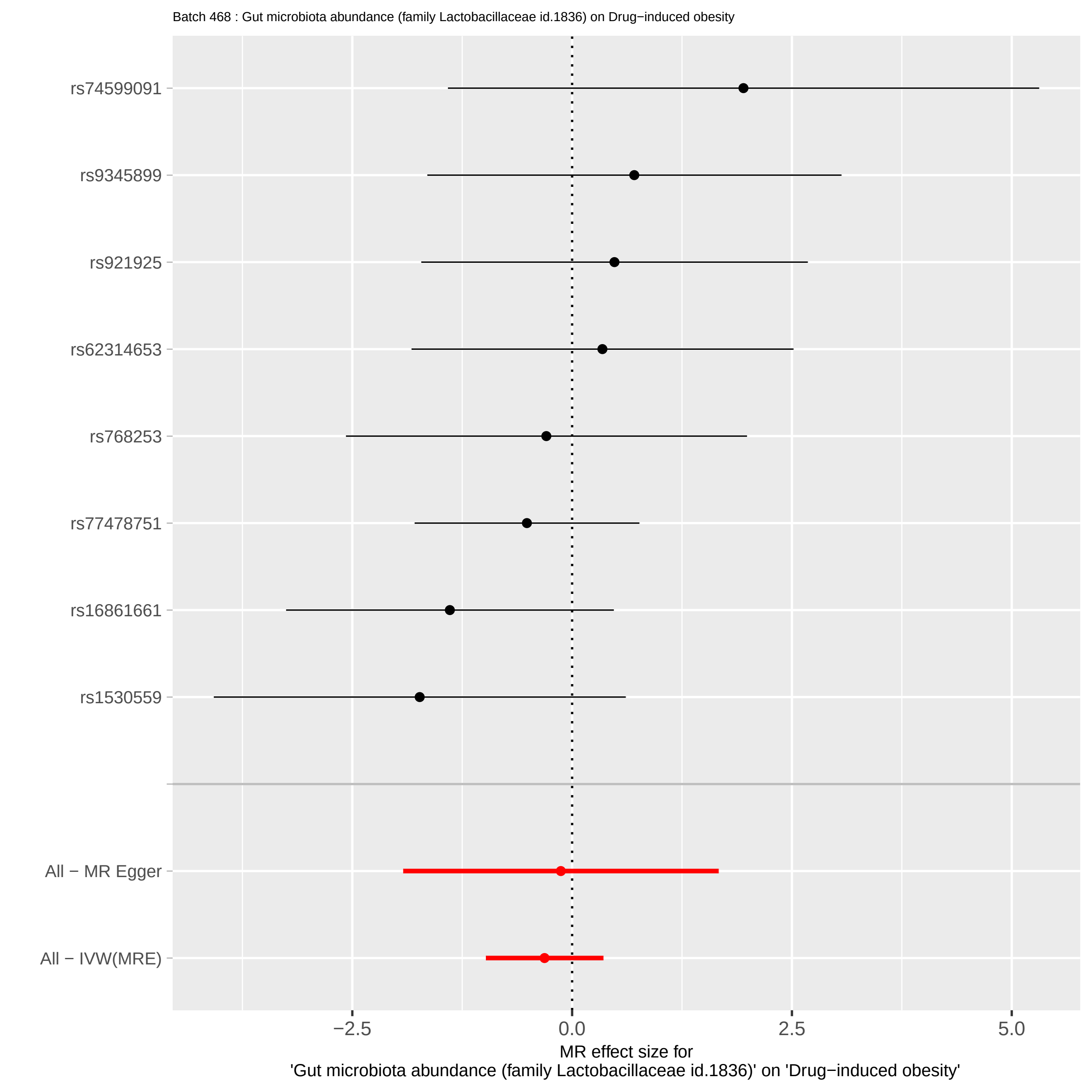

Batch 468 : Gut microbiota abundance (family Lactobacillaceae id.1836) on Drug−induced obesity
rs74599091
rs9345899
rs921925
rs62314653
rs768253
rs77478751
rs16861661
rs1530559
All − MR Egger
All − IVW(MRE)
−2.5
0.0
2.5
5.0
MR effect size for
'Gut microbiota abundance (family Lactobacillaceae id.1836)' on 'Drug−induced obesity'

Batch 469 : Gut microbiota abundance (family Methanobacteriaceae id.121) on Drug−induced obesity

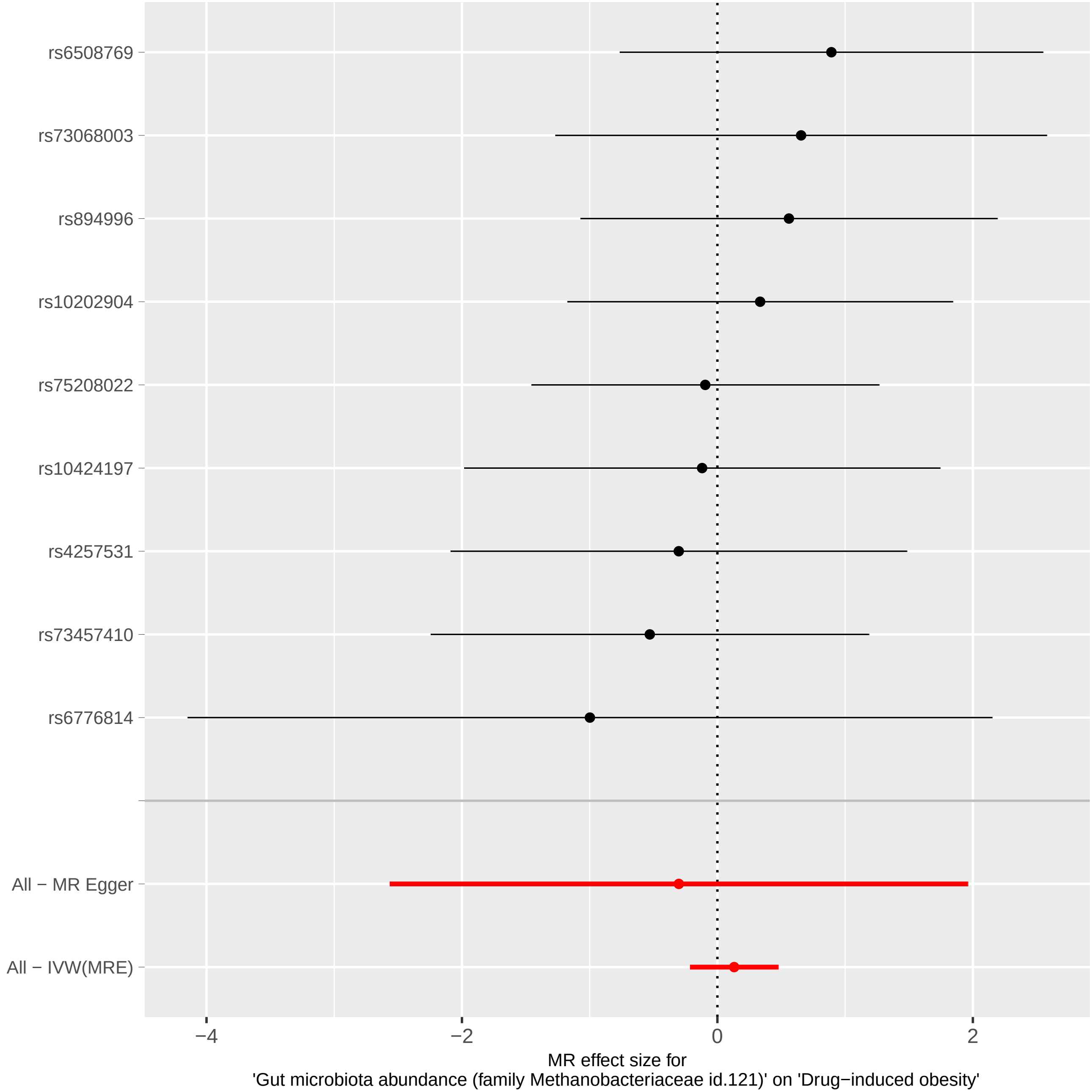

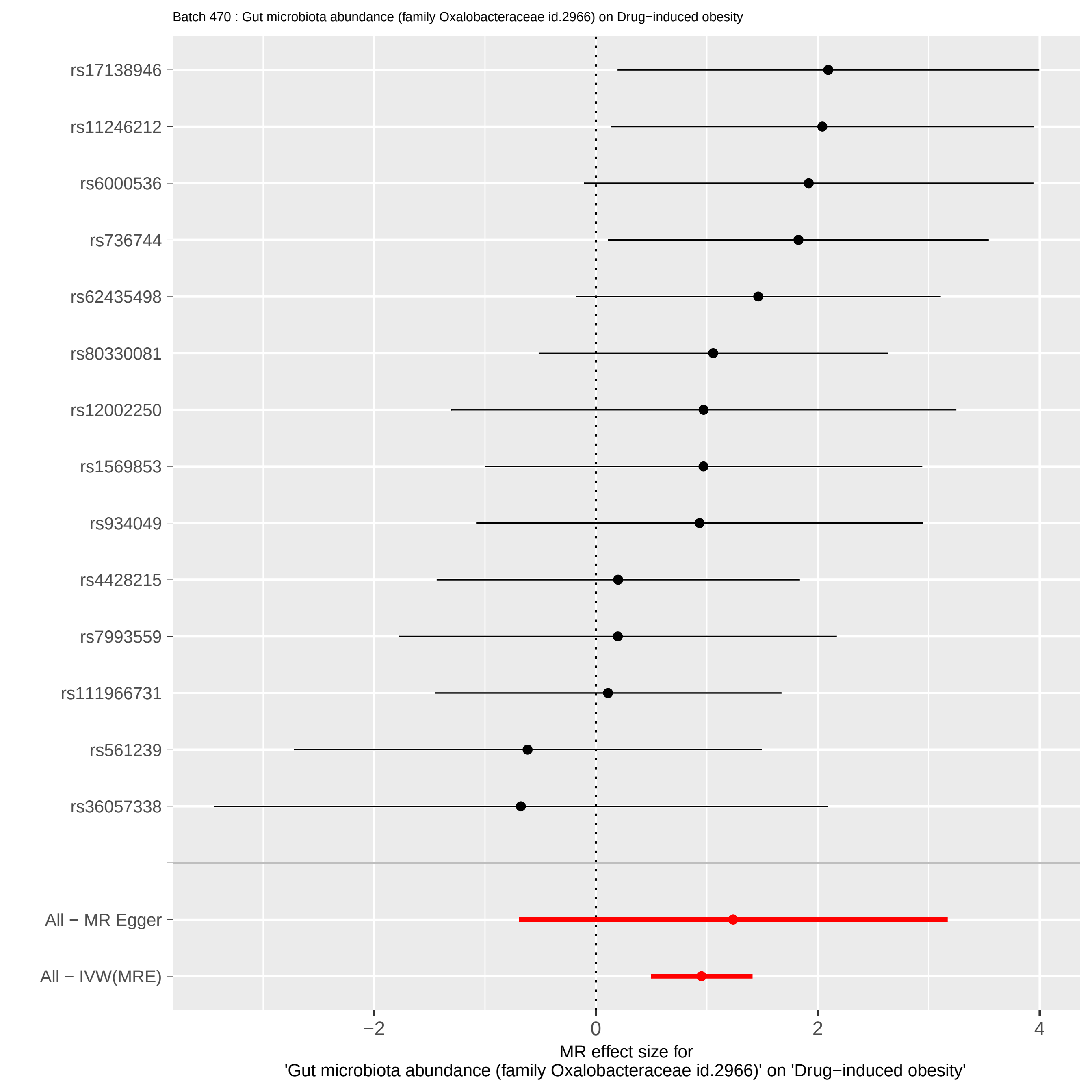
Batch 470 : Gut microbiota abundance (family Oxalobacteraceae id.2966) on Drug−induced obesity
rs17138946
rs11246212
rs6000536
rs736744
rs62435498
rs80330081
rs12002250
rs1569853
rs934049
rs4428215
rs7993559
rs111966731
rs561239
rs36057338
All − MR Egger
All − IVW(MRE)
−2
0
2
4
MR effect size for
'Gut microbiota abundance (family Oxalobacteraceae id.2966)' on 'Drug−induced obesity'

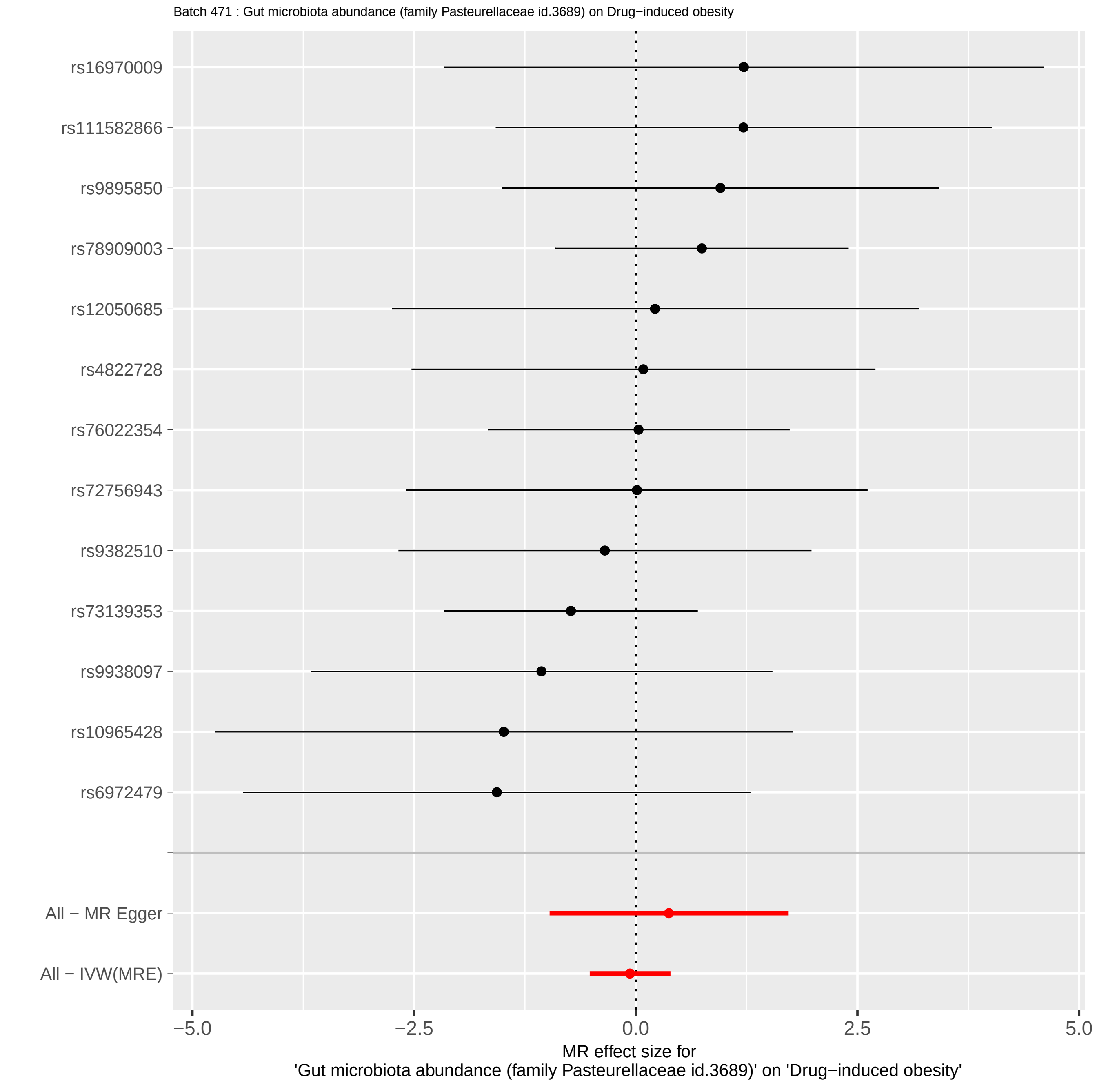
Batch 471 : Gut microbiota abundance (family Pasteurellaceae id.3689) on Drug−induced obesity
rs16970009
rs111582866
rs9895850
rs78909003
rs12050685
rs4822728
rs76022354
rs72756943
rs9382510
rs73139353
rs9938097
rs10965428
rs6972479
All − MR Egger
All − IVW(MRE)
−5.0
−2.5
0.0
2.5
5.0
MR effect size for
'Gut microbiota abundance (family Pasteurellaceae id.3689)' on 'Drug−induced obesity'

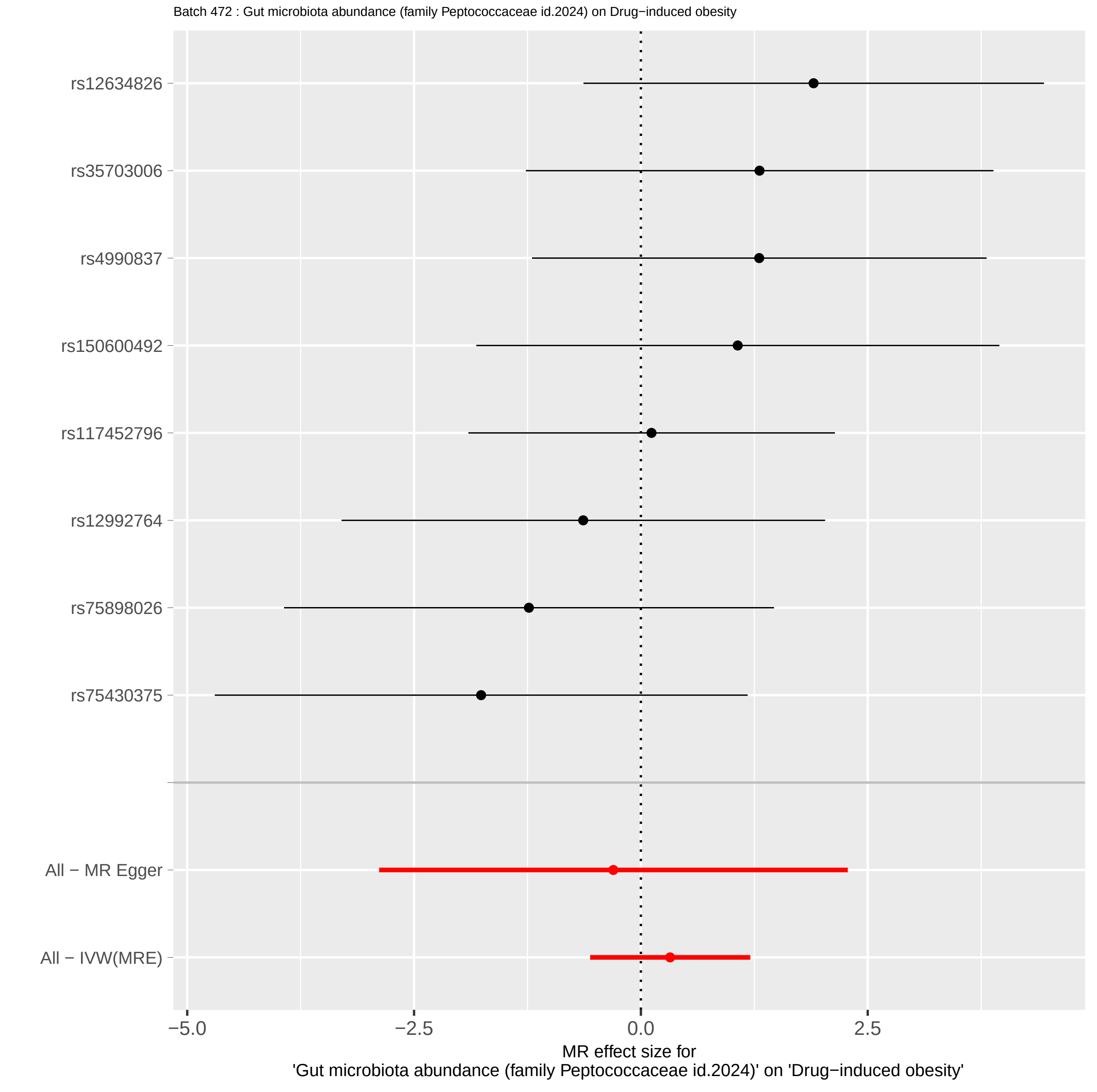
Batch 472 : Gut microbiota abundance (family Peptococcaceae id.2024) on Drug−induced obesity
rs12634826
rs35703006
rs4990837
rs150600492
rs117452796
rs12992764
rs75898026
rs75430375
All − MR Egger
All − IVW(MRE)
−5.0
−2.5
0.0
2.5
MR effect size for
'Gut microbiota abundance (family Peptococcaceae id.2024)' on 'Drug−induced obesity'

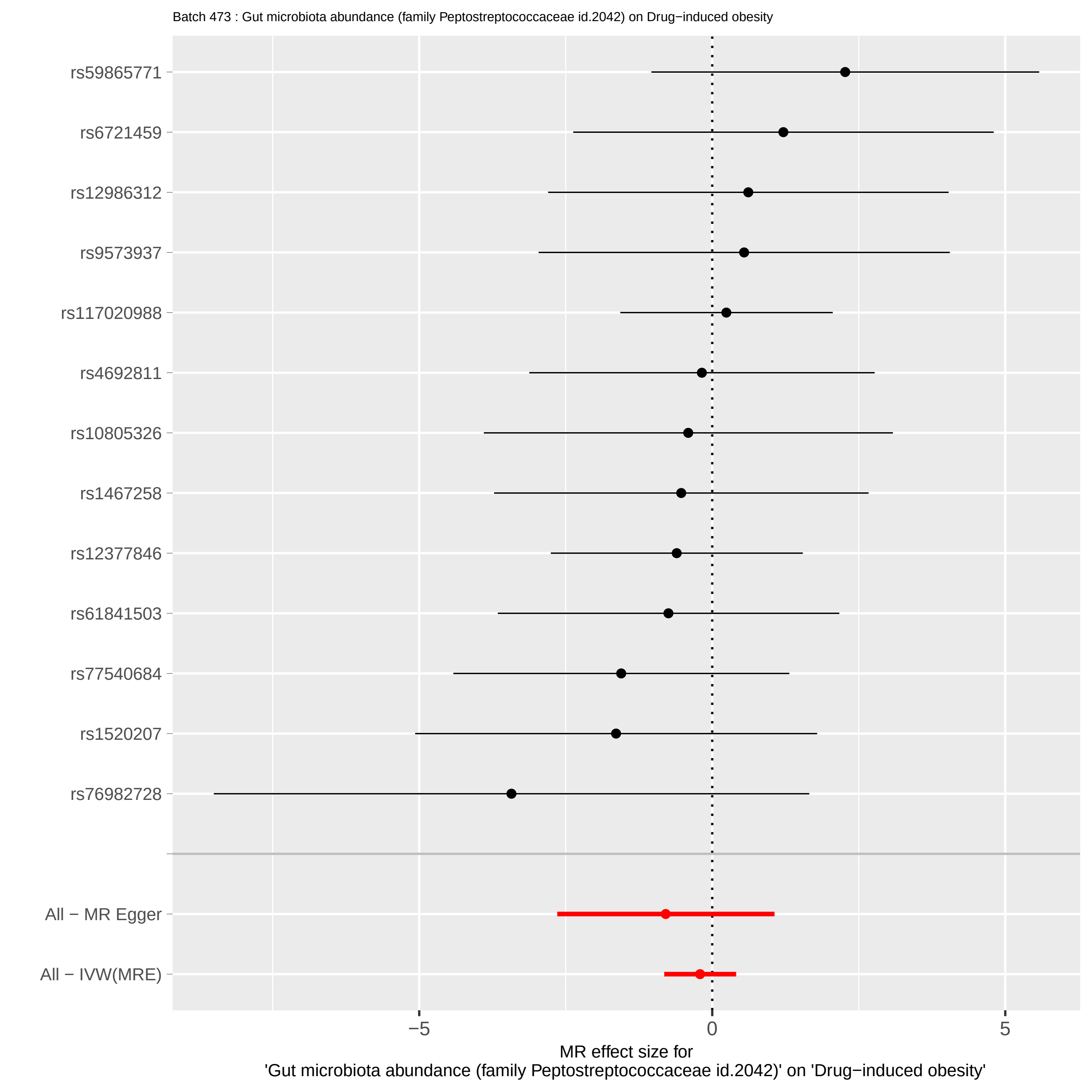

Batch 473 : Gut microbiota abundance (family Peptostreptococcaceae id.2042) on Drug−induced obesity
rs59865771
rs6721459
rs12986312
rs9573937
rs117020988
rs4692811
rs10805326
rs1467258
rs12377846
rs61841503
rs77540684
rs1520207
rs76982728
All − MR Egger
All − IVW(MRE)
−5
0
5
MR effect size for
'Gut microbiota abundance (family Peptostreptococcaceae id.2042)' on 'Drug−induced obesity'

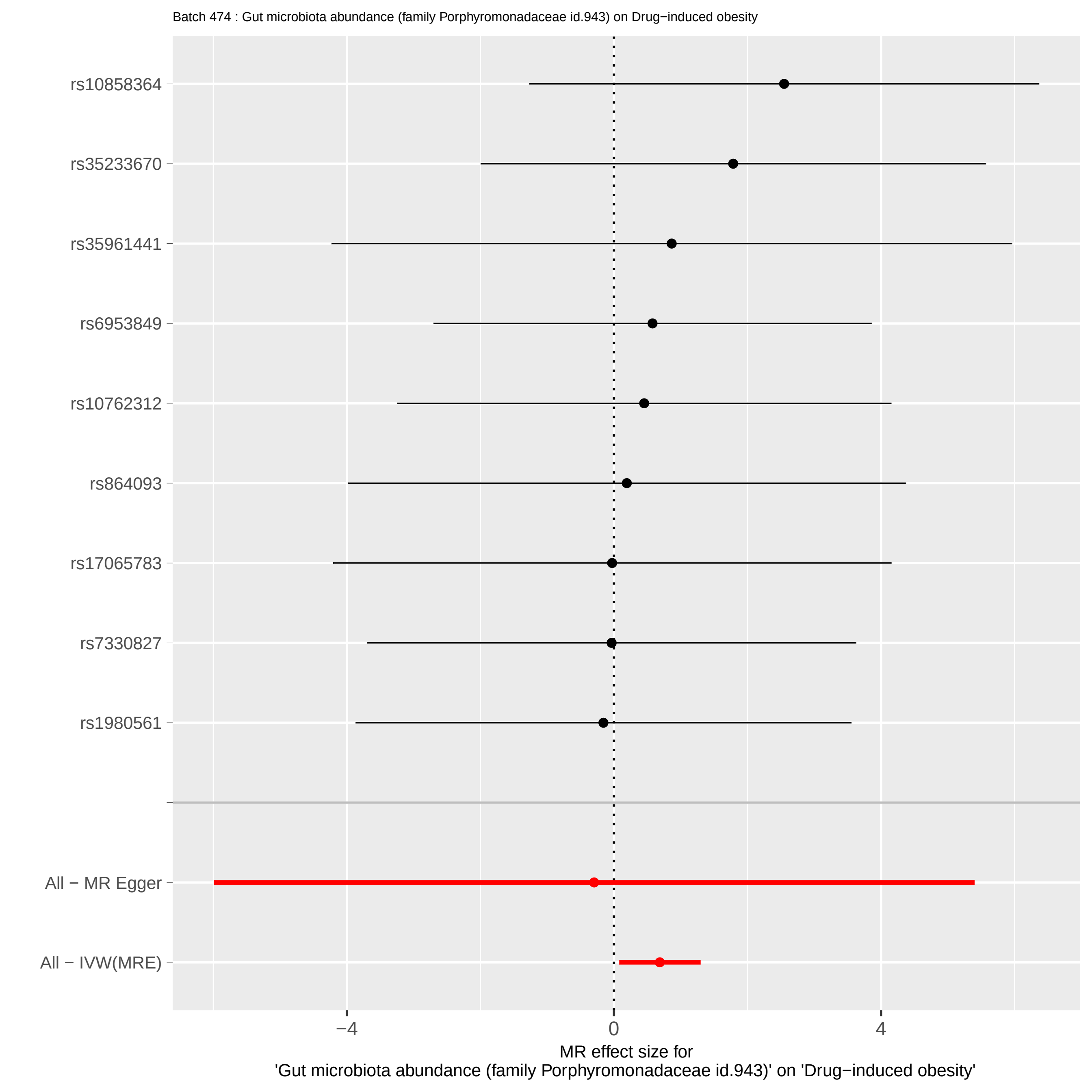
Batch 474 : Gut microbiota abundance (family Porphyromonadaceae id.943) on Drug−induced obesity
rs10858364
rs35233670
rs35961441
rs6953849
rs10762312
rs864093
rs17065783
rs7330827
rs1980561
All − MR Egger
All − IVW(MRE)
−4
0
4
MR effect size for
'Gut microbiota abundance (family Porphyromonadaceae id.943)' on 'Drug−induced obesity'

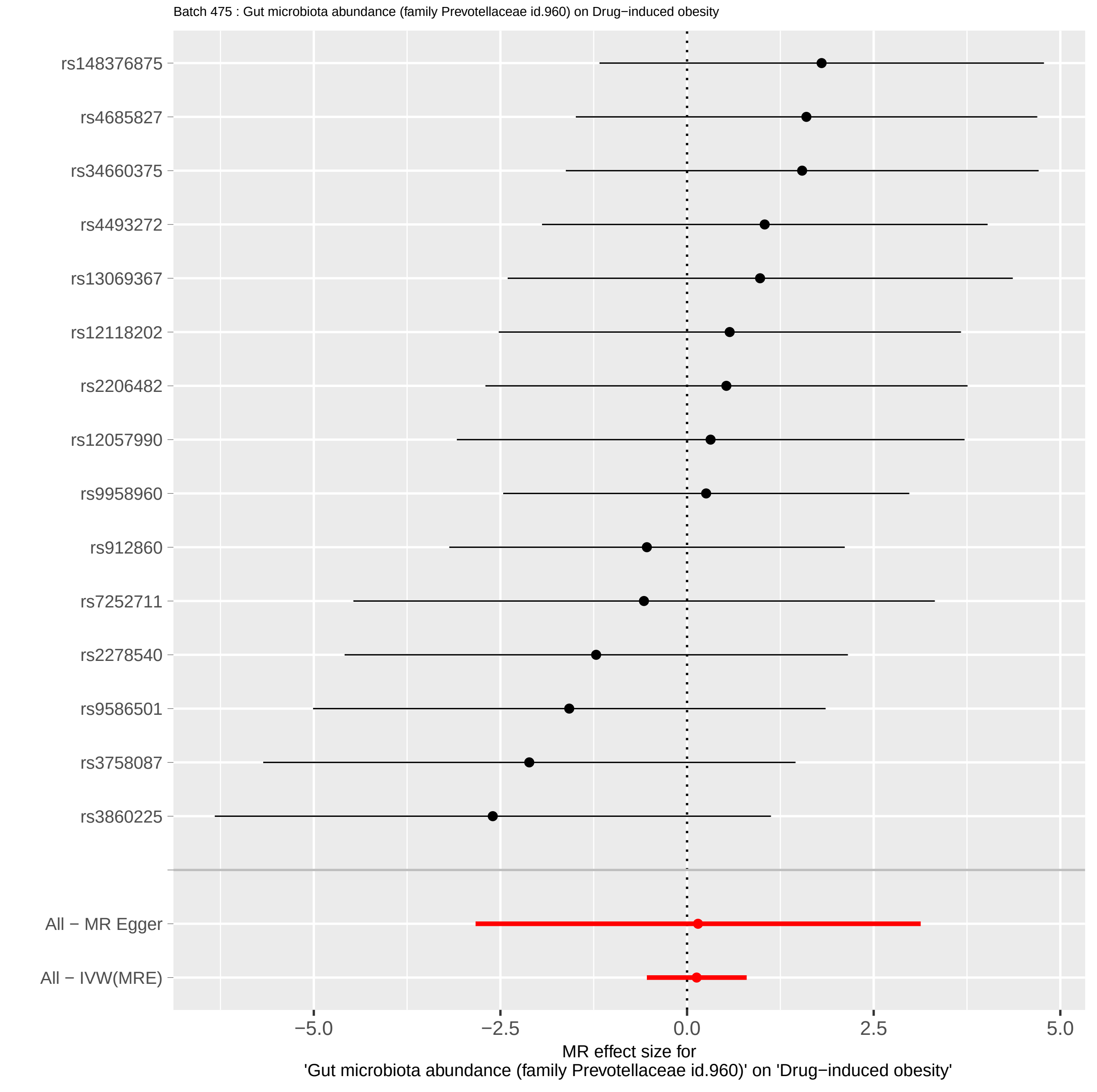

Batch 475 : Gut microbiota abundance (family Prevotellaceae id.960) on Drug−induced obesity
rs148376875
rs4685827
rs34660375
rs4493272
rs13069367
rs12118202
rs2206482
rs12057990
rs9958960
rs912860
rs7252711
rs2278540
rs9586501
rs3758087
rs3860225
All − MR Egger
All − IVW(MRE)
−5.0
−2.5
0.0
2.5
5.0
MR effect size for
'Gut microbiota abundance (family Prevotellaceae id.960)' on 'Drug−induced obesity'

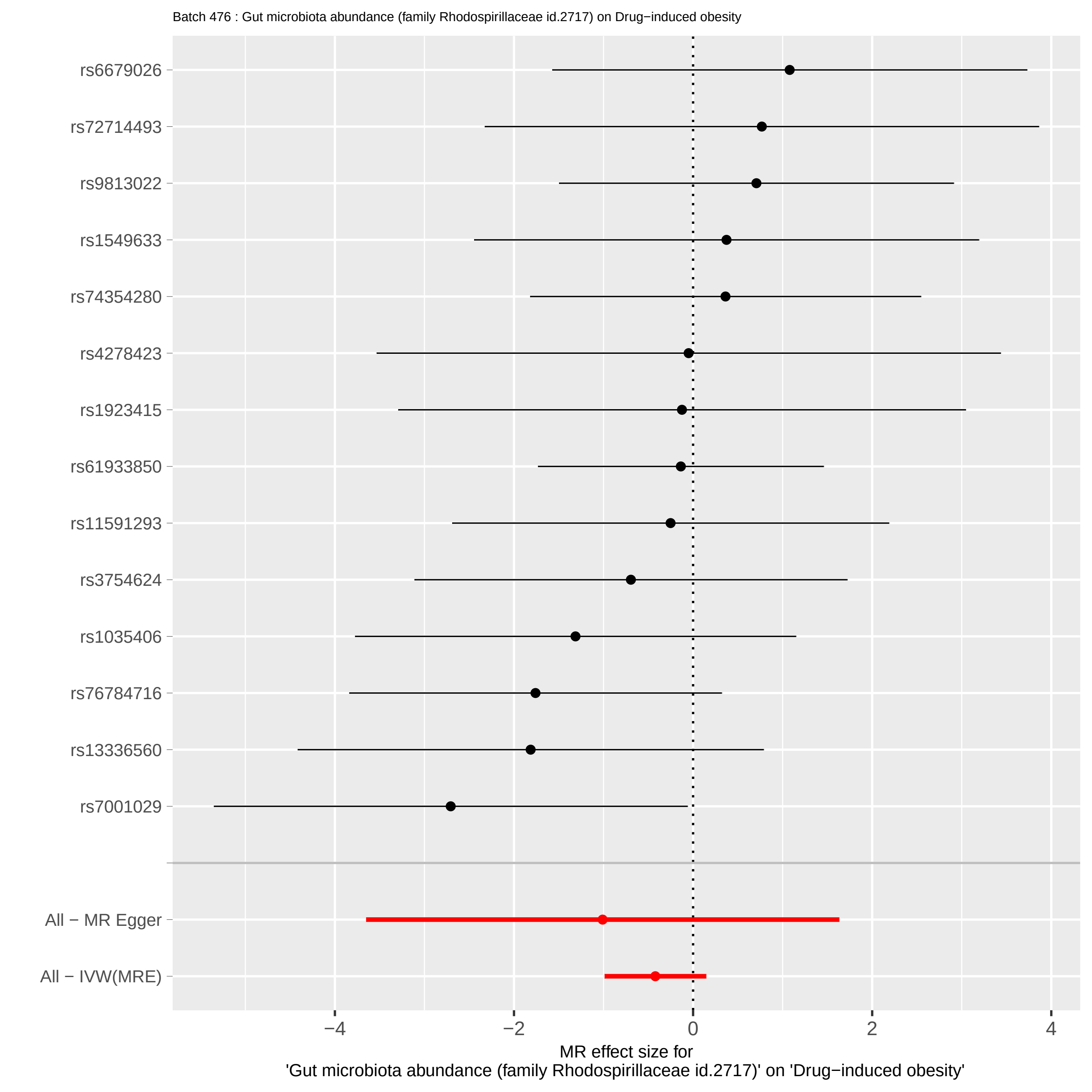

Batch 476 : Gut microbiota abundance (family Rhodospirillaceae id.2717) on Drug−induced obesity
rs6679026
rs72714493
rs9813022
rs1549633
rs74354280
rs4278423
rs1923415
rs61933850
rs11591293
rs3754624
rs1035406
rs76784716
rs13336560
rs7001029
All − MR Egger
All − IVW(MRE)
−4
−2
0
2
4
MR effect size for
'Gut microbiota abundance (family Rhodospirillaceae id.2717)' on 'Drug−induced obesity'

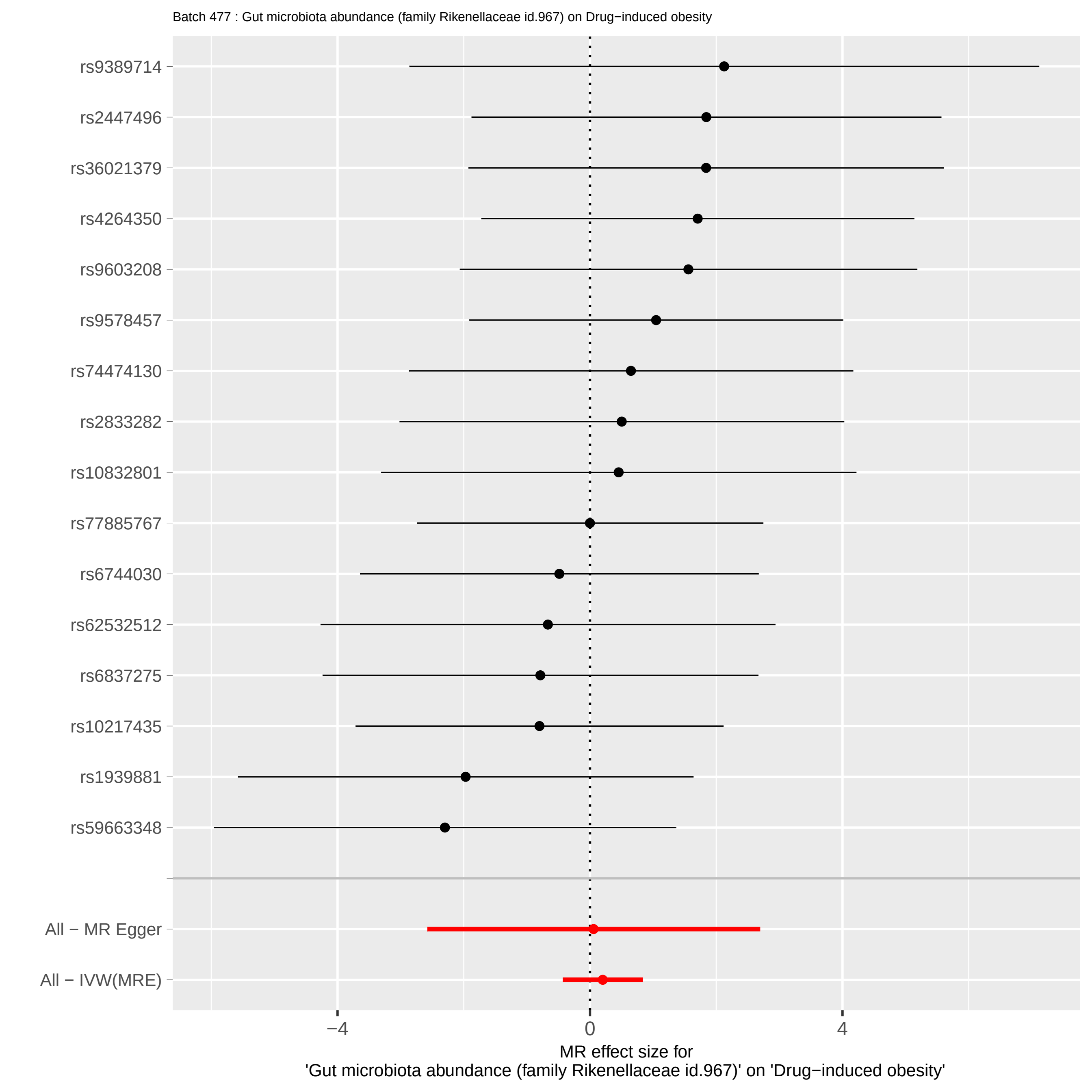
Batch 477 : Gut microbiota abundance (family Rikenellaceae id.967) on Drug−induced obesity
rs9389714
rs2447496
rs36021379
rs4264350
rs9603208
rs9578457
rs74474130
rs2833282
rs10832801
rs77885767
rs6744030
rs62532512
rs6837275
rs10217435
rs1939881
rs59663348
All − MR Egger
All − IVW(MRE)
−4
0
4
MR effect size for
'Gut microbiota abundance (family Rikenellaceae id.967)' on 'Drug−induced obesity'

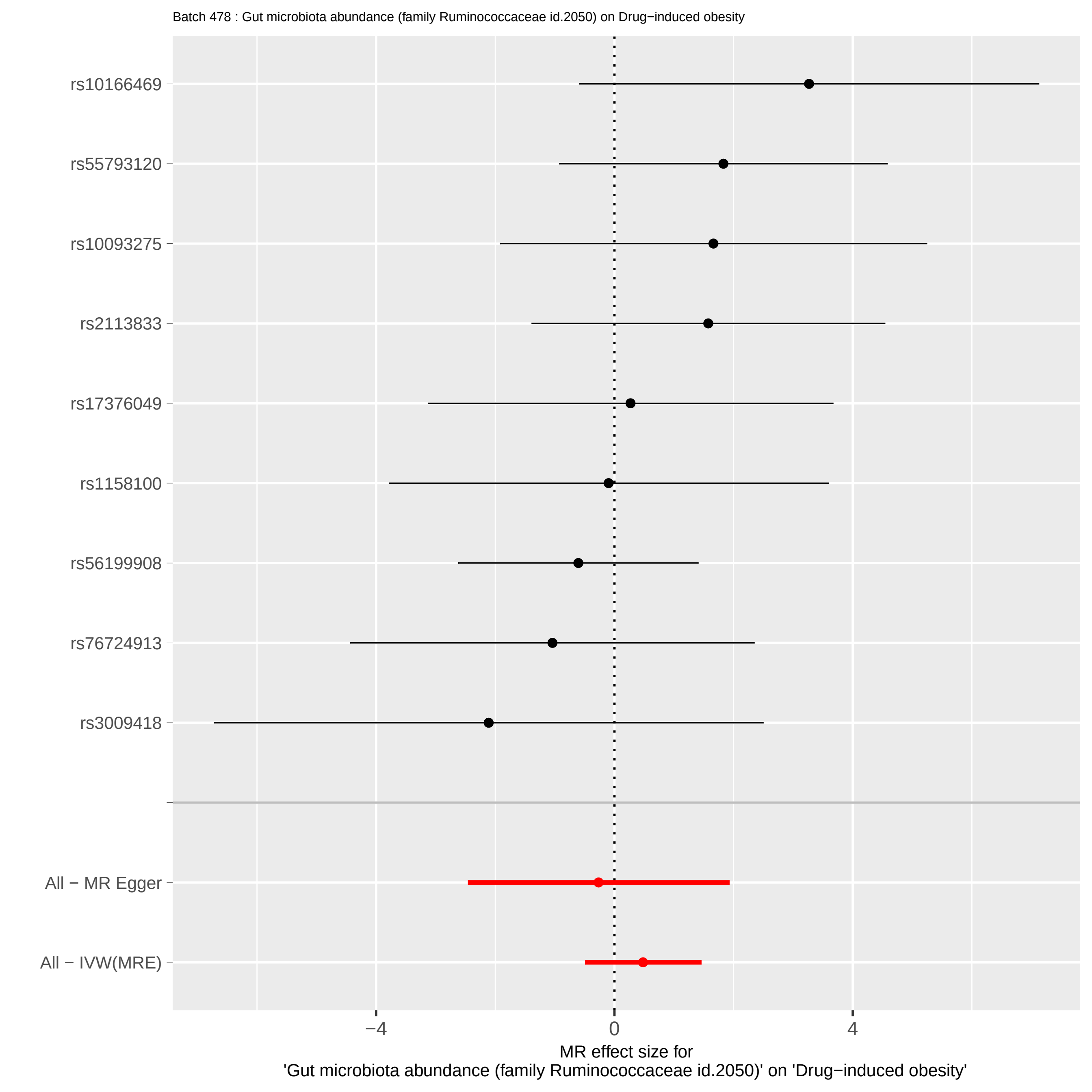

Batch 478 : Gut microbiota abundance (family Ruminococcaceae id.2050) on Drug−induced obesity
rs10166469
rs55793120
rs10093275
rs2113833
rs17376049
rs1158100
rs56199908
rs76724913
rs3009418
All − MR Egger
All − IVW(MRE)
−4
0
4
MR effect size for
'Gut microbiota abundance (family Ruminococcaceae id.2050)' on 'Drug−induced obesity'

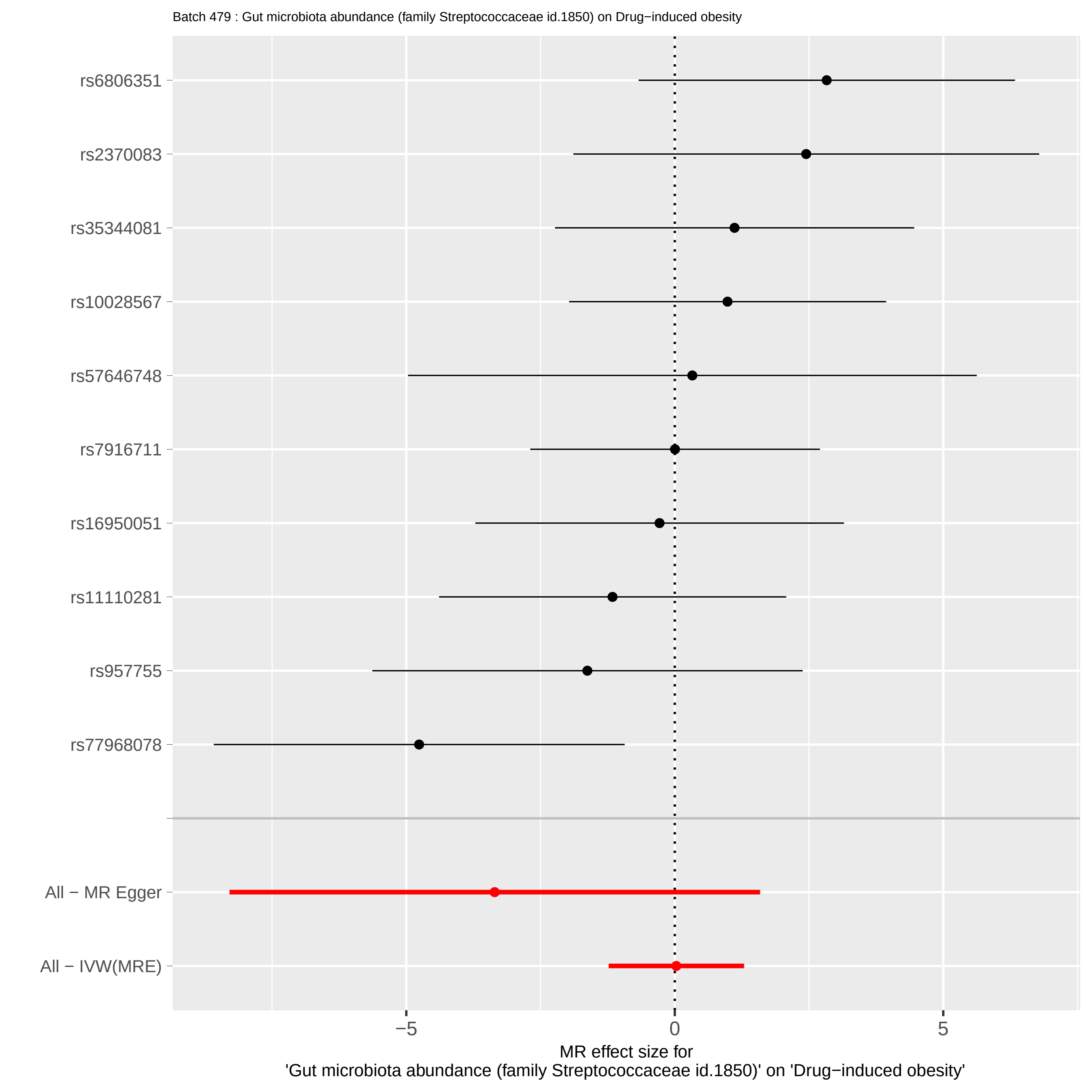

Batch 479 : Gut microbiota abundance (family Streptococcaceae id.1850) on Drug−induced obesity
rs6806351
rs2370083
rs35344081
rs10028567
rs57646748
rs7916711
rs16950051
rs11110281
rs957755
rs77968078
All − MR Egger
All − IVW(MRE)
−5
0
5
MR effect size for
'Gut microbiota abundance (family Streptococcaceae id.1850)' on 'Drug−induced obesity'

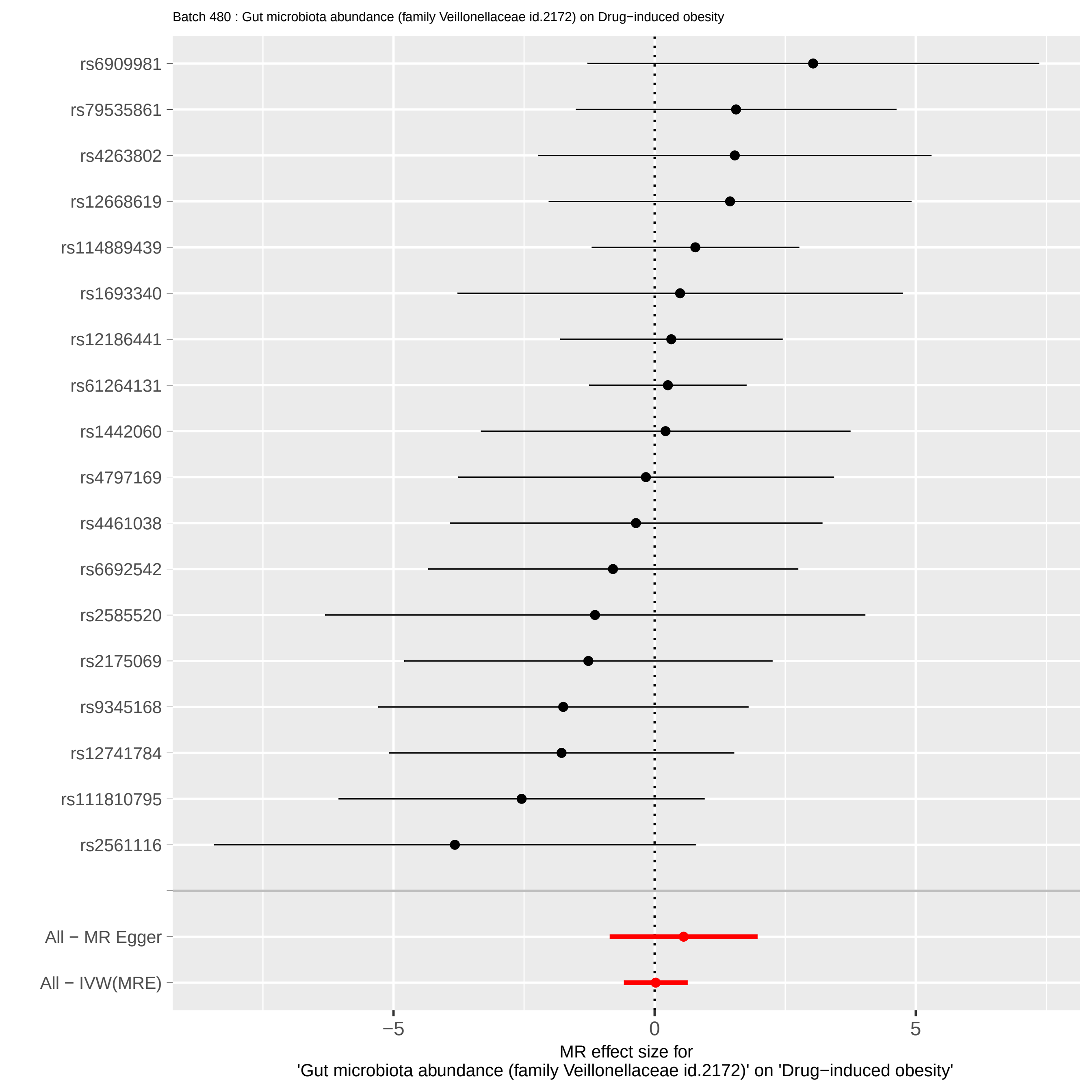

Batch 480 : Gut microbiota abundance (family Veillonellaceae id.2172) on Drug−induced obesity
rs6909981
rs79535861
rs4263802
rs12668619
rs114889439
rs1693340
rs12186441
rs61264131
rs1442060
rs4797169
rs4461038
rs6692542
rs2585520
rs2175069
rs9345168
rs12741784
rs111810795
rs2561116
All − MR Egger
All − IVW(MRE)
−5
0
5
MR effect size for
'Gut microbiota abundance (family Veillonellaceae id.2172)' on 'Drug−induced obesity'

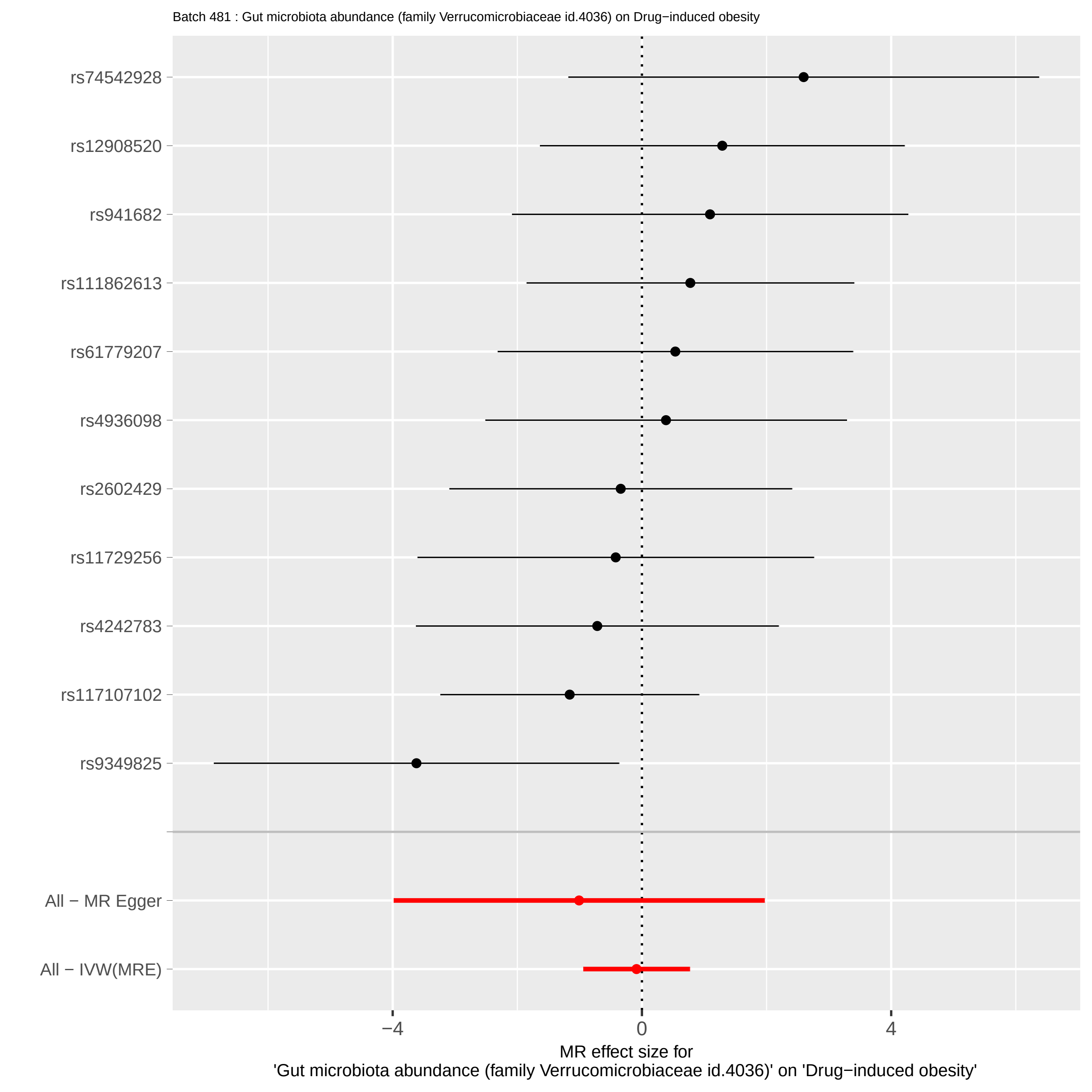

Batch 481 : Gut microbiota abundance (family Verrucomicrobiaceae id.4036) on Drug−induced obesity
rs74542928
rs12908520
rs941682
rs111862613
rs61779207
rs4936098
rs2602429
rs11729256
rs4242783
rs117107102
rs9349825
All − MR Egger
All − IVW(MRE)
−4
0
4
MR effect size for
'Gut microbiota abundance (family Verrucomicrobiaceae id.4036)' on 'Drug−induced obesity'

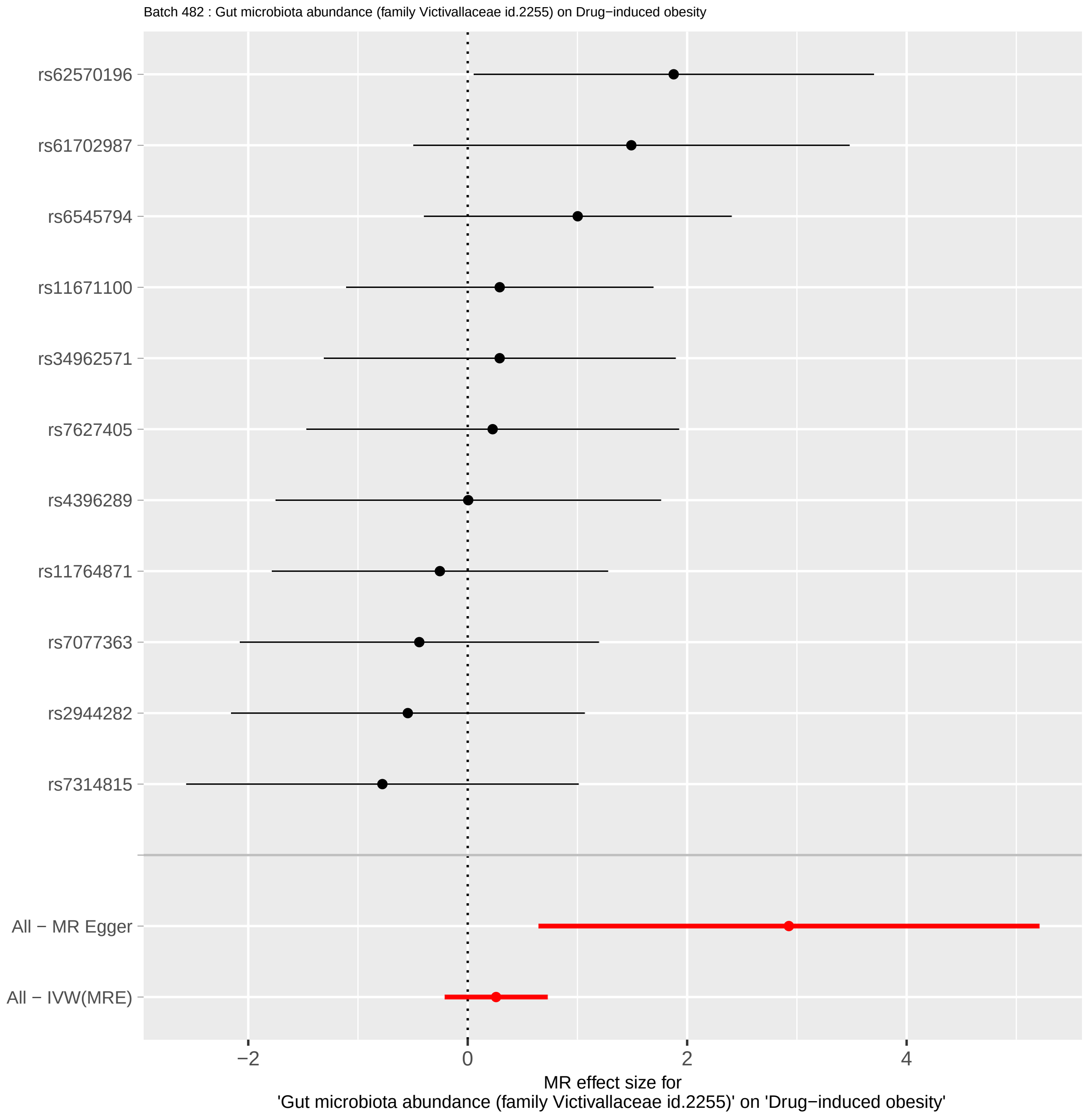
Batch 482 : Gut microbiota abundance (family Victivallaceae id.2255) on Drug−induced obesity
rs62570196
rs61702987
rs6545794
rs11671100
rs34962571
rs7627405
rs4396289
rs11764871
rs7077363
rs2944282
rs7314815
All − MR Egger
All − IVW(MRE)
−2
0
2
4
MR effect size for
'Gut microbiota abundance (family Victivallaceae id.2255)' on 'Drug−induced obesity'

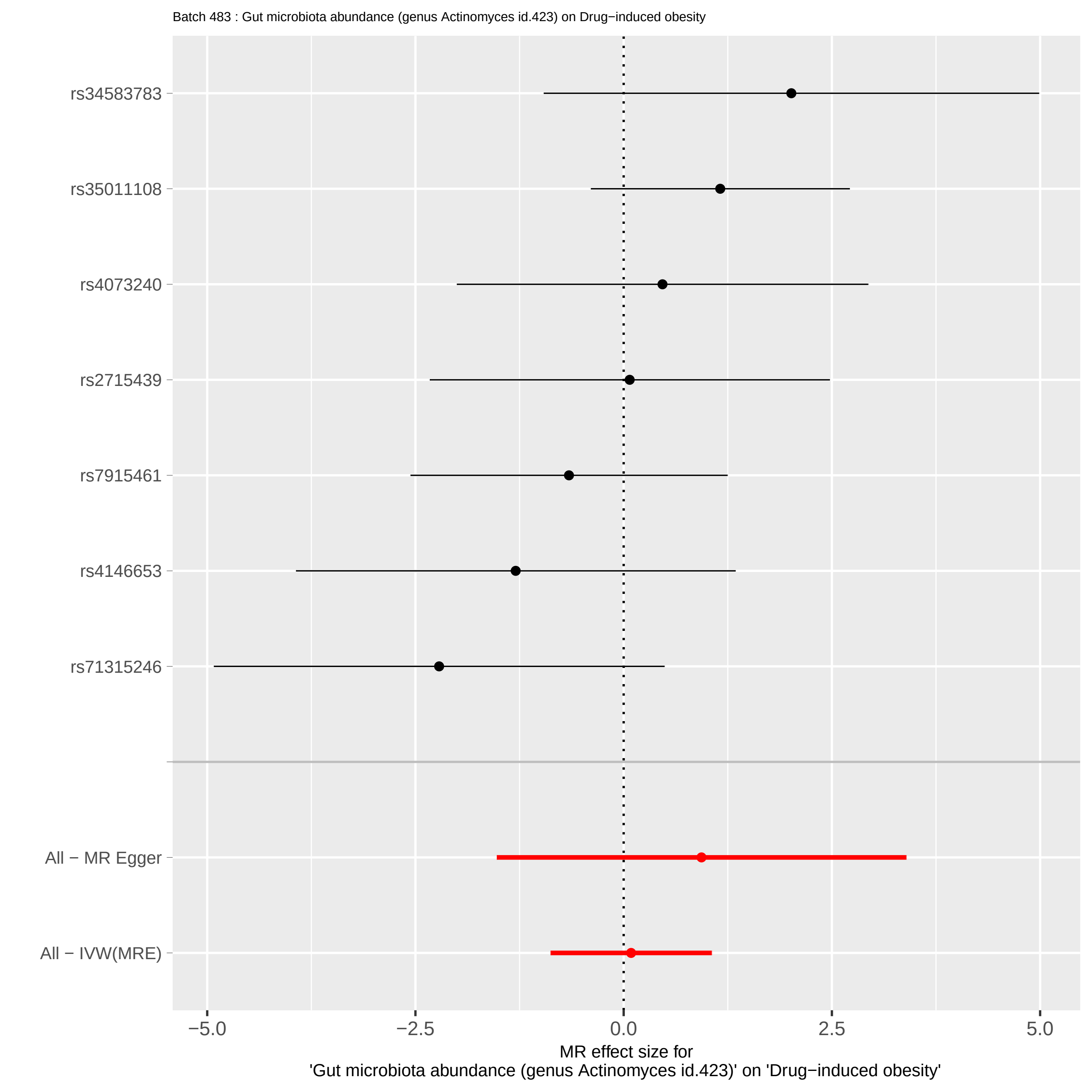

Batch 483 : Gut microbiota abundance (genus Actinomyces id.423) on Drug−induced obesity
rs34583783
rs35011108
rs4073240
rs2715439
rs7915461
rs4146653
rs71315246
All − MR Egger
All − IVW(MRE)
−5.0
−2.5
0.0
2.5
5.0
MR effect size for
'Gut microbiota abundance (genus Actinomyces id.423)' on 'Drug−induced obesity'

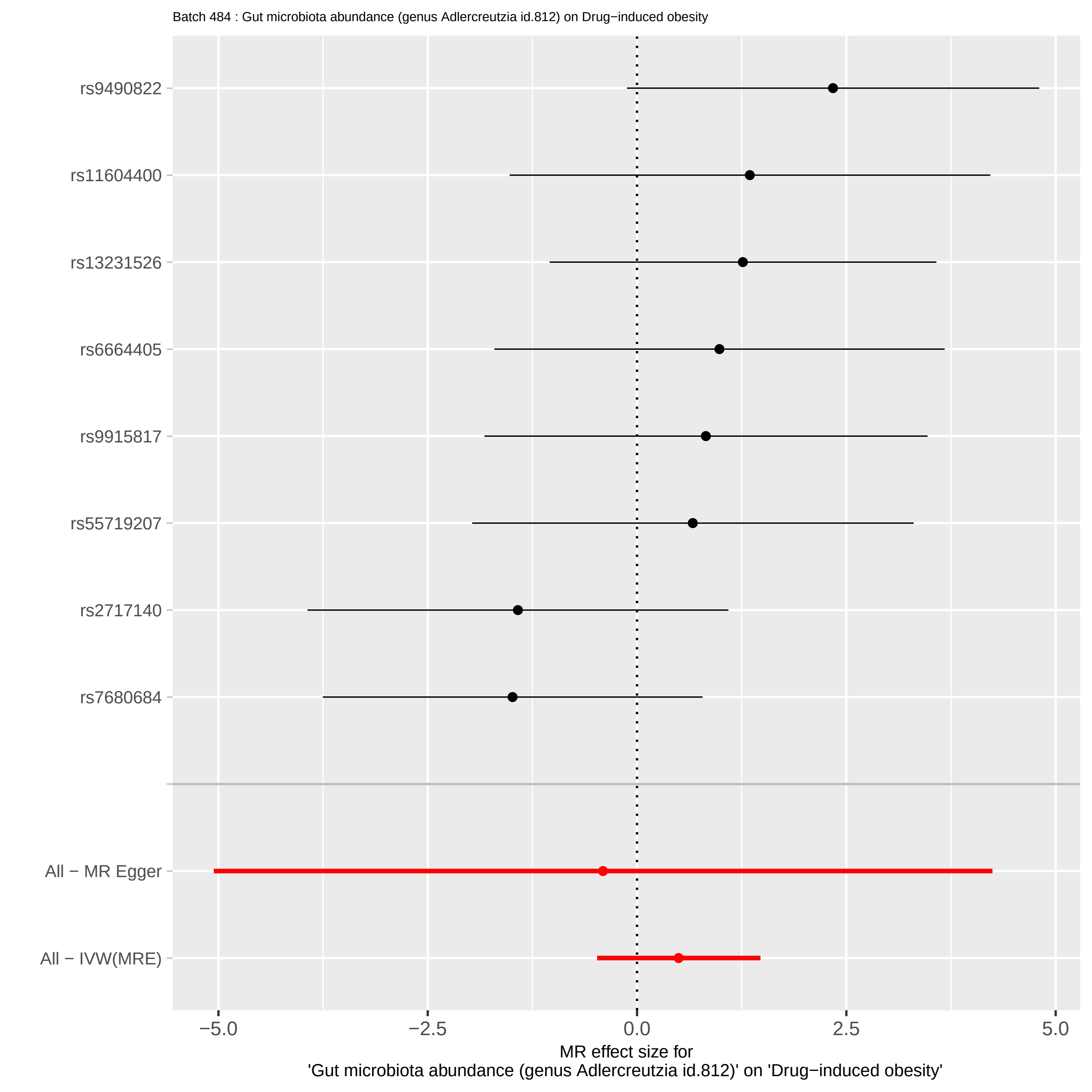

Batch 484 : Gut microbiota abundance (genus Adlercreutzia id.812) on Drug−induced obesity
rs9490822
rs11604400
rs13231526
rs6664405
rs9915817
rs55719207
rs2717140
rs7680684
All − MR Egger
All − IVW(MRE)
−5.0
−2.5
0.0
2.5
5.0
MR effect size for
'Gut microbiota abundance (genus Adlercreutzia id.812)' on 'Drug−induced obesity'

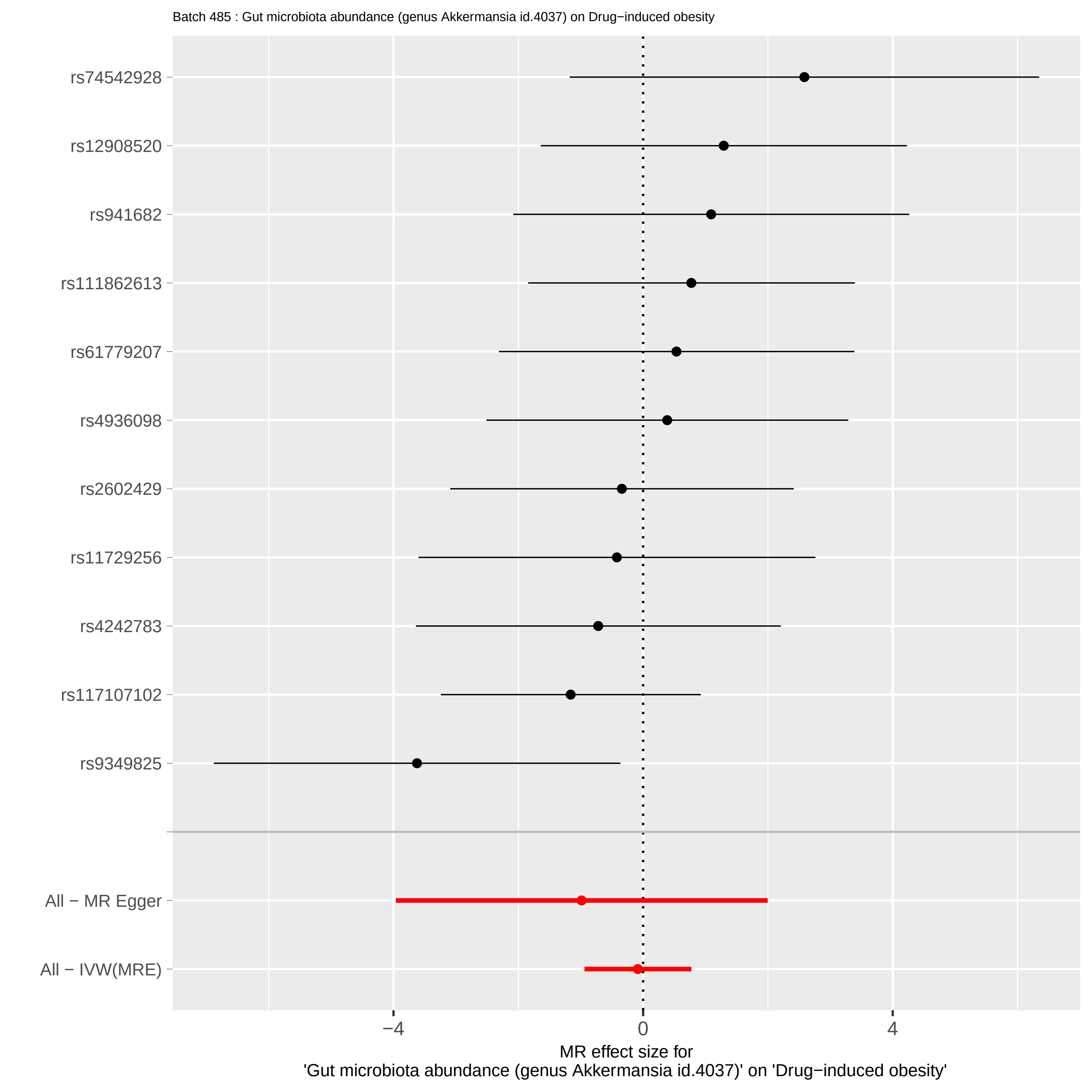

Batch 485 : Gut microbiota abundance (genus Akkermansia id.4037) on Drug−induced obesity
rs74542928
rs12908520
rs941682
rs111862613
rs61779207
rs4936098
rs2602429
rs11729256
rs4242783
rs117107102
rs9349825
All − MR Egger
All − IVW(MRE)
−4
0
4
MR effect size for
'Gut microbiota abundance (genus Akkermansia id.4037)' on 'Drug−induced obesity'

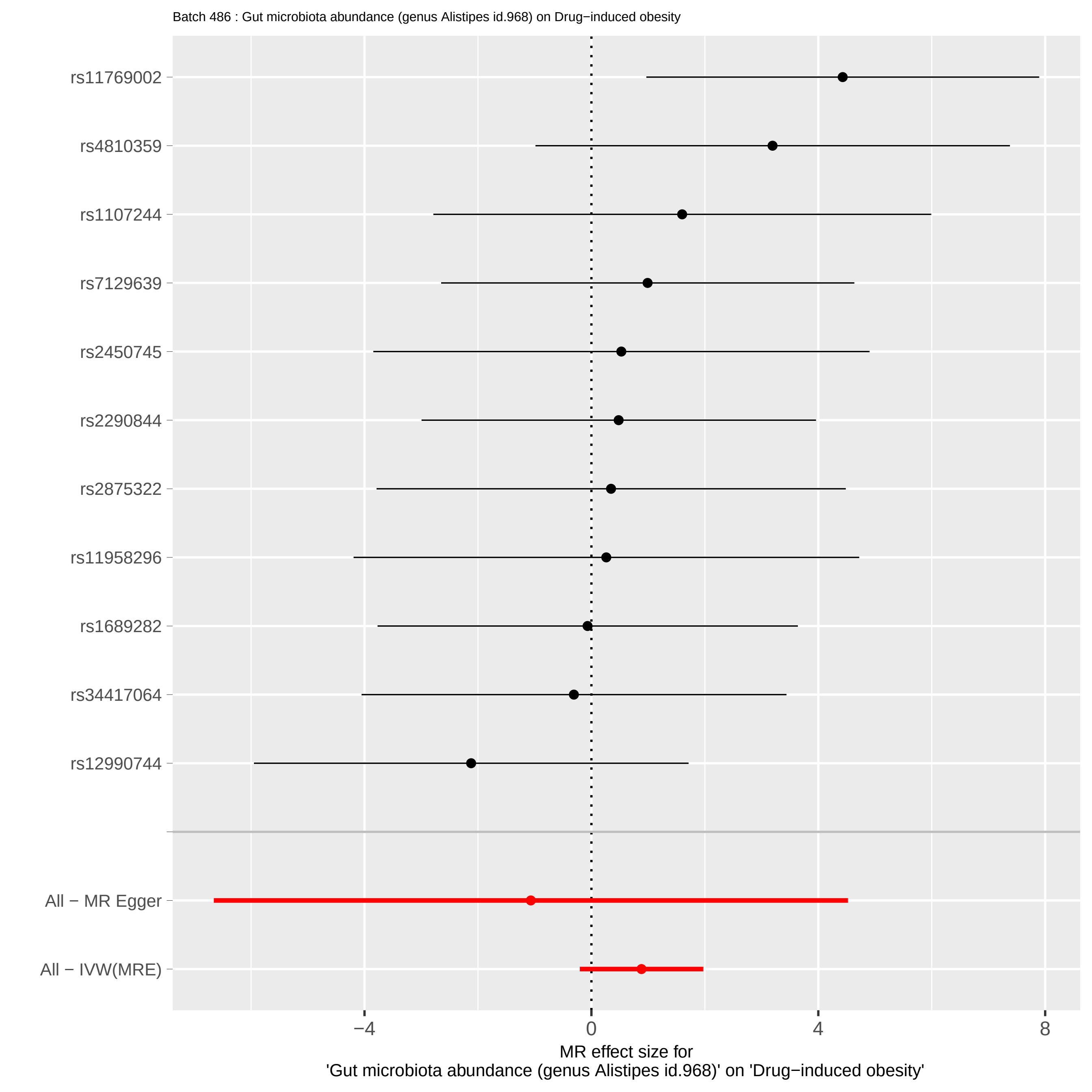
Batch 486 : Gut microbiota abundance (genus Alistipes id.968) on Drug−induced obesity
rs11769002
rs4810359
rs1107244
rs7129639
rs2450745
rs2290844
rs2875322
rs11958296
rs1689282
rs34417064
rs12990744
All − MR Egger
All − IVW(MRE)
−4
0
4
8
MR effect size for
'Gut microbiota abundance (genus Alistipes id.968)' on 'Drug−induced obesity'

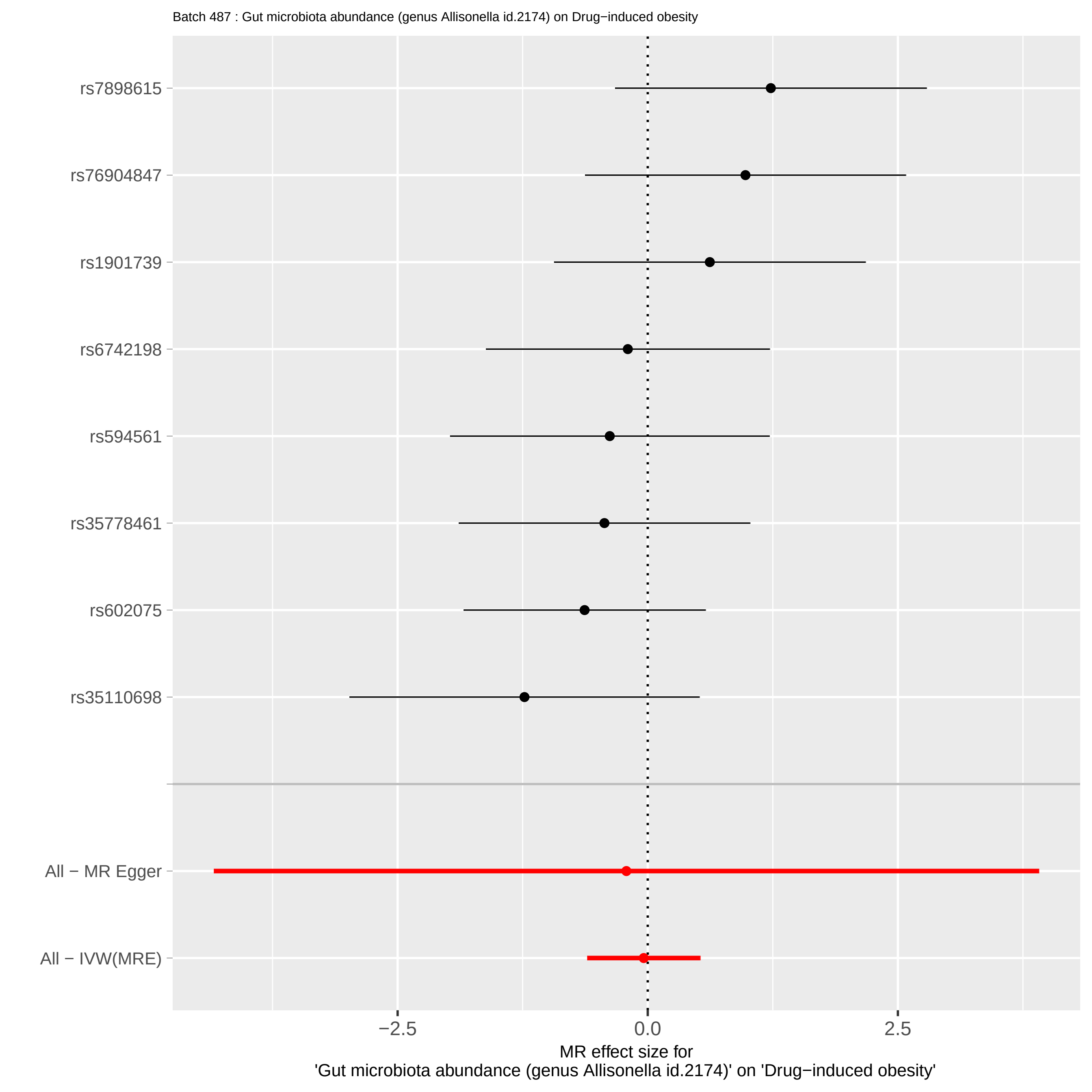

Batch 487 : Gut microbiota abundance (genus Allisonella id.2174) on Drug−induced obesity
rs7898615
rs76904847
rs1901739
rs6742198
rs594561
rs35778461
rs602075
rs35110698
All − MR Egger
All − IVW(MRE)
−2.5
0.0
2.5
MR effect size for
'Gut microbiota abundance (genus Allisonella id.2174)' on 'Drug−induced obesity'

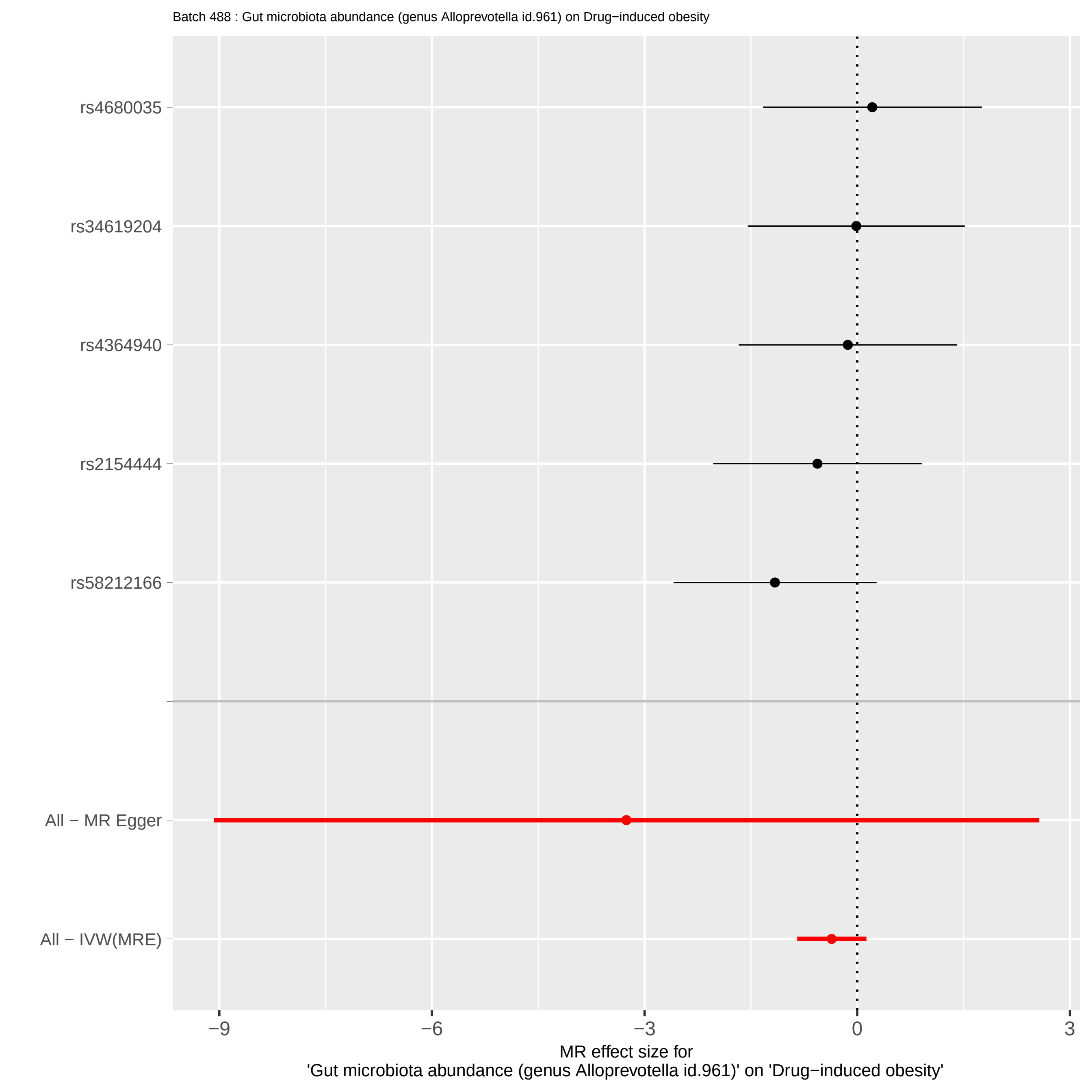

Batch 488 : Gut microbiota abundance (genus Alloprevotella id.961) on Drug−induced obesity
rs4680035
rs34619204
rs4364940
rs2154444
rs58212166
All − MR Egger
All − IVW(MRE)
−9
−6
−3
0
3
MR effect size for
'Gut microbiota abundance (genus Alloprevotella id.961)' on 'Drug−induced obesity'

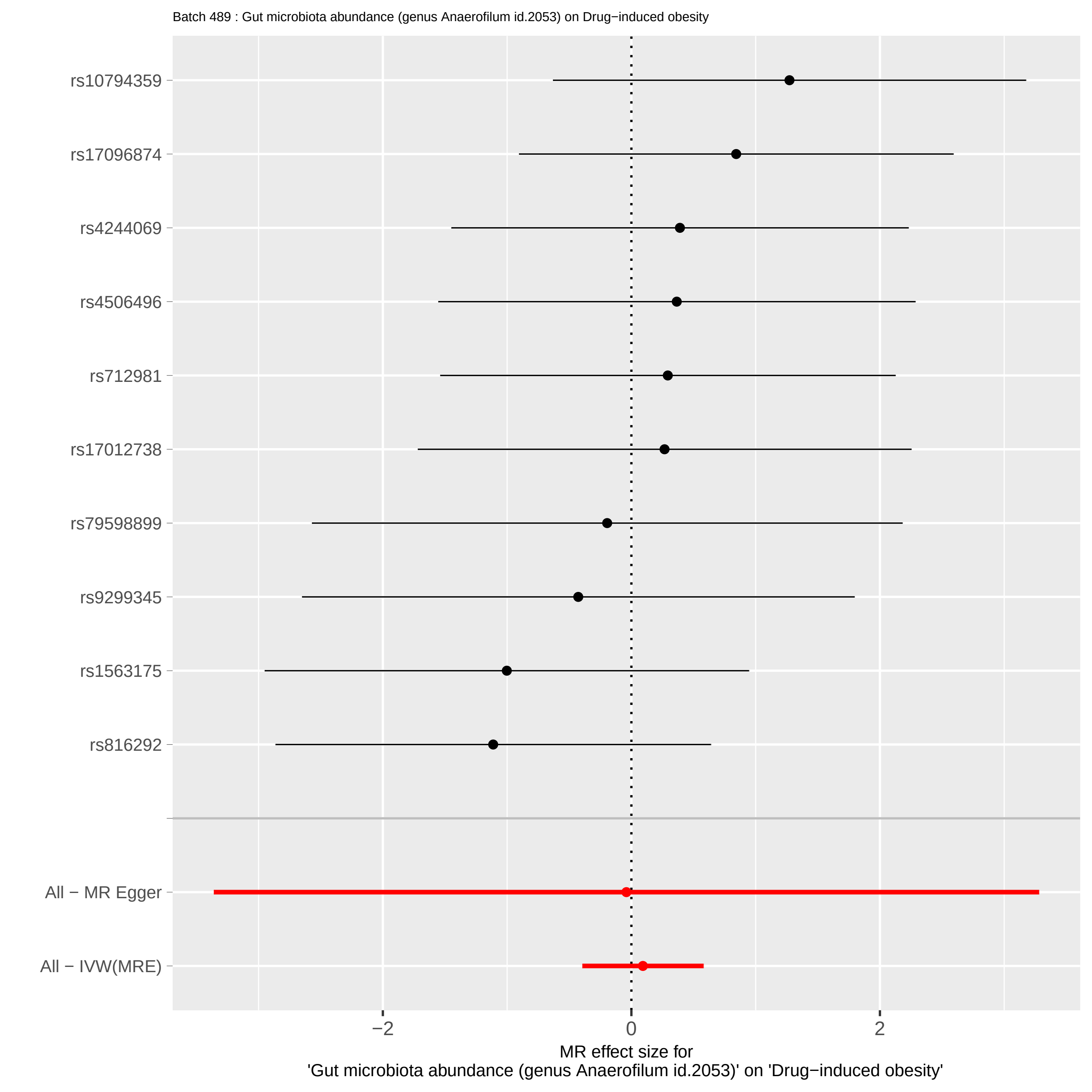

Batch 489 : Gut microbiota abundance (genus Anaerofilum id.2053) on Drug−induced obesity
rs10794359
rs17096874
rs4244069
rs4506496
rs712981
rs17012738
rs79598899
rs9299345
rs1563175
rs816292
All − MR Egger
All − IVW(MRE)
−2
0
2
MR effect size for
'Gut microbiota abundance (genus Anaerofilum id.2053)' on 'Drug−induced obesity'

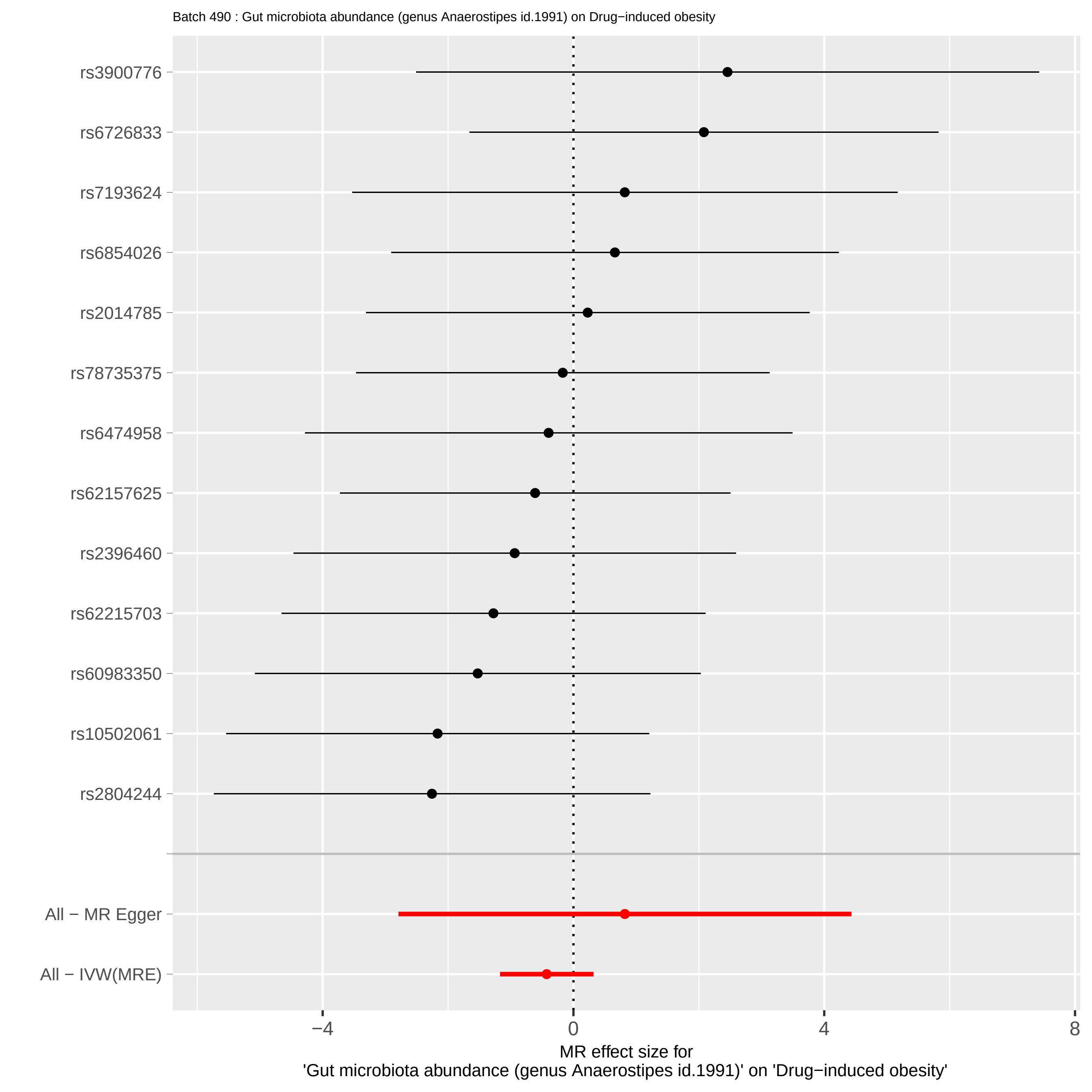

Batch 490 : Gut microbiota abundance (genus Anaerostipes id.1991) on Drug−induced obesity
rs3900776
rs6726833
rs7193624
rs6854026
rs2014785
rs78735375
rs6474958
rs62157625
rs2396460
rs62215703
rs60983350
rs10502061
rs2804244
All − MR Egger
All − IVW(MRE)
−4
0
4
8
MR effect size for
'Gut microbiota abundance (genus Anaerostipes id.1991)' on 'Drug−induced obesity'

Batch 491 : Gut microbiota abundance (genus Anaerotruncus id.2054) on Drug−induced obesity

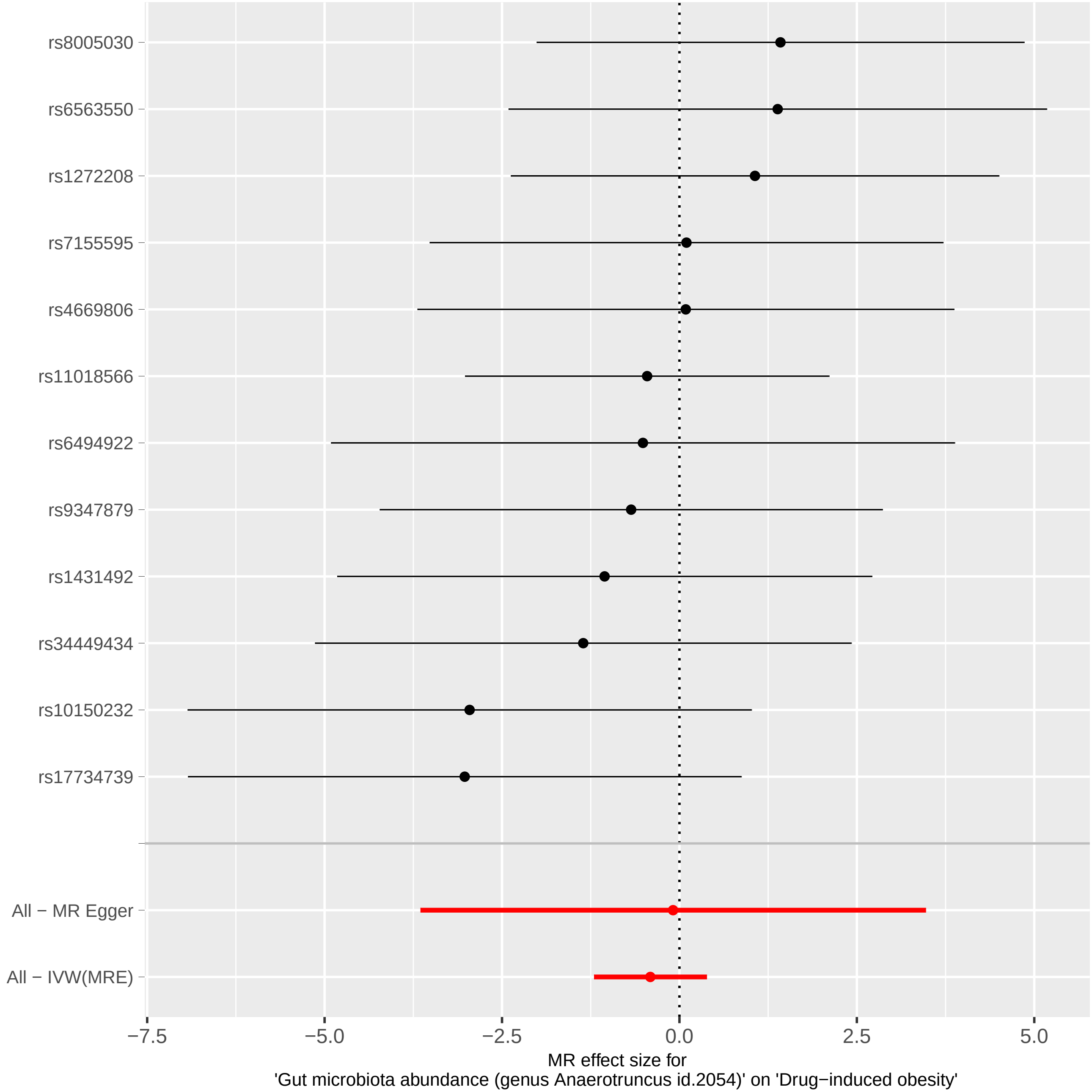

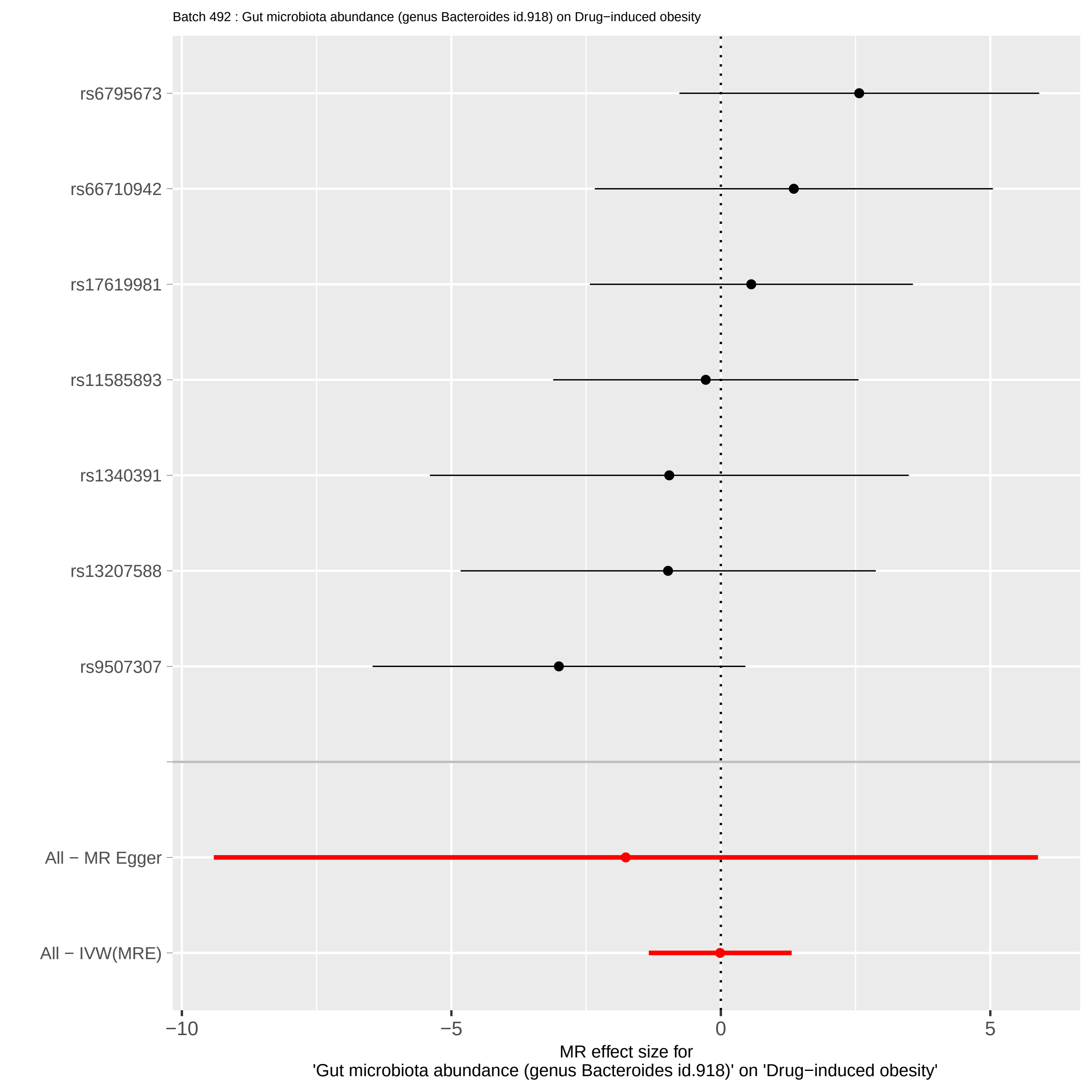
Batch 492 : Gut microbiota abundance (genus Bacteroides id.918) on Drug−induced obesity
rs6795673
rs66710942
rs17619981
rs11585893
rs1340391
rs13207588
rs9507307
All − MR Egger
All − IVW(MRE)
−10
−5
0
5
MR effect size for
'Gut microbiota abundance (genus Bacteroides id.918)' on 'Drug−induced obesity'

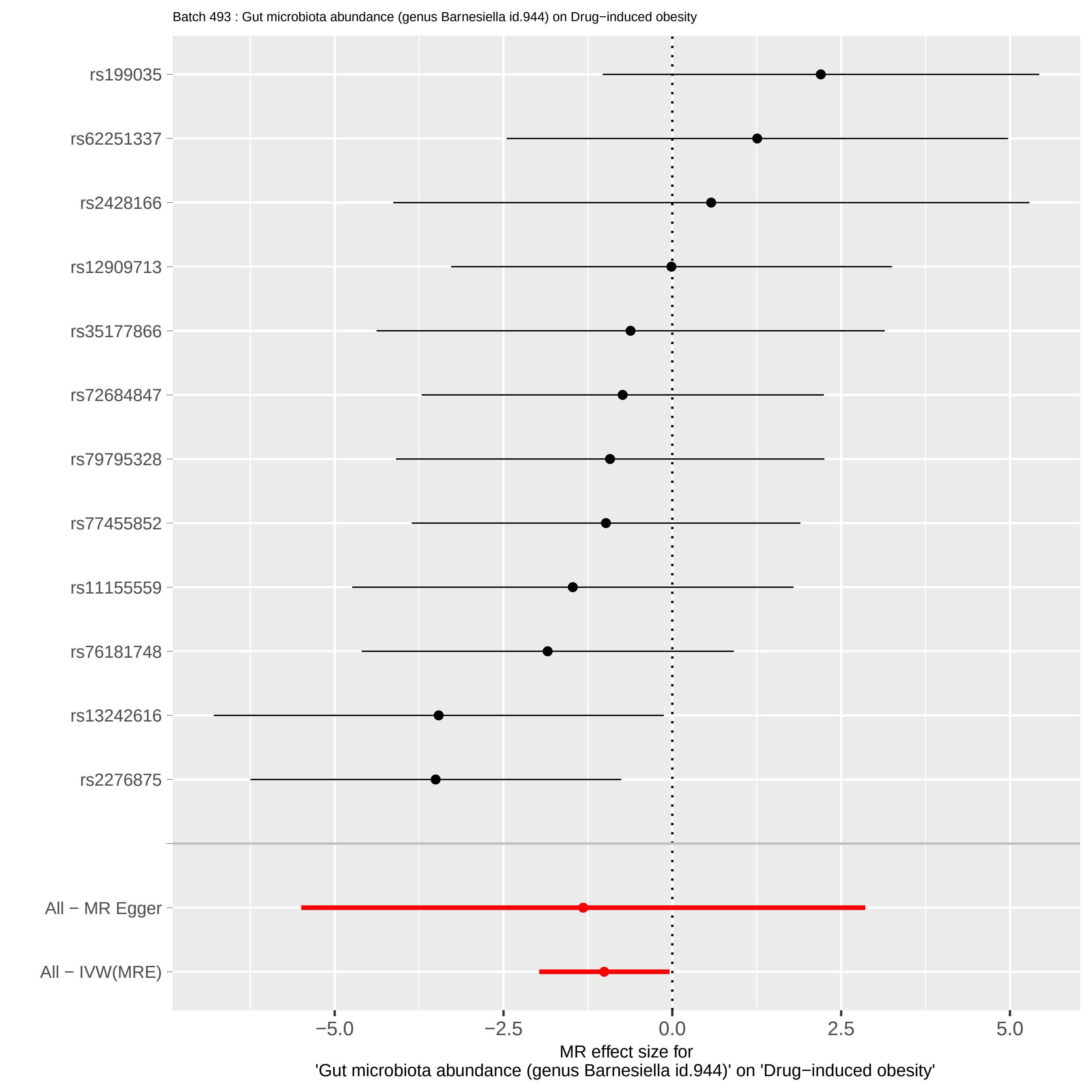

Batch 493 : Gut microbiota abundance (genus Barnesiella id.944) on Drug−induced obesity
rs199035
rs62251337
rs2428166
rs12909713
rs35177866
rs72684847
rs79795328
rs77455852
rs11155559
rs76181748
rs13242616
rs2276875
All − MR Egger
All − IVW(MRE)
−5.0
−2.5
0.0
2.5
5.0
MR effect size for
'Gut microbiota abundance (genus Barnesiella id.944)' on 'Drug−induced obesity'

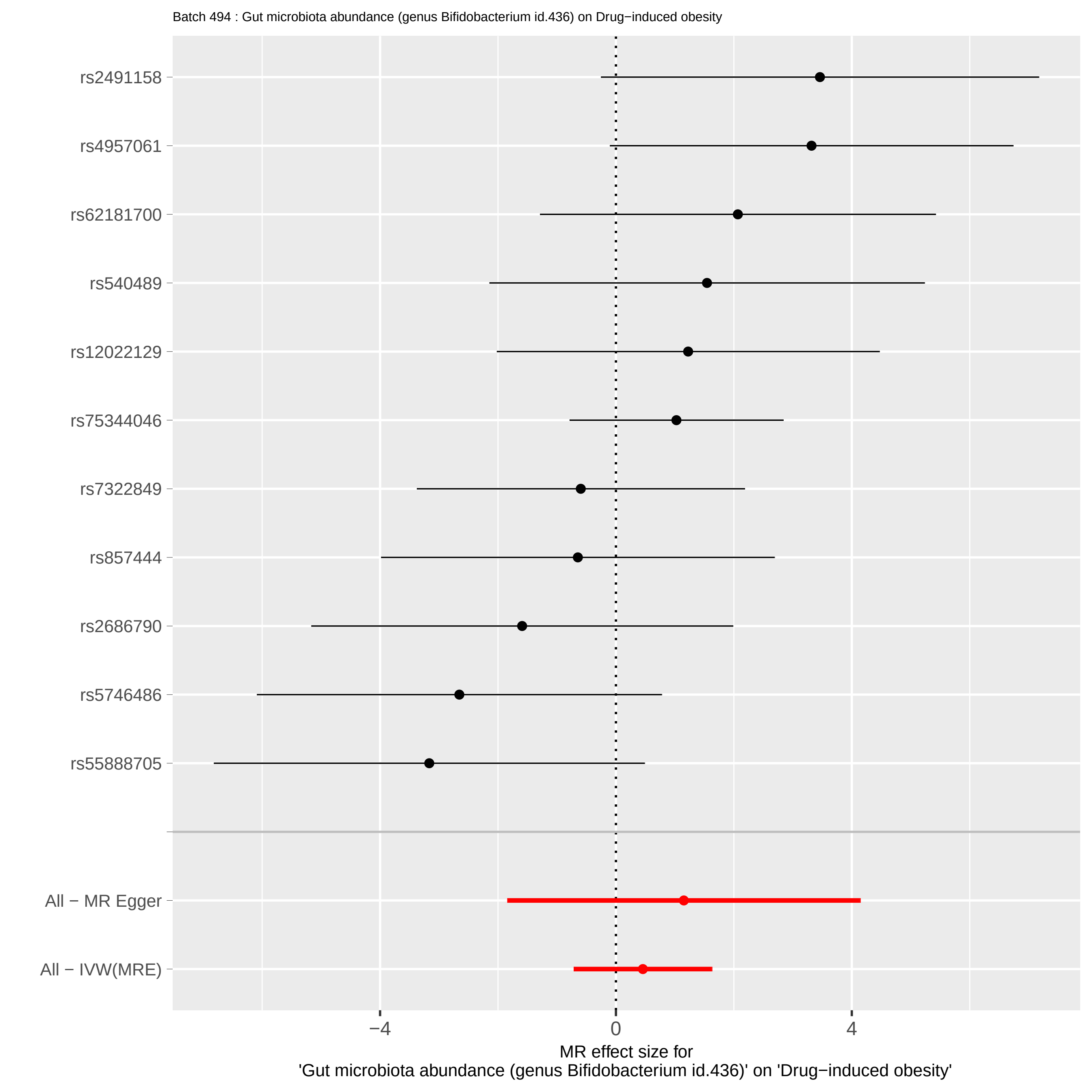
Batch 494 : Gut microbiota abundance (genus Bifidobacterium id.436) on Drug−induced obesity
rs2491158
rs4957061
rs62181700
rs540489
rs12022129
rs75344046
rs7322849
rs857444
rs2686790
rs5746486
rs55888705
All − MR Egger
All − IVW(MRE)
−4
0
4
MR effect size for
'Gut microbiota abundance (genus Bifidobacterium id.436)' on 'Drug−induced obesity'

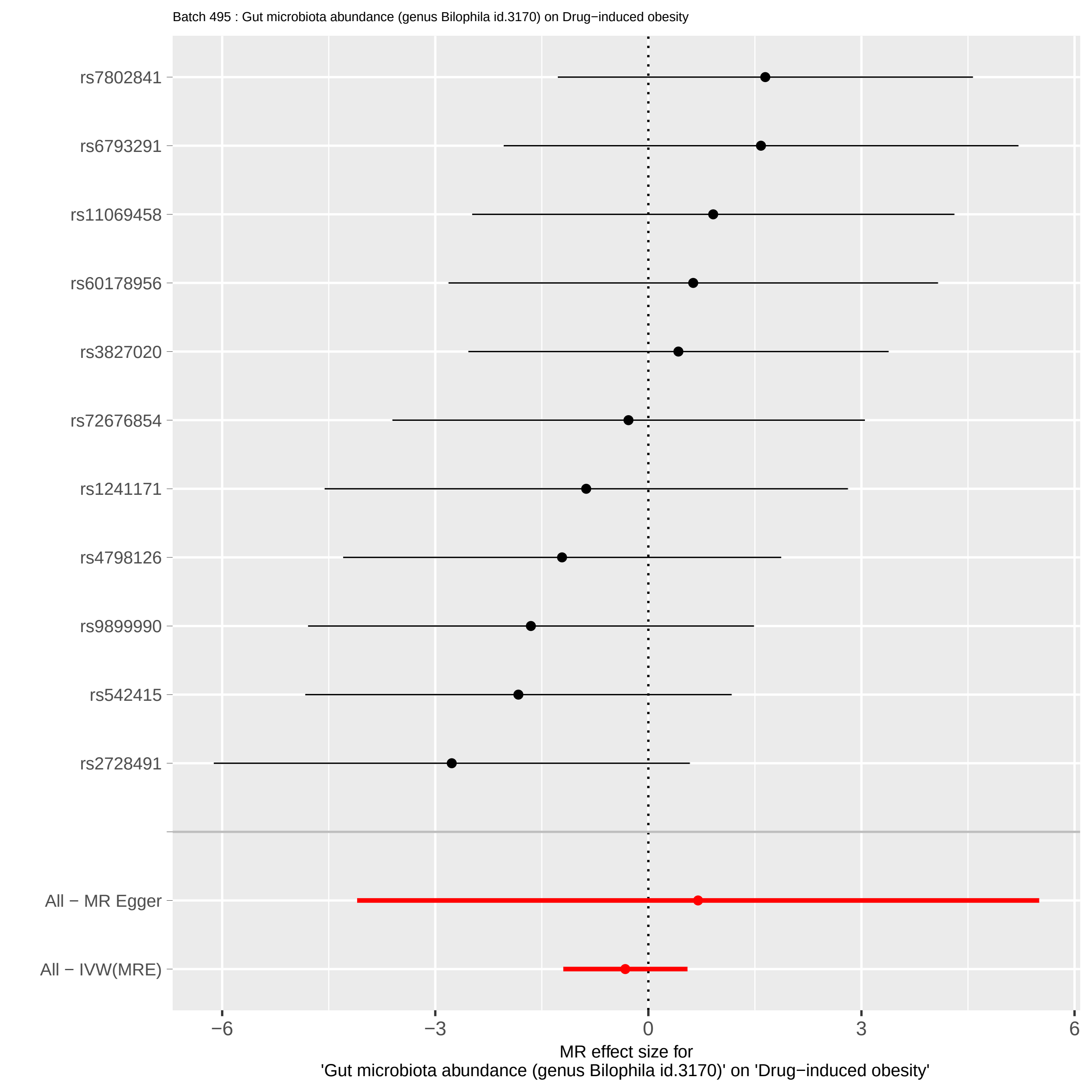

Batch 495 : Gut microbiota abundance (genus Bilophila id.3170) on Drug−induced obesity
rs7802841
rs6793291
rs11069458
rs60178956
rs3827020
rs72676854
rs1241171
rs4798126
rs9899990
rs542415
rs2728491
All − MR Egger
All − IVW(MRE)
−6
−3
0
3
6
MR effect size for
'Gut microbiota abundance (genus Bilophila id.3170)' on 'Drug−induced obesity'

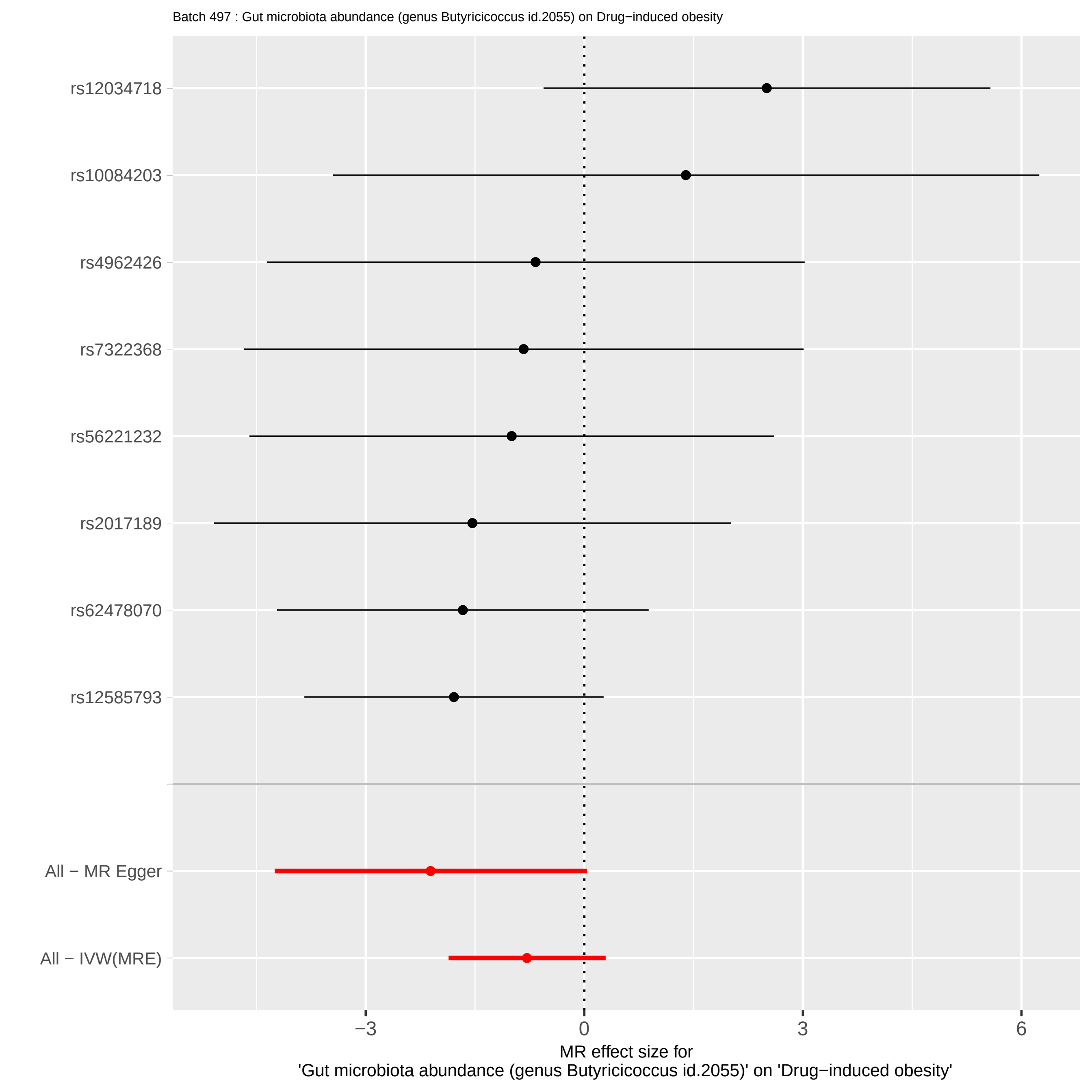

Batch 497 : Gut microbiota abundance (genus Butyricicoccus id.2055) on Drug−induced obesity
rs12034718
rs10084203
rs4962426
rs7322368
rs56221232
rs2017189
rs62478070
rs12585793
All − MR Egger
All − IVW(MRE)
−3
0
3
6
MR effect size for
'Gut microbiota abundance (genus Butyricicoccus id.2055)' on 'Drug−induced obesity'

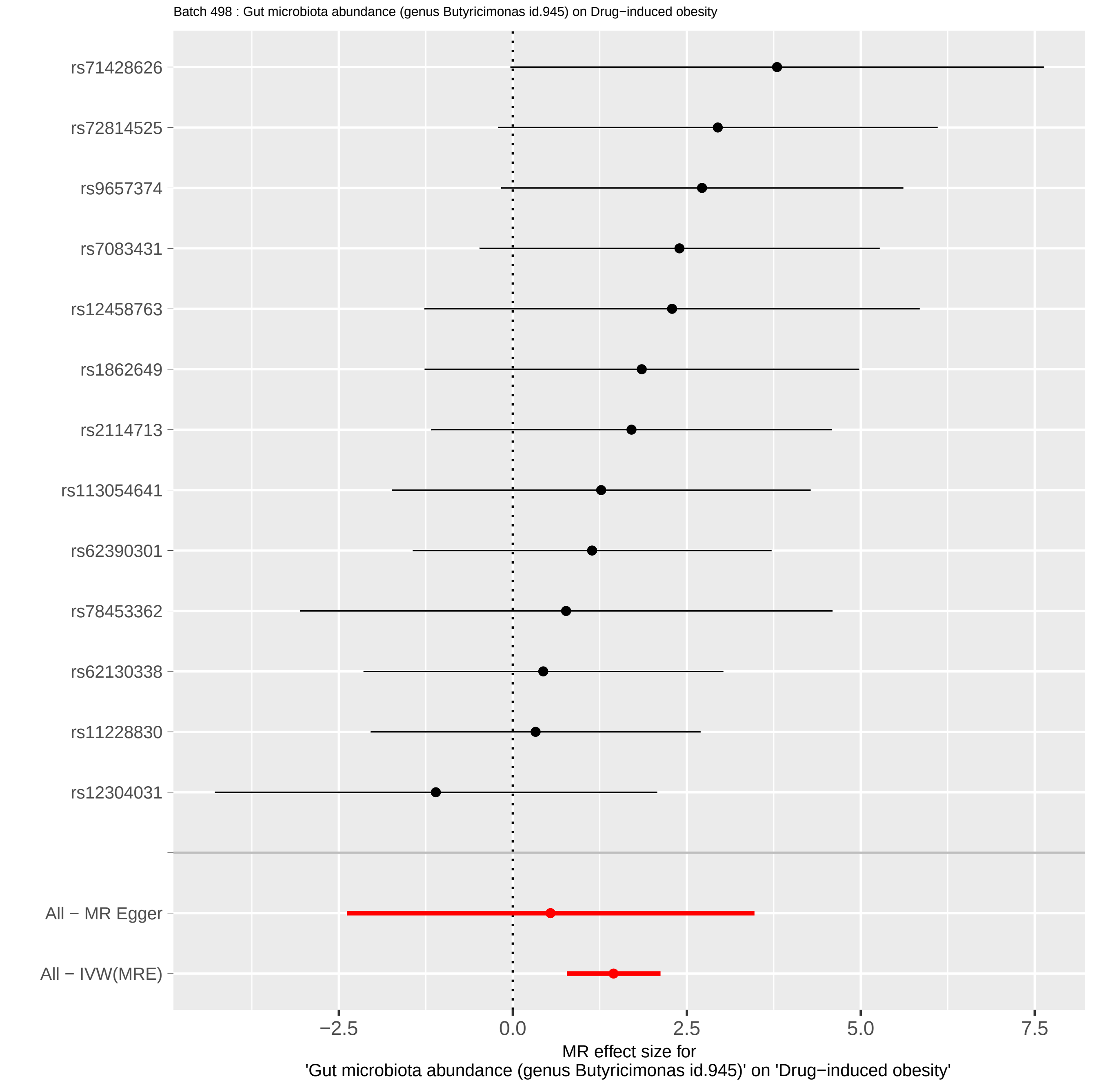

Batch 498 : Gut microbiota abundance (genus Butyricimonas id.945) on Drug−induced obesity
rs71428626
rs72814525
rs9657374
rs7083431
rs12458763
rs1862649
rs2114713
rs113054641
rs62390301
rs78453362
rs62130338
rs11228830
rs12304031
All − MR Egger
All − IVW(MRE)
−2.5
0.0
2.5
5.0
7.5
MR effect size for
'Gut microbiota abundance (genus Butyricimonas id.945)' on 'Drug−induced obesity'

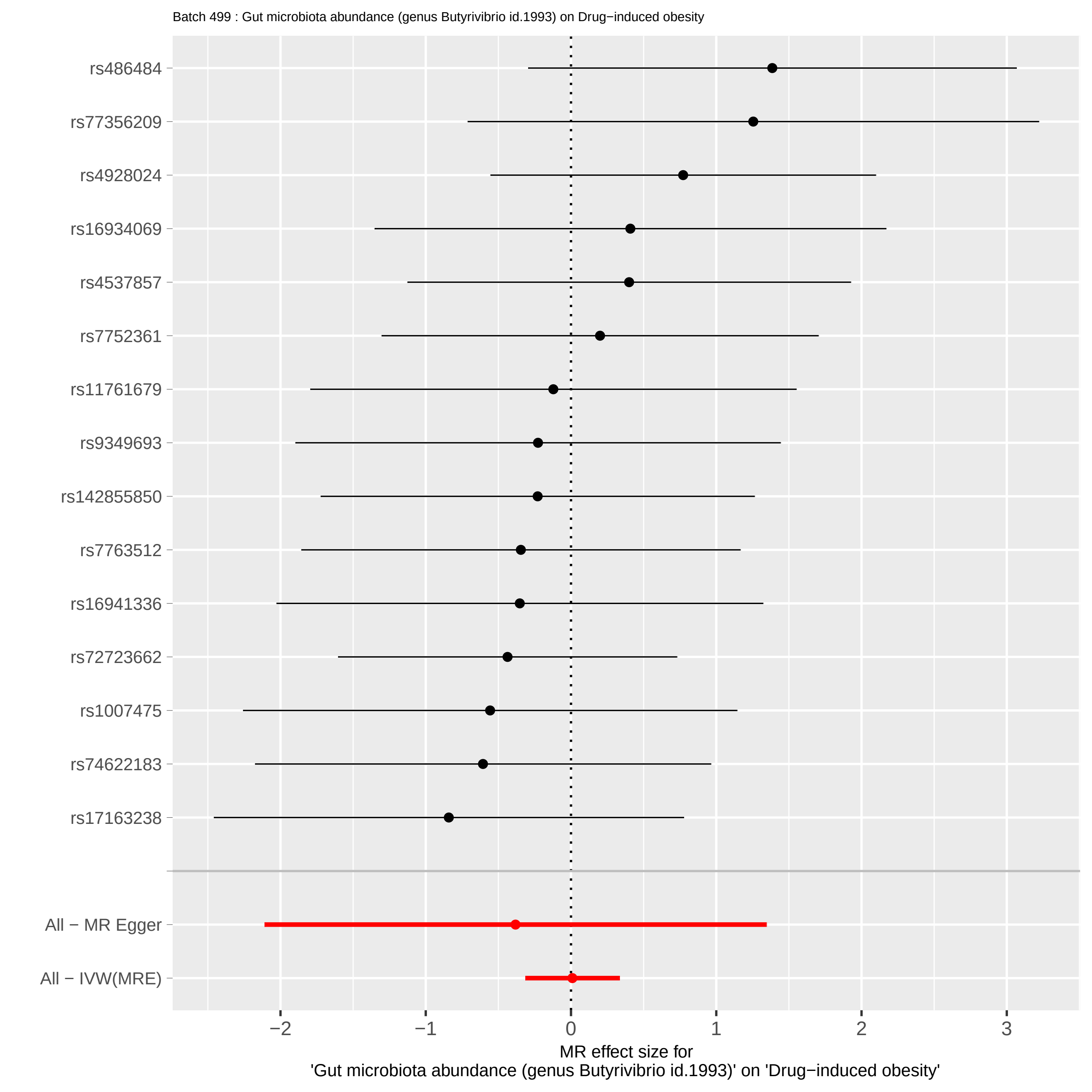
Batch 499 : Gut microbiota abundance (genus Butyrivibrio id.1993) on Drug−induced obesity
rs486484
rs77356209
rs4928024
rs16934069
rs4537857
rs7752361
rs11761679
rs9349693
rs142855850
rs7763512
rs16941336
rs72723662
rs1007475
rs74622183
rs17163238
All − MR Egger
All − IVW(MRE)
−2
−1
0
1
2
3
MR effect size for
'Gut microbiota abundance (genus Butyrivibrio id.1993)' on 'Drug−induced obesity'

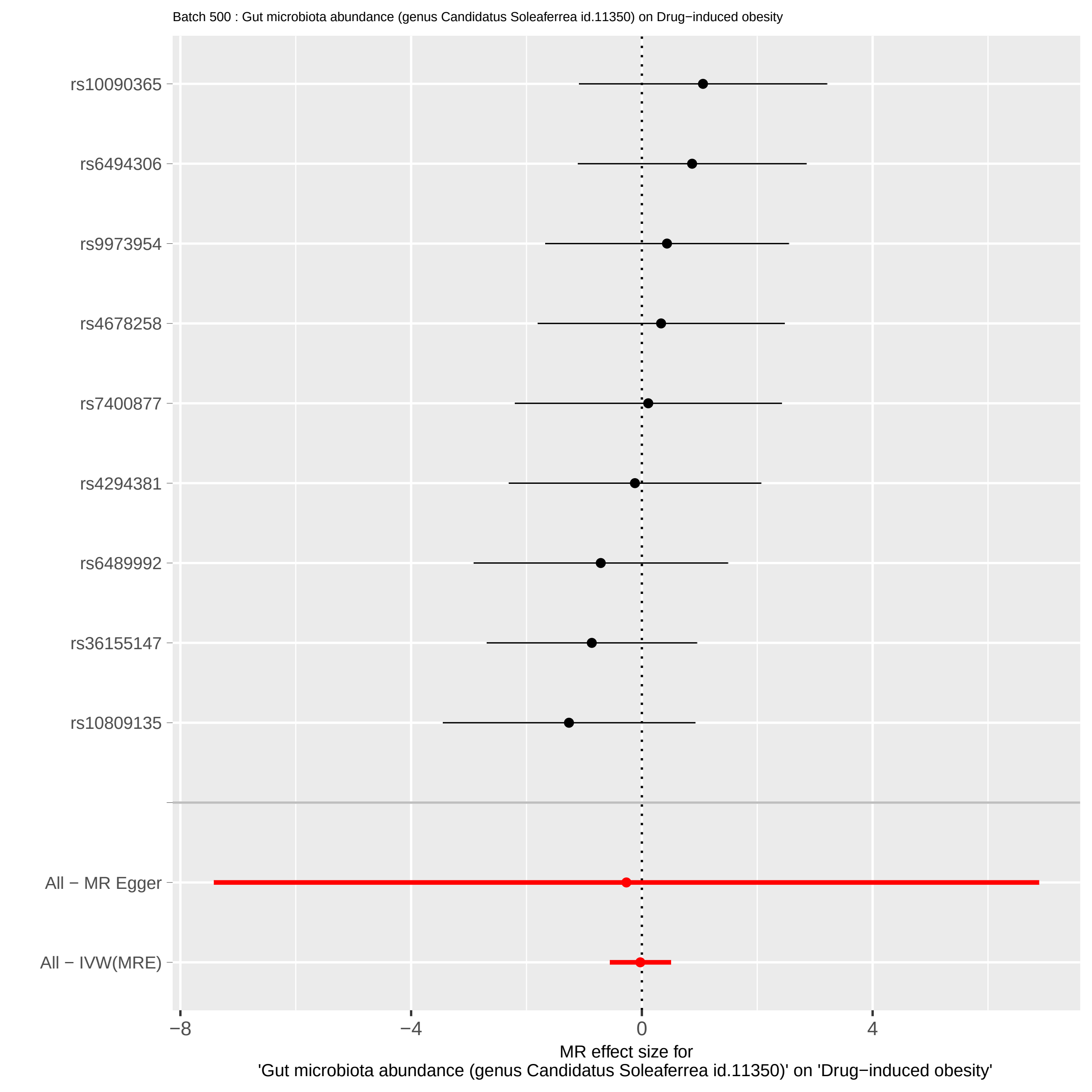

Batch 500 : Gut microbiota abundance (genus Candidatus Soleaferrea id.11350) on Drug−induced obesity
rs10090365
rs6494306
rs9973954
rs4678258
rs7400877
rs4294381
rs6489992
rs36155147
rs10809135
All − MR Egger
All − IVW(MRE)
−8
−4
0
4
MR effect size for
'Gut microbiota abundance (genus Candidatus Soleaferrea id.11350)' on 'Drug−induced obesity'

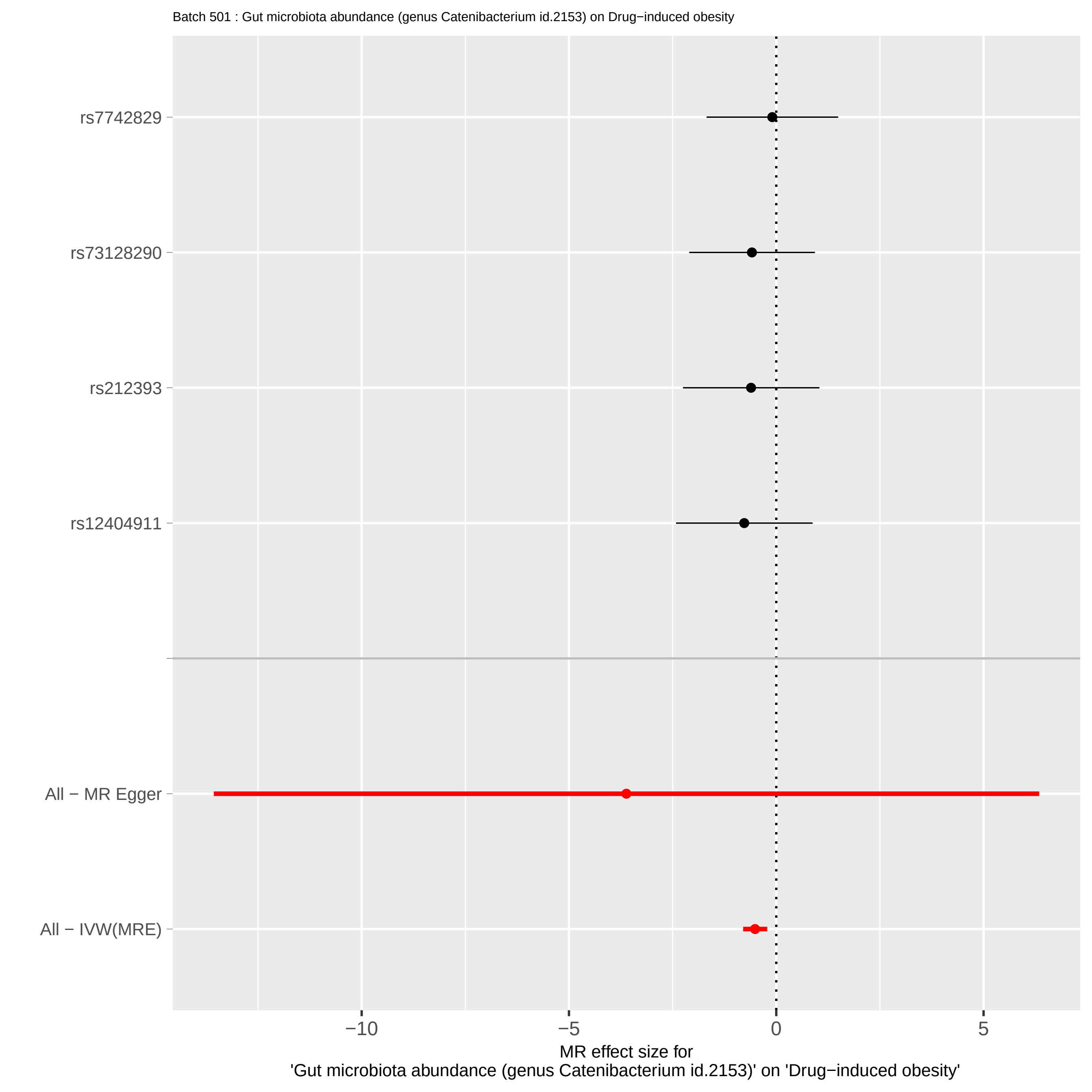

Batch 501 : Gut microbiota abundance (genus Catenibacterium id.2153) on Drug−induced obesity
rs7742829
rs73128290
rs212393
rs12404911
All − MR Egger
All − IVW(MRE)
−10
−5
0
5
MR effect size for
'Gut microbiota abundance (genus Catenibacterium id.2153)' on 'Drug−induced obesity'

Batch 502 : Gut microbiota abundance (genus Christensenellaceae R 7group id.11283) on Drug−induced obesity

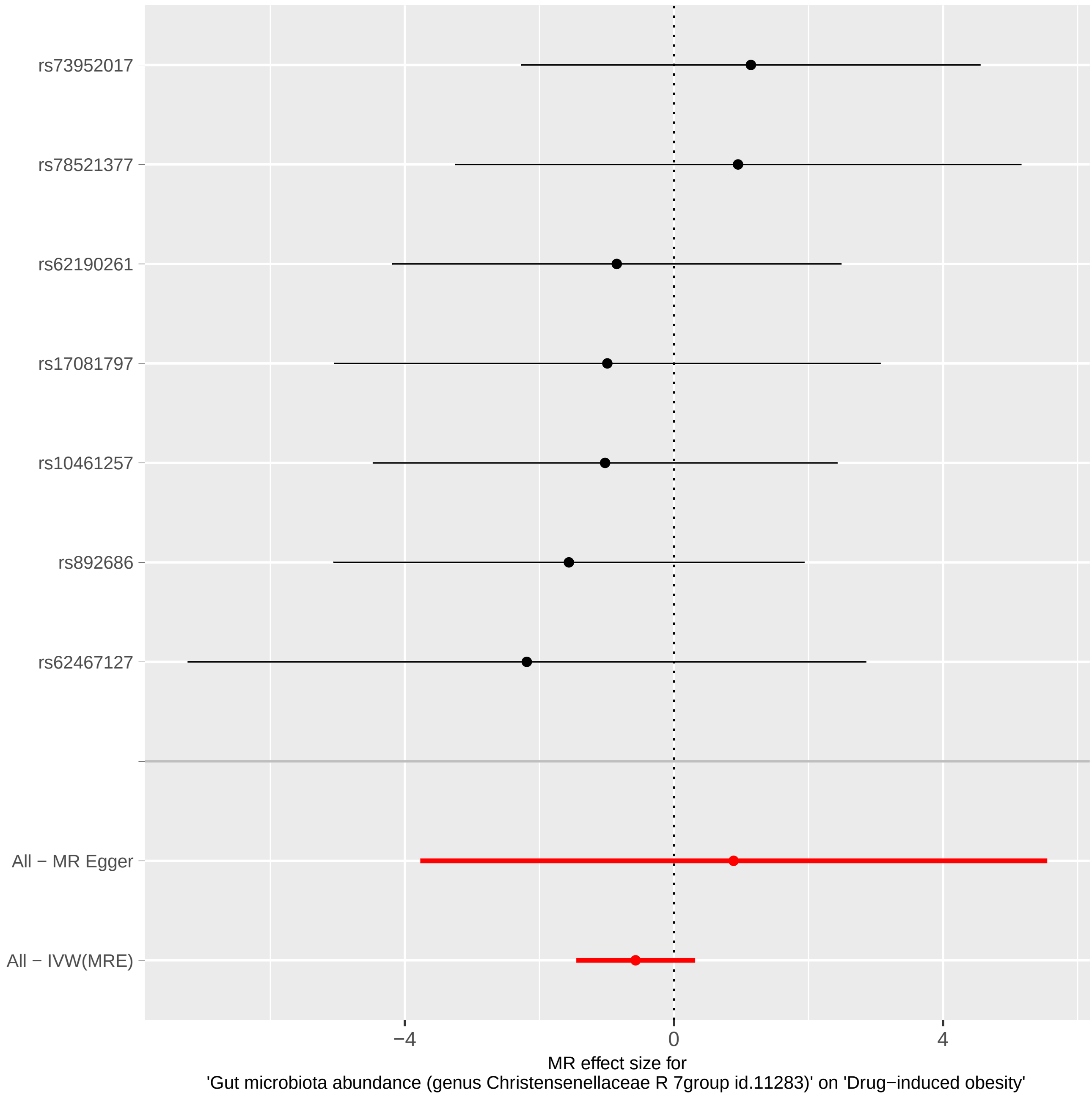

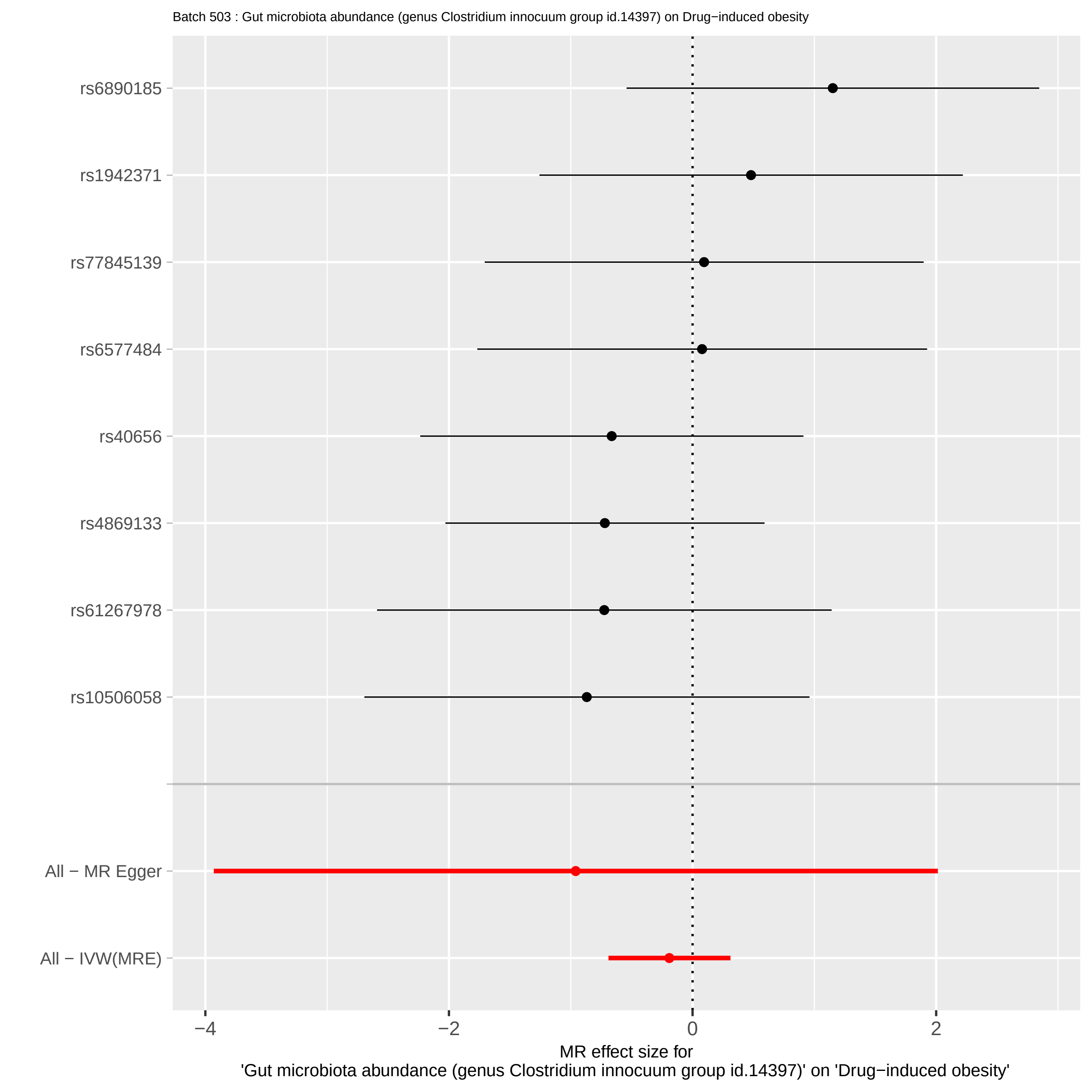
Batch 503 : Gut microbiota abundance (genus Clostridium innocuum group id.14397) on Drug−induced obesity
rs6890185
rs1942371
rs77845139
rs6577484
rs40656
rs4869133
rs61267978
rs10506058
All − MR Egger
All − IVW(MRE)
−4
−2
0
2
MR effect size for
'Gut microbiota abundance (genus Clostridium innocuum group id.14397)' on 'Drug−induced obesity'

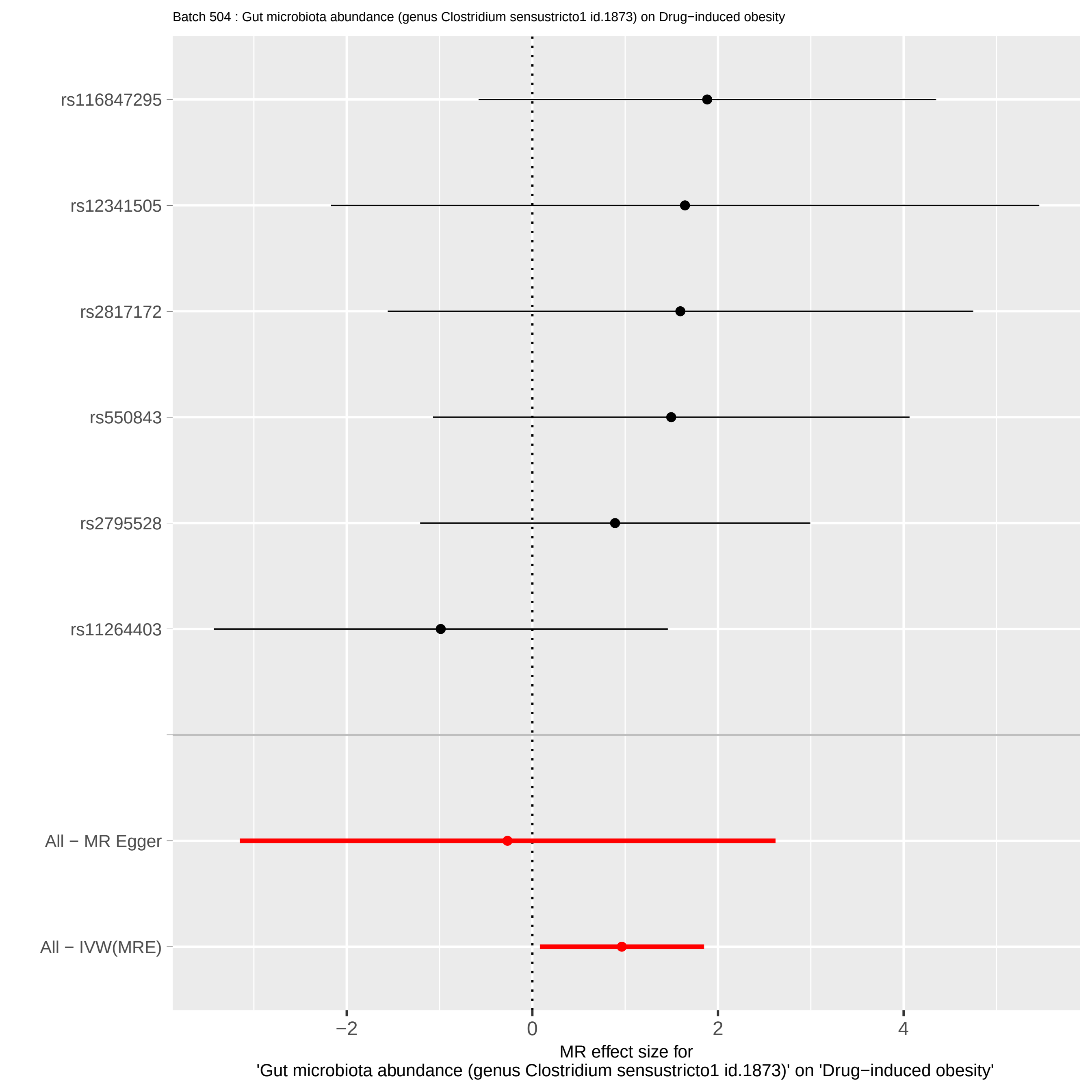
Batch 504 : Gut microbiota abundance (genus Clostridium sensustricto1 id.1873) on Drug−induced obesity
rs116847295
rs12341505
rs2817172
rs550843
rs2795528
rs11264403
All − MR Egger
All − IVW(MRE)
−2
0
2
4
MR effect size for
'Gut microbiota abundance (genus Clostridium sensustricto1 id.1873)' on 'Drug−induced obesity'

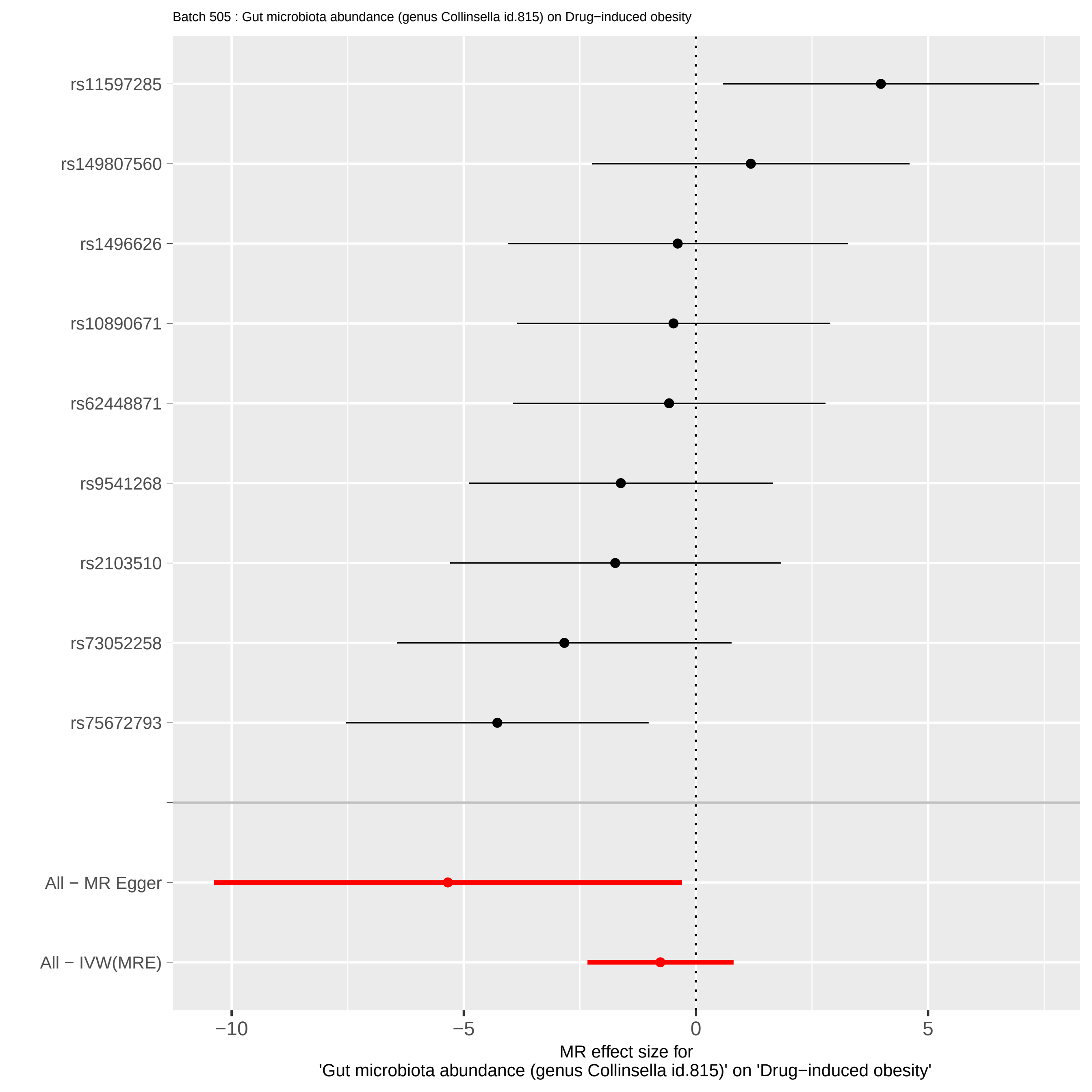

Batch 505 : Gut microbiota abundance (genus Collinsella id.815) on Drug−induced obesity
rs11597285
rs149807560
rs1496626
rs10890671
rs62448871
rs9541268
rs2103510
rs73052258
rs75672793
All − MR Egger
All − IVW(MRE)
−10
−5
0
5
MR effect size for
'Gut microbiota abundance (genus Collinsella id.815)' on 'Drug−induced obesity'

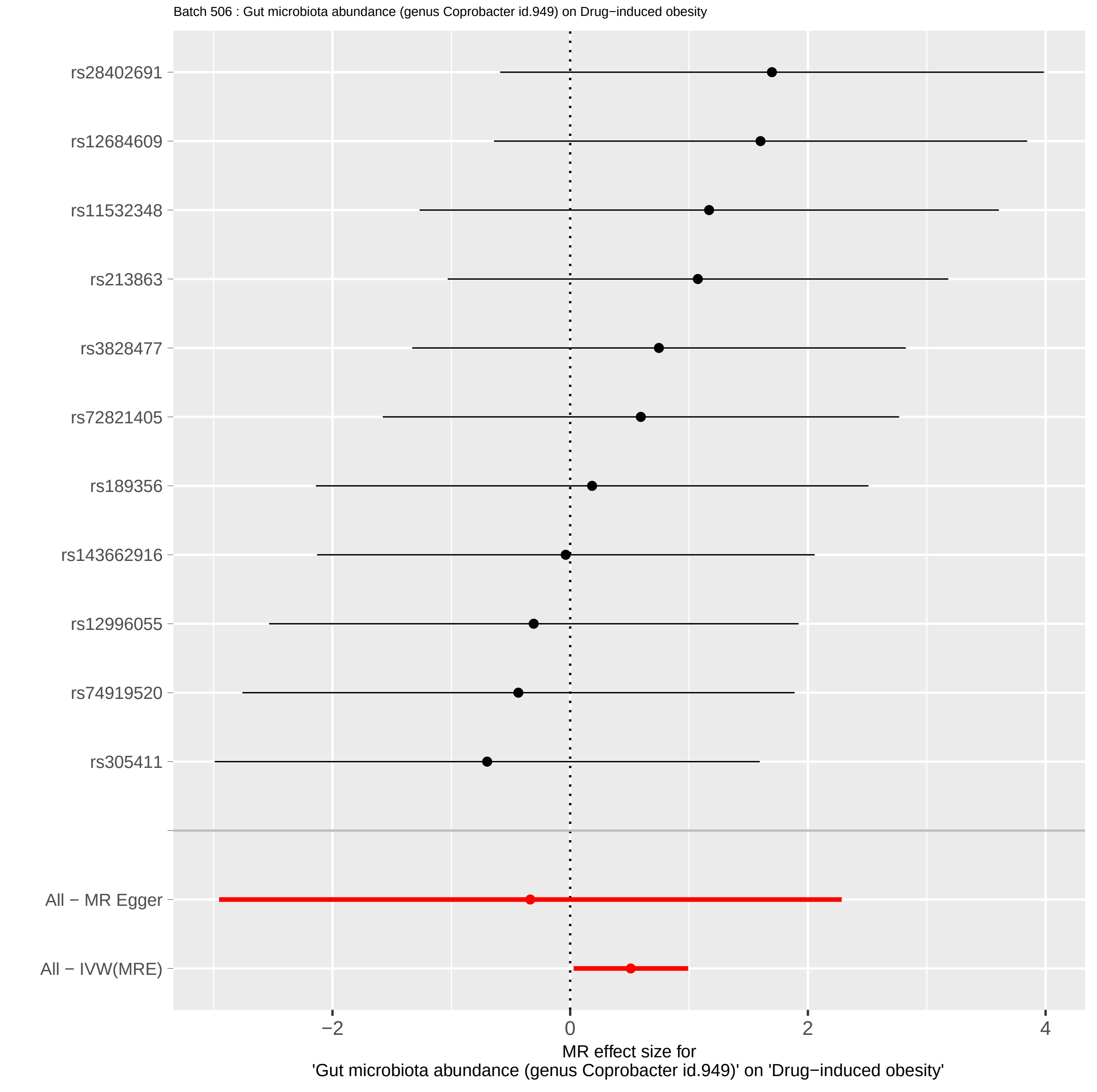

Batch 506 : Gut microbiota abundance (genus Coprobacter id.949) on Drug−induced obesity
rs28402691
rs12684609
rs11532348
rs213863
rs3828477
rs72821405
rs189356
rs143662916
rs12996055
rs74919520
rs305411
All − MR Egger
All − IVW(MRE)
−2
0
2
4
MR effect size for
'Gut microbiota abundance (genus Coprobacter id.949)' on 'Drug−induced obesity'

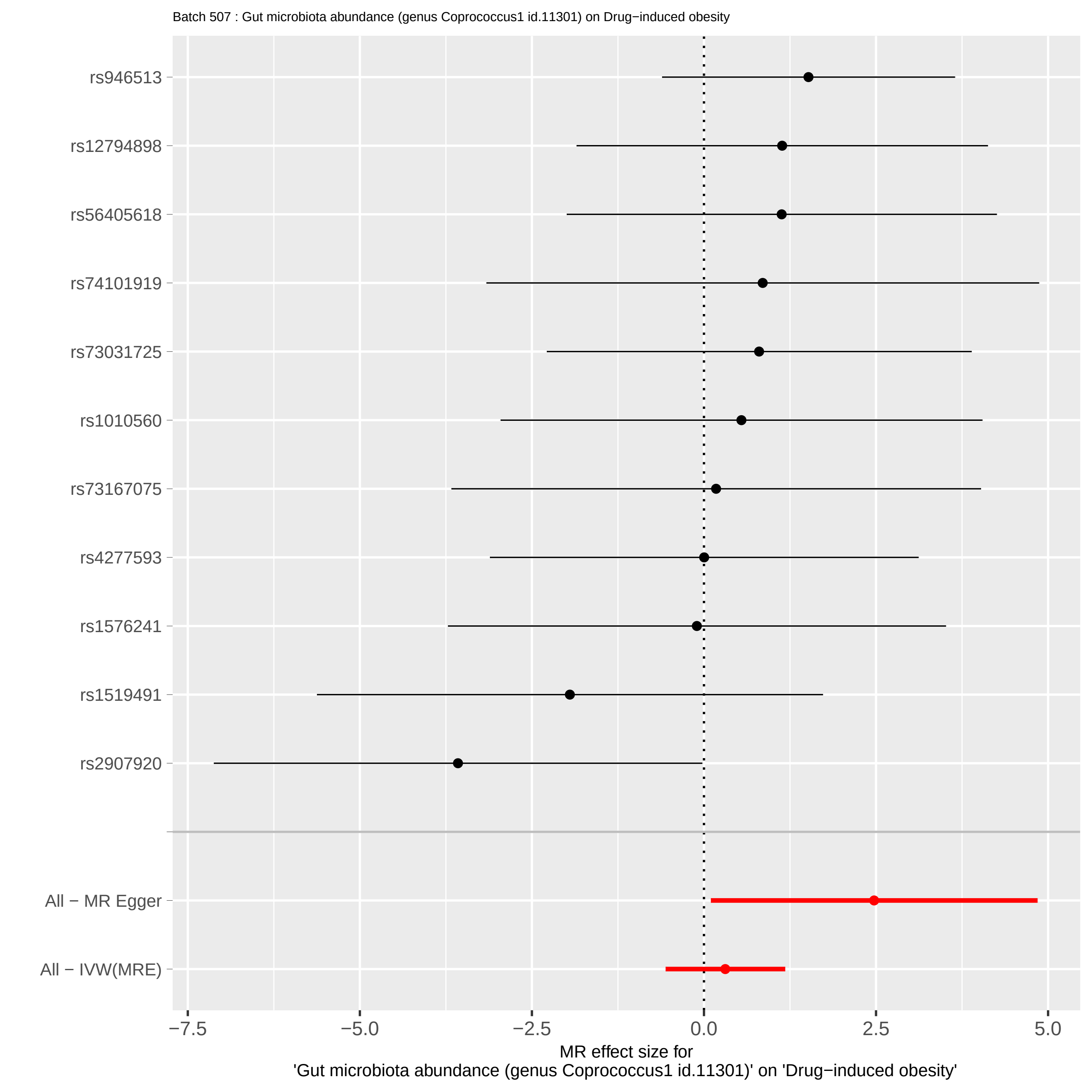
Batch 507 : Gut microbiota abundance (genus Coprococcus1 id.11301) on Drug−induced obesity
rs946513
rs12794898
rs56405618
rs74101919
rs73031725
rs1010560
rs73167075
rs4277593
rs1576241
rs1519491
rs2907920
All − MR Egger
All − IVW(MRE)
−7.5
−5.0
−2.5
0.0
2.5
5.0
MR effect size for
'Gut microbiota abundance (genus Coprococcus1 id.11301)' on 'Drug−induced obesity'

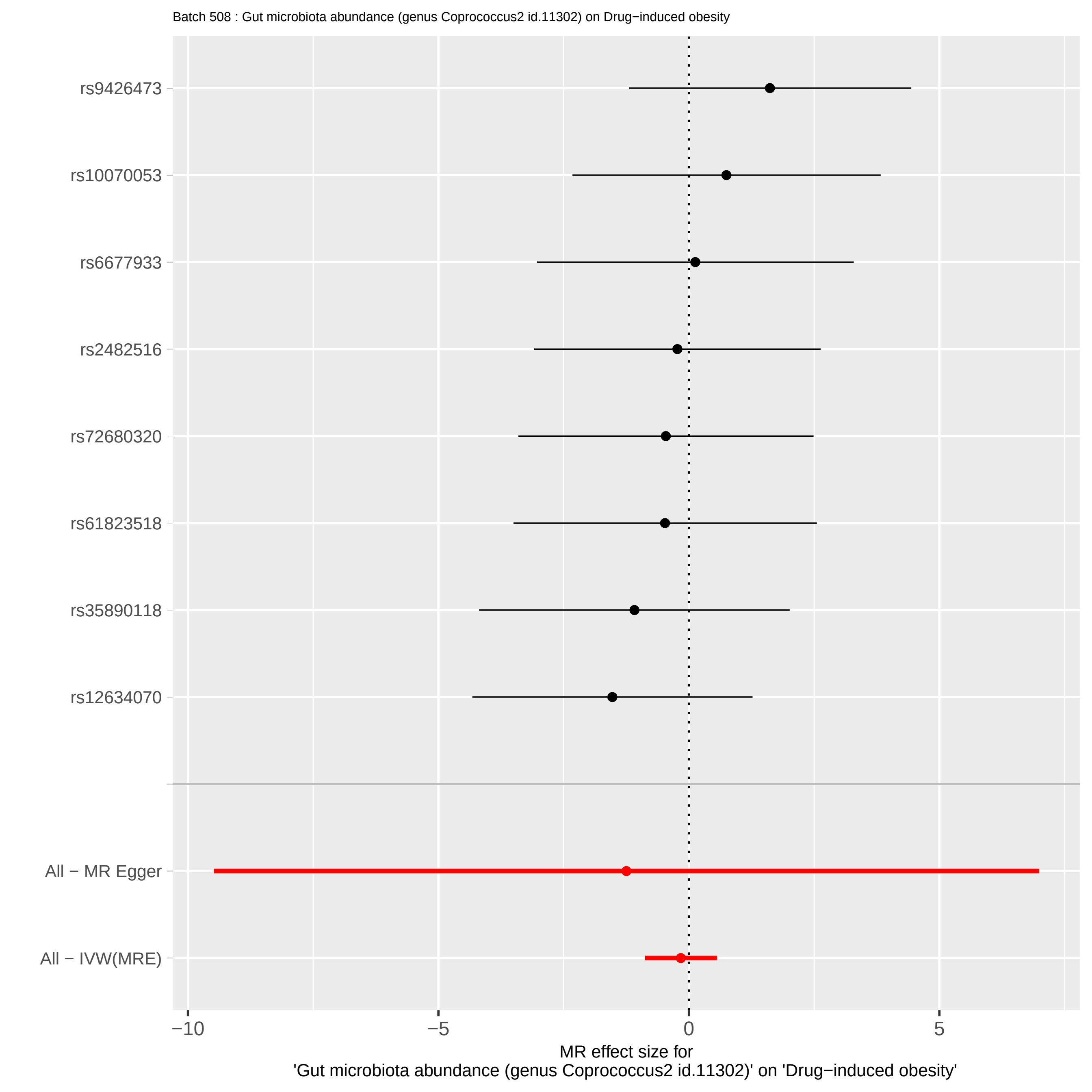

Batch 508 : Gut microbiota abundance (genus Coprococcus2 id.11302) on Drug−induced obesity
rs9426473
rs10070053
rs6677933
rs2482516
rs72680320
rs61823518
rs35890118
rs12634070
All − MR Egger
All − IVW(MRE)
−10
−5
0
5
MR effect size for
'Gut microbiota abundance (genus Coprococcus2 id.11302)' on 'Drug−induced obesity'

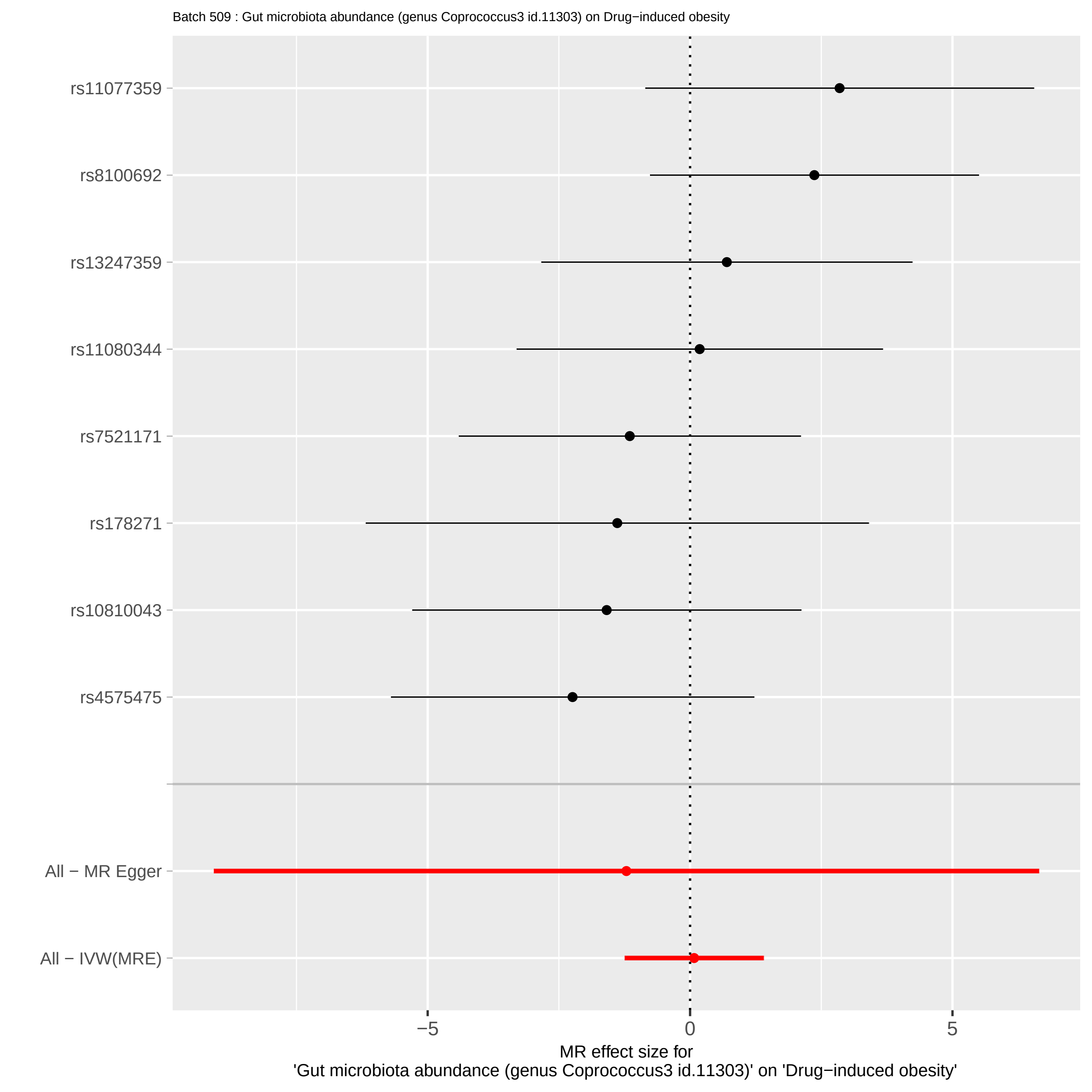

Batch 509 : Gut microbiota abundance (genus Coprococcus3 id.11303) on Drug−induced obesity
rs11077359
rs8100692
rs13247359
rs11080344
rs7521171
rs178271
rs10810043
rs4575475
All − MR Egger
All − IVW(MRE)
−5
0
5
MR effect size for
'Gut microbiota abundance (genus Coprococcus3 id.11303)' on 'Drug−induced obesity'

Batch 510 : Gut microbiota abundance (genus Defluviitaleaceae UCG011 id.11287) on Drug−induced obesity

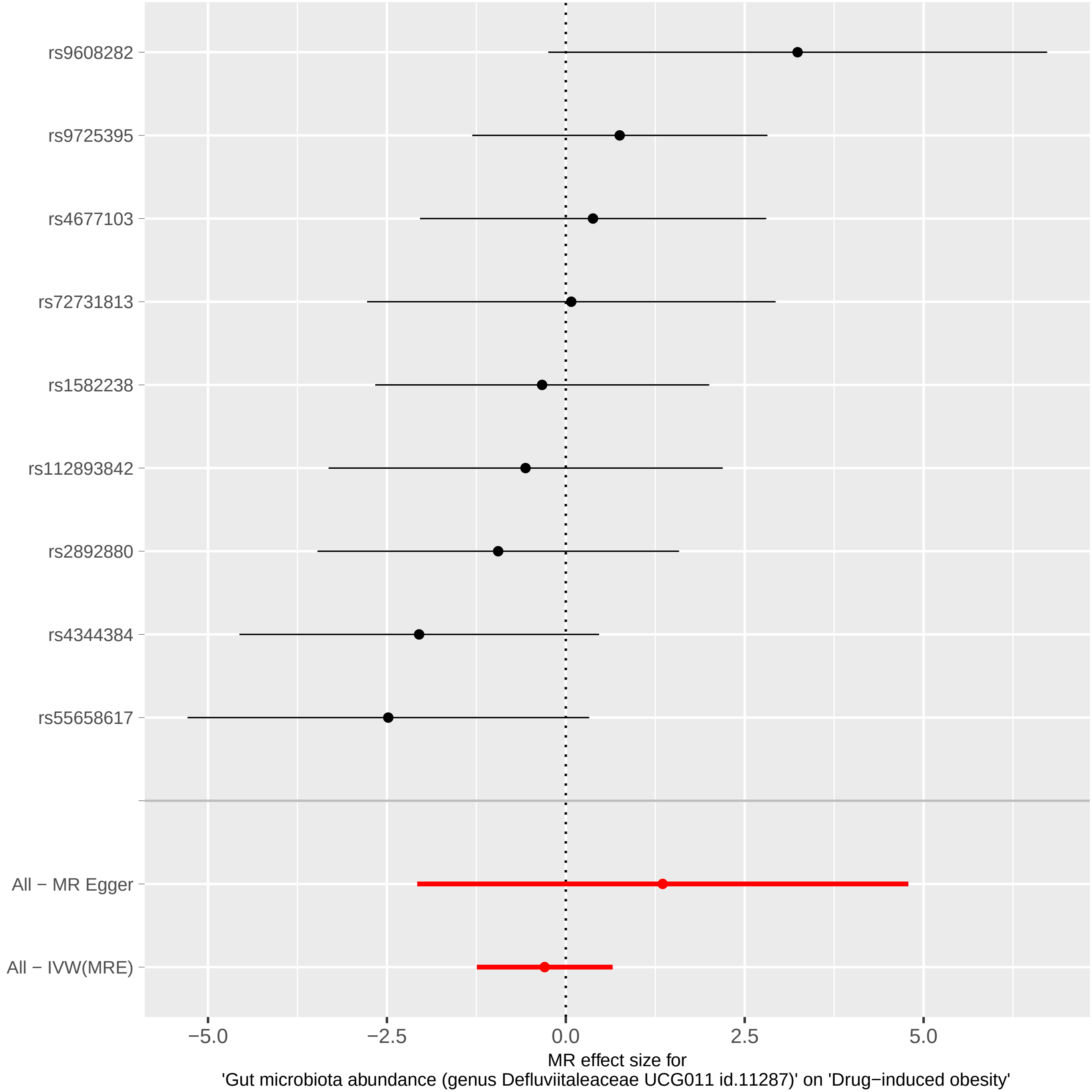

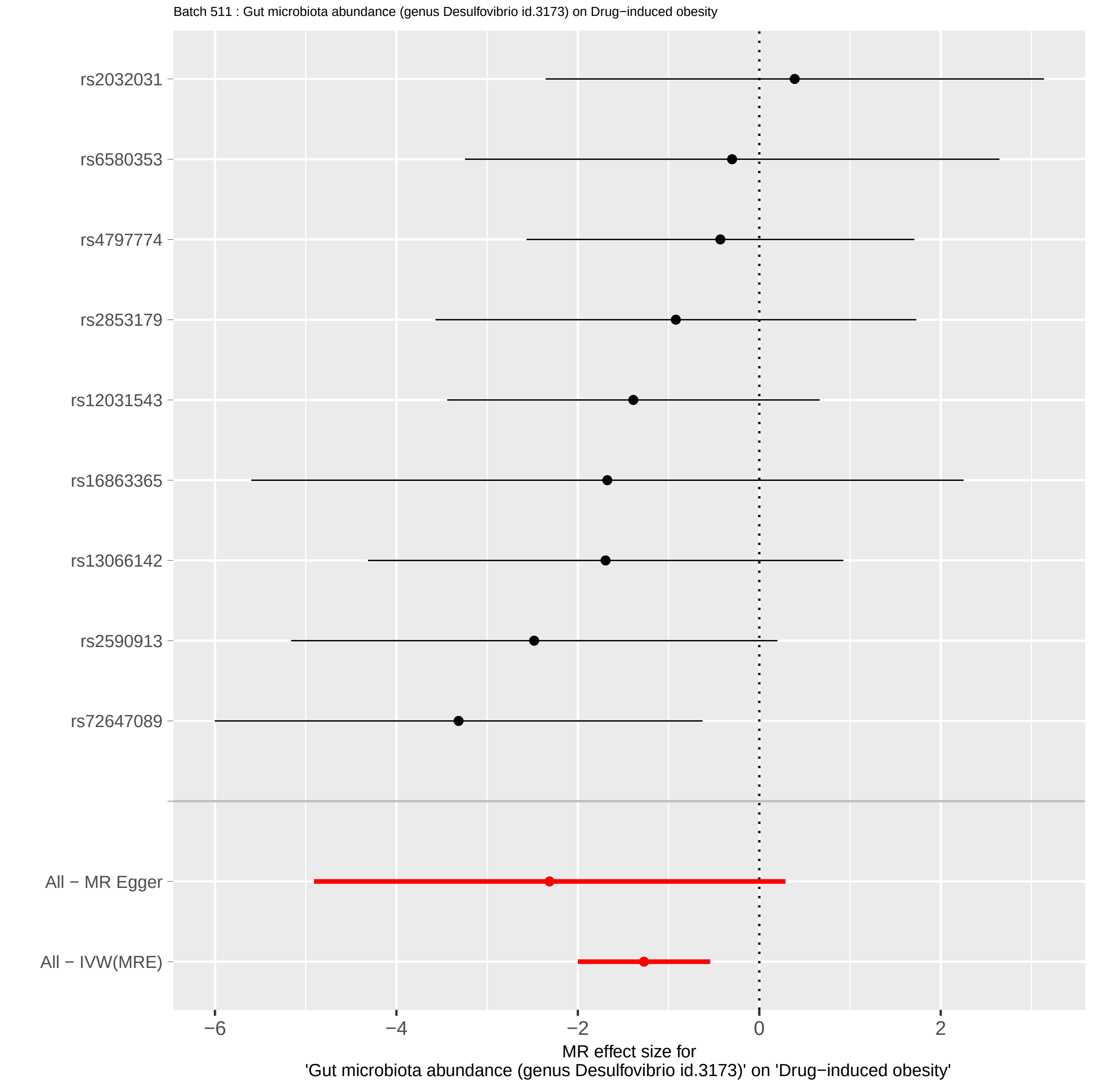

Batch 511 : Gut microbiota abundance (genus Desulfovibrio id.3173) on Drug−induced obesity
rs2032031
rs6580353
rs4797774
rs2853179
rs12031543
rs16863365
rs13066142
rs2590913
rs72647089
All − MR Egger
All − IVW(MRE)
−6
−4
−2
0
2
MR effect size for
'Gut microbiota abundance (genus Desulfovibrio id.3173)' on 'Drug−induced obesity'

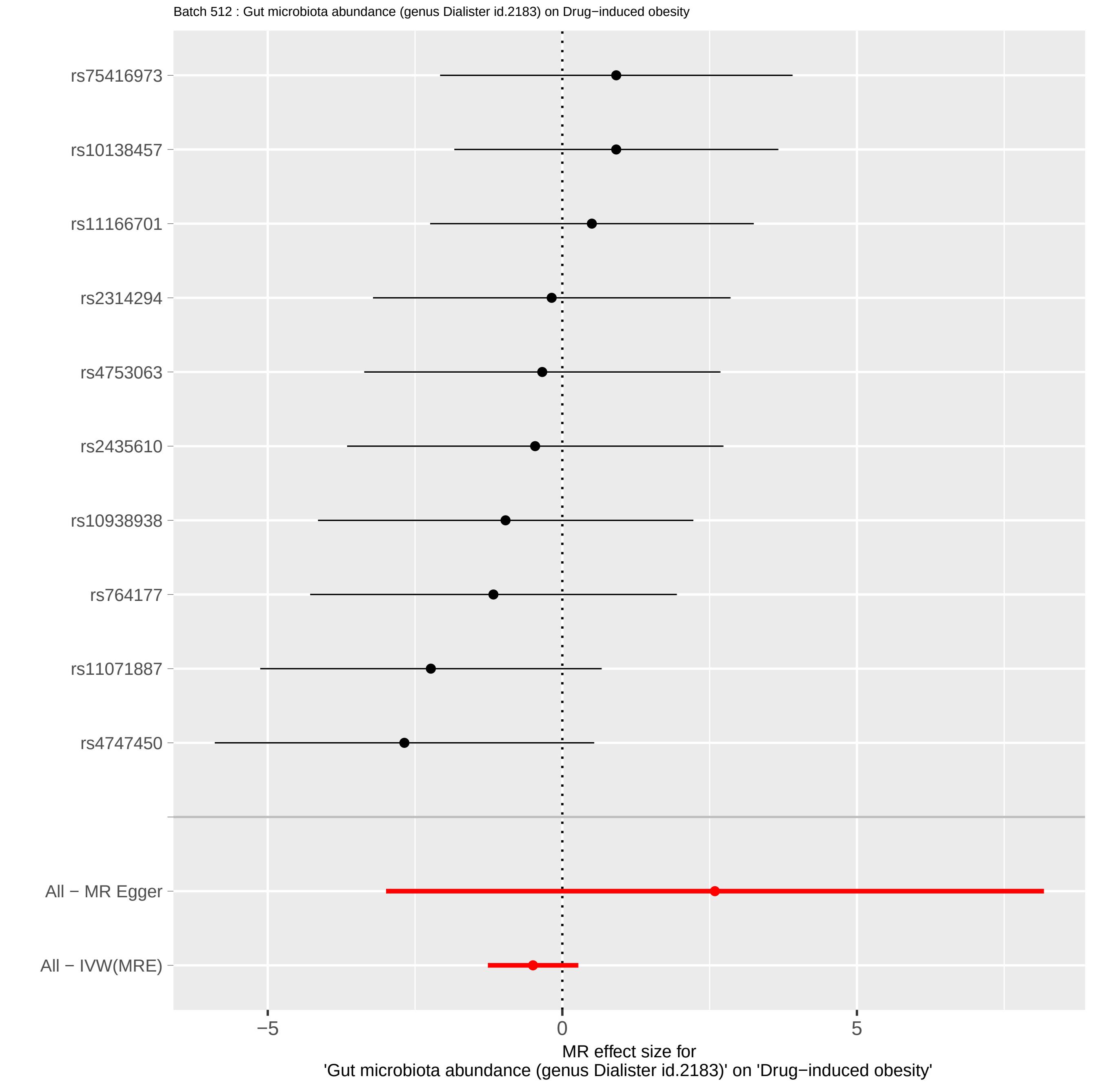
Batch 512 : Gut microbiota abundance (genus Dialister id.2183) on Drug−induced obesity
rs75416973
rs10138457
rs11166701
rs2314294
rs4753063
rs2435610
rs10938938
rs764177
rs11071887
rs4747450
All − MR Egger
All − IVW(MRE)
−5
0
5
MR effect size for
'Gut microbiota abundance (genus Dialister id.2183)' on 'Drug−induced obesity'

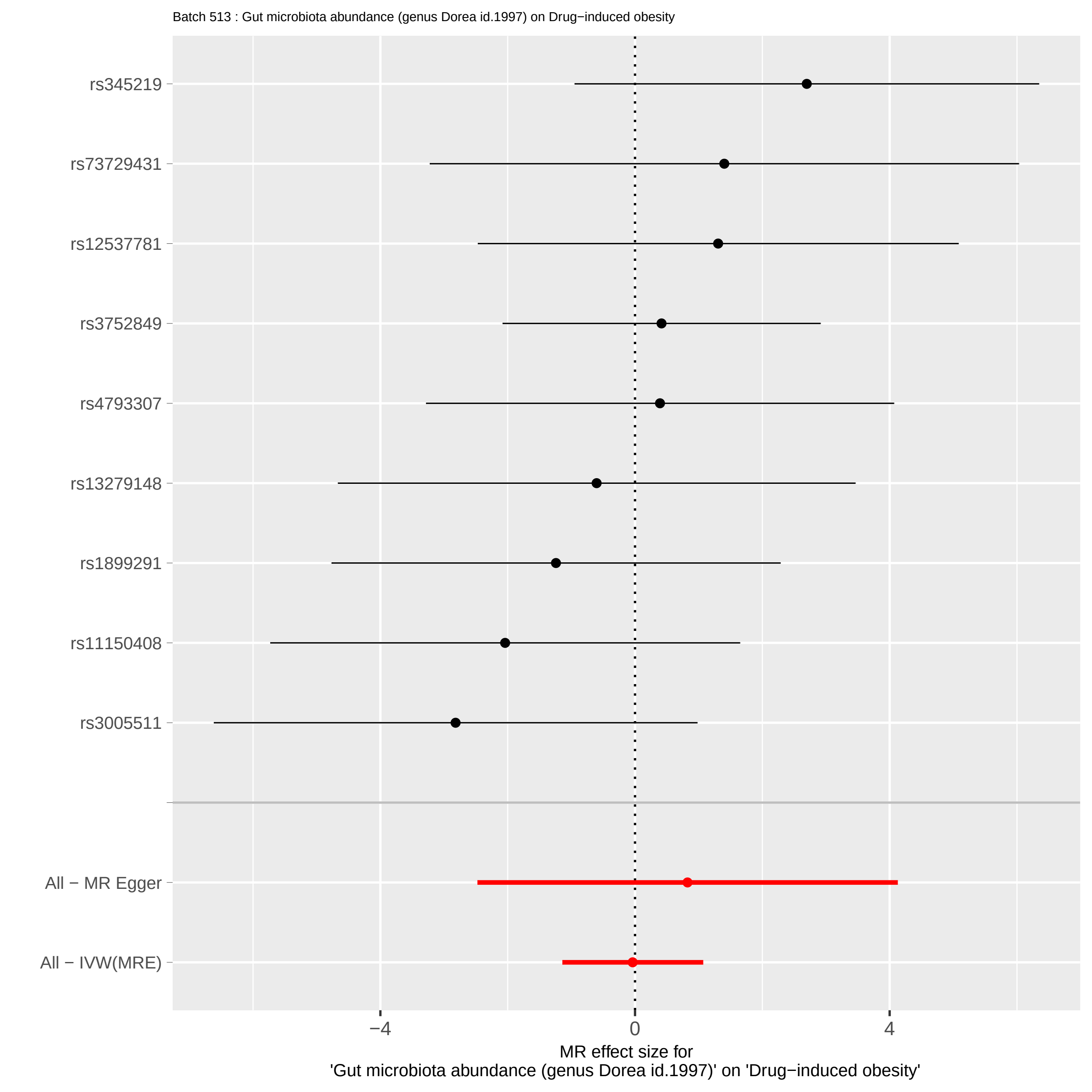

Batch 513 : Gut microbiota abundance (genus Dorea id.1997) on Drug−induced obesity
rs345219
rs73729431
rs12537781
rs3752849
rs4793307
rs13279148
rs1899291
rs11150408
rs3005511
All − MR Egger
All − IVW(MRE)
−4
0
4
MR effect size for
'Gut microbiota abundance (genus Dorea id.1997)' on 'Drug−induced obesity'

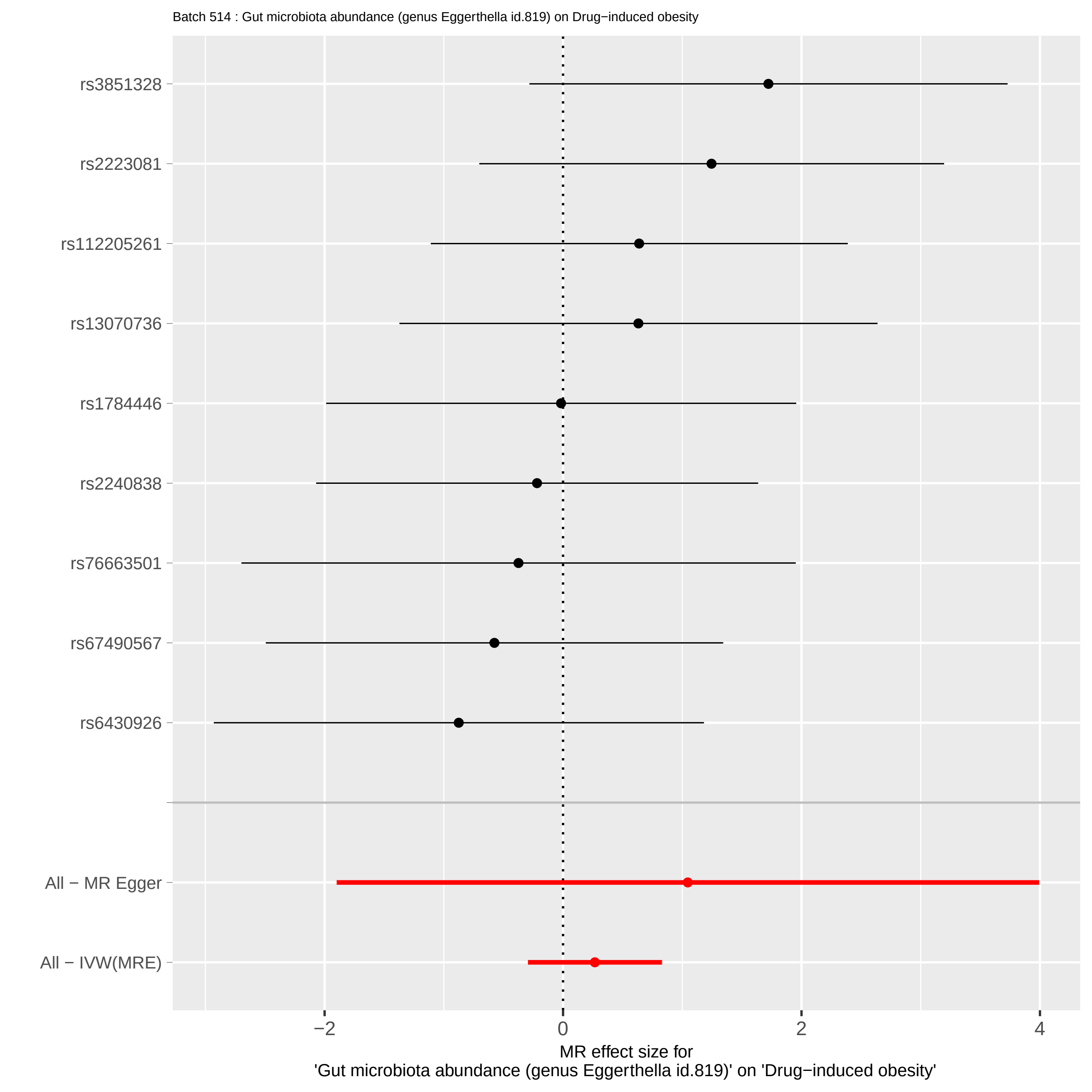

Batch 514 : Gut microbiota abundance (genus Eggerthella id.819) on Drug−induced obesity
rs3851328
rs2223081
rs112205261
rs13070736
rs1784446
rs2240838
rs76663501
rs67490567
rs6430926
All − MR Egger
All − IVW(MRE)
−2
0
2
4
MR effect size for
'Gut microbiota abundance (genus Eggerthella id.819)' on 'Drug−induced obesity'

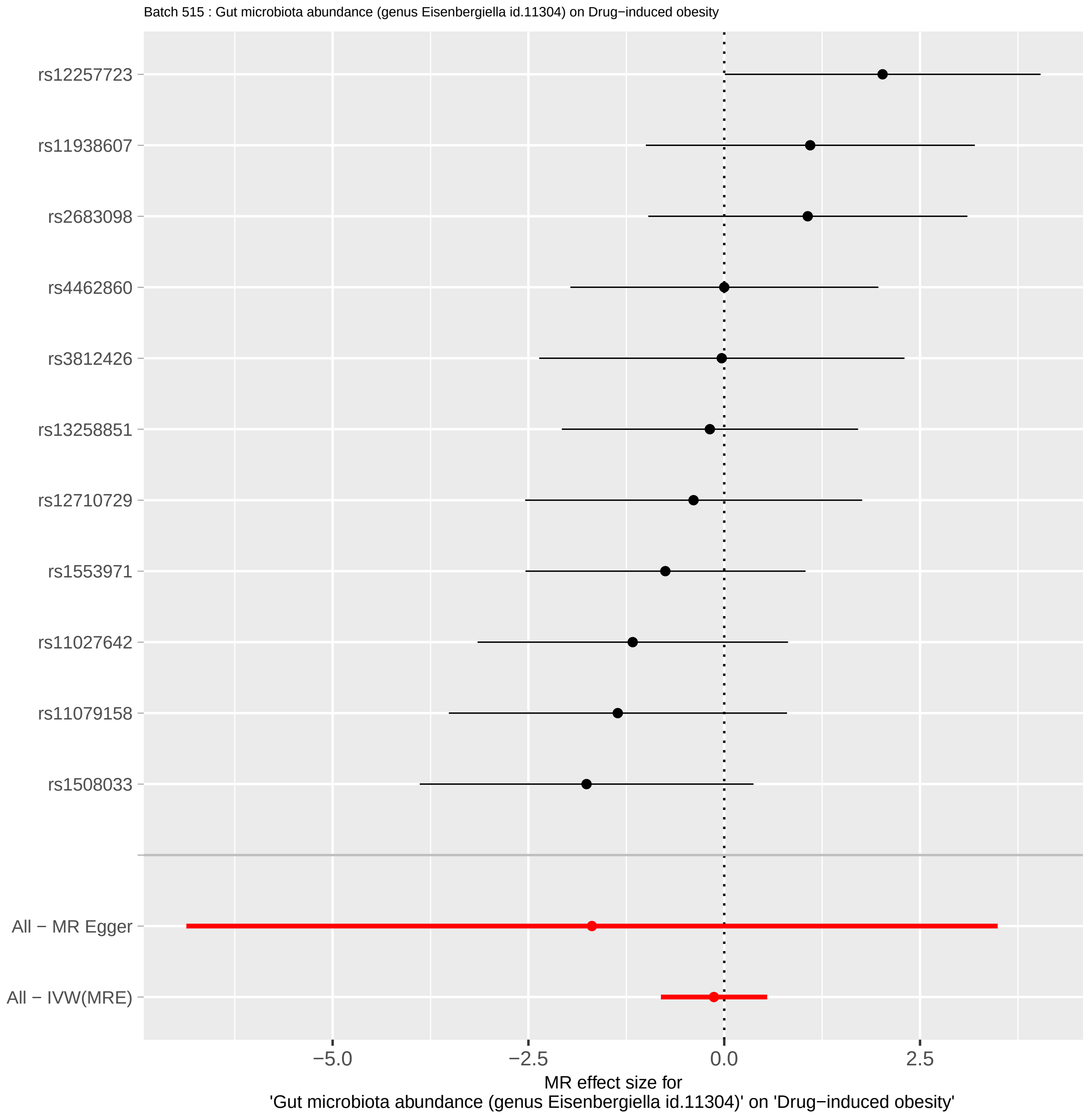

Batch 515 : Gut microbiota abundance (genus Eisenbergiella id.11304) on Drug−induced obesity
rs12257723
rs11938607
rs2683098
rs4462860
rs3812426
rs13258851
rs12710729
rs1553971
rs11027642
rs11079158
rs1508033
All − MR Egger
All − IVW(MRE)
−5.0
−2.5
0.0
2.5
MR effect size for
'Gut microbiota abundance (genus Eisenbergiella id.11304)' on 'Drug−induced obesity'

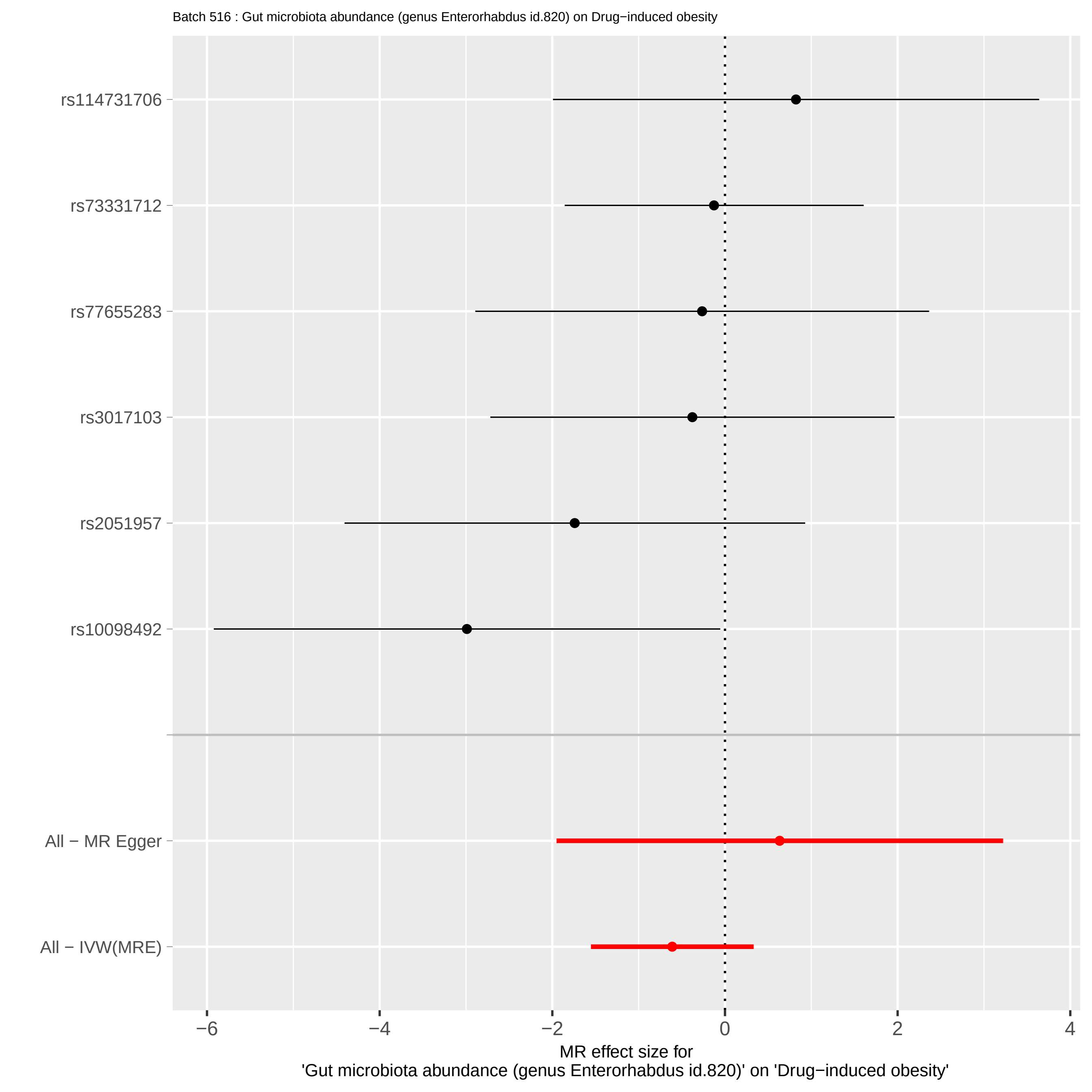

Batch 516 : Gut microbiota abundance (genus Enterorhabdus id.820) on Drug−induced obesity
rs114731706
rs73331712
rs77655283
rs3017103
rs2051957
rs10098492
All − MR Egger
All − IVW(MRE)
−6
−4
−2
0
2
4
MR effect size for
'Gut microbiota abundance (genus Enterorhabdus id.820)' on 'Drug−induced obesity'

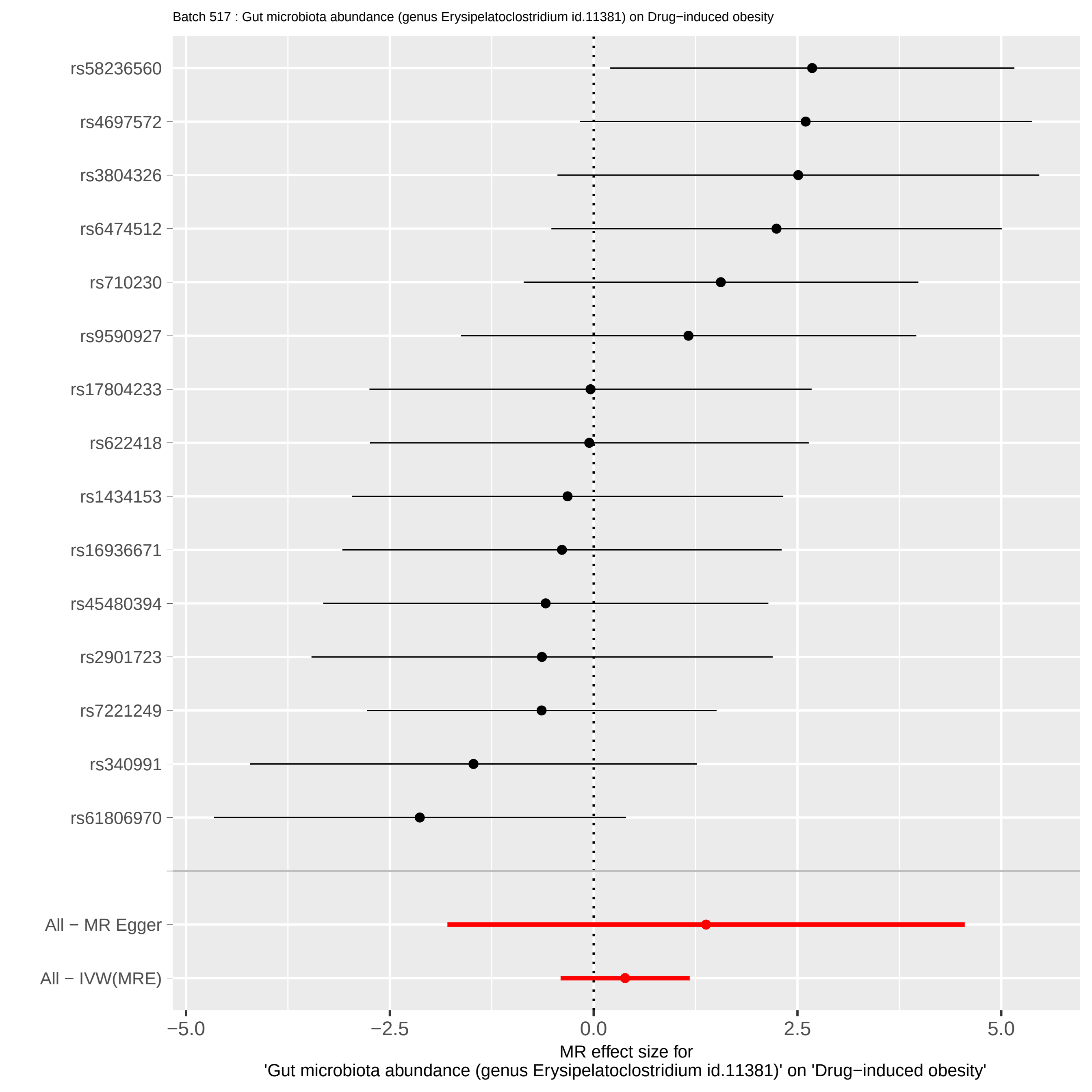

Batch 517 : Gut microbiota abundance (genus Erysipelatoclostridium id.11381) on Drug−induced obesity
rs58236560
rs4697572
rs3804326
rs6474512
rs710230
rs9590927
rs17804233
rs622418
rs1434153
rs16936671
rs45480394
rs2901723
rs7221249
rs340991
rs61806970
All − MR Egger
All − IVW(MRE)
−5.0
−2.5
0.0
2.5
5.0
MR effect size for
'Gut microbiota abundance (genus Erysipelatoclostridium id.11381)' on 'Drug−induced obesity'

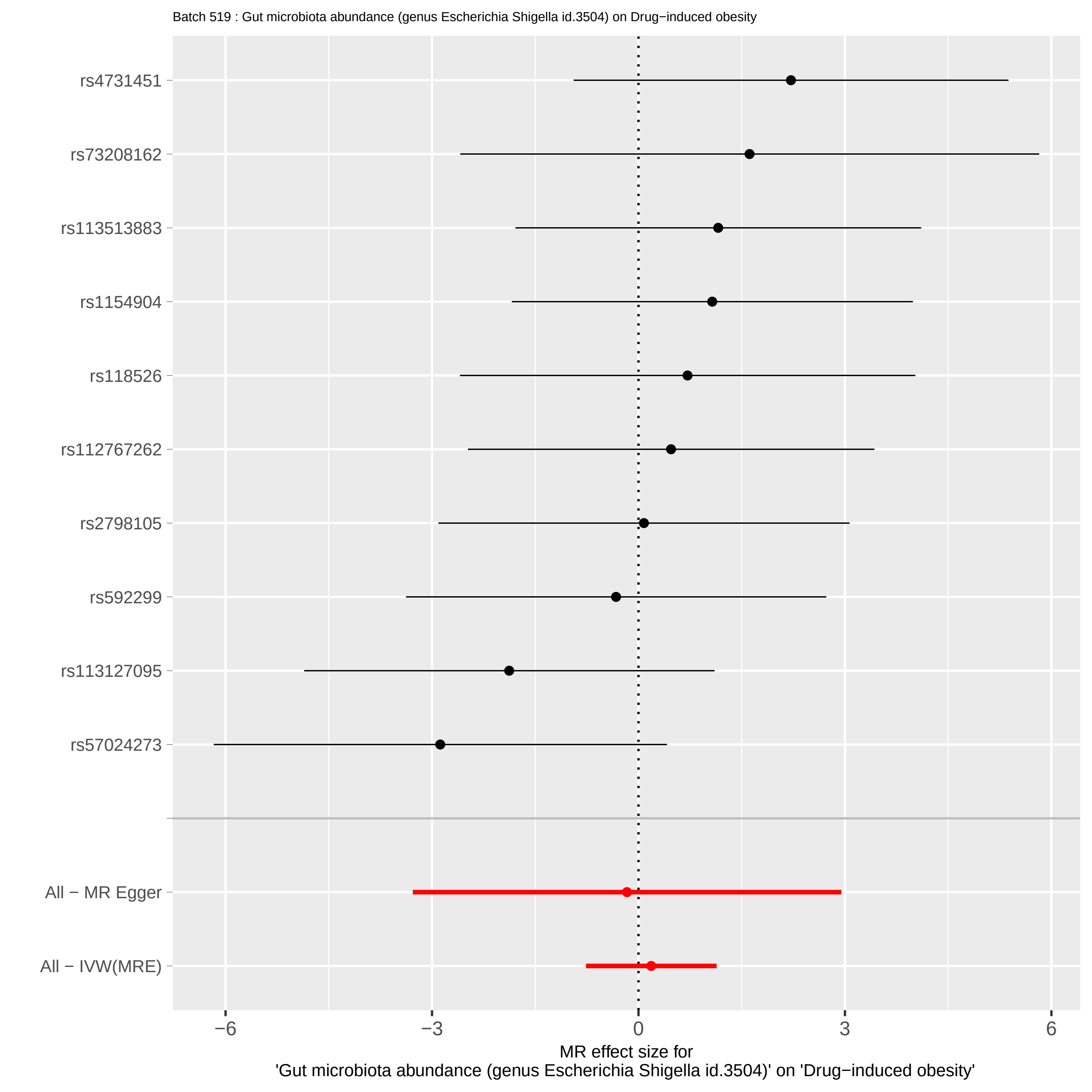

Batch 519 : Gut microbiota abundance (genus Escherichia Shigella id.3504) on Drug−induced obesity
rs4731451
rs73208162
rs113513883
rs1154904
rs118526
rs112767262
rs2798105
rs592299
rs113127095
rs57024273
All − MR Egger
All − IVW(MRE)
−6
−3
0
3
6
MR effect size for
'Gut microbiota abundance (genus Escherichia Shigella id.3504)' on 'Drug−induced obesity'

Batch 520 : Gut microbiota abundance (genus Eubacterium brachy group id.11296) on Drug−induced obesity

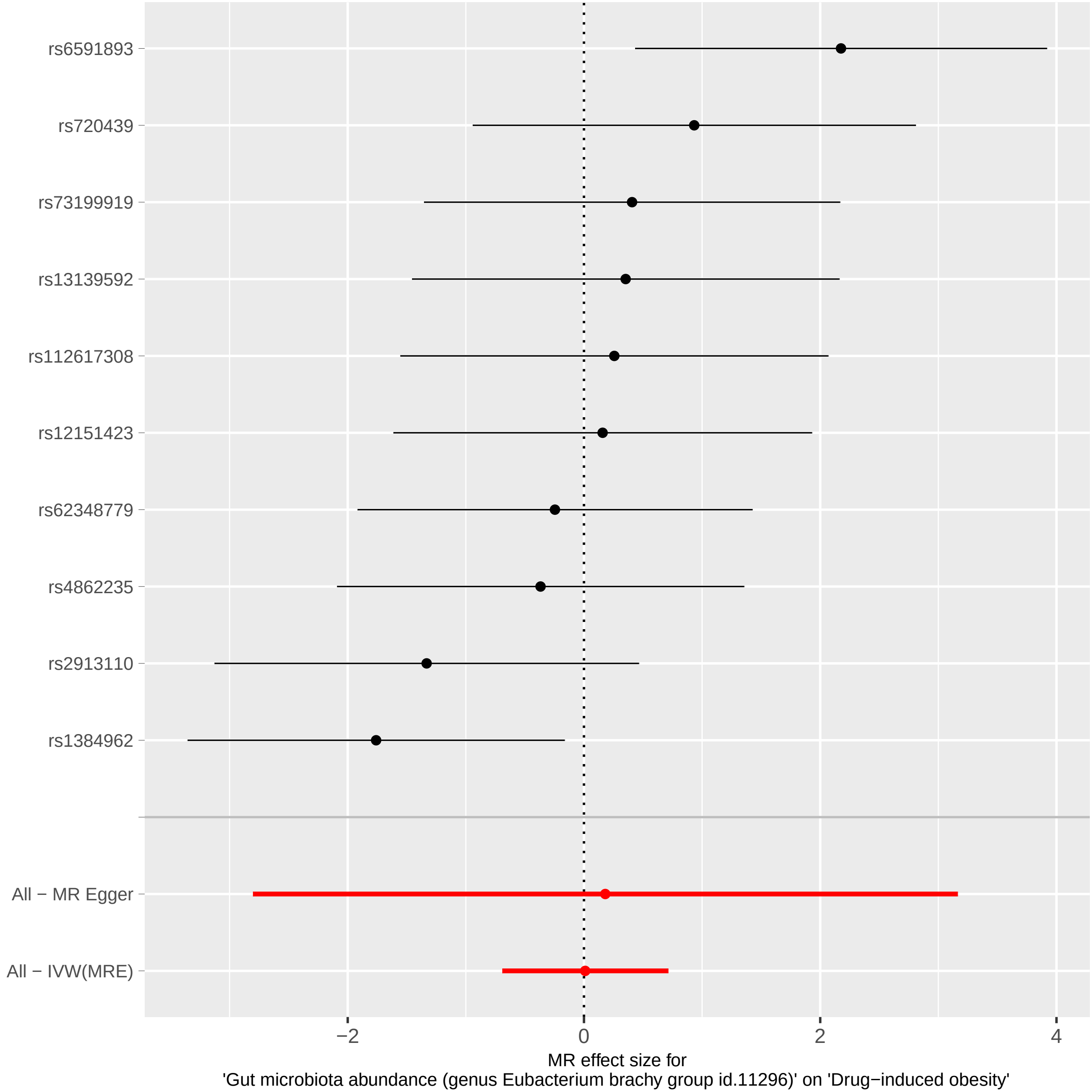

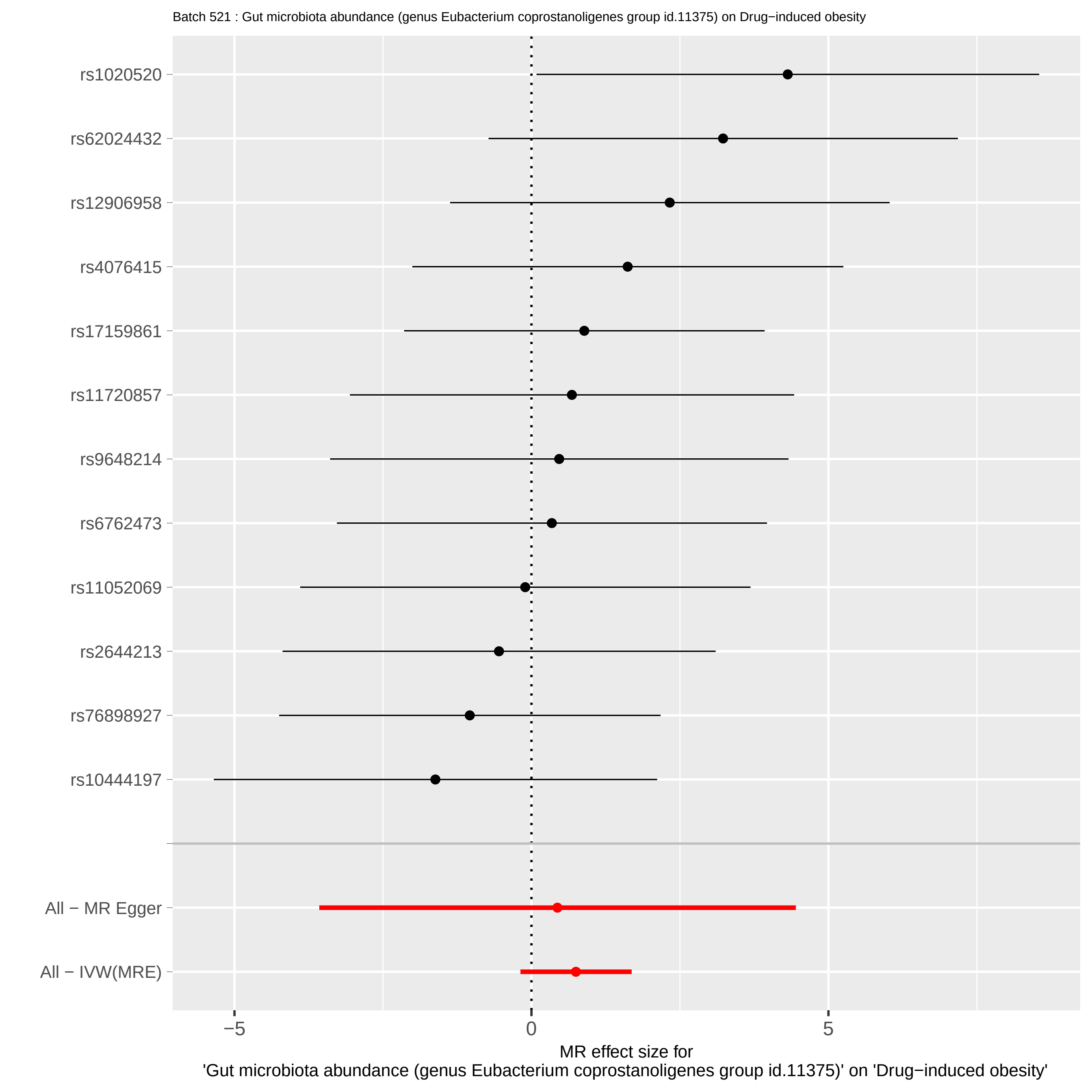
Batch 521 : Gut microbiota abundance (genus Eubacterium coprostanoligenes group id.11375) on Drug−induced obesity
rs1020520
rs62024432
rs12906958
rs4076415
rs17159861
rs11720857
rs9648214
rs6762473
rs11052069
rs2644213
rs76898927
rs10444197
All − MR Egger
All − IVW(MRE)
−5
0
5
MR effect size for
'Gut microbiota abundance (genus Eubacterium coprostanoligenes group id.11375)' on 'Drug−induced obesity'

Batch 522 : Gut microbiota abundance (genus Eubacterium eligens group id.14372) on Drug−induced obesity

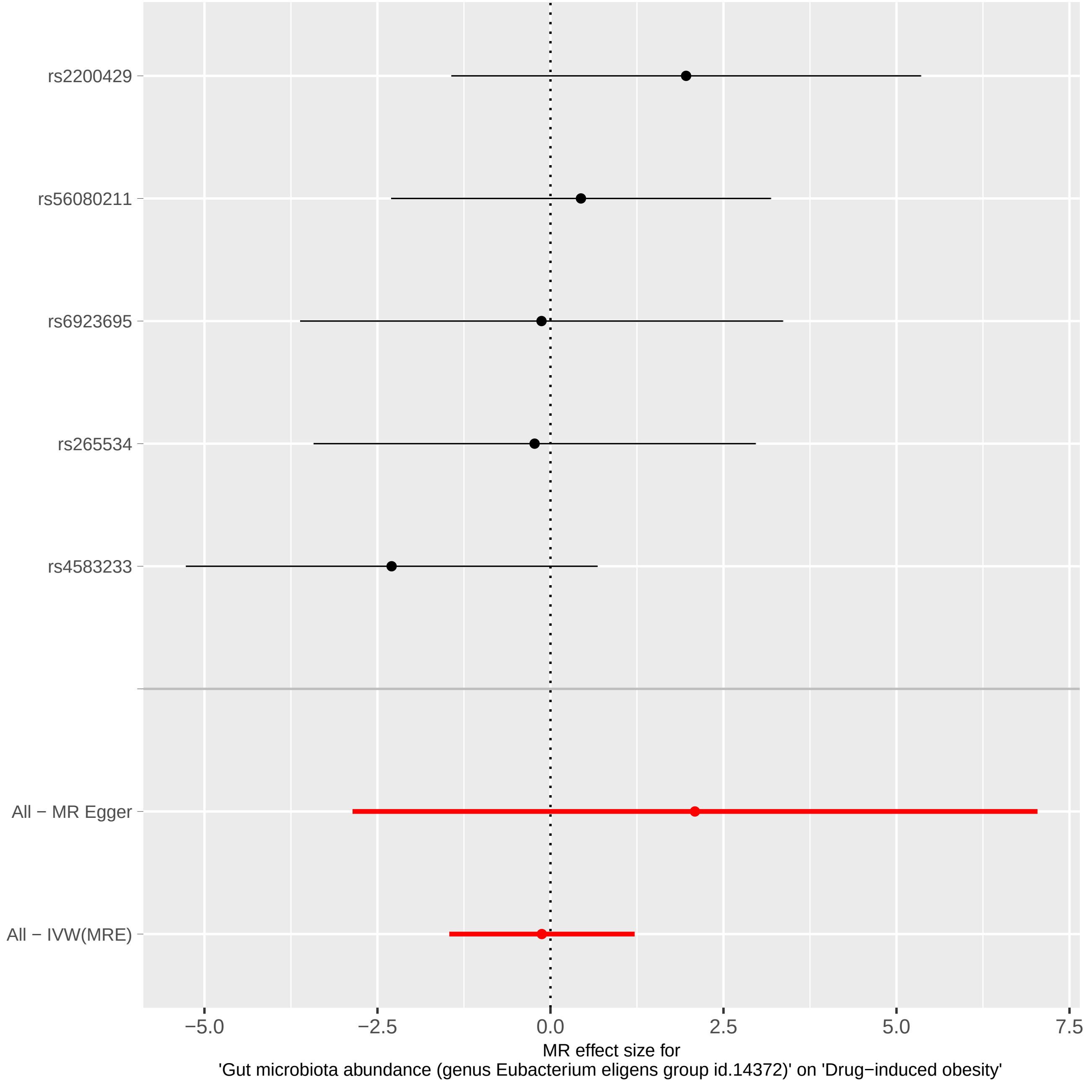

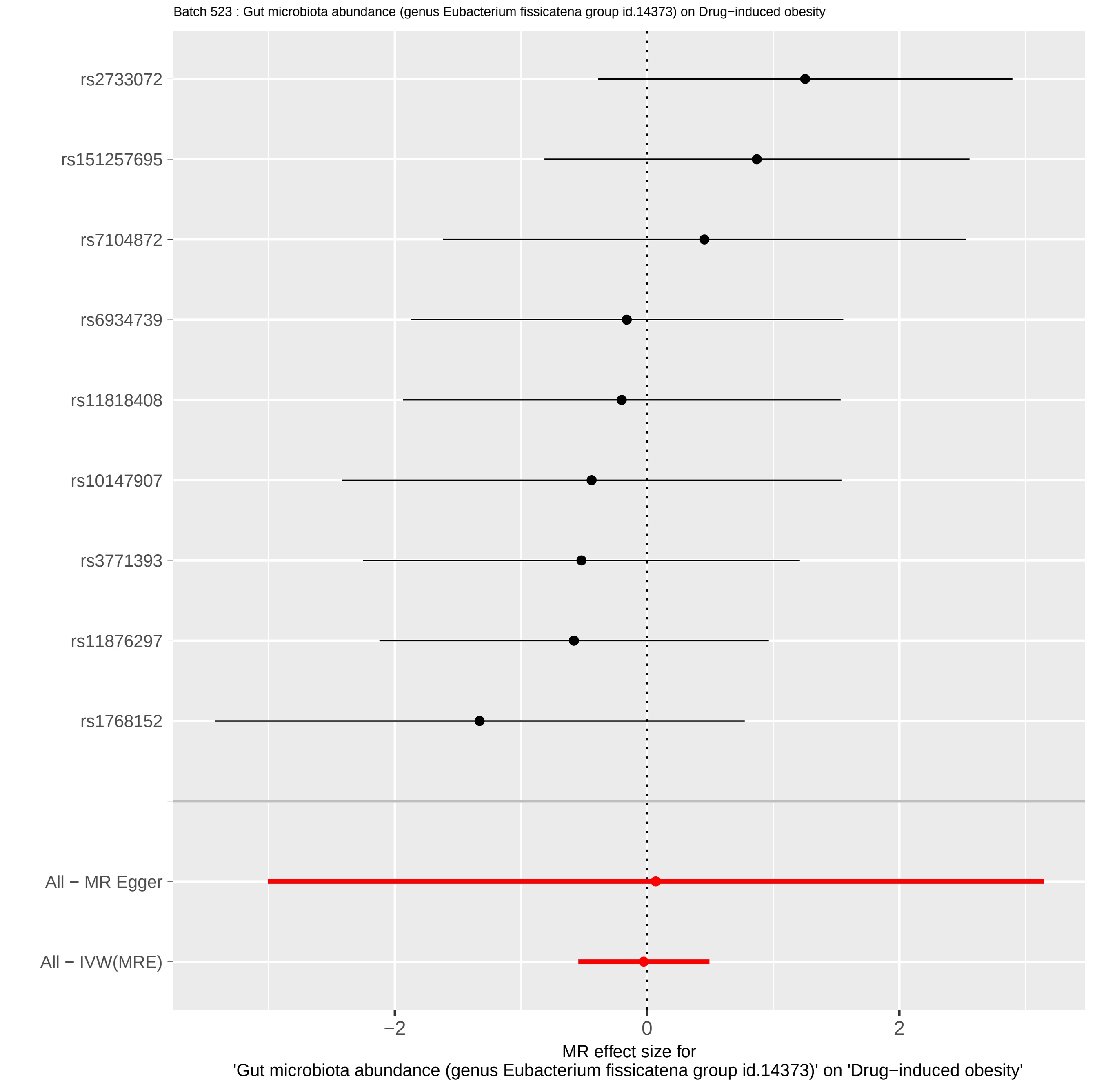

Batch 523 : Gut microbiota abundance (genus Eubacterium fissicatena group id.14373) on Drug−induced obesity
rs2733072
rs151257695
rs7104872
rs6934739
rs11818408
rs10147907
rs3771393
rs11876297
rs1768152
All − MR Egger
All − IVW(MRE)
−2
0
2
MR effect size for
'Gut microbiota abundance (genus Eubacterium fissicatena group id.14373)' on 'Drug−induced obesity'

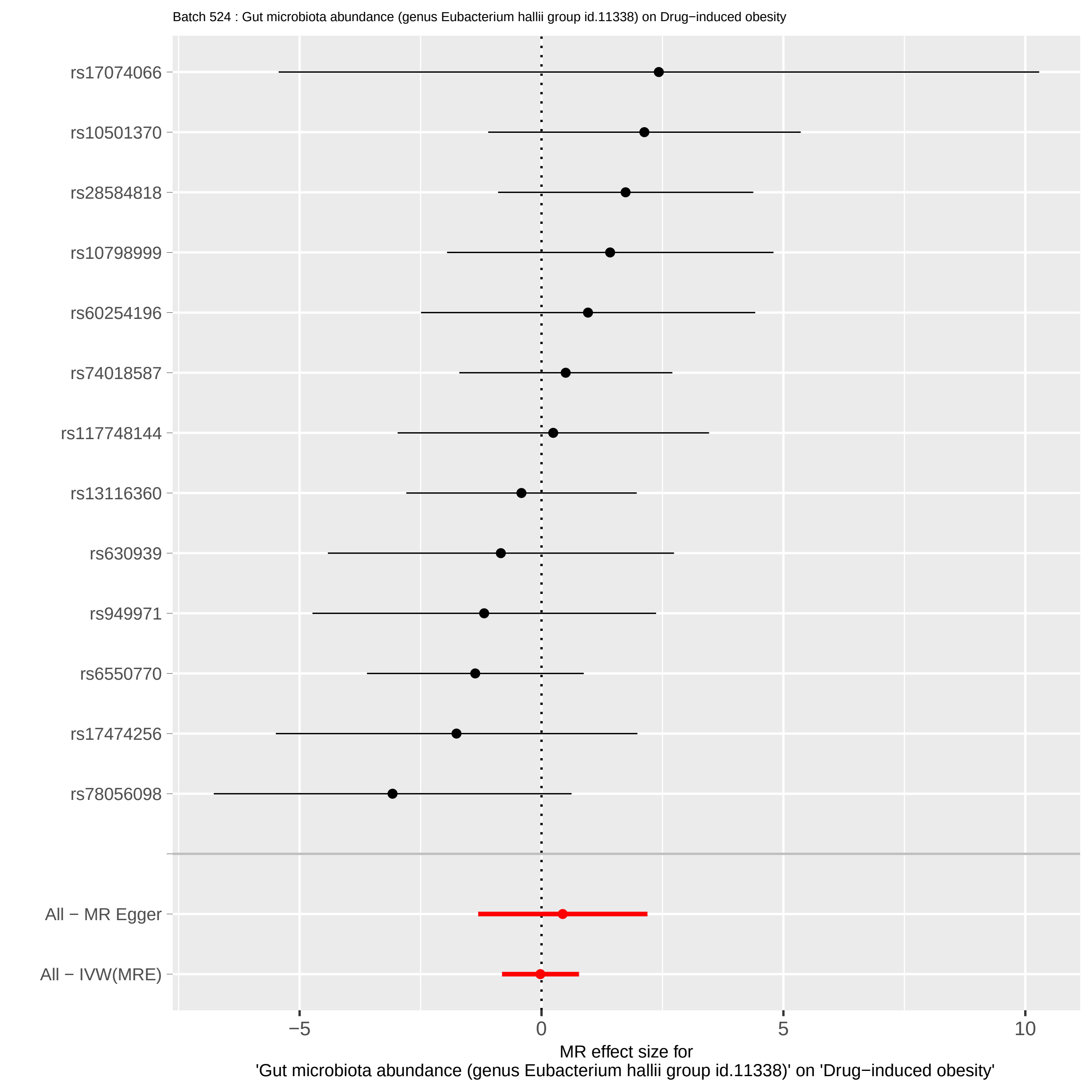

Batch 524 : Gut microbiota abundance (genus Eubacterium hallii group id.11338) on Drug−induced obesity
rs17074066
rs10501370
rs28584818
rs10798999
rs60254196
rs74018587
rs117748144
rs13116360
rs630939
rs949971
rs6550770
rs17474256
rs78056098
All − MR Egger
All − IVW(MRE)
−5
0
5
10
MR effect size for
'Gut microbiota abundance (genus Eubacterium hallii group id.11338)' on 'Drug−induced obesity'

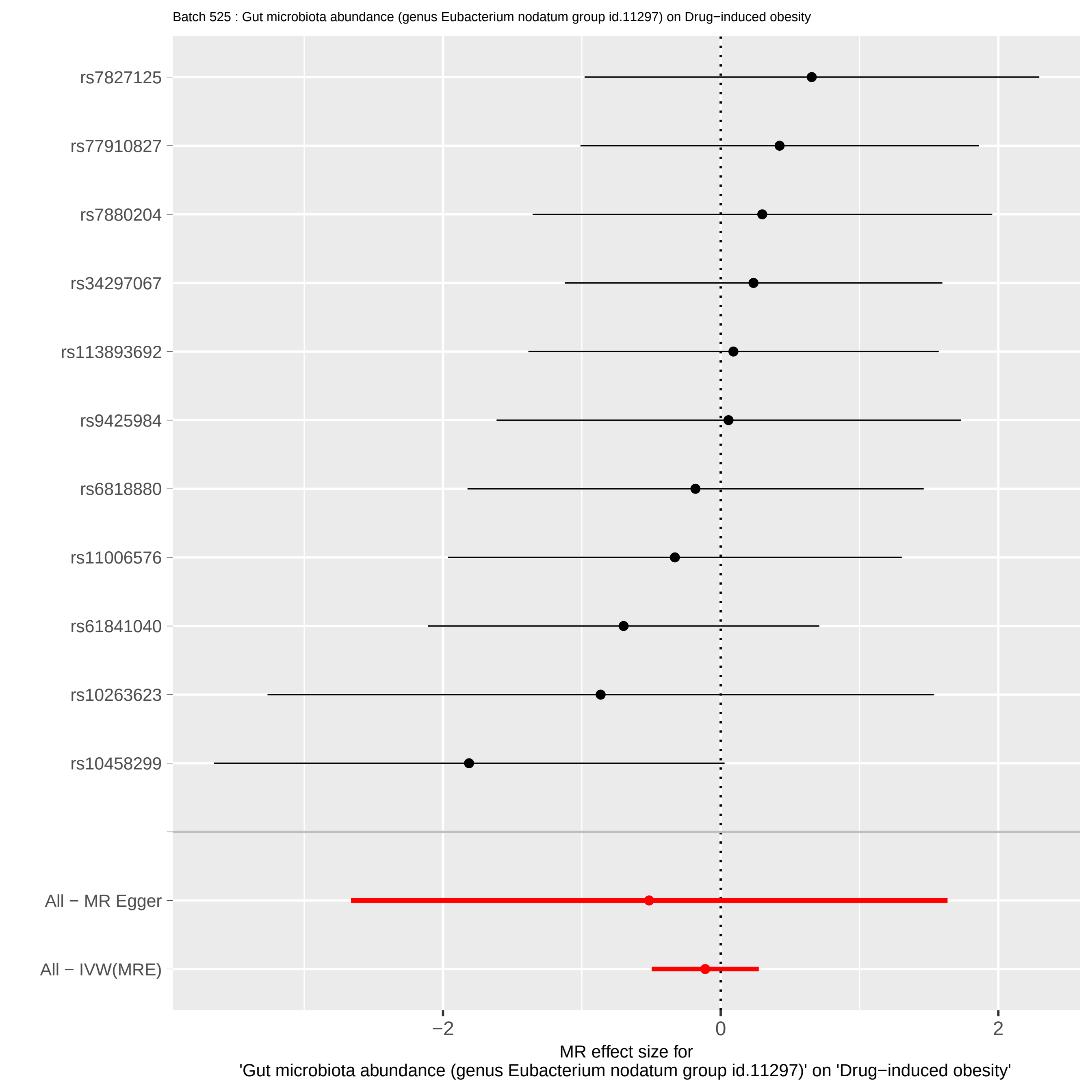

Batch 525 : Gut microbiota abundance (genus Eubacterium nodatum group id.11297) on Drug−induced obesity
rs7827125
rs77910827
rs7880204
rs34297067
rs113893692
rs9425984
rs6818880
rs11006576
rs61841040
rs10263623
rs10458299
All − MR Egger
All − IVW(MRE)
−2
0
2
MR effect size for
'Gut microbiota abundance (genus Eubacterium nodatum group id.11297)' on 'Drug−induced obesity'

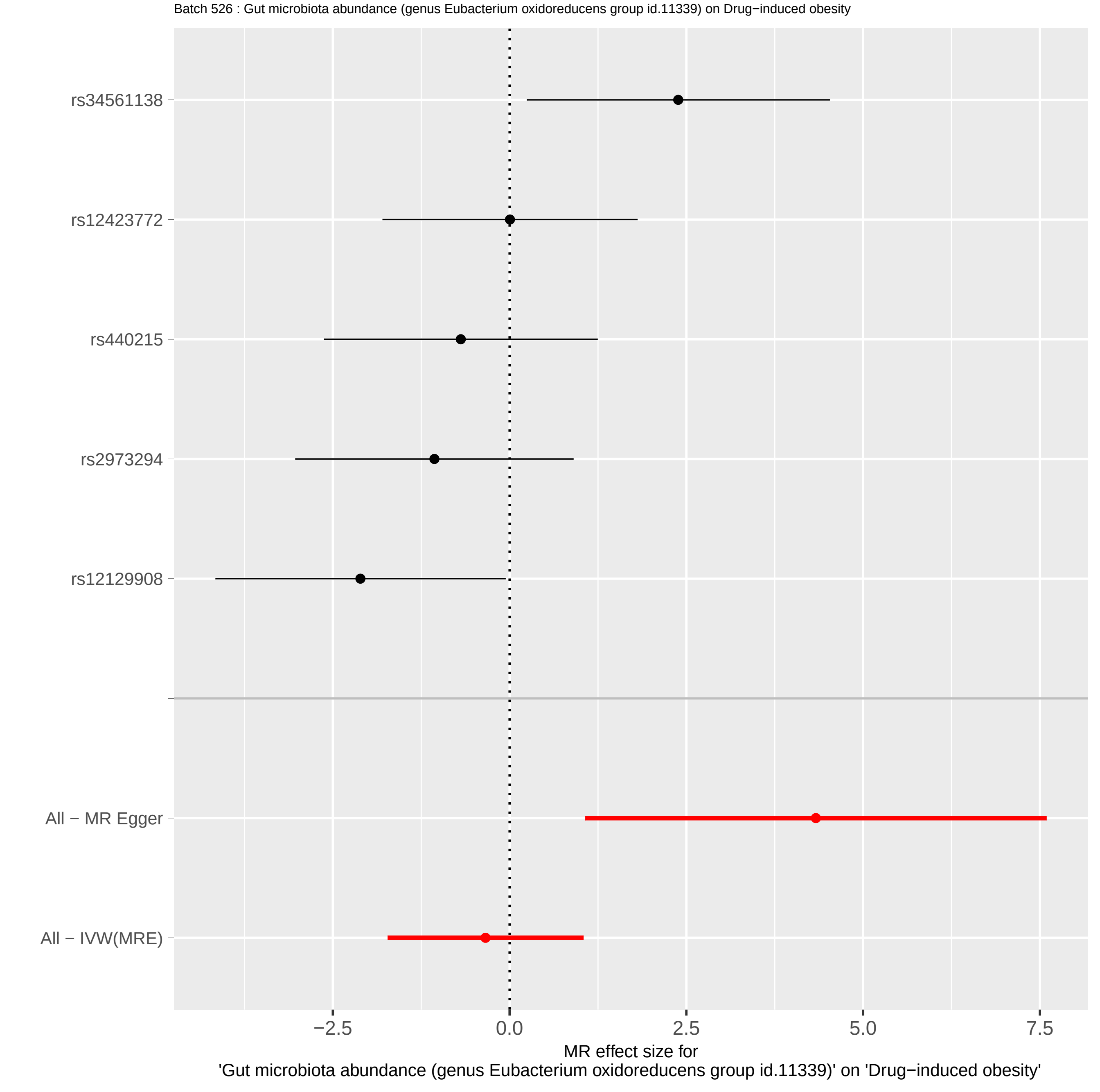
Batch 526 : Gut microbiota abundance (genus Eubacterium oxidoreducens group id.11339) on Drug−induced obesity
rs34561138
rs12423772
rs440215
rs2973294
rs12129908
All − MR Egger
All − IVW(MRE)
−2.5
0.0
2.5
5.0
7.5
MR effect size for
'Gut microbiota abundance (genus Eubacterium oxidoreducens group id.11339)' on 'Drug−induced obesity'

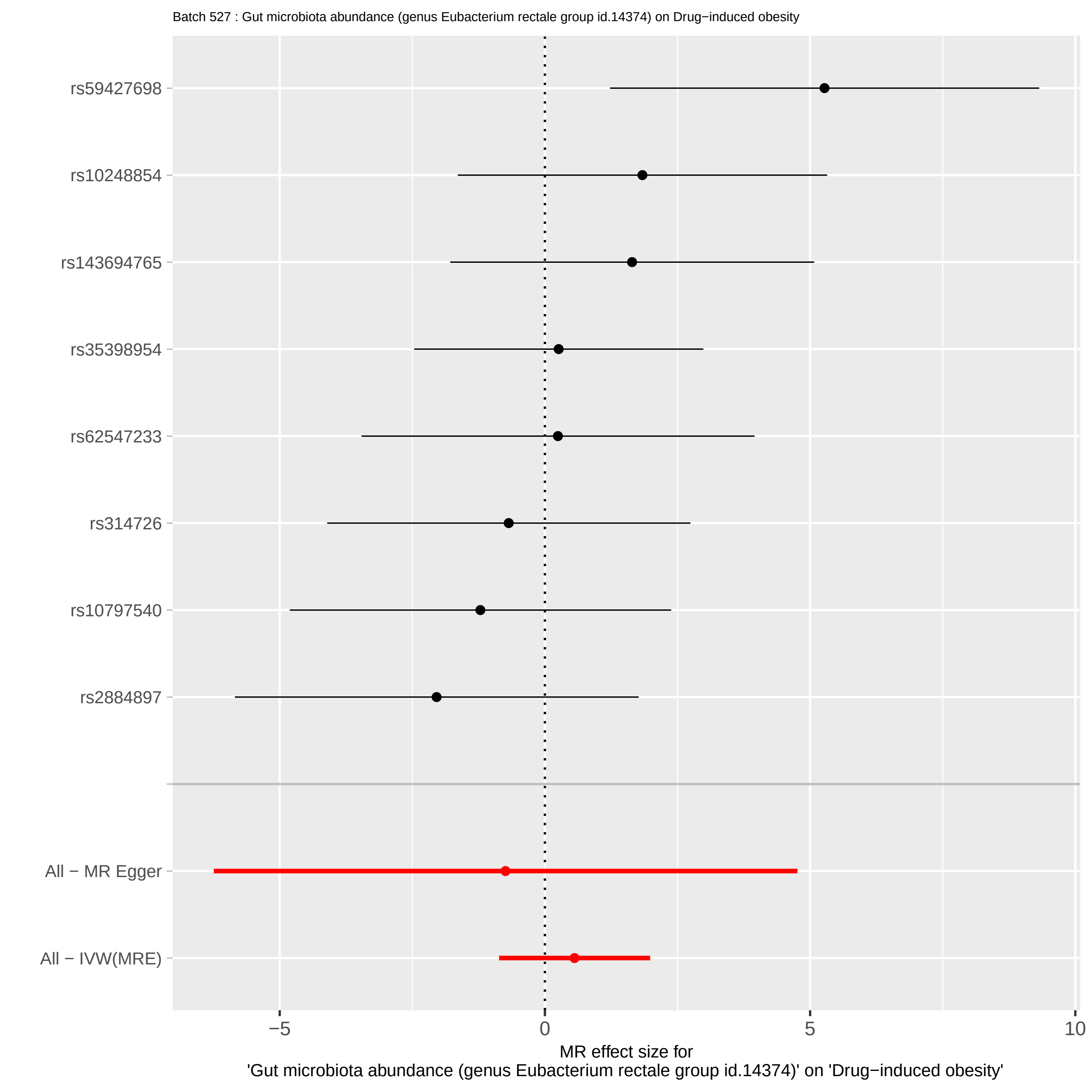

Batch 527 : Gut microbiota abundance (genus Eubacterium rectale group id.14374) on Drug−induced obesity
rs59427698
rs10248854
rs143694765
rs35398954
rs62547233
rs314726
rs10797540
rs2884897
All − MR Egger
All − IVW(MRE)
−5
0
5
10
MR effect size for
'Gut microbiota abundance (genus Eubacterium rectale group id.14374)' on 'Drug−induced obesity'

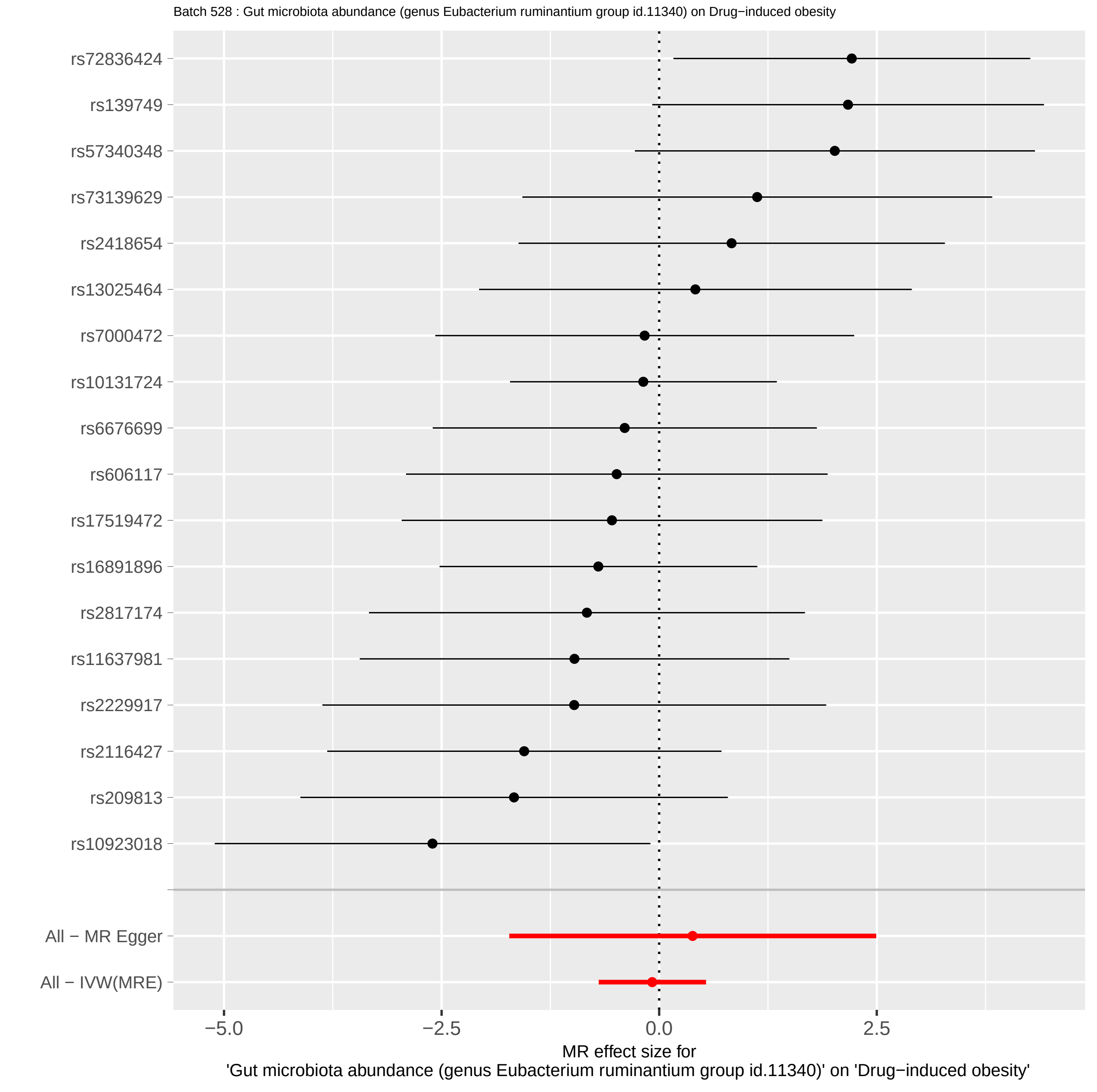

Batch 528 : Gut microbiota abundance (genus Eubacterium ruminantium group id.11340) on Drug−induced obesity
rs72836424
rs139749
rs57340348
rs73139629
rs2418654
rs13025464
rs7000472
rs10131724
rs6676699
rs606117
rs17519472
rs16891896
rs2817174
rs11637981
rs2229917
rs2116427
rs209813
rs10923018
All − MR Egger
All − IVW(MRE)
−5.0
−2.5
0.0
2.5
MR effect size for
'Gut microbiota abundance (genus Eubacterium ruminantium group id.11340)' on 'Drug−induced obesity'

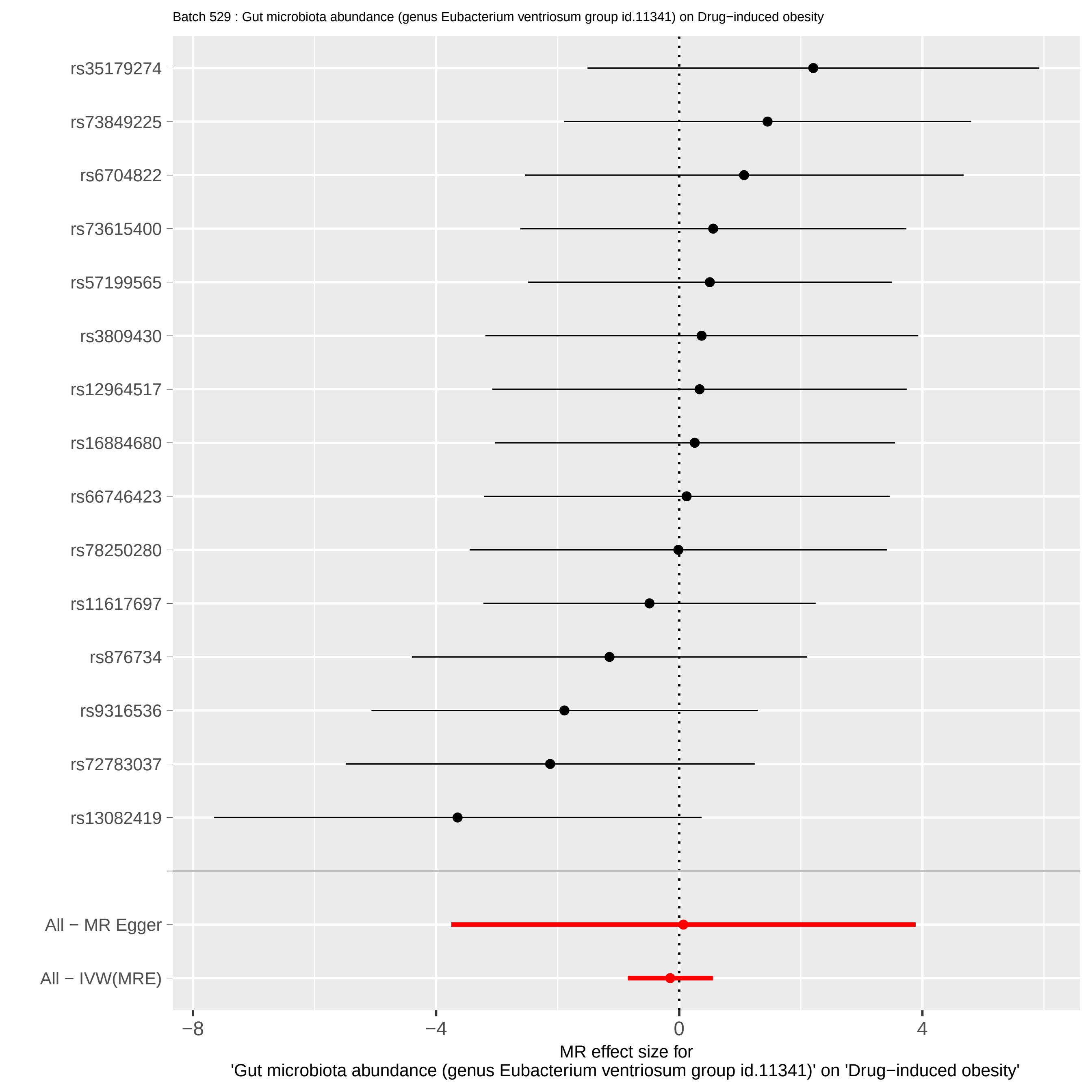
Batch 529 : Gut microbiota abundance (genus Eubacterium ventriosum group id.11341) on Drug−induced obesity
rs35179274
rs73849225
rs6704822
rs73615400
rs57199565
rs3809430
rs12964517
rs16884680
rs66746423
rs78250280
rs11617697
rs876734
rs9316536
rs72783037
rs13082419
All − MR Egger
All − IVW(MRE)
−8
−4
0
4
MR effect size for
'Gut microbiota abundance (genus Eubacterium ventriosum group id.11341)' on 'Drug−induced obesity'

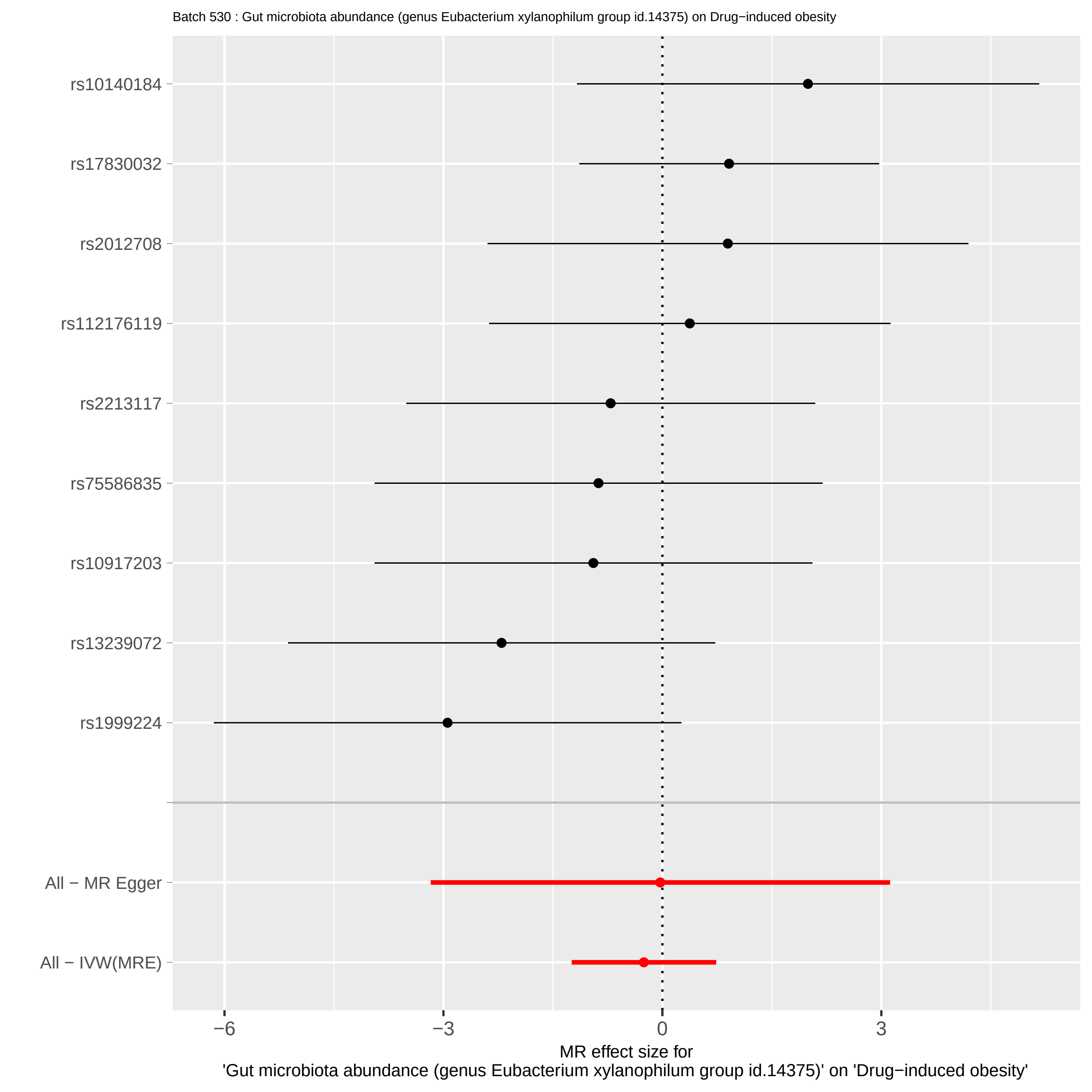

Batch 530 : Gut microbiota abundance (genus Eubacterium xylanophilum group id.14375) on Drug−induced obesity
rs10140184
rs17830032
rs2012708
rs112176119
rs2213117
rs75586835
rs10917203
rs13239072
rs1999224
All − MR Egger
All − IVW(MRE)
−6
−3
0
3
MR effect size for
'Gut microbiota abundance (genus Eubacterium xylanophilum group id.14375)' on 'Drug−induced obesity'

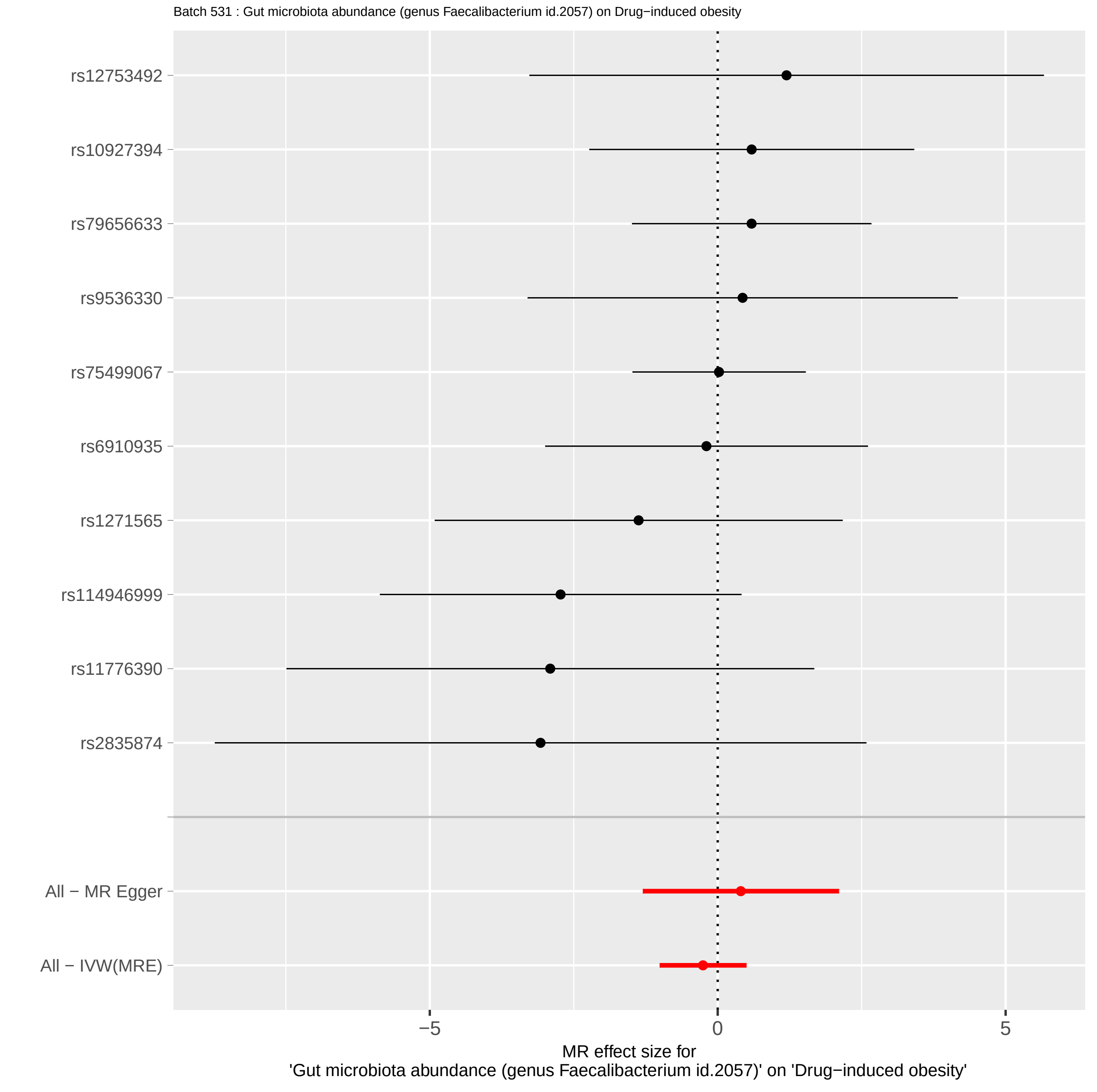
Batch 531 : Gut microbiota abundance (genus Faecalibacterium id.2057) on Drug−induced obesity
rs12753492
rs10927394
rs79656633
rs9536330
rs75499067
rs6910935
rs1271565
rs114946999
rs11776390
rs2835874
All − MR Egger
All − IVW(MRE)
−5
0
5
MR effect size for
'Gut microbiota abundance (genus Faecalibacterium id.2057)' on 'Drug−induced obesity'

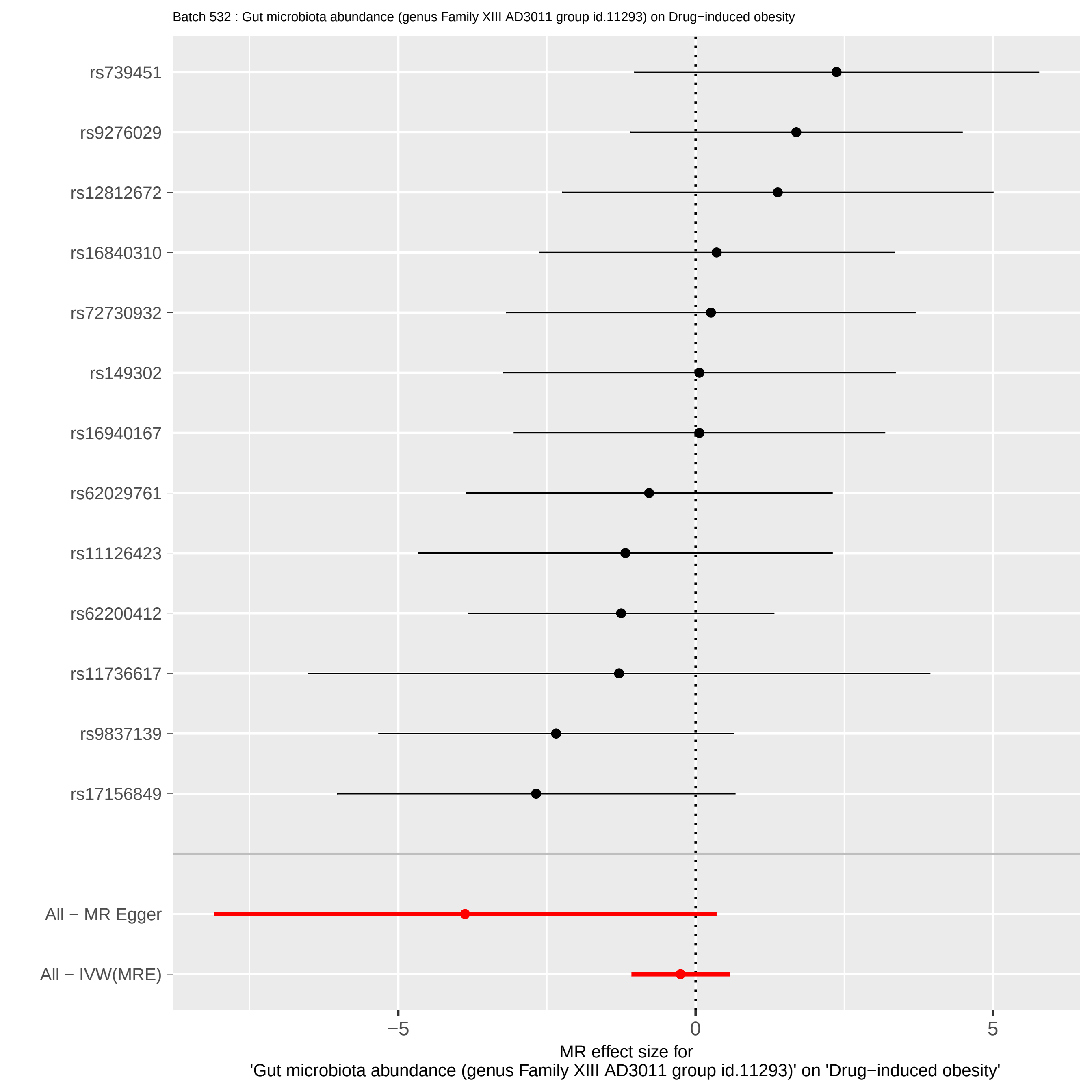

Batch 532 : Gut microbiota abundance (genus Family XIII AD3011 group id.11293) on Drug−induced obesity
rs739451
rs9276029
rs12812672
rs16840310
rs72730932
rs149302
rs16940167
rs62029761
rs11126423
rs62200412
rs11736617
rs9837139
rs17156849
All − MR Egger
All − IVW(MRE)
−5
0
5
MR effect size for
'Gut microbiota abundance (genus Family XIII AD3011 group id.11293)' on 'Drug−induced obesity'

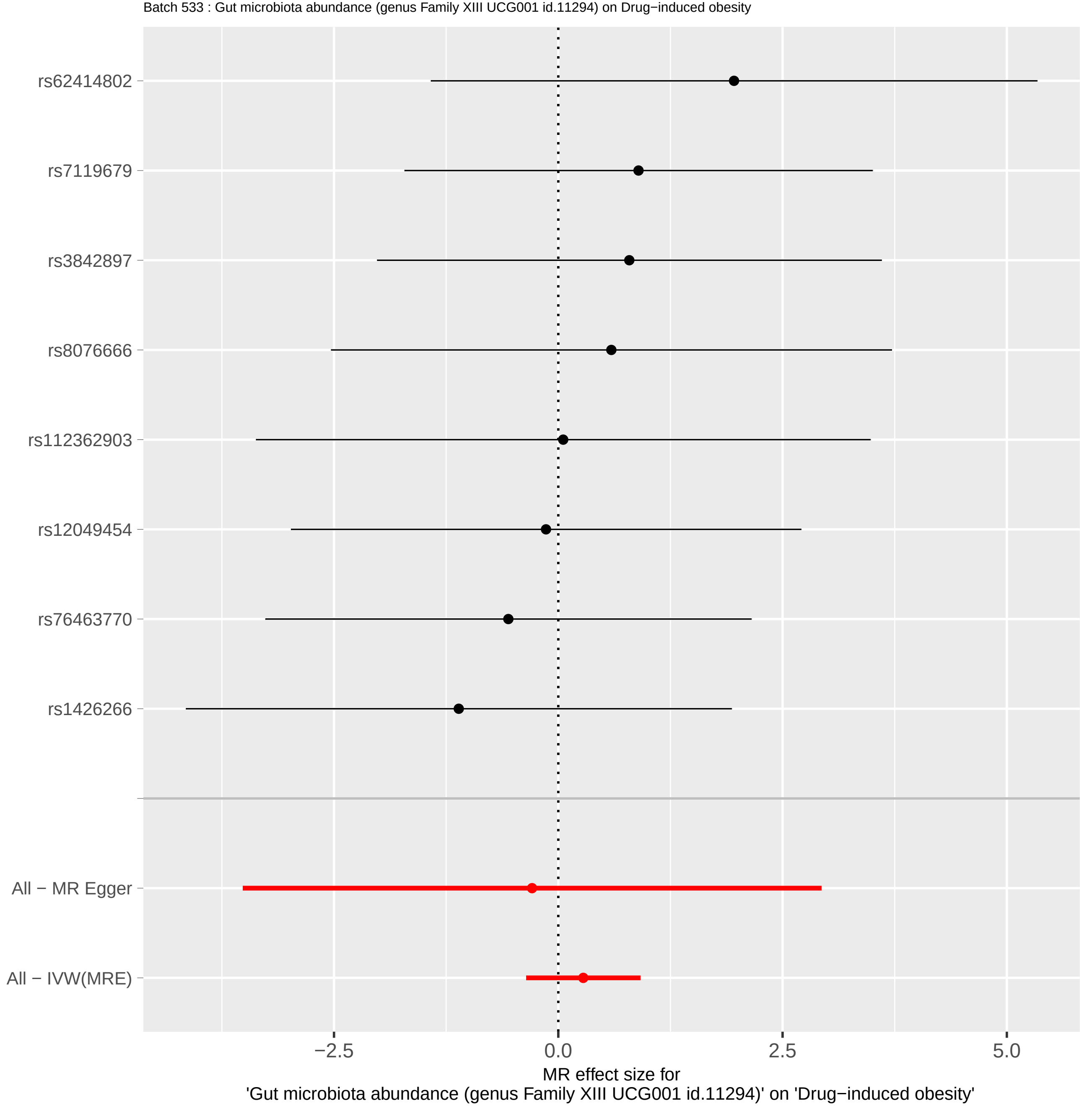
Batch 533 : Gut microbiota abundance (genus Family XIII UCG001 id.11294) on Drug−induced obesity
rs62414802
rs7119679
rs3842897
rs8076666
rs112362903
rs12049454
rs76463770
rs1426266
All − MR Egger
All − IVW(MRE)
−2.5
0.0
2.5
5.0
MR effect size for
'Gut microbiota abundance (genus Family XIII UCG001 id.11294)' on 'Drug−induced obesity'

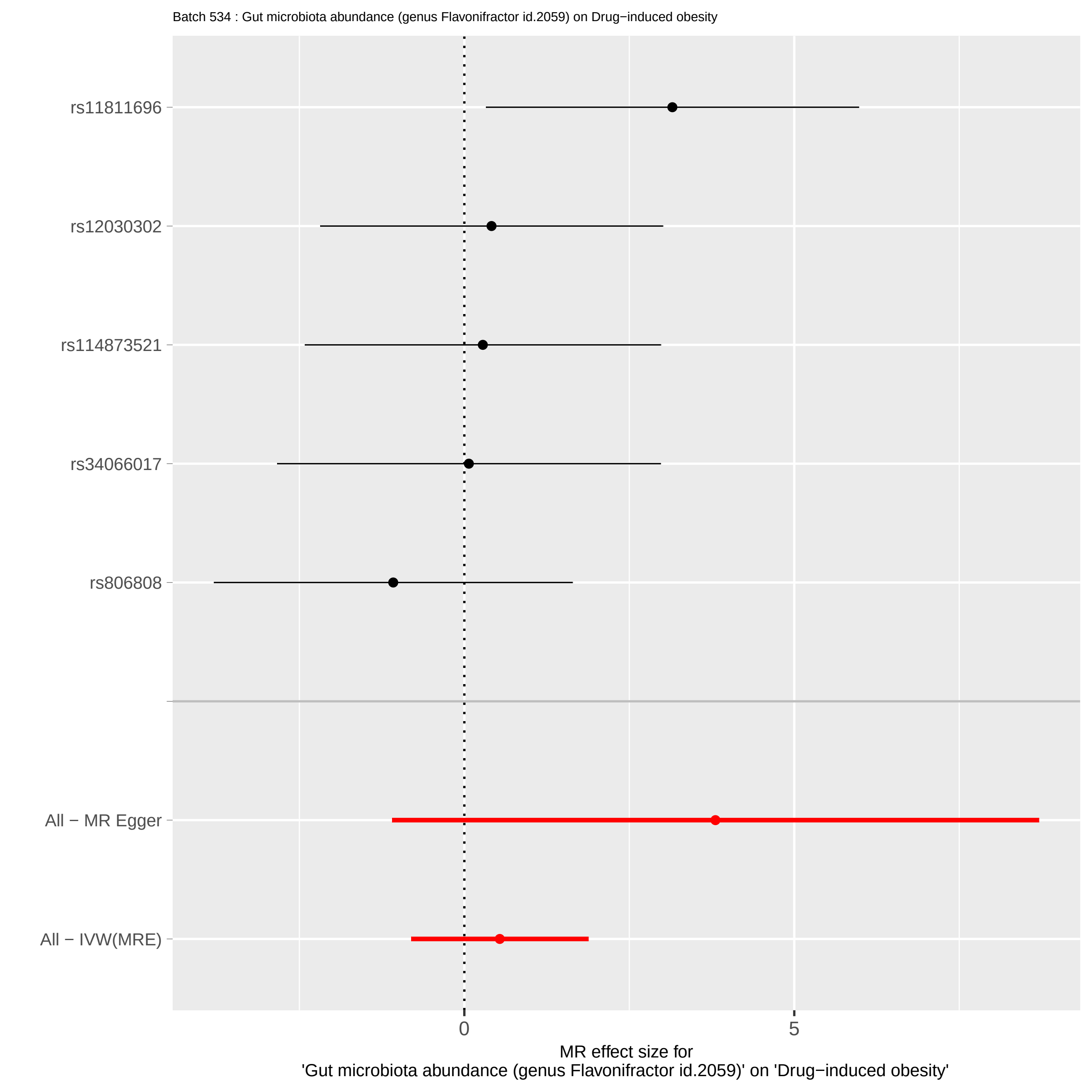
Batch 534 : Gut microbiota abundance (genus Flavonifractor id.2059) on Drug−induced obesity
rs11811696
rs12030302
rs114873521
rs34066017
rs806808
All − MR Egger
All − IVW(MRE)
0
5
MR effect size for
'Gut microbiota abundance (genus Flavonifractor id.2059)' on 'Drug−induced obesity'

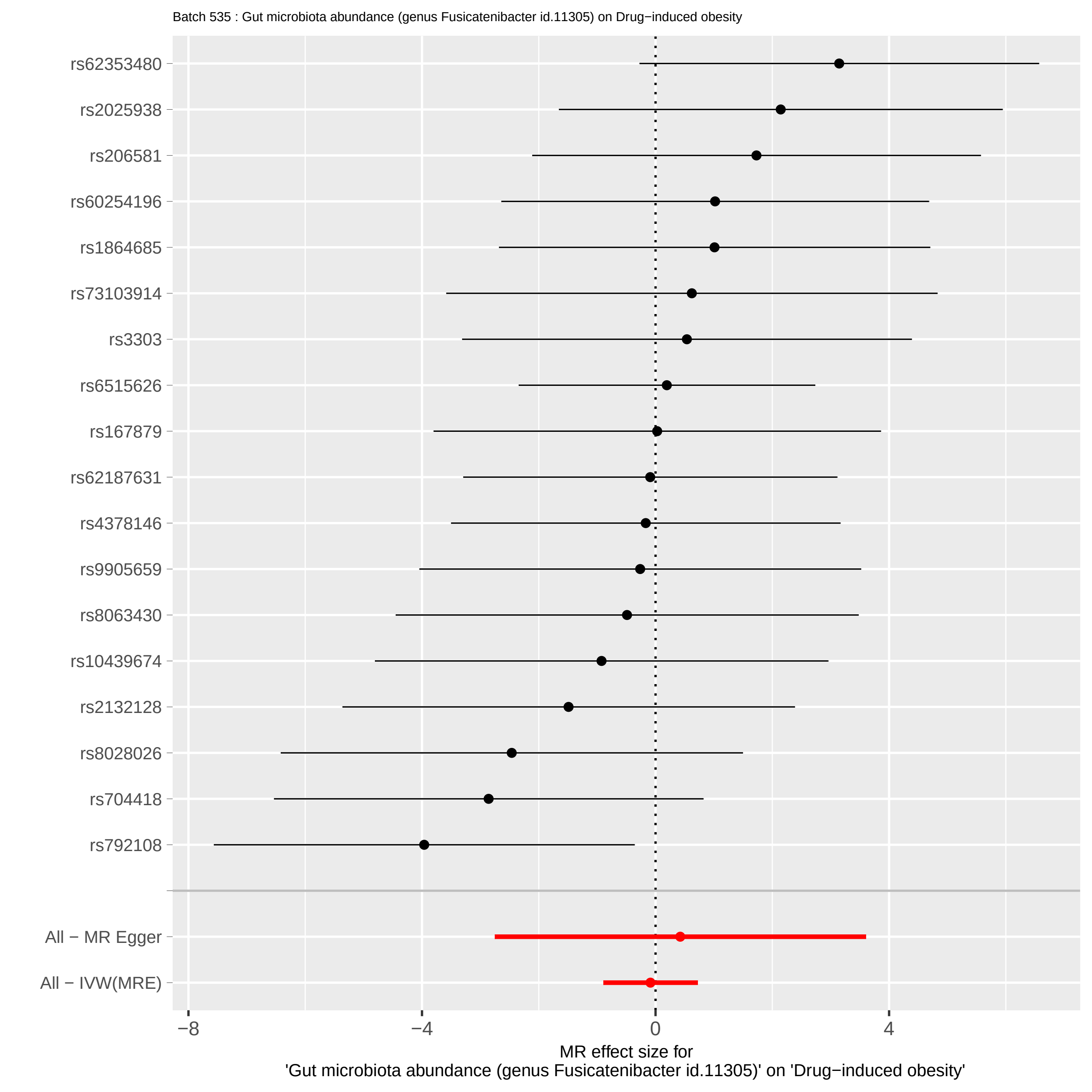

Batch 535 : Gut microbiota abundance (genus Fusicatenibacter id.11305) on Drug−induced obesity
rs62353480
rs2025938
rs206581
rs60254196
rs1864685
rs73103914
rs3303
rs6515626
rs167879
rs62187631
rs4378146
rs9905659
rs8063430
rs10439674
rs2132128
rs8028026
rs704418
rs792108
All − MR Egger
All − IVW(MRE)
−8
−4
0
4
MR effect size for
'Gut microbiota abundance (genus Fusicatenibacter id.11305)' on 'Drug−induced obesity'

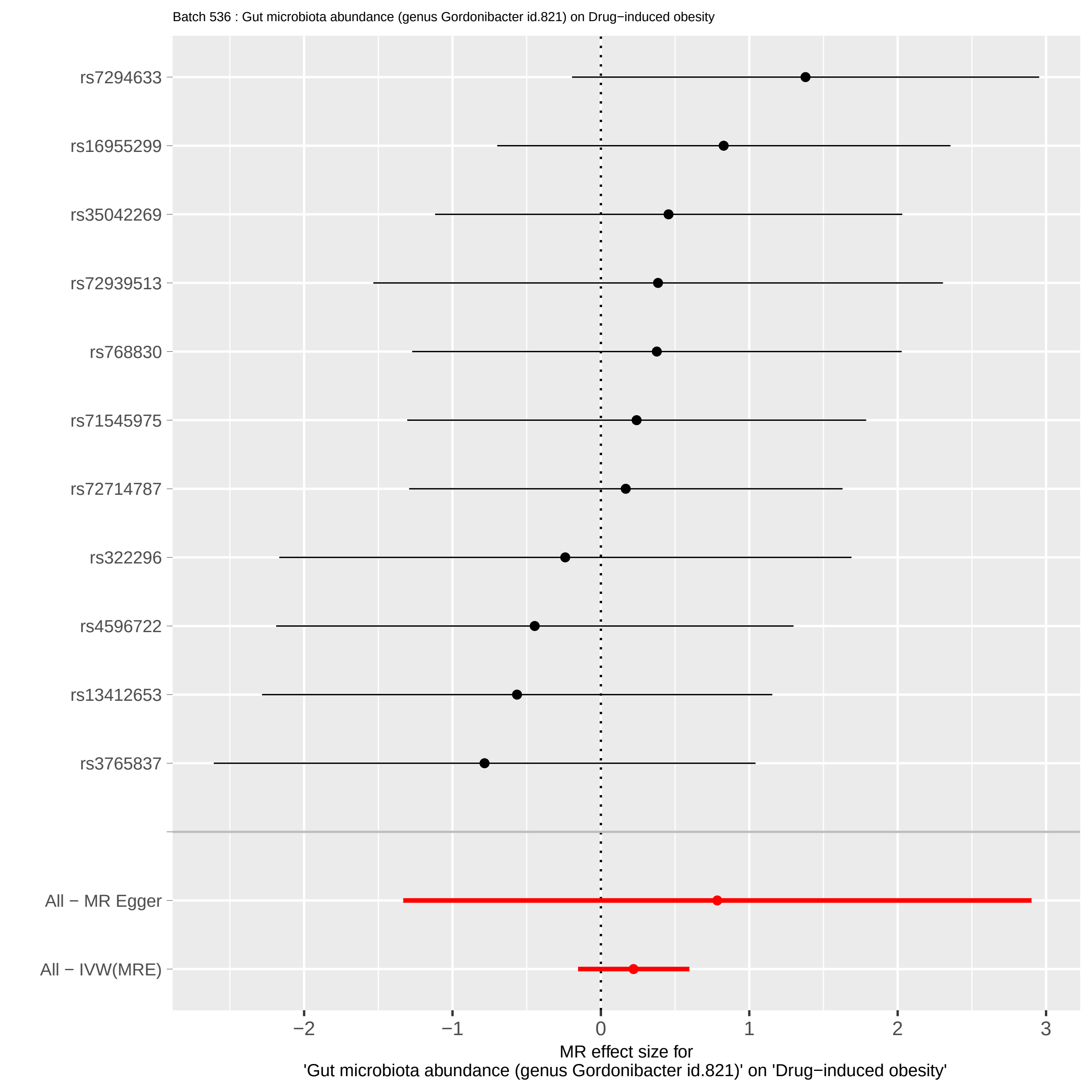
Batch 536 : Gut microbiota abundance (genus Gordonibacter id.821) on Drug−induced obesity
rs7294633
rs16955299
rs35042269
rs72939513
rs768830
rs71545975
rs72714787
rs322296
rs4596722
rs13412653
rs3765837
All − MR Egger
All − IVW(MRE)
−2
−1
0
1
2
3
MR effect size for
'Gut microbiota abundance (genus Gordonibacter id.821)' on 'Drug−induced obesity'

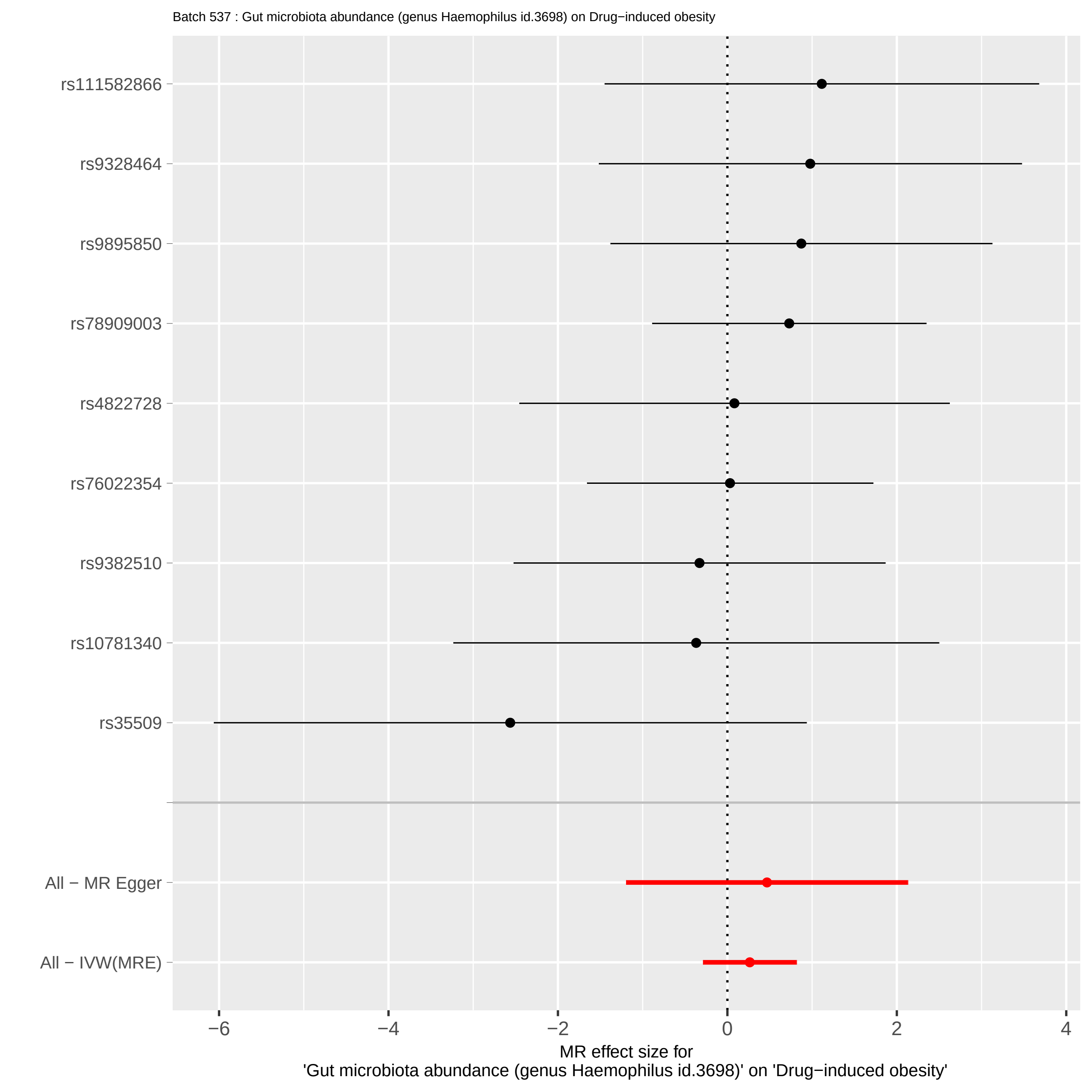
Batch 537 : Gut microbiota abundance (genus Haemophilus id.3698) on Drug−induced obesity
rs111582866
rs9328464
rs9895850
rs78909003
rs4822728
rs76022354
rs9382510
rs10781340
rs35509
All − MR Egger
All − IVW(MRE)
−6
−4
−2
0
2
4
MR effect size for
'Gut microbiota abundance (genus Haemophilus id.3698)' on 'Drug−induced obesity'

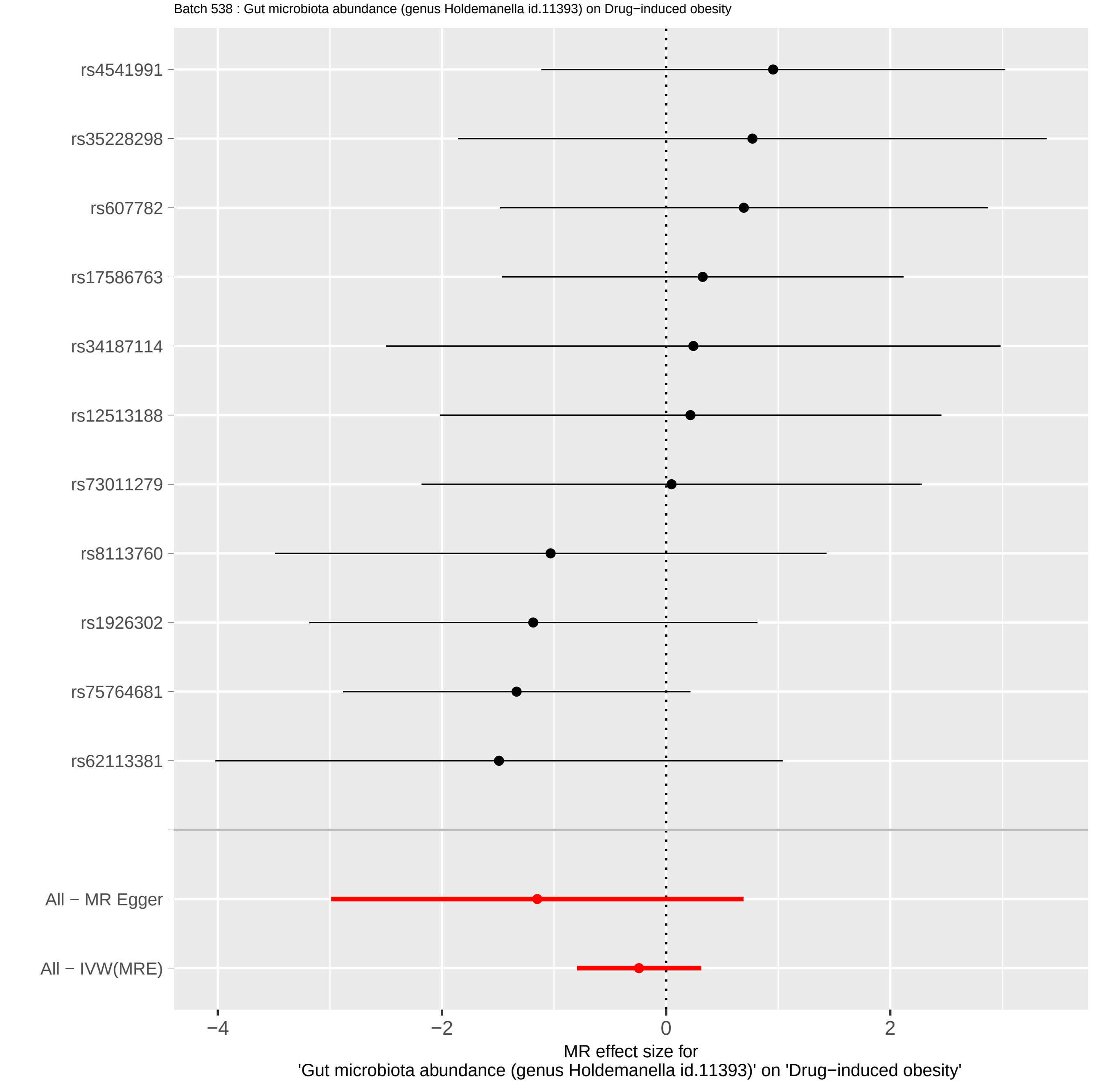

Batch 538 : Gut microbiota abundance (genus Holdemanella id.11393) on Drug−induced obesity
rs4541991
rs35228298
rs607782
rs17586763
rs34187114
rs12513188
rs73011279
rs8113760
rs1926302
rs75764681
rs62113381
All − MR Egger
All − IVW(MRE)
−4
−2
0
2
MR effect size for
'Gut microbiota abundance (genus Holdemanella id.11393)' on 'Drug−induced obesity'

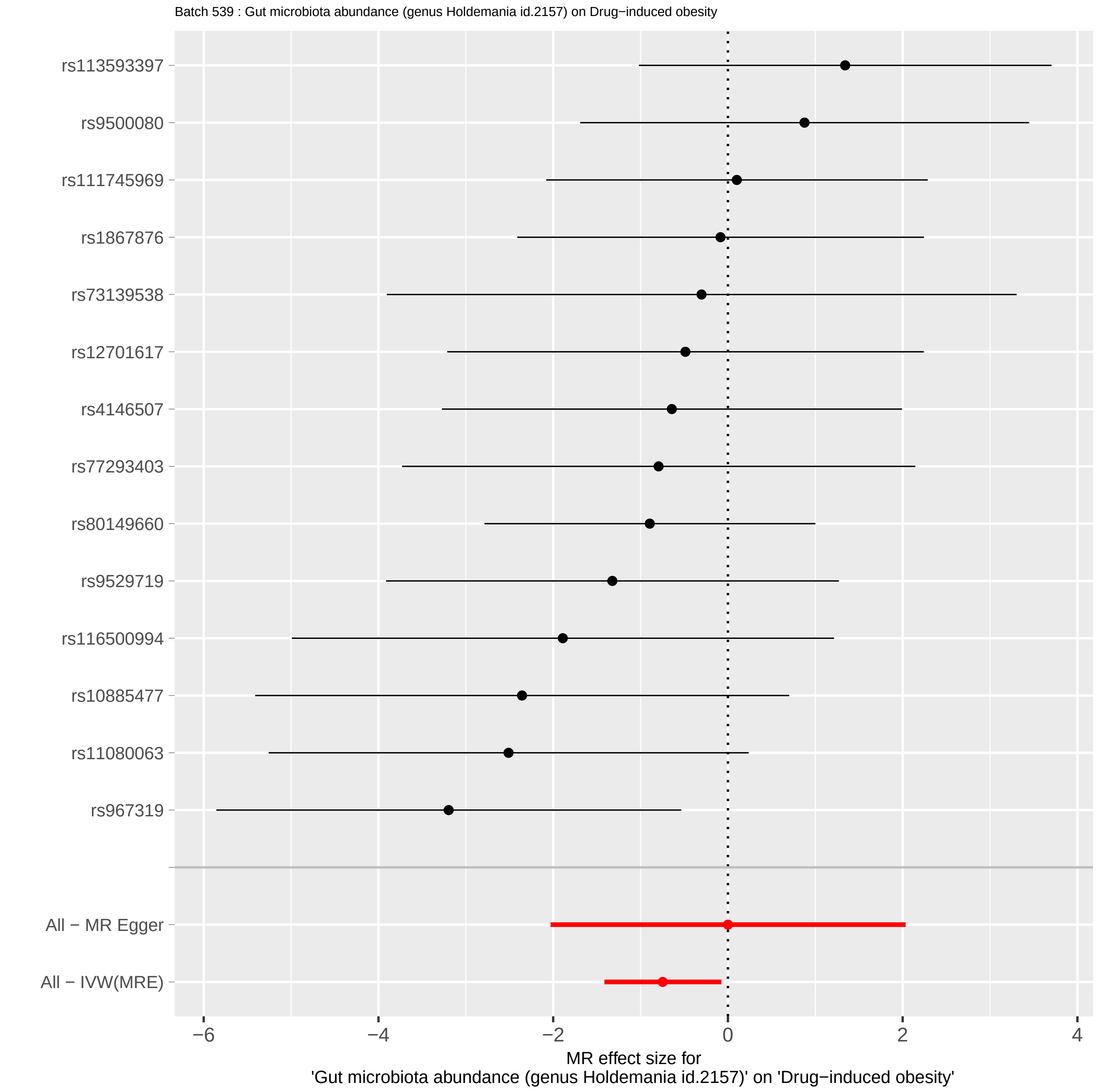
Batch 539 : Gut microbiota abundance (genus Holdemania id.2157) on Drug−induced obesity
rs113593397
rs9500080
rs111745969
rs1867876
rs73139538
rs12701617
rs4146507
rs77293403
rs80149660
rs9529719
rs116500994
rs10885477
rs11080063
rs967319
All − MR Egger
All − IVW(MRE)
−6
−4
−2
0
2
4
MR effect size for
'Gut microbiota abundance (genus Holdemania id.2157)' on 'Drug−induced obesity'

Batch 540 : Gut microbiota abundance (genus Howardella id.2000) on Drug−induced obesity

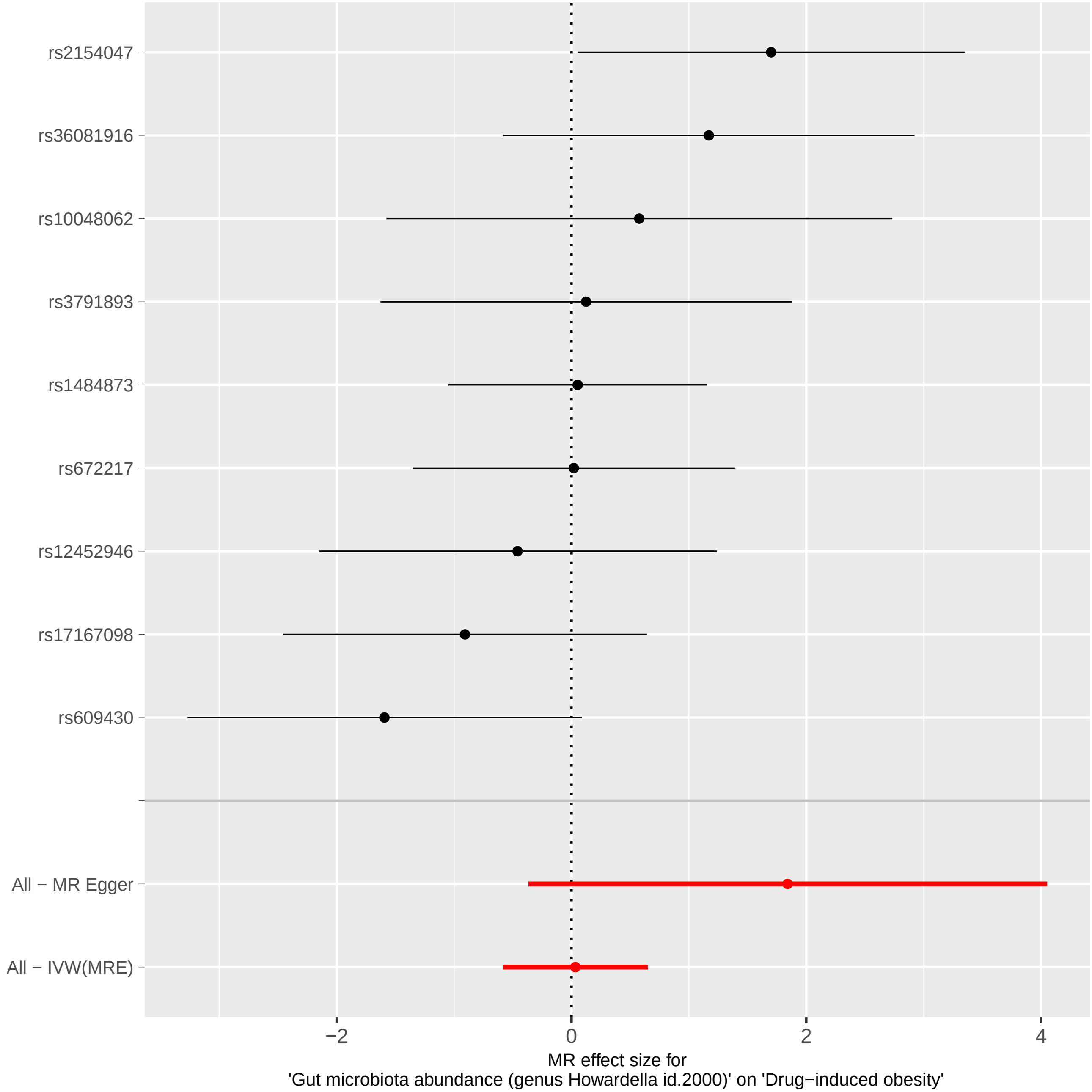

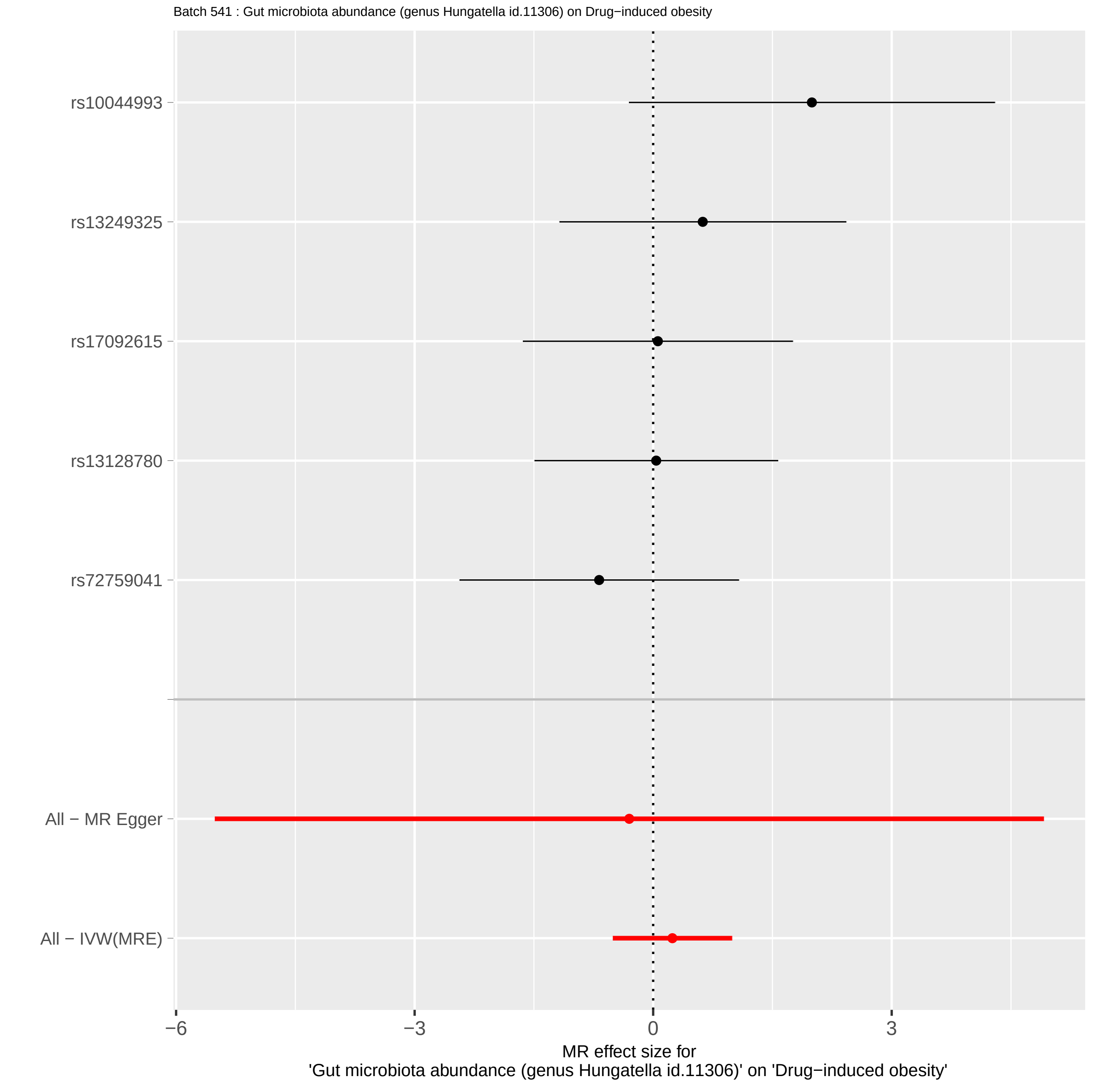
Batch 541 : Gut microbiota abundance (genus Hungatella id.11306) on Drug−induced obesity
rs10044993
rs13249325
rs17092615
rs13128780
rs72759041
All − MR Egger
All − IVW(MRE)
−6
−3
0
3
MR effect size for
'Gut microbiota abundance (genus Hungatella id.11306)' on 'Drug−induced obesity'

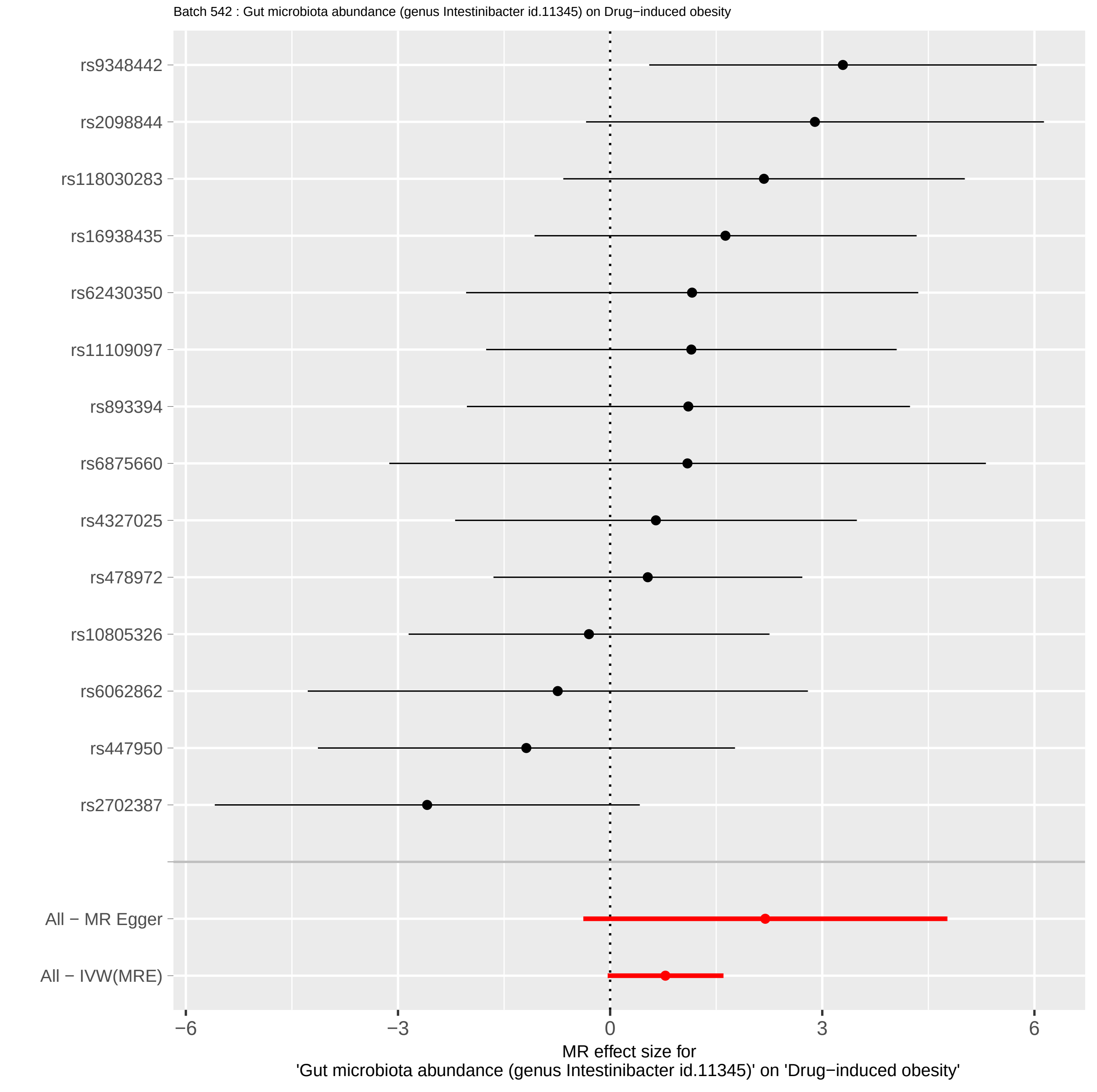
Batch 542 : Gut microbiota abundance (genus Intestinibacter id.11345) on Drug−induced obesity
rs9348442
rs2098844
rs118030283
rs16938435
rs62430350
rs11109097
rs893394
rs6875660
rs4327025
rs478972
rs10805326
rs6062862
rs447950
rs2702387
All − MR Egger
All − IVW(MRE)
−6
−3
0
3
6
MR effect size for
'Gut microbiota abundance (genus Intestinibacter id.11345)' on 'Drug−induced obesity'

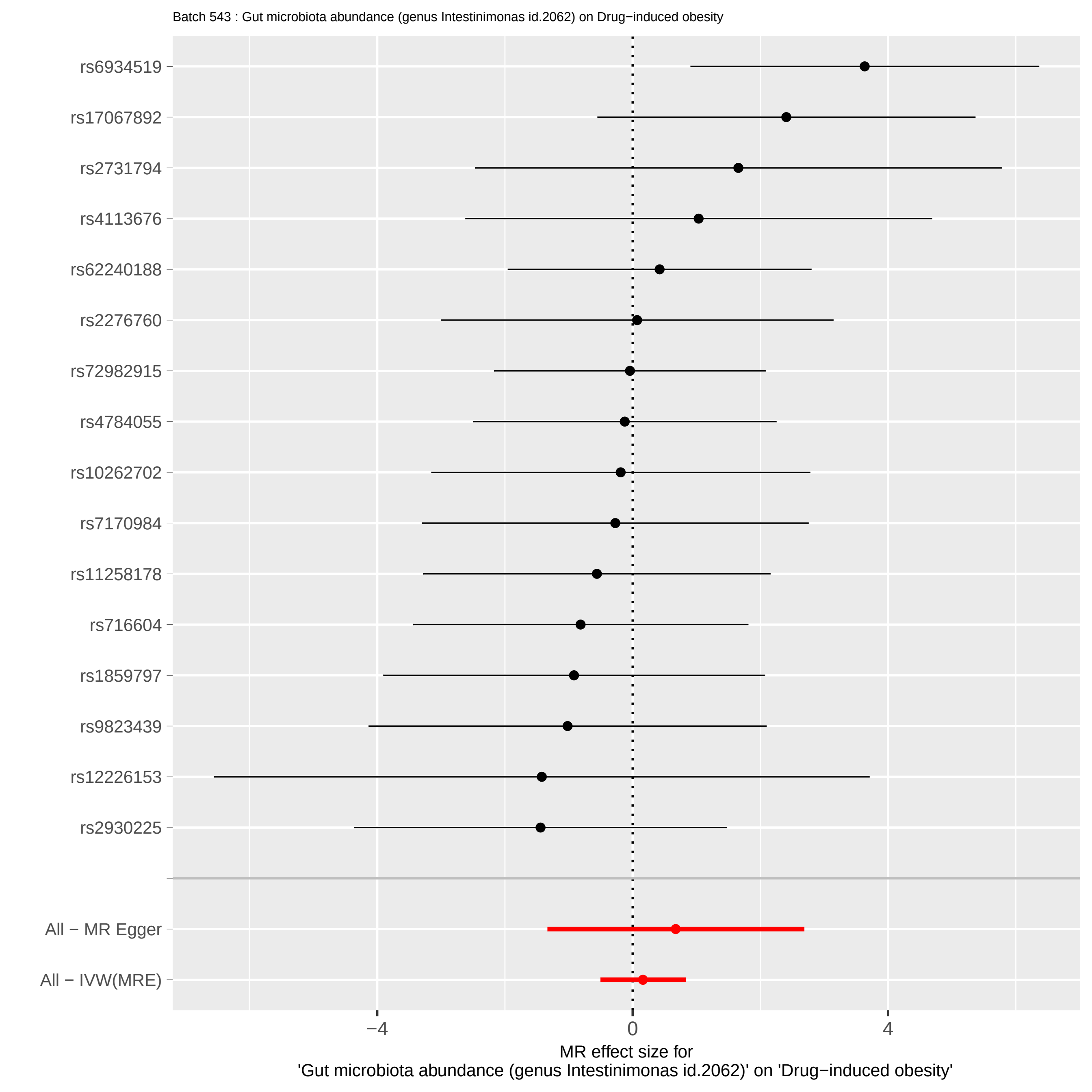

Batch 543 : Gut microbiota abundance (genus Intestinimonas id.2062) on Drug−induced obesity
rs6934519
rs17067892
rs2731794
rs4113676
rs62240188
rs2276760
rs72982915
rs4784055
rs10262702
rs7170984
rs11258178
rs716604
rs1859797
rs9823439
rs12226153
rs2930225
All − MR Egger
All − IVW(MRE)
−4
0
4
MR effect size for
'Gut microbiota abundance (genus Intestinimonas id.2062)' on 'Drug−induced obesity'

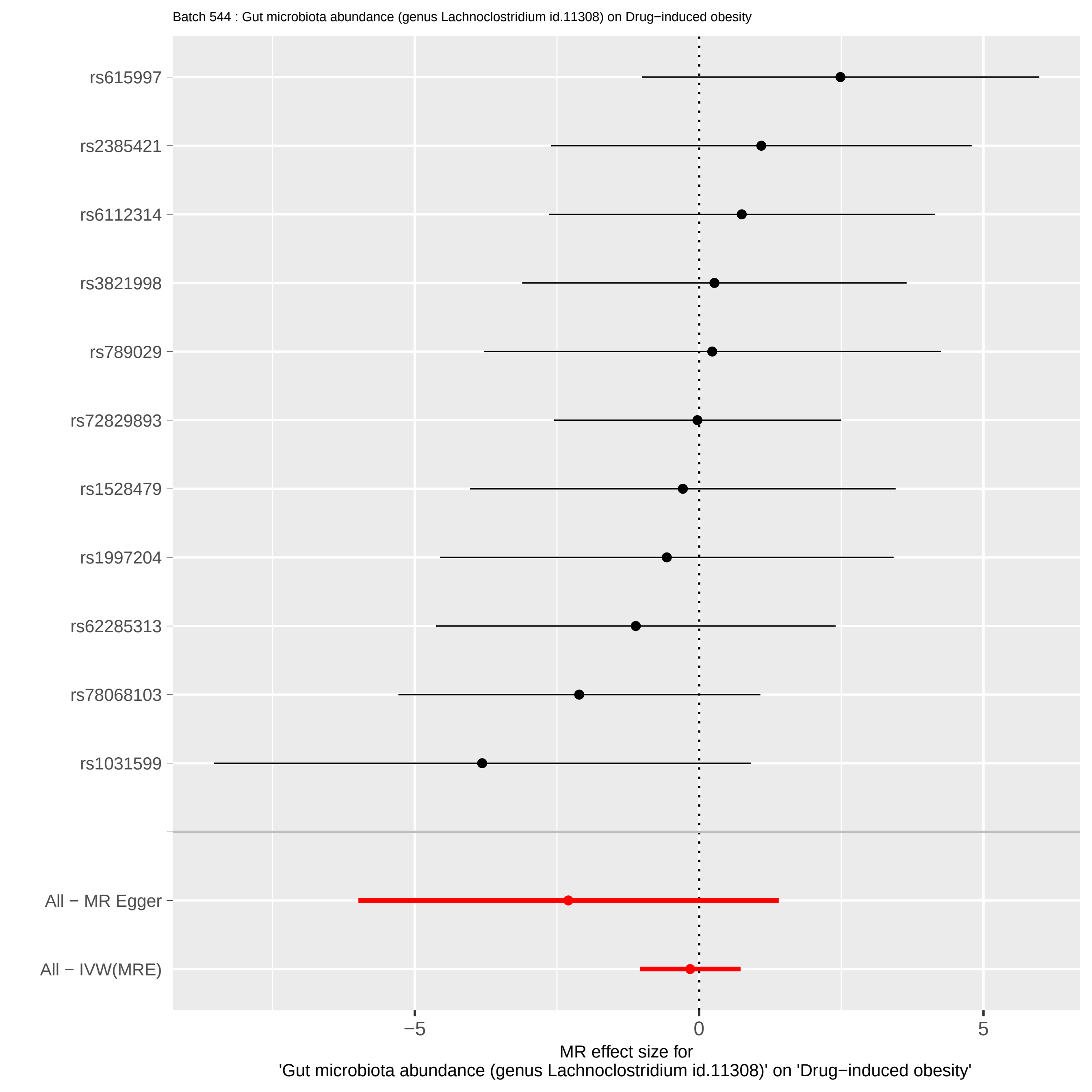

Batch 544 : Gut microbiota abundance (genus Lachnoclostridium id.11308) on Drug−induced obesity
rs615997
rs2385421
rs6112314
rs3821998
rs789029
rs72829893
rs1528479
rs1997204
rs62285313
rs78068103
rs1031599
All − MR Egger
All − IVW(MRE)
−5
0
5
MR effect size for
'Gut microbiota abundance (genus Lachnoclostridium id.11308)' on 'Drug−induced obesity'

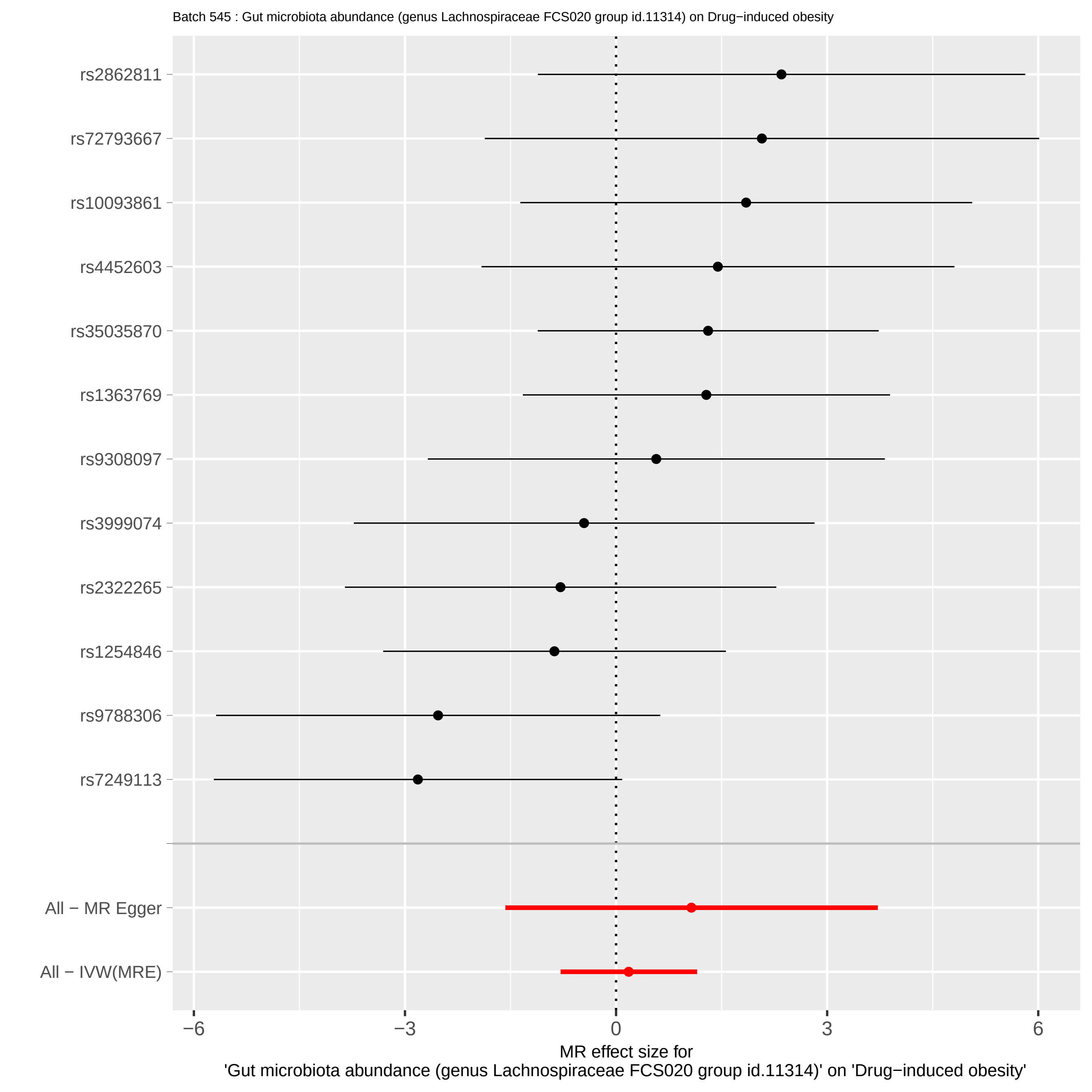
Batch 545 : Gut microbiota abundance (genus Lachnospiraceae FCS020 group id.11314) on Drug−induced obesity
rs2862811
rs72793667
rs10093861
rs4452603
rs35035870
rs1363769
rs9308097
rs3999074
rs2322265
rs1254846
rs9788306
rs7249113
All − MR Egger
All − IVW(MRE)
−6
−3
0
3
6
MR effect size for
'Gut microbiota abundance (genus Lachnospiraceae FCS020 group id.11314)' on 'Drug−induced obesity'

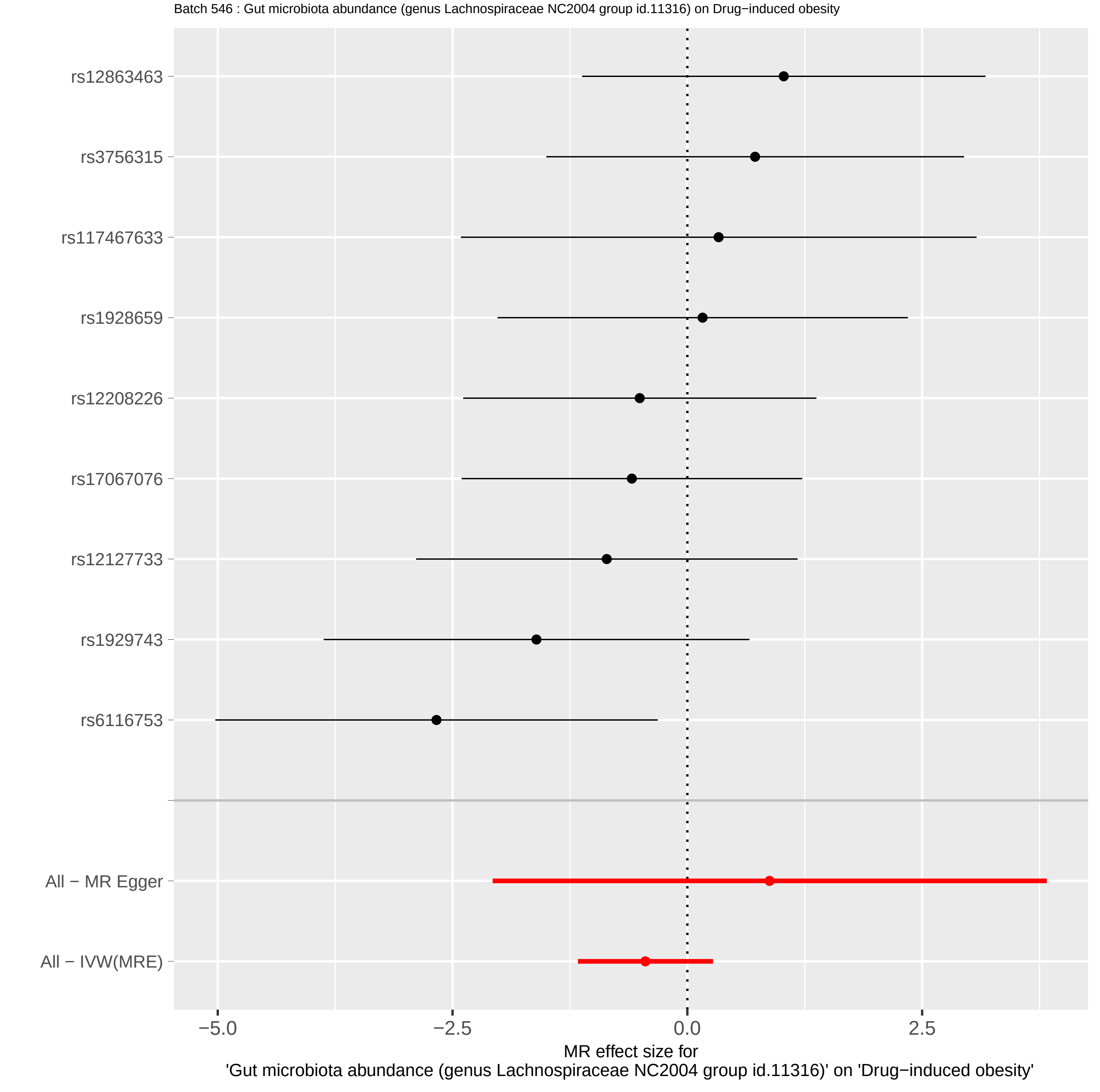
Batch 546 : Gut microbiota abundance (genus Lachnospiraceae NC2004 group id.11316) on Drug−induced obesity
rs12863463
rs3756315
rs117467633
rs1928659
rs12208226
rs17067076
rs12127733
rs1929743
rs6116753
All − MR Egger
All − IVW(MRE)
−5.0
−2.5
0.0
2.5
MR effect size for
'Gut microbiota abundance (genus Lachnospiraceae NC2004 group id.11316)' on 'Drug−induced obesity'

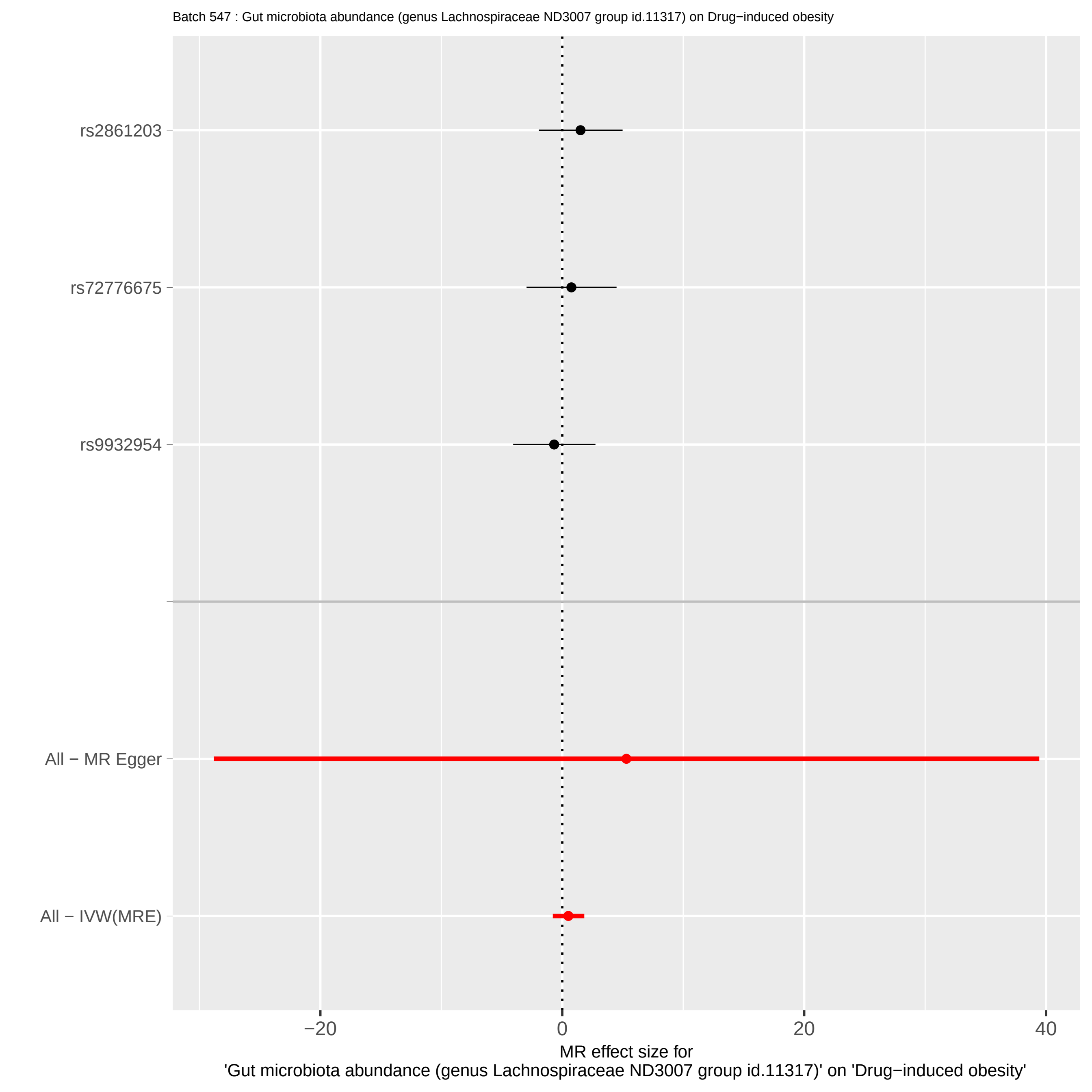

Batch 547 : Gut microbiota abundance (genus Lachnospiraceae ND3007 group id.11317) on Drug−induced obesity
rs2861203
rs72776675
rs9932954
All − MR Egger
All − IVW(MRE)
−20
0
20
40
MR effect size for
'Gut microbiota abundance (genus Lachnospiraceae ND3007 group id.11317)' on 'Drug−induced obesity'

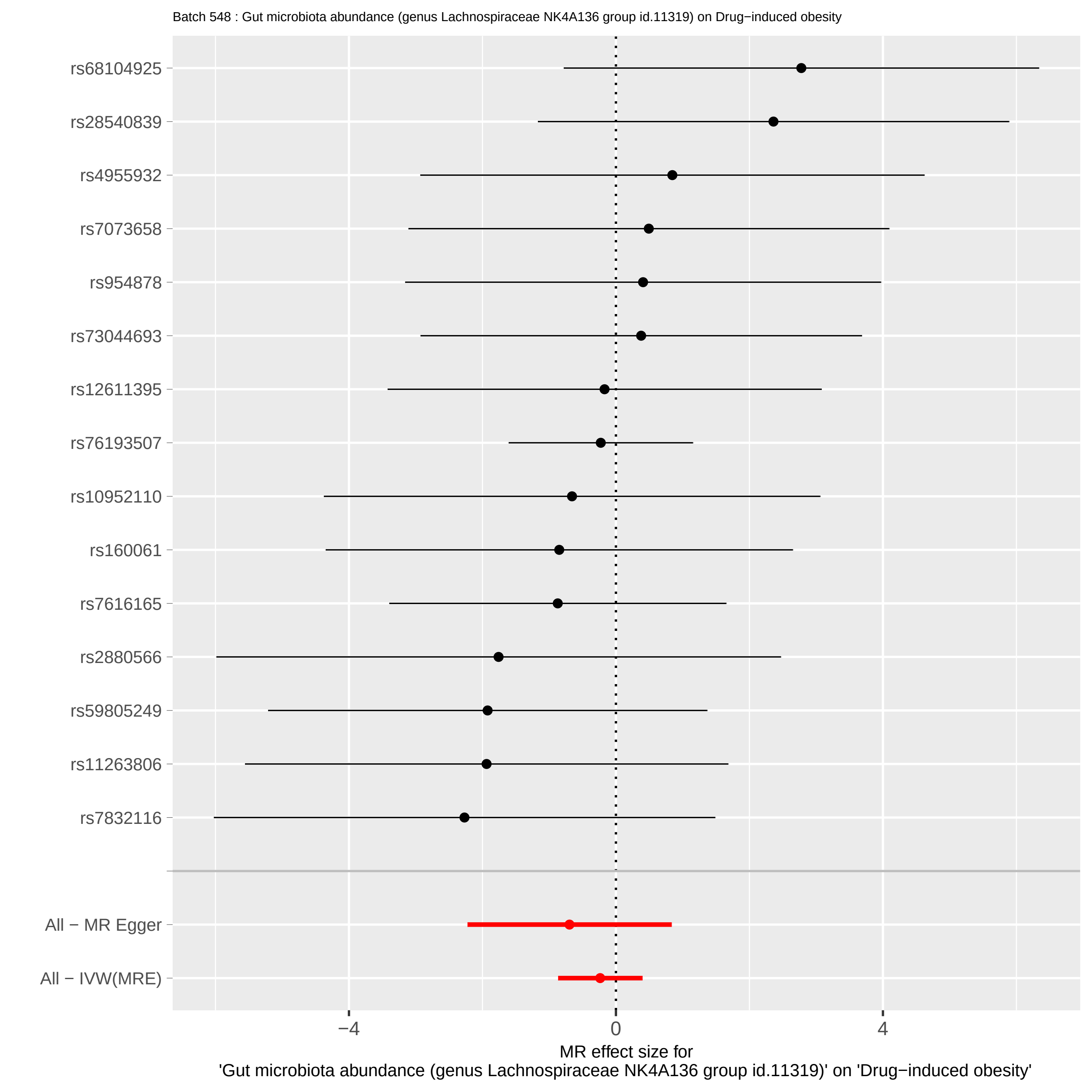
Batch 548 : Gut microbiota abundance (genus Lachnospiraceae NK4A136 group id.11319) on Drug−induced obesity
rs68104925
rs28540839
rs4955932
rs7073658
rs954878
rs73044693
rs12611395
rs76193507
rs10952110
rs160061
rs7616165
rs2880566
rs59805249
rs11263806
rs7832116
All − MR Egger
All − IVW(MRE)
−4
0
4
MR effect size for
'Gut microbiota abundance (genus Lachnospiraceae NK4A136 group id.11319)' on 'Drug−induced obesity'

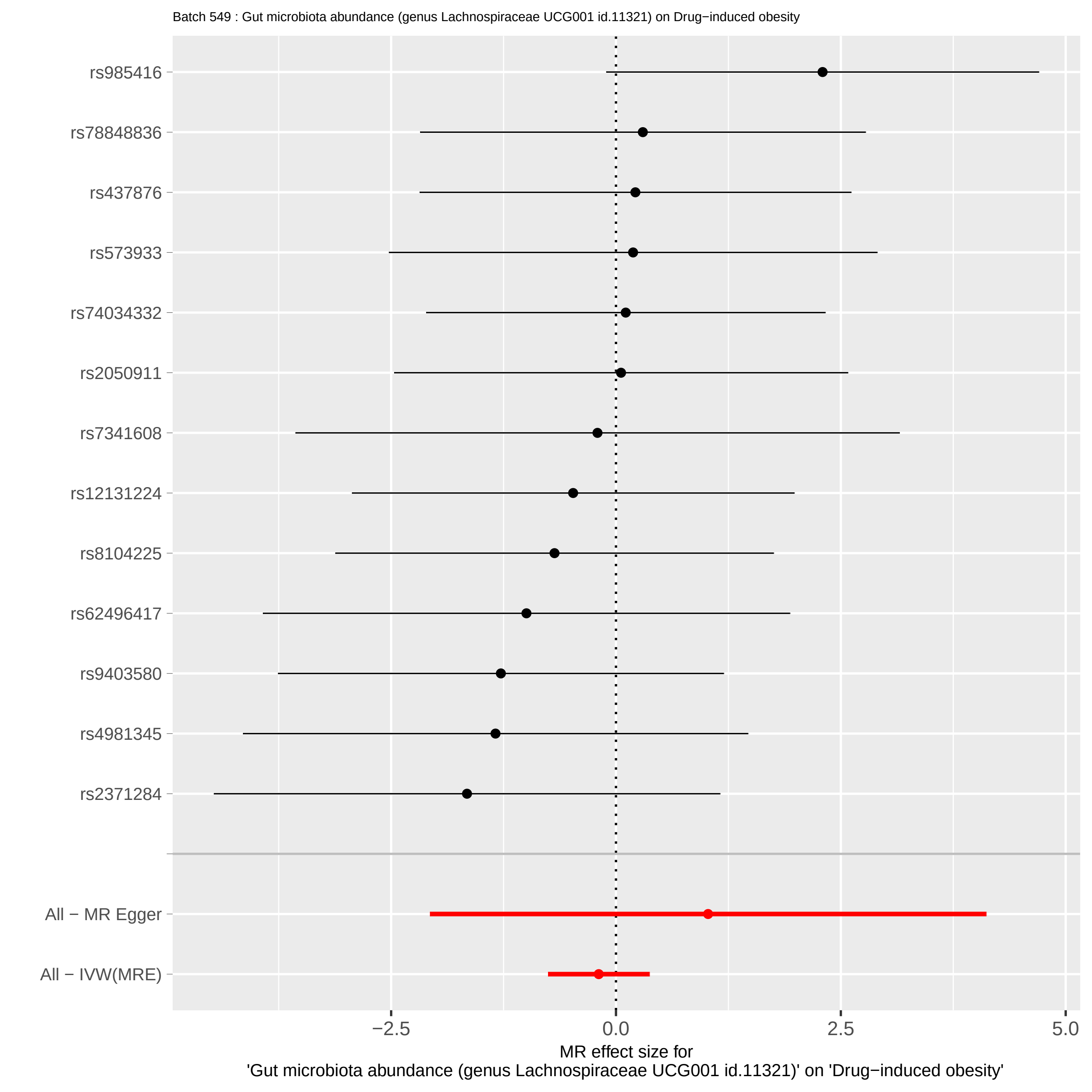

Batch 549 : Gut microbiota abundance (genus Lachnospiraceae UCG001 id.11321) on Drug−induced obesity
rs985416
rs78848836
rs437876
rs573933
rs74034332
rs2050911
rs7341608
rs12131224
rs8104225
rs62496417
rs9403580
rs4981345
rs2371284
All − MR Egger
All − IVW(MRE)
−2.5
0.0
2.5
5.0
MR effect size for
'Gut microbiota abundance (genus Lachnospiraceae UCG001 id.11321)' on 'Drug−induced obesity'

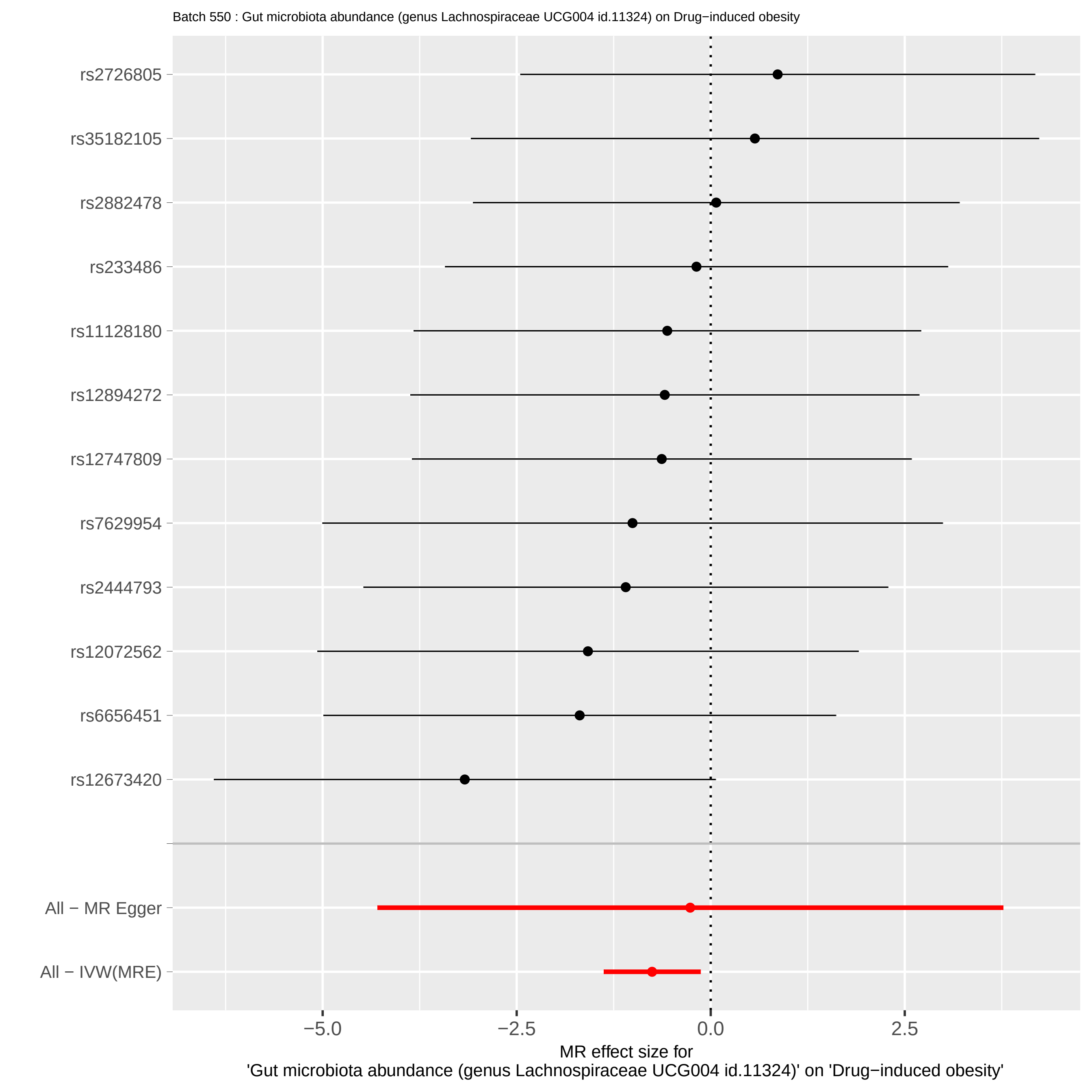

Batch 550 : Gut microbiota abundance (genus Lachnospiraceae UCG004 id.11324) on Drug−induced obesity
rs2726805
rs35182105
rs2882478
rs233486
rs11128180
rs12894272
rs12747809
rs7629954
rs2444793
rs12072562
rs6656451
rs12673420
All − MR Egger
All − IVW(MRE)
−5.0
−2.5
0.0
2.5
MR effect size for
'Gut microbiota abundance (genus Lachnospiraceae UCG004 id.11324)' on 'Drug−induced obesity'

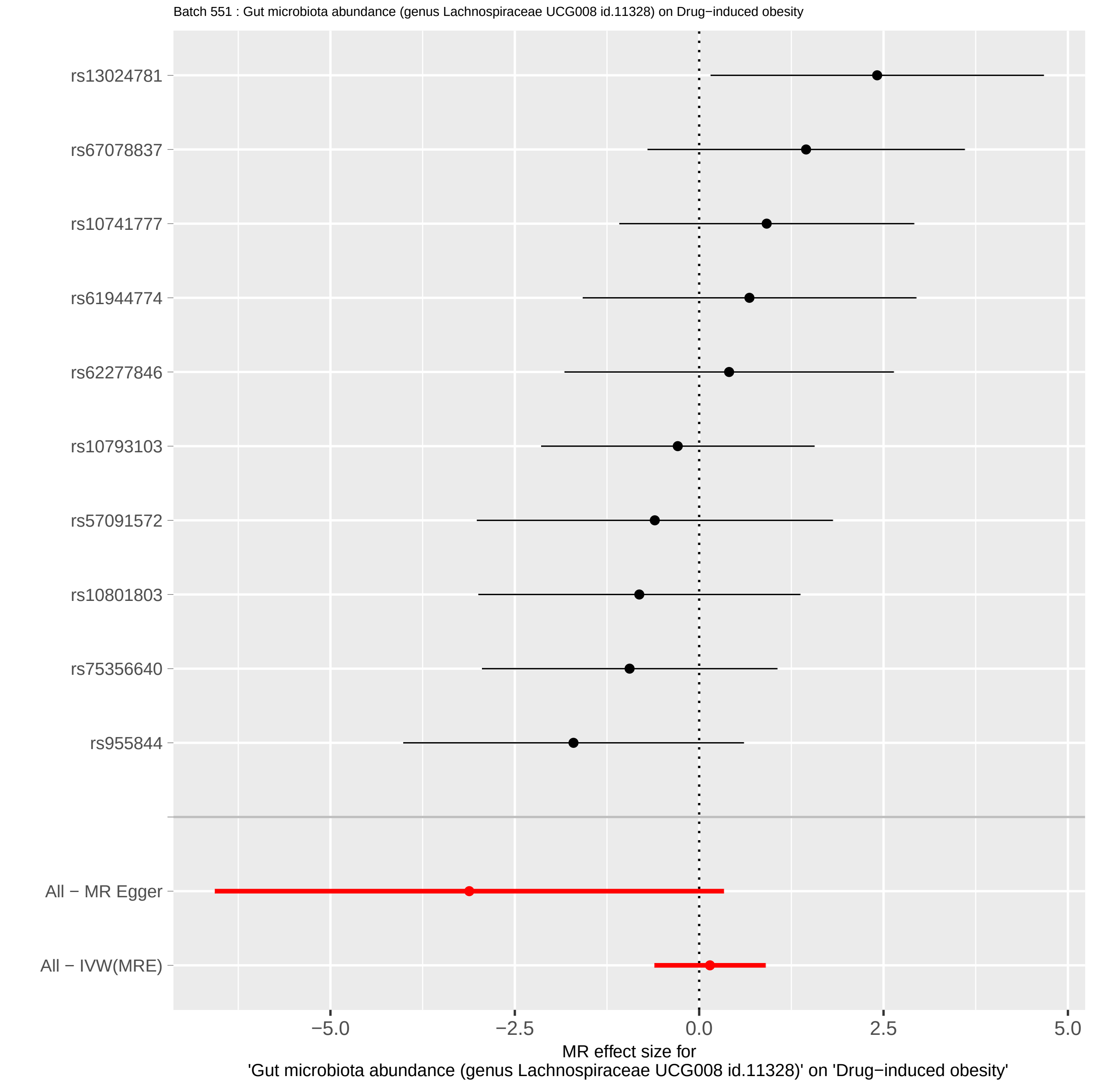

Batch 551 : Gut microbiota abundance (genus Lachnospiraceae UCG008 id.11328) on Drug−induced obesity
rs13024781
rs67078837
rs10741777
rs61944774
rs62277846
rs10793103
rs57091572
rs10801803
rs75356640
rs955844
All − MR Egger
All − IVW(MRE)
−5.0
−2.5
0.0
2.5
5.0
MR effect size for
'Gut microbiota abundance (genus Lachnospiraceae UCG008 id.11328)' on 'Drug−induced obesity'

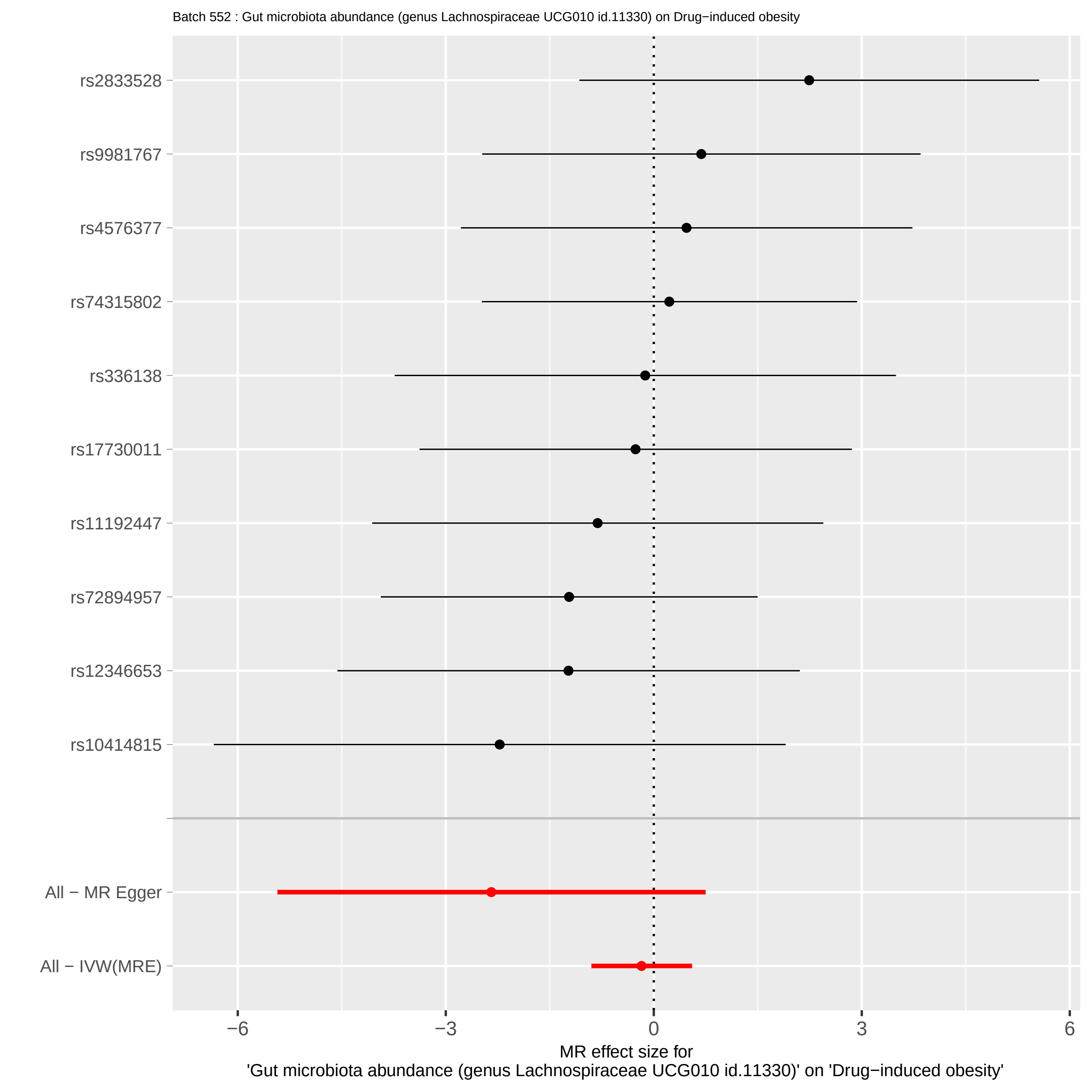

Batch 552 : Gut microbiota abundance (genus Lachnospiraceae UCG010 id.11330) on Drug−induced obesity
rs2833528
rs9981767
rs4576377
rs74315802
rs336138
rs17730011
rs11192447
rs72894957
rs12346653
rs10414815
All − MR Egger
All − IVW(MRE)
−6
−3
0
3
6
MR effect size for
'Gut microbiota abundance (genus Lachnospiraceae UCG010 id.11330)' on 'Drug−induced obesity'

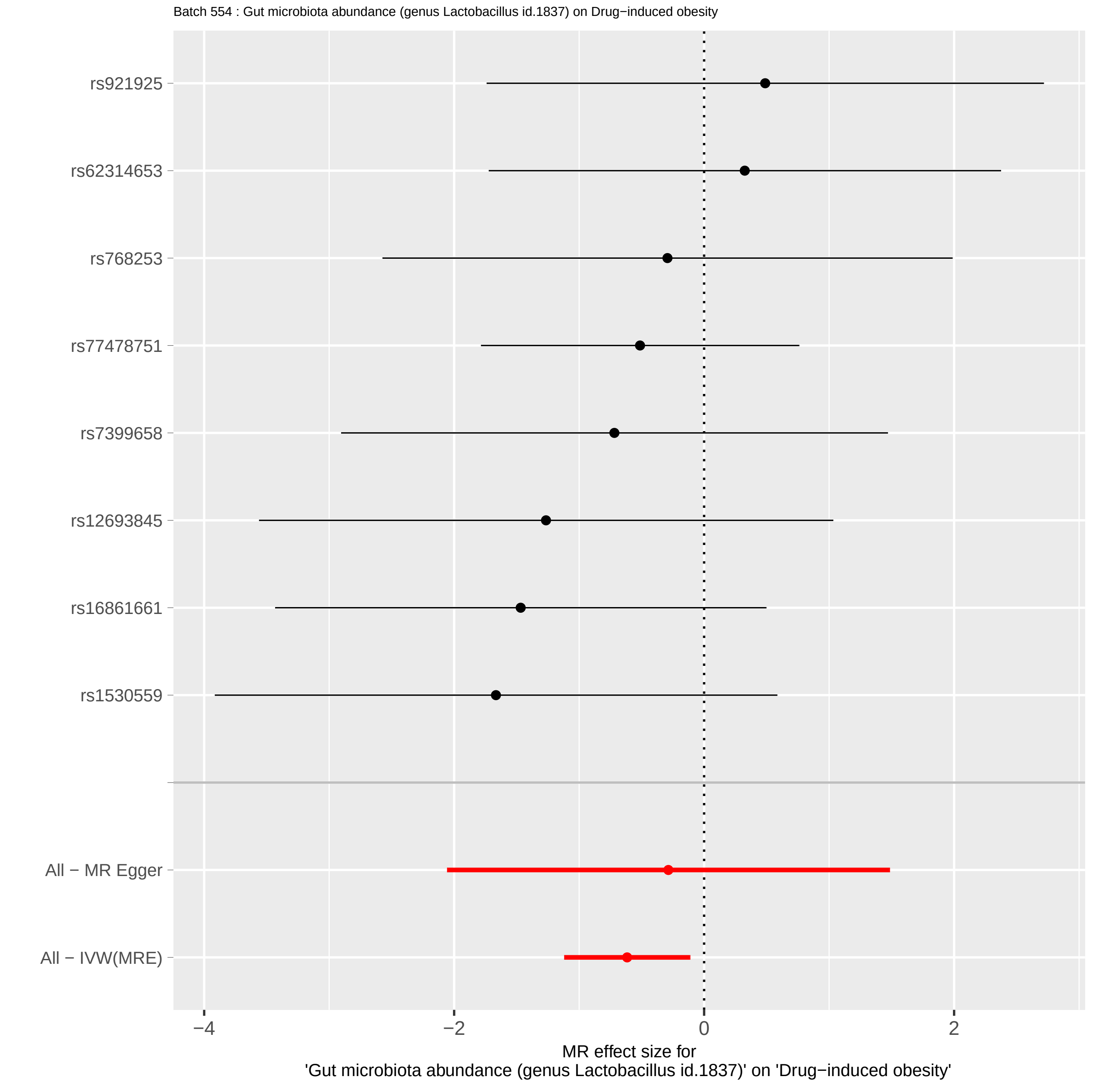

Batch 554 : Gut microbiota abundance (genus Lactobacillus id.1837) on Drug−induced obesity
rs921925
rs62314653
rs768253
rs77478751
rs7399658
rs12693845
rs16861661
rs1530559
All − MR Egger
All − IVW(MRE)
−4
−2
0
2
MR effect size for
'Gut microbiota abundance (genus Lactobacillus id.1837)' on 'Drug−induced obesity'

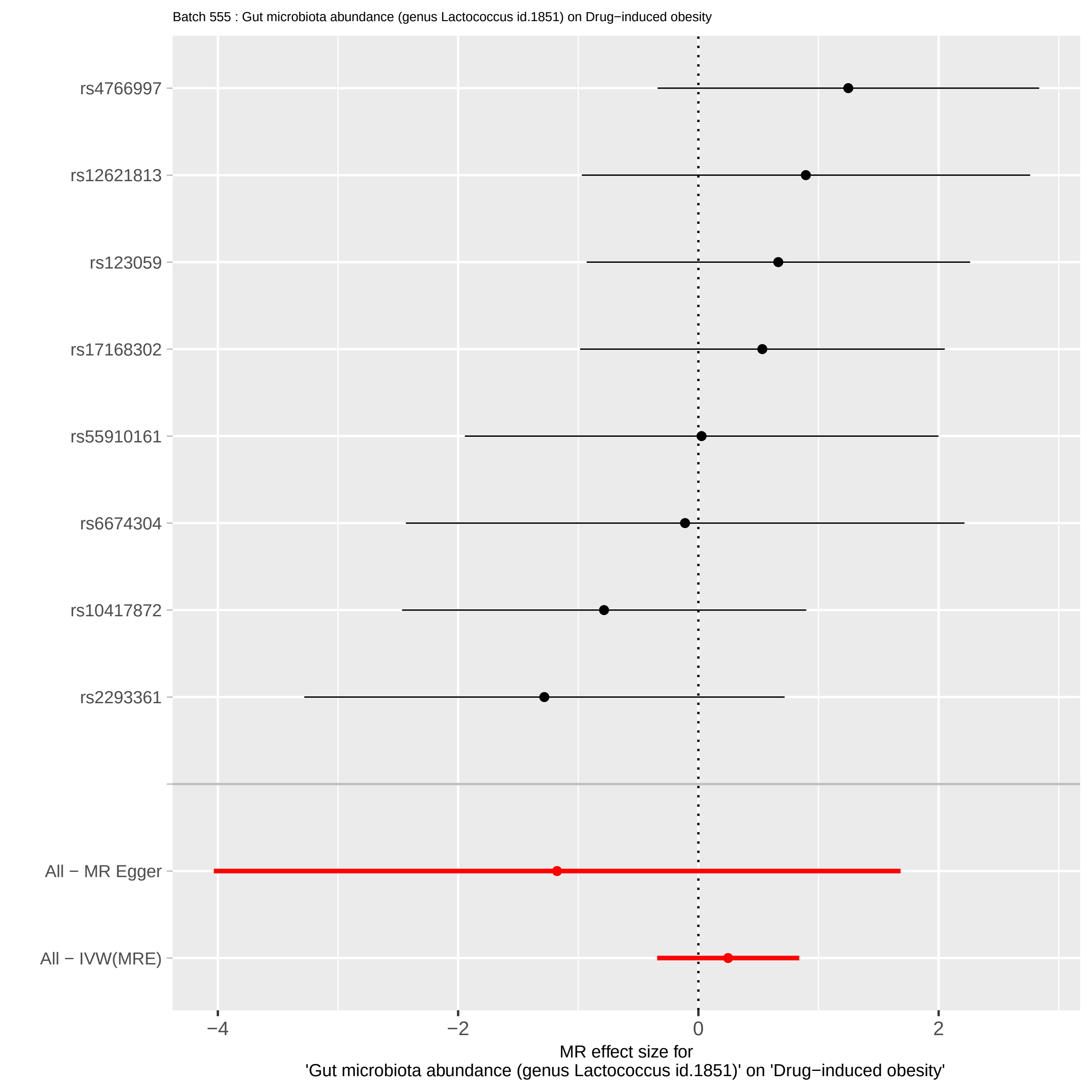
Batch 555 : Gut microbiota abundance (genus Lactococcus id.1851) on Drug−induced obesity
rs4766997
rs12621813
rs123059
rs17168302
rs55910161
rs6674304
rs10417872
rs2293361
All − MR Egger
All − IVW(MRE)
−4
−2
0
2
MR effect size for
'Gut microbiota abundance (genus Lactococcus id.1851)' on 'Drug−induced obesity'

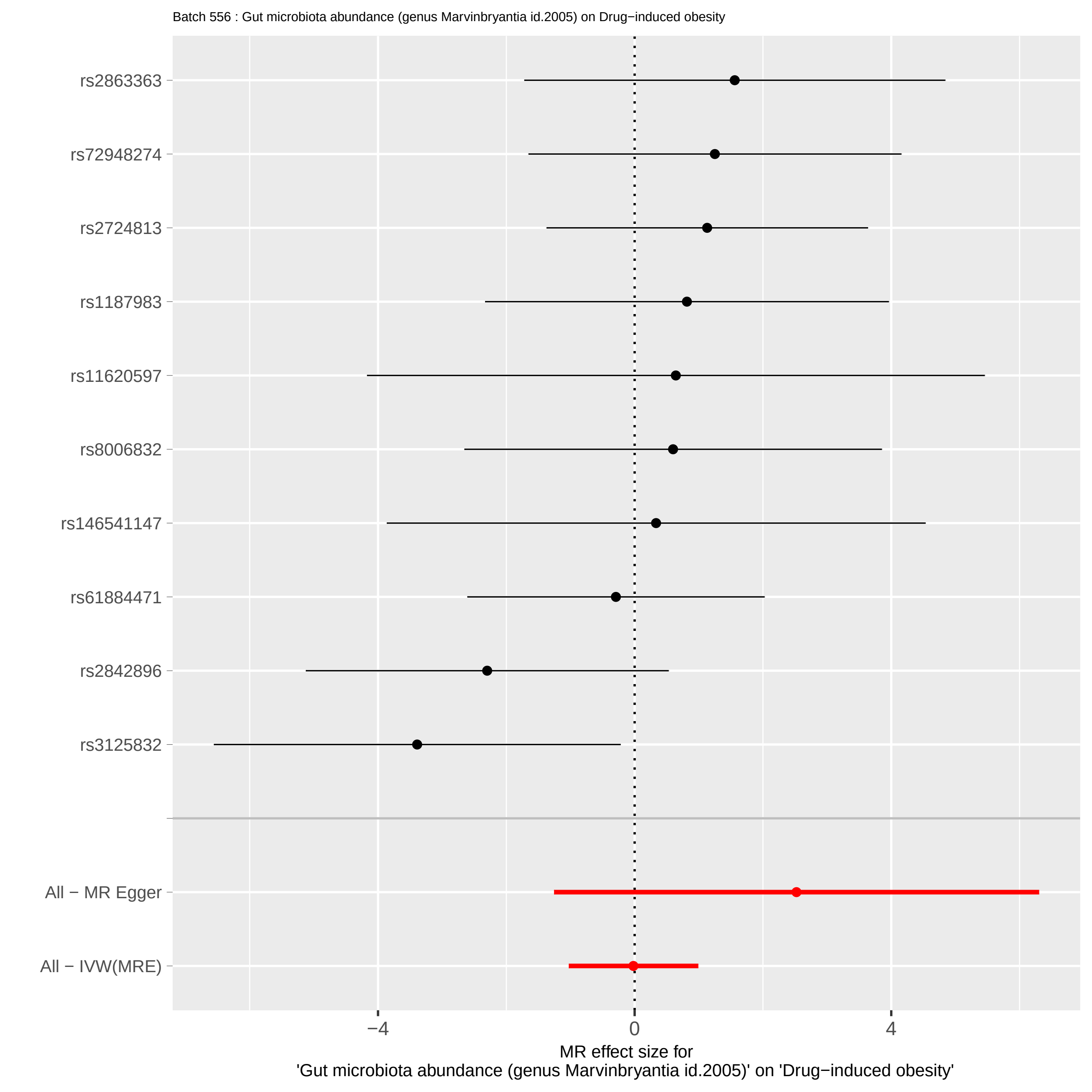

Batch 556 : Gut microbiota abundance (genus Marvinbryantia id.2005) on Drug−induced obesity
rs2863363
rs72948274
rs2724813
rs1187983
rs11620597
rs8006832
rs146541147
rs61884471
rs2842896
rs3125832
All − MR Egger
All − IVW(MRE)
−4
0
4
MR effect size for
'Gut microbiota abundance (genus Marvinbryantia id.2005)' on 'Drug−induced obesity'

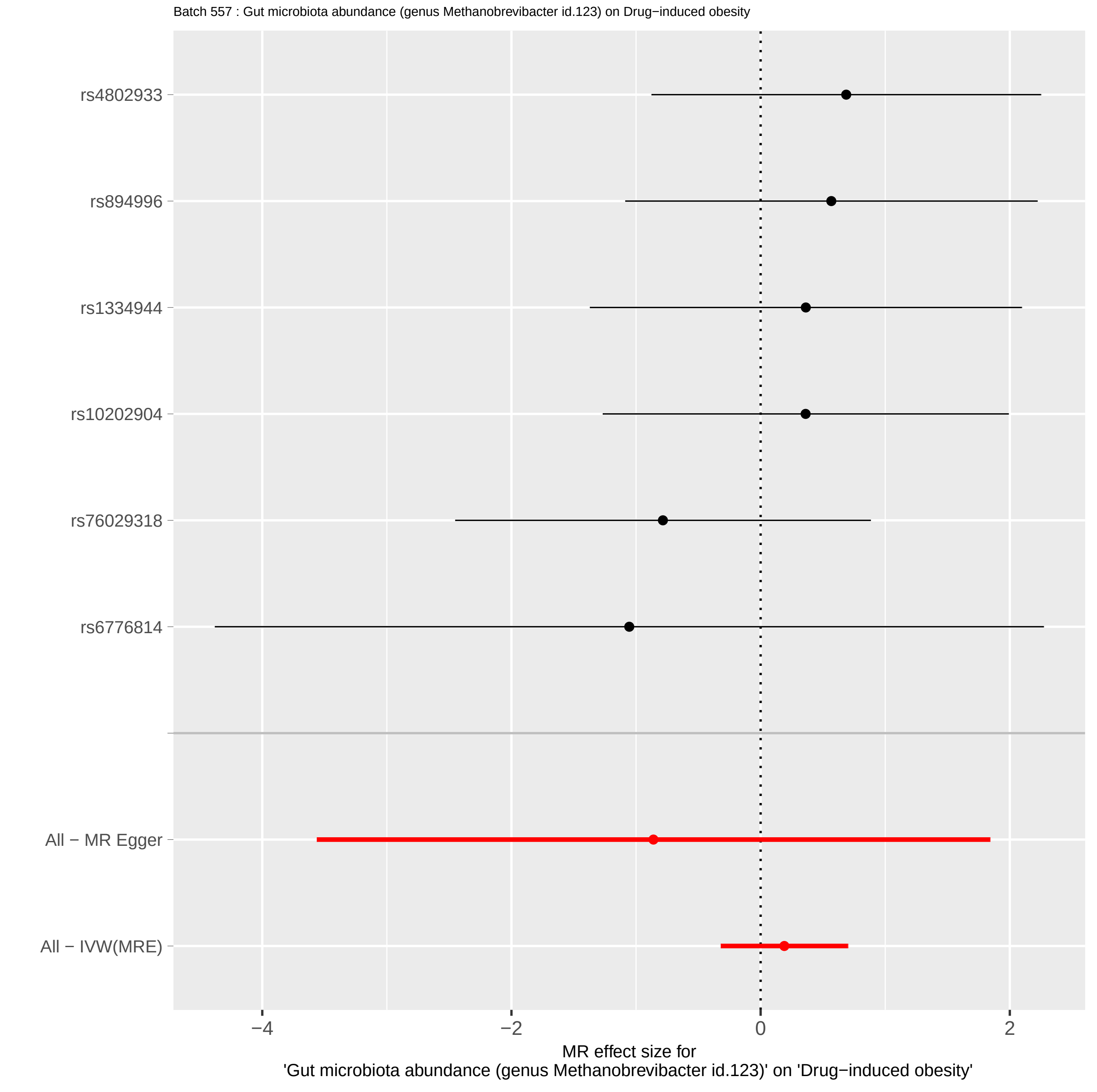
Batch 557 : Gut microbiota abundance (genus Methanobrevibacter id.123) on Drug−induced obesity
rs4802933
rs894996
rs1334944
rs10202904
rs76029318
rs6776814
All − MR Egger
All − IVW(MRE)
−4
−2
0
2
MR effect size for
'Gut microbiota abundance (genus Methanobrevibacter id.123)' on 'Drug−induced obesity'

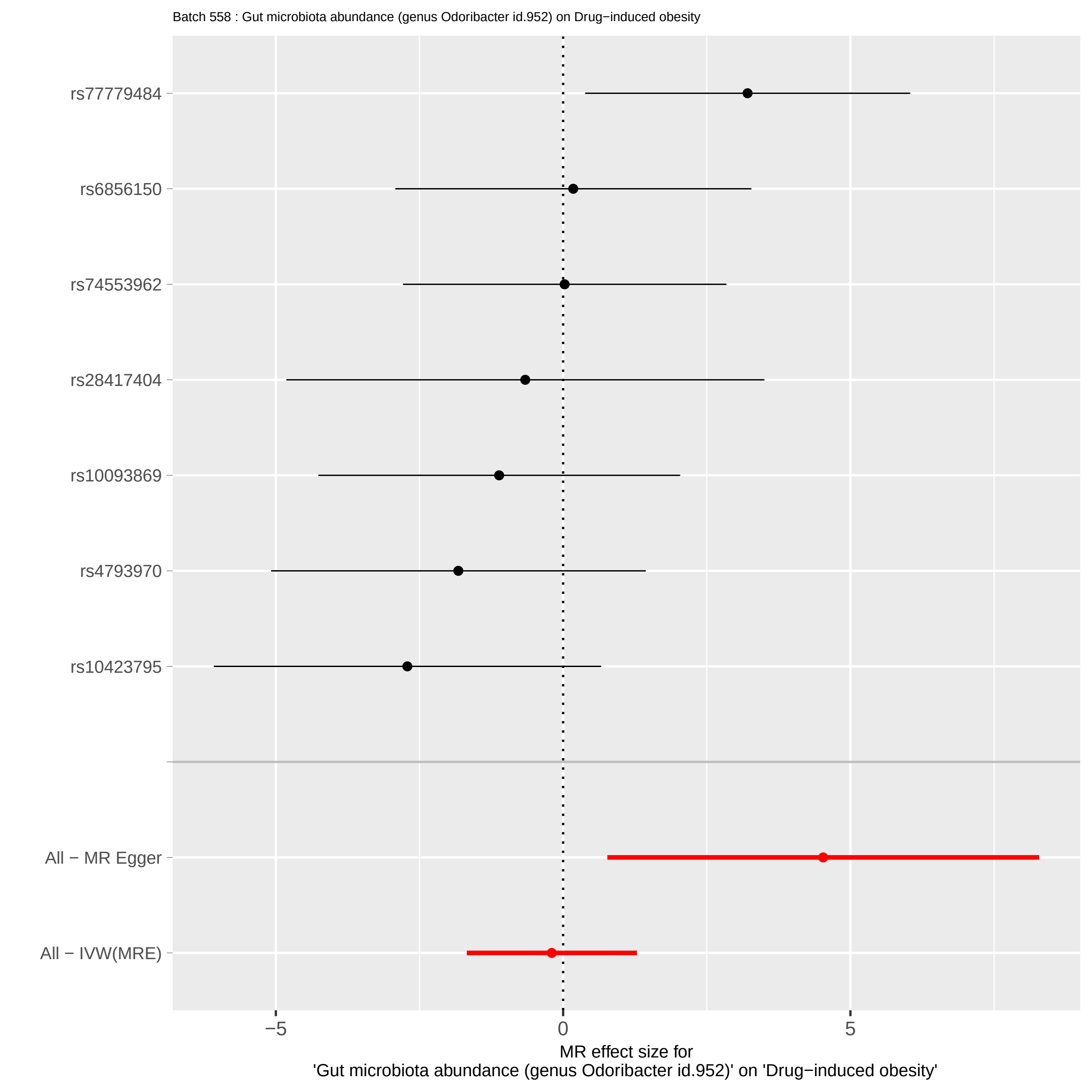
Batch 558 : Gut microbiota abundance (genus Odoribacter id.952) on Drug−induced obesity
rs77779484
rs6856150
rs74553962
rs28417404
rs10093869
rs4793970
rs10423795
All − MR Egger
All − IVW(MRE)
−5
0
5
MR effect size for
'Gut microbiota abundance (genus Odoribacter id.952)' on 'Drug−induced obesity'

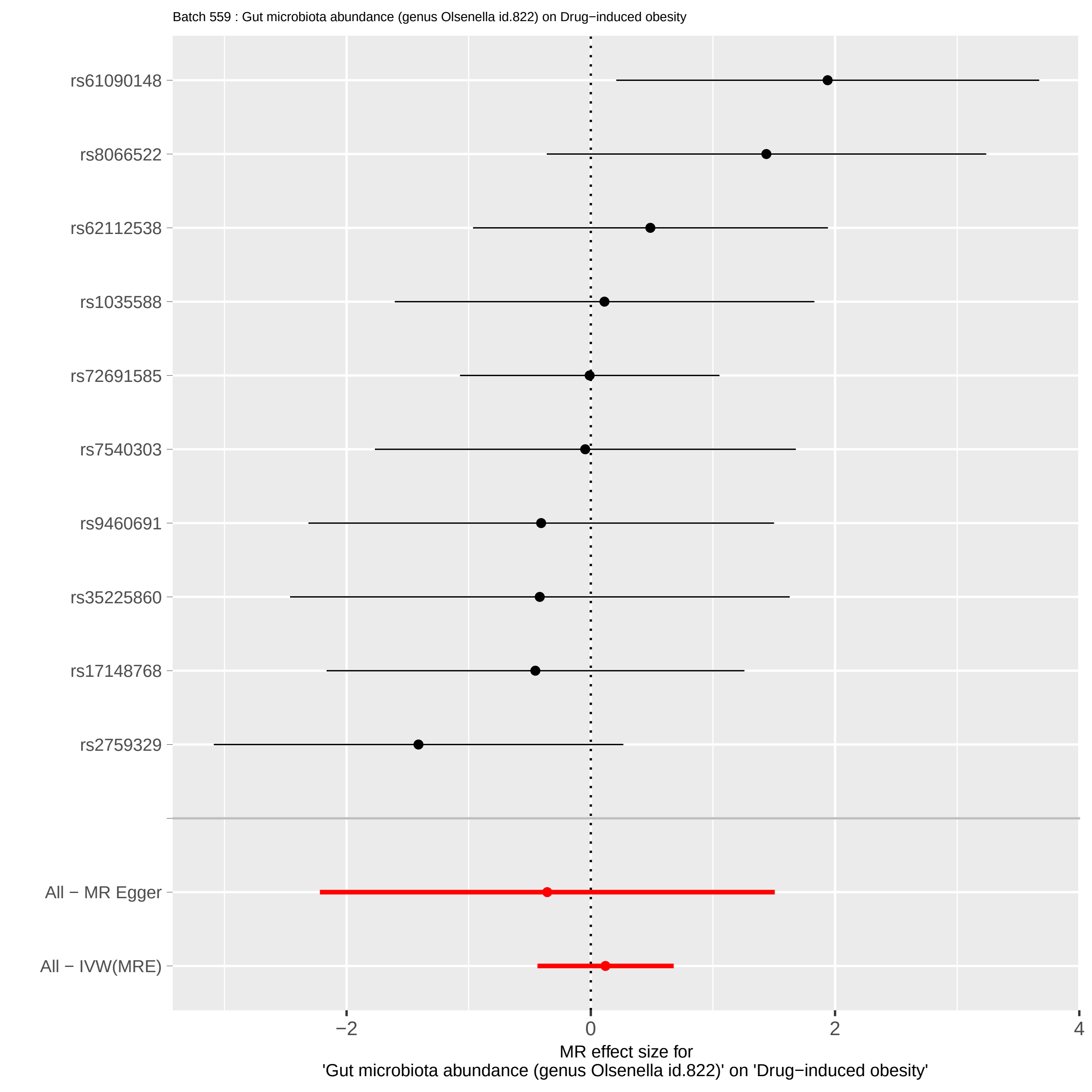

Batch 559 : Gut microbiota abundance (genus Olsenella id.822) on Drug−induced obesity
rs61090148
rs8066522
rs62112538
rs1035588
rs72691585
rs7540303
rs9460691
rs35225860
rs17148768
rs2759329
All − MR Egger
All − IVW(MRE)
−2
0
2
4
MR effect size for
'Gut microbiota abundance (genus Olsenella id.822)' on 'Drug−induced obesity'

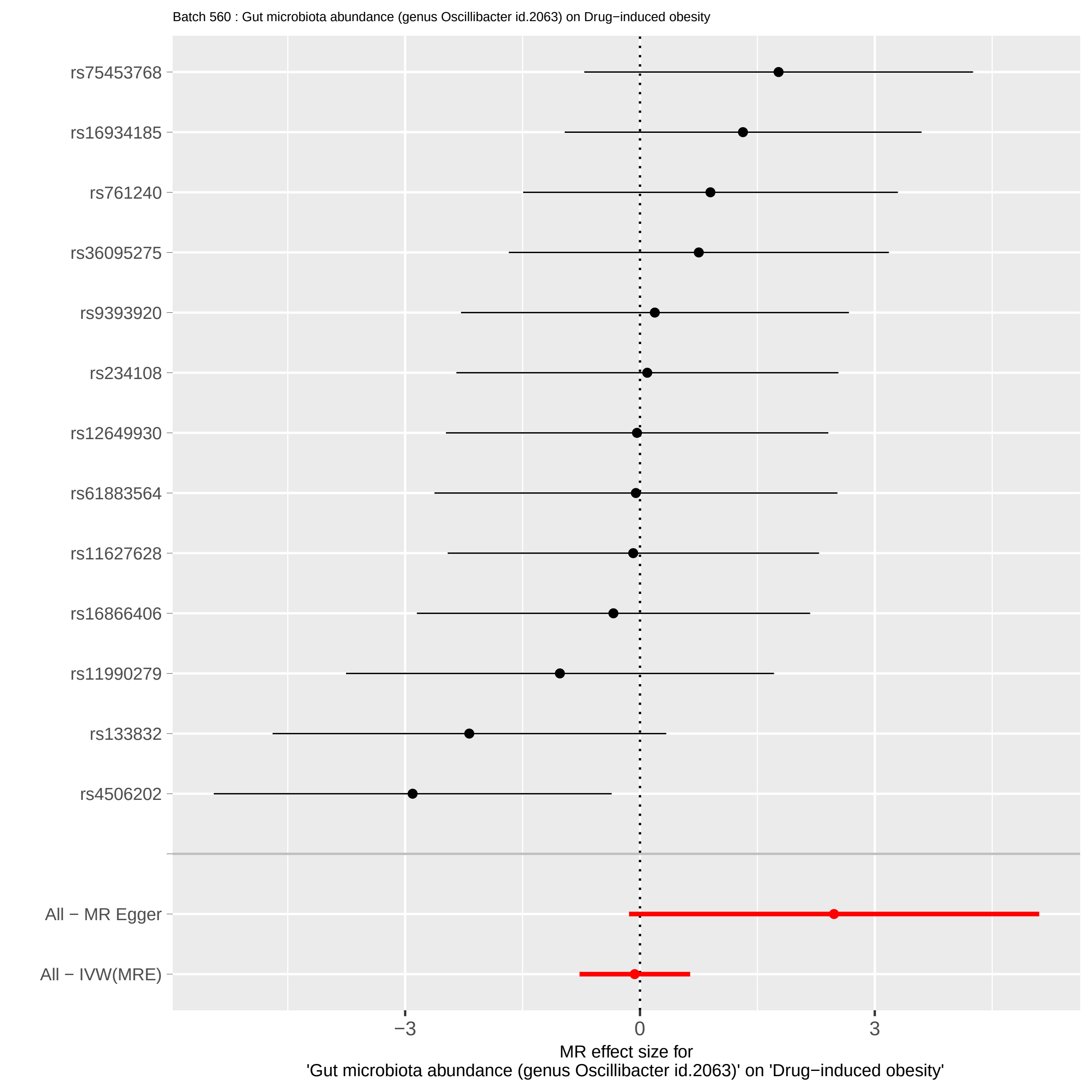
Batch 560 : Gut microbiota abundance (genus Oscillibacter id.2063) on Drug−induced obesity
rs75453768
rs16934185
rs761240
rs36095275
rs9393920
rs234108
rs12649930
rs61883564
rs11627628
rs16866406
rs11990279
rs133832
rs4506202
All − MR Egger
All − IVW(MRE)
−3
0
3
MR effect size for
'Gut microbiota abundance (genus Oscillibacter id.2063)' on 'Drug−induced obesity'

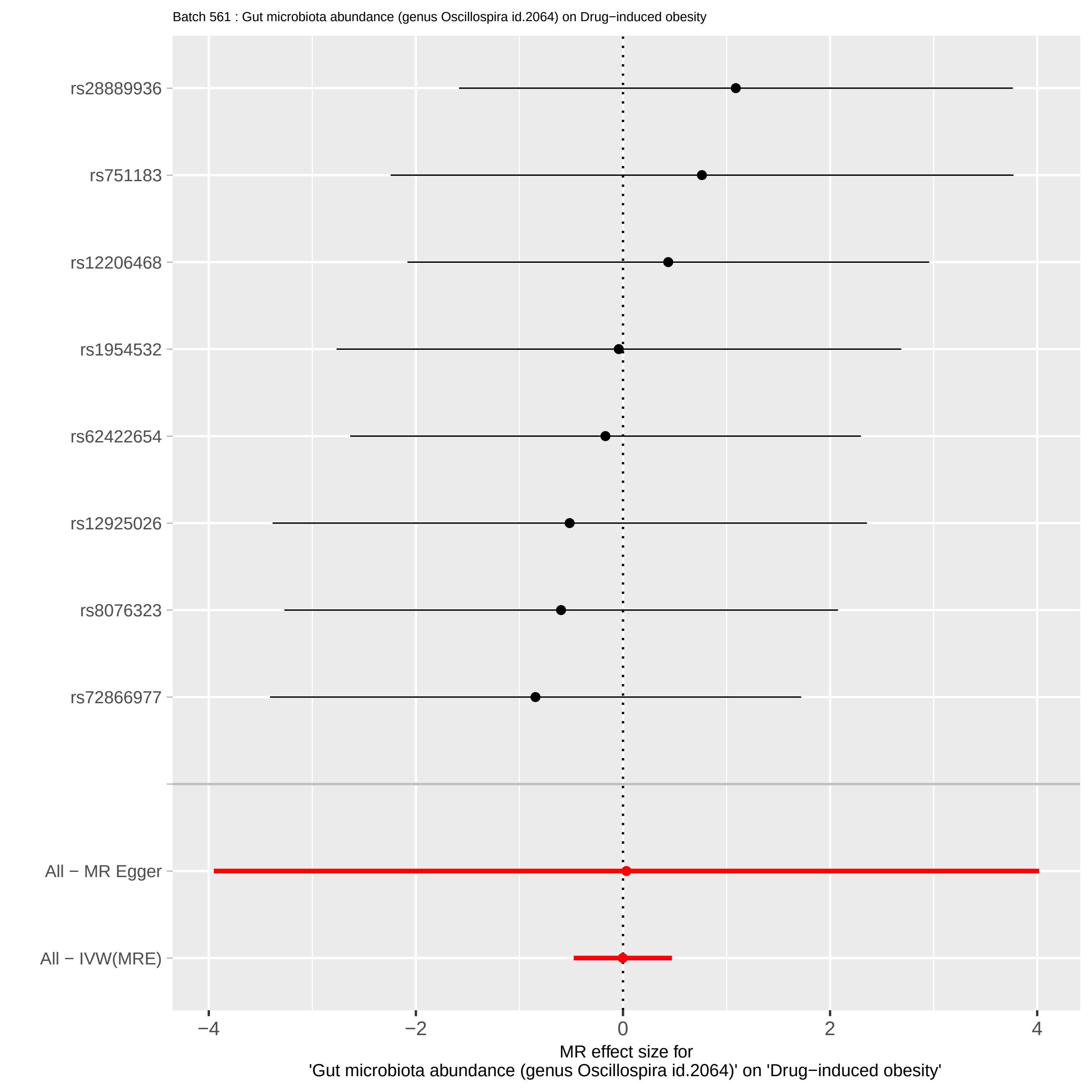

Batch 561 : Gut microbiota abundance (genus Oscillospira id.2064) on Drug−induced obesity
rs28889936
rs751183
rs12206468
rs1954532
rs62422654
rs12925026
rs8076323
rs72866977
All − MR Egger
All − IVW(MRE)
−4
−2
0
2
4
MR effect size for
'Gut microbiota abundance (genus Oscillospira id.2064)' on 'Drug−induced obesity'

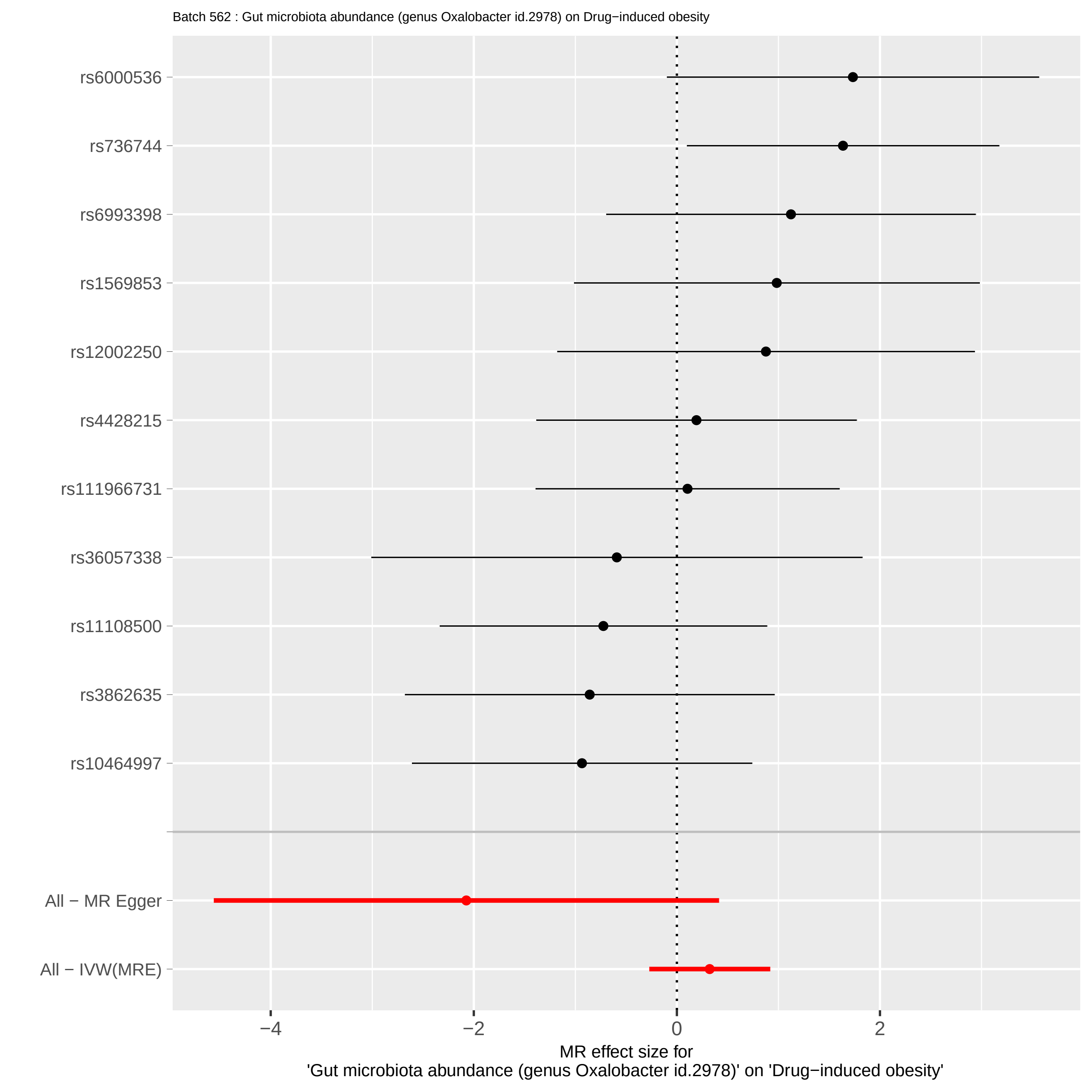

Batch 562 : Gut microbiota abundance (genus Oxalobacter id.2978) on Drug−induced obesity
rs6000536
rs736744
rs6993398
rs1569853
rs12002250
rs4428215
rs111966731
rs36057338
rs11108500
rs3862635
rs10464997
All − MR Egger
All − IVW(MRE)
−4
−2
0
2
MR effect size for
'Gut microbiota abundance (genus Oxalobacter id.2978)' on 'Drug−induced obesity'

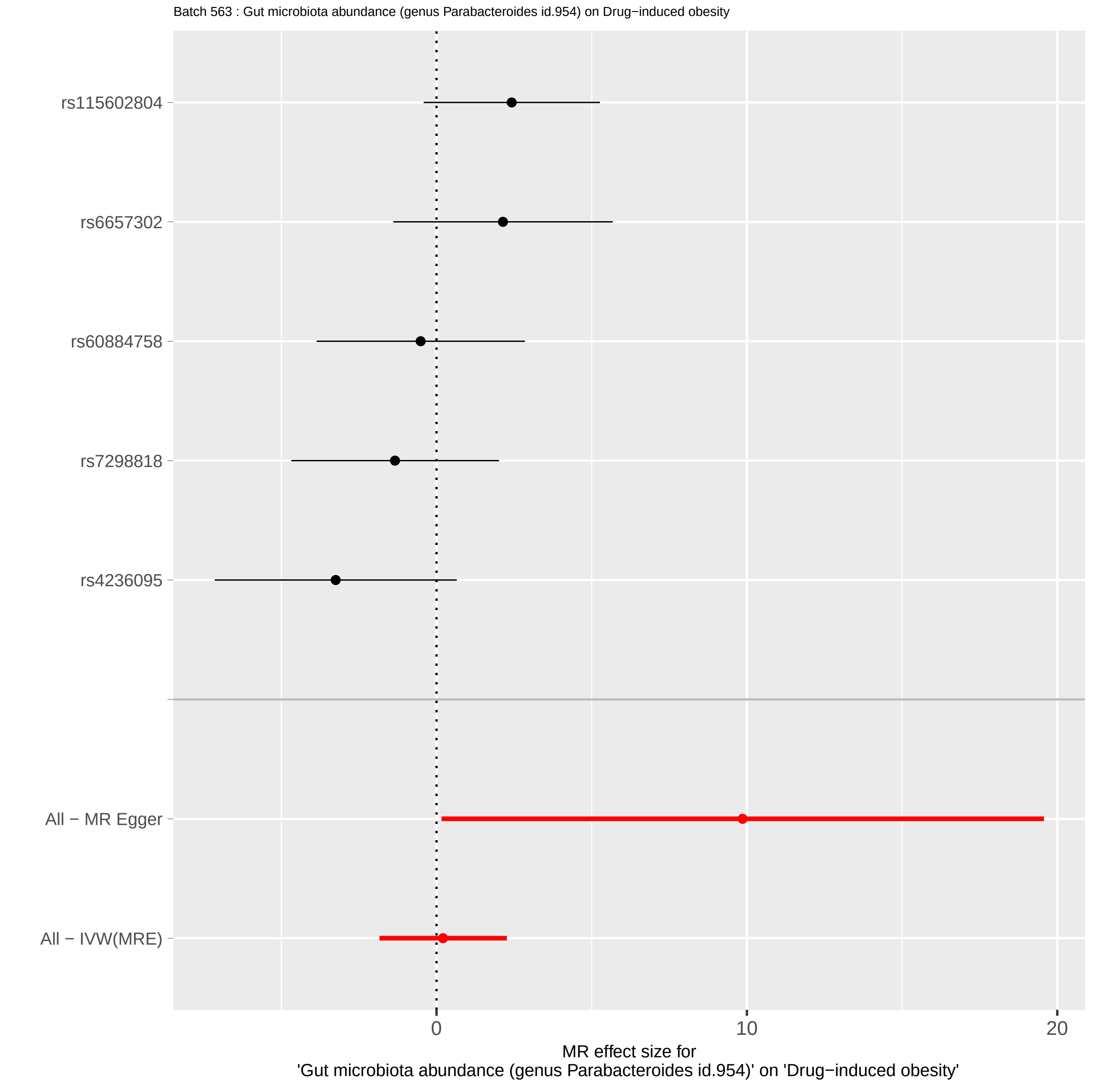

Batch 563 : Gut microbiota abundance (genus Parabacteroides id.954) on Drug−induced obesity
rs115602804
rs6657302
rs60884758
rs7298818
rs4236095
All − MR Egger
All − IVW(MRE)
0
10
20
MR effect size for
'Gut microbiota abundance (genus Parabacteroides id.954)' on 'Drug−induced obesity'

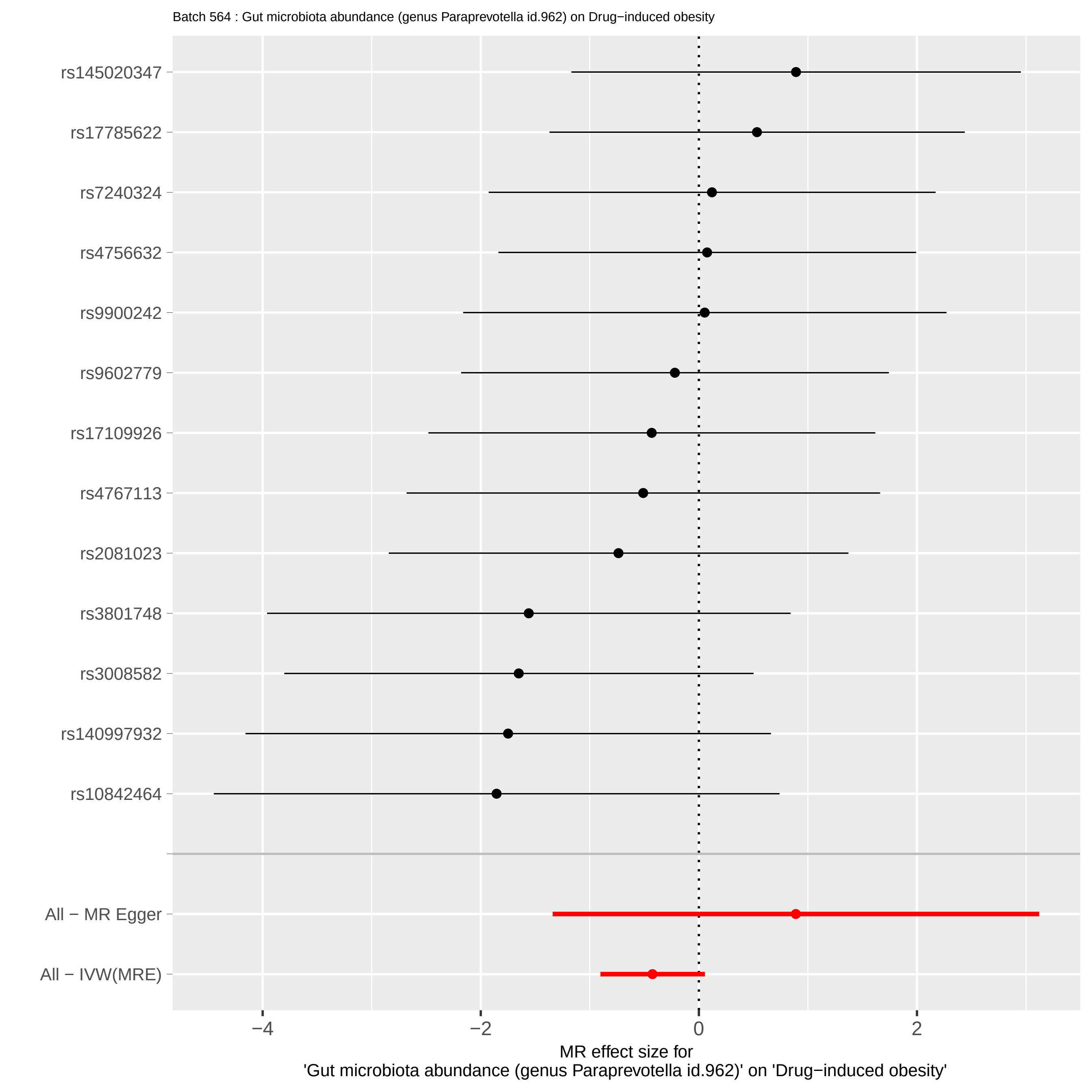
Batch 564 : Gut microbiota abundance (genus Paraprevotella id.962) on Drug−induced obesity
rs145020347
rs17785622
rs7240324
rs4756632
rs9900242
rs9602779
rs17109926
rs4767113
rs2081023
rs3801748
rs3008582
rs140997932
rs10842464
All − MR Egger
All − IVW(MRE)
−4
−2
0
2
MR effect size for
'Gut microbiota abundance (genus Paraprevotella id.962)' on 'Drug−induced obesity'

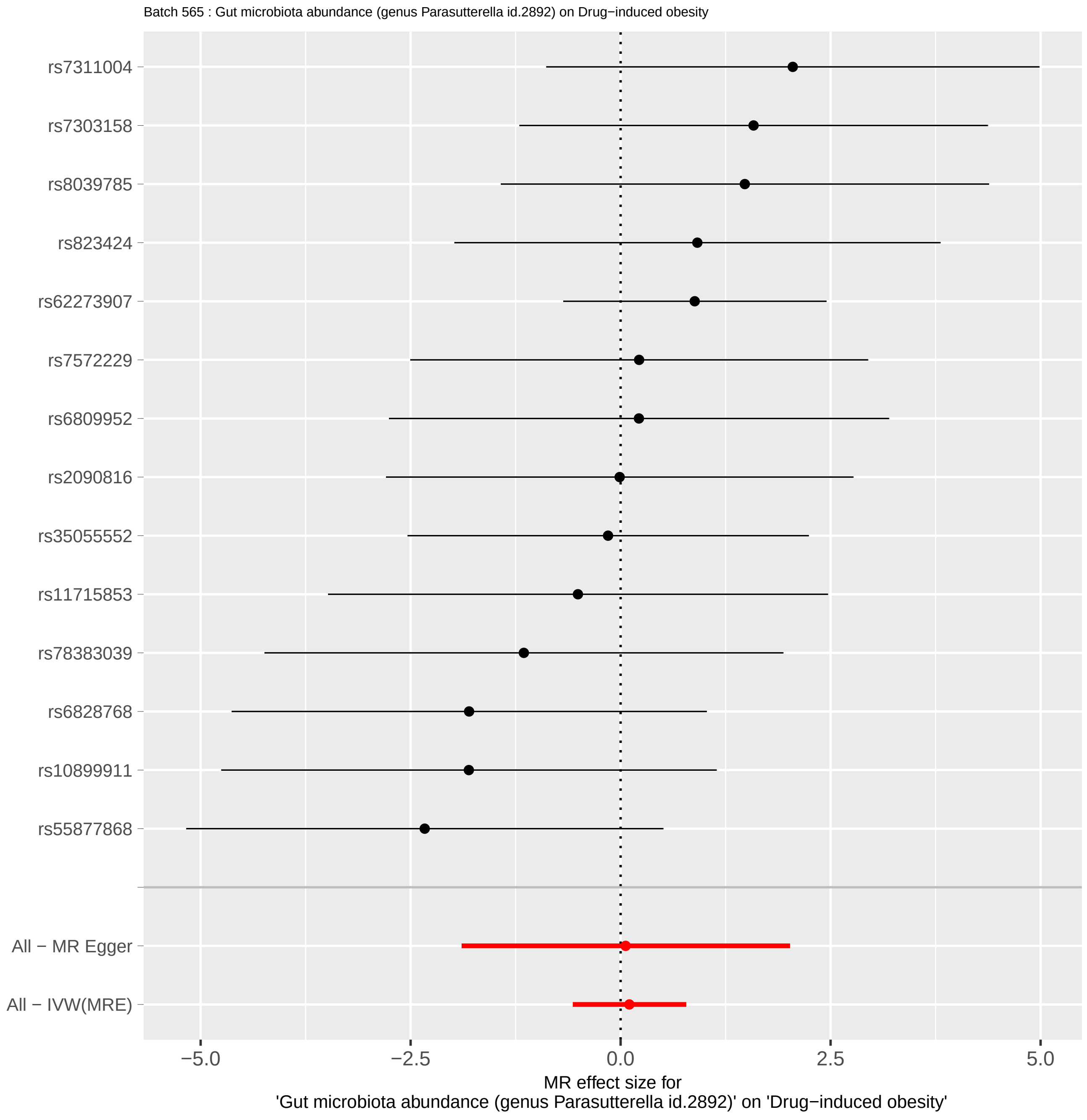

Batch 565 : Gut microbiota abundance (genus Parasutterella id.2892) on Drug−induced obesity
rs7311004
rs7303158
rs8039785
rs823424
rs62273907
rs7572229
rs6809952
rs2090816
rs35055552
rs11715853
rs78383039
rs6828768
rs10899911
rs55877868
All − MR Egger
All − IVW(MRE)
−5.0
−2.5
0.0
2.5
5.0
MR effect size for
'Gut microbiota abundance (genus Parasutterella id.2892)' on 'Drug−induced obesity'

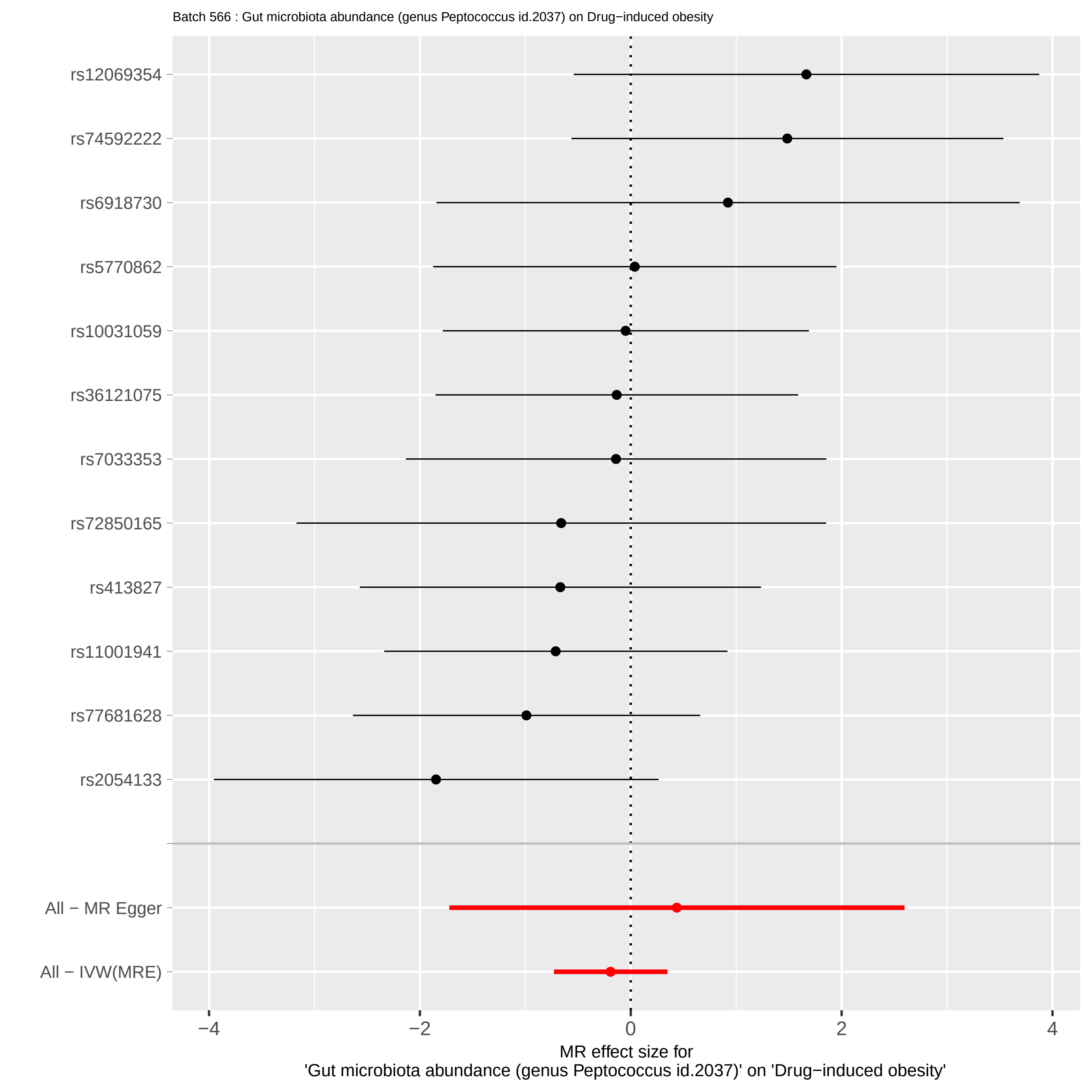

Batch 566 : Gut microbiota abundance (genus Peptococcus id.2037) on Drug−induced obesity
rs12069354
rs74592222
rs6918730
rs5770862
rs10031059
rs36121075
rs7033353
rs72850165
rs413827
rs11001941
rs77681628
rs2054133
All − MR Egger
All − IVW(MRE)
−4
−2
0
2
4
MR effect size for
'Gut microbiota abundance (genus Peptococcus id.2037)' on 'Drug−induced obesity'

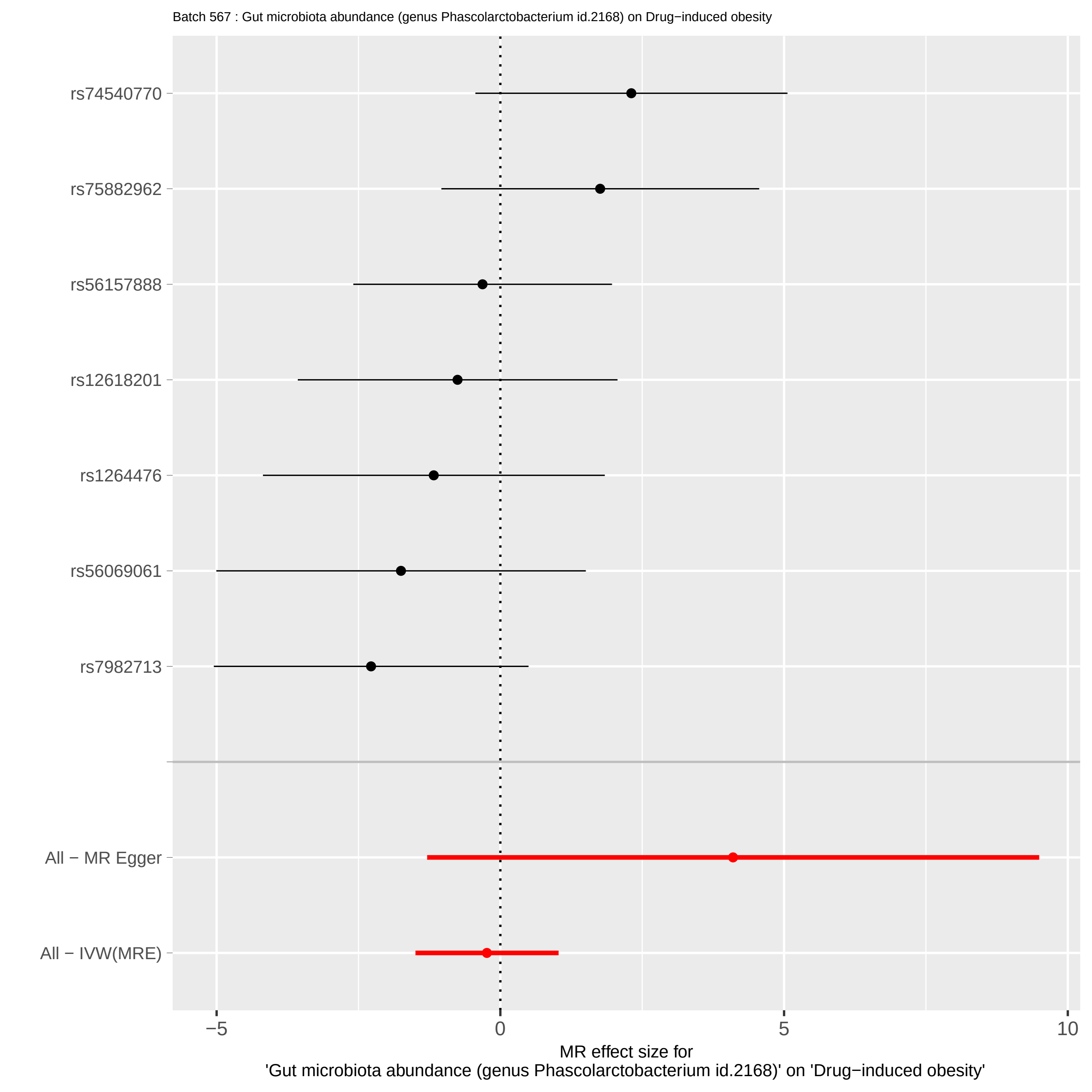

Batch 567 : Gut microbiota abundance (genus Phascolarctobacterium id.2168) on Drug−induced obesity
rs74540770
rs75882962
rs56157888
rs12618201
rs1264476
rs56069061
rs7982713
All − MR Egger
All − IVW(MRE)
−5
0
5
10
MR effect size for
'Gut microbiota abundance (genus Phascolarctobacterium id.2168)' on 'Drug−induced obesity'

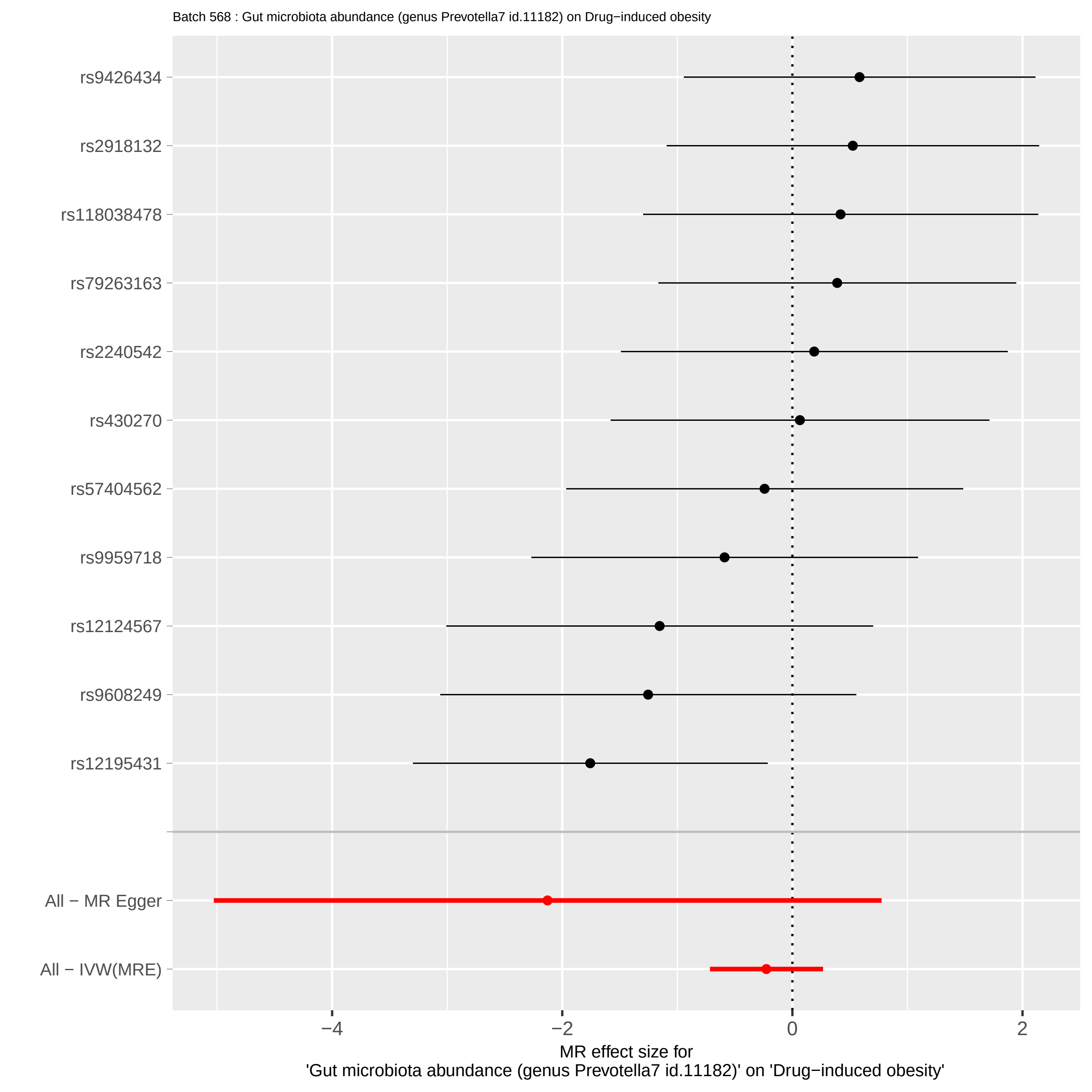

Batch 568 : Gut microbiota abundance (genus Prevotella7 id.11182) on Drug−induced obesity
rs9426434
rs2918132
rs118038478
rs79263163
rs2240542
rs430270
rs57404562
rs9959718
rs12124567
rs9608249
rs12195431
All − MR Egger
All − IVW(MRE)
−4
−2
0
2
MR effect size for
'Gut microbiota abundance (genus Prevotella7 id.11182)' on 'Drug−induced obesity'

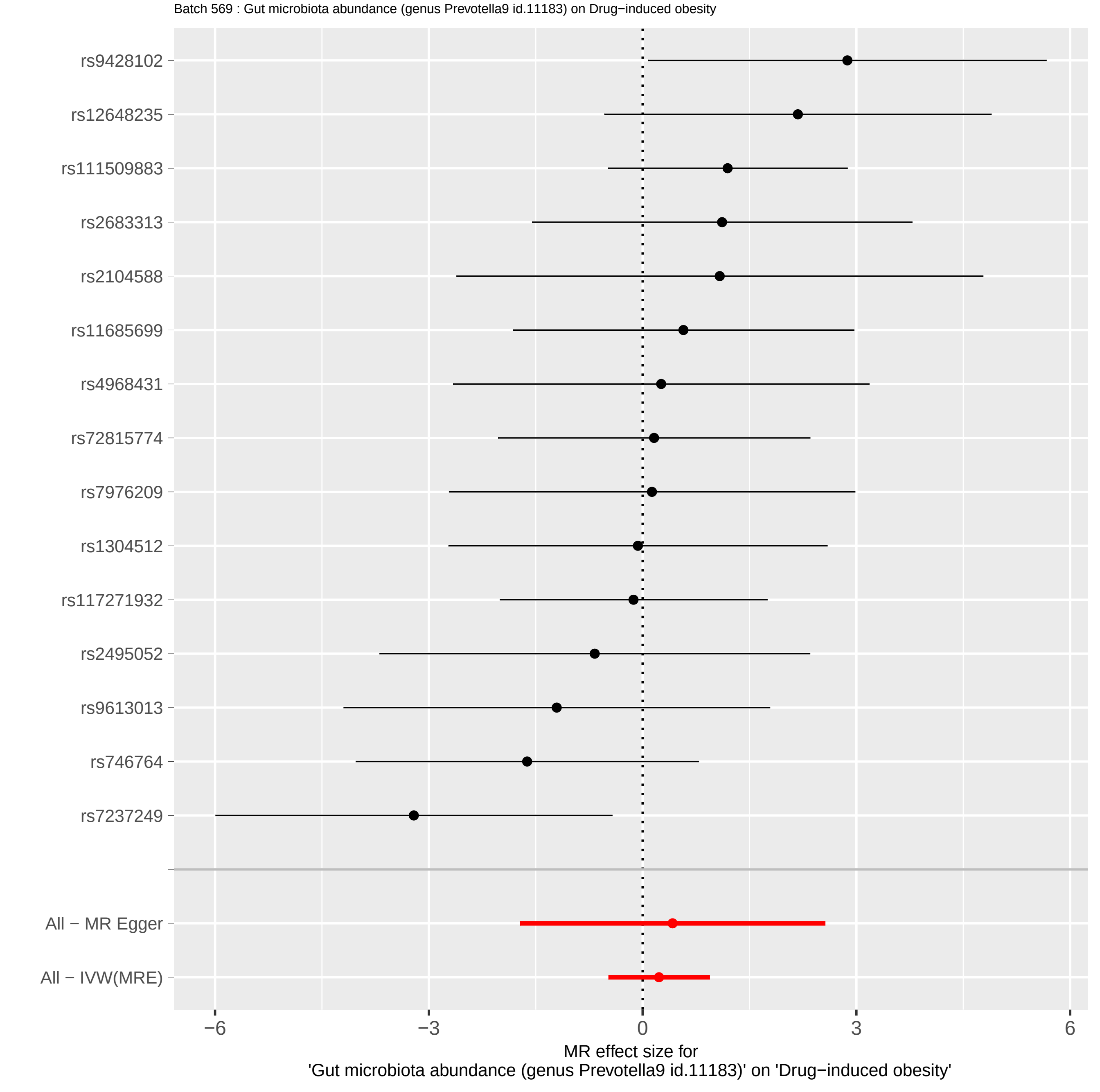

Batch 569 : Gut microbiota abundance (genus Prevotella9 id.11183) on Drug−induced obesity
rs9428102
rs12648235
rs111509883
rs2683313
rs2104588
rs11685699
rs4968431
rs72815774
rs7976209
rs1304512
rs117271932
rs2495052
rs9613013
rs746764
rs7237249
All − MR Egger
All − IVW(MRE)
−6
−3
0
3
6
MR effect size for
'Gut microbiota abundance (genus Prevotella9 id.11183)' on 'Drug−induced obesity'

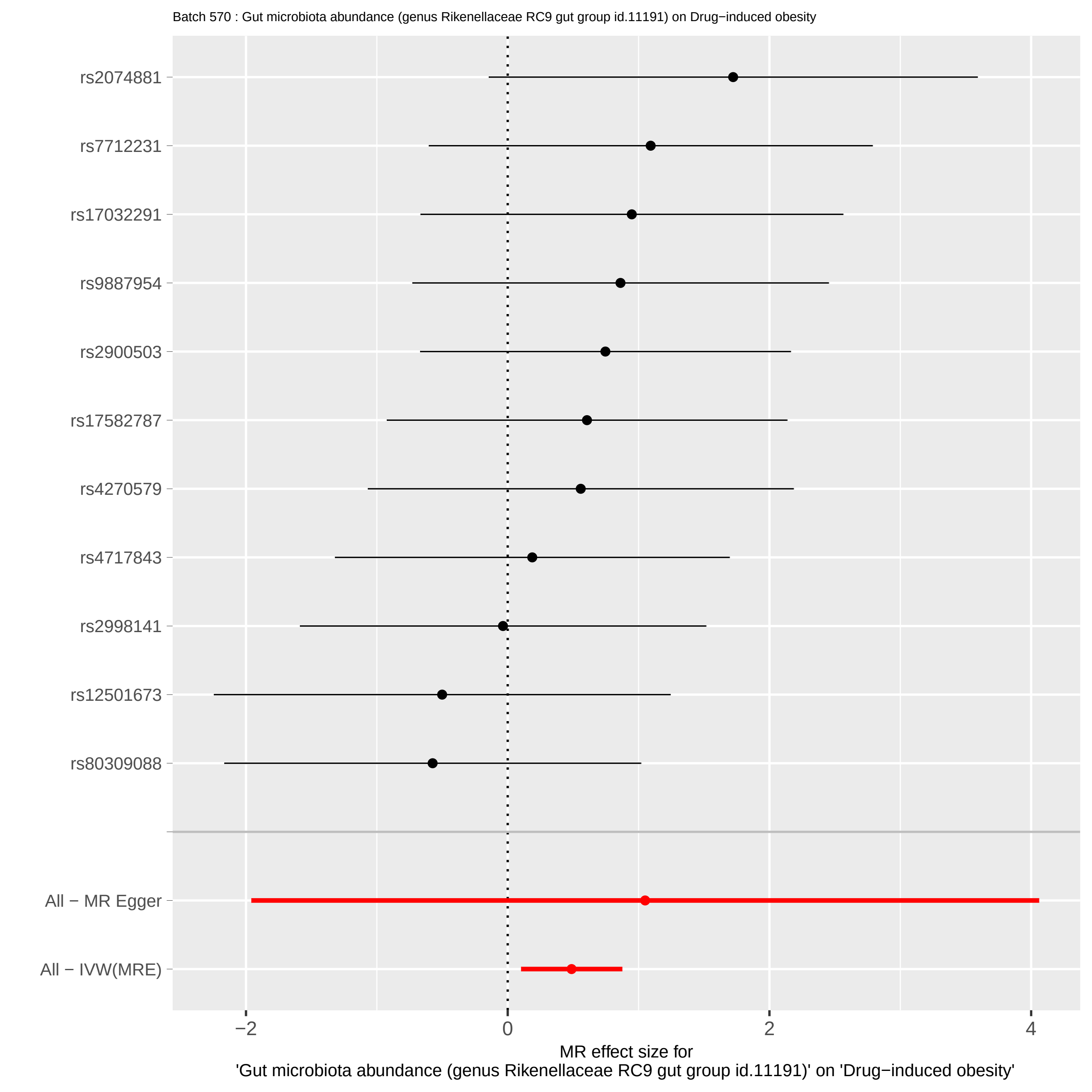

Batch 570 : Gut microbiota abundance (genus Rikenellaceae RC9 gut group id.11191) on Drug−induced obesity
rs2074881
rs7712231
rs17032291
rs9887954
rs2900503
rs17582787
rs4270579
rs4717843
rs2998141
rs12501673
rs80309088
All − MR Egger
All − IVW(MRE)
−2
0
2
4
MR effect size for
'Gut microbiota abundance (genus Rikenellaceae RC9 gut group id.11191)' on 'Drug−induced obesity'

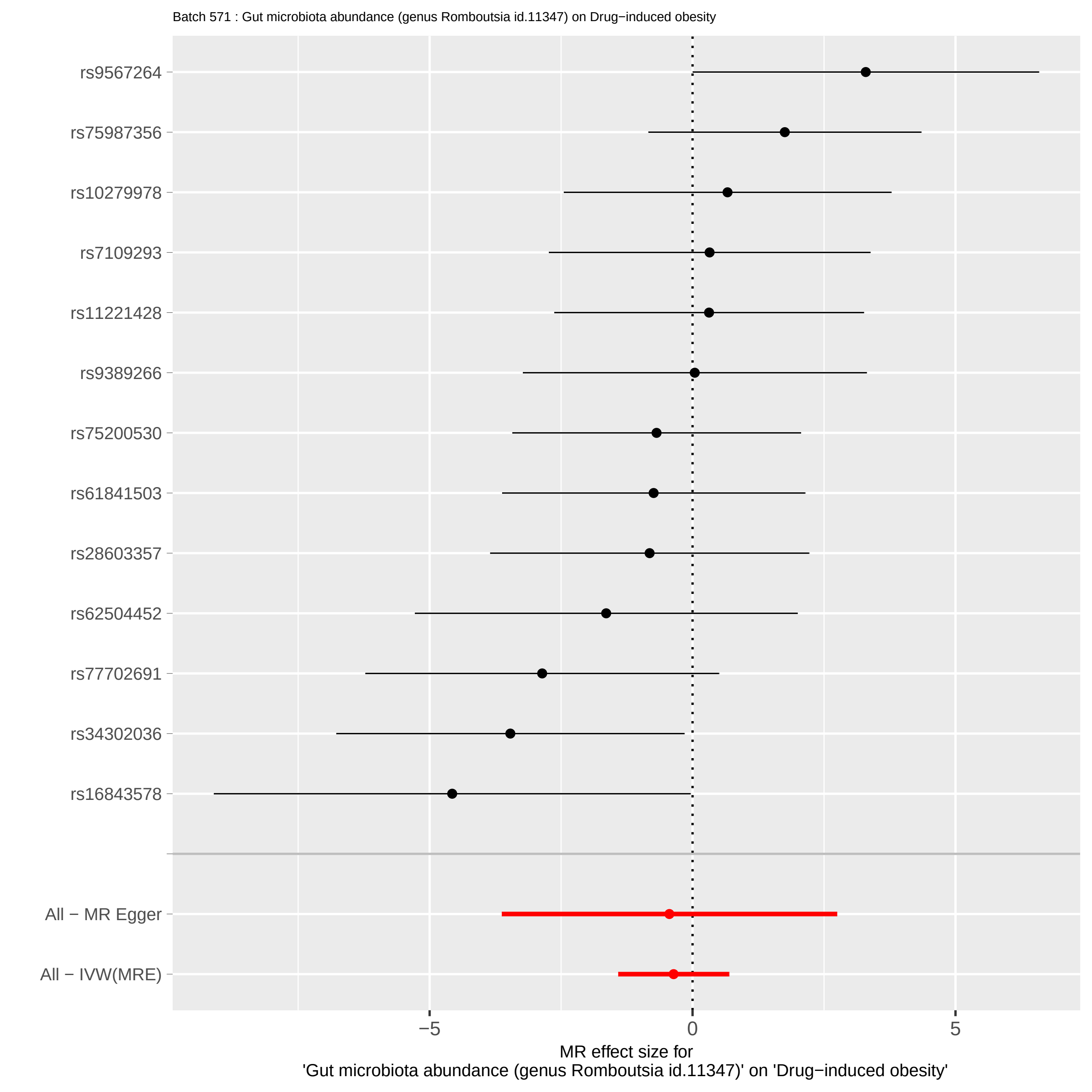

Batch 571 : Gut microbiota abundance (genus Romboutsia id.11347) on Drug−induced obesity
rs9567264
rs75987356
rs10279978
rs7109293
rs11221428
rs9389266
rs75200530
rs61841503
rs28603357
rs62504452
rs77702691
rs34302036
rs16843578
All − MR Egger
All − IVW(MRE)
−5
0
5
MR effect size for
'Gut microbiota abundance (genus Romboutsia id.11347)' on 'Drug−induced obesity'

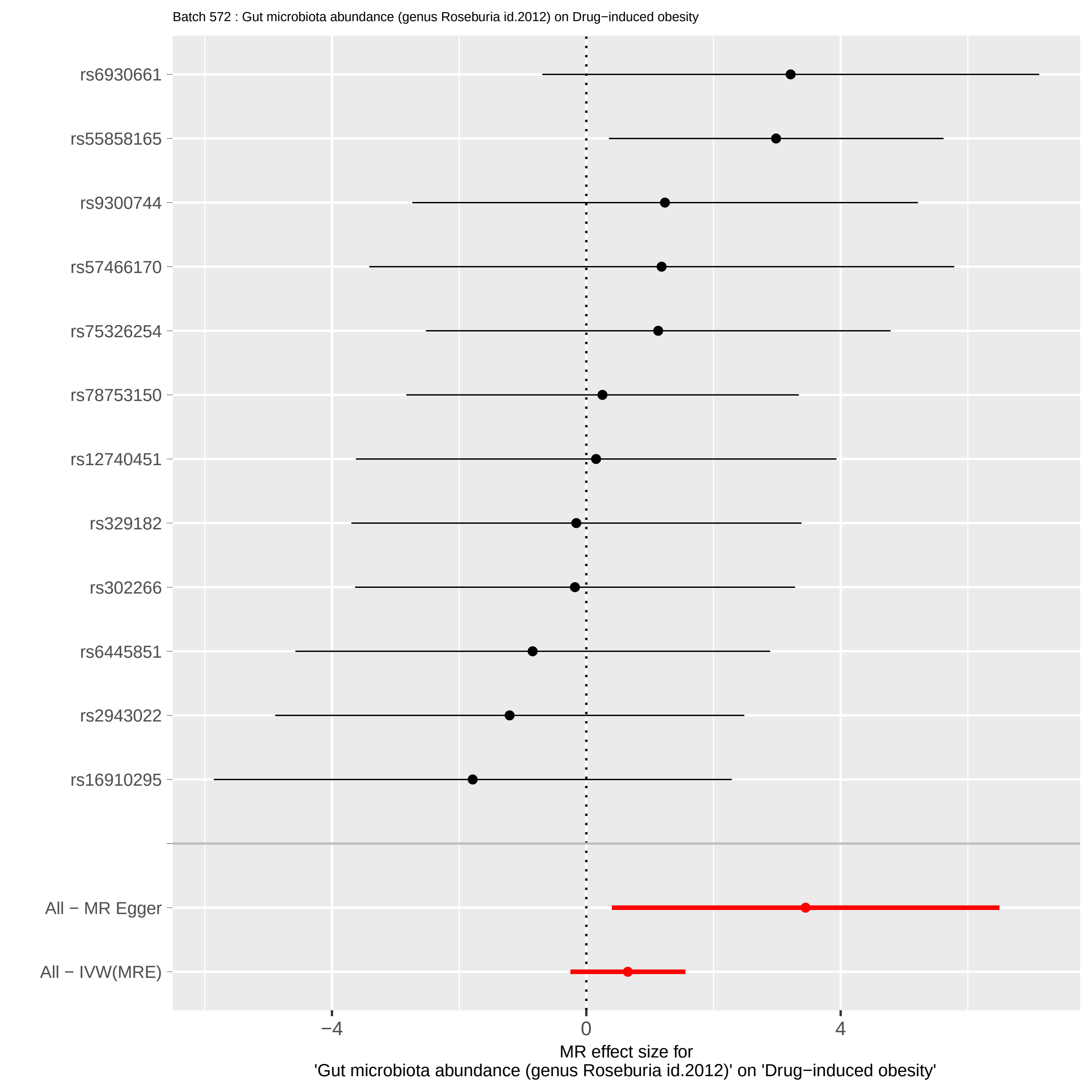

Batch 572 : Gut microbiota abundance (genus Roseburia id.2012) on Drug−induced obesity
rs6930661
rs55858165
rs9300744
rs57466170
rs75326254
rs78753150
rs12740451
rs329182
rs302266
rs6445851
rs2943022
rs16910295
All − MR Egger
All − IVW(MRE)
−4
0
4
MR effect size for
'Gut microbiota abundance (genus Roseburia id.2012)' on 'Drug−induced obesity'

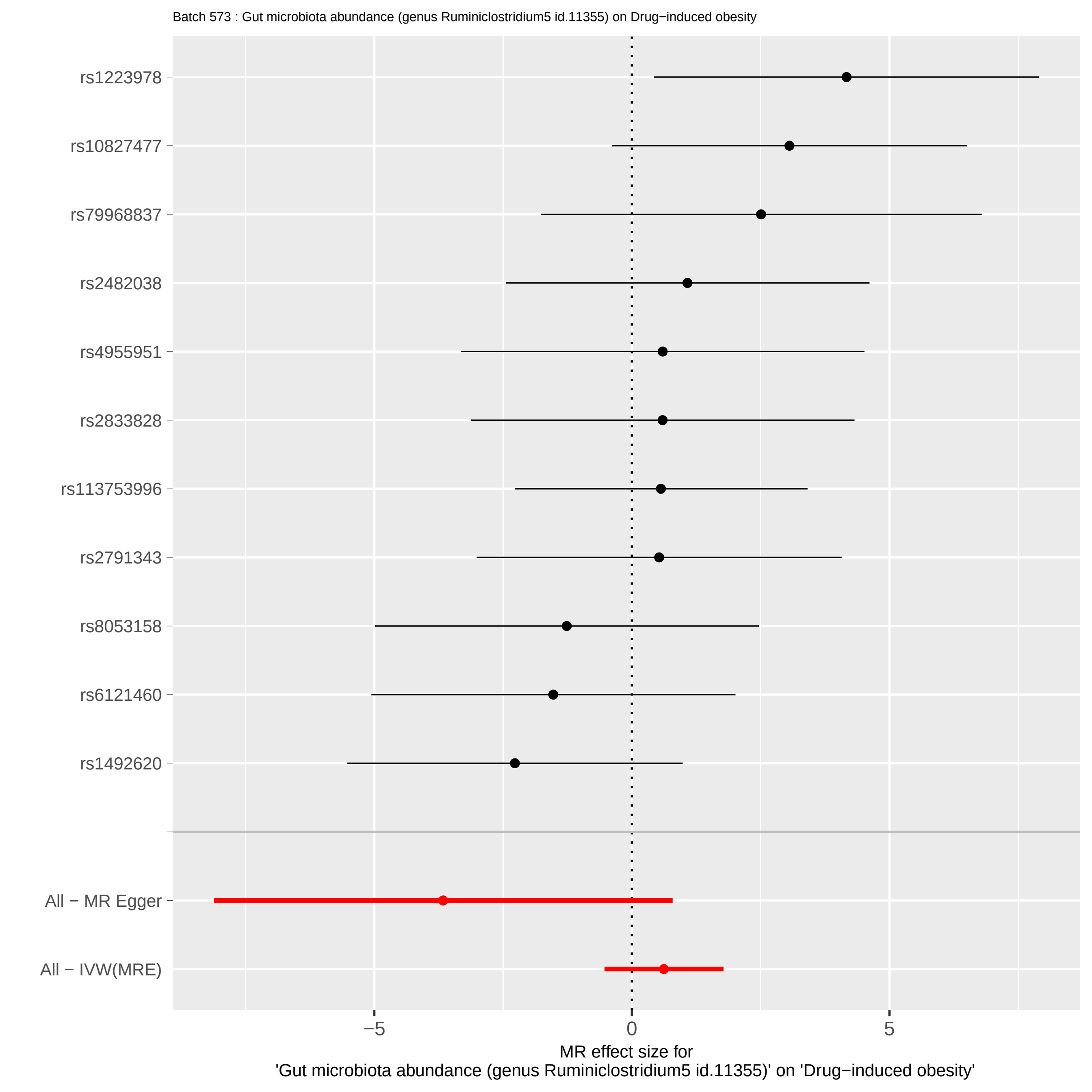
Batch 573 : Gut microbiota abundance (genus Ruminiclostridium5 id.11355) on Drug−induced obesity
rs1223978
rs10827477
rs79968837
rs2482038
rs4955951
rs2833828
rs113753996
rs2791343
rs8053158
rs6121460
rs1492620
All − MR Egger
All − IVW(MRE)
−5
0
5
MR effect size for
'Gut microbiota abundance (genus Ruminiclostridium5 id.11355)' on 'Drug−induced obesity'

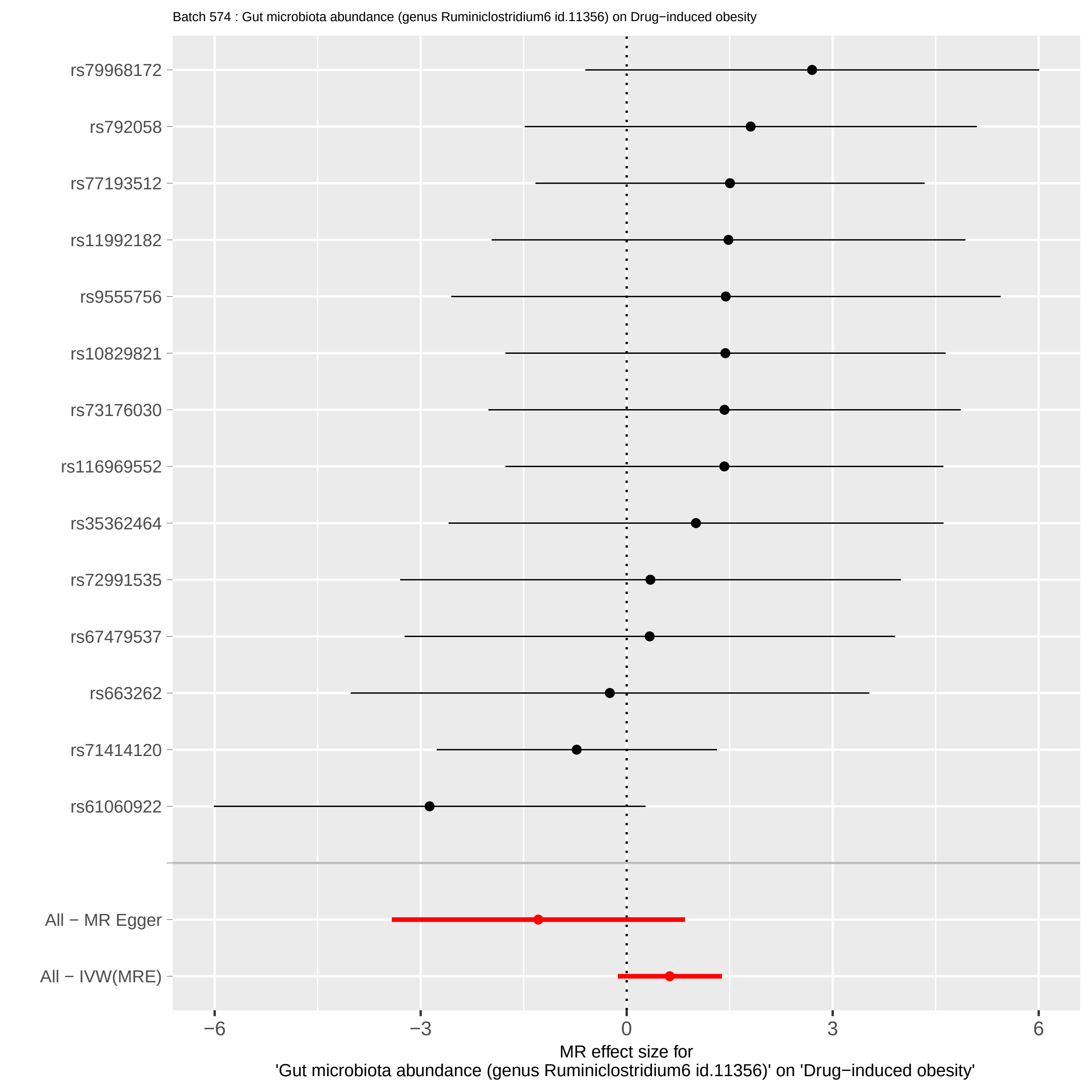

Batch 574 : Gut microbiota abundance (genus Ruminiclostridium6 id.11356) on Drug−induced obesity
rs79968172
rs792058
rs77193512
rs11992182
rs9555756
rs10829821
rs73176030
rs116969552
rs35362464
rs72991535
rs67479537
rs663262
rs71414120
rs61060922
All − MR Egger
All − IVW(MRE)
−6
−3
0
3
6
MR effect size for
'Gut microbiota abundance (genus Ruminiclostridium6 id.11356)' on 'Drug−induced obesity'

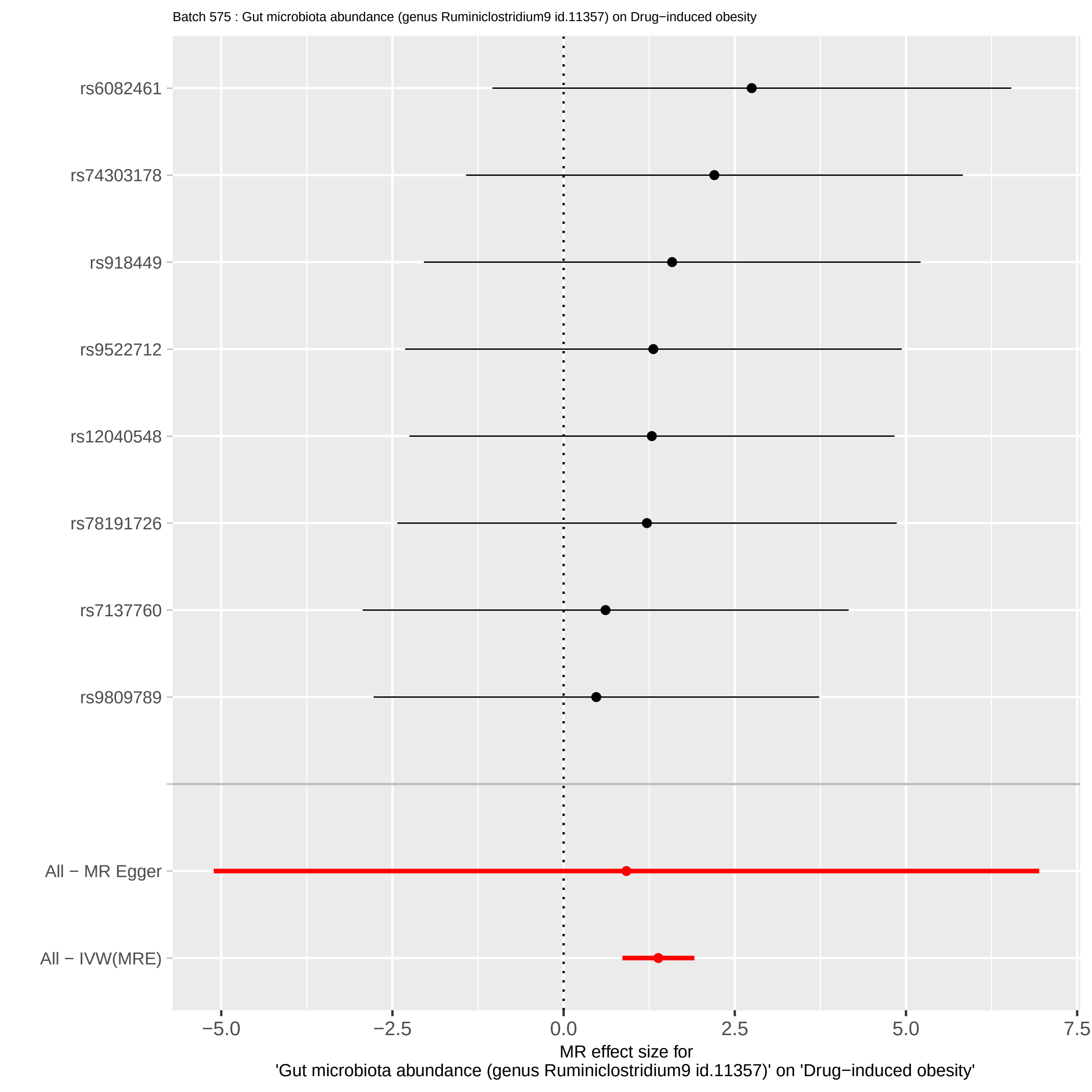

Batch 575 : Gut microbiota abundance (genus Ruminiclostridium9 id.11357) on Drug−induced obesity
rs6082461
rs74303178
rs918449
rs9522712
rs12040548
rs78191726
rs7137760
rs9809789
All − MR Egger
All − IVW(MRE)
−5.0
−2.5
0.0
2.5
5.0
7.5
MR effect size for
'Gut microbiota abundance (genus Ruminiclostridium9 id.11357)' on 'Drug−induced obesity'

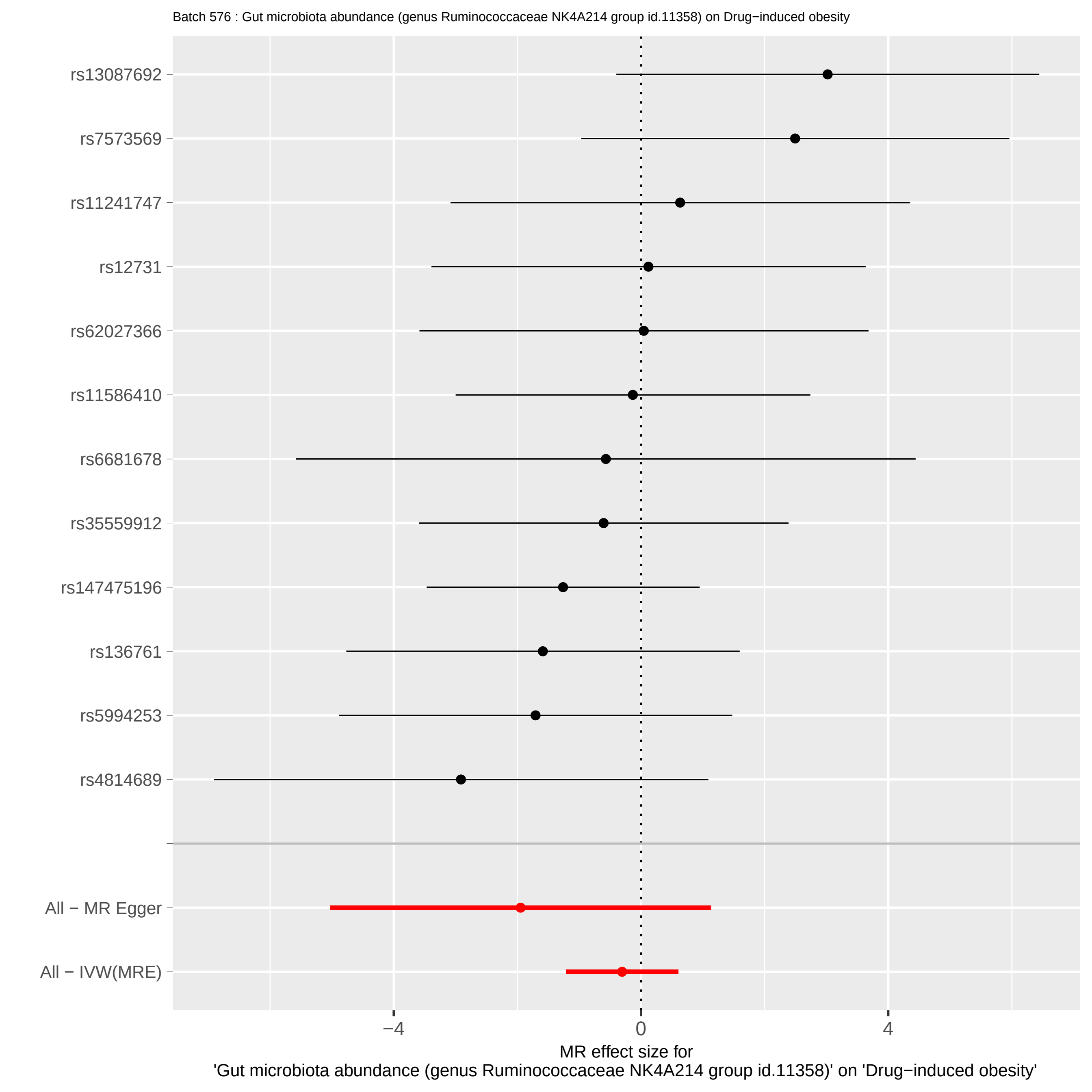
Batch 576 : Gut microbiota abundance (genus Ruminococcaceae NK4A214 group id.11358) on Drug−induced obesity
rs13087692
rs7573569
rs11241747
rs12731
rs62027366
rs11586410
rs6681678
rs35559912
rs147475196
rs136761
rs5994253
rs4814689
All − MR Egger
All − IVW(MRE)
−4
0
4
MR effect size for
'Gut microbiota abundance (genus Ruminococcaceae NK4A214 group id.11358)' on 'Drug−induced obesity'

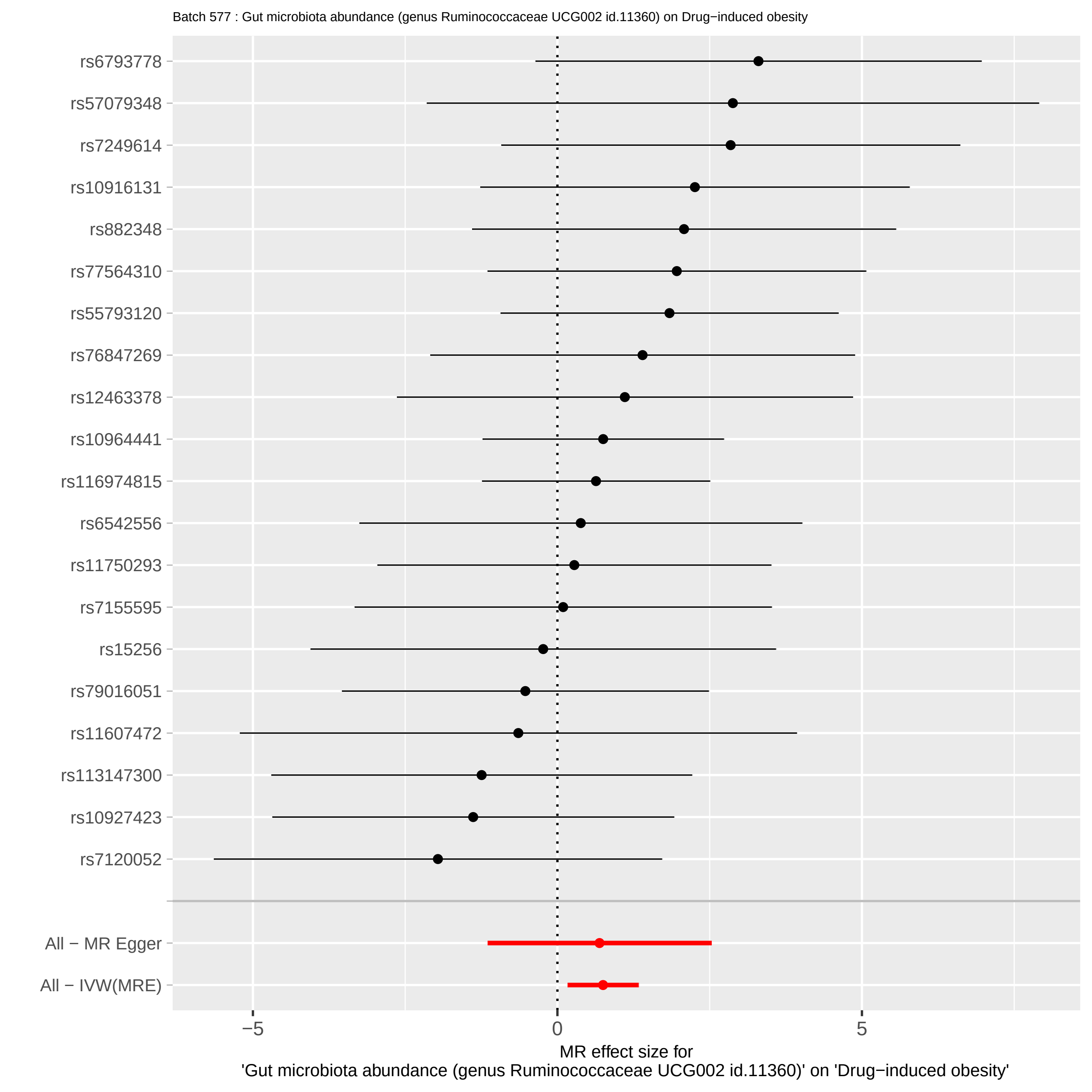

Batch 577 : Gut microbiota abundance (genus Ruminococcaceae UCG002 id.11360) on Drug−induced obesity
rs6793778
rs57079348
rs7249614
rs10916131
rs882348
rs77564310
rs55793120
rs76847269
rs12463378
rs10964441
rs116974815
rs6542556
rs11750293
rs7155595
rs15256
rs79016051
rs11607472
rs113147300
rs10927423
rs7120052
All − MR Egger
All − IVW(MRE)
−5
0
5
MR effect size for
'Gut microbiota abundance (genus Ruminococcaceae UCG002 id.11360)' on 'Drug−induced obesity'

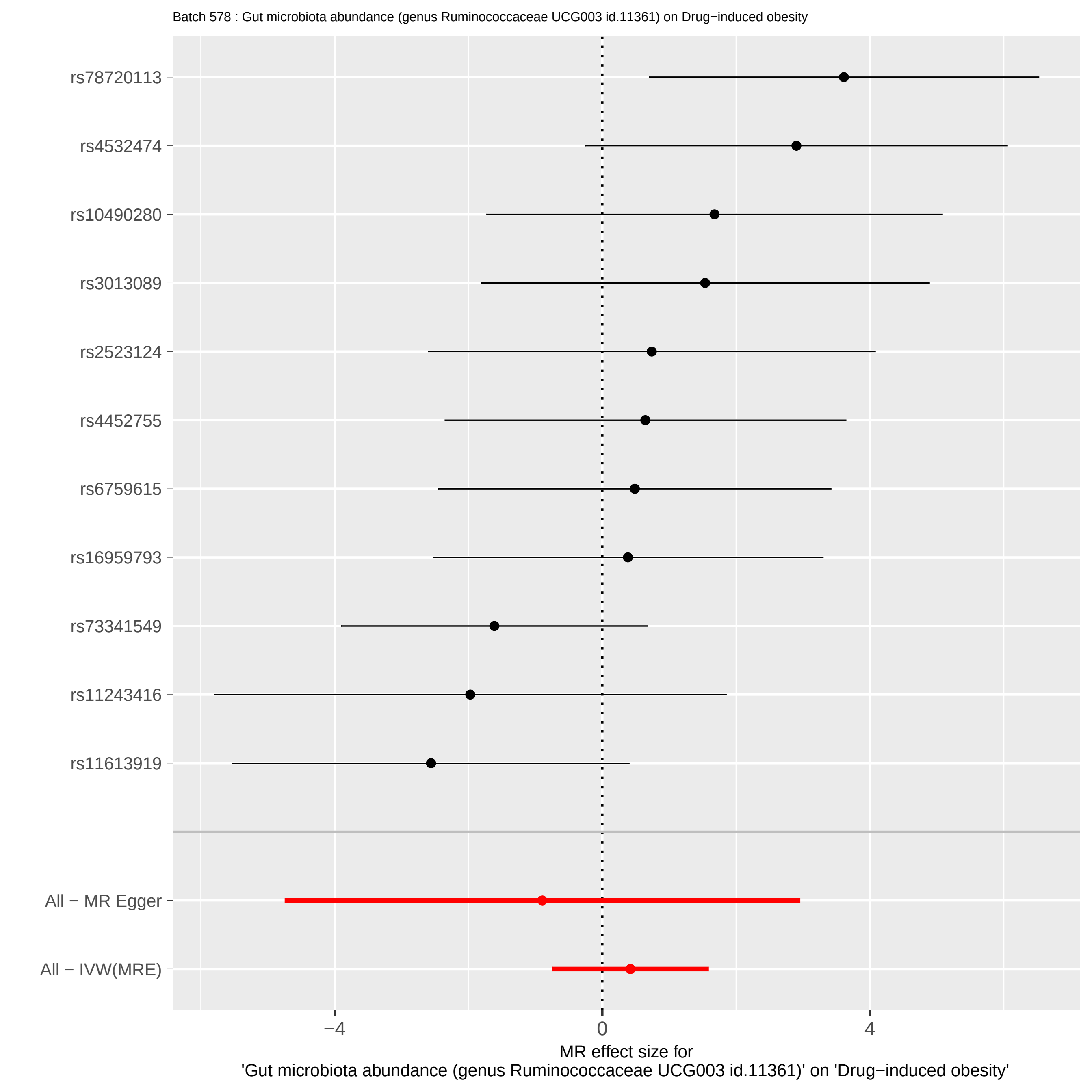

Batch 578 : Gut microbiota abundance (genus Ruminococcaceae UCG003 id.11361) on Drug−induced obesity
rs78720113
rs4532474
rs10490280
rs3013089
rs2523124
rs4452755
rs6759615
rs16959793
rs73341549
rs11243416
rs11613919
All − MR Egger
All − IVW(MRE)
−4
0
4
MR effect size for
'Gut microbiota abundance (genus Ruminococcaceae UCG003 id.11361)' on 'Drug−induced obesity'

Batch 579 : Gut microbiota abundance (genus Ruminococcaceae UCG004 id.11362) on Drug−induced obesity

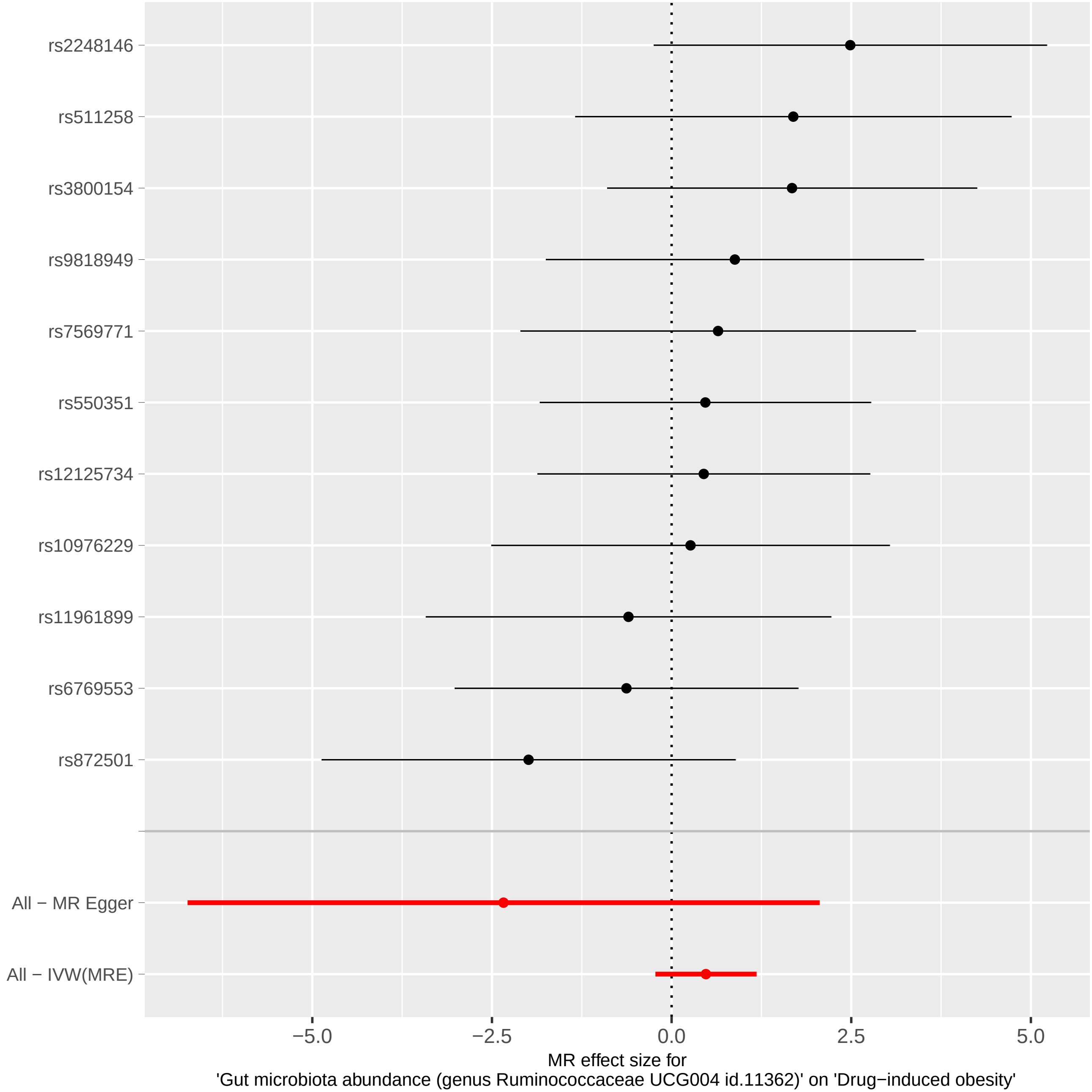

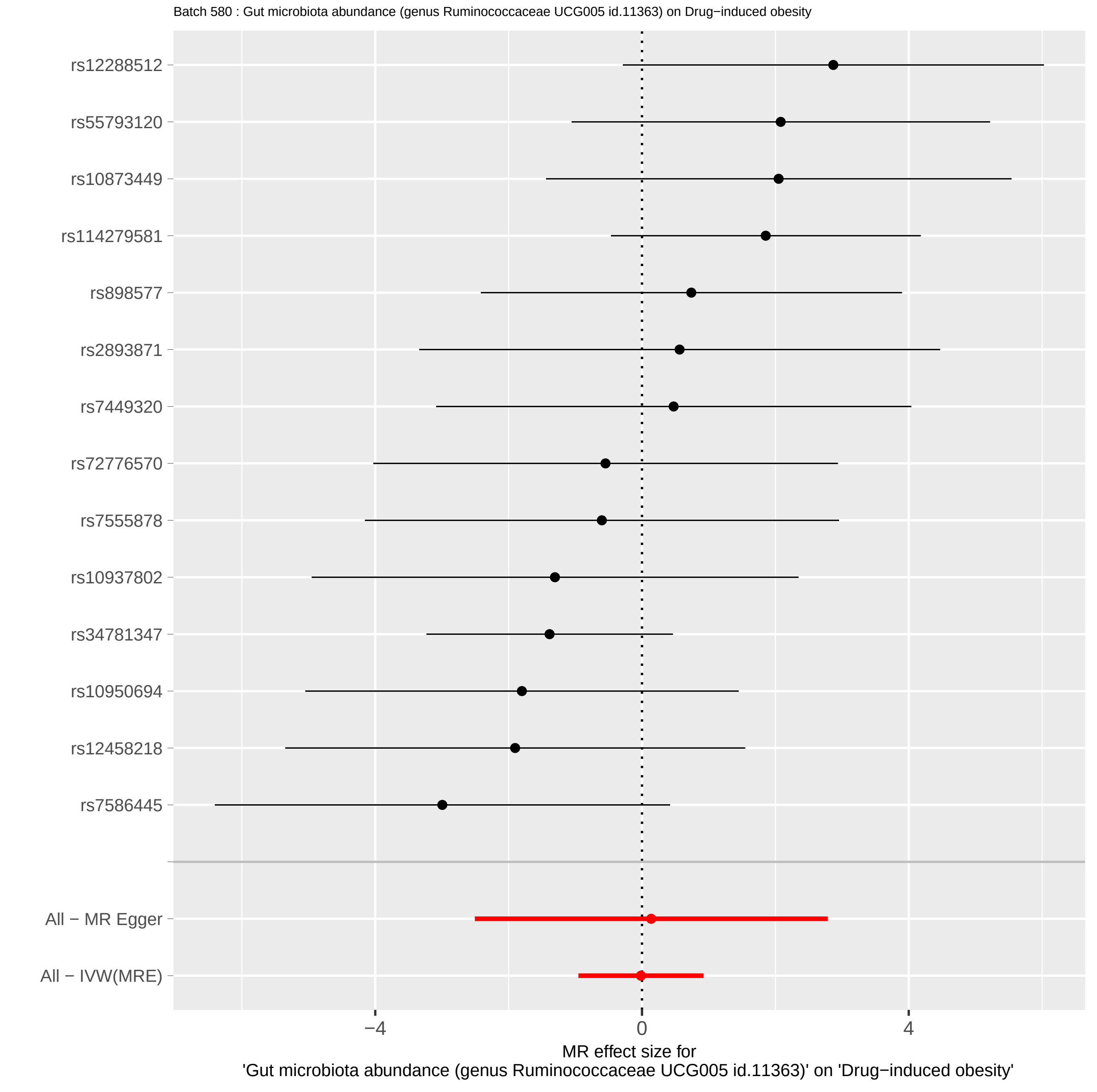
Batch 580 : Gut microbiota abundance (genus Ruminococcaceae UCG005 id.11363) on Drug−induced obesity
rs12288512
rs55793120
rs10873449
rs114279581
rs898577
rs2893871
rs7449320
rs72776570
rs7555878
rs10937802
rs34781347
rs10950694
rs12458218
rs7586445
All − MR Egger
All − IVW(MRE)
−4
0
4
MR effect size for
'Gut microbiota abundance (genus Ruminococcaceae UCG005 id.11363)' on 'Drug−induced obesity'

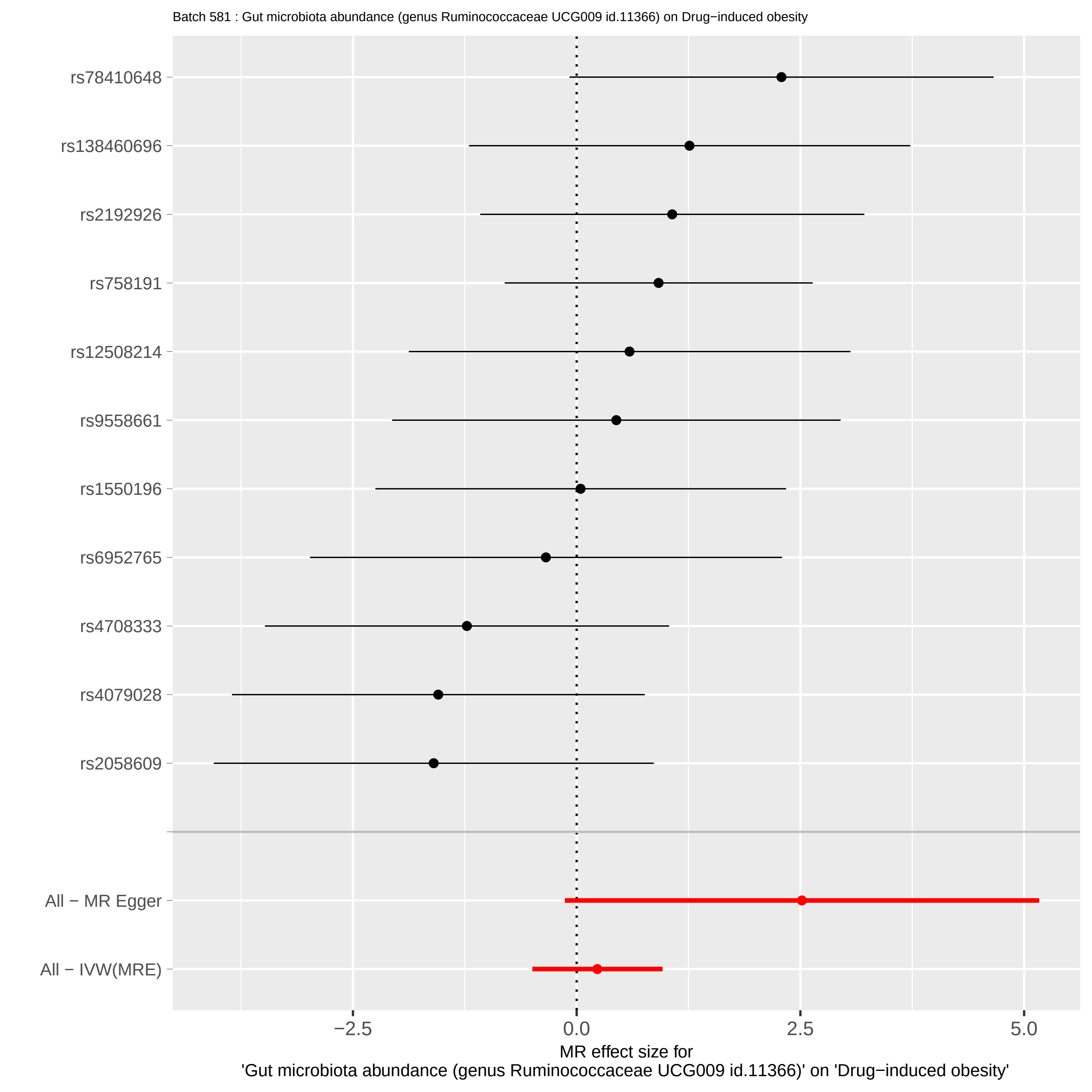

Batch 581 : Gut microbiota abundance (genus Ruminococcaceae UCG009 id.11366) on Drug−induced obesity
rs78410648
rs138460696
rs2192926
rs758191
rs12508214
rs9558661
rs1550196
rs6952765
rs4708333
rs4079028
rs2058609
All − MR Egger
All − IVW(MRE)
−2.5
0.0
2.5
5.0
MR effect size for
'Gut microbiota abundance (genus Ruminococcaceae UCG009 id.11366)' on 'Drug−induced obesity'

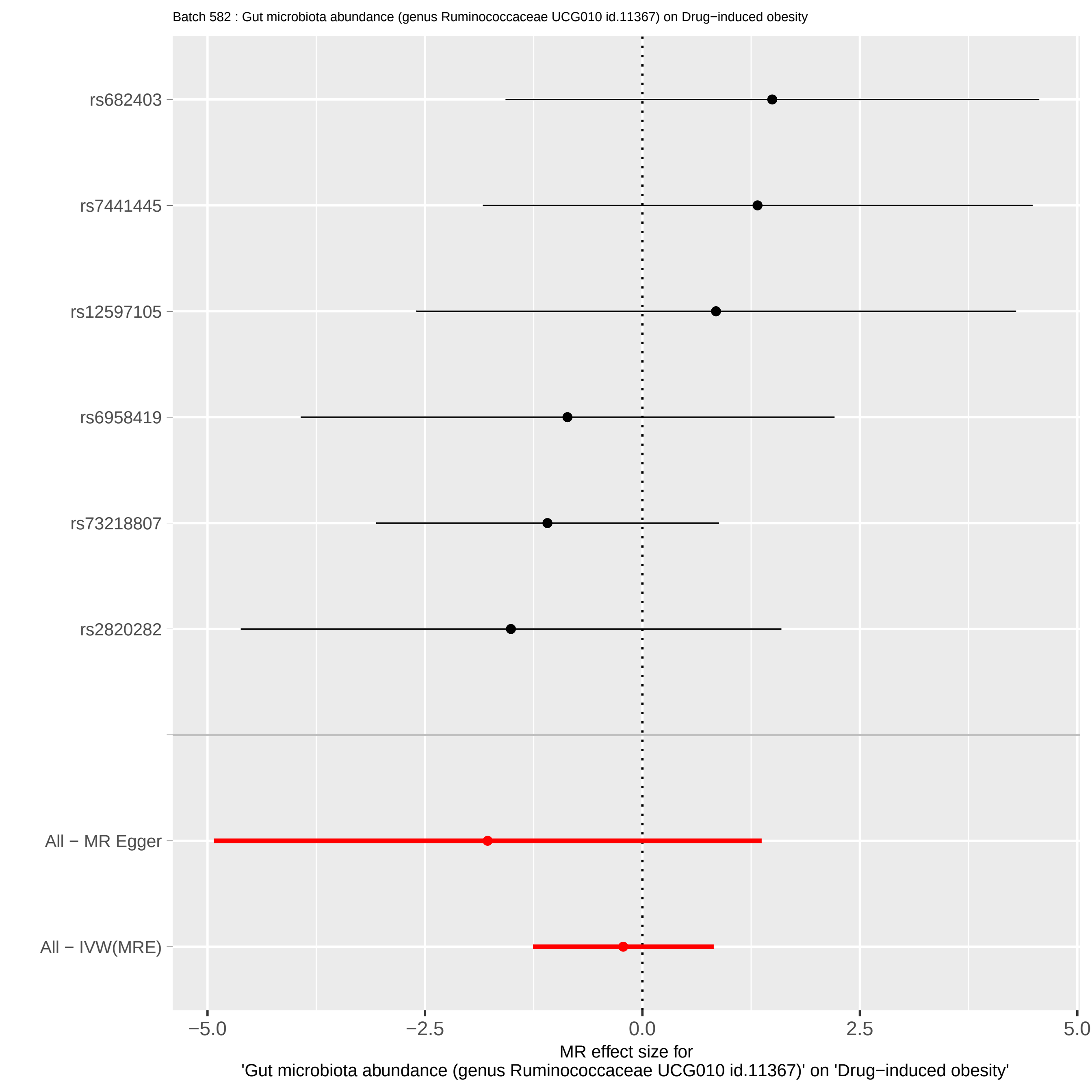

Batch 582 : Gut microbiota abundance (genus Ruminococcaceae UCG010 id.11367) on Drug−induced obesity
rs682403
rs7441445
rs12597105
rs6958419
rs73218807
rs2820282
All − MR Egger
All − IVW(MRE)
−5.0
−2.5
0.0
2.5
5.0
MR effect size for
'Gut microbiota abundance (genus Ruminococcaceae UCG010 id.11367)' on 'Drug−induced obesity'

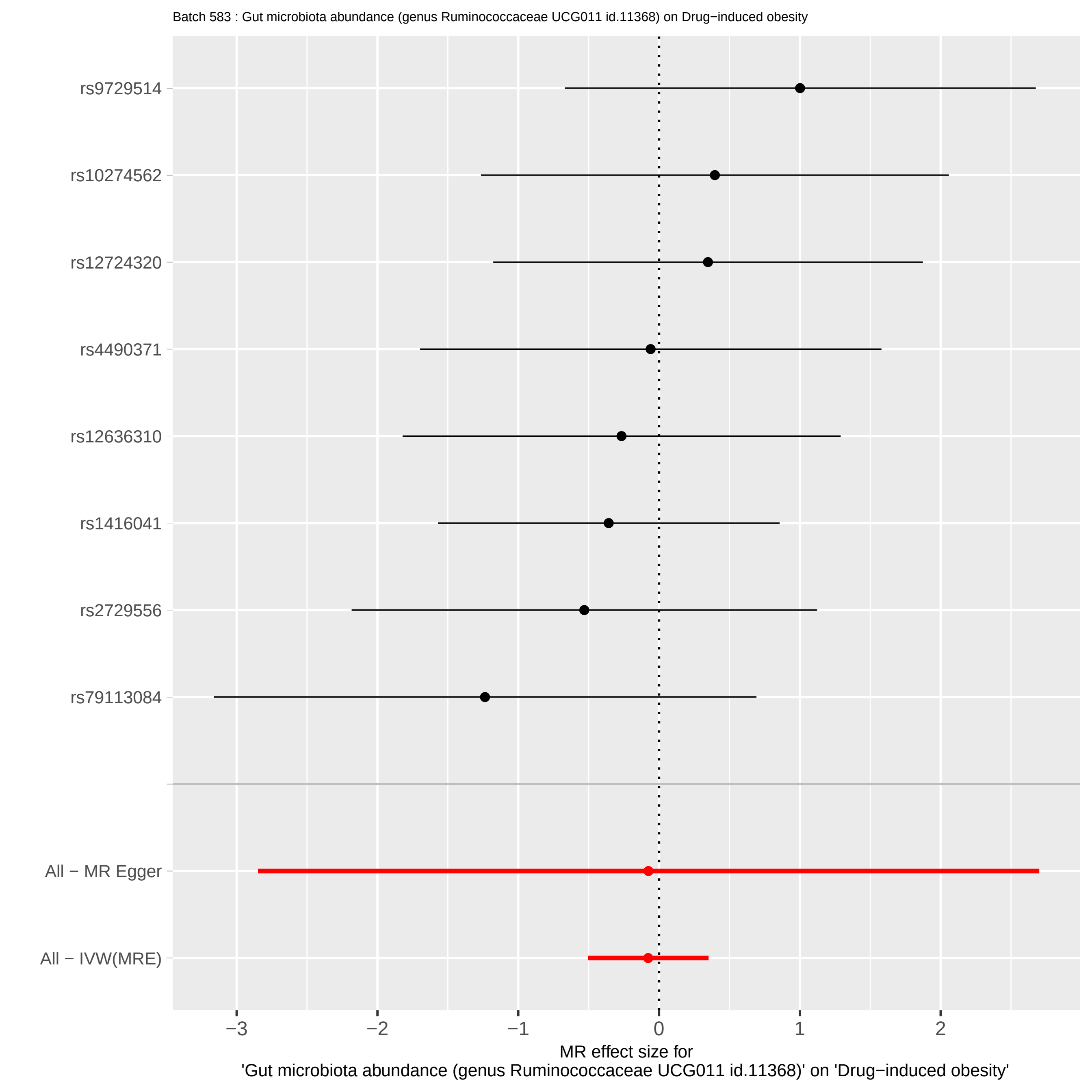

Batch 583 : Gut microbiota abundance (genus Ruminococcaceae UCG011 id.11368) on Drug−induced obesity
rs9729514
rs10274562
rs12724320
rs4490371
rs12636310
rs1416041
rs2729556
rs79113084
All − MR Egger
All − IVW(MRE)
−3
−2
−1
0
1
2
MR effect size for
'Gut microbiota abundance (genus Ruminococcaceae UCG011 id.11368)' on 'Drug−induced obesity'

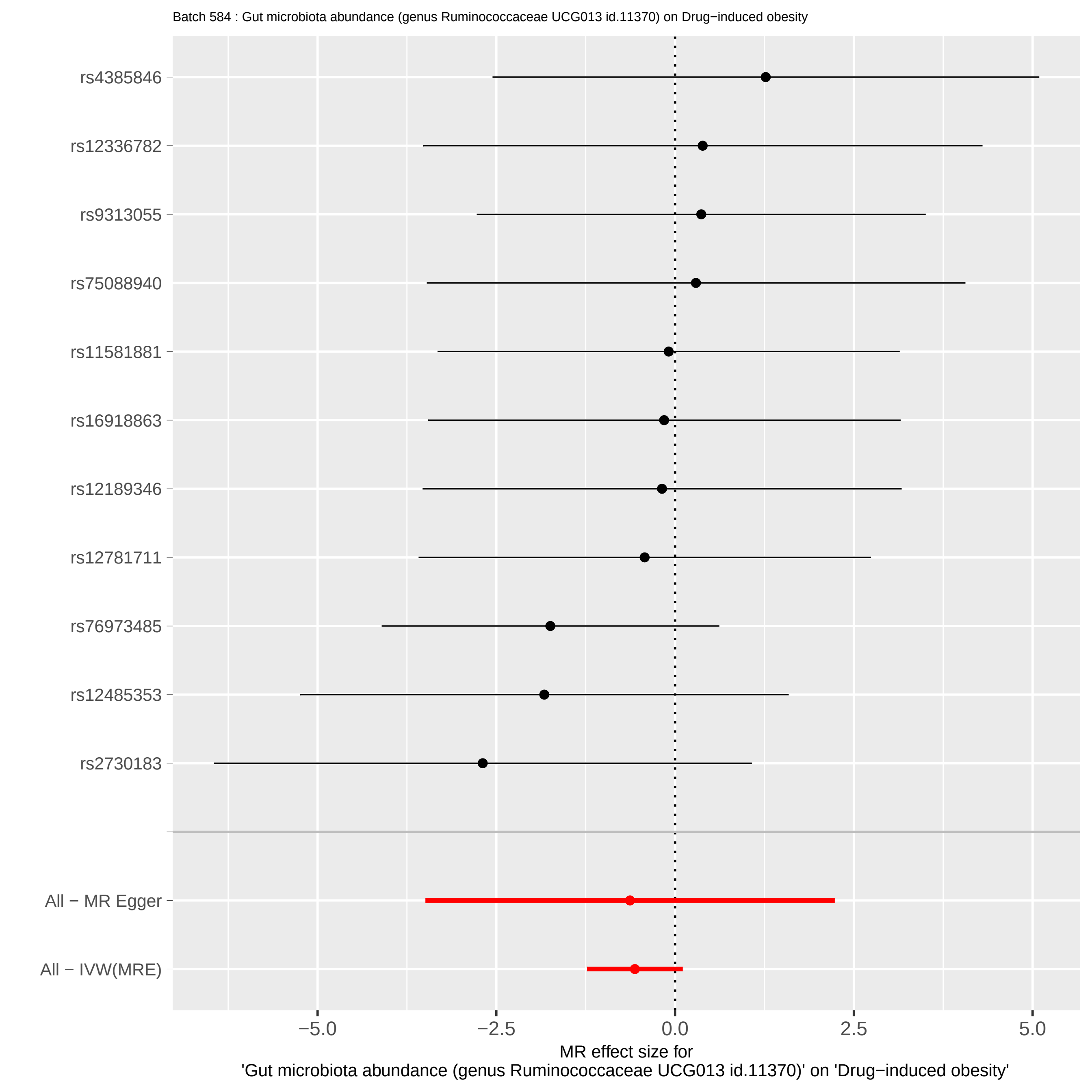
Batch 584 : Gut microbiota abundance (genus Ruminococcaceae UCG013 id.11370) on Drug−induced obesity
rs4385846
rs12336782
rs9313055
rs75088940
rs11581881
rs16918863
rs12189346
rs12781711
rs76973485
rs12485353
rs2730183
All − MR Egger
All − IVW(MRE)
−5.0
−2.5
0.0
2.5
5.0
MR effect size for
'Gut microbiota abundance (genus Ruminococcaceae UCG013 id.11370)' on 'Drug−induced obesity'

Batch 585 : Gut microbiota abundance (genus Ruminococcaceae UCG014 id.11371) on Drug−induced obesity

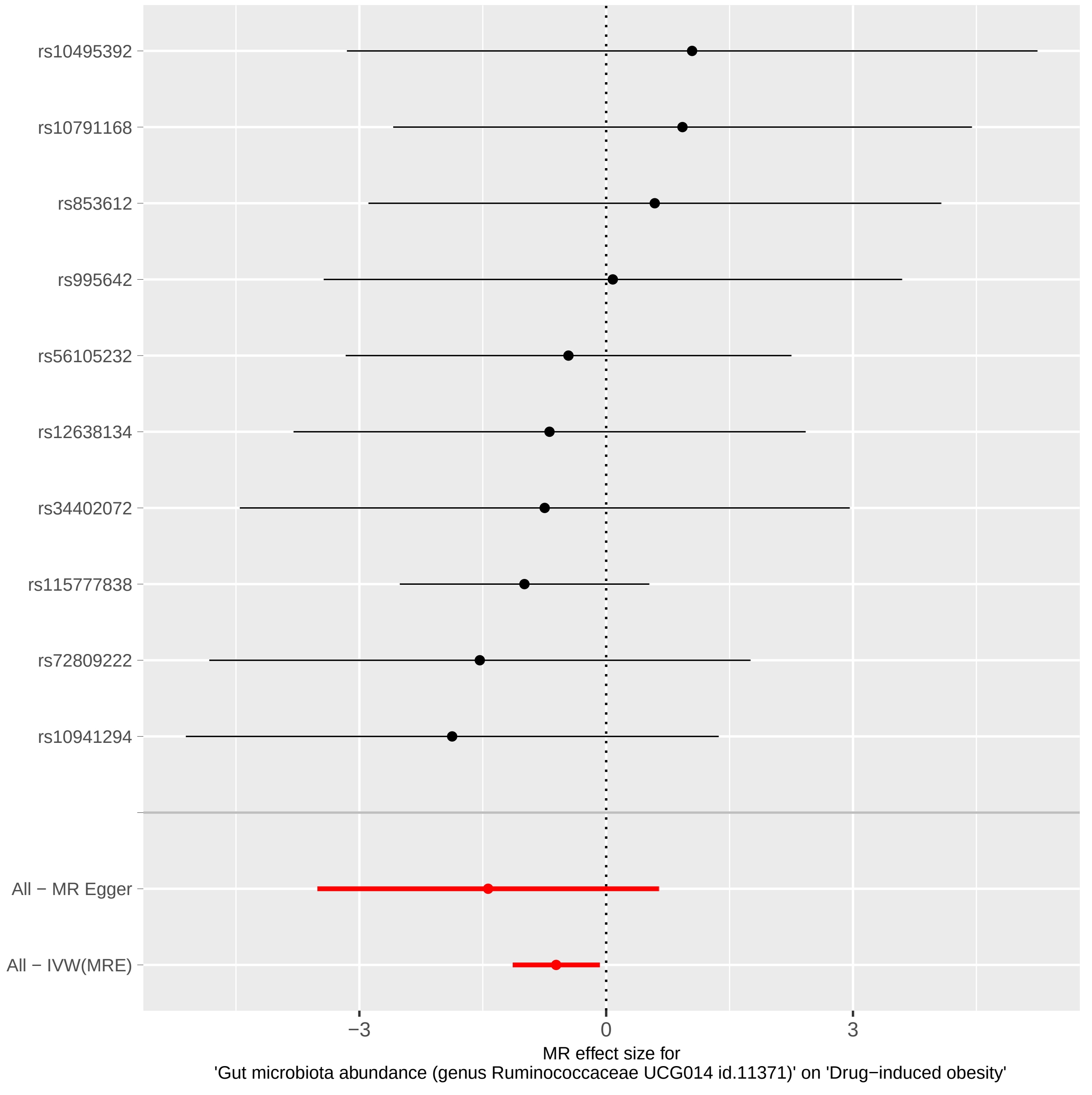

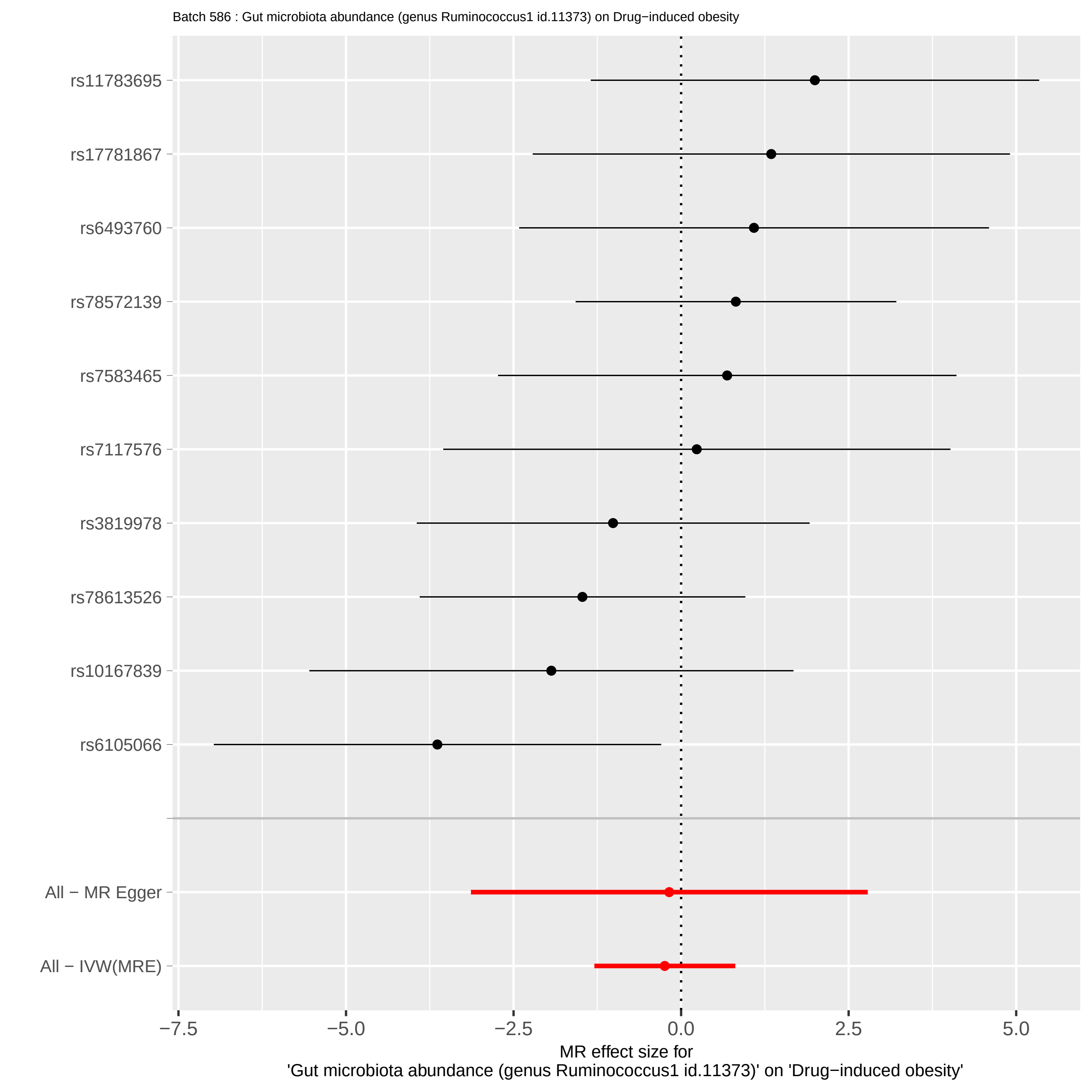
Batch 586 : Gut microbiota abundance (genus Ruminococcus1 id.11373) on Drug−induced obesity
rs11783695
rs17781867
rs6493760
rs78572139
rs7583465
rs7117576
rs3819978
rs78613526
rs10167839
rs6105066
All − MR Egger
All − IVW(MRE)
−7.5
−5.0
−2.5
0.0
2.5
5.0
MR effect size for
'Gut microbiota abundance (genus Ruminococcus1 id.11373)' on 'Drug−induced obesity'

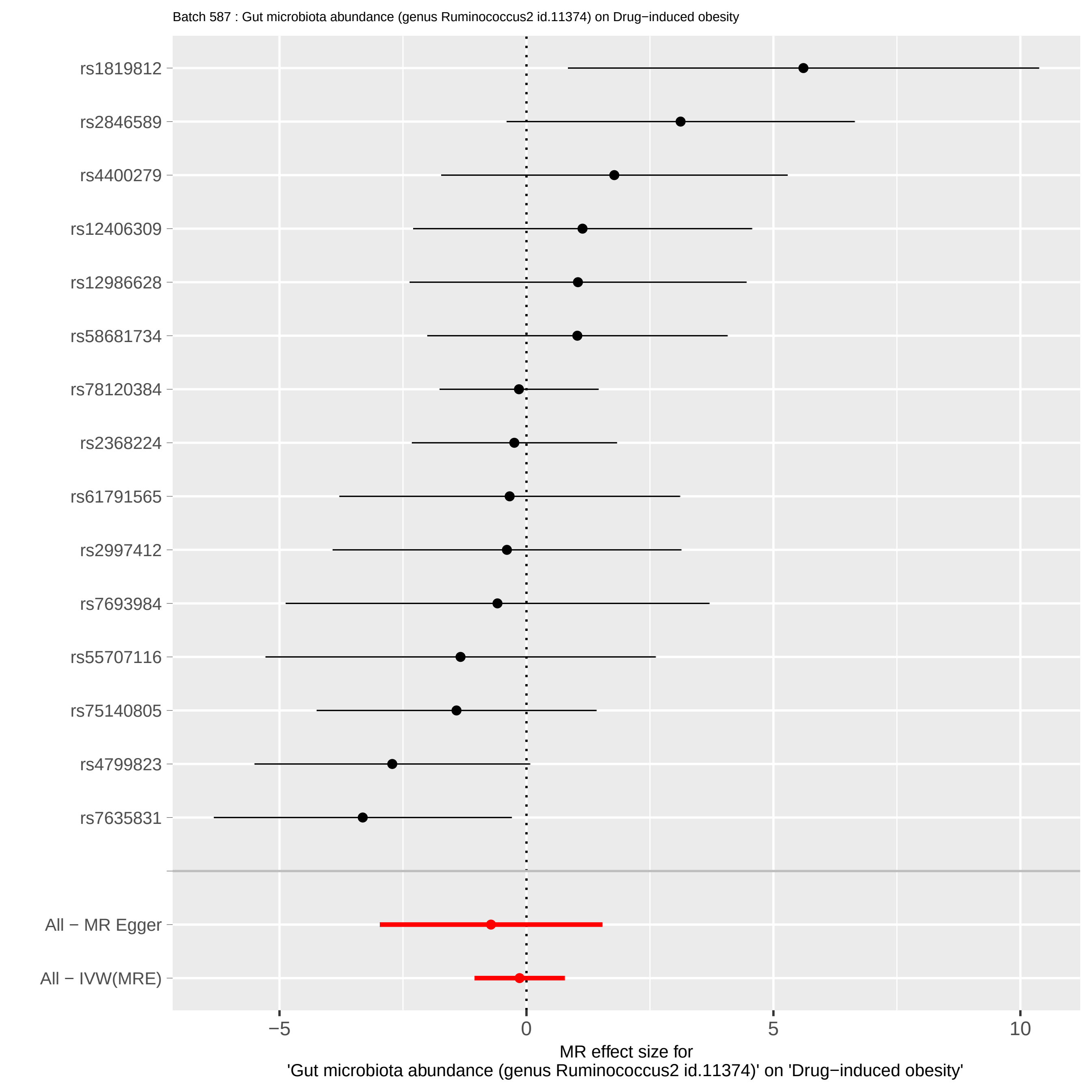

Batch 587 : Gut microbiota abundance (genus Ruminococcus2 id.11374) on Drug−induced obesity
rs1819812
rs2846589
rs4400279
rs12406309
rs12986628
rs58681734
rs78120384
rs2368224
rs61791565
rs2997412
rs7693984
rs55707116
rs75140805
rs4799823
rs7635831
All − MR Egger
All − IVW(MRE)
−5
0
5
10
MR effect size for
'Gut microbiota abundance (genus Ruminococcus2 id.11374)' on 'Drug−induced obesity'

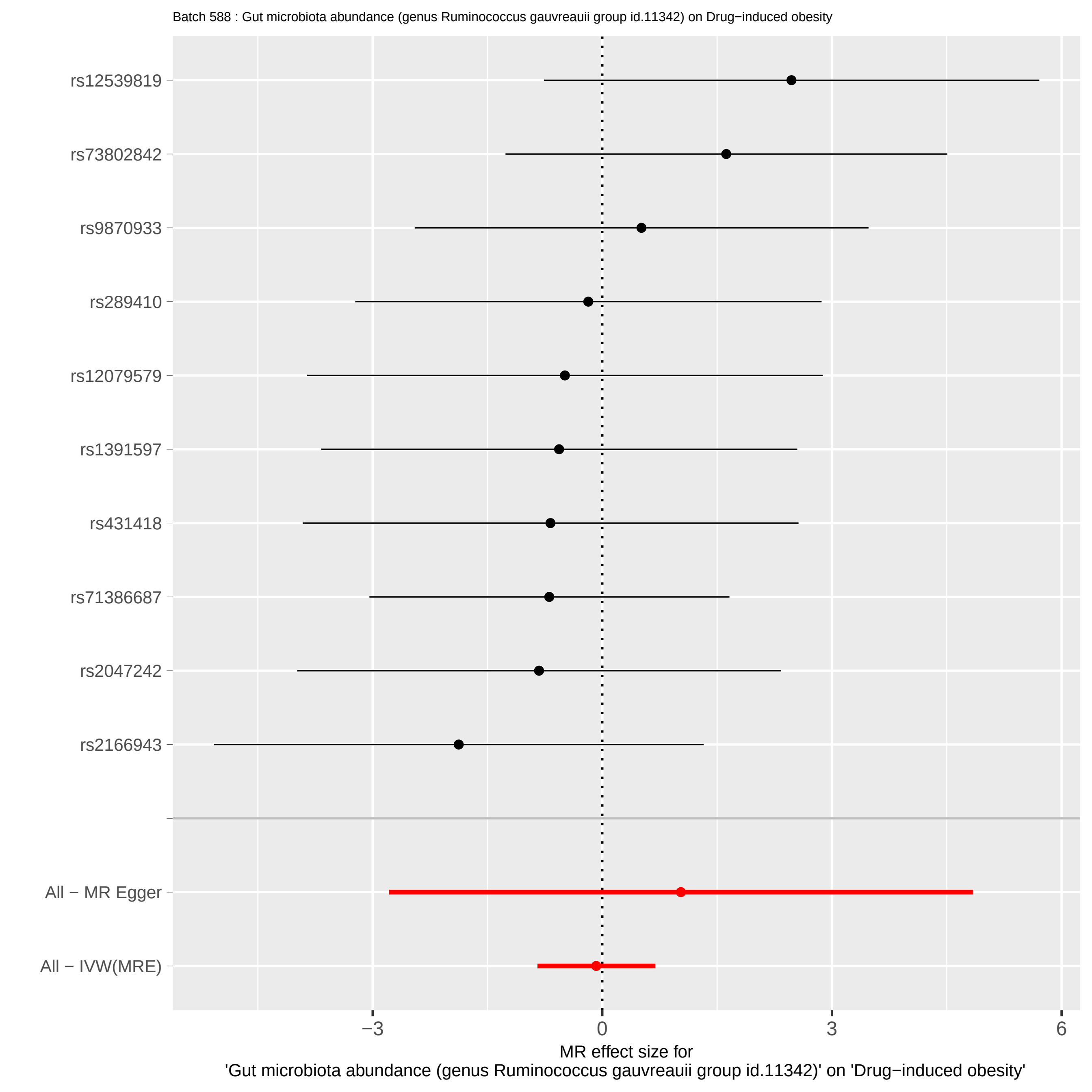
Batch 588 : Gut microbiota abundance (genus Ruminococcus gauvreauii group id.11342) on Drug−induced obesity
rs12539819
rs73802842
rs9870933
rs289410
rs12079579
rs1391597
rs431418
rs71386687
rs2047242
rs2166943
All − MR Egger
All − IVW(MRE)
−3
0
3
6
MR effect size for
'Gut microbiota abundance (genus Ruminococcus gauvreauii group id.11342)' on 'Drug−induced obesity'

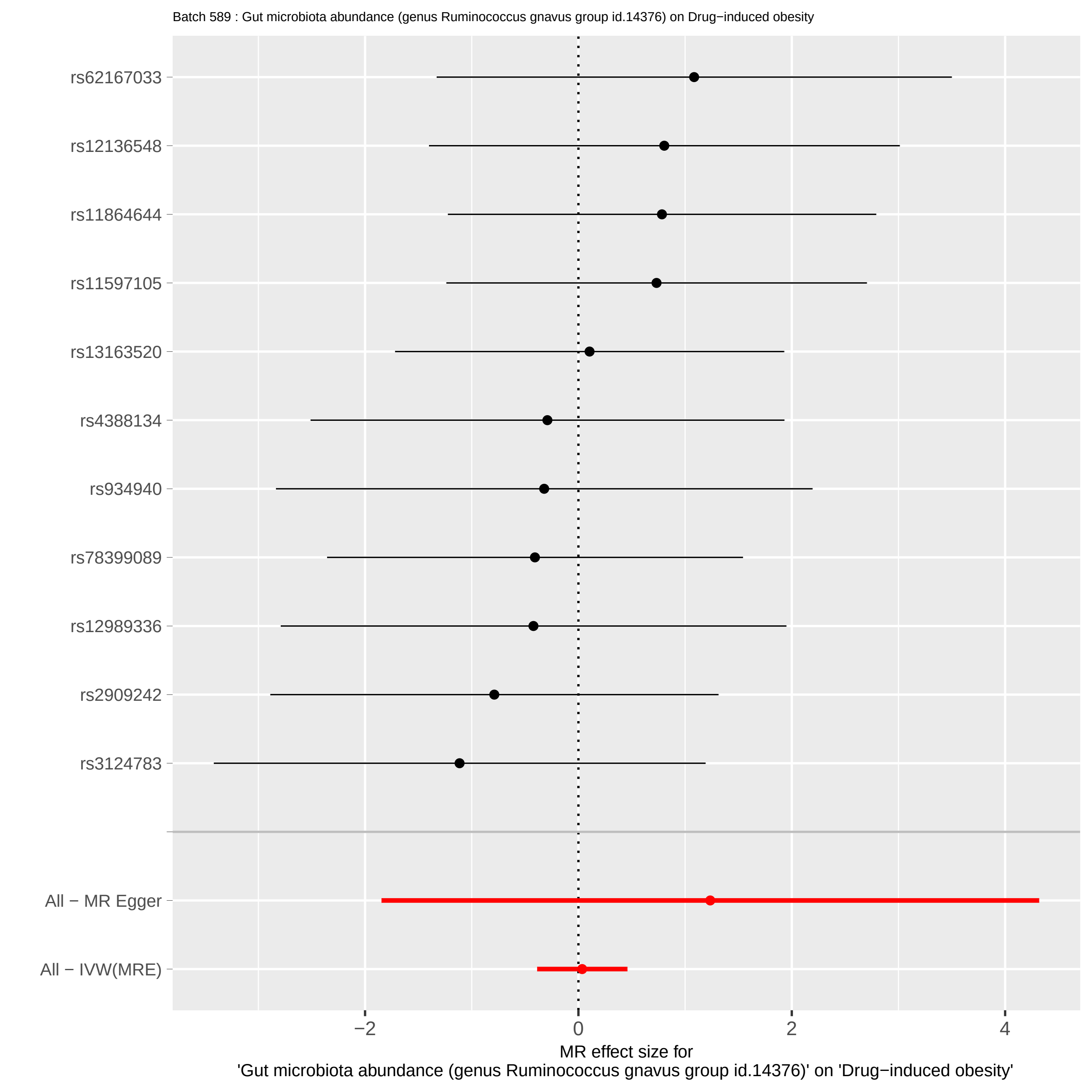

Batch 589 : Gut microbiota abundance (genus Ruminococcus gnavus group id.14376) on Drug−induced obesity
rs62167033
rs12136548
rs11864644
rs11597105
rs13163520
rs4388134
rs934940
rs78399089
rs12989336
rs2909242
rs3124783
All − MR Egger
All − IVW(MRE)
−2
0
2
4
MR effect size for
'Gut microbiota abundance (genus Ruminococcus gnavus group id.14376)' on 'Drug−induced obesity'

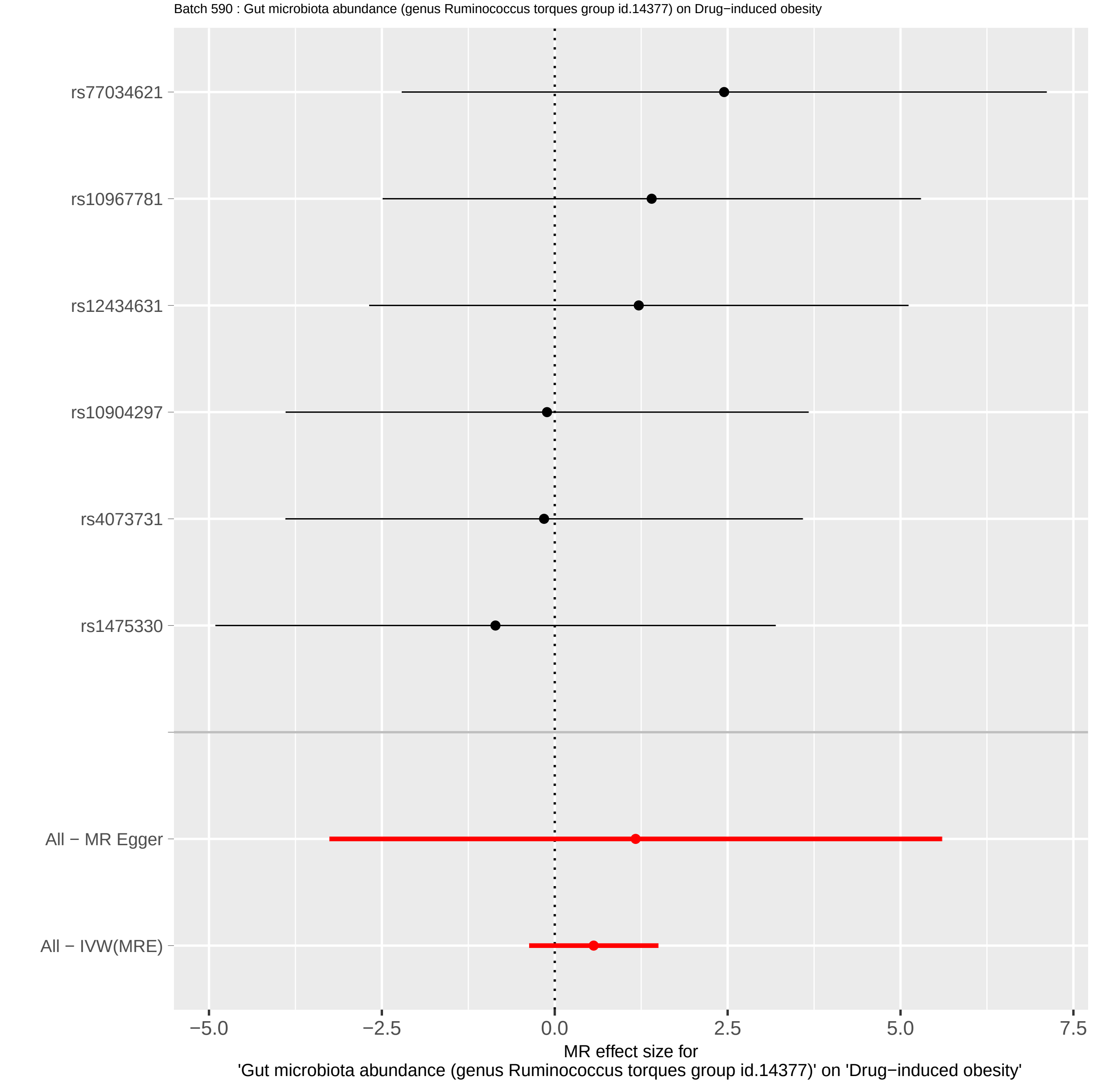

Batch 590 : Gut microbiota abundance (genus Ruminococcus torques group id.14377) on Drug−induced obesity
rs77034621
rs10967781
rs12434631
rs10904297
rs4073731
rs1475330
All − MR Egger
All − IVW(MRE)
−5.0
−2.5
0.0
2.5
5.0
7.5
MR effect size for
'Gut microbiota abundance (genus Ruminococcus torques group id.14377)' on 'Drug−induced obesity'

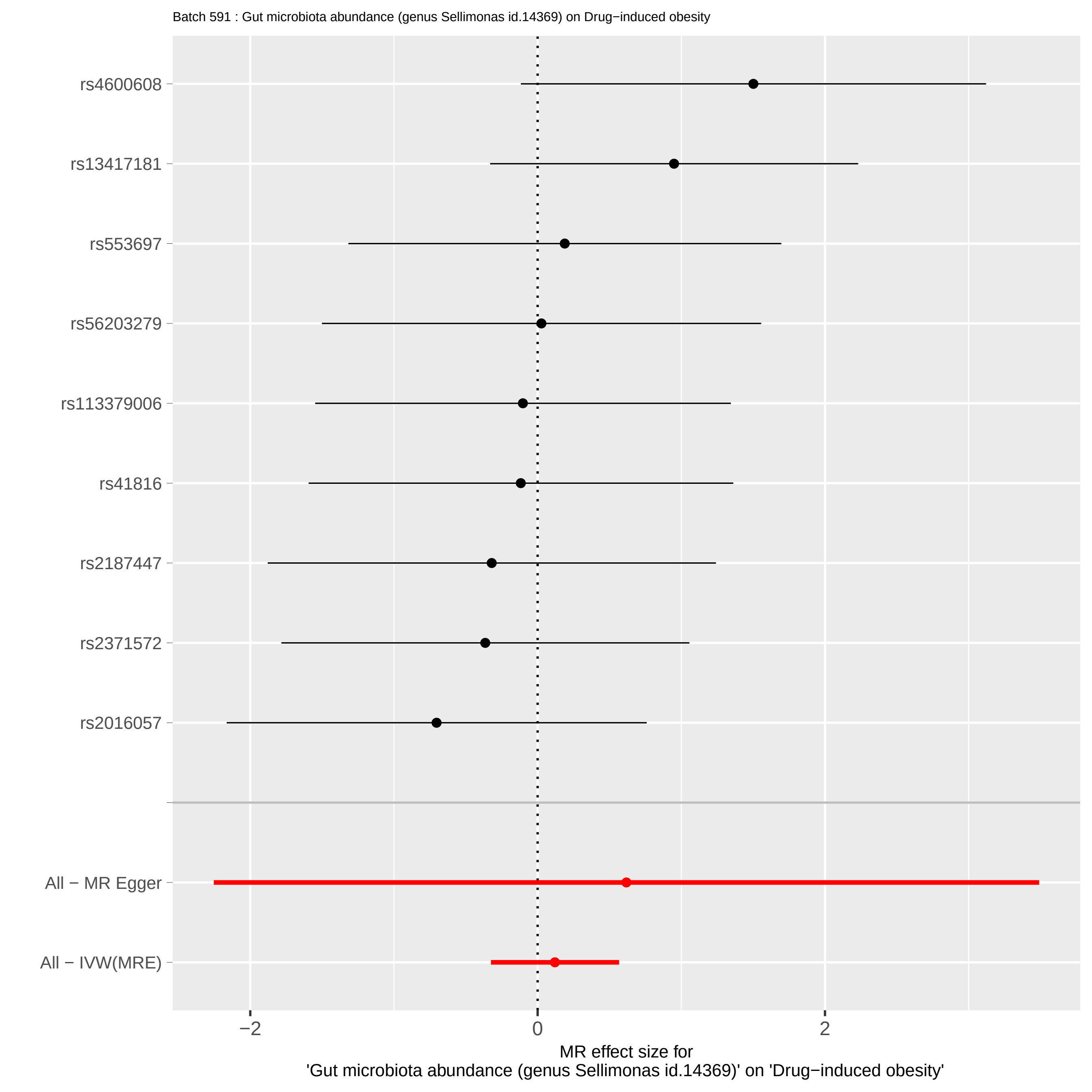

Batch 591 : Gut microbiota abundance (genus Sellimonas id.14369) on Drug−induced obesity
rs4600608
rs13417181
rs553697
rs56203279
rs113379006
rs41816
rs2187447
rs2371572
rs2016057
All − MR Egger
All − IVW(MRE)
−2
0
2
MR effect size for
'Gut microbiota abundance (genus Sellimonas id.14369)' on 'Drug−induced obesity'

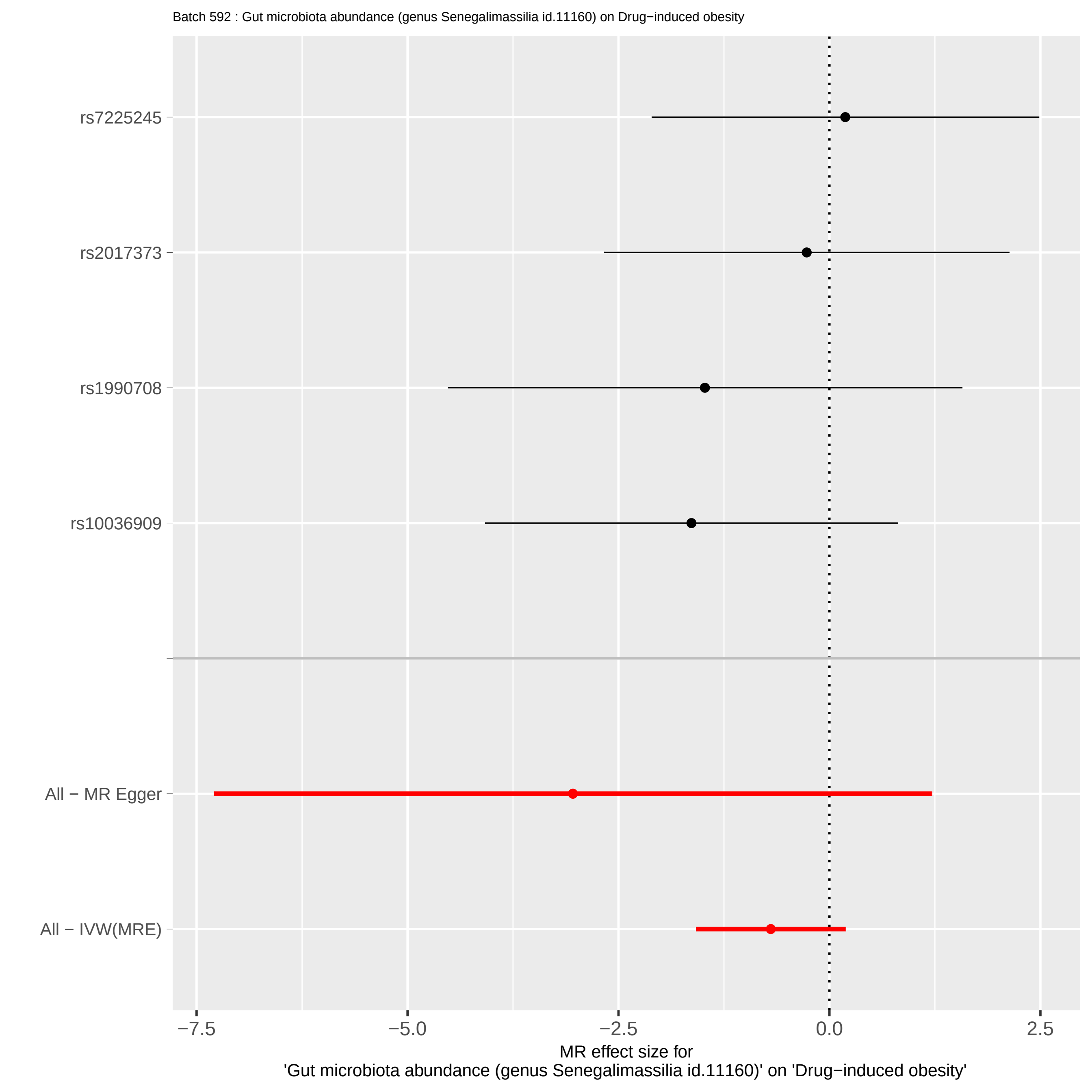
Batch 592 : Gut microbiota abundance (genus Senegalimassilia id.11160) on Drug−induced obesity
rs7225245
rs2017373
rs1990708
rs10036909
All − MR Egger
All − IVW(MRE)
−7.5
−5.0
−2.5
0.0
2.5
MR effect size for
'Gut microbiota abundance (genus Senegalimassilia id.11160)' on 'Drug−induced obesity'

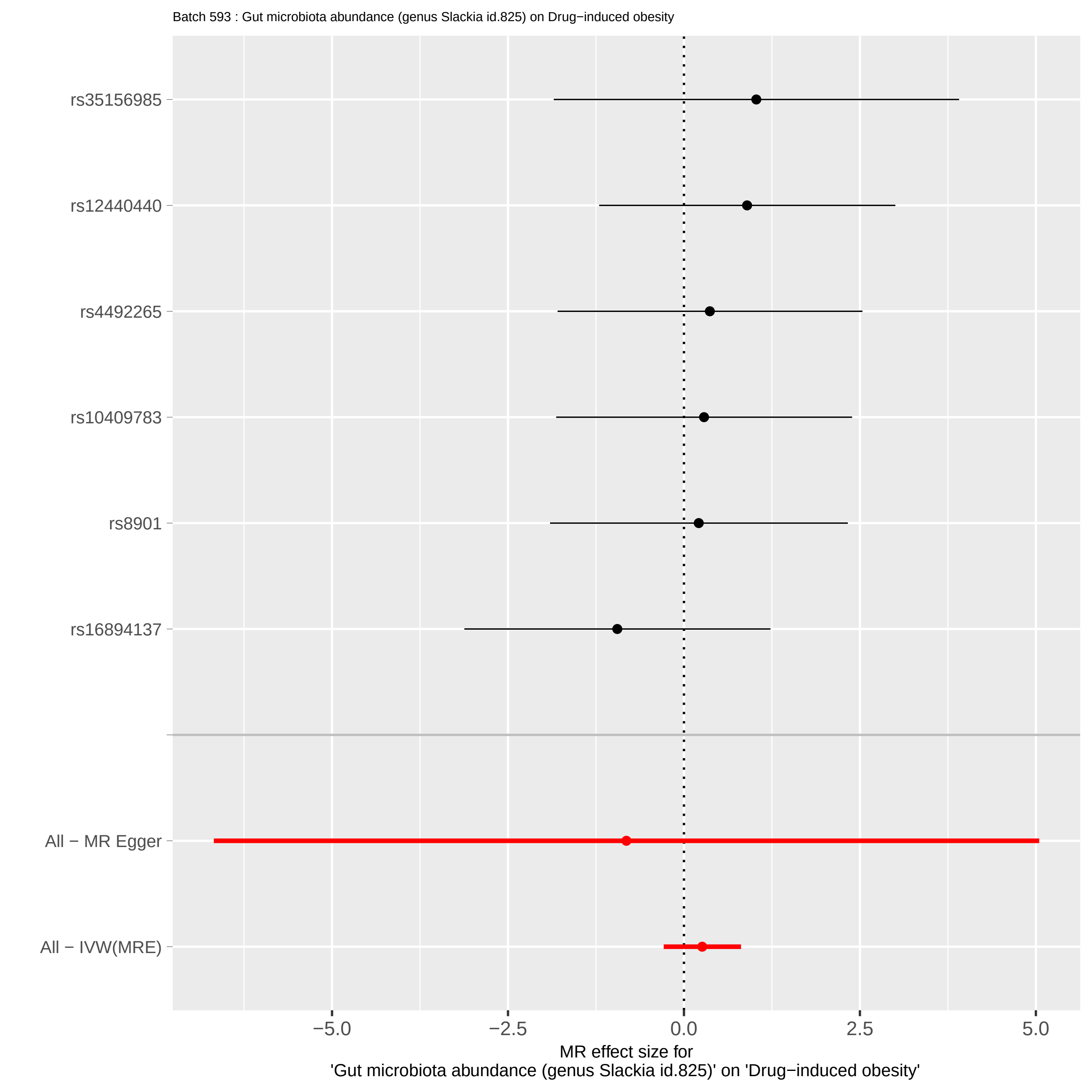

Batch 593 : Gut microbiota abundance (genus Slackia id.825) on Drug−induced obesity
rs35156985
rs12440440
rs4492265
rs10409783
rs8901
rs16894137
All − MR Egger
All − IVW(MRE)
−5.0
−2.5
0.0
2.5
5.0
MR effect size for
'Gut microbiota abundance (genus Slackia id.825)' on 'Drug−induced obesity'

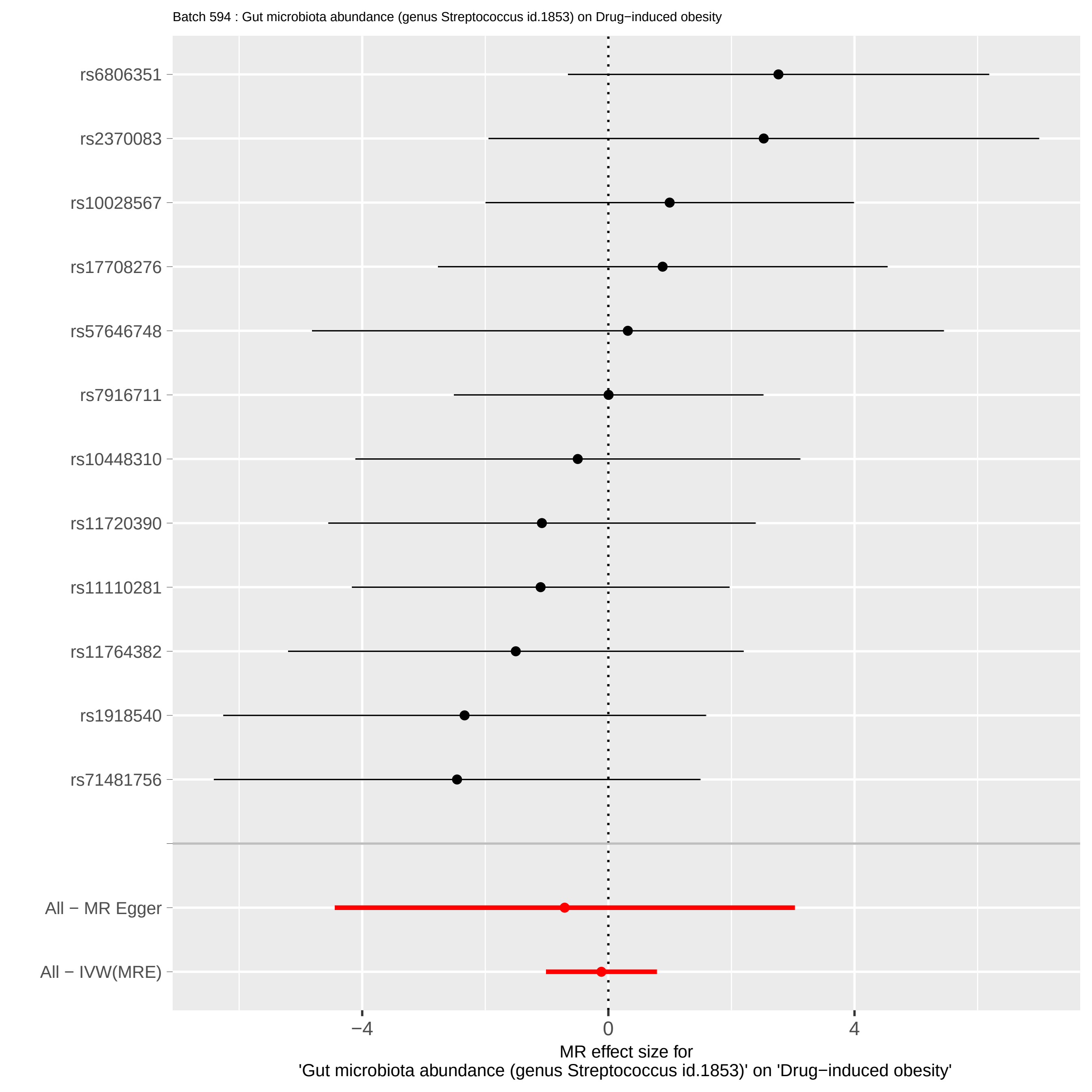
Batch 594 : Gut microbiota abundance (genus Streptococcus id.1853) on Drug−induced obesity
rs6806351
rs2370083
rs10028567
rs17708276
rs57646748
rs7916711
rs10448310
rs11720390
rs11110281
rs11764382
rs1918540
rs71481756
All − MR Egger
All − IVW(MRE)
−4
0
4
MR effect size for
'Gut microbiota abundance (genus Streptococcus id.1853)' on 'Drug−induced obesity'

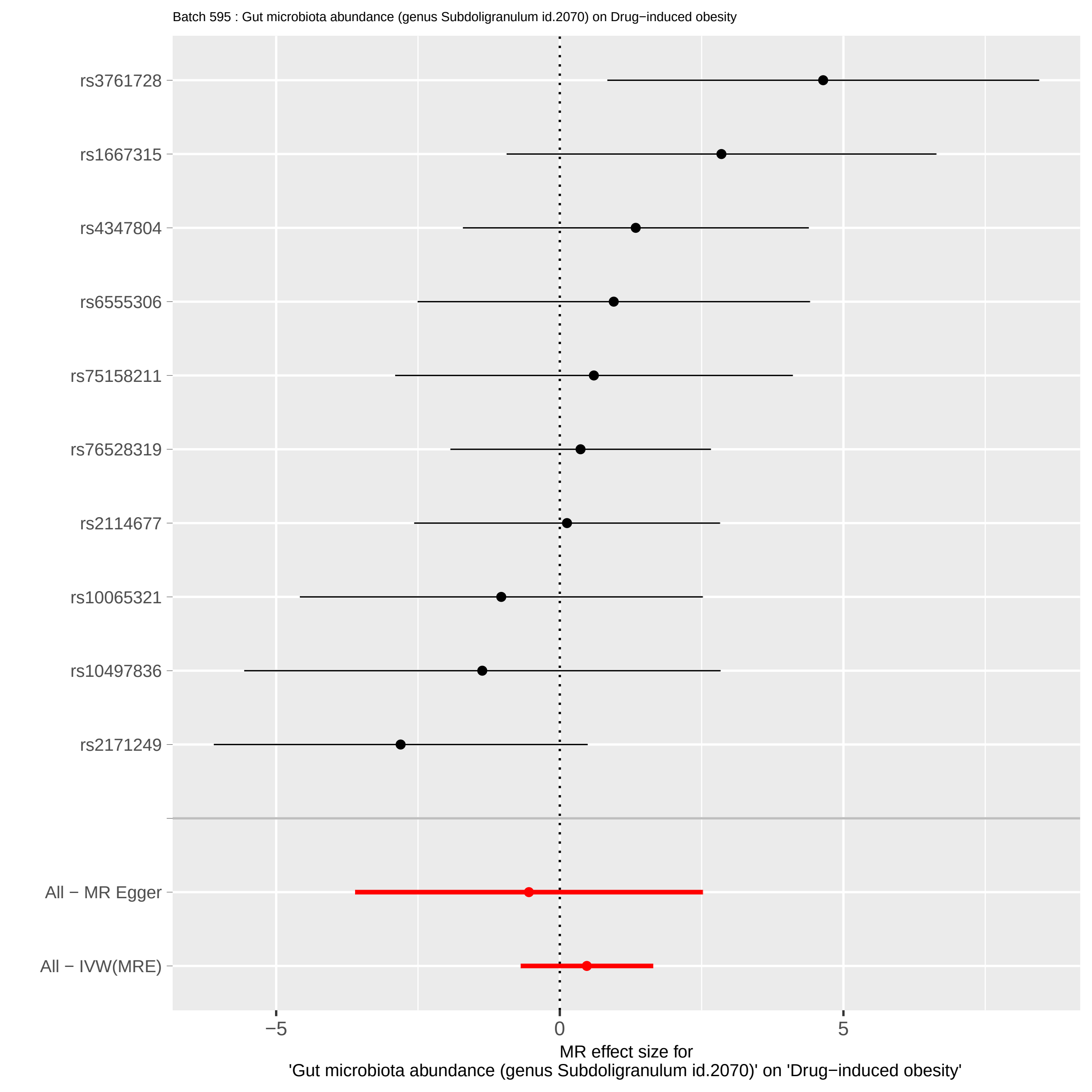
Batch 595 : Gut microbiota abundance (genus Subdoligranulum id.2070) on Drug−induced obesity
rs3761728
rs1667315
rs4347804
rs6555306
rs75158211
rs76528319
rs2114677
rs10065321
rs10497836
rs2171249
All − MR Egger
All − IVW(MRE)
−5
0
5
MR effect size for
'Gut microbiota abundance (genus Subdoligranulum id.2070)' on 'Drug−induced obesity'

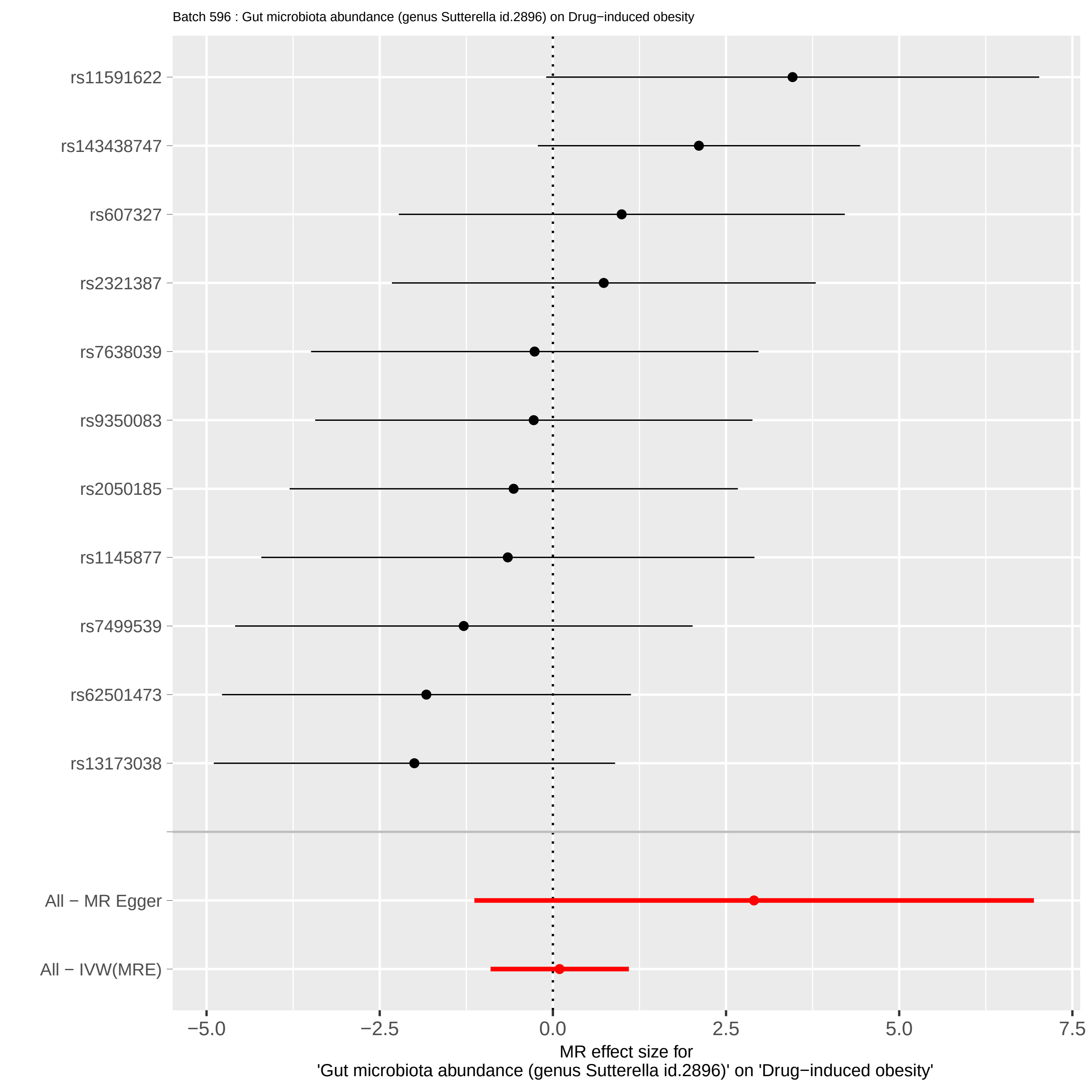
Batch 596 : Gut microbiota abundance (genus Sutterella id.2896) on Drug−induced obesity
rs11591622
rs143438747
rs607327
rs2321387
rs7638039
rs9350083
rs2050185
rs1145877
rs7499539
rs62501473
rs13173038
All − MR Egger
All − IVW(MRE)
−5.0
−2.5
0.0
2.5
5.0
7.5
MR effect size for
'Gut microbiota abundance (genus Sutterella id.2896)' on 'Drug−induced obesity'

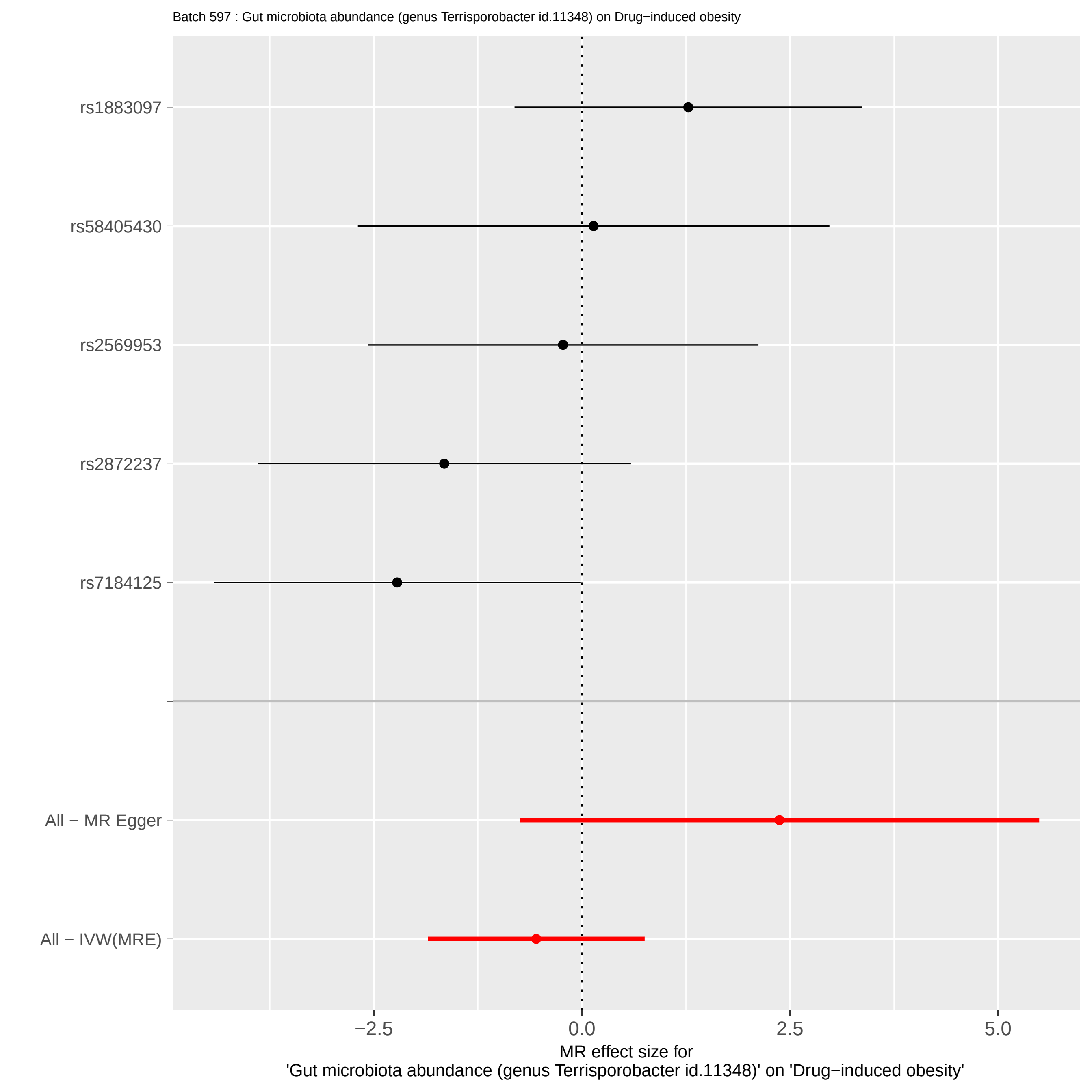
Batch 597 : Gut microbiota abundance (genus Terrisporobacter id.11348) on Drug−induced obesity
rs1883097
rs58405430
rs2569953
rs2872237
rs7184125
All − MR Egger
All − IVW(MRE)
−2.5
0.0
2.5
5.0
MR effect size for
'Gut microbiota abundance (genus Terrisporobacter id.11348)' on 'Drug−induced obesity'

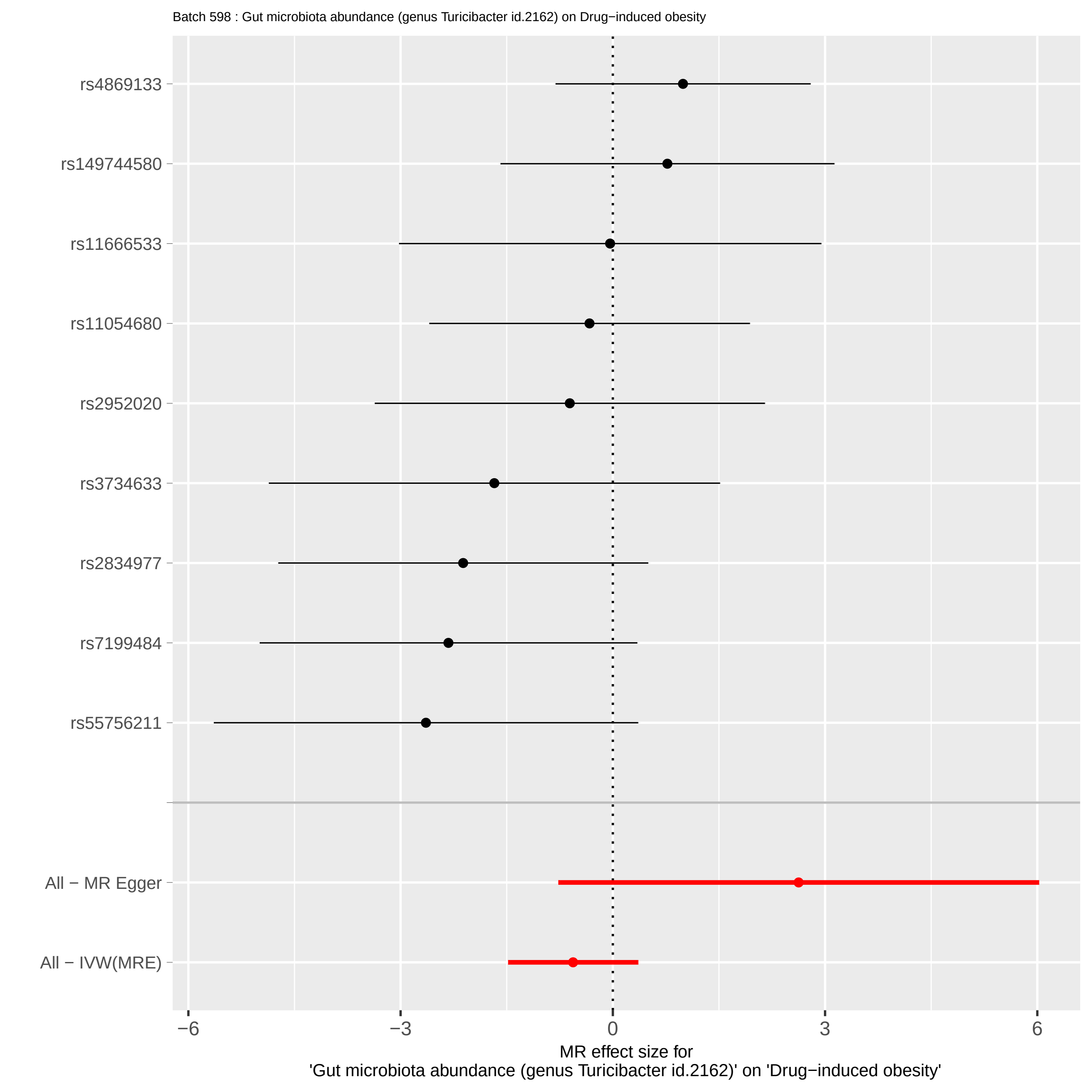

Batch 598 : Gut microbiota abundance (genus Turicibacter id.2162) on Drug−induced obesity
rs4869133
rs149744580
rs11666533
rs11054680
rs2952020
rs3734633
rs2834977
rs7199484
rs55756211
All − MR Egger
All − IVW(MRE)
−6
−3
0
3
6
MR effect size for
'Gut microbiota abundance (genus Turicibacter id.2162)' on 'Drug−induced obesity'

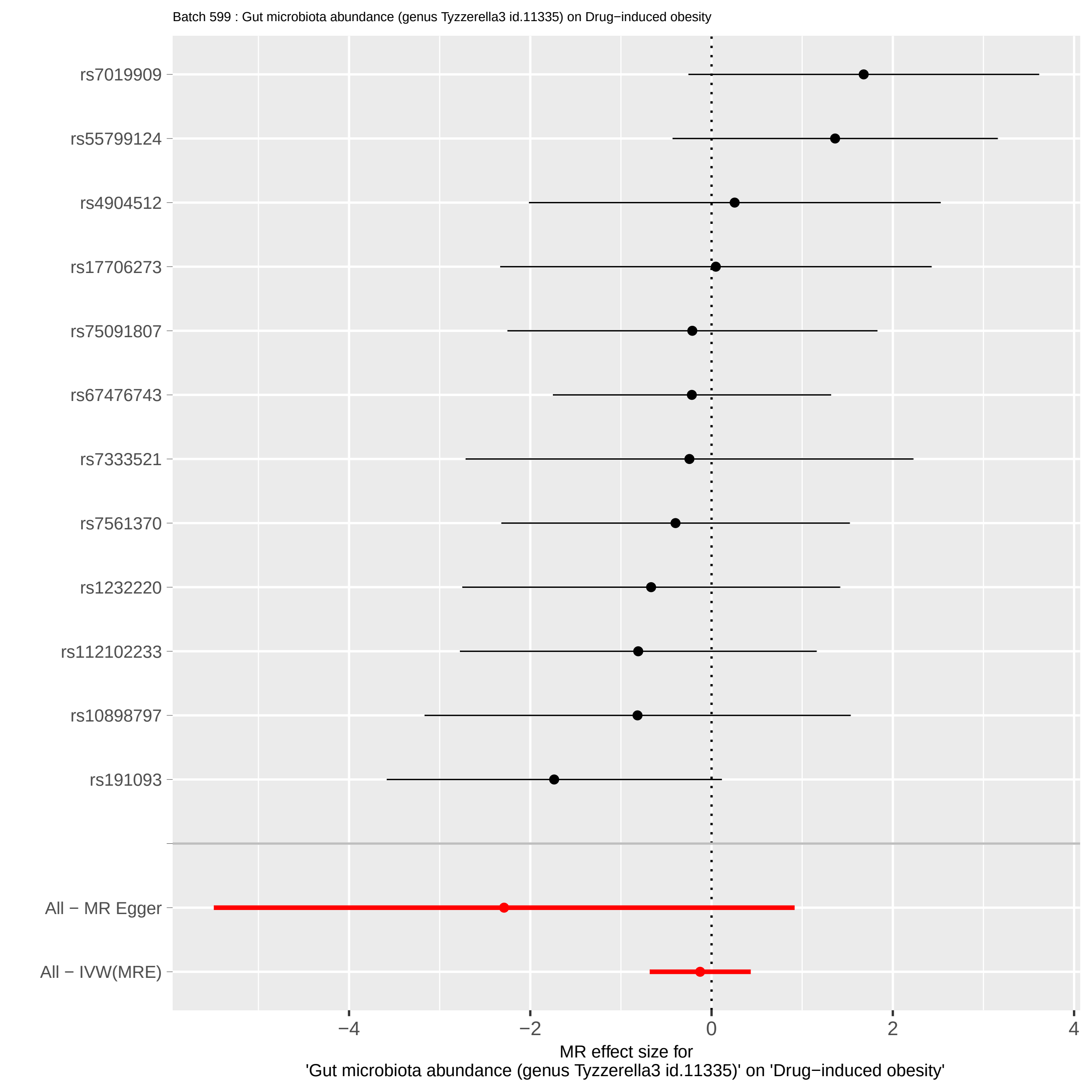

Batch 599 : Gut microbiota abundance (genus Tyzzerella3 id.11335) on Drug−induced obesity
rs7019909
rs55799124
rs4904512
rs17706273
rs75091807
rs67476743
rs7333521
rs7561370
rs1232220
rs112102233
rs10898797
rs191093
All − MR Egger
All − IVW(MRE)
−4
−2
0
2
4
MR effect size for
'Gut microbiota abundance (genus Tyzzerella3 id.11335)' on 'Drug−induced obesity'

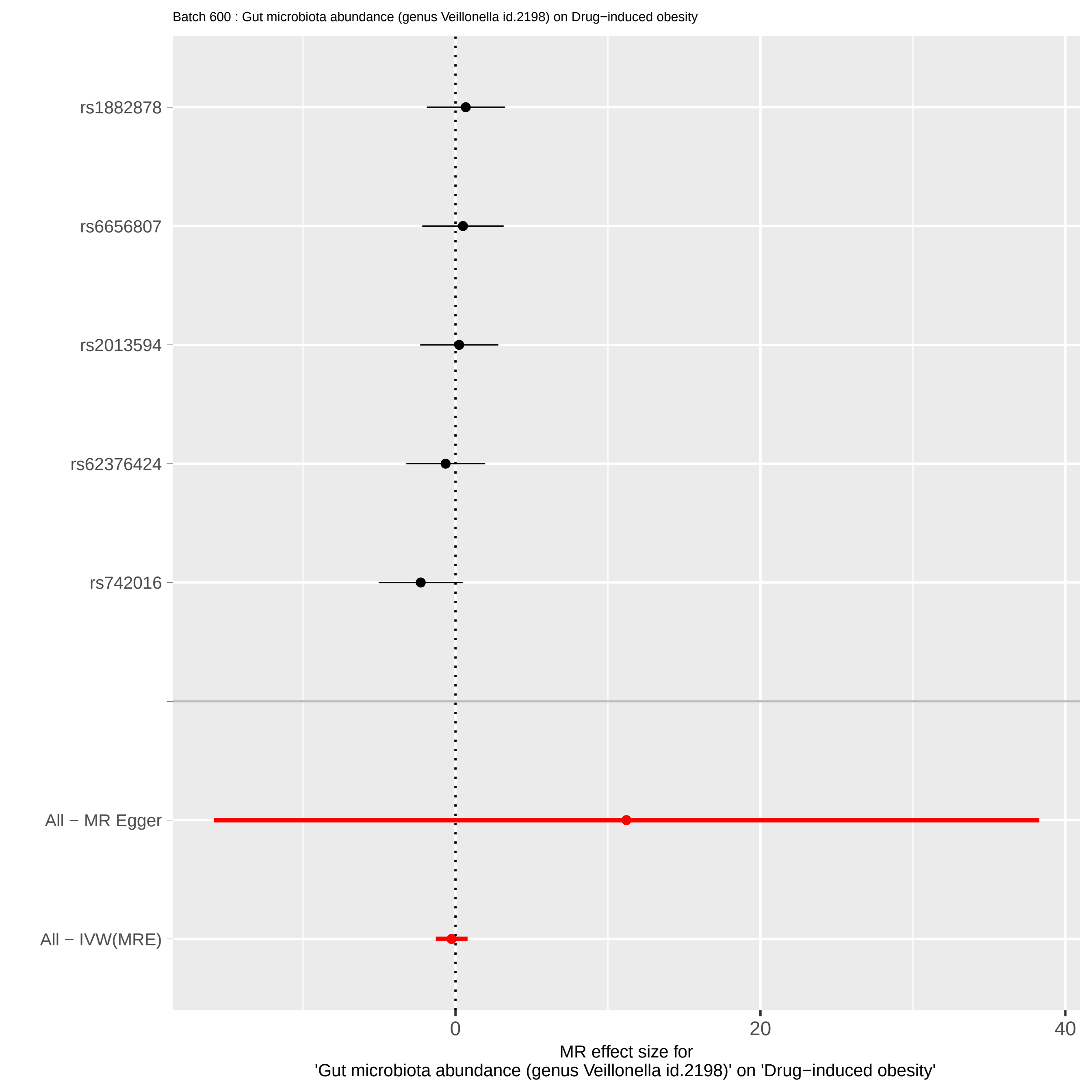

Batch 600 : Gut microbiota abundance (genus Veillonella id.2198) on Drug−induced obesity
rs1882878
rs6656807
rs2013594
rs62376424
rs742016
All − MR Egger
All − IVW(MRE)
0
20
40
MR effect size for
'Gut microbiota abundance (genus Veillonella id.2198)' on 'Drug−induced obesity'

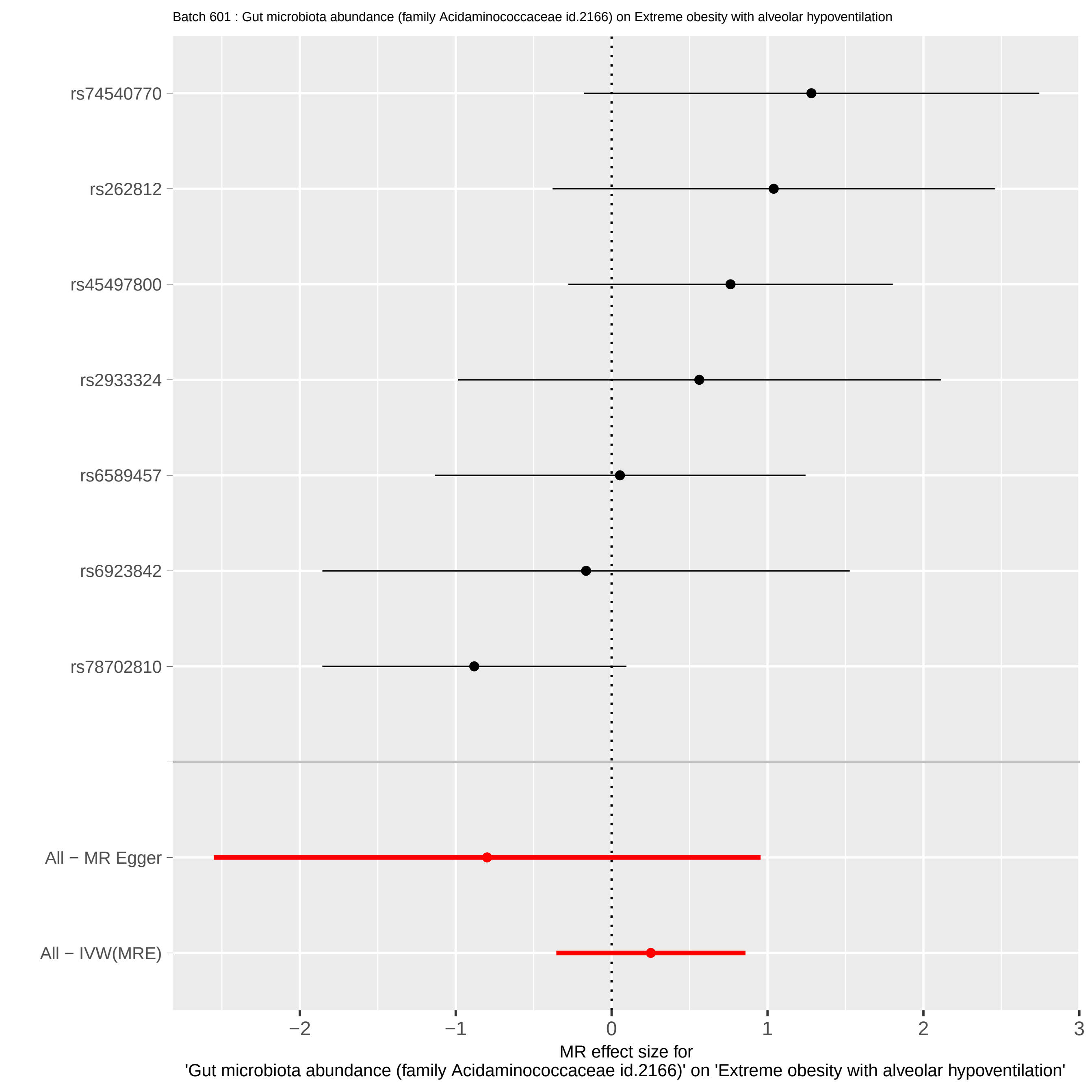

Batch 601 : Gut microbiota abundance (family Acidaminococcaceae id.2166) on Extreme obesity with alveolar hypoventilation
rs74540770
rs262812
rs45497800
rs2933324
rs6589457
rs6923842
rs78702810
All − MR Egger
All − IVW(MRE)
−2
−1
0
1
2
3
MR effect size for
'Gut microbiota abundance (family Acidaminococcaceae id.2166)' on 'Extreme obesity with alveolar hypoventilation'

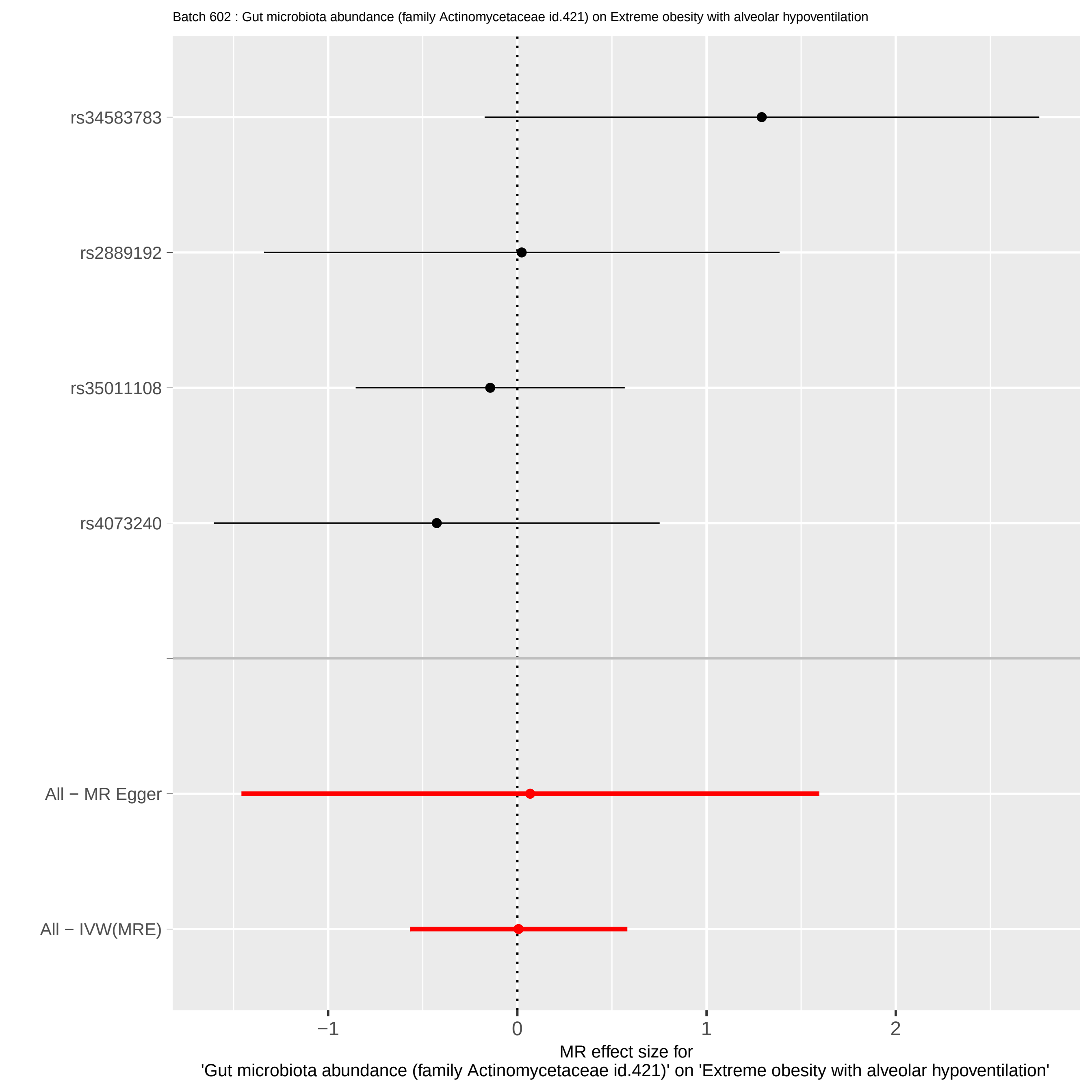

Batch 602 : Gut microbiota abundance (family Actinomycetaceae id.421) on Extreme obesity with alveolar hypoventilation
rs34583783
rs2889192
rs35011108
rs4073240
All − MR Egger
All − IVW(MRE)
−1
0
1
2
MR effect size for
'Gut microbiota abundance (family Actinomycetaceae id.421)' on 'Extreme obesity with alveolar hypoventilation'

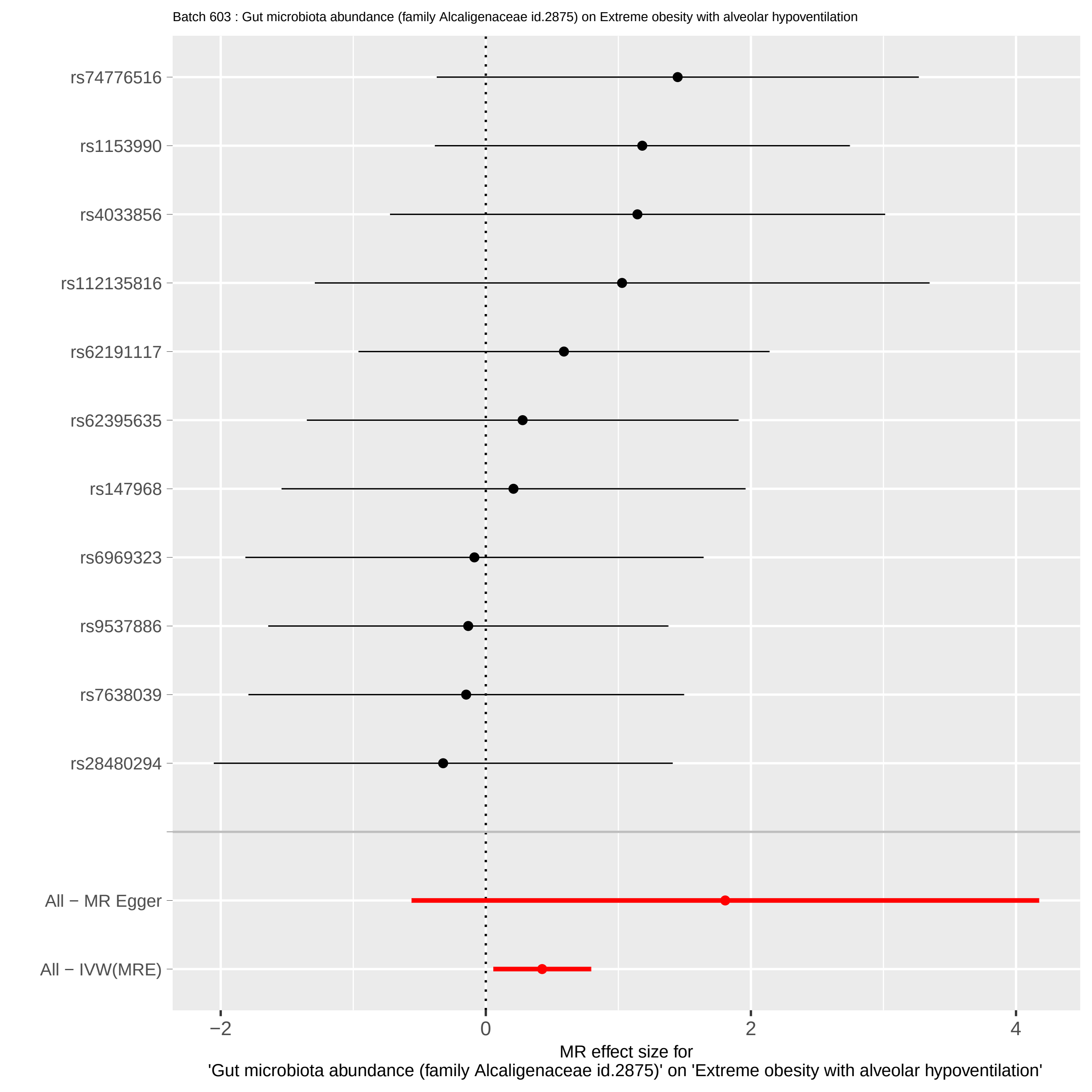
Batch 603 : Gut microbiota abundance (family Alcaligenaceae id.2875) on Extreme obesity with alveolar hypoventilation
rs74776516
rs1153990
rs4033856
rs112135816
rs62191117
rs62395635
rs147968
rs6969323
rs9537886
rs7638039
rs28480294
All − MR Egger
All − IVW(MRE)
−2
0
2
4
MR effect size for
'Gut microbiota abundance (family Alcaligenaceae id.2875)' on 'Extreme obesity with alveolar hypoventilation'

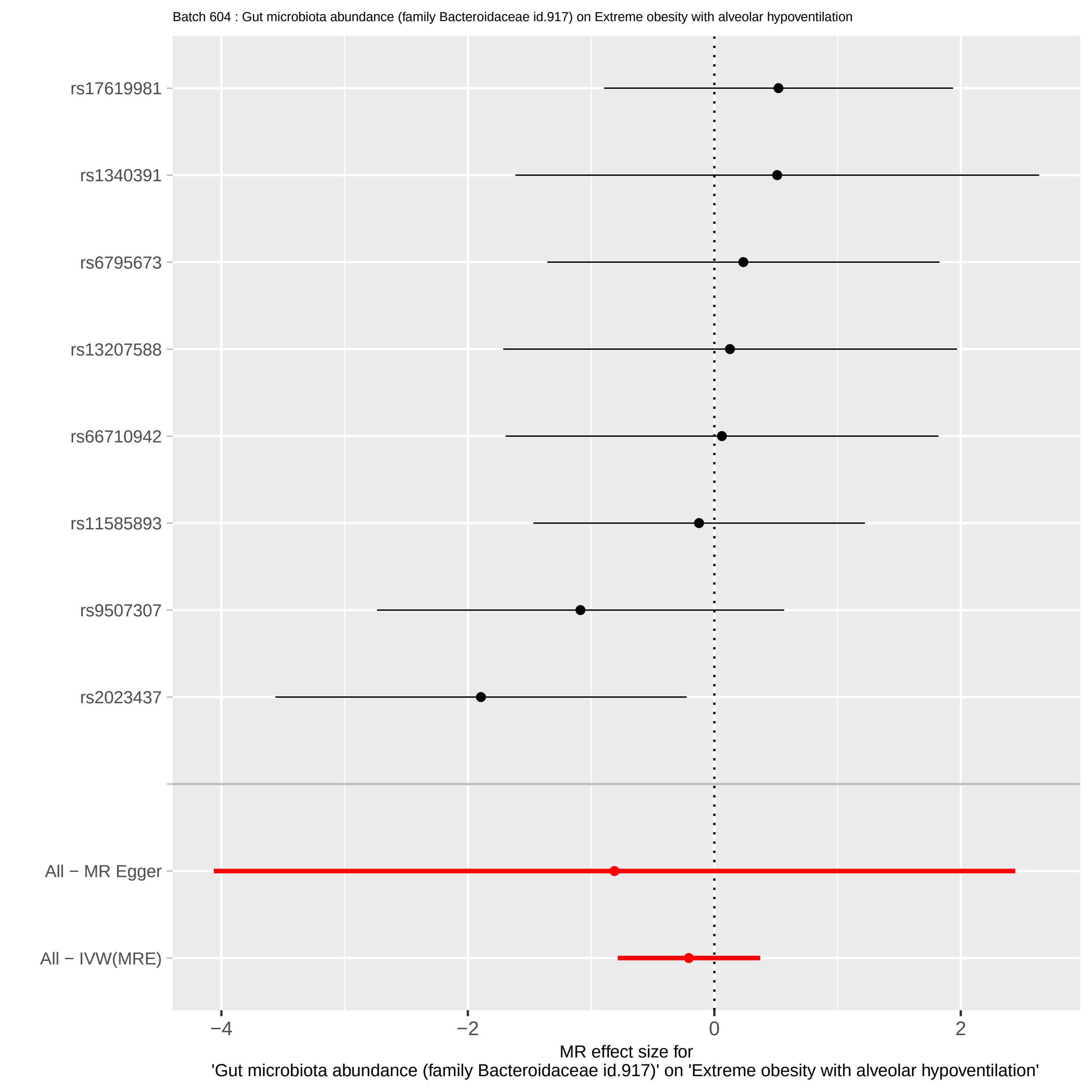

Batch 604 : Gut microbiota abundance (family Bacteroidaceae id.917) on Extreme obesity with alveolar hypoventilation
rs17619981
rs1340391
rs6795673
rs13207588
rs66710942
rs11585893
rs9507307
rs2023437
All − MR Egger
All − IVW(MRE)
−4
−2
0
2
MR effect size for
'Gut microbiota abundance (family Bacteroidaceae id.917)' on 'Extreme obesity with alveolar hypoventilation'

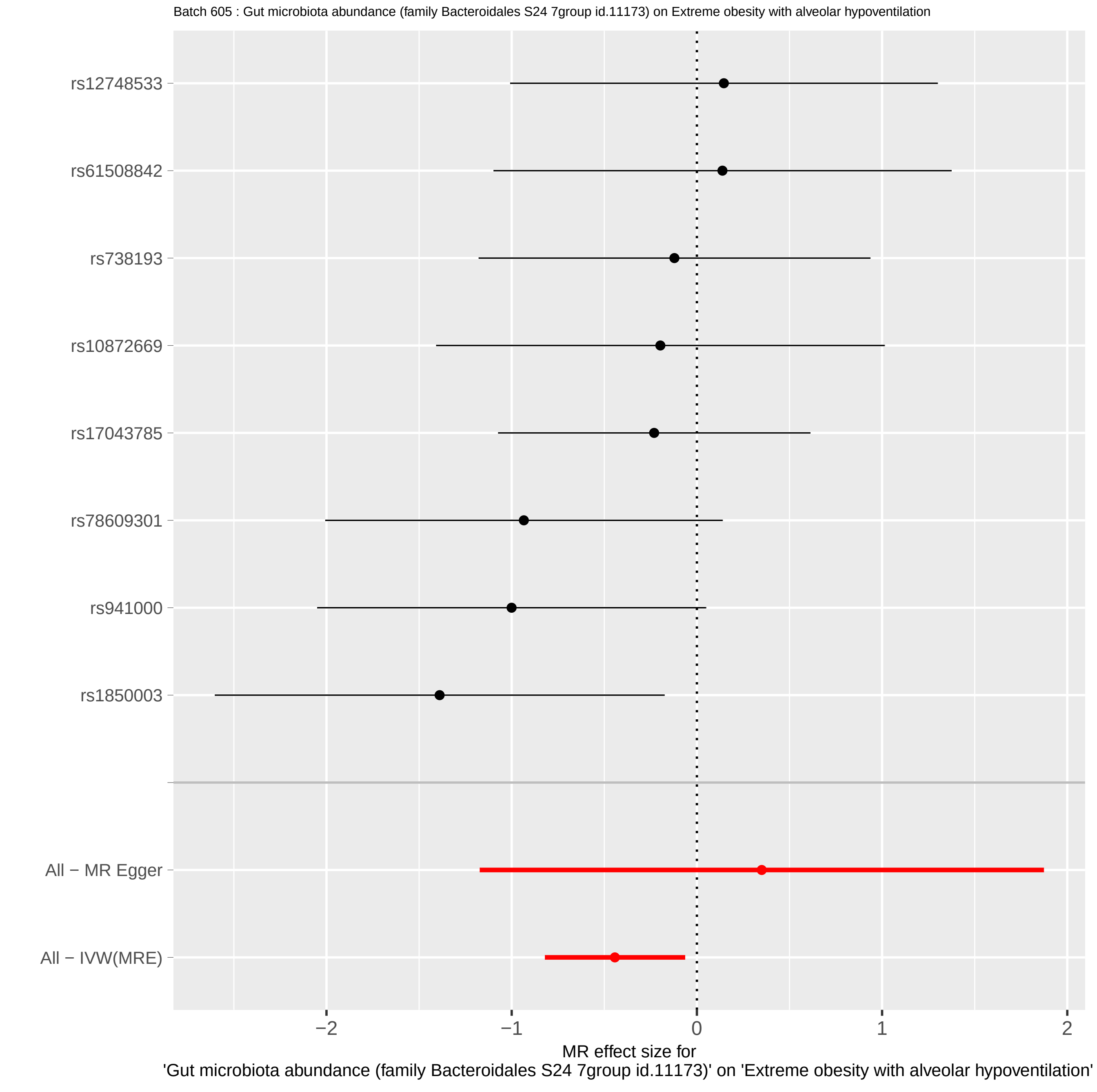

Batch 605 : Gut microbiota abundance (family Bacteroidales S24 7group id.11173) on Extreme obesity with alveolar hypoventilation
rs12748533
rs61508842
rs738193
rs10872669
rs17043785
rs78609301
rs941000
rs1850003
All − MR Egger
All − IVW(MRE)
−2
−1
0
1
2
MR effect size for
'Gut microbiota abundance (family Bacteroidales S24 7group id.11173)' on 'Extreme obesity with alveolar hypoventilation'

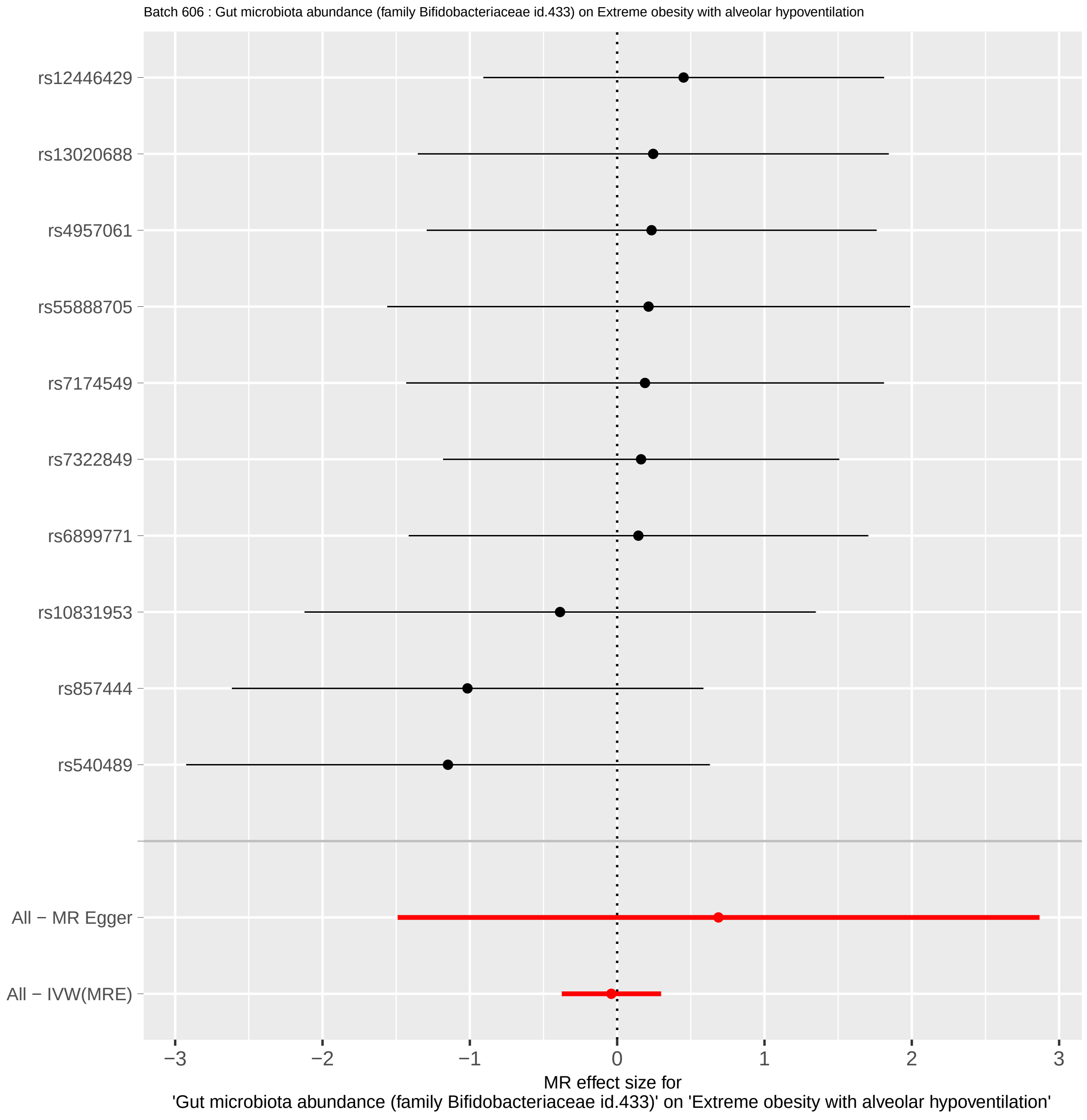

Batch 606 : Gut microbiota abundance (family Bifidobacteriaceae id.433) on Extreme obesity with alveolar hypoventilation
rs12446429
rs13020688
rs4957061
rs55888705
rs7174549
rs7322849
rs6899771
rs10831953
rs857444
rs540489
All − MR Egger
All − IVW(MRE)
−3
−2
−1
0
1
2
3
MR effect size for
'Gut microbiota abundance (family Bifidobacteriaceae id.433)' on 'Extreme obesity with alveolar hypoventilation'

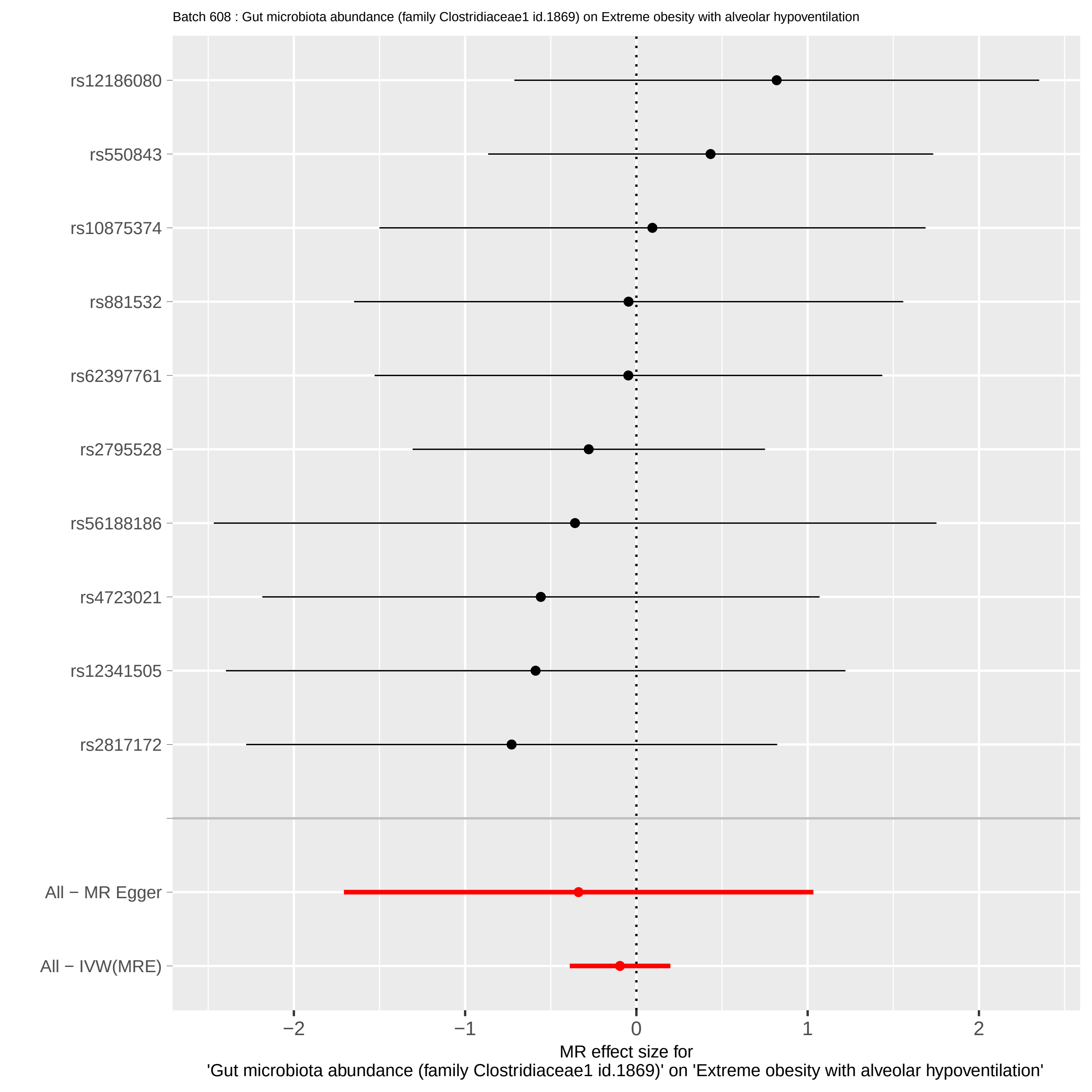
Batch 608 : Gut microbiota abundance (family Clostridiaceae1 id.1869) on Extreme obesity with alveolar hypoventilation
rs12186080
rs550843
rs10875374
rs881532
rs62397761
rs2795528
rs56188186
rs4723021
rs12341505
rs2817172
All − MR Egger
All − IVW(MRE)
−2
−1
0
1
2
MR effect size for
'Gut microbiota abundance (family Clostridiaceae1 id.1869)' on 'Extreme obesity with alveolar hypoventilation'

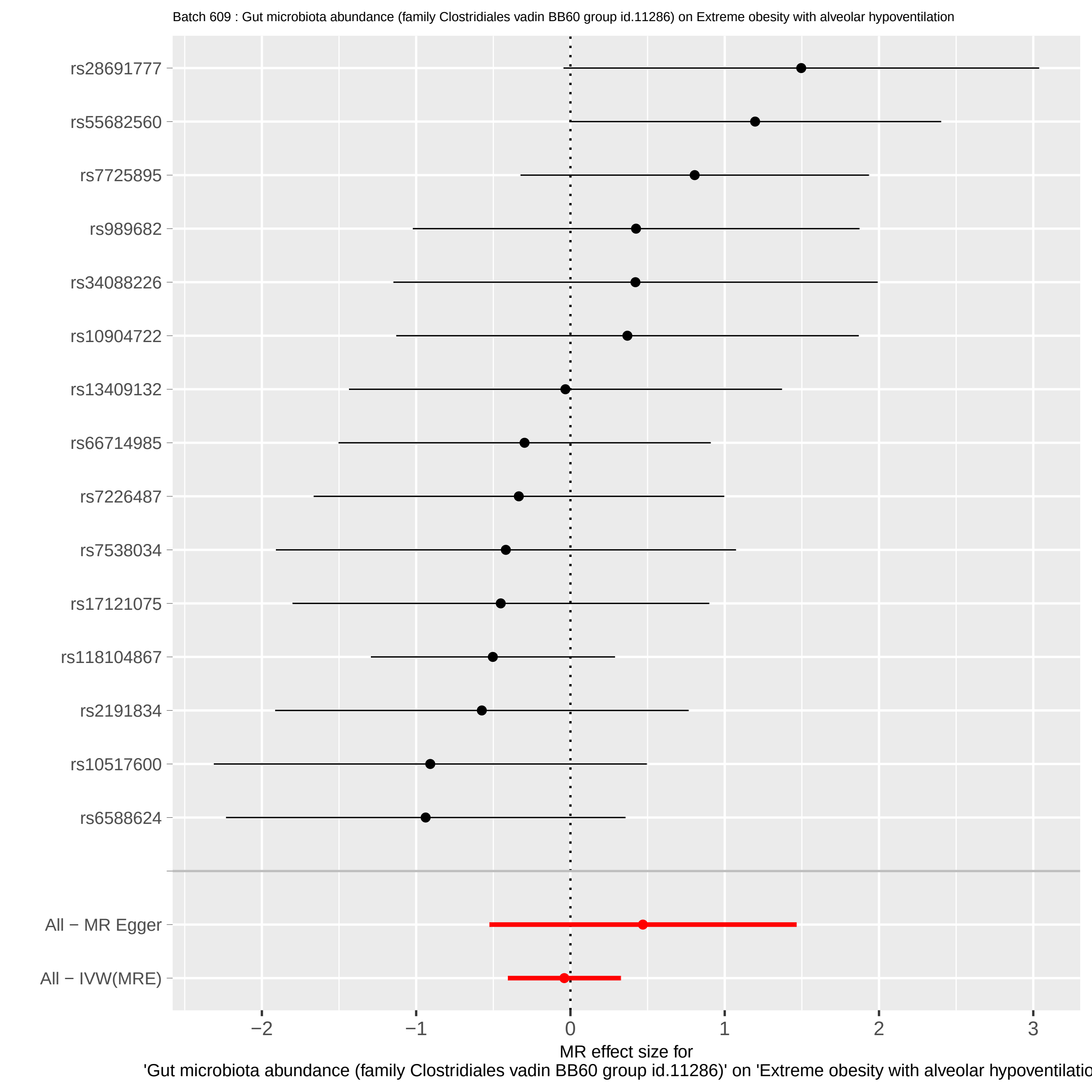

Batch 609 : Gut microbiota abundance (family Clostridiales vadin BB60 group id.11286) on Extreme obesity with alveolar hypoventilation
rs28691777
rs55682560
rs7725895
rs989682
rs34088226
rs10904722
rs13409132
rs66714985
rs7226487
rs7538034
rs17121075
rs118104867
rs2191834
rs10517600
rs6588624
All − MR Egger
All − IVW(MRE)
−2
−1
0
1
2
3
MR effect size for
'Gut microbiota abundance (family Clostridiales vadin BB60 group id.11286)' on 'Extreme obesity with alveolar hypoventilatio

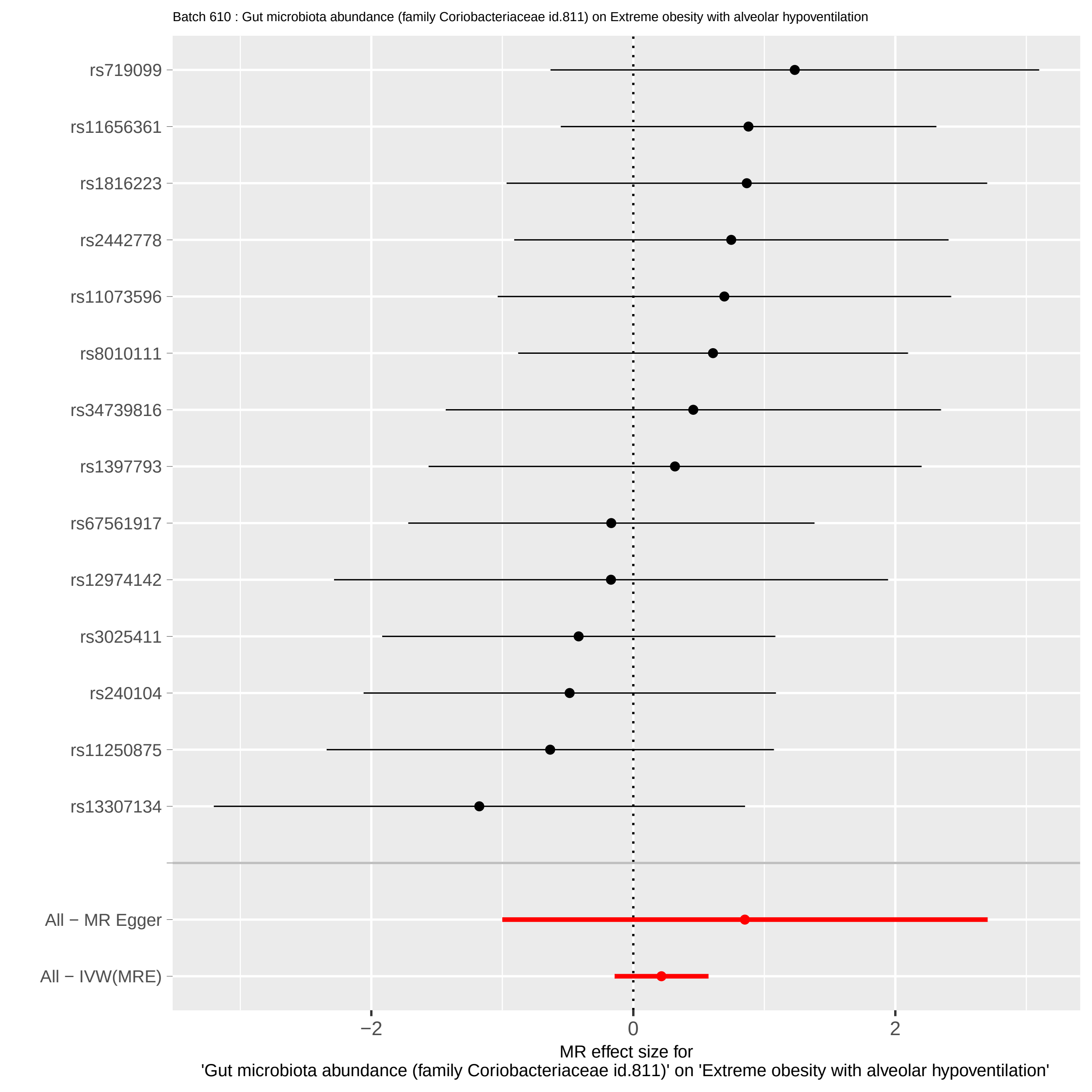
Batch 610 : Gut microbiota abundance (family Coriobacteriaceae id.811) on Extreme obesity with alveolar hypoventilation
rs719099
rs11656361
rs1816223
rs2442778
rs11073596
rs8010111
rs34739816
rs1397793
rs67561917
rs12974142
rs3025411
rs240104
rs11250875
rs13307134
All − MR Egger
All − IVW(MRE)
−2
0
2
MR effect size for
'Gut microbiota abundance (family Coriobacteriaceae id.811)' on 'Extreme obesity with alveolar hypoventilation'

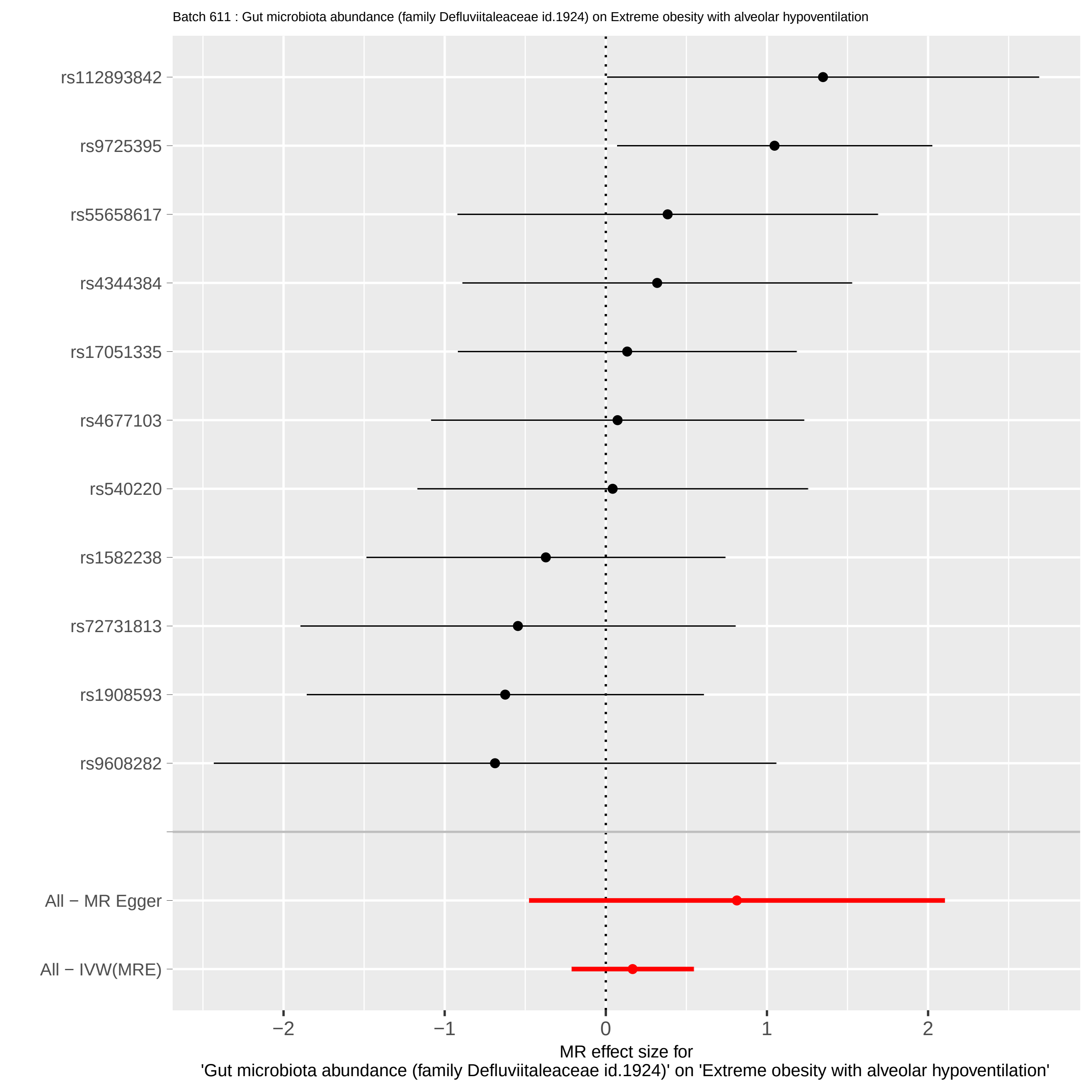
Batch 611 : Gut microbiota abundance (family Defluviitaleaceae id.1924) on Extreme obesity with alveolar hypoventilation
rs112893842
rs9725395
rs55658617
rs4344384
rs17051335
rs4677103
rs540220
rs1582238
rs72731813
rs1908593
rs9608282
All − MR Egger
All − IVW(MRE)
−2
−1
0
1
2
MR effect size for
'Gut microbiota abundance (family Defluviitaleaceae id.1924)' on 'Extreme obesity with alveolar hypoventilation'

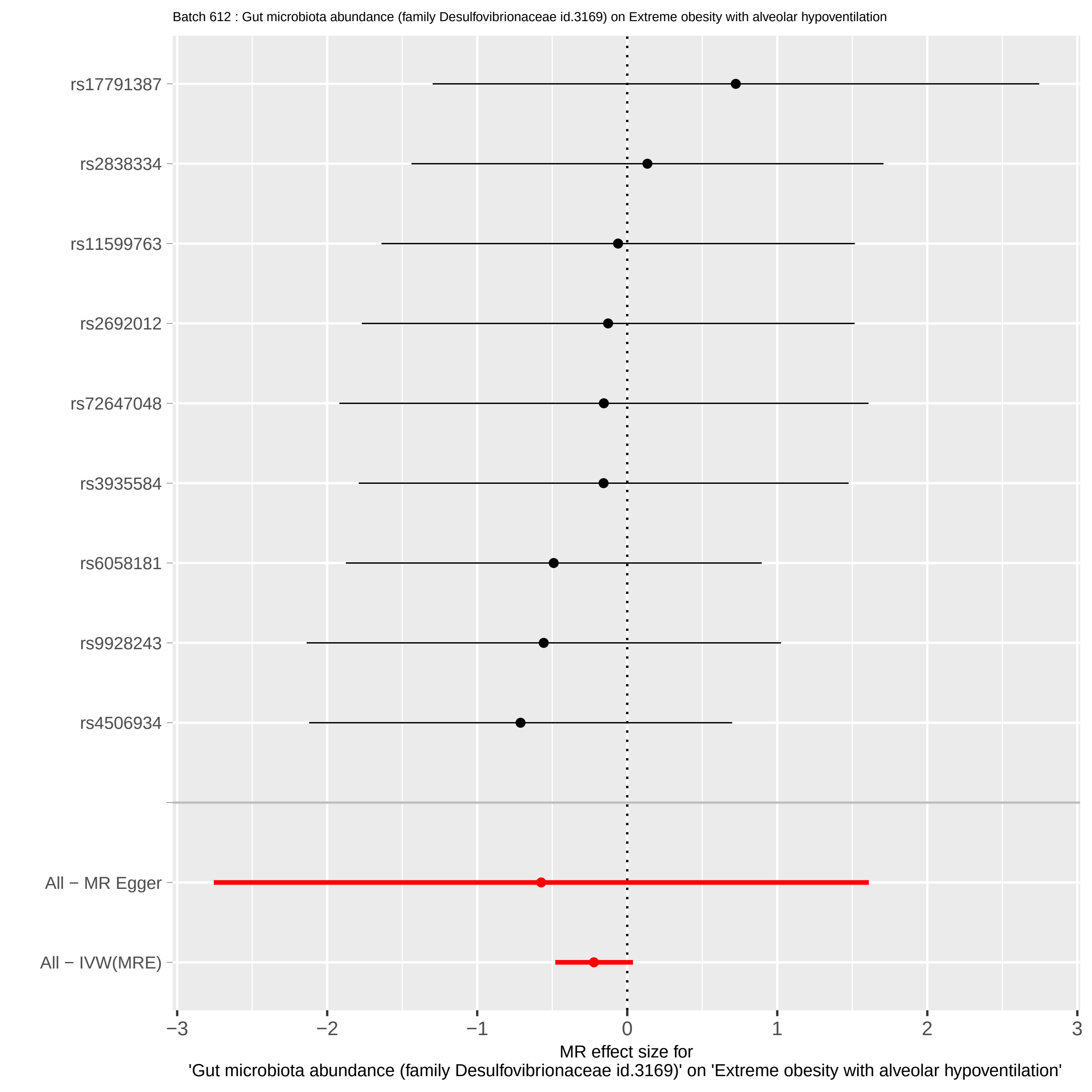

Batch 612 : Gut microbiota abundance (family Desulfovibrionaceae id.3169) on Extreme obesity with alveolar hypoventilation
rs17791387
rs2838334
rs11599763
rs2692012
rs72647048
rs3935584
rs6058181
rs9928243
rs4506934
All − MR Egger
All − IVW(MRE)
−3
−2
−1
0
1
2
3
MR effect size for
'Gut microbiota abundance (family Desulfovibrionaceae id.3169)' on 'Extreme obesity with alveolar hypoventilation'

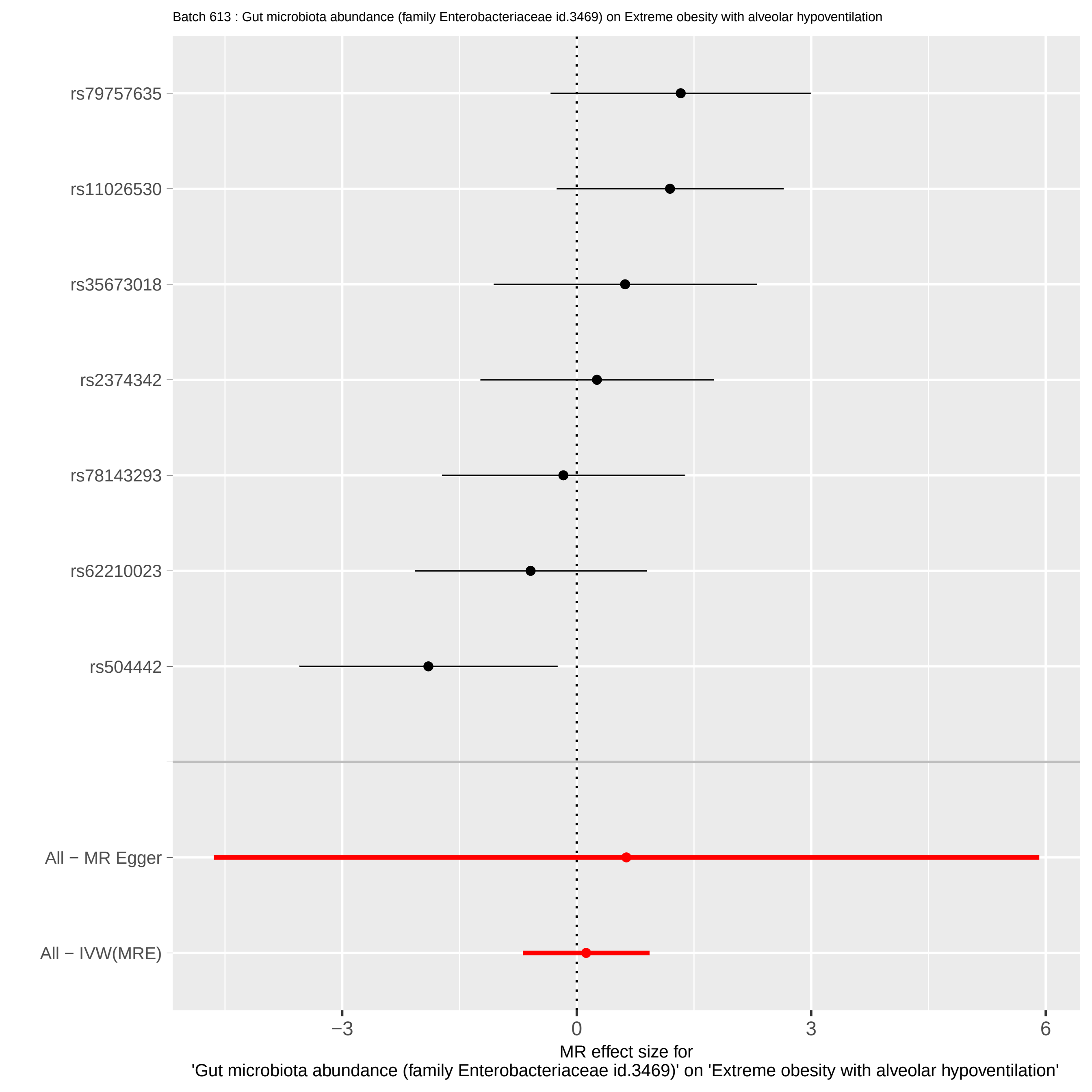

Batch 613 : Gut microbiota abundance (family Enterobacteriaceae id.3469) on Extreme obesity with alveolar hypoventilation
rs79757635
rs11026530
rs35673018
rs2374342
rs78143293
rs62210023
rs504442
All − MR Egger
All − IVW(MRE)
−3
0
3
6
MR effect size for
'Gut microbiota abundance (family Enterobacteriaceae id.3469)' on 'Extreme obesity with alveolar hypoventilation'

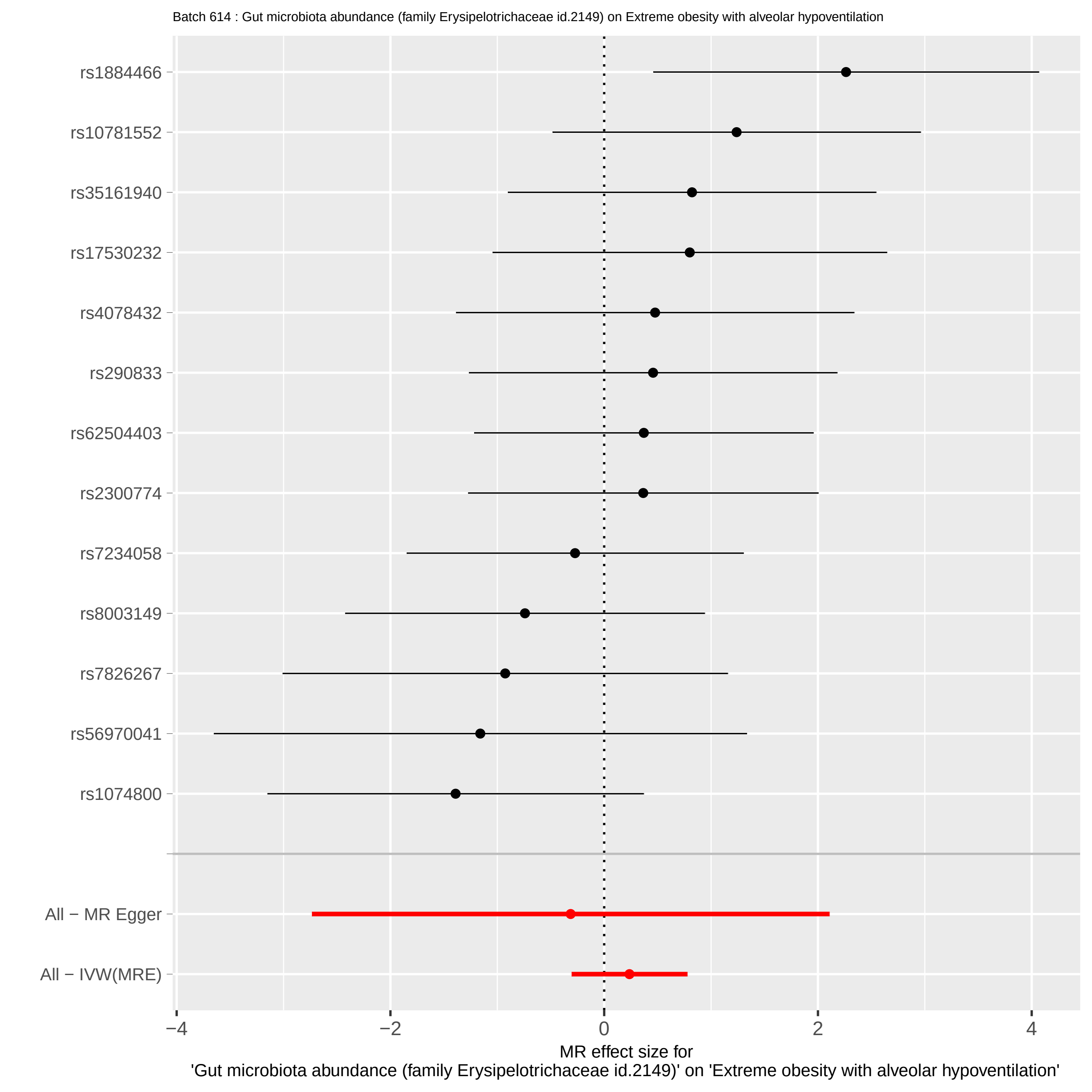

Batch 614 : Gut microbiota abundance (family Erysipelotrichaceae id.2149) on Extreme obesity with alveolar hypoventilation
rs1884466
rs10781552
rs35161940
rs17530232
rs4078432
rs290833
rs62504403
rs2300774
rs7234058
rs8003149
rs7826267
rs56970041
rs1074800
All − MR Egger
All − IVW(MRE)
−4
−2
0
2
4
MR effect size for
'Gut microbiota abundance (family Erysipelotrichaceae id.2149)' on 'Extreme obesity with alveolar hypoventilation'

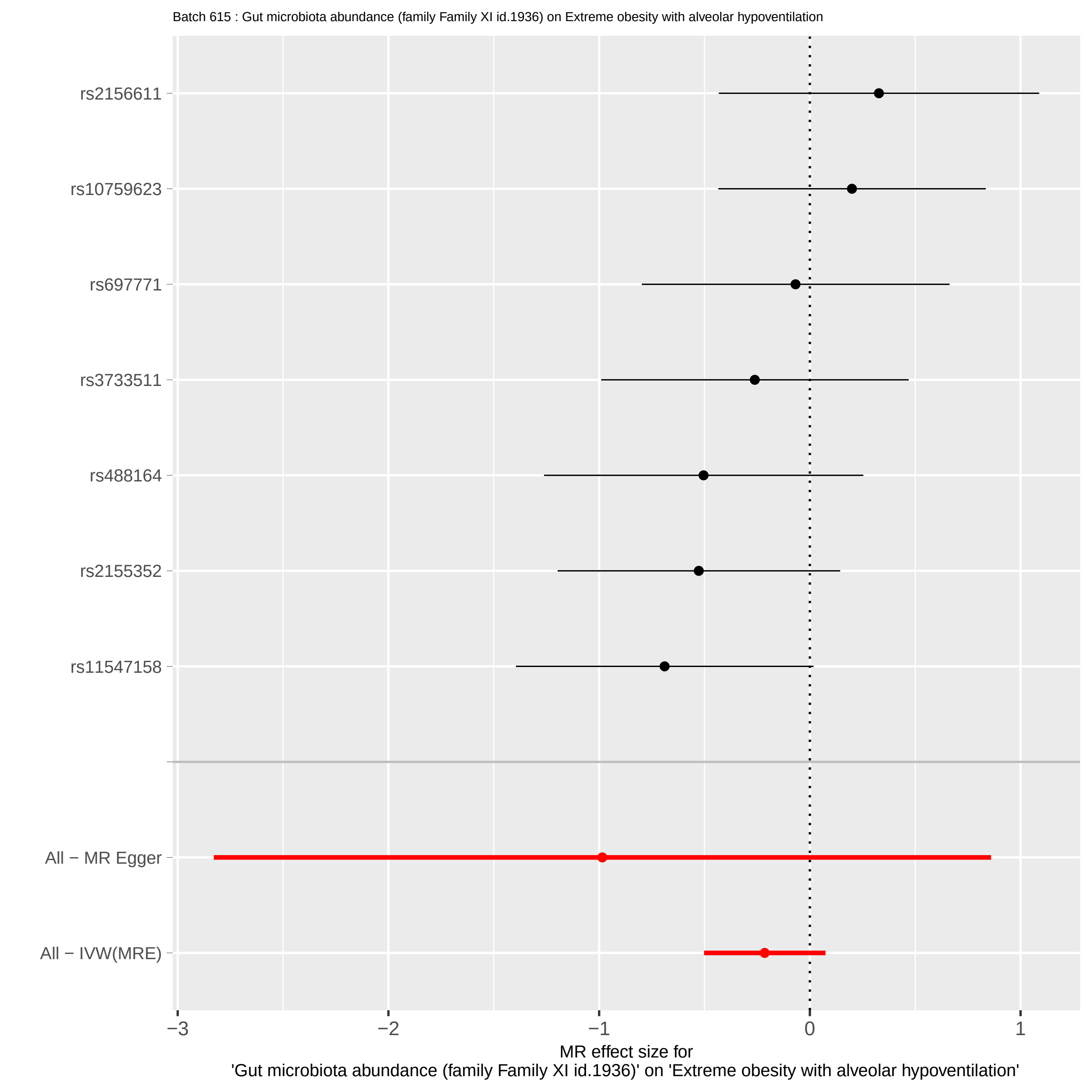

Batch 615 : Gut microbiota abundance (family Family XI id.1936) on Extreme obesity with alveolar hypoventilation
rs2156611
rs10759623
rs697771
rs3733511
rs488164
rs2155352
rs11547158
All − MR Egger
All − IVW(MRE)
−3
−2
−1
0
1
MR effect size for
'Gut microbiota abundance (family Family XI id.1936)' on 'Extreme obesity with alveolar hypoventilation'

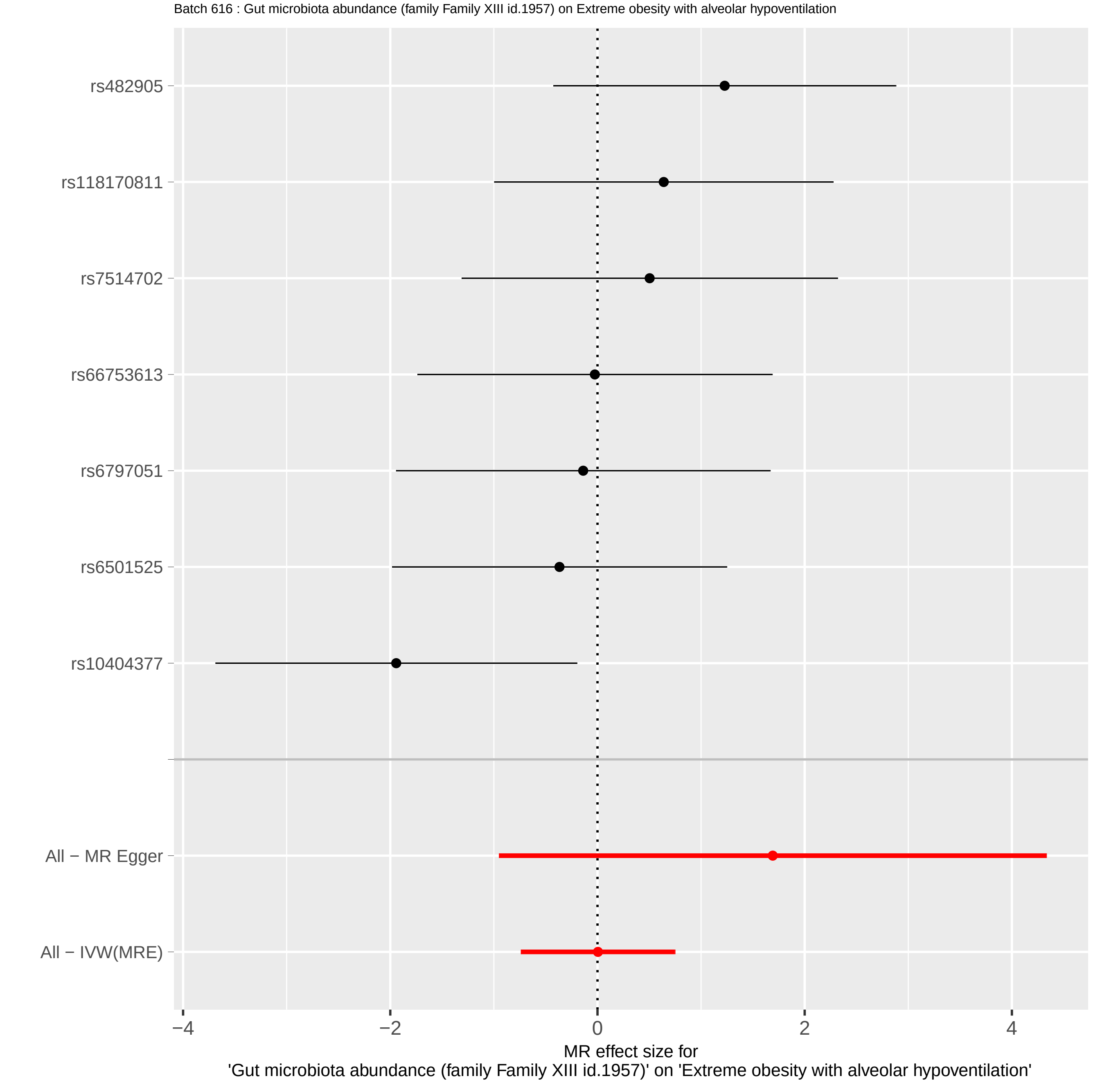
Batch 616 : Gut microbiota abundance (family Family XIII id.1957) on Extreme obesity with alveolar hypoventilation
rs482905
rs118170811
rs7514702
rs66753613
rs6797051
rs6501525
rs10404377
All − MR Egger
All − IVW(MRE)
−4
−2
0
2
4
MR effect size for
'Gut microbiota abundance (family Family XIII id.1957)' on 'Extreme obesity with alveolar hypoventilation'

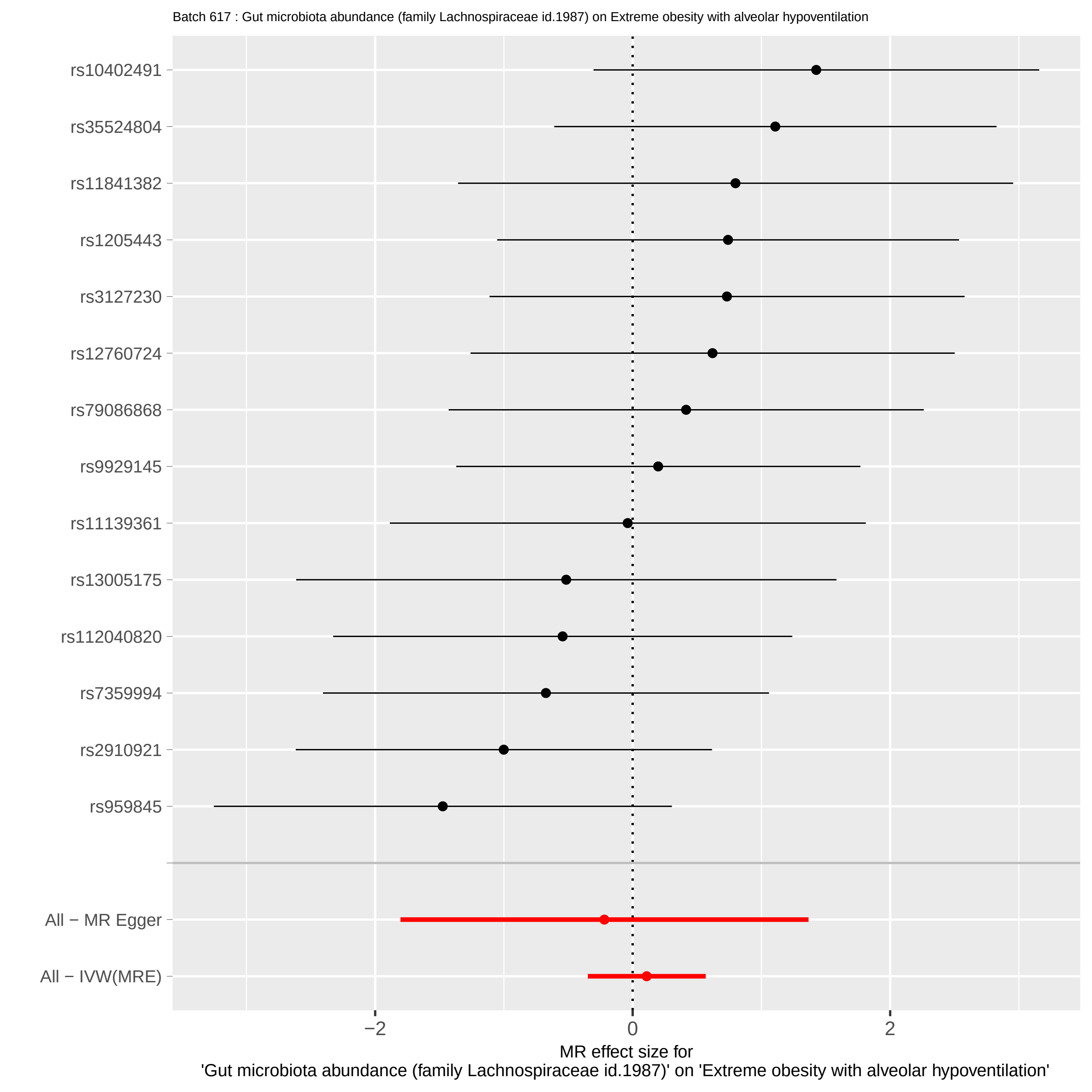

Batch 617 : Gut microbiota abundance (family Lachnospiraceae id.1987) on Extreme obesity with alveolar hypoventilation
rs10402491
rs35524804
rs11841382
rs1205443
rs3127230
rs12760724
rs79086868
rs9929145
rs11139361
rs13005175
rs112040820
rs7359994
rs2910921
rs959845
All − MR Egger
All − IVW(MRE)
−2
0
2
MR effect size for
'Gut microbiota abundance (family Lachnospiraceae id.1987)' on 'Extreme obesity with alveolar hypoventilation'

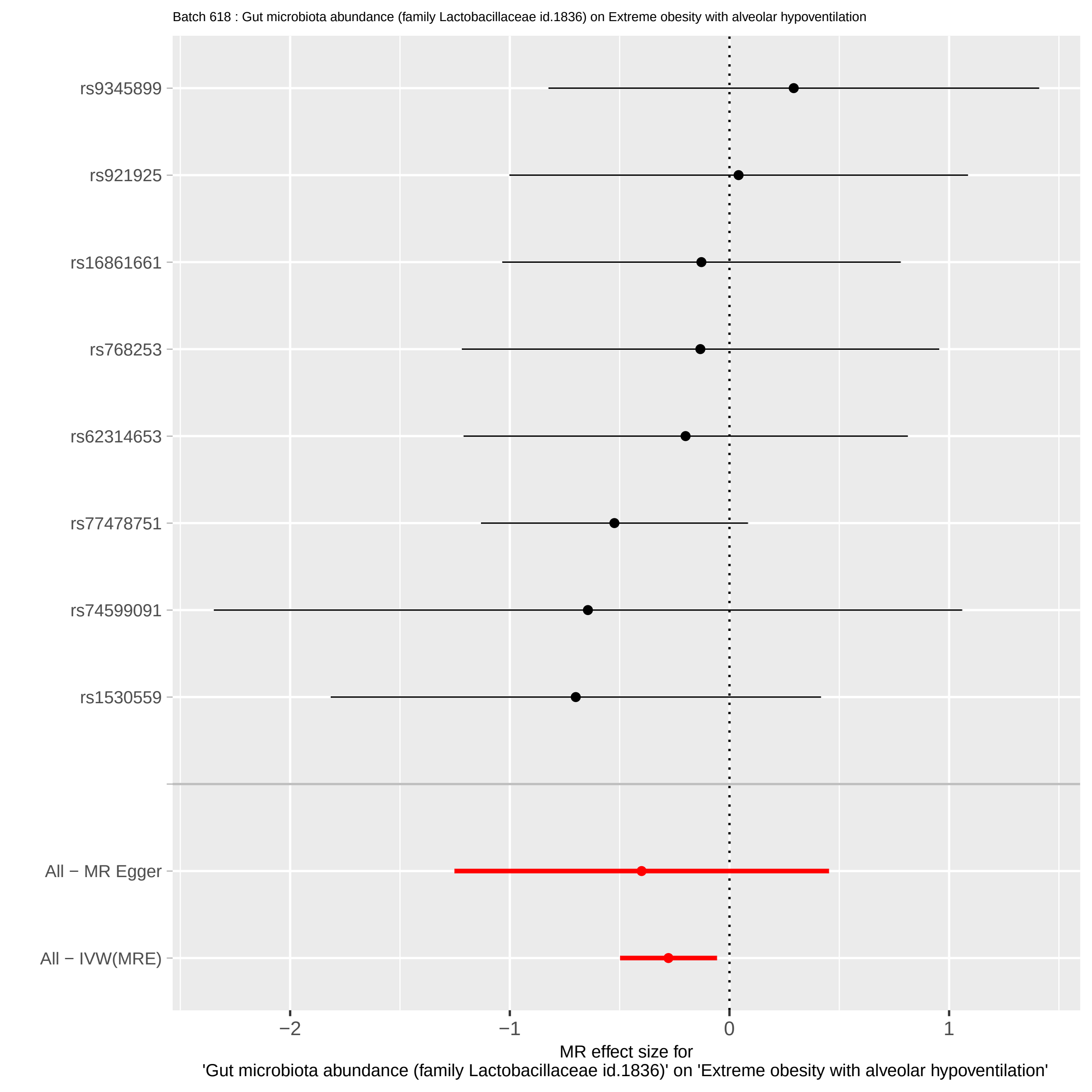

Batch 618 : Gut microbiota abundance (family Lactobacillaceae id.1836) on Extreme obesity with alveolar hypoventilation
rs9345899
rs921925
rs16861661
rs768253
rs62314653
rs77478751
rs74599091
rs1530559
All − MR Egger
All − IVW(MRE)
−2
−1
0
1
MR effect size for
'Gut microbiota abundance (family Lactobacillaceae id.1836)' on 'Extreme obesity with alveolar hypoventilation'

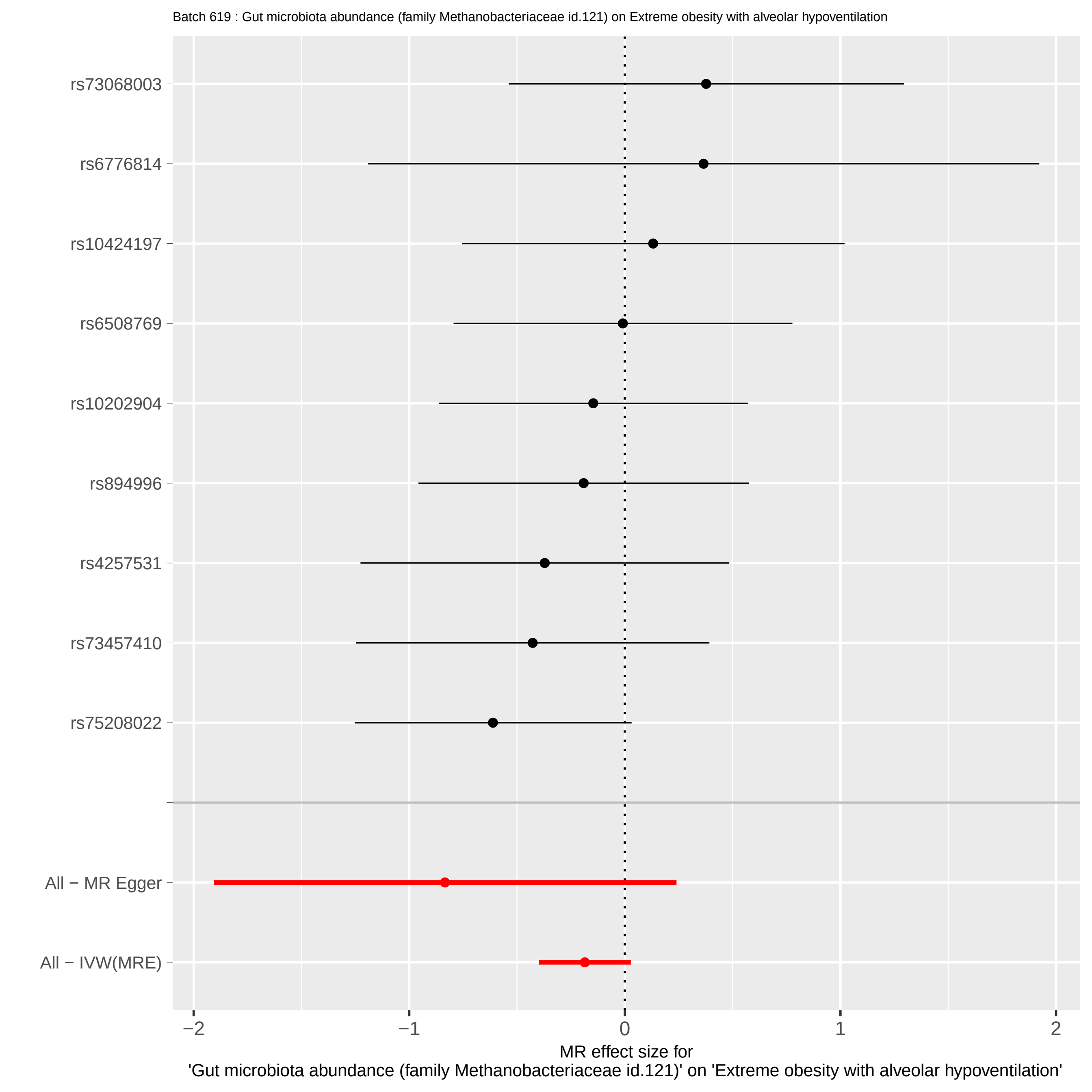

Batch 619 : Gut microbiota abundance (family Methanobacteriaceae id.121) on Extreme obesity with alveolar hypoventilation
rs73068003
rs6776814
rs10424197
rs6508769
rs10202904
rs894996
rs4257531
rs73457410
rs75208022
All − MR Egger
All − IVW(MRE)
−2
−1
0
1
2
MR effect size for
'Gut microbiota abundance (family Methanobacteriaceae id.121)' on 'Extreme obesity with alveolar hypoventilation'

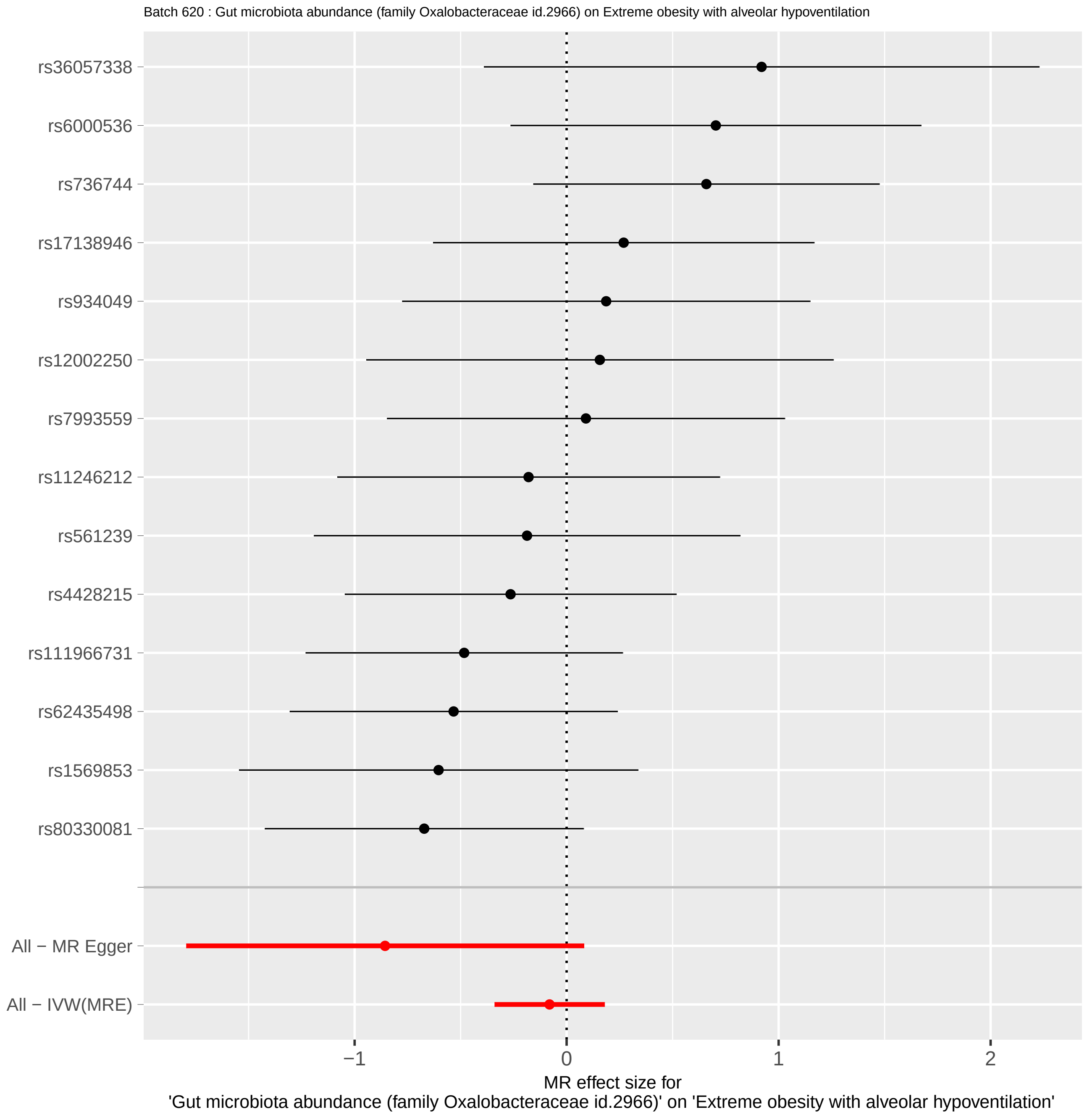

Batch 620 : Gut microbiota abundance (family Oxalobacteraceae id.2966) on Extreme obesity with alveolar hypoventilation
rs36057338
rs6000536
rs736744
rs17138946
rs934049
rs12002250
rs7993559
rs11246212
rs561239
rs4428215
rs111966731
rs62435498
rs1569853
rs80330081
All − MR Egger
All − IVW(MRE)
−1
0
1
2
MR effect size for
'Gut microbiota abundance (family Oxalobacteraceae id.2966)' on 'Extreme obesity with alveolar hypoventilation'

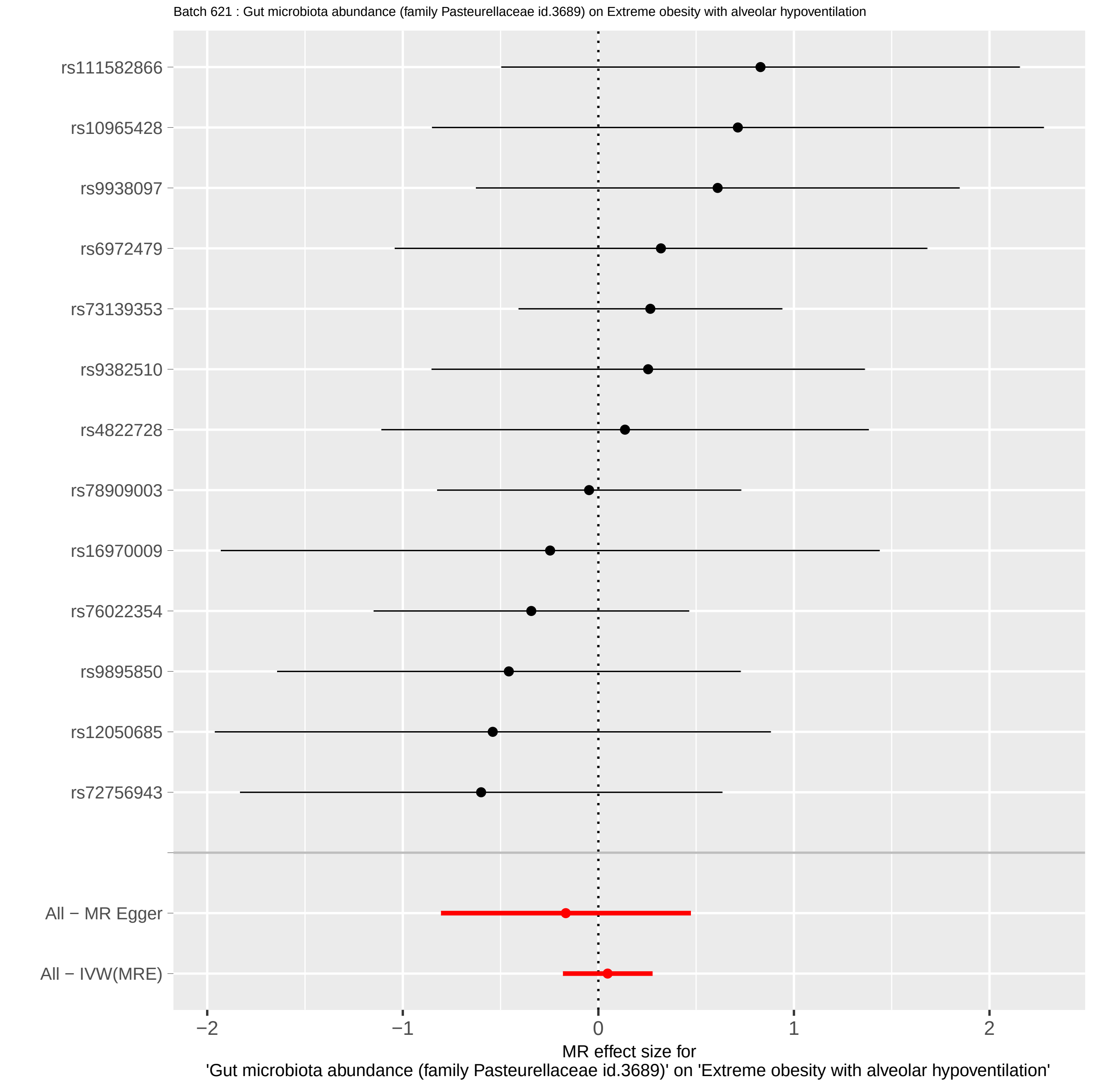

Batch 621 : Gut microbiota abundance (family Pasteurellaceae id.3689) on Extreme obesity with alveolar hypoventilation
rs111582866
rs10965428
rs9938097
rs6972479
rs73139353
rs9382510
rs4822728
rs78909003
rs16970009
rs76022354
rs9895850
rs12050685
rs72756943
All − MR Egger
All − IVW(MRE)
−2
−1
0
1
2
MR effect size for
'Gut microbiota abundance (family Pasteurellaceae id.3689)' on 'Extreme obesity with alveolar hypoventilation'

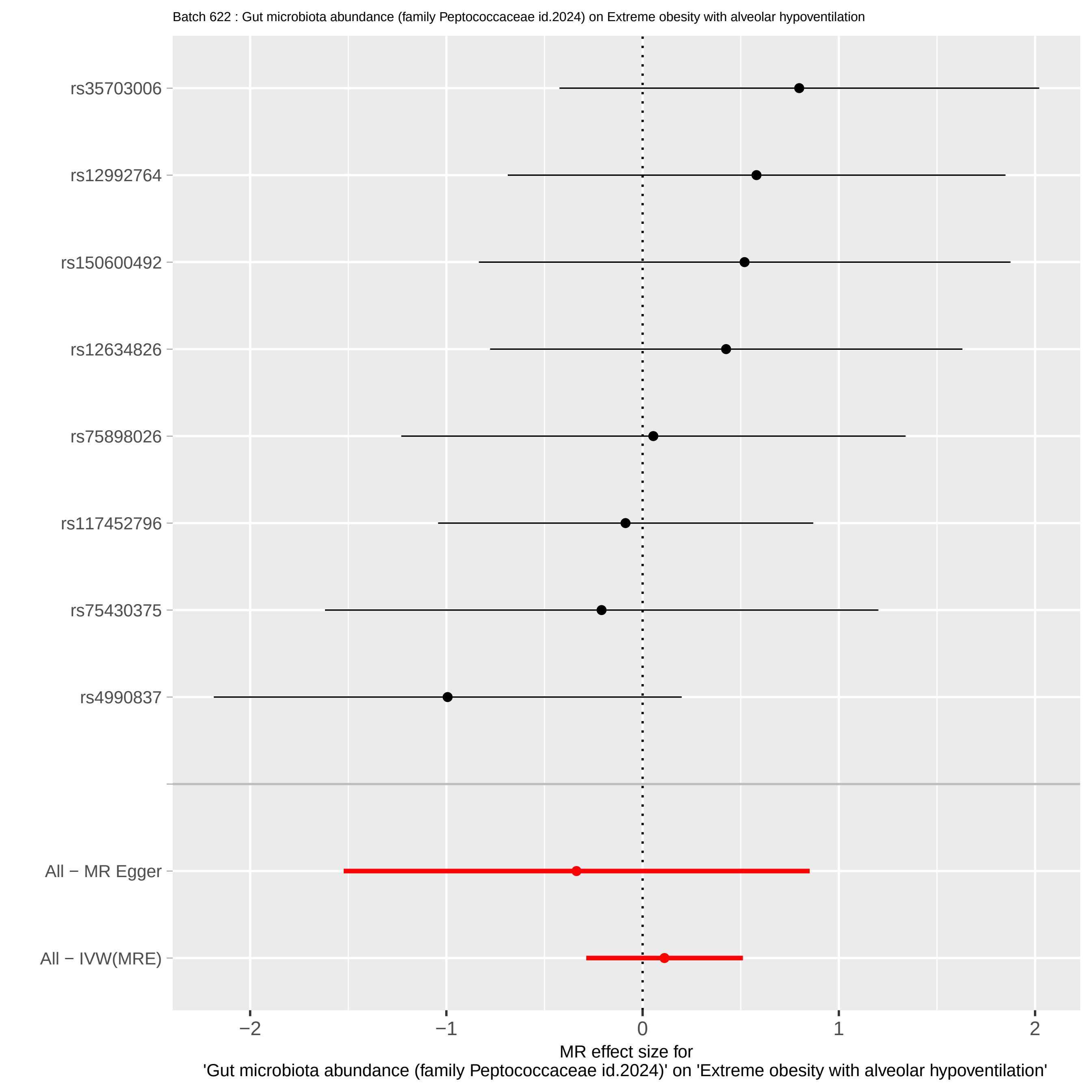

Batch 622 : Gut microbiota abundance (family Peptococcaceae id.2024) on Extreme obesity with alveolar hypoventilation
rs35703006
rs12992764
rs150600492
rs12634826
rs75898026
rs117452796
rs75430375
rs4990837
All − MR Egger
All − IVW(MRE)
−2
−1
0
1
2
MR effect size for
'Gut microbiota abundance (family Peptococcaceae id.2024)' on 'Extreme obesity with alveolar hypoventilation'

Batch 623 : Gut microbiota abundance (family Peptostreptococcaceae id.2042) on Extreme obesity with alveolar hypoventilation

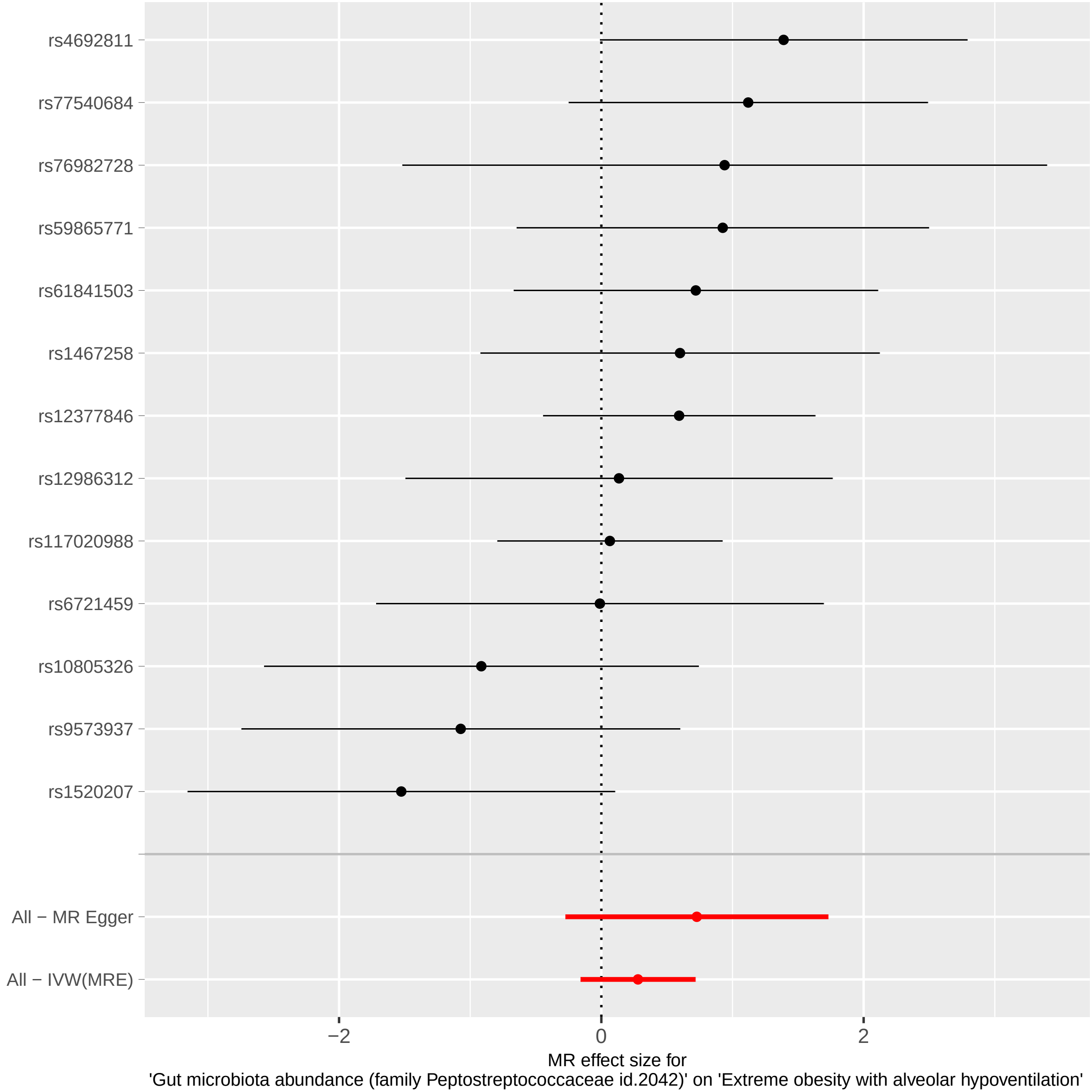

Batch 624 : Gut microbiota abundance (family Porphyromonadaceae id.943) on Extreme obesity with alveolar hypoventilation

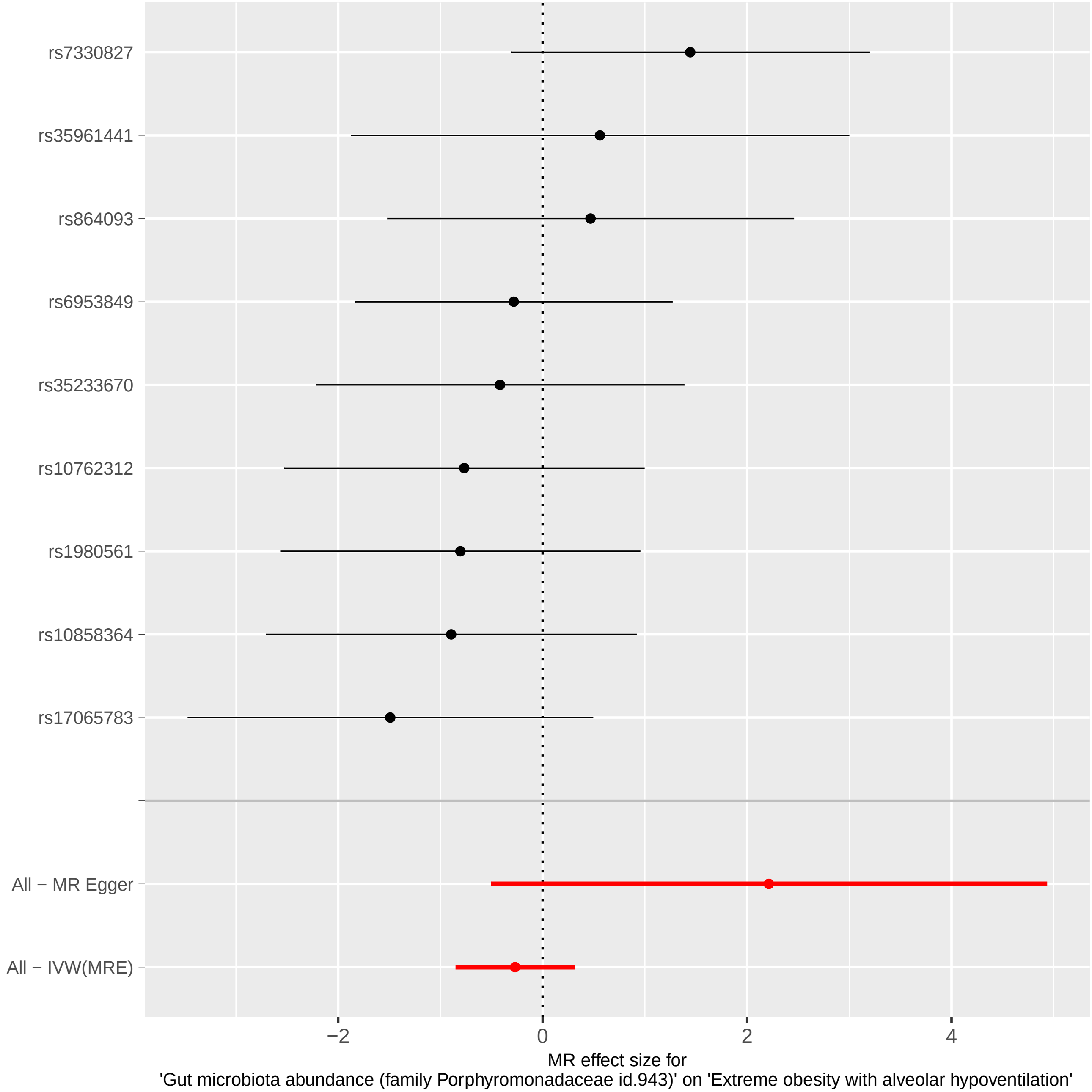

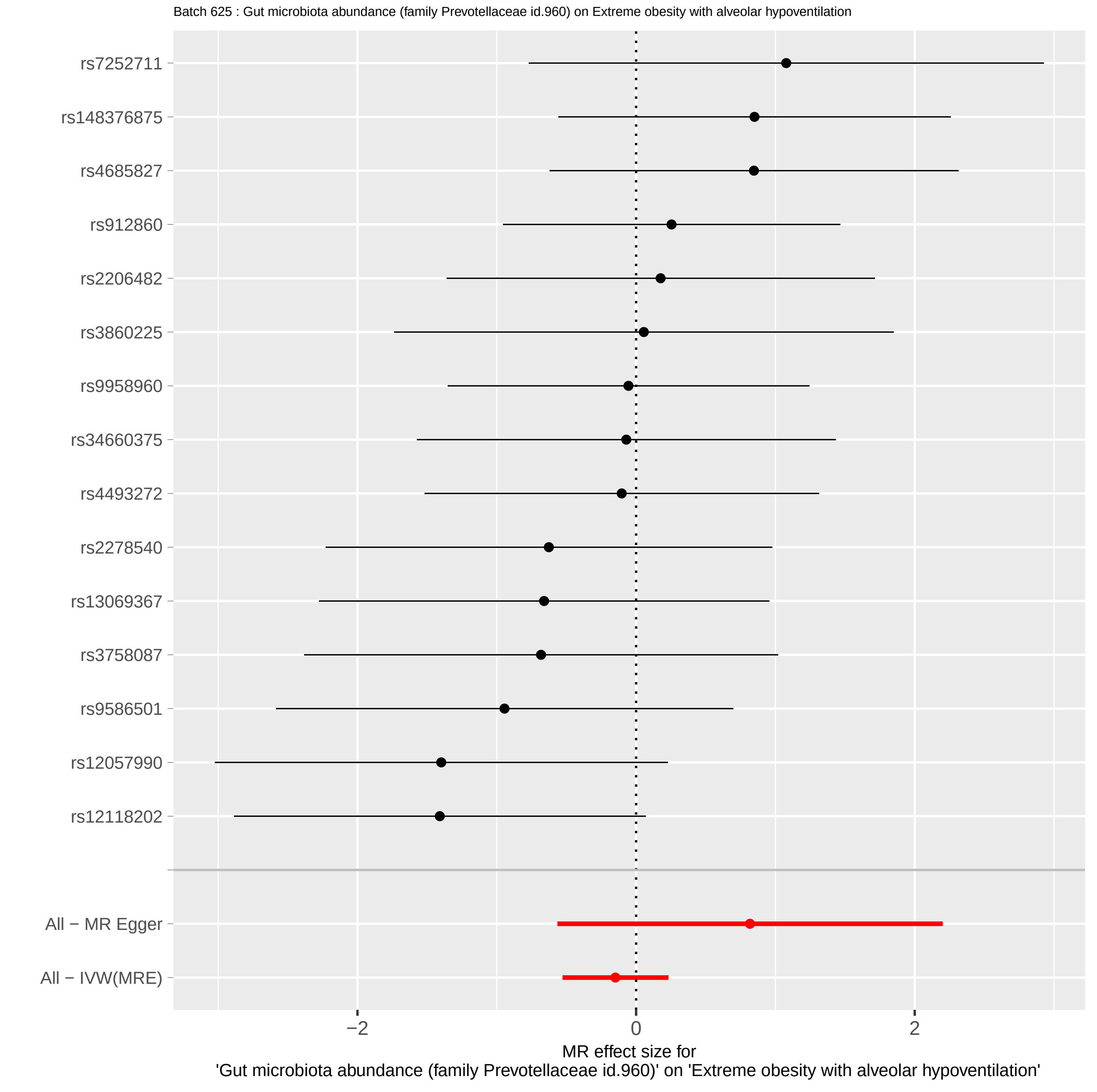

Batch 625 : Gut microbiota abundance (family Prevotellaceae id.960) on Extreme obesity with alveolar hypoventilation
rs7252711
rs148376875
rs4685827
rs912860
rs2206482
rs3860225
rs9958960
rs34660375
rs4493272
rs2278540
rs13069367
rs3758087
rs9586501
rs12057990
rs12118202
All − MR Egger
All − IVW(MRE)
−2
0
2
MR effect size for
'Gut microbiota abundance (family Prevotellaceae id.960)' on 'Extreme obesity with alveolar hypoventilation'

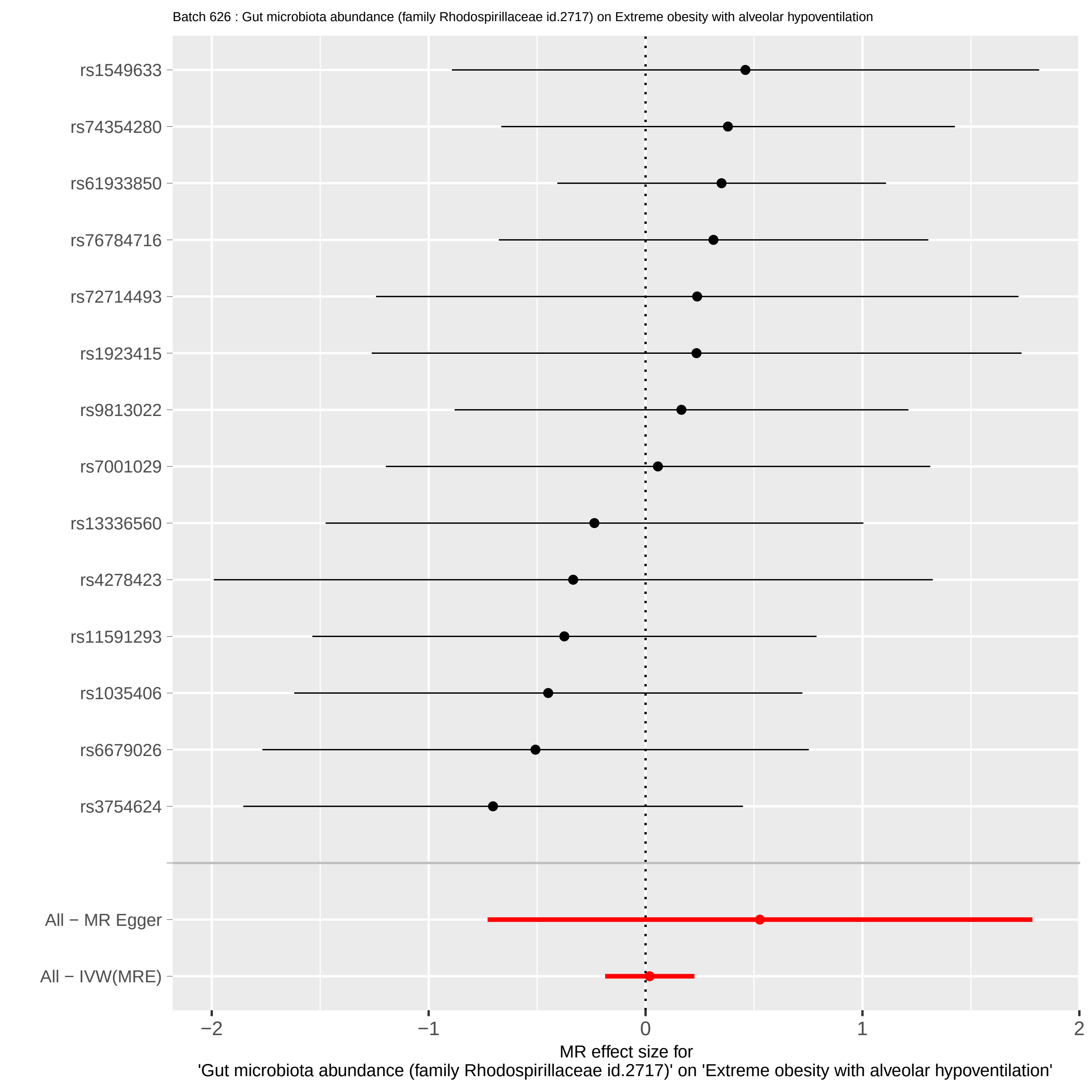

Batch 626 : Gut microbiota abundance (family Rhodospirillaceae id.2717) on Extreme obesity with alveolar hypoventilation
rs1549633
rs74354280
rs61933850
rs76784716
rs72714493
rs1923415
rs9813022
rs7001029
rs13336560
rs4278423
rs11591293
rs1035406
rs6679026
rs3754624
All − MR Egger
All − IVW(MRE)
−2
−1
0
1
2
MR effect size for
'Gut microbiota abundance (family Rhodospirillaceae id.2717)' on 'Extreme obesity with alveolar hypoventilation'

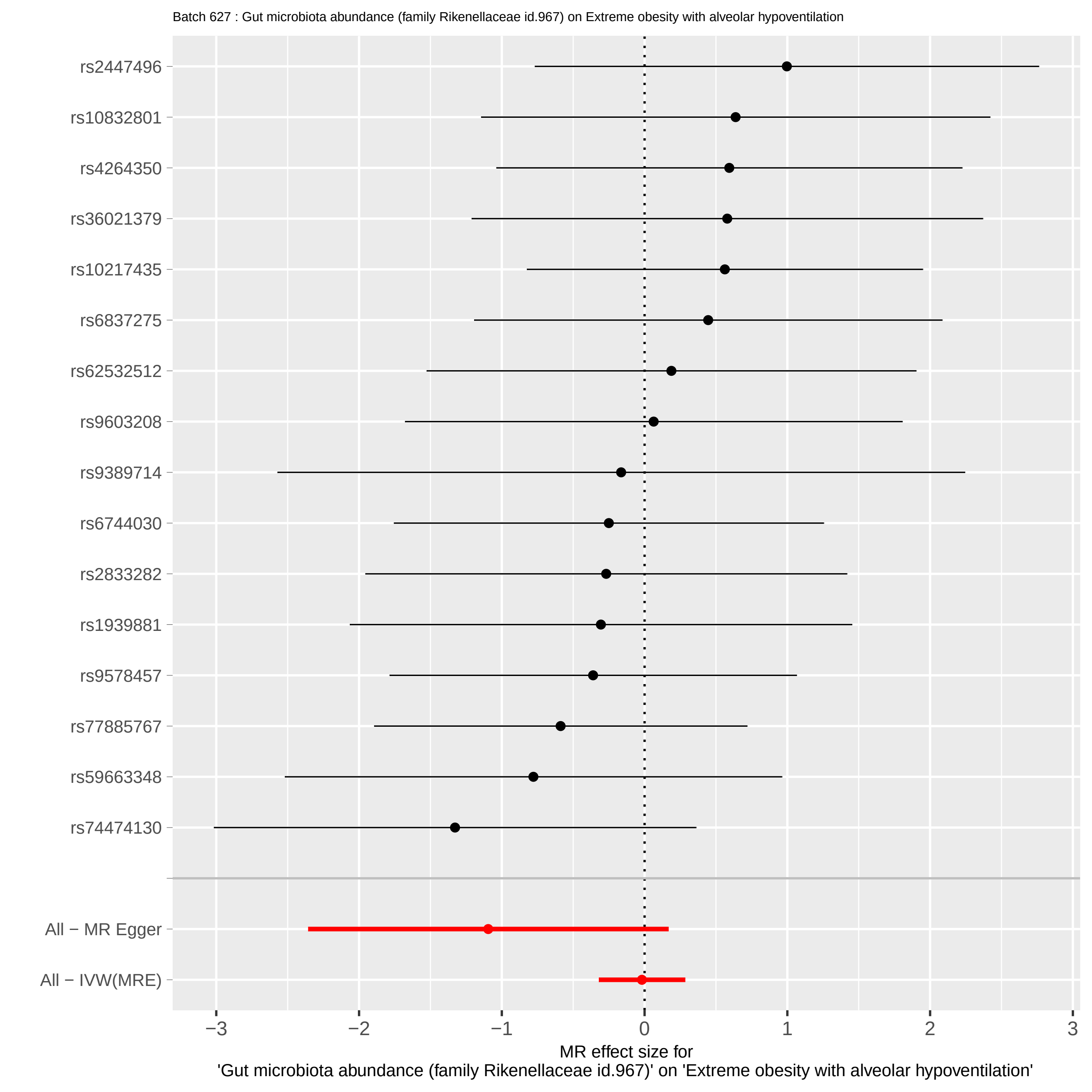
Batch 627 : Gut microbiota abundance (family Rikenellaceae id.967) on Extreme obesity with alveolar hypoventilation
rs2447496
rs10832801
rs4264350
rs36021379
rs10217435
rs6837275
rs62532512
rs9603208
rs9389714
rs6744030
rs2833282
rs1939881
rs9578457
rs77885767
rs59663348
rs74474130
All − MR Egger
All − IVW(MRE)
−3
−2
−1
0
1
2
3
MR effect size for
'Gut microbiota abundance (family Rikenellaceae id.967)' on 'Extreme obesity with alveolar hypoventilation'

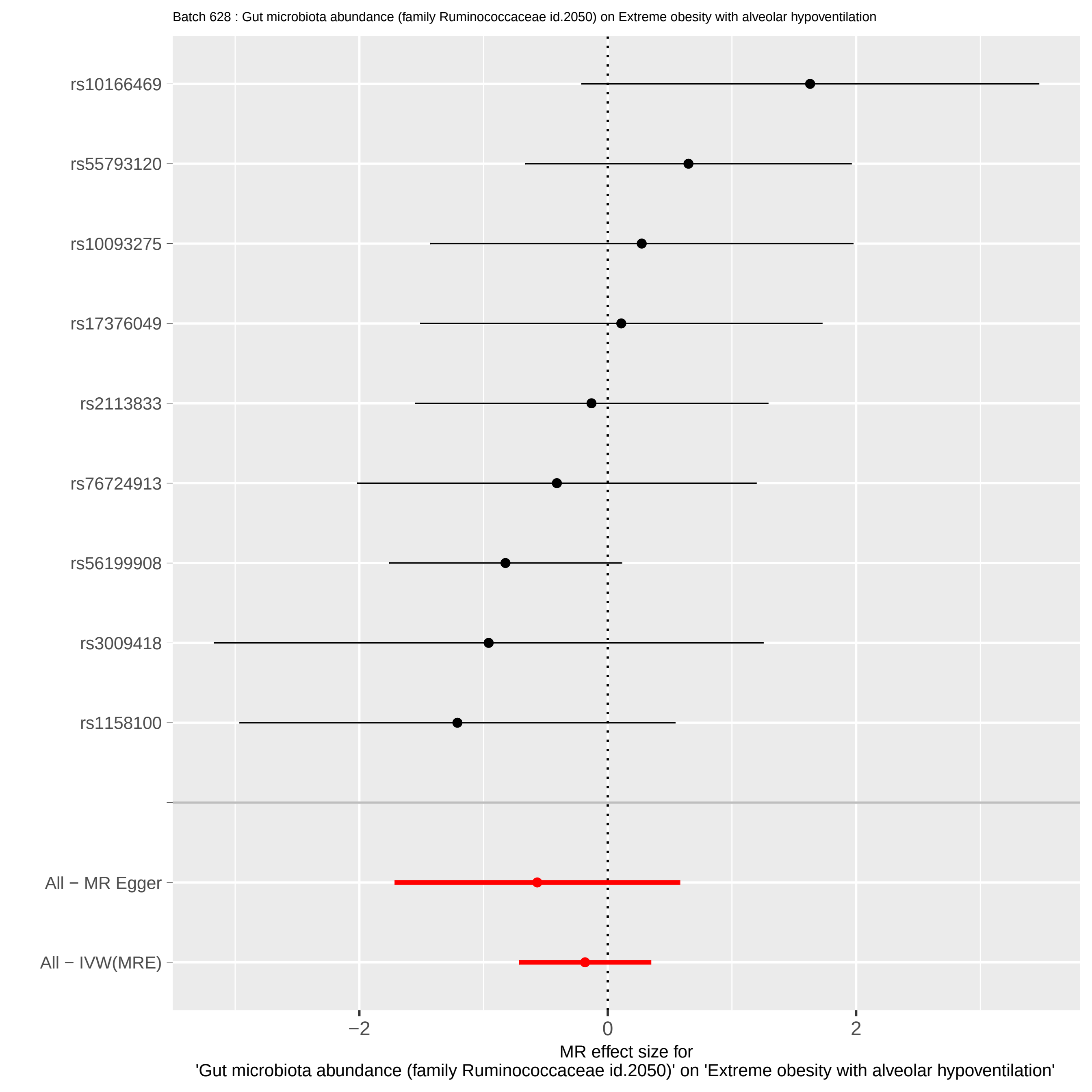
Batch 628 : Gut microbiota abundance (family Ruminococcaceae id.2050) on Extreme obesity with alveolar hypoventilation
rs10166469
rs55793120
rs10093275
rs17376049
rs2113833
rs76724913
rs56199908
rs3009418
rs1158100
All − MR Egger
All − IVW(MRE)
−2
0
2
MR effect size for
'Gut microbiota abundance (family Ruminococcaceae id.2050)' on 'Extreme obesity with alveolar hypoventilation'

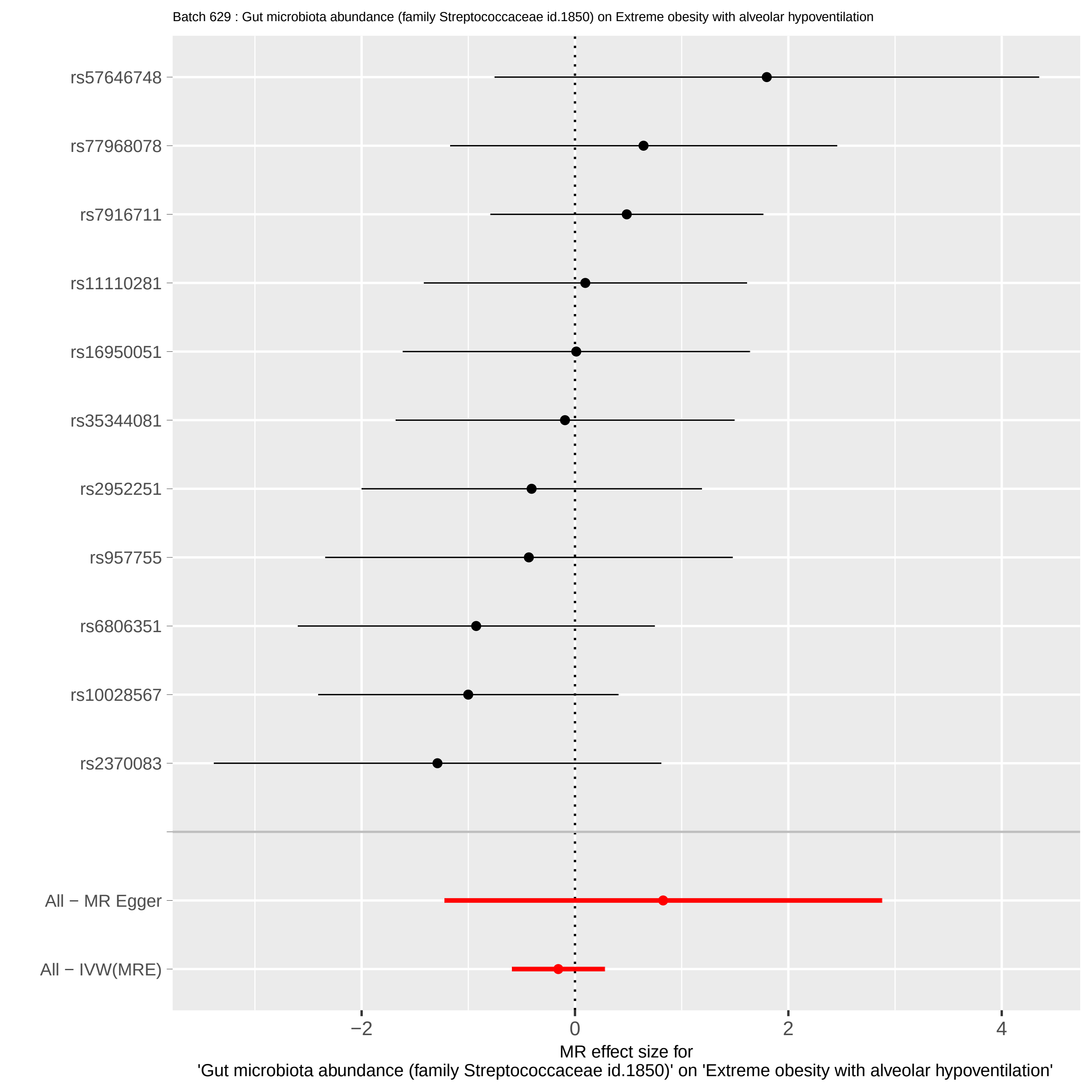

Batch 629 : Gut microbiota abundance (family Streptococcaceae id.1850) on Extreme obesity with alveolar hypoventilation
rs57646748
rs77968078
rs7916711
rs11110281
rs16950051
rs35344081
rs2952251
rs957755
rs6806351
rs10028567
rs2370083
All − MR Egger
All − IVW(MRE)
−2
0
2
4
MR effect size for
'Gut microbiota abundance (family Streptococcaceae id.1850)' on 'Extreme obesity with alveolar hypoventilation'

Batch 630 : Gut microbiota abundance (family Veillonellaceae id.2172) on Extreme obesity with alveolar hypoventilation

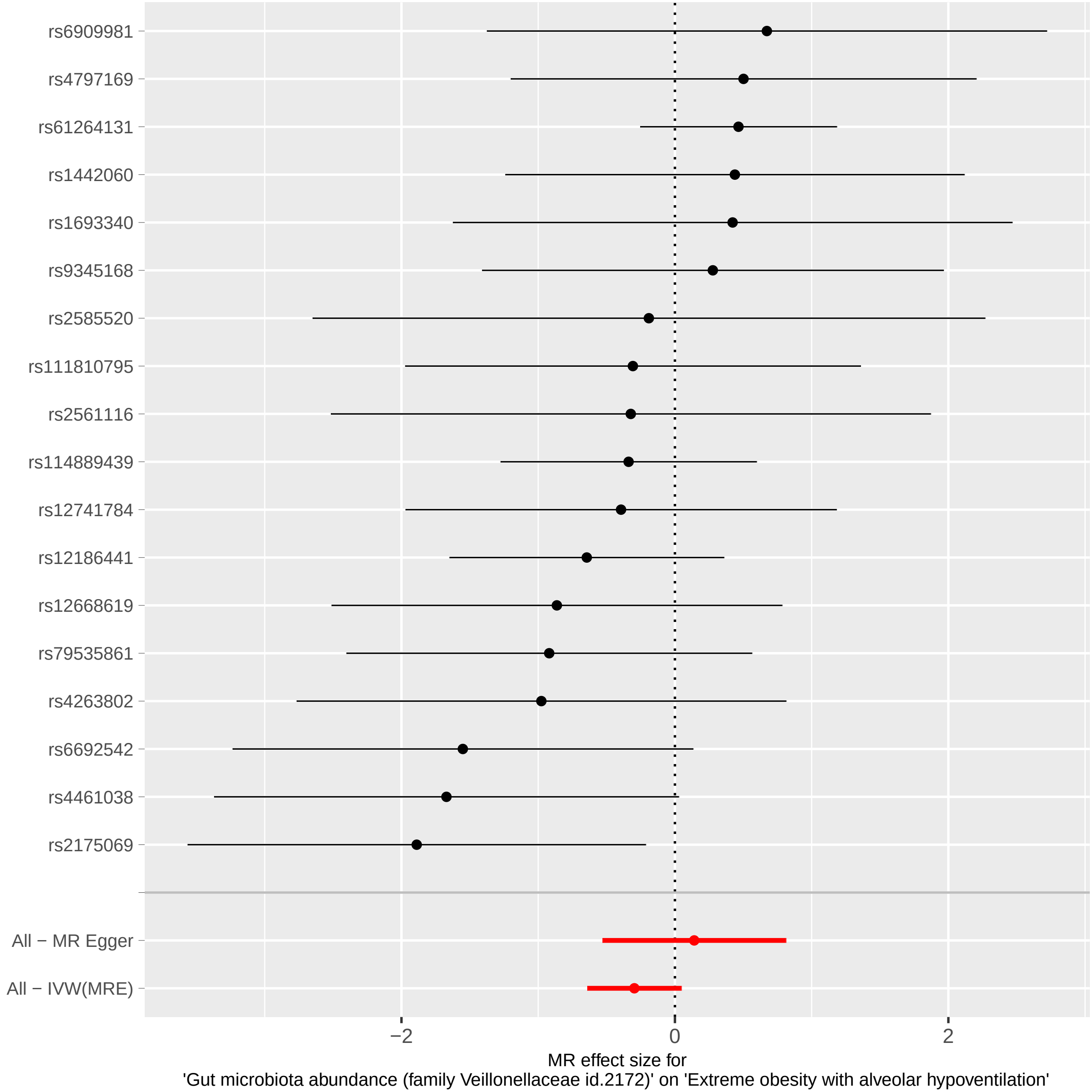

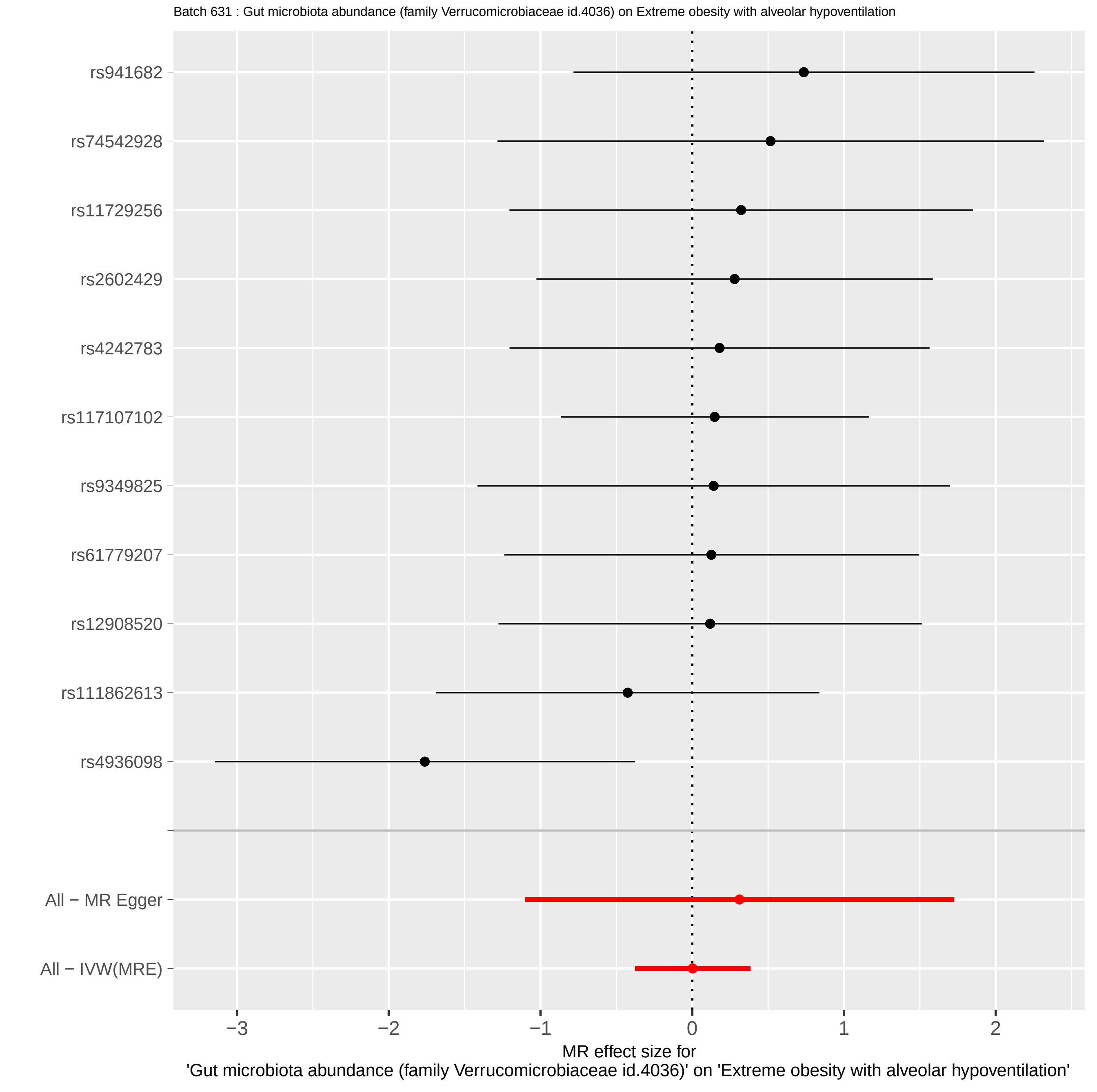

Batch 631 : Gut microbiota abundance (family Verrucomicrobiaceae id.4036) on Extreme obesity with alveolar hypoventilation
rs941682
rs74542928
rs11729256
rs2602429
rs4242783
rs117107102
rs9349825
rs61779207
rs12908520
rs111862613
rs4936098
All − MR Egger
All − IVW(MRE)
−3
−2
−1
0
1
2
MR effect size for
'Gut microbiota abundance (family Verrucomicrobiaceae id.4036)' on 'Extreme obesity with alveolar hypoventilation'

Batch 632 : Gut microbiota abundance (family Victivallaceae id.2255) on Extreme obesity with alveolar hypoventilation

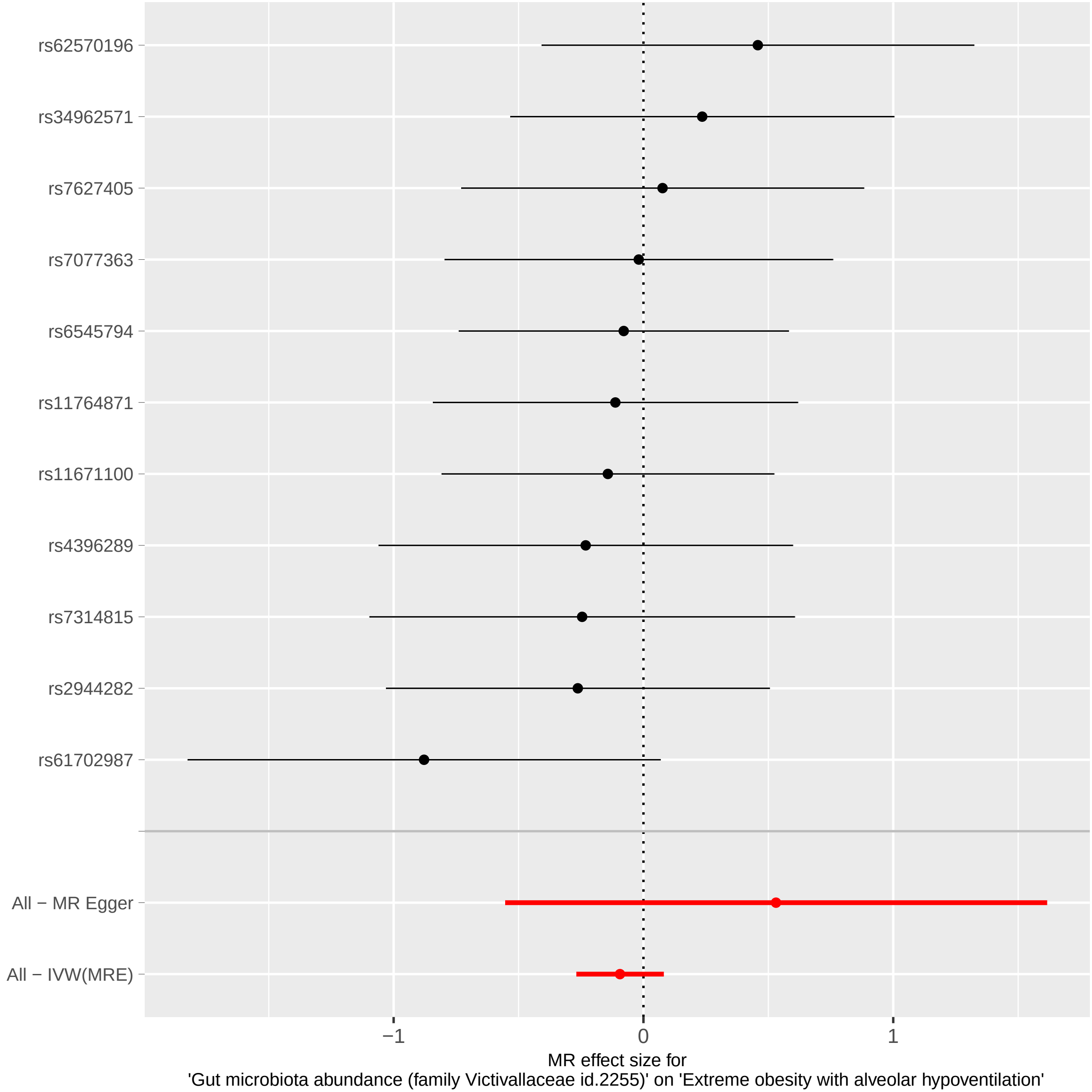

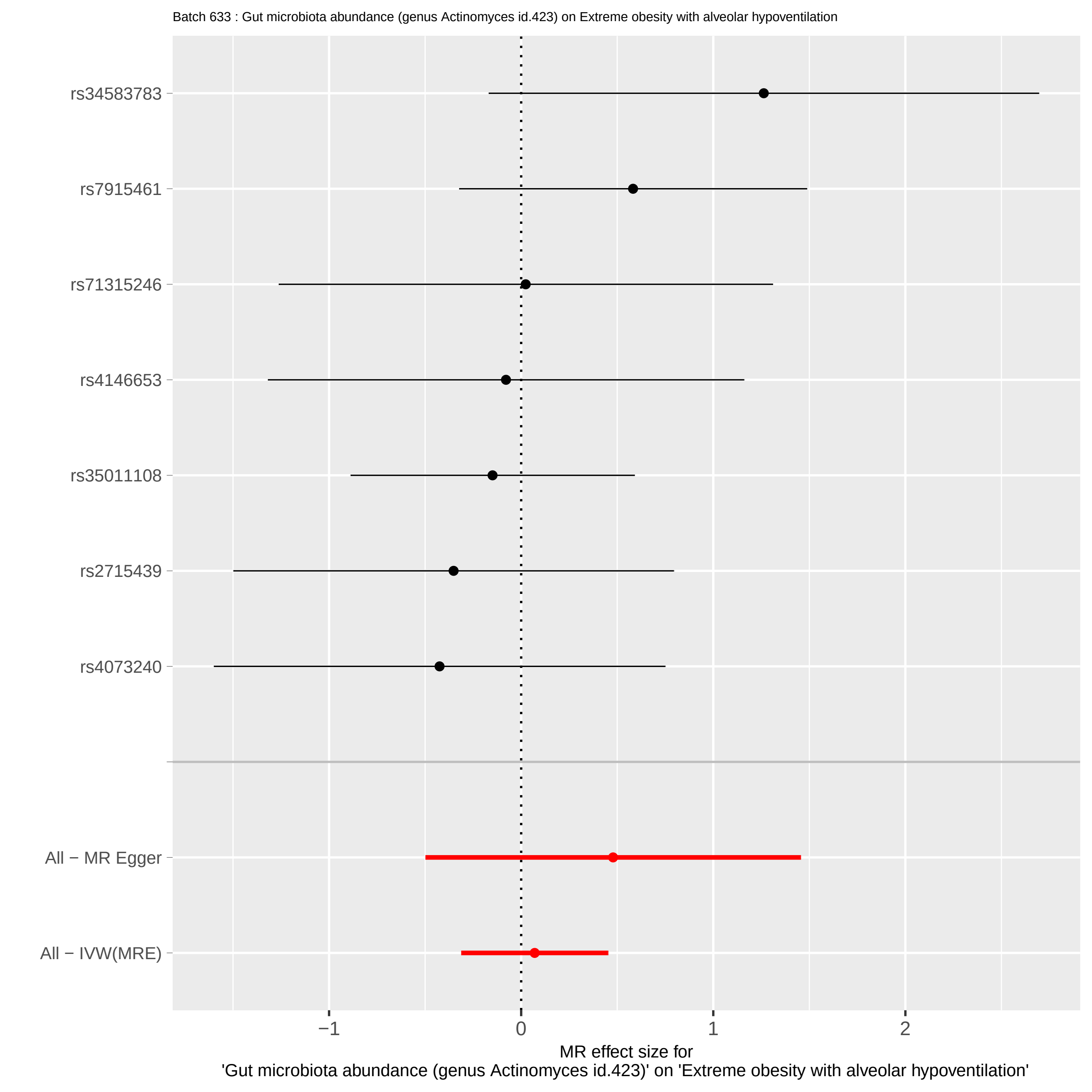
Batch 633 : Gut microbiota abundance (genus Actinomyces id.423) on Extreme obesity with alveolar hypoventilation
rs34583783
rs7915461
rs71315246
rs4146653
rs35011108
rs2715439
rs4073240
All − MR Egger
All − IVW(MRE)
−1
0
1
2
MR effect size for
'Gut microbiota abundance (genus Actinomyces id.423)' on 'Extreme obesity with alveolar hypoventilation'

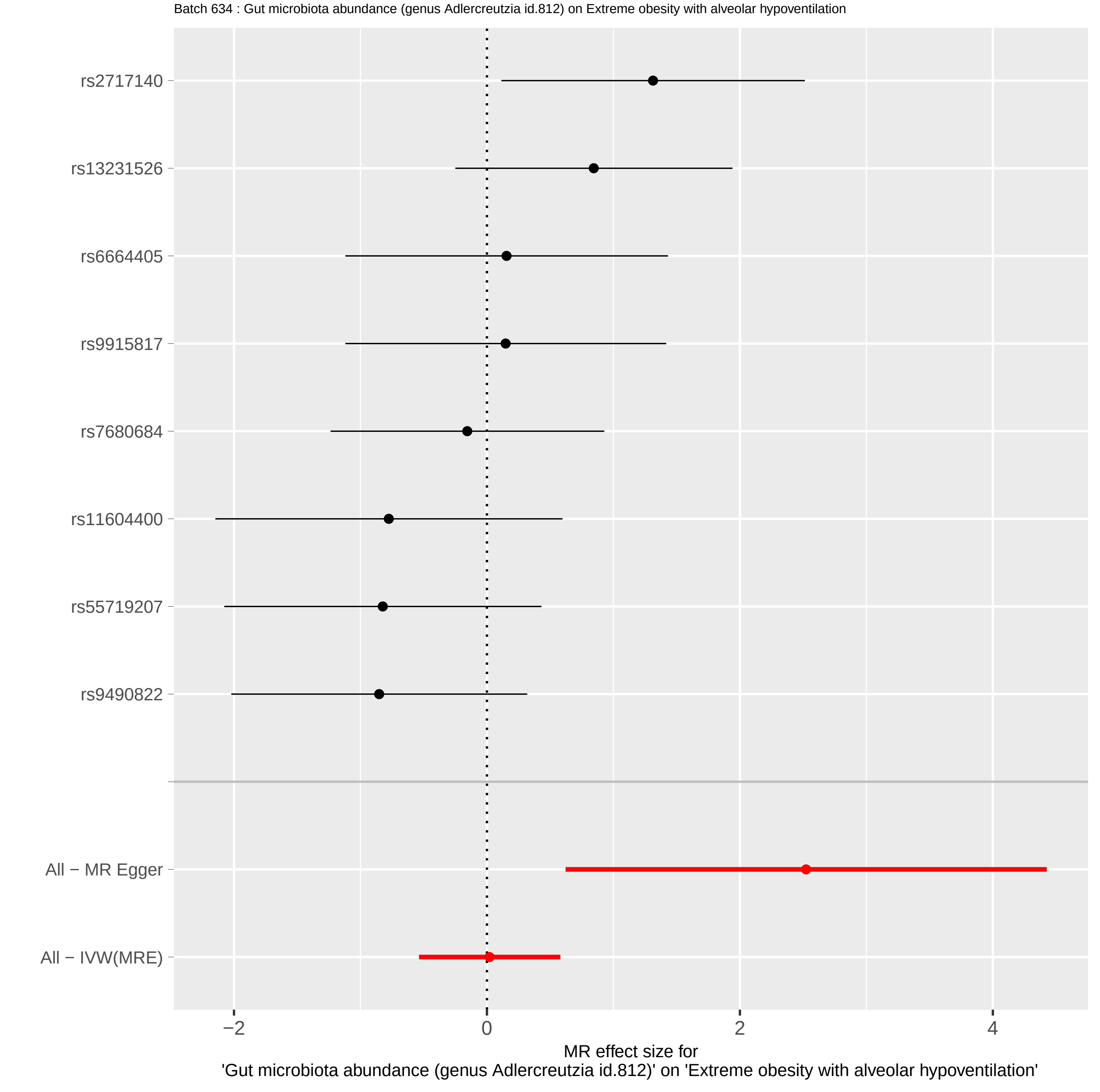
Batch 634 : Gut microbiota abundance (genus Adlercreutzia id.812) on Extreme obesity with alveolar hypoventilation
rs2717140
rs13231526
rs6664405
rs9915817
rs7680684
rs11604400
rs55719207
rs9490822
All − MR Egger
All − IVW(MRE)
−2
0
2
4
MR effect size for
'Gut microbiota abundance (genus Adlercreutzia id.812)' on 'Extreme obesity with alveolar hypoventilation'

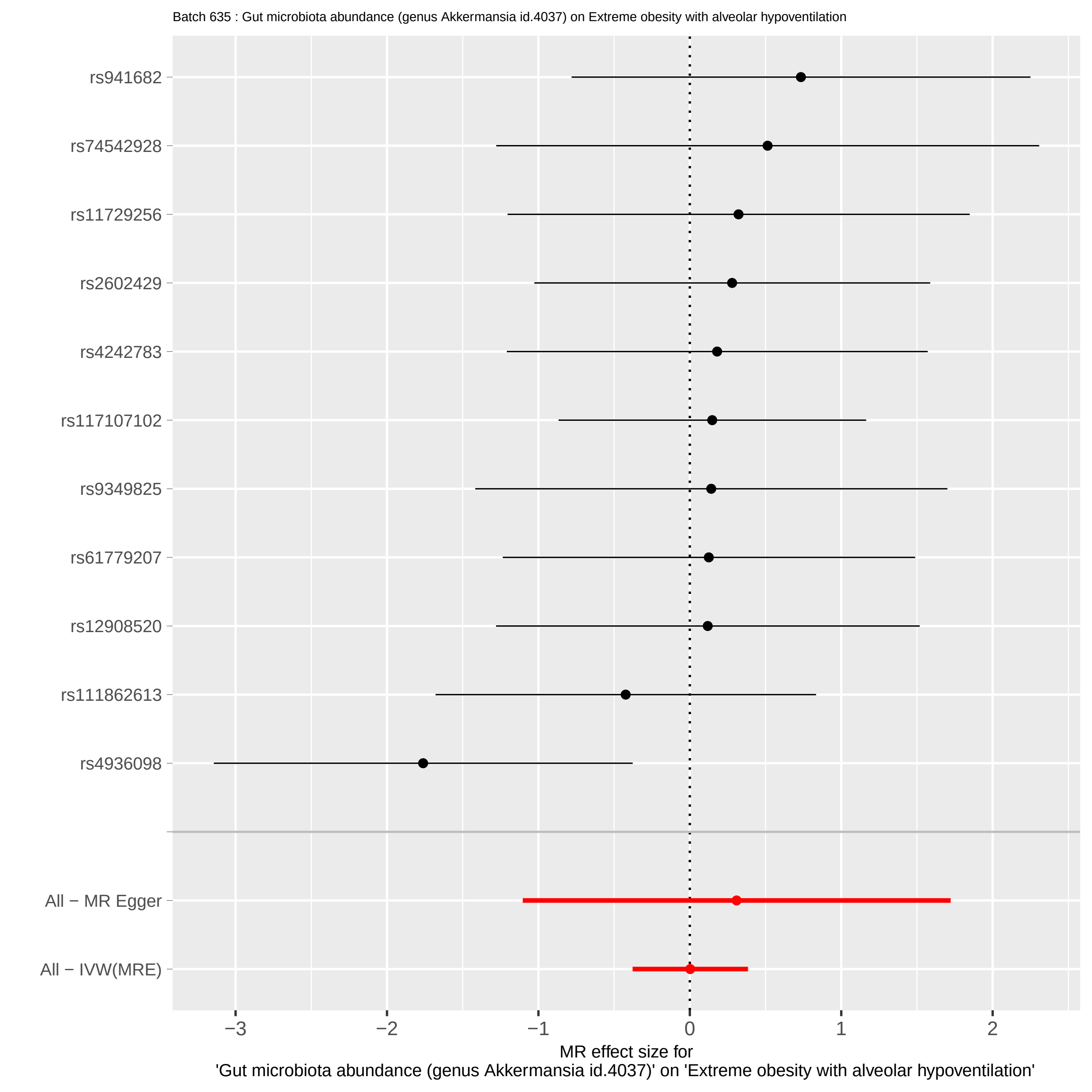

Batch 635 : Gut microbiota abundance (genus Akkermansia id.4037) on Extreme obesity with alveolar hypoventilation
rs941682
rs74542928
rs11729256
rs2602429
rs4242783
rs117107102
rs9349825
rs61779207
rs12908520
rs111862613
rs4936098
All − MR Egger
All − IVW(MRE)
−3
−2
−1
0
1
2
MR effect size for
'Gut microbiota abundance (genus Akkermansia id.4037)' on 'Extreme obesity with alveolar hypoventilation'

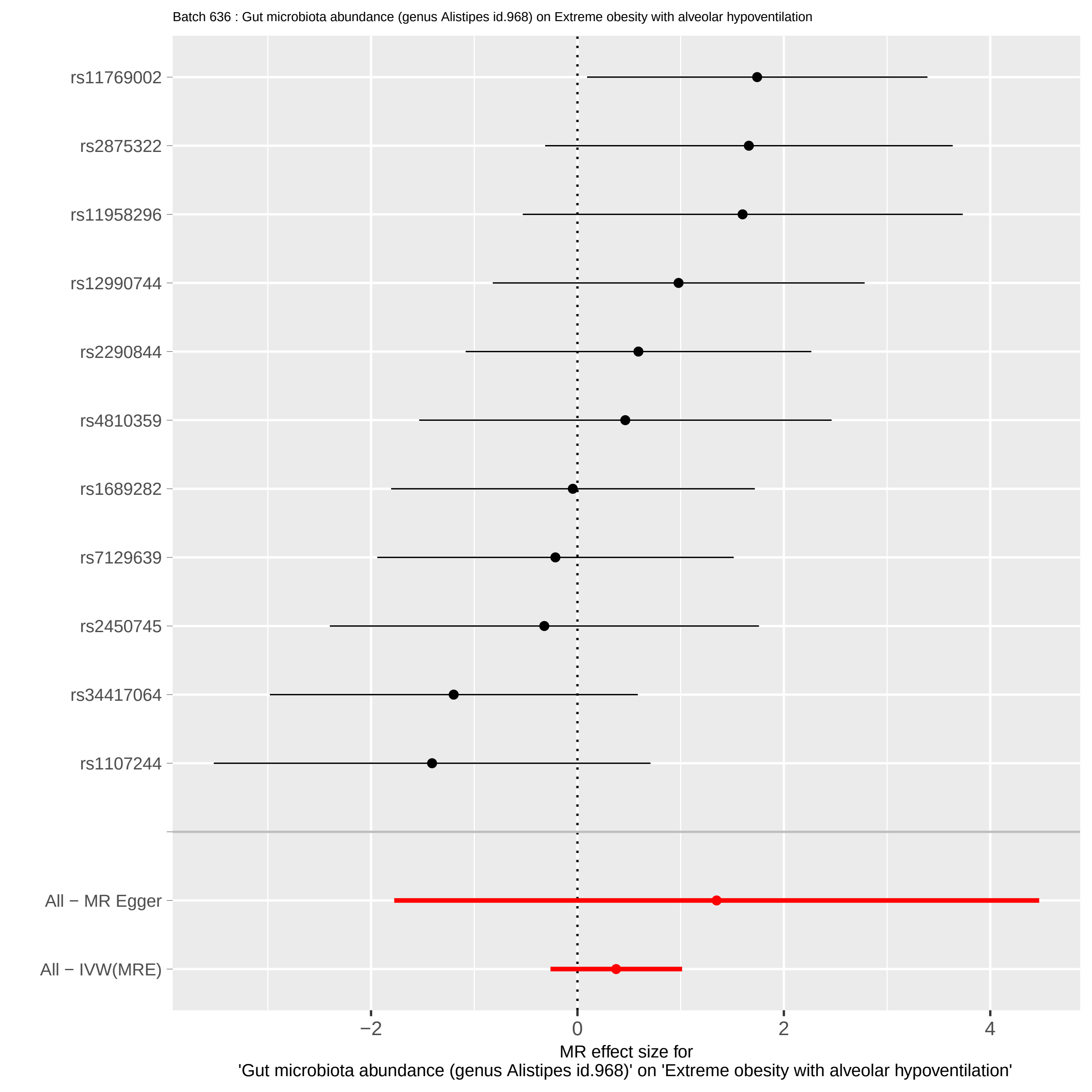
Batch 636 : Gut microbiota abundance (genus Alistipes id.968) on Extreme obesity with alveolar hypoventilation
rs11769002
rs2875322
rs11958296
rs12990744
rs2290844
rs4810359
rs1689282
rs7129639
rs2450745
rs34417064
rs1107244
All − MR Egger
All − IVW(MRE)
−2
0
2
4
MR effect size for
'Gut microbiota abundance (genus Alistipes id.968)' on 'Extreme obesity with alveolar hypoventilation'

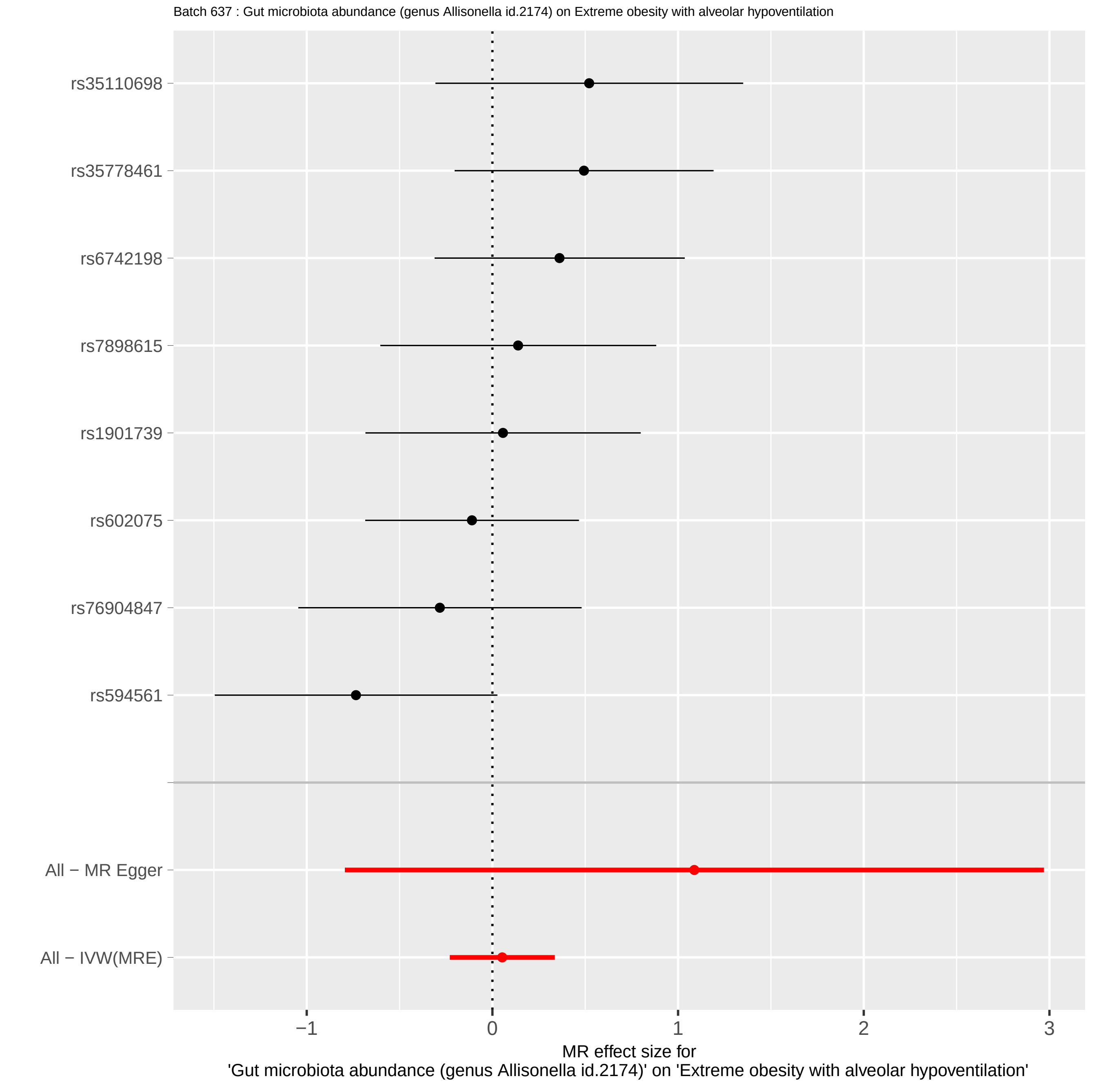

Batch 637 : Gut microbiota abundance (genus Allisonella id.2174) on Extreme obesity with alveolar hypoventilation
rs35110698
rs35778461
rs6742198
rs7898615
rs1901739
rs602075
rs76904847
rs594561
All − MR Egger
All − IVW(MRE)
−1
0
1
2
3
MR effect size for
'Gut microbiota abundance (genus Allisonella id.2174)' on 'Extreme obesity with alveolar hypoventilation'

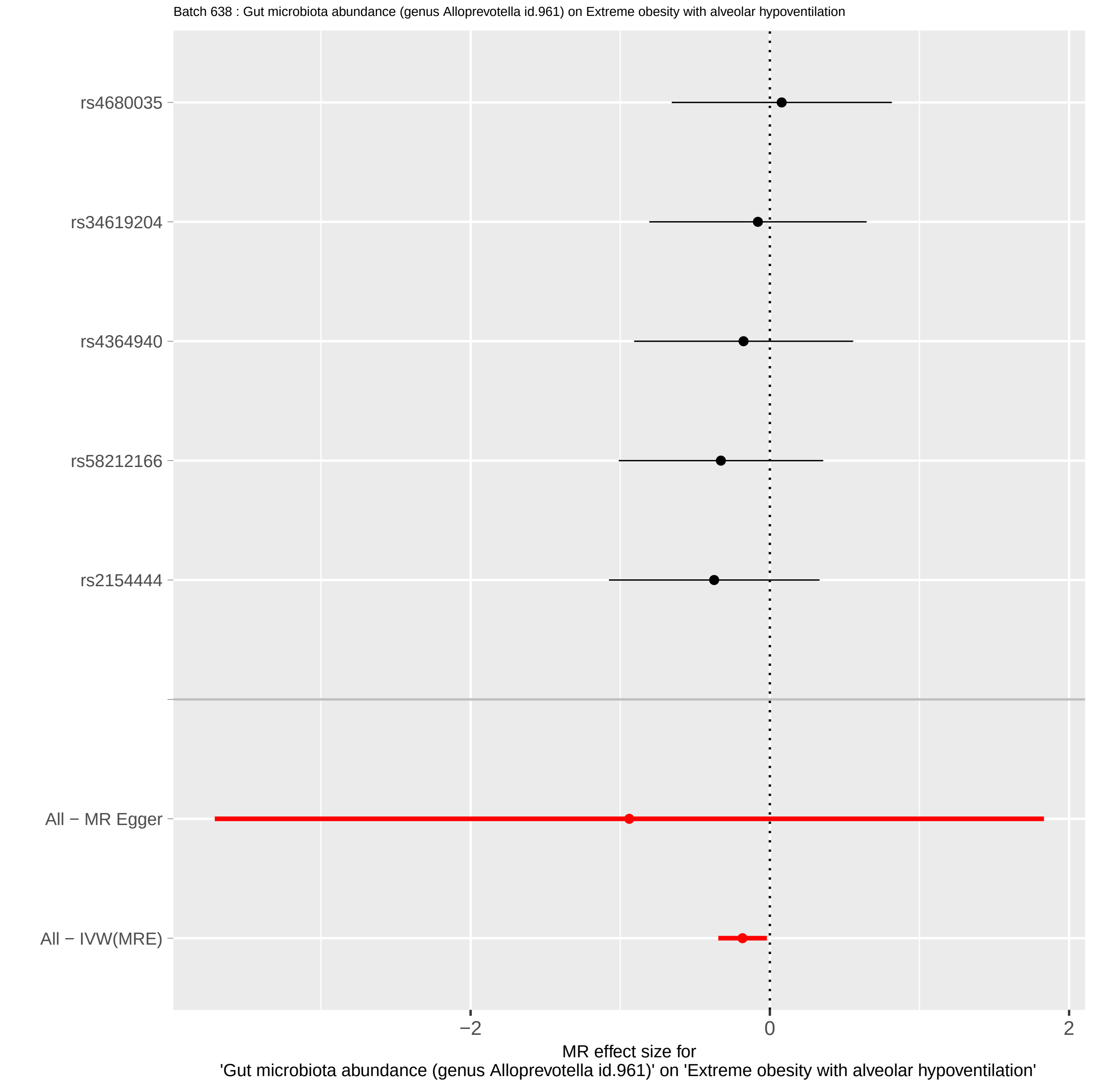
Batch 638 : Gut microbiota abundance (genus Alloprevotella id.961) on Extreme obesity with alveolar hypoventilation
rs4680035
rs34619204
rs4364940
rs58212166
rs2154444
All − MR Egger
All − IVW(MRE)
−2
0
2
MR effect size for
'Gut microbiota abundance (genus Alloprevotella id.961)' on 'Extreme obesity with alveolar hypoventilation'

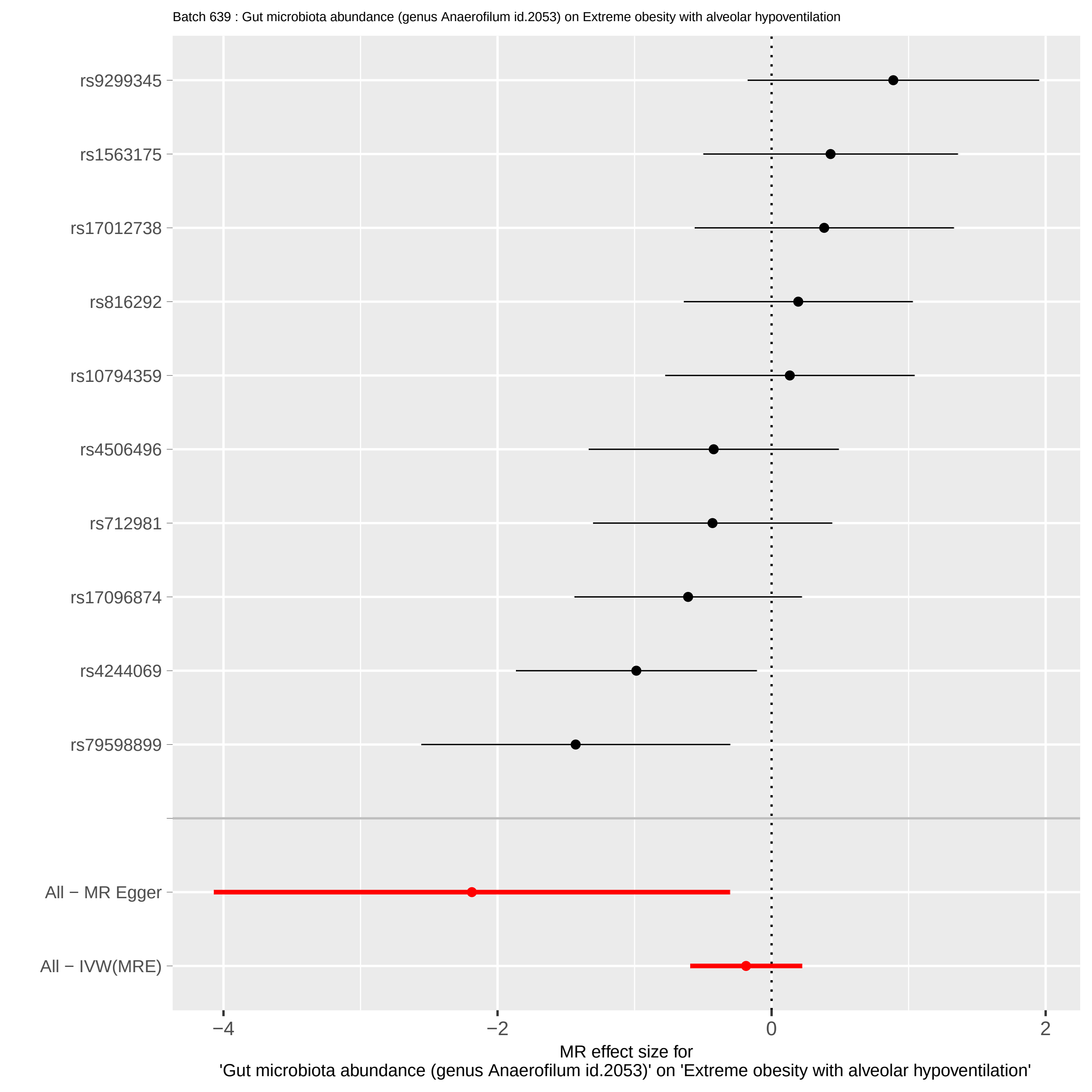
Batch 639 : Gut microbiota abundance (genus Anaerofilum id.2053) on Extreme obesity with alveolar hypoventilation
rs9299345
rs1563175
rs17012738
rs816292
rs10794359
rs4506496
rs712981
rs17096874
rs4244069
rs79598899
All − MR Egger
All − IVW(MRE)
−4
−2
0
2
MR effect size for
'Gut microbiota abundance (genus Anaerofilum id.2053)' on 'Extreme obesity with alveolar hypoventilation'

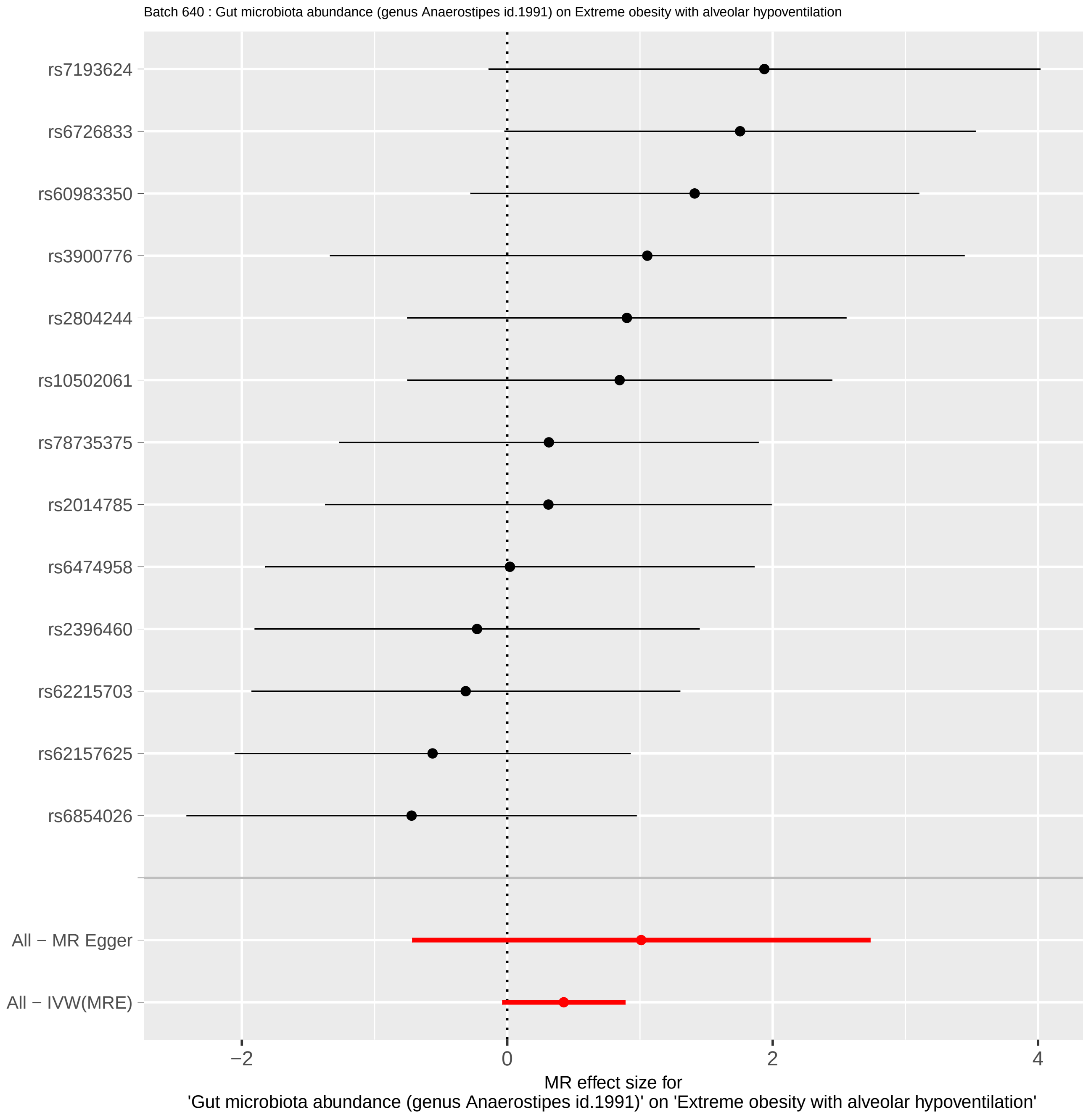

Batch 640 : Gut microbiota abundance (genus Anaerostipes id.1991) on Extreme obesity with alveolar hypoventilation
rs7193624
rs6726833
rs60983350
rs3900776
rs2804244
rs10502061
rs78735375
rs2014785
rs6474958
rs2396460
rs62215703
rs62157625
rs6854026
All − MR Egger
All − IVW(MRE)
−2
0
2
4
MR effect size for
'Gut microbiota abundance (genus Anaerostipes id.1991)' on 'Extreme obesity with alveolar hypoventilation'

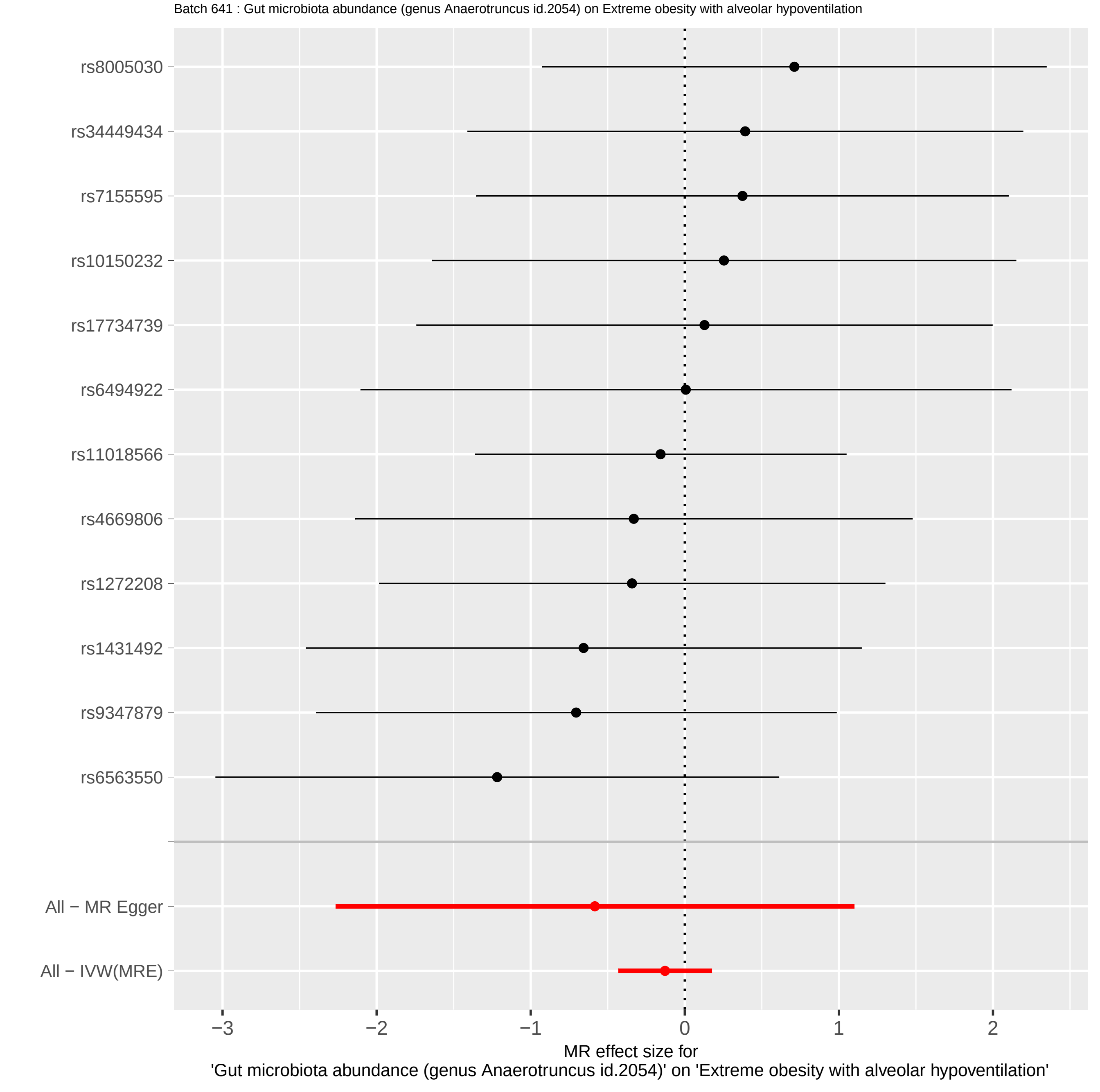
Batch 641 : Gut microbiota abundance (genus Anaerotruncus id.2054) on Extreme obesity with alveolar hypoventilation
rs8005030
rs34449434
rs7155595
rs10150232
rs17734739
rs6494922
rs11018566
rs4669806
rs1272208
rs1431492
rs9347879
rs6563550
All − MR Egger
All − IVW(MRE)
−3
−2
−1
0
1
2
MR effect size for
'Gut microbiota abundance (genus Anaerotruncus id.2054)' on 'Extreme obesity with alveolar hypoventilation'

Batch 642 : Gut microbiota abundance (genus Bacteroides id.918) on Extreme obesity with alveolar hypoventilation

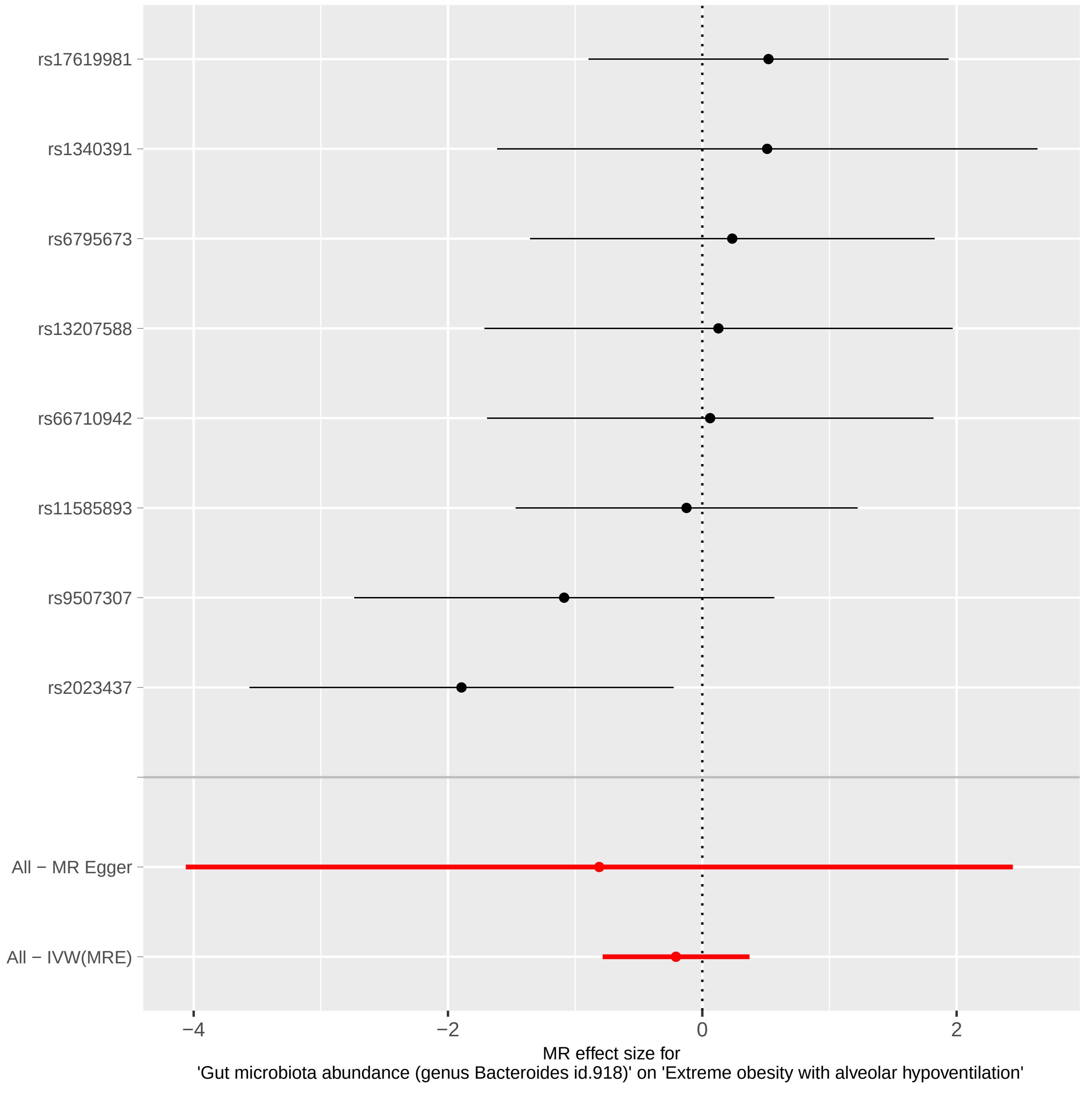

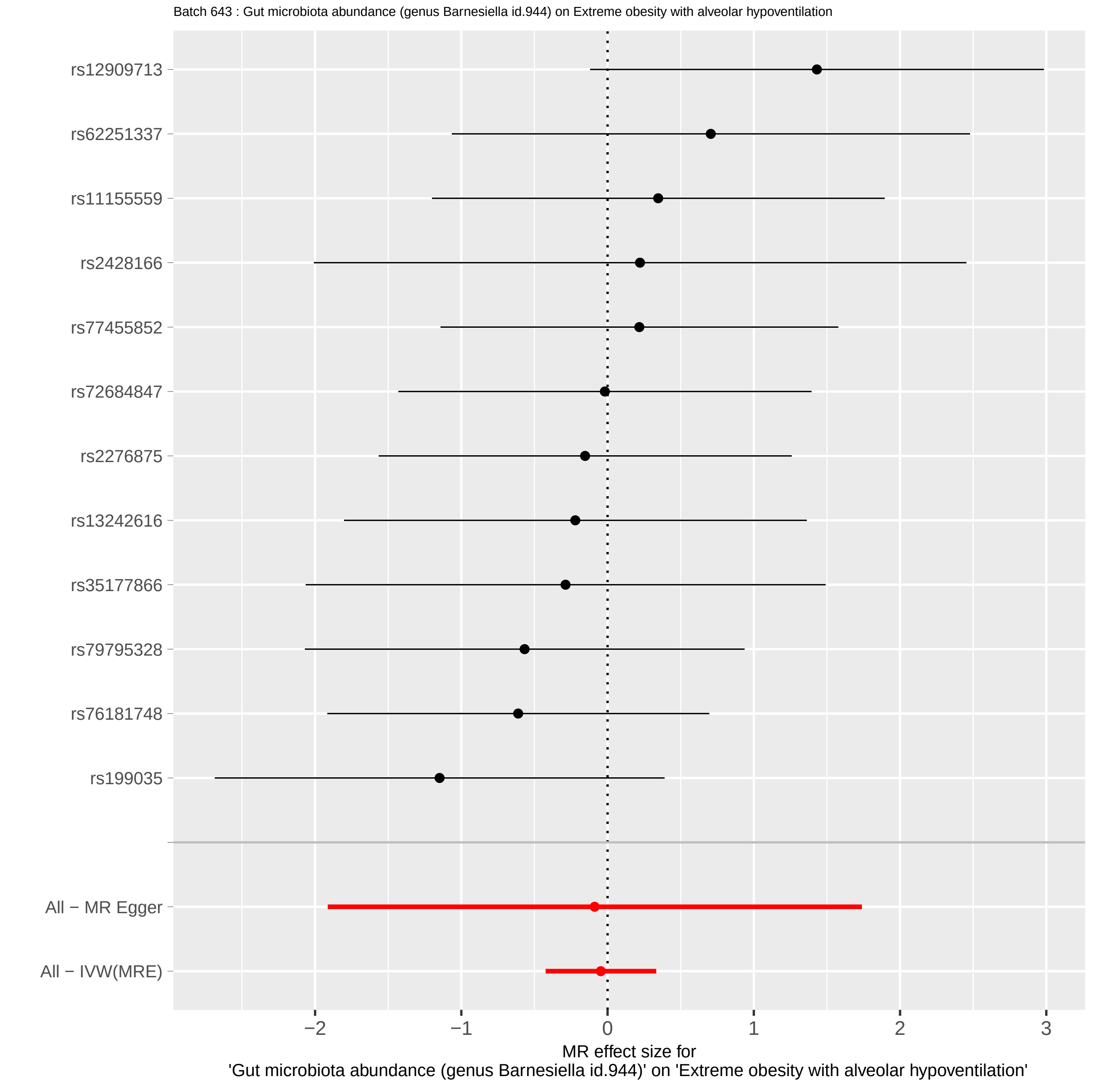

Batch 643 : Gut microbiota abundance (genus Barnesiella id.944) on Extreme obesity with alveolar hypoventilation
rs12909713
rs62251337
rs11155559
rs2428166
rs77455852
rs72684847
rs2276875
rs13242616
rs35177866
rs79795328
rs76181748
rs199035
All − MR Egger
All − IVW(MRE)
−2
−1
0
1
2
3
MR effect size for
'Gut microbiota abundance (genus Barnesiella id.944)' on 'Extreme obesity with alveolar hypoventilation'

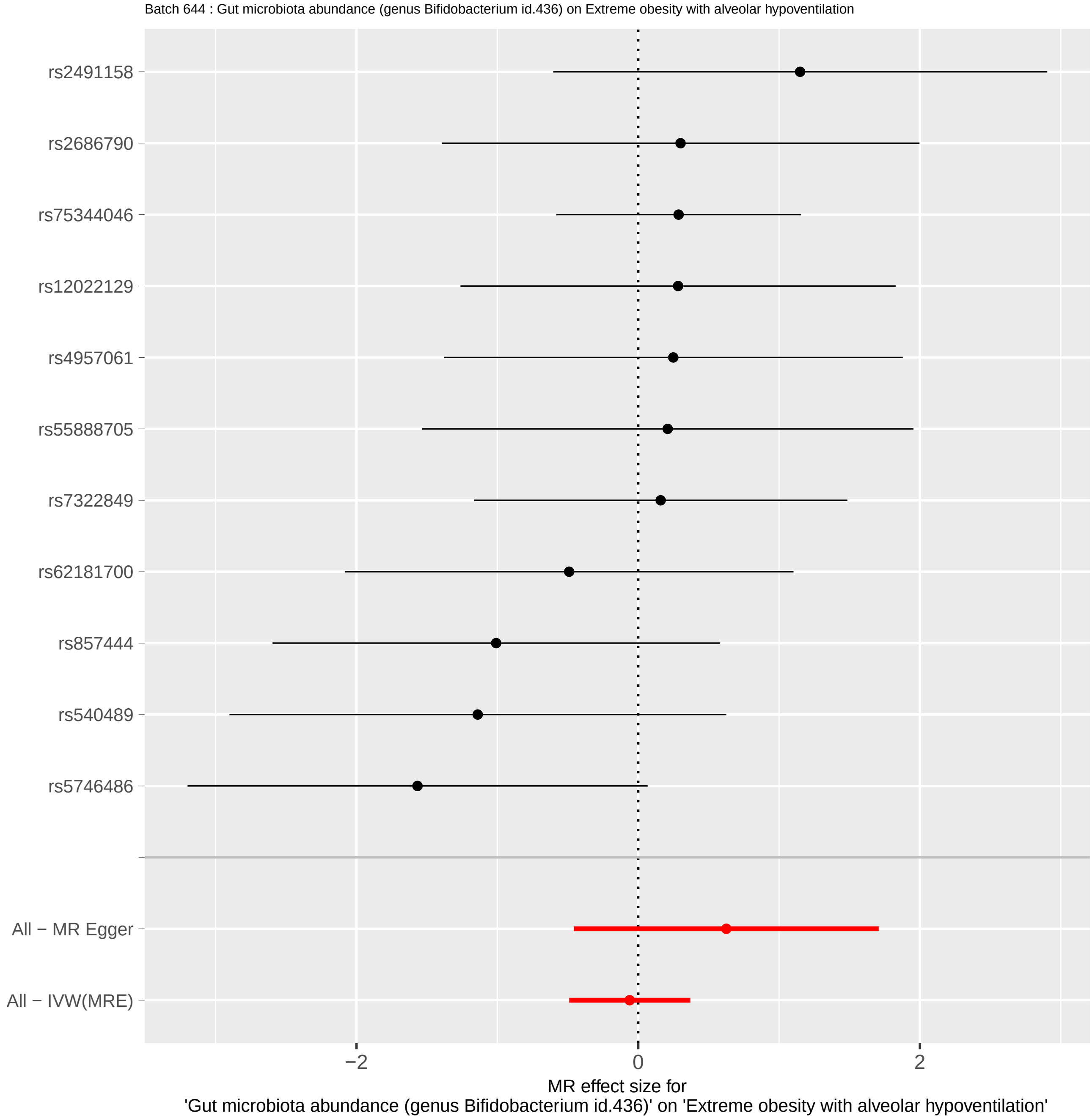

Batch 644 : Gut microbiota abundance (genus Bifidobacterium id.436) on Extreme obesity with alveolar hypoventilation
rs2491158
rs2686790
rs75344046
rs12022129
rs4957061
rs55888705
rs7322849
rs62181700
rs857444
rs540489
rs5746486
All − MR Egger
All − IVW(MRE)
−2
0
2
MR effect size for
'Gut microbiota abundance (genus Bifidobacterium id.436)' on 'Extreme obesity with alveolar hypoventilation'

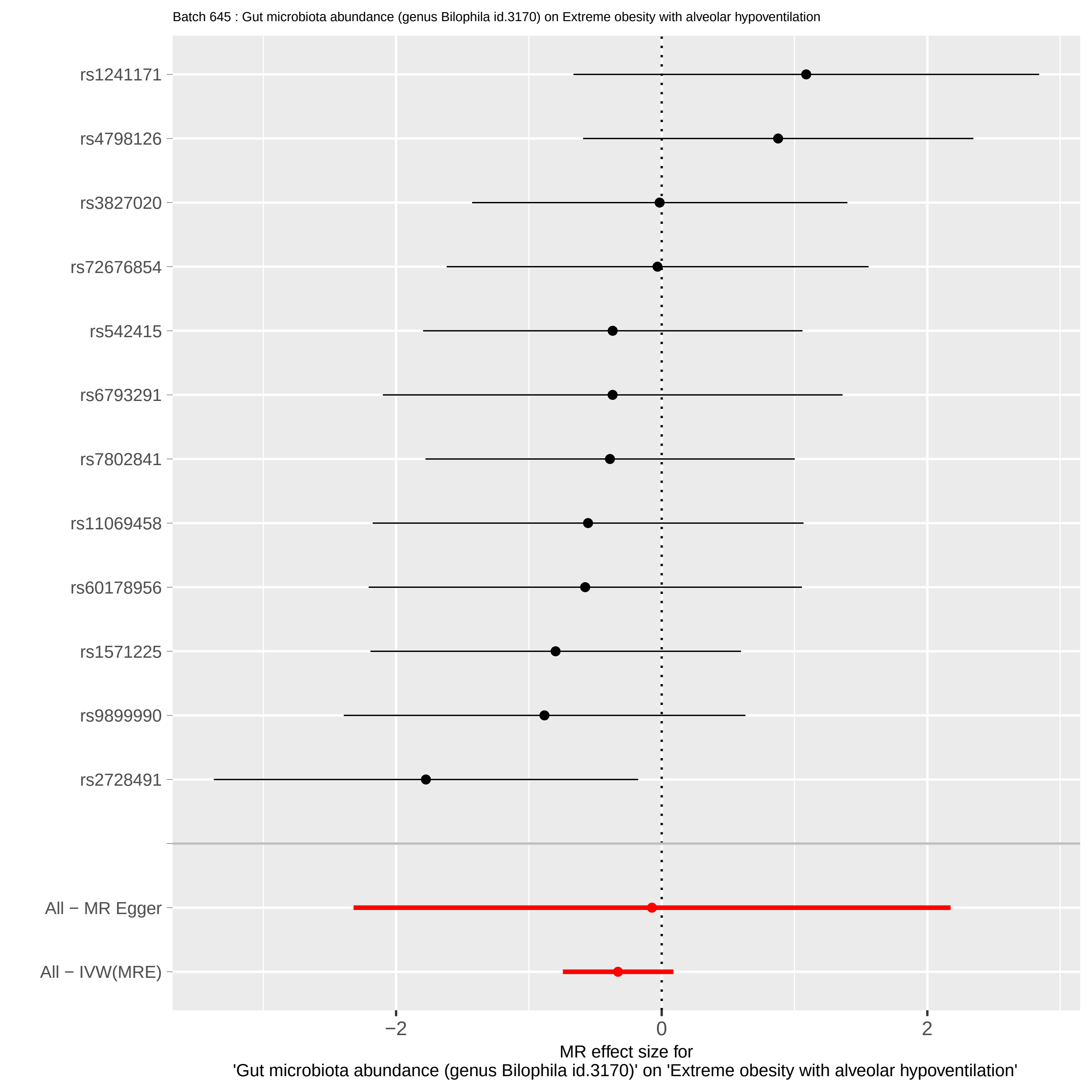
Batch 645 : Gut microbiota abundance (genus Bilophila id.3170) on Extreme obesity with alveolar hypoventilation
rs1241171
rs4798126
rs3827020
rs72676854
rs542415
rs6793291
rs7802841
rs11069458
rs60178956
rs1571225
rs9899990
rs2728491
All − MR Egger
All − IVW(MRE)
−2
0
2
MR effect size for
'Gut microbiota abundance (genus Bilophila id.3170)' on 'Extreme obesity with alveolar hypoventilation'

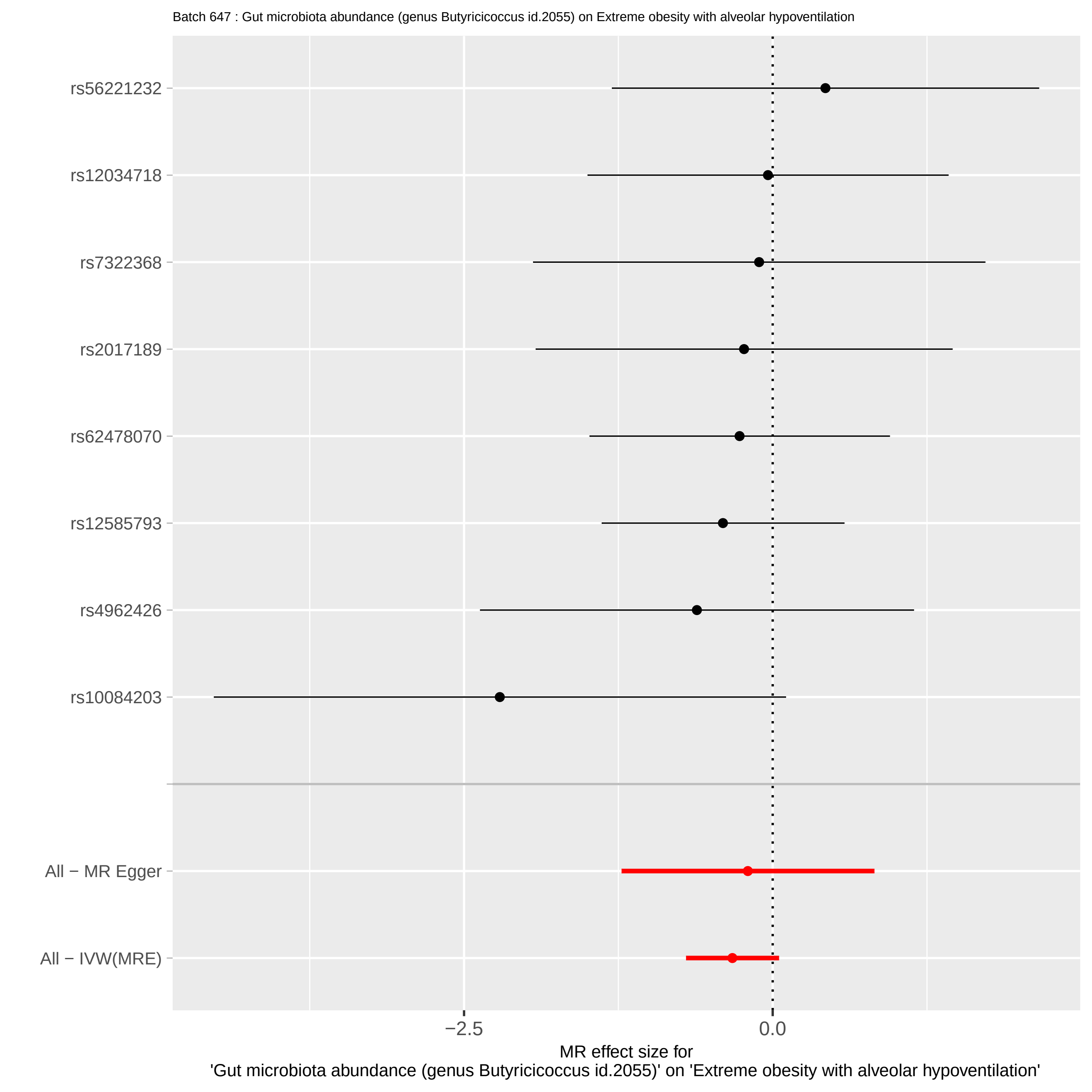

Batch 647 : Gut microbiota abundance (genus Butyricicoccus id.2055) on Extreme obesity with alveolar hypoventilation
rs56221232
rs12034718
rs7322368
rs2017189
rs62478070
rs12585793
rs4962426
rs10084203
All − MR Egger
All − IVW(MRE)
−2.5
0.0
MR effect size for
'Gut microbiota abundance (genus Butyricicoccus id.2055)' on 'Extreme obesity with alveolar hypoventilation'

Batch 648 : Gut microbiota abundance (genus Butyricimonas id.945) on Extreme obesity with alveolar hypoventilation

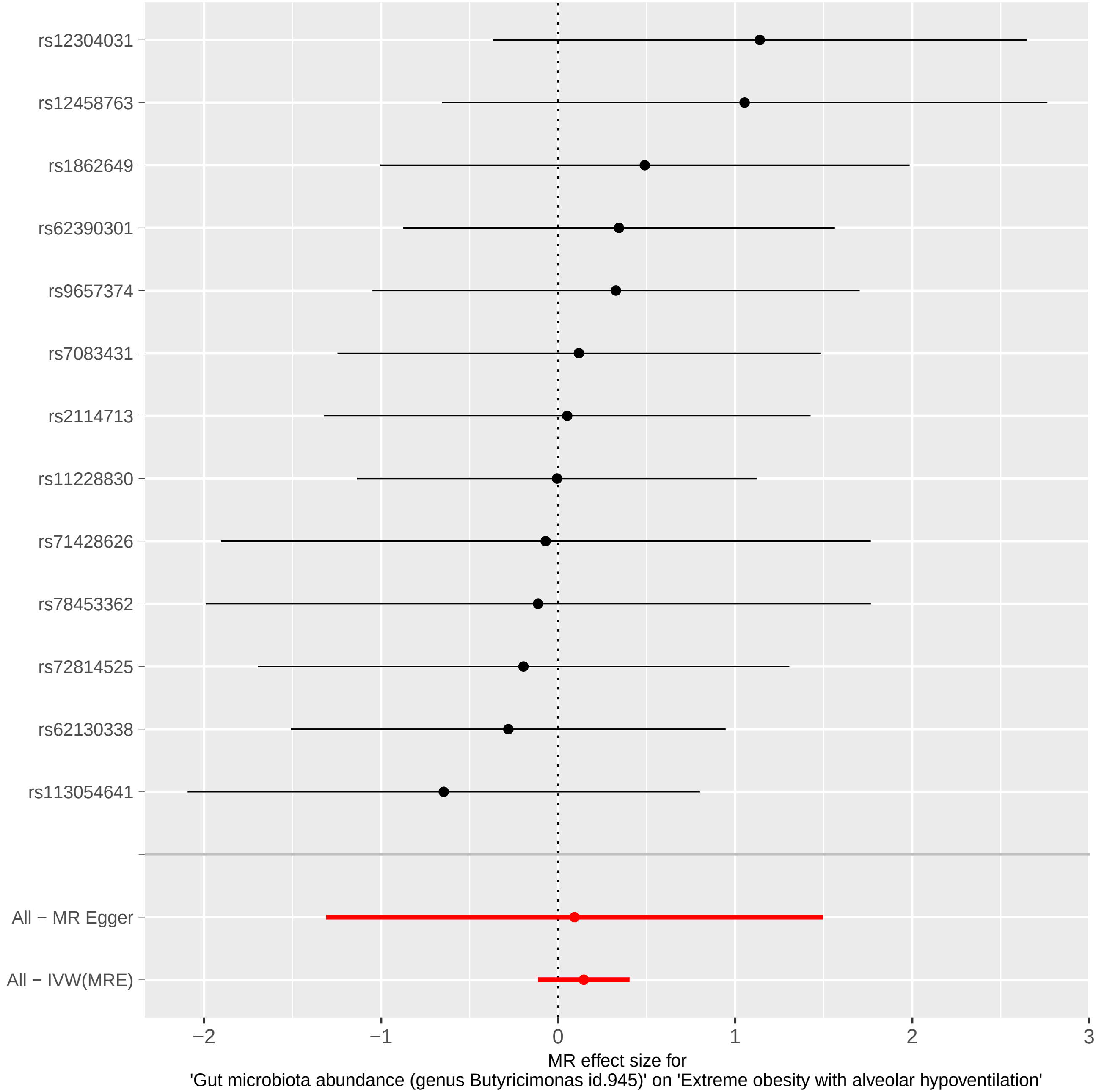

Batch 649 : Gut microbiota abundance (genus Butyrivibrio id.1993) on Extreme obesity with alveolar hypoventilation

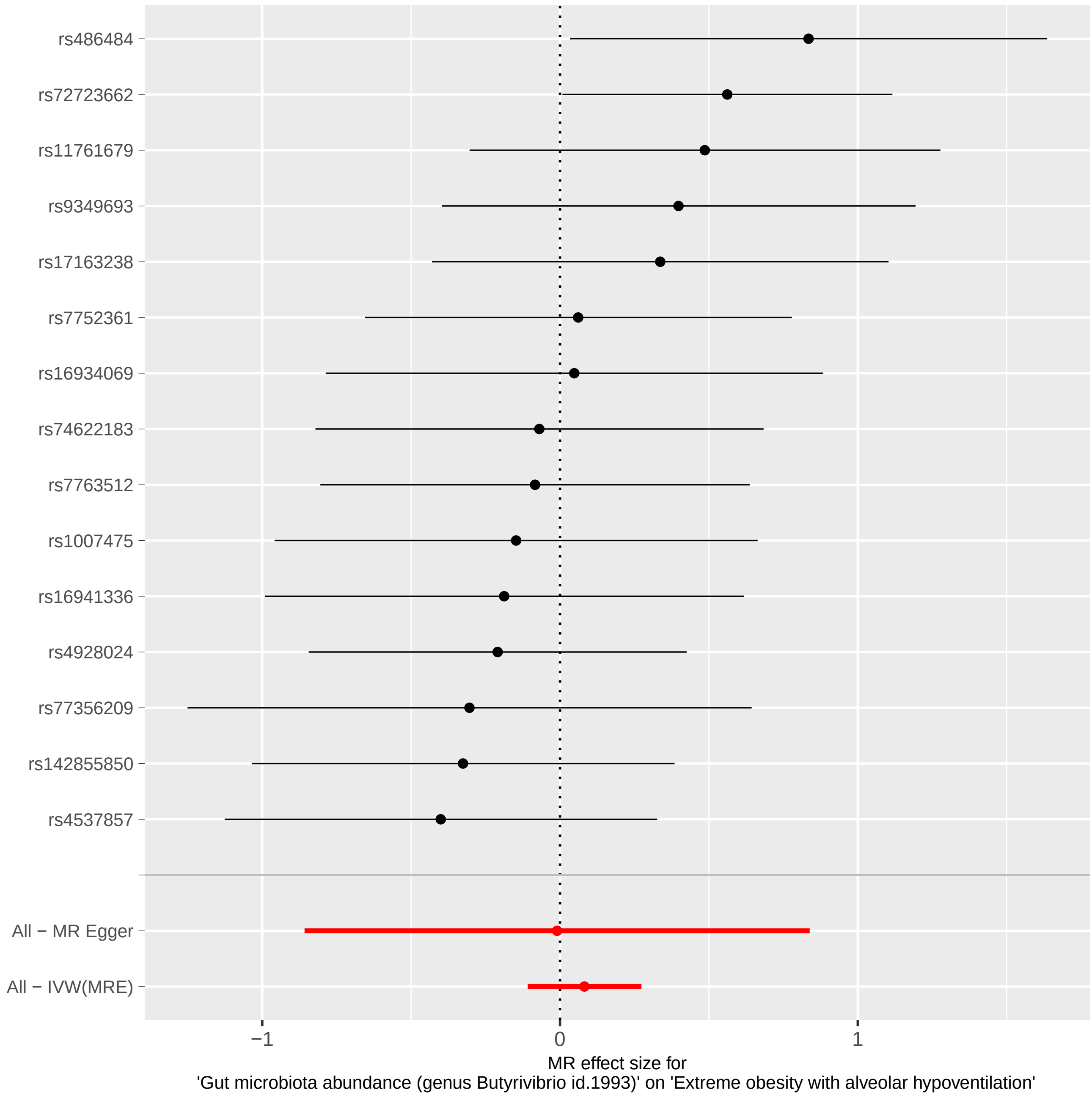

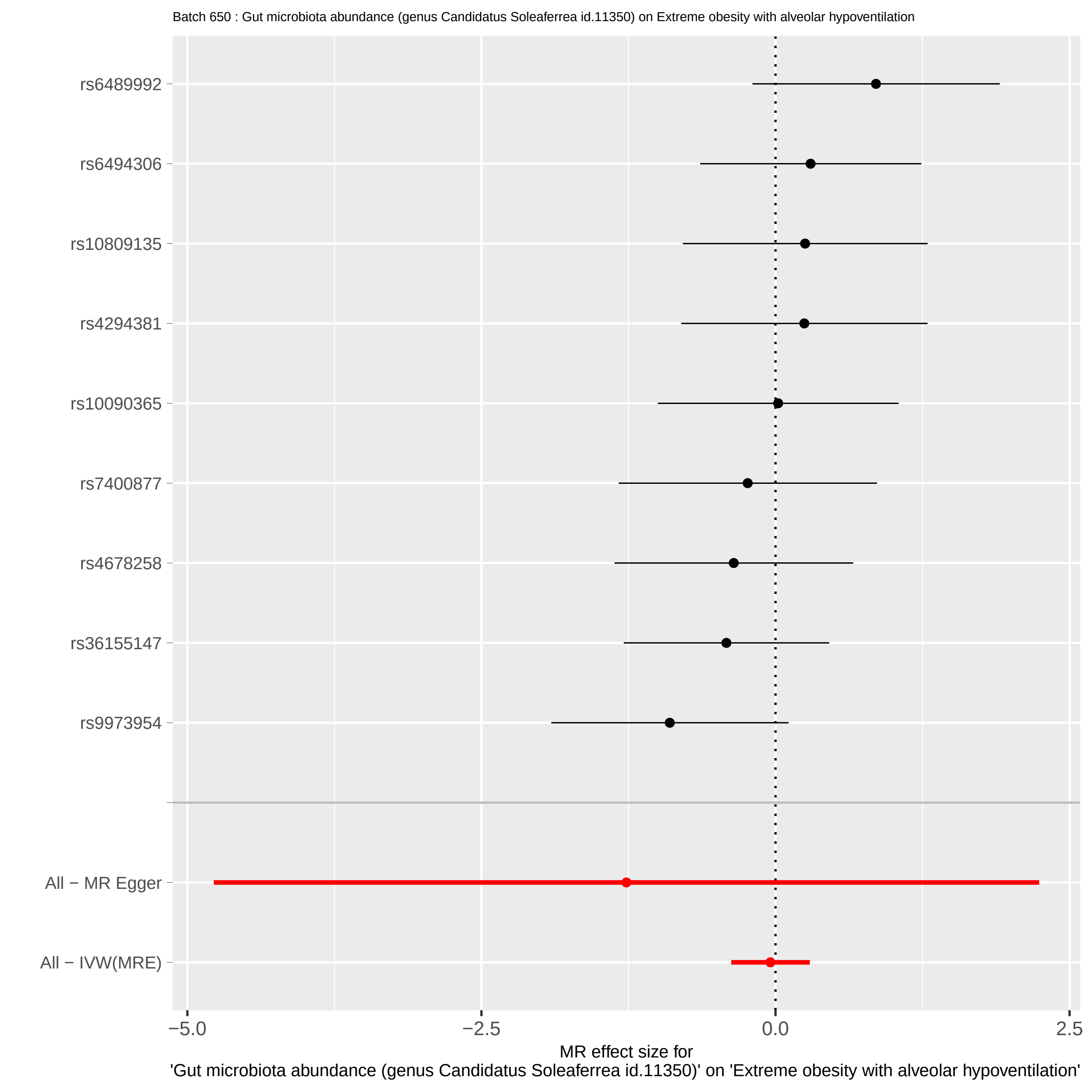

Batch 650 : Gut microbiota abundance (genus Candidatus Soleaferrea id.11350) on Extreme obesity with alveolar hypoventilation
rs6489992
rs6494306
rs10809135
rs4294381
rs10090365
rs7400877
rs4678258
rs36155147
rs9973954
All − MR Egger
All − IVW(MRE)
−5.0
−2.5
0.0
2.5
MR effect size for
'Gut microbiota abundance (genus Candidatus Soleaferrea id.11350)' on 'Extreme obesity with alveolar hypoventilation'

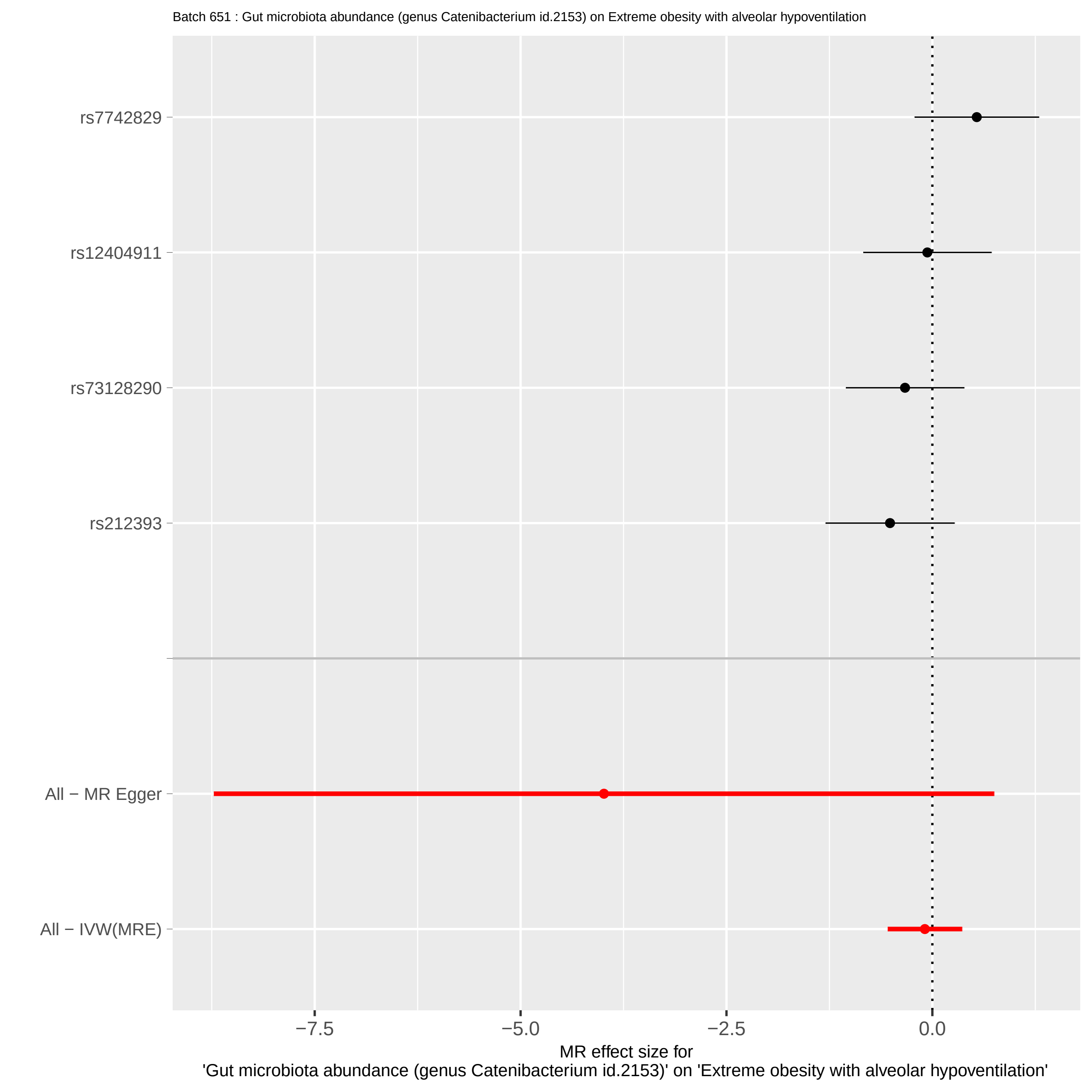
Batch 651 : Gut microbiota abundance (genus Catenibacterium id.2153) on Extreme obesity with alveolar hypoventilation
rs7742829
rs12404911
rs73128290
rs212393
All − MR Egger
All − IVW(MRE)
−7.5
−5.0
−2.5
0.0
MR effect size for
'Gut microbiota abundance (genus Catenibacterium id.2153)' on 'Extreme obesity with alveolar hypoventilation'

Batch 652 : Gut microbiota abundance (genus Christensenellaceae R 7group id.11283) on Extreme obesity with alveolar hypoventilation

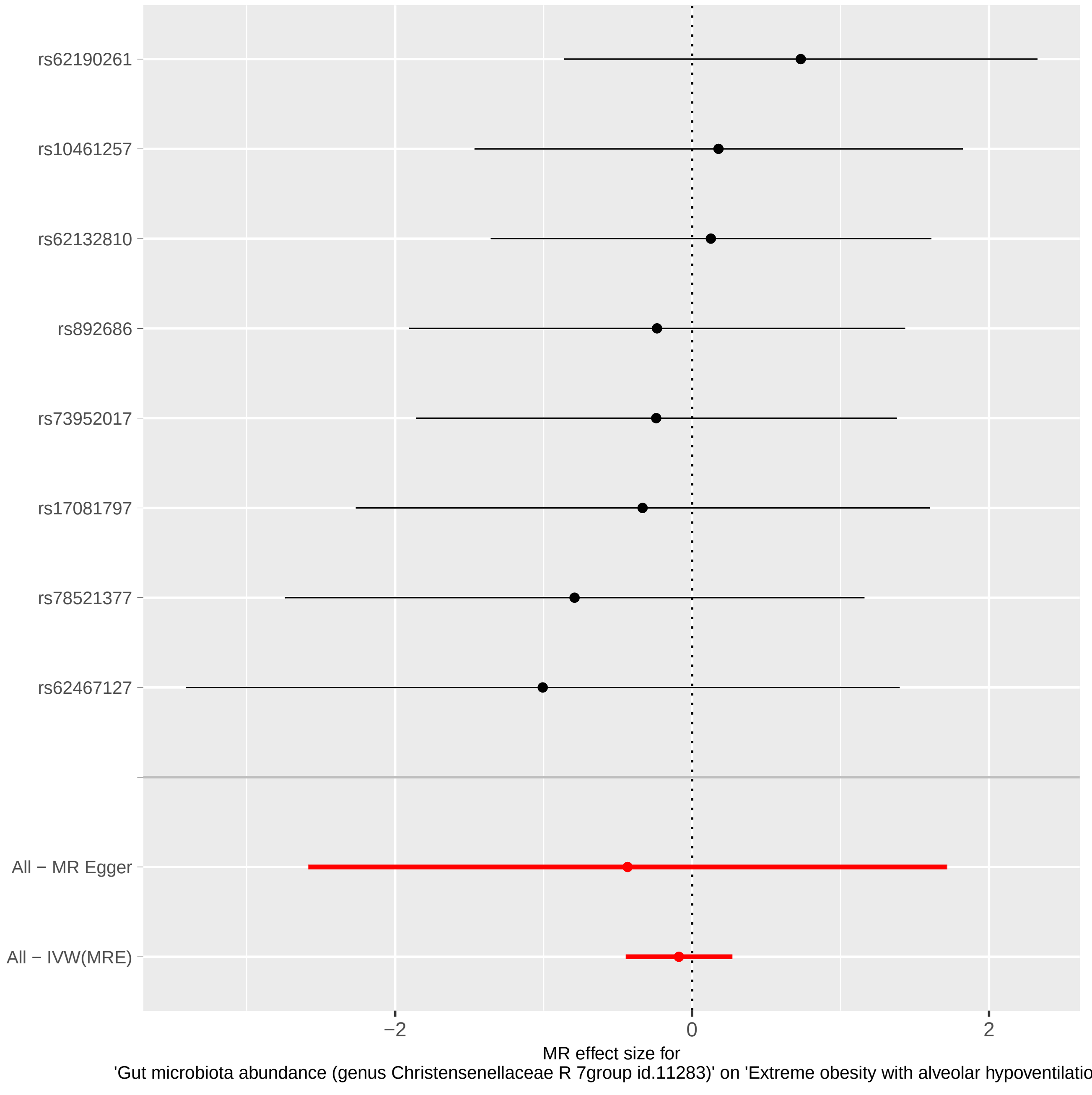

Batch 653 : Gut microbiota abundance (genus Clostridium innocuum group id.14397) on Extreme obesity with alveolar hypoventilation

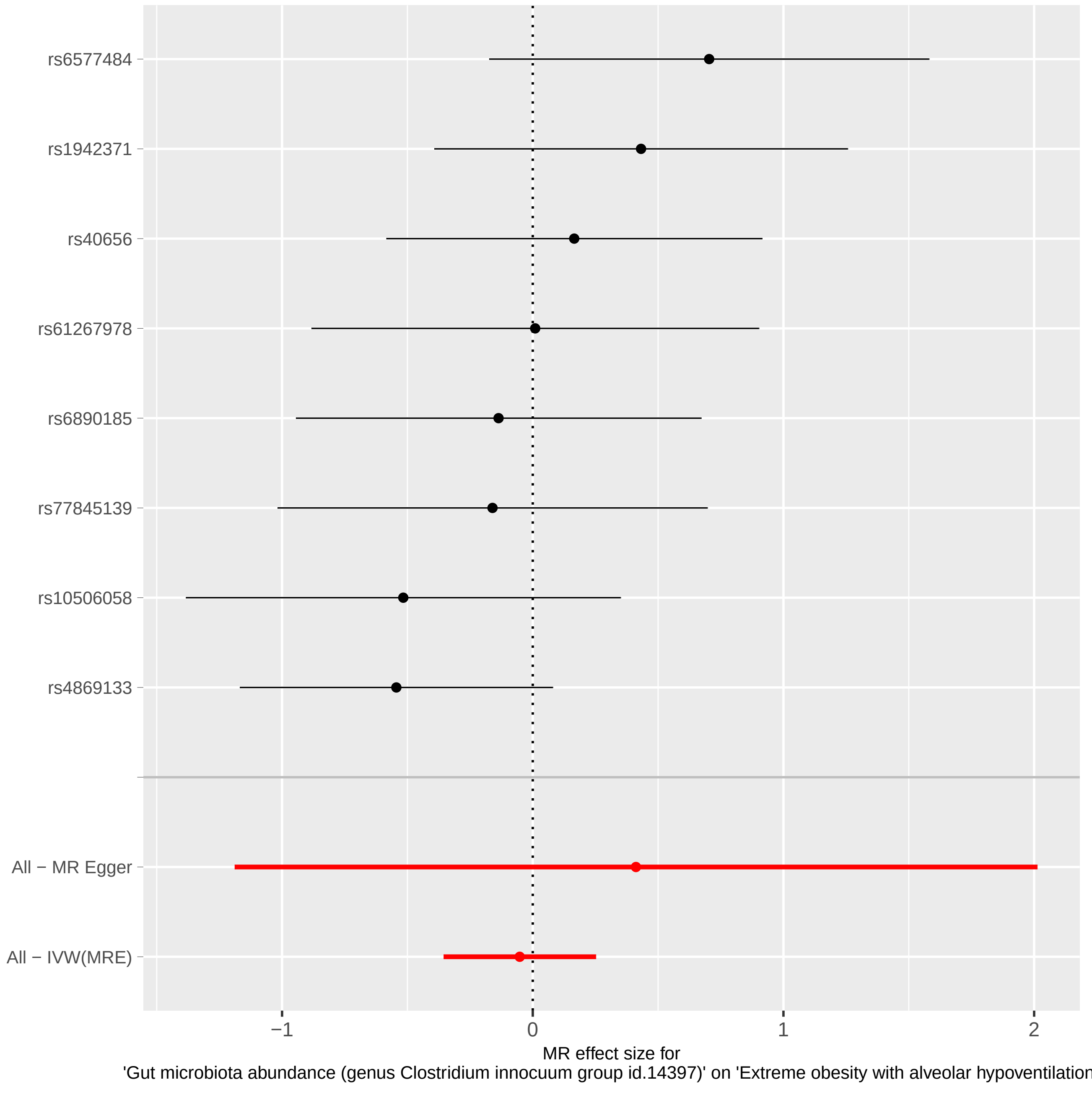

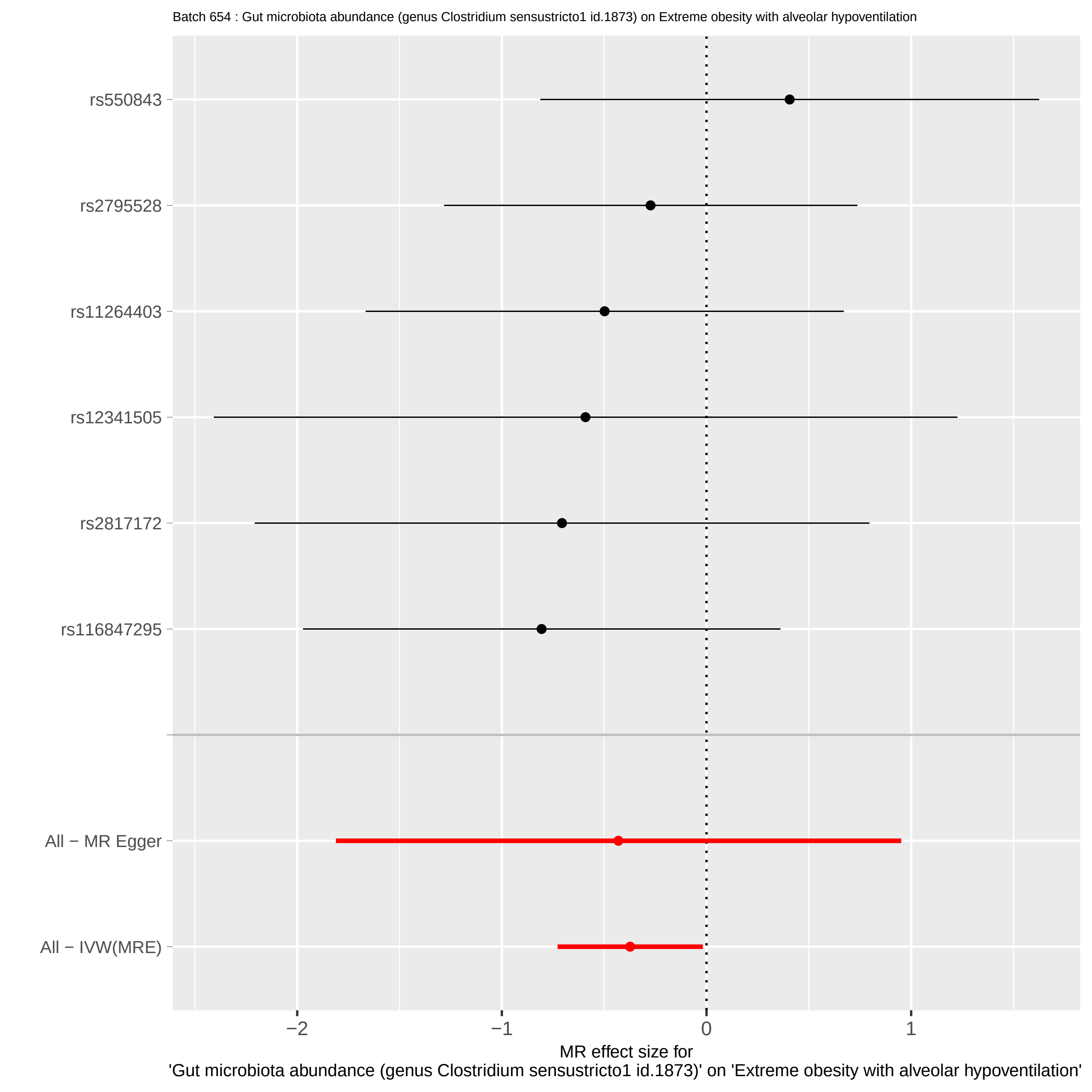

Batch 654 : Gut microbiota abundance (genus Clostridium sensustricto1 id.1873) on Extreme obesity with alveolar hypoventilation
rs550843
rs2795528
rs11264403
rs12341505
rs2817172
rs116847295
All − MR Egger
All − IVW(MRE)
−2
−1
0
1
MR effect size for
'Gut microbiota abundance (genus Clostridium sensustricto1 id.1873)' on 'Extreme obesity with alveolar hypoventilation'

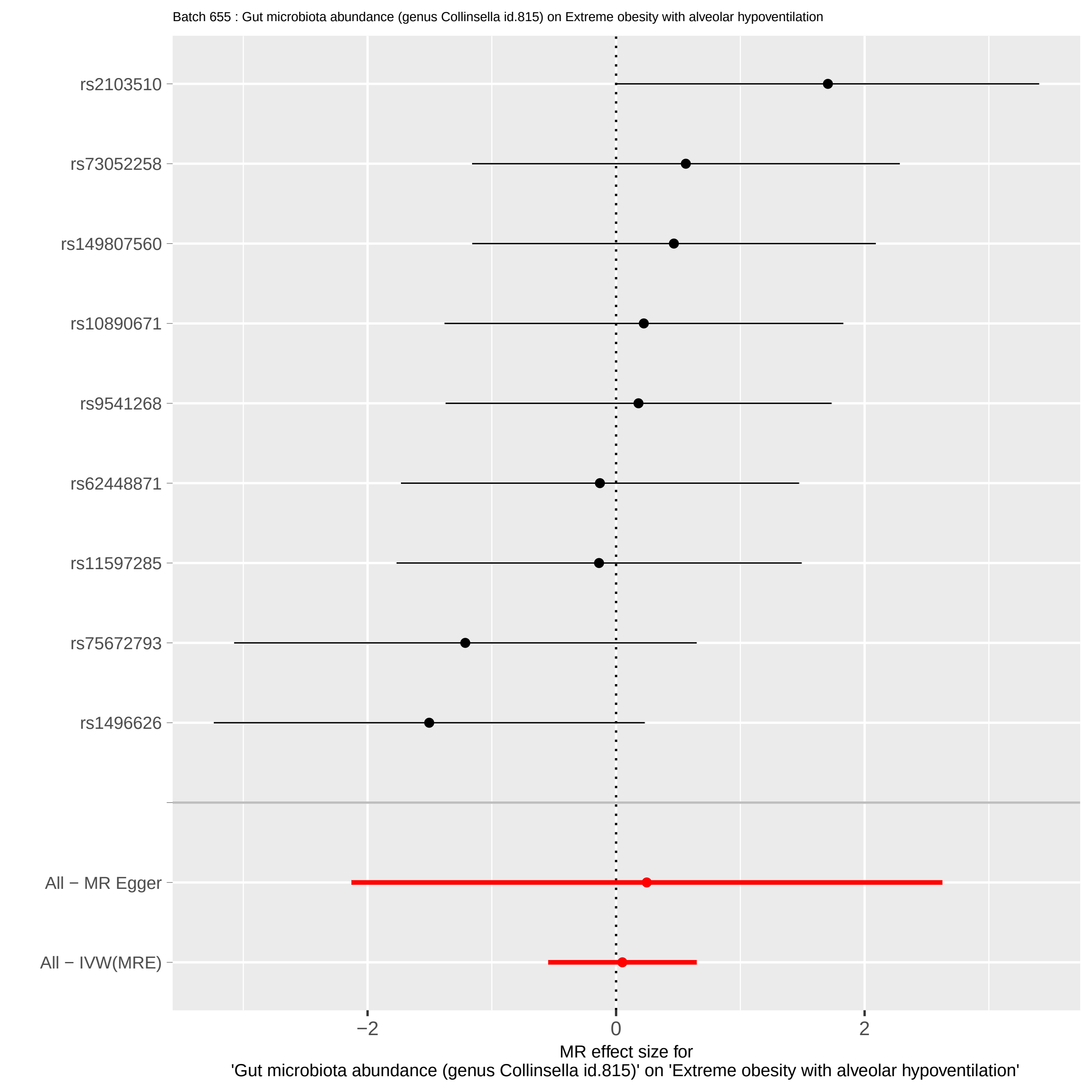
Batch 655 : Gut microbiota abundance (genus Collinsella id.815) on Extreme obesity with alveolar hypoventilation
rs2103510
rs73052258
rs149807560
rs10890671
rs9541268
rs62448871
rs11597285
rs75672793
rs1496626
All − MR Egger
All − IVW(MRE)
−2
0
2
MR effect size for
'Gut microbiota abundance (genus Collinsella id.815)' on 'Extreme obesity with alveolar hypoventilation'

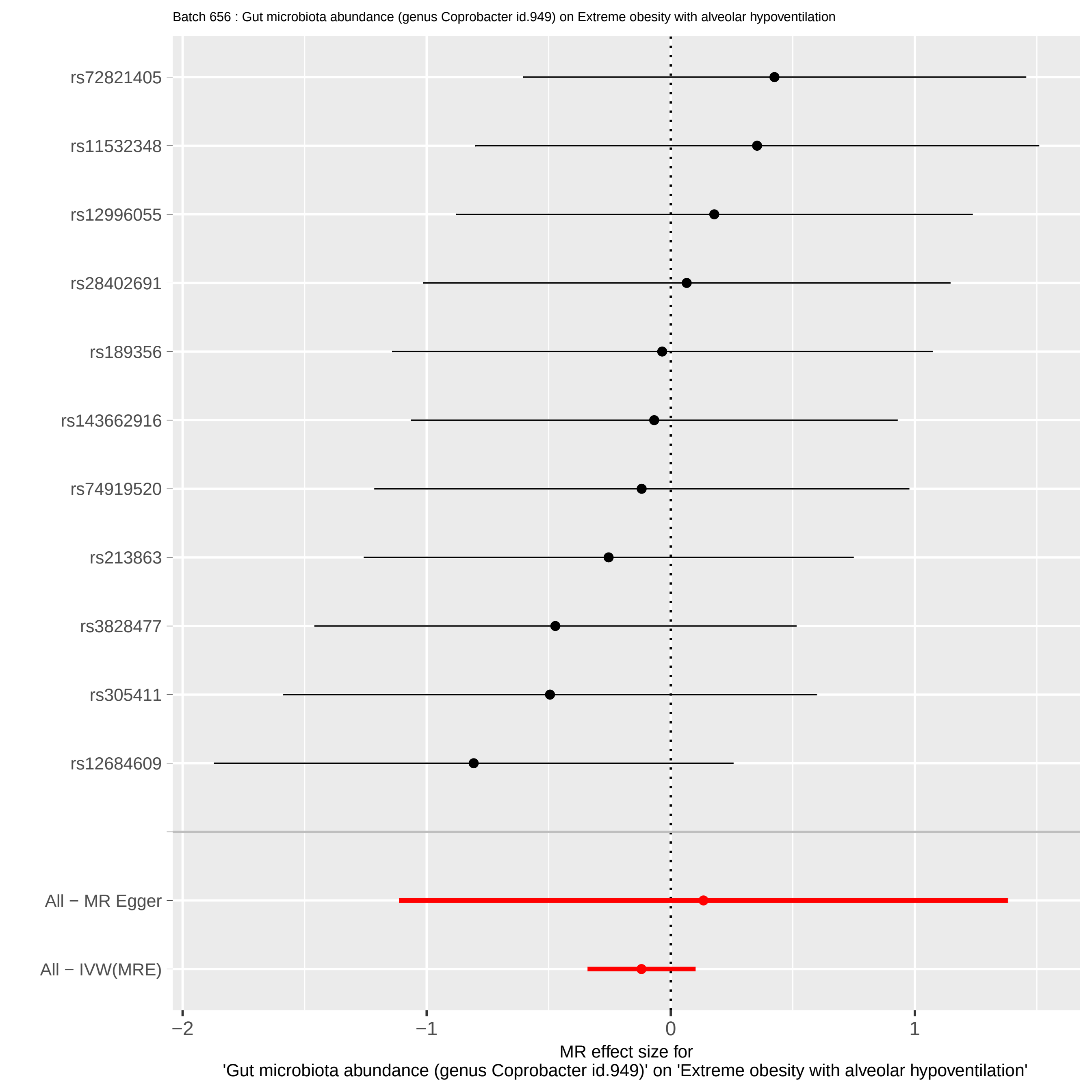

Batch 656 : Gut microbiota abundance (genus Coprobacter id.949) on Extreme obesity with alveolar hypoventilation
rs72821405
rs11532348
rs12996055
rs28402691
rs189356
rs143662916
rs74919520
rs213863
rs3828477
rs305411
rs12684609
All − MR Egger
All − IVW(MRE)
−2
−1
0
1
MR effect size for
'Gut microbiota abundance (genus Coprobacter id.949)' on 'Extreme obesity with alveolar hypoventilation'

Batch 657 : Gut microbiota abundance (genus Coprococcus1 id.11301) on Extreme obesity with alveolar hypoventilation

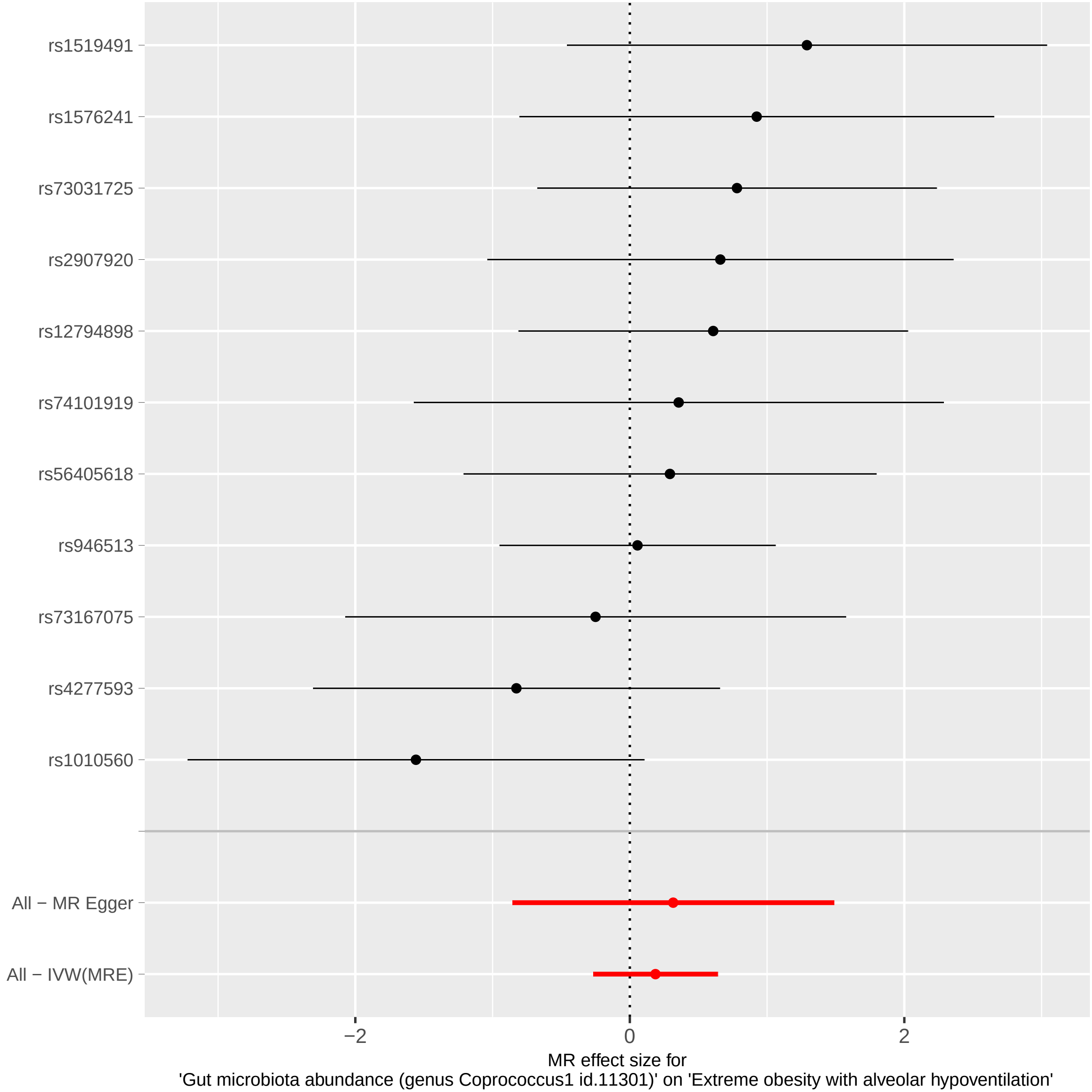

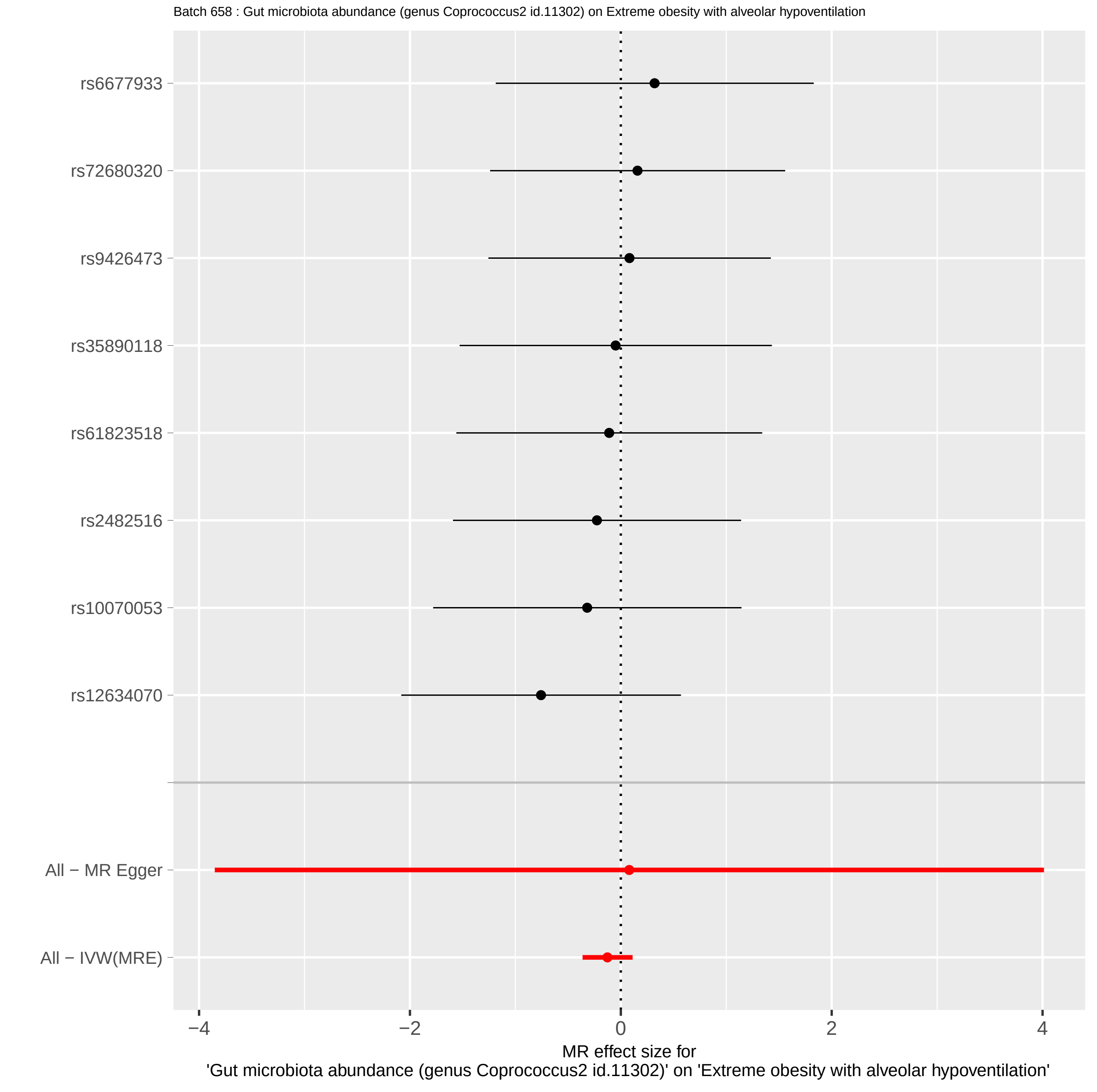
Batch 658 : Gut microbiota abundance (genus Coprococcus2 id.11302) on Extreme obesity with alveolar hypoventilation
rs6677933
rs72680320
rs9426473
rs35890118
rs61823518
rs2482516
rs10070053
rs12634070
All − MR Egger
All − IVW(MRE)
−4
−2
0
2
4
MR effect size for
'Gut microbiota abundance (genus Coprococcus2 id.11302)' on 'Extreme obesity with alveolar hypoventilation'

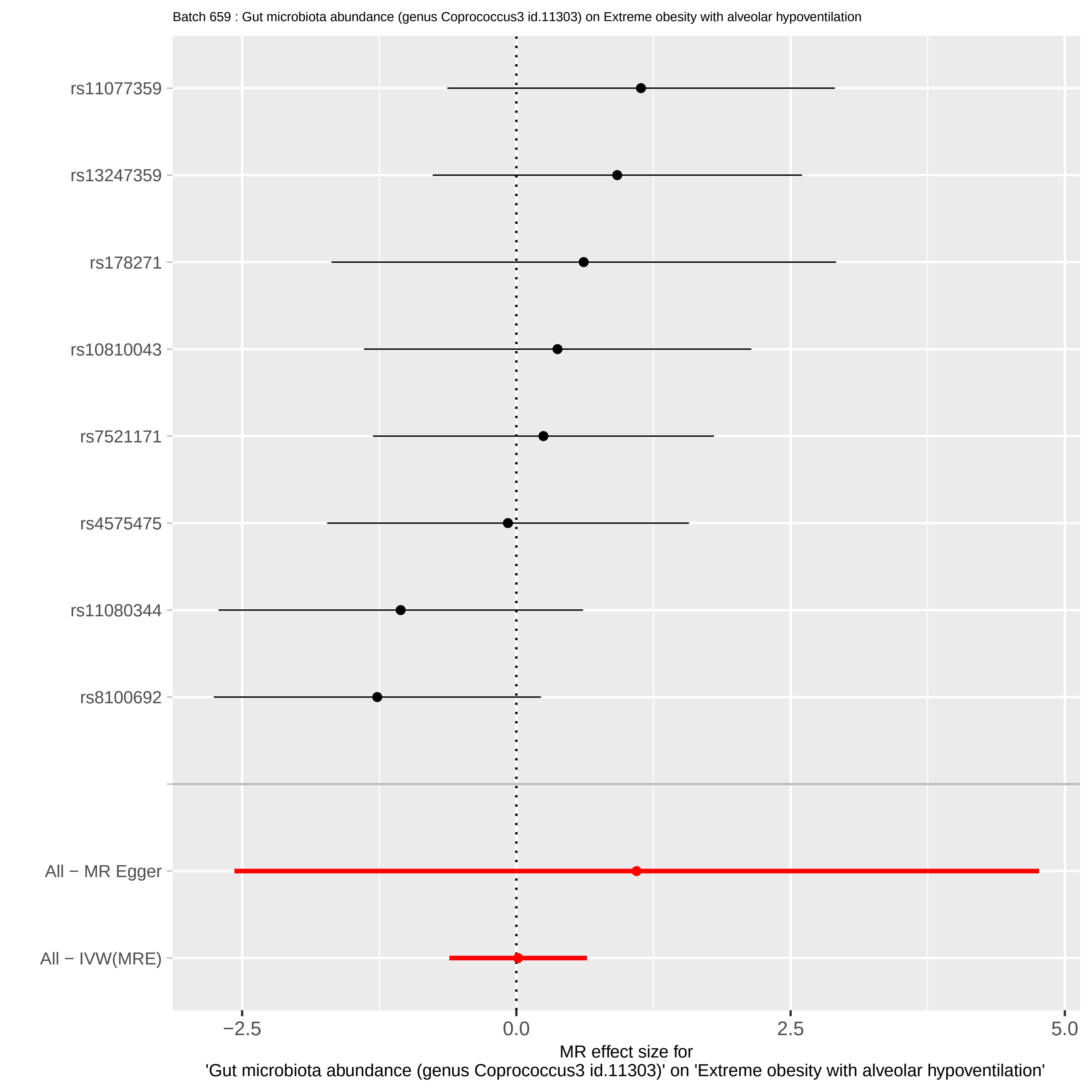

Batch 659 : Gut microbiota abundance (genus Coprococcus3 id.11303) on Extreme obesity with alveolar hypoventilation
rs11077359
rs13247359
rs178271
rs10810043
rs7521171
rs4575475
rs11080344
rs8100692
All − MR Egger
All − IVW(MRE)
−2.5
0.0
2.5
5.0
MR effect size for
'Gut microbiota abundance (genus Coprococcus3 id.11303)' on 'Extreme obesity with alveolar hypoventilation'

Batch 660 : Gut microbiota abundance (genus Defluviitaleaceae UCG011 id.11287) on Extreme obesity with alveolar hypoventilation

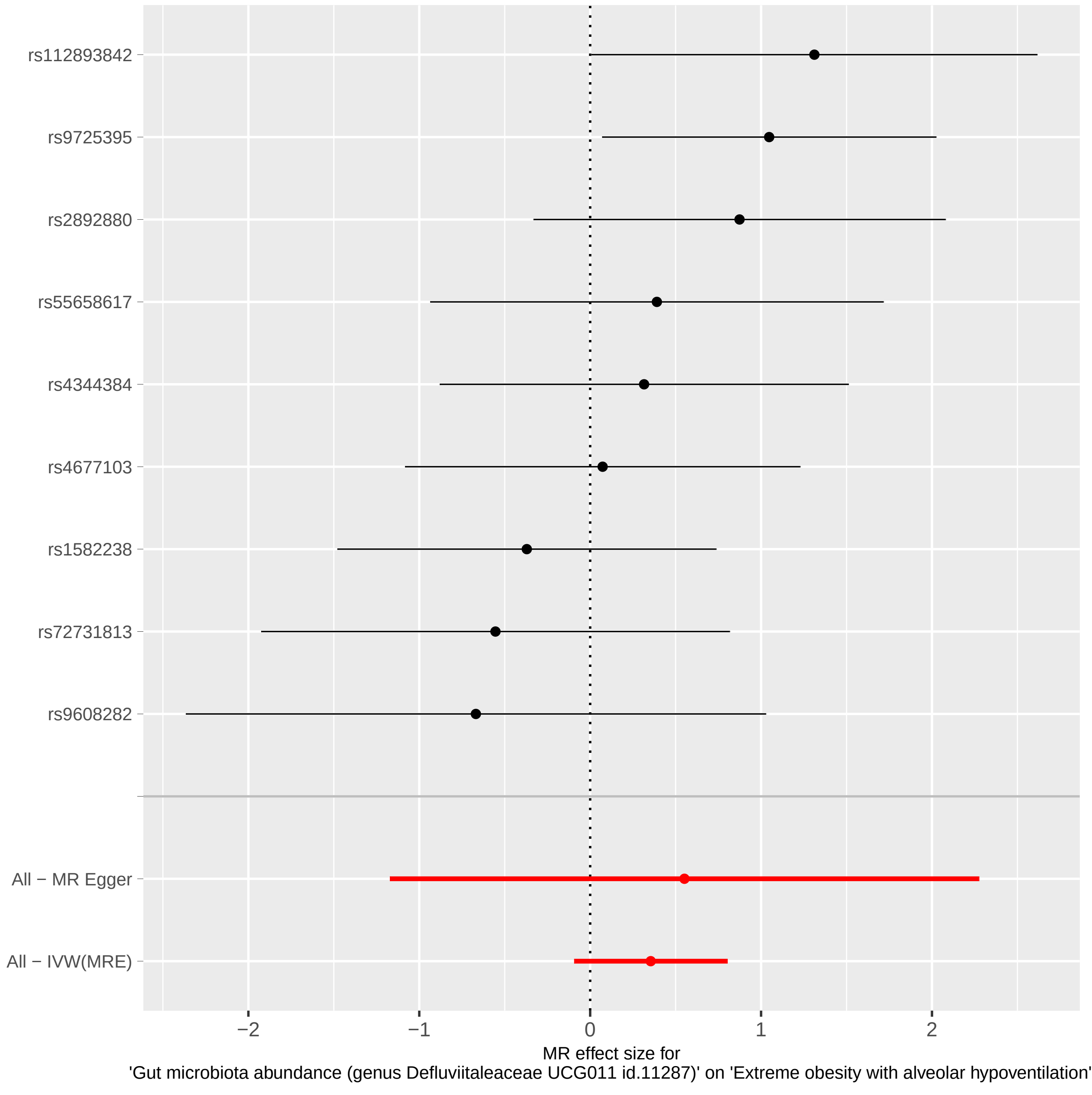

Batch 661 : Gut microbiota abundance (genus Desulfovibrio id.3173) on Extreme obesity with alveolar hypoventilation

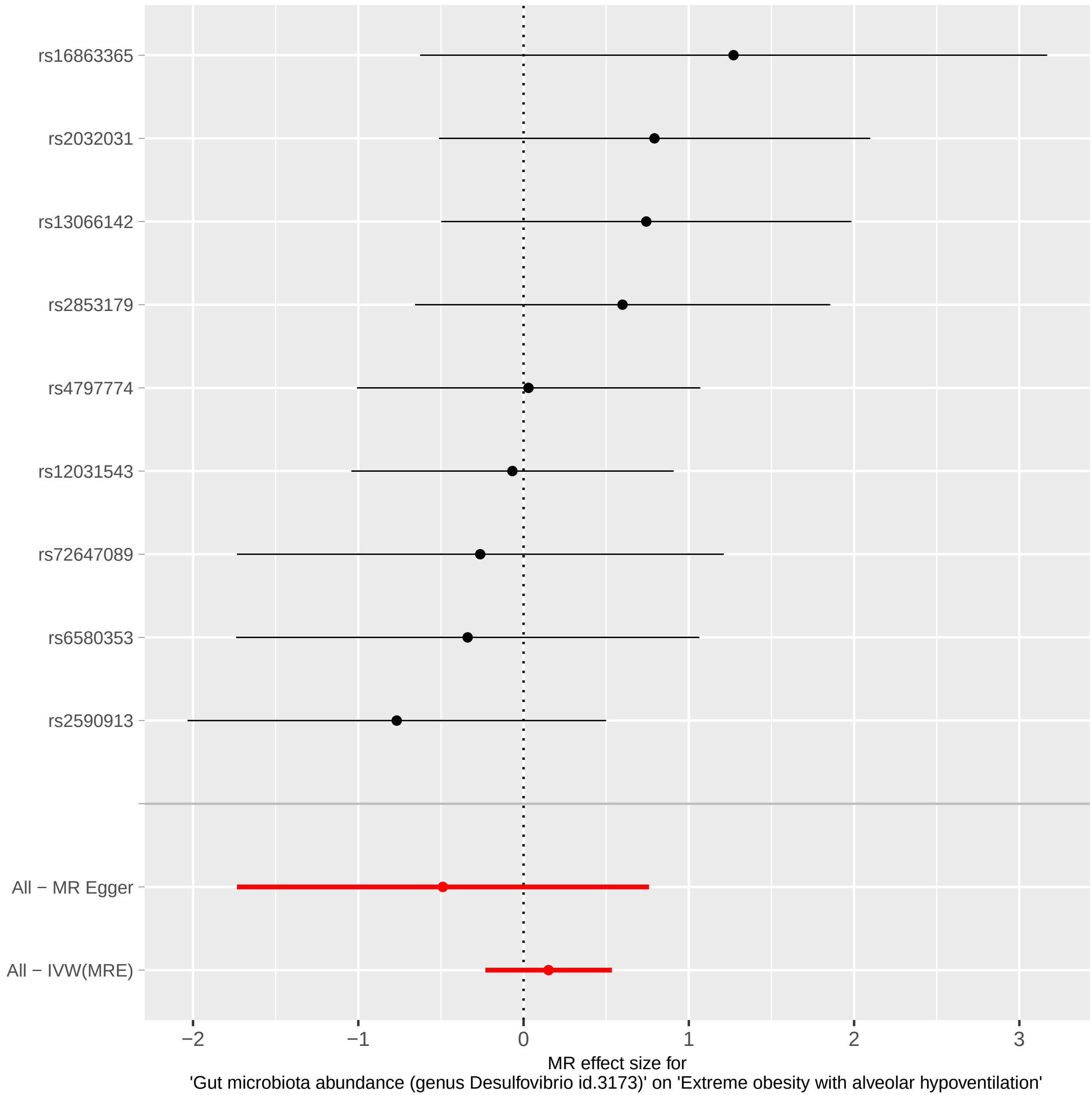

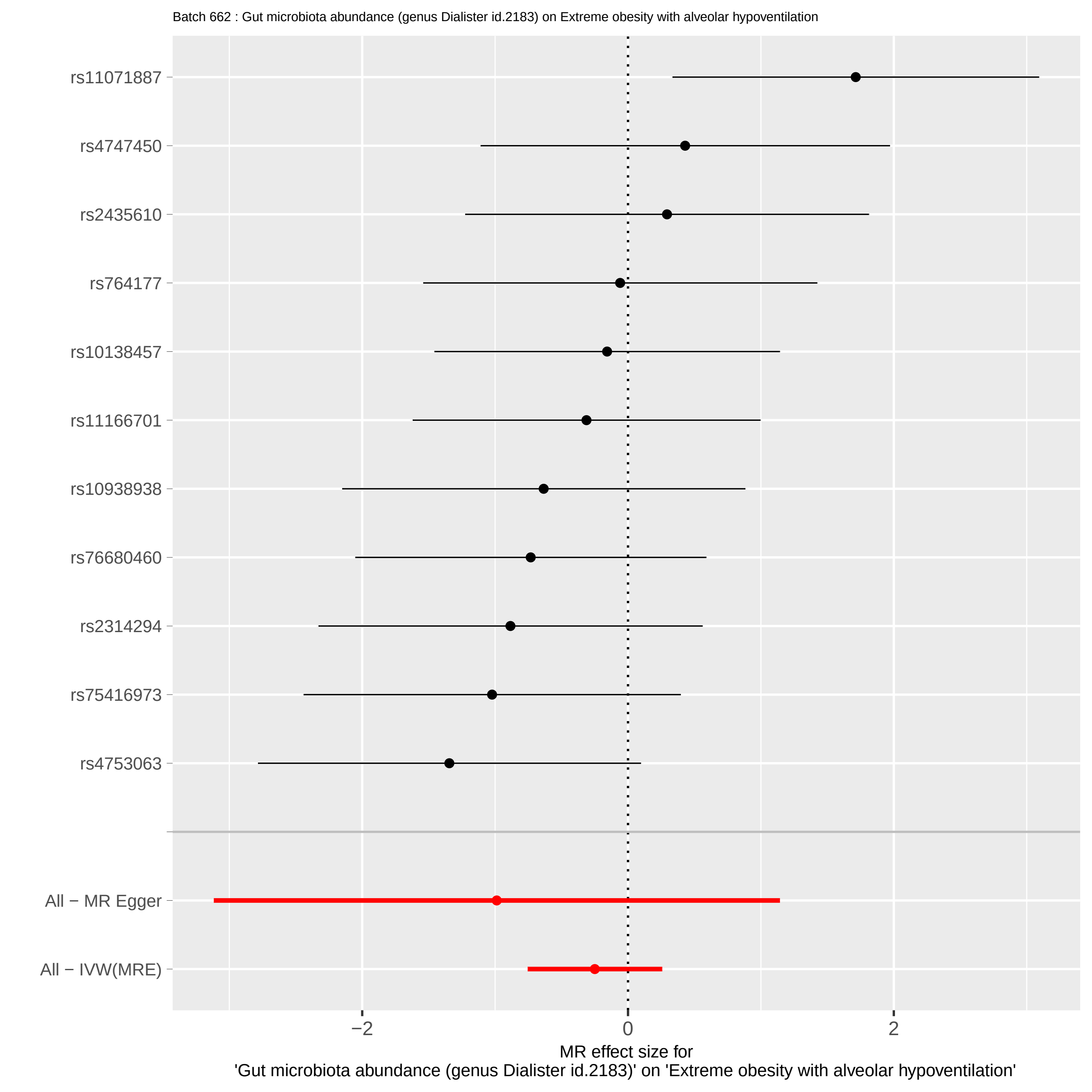

Batch 662 : Gut microbiota abundance (genus Dialister id.2183) on Extreme obesity with alveolar hypoventilation
rs11071887
rs4747450
rs2435610
rs764177
rs10138457
rs11166701
rs10938938
rs76680460
rs2314294
rs75416973
rs4753063
All − MR Egger
All − IVW(MRE)
−2
0
2
MR effect size for
'Gut microbiota abundance (genus Dialister id.2183)' on 'Extreme obesity with alveolar hypoventilation'

Batch 663 : Gut microbiota abundance (genus Dorea id.1997) on Extreme obesity with alveolar hypoventilation

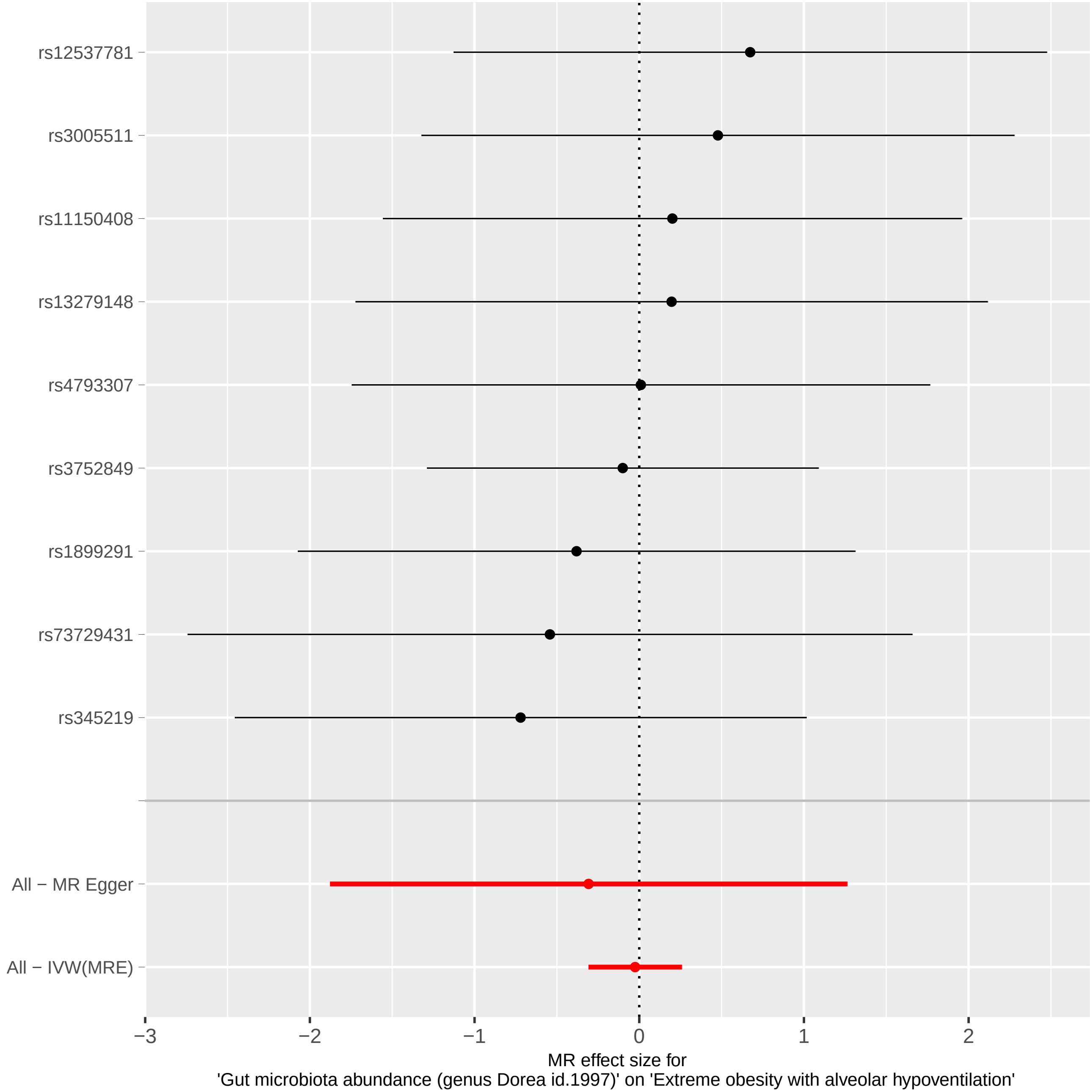

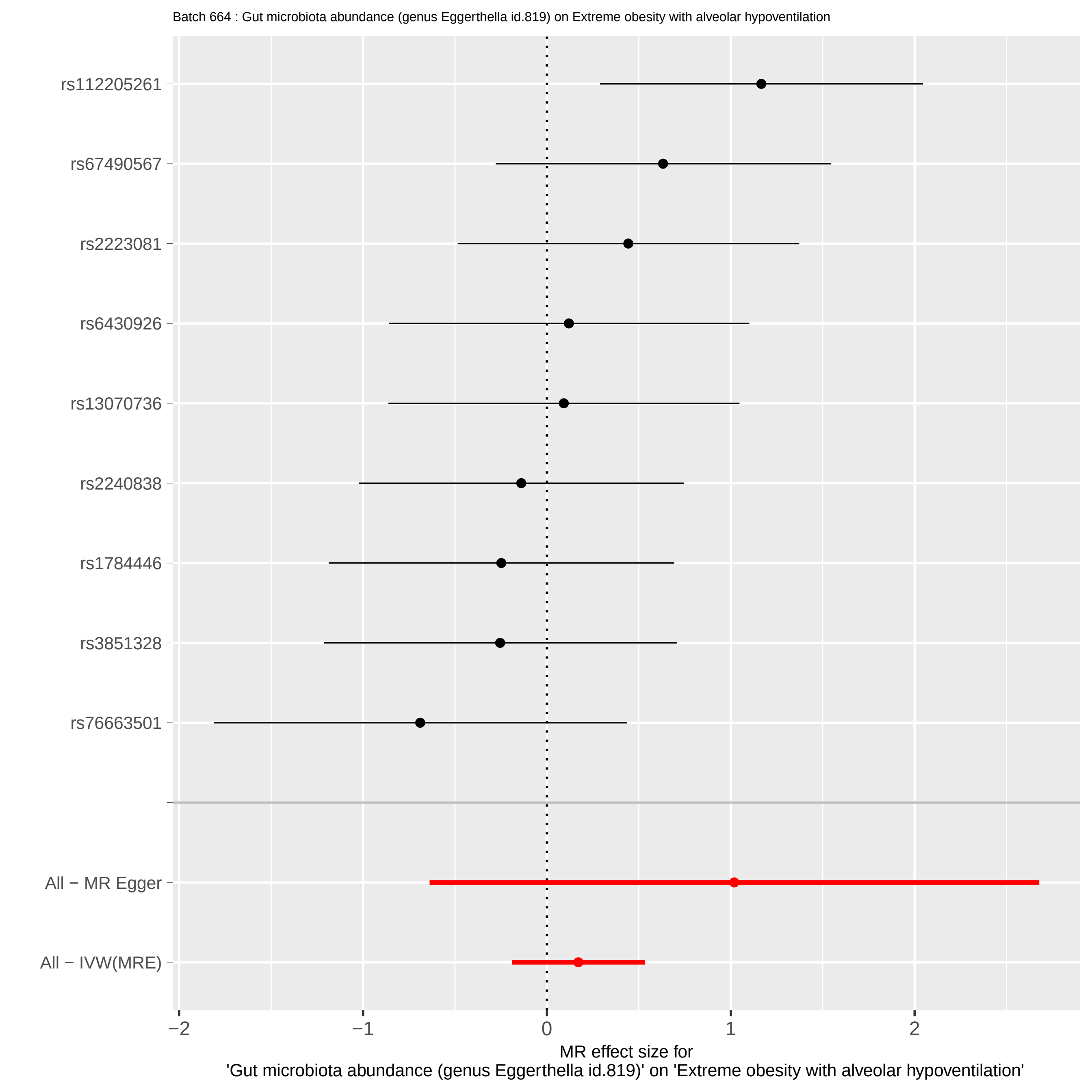
Batch 664 : Gut microbiota abundance (genus Eggerthella id.819) on Extreme obesity with alveolar hypoventilation
rs112205261
rs67490567
rs2223081
rs6430926
rs13070736
rs2240838
rs1784446
rs3851328
rs76663501
All − MR Egger
All − IVW(MRE)
−2
−1
0
1
2
MR effect size for
'Gut microbiota abundance (genus Eggerthella id.819)' on 'Extreme obesity with alveolar hypoventilation'

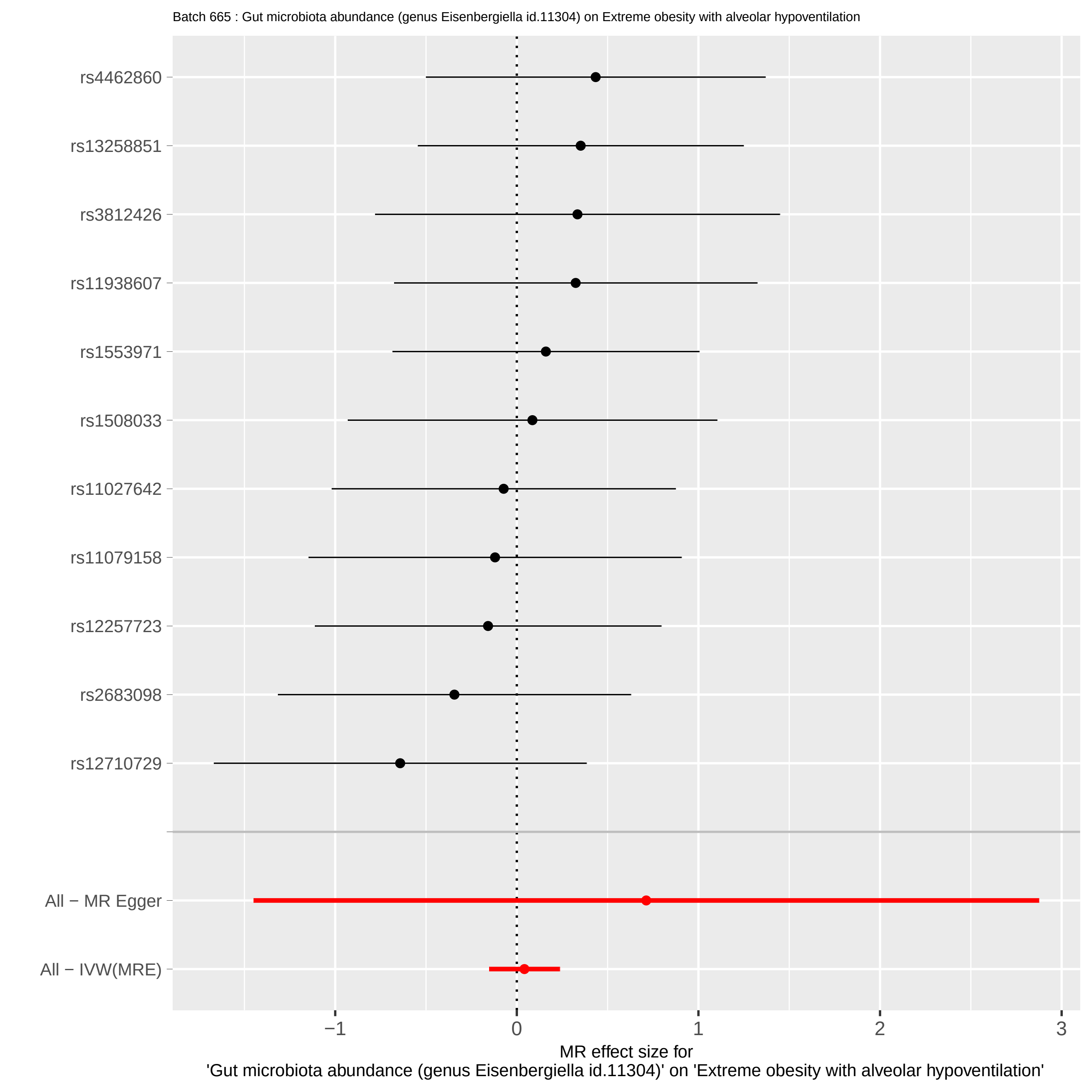
Batch 665 : Gut microbiota abundance (genus Eisenbergiella id.11304) on Extreme obesity with alveolar hypoventilation
rs4462860
rs13258851
rs3812426
rs11938607
rs1553971
rs1508033
rs11027642
rs11079158
rs12257723
rs2683098
rs12710729
All − MR Egger
All − IVW(MRE)
−1
0
1
2
3
MR effect size for
'Gut microbiota abundance (genus Eisenbergiella id.11304)' on 'Extreme obesity with alveolar hypoventilation'

Batch 666 : Gut microbiota abundance (genus Enterorhabdus id.820) on Extreme obesity with alveolar hypoventilation

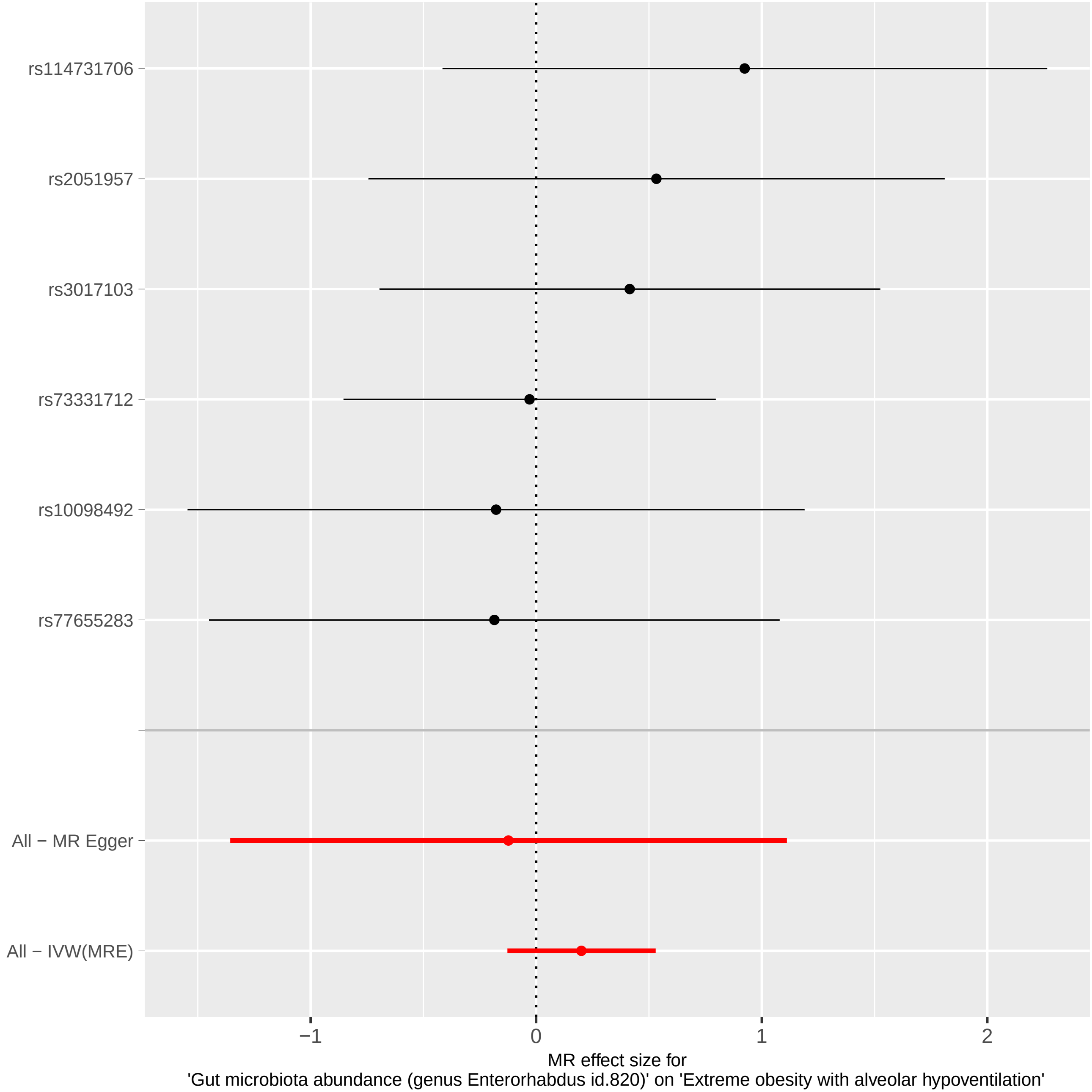

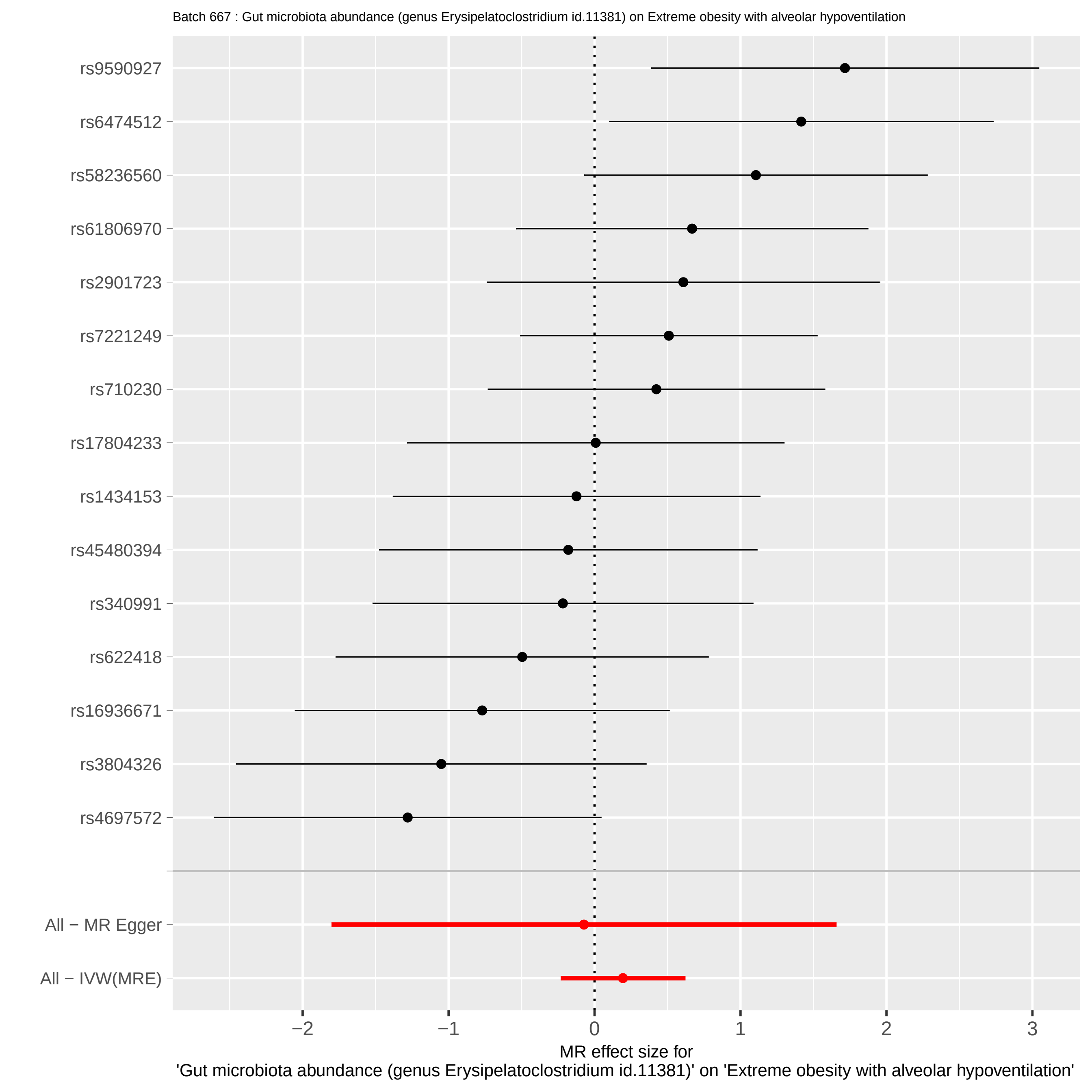

Batch 667 : Gut microbiota abundance (genus Erysipelatoclostridium id.11381) on Extreme obesity with alveolar hypoventilation
rs9590927
rs6474512
rs58236560
rs61806970
rs2901723
rs7221249
rs710230
rs17804233
rs1434153
rs45480394
rs340991
rs622418
rs16936671
rs3804326
rs4697572
All − MR Egger
All − IVW(MRE)
−2
−1
0
1
2
3
MR effect size for
'Gut microbiota abundance (genus Erysipelatoclostridium id.11381)' on 'Extreme obesity with alveolar hypoventilation'

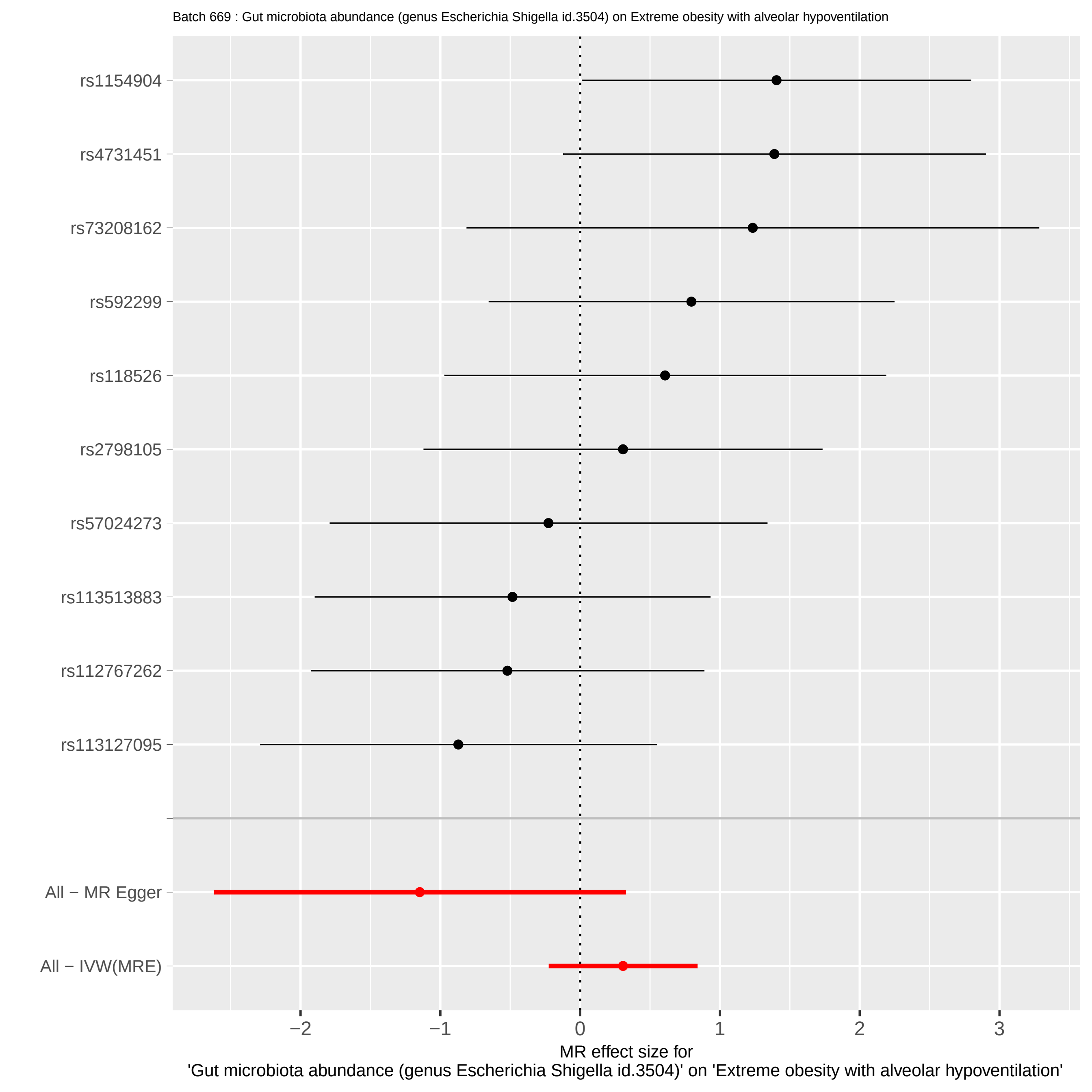

Batch 669 : Gut microbiota abundance (genus Escherichia Shigella id.3504) on Extreme obesity with alveolar hypoventilation
rs1154904
rs4731451
rs73208162
rs592299
rs118526
rs2798105
rs57024273
rs113513883
rs112767262
rs113127095
All − MR Egger
All − IVW(MRE)
−2
−1
0
1
2
3
MR effect size for
'Gut microbiota abundance (genus Escherichia Shigella id.3504)' on 'Extreme obesity with alveolar hypoventilation'

Batch 670 : Gut microbiota abundance (genus Eubacterium brachy group id.11296) on Extreme obesity with alveolar hypoventilation

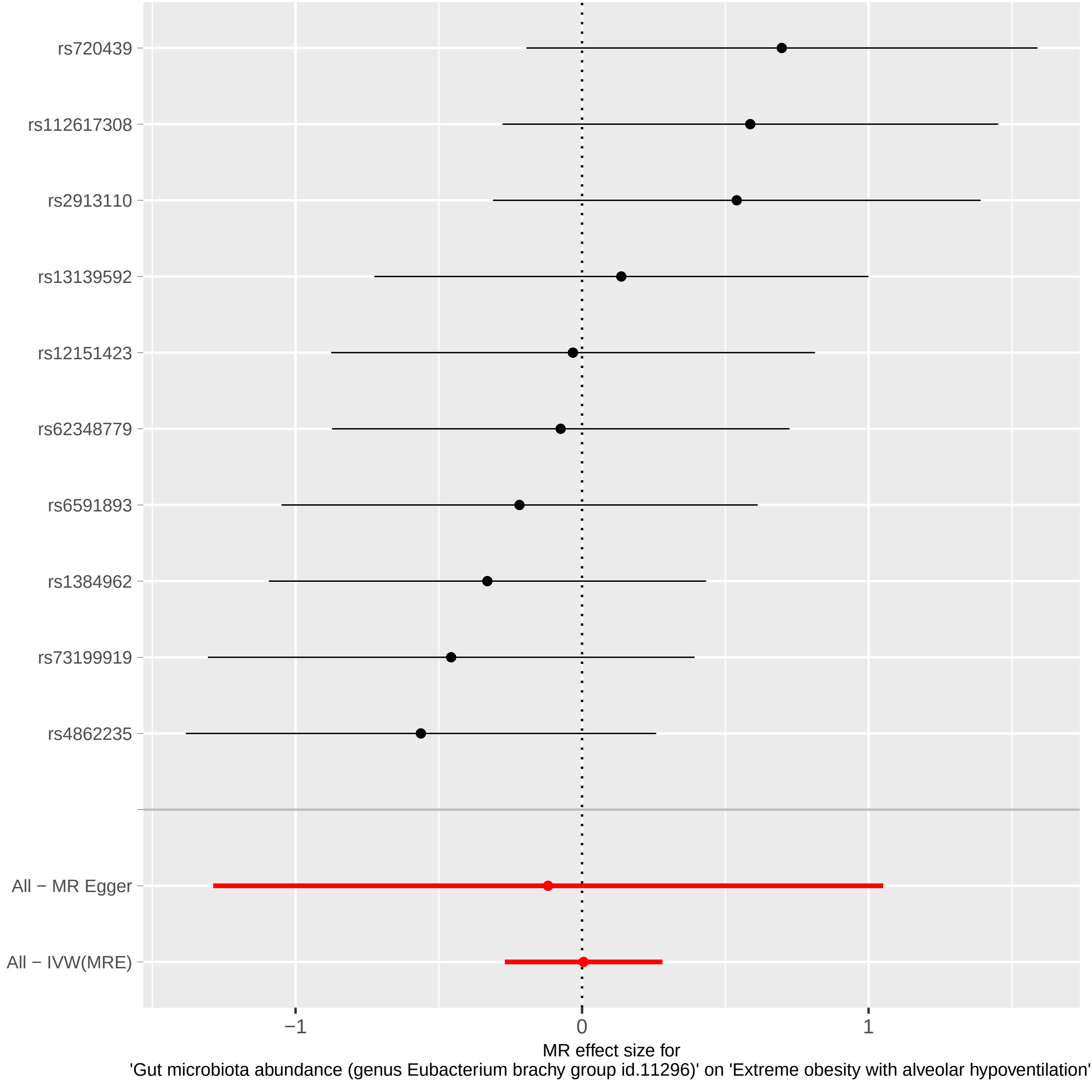

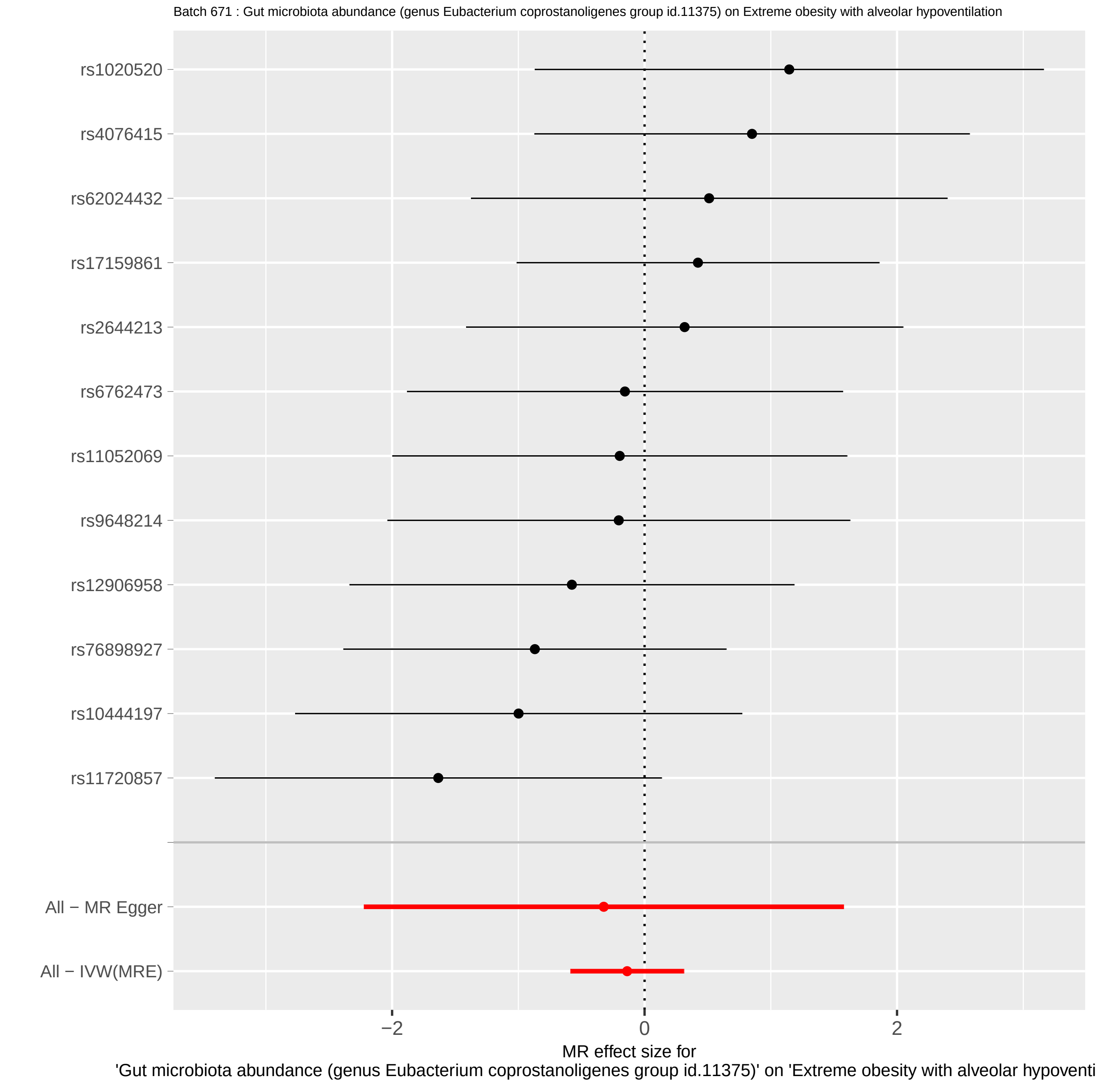

Batch 671 : Gut microbiota abundance (genus Eubacterium coprostanoligenes group id.11375) on Extreme obesity with alveolar hypoventilation
rs1020520
rs4076415
rs62024432
rs17159861
rs2644213
rs6762473
rs11052069
rs9648214
rs12906958
rs76898927
rs10444197
rs11720857
All − MR Egger
All − IVW(MRE)
−2
0
2
MR effect size for
'Gut microbiota abundance (genus Eubacterium coprostanoligenes group id.11375)' on 'Extreme obesity with alveolar hypoventi

Batch 672 : Gut microbiota abundance (genus Eubacterium eligens group id.14372) on Extreme obesity with alveolar hypoventilation

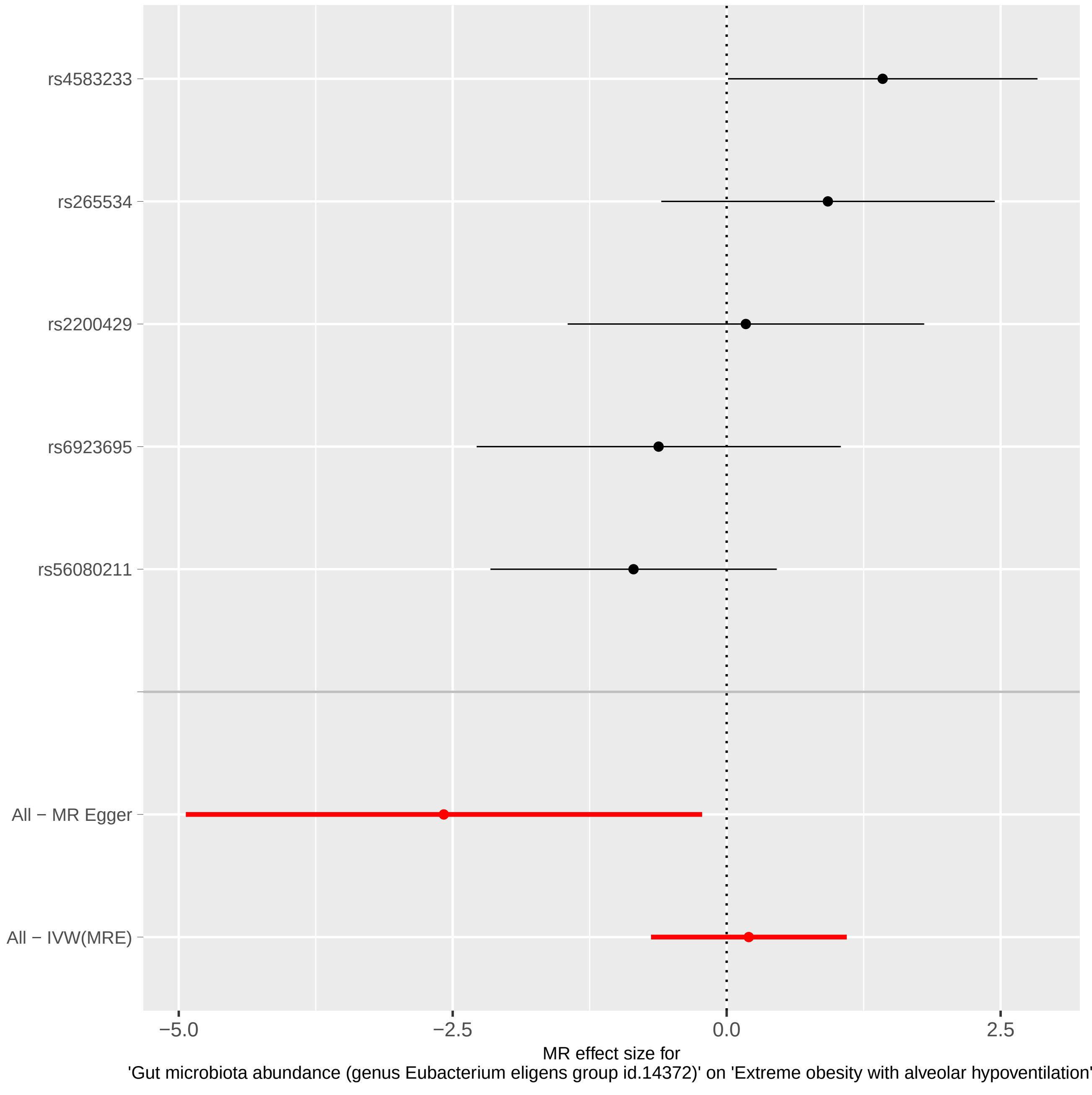

Batch 673 : Gut microbiota abundance (genus Eubacterium fissicatena group id.14373) on Extreme obesity with alveolar hypoventilation

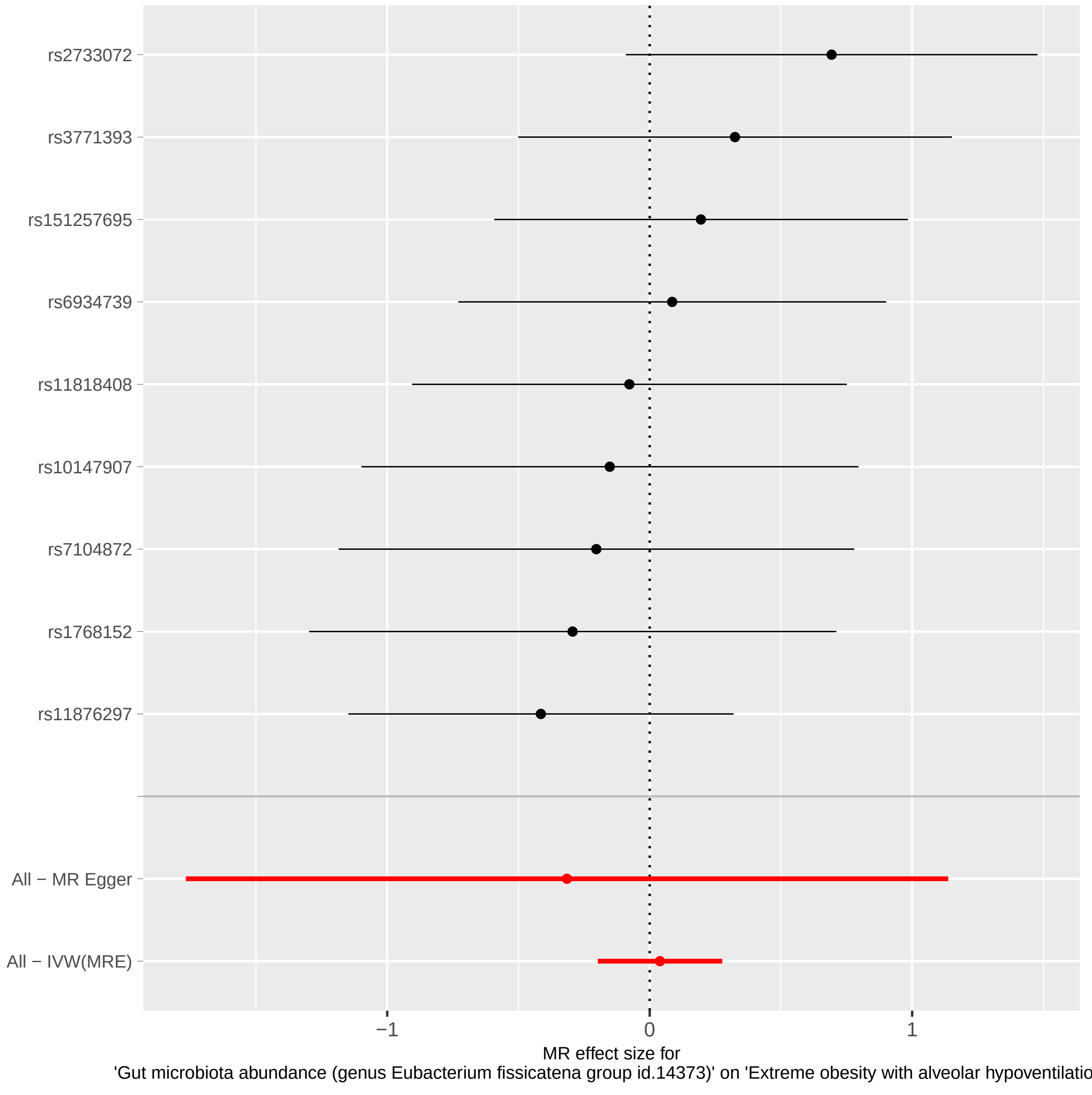

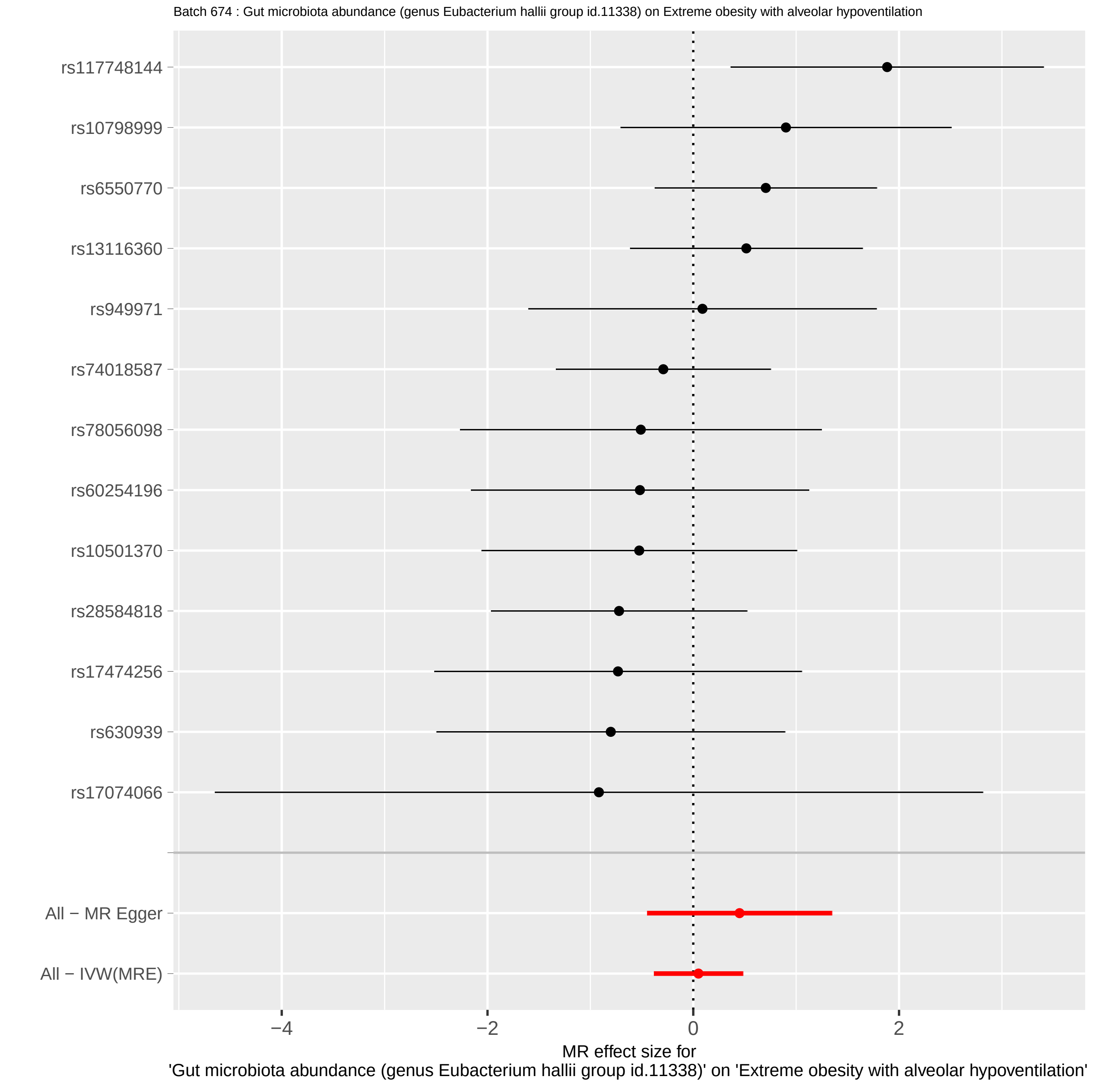

Batch 674 : Gut microbiota abundance (genus Eubacterium hallii group id.11338) on Extreme obesity with alveolar hypoventilation
rs117748144
rs10798999
rs6550770
rs13116360
rs949971
rs74018587
rs78056098
rs60254196
rs10501370
rs28584818
rs17474256
rs630939
rs17074066
All − MR Egger
All − IVW(MRE)
−4
−2
0
2
MR effect size for
'Gut microbiota abundance (genus Eubacterium hallii group id.11338)' on 'Extreme obesity with alveolar hypoventilation'

Batch 675 : Gut microbiota abundance (genus Eubacterium nodatum group id.11297) on Extreme obesity with alveolar hypoventilation

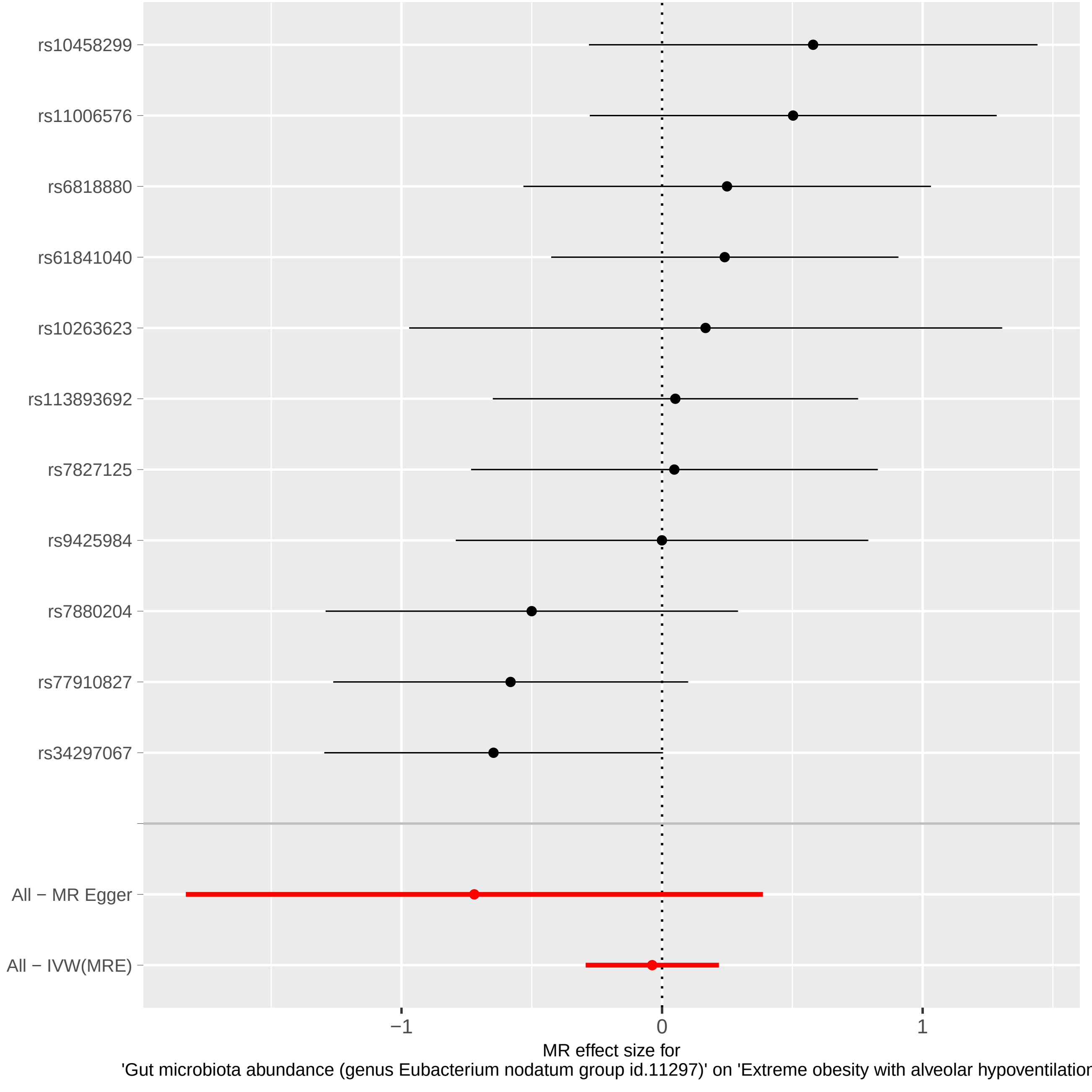

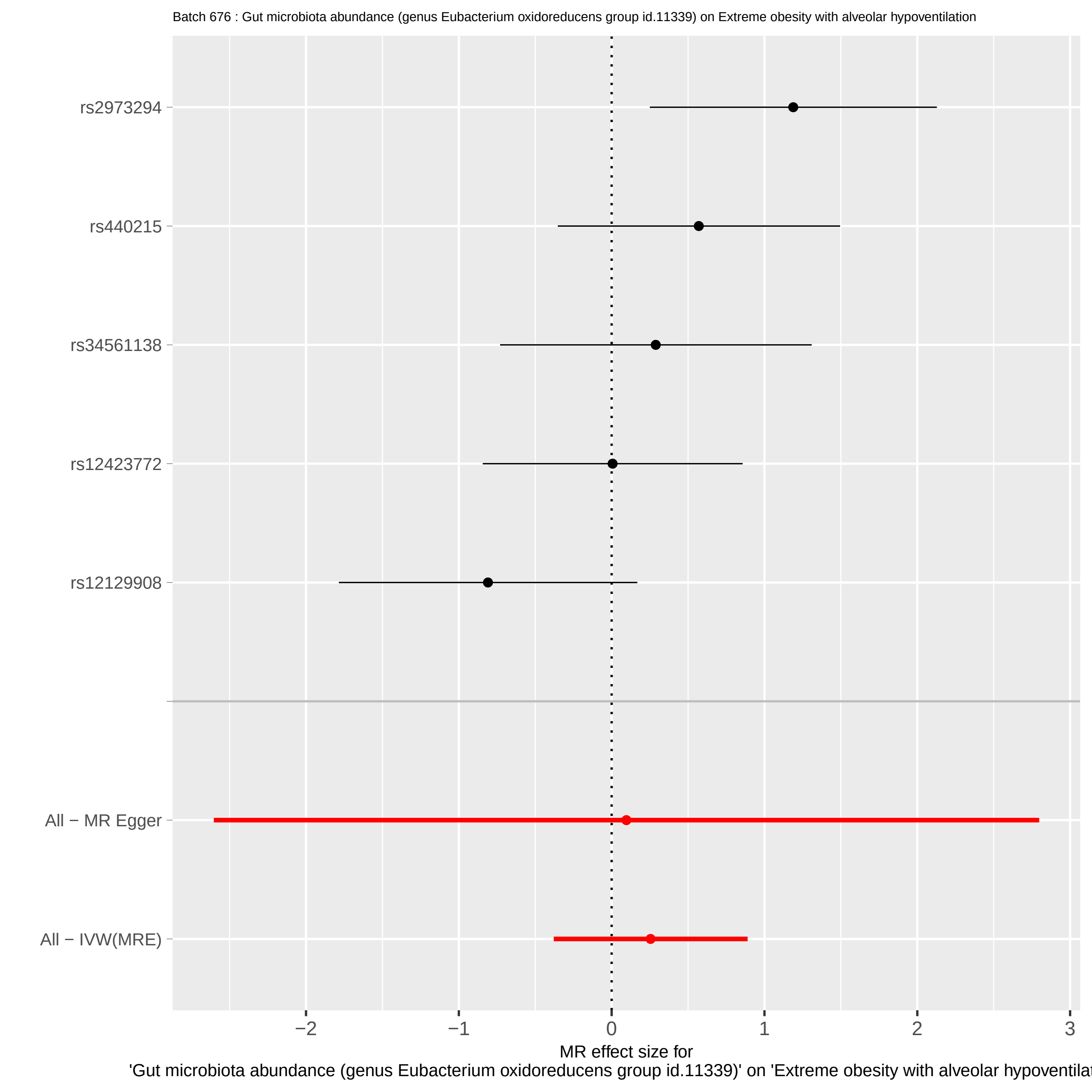

Batch 676 : Gut microbiota abundance (genus Eubacterium oxidoreducens group id.11339) on Extreme obesity with alveolar hypoventilation
rs2973294
rs440215
rs34561138
rs12423772
rs12129908
All − MR Egger
All − IVW(MRE)
−2
−1
0
1
2
3
MR effect size for
'Gut microbiota abundance (genus Eubacterium oxidoreducens group id.11339)' on 'Extreme obesity with alveolar hypoventilat

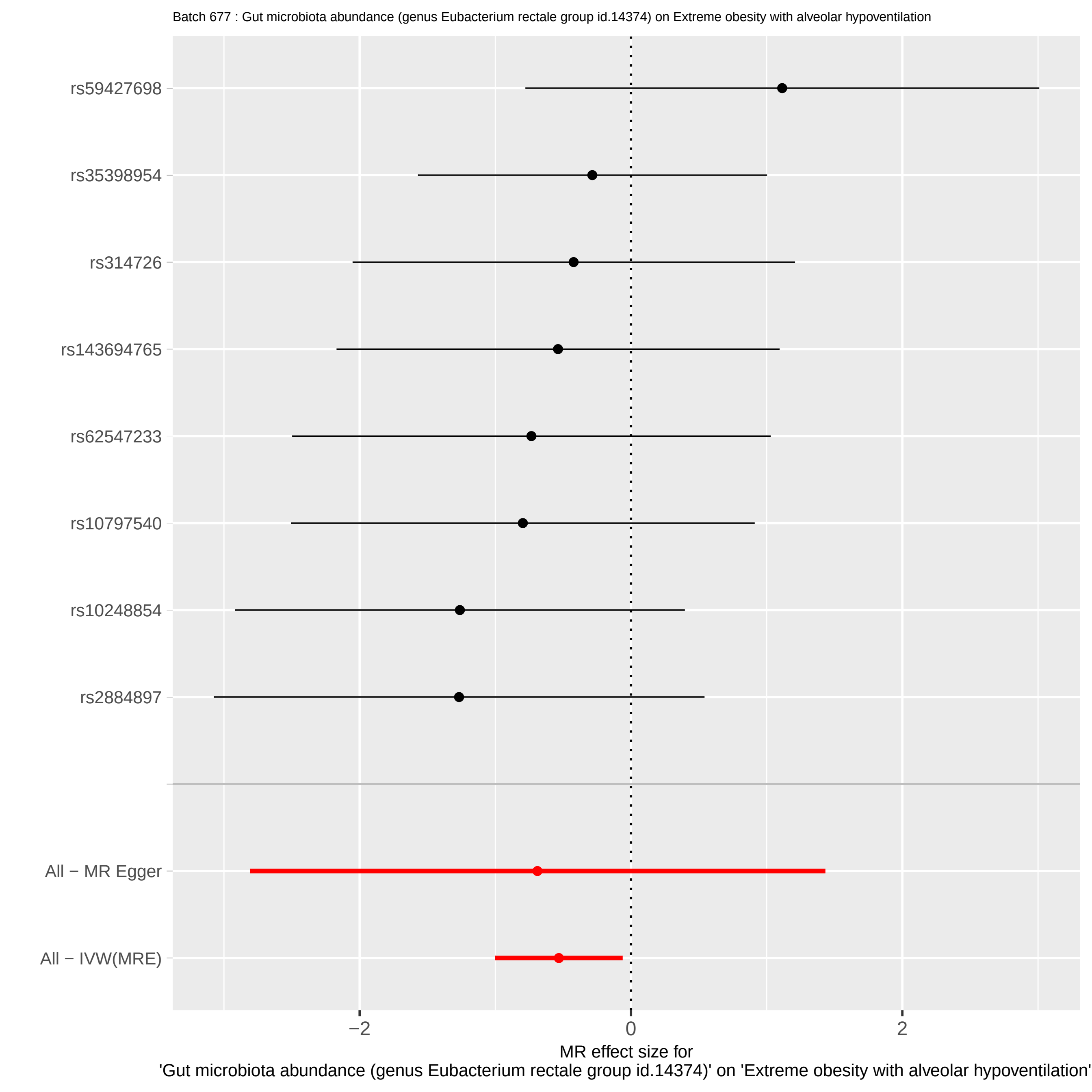
Batch 677 : Gut microbiota abundance (genus Eubacterium rectale group id.14374) on Extreme obesity with alveolar hypoventilation
rs59427698
rs35398954
rs314726
rs143694765
rs62547233
rs10797540
rs10248854
rs2884897
All − MR Egger
All − IVW(MRE)
−2
0
2
MR effect size for
'Gut microbiota abundance (genus Eubacterium rectale group id.14374)' on 'Extreme obesity with alveolar hypoventilation'

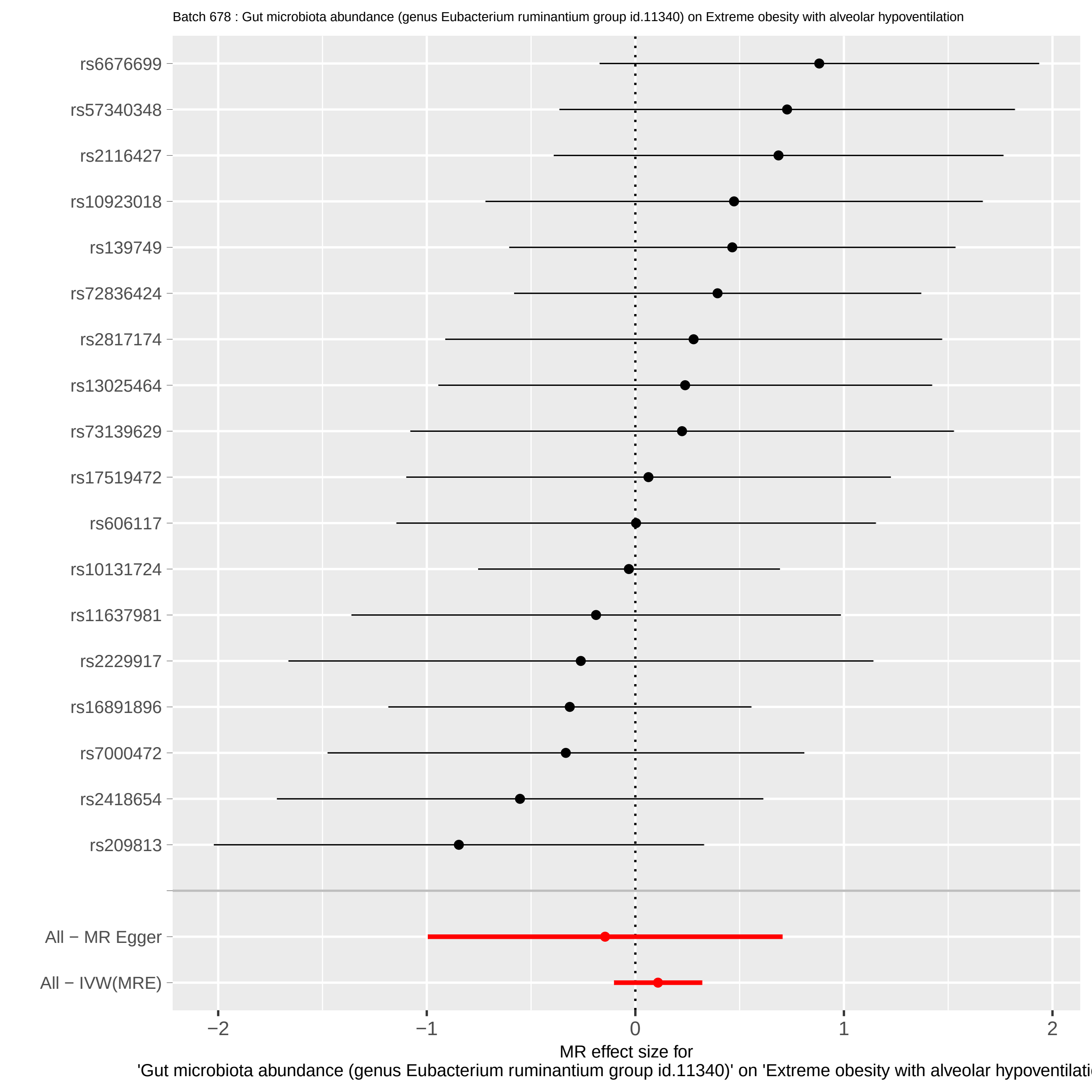

Batch 678 : Gut microbiota abundance (genus Eubacterium ruminantium group id.11340) on Extreme obesity with alveolar hypoventilation
rs6676699
rs57340348
rs2116427
rs10923018
rs139749
rs72836424
rs2817174
rs13025464
rs73139629
rs17519472
rs606117
rs10131724
rs11637981
rs2229917
rs16891896
rs7000472
rs2418654
rs209813
All − MR Egger
All − IVW(MRE)
−2
−1
0
1
2
MR effect size for
'Gut microbiota abundance (genus Eubacterium ruminantium group id.11340)' on 'Extreme obesity with alveolar hypoventilati

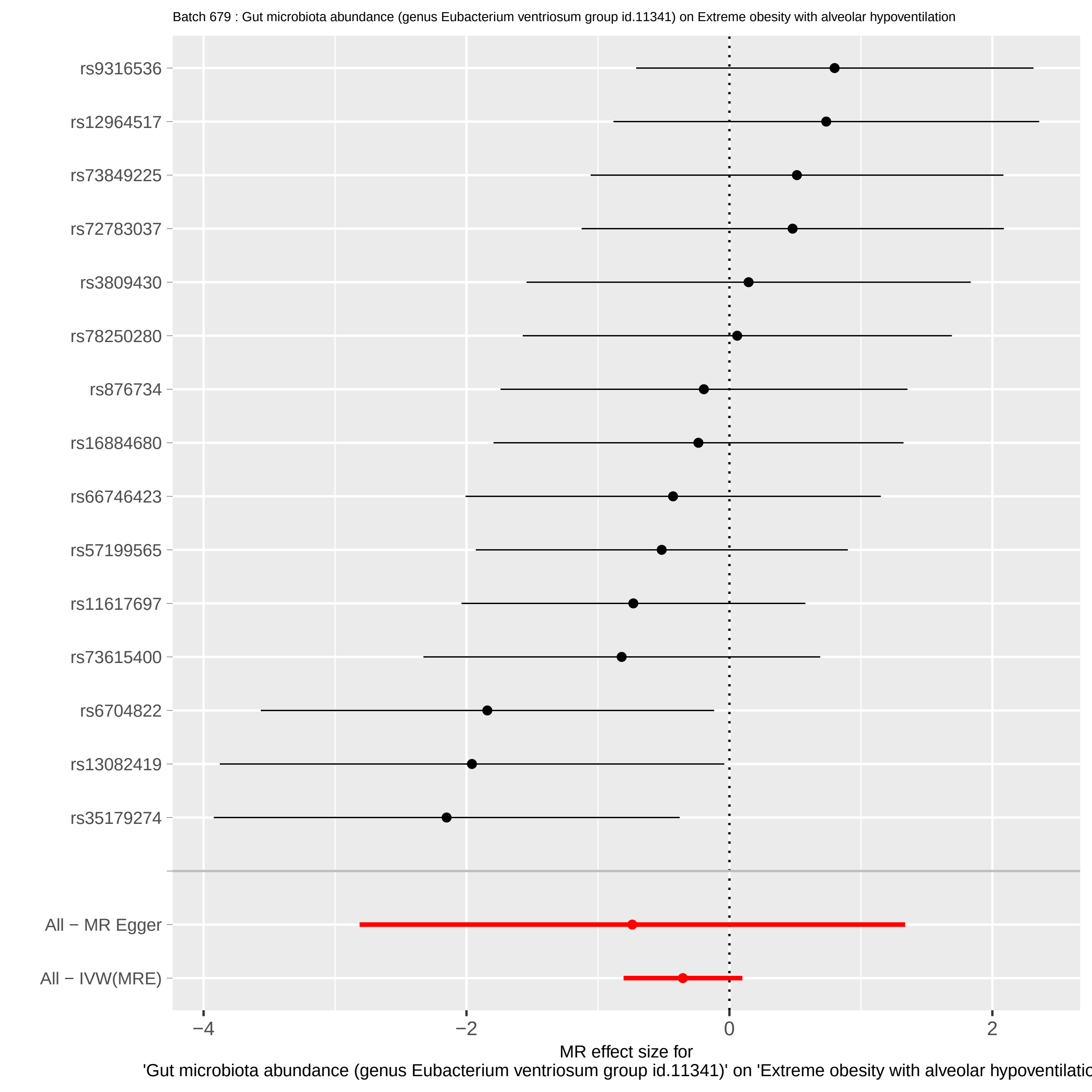

Batch 679 : Gut microbiota abundance (genus Eubacterium ventriosum group id.11341) on Extreme obesity with alveolar hypoventilation
rs9316536
rs12964517
rs73849225
rs72783037
rs3809430
rs78250280
rs876734
rs16884680
rs66746423
rs57199565
rs11617697
rs73615400
rs6704822
rs13082419
rs35179274
All − MR Egger
All − IVW(MRE)
−4
−2
0
2
MR effect size for
'Gut microbiota abundance (genus Eubacterium ventriosum group id.11341)' on 'Extreme obesity with alveolar hypoventilatio

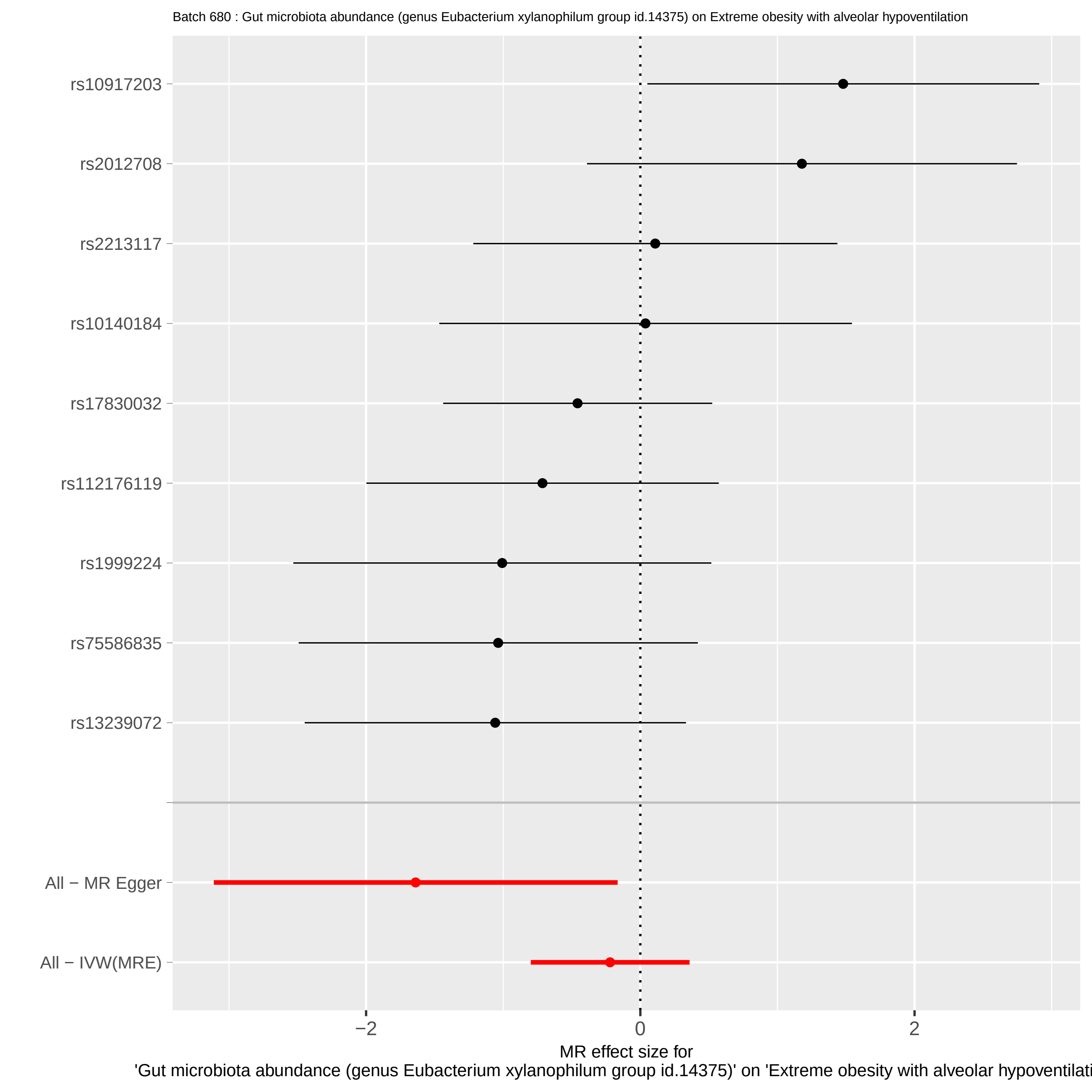
Batch 680 : Gut microbiota abundance (genus Eubacterium xylanophilum group id.14375) on Extreme obesity with alveolar hypoventilation
rs10917203
rs2012708
rs2213117
rs10140184
rs17830032
rs112176119
rs1999224
rs75586835
rs13239072
All − MR Egger
All − IVW(MRE)
−2
0
2
MR effect size for
'Gut microbiota abundance (genus Eubacterium xylanophilum group id.14375)' on 'Extreme obesity with alveolar hypoventilati

Batch 681 : Gut microbiota abundance (genus Faecalibacterium id.2057) on Extreme obesity with alveolar hypoventilation

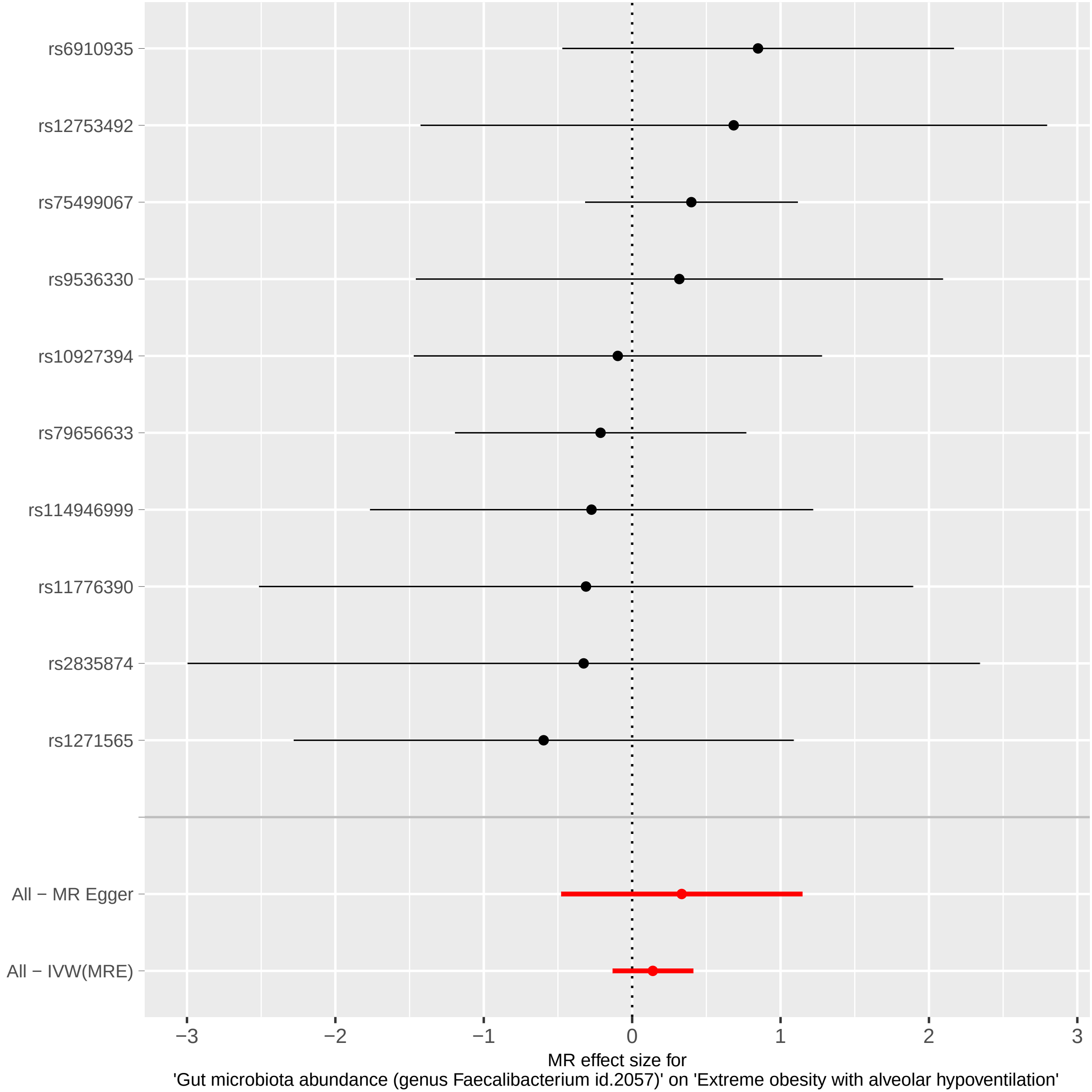

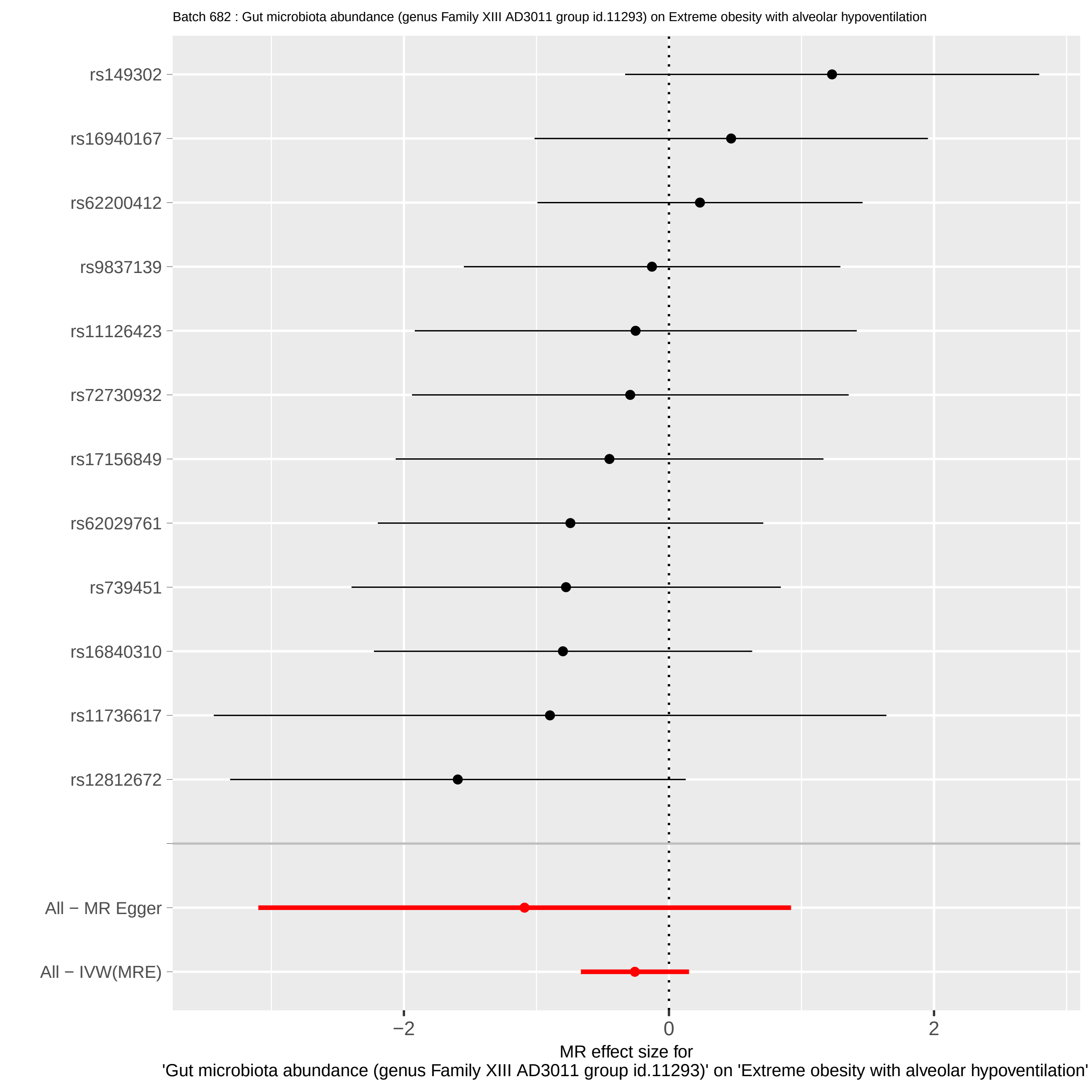

Batch 682 : Gut microbiota abundance (genus Family XIII AD3011 group id.11293) on Extreme obesity with alveolar hypoventilation
rs149302
rs16940167
rs62200412
rs9837139
rs11126423
rs72730932
rs17156849
rs62029761
rs739451
rs16840310
rs11736617
rs12812672
All − MR Egger
All − IVW(MRE)
−2
0
2
MR effect size for
'Gut microbiota abundance (genus Family XIII AD3011 group id.11293)' on 'Extreme obesity with alveolar hypoventilation'

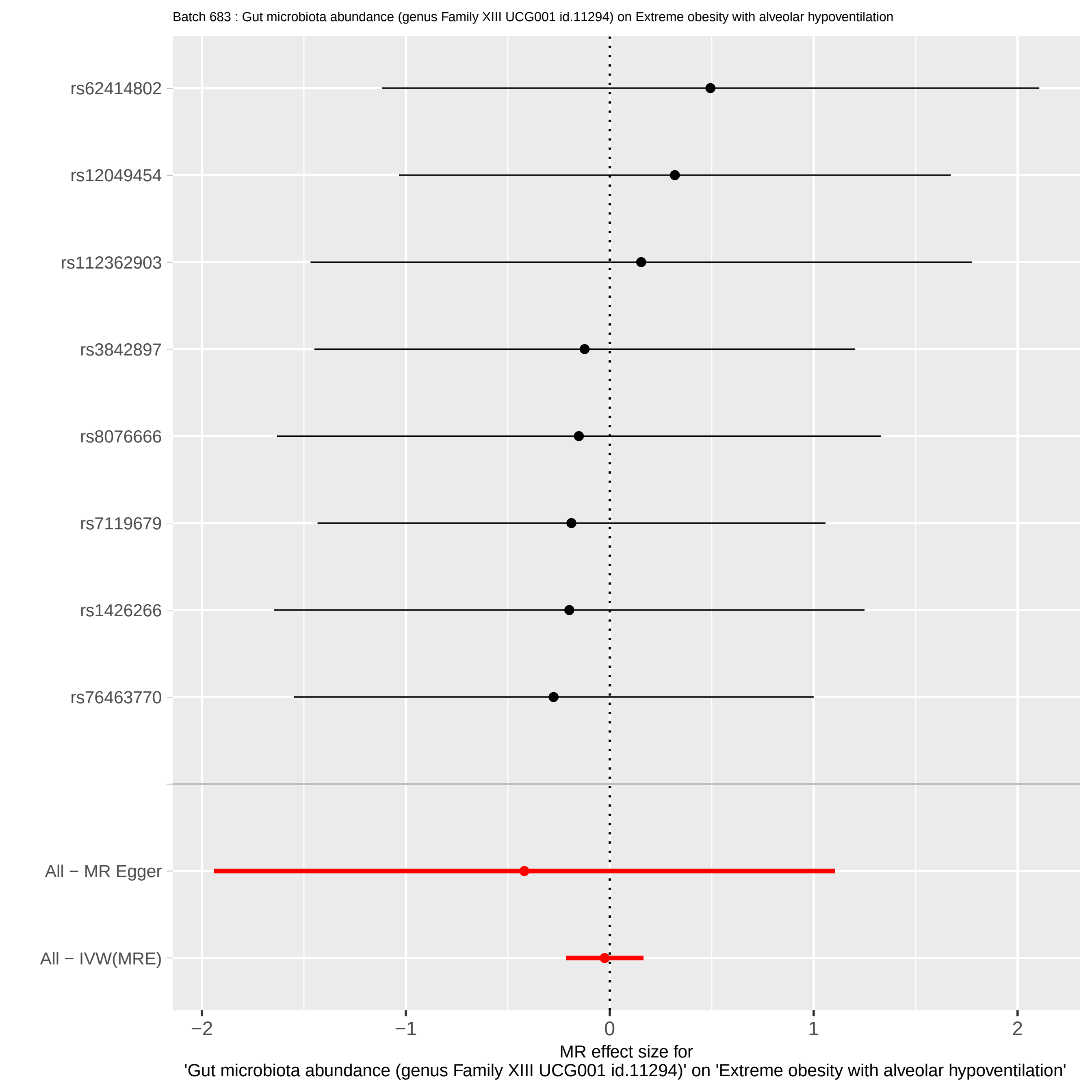

Batch 683 : Gut microbiota abundance (genus Family XIII UCG001 id.11294) on Extreme obesity with alveolar hypoventilation
rs62414802
rs12049454
rs112362903
rs3842897
rs8076666
rs7119679
rs1426266
rs76463770
All − MR Egger
All − IVW(MRE)
−2
−1
0
1
2
MR effect size for
'Gut microbiota abundance (genus Family XIII UCG001 id.11294)' on 'Extreme obesity with alveolar hypoventilation'

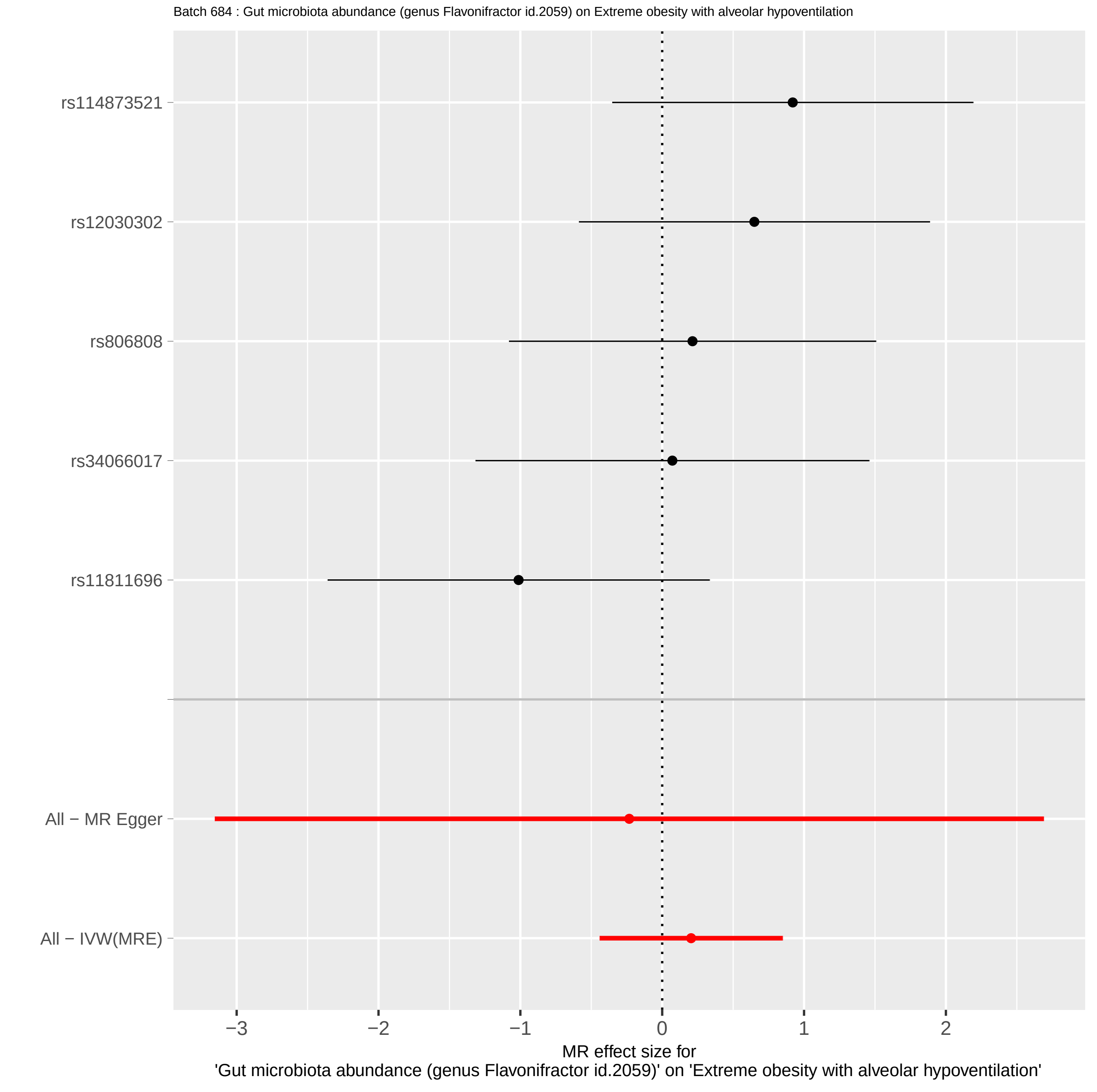
Batch 684 : Gut microbiota abundance (genus Flavonifractor id.2059) on Extreme obesity with alveolar hypoventilation
rs114873521
rs12030302
rs806808
rs34066017
rs11811696
All − MR Egger
All − IVW(MRE)
−3
−2
−1
0
1
2
MR effect size for
'Gut microbiota abundance (genus Flavonifractor id.2059)' on 'Extreme obesity with alveolar hypoventilation'

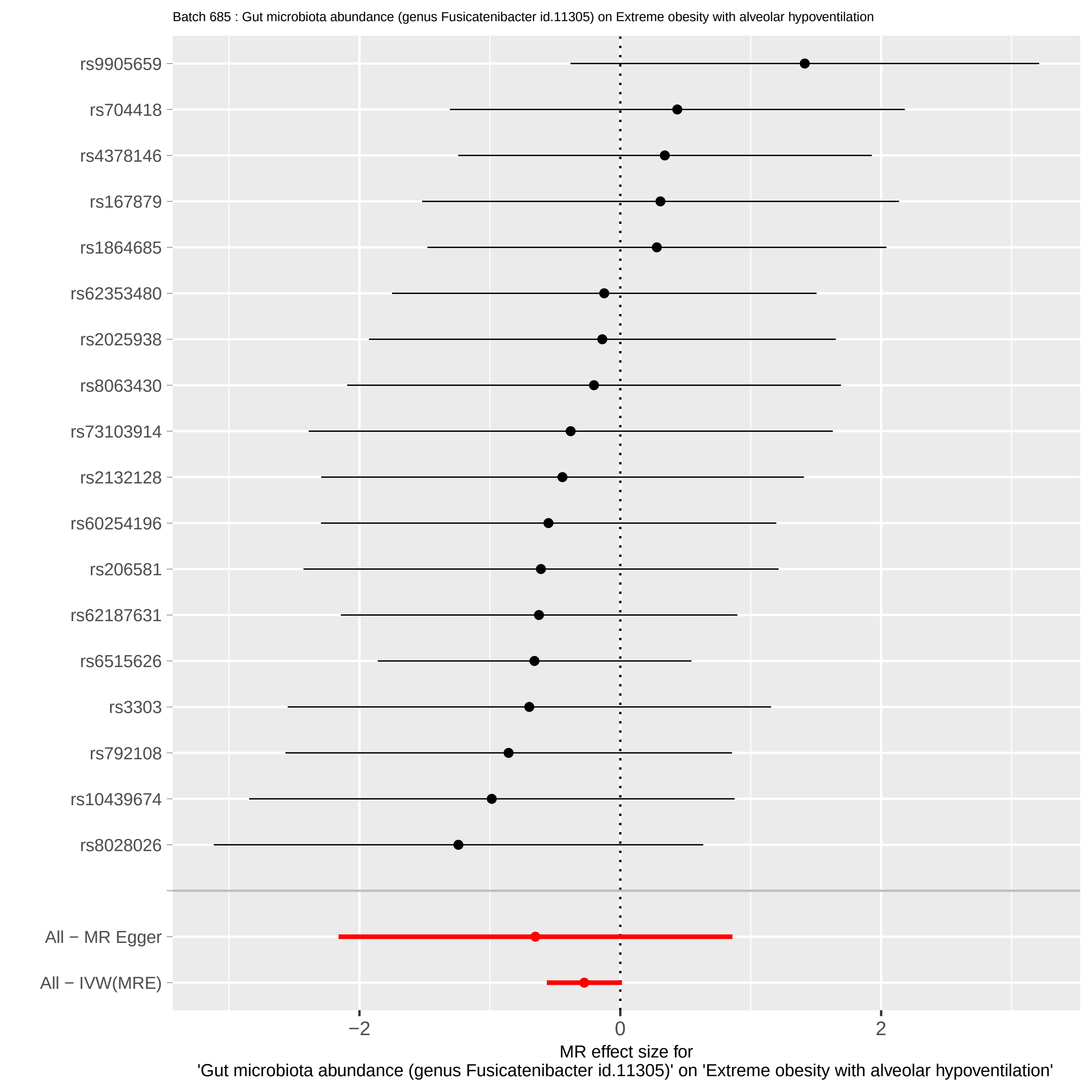

Batch 685 : Gut microbiota abundance (genus Fusicatenibacter id.11305) on Extreme obesity with alveolar hypoventilation
rs9905659
rs704418
rs4378146
rs167879
rs1864685
rs62353480
rs2025938
rs8063430
rs73103914
rs2132128
rs60254196
rs206581
rs62187631
rs6515626
rs3303
rs792108
rs10439674
rs8028026
All − MR Egger
All − IVW(MRE)
−2
0
2
MR effect size for
'Gut microbiota abundance (genus Fusicatenibacter id.11305)' on 'Extreme obesity with alveolar hypoventilation'

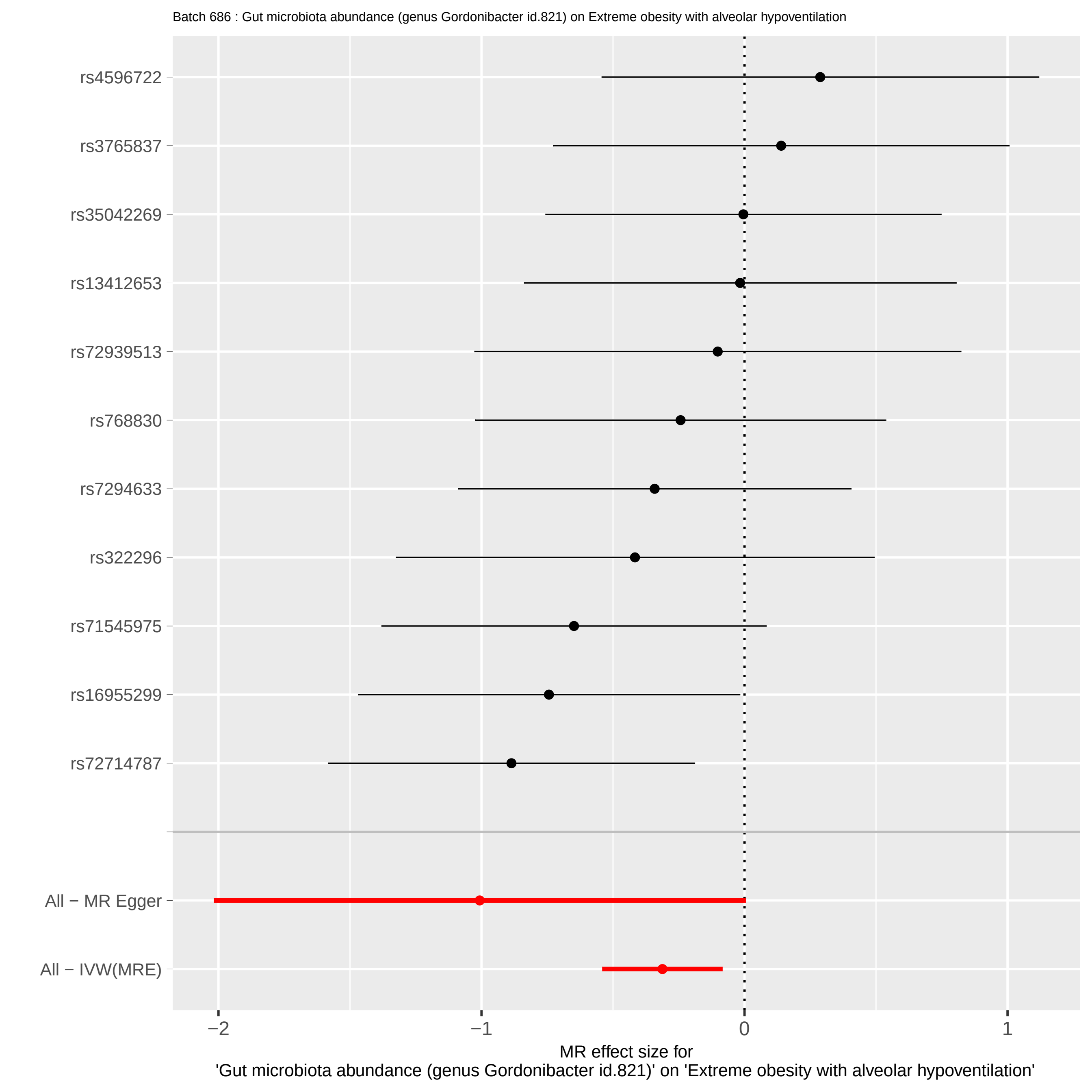
Batch 686 : Gut microbiota abundance (genus Gordonibacter id.821) on Extreme obesity with alveolar hypoventilation
rs4596722
rs3765837
rs35042269
rs13412653
rs72939513
rs768830
rs7294633
rs322296
rs71545975
rs16955299
rs72714787
All − MR Egger
All − IVW(MRE)
−2
−1
0
1
MR effect size for
'Gut microbiota abundance (genus Gordonibacter id.821)' on 'Extreme obesity with alveolar hypoventilation'

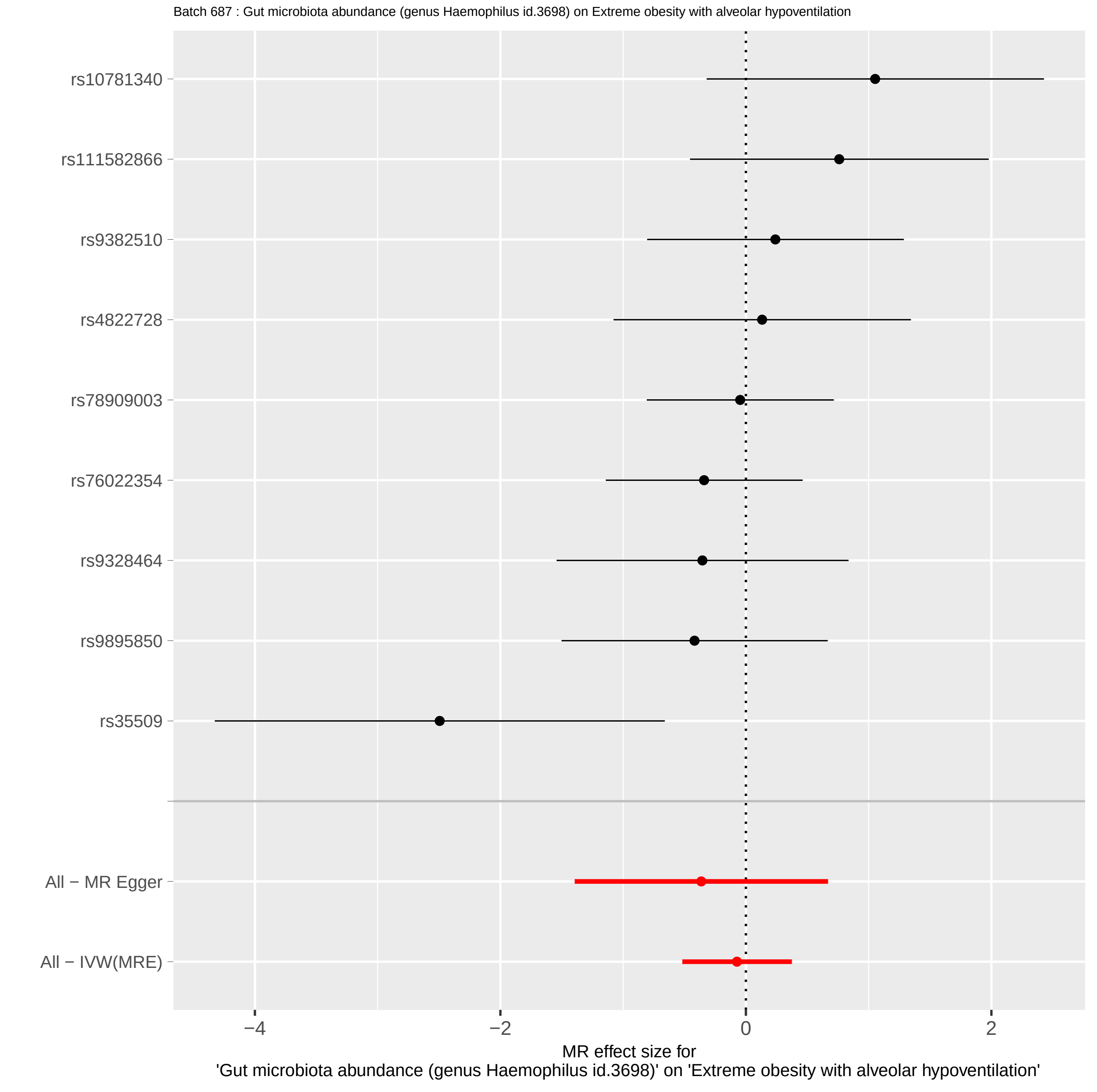

Batch 687 : Gut microbiota abundance (genus Haemophilus id.3698) on Extreme obesity with alveolar hypoventilation
rs10781340
rs111582866
rs9382510
rs4822728
rs78909003
rs76022354
rs9328464
rs9895850
rs35509
All − MR Egger
All − IVW(MRE)
−4
−2
0
2
MR effect size for
'Gut microbiota abundance (genus Haemophilus id.3698)' on 'Extreme obesity with alveolar hypoventilation'

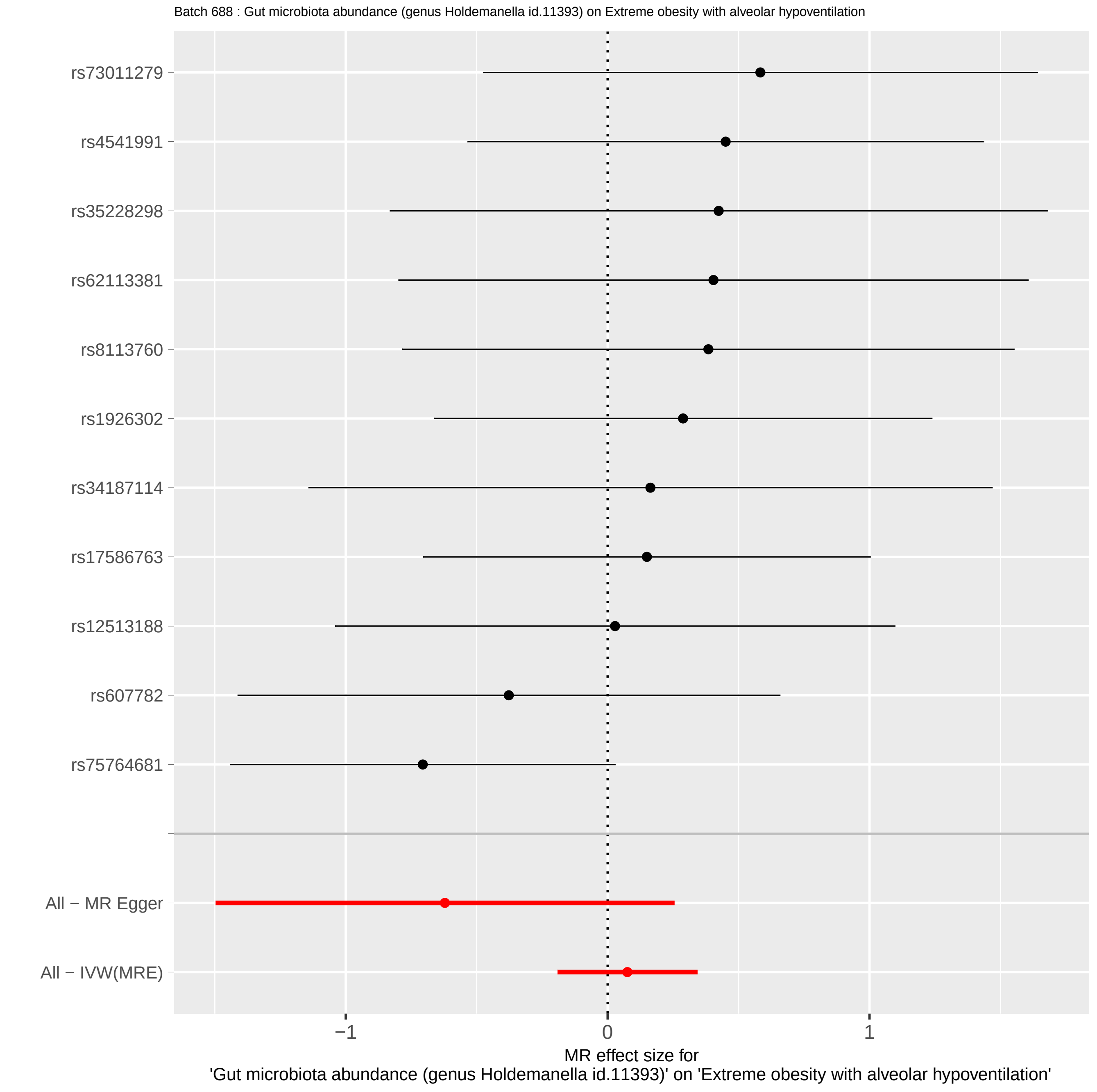

Batch 688 : Gut microbiota abundance (genus Holdemanella id.11393) on Extreme obesity with alveolar hypoventilation
rs73011279
rs4541991
rs35228298
rs62113381
rs8113760
rs1926302
rs34187114
rs17586763
rs12513188
rs607782
rs75764681
All − MR Egger
All − IVW(MRE)
−1
0
1
MR effect size for
'Gut microbiota abundance (genus Holdemanella id.11393)' on 'Extreme obesity with alveolar hypoventilation'

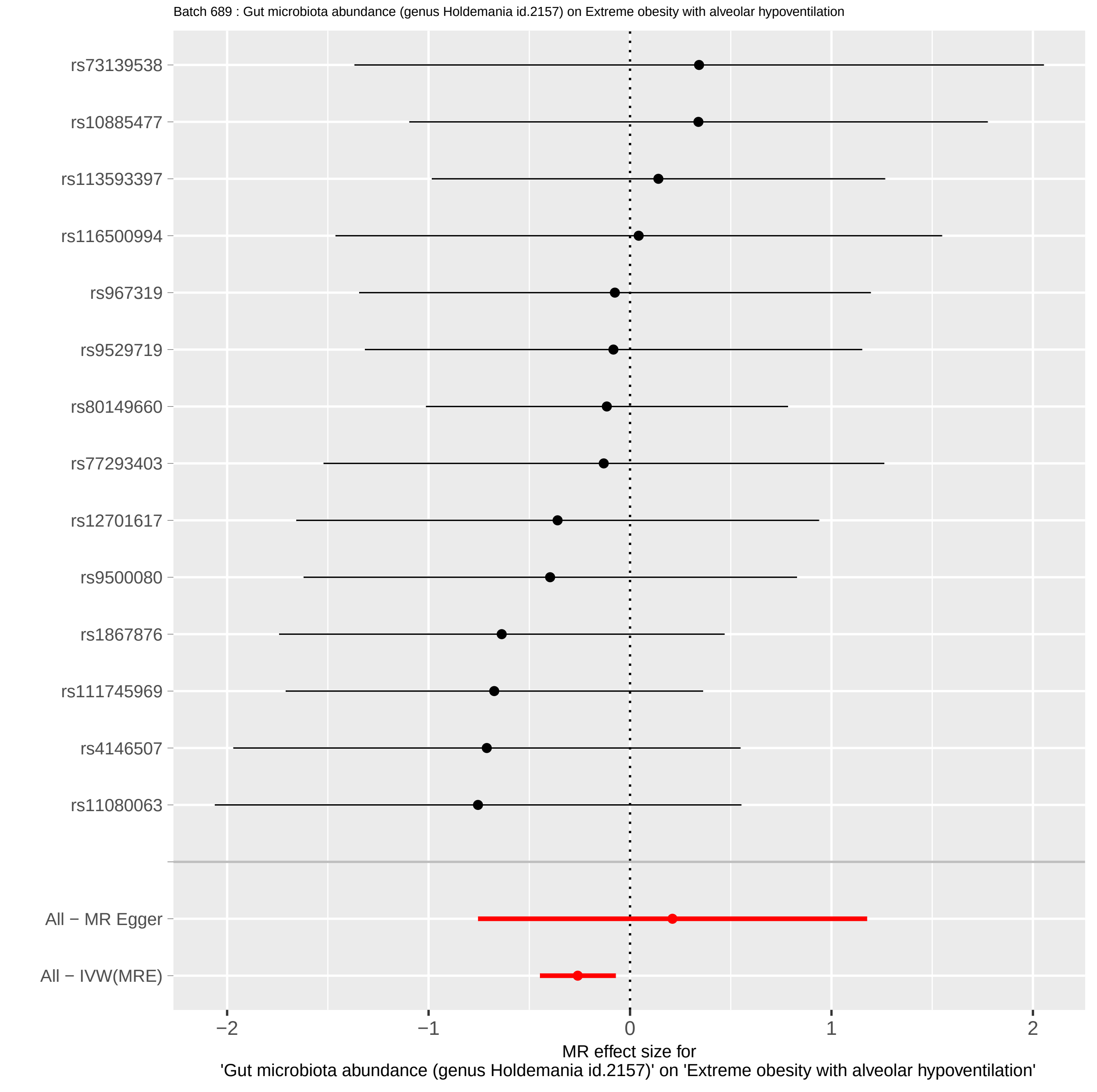

Batch 689 : Gut microbiota abundance (genus Holdemania id.2157) on Extreme obesity with alveolar hypoventilation
rs73139538
rs10885477
rs113593397
rs116500994
rs967319
rs9529719
rs80149660
rs77293403
rs12701617
rs9500080
rs1867876
rs111745969
rs4146507
rs11080063
All − MR Egger
All − IVW(MRE)
−2
−1
0
1
2
MR effect size for
'Gut microbiota abundance (genus Holdemania id.2157)' on 'Extreme obesity with alveolar hypoventilation'

Batch 690 : Gut microbiota abundance (genus Howardella id.2000) on Extreme obesity with alveolar hypoventilation

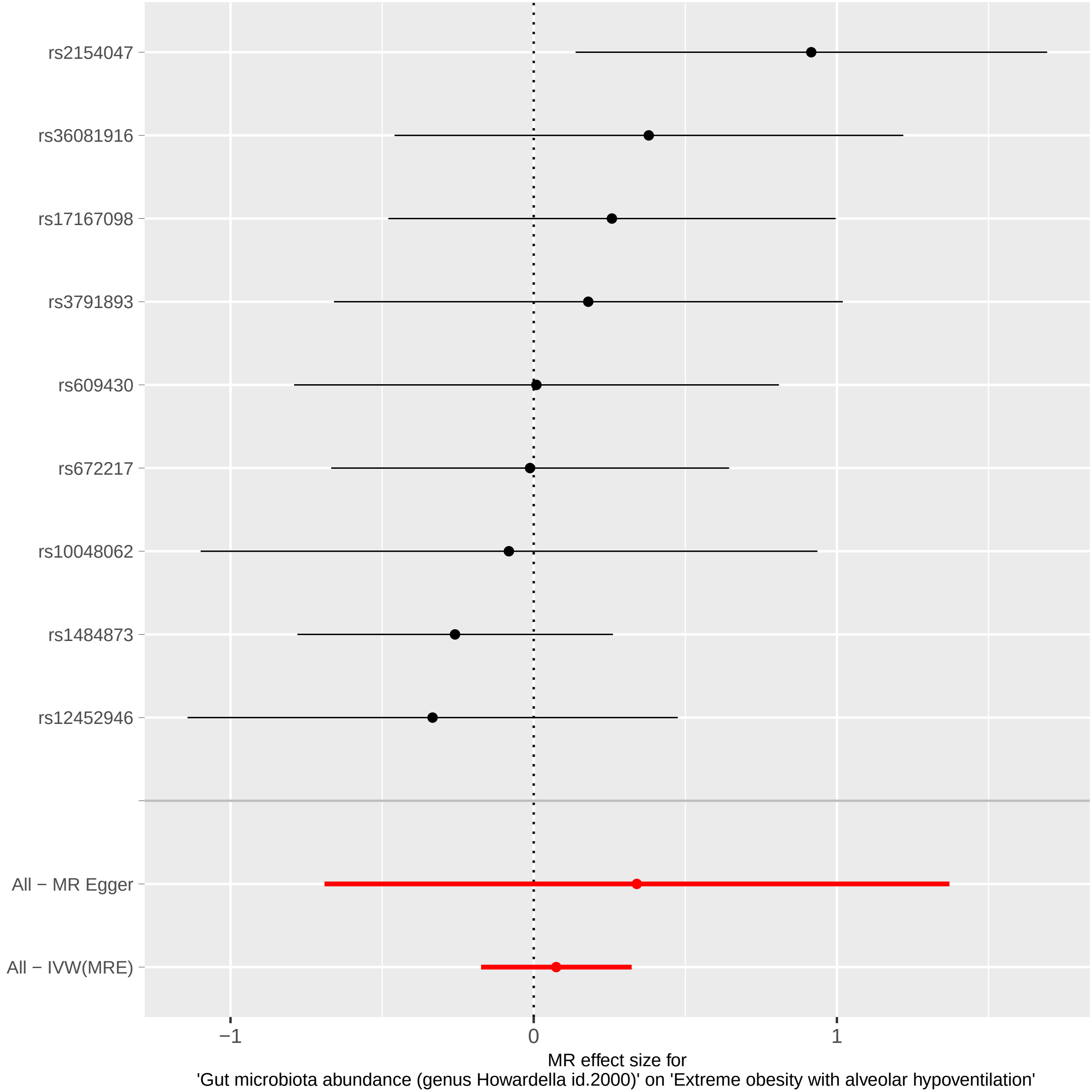

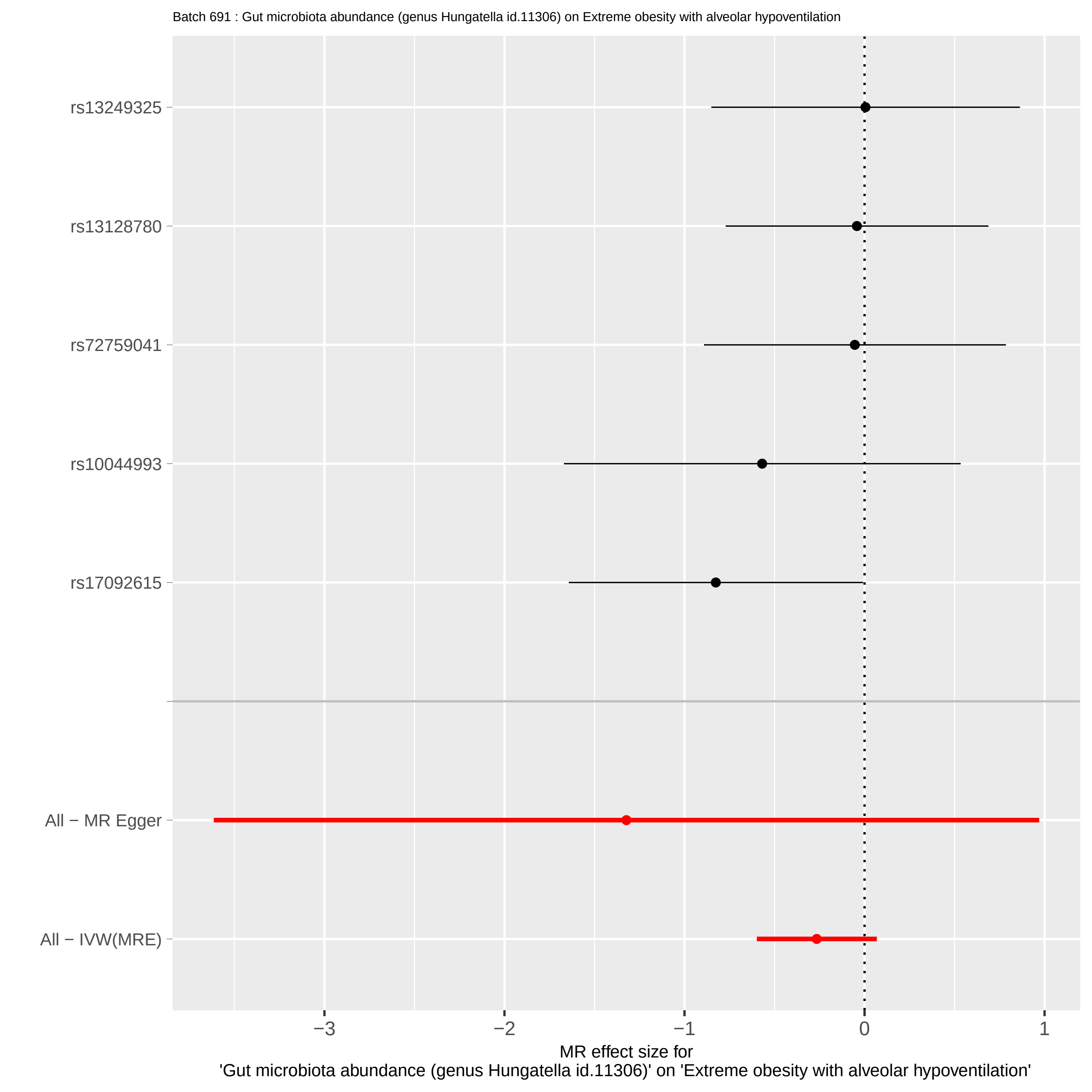

Batch 691 : Gut microbiota abundance (genus Hungatella id.11306) on Extreme obesity with alveolar hypoventilation
rs13249325
rs13128780
rs72759041
rs10044993
rs17092615
All − MR Egger
All − IVW(MRE)
−3
−2
−1
0
1
MR effect size for
'Gut microbiota abundance (genus Hungatella id.11306)' on 'Extreme obesity with alveolar hypoventilation'

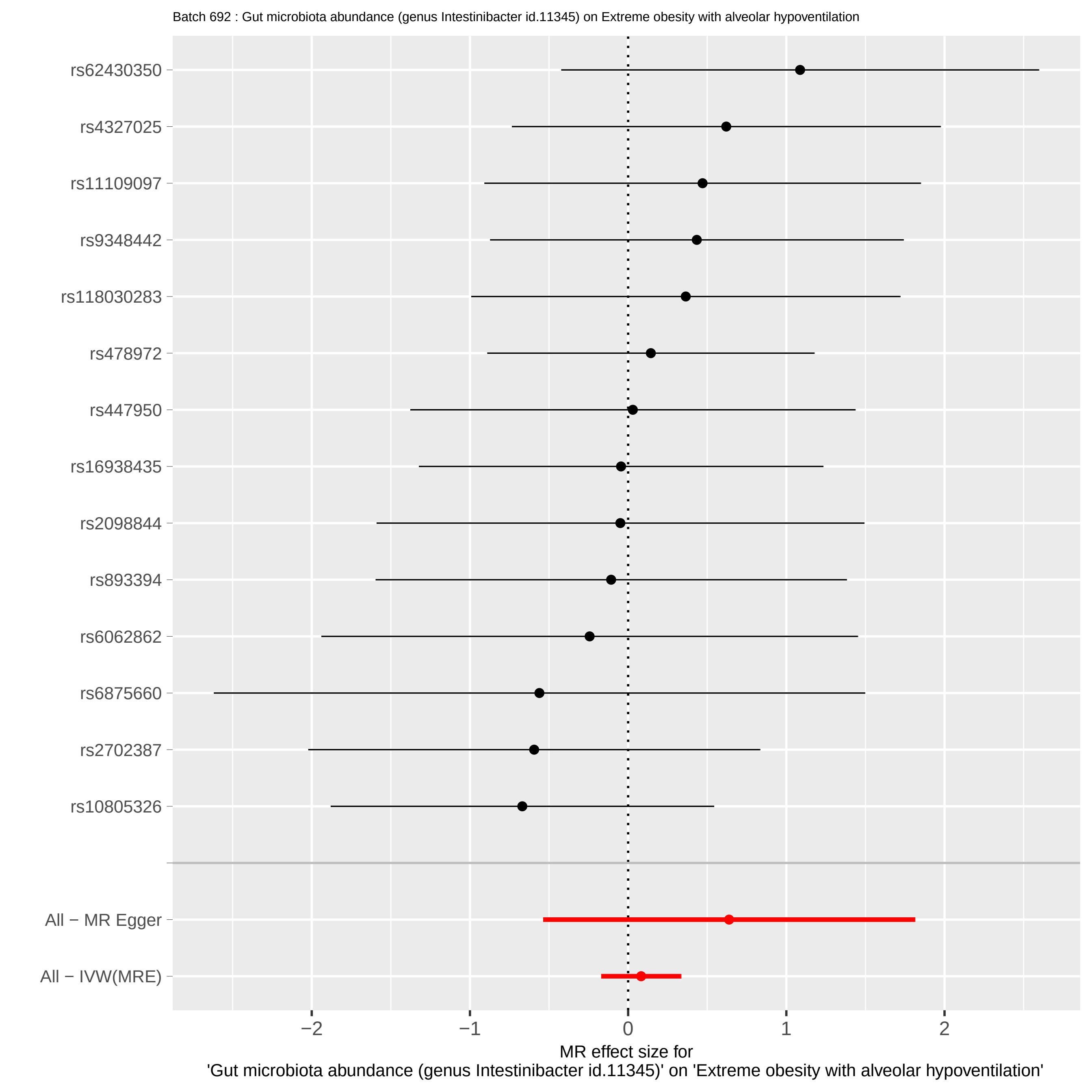
Batch 692 : Gut microbiota abundance (genus Intestinibacter id.11345) on Extreme obesity with alveolar hypoventilation
rs62430350
rs4327025
rs11109097
rs9348442
rs118030283
rs478972
rs447950
rs16938435
rs2098844
rs893394
rs6062862
rs6875660
rs2702387
rs10805326
All − MR Egger
All − IVW(MRE)
−2
−1
0
1
2
MR effect size for
'Gut microbiota abundance (genus Intestinibacter id.11345)' on 'Extreme obesity with alveolar hypoventilation'

Batch 693 : Gut microbiota abundance (genus Intestinimonas id.2062) on Extreme obesity with alveolar hypoventilation

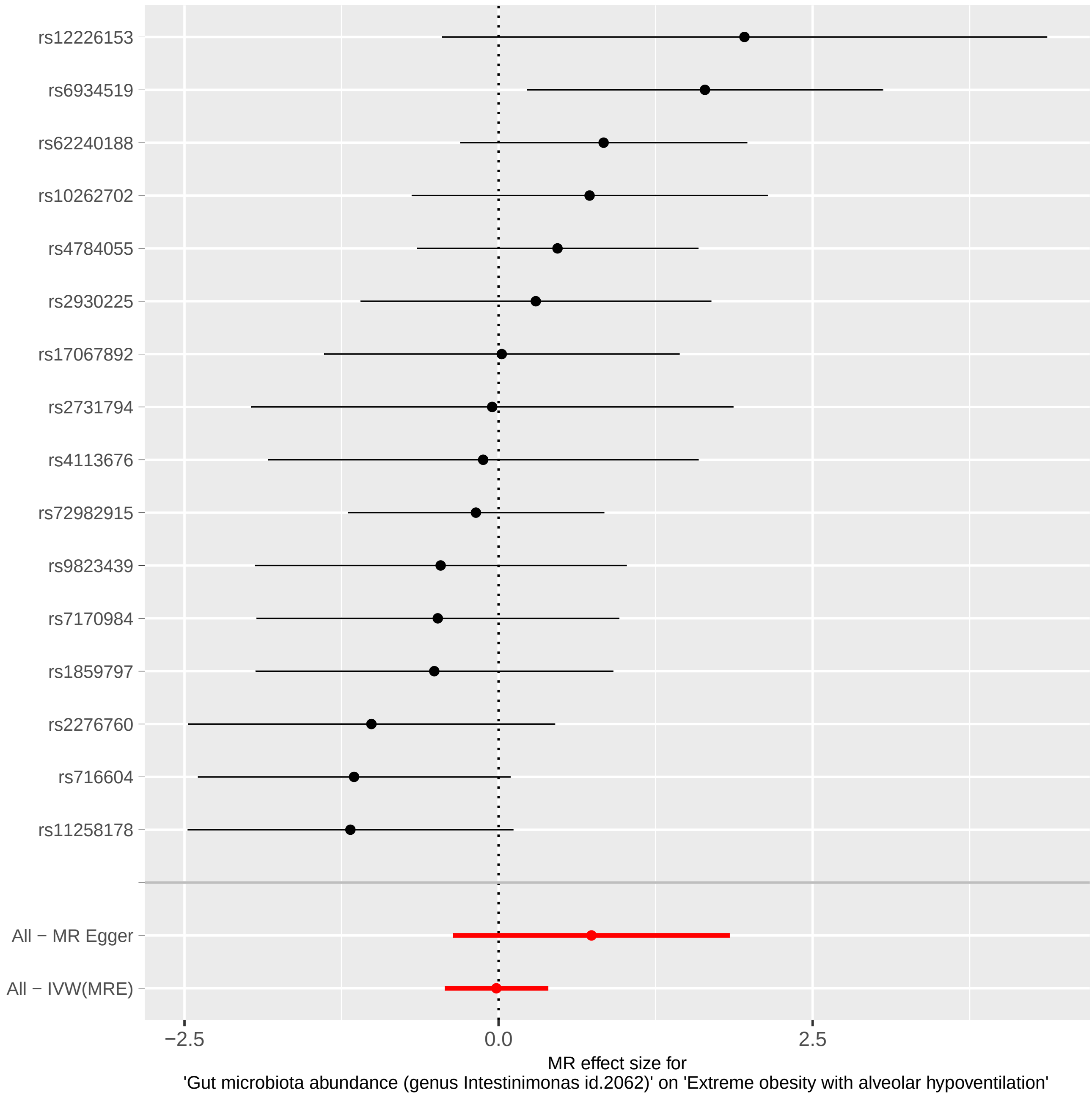

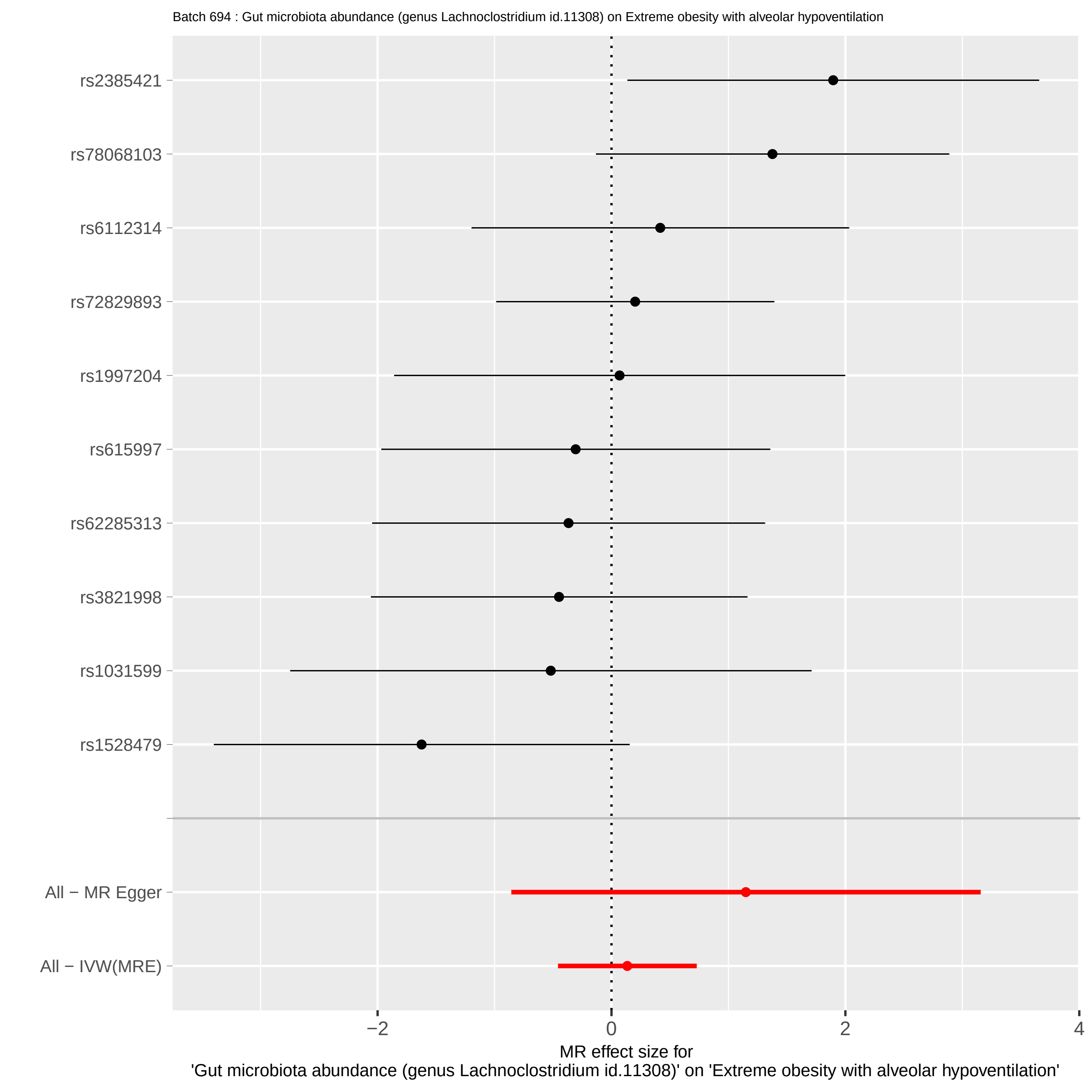

Batch 694 : Gut microbiota abundance (genus Lachnoclostridium id.11308) on Extreme obesity with alveolar hypoventilation
rs2385421
rs78068103
rs6112314
rs72829893
rs1997204
rs615997
rs62285313
rs3821998
rs1031599
rs1528479
All − MR Egger
All − IVW(MRE)
−2
0
2
4
MR effect size for
'Gut microbiota abundance (genus Lachnoclostridium id.11308)' on 'Extreme obesity with alveolar hypoventilation'

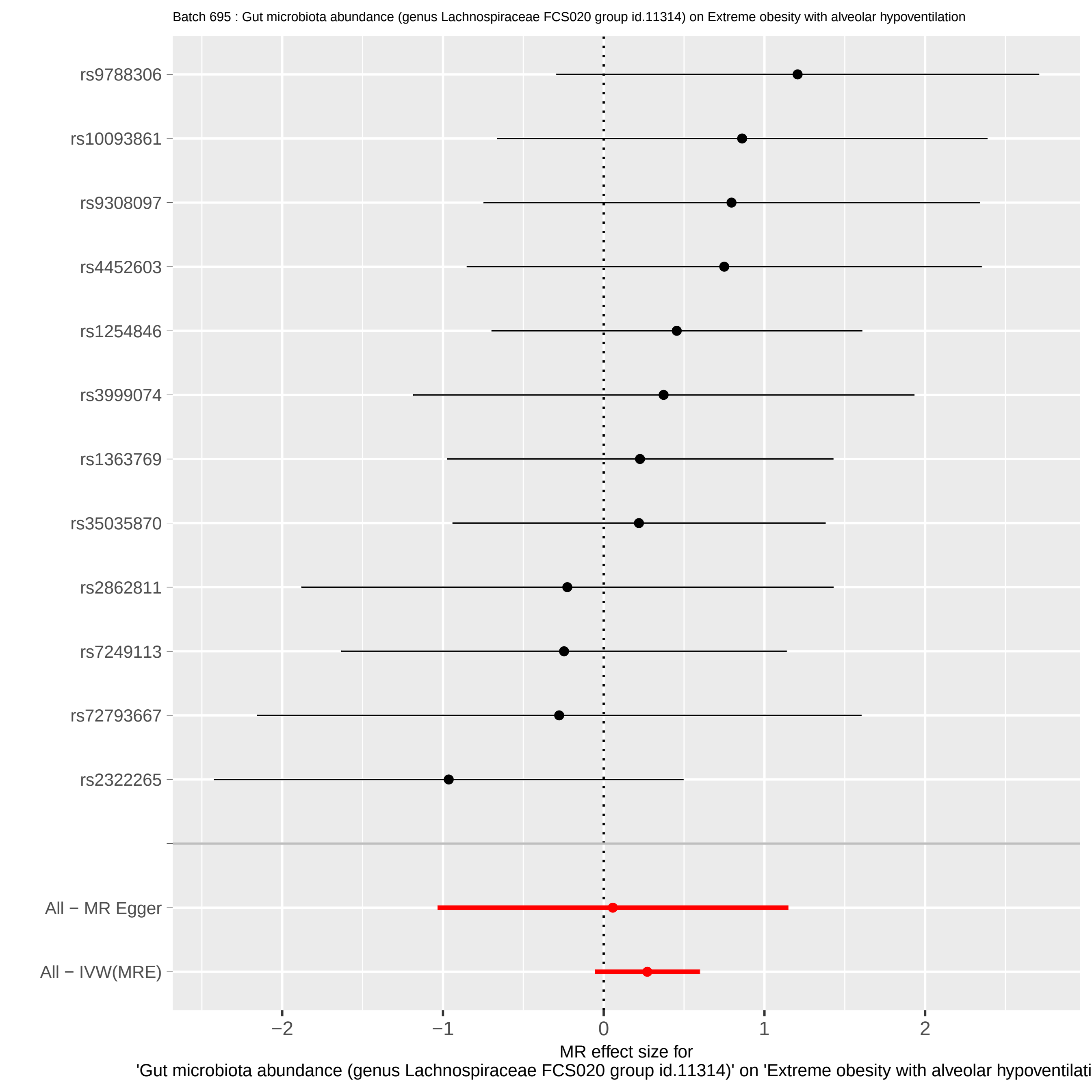

Batch 695 : Gut microbiota abundance (genus Lachnospiraceae FCS020 group id.11314) on Extreme obesity with alveolar hypoventilation
rs9788306
rs10093861
rs9308097
rs4452603
rs1254846
rs3999074
rs1363769
rs35035870
rs2862811
rs7249113
rs72793667
rs2322265
All − MR Egger
All − IVW(MRE)
−2
−1
0
1
2
MR effect size for
'Gut microbiota abundance (genus Lachnospiraceae FCS020 group id.11314)' on 'Extreme obesity with alveolar hypoventilati

Batch 696 : Gut microbiota abundance (genus Lachnospiraceae NC2004 group id.11316) on Extreme obesity with alveolar hypoventilation

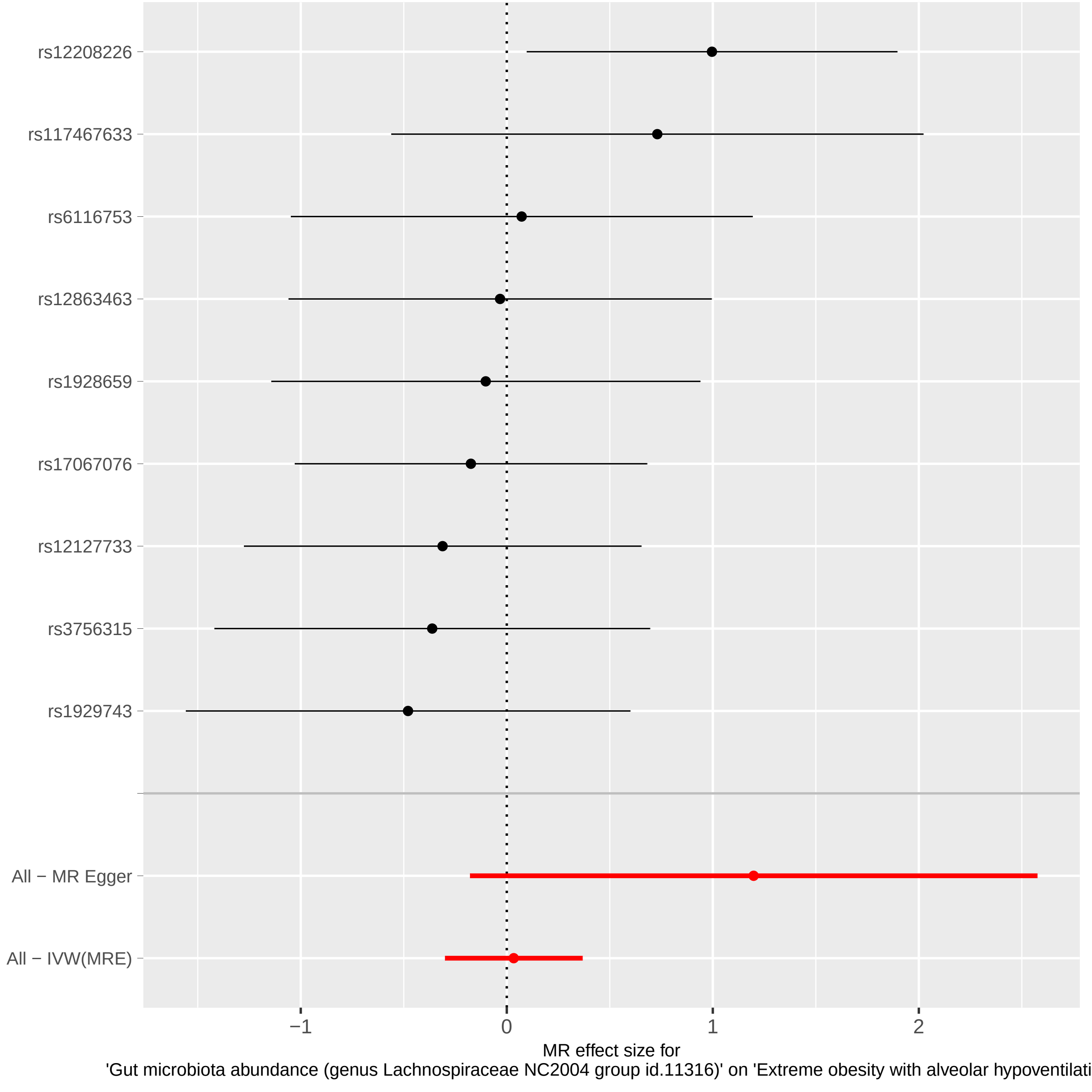

Batch 697 : Gut microbiota abundance (genus Lachnospiraceae ND3007 group id.11317) on Extreme obesity with alveolar hypoventilation

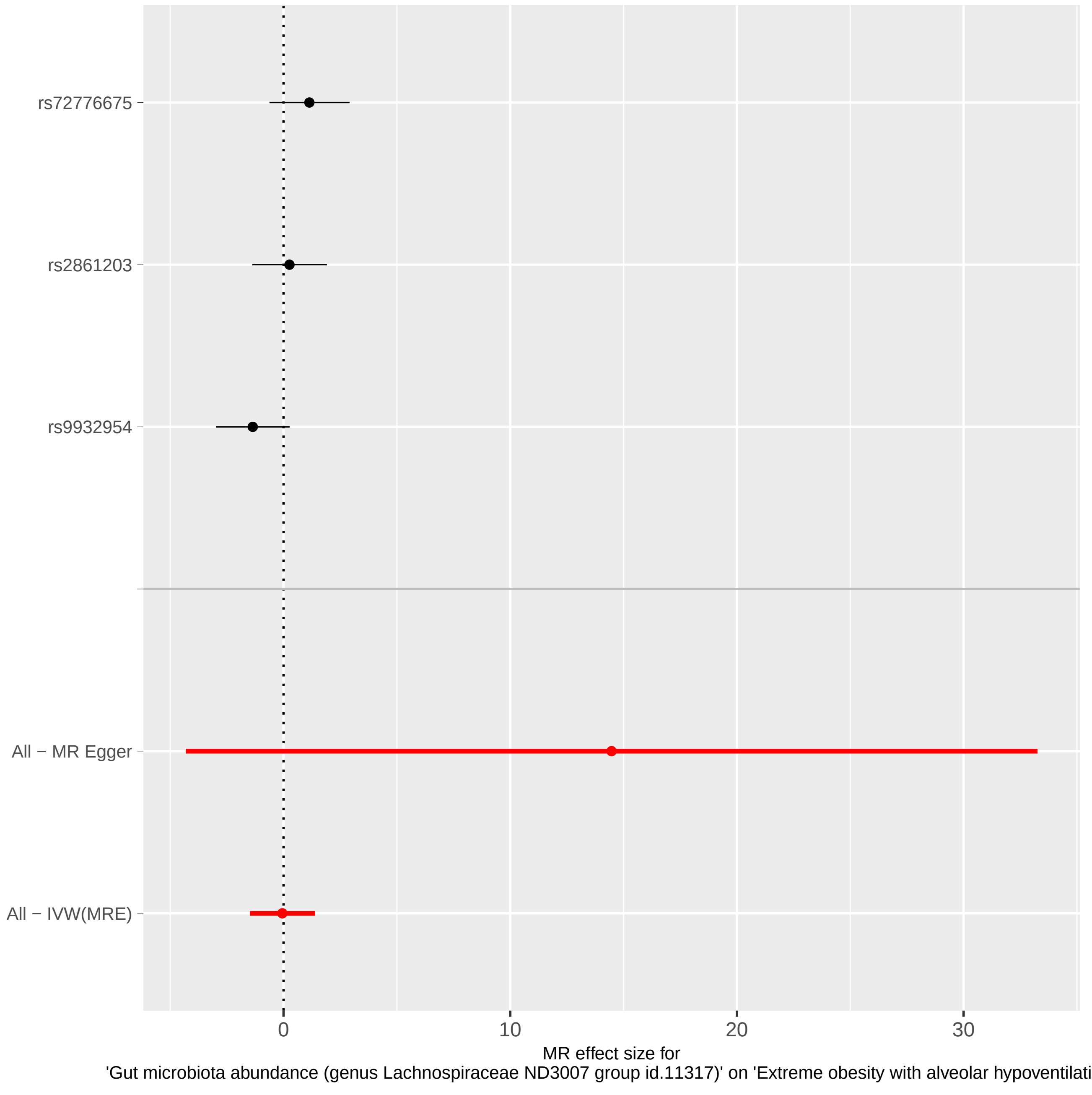

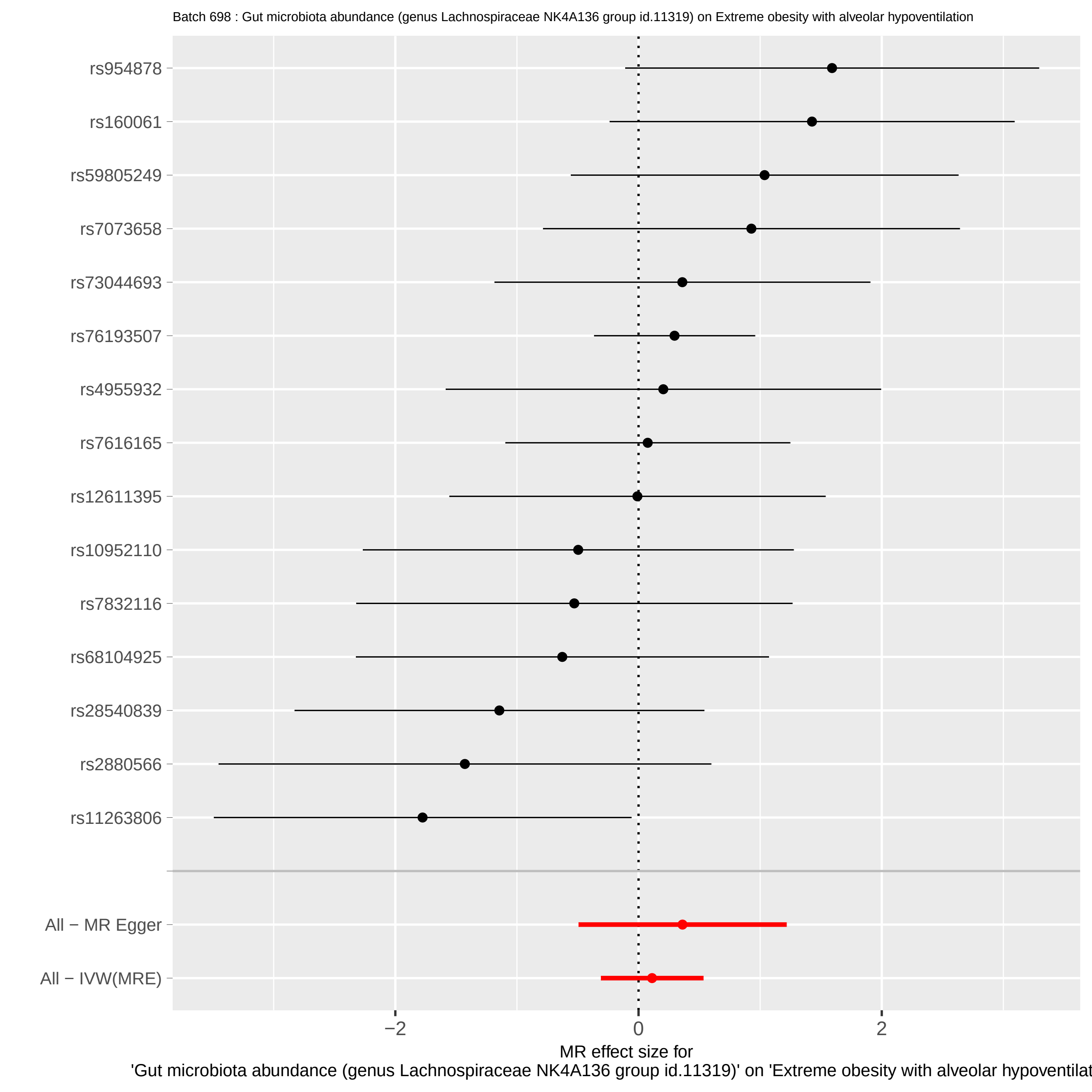

Batch 698 : Gut microbiota abundance (genus Lachnospiraceae NK4A136 group id.11319) on Extreme obesity with alveolar hypoventilation
rs954878
rs160061
rs59805249
rs7073658
rs73044693
rs76193507
rs4955932
rs7616165
rs12611395
rs10952110
rs7832116
rs68104925
rs28540839
rs2880566
rs11263806
All − MR Egger
All − IVW(MRE)
−2
0
2
MR effect size for
'Gut microbiota abundance (genus Lachnospiraceae NK4A136 group id.11319)' on 'Extreme obesity with alveolar hypoventilat

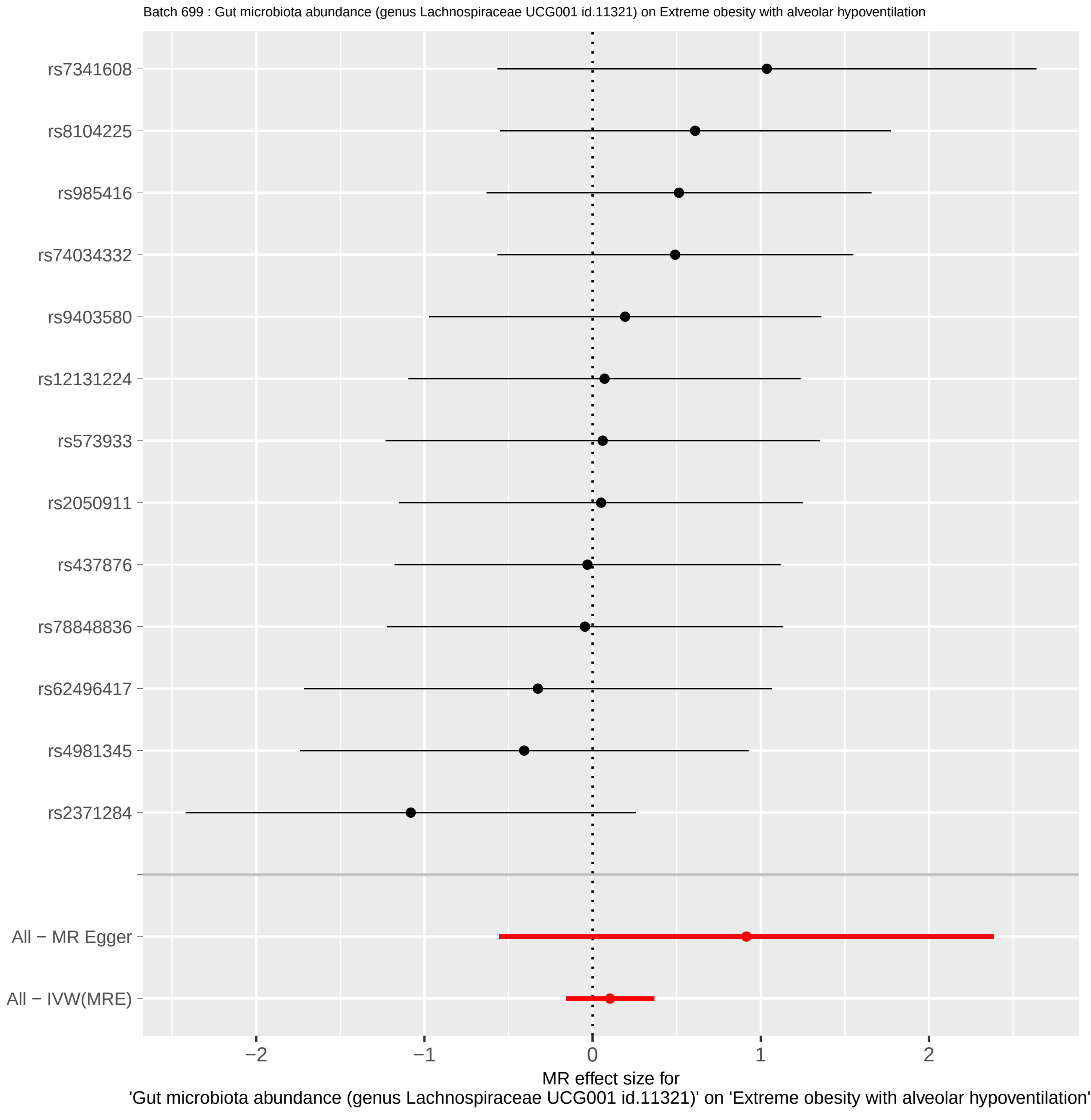

Batch 699 : Gut microbiota abundance (genus Lachnospiraceae UCG001 id.11321) on Extreme obesity with alveolar hypoventilation
rs7341608
rs8104225
rs985416
rs74034332
rs9403580
rs12131224
rs573933
rs2050911
rs437876
rs78848836
rs62496417
rs4981345
rs2371284
All − MR Egger
All − IVW(MRE)
−2
−1
0
1
2
MR effect size for
'Gut microbiota abundance (genus Lachnospiraceae UCG001 id.11321)' on 'Extreme obesity with alveolar hypoventilation'

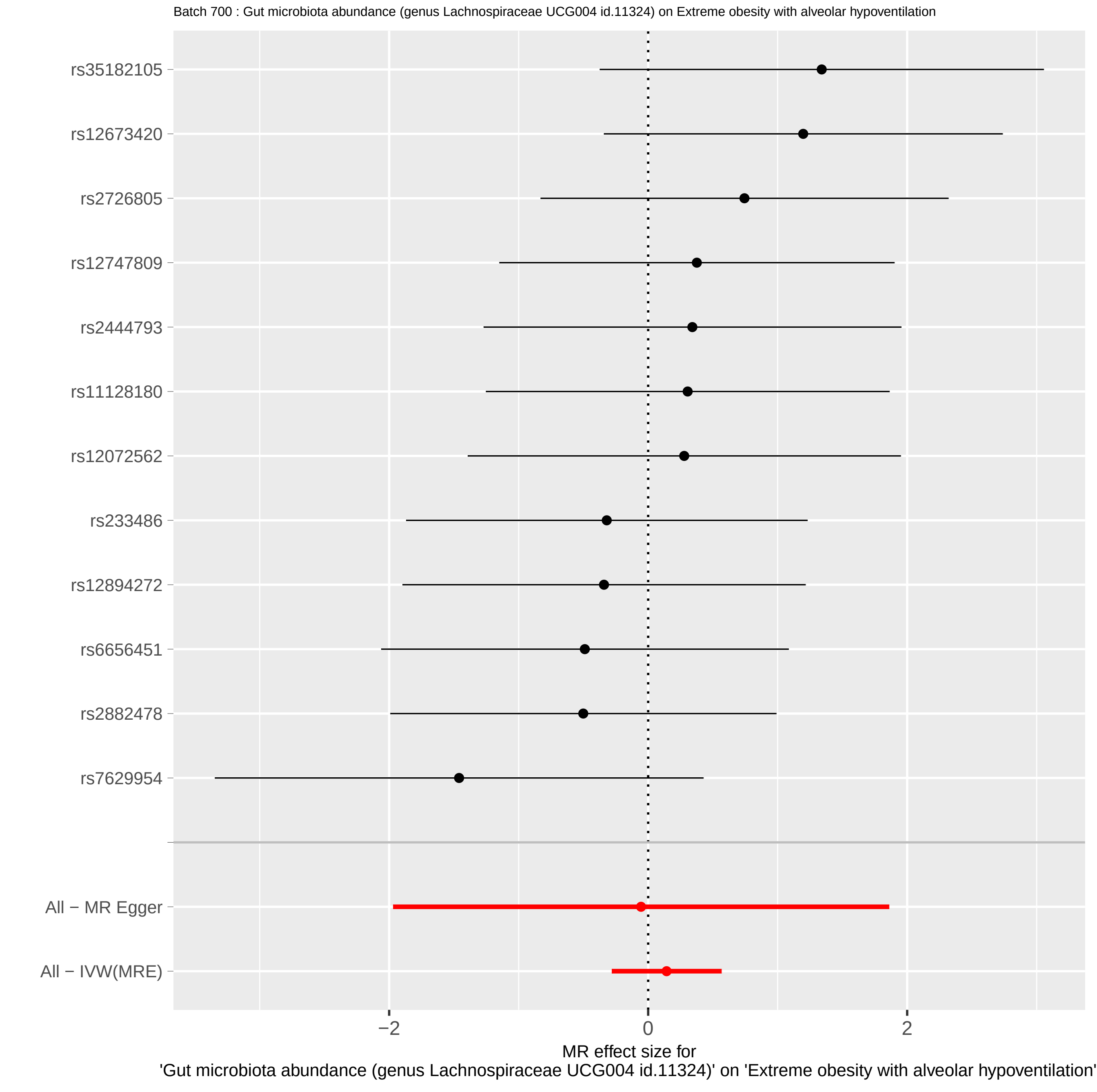

Batch 700 : Gut microbiota abundance (genus Lachnospiraceae UCG004 id.11324) on Extreme obesity with alveolar hypoventilation
rs35182105
rs12673420
rs2726805
rs12747809
rs2444793
rs11128180
rs12072562
rs233486
rs12894272
rs6656451
rs2882478
rs7629954
All − MR Egger
All − IVW(MRE)
−2
0
2
MR effect size for
'Gut microbiota abundance (genus Lachnospiraceae UCG004 id.11324)' on 'Extreme obesity with alveolar hypoventilation'

Batch 701 : Gut microbiota abundance (genus Lachnospiraceae UCG008 id.11328) on Extreme obesity with alveolar hypoventilation

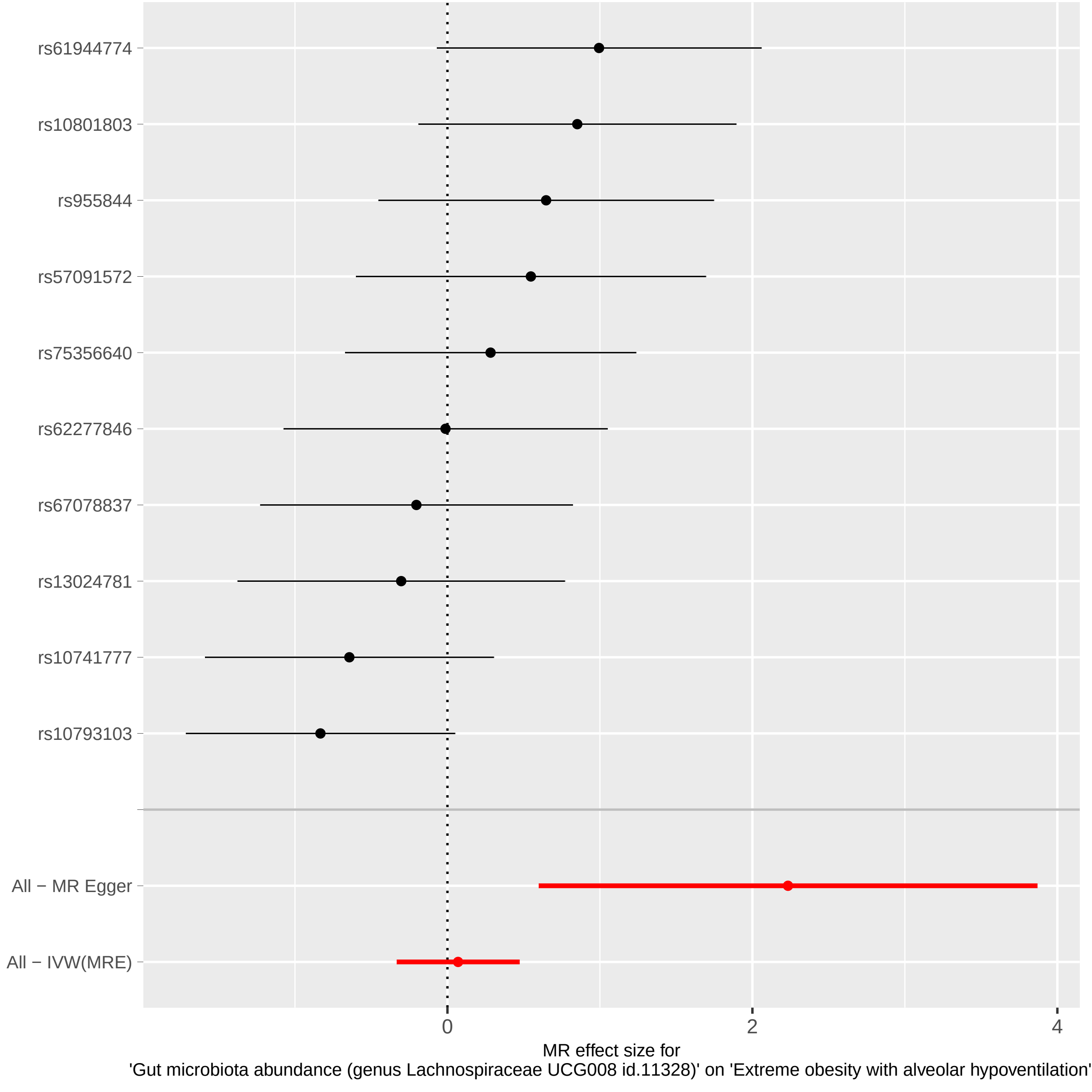

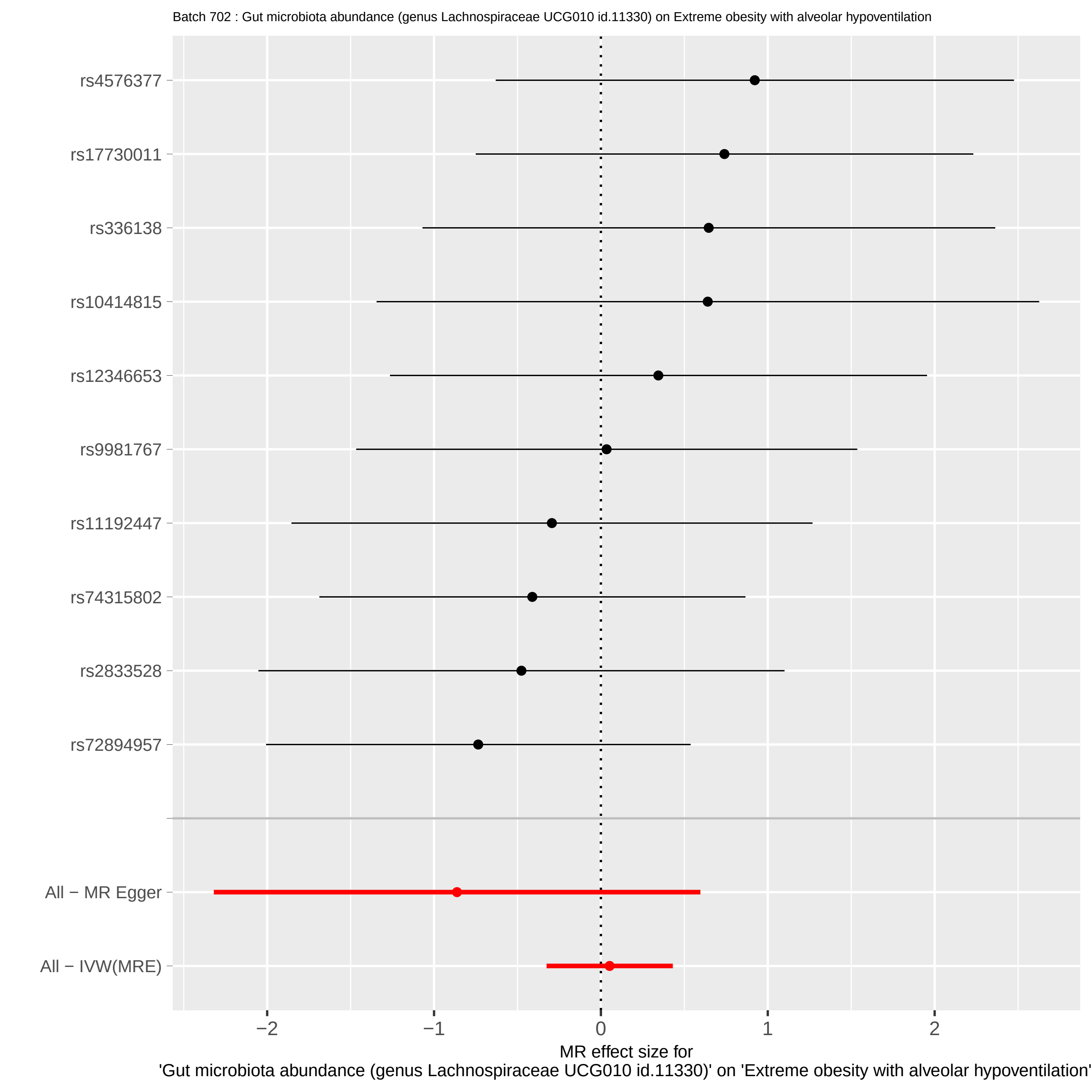
Batch 702 : Gut microbiota abundance (genus Lachnospiraceae UCG010 id.11330) on Extreme obesity with alveolar hypoventilation
rs4576377
rs17730011
rs336138
rs10414815
rs12346653
rs9981767
rs11192447
rs74315802
rs2833528
rs72894957
All − MR Egger
All − IVW(MRE)
−2
−1
0
1
2
MR effect size for
'Gut microbiota abundance (genus Lachnospiraceae UCG010 id.11330)' on 'Extreme obesity with alveolar hypoventilation'

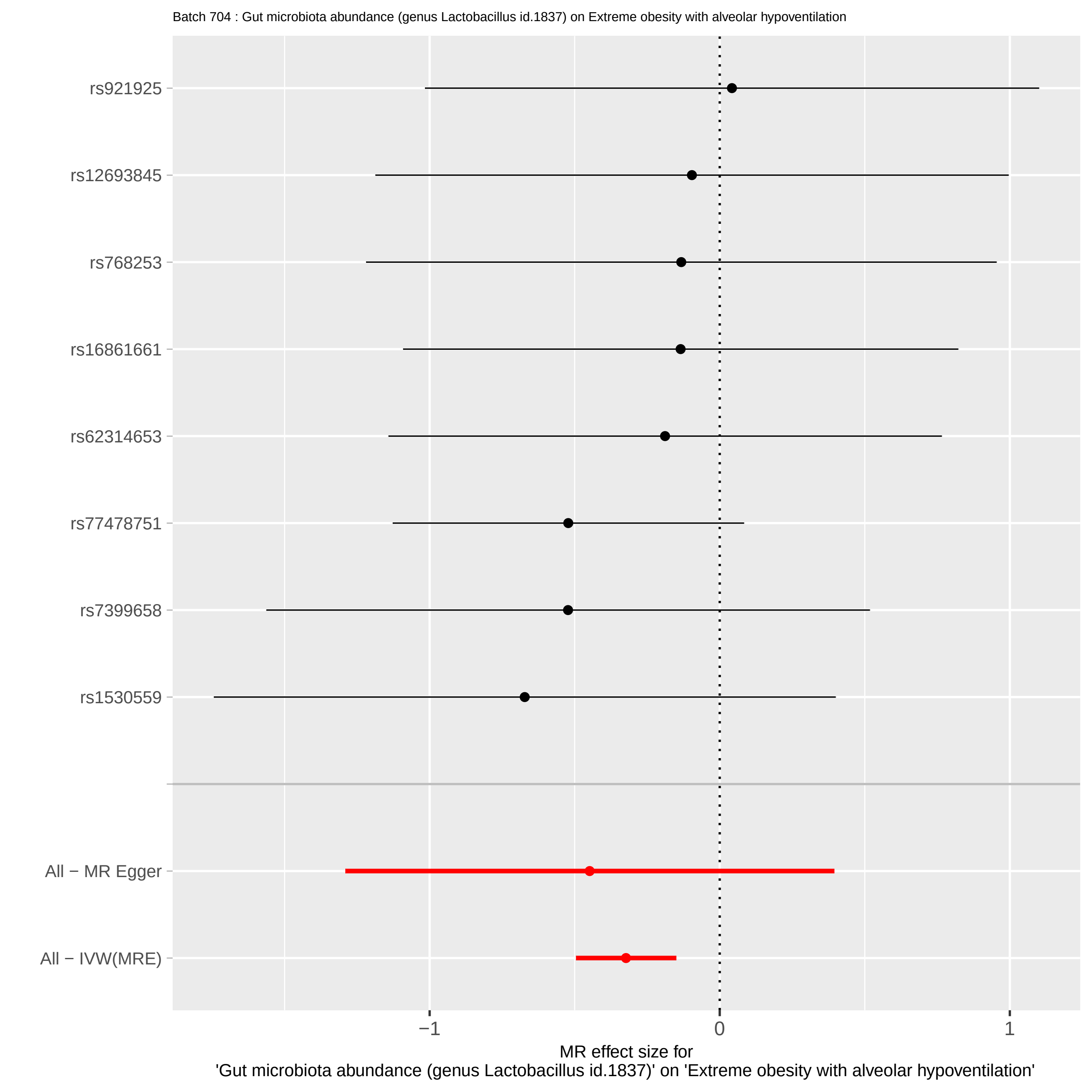
Batch 704 : Gut microbiota abundance (genus Lactobacillus id.1837) on Extreme obesity with alveolar hypoventilation
rs921925
rs12693845
rs768253
rs16861661
rs62314653
rs77478751
rs7399658
rs1530559
All − MR Egger
All − IVW(MRE)
−1
0
1
MR effect size for
'Gut microbiota abundance (genus Lactobacillus id.1837)' on 'Extreme obesity with alveolar hypoventilation'

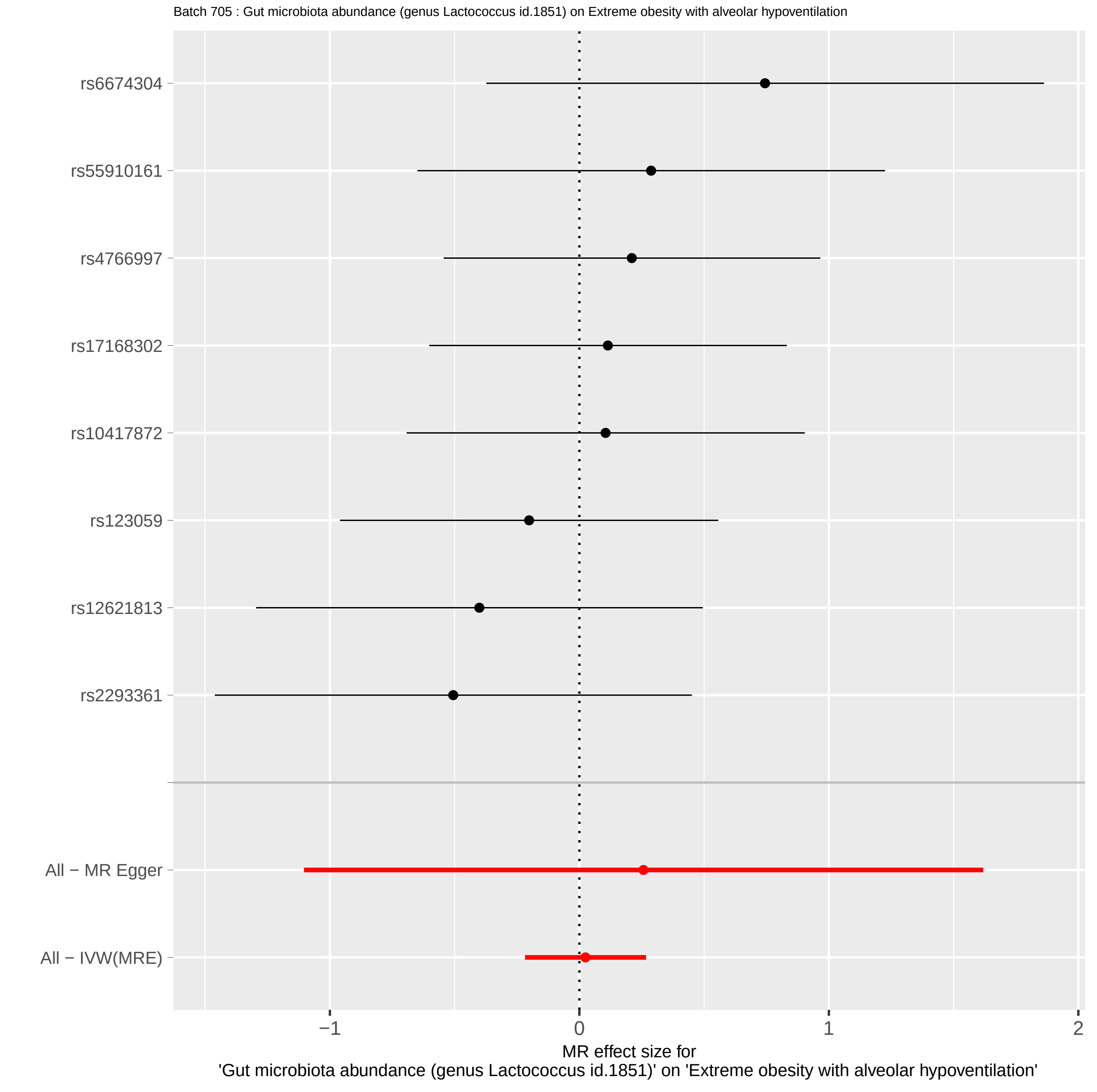

Batch 705 : Gut microbiota abundance (genus Lactococcus id.1851) on Extreme obesity with alveolar hypoventilation
rs6674304
rs55910161
rs4766997
rs17168302
rs10417872
rs123059
rs12621813
rs2293361
All − MR Egger
All − IVW(MRE)
−1
0
1
2
MR effect size for
'Gut microbiota abundance (genus Lactococcus id.1851)' on 'Extreme obesity with alveolar hypoventilation'

Batch 706 : Gut microbiota abundance (genus Marvinbryantia id.2005) on Extreme obesity with alveolar hypoventilation

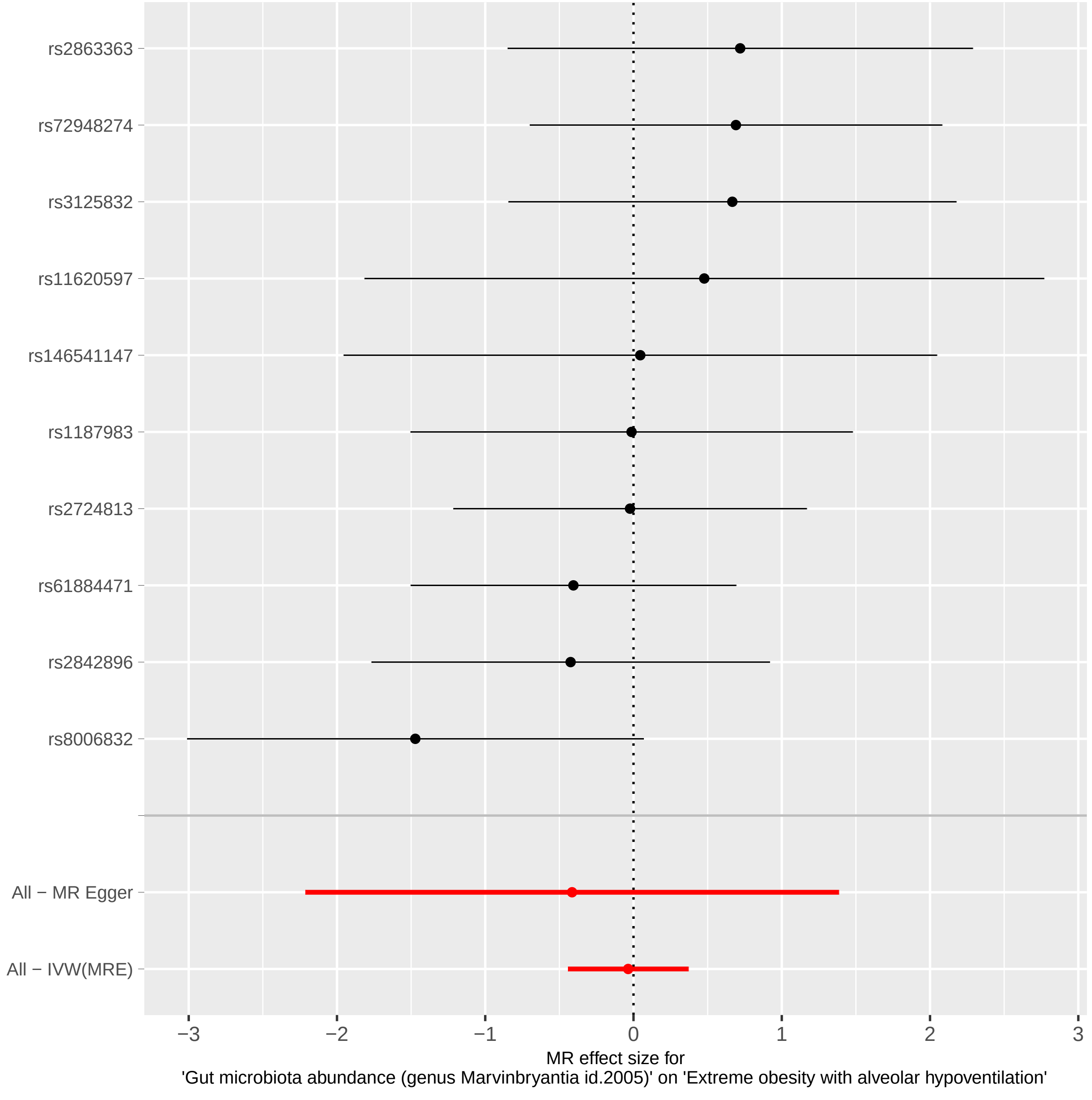

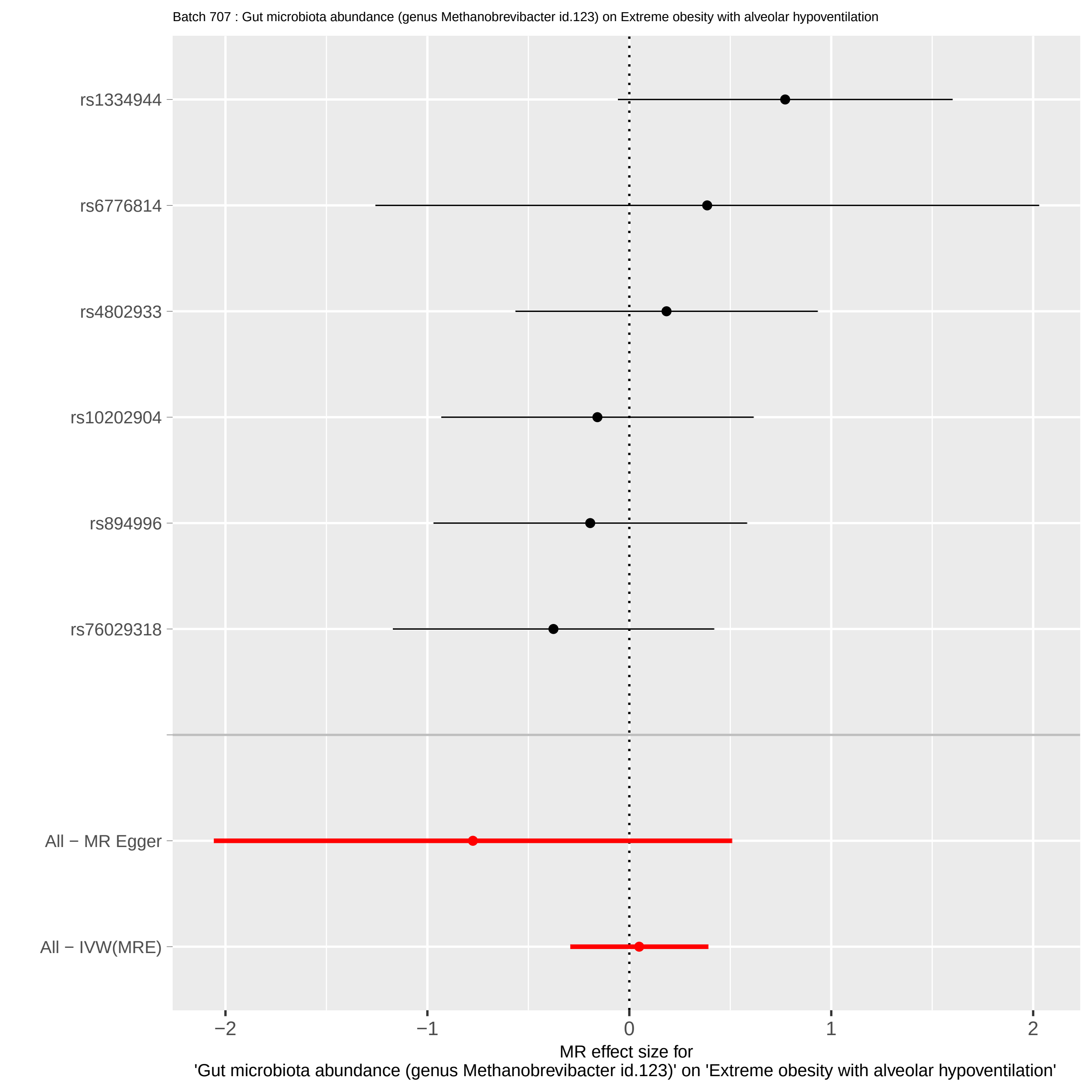

Batch 707 : Gut microbiota abundance (genus Methanobrevibacter id.123) on Extreme obesity with alveolar hypoventilation
rs1334944
rs6776814
rs4802933
rs10202904
rs894996
rs76029318
All − MR Egger
All − IVW(MRE)
−2
−1
0
1
2
MR effect size for
'Gut microbiota abundance (genus Methanobrevibacter id.123)' on 'Extreme obesity with alveolar hypoventilation'

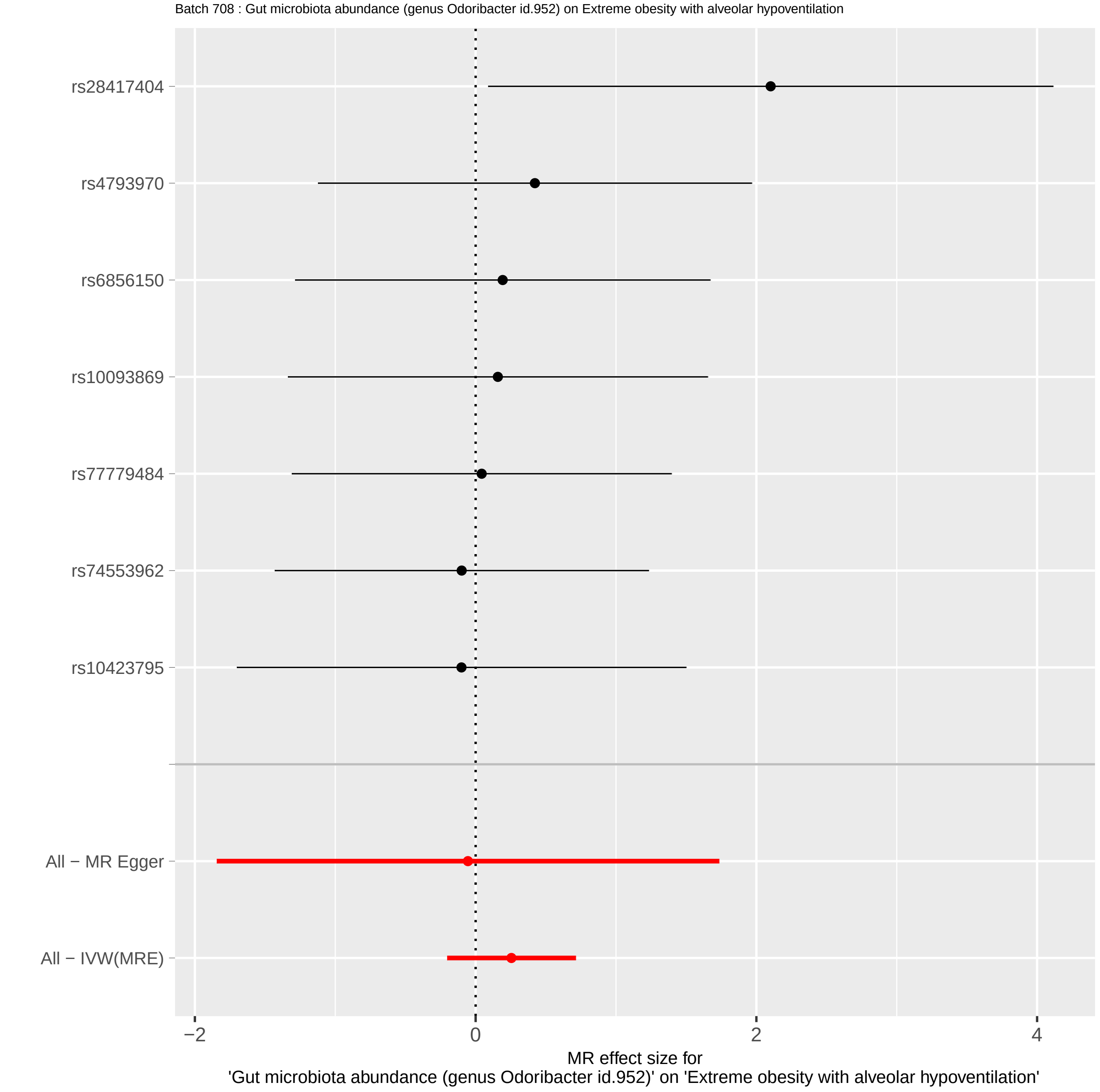
Batch 708 : Gut microbiota abundance (genus Odoribacter id.952) on Extreme obesity with alveolar hypoventilation
rs28417404
rs4793970
rs6856150
rs10093869
rs77779484
rs74553962
rs10423795
All − MR Egger
All − IVW(MRE)
−2
0
2
4
MR effect size for
'Gut microbiota abundance (genus Odoribacter id.952)' on 'Extreme obesity with alveolar hypoventilation'

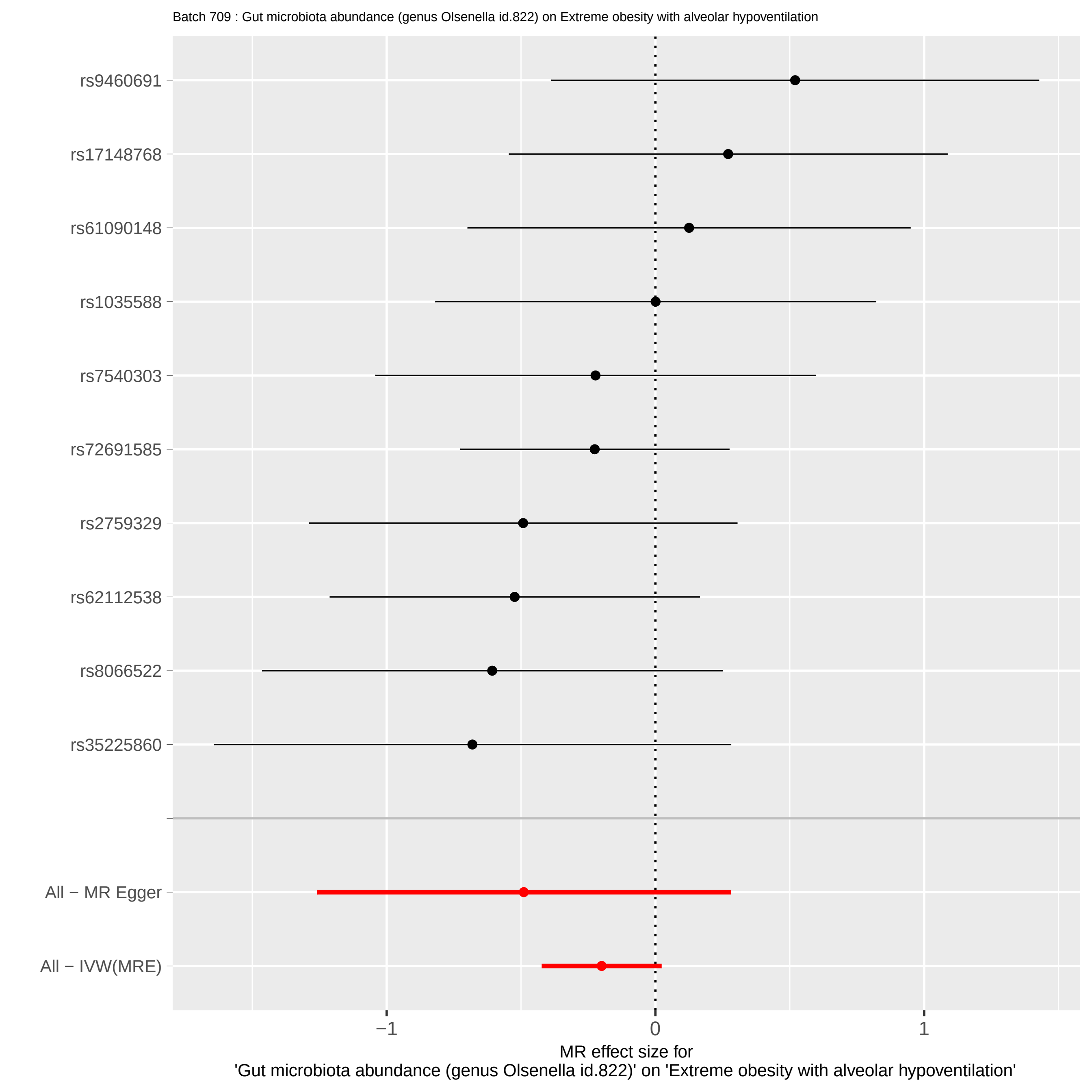
Batch 709 : Gut microbiota abundance (genus Olsenella id.822) on Extreme obesity with alveolar hypoventilation
rs9460691
rs17148768
rs61090148
rs1035588
rs7540303
rs72691585
rs2759329
rs62112538
rs8066522
rs35225860
All − MR Egger
All − IVW(MRE)
−1
0
1
MR effect size for
'Gut microbiota abundance (genus Olsenella id.822)' on 'Extreme obesity with alveolar hypoventilation'

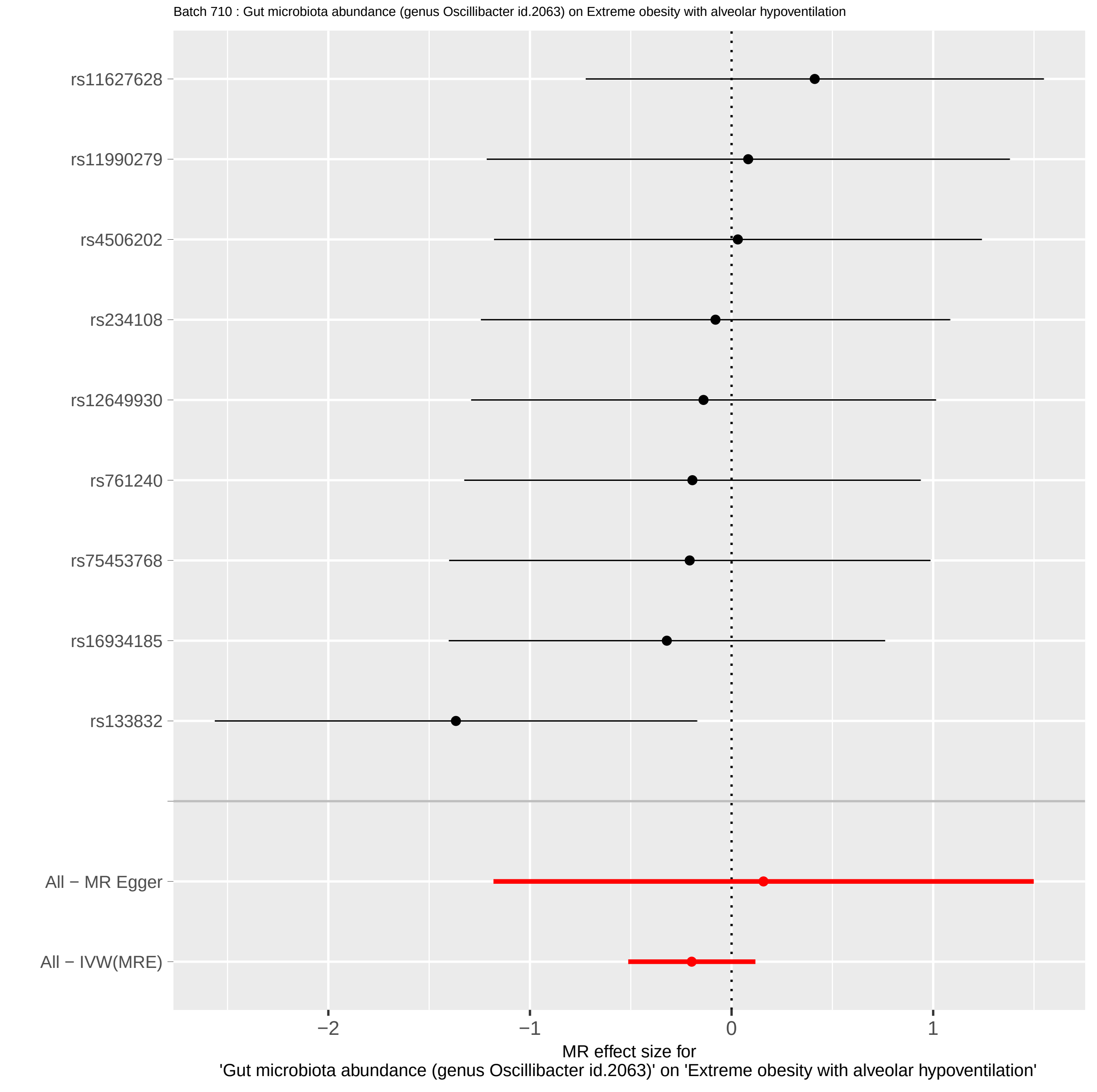

Batch 710 : Gut microbiota abundance (genus Oscillibacter id.2063) on Extreme obesity with alveolar hypoventilation
rs11627628
rs11990279
rs4506202
rs234108
rs12649930
rs761240
rs75453768
rs16934185
rs133832
All − MR Egger
All − IVW(MRE)
−2
−1
0
1
MR effect size for
'Gut microbiota abundance (genus Oscillibacter id.2063)' on 'Extreme obesity with alveolar hypoventilation'

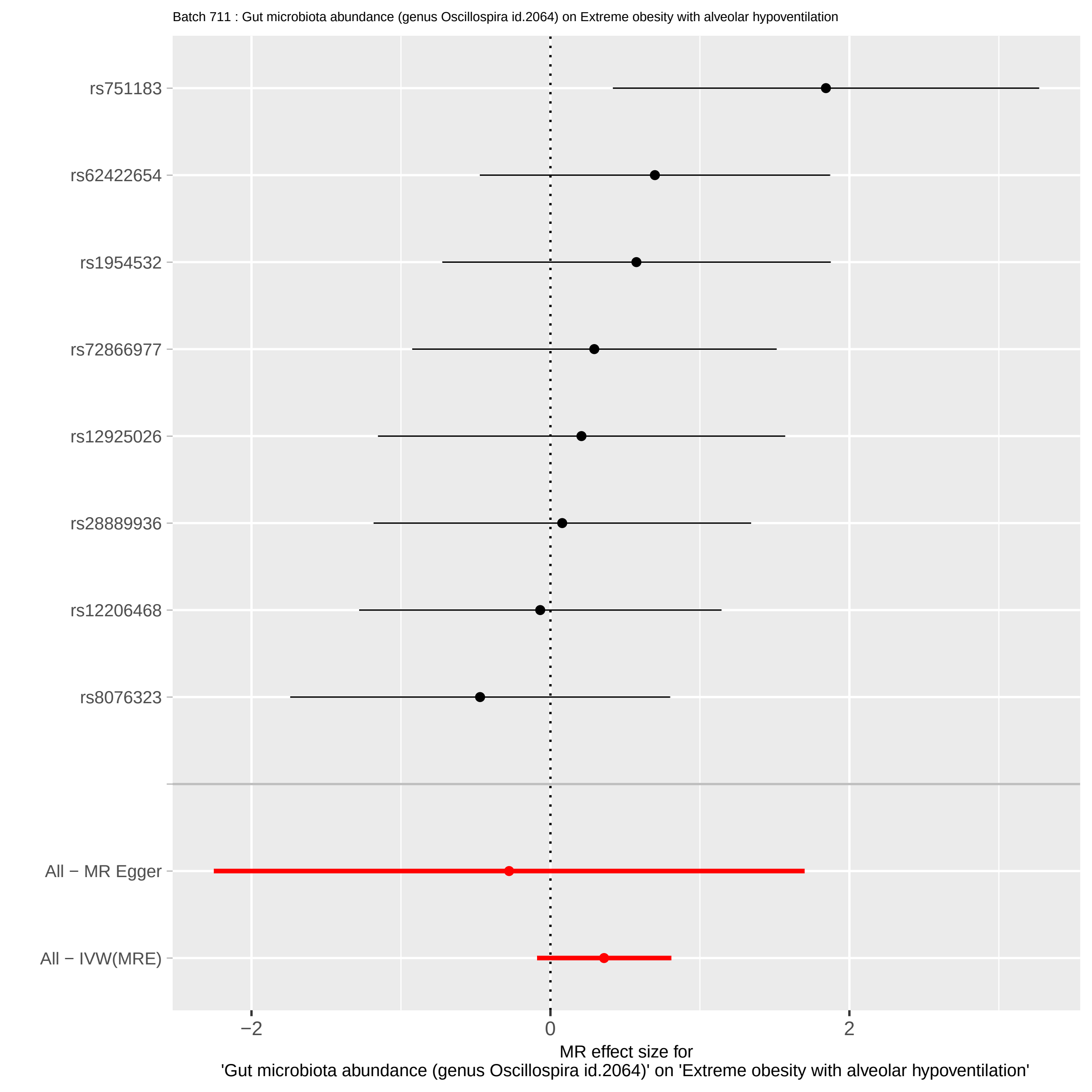

Batch 711 : Gut microbiota abundance (genus Oscillospira id.2064) on Extreme obesity with alveolar hypoventilation
rs751183
rs62422654
rs1954532
rs72866977
rs12925026
rs28889936
rs12206468
rs8076323
All − MR Egger
All − IVW(MRE)
−2
0
2
MR effect size for
'Gut microbiota abundance (genus Oscillospira id.2064)' on 'Extreme obesity with alveolar hypoventilation'

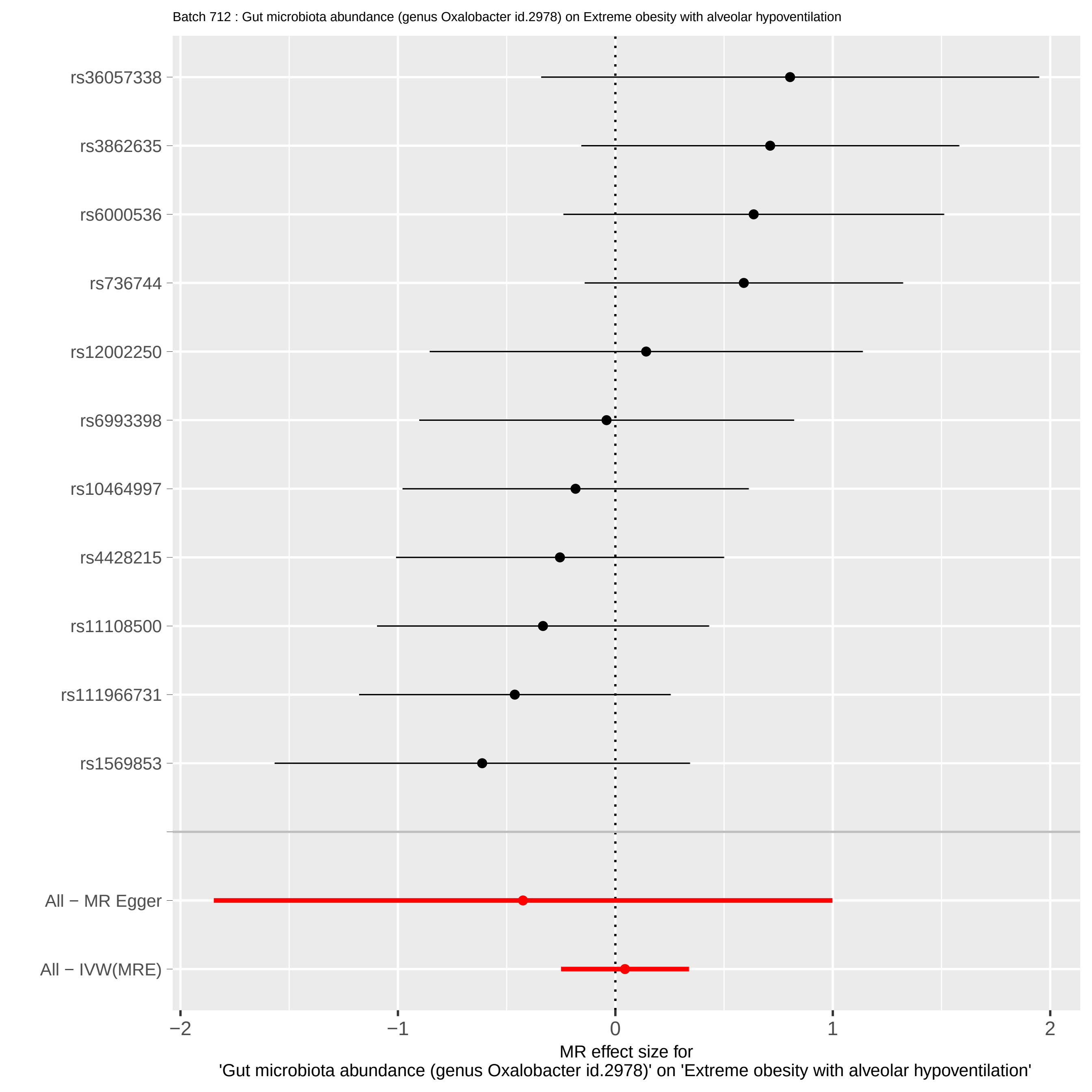

Batch 712 : Gut microbiota abundance (genus Oxalobacter id.2978) on Extreme obesity with alveolar hypoventilation
rs36057338
rs3862635
rs6000536
rs736744
rs12002250
rs6993398
rs10464997
rs4428215
rs11108500
rs111966731
rs1569853
All − MR Egger
All − IVW(MRE)
−2
−1
0
1
2
MR effect size for
'Gut microbiota abundance (genus Oxalobacter id.2978)' on 'Extreme obesity with alveolar hypoventilation'

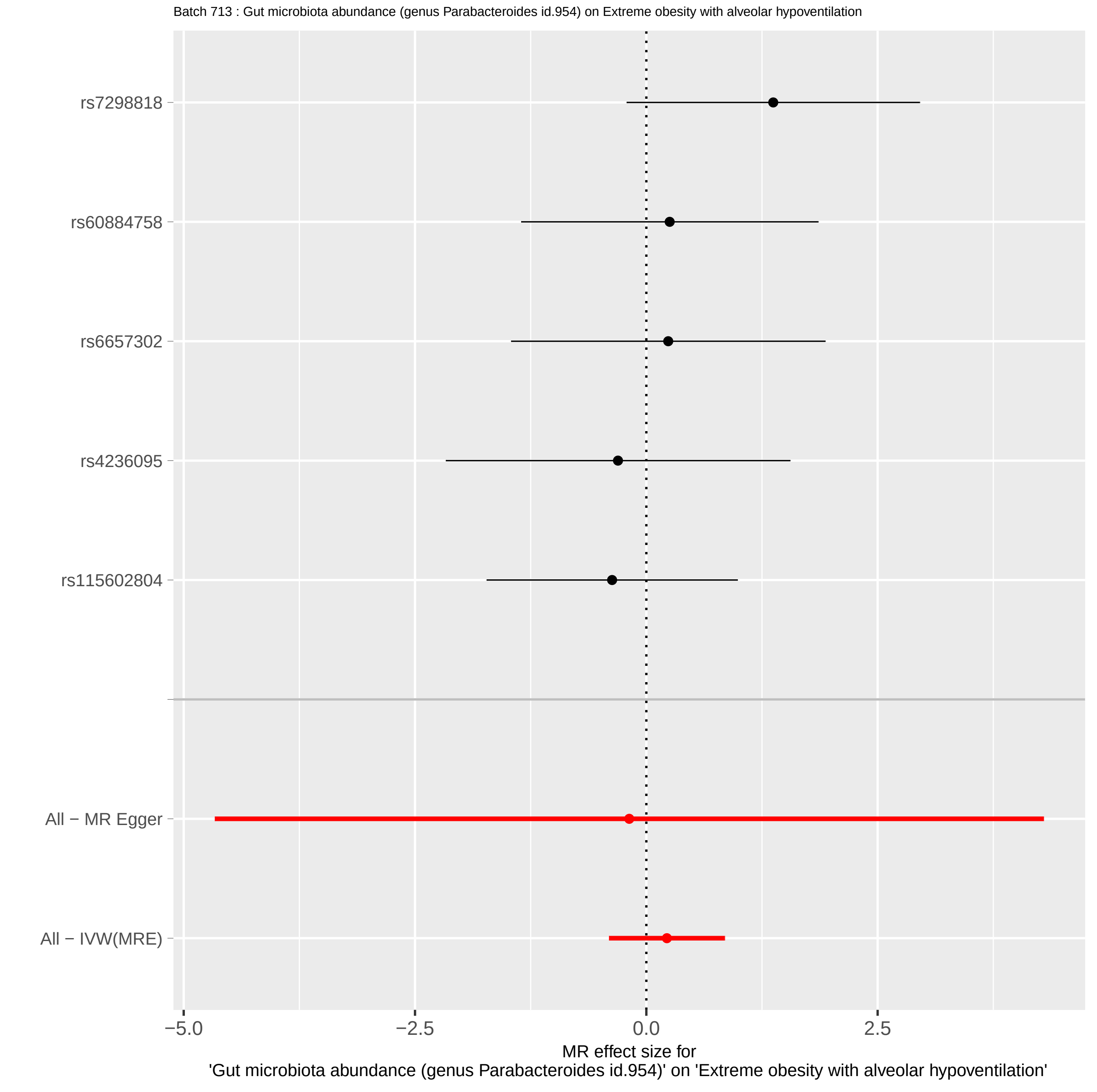

Batch 713 : Gut microbiota abundance (genus Parabacteroides id.954) on Extreme obesity with alveolar hypoventilation
rs7298818
rs60884758
rs6657302
rs4236095
rs115602804
All − MR Egger
All − IVW(MRE)
−5.0
−2.5
0.0
2.5
MR effect size for
'Gut microbiota abundance (genus Parabacteroides id.954)' on 'Extreme obesity with alveolar hypoventilation'

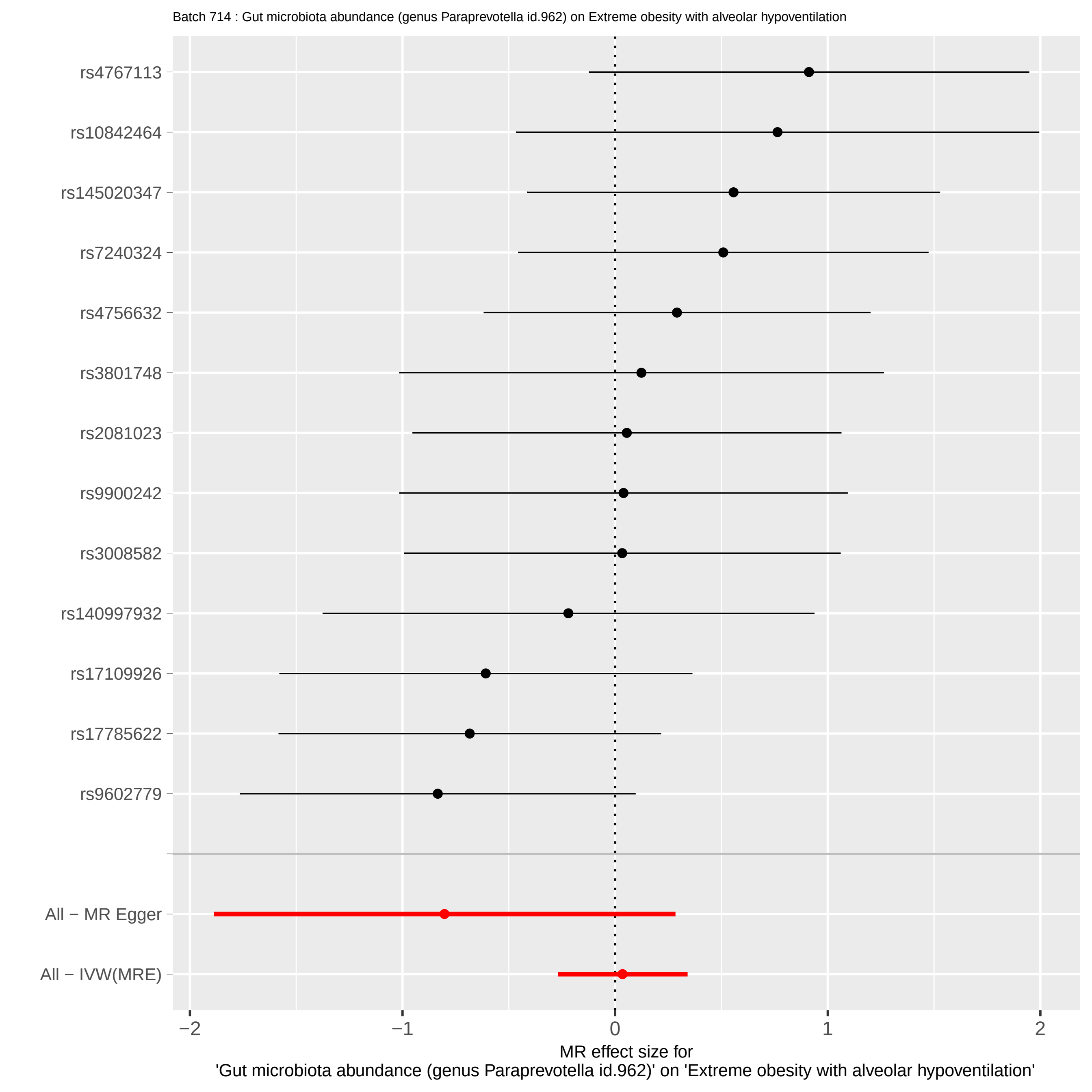
Batch 714 : Gut microbiota abundance (genus Paraprevotella id.962) on Extreme obesity with alveolar hypoventilation
rs4767113
rs10842464
rs145020347
rs7240324
rs4756632
rs3801748
rs2081023
rs9900242
rs3008582
rs140997932
rs17109926
rs17785622
rs9602779
All − MR Egger
All − IVW(MRE)
−2
−1
0
1
2
MR effect size for
'Gut microbiota abundance (genus Paraprevotella id.962)' on 'Extreme obesity with alveolar hypoventilation'

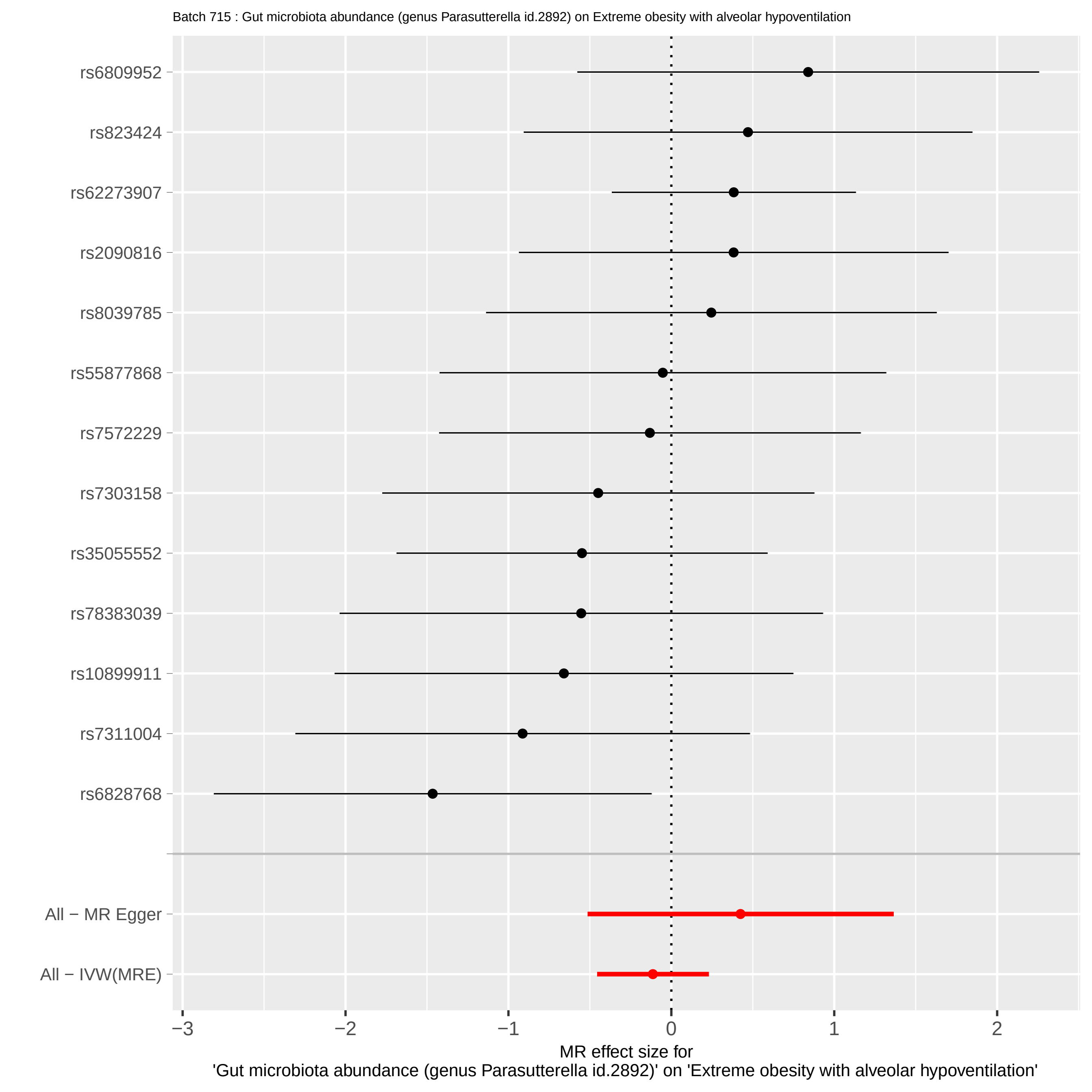

Batch 715 : Gut microbiota abundance (genus Parasutterella id.2892) on Extreme obesity with alveolar hypoventilation
rs6809952
rs823424
rs62273907
rs2090816
rs8039785
rs55877868
rs7572229
rs7303158
rs35055552
rs78383039
rs10899911
rs7311004
rs6828768
All − MR Egger
All − IVW(MRE)
−3
−2
−1
0
1
2
MR effect size for
'Gut microbiota abundance (genus Parasutterella id.2892)' on 'Extreme obesity with alveolar hypoventilation'

Batch 716 : Gut microbiota abundance (genus Peptococcus id.2037) on Extreme obesity with alveolar hypoventilation

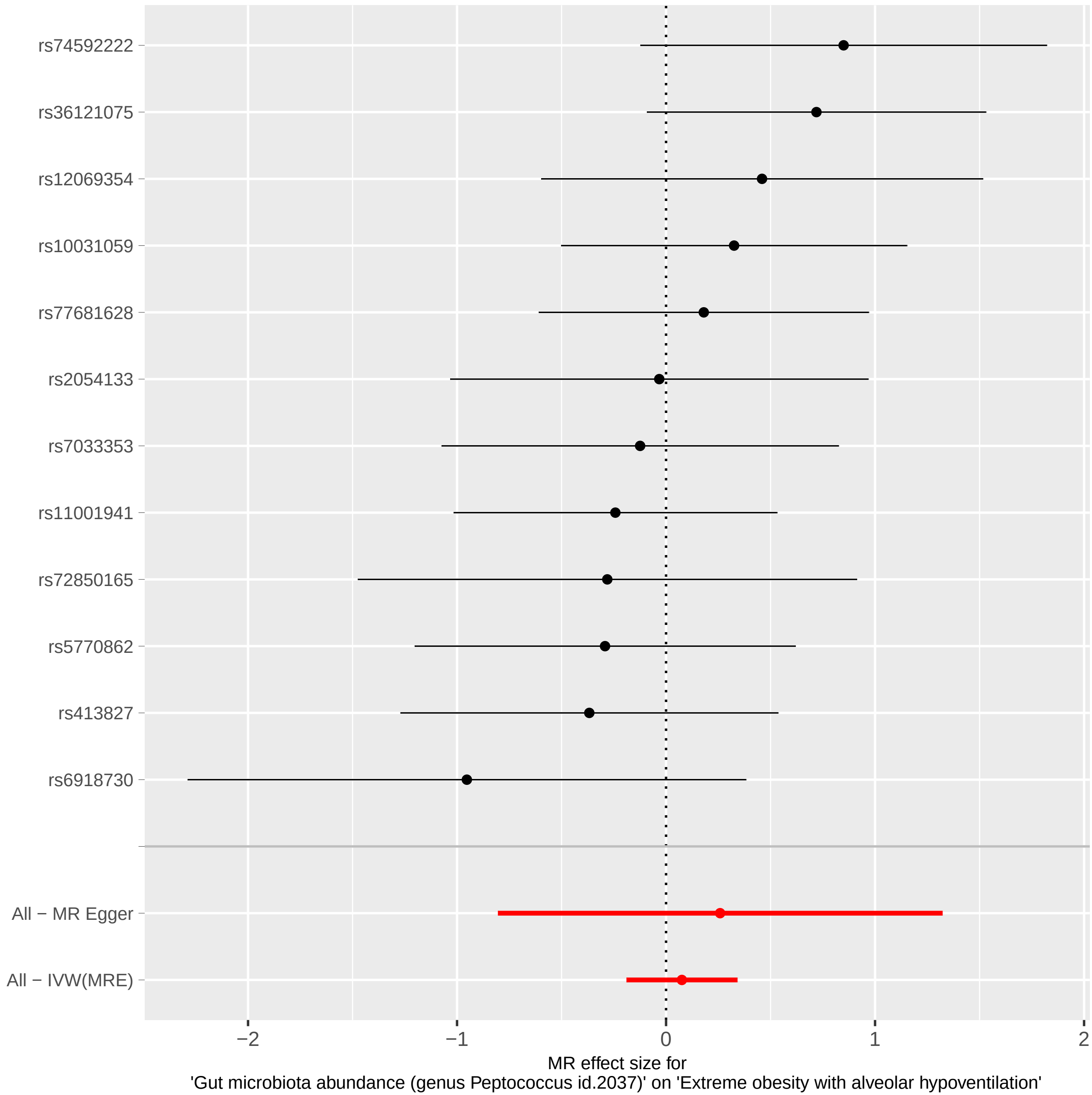

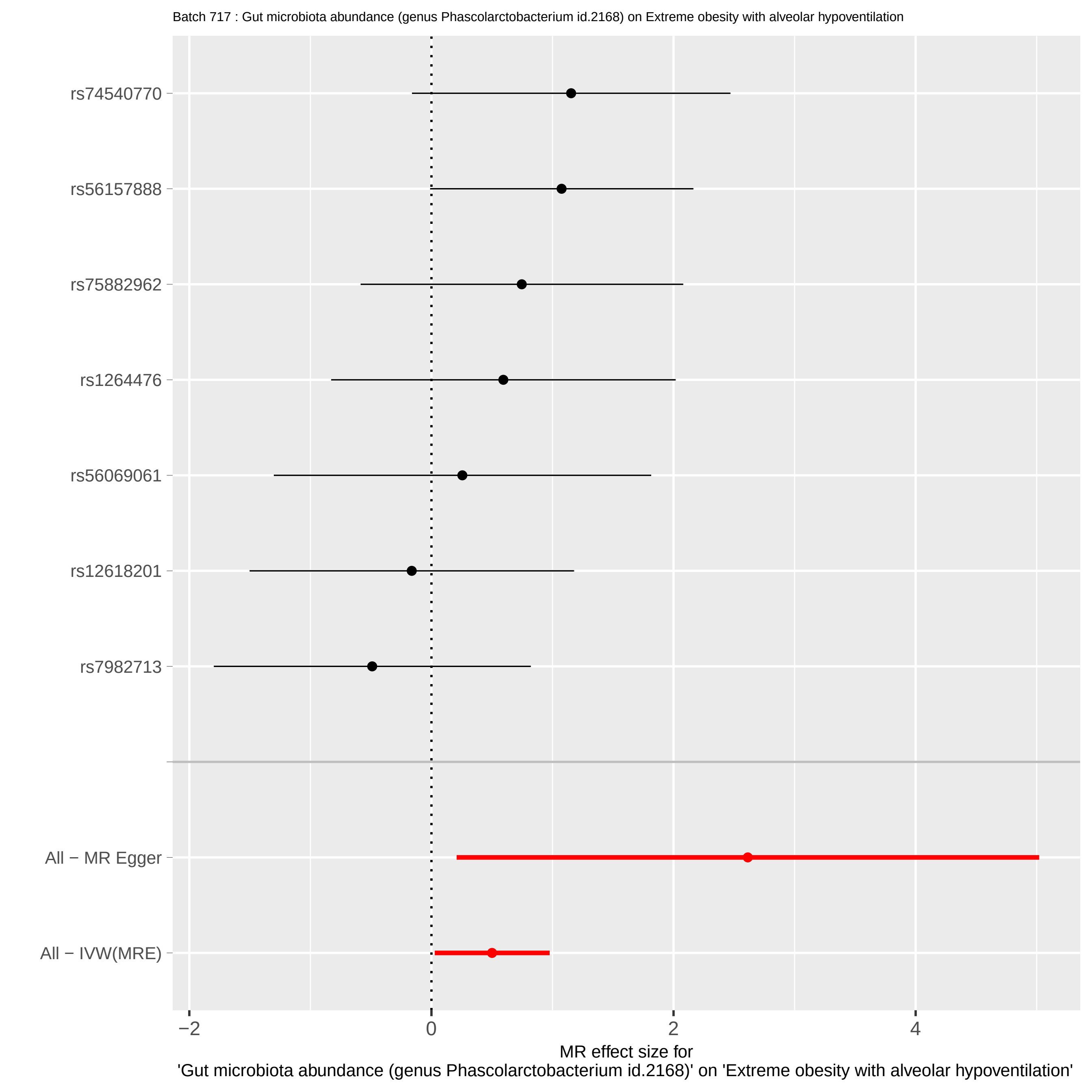

Batch 717 : Gut microbiota abundance (genus Phascolarctobacterium id.2168) on Extreme obesity with alveolar hypoventilation
rs74540770
rs56157888
rs75882962
rs1264476
rs56069061
rs12618201
rs7982713
All − MR Egger
All − IVW(MRE)
−2
0
2
4
MR effect size for
'Gut microbiota abundance (genus Phascolarctobacterium id.2168)' on 'Extreme obesity with alveolar hypoventilation'

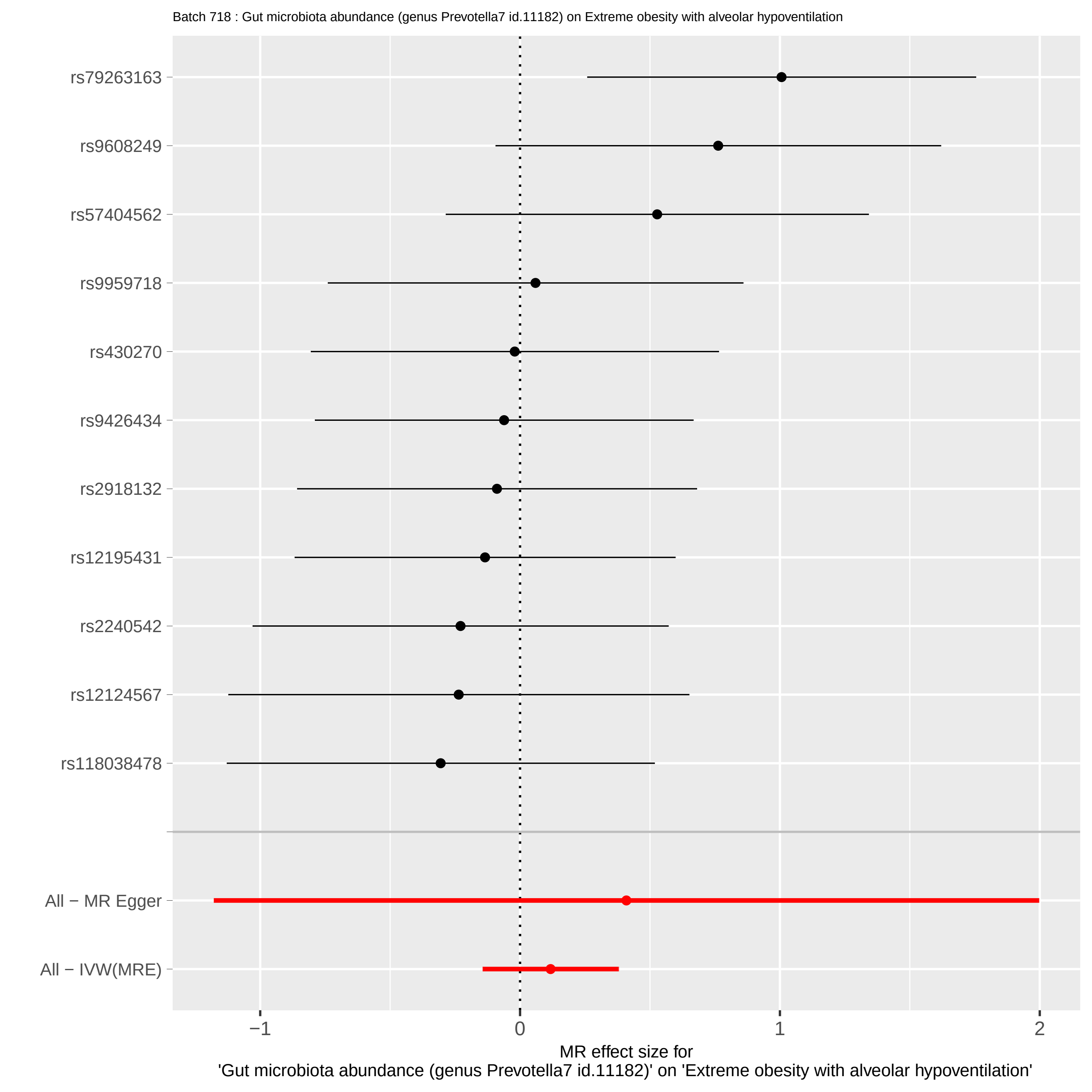

Batch 718 : Gut microbiota abundance (genus Prevotella7 id.11182) on Extreme obesity with alveolar hypoventilation
rs79263163
rs9608249
rs57404562
rs9959718
rs430270
rs9426434
rs2918132
rs12195431
rs2240542
rs12124567
rs118038478
All − MR Egger
All − IVW(MRE)
−1
0
1
2
MR effect size for
'Gut microbiota abundance (genus Prevotella7 id.11182)' on 'Extreme obesity with alveolar hypoventilation'

Batch 719 : Gut microbiota abundance (genus Prevotella9 id.11183) on Extreme obesity with alveolar hypoventilation

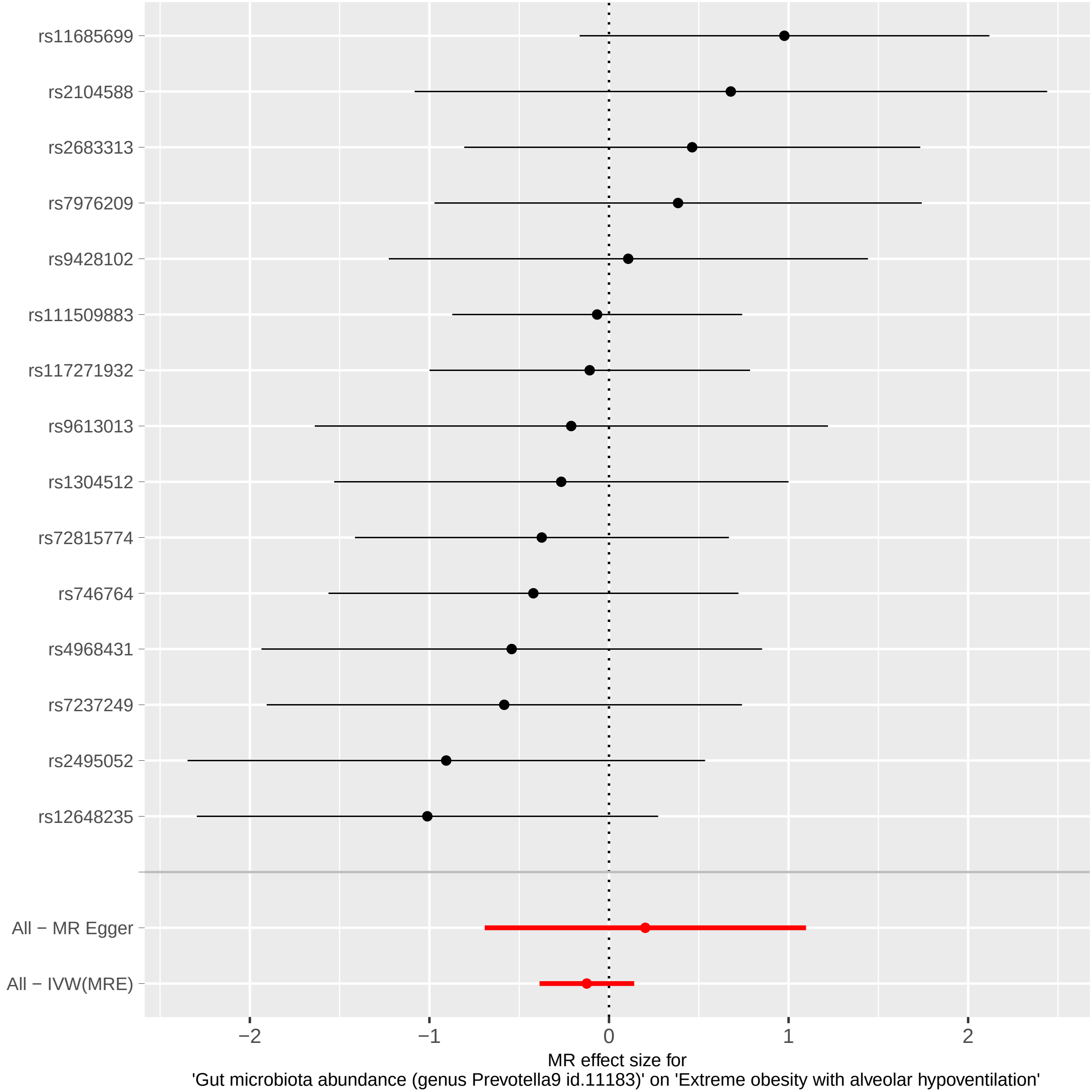

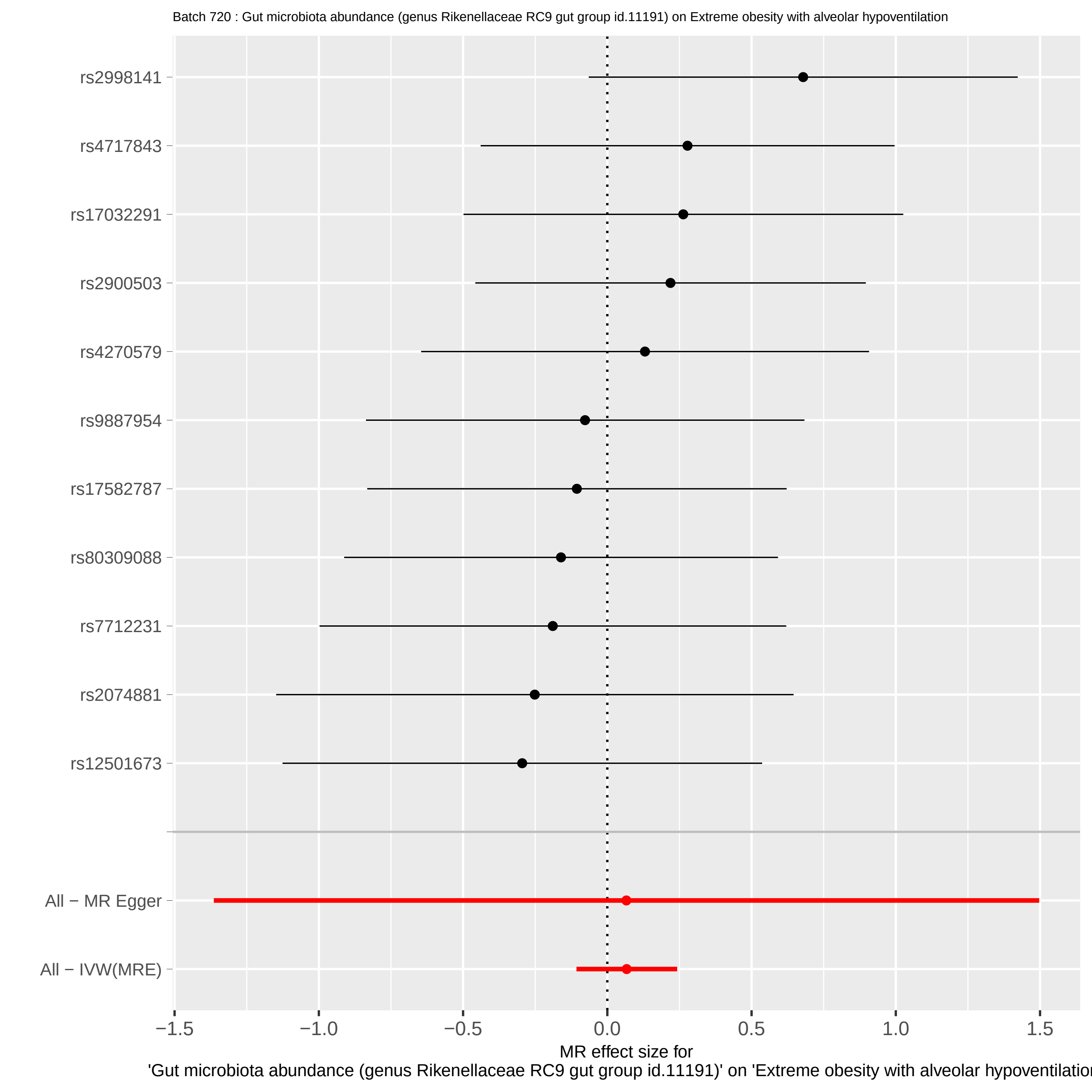

Batch 720 : Gut microbiota abundance (genus Rikenellaceae RC9 gut group id.11191) on Extreme obesity with alveolar hypoventilation
rs2998141
rs4717843
rs17032291
rs2900503
rs4270579
rs9887954
rs17582787
rs80309088
rs7712231
rs2074881
rs12501673
All − MR Egger
All − IVW(MRE)
−1.5
−1.0
−0.5
0.0
0.5
1.0
1.5
MR effect size for
'Gut microbiota abundance (genus Rikenellaceae RC9 gut group id.11191)' on 'Extreme obesity with alveolar hypoventilatio

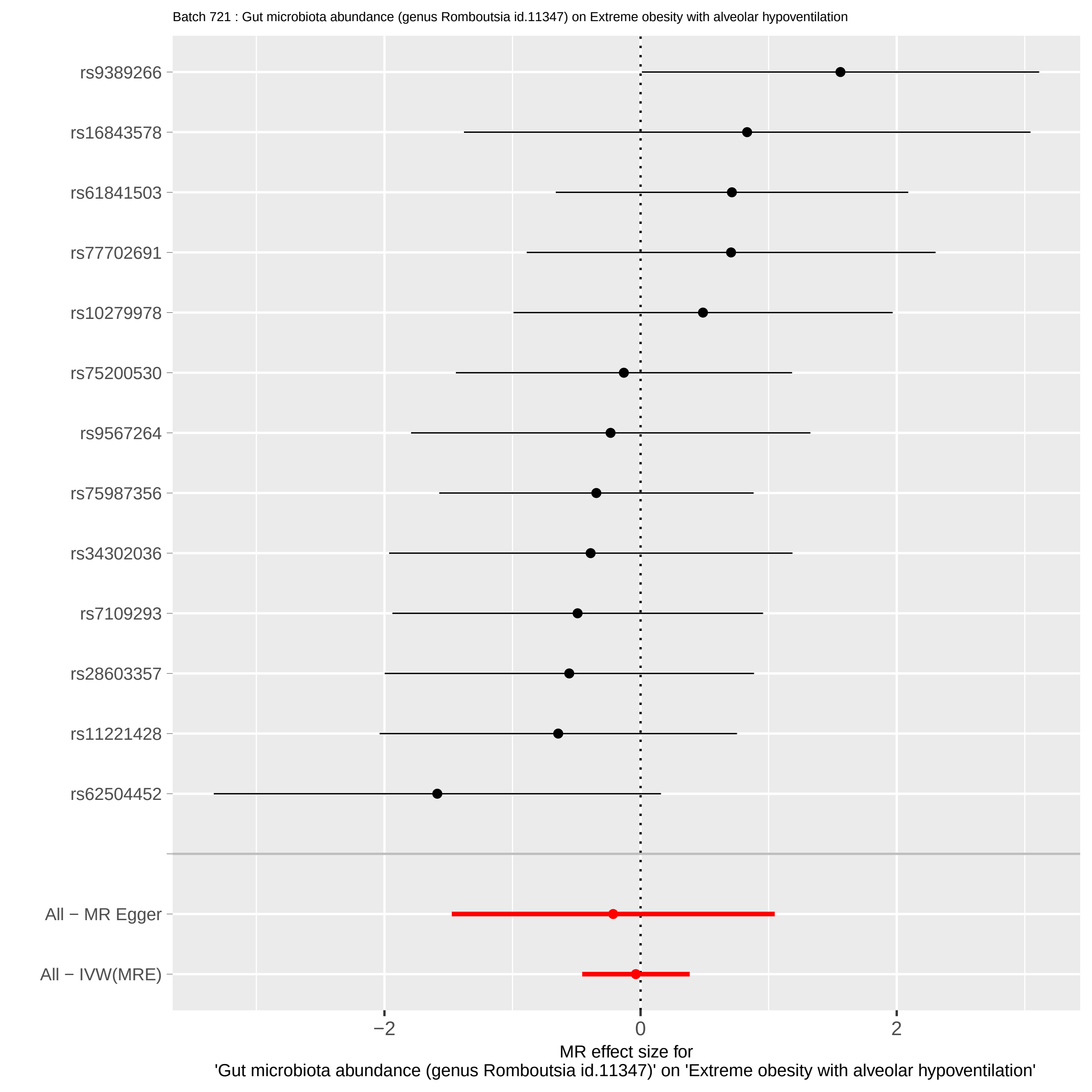
Batch 721 : Gut microbiota abundance (genus Romboutsia id.11347) on Extreme obesity with alveolar hypoventilation
rs9389266
rs16843578
rs61841503
rs77702691
rs10279978
rs75200530
rs9567264
rs75987356
rs34302036
rs7109293
rs28603357
rs11221428
rs62504452
All − MR Egger
All − IVW(MRE)
−2
0
2
MR effect size for
'Gut microbiota abundance (genus Romboutsia id.11347)' on 'Extreme obesity with alveolar hypoventilation'

Batch 722 : Gut microbiota abundance (genus Roseburia id.2012) on Extreme obesity with alveolar hypoventilation

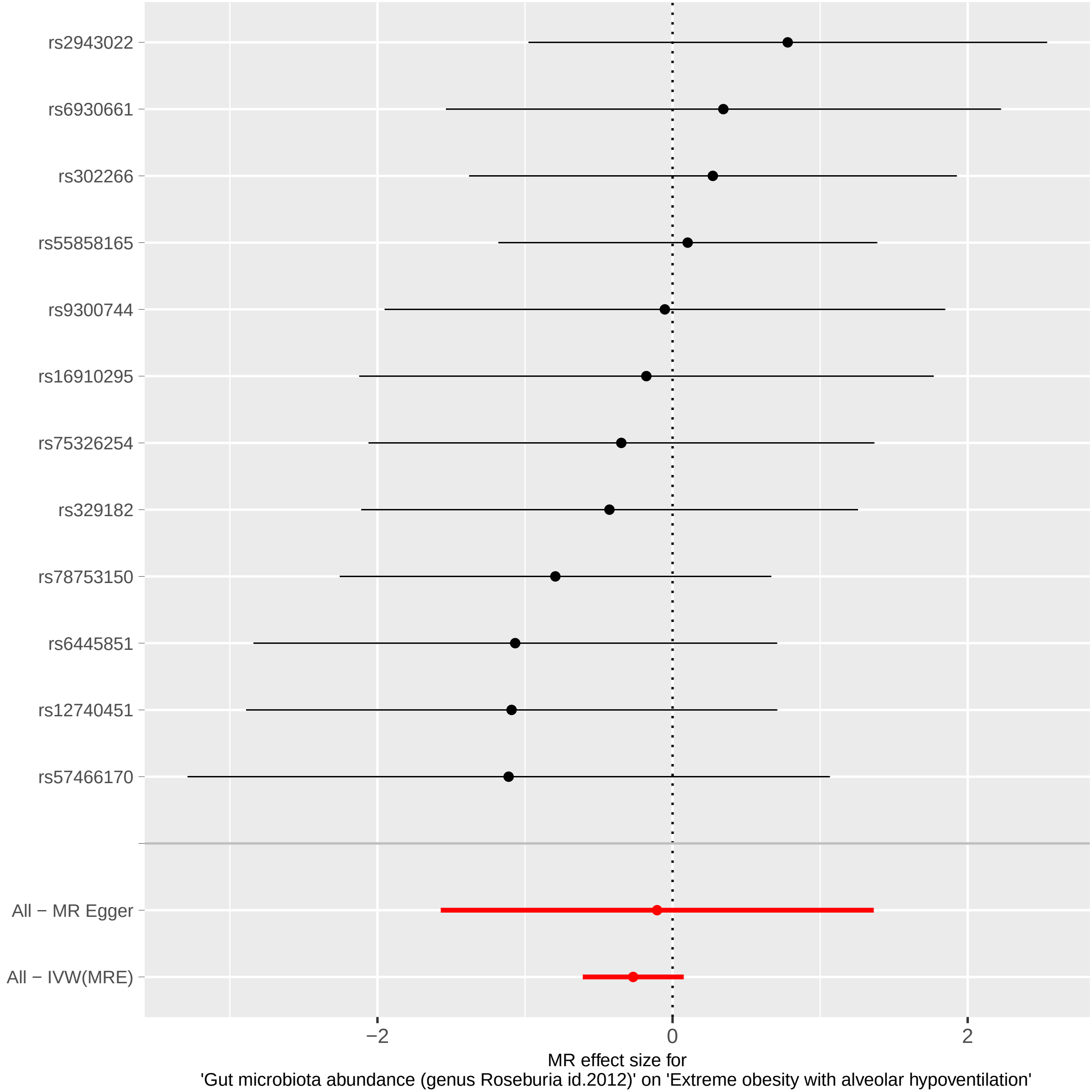

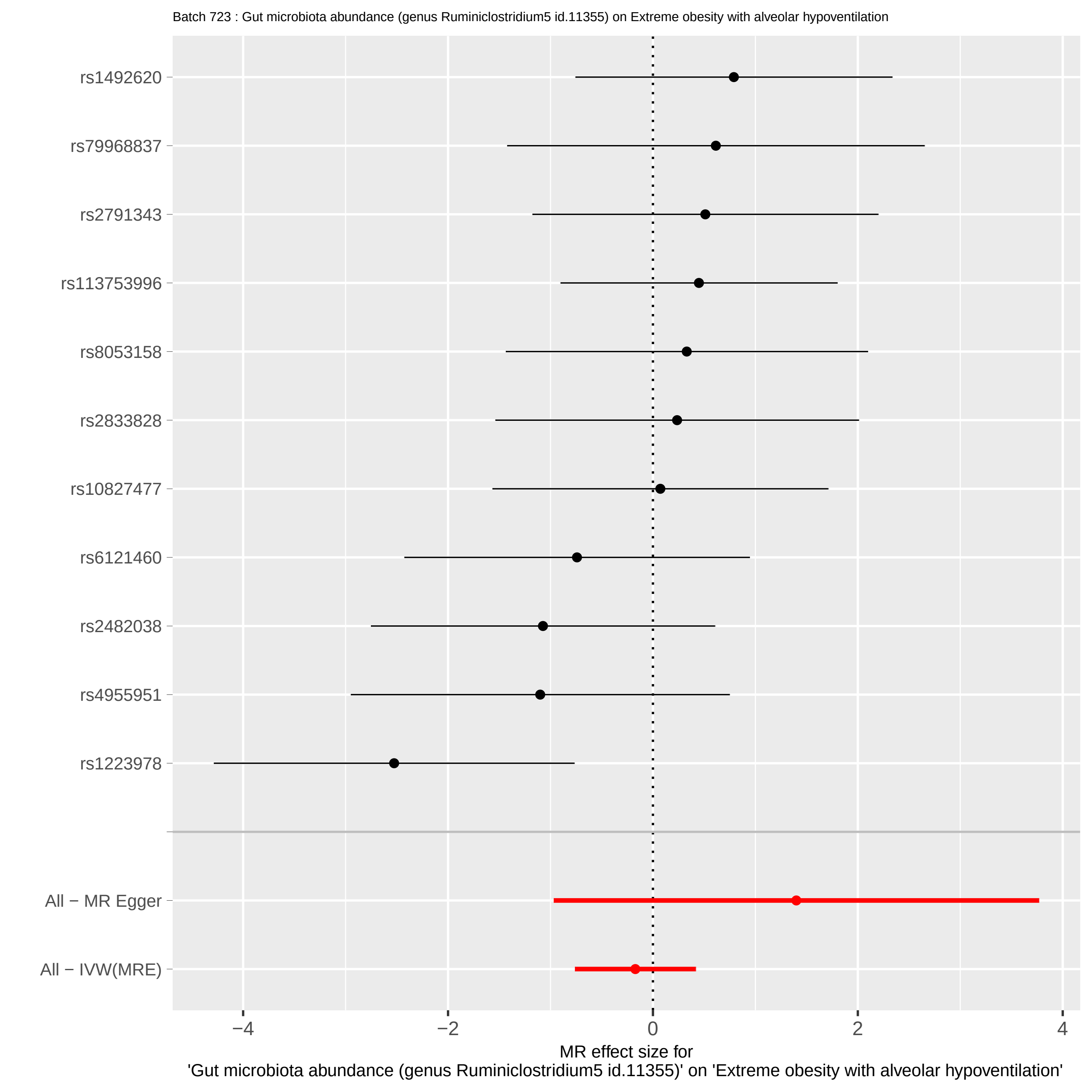

Batch 723 : Gut microbiota abundance (genus Ruminiclostridium5 id.11355) on Extreme obesity with alveolar hypoventilation
rs1492620
rs79968837
rs2791343
rs113753996
rs8053158
rs2833828
rs10827477
rs6121460
rs2482038
rs4955951
rs1223978
All − MR Egger
All − IVW(MRE)
−4
−2
0
2
4
MR effect size for
'Gut microbiota abundance (genus Ruminiclostridium5 id.11355)' on 'Extreme obesity with alveolar hypoventilation'

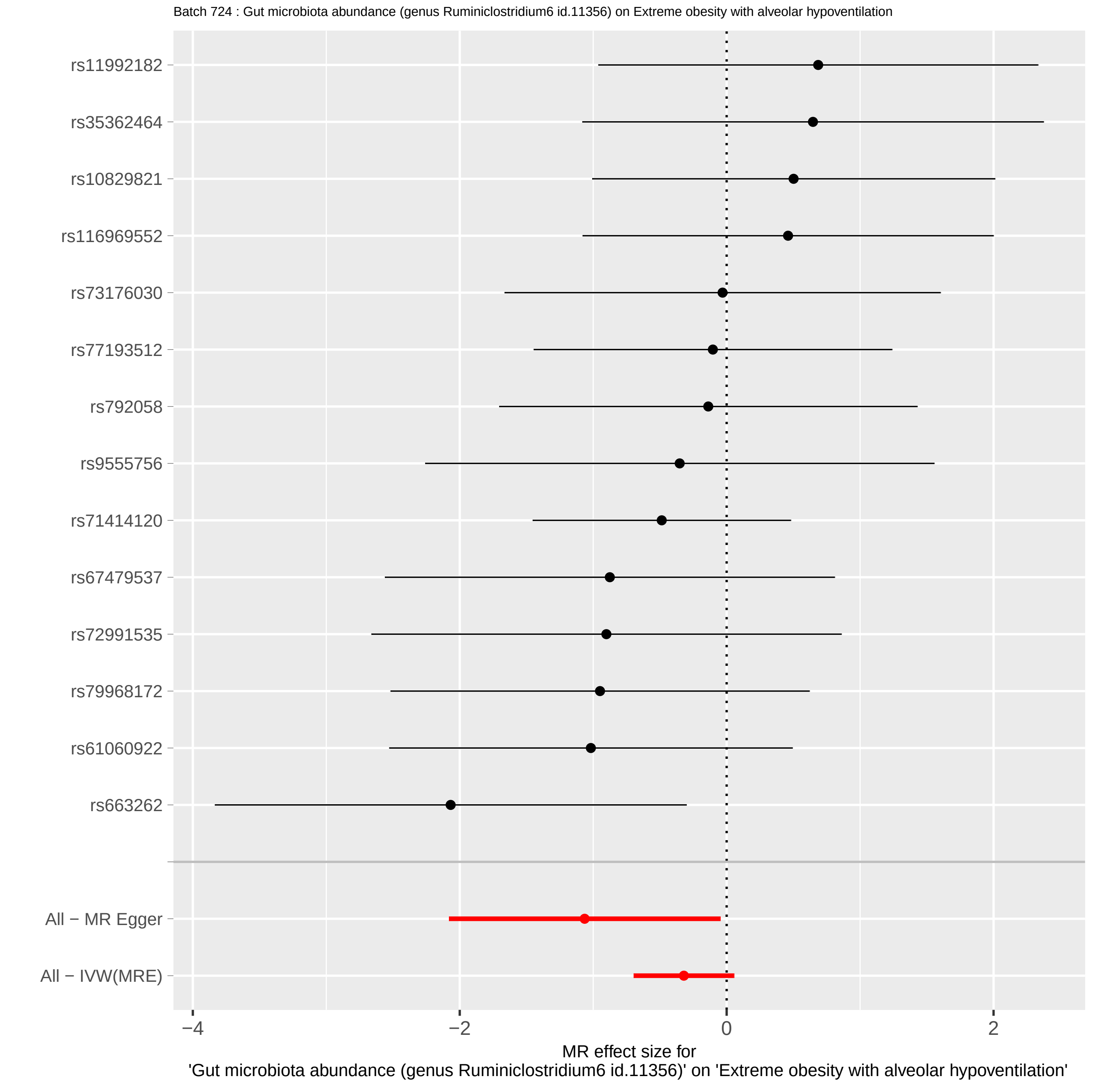

Batch 724 : Gut microbiota abundance (genus Ruminiclostridium6 id.11356) on Extreme obesity with alveolar hypoventilation
rs11992182
rs35362464
rs10829821
rs116969552
rs73176030
rs77193512
rs792058
rs9555756
rs71414120
rs67479537
rs72991535
rs79968172
rs61060922
rs663262
All − MR Egger
All − IVW(MRE)
−4
−2
0
2
MR effect size for
'Gut microbiota abundance (genus Ruminiclostridium6 id.11356)' on 'Extreme obesity with alveolar hypoventilation'

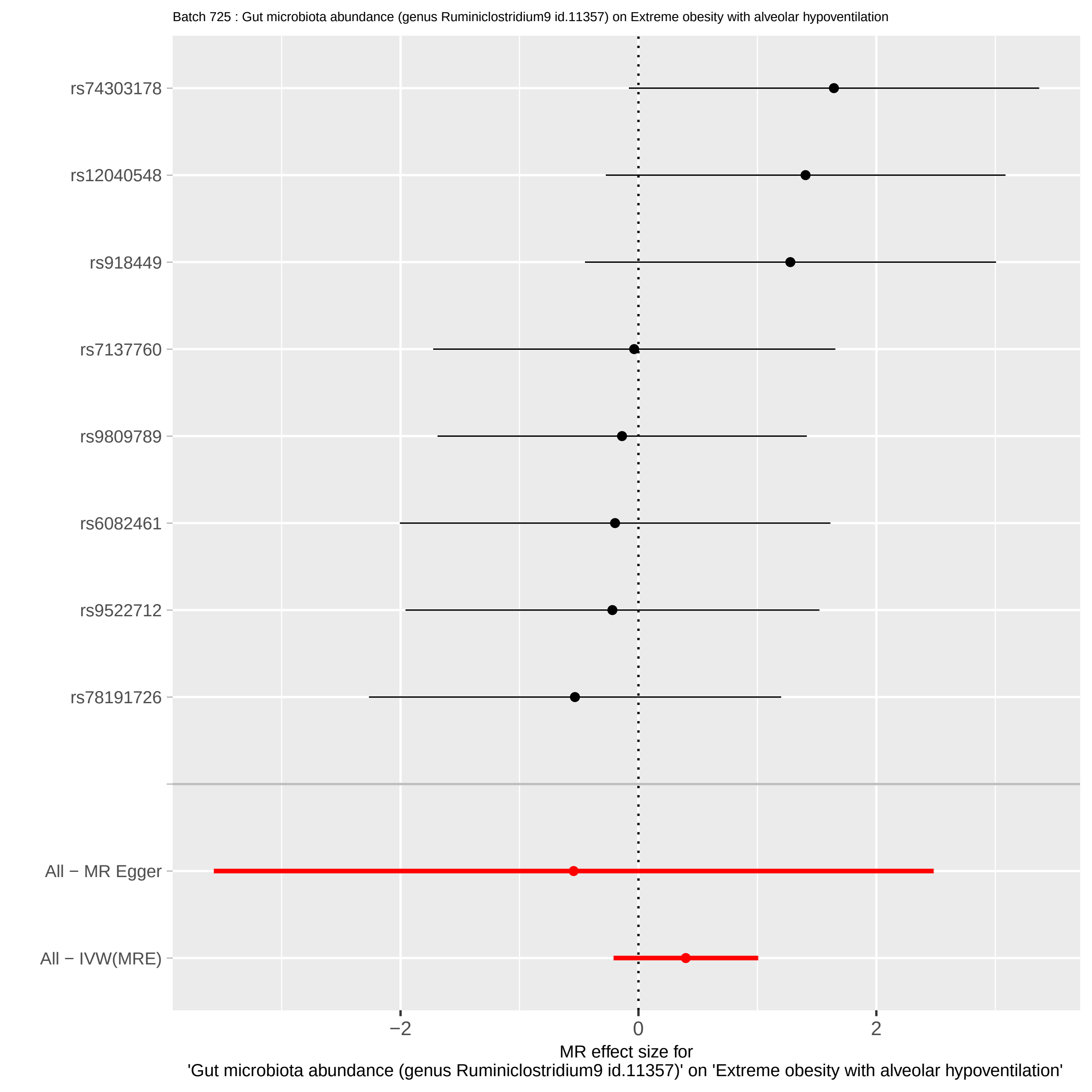
Batch 725 : Gut microbiota abundance (genus Ruminiclostridium9 id.11357) on Extreme obesity with alveolar hypoventilation
rs74303178
rs12040548
rs918449
rs7137760
rs9809789
rs6082461
rs9522712
rs78191726
All − MR Egger
All − IVW(MRE)
−2
0
2
MR effect size for
'Gut microbiota abundance (genus Ruminiclostridium9 id.11357)' on 'Extreme obesity with alveolar hypoventilation'

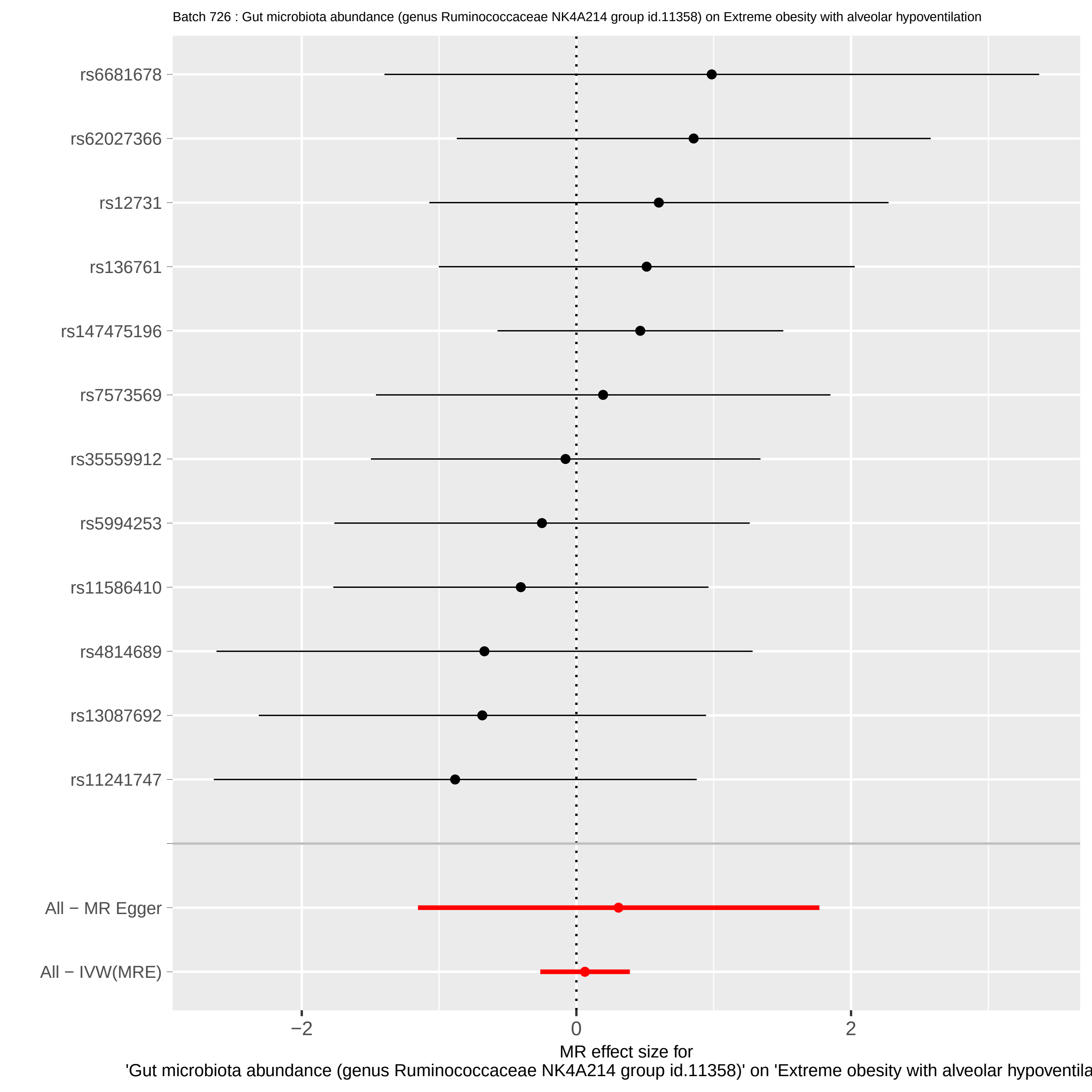

Batch 726 : Gut microbiota abundance (genus Ruminococcaceae NK4A214 group id.11358) on Extreme obesity with alveolar hypoventilation
rs6681678
rs62027366
rs12731
rs136761
rs147475196
rs7573569
rs35559912
rs5994253
rs11586410
rs4814689
rs13087692
rs11241747
All − MR Egger
All − IVW(MRE)
−2
0
2
MR effect size for
'Gut microbiota abundance (genus Ruminococcaceae NK4A214 group id.11358)' on 'Extreme obesity with alveolar hypoventila

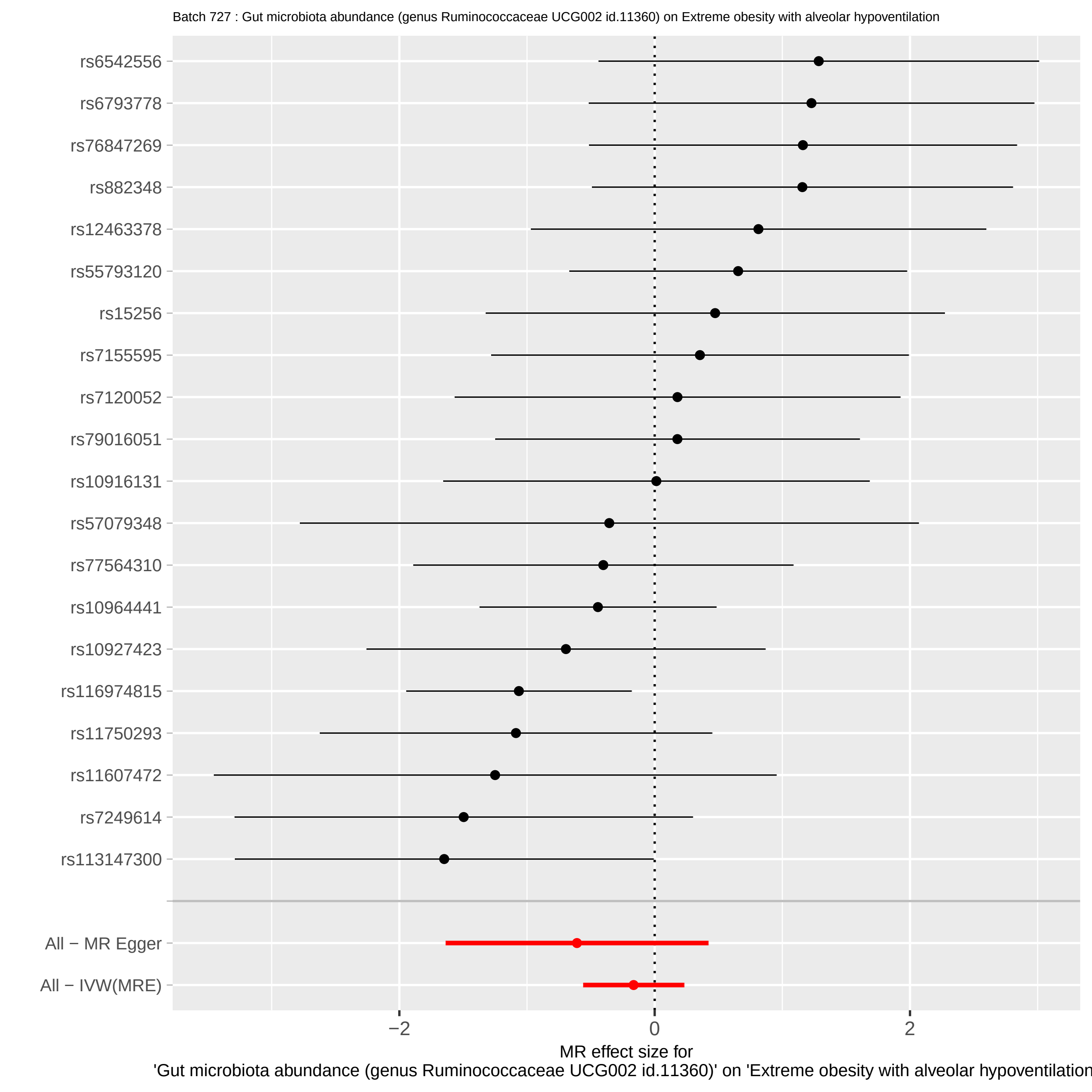

Batch 727 : Gut microbiota abundance (genus Ruminococcaceae UCG002 id.11360) on Extreme obesity with alveolar hypoventilation
rs6542556
rs6793778
rs76847269
rs882348
rs12463378
rs55793120
rs15256
rs7155595
rs7120052
rs79016051
rs10916131
rs57079348
rs77564310
rs10964441
rs10927423
rs116974815
rs11750293
rs11607472
rs7249614
rs113147300
All − MR Egger
All − IVW(MRE)
−2
0
2
MR effect size for
'Gut microbiota abundance (genus Ruminococcaceae UCG002 id.11360)' on 'Extreme obesity with alveolar hypoventilation

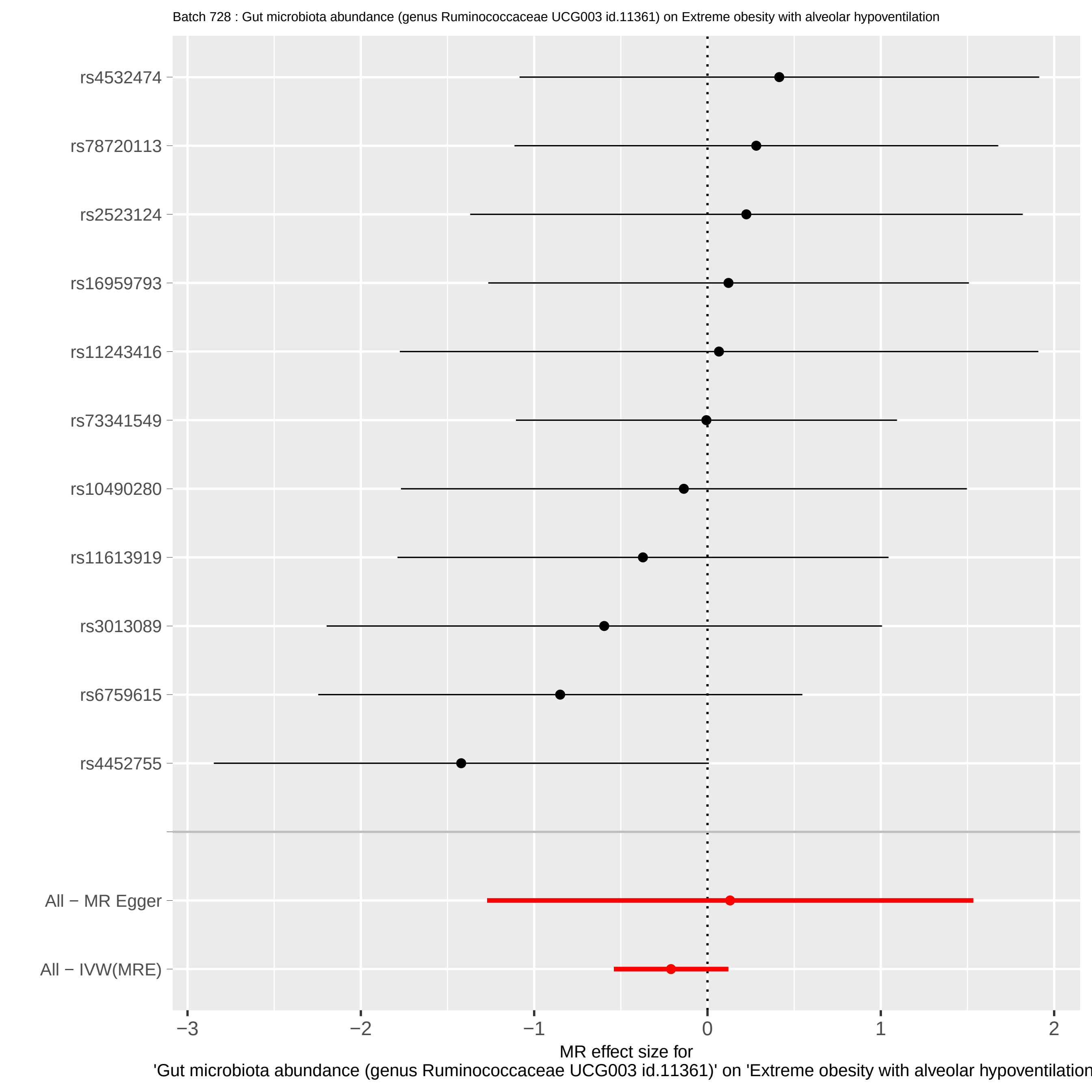

Batch 728 : Gut microbiota abundance (genus Ruminococcaceae UCG003 id.11361) on Extreme obesity with alveolar hypoventilation
rs4532474
rs78720113
rs2523124
rs16959793
rs11243416
rs73341549
rs10490280
rs11613919
rs3013089
rs6759615
rs4452755
All − MR Egger
All − IVW(MRE)
−3
−2
−1
0
1
2
MR effect size for
'Gut microbiota abundance (genus Ruminococcaceae UCG003 id.11361)' on 'Extreme obesity with alveolar hypoventilation

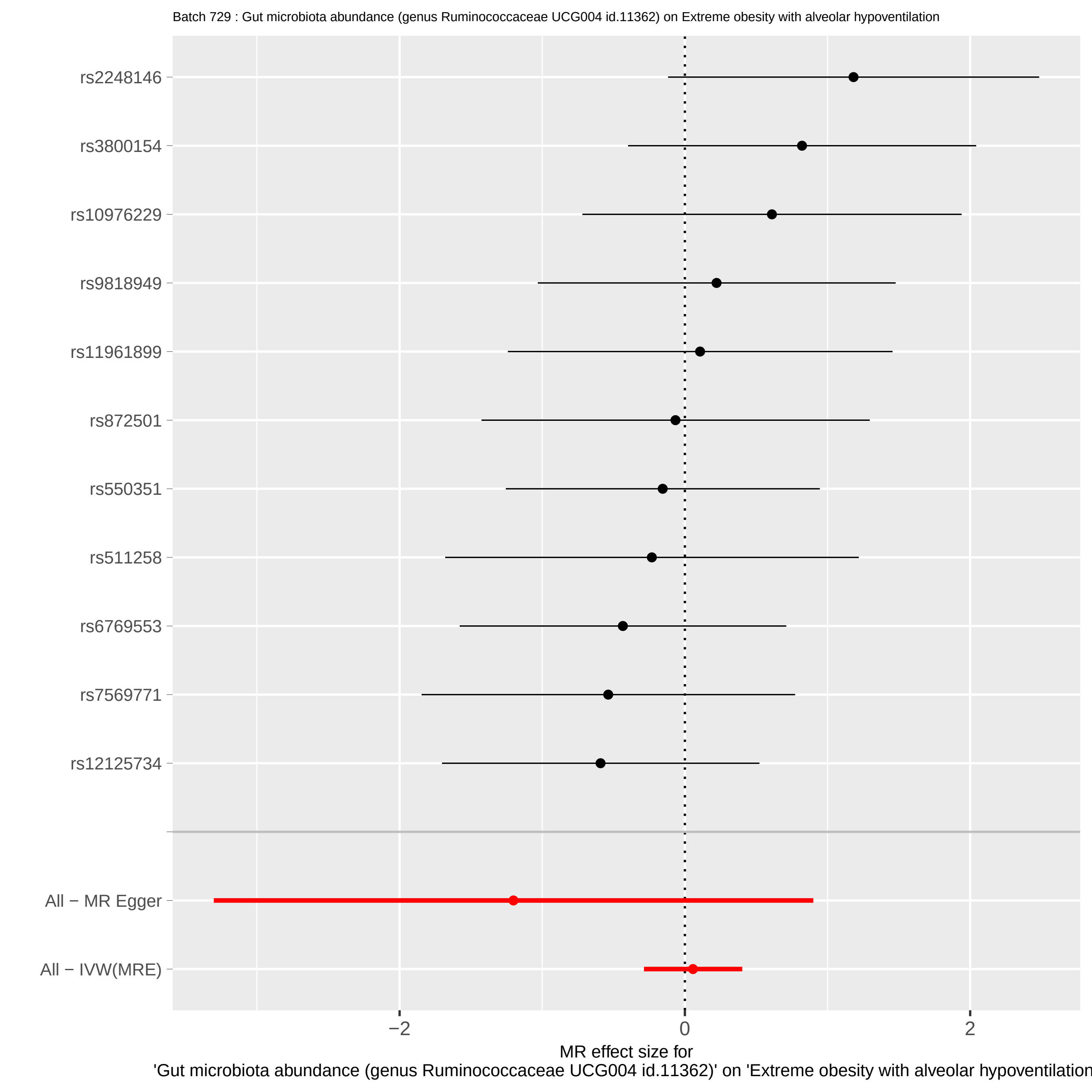
Batch 729 : Gut microbiota abundance (genus Ruminococcaceae UCG004 id.11362) on Extreme obesity with alveolar hypoventilation
rs2248146
rs3800154
rs10976229
rs9818949
rs11961899
rs872501
rs550351
rs511258
rs6769553
rs7569771
rs12125734
All − MR Egger
All − IVW(MRE)
−2
0
2
MR effect size for
'Gut microbiota abundance (genus Ruminococcaceae UCG004 id.11362)' on 'Extreme obesity with alveolar hypoventilation

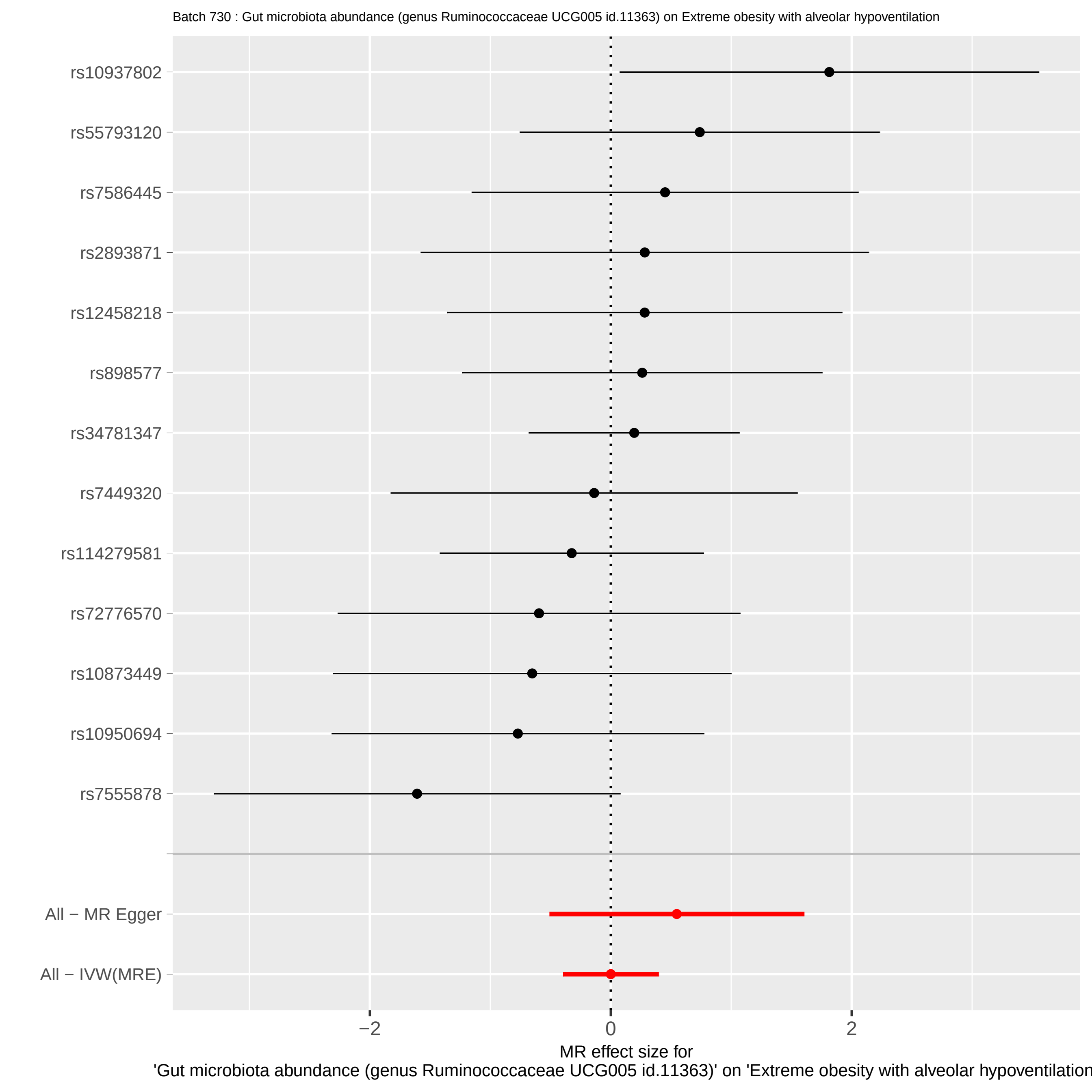

Batch 730 : Gut microbiota abundance (genus Ruminococcaceae UCG005 id.11363) on Extreme obesity with alveolar hypoventilation
rs10937802
rs55793120
rs7586445
rs2893871
rs12458218
rs898577
rs34781347
rs7449320
rs114279581
rs72776570
rs10873449
rs10950694
rs7555878
All − MR Egger
All − IVW(MRE)
−2
0
2
MR effect size for
'Gut microbiota abundance (genus Ruminococcaceae UCG005 id.11363)' on 'Extreme obesity with alveolar hypoventilation

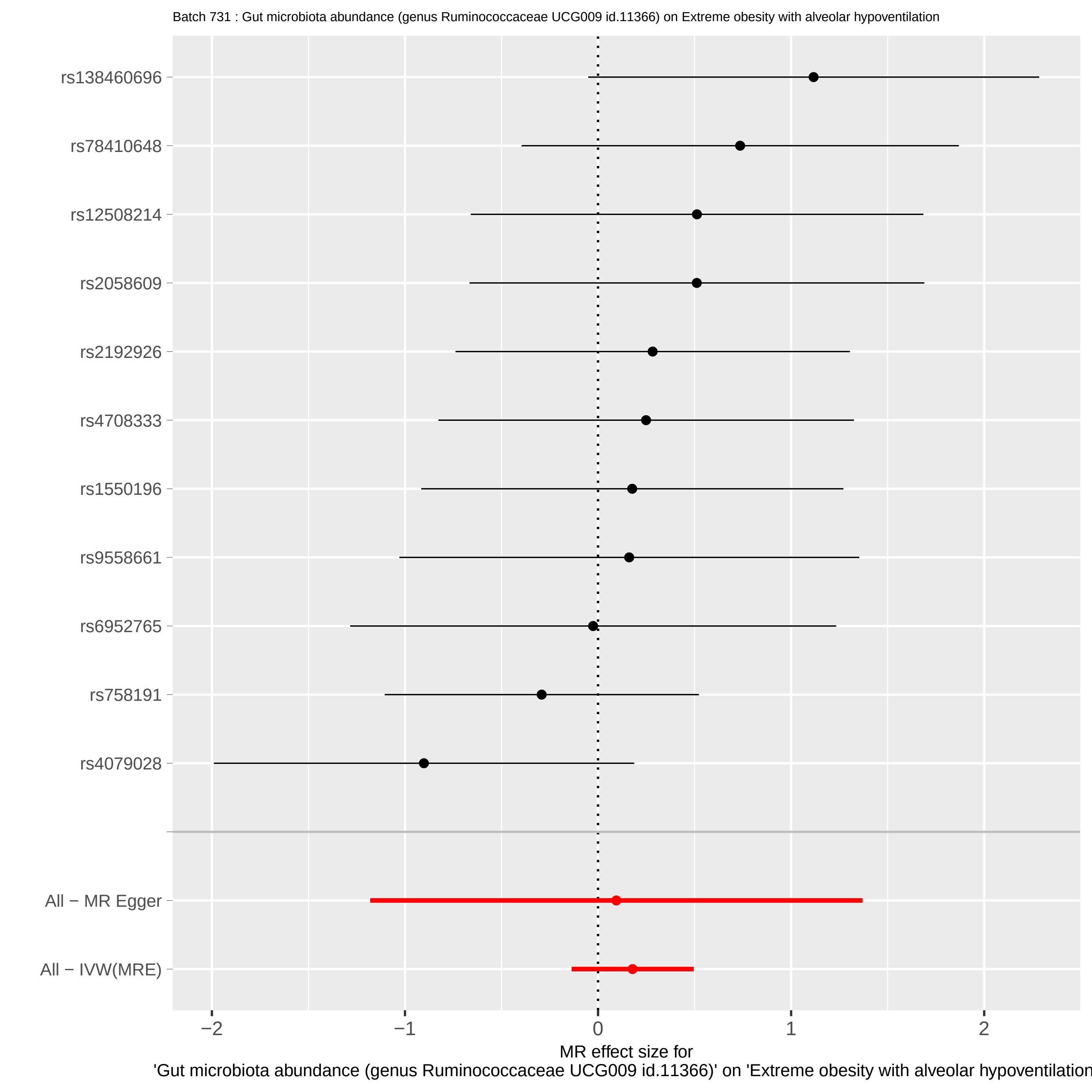

Batch 731 : Gut microbiota abundance (genus Ruminococcaceae UCG009 id.11366) on Extreme obesity with alveolar hypoventilation
rs138460696
rs78410648
rs12508214
rs2058609
rs2192926
rs4708333
rs1550196
rs9558661
rs6952765
rs758191
rs4079028
All − MR Egger
All − IVW(MRE)
−2
−1
0
1
2
MR effect size for
'Gut microbiota abundance (genus Ruminococcaceae UCG009 id.11366)' on 'Extreme obesity with alveolar hypoventilation

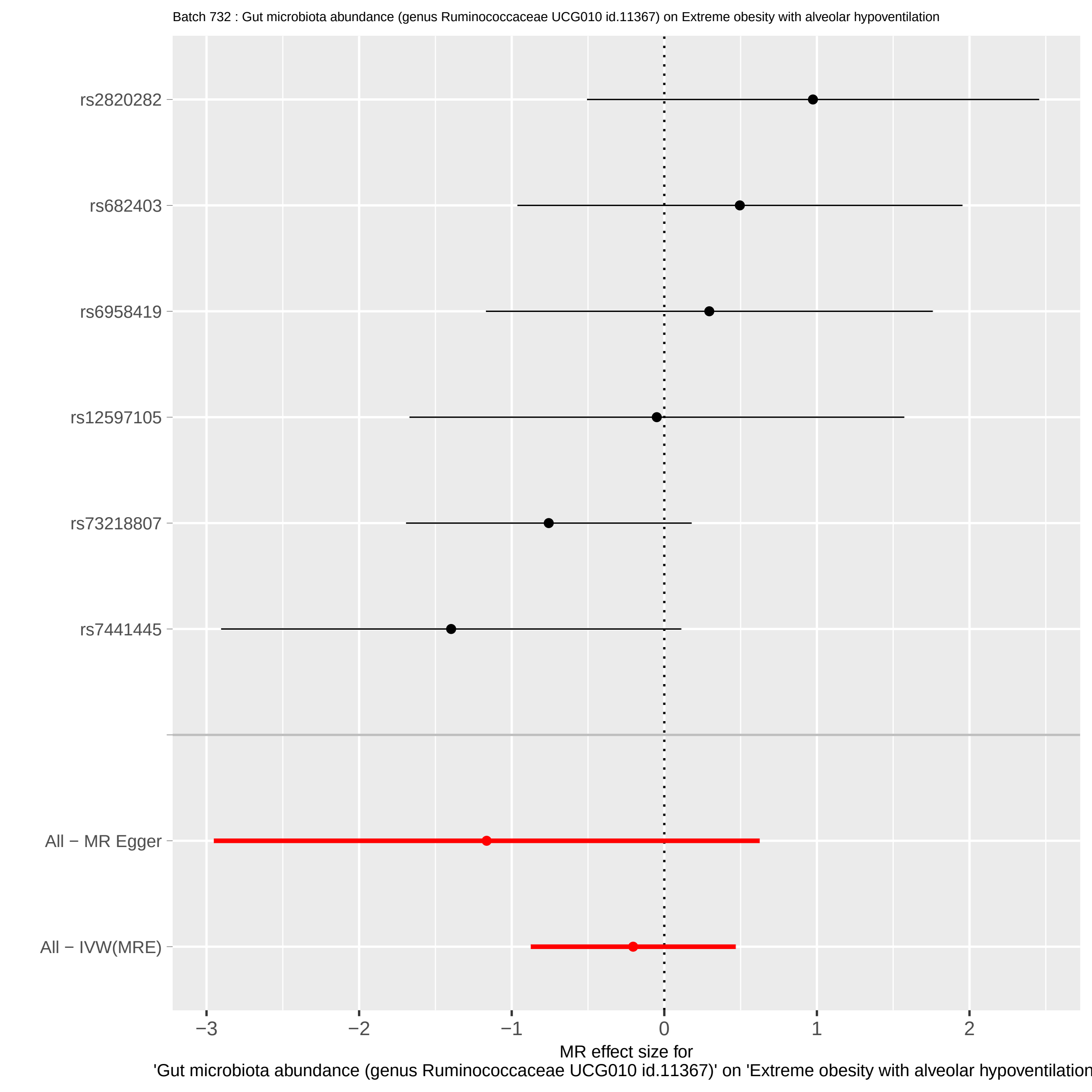
Batch 732 : Gut microbiota abundance (genus Ruminococcaceae UCG010 id.11367) on Extreme obesity with alveolar hypoventilation
rs2820282
rs682403
rs6958419
rs12597105
rs73218807
rs7441445
All − MR Egger
All − IVW(MRE)
−3
−2
−1
0
1
2
MR effect size for
'Gut microbiota abundance (genus Ruminococcaceae UCG010 id.11367)' on 'Extreme obesity with alveolar hypoventilation

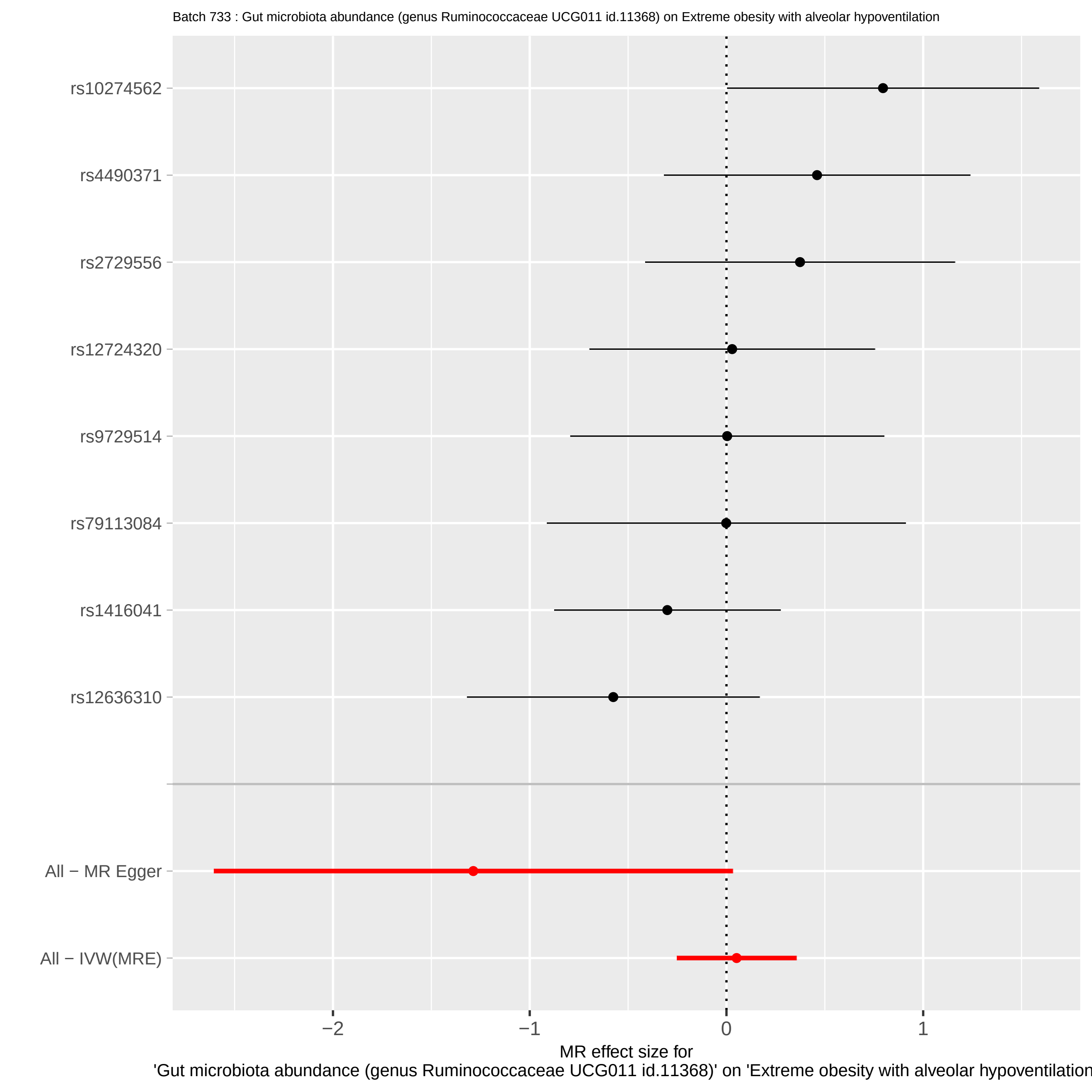

Batch 733 : Gut microbiota abundance (genus Ruminococcaceae UCG011 id.11368) on Extreme obesity with alveolar hypoventilation
rs10274562
rs4490371
rs2729556
rs12724320
rs9729514
rs79113084
rs1416041
rs12636310
All − MR Egger
All − IVW(MRE)
−2
−1
0
1
MR effect size for
'Gut microbiota abundance (genus Ruminococcaceae UCG011 id.11368)' on 'Extreme obesity with alveolar hypoventilation

Batch 734 : Gut microbiota abundance (genus Ruminococcaceae UCG013 id.11370) on Extreme obesity with alveolar hypoventilation

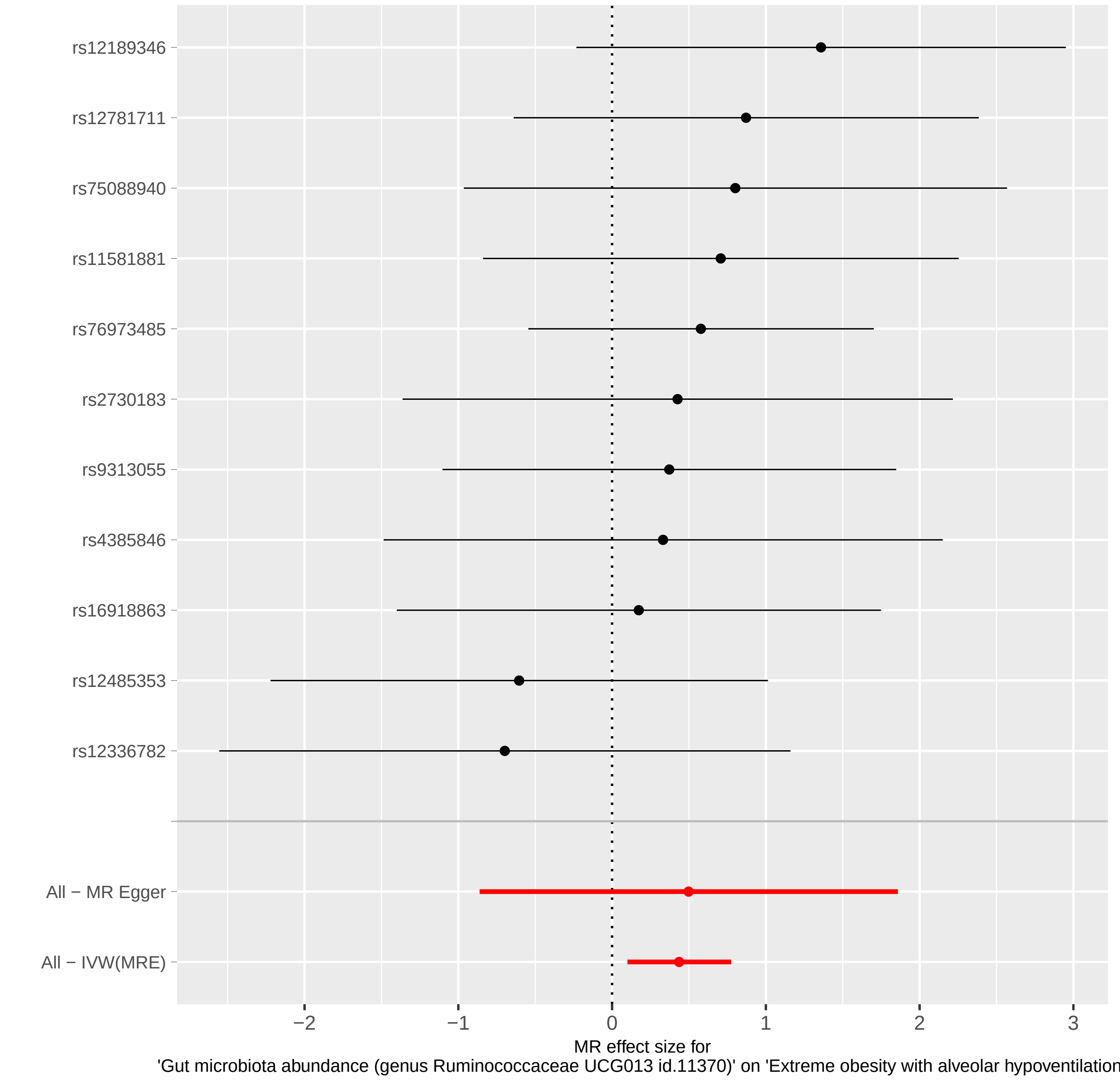

Batch 735 : Gut microbiota abundance (genus Ruminococcaceae UCG014 id.11371) on Extreme obesity with alveolar hypoventilation

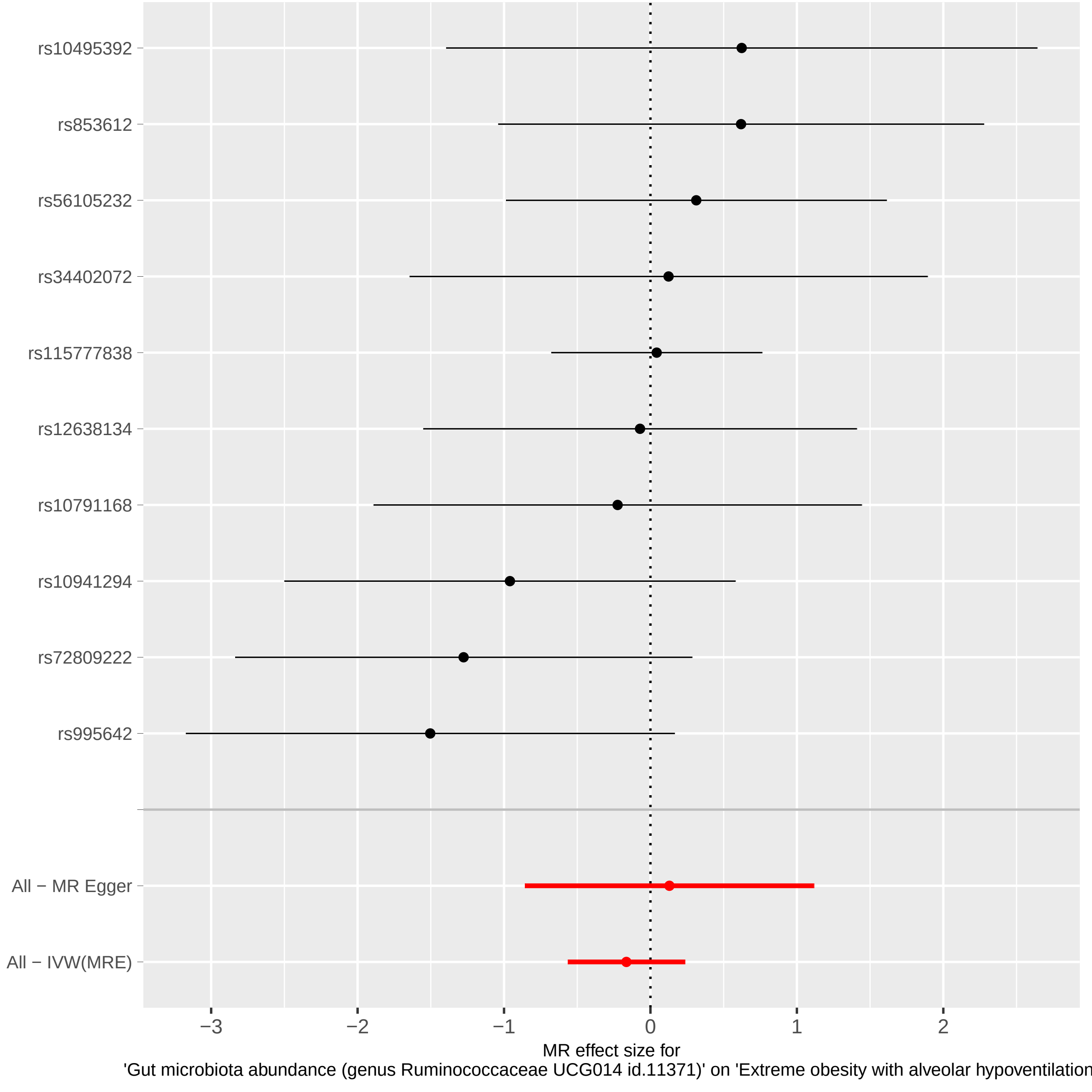

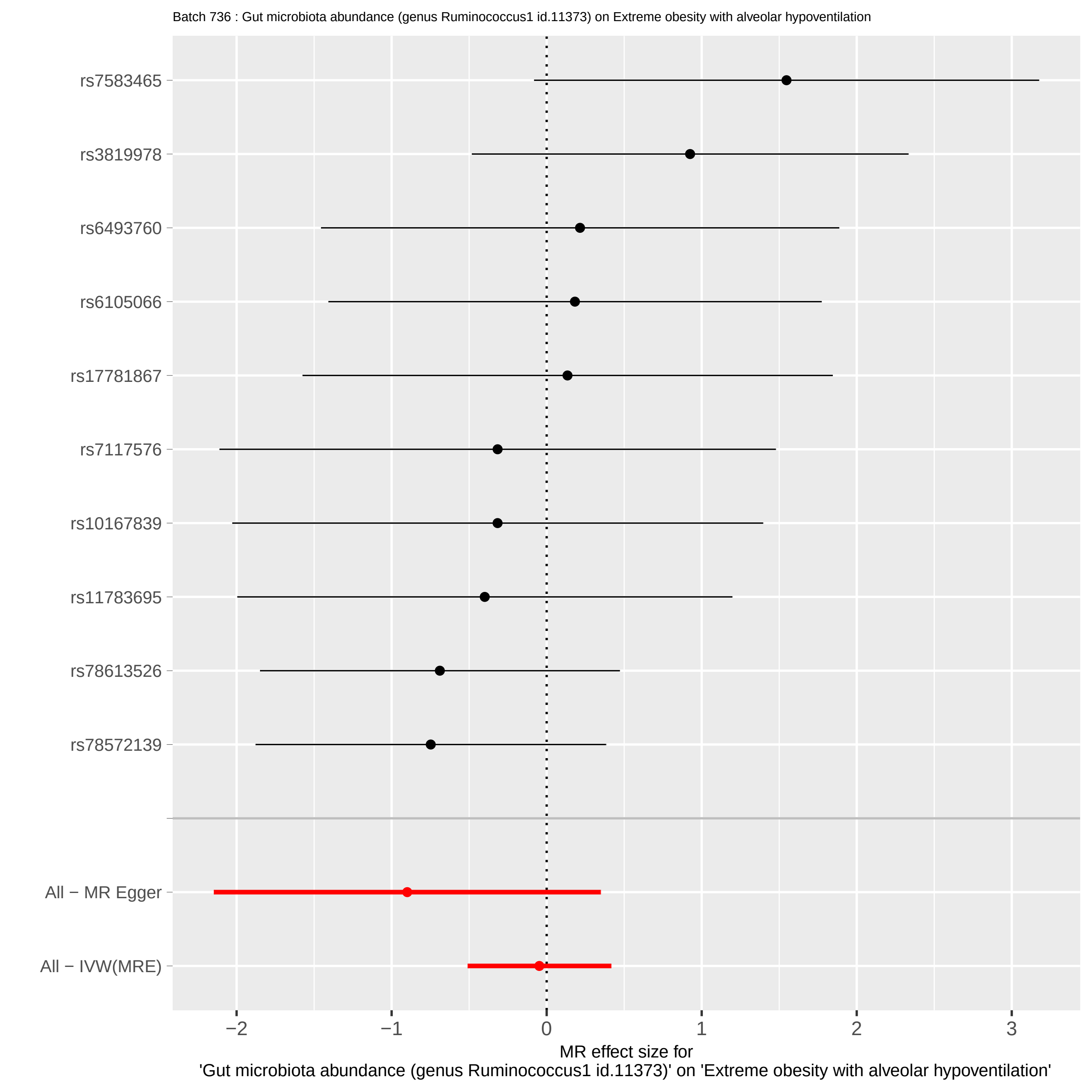
Batch 736 : Gut microbiota abundance (genus Ruminococcus1 id.11373) on Extreme obesity with alveolar hypoventilation
rs7583465
rs3819978
rs6493760
rs6105066
rs17781867
rs7117576
rs10167839
rs11783695
rs78613526
rs78572139
All − MR Egger
All − IVW(MRE)
−2
−1
0
1
2
3
MR effect size for
'Gut microbiota abundance (genus Ruminococcus1 id.11373)' on 'Extreme obesity with alveolar hypoventilation'

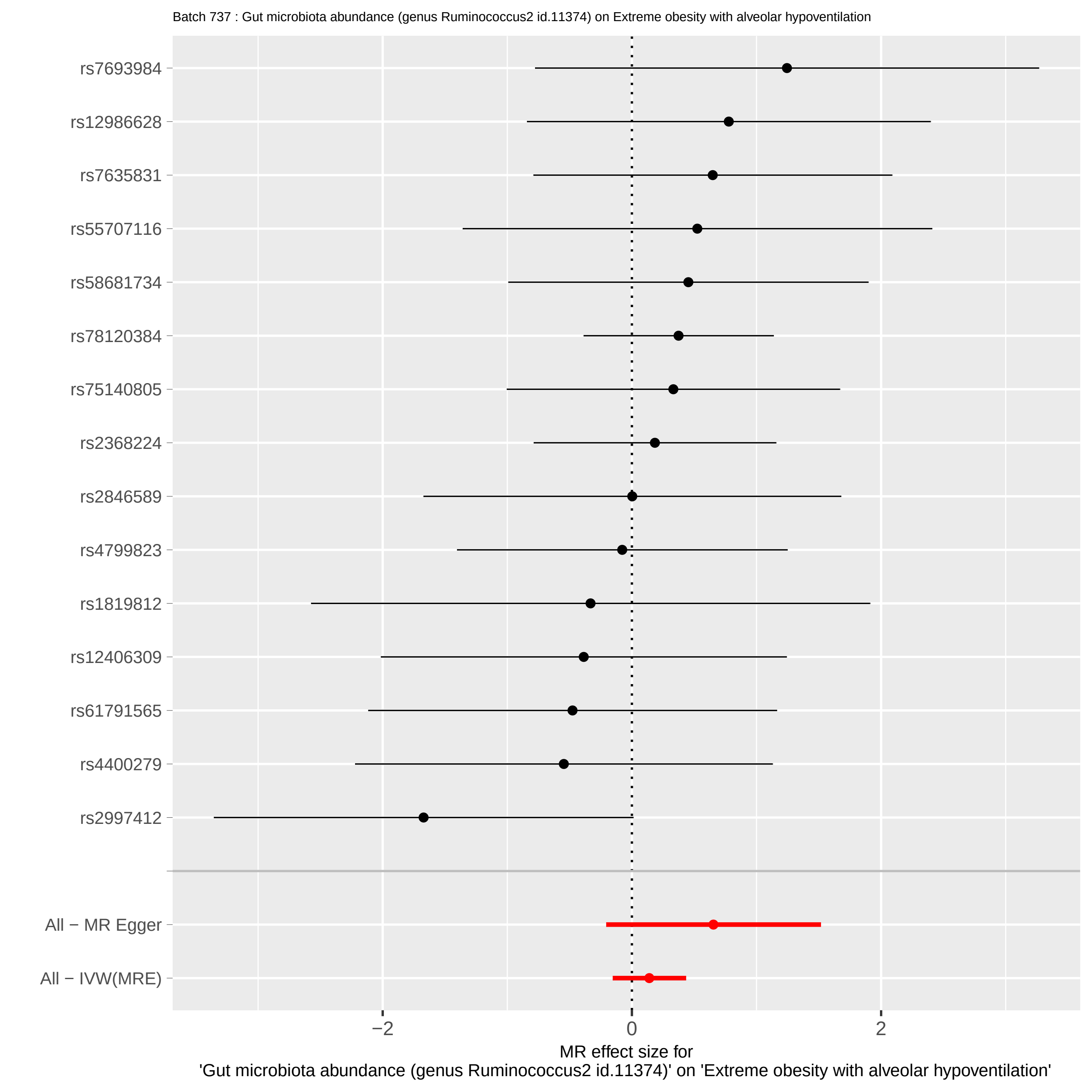
Batch 737 : Gut microbiota abundance (genus Ruminococcus2 id.11374) on Extreme obesity with alveolar hypoventilation
rs7693984
rs12986628
rs7635831
rs55707116
rs58681734
rs78120384
rs75140805
rs2368224
rs2846589
rs4799823
rs1819812
rs12406309
rs61791565
rs4400279
rs2997412
All − MR Egger
All − IVW(MRE)
−2
0
2
MR effect size for
'Gut microbiota abundance (genus Ruminococcus2 id.11374)' on 'Extreme obesity with alveolar hypoventilation'

Batch 738 : Gut microbiota abundance (genus Ruminococcus gauvreauii group id.11342) on Extreme obesity with alveolar hypoventilation

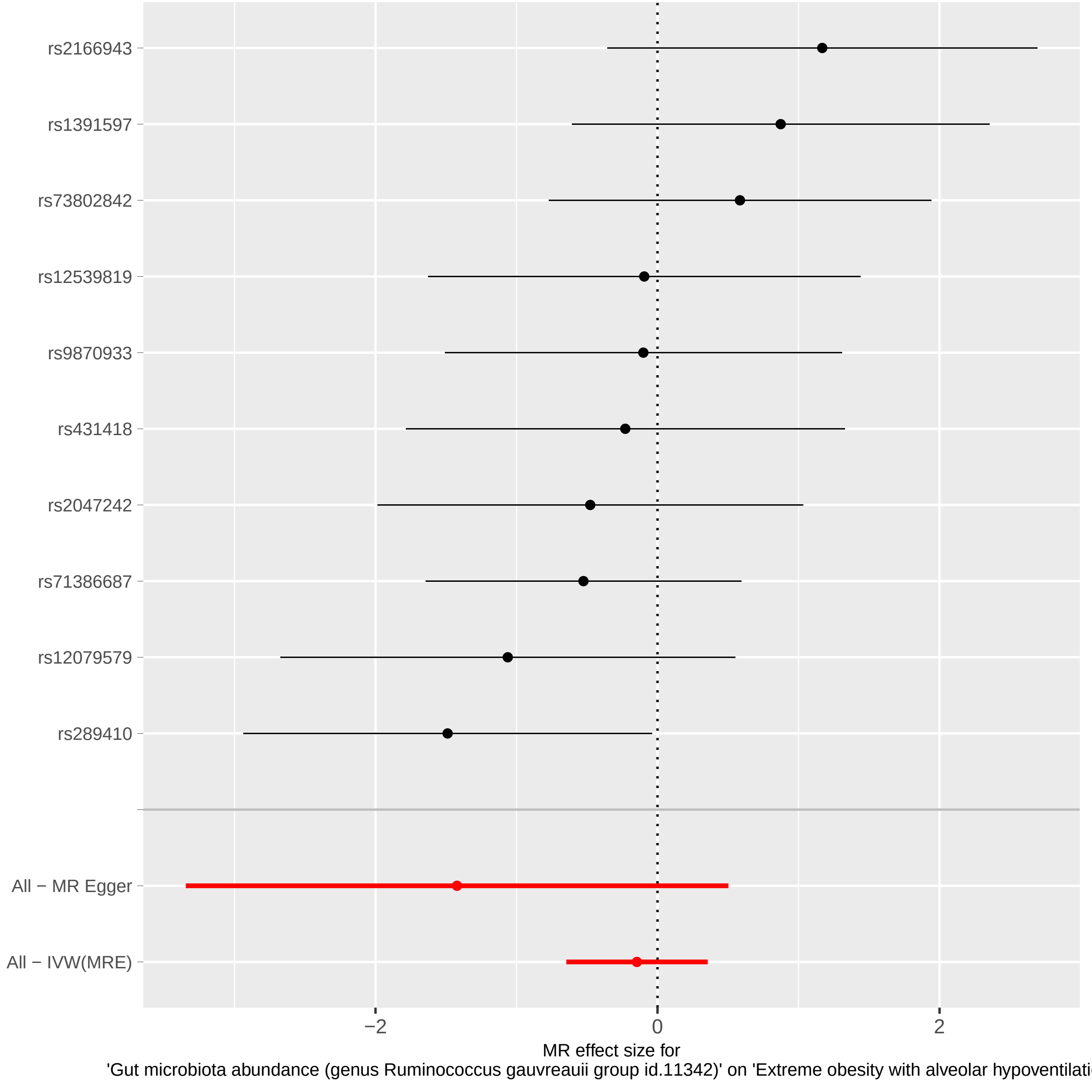

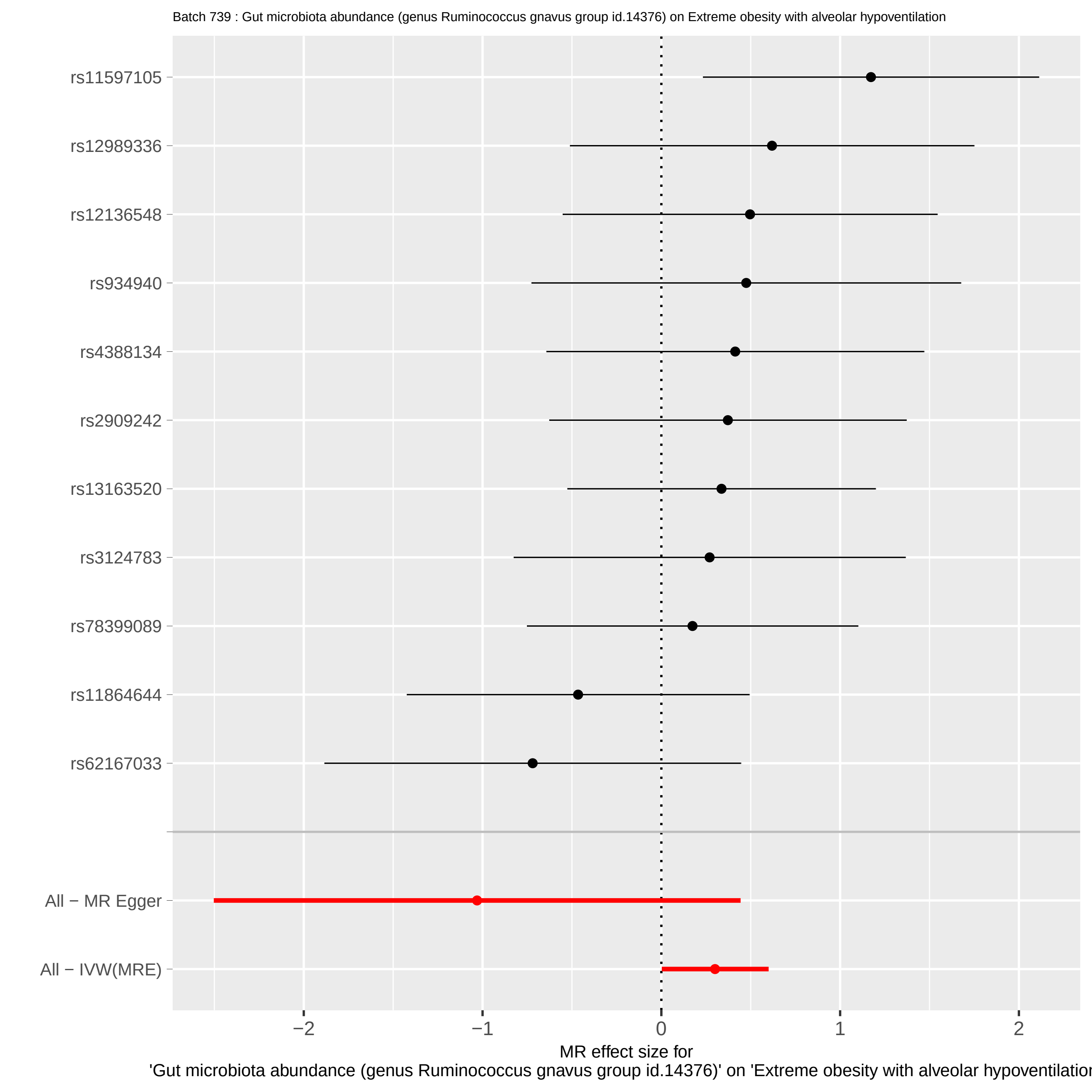

Batch 739 : Gut microbiota abundance (genus Ruminococcus gnavus group id.14376) on Extreme obesity with alveolar hypoventilation
rs11597105
rs12989336
rs12136548
rs934940
rs4388134
rs2909242
rs13163520
rs3124783
rs78399089
rs11864644
rs62167033
All − MR Egger
All − IVW(MRE)
−2
−1
0
1
2
MR effect size for
'Gut microbiota abundance (genus Ruminococcus gnavus group id.14376)' on 'Extreme obesity with alveolar hypoventilation

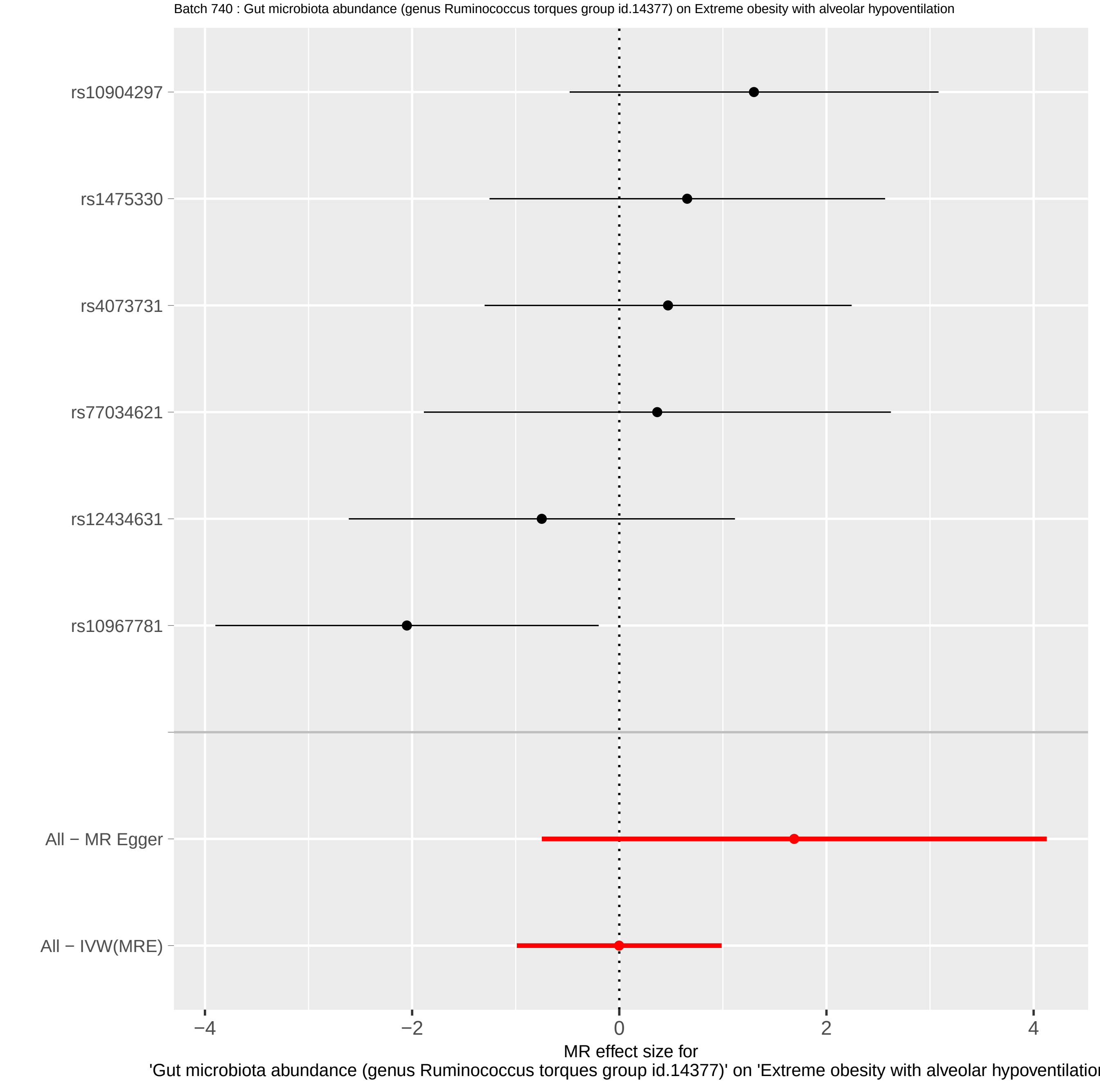
Batch 740 : Gut microbiota abundance (genus Ruminococcus torques group id.14377) on Extreme obesity with alveolar hypoventilation
rs10904297
rs1475330
rs4073731
rs77034621
rs12434631
rs10967781
All − MR Egger
All − IVW(MRE)
−4
−2
0
2
4
MR effect size for
'Gut microbiota abundance (genus Ruminococcus torques group id.14377)' on 'Extreme obesity with alveolar hypoventilatio

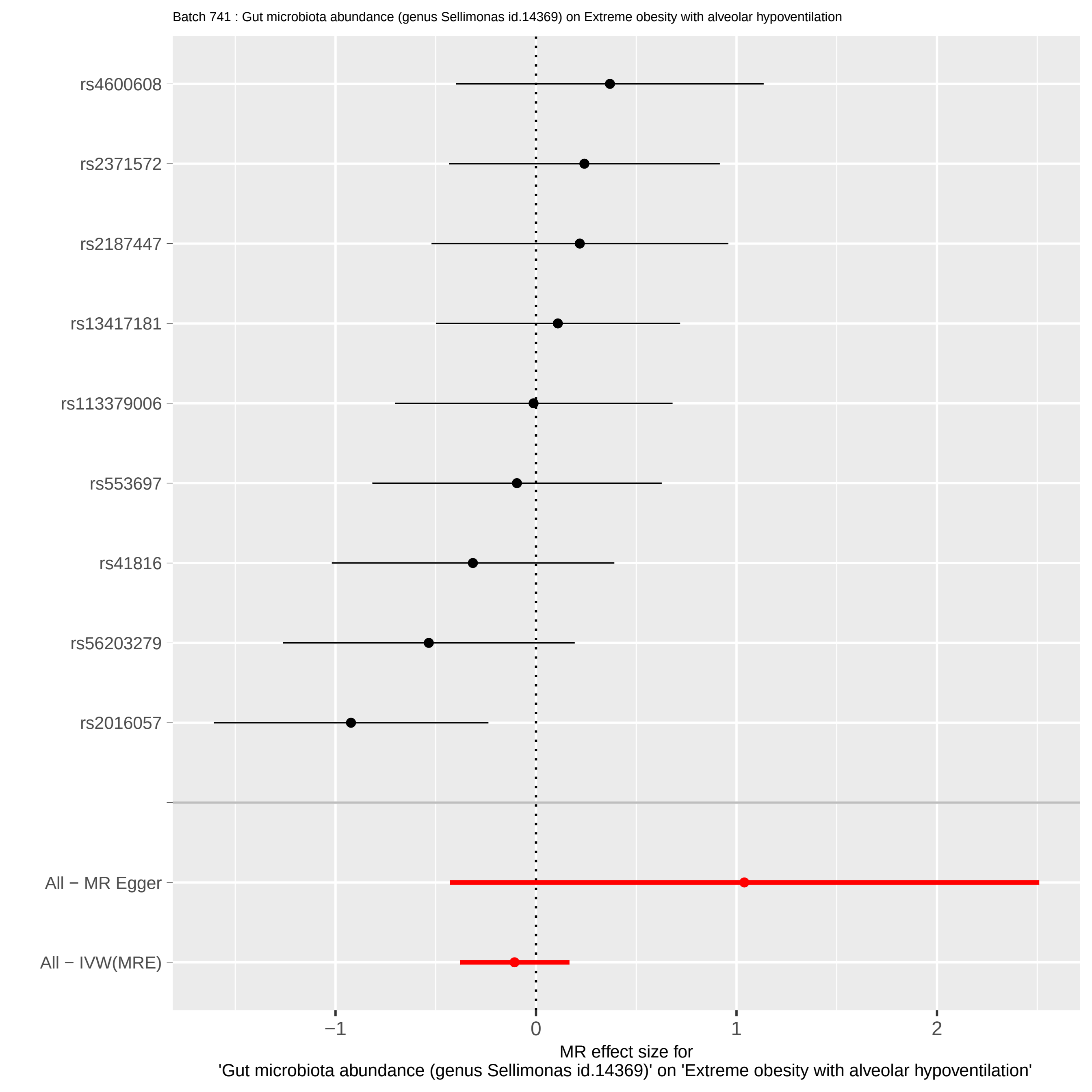
Batch 741 : Gut microbiota abundance (genus Sellimonas id.14369) on Extreme obesity with alveolar hypoventilation
rs4600608
rs2371572
rs2187447
rs13417181
rs113379006
rs553697
rs41816
rs56203279
rs2016057
All − MR Egger
All − IVW(MRE)
−1
0
1
2
MR effect size for
'Gut microbiota abundance (genus Sellimonas id.14369)' on 'Extreme obesity with alveolar hypoventilation'

Batch 742 : Gut microbiota abundance (genus Senegalimassilia id.11160) on Extreme obesity with alveolar hypoventilation

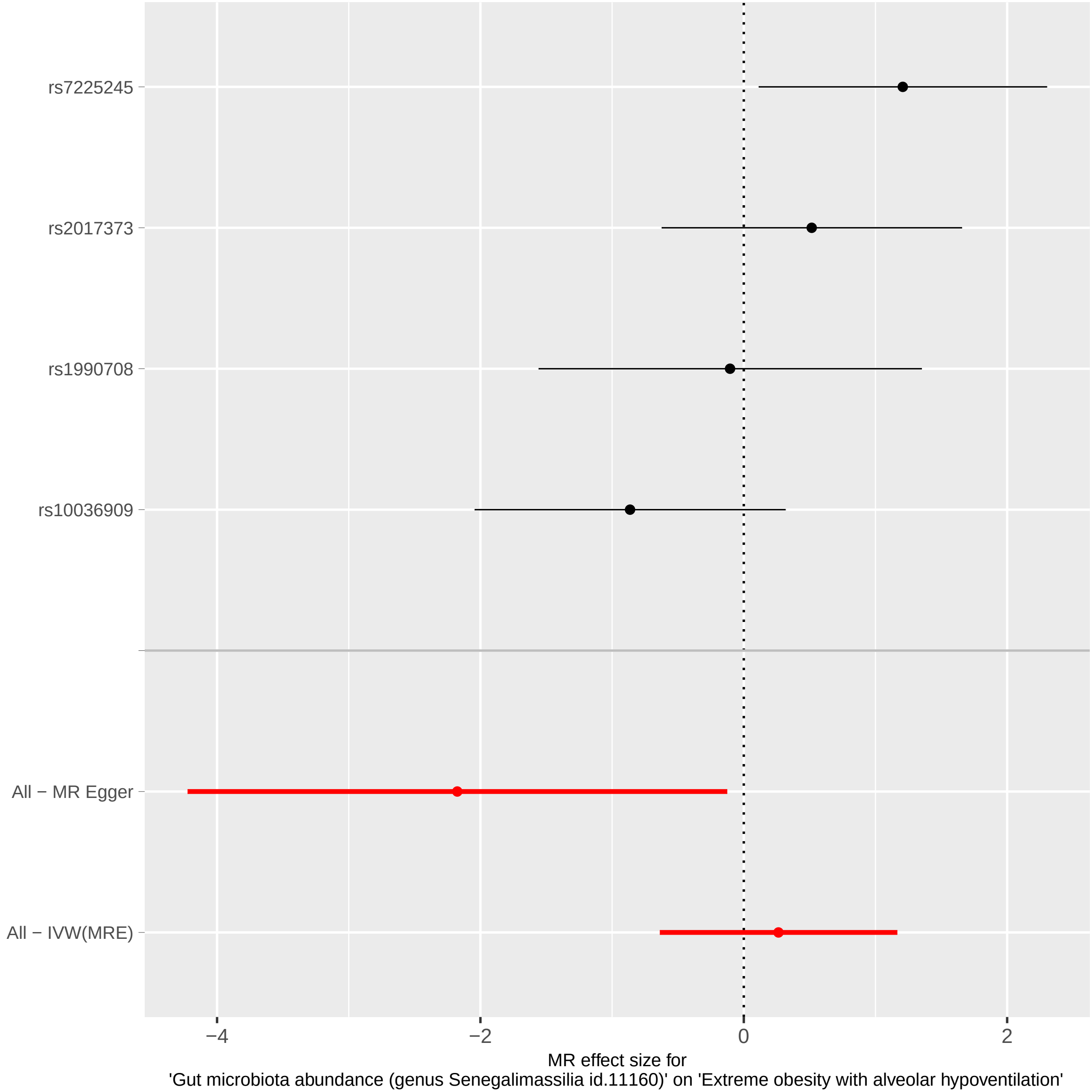

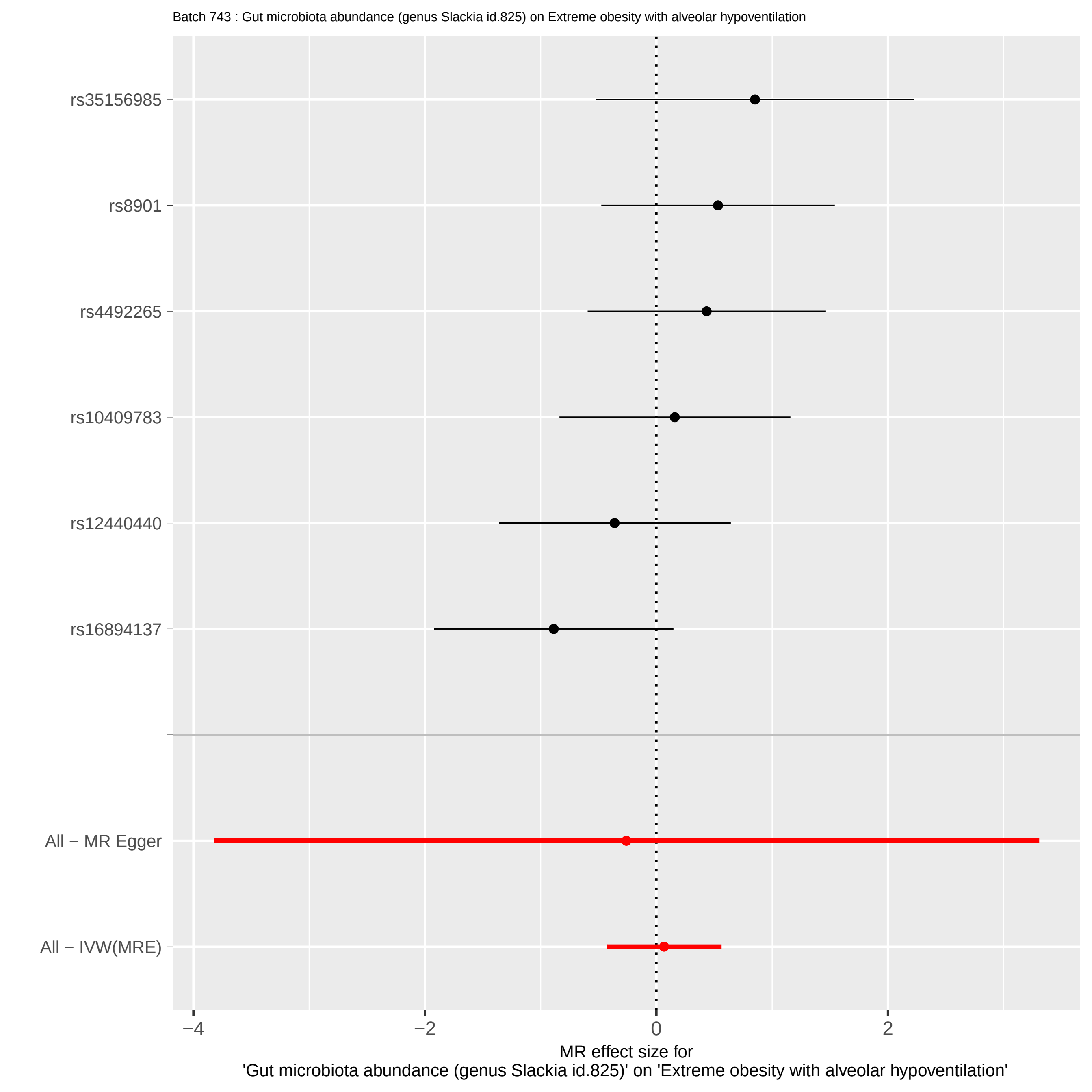
Batch 743 : Gut microbiota abundance (genus Slackia id.825) on Extreme obesity with alveolar hypoventilation
rs35156985
rs8901
rs4492265
rs10409783
rs12440440
rs16894137
All − MR Egger
All − IVW(MRE)
−4
−2
0
2
MR effect size for
'Gut microbiota abundance (genus Slackia id.825)' on 'Extreme obesity with alveolar hypoventilation'

Batch 744 : Gut microbiota abundance (genus Streptococcus id.1853) on Extreme obesity with alveolar hypoventilation

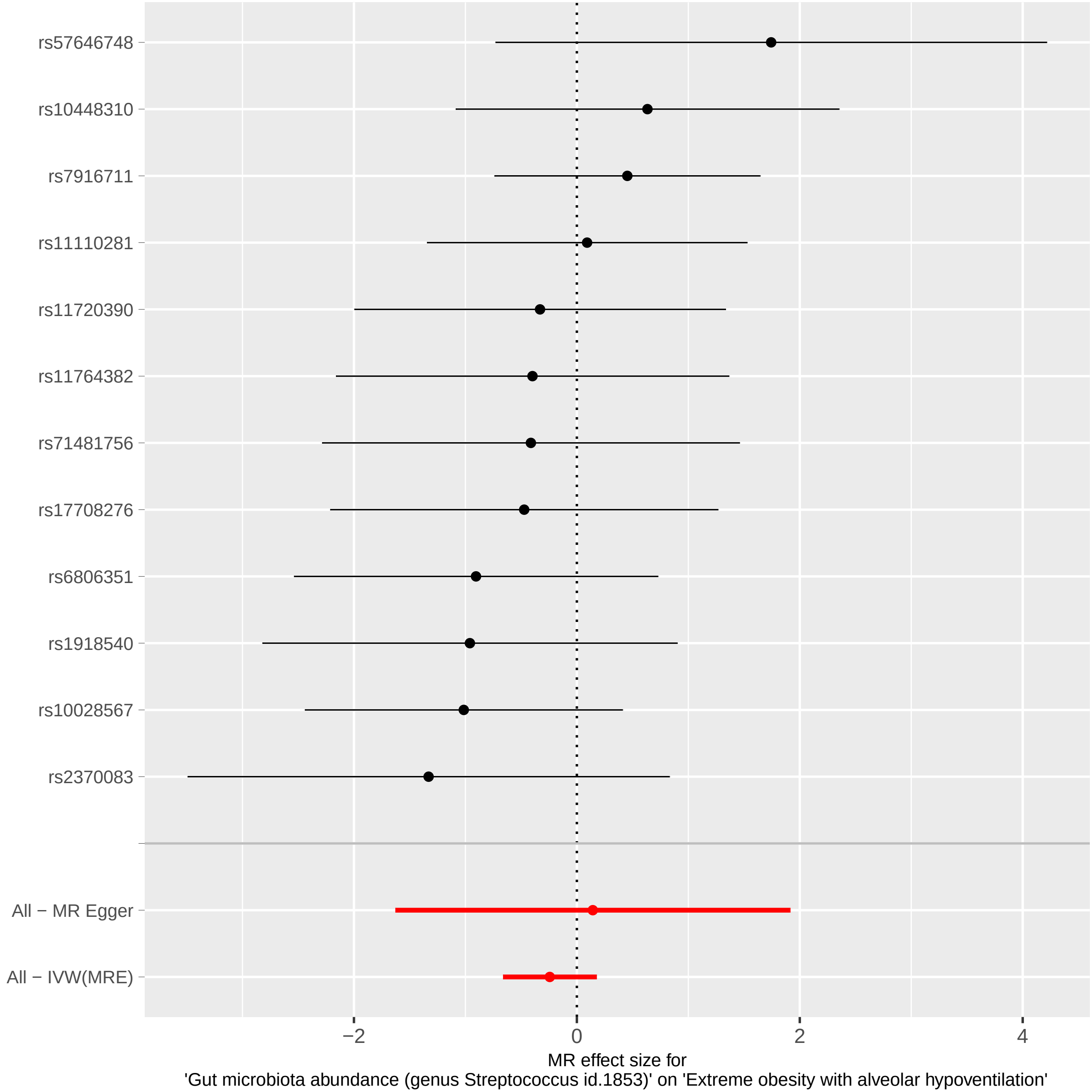

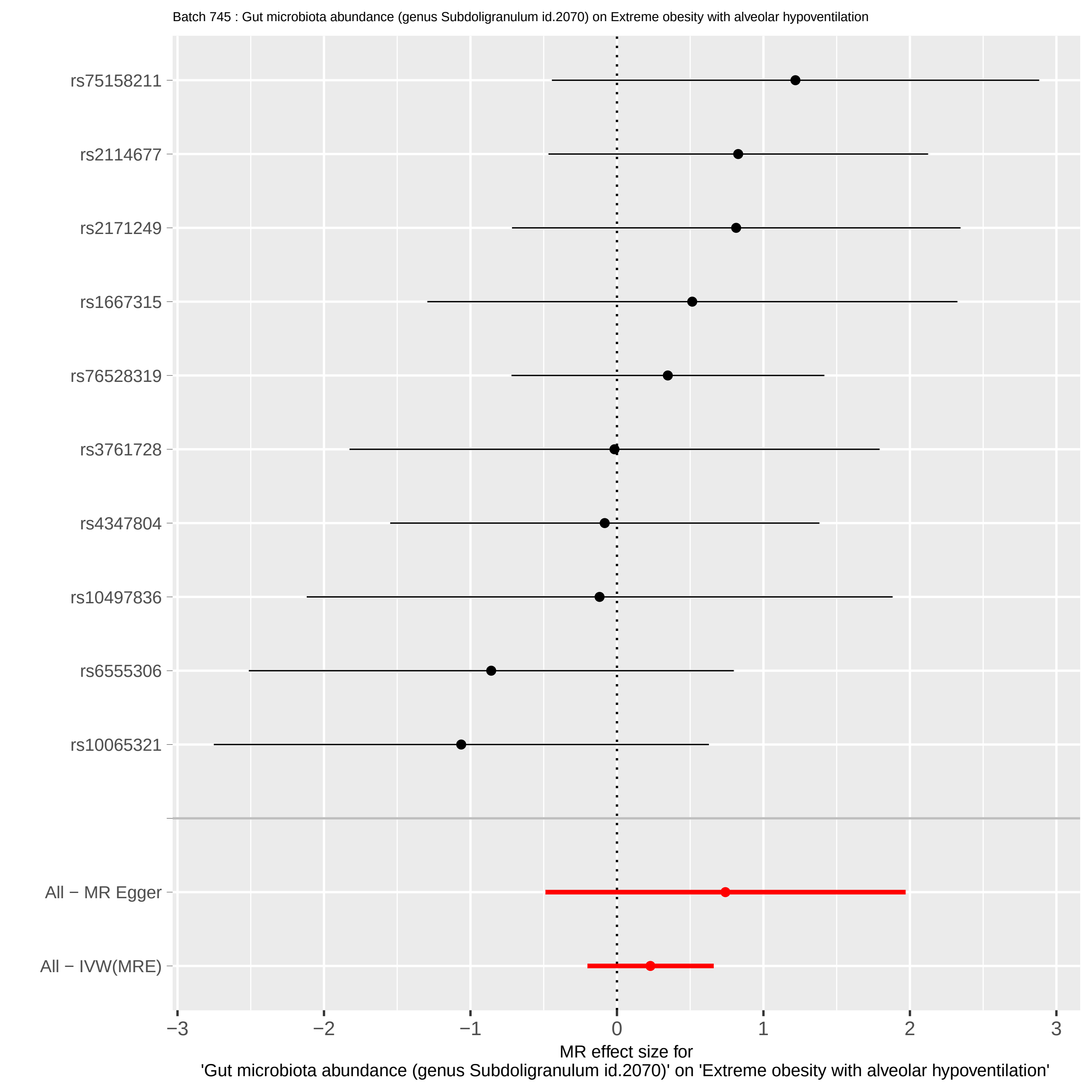
Batch 745 : Gut microbiota abundance (genus Subdoligranulum id.2070) on Extreme obesity with alveolar hypoventilation
rs75158211
rs2114677
rs2171249
rs1667315
rs76528319
rs3761728
rs4347804
rs10497836
rs6555306
rs10065321
All − MR Egger
All − IVW(MRE)
−3
−2
−1
0
1
2
3
MR effect size for
'Gut microbiota abundance (genus Subdoligranulum id.2070)' on 'Extreme obesity with alveolar hypoventilation'

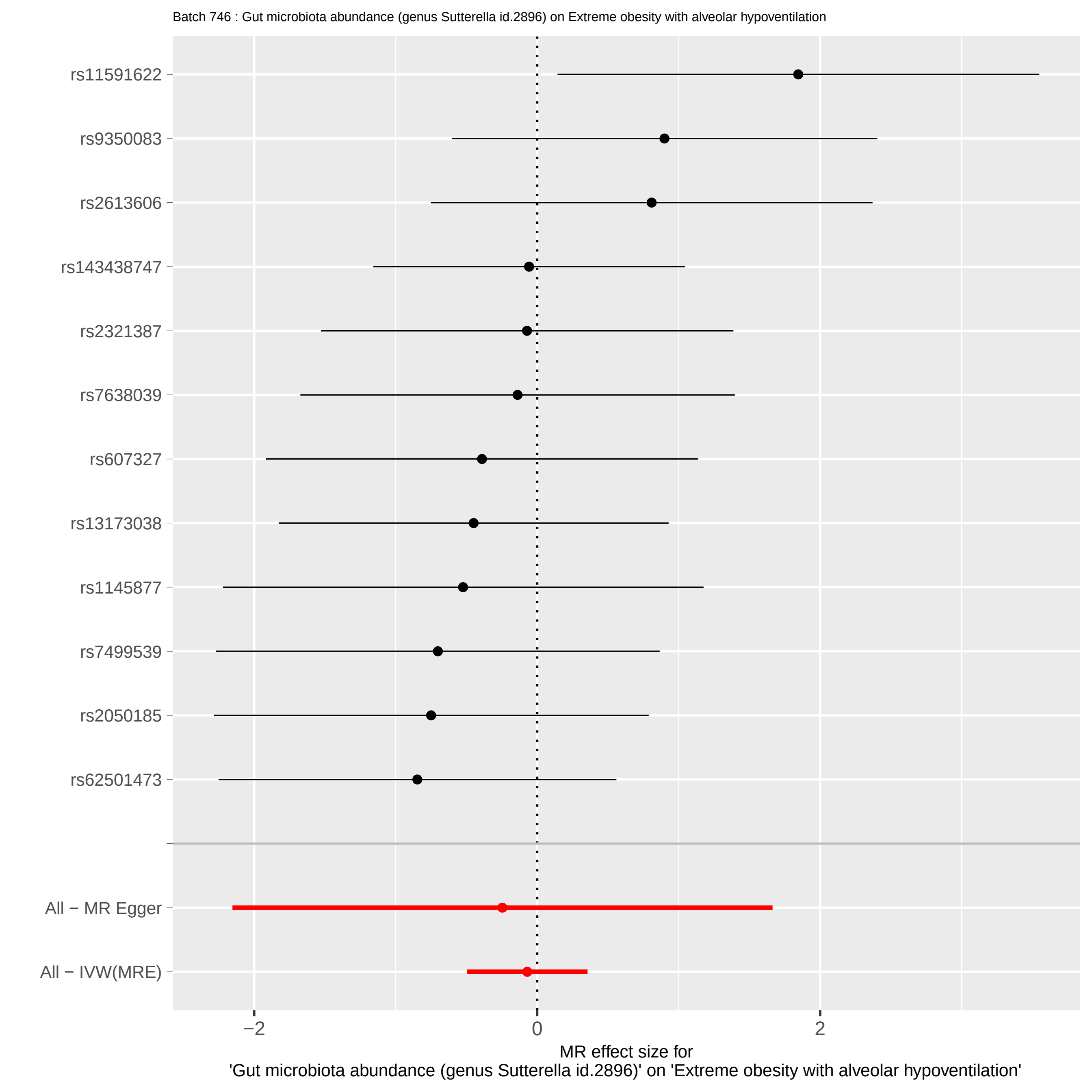
Batch 746 : Gut microbiota abundance (genus Sutterella id.2896) on Extreme obesity with alveolar hypoventilation
rs11591622
rs9350083
rs2613606
rs143438747
rs2321387
rs7638039
rs607327
rs13173038
rs1145877
rs7499539
rs2050185
rs62501473
All − MR Egger
All − IVW(MRE)
−2
0
2
MR effect size for
'Gut microbiota abundance (genus Sutterella id.2896)' on 'Extreme obesity with alveolar hypoventilation'

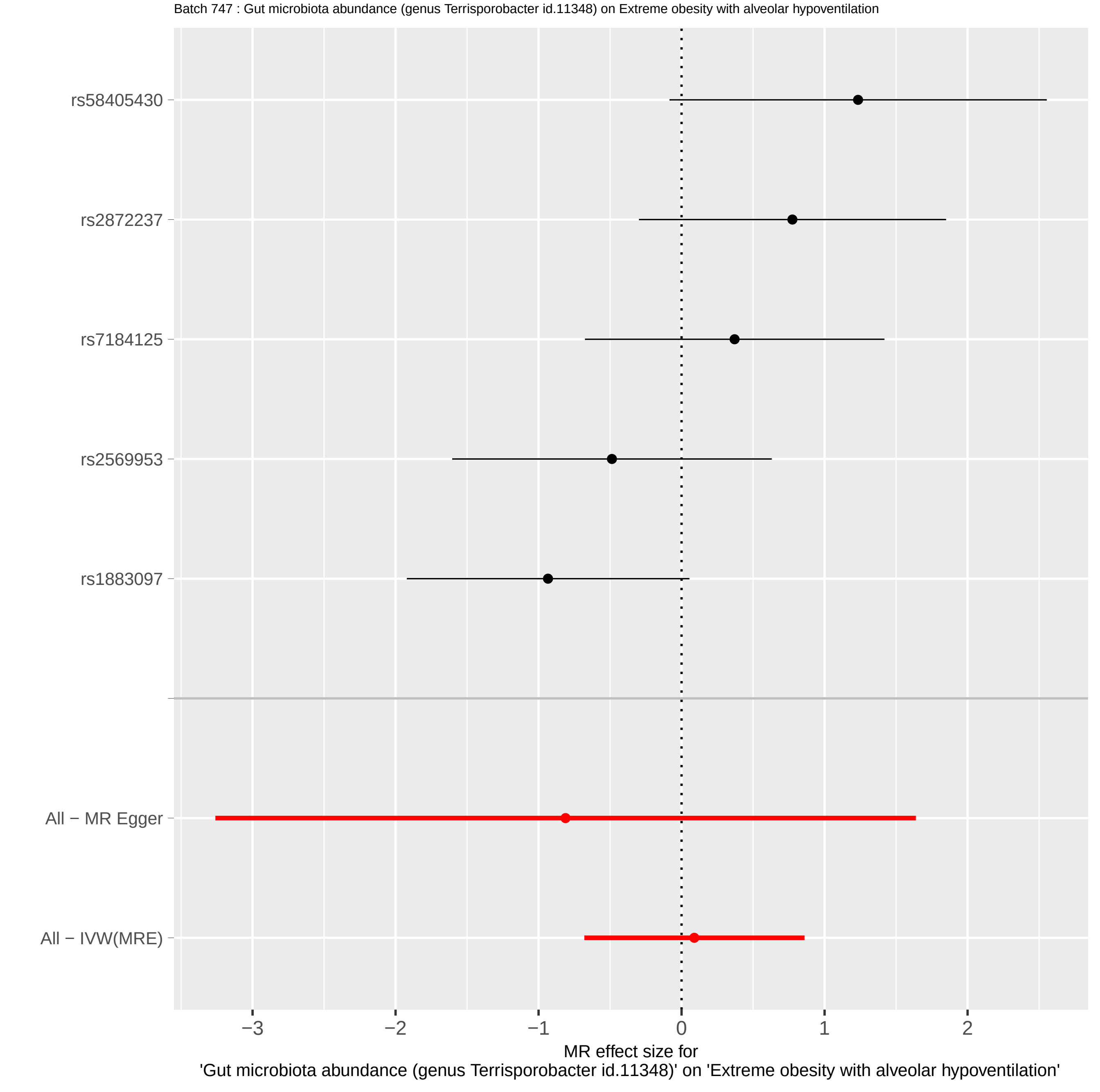
Batch 747 : Gut microbiota abundance (genus Terrisporobacter id.11348) on Extreme obesity with alveolar hypoventilation
rs58405430
rs2872237
rs7184125
rs2569953
rs1883097
All − MR Egger
All − IVW(MRE)
−3
−2
−1
0
1
2
MR effect size for
'Gut microbiota abundance (genus Terrisporobacter id.11348)' on 'Extreme obesity with alveolar hypoventilation'

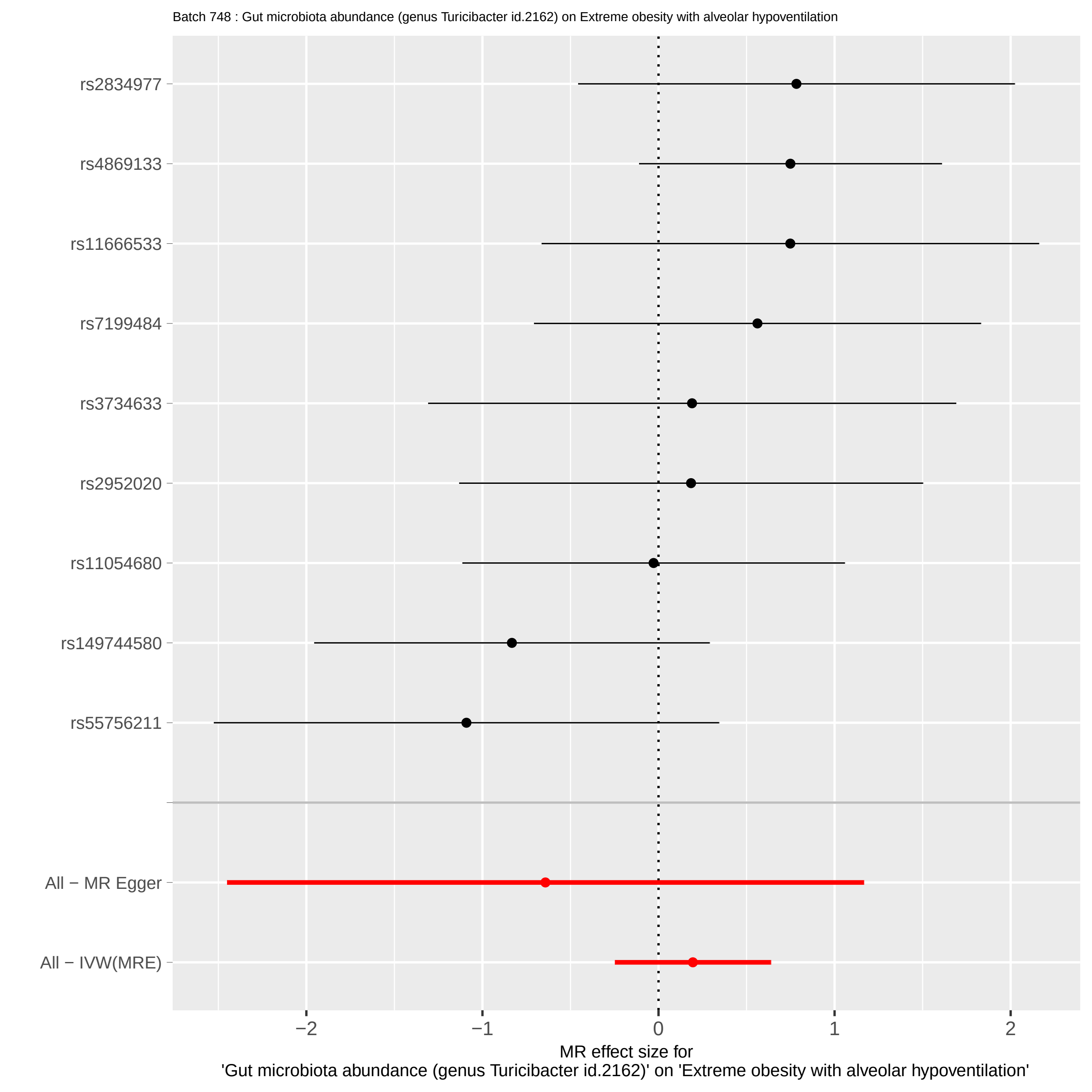

Batch 748 : Gut microbiota abundance (genus Turicibacter id.2162) on Extreme obesity with alveolar hypoventilation
rs2834977
rs4869133
rs11666533
rs7199484
rs3734633
rs2952020
rs11054680
rs149744580
rs55756211
All − MR Egger
All − IVW(MRE)
−2
−1
0
1
2
MR effect size for
'Gut microbiota abundance (genus Turicibacter id.2162)' on 'Extreme obesity with alveolar hypoventilation'

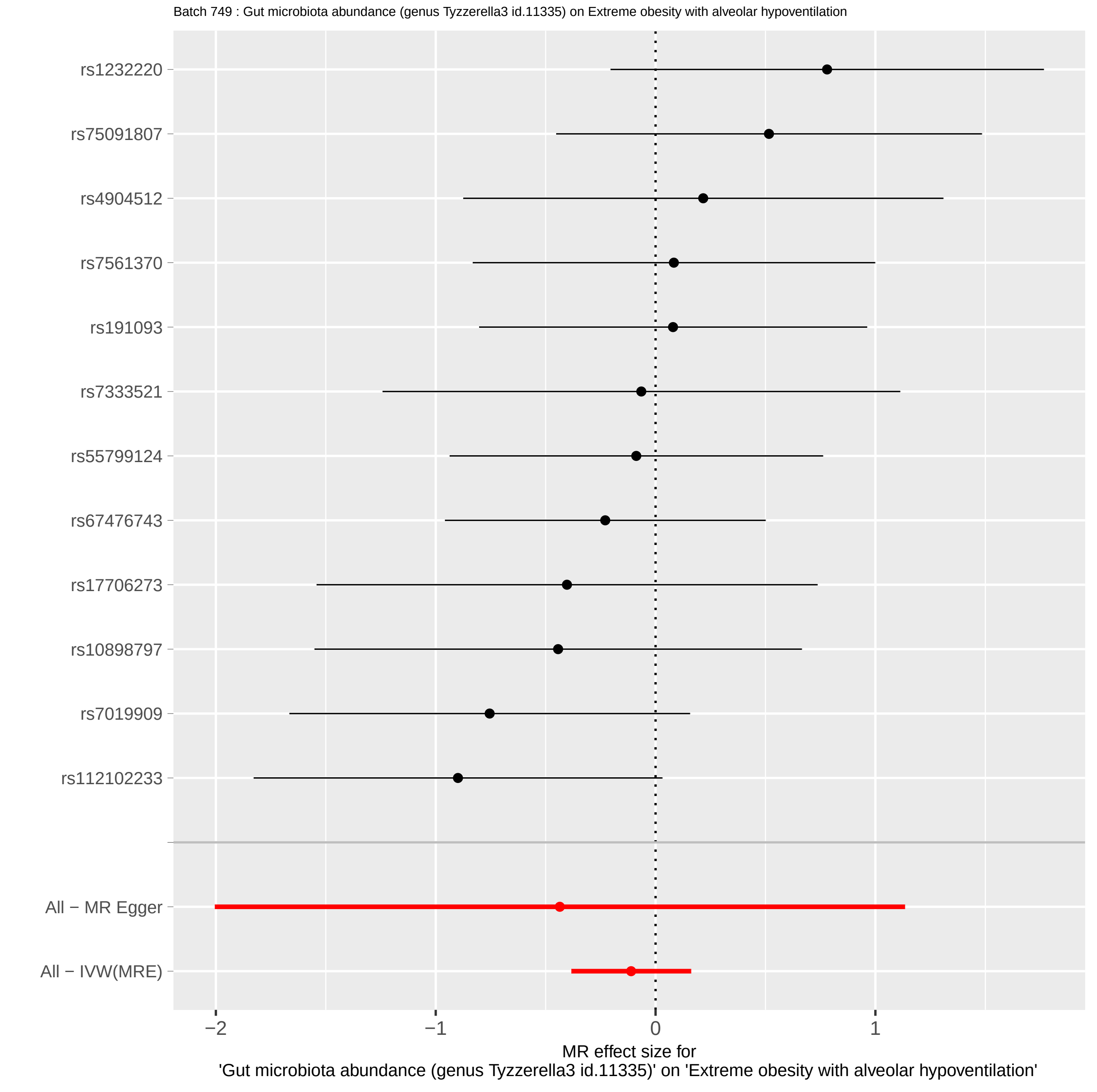
Batch 749 : Gut microbiota abundance (genus Tyzzerella3 id.11335) on Extreme obesity with alveolar hypoventilation
rs1232220
rs75091807
rs4904512
rs7561370
rs191093
rs7333521
rs55799124
rs67476743
rs17706273
rs10898797
rs7019909
rs112102233
All − MR Egger
All − IVW(MRE)
−2
−1
0
1
MR effect size for
'Gut microbiota abundance (genus Tyzzerella3 id.11335)' on 'Extreme obesity with alveolar hypoventilation'

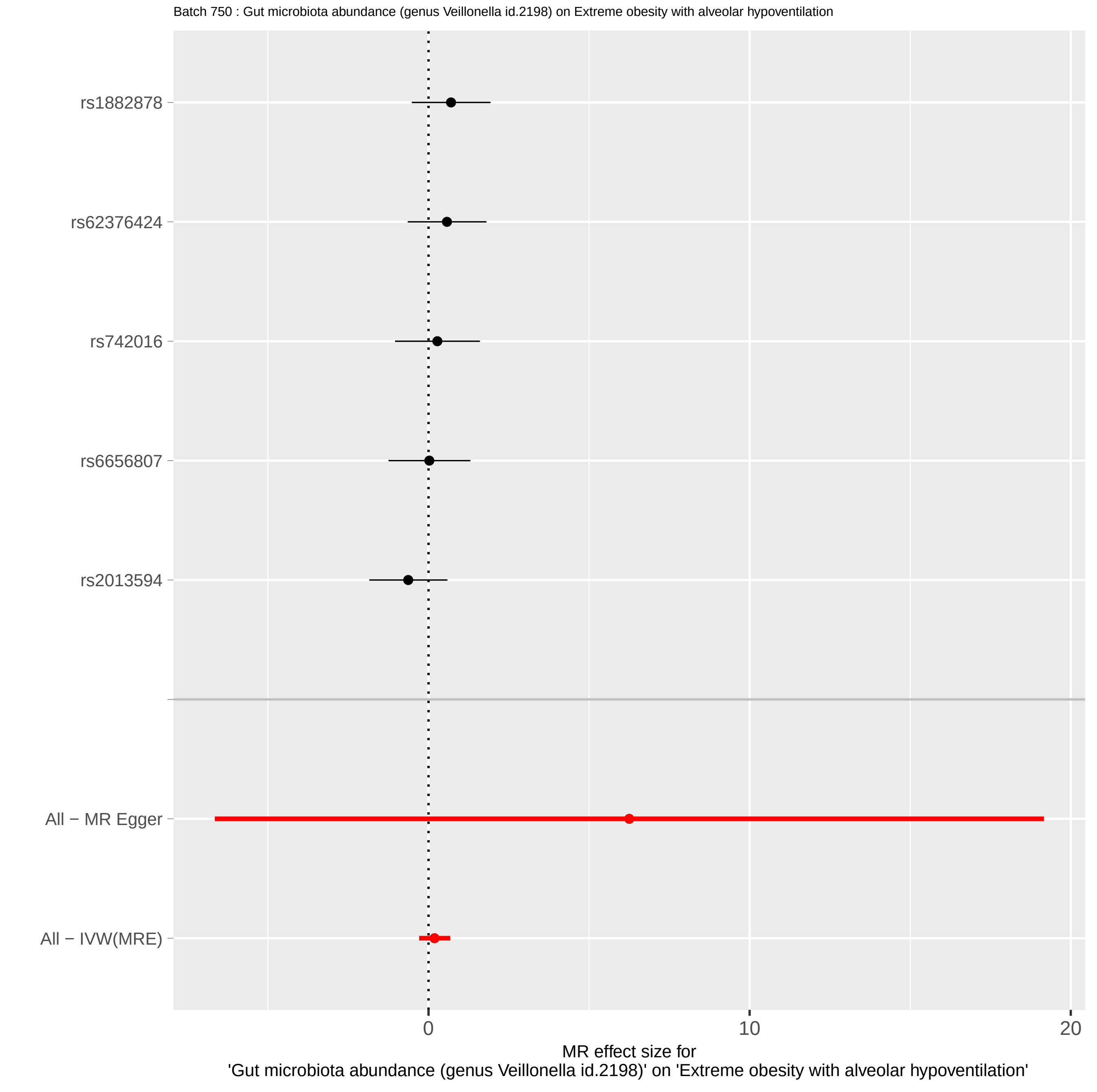
Batch 750 : Gut microbiota abundance (genus Veillonella id.2198) on Extreme obesity with alveolar hypoventilation
rs1882878
rs62376424
rs742016
rs6656807
rs2013594
All − MR Egger
All − IVW(MRE)
0
10
20
MR effect size for
'Gut microbiota abundance (genus Veillonella id.2198)' on 'Extreme obesity with alveolar hypoventilation'
