# Supplementary material for: Unraveling the mystery: a Mendelian randomized exploration of gut microbiota and different types of obesity
Source: Front Cell Infect Microbiol. 2024 Feb 5;14:1352109. doi: 10.3389/fcimb.2024.1352109 (PMC10875079; doi:10.3389/fcimb.2024.1352109)
Supplement: Supplementary file 2 [file DataSheet_2.pdf]

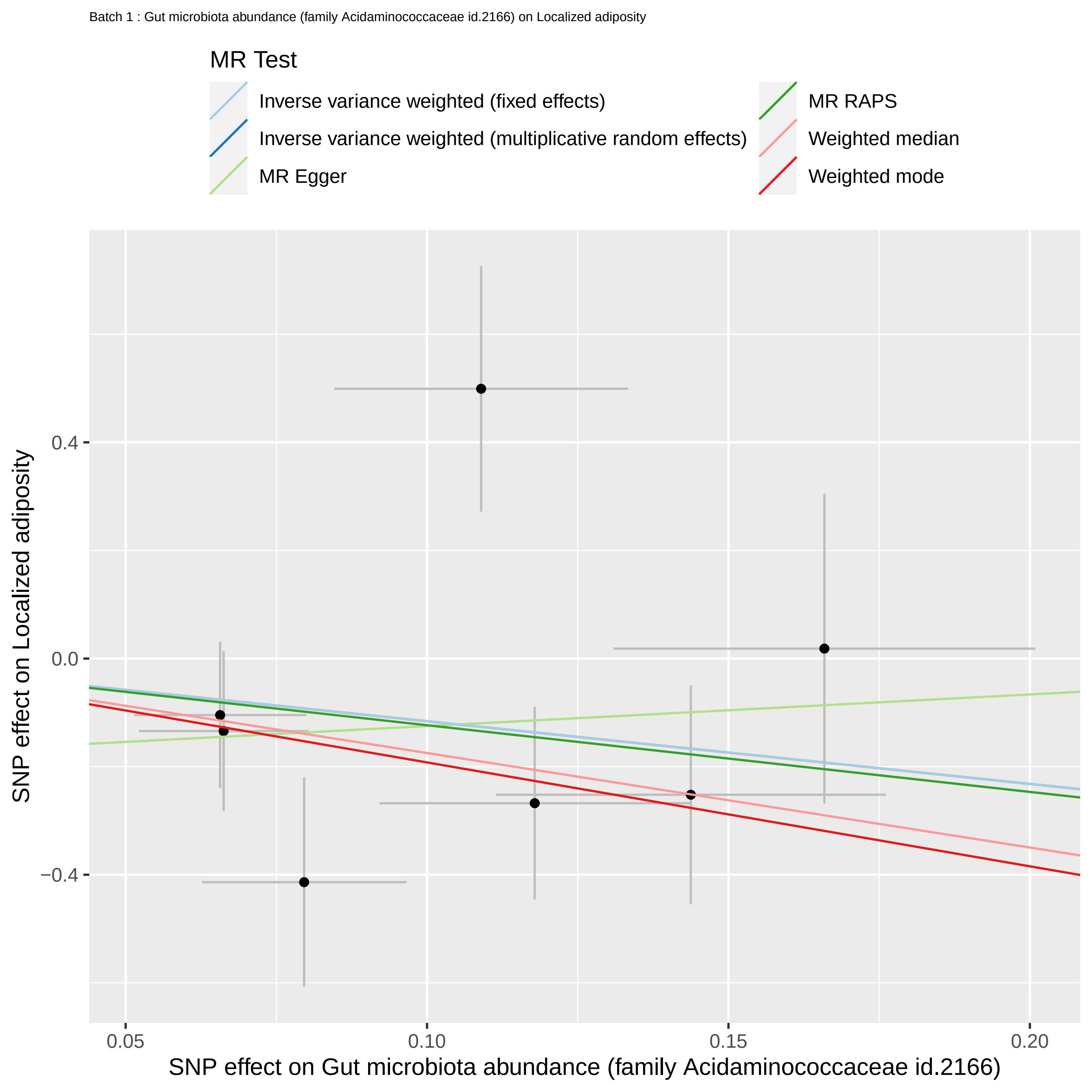
Batch 1 : Gut microbiota abundance (family Acidaminococcaceae id.2166) on Localized adiposity
MR Test
Inverse variance weighted (fixed effects)
Inverse variance weighted (multiplicative random effects)
MR Egger
MR RAPS
Weighted median
Weighted mode
0.4
0.0
−0.4
0.05
0.10
0.15
0.20
SNP effect on Localized adiposity
SNP effect on Gut microbiota abundance (family Acidaminococcaceae id.2166)

Batch 2 : Gut microbiota abundance (family Actinomycetaceae id.421) on Localized adiposity

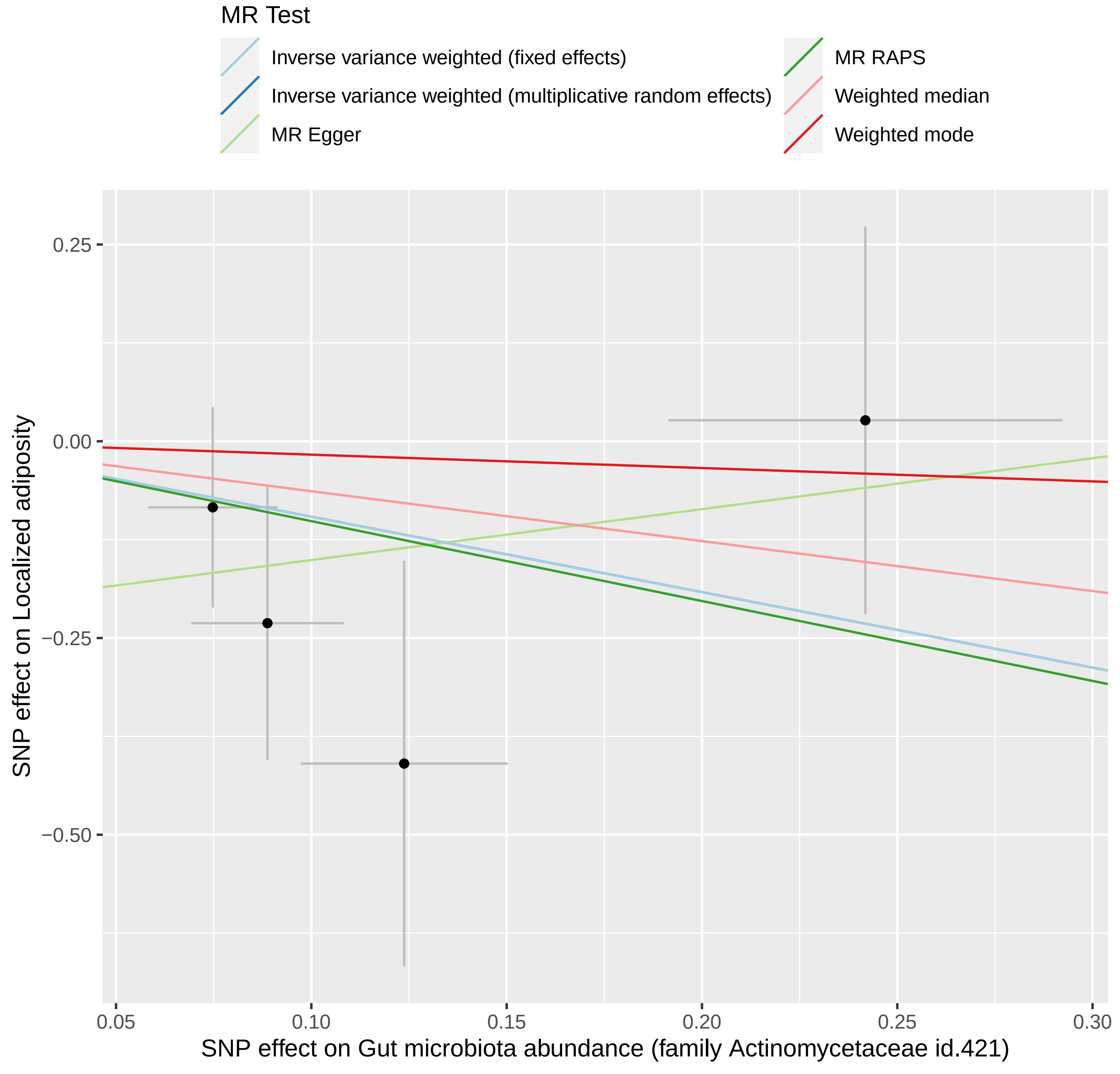

Batch 3 : Gut microbiota abundance (family Alcaligenaceae id.2875) on Localized adiposity

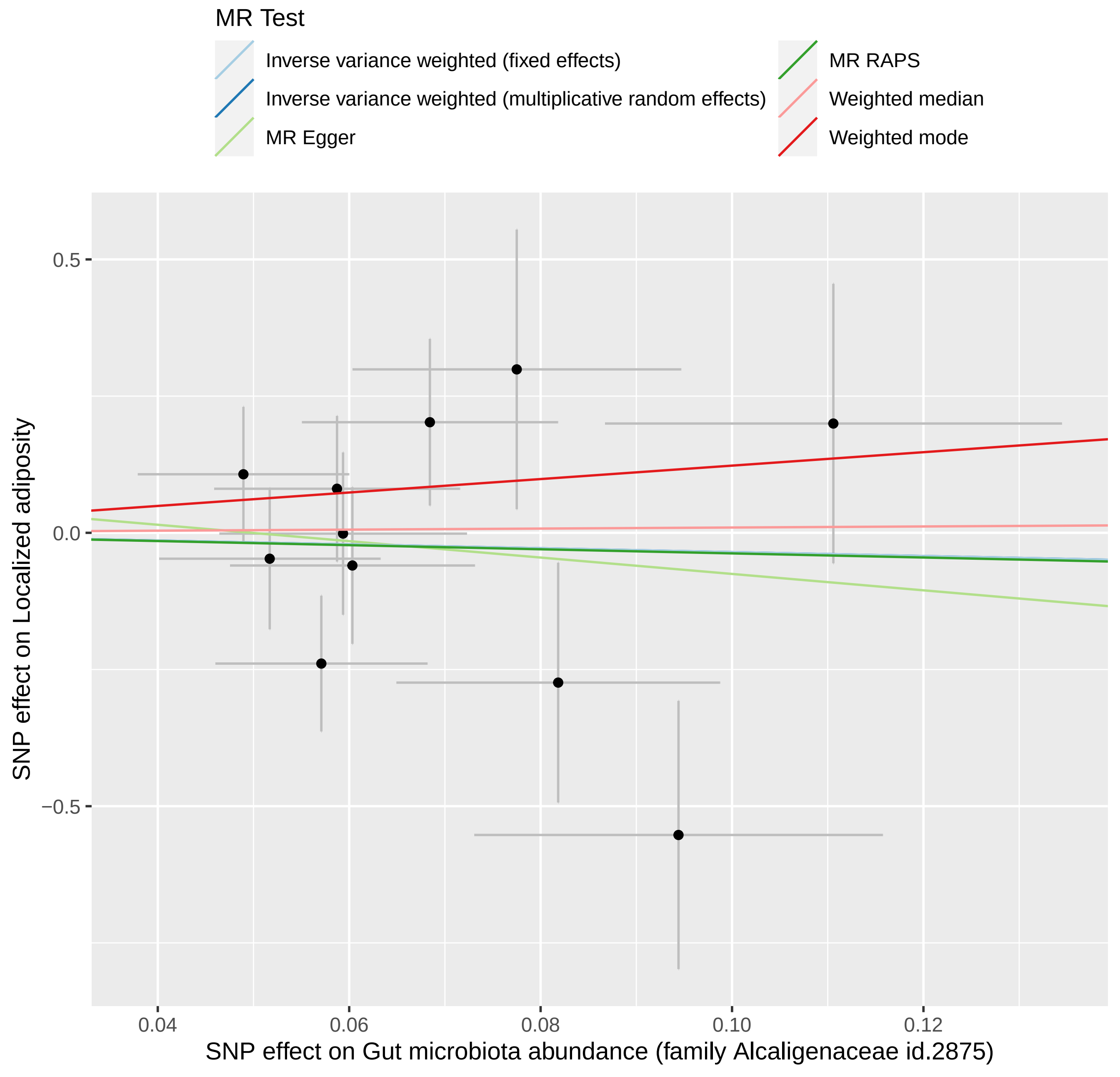

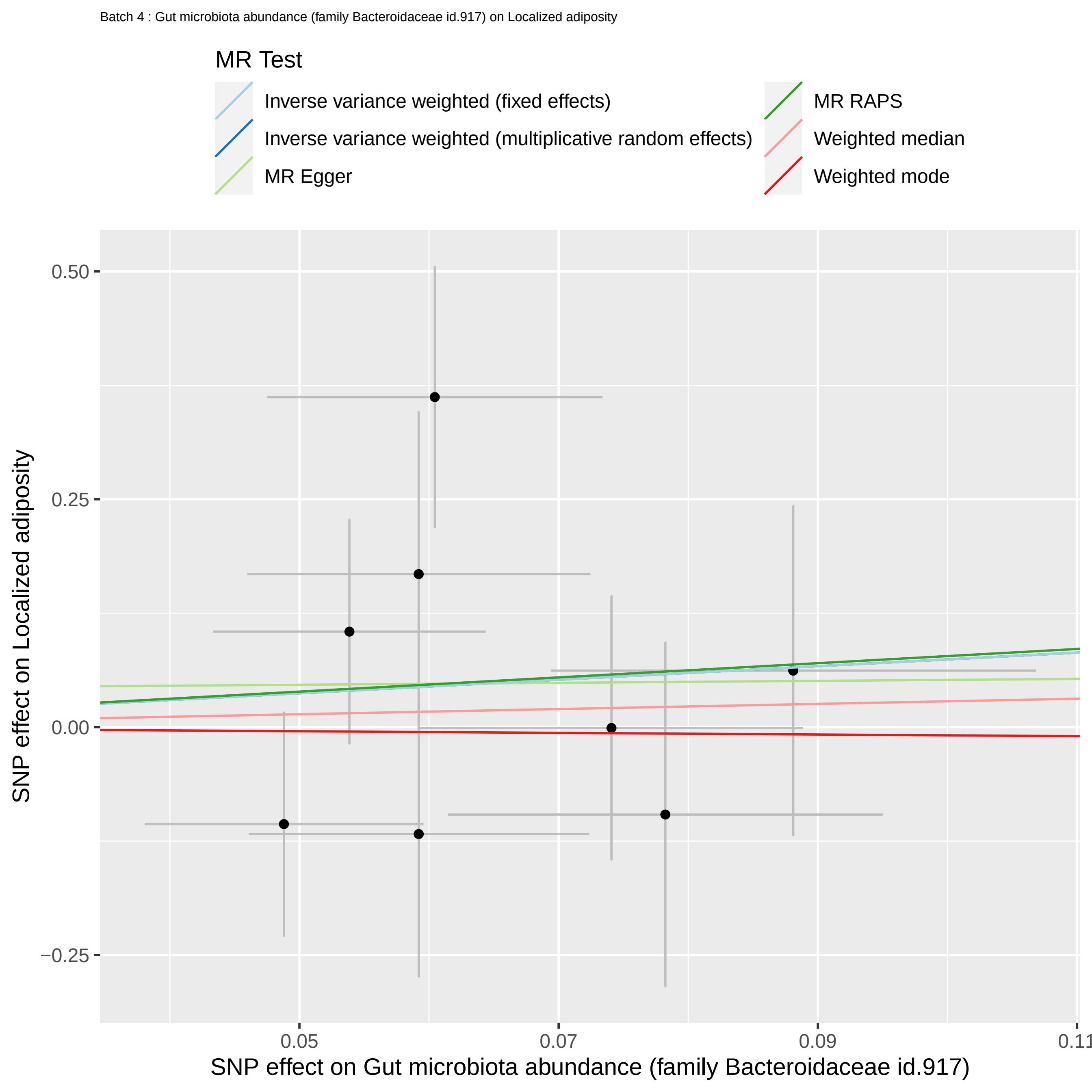
Batch 4 : Gut microbiota abundance (family Bacteroidaceae id.917) on Localized adiposity
MR Test
Inverse variance weighted (fixed effects)
Inverse variance weighted (multiplicative random effects)
MR Egger
MR RAPS
Weighted median
Weighted mode
0.50
0.25
0.00
−0.25
0.05
0.07
0.09
0.11
SNP effect on Localized adiposity
SNP effect on Gut microbiota abundance (family Bacteroidaceae id.917)

Batch 5 : Gut microbiota abundance (family Bacteroidales S24 7group id.11173) on Localized adiposity

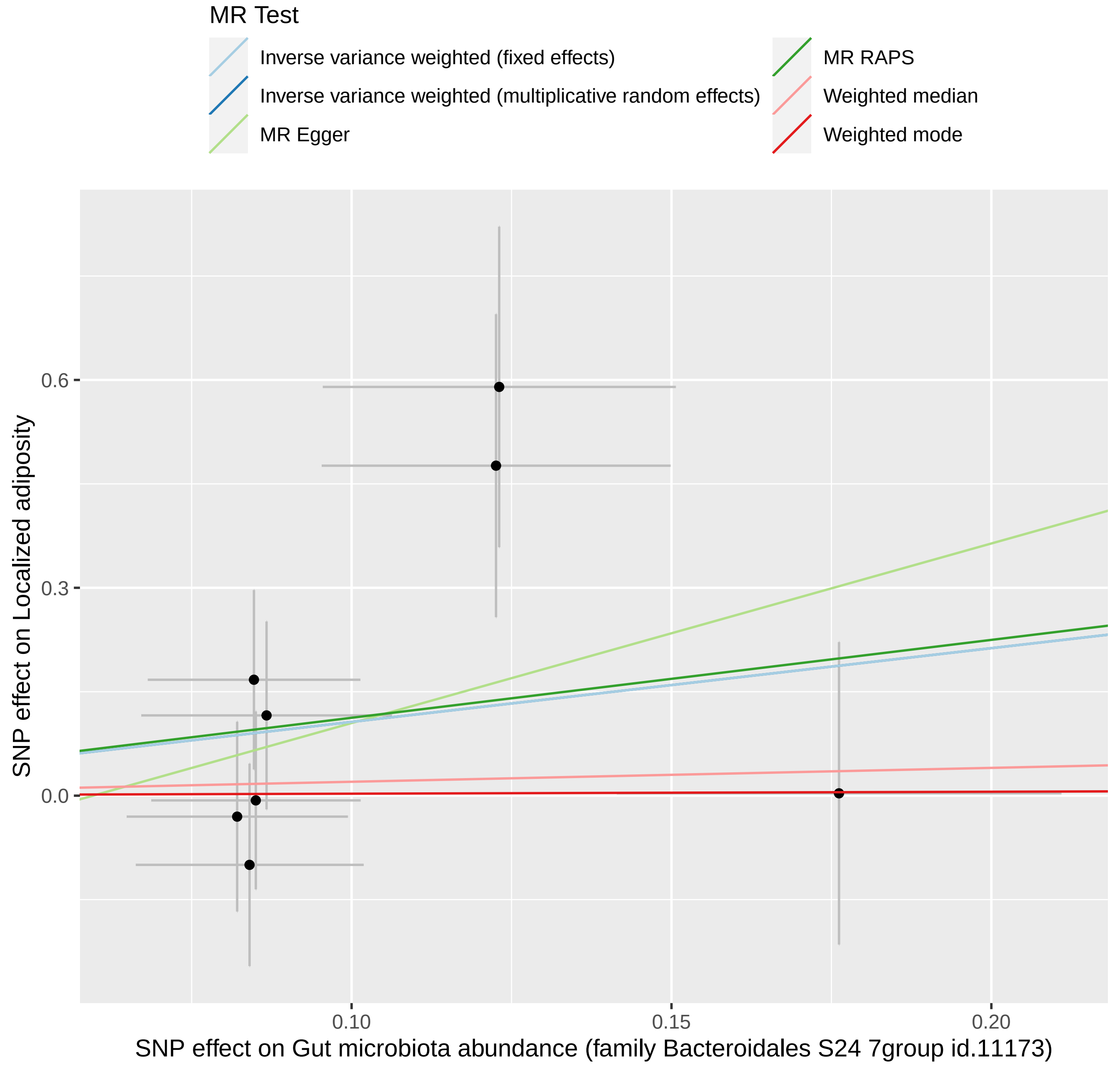

Batch 6 : Gut microbiota abundance (family Bifidobacteriaceae id.433) on Localized adiposity

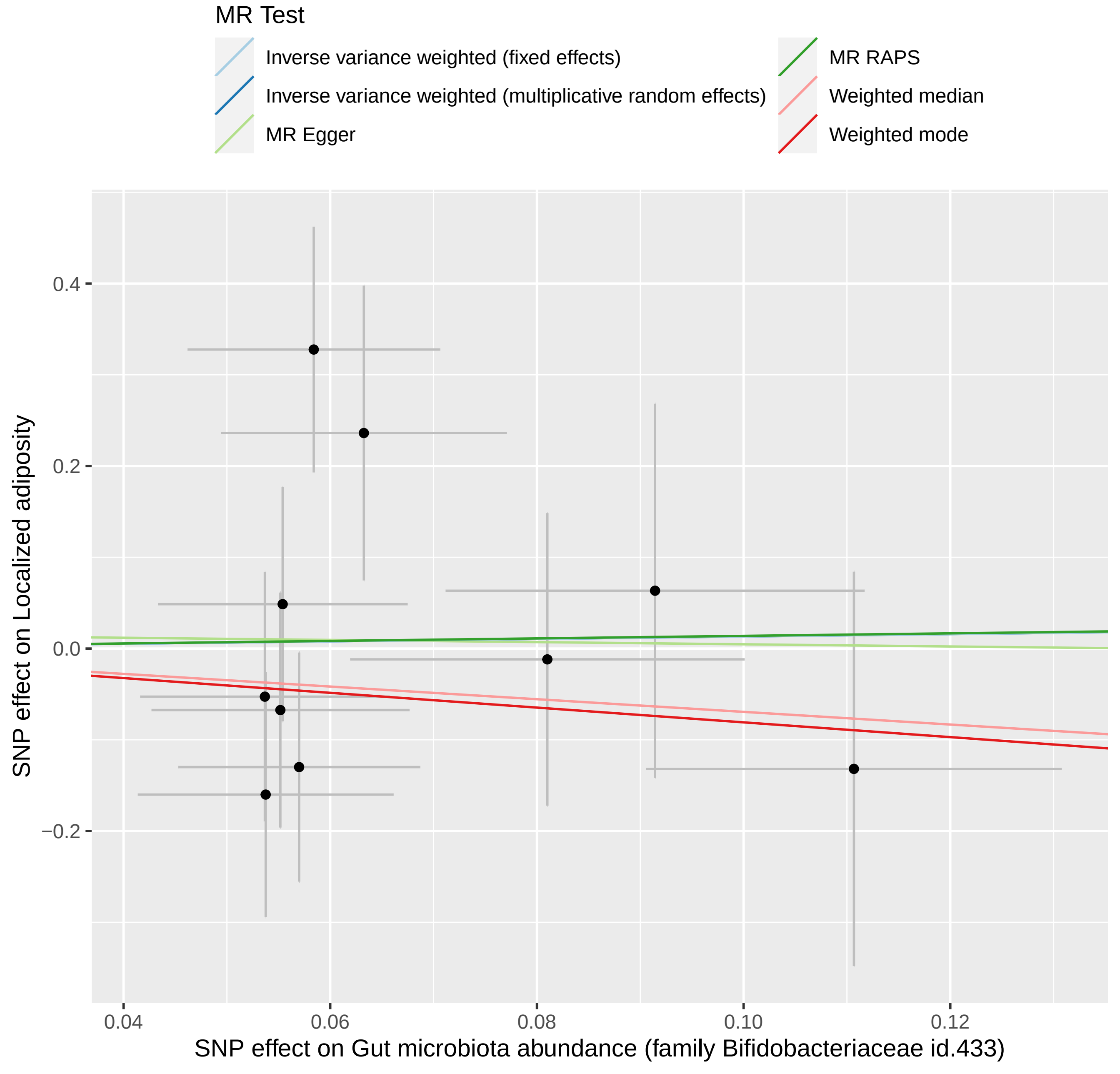

Batch 8 : Gut microbiota abundance (family Clostridiaceae1 id.1869) on Localized adiposity

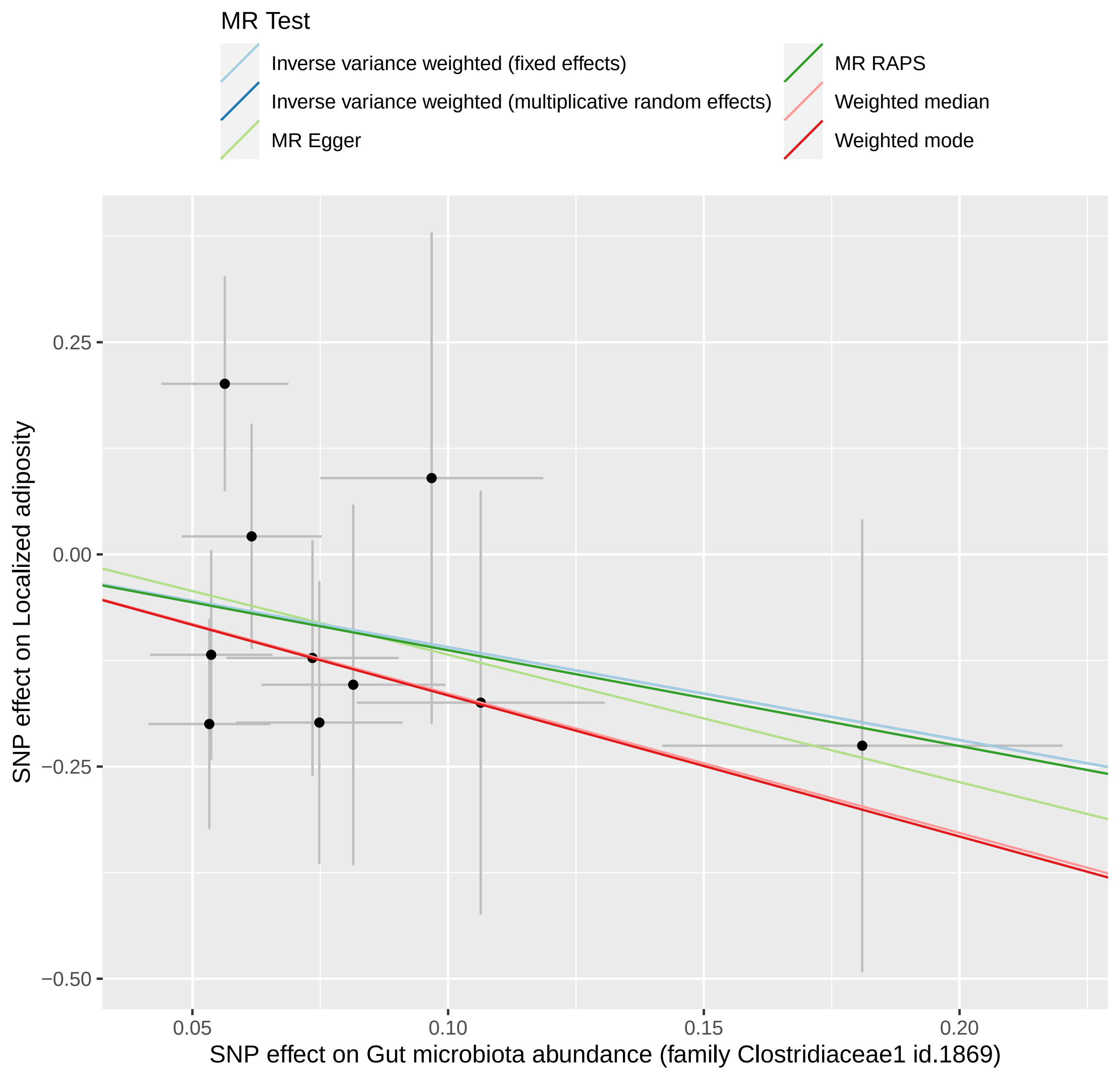

Batch 9 : Gut microbiota abundance (family Clostridiales vadin BB60 group id.11286) on Localized adiposity

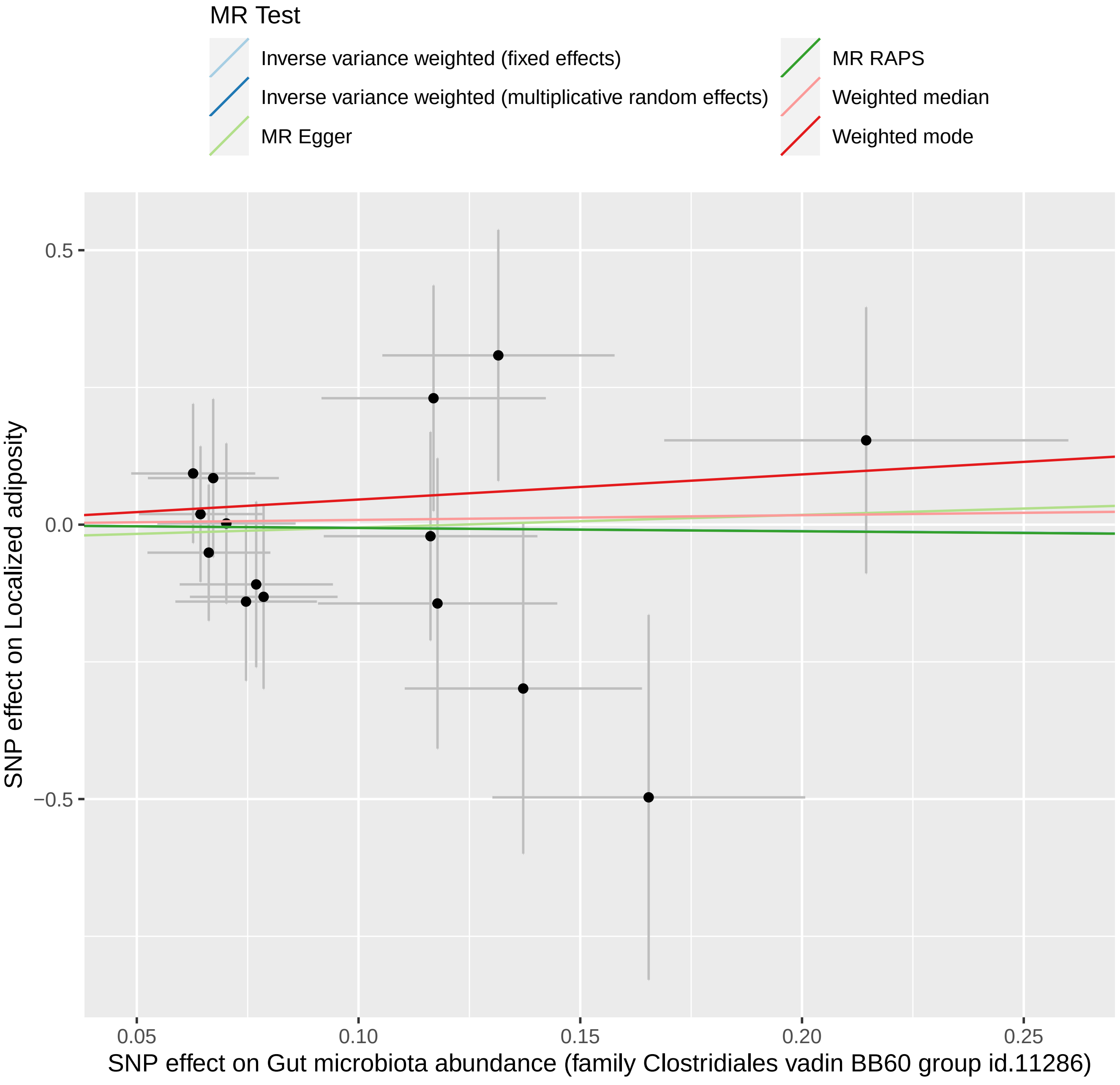

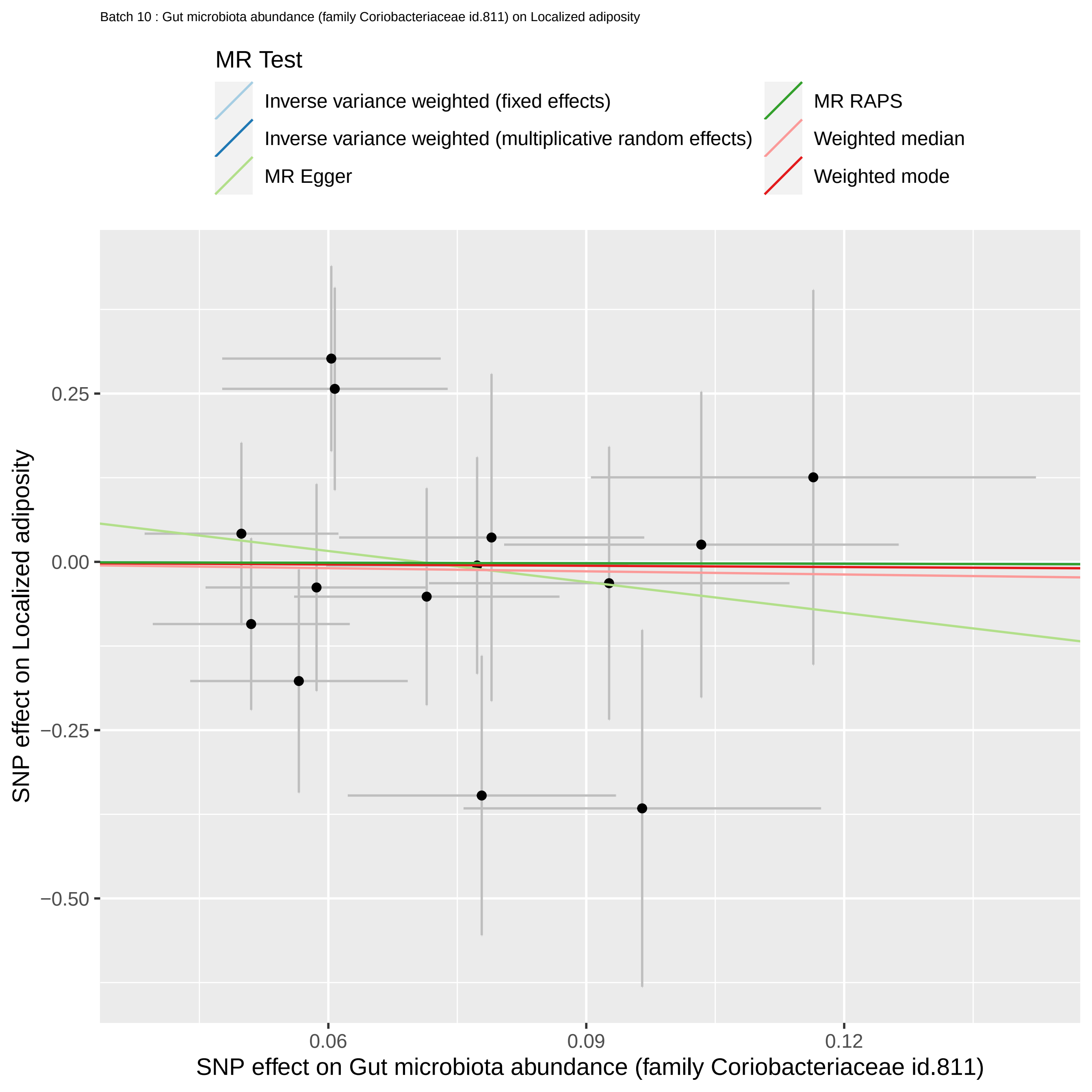

Batch 10 : Gut microbiota abundance (family Coriobacteriaceae id.811) on Localized adiposity
MR Test
Inverse variance weighted (fixed effects)
Inverse variance weighted (multiplicative random effects)
MR Egger
MR RAPS
Weighted median
Weighted mode
0.25
0.00
−0.25
−0.50
0.06
0.09
0.12
SNP effect on Localized adiposity
SNP effect on Gut microbiota abundance (family Coriobacteriaceae id.811)

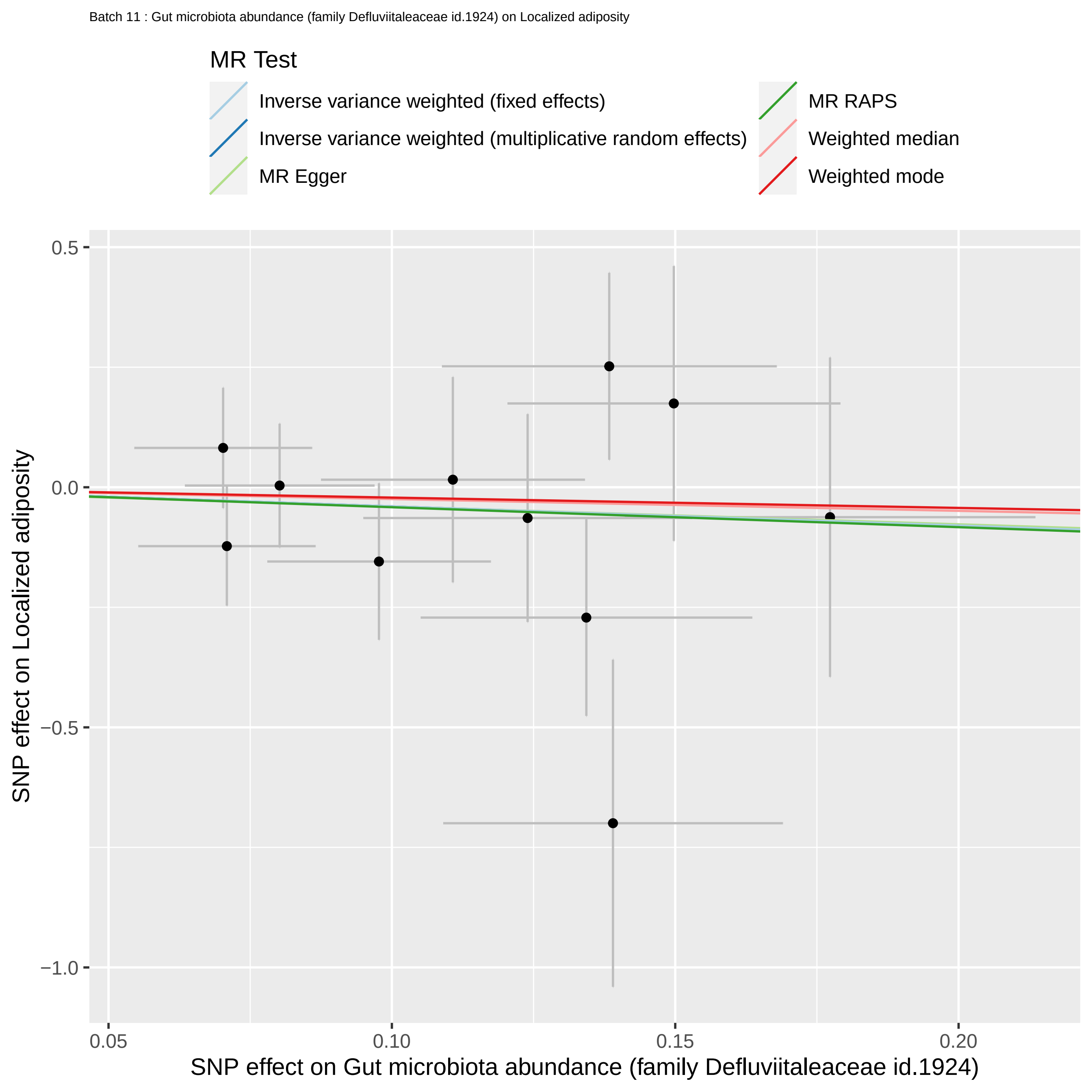
Batch 11 : Gut microbiota abundance (family Defluviitaleaceae id.1924) on Localized adiposity
MR Test
Inverse variance weighted (fixed effects)
Inverse variance weighted (multiplicative random effects)
MR Egger
MR RAPS
Weighted median
Weighted mode
0.5
0.0
−0.5
−1.0
0.05
0.10
0.15
0.20
SNP effect on Localized adiposity
SNP effect on Gut microbiota abundance (family Defluviitaleaceae id.1924)

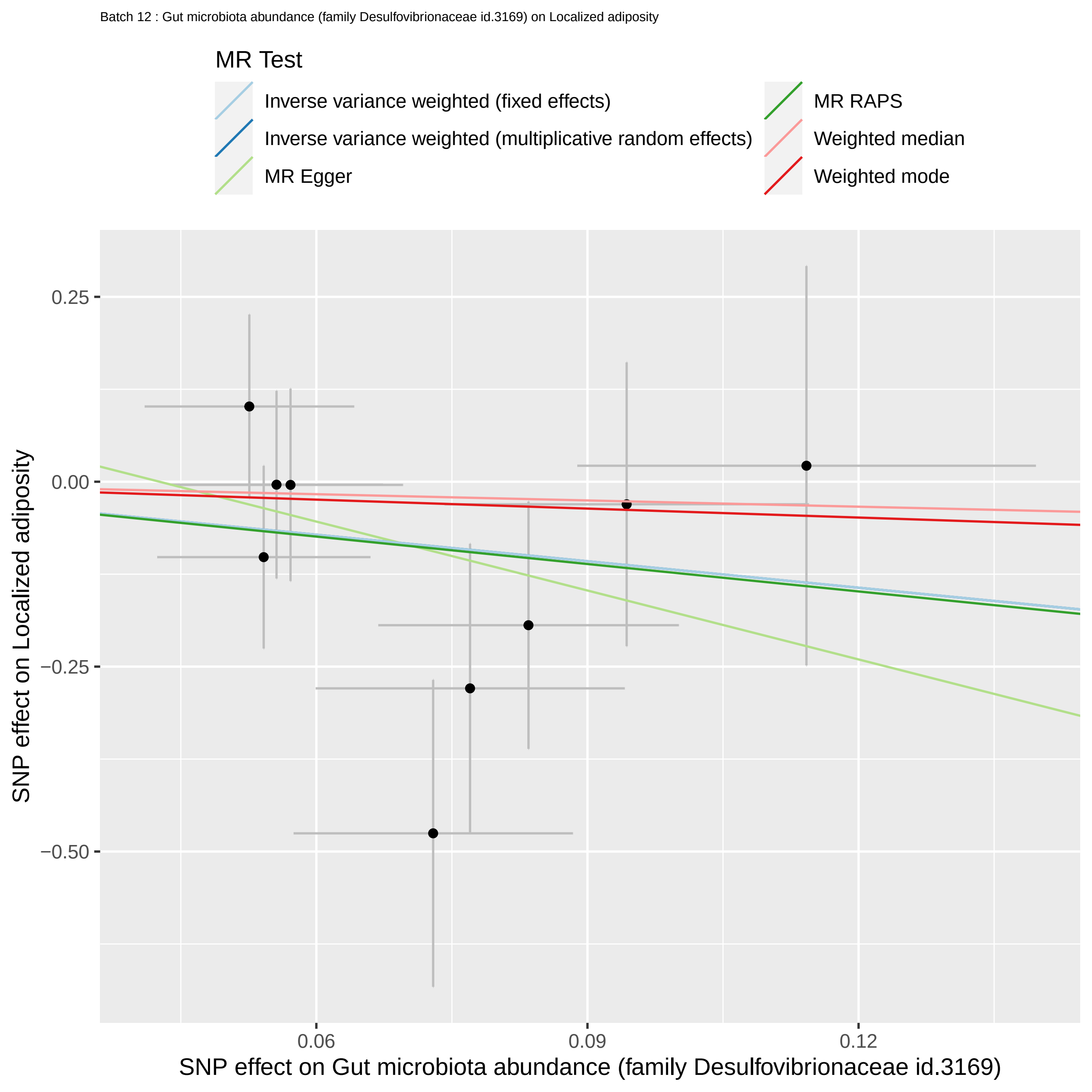
Batch 12 : Gut microbiota abundance (family Desulfovibrionaceae id.3169) on Localized adiposity
MR Test
Inverse variance weighted (fixed effects)
Inverse variance weighted (multiplicative random effects)
MR Egger
MR RAPS
Weighted median
Weighted mode
0.25
0.00
−0.25
−0.50
0.06
0.09
0.12
SNP effect on Localized adiposity
SNP effect on Gut microbiota abundance (family Desulfovibrionaceae id.3169)

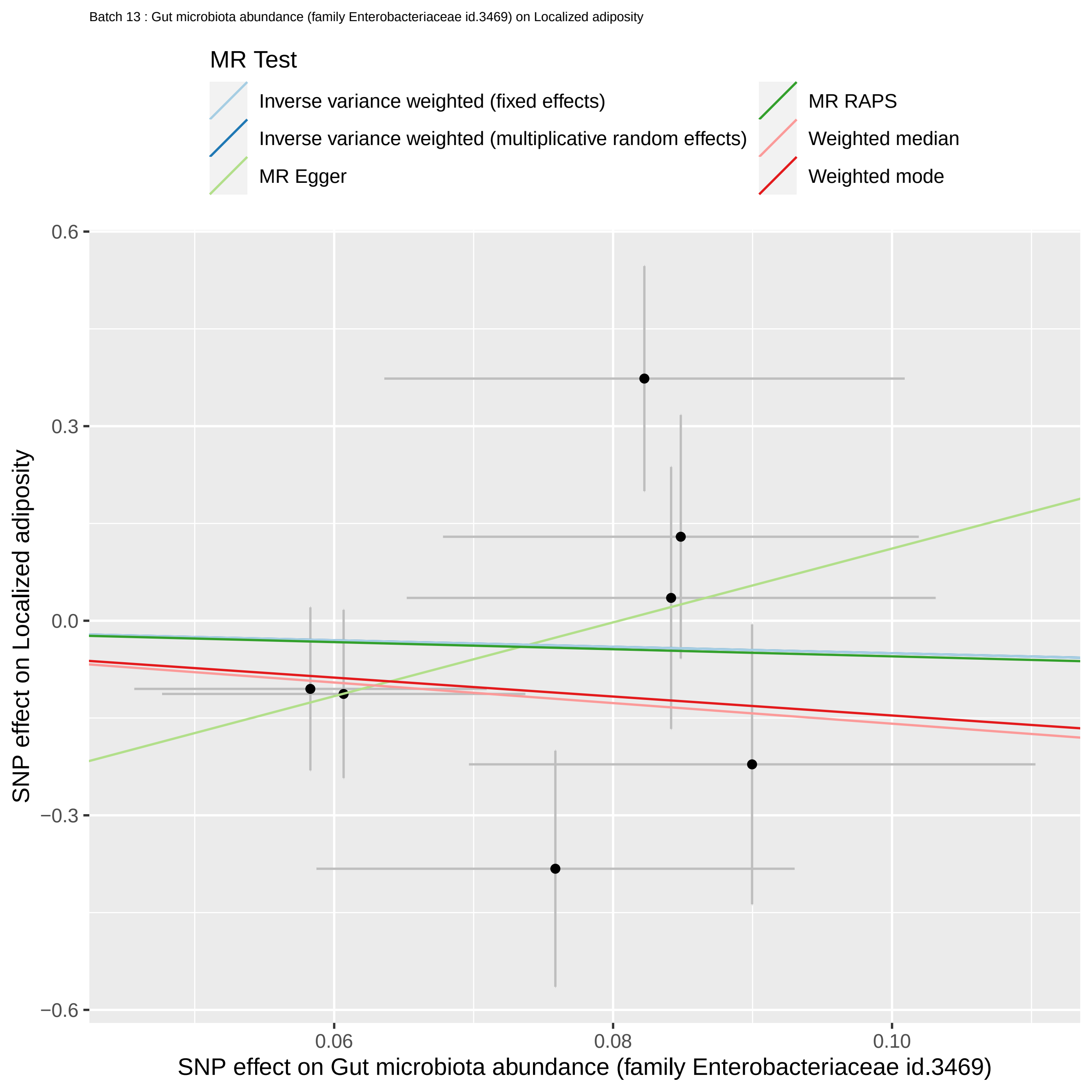
Batch 13 : Gut microbiota abundance (family Enterobacteriaceae id.3469) on Localized adiposity
MR Test
Inverse variance weighted (fixed effects)
Inverse variance weighted (multiplicative random effects)
MR Egger
MR RAPS
Weighted median
Weighted mode
0.6
0.3
0.0
−0.3
−0.6
SNP effect on Localized adiposity
0.06
0.08
0.10
SNP effect on Gut microbiota abundance (family Enterobacteriaceae id.3469)

Batch 14 : Gut microbiota abundance (family Erysipelotrichaceae id.2149) on Localized adiposity

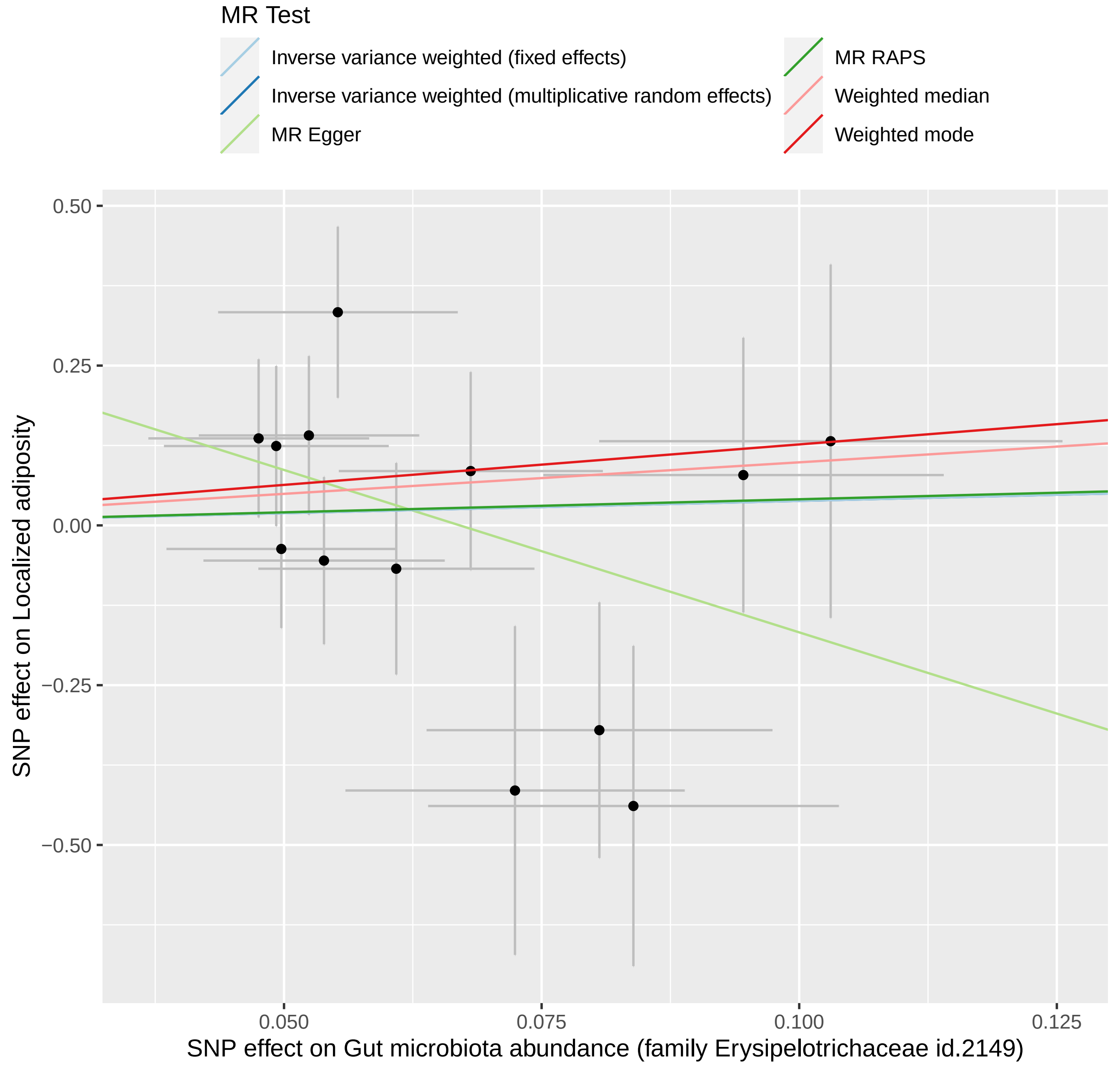

Batch 15 : Gut microbiota abundance (family Family XI id.1936) on Localized adiposity

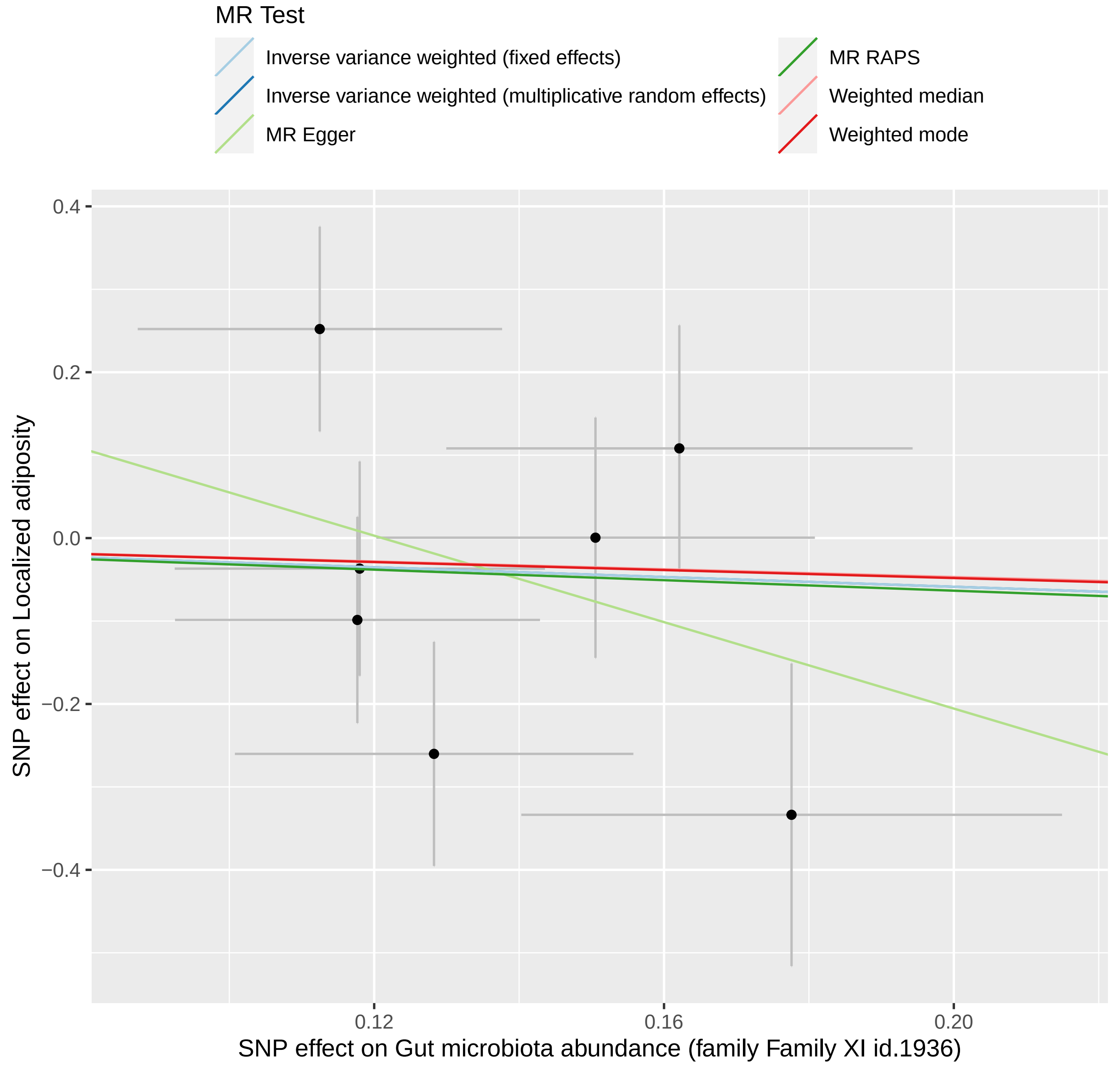

Batch 16 : Gut microbiota abundance (family Family XIII id.1957) on Localized adiposity

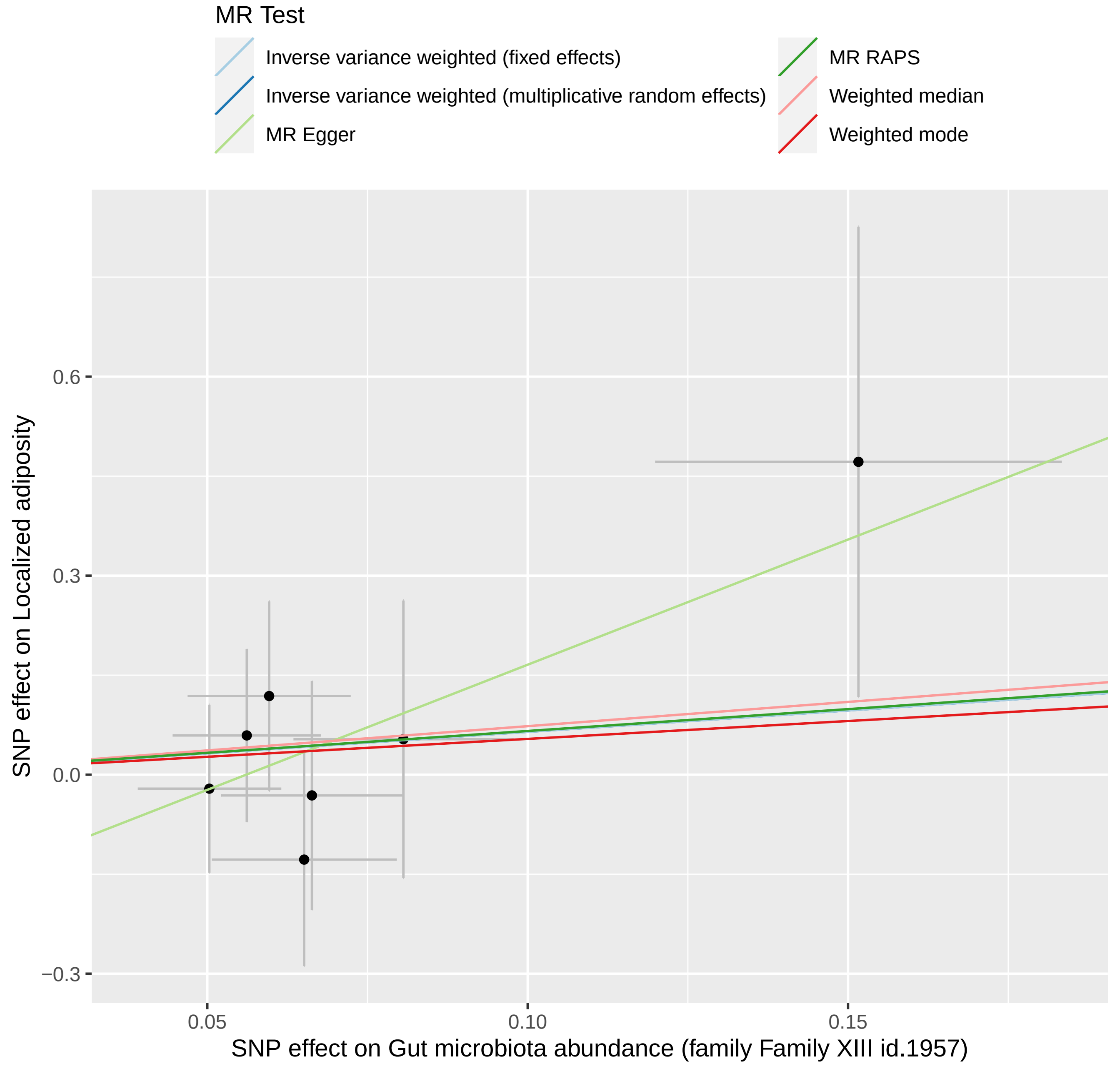

Batch 17 : Gut microbiota abundance (family Lachnospiraceae id.1987) on Localized adiposity

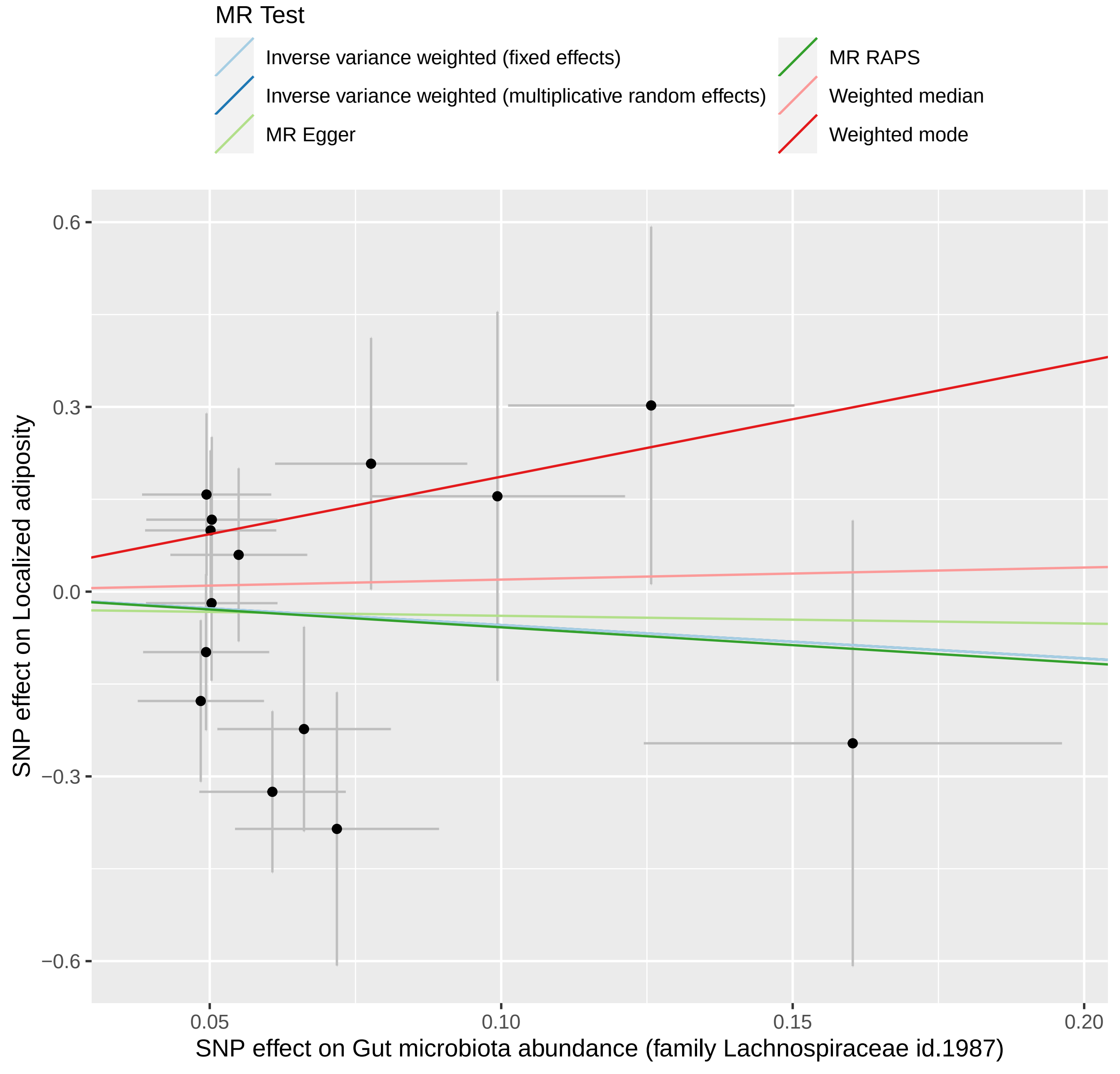

Batch 18 : Gut microbiota abundance (family Lactobacillaceae id.1836) on Localized adiposity

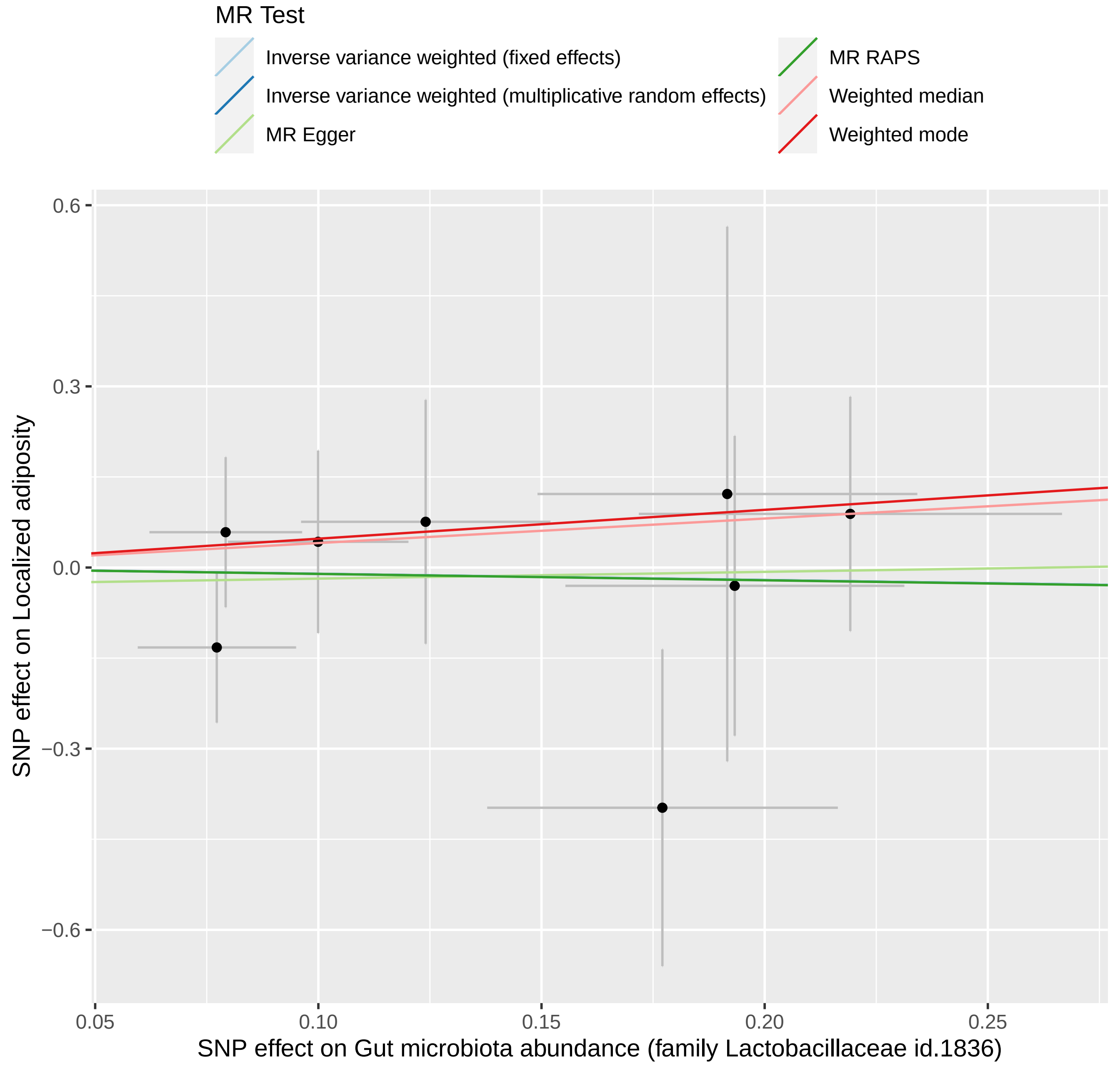

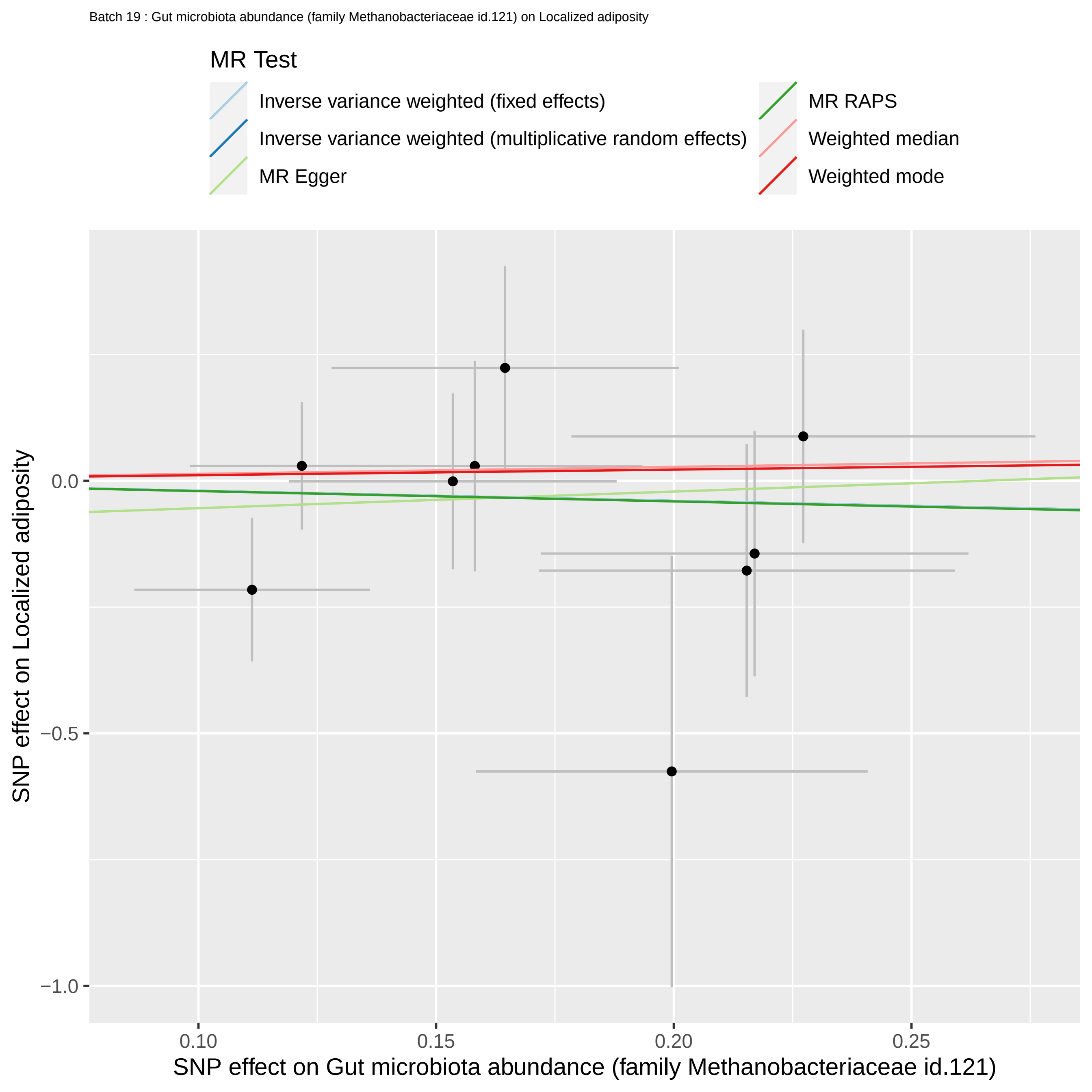

Batch 19 : Gut microbiota abundance (family Methanobacteriaceae id.121) on Localized adiposity
MR Test
Inverse variance weighted (fixed effects)
Inverse variance weighted (multiplicative random effects)
MR Egger
MR RAPS
Weighted median
Weighted mode
0.0
−0.5
−1.0
0.10
0.15
0.20
0.25
SNP effect on Gut microbiota abundance (family Methanobacteriaceae id.121)
SNP effect on Localized adiposity

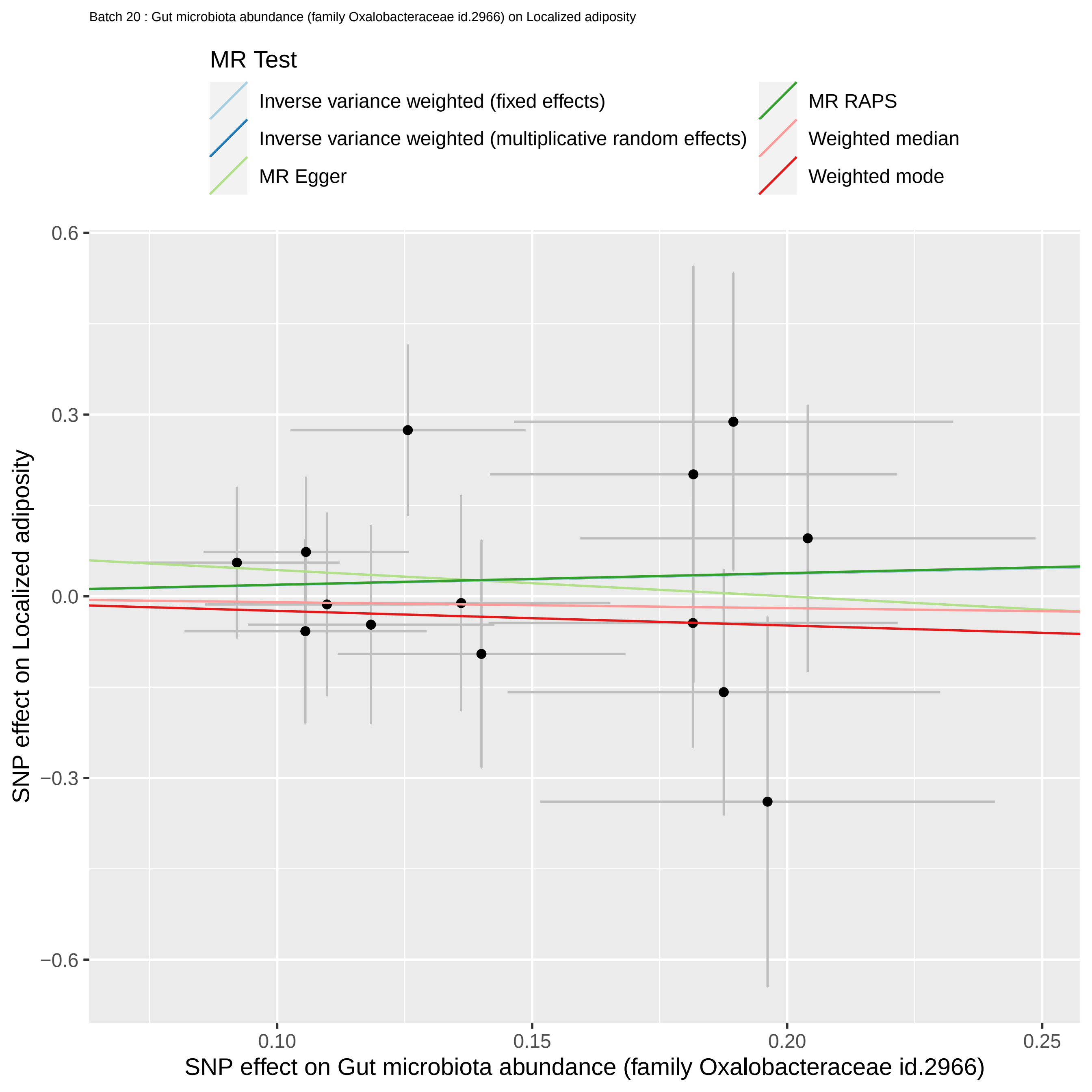

Batch 20 : Gut microbiota abundance (family Oxalobacteraceae id.2966) on Localized adiposity
MR Test
Inverse variance weighted (fixed effects)
Inverse variance weighted (multiplicative random effects)
MR Egger
MR RAPS
Weighted median
Weighted mode
0.6
0.3
0.0
−0.3
−0.6
SNP effect on Localized adiposity
0.10
0.15
0.20
0.25
SNP effect on Gut microbiota abundance (family Oxalobacteraceae id.2966)

Batch 21 : Gut microbiota abundance (family Pasteurellaceae id.3689) on Localized adiposity

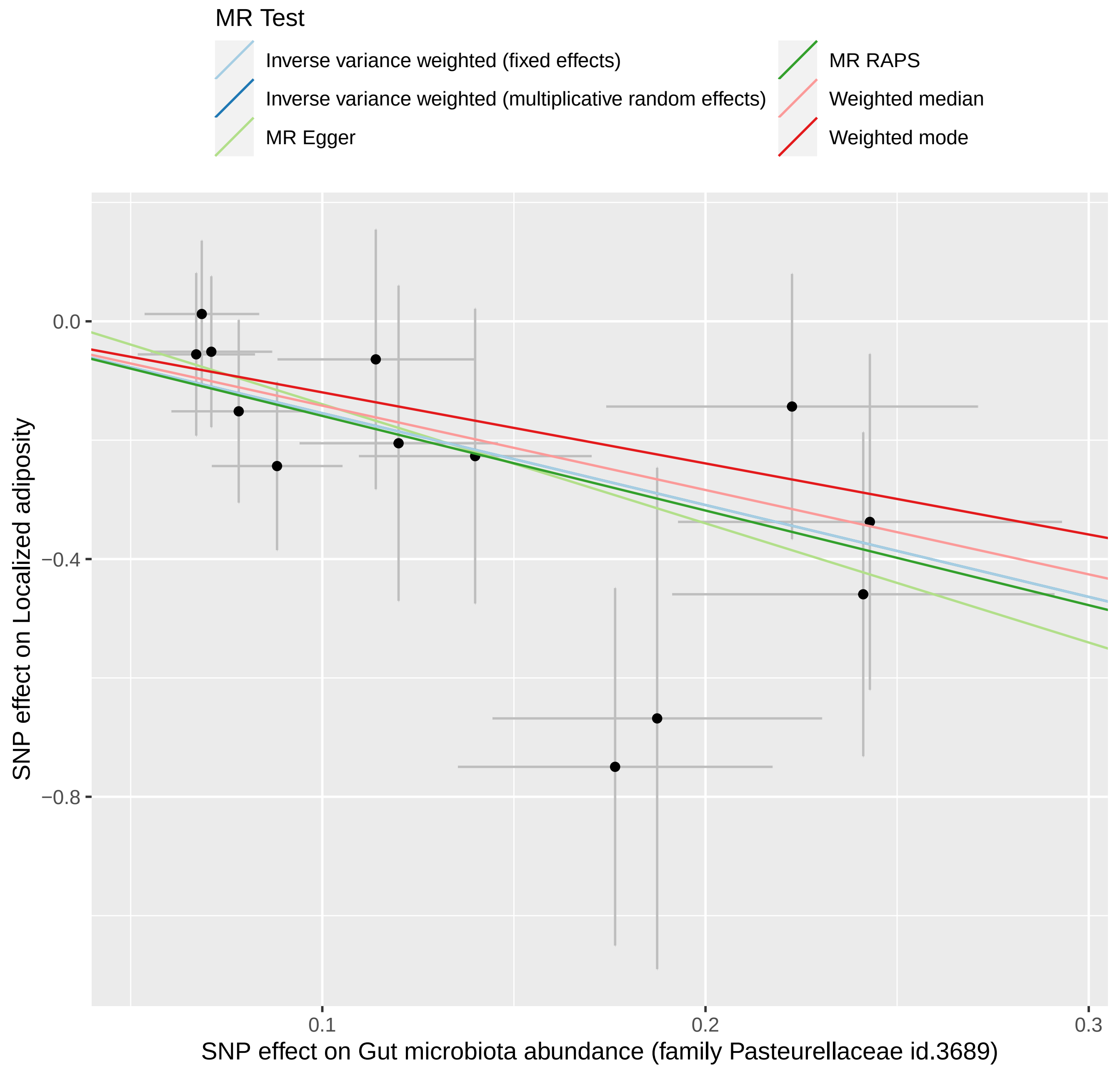

Batch 22 : Gut microbiota abundance (family Peptococcaceae id.2024) on Localized adiposity

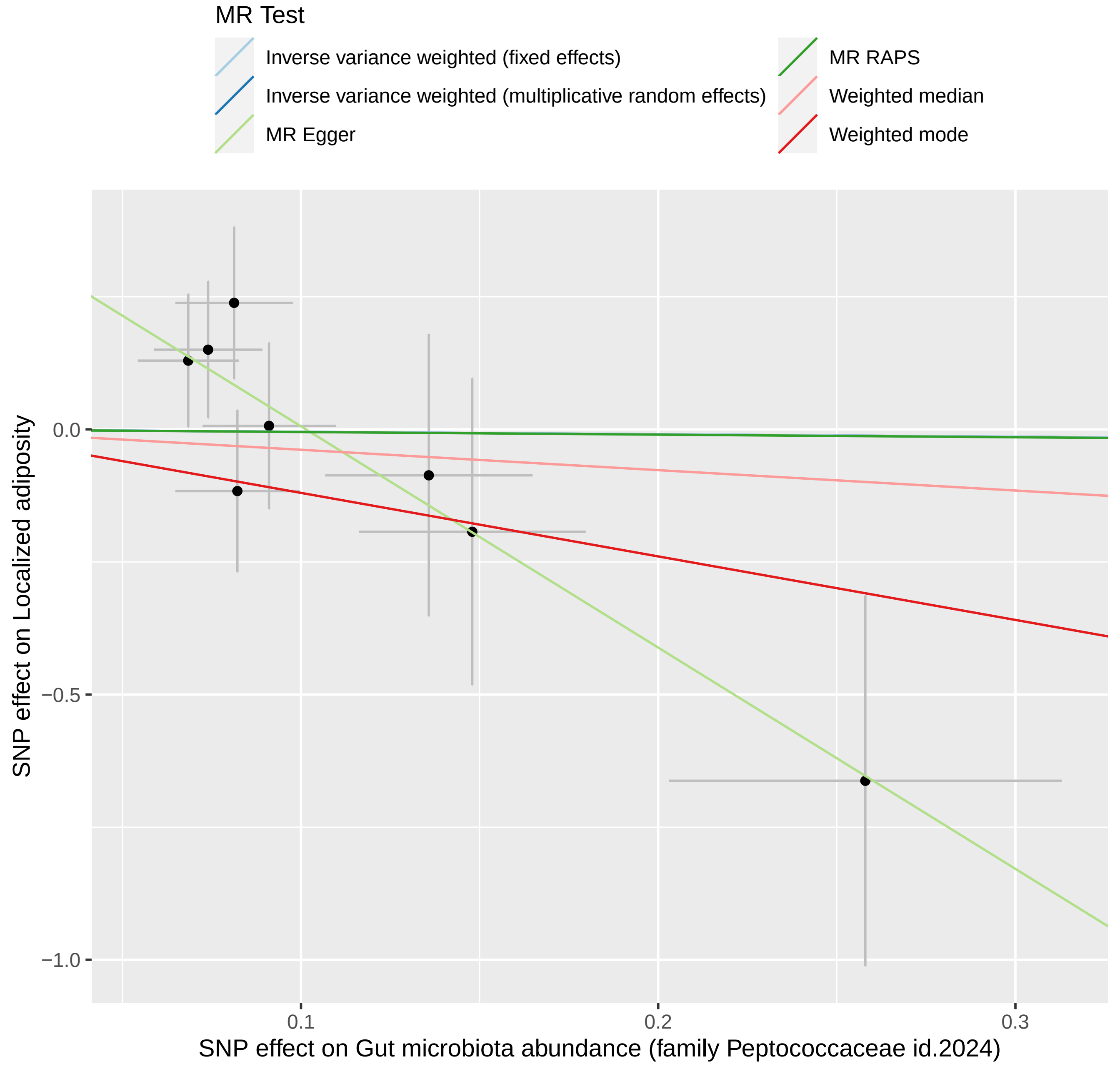

Batch 23 : Gut microbiota abundance (family Peptostreptococcaceae id.2042) on Localized adiposity

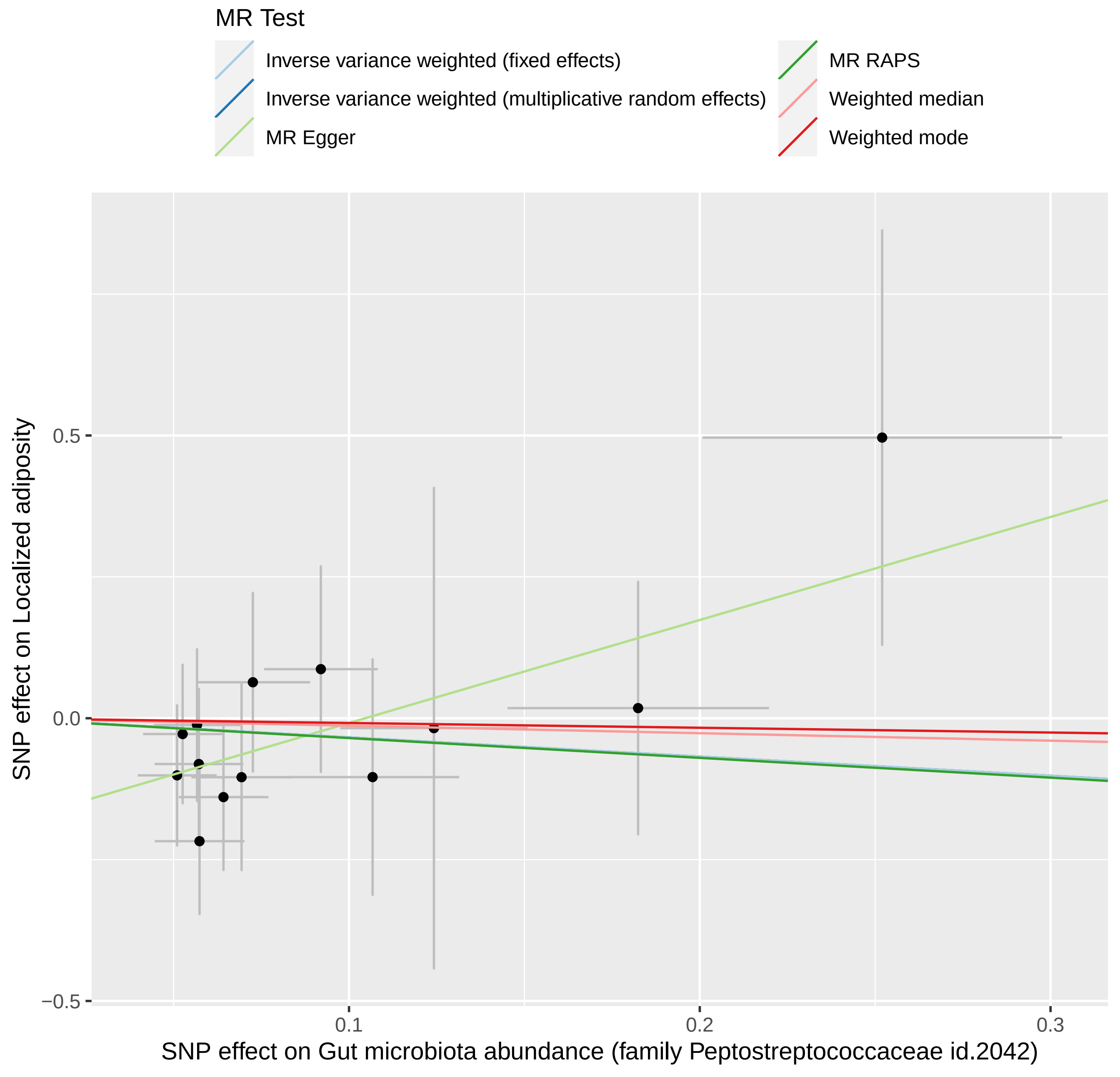

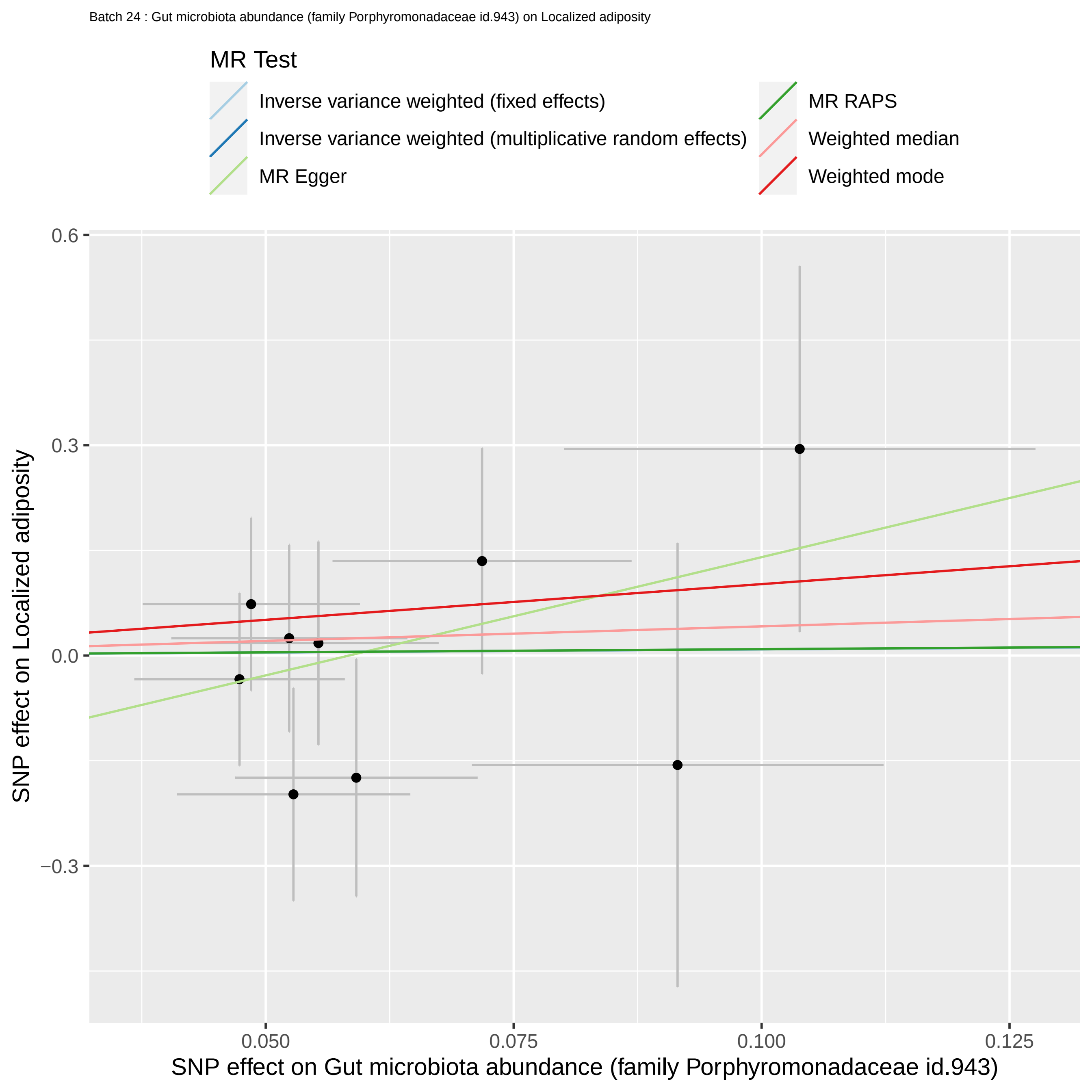

Batch 24 : Gut microbiota abundance (family Porphyromonadaceae id.943) on Localized adiposity
MR Test
Inverse variance weighted (fixed effects)
Inverse variance weighted (multiplicative random effects)
MR Egger
MR RAPS
Weighted median
Weighted mode
0.6
0.3
0.0
−0.3
0.050
0.075
0.100
0.125
SNP effect on Localized adiposity
SNP effect on Gut microbiota abundance (family Porphyromonadaceae id.943)

Batch 25 : Gut microbiota abundance (family Prevotellaceae id.960) on Localized adiposity

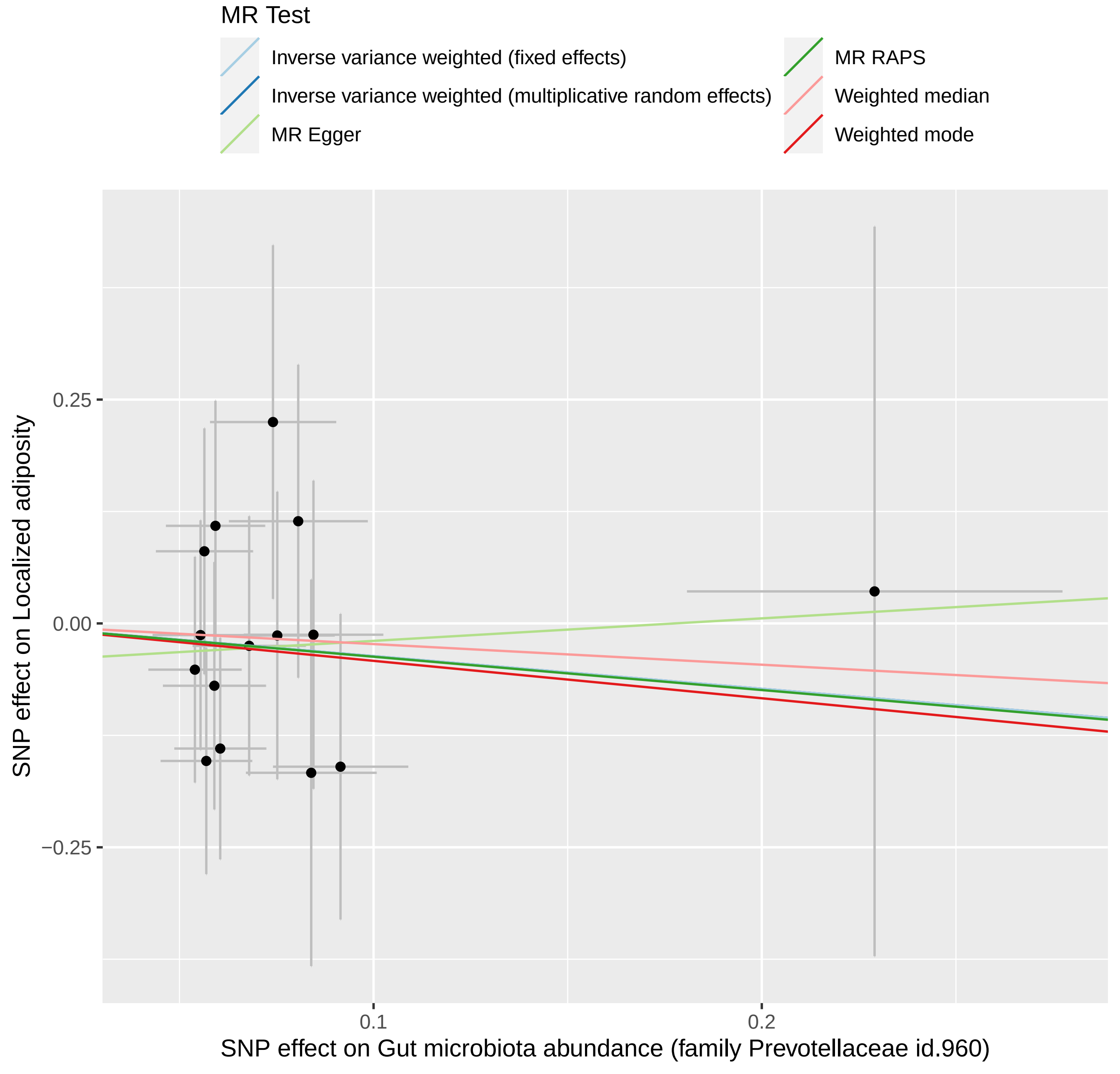

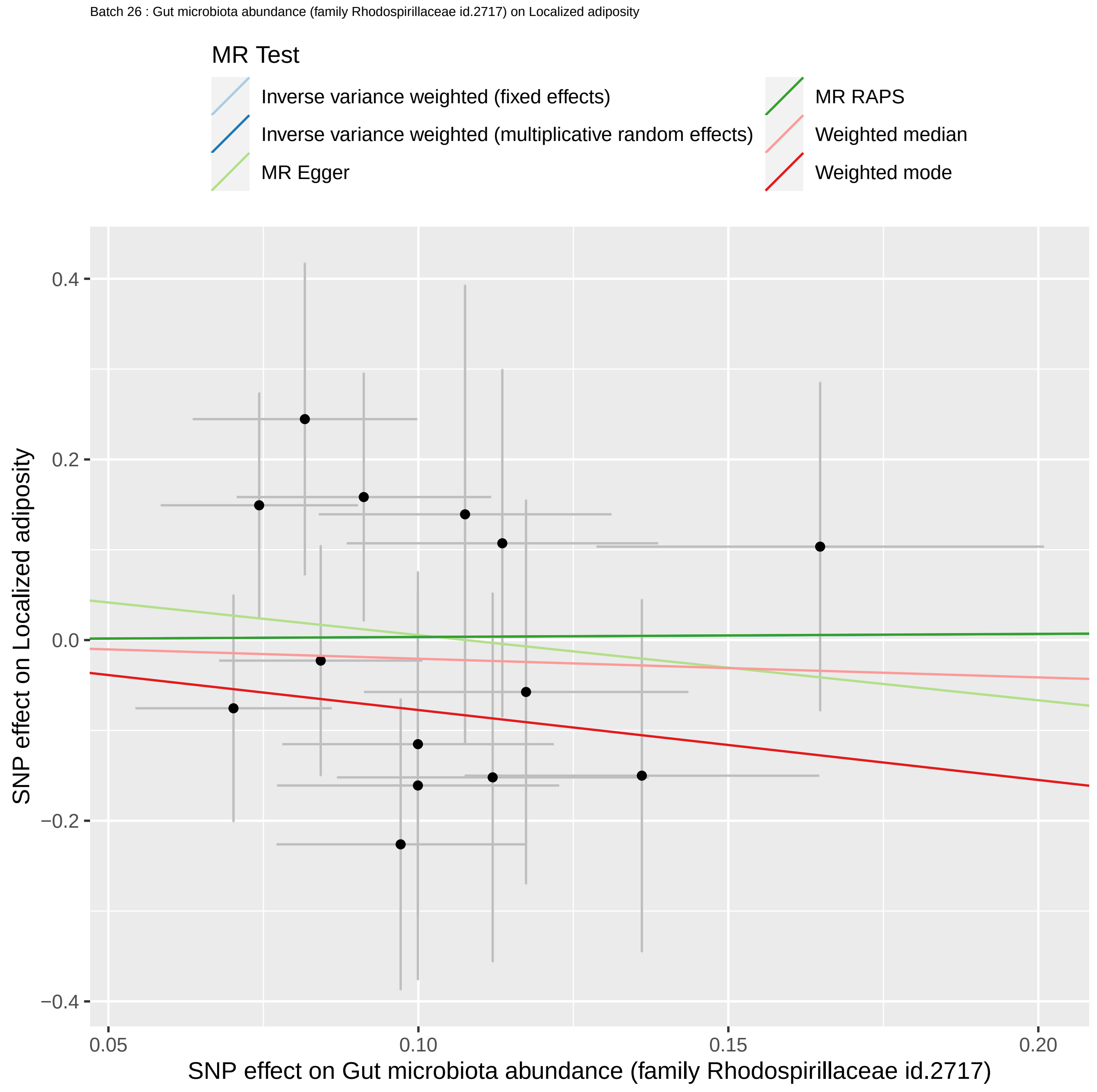
Batch 26 : Gut microbiota abundance (family Rhodospirillaceae id.2717) on Localized adiposity
MR Test
Inverse variance weighted (fixed effects)
Inverse variance weighted (multiplicative random effects)
MR Egger
MR RAPS
Weighted median
Weighted mode
0.4
0.2
0.0
−0.2
−0.4
SNP effect on Localized adiposity
0.05
0.10
0.15
0.20
SNP effect on Gut microbiota abundance (family Rhodospirillaceae id.2717)

Batch 27 : Gut microbiota abundance (family Rikenellaceae id.967) on Localized adiposity

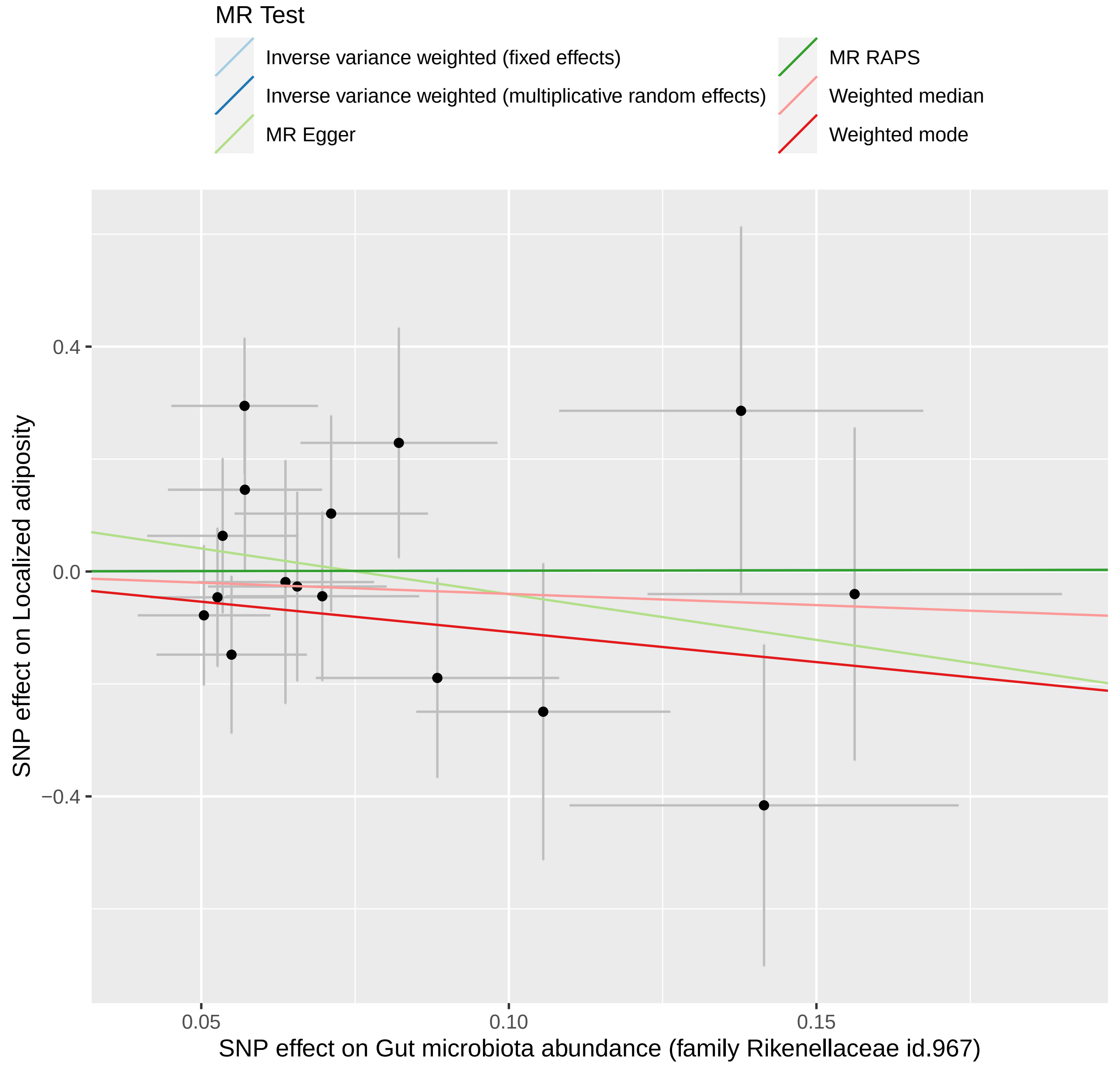

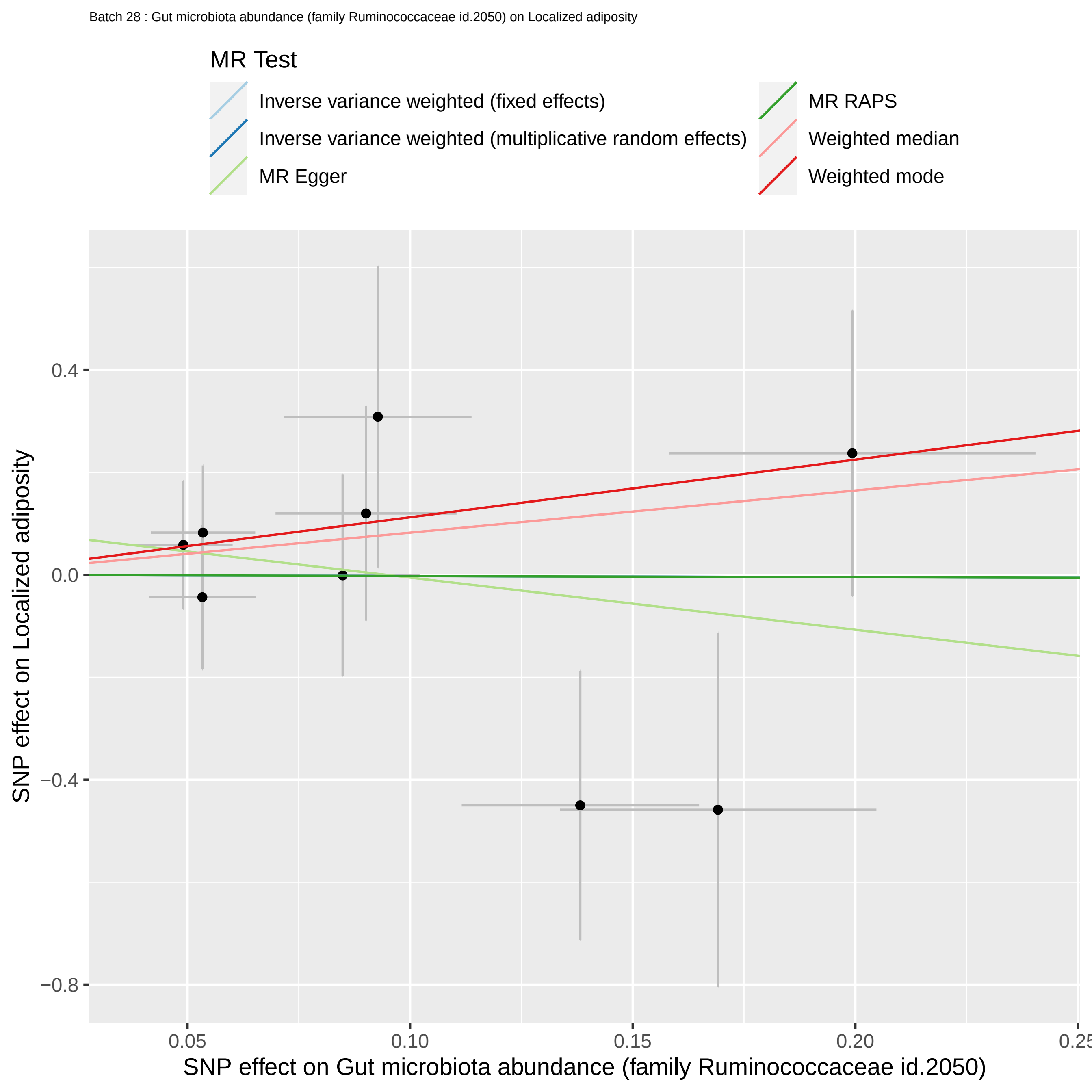
Batch 28 : Gut microbiota abundance (family Ruminococcaceae id.2050) on Localized adiposity
MR Test
Inverse variance weighted (fixed effects)
Inverse variance weighted (multiplicative random effects)
MR Egger
MR RAPS
Weighted median
Weighted mode
0.4
0.0
−0.4
−0.8
SNP effect on Localized adiposity
0.05
0.10
0.15
0.20
0.25
SNP effect on Gut microbiota abundance (family Ruminococcaceae id.2050)

Batch 29 : Gut microbiota abundance (family Streptococcaceae id.1850) on Localized adiposity

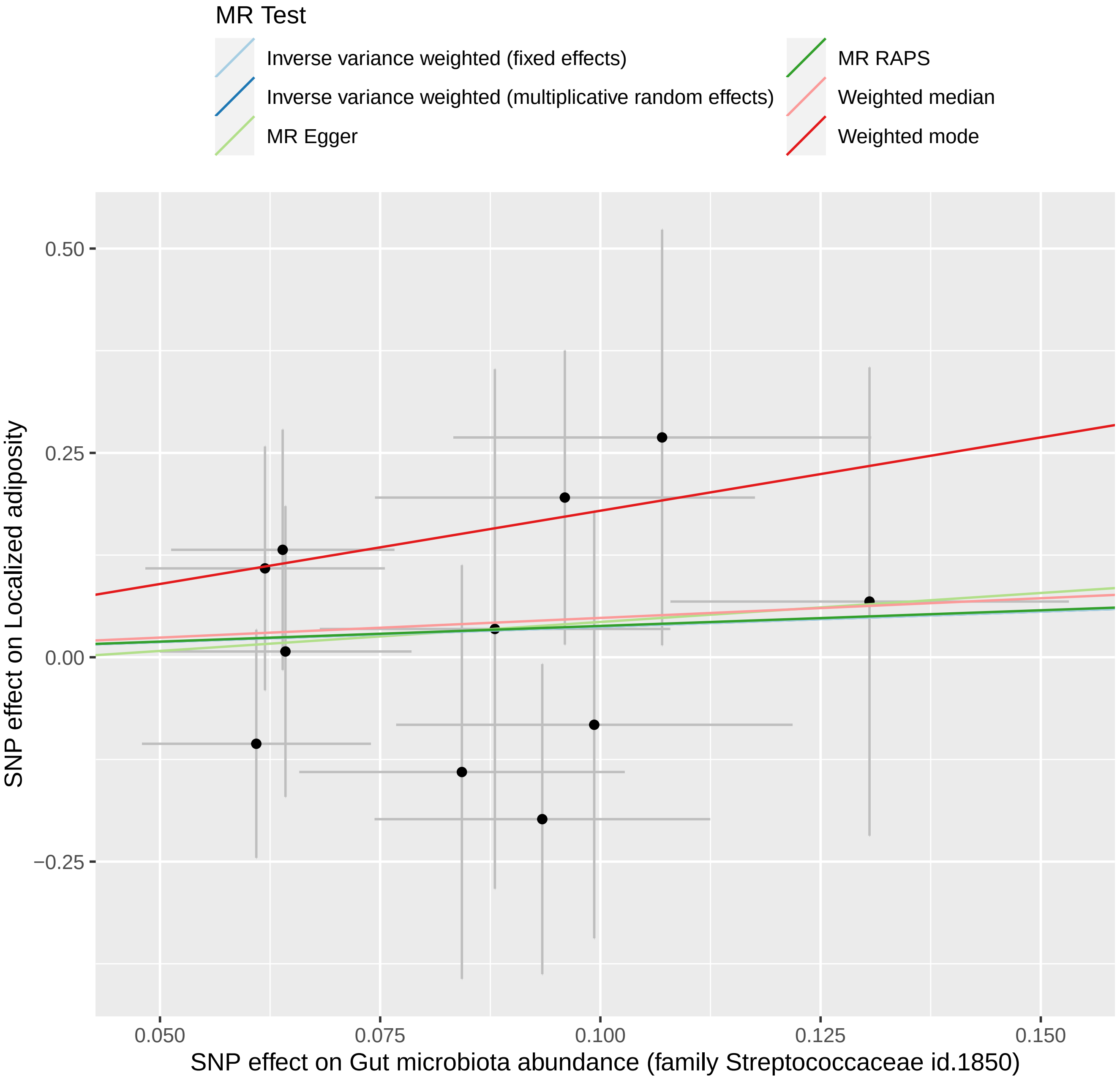

Batch 30 : Gut microbiota abundance (family Veillonellaceae id.2172) on Localized adiposity

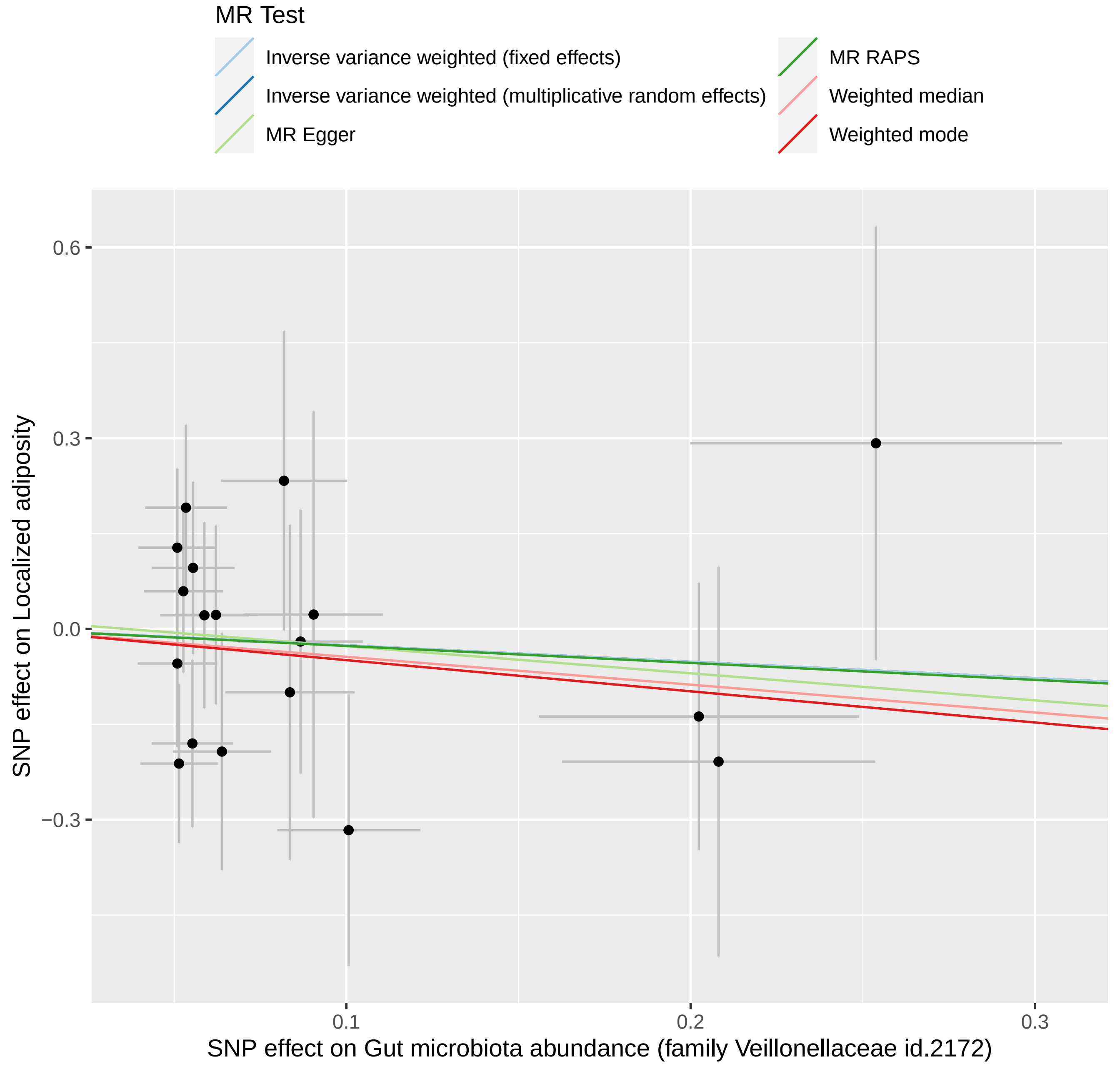

Batch 31 : Gut microbiota abundance (family Verrucomicrobiaceae id.4036) on Localized adiposity

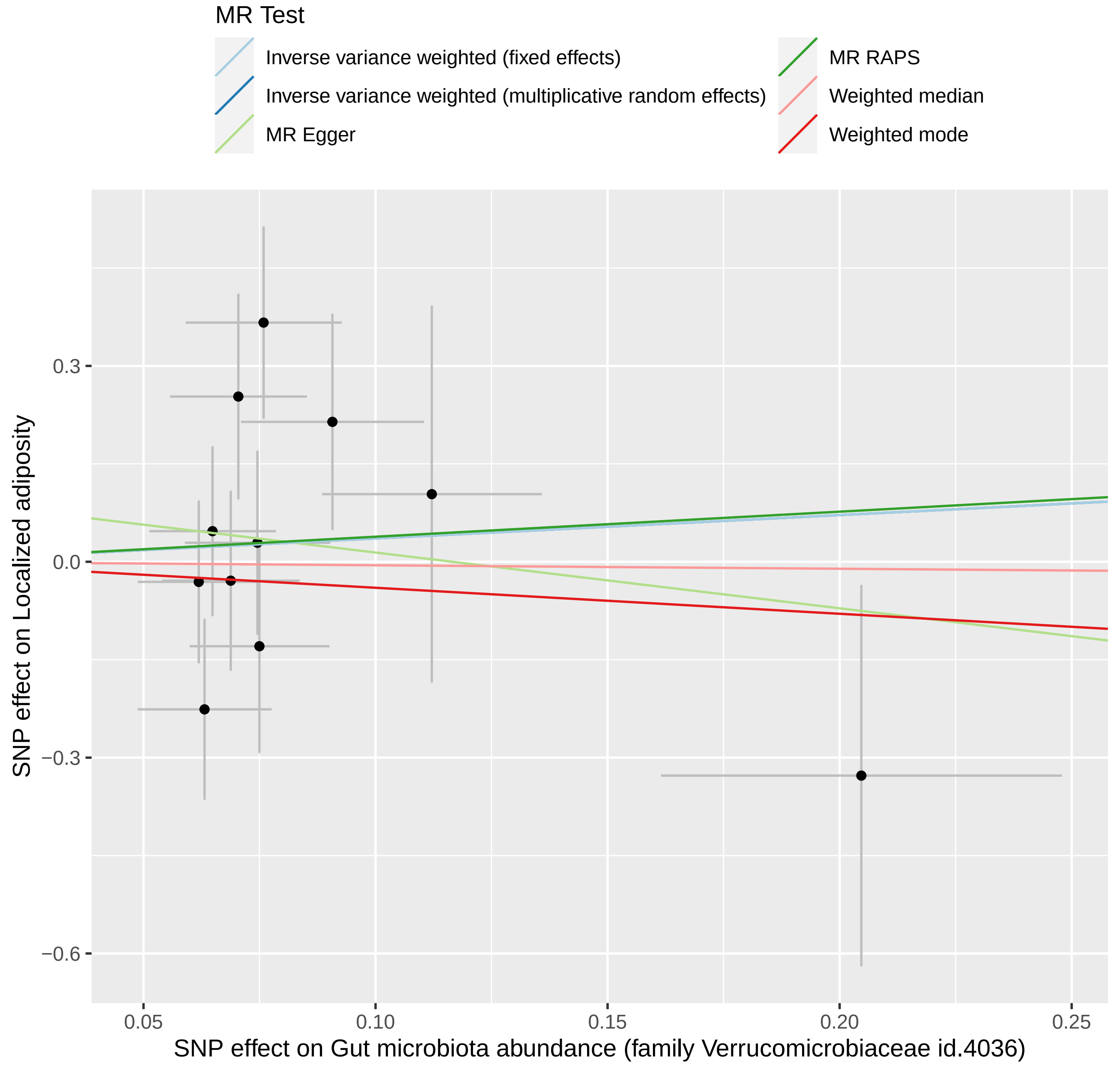

Batch 32 : Gut microbiota abundance (family Victivallaceae id.2255) on Localized adiposity

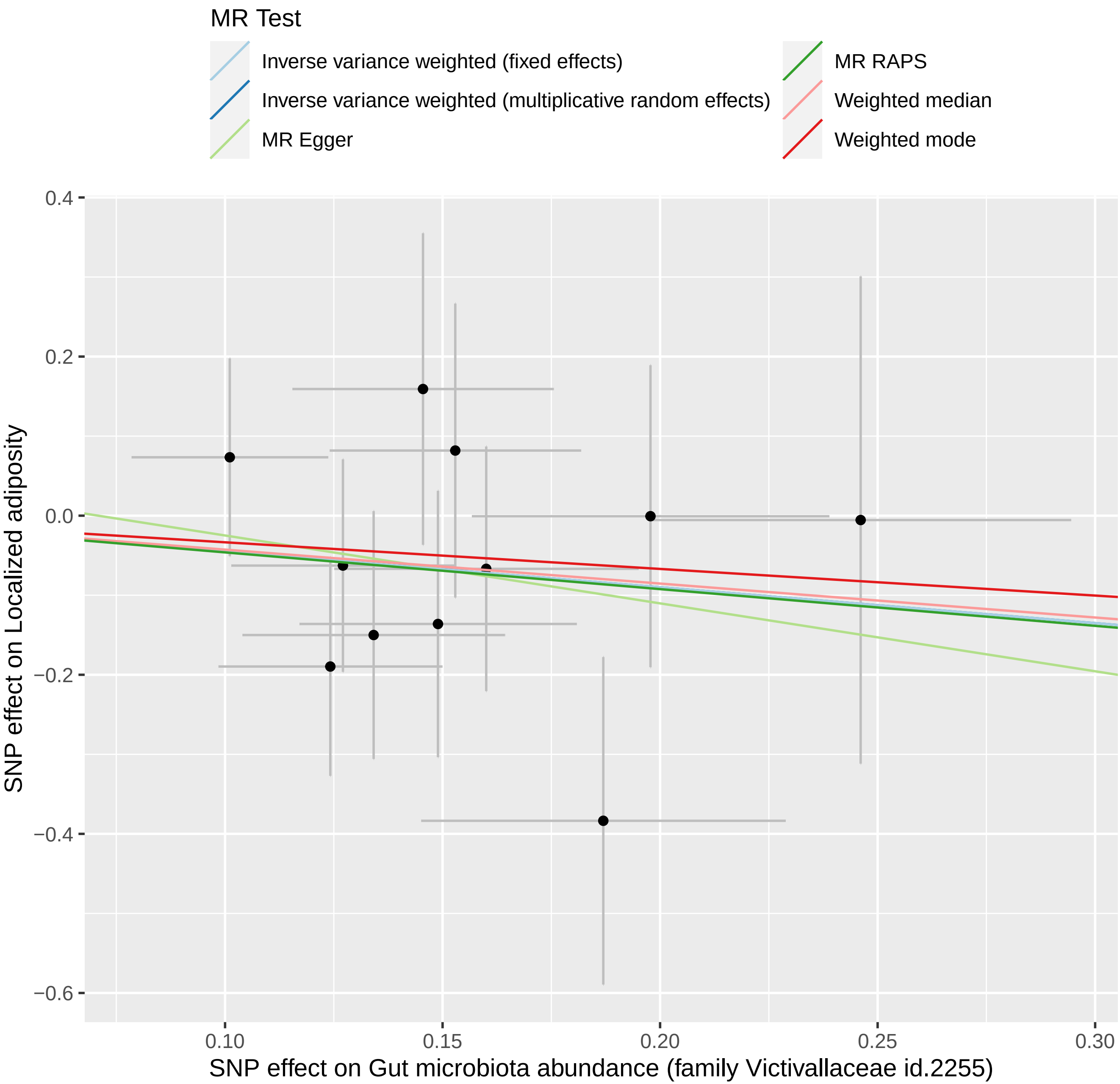

Batch 33 : Gut microbiota abundance (genus Actinomyces id.423) on Localized adiposity

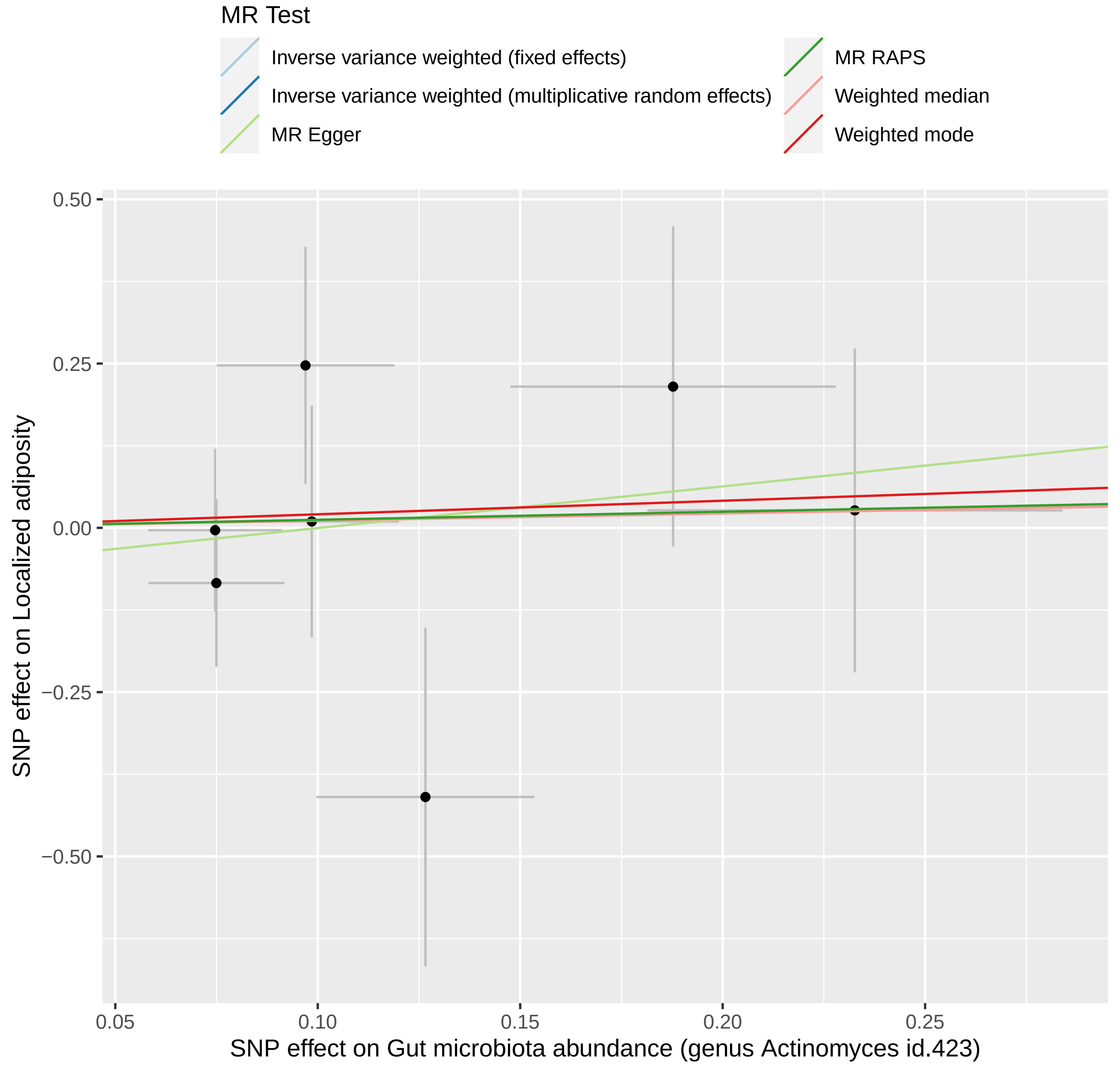

Batch 34 : Gut microbiota abundance (genus Adlercreutzia id.812) on Localized adiposity

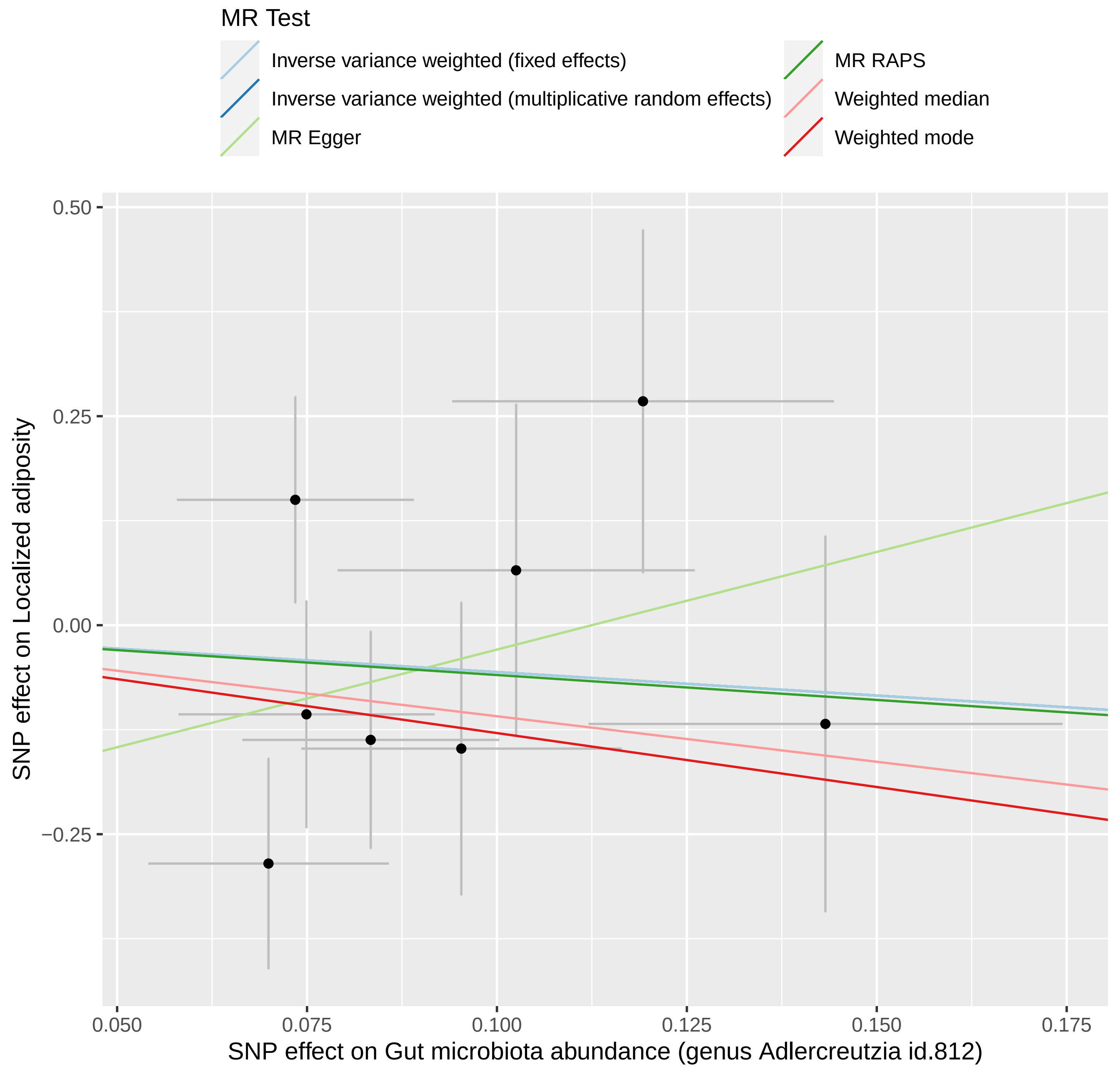

Batch 35 : Gut microbiota abundance (genus Akkermansia id.4037) on Localized adiposity

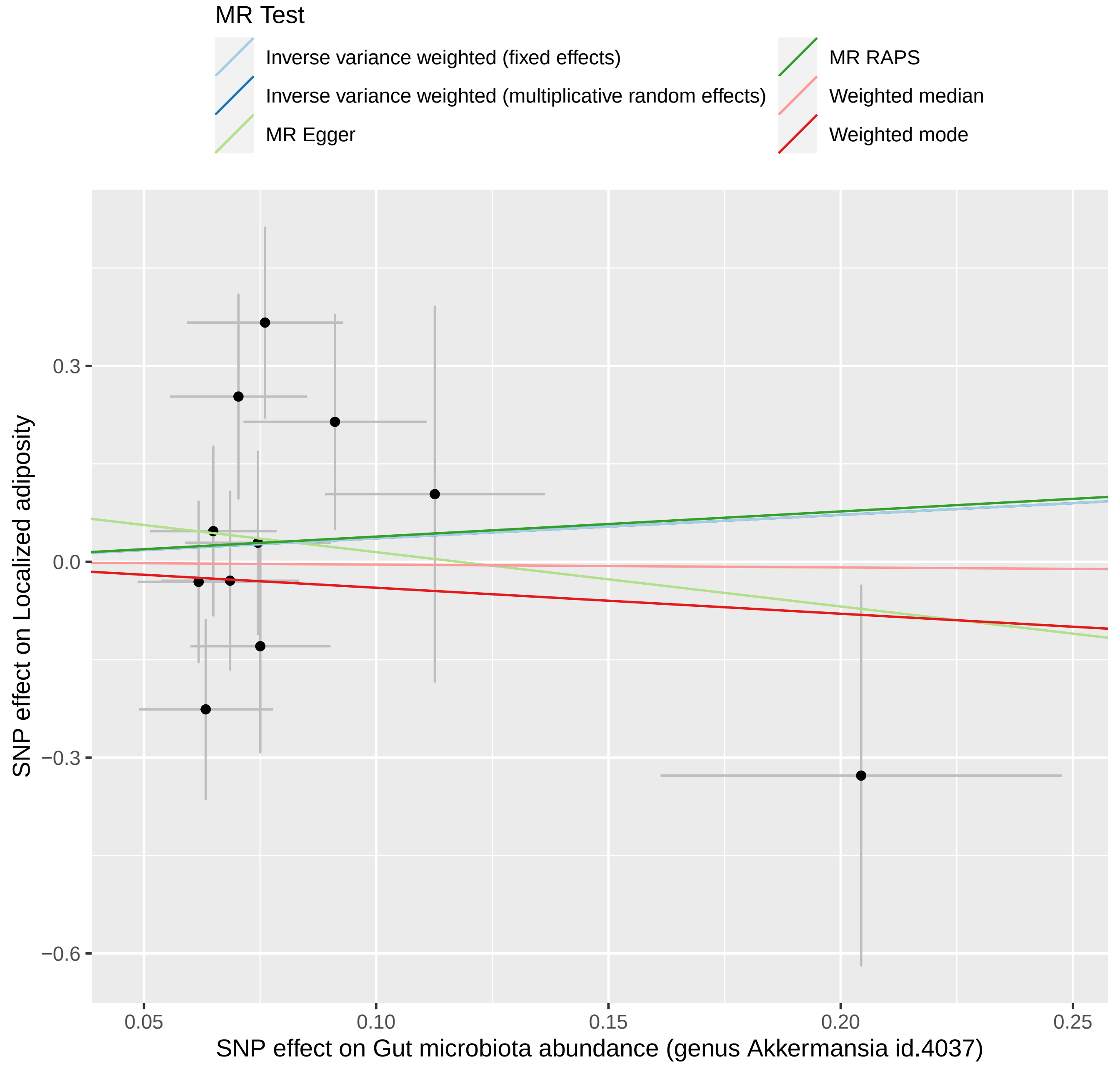

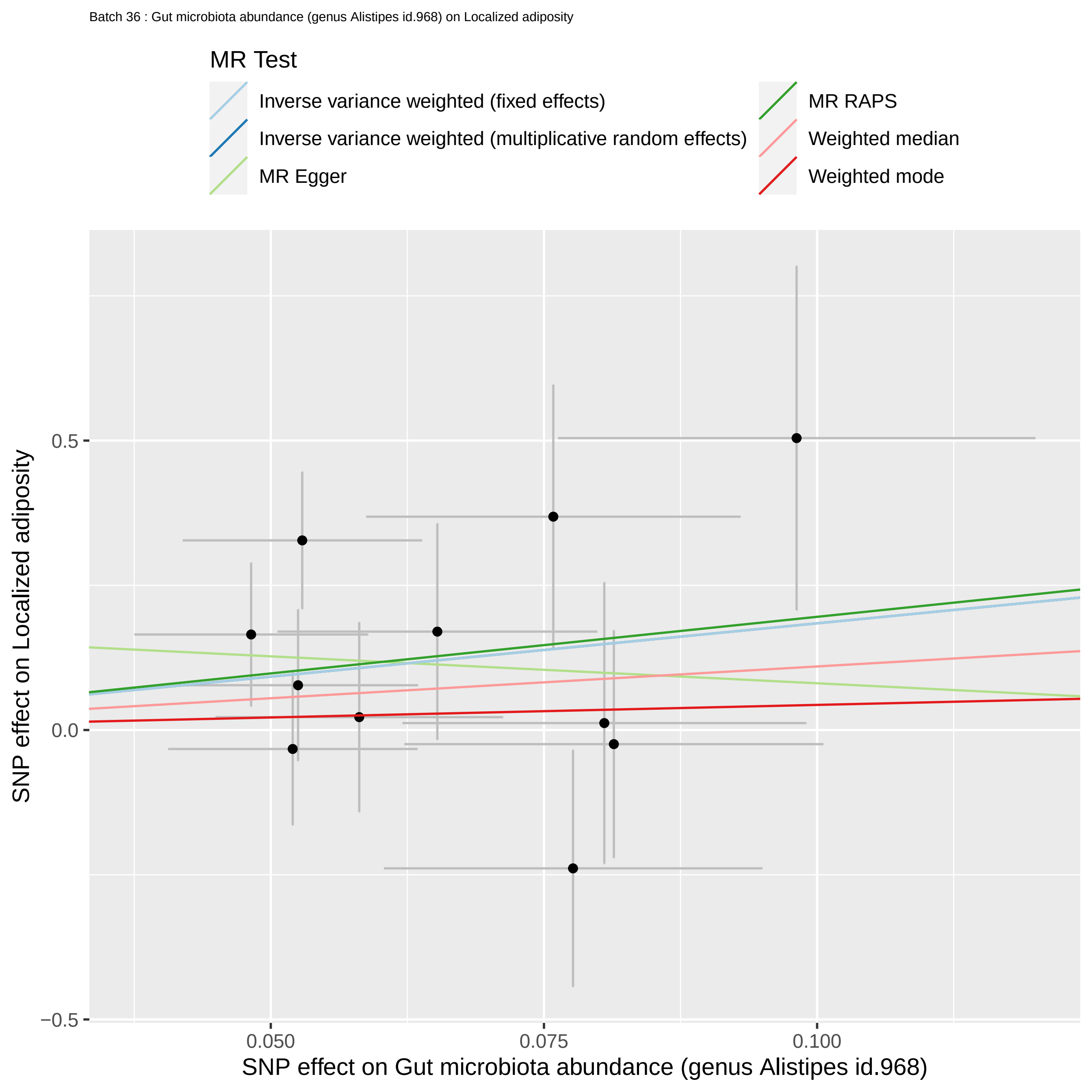
Batch 36 : Gut microbiota abundance (genus Alistipes id.968) on Localized adiposity
MR Test
Inverse variance weighted (fixed effects)
Inverse variance weighted (multiplicative random effects)
MR Egger
MR RAPS
Weighted median
Weighted mode
SNP effect on Localized adiposity
0.5
0.0
−0.5
0.050
0.075
0.100
SNP effect on Gut microbiota abundance (genus Alistipes id.968)

Batch 37 : Gut microbiota abundance (genus Allisonella id.2174) on Localized adiposity

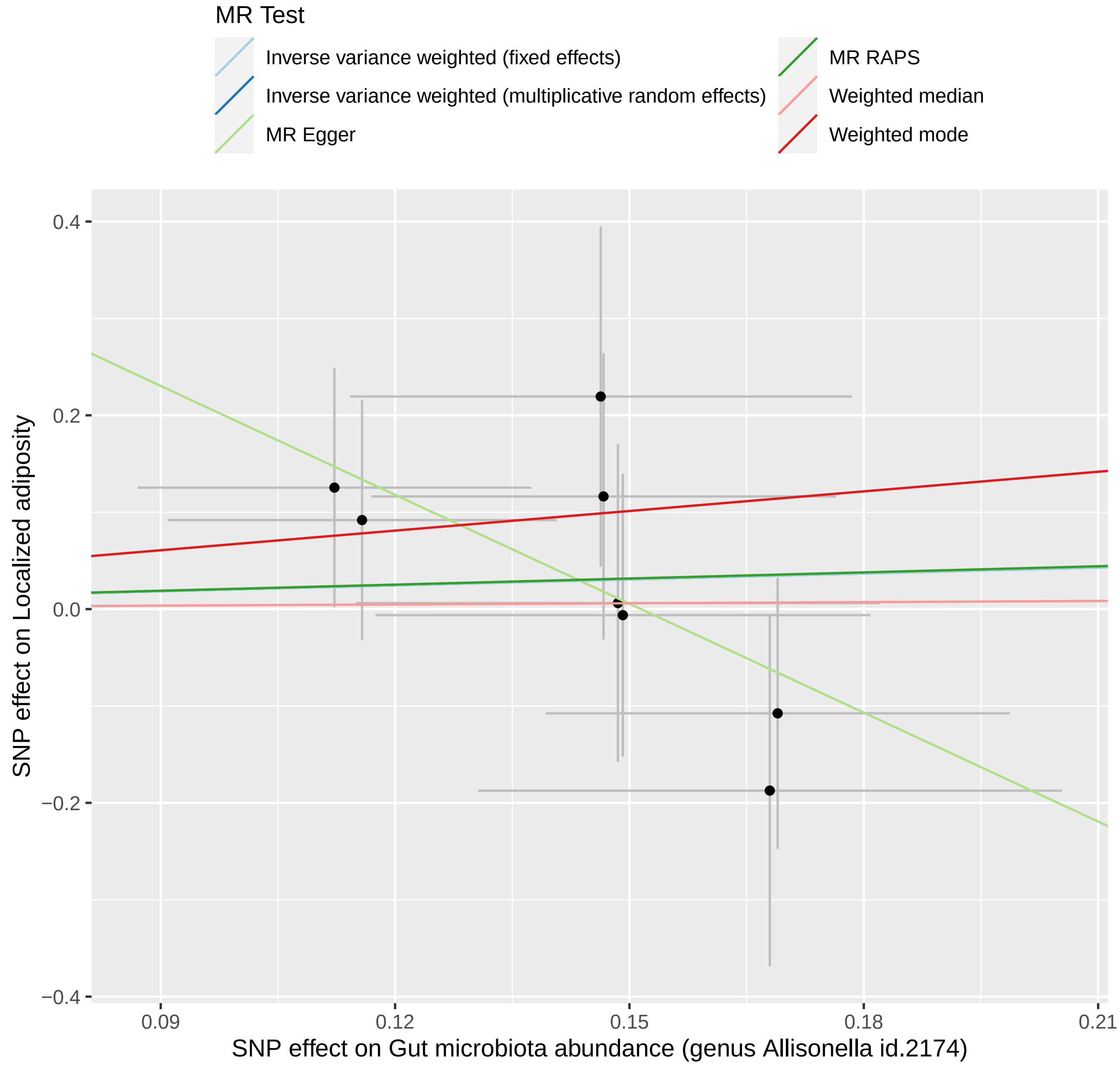

Batch 38 : Gut microbiota abundance (genus Alloprevotella id.961) on Localized adiposity

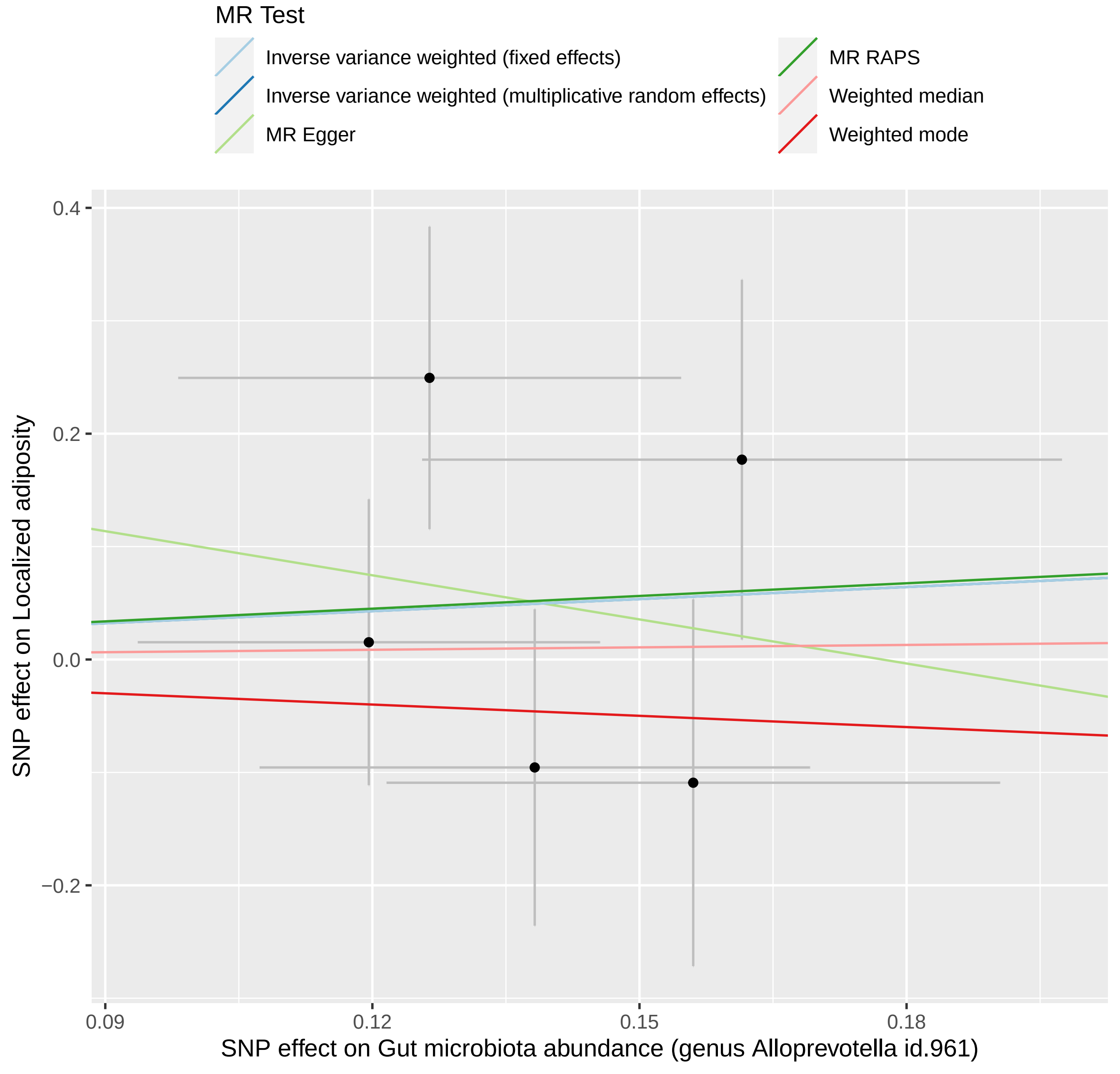

Batch 39 : Gut microbiota abundance (genus Anaerofilum id.2053) on Localized adiposity

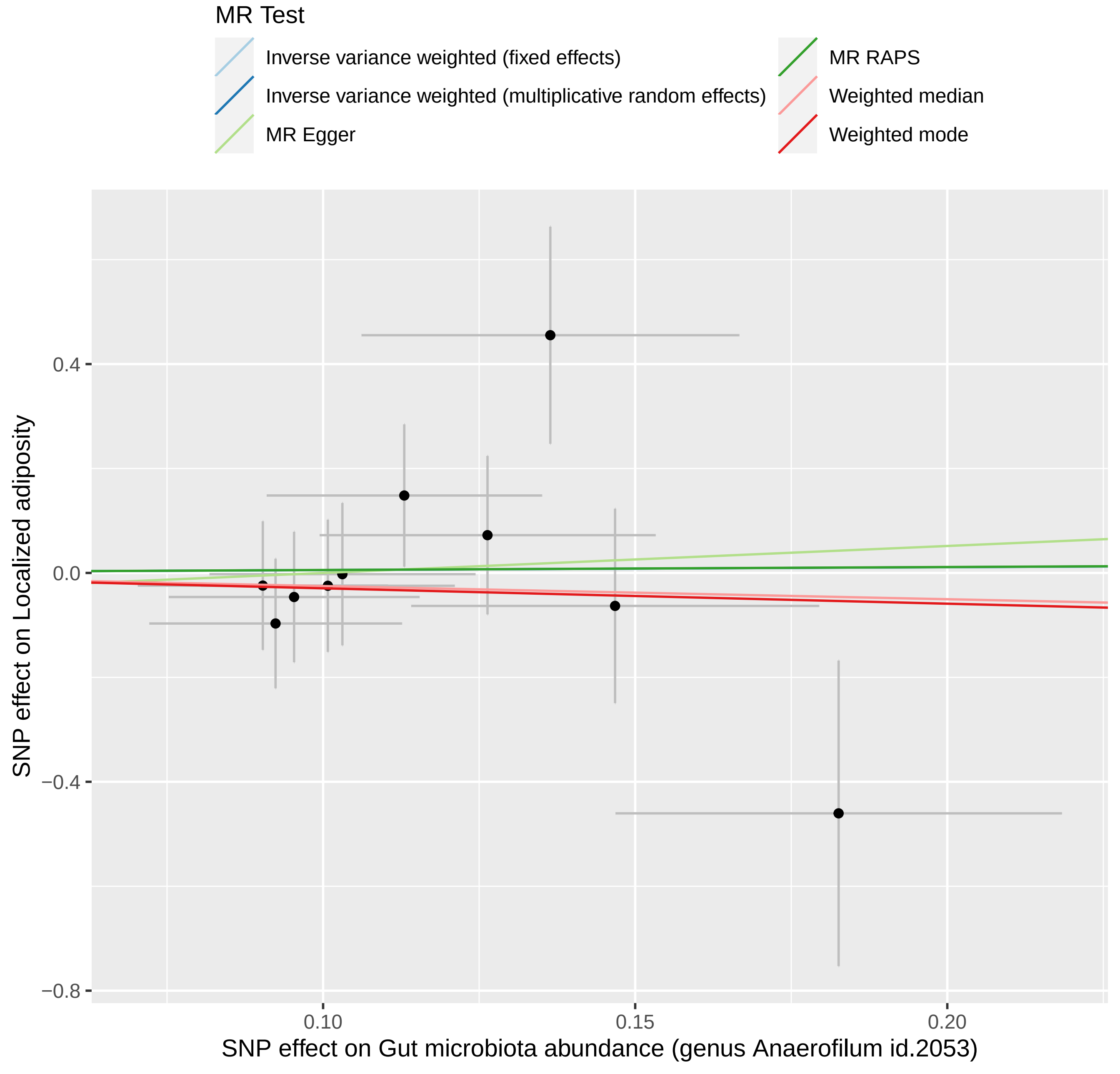

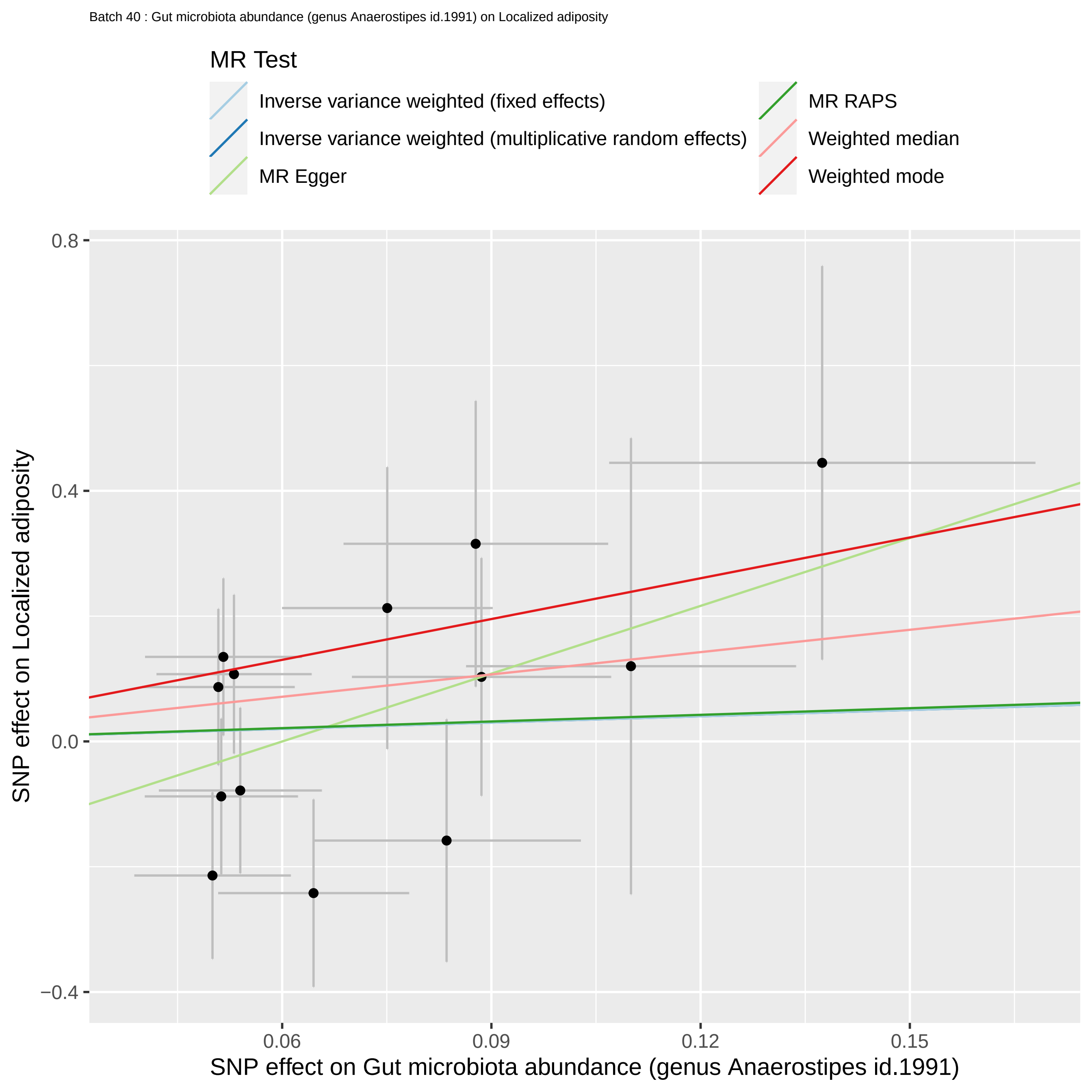
Batch 40 : Gut microbiota abundance (genus Anaerostipes id.1991) on Localized adiposity
MR Test
Inverse variance weighted (fixed effects)
Inverse variance weighted (multiplicative random effects)
MR Egger
MR RAPS
Weighted median
Weighted mode
0.8
0.4
0.0
−0.4
0.06
0.09
0.12
0.15
SNP effect on Localized adiposity
SNP effect on Gut microbiota abundance (genus Anaerostipes id.1991)

Batch 41 : Gut microbiota abundance (genus Anaerotruncus id.2054) on Localized adiposity

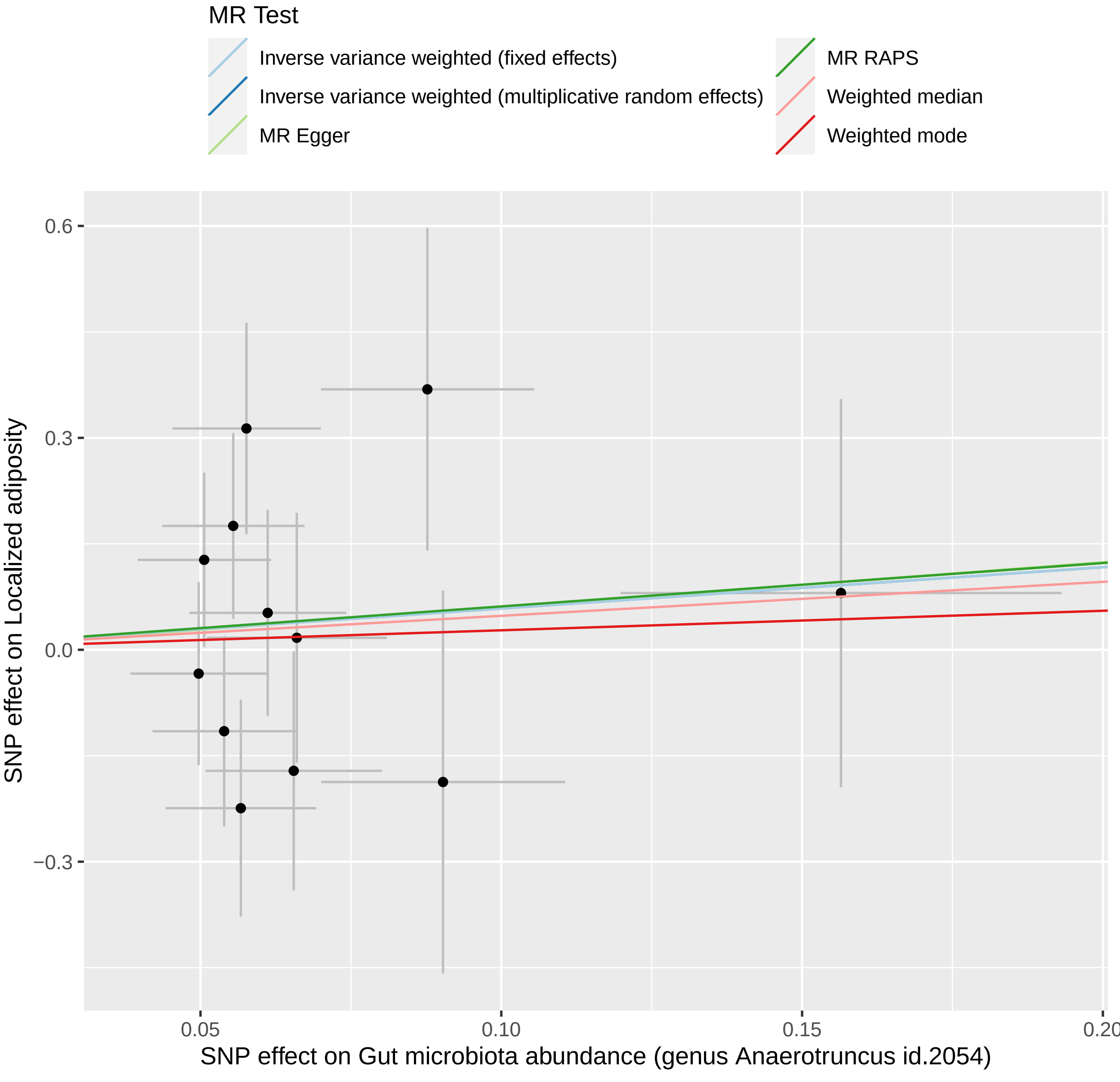

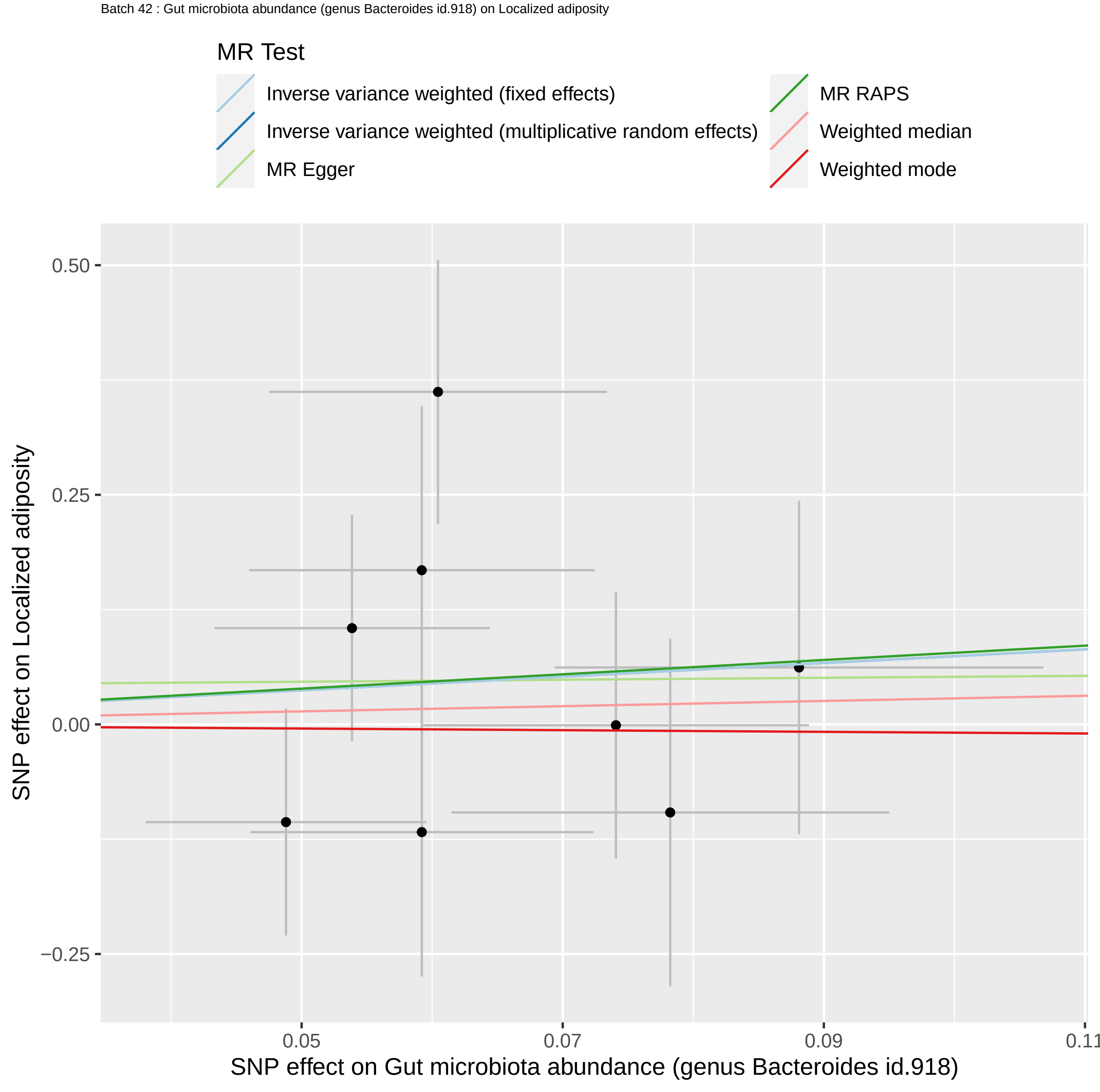

Batch 42 : Gut microbiota abundance (genus Bacteroides id.918) on Localized adiposity
MR Test
Inverse variance weighted (fixed effects)
Inverse variance weighted (multiplicative random effects)
MR Egger
MR RAPS
Weighted median
Weighted mode
0.50
0.25
0.00
−0.25
0.05
0.07
0.09
0.11
SNP effect on Localized adiposity
SNP effect on Gut microbiota abundance (genus Bacteroides id.918)

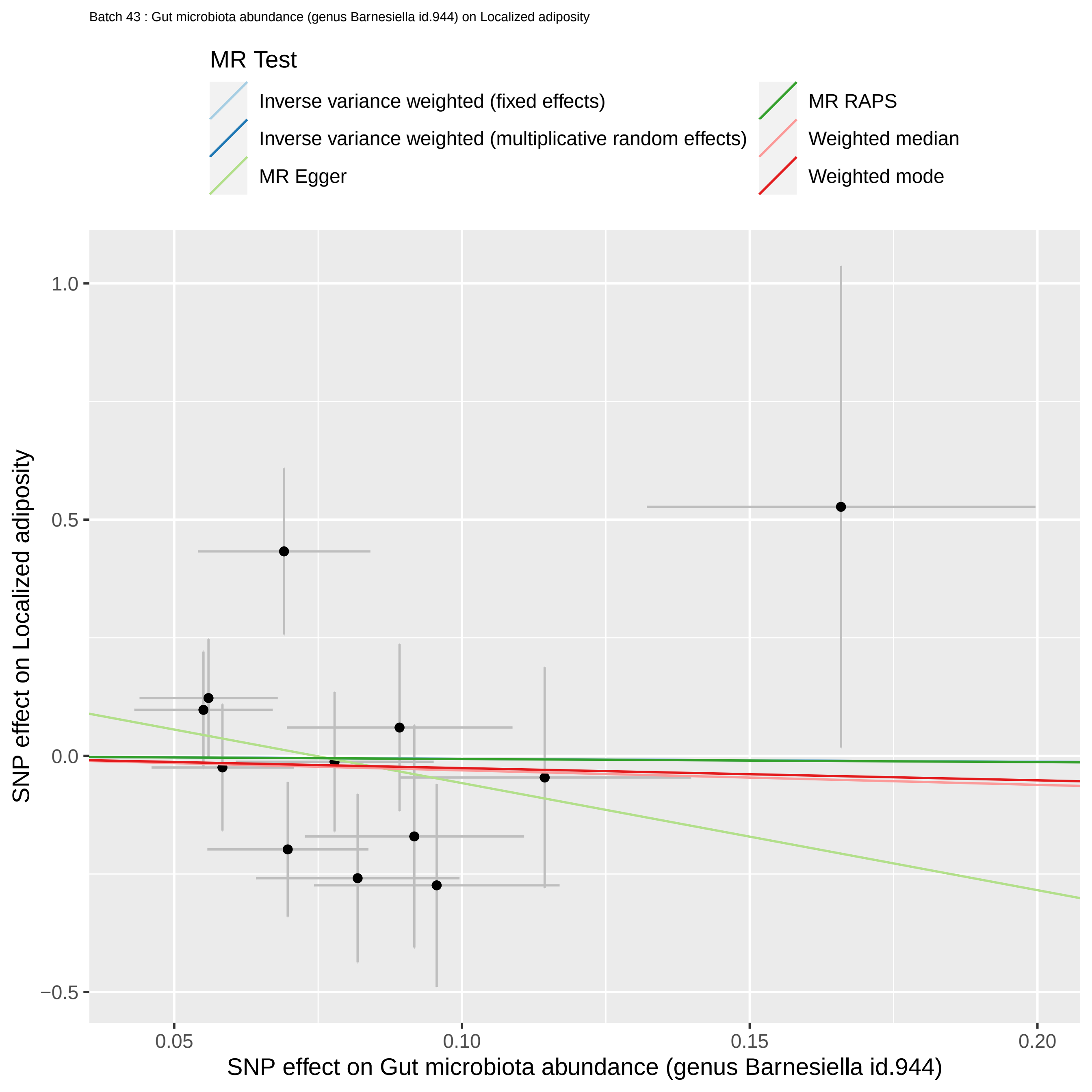
Batch 43 : Gut microbiota abundance (genus Barnesiella id.944) on Localized adiposity
MR Test
Inverse variance weighted (fixed effects)
Inverse variance weighted (multiplicative random effects)
MR Egger
MR RAPS
Weighted median
Weighted mode
1.0
0.5
0.0
−0.5
0.05
0.10
0.15
0.20
SNP effect on Localized adiposity
SNP effect on Gut microbiota abundance (genus Barnesiella id.944)

Batch 44 : Gut microbiota abundance (genus Bifidobacterium id.436) on Localized adiposity

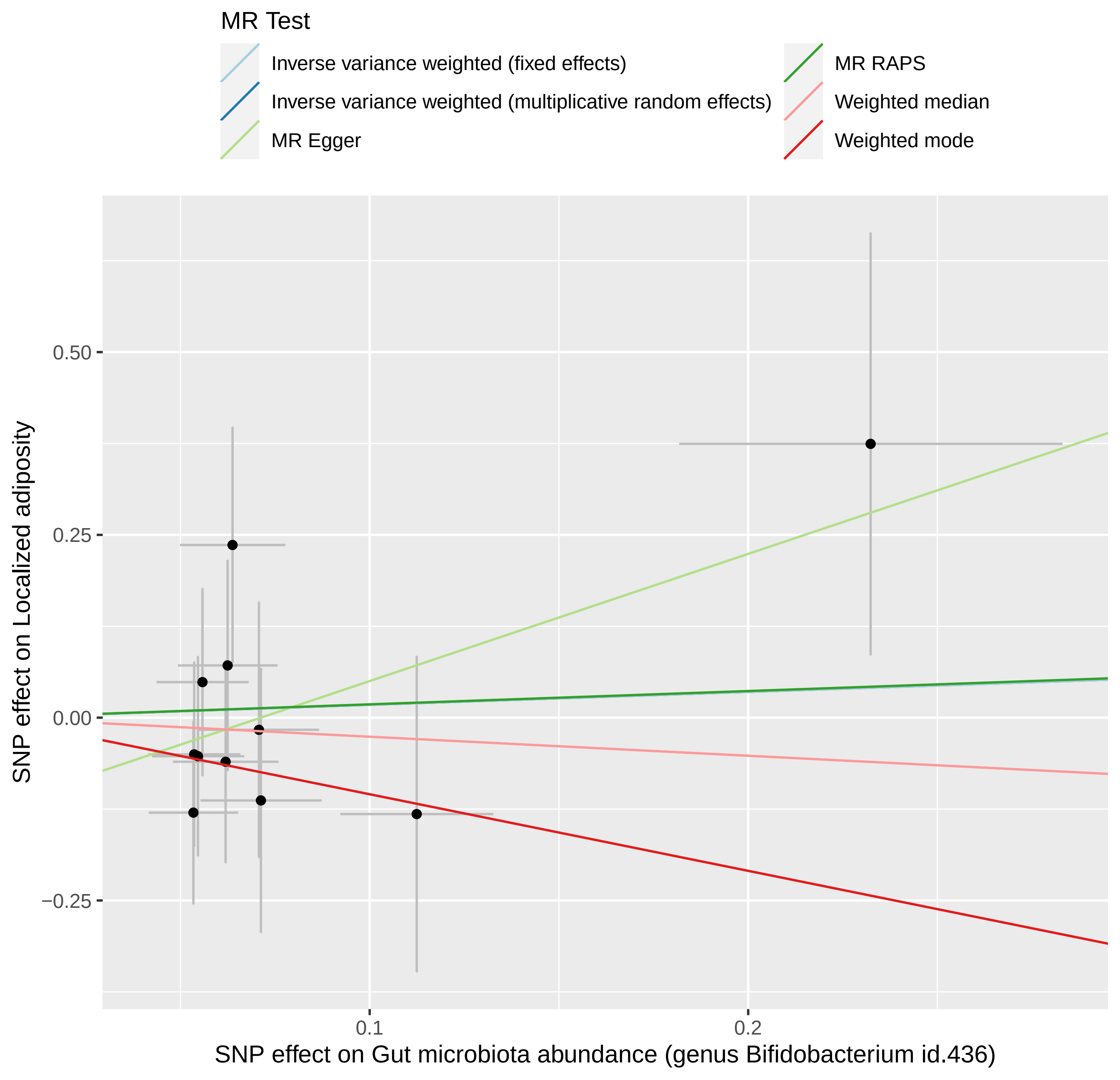

Batch 45 : Gut microbiota abundance (genus Bilophila id.3170) on Localized adiposity

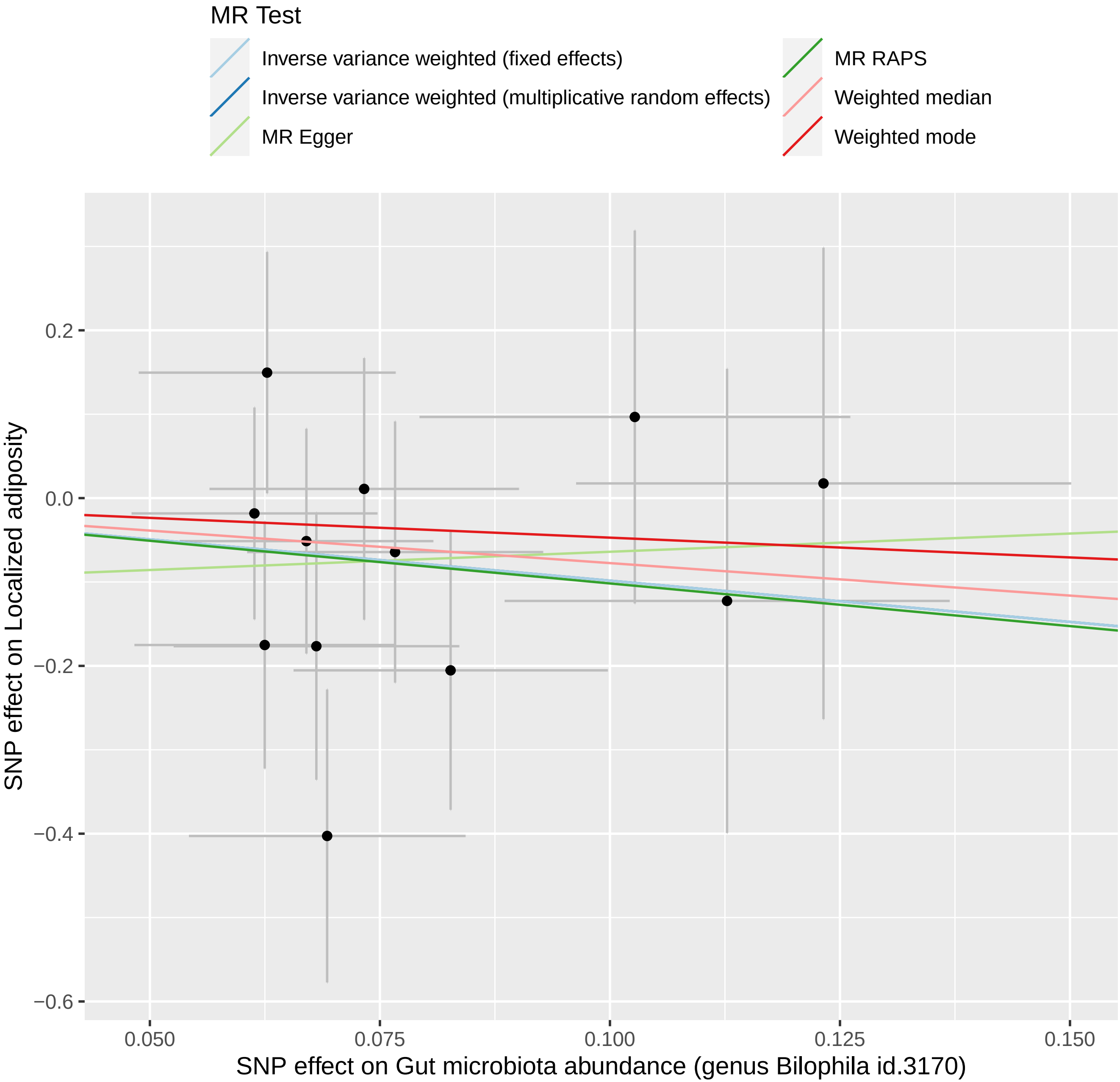

Batch 46 : Gut microbiota abundance (genus Blautia id.1992) on Localized adiposity

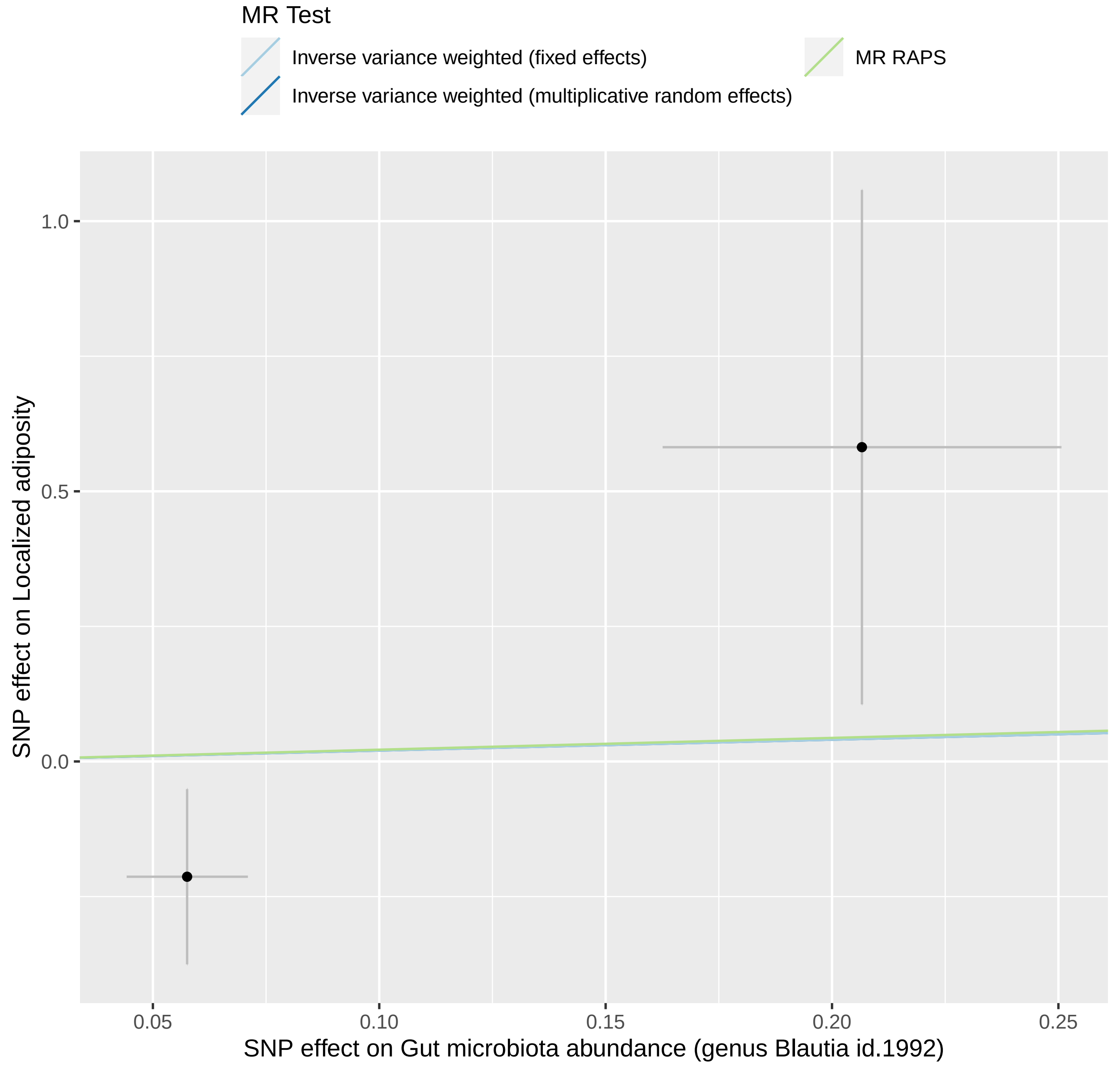

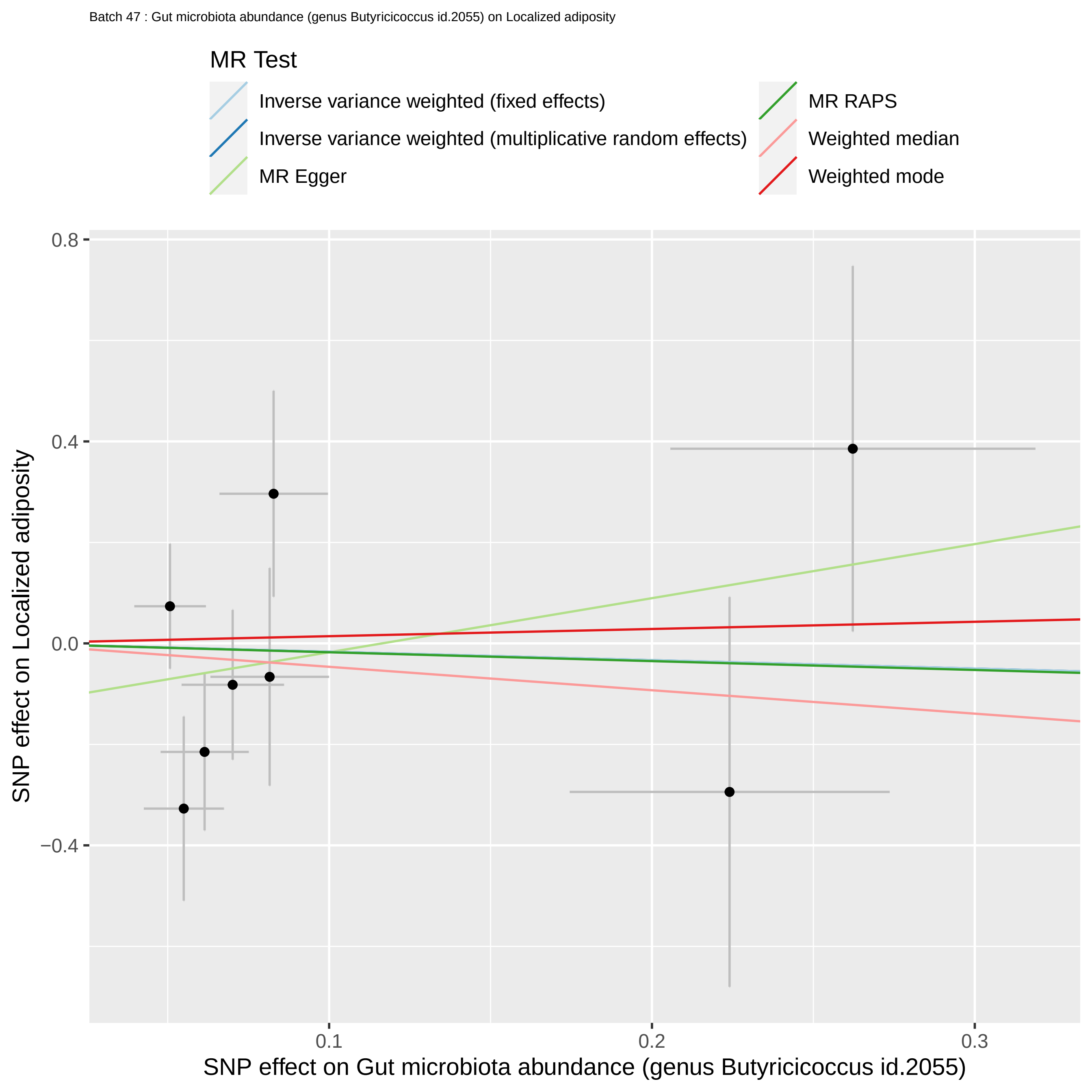
Batch 47 : Gut microbiota abundance (genus Butyricicoccus id.2055) on Localized adiposity
MR Test
Inverse variance weighted (fixed effects)
Inverse variance weighted (multiplicative random effects)
MR Egger
MR RAPS
Weighted median
Weighted mode
0.8
0.4
0.0
−0.4
SNP effect on Localized adiposity
0.1
0.2
0.3
SNP effect on Gut microbiota abundance (genus Butyricicoccus id.2055)

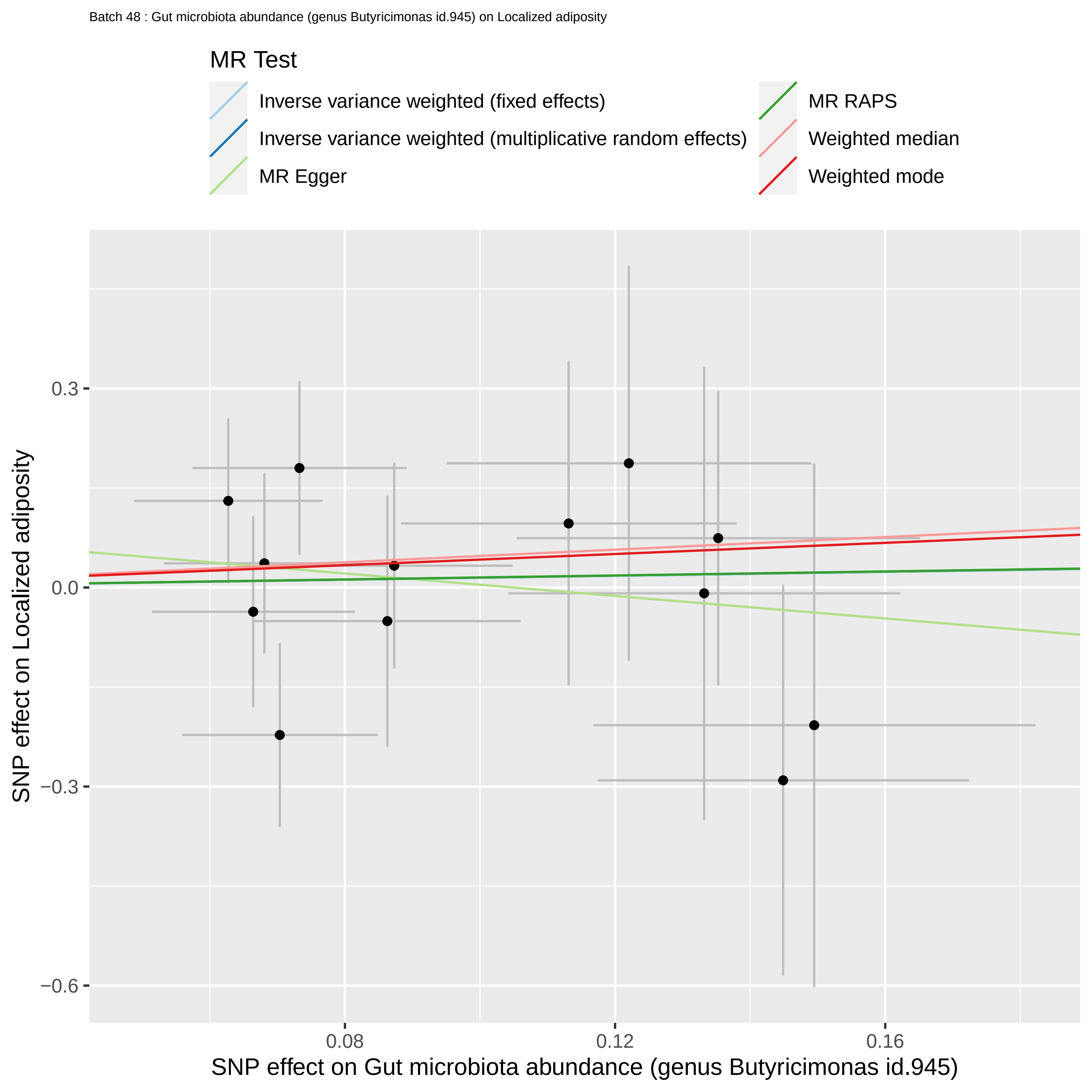
Batch 48 : Gut microbiota abundance (genus Butyricimonas id.945) on Localized adiposity
MR Test
Inverse variance weighted (fixed effects)
Inverse variance weighted (multiplicative random effects)
MR Egger
MR RAPS
Weighted median
Weighted mode
0.3
0.0
−0.3
−0.6
0.08
0.12
0.16
SNP effect on Localized adiposity
SNP effect on Gut microbiota abundance (genus Butyricimonas id.945)

Batch 49 : Gut microbiota abundance (genus Butyrivibrio id.1993) on Localized adiposity

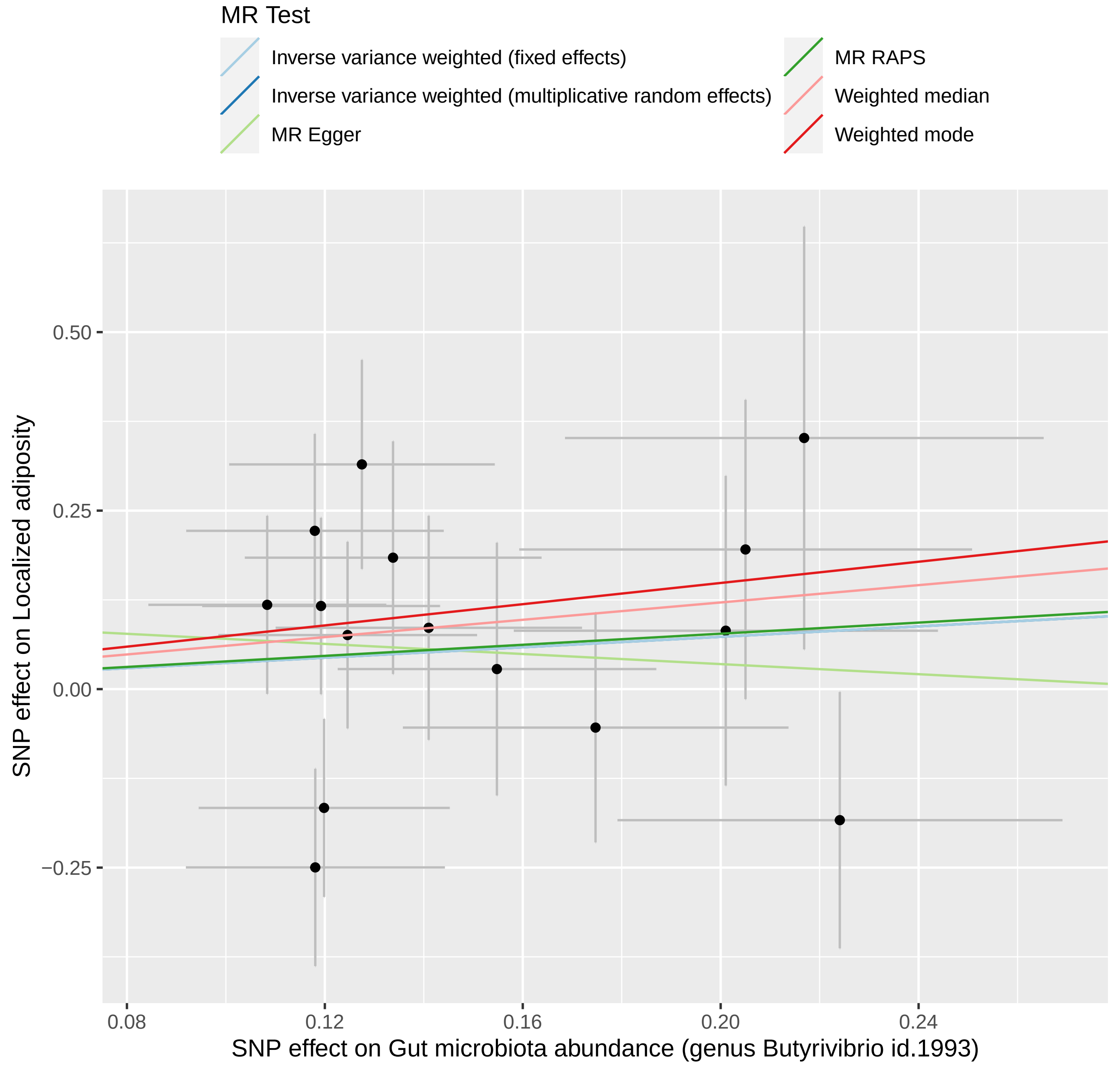

Batch 50 : Gut microbiota abundance (genus Candidatus Soleaferrea id.11350) on Localized adiposity

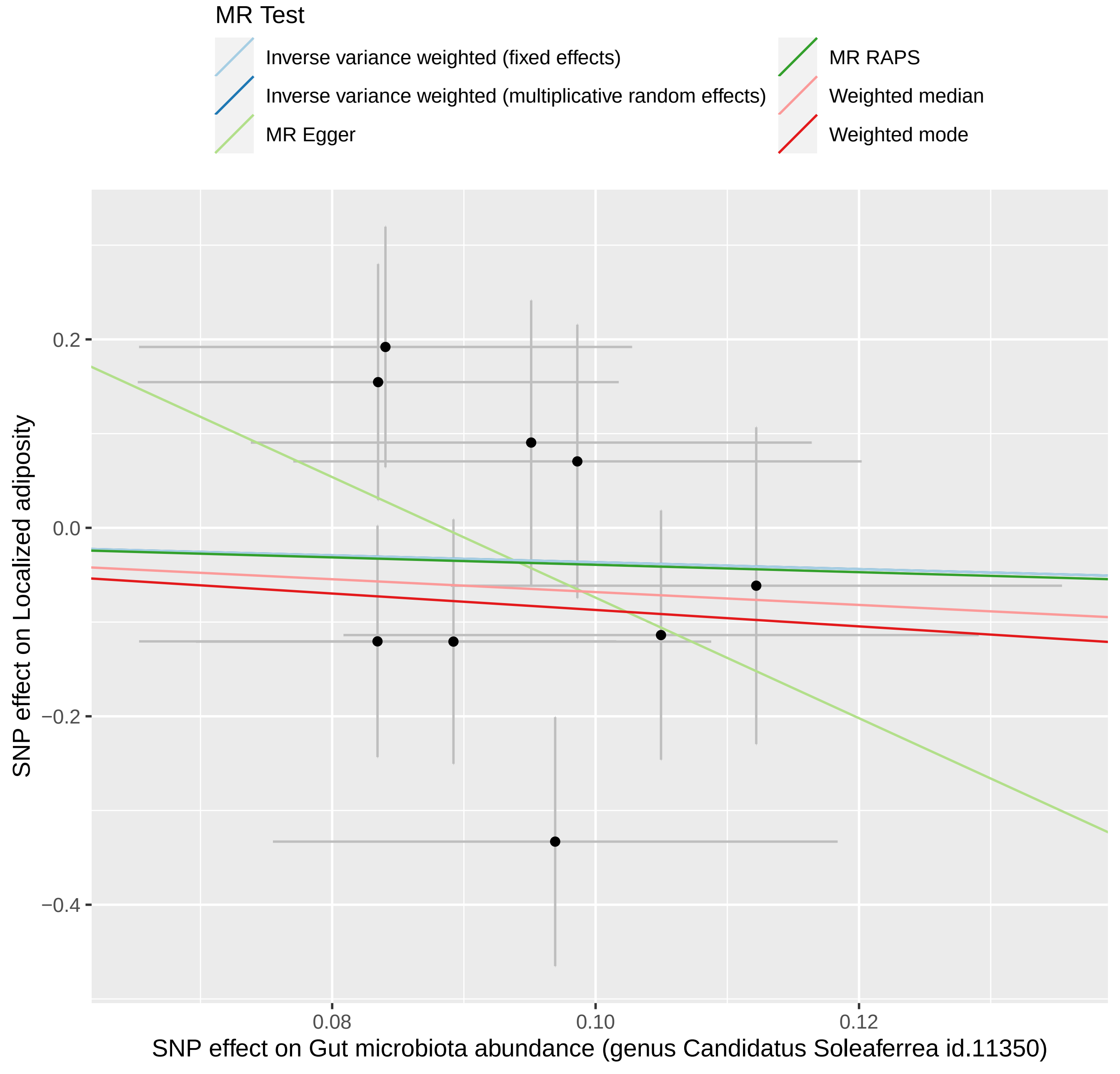

Batch 51 : Gut microbiota abundance (genus Catenibacterium id.2153) on Localized adiposity

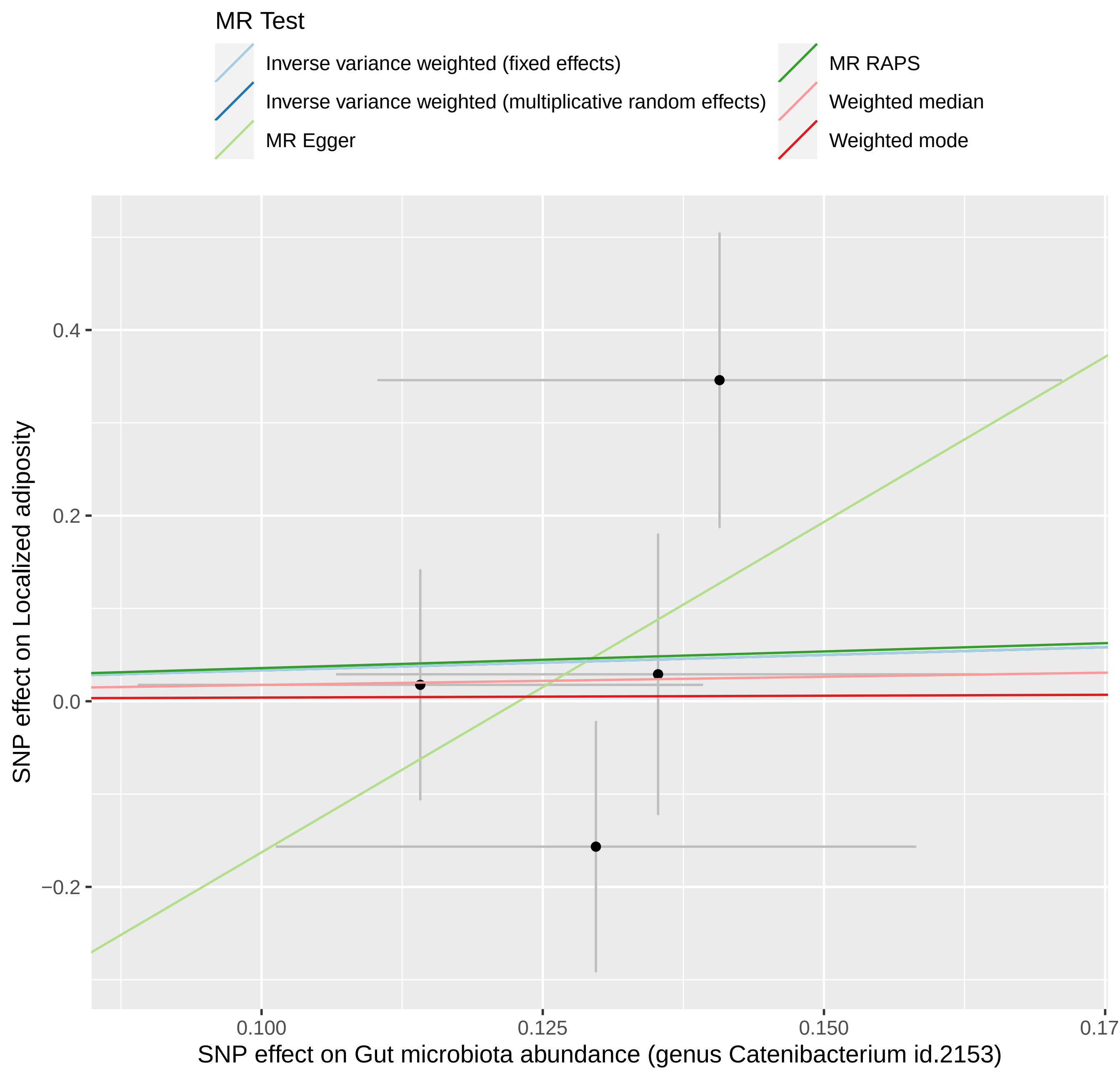

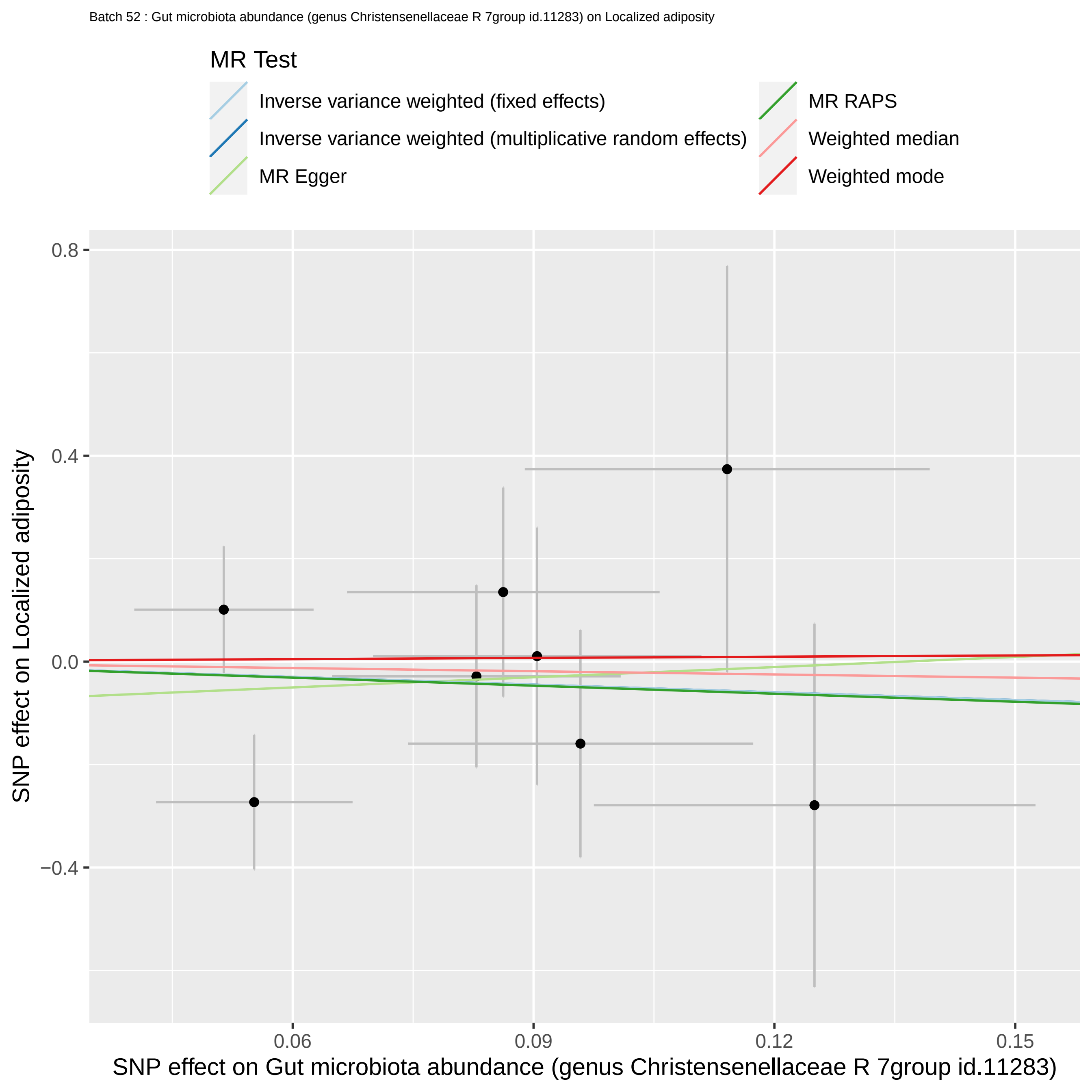

Batch 52 : Gut microbiota abundance (genus Christensenellaceae R 7group id.11283) on Localized adiposity
MR Test
Inverse variance weighted (fixed effects)
Inverse variance weighted (multiplicative random effects)
MR Egger
MR RAPS
Weighted median
Weighted mode
0.8
0.4
0.0
−0.4
0.06
0.09
0.12
0.15
SNP effect on Localized adiposity
SNP effect on Gut microbiota abundance (genus Christensenellaceae R 7group id.11283)

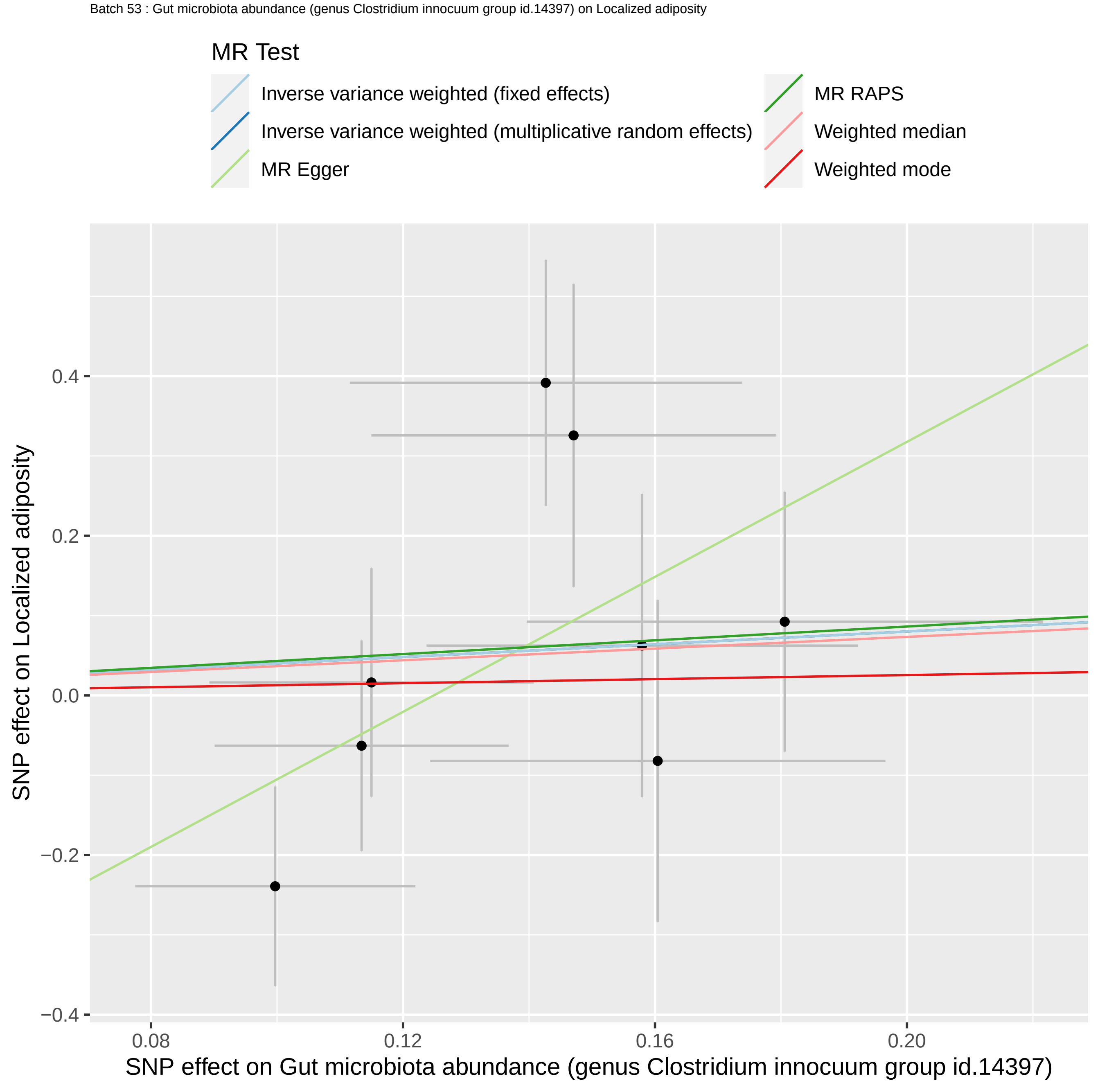

Batch 53 : Gut microbiota abundance (genus Clostridium innocuum group id.14397) on Localized adiposity
MR Test
Inverse variance weighted (fixed effects)
Inverse variance weighted (multiplicative random effects)
MR Egger
MR RAPS
Weighted median
Weighted mode
0.4
0.2
0.0
−0.2
−0.4
SNP effect on Localized adiposity
0.08
0.12
0.16
0.20
SNP effect on Gut microbiota abundance (genus Clostridium innocuum group id.14397)

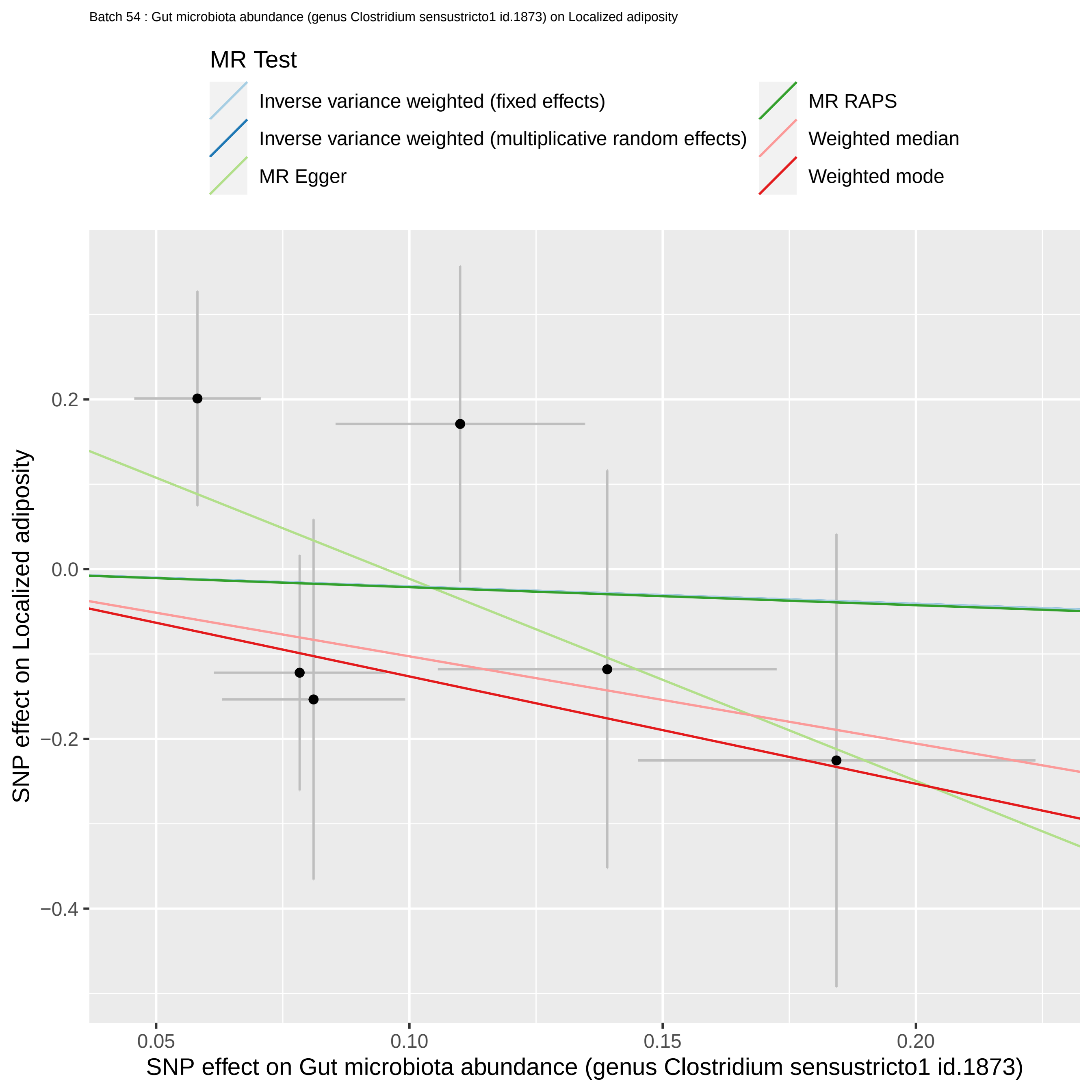
Batch 54 : Gut microbiota abundance (genus Clostridium sensustricto1 id.1873) on Localized adiposity
MR Test
Inverse variance weighted (fixed effects)
Inverse variance weighted (multiplicative random effects)
MR Egger
MR RAPS
Weighted median
Weighted mode
0.2
0.0
−0.2
−0.4
0.05
0.10
0.15
0.20
SNP effect on Localized adiposity
SNP effect on Gut microbiota abundance (genus Clostridium sensustricto1 id.1873)

Batch 55 : Gut microbiota abundance (genus Collinsella id.815) on Localized adiposity

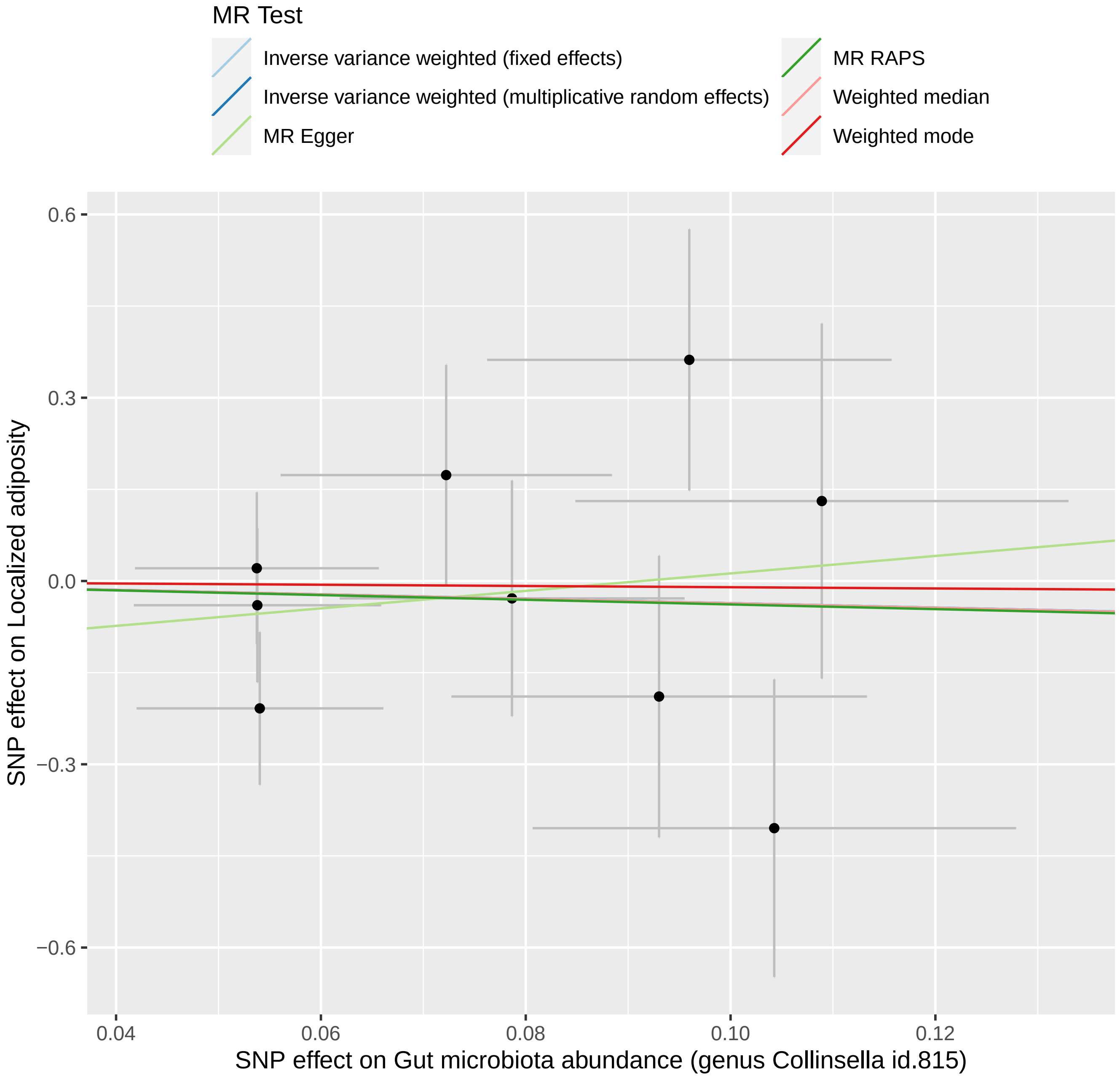

Batch 56 : Gut microbiota abundance (genus Coprobacter id.949) on Localized adiposity

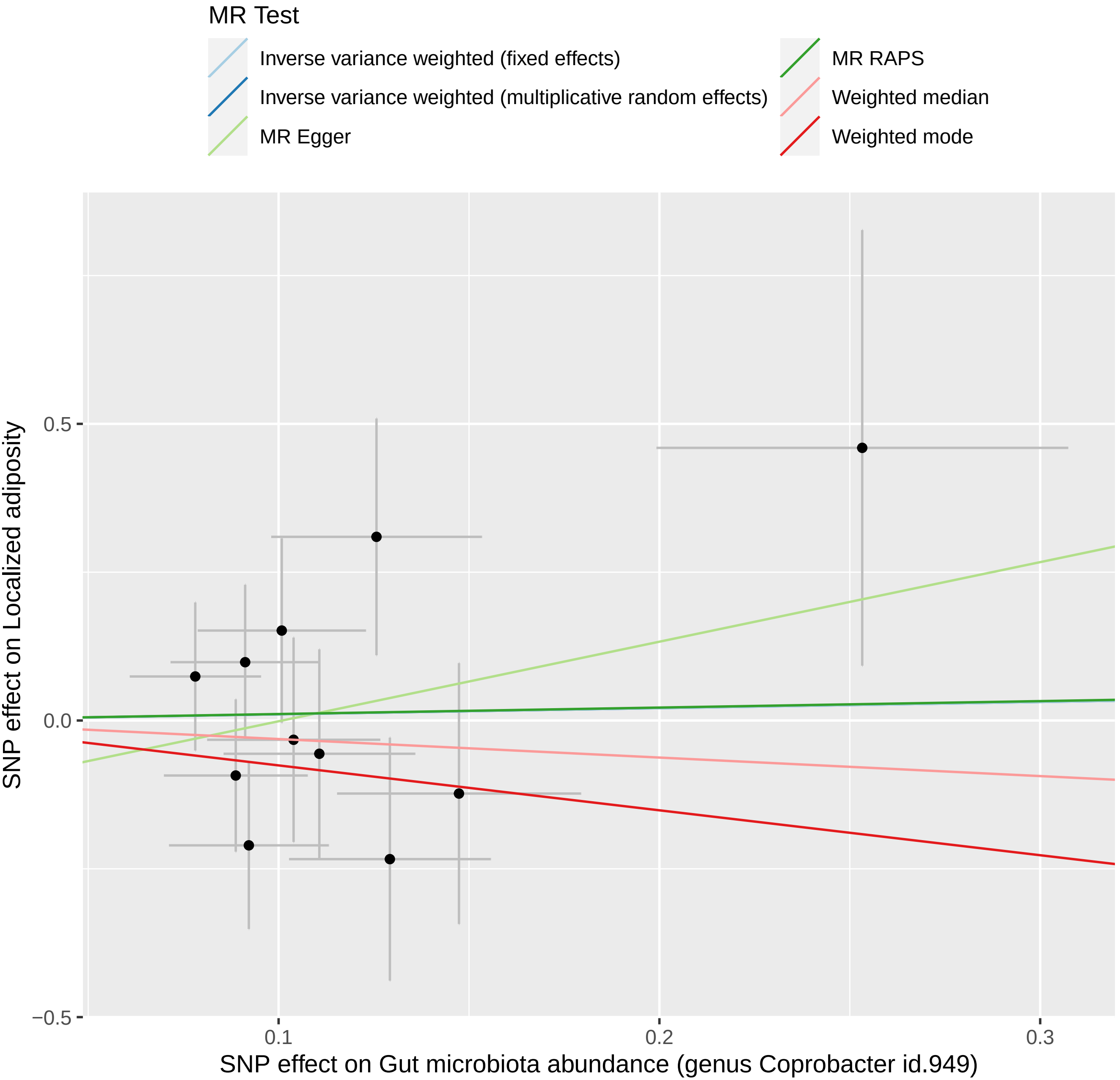

Batch 57 : Gut microbiota abundance (genus Coprococcus1 id.11301) on Localized adiposity

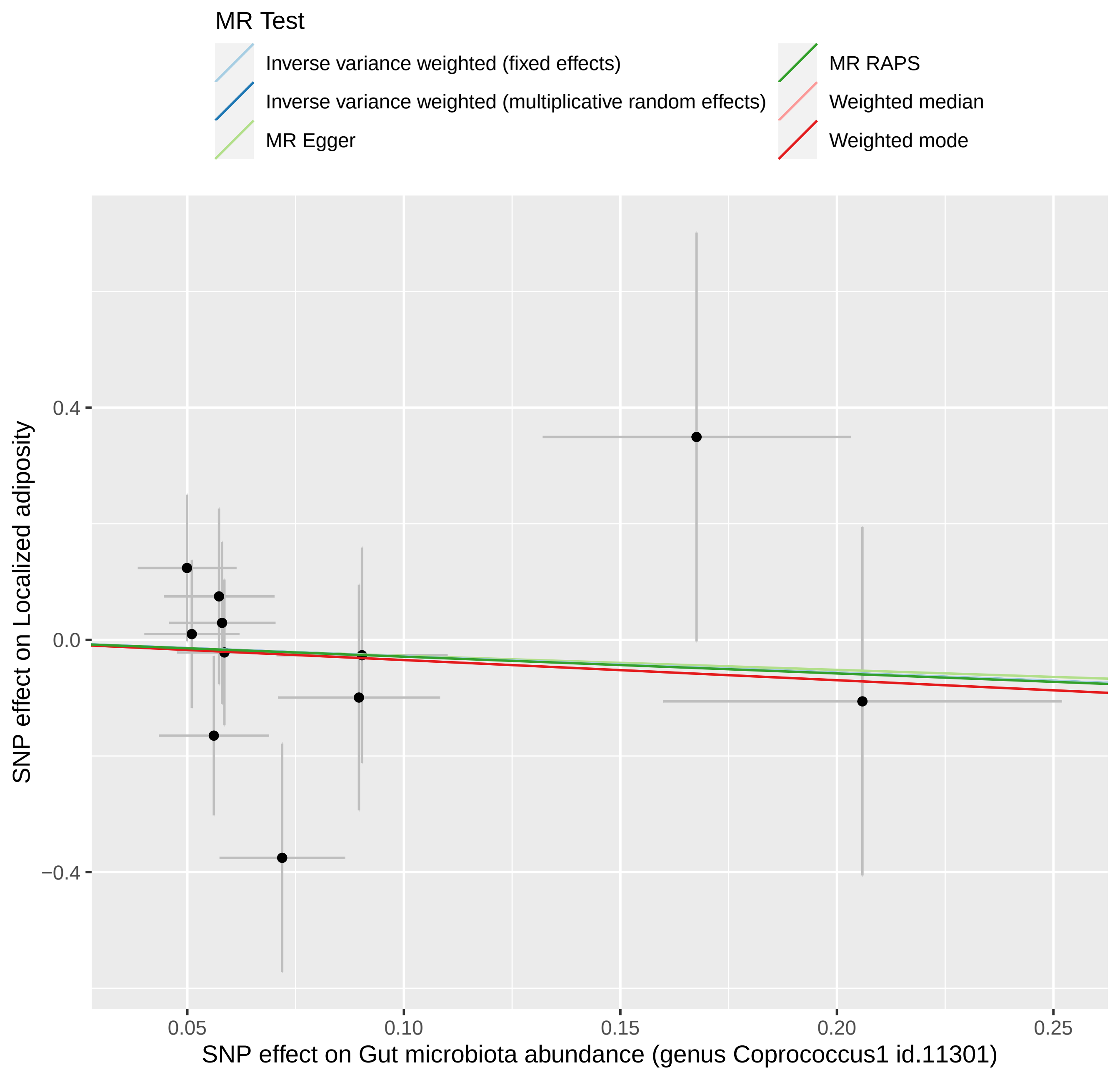

Batch 58 : Gut microbiota abundance (genus Coprococcus2 id.11302) on Localized adiposity

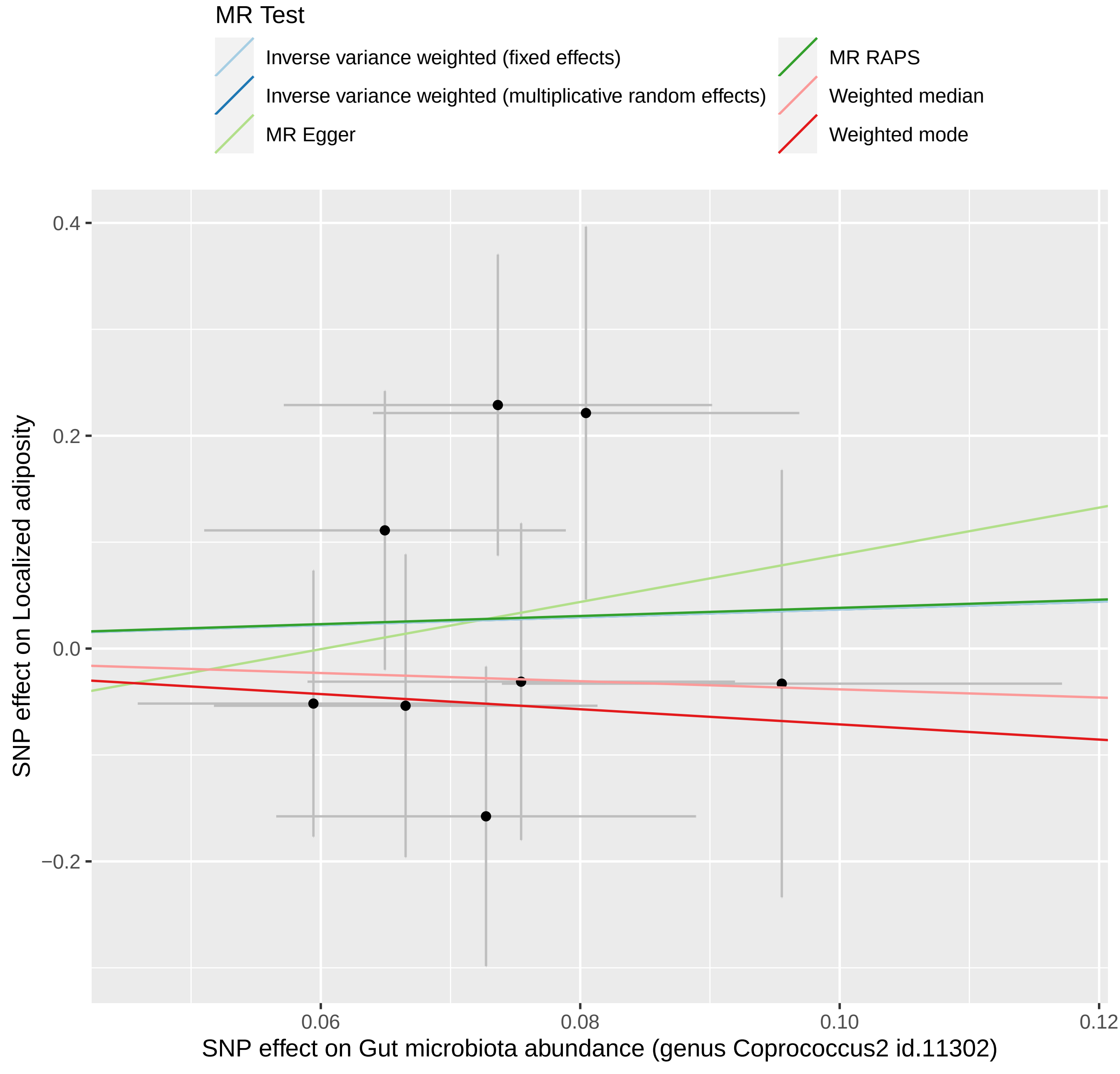

Batch 59 : Gut microbiota abundance (genus Coprococcus3 id.11303) on Localized adiposity

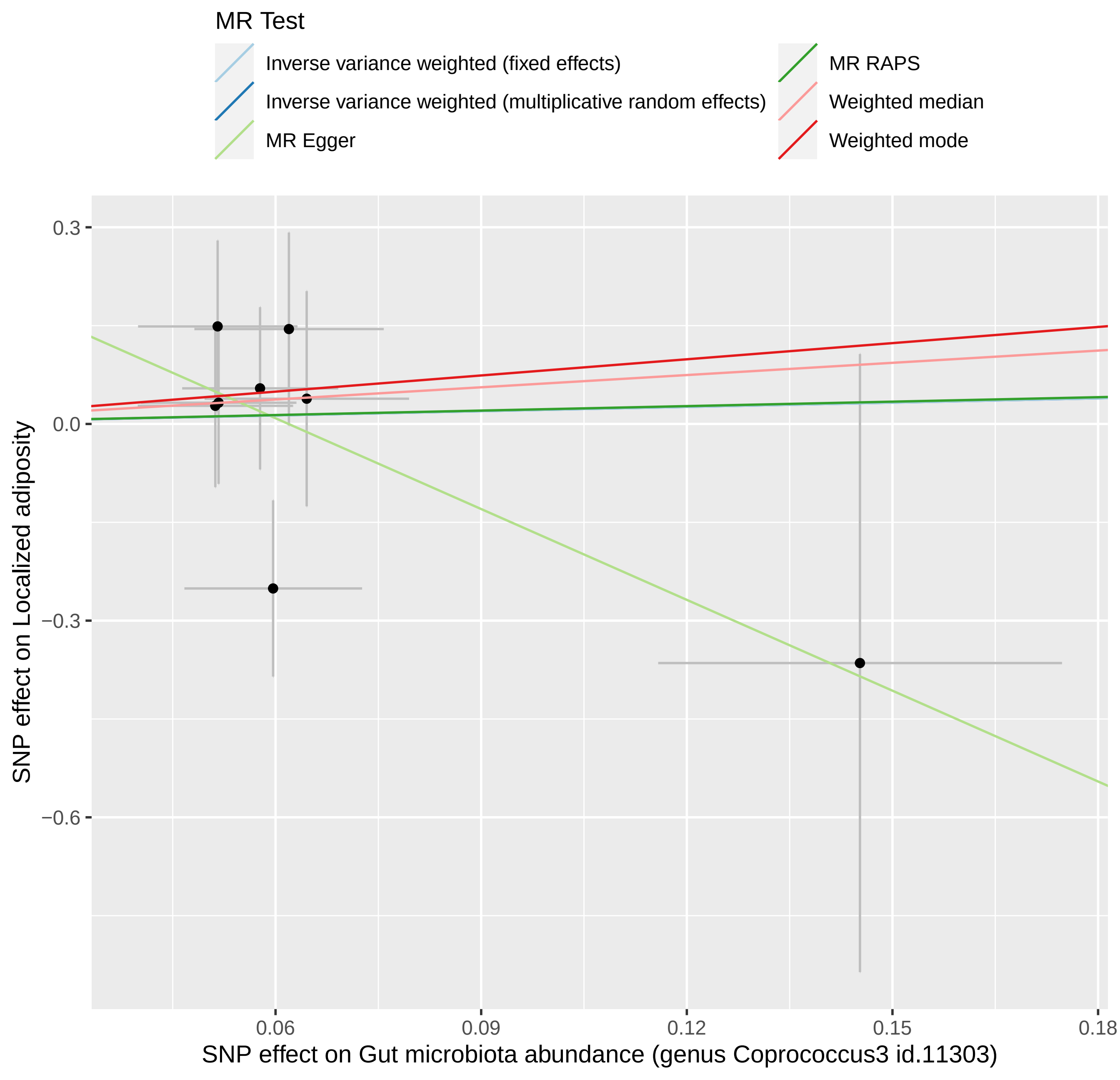

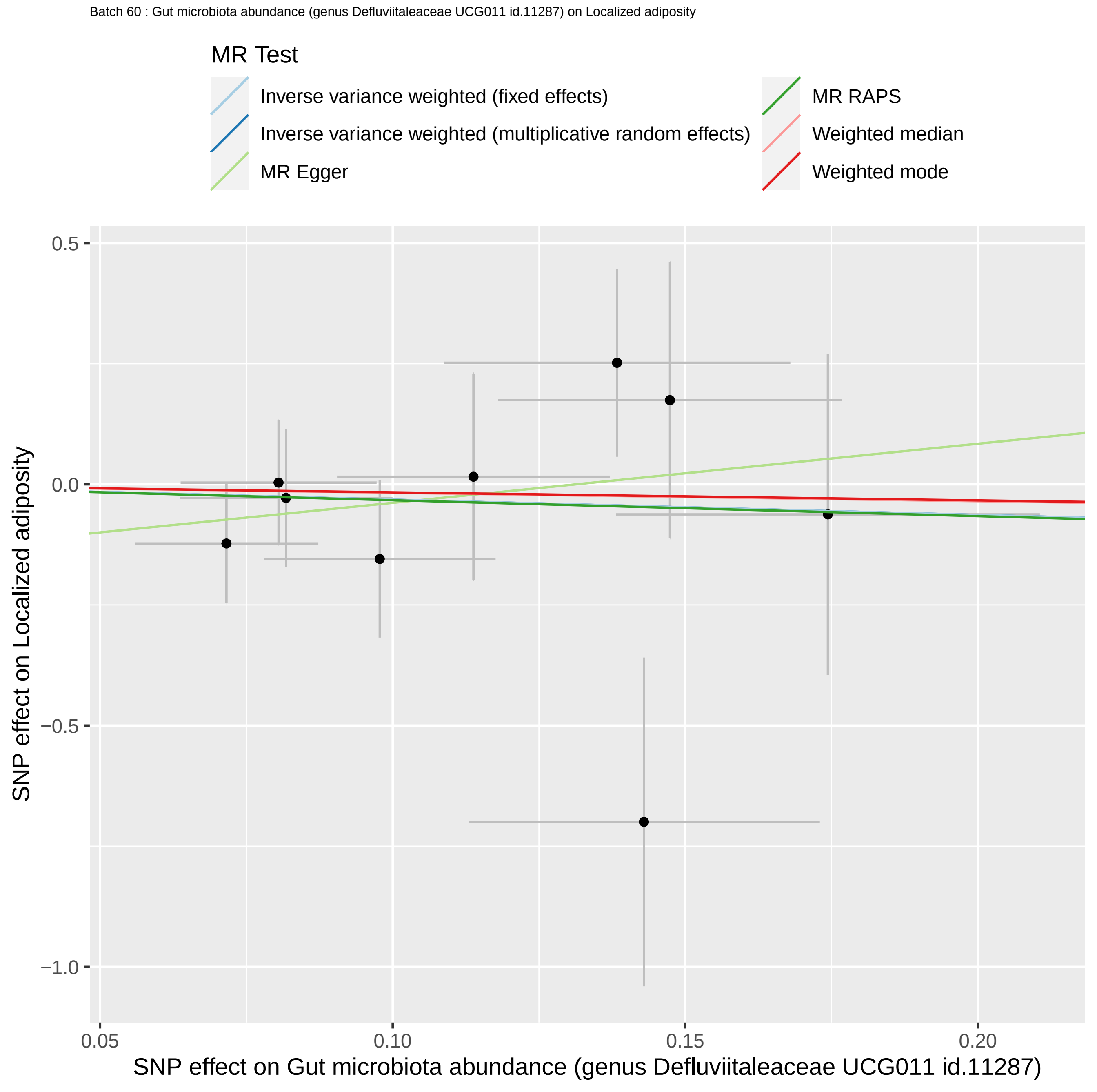
Batch 60 : Gut microbiota abundance (genus Defluviitaleaceae UCG011 id.11287) on Localized adiposity
MR Test
Inverse variance weighted (fixed effects)
Inverse variance weighted (multiplicative random effects)
MR Egger
MR RAPS
Weighted median
Weighted mode
0.5
0.0
−0.5
−1.0
0.05
0.10
0.15
0.20
SNP effect on Localized adiposity
SNP effect on Gut microbiota abundance (genus Defluviitaleaceae UCG011 id.11287)

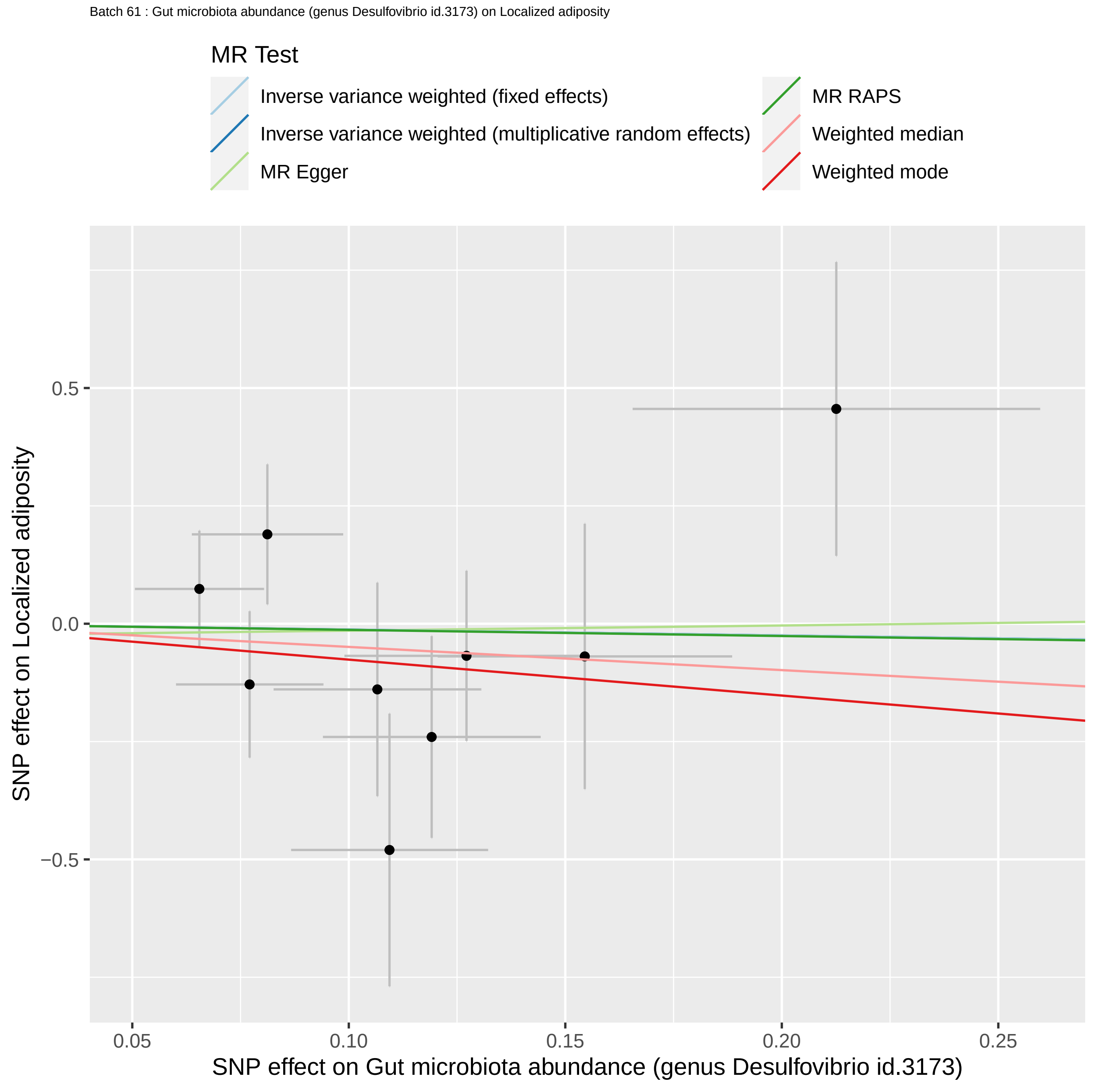

Batch 61 : Gut microbiota abundance (genus Desulfovibrio id.3173) on Localized adiposity
MR Test
Inverse variance weighted (fixed effects)
Inverse variance weighted (multiplicative random effects)
MR Egger
MR RAPS
Weighted median
Weighted mode
0.5
0.0
−0.5
0.05
0.10
0.15
0.20
0.25
SNP effect on Localized adiposity
SNP effect on Gut microbiota abundance (genus Desulfovibrio id.3173)

Batch 62 : Gut microbiota abundance (genus Dialister id.2183) on Localized adiposity

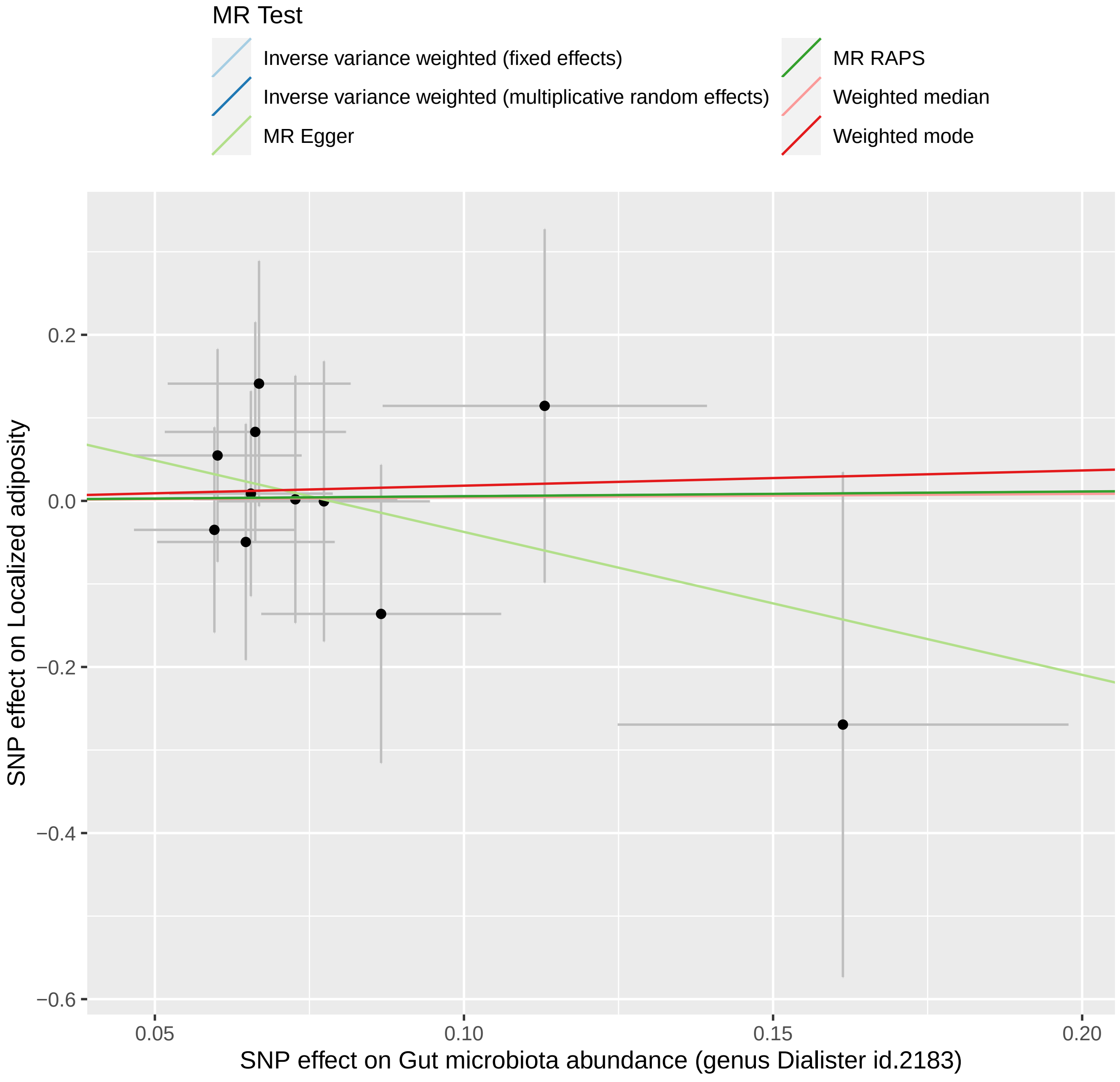

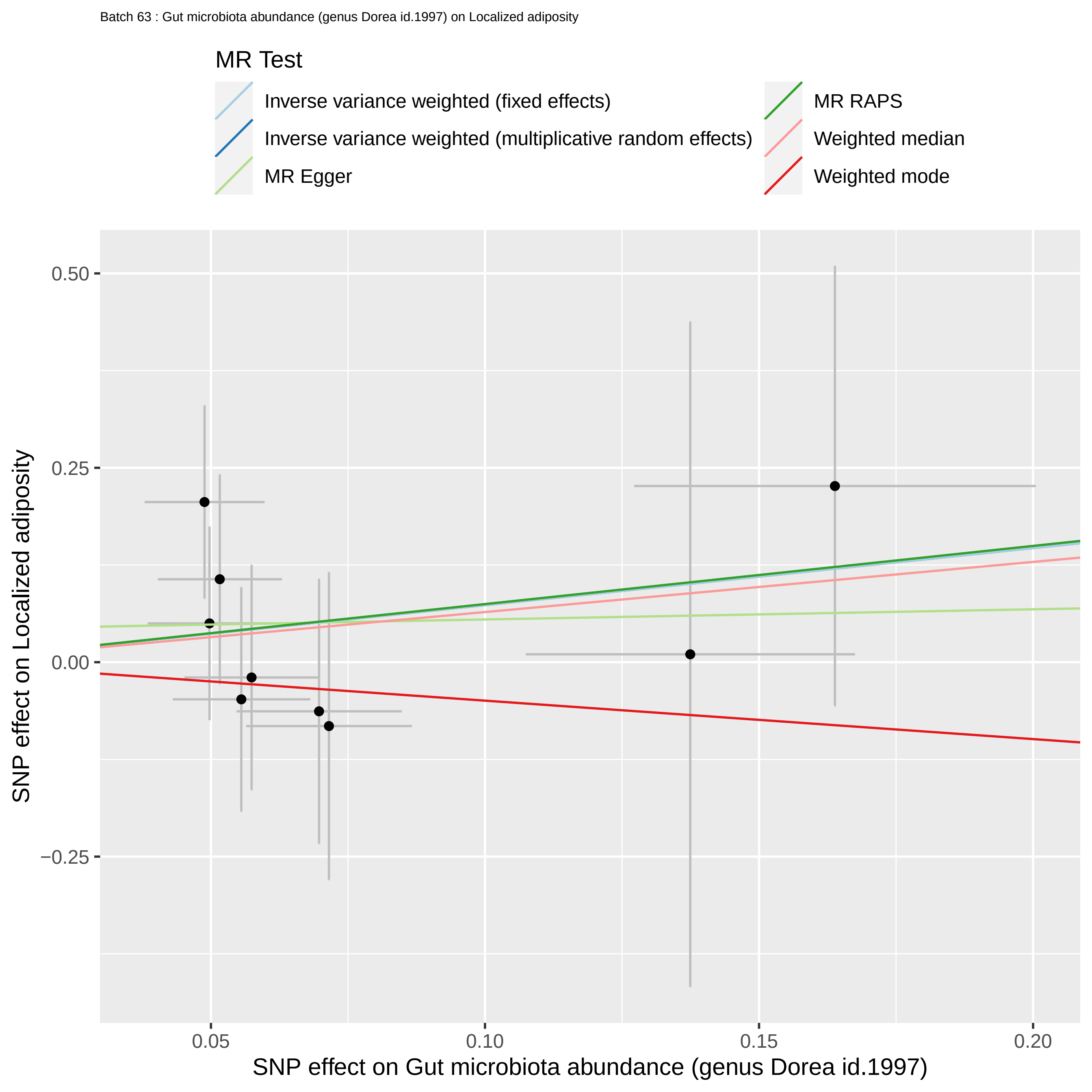

Batch 63 : Gut microbiota abundance (genus Dorea id.1997) on Localized adiposity
MR Test
Inverse variance weighted (fixed effects)
Inverse variance weighted (multiplicative random effects)
MR Egger
MR RAPS
Weighted median
Weighted mode
0.50
0.25
0.00
−0.25
0.05
0.10
0.15
0.20
SNP effect on Localized adiposity
SNP effect on Gut microbiota abundance (genus Dorea id.1997)

Batch 64 : Gut microbiota abundance (genus Eggerthella id.819) on Localized adiposity

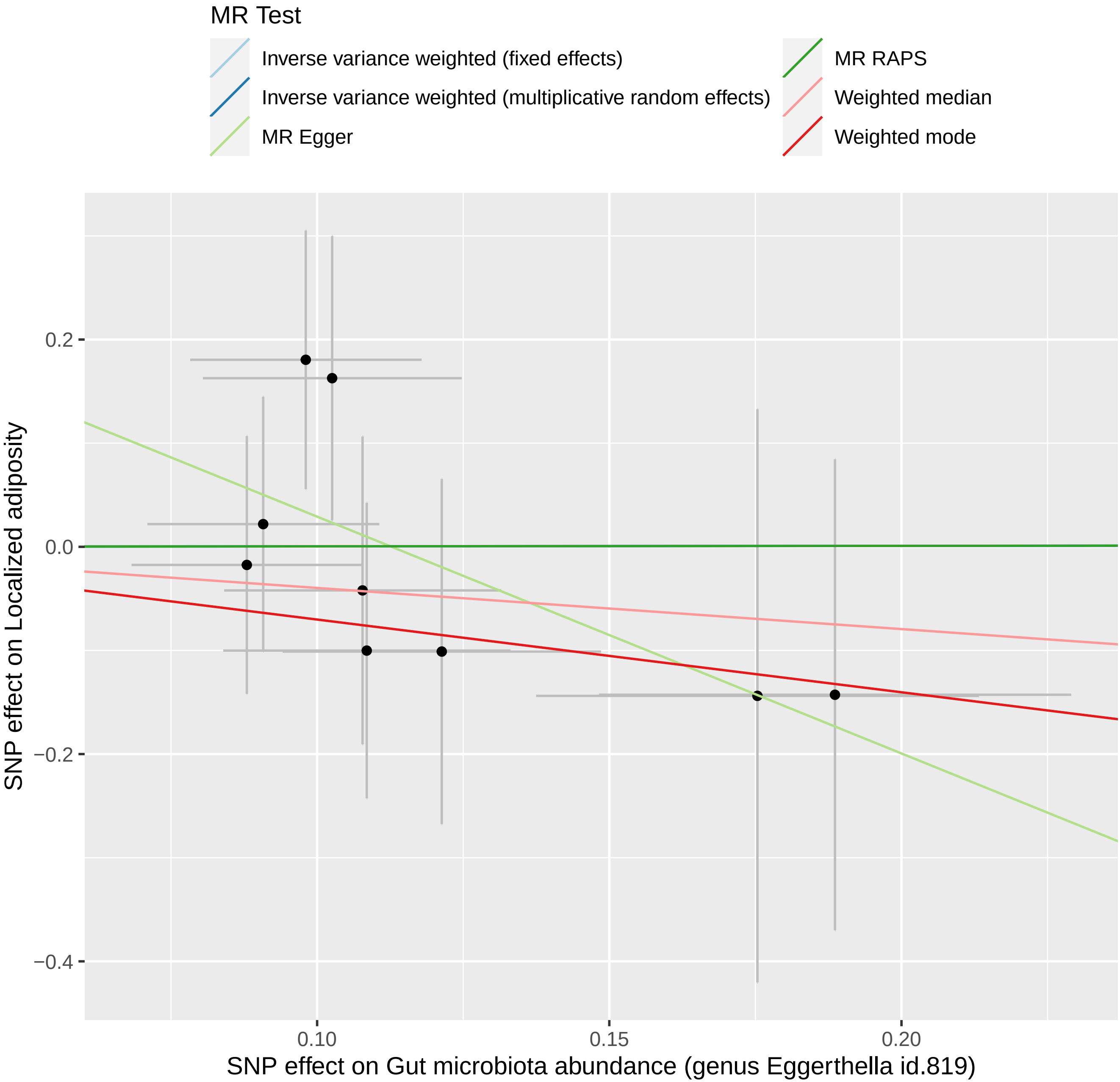

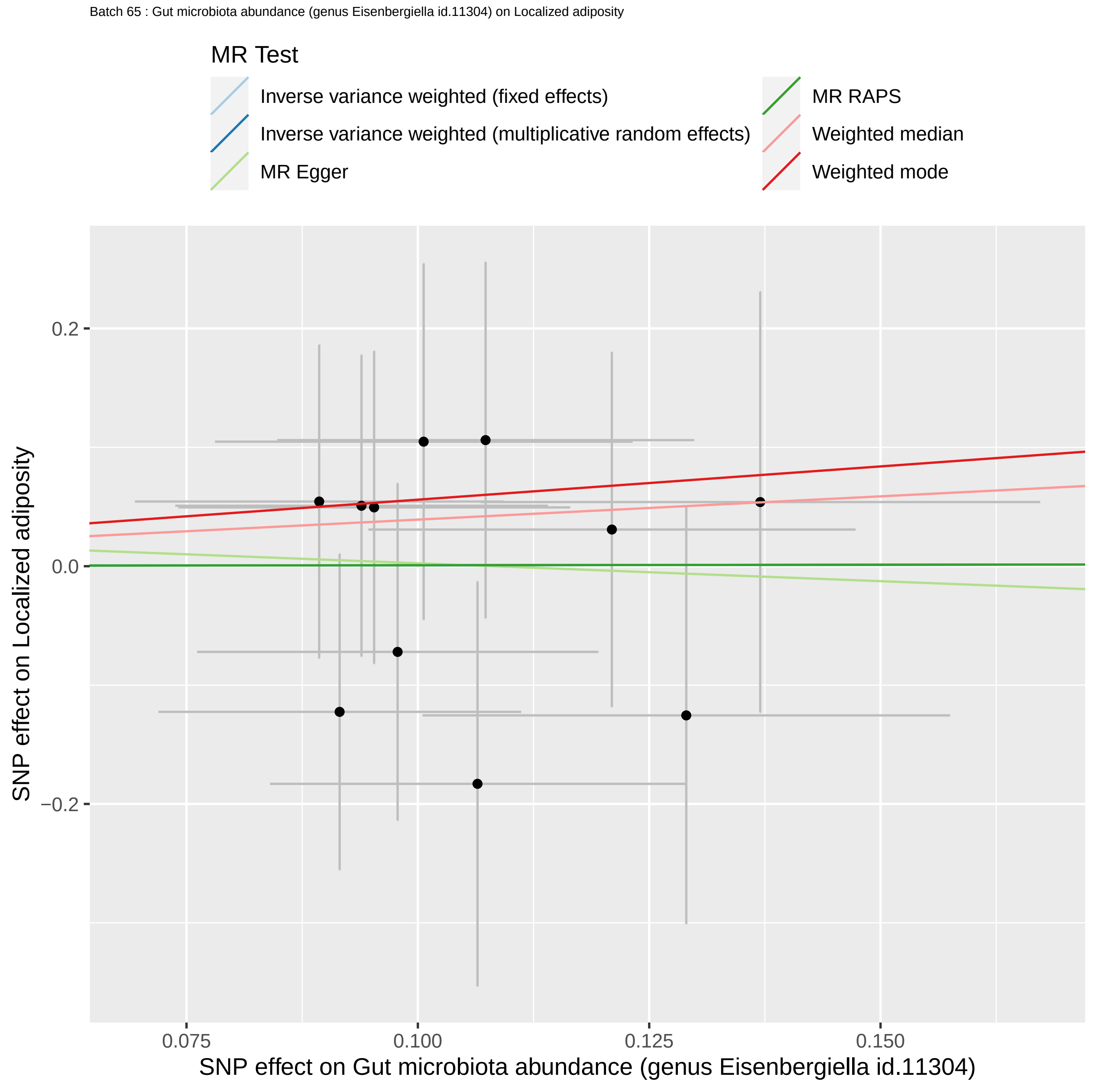
Batch 65 : Gut microbiota abundance (genus Eisenbergiella id.11304) on Localized adiposity
MR Test
Inverse variance weighted (fixed effects)
Inverse variance weighted (multiplicative random effects)
MR Egger
MR RAPS
Weighted median
Weighted mode
0.2
0.0
−0.2
0.075
0.100
0.125
0.150
SNP effect on Localized adiposity
SNP effect on Gut microbiota abundance (genus Eisenbergiella id.11304)

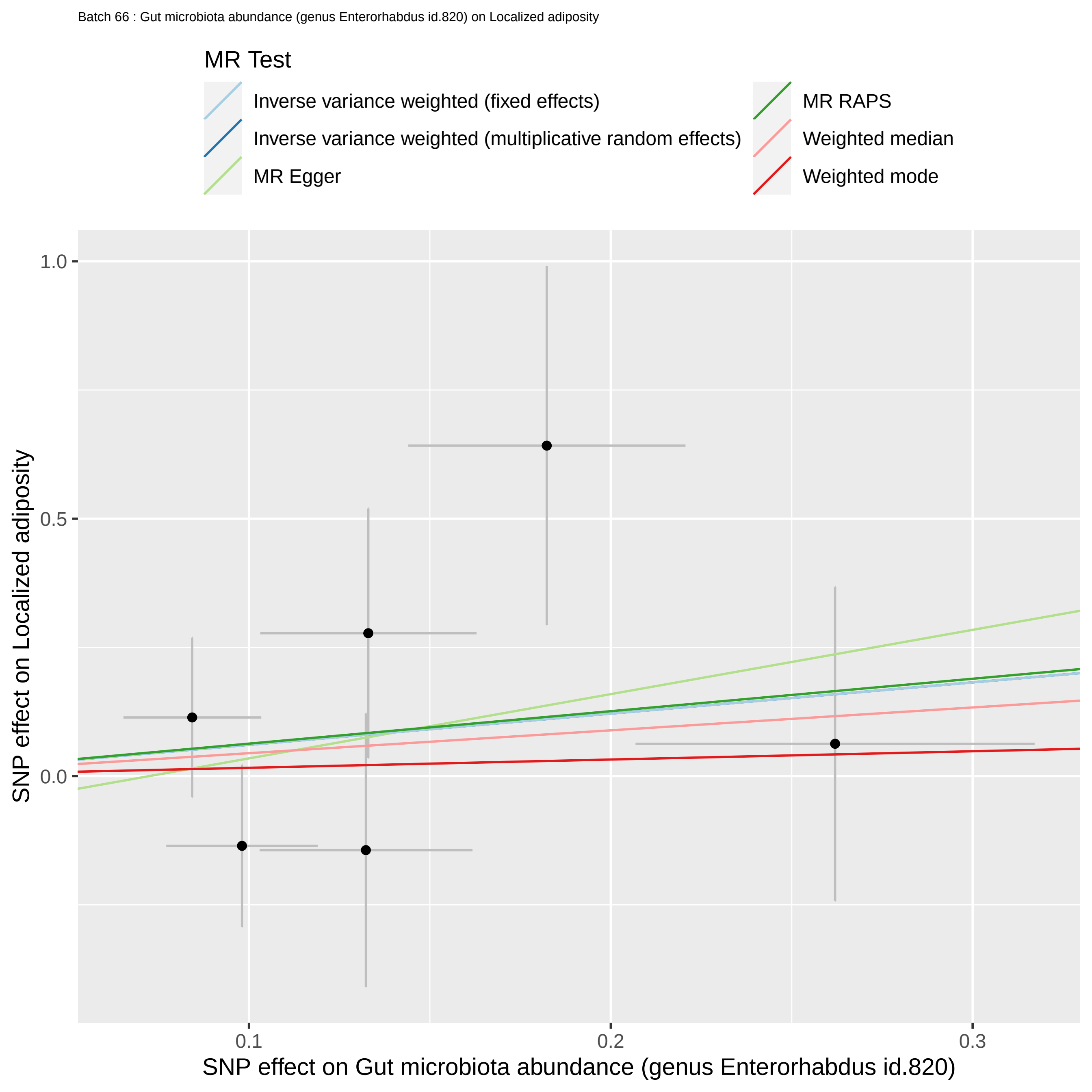

Batch 66 : Gut microbiota abundance (genus Enterorhabdus id.820) on Localized adiposity
MR Test
Inverse variance weighted (fixed effects)
Inverse variance weighted (multiplicative random effects)
MR Egger
MR RAPS
Weighted median
Weighted mode
SNP effect on Localized adiposity
1.0
0.5
0.0
0.1
0.2
0.3
SNP effect on Gut microbiota abundance (genus Enterorhabdus id.820)

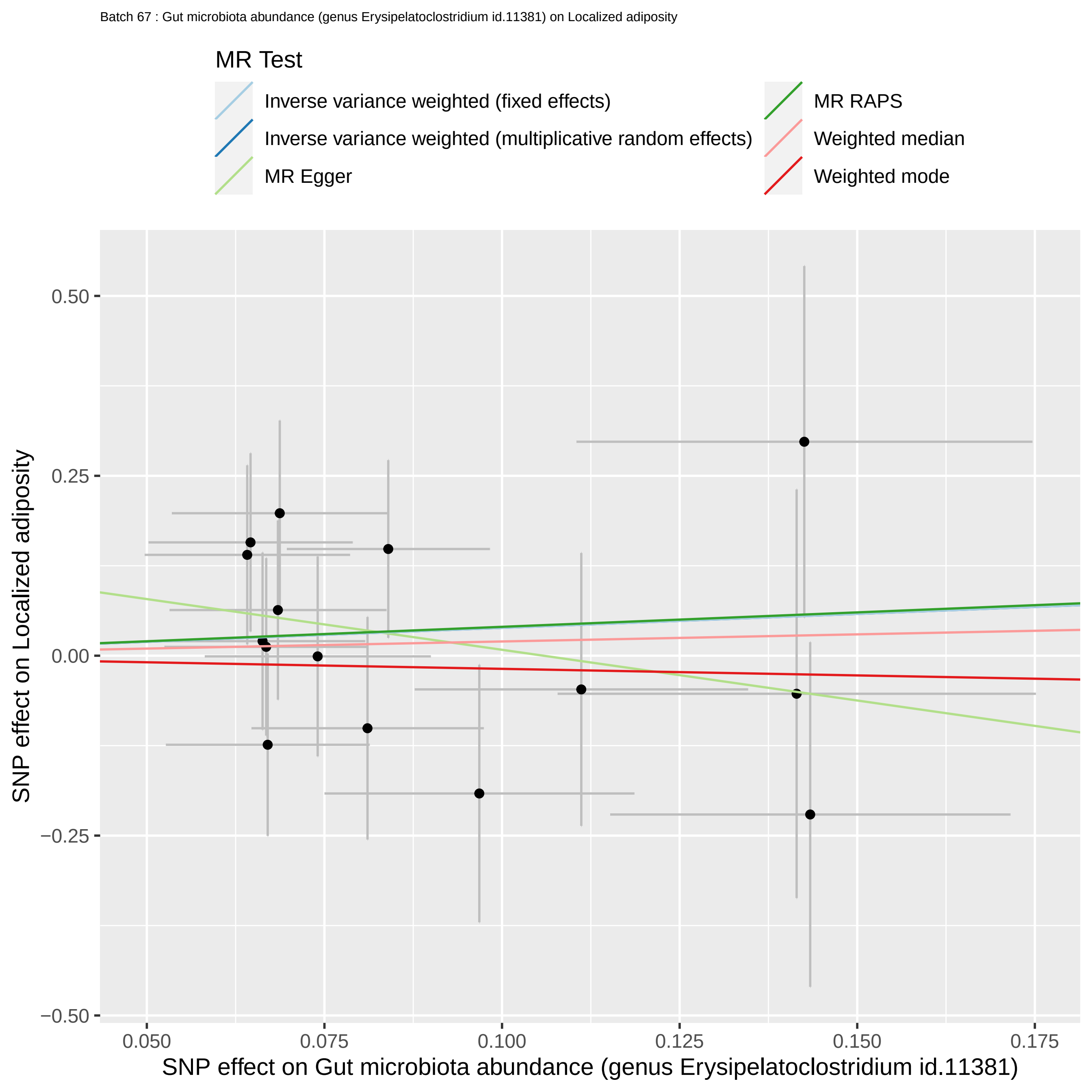
Batch 67 : Gut microbiota abundance (genus Erysipelatoclostridium id.11381) on Localized adiposity
MR Test
Inverse variance weighted (fixed effects)
Inverse variance weighted (multiplicative random effects)
MR Egger
MR RAPS
Weighted median
Weighted mode
0.50
0.25
0.00
−0.25
−0.50
0.050
0.075
0.100
0.125
0.150
0.175
SNP effect on Localized adiposity
SNP effect on Gut microbiota abundance (genus Erysipelatoclostridium id.11381)

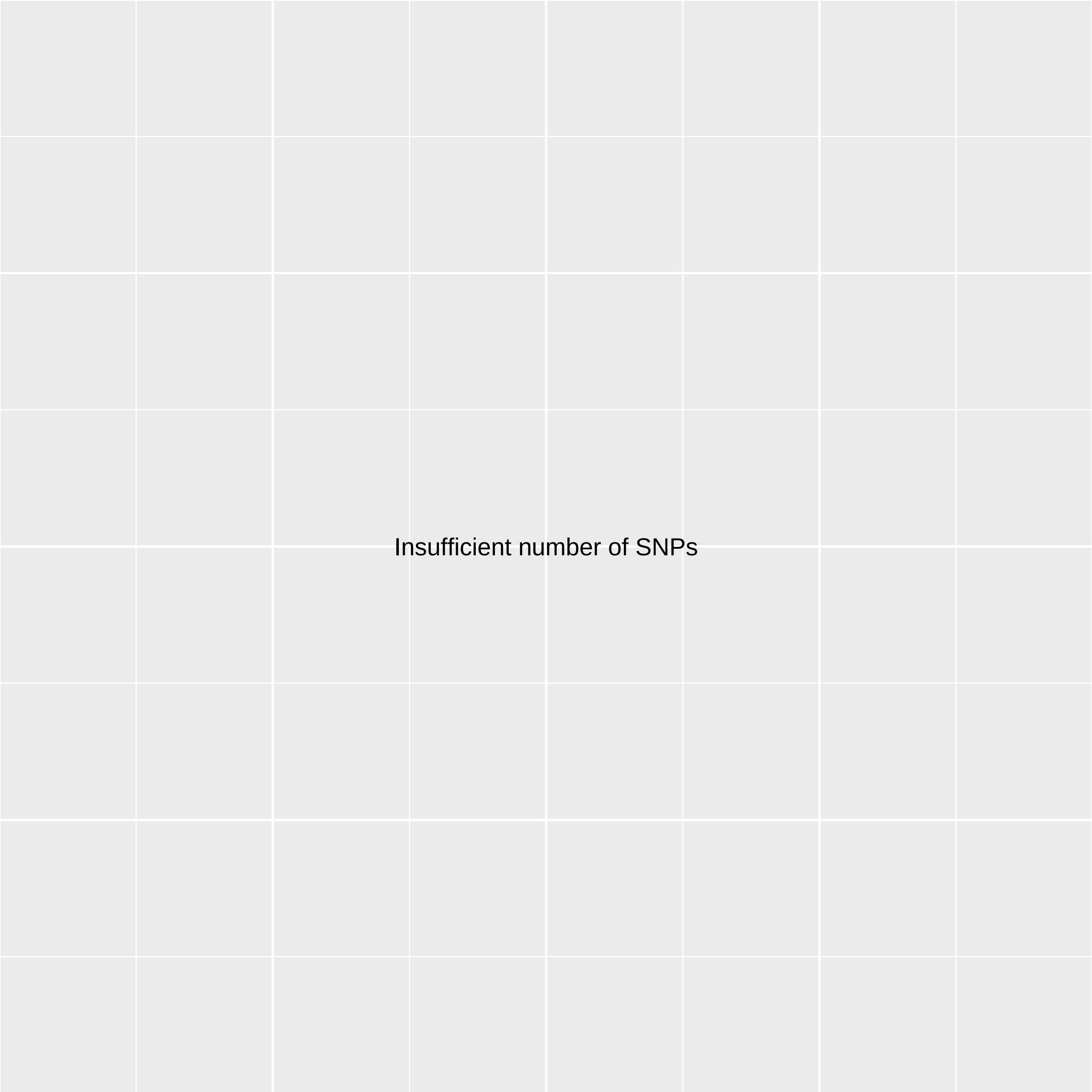
Insufficient number of SNPs

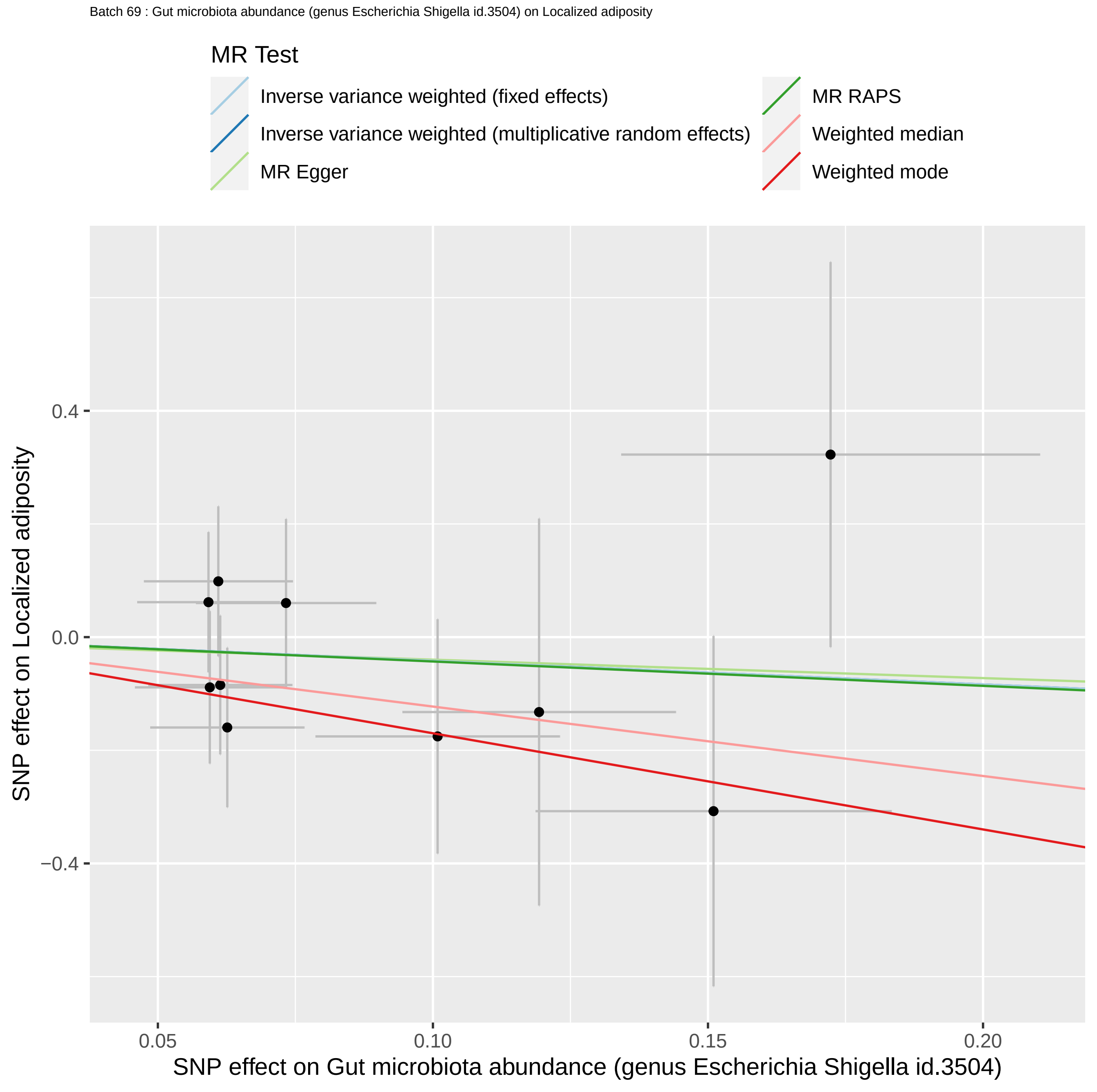
Batch 69 : Gut microbiota abundance (genus Escherichia Shigella id.3504) on Localized adiposity
MR Test
Inverse variance weighted (fixed effects)
Inverse variance weighted (multiplicative random effects)
MR Egger
MR RAPS
Weighted median
Weighted mode
0.4
0.0
−0.4
SNP effect on Localized adiposity
0.05
0.10
0.15
0.20
SNP effect on Gut microbiota abundance (genus Escherichia Shigella id.3504)

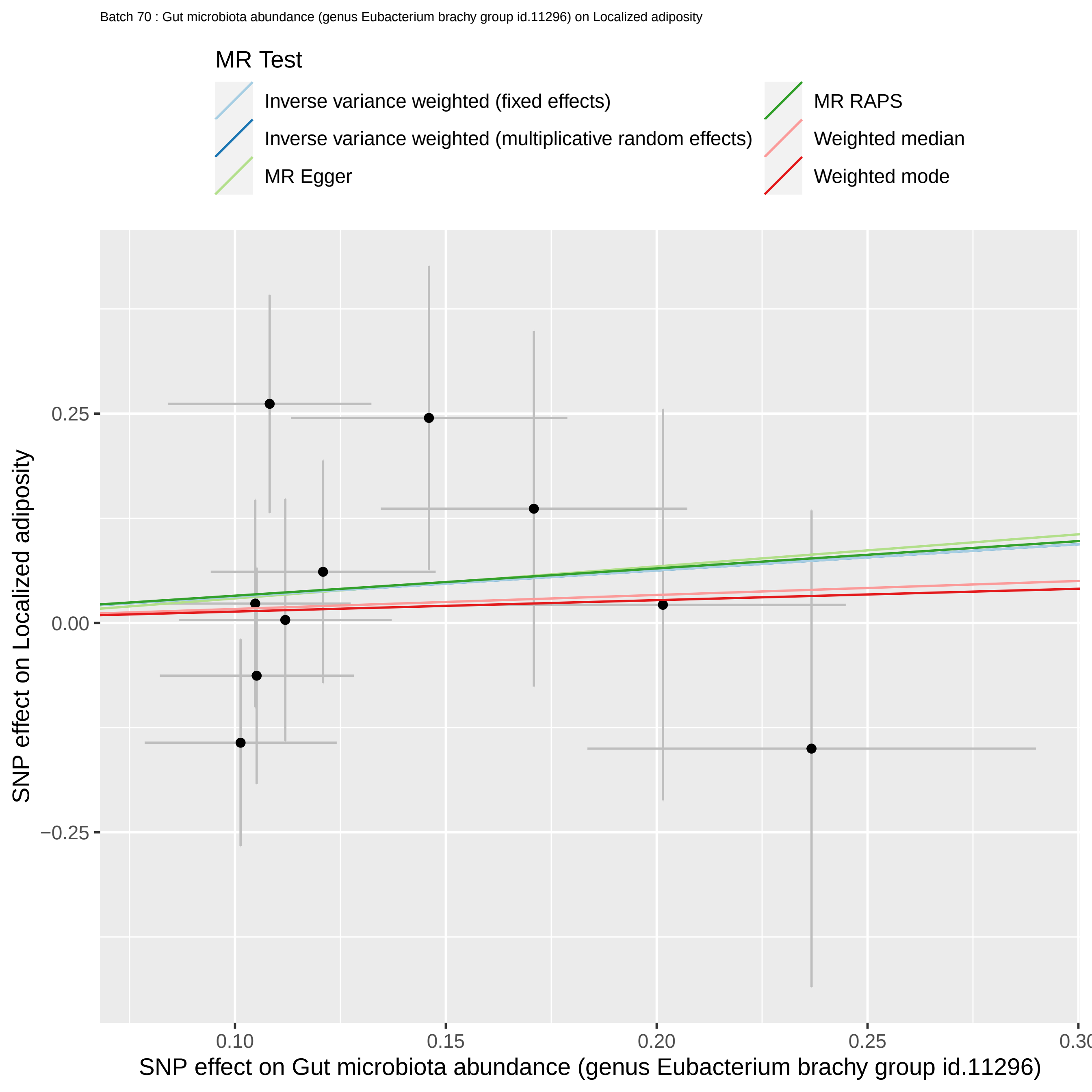
Batch 70 : Gut microbiota abundance (genus Eubacterium brachy group id.11296) on Localized adiposity
MR Test
Inverse variance weighted (fixed effects)
Inverse variance weighted (multiplicative random effects)
MR Egger
MR RAPS
Weighted median
Weighted mode
0.25
0.00
−0.25
0.10
0.15
0.20
0.25
0.30
SNP effect on Gut microbiota abundance (genus Eubacterium brachy group id.11296)
SNP effect on Localized adiposity

Batch 71 : Gut microbiota abundance (genus Eubacterium coprostanoligenes group id.11375) on Localized adiposity

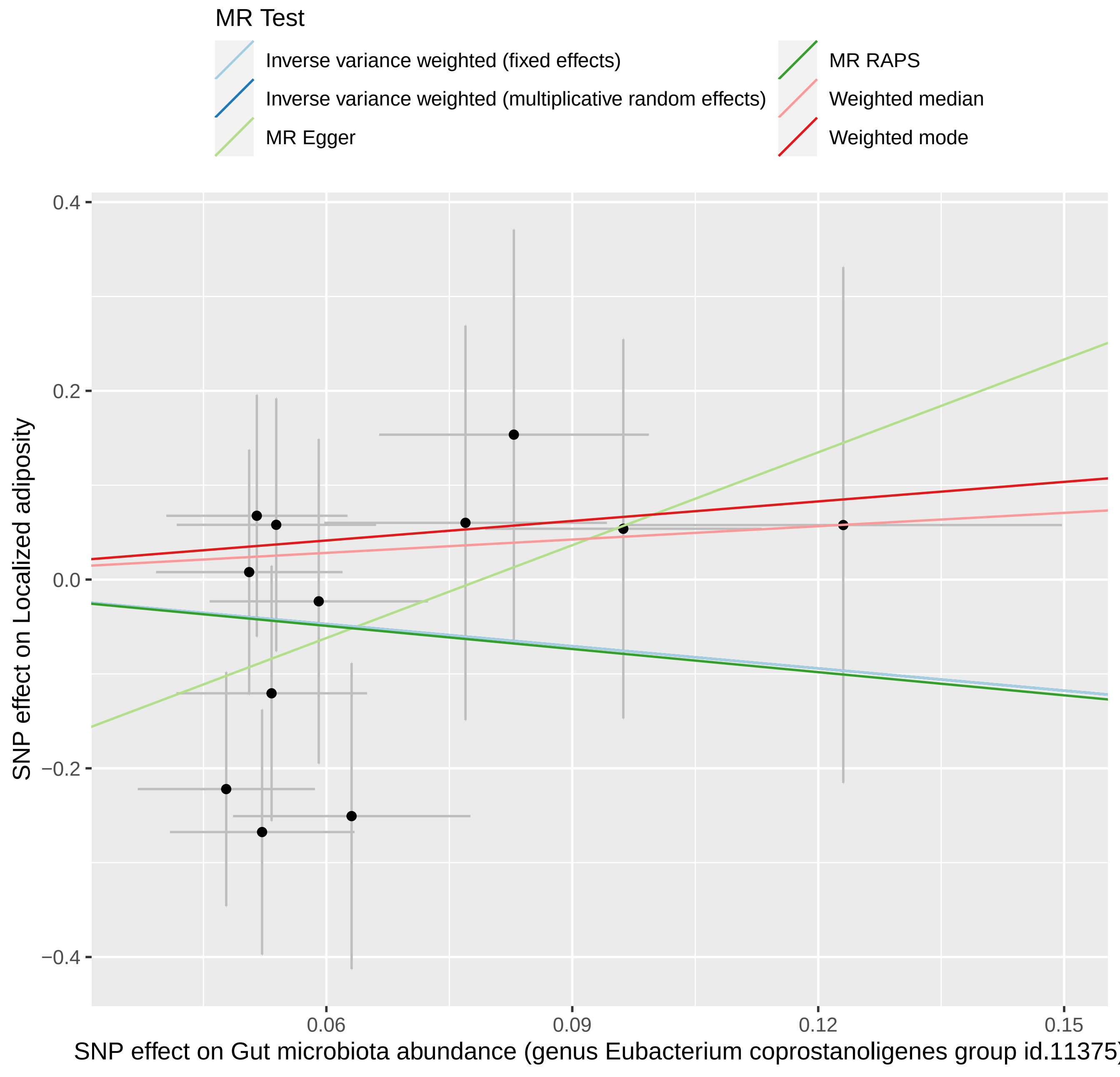

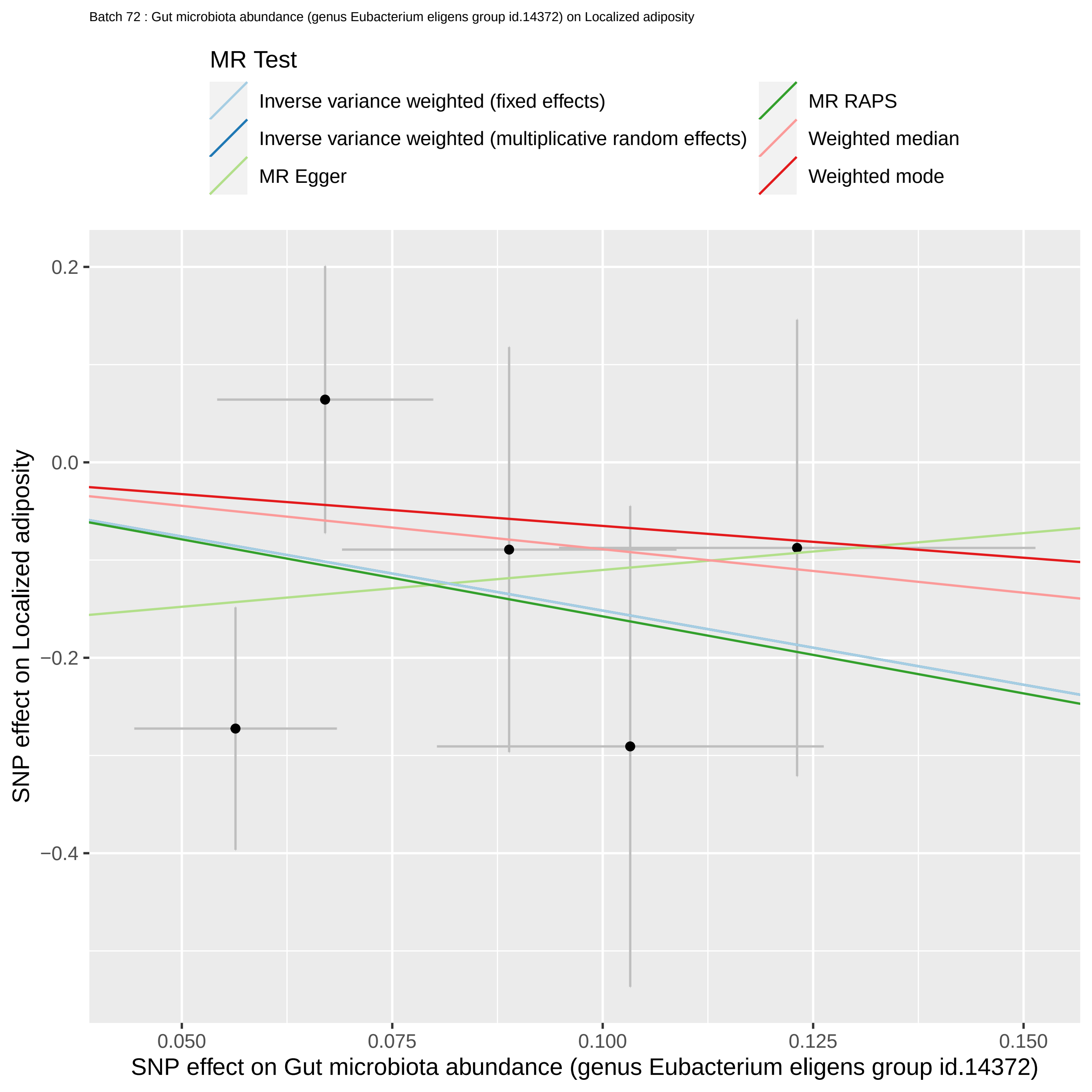

Batch 72 : Gut microbiota abundance (genus Eubacterium eligens group id.14372) on Localized adiposity
MR Test
Inverse variance weighted (fixed effects)
Inverse variance weighted (multiplicative random effects)
MR Egger
MR RAPS
Weighted median
Weighted mode
0.2
0.0
−0.2
−0.4
SNP effect on Localized adiposity
0.050
0.075
0.100
0.125
0.150
SNP effect on Gut microbiota abundance (genus Eubacterium eligens group id.14372)

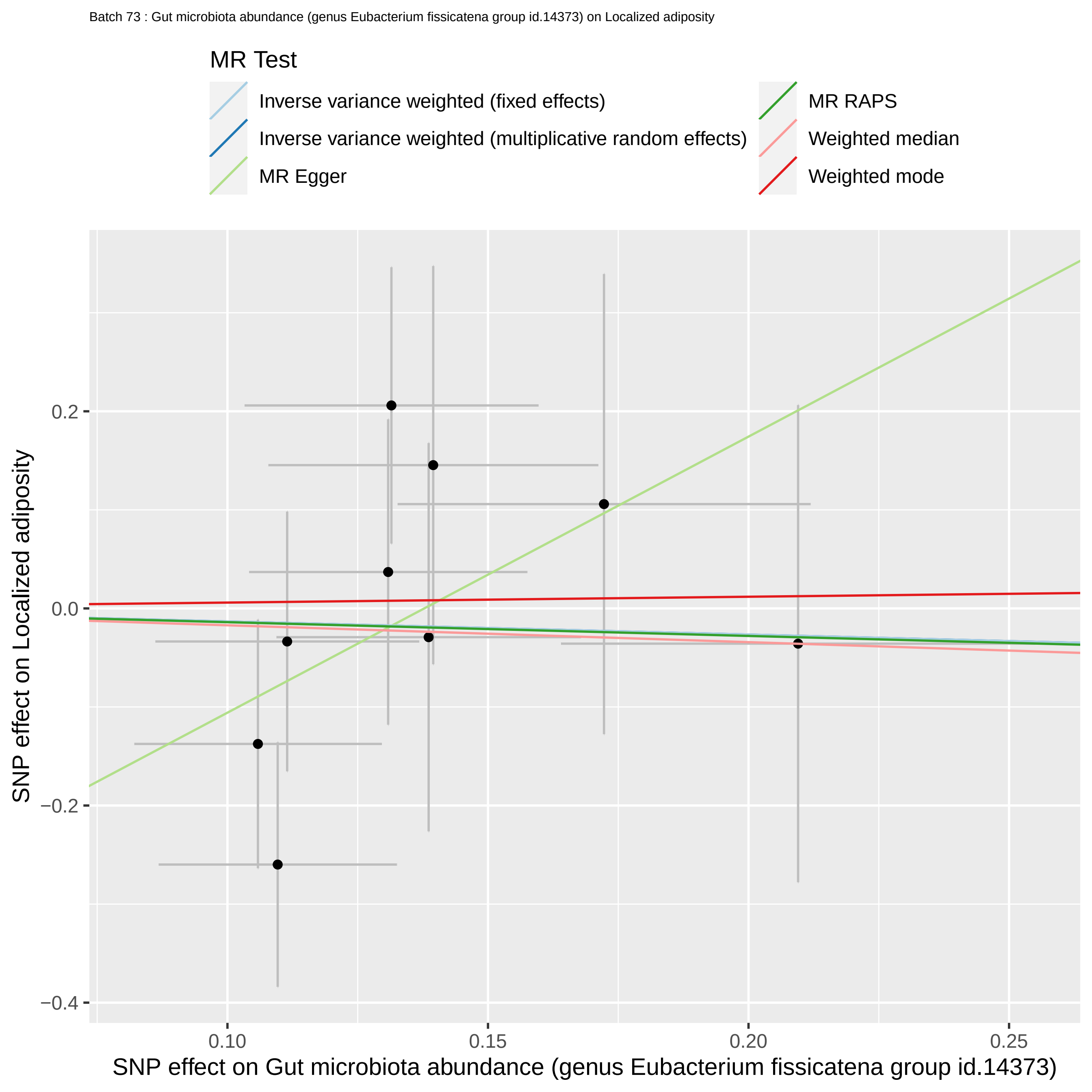

Batch 73 : Gut microbiota abundance (genus Eubacterium fissicatena group id.14373) on Localized adiposity
MR Test
Inverse variance weighted (fixed effects)
Inverse variance weighted (multiplicative random effects)
MR Egger
MR RAPS
Weighted median
Weighted mode
SNP effect on Localized adiposity
0.2
0.0
−0.2
−0.4
0.10
0.15
0.20
0.25
SNP effect on Gut microbiota abundance (genus Eubacterium fissicatena group id.14373)

Batch 74 : Gut microbiota abundance (genus Eubacterium hallii group id.11338) on Localized adiposity

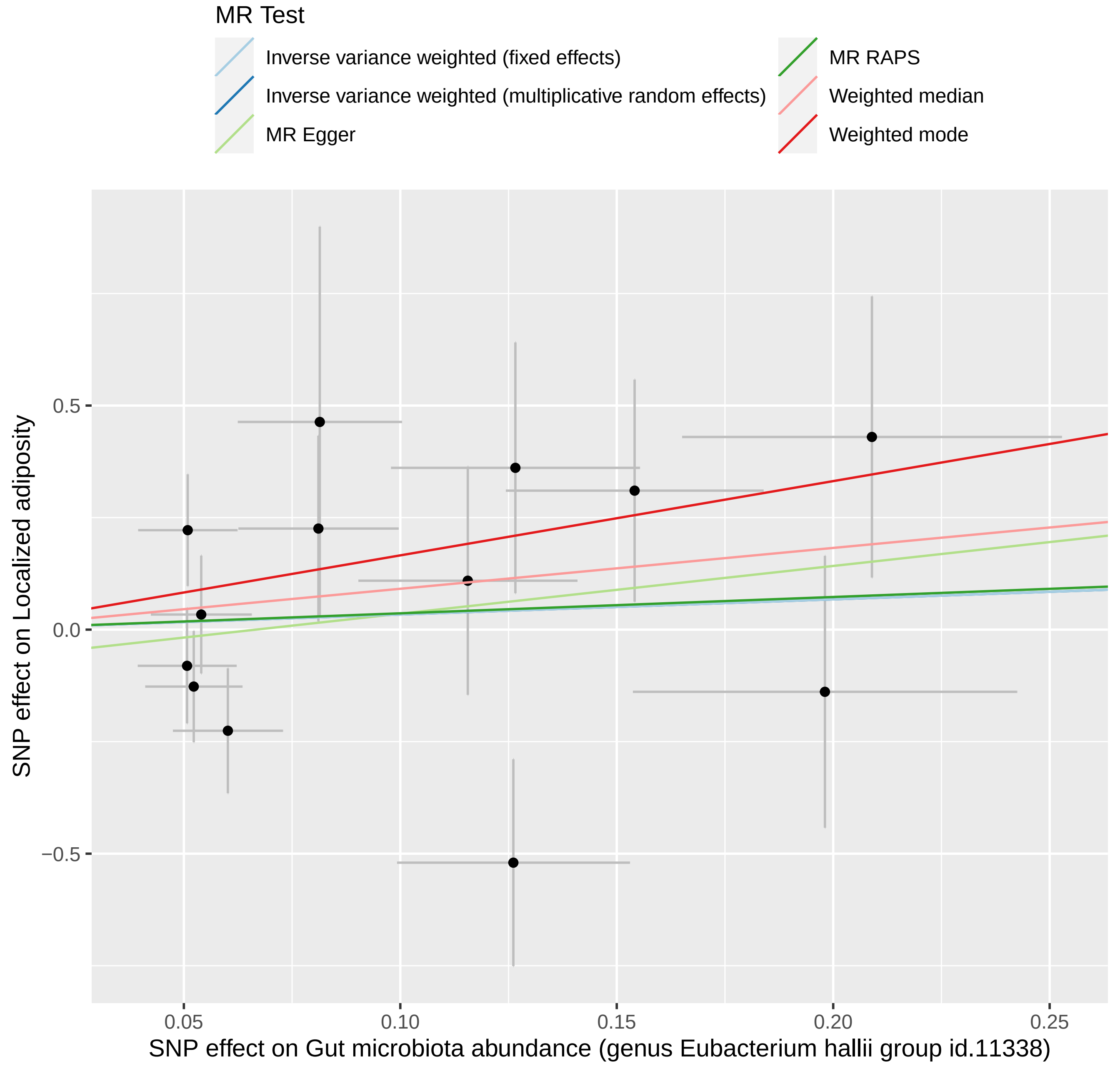

Batch 75 : Gut microbiota abundance (genus Eubacterium nodatum group id.11297) on Localized adiposity

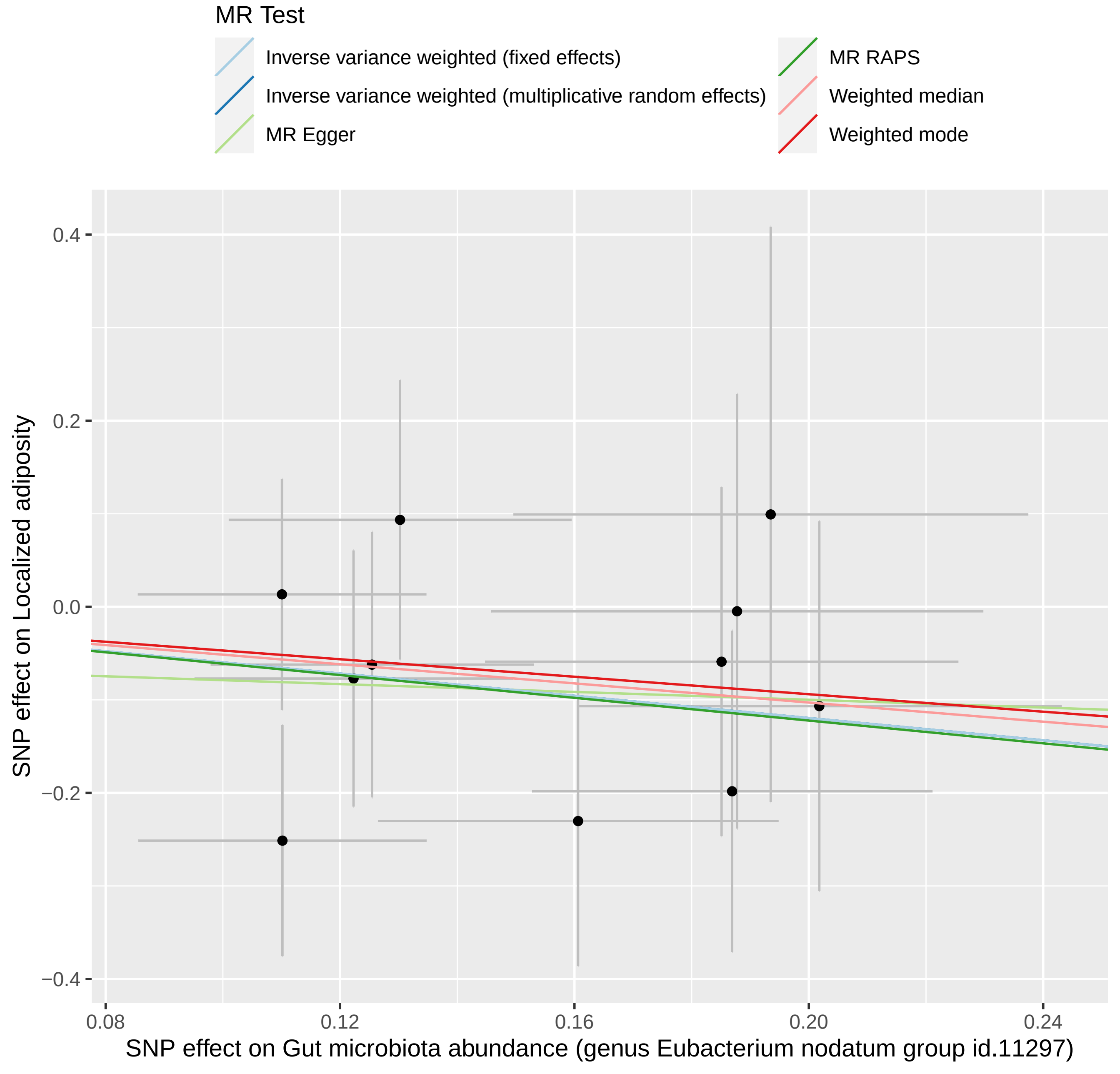

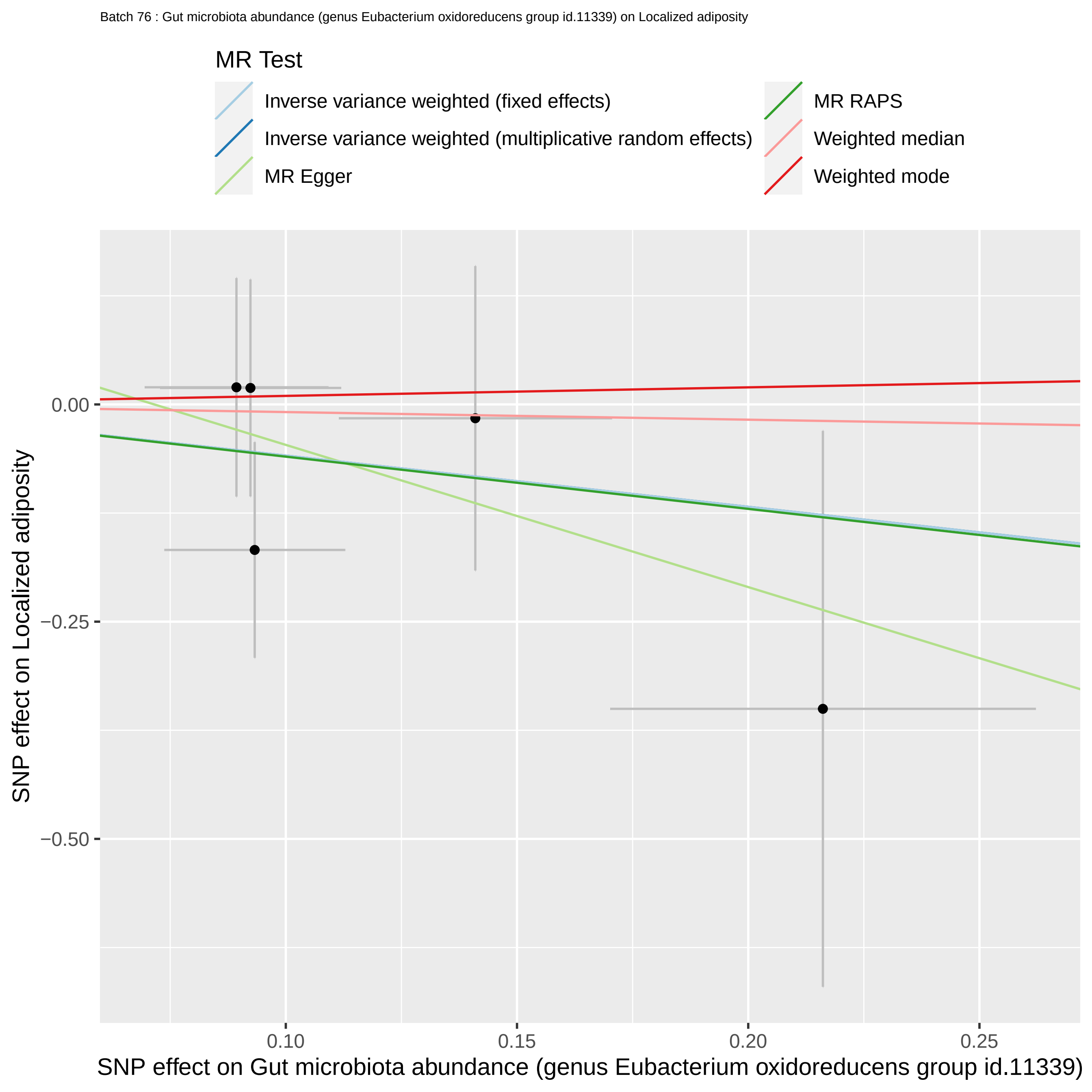
Batch 76 : Gut microbiota abundance (genus Eubacterium oxidoreducens group id.11339) on Localized adiposity
MR Test
Inverse variance weighted (fixed effects)
Inverse variance weighted (multiplicative random effects)
MR Egger
MR RAPS
Weighted median
Weighted mode
0.00
−0.25
−0.50
0.10
0.15
0.20
0.25
SNP effect on Localized adiposity
SNP effect on Gut microbiota abundance (genus Eubacterium oxidoreducens group id.11339)

Batch 77 : Gut microbiota abundance (genus Eubacterium rectale group id.14374) on Localized adiposity

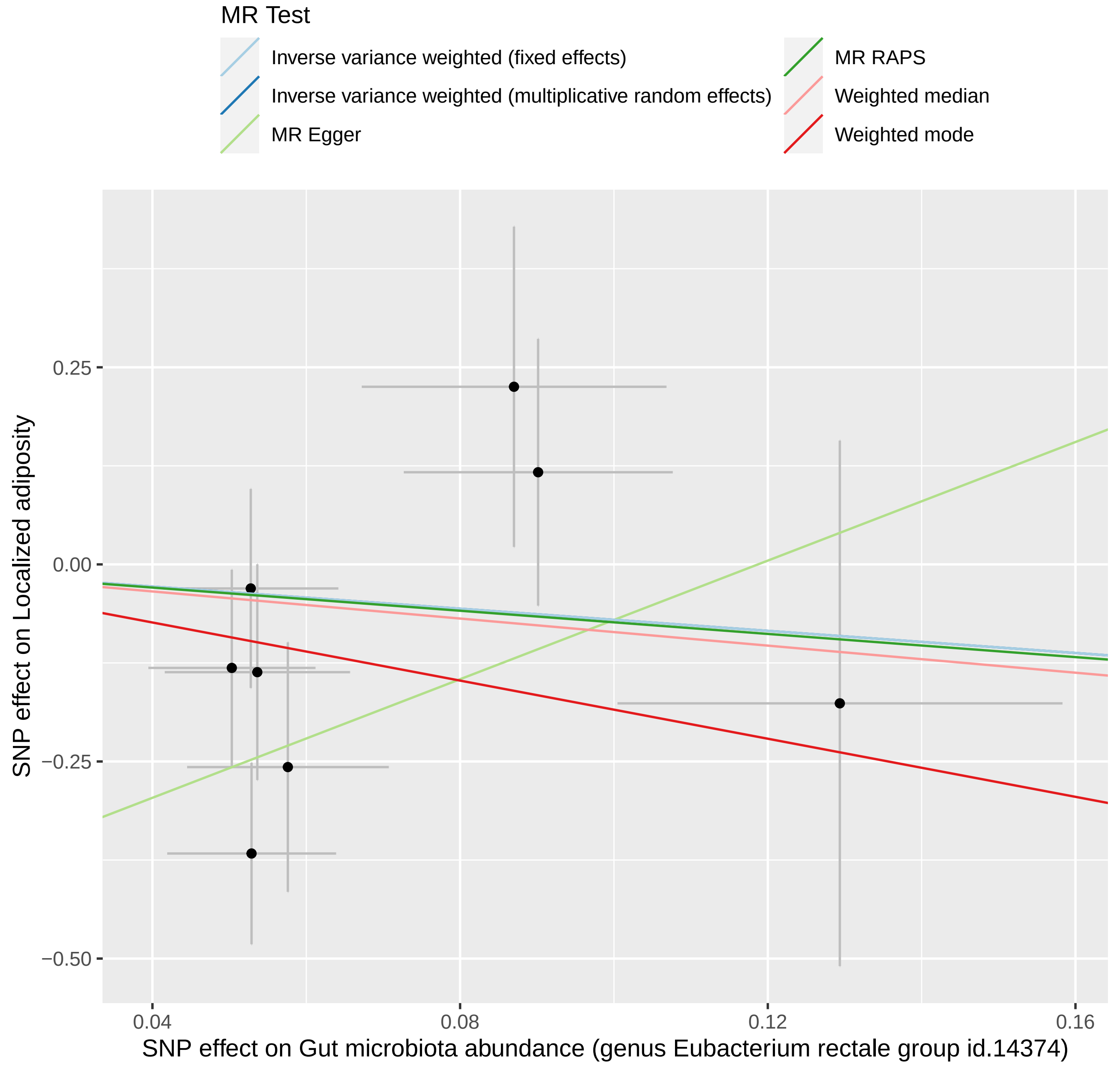

Batch 78 : Gut microbiota abundance (genus Eubacterium ruminantium group id.11340) on Localized adiposity

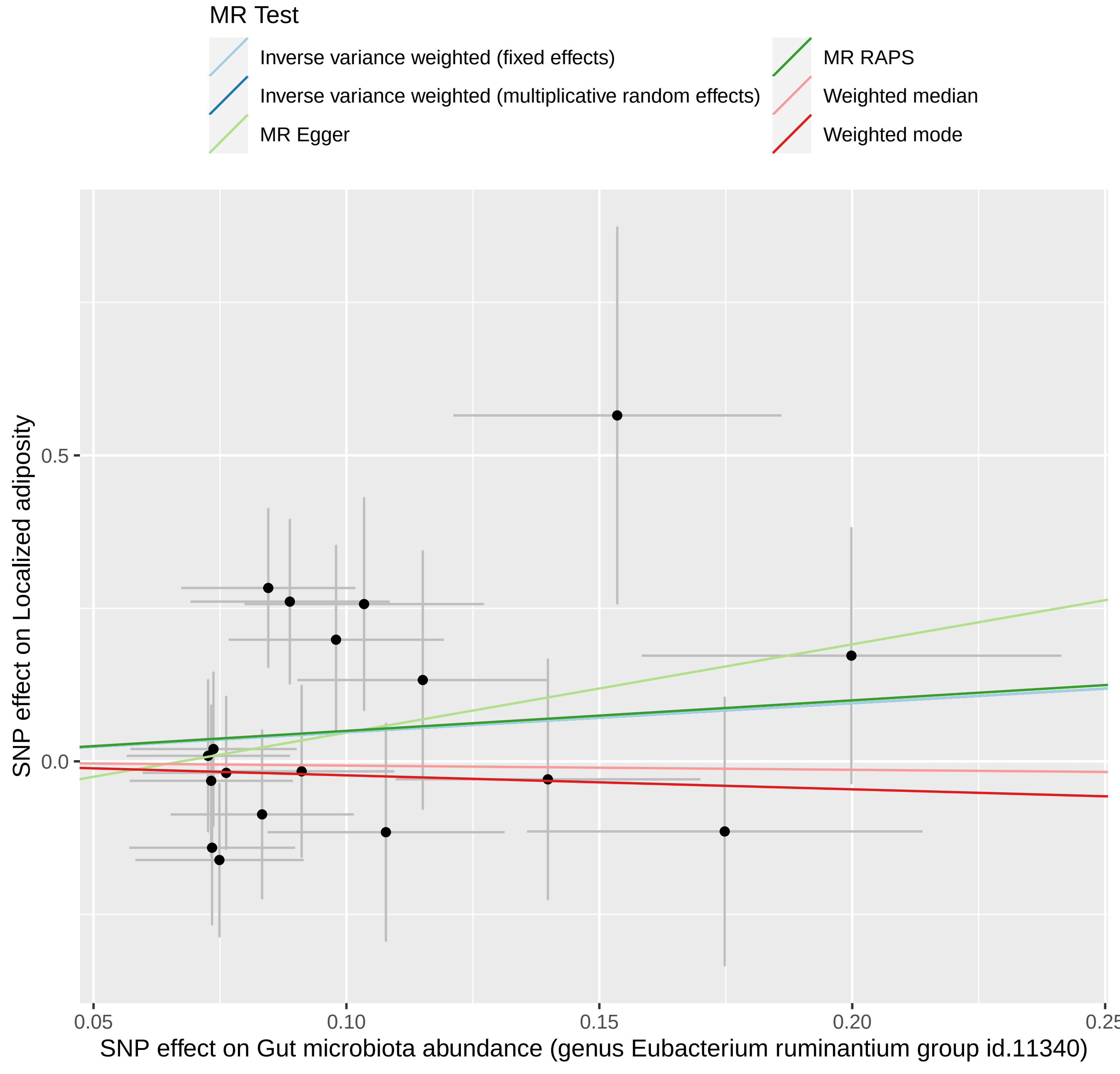

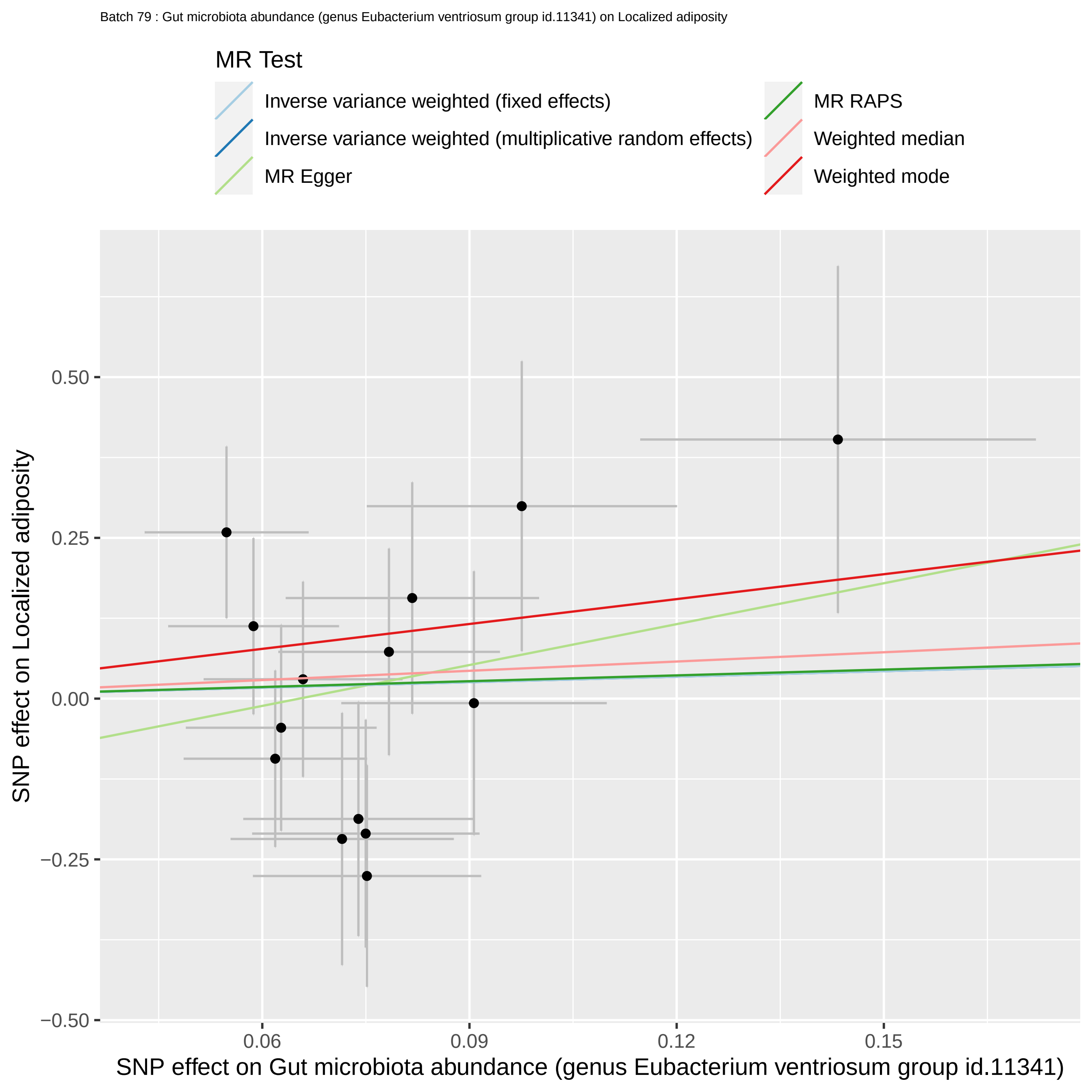

Batch 79 : Gut microbiota abundance (genus Eubacterium ventriosum group id.11341) on Localized adiposity
MR Test
Inverse variance weighted (fixed effects)
Inverse variance weighted (multiplicative random effects)
MR Egger
MR RAPS
Weighted median
Weighted mode
0.50
0.25
0.00
−0.25
−0.50
0.06
0.09
0.12
0.15
SNP effect on Localized adiposity
SNP effect on Gut microbiota abundance (genus Eubacterium ventriosum group id.11341)

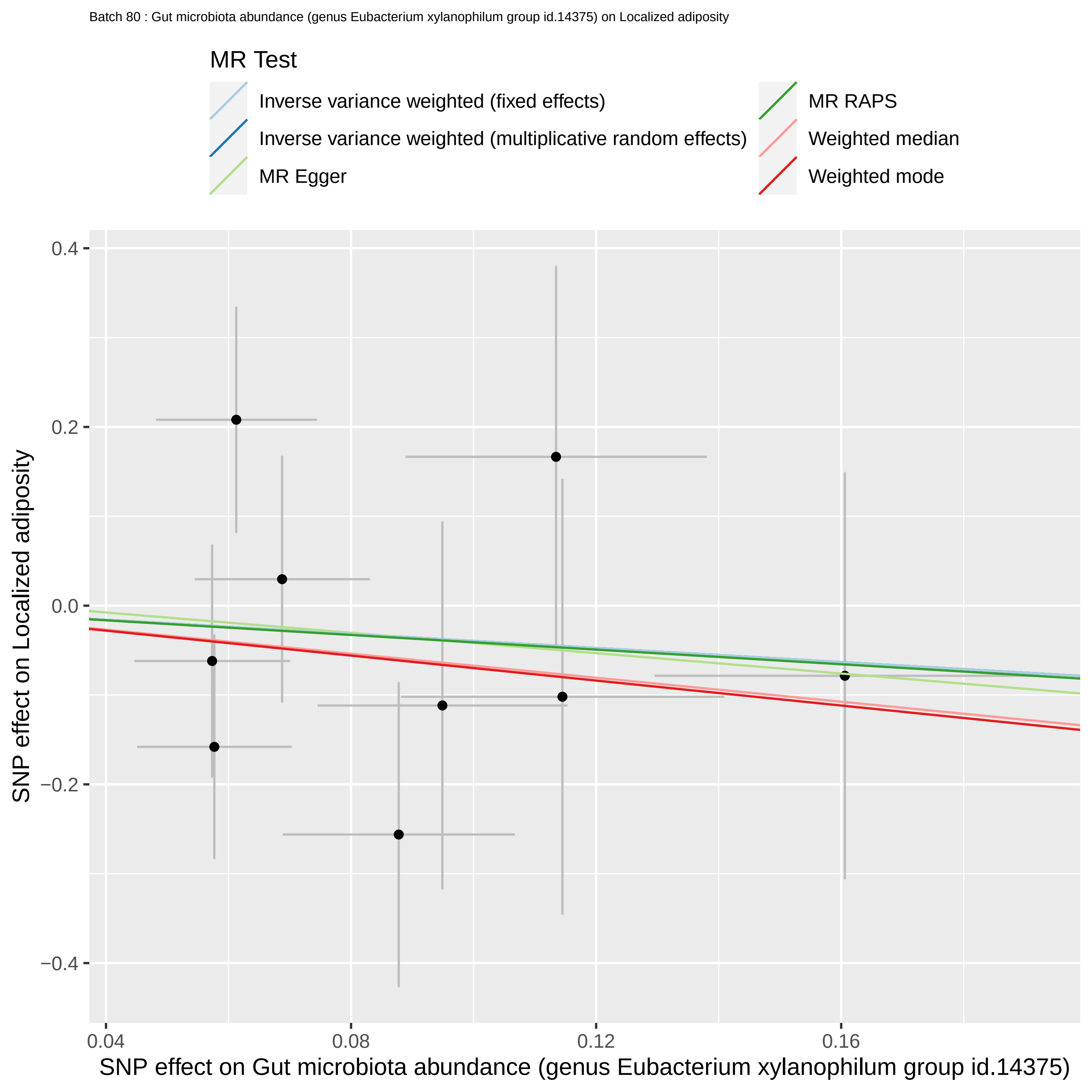
Batch 80 : Gut microbiota abundance (genus Eubacterium xylanophilum group id.14375) on Localized adiposity
MR Test
Inverse variance weighted (fixed effects)
Inverse variance weighted (multiplicative random effects)
MR Egger
MR RAPS
Weighted median
Weighted mode
0.4
0.2
0.0
−0.2
−0.4
0.04
0.08
0.12
0.16
SNP effect on Localized adiposity
SNP effect on Gut microbiota abundance (genus Eubacterium xylanophilum group id.14375)

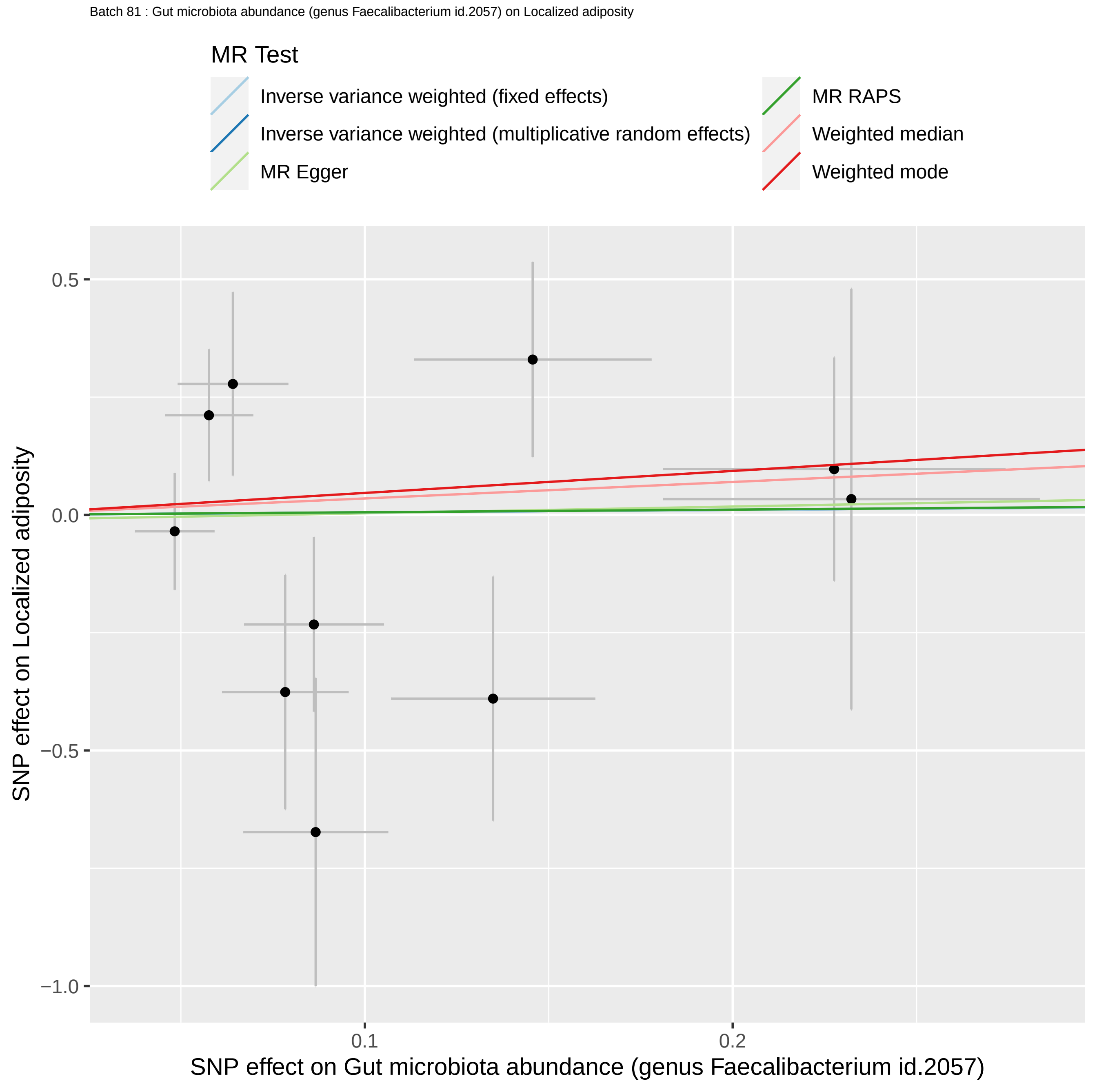

Batch 81 : Gut microbiota abundance (genus Faecalibacterium id.2057) on Localized adiposity
MR Test
Inverse variance weighted (fixed effects)
Inverse variance weighted (multiplicative random effects)
MR Egger
MR RAPS
Weighted median
Weighted mode
0.5
0.0
−0.5
−1.0
0.1
0.2
SNP effect on Localized adiposity
SNP effect on Gut microbiota abundance (genus Faecalibacterium id.2057)

Batch 82 : Gut microbiota abundance (genus Family XIII AD3011 group id.11293) on Localized adiposity

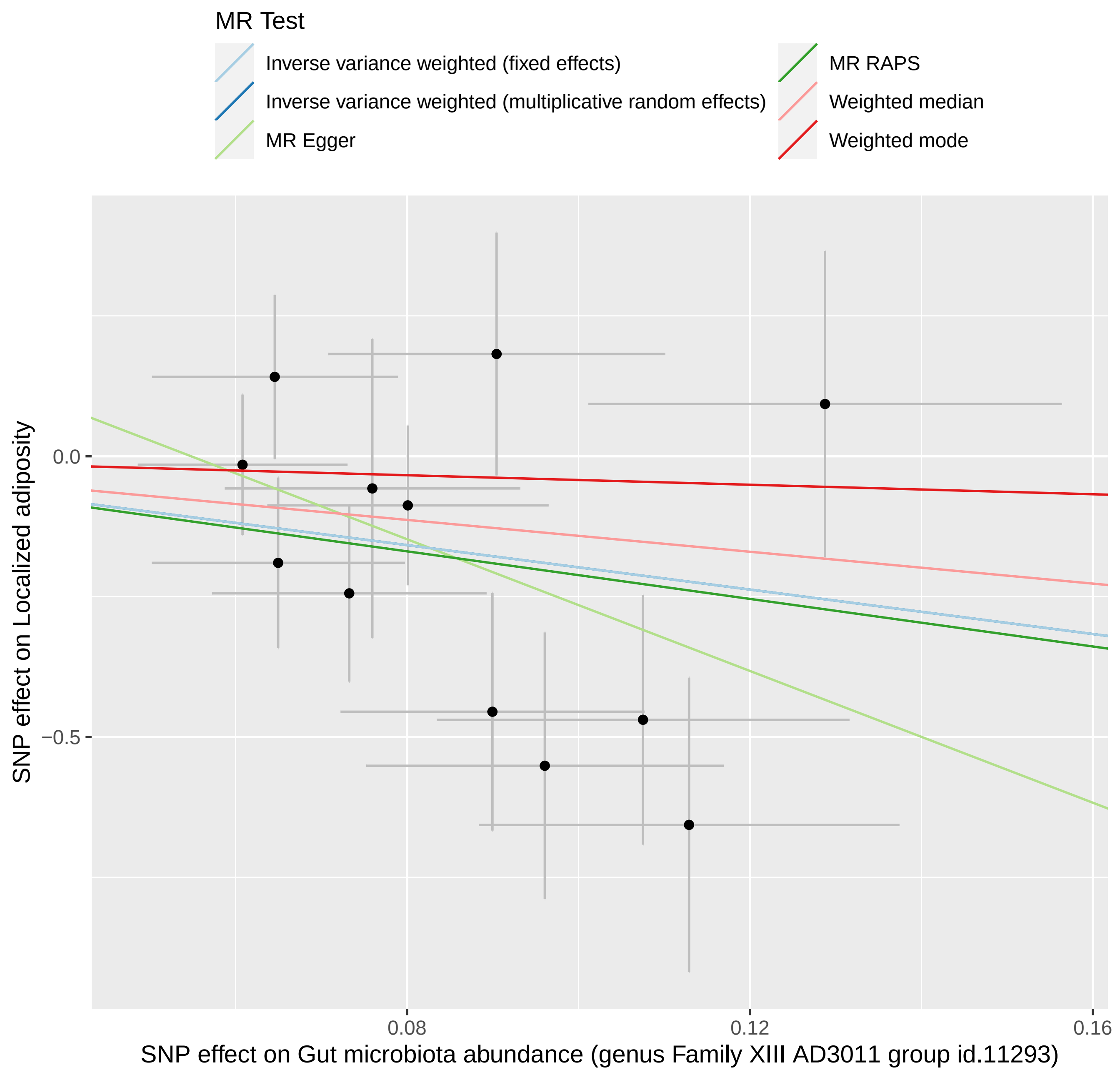

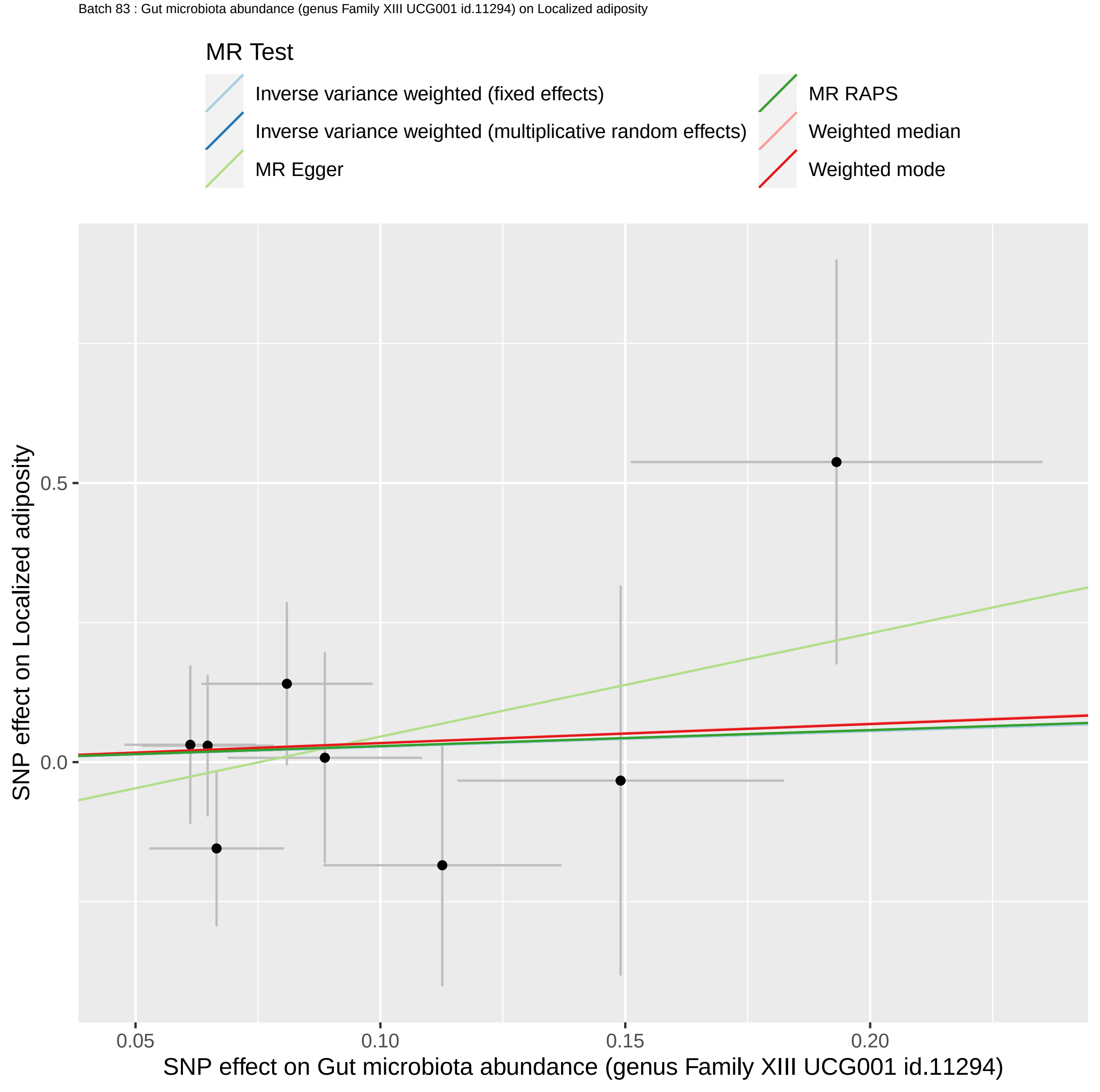
Batch 83 : Gut microbiota abundance (genus Family XIII UCG001 id.11294) on Localized adiposity
MR Test
Inverse variance weighted (fixed effects)
Inverse variance weighted (multiplicative random effects)
MR Egger
MR RAPS
Weighted median
Weighted mode
SNP effect on Localized adiposity
0.5
0.0
0.05
0.10
0.15
0.20
SNP effect on Gut microbiota abundance (genus Family XIII UCG001 id.11294)

Batch 84 : Gut microbiota abundance (genus Flavonifractor id.2059) on Localized adiposity

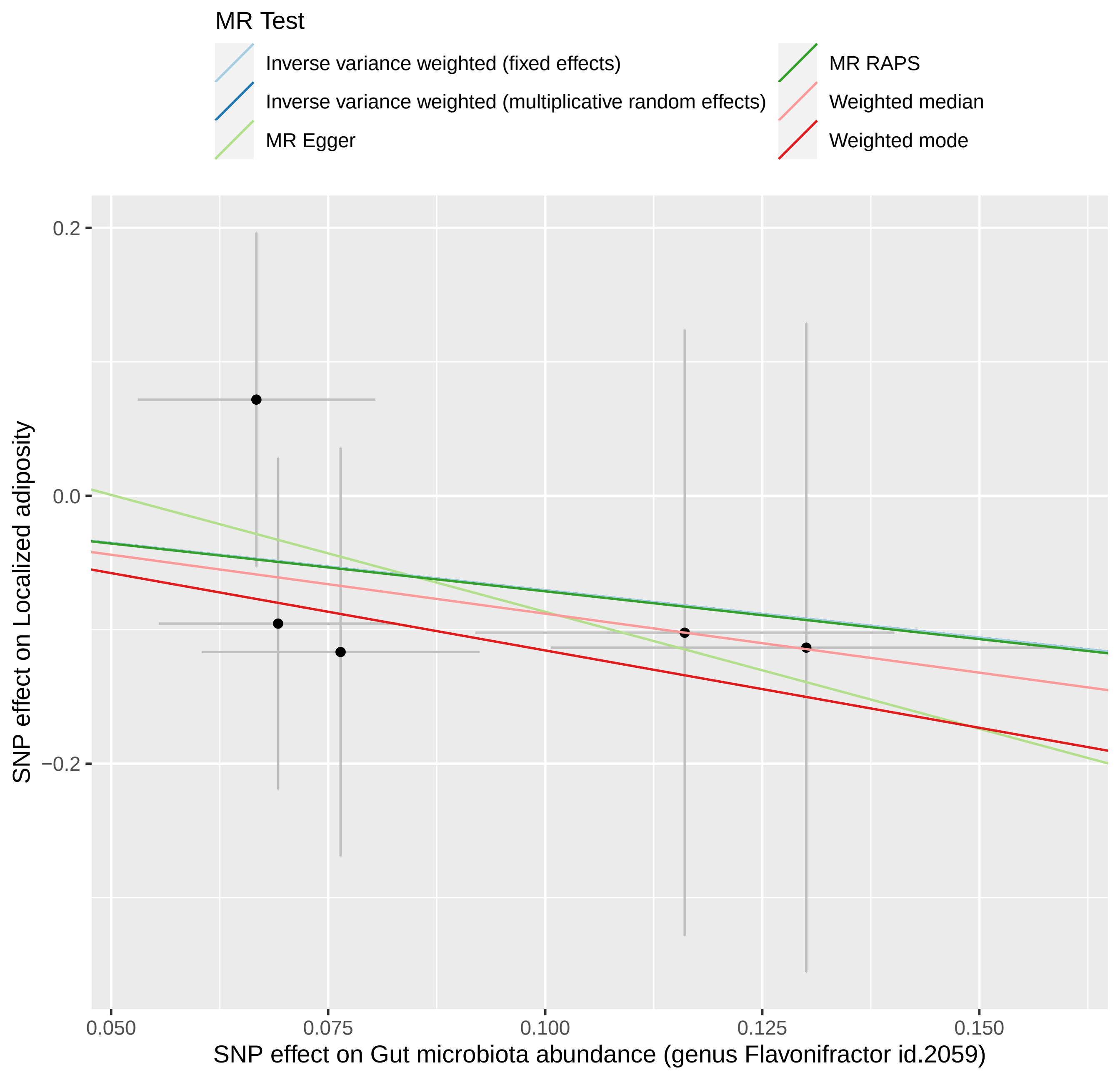

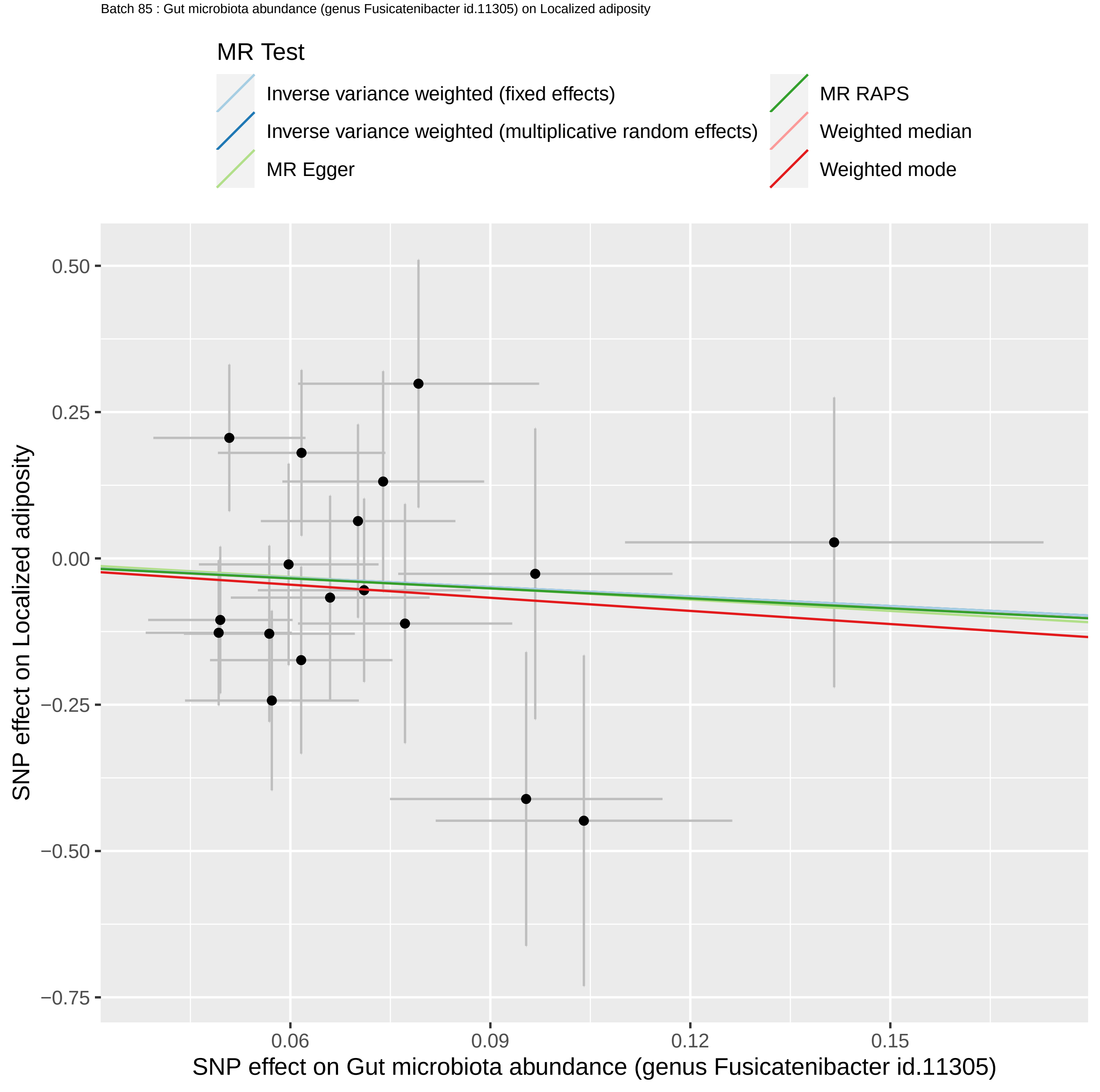
Batch 85 : Gut microbiota abundance (genus Fusicatenibacter id.11305) on Localized adiposity
MR Test
Inverse variance weighted (fixed effects)
Inverse variance weighted (multiplicative random effects)
MR Egger
MR RAPS
Weighted median
Weighted mode
0.50
0.25
0.00
−0.25
−0.50
−0.75
0.06
0.09
0.12
0.15
SNP effect on Localized adiposity
SNP effect on Gut microbiota abundance (genus Fusicatenibacter id.11305)

Batch 86 : Gut microbiota abundance (genus Gordonibacter id.821) on Localized adiposity

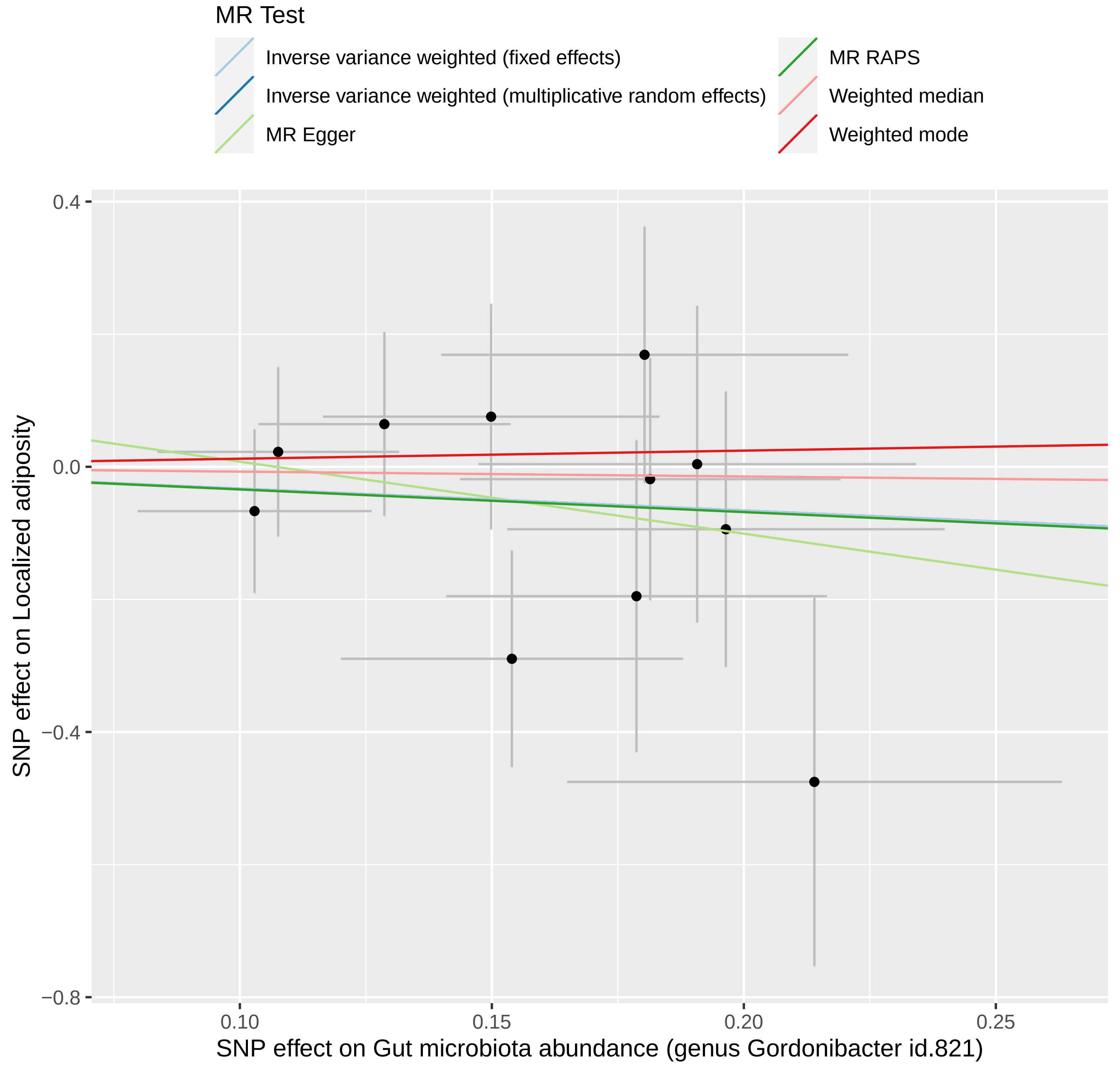

Batch 87 : Gut microbiota abundance (genus Haemophilus id.3698) on Localized adiposity

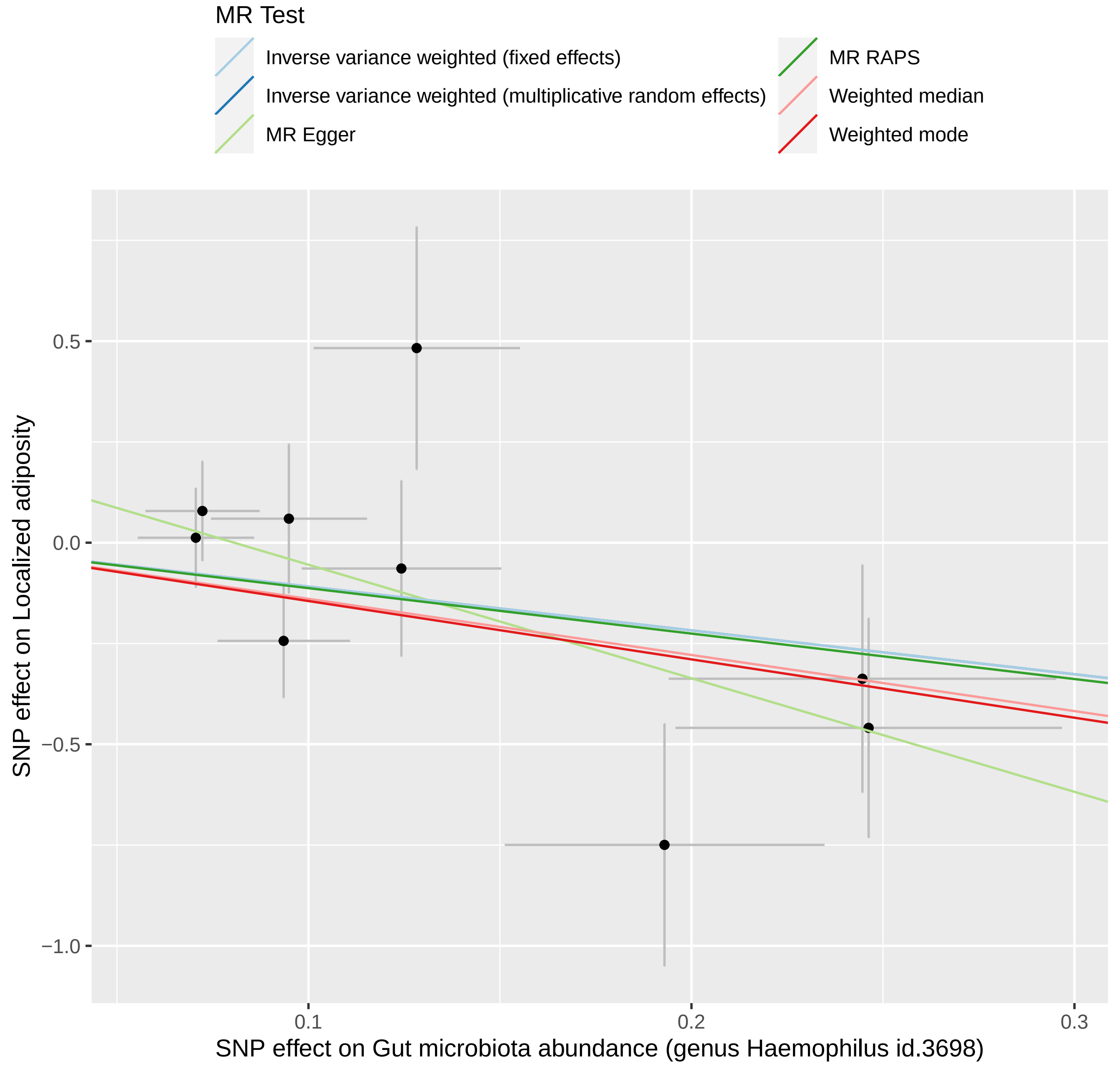

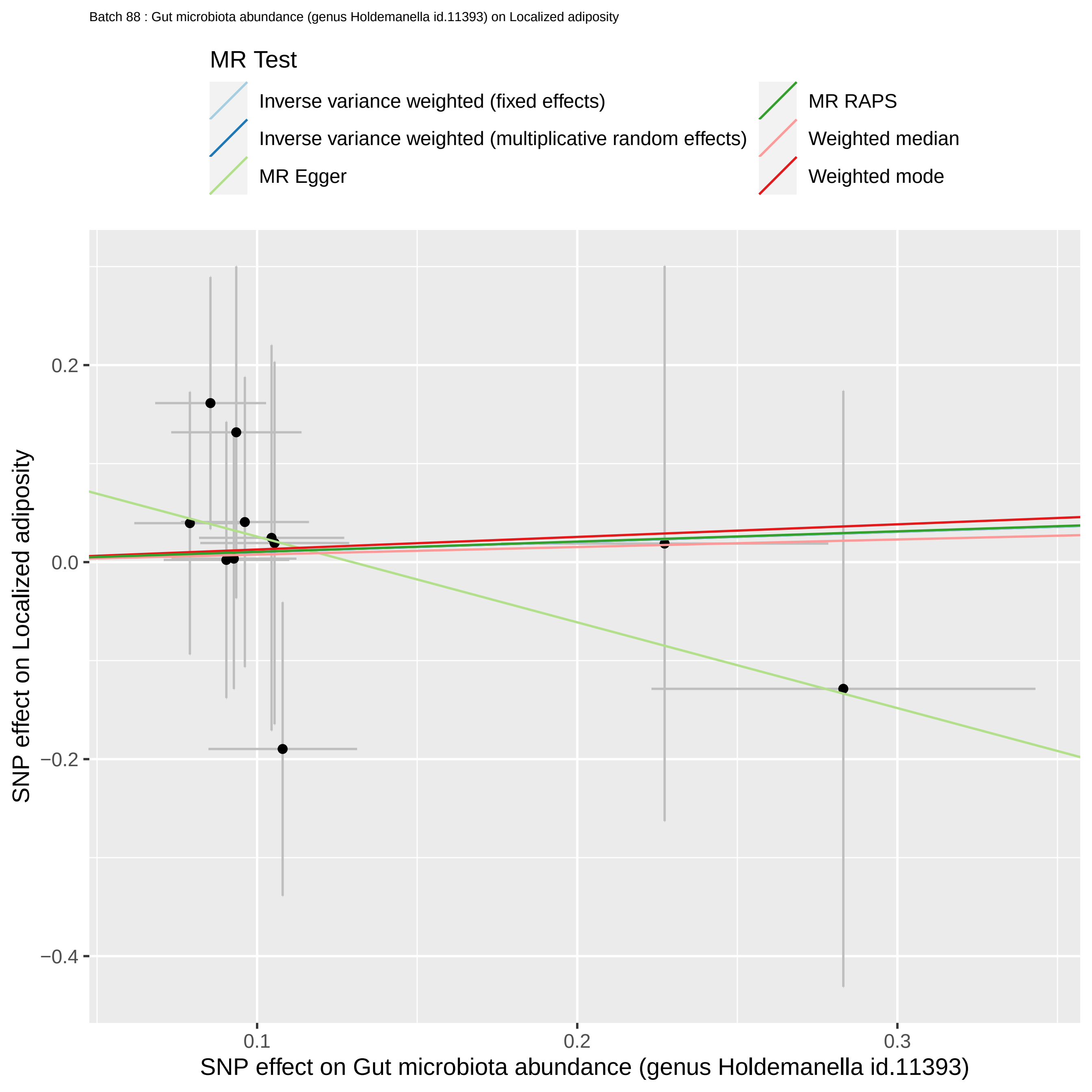
Batch 88 : Gut microbiota abundance (genus Holdemanella id.11393) on Localized adiposity
MR Test
Inverse variance weighted (fixed effects)
Inverse variance weighted (multiplicative random effects)
MR Egger
MR RAPS
Weighted median
Weighted mode
0.2
0.0
−0.2
−0.4
0.1
0.2
0.3
SNP effect on Localized adiposity
SNP effect on Gut microbiota abundance (genus Holdemanella id.11393)

Batch 89 : Gut microbiota abundance (genus Holdemania id.2157) on Localized adiposity

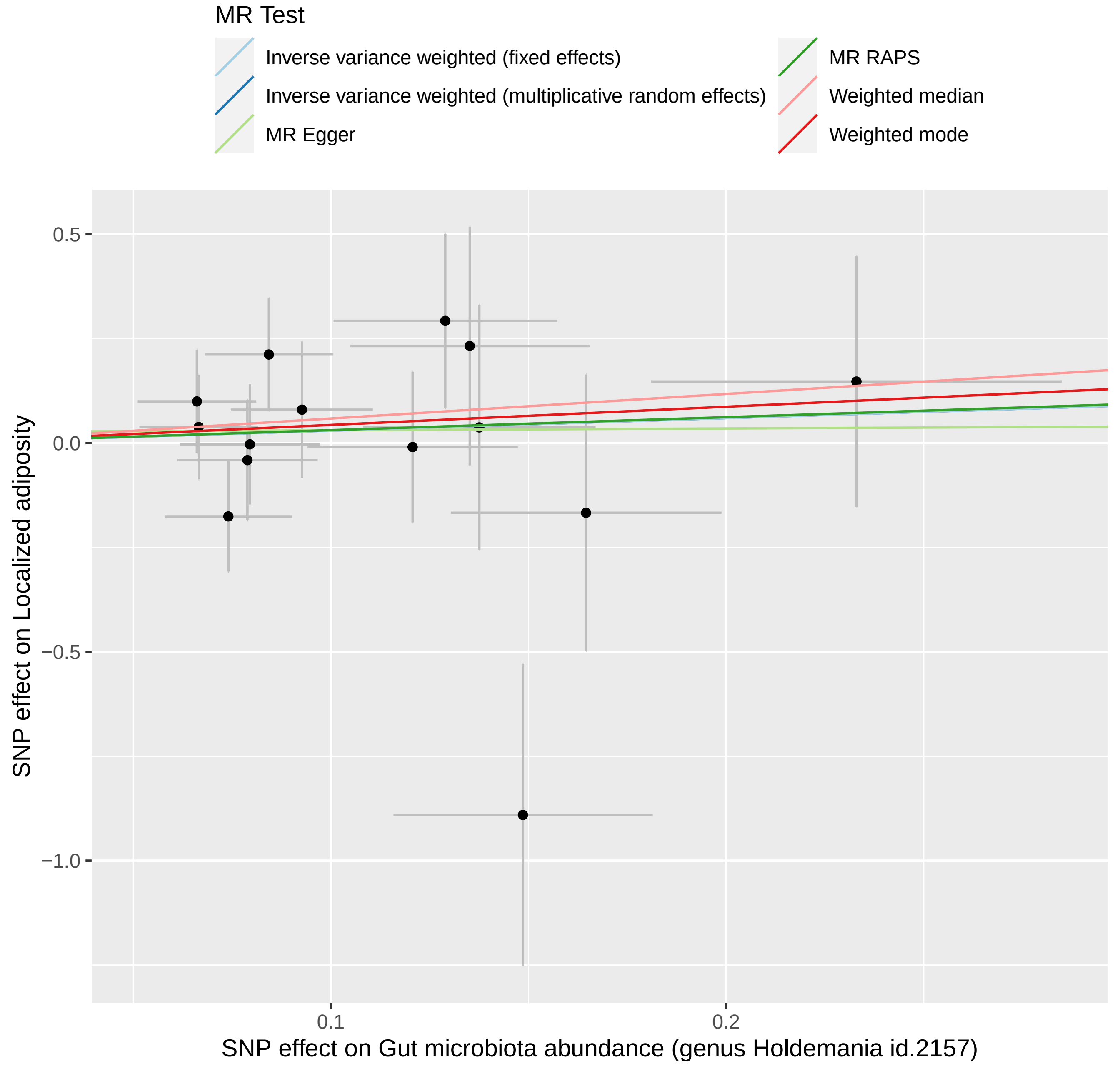

Batch 90 : Gut microbiota abundance (genus Howardella id.2000) on Localized adiposity

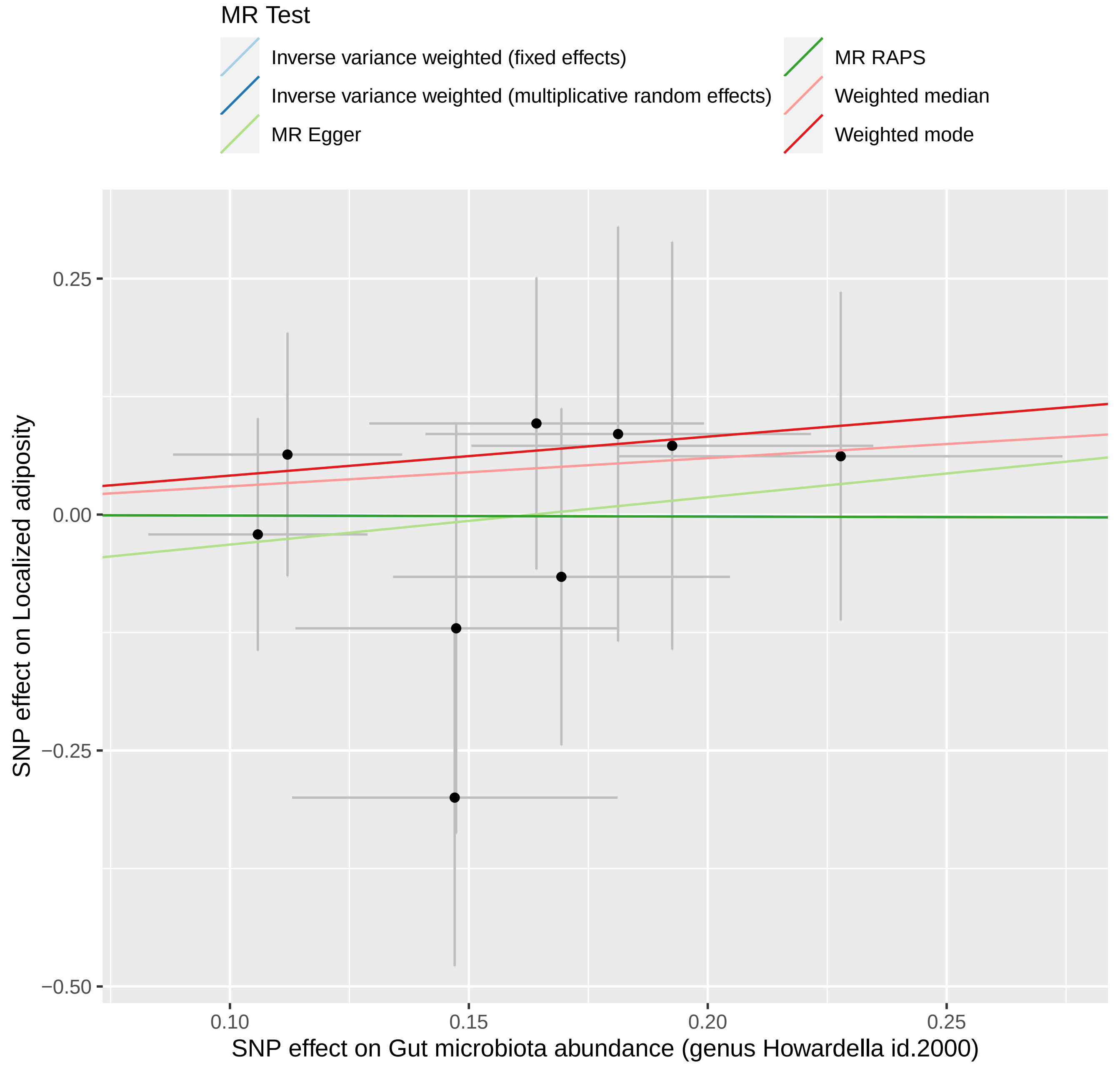

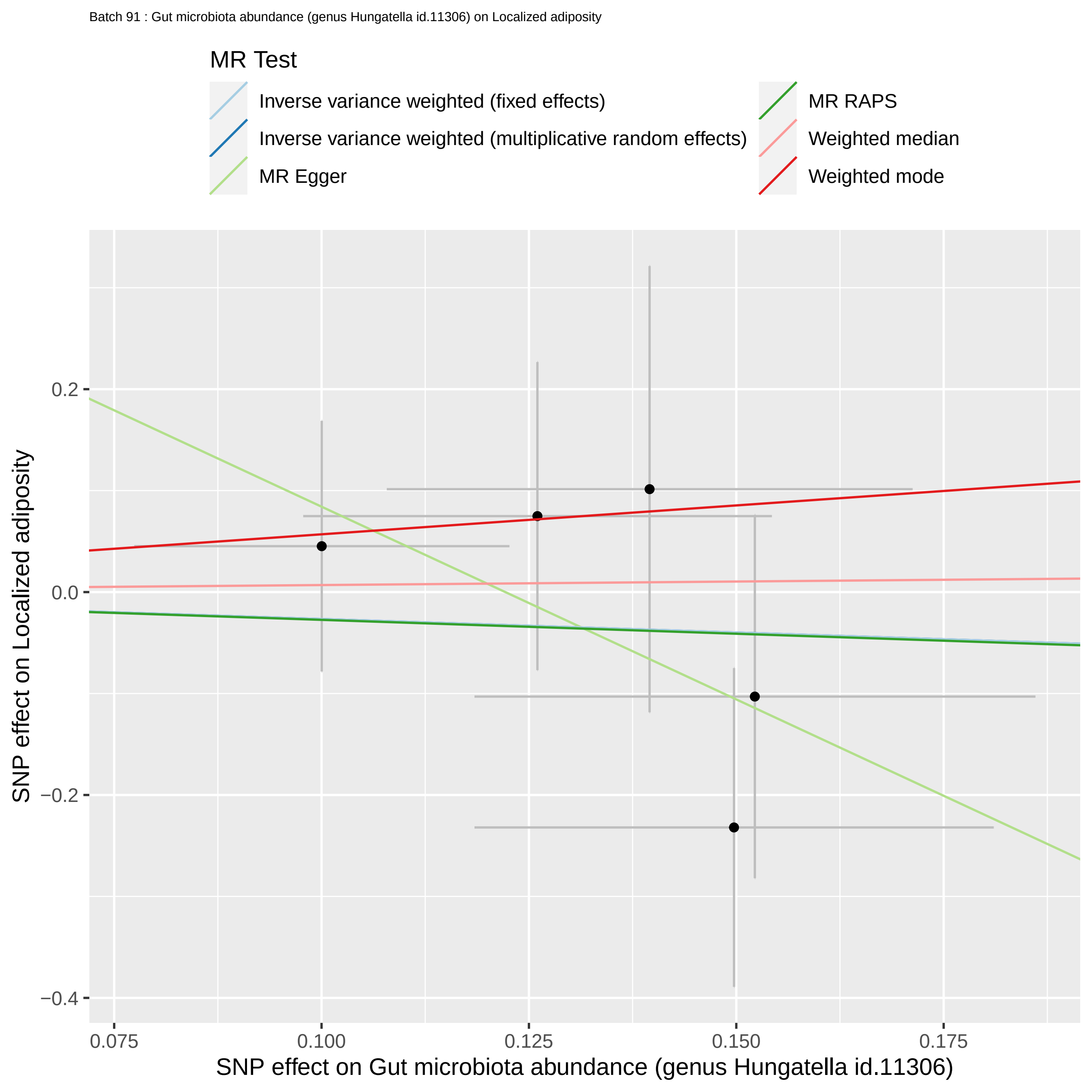

Batch 91 : Gut microbiota abundance (genus Hungatella id.11306) on Localized adiposity
MR Test
Inverse variance weighted (fixed effects)
Inverse variance weighted (multiplicative random effects)
MR Egger
MR RAPS
Weighted median
Weighted mode
SNP effect on Localized adiposity
0.2
0.0
−0.2
−0.4
0.075
0.100
0.125
0.150
0.175
SNP effect on Gut microbiota abundance (genus Hungatella id.11306)

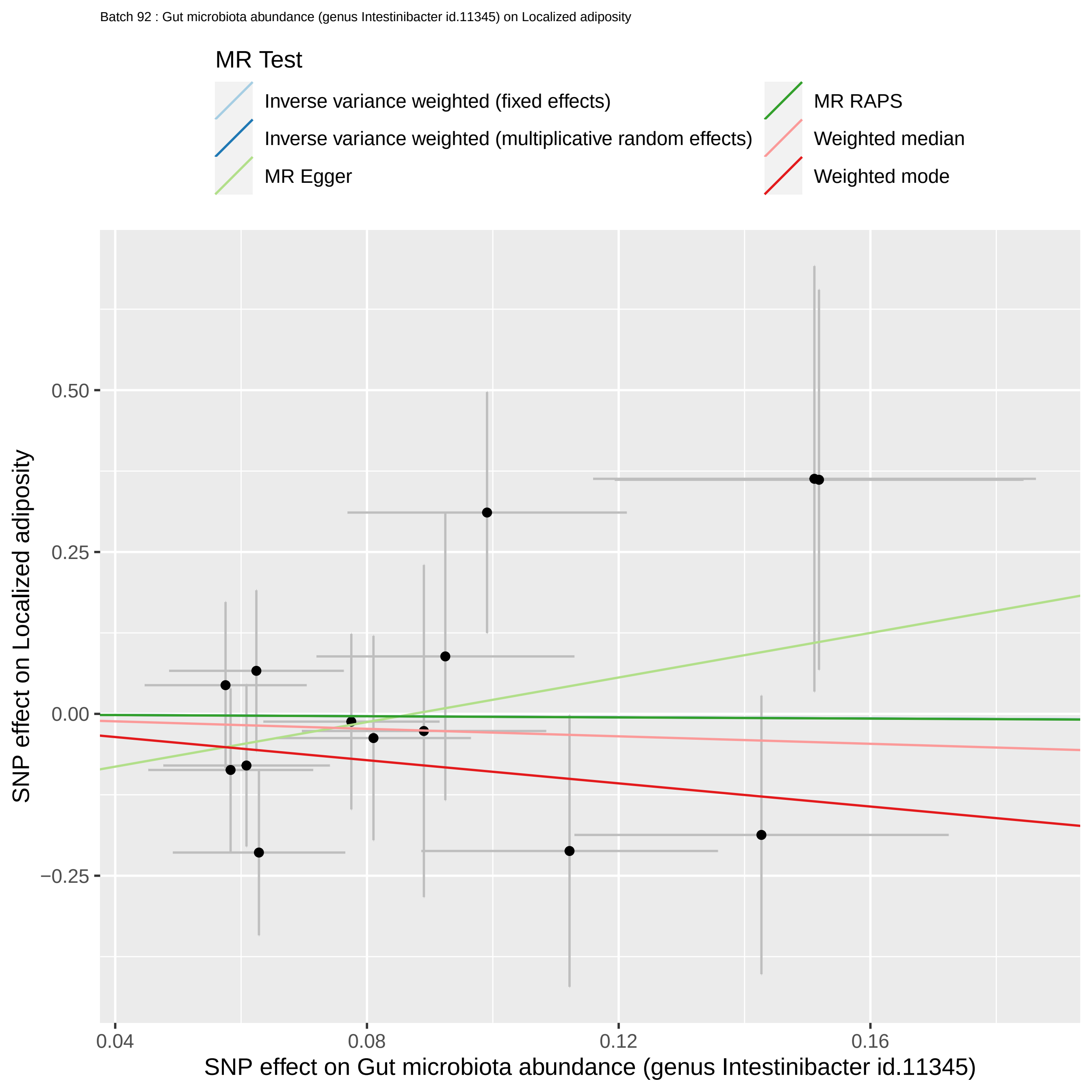
Batch 92 : Gut microbiota abundance (genus Intestinibacter id.11345) on Localized adiposity
MR Test
Inverse variance weighted (fixed effects)
Inverse variance weighted (multiplicative random effects)
MR Egger
MR RAPS
Weighted median
Weighted mode
0.50
0.25
0.00
−0.25
0.04
0.08
0.12
0.16
SNP effect on Localized adiposity
SNP effect on Gut microbiota abundance (genus Intestinibacter id.11345)

Batch 93 : Gut microbiota abundance (genus Intestinimonas id.2062) on Localized adiposity

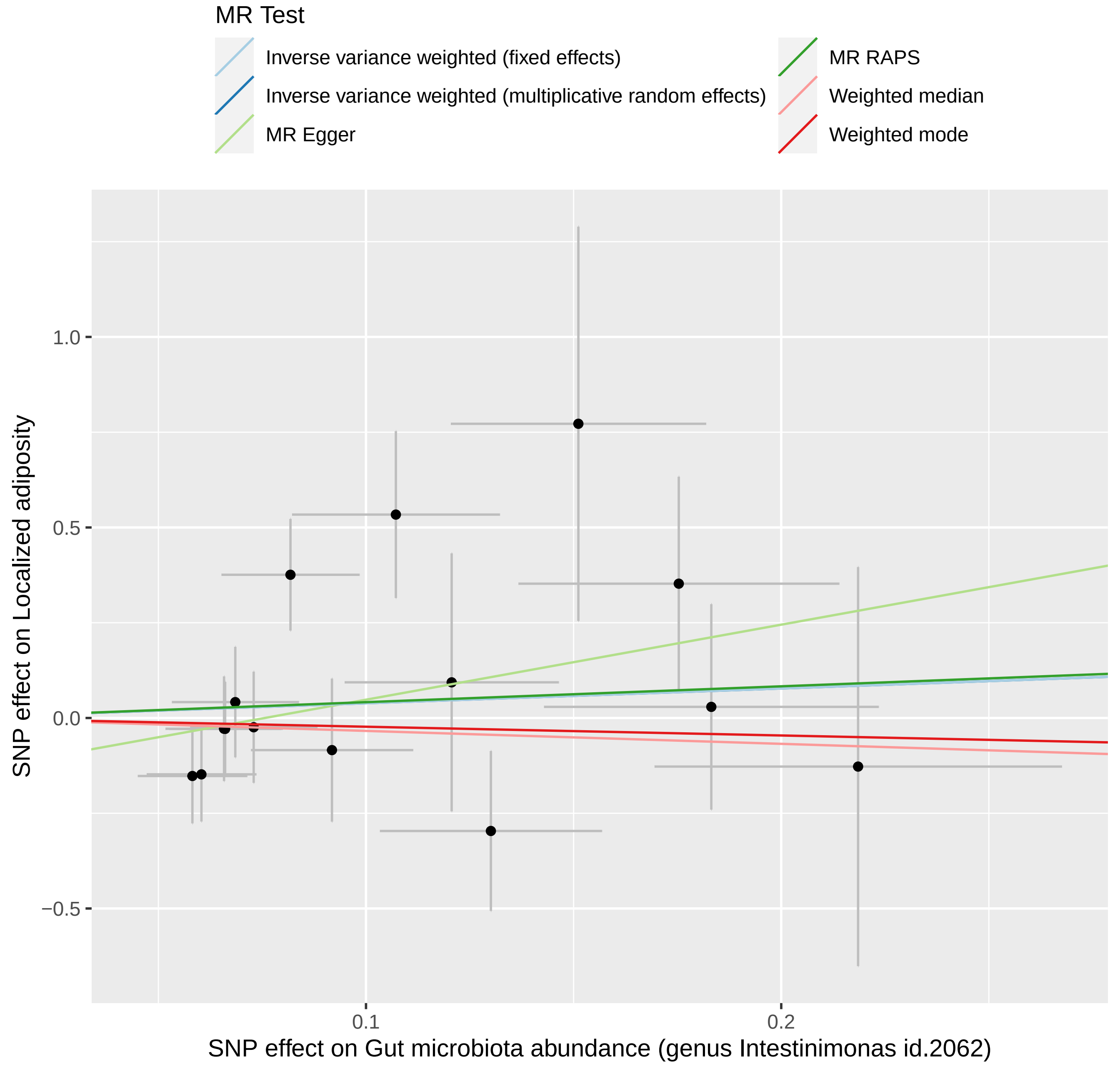

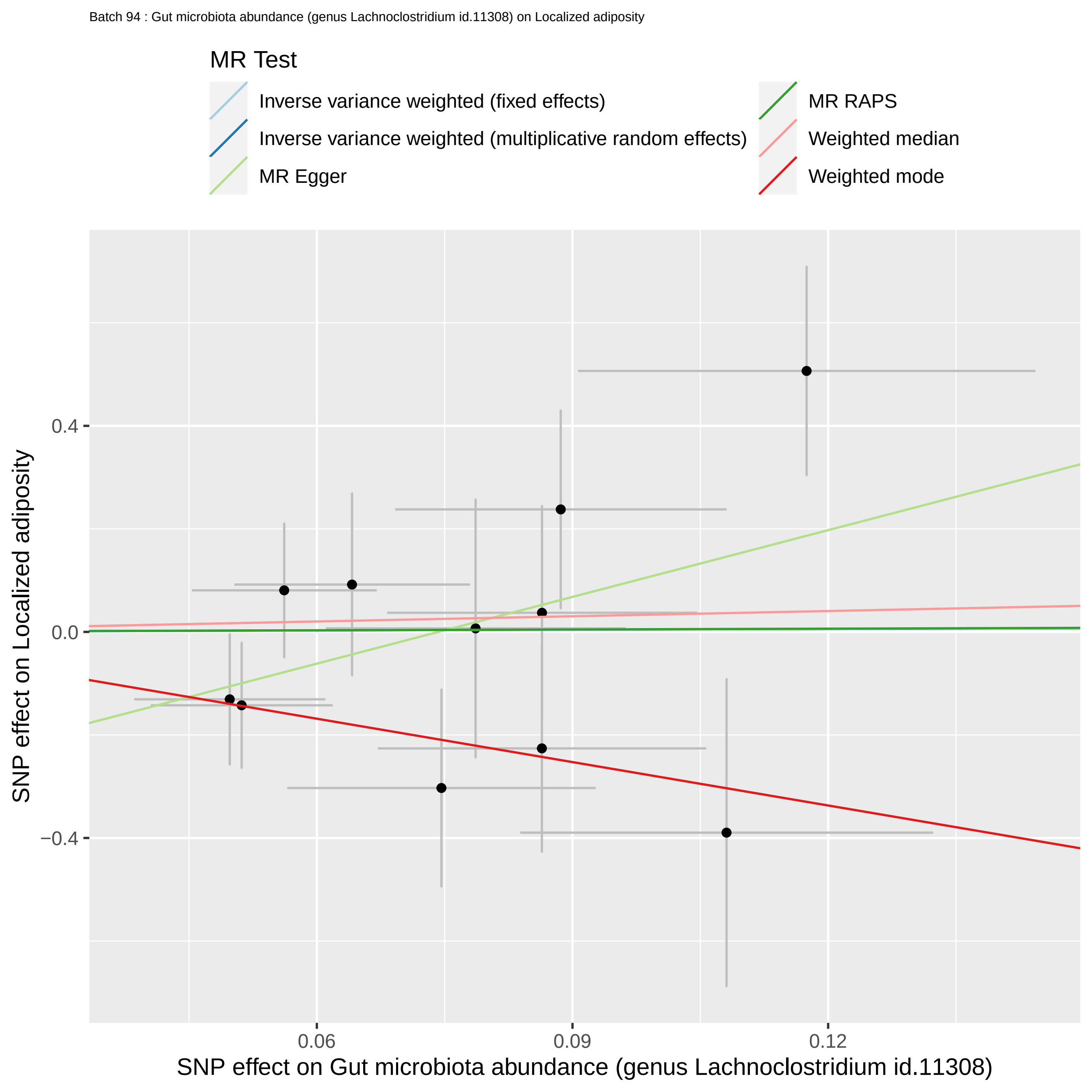

Batch 94 : Gut microbiota abundance (genus Lachnoclostridium id.11308) on Localized adiposity
MR Test
Inverse variance weighted (fixed effects)
Inverse variance weighted (multiplicative random effects)
MR Egger
MR RAPS
Weighted median
Weighted mode
0.4
0.0
−0.4
0.06
0.09
0.12
SNP effect on Localized adiposity
SNP effect on Gut microbiota abundance (genus Lachnoclostridium id.11308)

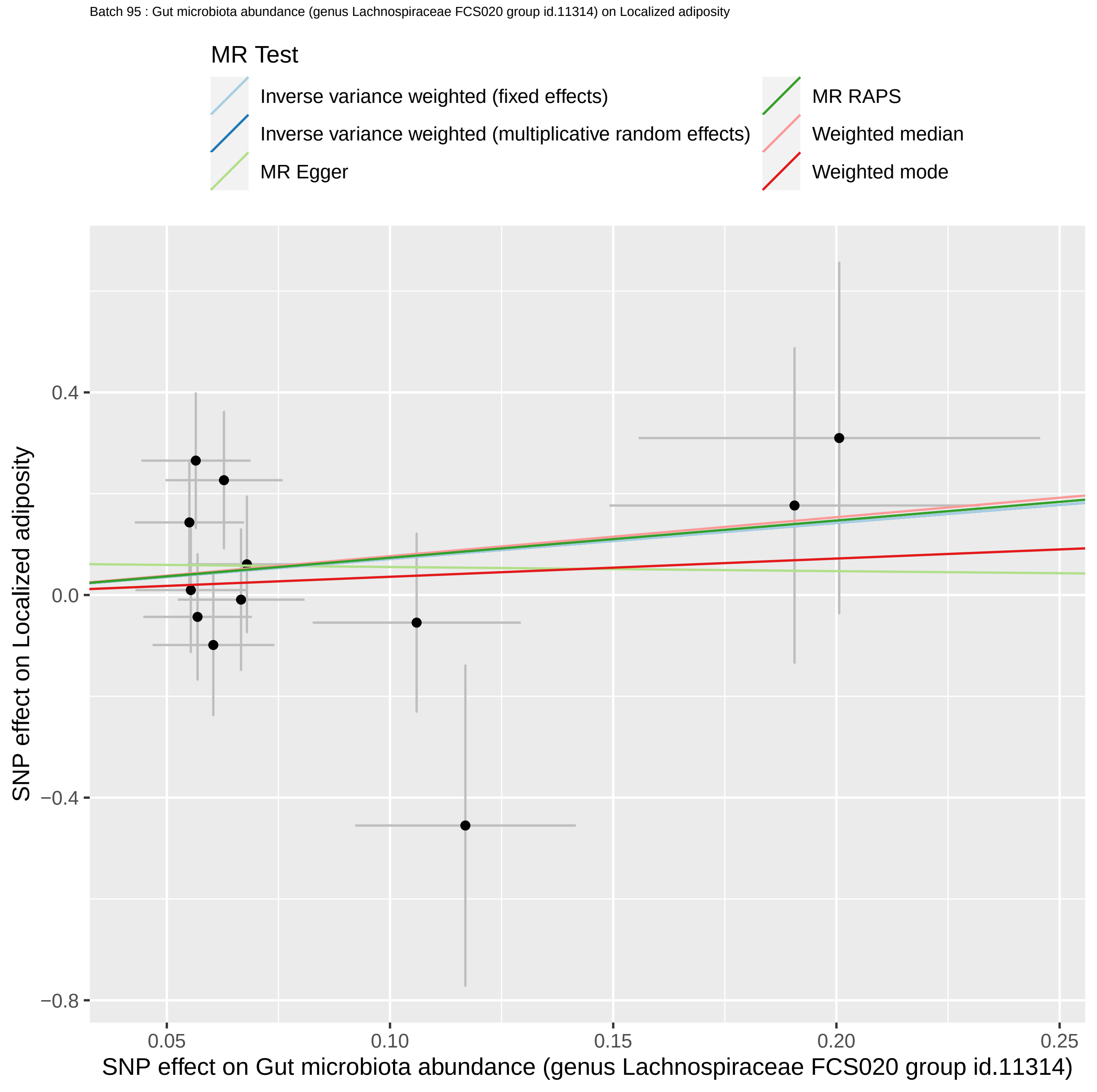
Batch 95 : Gut microbiota abundance (genus Lachnospiraceae FCS020 group id.11314) on Localized adiposity
MR Test
Inverse variance weighted (fixed effects)
Inverse variance weighted (multiplicative random effects)
MR Egger
MR RAPS
Weighted median
Weighted mode
0.4
0.0
−0.4
−0.8
0.05
0.10
0.15
0.20
0.25
SNP effect on Localized adiposity
SNP effect on Gut microbiota abundance (genus Lachnospiraceae FCS020 group id.11314)

Batch 96 : Gut microbiota abundance (genus Lachnospiraceae NC2004 group id.11316) on Localized adiposity

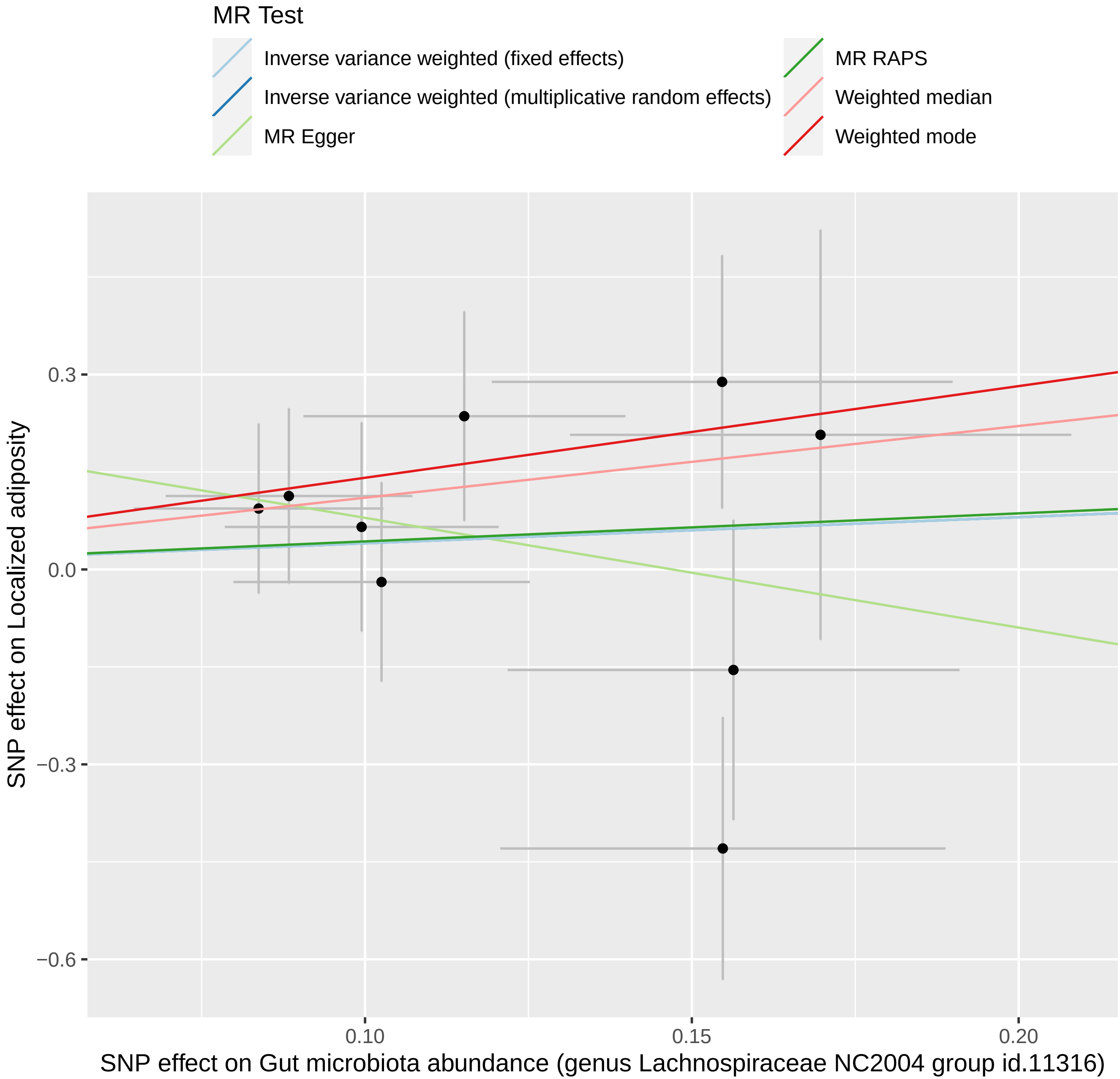

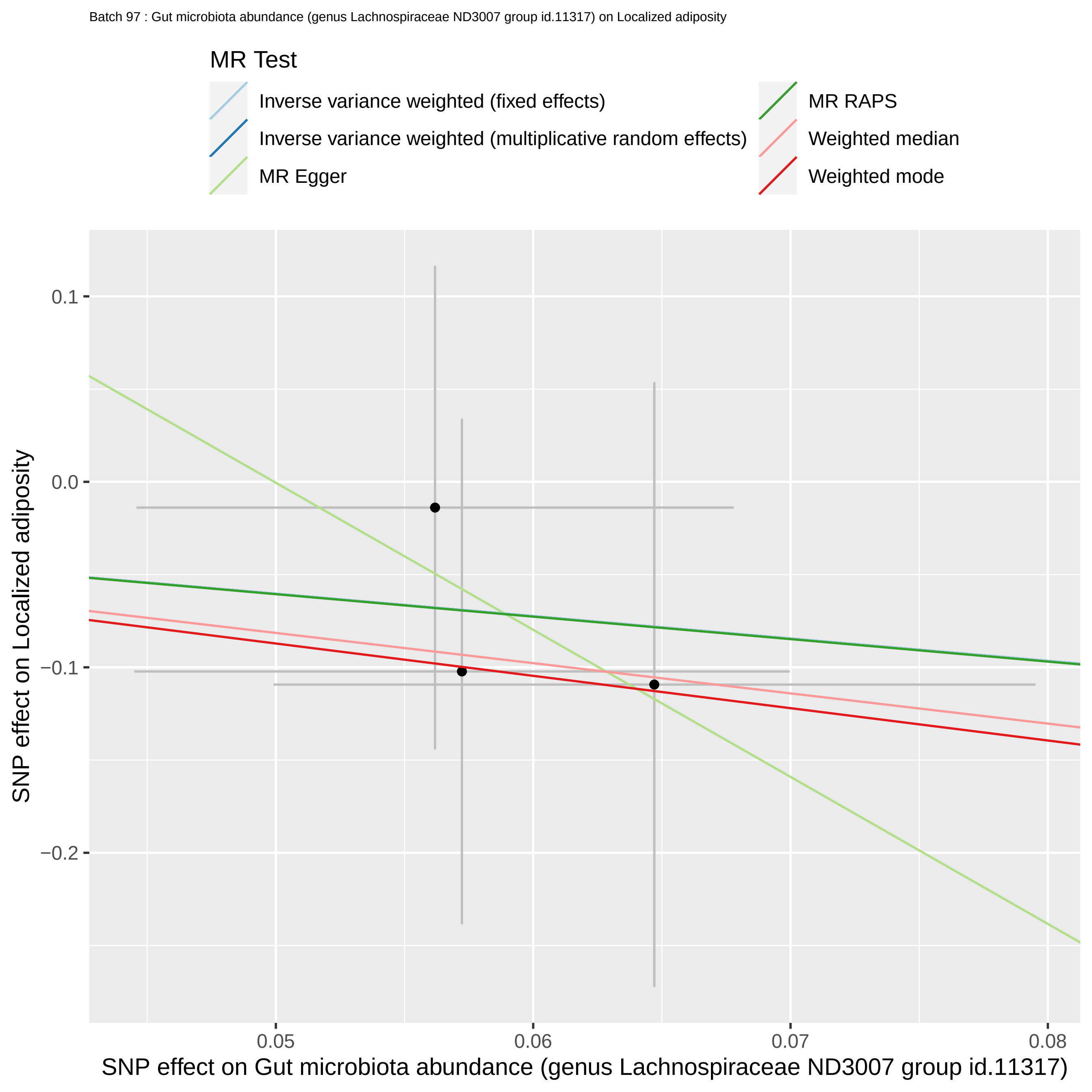

Batch 97 : Gut microbiota abundance (genus Lachnospiraceae ND3007 group id.11317) on Localized adiposity
MR Test
Inverse variance weighted (fixed effects)
Inverse variance weighted (multiplicative random effects)
MR Egger
MR RAPS
Weighted median
Weighted mode
0.1
0.0
−0.1
−0.2
SNP effect on Localized adiposity
0.05
0.06
0.07
0.08
SNP effect on Gut microbiota abundance (genus Lachnospiraceae ND3007 group id.11317)

Batch 98 : Gut microbiota abundance (genus Lachnospiraceae NK4A136 group id.11319) on Localized adiposity

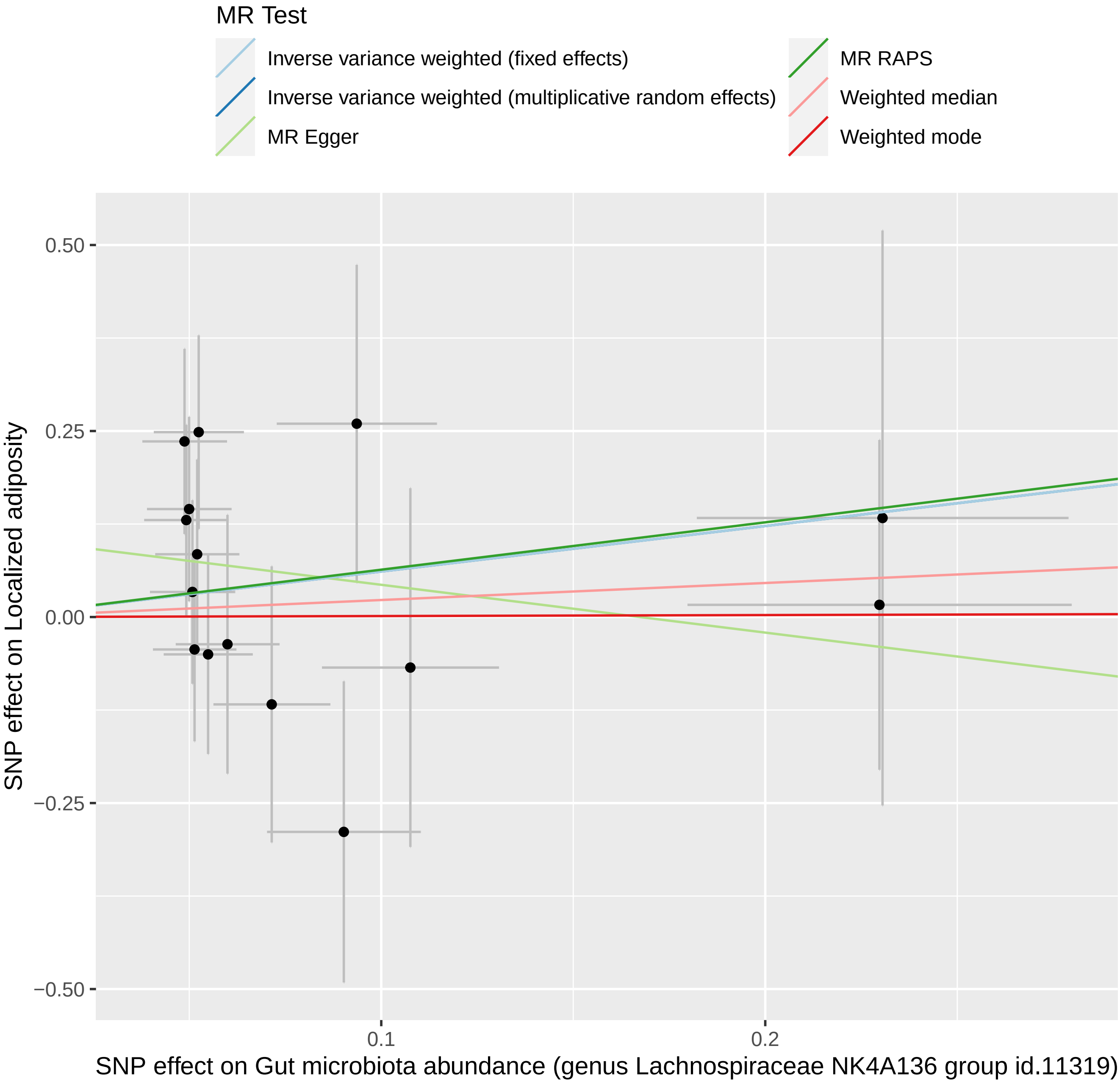

Batch 99 : Gut microbiota abundance (genus Lachnospiraceae UCG001 id.11321) on Localized adiposity

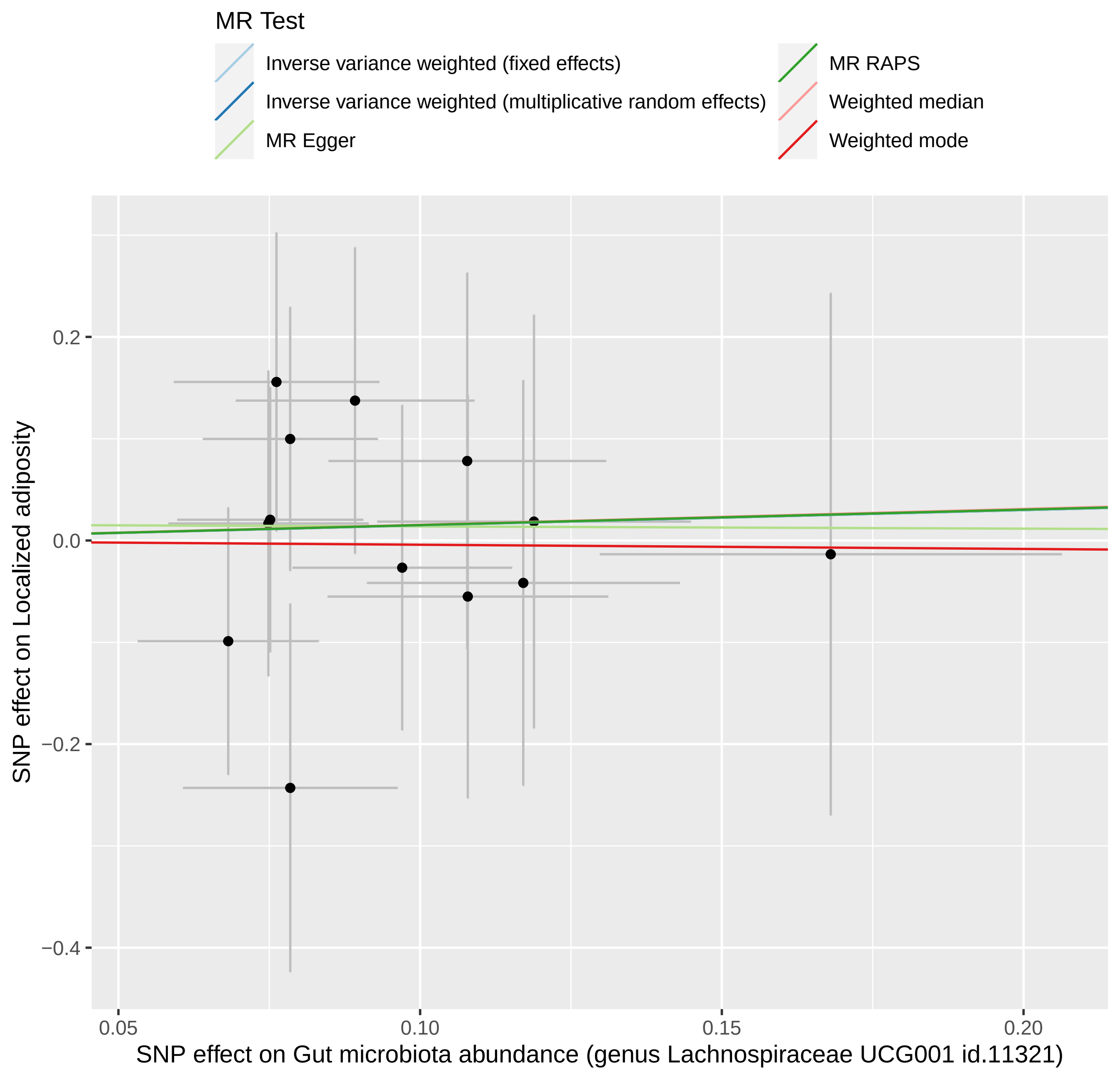

Batch 100 : Gut microbiota abundance (genus Lachnospiraceae UCG004 id.11324) on Localized adiposity

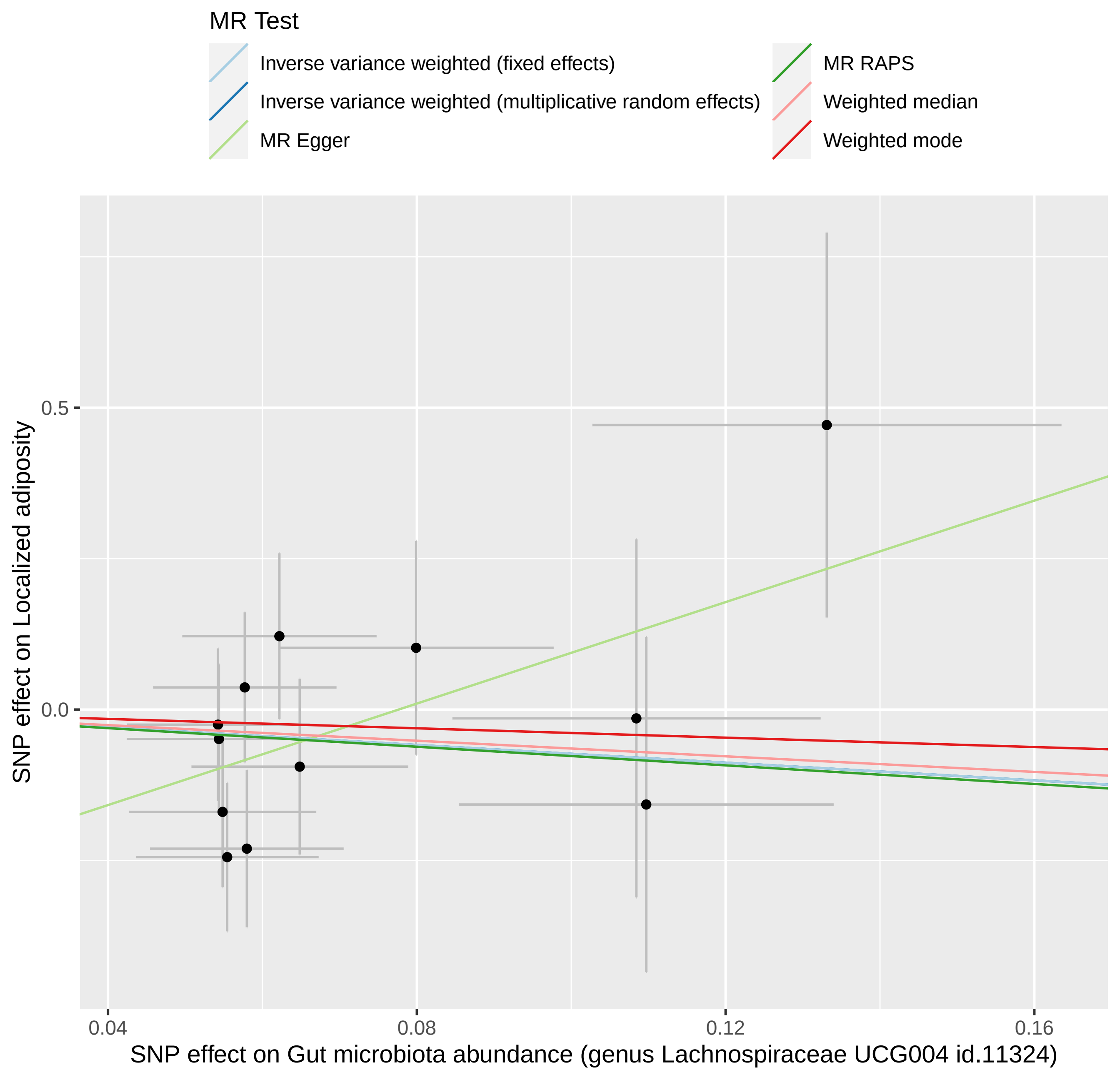

Batch 101 : Gut microbiota abundance (genus Lachnospiraceae UCG008 id.11328) on Localized adiposity

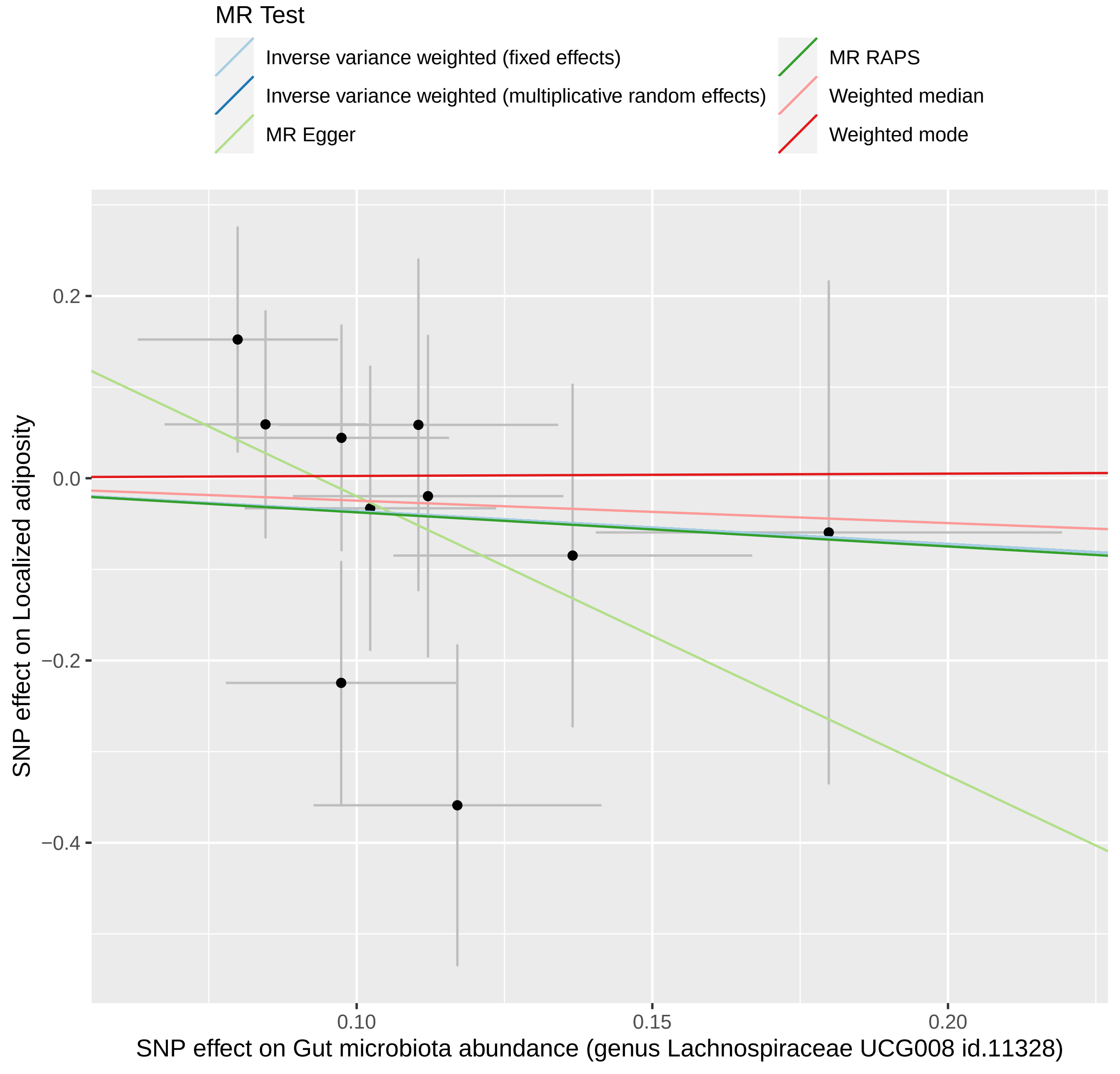

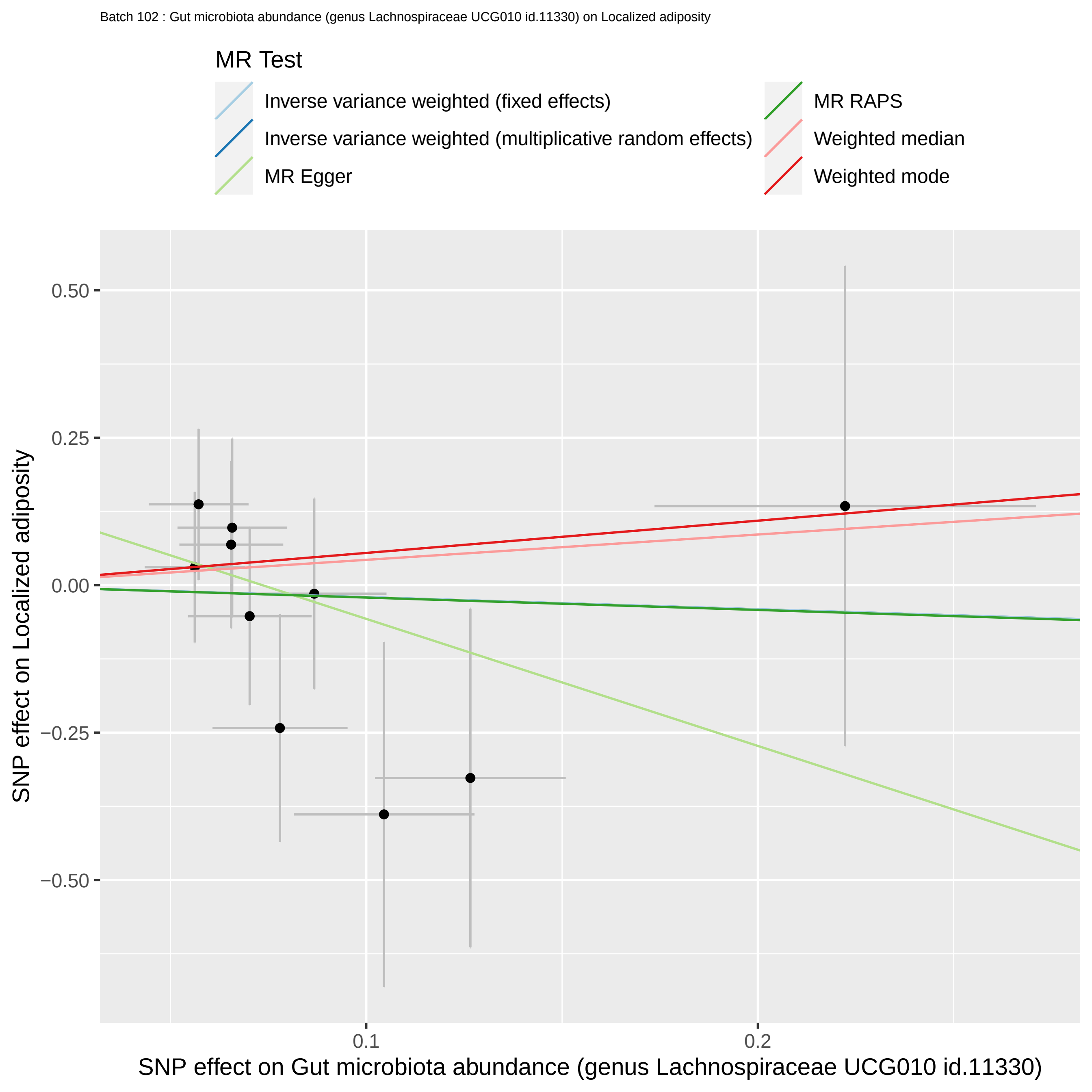

Batch 102 : Gut microbiota abundance (genus Lachnospiraceae UCG010 id.11330) on Localized adiposity
MR Test
Inverse variance weighted (fixed effects)
Inverse variance weighted (multiplicative random effects)
MR Egger
MR RAPS
Weighted median
Weighted mode
0.50
0.25
0.00
−0.25
−0.50
0.1
0.2
SNP effect on Localized adiposity
SNP effect on Gut microbiota abundance (genus Lachnospiraceae UCG010 id.11330)

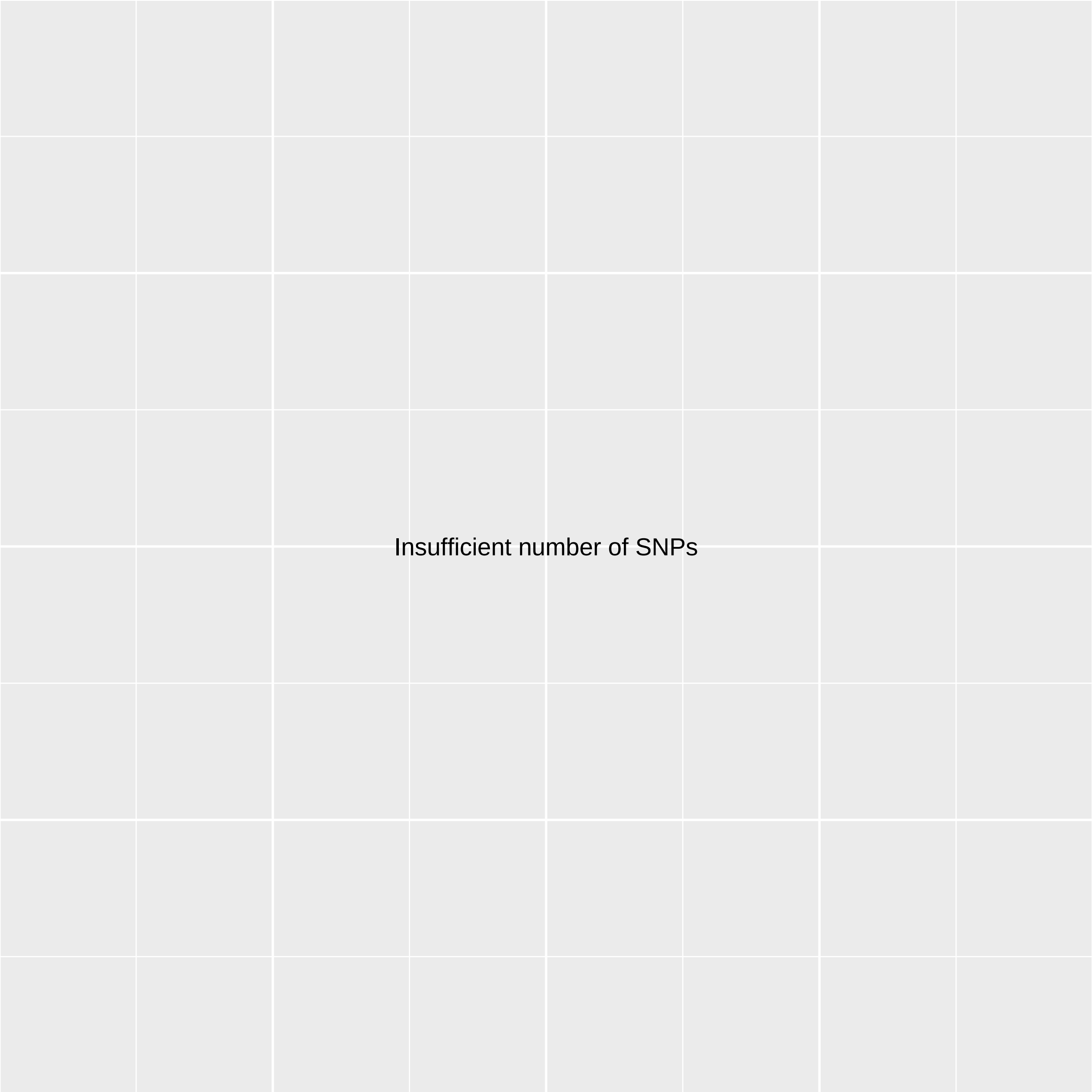
Insufficient number of SNPs

Batch 104 : Gut microbiota abundance (genus Lactobacillus id.1837) on Localized adiposity

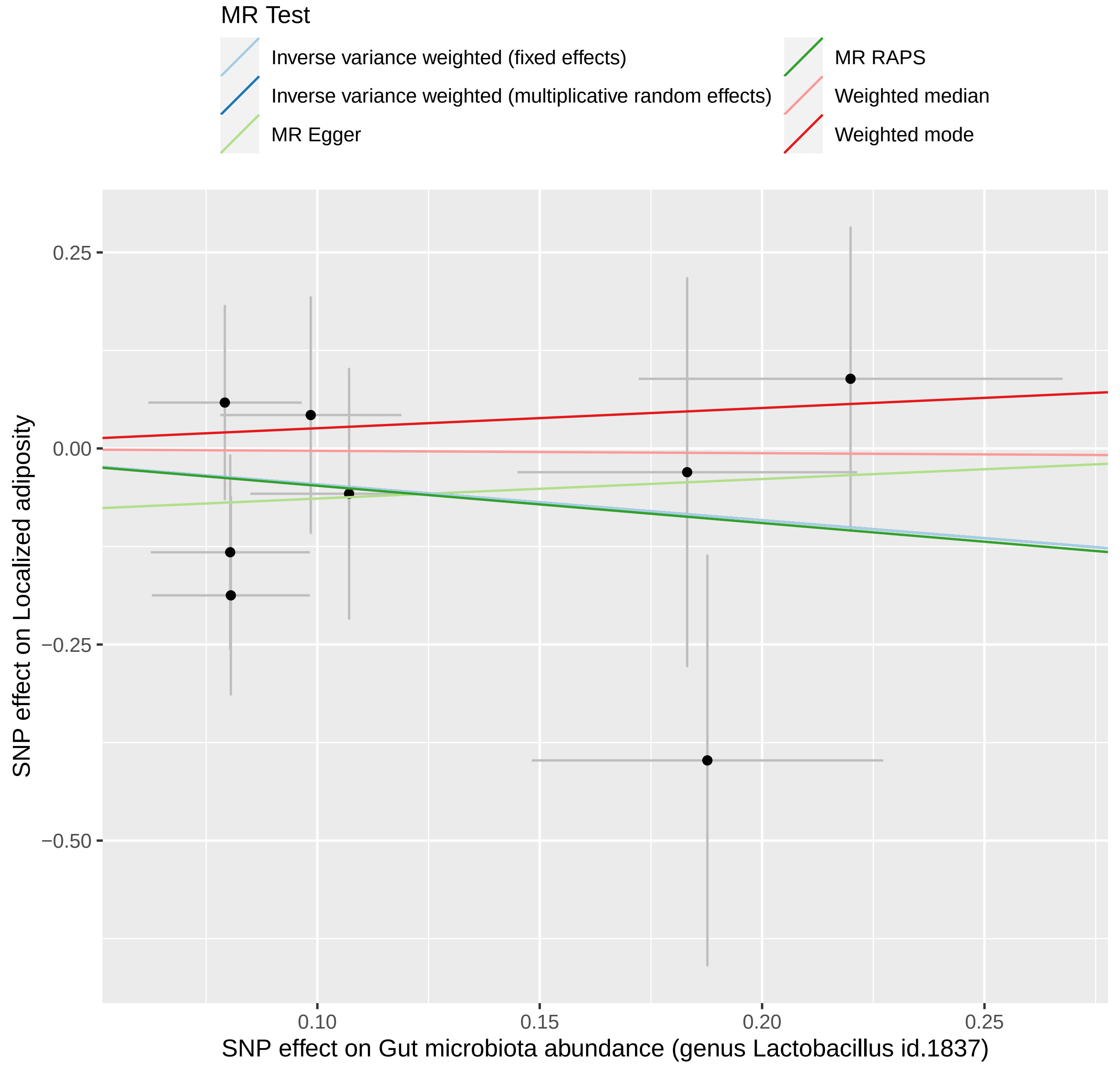

Batch 105 : Gut microbiota abundance (genus Lactococcus id.1851) on Localized adiposity

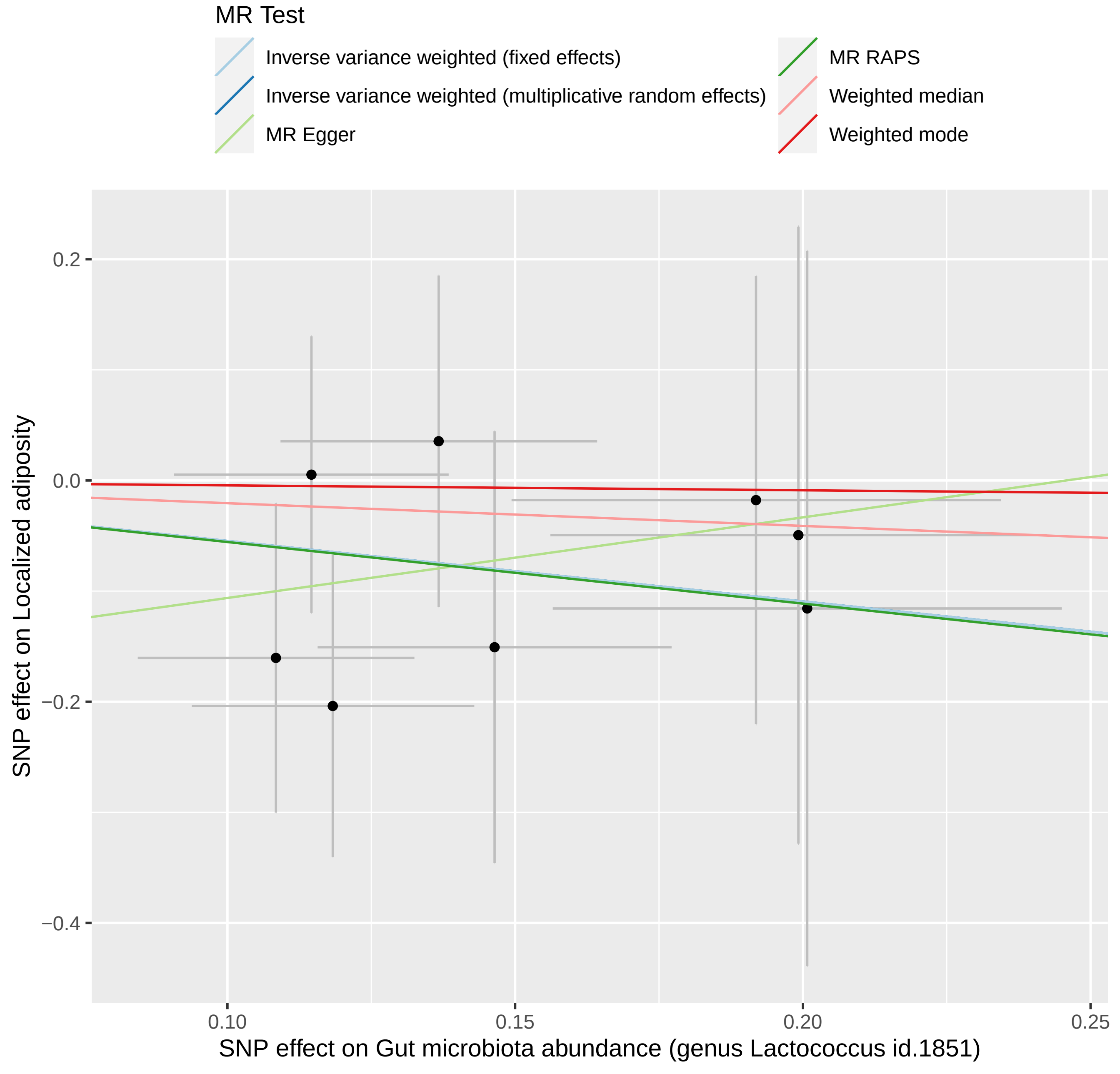

Batch 106 : Gut microbiota abundance (genus Marvinbryantia id.2005) on Localized adiposity

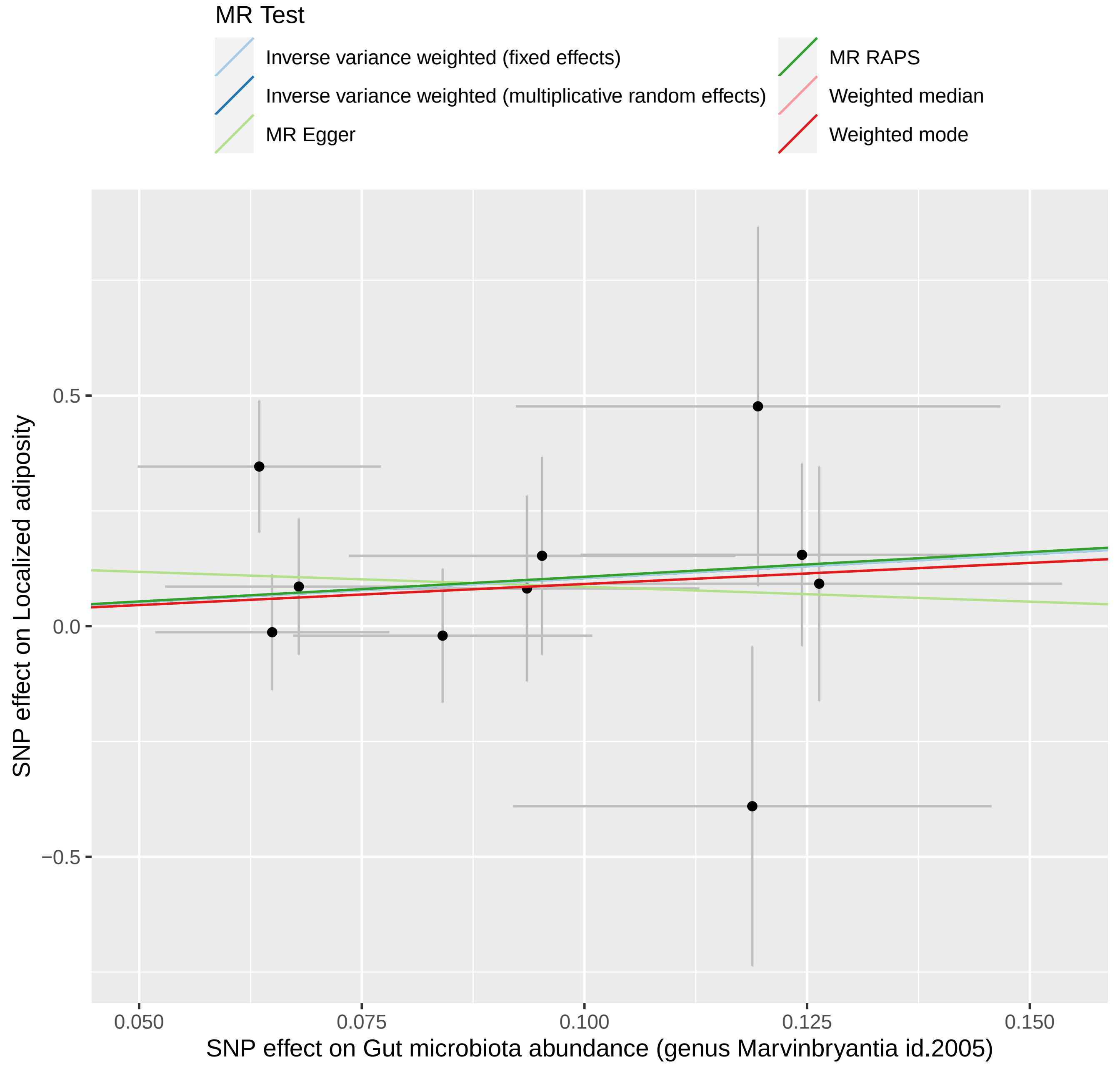

Batch 107 : Gut microbiota abundance (genus Methanobrevibacter id.123) on Localized adiposity

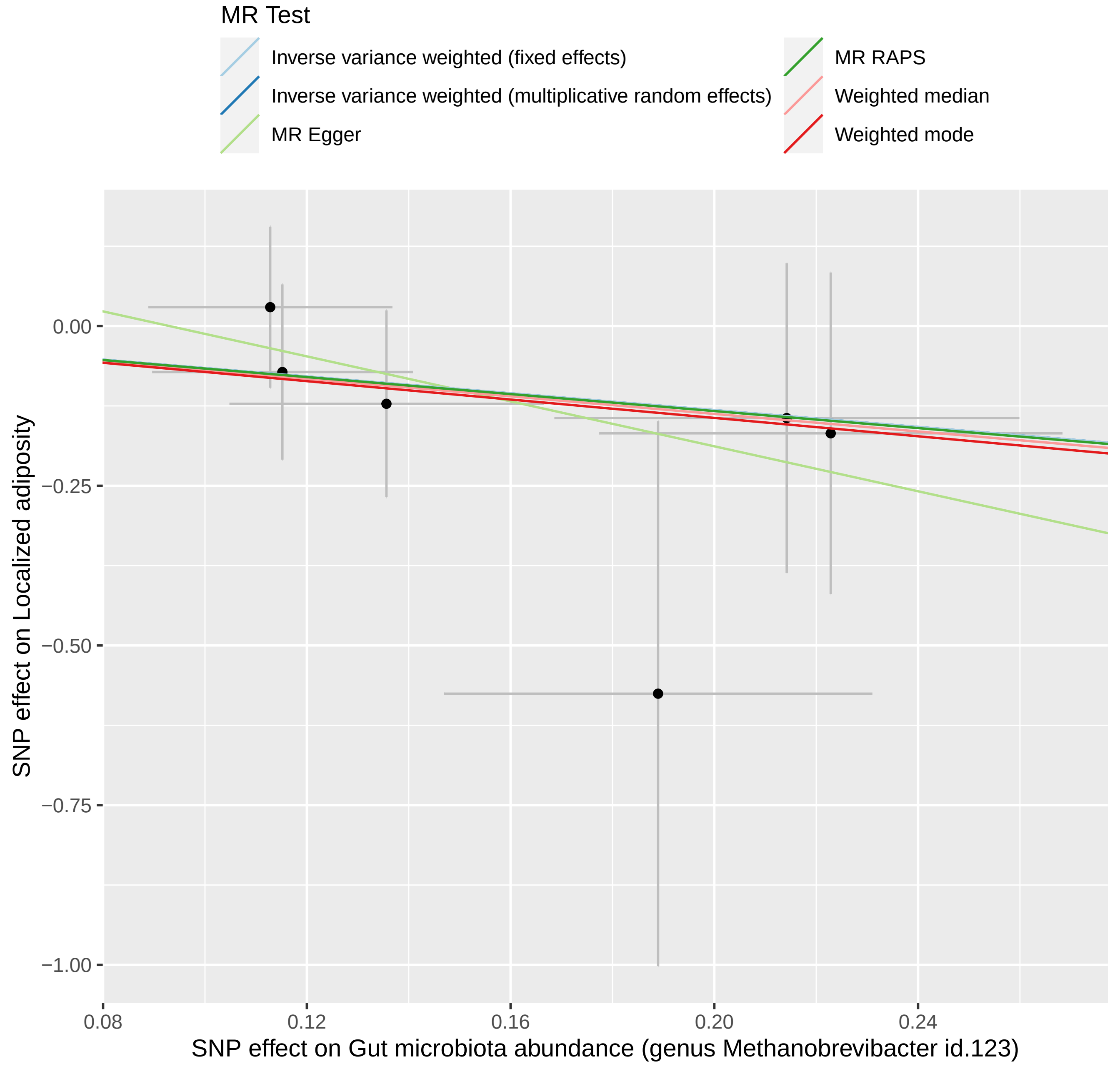

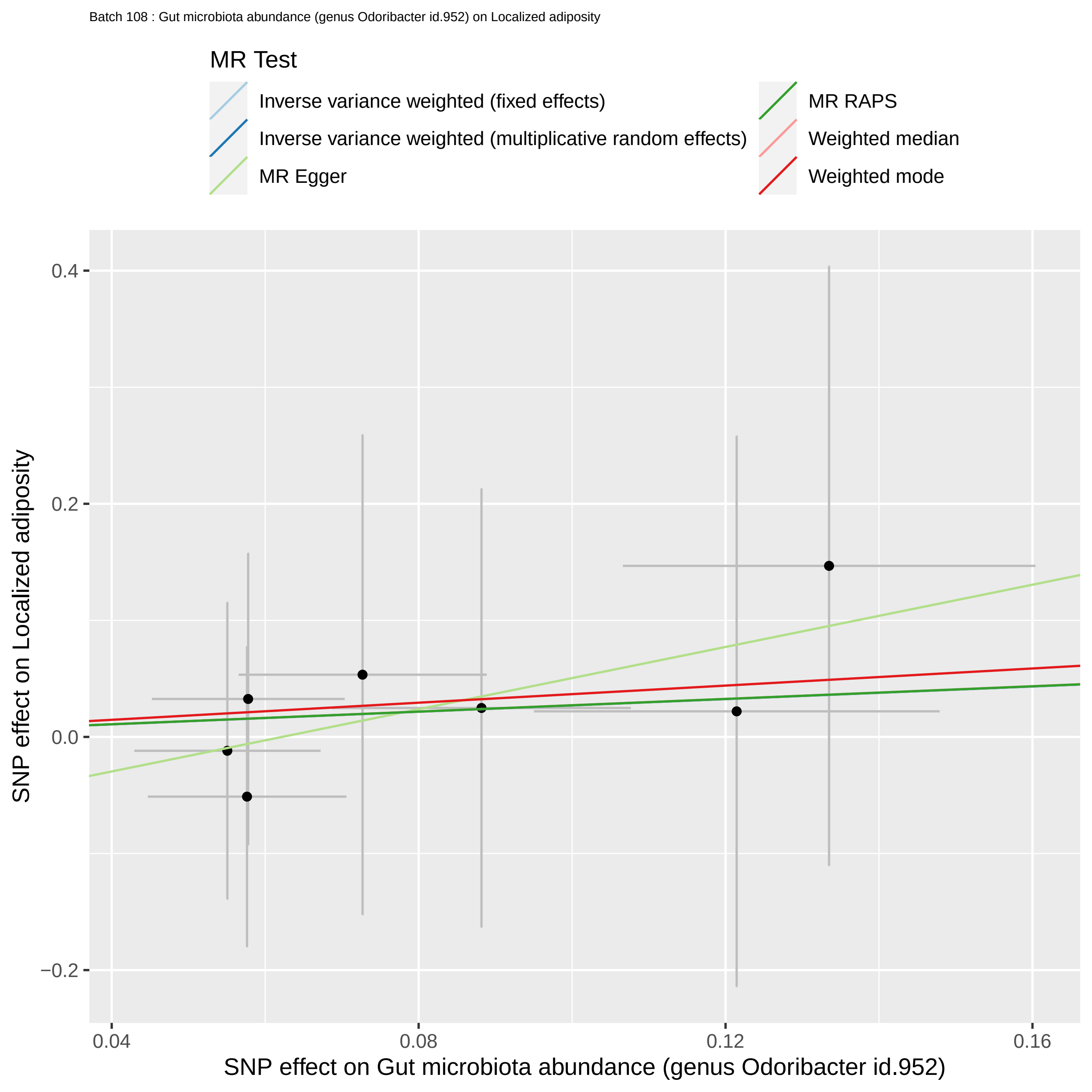
Batch 108 : Gut microbiota abundance (genus Odoribacter id.952) on Localized adiposity
MR Test
Inverse variance weighted (fixed effects)
Inverse variance weighted (multiplicative random effects)
MR Egger
MR RAPS
Weighted median
Weighted mode
0.4
0.2
0.0
−0.2
0.04
0.08
0.12
0.16
SNP effect on Localized adiposity
SNP effect on Gut microbiota abundance (genus Odoribacter id.952)

Batch 109 : Gut microbiota abundance (genus Olsenella id.822) on Localized adiposity

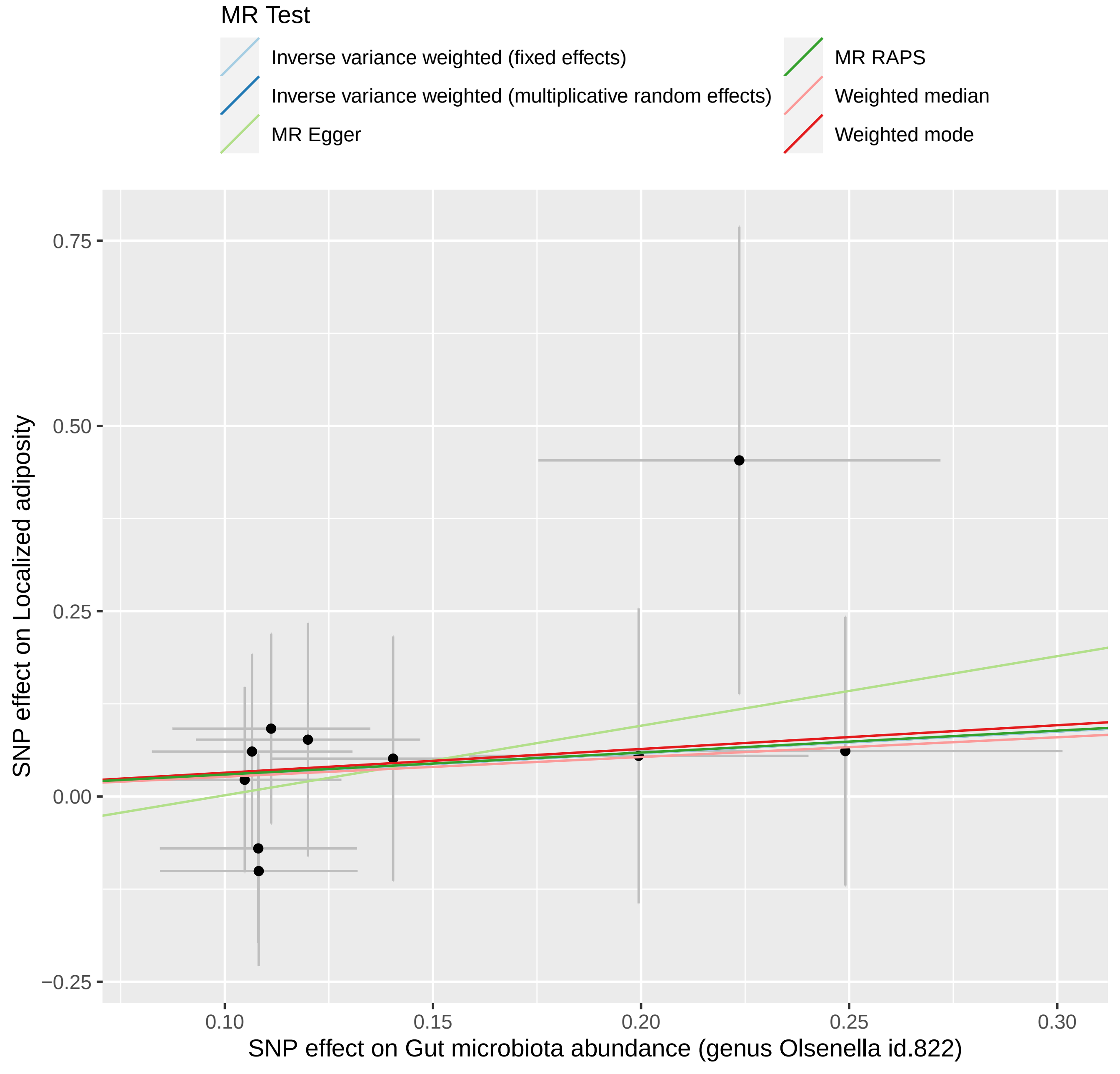

Batch 110 : Gut microbiota abundance (genus Oscillibacter id.2063) on Localized adiposity

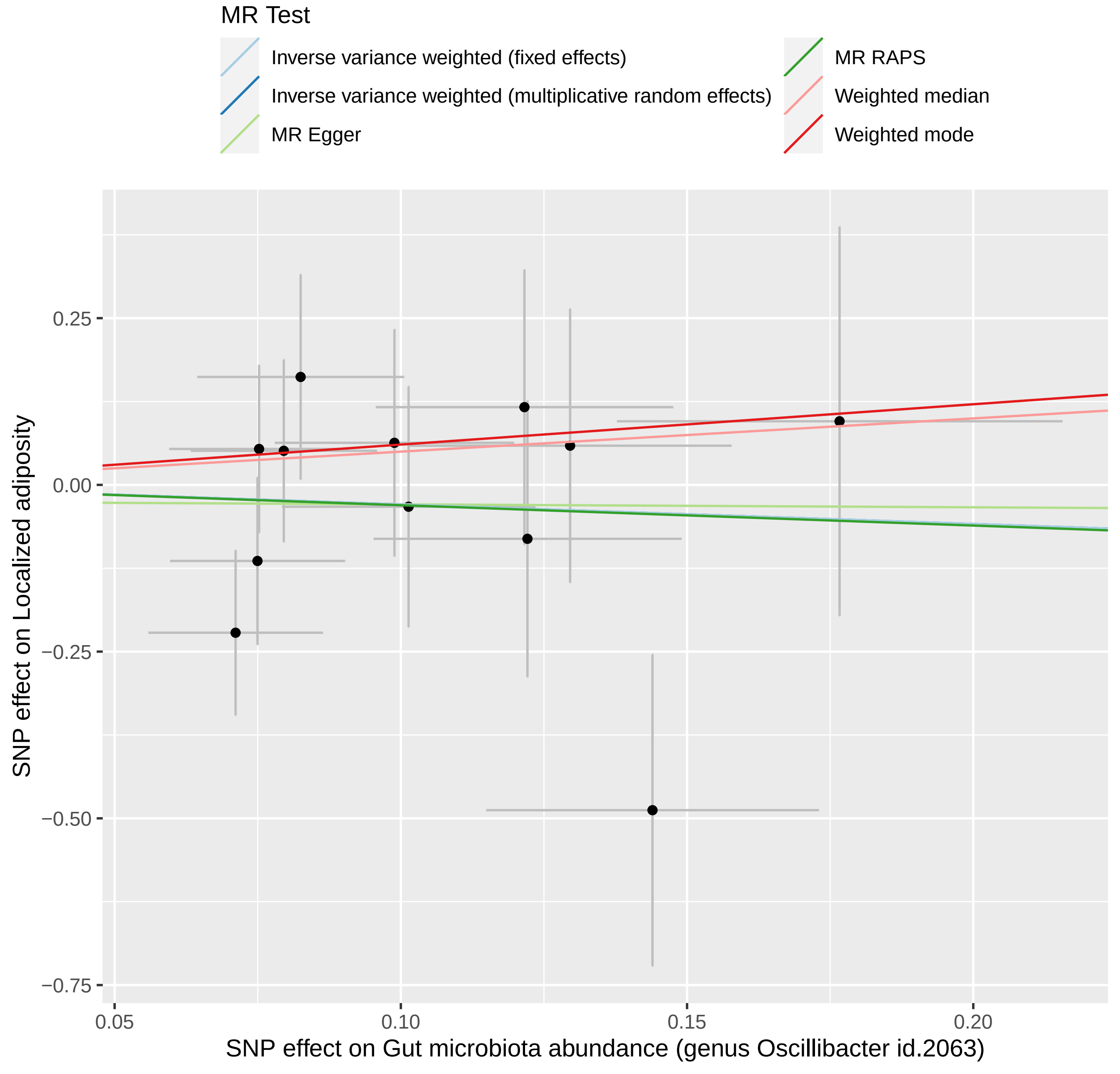

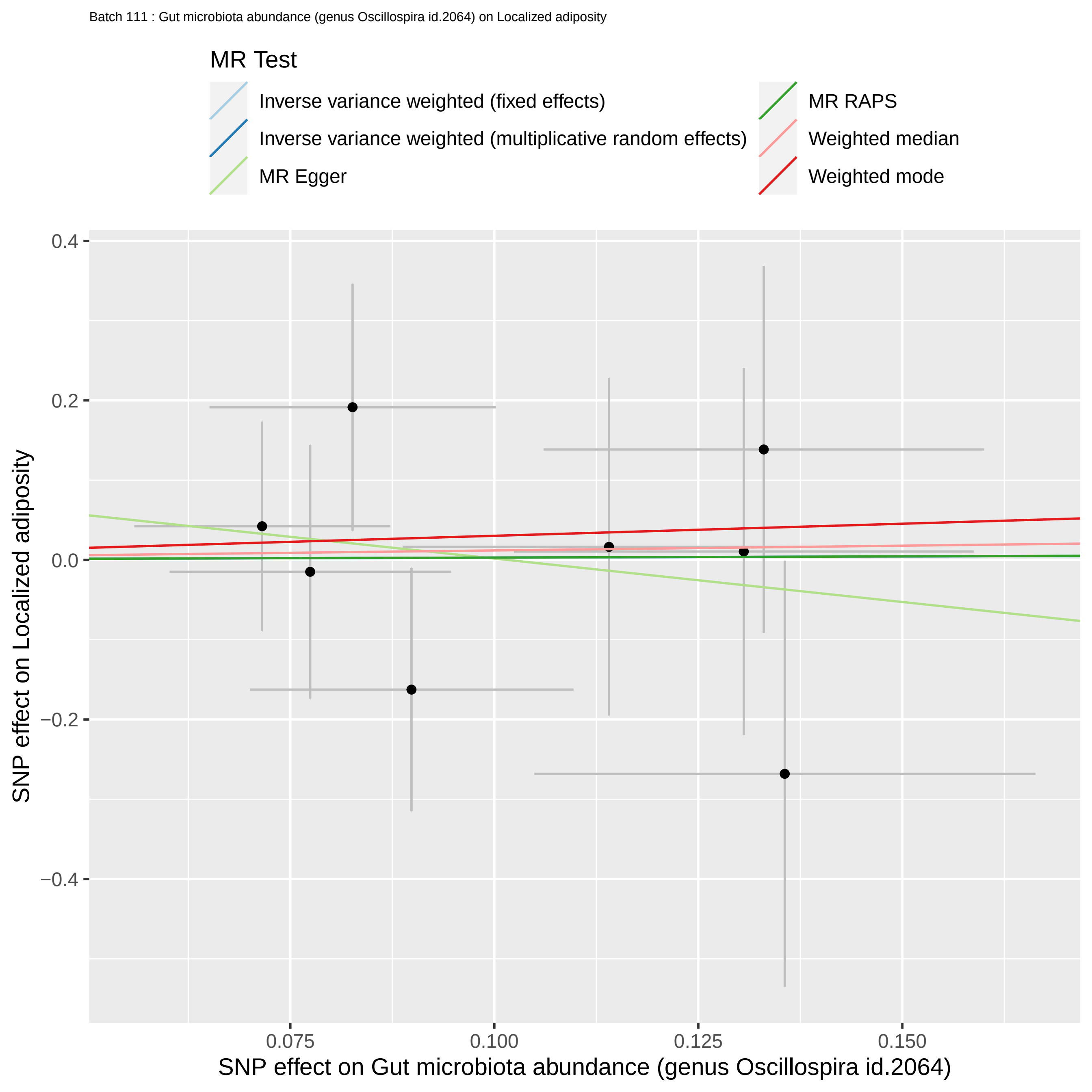
Batch 111 : Gut microbiota abundance (genus Oscillospira id.2064) on Localized adiposity
MR Test
Inverse variance weighted (fixed effects)
Inverse variance weighted (multiplicative random effects)
MR Egger
MR RAPS
Weighted median
Weighted mode
0.4
0.2
0.0
−0.2
−0.4
0.075
0.100
0.125
0.150
SNP effect on Localized adiposity
SNP effect on Gut microbiota abundance (genus Oscillospira id.2064)

Batch 112 : Gut microbiota abundance (genus Oxalobacter id.2978) on Localized adiposity

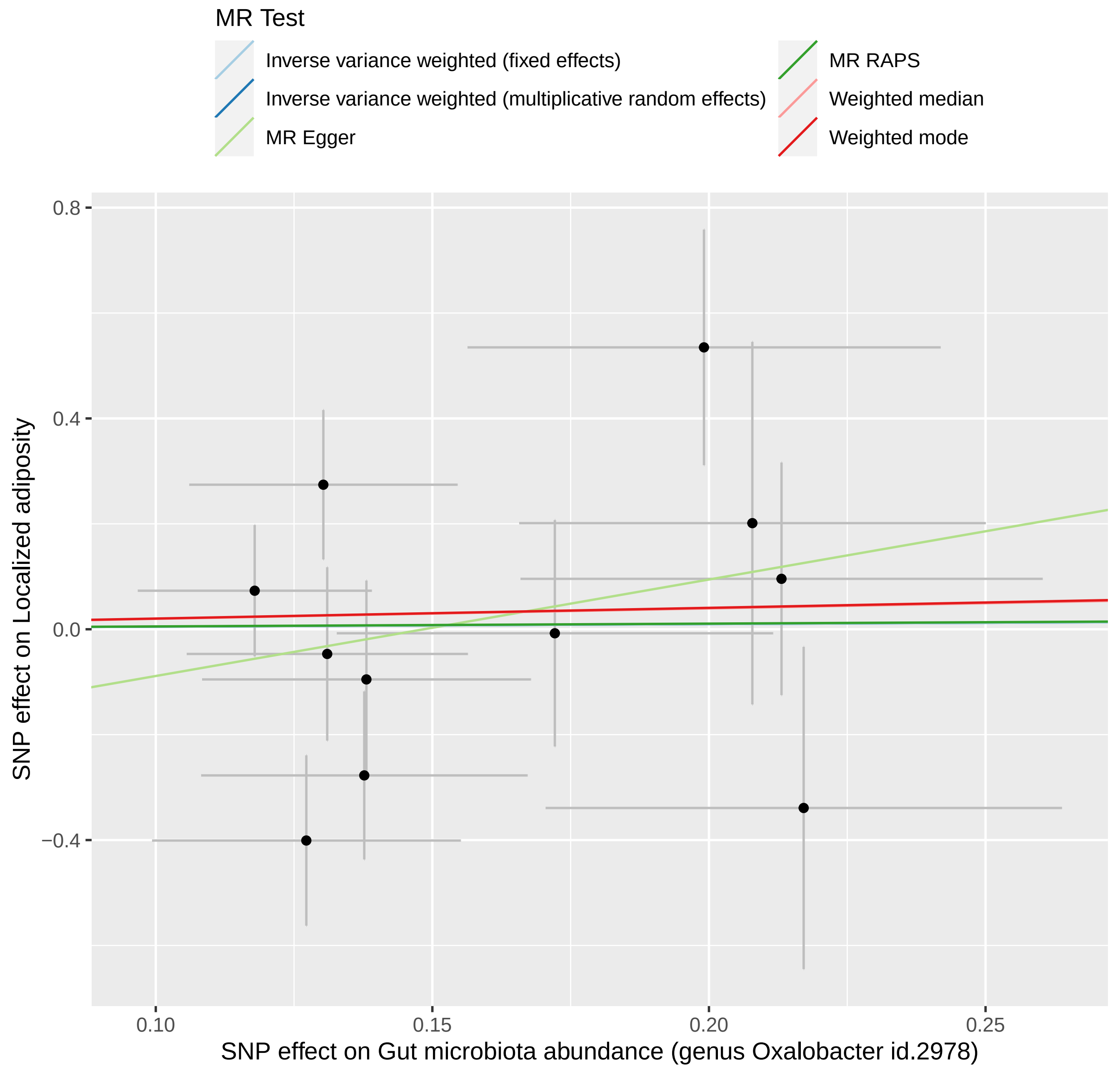

Batch 113 : Gut microbiota abundance (genus Parabacteroides id.954) on Localized adiposity

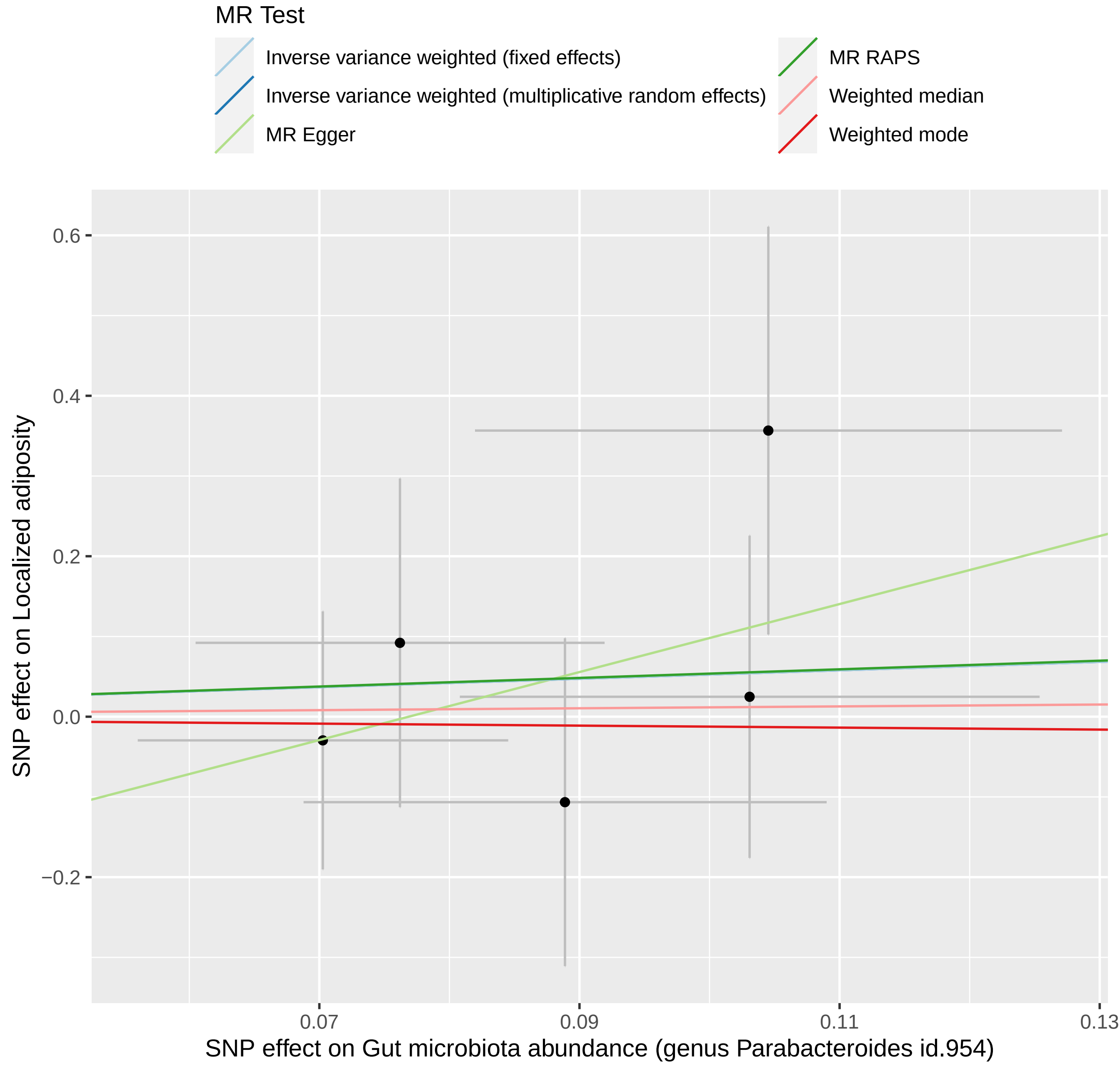

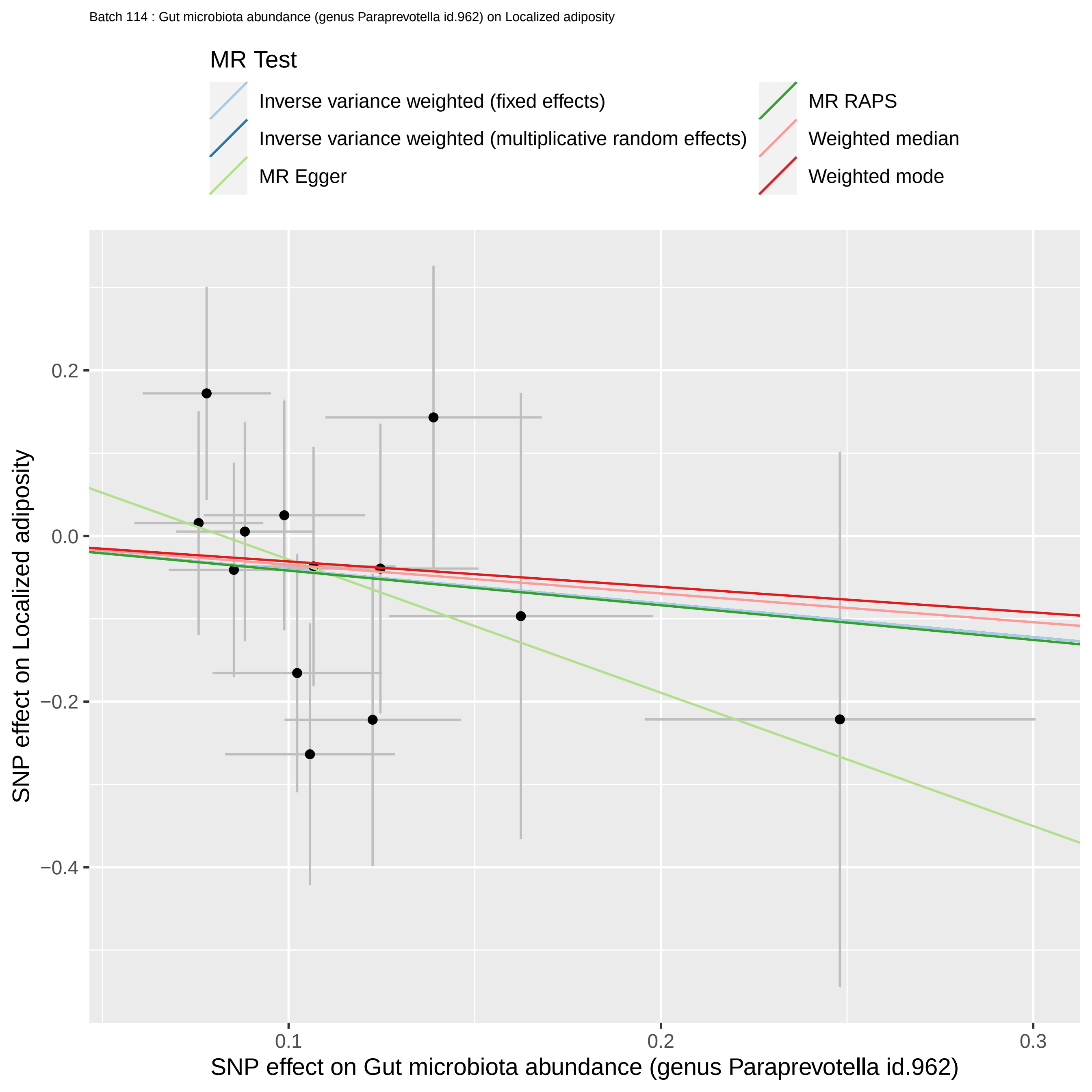
Batch 114 : Gut microbiota abundance (genus Paraprevotella id.962) on Localized adiposity
MR Test
Inverse variance weighted (fixed effects)
Inverse variance weighted (multiplicative random effects)
MR Egger
MR RAPS
Weighted median
Weighted mode
SNP effect on Localized adiposity
0.2
0.0
−0.2
−0.4
0.1
0.2
0.3
SNP effect on Gut microbiota abundance (genus Paraprevotella id.962)

Batch 115 : Gut microbiota abundance (genus Parasutterella id.2892) on Localized adiposity

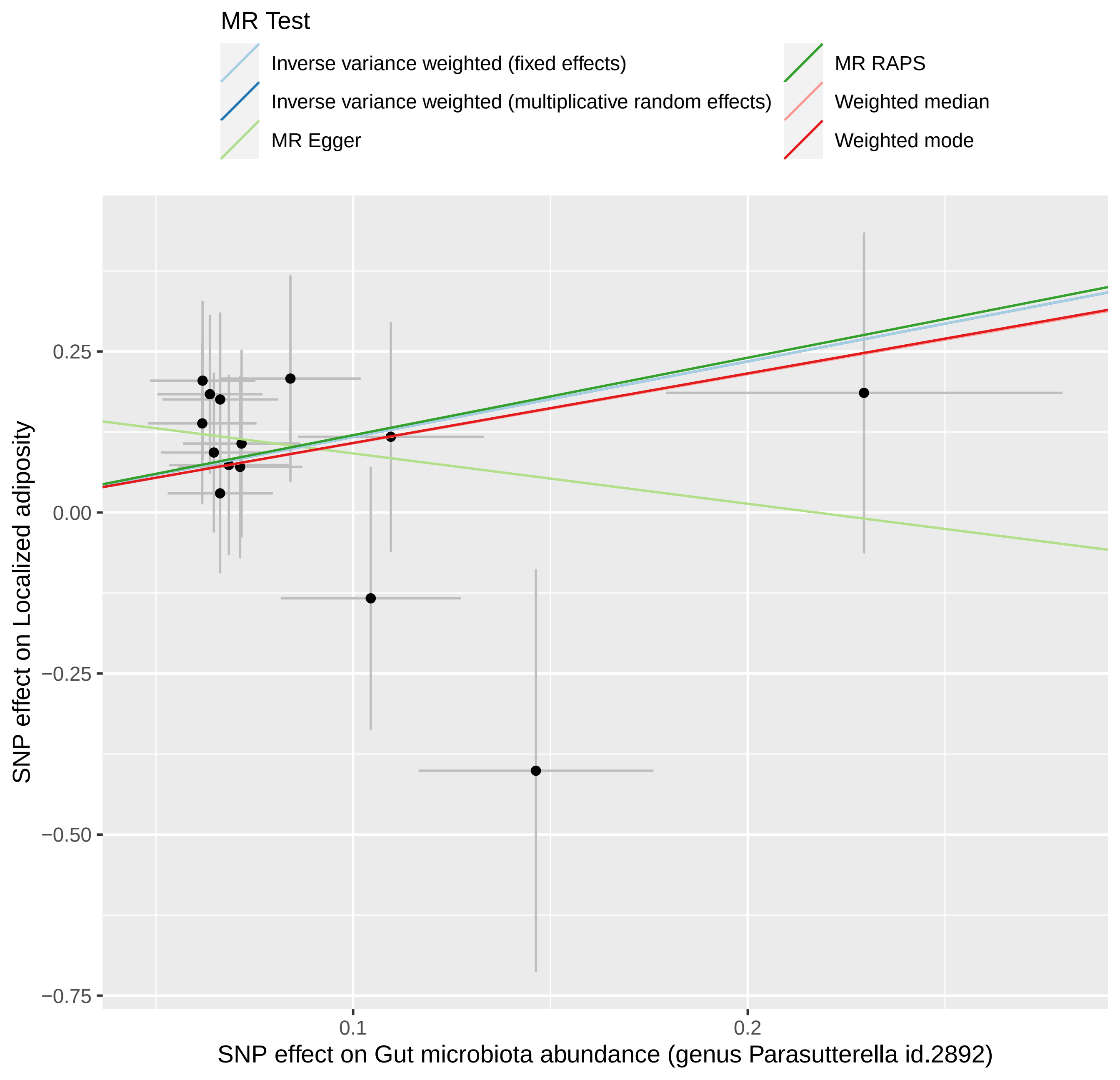

Batch 116 : Gut microbiota abundance (genus Peptococcus id.2037) on Localized adiposity

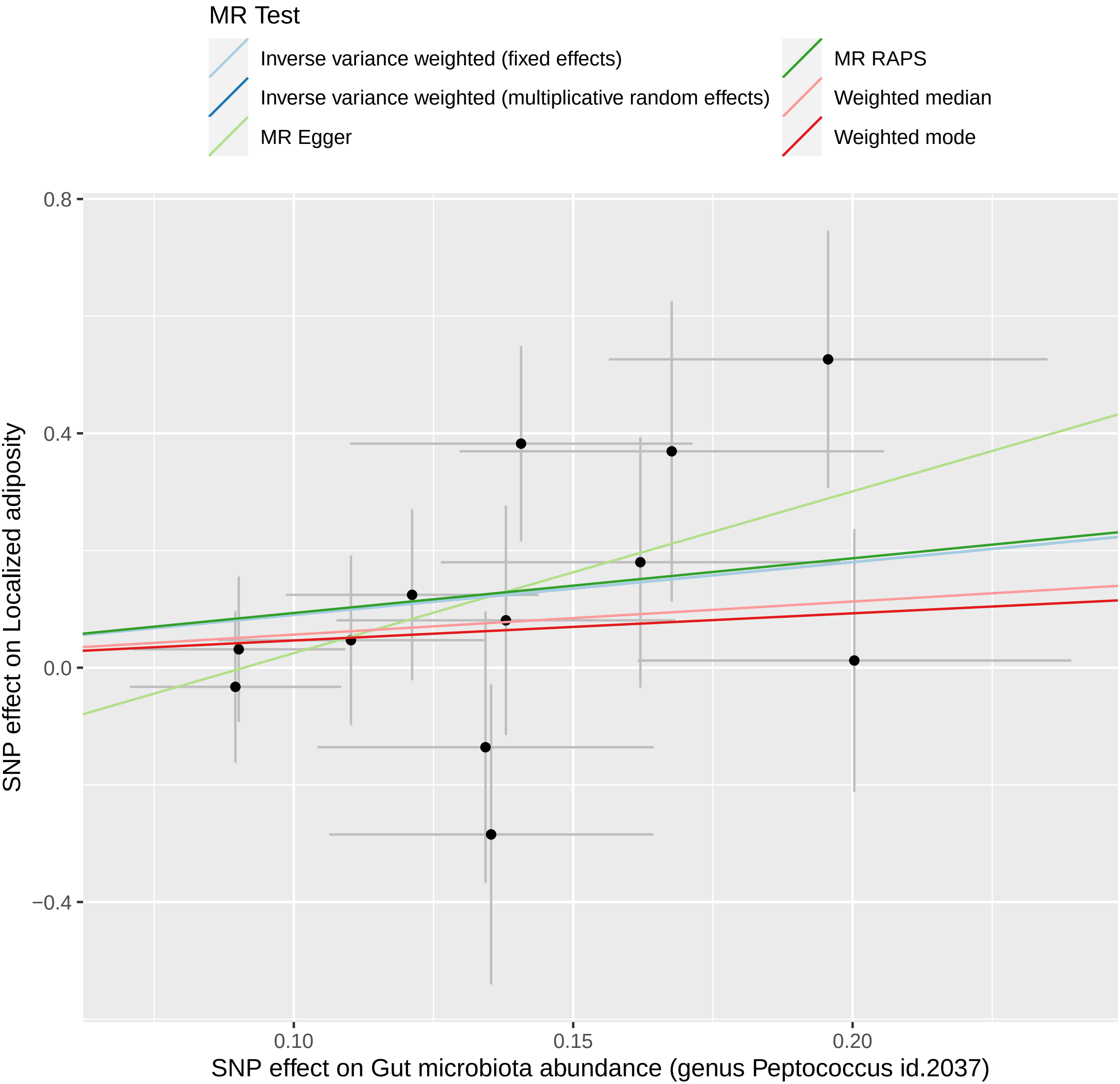

Batch 117 : Gut microbiota abundance (genus Phascolarctobacterium id.2168) on Localized adiposity

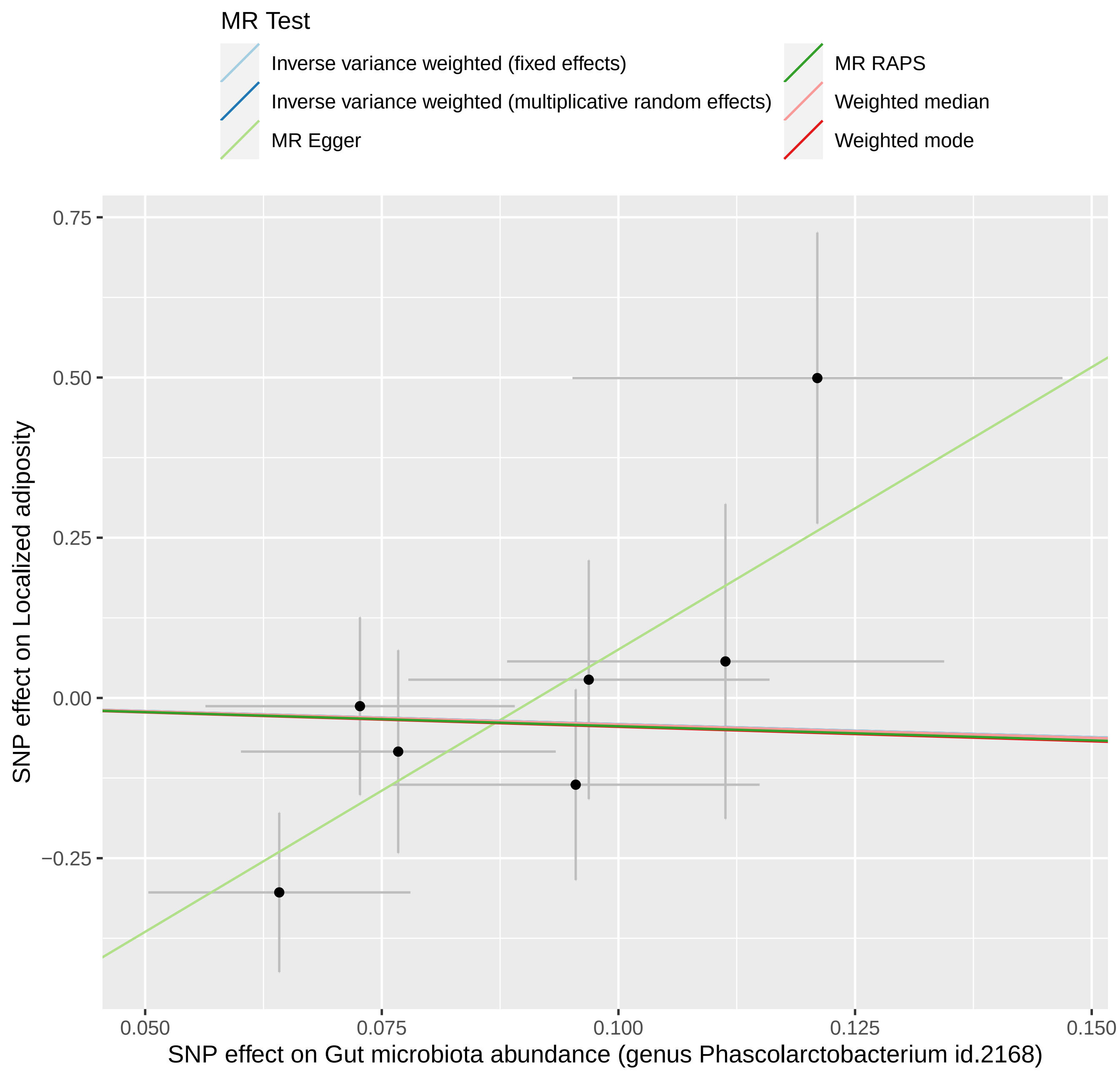

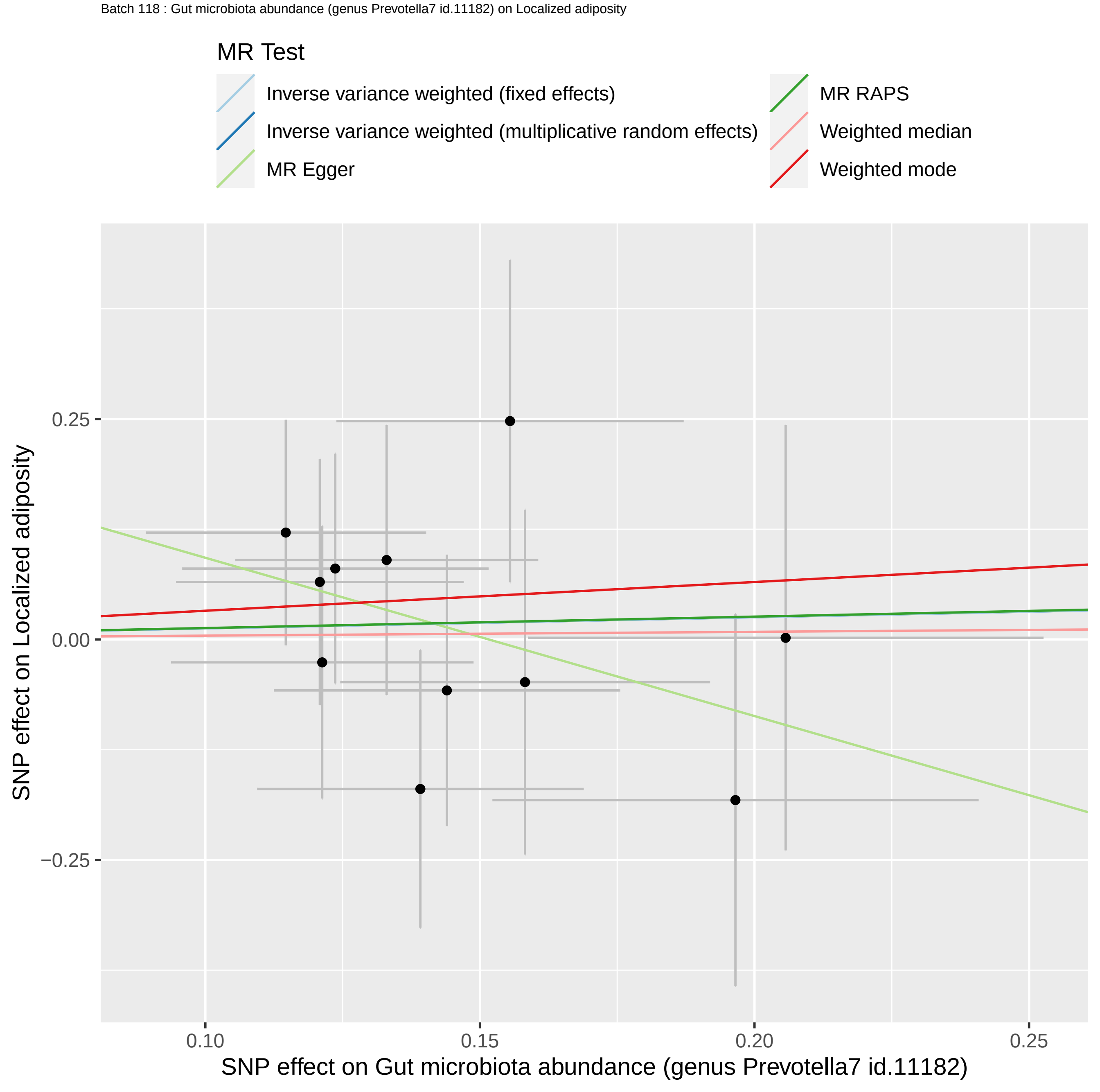

Batch 118 : Gut microbiota abundance (genus Prevotella7 id.11182) on Localized adiposity
MR Test
Inverse variance weighted (fixed effects)
Inverse variance weighted (multiplicative random effects)
MR Egger
MR RAPS
Weighted median
Weighted mode
0.25
0.00
−0.25
0.10
0.15
0.20
0.25
SNP effect on Localized adiposity
SNP effect on Gut microbiota abundance (genus Prevotella7 id.11182)

Batch 119 : Gut microbiota abundance (genus Prevotella9 id.11183) on Localized adiposity

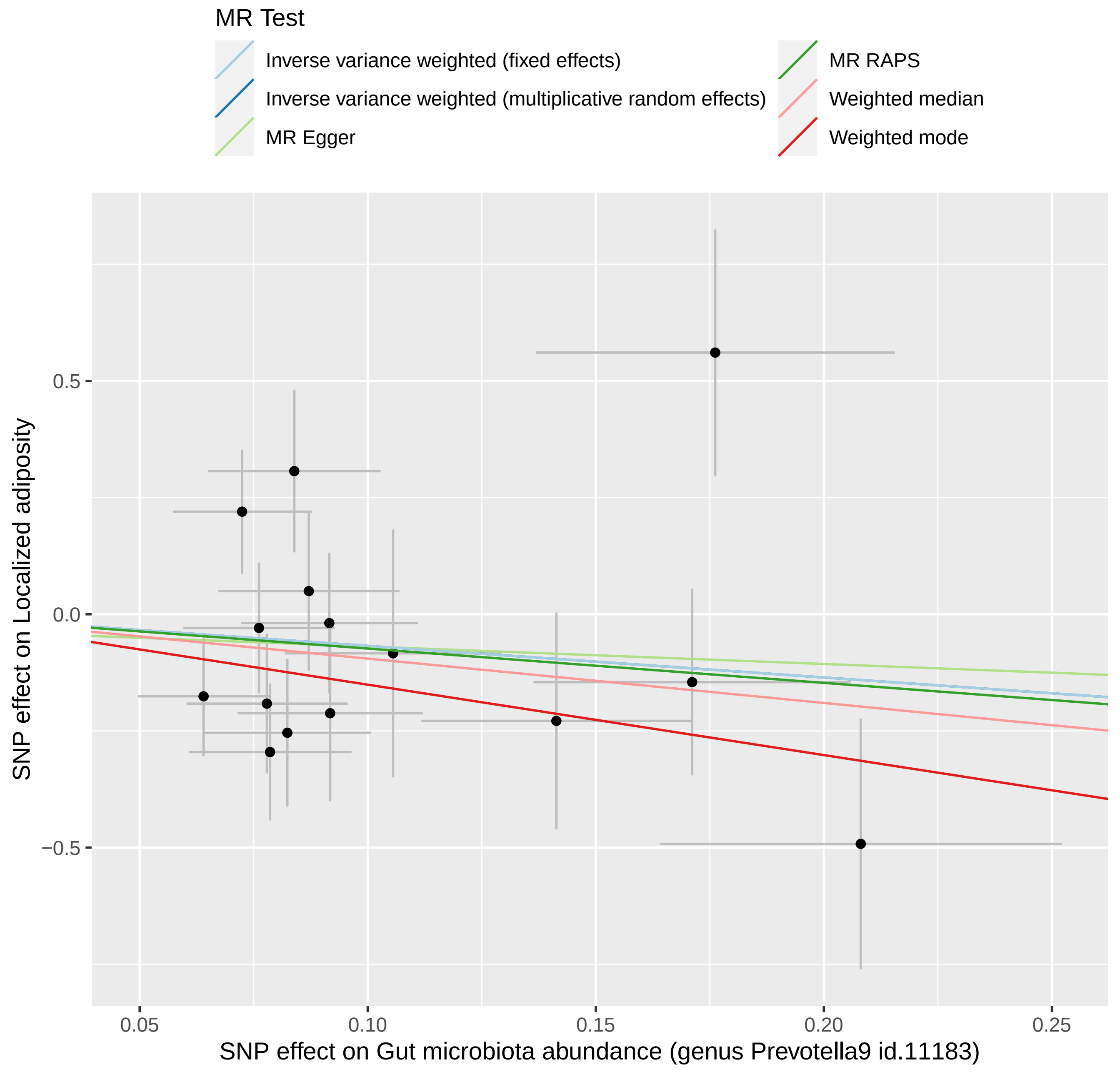

Batch 120 : Gut microbiota abundance (genus Rikenellaceae RC9 gut group id.11191) on Localized adiposity

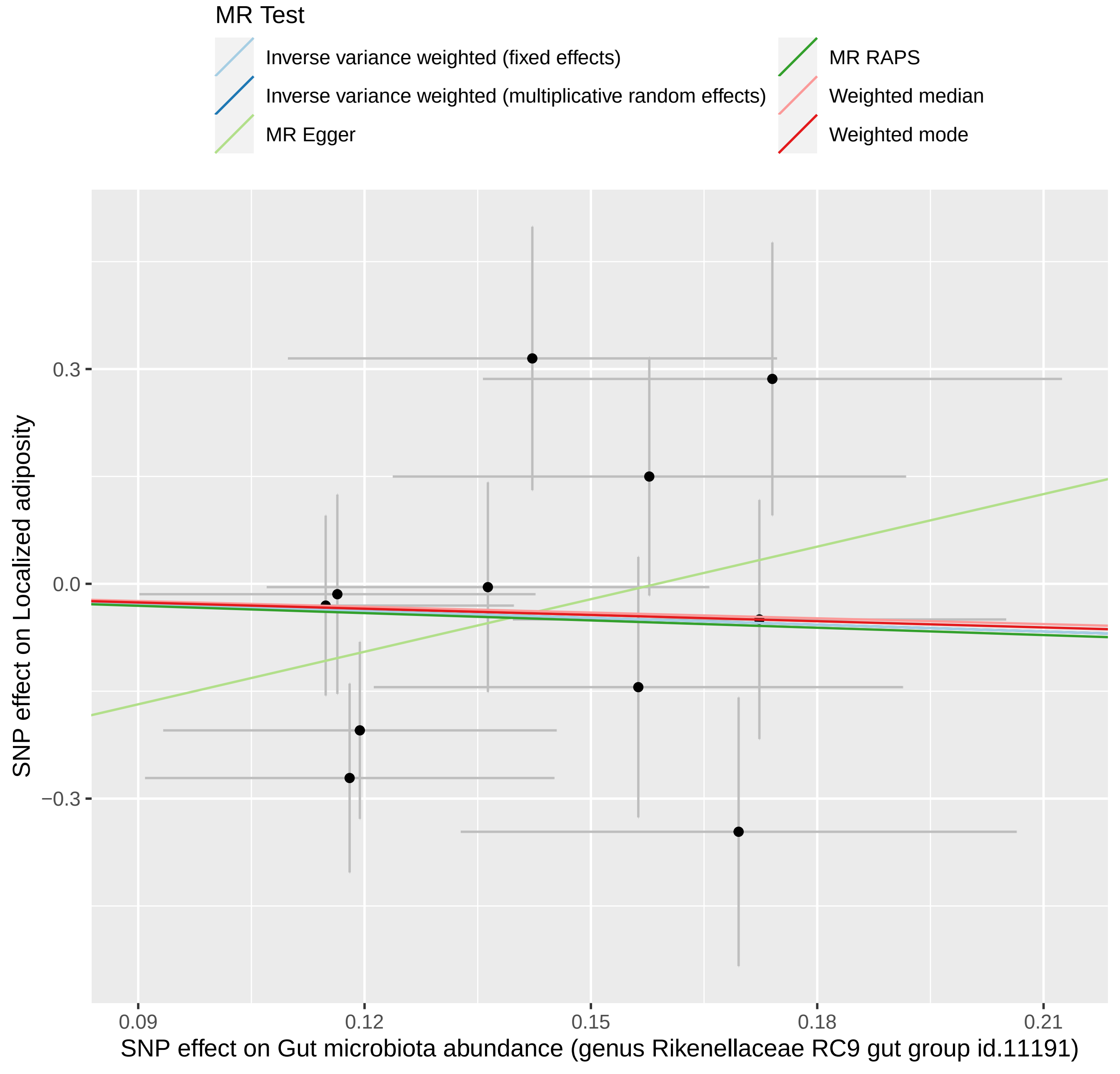

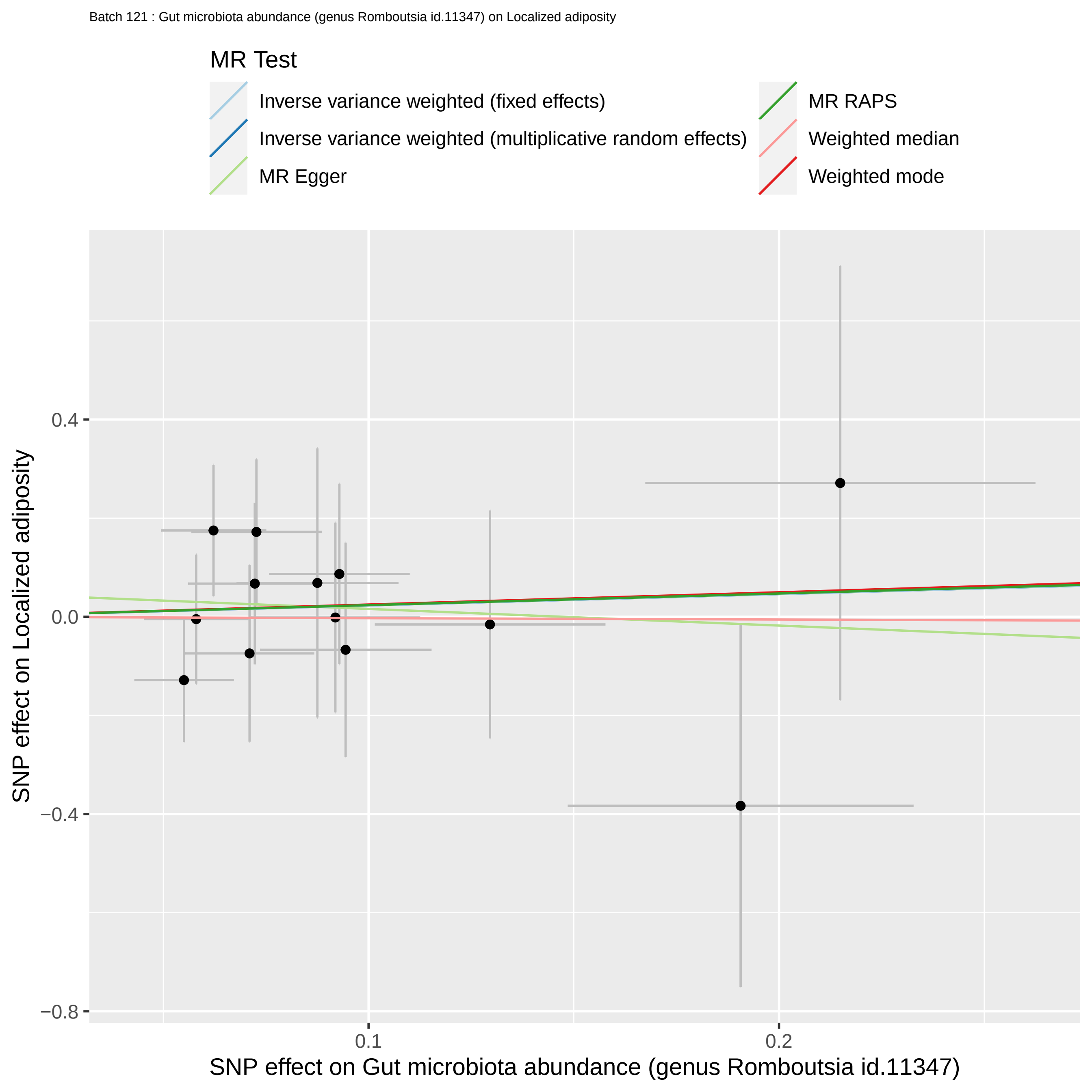

Batch 121 : Gut microbiota abundance (genus Romboutsia id.11347) on Localized adiposity
MR Test
Inverse variance weighted (fixed effects)
Inverse variance weighted (multiplicative random effects)
MR Egger
MR RAPS
Weighted median
Weighted mode
0.4
0.0
−0.4
−0.8
0.1
0.2
SNP effect on Localized adiposity
SNP effect on Gut microbiota abundance (genus Romboutsia id.11347)

Batch 122 : Gut microbiota abundance (genus Roseburia id.2012) on Localized adiposity

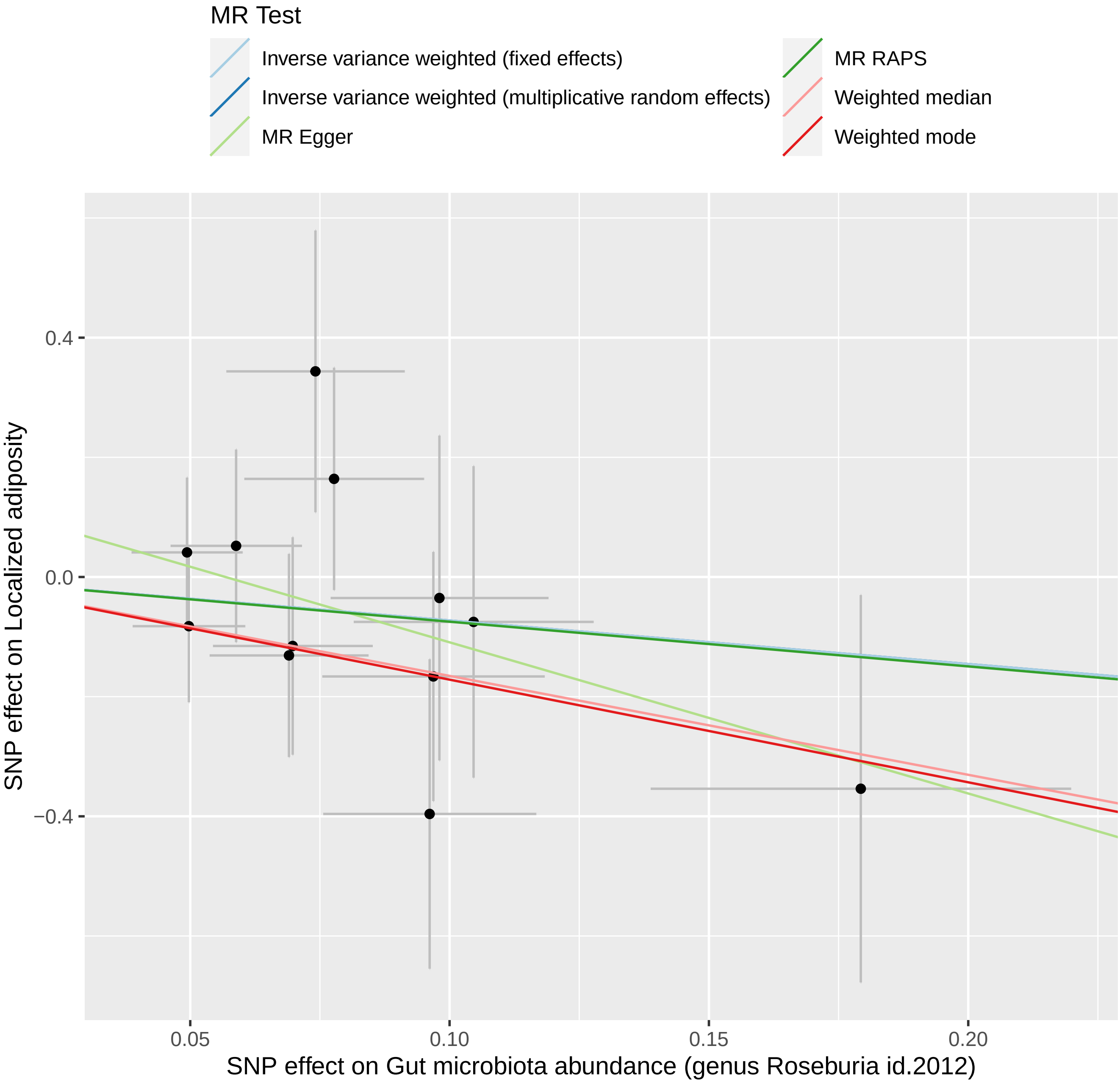

Batch 123 : Gut microbiota abundance (genus Ruminiclostridium5 id.11355) on Localized adiposity

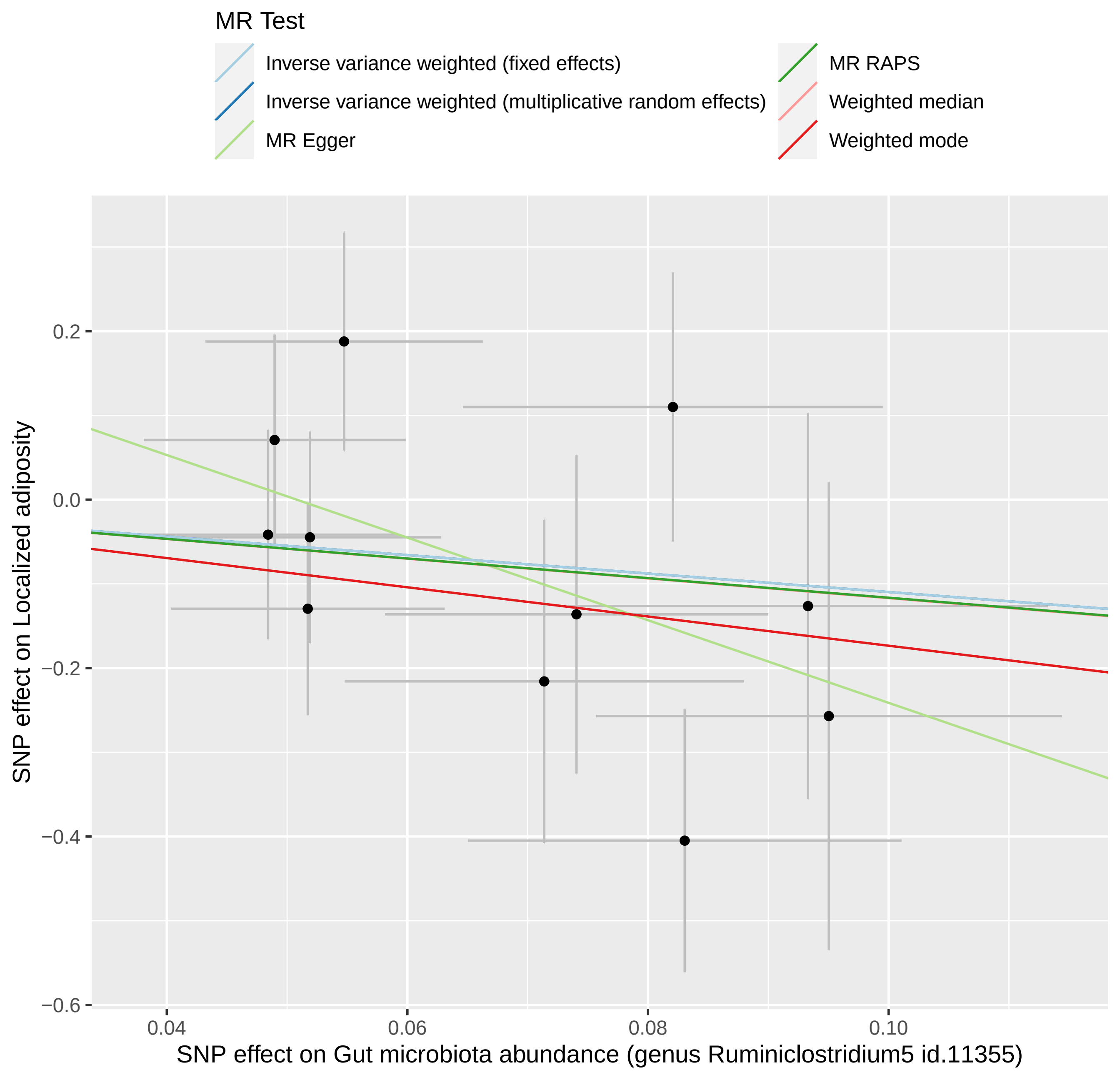

Batch 124 : Gut microbiota abundance (genus Ruminiclostridium6 id.11356) on Localized adiposity

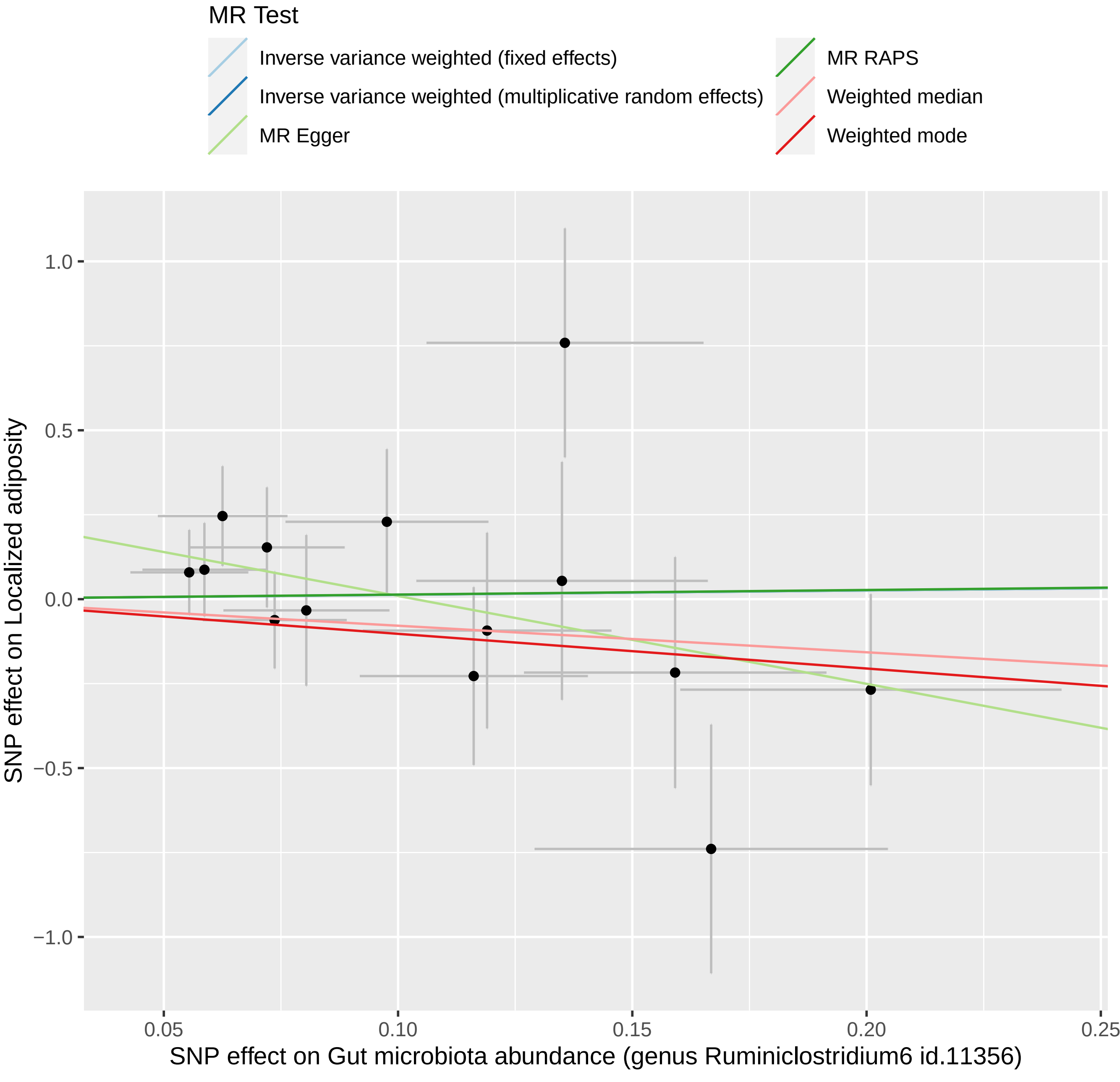

Batch 125 : Gut microbiota abundance (genus Ruminiclostridium9 id.11357) on Localized adiposity

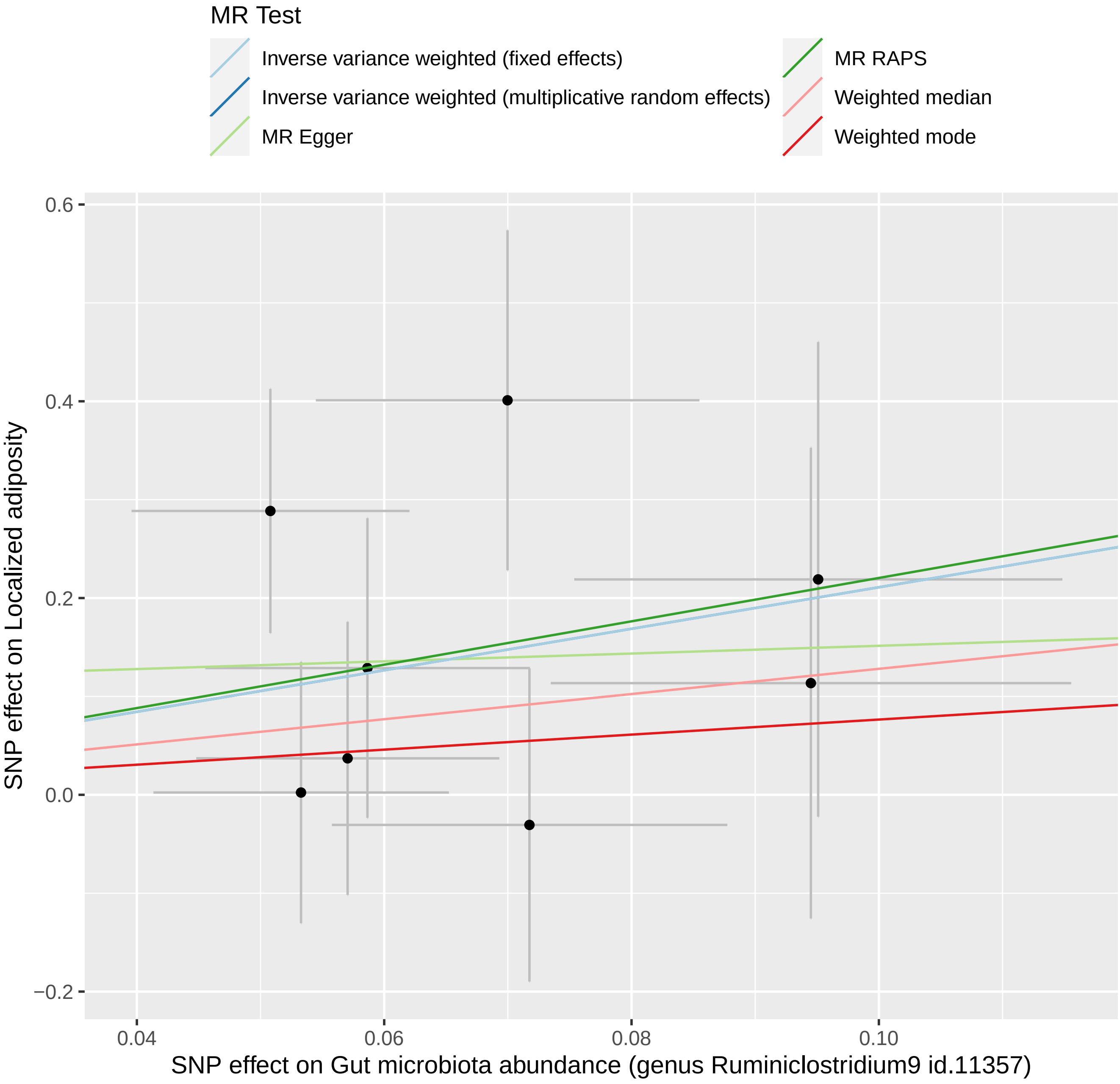

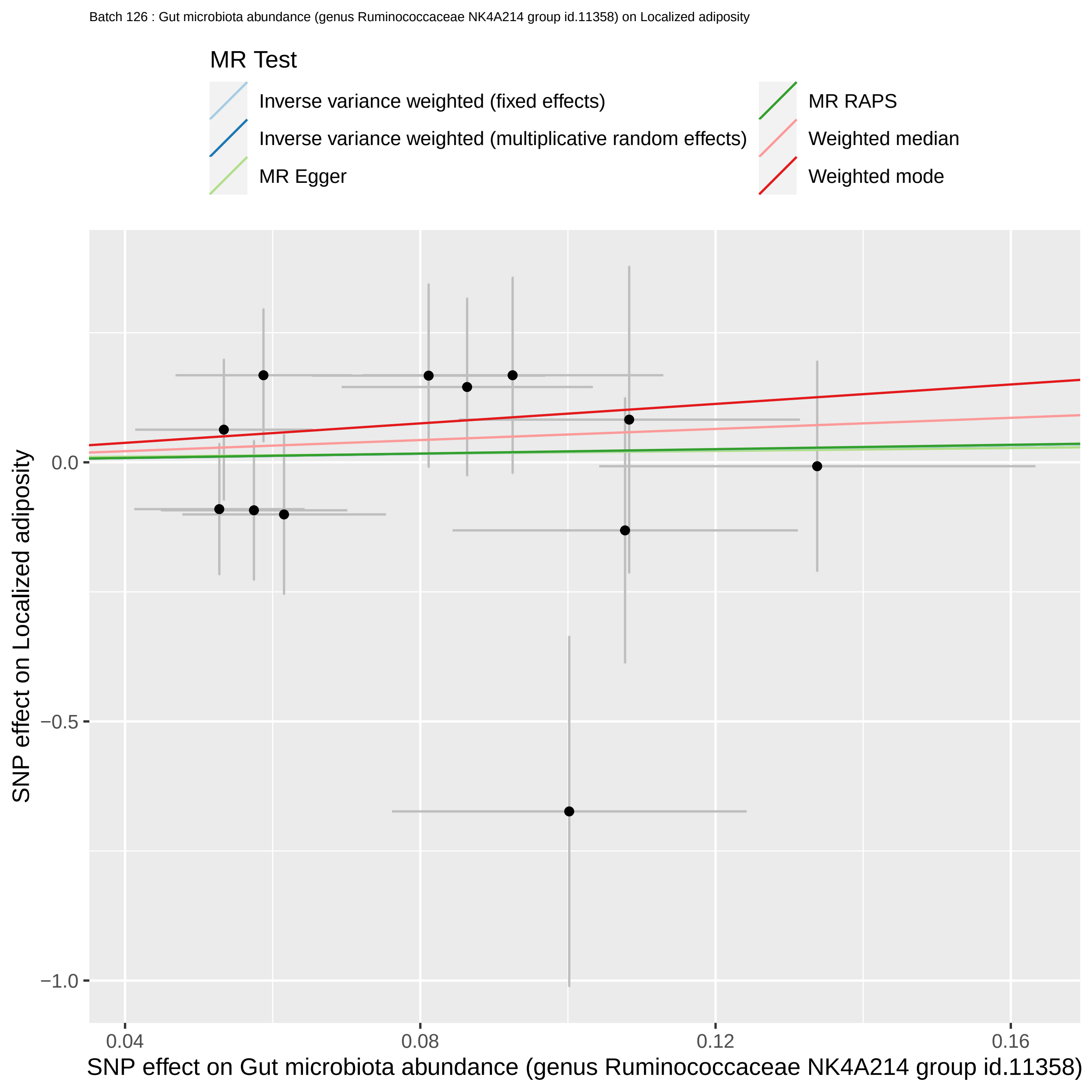
Batch 126 : Gut microbiota abundance (genus Ruminococcaceae NK4A214 group id.11358) on Localized adiposity
MR Test
Inverse variance weighted (fixed effects)
Inverse variance weighted (multiplicative random effects)
MR Egger
MR RAPS
Weighted median
Weighted mode
SNP effect on Localized adiposity
0.0
−0.5
−1.0
0.04
0.08
0.12
0.16
SNP effect on Gut microbiota abundance (genus Ruminococcaceae NK4A214 group id.11358)

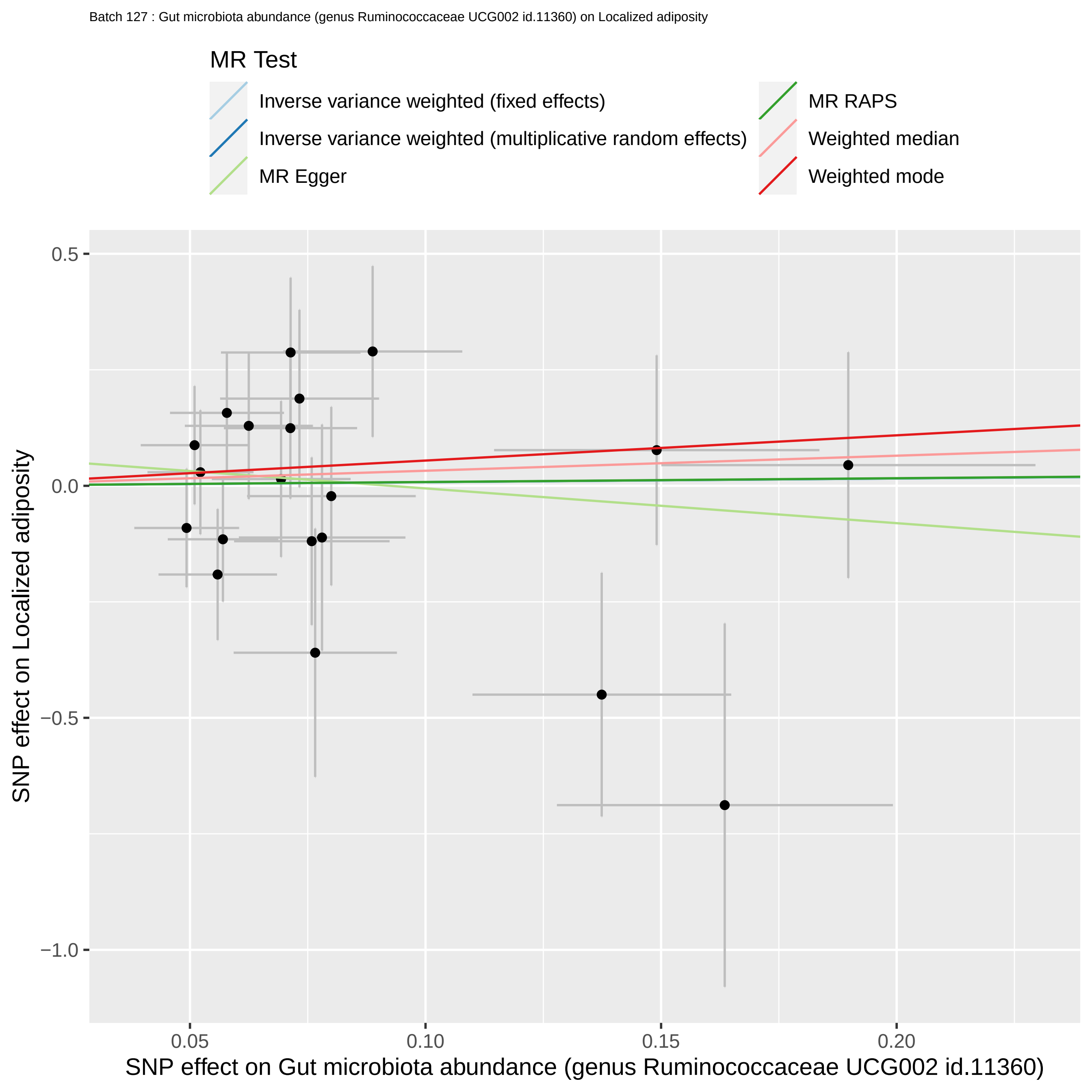
Batch 127 : Gut microbiota abundance (genus Ruminococcaceae UCG002 id.11360) on Localized adiposity
MR Test
Inverse variance weighted (fixed effects)
Inverse variance weighted (multiplicative random effects)
MR Egger
MR RAPS
Weighted median
Weighted mode
0.5
0.0
−0.5
−1.0
0.05
0.10
0.15
0.20
SNP effect on Localized adiposity
SNP effect on Gut microbiota abundance (genus Ruminococcaceae UCG002 id.11360)

Batch 128 : Gut microbiota abundance (genus Ruminococcaceae UCG003 id.11361) on Localized adiposity

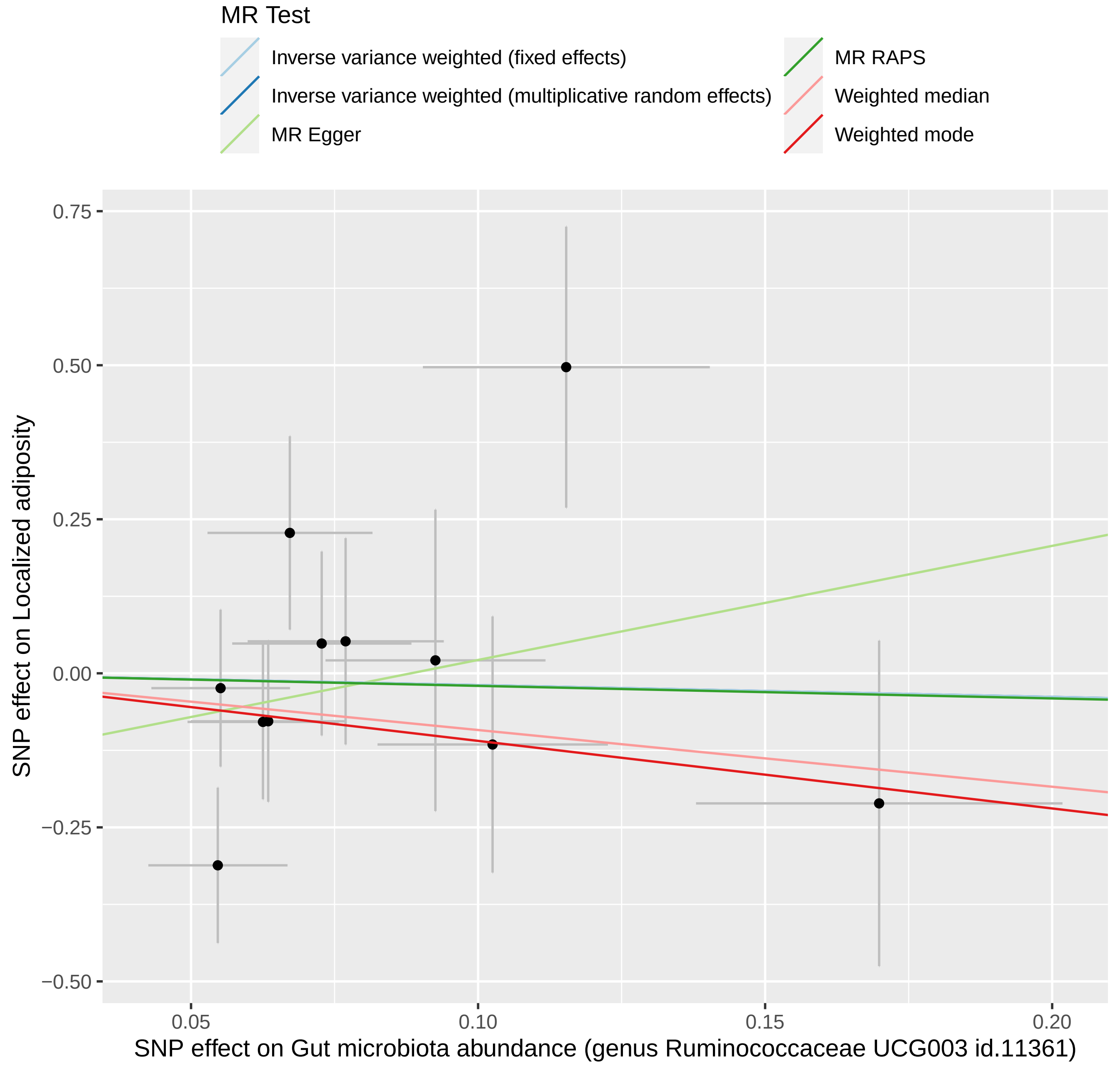

Batch 129 : Gut microbiota abundance (genus Ruminococcaceae UCG004 id.11362) on Localized adiposity

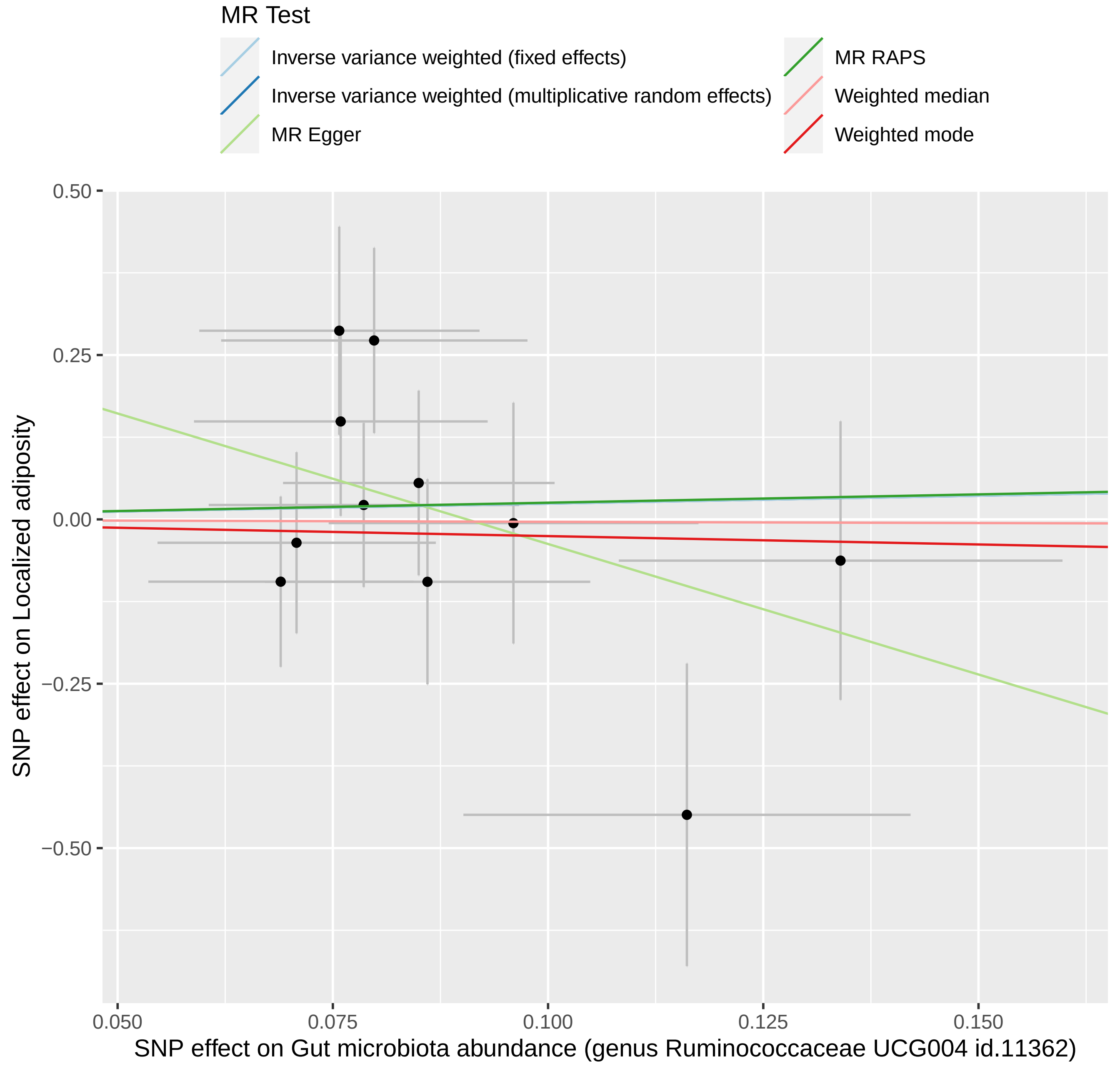

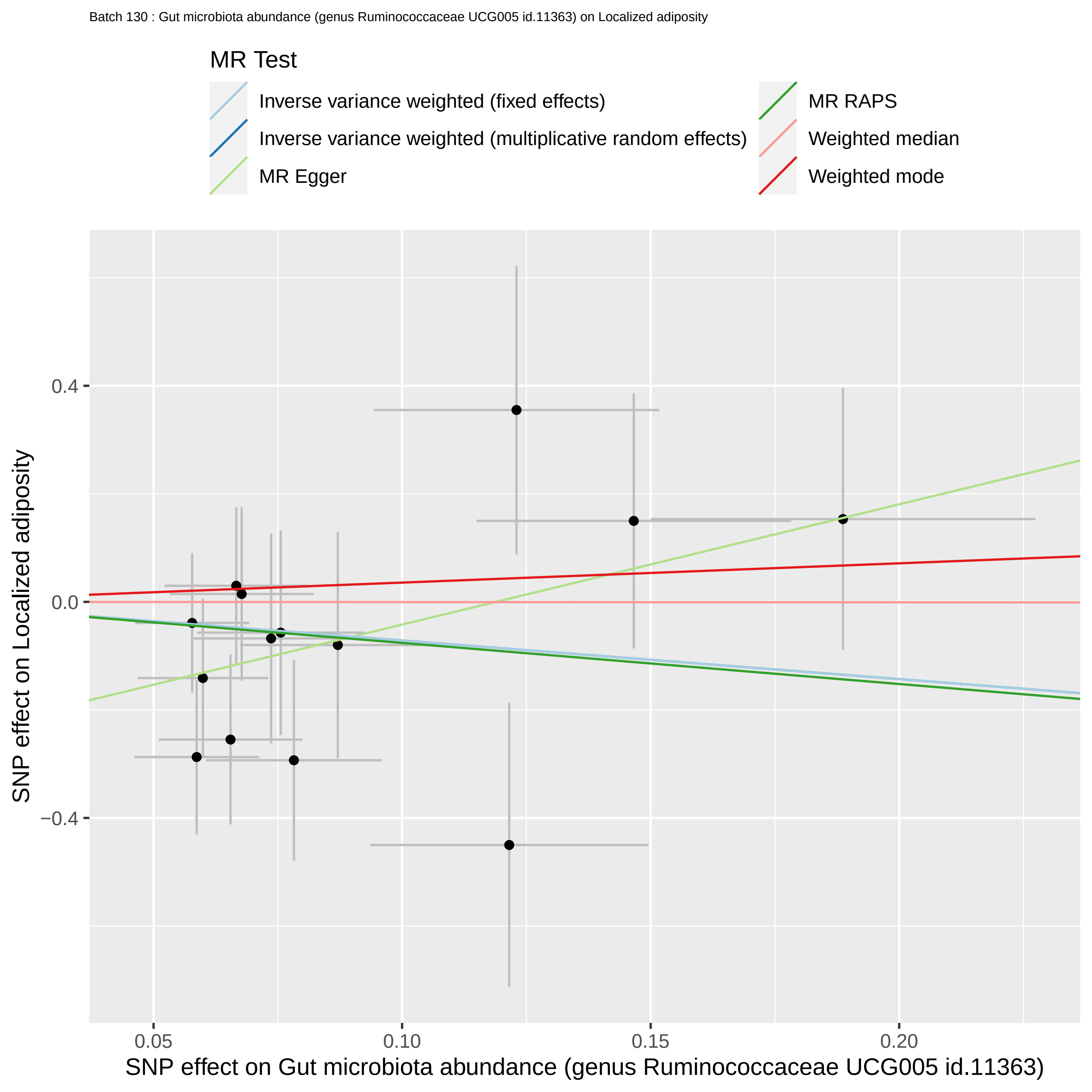

Batch 130 : Gut microbiota abundance (genus Ruminococcaceae UCG005 id.11363) on Localized adiposity
MR Test
Inverse variance weighted (fixed effects)
Inverse variance weighted (multiplicative random effects)
MR Egger
MR RAPS
Weighted median
Weighted mode
SNP effect on Localized adiposity
0.4
0.0
−0.4
0.05
0.10
0.15
0.20
SNP effect on Gut microbiota abundance (genus Ruminococcaceae UCG005 id.11363)

Batch 131 : Gut microbiota abundance (genus Ruminococcaceae UCG009 id.11366) on Localized adiposity

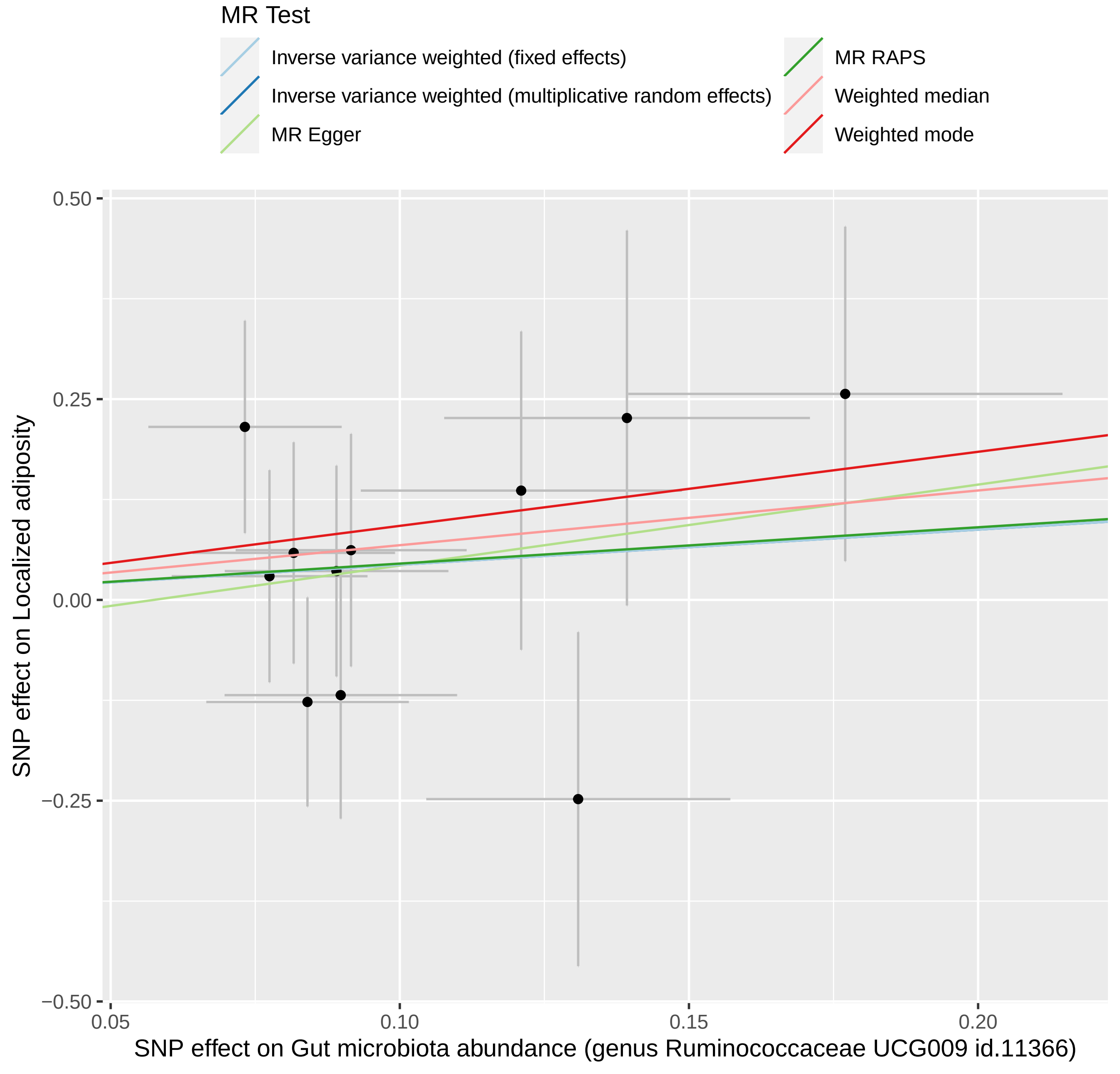

Batch 132 : Gut microbiota abundance (genus Ruminococcaceae UCG010 id.11367) on Localized adiposity

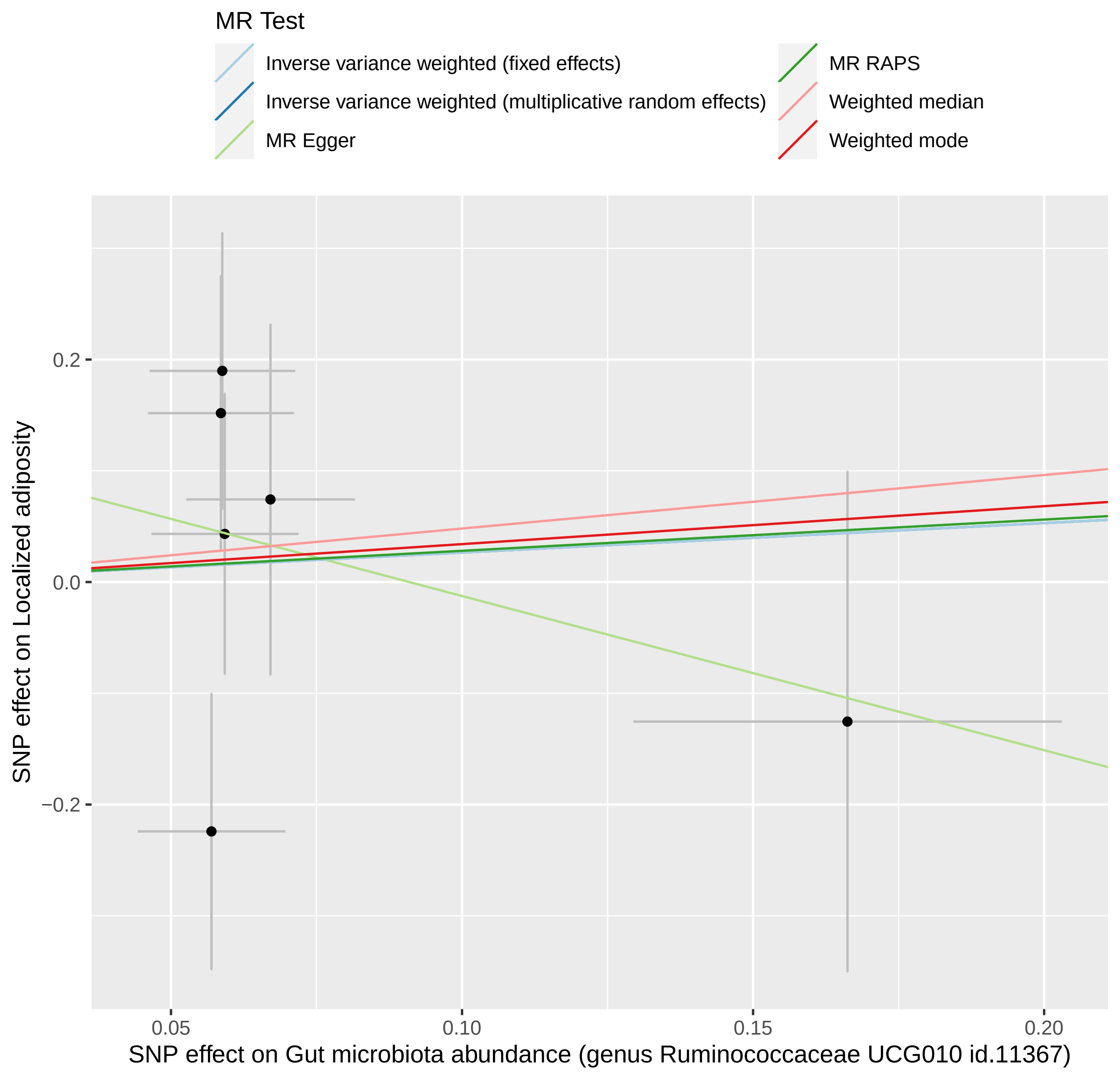

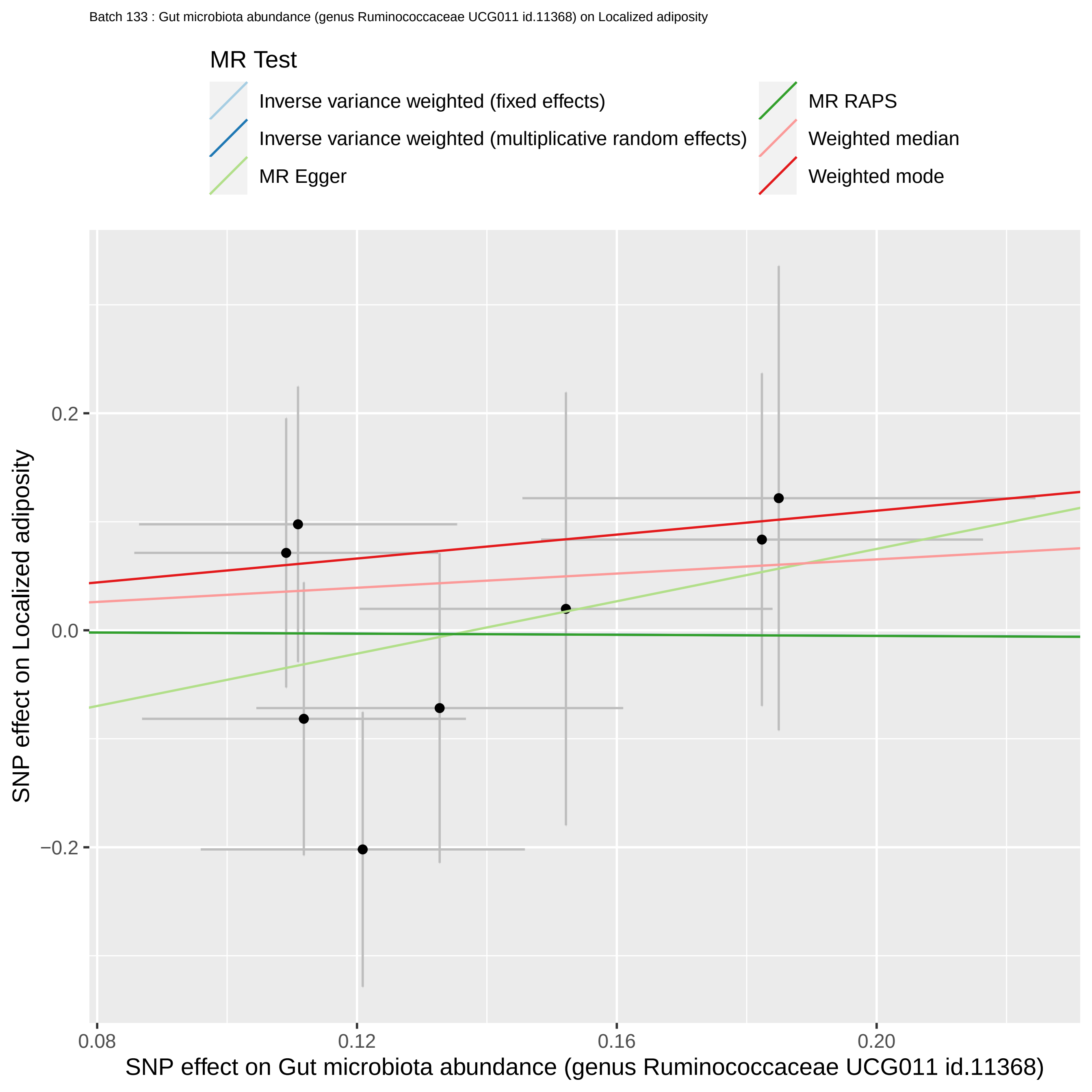

Batch 133 : Gut microbiota abundance (genus Ruminococcaceae UCG011 id.11368) on Localized adiposity
MR Test
Inverse variance weighted (fixed effects)
Inverse variance weighted (multiplicative random effects)
MR Egger
MR RAPS
Weighted median
Weighted mode
0.2
0.0
−0.2
0.08
0.12
0.16
0.20
SNP effect on Localized adiposity
SNP effect on Gut microbiota abundance (genus Ruminococcaceae UCG011 id.11368)

Batch 134 : Gut microbiota abundance (genus Ruminococcaceae UCG013 id.11370) on Localized adiposity

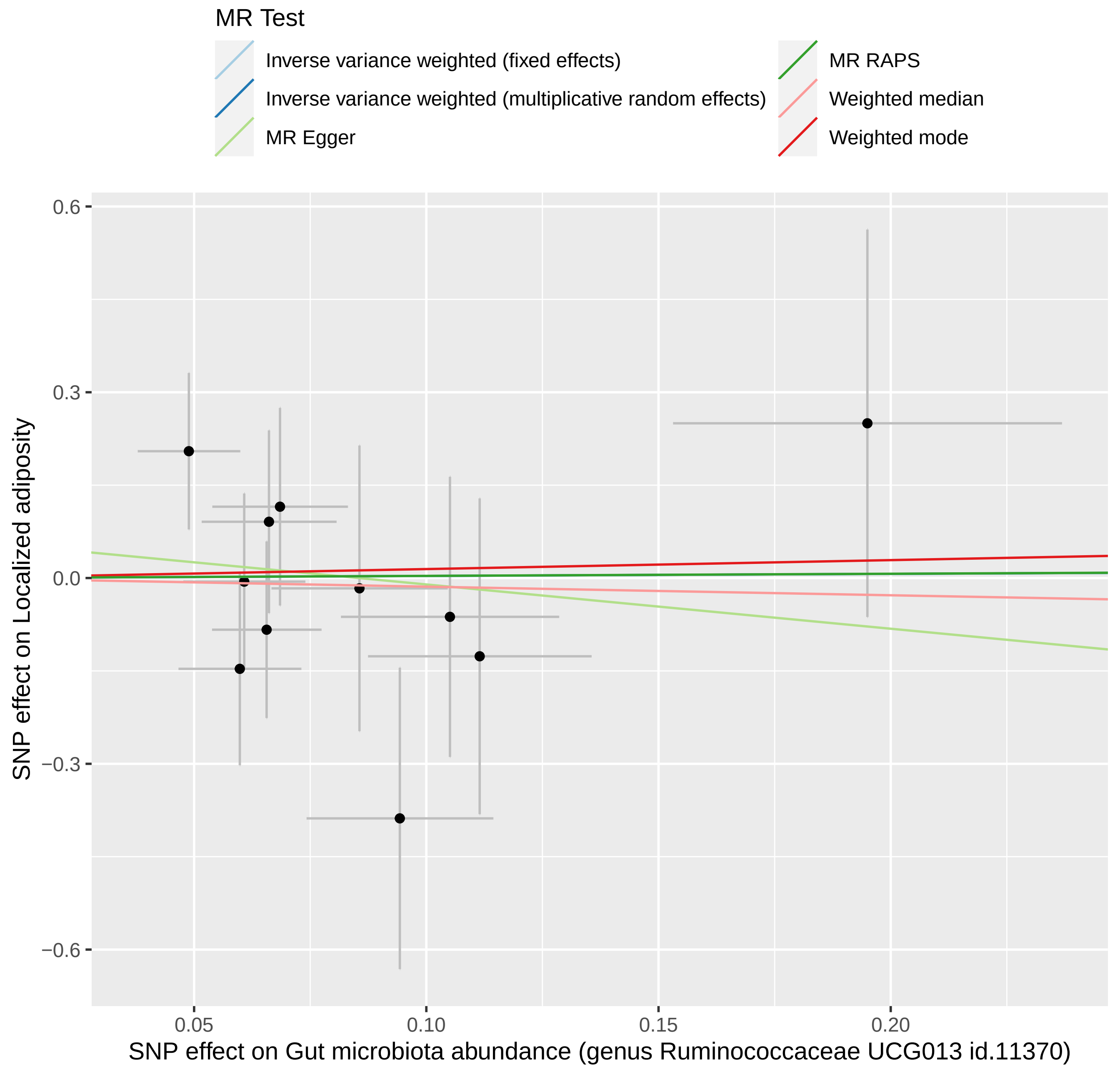

Batch 135 : Gut microbiota abundance (genus Ruminococcaceae UCG014 id.11371) on Localized adiposity

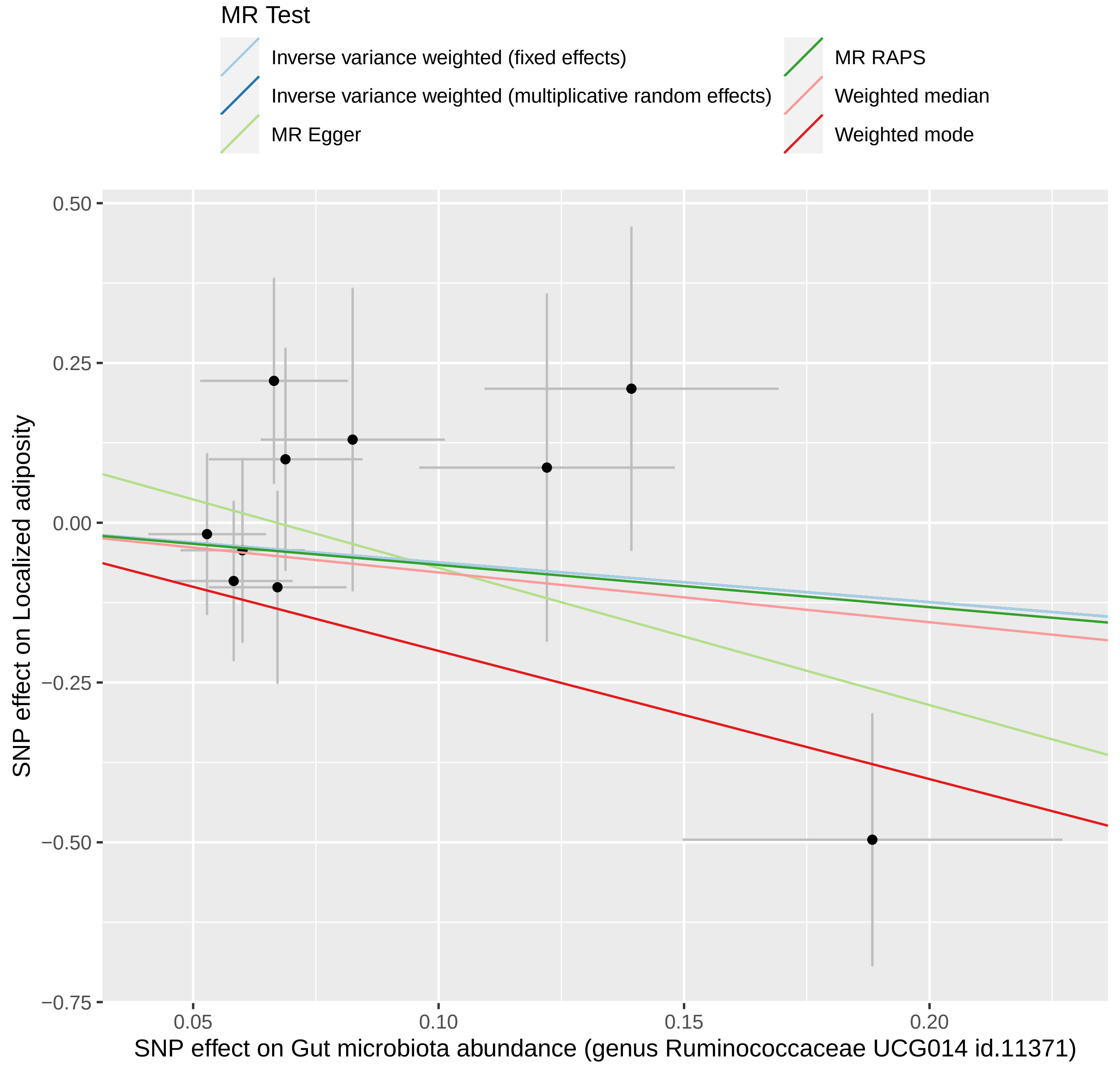

Batch 136 : Gut microbiota abundance (genus Ruminococcus1 id.11373) on Localized adiposity

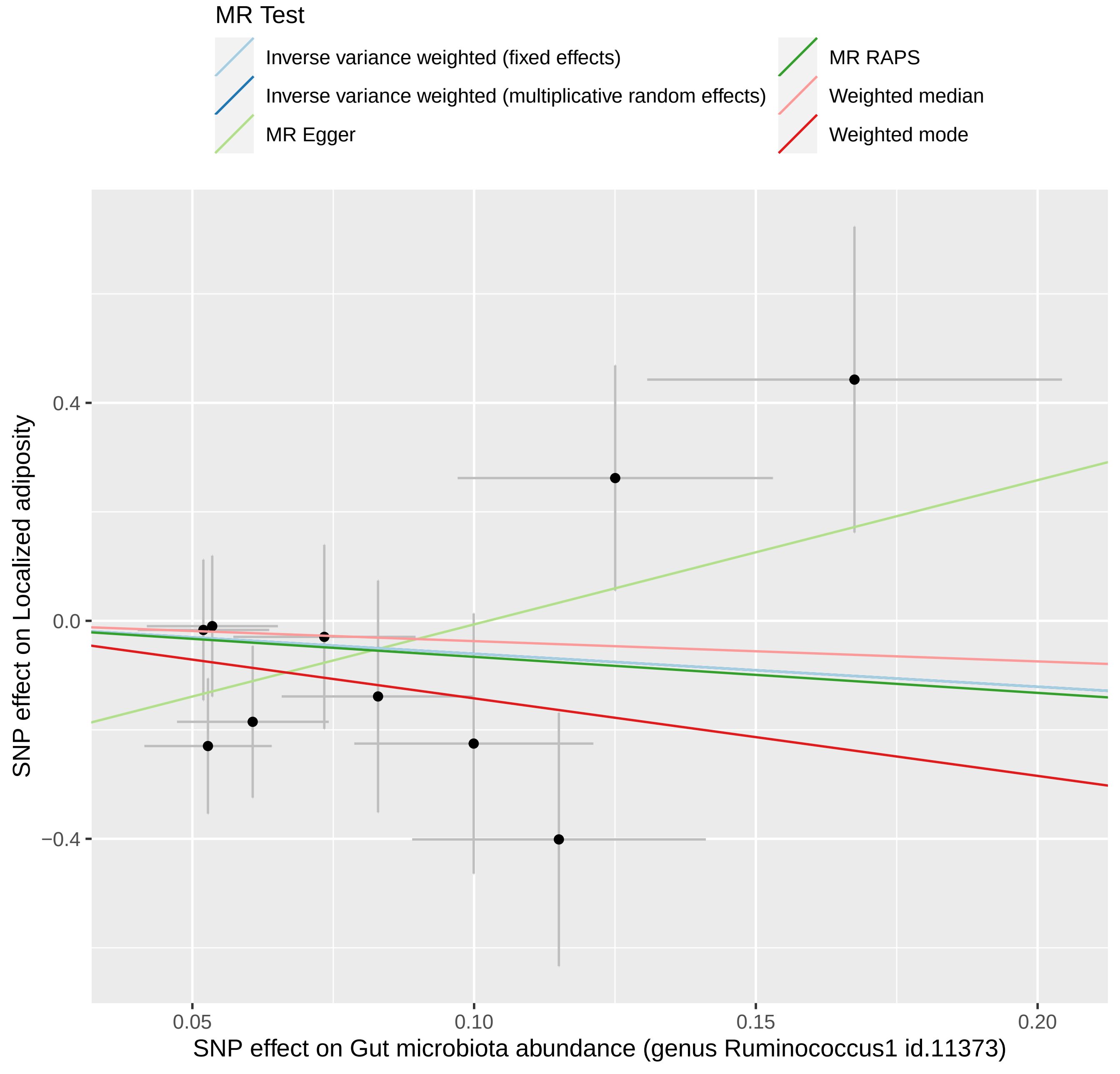

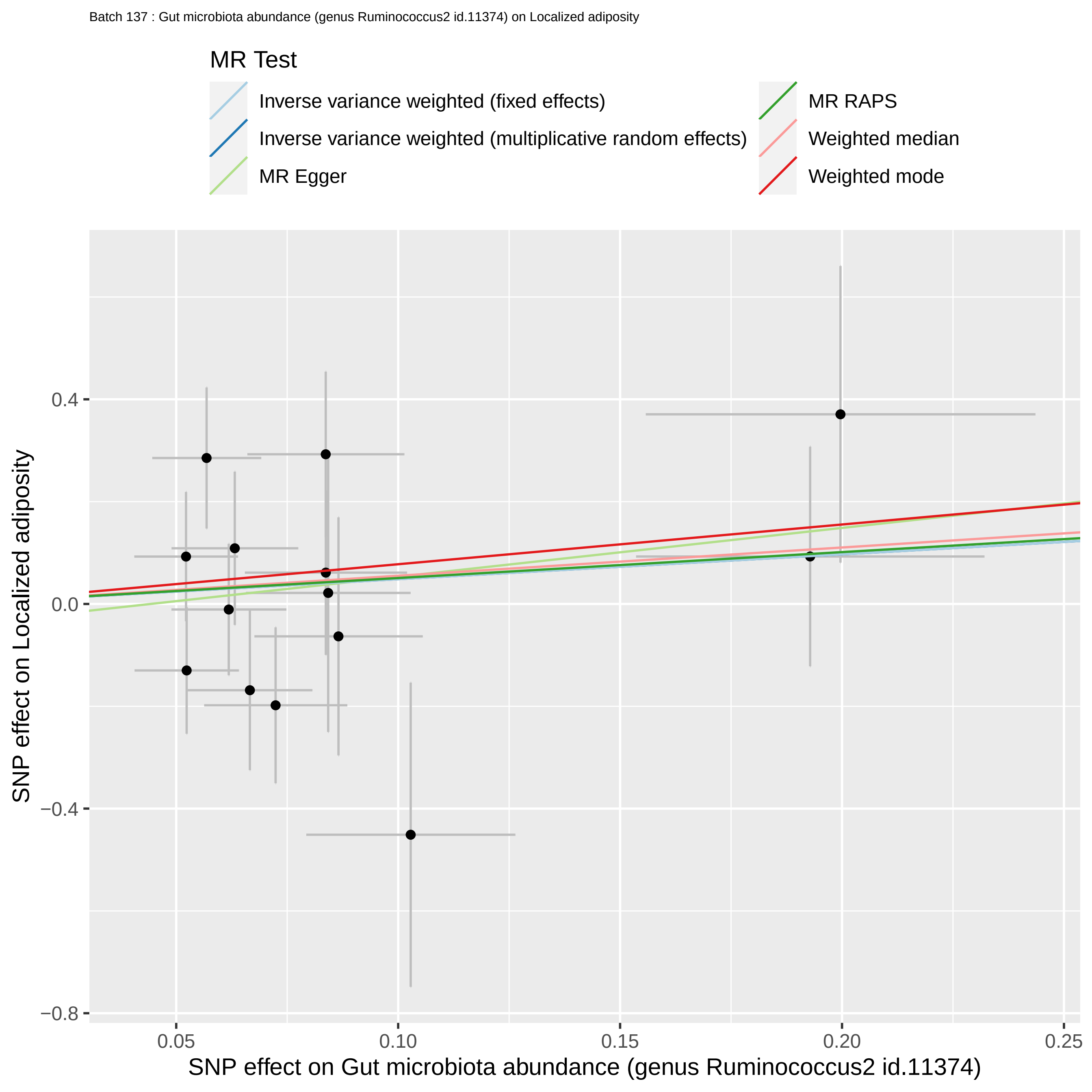

Batch 137 : Gut microbiota abundance (genus Ruminococcus2 id.11374) on Localized adiposity
MR Test
Inverse variance weighted (fixed effects)
Inverse variance weighted (multiplicative random effects)
MR Egger
MR RAPS
Weighted median
Weighted mode
0.4
0.0
−0.4
−0.8
0.05
0.10
0.15
0.20
0.25
SNP effect on Localized adiposity
SNP effect on Gut microbiota abundance (genus Ruminococcus2 id.11374)

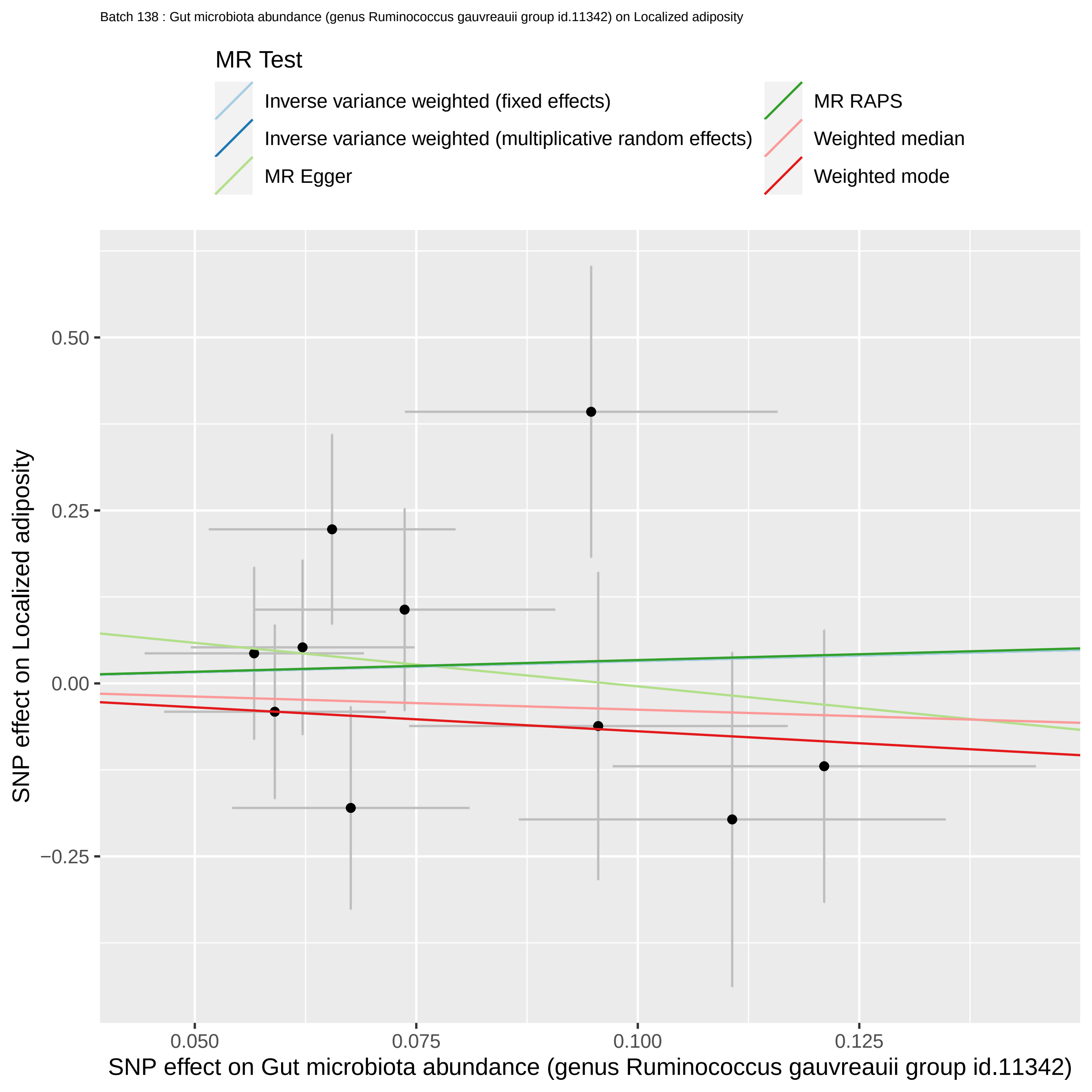

Batch 138 : Gut microbiota abundance (genus Ruminococcus gauvreauii group id.11342) on Localized adiposity
MR Test
Inverse variance weighted (fixed effects)
Inverse variance weighted (multiplicative random effects)
MR Egger
MR RAPS
Weighted median
Weighted mode
0.50
0.25
0.00
−0.25
0.050
0.075
0.100
0.125
SNP effect on Localized adiposity
SNP effect on Gut microbiota abundance (genus Ruminococcus gauvreauii group id.11342)

Batch 139 : Gut microbiota abundance (genus Ruminococcus gnavus group id.14376) on Localized adiposity

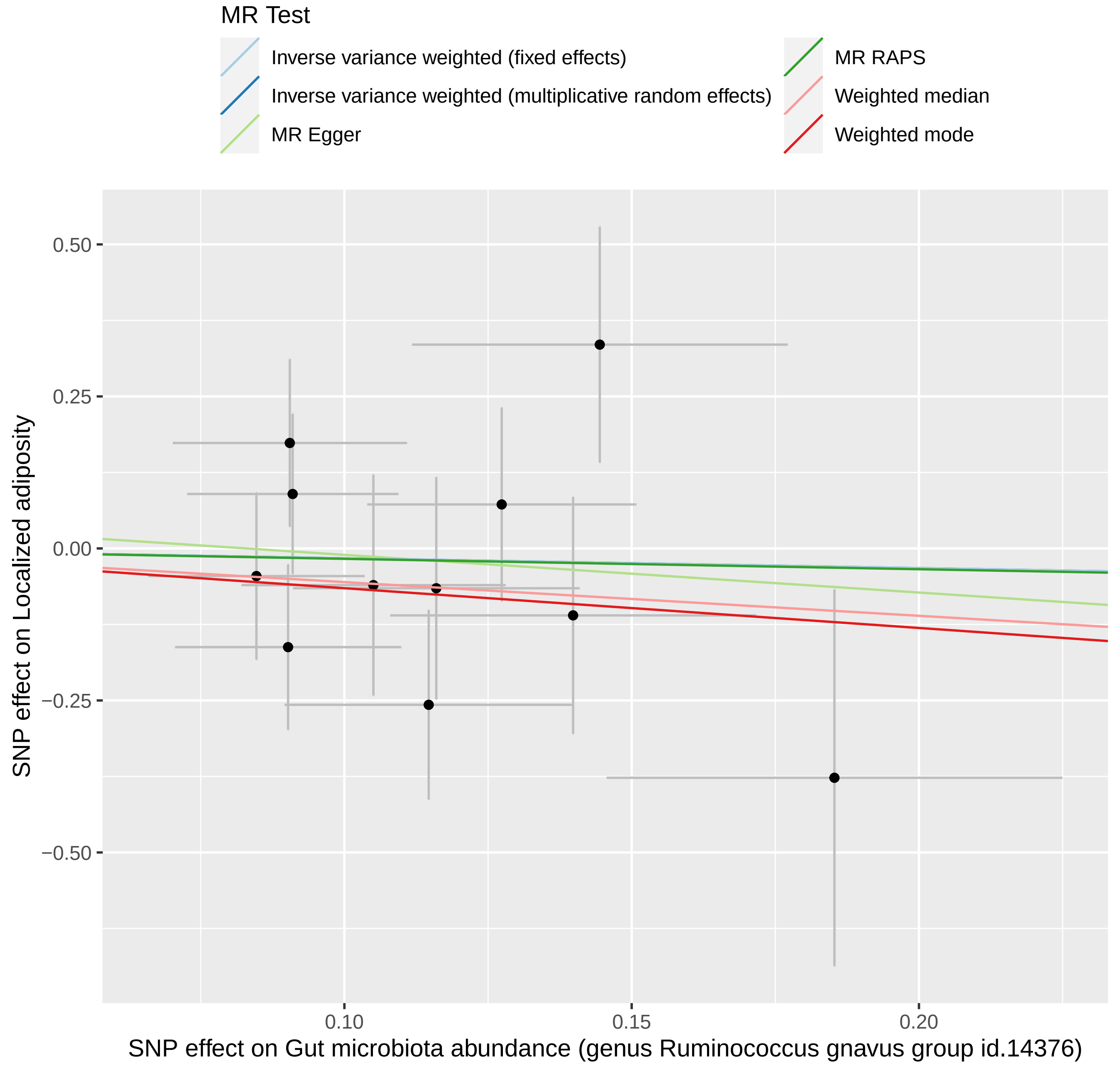

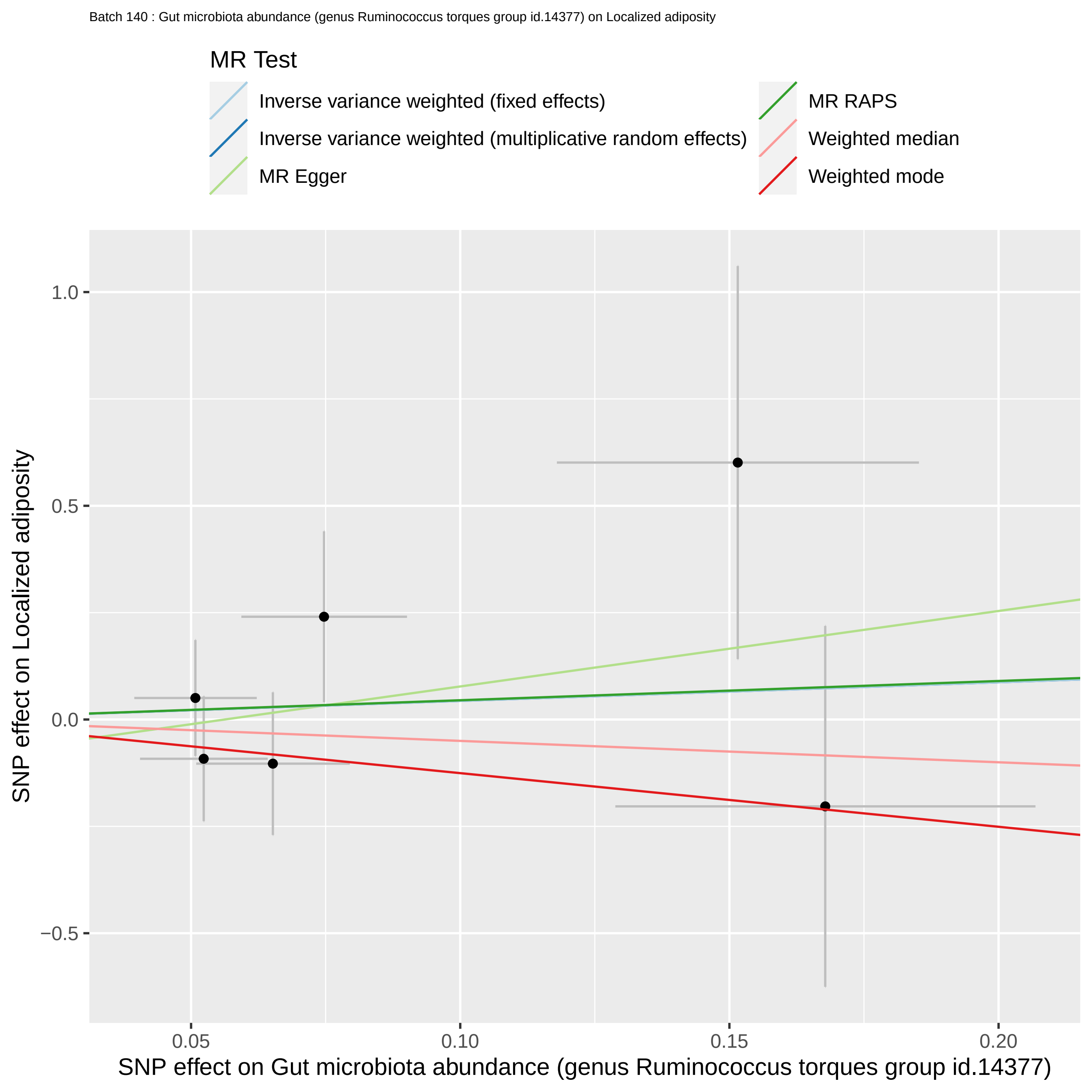

Batch 140 : Gut microbiota abundance (genus Ruminococcus torques group id.14377) on Localized adiposity
MR Test
Inverse variance weighted (fixed effects)
Inverse variance weighted (multiplicative random effects)
MR Egger
MR RAPS
Weighted median
Weighted mode
1.0
0.5
0.0
−0.5
0.05
0.10
0.15
0.20
SNP effect on Localized adiposity
SNP effect on Gut microbiota abundance (genus Ruminococcus torques group id.14377)

Batch 141 : Gut microbiota abundance (genus Sellimonas id.14369) on Localized adiposity

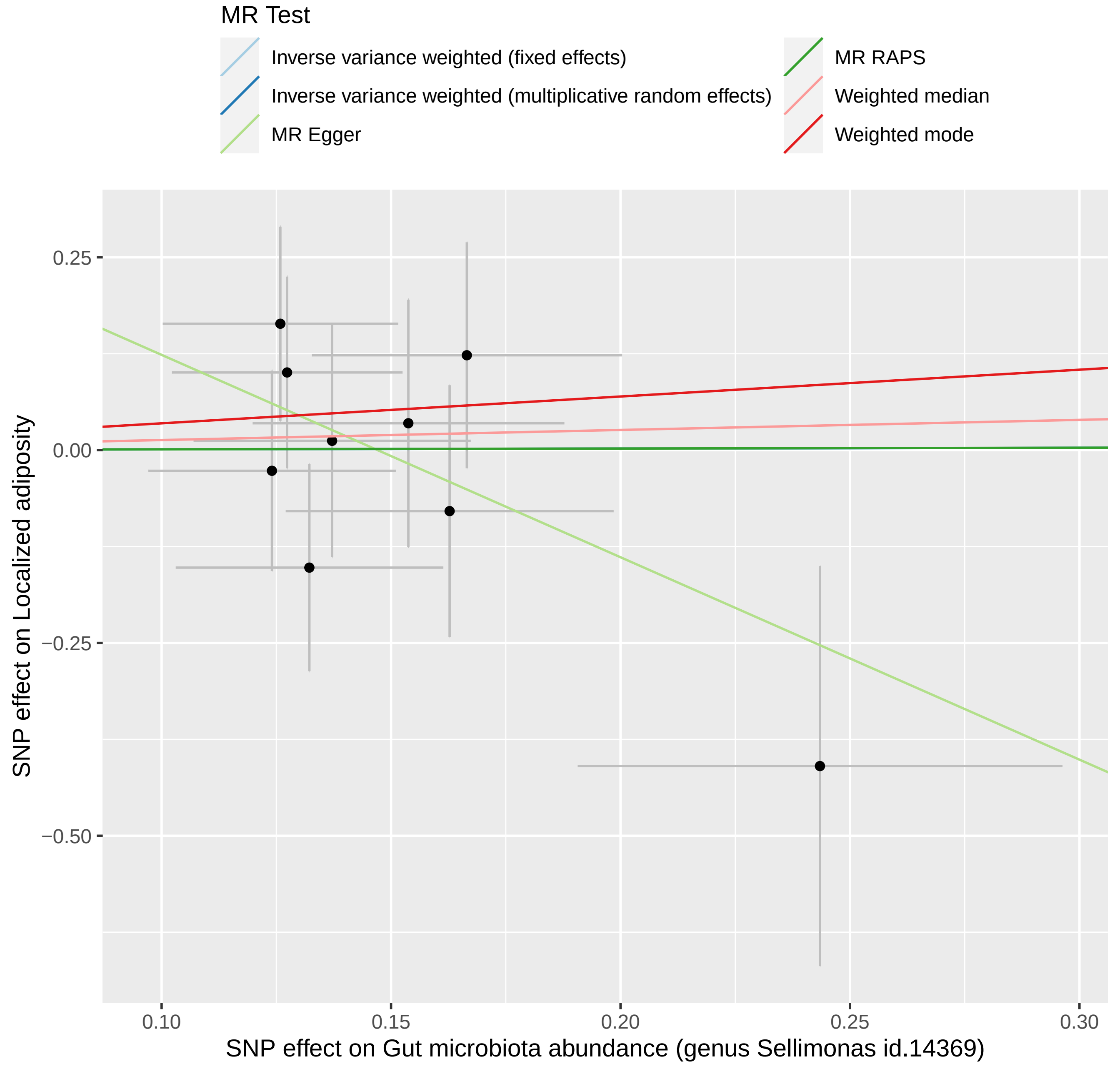

Batch 142 : Gut microbiota abundance (genus Senegalimassilia id.11160) on Localized adiposity

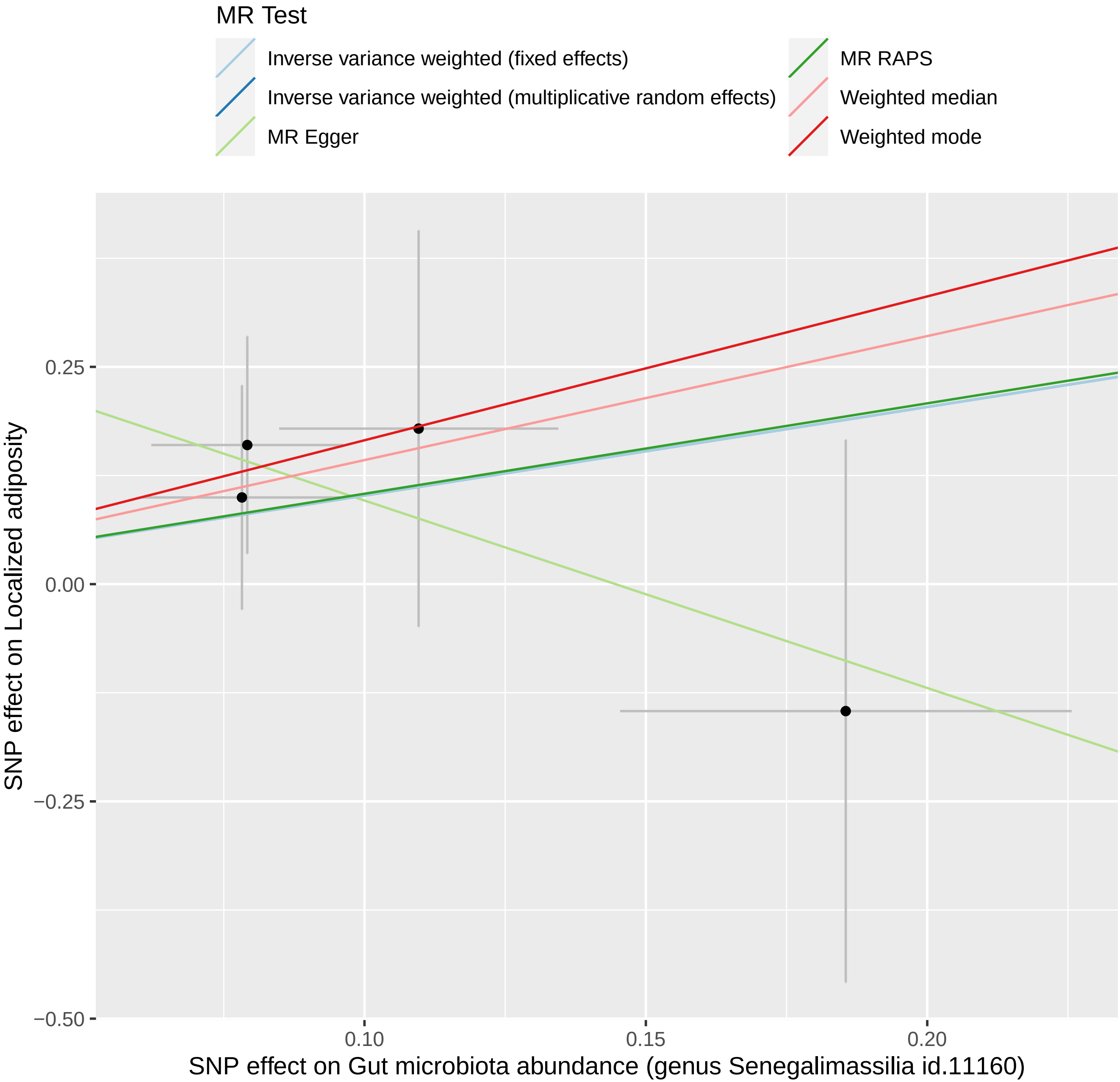

Batch 143 : Gut microbiota abundance (genus Slackia id.825) on Localized adiposity

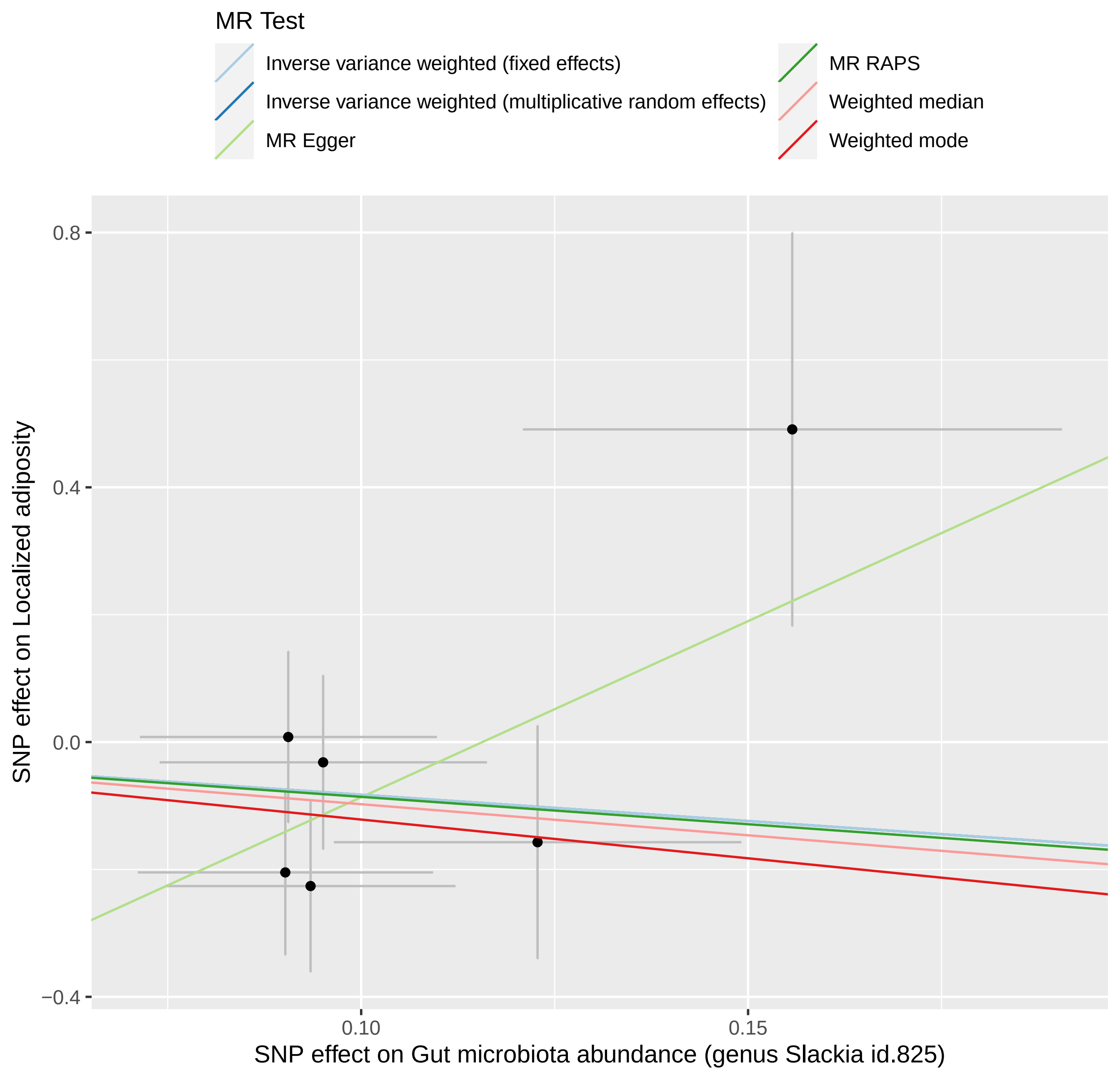

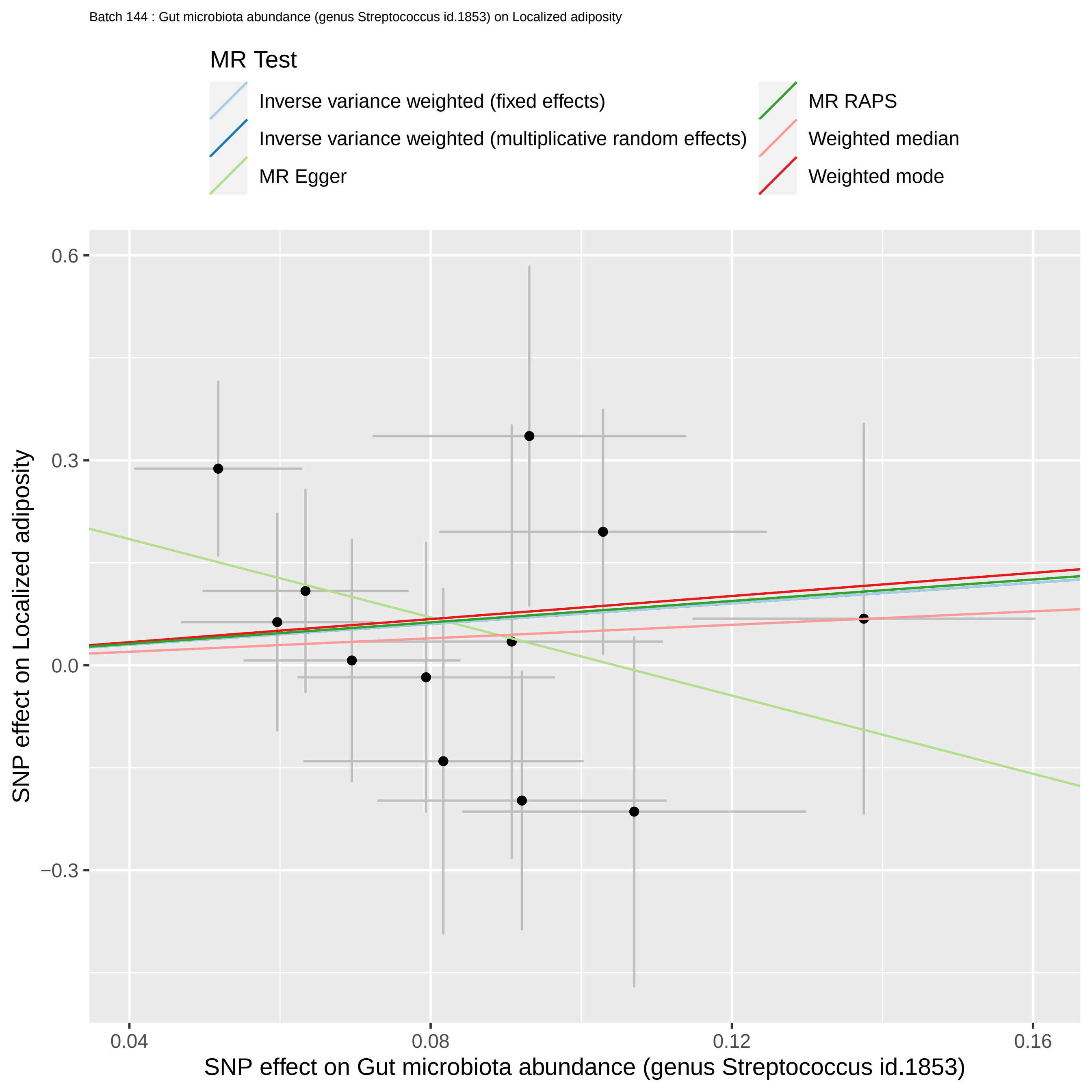

Batch 144 : Gut microbiota abundance (genus Streptococcus id.1853) on Localized adiposity
MR Test
Inverse variance weighted (fixed effects)
Inverse variance weighted (multiplicative random effects)
MR Egger
MR RAPS
Weighted median
Weighted mode
0.6
0.3
0.0
−0.3
0.04
0.08
0.12
0.16
SNP effect on Localized adiposity
SNP effect on Gut microbiota abundance (genus Streptococcus id.1853)

Batch 145 : Gut microbiota abundance (genus Subdoligranulum id.2070) on Localized adiposity

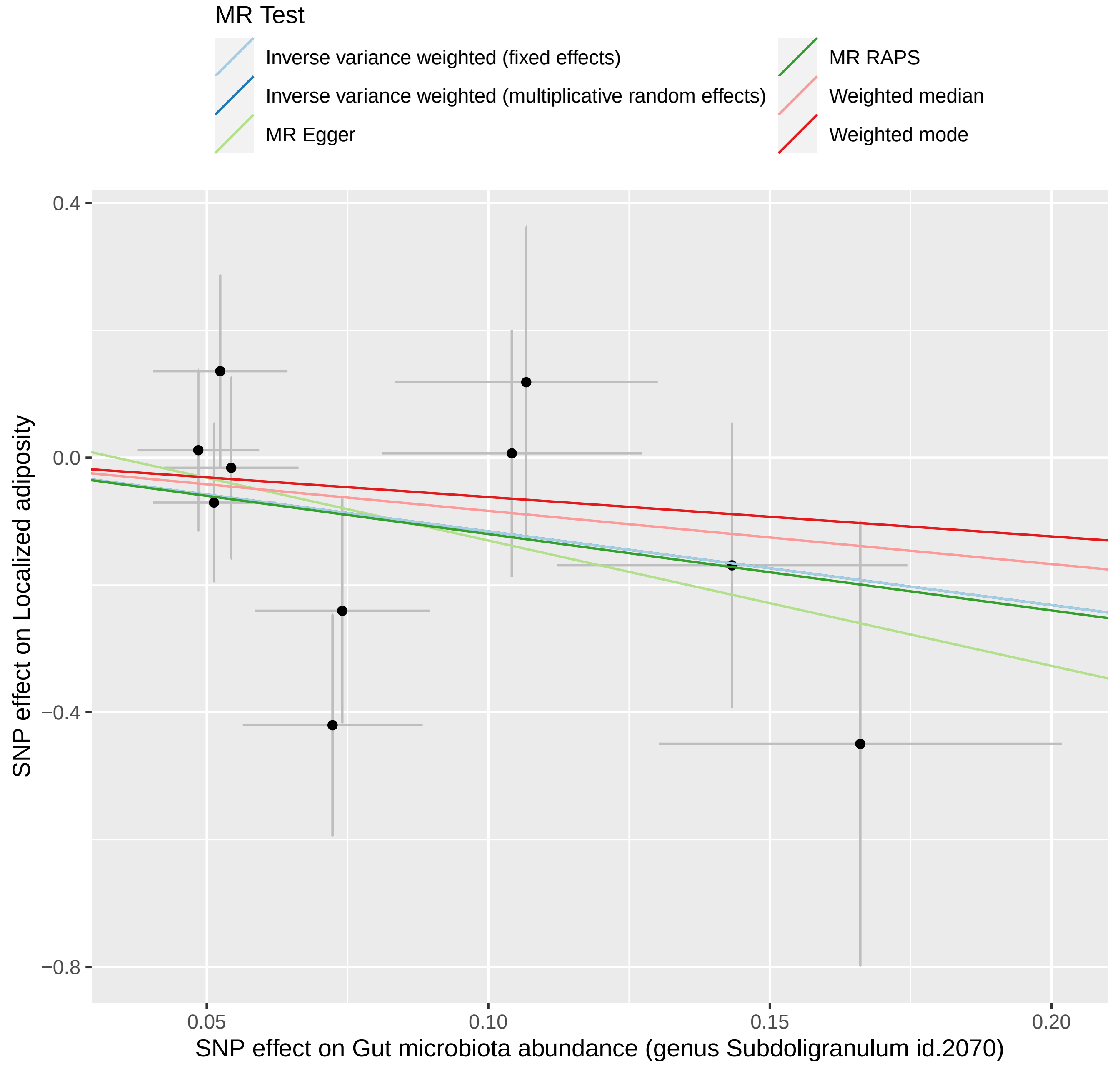

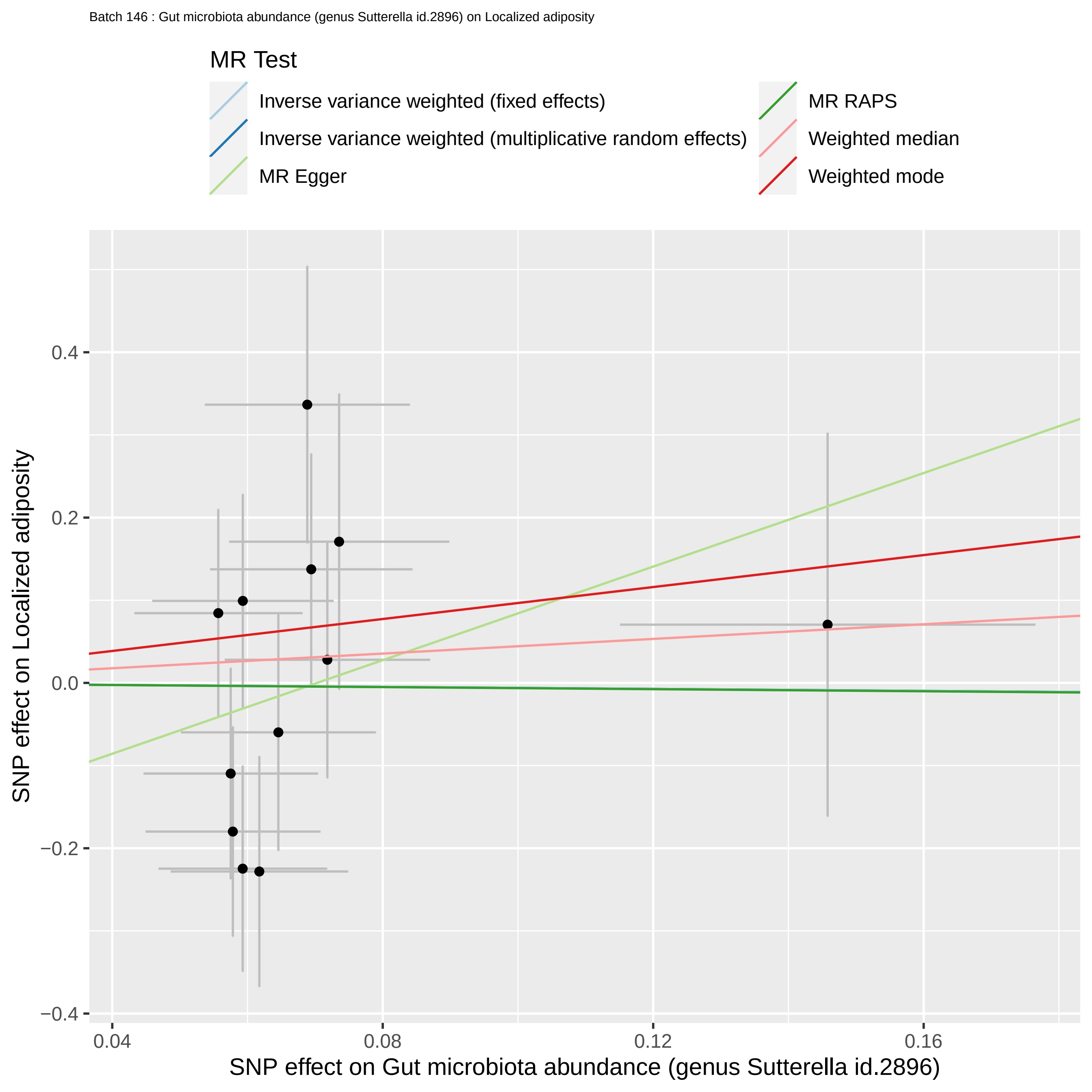
Batch 146 : Gut microbiota abundance (genus Sutterella id.2896) on Localized adiposity
MR Test
Inverse variance weighted (fixed effects)
Inverse variance weighted (multiplicative random effects)
MR Egger
MR RAPS
Weighted median
Weighted mode
SNP effect on Localized adiposity
0.4
0.2
0.0
−0.2
−0.4
0.04
0.08
0.12
0.16
SNP effect on Gut microbiota abundance (genus Sutterella id.2896)

Batch 147 : Gut microbiota abundance (genus Terrisporobacter id.11348) on Localized adiposity

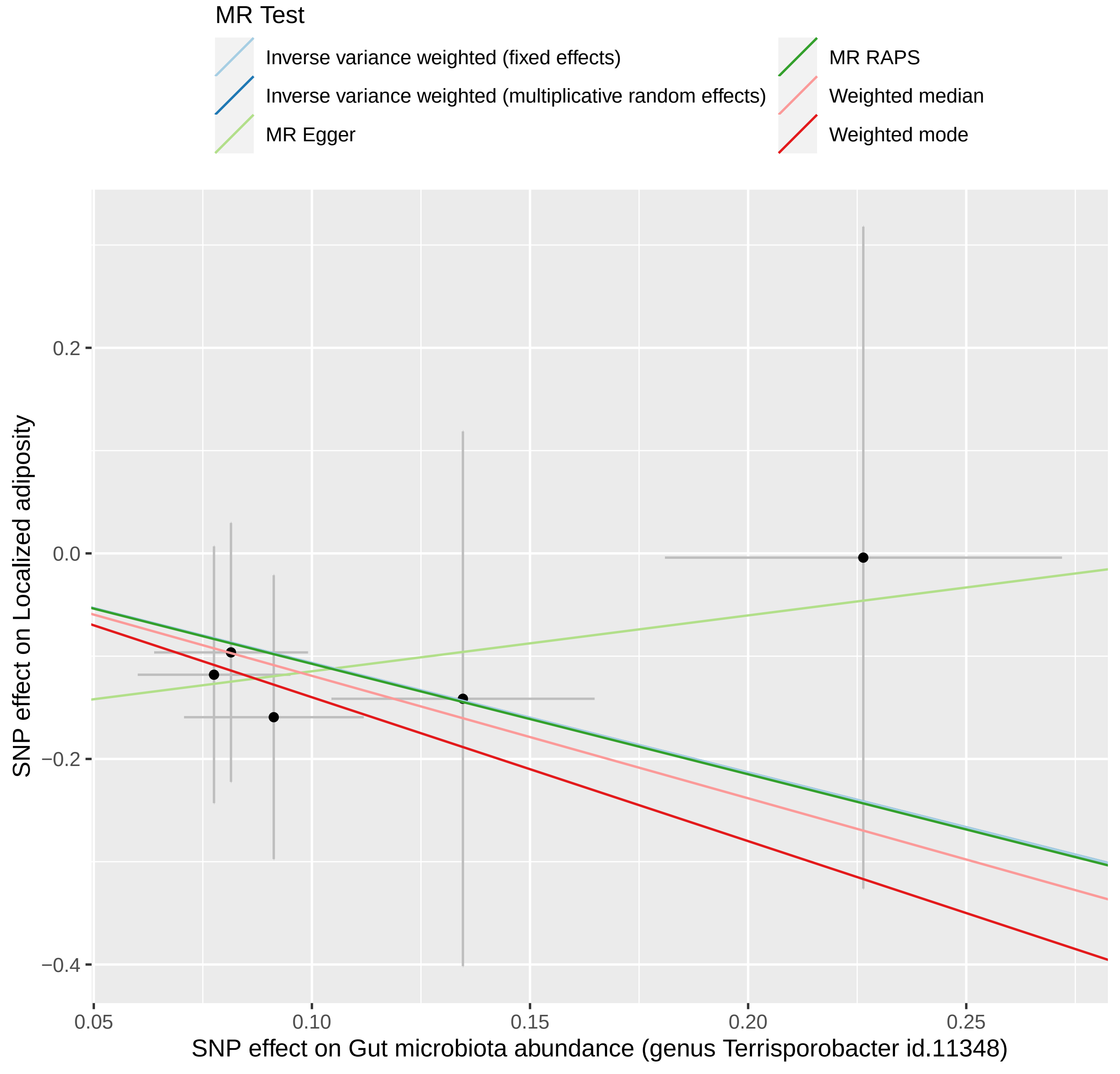

Batch 148 : Gut microbiota abundance (genus Turicibacter id.2162) on Localized adiposity

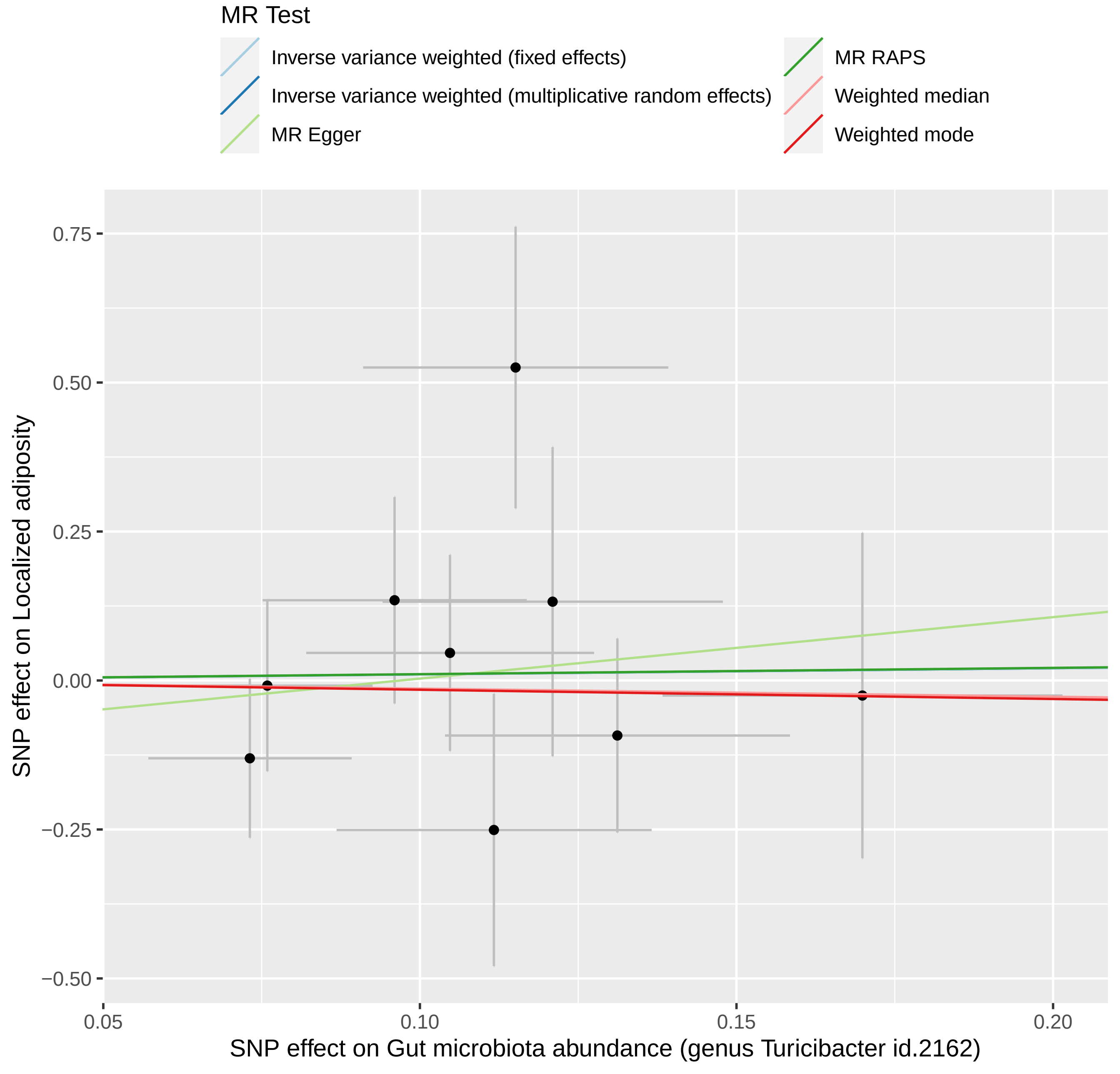

Batch 149 : Gut microbiota abundance (genus Tyzzerella3 id.11335) on Localized adiposity

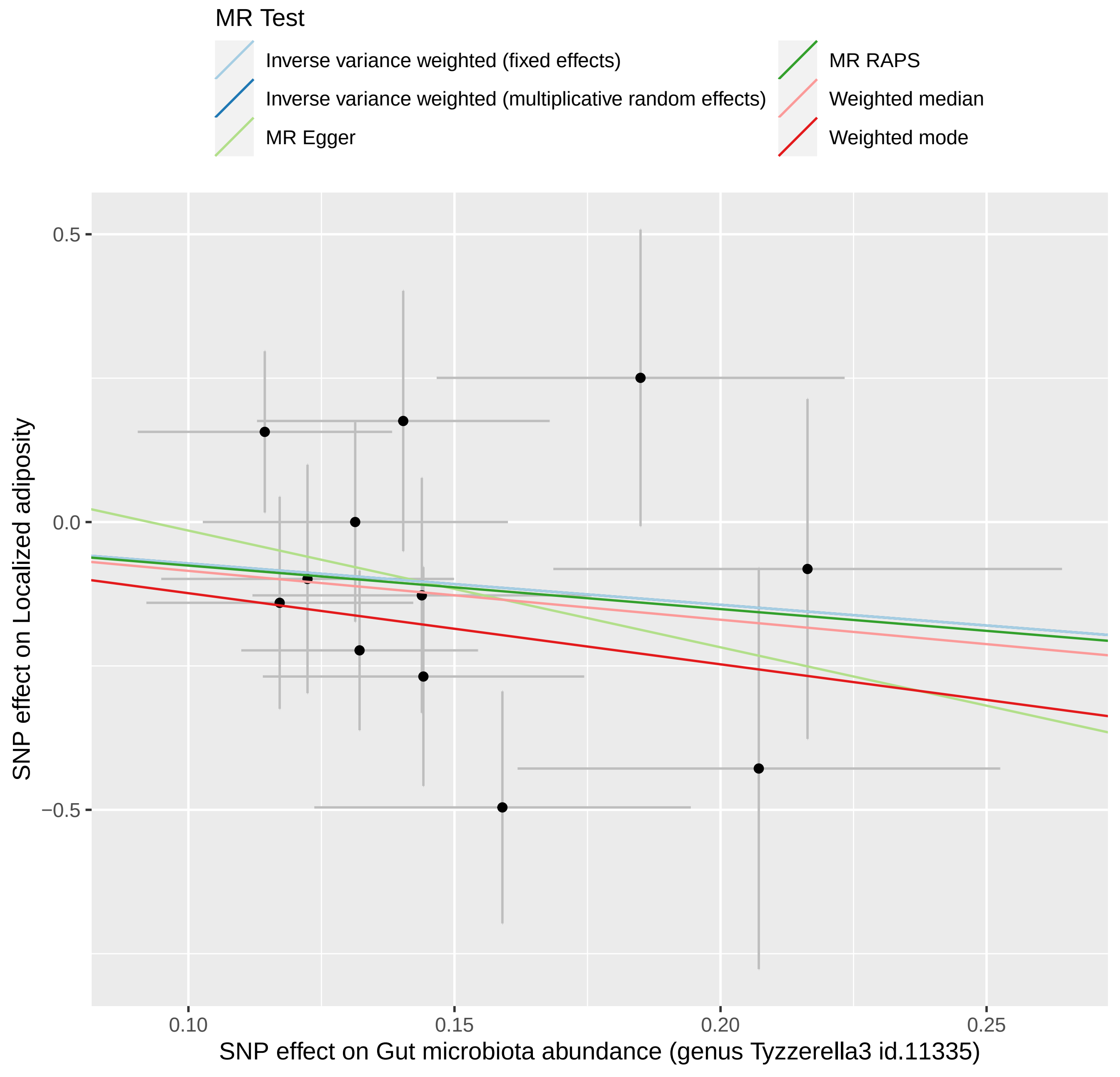

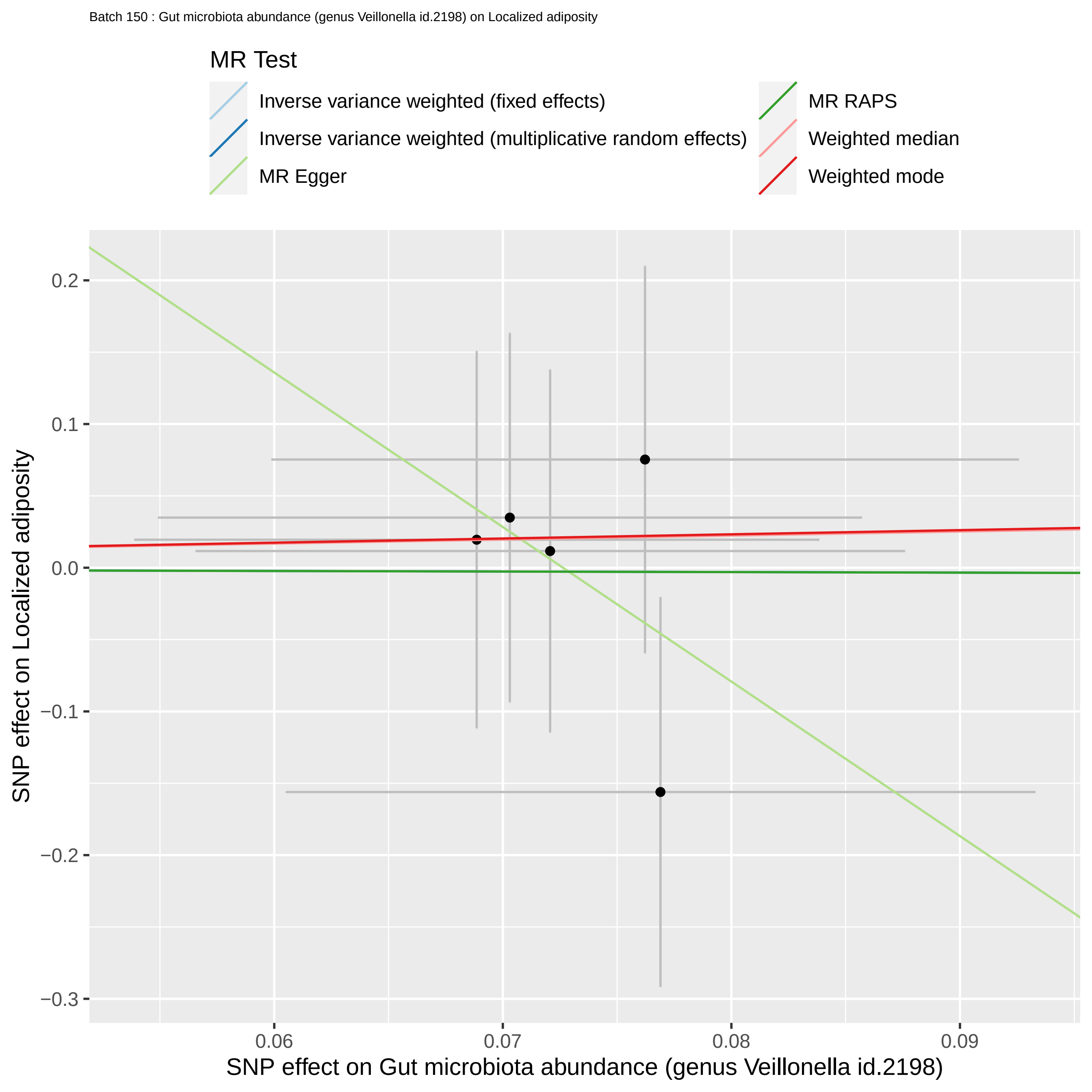
Batch 150 : Gut microbiota abundance (genus Veillonella id.2198) on Localized adiposity
MR Test
Inverse variance weighted (fixed effects)
Inverse variance weighted (multiplicative random effects)
MR Egger
MR RAPS
Weighted median
Weighted mode
0.2
0.1
0.0
−0.1
−0.2
−0.3
0.06
0.07
0.08
0.09
SNP effect on Localized adiposity
SNP effect on Gut microbiota abundance (genus Veillonella id.2198)

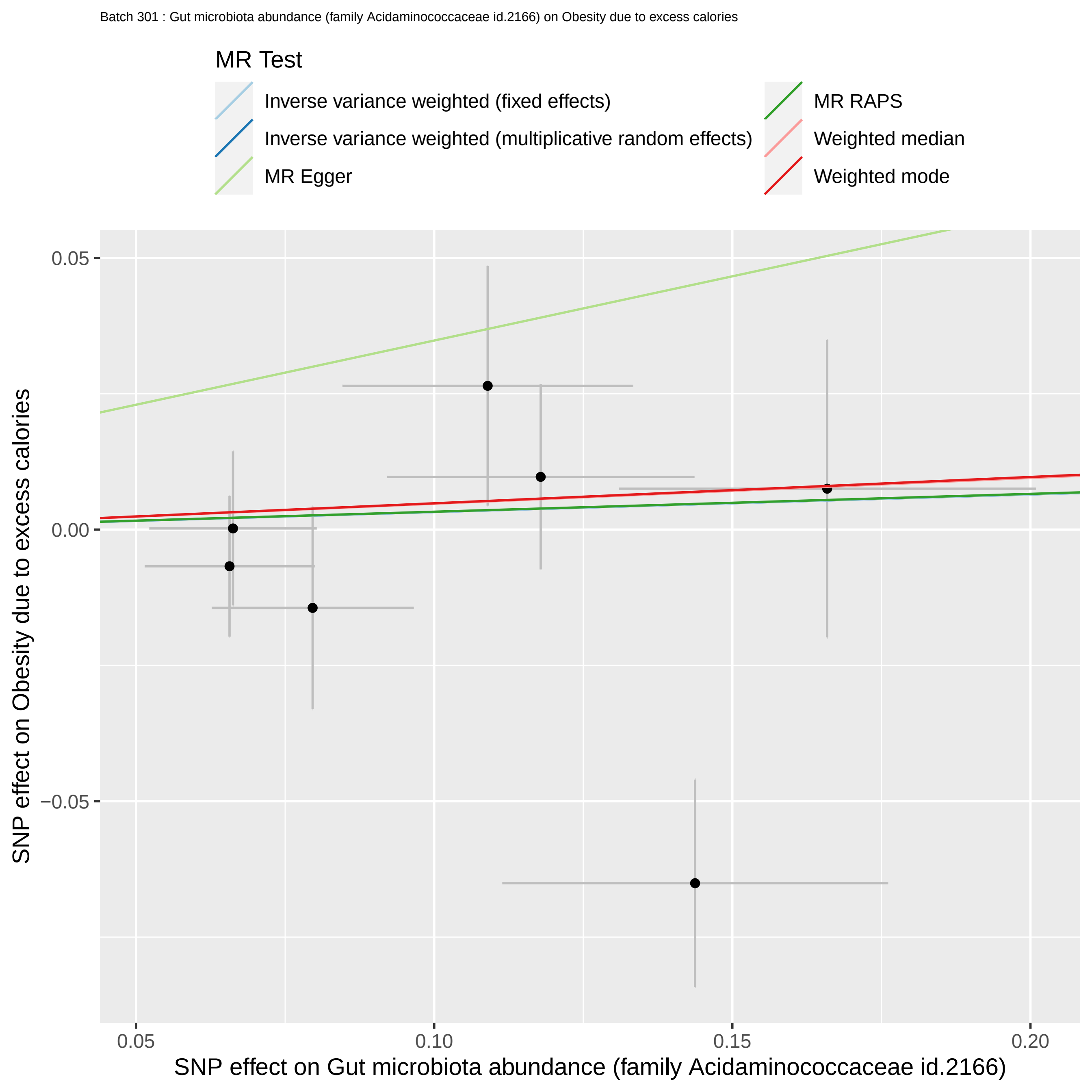
Batch 301 : Gut microbiota abundance (family Acidaminococcaceae id.2166) on Obesity due to excess calories
MR Test
Inverse variance weighted (fixed effects)
Inverse variance weighted (multiplicative random effects)
MR Egger
MR RAPS
Weighted median
Weighted mode
0.05
0.00
−0.05
0.05
0.10
0.15
0.20
SNP effect on Obesity due to excess calories
SNP effect on Gut microbiota abundance (family Acidaminococcaceae id.2166)

Batch 302 : Gut microbiota abundance (family Actinomycetaceae id.421) on Obesity due to excess calories

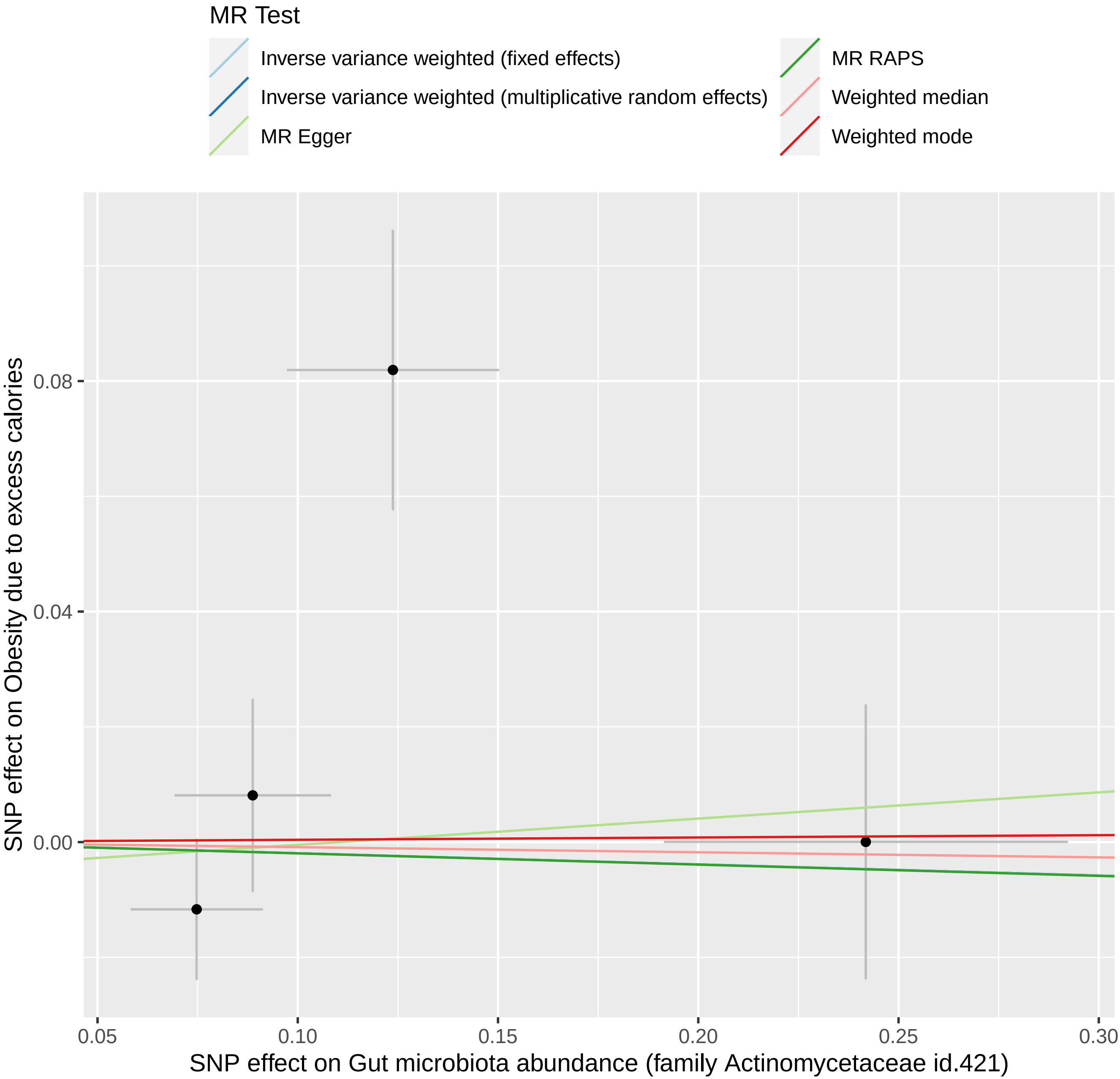

Batch 303 : Gut microbiota abundance (family Alcaligenaceae id.2875) on Obesity due to excess calories

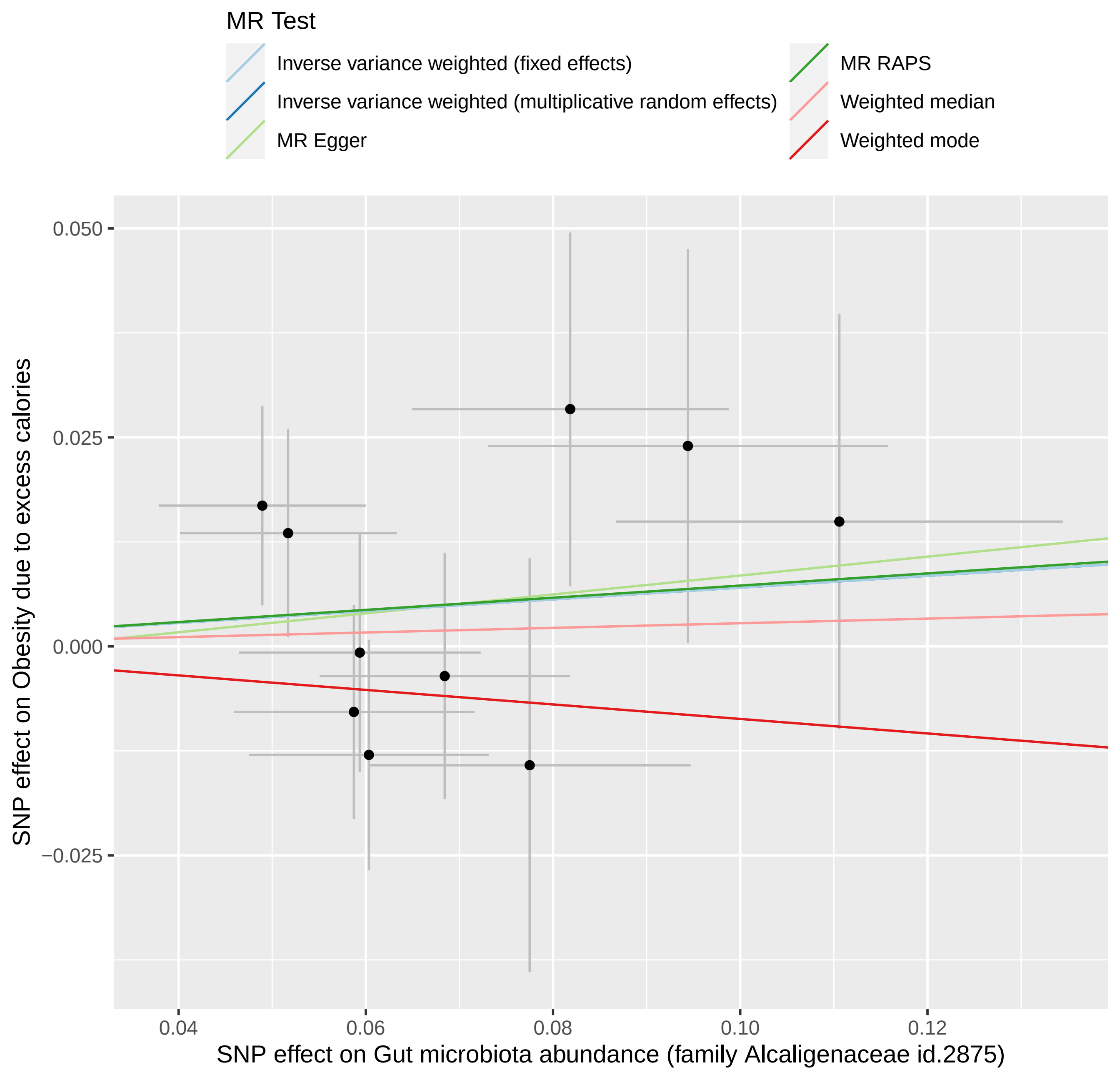

Batch 304 : Gut microbiota abundance (family Bacteroidaceae id.917) on Obesity due to excess calories

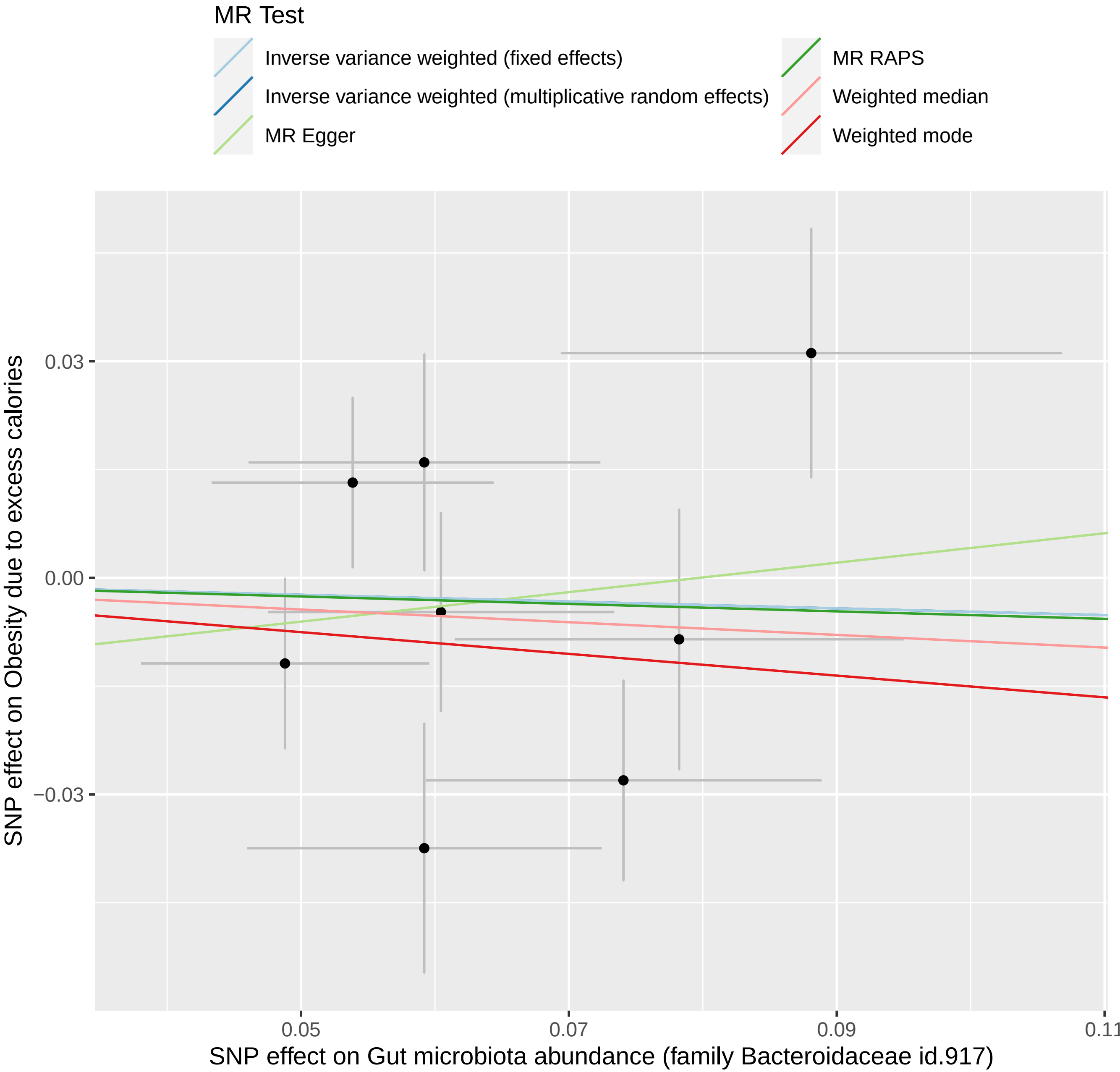

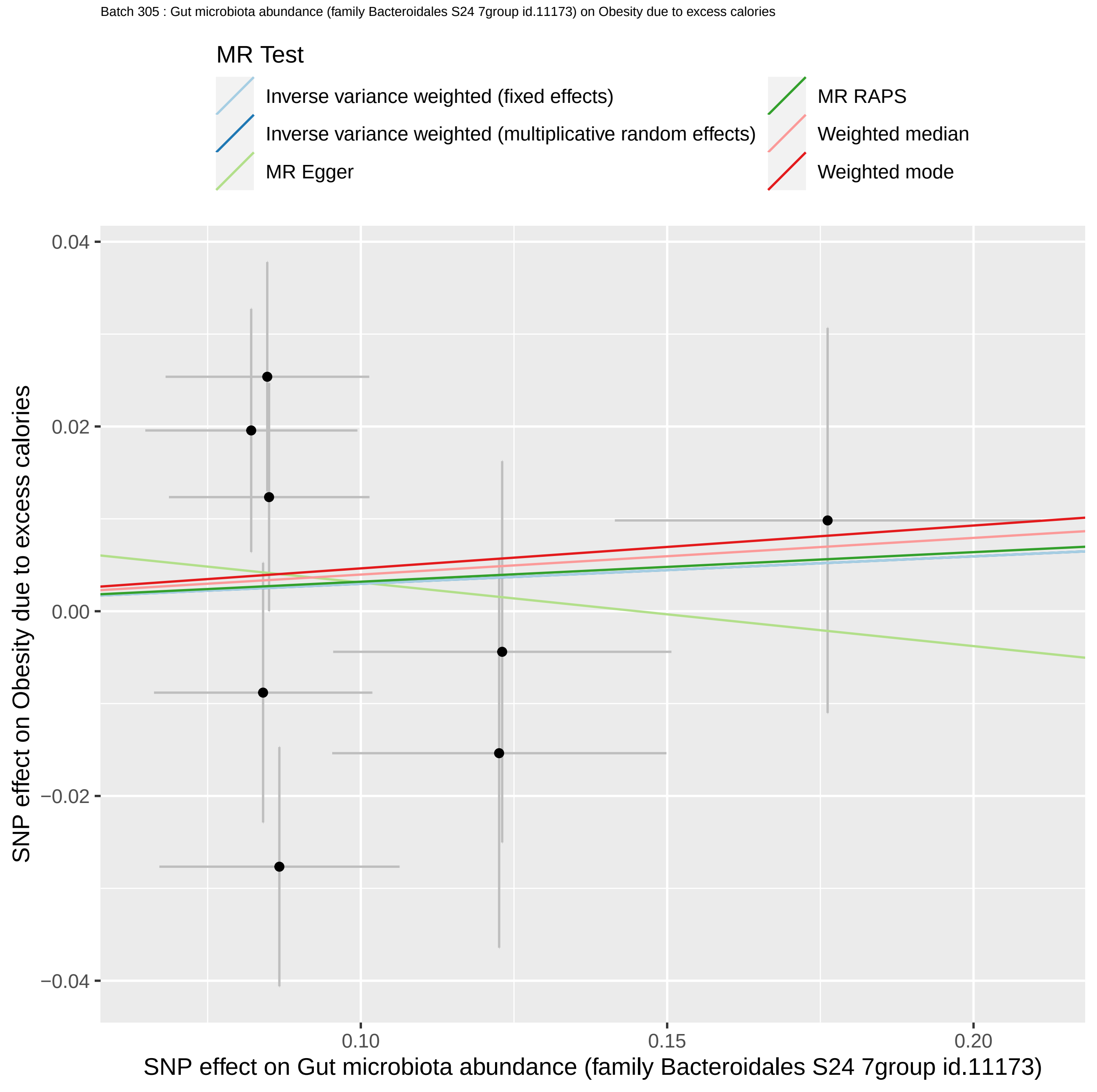

Batch 305 : Gut microbiota abundance (family Bacteroidales S24 7group id.11173) on Obesity due to excess calories
MR Test
Inverse variance weighted (fixed effects)
Inverse variance weighted (multiplicative random effects)
MR Egger
MR RAPS
Weighted median
Weighted mode
0.04
0.02
0.00
−0.02
−0.04
0.10
0.15
0.20
SNP effect on Obesity due to excess calories
SNP effect on Gut microbiota abundance (family Bacteroidales S24 7group id.11173)

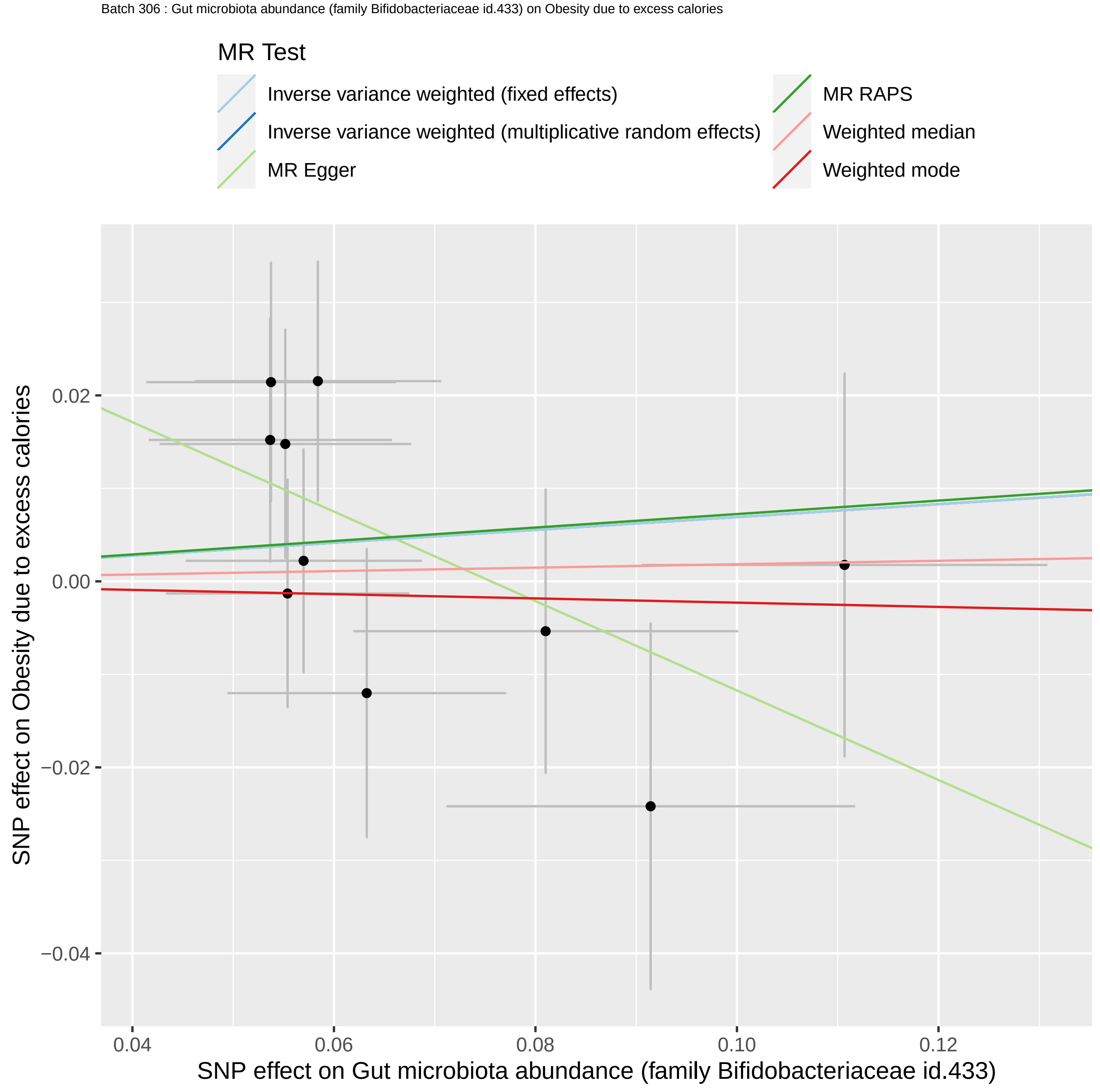

Batch 306 : Gut microbiota abundance (family Bifidobacteriaceae id.433) on Obesity due to excess calories
MR Test
Inverse variance weighted (fixed effects)
Inverse variance weighted (multiplicative random effects)
MR Egger
MR RAPS
Weighted median
Weighted mode
SNP effect on Obesity due to excess calories
0.02
0.00
−0.02
−0.04
0.04
0.06
0.08
0.10
0.12
SNP effect on Gut microbiota abundance (family Bifidobacteriaceae id.433)

Batch 308 : Gut microbiota abundance (family Clostridiaceae1 id.1869) on Obesity due to excess calories

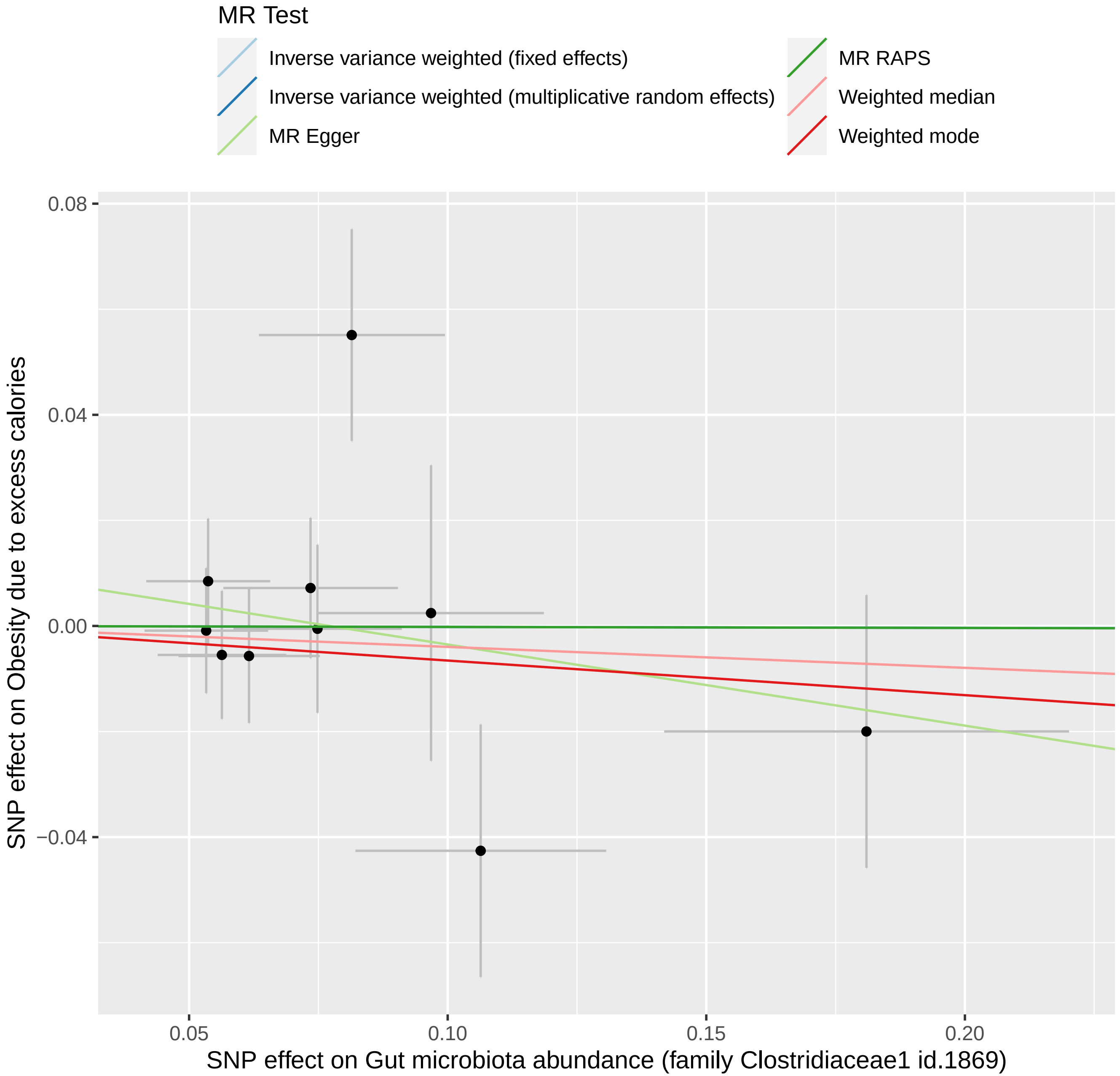

Batch 309 : Gut microbiota abundance (family Clostridiales vadin BB60 group id.11286) on Obesity due to excess calories

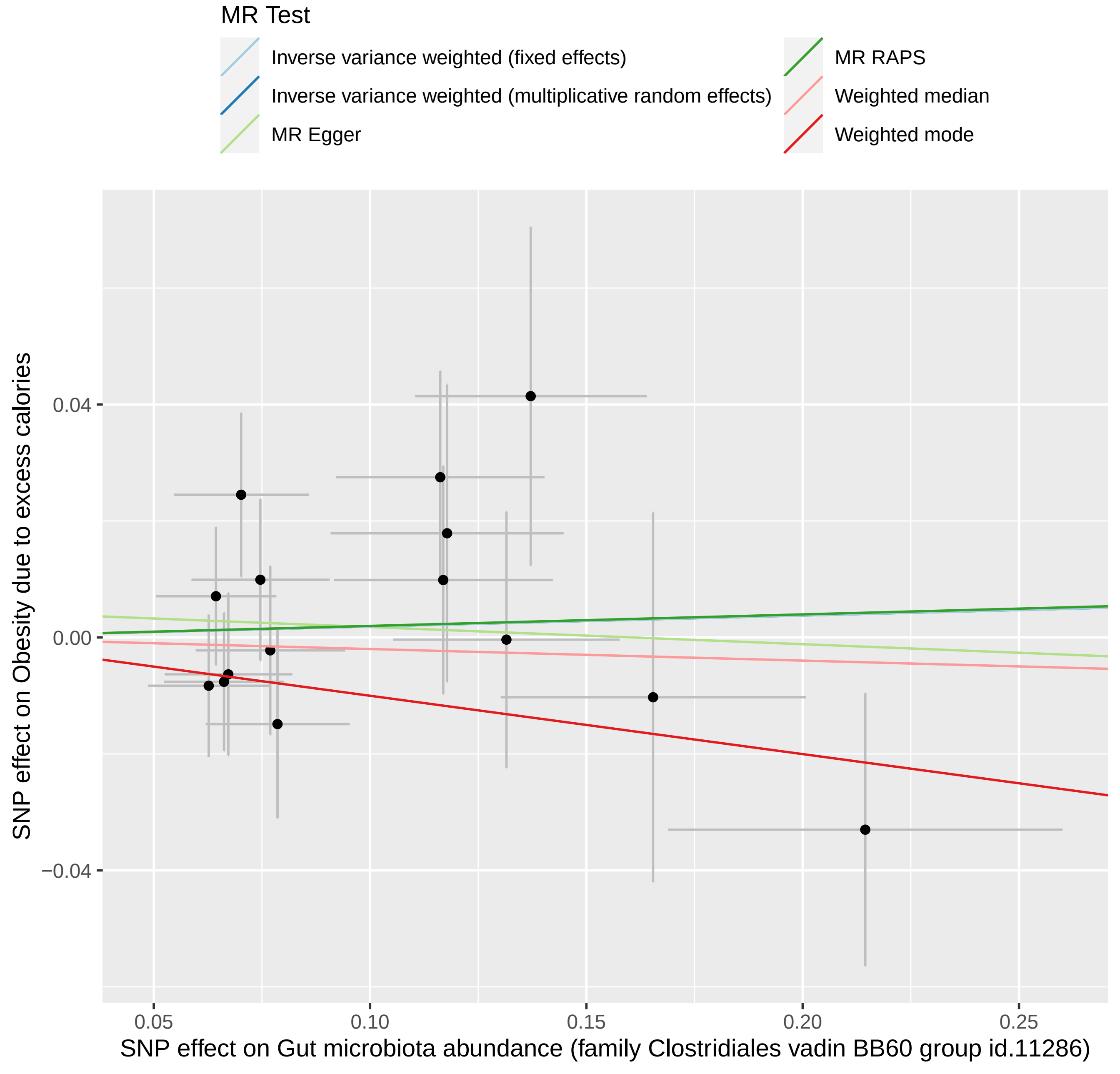

Batch 310 : Gut microbiota abundance (family Coriobacteriaceae id.811) on Obesity due to excess calories

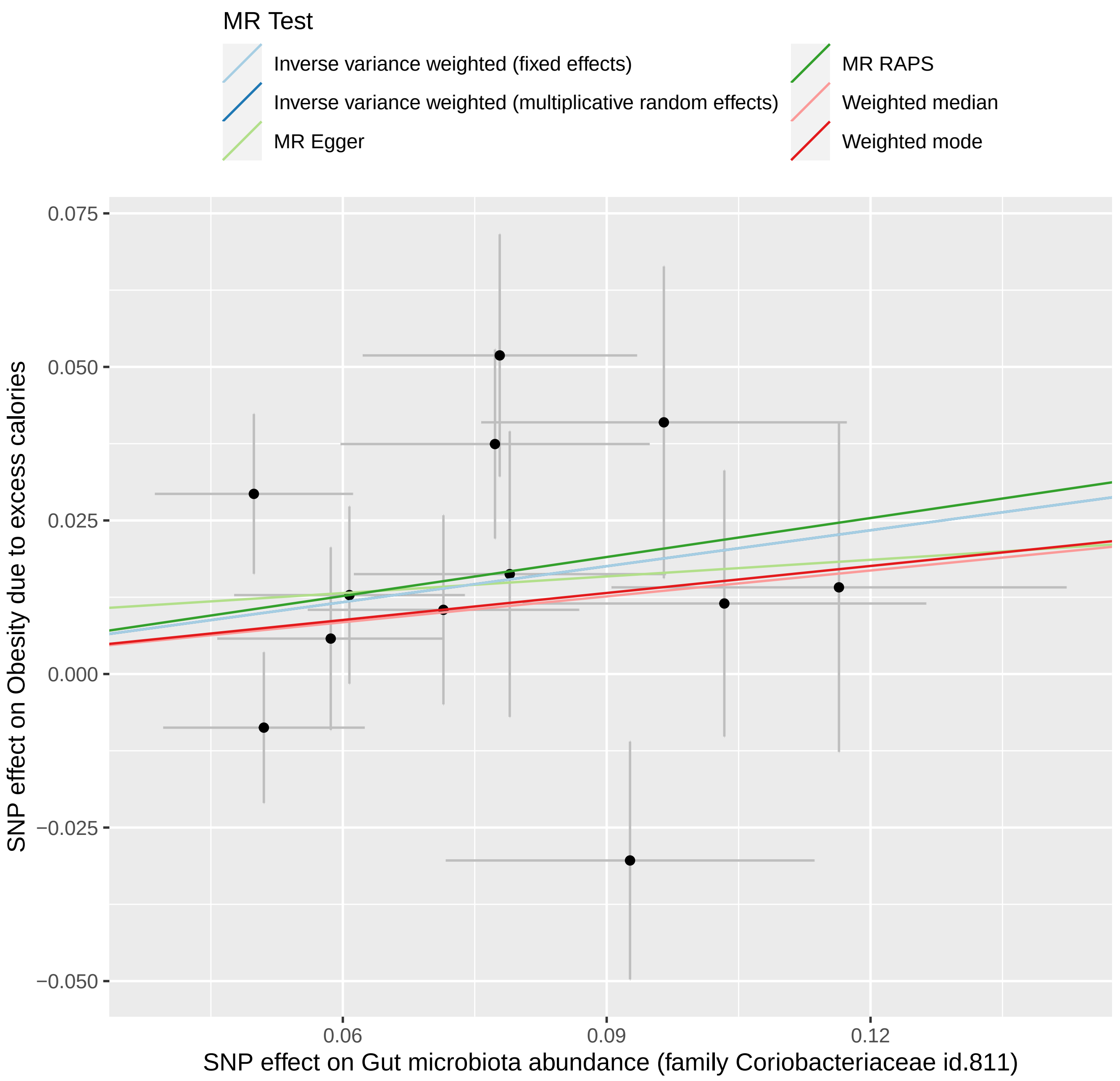

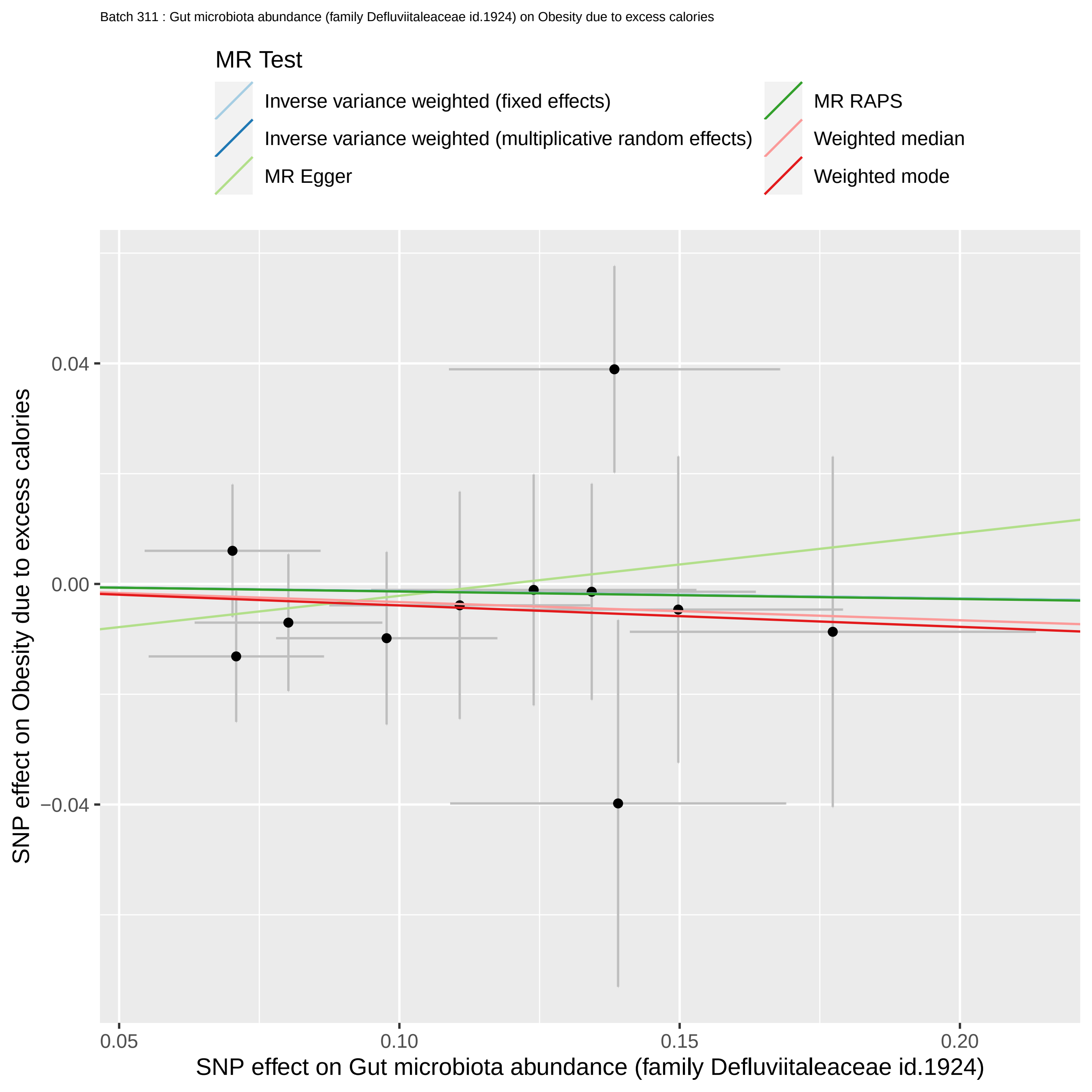
Batch 311 : Gut microbiota abundance (family Defluviitaleaceae id.1924) on Obesity due to excess calories
MR Test
Inverse variance weighted (fixed effects)
Inverse variance weighted (multiplicative random effects)
MR Egger
MR RAPS
Weighted median
Weighted mode
0.04
0.00
−0.04
0.05
0.10
0.15
0.20
SNP effect on Obesity due to excess calories
SNP effect on Gut microbiota abundance (family Defluviitaleaceae id.1924)

Batch 312 : Gut microbiota abundance (family Desulfovibrionaceae id.3169) on Obesity due to excess calories

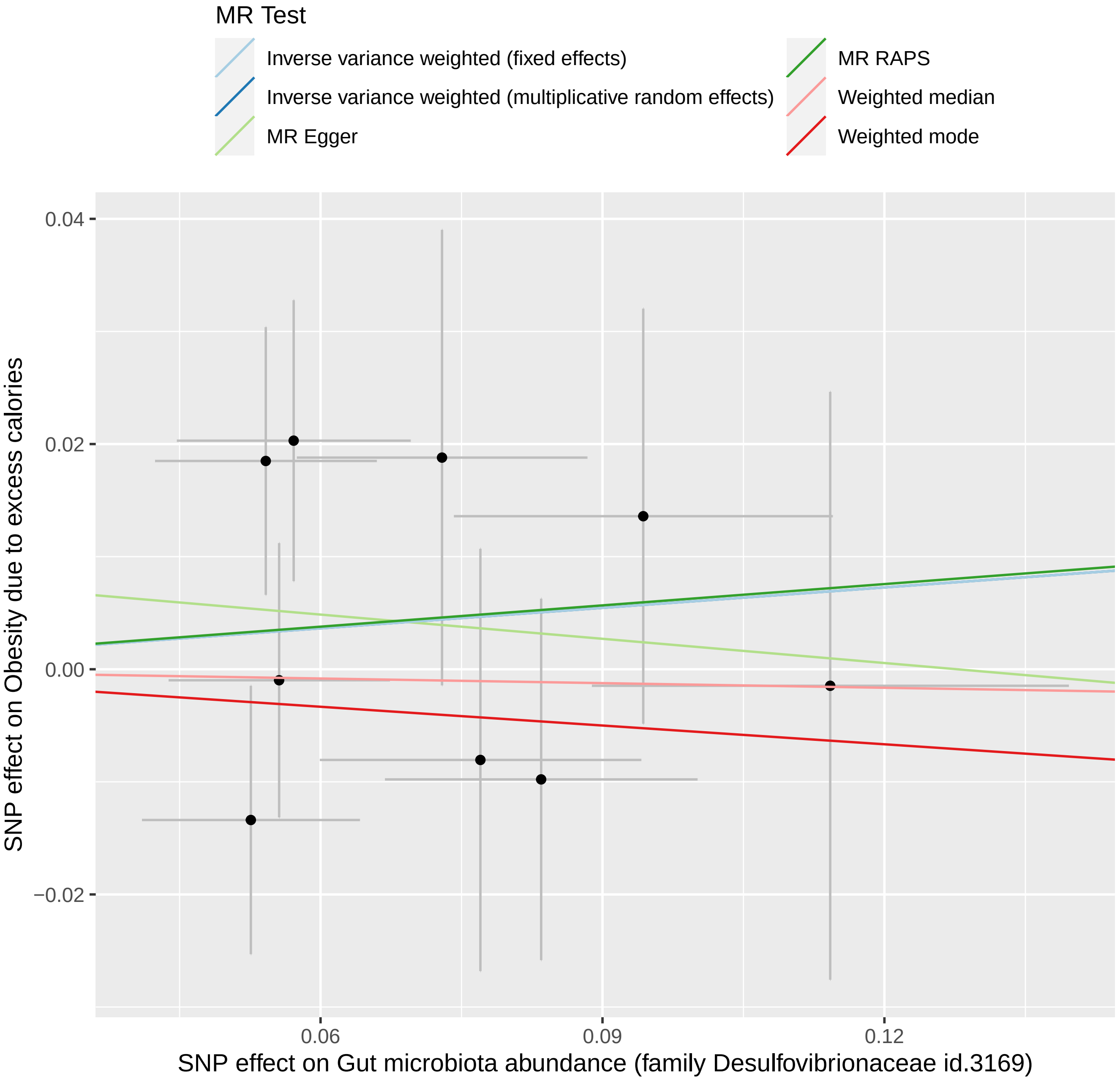

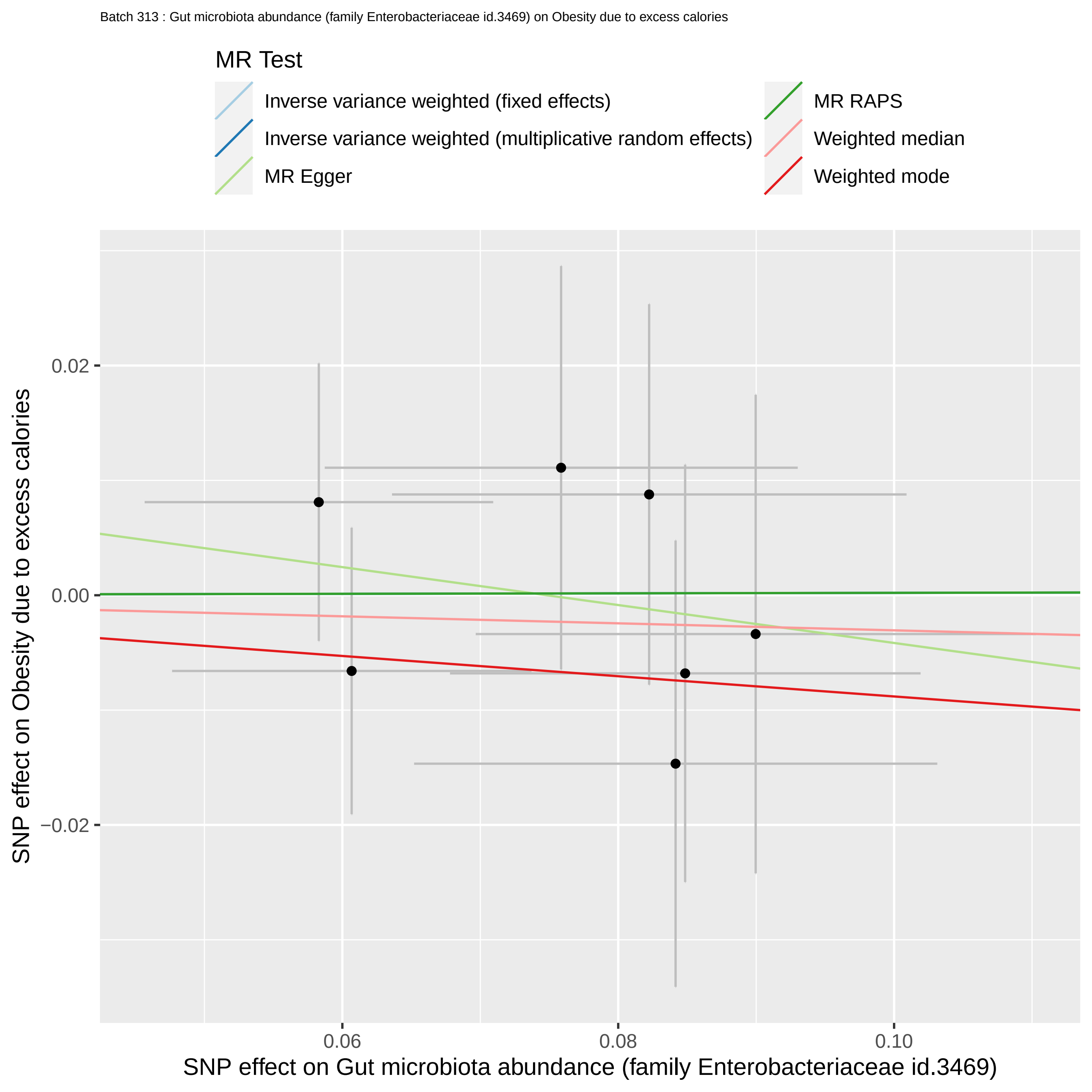
Batch 313 : Gut microbiota abundance (family Enterobacteriaceae id.3469) on Obesity due to excess calories
MR Test
Inverse variance weighted (fixed effects)
Inverse variance weighted (multiplicative random effects)
MR Egger
MR RAPS
Weighted median
Weighted mode
0.02
0.00
−0.02
0.06
0.08
0.10
SNP effect on Obesity due to excess calories
SNP effect on Gut microbiota abundance (family Enterobacteriaceae id.3469)

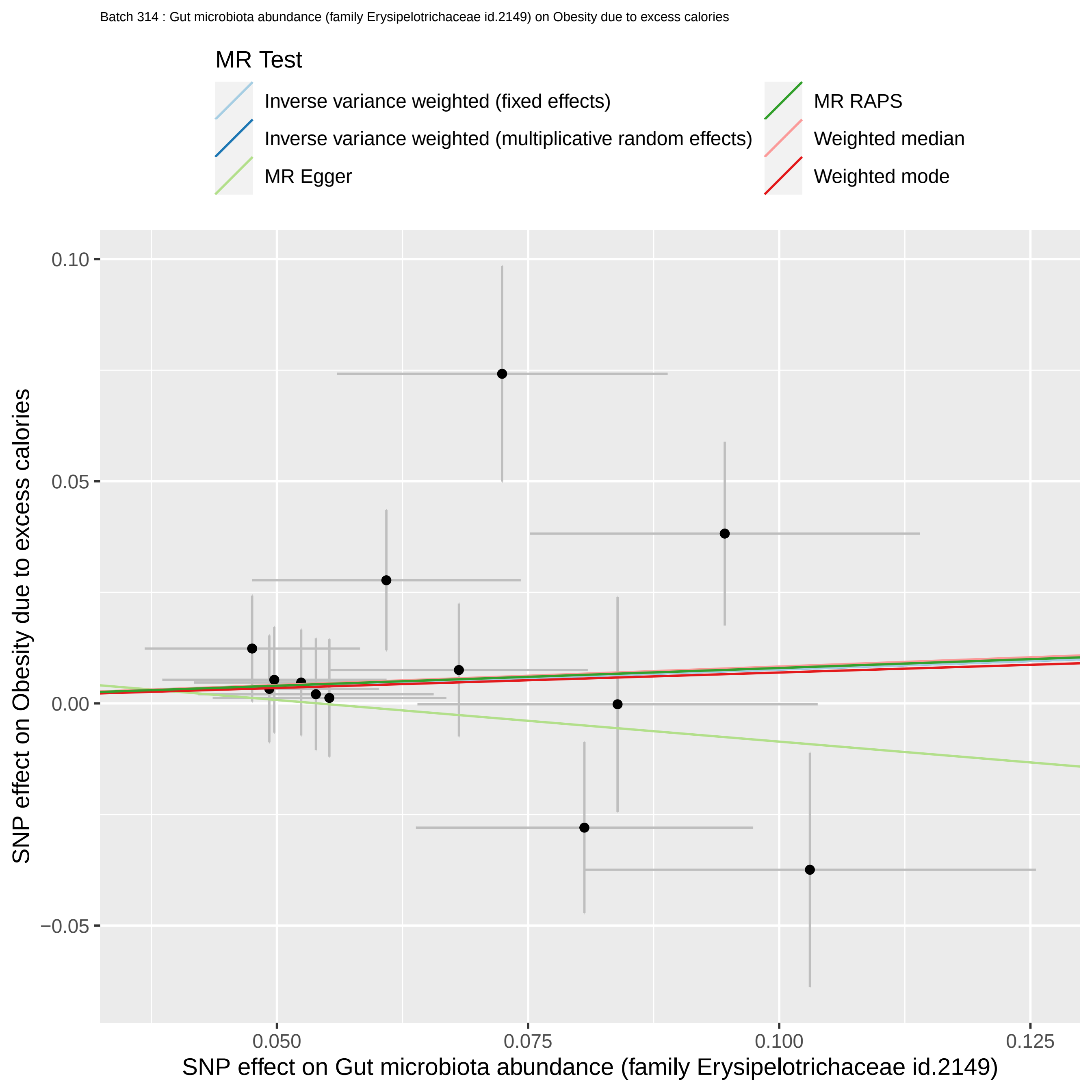
Batch 314 : Gut microbiota abundance (family Erysipelotrichaceae id.2149) on Obesity due to excess calories
MR Test
Inverse variance weighted (fixed effects)
Inverse variance weighted (multiplicative random effects)
MR Egger
MR RAPS
Weighted median
Weighted mode
0.10
0.05
0.00
−0.05
SNP effect on Obesity due to excess calories
0.050
0.075
0.100
0.125
SNP effect on Gut microbiota abundance (family Erysipelotrichaceae id.2149)

Batch 315 : Gut microbiota abundance (family Family XI id.1936) on Obesity due to excess calories

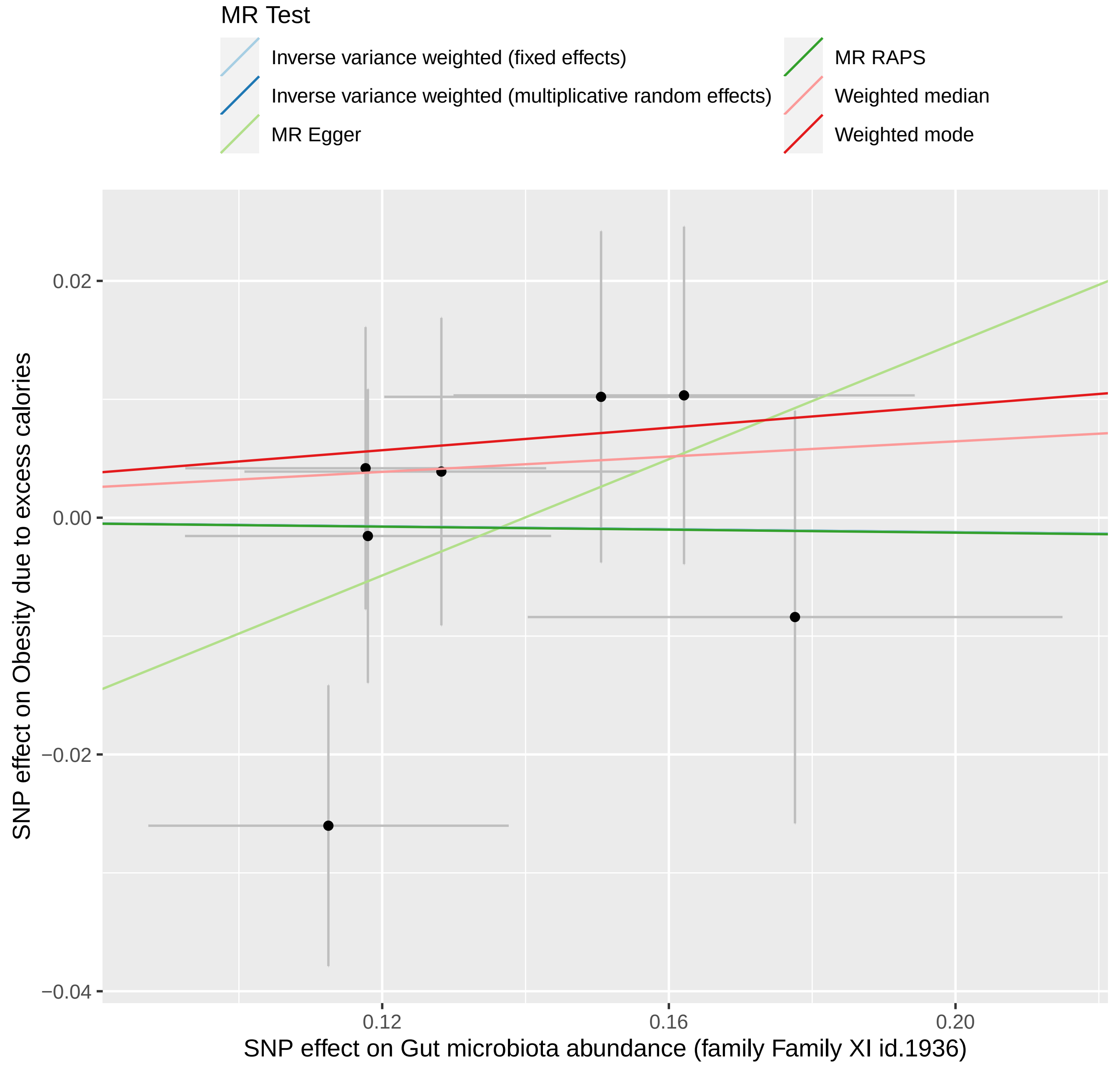

Batch 316 : Gut microbiota abundance (family Family XIII id.1957) on Obesity due to excess calories

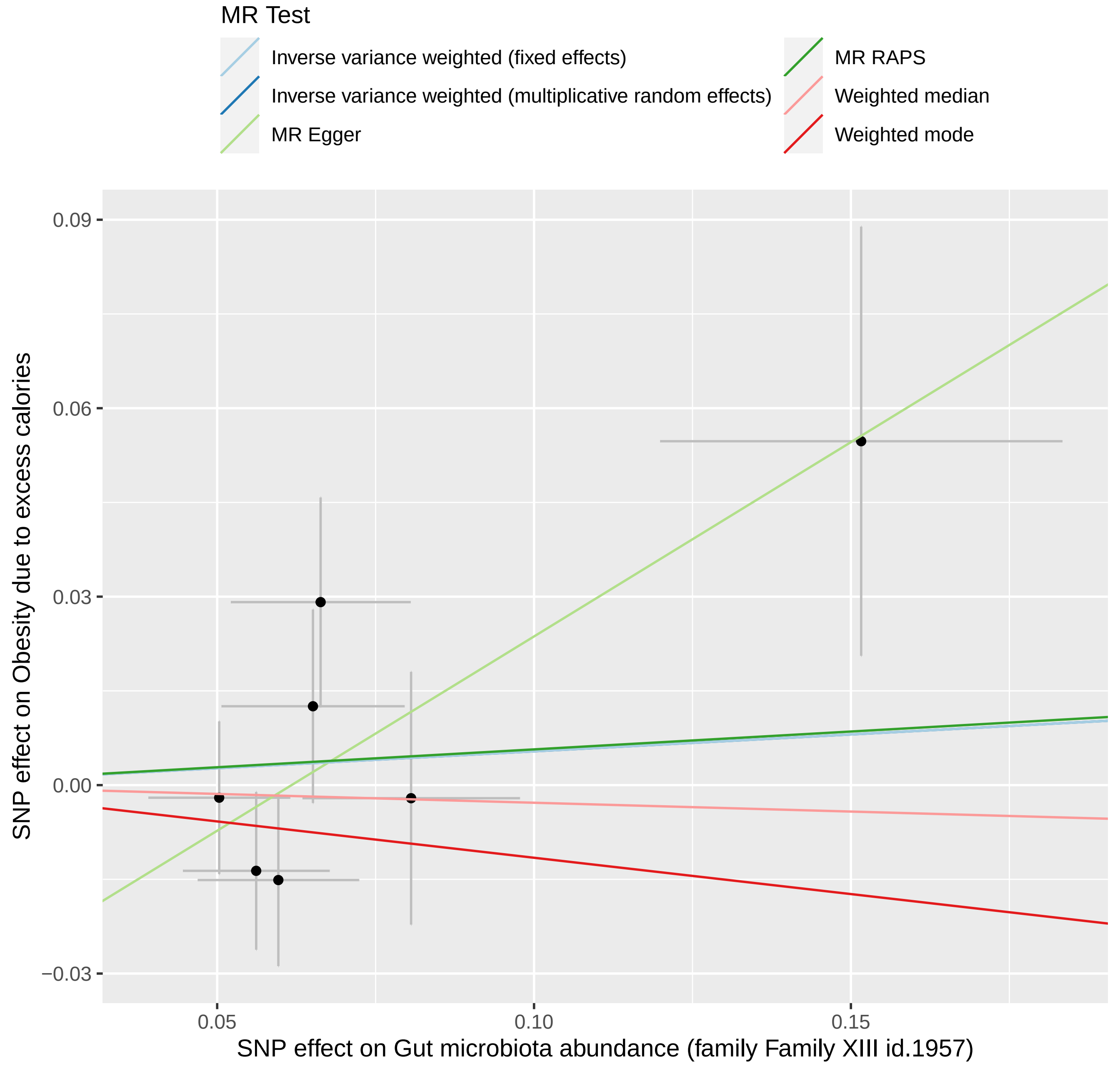

Batch 317 : Gut microbiota abundance (family Lachnospiraceae id.1987) on Obesity due to excess calories

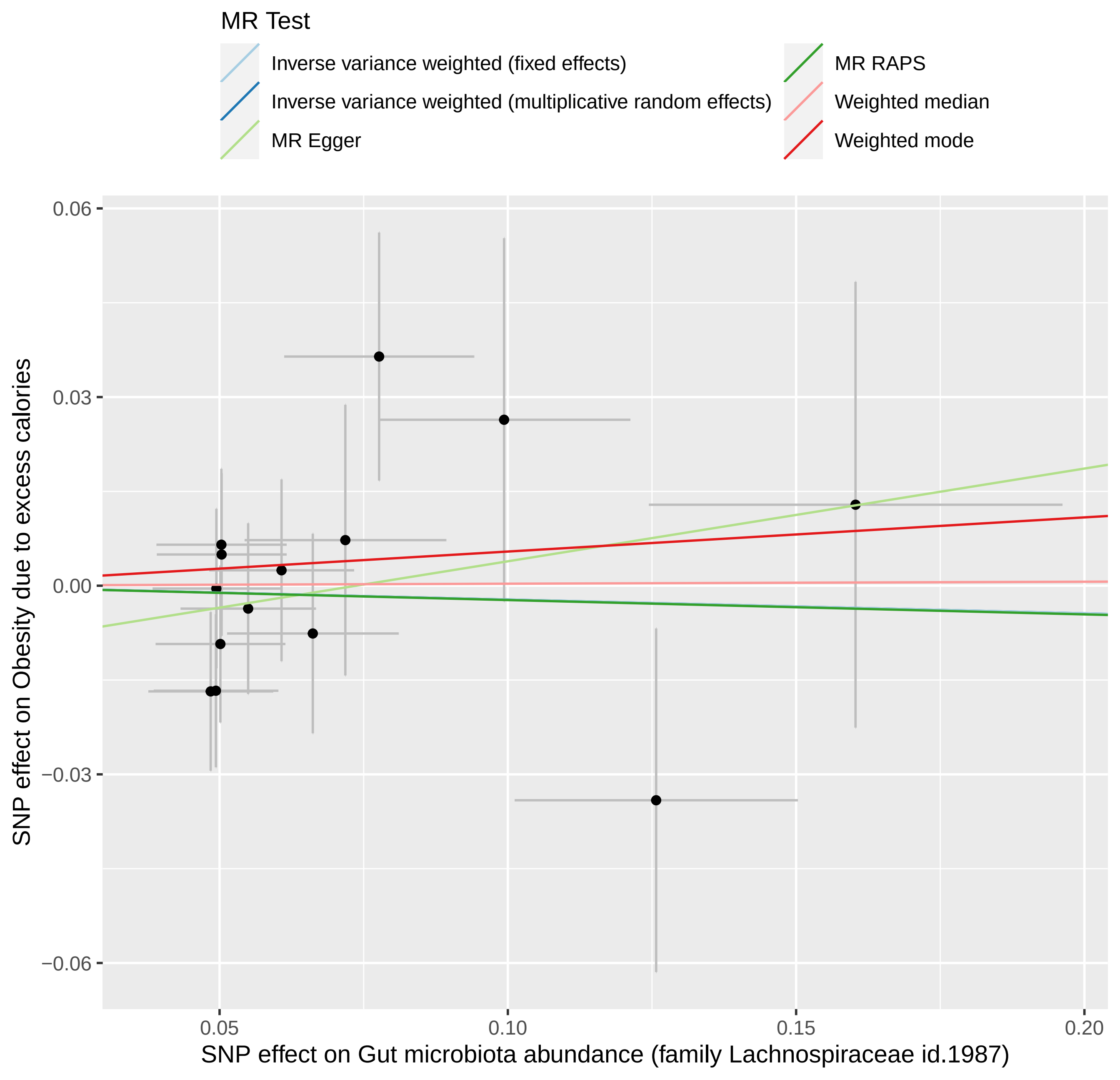

Batch 318 : Gut microbiota abundance (family Lactobacillaceae id.1836) on Obesity due to excess calories

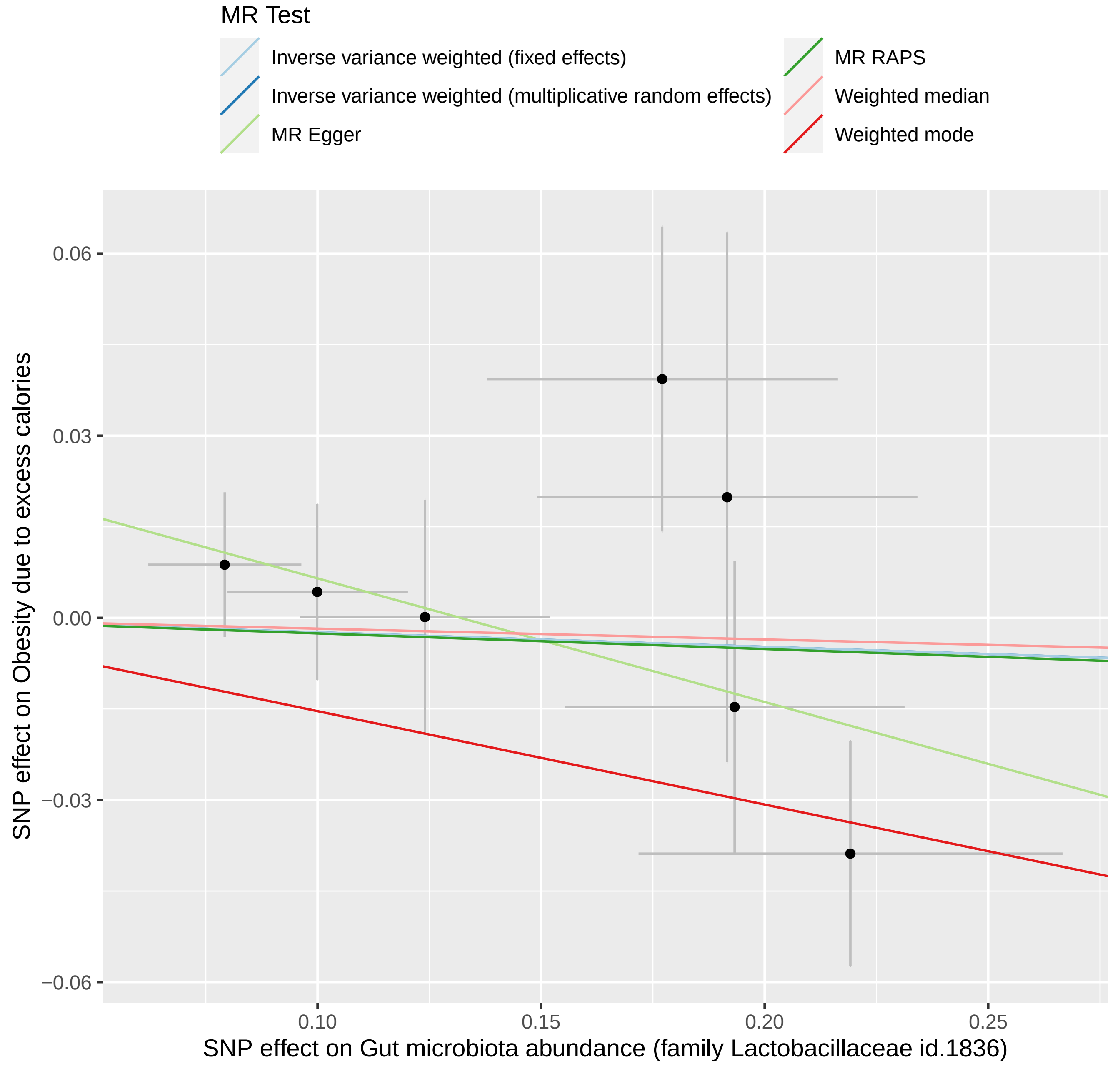

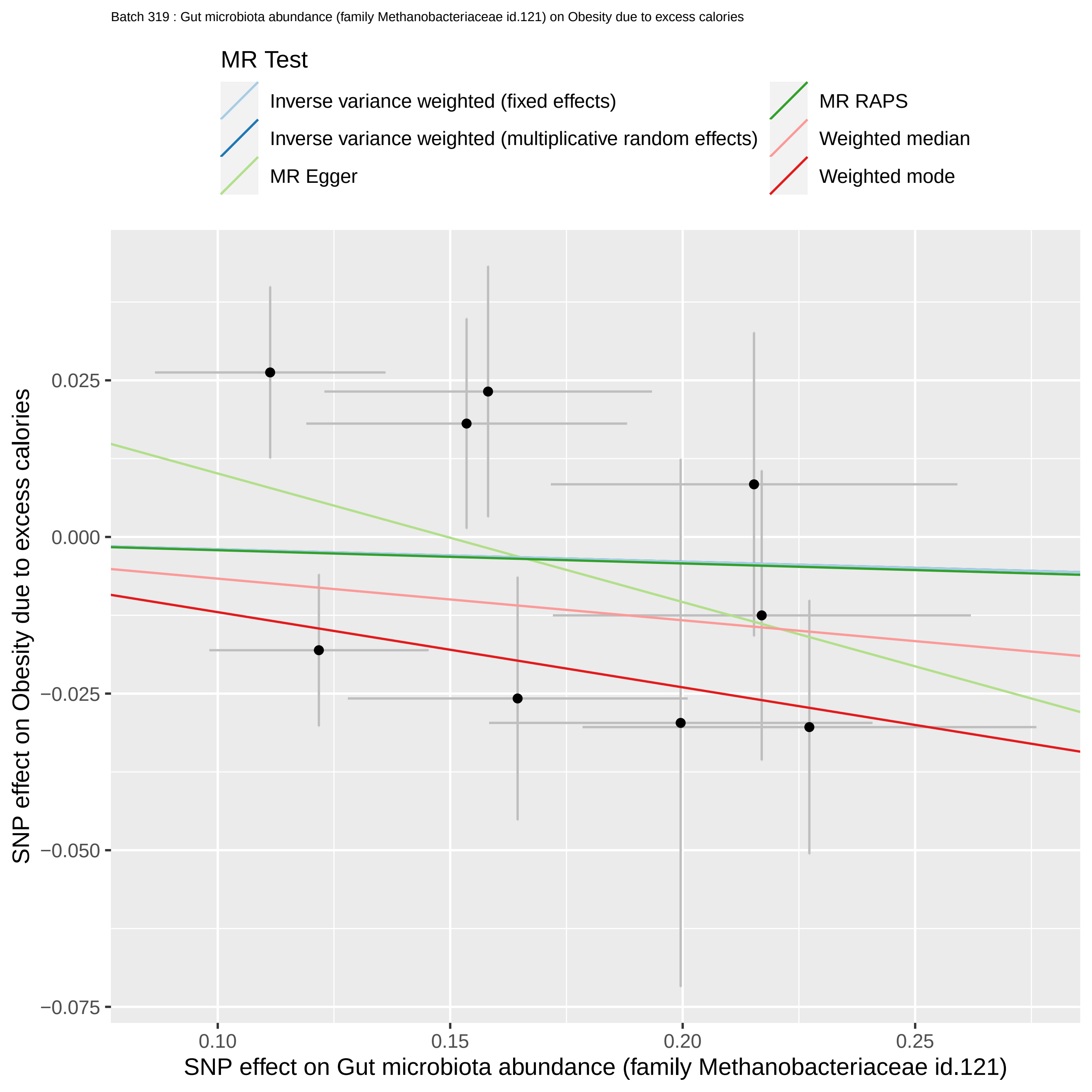
Batch 319 : Gut microbiota abundance (family Methanobacteriaceae id.121) on Obesity due to excess calories
MR Test
Inverse variance weighted (fixed effects)
Inverse variance weighted (multiplicative random effects)
MR Egger
MR RAPS
Weighted median
Weighted mode
SNP effect on Obesity due to excess calories
0.025
0.000
−0.025
−0.050
−0.075
0.10
0.15
0.20
0.25
SNP effect on Gut microbiota abundance (family Methanobacteriaceae id.121)

Batch 320 : Gut microbiota abundance (family Oxalobacteraceae id.2966) on Obesity due to excess calories

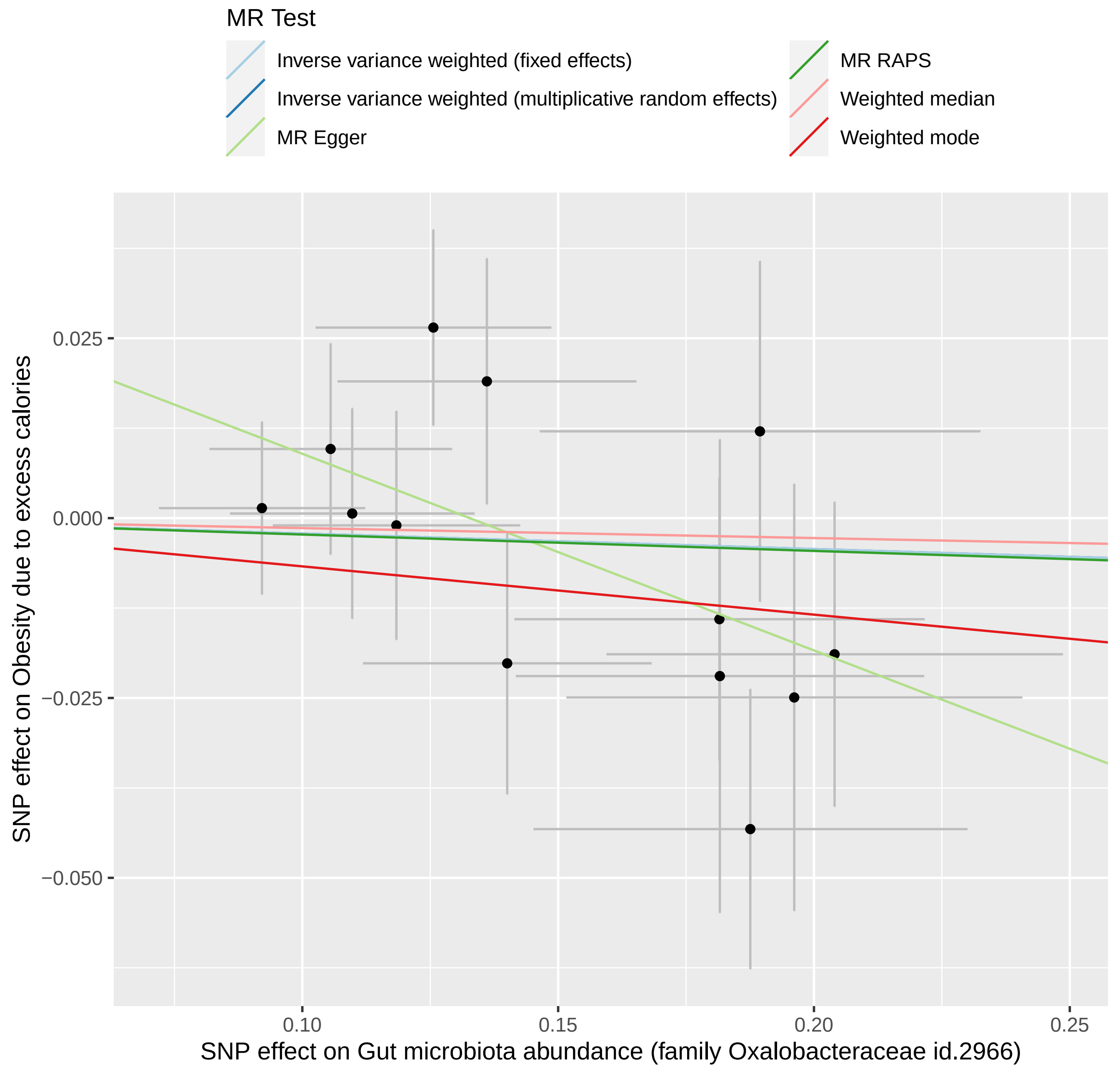

Batch 321 : Gut microbiota abundance (family Pasteurellaceae id.3689) on Obesity due to excess calories

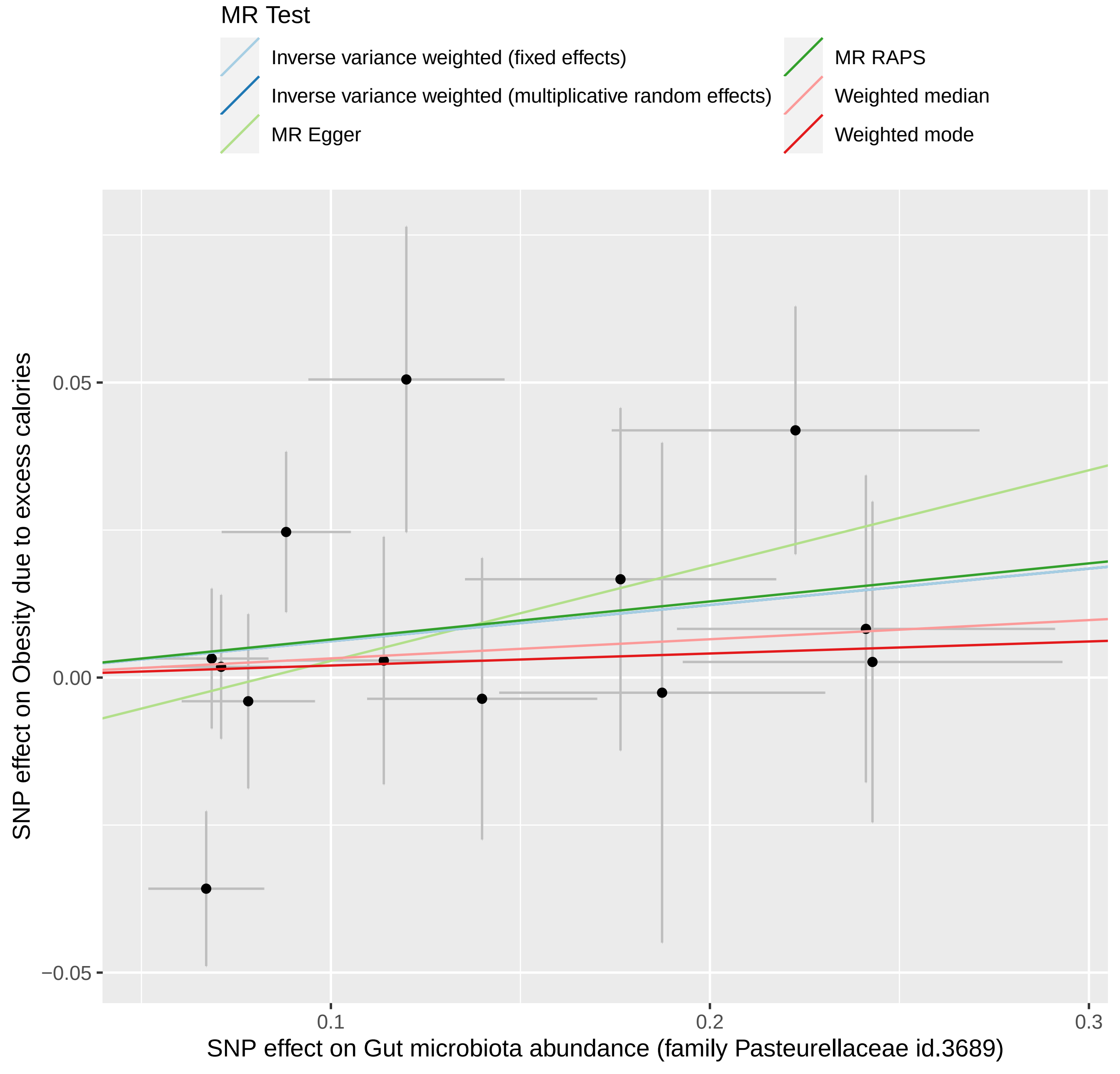

Batch 322 : Gut microbiota abundance (family Peptococcaceae id.2024) on Obesity due to excess calories

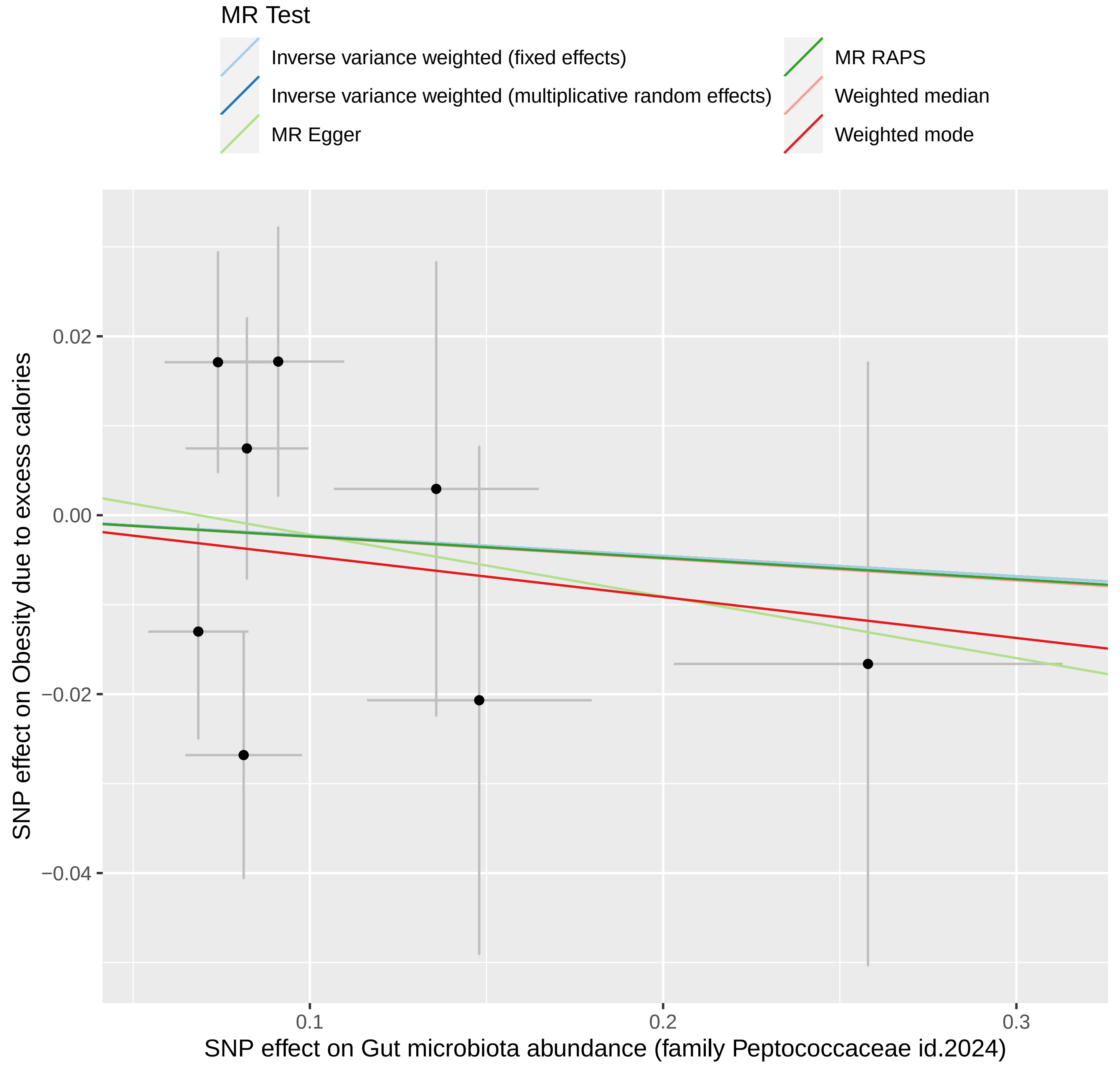

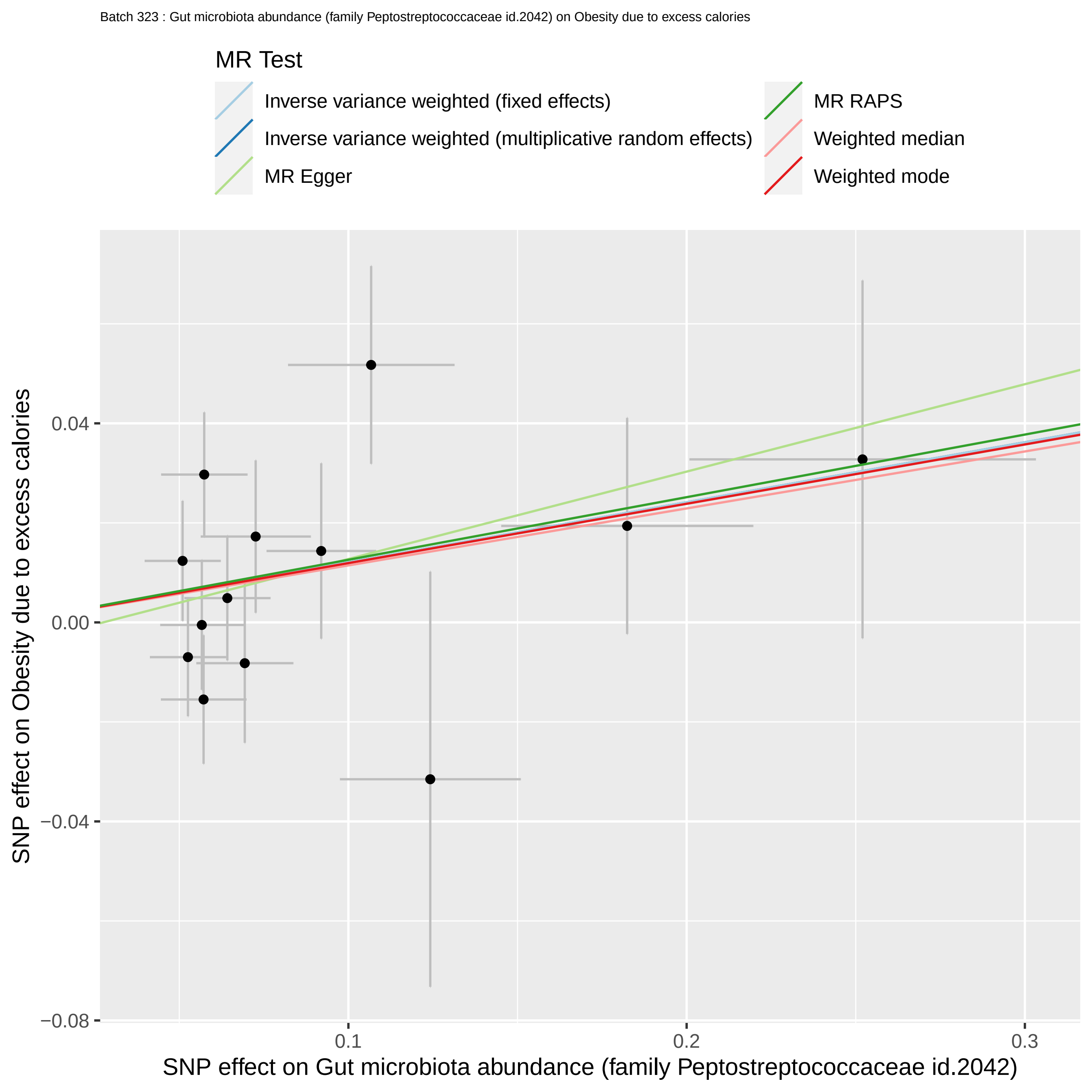
Batch 323 : Gut microbiota abundance (family Peptostreptococcaceae id.2042) on Obesity due to excess calories
MR Test
Inverse variance weighted (fixed effects)
Inverse variance weighted (multiplicative random effects)
MR Egger
MR RAPS
Weighted median
Weighted mode
SNP effect on Obesity due to excess calories
0.04
0.00
−0.04
−0.08
0.1
0.2
0.3
SNP effect on Gut microbiota abundance (family Peptostreptococcaceae id.2042)

Batch 324 : Gut microbiota abundance (family Porphyromonadaceae id.943) on Obesity due to excess calories

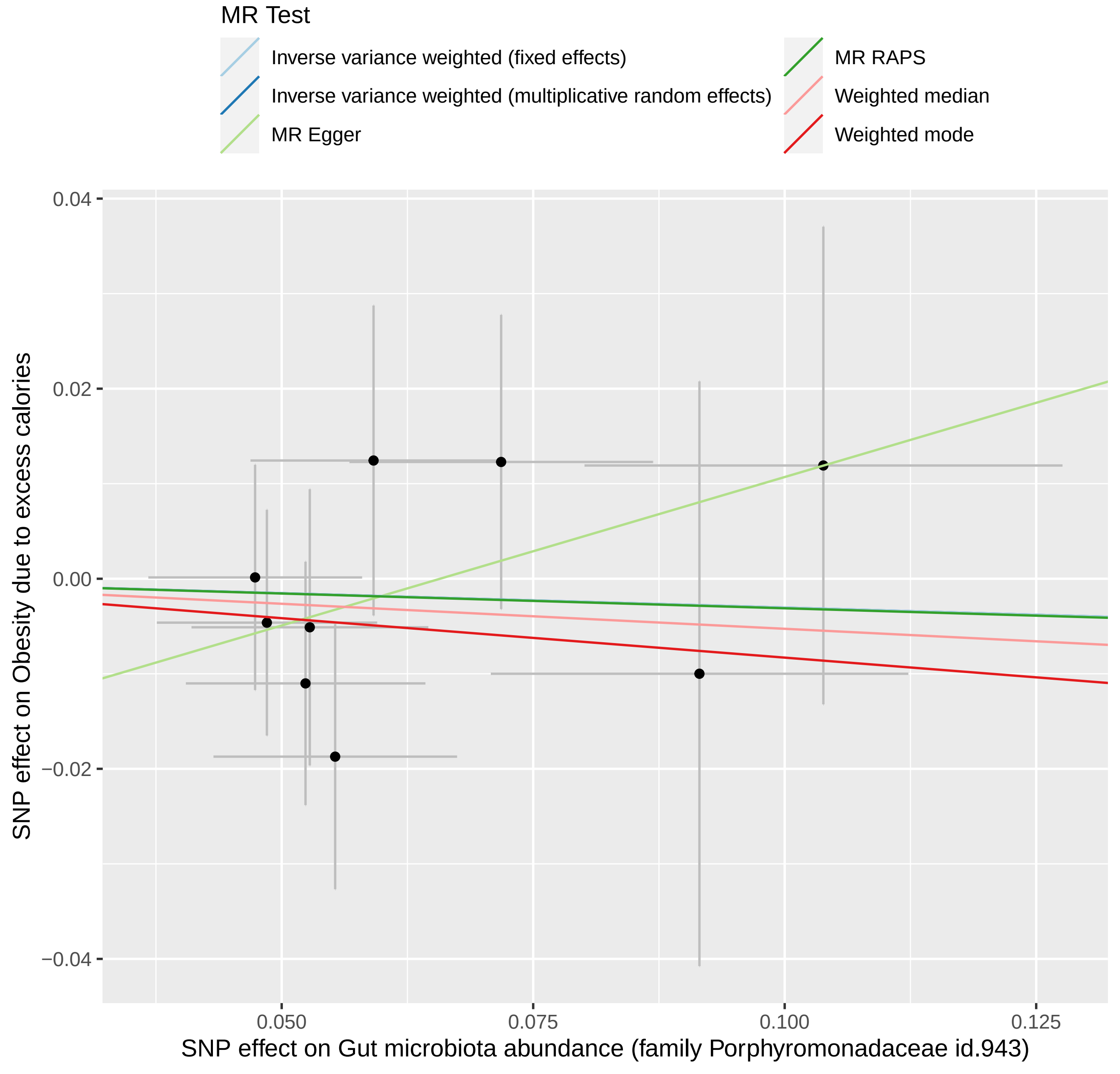

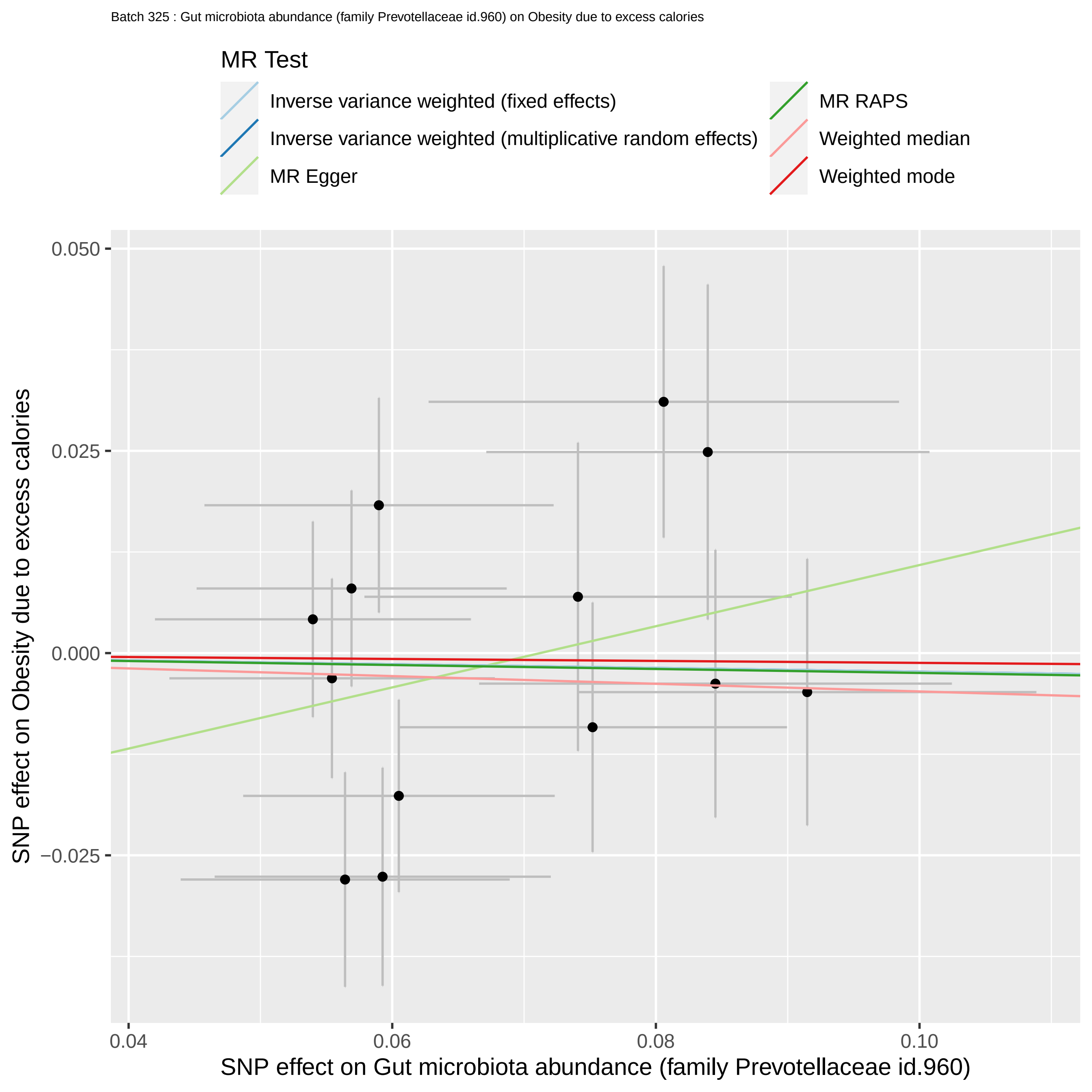

Batch 325 : Gut microbiota abundance (family Prevotellaceae id.960) on Obesity due to excess calories
MR Test
Inverse variance weighted (fixed effects)
Inverse variance weighted (multiplicative random effects)
MR Egger
MR RAPS
Weighted median
Weighted mode
0.050
0.025
0.000
−0.025
SNP effect on Obesity due to excess calories
0.04
0.06
0.08
0.10
SNP effect on Gut microbiota abundance (family Prevotellaceae id.960)

Batch 326 : Gut microbiota abundance (family Rhodospirillaceae id.2717) on Obesity due to excess calories

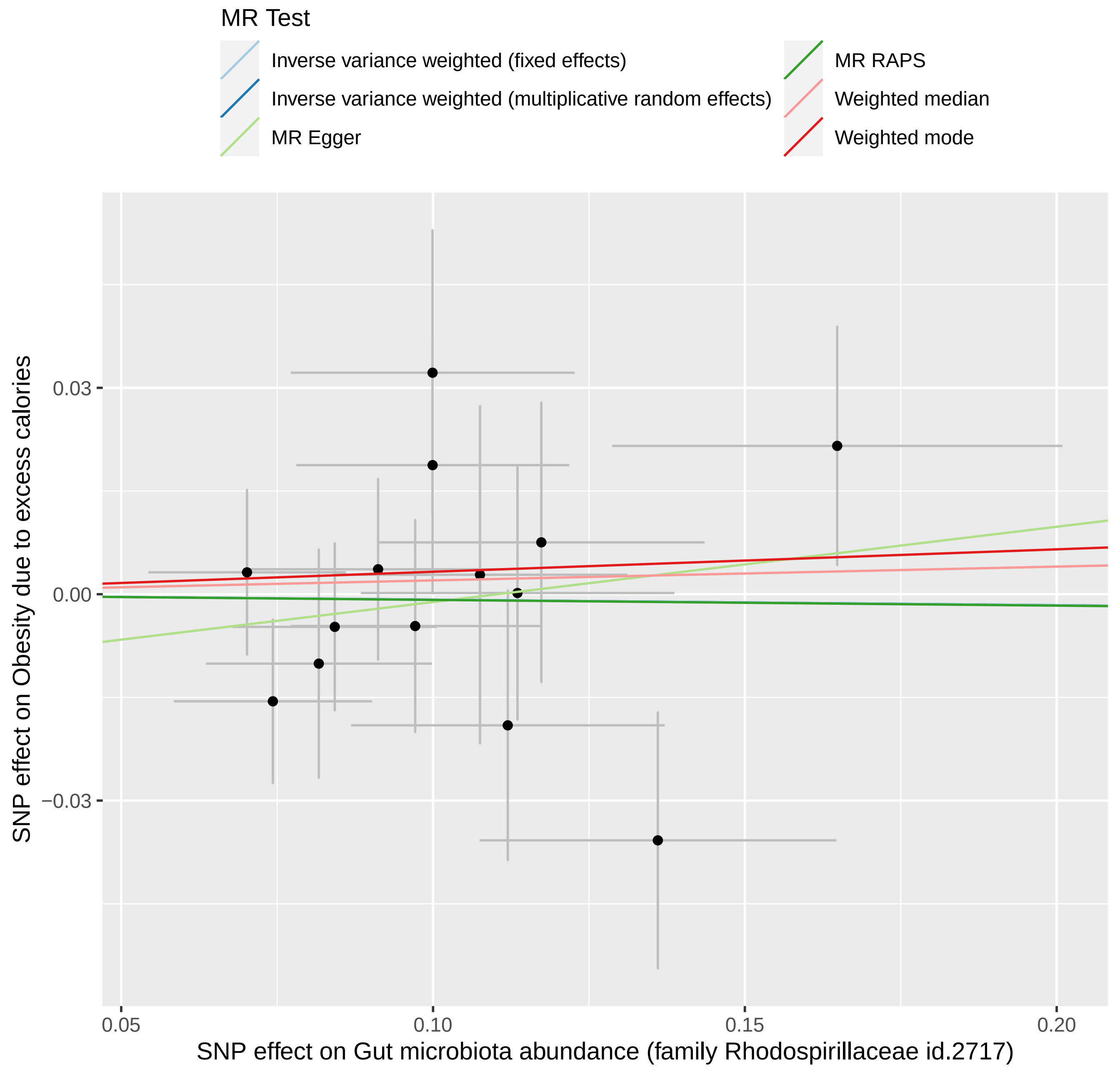

Batch 327 : Gut microbiota abundance (family Rikenellaceae id.967) on Obesity due to excess calories

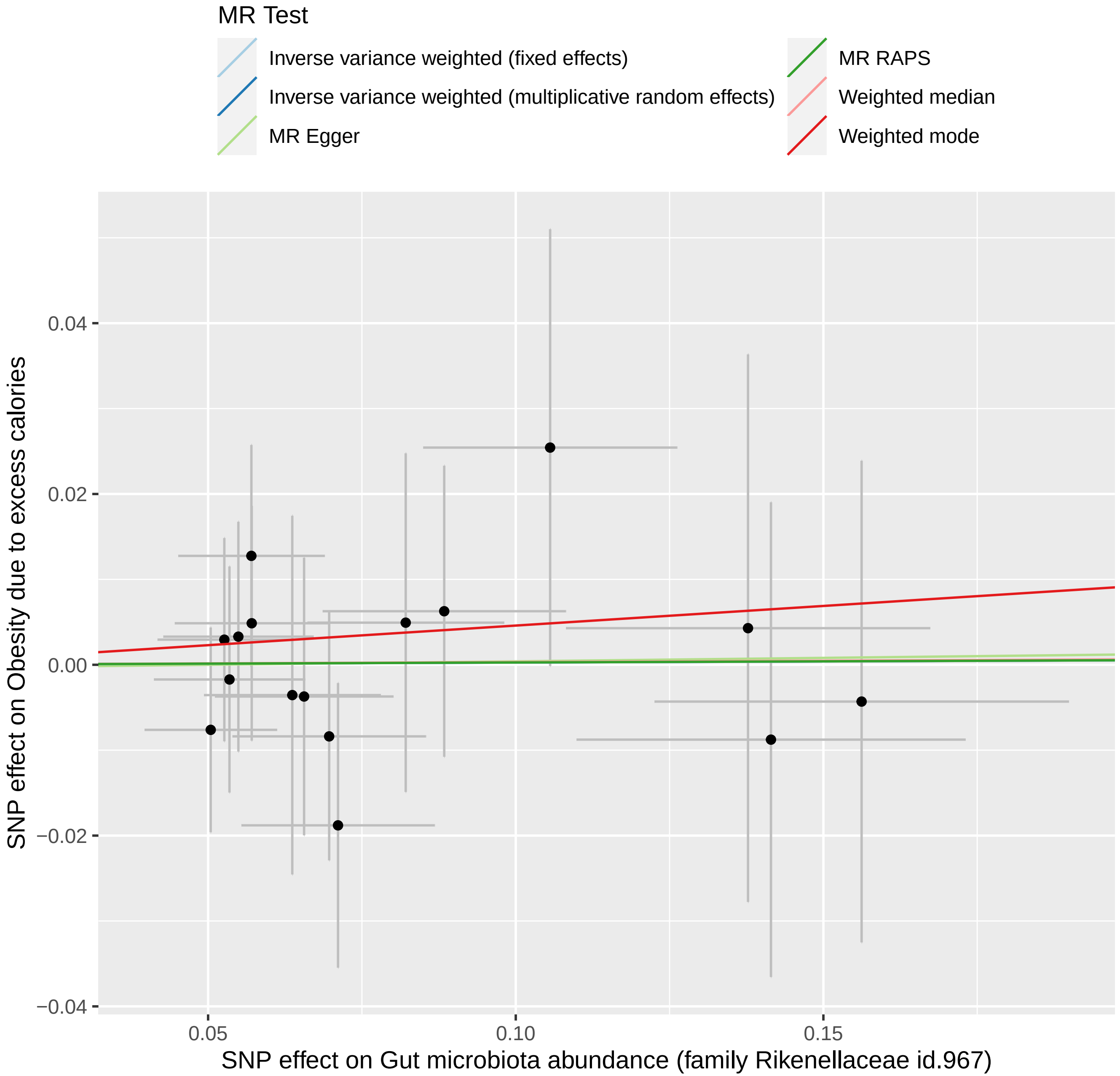

Batch 328 : Gut microbiota abundance (family Ruminococcaceae id.2050) on Obesity due to excess calories

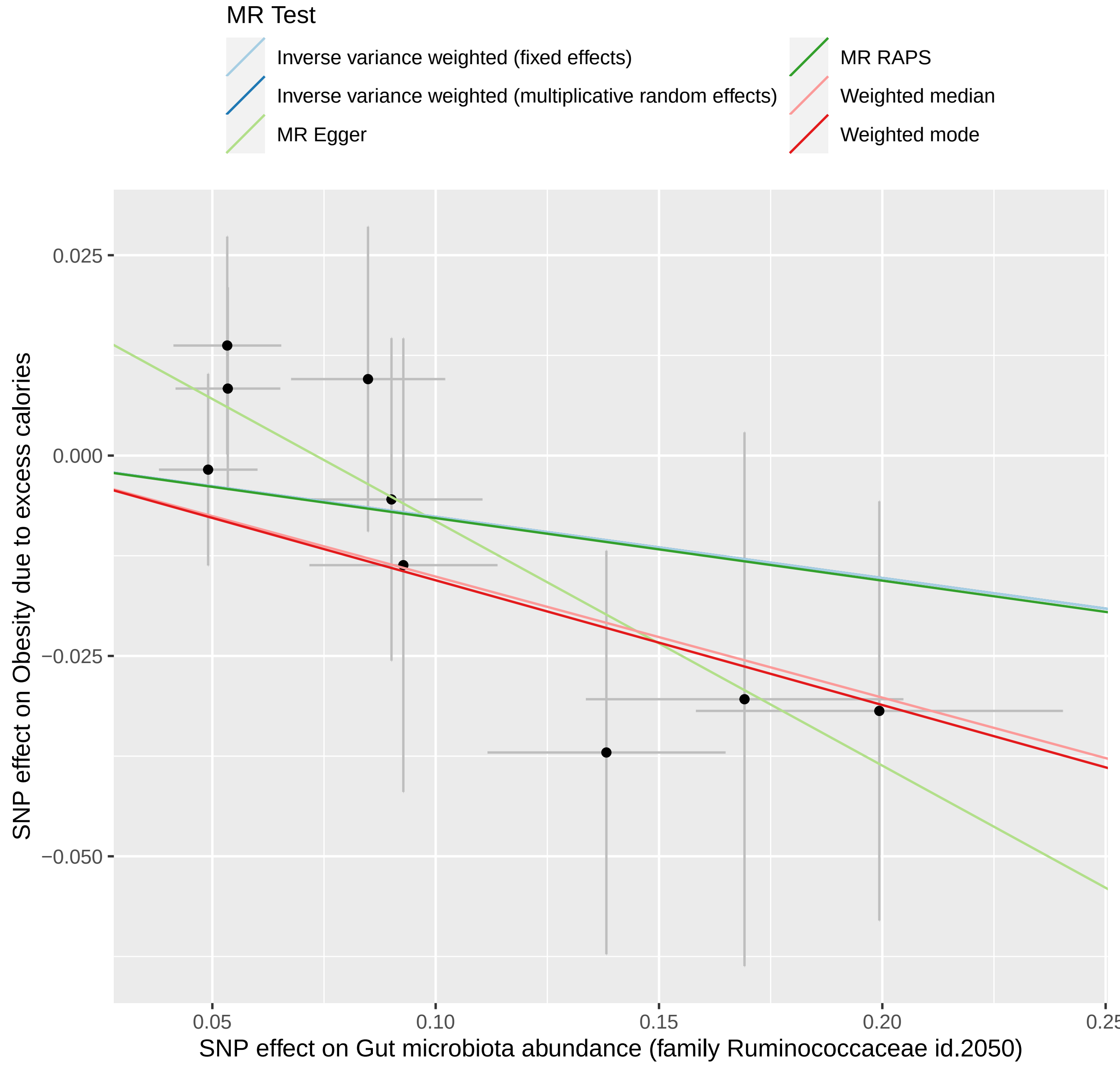

Batch 329 : Gut microbiota abundance (family Streptococcaceae id.1850) on Obesity due to excess calories

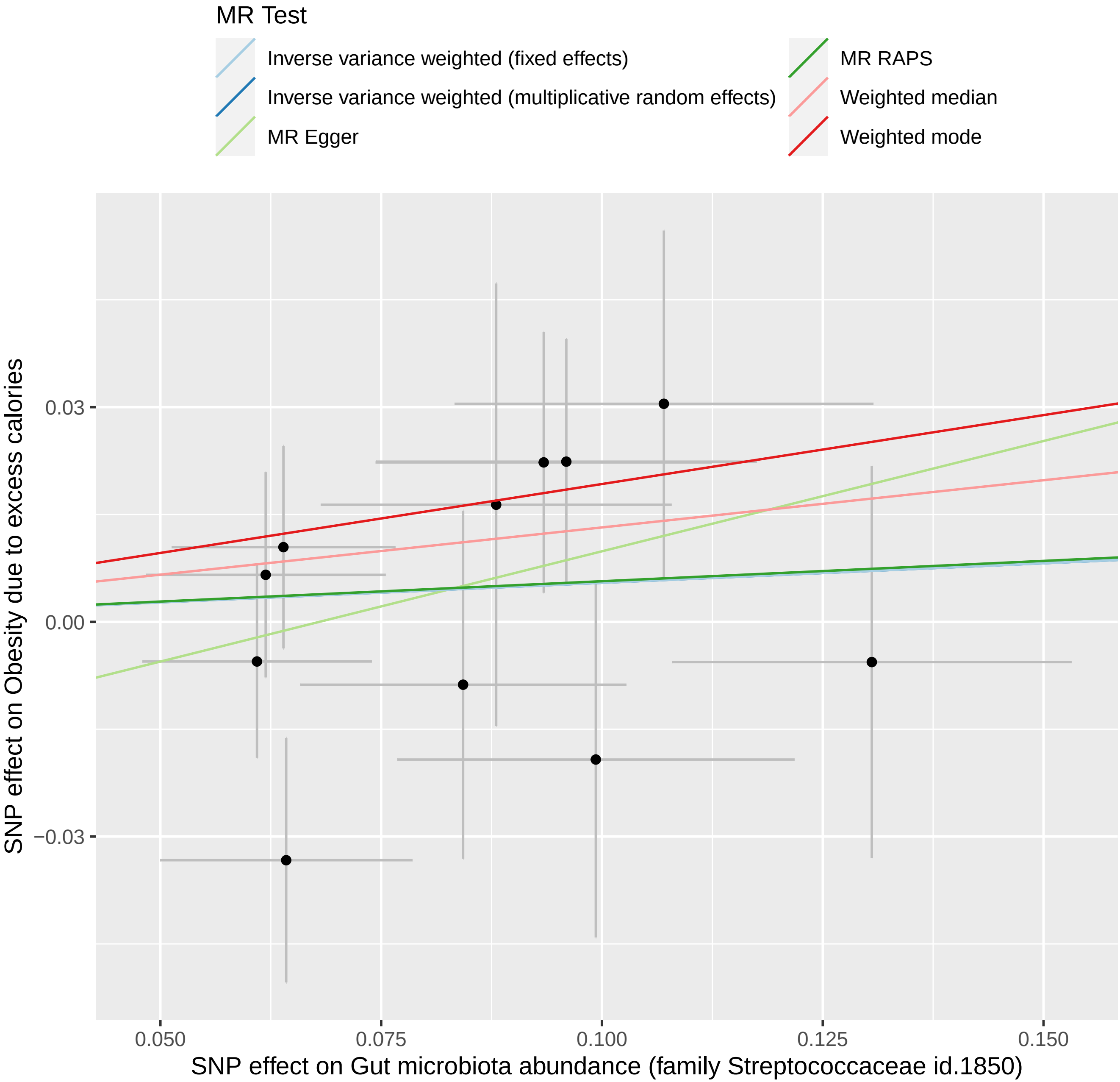

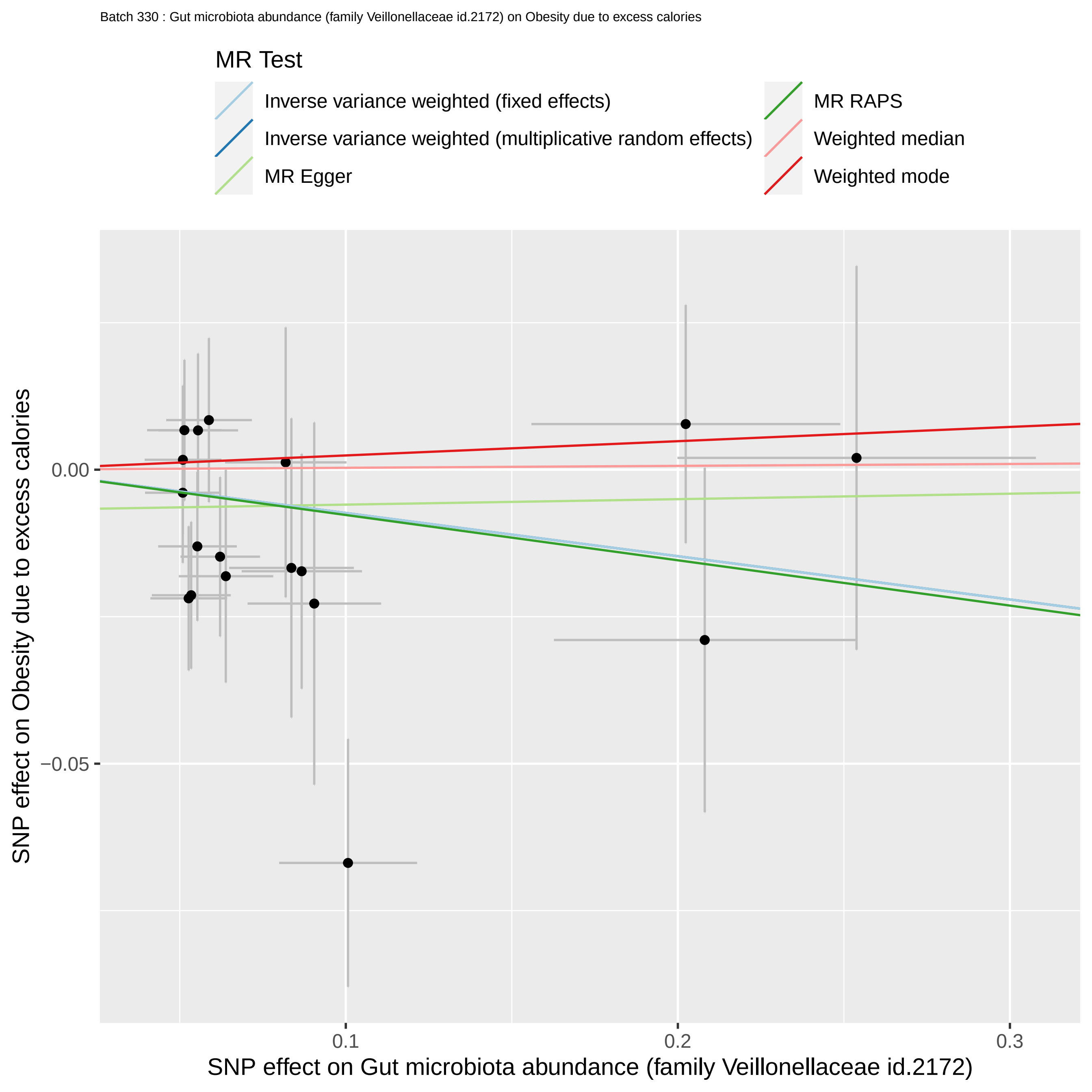
Batch 330 : Gut microbiota abundance (family Veillonellaceae id.2172) on Obesity due to excess calories
MR Test
Inverse variance weighted (fixed effects)
Inverse variance weighted (multiplicative random effects)
MR Egger
MR RAPS
Weighted median
Weighted mode
0.00
−0.05
0.1
0.2
0.3
SNP effect on Obesity due to excess calories
SNP effect on Gut microbiota abundance (family Veillonellaceae id.2172)

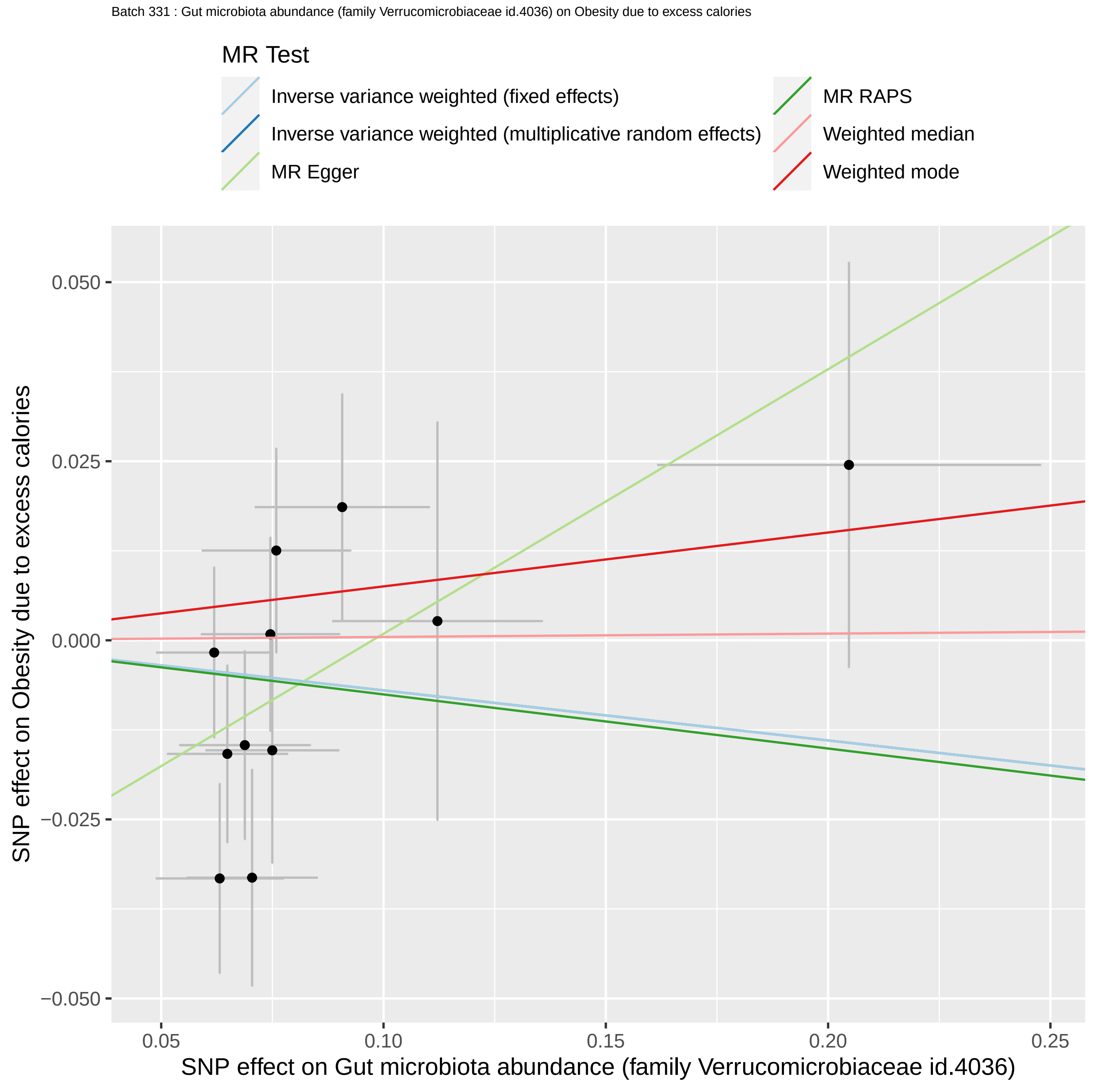
Batch 331 : Gut microbiota abundance (family Verrucomicrobiaceae id.4036) on Obesity due to excess calories
MR Test
Inverse variance weighted (fixed effects)
Inverse variance weighted (multiplicative random effects)
MR Egger
MR RAPS
Weighted median
Weighted mode
0.050
0.025
0.000
−0.025
−0.050
0.05
0.10
0.15
0.20
0.25
SNP effect on Obesity due to excess calories
SNP effect on Gut microbiota abundance (family Verrucomicrobiaceae id.4036)

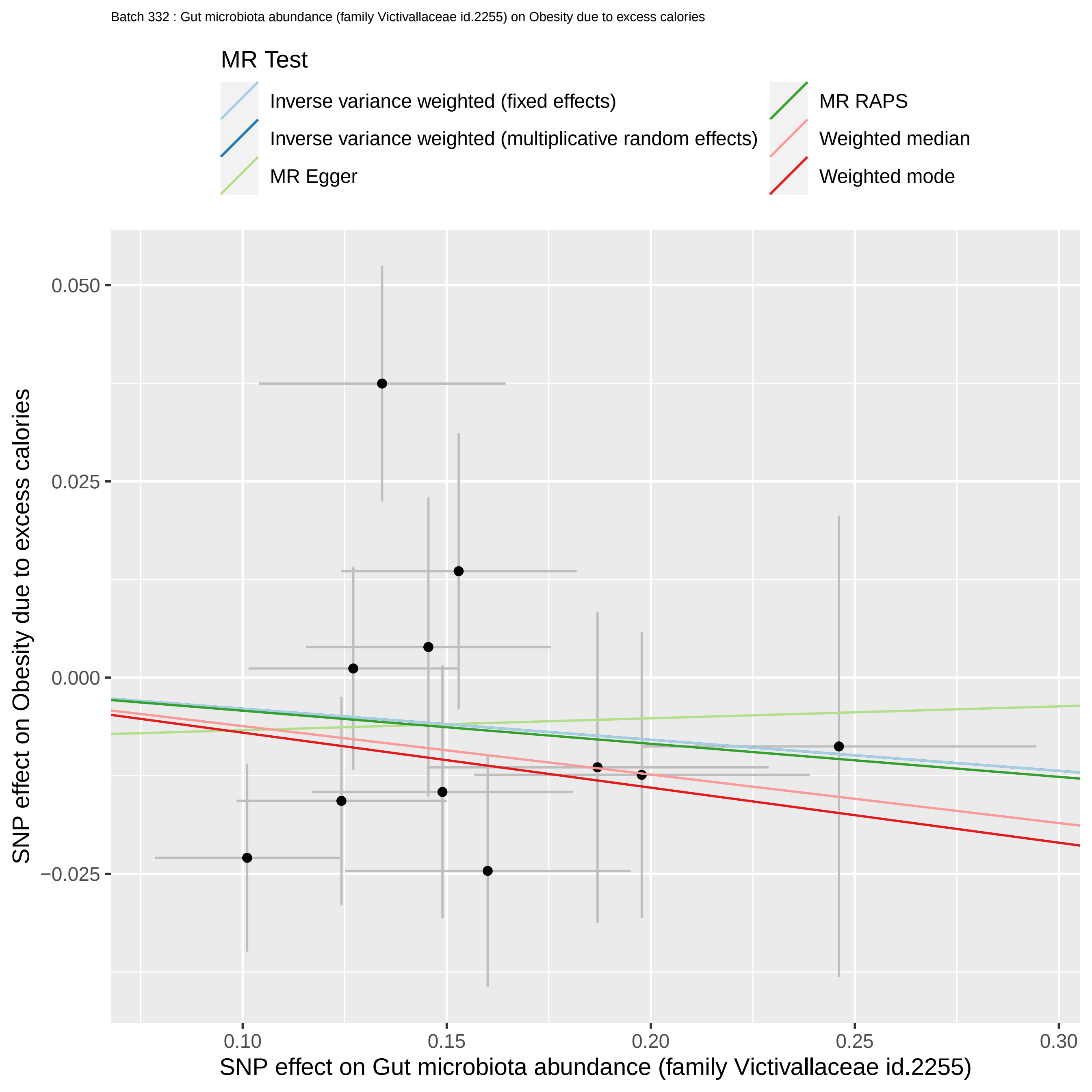

Batch 332 : Gut microbiota abundance (family Victivallaceae id.2255) on Obesity due to excess calories
MR Test
Inverse variance weighted (fixed effects)
Inverse variance weighted (multiplicative random effects)
MR Egger
MR RAPS
Weighted median
Weighted mode
0.050
0.025
0.000
−0.025
0.10
0.15
0.20
0.25
0.30
SNP effect on Obesity due to excess calories
SNP effect on Gut microbiota abundance (family Victivallaceae id.2255)

Batch 333 : Gut microbiota abundance (genus Actinomyces id.423) on Obesity due to excess calories

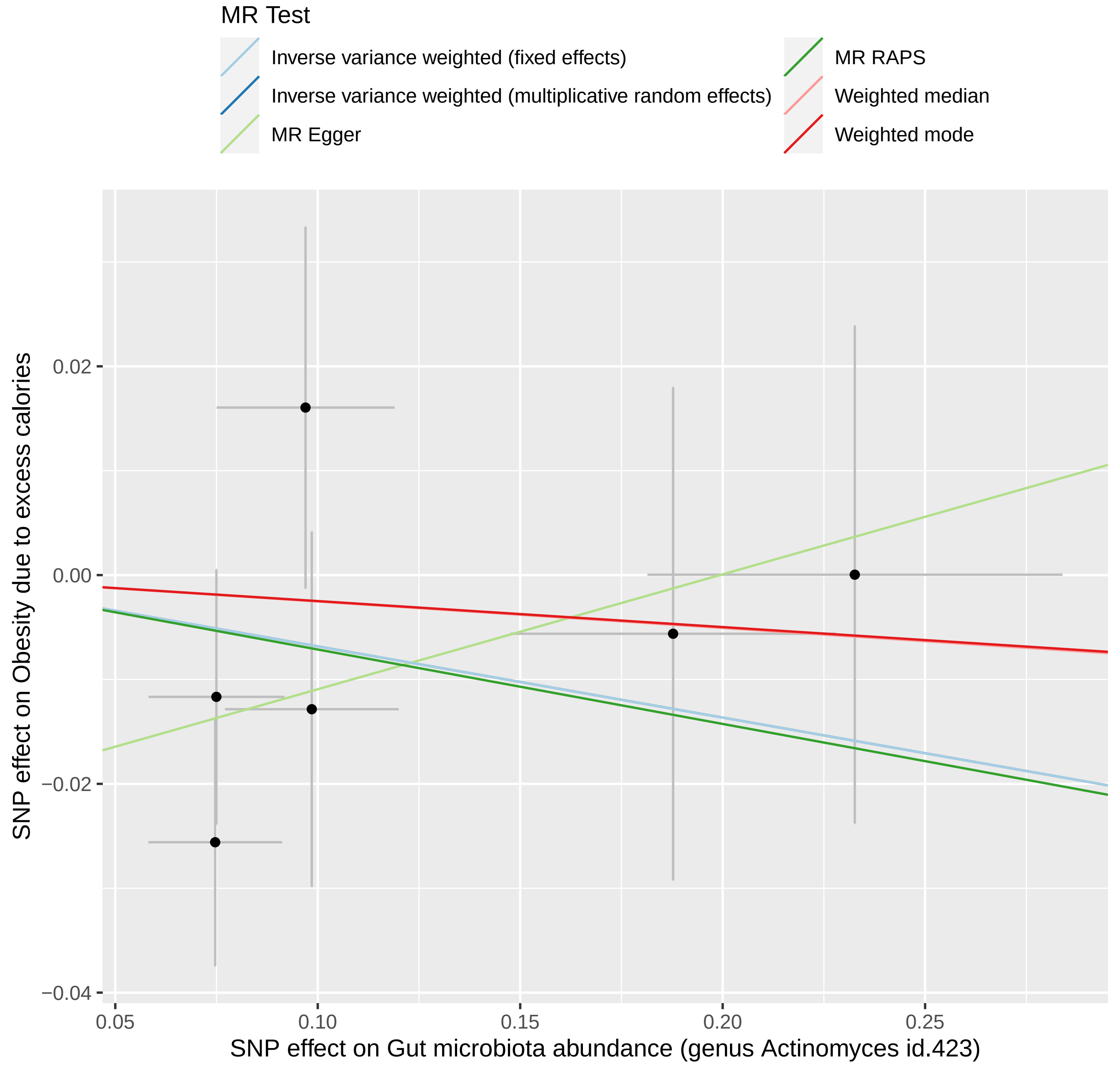

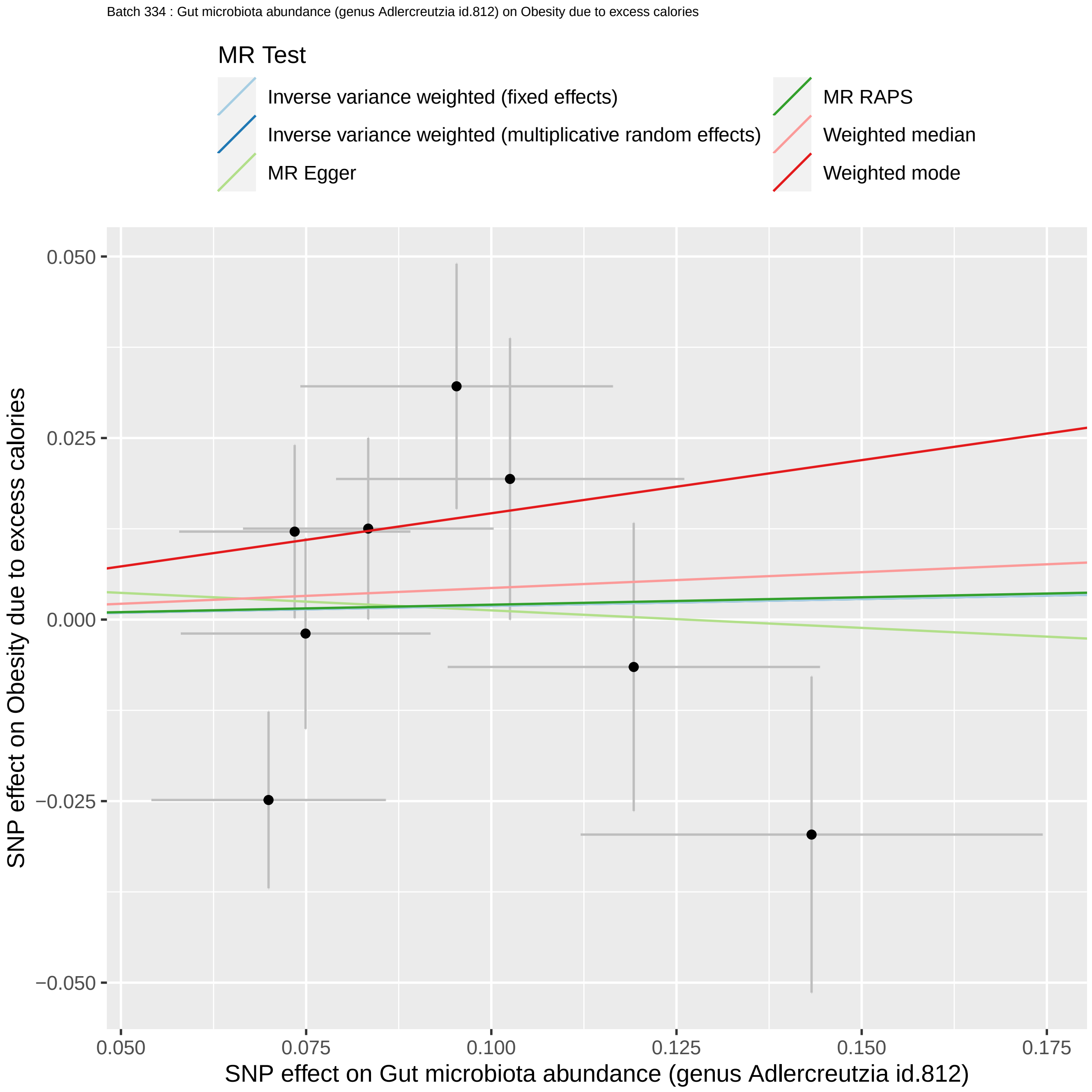

Batch 334 : Gut microbiota abundance (genus Adlercreutzia id.812) on Obesity due to excess calories
MR Test
Inverse variance weighted (fixed effects)
Inverse variance weighted (multiplicative random effects)
MR Egger
MR RAPS
Weighted median
Weighted mode
0.050
0.025
0.000
−0.025
−0.050
SNP effect on Obesity due to excess calories
0.050
0.075
0.100
0.125
0.150
0.175
SNP effect on Gut microbiota abundance (genus Adlercreutzia id.812)

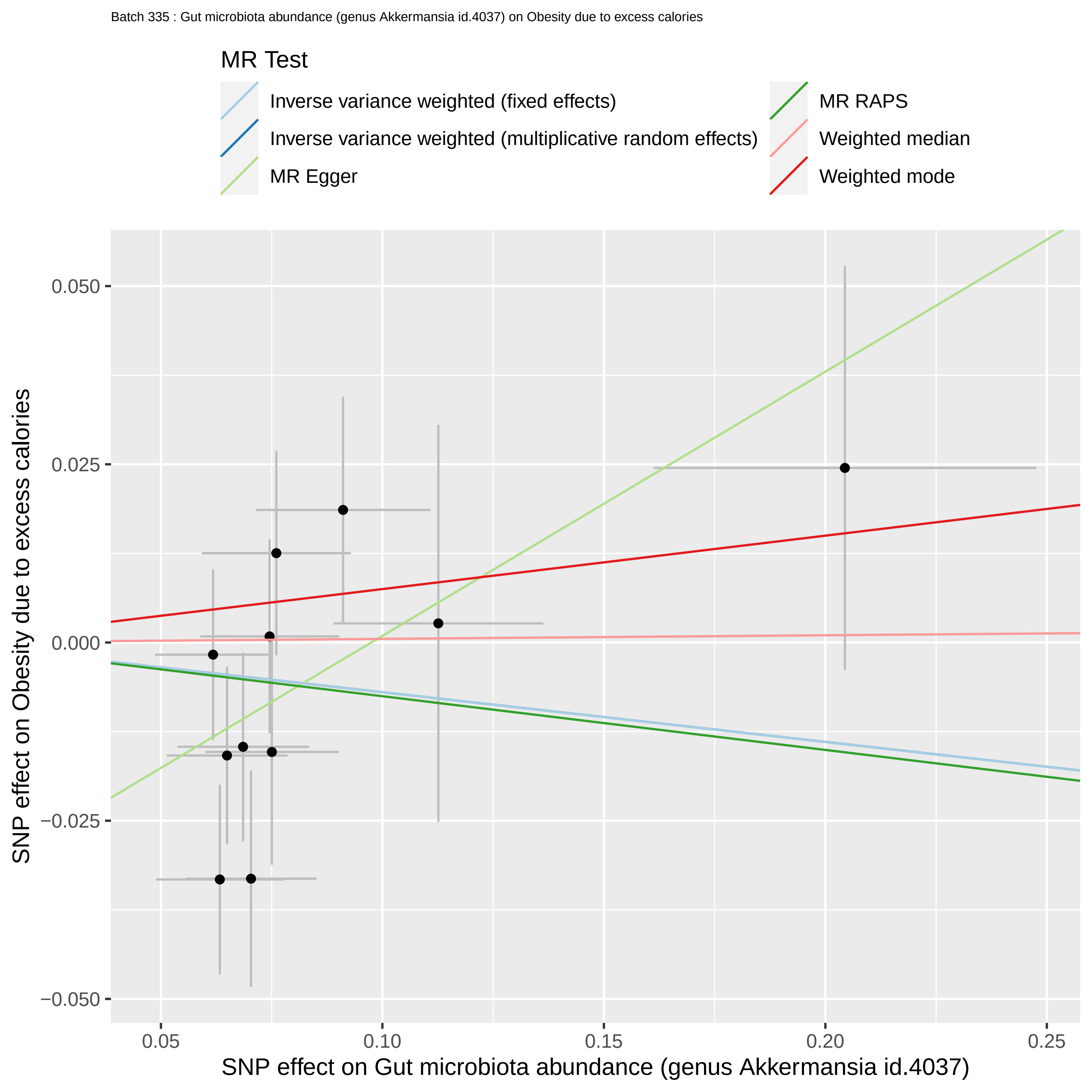

Batch 335 : Gut microbiota abundance (genus Akkermansia id.4037) on Obesity due to excess calories
MR Test
Inverse variance weighted (fixed effects)
Inverse variance weighted (multiplicative random effects)
MR Egger
MR RAPS
Weighted median
Weighted mode
SNP effect on Obesity due to excess calories
0.050
0.025
0.000
−0.025
−0.050
0.05
0.10
0.15
0.20
0.25
SNP effect on Gut microbiota abundance (genus Akkermansia id.4037)

Batch 336 : Gut microbiota abundance (genus Alistipes id.968) on Obesity due to excess calories

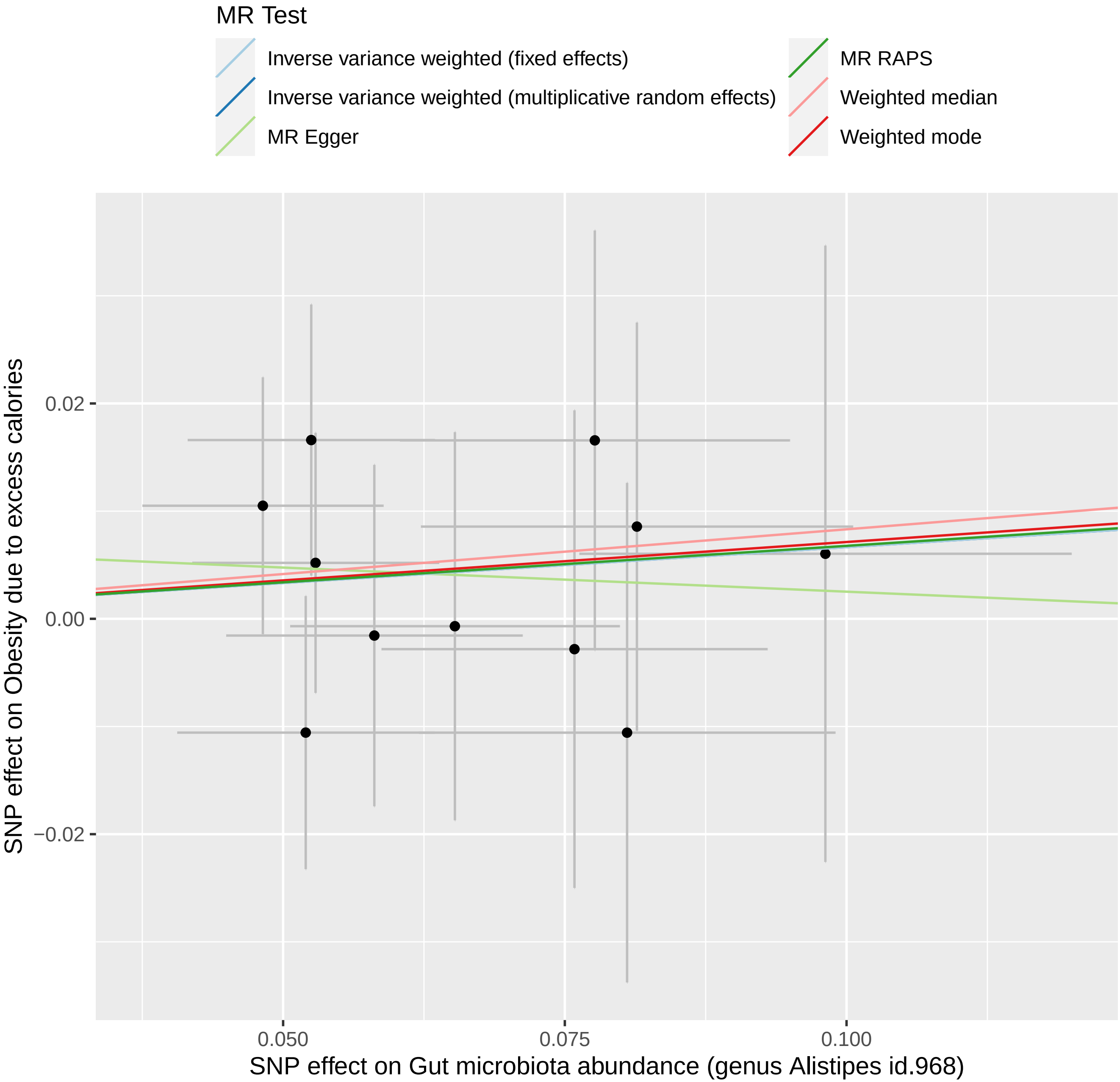

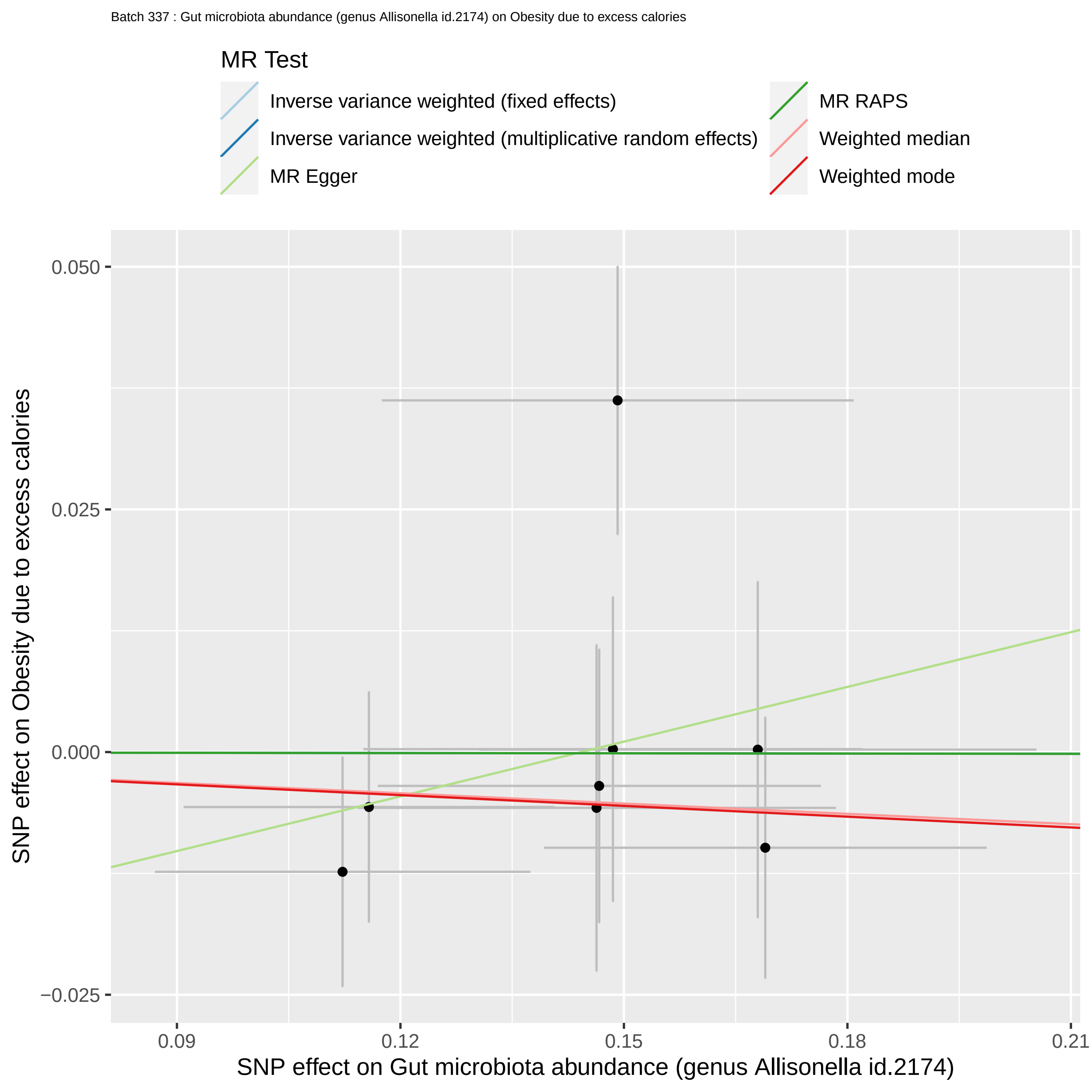

Batch 337 : Gut microbiota abundance (genus Allisonella id.2174) on Obesity due to excess calories
MR Test
Inverse variance weighted (fixed effects)
Inverse variance weighted (multiplicative random effects)
MR Egger
MR RAPS
Weighted median
Weighted mode
0.050
0.025
0.000
−0.025
0.09
0.12
0.15
0.18
0.21
SNP effect on Obesity due to excess calories
SNP effect on Gut microbiota abundance (genus Allisonella id.2174)

Batch 338 : Gut microbiota abundance (genus Alloprevotella id.961) on Obesity due to excess calories

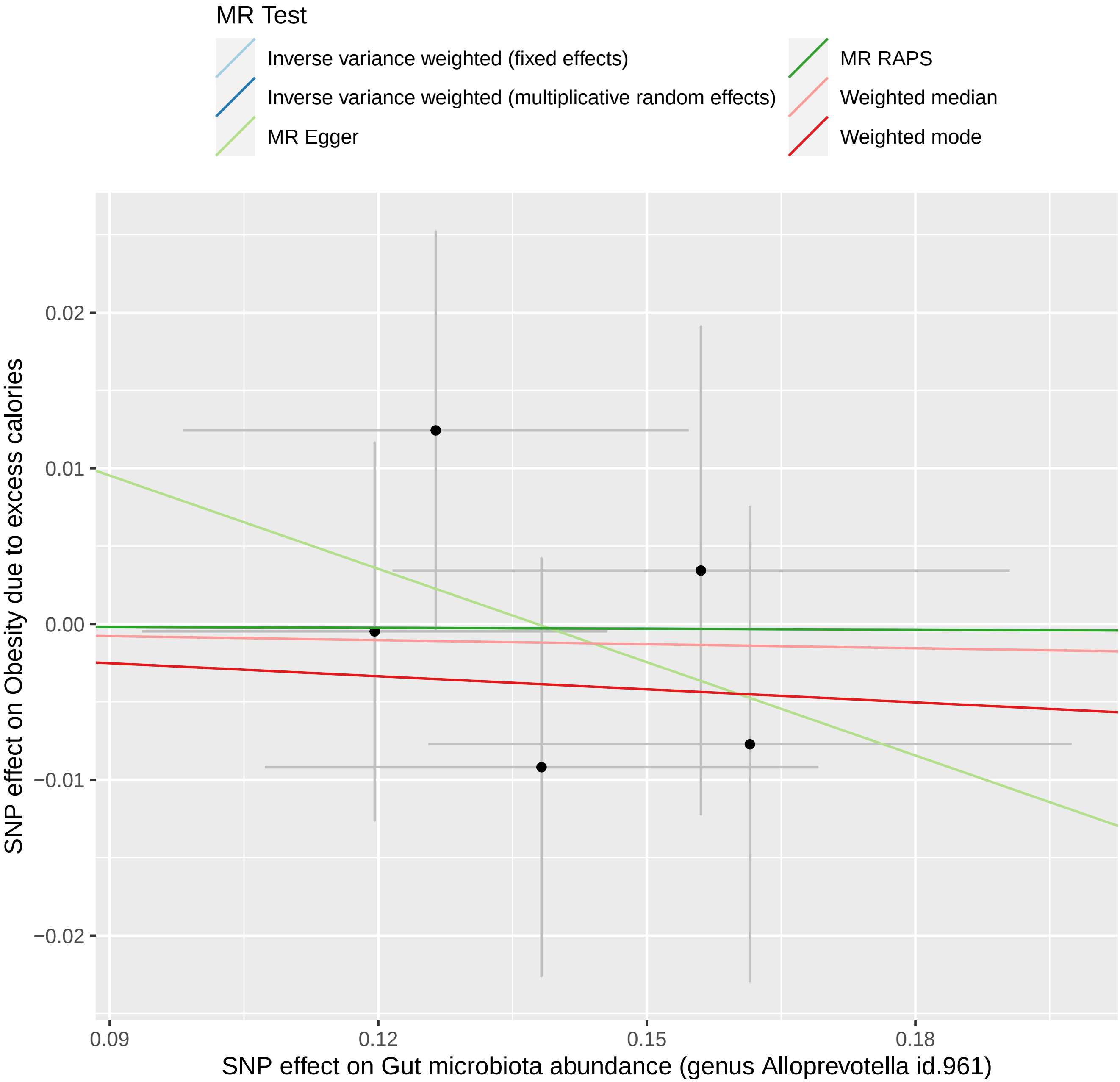

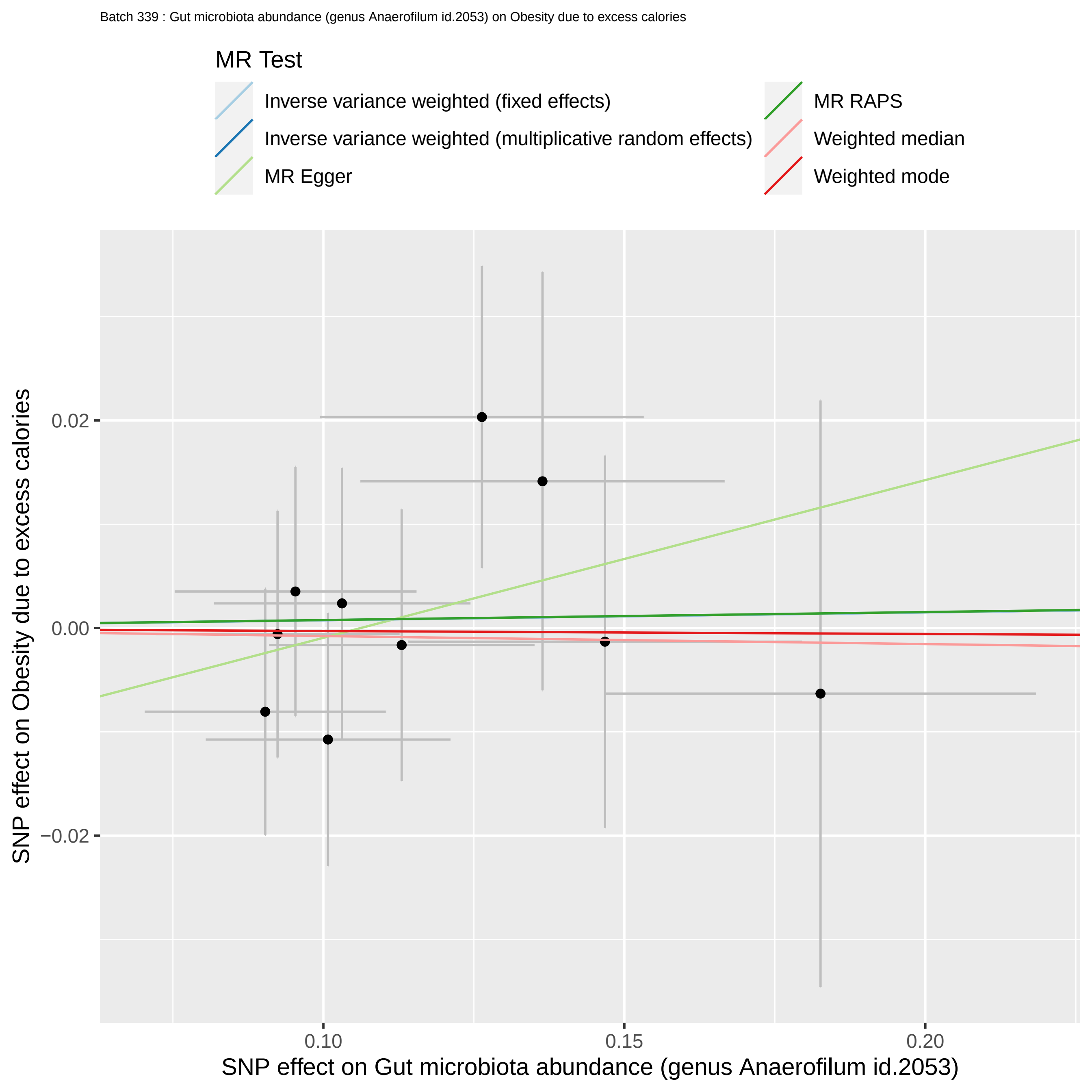
Batch 339 : Gut microbiota abundance (genus Anaerofilum id.2053) on Obesity due to excess calories
MR Test
Inverse variance weighted (fixed effects)
Inverse variance weighted (multiplicative random effects)
MR Egger
MR RAPS
Weighted median
Weighted mode
0.02
0.00
−0.02
0.10
0.15
0.20
SNP effect on Obesity due to excess calories
SNP effect on Gut microbiota abundance (genus Anaerofilum id.2053)

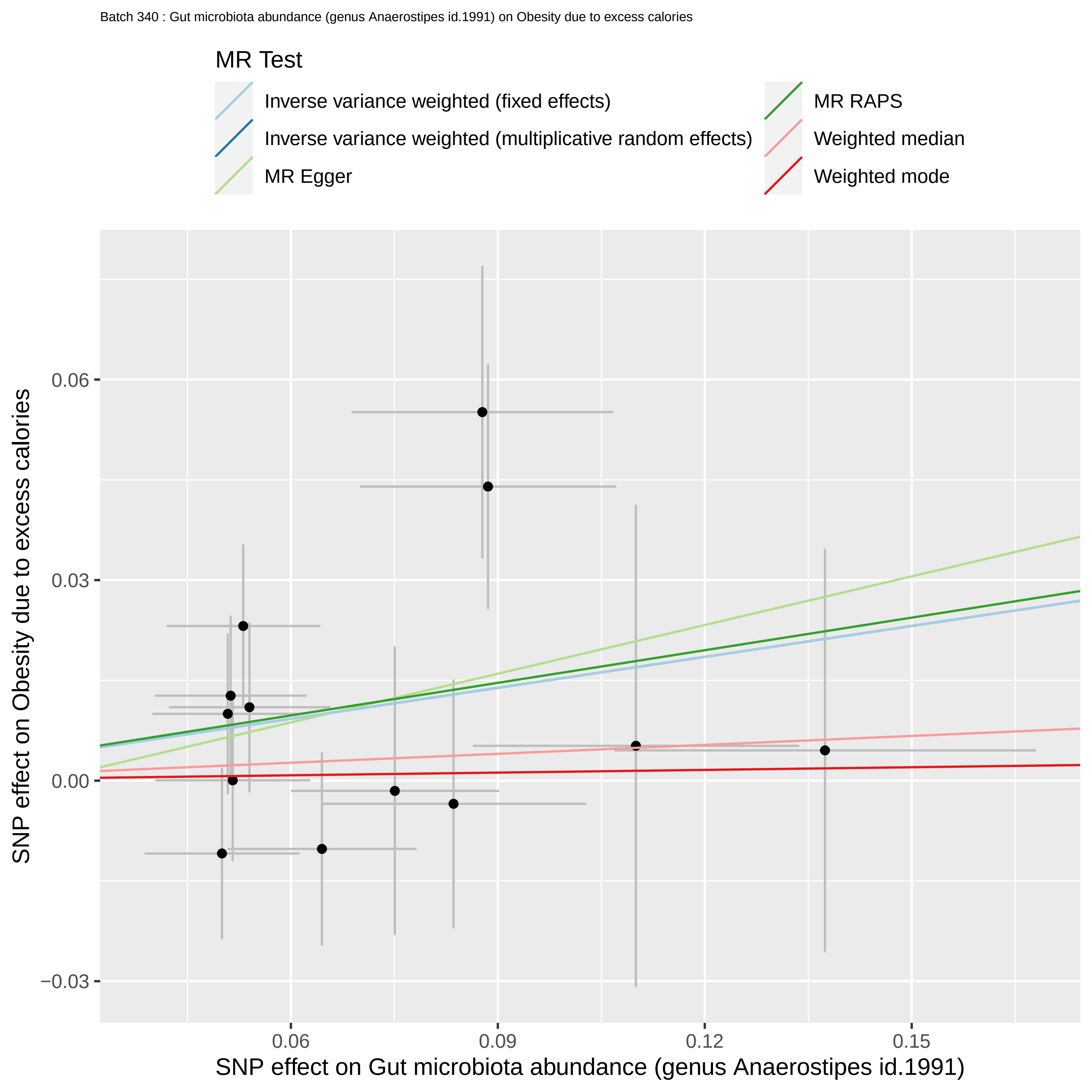

Batch 340 : Gut microbiota abundance (genus Anaerostipes id.1991) on Obesity due to excess calories
MR Test
Inverse variance weighted (fixed effects)
Inverse variance weighted (multiplicative random effects)
MR Egger
MR RAPS
Weighted median
Weighted mode
SNP effect on Obesity due to excess calories
0.06
0.03
0.00
−0.03
0.06
0.09
0.12
0.15
SNP effect on Gut microbiota abundance (genus Anaerostipes id.1991)

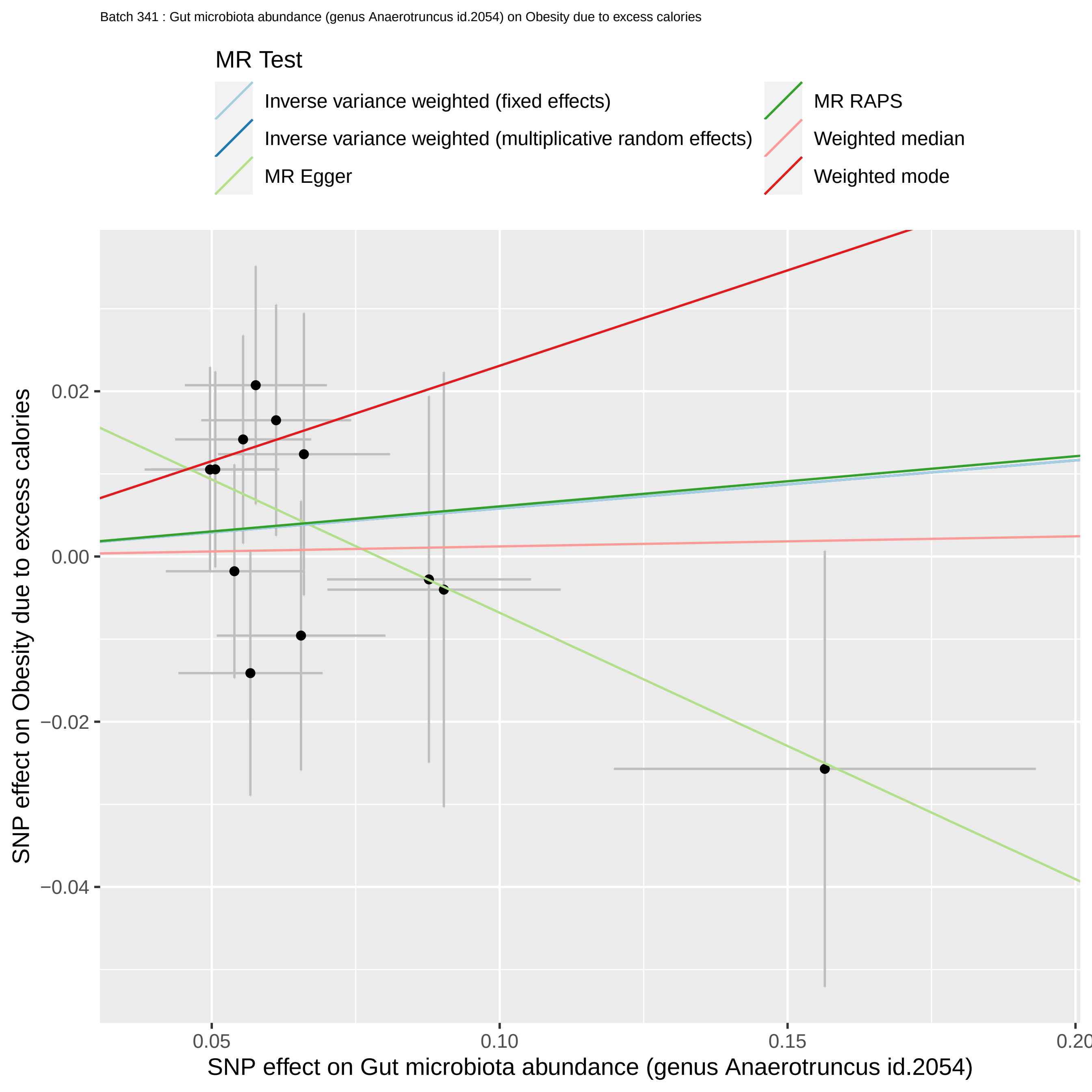

Batch 341 : Gut microbiota abundance (genus Anaerotruncus id.2054) on Obesity due to excess calories
MR Test
Inverse variance weighted (fixed effects)
Inverse variance weighted (multiplicative random effects)
MR Egger
MR RAPS
Weighted median
Weighted mode
0.02
0.00
−0.02
−0.04
0.05
0.10
0.15
0.20
SNP effect on Obesity due to excess calories
SNP effect on Gut microbiota abundance (genus Anaerotruncus id.2054)

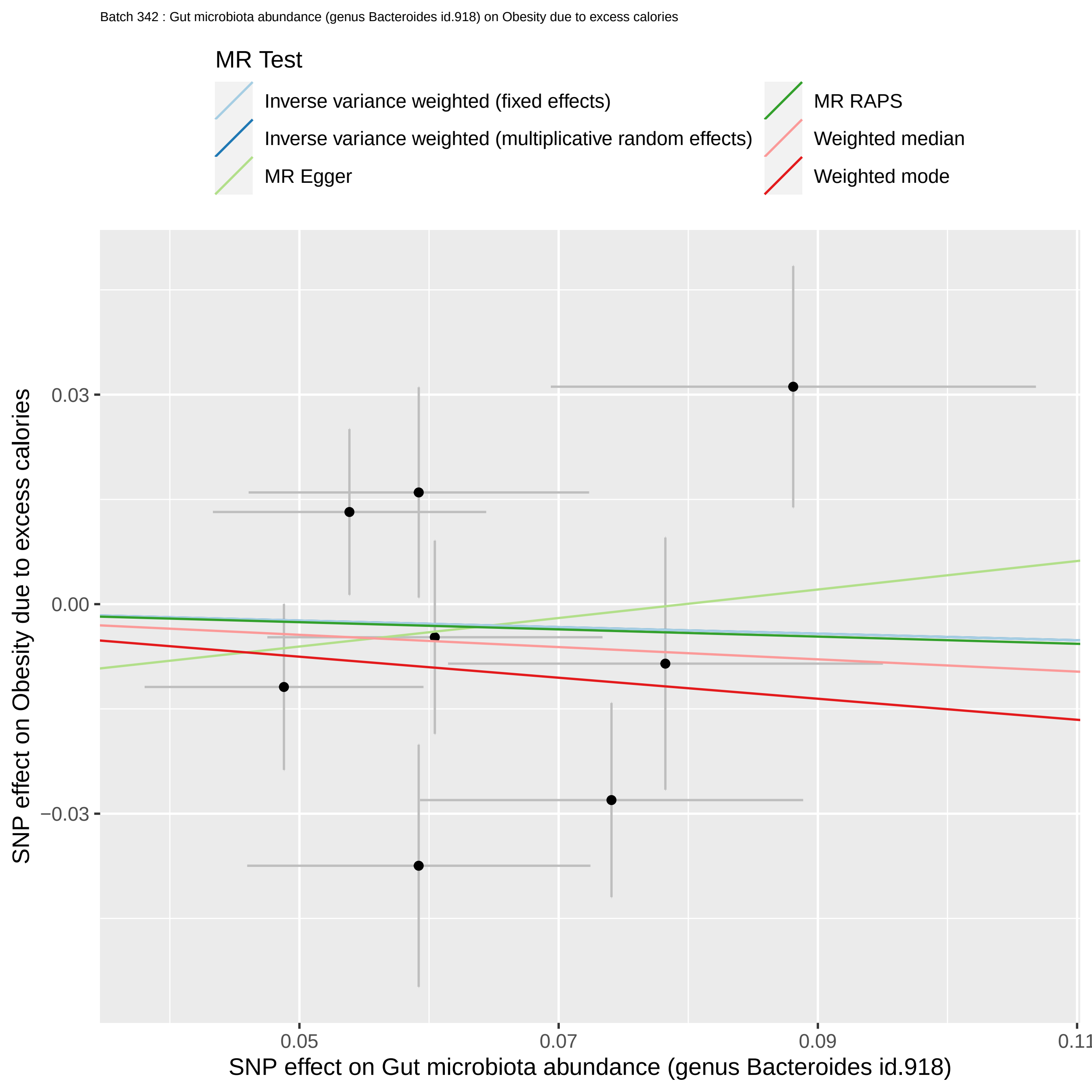

Batch 342 : Gut microbiota abundance (genus Bacteroides id.918) on Obesity due to excess calories
MR Test
Inverse variance weighted (fixed effects)
Inverse variance weighted (multiplicative random effects)
MR Egger
MR RAPS
Weighted median
Weighted mode
0.03
0.00
−0.03
0.05
0.07
0.09
0.11
SNP effect on Obesity due to excess calories
SNP effect on Gut microbiota abundance (genus Bacteroides id.918)

Batch 343 : Gut microbiota abundance (genus Barnesiella id.944) on Obesity due to excess calories

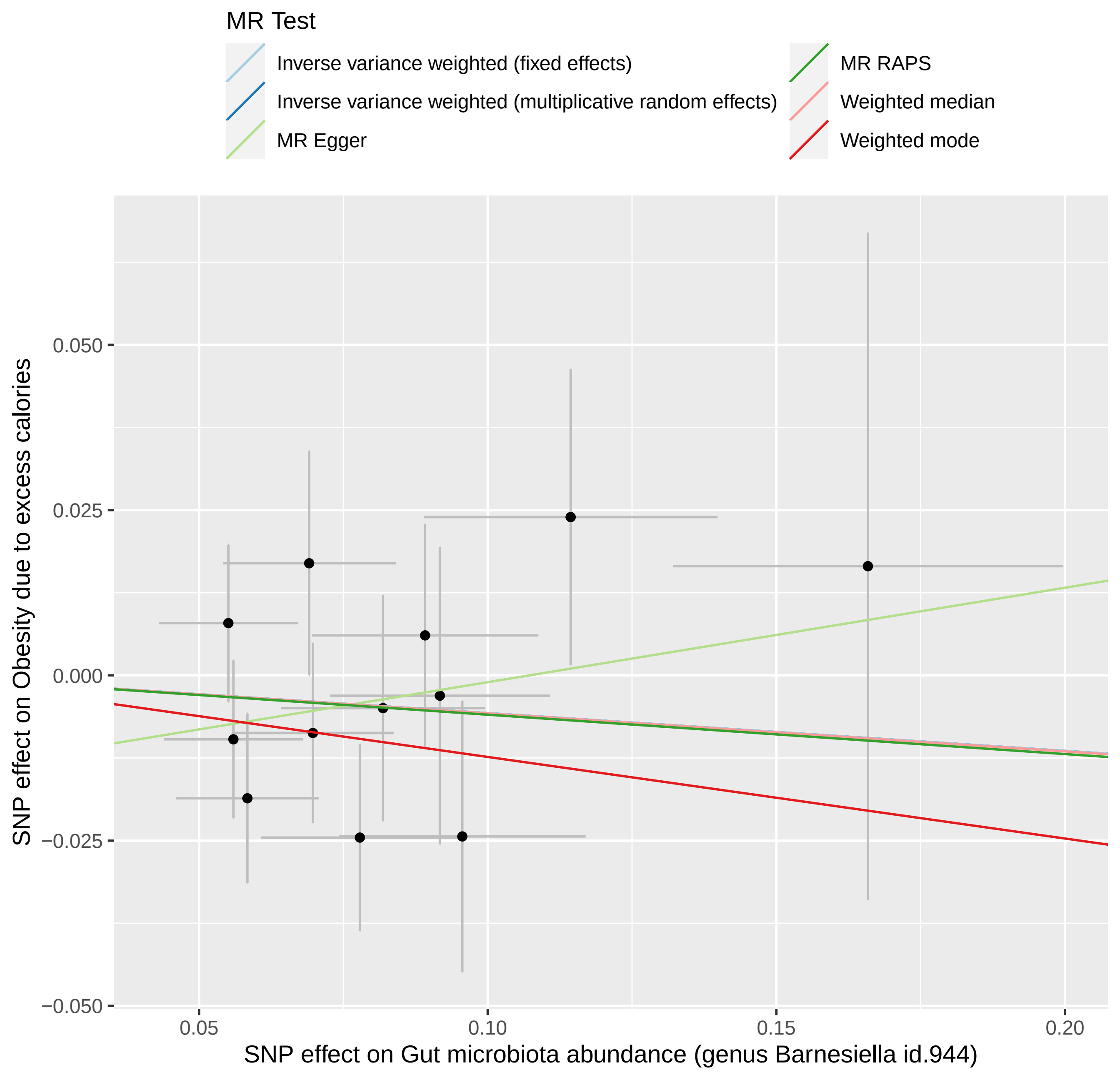

Batch 344 : Gut microbiota abundance (genus Bifidobacterium id.436) on Obesity due to excess calories

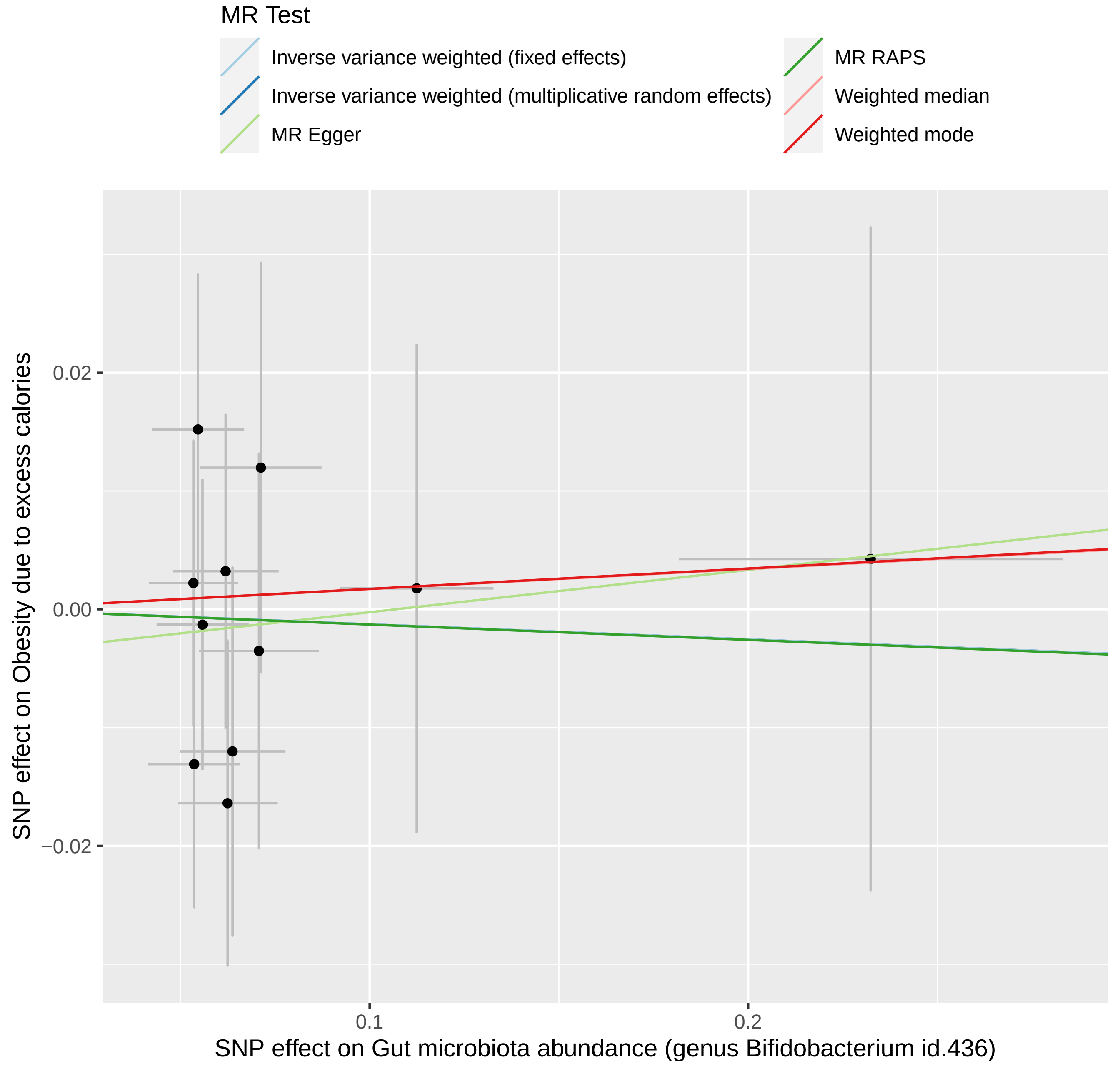

Batch 345 : Gut microbiota abundance (genus Bilophila id.3170) on Obesity due to excess calories

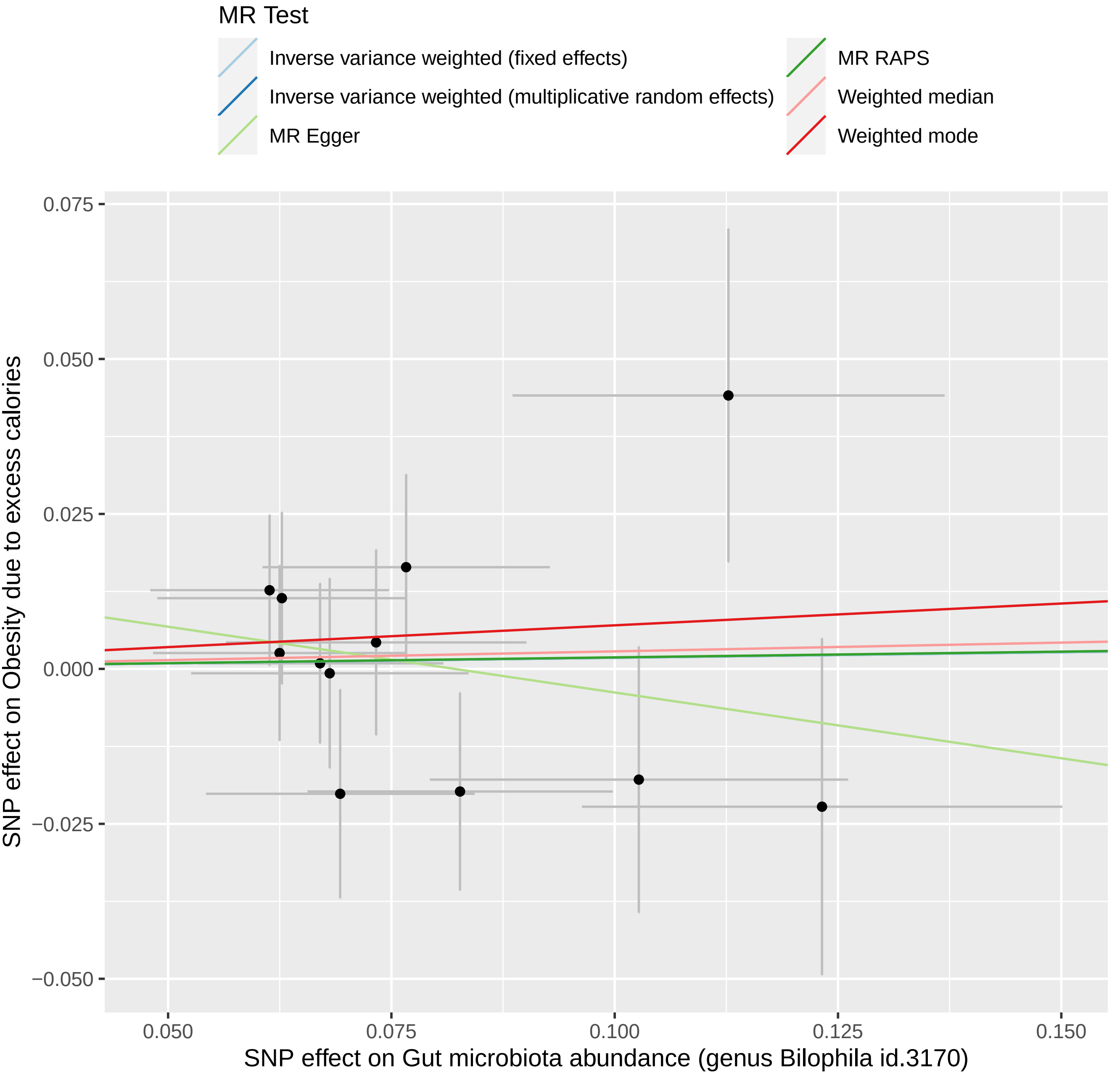

Batch 346 : Gut microbiota abundance (genus Blautia id.1992) on Obesity due to excess calories

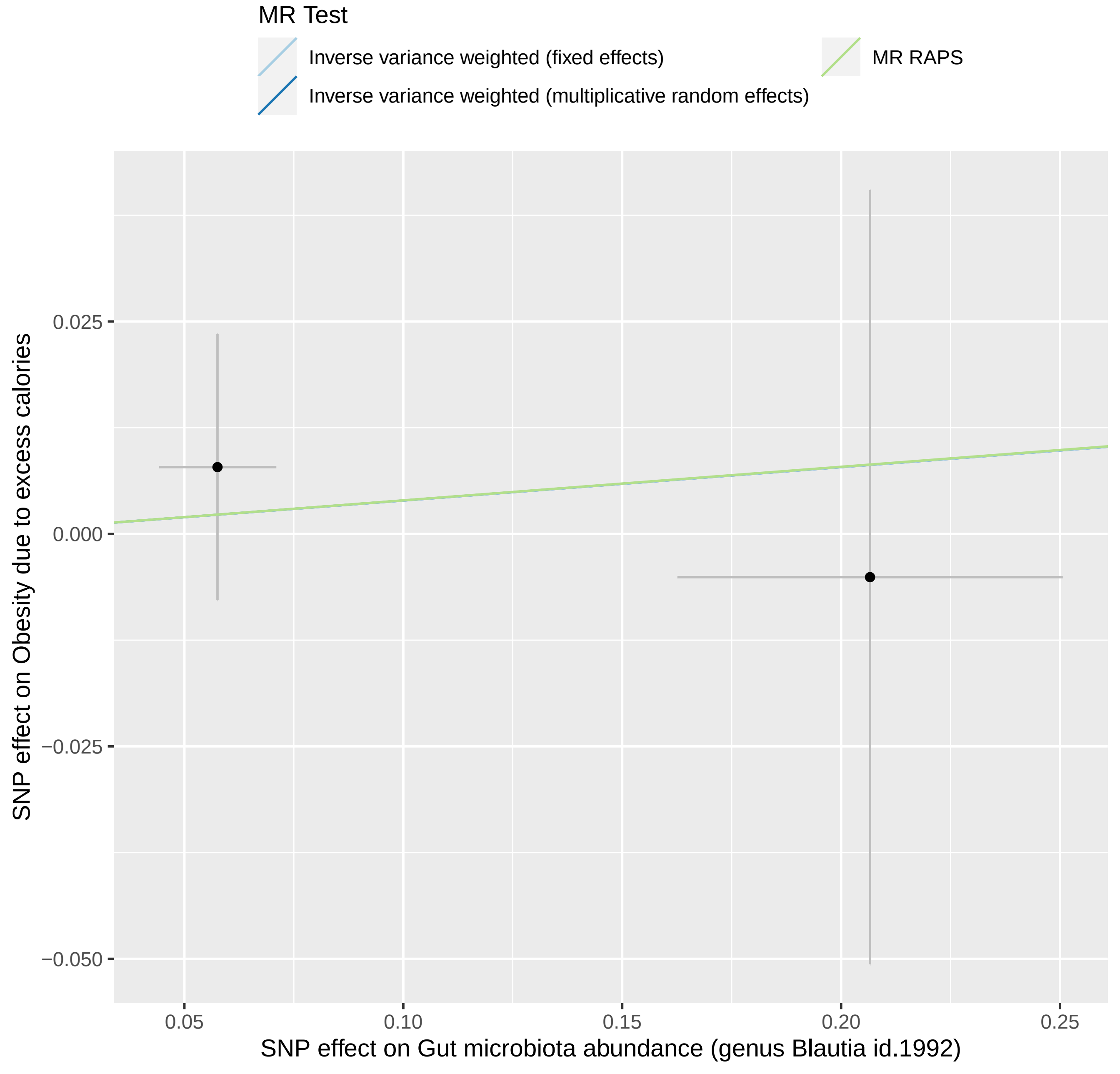

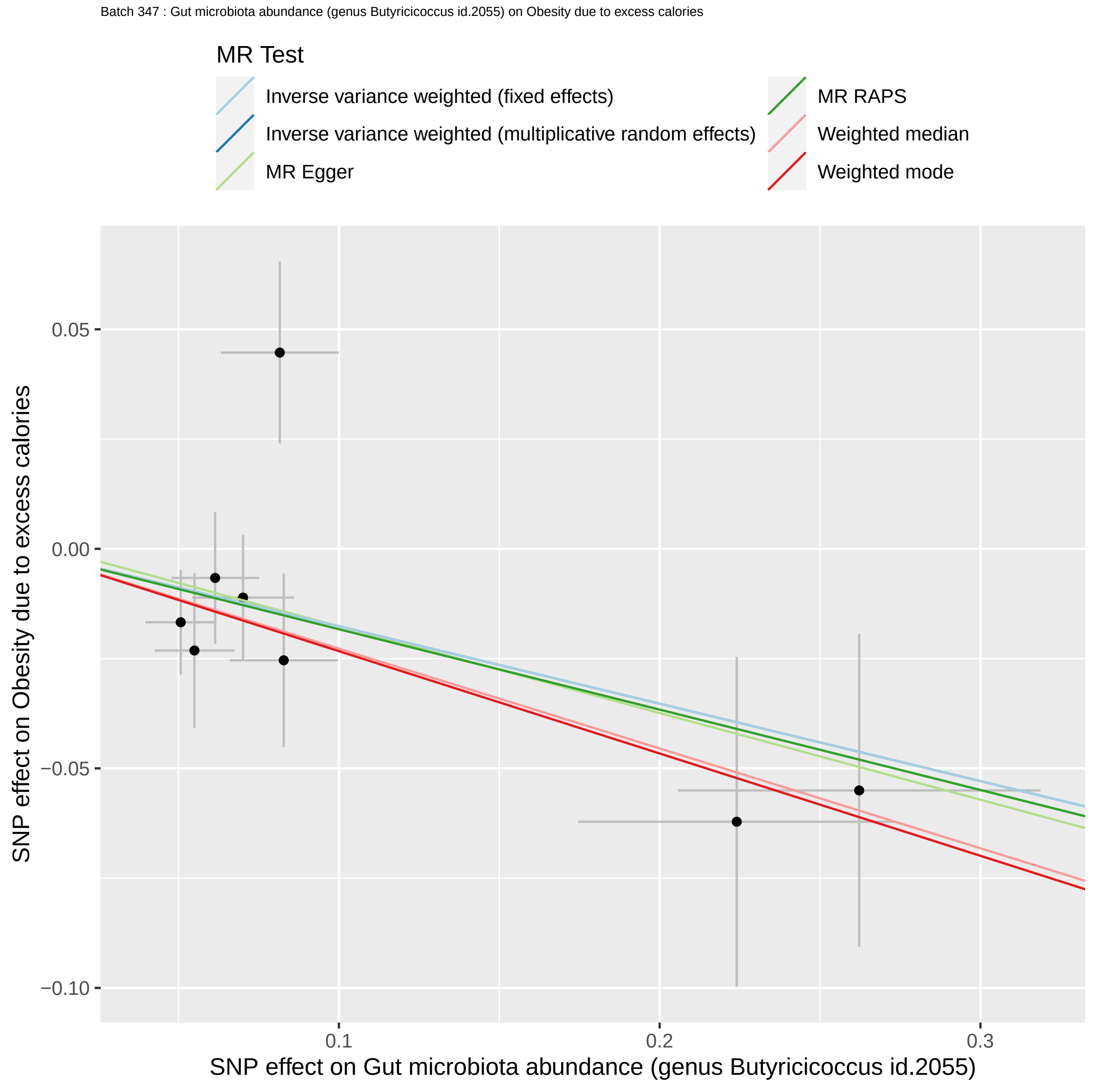

Batch 347 : Gut microbiota abundance (genus Butyricicoccus id.2055) on Obesity due to excess calories
MR Test
Inverse variance weighted (fixed effects)
Inverse variance weighted (multiplicative random effects)
MR Egger
MR RAPS
Weighted median
Weighted mode
0.05
0.00
−0.05
−0.10
0.1
0.2
0.3
SNP effect on Obesity due to excess calories
SNP effect on Gut microbiota abundance (genus Butyricicoccus id.2055)

Batch 348 : Gut microbiota abundance (genus Butyricimonas id.945) on Obesity due to excess calories

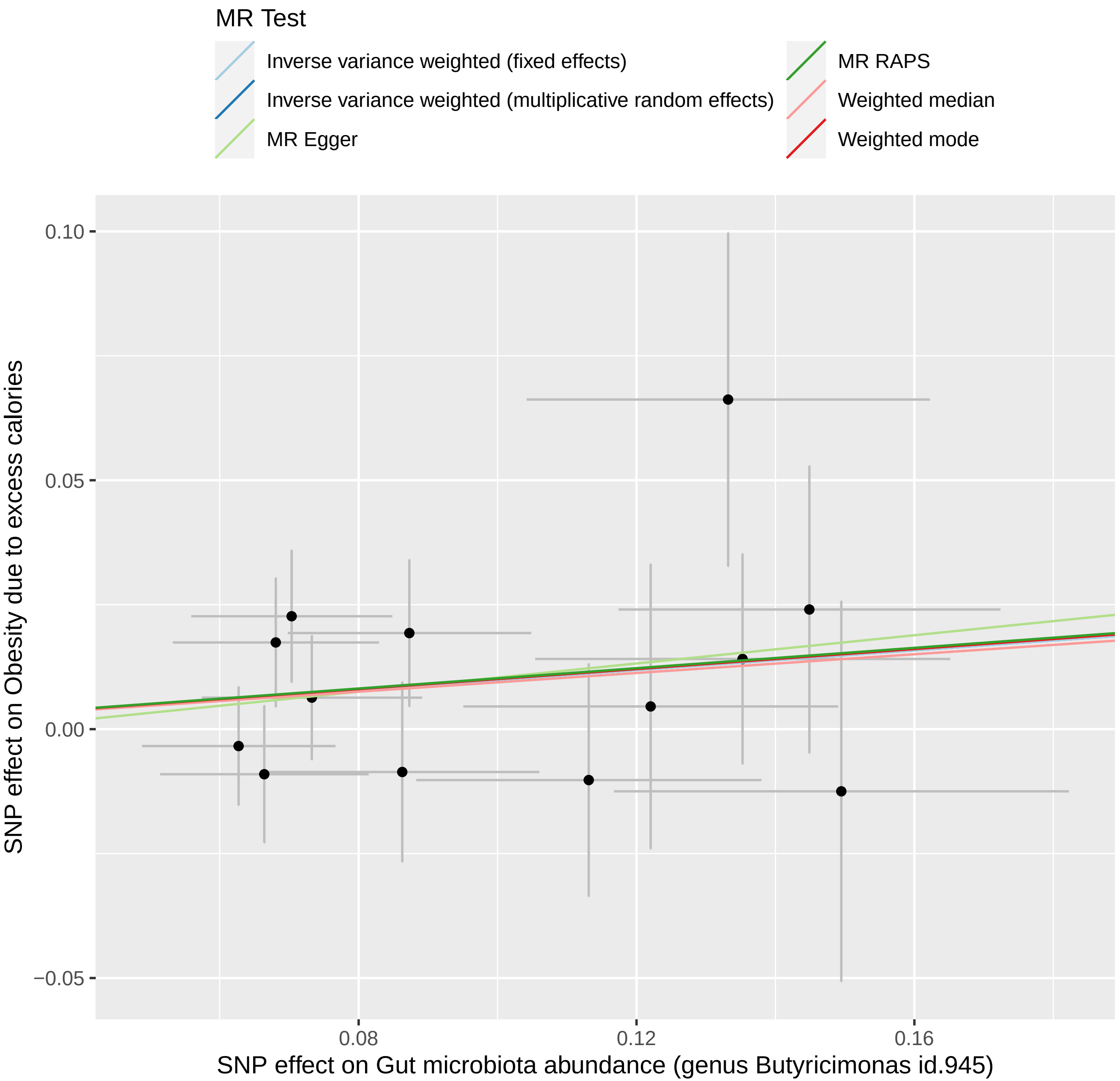

Batch 349 : Gut microbiota abundance (genus Butyrivibrio id.1993) on Obesity due to excess calories

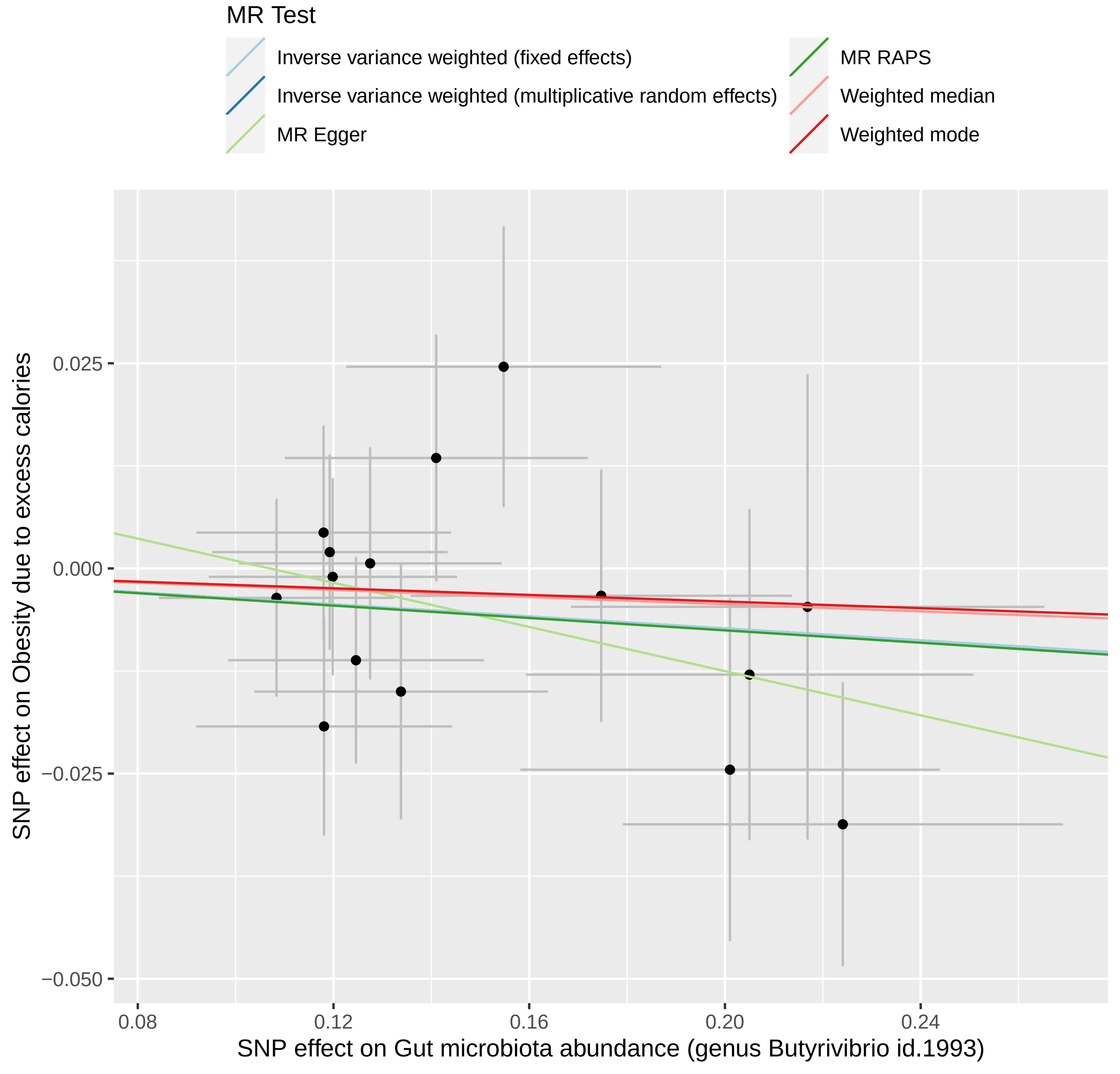

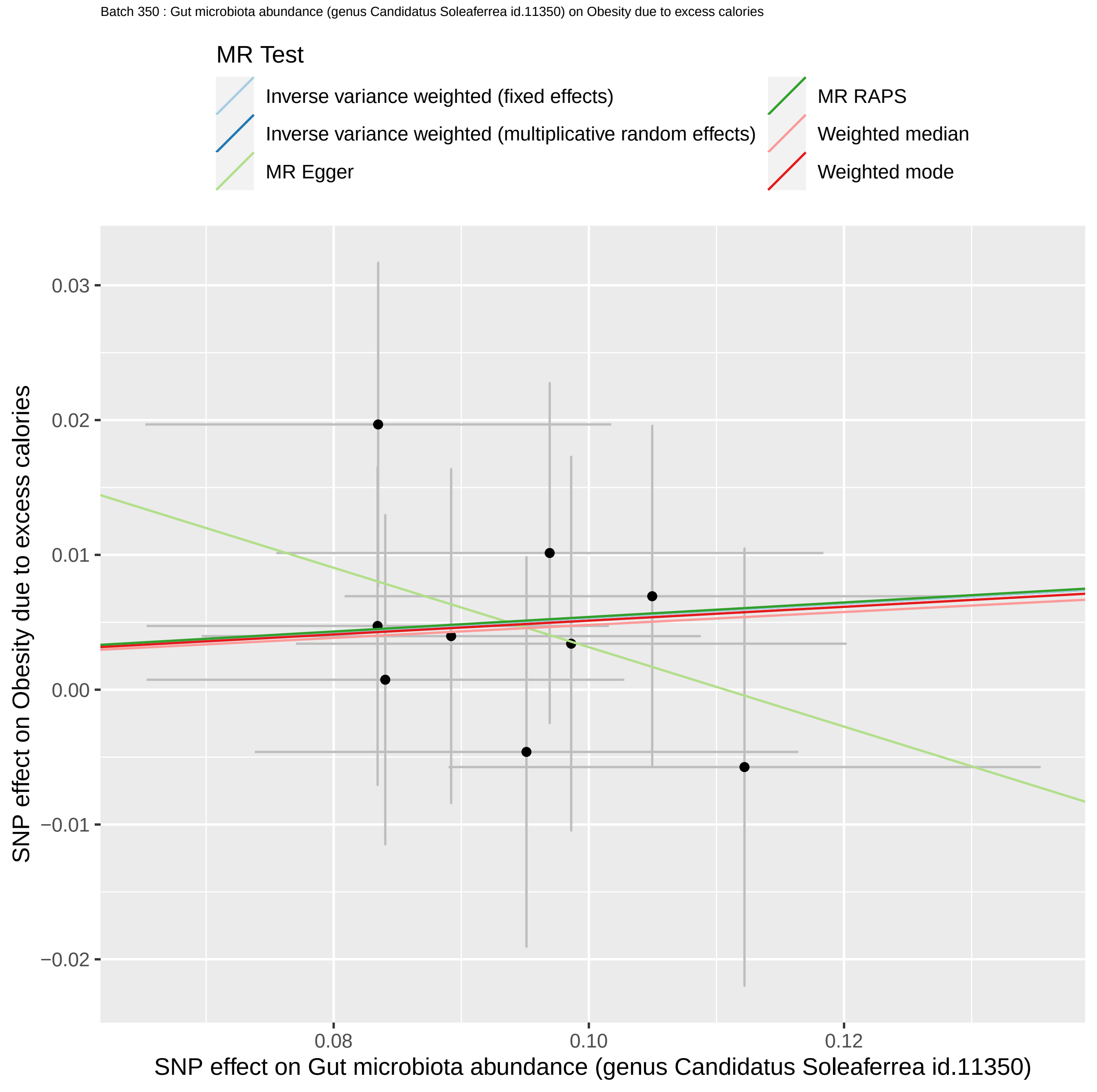

Batch 350 : Gut microbiota abundance (genus Candidatus Soleaferrea id.11350) on Obesity due to excess calories
MR Test
Inverse variance weighted (fixed effects)
Inverse variance weighted (multiplicative random effects)
MR Egger
MR RAPS
Weighted median
Weighted mode
0.03
0.02
0.01
0.00
−0.01
−0.02
0.08
0.10
0.12
SNP effect on Obesity due to excess calories
SNP effect on Gut microbiota abundance (genus Candidatus Soleaferrea id.11350)

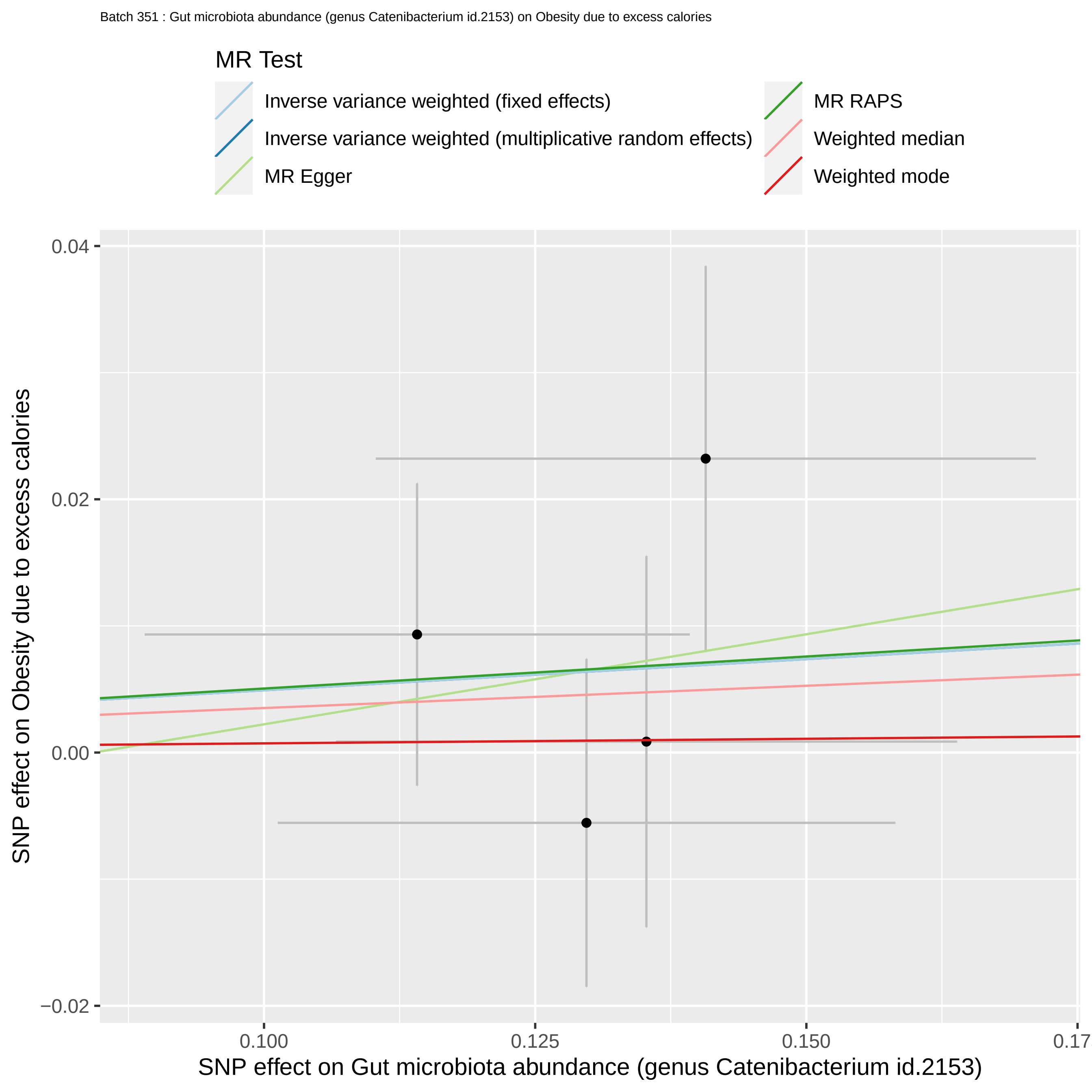

Batch 351 : Gut microbiota abundance (genus Catenibacterium id.2153) on Obesity due to excess calories
MR Test
Inverse variance weighted (fixed effects)
Inverse variance weighted (multiplicative random effects)
MR Egger
MR RAPS
Weighted median
Weighted mode
0.04
0.02
0.00
−0.02
0.100
0.125
0.150
0.17
SNP effect on Obesity due to excess calories
SNP effect on Gut microbiota abundance (genus Catenibacterium id.2153)

Batch 352 : Gut microbiota abundance (genus Christensenellaceae R 7group id.11283) on Obesity due to excess calories

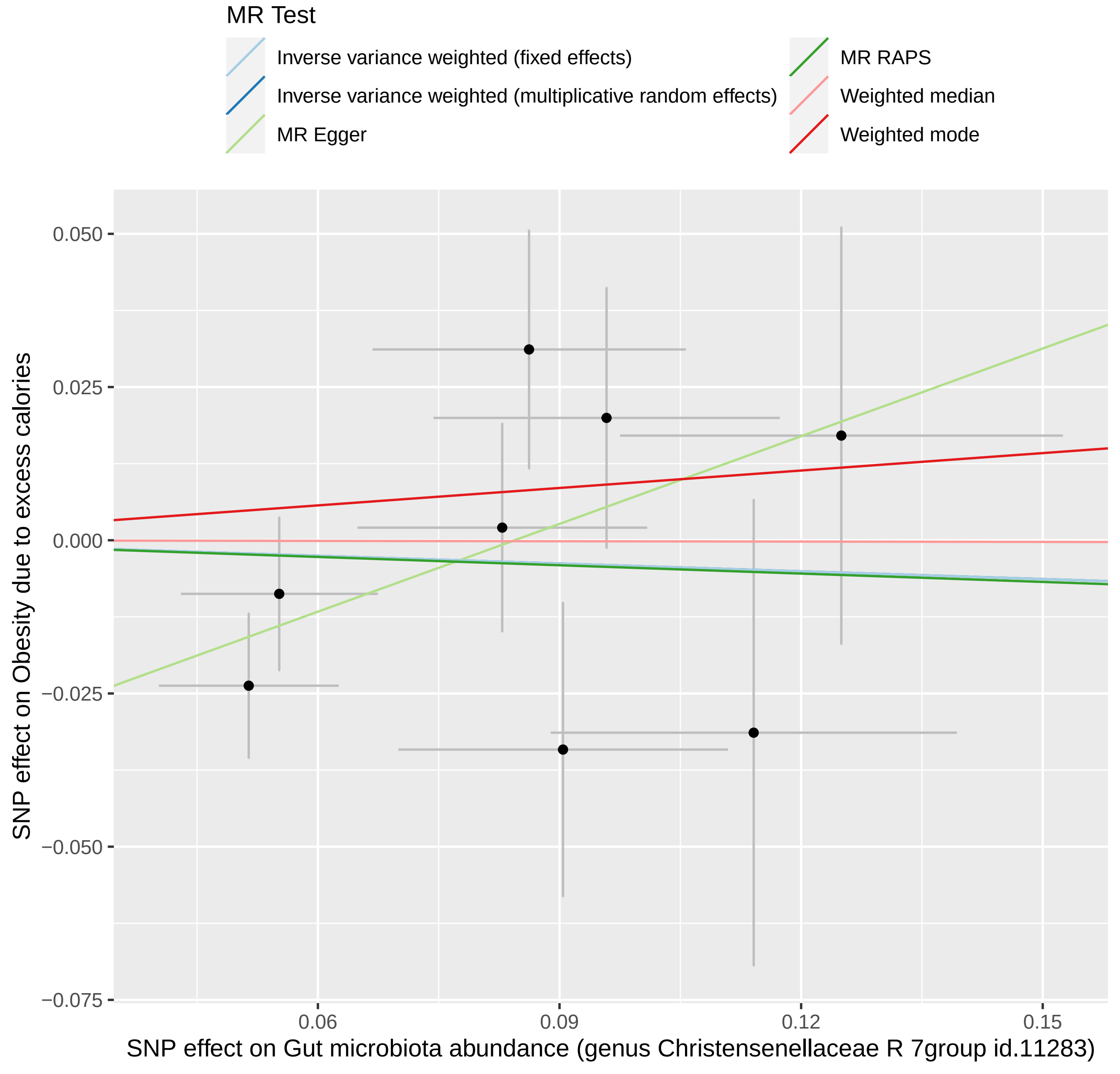

Batch 353 : Gut microbiota abundance (genus Clostridium innocuum group id.14397) on Obesity due to excess calories

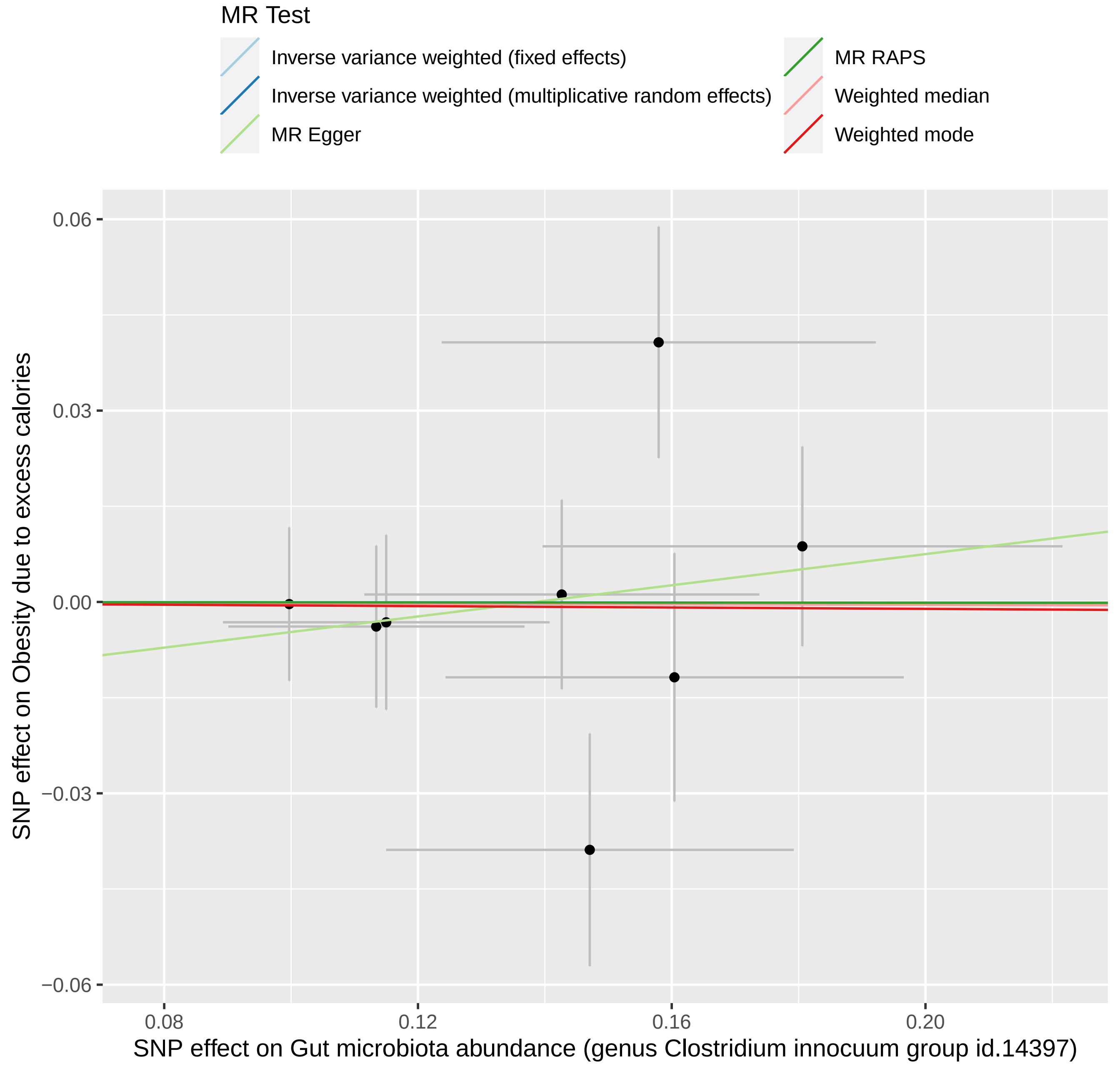

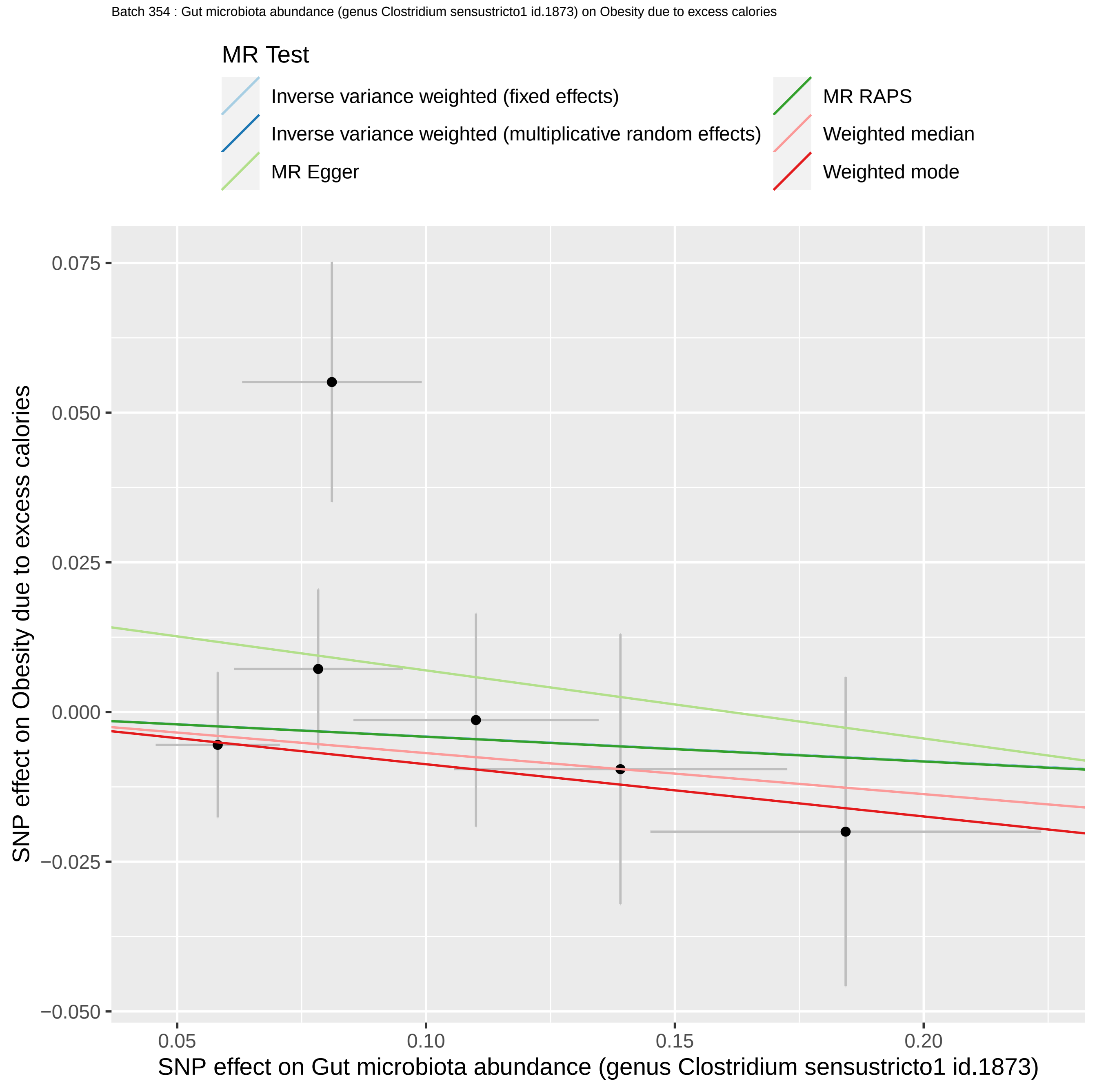

Batch 354 : Gut microbiota abundance (genus Clostridium sensustricto1 id.1873) on Obesity due to excess calories
MR Test
Inverse variance weighted (fixed effects)
Inverse variance weighted (multiplicative random effects)
MR Egger
MR RAPS
Weighted median
Weighted mode
0.075
0.050
0.025
0.000
−0.025
−0.050
0.05
0.10
0.15
0.20
SNP effect on Obesity due to excess calories
SNP effect on Gut microbiota abundance (genus Clostridium sensustricto1 id.1873)

Batch 355 : Gut microbiota abundance (genus Collinsella id.815) on Obesity due to excess calories

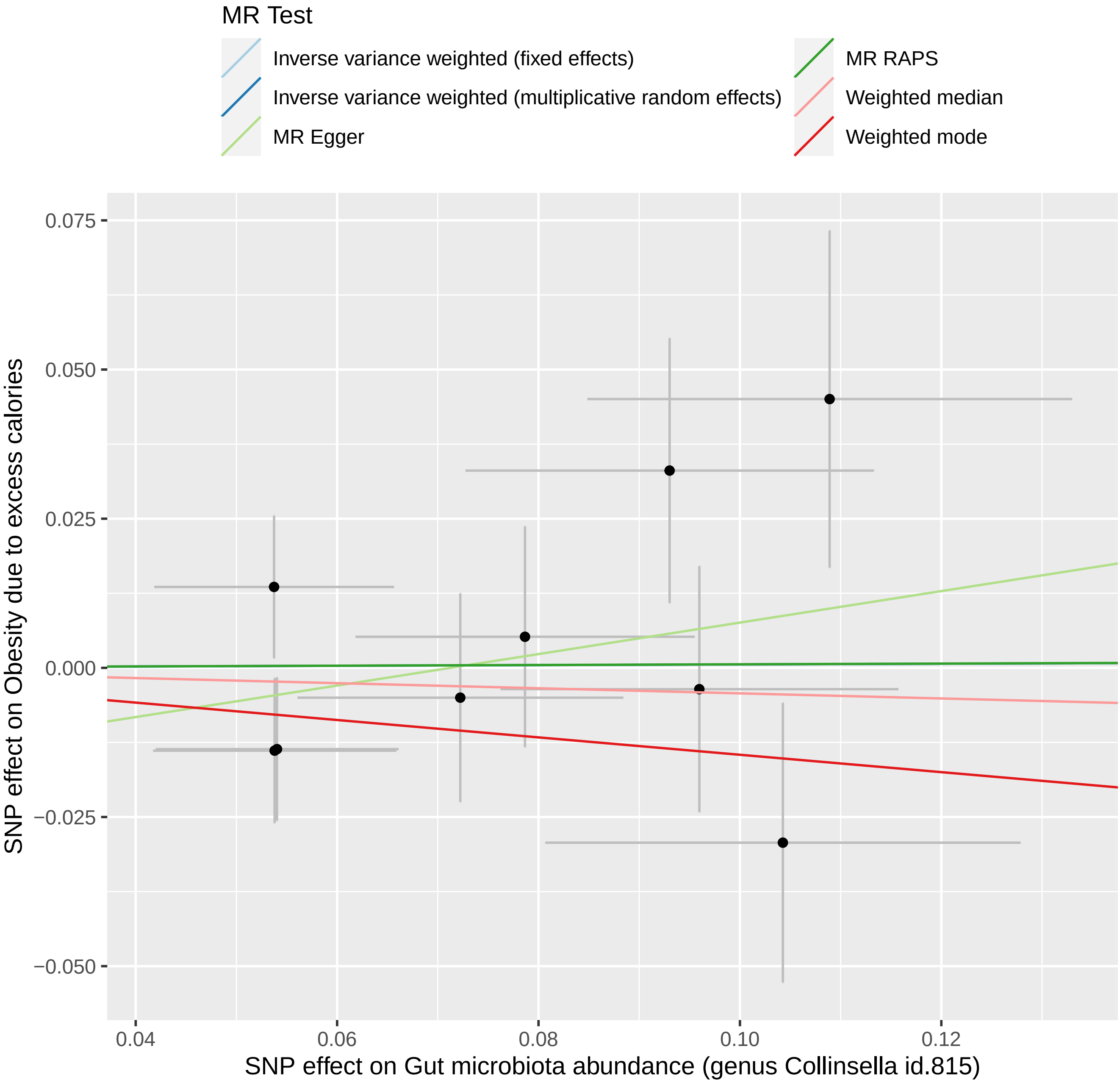

Batch 356 : Gut microbiota abundance (genus Coprobacter id.949) on Obesity due to excess calories

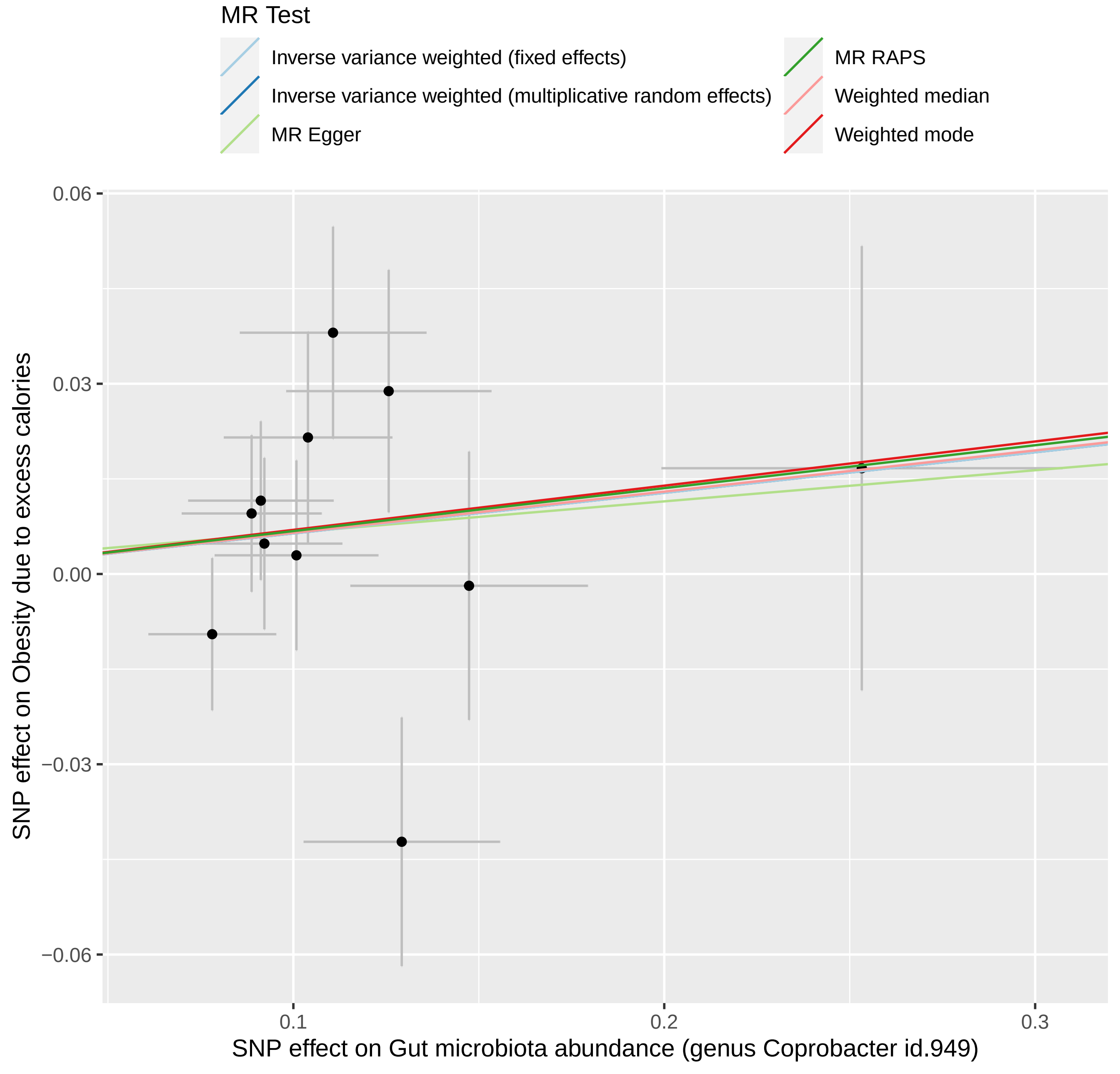

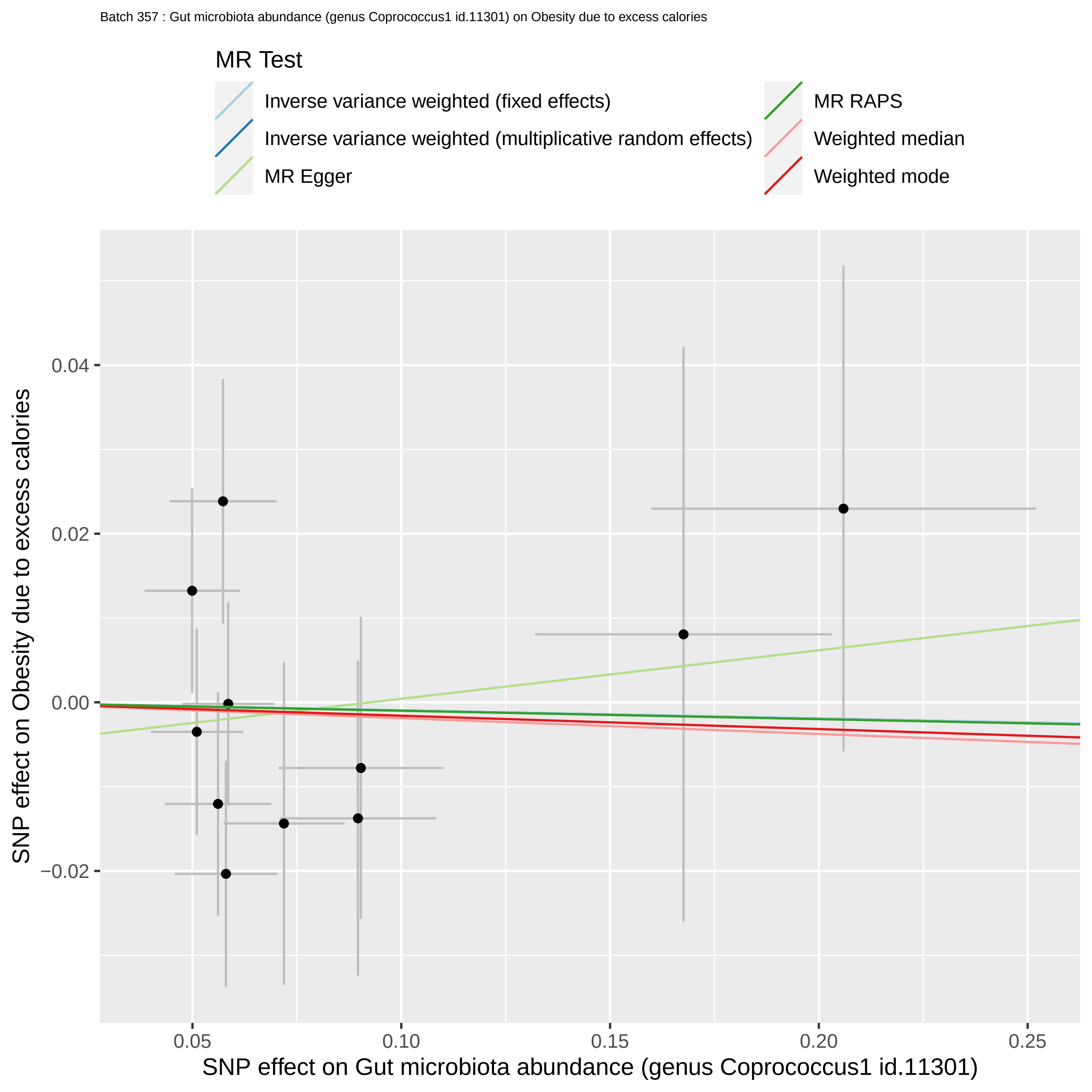

Batch 357 : Gut microbiota abundance (genus Coprococcus1 id.11301) on Obesity due to excess calories
MR Test
Inverse variance weighted (fixed effects)
Inverse variance weighted (multiplicative random effects)
MR Egger
MR RAPS
Weighted median
Weighted mode
0.04
0.02
0.00
−0.02
SNP effect on Obesity due to excess calories
0.05
0.10
0.15
0.20
0.25
SNP effect on Gut microbiota abundance (genus Coprococcus1 id.11301)

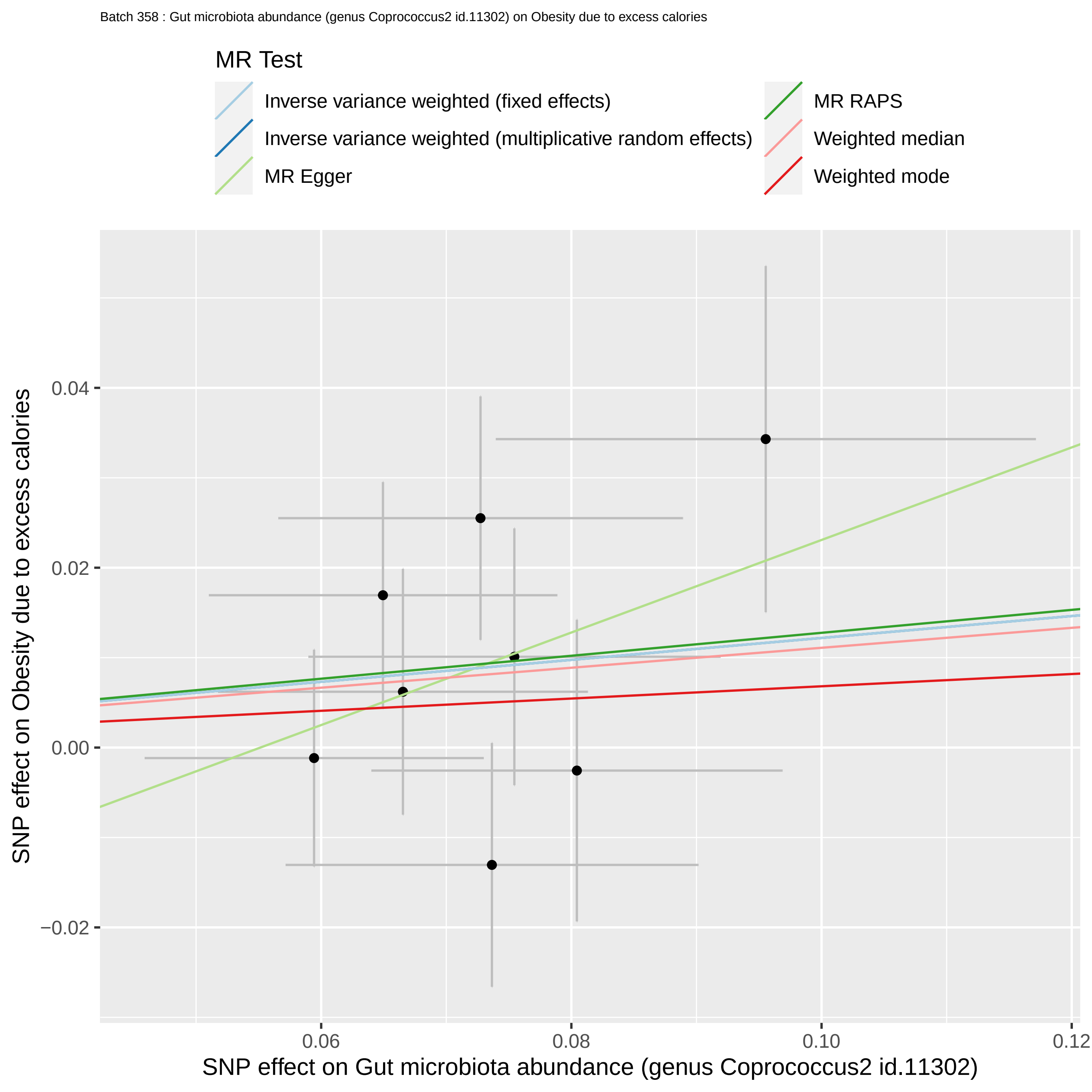
Batch 358 : Gut microbiota abundance (genus Coprococcus2 id.11302) on Obesity due to excess calories
MR Test
Inverse variance weighted (fixed effects)
Inverse variance weighted (multiplicative random effects)
MR Egger
MR RAPS
Weighted median
Weighted mode
0.04
0.02
0.00
−0.02
0.06
0.08
0.10
0.12
SNP effect on Obesity due to excess calories
SNP effect on Gut microbiota abundance (genus Coprococcus2 id.11302)

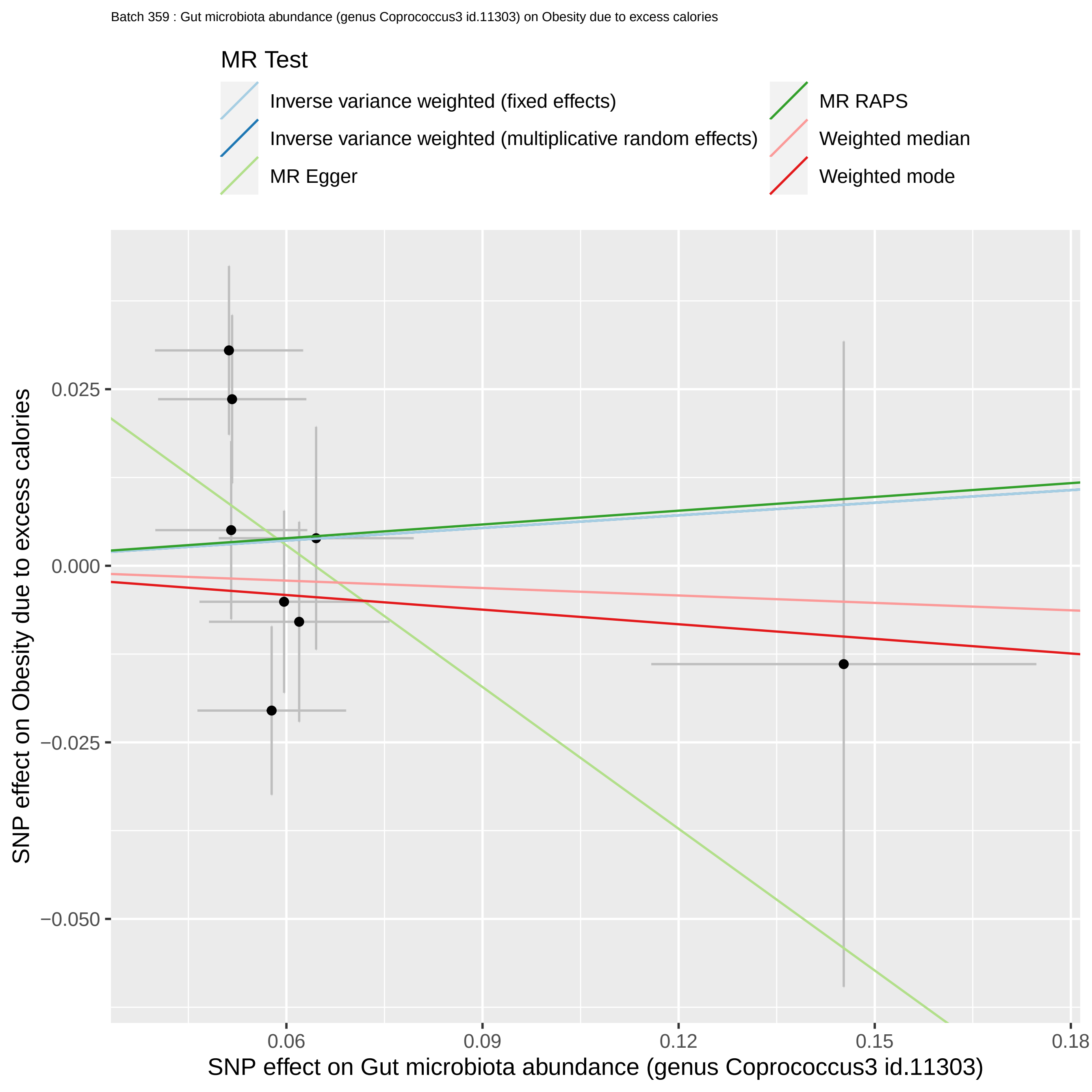
Batch 359 : Gut microbiota abundance (genus Coprococcus3 id.11303) on Obesity due to excess calories
MR Test
Inverse variance weighted (fixed effects)
Inverse variance weighted (multiplicative random effects)
MR Egger
MR RAPS
Weighted median
Weighted mode
0.025
0.000
−0.025
−0.050
0.06
0.09
0.12
0.15
0.18
SNP effect on Gut microbiota abundance (genus Coprococcus3 id.11303)
SNP effect on Obesity due to excess calories

Batch 360 : Gut microbiota abundance (genus Defluviitaleaceae UCG011 id.11287) on Obesity due to excess calories

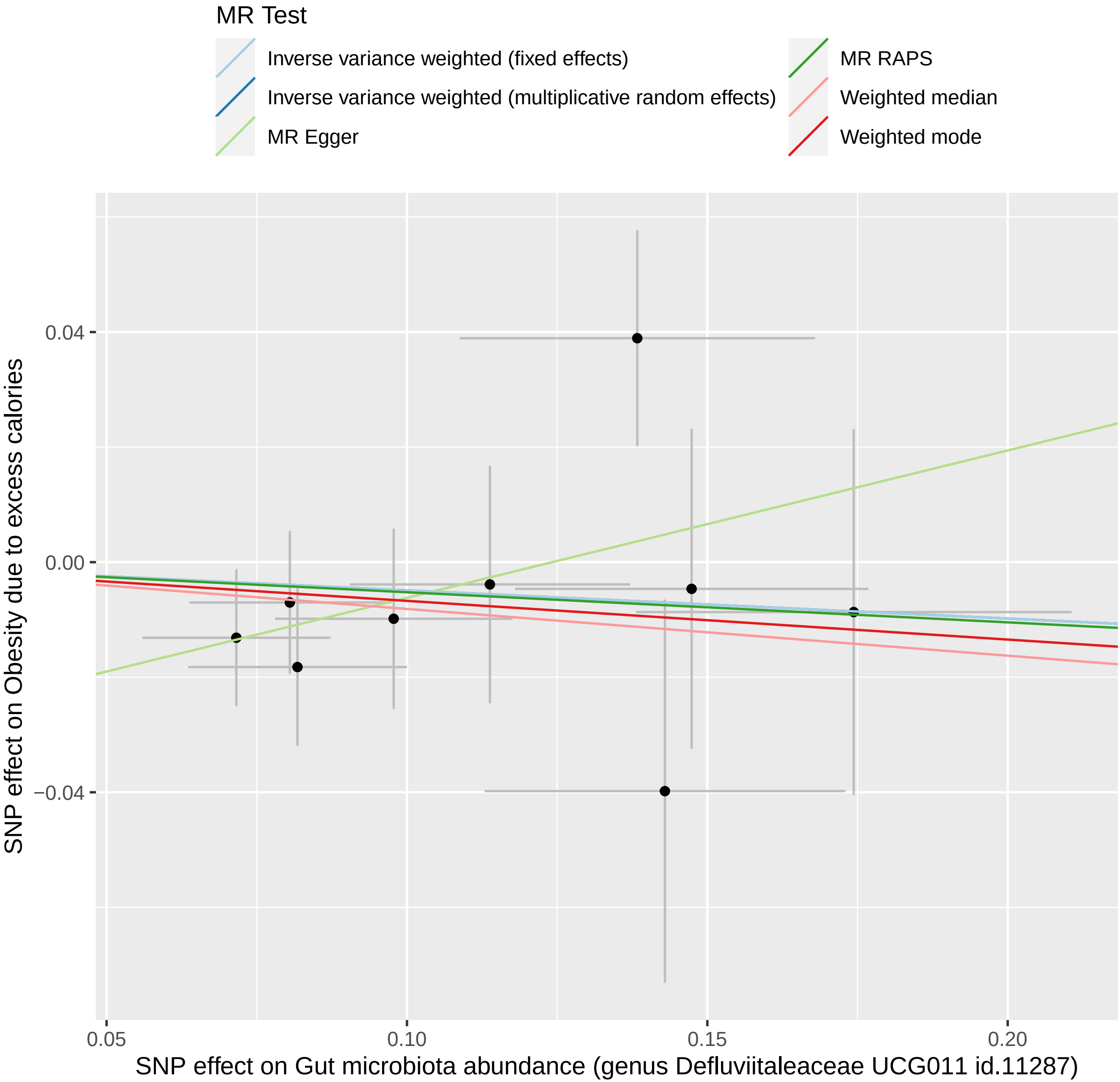

Batch 361 : Gut microbiota abundance (genus Desulfovibrio id.3173) on Obesity due to excess calories

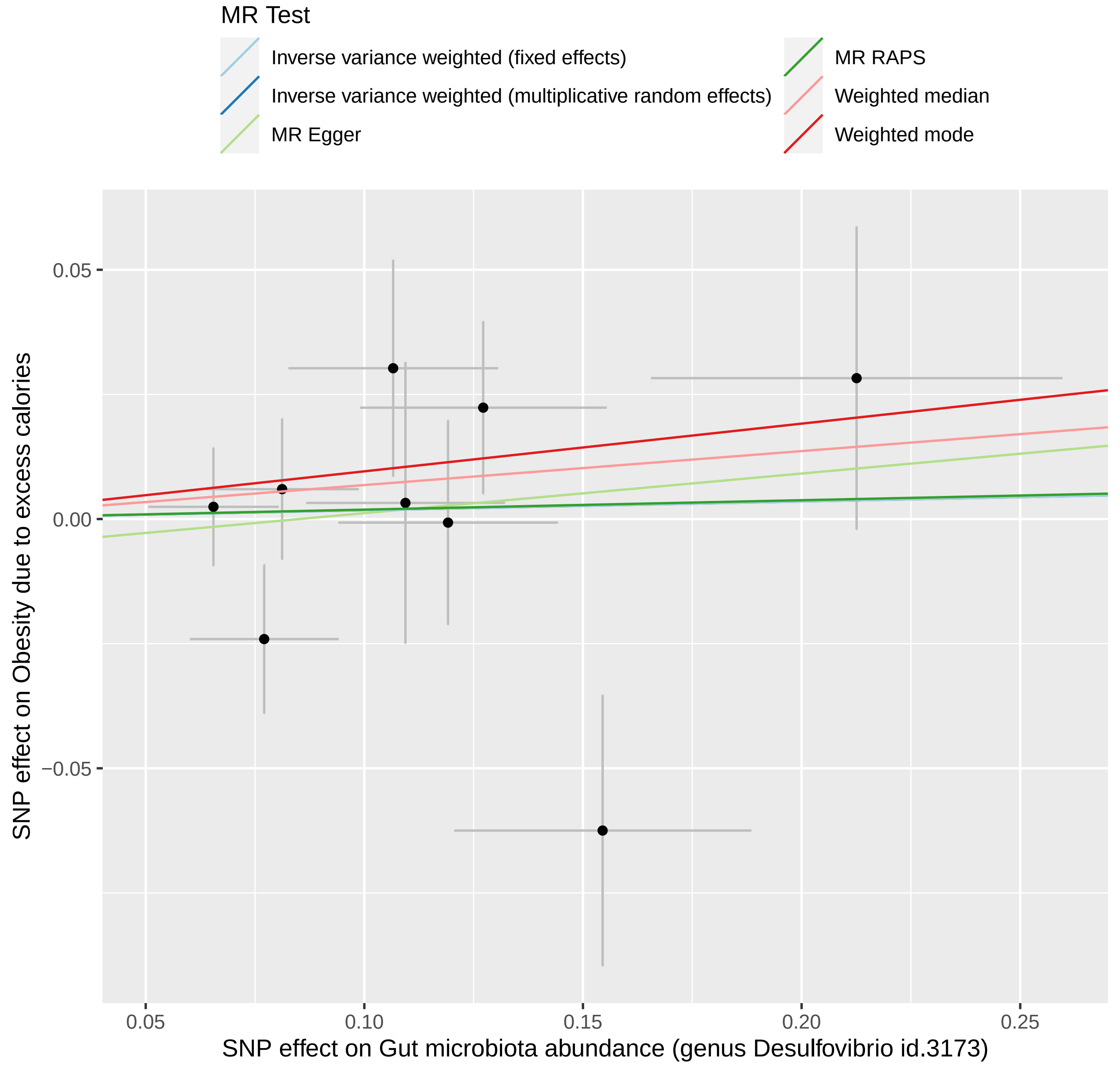

Batch 362 : Gut microbiota abundance (genus Dialister id.2183) on Obesity due to excess calories

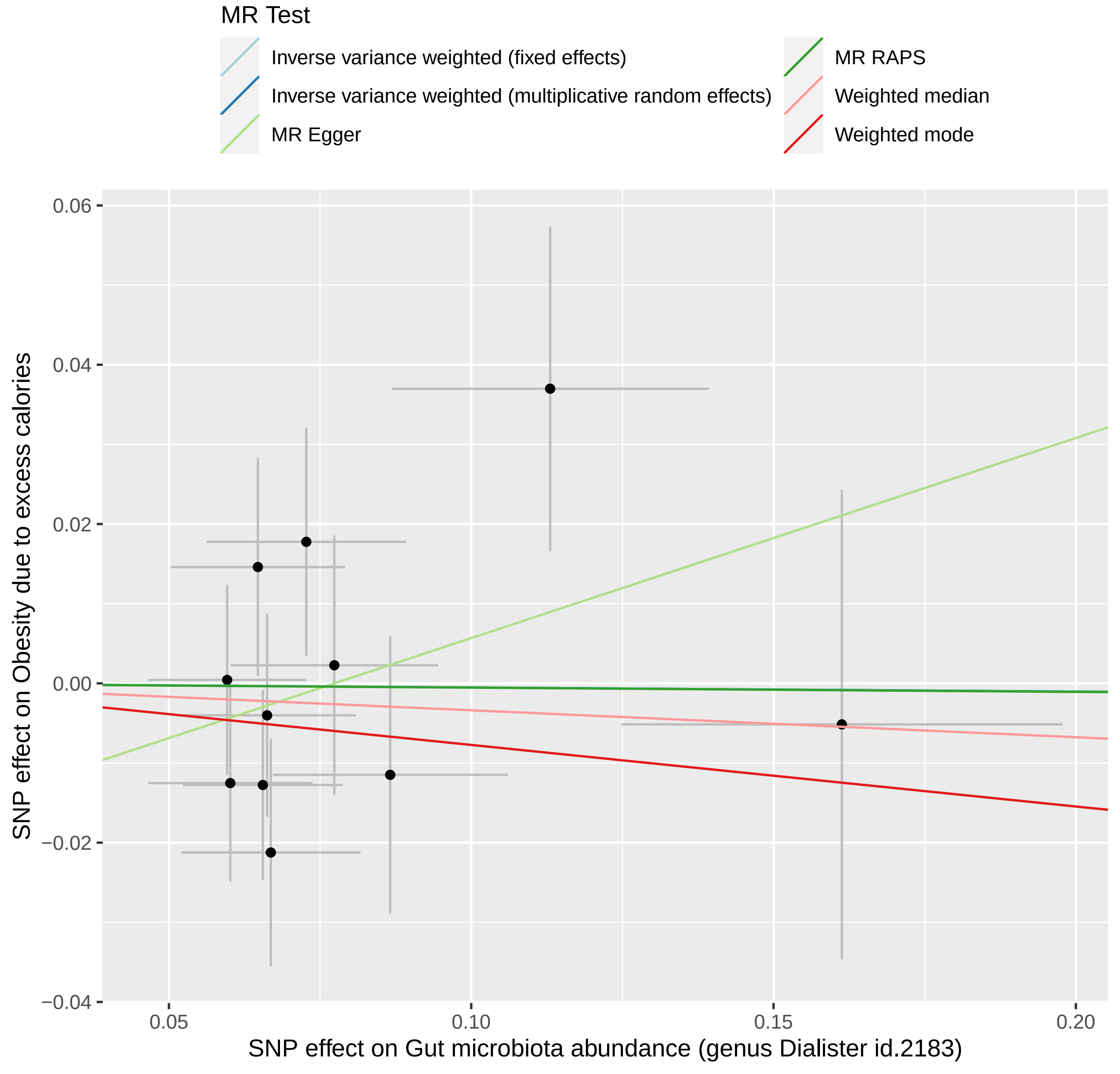

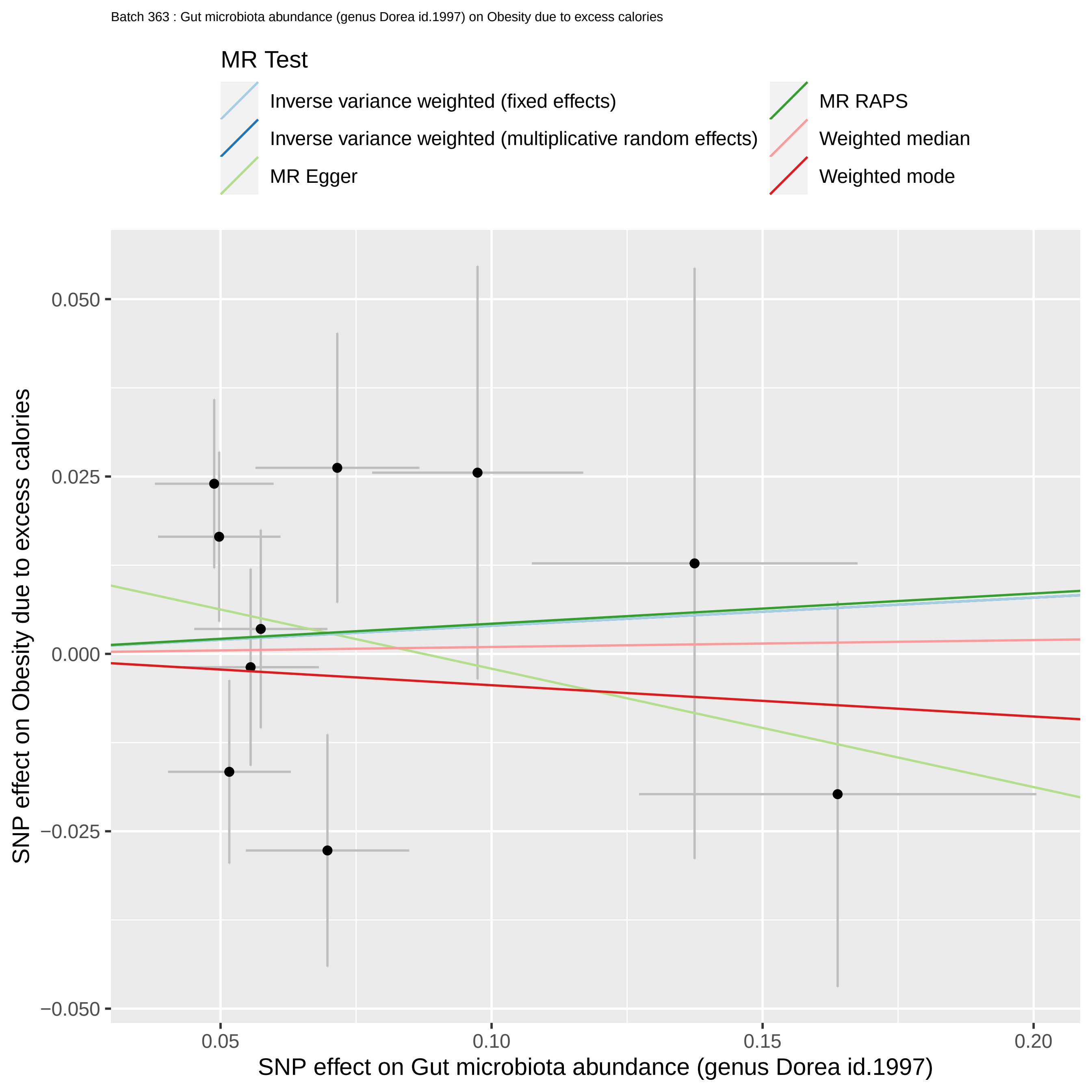

Batch 363 : Gut microbiota abundance (genus Dorea id.1997) on Obesity due to excess calories
MR Test
Inverse variance weighted (fixed effects)
Inverse variance weighted (multiplicative random effects)
MR Egger
MR RAPS
Weighted median
Weighted mode
0.050
0.025
0.000
−0.025
−0.050
0.05
0.10
0.15
0.20
SNP effect on Obesity due to excess calories
SNP effect on Gut microbiota abundance (genus Dorea id.1997)

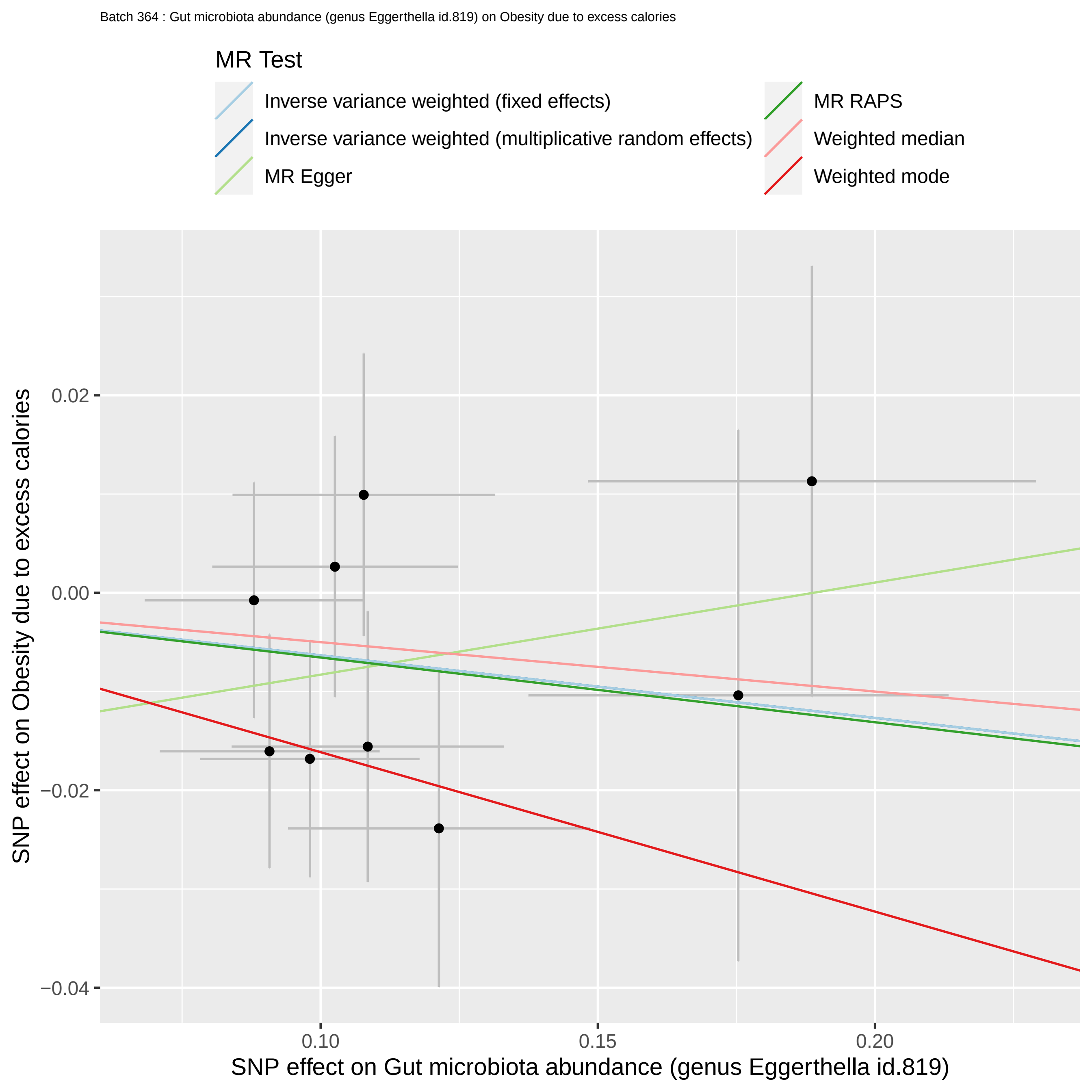

Batch 364 : Gut microbiota abundance (genus Eggerthella id.819) on Obesity due to excess calories
MR Test
Inverse variance weighted (fixed effects)
Inverse variance weighted (multiplicative random effects)
MR Egger
MR RAPS
Weighted median
Weighted mode
0.02
0.00
−0.02
−0.04
0.10
0.15
0.20
SNP effect on Obesity due to excess calories
SNP effect on Gut microbiota abundance (genus Eggerthella id.819)

Batch 365 : Gut microbiota abundance (genus Eisenbergiella id.11304) on Obesity due to excess calories

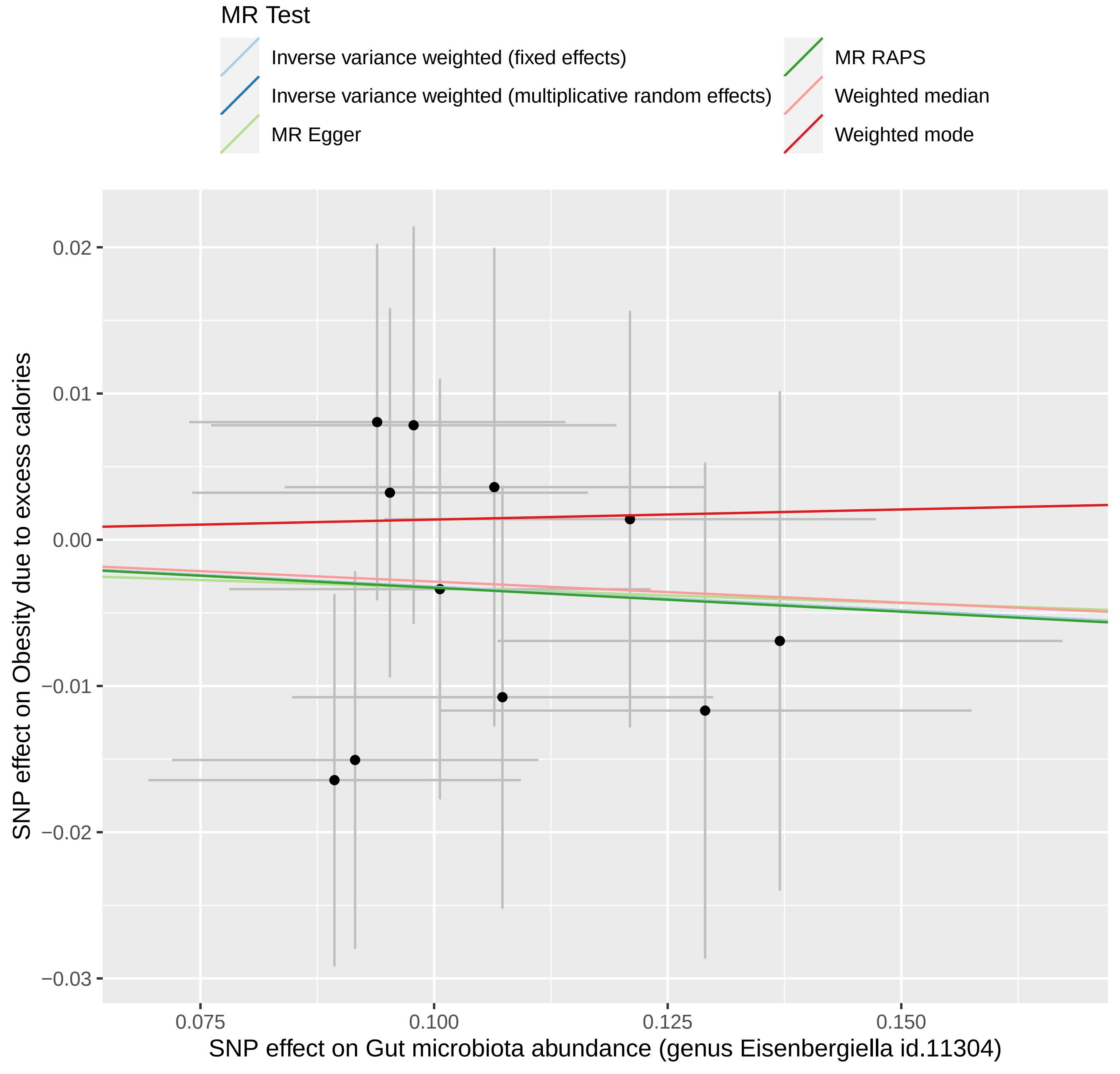

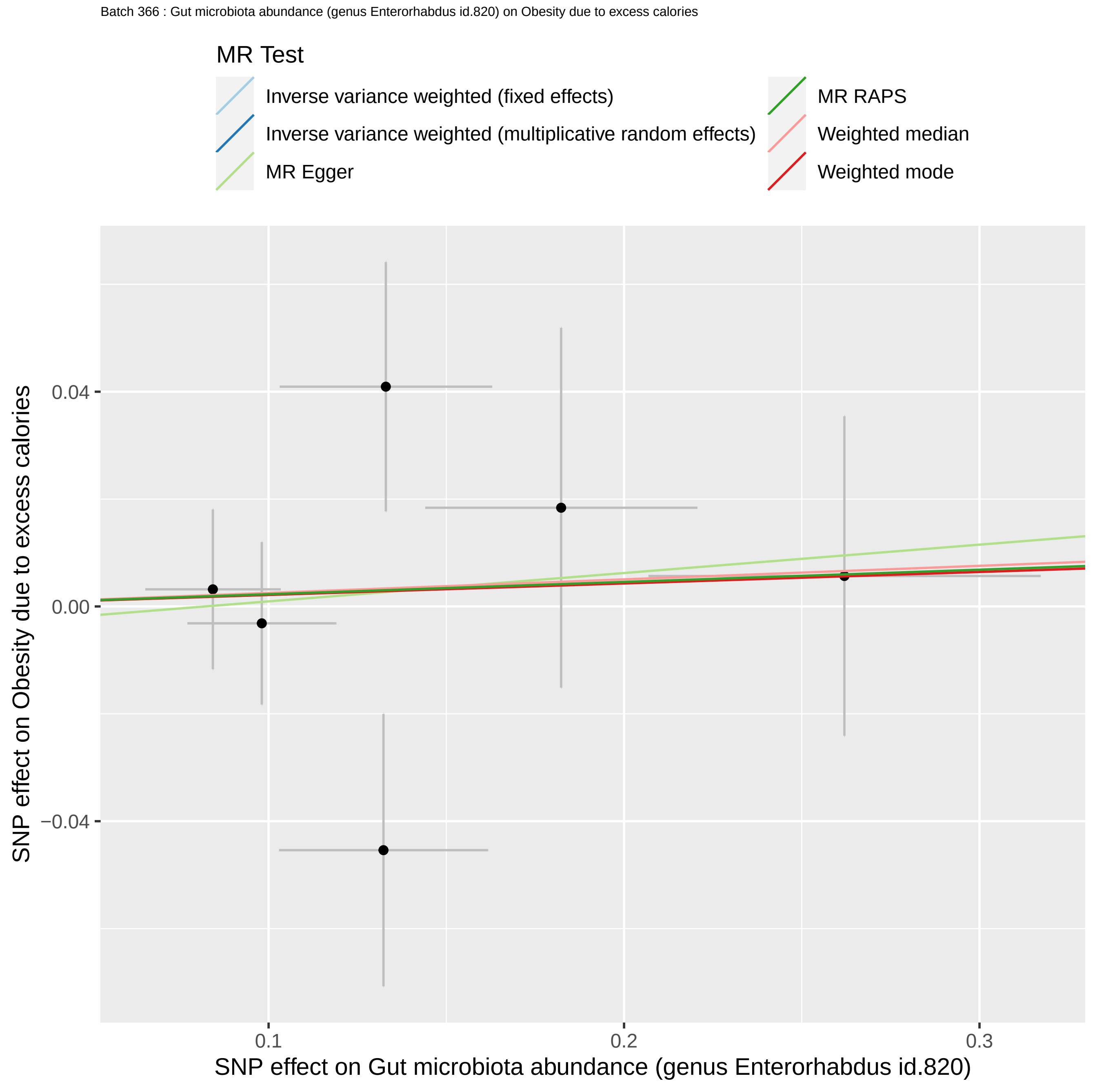

Batch 366 : Gut microbiota abundance (genus Enterorhabdus id.820) on Obesity due to excess calories
MR Test
Inverse variance weighted (fixed effects)
Inverse variance weighted (multiplicative random effects)
MR Egger
MR RAPS
Weighted median
Weighted mode
SNP effect on Obesity due to excess calories
0.04
0.00
−0.04
0.1
0.2
0.3
SNP effect on Gut microbiota abundance (genus Enterorhabdus id.820)

Batch 367 : Gut microbiota abundance (genus Erysipelatoclostridium id.11381) on Obesity due to excess calories

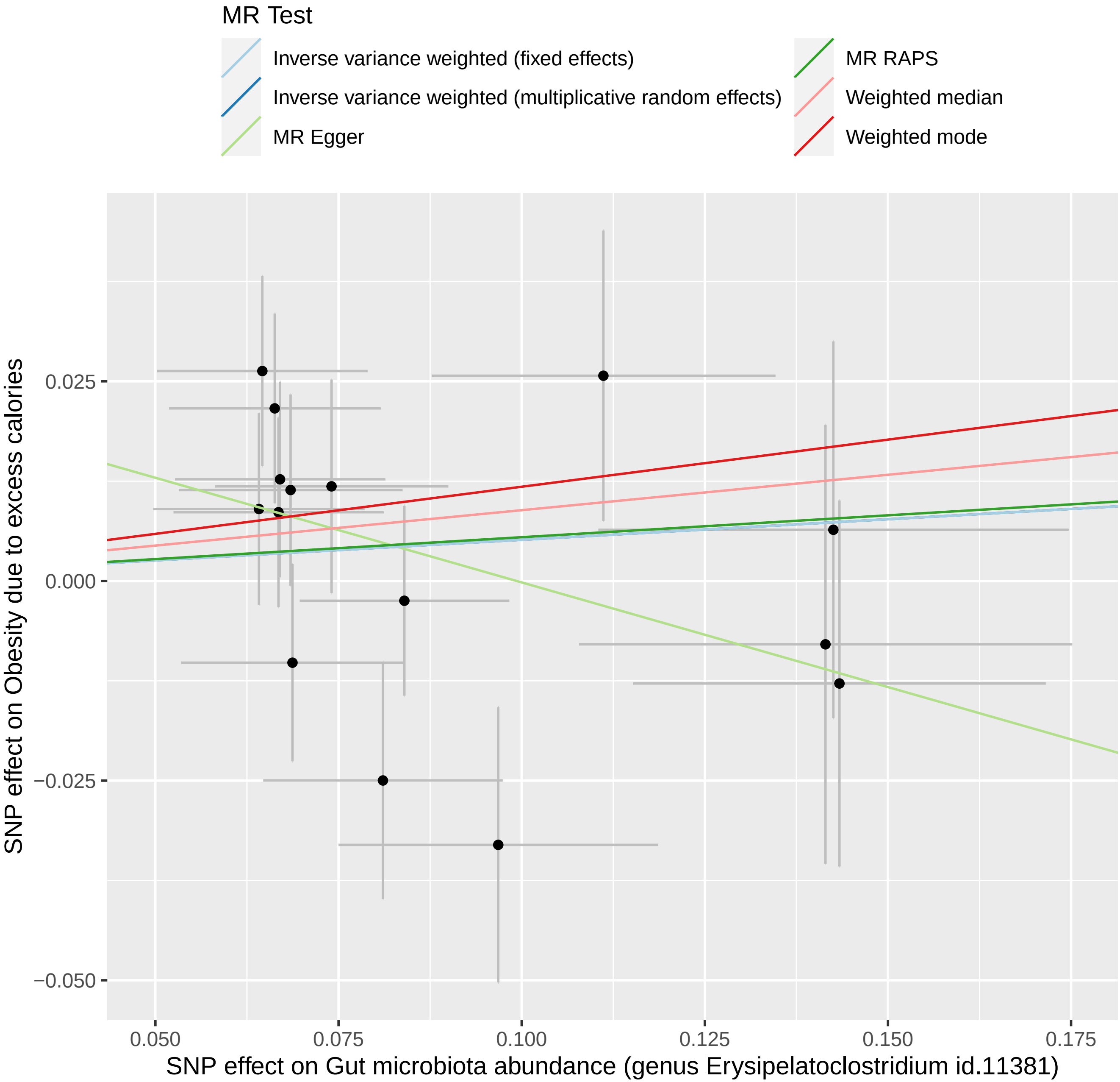

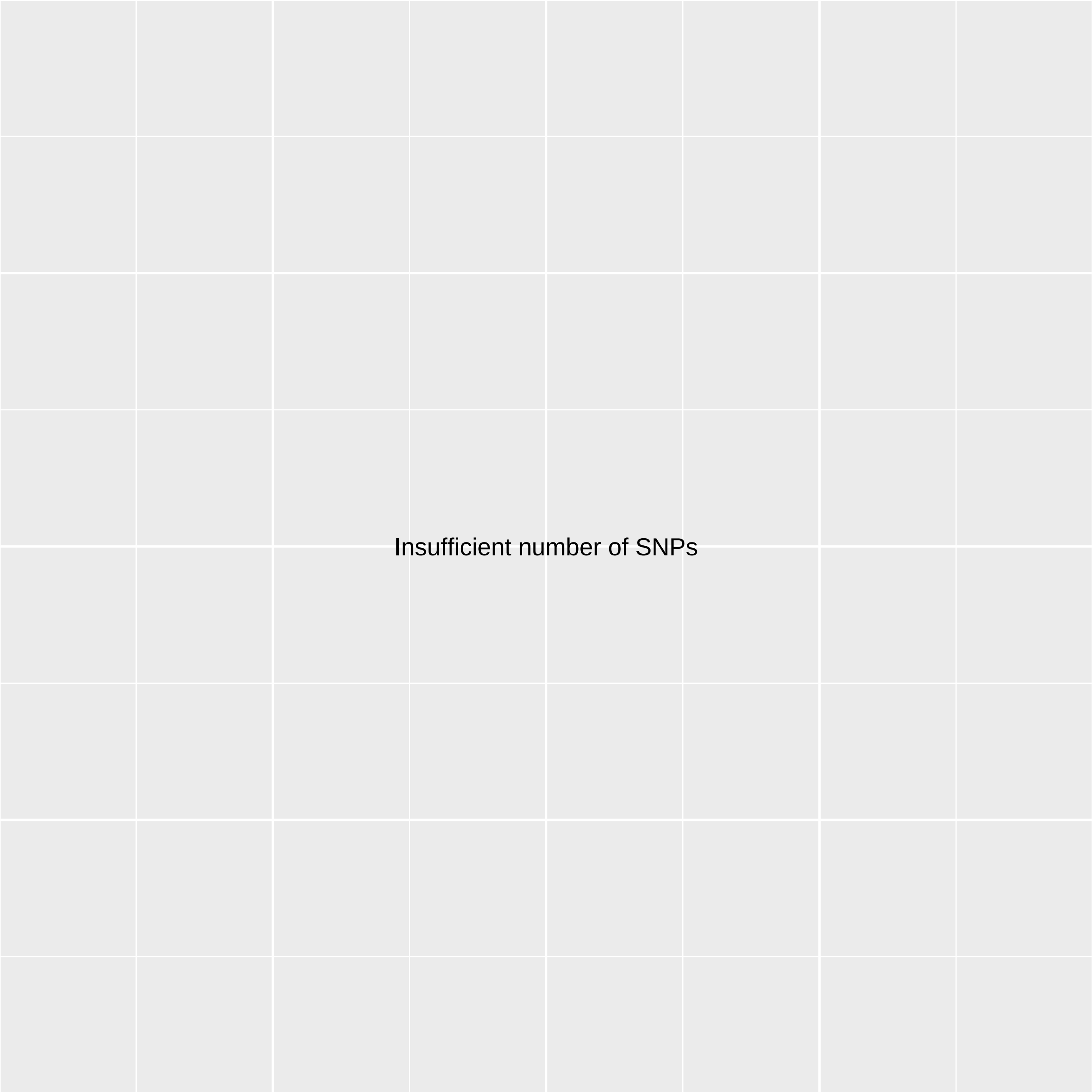
Insufficient number of SNPs

Batch 369 : Gut microbiota abundance (genus Escherichia Shigella id.3504) on Obesity due to excess calories

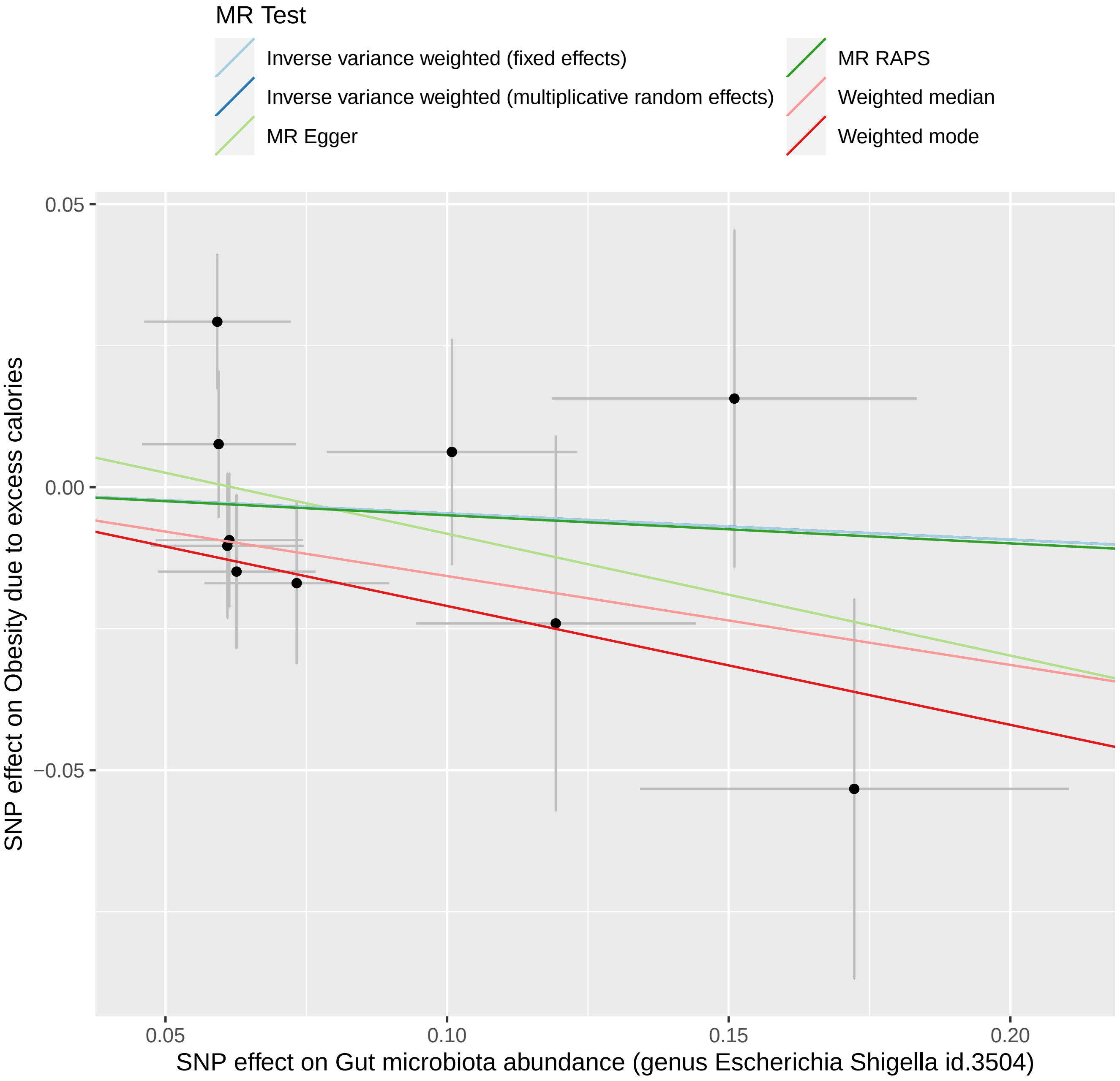

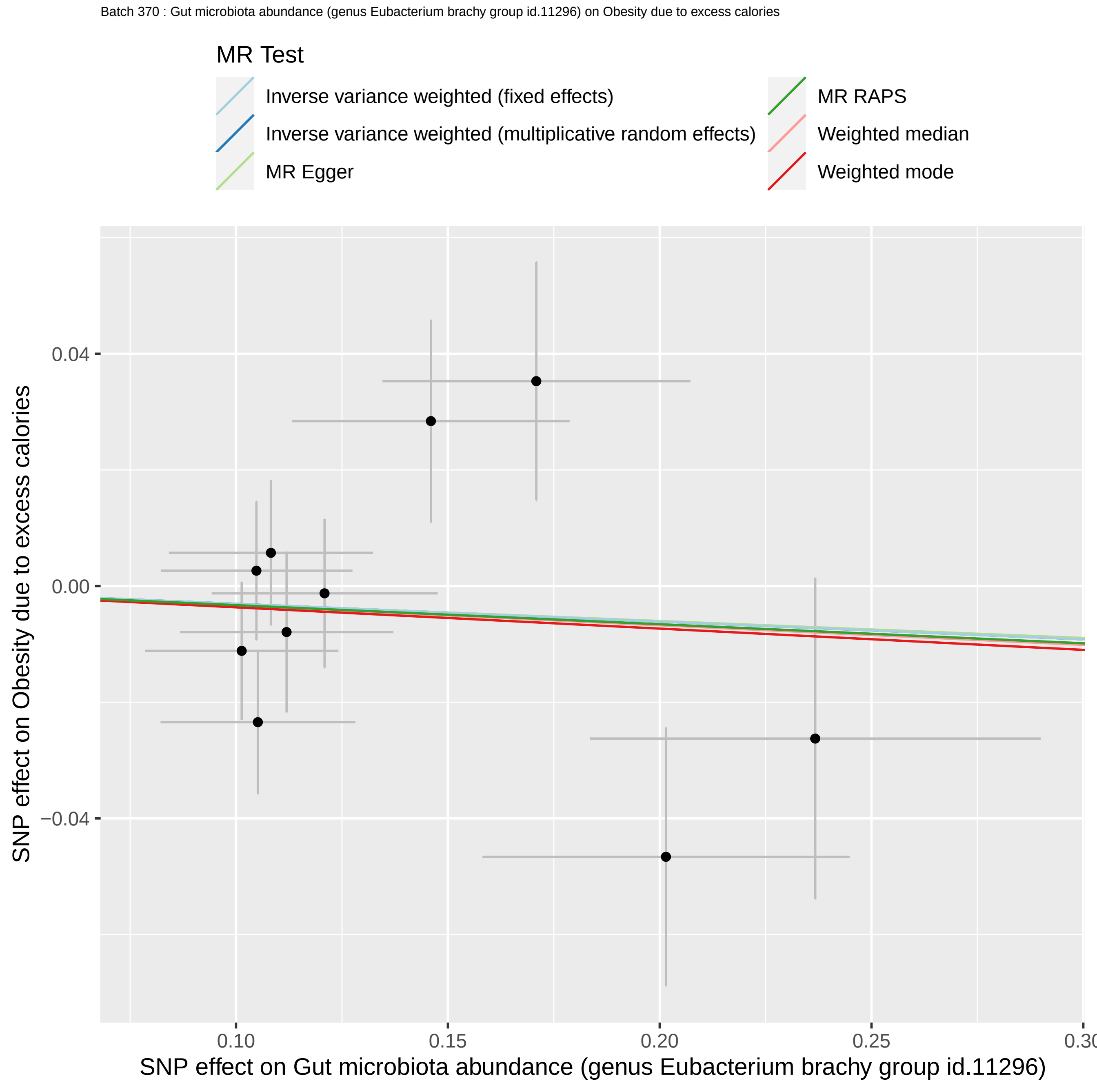
Batch 370 : Gut microbiota abundance (genus Eubacterium brachy group id.11296) on Obesity due to excess calories
MR Test
Inverse variance weighted (fixed effects)
Inverse variance weighted (multiplicative random effects)
MR Egger
MR RAPS
Weighted median
Weighted mode
0.04
0.00
−0.04
0.10
0.15
0.20
0.25
0.30
SNP effect on Obesity due to excess calories
SNP effect on Gut microbiota abundance (genus Eubacterium brachy group id.11296)

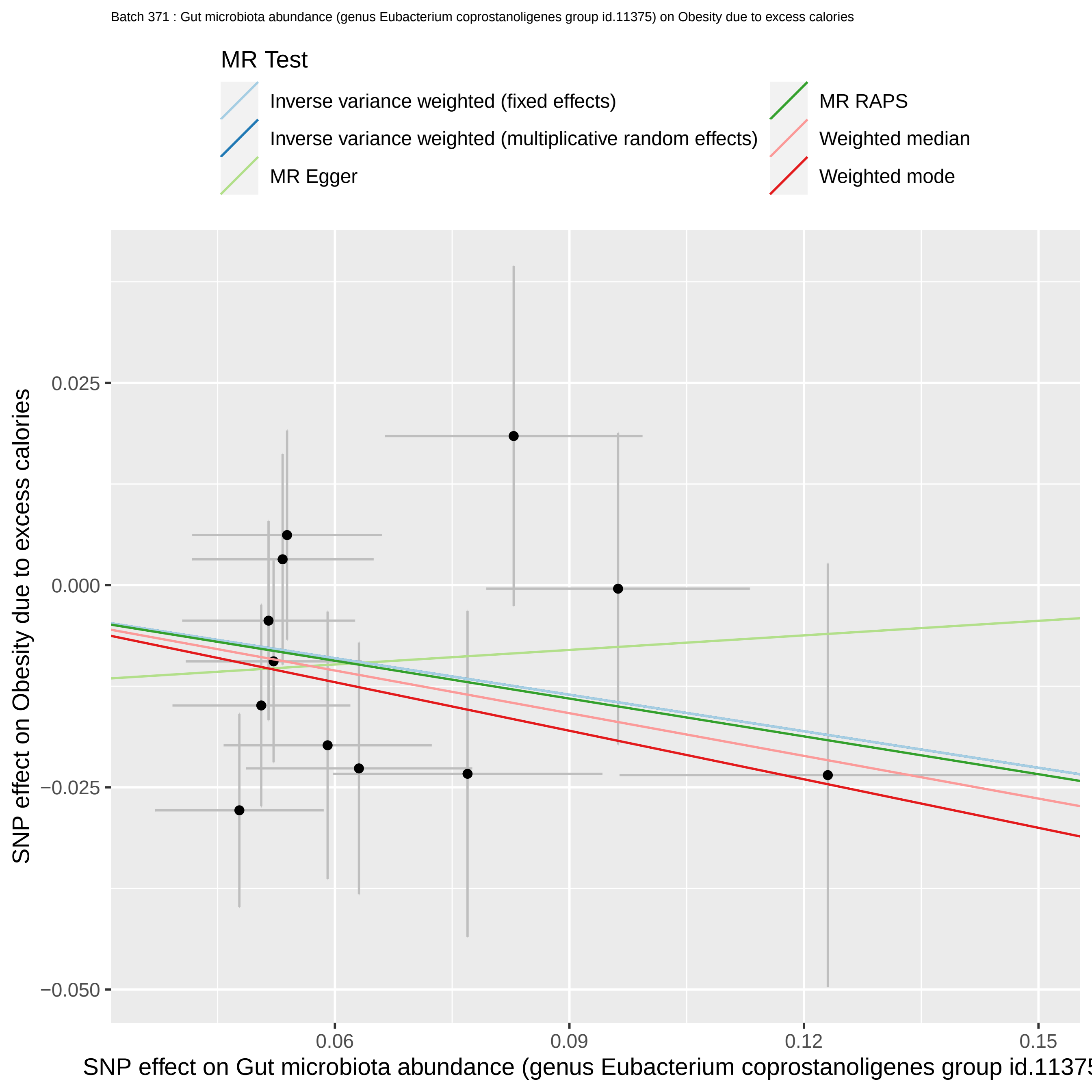

Batch 371 : Gut microbiota abundance (genus Eubacterium coprostanoligenes group id.11375) on Obesity due to excess calories
MR Test
Inverse variance weighted (fixed effects)
Inverse variance weighted (multiplicative random effects)
MR Egger
MR RAPS
Weighted median
Weighted mode
0.025
0.000
−0.025
−0.050
0.06
0.09
0.12
0.15
SNP effect on Obesity due to excess calories
SNP effect on Gut microbiota abundance (genus Eubacterium coprostanoligenes group id.11375

Batch 372 : Gut microbiota abundance (genus Eubacterium eligens group id.14372) on Obesity due to excess calories

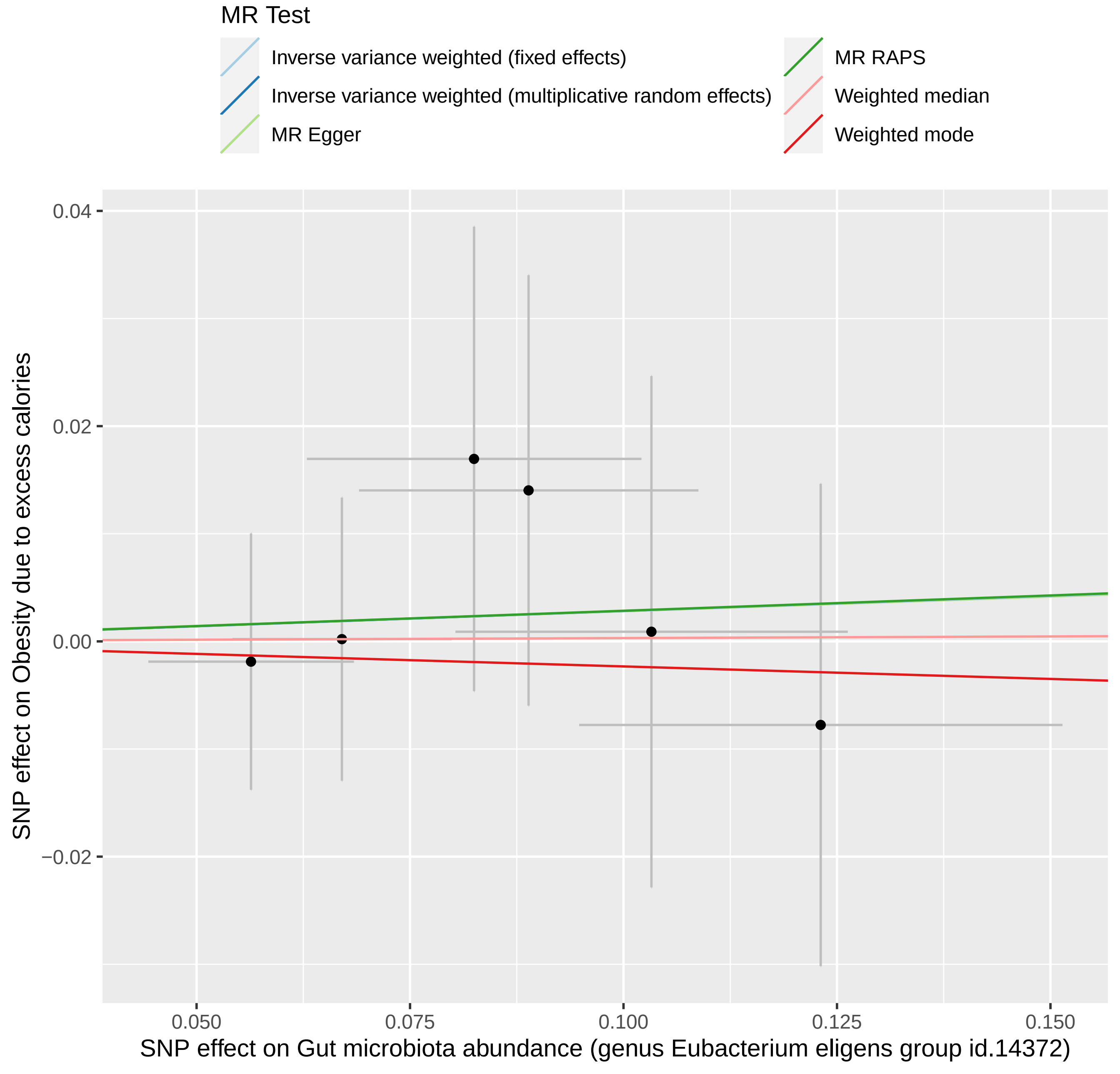

Batch 373 : Gut microbiota abundance (genus Eubacterium fissicatena group id.14373) on Obesity due to excess calories

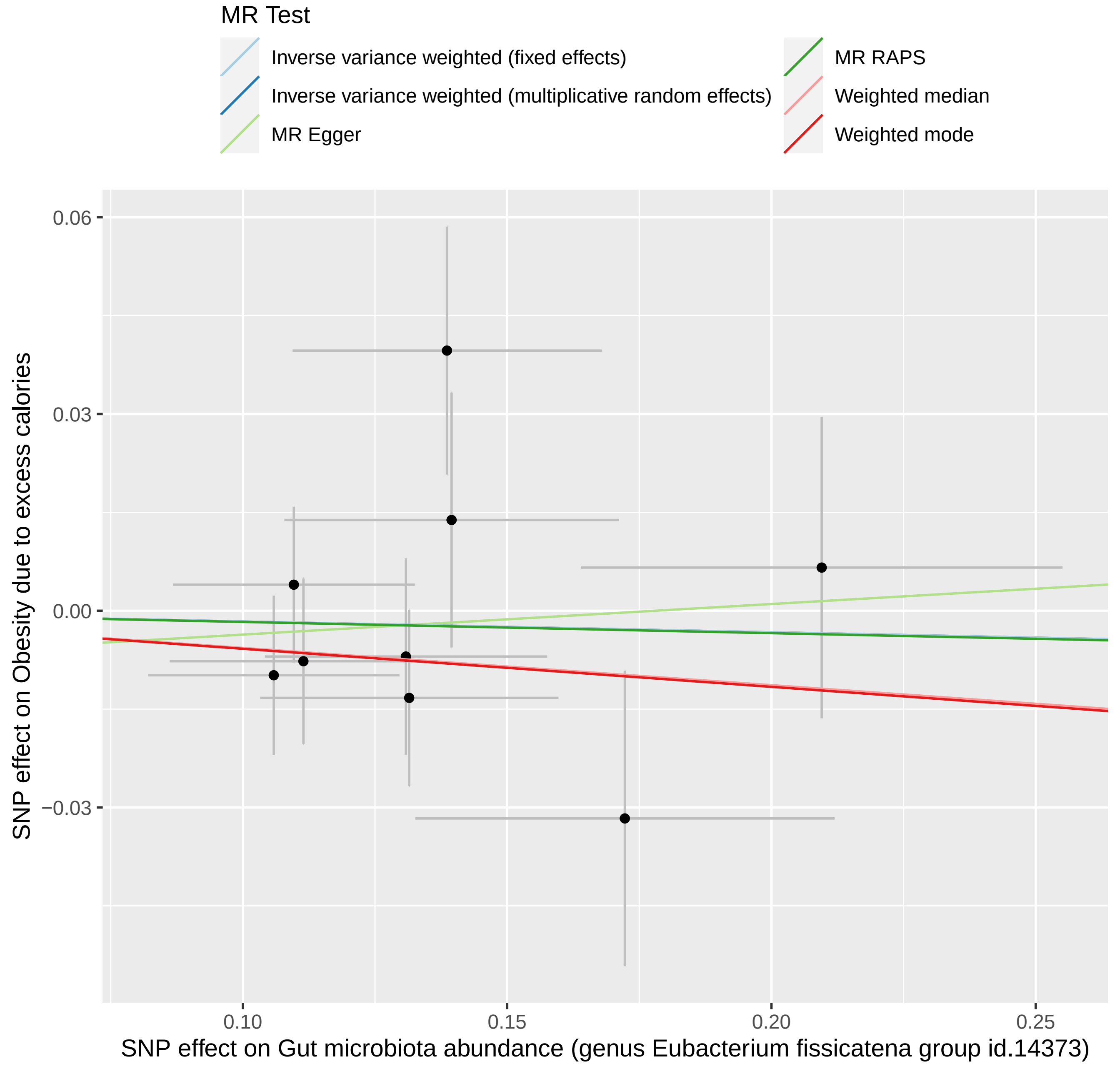

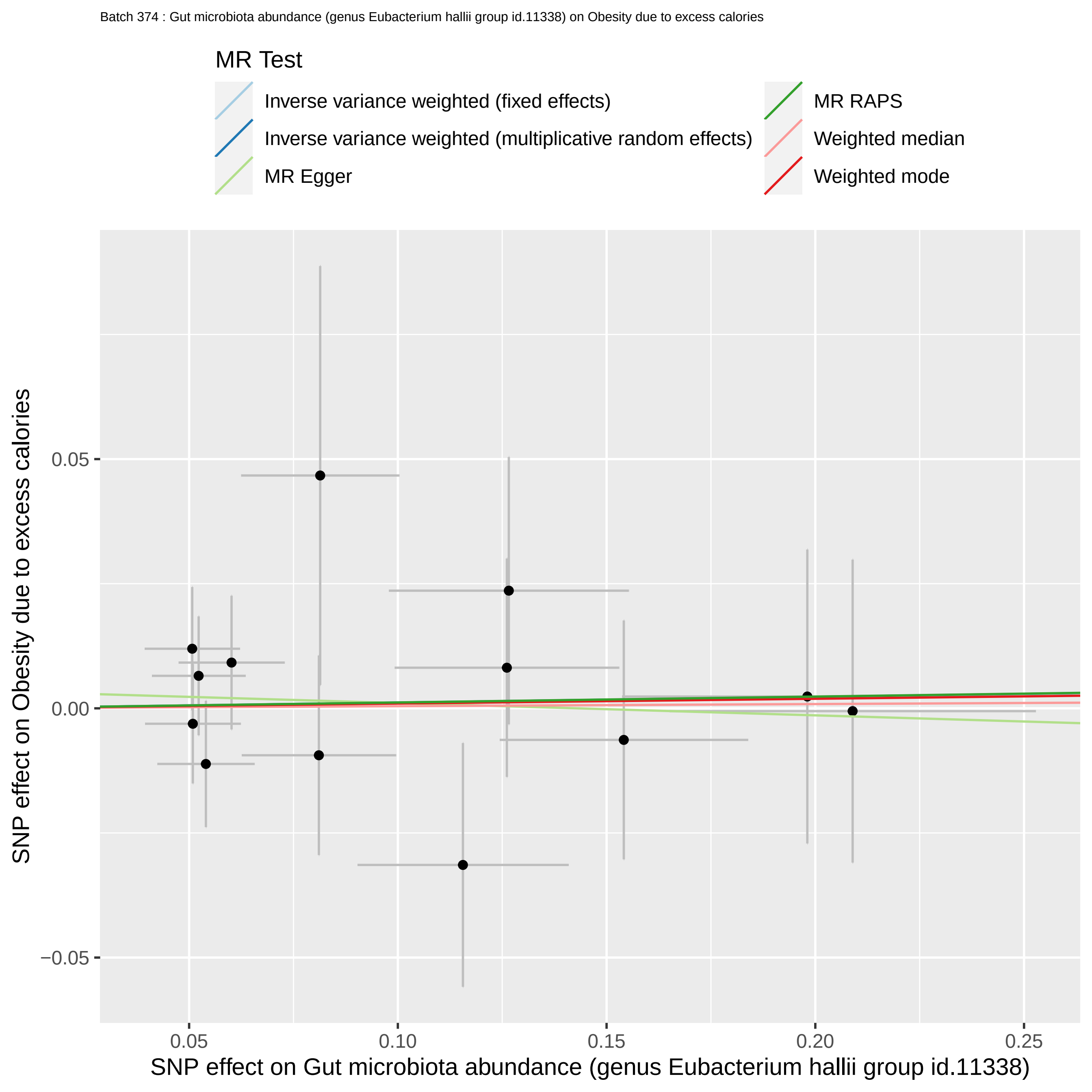
Batch 374 : Gut microbiota abundance (genus Eubacterium hallii group id.11338) on Obesity due to excess calories
MR Test
Inverse variance weighted (fixed effects)
Inverse variance weighted (multiplicative random effects)
MR Egger
MR RAPS
Weighted median
Weighted mode
SNP effect on Obesity due to excess calories
0.05
0.00
−0.05
0.05
0.10
0.15
0.20
0.25
SNP effect on Gut microbiota abundance (genus Eubacterium hallii group id.11338)

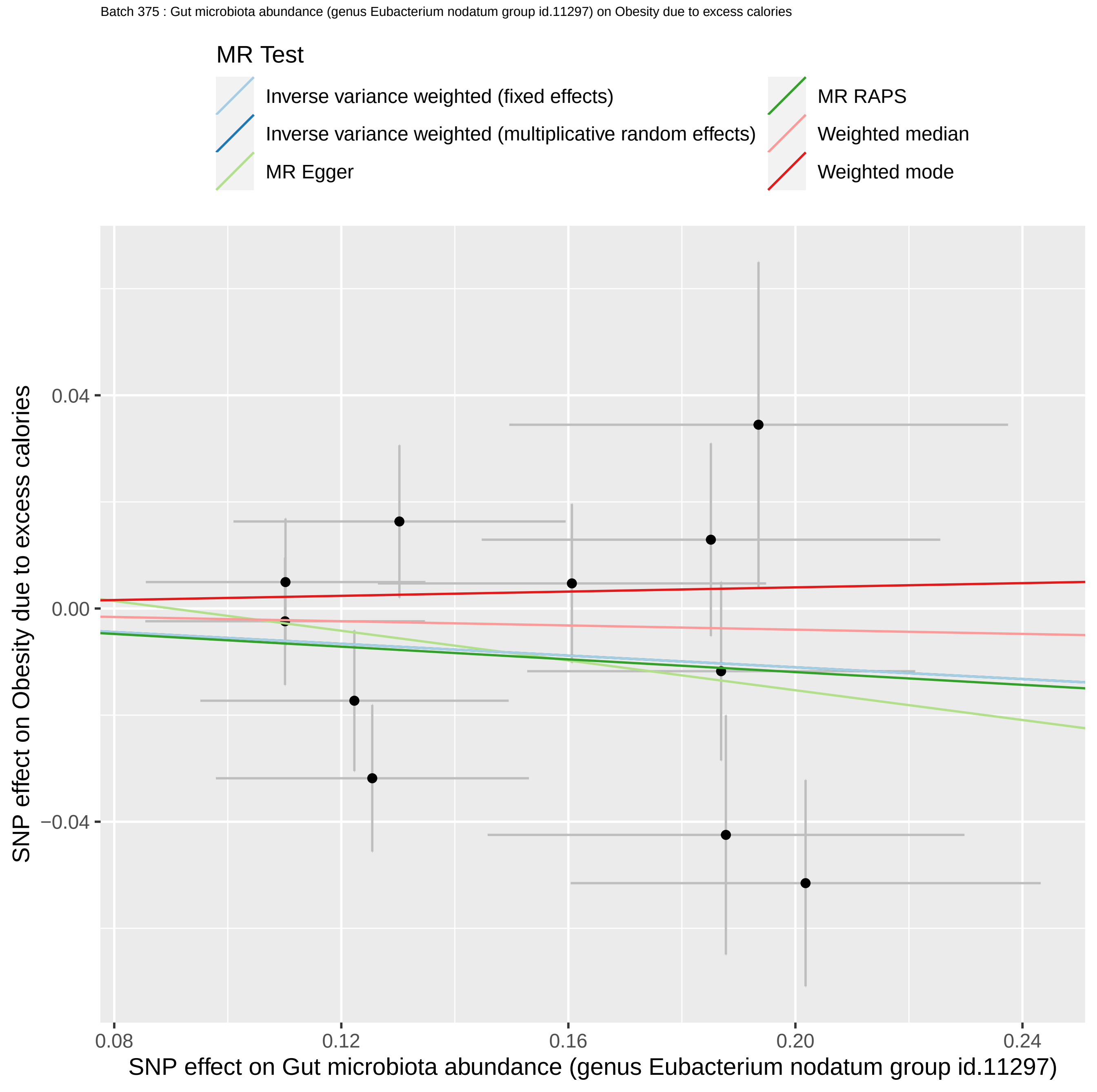
Batch 375 : Gut microbiota abundance (genus Eubacterium nodatum group id.11297) on Obesity due to excess calories
MR Test
Inverse variance weighted (fixed effects)
Inverse variance weighted (multiplicative random effects)
MR Egger
MR RAPS
Weighted median
Weighted mode
0.04
0.00
−0.04
0.08
0.12
0.16
0.20
0.24
SNP effect on Obesity due to excess calories
SNP effect on Gut microbiota abundance (genus Eubacterium nodatum group id.11297)

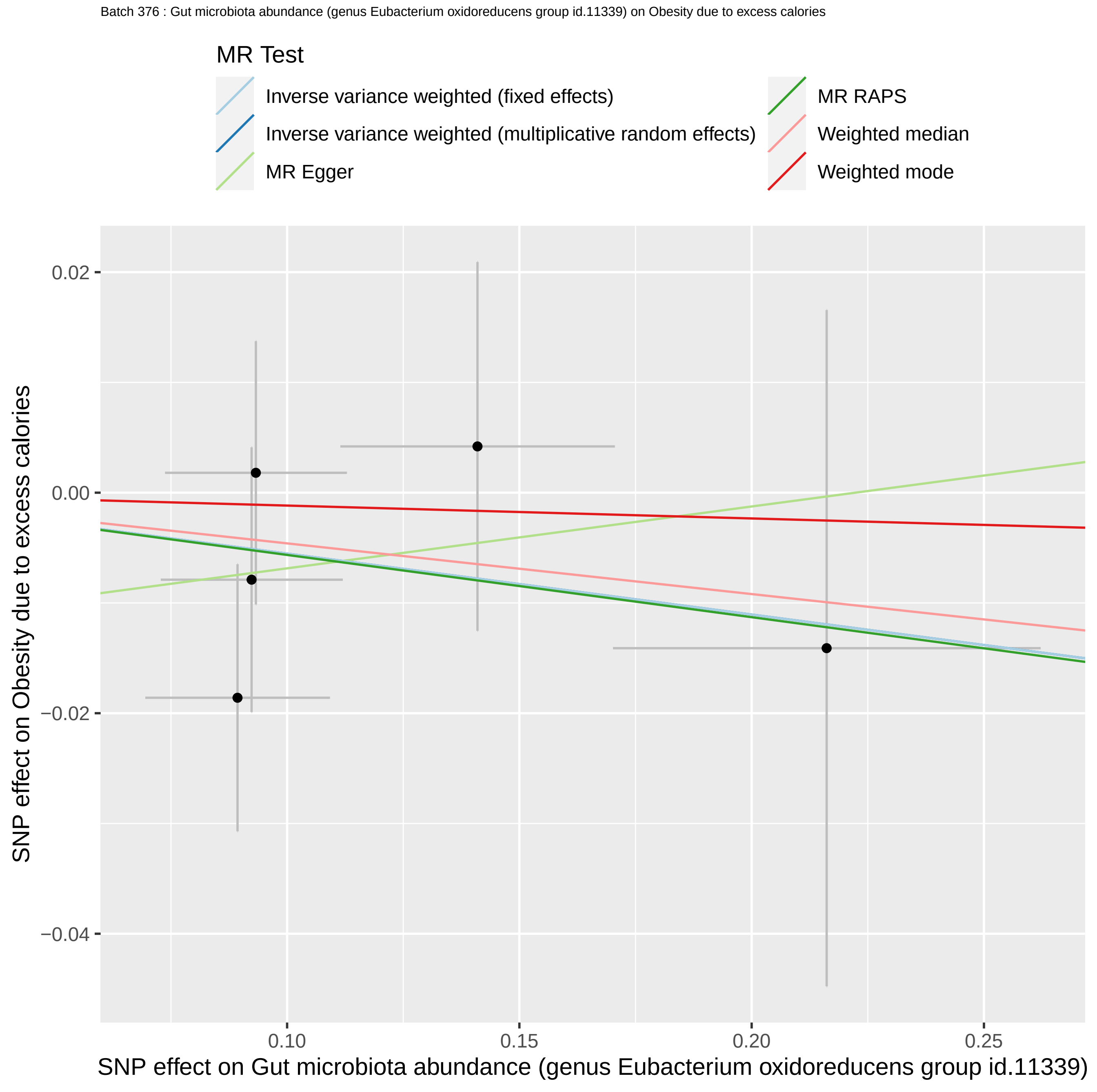

Batch 376 : Gut microbiota abundance (genus Eubacterium oxidoreducens group id.11339) on Obesity due to excess calories
MR Test
Inverse variance weighted (fixed effects)
Inverse variance weighted (multiplicative random effects)
MR Egger
MR RAPS
Weighted median
Weighted mode
0.02
0.00
−0.02
−0.04
0.10
0.15
0.20
0.25
SNP effect on Obesity due to excess calories
SNP effect on Gut microbiota abundance (genus Eubacterium oxidoreducens group id.11339)

Batch 377 : Gut microbiota abundance (genus Eubacterium rectale group id.14374) on Obesity due to excess calories

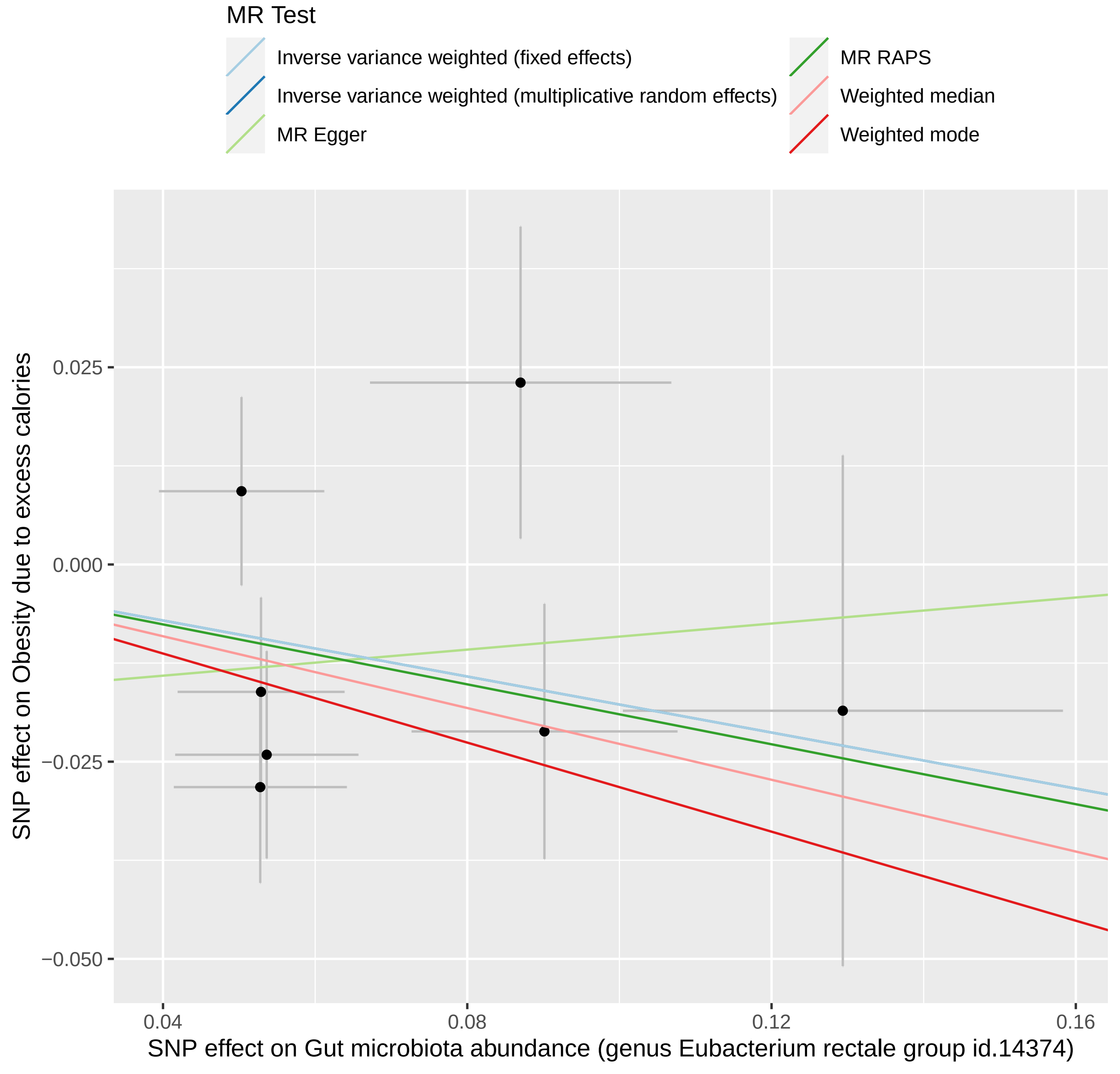

Batch 378 : Gut microbiota abundance (genus Eubacterium ruminantium group id.11340) on Obesity due to excess calories

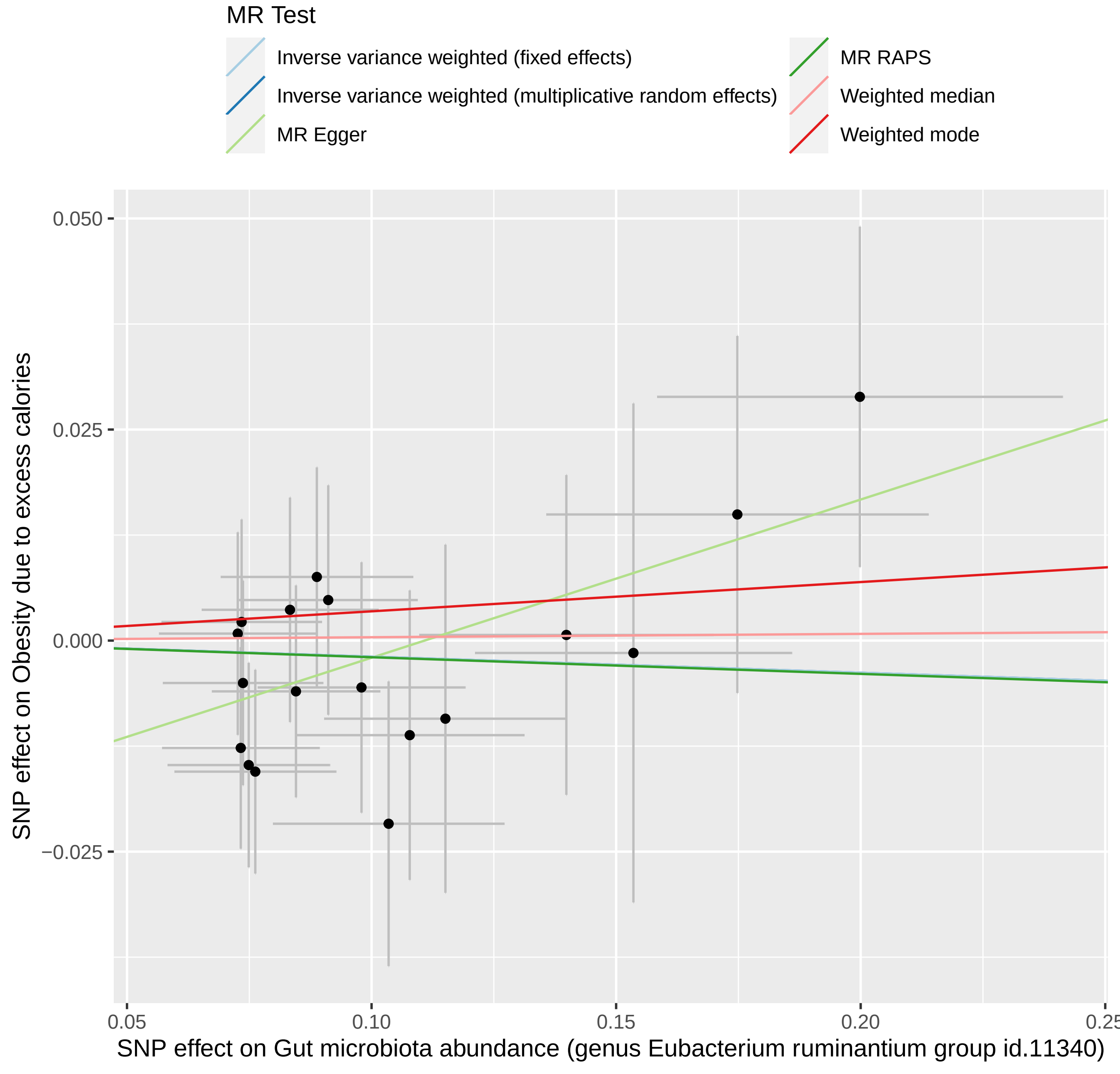

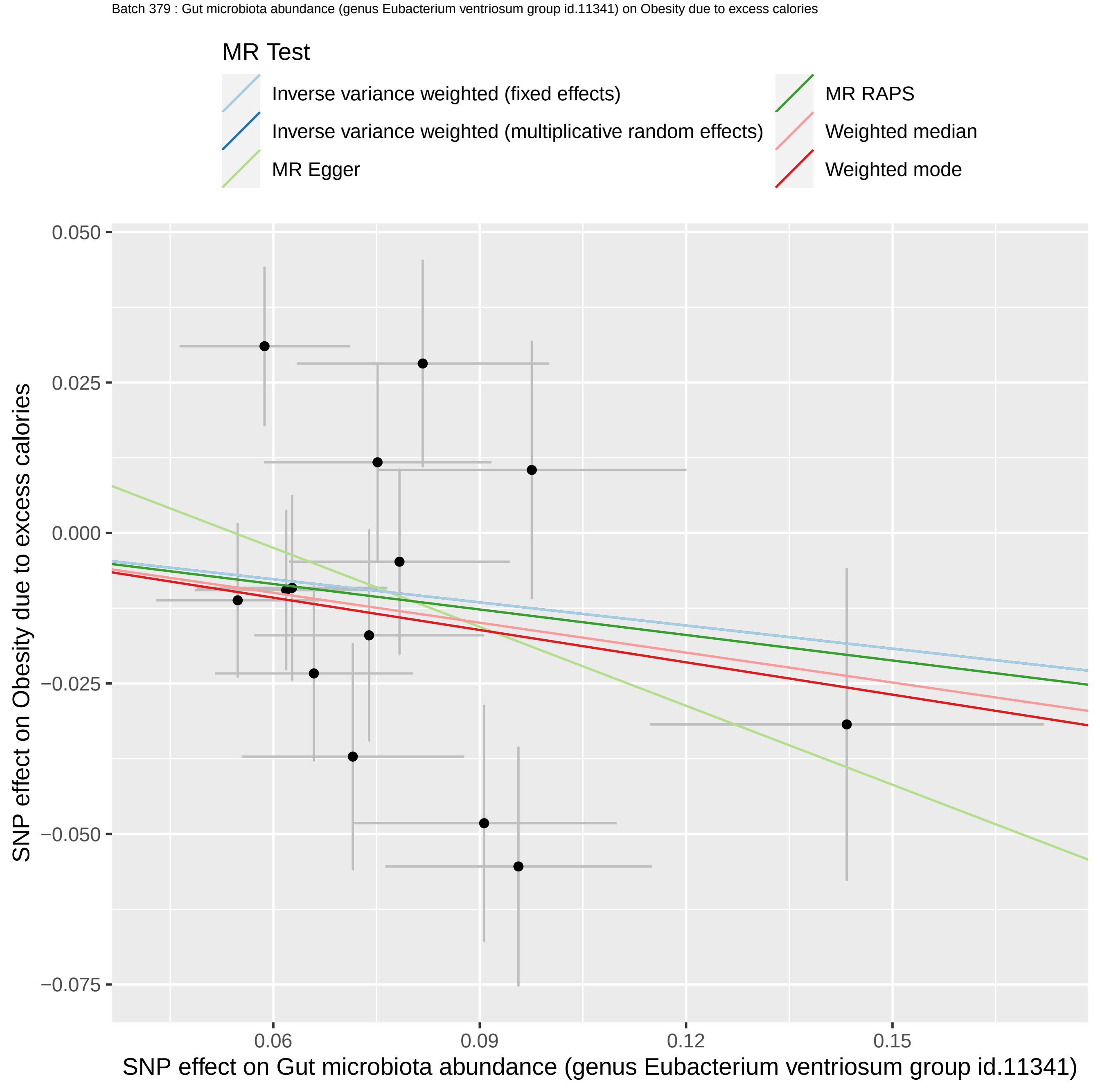

Batch 379 : Gut microbiota abundance (genus Eubacterium ventriosum group id.11341) on Obesity due to excess calories
MR Test
Inverse variance weighted (fixed effects)
Inverse variance weighted (multiplicative random effects)
MR Egger
MR RAPS
Weighted median
Weighted mode
0.050
0.025
0.000
−0.025
−0.050
−0.075
0.06
0.09
0.12
0.15
SNP effect on Obesity due to excess calories
SNP effect on Gut microbiota abundance (genus Eubacterium ventriosum group id.11341)

Batch 380 : Gut microbiota abundance (genus Eubacterium xylanophilum group id.14375) on Obesity due to excess calories

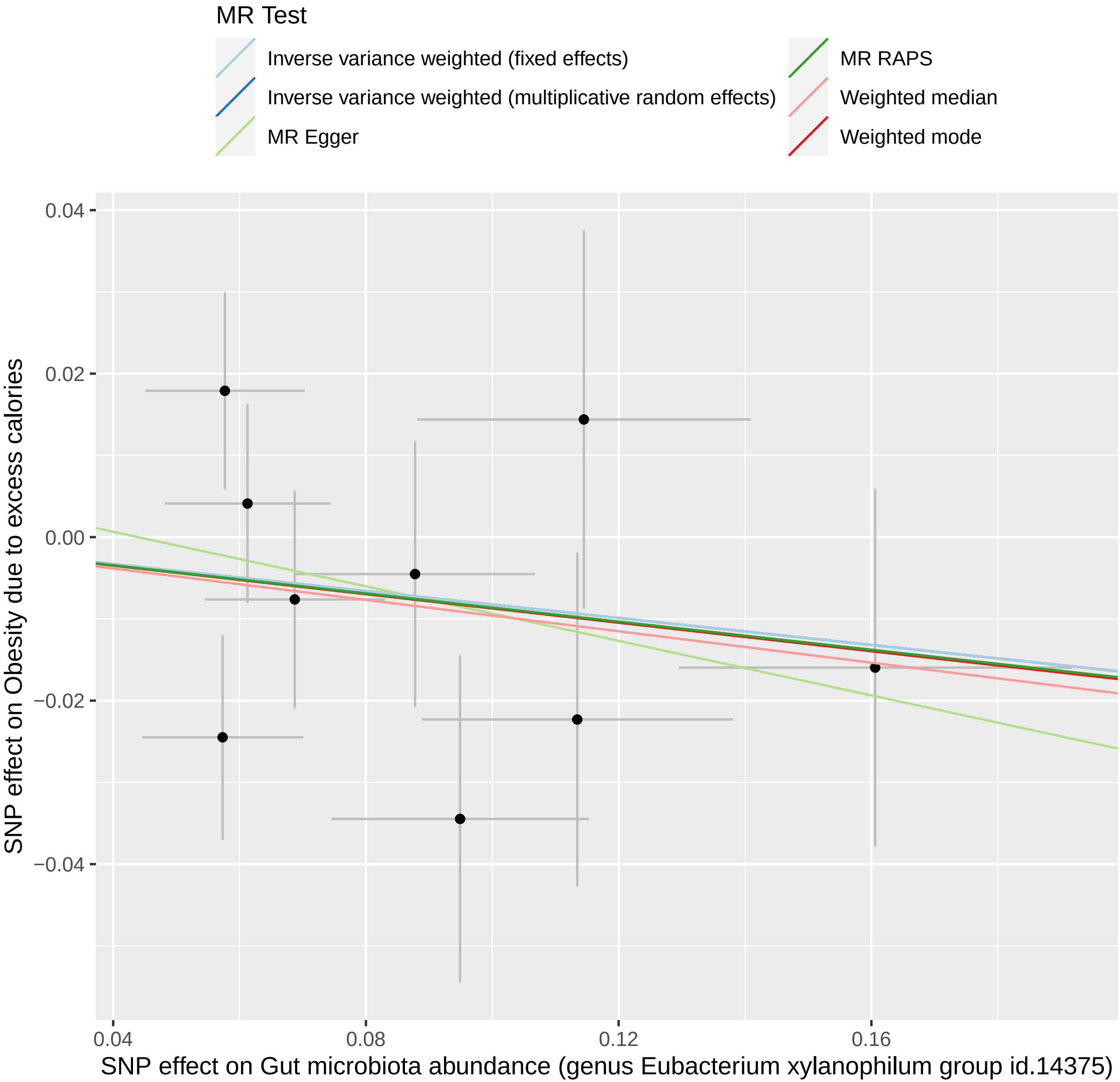

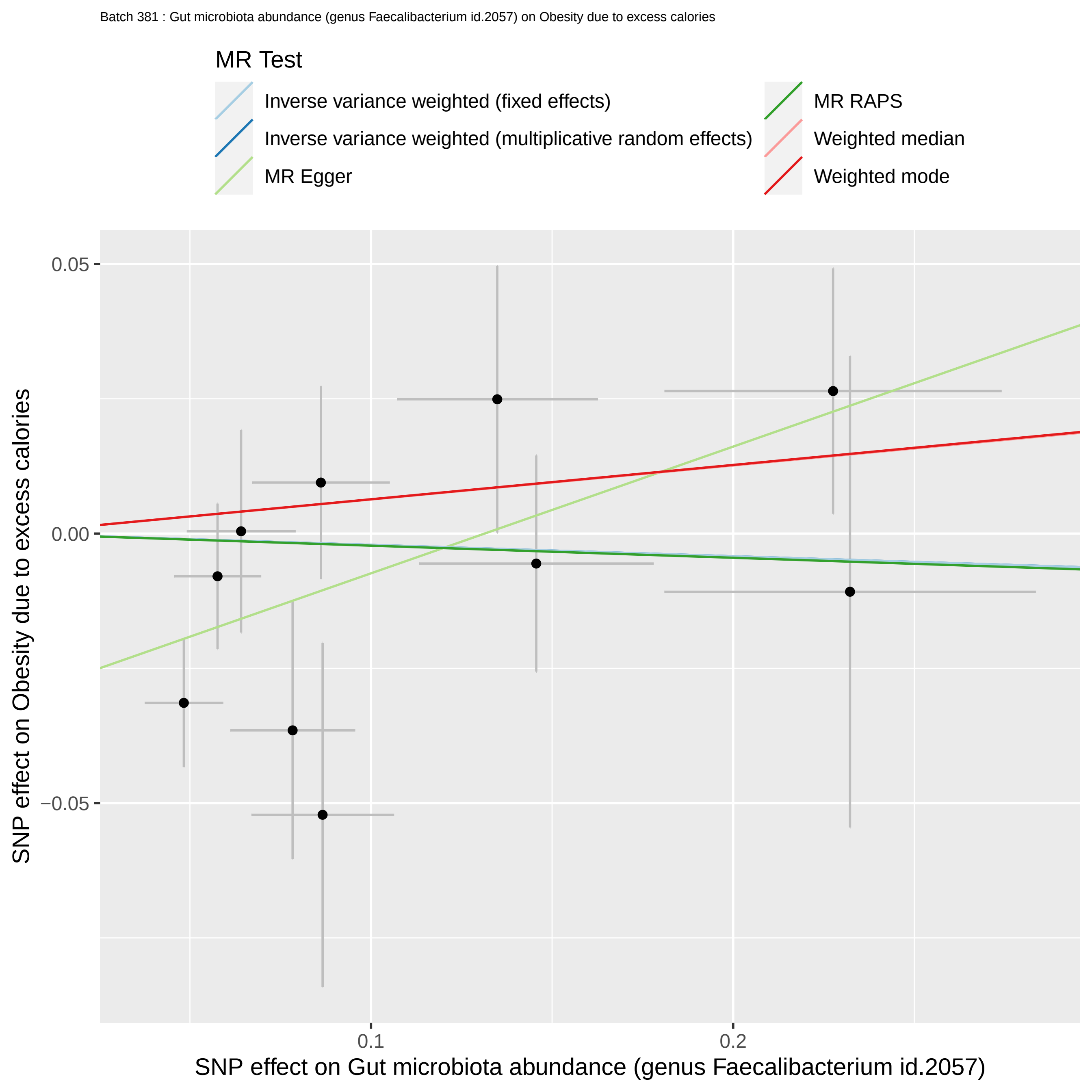

Batch 381 : Gut microbiota abundance (genus Faecalibacterium id.2057) on Obesity due to excess calories
MR Test
Inverse variance weighted (fixed effects)
Inverse variance weighted (multiplicative random effects)
MR Egger
MR RAPS
Weighted median
Weighted mode
0.05
0.00
−0.05
0.1
0.2
SNP effect on Obesity due to excess calories
SNP effect on Gut microbiota abundance (genus Faecalibacterium id.2057)

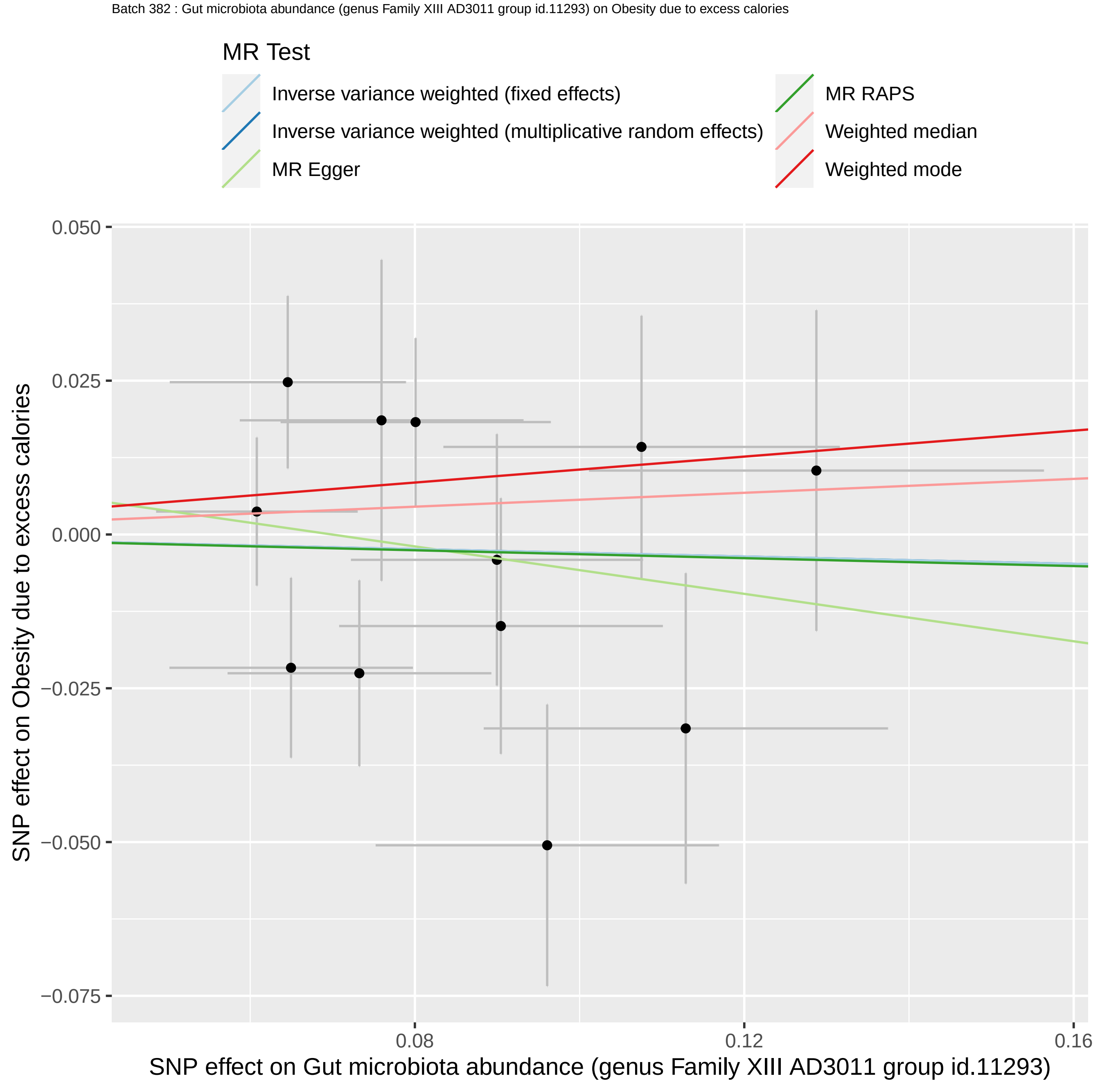

Batch 382 : Gut microbiota abundance (genus Family XIII AD3011 group id.11293) on Obesity due to excess calories
MR Test
Inverse variance weighted (fixed effects)
Inverse variance weighted (multiplicative random effects)
MR Egger
MR RAPS
Weighted median
Weighted mode
0.050
0.025
0.000
−0.025
−0.050
−0.075
0.08
0.12
0.16
SNP effect on Obesity due to excess calories
SNP effect on Gut microbiota abundance (genus Family XIII AD3011 group id.11293)

Batch 383 : Gut microbiota abundance (genus Family XIII UCG001 id.11294) on Obesity due to excess calories

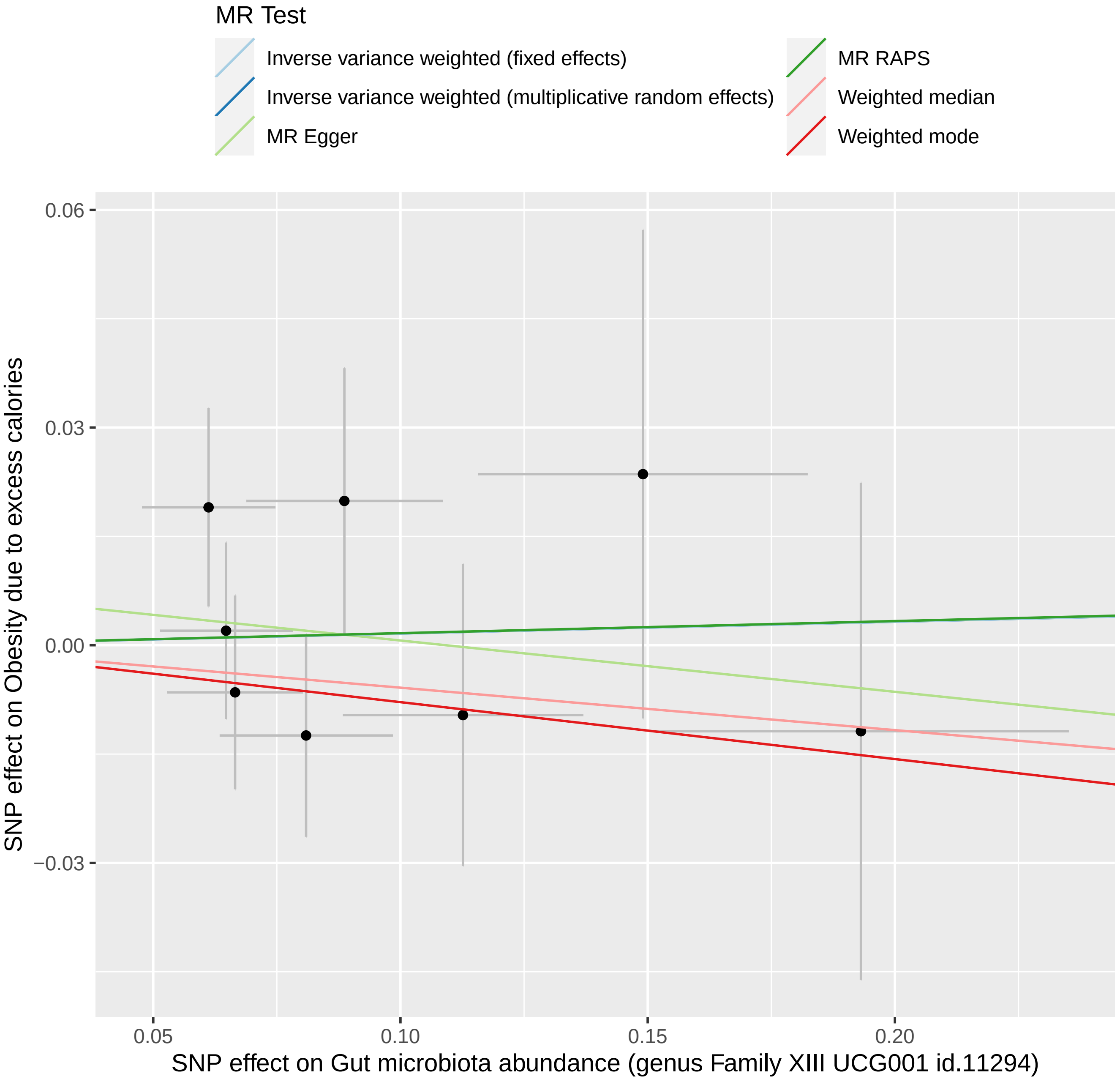

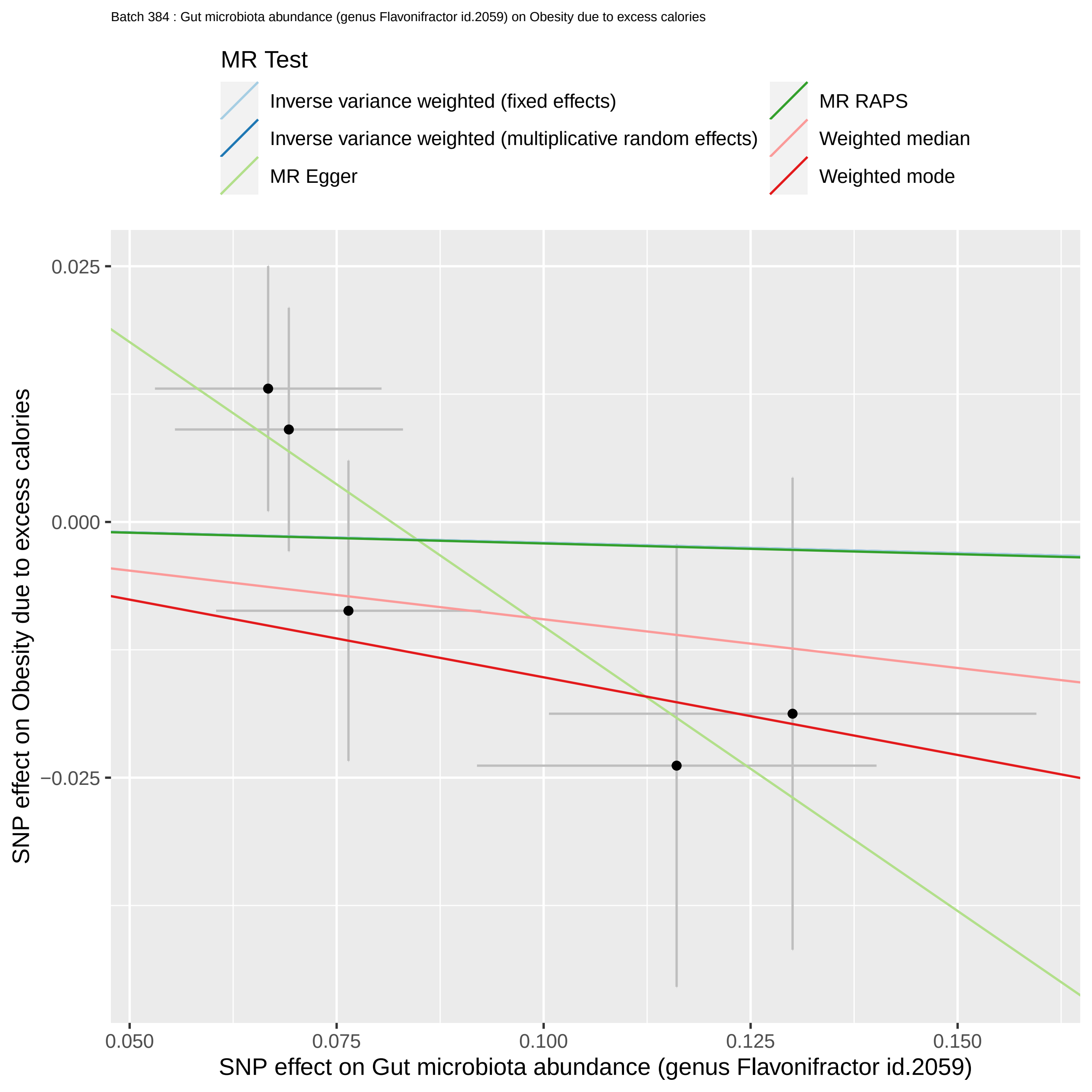

Batch 384 : Gut microbiota abundance (genus Flavonifractor id.2059) on Obesity due to excess calories
MR Test
Inverse variance weighted (fixed effects)
Inverse variance weighted (multiplicative random effects)
MR Egger
MR RAPS
Weighted median
Weighted mode
0.025
0.000
−0.025
0.050
0.075
0.100
0.125
0.150
SNP effect on Obesity due to excess calories
SNP effect on Gut microbiota abundance (genus Flavonifractor id.2059)

Batch 385 : Gut microbiota abundance (genus Fusicatenibacter id.11305) on Obesity due to excess calories

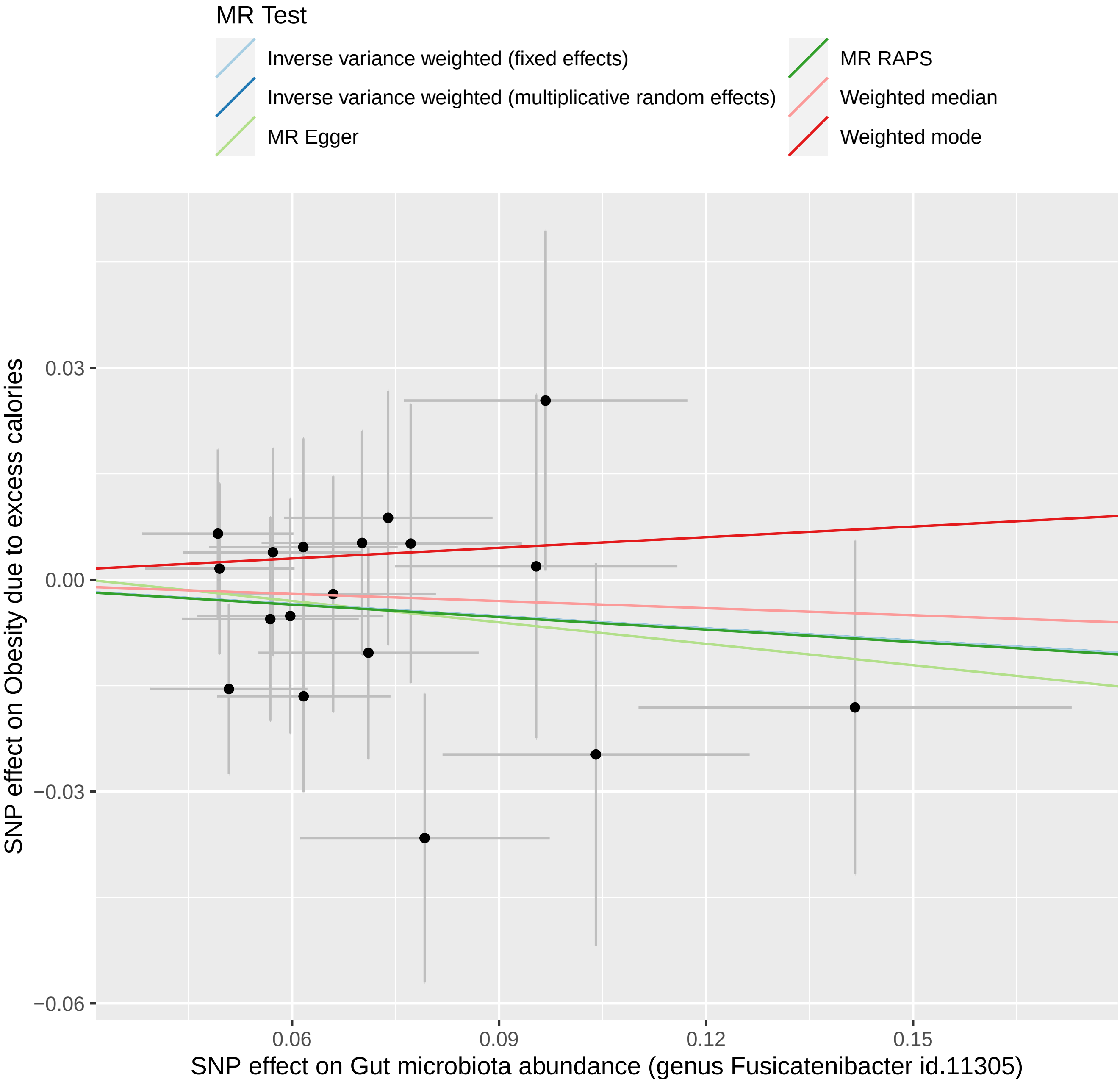

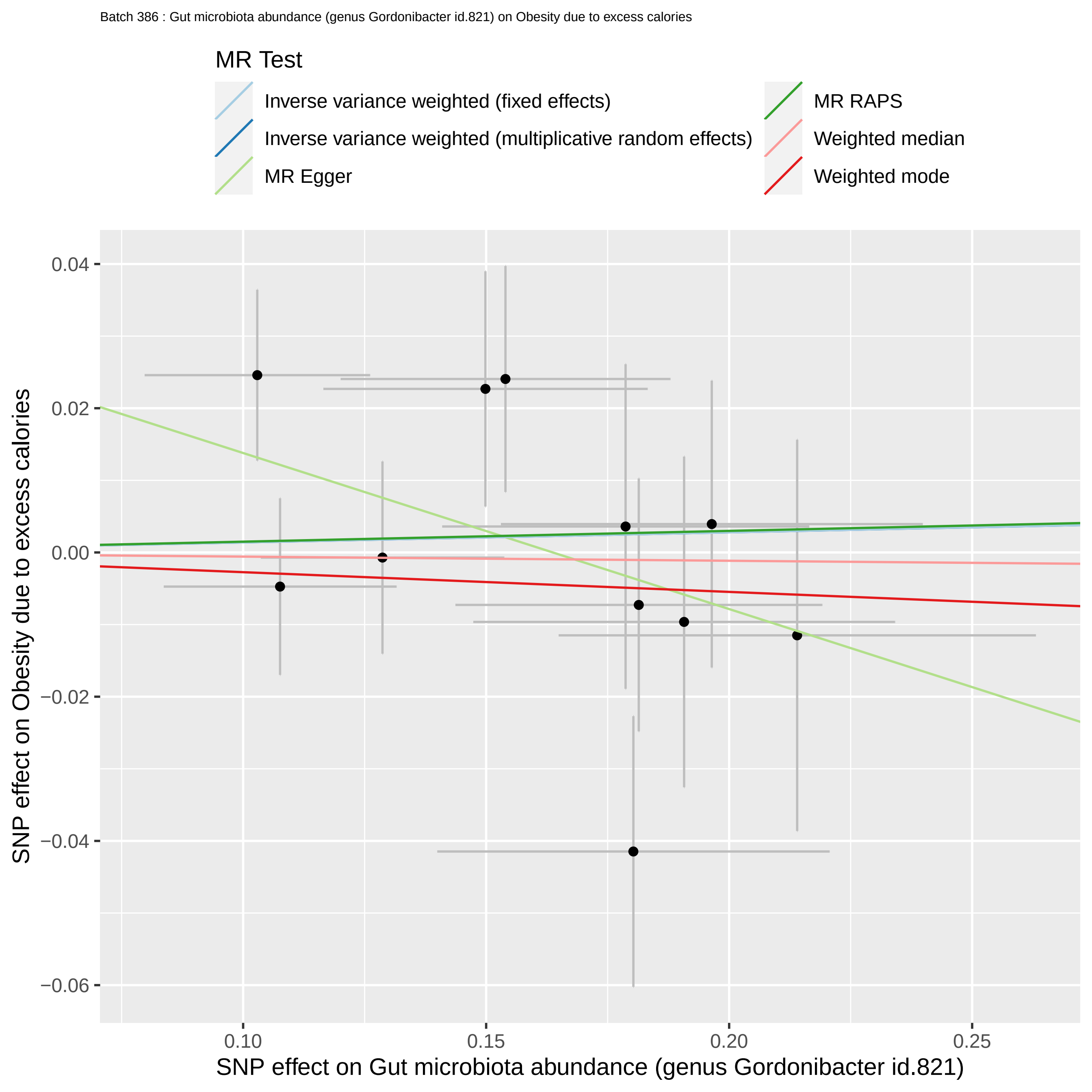
Batch 386 : Gut microbiota abundance (genus Gordonibacter id.821) on Obesity due to excess calories
MR Test
Inverse variance weighted (fixed effects)
Inverse variance weighted (multiplicative random effects)
MR Egger
MR RAPS
Weighted median
Weighted mode
0.04
0.02
0.00
−0.02
−0.04
−0.06
0.10
0.15
0.20
0.25
SNP effect on Obesity due to excess calories
SNP effect on Gut microbiota abundance (genus Gordonibacter id.821)

Batch 387 : Gut microbiota abundance (genus Haemophilus id.3698) on Obesity due to excess calories

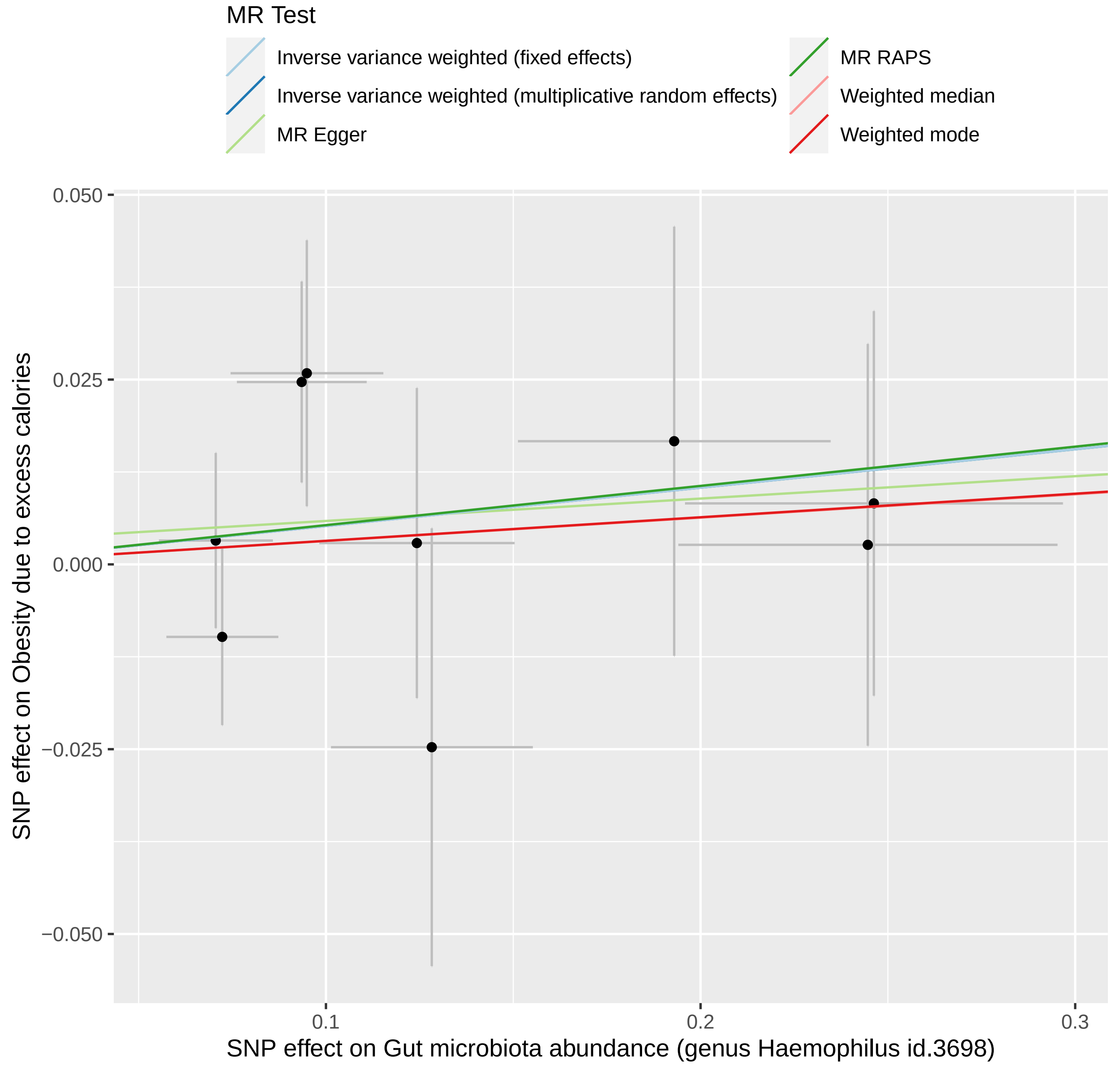

Batch 388 : Gut microbiota abundance (genus Holdemanella id.11393) on Obesity due to excess calories

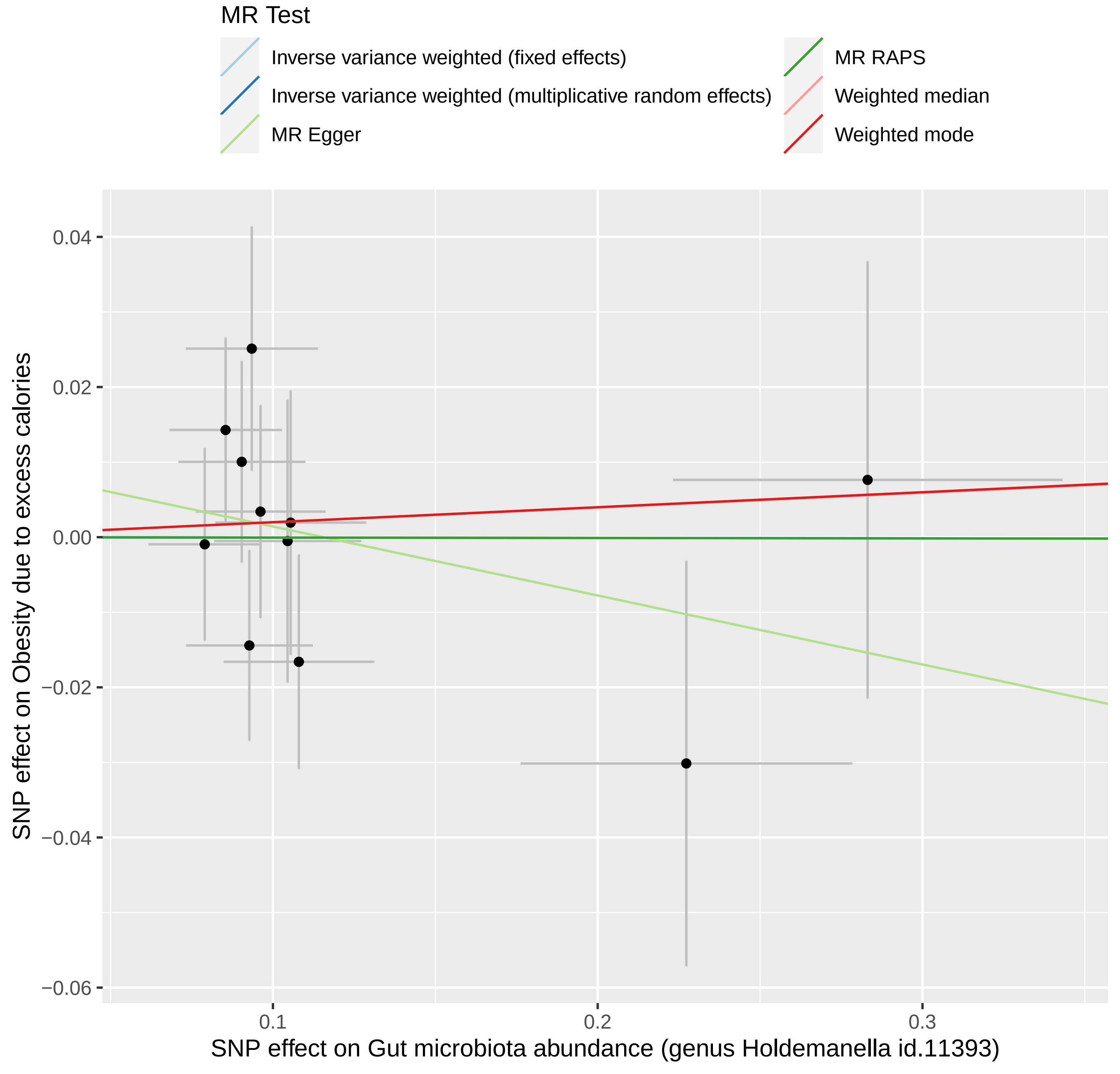

Batch 389 : Gut microbiota abundance (genus Holdemania id.2157) on Obesity due to excess calories

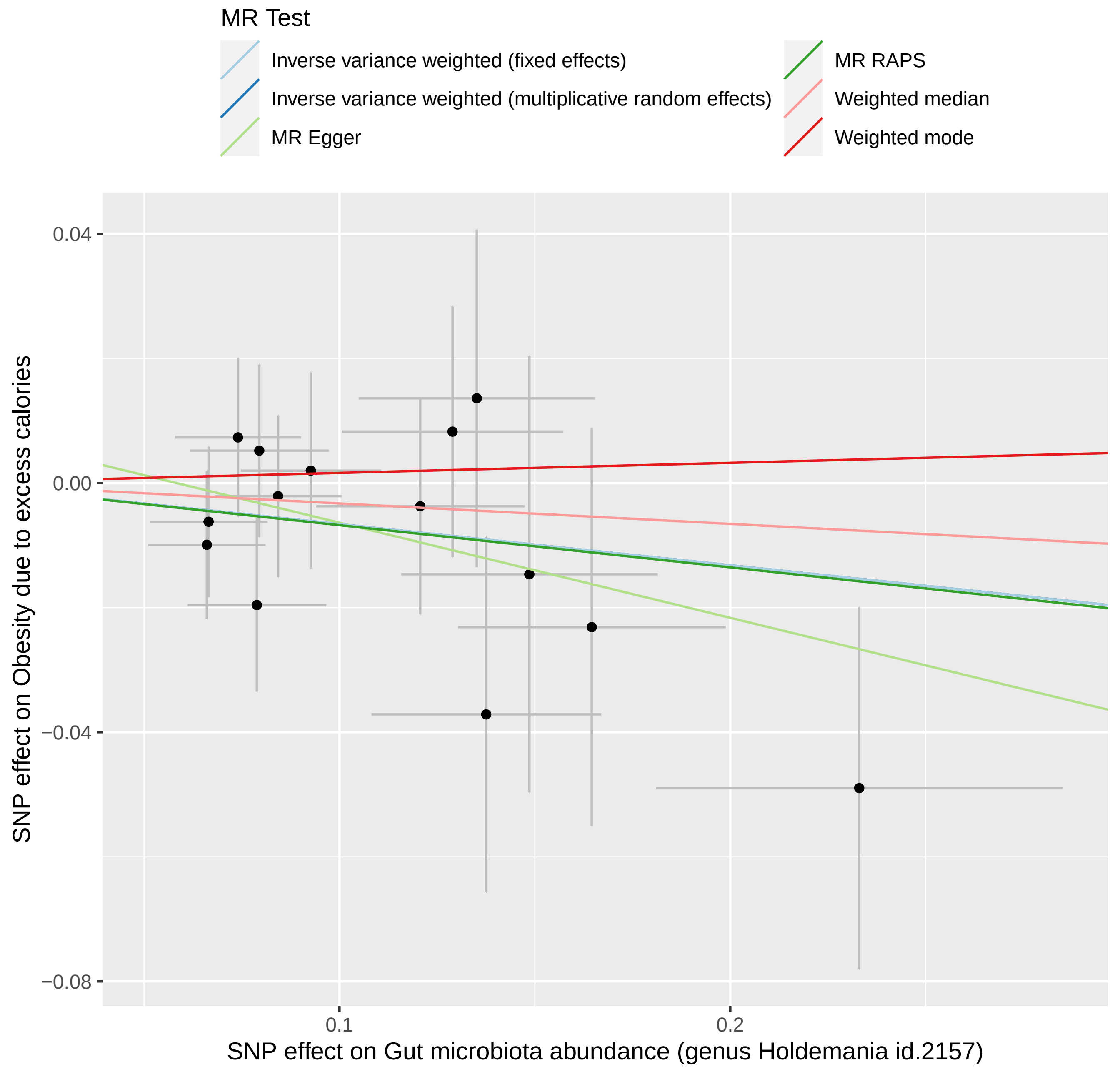

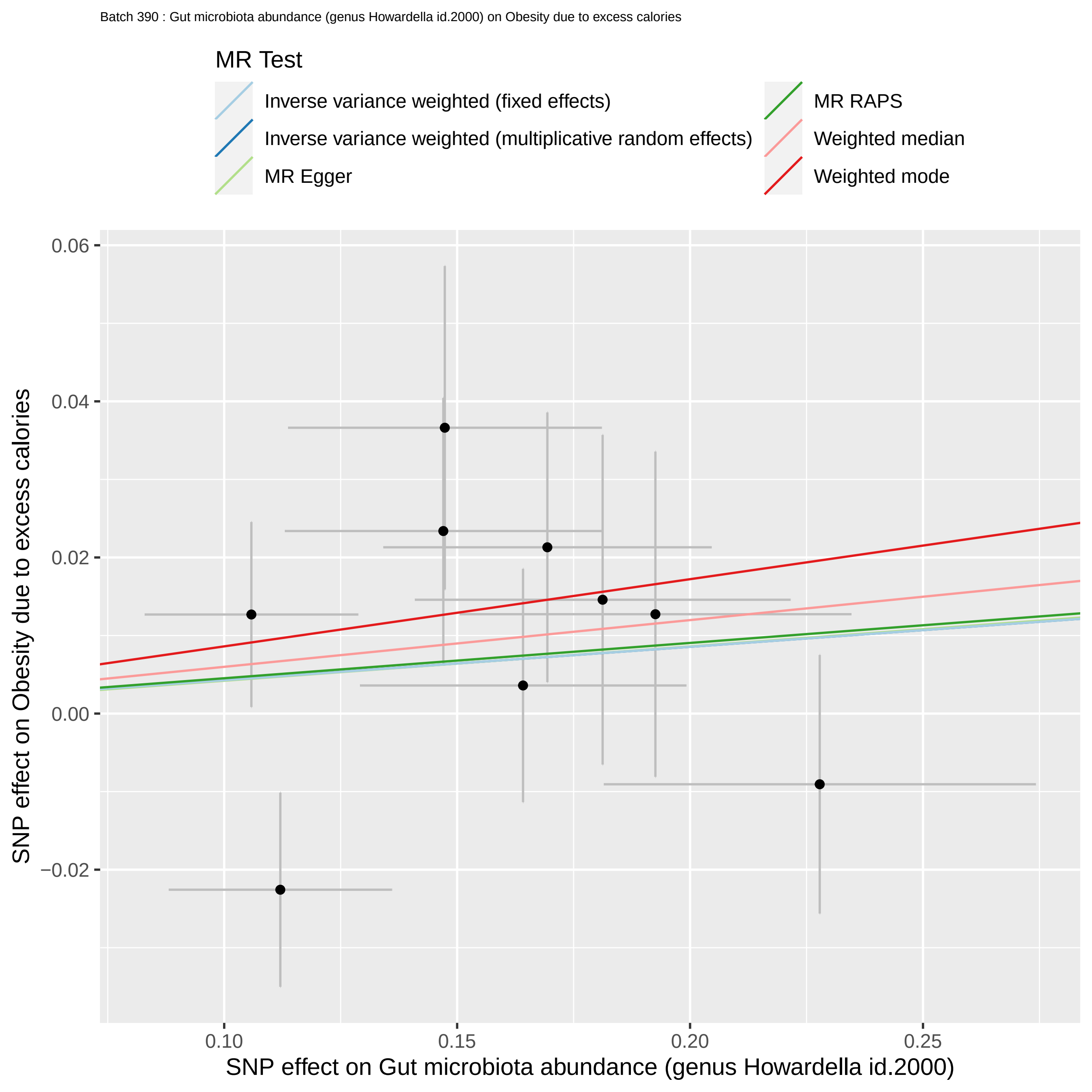
Batch 390 : Gut microbiota abundance (genus Howardella id.2000) on Obesity due to excess calories
MR Test
Inverse variance weighted (fixed effects)
Inverse variance weighted (multiplicative random effects)
MR Egger
MR RAPS
Weighted median
Weighted mode
0.06
0.04
0.02
0.00
−0.02
0.10
0.15
0.20
0.25
SNP effect on Obesity due to excess calories
SNP effect on Gut microbiota abundance (genus Howardella id.2000)

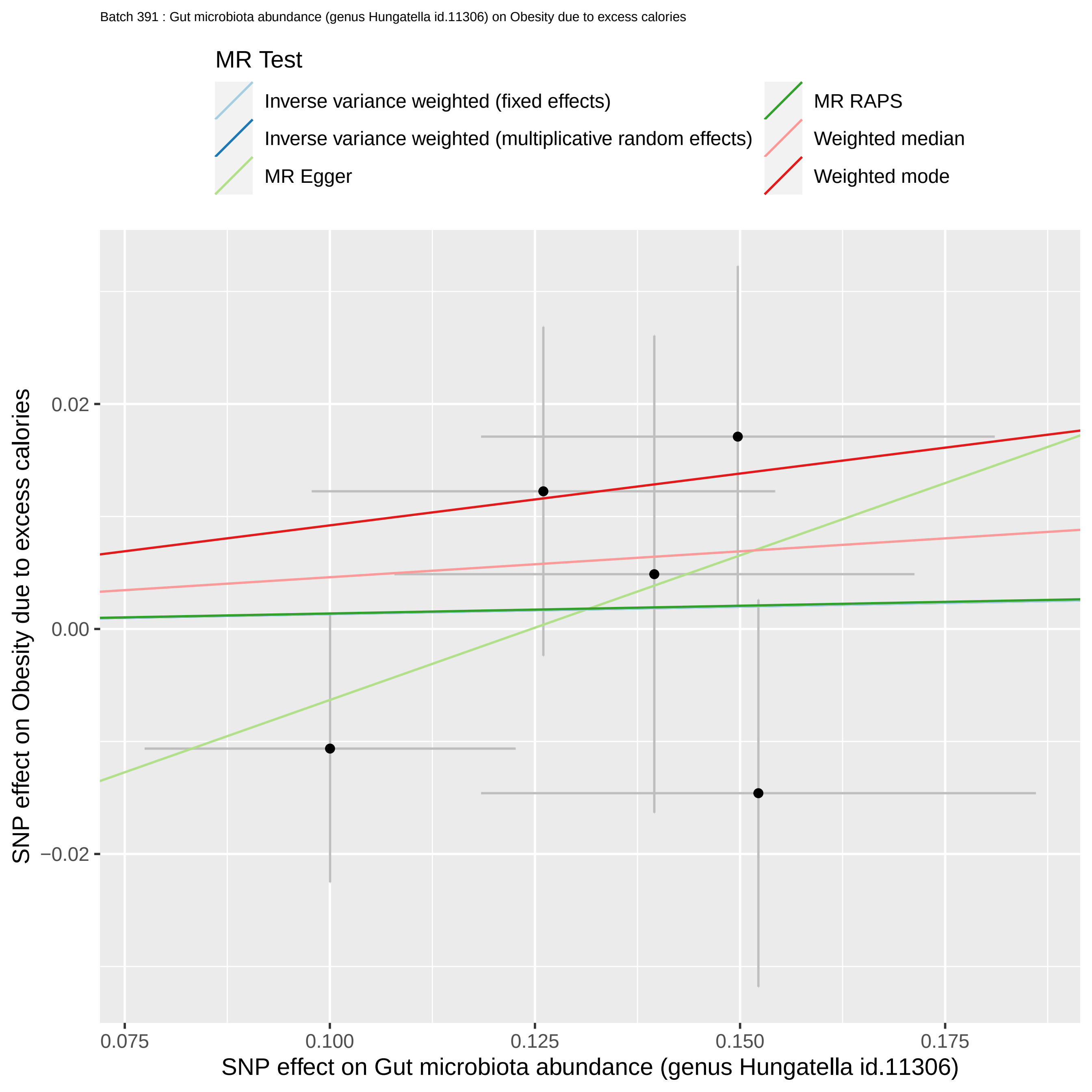
Batch 391 : Gut microbiota abundance (genus Hungatella id.11306) on Obesity due to excess calories
MR Test
Inverse variance weighted (fixed effects)
Inverse variance weighted (multiplicative random effects)
MR Egger
MR RAPS
Weighted median
Weighted mode
0.02
0.00
−0.02
0.075
0.100
0.125
0.150
0.175
SNP effect on Obesity due to excess calories
SNP effect on Gut microbiota abundance (genus Hungatella id.11306)

Batch 392 : Gut microbiota abundance (genus Intestinibacter id.11345) on Obesity due to excess calories

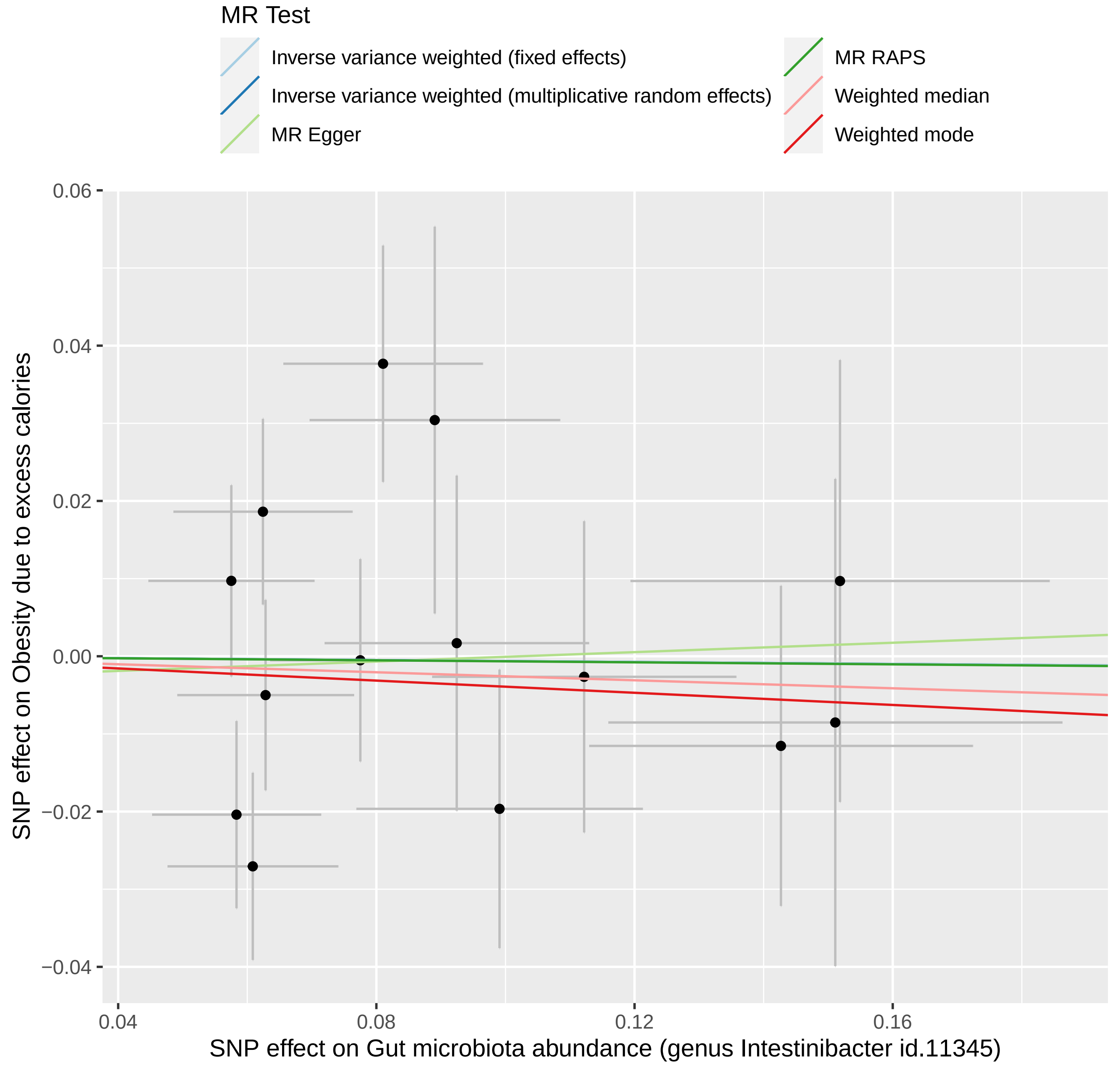

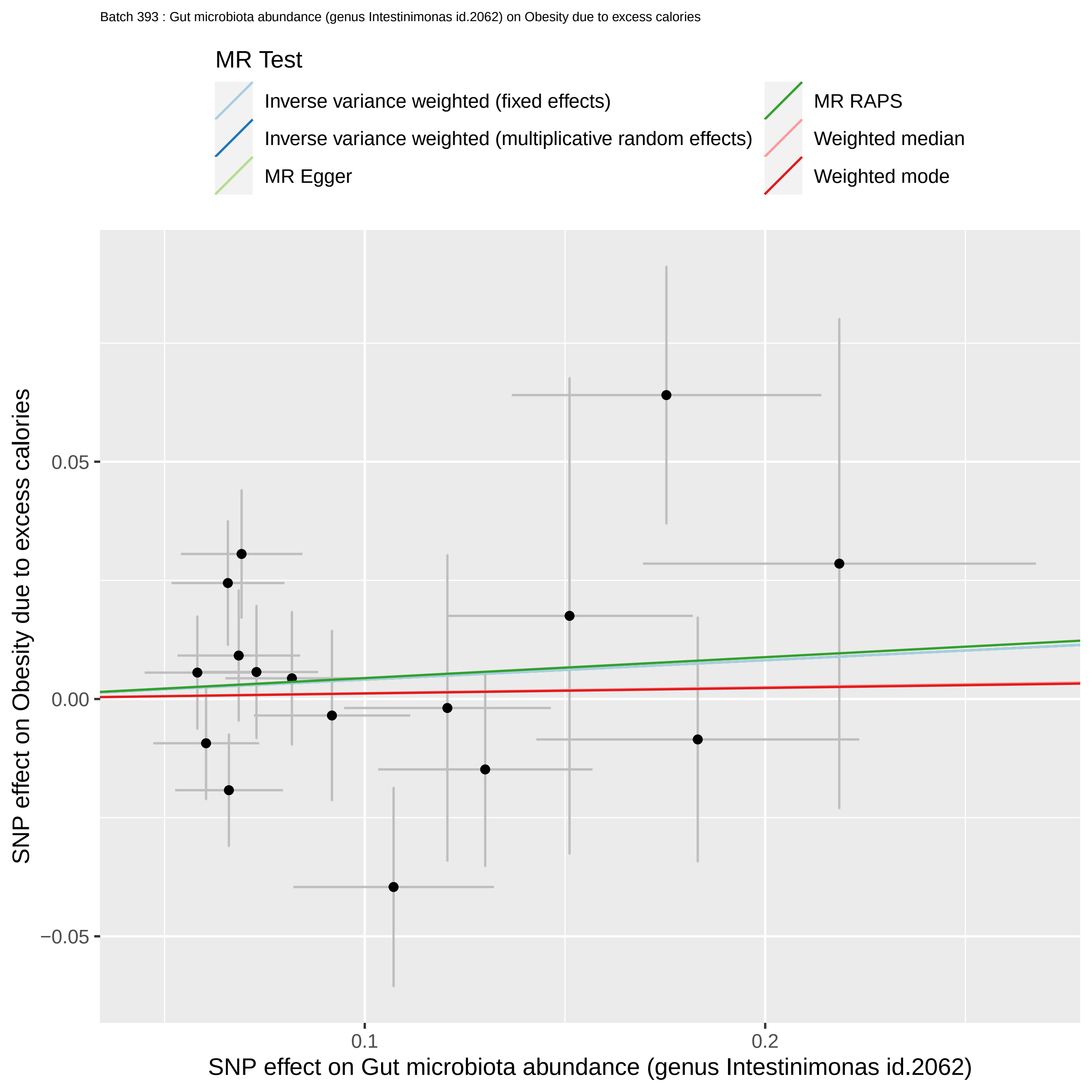
Batch 393 : Gut microbiota abundance (genus Intestinimonas id.2062) on Obesity due to excess calories
MR Test
Inverse variance weighted (fixed effects)
Inverse variance weighted (multiplicative random effects)
MR Egger
MR RAPS
Weighted median
Weighted mode
SNP effect on Obesity due to excess calories
0.05
0.00
−0.05
0.1
0.2
SNP effect on Gut microbiota abundance (genus Intestinimonas id.2062)

Batch 394 : Gut microbiota abundance (genus Lachnoclostridium id.11308) on Obesity due to excess calories

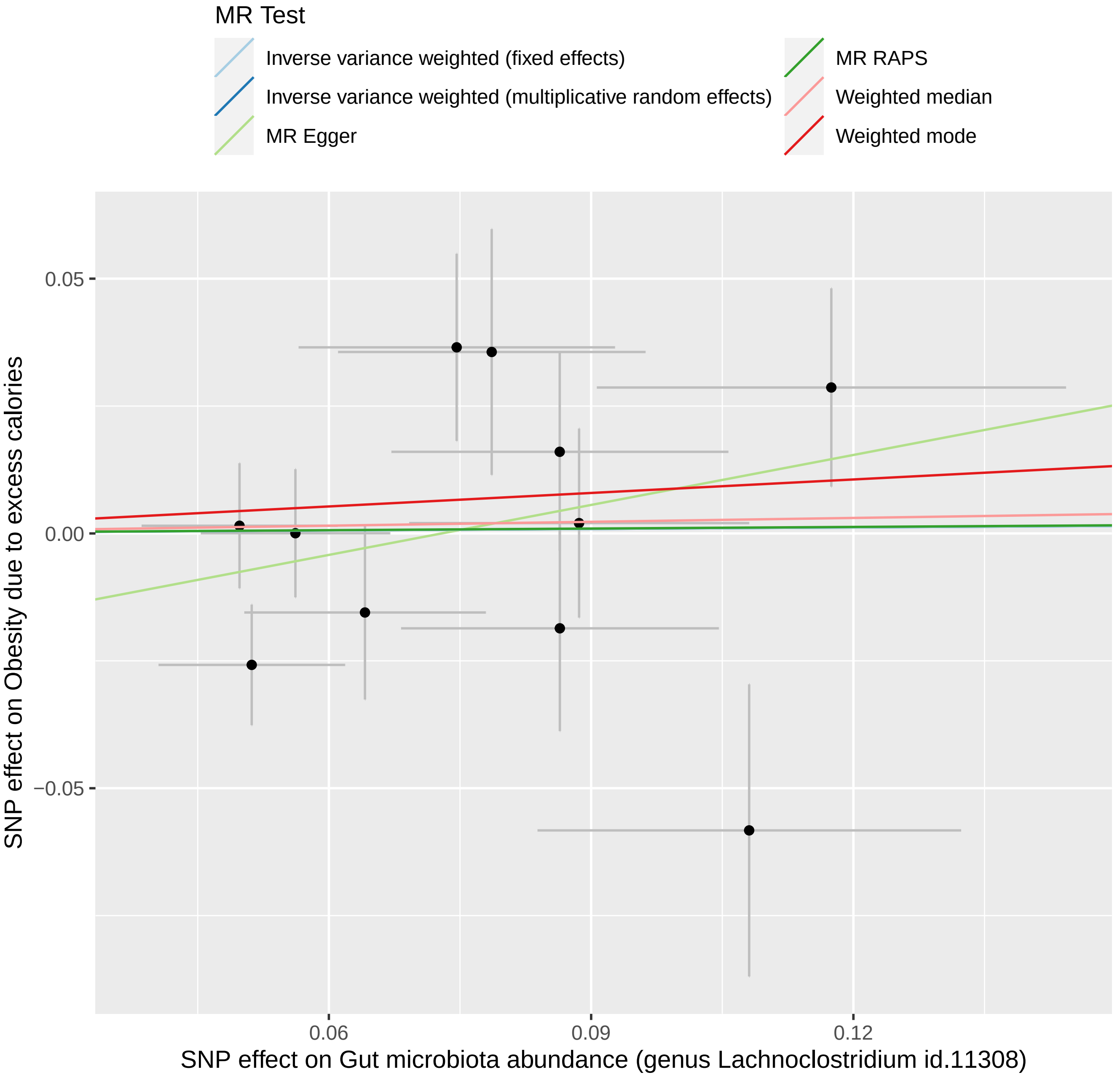

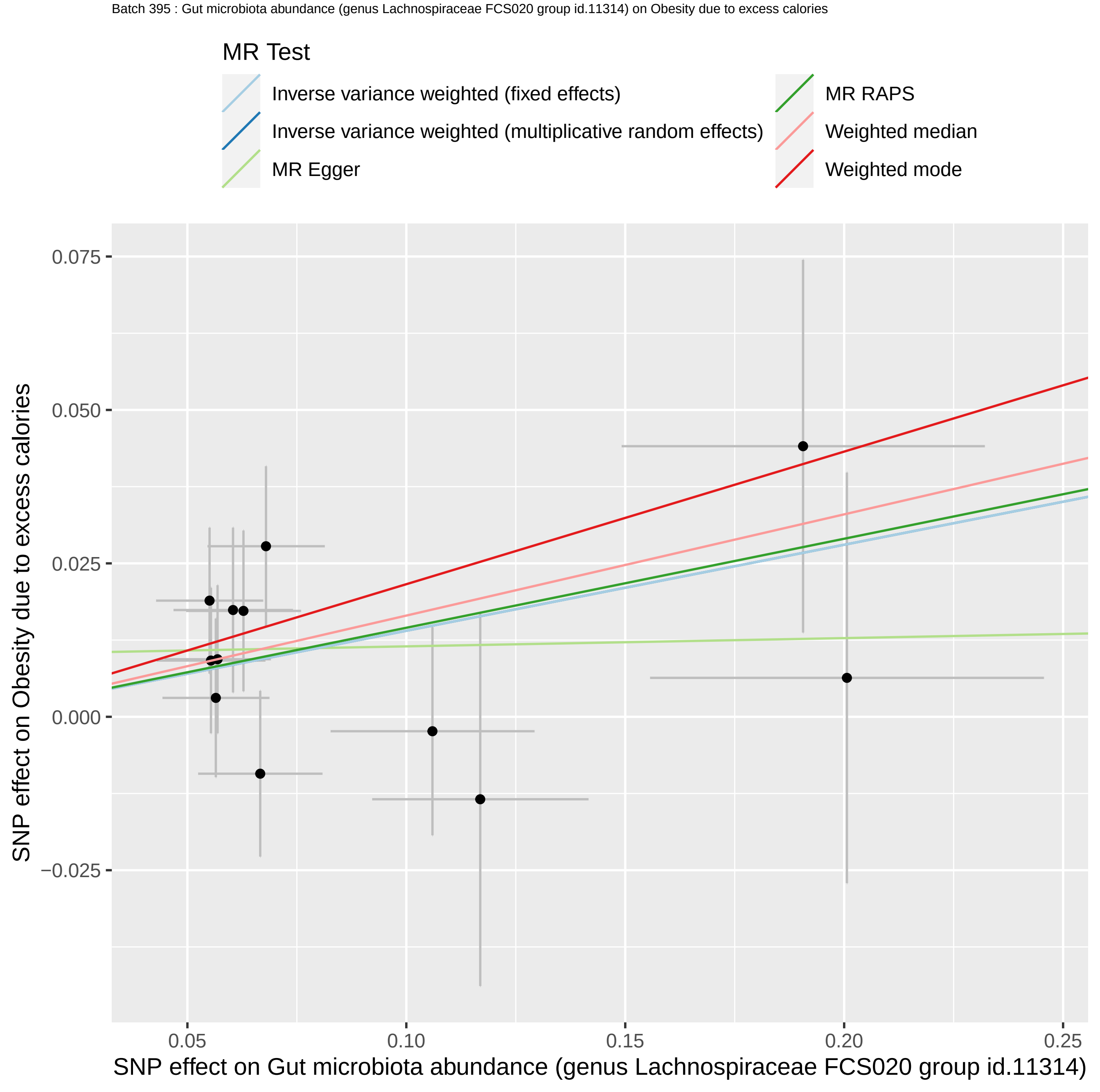

Batch 395 : Gut microbiota abundance (genus Lachnospiraceae FCS020 group id.11314) on Obesity due to excess calories
MR Test
Inverse variance weighted (fixed effects)
Inverse variance weighted (multiplicative random effects)
MR Egger
MR RAPS
Weighted median
Weighted mode
0.075
0.050
0.025
0.000
−0.025
0.05
0.10
0.15
0.20
0.25
SNP effect on Obesity due to excess calories
SNP effect on Gut microbiota abundance (genus Lachnospiraceae FCS020 group id.11314)

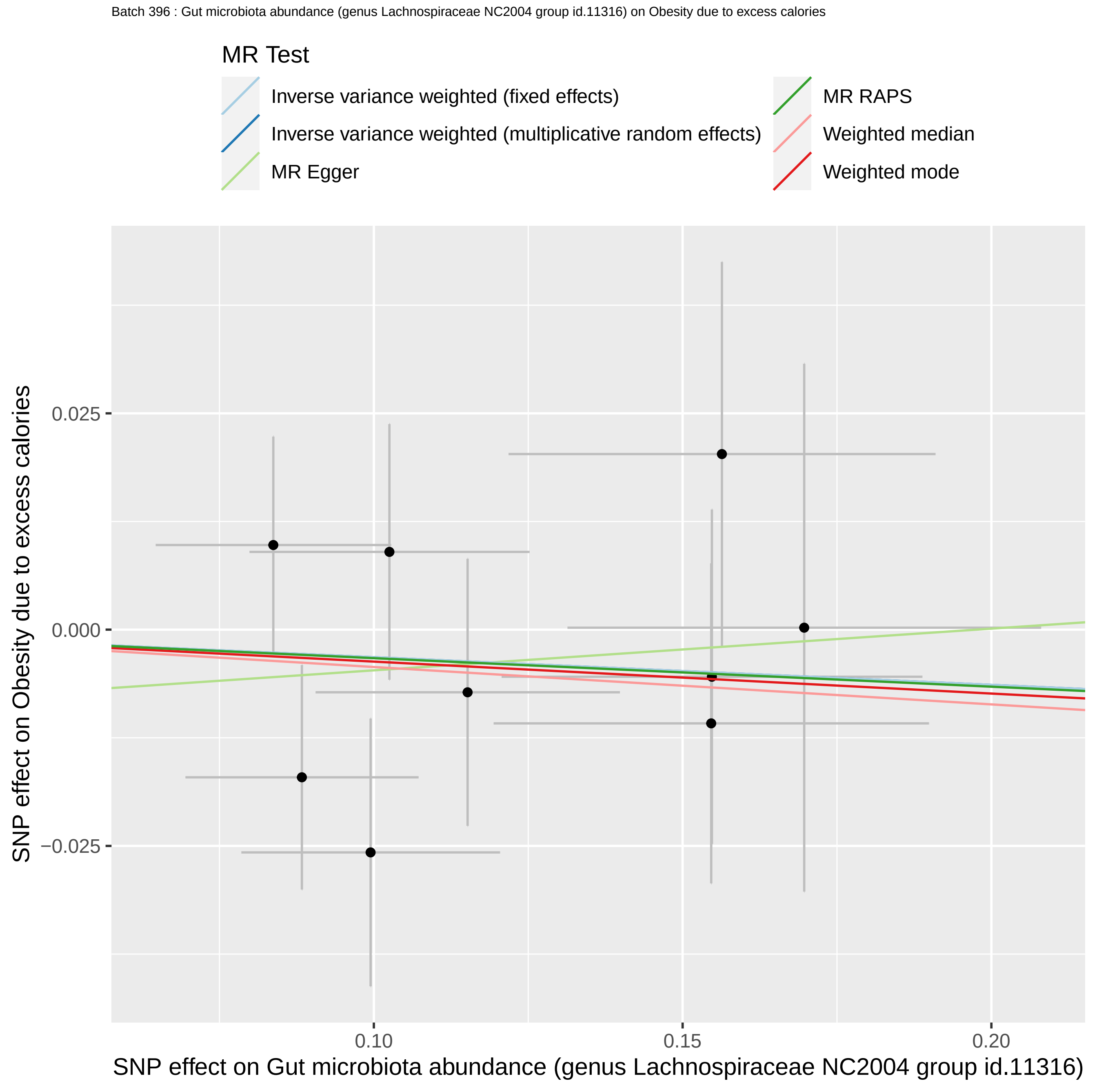

Batch 396 : Gut microbiota abundance (genus Lachnospiraceae NC2004 group id.11316) on Obesity due to excess calories
MR Test
Inverse variance weighted (fixed effects)
Inverse variance weighted (multiplicative random effects)
MR Egger
MR RAPS
Weighted median
Weighted mode
SNP effect on Obesity due to excess calories
0.025
0.000
−0.025
0.10
0.15
0.20
SNP effect on Gut microbiota abundance (genus Lachnospiraceae NC2004 group id.11316)

Batch 397 : Gut microbiota abundance (genus Lachnospiraceae ND3007 group id.11317) on Obesity due to excess calories

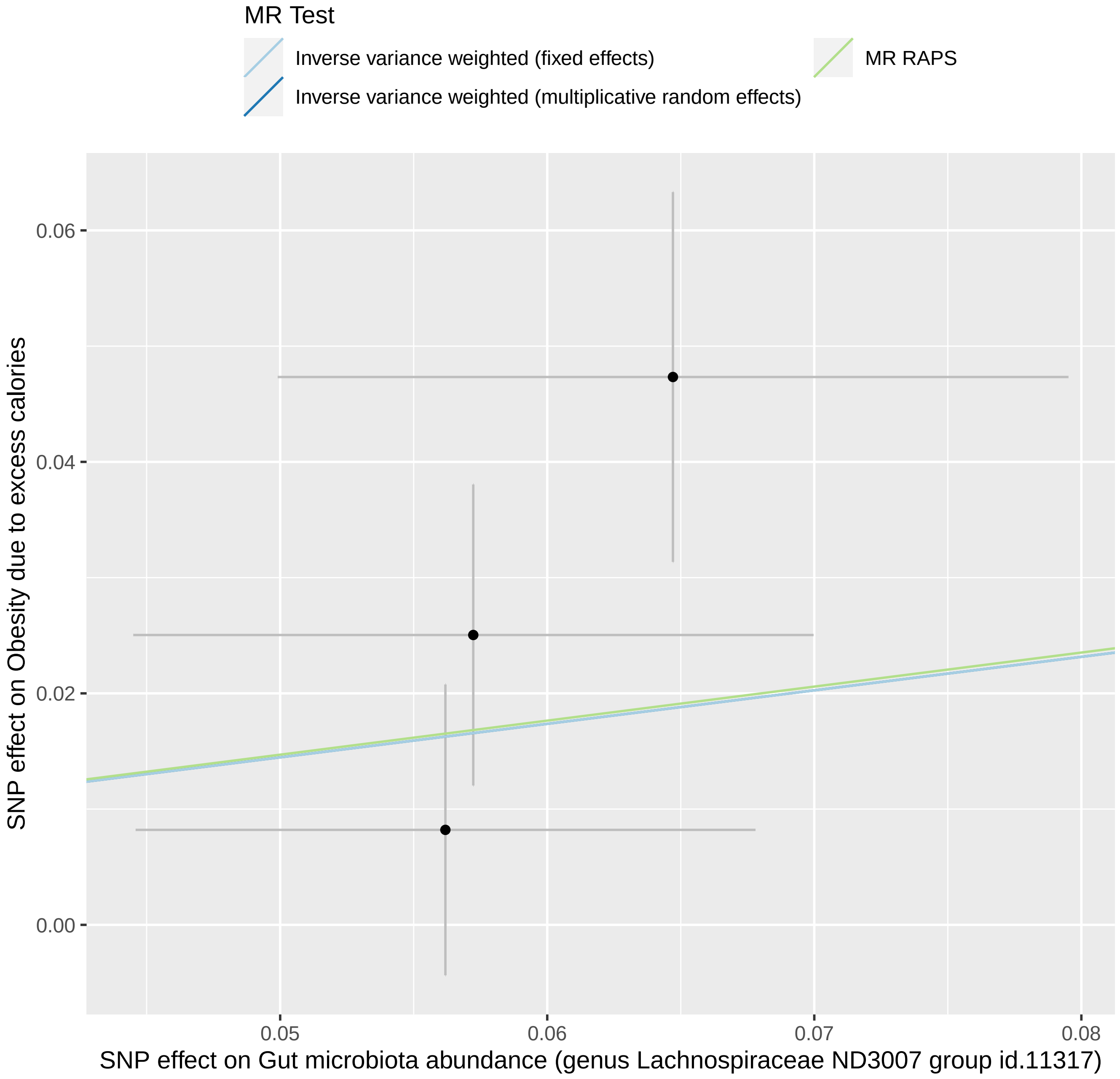

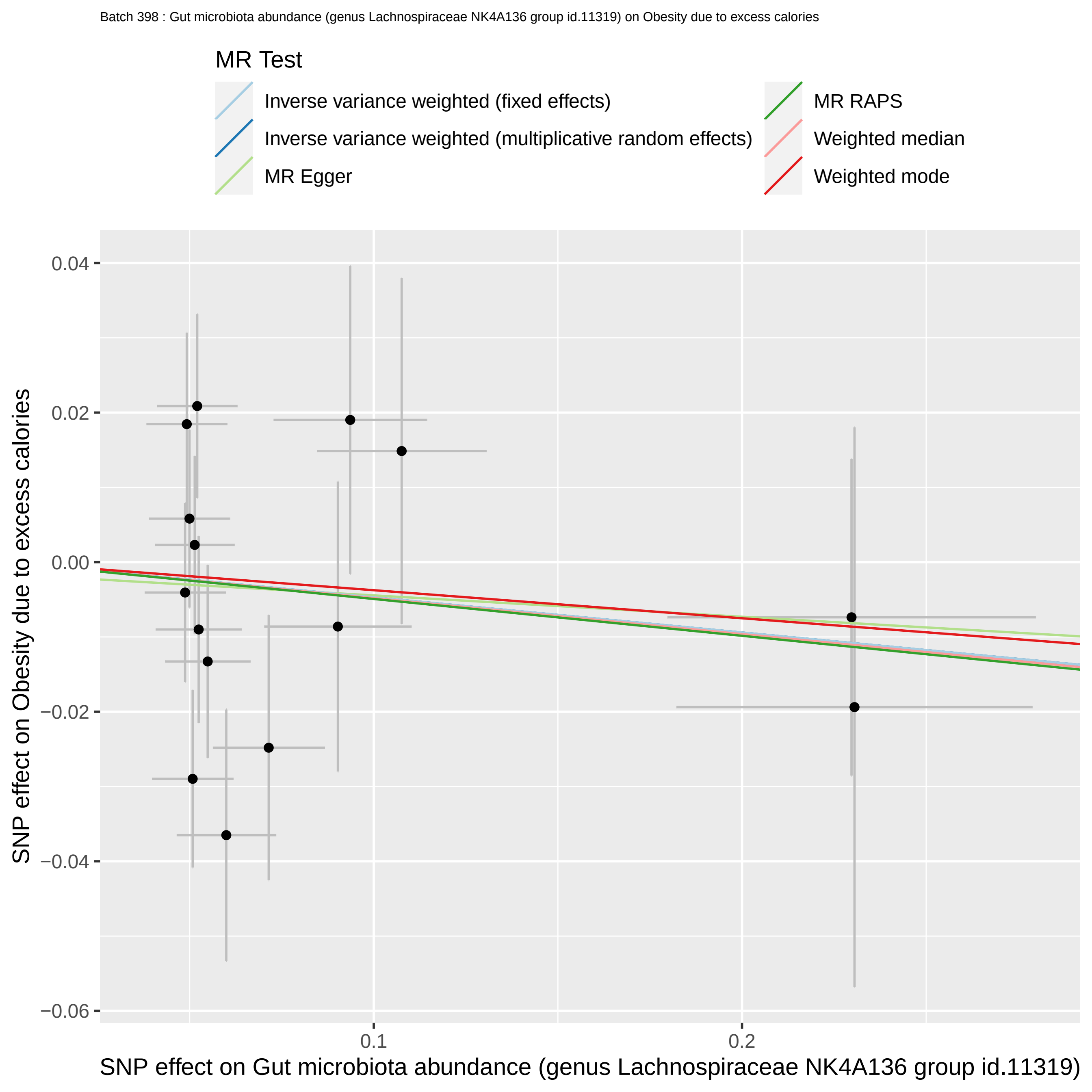

Batch 398 : Gut microbiota abundance (genus Lachnospiraceae NK4A136 group id.11319) on Obesity due to excess calories
MR Test
Inverse variance weighted (fixed effects)
Inverse variance weighted (multiplicative random effects)
MR Egger
MR RAPS
Weighted median
Weighted mode
0.04
0.02
0.00
−0.02
−0.04
−0.06
0.1
0.2
SNP effect on Obesity due to excess calories
SNP effect on Gut microbiota abundance (genus Lachnospiraceae NK4A136 group id.11319)

Batch 399 : Gut microbiota abundance (genus Lachnospiraceae UCG001 id.11321) on Obesity due to excess calories

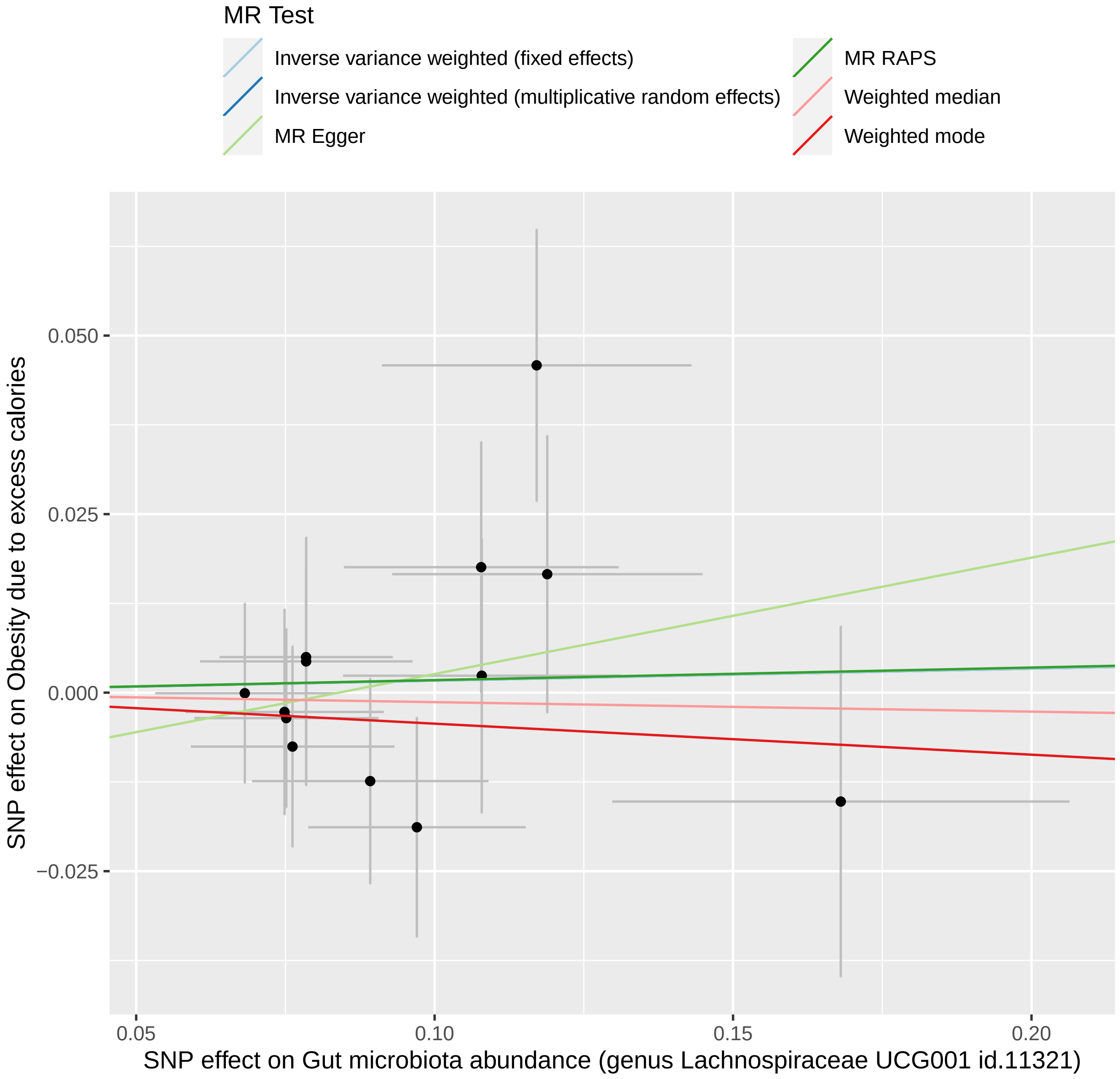

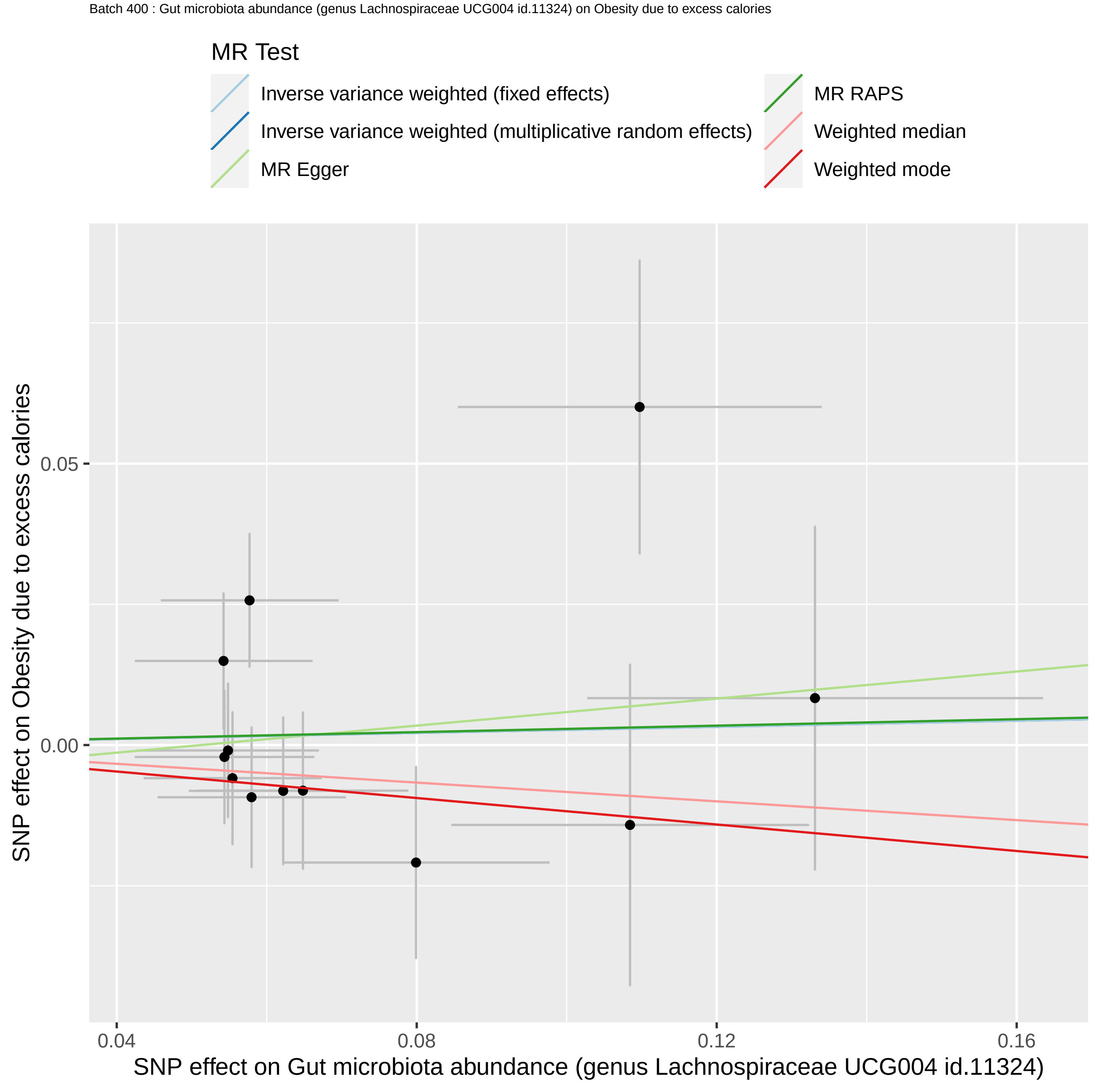

Batch 400 : Gut microbiota abundance (genus Lachnospiraceae UCG004 id.11324) on Obesity due to excess calories
MR Test
Inverse variance weighted (fixed effects)
Inverse variance weighted (multiplicative random effects)
MR Egger
MR RAPS
Weighted median
Weighted mode
0.05
0.00
0.04
0.08
0.12
0.16
SNP effect on Obesity due to excess calories
SNP effect on Gut microbiota abundance (genus Lachnospiraceae UCG004 id.11324)

Batch 401 : Gut microbiota abundance (genus Lachnospiraceae UCG008 id.11328) on Obesity due to excess calories

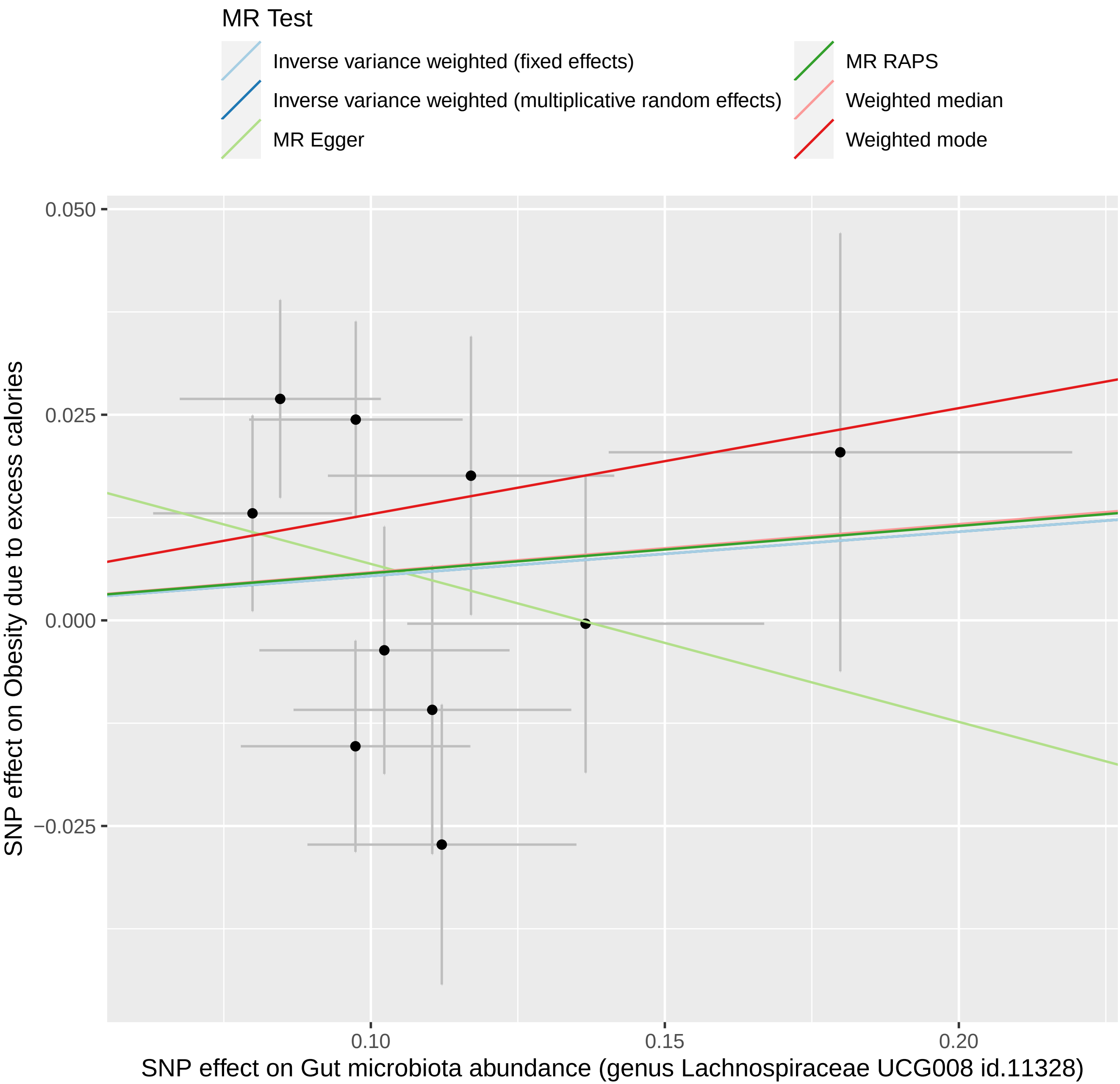

Batch 402 : Gut microbiota abundance (genus Lachnospiraceae UCG010 id.11330) on Obesity due to excess calories

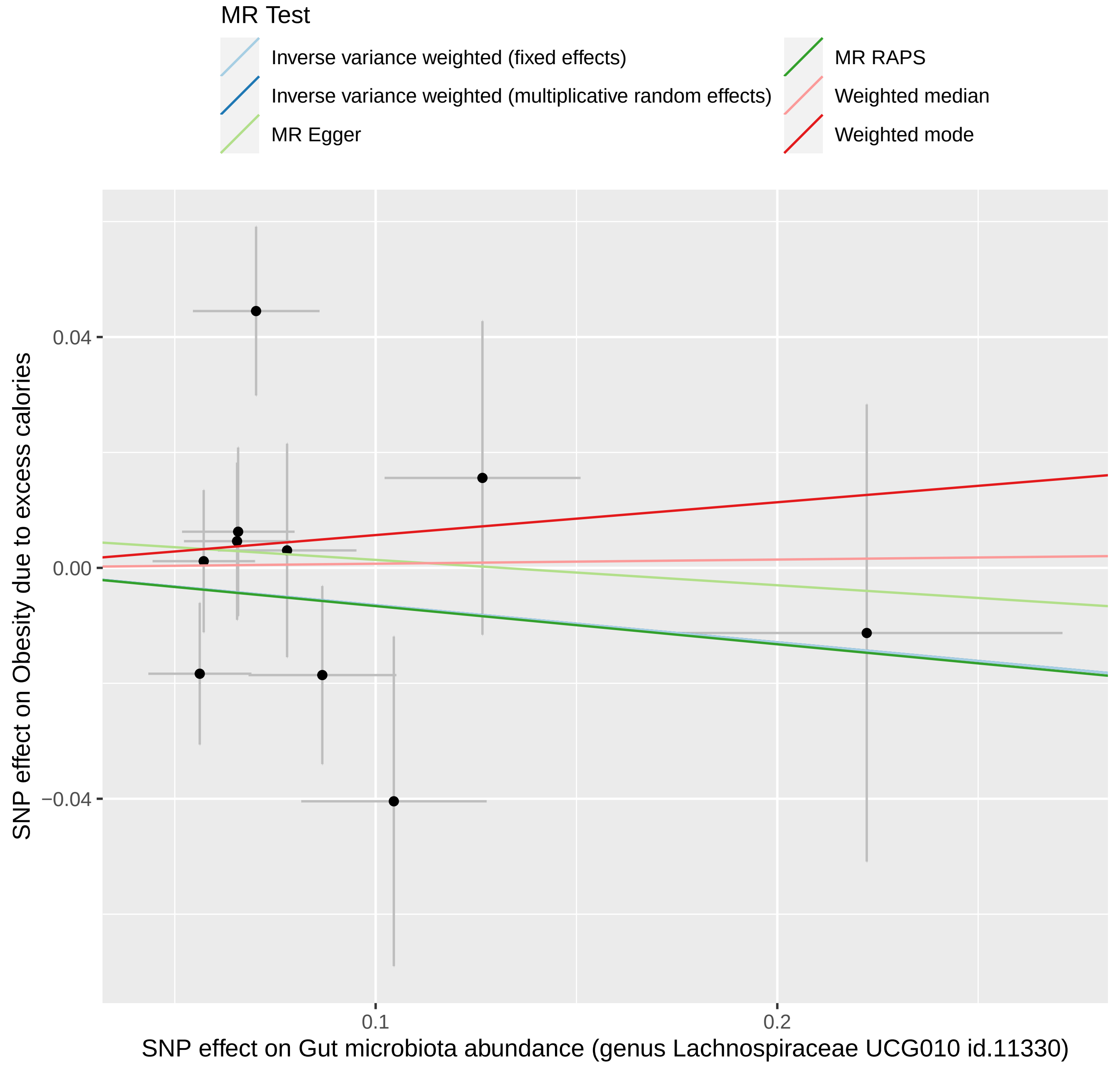

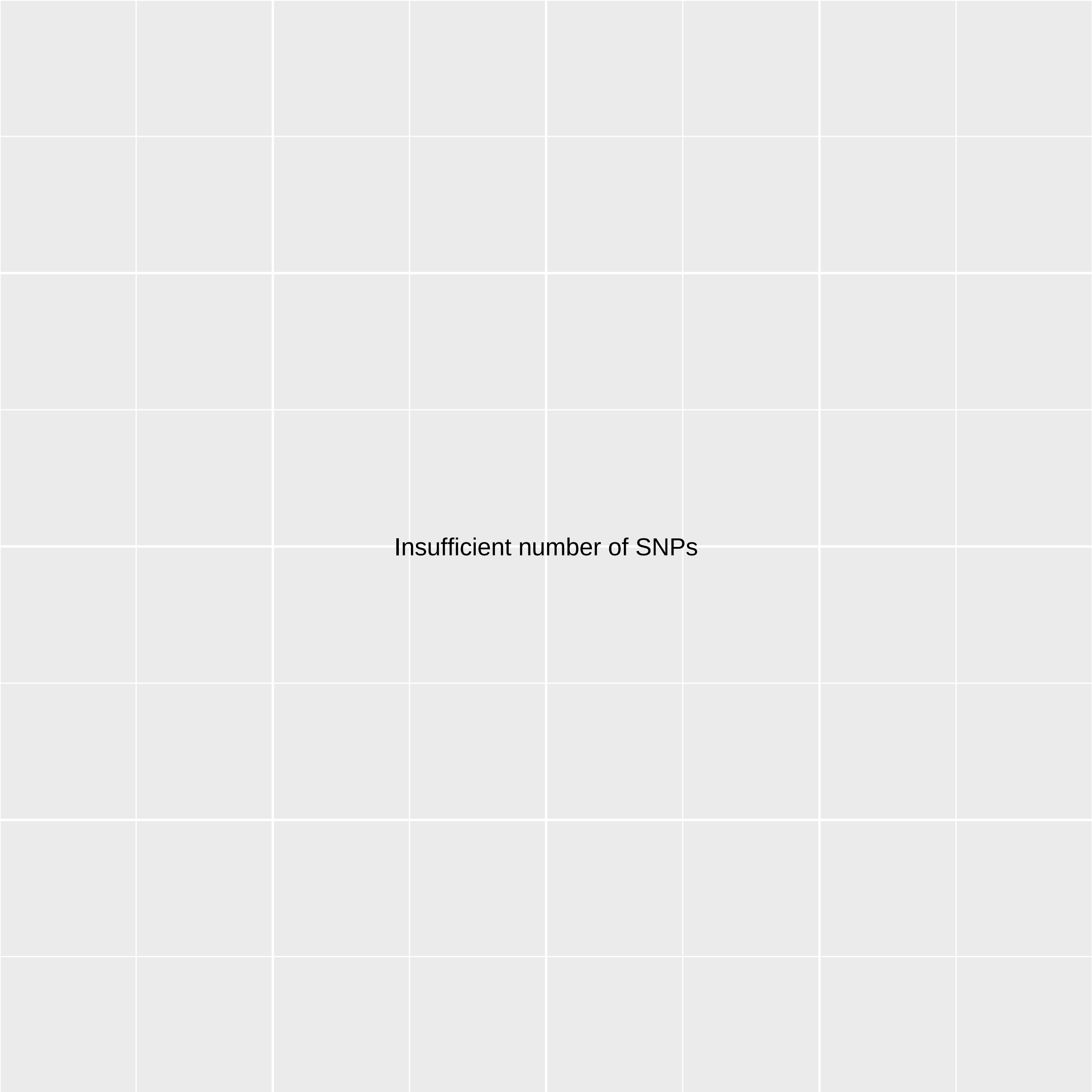
Insufficient number of SNPs

Batch 404 : Gut microbiota abundance (genus Lactobacillus id.1837) on Obesity due to excess calories

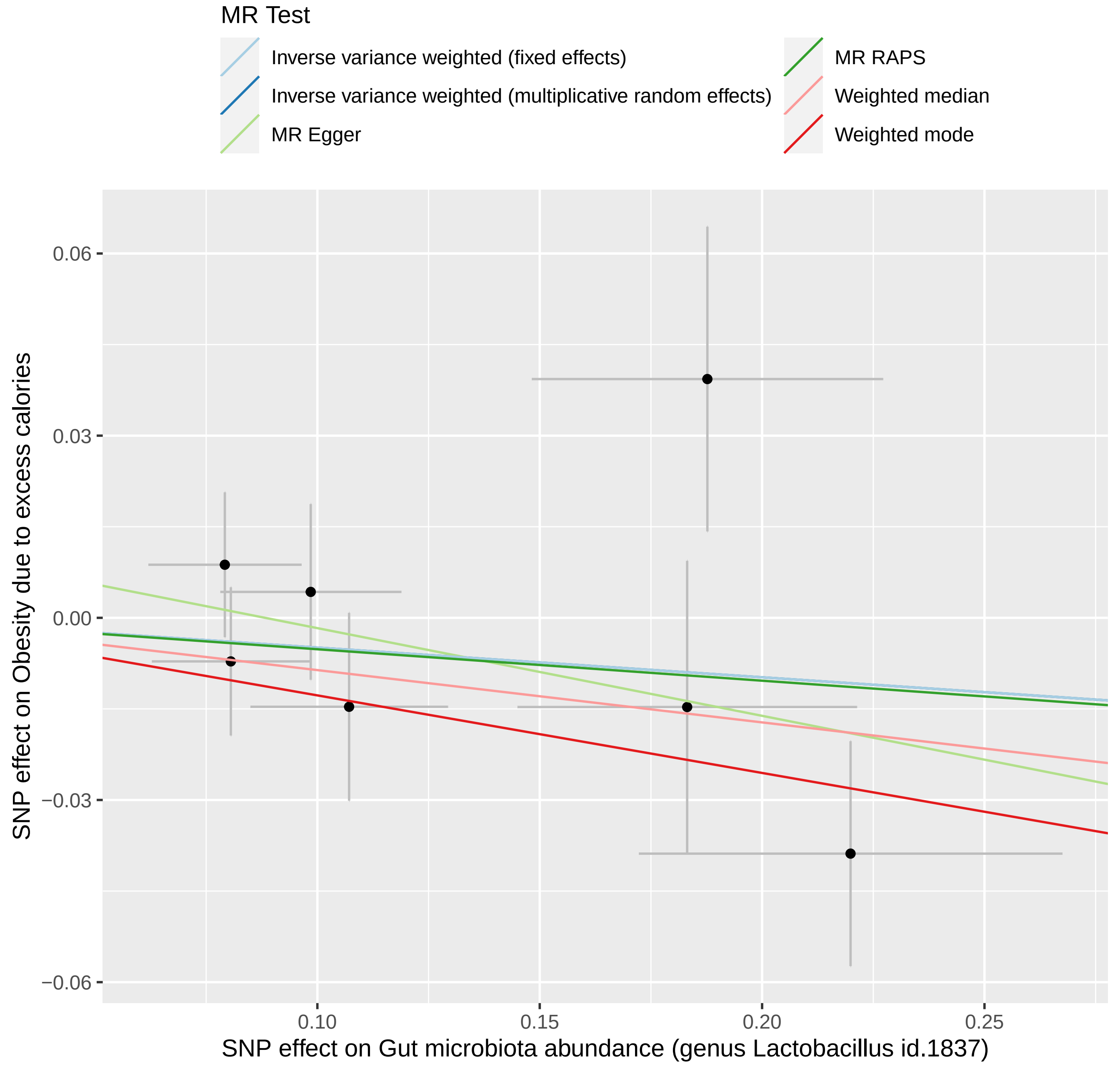

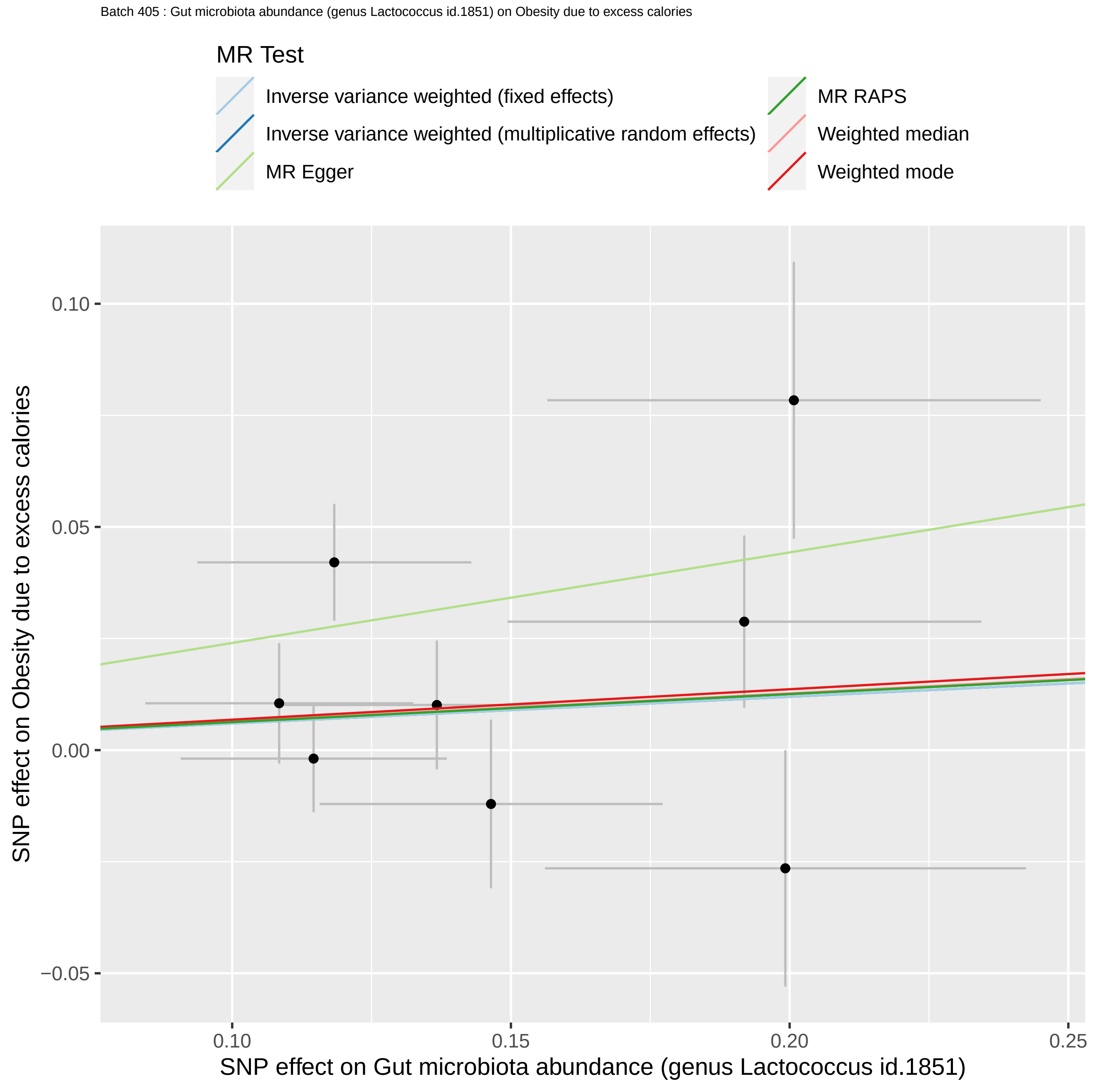
Batch 405 : Gut microbiota abundance (genus Lactococcus id.1851) on Obesity due to excess calories
MR Test
Inverse variance weighted (fixed effects)
Inverse variance weighted (multiplicative random effects)
MR Egger
MR RAPS
Weighted median
Weighted mode
SNP effect on Obesity due to excess calories
0.10
0.05
0.00
−0.05
0.10
0.15
0.20
0.25
SNP effect on Gut microbiota abundance (genus Lactococcus id.1851)

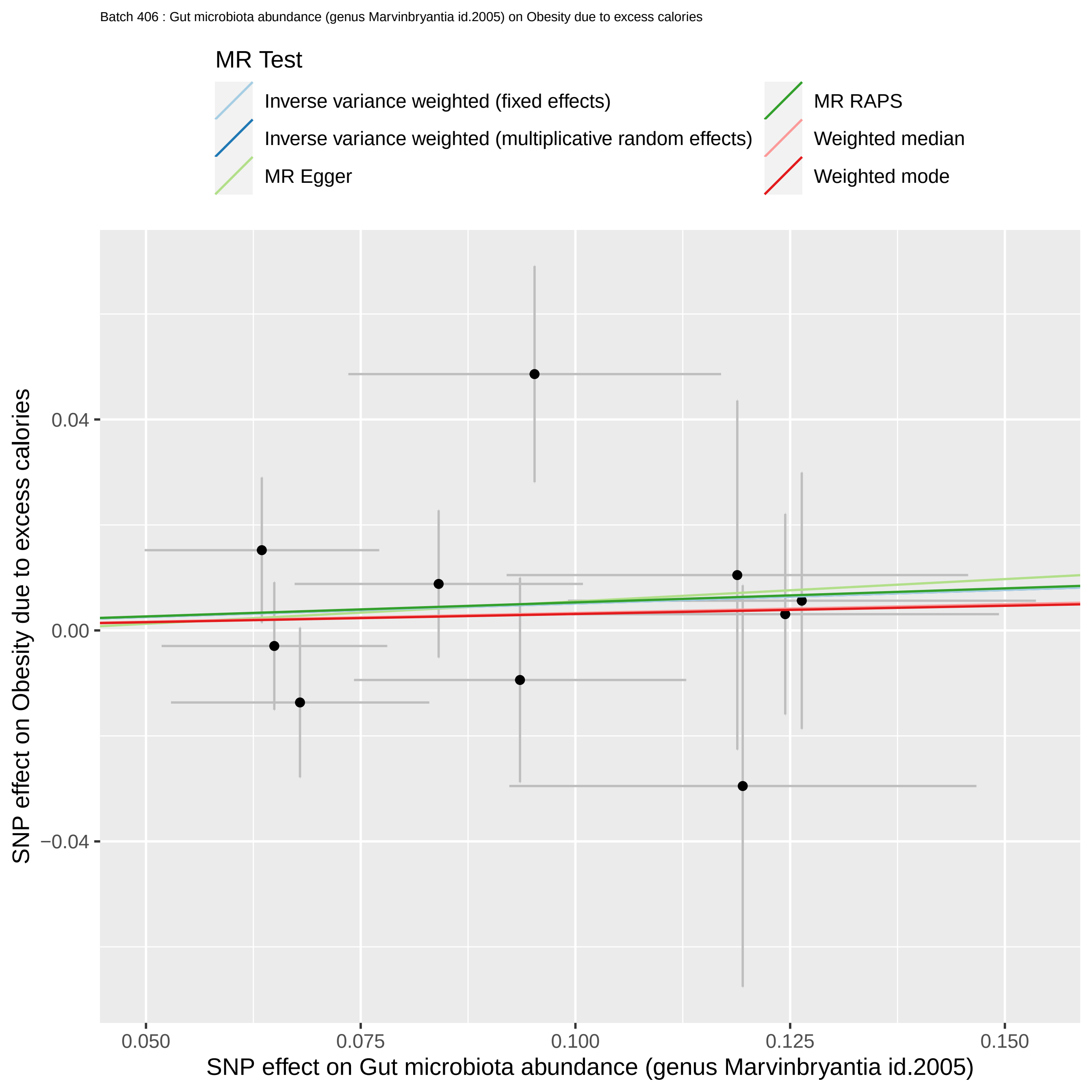
Batch 406 : Gut microbiota abundance (genus Marvinbryantia id.2005) on Obesity due to excess calories
MR Test
Inverse variance weighted (fixed effects)
Inverse variance weighted (multiplicative random effects)
MR Egger
MR RAPS
Weighted median
Weighted mode
0.04
0.00
−0.04
0.050
0.075
0.100
0.125
0.150
SNP effect on Obesity due to excess calories
SNP effect on Gut microbiota abundance (genus Marvinbryantia id.2005)

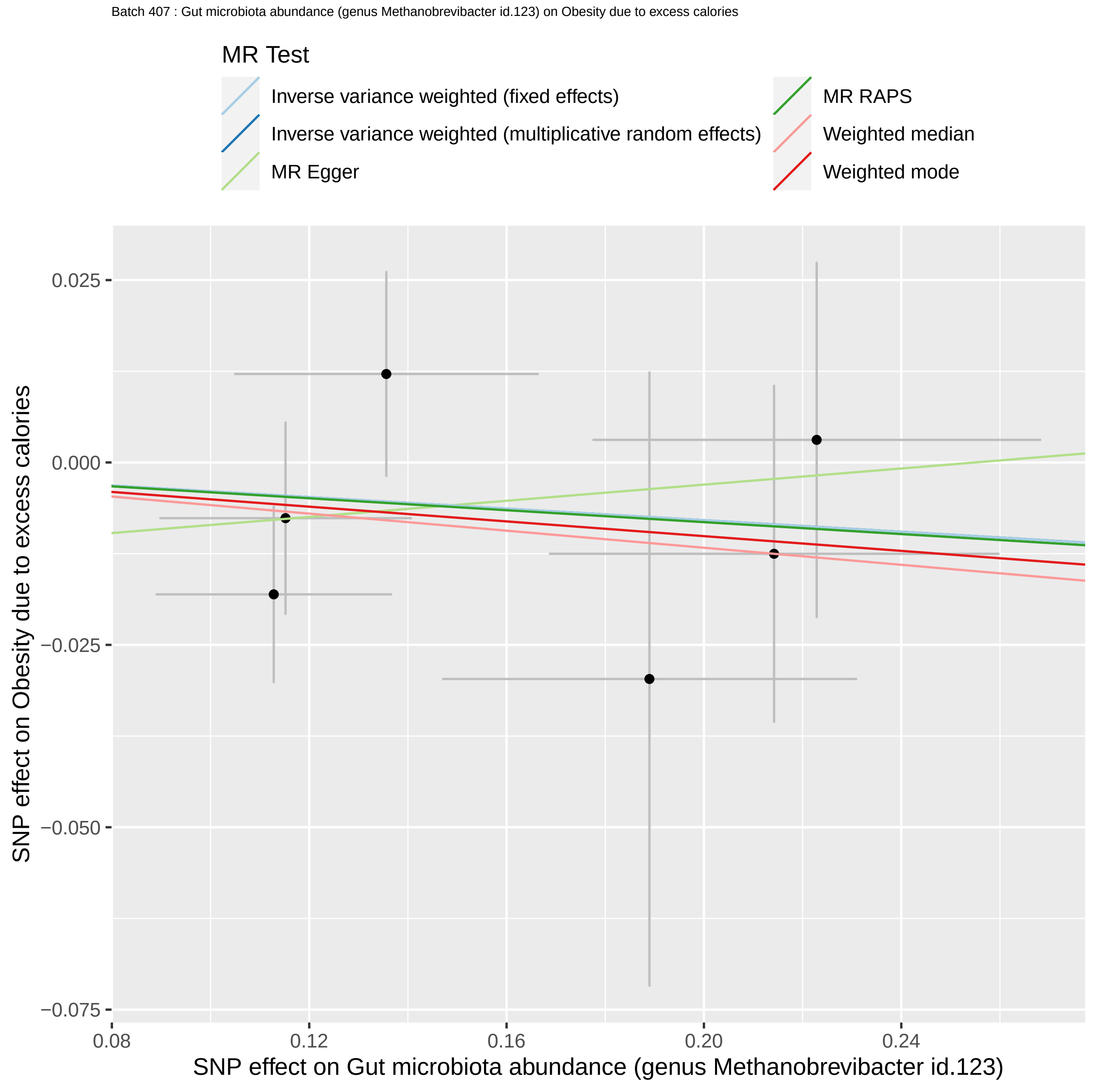

Batch 407 : Gut microbiota abundance (genus Methanobrevibacter id.123) on Obesity due to excess calories
MR Test
Inverse variance weighted (fixed effects)
Inverse variance weighted (multiplicative random effects)
MR Egger
MR RAPS
Weighted median
Weighted mode
0.025
0.000
−0.025
−0.050
−0.075
0.08
0.12
0.16
0.20
0.24
SNP effect on Obesity due to excess calories
SNP effect on Gut microbiota abundance (genus Methanobrevibacter id.123)

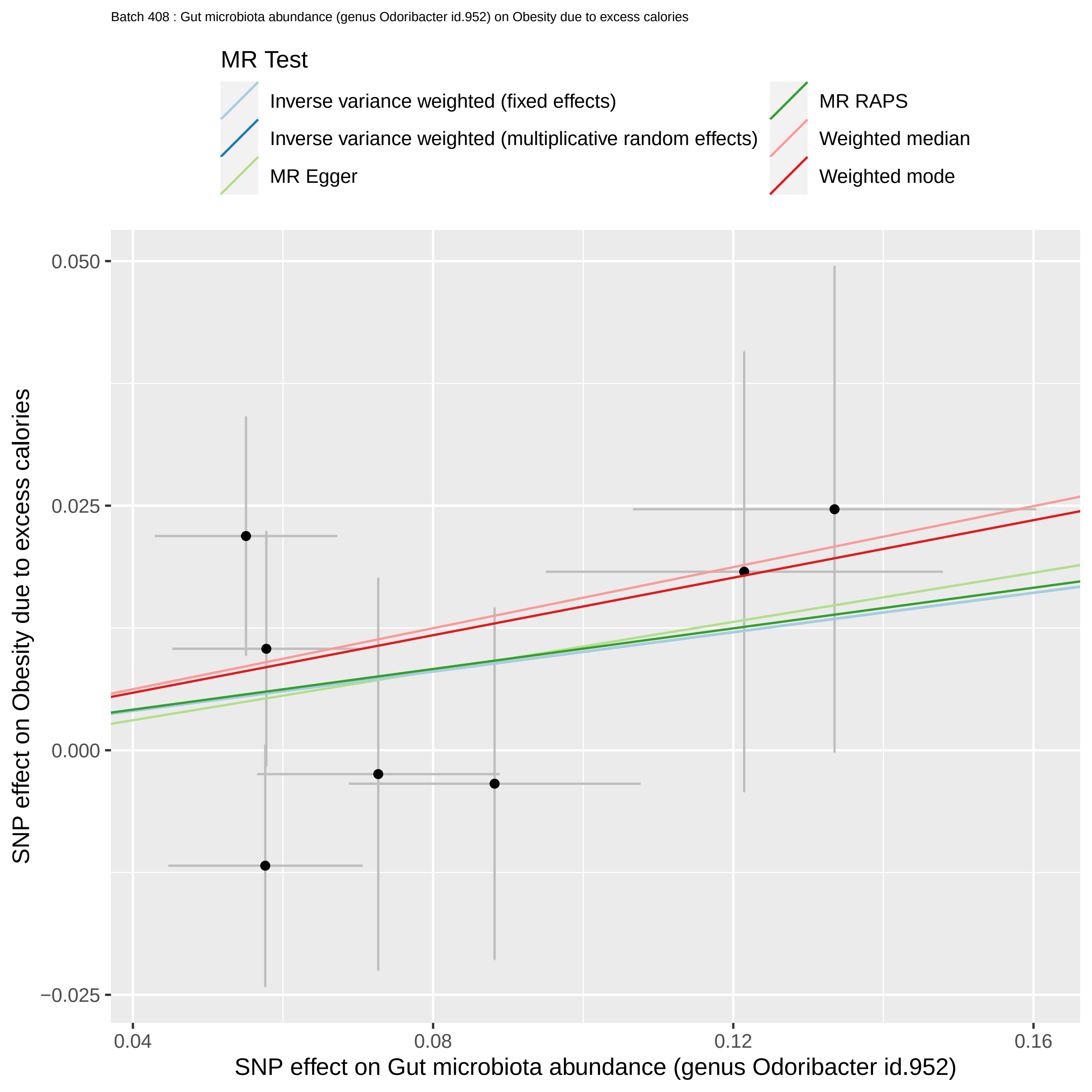
Batch 408 : Gut microbiota abundance (genus Odoribacter id.952) on Obesity due to excess calories
MR Test
Inverse variance weighted (fixed effects)
Inverse variance weighted (multiplicative random effects)
MR Egger
MR RAPS
Weighted median
Weighted mode
SNP effect on Obesity due to excess calories
0.050
0.025
0.000
−0.025
0.04
0.08
0.12
0.16
SNP effect on Gut microbiota abundance (genus Odoribacter id.952)

Batch 409 : Gut microbiota abundance (genus Olsenella id.822) on Obesity due to excess calories

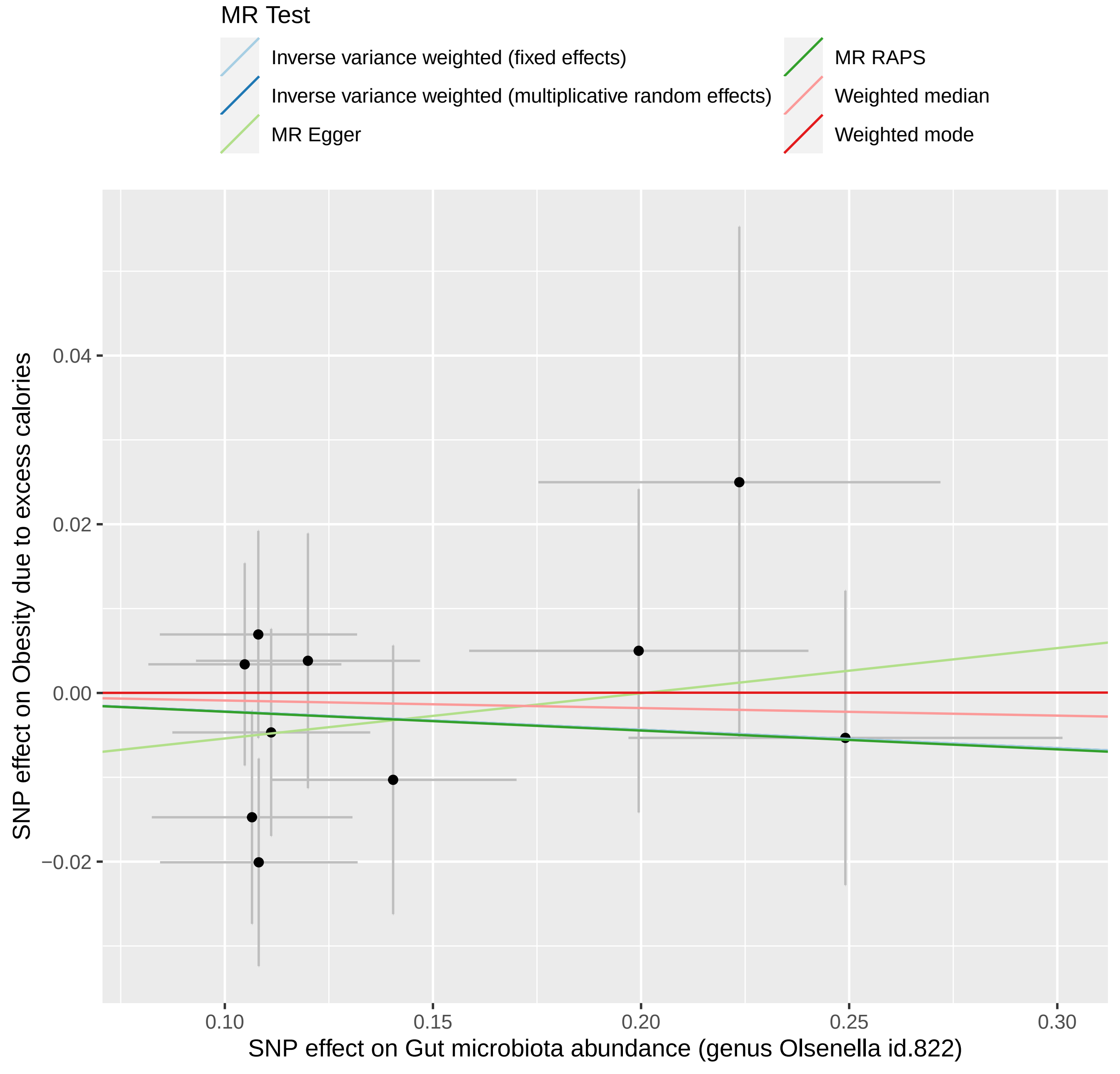

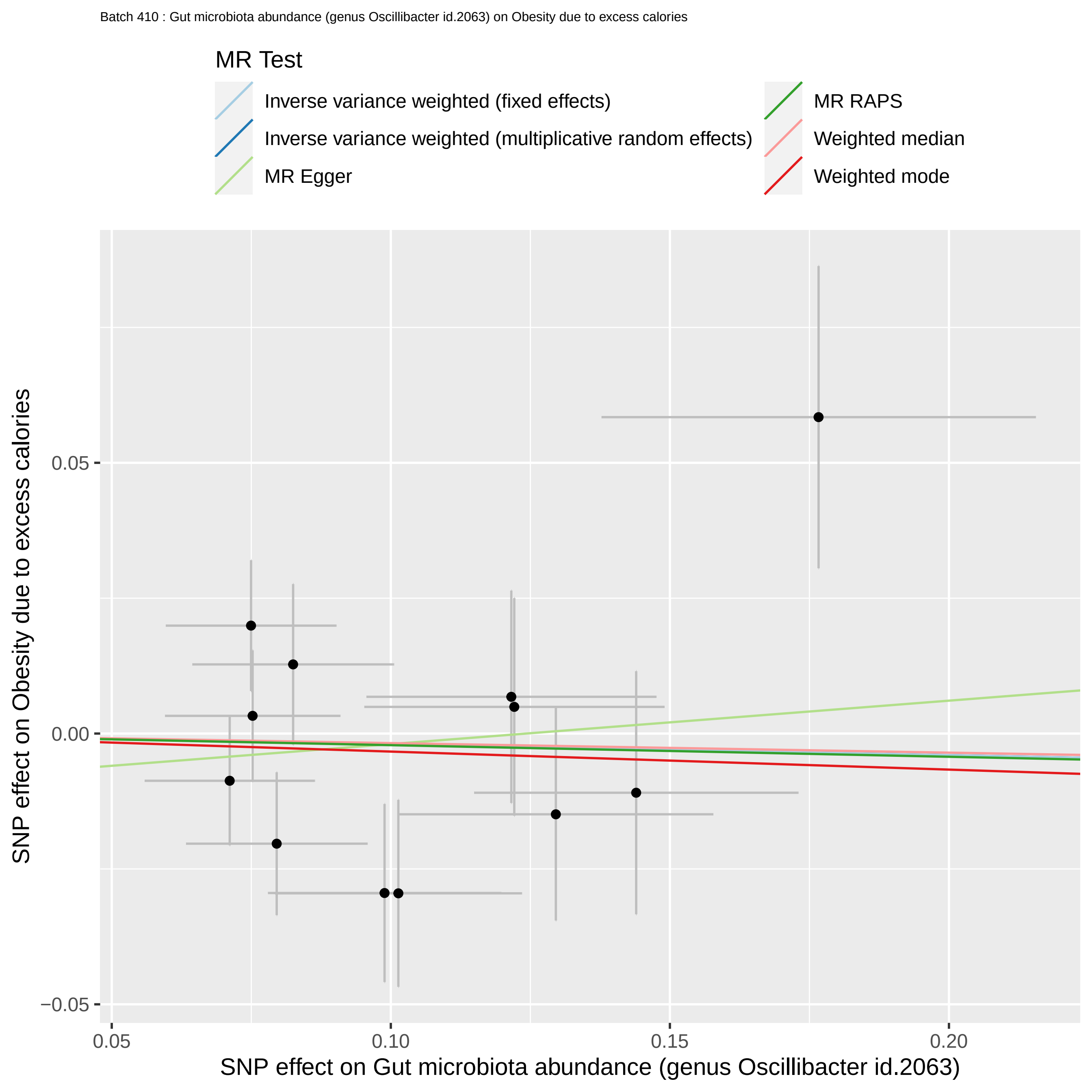
Batch 410 : Gut microbiota abundance (genus Oscillibacter id.2063) on Obesity due to excess calories
MR Test
Inverse variance weighted (fixed effects)
Inverse variance weighted (multiplicative random effects)
MR Egger
MR RAPS
Weighted median
Weighted mode
0.05
0.00
−0.05
0.05
0.10
0.15
0.20
SNP effect on Obesity due to excess calories
SNP effect on Gut microbiota abundance (genus Oscillibacter id.2063)

Batch 411 : Gut microbiota abundance (genus Oscillospira id.2064) on Obesity due to excess calories

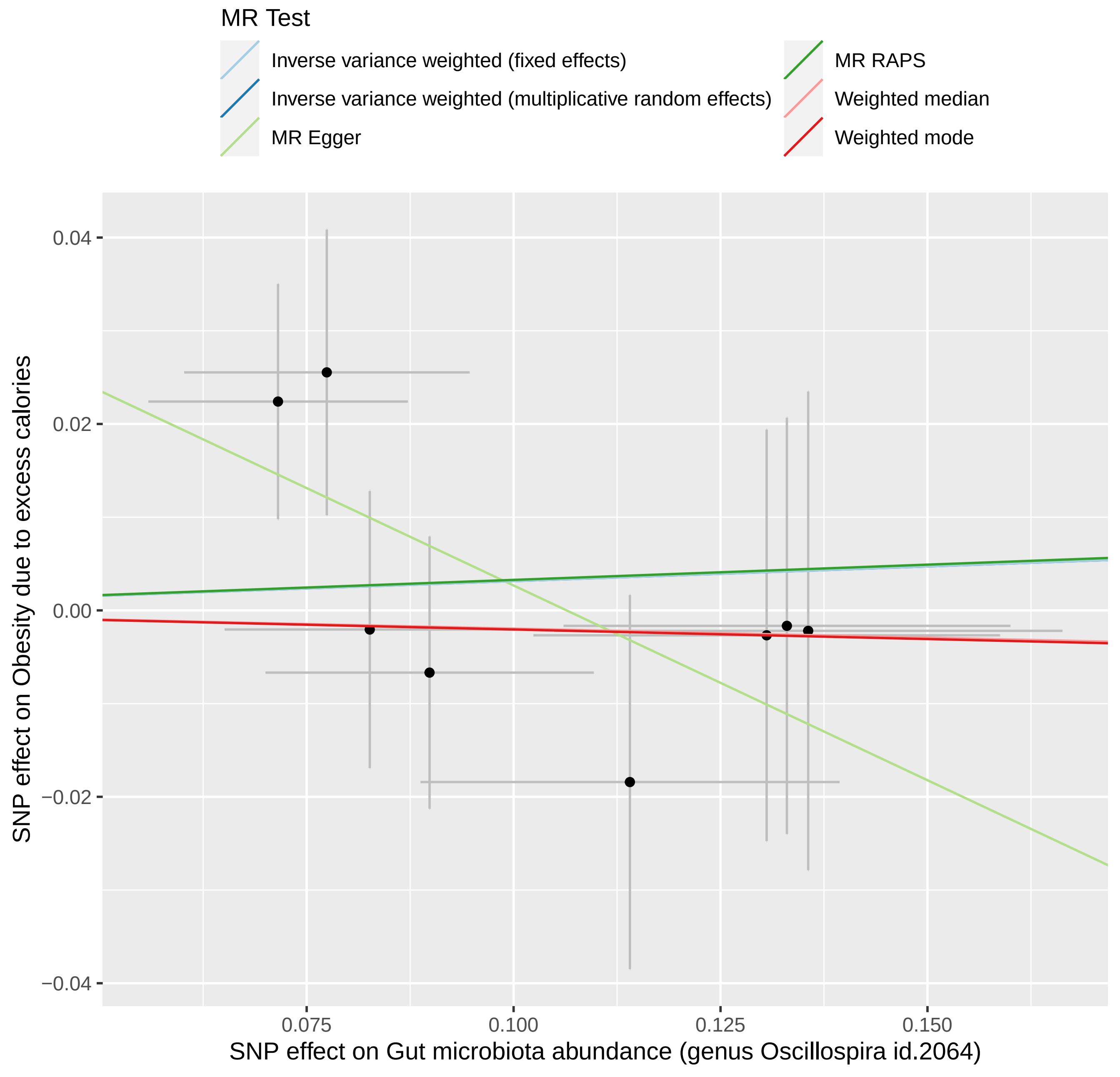

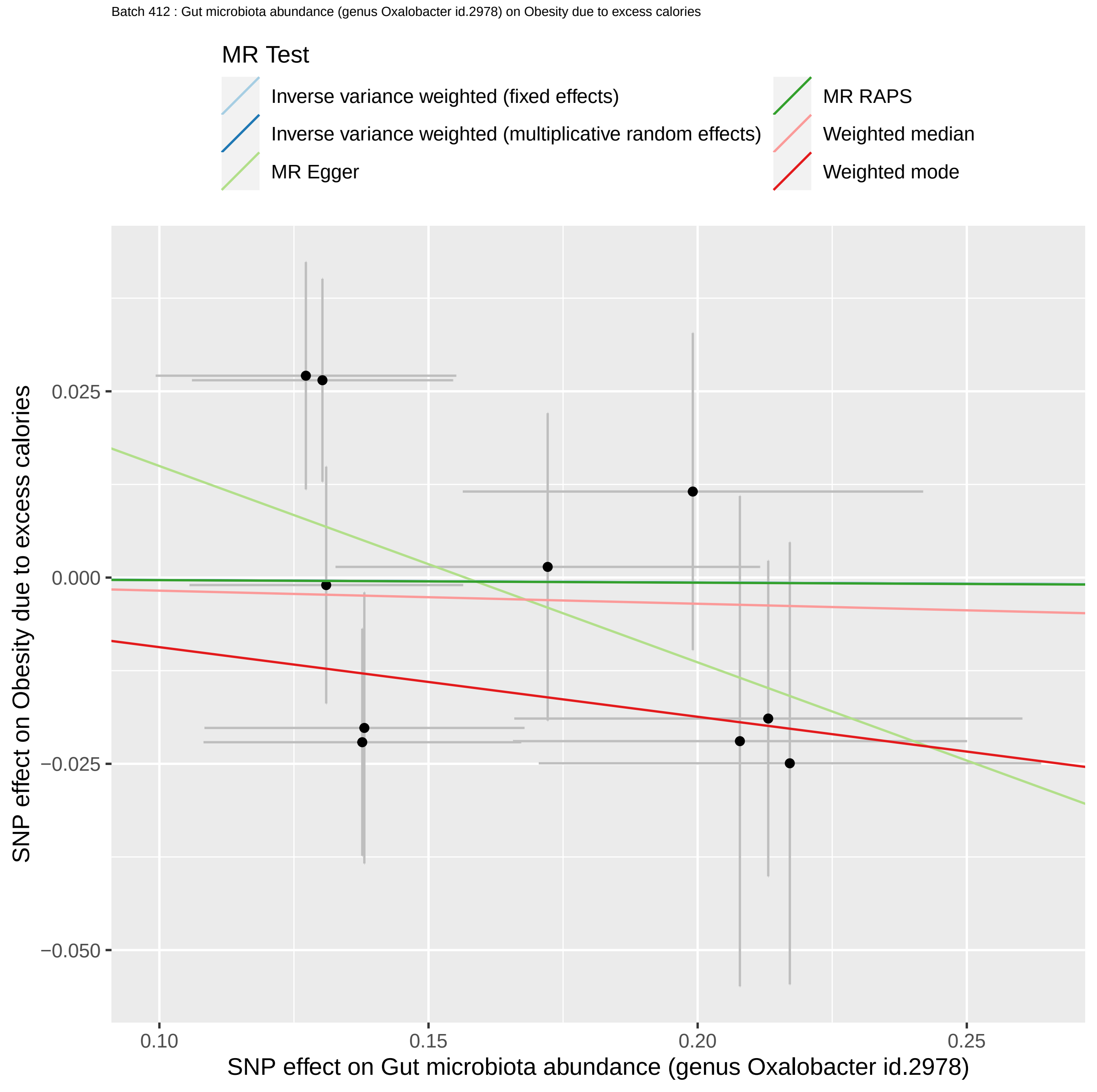
Batch 412 : Gut microbiota abundance (genus Oxalobacter id.2978) on Obesity due to excess calories
MR Test
Inverse variance weighted (fixed effects)
Inverse variance weighted (multiplicative random effects)
MR Egger
MR RAPS
Weighted median
Weighted mode
0.025
0.000
−0.025
−0.050
0.10
0.15
0.20
0.25
SNP effect on Obesity due to excess calories
SNP effect on Gut microbiota abundance (genus Oxalobacter id.2978)

Batch 413 : Gut microbiota abundance (genus Parabacteroides id.954) on Obesity due to excess calories

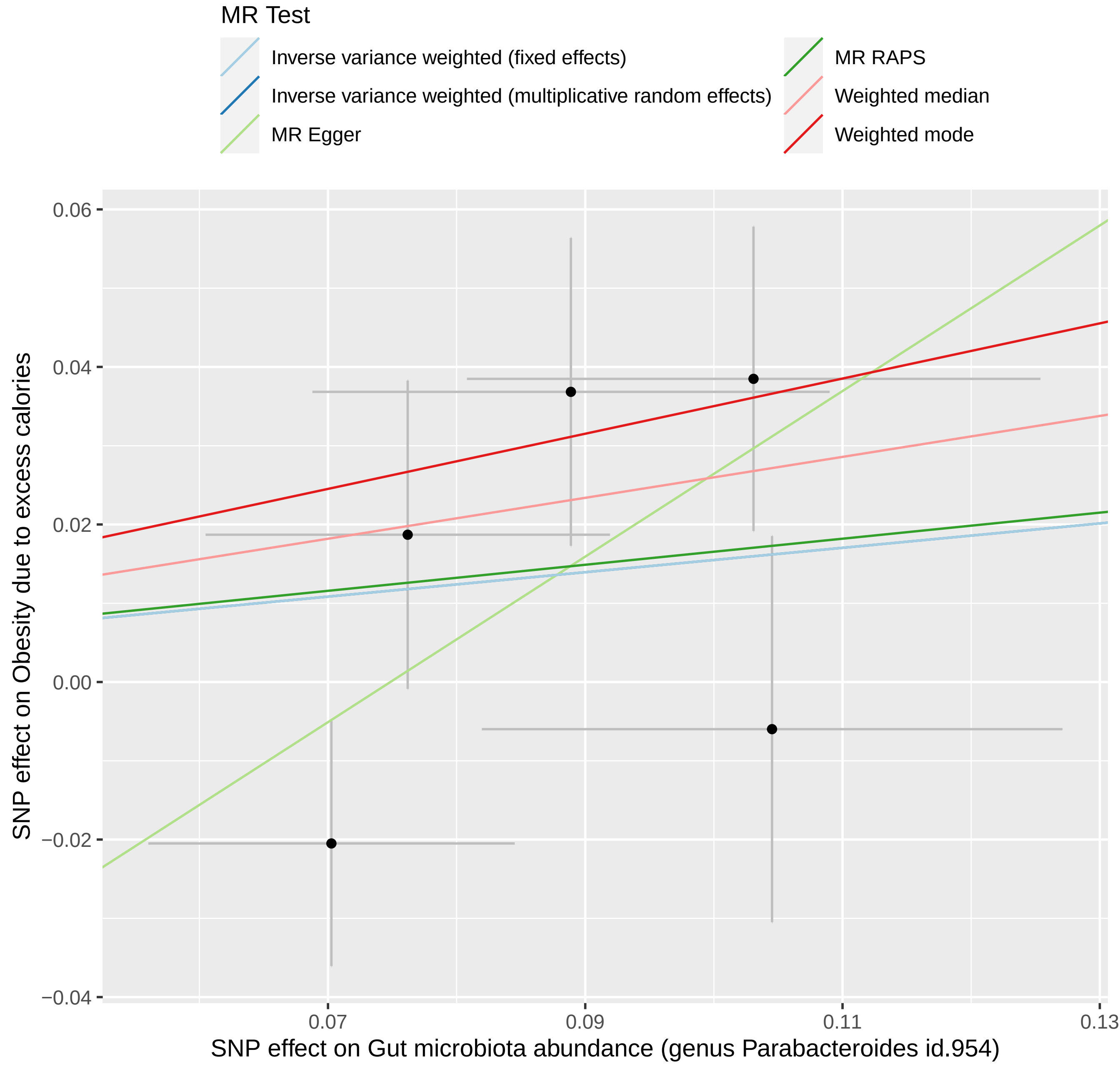

Batch 414 : Gut microbiota abundance (genus Paraprevotella id.962) on Obesity due to excess calories

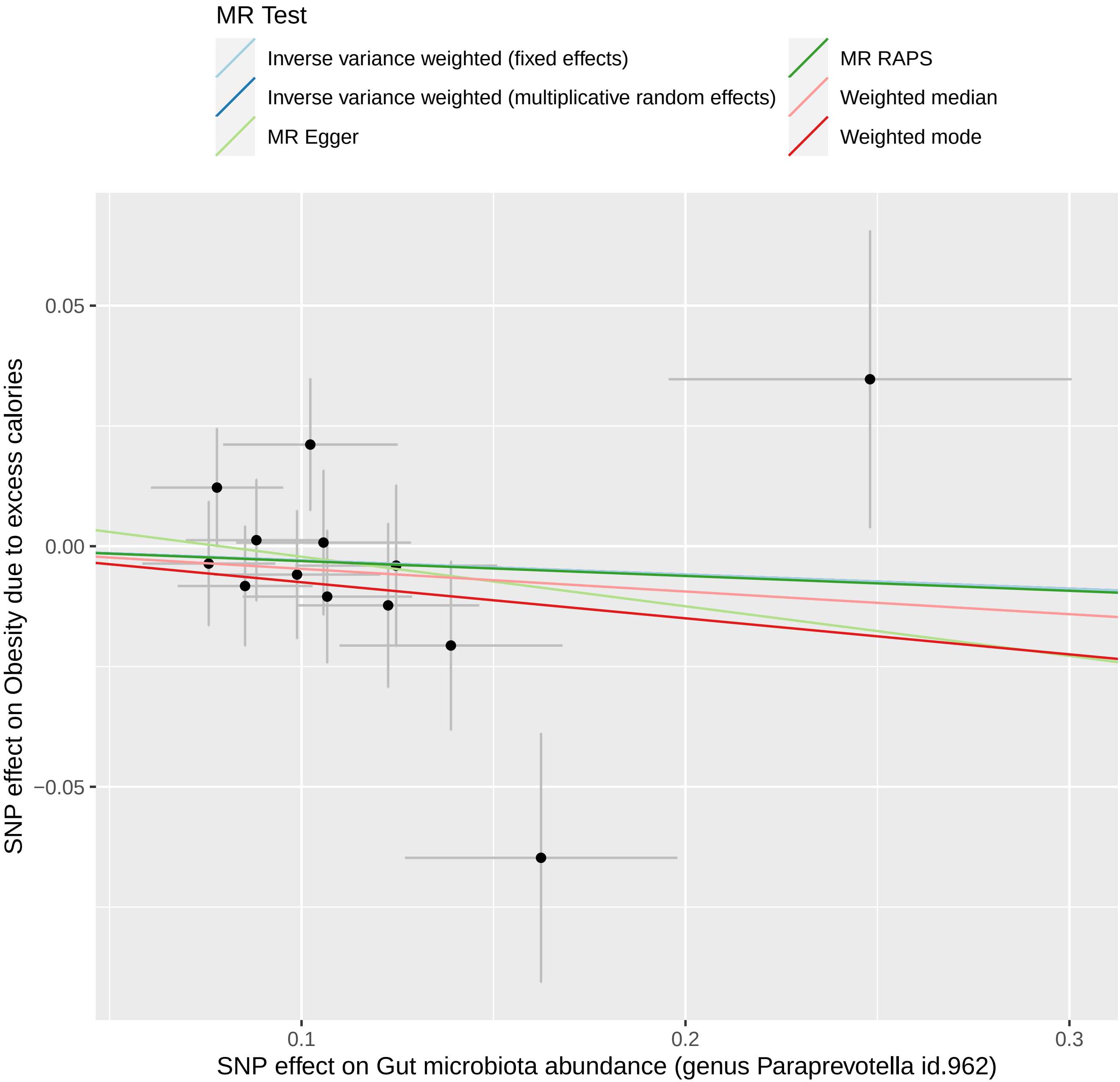

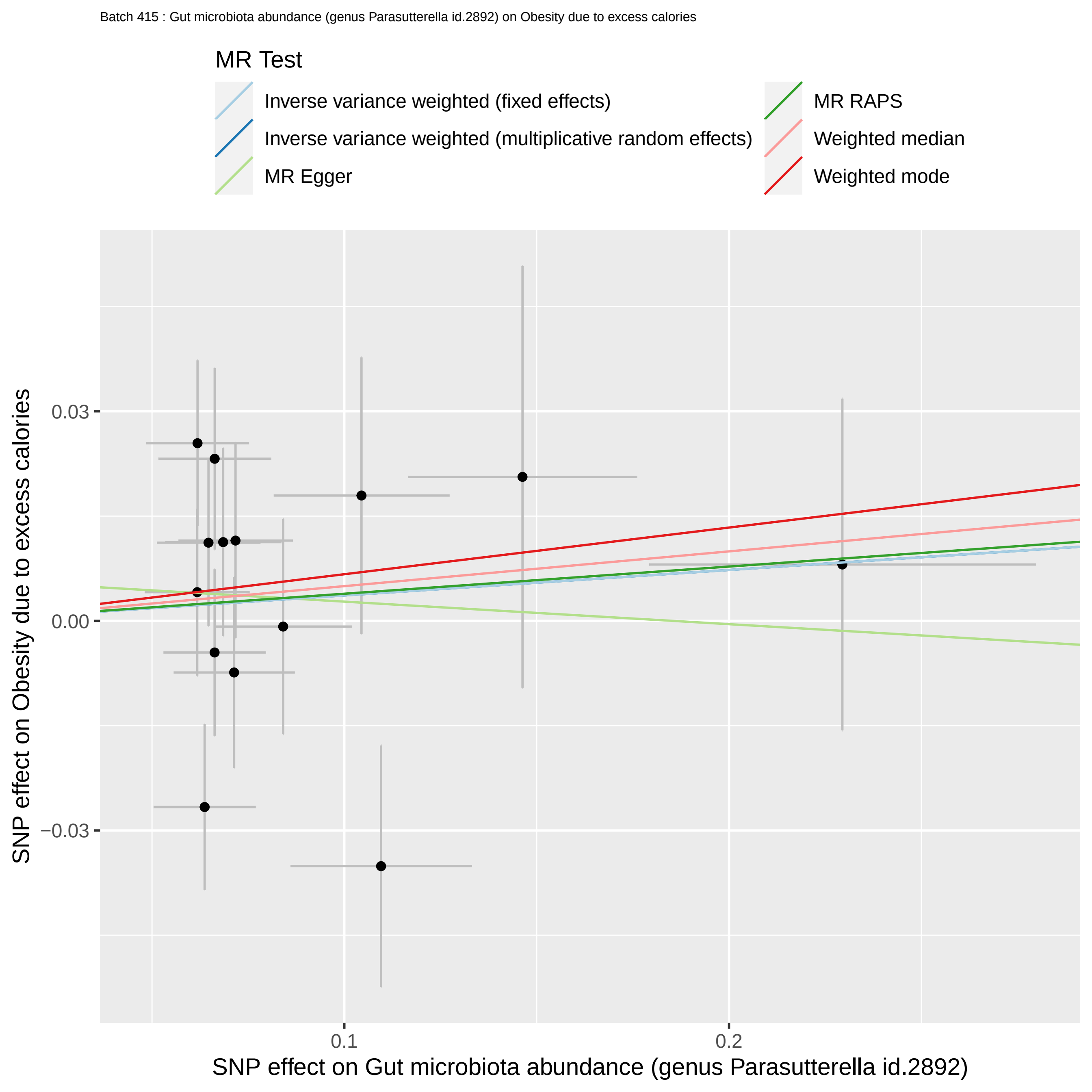

Batch 415 : Gut microbiota abundance (genus Parasutterella id.2892) on Obesity due to excess calories
MR Test
Inverse variance weighted (fixed effects)
Inverse variance weighted (multiplicative random effects)
MR Egger
MR RAPS
Weighted median
Weighted mode
0.03
0.00
−0.03
0.1
0.2
SNP effect on Obesity due to excess calories
SNP effect on Gut microbiota abundance (genus Parasutterella id.2892)

Batch 416 : Gut microbiota abundance (genus Peptococcus id.2037) on Obesity due to excess calories

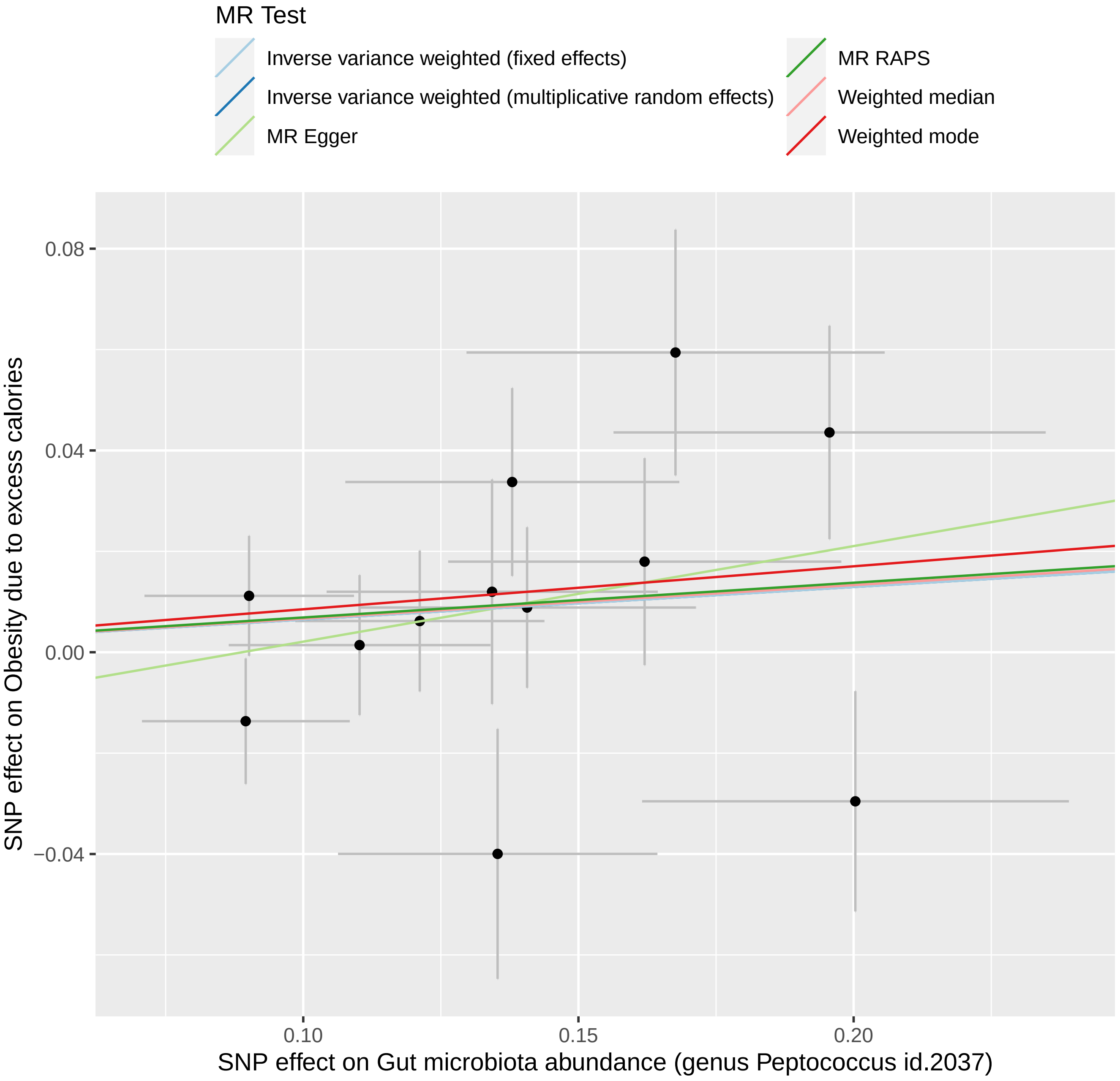

Batch 417 : Gut microbiota abundance (genus Phascolarctobacterium id.2168) on Obesity due to excess calories

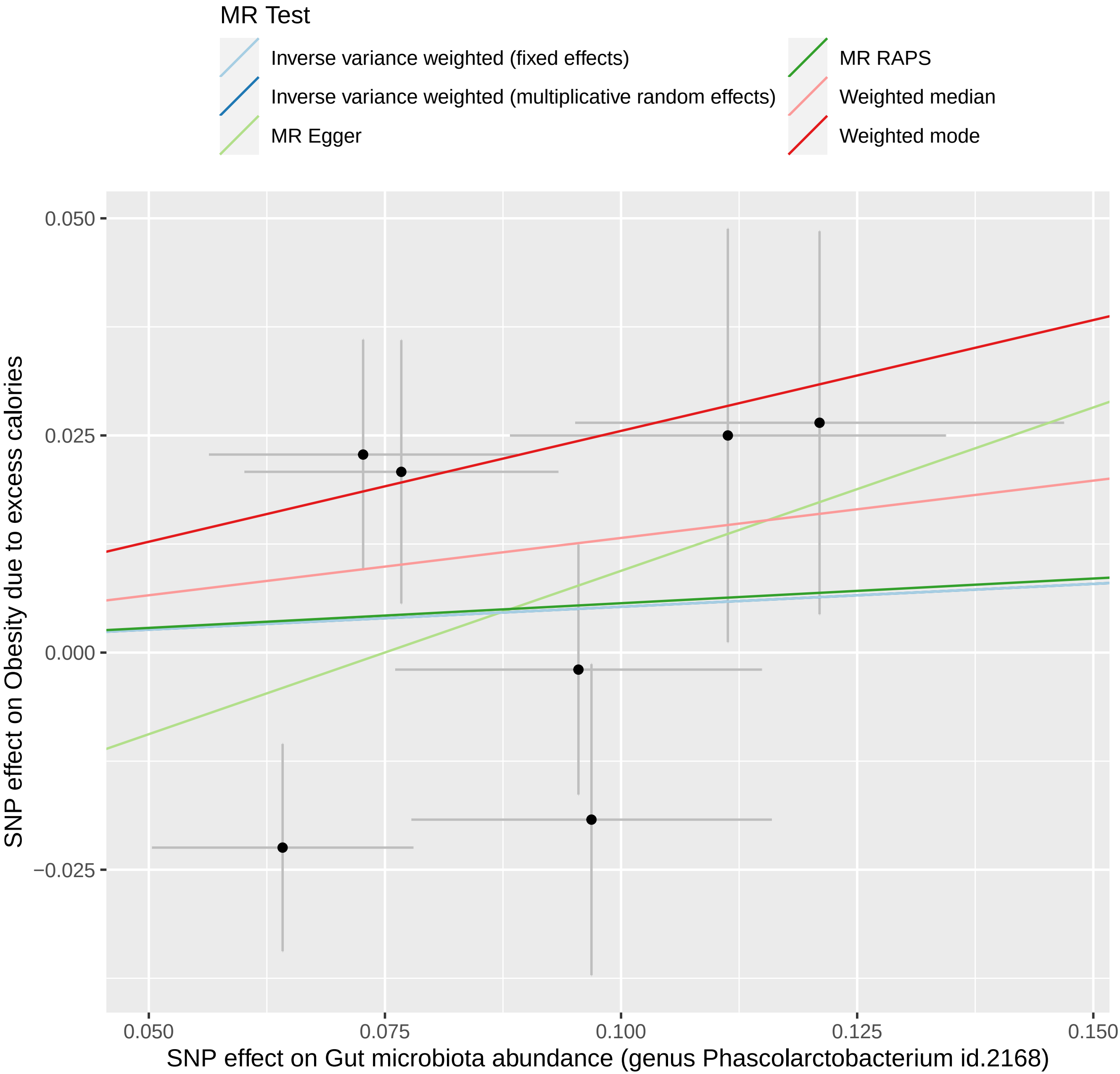

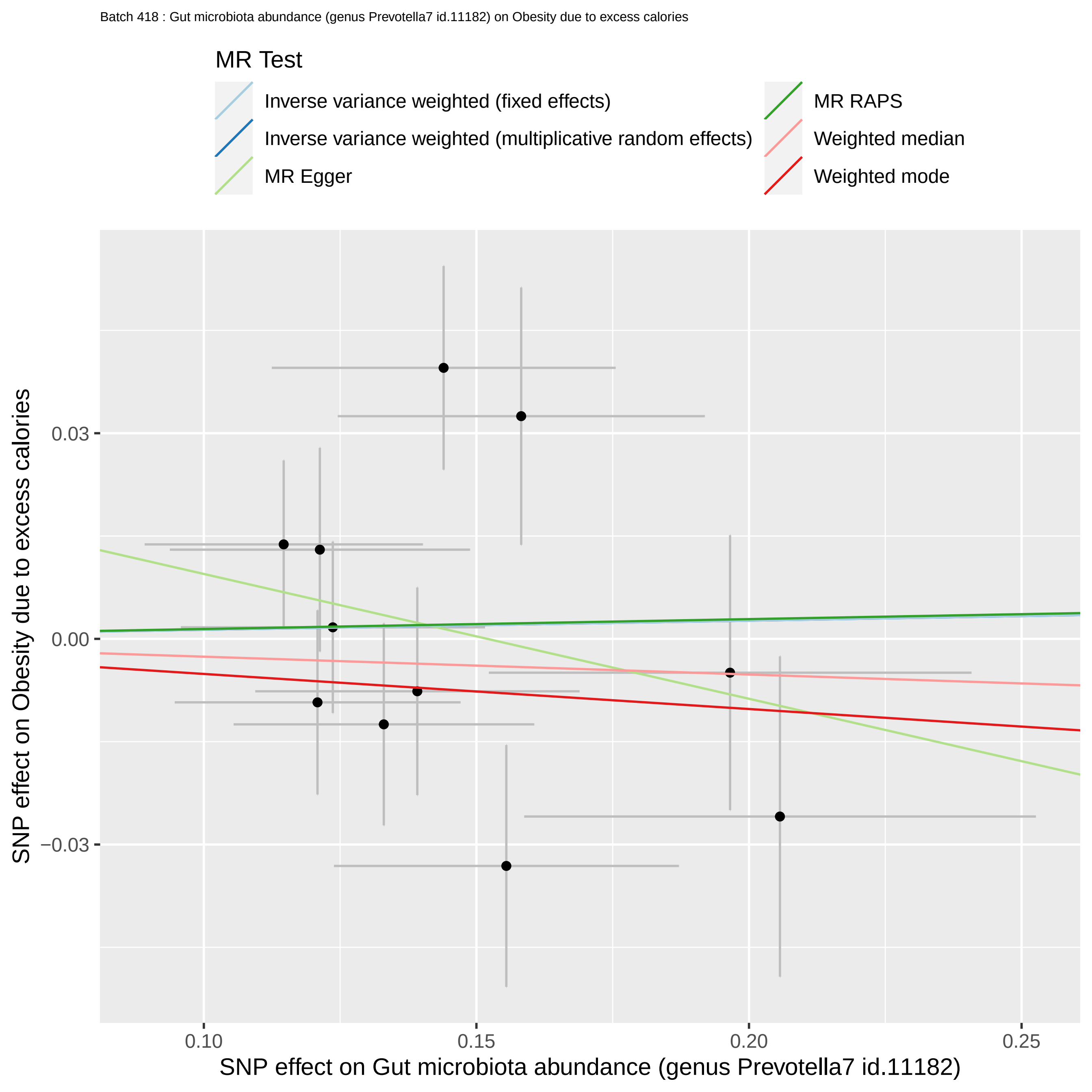

Batch 418 : Gut microbiota abundance (genus Prevotella7 id.11182) on Obesity due to excess calories
MR Test
Inverse variance weighted (fixed effects)
Inverse variance weighted (multiplicative random effects)
MR Egger
MR RAPS
Weighted median
Weighted mode
SNP effect on Obesity due to excess calories
0.03
0.00
−0.03
0.10
0.15
0.20
0.25
SNP effect on Gut microbiota abundance (genus Prevotella7 id.11182)

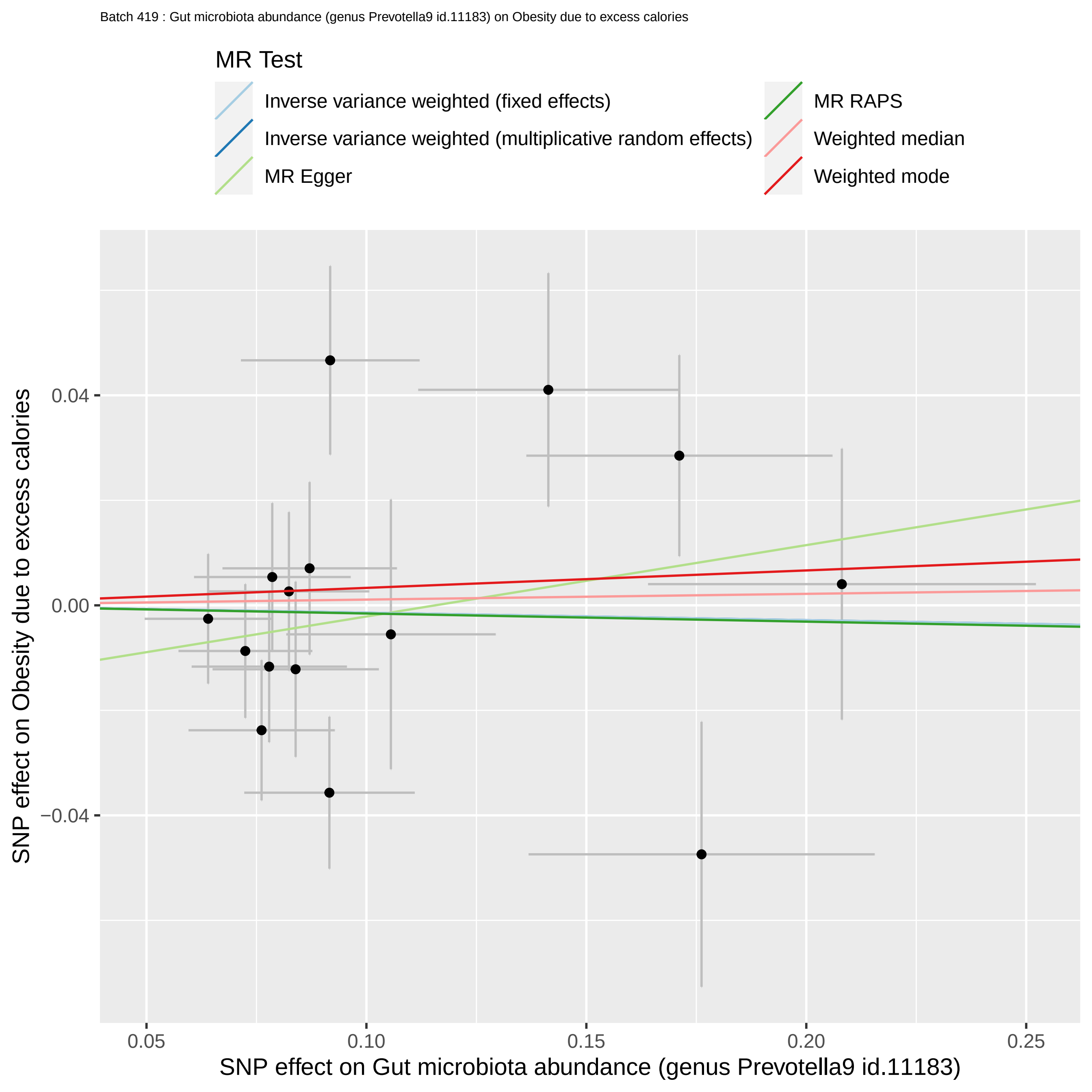

Batch 419 : Gut microbiota abundance (genus Prevotella9 id.11183) on Obesity due to excess calories
MR Test
Inverse variance weighted (fixed effects)
Inverse variance weighted (multiplicative random effects)
MR Egger
MR RAPS
Weighted median
Weighted mode
SNP effect on Obesity due to excess calories
0.04
0.00
−0.04
0.05
0.10
0.15
0.20
0.25
SNP effect on Gut microbiota abundance (genus Prevotella9 id.11183)

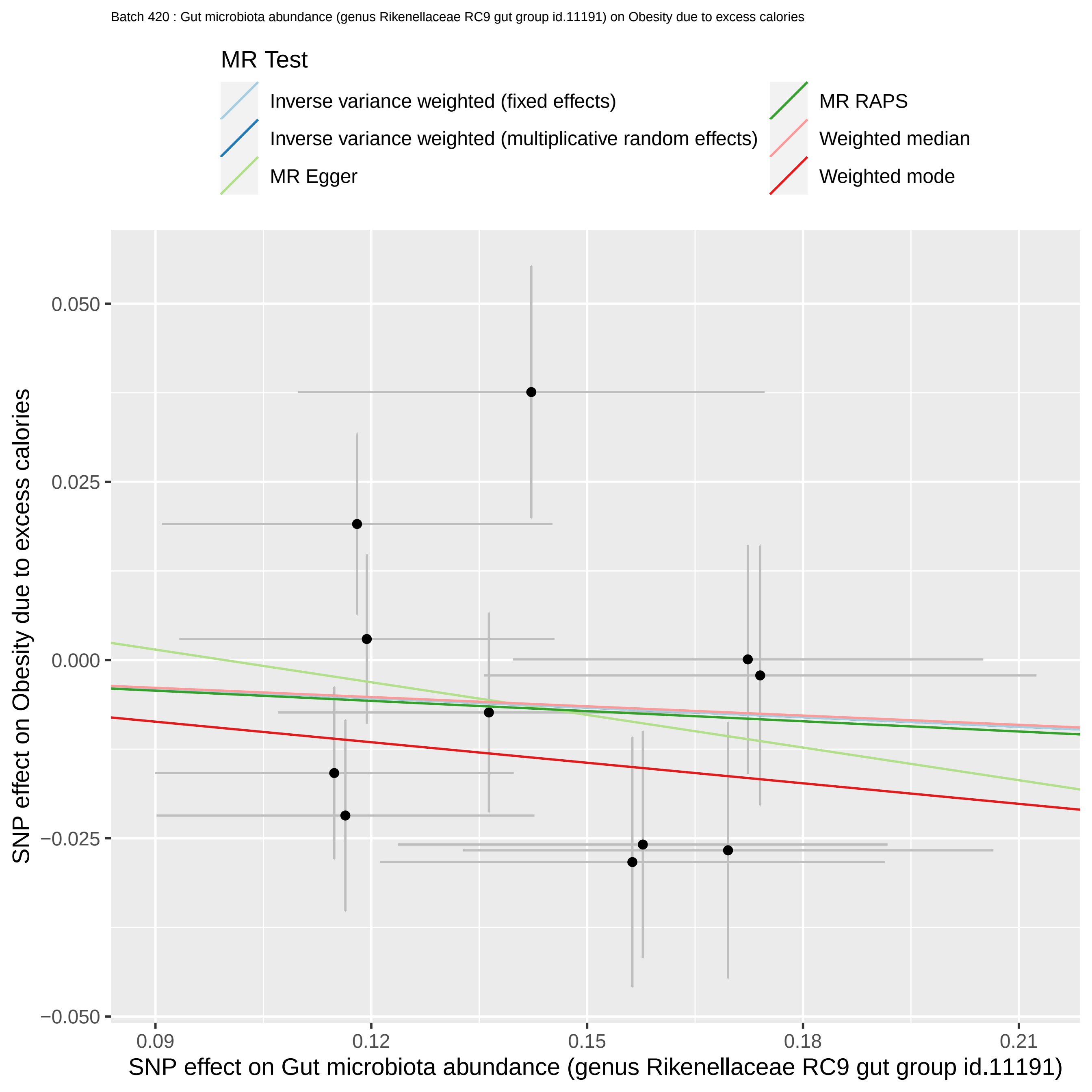

Batch 420 : Gut microbiota abundance (genus Rikenellaceae RC9 gut group id.11191) on Obesity due to excess calories
MR Test
Inverse variance weighted (fixed effects)
Inverse variance weighted (multiplicative random effects)
MR Egger
MR RAPS
Weighted median
Weighted mode
0.050
0.025
0.000
−0.025
−0.050
0.09
0.12
0.15
0.18
0.21
SNP effect on Obesity due to excess calories
SNP effect on Gut microbiota abundance (genus Rikenellaceae RC9 gut group id.11191)

Batch 421 : Gut microbiota abundance (genus Romboutsia id.11347) on Obesity due to excess calories

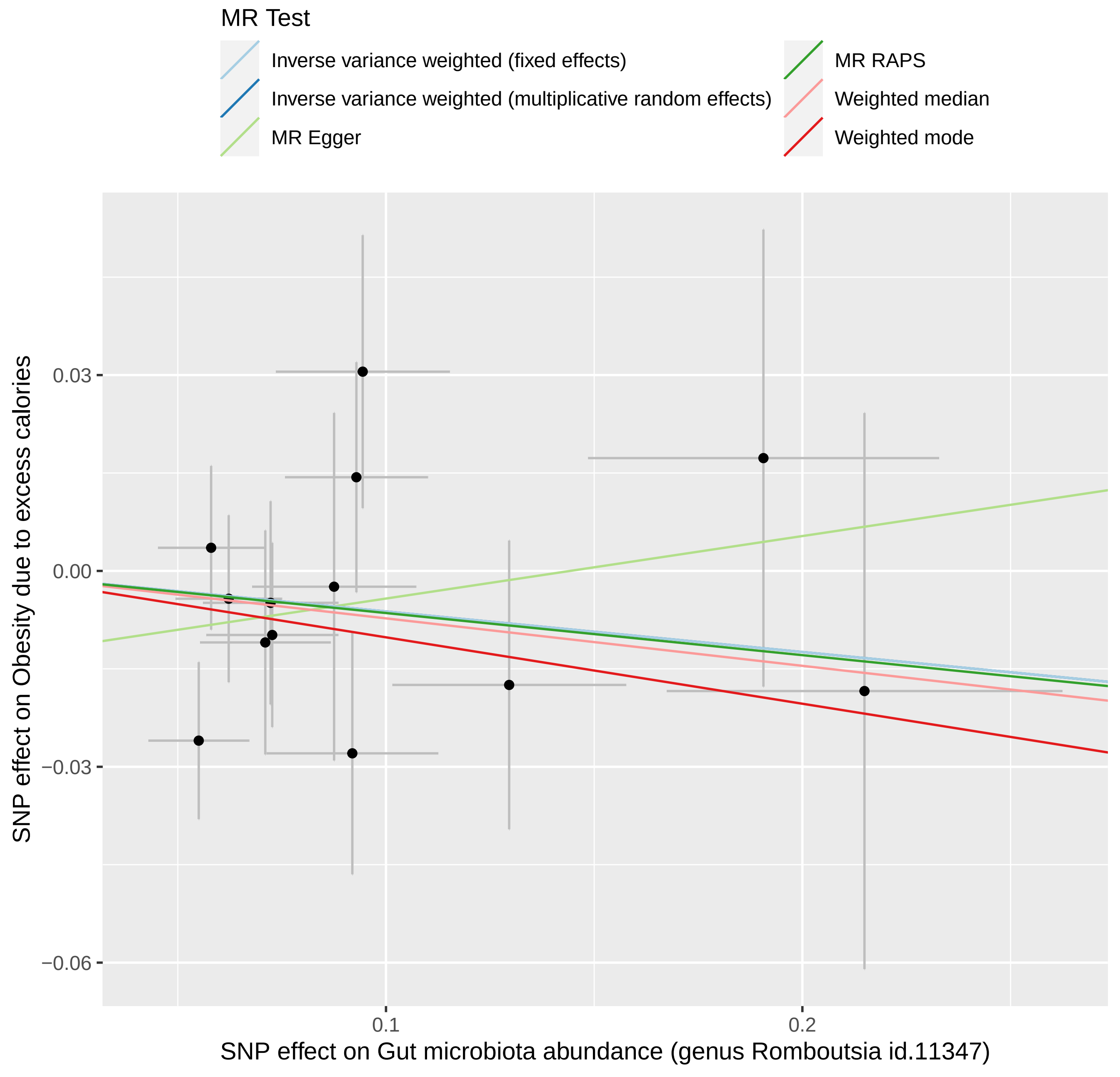

Batch 422 : Gut microbiota abundance (genus Roseburia id.2012) on Obesity due to excess calories

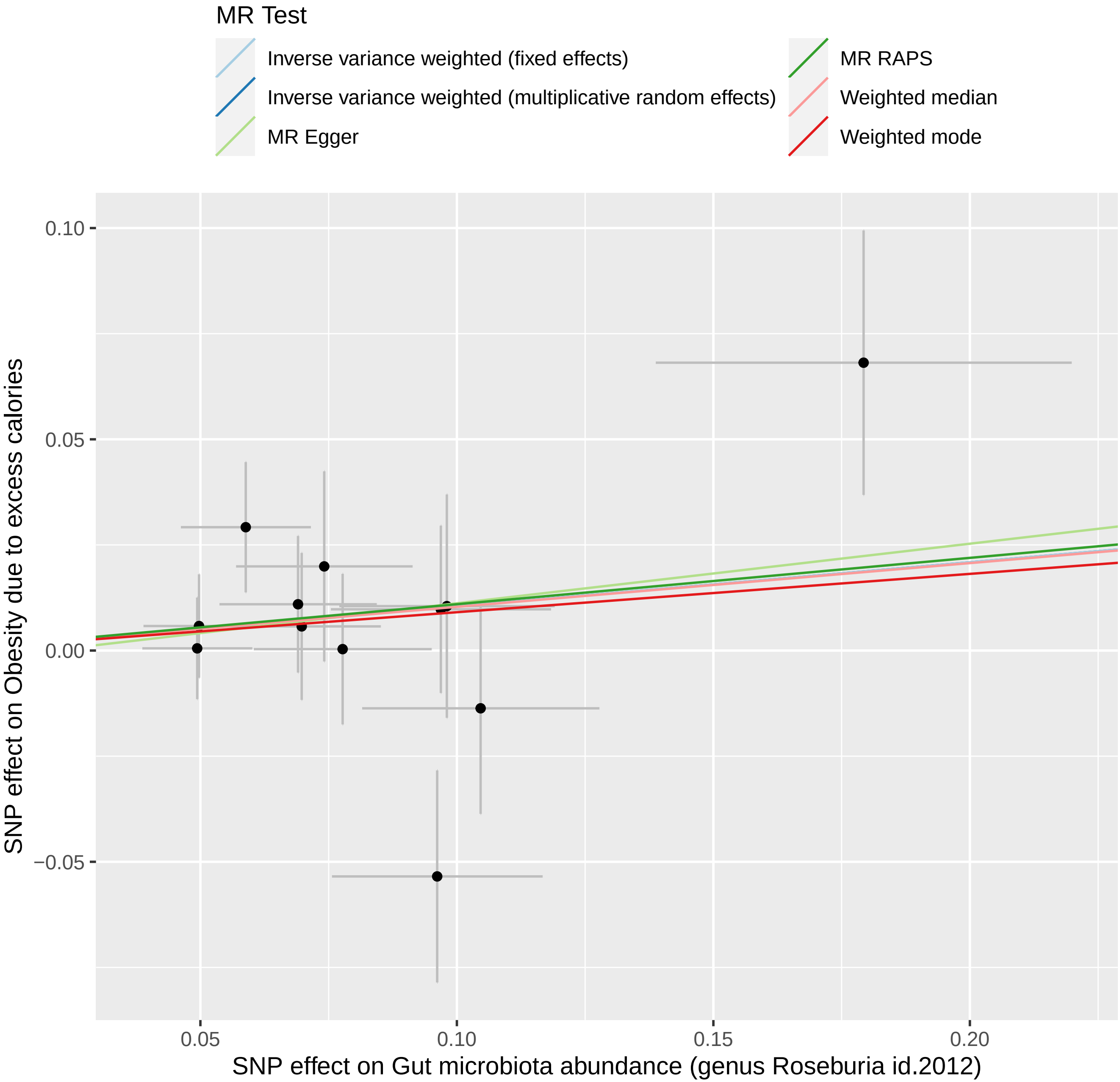

Batch 423 : Gut microbiota abundance (genus Ruminiclostridium5 id.11355) on Obesity due to excess calories

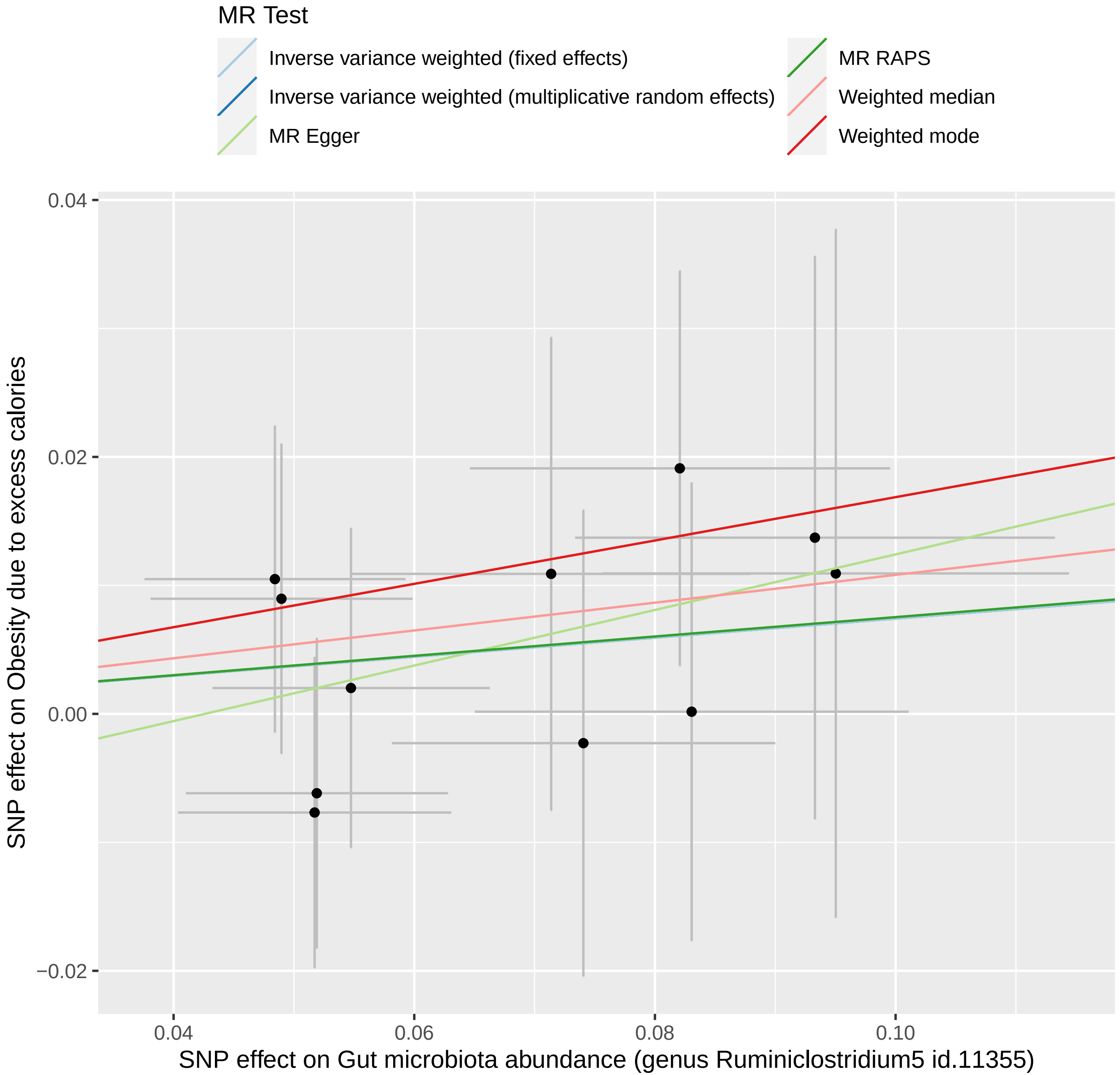

Batch 424 : Gut microbiota abundance (genus Ruminiclostridium6 id.11356) on Obesity due to excess calories

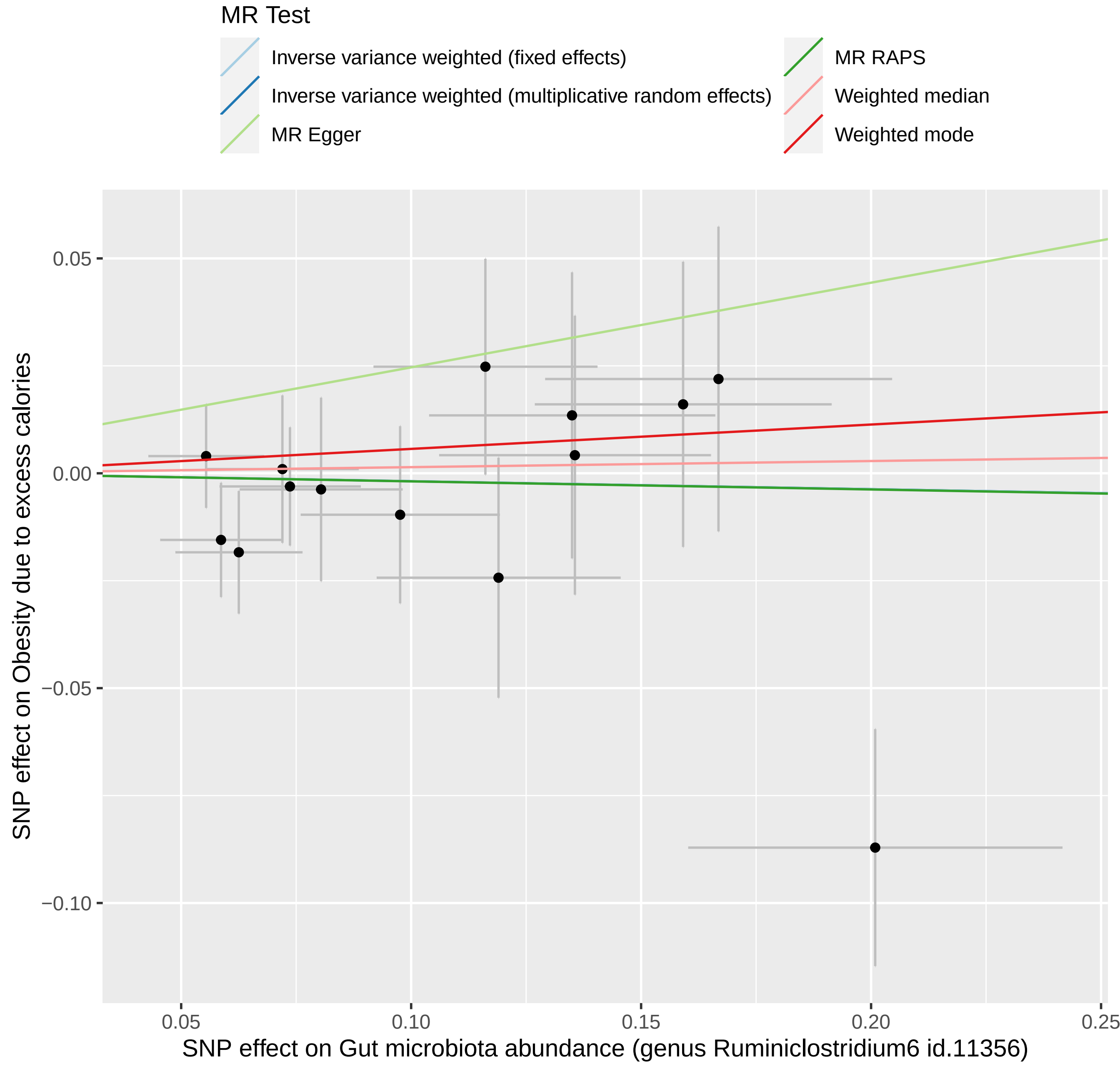

Batch 425 : Gut microbiota abundance (genus Ruminiclostridium9 id.11357) on Obesity due to excess calories

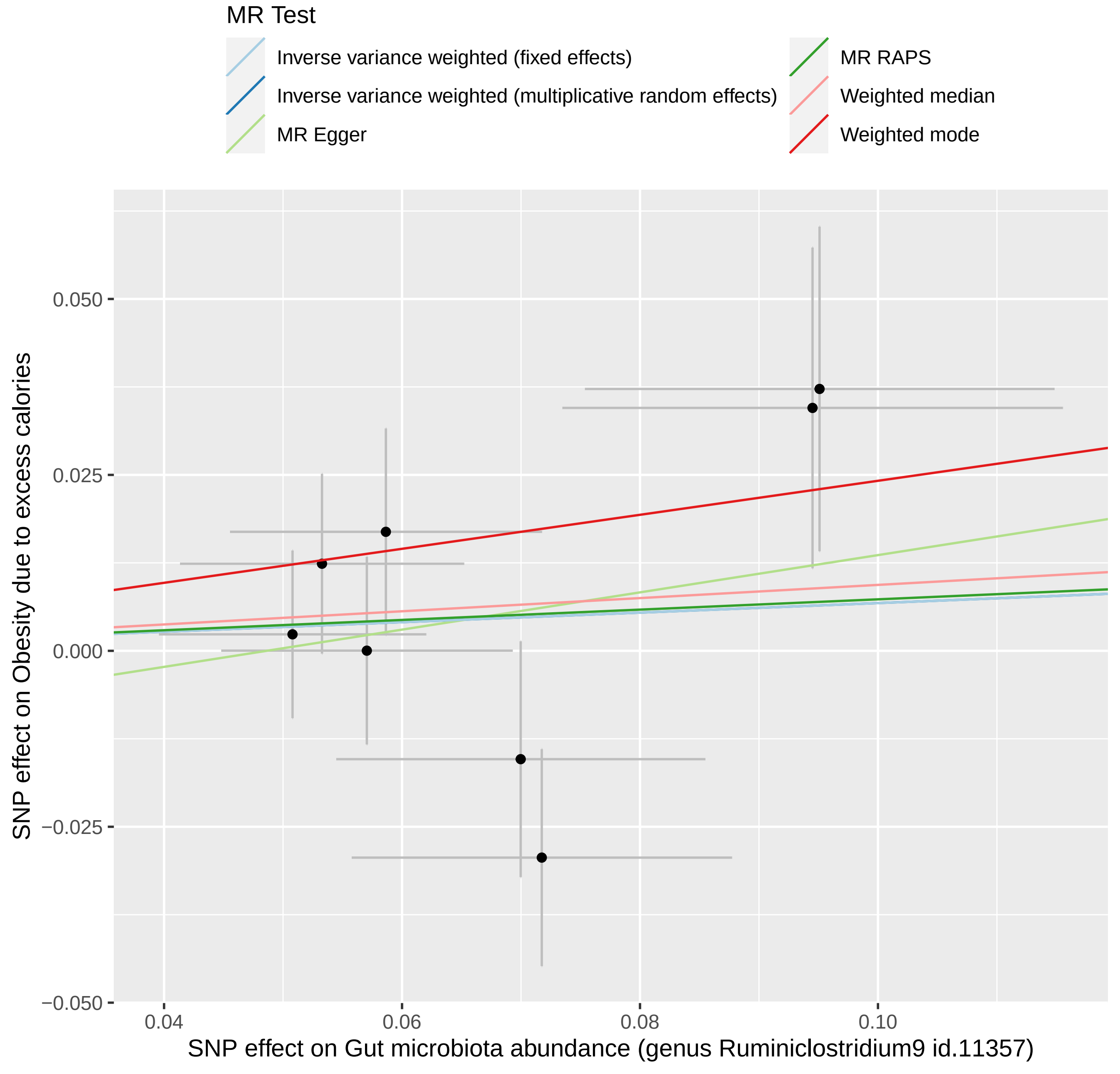

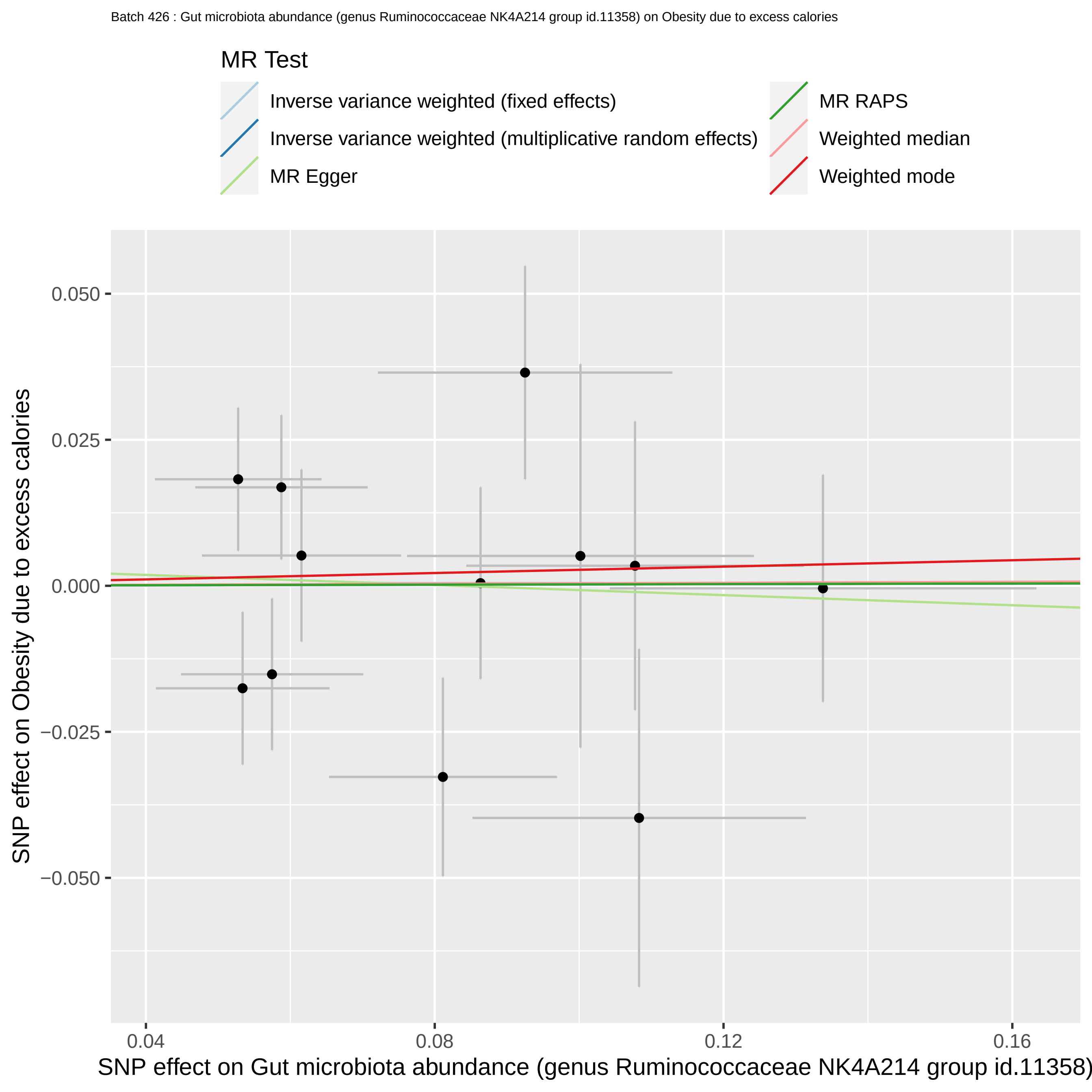
Batch 426 : Gut microbiota abundance (genus Ruminococcaceae NK4A214 group id.11358) on Obesity due to excess calories
MR Test
Inverse variance weighted (fixed effects)
Inverse variance weighted (multiplicative random effects)
MR Egger
MR RAPS
Weighted median
Weighted mode
SNP effect on Obesity due to excess calories
0.050
0.025
0.000
−0.025
−0.050
0.04
0.08
0.12
0.16
SNP effect on Gut microbiota abundance (genus Ruminococcaceae NK4A214 group id.11358)

Batch 427 : Gut microbiota abundance (genus Ruminococcaceae UCG002 id.11360) on Obesity due to excess calories

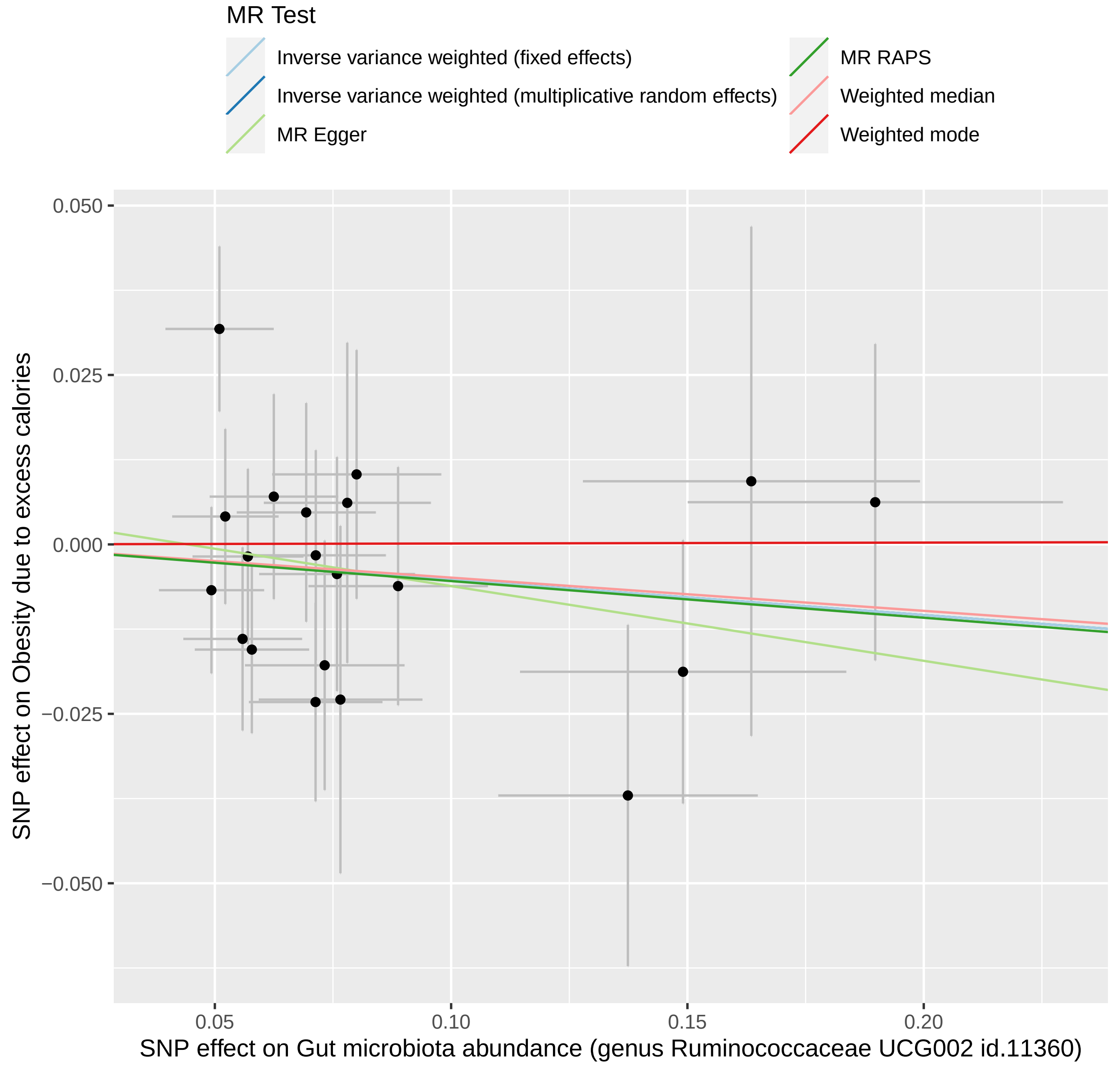

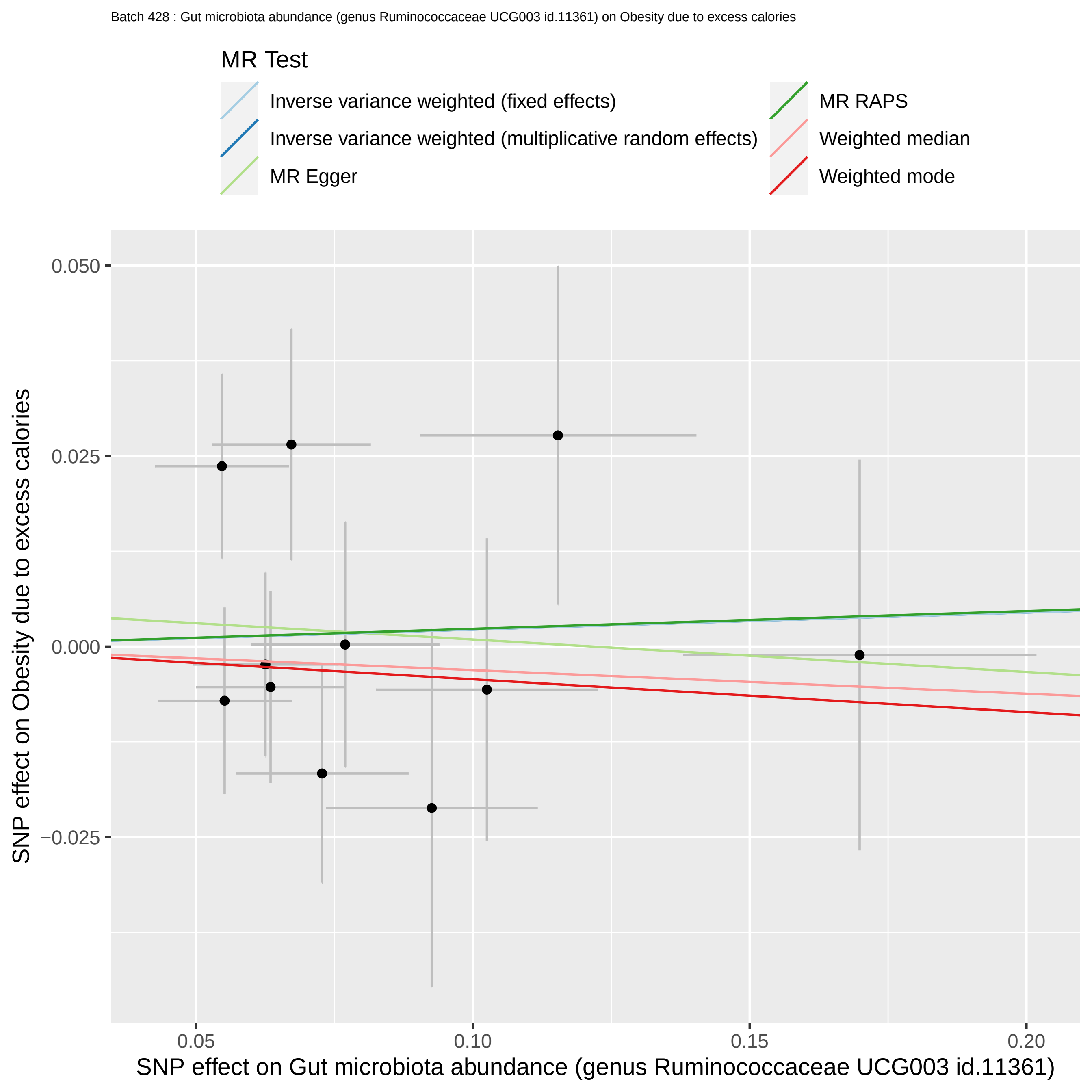

Batch 428 : Gut microbiota abundance (genus Ruminococcaceae UCG003 id.11361) on Obesity due to excess calories
MR Test
Inverse variance weighted (fixed effects)
Inverse variance weighted (multiplicative random effects)
MR Egger
MR RAPS
Weighted median
Weighted mode
0.050
0.025
0.000
−0.025
0.05
0.10
0.15
0.20
SNP effect on Obesity due to excess calories
SNP effect on Gut microbiota abundance (genus Ruminococcaceae UCG003 id.11361)

Batch 429 : Gut microbiota abundance (genus Ruminococcaceae UCG004 id.11362) on Obesity due to excess calories

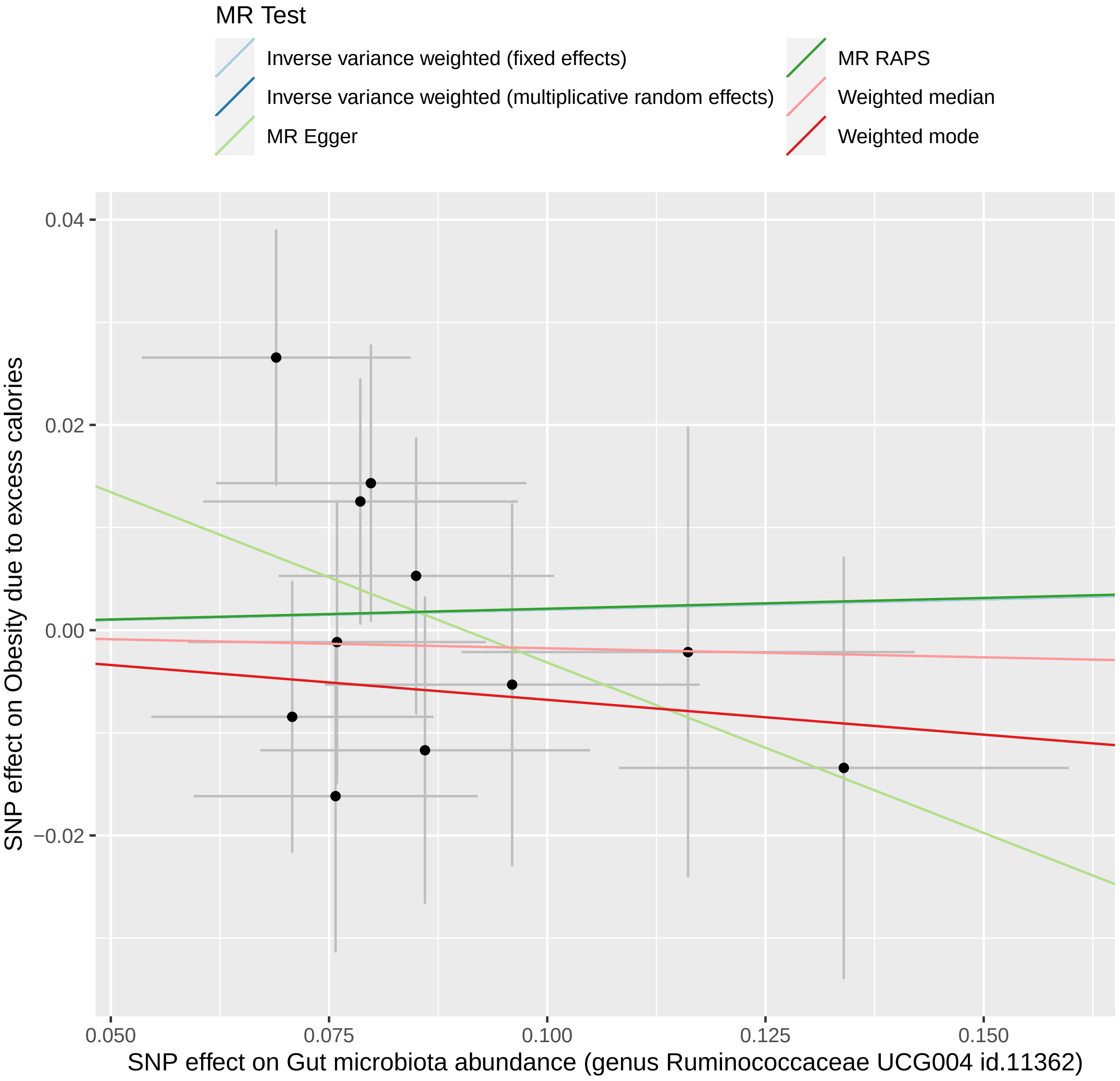

Batch 430 : Gut microbiota abundance (genus Ruminococcaceae UCG005 id.11363) on Obesity due to excess calories

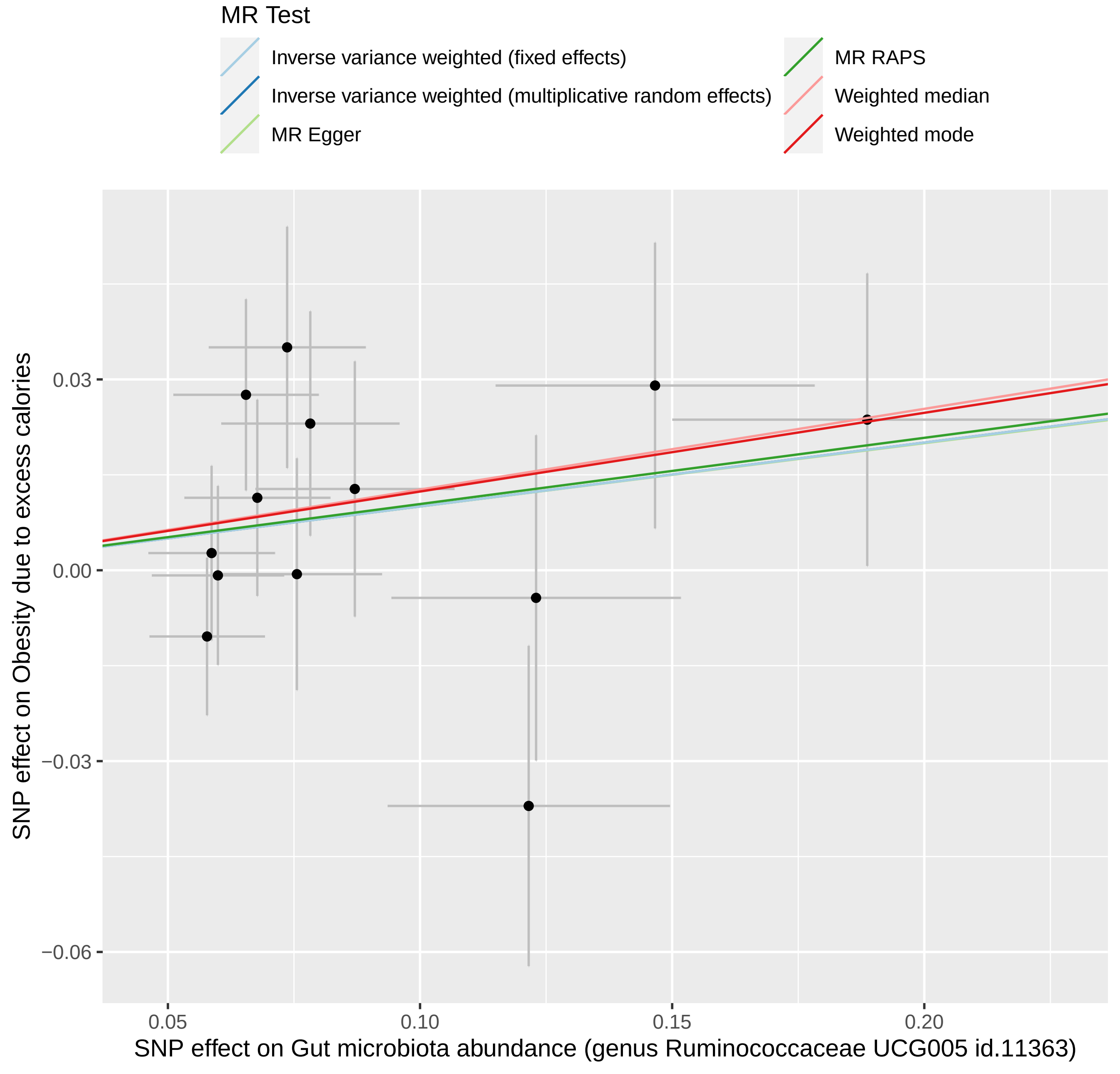

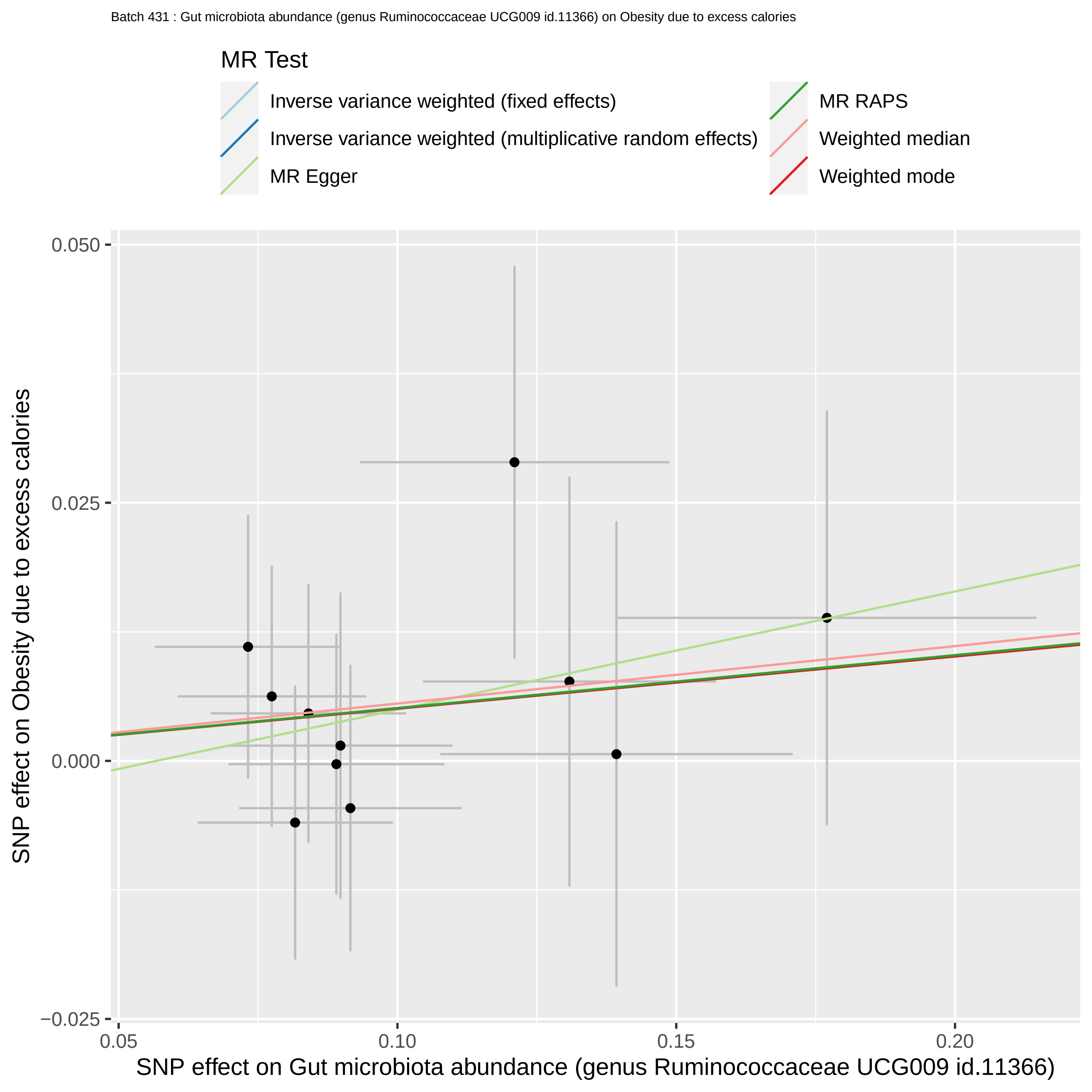

Batch 431 : Gut microbiota abundance (genus Ruminococcaceae UCG009 id.11366) on Obesity due to excess calories
MR Test
Inverse variance weighted (fixed effects)
Inverse variance weighted (multiplicative random effects)
MR Egger
MR RAPS
Weighted median
Weighted mode
0.050
0.025
0.000
−0.025
0.05
0.10
0.15
0.20
SNP effect on Obesity due to excess calories
SNP effect on Gut microbiota abundance (genus Ruminococcaceae UCG009 id.11366)

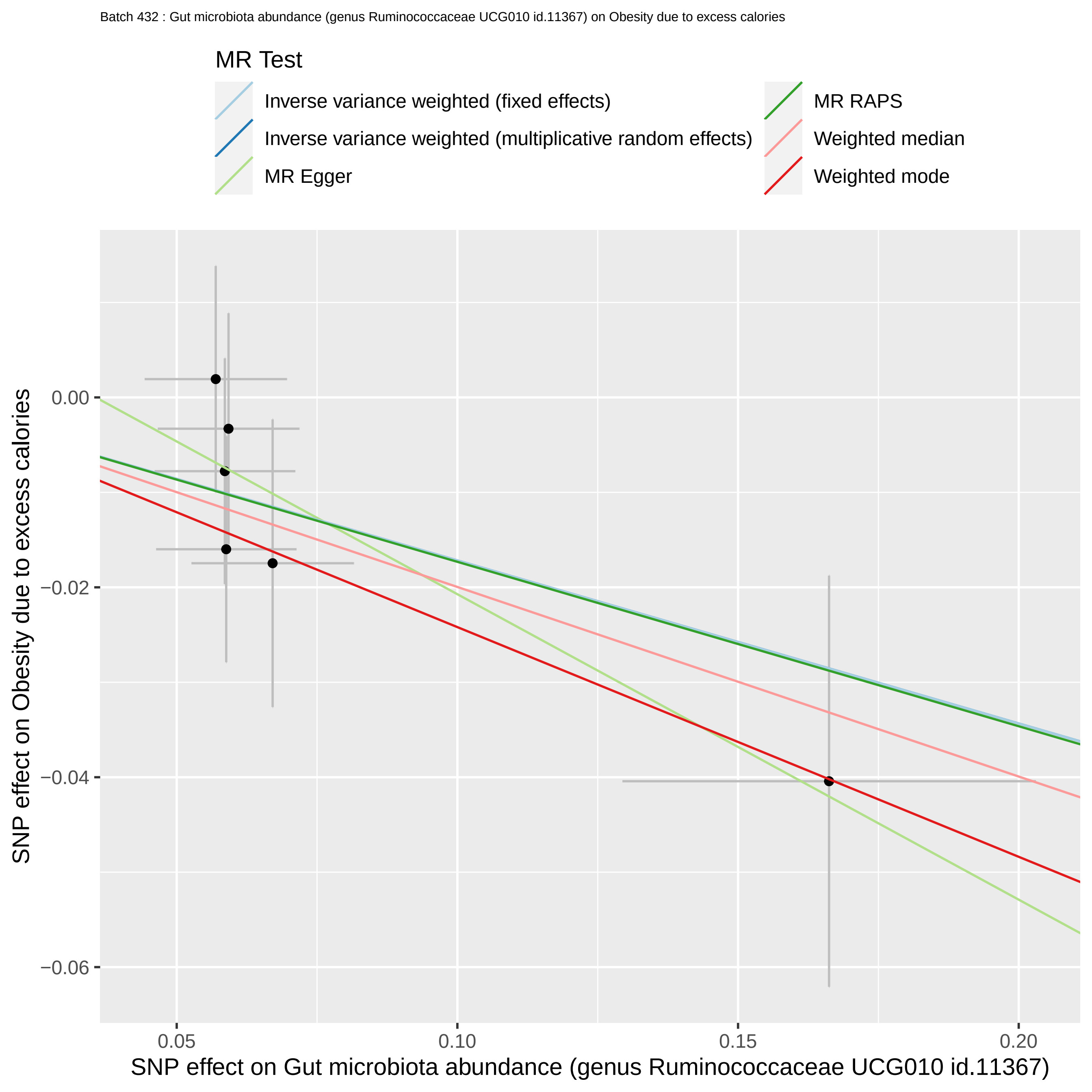

Batch 432 : Gut microbiota abundance (genus Ruminococcaceae UCG010 id.11367) on Obesity due to excess calories
MR Test
Inverse variance weighted (fixed effects)
Inverse variance weighted (multiplicative random effects)
MR Egger
MR RAPS
Weighted median
Weighted mode
SNP effect on Obesity due to excess calories
0.00
−0.02
−0.04
−0.06
0.05
0.10
0.15
0.20
SNP effect on Gut microbiota abundance (genus Ruminococcaceae UCG010 id.11367)

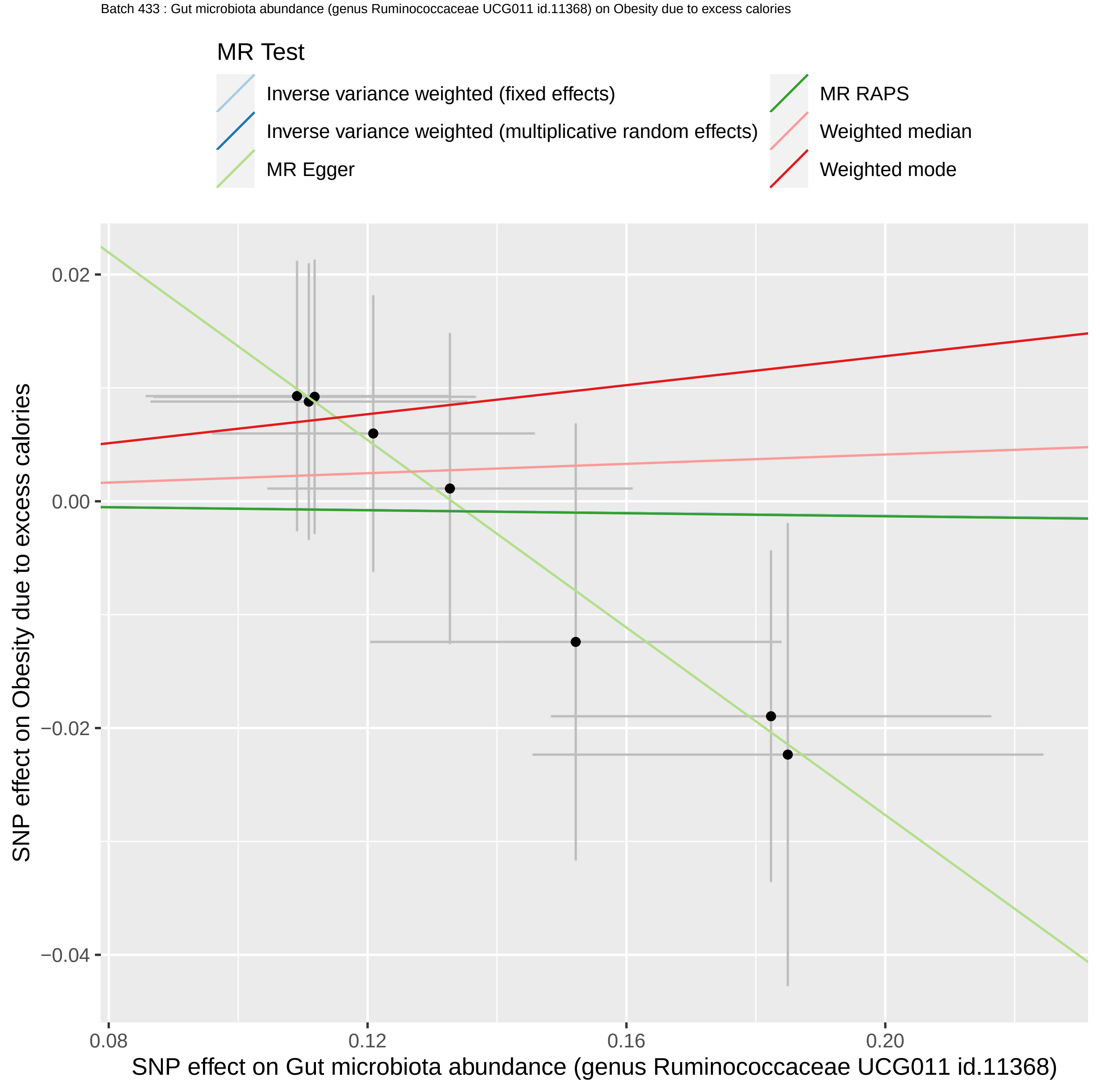
Batch 433 : Gut microbiota abundance (genus Ruminococcaceae UCG011 id.11368) on Obesity due to excess calories
MR Test
Inverse variance weighted (fixed effects)
Inverse variance weighted (multiplicative random effects)
MR Egger
MR RAPS
Weighted median
Weighted mode
0.02
0.00
−0.02
−0.04
0.08
0.12
0.16
0.20
SNP effect on Obesity due to excess calories
SNP effect on Gut microbiota abundance (genus Ruminococcaceae UCG011 id.11368)

Batch 434 : Gut microbiota abundance (genus Ruminococcaceae UCG013 id.11370) on Obesity due to excess calories

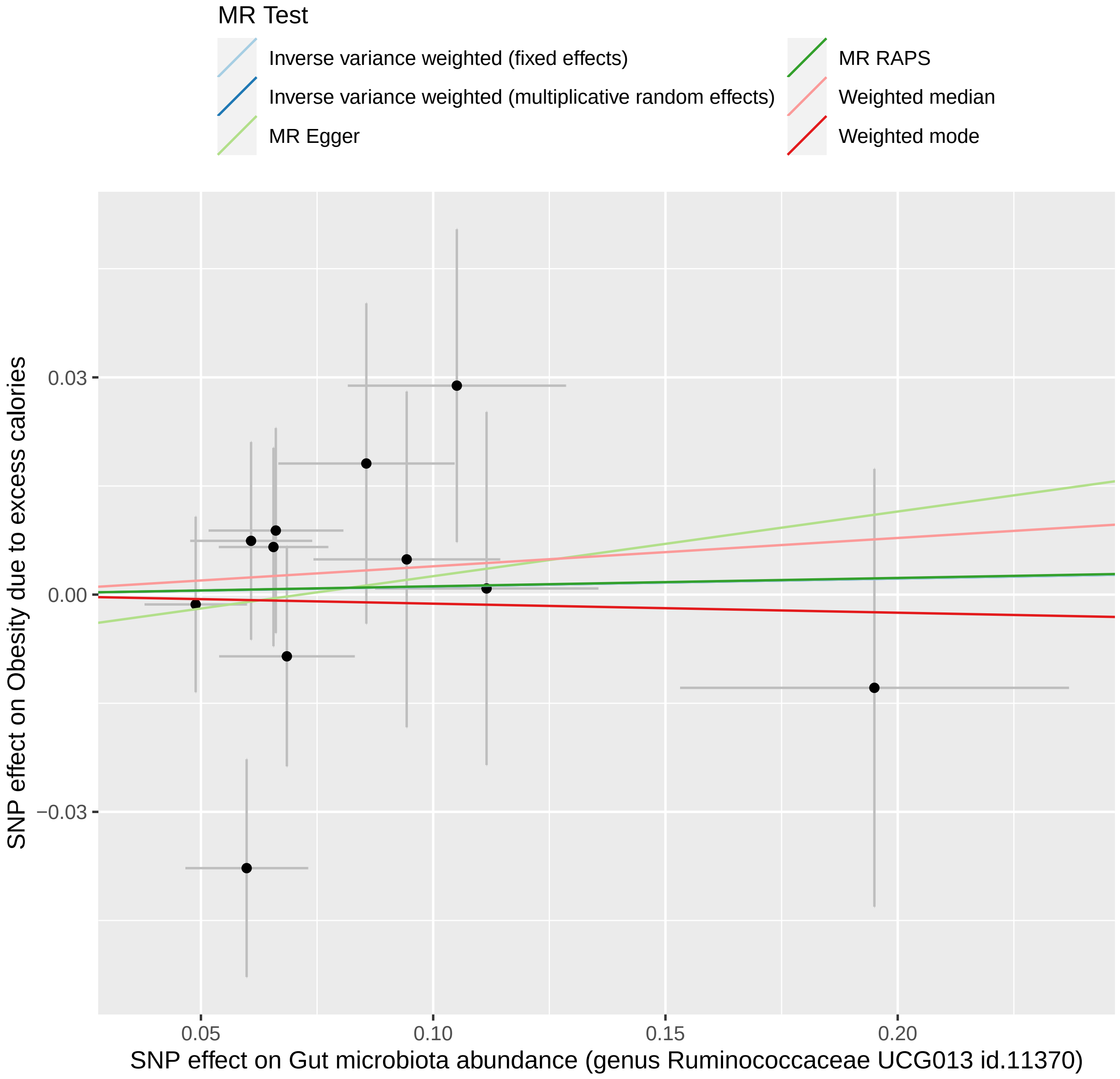

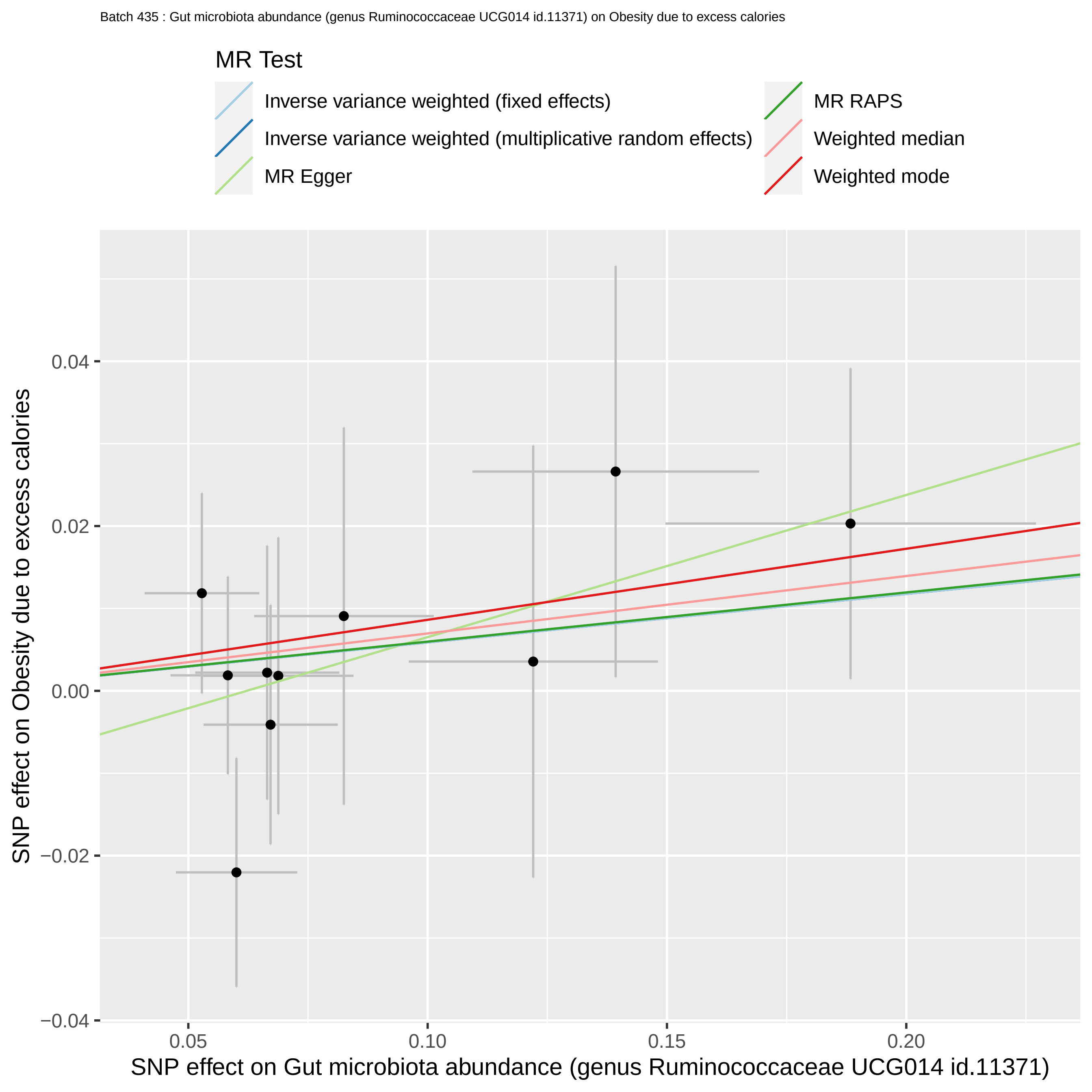

Batch 435 : Gut microbiota abundance (genus Ruminococcaceae UCG014 id.11371) on Obesity due to excess calories
MR Test
Inverse variance weighted (fixed effects)
Inverse variance weighted (multiplicative random effects)
MR Egger
MR RAPS
Weighted median
Weighted mode
SNP effect on Obesity due to excess calories
0.04
0.02
0.00
−0.02
−0.04
0.05
0.10
0.15
0.20
SNP effect on Gut microbiota abundance (genus Ruminococcaceae UCG014 id.11371)

Batch 436 : Gut microbiota abundance (genus Ruminococcus1 id.11373) on Obesity due to excess calories

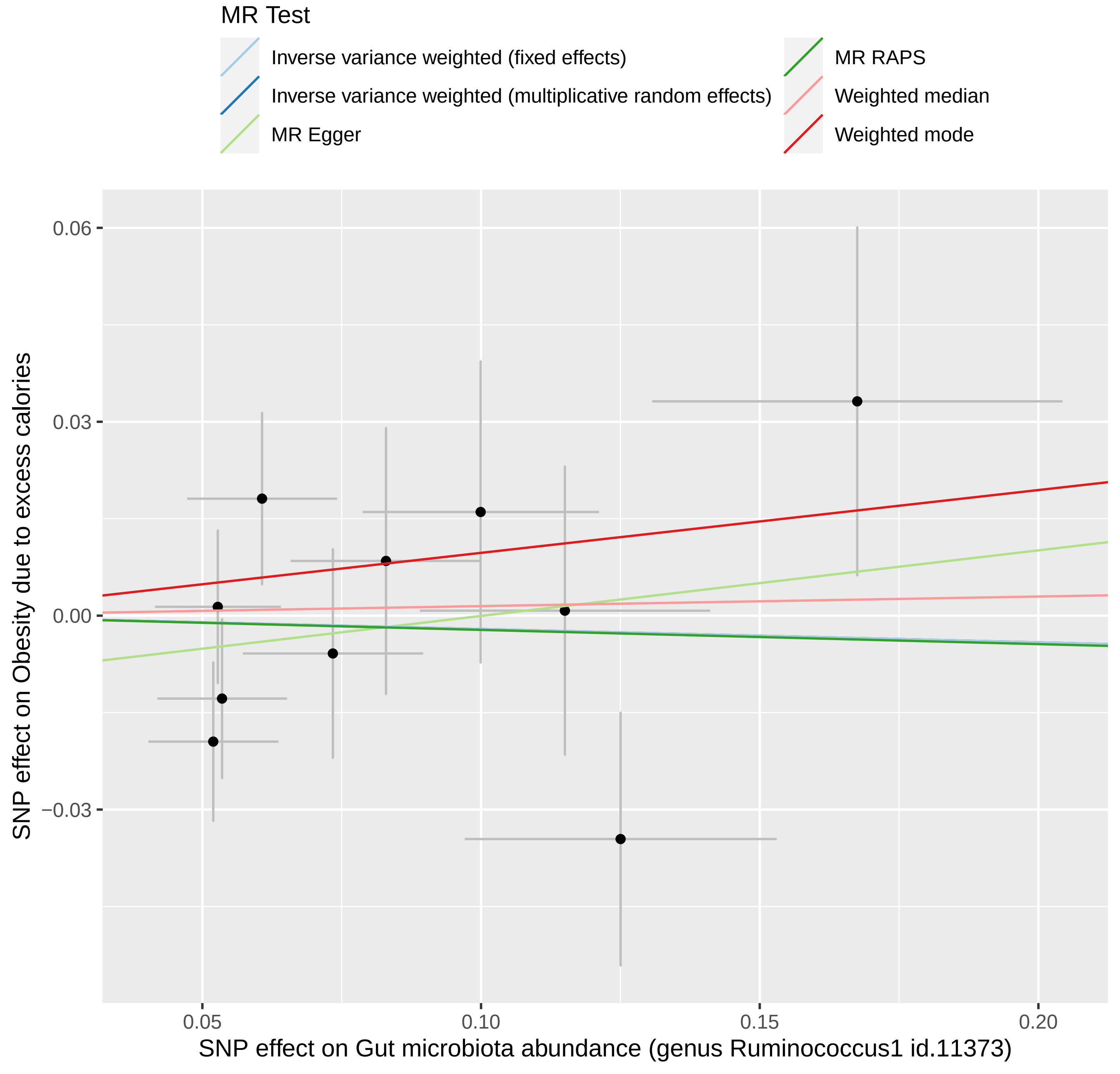

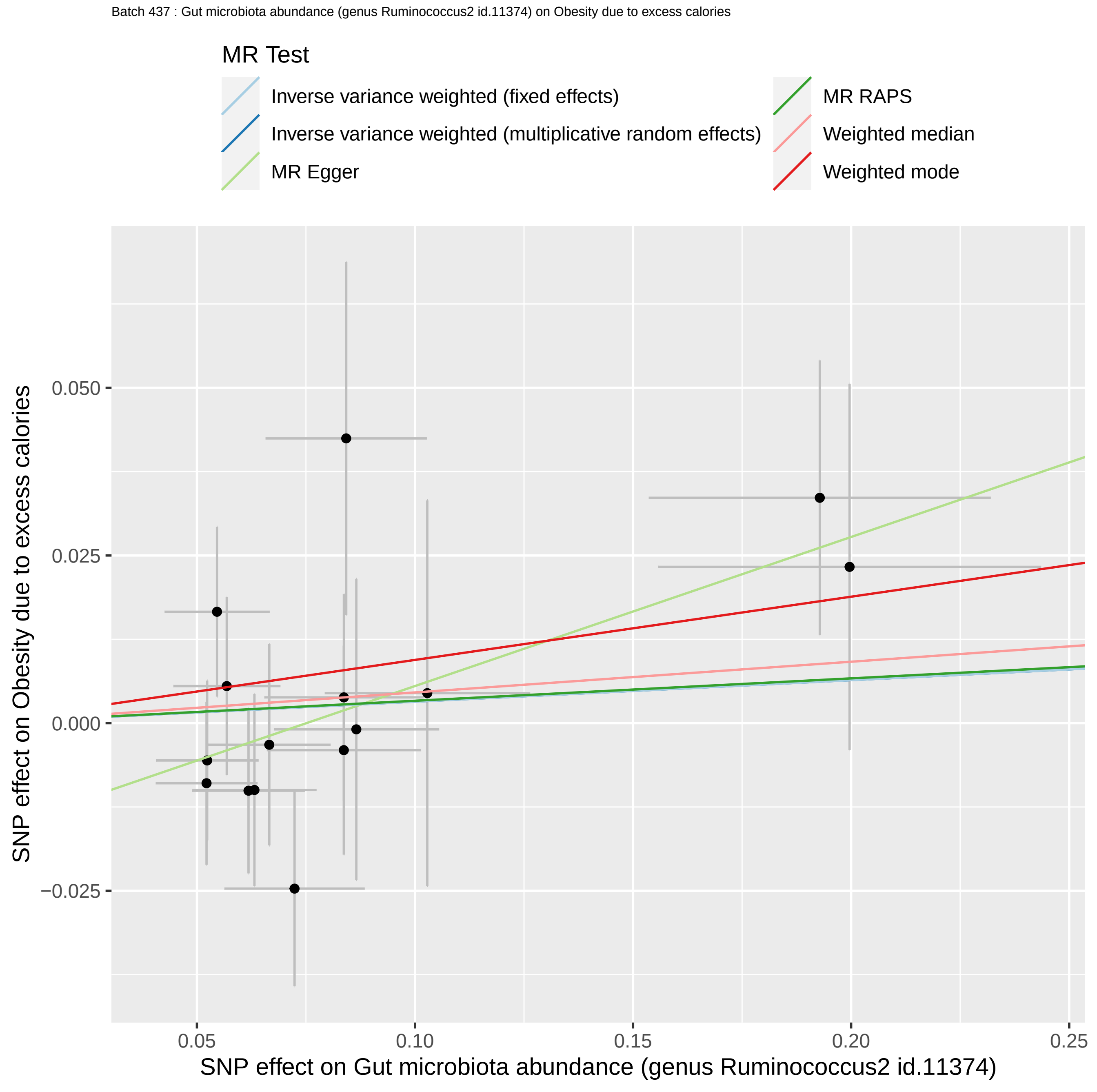
Batch 437 : Gut microbiota abundance (genus Ruminococcus2 id.11374) on Obesity due to excess calories
MR Test
Inverse variance weighted (fixed effects)
Inverse variance weighted (multiplicative random effects)
MR Egger
MR RAPS
Weighted median
Weighted mode
0.050
0.025
0.000
−0.025
0.05
0.10
0.15
0.20
0.25
SNP effect on Obesity due to excess calories
SNP effect on Gut microbiota abundance (genus Ruminococcus2 id.11374)

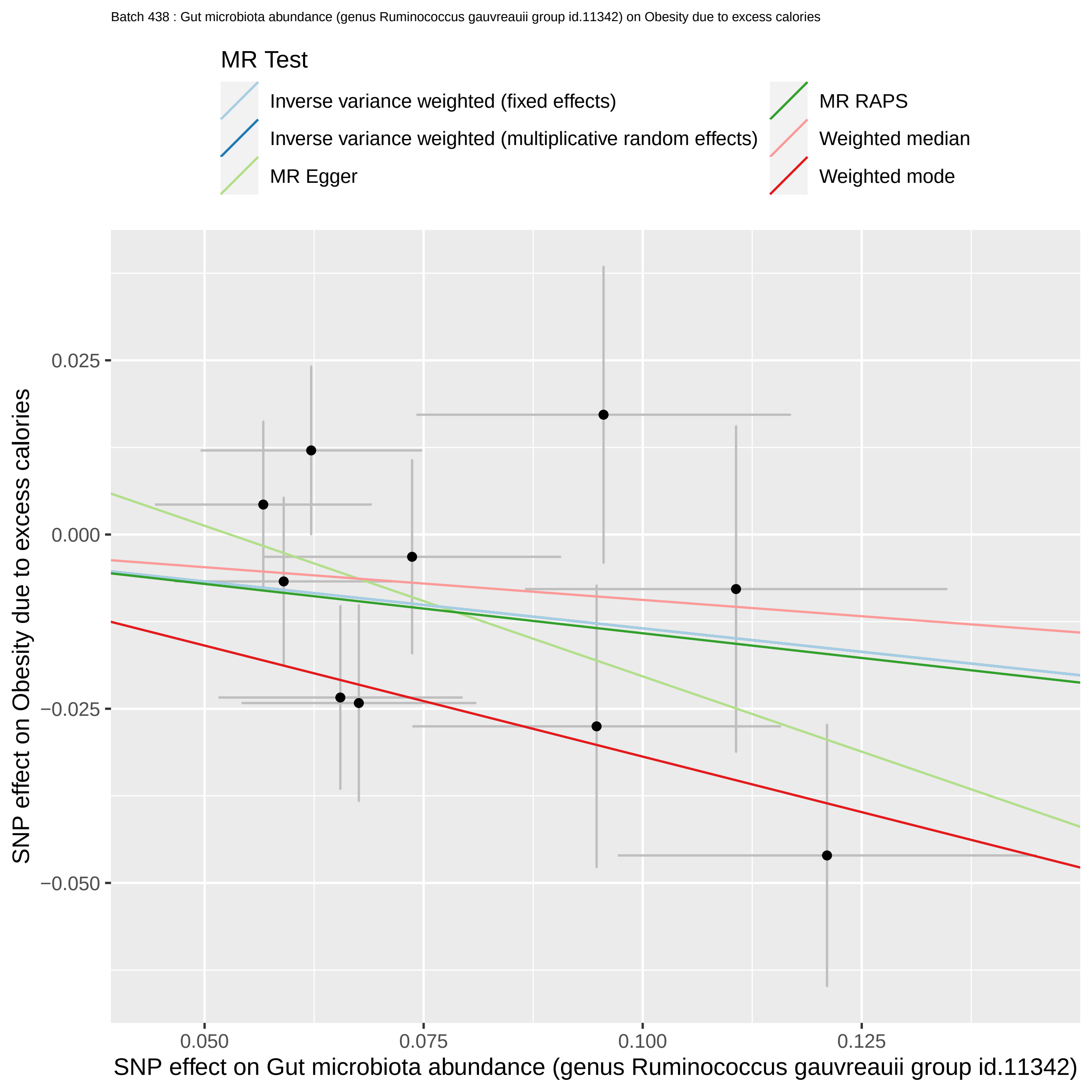

Batch 438 : Gut microbiota abundance (genus Ruminococcus gauvreauii group id.11342) on Obesity due to excess calories
MR Test
Inverse variance weighted (fixed effects)
Inverse variance weighted (multiplicative random effects)
MR Egger
MR RAPS
Weighted median
Weighted mode
0.025
0.000
−0.025
−0.050
0.050
0.075
0.100
0.125
SNP effect on Obesity due to excess calories
SNP effect on Gut microbiota abundance (genus Ruminococcus gauvreauii group id.11342)

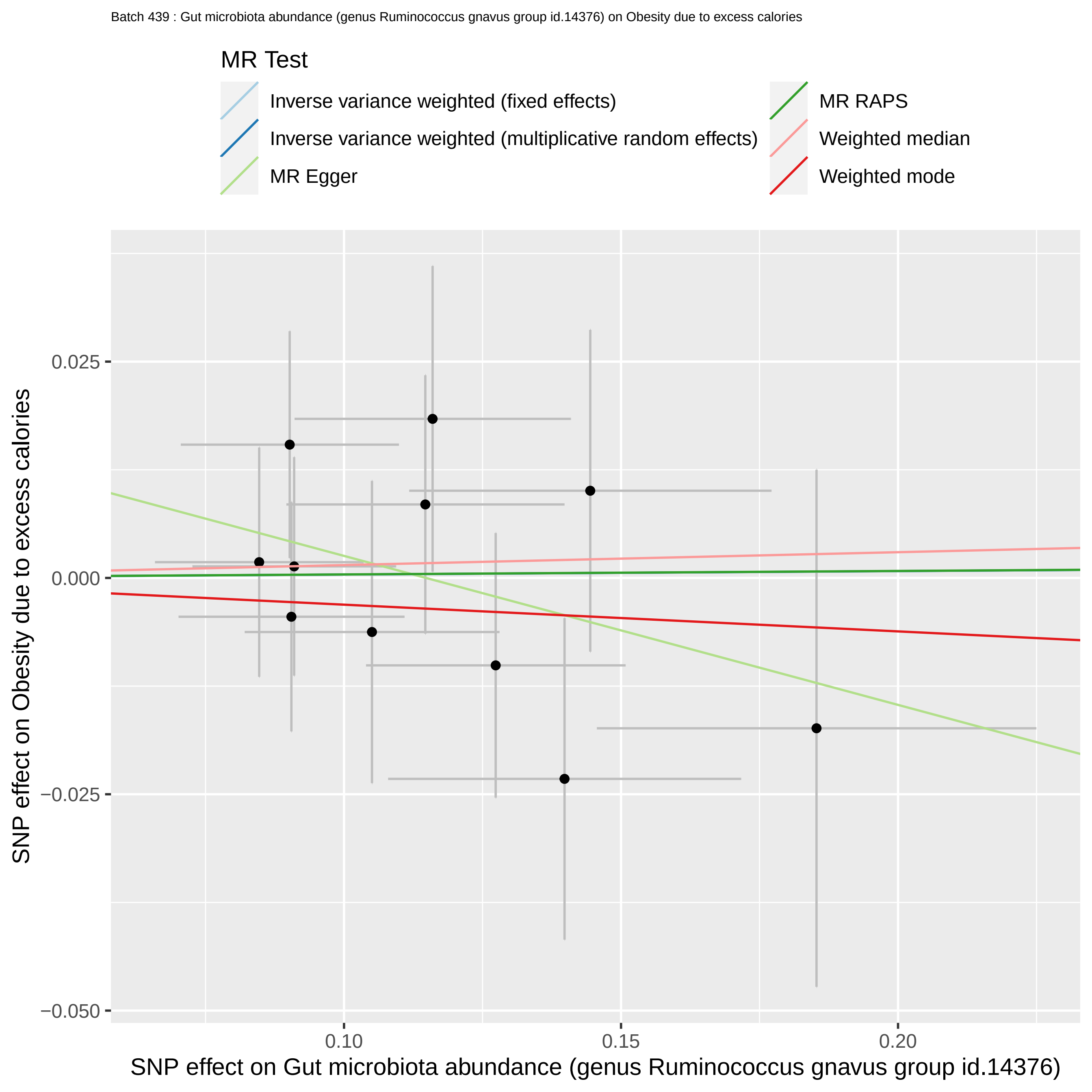
Batch 439 : Gut microbiota abundance (genus Ruminococcus gnavus group id.14376) on Obesity due to excess calories
MR Test
Inverse variance weighted (fixed effects)
Inverse variance weighted (multiplicative random effects)
MR Egger
MR RAPS
Weighted median
Weighted mode
0.025
0.000
−0.025
−0.050
0.10
0.15
0.20
SNP effect on Obesity due to excess calories
SNP effect on Gut microbiota abundance (genus Ruminococcus gnavus group id.14376)

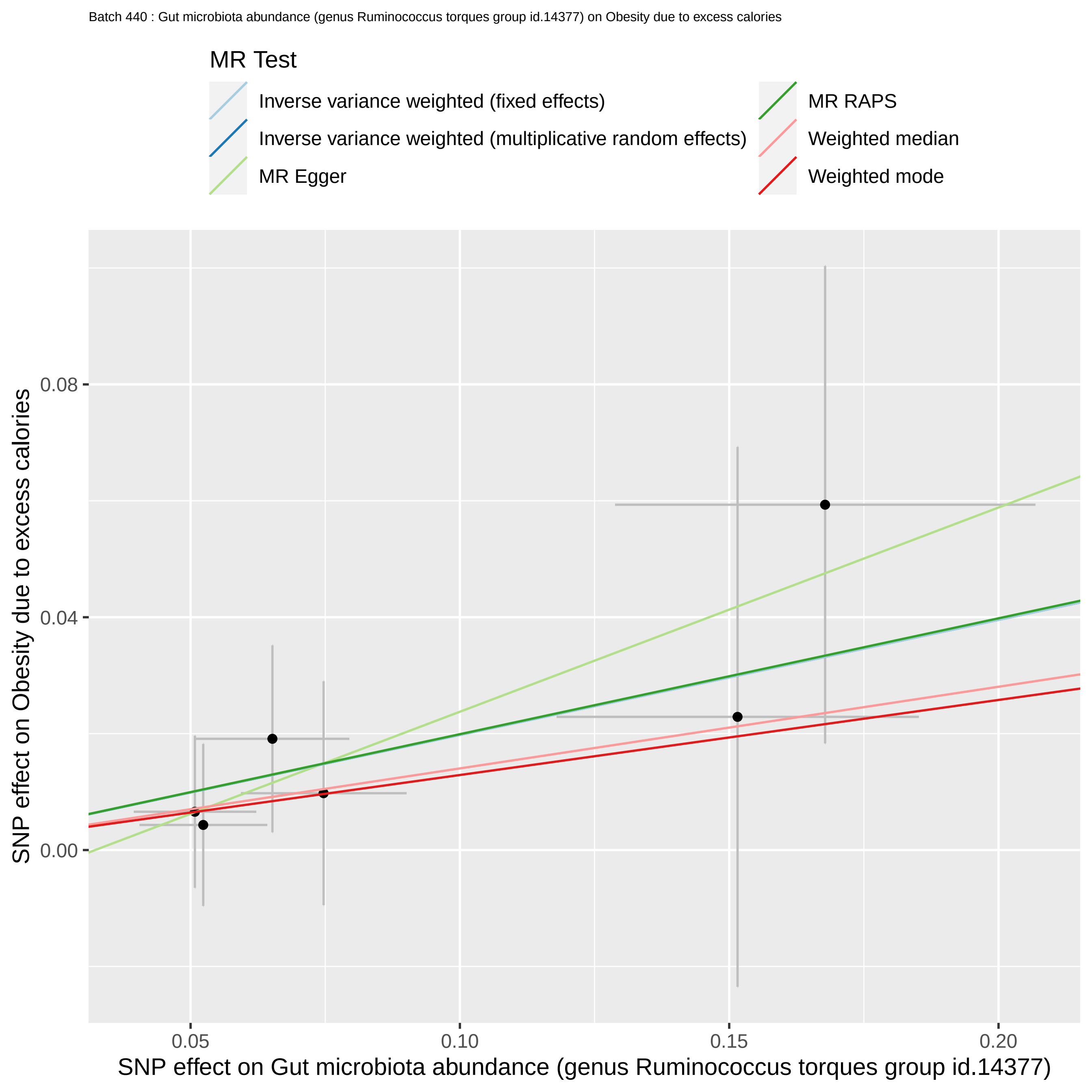

Batch 440 : Gut microbiota abundance (genus Ruminococcus torques group id.14377) on Obesity due to excess calories
MR Test
Inverse variance weighted (fixed effects)
Inverse variance weighted (multiplicative random effects)
MR Egger
MR RAPS
Weighted median
Weighted mode
0.08
0.04
0.00
0.05
0.10
0.15
0.20
SNP effect on Obesity due to excess calories
SNP effect on Gut microbiota abundance (genus Ruminococcus torques group id.14377)

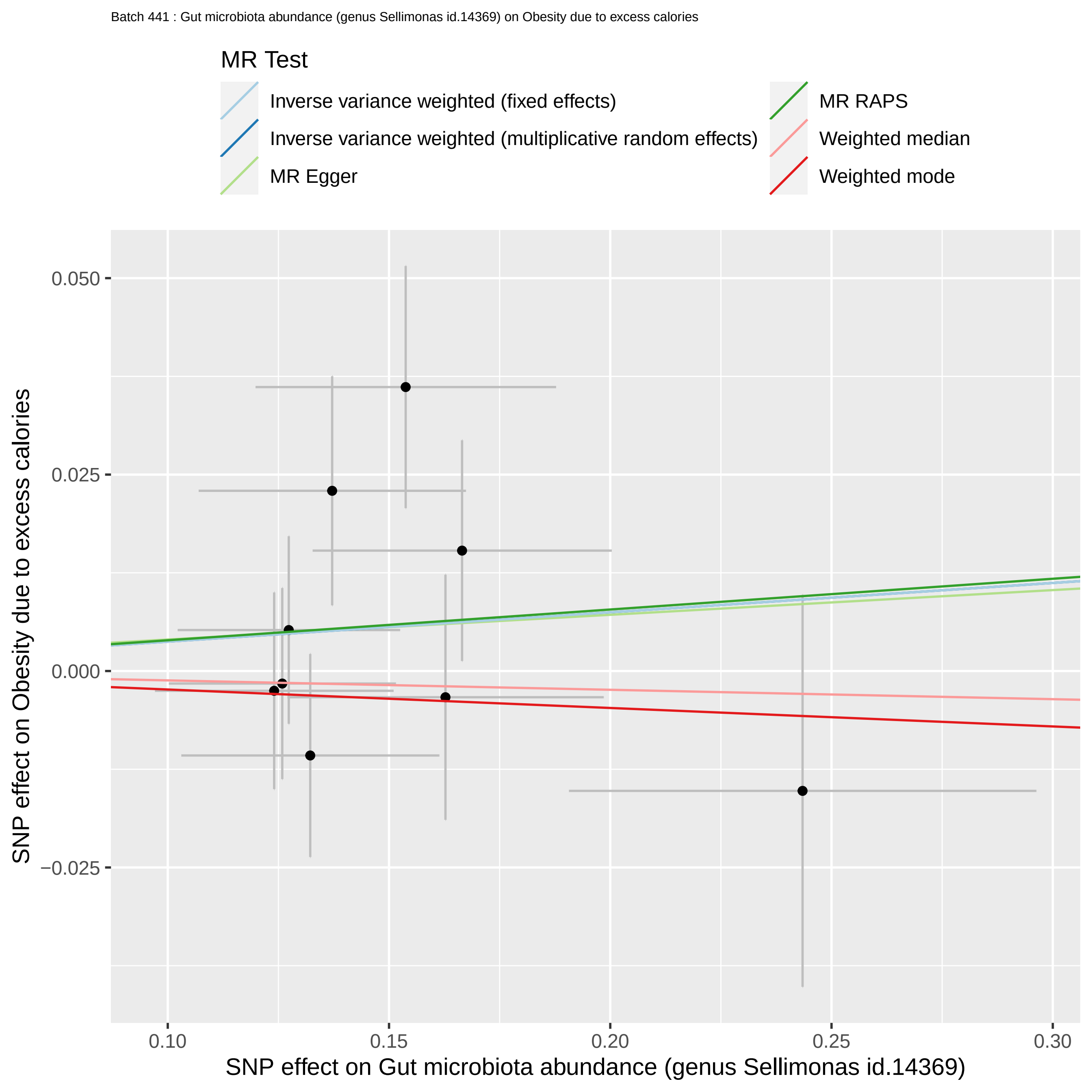

Batch 441 : Gut microbiota abundance (genus Sellimonas id.14369) on Obesity due to excess calories
MR Test
Inverse variance weighted (fixed effects)
Inverse variance weighted (multiplicative random effects)
MR Egger
MR RAPS
Weighted median
Weighted mode
0.050
0.025
0.000
−0.025
0.10
0.15
0.20
0.25
0.30
SNP effect on Obesity due to excess calories
SNP effect on Gut microbiota abundance (genus Sellimonas id.14369)

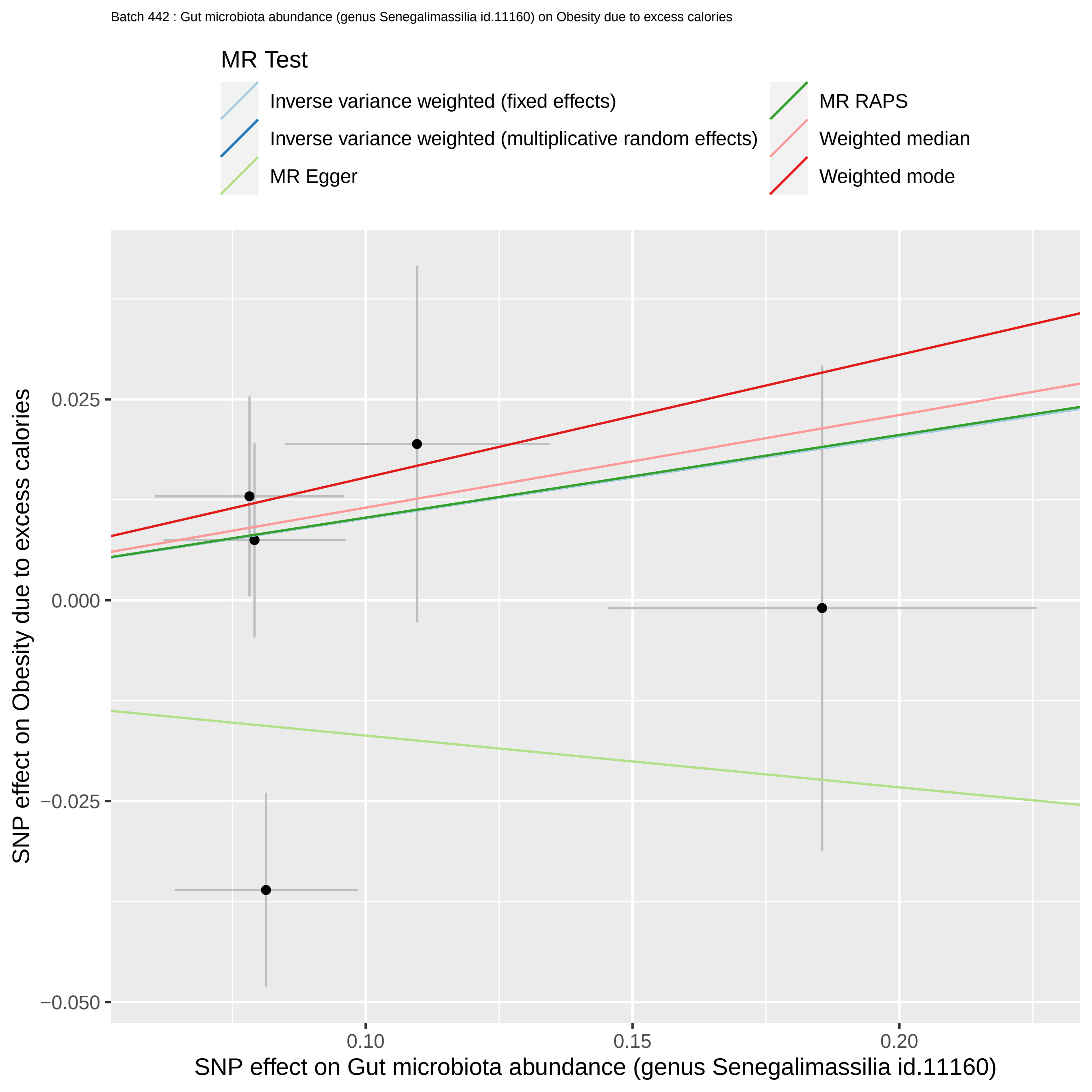

Batch 442 : Gut microbiota abundance (genus Senegalimassilia id.11160) on Obesity due to excess calories
MR Test
Inverse variance weighted (fixed effects)
Inverse variance weighted (multiplicative random effects)
MR Egger
MR RAPS
Weighted median
Weighted mode
SNP effect on Obesity due to excess calories
0.025
0.000
−0.025
−0.050
0.10
0.15
0.20
SNP effect on Gut microbiota abundance (genus Senegalimassilia id.11160)

Batch 443 : Gut microbiota abundance (genus Slackia id.825) on Obesity due to excess calories

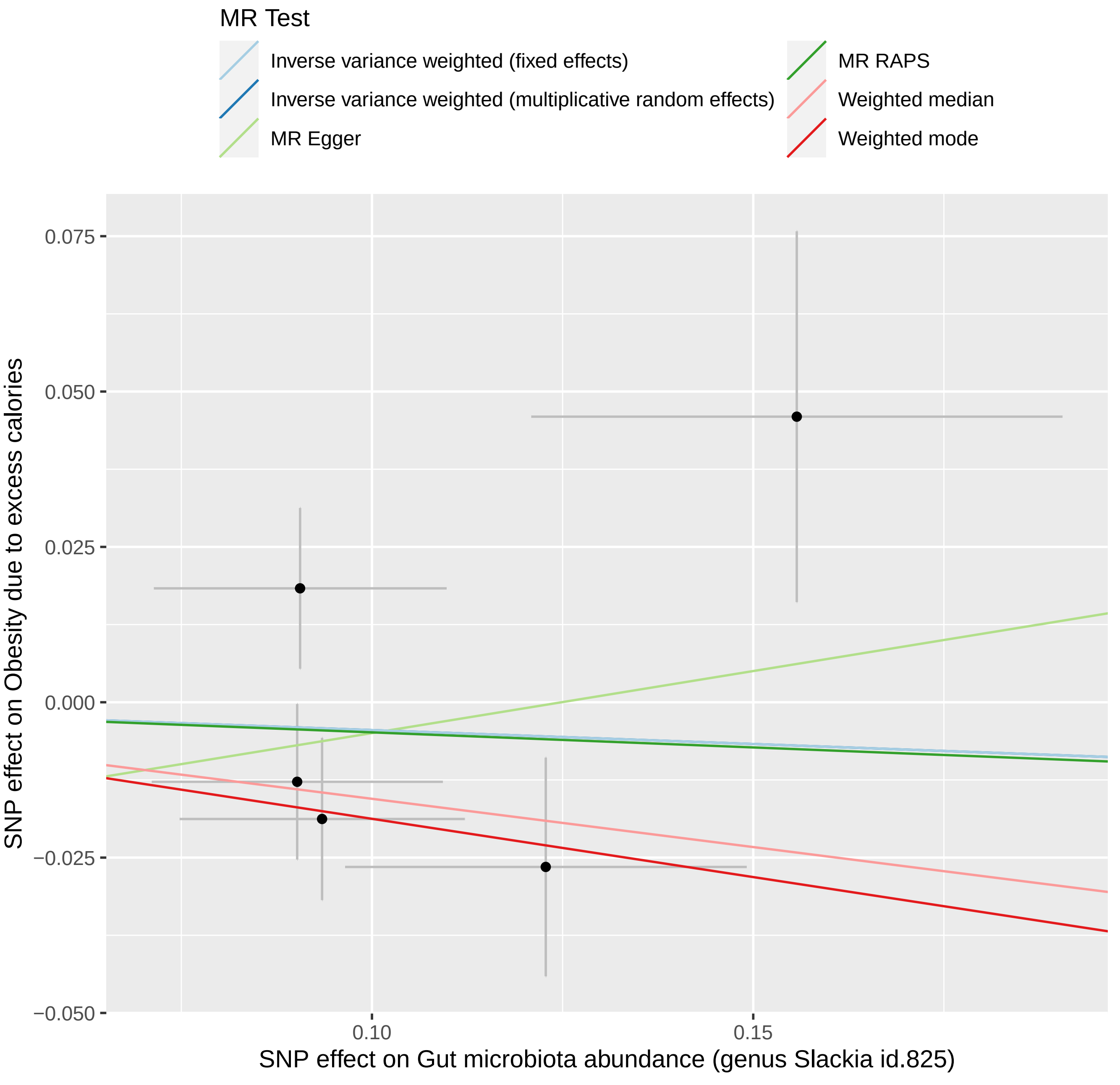

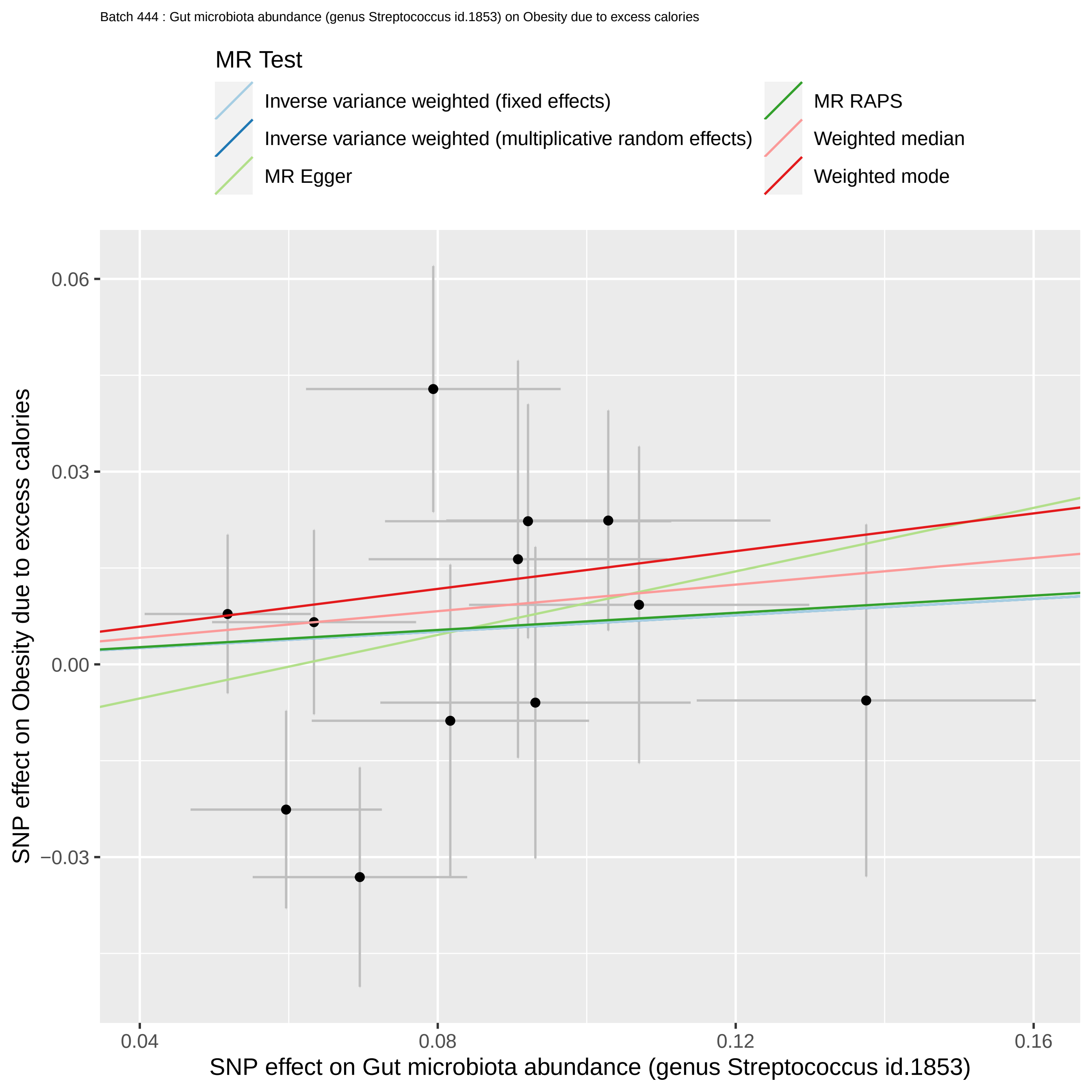
Batch 444 : Gut microbiota abundance (genus Streptococcus id.1853) on Obesity due to excess calories
MR Test
Inverse variance weighted (fixed effects)
Inverse variance weighted (multiplicative random effects)
MR Egger
MR RAPS
Weighted median
Weighted mode
0.06
0.03
0.00
−0.03
0.04
0.08
0.12
0.16
SNP effect on Obesity due to excess calories
SNP effect on Gut microbiota abundance (genus Streptococcus id.1853)

Batch 445 : Gut microbiota abundance (genus Subdoligranulum id.2070) on Obesity due to excess calories

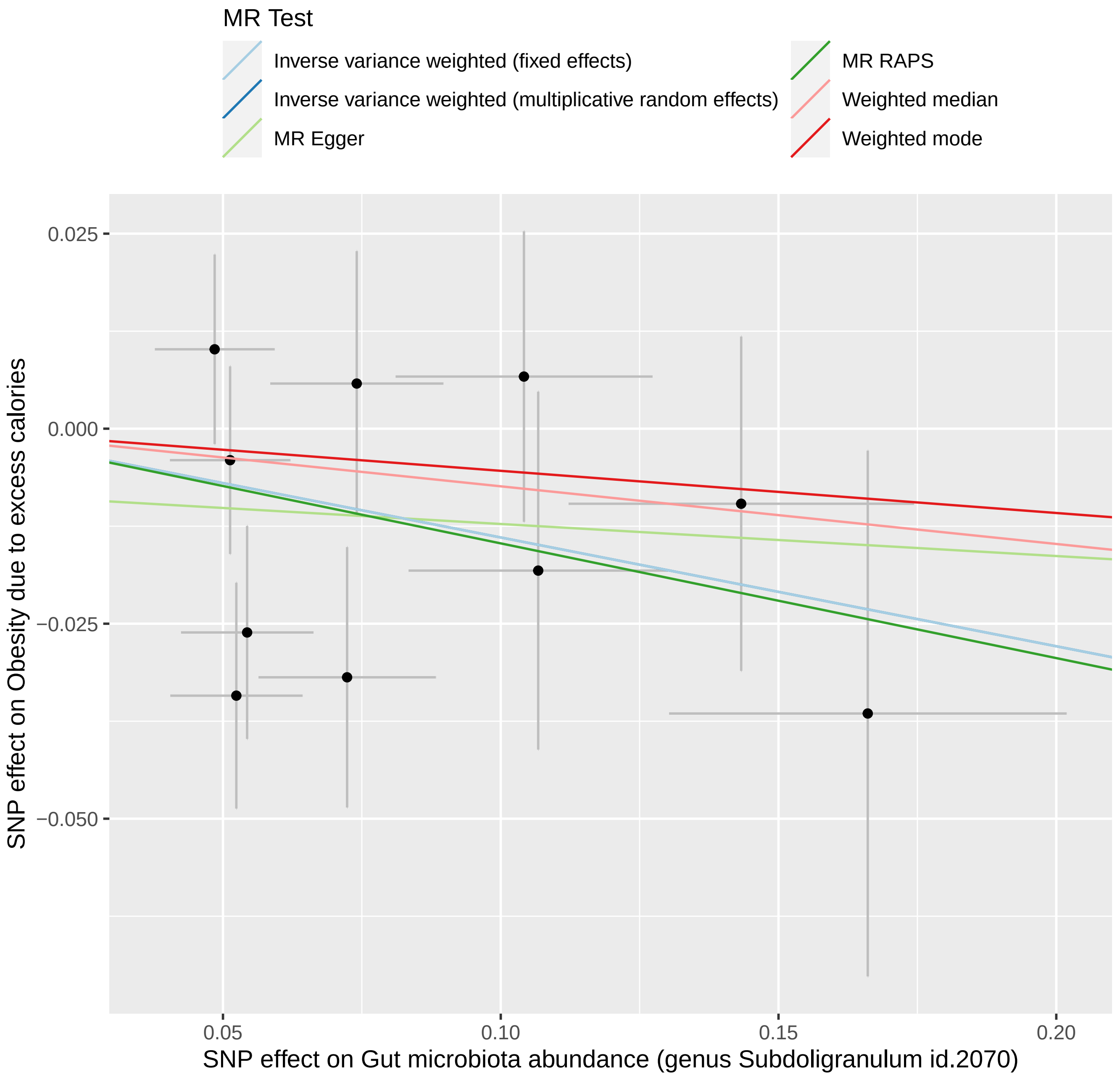

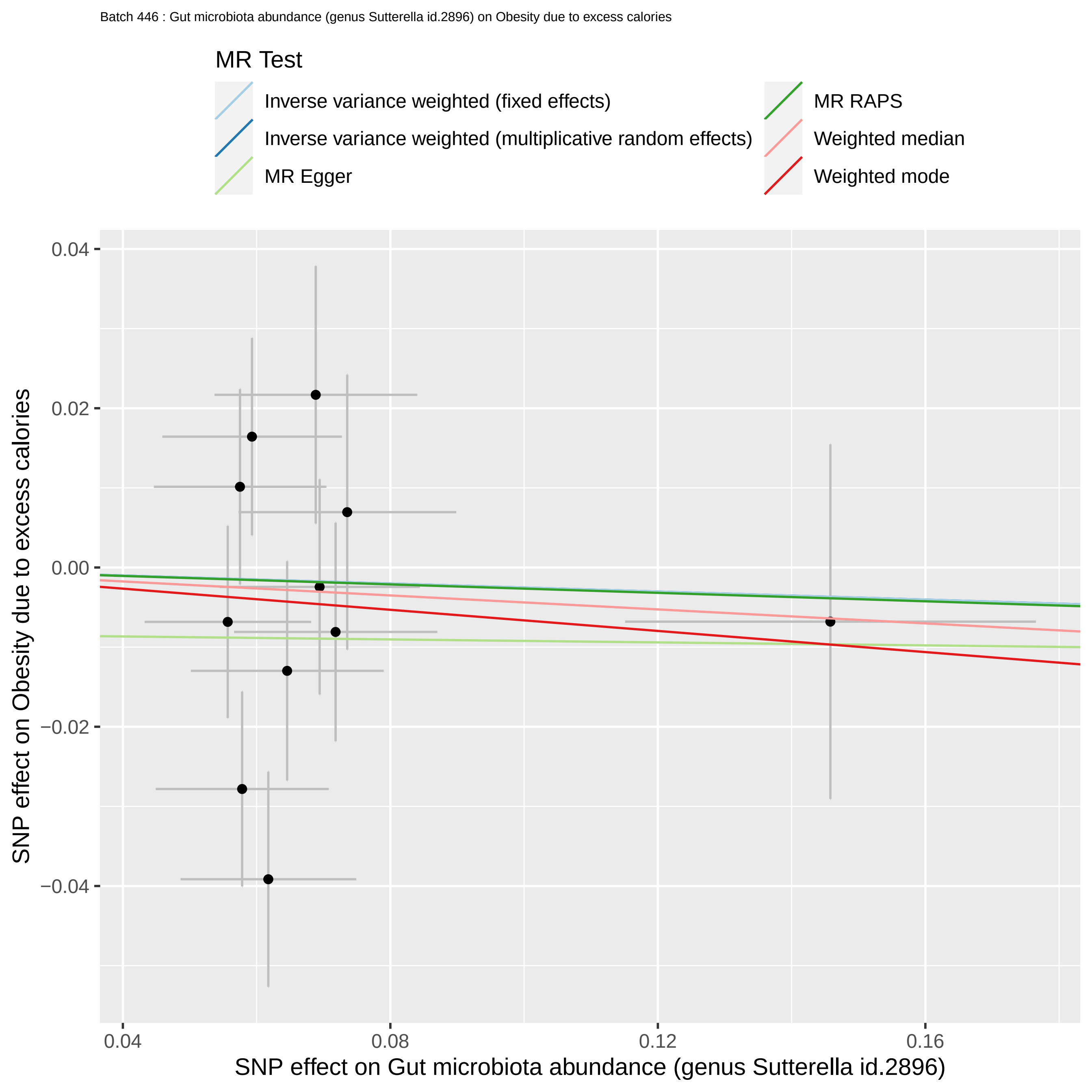

Batch 446 : Gut microbiota abundance (genus Sutterella id.2896) on Obesity due to excess calories
MR Test
Inverse variance weighted (fixed effects)
Inverse variance weighted (multiplicative random effects)
MR Egger
MR RAPS
Weighted median
Weighted mode
0.04
0.02
0.00
−0.02
−0.04
0.04
0.08
0.12
0.16
SNP effect on Obesity due to excess calories
SNP effect on Gut microbiota abundance (genus Sutterella id.2896)

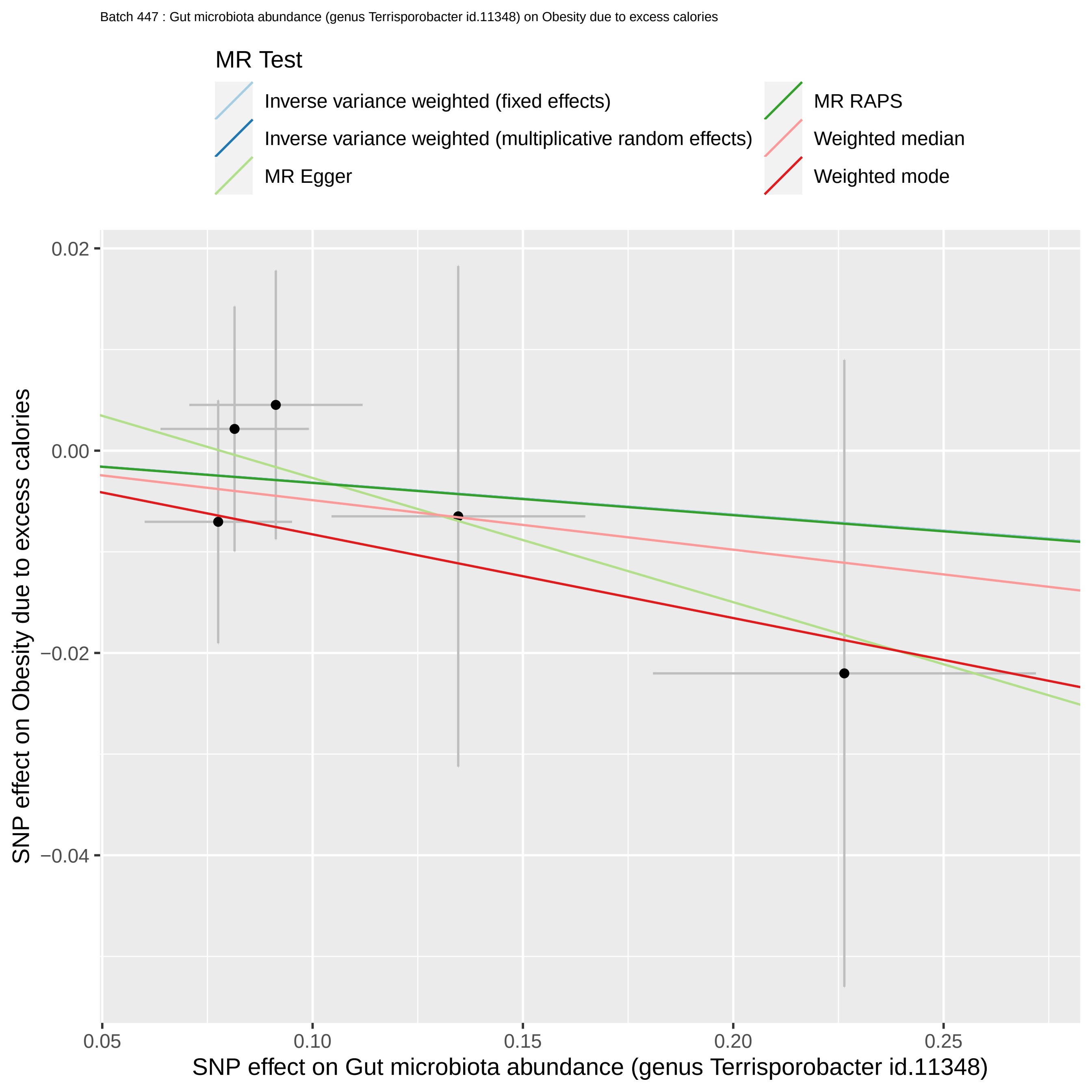

Batch 447 : Gut microbiota abundance (genus Terrisporobacter id.11348) on Obesity due to excess calories
MR Test
Inverse variance weighted (fixed effects)
Inverse variance weighted (multiplicative random effects)
MR Egger
MR RAPS
Weighted median
Weighted mode
0.02
0.00
−0.02
−0.04
0.05
0.10
0.15
0.20
0.25
SNP effect on Obesity due to excess calories
SNP effect on Gut microbiota abundance (genus Terrisporobacter id.11348)

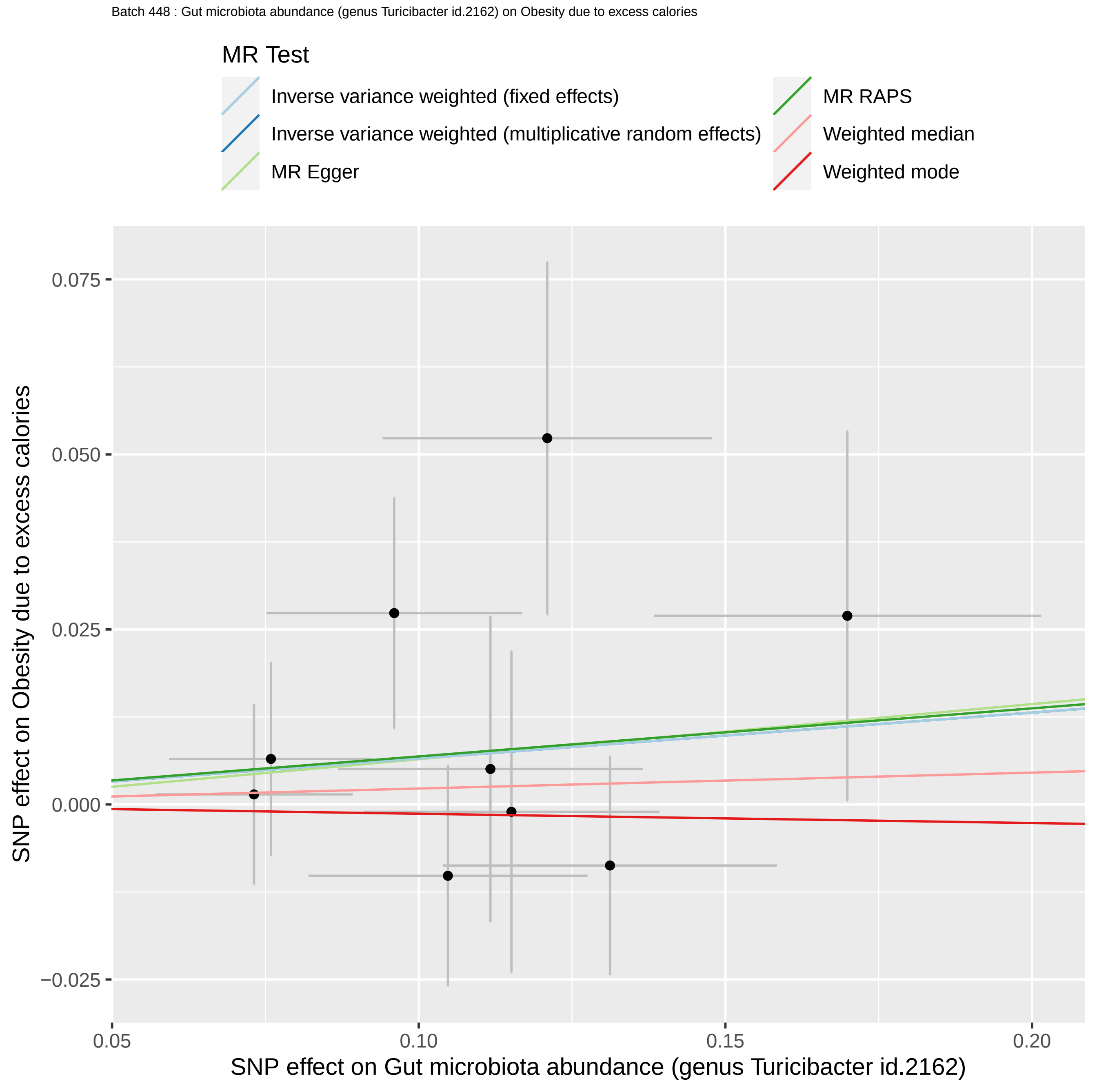

Batch 448 : Gut microbiota abundance (genus Turicibacter id.2162) on Obesity due to excess calories
MR Test
Inverse variance weighted (fixed effects)
Inverse variance weighted (multiplicative random effects)
MR Egger
MR RAPS
Weighted median
Weighted mode
0.075
0.050
0.025
0.000
−0.025
0.05
0.10
0.15
0.20
SNP effect on Obesity due to excess calories
SNP effect on Gut microbiota abundance (genus Turicibacter id.2162)

Batch 449 : Gut microbiota abundance (genus Tyzzerella3 id.11335) on Obesity due to excess calories

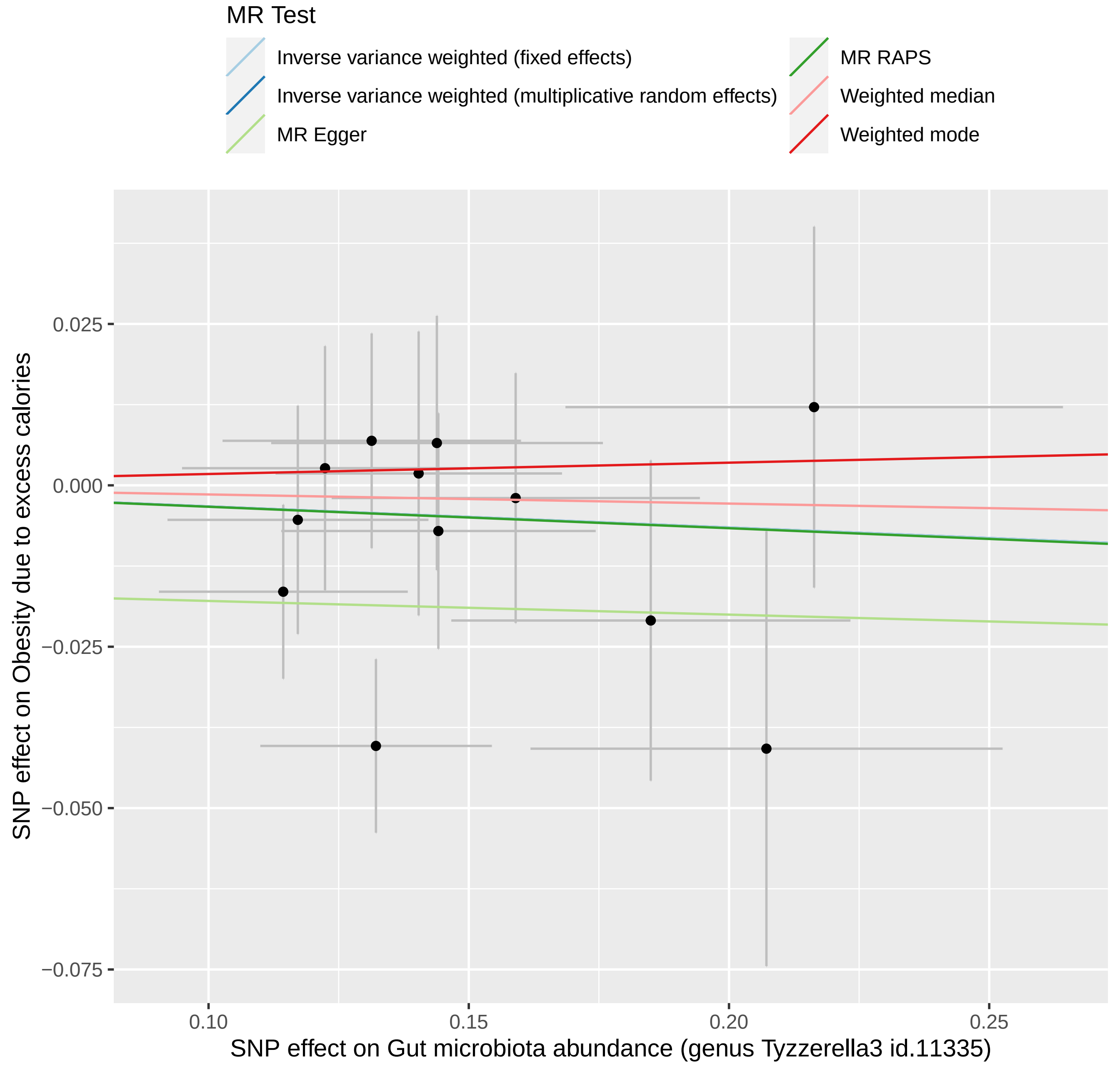

Batch 450 : Gut microbiota abundance (genus Veillonella id.2198) on Obesity due to excess calories

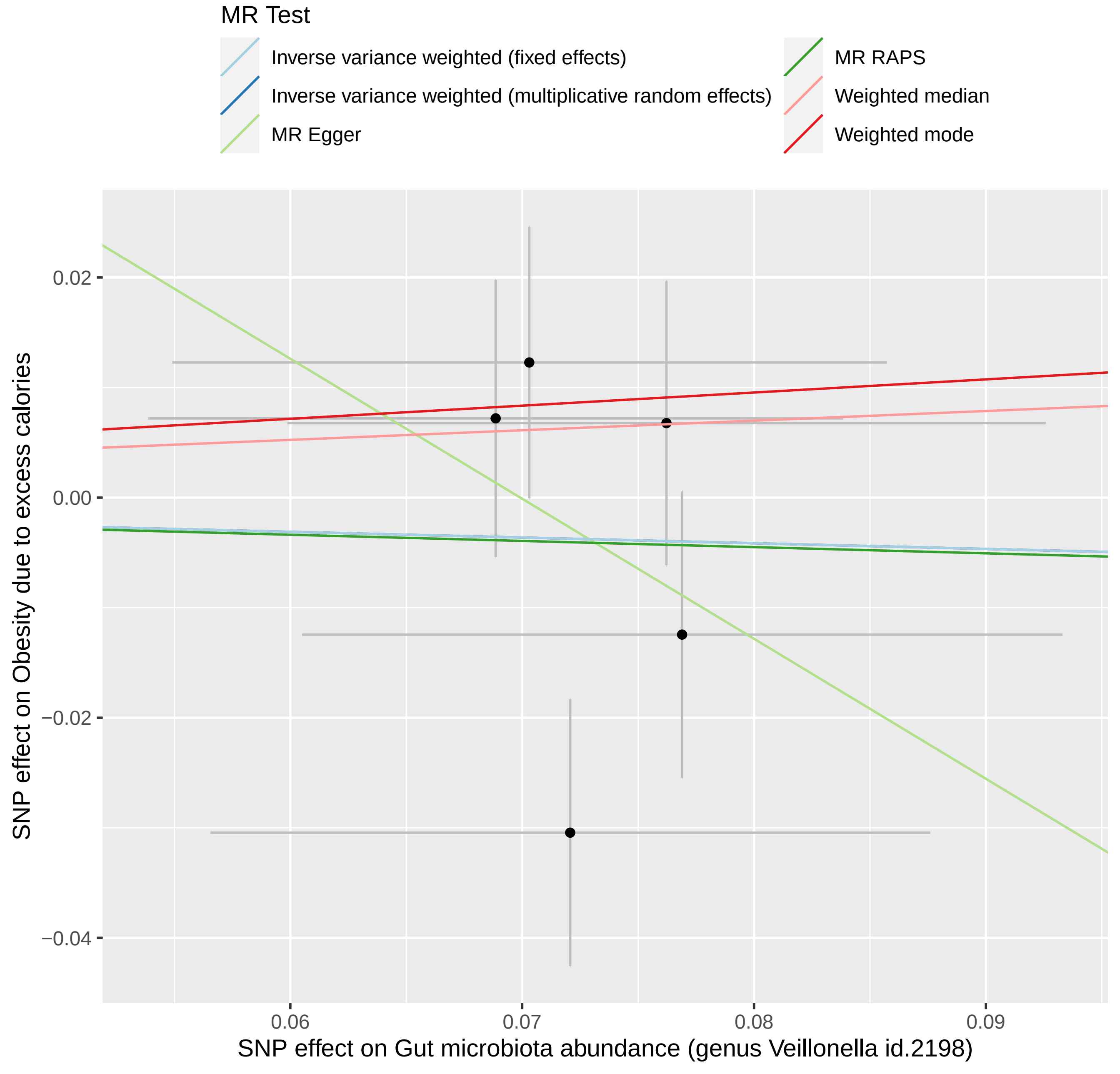

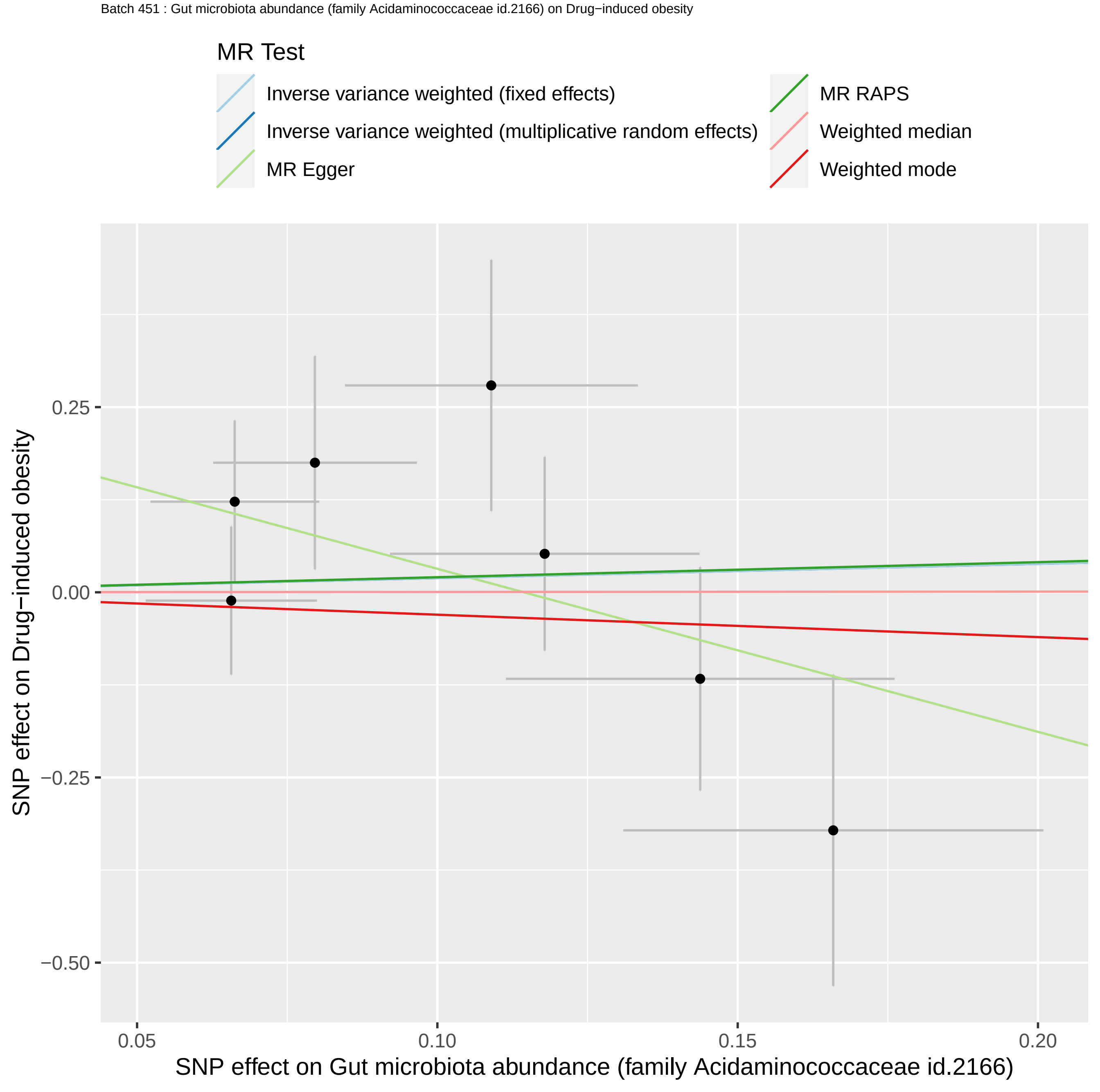
Batch 451 : Gut microbiota abundance (family Acidaminococcaceae id.2166) on Drug−induced obesity
MR Test
Inverse variance weighted (fixed effects)
Inverse variance weighted (multiplicative random effects)
MR Egger
MR RAPS
Weighted median
Weighted mode
0.25
0.00
−0.25
−0.50
0.05
0.10
0.15
0.20
SNP effect on Drug−induced obesity
SNP effect on Gut microbiota abundance (family Acidaminococcaceae id.2166)

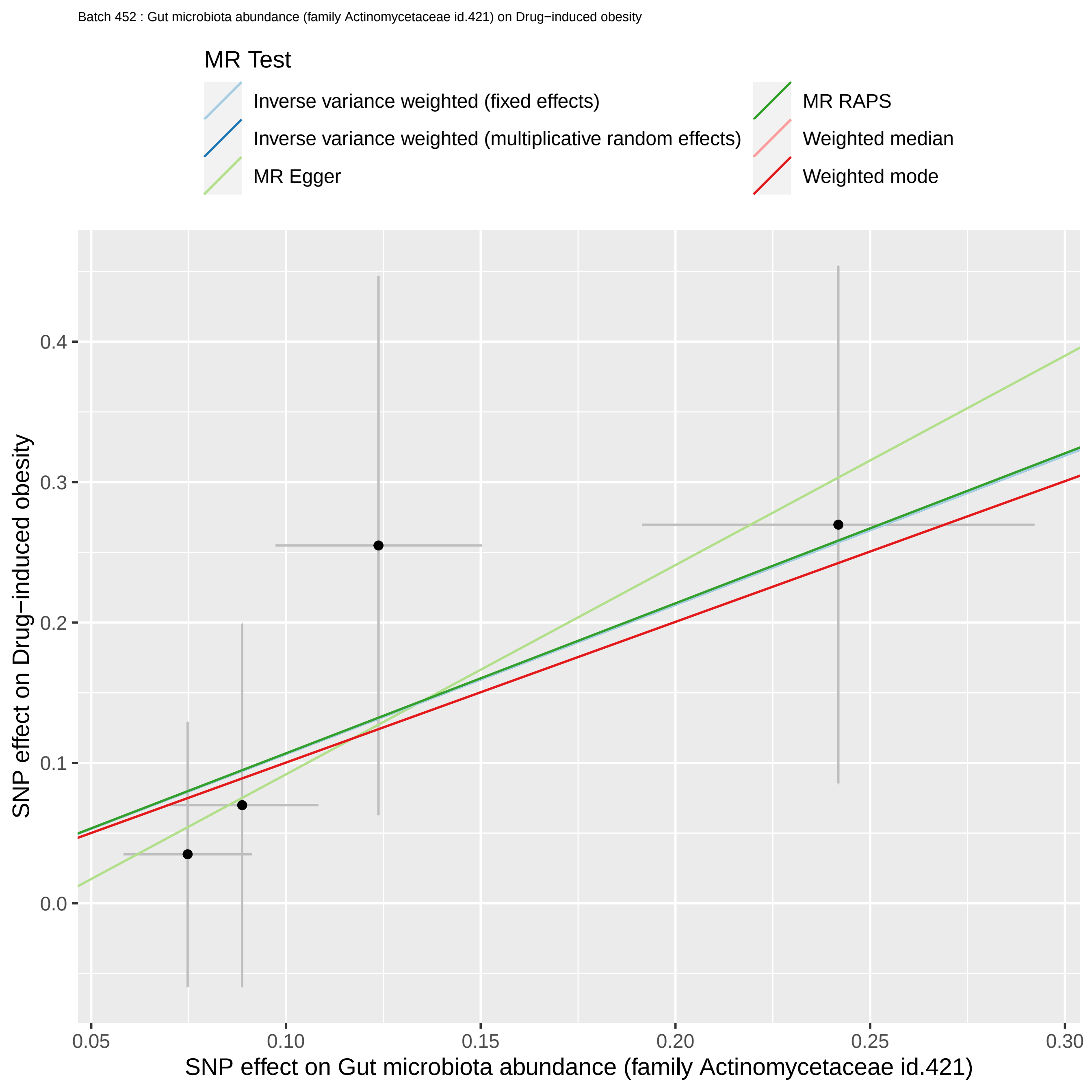

Batch 452 : Gut microbiota abundance (family Actinomycetaceae id.421) on Drug−induced obesity
MR Test
Inverse variance weighted (fixed effects)
Inverse variance weighted (multiplicative random effects)
MR Egger
MR RAPS
Weighted median
Weighted mode
SNP effect on Drug−induced obesity
0.0
0.1
0.2
0.3
0.4
0.05
0.10
0.15
0.20
0.25
0.30
SNP effect on Gut microbiota abundance (family Actinomycetaceae id.421)

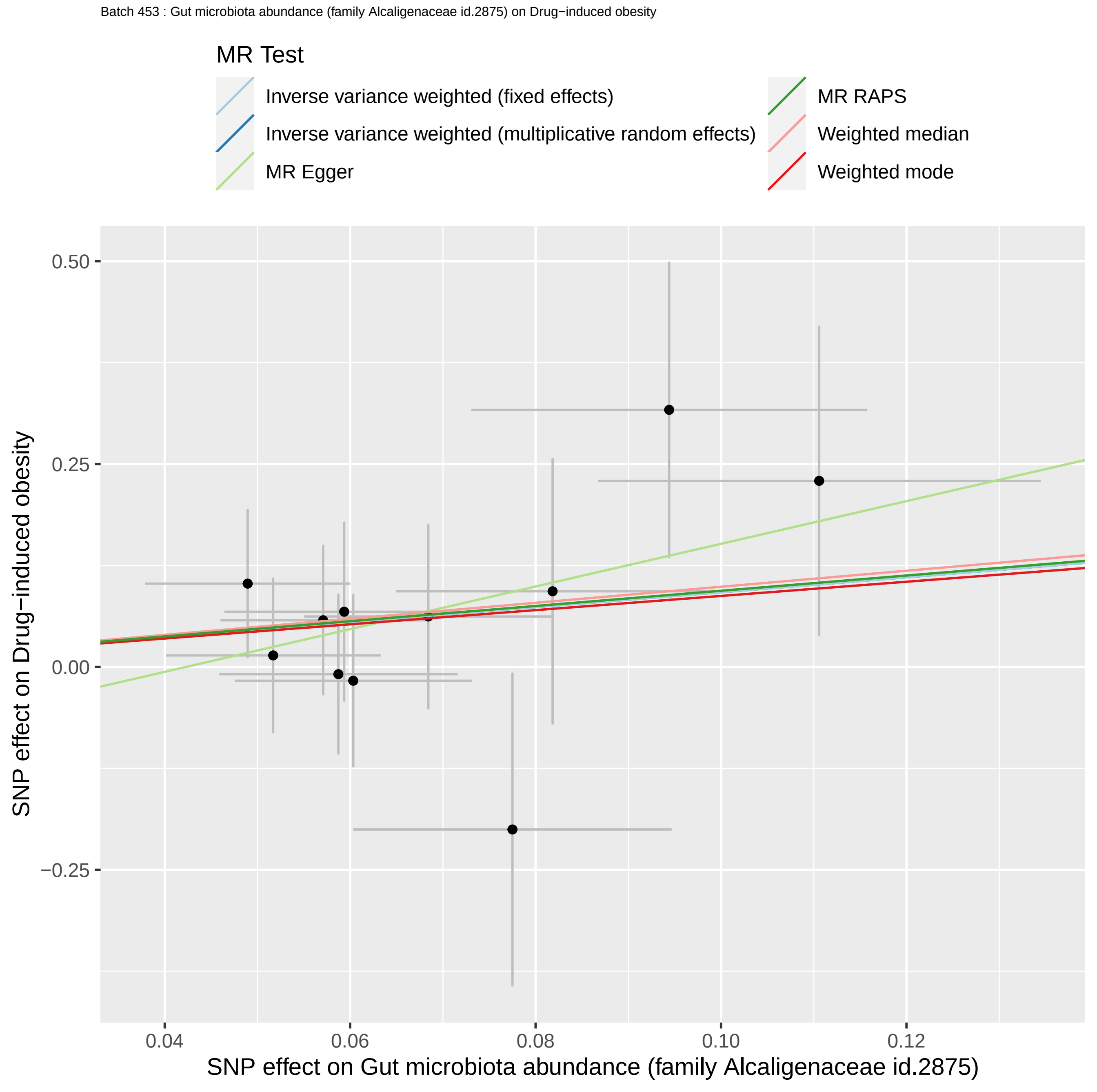

Batch 453 : Gut microbiota abundance (family Alcaligenaceae id.2875) on Drug−induced obesity
MR Test
Inverse variance weighted (fixed effects)
Inverse variance weighted (multiplicative random effects)
MR Egger
MR RAPS
Weighted median
Weighted mode
0.50
0.25
0.00
−0.25
0.04
0.06
0.08
0.10
0.12
SNP effect on Drug−induced obesity
SNP effect on Gut microbiota abundance (family Alcaligenaceae id.2875)

Batch 454 : Gut microbiota abundance (family Bacteroidaceae id.917) on Drug−induced obesity

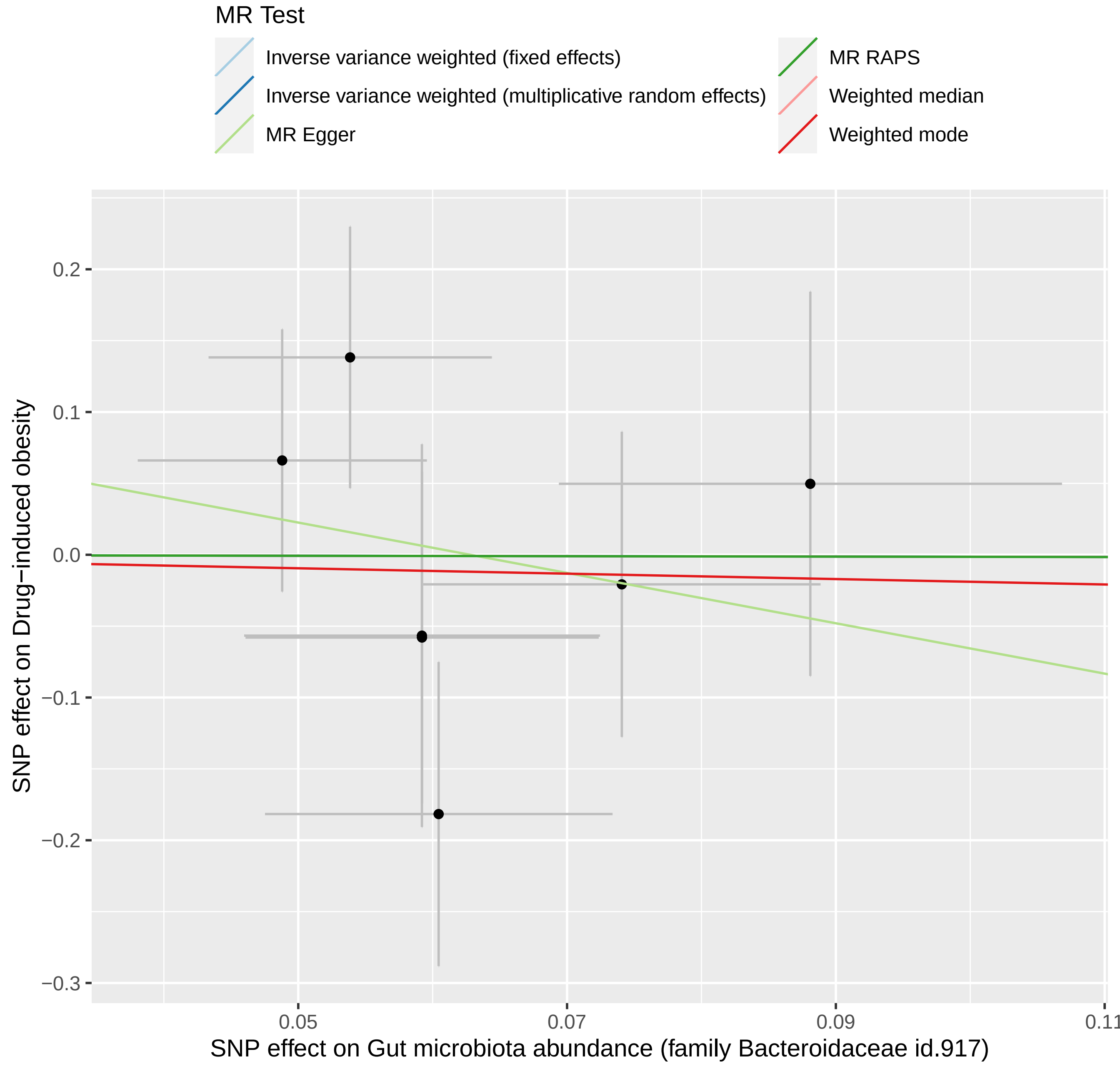

Batch 455 : Gut microbiota abundance (family Bacteroidales S24 7group id.11173) on Drug−induced obesity

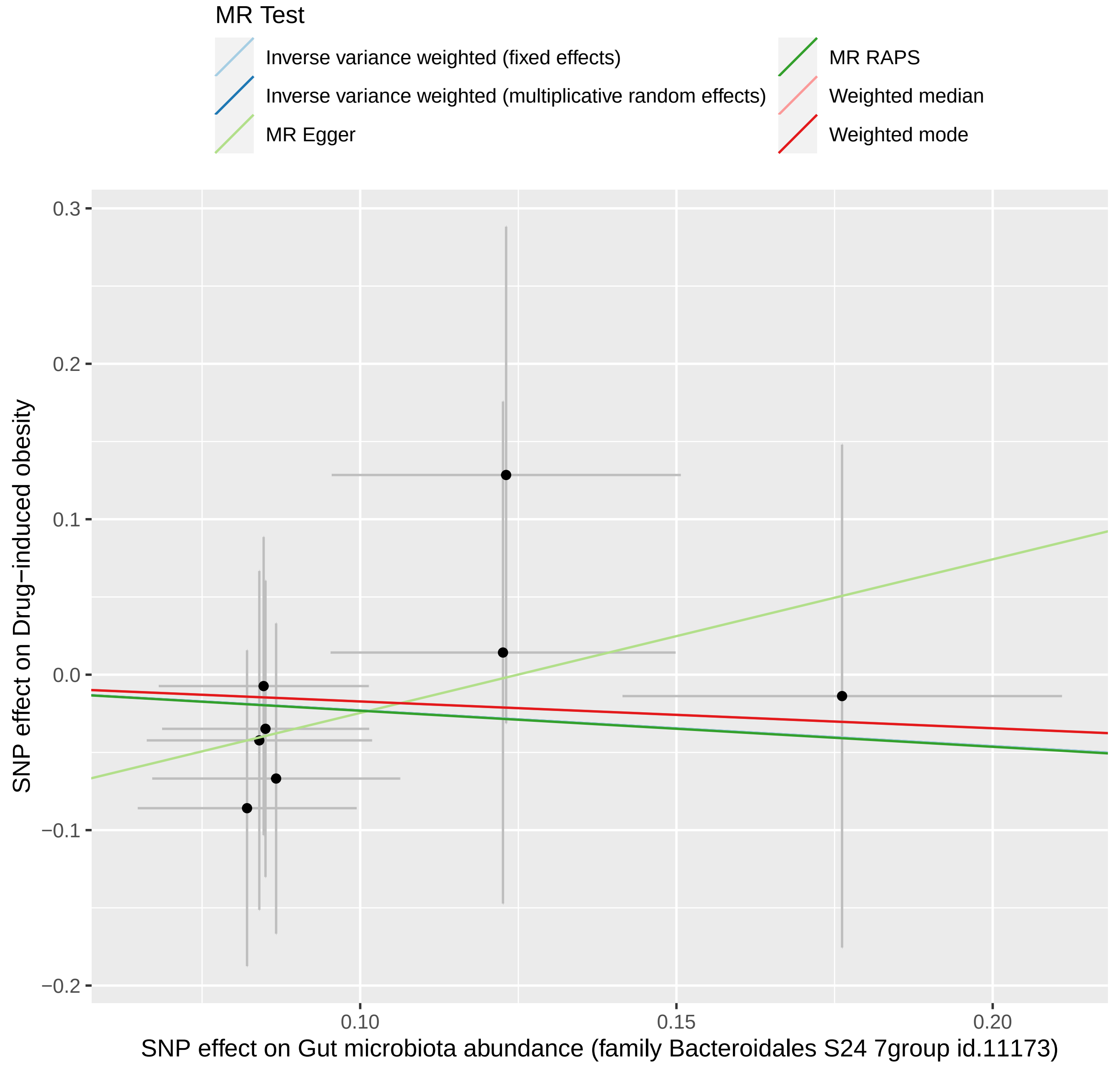

Batch 456 : Gut microbiota abundance (family Bifidobacteriaceae id.433) on Drug−induced obesity

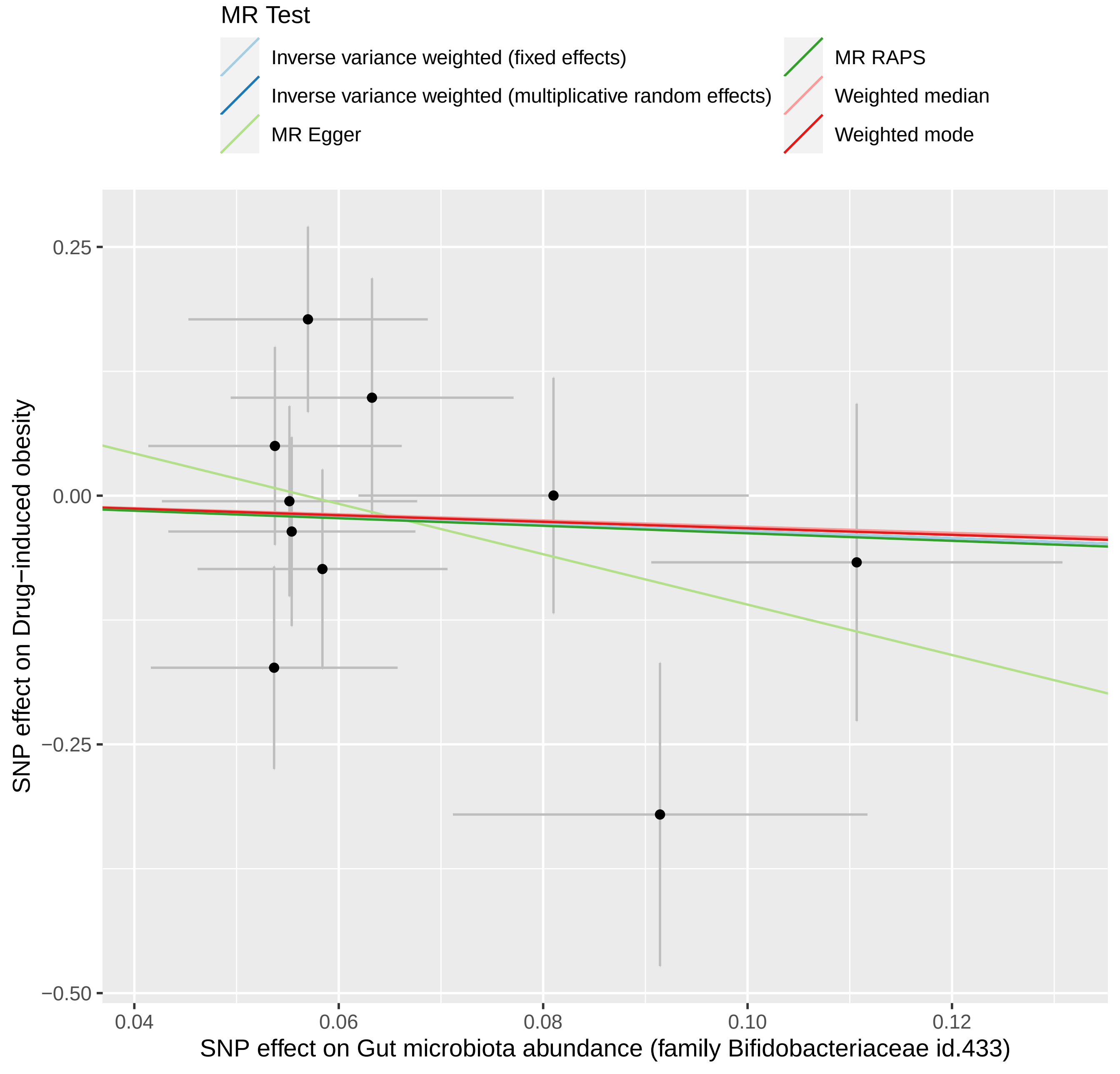

Batch 458 : Gut microbiota abundance (family Clostridiaceae1 id.1869) on Drug−induced obesity

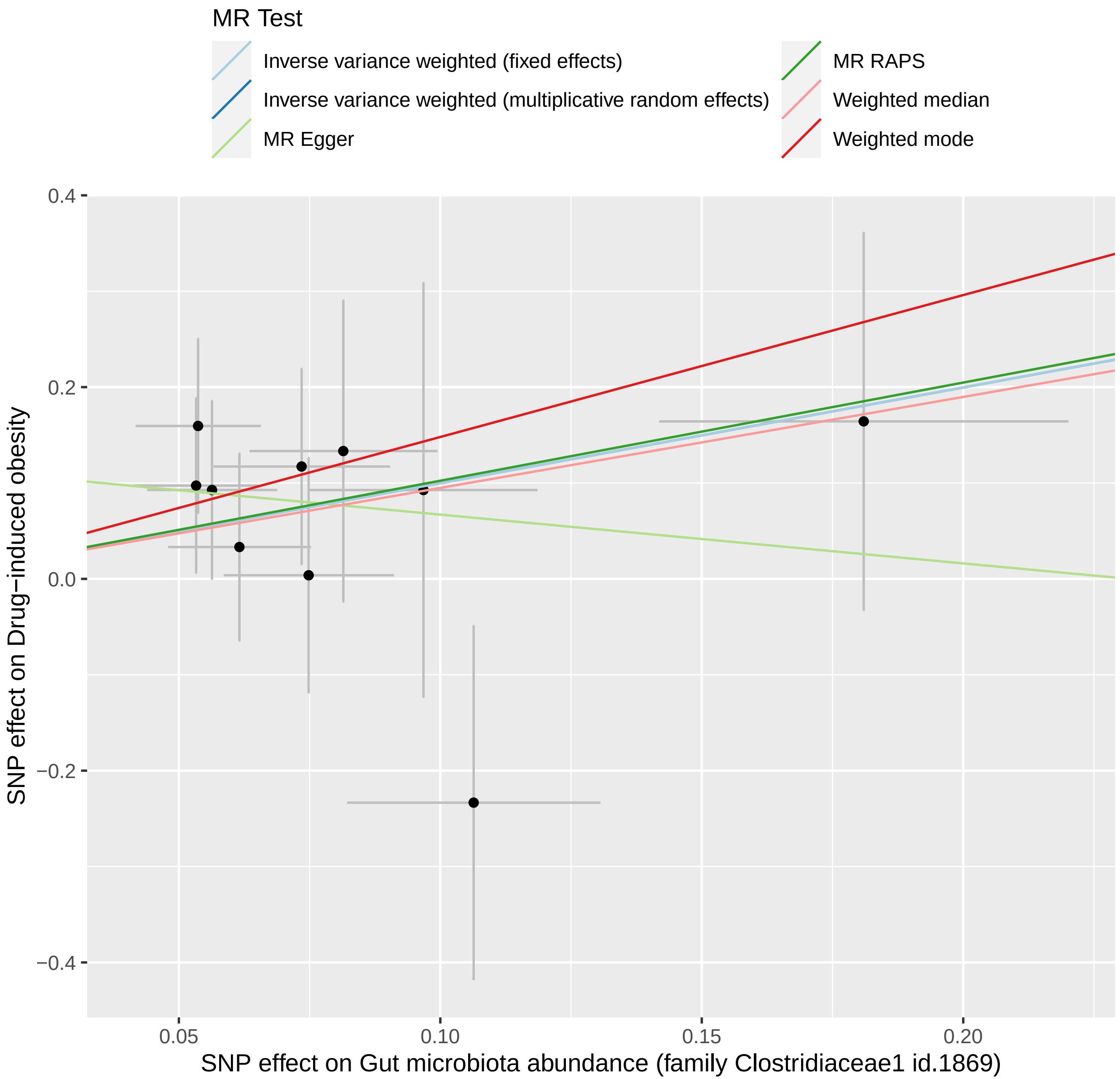

Batch 459 : Gut microbiota abundance (family Clostridiales vadin BB60 group id.11286) on Drug−induced obesity

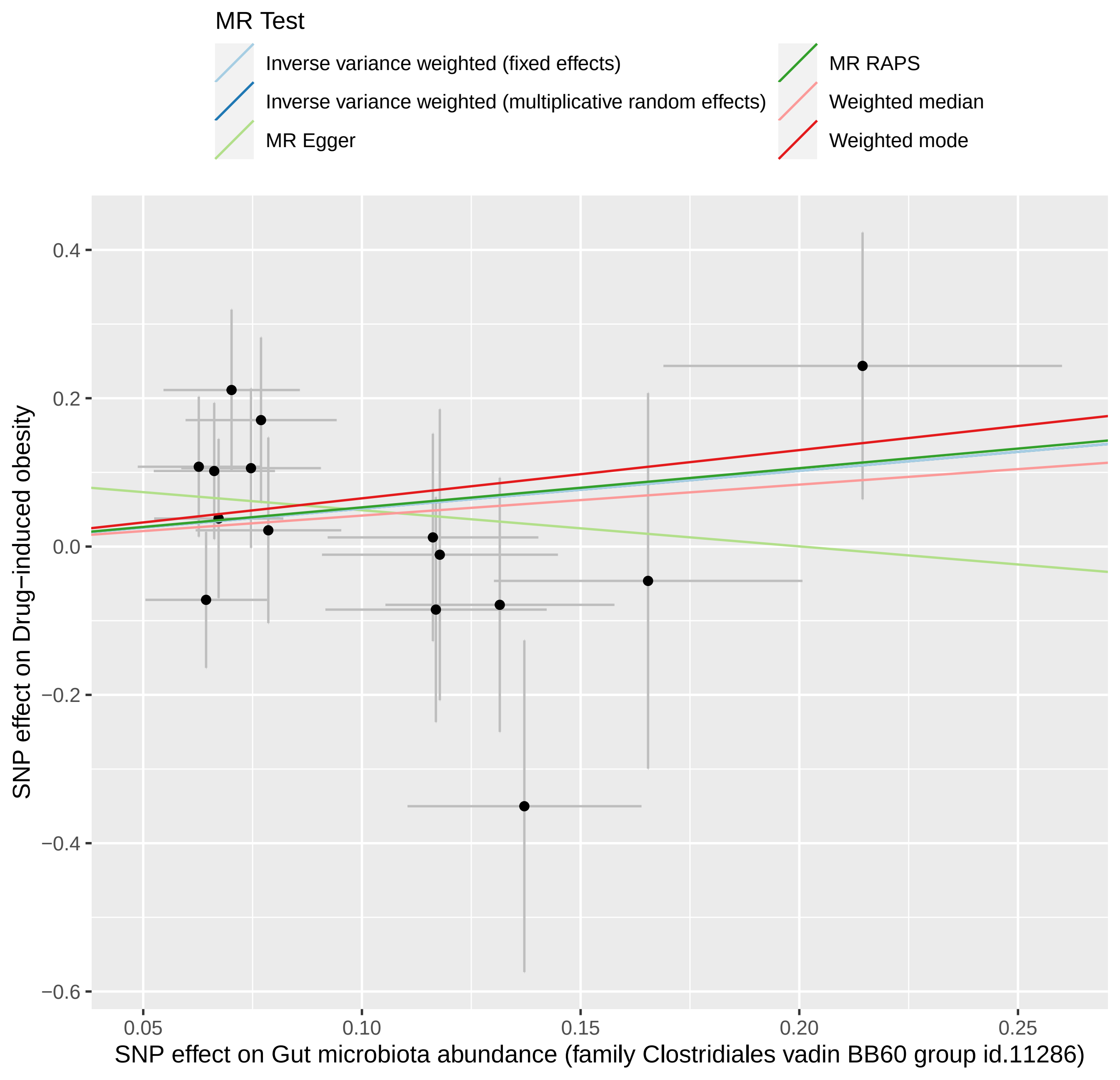

Batch 460 : Gut microbiota abundance (family Coriobacteriaceae id.811) on Drug−induced obesity

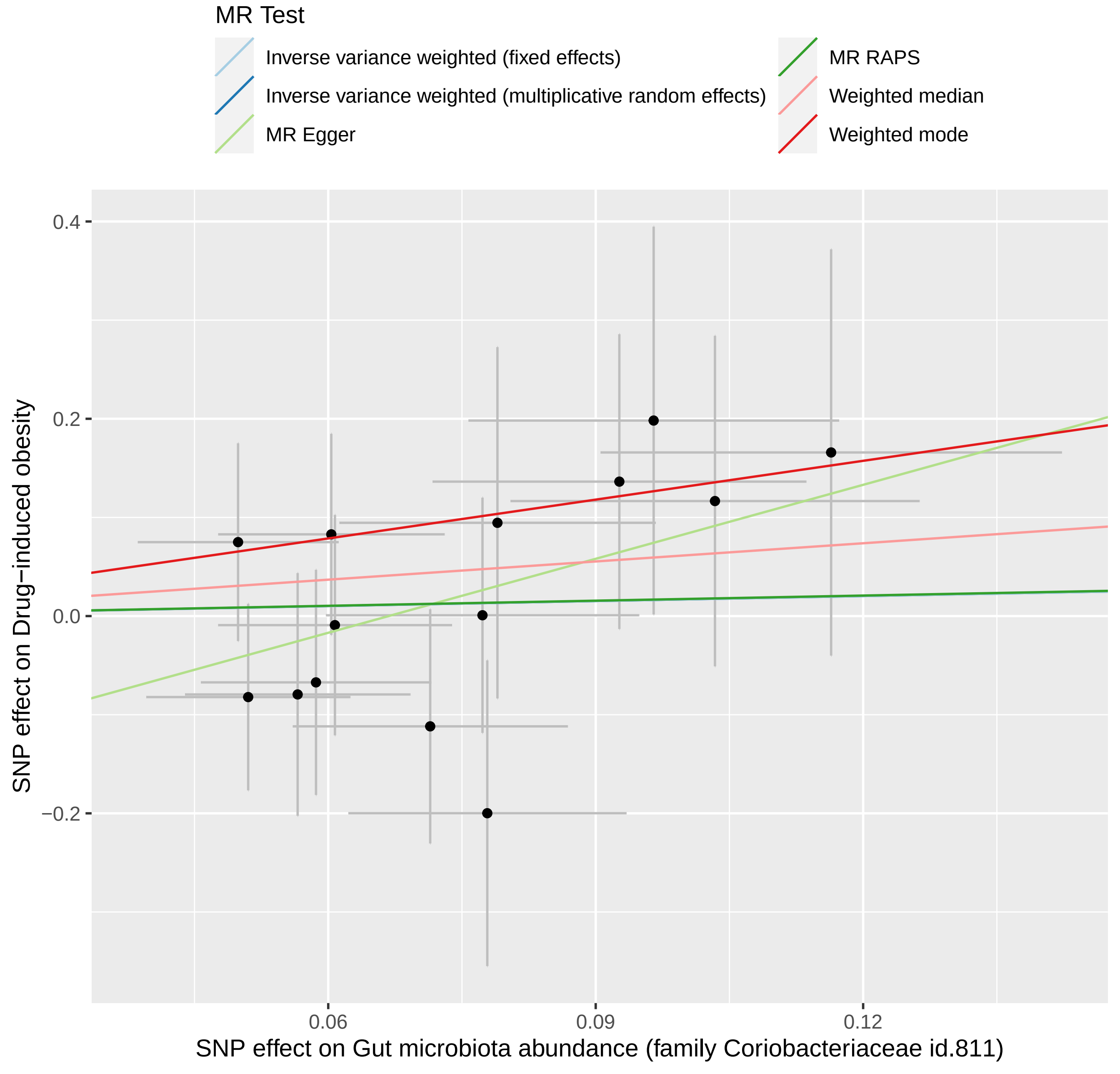

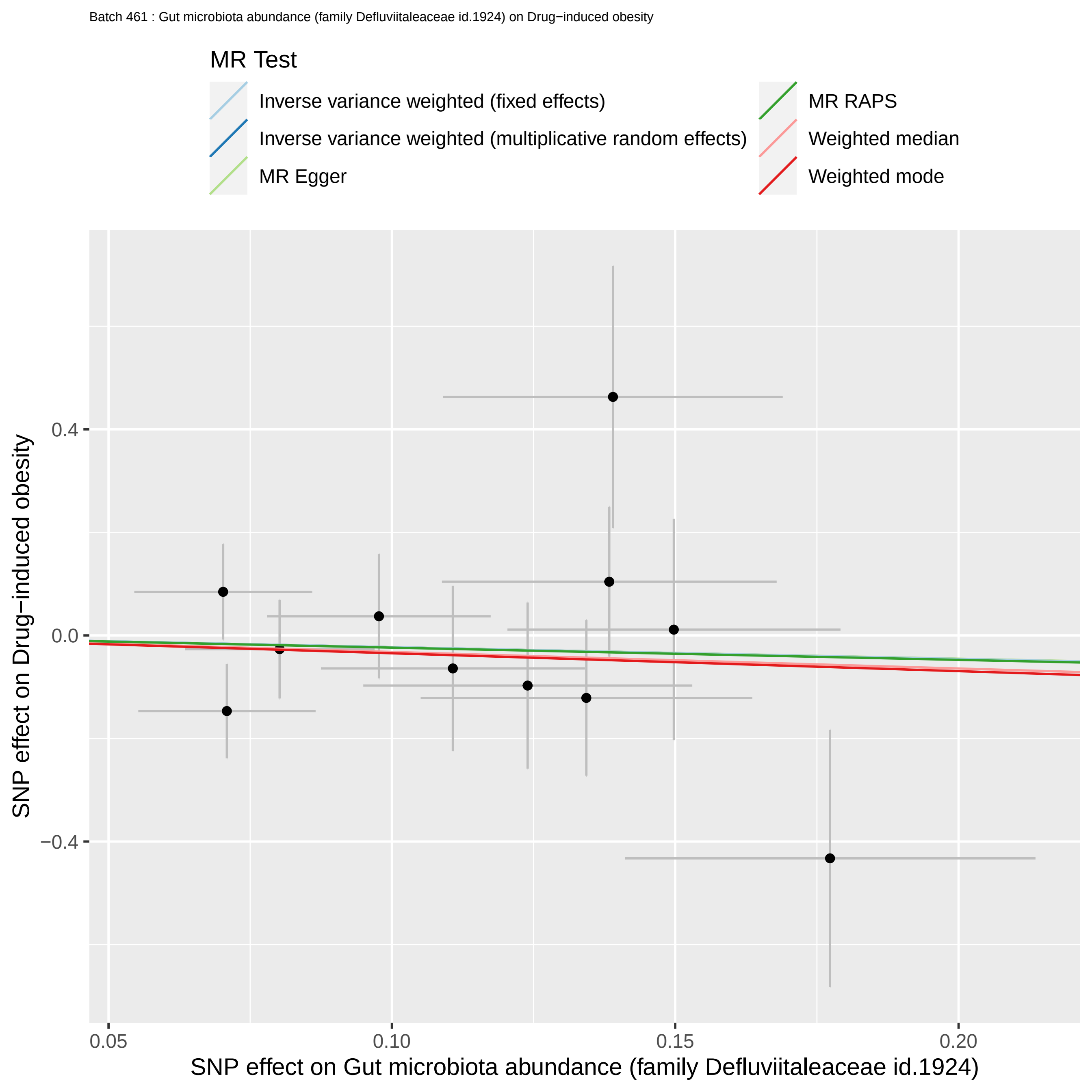

Batch 461 : Gut microbiota abundance (family Defluviitaleaceae id.1924) on Drug−induced obesity
MR Test
Inverse variance weighted (fixed effects)
Inverse variance weighted (multiplicative random effects)
MR Egger
MR RAPS
Weighted median
Weighted mode
0.4
0.0
−0.4
0.05
0.10
0.15
0.20
SNP effect on Drug−induced obesity
SNP effect on Gut microbiota abundance (family Defluviitaleaceae id.1924)

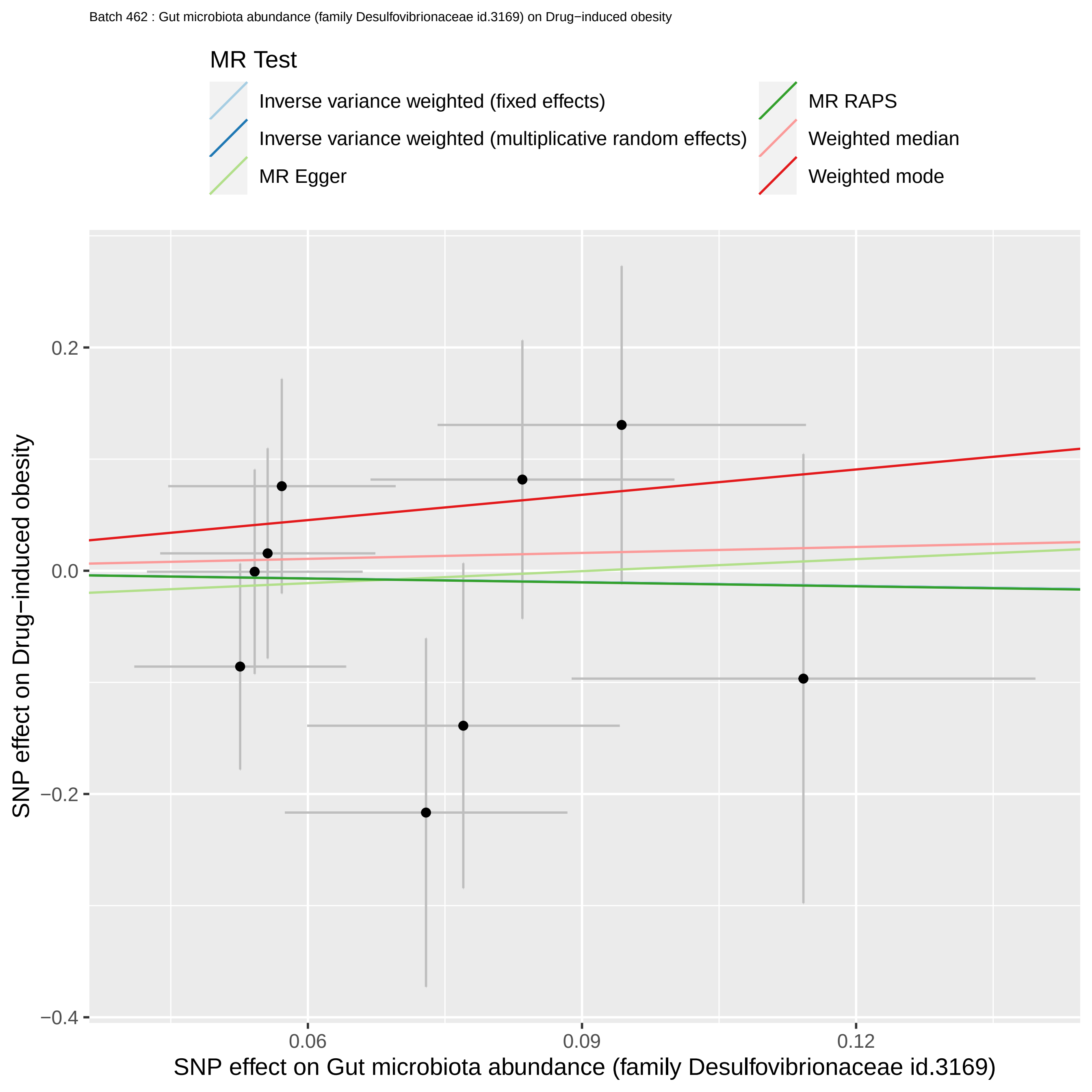
Batch 462 : Gut microbiota abundance (family Desulfovibrionaceae id.3169) on Drug−induced obesity
MR Test
Inverse variance weighted (fixed effects)
Inverse variance weighted (multiplicative random effects)
MR Egger
MR RAPS
Weighted median
Weighted mode
0.2
0.0
−0.2
−0.4
0.06
0.09
0.12
SNP effect on Drug−induced obesity
SNP effect on Gut microbiota abundance (family Desulfovibrionaceae id.3169)

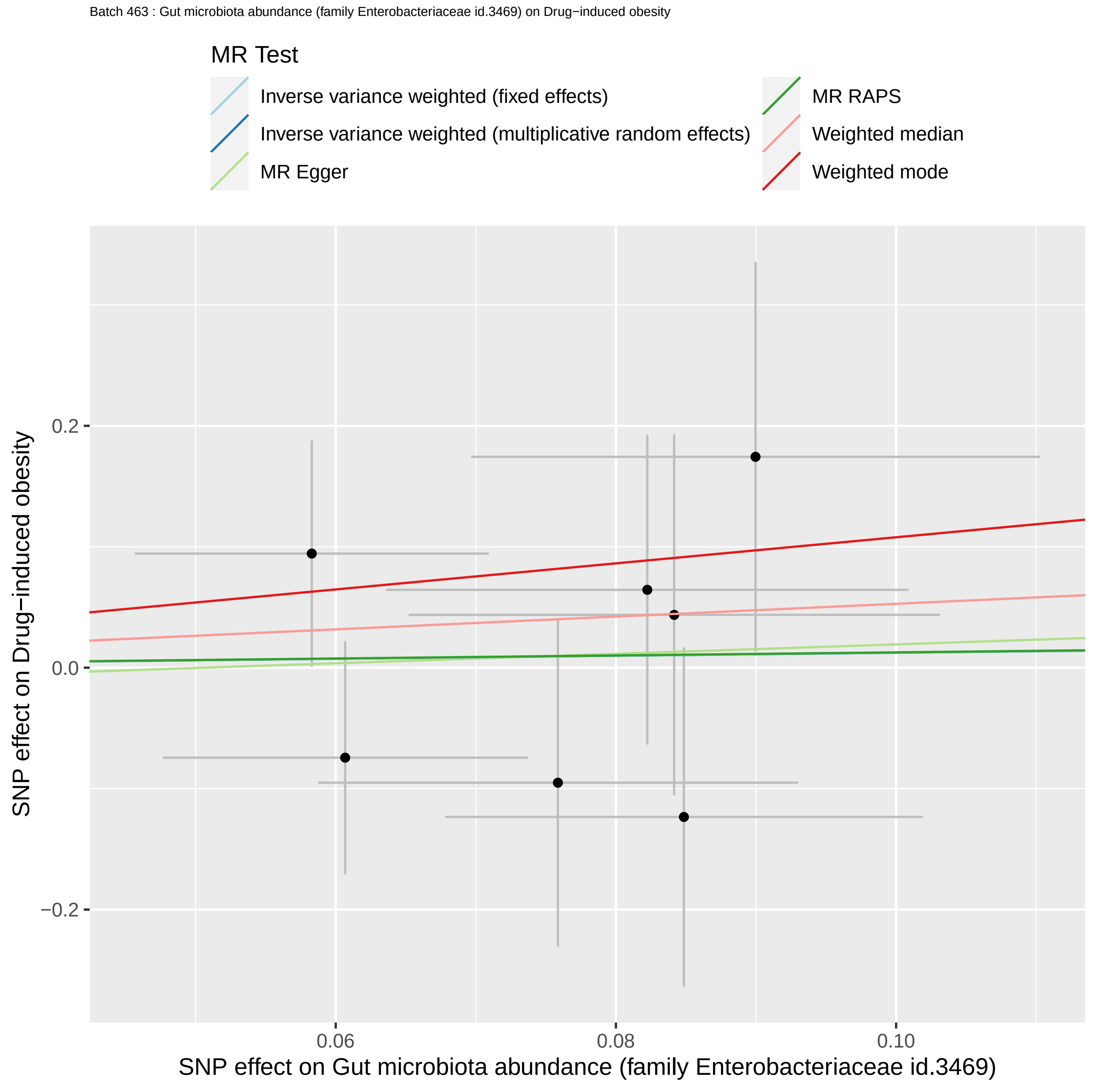
Batch 463 : Gut microbiota abundance (family Enterobacteriaceae id.3469) on Drug−induced obesity
MR Test
Inverse variance weighted (fixed effects)
Inverse variance weighted (multiplicative random effects)
MR Egger
MR RAPS
Weighted median
Weighted mode
0.2
0.0
−0.2
0.06
0.08
0.10
SNP effect on Drug−induced obesity
SNP effect on Gut microbiota abundance (family Enterobacteriaceae id.3469)

Batch 464 : Gut microbiota abundance (family Erysipelotrichaceae id.2149) on Drug−induced obesity

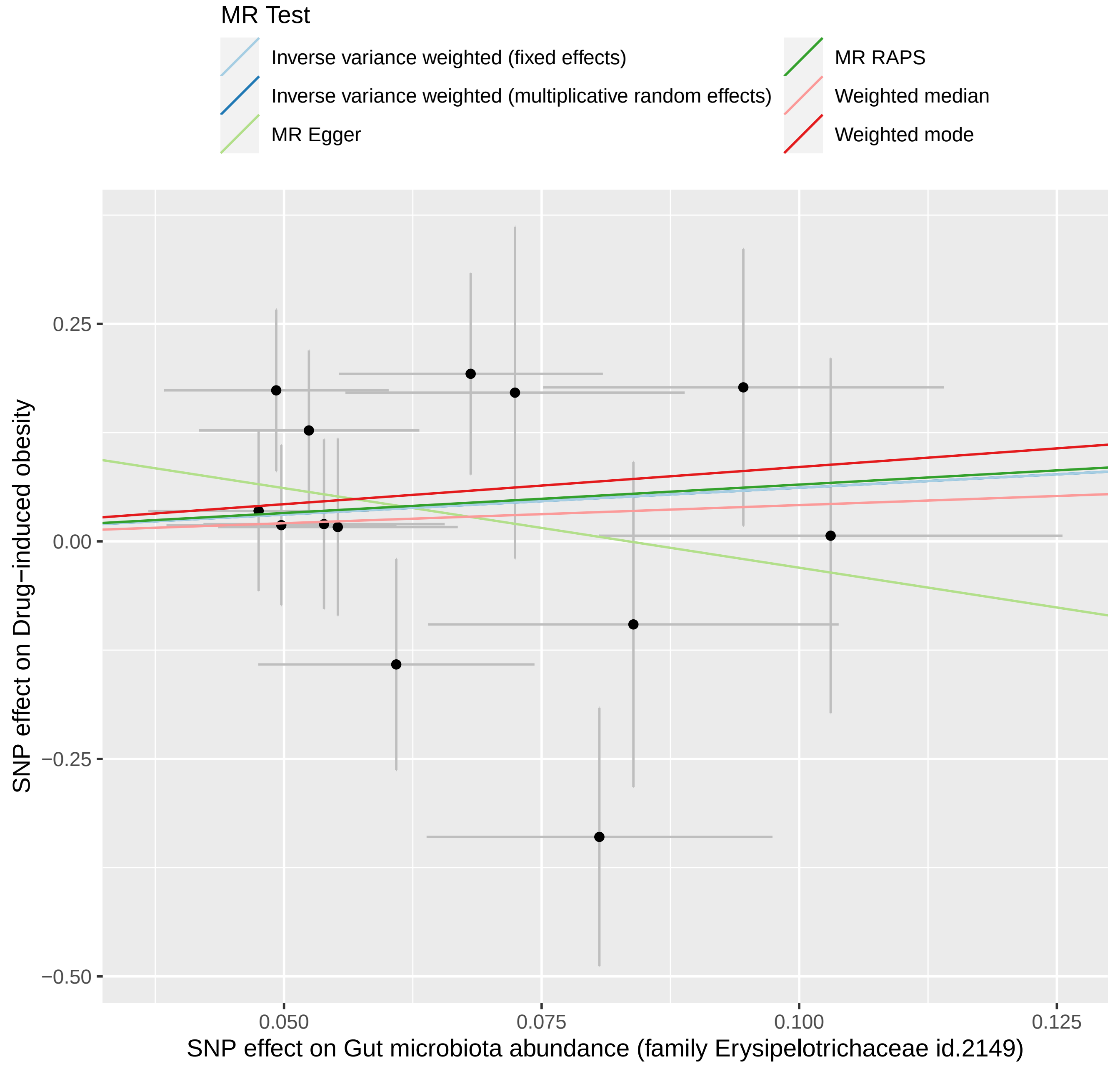

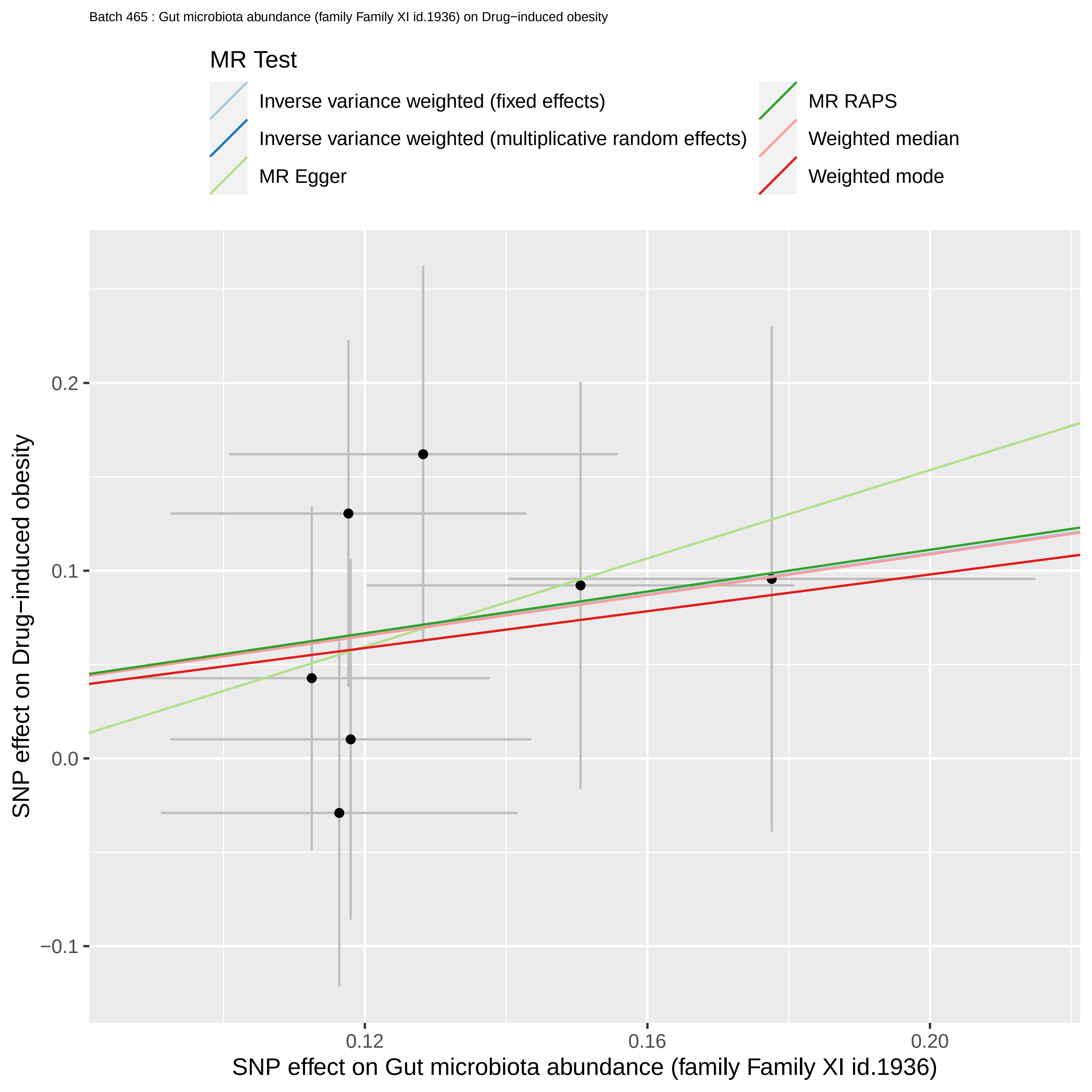

Batch 465 : Gut microbiota abundance (family Family XI id.1936) on Drug−induced obesity
MR Test
Inverse variance weighted (fixed effects)
Inverse variance weighted (multiplicative random effects)
MR Egger
MR RAPS
Weighted median
Weighted mode
0.2
0.1
0.0
−0.1
0.12
0.16
0.20
SNP effect on Gut microbiota abundance (family Family XI id.1936)
SNP effect on Drug−induced obesity

Batch 466 : Gut microbiota abundance (family Family XIII id.1957) on Drug−induced obesity

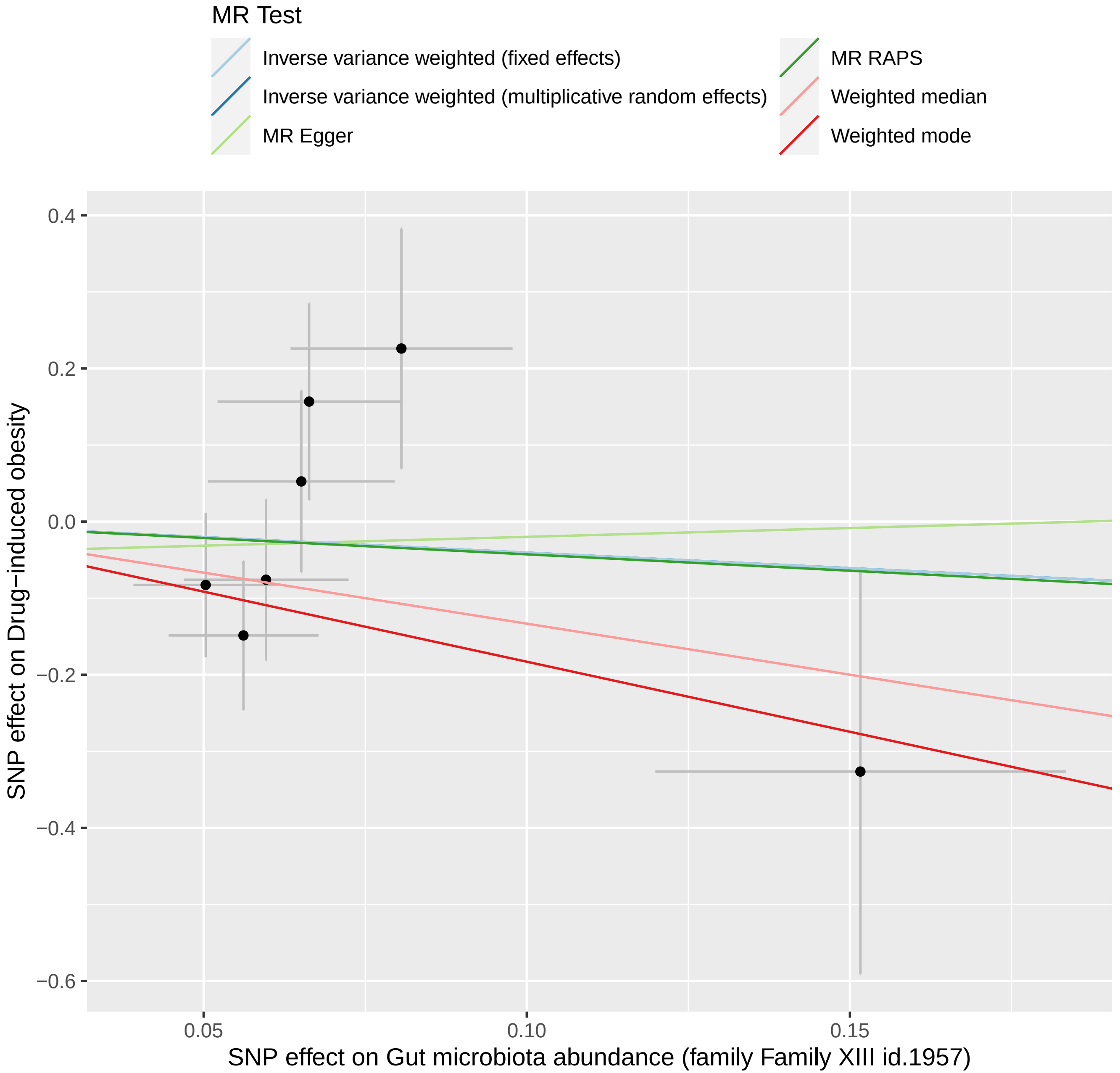

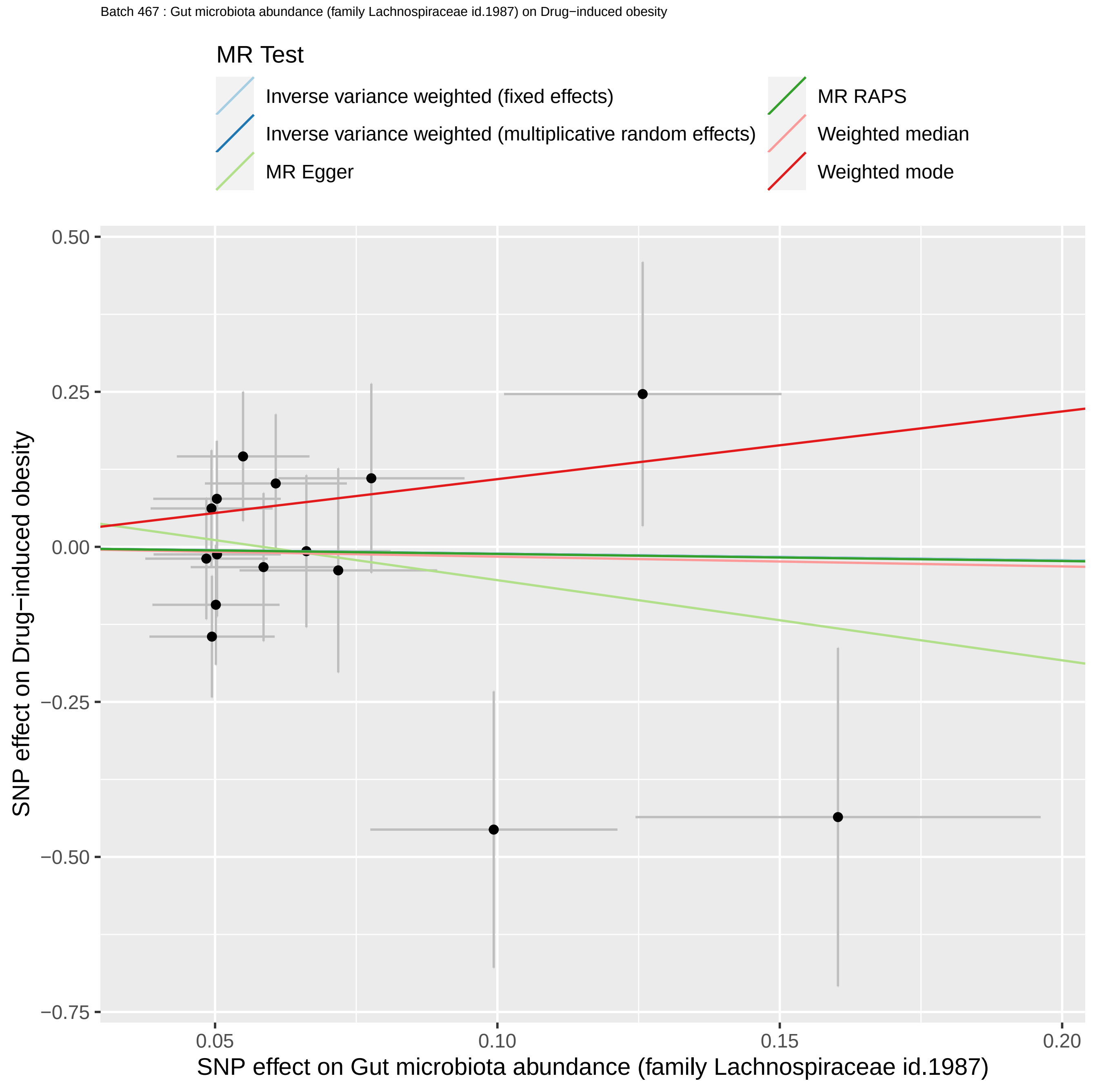

Batch 467 : Gut microbiota abundance (family Lachnospiraceae id.1987) on Drug−induced obesity
MR Test
Inverse variance weighted (fixed effects)
Inverse variance weighted (multiplicative random effects)
MR Egger
MR RAPS
Weighted median
Weighted mode
0.50
0.25
0.00
−0.25
−0.50
−0.75
0.05
0.10
0.15
0.20
SNP effect on Drug−induced obesity
SNP effect on Gut microbiota abundance (family Lachnospiraceae id.1987)

Batch 468 : Gut microbiota abundance (family Lactobacillaceae id.1836) on Drug−induced obesity

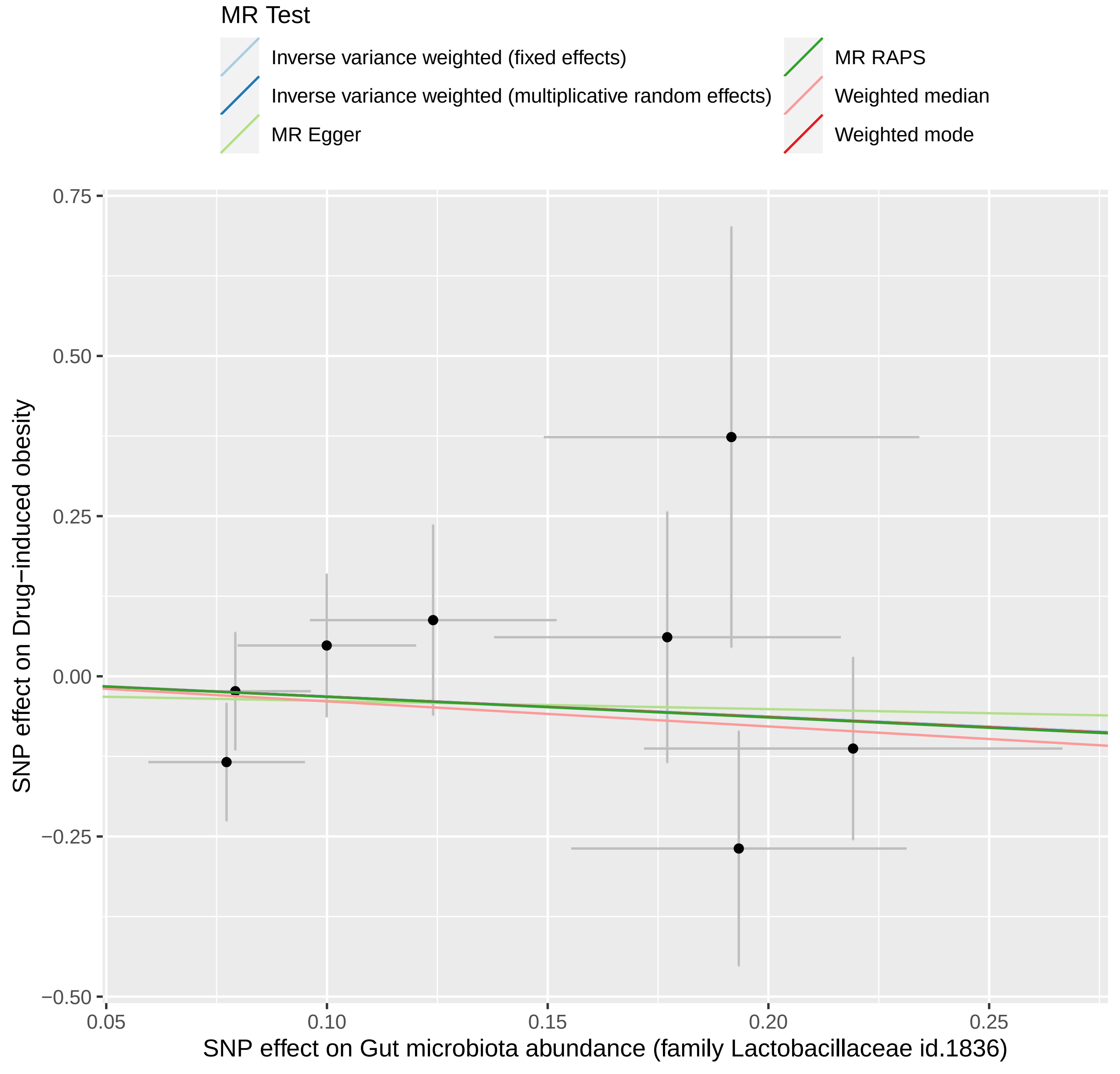

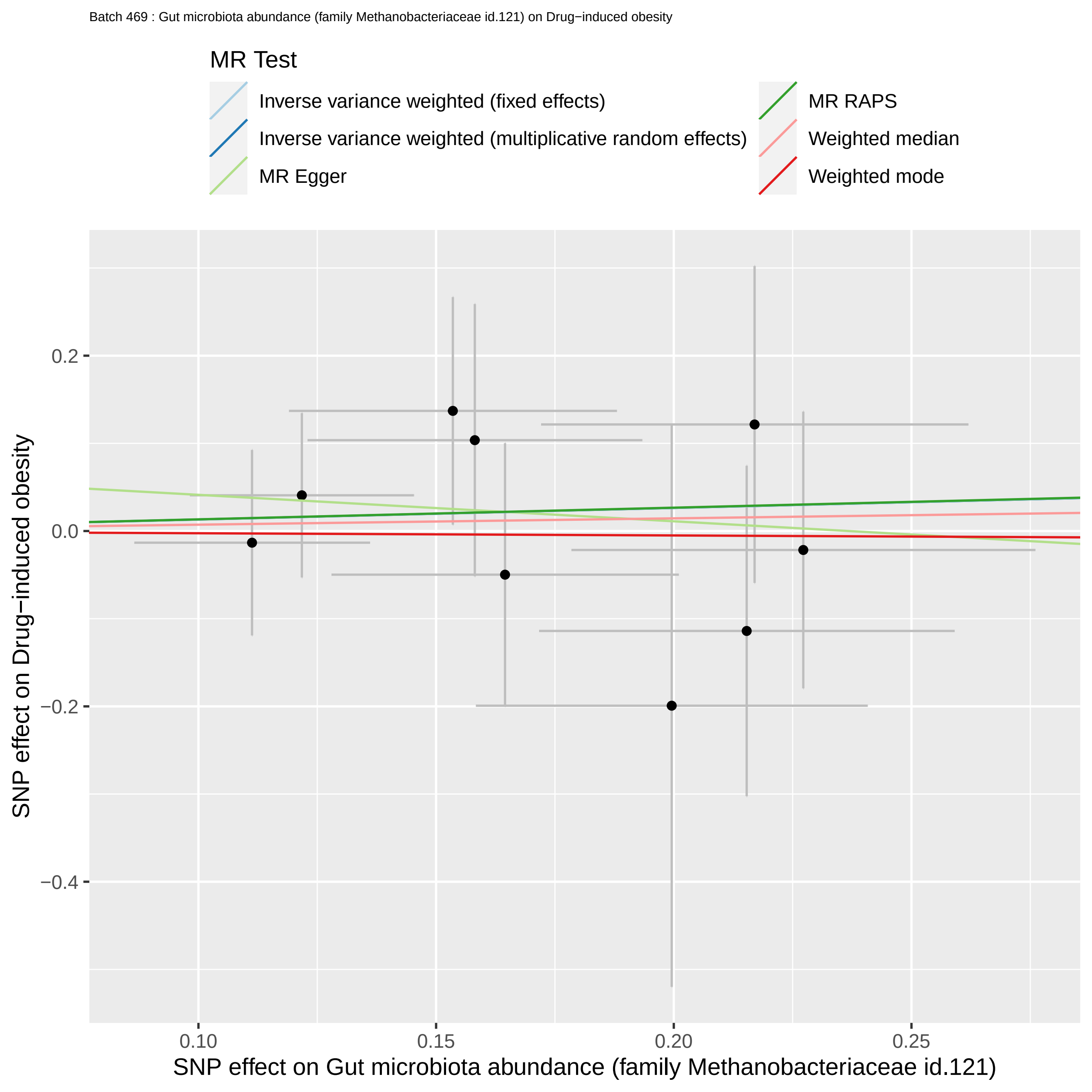
Batch 469 : Gut microbiota abundance (family Methanobacteriaceae id.121) on Drug−induced obesity
MR Test
Inverse variance weighted (fixed effects)
Inverse variance weighted (multiplicative random effects)
MR Egger
MR RAPS
Weighted median
Weighted mode
0.2
0.0
−0.2
−0.4
0.10
0.15
0.20
0.25
SNP effect on Drug−induced obesity
SNP effect on Gut microbiota abundance (family Methanobacteriaceae id.121)

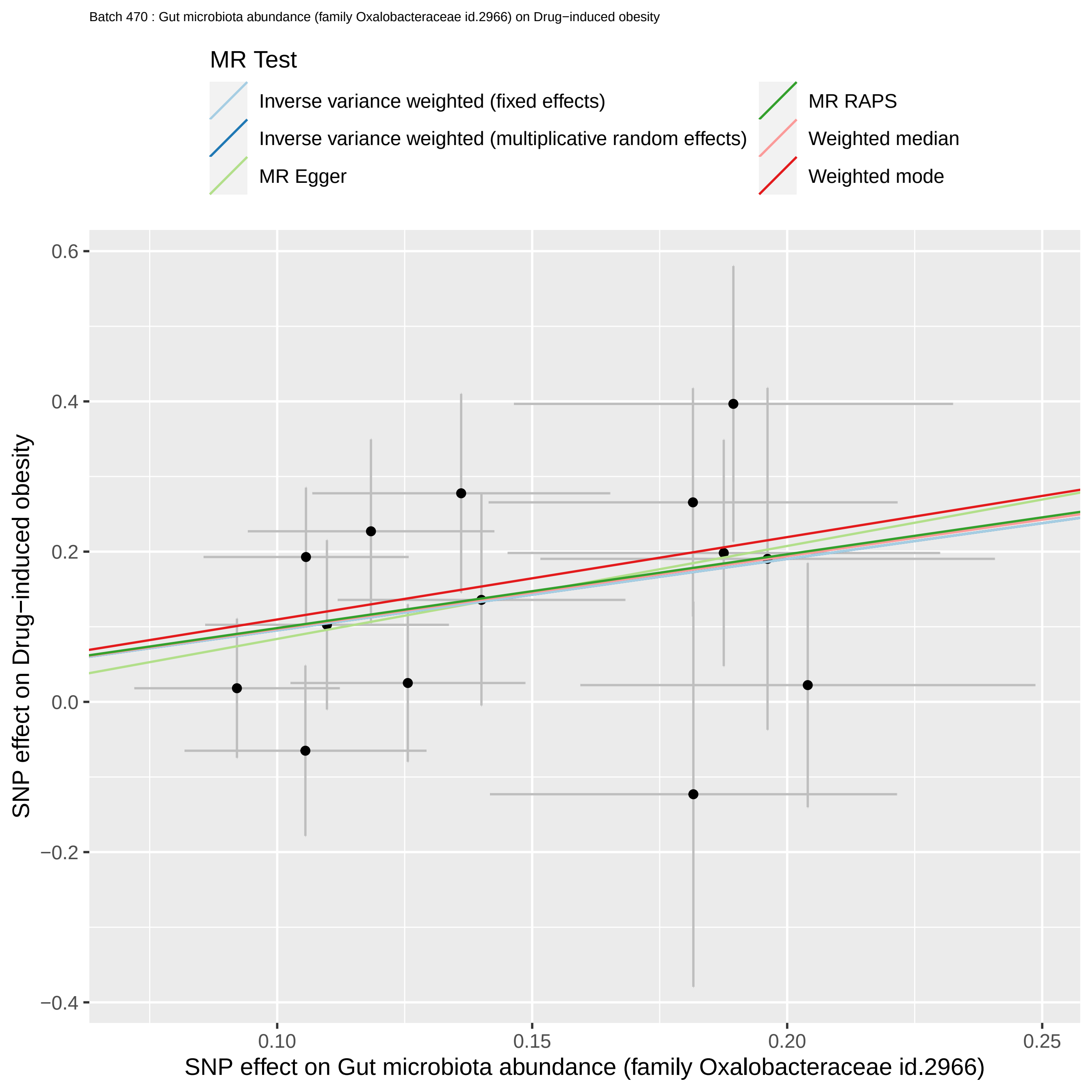
Batch 470 : Gut microbiota abundance (family Oxalobacteraceae id.2966) on Drug−induced obesity
MR Test
Inverse variance weighted (fixed effects)
Inverse variance weighted (multiplicative random effects)
MR Egger
MR RAPS
Weighted median
Weighted mode
SNP effect on Drug−induced obesity
0.6
0.4
0.2
0.0
−0.2
−0.4
0.10
0.15
0.20
0.25
SNP effect on Gut microbiota abundance (family Oxalobacteraceae id.2966)

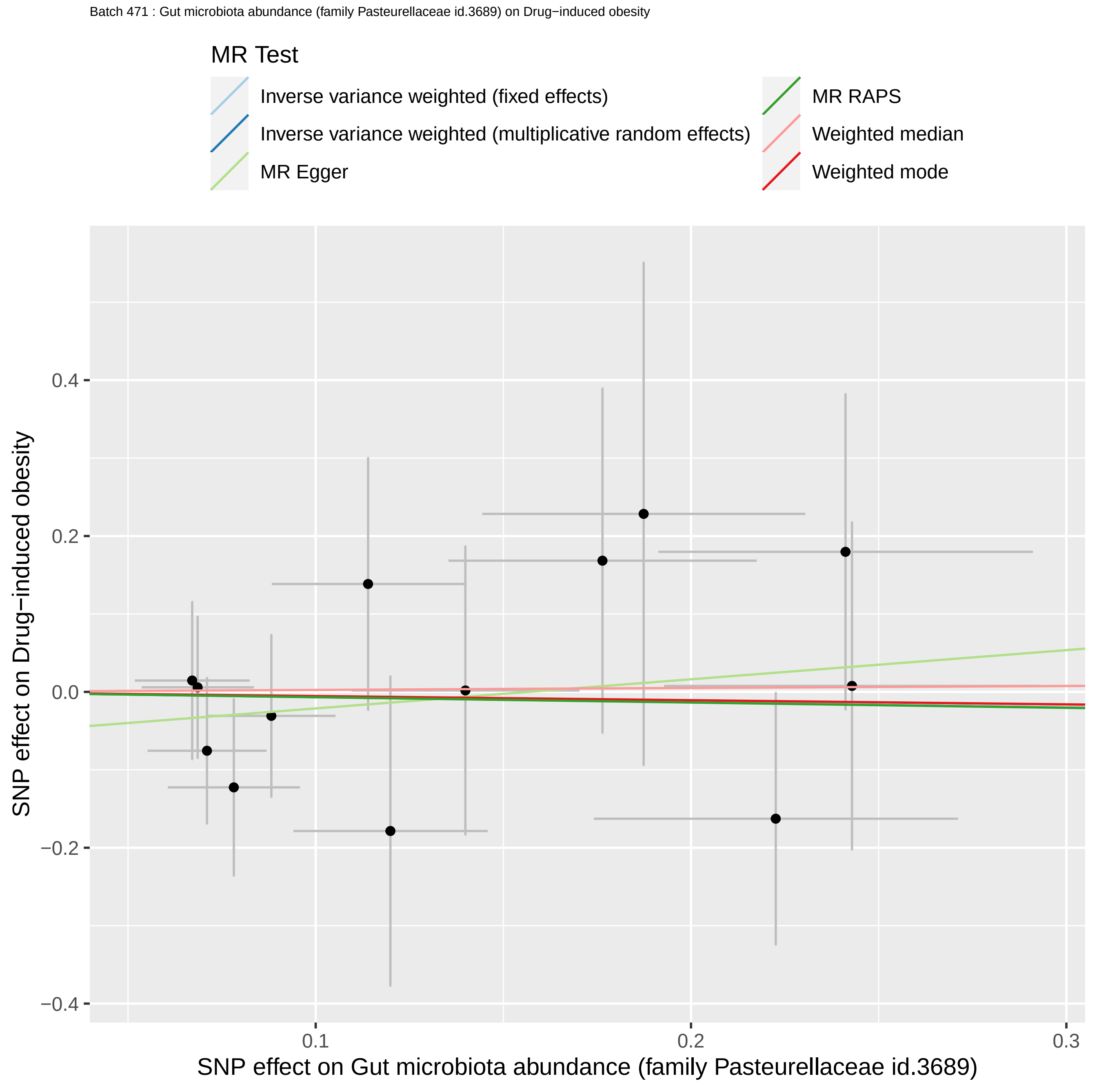
Batch 471 : Gut microbiota abundance (family Pasteurellaceae id.3689) on Drug−induced obesity
MR Test
Inverse variance weighted (fixed effects)
Inverse variance weighted (multiplicative random effects)
MR Egger
MR RAPS
Weighted median
Weighted mode
SNP effect on Drug−induced obesity
0.4
0.2
0.0
−0.2
−0.4
0.1
0.2
0.3
SNP effect on Gut microbiota abundance (family Pasteurellaceae id.3689)

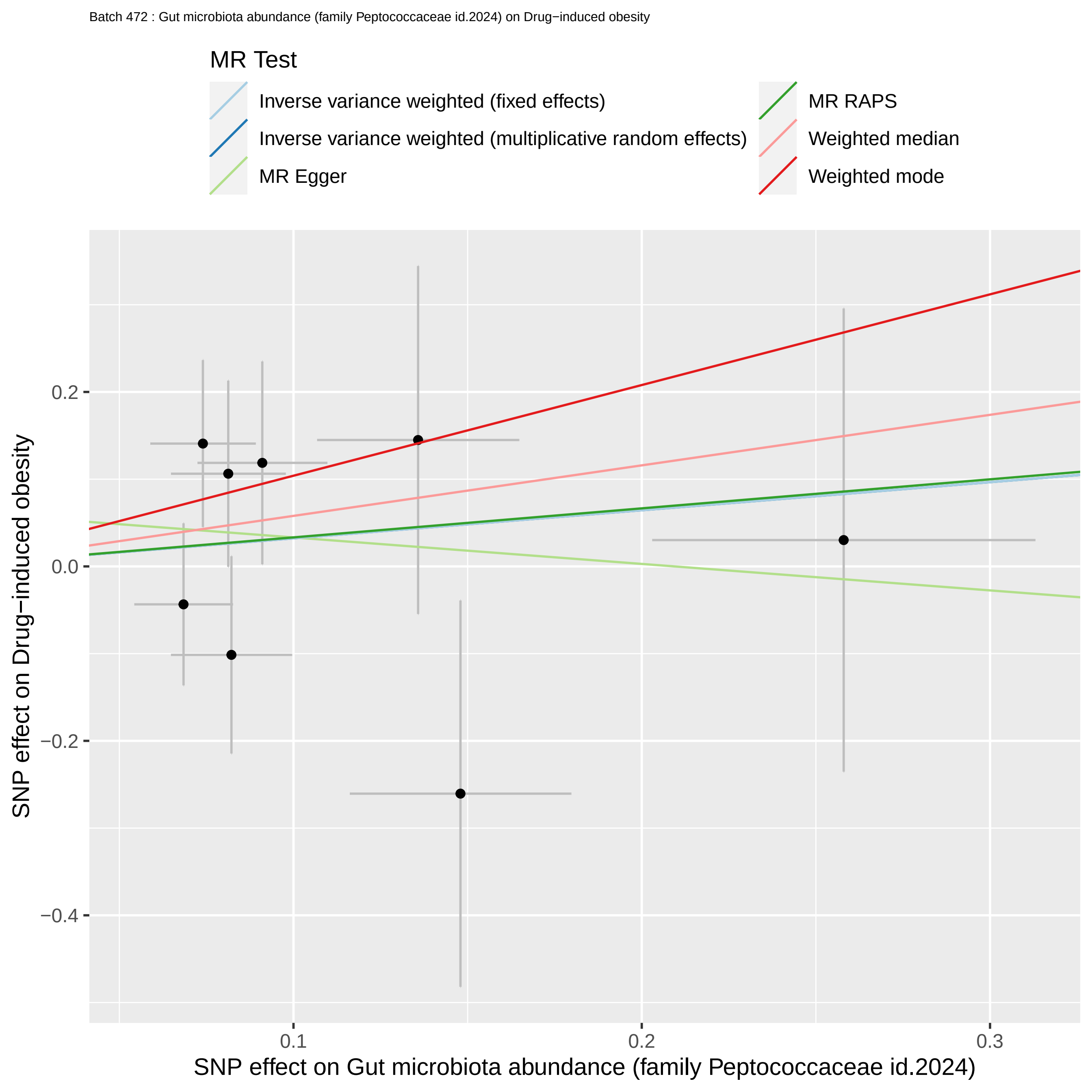
Batch 472 : Gut microbiota abundance (family Peptococcaceae id.2024) on Drug−induced obesity
MR Test
Inverse variance weighted (fixed effects)
Inverse variance weighted (multiplicative random effects)
MR Egger
MR RAPS
Weighted median
Weighted mode
0.2
0.0
−0.2
−0.4
0.1
0.2
0.3
SNP effect on Drug−induced obesity
SNP effect on Gut microbiota abundance (family Peptococcaceae id.2024)

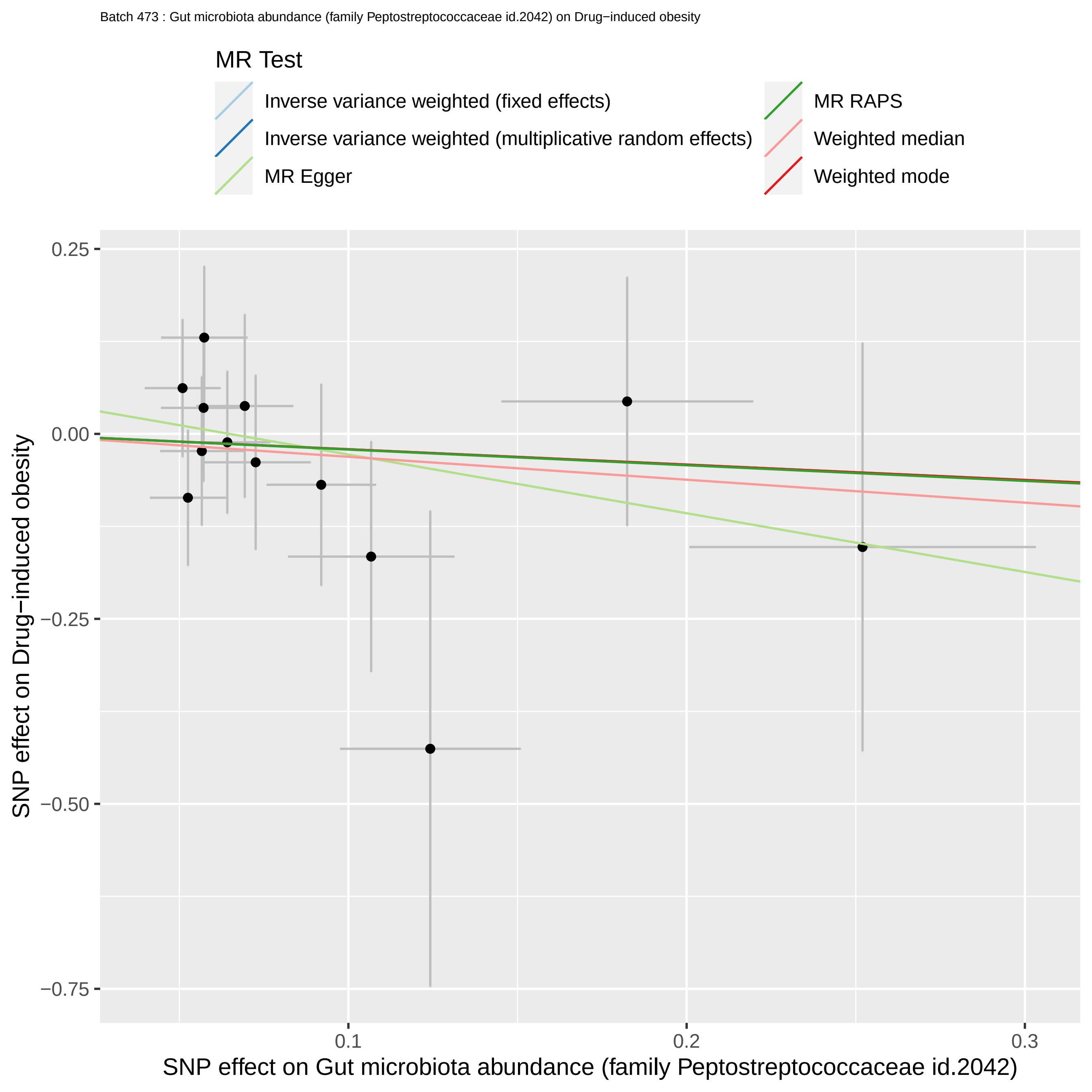

Batch 473 : Gut microbiota abundance (family Peptostreptococcaceae id.2042) on Drug−induced obesity
MR Test
Inverse variance weighted (fixed effects)
Inverse variance weighted (multiplicative random effects)
MR Egger
MR RAPS
Weighted median
Weighted mode
0.25
0.00
−0.25
−0.50
−0.75
0.1
0.2
0.3
SNP effect on Drug−induced obesity
SNP effect on Gut microbiota abundance (family Peptostreptococcaceae id.2042)

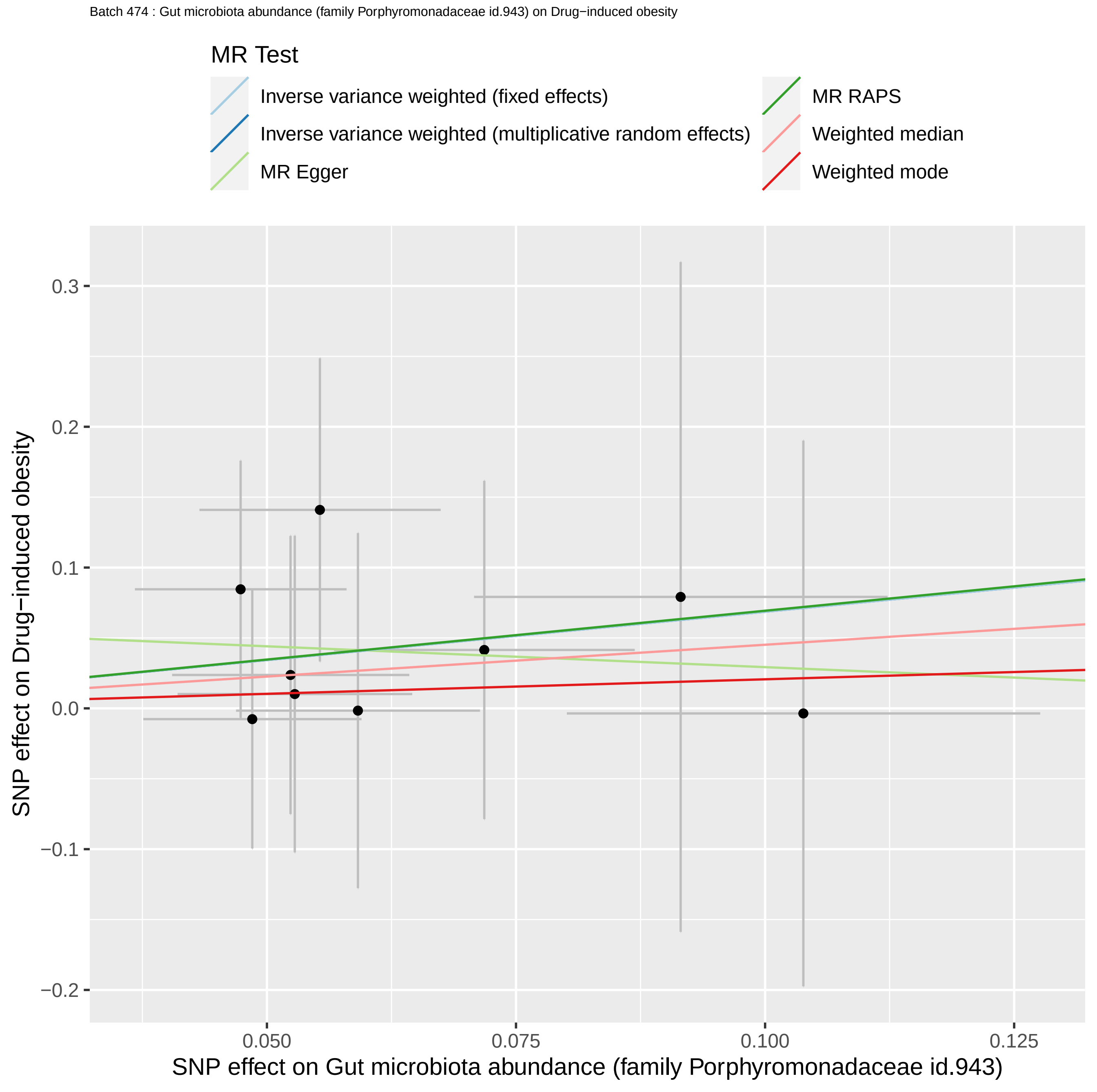

Batch 474 : Gut microbiota abundance (family Porphyromonadaceae id.943) on Drug−induced obesity
MR Test
Inverse variance weighted (fixed effects)
Inverse variance weighted (multiplicative random effects)
MR Egger
MR RAPS
Weighted median
Weighted mode
0.3
0.2
0.1
0.0
−0.1
−0.2
0.050
0.075
0.100
0.125
SNP effect on Drug−induced obesity
SNP effect on Gut microbiota abundance (family Porphyromonadaceae id.943)

Batch 475 : Gut microbiota abundance (family Prevotellaceae id.960) on Drug−induced obesity

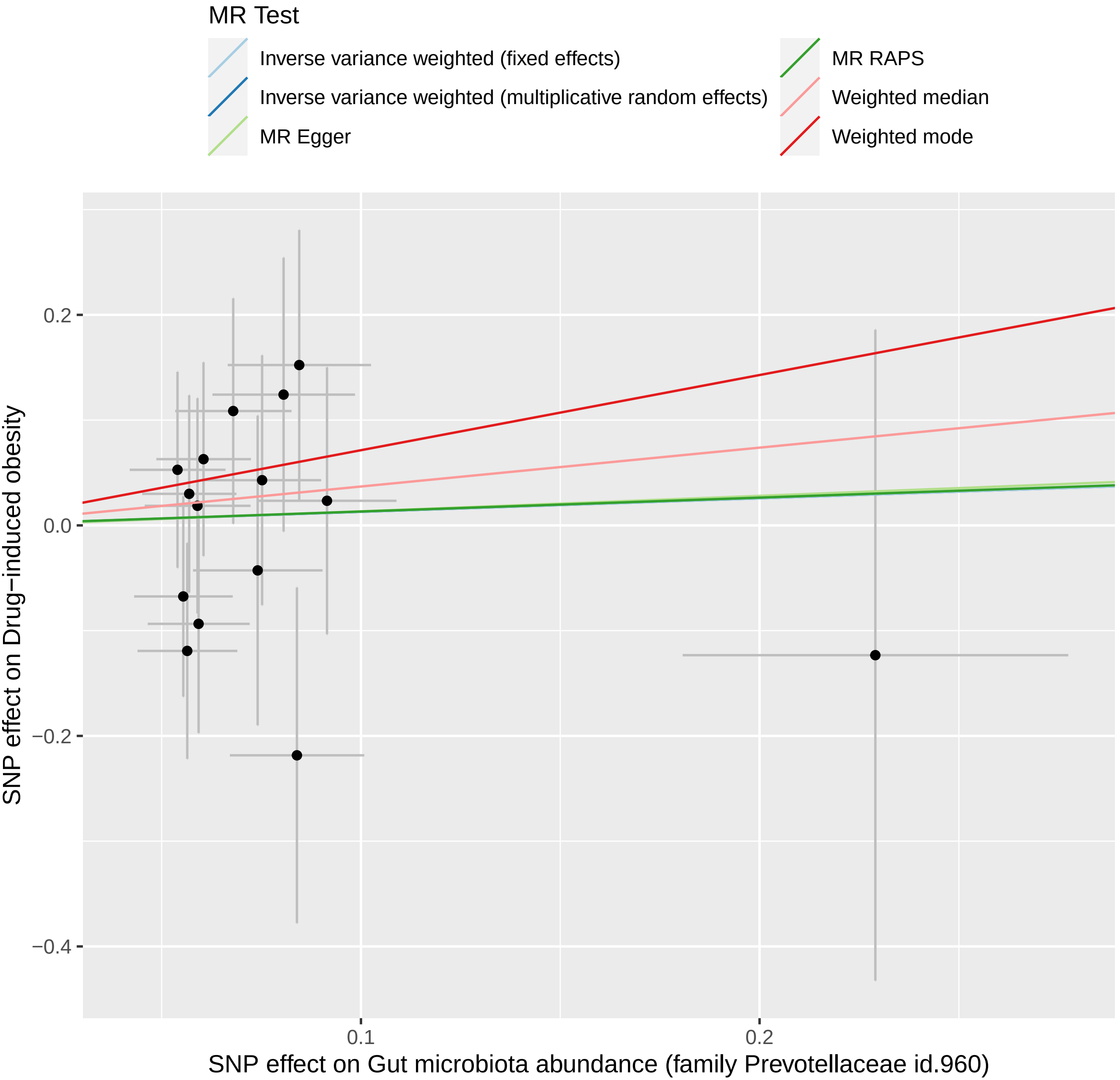

Batch 476 : Gut microbiota abundance (family Rhodospirillaceae id.2717) on Drug−induced obesity

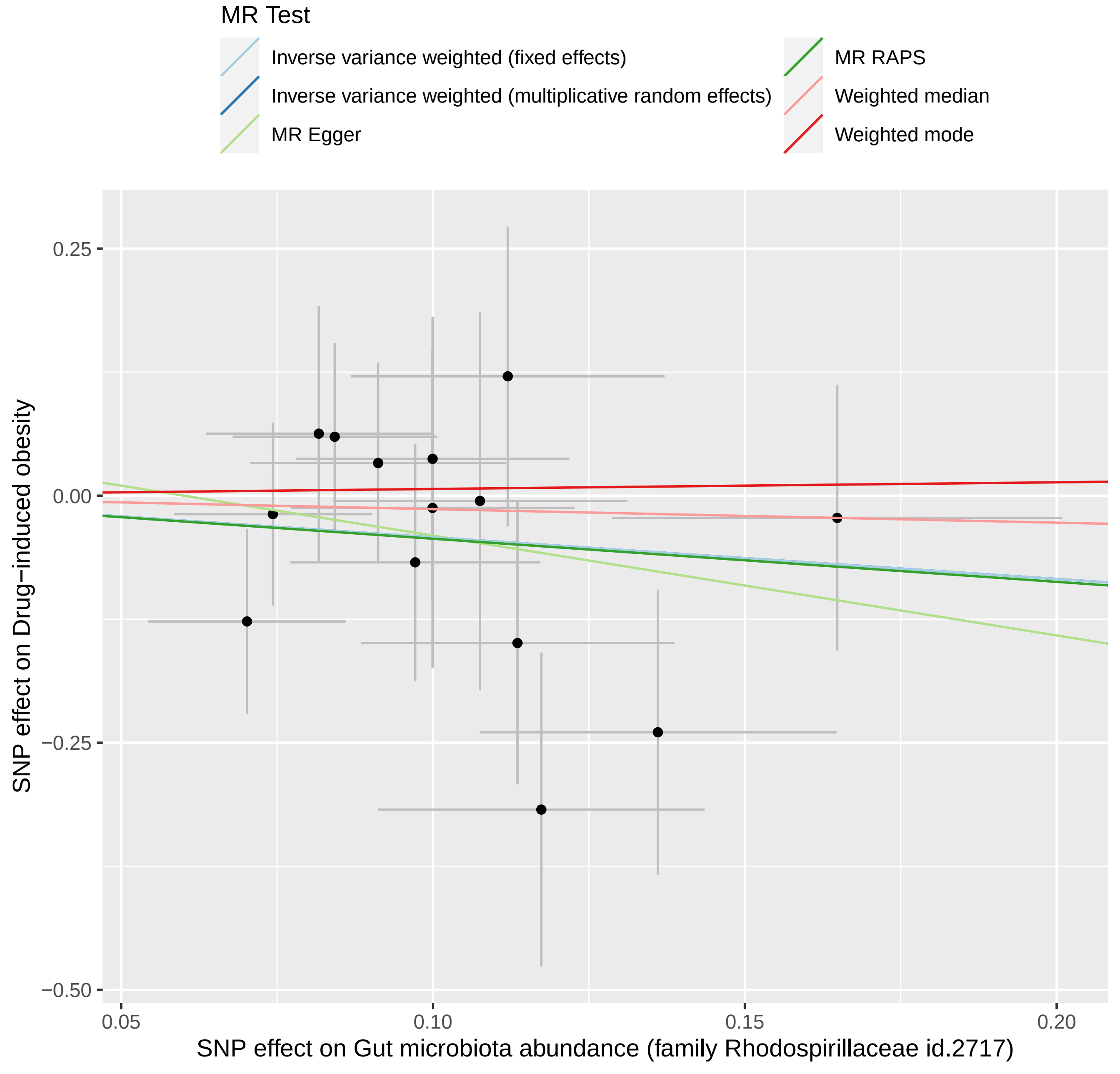

Batch 477 : Gut microbiota abundance (family Rikenellaceae id.967) on Drug−induced obesity

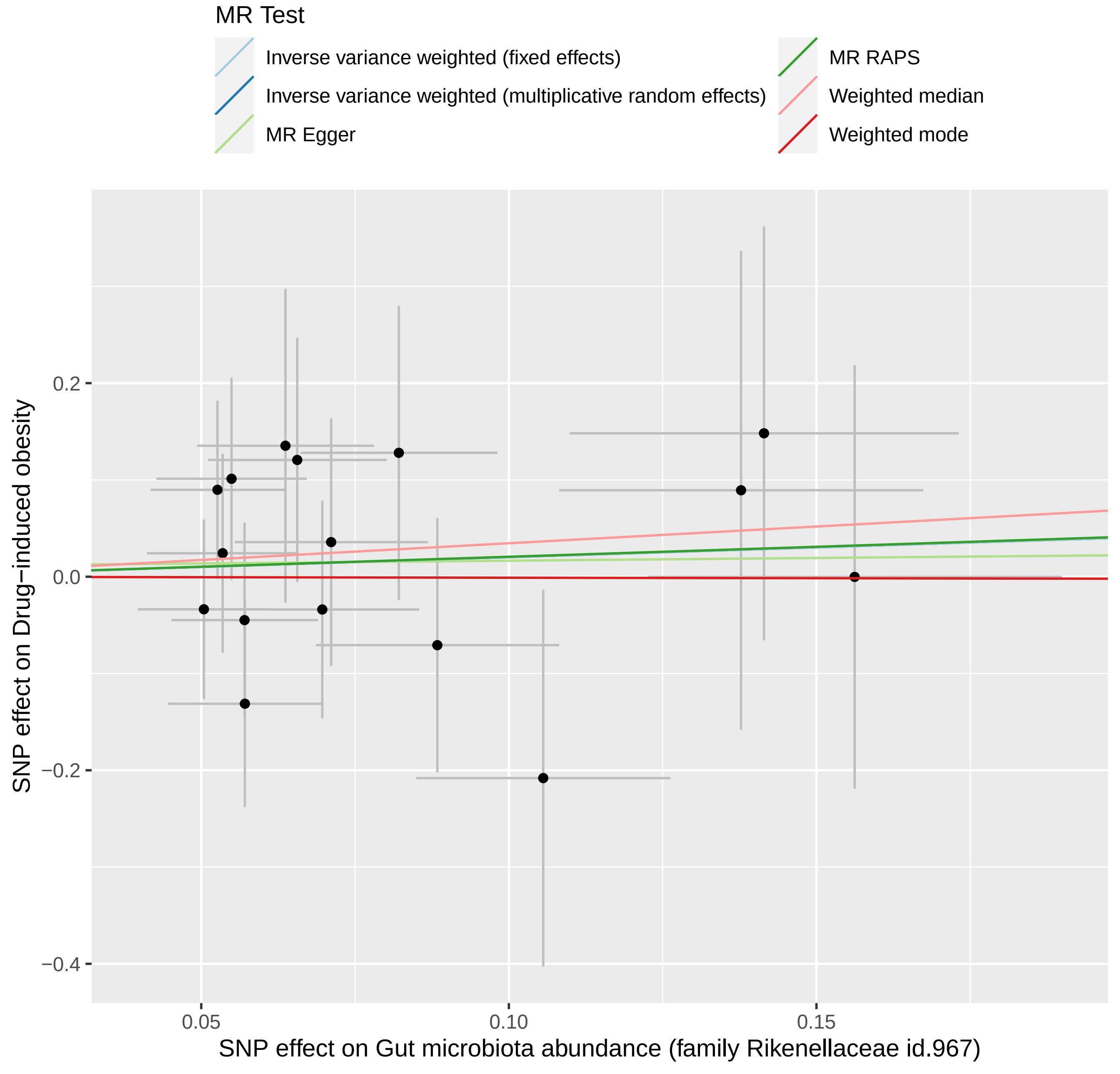

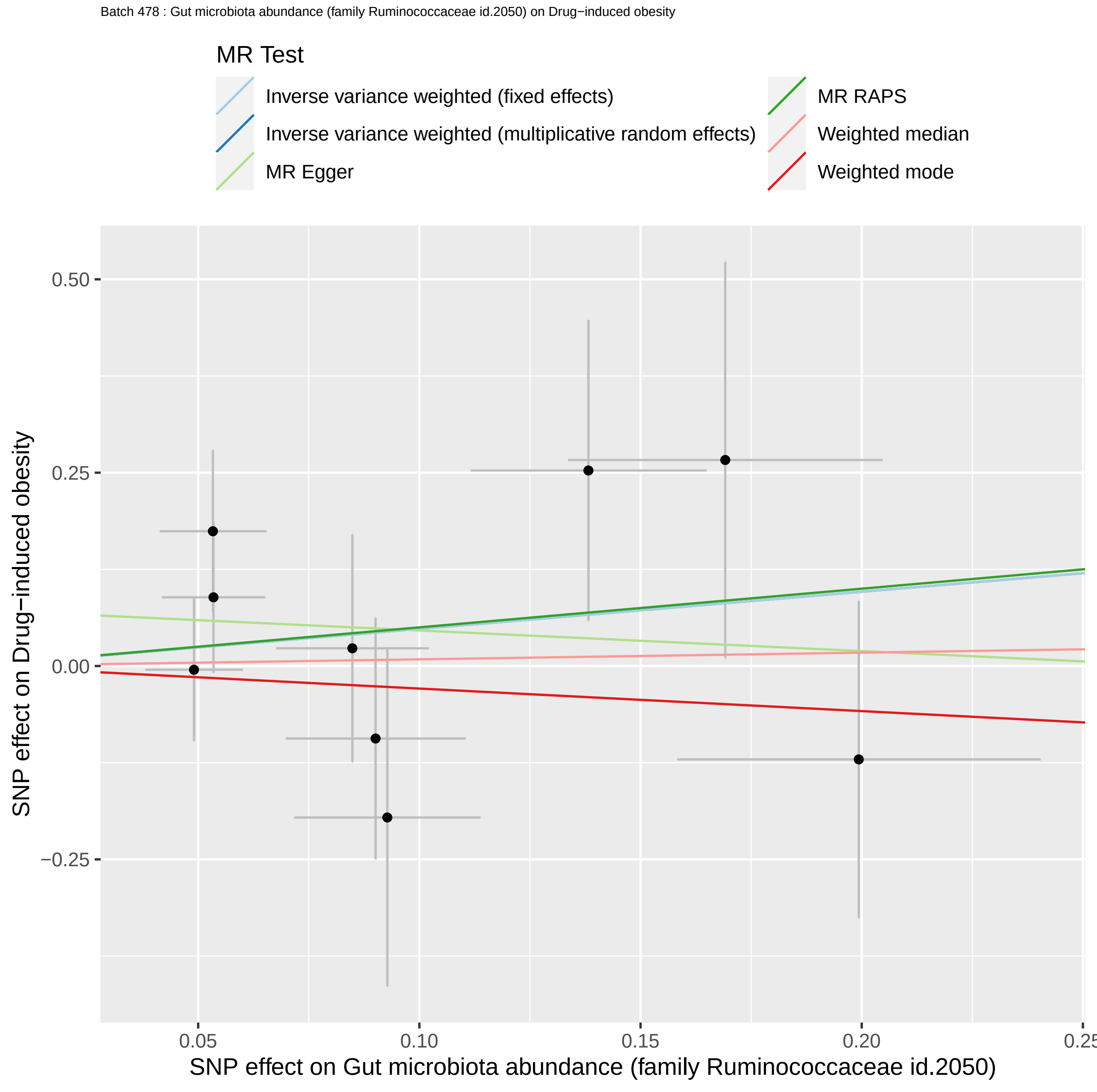

Batch 478 : Gut microbiota abundance (family Ruminococcaceae id.2050) on Drug−induced obesity
MR Test
Inverse variance weighted (fixed effects)
Inverse variance weighted (multiplicative random effects)
MR Egger
MR RAPS
Weighted median
Weighted mode
0.50
0.25
0.00
−0.25
0.05
0.10
0.15
0.20
SNP effect on Drug−induced obesity
SNP effect on Gut microbiota abundance (family Ruminococcaceae id.2050)

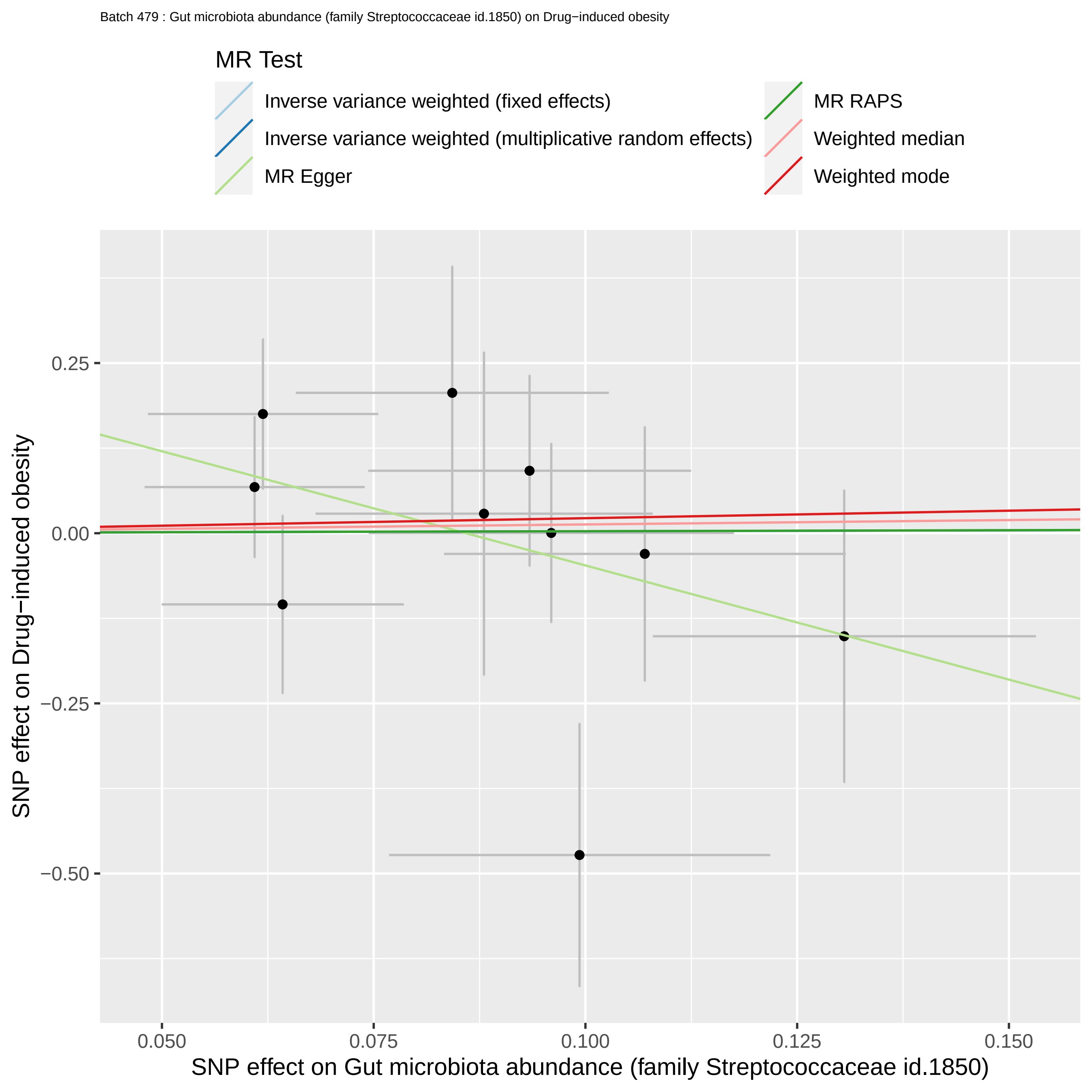
Batch 479 : Gut microbiota abundance (family Streptococcaceae id.1850) on Drug−induced obesity
MR Test
Inverse variance weighted (fixed effects)
Inverse variance weighted (multiplicative random effects)
MR Egger
MR RAPS
Weighted median
Weighted mode
SNP effect on Drug−induced obesity
0.25
0.00
−0.25
−0.50
0.050
0.075
0.100
0.125
0.150
SNP effect on Gut microbiota abundance (family Streptococcaceae id.1850)

Batch 480 : Gut microbiota abundance (family Veillonellaceae id.2172) on Drug−induced obesity

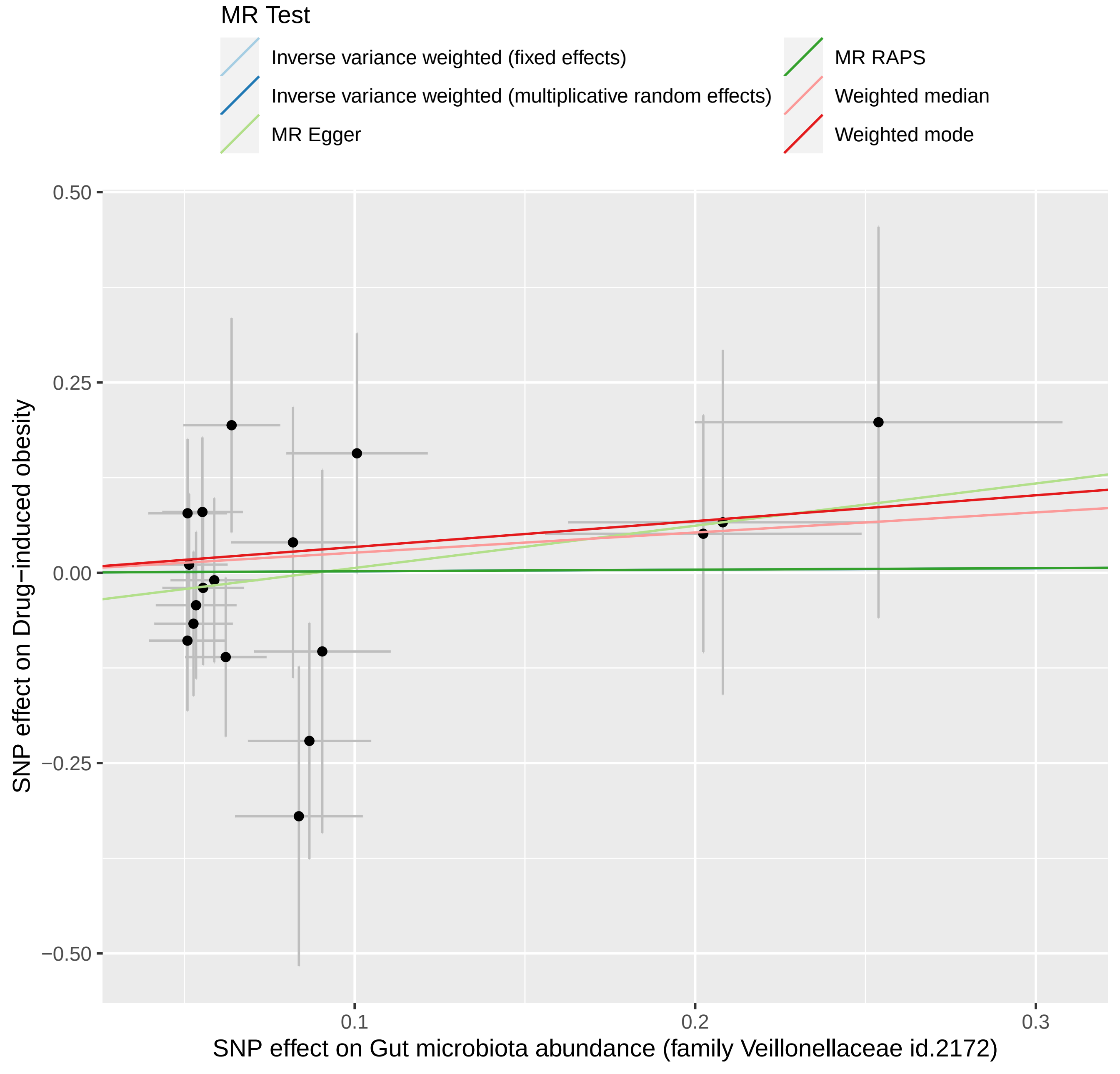

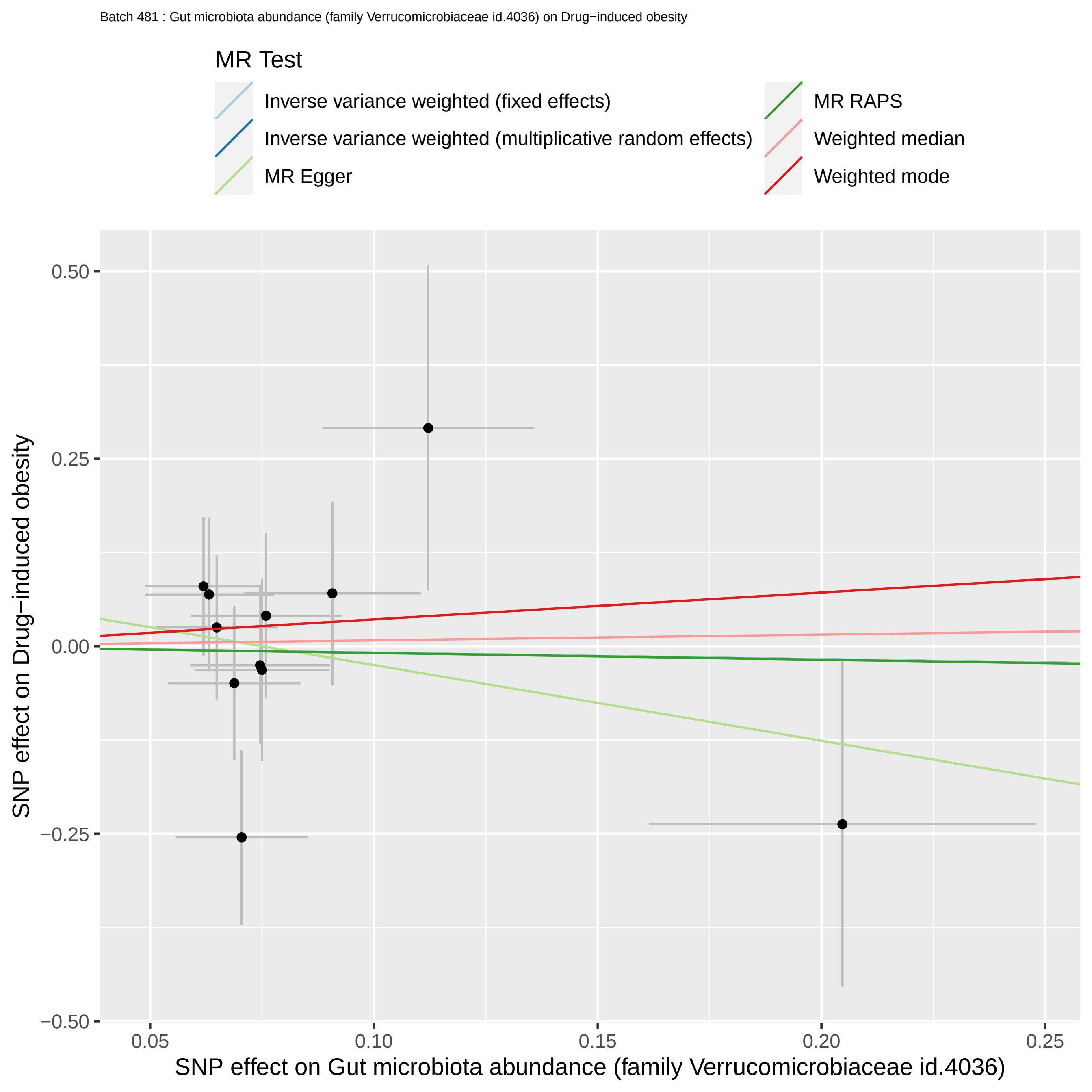
Batch 481 : Gut microbiota abundance (family Verrucomicrobiaceae id.4036) on Drug−induced obesity
MR Test
Inverse variance weighted (fixed effects)
Inverse variance weighted (multiplicative random effects)
MR Egger
MR RAPS
Weighted median
Weighted mode
0.50
0.25
0.00
−0.25
−0.50
0.05
0.10
0.15
0.20
0.25
SNP effect on Drug−induced obesity
SNP effect on Gut microbiota abundance (family Verrucomicrobiaceae id.4036)

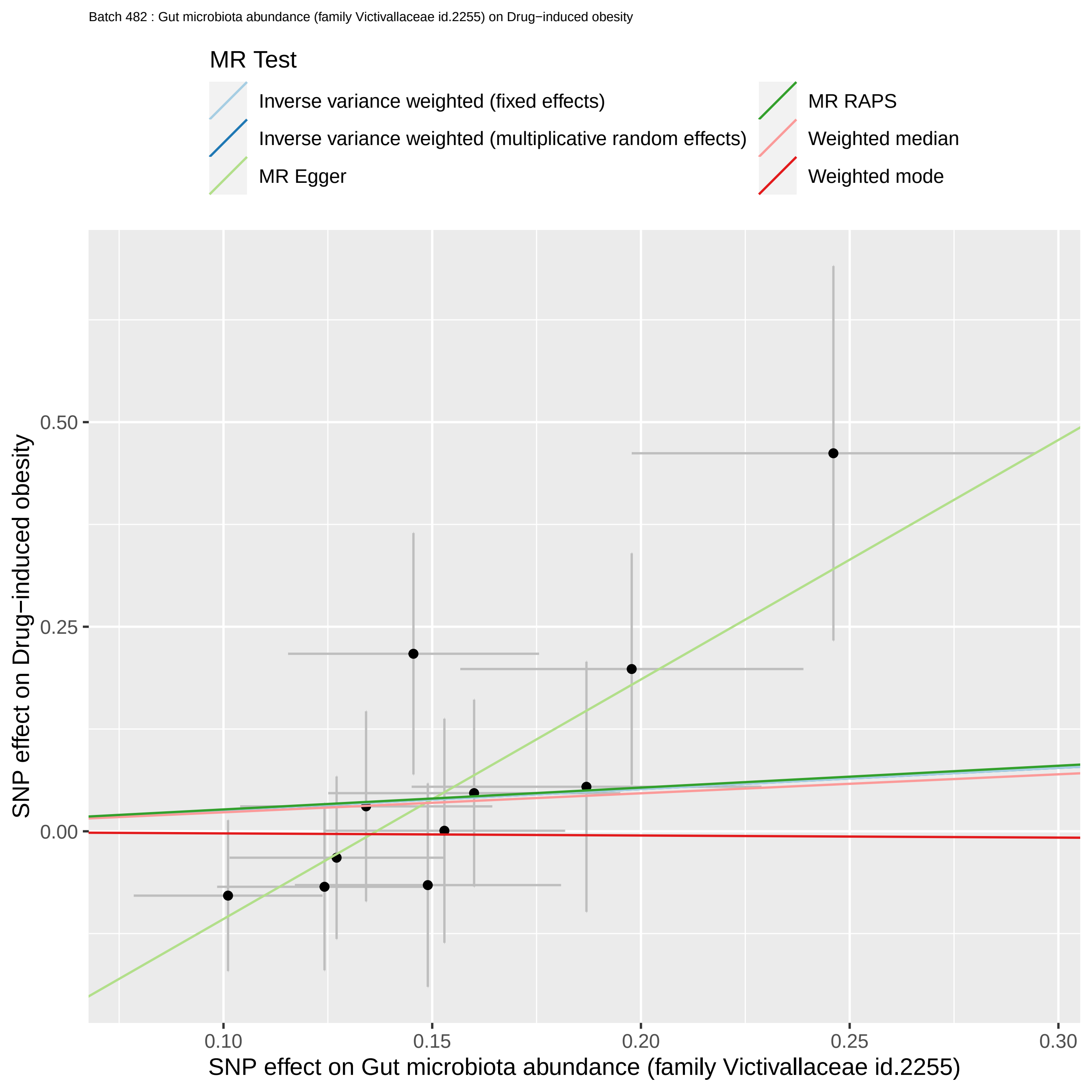
Batch 482 : Gut microbiota abundance (family Victivallaceae id.2255) on Drug−induced obesity
MR Test
Inverse variance weighted (fixed effects)
Inverse variance weighted (multiplicative random effects)
MR Egger
MR RAPS
Weighted median
Weighted mode
SNP effect on Drug−induced obesity
0.50
0.25
0.00
0.10
0.15
0.20
0.25
0.30
SNP effect on Gut microbiota abundance (family Victivallaceae id.2255)

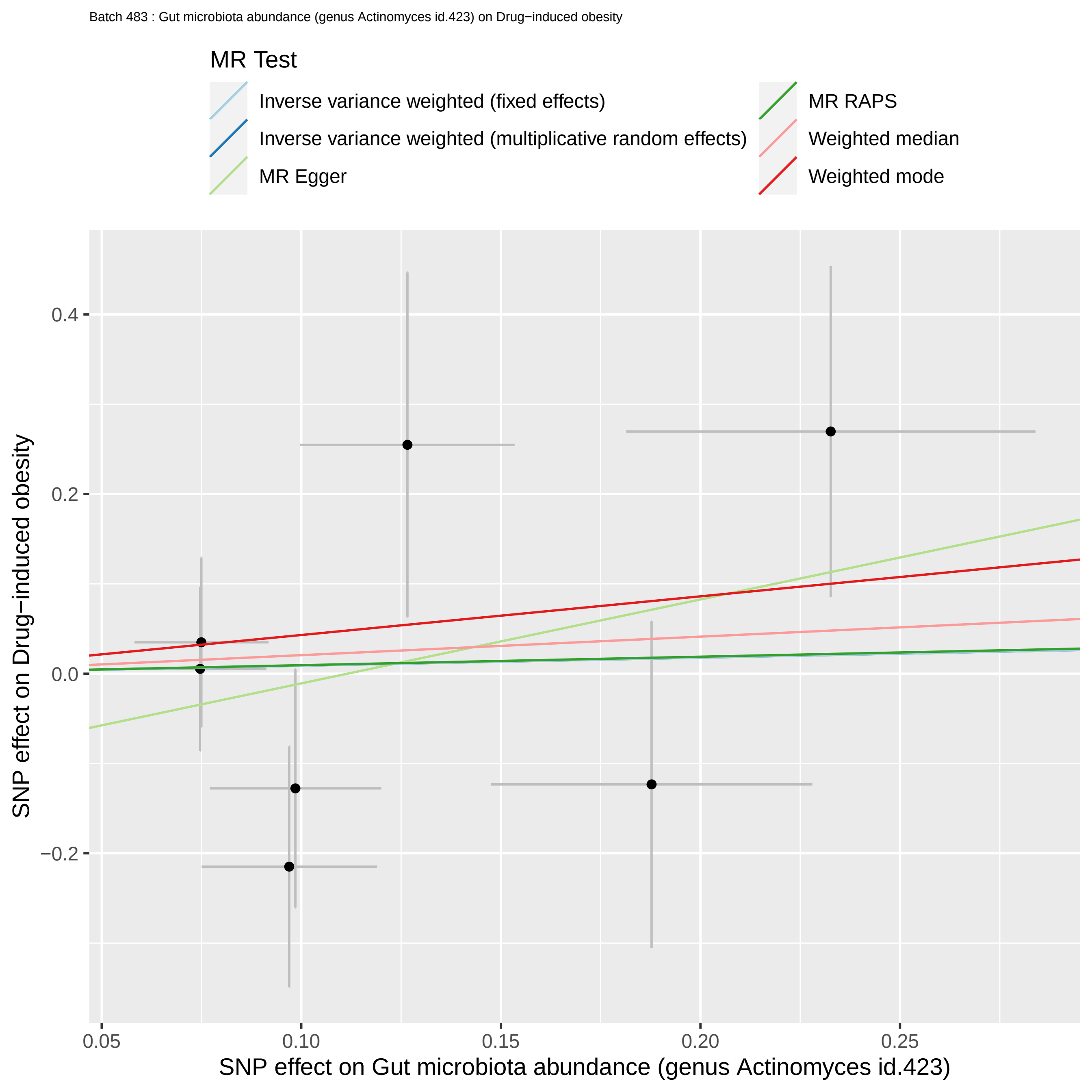
Batch 483 : Gut microbiota abundance (genus Actinomyces id.423) on Drug−induced obesity
MR Test
Inverse variance weighted (fixed effects)
Inverse variance weighted (multiplicative random effects)
MR Egger
MR RAPS
Weighted median
Weighted mode
0.4
0.2
0.0
−0.2
0.05
0.10
0.15
0.20
0.25
SNP effect on Drug−induced obesity
SNP effect on Gut microbiota abundance (genus Actinomyces id.423)

Batch 484 : Gut microbiota abundance (genus Adlercreutzia id.812) on Drug−induced obesity

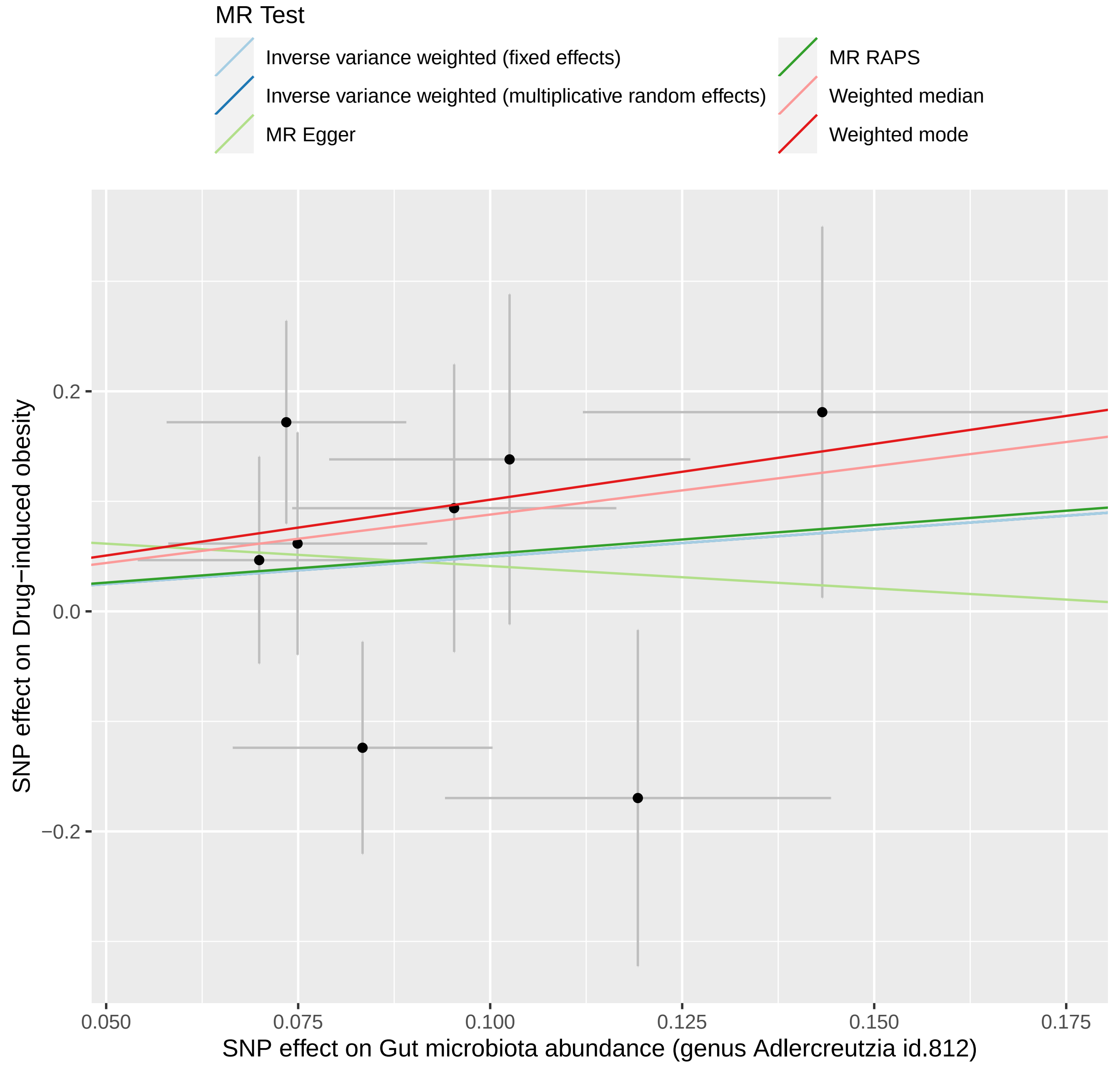

Batch 485 : Gut microbiota abundance (genus Akkermansia id.4037) on Drug−induced obesity

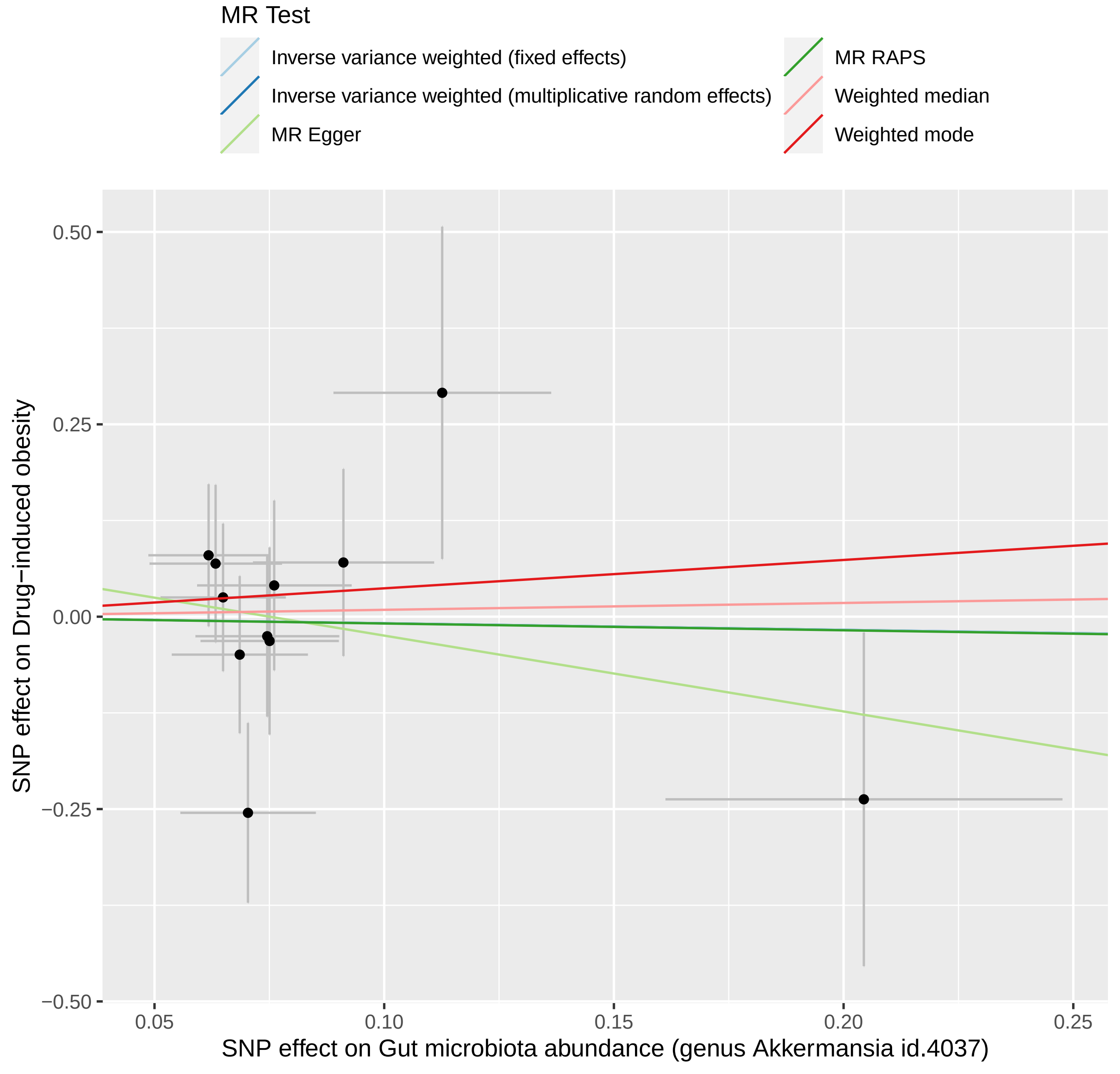

Batch 486 : Gut microbiota abundance (genus Alistipes id.968) on Drug−induced obesity

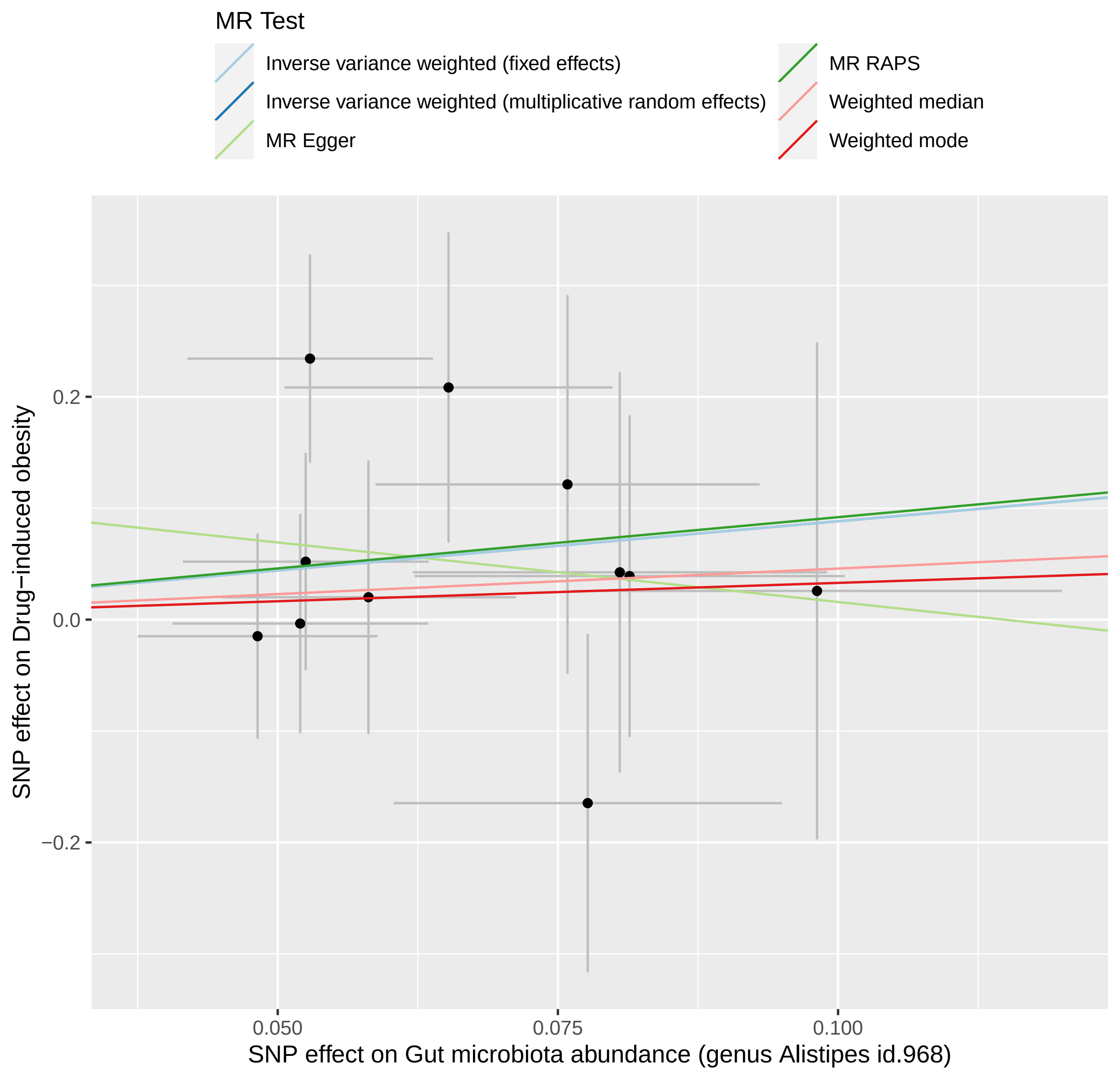

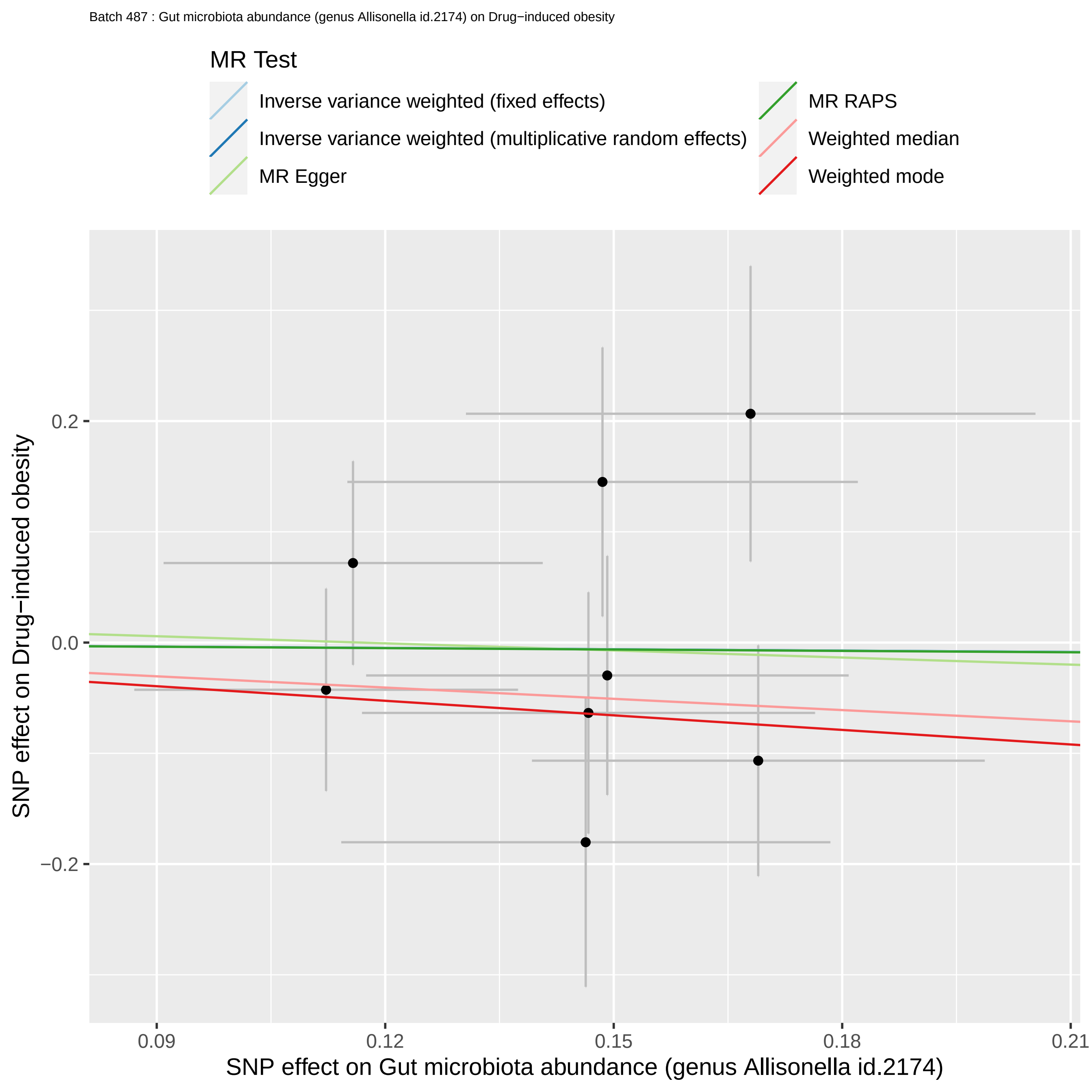

Batch 487 : Gut microbiota abundance (genus Allisonella id.2174) on Drug−induced obesity
MR Test
Inverse variance weighted (fixed effects)
Inverse variance weighted (multiplicative random effects)
MR Egger
MR RAPS
Weighted median
Weighted mode
0.2
0.0
−0.2
0.09
0.12
0.15
0.18
0.21
SNP effect on Drug−induced obesity
SNP effect on Gut microbiota abundance (genus Allisonella id.2174)

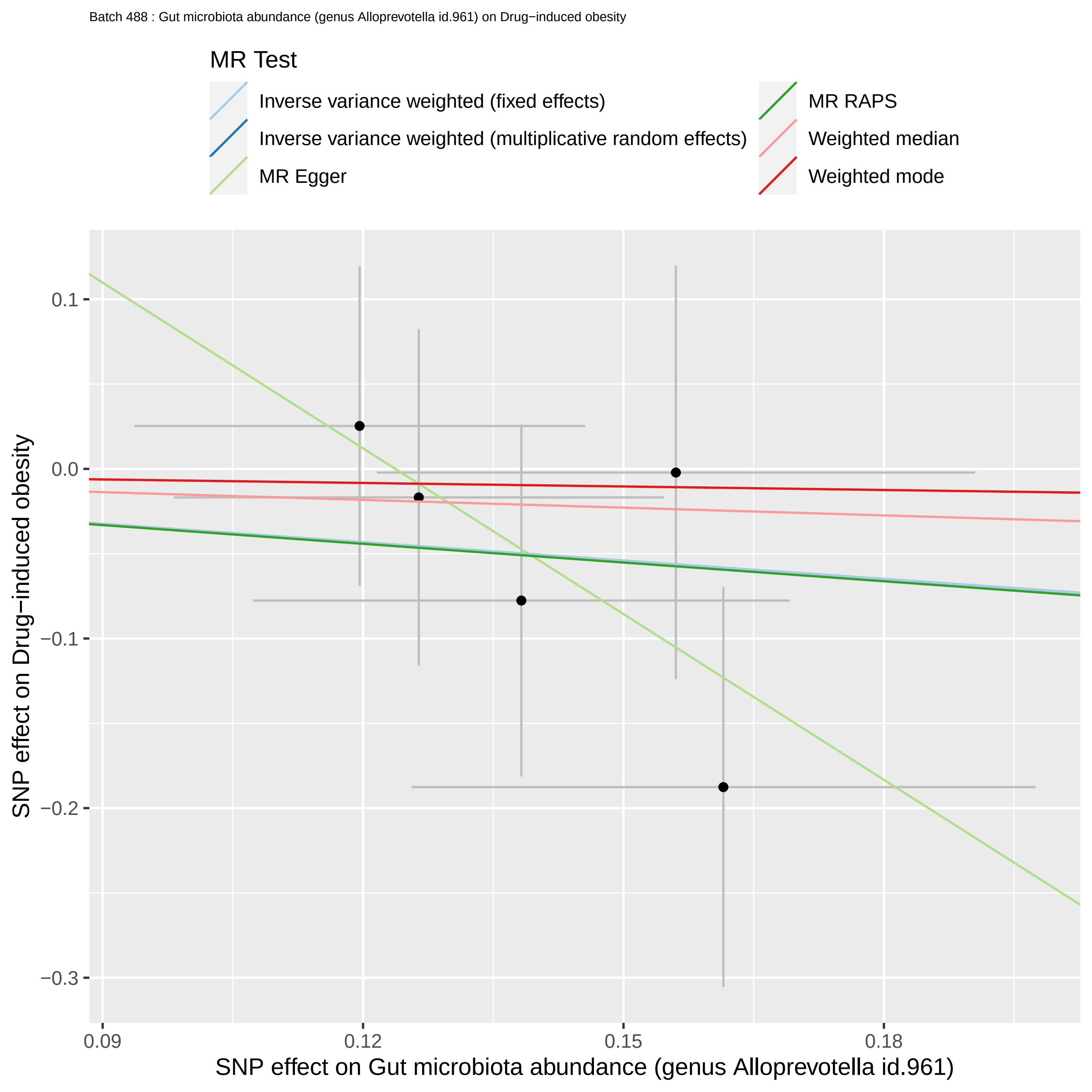

Batch 488 : Gut microbiota abundance (genus Alloprevotella id.961) on Drug−induced obesity
MR Test
Inverse variance weighted (fixed effects)
Inverse variance weighted (multiplicative random effects)
MR Egger
MR RAPS
Weighted median
Weighted mode
0.1
0.0
−0.1
−0.2
−0.3
SNP effect on Drug−induced obesity
0.09
0.12
0.15
0.18
SNP effect on Gut microbiota abundance (genus Alloprevotella id.961)

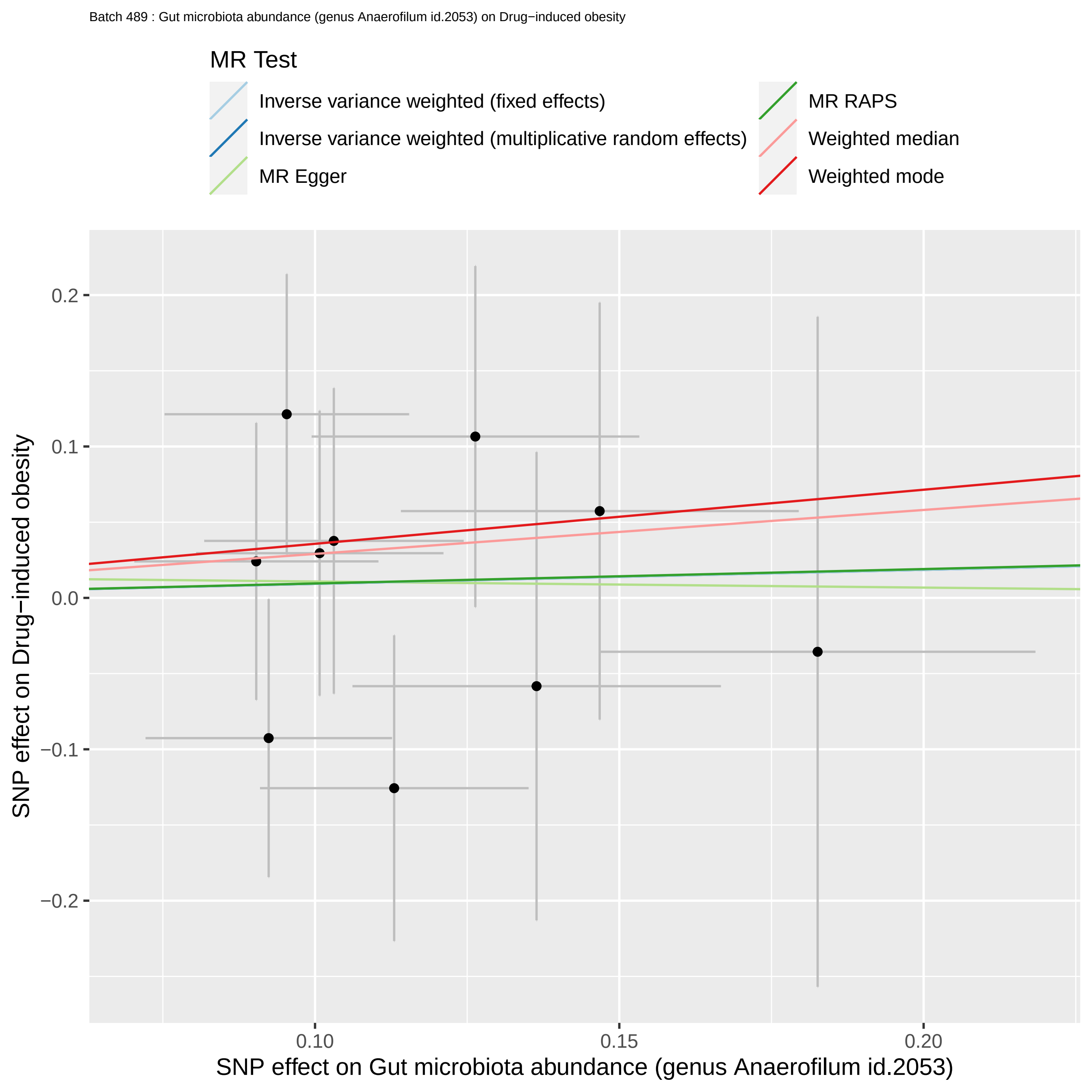

Batch 489 : Gut microbiota abundance (genus Anaerofilum id.2053) on Drug−induced obesity
MR Test
Inverse variance weighted (fixed effects)
Inverse variance weighted (multiplicative random effects)
MR Egger
MR RAPS
Weighted median
Weighted mode
SNP effect on Drug−induced obesity
0.2
0.1
0.0
−0.1
−0.2
0.10
0.15
0.20
SNP effect on Gut microbiota abundance (genus Anaerofilum id.2053)

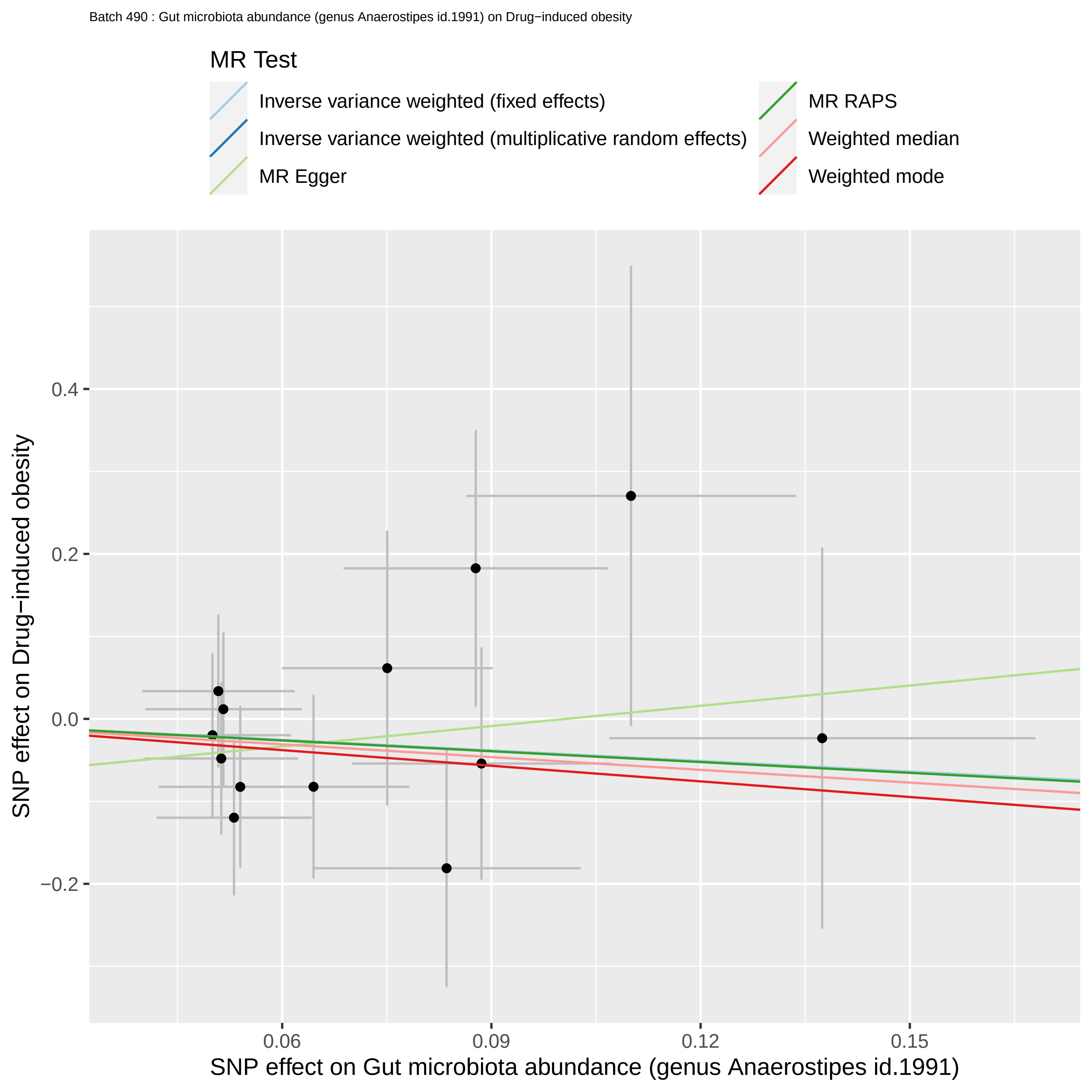

Batch 490 : Gut microbiota abundance (genus Anaerostipes id.1991) on Drug−induced obesity
MR Test
Inverse variance weighted (fixed effects)
Inverse variance weighted (multiplicative random effects)
MR Egger
MR RAPS
Weighted median
Weighted mode
SNP effect on Drug−induced obesity
0.4
0.2
0.0
−0.2
0.06
0.09
0.12
0.15
SNP effect on Gut microbiota abundance (genus Anaerostipes id.1991)

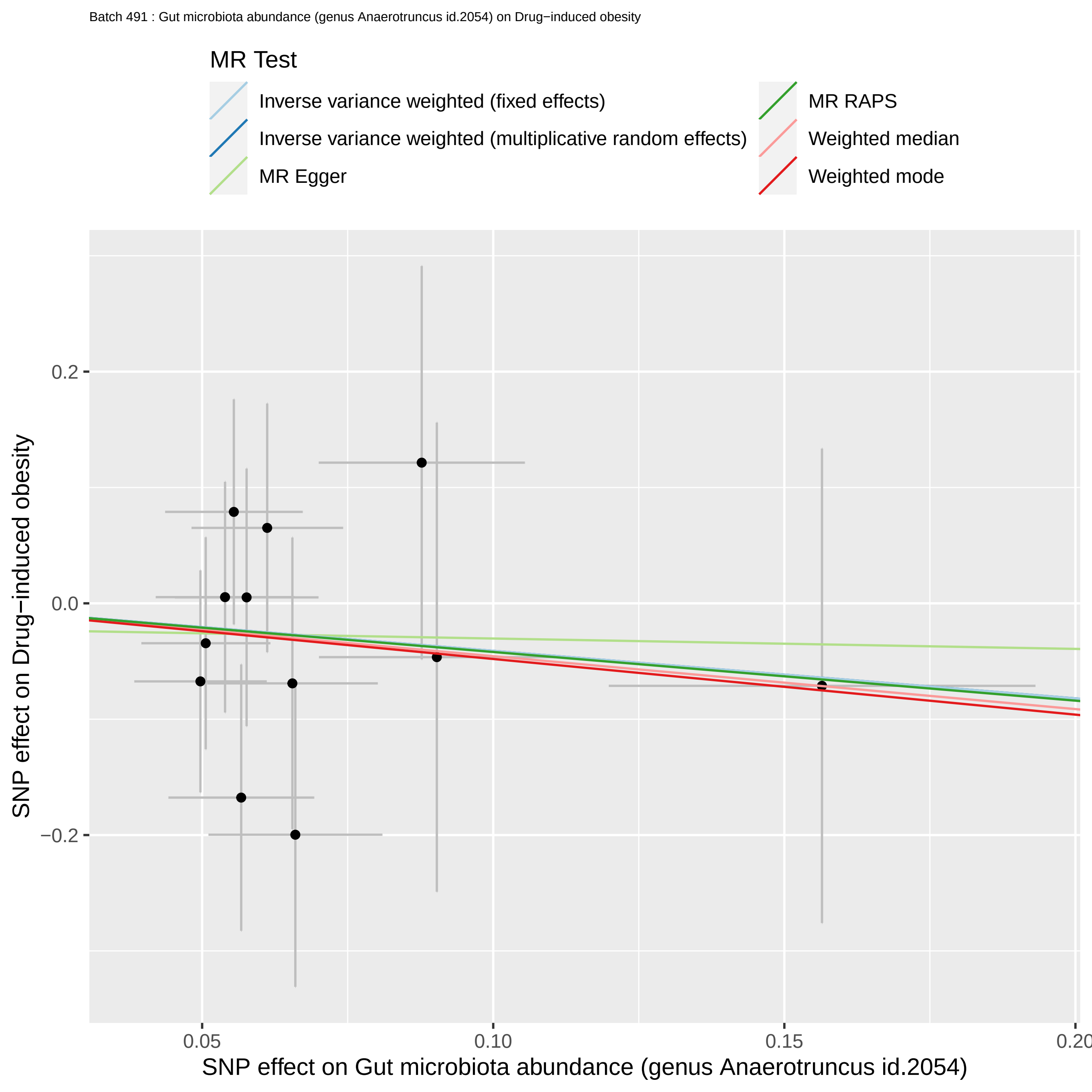

Batch 491 : Gut microbiota abundance (genus Anaerotruncus id.2054) on Drug−induced obesity
MR Test
Inverse variance weighted (fixed effects)
Inverse variance weighted (multiplicative random effects)
MR Egger
MR RAPS
Weighted median
Weighted mode
0.2
0.0
−0.2
0.05
0.10
0.15
0.20
SNP effect on Drug−induced obesity
SNP effect on Gut microbiota abundance (genus Anaerotruncus id.2054)

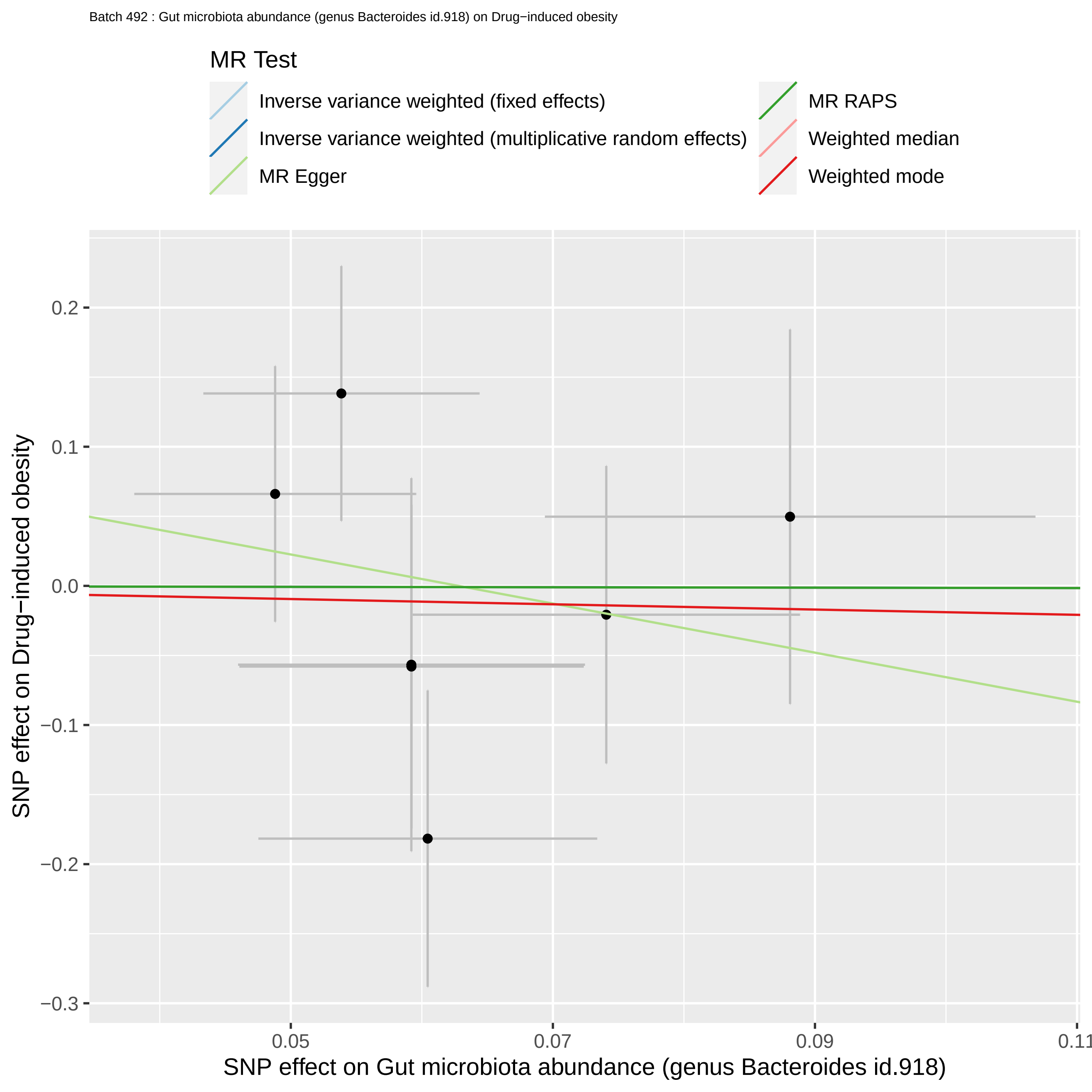
Batch 492 : Gut microbiota abundance (genus Bacteroides id.918) on Drug−induced obesity
MR Test
Inverse variance weighted (fixed effects)
Inverse variance weighted (multiplicative random effects)
MR Egger
MR RAPS
Weighted median
Weighted mode
0.2
0.1
0.0
−0.1
−0.2
−0.3
0.05
0.07
0.09
0.11
SNP effect on Drug−induced obesity
SNP effect on Gut microbiota abundance (genus Bacteroides id.918)

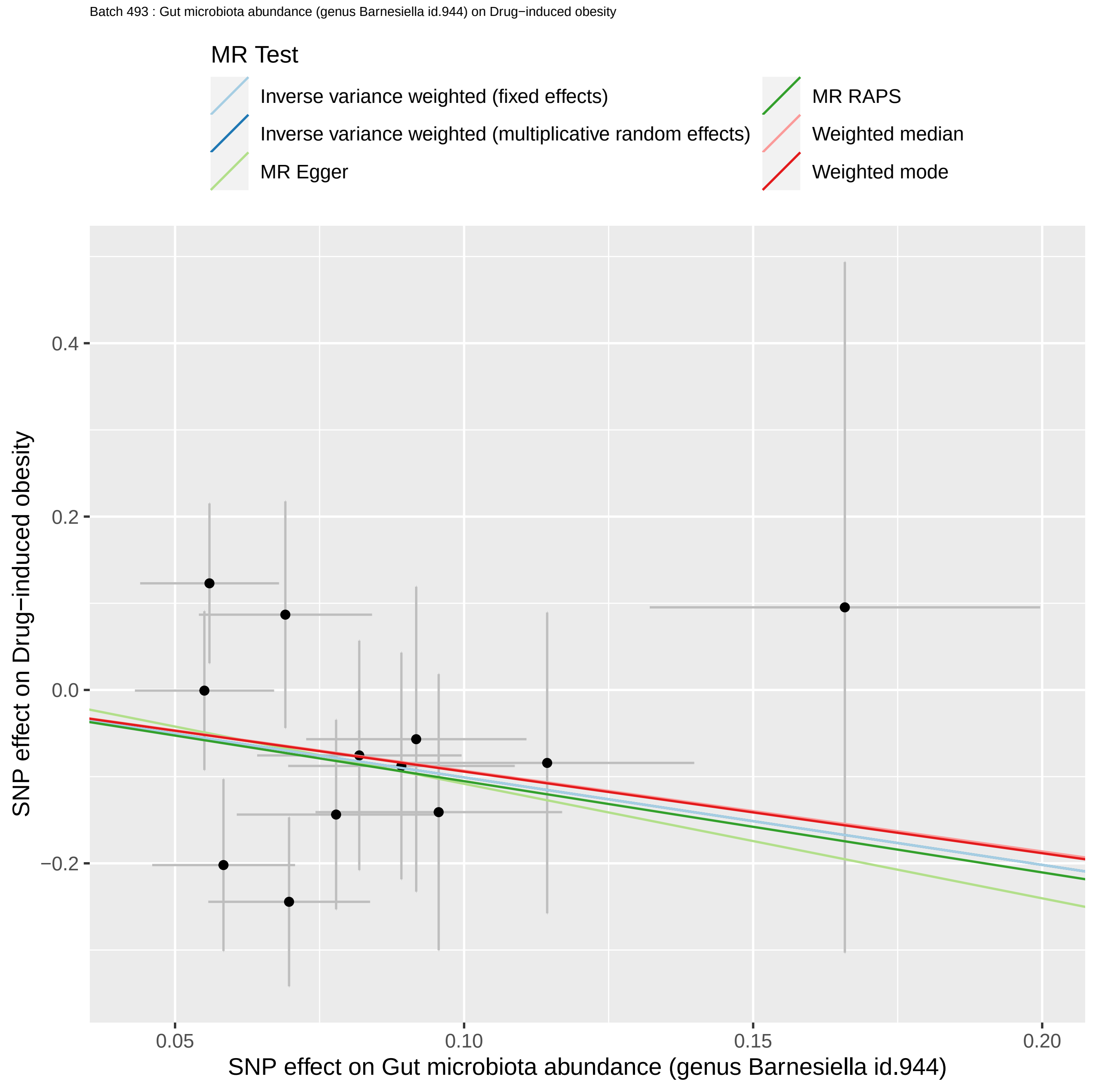
Batch 493 : Gut microbiota abundance (genus Barnesiella id.944) on Drug−induced obesity
MR Test
Inverse variance weighted (fixed effects)
Inverse variance weighted (multiplicative random effects)
MR Egger
MR RAPS
Weighted median
Weighted mode
0.4
0.2
0.0
−0.2
0.05
0.10
0.15
0.20
SNP effect on Drug−induced obesity
SNP effect on Gut microbiota abundance (genus Barnesiella id.944)

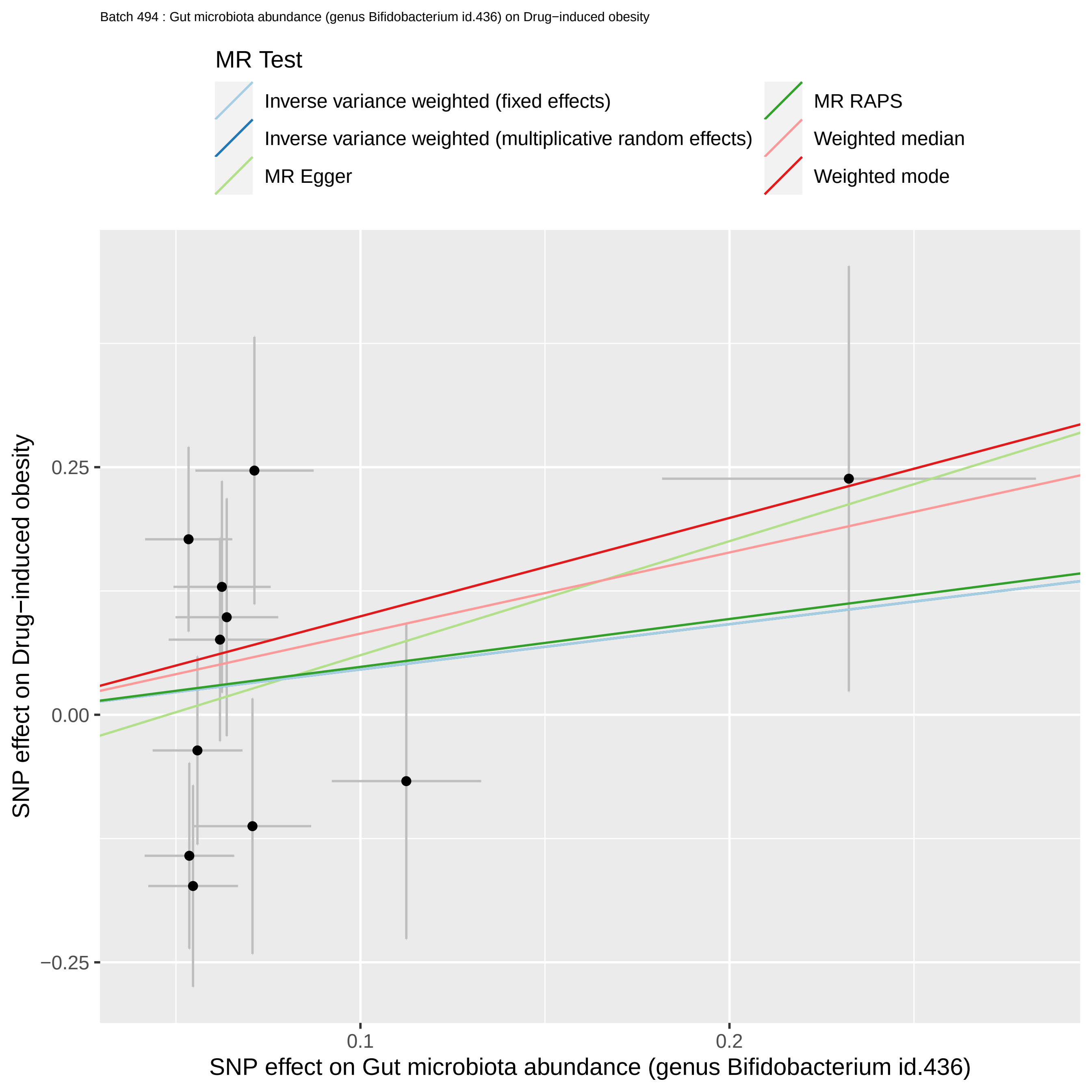

Batch 494 : Gut microbiota abundance (genus Bifidobacterium id.436) on Drug−induced obesity
MR Test
Inverse variance weighted (fixed effects)
Inverse variance weighted (multiplicative random effects)
MR Egger
MR RAPS
Weighted median
Weighted mode
0.25
0.00
−0.25
0.1
0.2
SNP effect on Drug−induced obesity
SNP effect on Gut microbiota abundance (genus Bifidobacterium id.436)

Batch 495 : Gut microbiota abundance (genus Bilophila id.3170) on Drug−induced obesity

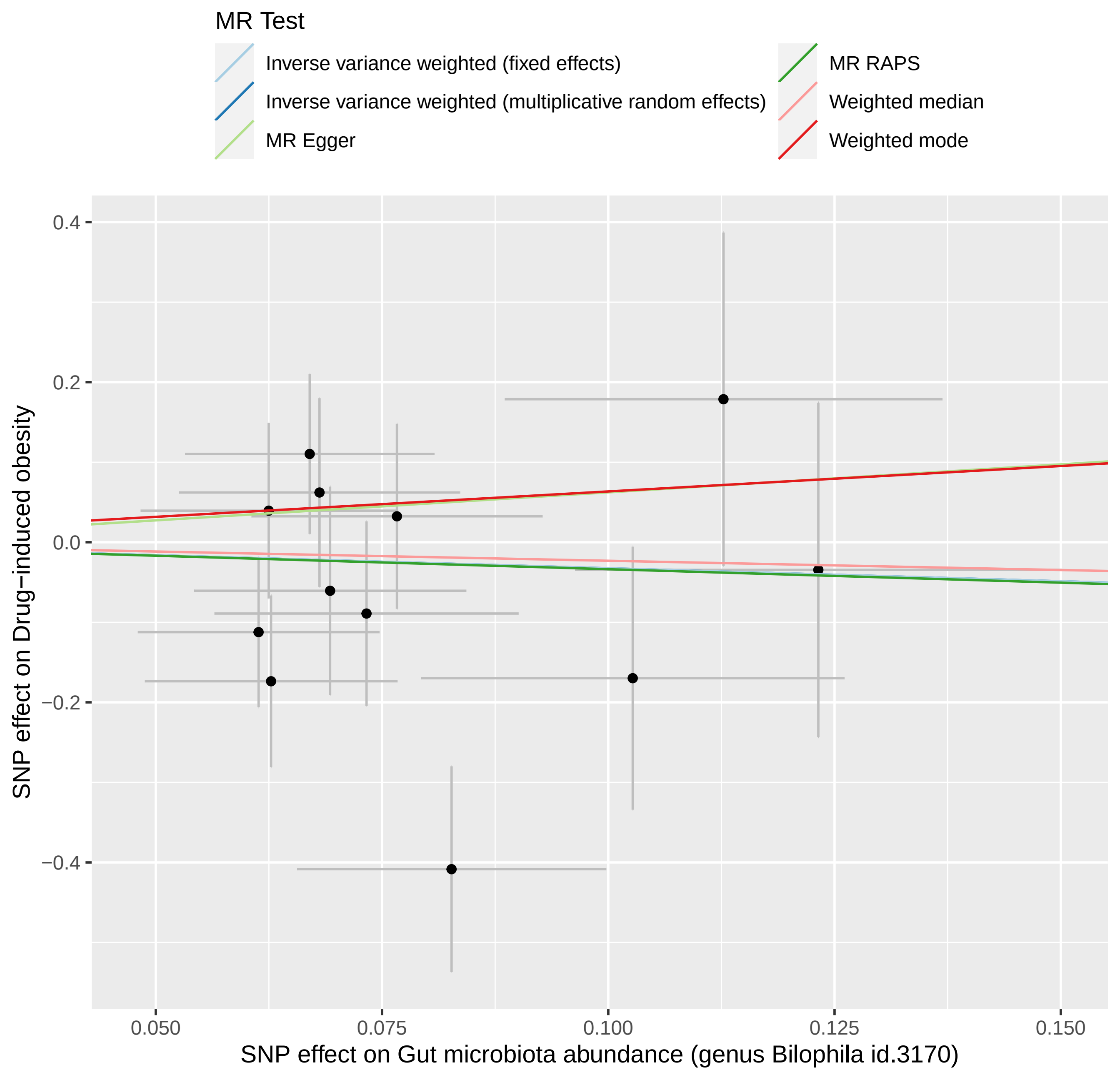

Batch 496 : Gut microbiota abundance (genus Blautia id.1992) on Drug−induced obesity

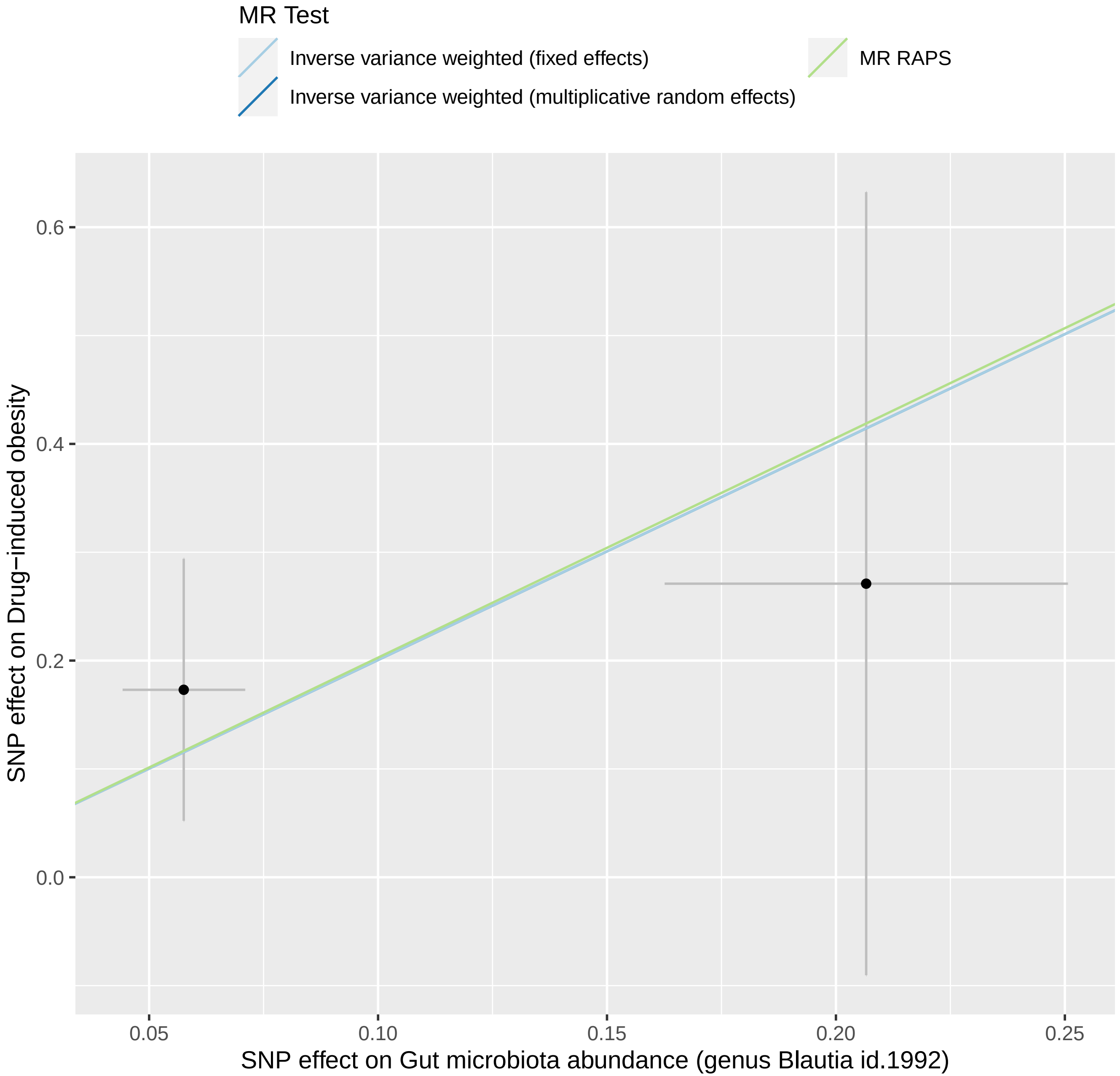

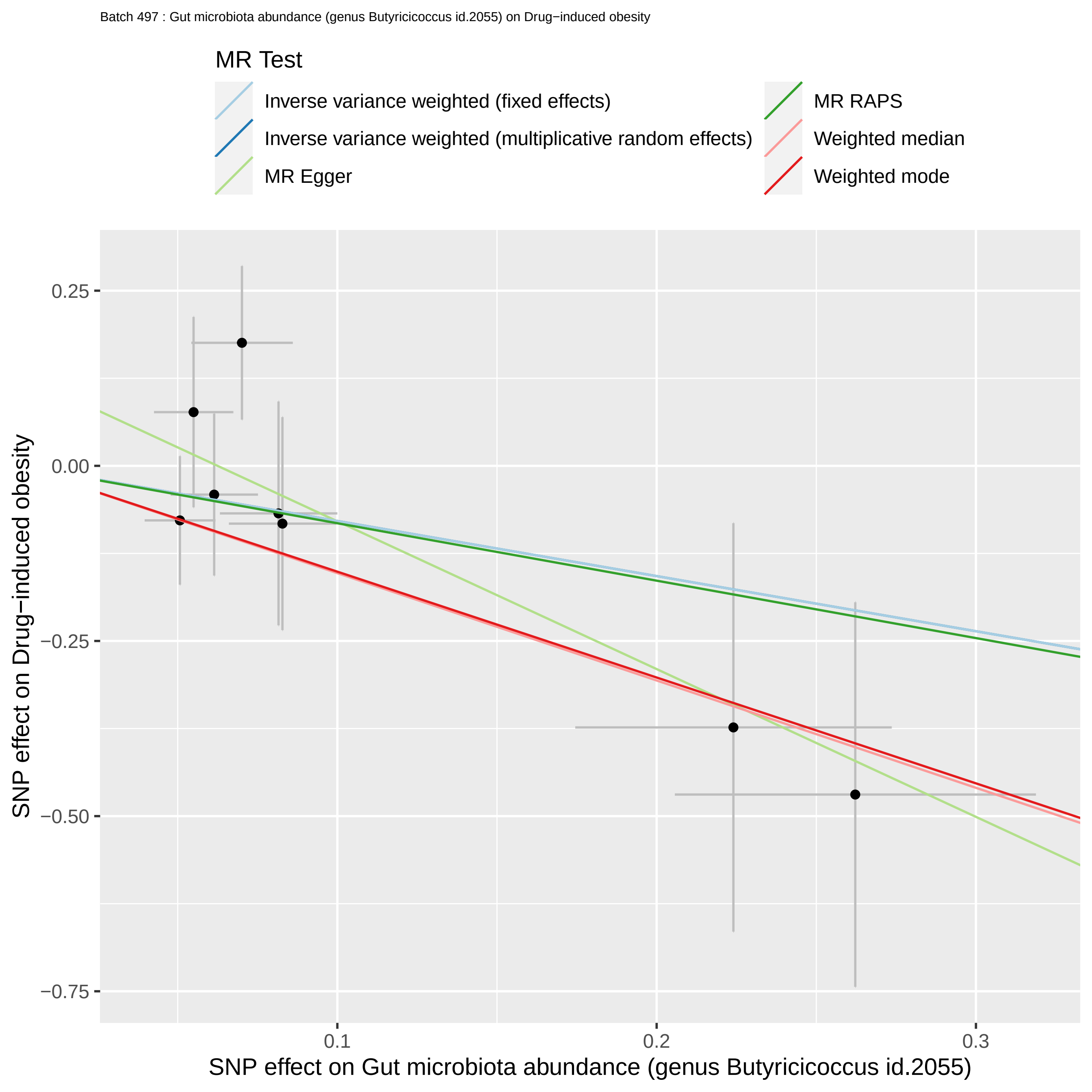

Batch 497 : Gut microbiota abundance (genus Butyricicoccus id.2055) on Drug−induced obesity
MR Test
Inverse variance weighted (fixed effects)
Inverse variance weighted (multiplicative random effects)
MR Egger
MR RAPS
Weighted median
Weighted mode
0.25
0.00
−0.25
−0.50
−0.75
0.1
0.2
0.3
SNP effect on Drug−induced obesity
SNP effect on Gut microbiota abundance (genus Butyricicoccus id.2055)

Batch 498 : Gut microbiota abundance (genus Butyricimonas id.945) on Drug−induced obesity

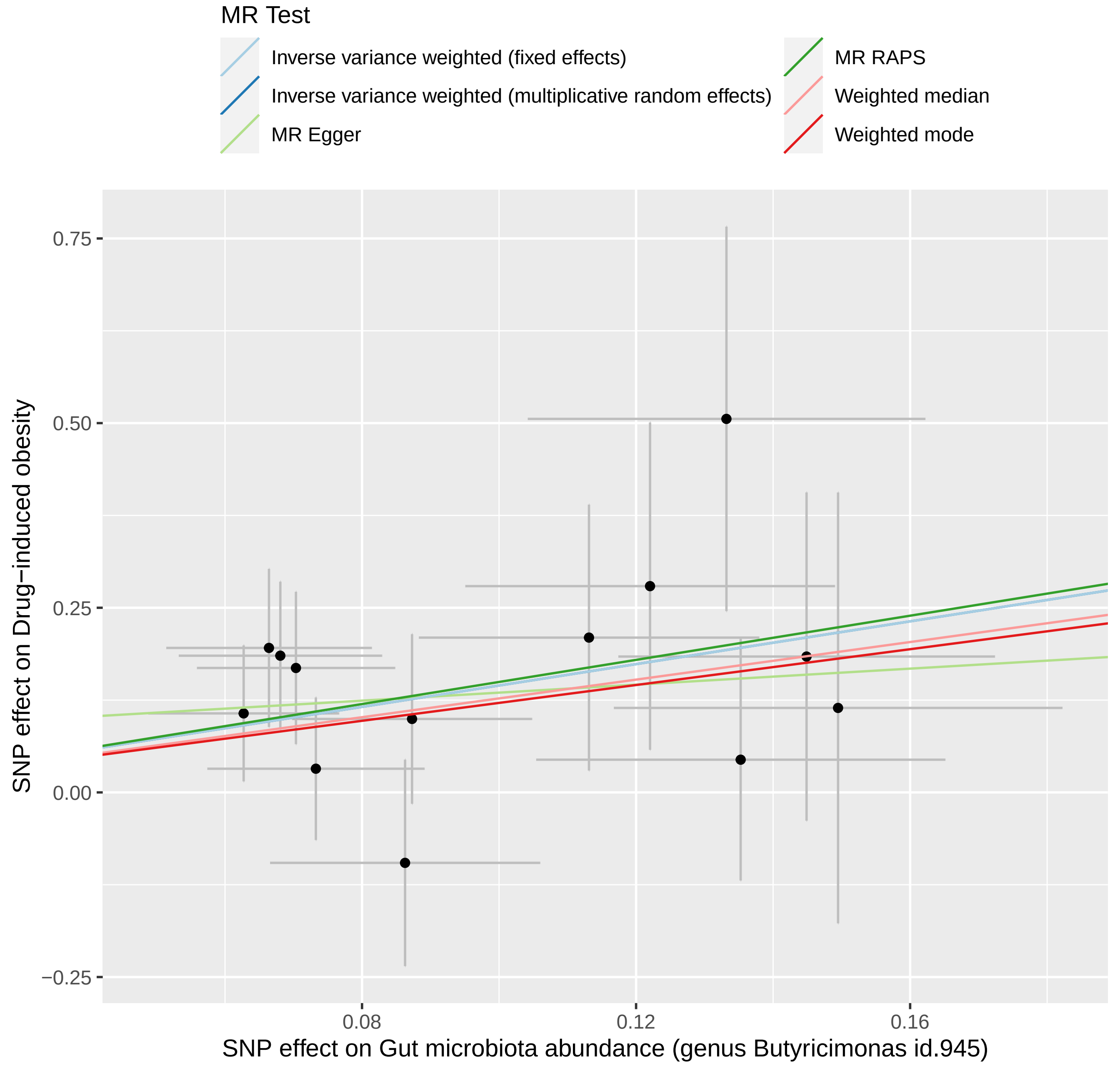

Batch 499 : Gut microbiota abundance (genus Butyrivibrio id.1993) on Drug−induced obesity

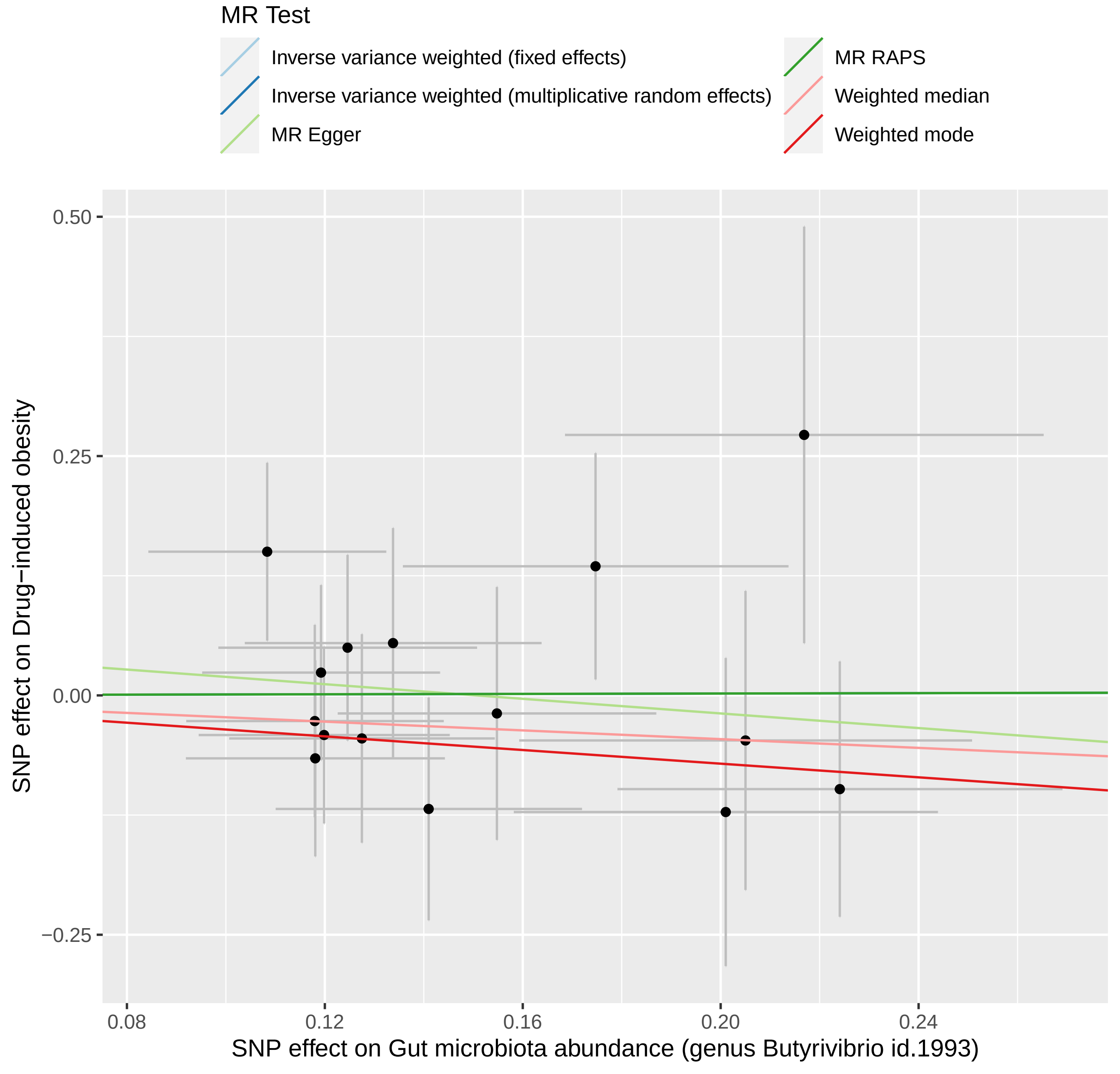

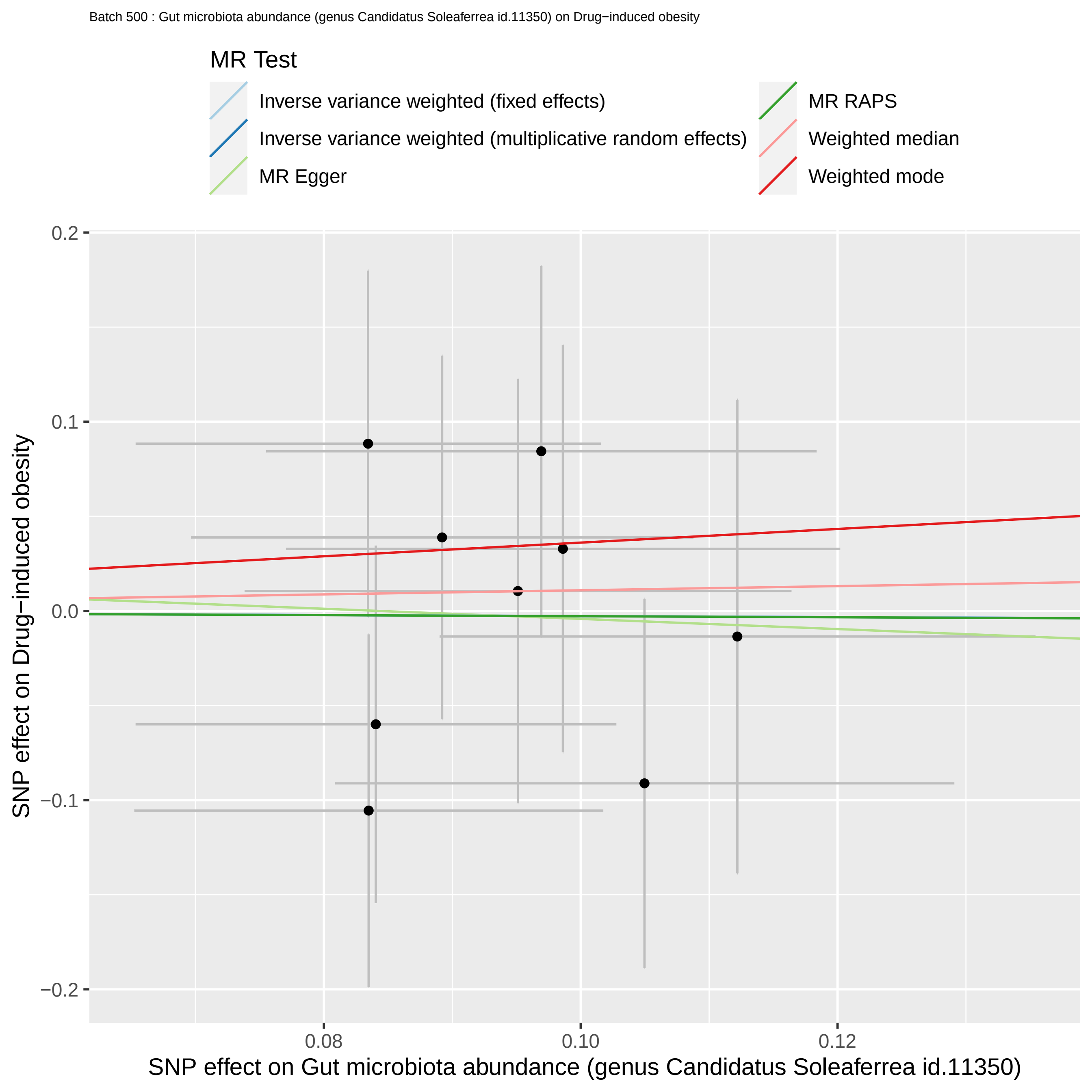

Batch 500 : Gut microbiota abundance (genus Candidatus Soleaferrea id.11350) on Drug−induced obesity
MR Test
Inverse variance weighted (fixed effects)
Inverse variance weighted (multiplicative random effects)
MR Egger
MR RAPS
Weighted median
Weighted mode
0.2
0.1
0.0
−0.1
−0.2
0.08
0.10
0.12
SNP effect on Drug−induced obesity
SNP effect on Gut microbiota abundance (genus Candidatus Soleaferrea id.11350)

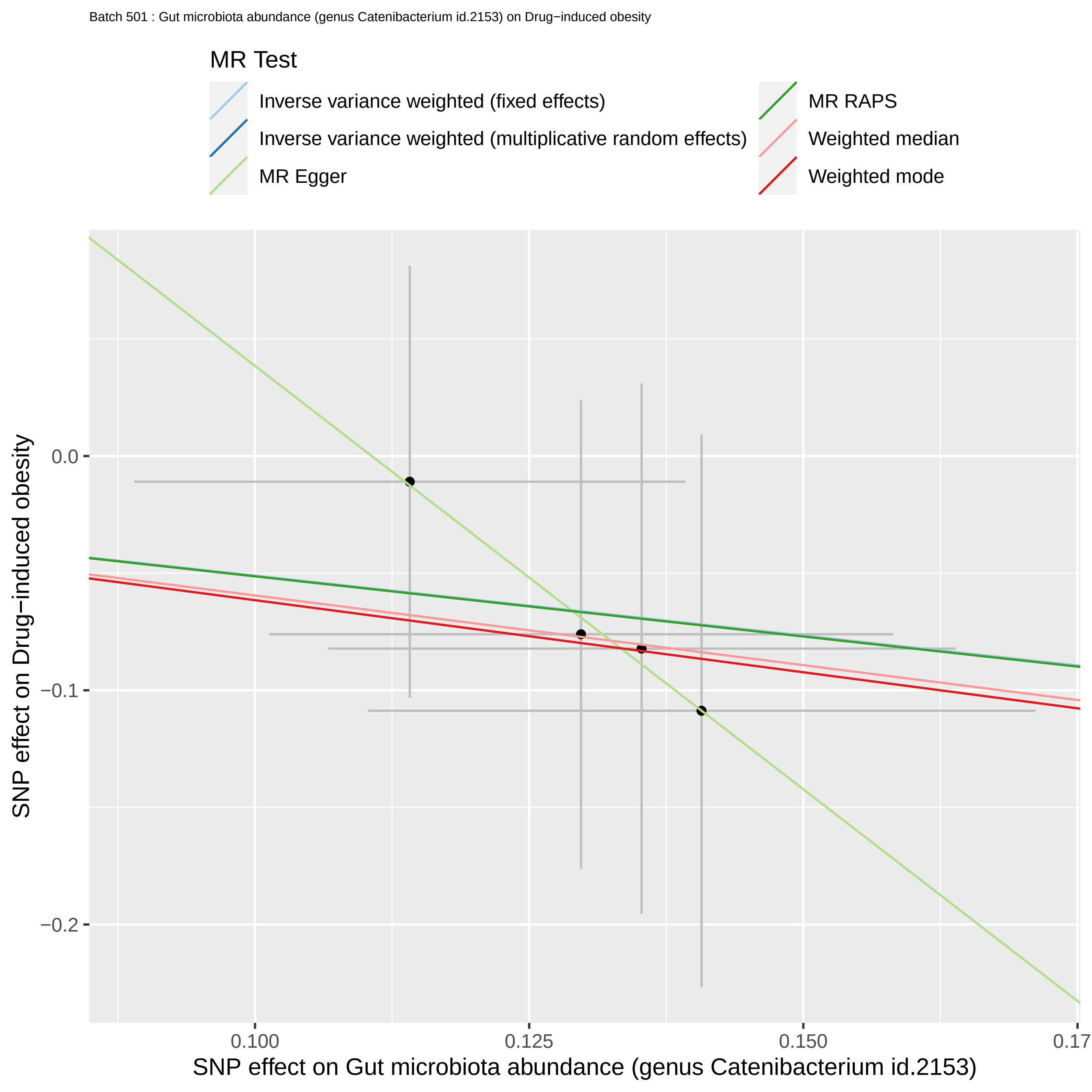

Batch 501 : Gut microbiota abundance (genus Catenibacterium id.2153) on Drug−induced obesity
MR Test
Inverse variance weighted (fixed effects)
Inverse variance weighted (multiplicative random effects)
MR Egger
MR RAPS
Weighted median
Weighted mode
SNP effect on Drug−induced obesity
0.0
−0.1
−0.2
0.100
0.125
0.150
0.17
SNP effect on Gut microbiota abundance (genus Catenibacterium id.2153)

Batch 502 : Gut microbiota abundance (genus Christensenellaceae R 7group id.11283) on Drug−induced obesity

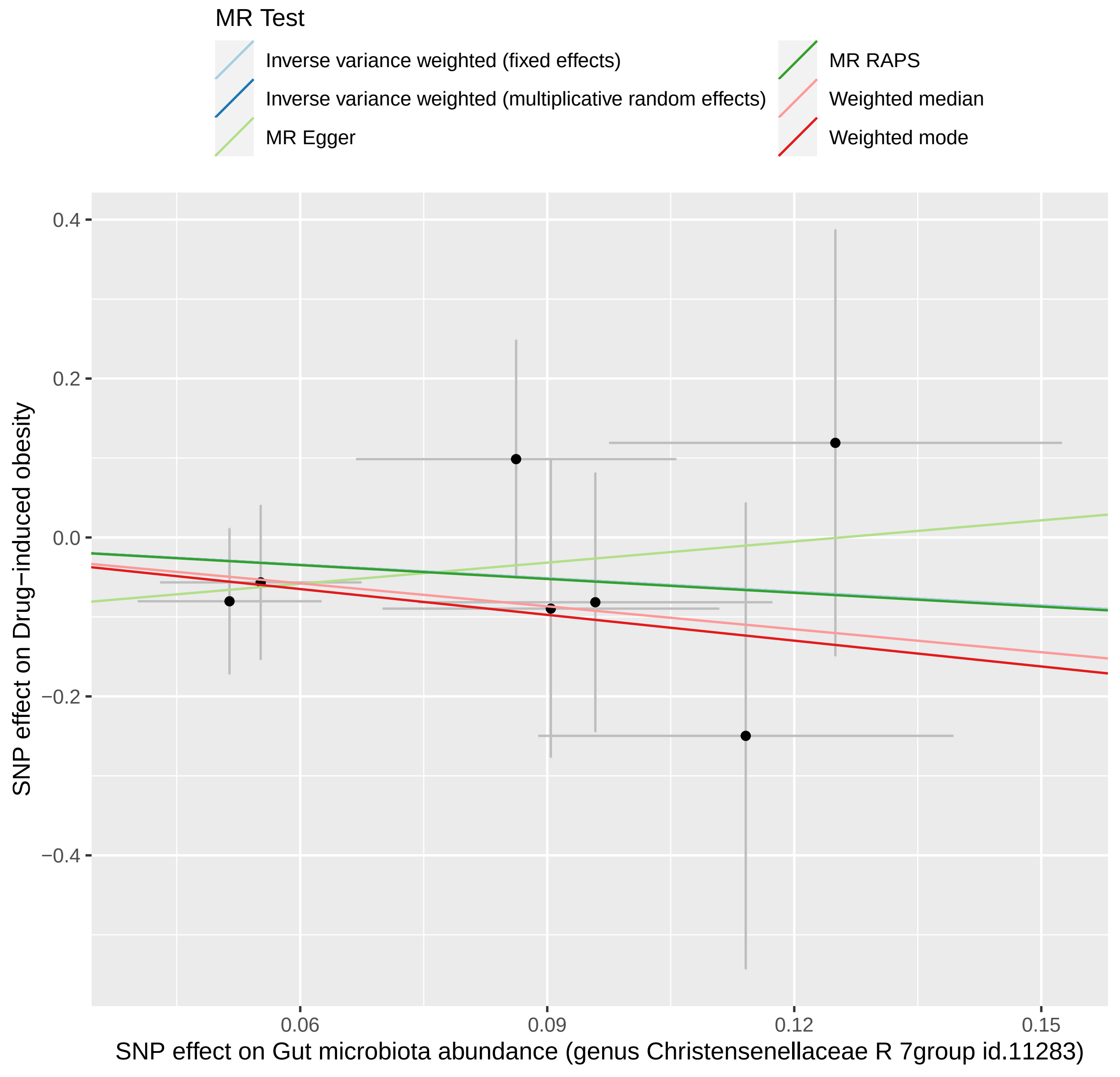

Batch 503 : Gut microbiota abundance (genus Clostridium innocuum group id.14397) on Drug−induced obesity

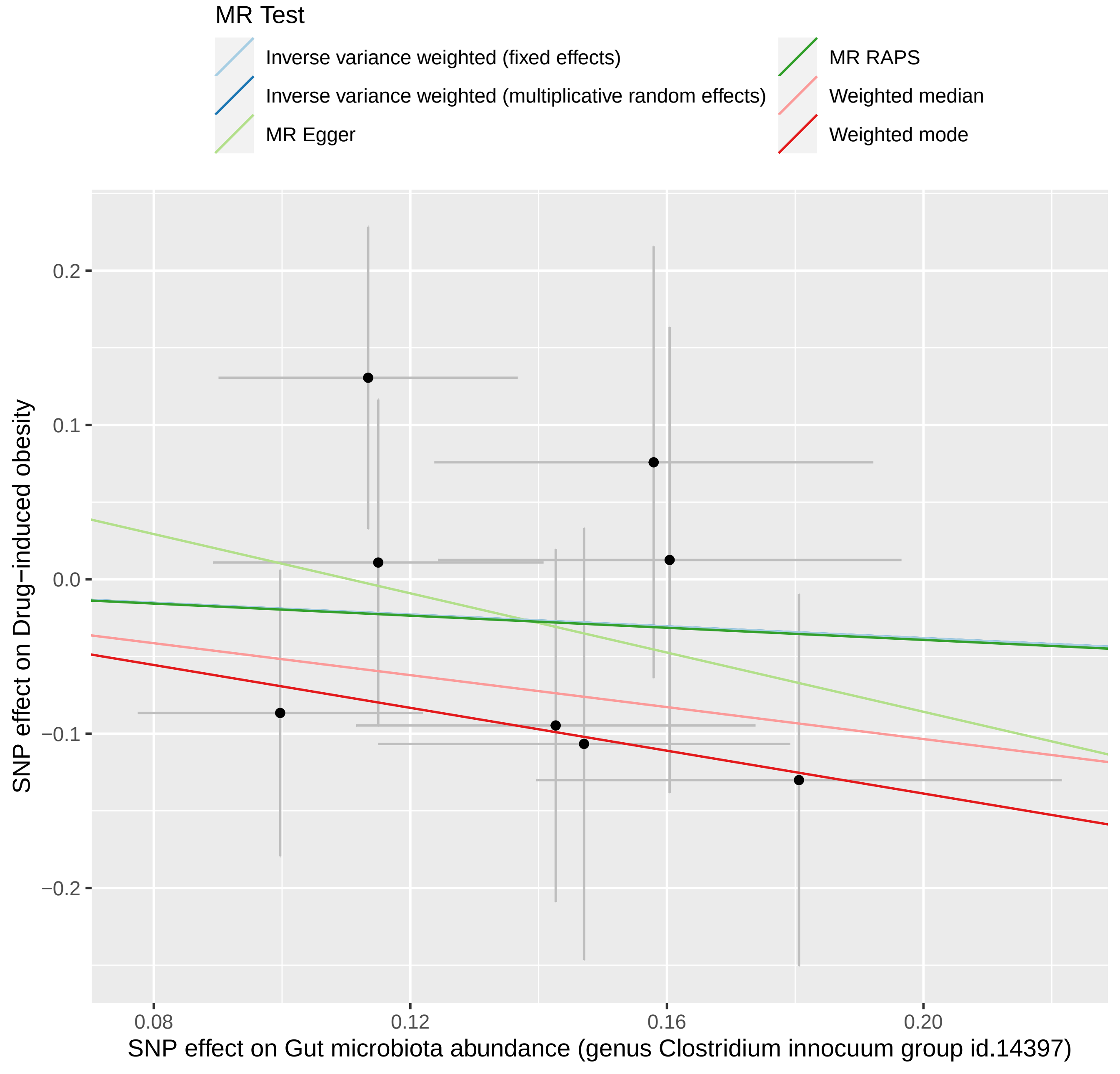

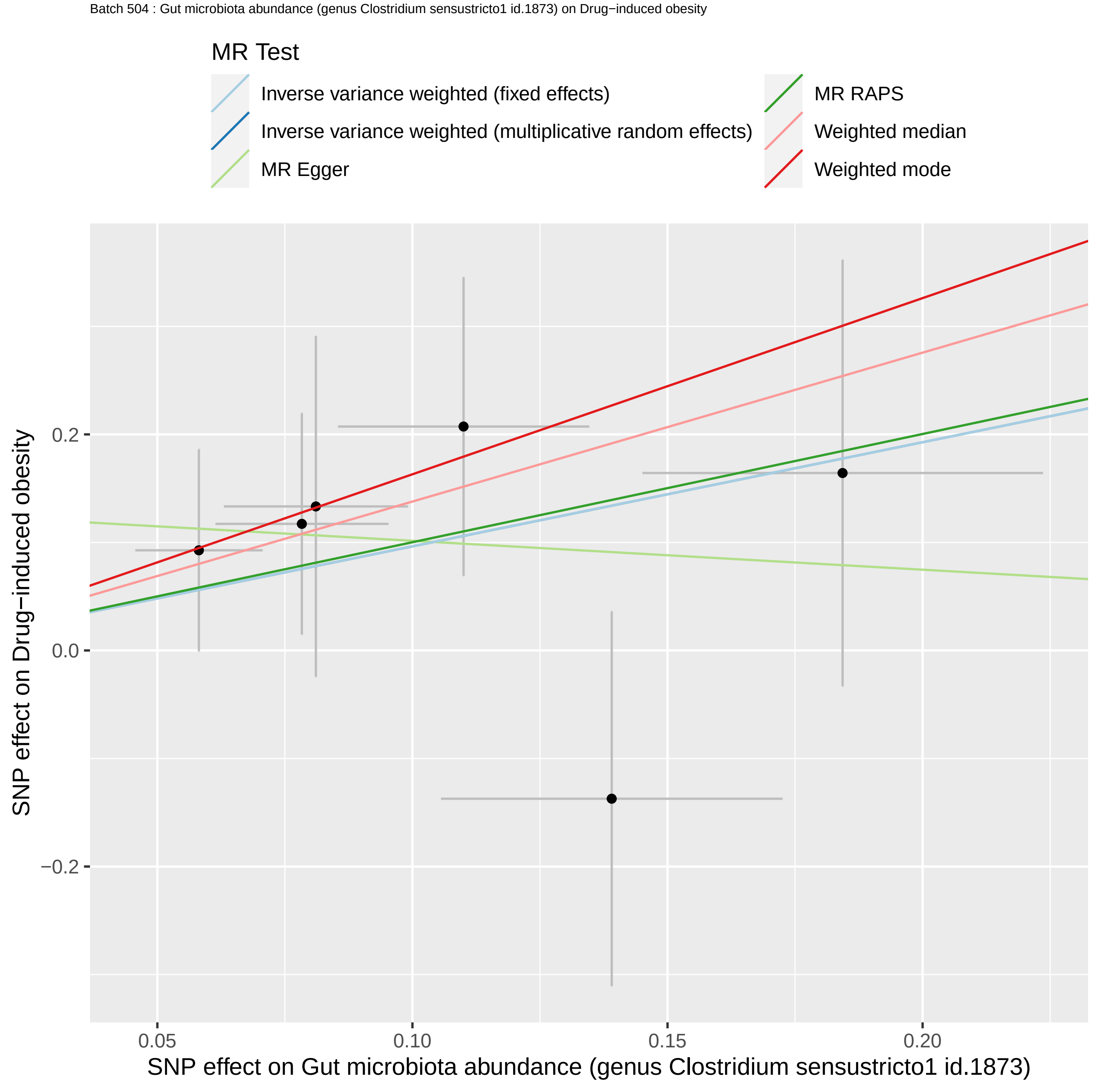

Batch 504 : Gut microbiota abundance (genus Clostridium sensustricto1 id.1873) on Drug−induced obesity
MR Test
Inverse variance weighted (fixed effects)
Inverse variance weighted (multiplicative random effects)
MR Egger
MR RAPS
Weighted median
Weighted mode
0.2
0.0
−0.2
0.05
0.10
0.15
0.20
SNP effect on Drug−induced obesity
SNP effect on Gut microbiota abundance (genus Clostridium sensustricto1 id.1873)

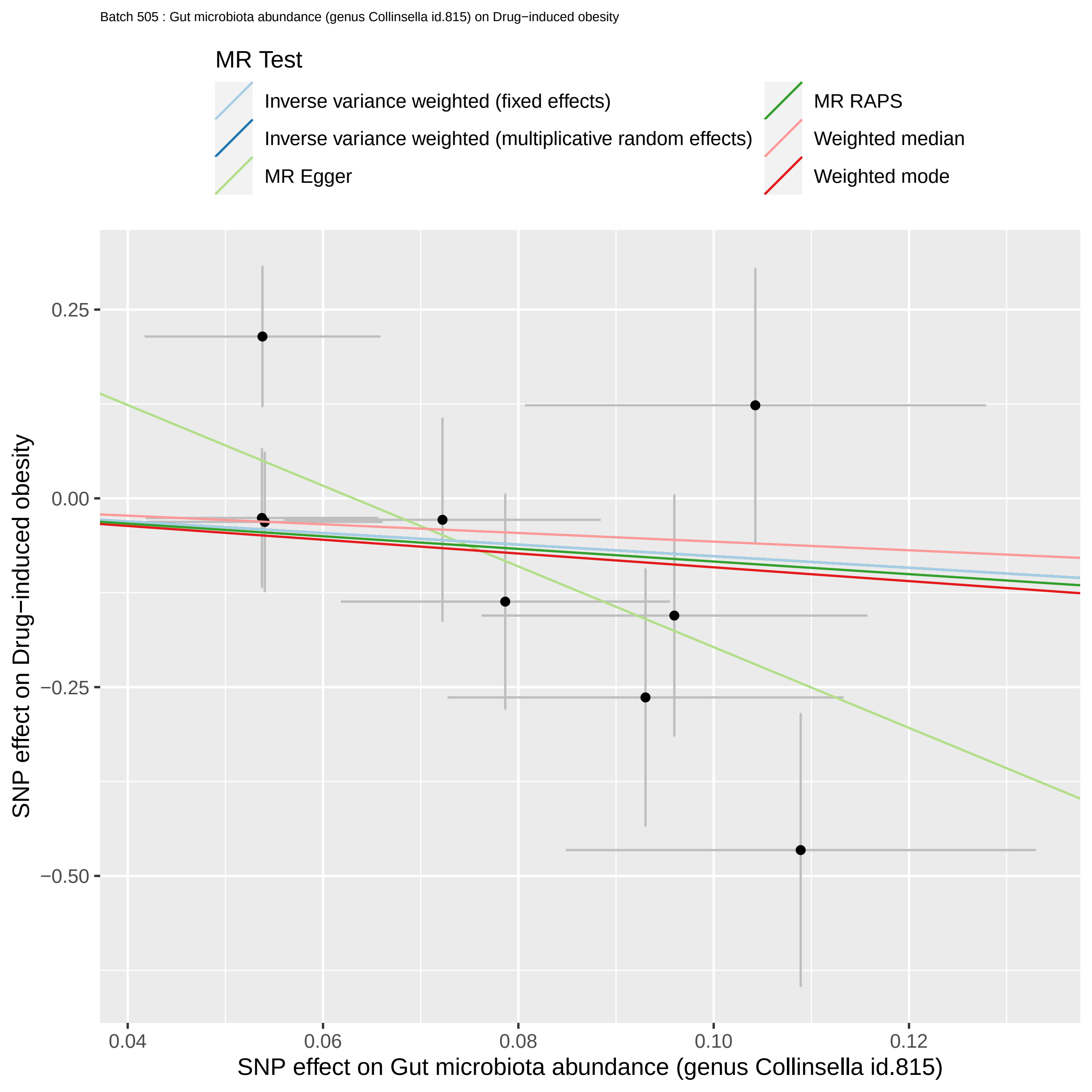

Batch 505 : Gut microbiota abundance (genus Collinsella id.815) on Drug−induced obesity
MR Test
Inverse variance weighted (fixed effects)
Inverse variance weighted (multiplicative random effects)
MR Egger
MR RAPS
Weighted median
Weighted mode
0.25
0.00
−0.25
−0.50
0.04
0.06
0.08
0.10
0.12
SNP effect on Drug−induced obesity
SNP effect on Gut microbiota abundance (genus Collinsella id.815)

Batch 506 : Gut microbiota abundance (genus Coprobacter id.949) on Drug−induced obesity

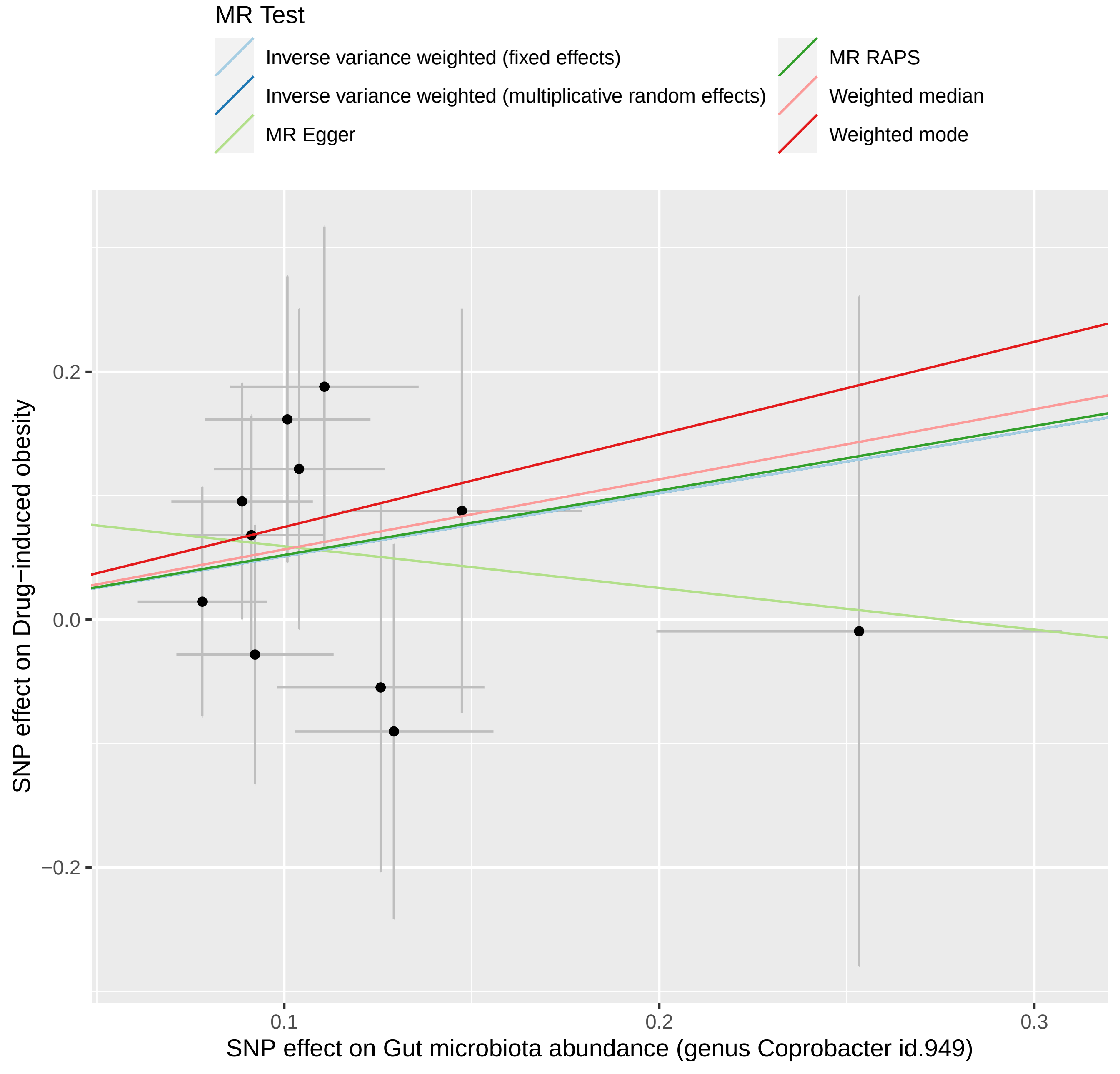

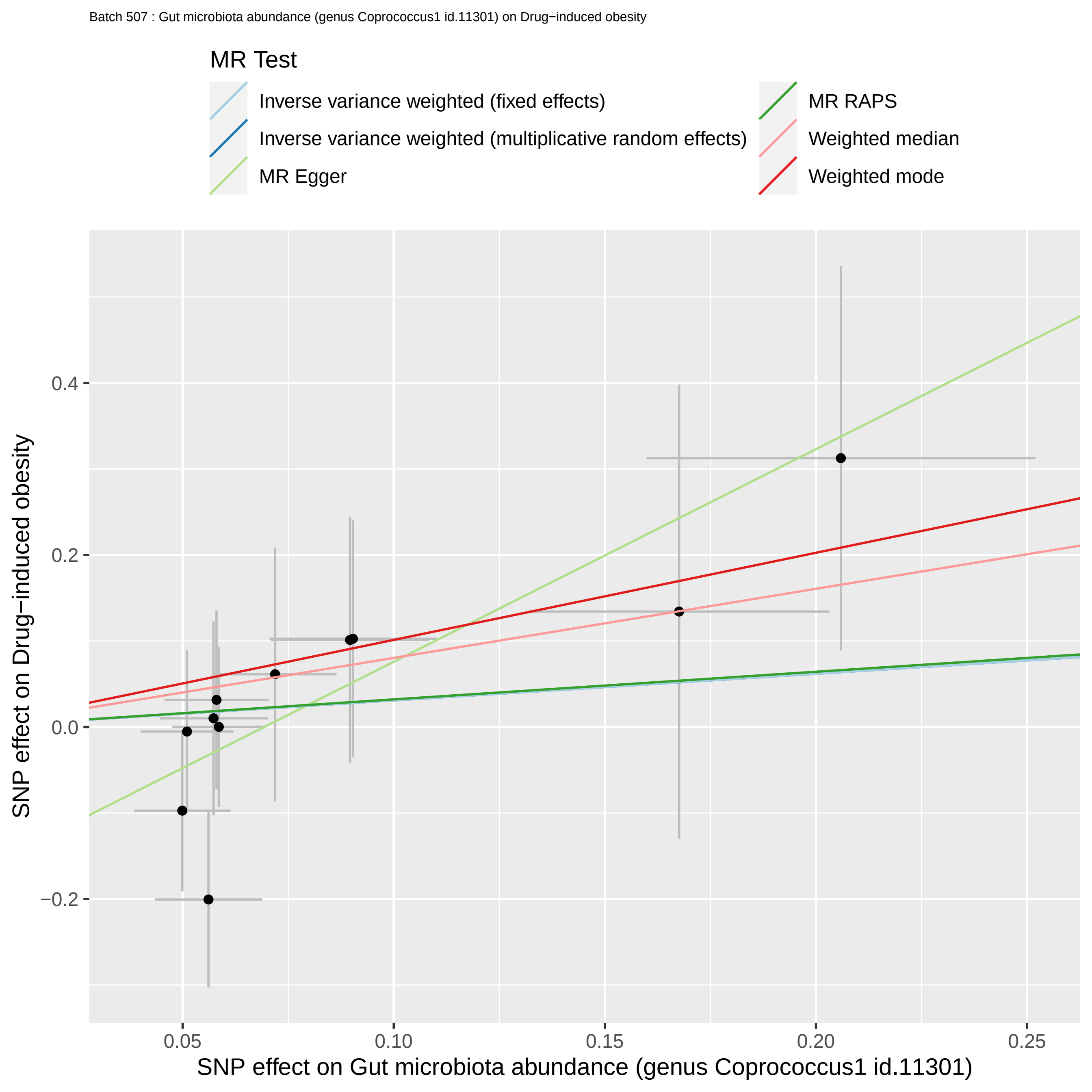

Batch 507 : Gut microbiota abundance (genus Coprococcus1 id.11301) on Drug−induced obesity
MR Test
Inverse variance weighted (fixed effects)
Inverse variance weighted (multiplicative random effects)
MR Egger
MR RAPS
Weighted median
Weighted mode
0.4
0.2
0.0
−0.2
0.05
0.10
0.15
0.20
0.25
SNP effect on Drug−induced obesity
SNP effect on Gut microbiota abundance (genus Coprococcus1 id.11301)

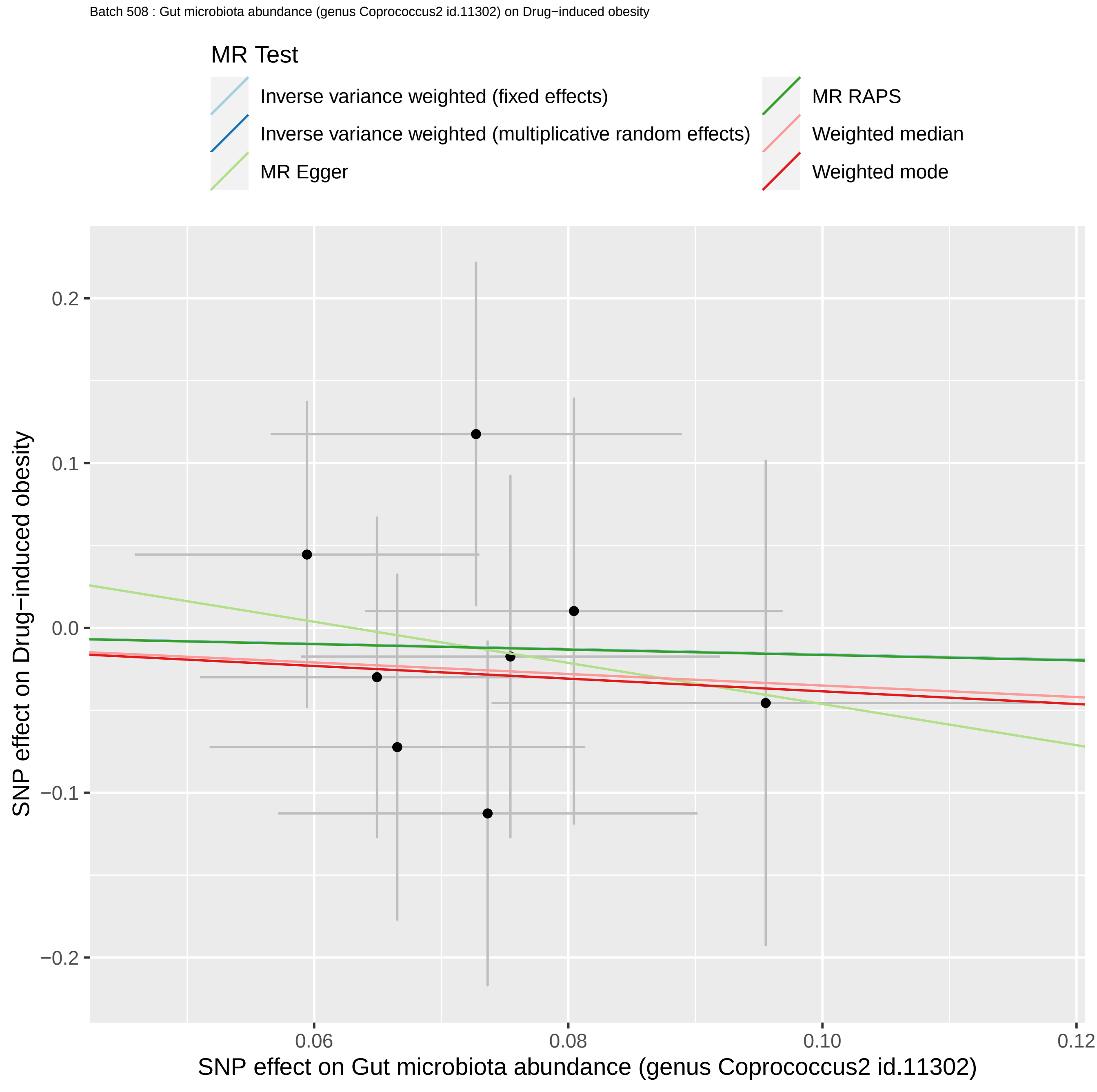
Batch 508 : Gut microbiota abundance (genus Coprococcus2 id.11302) on Drug−induced obesity
MR Test
Inverse variance weighted (fixed effects)
Inverse variance weighted (multiplicative random effects)
MR Egger
MR RAPS
Weighted median
Weighted mode
0.2
0.1
0.0
−0.1
−0.2
0.06
0.08
0.10
0.12
SNP effect on Drug−induced obesity
SNP effect on Gut microbiota abundance (genus Coprococcus2 id.11302)

Batch 509 : Gut microbiota abundance (genus Coprococcus3 id.11303) on Drug−induced obesity

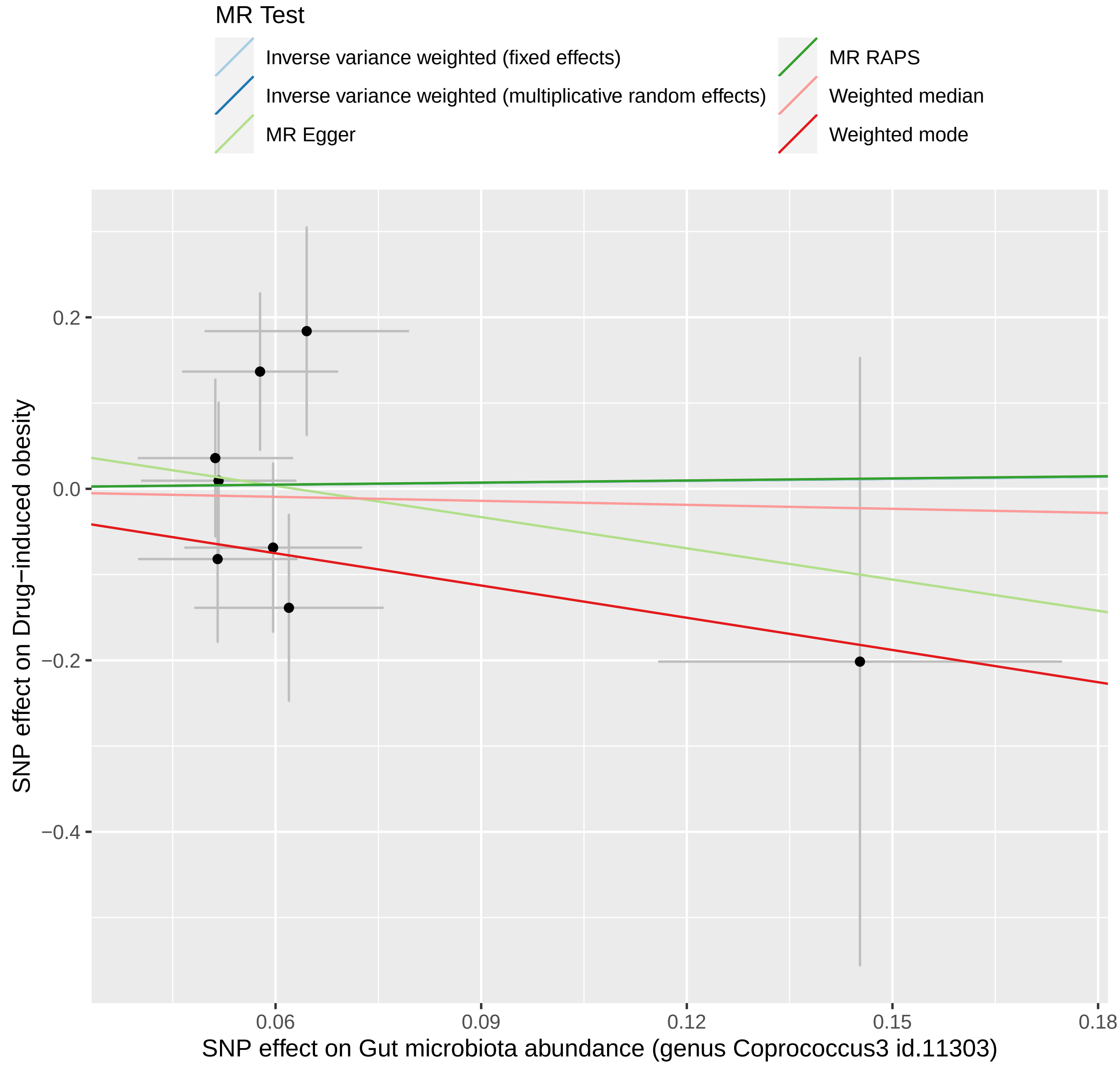

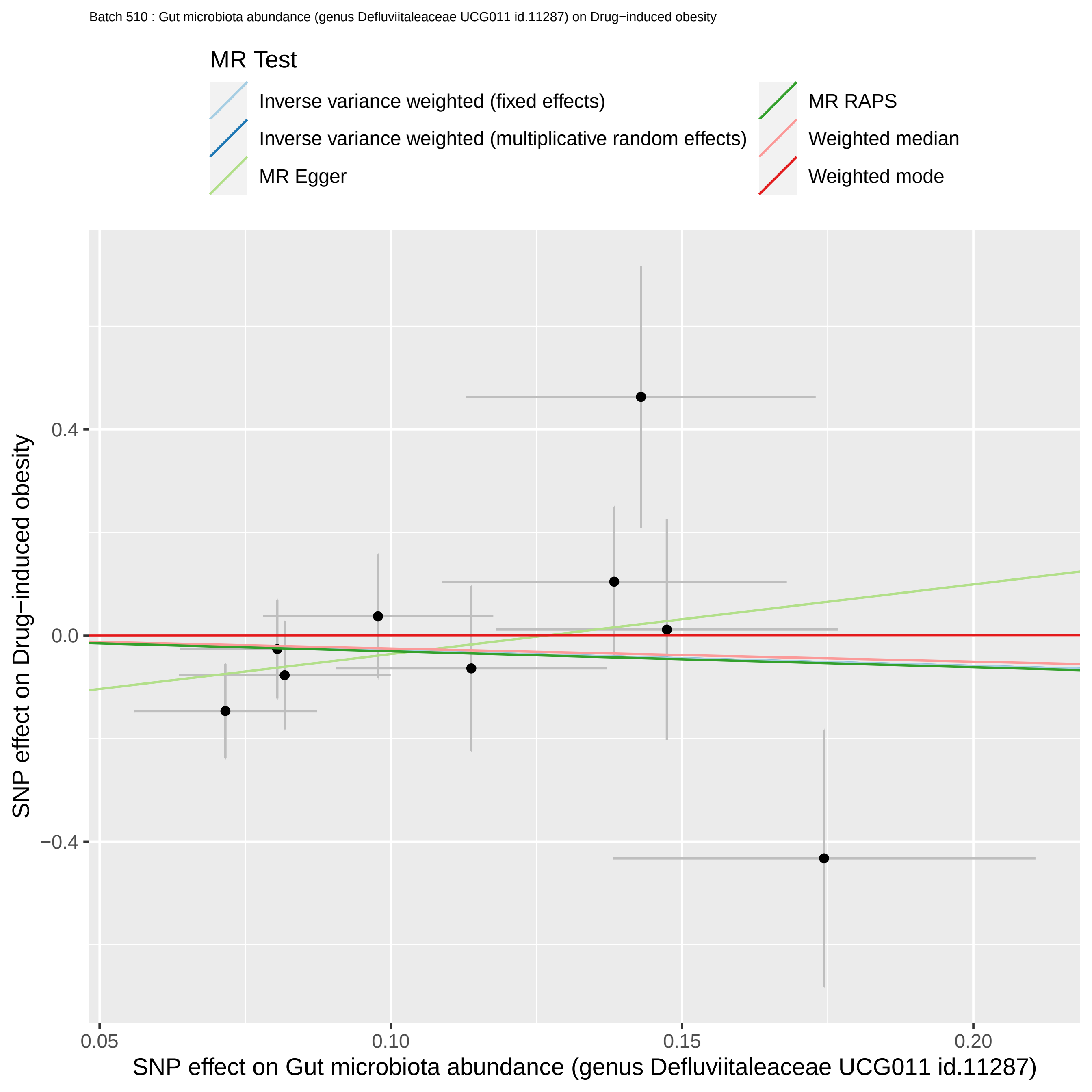
Batch 510 : Gut microbiota abundance (genus Defluviitaleaceae UCG011 id.11287) on Drug−induced obesity
MR Test
Inverse variance weighted (fixed effects)
Inverse variance weighted (multiplicative random effects)
MR Egger
MR RAPS
Weighted median
Weighted mode
0.4
0.0
−0.4
0.05
0.10
0.15
0.20
SNP effect on Drug−induced obesity
SNP effect on Gut microbiota abundance (genus Defluviitaleaceae UCG011 id.11287)

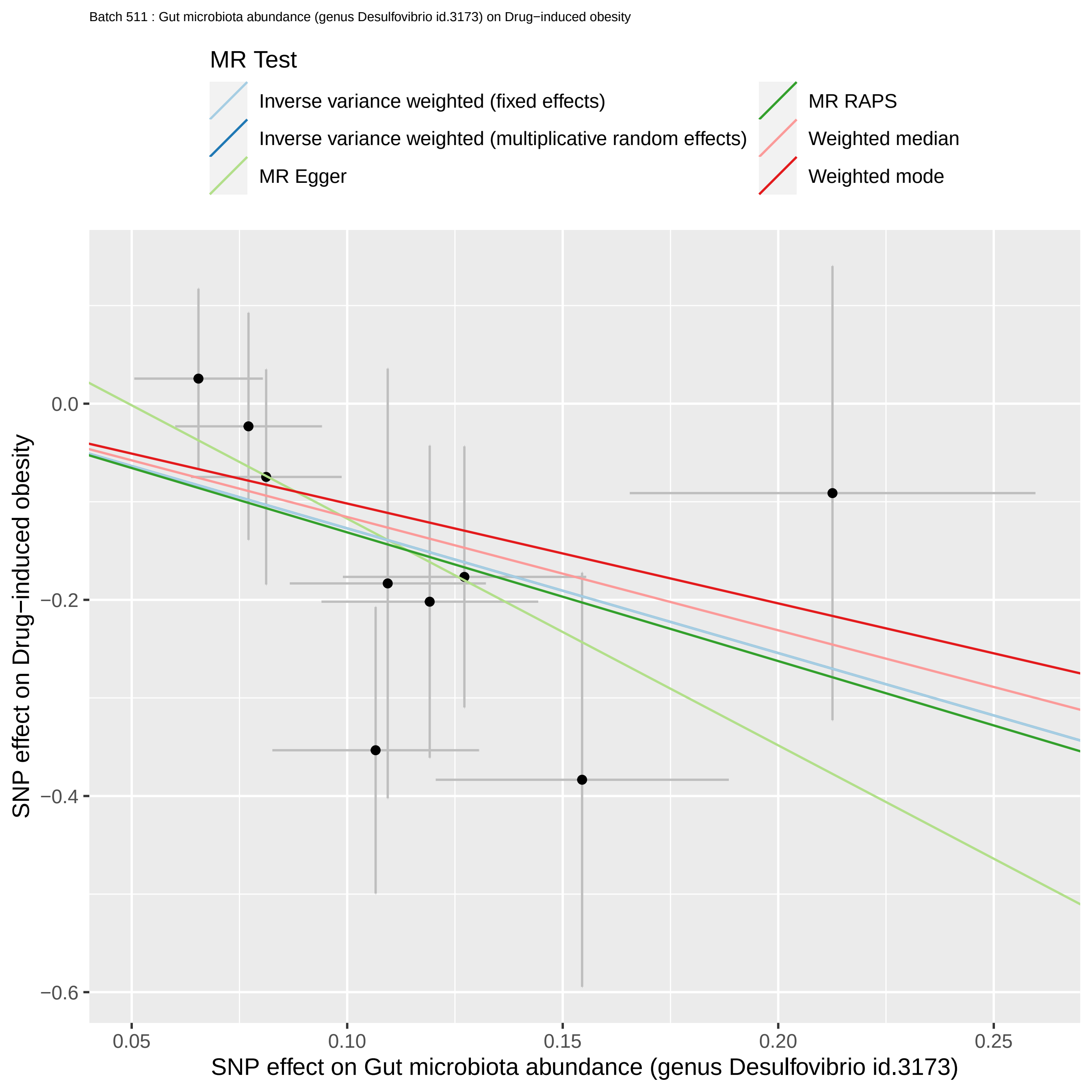

Batch 511 : Gut microbiota abundance (genus Desulfovibrio id.3173) on Drug−induced obesity
MR Test
Inverse variance weighted (fixed effects)
Inverse variance weighted (multiplicative random effects)
MR Egger
MR RAPS
Weighted median
Weighted mode
SNP effect on Drug−induced obesity
0.0
−0.2
−0.4
−0.6
0.05
0.10
0.15
0.20
0.25
SNP effect on Gut microbiota abundance (genus Desulfovibrio id.3173)

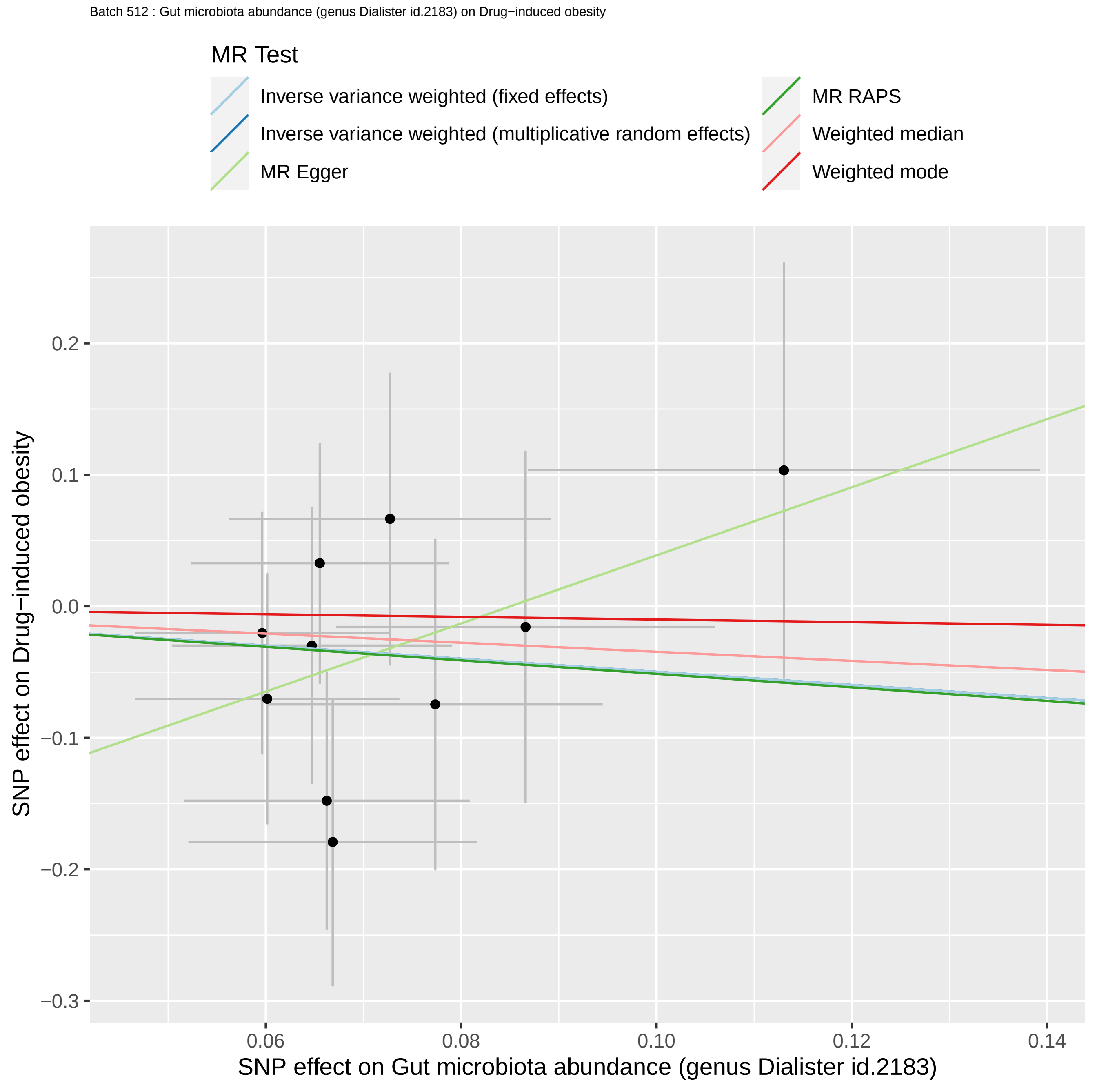
Batch 512 : Gut microbiota abundance (genus Dialister id.2183) on Drug−induced obesity
MR Test
Inverse variance weighted (fixed effects)
Inverse variance weighted (multiplicative random effects)
MR Egger
MR RAPS
Weighted median
Weighted mode
SNP effect on Drug−induced obesity
0.2
0.1
0.0
−0.1
−0.2
−0.3
0.06
0.08
0.10
0.12
0.14
SNP effect on Gut microbiota abundance (genus Dialister id.2183)

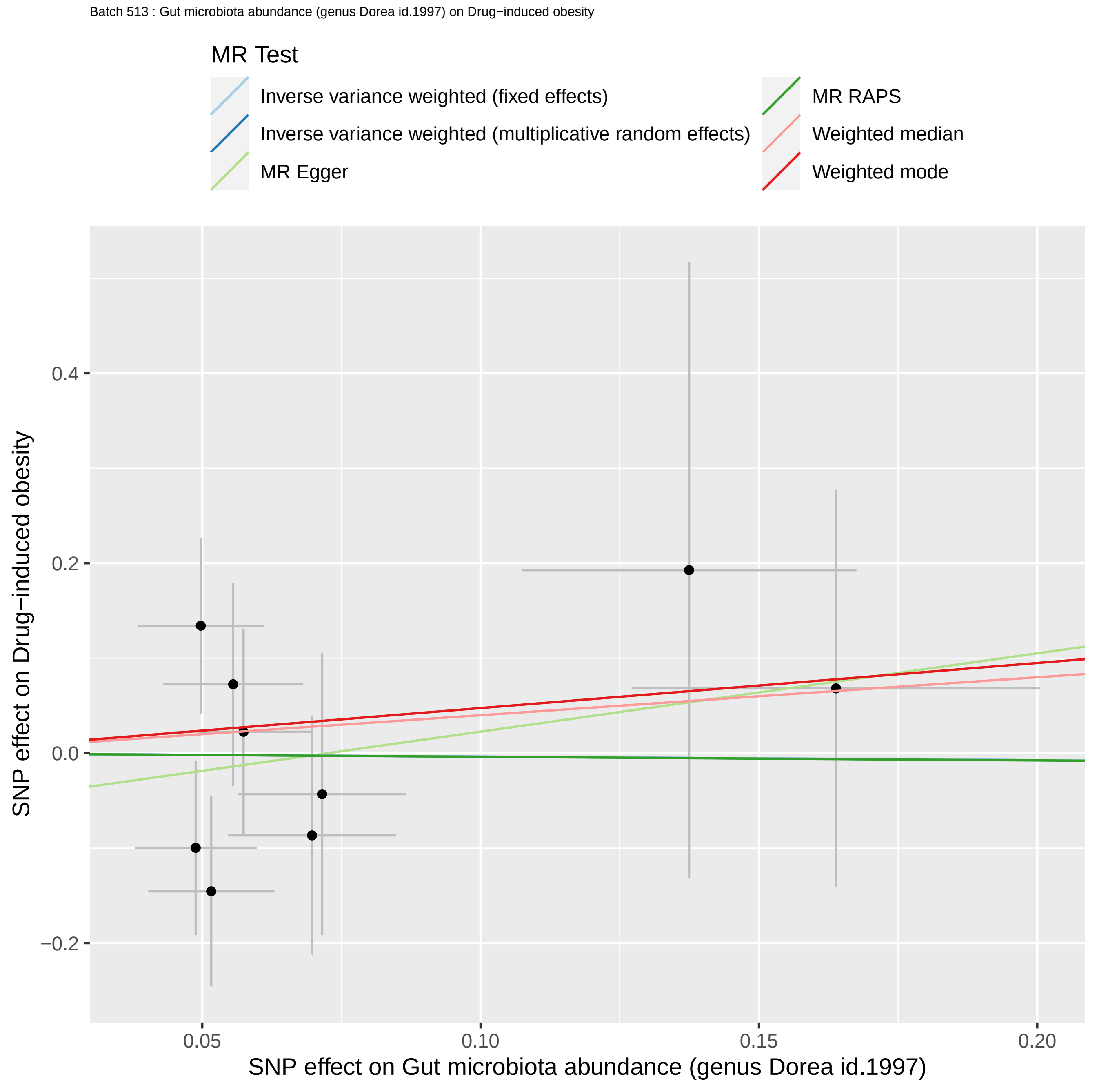

Batch 513 : Gut microbiota abundance (genus Dorea id.1997) on Drug−induced obesity
MR Test
Inverse variance weighted (fixed effects)
Inverse variance weighted (multiplicative random effects)
MR Egger
MR RAPS
Weighted median
Weighted mode
0.4
0.2
0.0
−0.2
0.05
0.10
0.15
0.20
SNP effect on Drug−induced obesity
SNP effect on Gut microbiota abundance (genus Dorea id.1997)

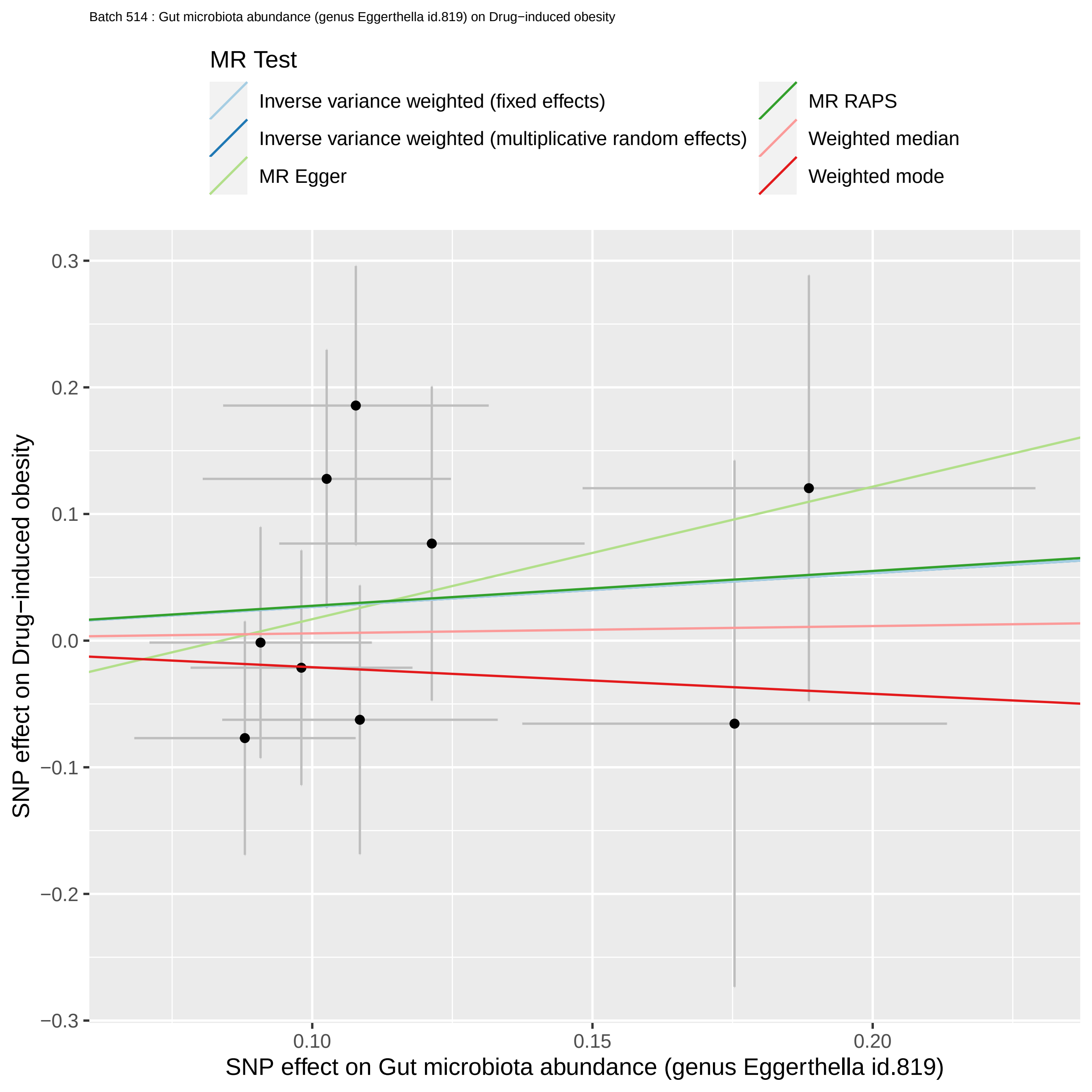
Batch 514 : Gut microbiota abundance (genus Eggerthella id.819) on Drug−induced obesity
MR Test
Inverse variance weighted (fixed effects)
Inverse variance weighted (multiplicative random effects)
MR Egger
MR RAPS
Weighted median
Weighted mode
0.3
0.2
0.1
0.0
−0.1
−0.2
−0.3
0.10
0.15
0.20
SNP effect on Drug−induced obesity
SNP effect on Gut microbiota abundance (genus Eggerthella id.819)

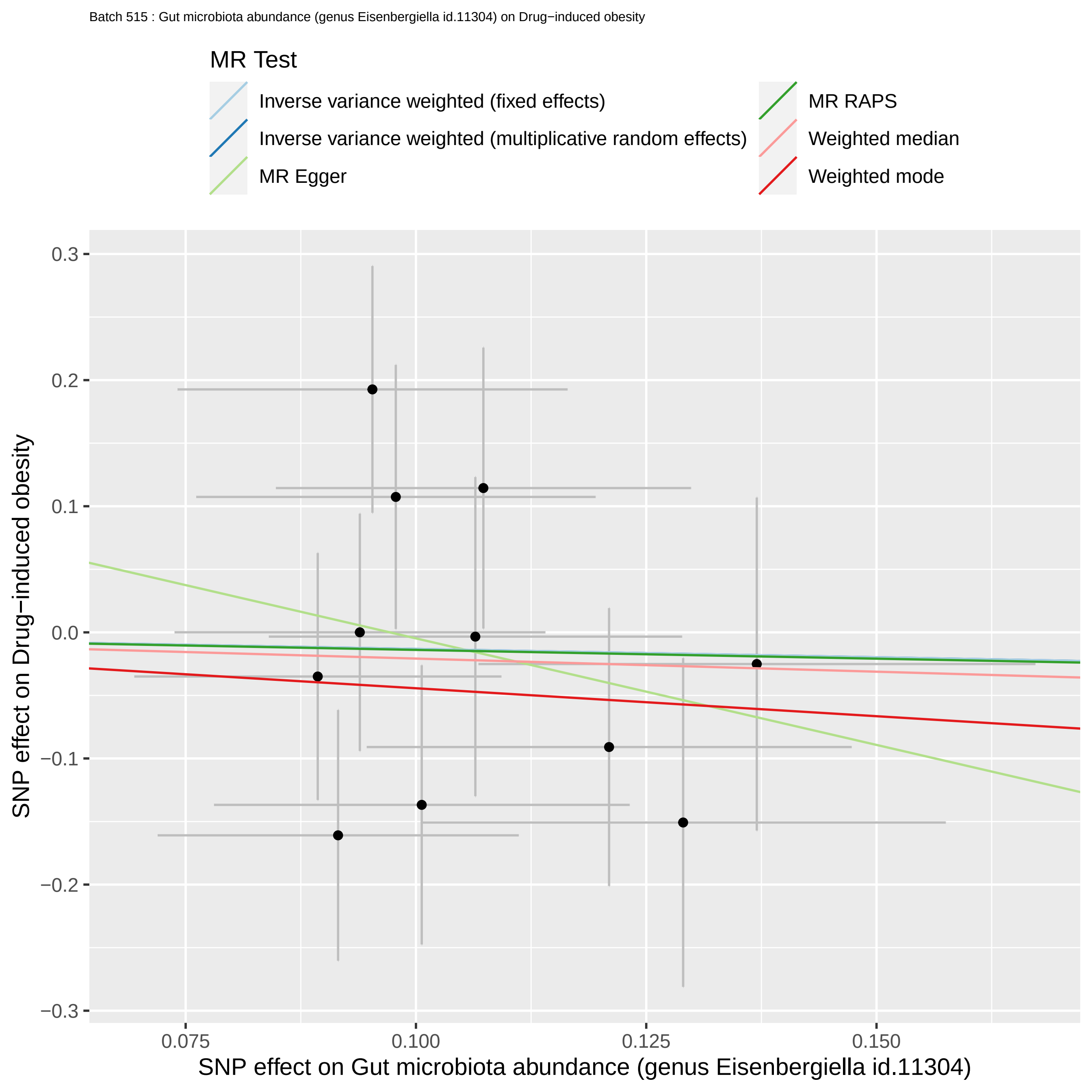

Batch 515 : Gut microbiota abundance (genus Eisenbergiella id.11304) on Drug−induced obesity
MR Test
Inverse variance weighted (fixed effects)
Inverse variance weighted (multiplicative random effects)
MR Egger
MR RAPS
Weighted median
Weighted mode
0.3
0.2
0.1
0.0
−0.1
−0.2
−0.3
0.075
0.100
0.125
0.150
SNP effect on Drug−induced obesity
SNP effect on Gut microbiota abundance (genus Eisenbergiella id.11304)

Batch 516 : Gut microbiota abundance (genus Enterorhabdus id.820) on Drug−induced obesity

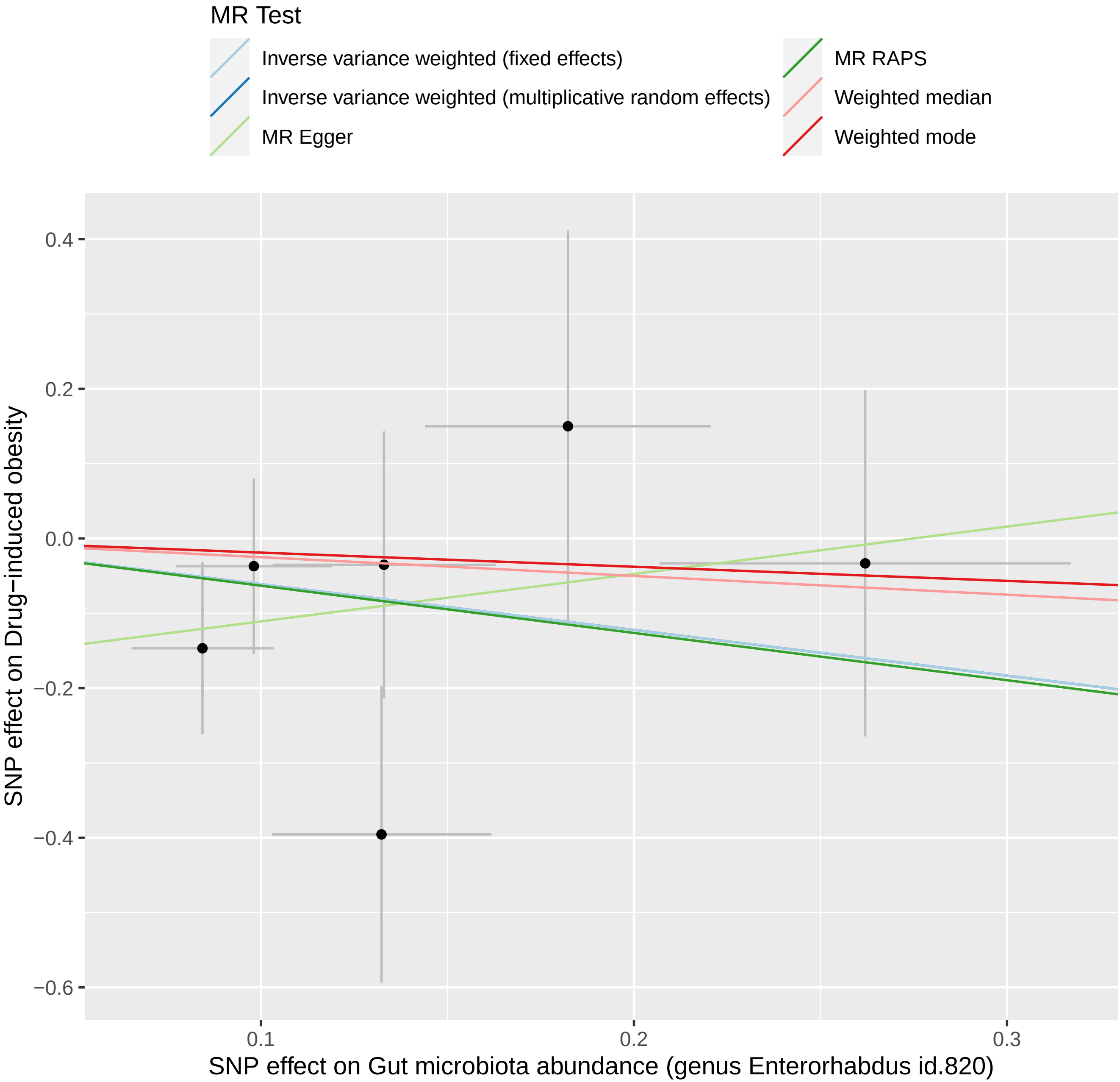

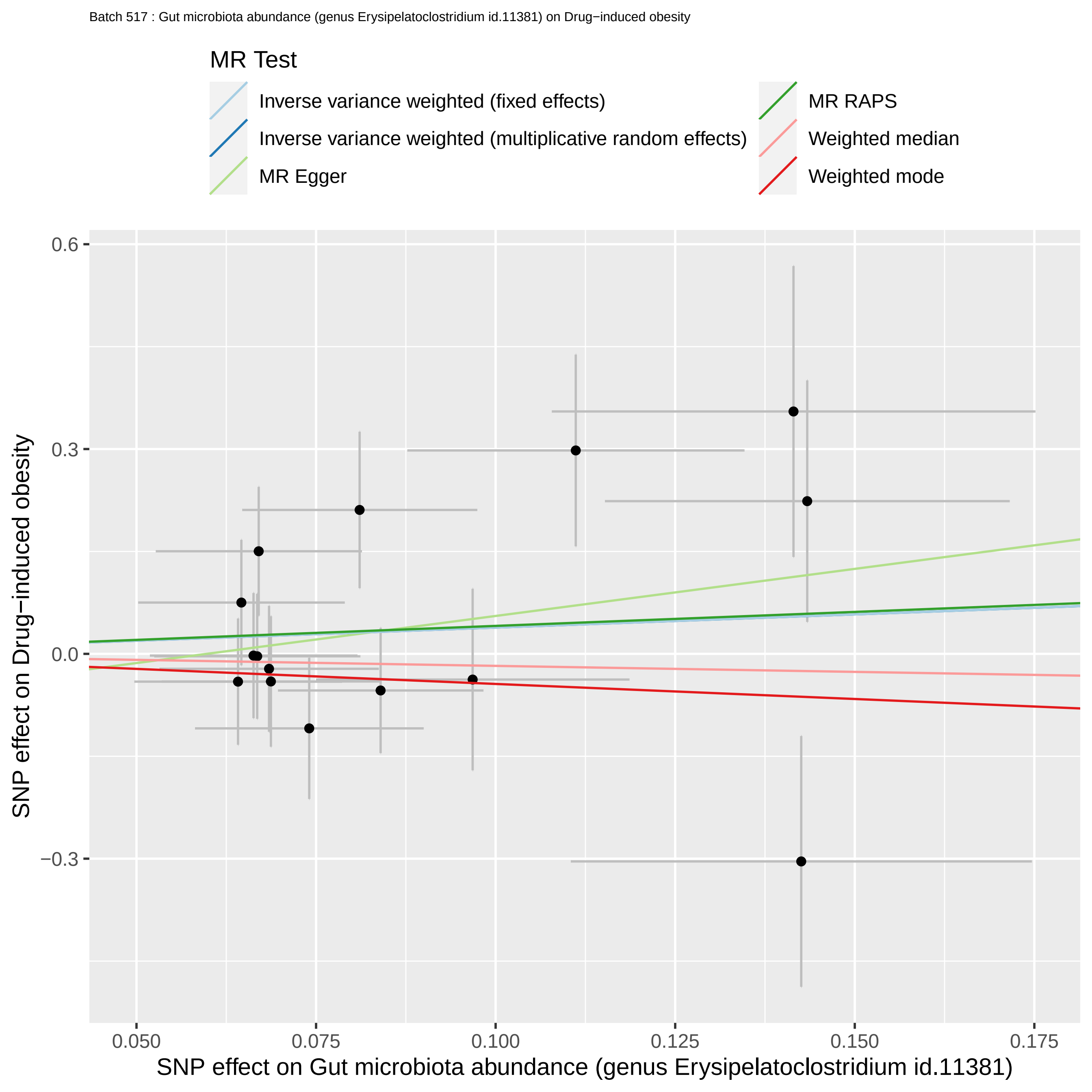

Batch 517 : Gut microbiota abundance (genus Erysipelatoclostridium id.11381) on Drug−induced obesity
MR Test
Inverse variance weighted (fixed effects)
Inverse variance weighted (multiplicative random effects)
MR Egger
MR RAPS
Weighted median
Weighted mode
0.6
0.3
0.0
−0.3
0.050
0.075
0.100
0.125
0.150
0.175
SNP effect on Gut microbiota abundance (genus Erysipelatoclostridium id.11381)
SNP effect on Drug−induced obesity

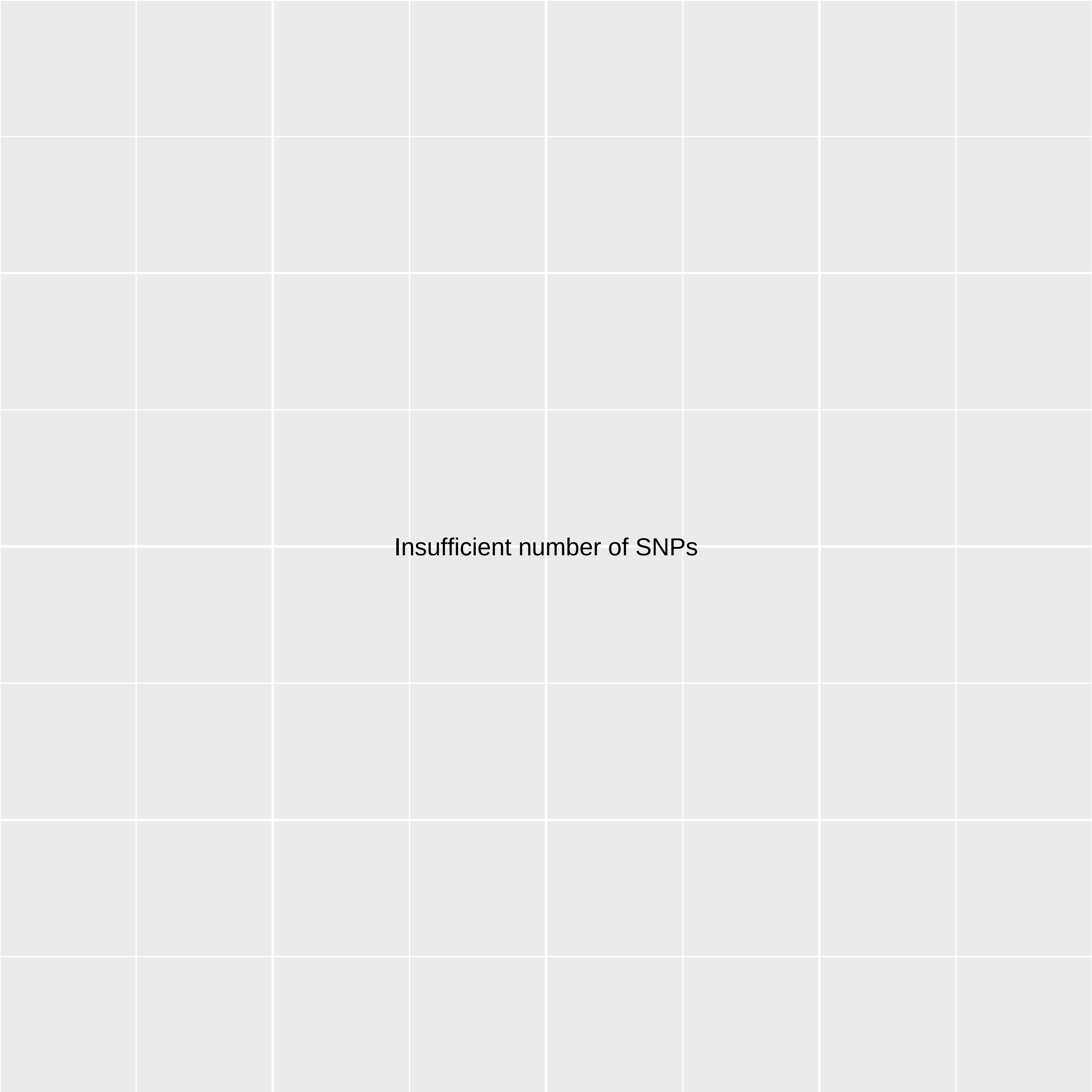
Insufficient number of SNPs

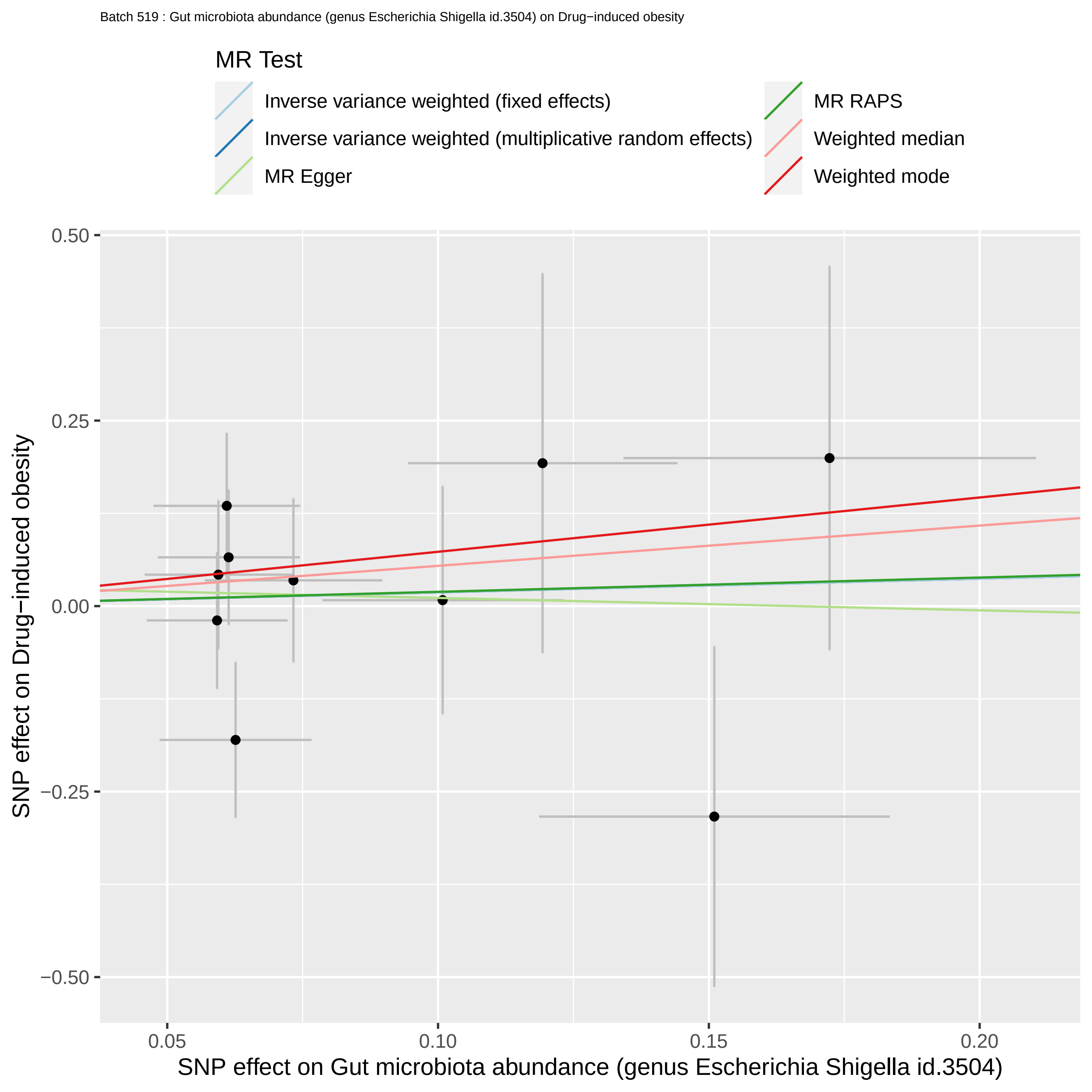

Batch 519 : Gut microbiota abundance (genus Escherichia Shigella id.3504) on Drug−induced obesity
MR Test
Inverse variance weighted (fixed effects)
Inverse variance weighted (multiplicative random effects)
MR Egger
MR RAPS
Weighted median
Weighted mode
0.50
0.25
0.00
−0.25
−0.50
0.05
0.10
0.15
0.20
SNP effect on Drug−induced obesity
SNP effect on Gut microbiota abundance (genus Escherichia Shigella id.3504)

Batch 520 : Gut microbiota abundance (genus Eubacterium brachy group id.11296) on Drug−induced obesity

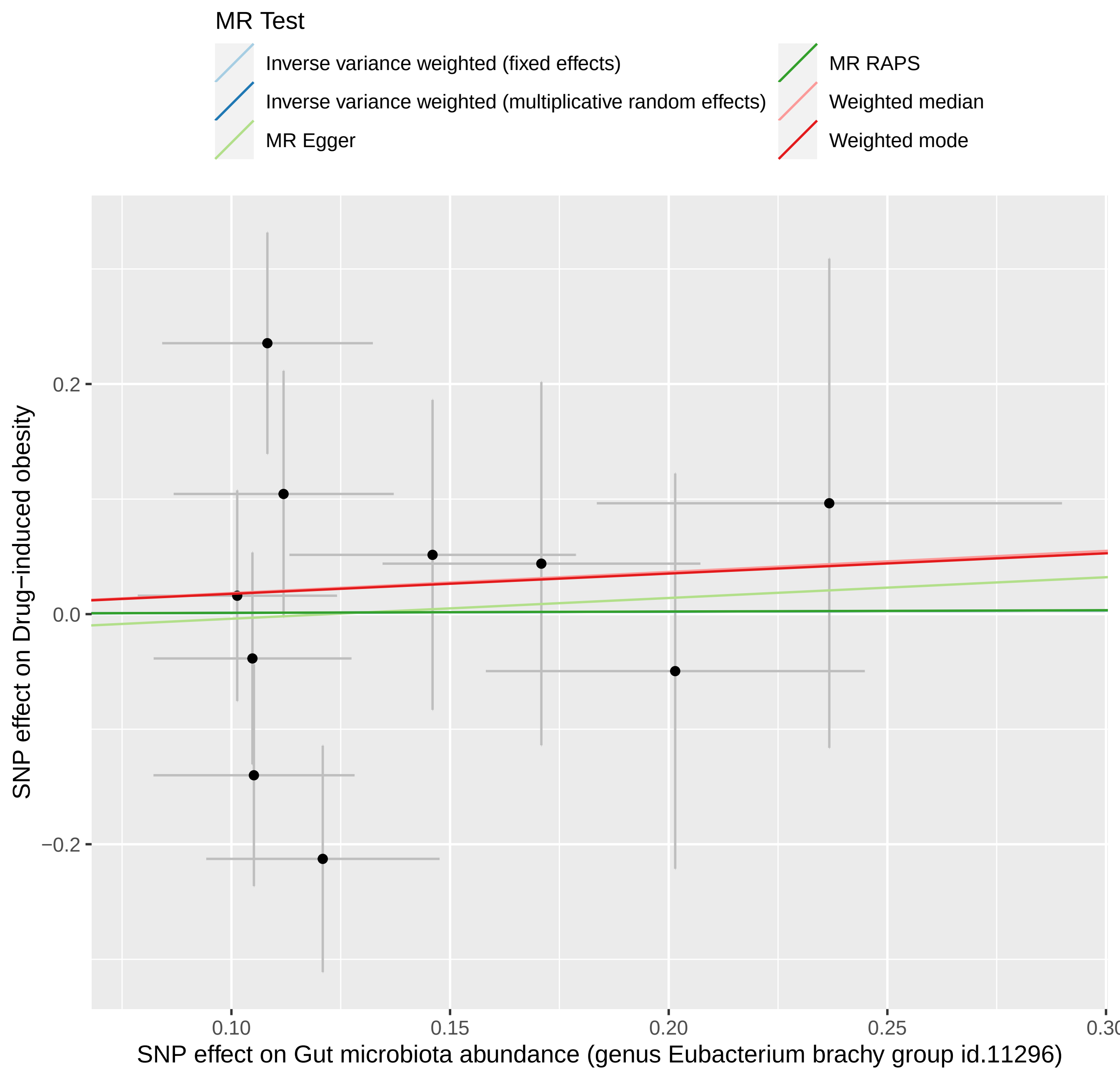

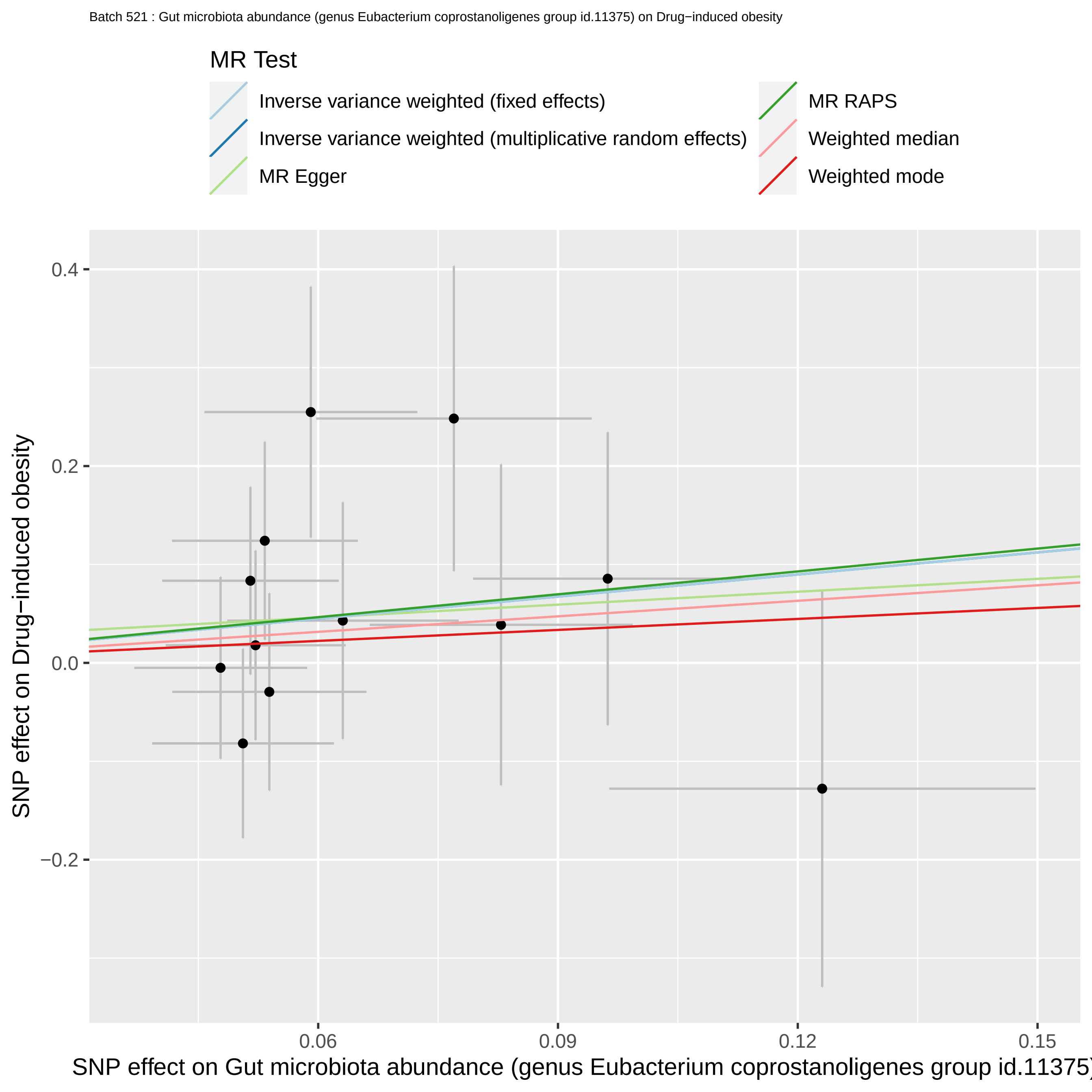
Batch 521 : Gut microbiota abundance (genus Eubacterium coprostanoligenes group id.11375) on Drug−induced obesity
MR Test
Inverse variance weighted (fixed effects)
Inverse variance weighted (multiplicative random effects)
MR Egger
MR RAPS
Weighted median
Weighted mode
SNP effect on Drug−induced obesity
0.4
0.2
0.0
−0.2
0.06
0.09
0.12
0.15
SNP effect on Gut microbiota abundance (genus Eubacterium coprostanoligenes group id.11375)

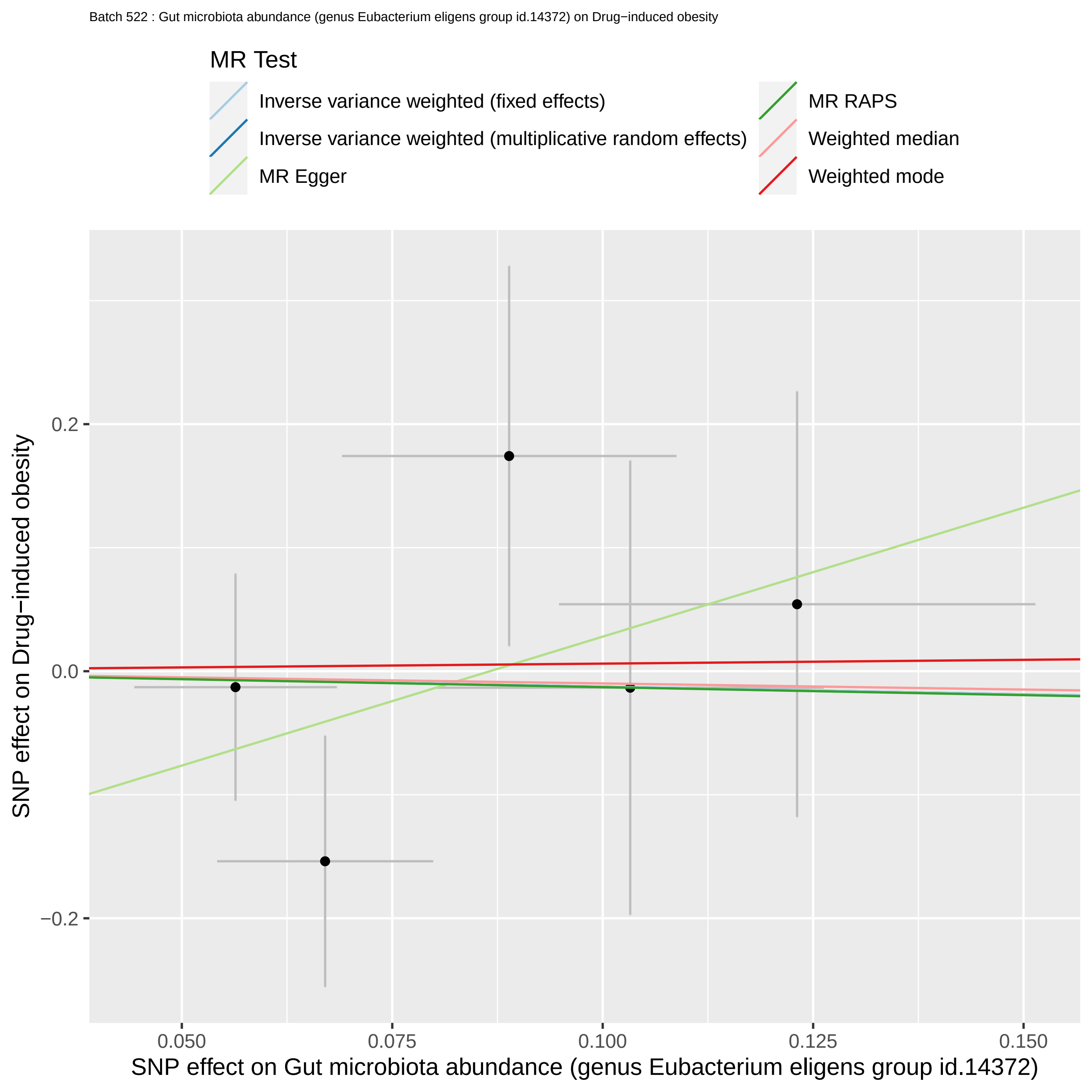
Batch 522 : Gut microbiota abundance (genus Eubacterium eligens group id.14372) on Drug−induced obesity
MR Test
Inverse variance weighted (fixed effects)
Inverse variance weighted (multiplicative random effects)
MR Egger
MR RAPS
Weighted median
Weighted mode
0.2
0.0
−0.2
0.050
0.075
0.100
0.125
0.150
SNP effect on Drug−induced obesity
SNP effect on Gut microbiota abundance (genus Eubacterium eligens group id.14372)

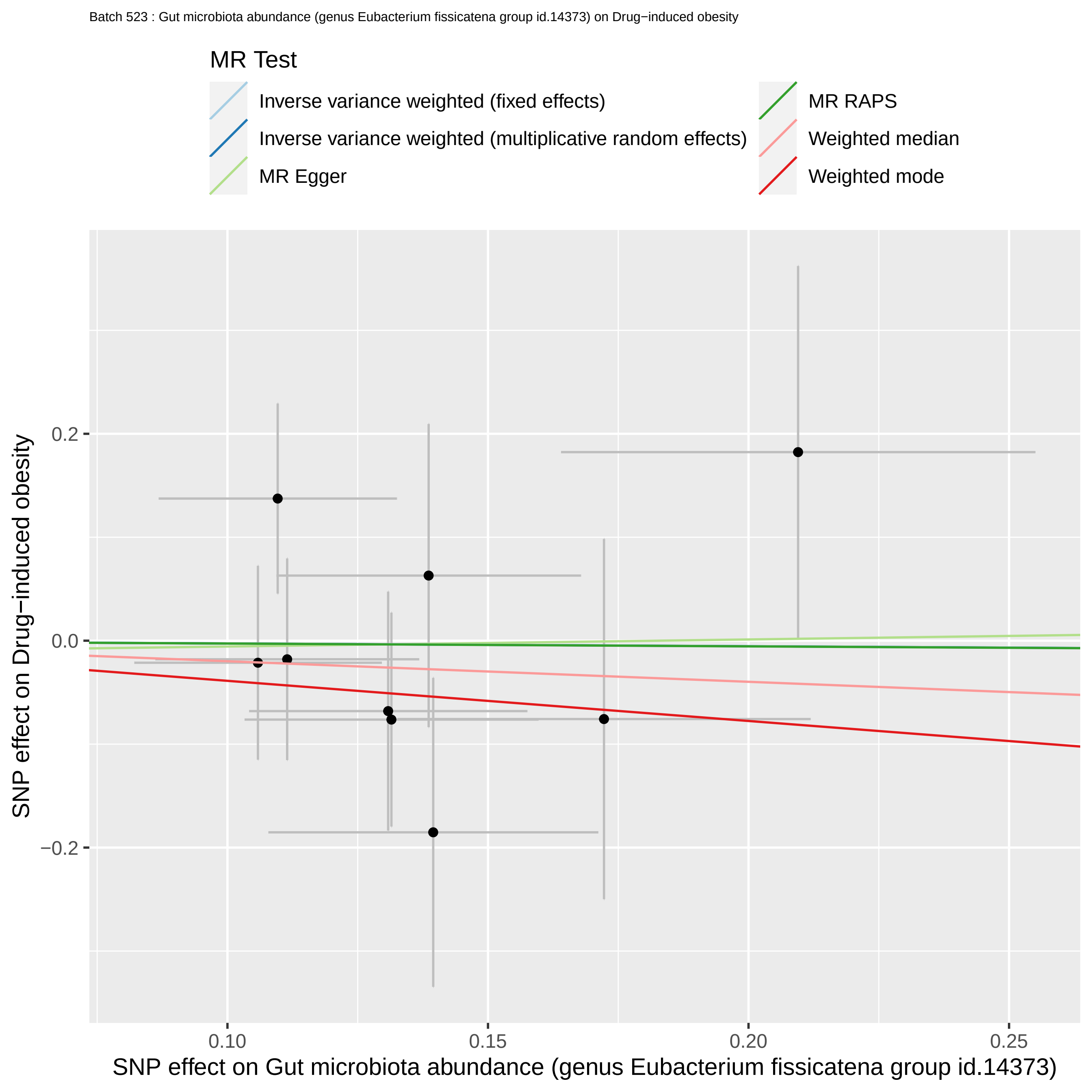

Batch 523 : Gut microbiota abundance (genus Eubacterium fissicatena group id.14373) on Drug−induced obesity
MR Test
Inverse variance weighted (fixed effects)
Inverse variance weighted (multiplicative random effects)
MR Egger
MR RAPS
Weighted median
Weighted mode
0.2
0.0
−0.2
SNP effect on Drug−induced obesity
0.10
0.15
0.20
0.25
SNP effect on Gut microbiota abundance (genus Eubacterium fissicatena group id.14373)

Batch 524 : Gut microbiota abundance (genus Eubacterium hallii group id.11338) on Drug−induced obesity

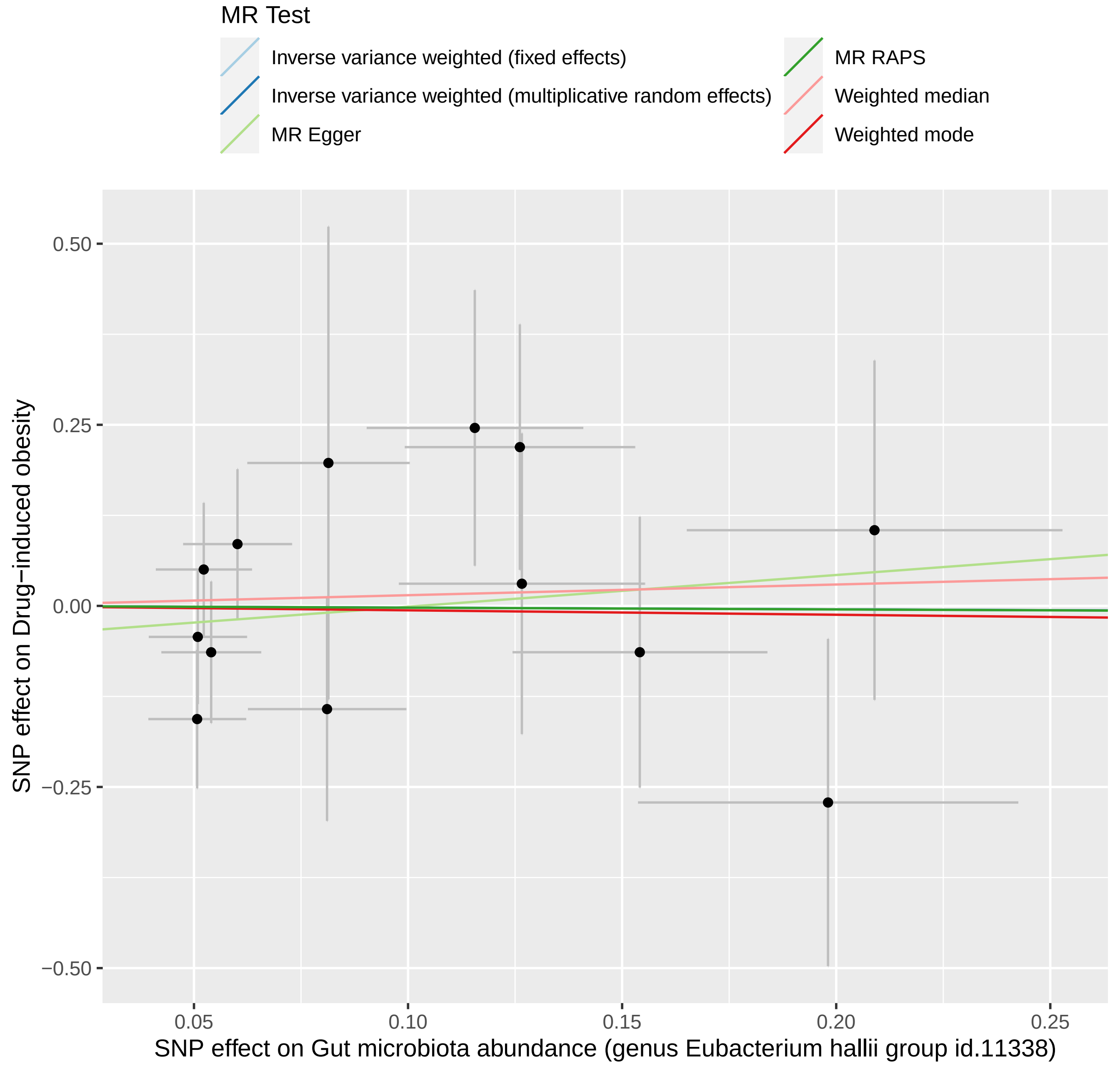

Batch 525 : Gut microbiota abundance (genus Eubacterium nodatum group id.11297) on Drug−induced obesity

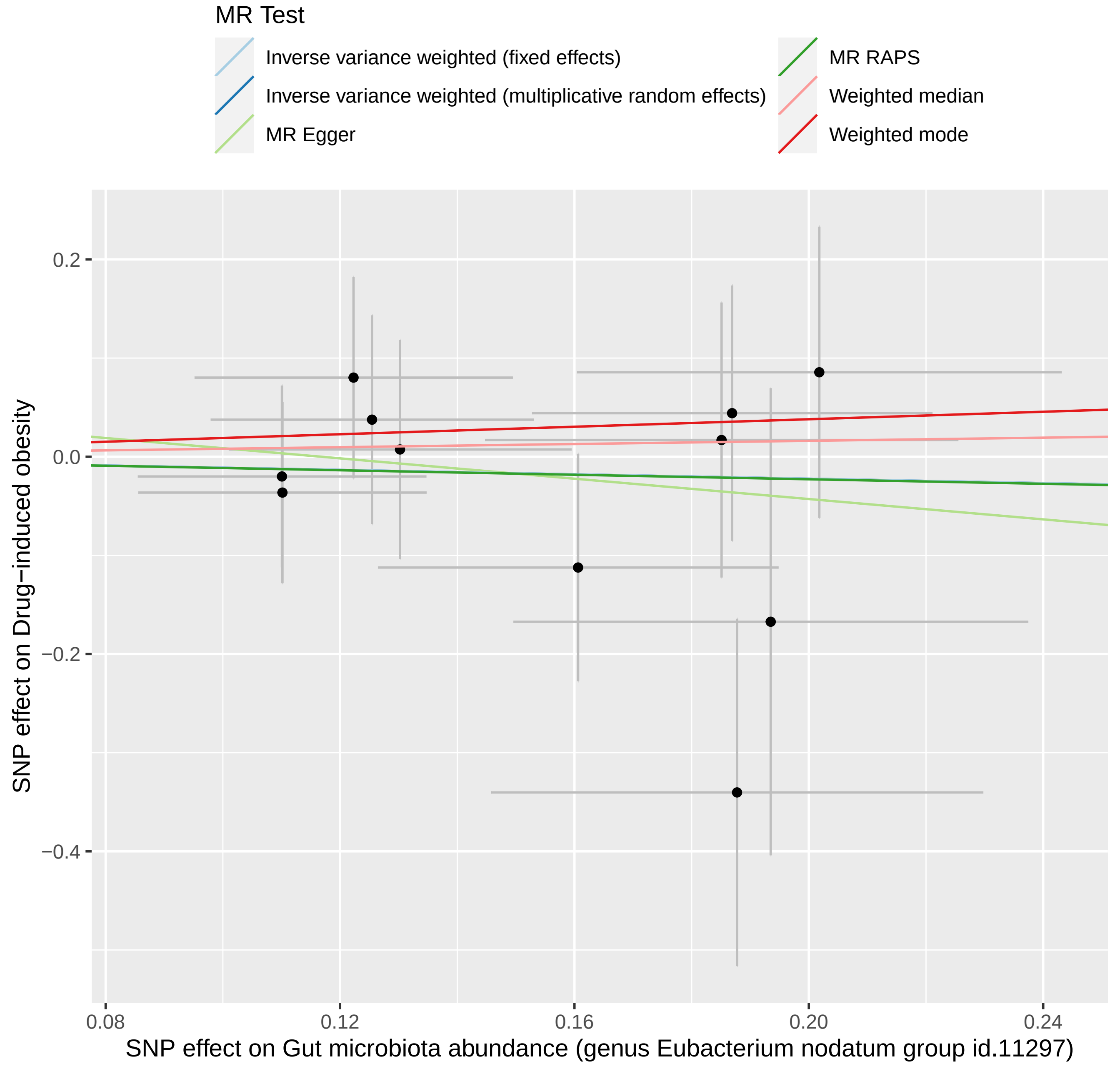

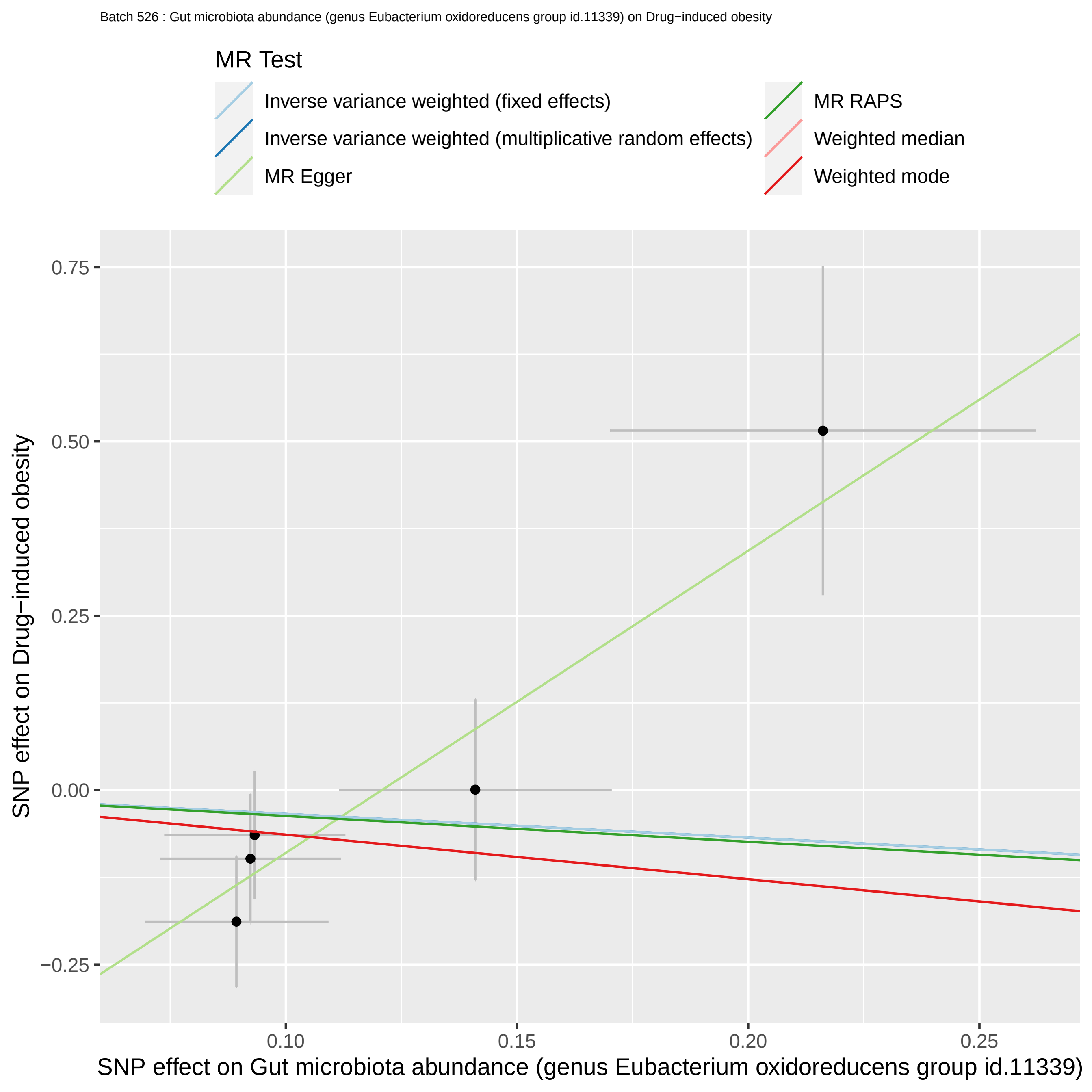
Batch 526 : Gut microbiota abundance (genus Eubacterium oxidoreducens group id.11339) on Drug−induced obesity
MR Test
Inverse variance weighted (fixed effects)
Inverse variance weighted (multiplicative random effects)
MR Egger
MR RAPS
Weighted median
Weighted mode
0.75
0.50
0.25
0.00
−0.25
0.10
0.15
0.20
0.25
SNP effect on Drug−induced obesity
SNP effect on Gut microbiota abundance (genus Eubacterium oxidoreducens group id.11339)

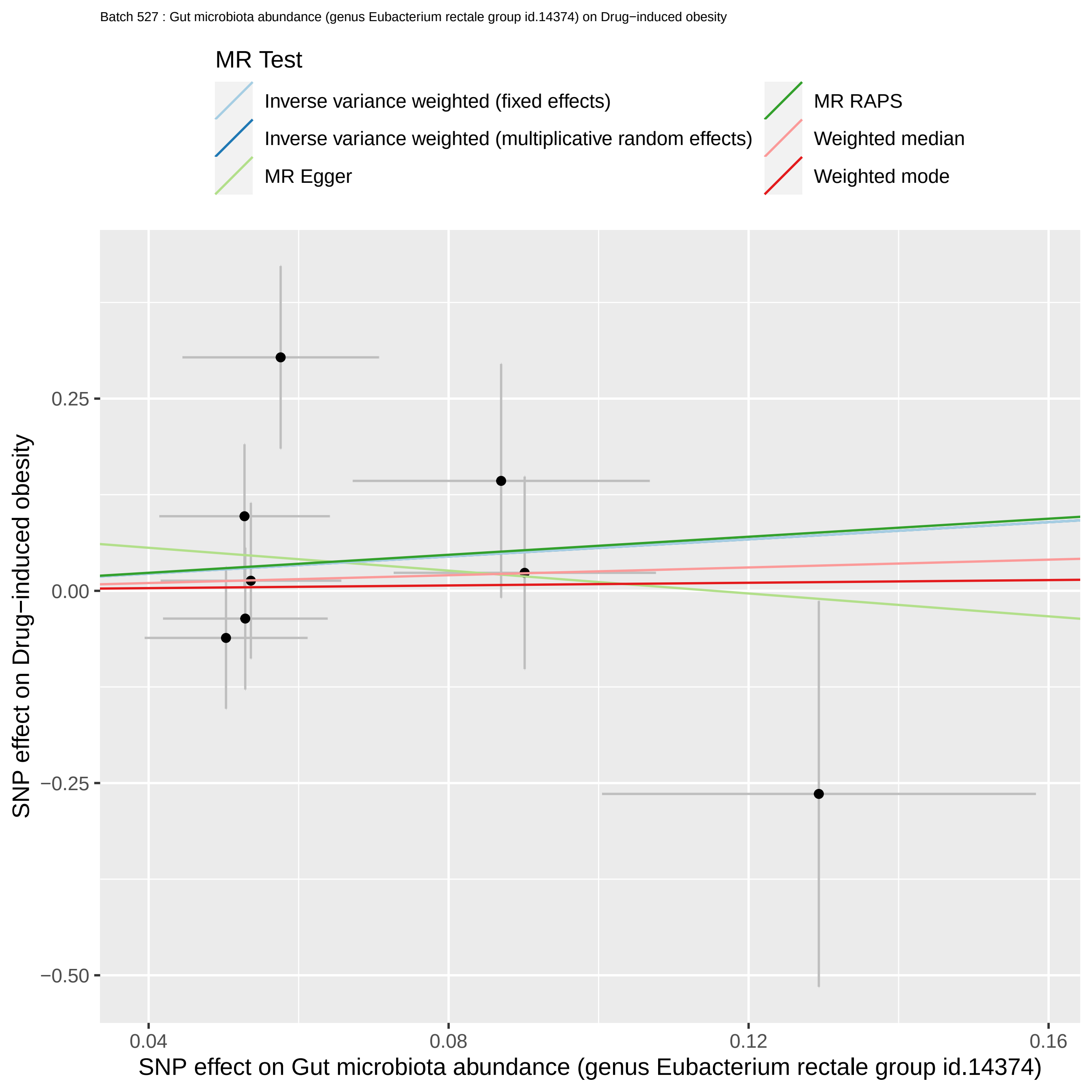
Batch 527 : Gut microbiota abundance (genus Eubacterium rectale group id.14374) on Drug−induced obesity
MR Test
Inverse variance weighted (fixed effects)
Inverse variance weighted (multiplicative random effects)
MR Egger
MR RAPS
Weighted median
Weighted mode
0.25
0.00
−0.25
−0.50
SNP effect on Drug−induced obesity
0.04
0.08
0.12
0.16
SNP effect on Gut microbiota abundance (genus Eubacterium rectale group id.14374)

Batch 528 : Gut microbiota abundance (genus Eubacterium ruminantium group id.11340) on Drug−induced obesity

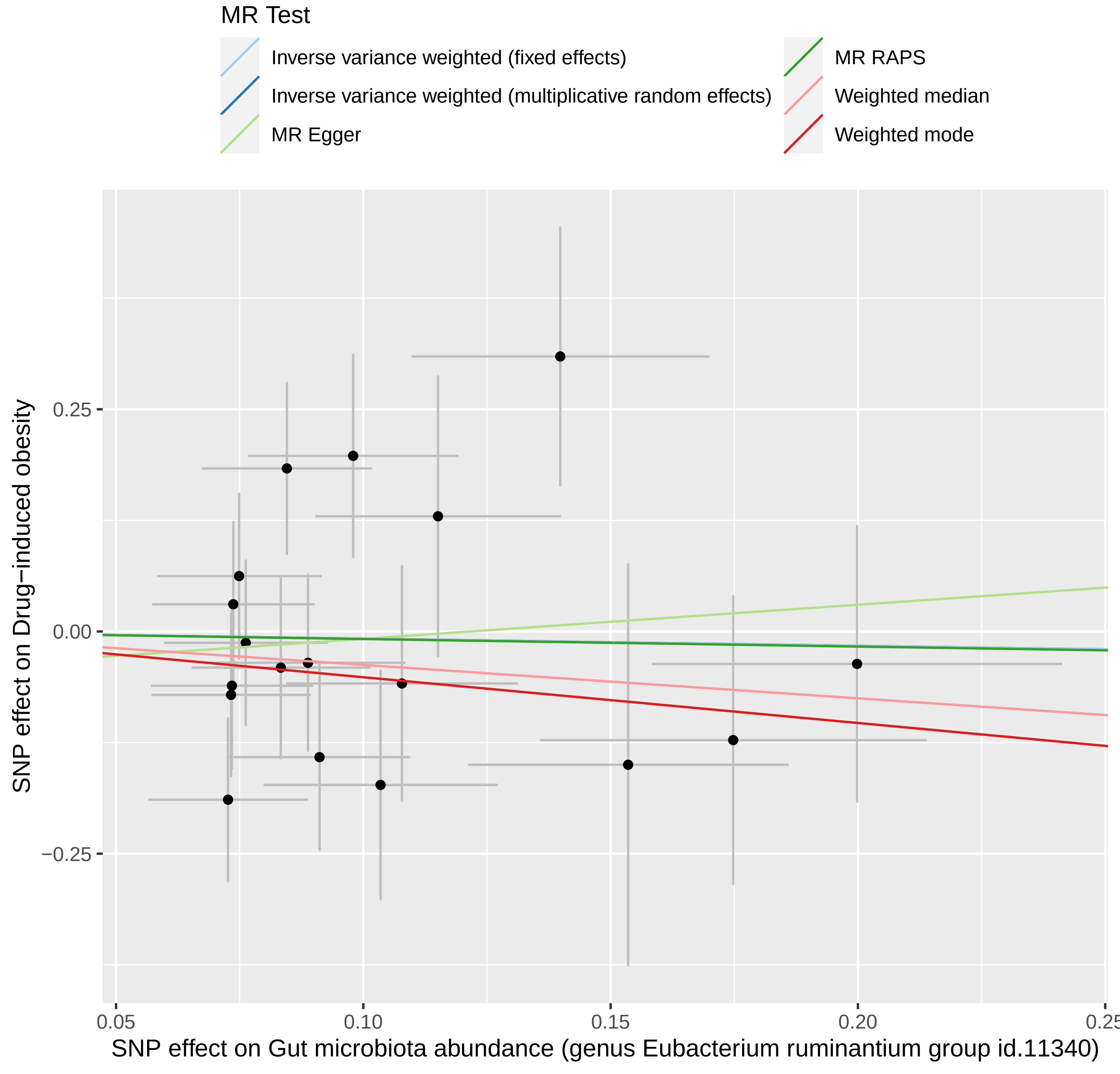

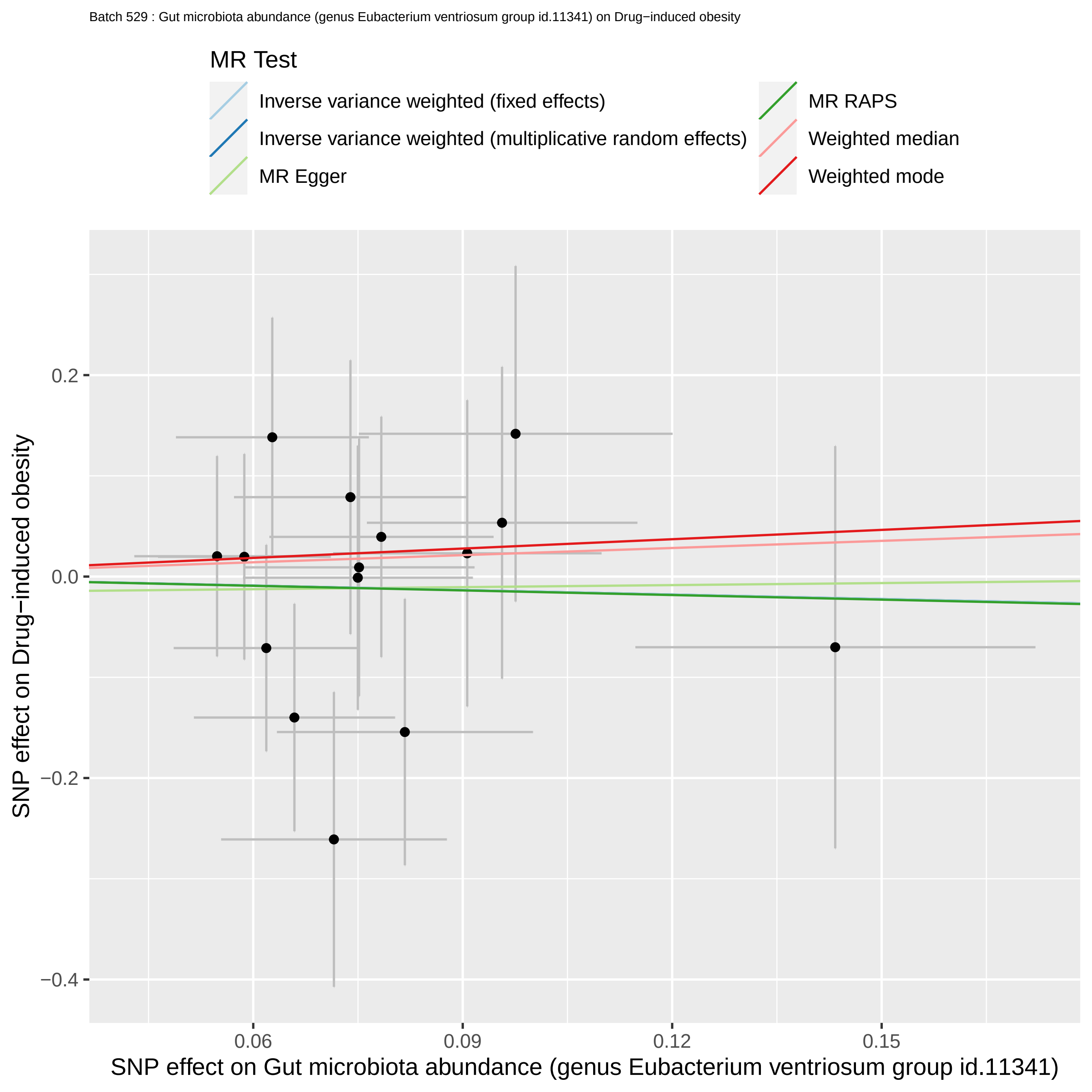

Batch 529 : Gut microbiota abundance (genus Eubacterium ventriosum group id.11341) on Drug−induced obesity
MR Test
Inverse variance weighted (fixed effects)
Inverse variance weighted (multiplicative random effects)
MR Egger
MR RAPS
Weighted median
Weighted mode
0.2
0.0
−0.2
−0.4
0.06
0.09
0.12
0.15
SNP effect on Drug−induced obesity
SNP effect on Gut microbiota abundance (genus Eubacterium ventriosum group id.11341)

Batch 530 : Gut microbiota abundance (genus Eubacterium xylanophilum group id.14375) on Drug−induced obesity

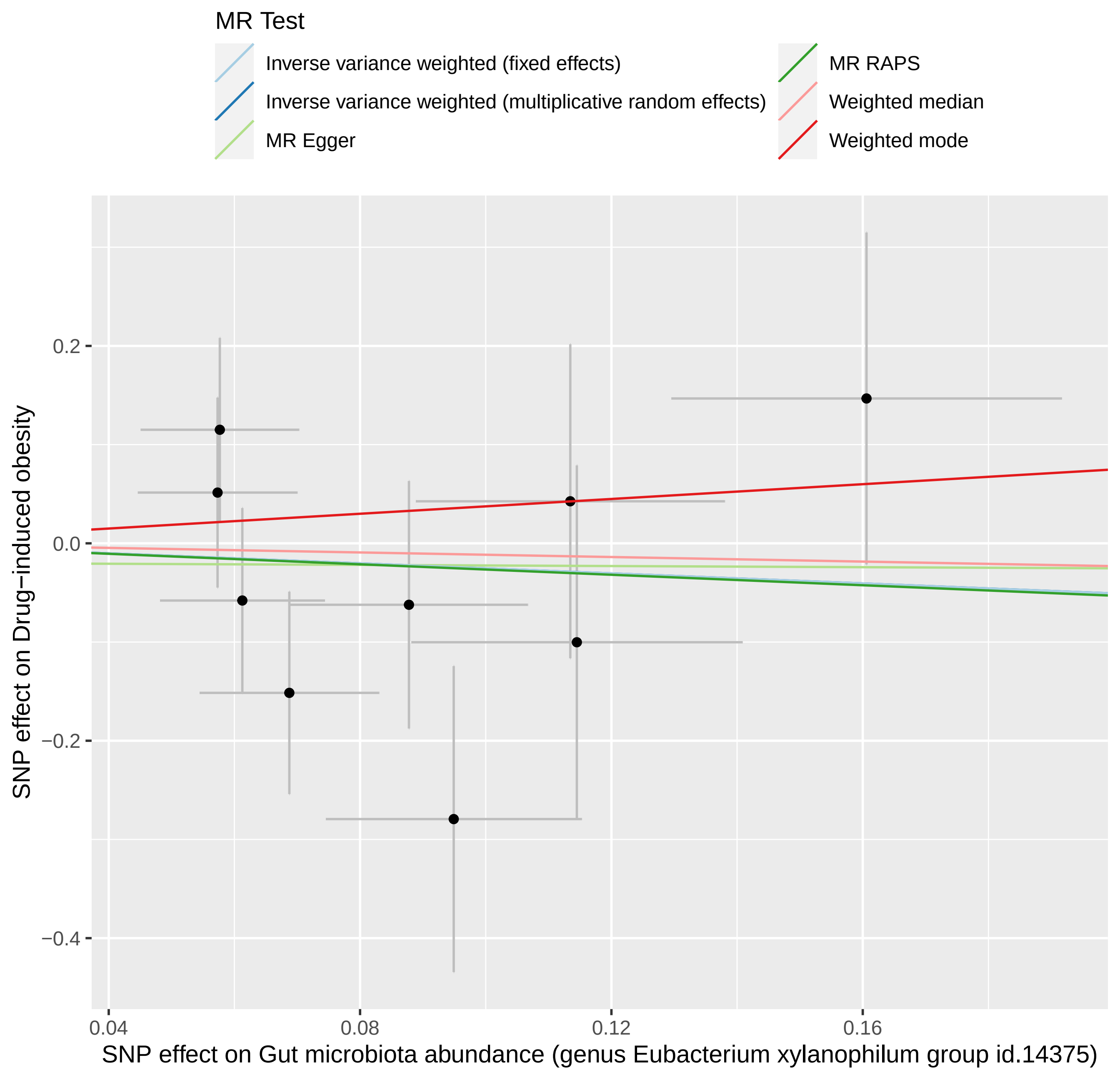

Batch 531 : Gut microbiota abundance (genus Faecalibacterium id.2057) on Drug−induced obesity

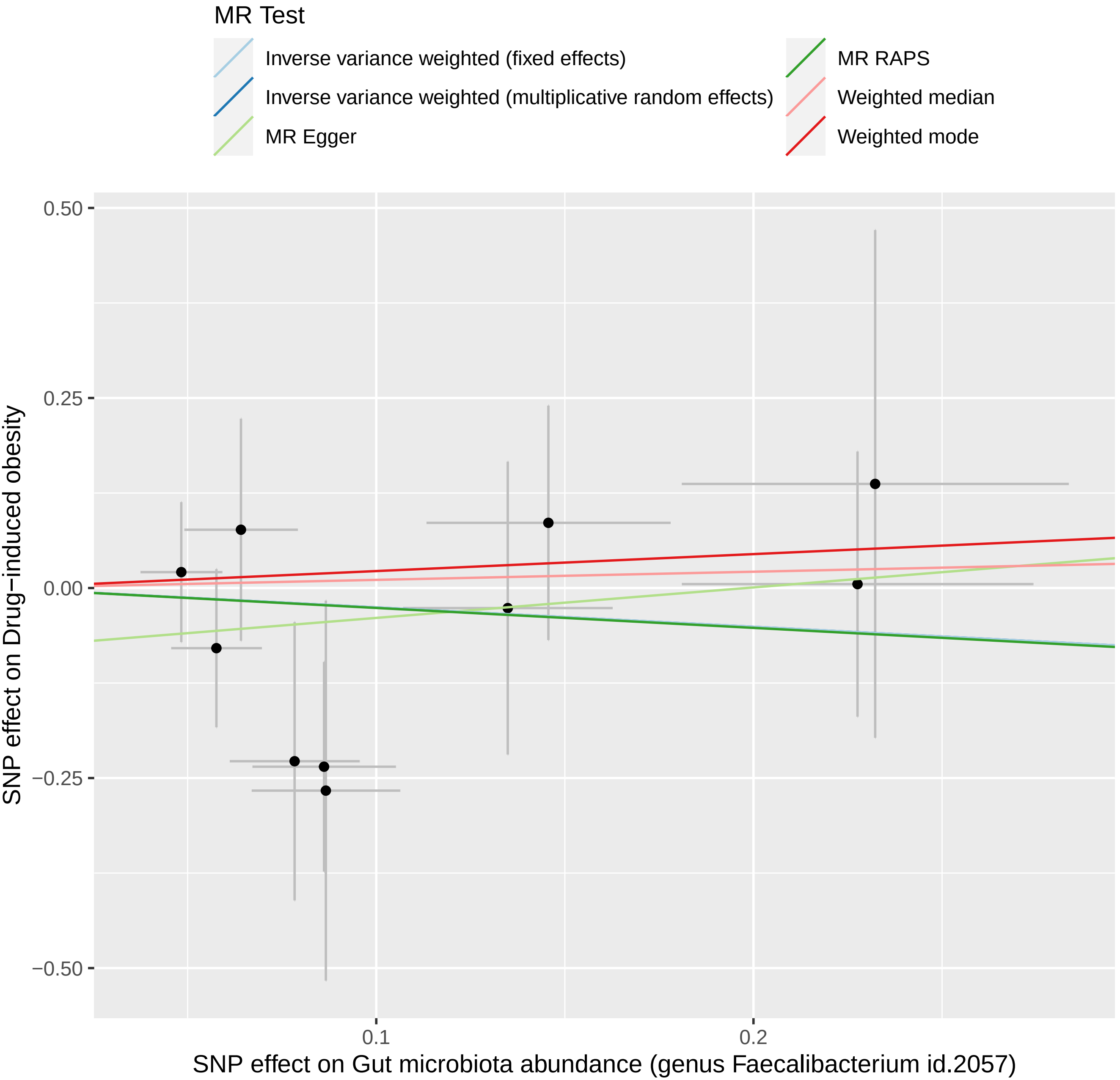

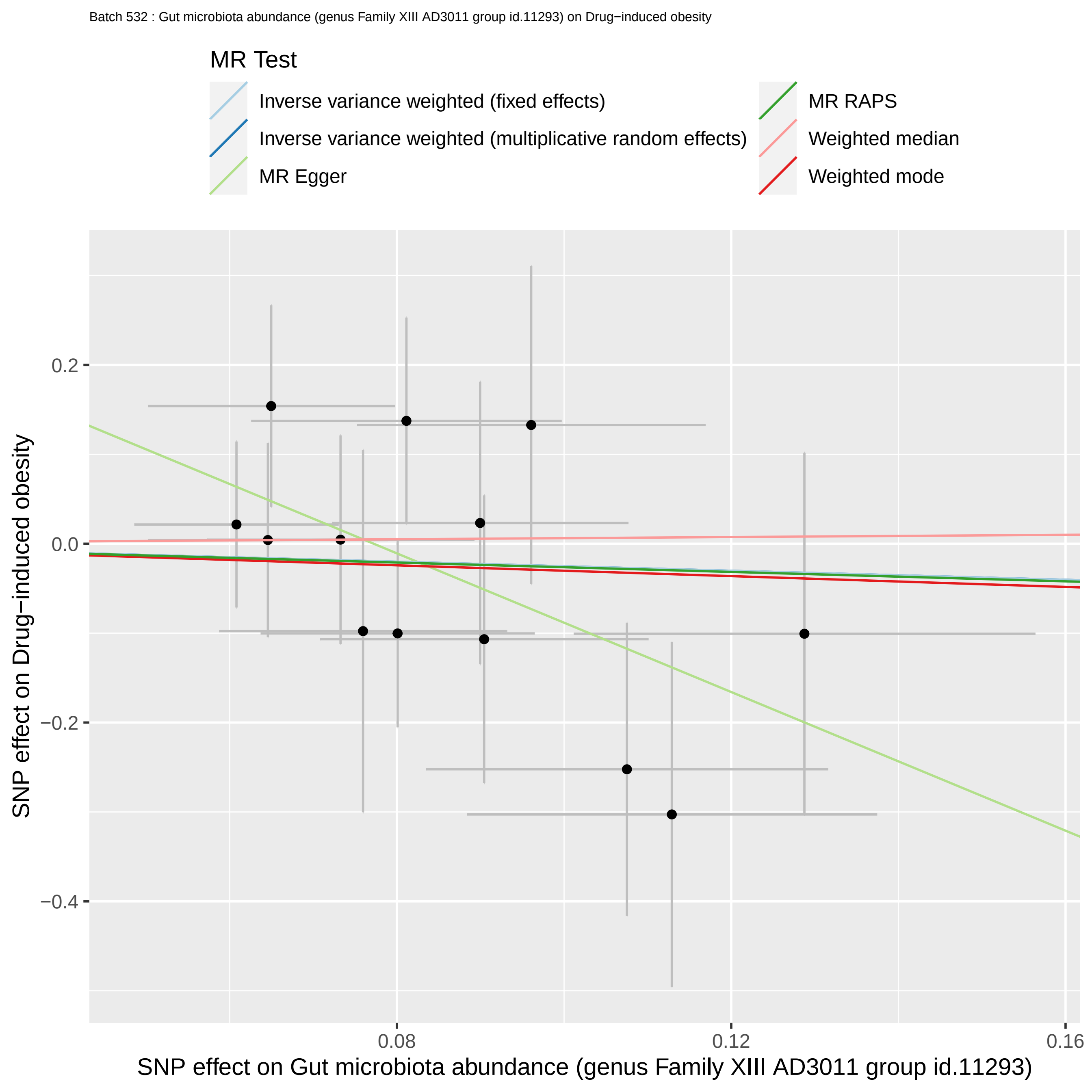
Batch 532 : Gut microbiota abundance (genus Family XIII AD3011 group id.11293) on Drug−induced obesity
MR Test
Inverse variance weighted (fixed effects)
Inverse variance weighted (multiplicative random effects)
MR Egger
MR RAPS
Weighted median
Weighted mode
0.2
0.0
−0.2
−0.4
0.08
0.12
0.16
SNP effect on Drug−induced obesity
SNP effect on Gut microbiota abundance (genus Family XIII AD3011 group id.11293)

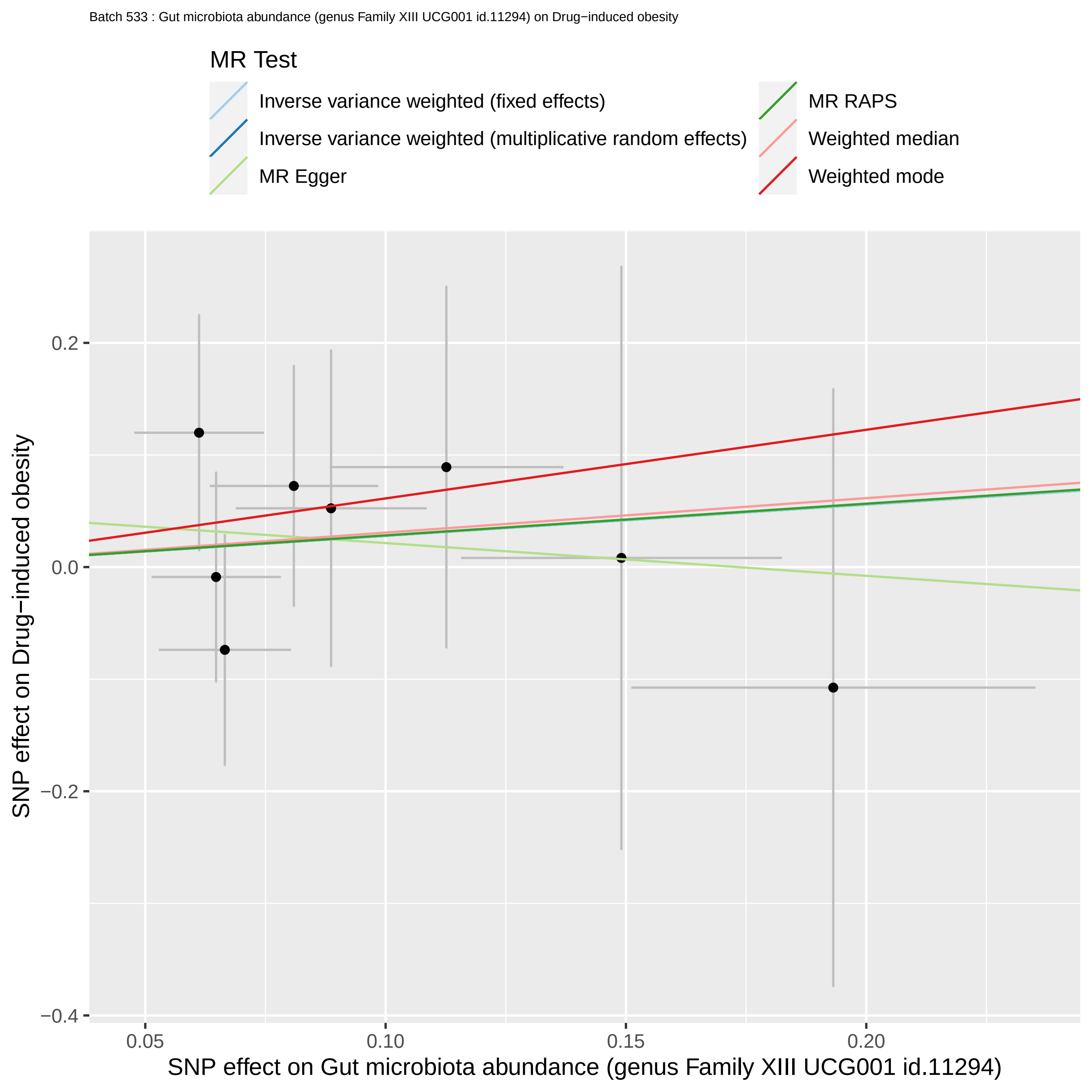
Batch 533 : Gut microbiota abundance (genus Family XIII UCG001 id.11294) on Drug−induced obesity
MR Test
Inverse variance weighted (fixed effects)
Inverse variance weighted (multiplicative random effects)
MR Egger
MR RAPS
Weighted median
Weighted mode
0.2
0.0
−0.2
−0.4
0.05
0.10
0.15
0.20
SNP effect on Drug−induced obesity
SNP effect on Gut microbiota abundance (genus Family XIII UCG001 id.11294)

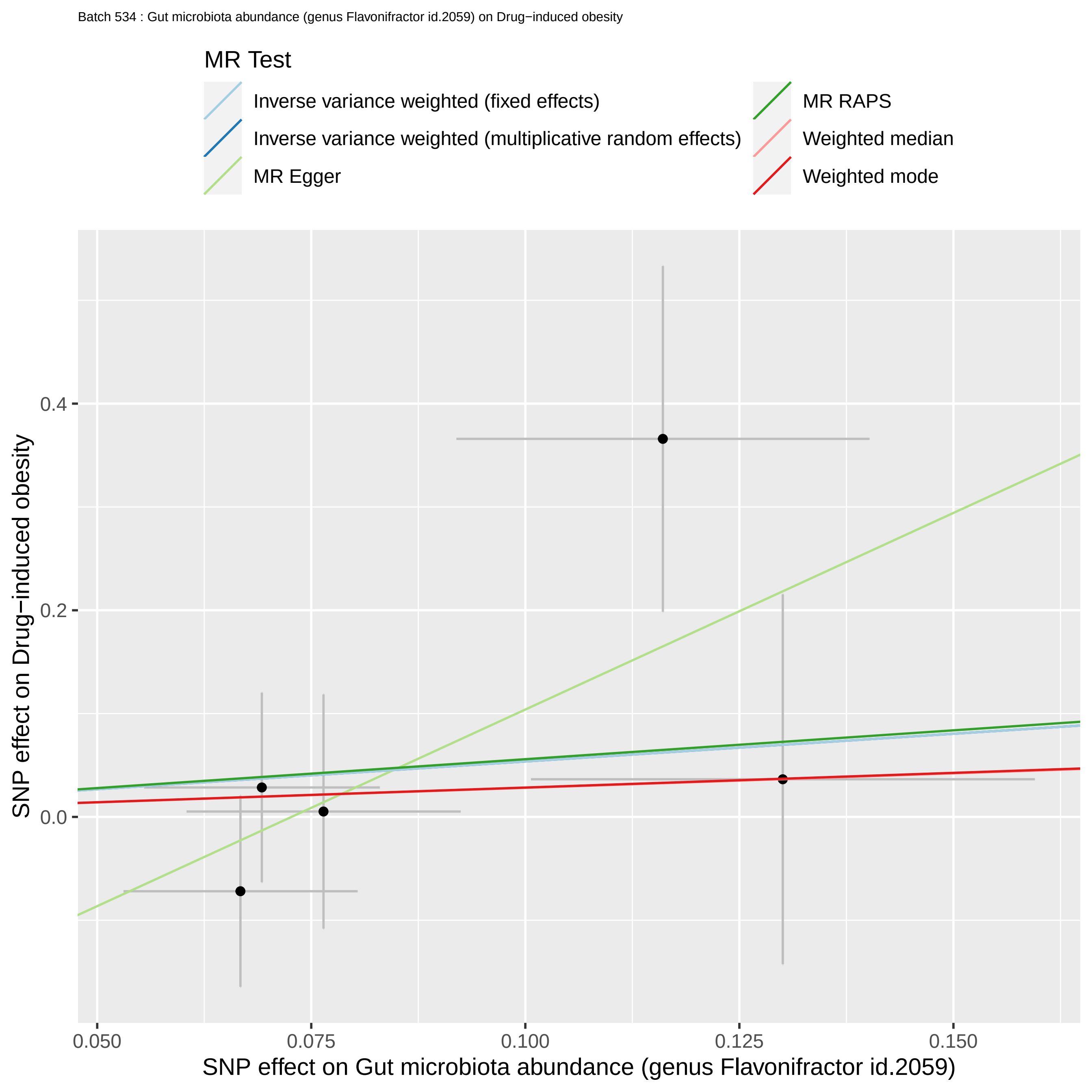
Batch 534 : Gut microbiota abundance (genus Flavonifractor id.2059) on Drug−induced obesity
MR Test
Inverse variance weighted (fixed effects)
Inverse variance weighted (multiplicative random effects)
MR Egger
MR RAPS
Weighted median
Weighted mode
0.4
0.2
0.0
SNP effect on Drug−induced obesity
0.050
0.075
0.100
0.125
0.150
SNP effect on Gut microbiota abundance (genus Flavonifractor id.2059)

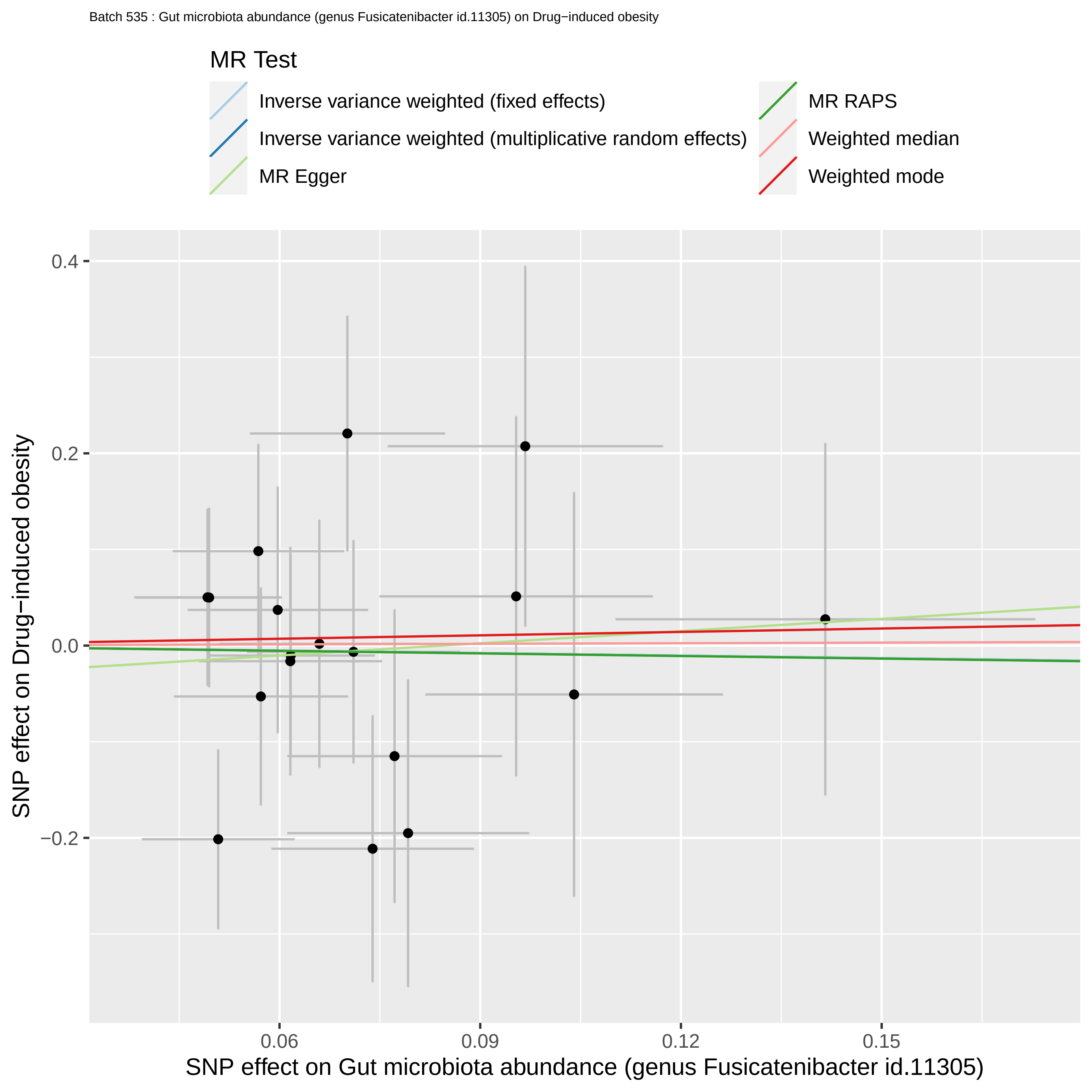

Batch 535 : Gut microbiota abundance (genus Fusicatenibacter id.11305) on Drug−induced obesity
MR Test
Inverse variance weighted (fixed effects)
Inverse variance weighted (multiplicative random effects)
MR Egger
MR RAPS
Weighted median
Weighted mode
0.4
0.2
0.0
−0.2
0.06
0.09
0.12
0.15
SNP effect on Drug−induced obesity
SNP effect on Gut microbiota abundance (genus Fusicatenibacter id.11305)

Batch 536 : Gut microbiota abundance (genus Gordonibacter id.821) on Drug−induced obesity

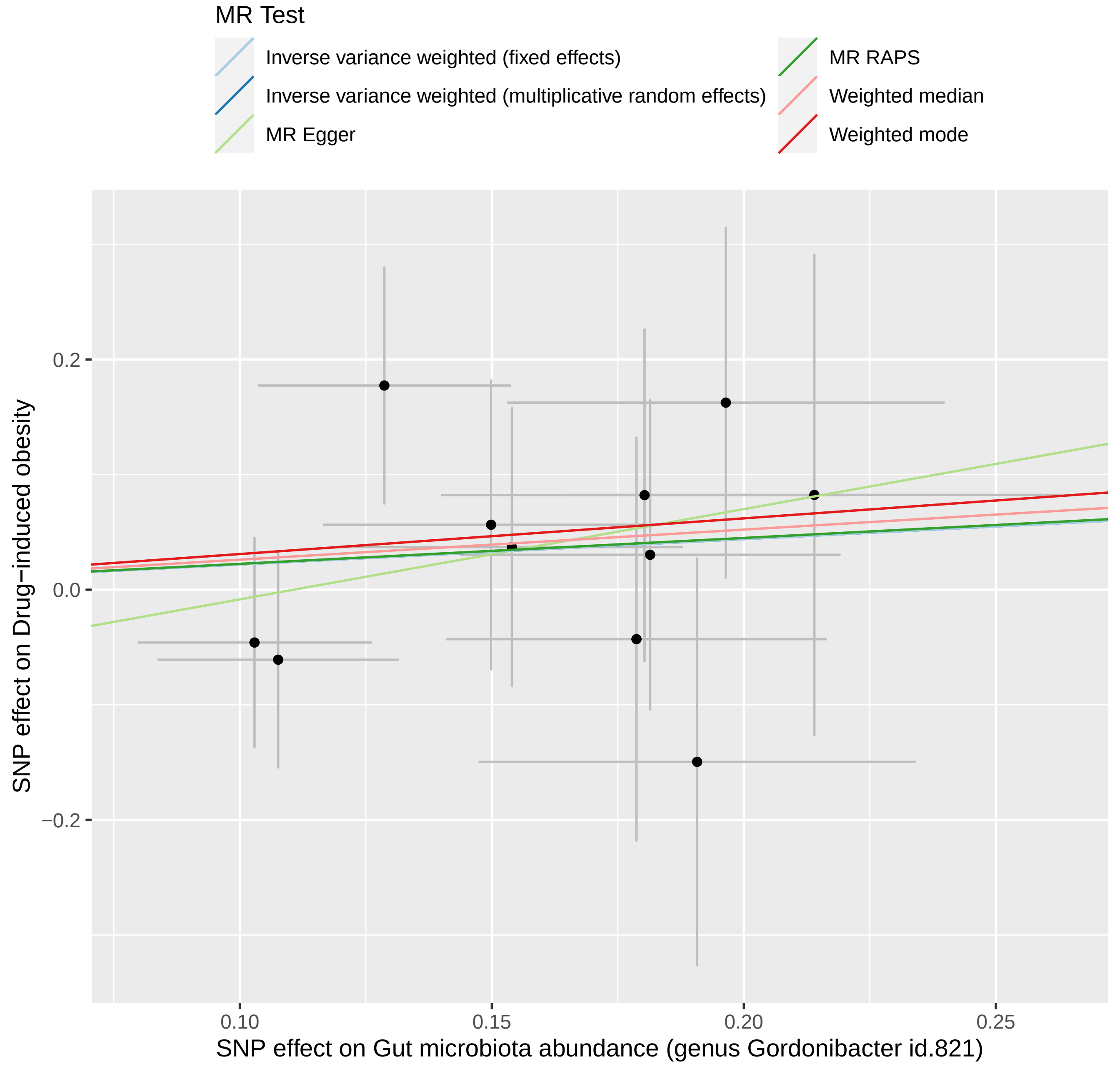

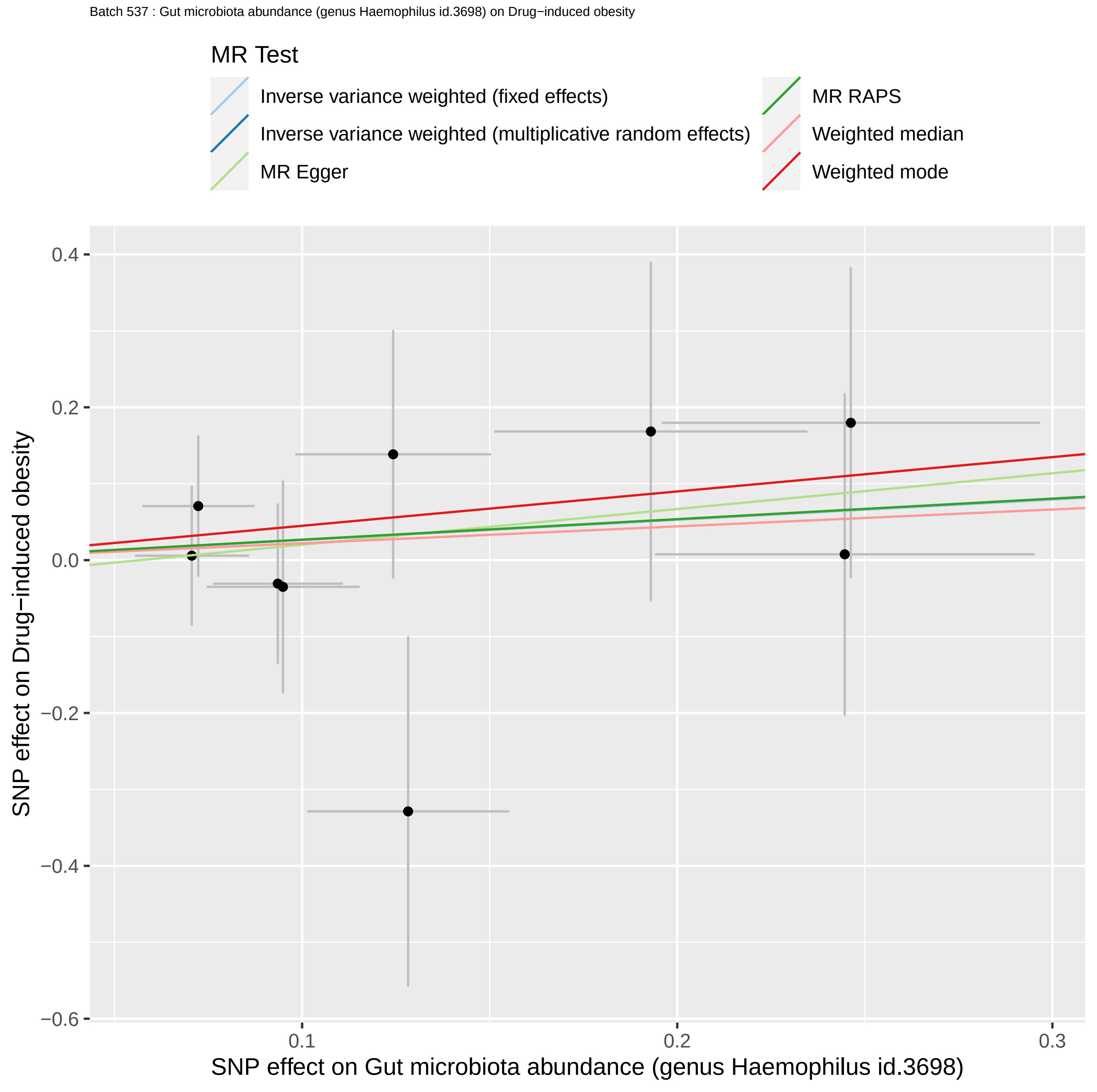
Batch 537 : Gut microbiota abundance (genus Haemophilus id.3698) on Drug−induced obesity
MR Test
Inverse variance weighted (fixed effects)
Inverse variance weighted (multiplicative random effects)
MR Egger
MR RAPS
Weighted median
Weighted mode
0.4
0.2
0.0
−0.2
−0.4
−0.6
0.1
0.2
0.3
SNP effect on Drug−induced obesity
SNP effect on Gut microbiota abundance (genus Haemophilus id.3698)

Batch 538 : Gut microbiota abundance (genus Holdemanella id.11393) on Drug−induced obesity

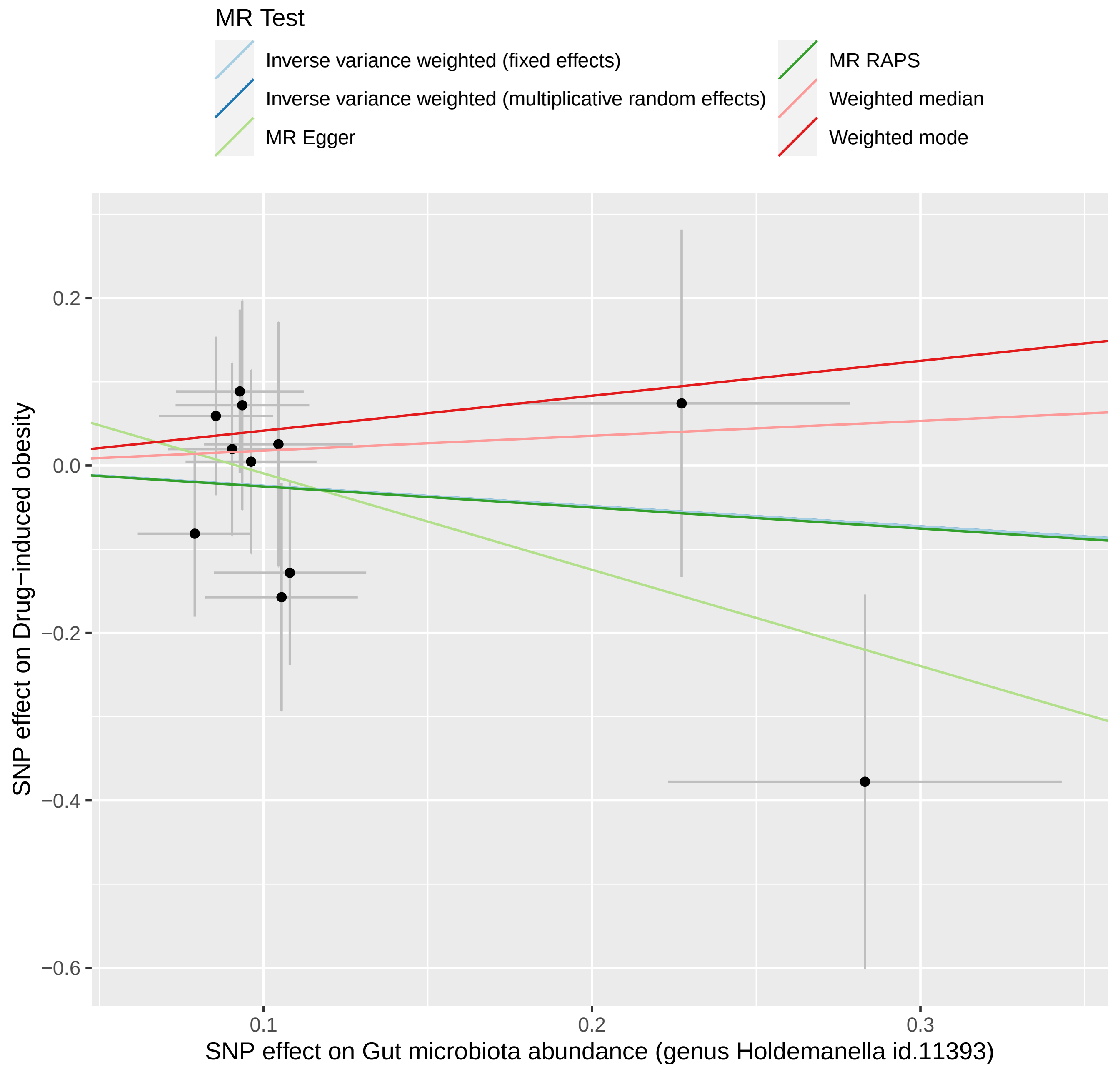

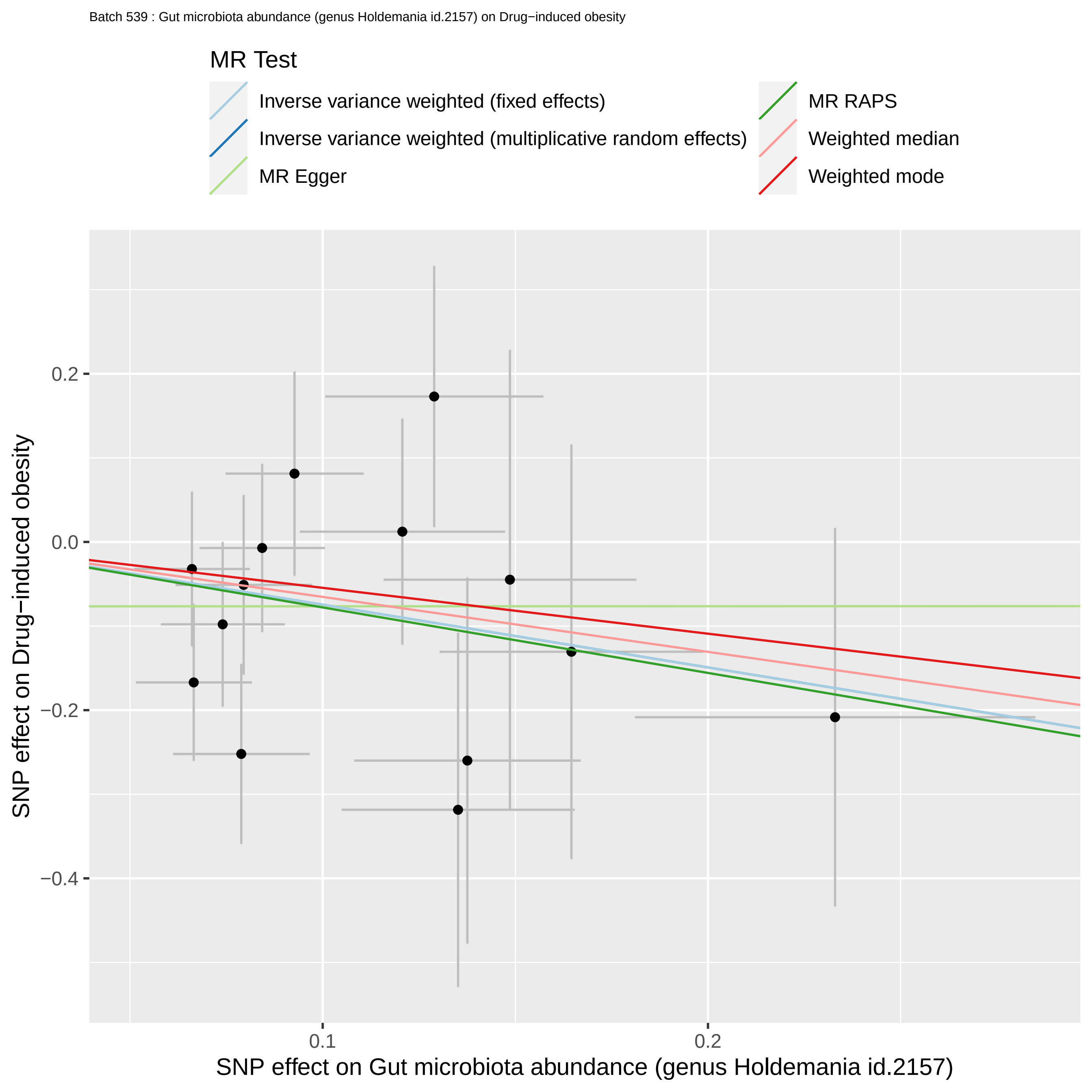

Batch 539 : Gut microbiota abundance (genus Holdemania id.2157) on Drug−induced obesity
MR Test
Inverse variance weighted (fixed effects)
Inverse variance weighted (multiplicative random effects)
MR Egger
MR RAPS
Weighted median
Weighted mode
0.2
0.0
−0.2
−0.4
0.1
0.2
SNP effect on Drug−induced obesity
SNP effect on Gut microbiota abundance (genus Holdemania id.2157)

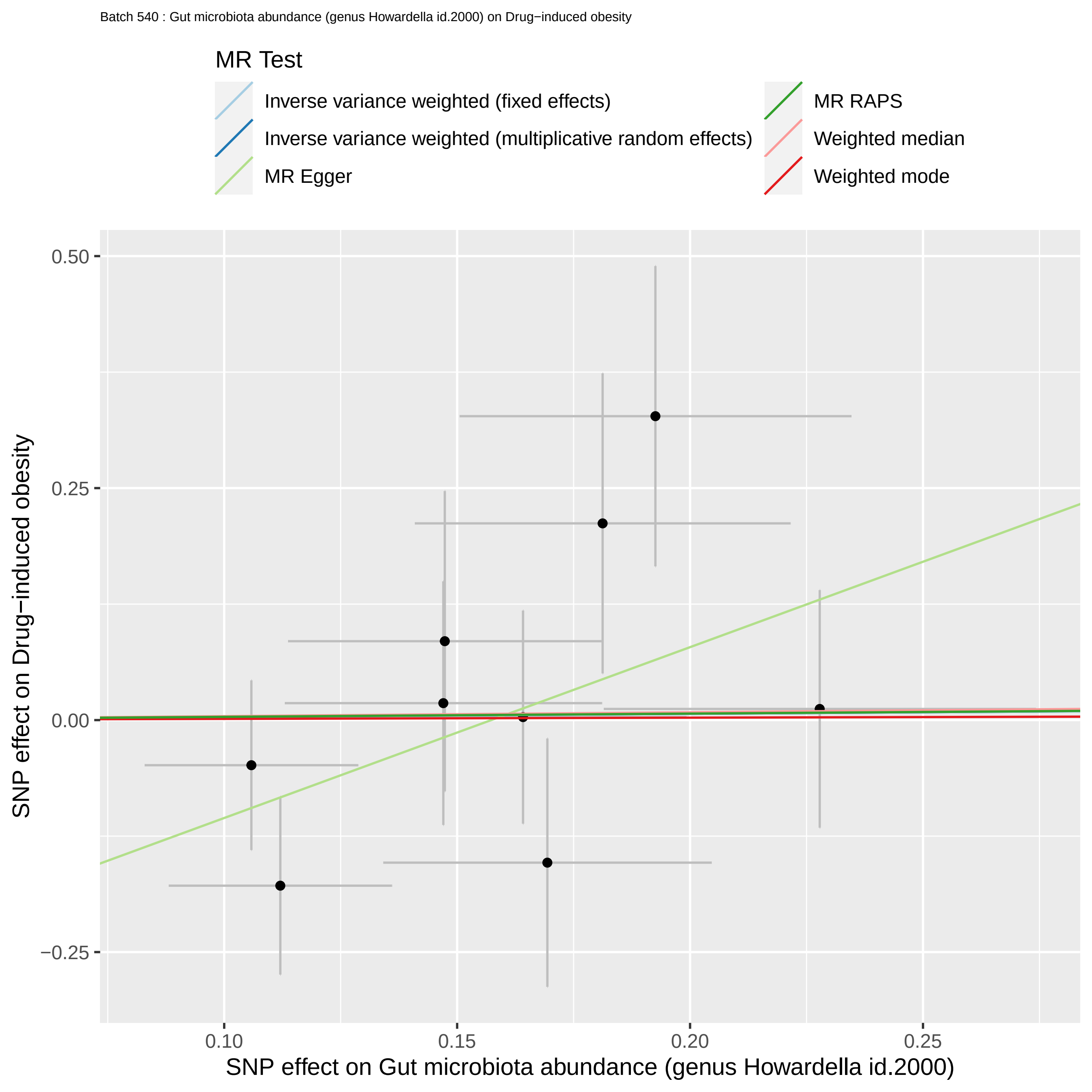

Batch 540 : Gut microbiota abundance (genus Howardella id.2000) on Drug−induced obesity
MR Test
Inverse variance weighted (fixed effects)
Inverse variance weighted (multiplicative random effects)
MR Egger
MR RAPS
Weighted median
Weighted mode
0.50
0.25
0.00
−0.25
0.10
0.15
0.20
0.25
SNP effect on Drug−induced obesity
SNP effect on Gut microbiota abundance (genus Howardella id.2000)

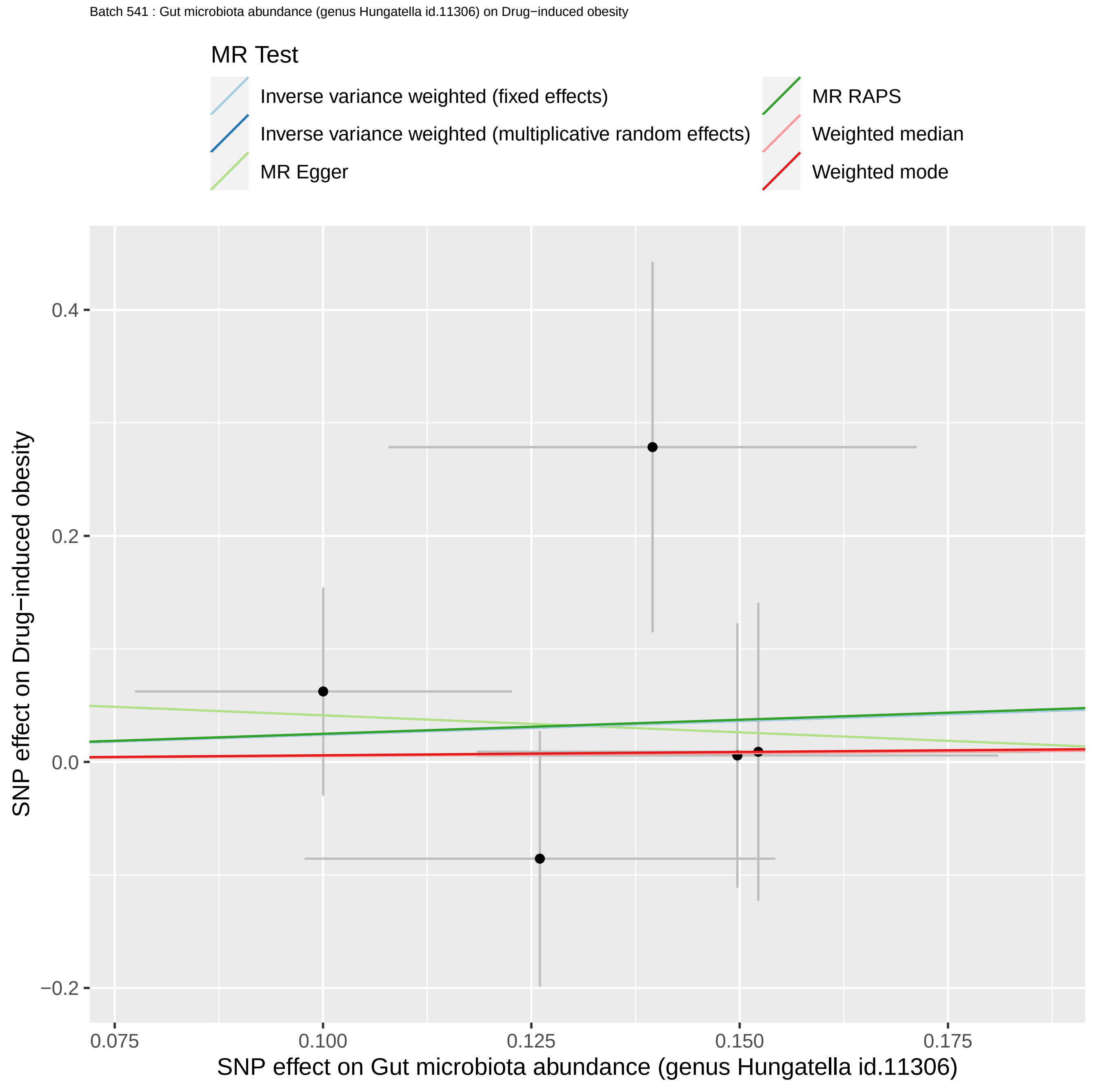
Batch 541 : Gut microbiota abundance (genus Hungatella id.11306) on Drug−induced obesity
MR Test
Inverse variance weighted (fixed effects)
Inverse variance weighted (multiplicative random effects)
MR Egger
MR RAPS
Weighted median
Weighted mode
0.4
0.2
0.0
−0.2
0.075
0.100
0.125
0.150
0.175
SNP effect on Drug−induced obesity
SNP effect on Gut microbiota abundance (genus Hungatella id.11306)

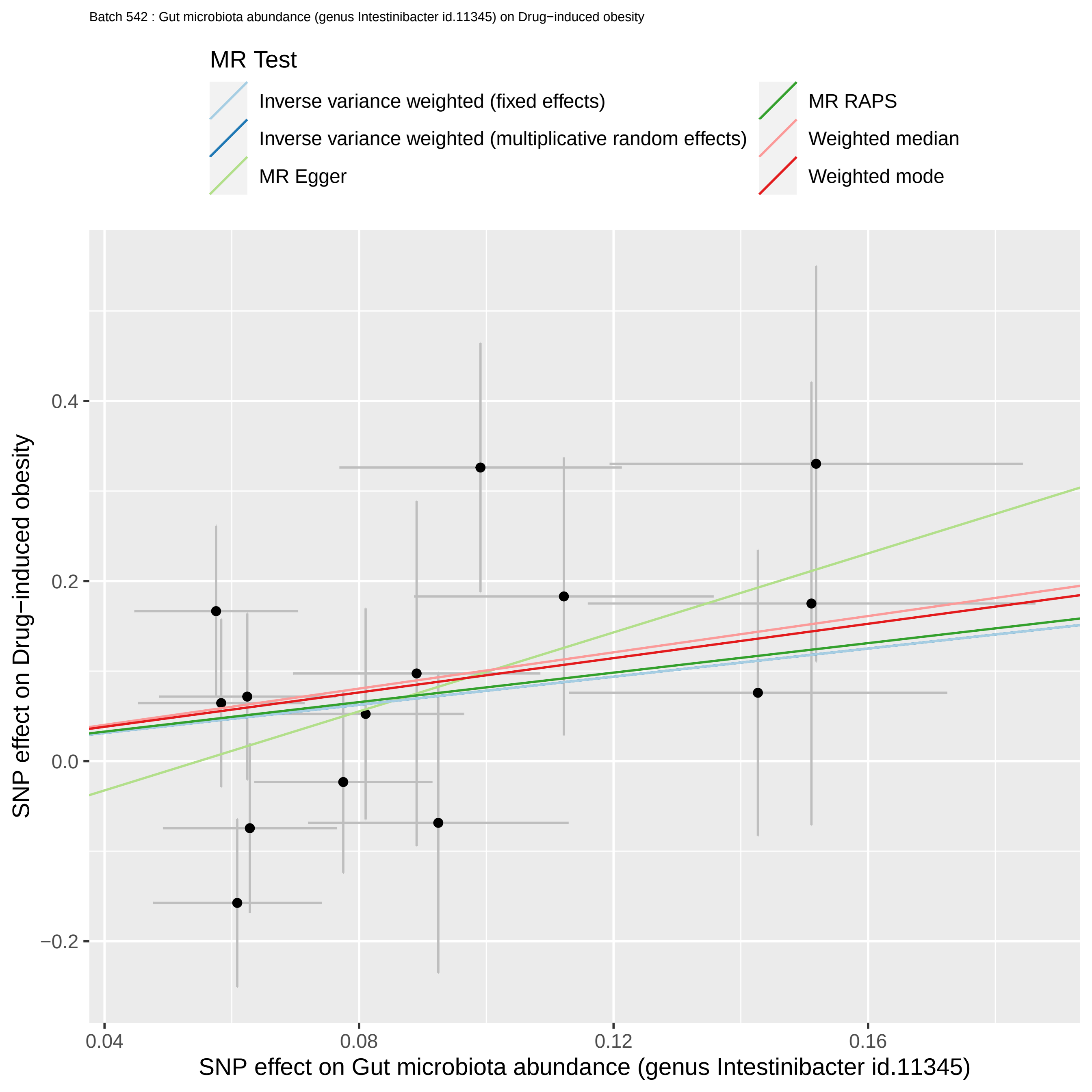

Batch 542 : Gut microbiota abundance (genus Intestinibacter id.11345) on Drug−induced obesity
MR Test
Inverse variance weighted (fixed effects)
Inverse variance weighted (multiplicative random effects)
MR Egger
MR RAPS
Weighted median
Weighted mode
0.4
0.2
0.0
−0.2
0.04
0.08
0.12
0.16
SNP effect on Drug−induced obesity
SNP effect on Gut microbiota abundance (genus Intestinibacter id.11345)

Batch 543 : Gut microbiota abundance (genus Intestinimonas id.2062) on Drug−induced obesity

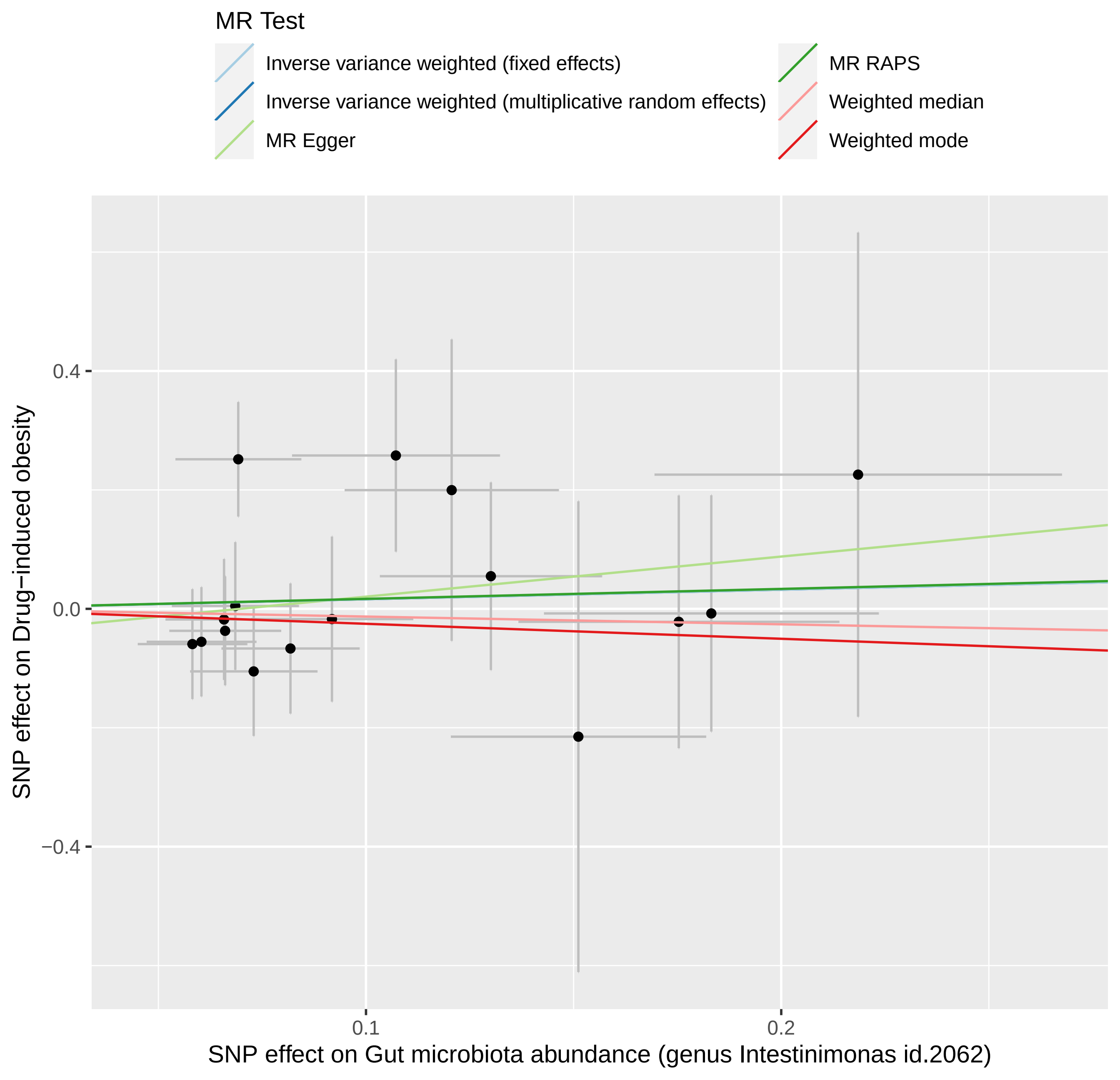

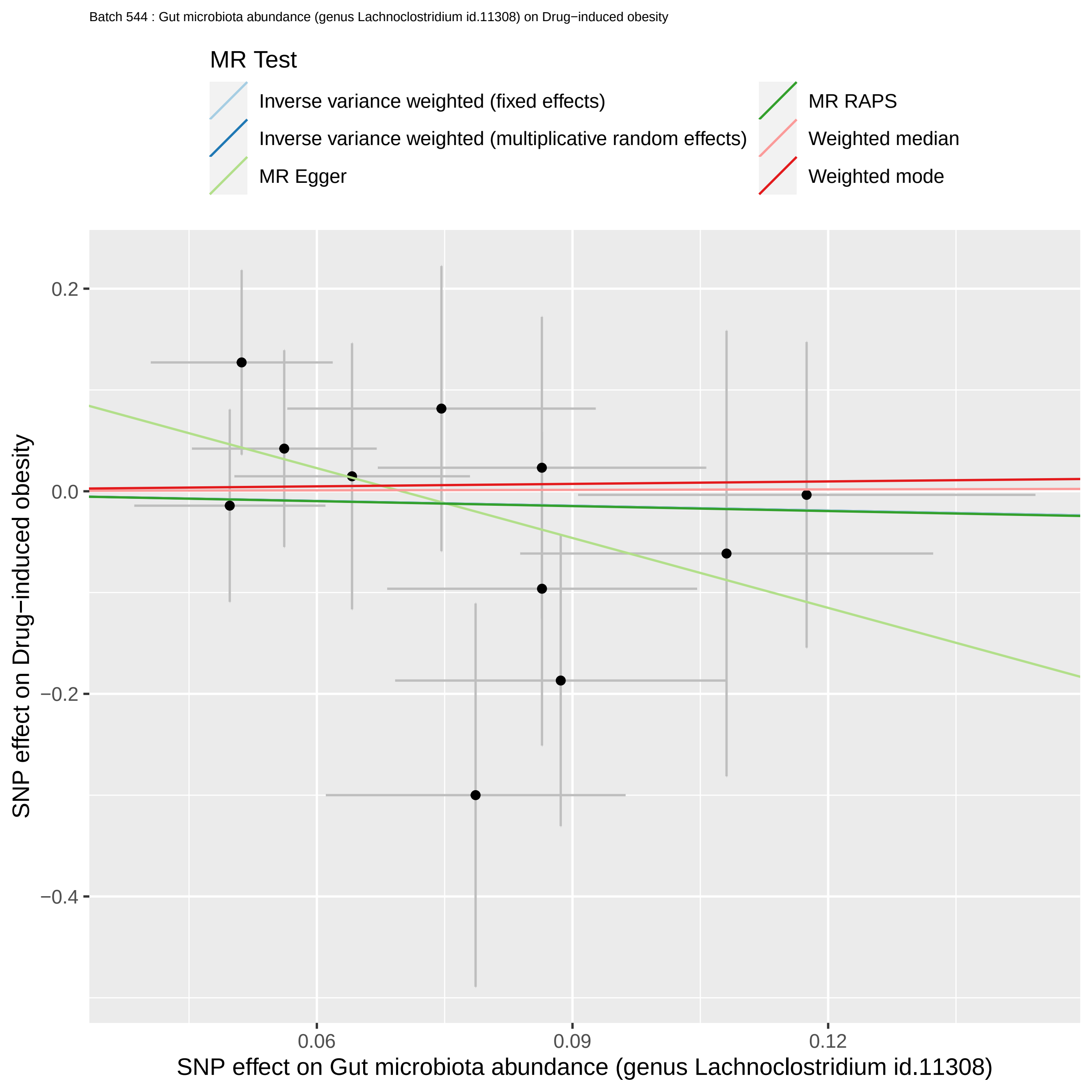
Batch 544 : Gut microbiota abundance (genus Lachnoclostridium id.11308) on Drug−induced obesity
MR Test
Inverse variance weighted (fixed effects)
Inverse variance weighted (multiplicative random effects)
MR Egger
MR RAPS
Weighted median
Weighted mode
SNP effect on Drug−induced obesity
0.2
0.0
−0.2
−0.4
0.06
0.09
0.12
SNP effect on Gut microbiota abundance (genus Lachnoclostridium id.11308)

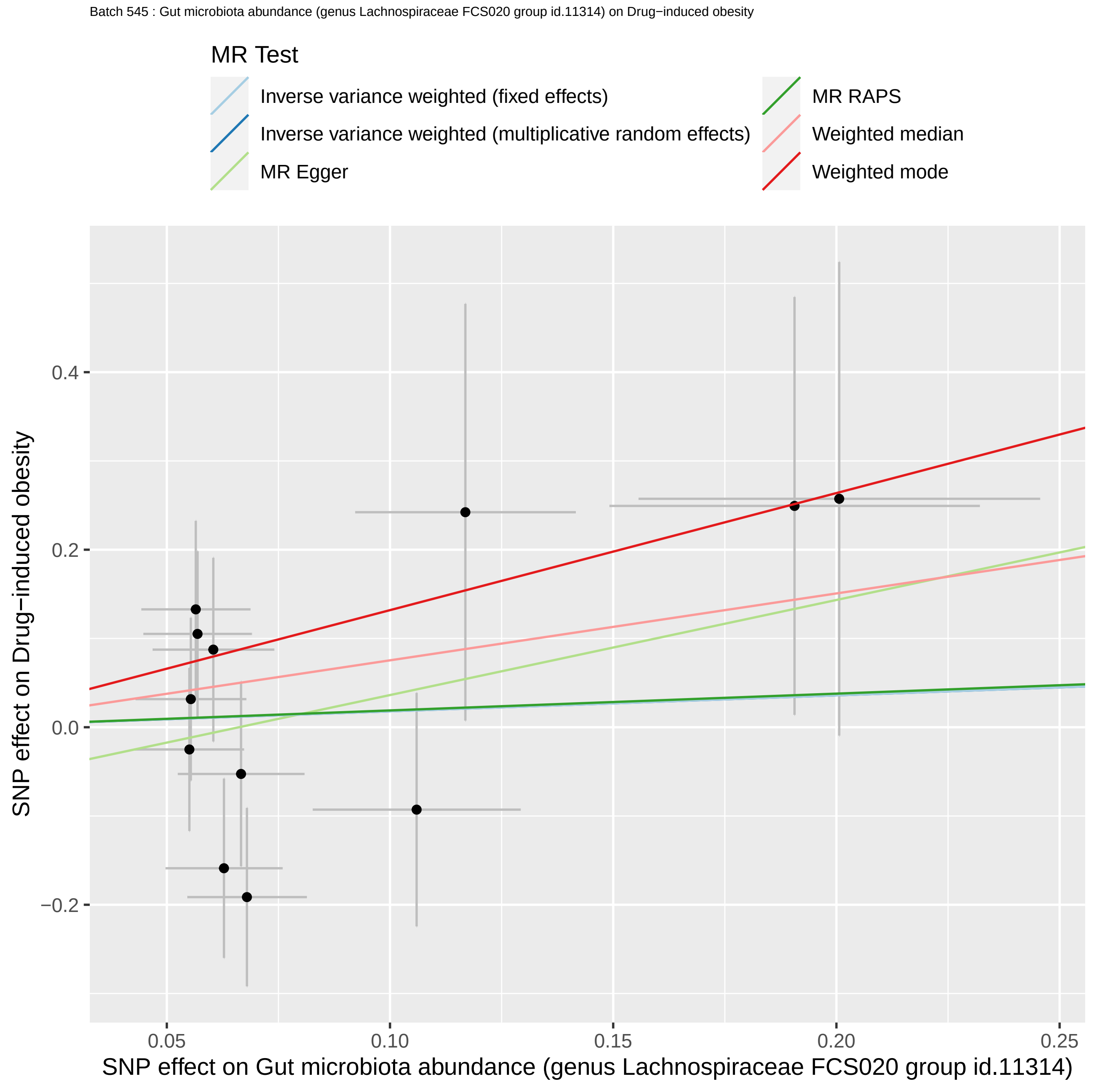
Batch 545 : Gut microbiota abundance (genus Lachnospiraceae FCS020 group id.11314) on Drug−induced obesity
MR Test
Inverse variance weighted (fixed effects)
Inverse variance weighted (multiplicative random effects)
MR Egger
MR RAPS
Weighted median
Weighted mode
0.4
0.2
0.0
−0.2
0.05
0.10
0.15
0.20
0.25
SNP effect on Drug−induced obesity
SNP effect on Gut microbiota abundance (genus Lachnospiraceae FCS020 group id.11314)

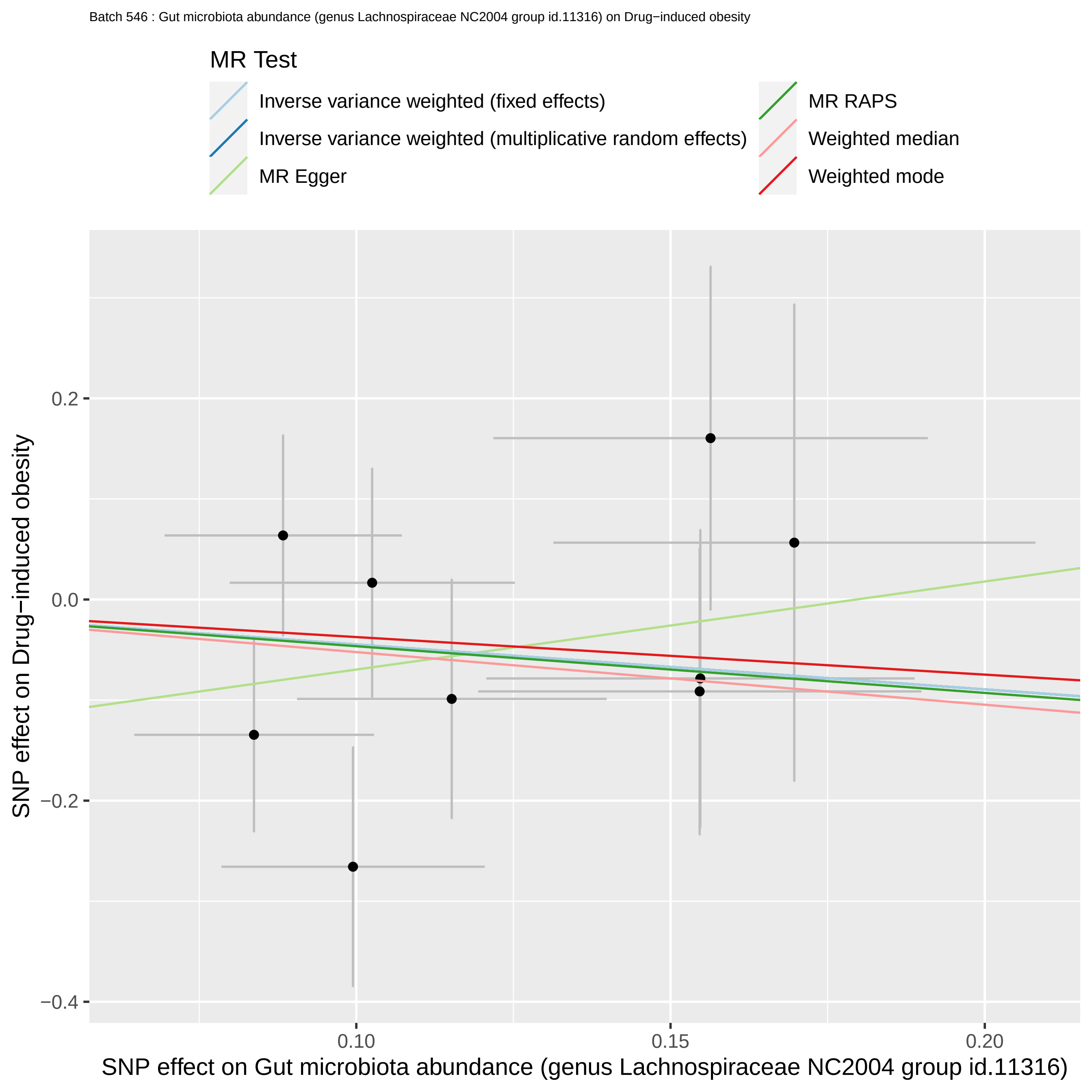
Batch 546 : Gut microbiota abundance (genus Lachnospiraceae NC2004 group id.11316) on Drug−induced obesity
MR Test
Inverse variance weighted (fixed effects)
Inverse variance weighted (multiplicative random effects)
MR Egger
MR RAPS
Weighted median
Weighted mode
0.2
0.0
−0.2
−0.4
0.10
0.15
0.20
SNP effect on Drug−induced obesity
SNP effect on Gut microbiota abundance (genus Lachnospiraceae NC2004 group id.11316)

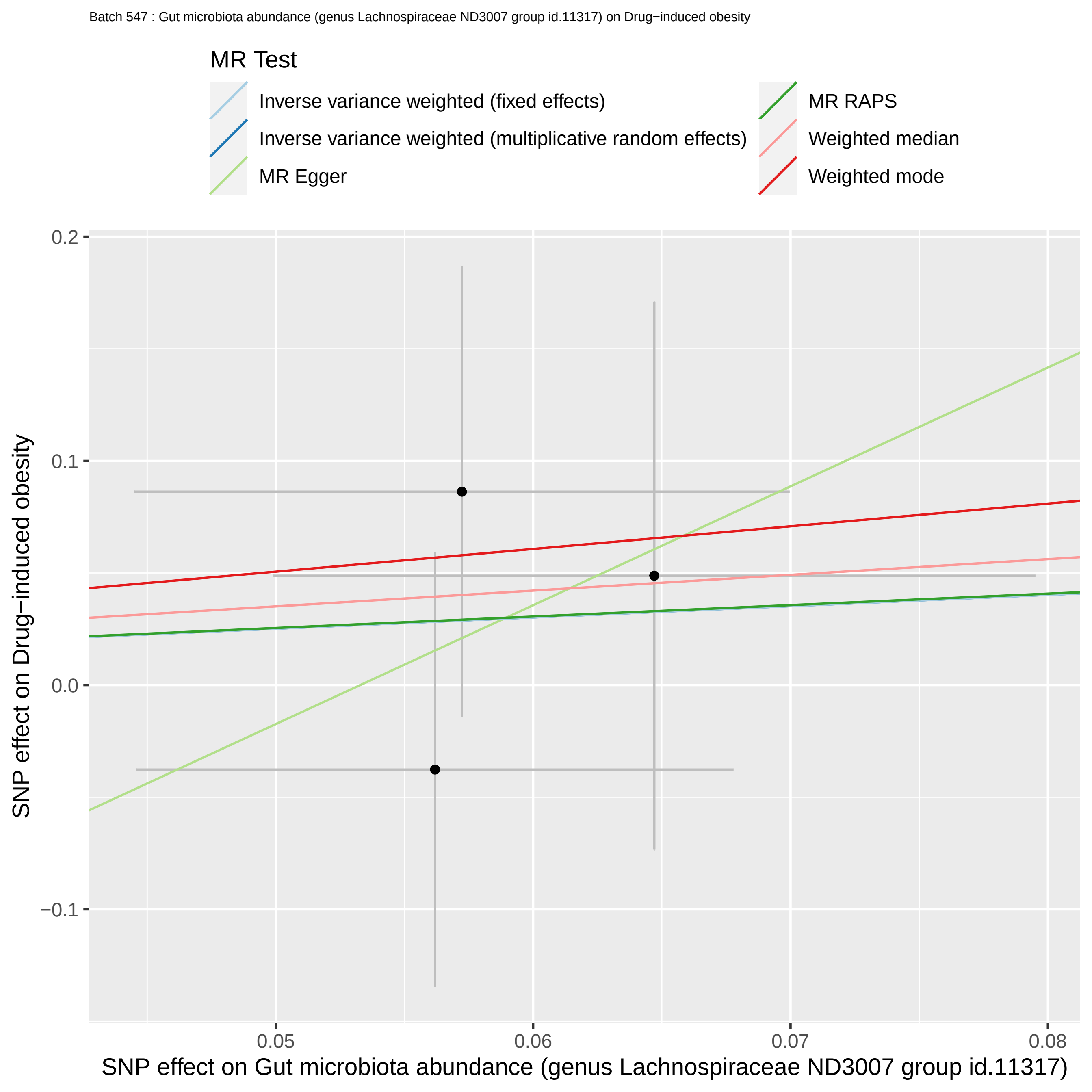
Batch 547 : Gut microbiota abundance (genus Lachnospiraceae ND3007 group id.11317) on Drug−induced obesity
MR Test
Inverse variance weighted (fixed effects)
Inverse variance weighted (multiplicative random effects)
MR Egger
MR RAPS
Weighted median
Weighted mode
0.2
0.1
0.0
−0.1
0.05
0.06
0.07
0.08
SNP effect on Drug−induced obesity
SNP effect on Gut microbiota abundance (genus Lachnospiraceae ND3007 group id.11317)

Batch 548 : Gut microbiota abundance (genus Lachnospiraceae NK4A136 group id.11319) on Drug−induced obesity

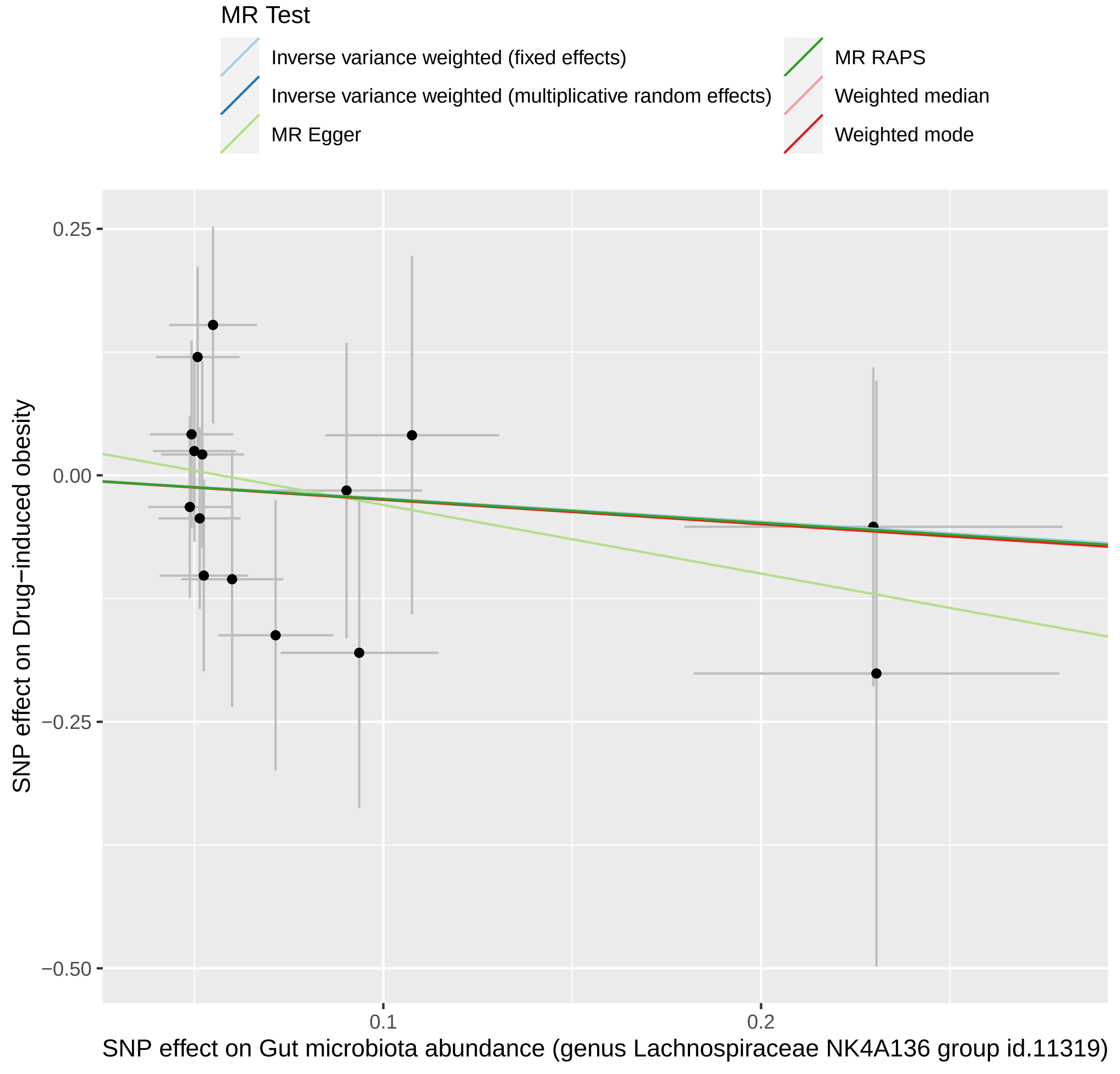

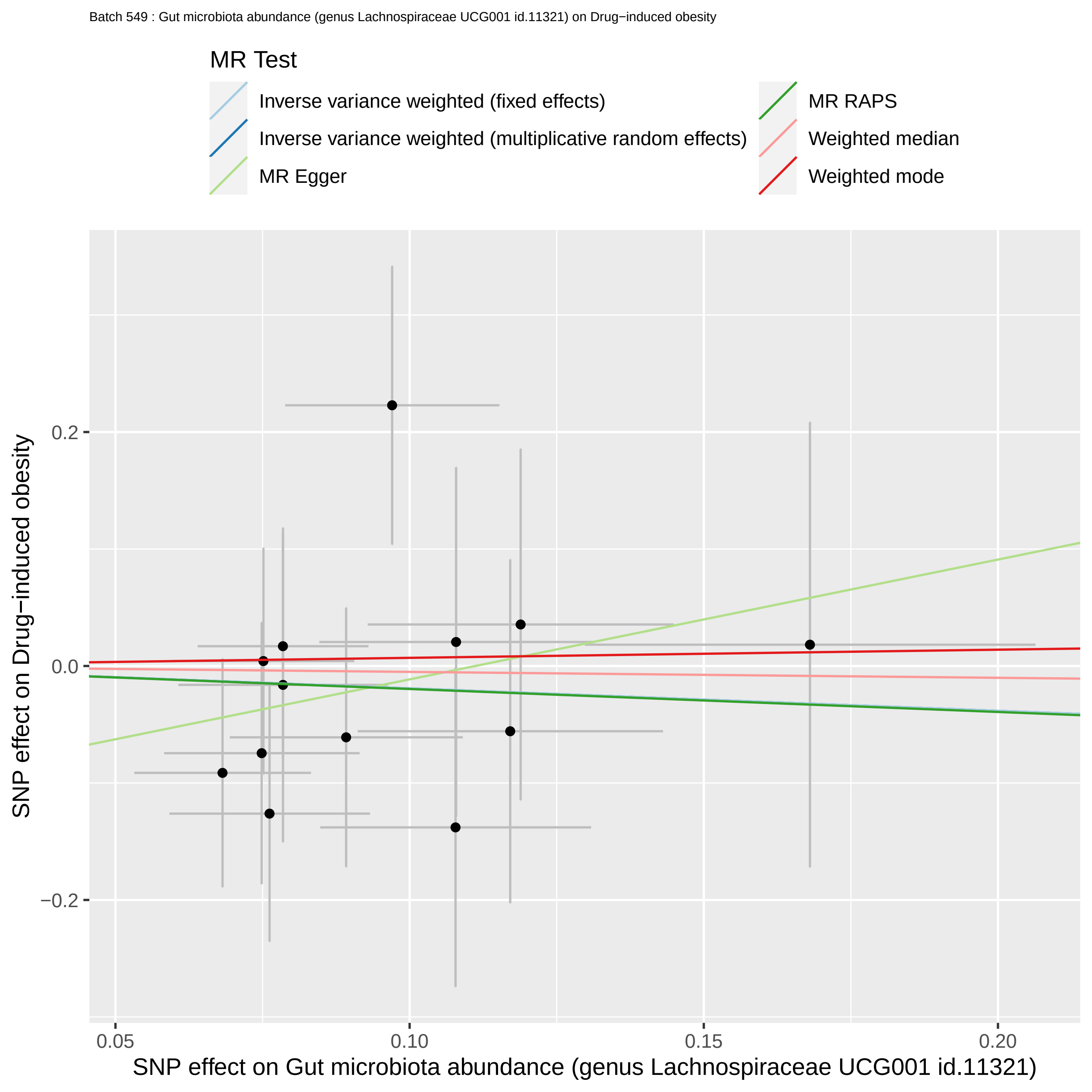

Batch 549 : Gut microbiota abundance (genus Lachnospiraceae UCG001 id.11321) on Drug−induced obesity
MR Test
Inverse variance weighted (fixed effects)
Inverse variance weighted (multiplicative random effects)
MR Egger
MR RAPS
Weighted median
Weighted mode
0.2
0.0
−0.2
0.05
0.10
0.15
0.20
SNP effect on Drug−induced obesity
SNP effect on Gut microbiota abundance (genus Lachnospiraceae UCG001 id.11321)

Batch 550 : Gut microbiota abundance (genus Lachnospiraceae UCG004 id.11324) on Drug−induced obesity

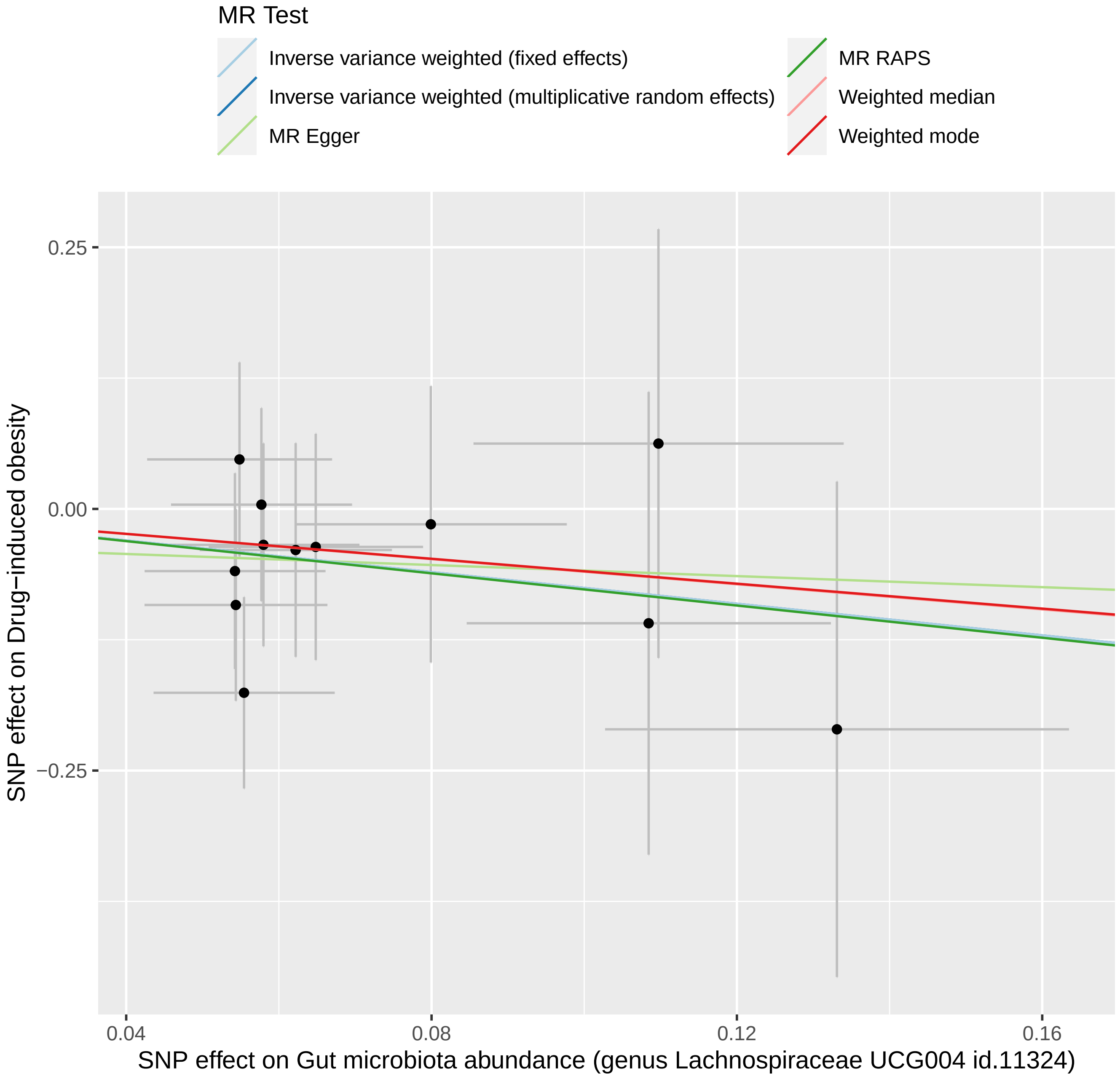

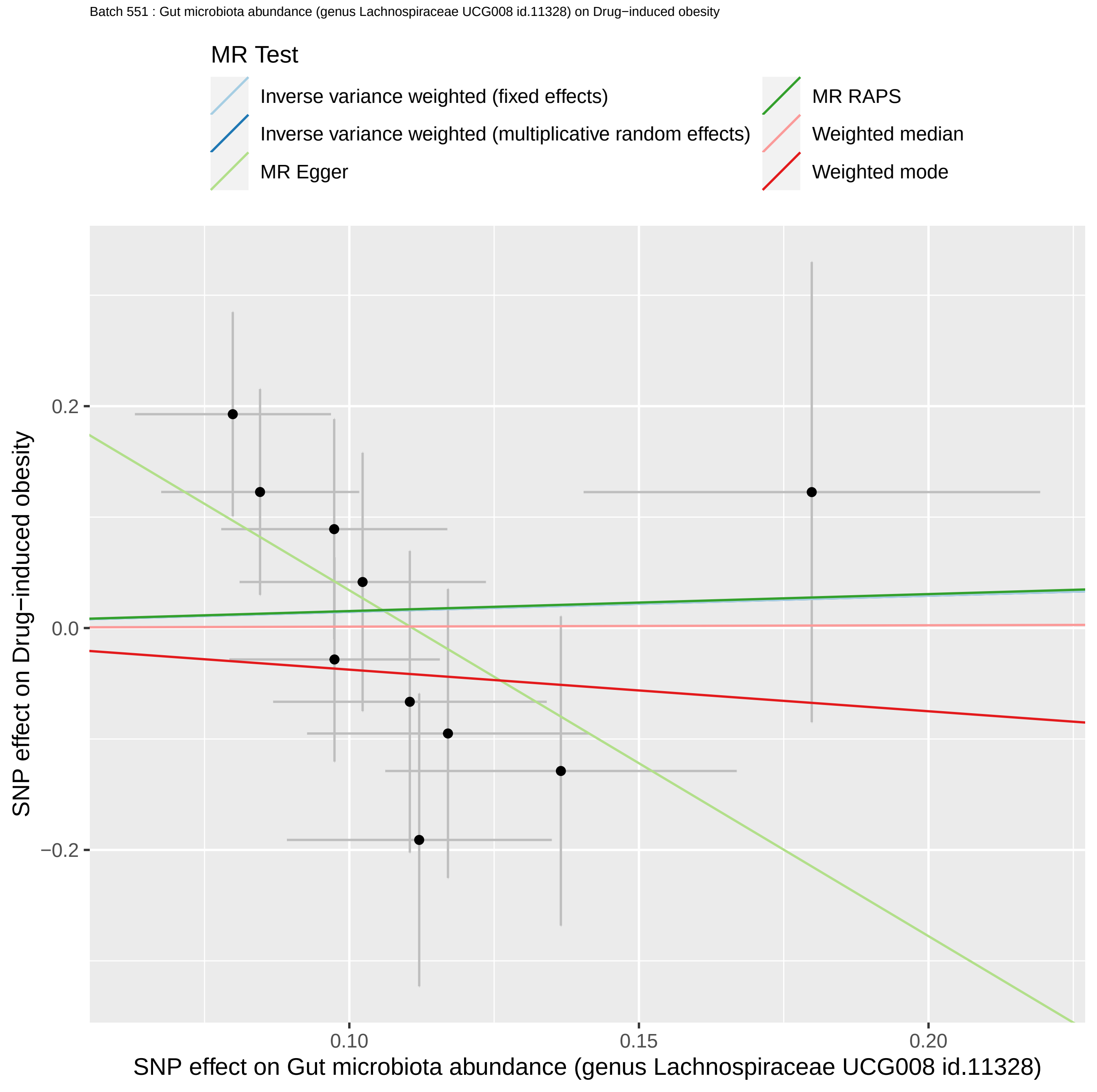
Batch 551 : Gut microbiota abundance (genus Lachnospiraceae UCG008 id.11328) on Drug−induced obesity
MR Test
Inverse variance weighted (fixed effects)
Inverse variance weighted (multiplicative random effects)
MR Egger
MR RAPS
Weighted median
Weighted mode
0.2
0.0
−0.2
0.10
0.15
0.20
SNP effect on Drug−induced obesity
SNP effect on Gut microbiota abundance (genus Lachnospiraceae UCG008 id.11328)

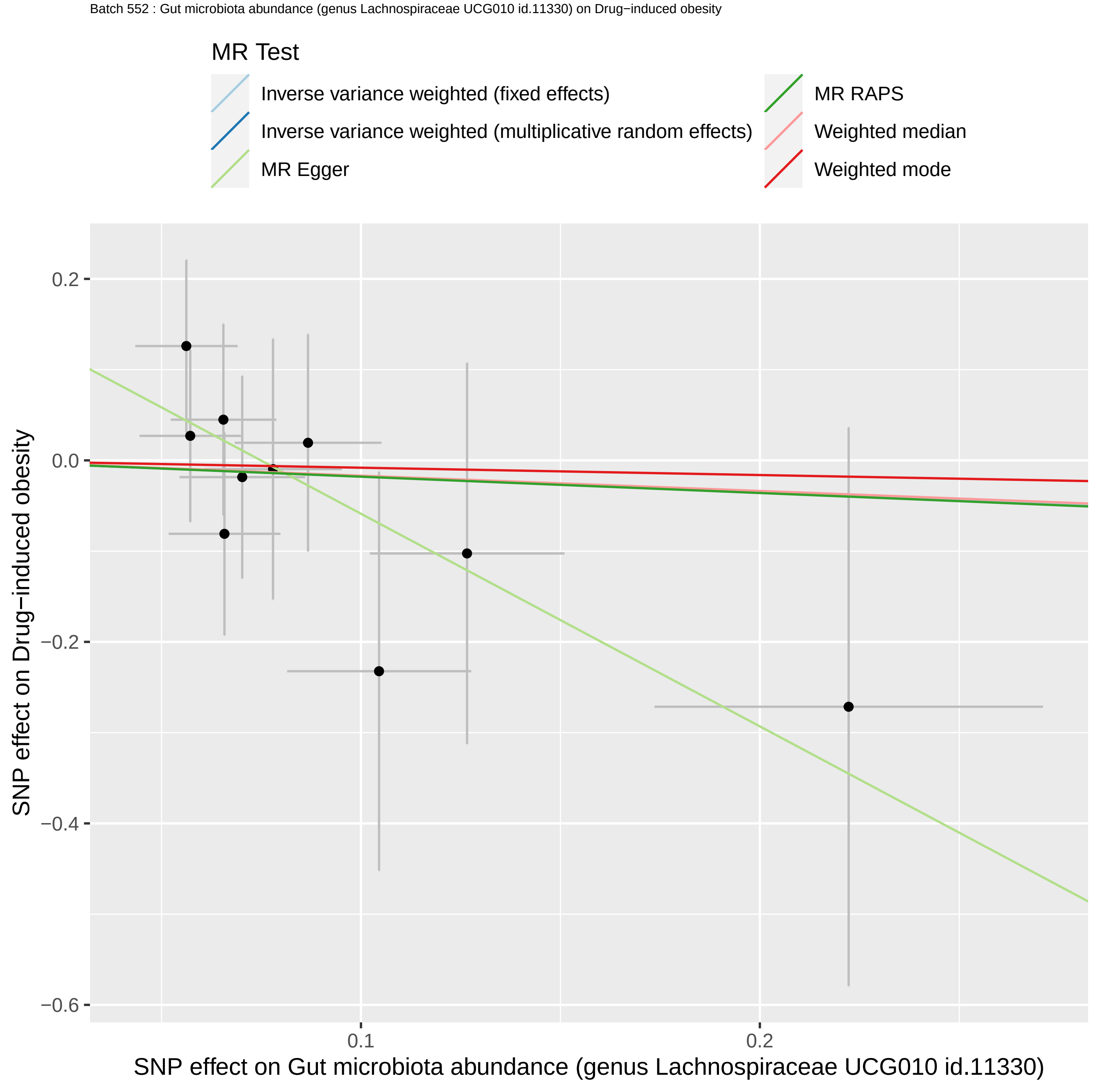

Batch 552 : Gut microbiota abundance (genus Lachnospiraceae UCG010 id.11330) on Drug−induced obesity
MR Test
Inverse variance weighted (fixed effects)
Inverse variance weighted (multiplicative random effects)
MR Egger
MR RAPS
Weighted median
Weighted mode
0.2
0.0
−0.2
−0.4
−0.6
0.1
0.2
SNP effect on Drug−induced obesity
SNP effect on Gut microbiota abundance (genus Lachnospiraceae UCG010 id.11330)

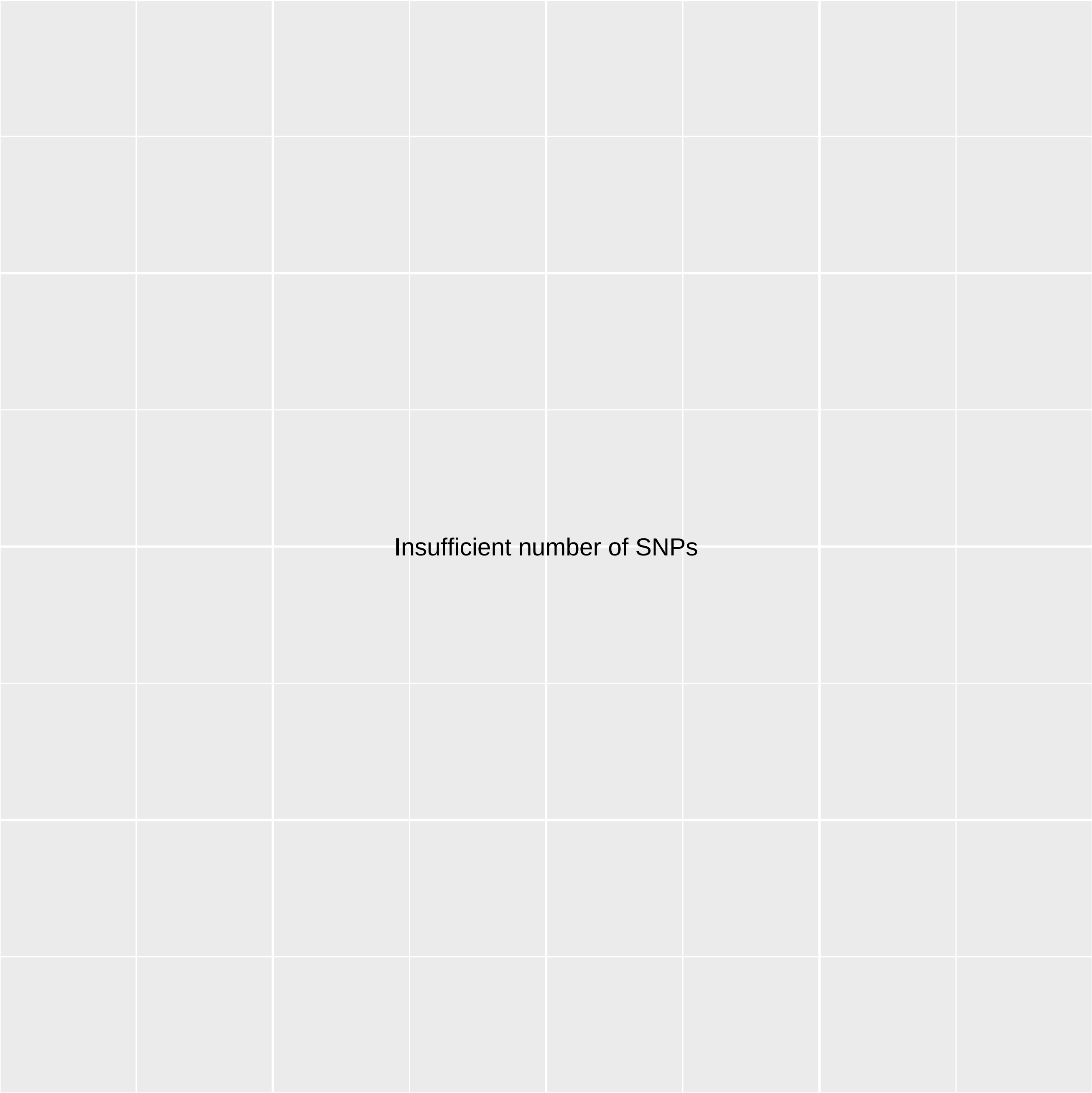
Insufficient number of SNPs

Batch 554 : Gut microbiota abundance (genus Lactobacillus id.1837) on Drug−induced obesity

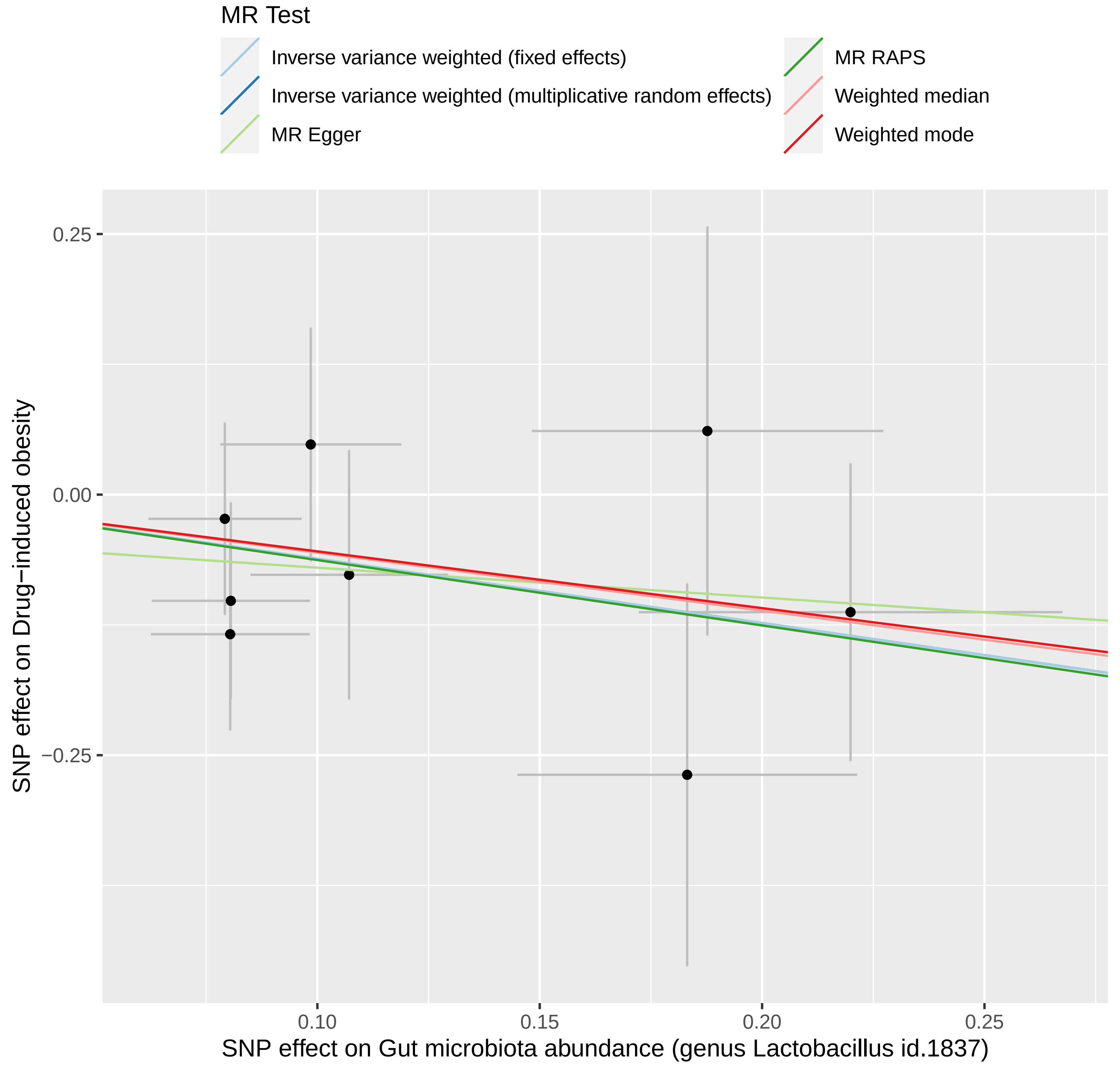

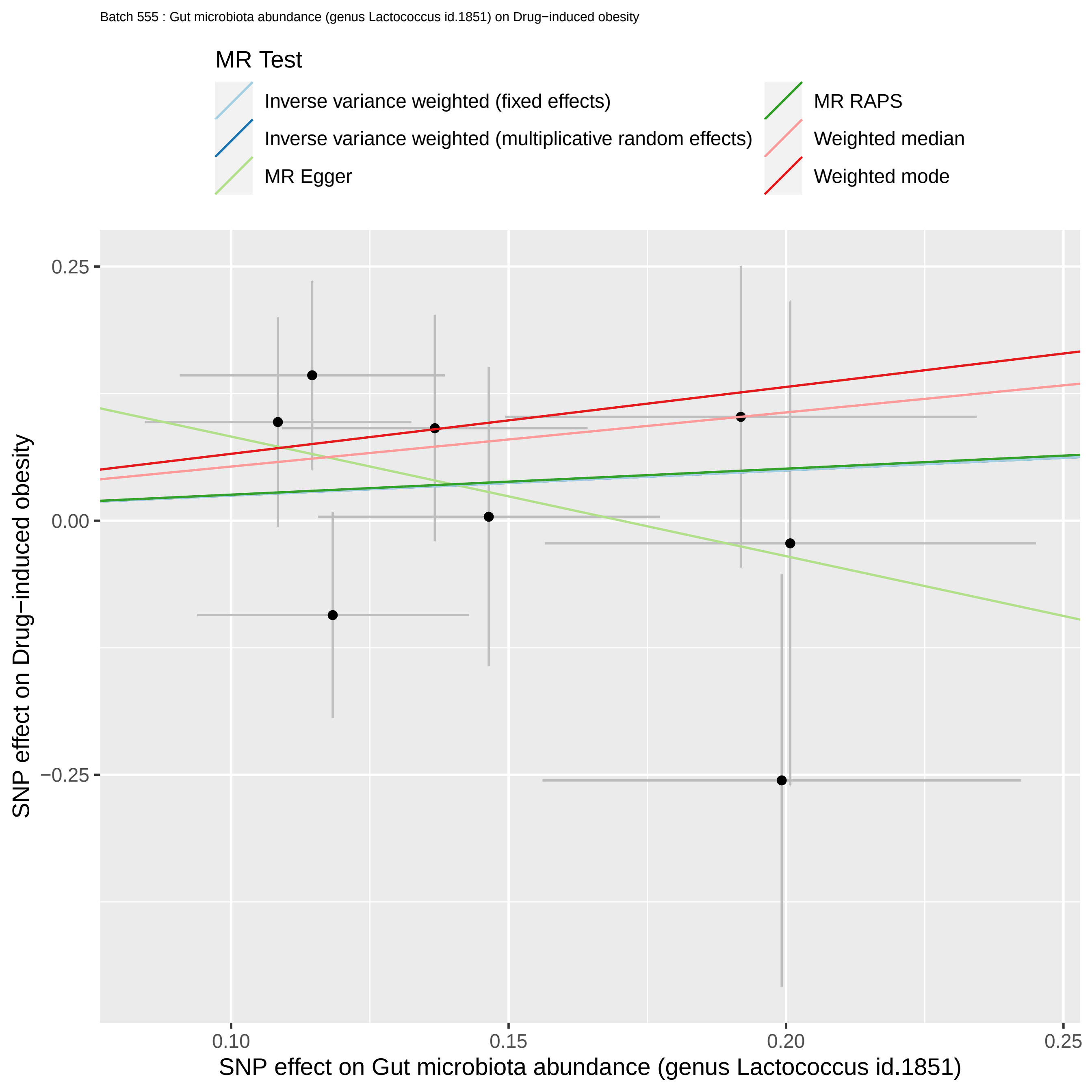

Batch 555 : Gut microbiota abundance (genus Lactococcus id.1851) on Drug−induced obesity
MR Test
Inverse variance weighted (fixed effects)
Inverse variance weighted (multiplicative random effects)
MR Egger
MR RAPS
Weighted median
Weighted mode
0.25
0.00
−0.25
0.10
0.15
0.20
0.25
SNP effect on Drug−induced obesity
SNP effect on Gut microbiota abundance (genus Lactococcus id.1851)

Batch 556 : Gut microbiota abundance (genus Marvinbryantia id.2005) on Drug−induced obesity

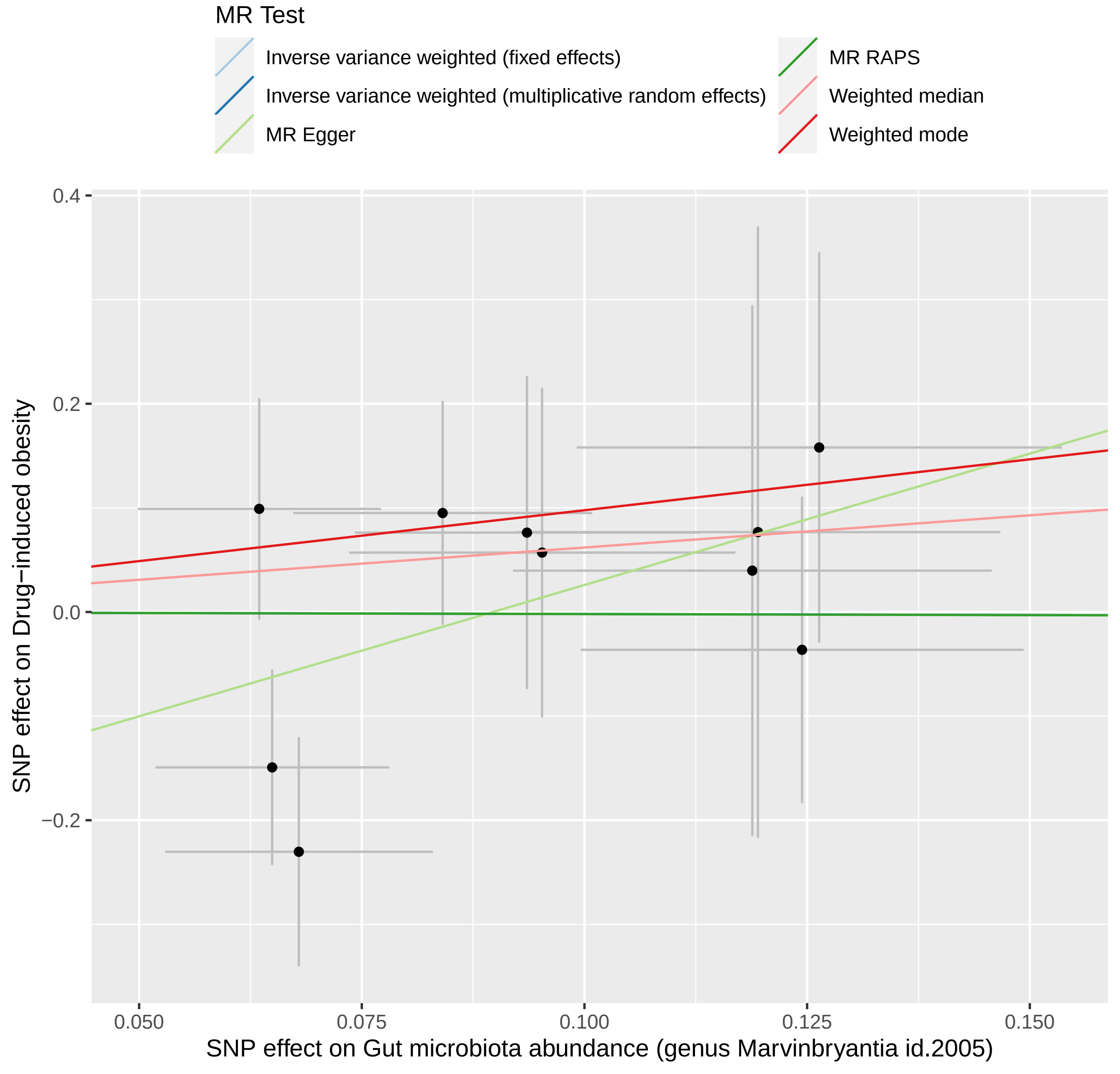

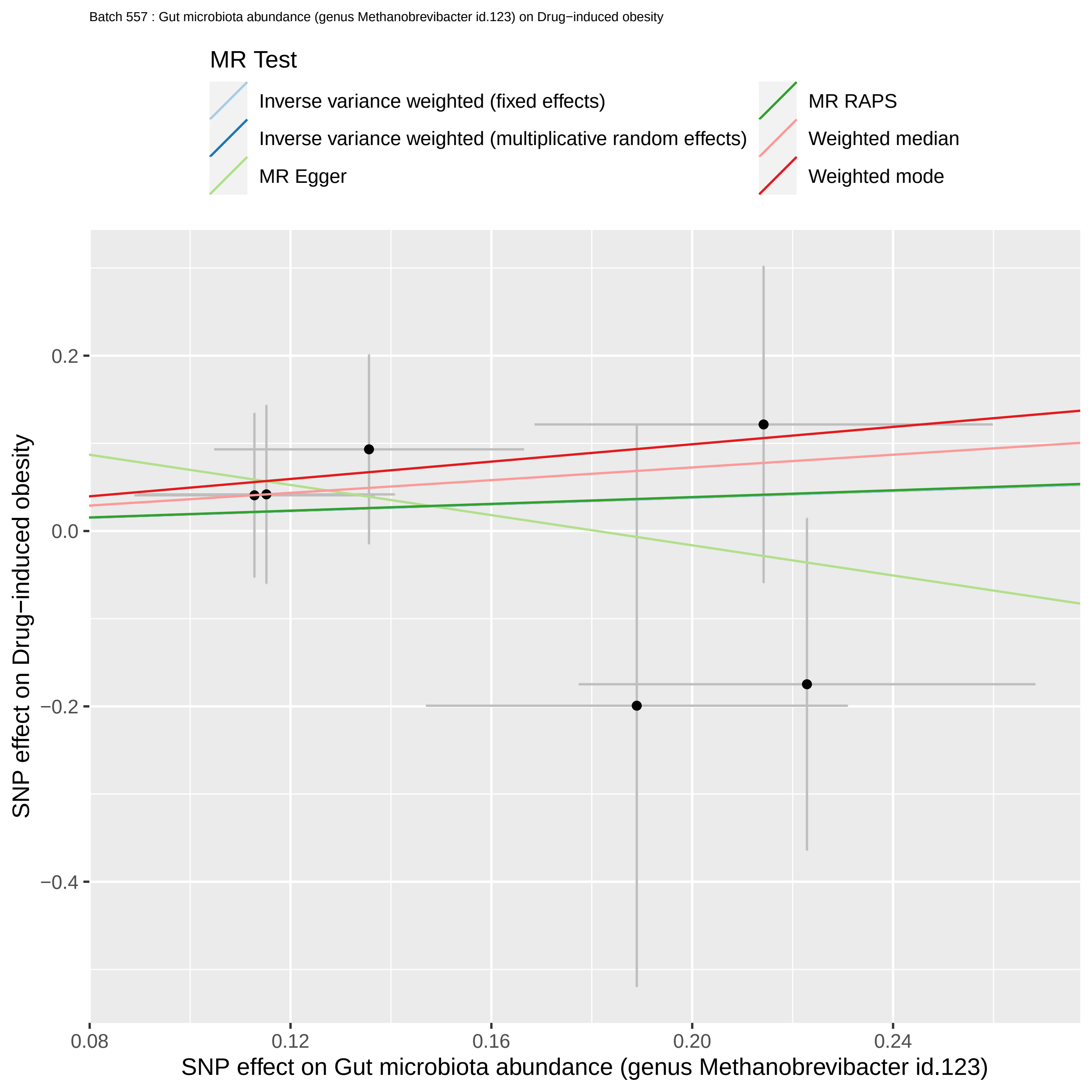

Batch 557 : Gut microbiota abundance (genus Methanobrevibacter id.123) on Drug−induced obesity
MR Test
Inverse variance weighted (fixed effects)
Inverse variance weighted (multiplicative random effects)
MR Egger
MR RAPS
Weighted median
Weighted mode
0.2
0.0
−0.2
−0.4
0.08
0.12
0.16
0.20
0.24
SNP effect on Drug−induced obesity
SNP effect on Gut microbiota abundance (genus Methanobrevibacter id.123)

Batch 558 : Gut microbiota abundance (genus Odoribacter id.952) on Drug−induced obesity

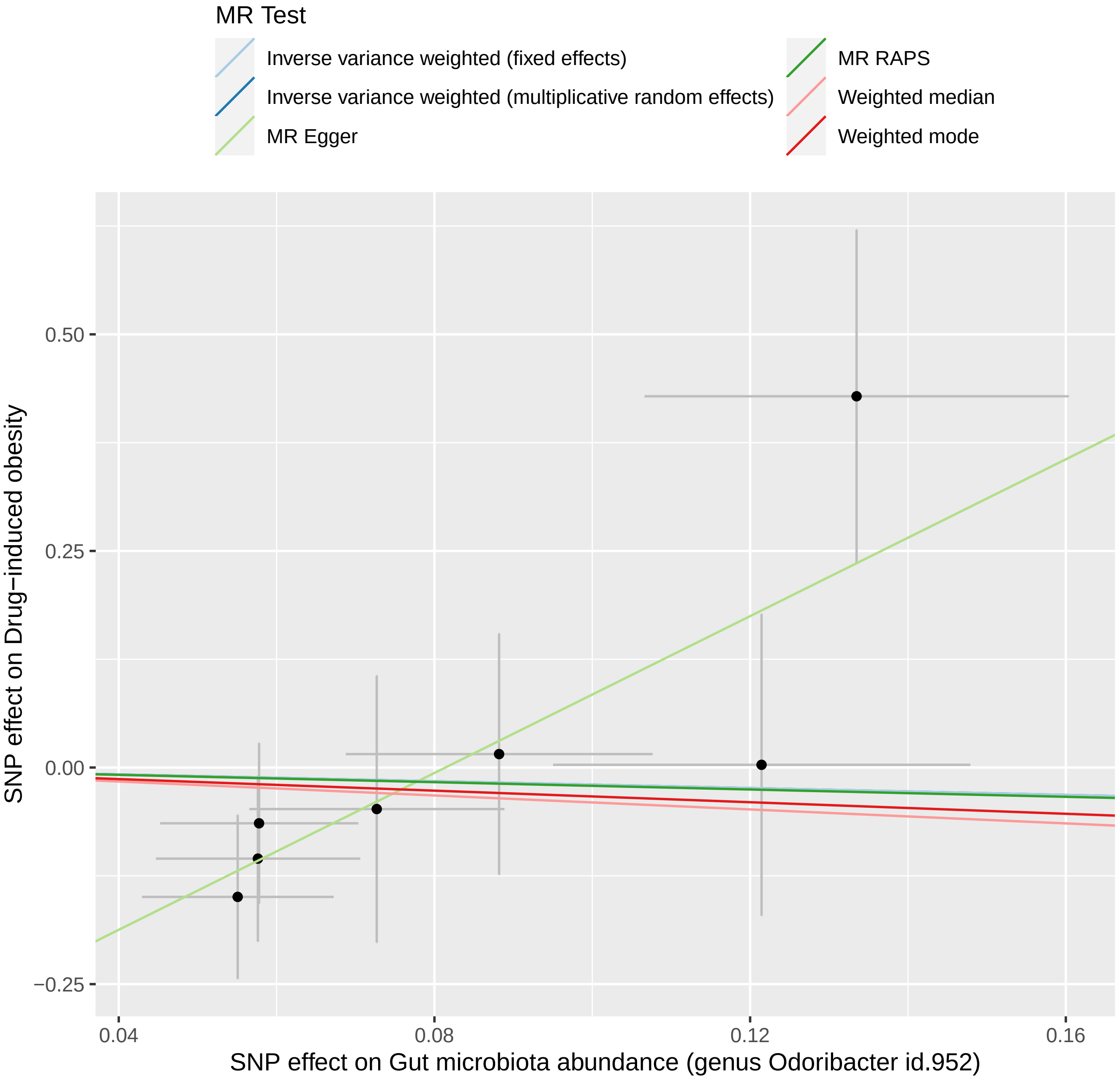

Batch 559 : Gut microbiota abundance (genus Olsenella id.822) on Drug−induced obesity

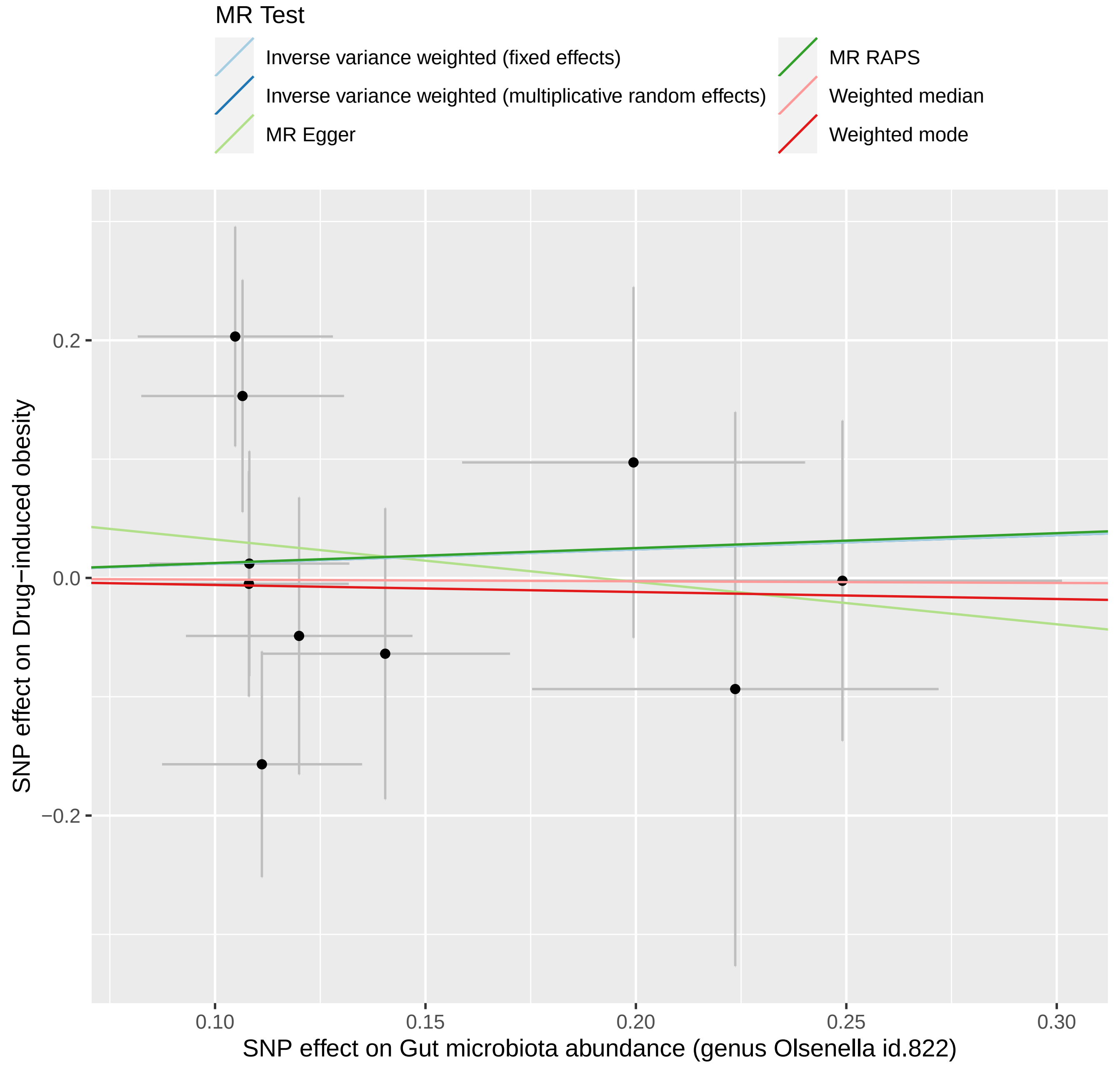

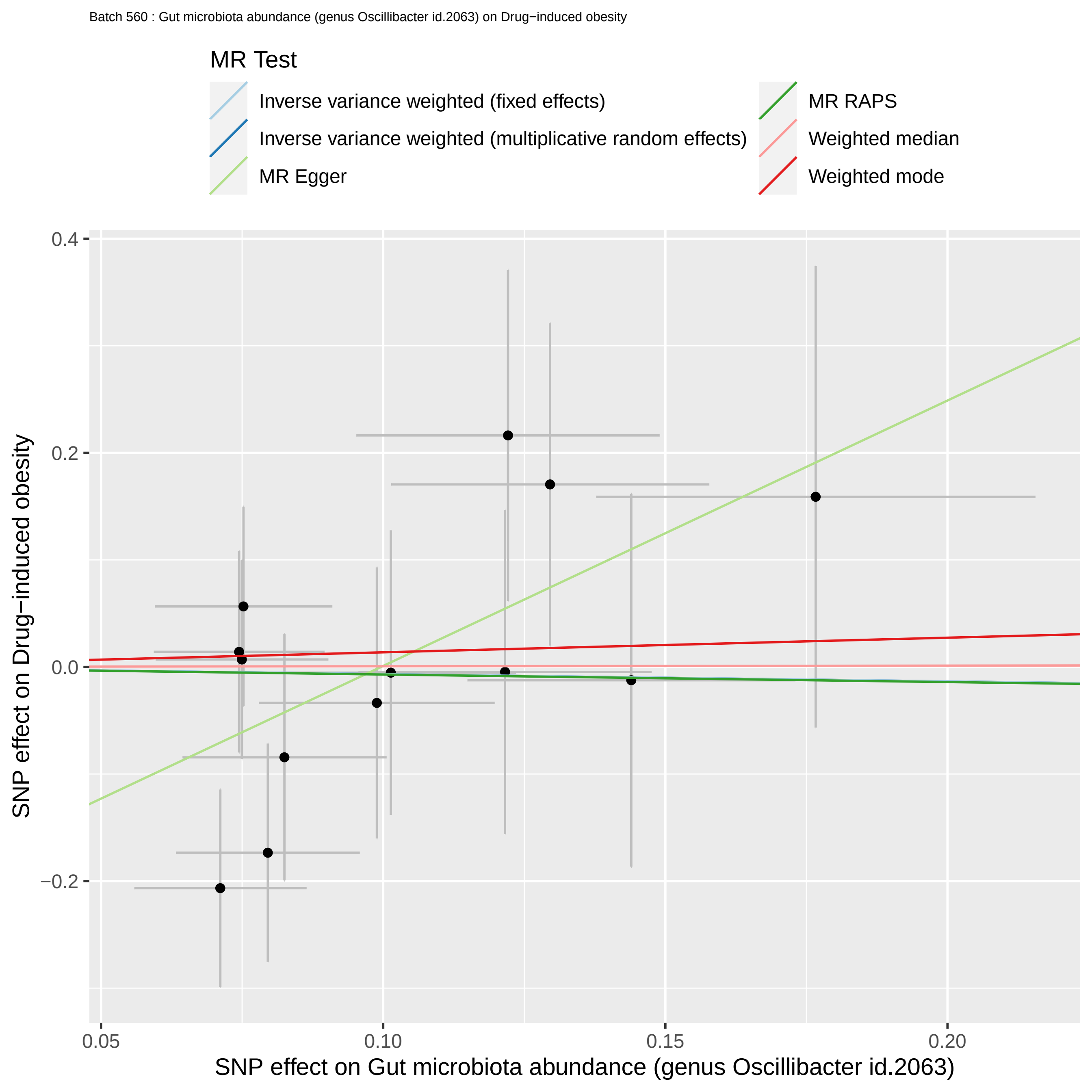

Batch 560 : Gut microbiota abundance (genus Oscillibacter id.2063) on Drug−induced obesity
MR Test
Inverse variance weighted (fixed effects)
Inverse variance weighted (multiplicative random effects)
MR Egger
MR RAPS
Weighted median
Weighted mode
SNP effect on Drug−induced obesity
0.4
0.2
0.0
−0.2
0.05
0.10
0.15
0.20
SNP effect on Gut microbiota abundance (genus Oscillibacter id.2063)

Batch 561 : Gut microbiota abundance (genus Oscillospira id.2064) on Drug−induced obesity

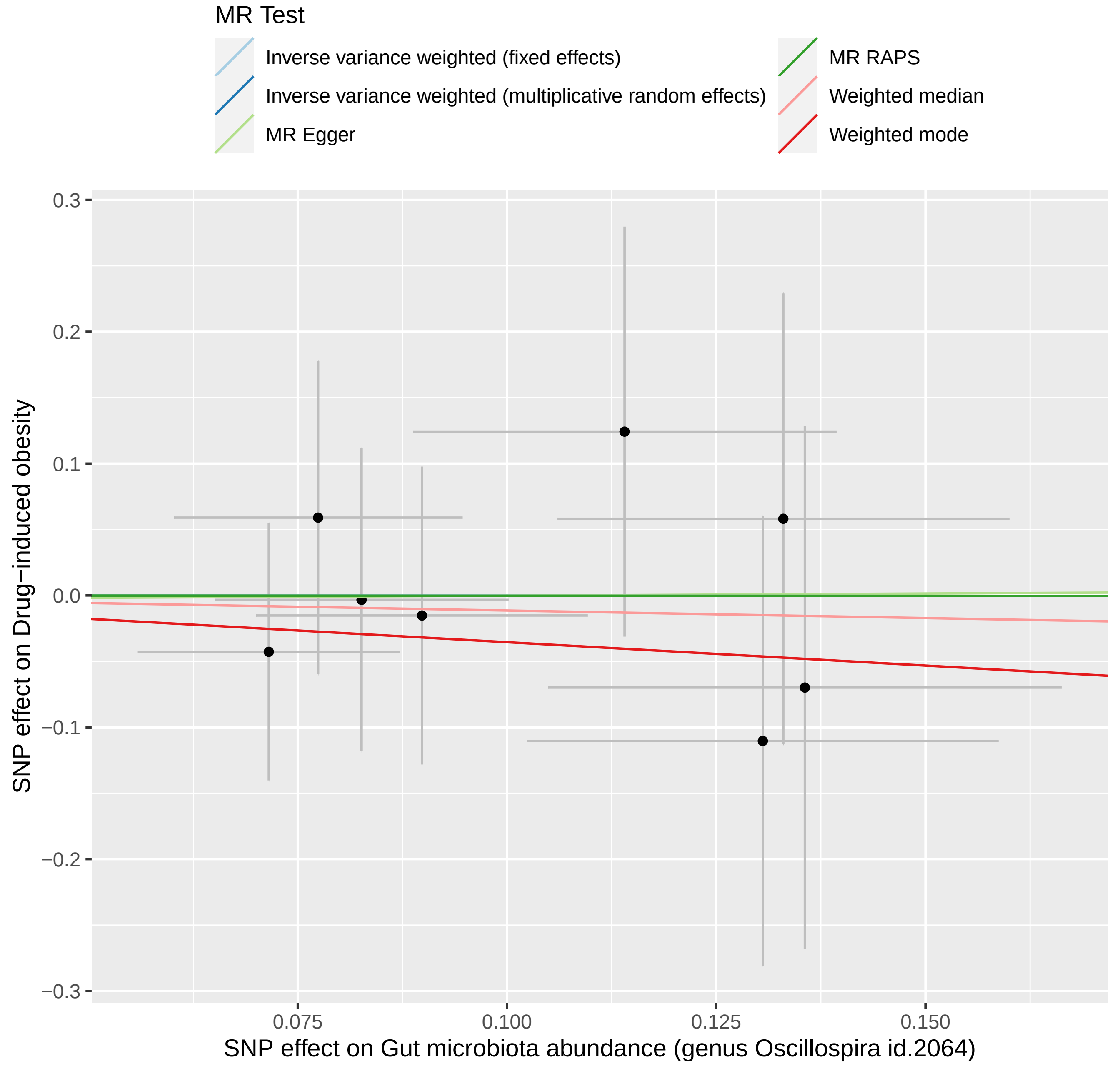

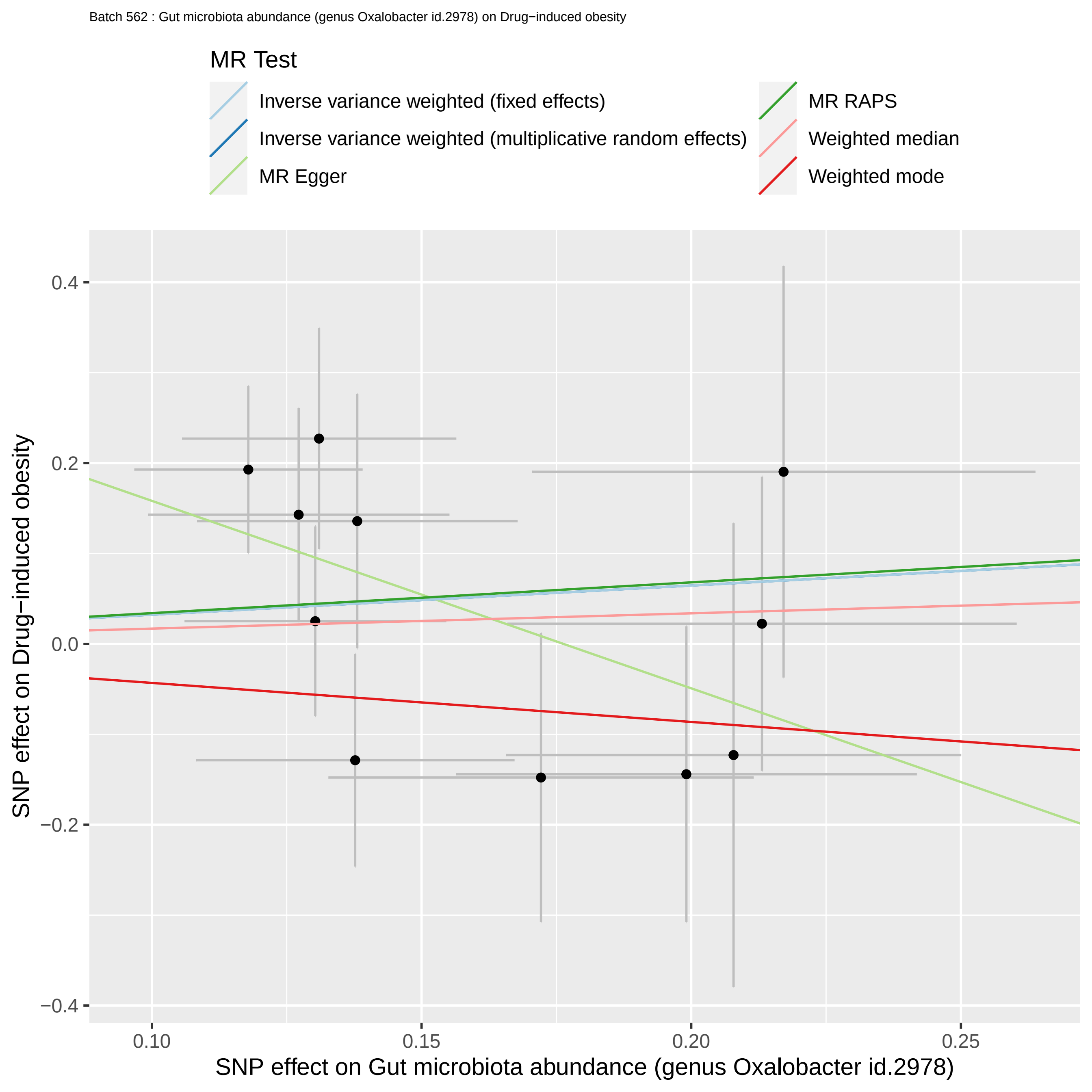

Batch 562 : Gut microbiota abundance (genus Oxalobacter id.2978) on Drug−induced obesity
MR Test
Inverse variance weighted (fixed effects)
Inverse variance weighted (multiplicative random effects)
MR Egger
MR RAPS
Weighted median
Weighted mode
0.4
0.2
0.0
−0.2
−0.4
SNP effect on Drug−induced obesity
0.10
0.15
0.20
0.25
SNP effect on Gut microbiota abundance (genus Oxalobacter id.2978)

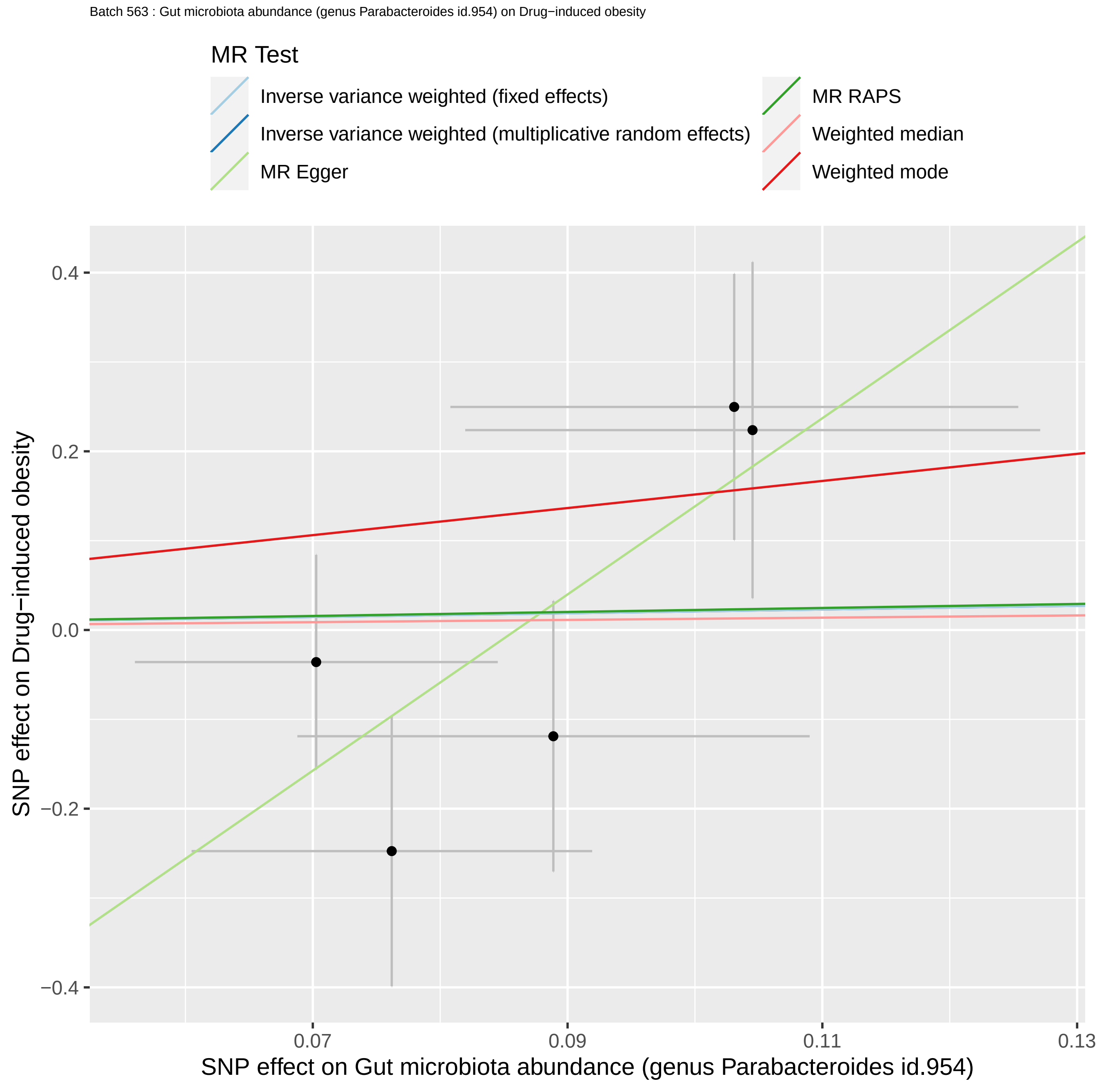
Batch 563 : Gut microbiota abundance (genus Parabacteroides id.954) on Drug−induced obesity
MR Test
Inverse variance weighted (fixed effects)
Inverse variance weighted (multiplicative random effects)
MR Egger
MR RAPS
Weighted median
Weighted mode
0.4
0.2
0.0
−0.2
−0.4
0.07
0.09
0.11
0.13
SNP effect on Drug−induced obesity
SNP effect on Gut microbiota abundance (genus Parabacteroides id.954)

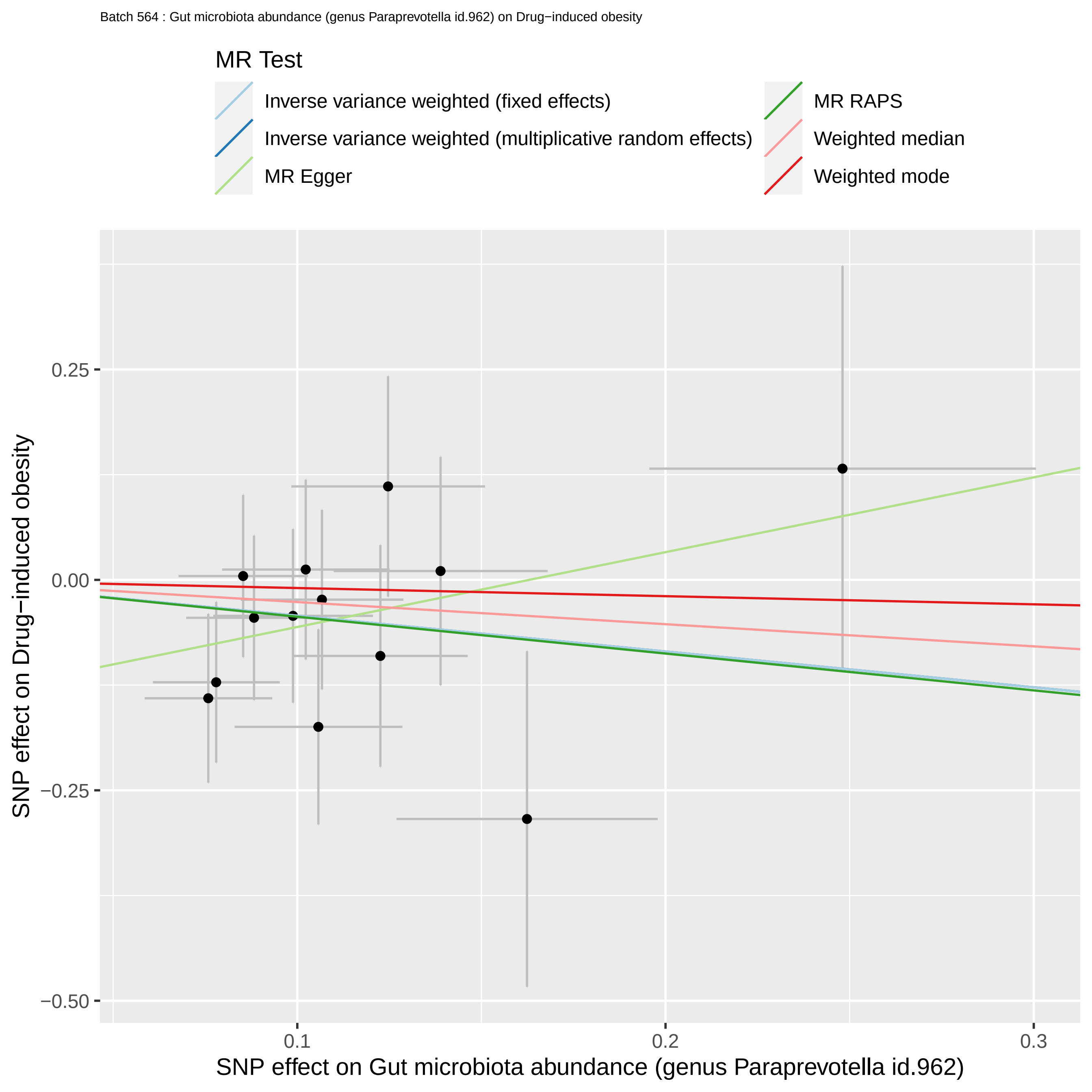
Batch 564 : Gut microbiota abundance (genus Paraprevotella id.962) on Drug−induced obesity
MR Test
Inverse variance weighted (fixed effects)
Inverse variance weighted (multiplicative random effects)
MR Egger
MR RAPS
Weighted median
Weighted mode
0.25
0.00
−0.25
−0.50
0.1
0.2
0.3
SNP effect on Drug−induced obesity
SNP effect on Gut microbiota abundance (genus Paraprevotella id.962)

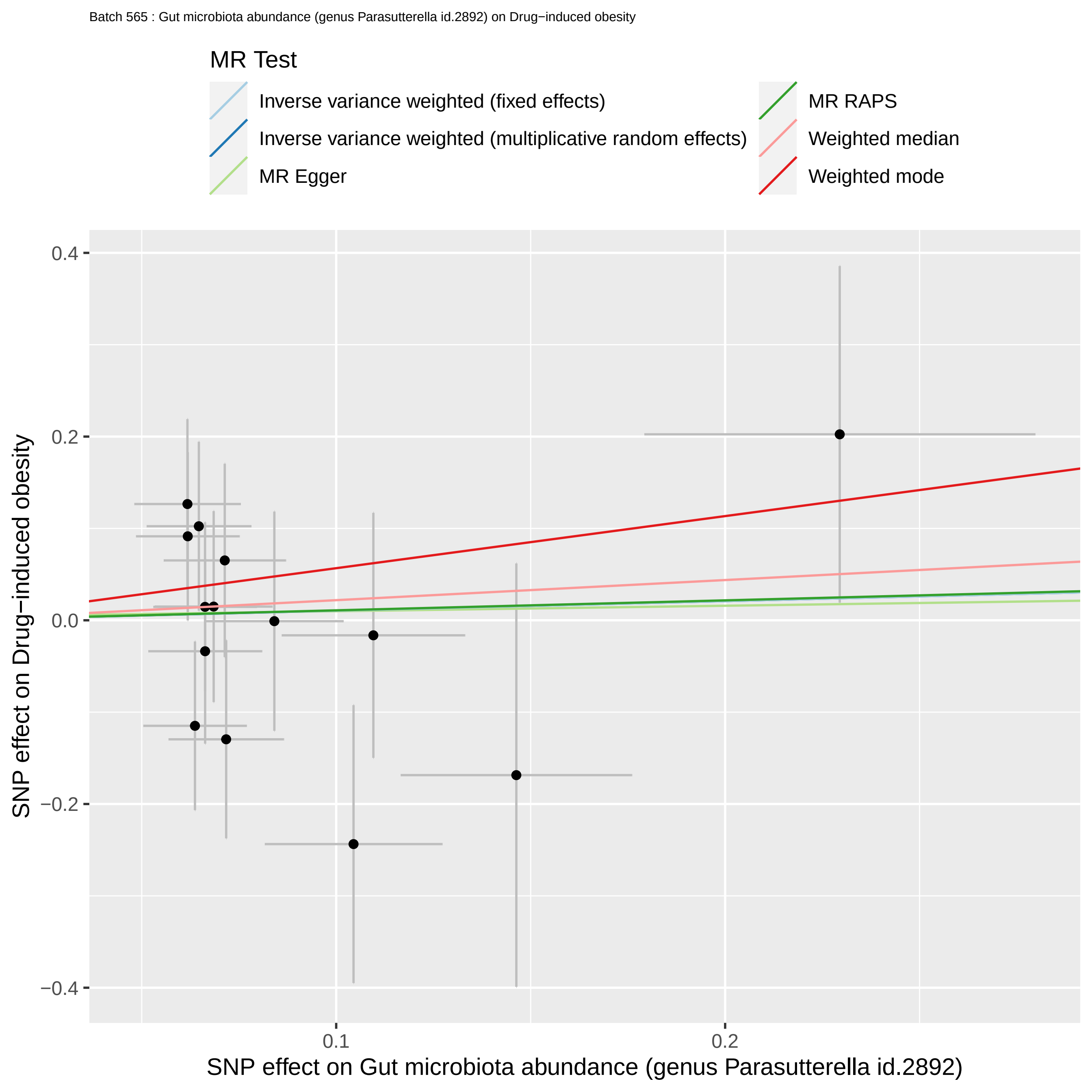

Batch 565 : Gut microbiota abundance (genus Parasutterella id.2892) on Drug−induced obesity
MR Test
Inverse variance weighted (fixed effects)
Inverse variance weighted (multiplicative random effects)
MR Egger
MR RAPS
Weighted median
Weighted mode
0.4
0.2
0.0
−0.2
−0.4
0.1
0.2
SNP effect on Drug−induced obesity
SNP effect on Gut microbiota abundance (genus Parasutterella id.2892)

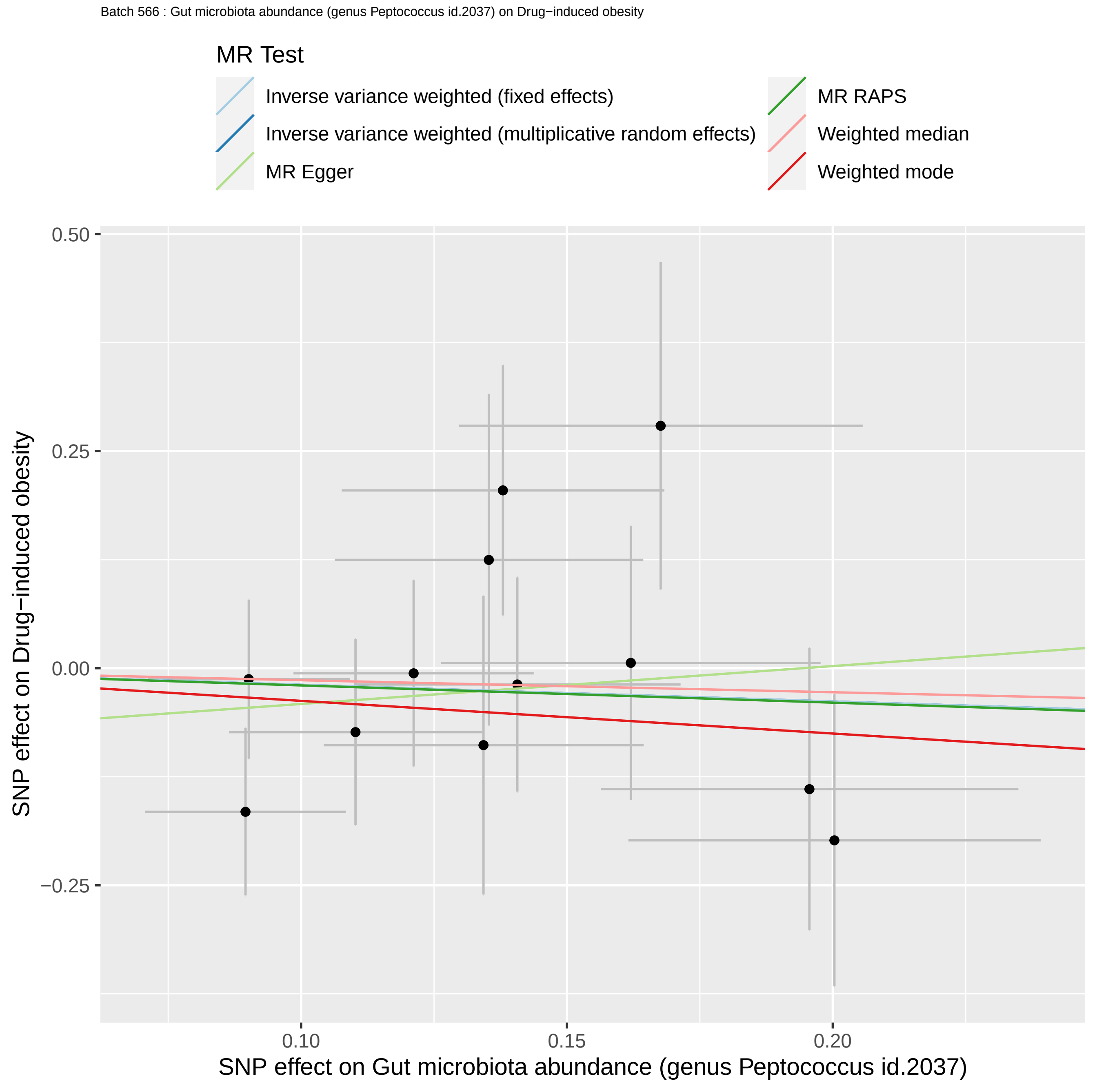

Batch 566 : Gut microbiota abundance (genus Peptococcus id.2037) on Drug−induced obesity
MR Test
Inverse variance weighted (fixed effects)
Inverse variance weighted (multiplicative random effects)
MR Egger
MR RAPS
Weighted median
Weighted mode
0.50
0.25
0.00
−0.25
SNP effect on Drug−induced obesity
0.10
0.15
0.20
SNP effect on Gut microbiota abundance (genus Peptococcus id.2037)

Batch 567 : Gut microbiota abundance (genus Phascolarctobacterium id.2168) on Drug−induced obesity

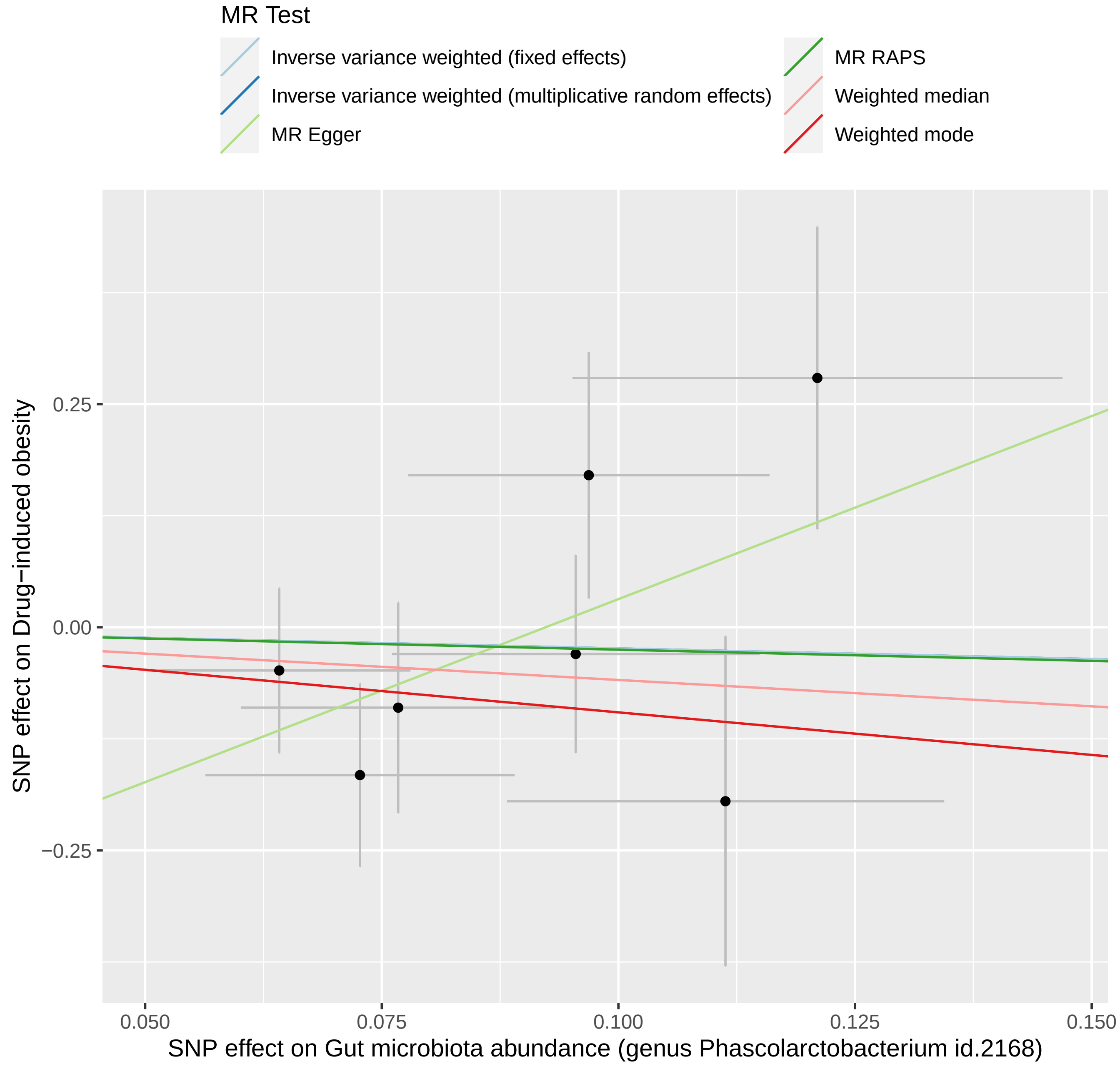

Batch 568 : Gut microbiota abundance (genus Prevotella7 id.11182) on Drug−induced obesity

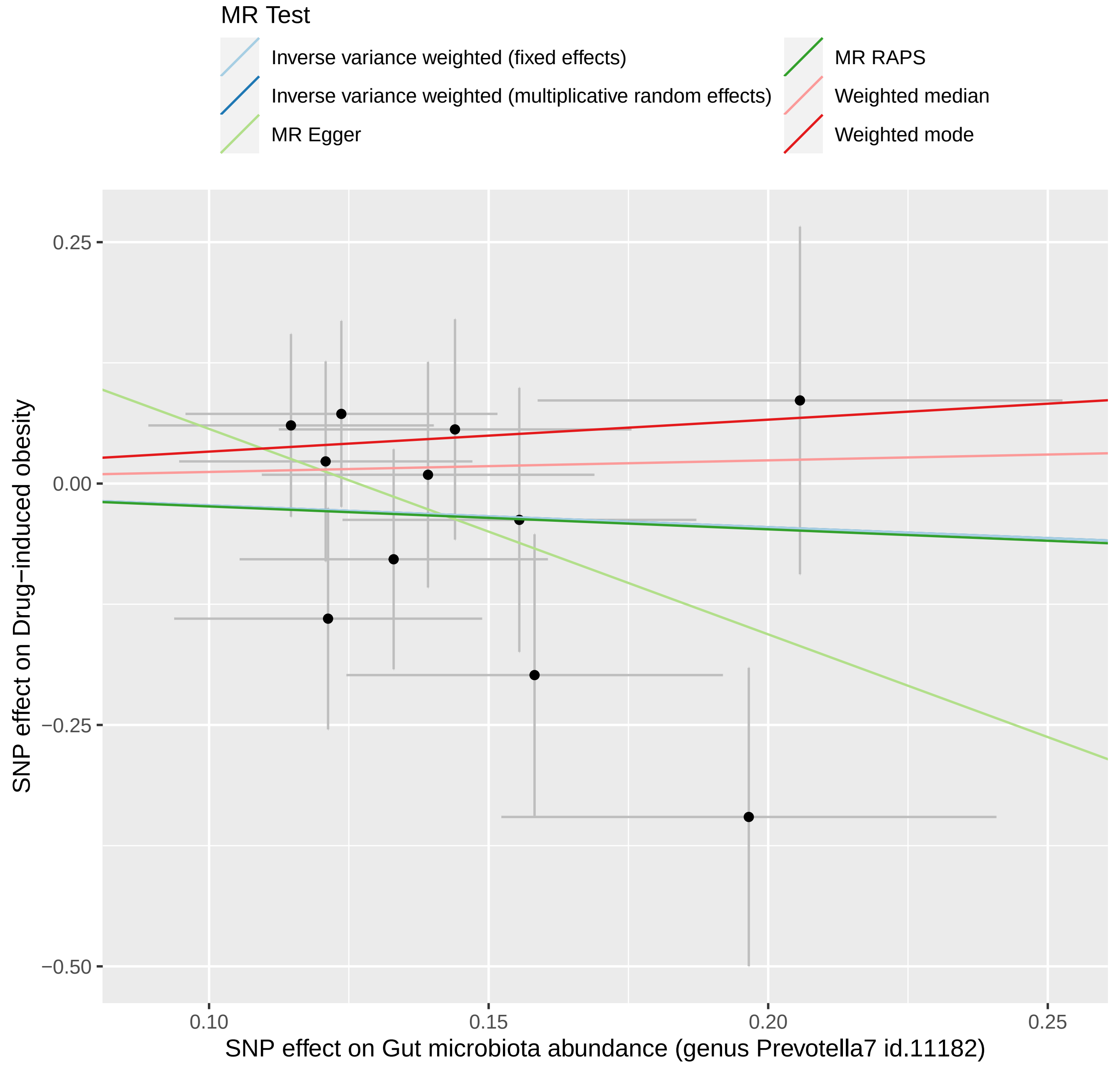

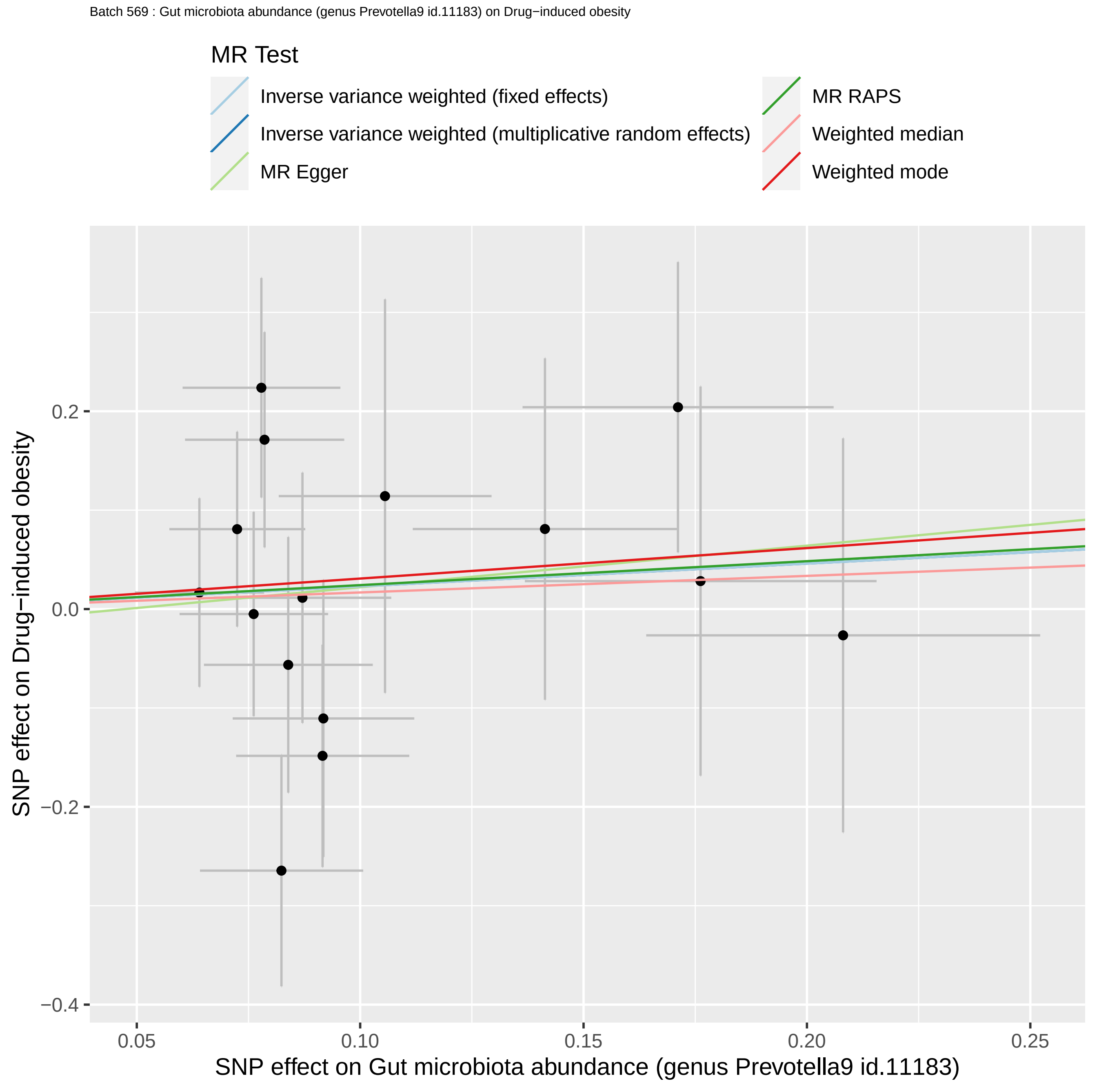

Batch 569 : Gut microbiota abundance (genus Prevotella9 id.11183) on Drug−induced obesity
MR Test
Inverse variance weighted (fixed effects)
Inverse variance weighted (multiplicative random effects)
MR Egger
MR RAPS
Weighted median
Weighted mode
SNP effect on Drug−induced obesity
0.2
0.0
−0.2
−0.4
0.05
0.10
0.15
0.20
0.25
SNP effect on Gut microbiota abundance (genus Prevotella9 id.11183)

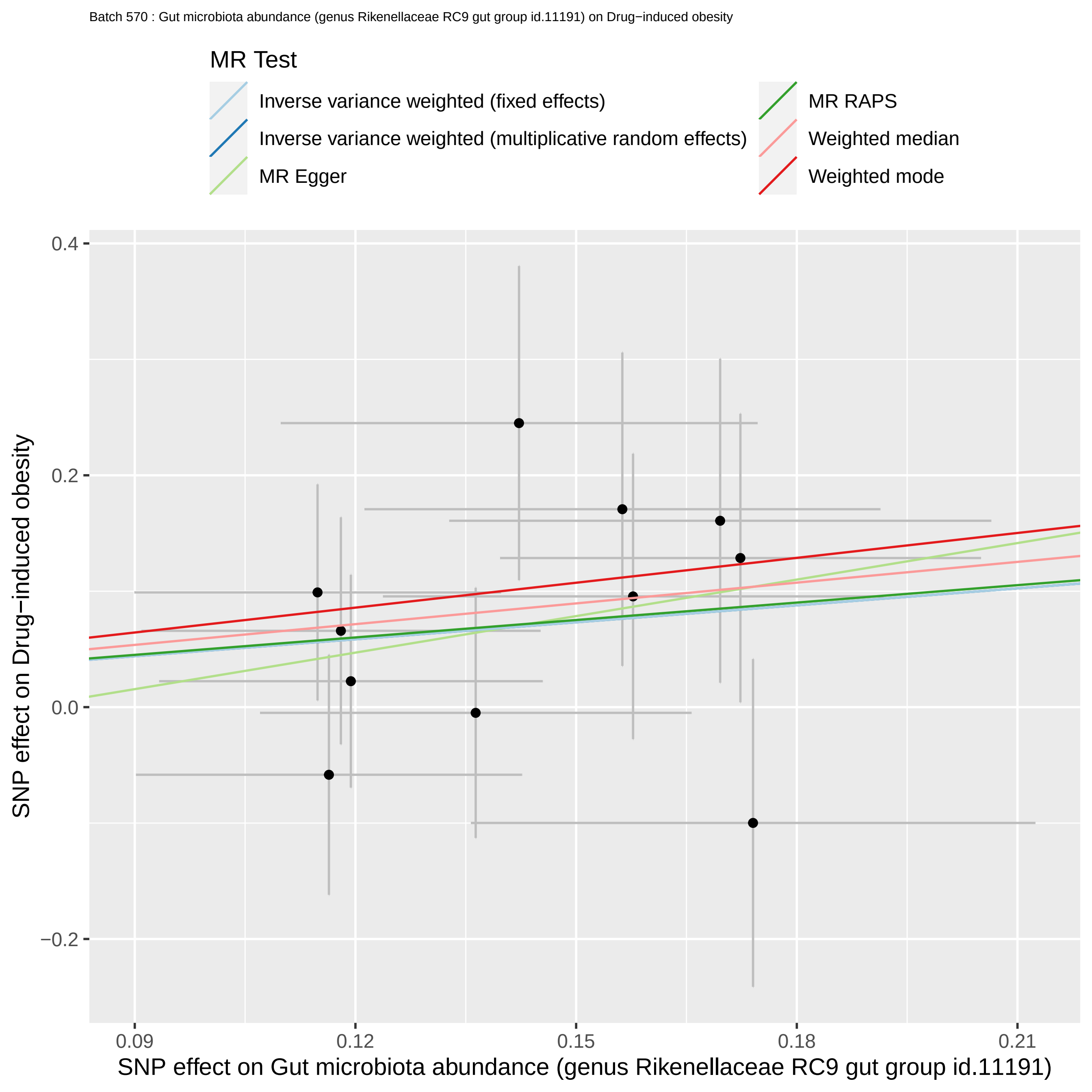

Batch 570 : Gut microbiota abundance (genus Rikenellaceae RC9 gut group id.11191) on Drug−induced obesity
MR Test
Inverse variance weighted (fixed effects)
Inverse variance weighted (multiplicative random effects)
MR Egger
MR RAPS
Weighted median
Weighted mode
0.4
0.2
0.0
−0.2
0.09
0.12
0.15
0.18
0.21
SNP effect on Drug−induced obesity
SNP effect on Gut microbiota abundance (genus Rikenellaceae RC9 gut group id.11191)

Batch 571 : Gut microbiota abundance (genus Romboutsia id.11347) on Drug−induced obesity

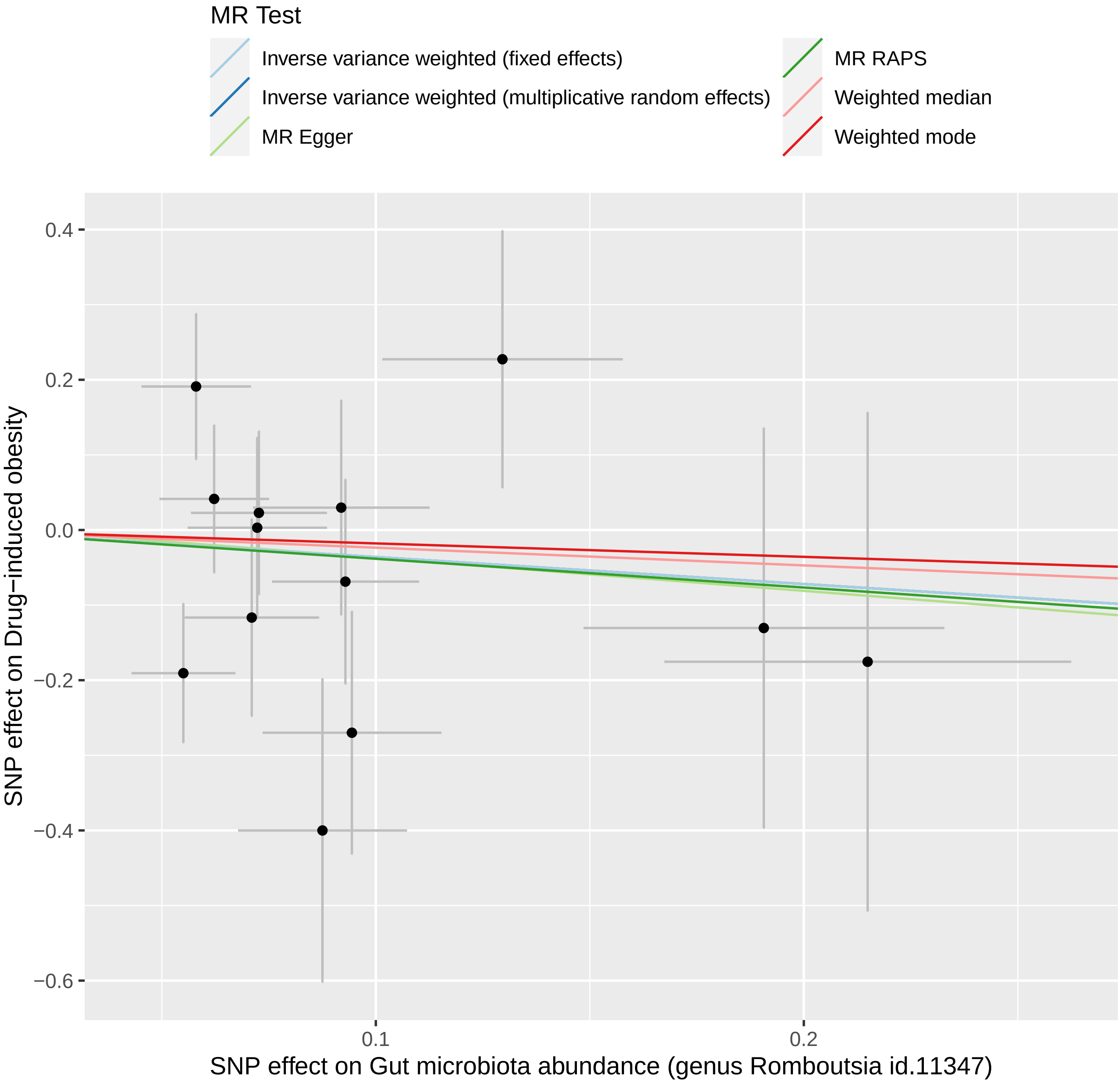

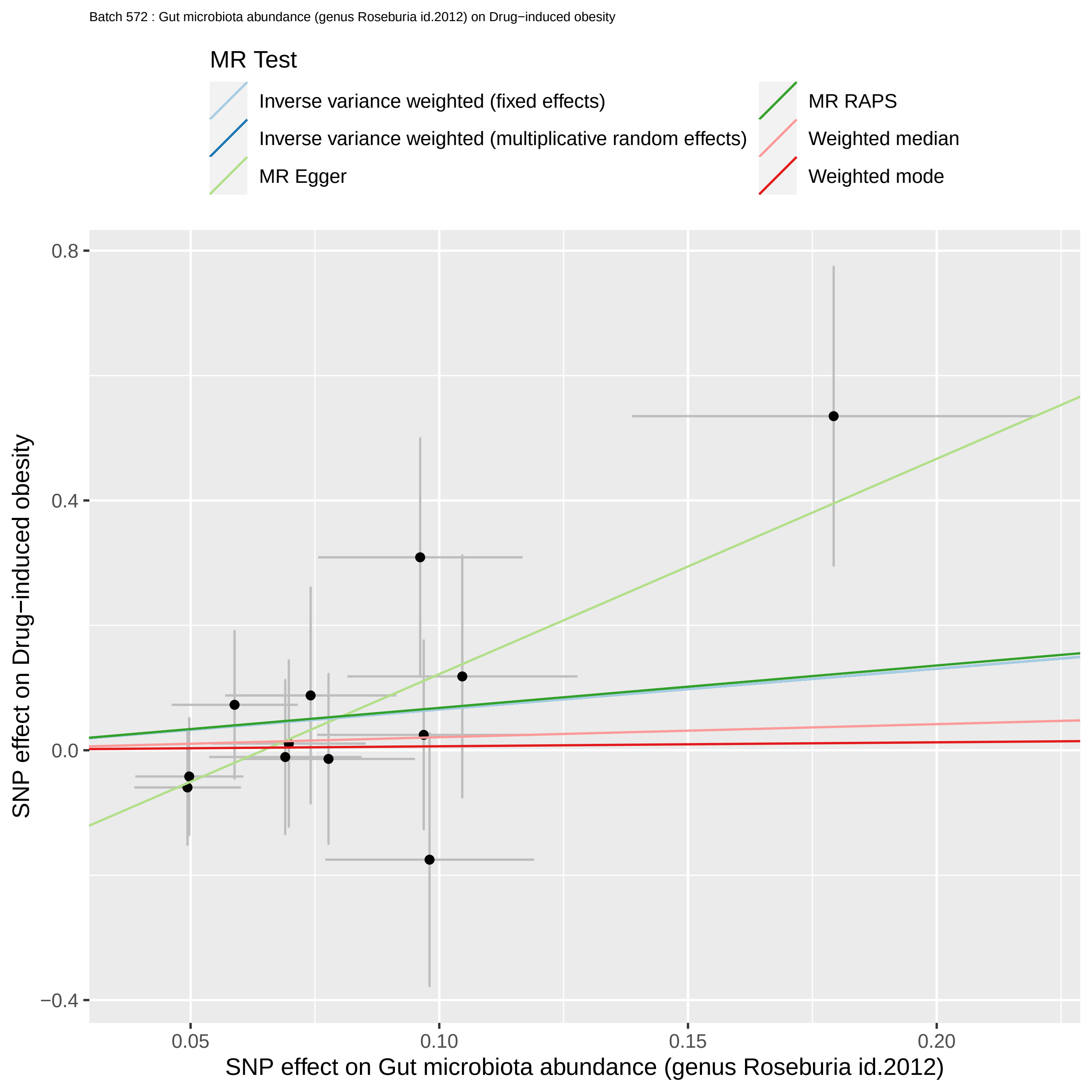

Batch 572 : Gut microbiota abundance (genus Roseburia id.2012) on Drug−induced obesity
MR Test
Inverse variance weighted (fixed effects)
Inverse variance weighted (multiplicative random effects)
MR Egger
MR RAPS
Weighted median
Weighted mode
0.8
0.4
0.0
−0.4
0.05
0.10
0.15
0.20
SNP effect on Drug−induced obesity
SNP effect on Gut microbiota abundance (genus Roseburia id.2012)

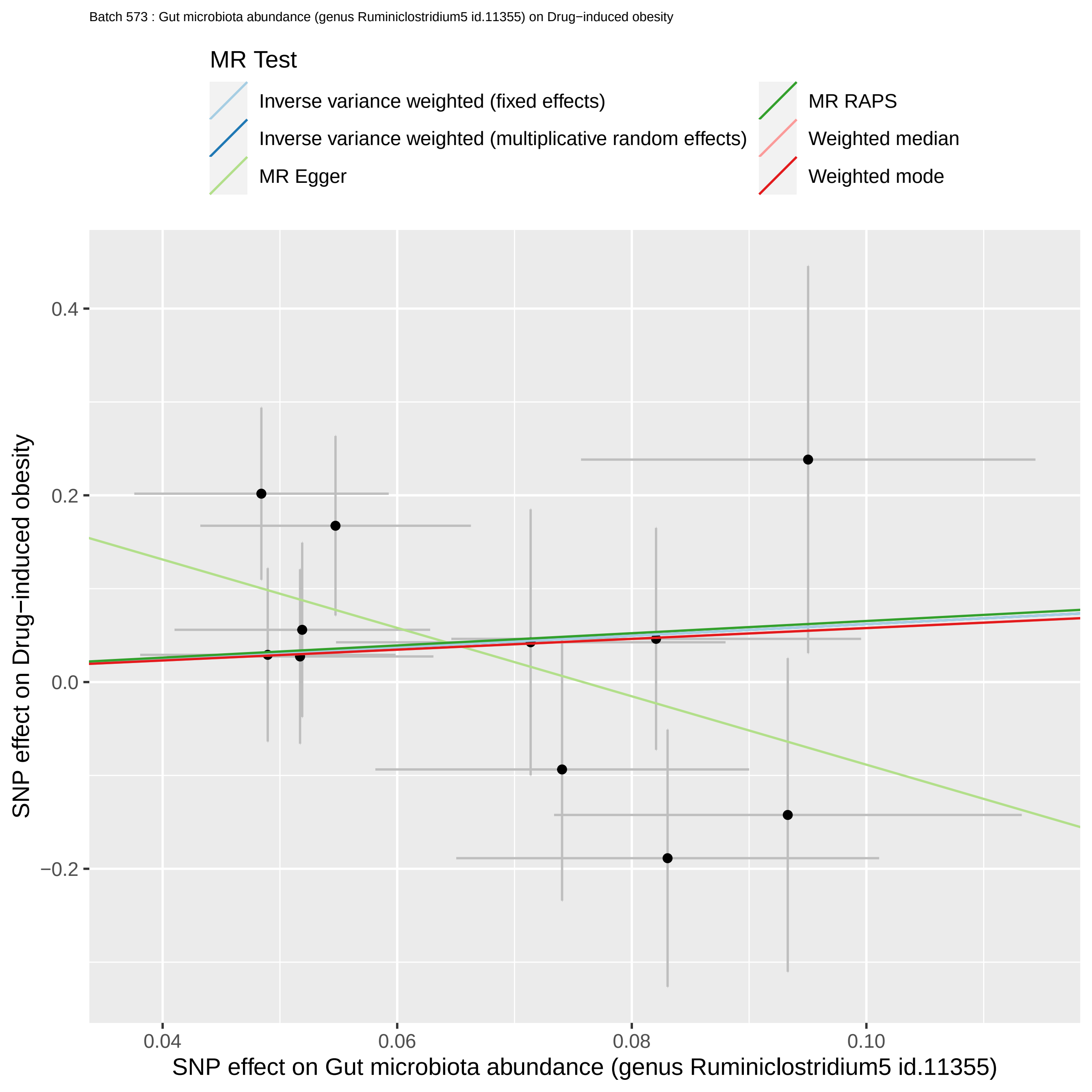
Batch 573 : Gut microbiota abundance (genus Ruminiclostridium5 id.11355) on Drug−induced obesity
MR Test
Inverse variance weighted (fixed effects)
Inverse variance weighted (multiplicative random effects)
MR Egger
MR RAPS
Weighted median
Weighted mode
0.4
0.2
0.0
−0.2
0.04
0.06
0.08
0.10
SNP effect on Drug−induced obesity
SNP effect on Gut microbiota abundance (genus Ruminiclostridium5 id.11355)

Batch 574 : Gut microbiota abundance (genus Ruminiclostridium6 id.11356) on Drug−induced obesity

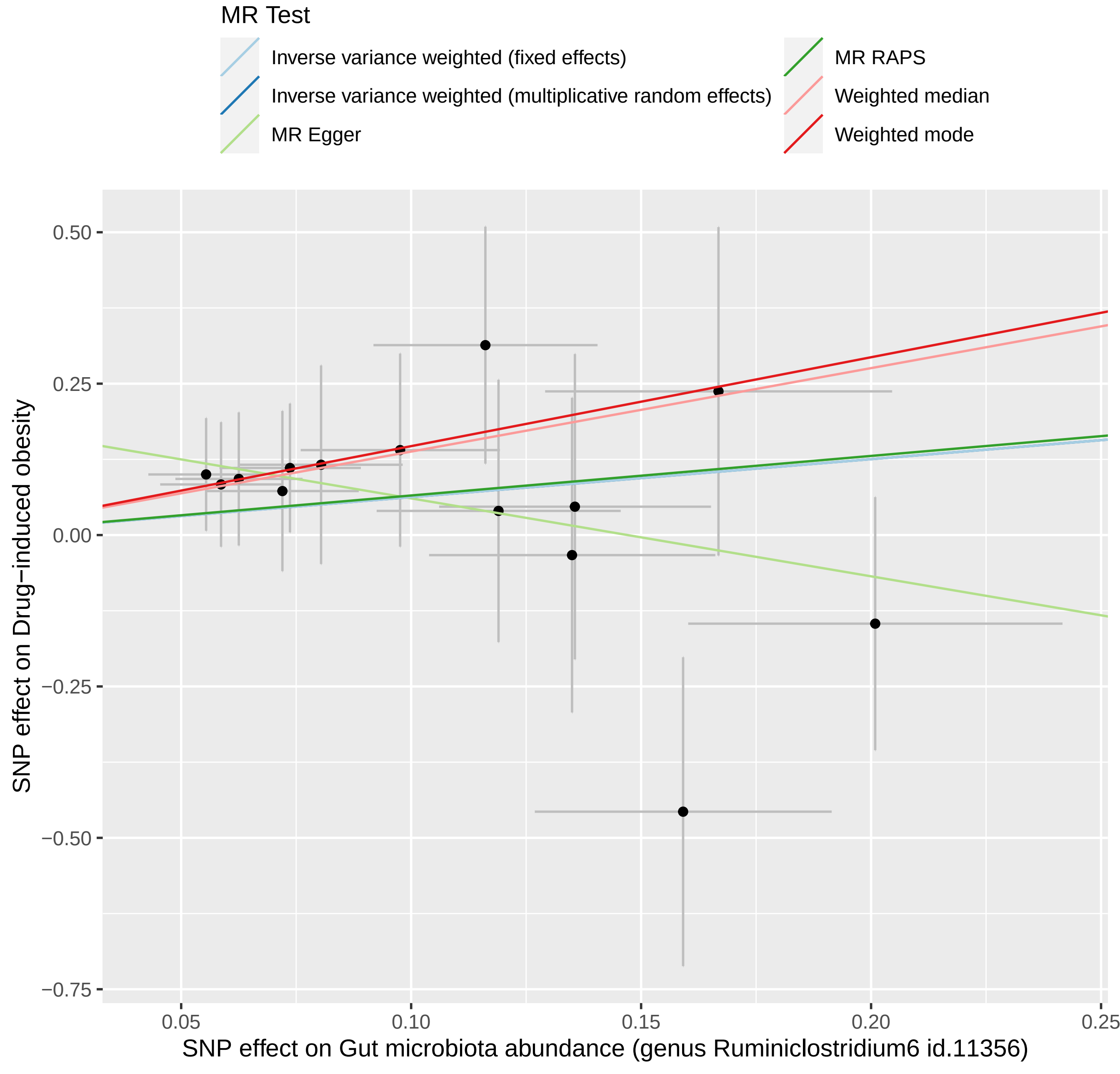

Batch 575 : Gut microbiota abundance (genus Ruminiclostridium9 id.11357) on Drug−induced obesity

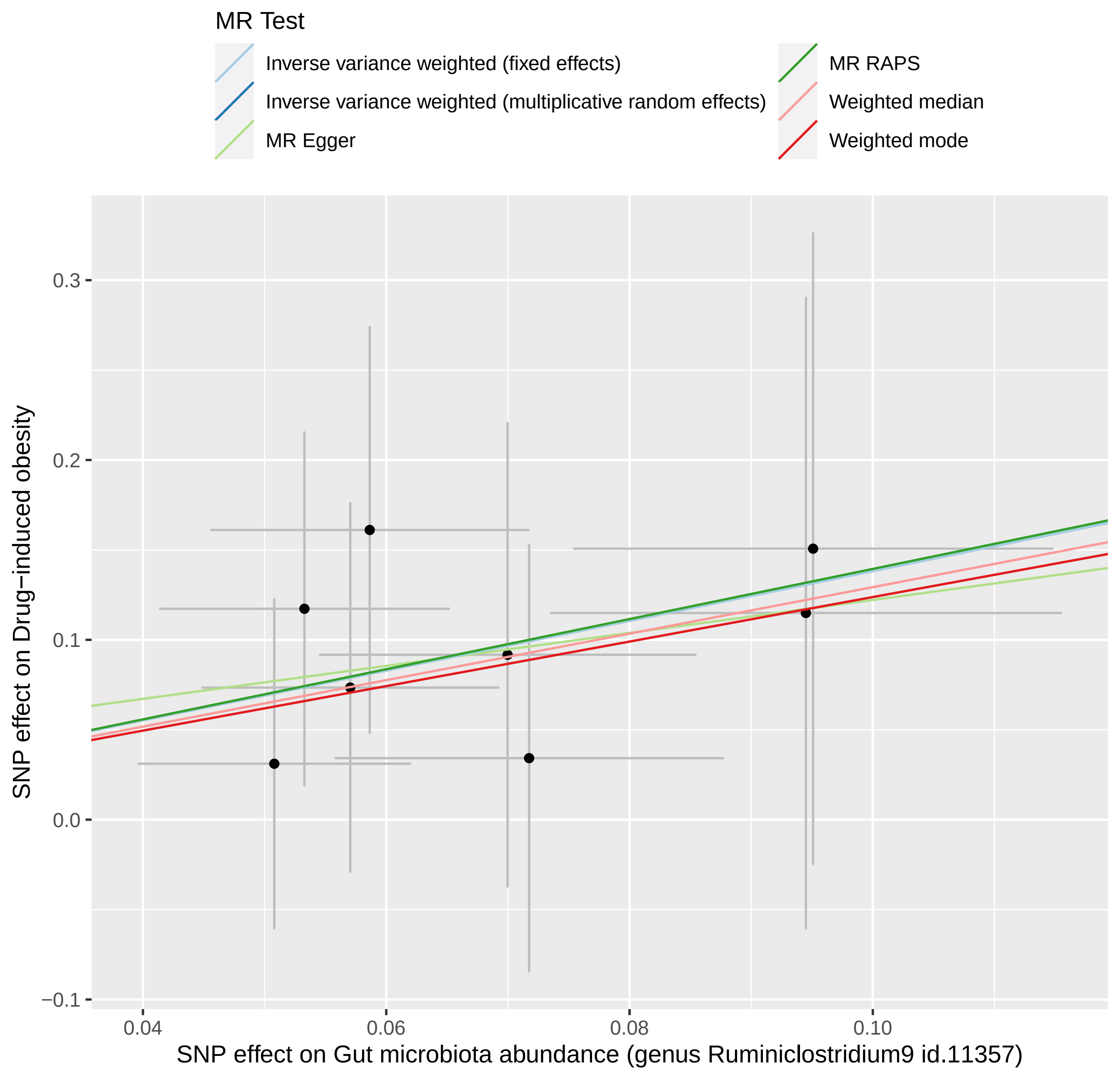

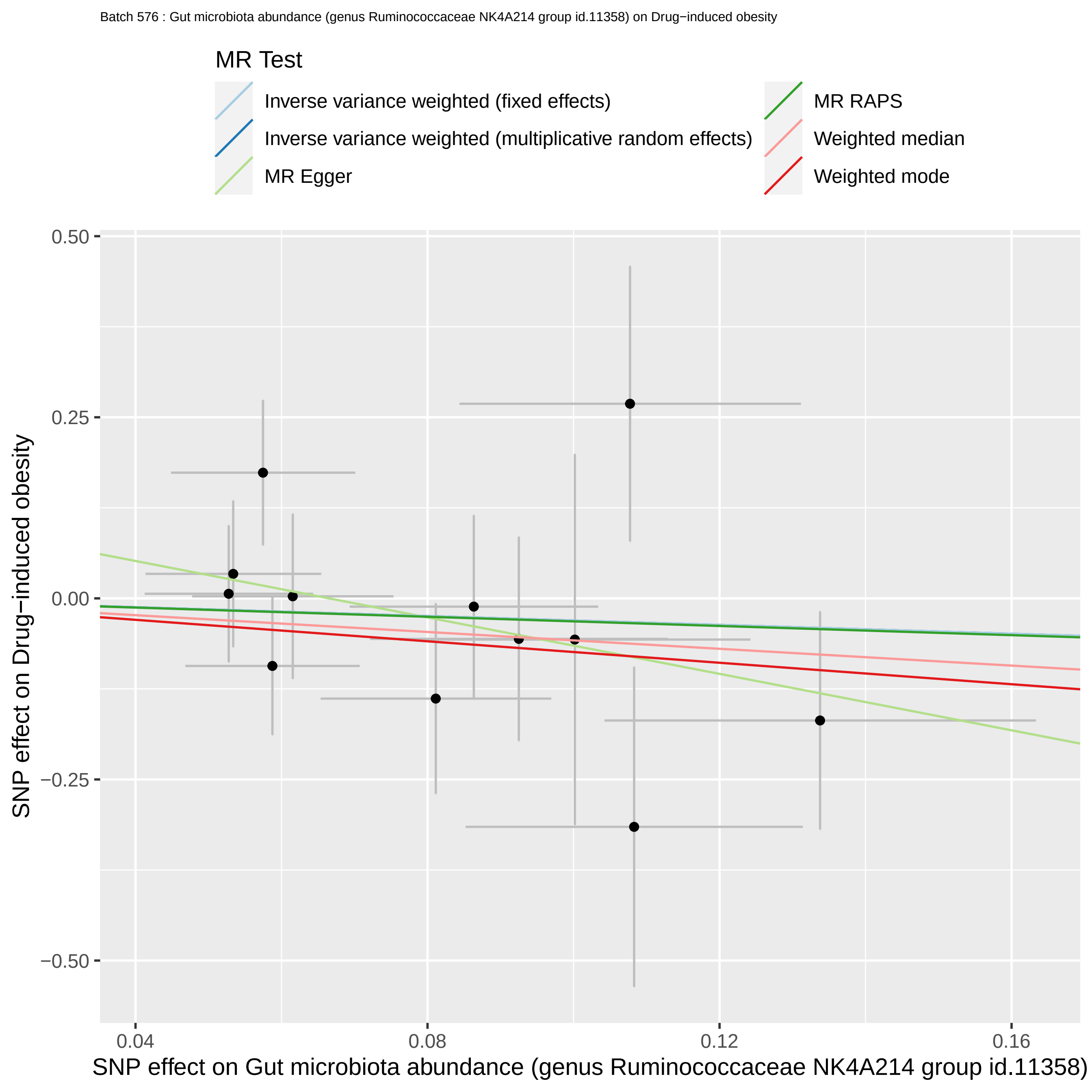

Batch 576 : Gut microbiota abundance (genus Ruminococcaceae NK4A214 group id.11358) on Drug−induced obesity
MR Test
Inverse variance weighted (fixed effects)
Inverse variance weighted (multiplicative random effects)
MR Egger
MR RAPS
Weighted median
Weighted mode
0.50
0.25
0.00
−0.25
−0.50
SNP effect on Drug−induced obesity
0.04
0.08
0.12
0.16
SNP effect on Gut microbiota abundance (genus Ruminococcaceae NK4A214 group id.11358)

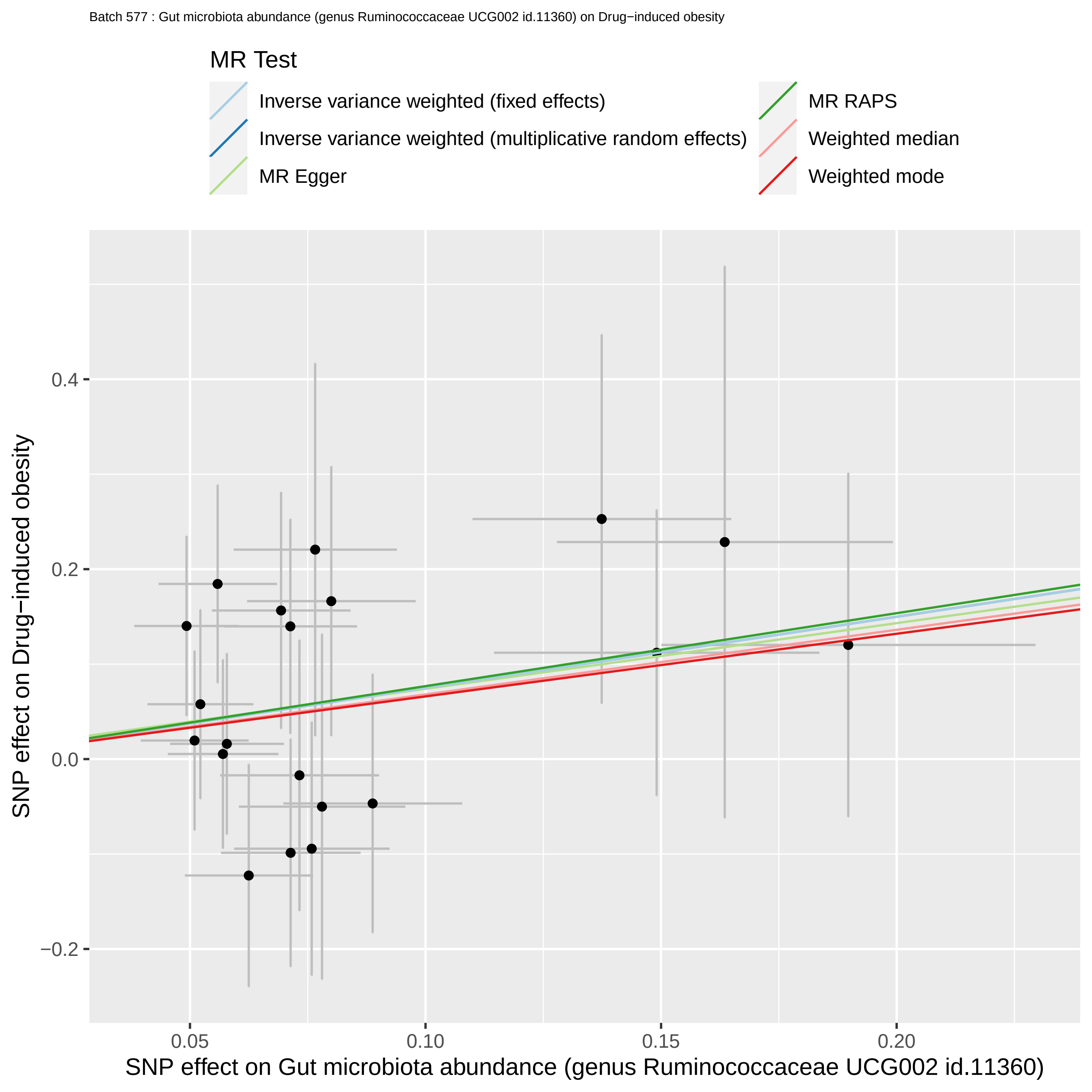
Batch 577 : Gut microbiota abundance (genus Ruminococcaceae UCG002 id.11360) on Drug−induced obesity
MR Test
Inverse variance weighted (fixed effects)
Inverse variance weighted (multiplicative random effects)
MR Egger
MR RAPS
Weighted median
Weighted mode
SNP effect on Drug−induced obesity
0.4
0.2
0.0
−0.2
0.05
0.10
0.15
0.20
SNP effect on Gut microbiota abundance (genus Ruminococcaceae UCG002 id.11360)

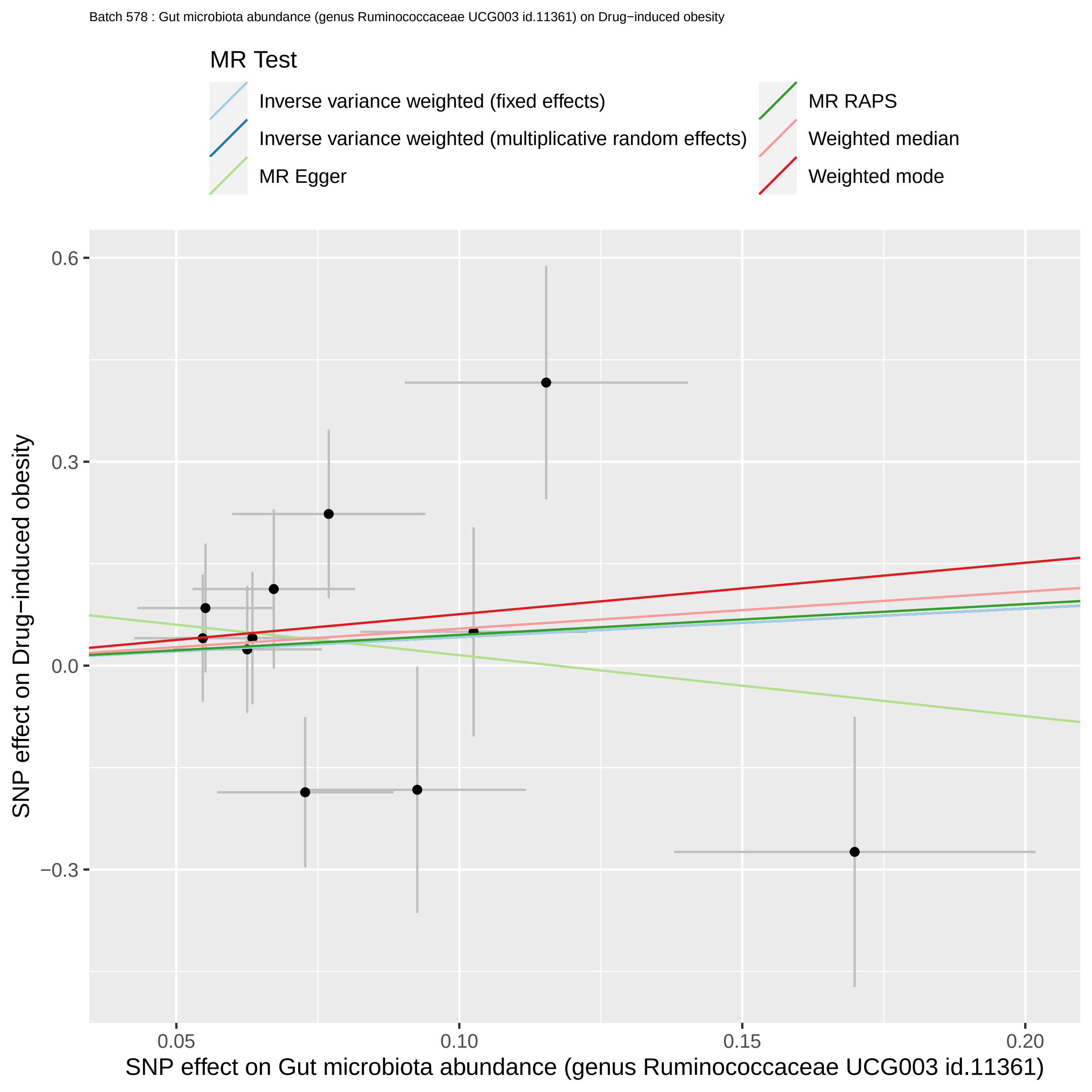

Batch 578 : Gut microbiota abundance (genus Ruminococcaceae UCG003 id.11361) on Drug−induced obesity
MR Test
Inverse variance weighted (fixed effects)
Inverse variance weighted (multiplicative random effects)
MR Egger
MR RAPS
Weighted median
Weighted mode
0.6
0.3
0.0
−0.3
0.05
0.10
0.15
0.20
SNP effect on Drug−induced obesity
SNP effect on Gut microbiota abundance (genus Ruminococcaceae UCG003 id.11361)

Batch 579 : Gut microbiota abundance (genus Ruminococcaceae UCG004 id.11362) on Drug−induced obesity

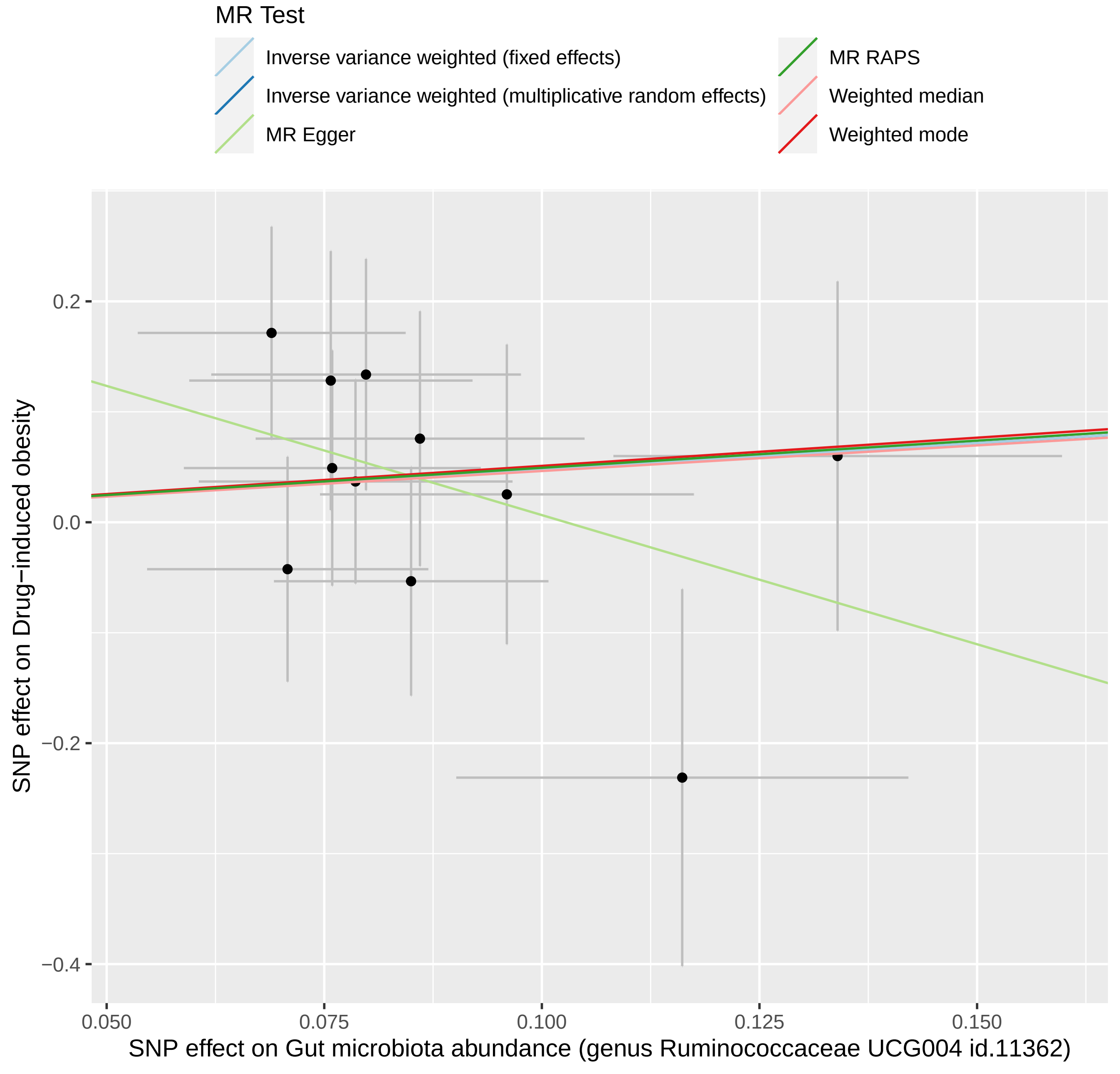

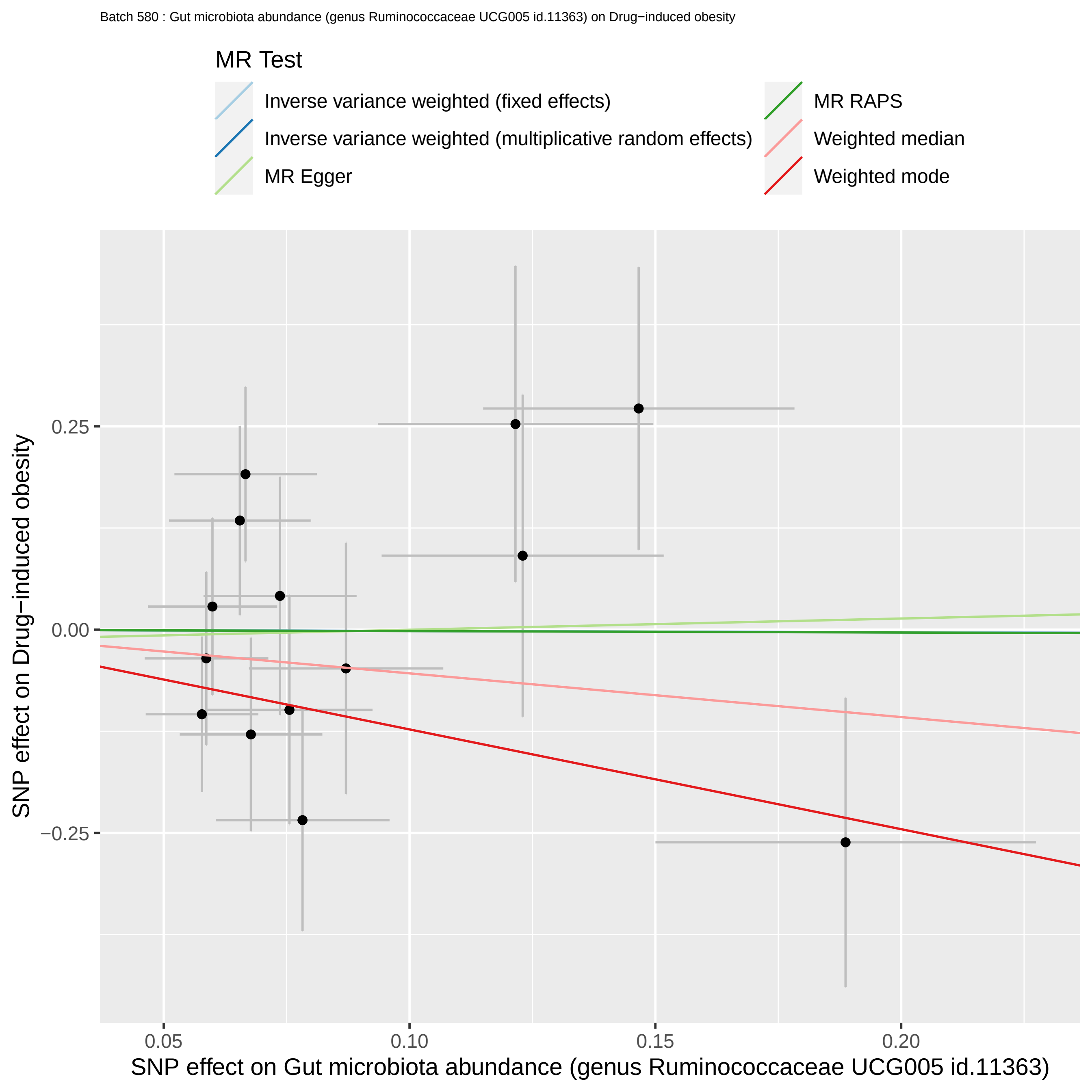

Batch 580 : Gut microbiota abundance (genus Ruminococcaceae UCG005 id.11363) on Drug−induced obesity
MR Test
Inverse variance weighted (fixed effects)
Inverse variance weighted (multiplicative random effects)
MR Egger
MR RAPS
Weighted median
Weighted mode
SNP effect on Drug−induced obesity
0.25
0.00
−0.25
0.05
0.10
0.15
0.20
SNP effect on Gut microbiota abundance (genus Ruminococcaceae UCG005 id.11363)

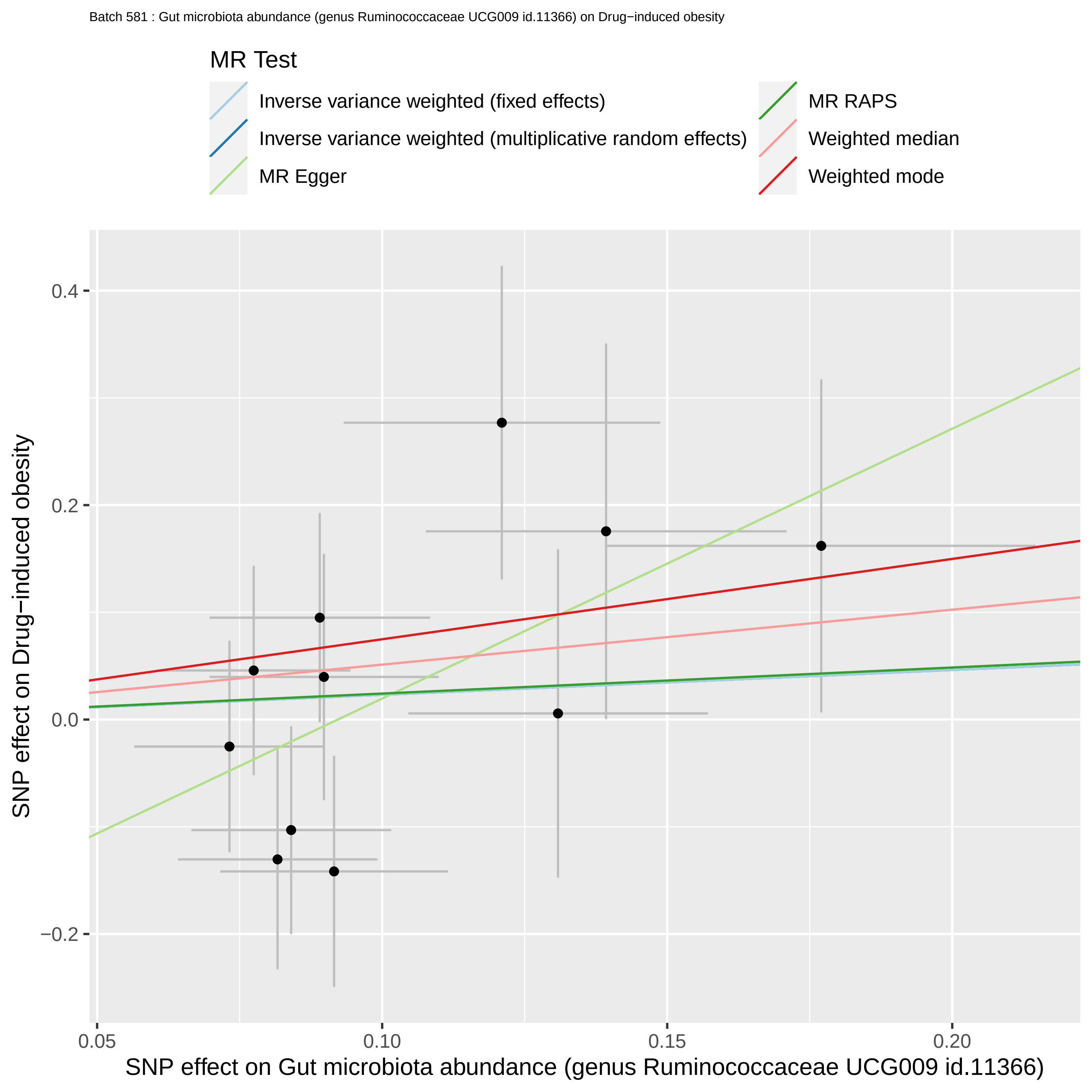
Batch 581 : Gut microbiota abundance (genus Ruminococcaceae UCG009 id.11366) on Drug−induced obesity
MR Test
Inverse variance weighted (fixed effects)
Inverse variance weighted (multiplicative random effects)
MR Egger
MR RAPS
Weighted median
Weighted mode
0.4
0.2
0.0
−0.2
0.05
0.10
0.15
0.20
SNP effect on Drug−induced obesity
SNP effect on Gut microbiota abundance (genus Ruminococcaceae UCG009 id.11366)

Batch 582 : Gut microbiota abundance (genus Ruminococcaceae UCG010 id.11367) on Drug−induced obesity

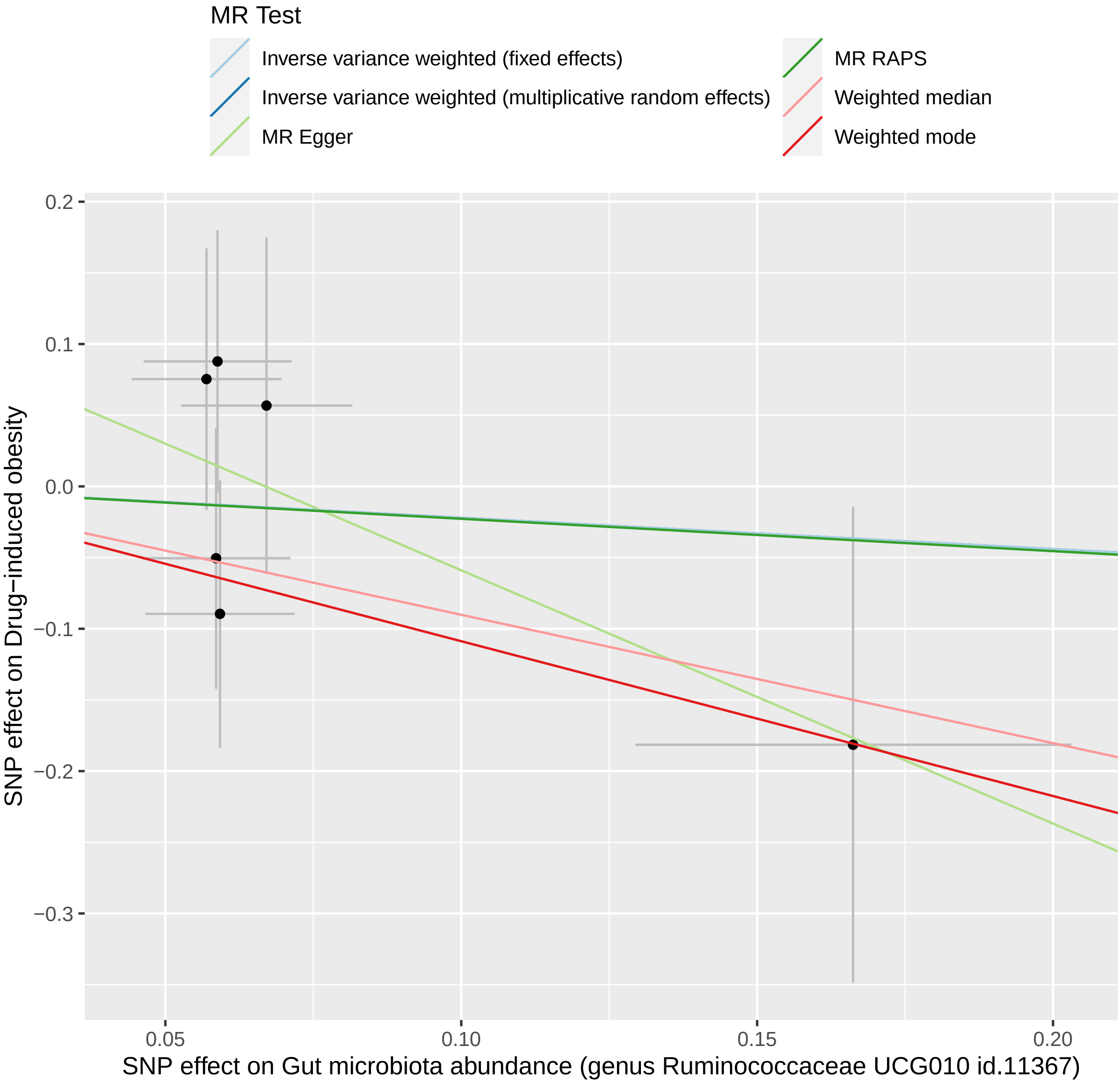

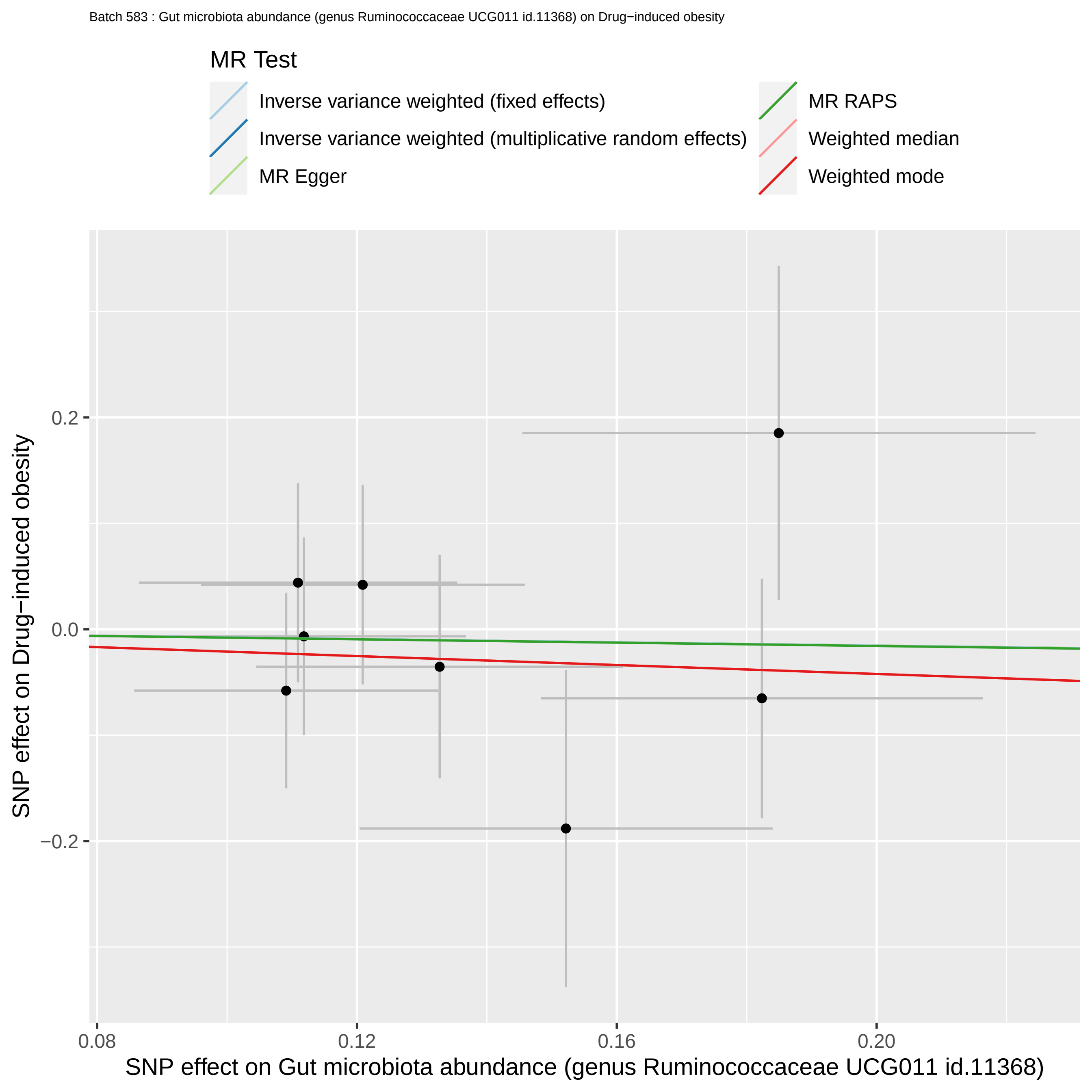
Batch 583 : Gut microbiota abundance (genus Ruminococcaceae UCG011 id.11368) on Drug−induced obesity
MR Test
Inverse variance weighted (fixed effects)
Inverse variance weighted (multiplicative random effects)
MR Egger
MR RAPS
Weighted median
Weighted mode
0.2
0.0
−0.2
0.08
0.12
0.16
0.20
SNP effect on Drug−induced obesity
SNP effect on Gut microbiota abundance (genus Ruminococcaceae UCG011 id.11368)

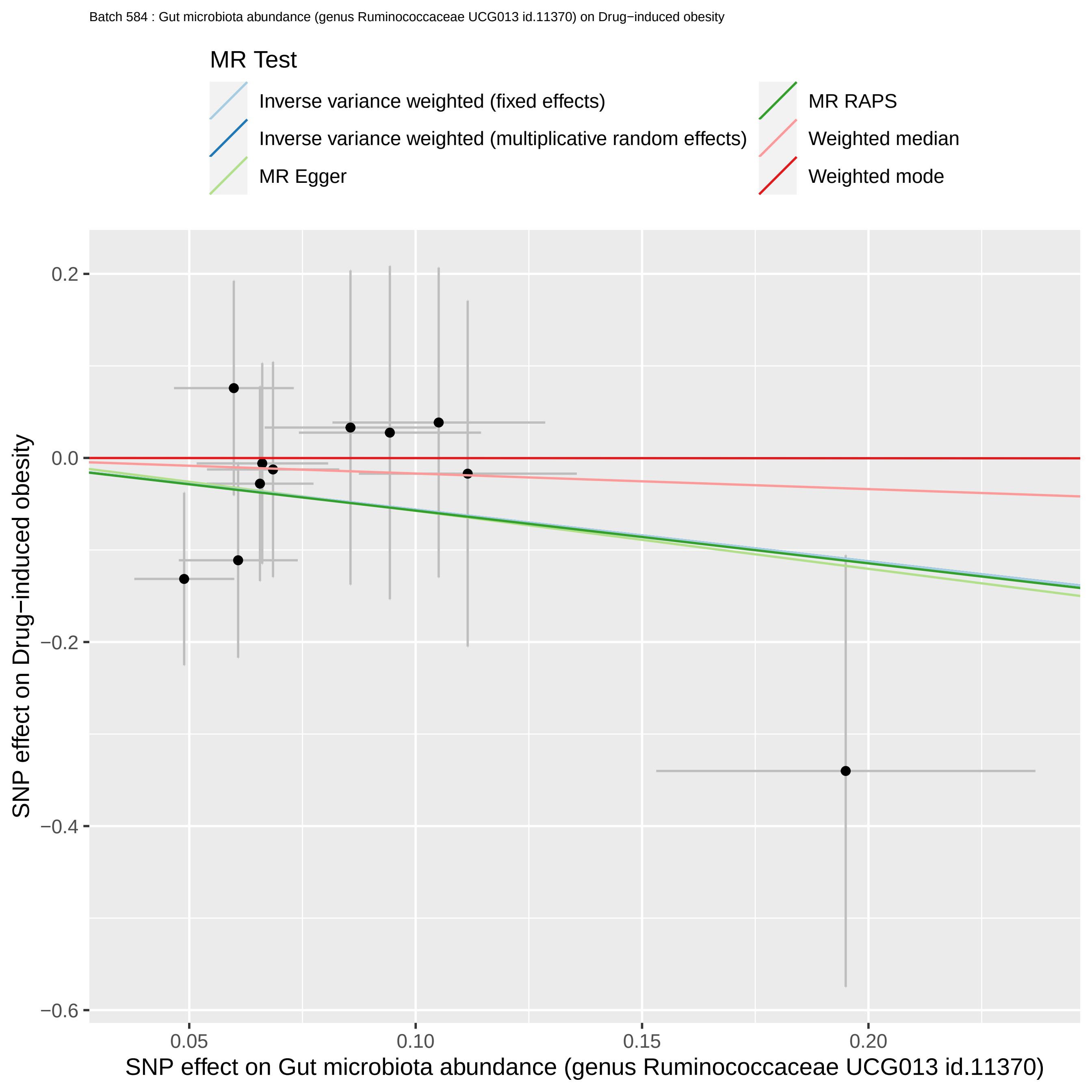
Batch 584 : Gut microbiota abundance (genus Ruminococcaceae UCG013 id.11370) on Drug−induced obesity
MR Test
Inverse variance weighted (fixed effects)
Inverse variance weighted (multiplicative random effects)
MR Egger
MR RAPS
Weighted median
Weighted mode
0.2
0.0
−0.2
−0.4
−0.6
0.05
0.10
0.15
0.20
SNP effect on Drug−induced obesity
SNP effect on Gut microbiota abundance (genus Ruminococcaceae UCG013 id.11370)

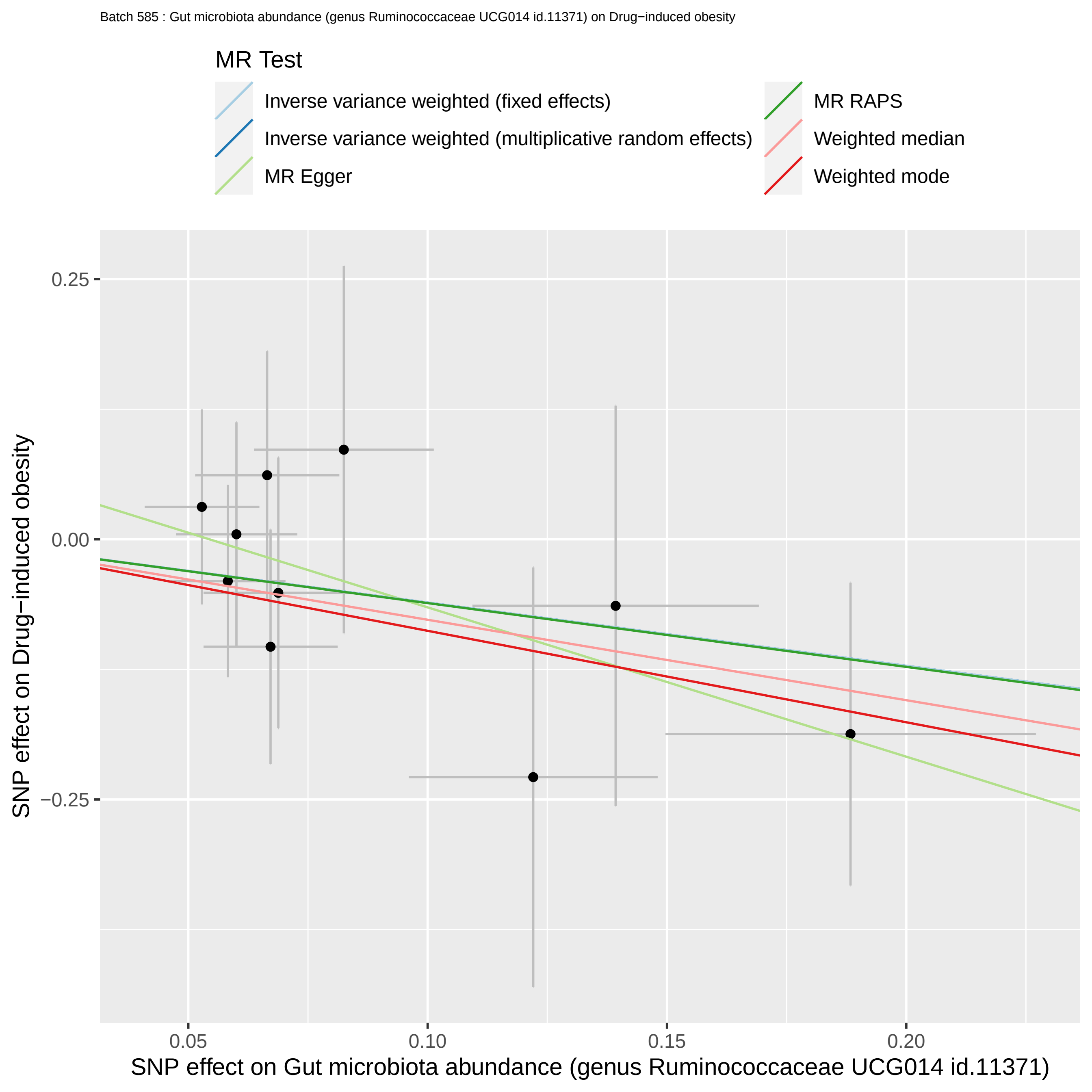
Batch 585 : Gut microbiota abundance (genus Ruminococcaceae UCG014 id.11371) on Drug−induced obesity
MR Test
Inverse variance weighted (fixed effects)
Inverse variance weighted (multiplicative random effects)
MR Egger
MR RAPS
Weighted median
Weighted mode
0.25
0.00
−0.25
0.05
0.10
0.15
0.20
SNP effect on Drug−induced obesity
SNP effect on Gut microbiota abundance (genus Ruminococcaceae UCG014 id.11371)

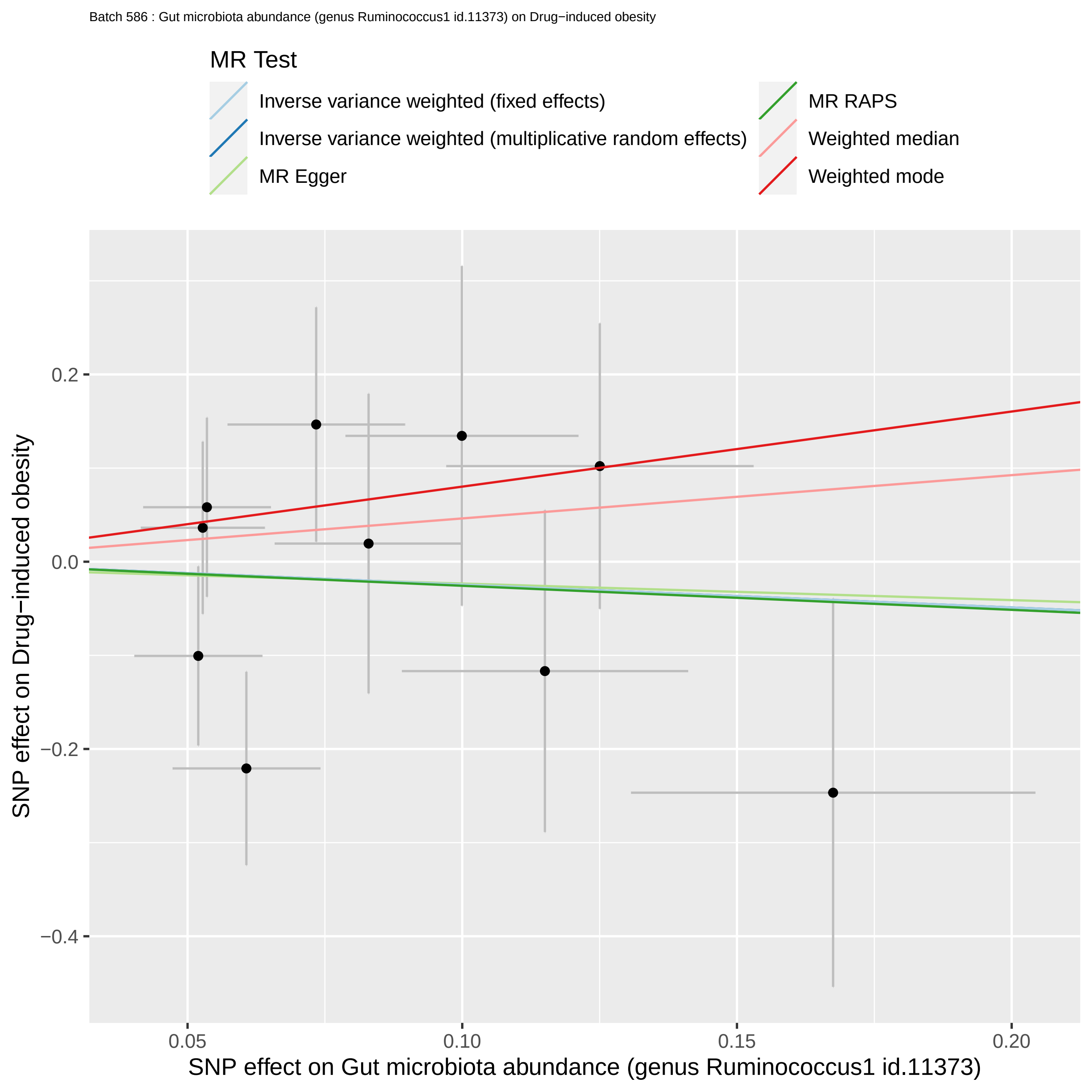

Batch 586 : Gut microbiota abundance (genus Ruminococcus1 id.11373) on Drug−induced obesity
MR Test
Inverse variance weighted (fixed effects)
Inverse variance weighted (multiplicative random effects)
MR Egger
MR RAPS
Weighted median
Weighted mode
SNP effect on Drug−induced obesity
0.2
0.0
−0.2
−0.4
0.05
0.10
0.15
0.20
SNP effect on Gut microbiota abundance (genus Ruminococcus1 id.11373)

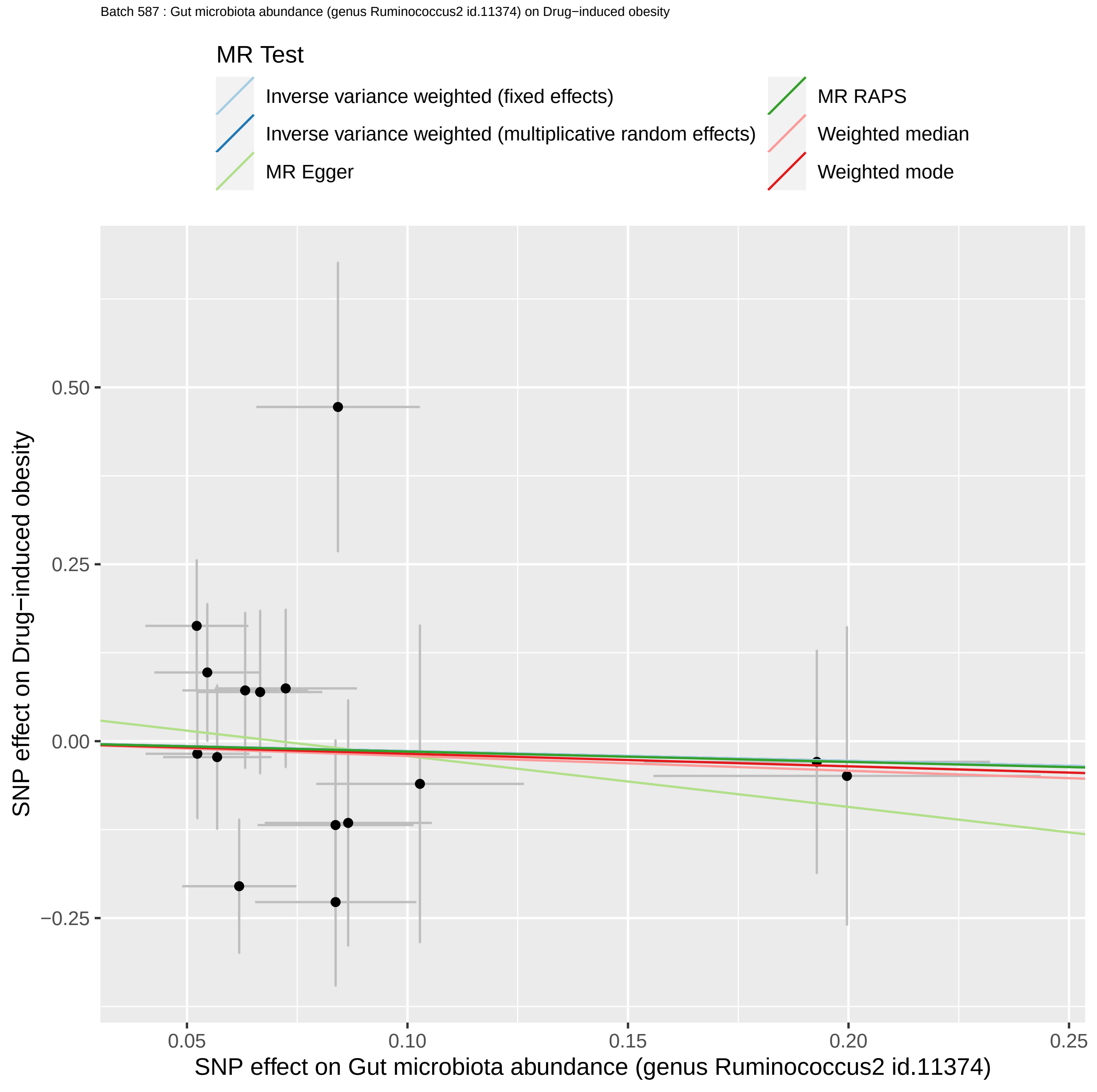

Batch 587 : Gut microbiota abundance (genus Ruminococcus2 id.11374) on Drug−induced obesity
MR Test
Inverse variance weighted (fixed effects)
Inverse variance weighted (multiplicative random effects)
MR Egger
MR RAPS
Weighted median
Weighted mode
0.50
0.25
0.00
−0.25
0.05
0.10
0.15
0.20
0.25
SNP effect on Drug−induced obesity
SNP effect on Gut microbiota abundance (genus Ruminococcus2 id.11374)

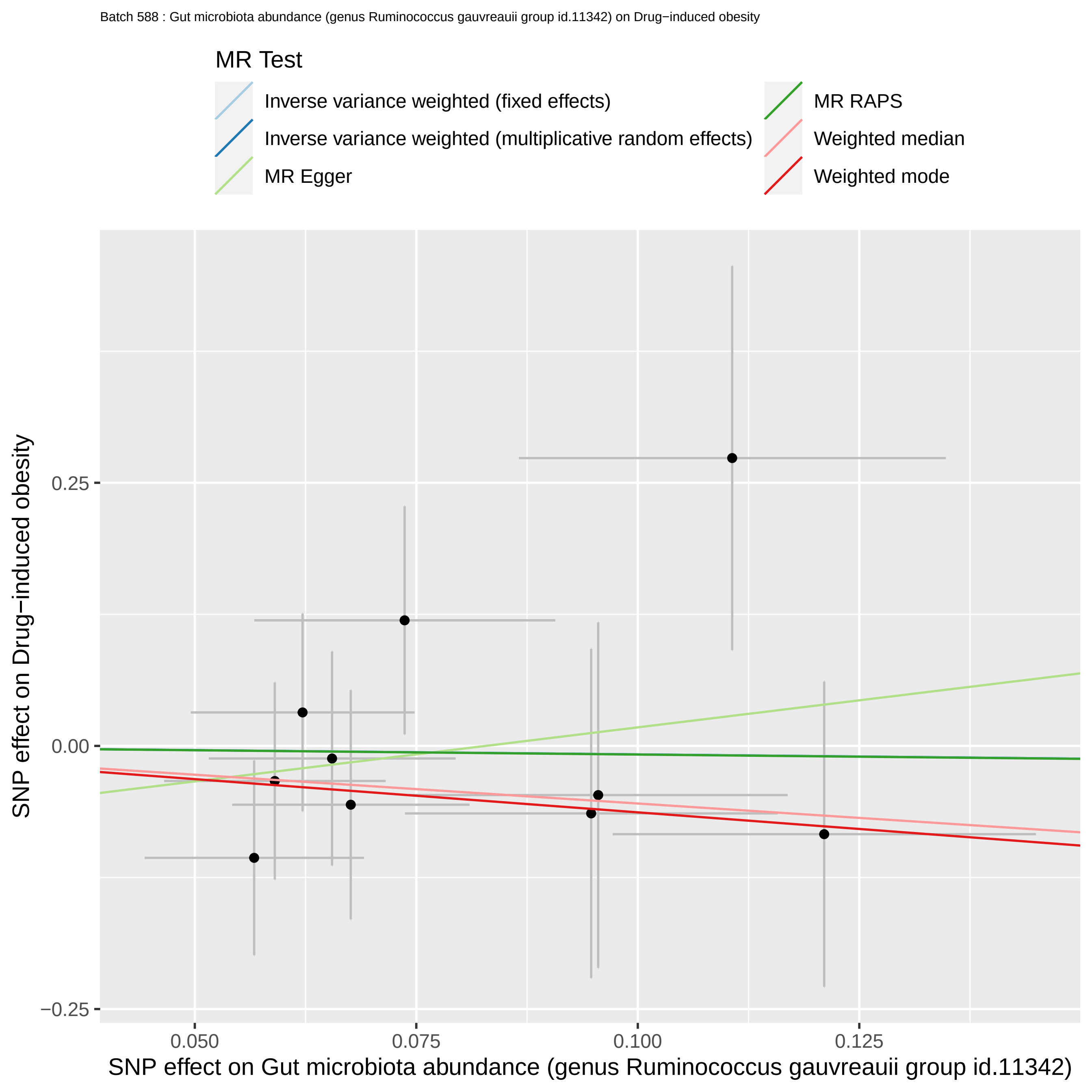
Batch 588 : Gut microbiota abundance (genus Ruminococcus gauvreauii group id.11342) on Drug−induced obesity
MR Test
Inverse variance weighted (fixed effects)
Inverse variance weighted (multiplicative random effects)
MR Egger
MR RAPS
Weighted median
Weighted mode
0.25
0.00
−0.25
0.050
0.075
0.100
0.125
SNP effect on Drug−induced obesity
SNP effect on Gut microbiota abundance (genus Ruminococcus gauvreauii group id.11342)

Batch 589 : Gut microbiota abundance (genus Ruminococcus gnavus group id.14376) on Drug−induced obesity

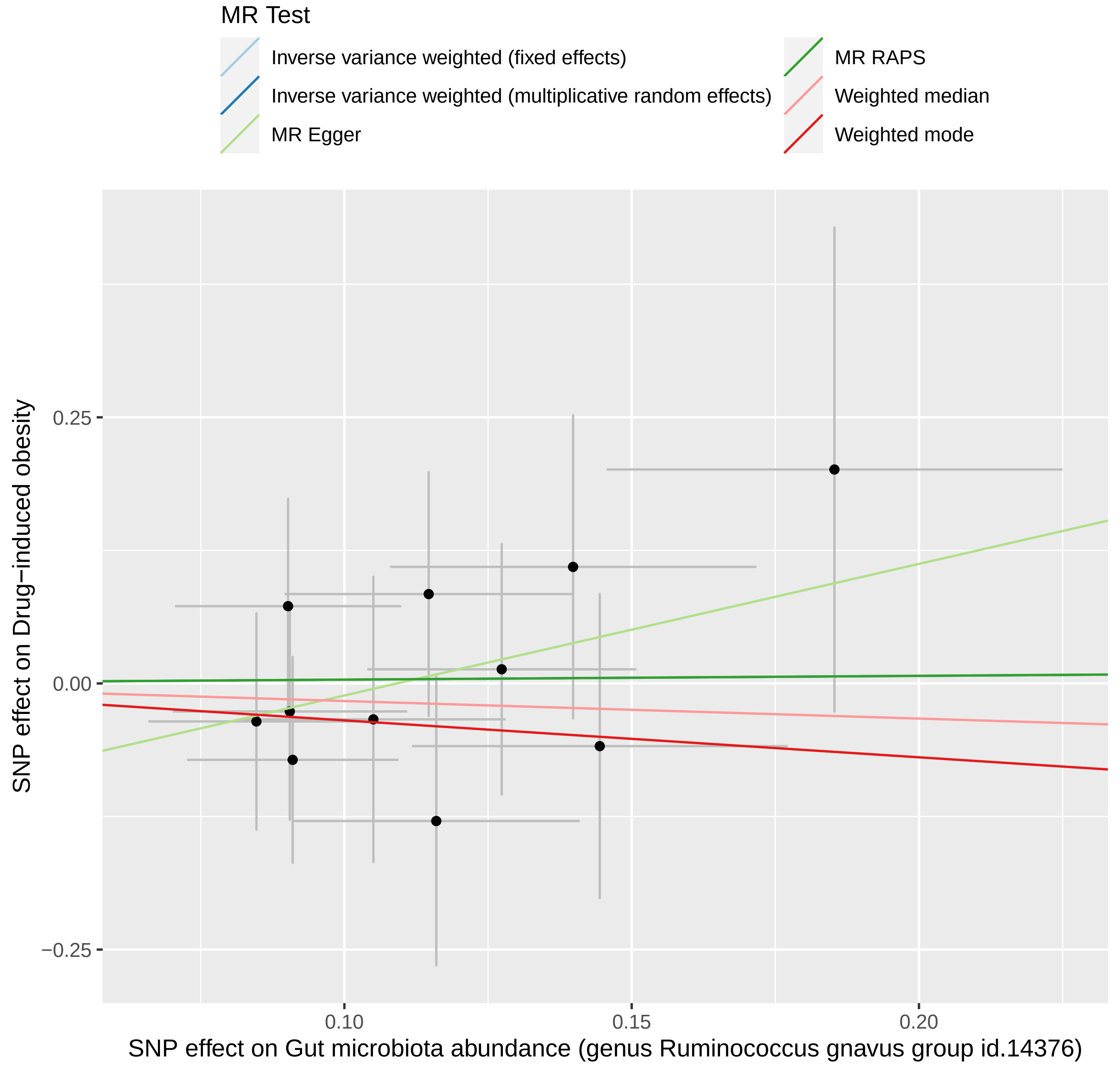

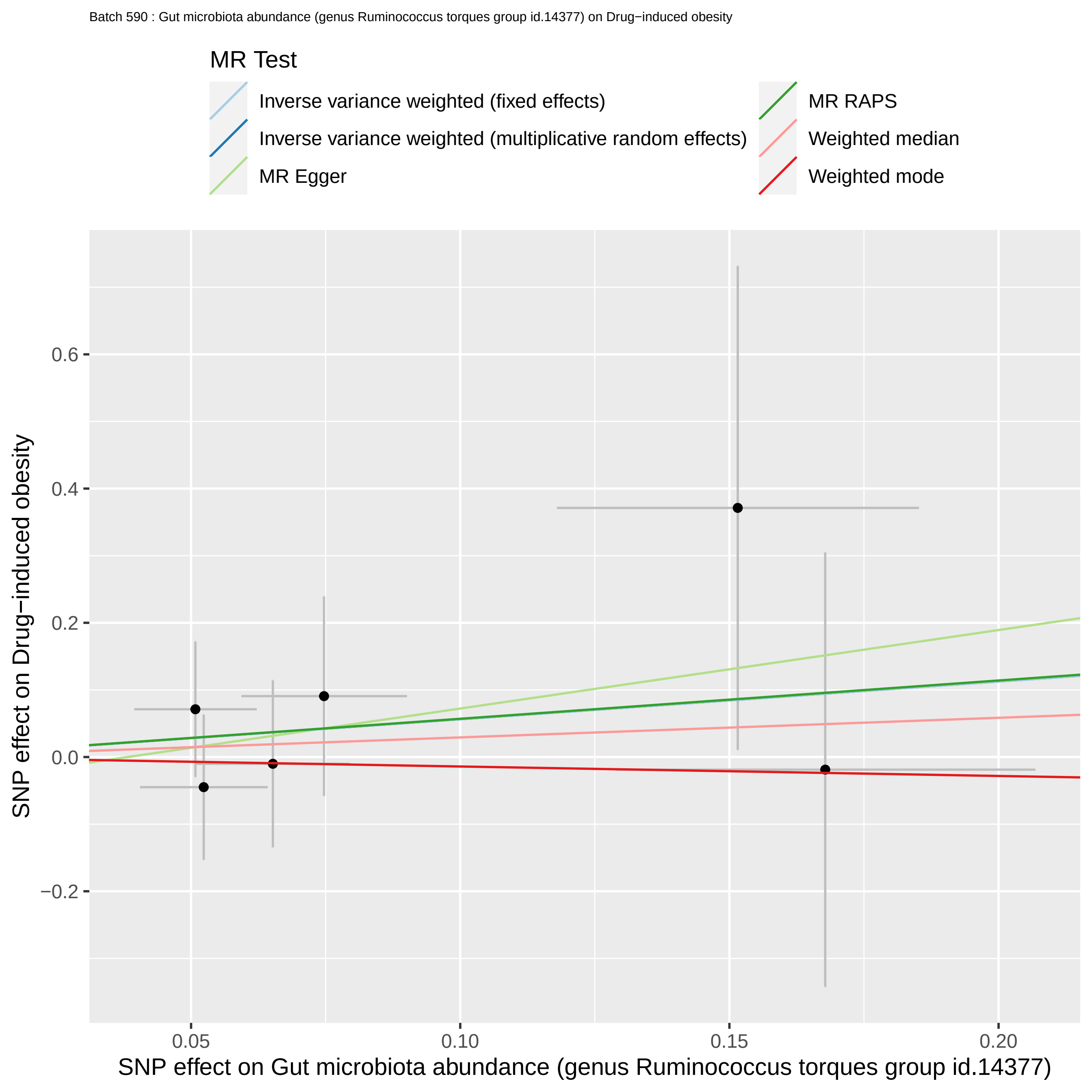

Batch 590 : Gut microbiota abundance (genus Ruminococcus torques group id.14377) on Drug−induced obesity
MR Test
Inverse variance weighted (fixed effects)
Inverse variance weighted (multiplicative random effects)
MR Egger
MR RAPS
Weighted median
Weighted mode
0.6
0.4
0.2
0.0
−0.2
0.05
0.10
0.15
0.20
SNP effect on Drug−induced obesity
SNP effect on Gut microbiota abundance (genus Ruminococcus torques group id.14377)

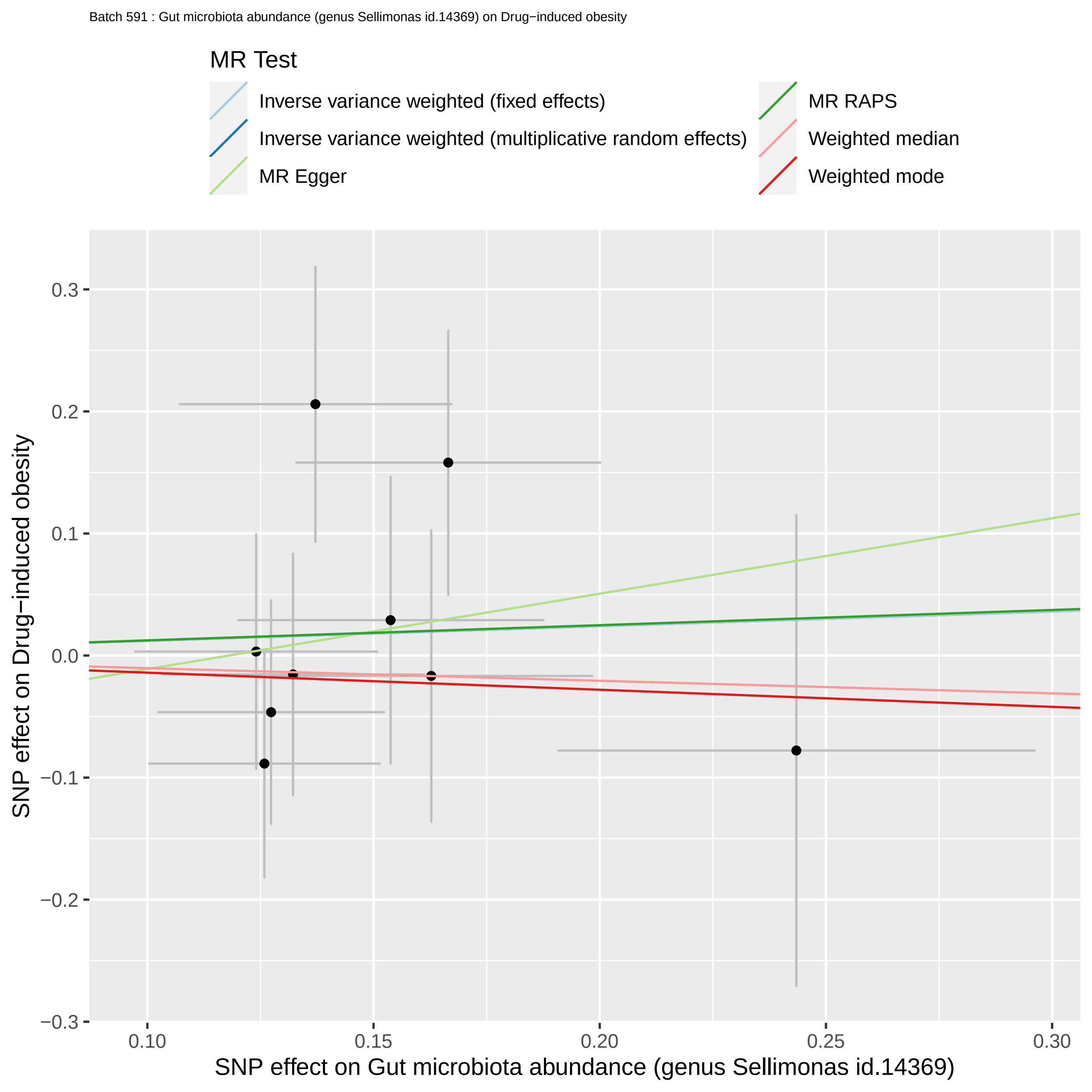
Batch 591 : Gut microbiota abundance (genus Sellimonas id.14369) on Drug−induced obesity
MR Test
Inverse variance weighted (fixed effects)
Inverse variance weighted (multiplicative random effects)
MR Egger
MR RAPS
Weighted median
Weighted mode
0.3
0.2
0.1
0.0
−0.1
−0.2
−0.3
0.10
0.15
0.20
0.25
0.30
SNP effect on Drug−induced obesity
SNP effect on Gut microbiota abundance (genus Sellimonas id.14369)

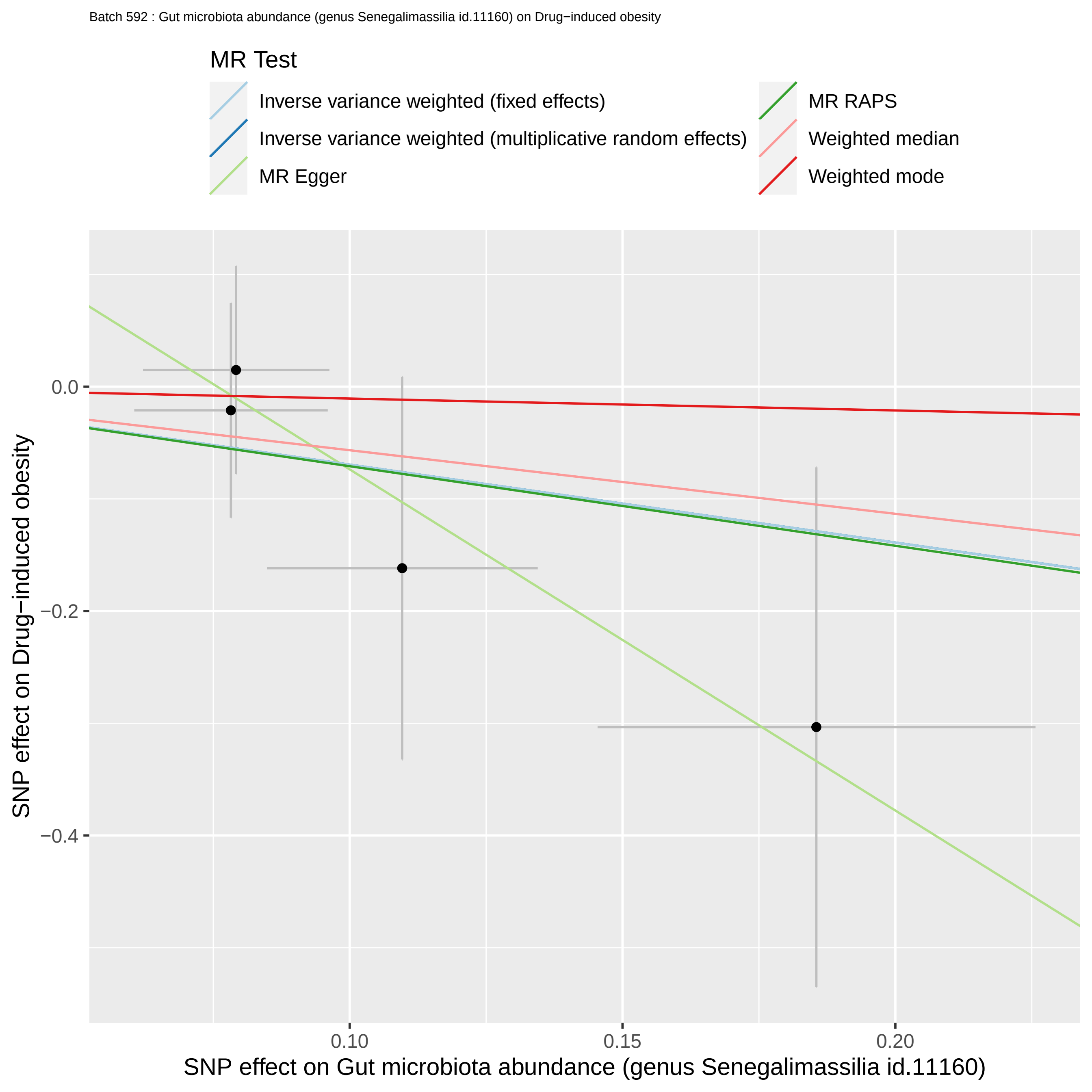
Batch 592 : Gut microbiota abundance (genus Senegalimassilia id.11160) on Drug−induced obesity
MR Test
Inverse variance weighted (fixed effects)
Inverse variance weighted (multiplicative random effects)
MR Egger
MR RAPS
Weighted median
Weighted mode
0.0
−0.2
−0.4
0.10
0.15
0.20
SNP effect on Drug−induced obesity
SNP effect on Gut microbiota abundance (genus Senegalimassilia id.11160)

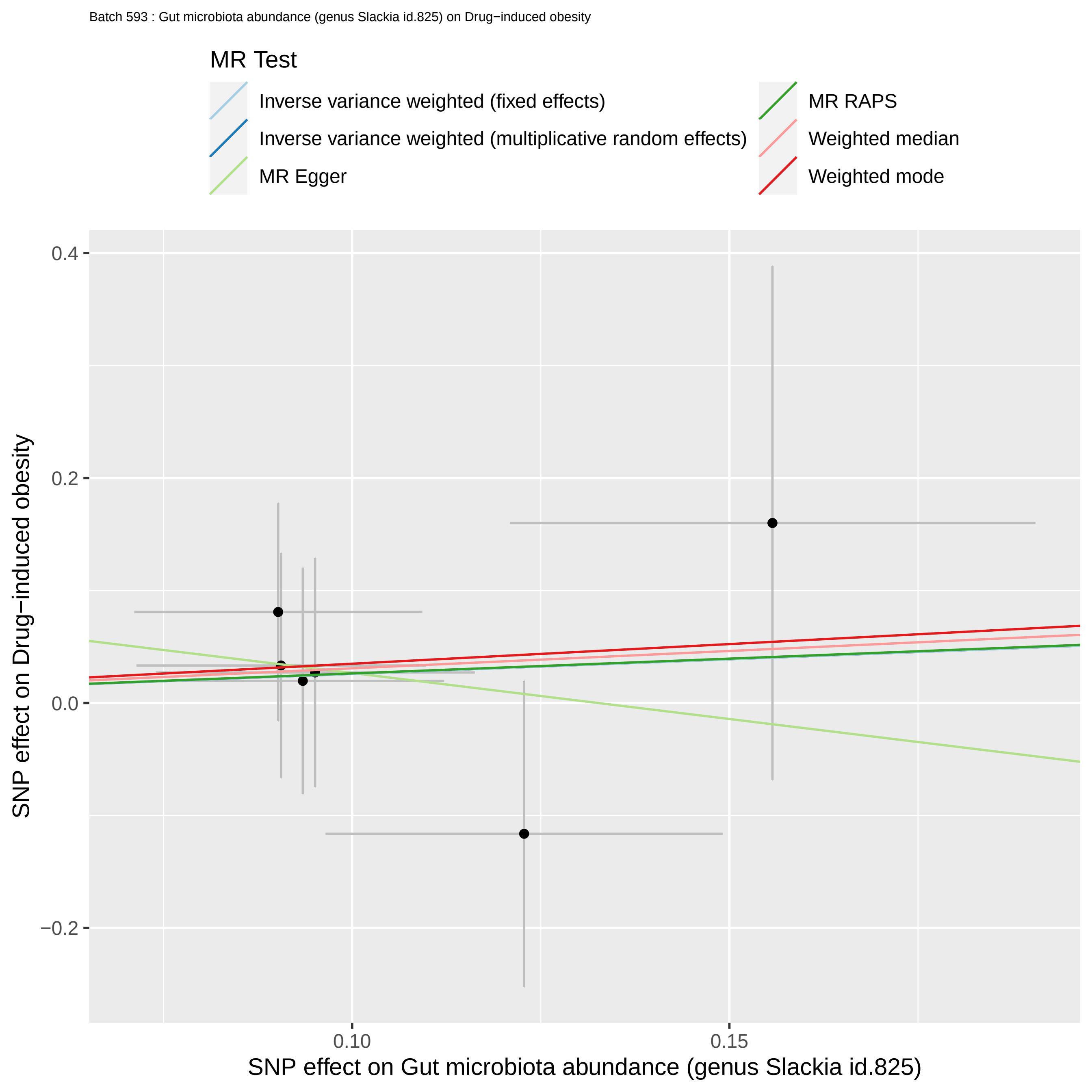
Batch 593 : Gut microbiota abundance (genus Slackia id.825) on Drug−induced obesity
MR Test
Inverse variance weighted (fixed effects)
Inverse variance weighted (multiplicative random effects)
MR Egger
MR RAPS
Weighted median
Weighted mode
0.4
0.2
0.0
−0.2
0.10
0.15
SNP effect on Drug−induced obesity
SNP effect on Gut microbiota abundance (genus Slackia id.825)

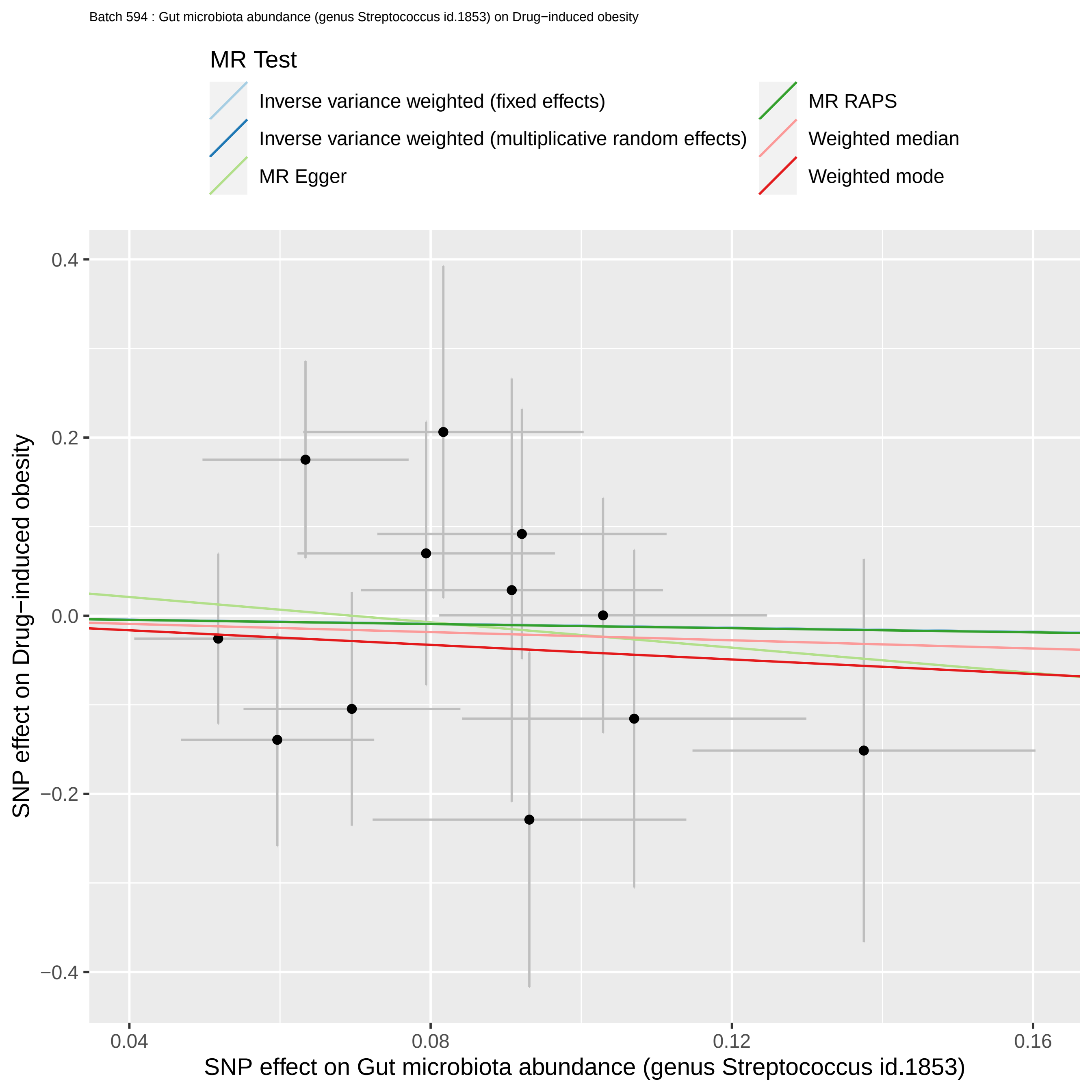

Batch 594 : Gut microbiota abundance (genus Streptococcus id.1853) on Drug−induced obesity
MR Test
Inverse variance weighted (fixed effects)
Inverse variance weighted (multiplicative random effects)
MR Egger
MR RAPS
Weighted median
Weighted mode
SNP effect on Drug−induced obesity
0.4
0.2
0.0
−0.2
−0.4
0.04
0.08
0.12
0.16
SNP effect on Gut microbiota abundance (genus Streptococcus id.1853)

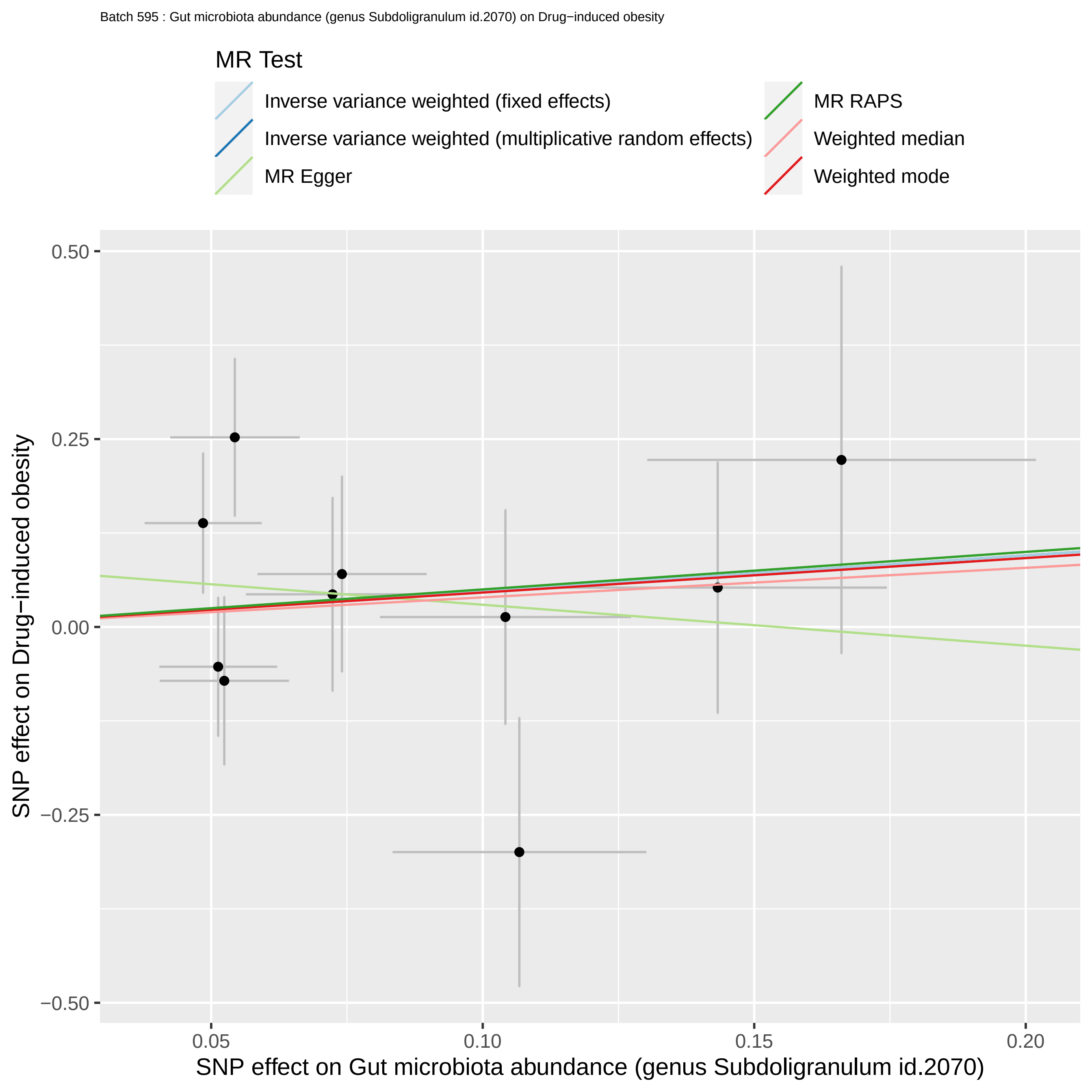

Batch 595 : Gut microbiota abundance (genus Subdoligranulum id.2070) on Drug−induced obesity
MR Test
Inverse variance weighted (fixed effects)
Inverse variance weighted (multiplicative random effects)
MR Egger
MR RAPS
Weighted median
Weighted mode
SNP effect on Drug−induced obesity
0.50
0.25
0.00
−0.25
−0.50
0.05
0.10
0.15
0.20
SNP effect on Gut microbiota abundance (genus Subdoligranulum id.2070)

Batch 596 : Gut microbiota abundance (genus Sutterella id.2896) on Drug−induced obesity

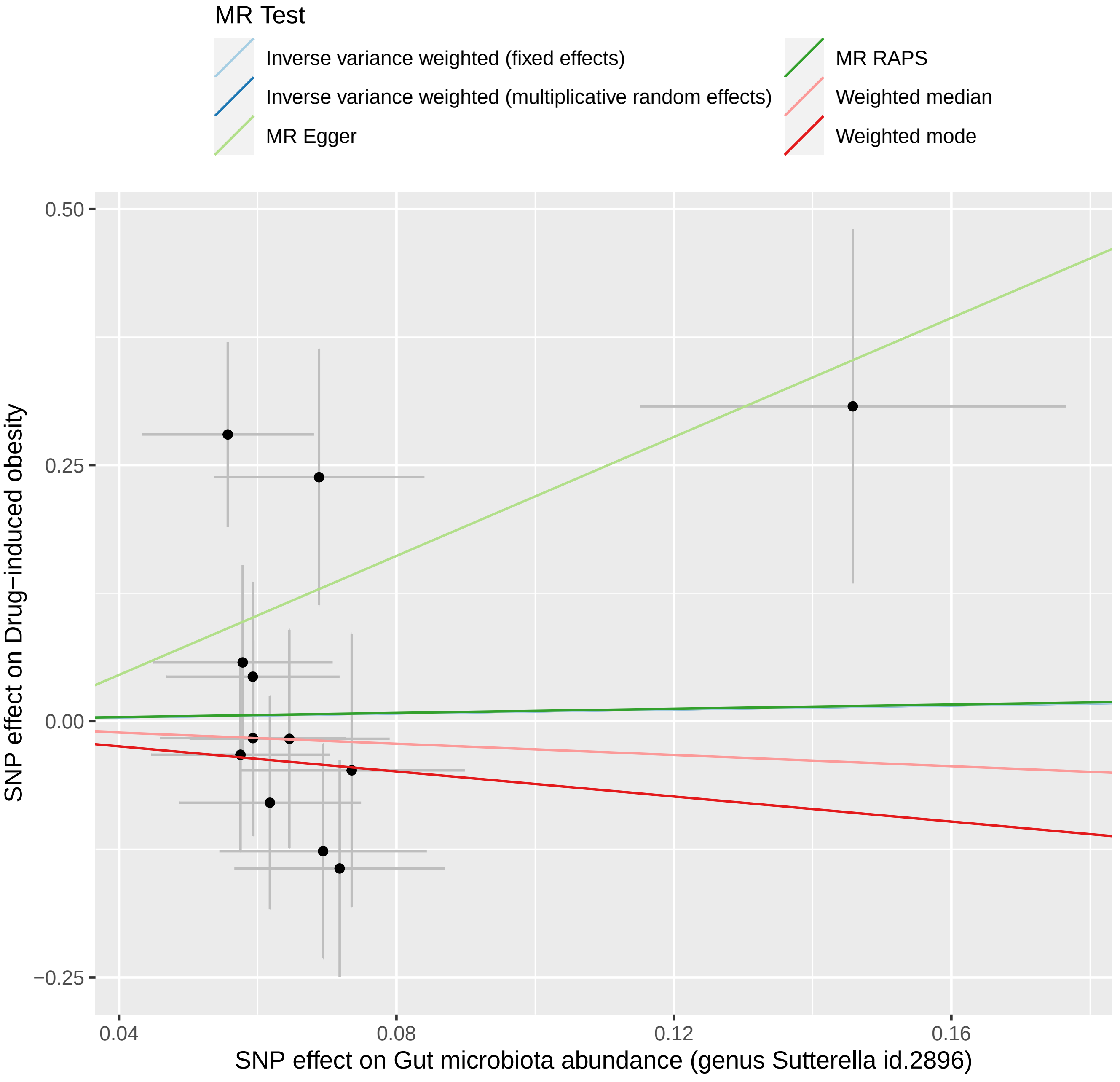

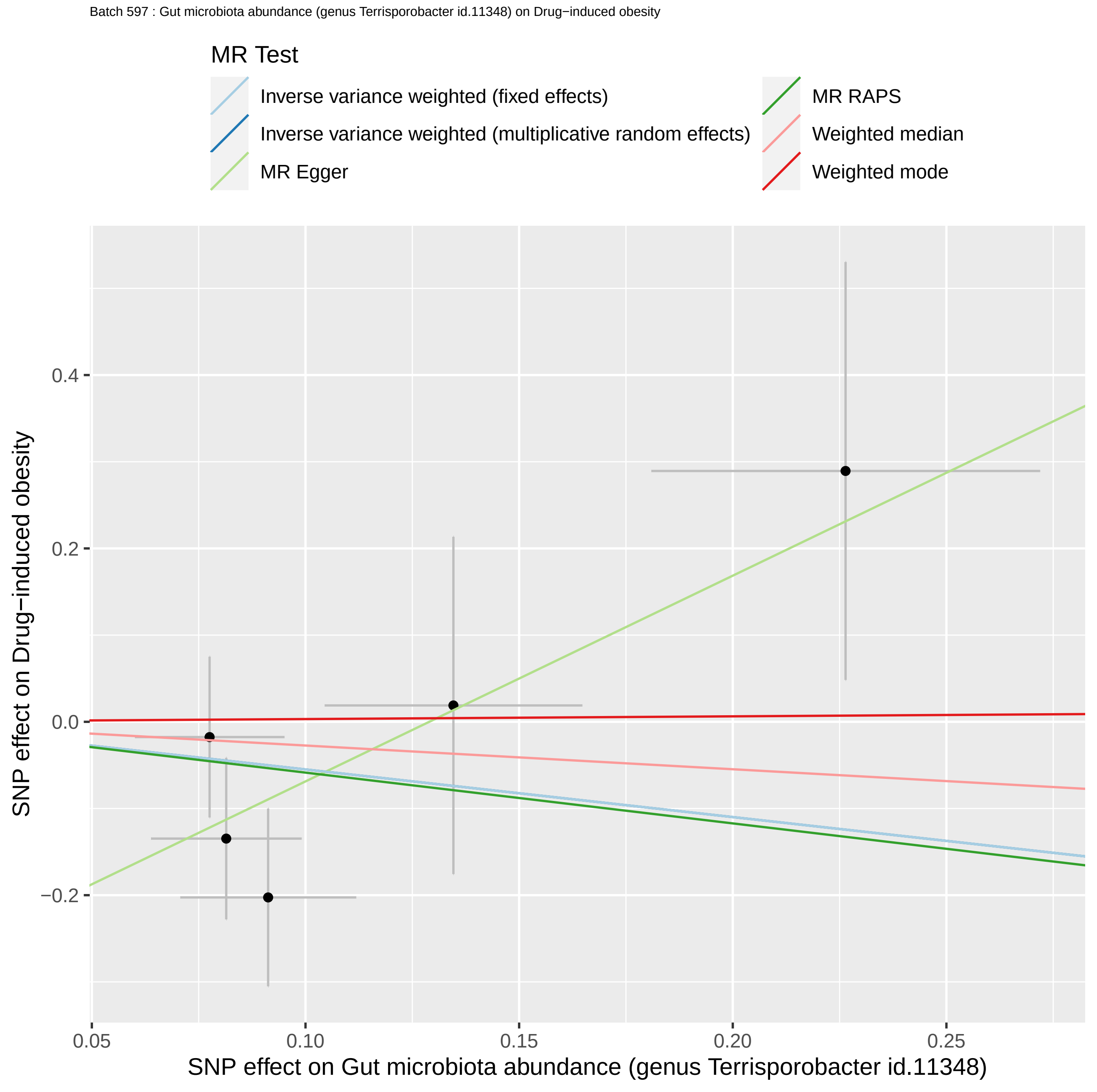

Batch 597 : Gut microbiota abundance (genus Terrisporobacter id.11348) on Drug−induced obesity
MR Test
Inverse variance weighted (fixed effects)
Inverse variance weighted (multiplicative random effects)
MR Egger
MR RAPS
Weighted median
Weighted mode
SNP effect on Drug−induced obesity
0.4
0.2
0.0
−0.2
0.05
0.10
0.15
0.20
0.25
SNP effect on Gut microbiota abundance (genus Terrisporobacter id.11348)

Batch 598 : Gut microbiota abundance (genus Turicibacter id.2162) on Drug−induced obesity

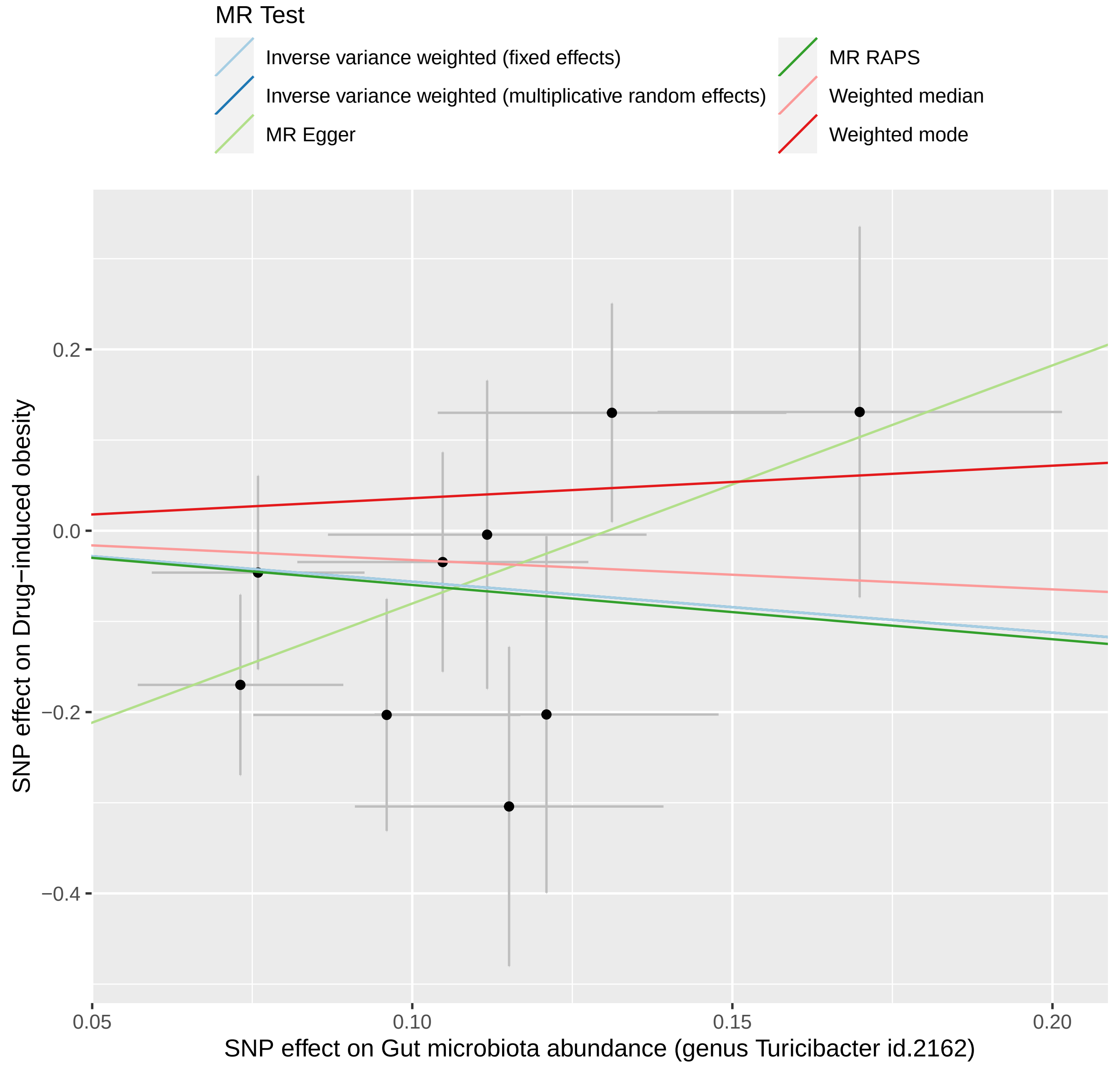

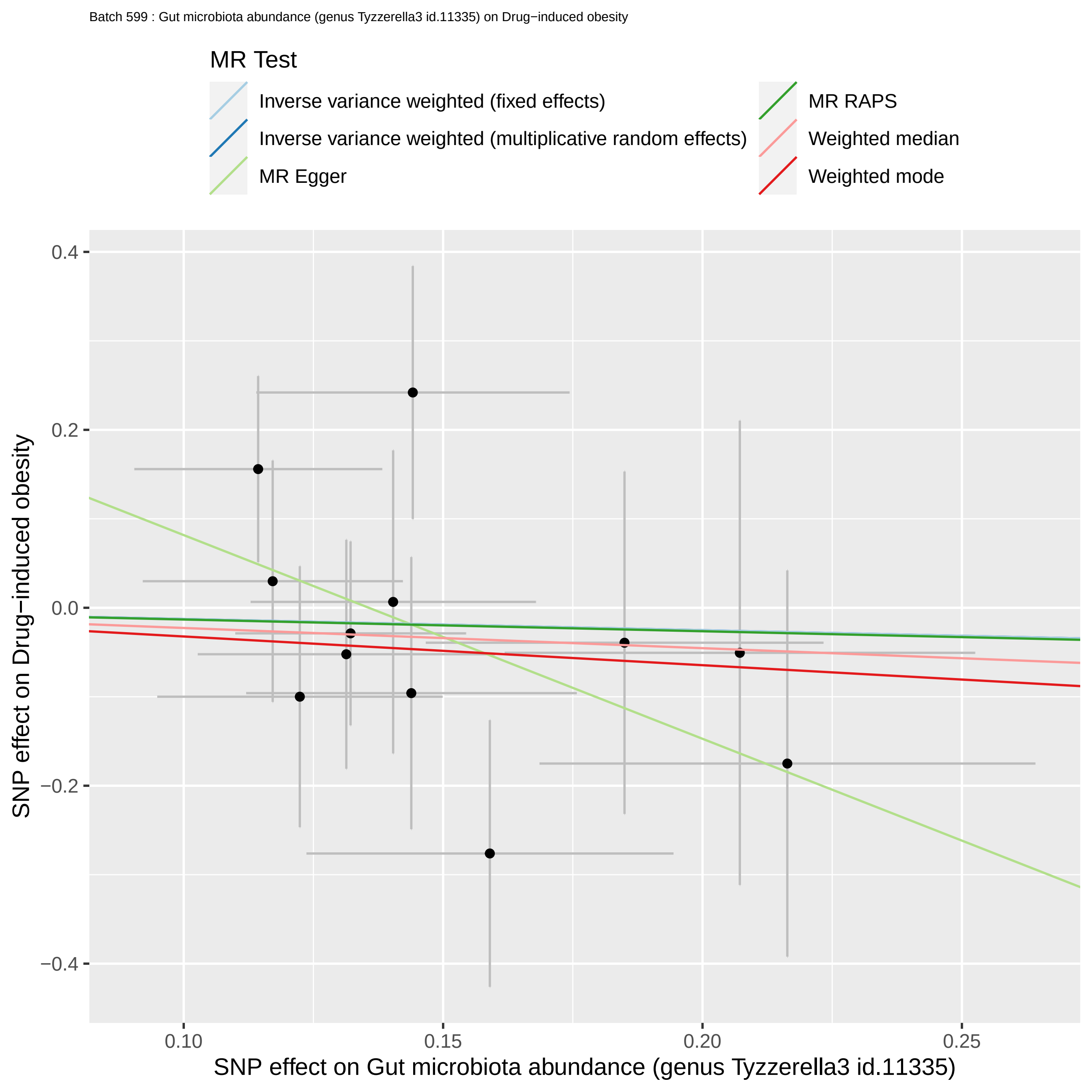

Batch 599 : Gut microbiota abundance (genus Tyzzerella3 id.11335) on Drug−induced obesity
MR Test
Inverse variance weighted (fixed effects)
Inverse variance weighted (multiplicative random effects)
MR Egger
MR RAPS
Weighted median
Weighted mode
SNP effect on Drug−induced obesity
0.4
0.2
0.0
−0.2
−0.4
0.10
0.15
0.20
0.25
SNP effect on Gut microbiota abundance (genus Tyzzerella3 id.11335)

Batch 600 : Gut microbiota abundance (genus Veillonella id.2198) on Drug−induced obesity

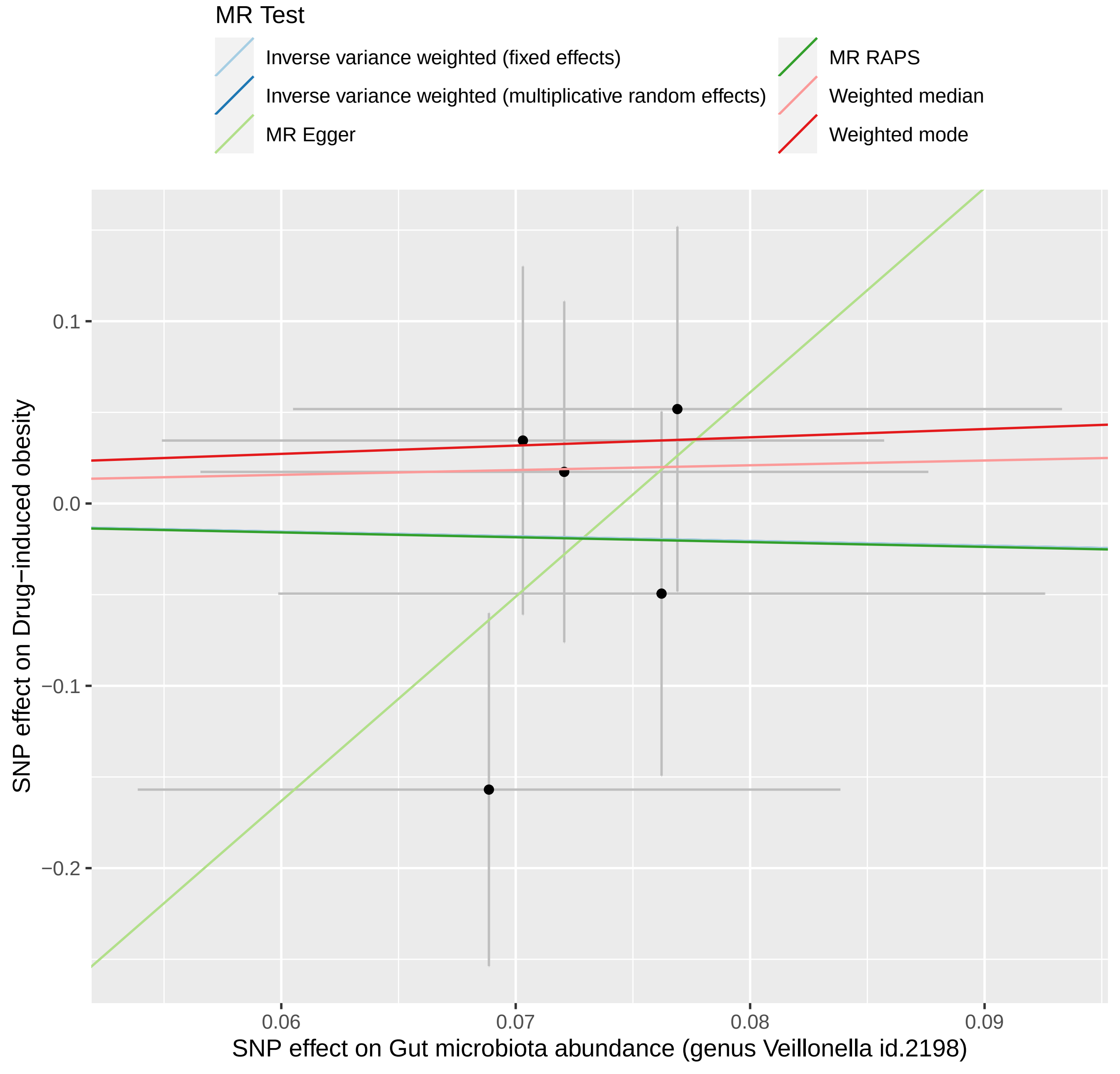

Batch 601 : Gut microbiota abundance (family Acidaminococcaceae id.2166) on Extreme obesity with alveolar hypoventilation

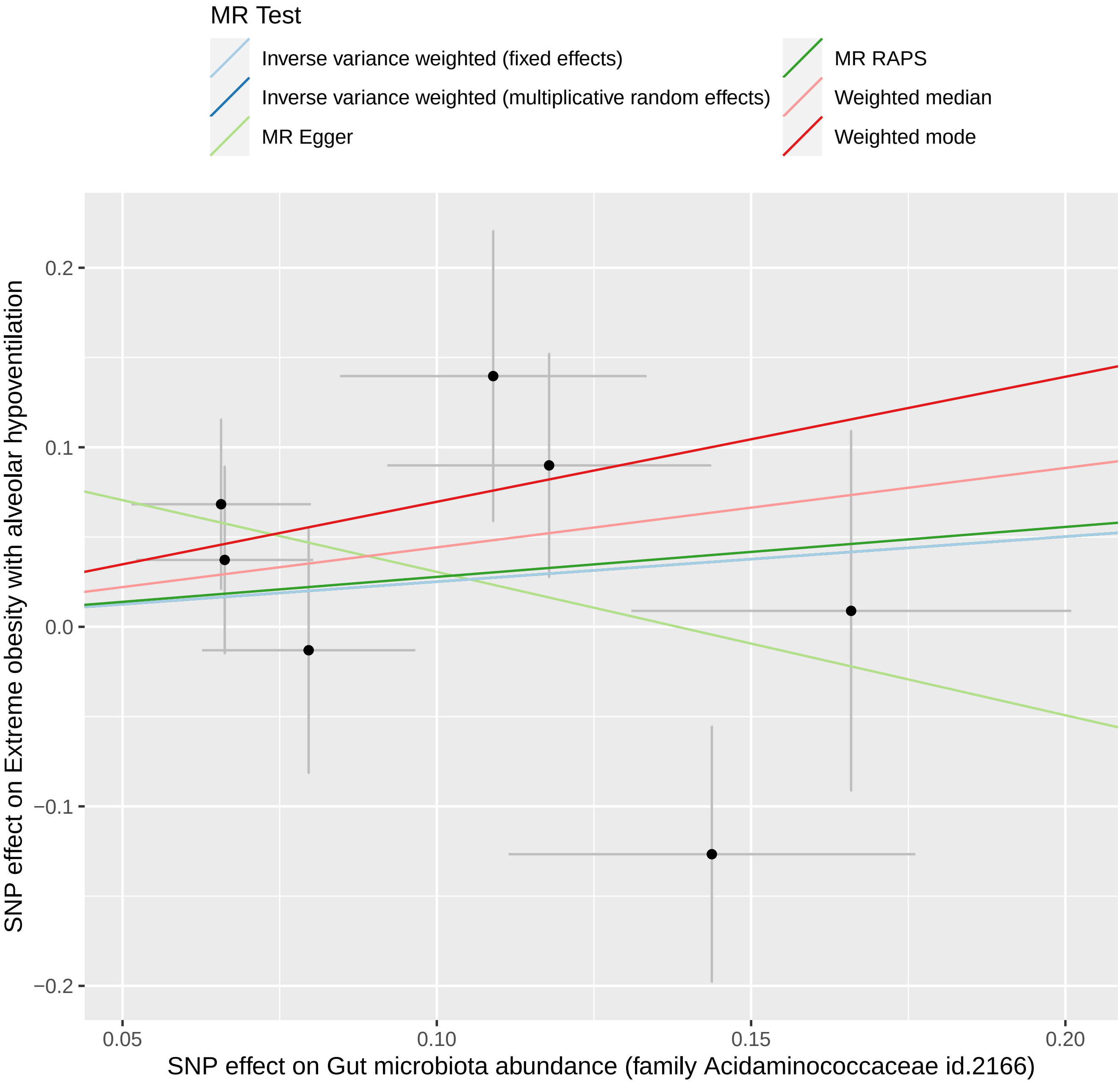

Batch 602 : Gut microbiota abundance (family Actinomycetaceae id.421) on Extreme obesity with alveolar hypoventilation

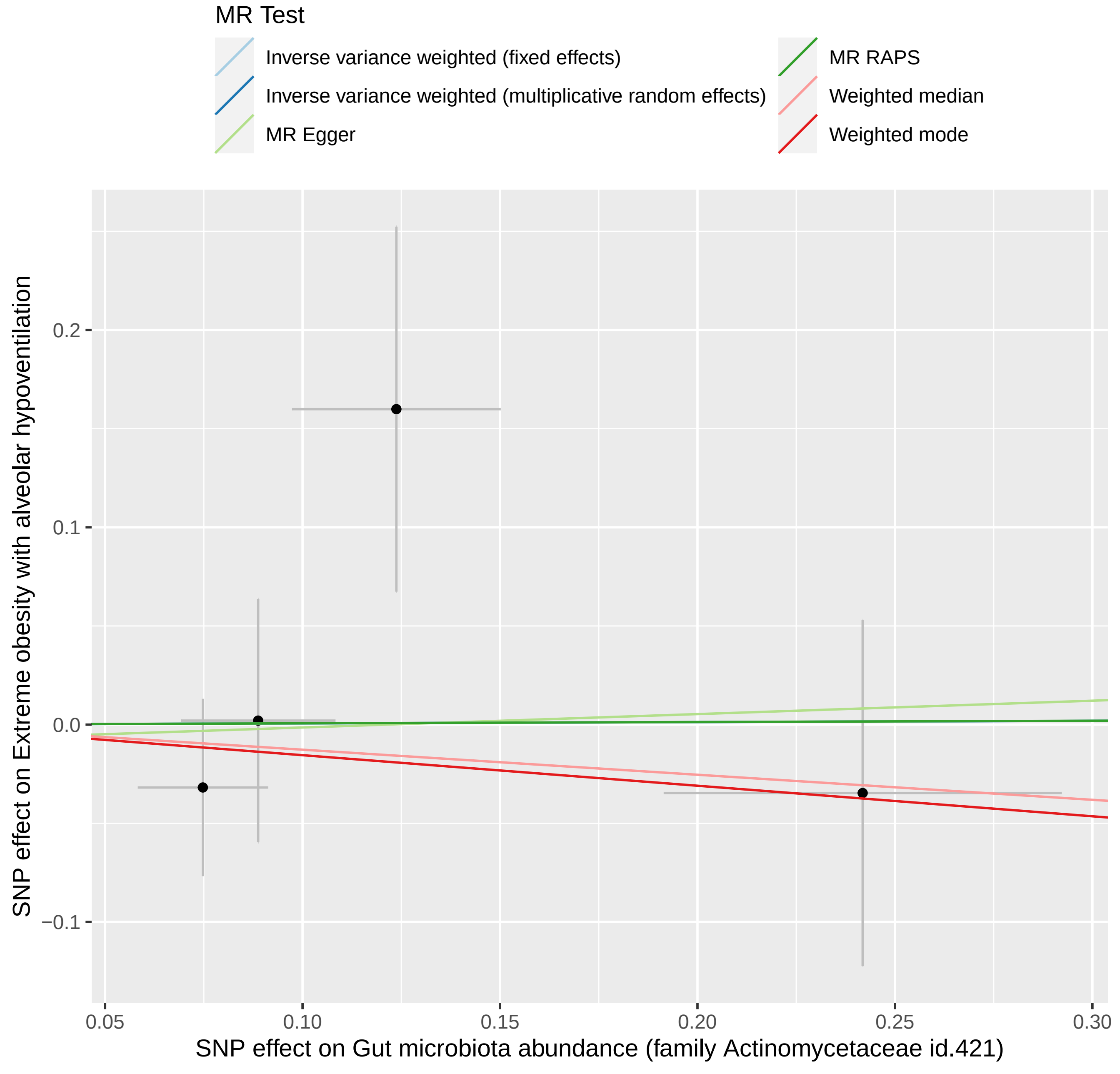

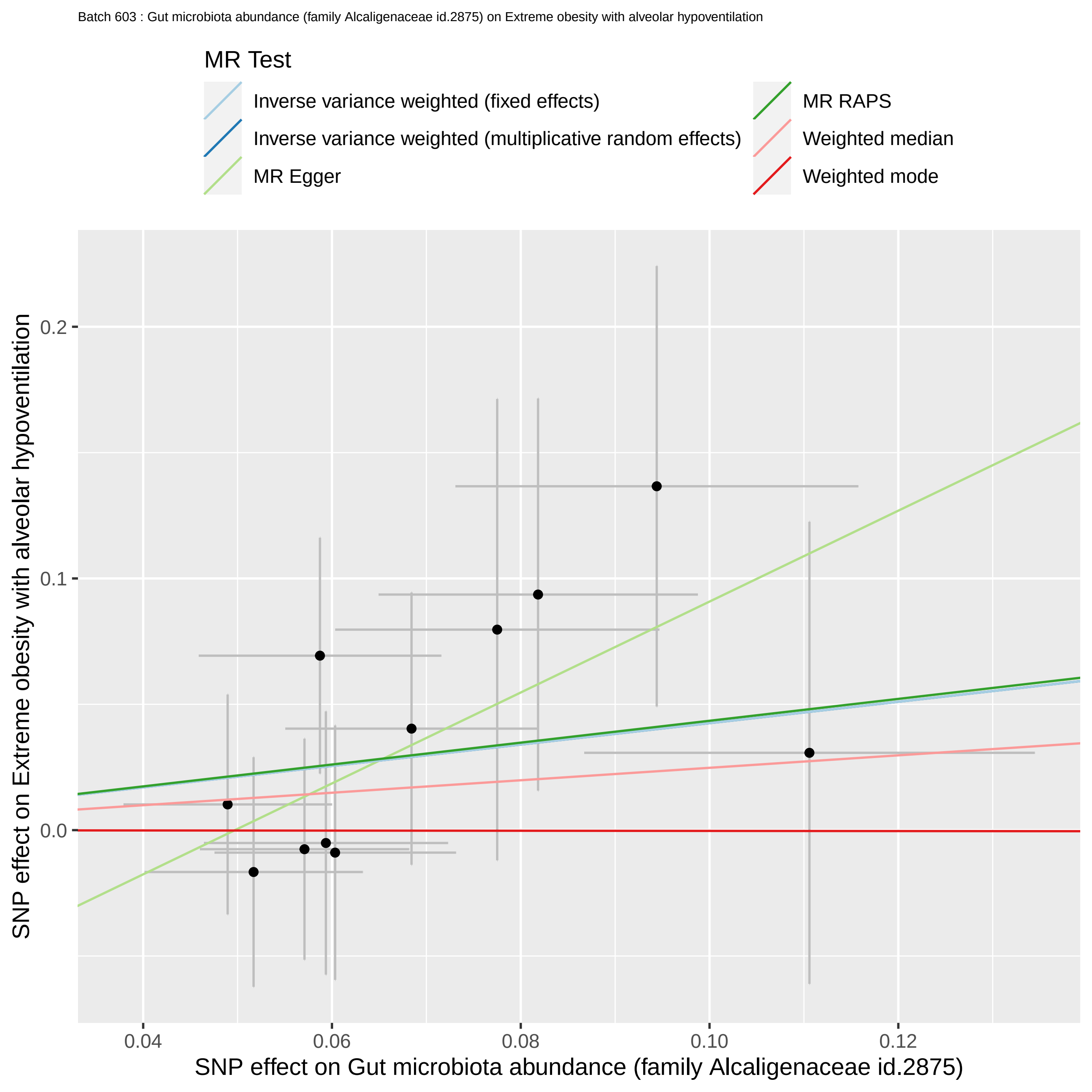
Batch 603 : Gut microbiota abundance (family Alcaligenaceae id.2875) on Extreme obesity with alveolar hypoventilation
MR Test
Inverse variance weighted (fixed effects)
Inverse variance weighted (multiplicative random effects)
MR Egger
MR RAPS
Weighted median
Weighted mode
SNP effect on Extreme obesity with alveolar hypoventilation
0.2
0.1
0.0
0.04
0.06
0.08
0.10
0.12
SNP effect on Gut microbiota abundance (family Alcaligenaceae id.2875)

Batch 604 : Gut microbiota abundance (family Bacteroidaceae id.917) on Extreme obesity with alveolar hypoventilation

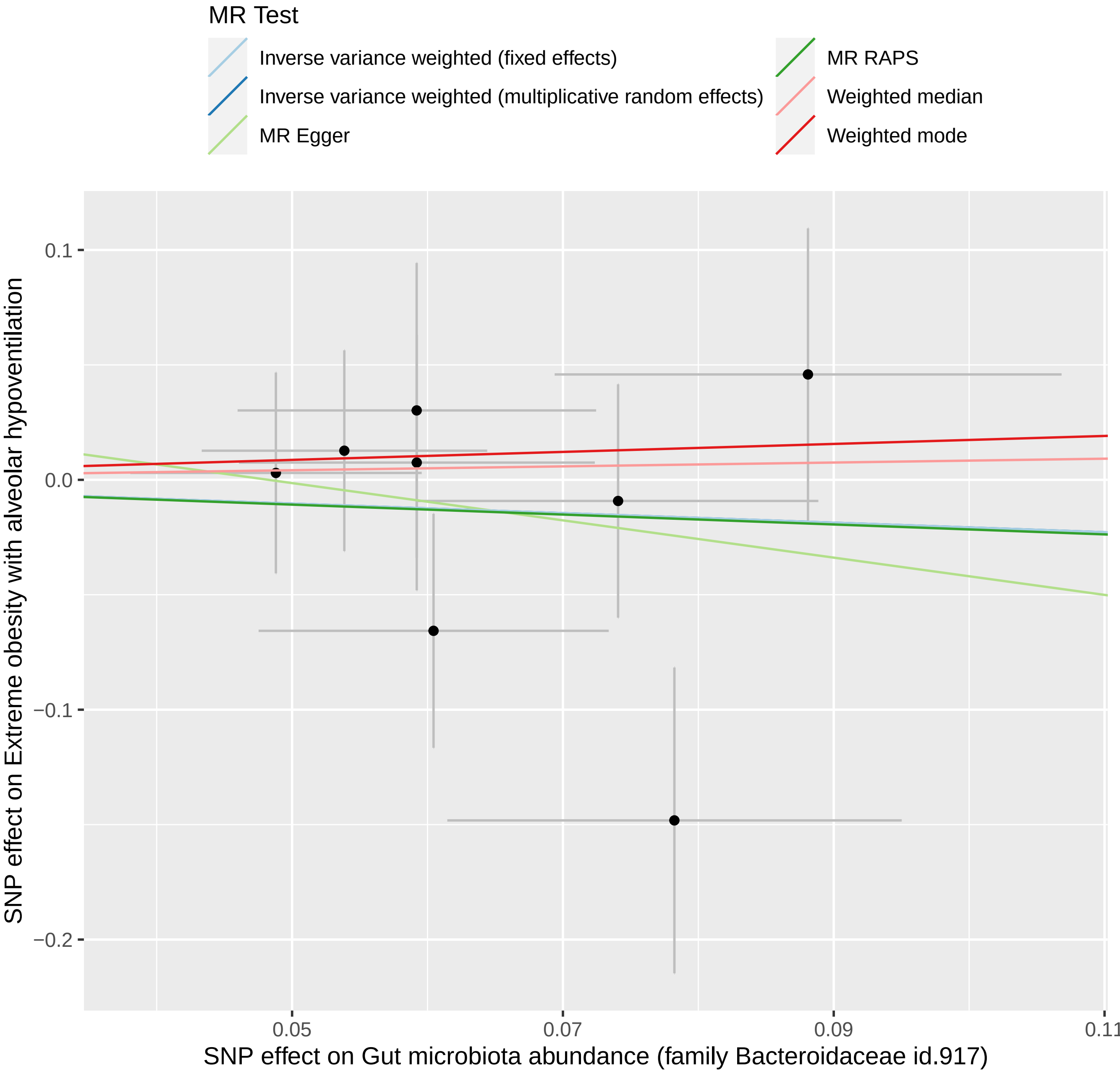

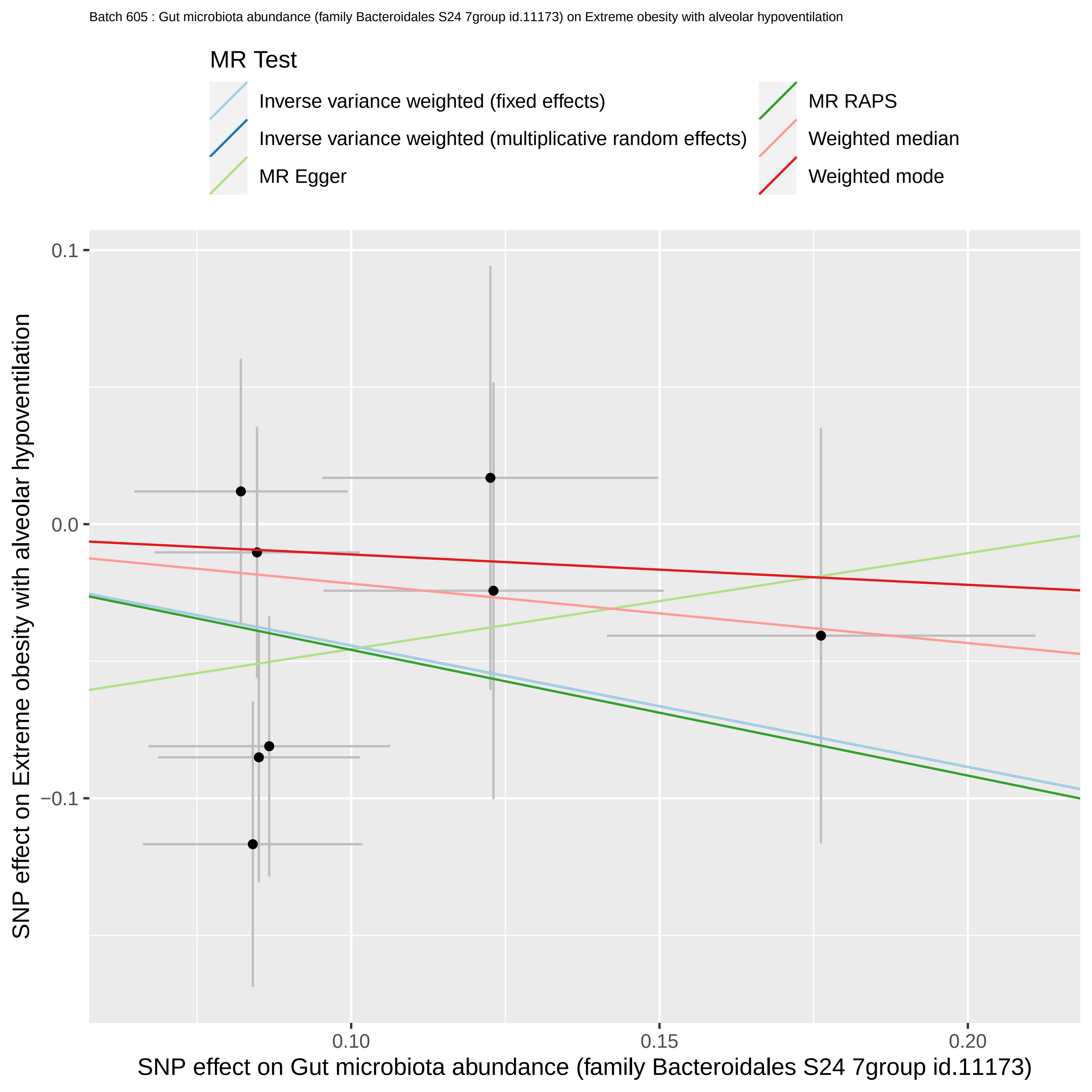

Batch 605 : Gut microbiota abundance (family Bacteroidales S24 7group id.11173) on Extreme obesity with alveolar hypoventilation
MR Test
Inverse variance weighted (fixed effects)
Inverse variance weighted (multiplicative random effects)
MR Egger
MR RAPS
Weighted median
Weighted mode
0.1
0.0
−0.1
0.10
0.15
0.20
SNP effect on Extreme obesity with alveolar hypoventilation
SNP effect on Gut microbiota abundance (family Bacteroidales S24 7group id.11173)

Batch 606 : Gut microbiota abundance (family Bifidobacteriaceae id.433) on Extreme obesity with alveolar hypoventilation

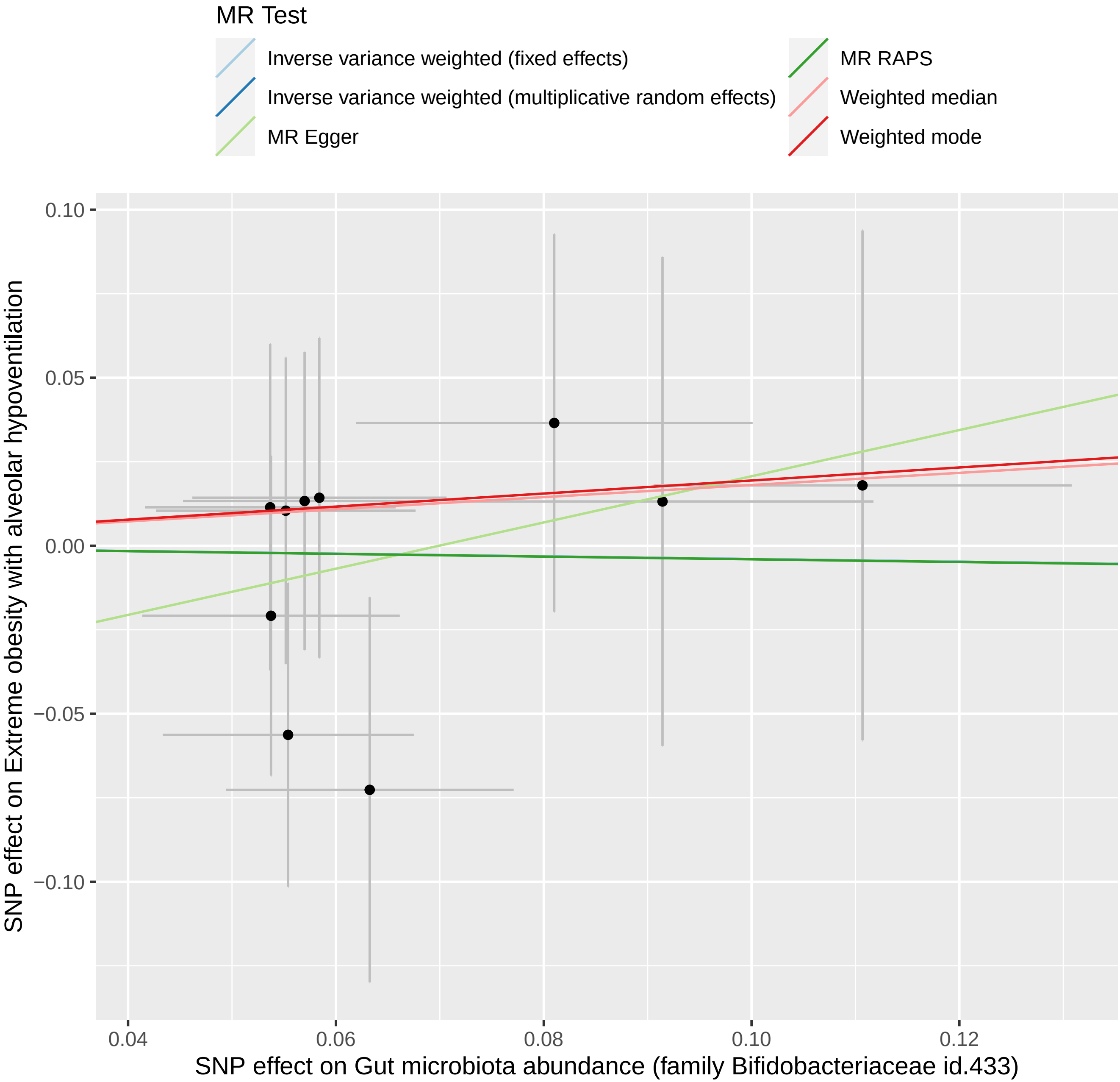

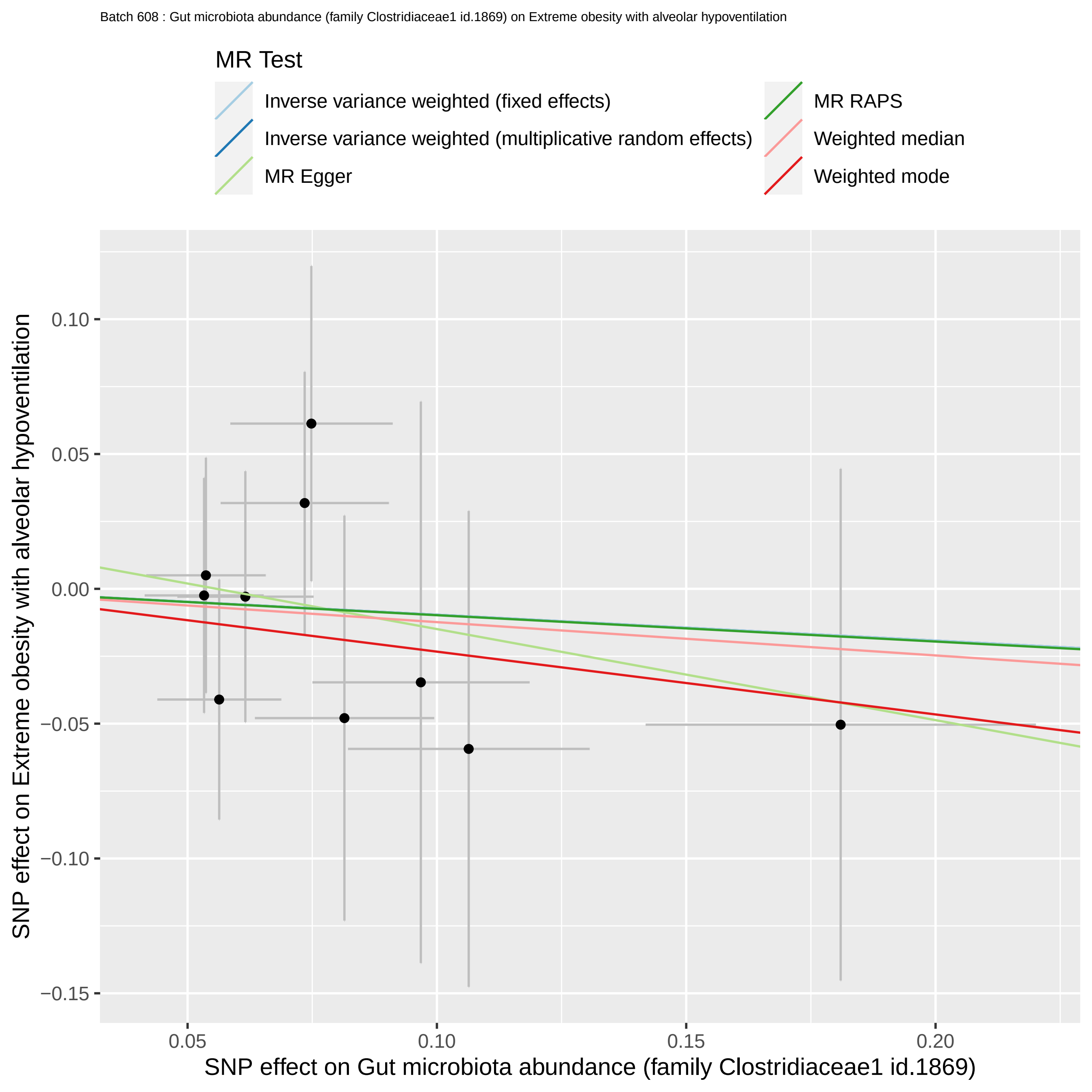
Batch 608 : Gut microbiota abundance (family Clostridiaceae1 id.1869) on Extreme obesity with alveolar hypoventilation
MR Test
Inverse variance weighted (fixed effects)
Inverse variance weighted (multiplicative random effects)
MR Egger
MR RAPS
Weighted median
Weighted mode
0.10
0.05
0.00
−0.05
−0.10
−0.15
SNP effect on Extreme obesity with alveolar hypoventilation
0.05
0.10
0.15
0.20
SNP effect on Gut microbiota abundance (family Clostridiaceae1 id.1869)

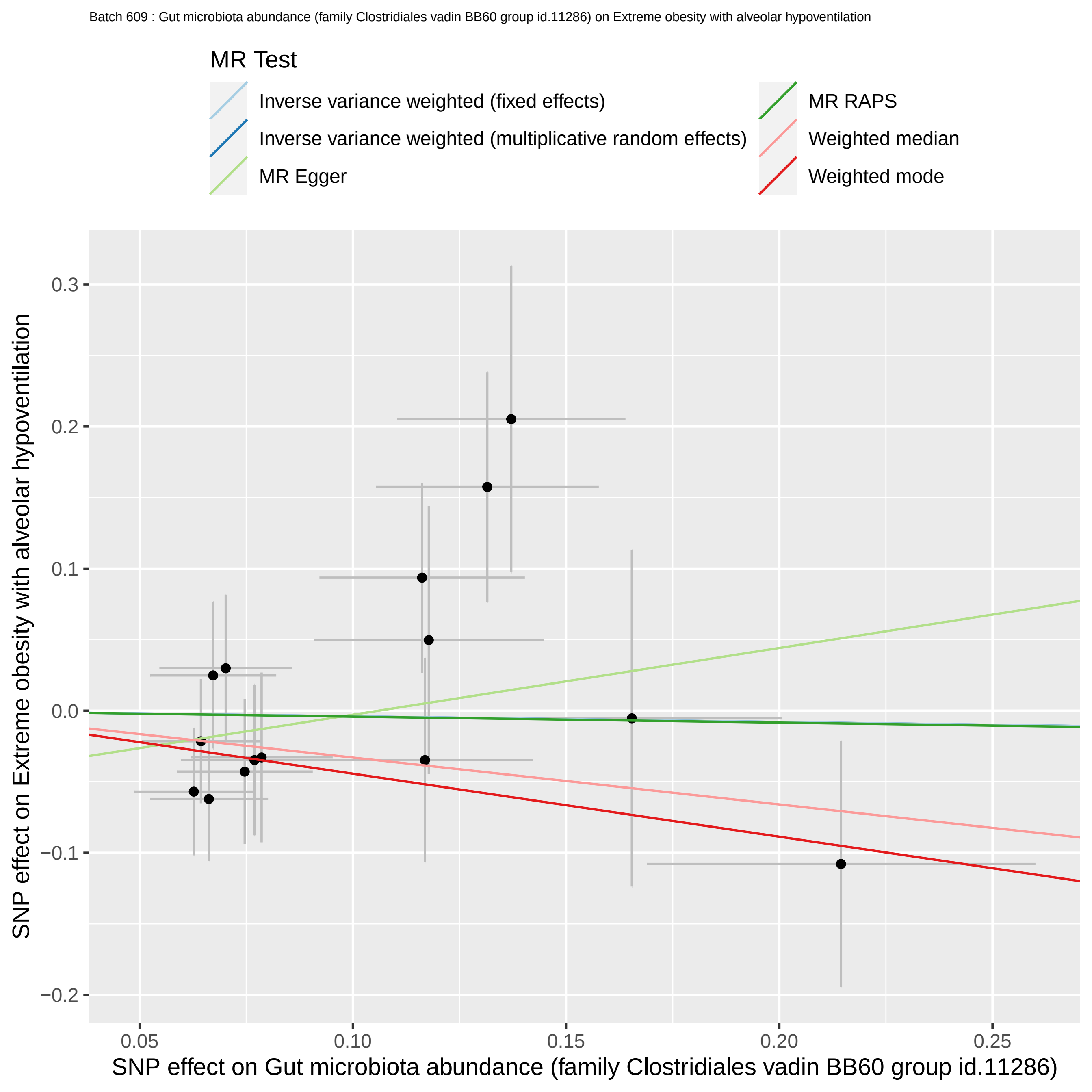

Batch 609 : Gut microbiota abundance (family Clostridiales vadin BB60 group id.11286) on Extreme obesity with alveolar hypoventilation
MR Test
Inverse variance weighted (fixed effects)
Inverse variance weighted (multiplicative random effects)
MR Egger
MR RAPS
Weighted median
Weighted mode
0.3
0.2
0.1
0.0
−0.1
−0.2
0.05
0.10
0.15
0.20
0.25
SNP effect on Extreme obesity with alveolar hypoventilation
SNP effect on Gut microbiota abundance (family Clostridiales vadin BB60 group id.11286)

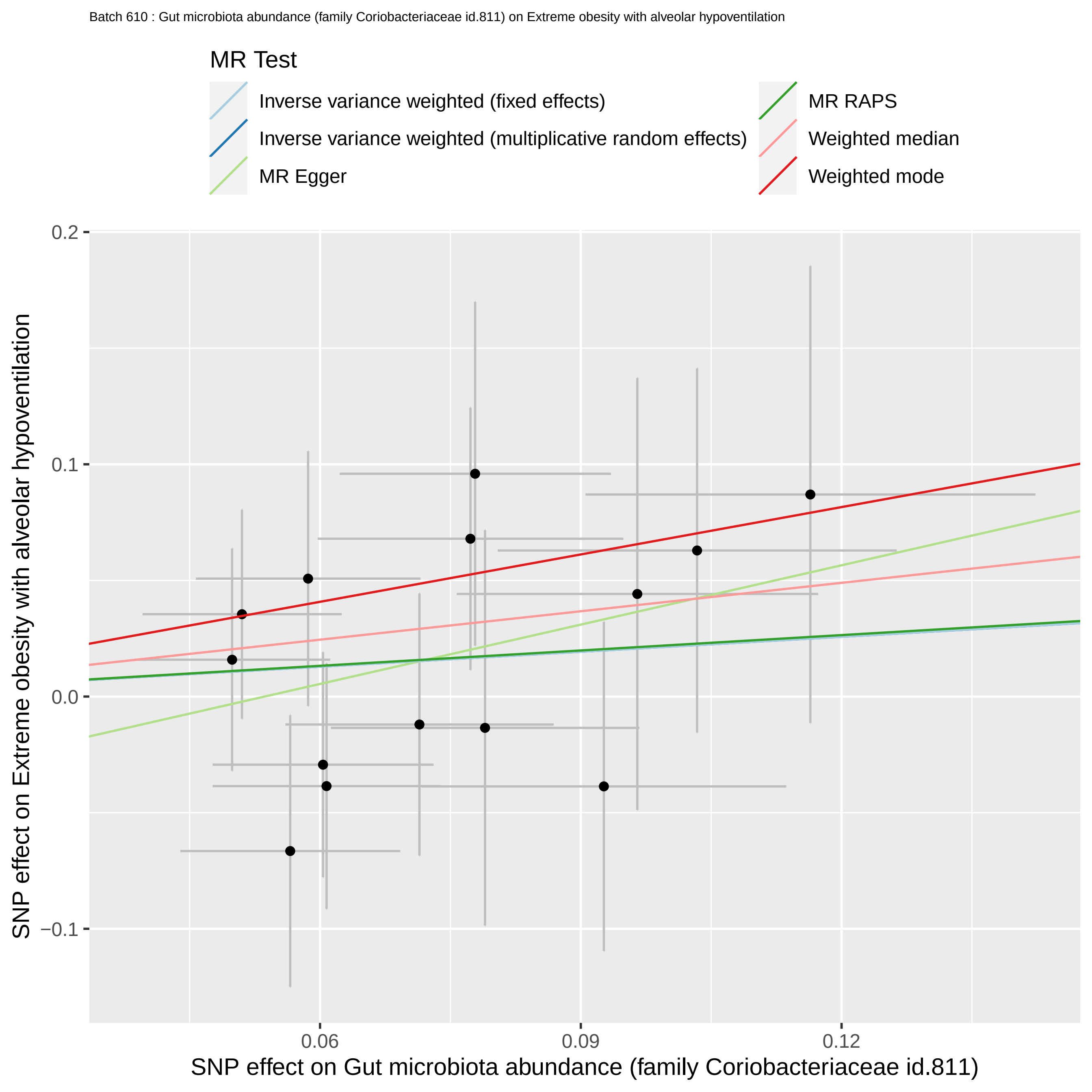

Batch 610 : Gut microbiota abundance (family Coriobacteriaceae id.811) on Extreme obesity with alveolar hypoventilation
MR Test
Inverse variance weighted (fixed effects)
Inverse variance weighted (multiplicative random effects)
MR Egger
MR RAPS
Weighted median
Weighted mode
0.2
0.1
0.0
−0.1
0.06
0.09
0.12
SNP effect on Extreme obesity with alveolar hypoventilation
SNP effect on Gut microbiota abundance (family Coriobacteriaceae id.811)

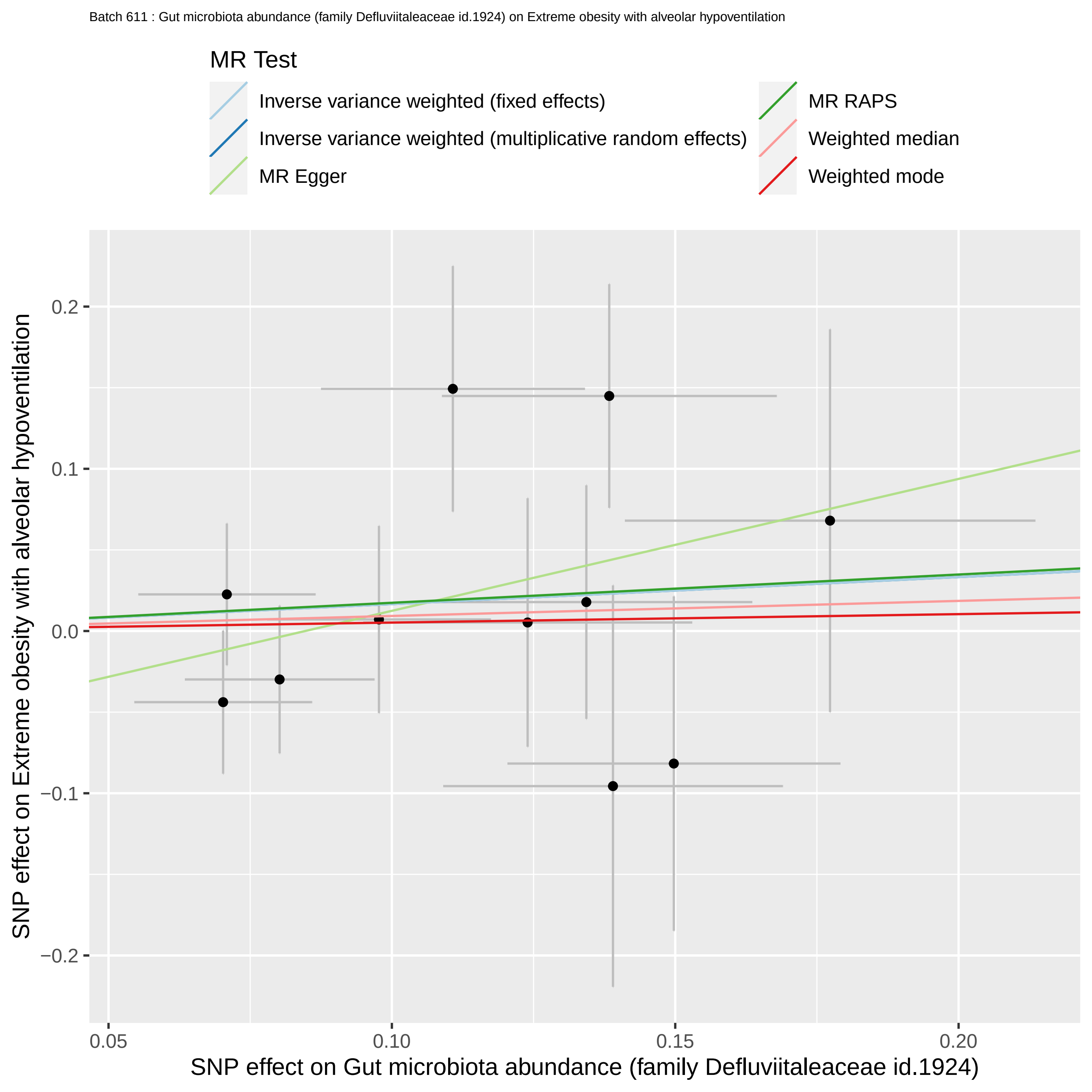

Batch 611 : Gut microbiota abundance (family Defluviitaleaceae id.1924) on Extreme obesity with alveolar hypoventilation
MR Test
Inverse variance weighted (fixed effects)
Inverse variance weighted (multiplicative random effects)
MR Egger
MR RAPS
Weighted median
Weighted mode
0.2
0.1
0.0
−0.1
−0.2
0.05
0.10
0.15
0.20
SNP effect on Extreme obesity with alveolar hypoventilation
SNP effect on Gut microbiota abundance (family Defluviitaleaceae id.1924)

Batch 612 : Gut microbiota abundance (family Desulfovibrionaceae id.3169) on Extreme obesity with alveolar hypoventilation

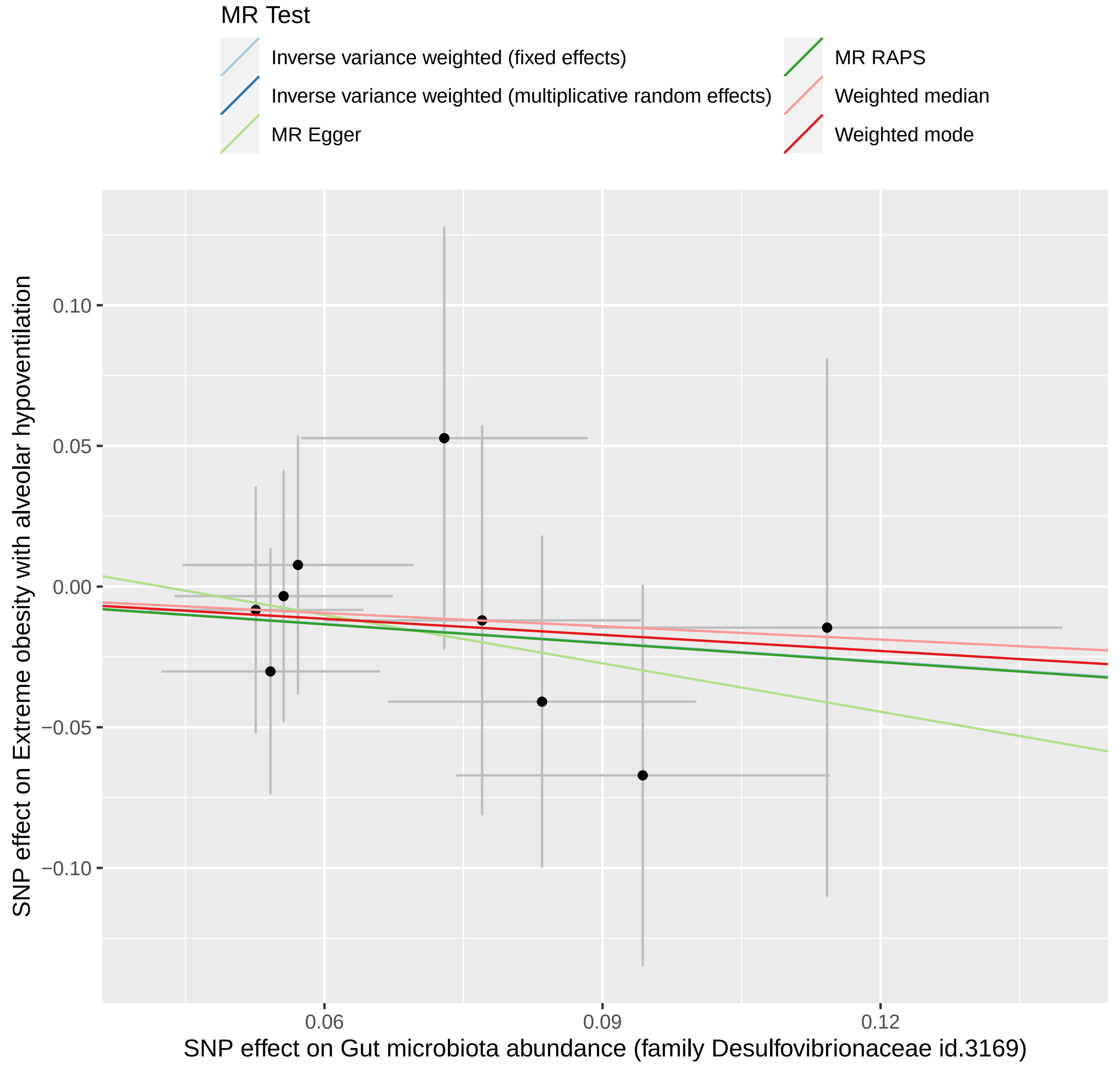

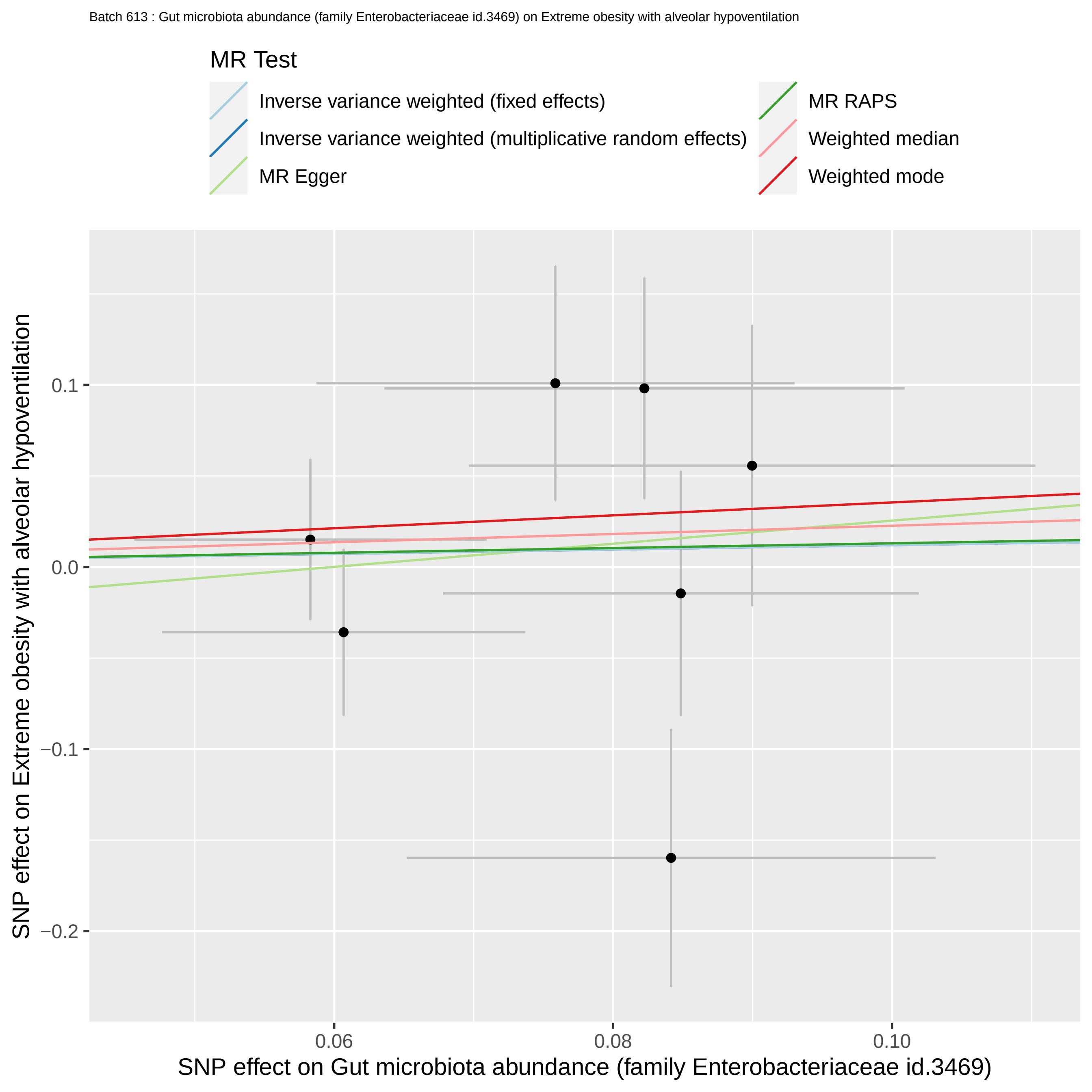
Batch 613 : Gut microbiota abundance (family Enterobacteriaceae id.3469) on Extreme obesity with alveolar hypoventilation
MR Test
Inverse variance weighted (fixed effects)
Inverse variance weighted (multiplicative random effects)
MR Egger
MR RAPS
Weighted median
Weighted mode
0.1
0.0
−0.1
−0.2
0.06
0.08
0.10
SNP effect on Extreme obesity with alveolar hypoventilation
SNP effect on Gut microbiota abundance (family Enterobacteriaceae id.3469)

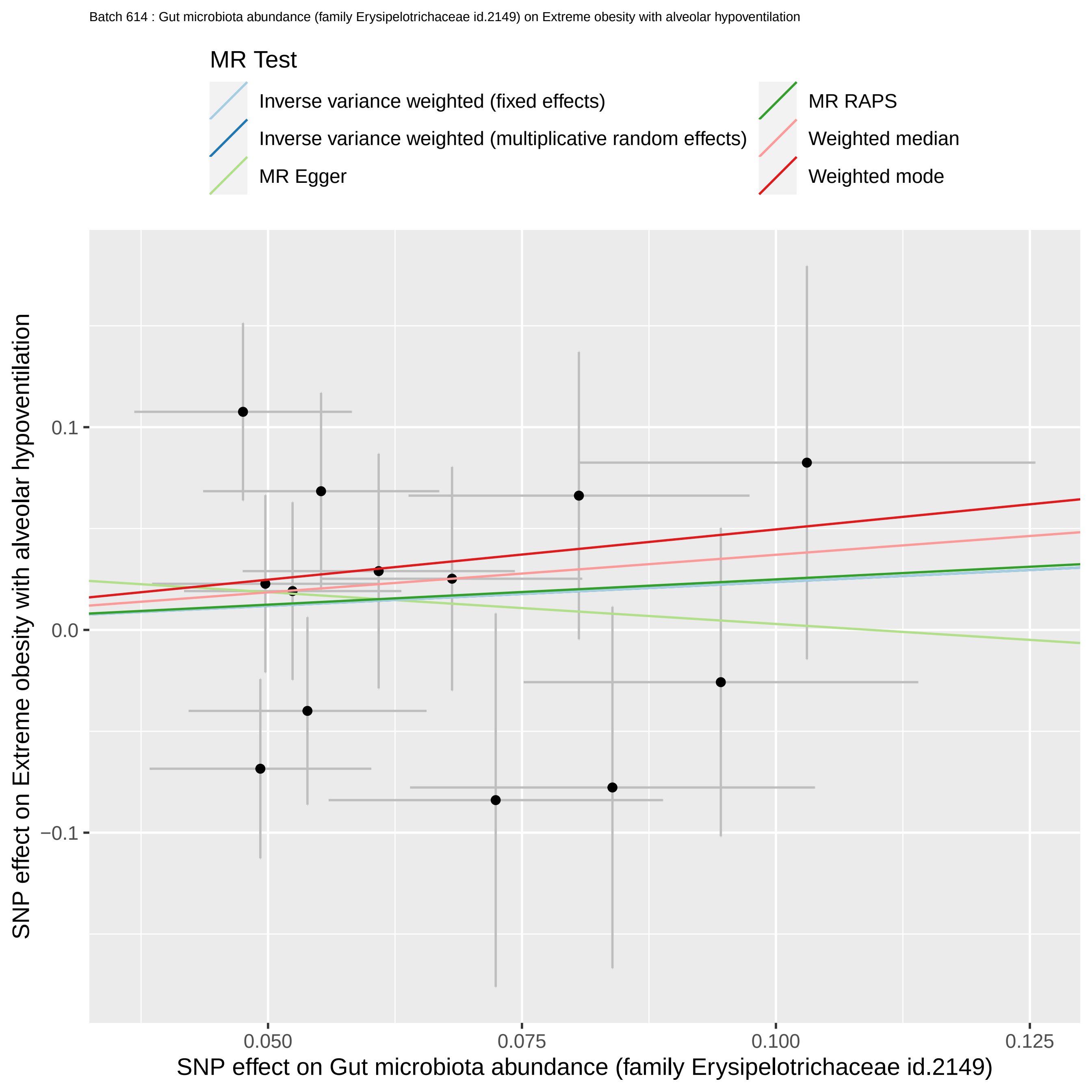

Batch 614 : Gut microbiota abundance (family Erysipelotrichaceae id.2149) on Extreme obesity with alveolar hypoventilation
MR Test
Inverse variance weighted (fixed effects)
Inverse variance weighted (multiplicative random effects)
MR Egger
MR RAPS
Weighted median
Weighted mode
0.1
0.0
−0.1
0.050
0.075
0.100
0.125
SNP effect on Extreme obesity with alveolar hypoventilation
SNP effect on Gut microbiota abundance (family Erysipelotrichaceae id.2149)

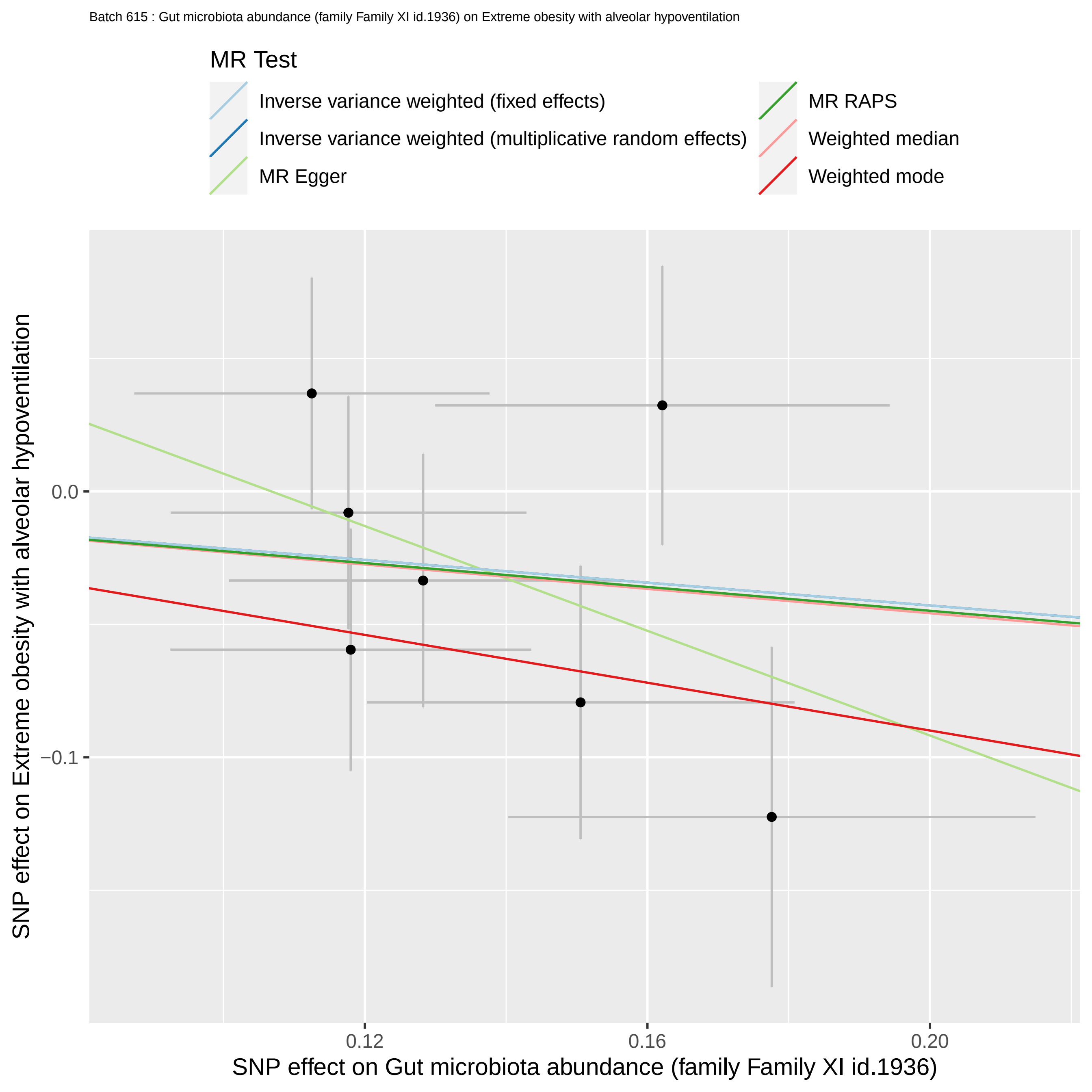
Batch 615 : Gut microbiota abundance (family Family XI id.1936) on Extreme obesity with alveolar hypoventilation
MR Test
Inverse variance weighted (fixed effects)
Inverse variance weighted (multiplicative random effects)
MR Egger
MR RAPS
Weighted median
Weighted mode
0.0
−0.1
0.12
0.16
0.20
SNP effect on Gut microbiota abundance (family Family XI id.1936)
SNP effect on Extreme obesity with alveolar hypoventilation

Batch 616 : Gut microbiota abundance (family Family XIII id.1957) on Extreme obesity with alveolar hypoventilation

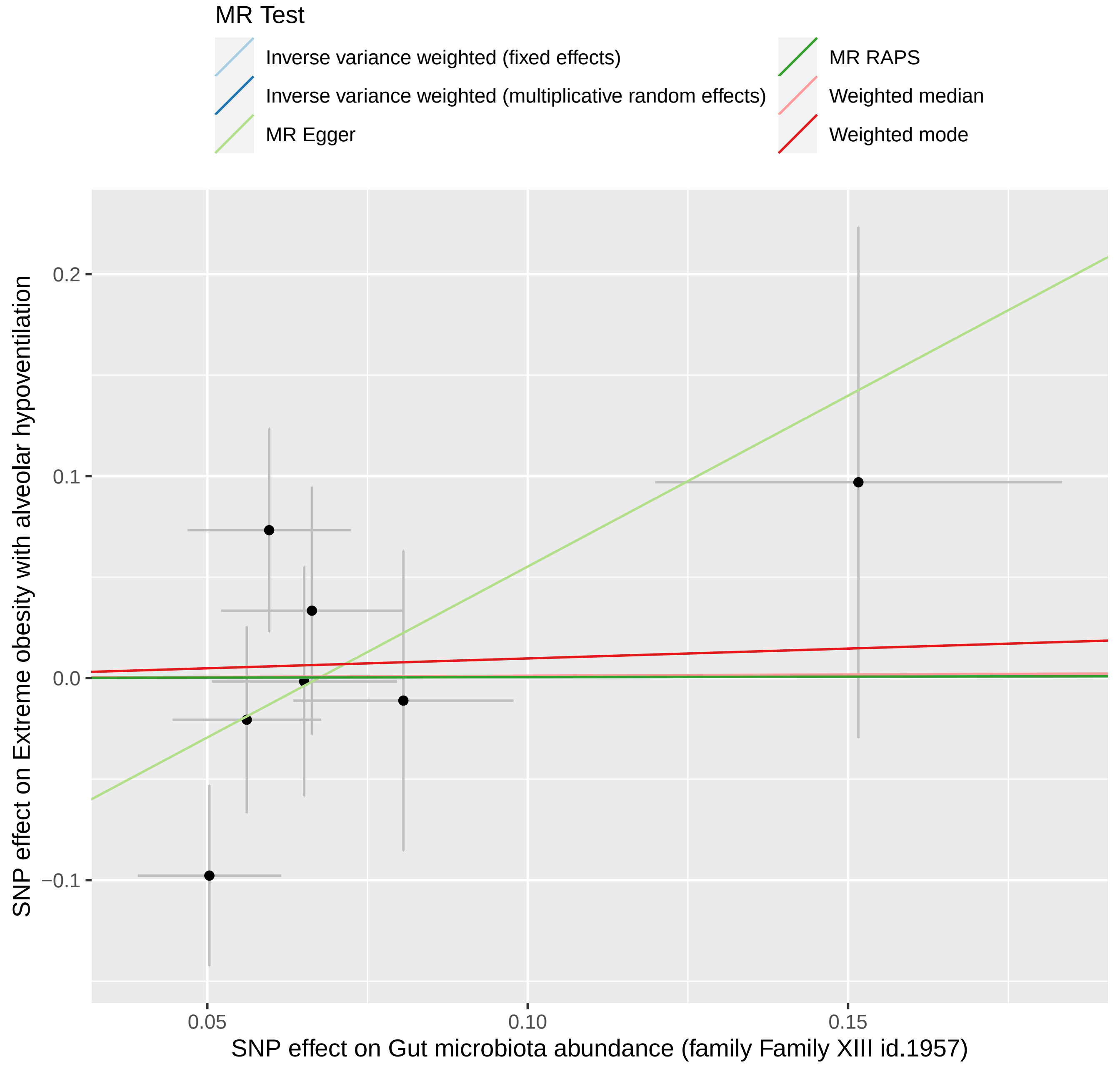

Batch 617 : Gut microbiota abundance (family Lachnospiraceae id.1987) on Extreme obesity with alveolar hypoventilation

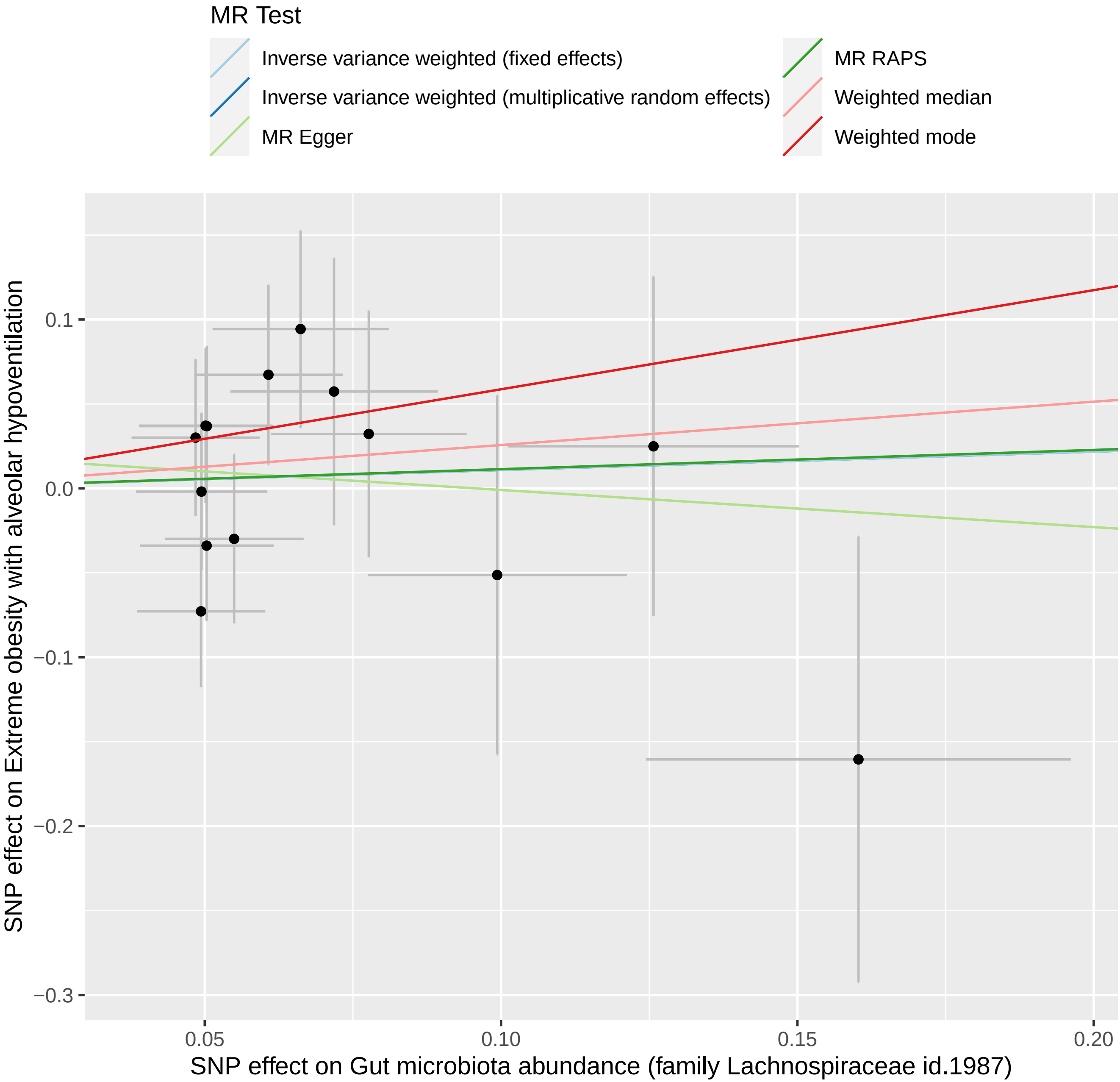

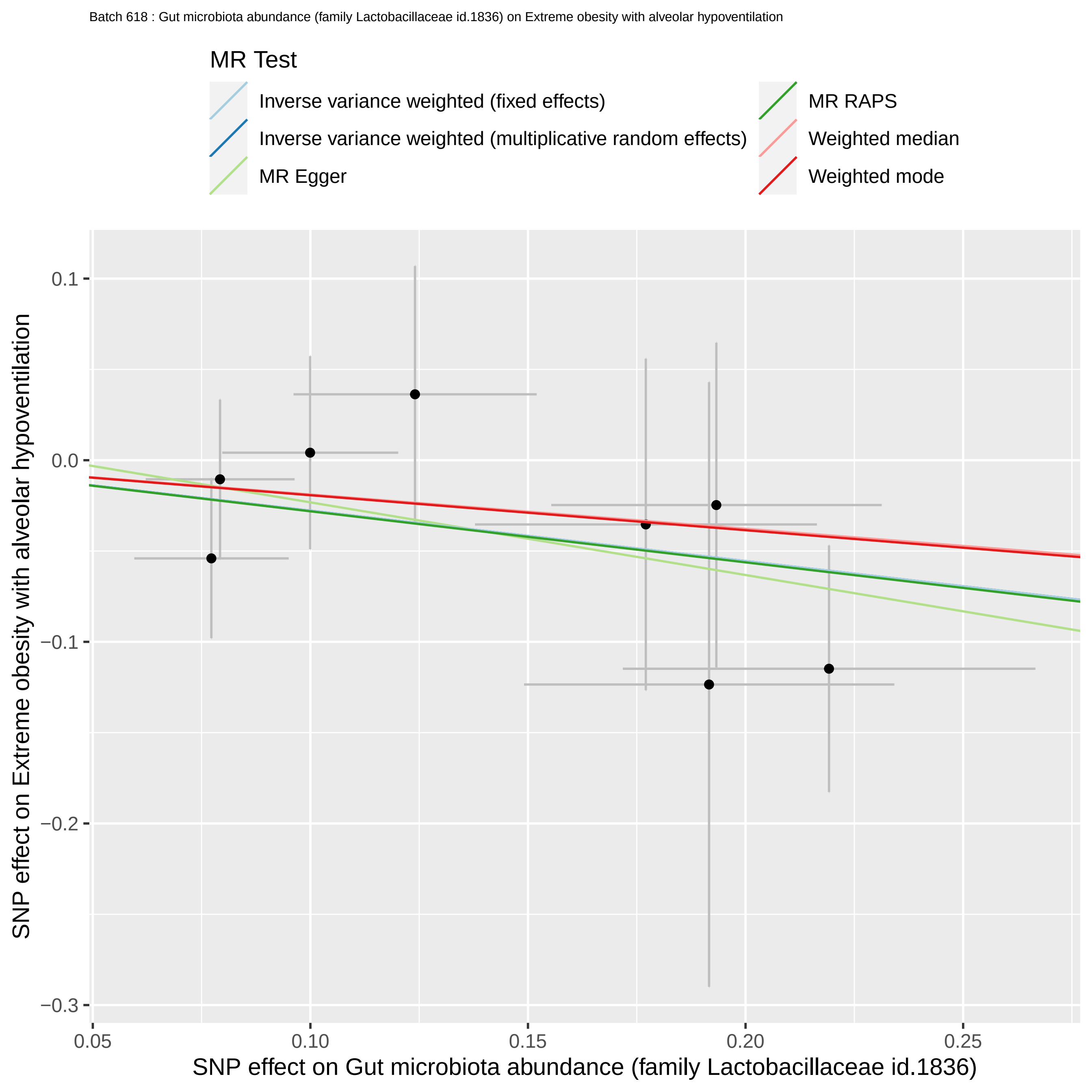
Batch 618 : Gut microbiota abundance (family Lactobacillaceae id.1836) on Extreme obesity with alveolar hypoventilation
MR Test
Inverse variance weighted (fixed effects)
Inverse variance weighted (multiplicative random effects)
MR Egger
MR RAPS
Weighted median
Weighted mode
SNP effect on Extreme obesity with alveolar hypoventilation
0.1
0.0
−0.1
−0.2
−0.3
0.05
0.10
0.15
0.20
0.25
SNP effect on Gut microbiota abundance (family Lactobacillaceae id.1836)

Batch 619 : Gut microbiota abundance (family Methanobacteriaceae id.121) on Extreme obesity with alveolar hypoventilation

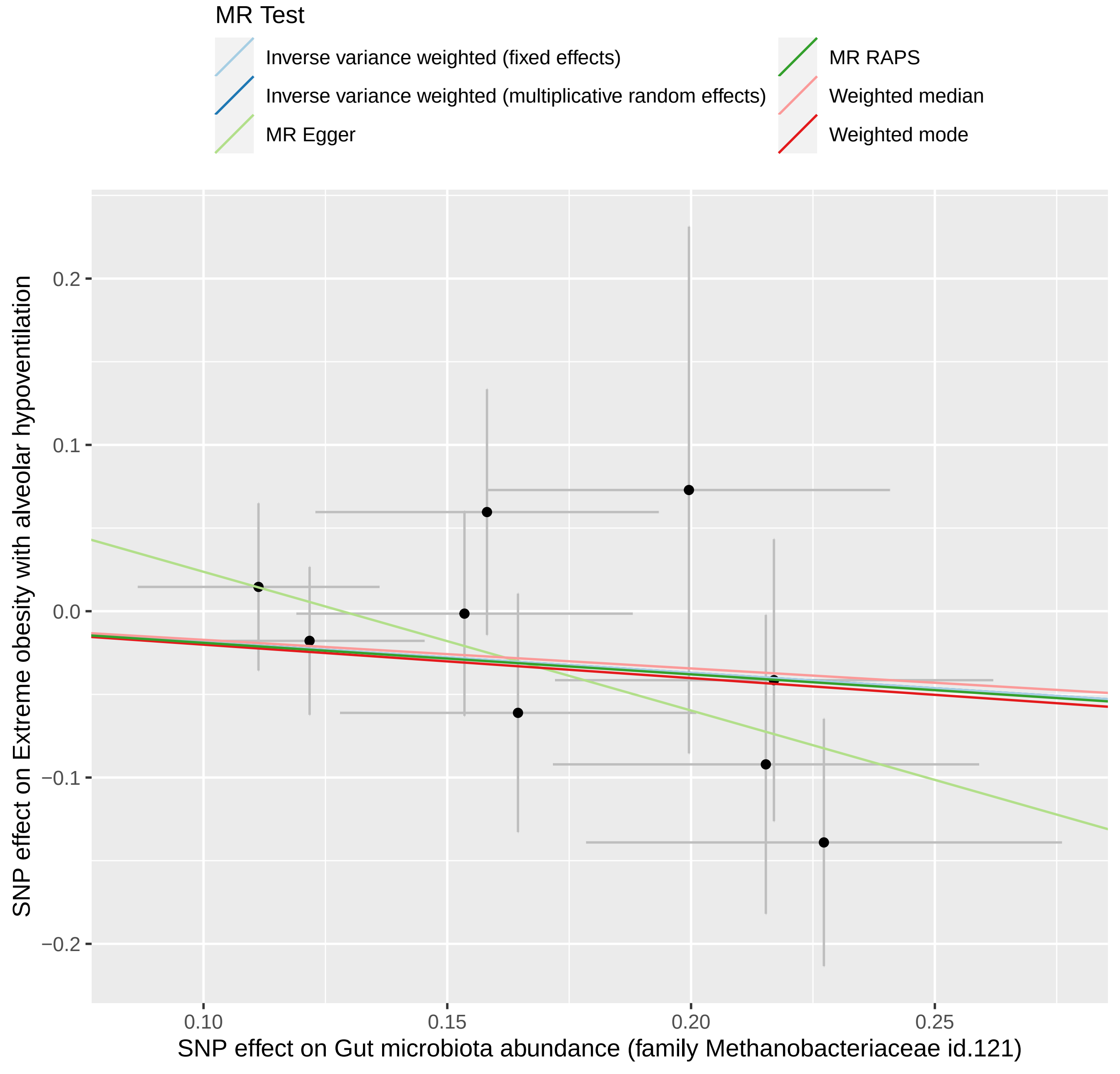

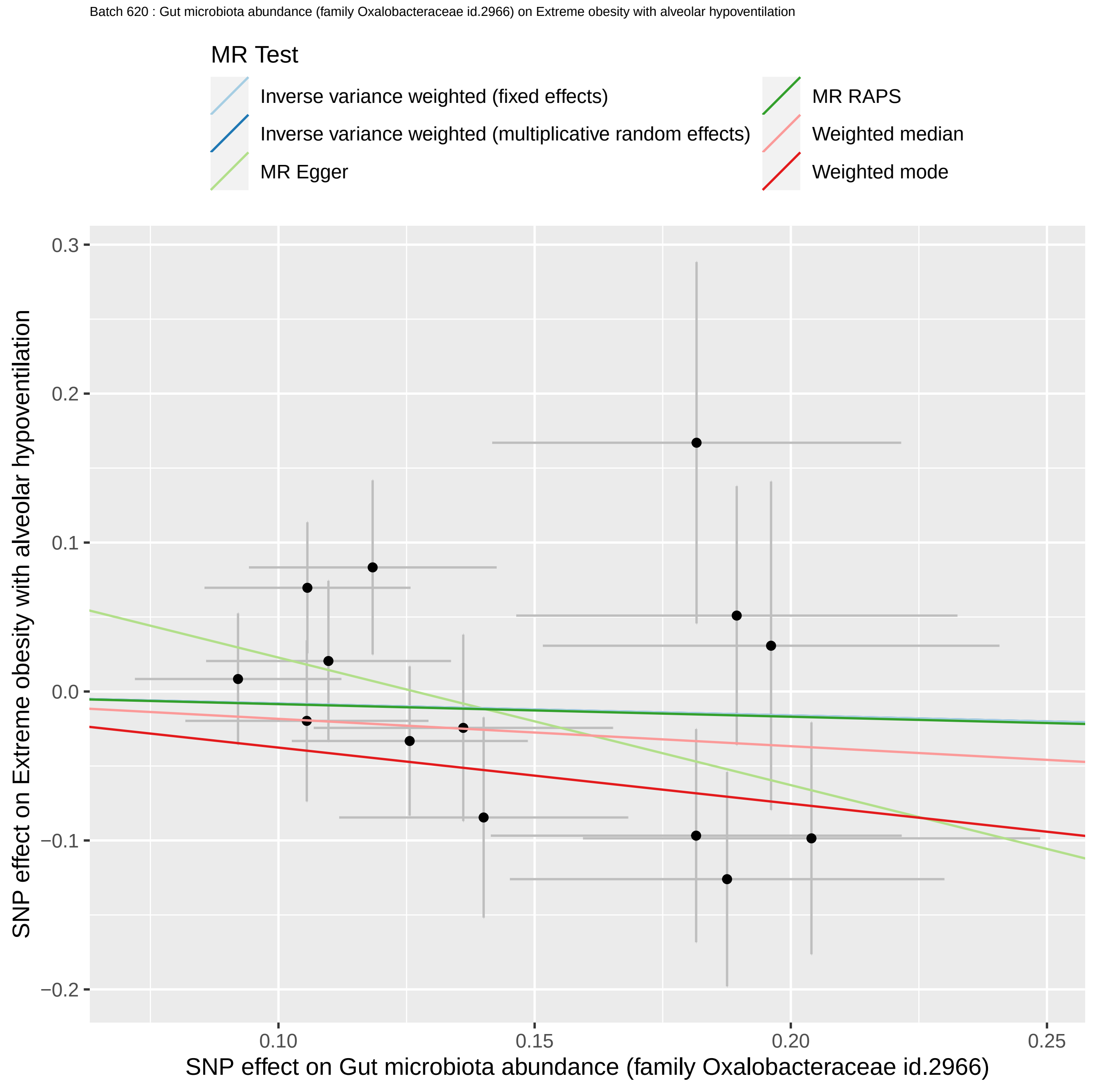
Batch 620 : Gut microbiota abundance (family Oxalobacteraceae id.2966) on Extreme obesity with alveolar hypoventilation
MR Test
Inverse variance weighted (fixed effects)
Inverse variance weighted (multiplicative random effects)
MR Egger
MR RAPS
Weighted median
Weighted mode
SNP effect on Extreme obesity with alveolar hypoventilation
0.3
0.2
0.1
0.0
−0.1
−0.2
0.10
0.15
0.20
0.25
SNP effect on Gut microbiota abundance (family Oxalobacteraceae id.2966)

Batch 621 : Gut microbiota abundance (family Pasteurellaceae id.3689) on Extreme obesity with alveolar hypoventilation

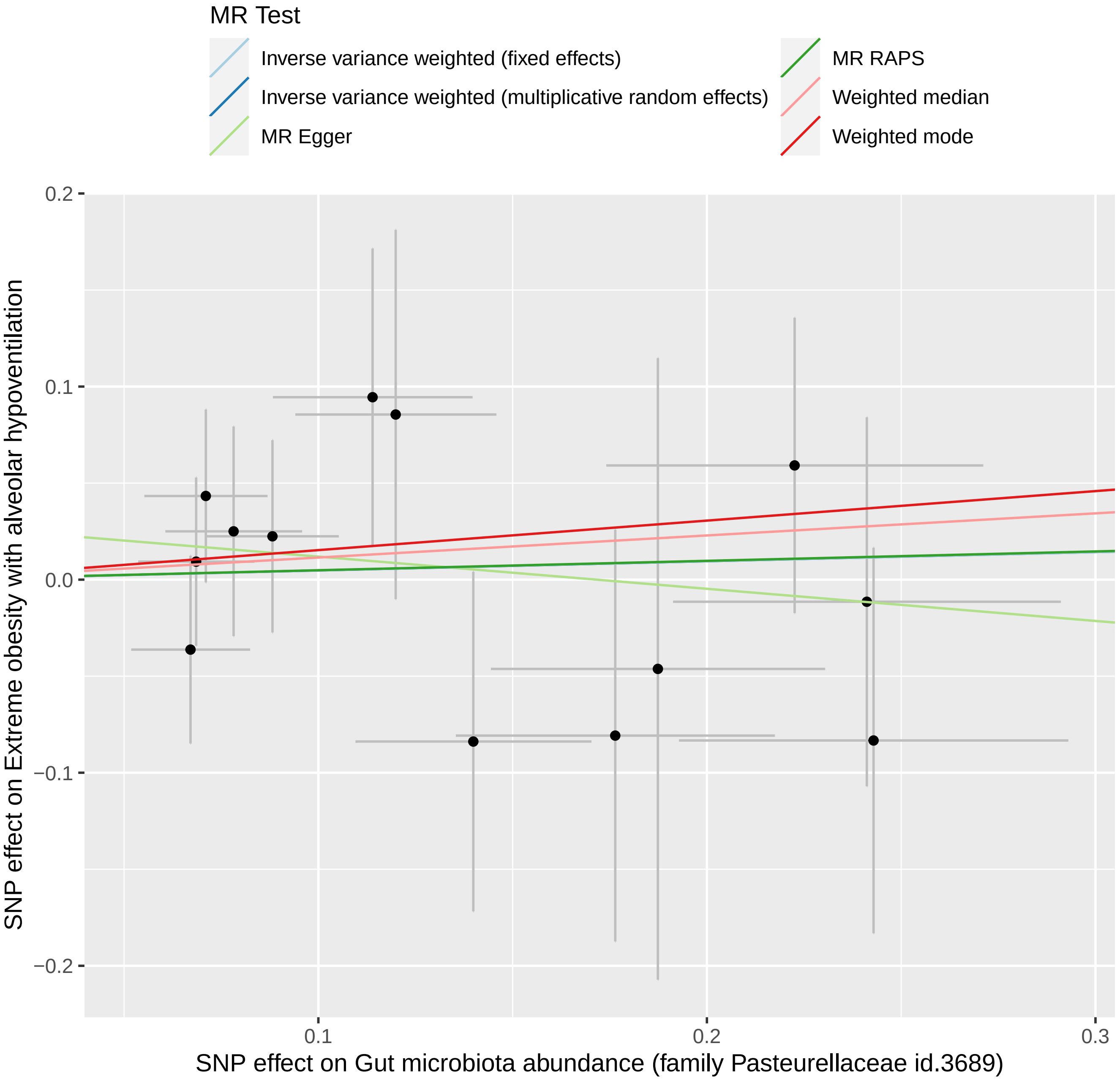

Batch 622 : Gut microbiota abundance (family Peptococcaceae id.2024) on Extreme obesity with alveolar hypoventilation

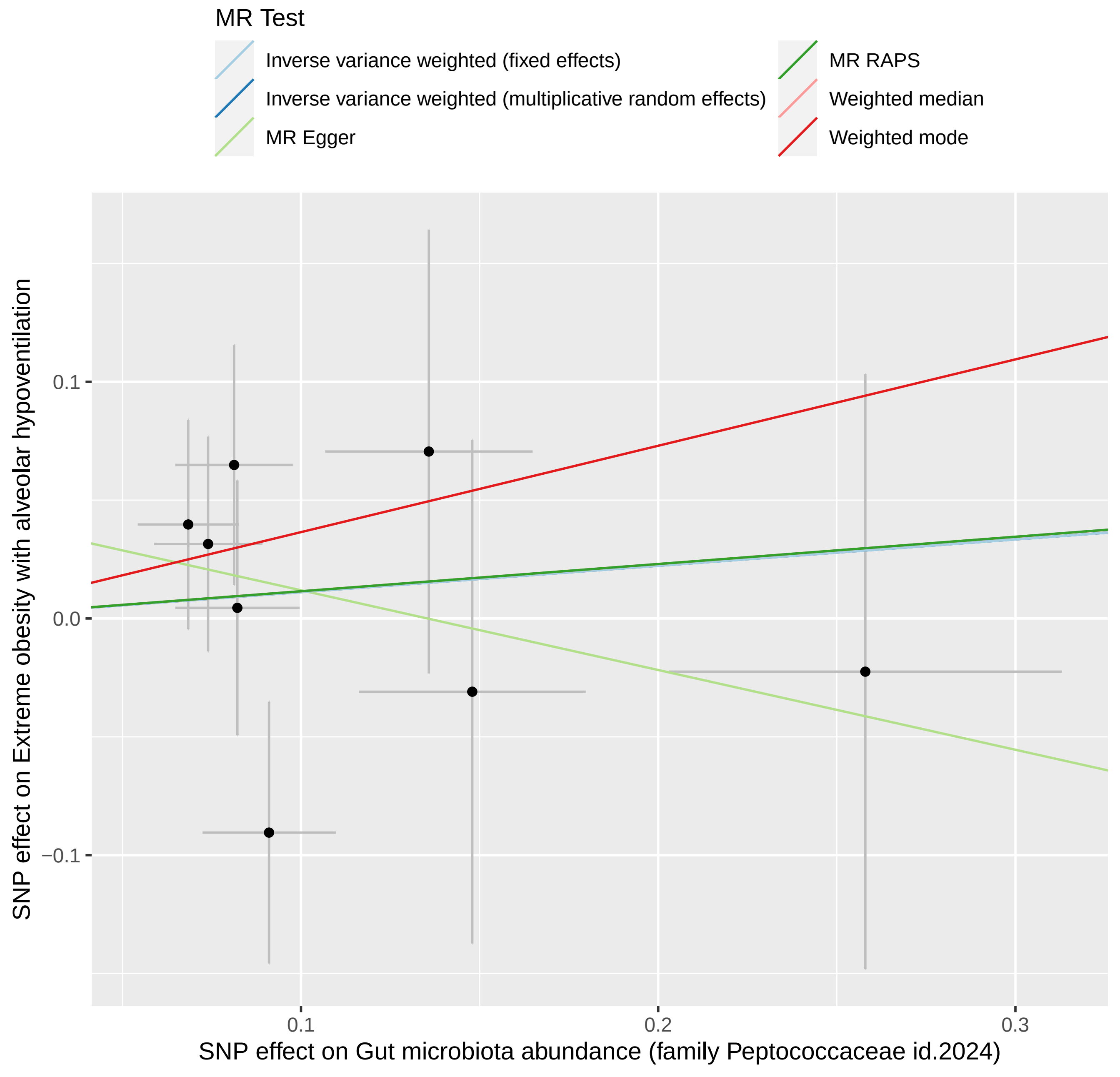

Batch 623 : Gut microbiota abundance (family Peptostreptococcaceae id.2042) on Extreme obesity with alveolar hypoventilation

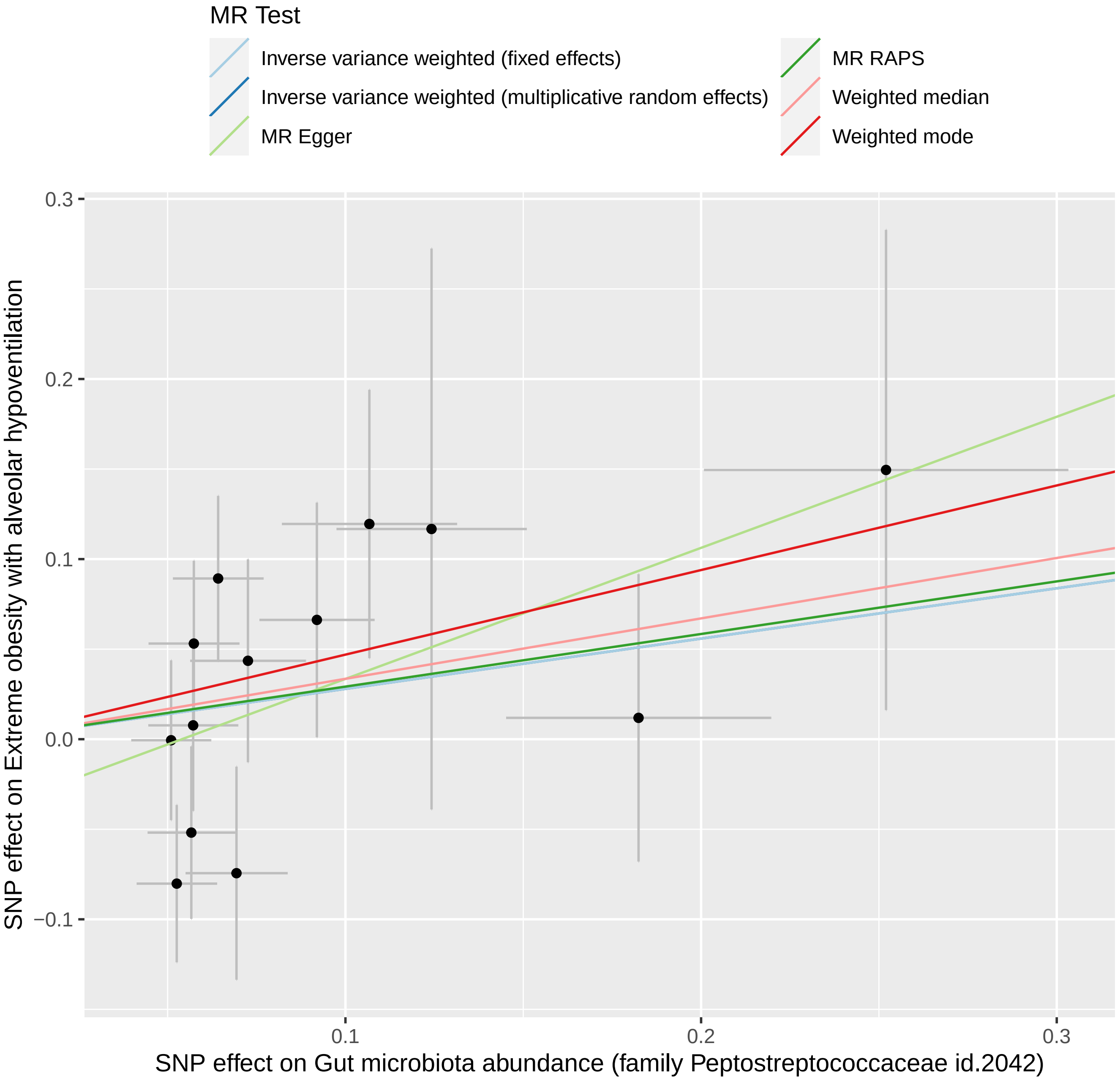

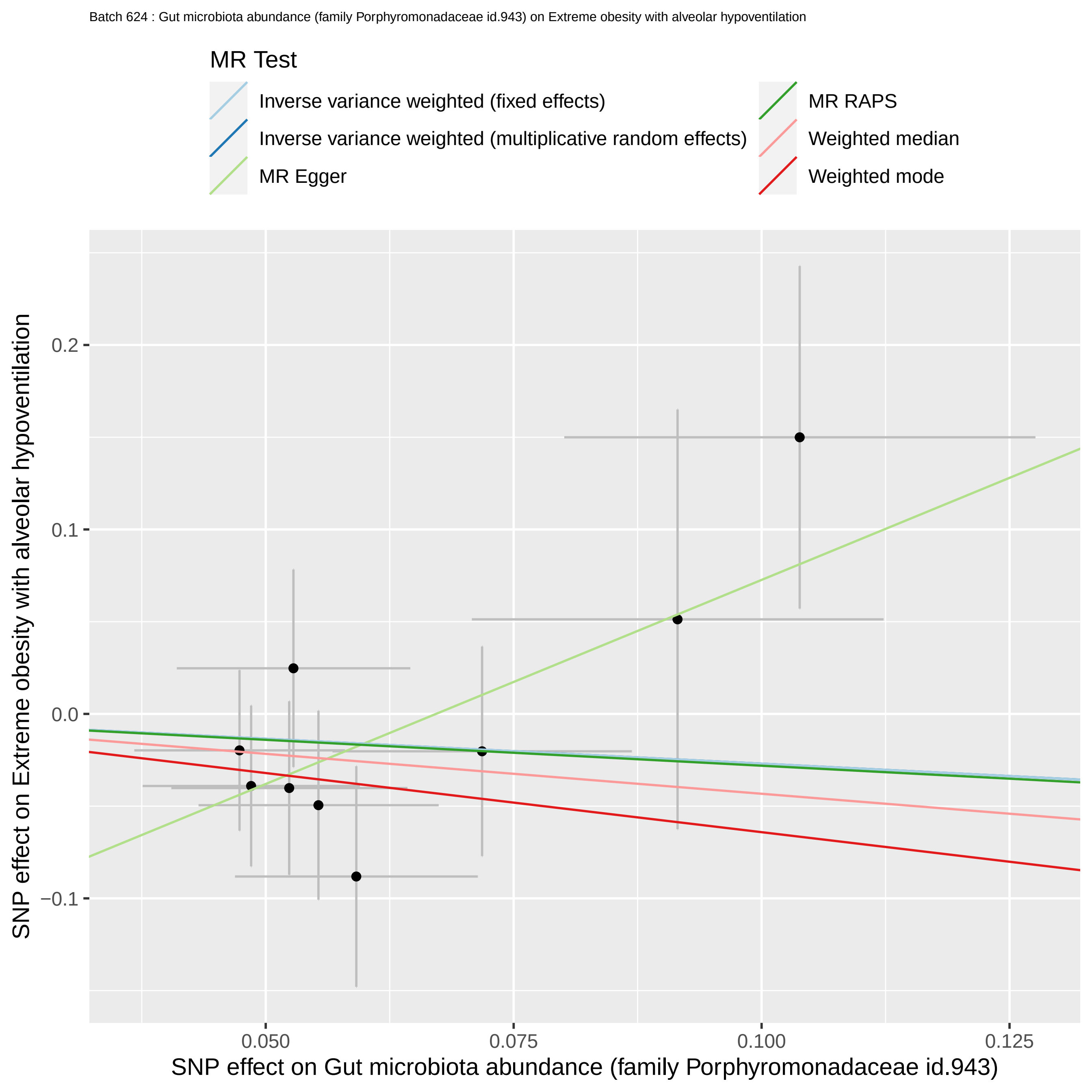
Batch 624 : Gut microbiota abundance (family Porphyromonadaceae id.943) on Extreme obesity with alveolar hypoventilation
MR Test
Inverse variance weighted (fixed effects)
Inverse variance weighted (multiplicative random effects)
MR Egger
MR RAPS
Weighted median
Weighted mode
0.2
0.1
0.0
−0.1
0.050
0.075
0.100
0.125
SNP effect on Extreme obesity with alveolar hypoventilation
SNP effect on Gut microbiota abundance (family Porphyromonadaceae id.943)

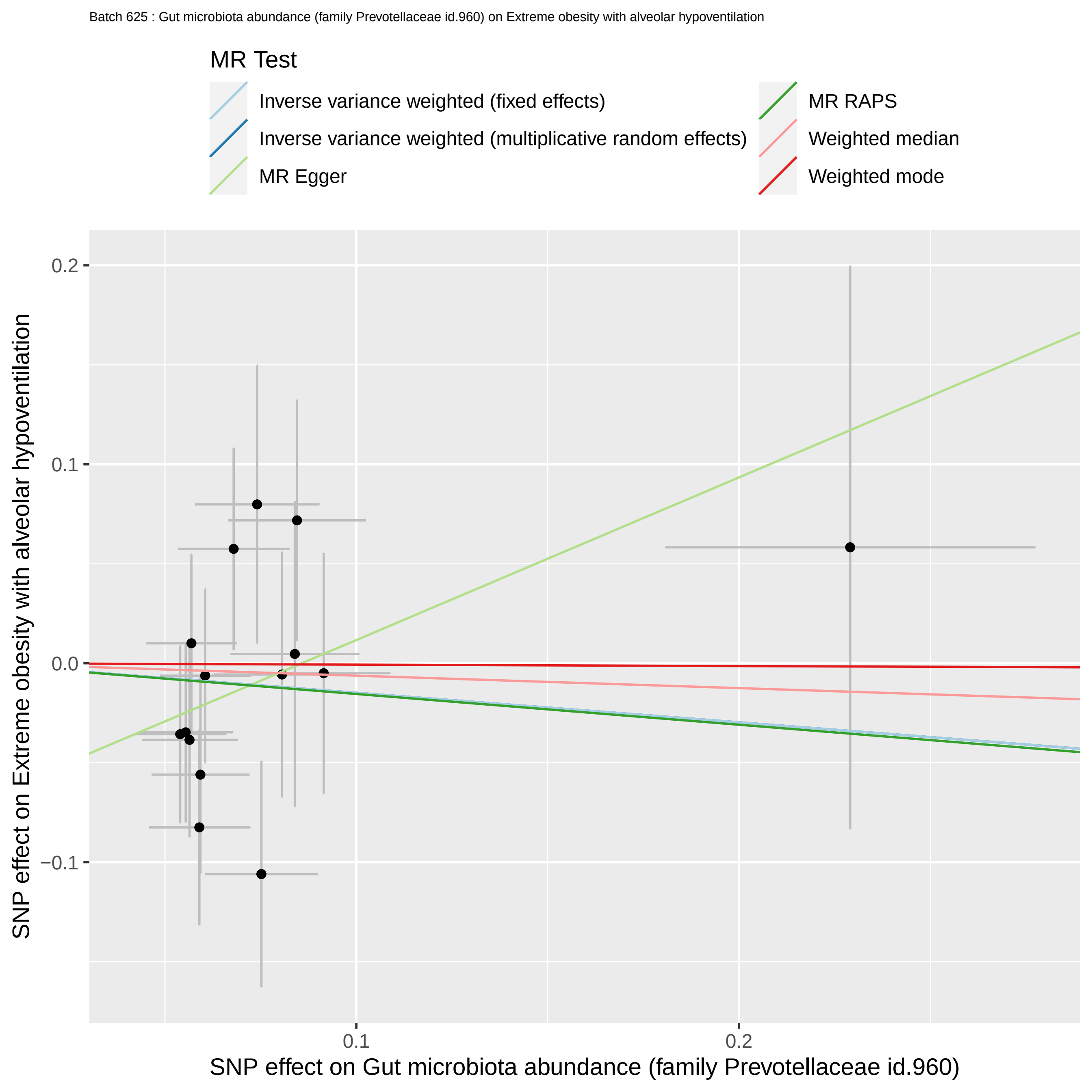
Batch 625 : Gut microbiota abundance (family Prevotellaceae id.960) on Extreme obesity with alveolar hypoventilation
MR Test
Inverse variance weighted (fixed effects)
Inverse variance weighted (multiplicative random effects)
MR Egger
MR RAPS
Weighted median
Weighted mode
0.2
0.1
0.0
−0.1
0.1
0.2
SNP effect on Extreme obesity with alveolar hypoventilation
SNP effect on Gut microbiota abundance (family Prevotellaceae id.960)

Batch 626 : Gut microbiota abundance (family Rhodospirillaceae id.2717) on Extreme obesity with alveolar hypoventilation

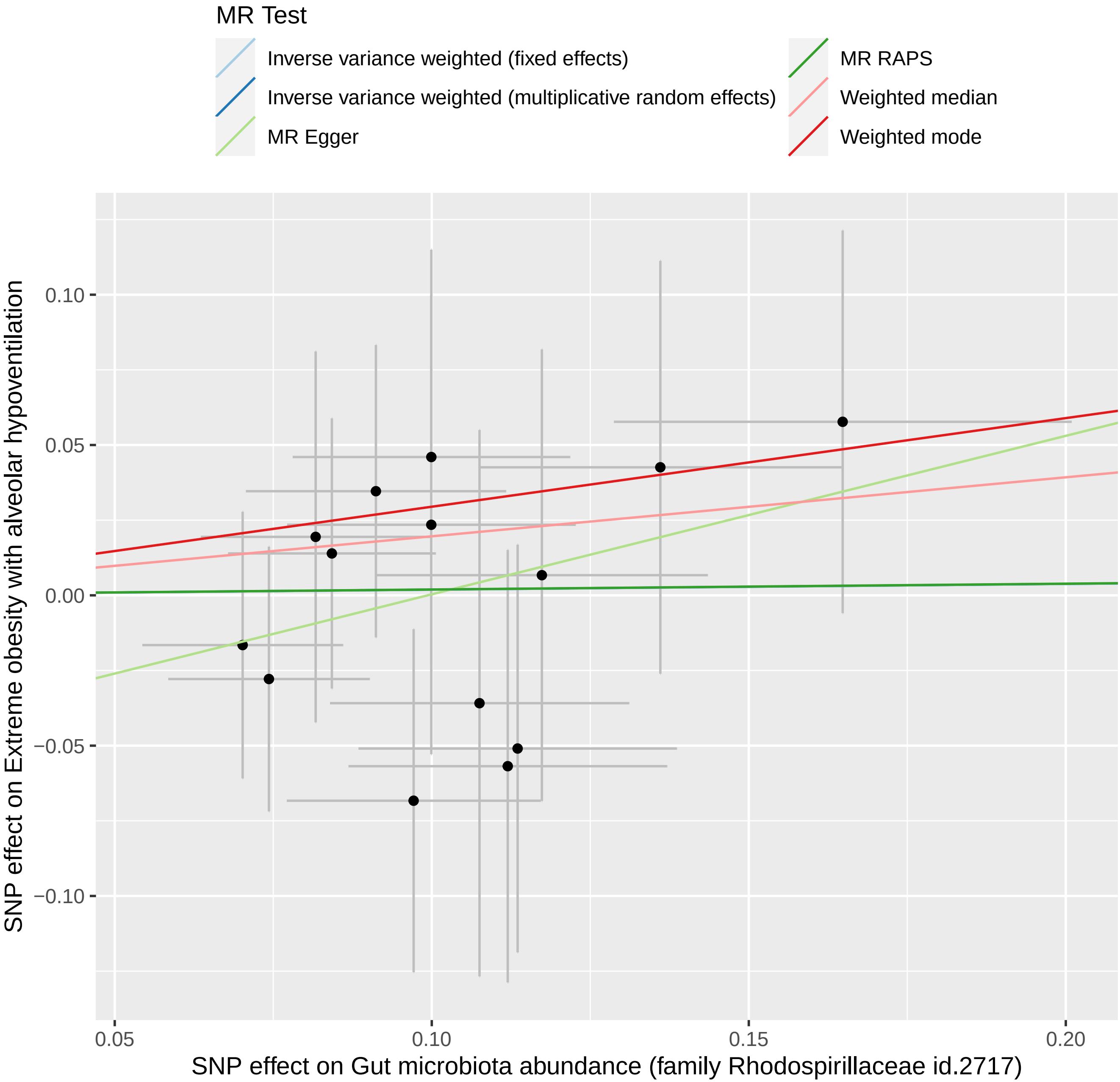

Batch 627 : Gut microbiota abundance (family Rikenellaceae id.967) on Extreme obesity with alveolar hypoventilation

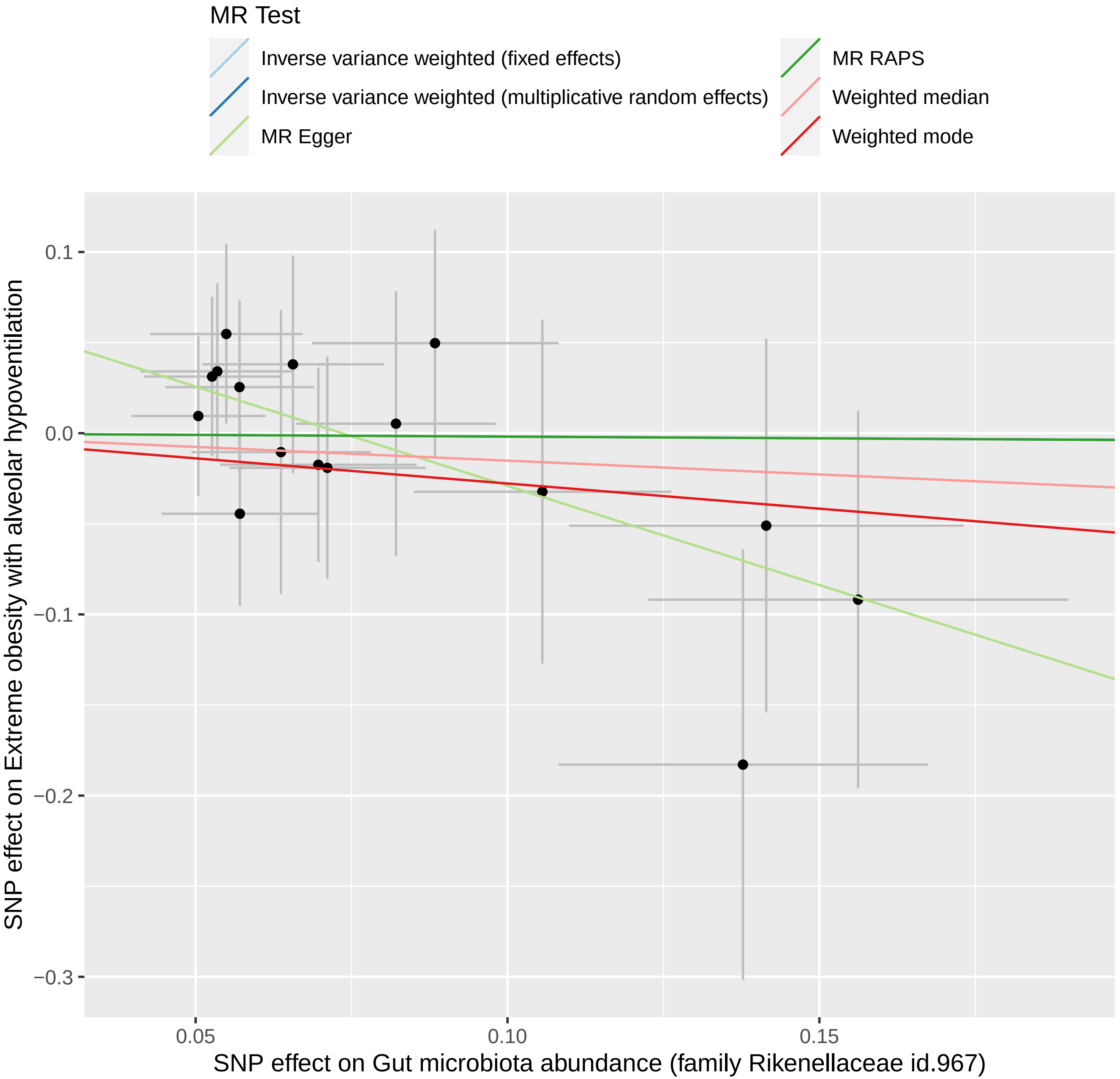

Batch 628 : Gut microbiota abundance (family Ruminococcaceae id.2050) on Extreme obesity with alveolar hypoventilation

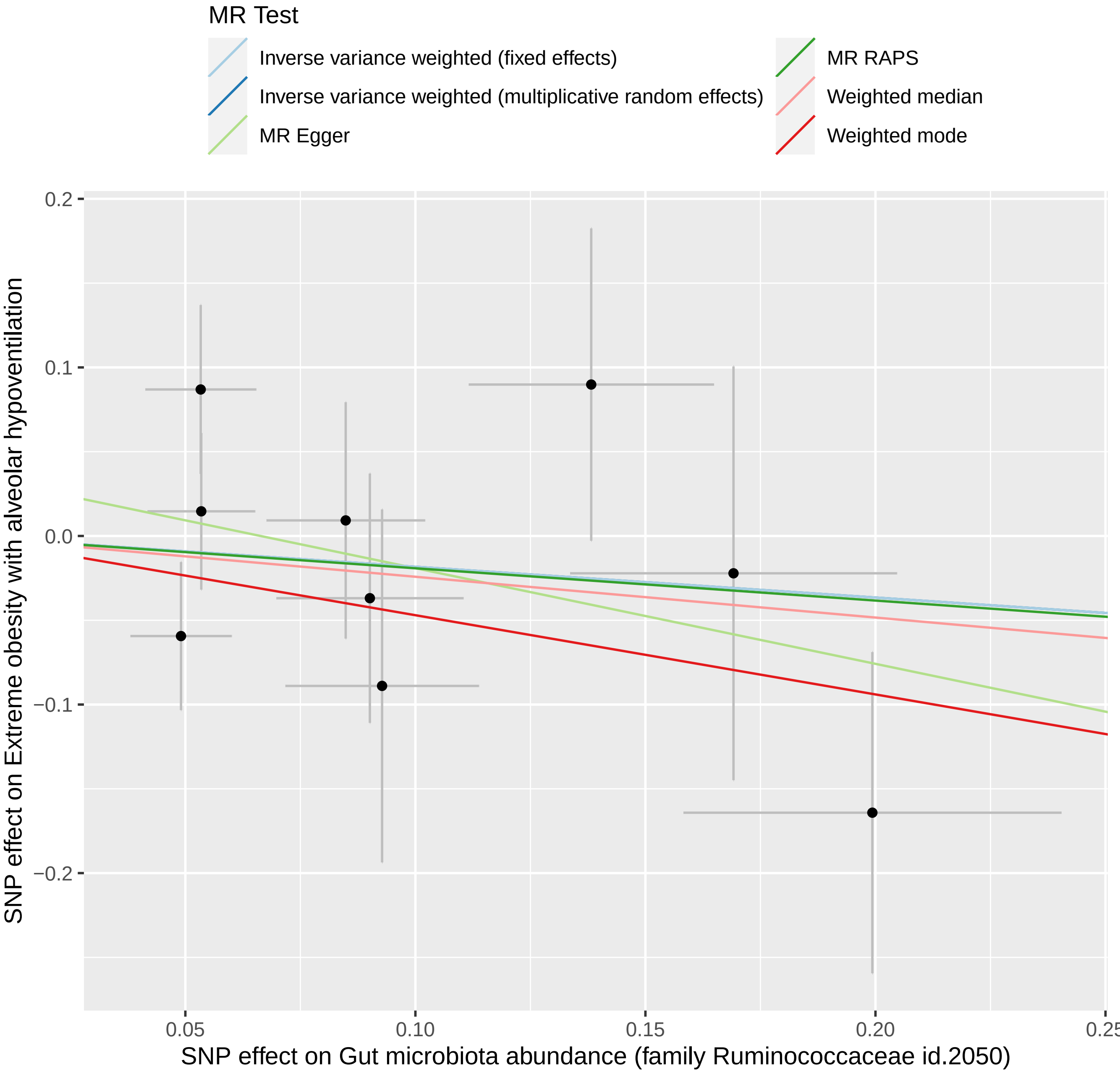

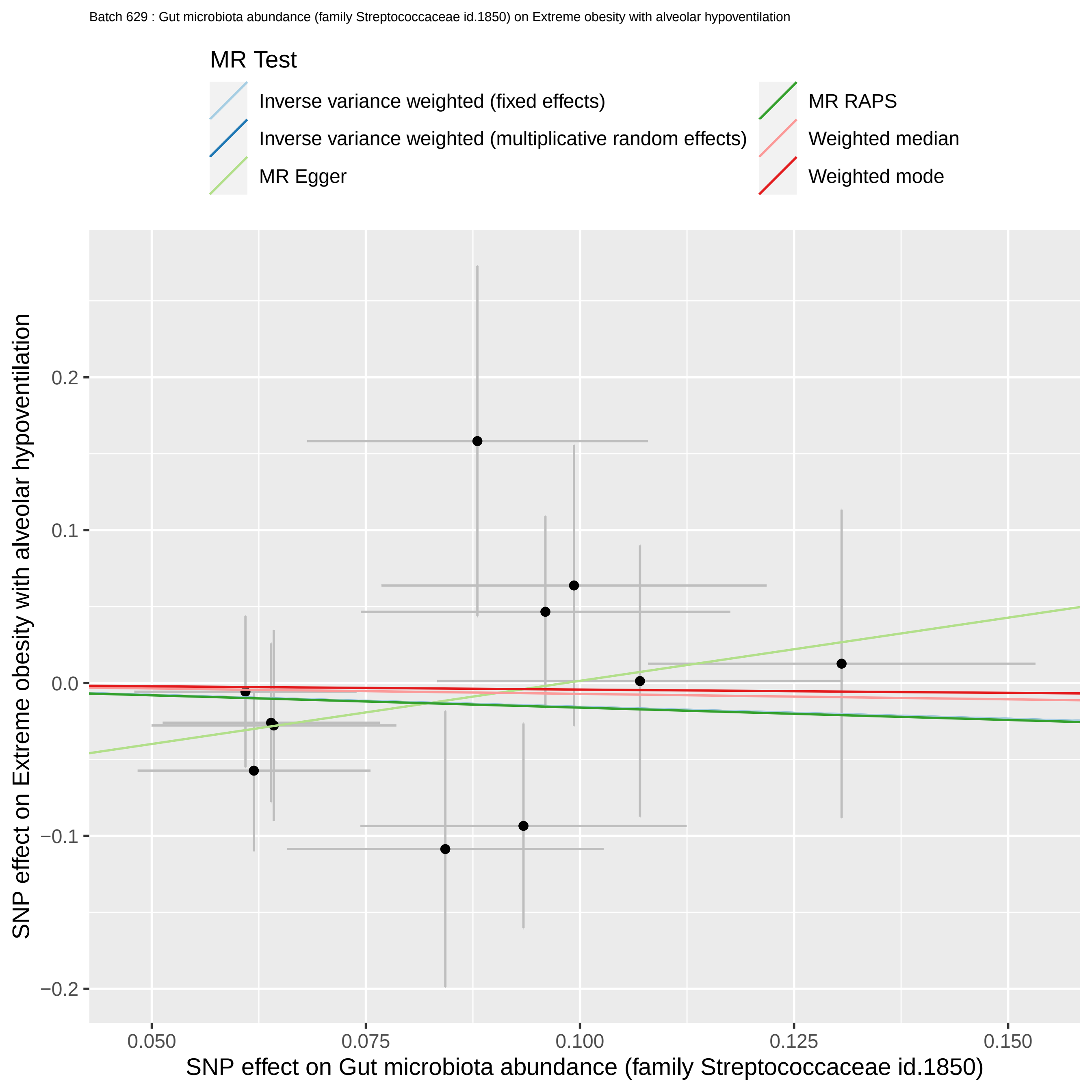

Batch 629 : Gut microbiota abundance (family Streptococcaceae id.1850) on Extreme obesity with alveolar hypoventilation
MR Test
Inverse variance weighted (fixed effects)
Inverse variance weighted (multiplicative random effects)
MR Egger
MR RAPS
Weighted median
Weighted mode
0.2
0.1
0.0
−0.1
−0.2
0.050
0.075
0.100
0.125
0.150
SNP effect on Extreme obesity with alveolar hypoventilation
SNP effect on Gut microbiota abundance (family Streptococcaceae id.1850)

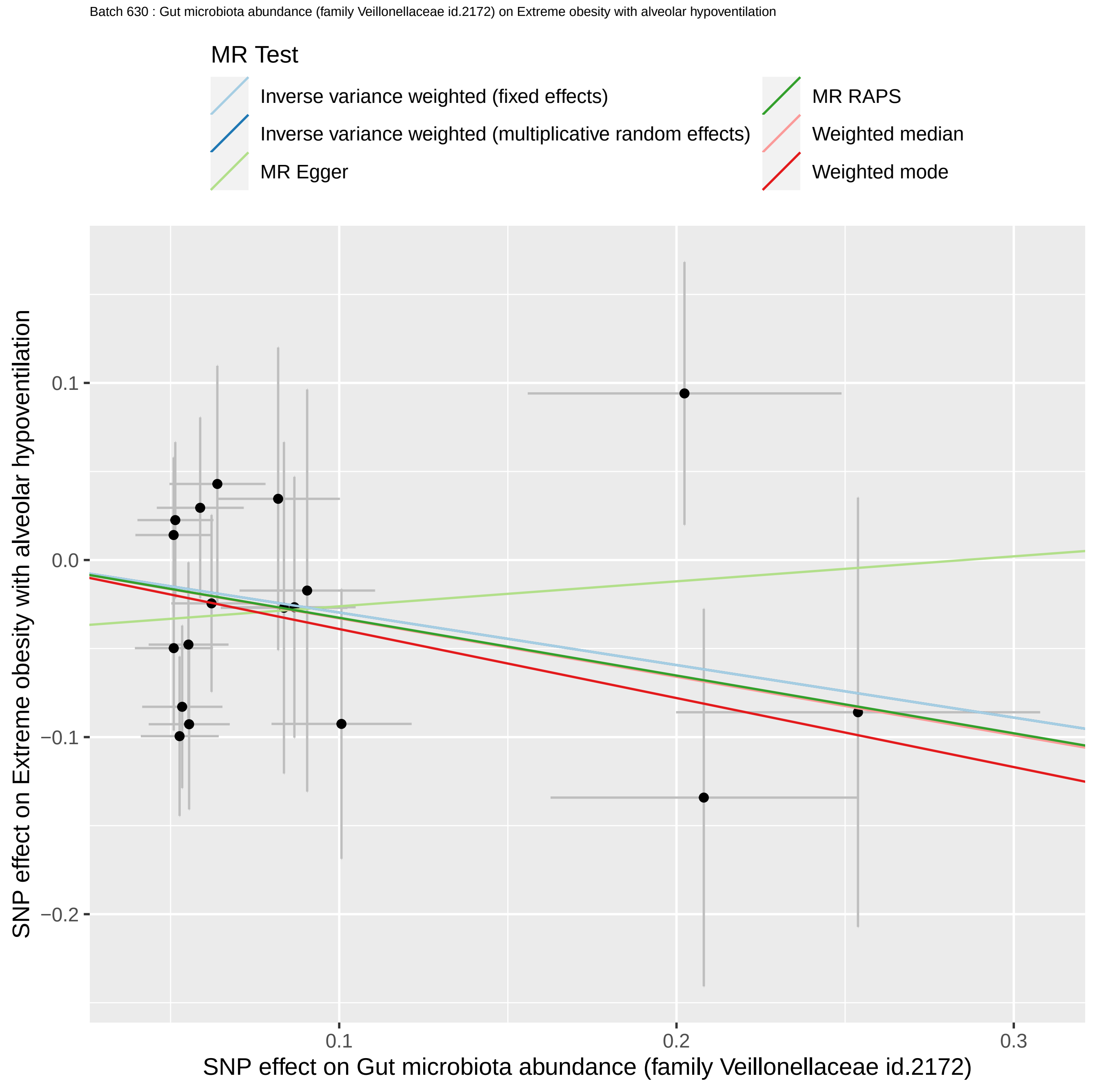
Batch 630 : Gut microbiota abundance (family Veillonellaceae id.2172) on Extreme obesity with alveolar hypoventilation
MR Test
Inverse variance weighted (fixed effects)
Inverse variance weighted (multiplicative random effects)
MR Egger
MR RAPS
Weighted median
Weighted mode
SNP effect on Extreme obesity with alveolar hypoventilation
0.1
0.0
−0.1
−0.2
0.1
0.2
0.3
SNP effect on Gut microbiota abundance (family Veillonellaceae id.2172)

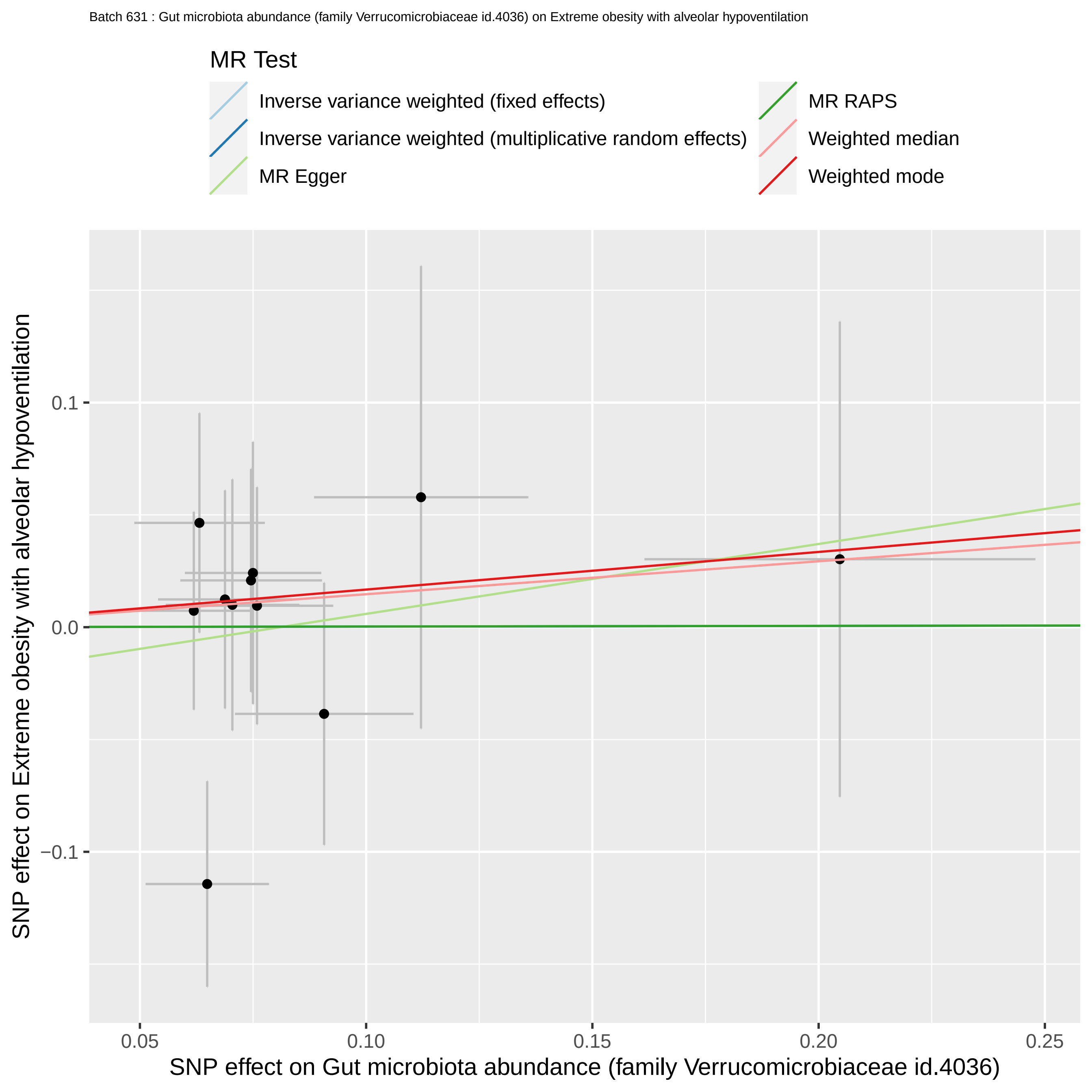

Batch 631 : Gut microbiota abundance (family Verrucomicrobiaceae id.4036) on Extreme obesity with alveolar hypoventilation
MR Test
Inverse variance weighted (fixed effects)
Inverse variance weighted (multiplicative random effects)
MR Egger
MR RAPS
Weighted median
Weighted mode
SNP effect on Extreme obesity with alveolar hypoventilation
0.1
0.0
−0.1
0.05
0.10
0.15
0.20
0.25
SNP effect on Gut microbiota abundance (family Verrucomicrobiaceae id.4036)

Batch 632 : Gut microbiota abundance (family Victivallaceae id.2255) on Extreme obesity with alveolar hypoventilation

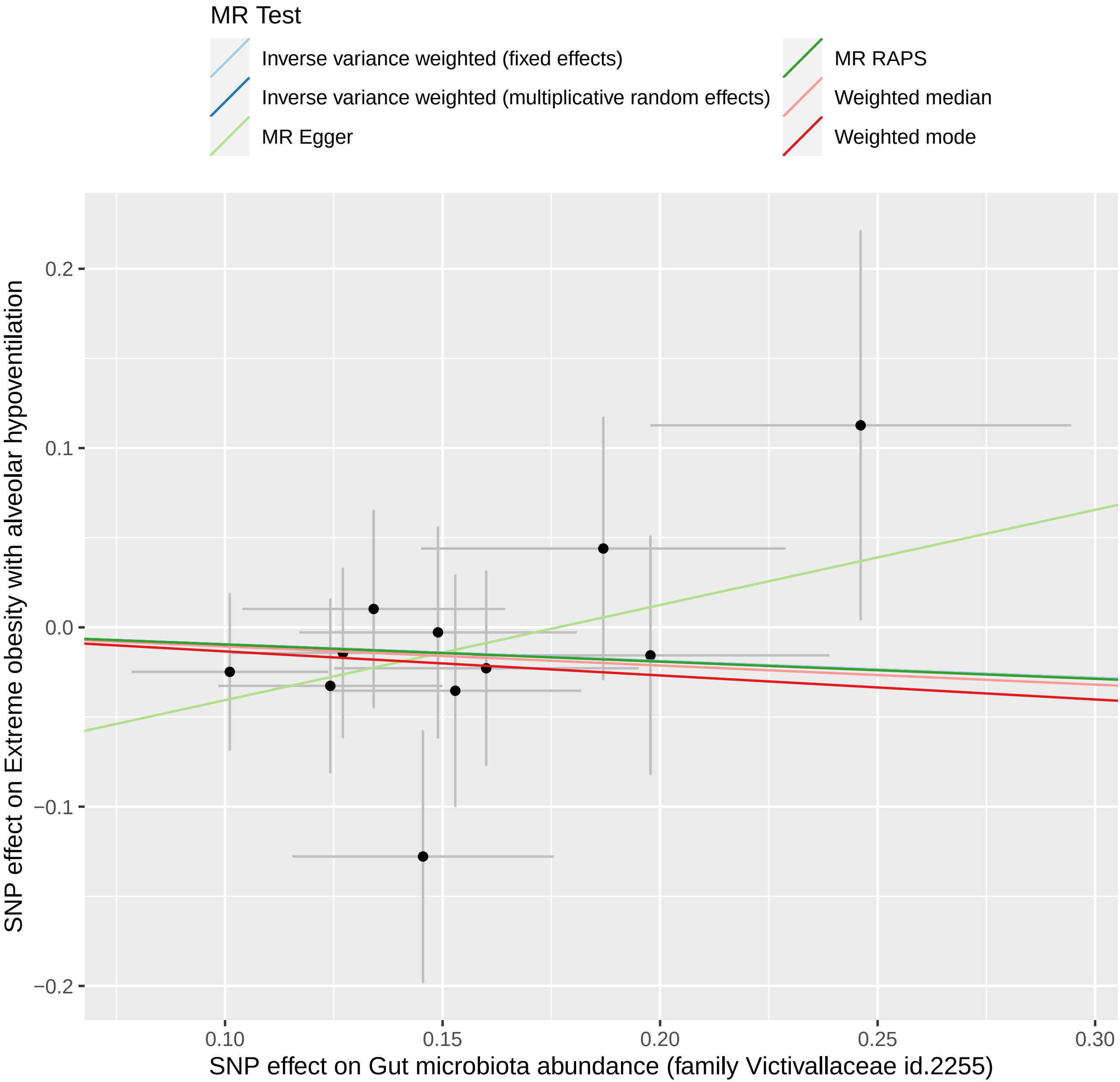

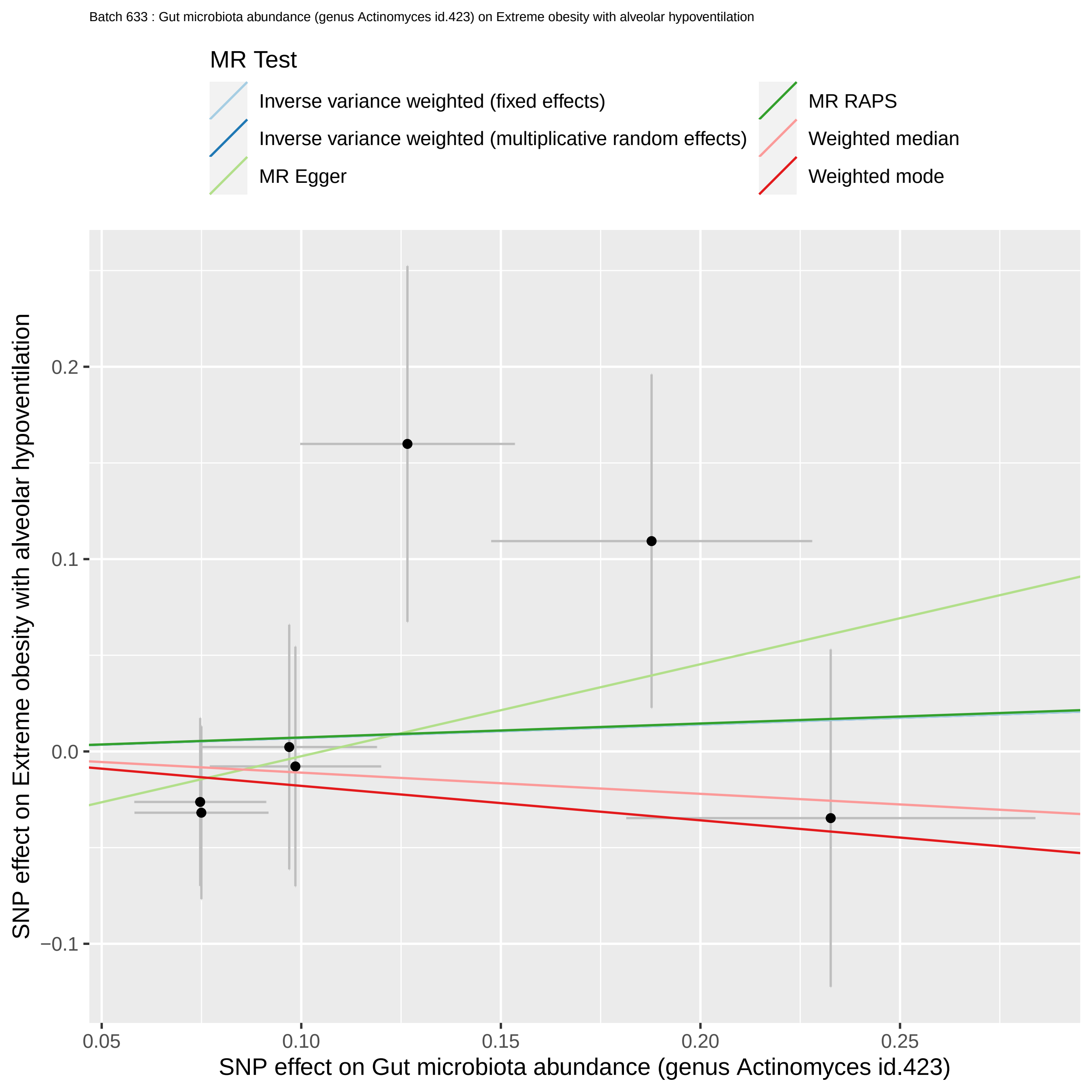

Batch 633 : Gut microbiota abundance (genus Actinomyces id.423) on Extreme obesity with alveolar hypoventilation
MR Test
Inverse variance weighted (fixed effects)
Inverse variance weighted (multiplicative random effects)
MR Egger
MR RAPS
Weighted median
Weighted mode
SNP effect on Extreme obesity with alveolar hypoventilation
0.2
0.1
0.0
−0.1
0.05
0.10
0.15
0.20
0.25
SNP effect on Gut microbiota abundance (genus Actinomyces id.423)

Batch 634 : Gut microbiota abundance (genus Adlercreutzia id.812) on Extreme obesity with alveolar hypoventilation

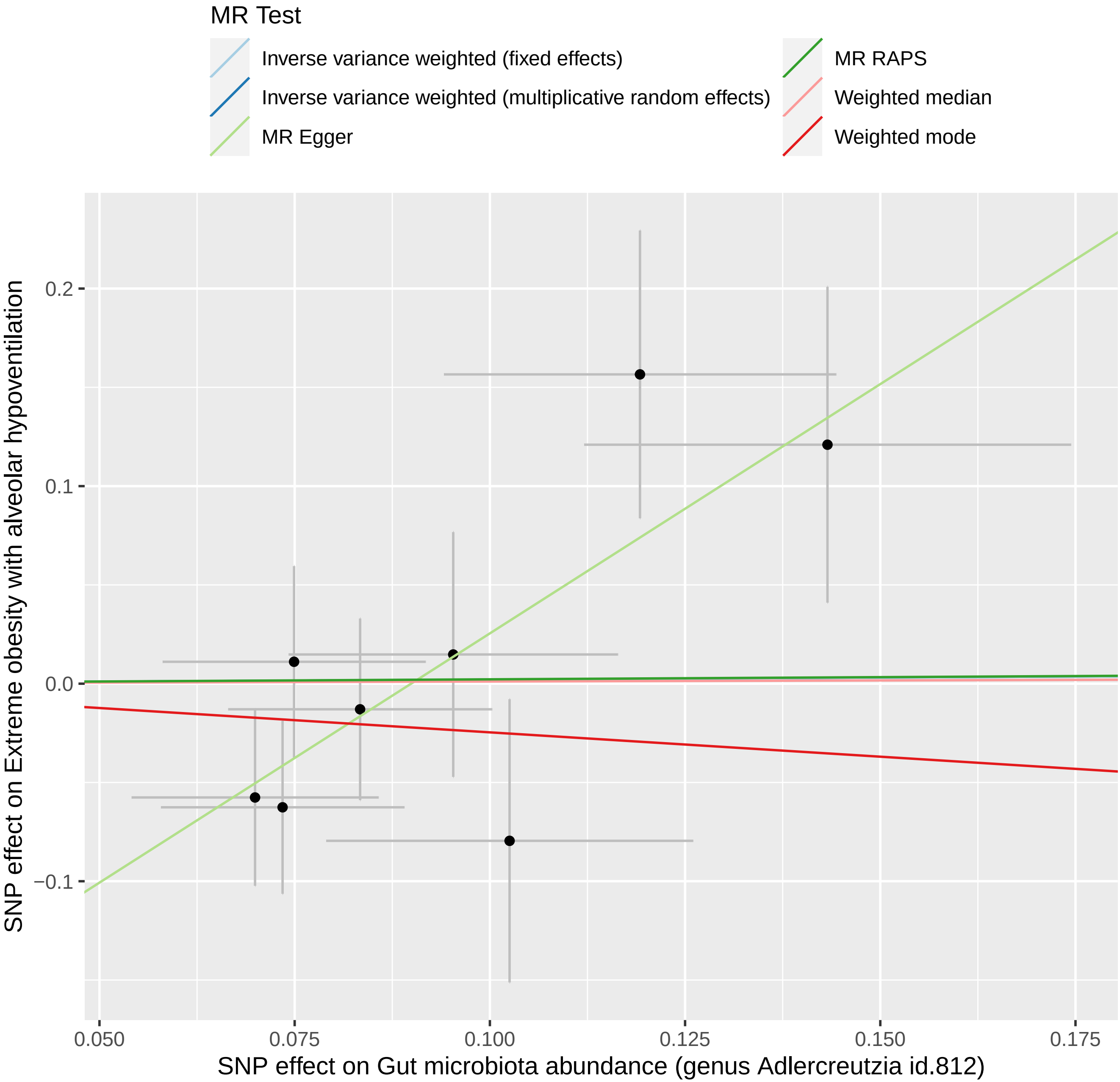

Batch 635 : Gut microbiota abundance (genus Akkermansia id.4037) on Extreme obesity with alveolar hypoventilation

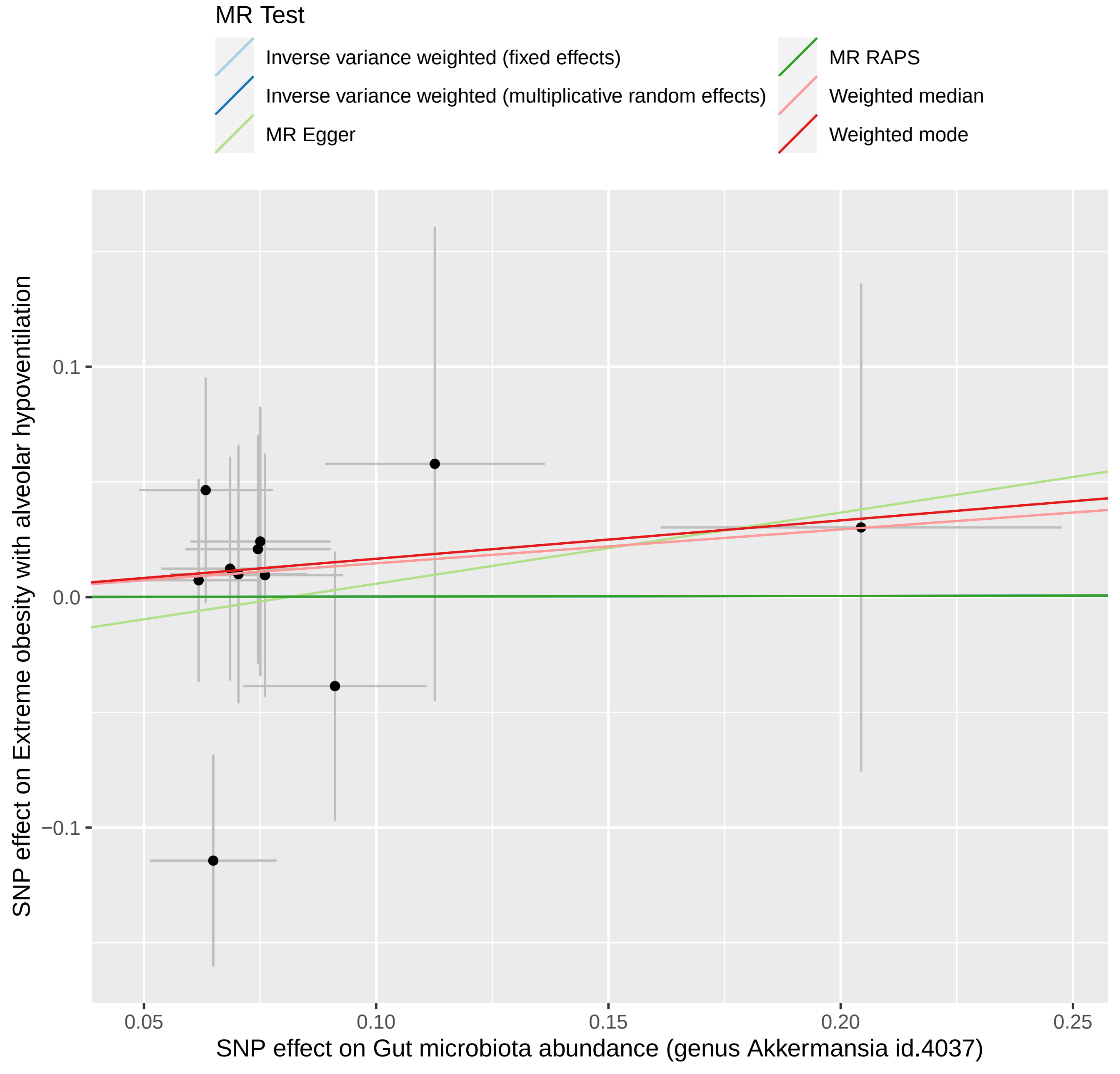

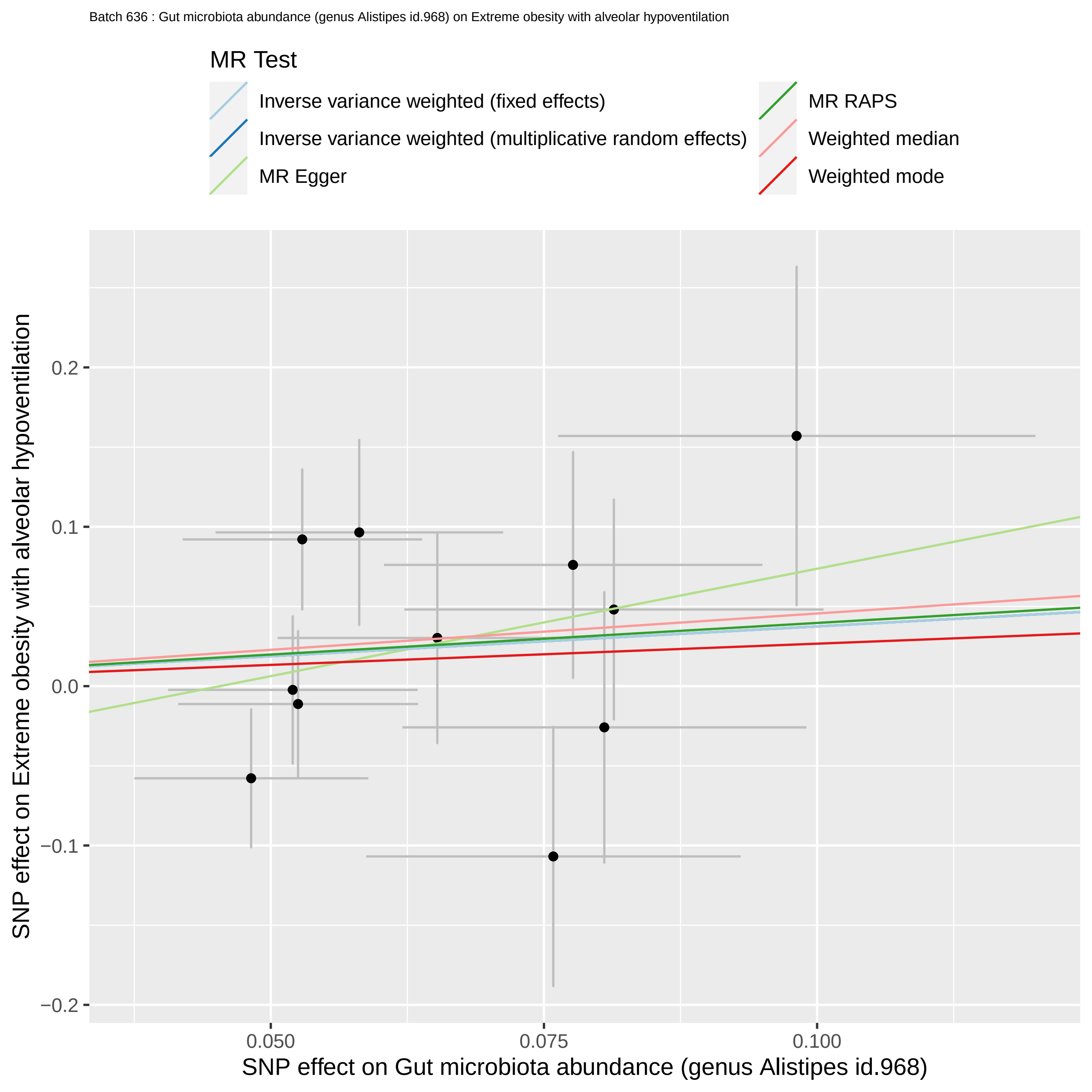
Batch 636 : Gut microbiota abundance (genus Alistipes id.968) on Extreme obesity with alveolar hypoventilation
MR Test
Inverse variance weighted (fixed effects)
Inverse variance weighted (multiplicative random effects)
MR Egger
MR RAPS
Weighted median
Weighted mode
SNP effect on Extreme obesity with alveolar hypoventilation
0.2
0.1
0.0
−0.1
−0.2
0.050
0.075
0.100
SNP effect on Gut microbiota abundance (genus Alistipes id.968)

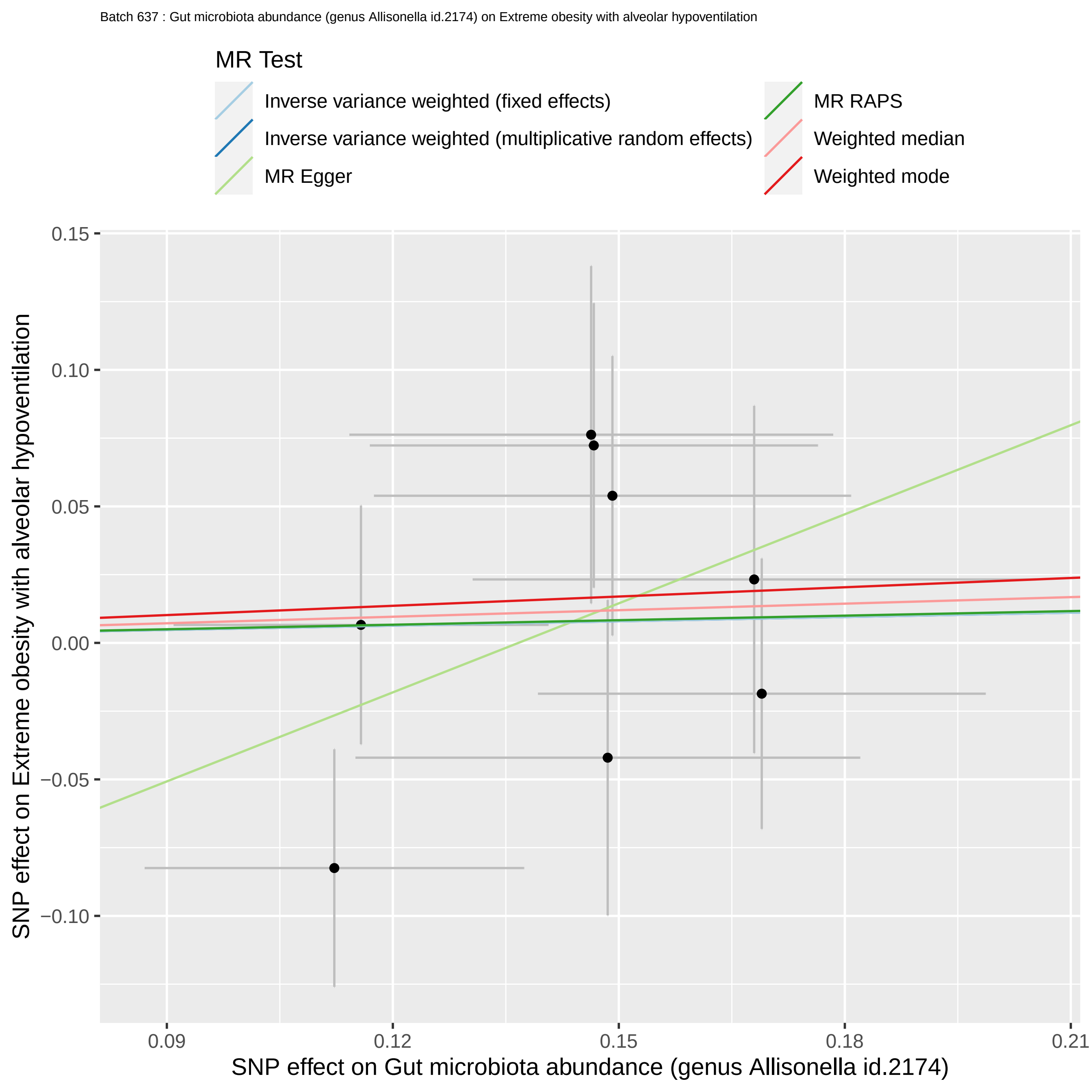
Batch 637 : Gut microbiota abundance (genus Allisonella id.2174) on Extreme obesity with alveolar hypoventilation
MR Test
Inverse variance weighted (fixed effects)
Inverse variance weighted (multiplicative random effects)
MR Egger
MR RAPS
Weighted median
Weighted mode
0.15
0.10
0.05
0.00
−0.05
−0.10
0.09
0.12
0.15
0.18
0.21
SNP effect on Extreme obesity with alveolar hypoventilation
SNP effect on Gut microbiota abundance (genus Allisonella id.2174)

Batch 638 : Gut microbiota abundance (genus Alloprevotella id.961) on Extreme obesity with alveolar hypoventilation

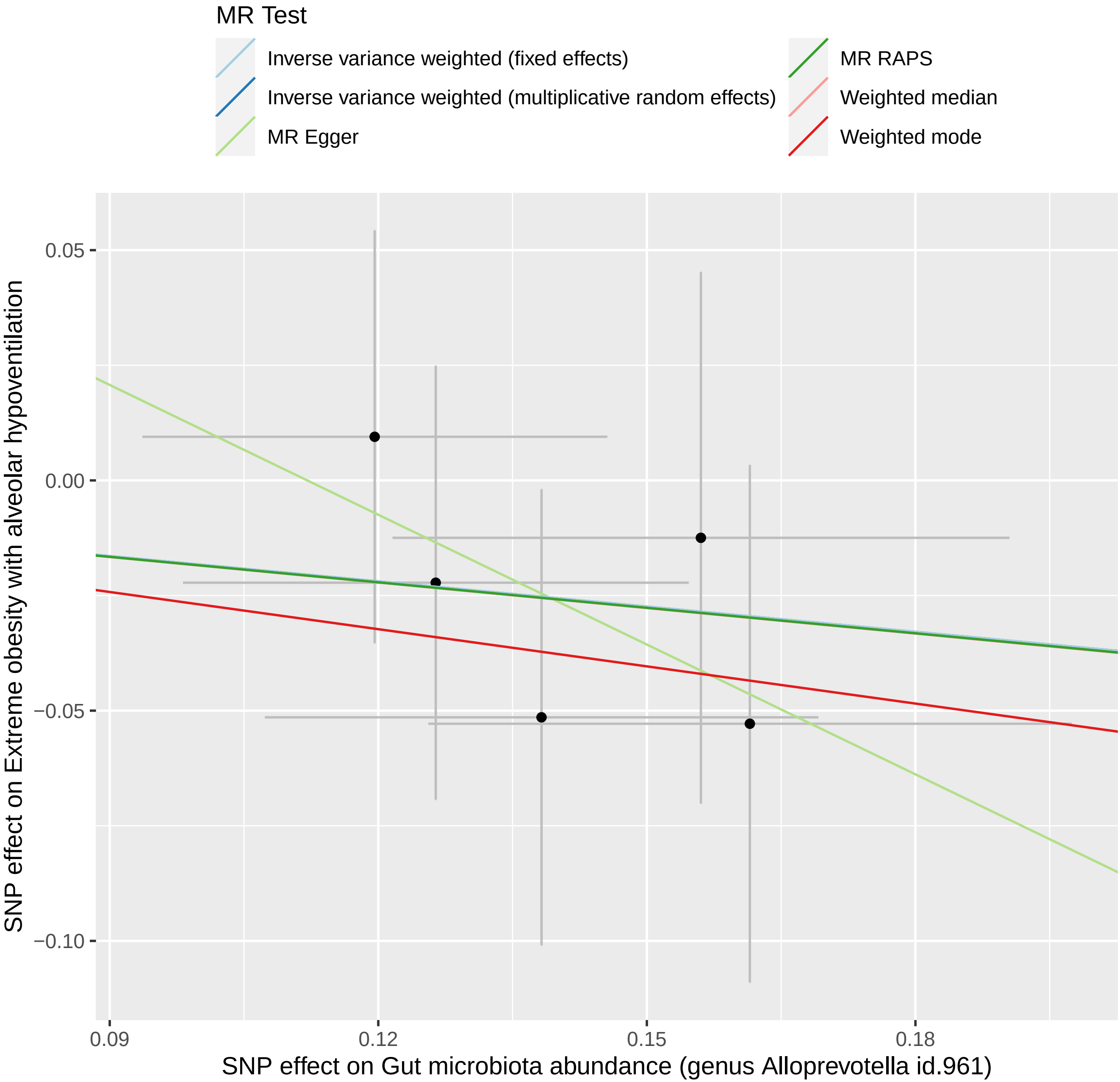

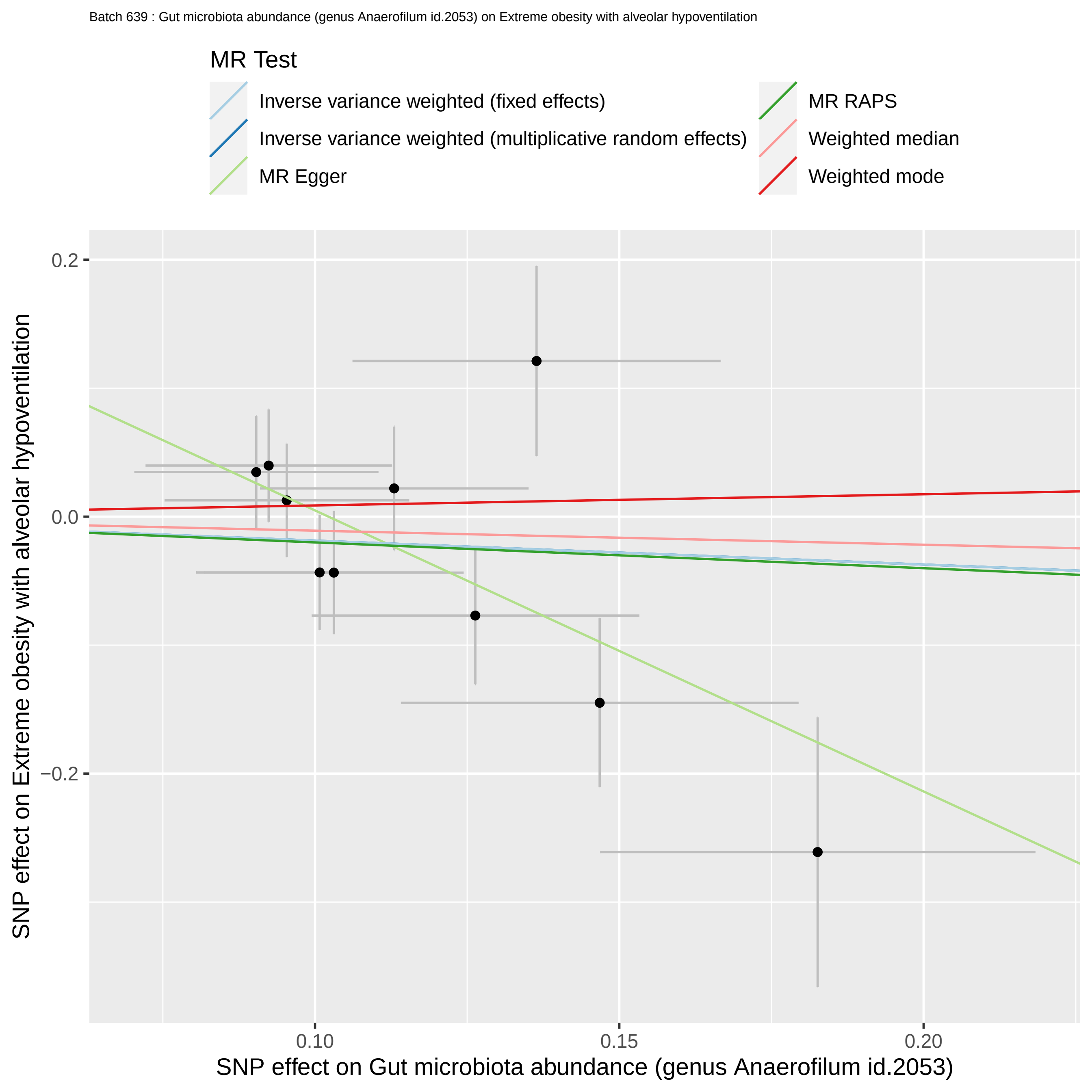
Batch 639 : Gut microbiota abundance (genus Anaerofilum id.2053) on Extreme obesity with alveolar hypoventilation
MR Test
Inverse variance weighted (fixed effects)
Inverse variance weighted (multiplicative random effects)
MR Egger
MR RAPS
Weighted median
Weighted mode
0.2
0.0
−0.2
0.10
0.15
0.20
SNP effect on Extreme obesity with alveolar hypoventilation
SNP effect on Gut microbiota abundance (genus Anaerofilum id.2053)

Batch 640 : Gut microbiota abundance (genus Anaerostipes id.1991) on Extreme obesity with alveolar hypoventilation

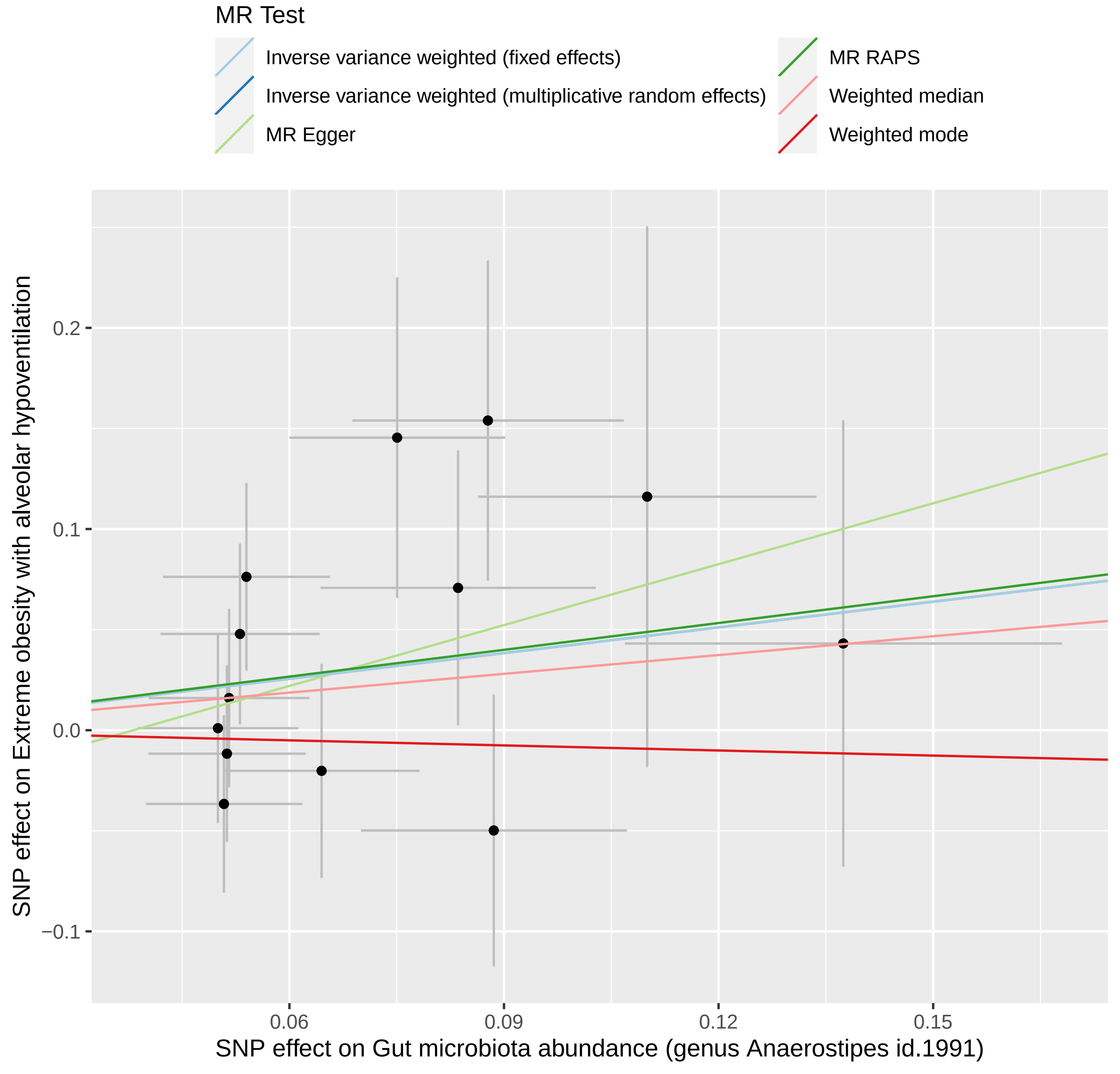

Batch 641 : Gut microbiota abundance (genus Anaerotruncus id.2054) on Extreme obesity with alveolar hypoventilation

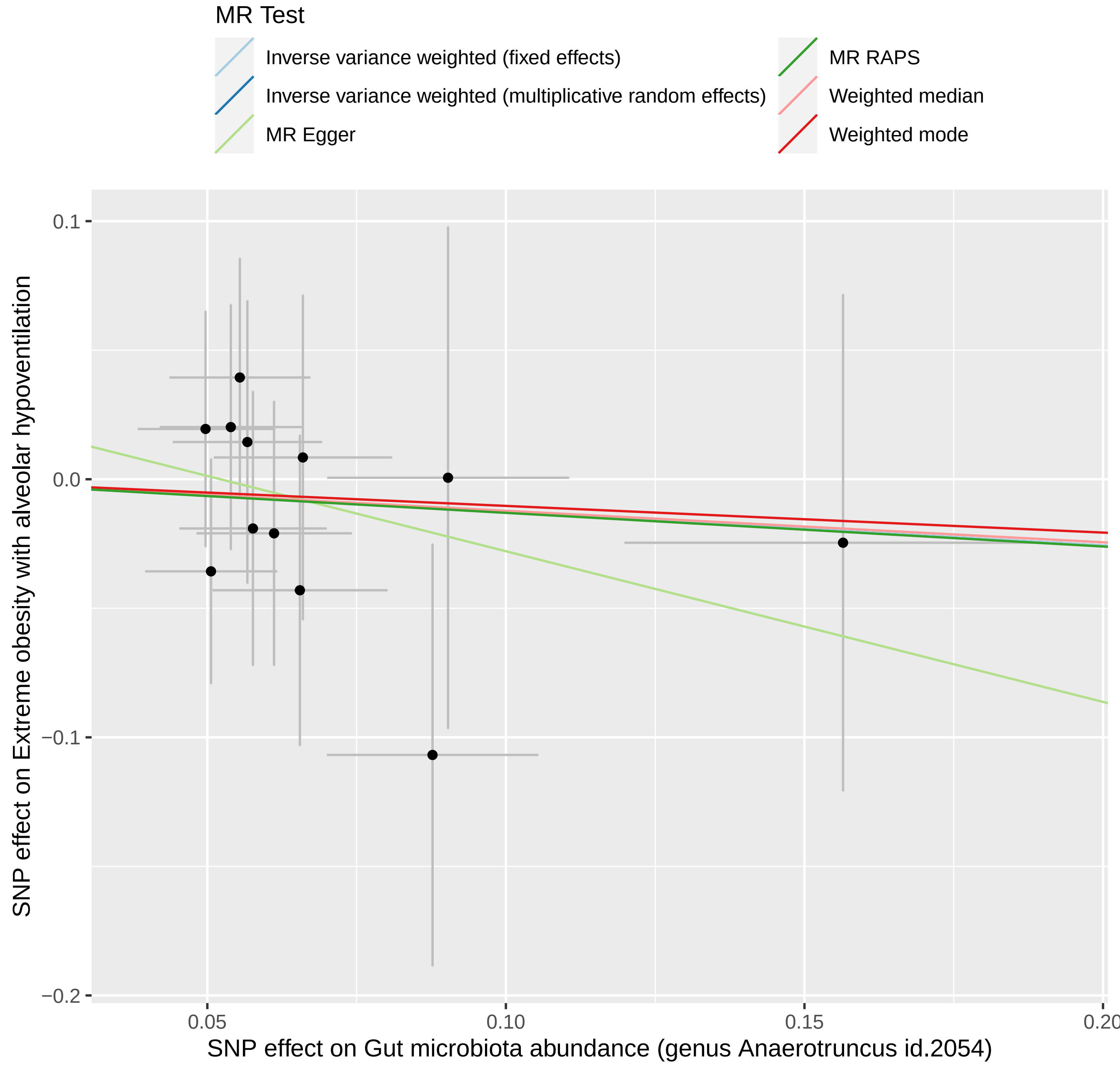

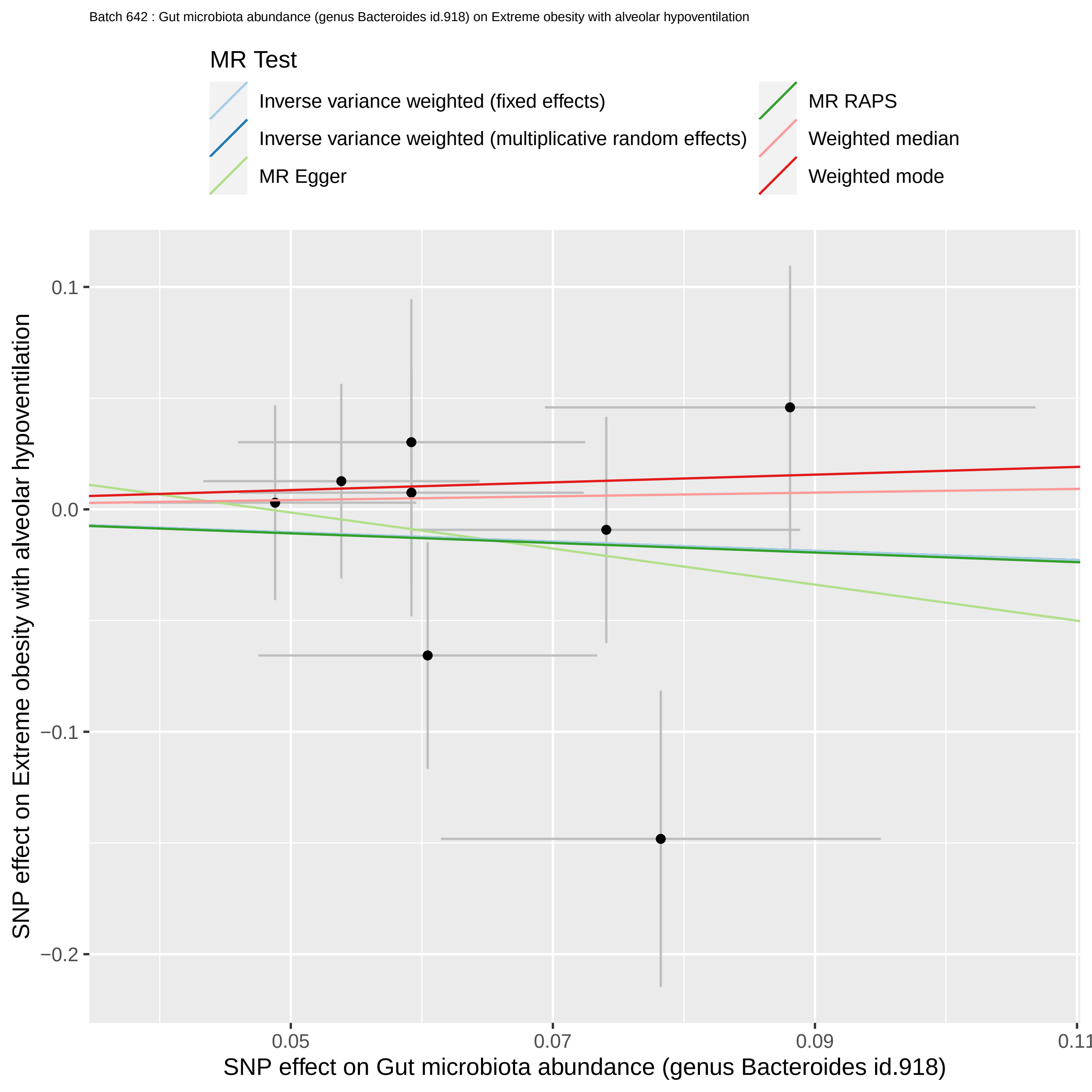

Batch 642 : Gut microbiota abundance (genus Bacteroides id.918) on Extreme obesity with alveolar hypoventilation
MR Test
Inverse variance weighted (fixed effects)
Inverse variance weighted (multiplicative random effects)
MR Egger
MR RAPS
Weighted median
Weighted mode
0.1
0.0
−0.1
−0.2
0.05
0.07
0.09
0.11
SNP effect on Extreme obesity with alveolar hypoventilation
SNP effect on Gut microbiota abundance (genus Bacteroides id.918)

Batch 643 : Gut microbiota abundance (genus Barnesiella id.944) on Extreme obesity with alveolar hypoventilation

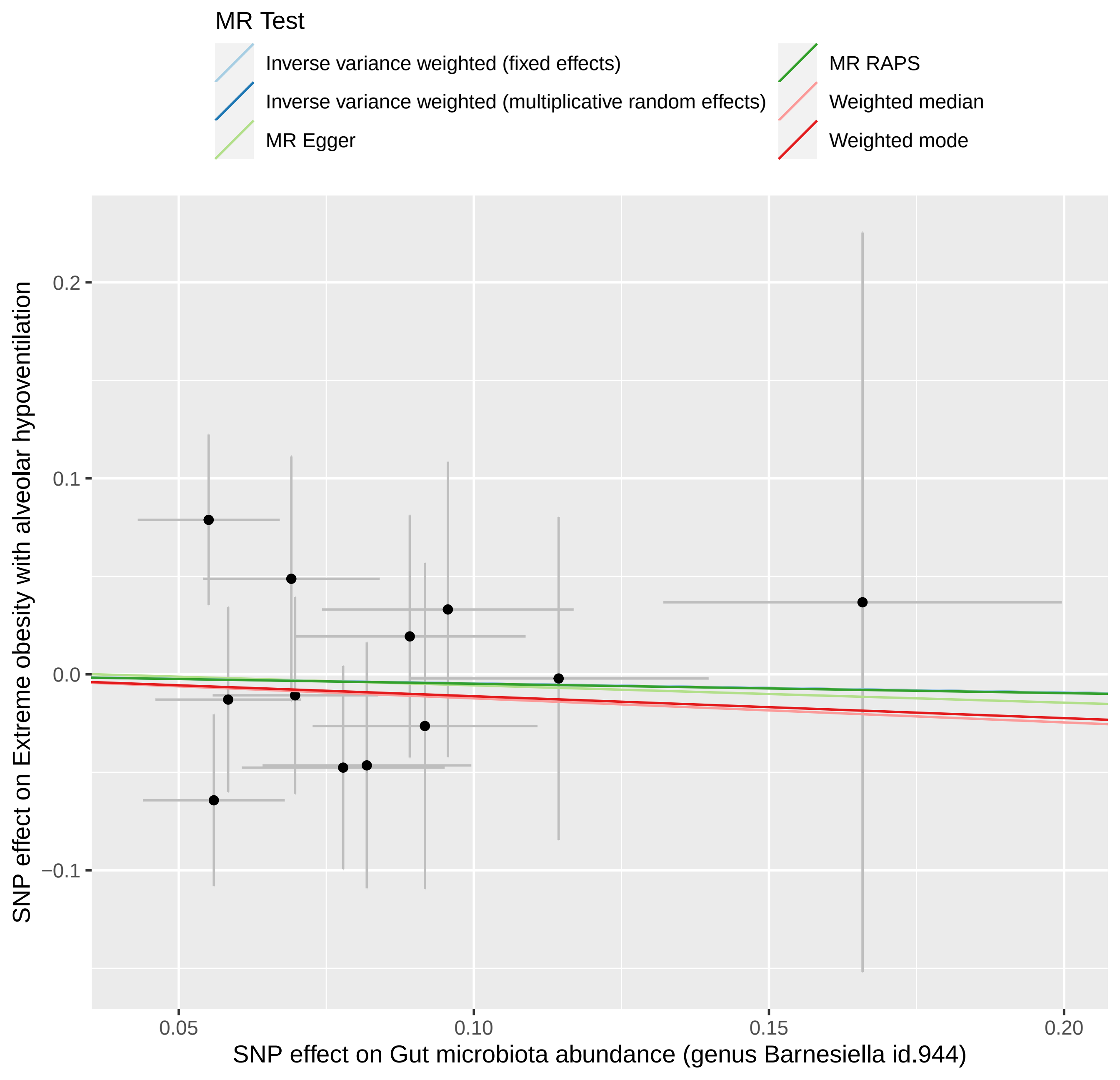

Batch 644 : Gut microbiota abundance (genus Bifidobacterium id.436) on Extreme obesity with alveolar hypoventilation

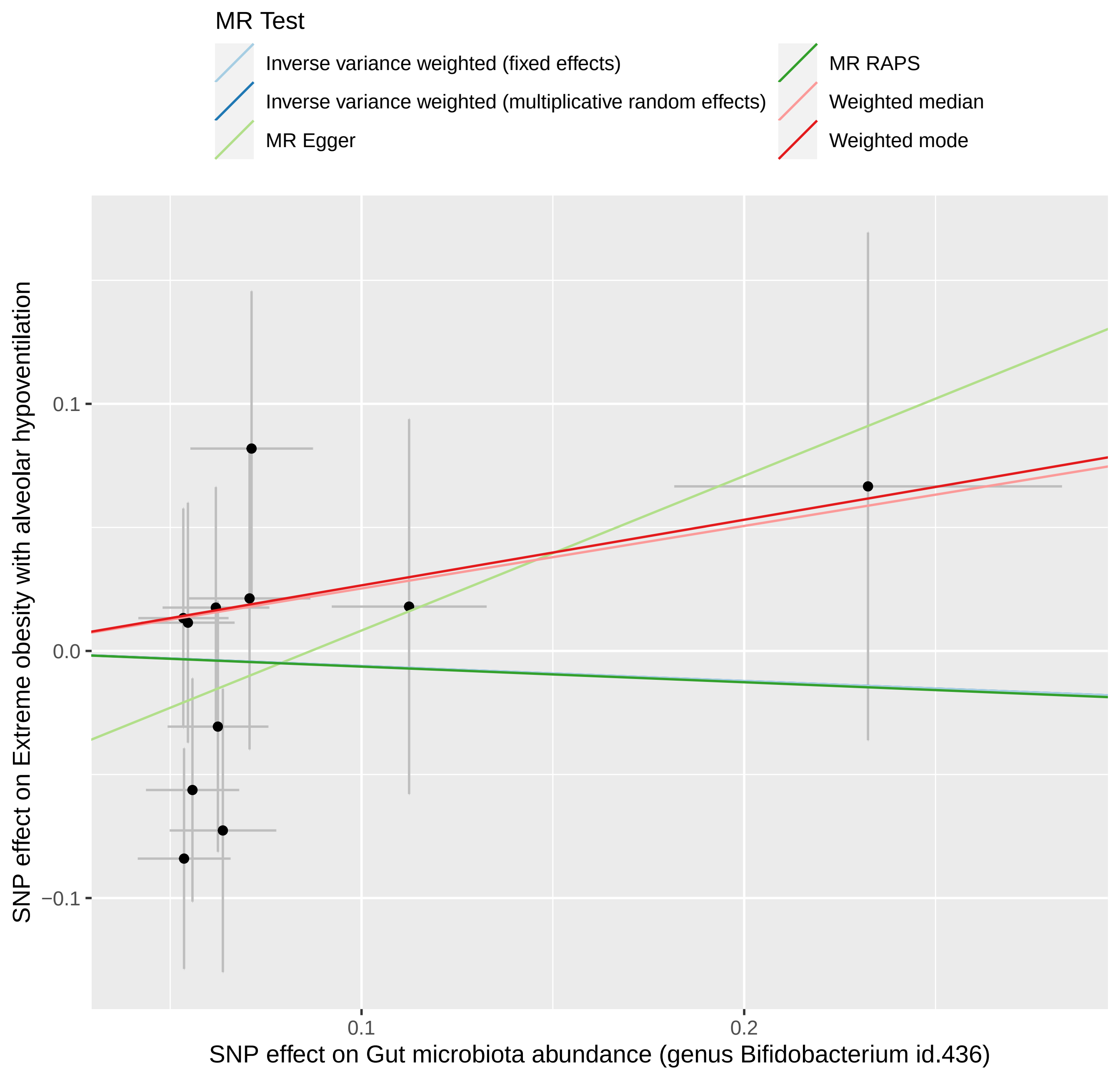

Batch 645 : Gut microbiota abundance (genus Bilophila id.3170) on Extreme obesity with alveolar hypoventilation

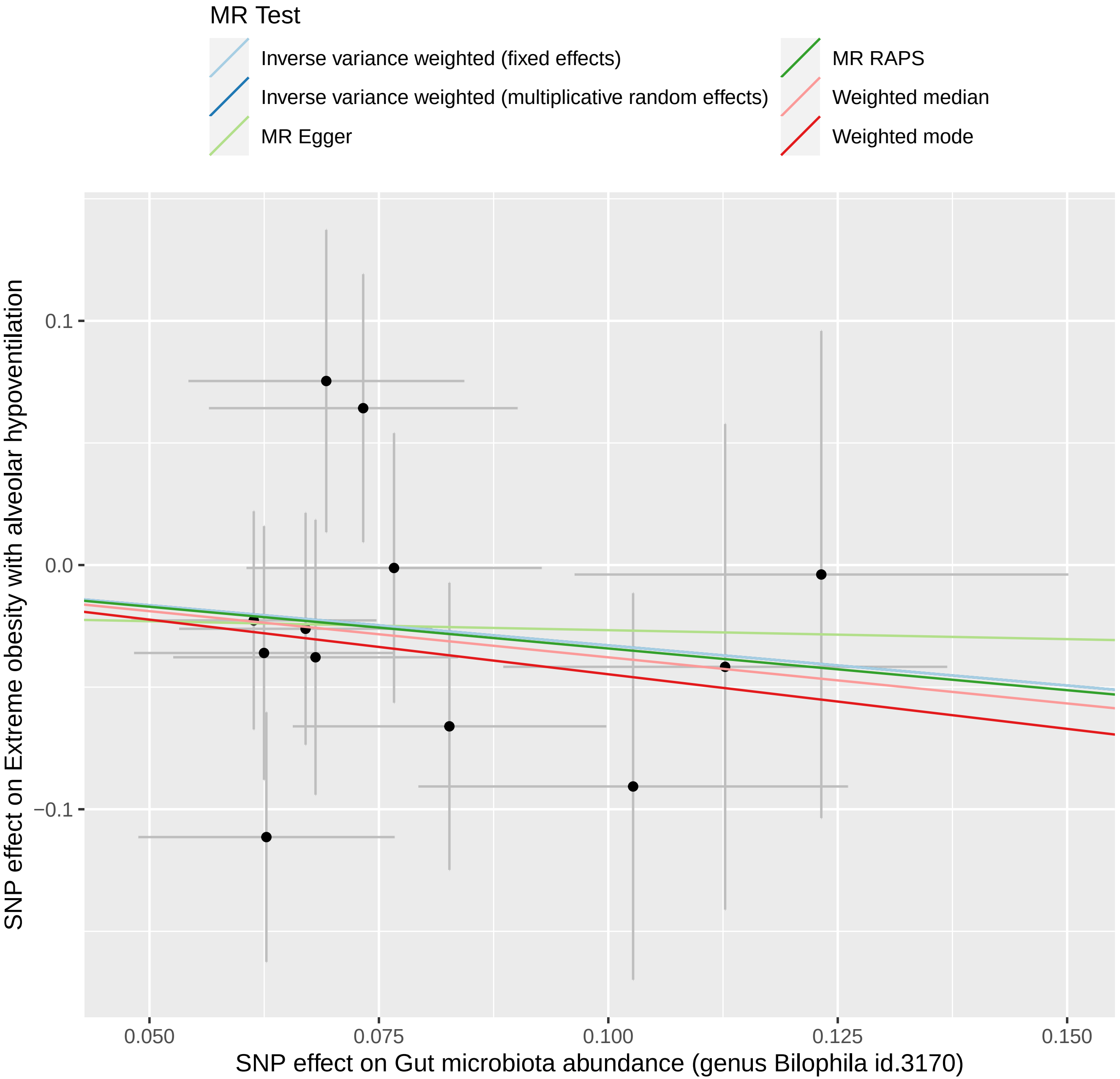

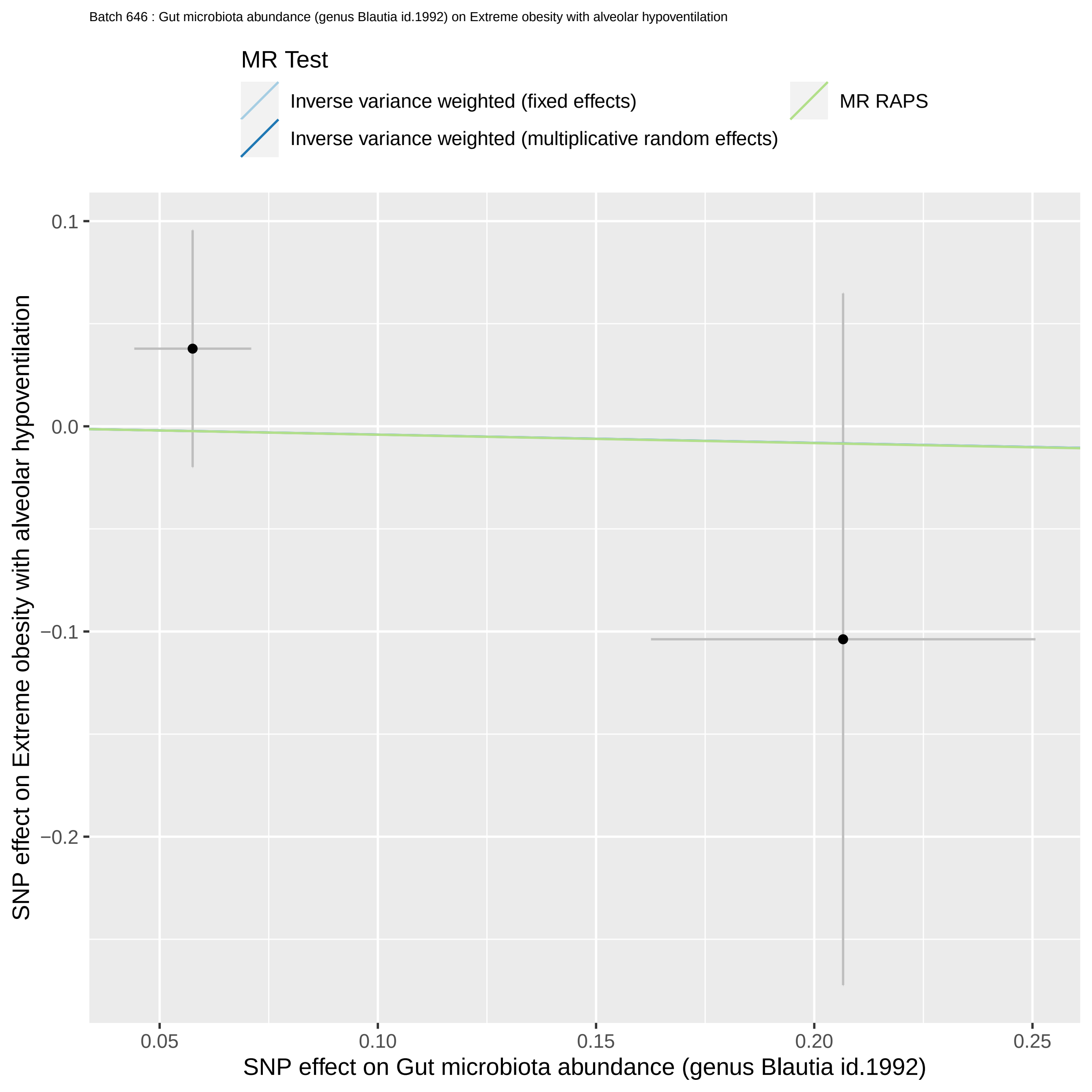

Batch 646 : Gut microbiota abundance (genus Blautia id.1992) on Extreme obesity with alveolar hypoventilation
MR Test
Inverse variance weighted (fixed effects)
Inverse variance weighted (multiplicative random effects)
MR RAPS
0.1
0.0
−0.1
−0.2
0.05
0.10
0.15
0.20
0.25
SNP effect on Extreme obesity with alveolar hypoventilation
SNP effect on Gut microbiota abundance (genus Blautia id.1992)

Batch 647 : Gut microbiota abundance (genus Butyricicoccus id.2055) on Extreme obesity with alveolar hypoventilation

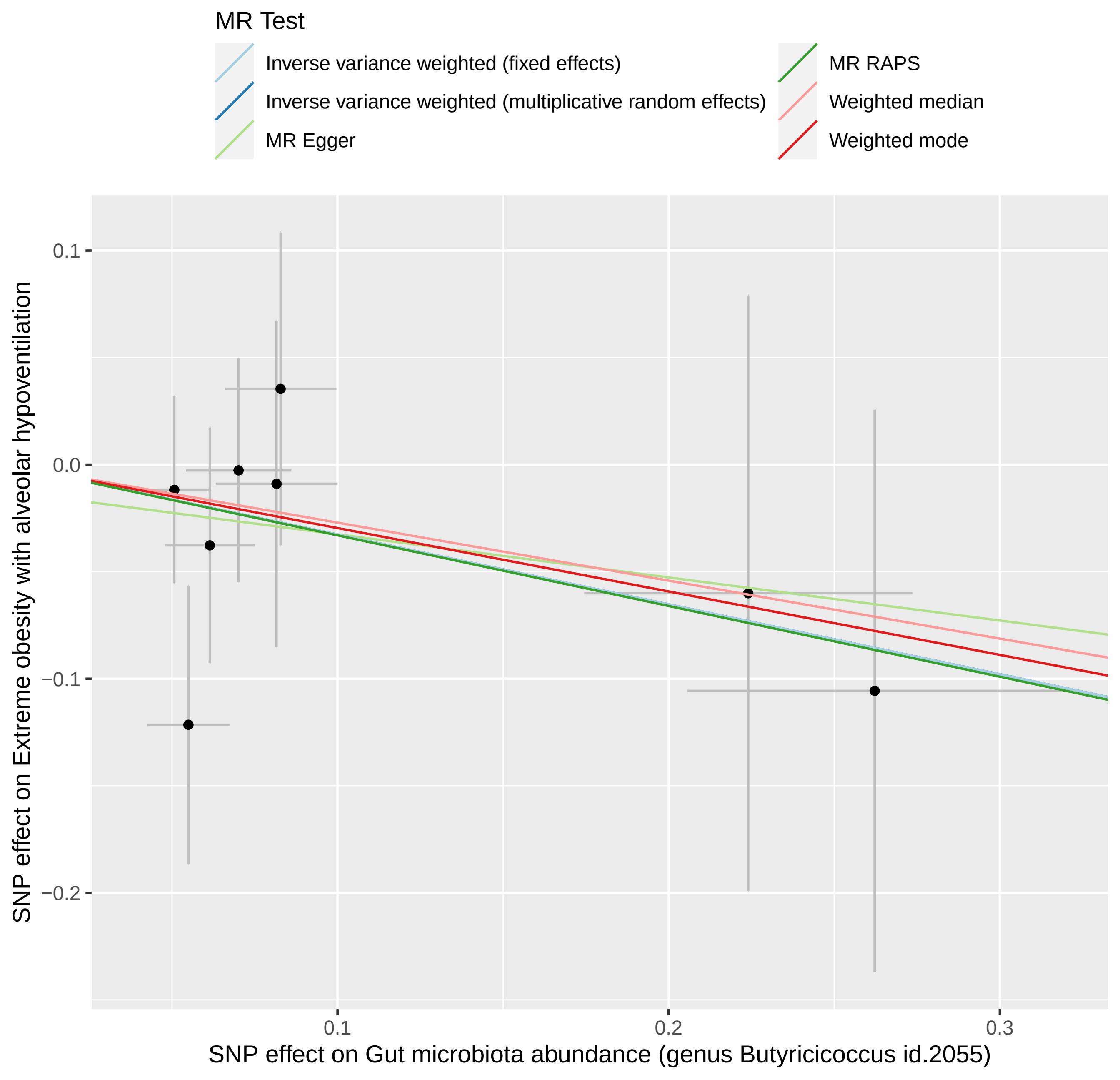

Batch 648 : Gut microbiota abundance (genus Butyricimonas id.945) on Extreme obesity with alveolar hypoventilation

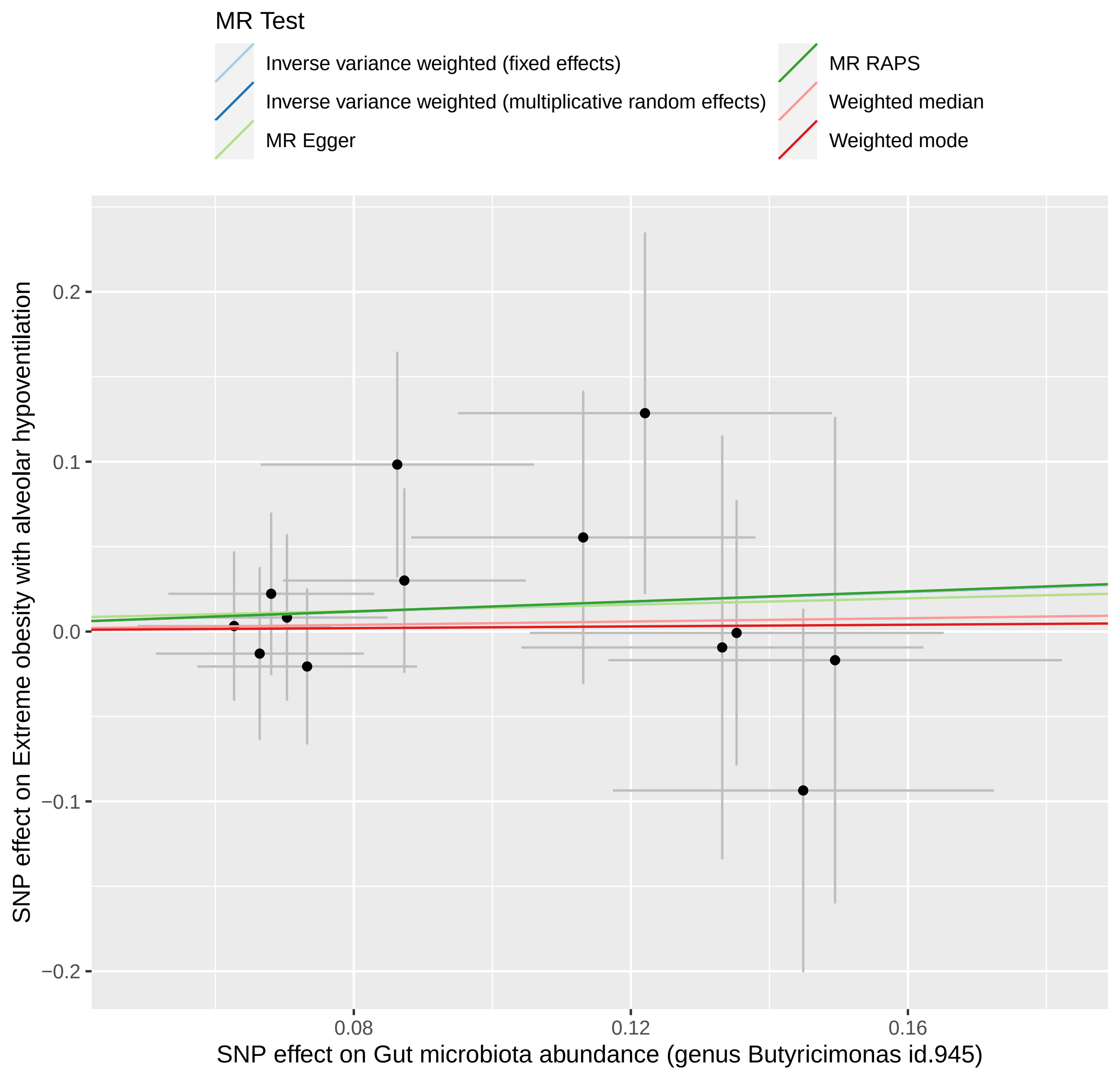

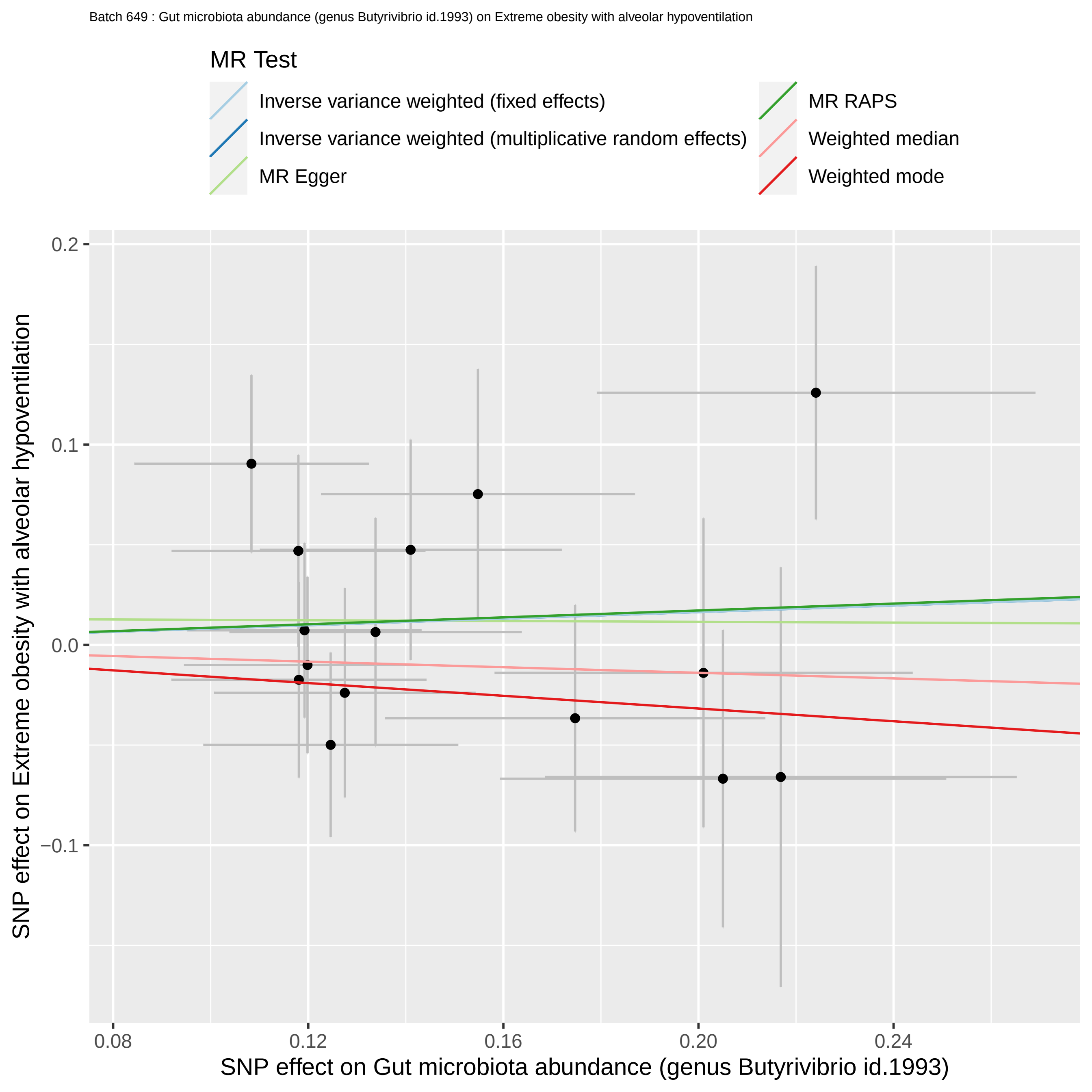

Batch 649 : Gut microbiota abundance (genus Butyrivibrio id.1993) on Extreme obesity with alveolar hypoventilation
MR Test
Inverse variance weighted (fixed effects)
Inverse variance weighted (multiplicative random effects)
MR Egger
MR RAPS
Weighted median
Weighted mode
0.2
0.1
0.0
−0.1
0.08
0.12
0.16
0.20
0.24
SNP effect on Extreme obesity with alveolar hypoventilation
SNP effect on Gut microbiota abundance (genus Butyrivibrio id.1993)

Batch 650 : Gut microbiota abundance (genus Candidatus Soleaferrea id.11350) on Extreme obesity with alveolar hypoventilation

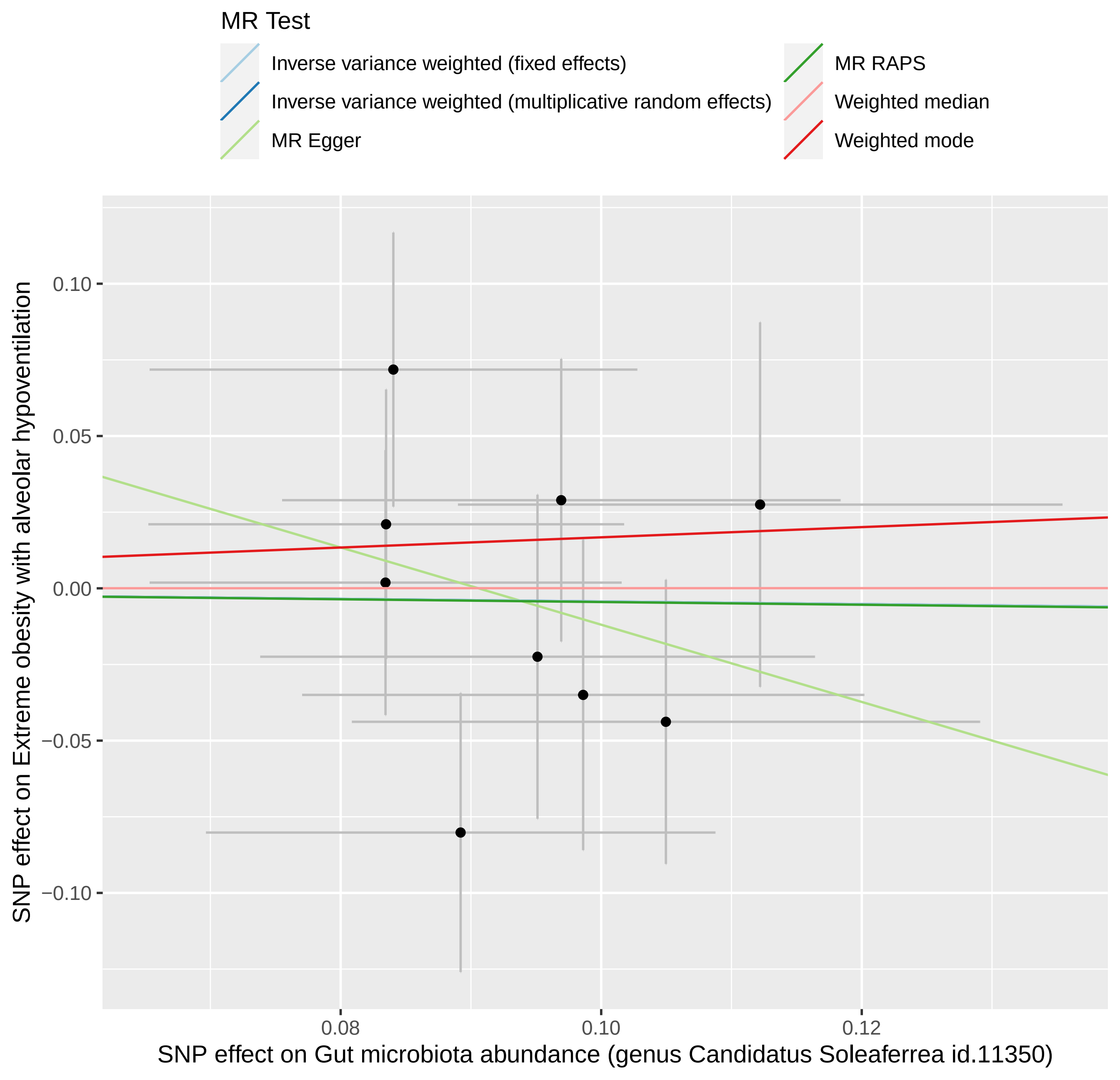

Batch 651 : Gut microbiota abundance (genus Catenibacterium id.2153) on Extreme obesity with alveolar hypoventilation

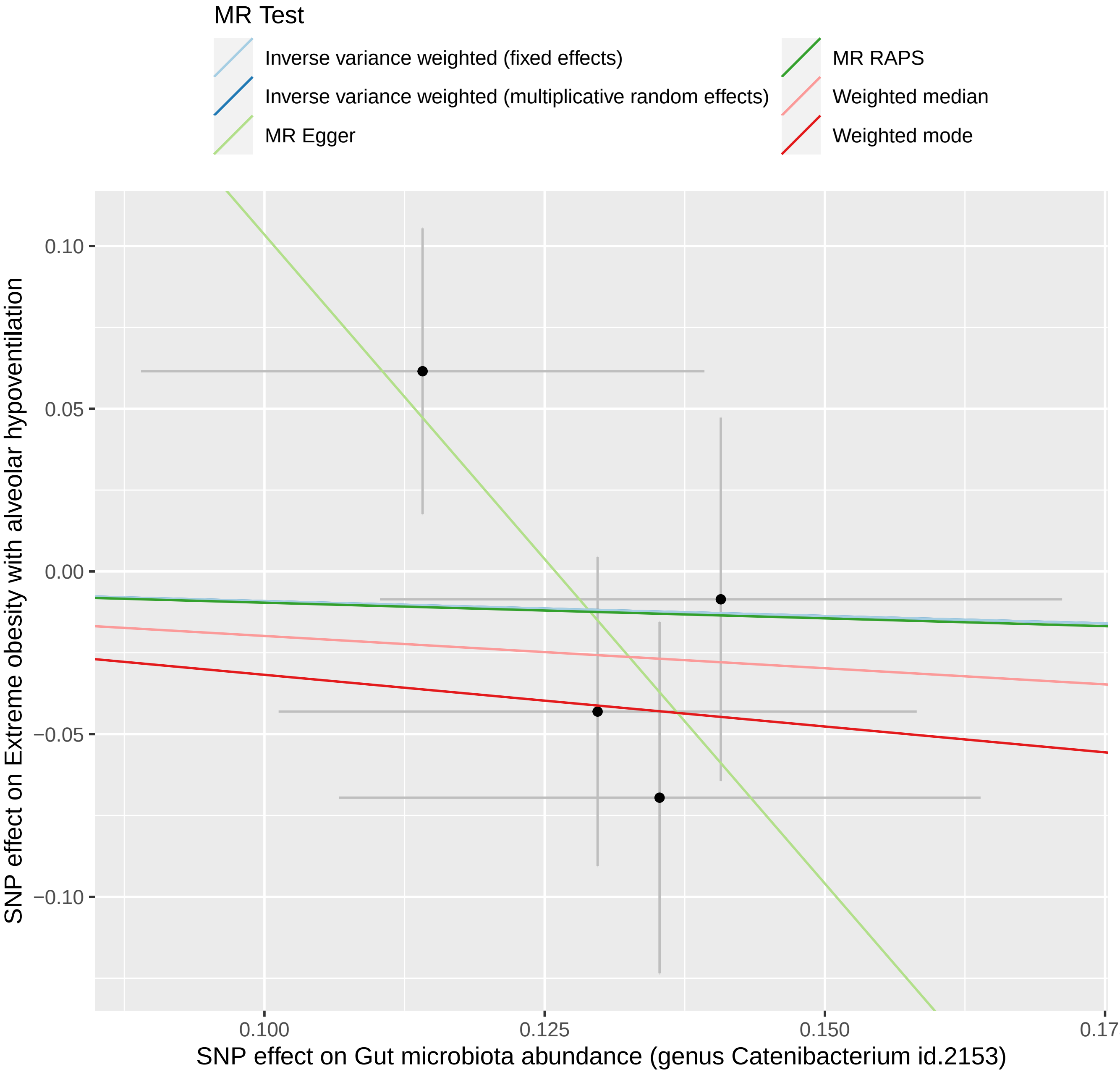

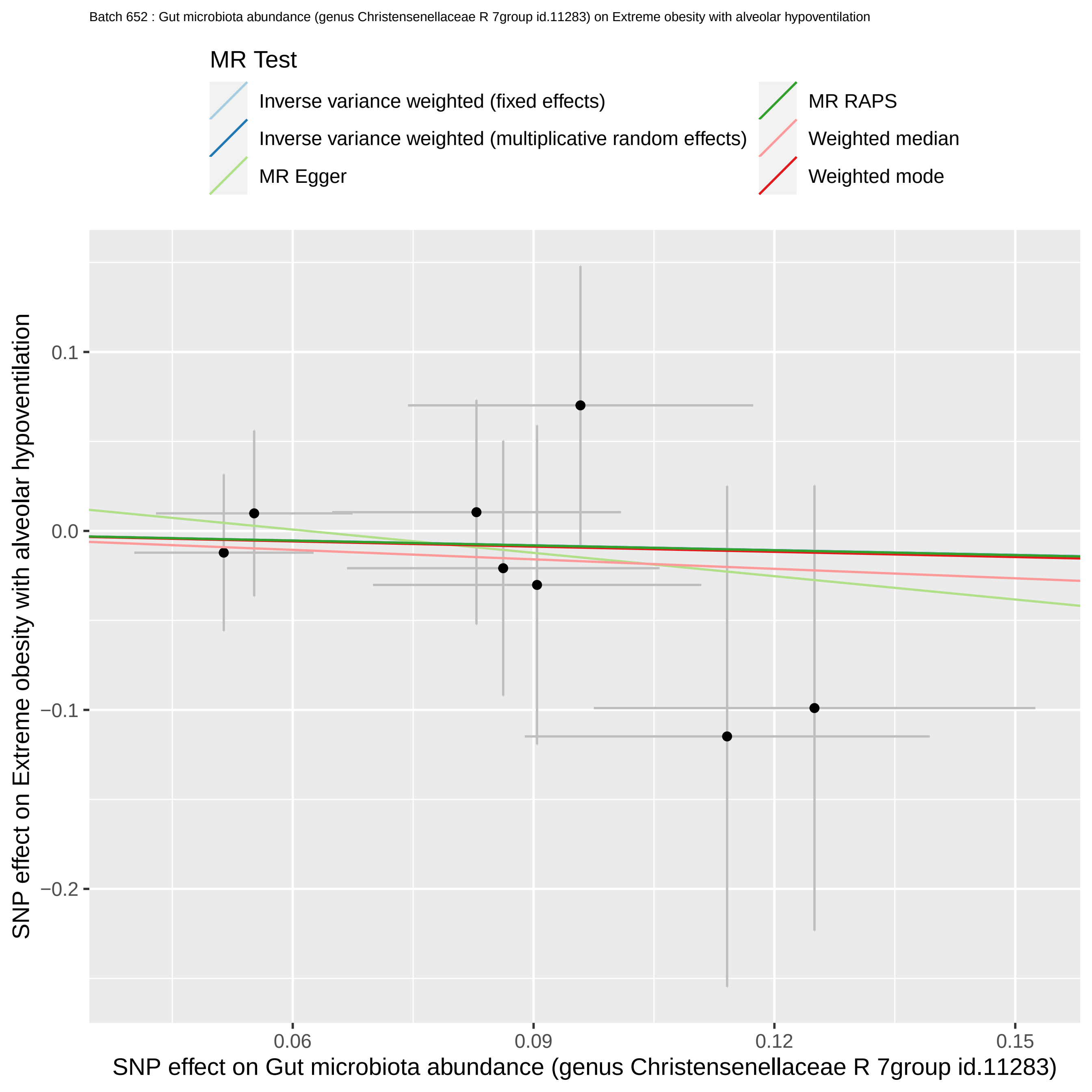
Batch 652 : Gut microbiota abundance (genus Christensenellaceae R 7group id.11283) on Extreme obesity with alveolar hypoventilation
MR Test
Inverse variance weighted (fixed effects)
Inverse variance weighted (multiplicative random effects)
MR Egger
MR RAPS
Weighted median
Weighted mode
0.1
0.0
−0.1
−0.2
0.06
0.09
0.12
0.15
SNP effect on Extreme obesity with alveolar hypoventilation
SNP effect on Gut microbiota abundance (genus Christensenellaceae R 7group id.11283)

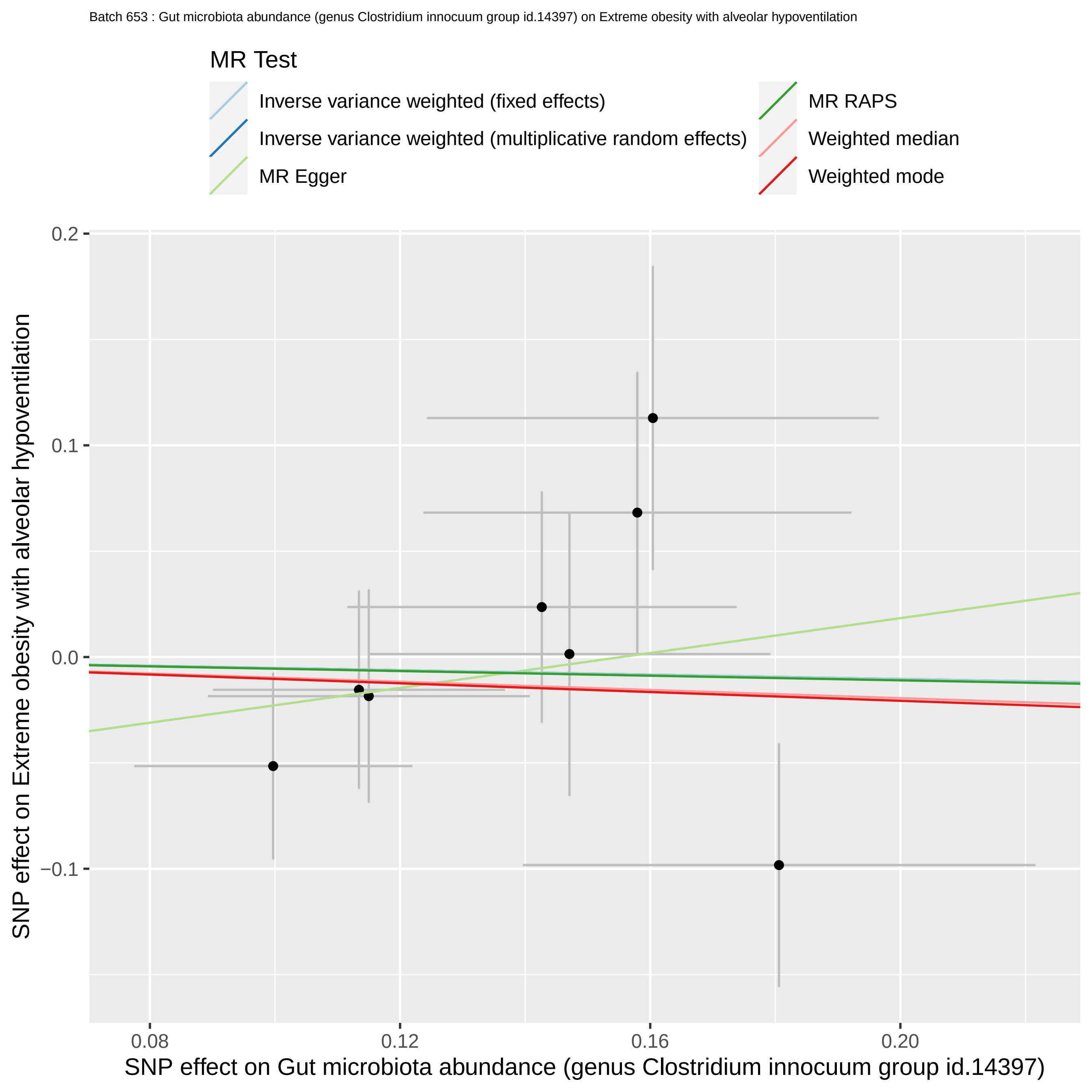

Batch 653 : Gut microbiota abundance (genus Clostridium innocuum group id.14397) on Extreme obesity with alveolar hypoventilation
MR Test
Inverse variance weighted (fixed effects)
Inverse variance weighted (multiplicative random effects)
MR Egger
MR RAPS
Weighted median
Weighted mode
0.2
0.1
0.0
−0.1
0.08
0.12
0.16
0.20
SNP effect on Extreme obesity with alveolar hypoventilation
SNP effect on Gut microbiota abundance (genus Clostridium innocuum group id.14397)

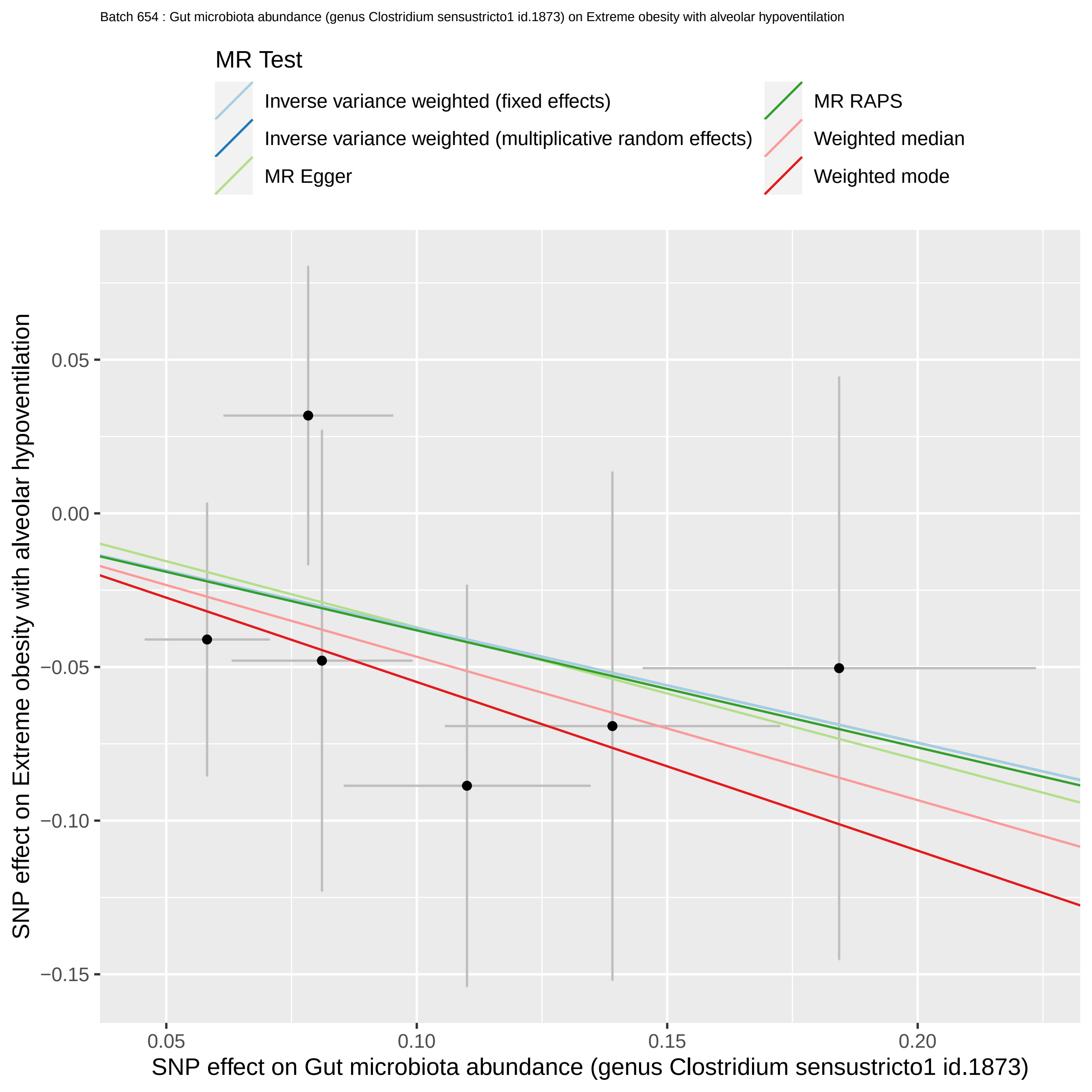

Batch 654 : Gut microbiota abundance (genus Clostridium sensustricto1 id.1873) on Extreme obesity with alveolar hypoventilation
MR Test
Inverse variance weighted (fixed effects)
Inverse variance weighted (multiplicative random effects)
MR Egger
MR RAPS
Weighted median
Weighted mode
0.05
0.00
−0.05
−0.10
−0.15
0.05
0.10
0.15
0.20
SNP effect on Extreme obesity with alveolar hypoventilation
SNP effect on Gut microbiota abundance (genus Clostridium sensustricto1 id.1873)

Batch 655 : Gut microbiota abundance (genus Collinsella id.815) on Extreme obesity with alveolar hypoventilation

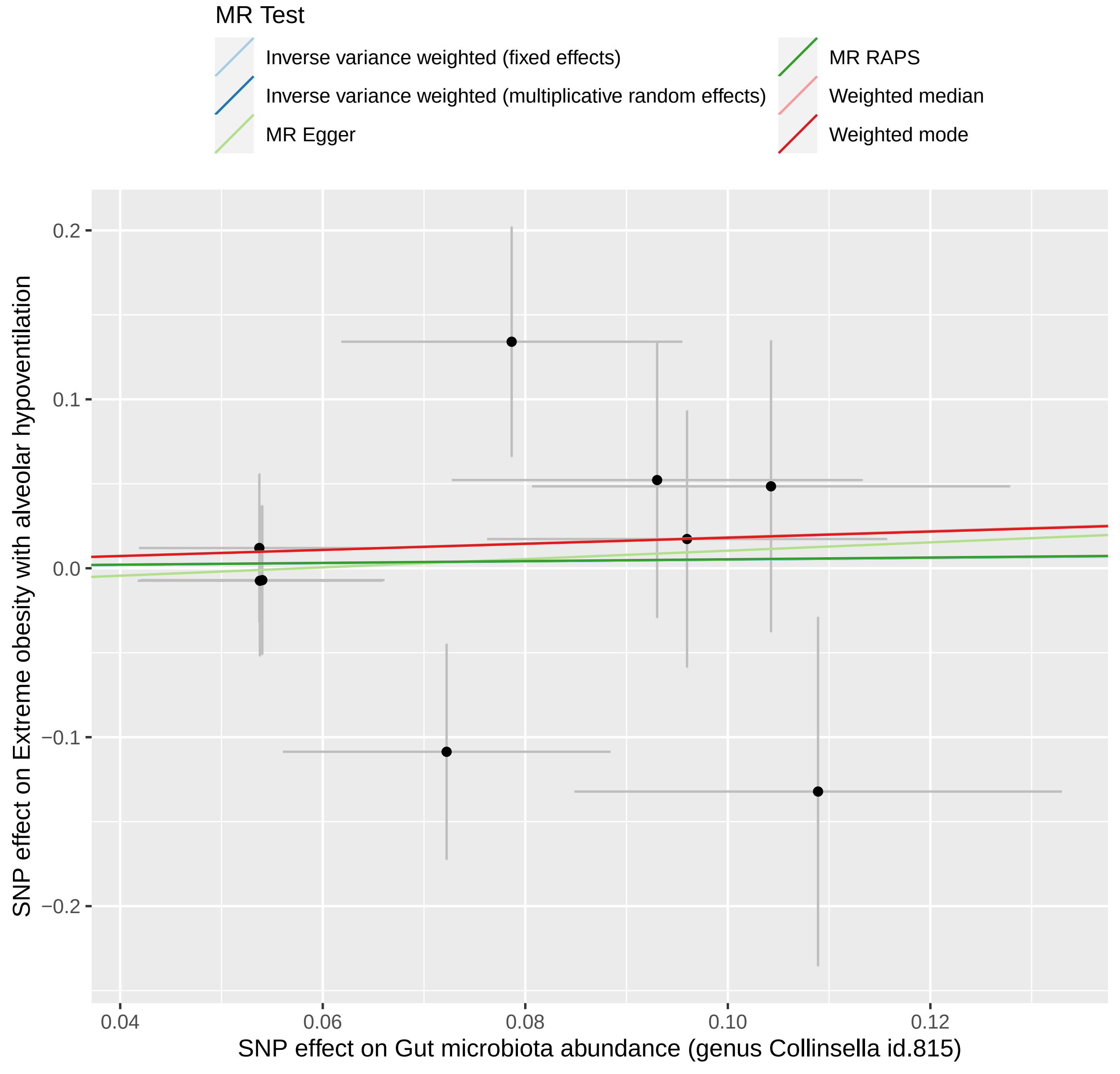

Batch 656 : Gut microbiota abundance (genus Coprobacter id.949) on Extreme obesity with alveolar hypoventilation

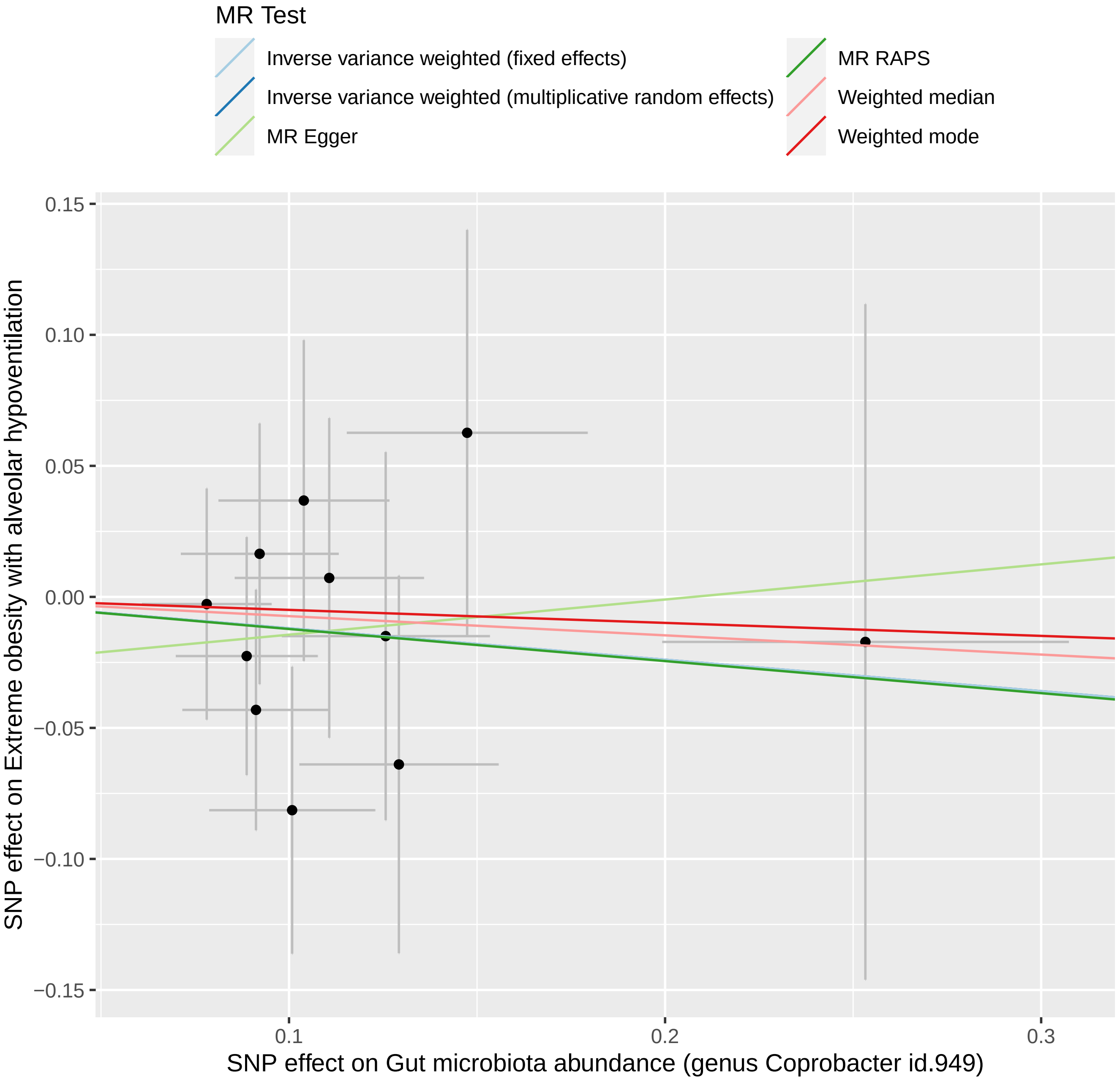

Batch 657 : Gut microbiota abundance (genus Coprococcus1 id.11301) on Extreme obesity with alveolar hypoventilation

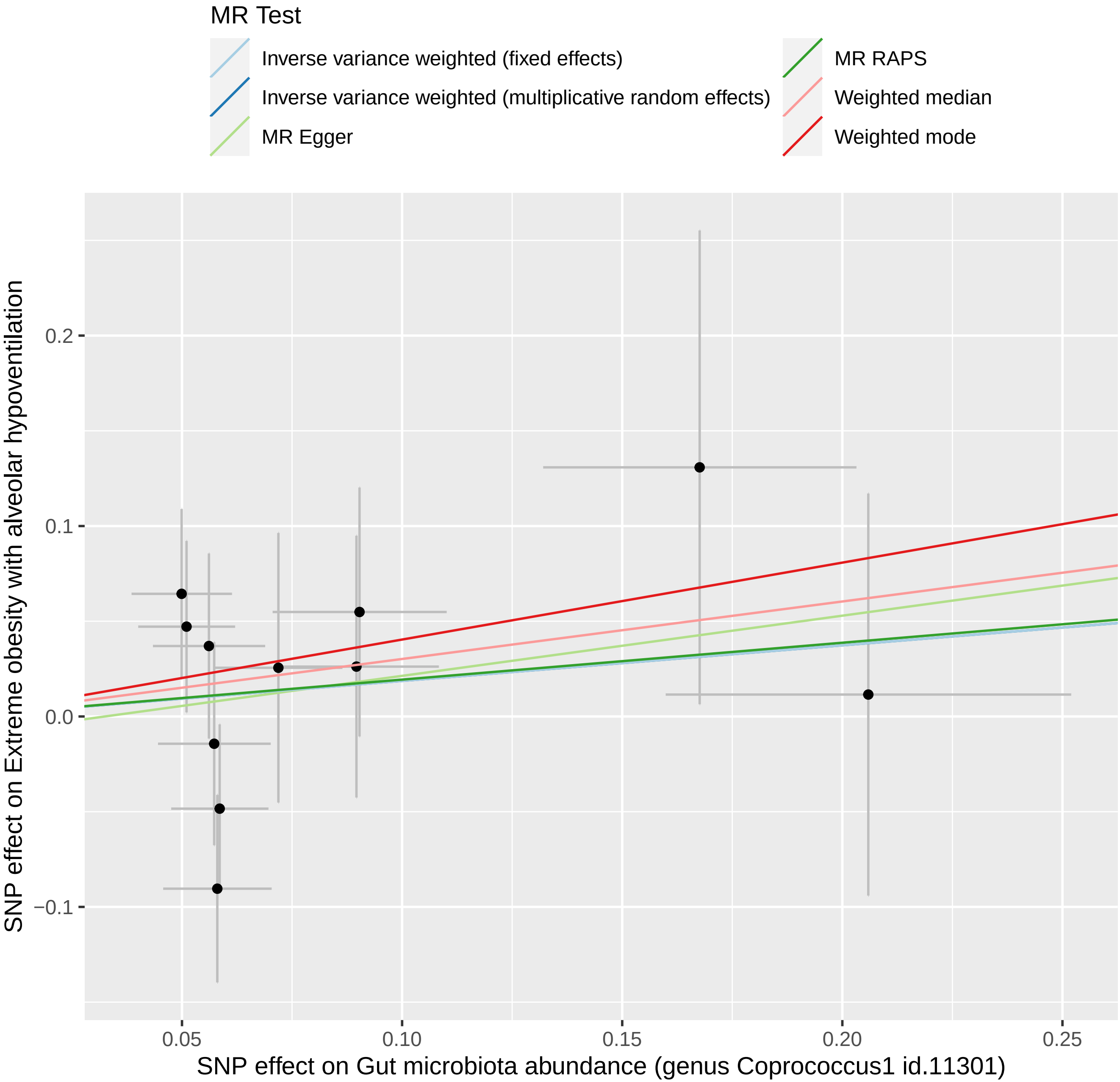

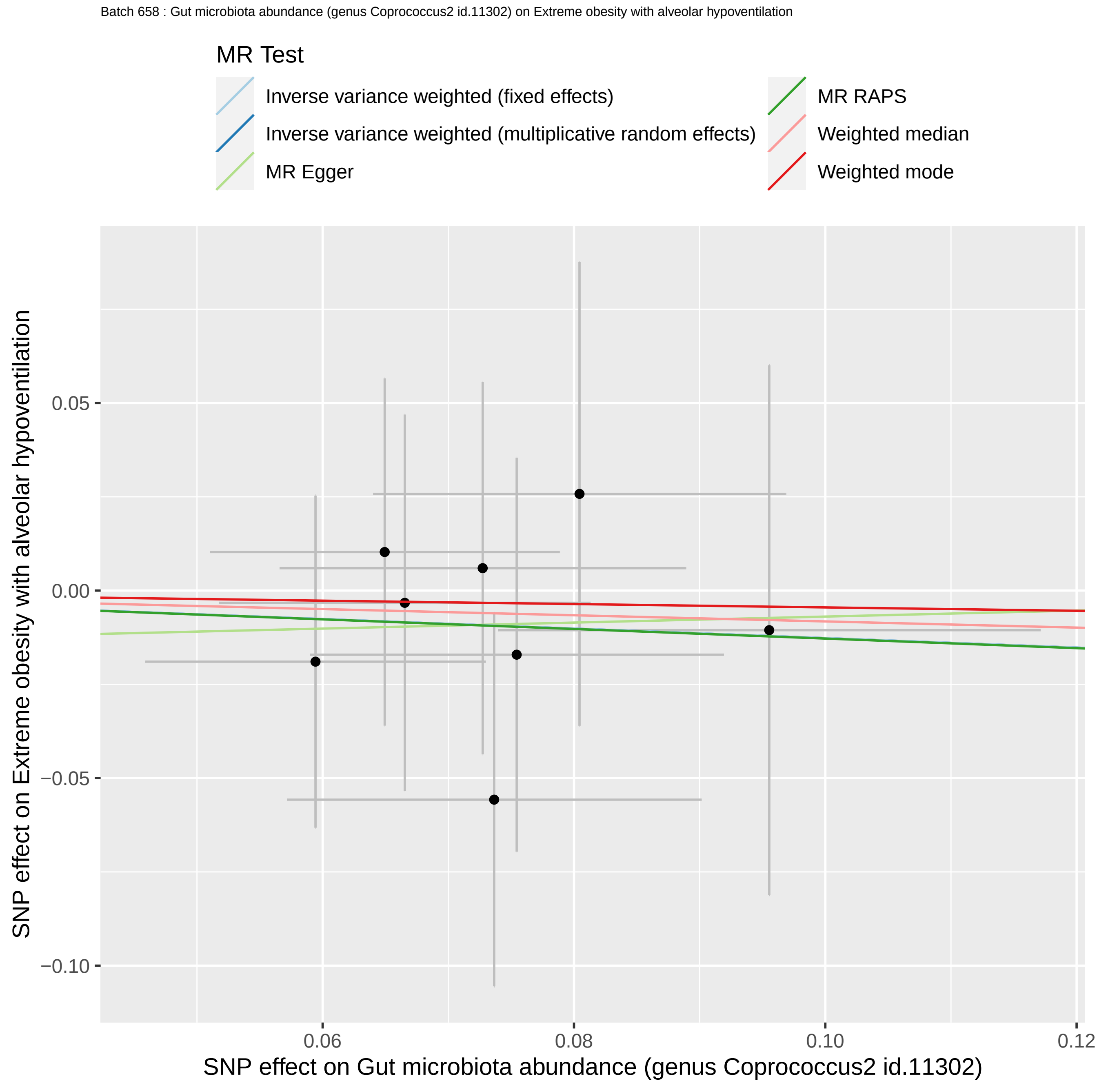
Batch 658 : Gut microbiota abundance (genus Coprococcus2 id.11302) on Extreme obesity with alveolar hypoventilation
MR Test
Inverse variance weighted (fixed effects)
Inverse variance weighted (multiplicative random effects)
MR Egger
MR RAPS
Weighted median
Weighted mode
SNP effect on Extreme obesity with alveolar hypoventilation
0.05
0.00
−0.05
−0.10
0.06
0.08
0.10
0.12
SNP effect on Gut microbiota abundance (genus Coprococcus2 id.11302)

Batch 659 : Gut microbiota abundance (genus Coprococcus3 id.11303) on Extreme obesity with alveolar hypoventilation

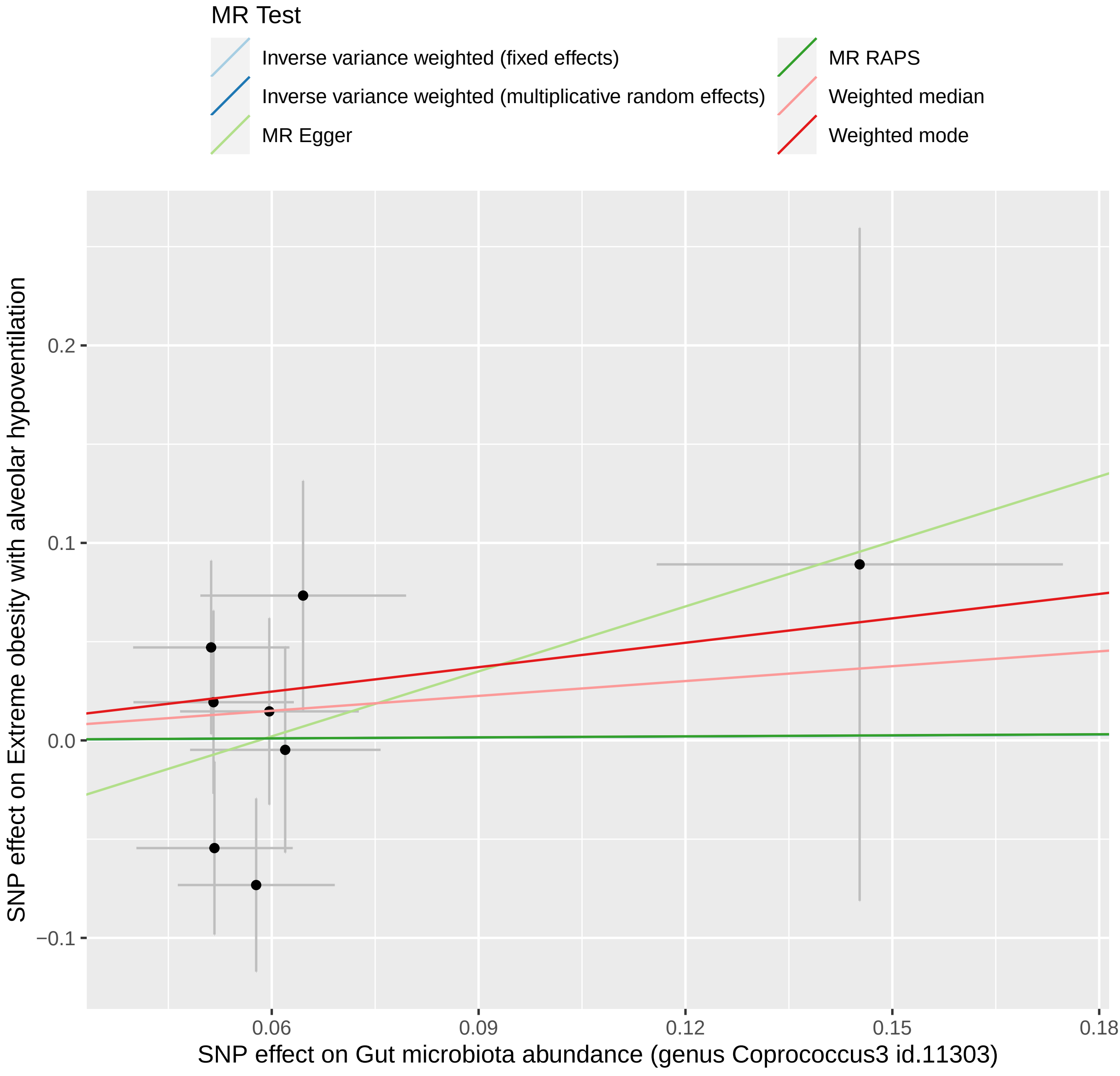

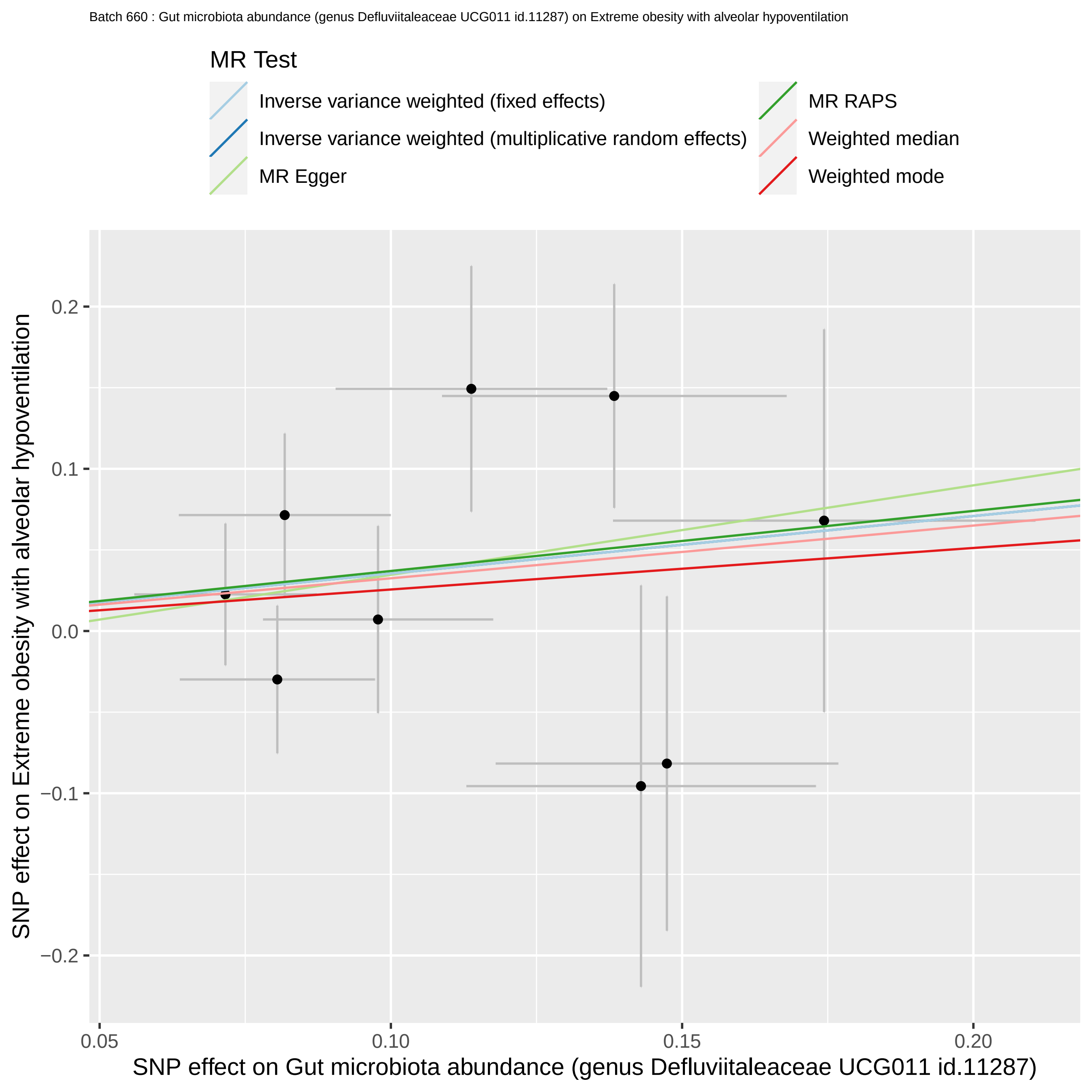
Batch 660 : Gut microbiota abundance (genus Defluviitaleaceae UCG011 id.11287) on Extreme obesity with alveolar hypoventilation
MR Test
Inverse variance weighted (fixed effects)
Inverse variance weighted (multiplicative random effects)
MR Egger
MR RAPS
Weighted median
Weighted mode
0.2
0.1
0.0
−0.1
−0.2
0.05
0.10
0.15
0.20
SNP effect on Extreme obesity with alveolar hypoventilation
SNP effect on Gut microbiota abundance (genus Defluviitaleaceae UCG011 id.11287)

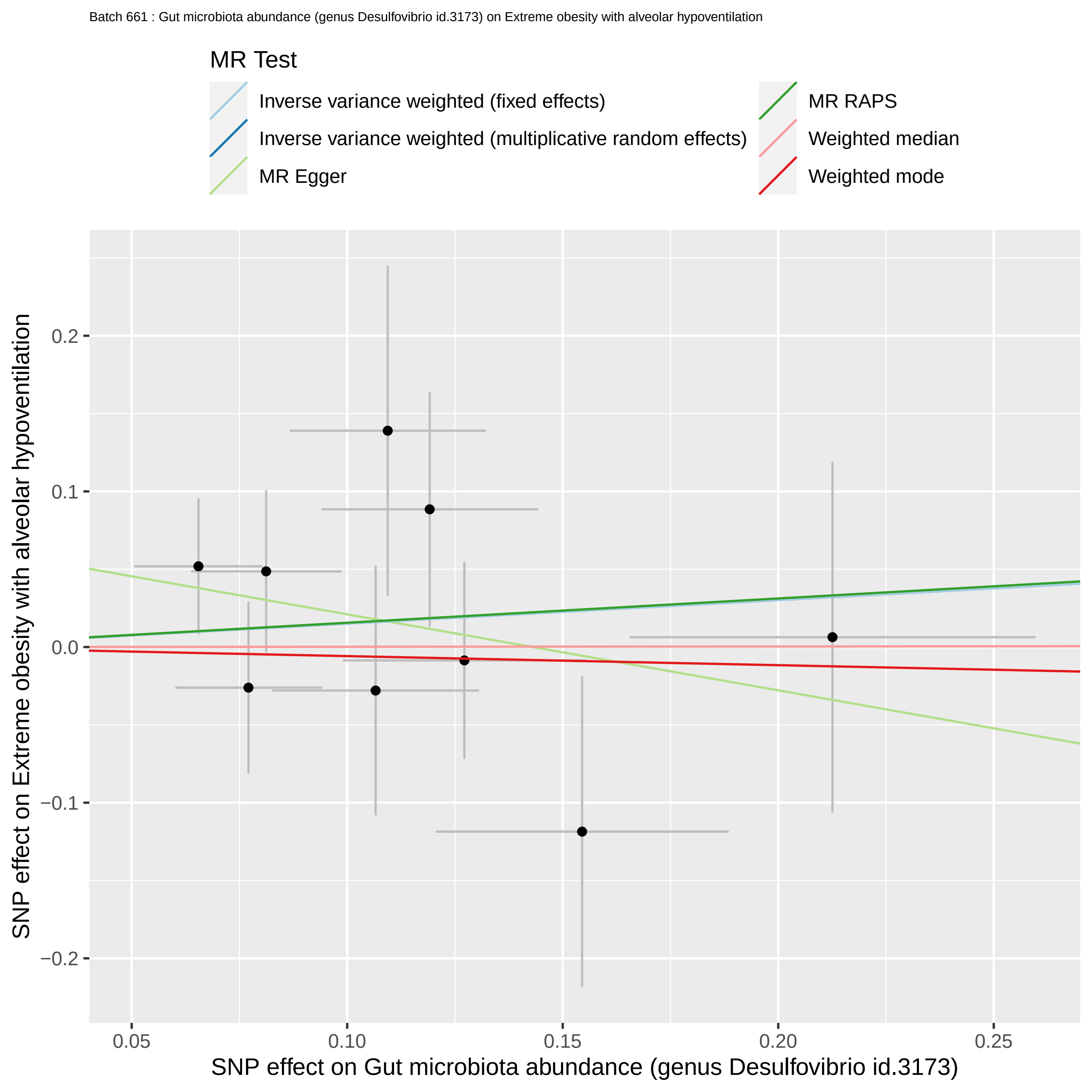
Batch 661 : Gut microbiota abundance (genus Desulfovibrio id.3173) on Extreme obesity with alveolar hypoventilation
MR Test
Inverse variance weighted (fixed effects)
Inverse variance weighted (multiplicative random effects)
MR Egger
MR RAPS
Weighted median
Weighted mode
SNP effect on Extreme obesity with alveolar hypoventilation
0.2
0.1
0.0
−0.1
−0.2
0.05
0.10
0.15
0.20
0.25
SNP effect on Gut microbiota abundance (genus Desulfovibrio id.3173)

Batch 662 : Gut microbiota abundance (genus Dialister id.2183) on Extreme obesity with alveolar hypoventilation

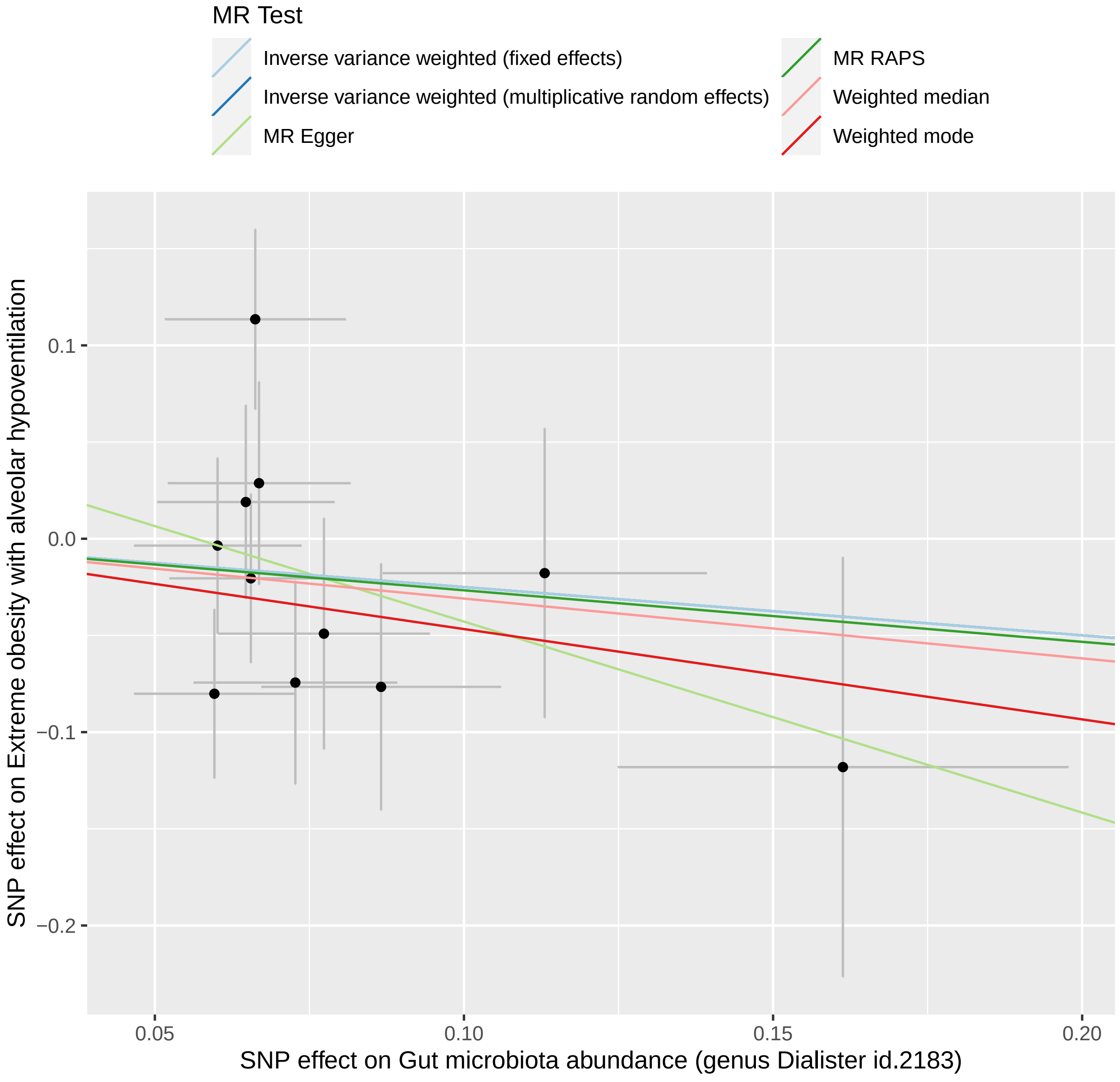

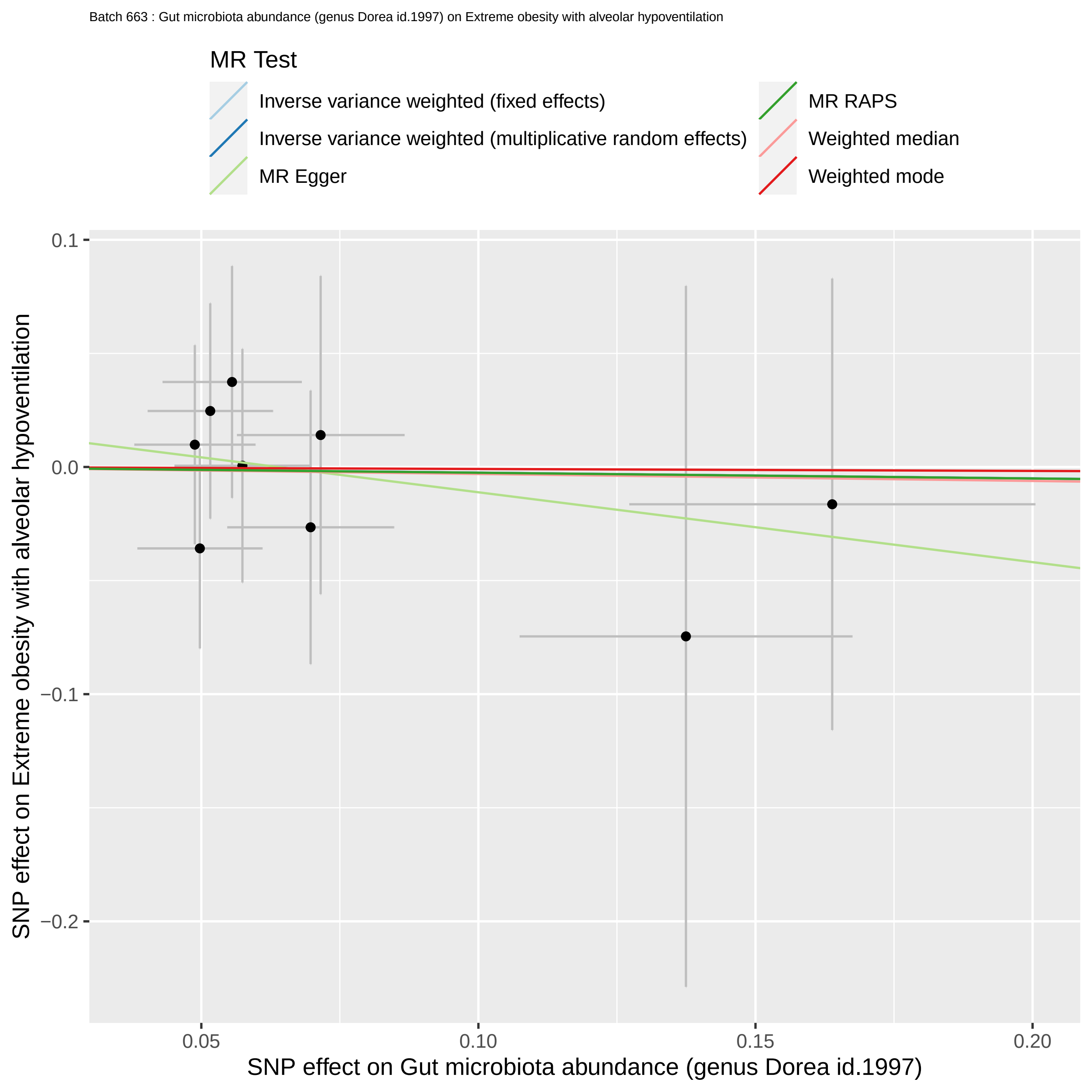

Batch 663 : Gut microbiota abundance (genus Dorea id.1997) on Extreme obesity with alveolar hypoventilation
MR Test
Inverse variance weighted (fixed effects)
Inverse variance weighted (multiplicative random effects)
MR Egger
MR RAPS
Weighted median
Weighted mode
0.1
0.0
−0.1
−0.2
0.05
0.10
0.15
0.20
SNP effect on Extreme obesity with alveolar hypoventilation
SNP effect on Gut microbiota abundance (genus Dorea id.1997)

Batch 664 : Gut microbiota abundance (genus Eggerthella id.819) on Extreme obesity with alveolar hypoventilation

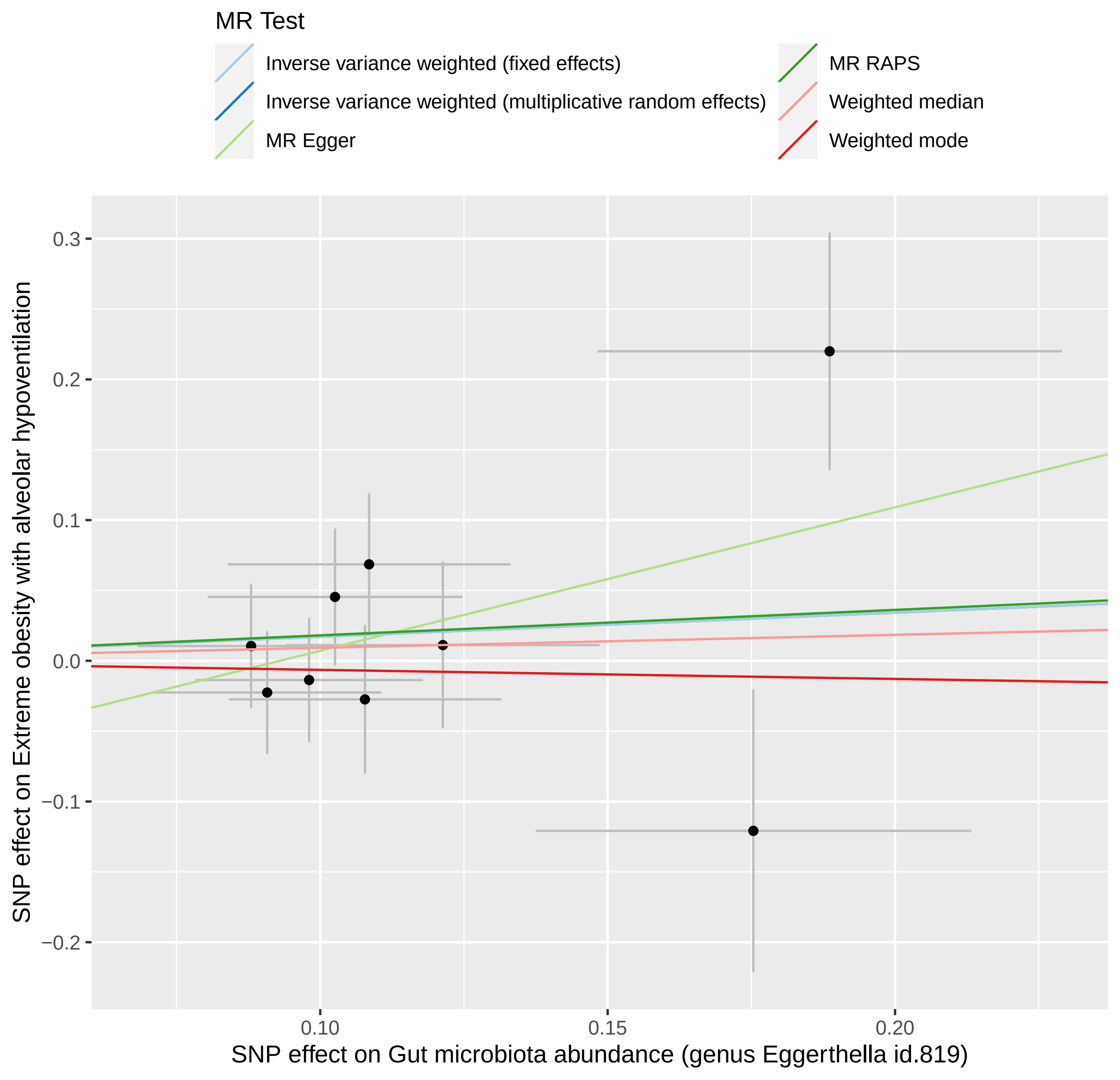

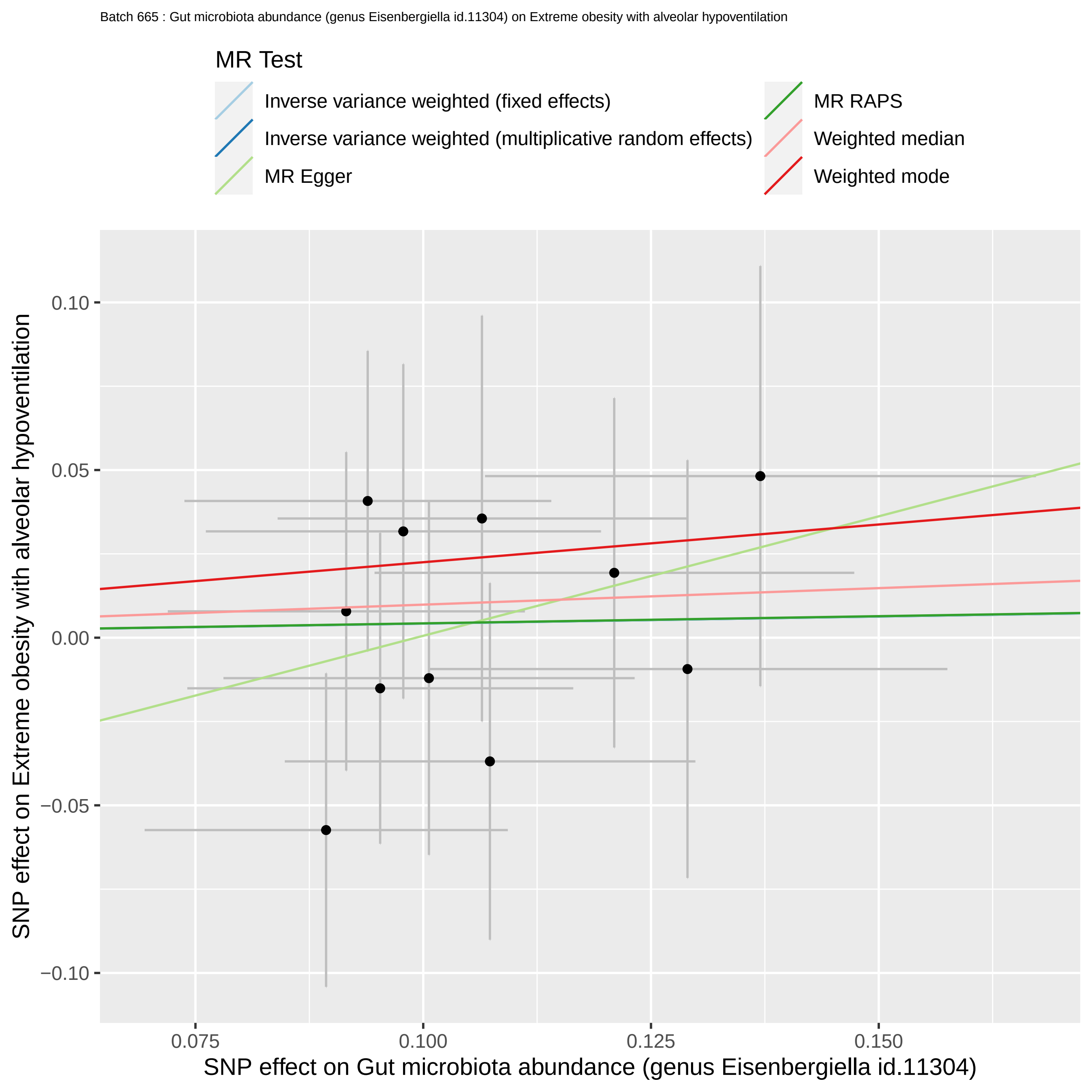

Batch 665 : Gut microbiota abundance (genus Eisenbergiella id.11304) on Extreme obesity with alveolar hypoventilation
MR Test
Inverse variance weighted (fixed effects)
Inverse variance weighted (multiplicative random effects)
MR Egger
MR RAPS
Weighted median
Weighted mode
0.10
0.05
0.00
−0.05
−0.10
0.075
0.100
0.125
0.150
SNP effect on Extreme obesity with alveolar hypoventilation
SNP effect on Gut microbiota abundance (genus Eisenbergiella id.11304)

Batch 666 : Gut microbiota abundance (genus Enterorhabdus id.820) on Extreme obesity with alveolar hypoventilation

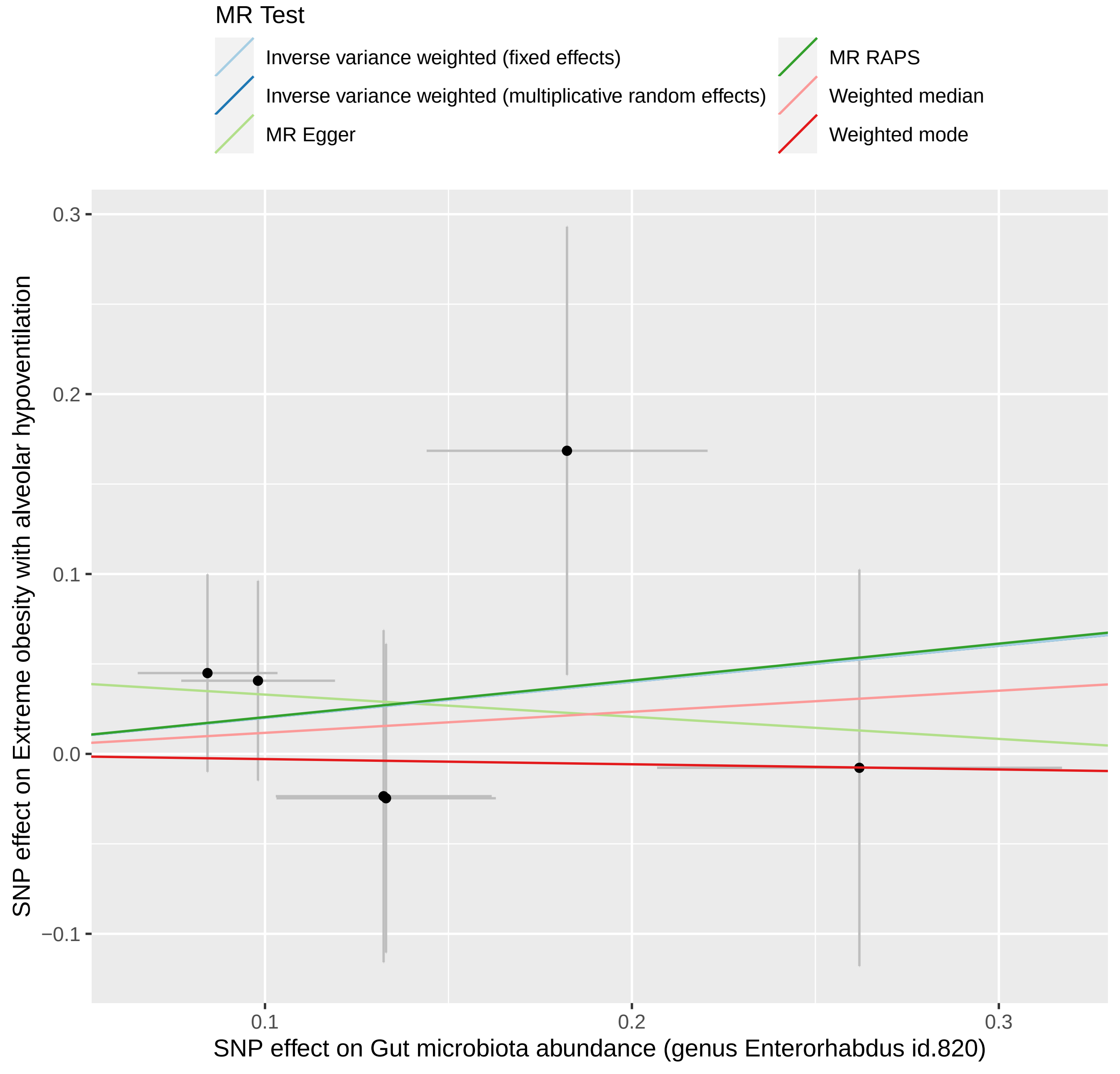

Batch 667 : Gut microbiota abundance (genus Erysipelatoclostridium id.11381) on Extreme obesity with alveolar hypoventilation

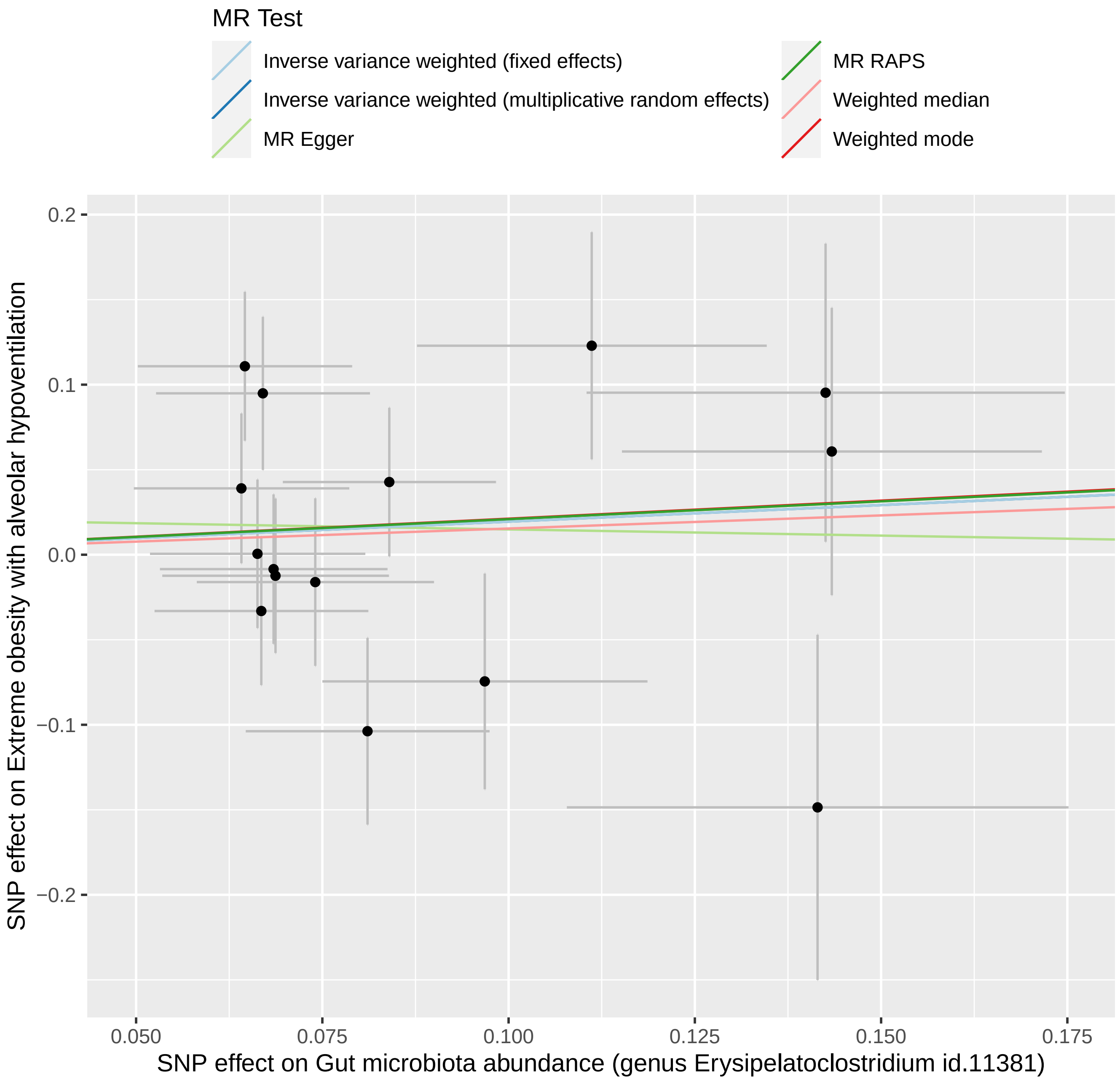

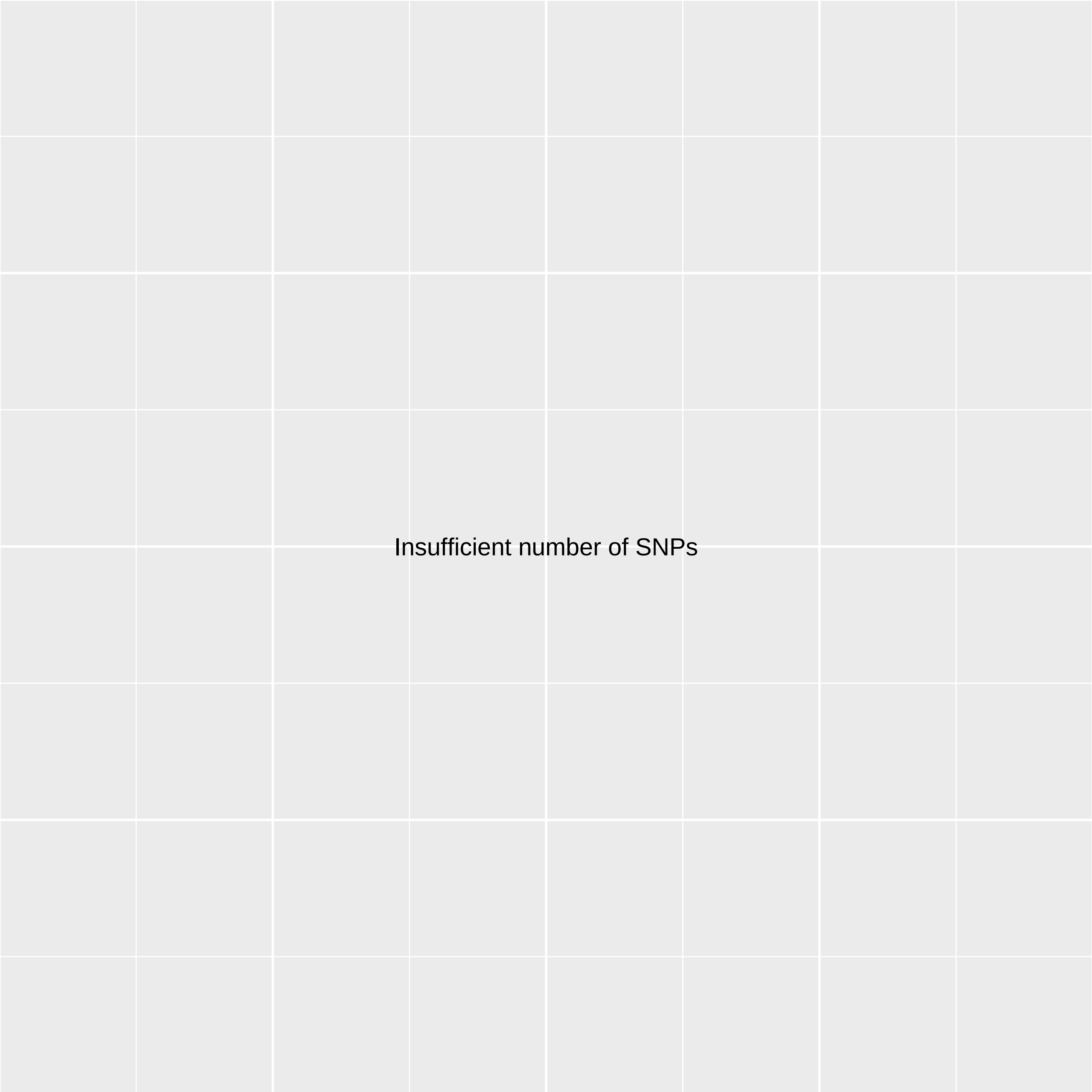
Insufficient number of SNPs

Batch 669 : Gut microbiota abundance (genus Escherichia Shigella id.3504) on Extreme obesity with alveolar hypoventilation

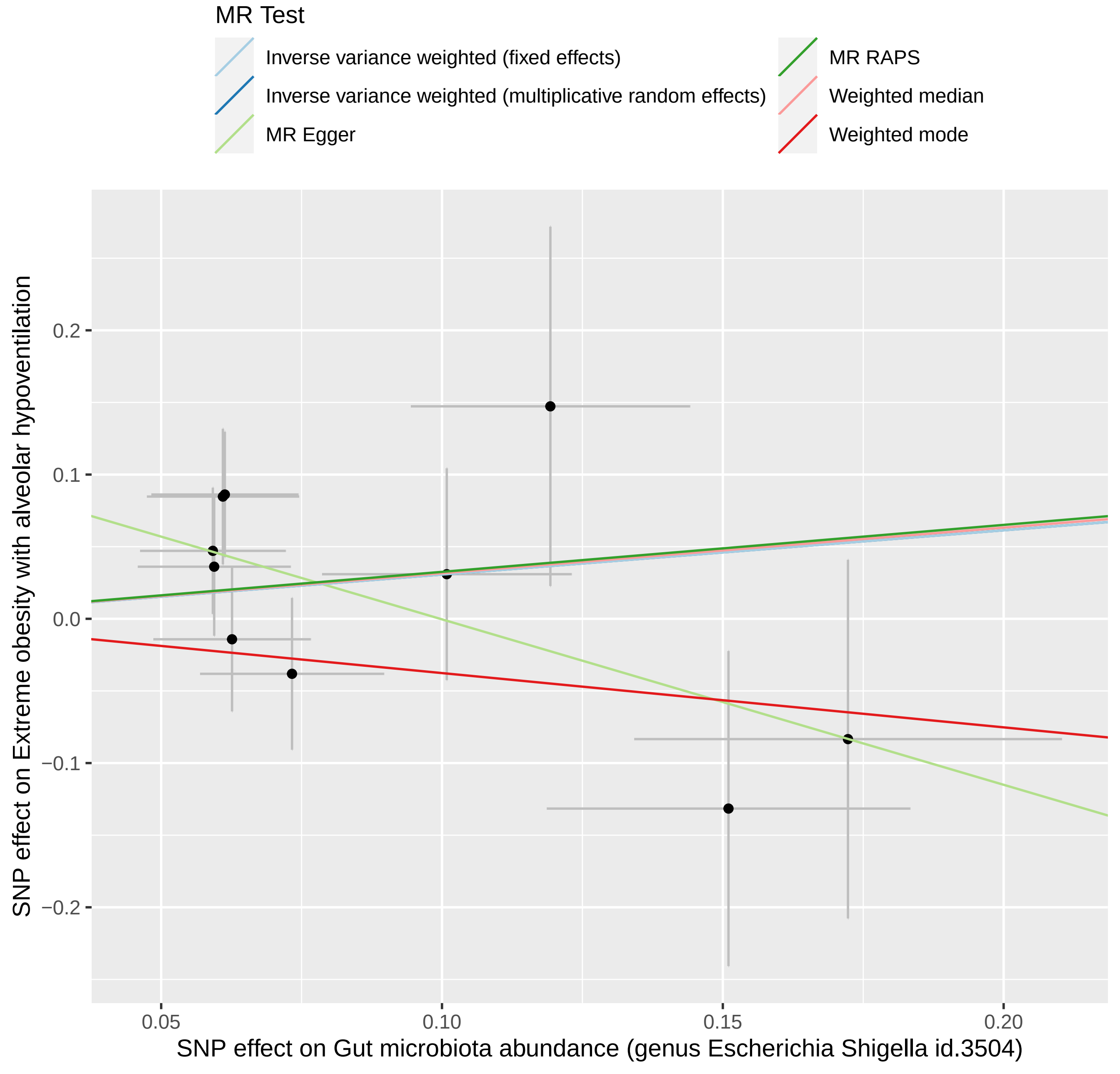

Batch 670 : Gut microbiota abundance (genus Eubacterium brachy group id.11296) on Extreme obesity with alveolar hypoventilation

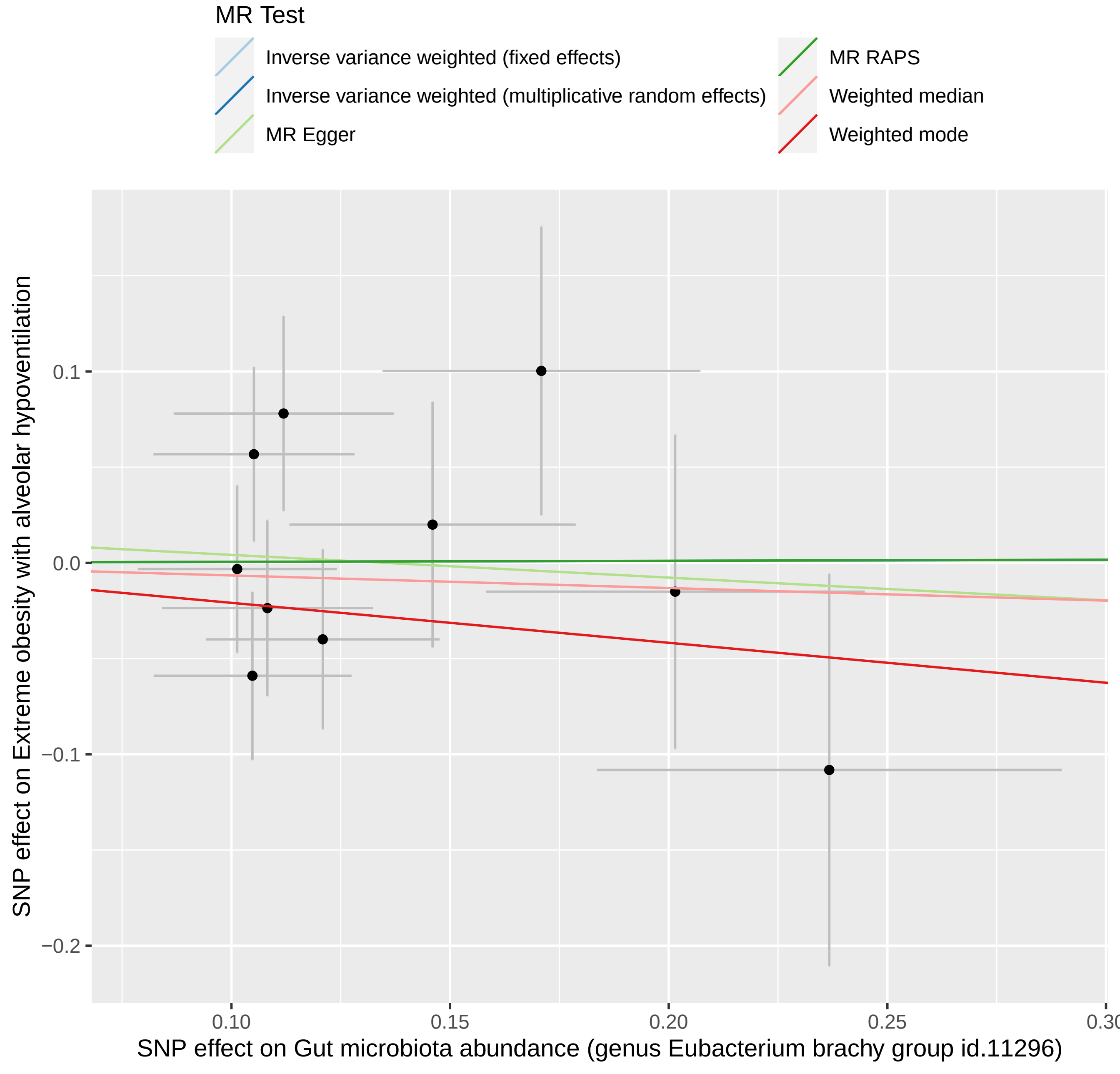

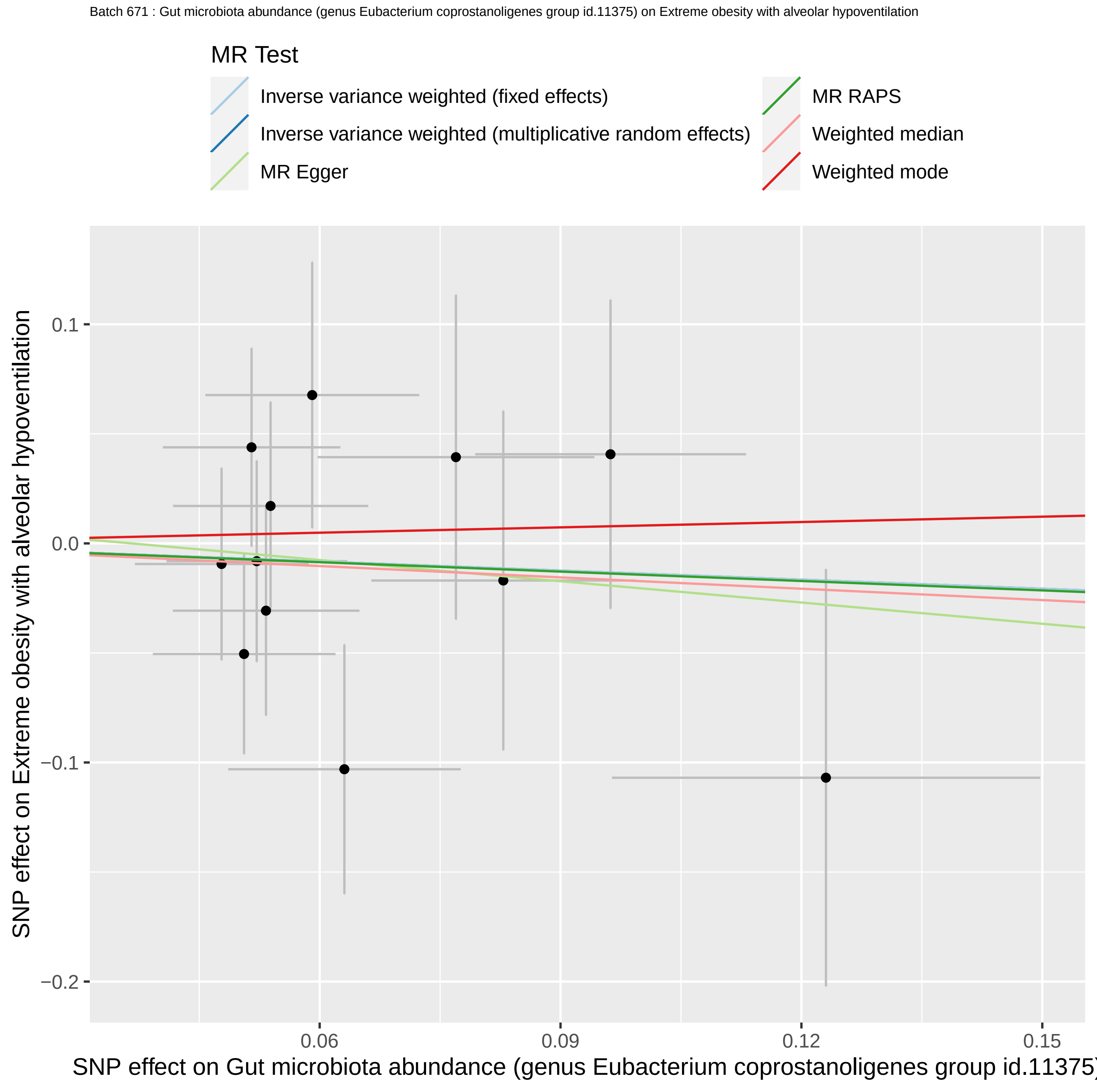

Batch 671 : Gut microbiota abundance (genus Eubacterium coprostanoligenes group id.11375) on Extreme obesity with alveolar hypoventilation
MR Test
Inverse variance weighted (fixed effects)
Inverse variance weighted (multiplicative random effects)
MR Egger
MR RAPS
Weighted median
Weighted mode
0.1
0.0
−0.1
−0.2
0.06
0.09
0.12
0.15
SNP effect on Extreme obesity with alveolar hypoventilation
SNP effect on Gut microbiota abundance (genus Eubacterium coprostanoligenes group id.11375)

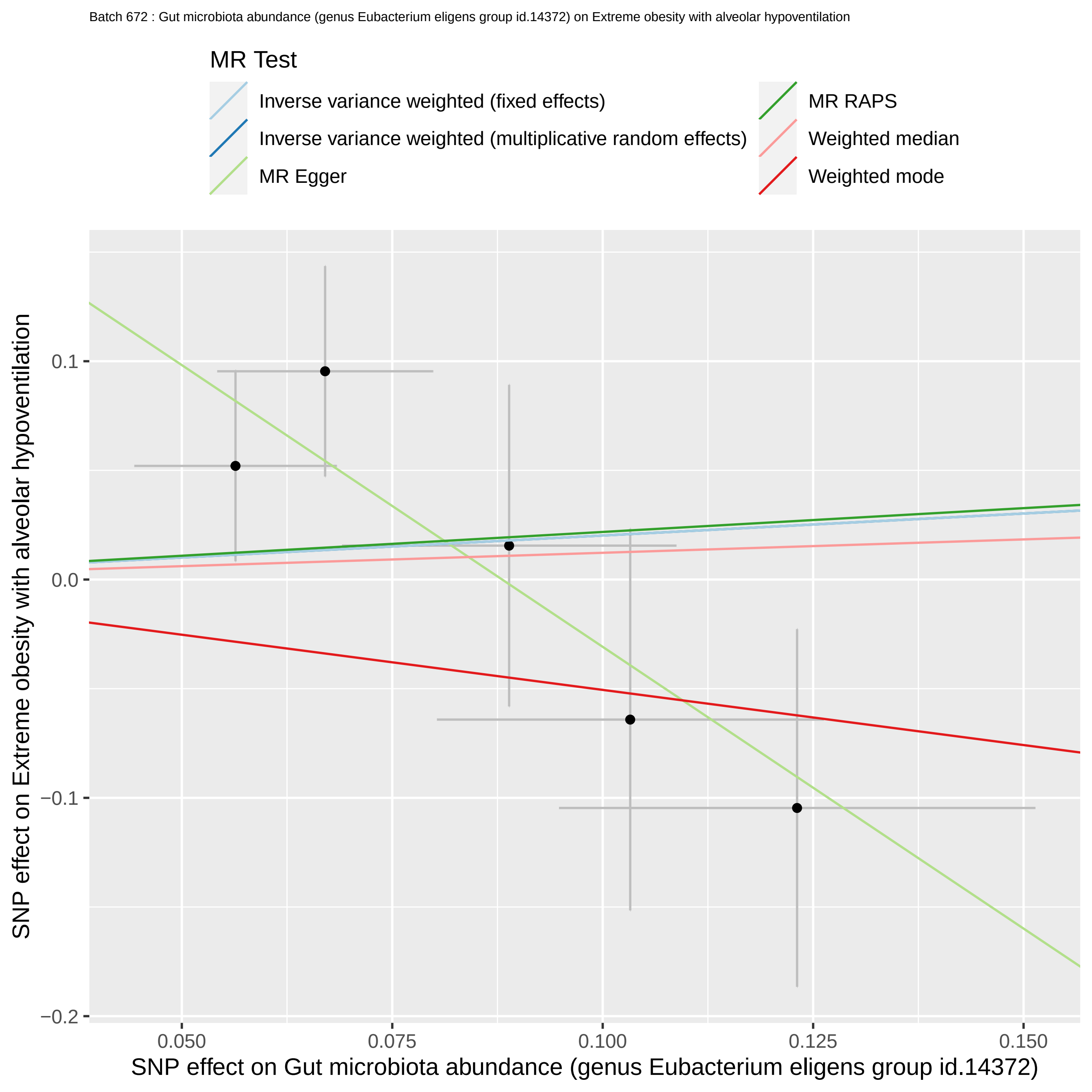

Batch 672 : Gut microbiota abundance (genus Eubacterium eligens group id.14372) on Extreme obesity with alveolar hypoventilation
MR Test
Inverse variance weighted (fixed effects)
Inverse variance weighted (multiplicative random effects)
MR Egger
MR RAPS
Weighted median
Weighted mode
0.1
0.0
−0.1
−0.2
0.050
0.075
0.100
0.125
0.150
SNP effect on Extreme obesity with alveolar hypoventilation
SNP effect on Gut microbiota abundance (genus Eubacterium eligens group id.14372)

Batch 673 : Gut microbiota abundance (genus Eubacterium fissicatena group id.14373) on Extreme obesity with alveolar hypoventilation

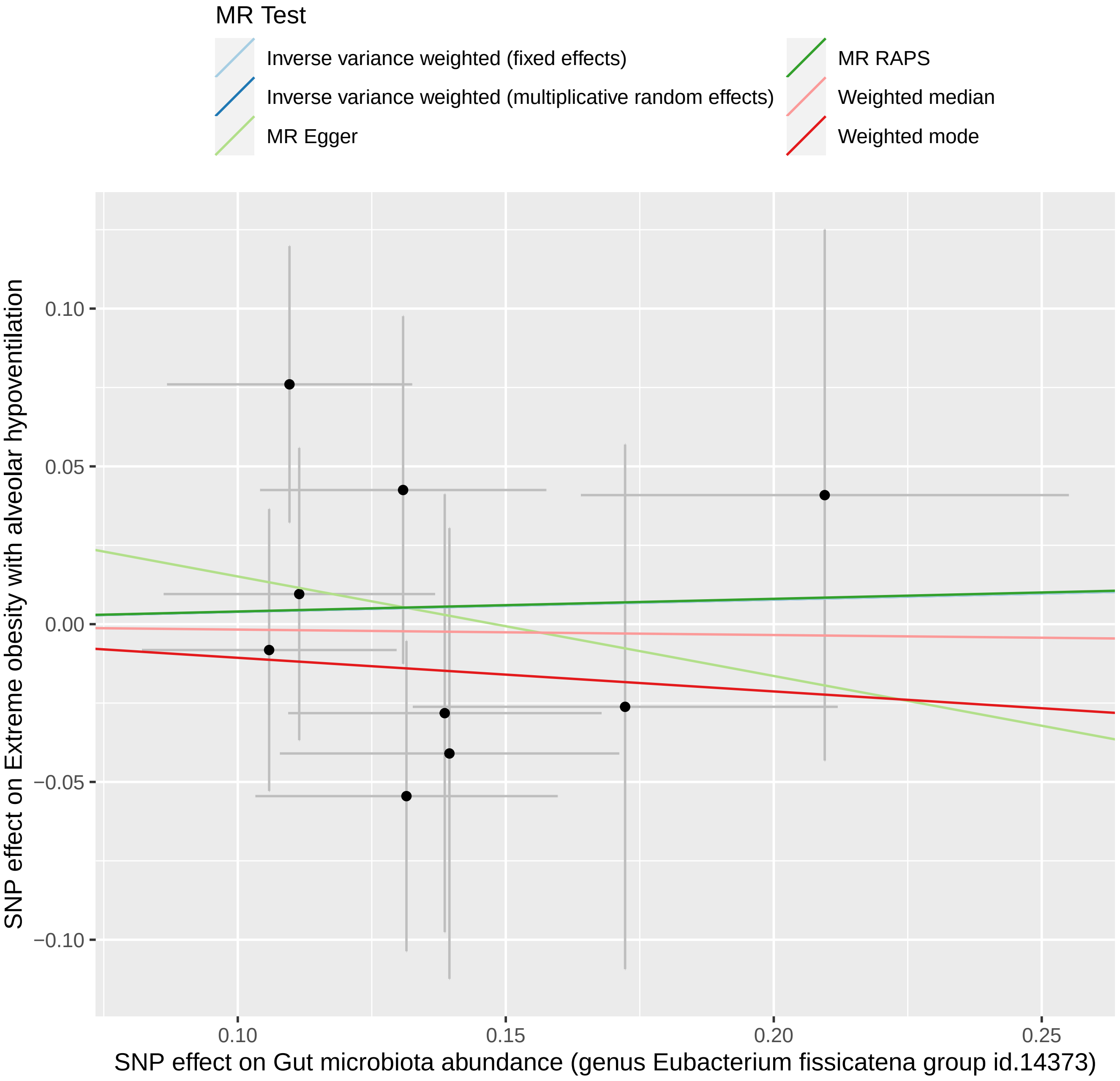

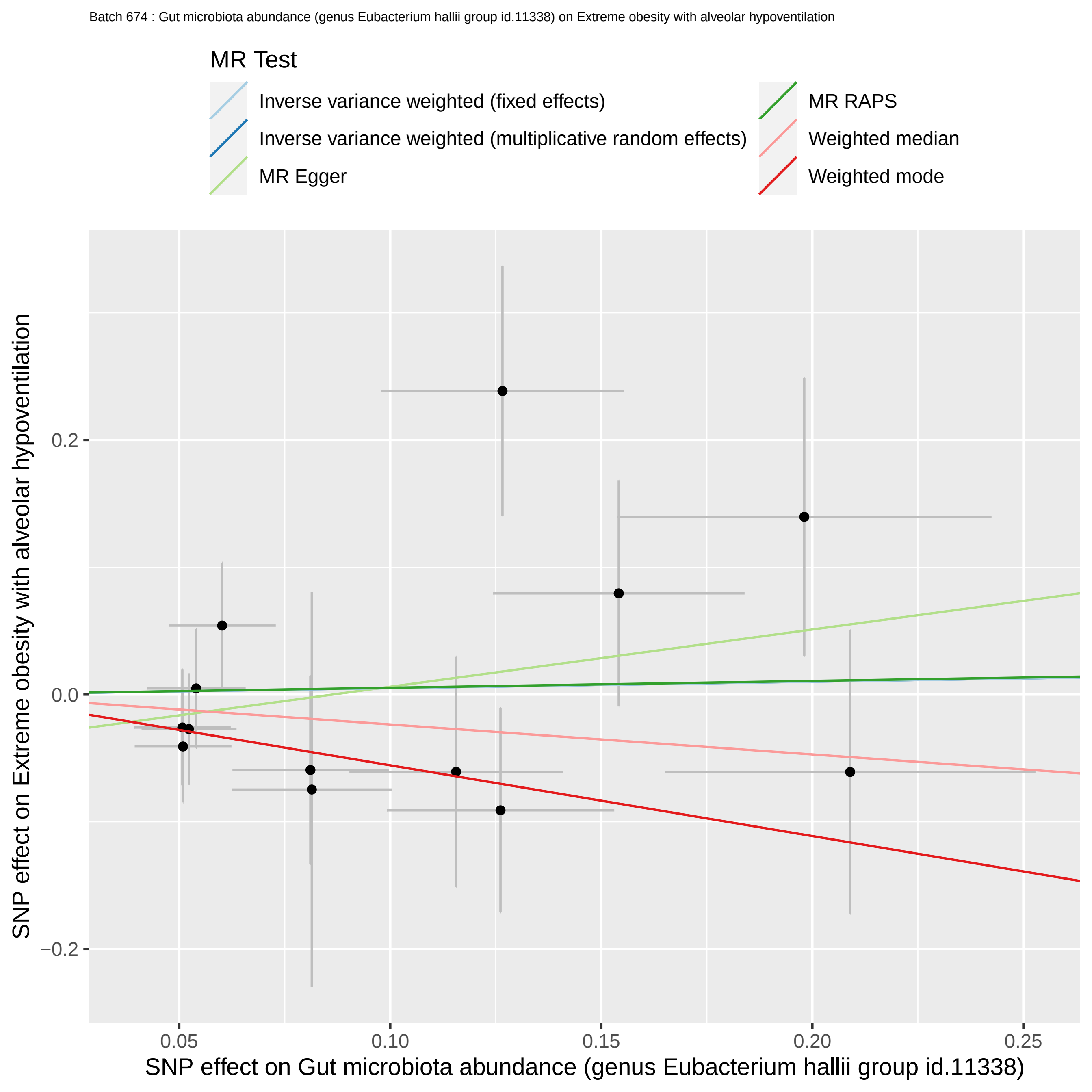
Batch 674 : Gut microbiota abundance (genus Eubacterium hallii group id.11338) on Extreme obesity with alveolar hypoventilation
MR Test
Inverse variance weighted (fixed effects)
Inverse variance weighted (multiplicative random effects)
MR Egger
MR RAPS
Weighted median
Weighted mode
0.2
0.0
−0.2
0.05
0.10
0.15
0.20
0.25
SNP effect on Extreme obesity with alveolar hypoventilation
SNP effect on Gut microbiota abundance (genus Eubacterium hallii group id.11338)

Batch 675 : Gut microbiota abundance (genus Eubacterium nodatum group id.11297) on Extreme obesity with alveolar hypoventilation

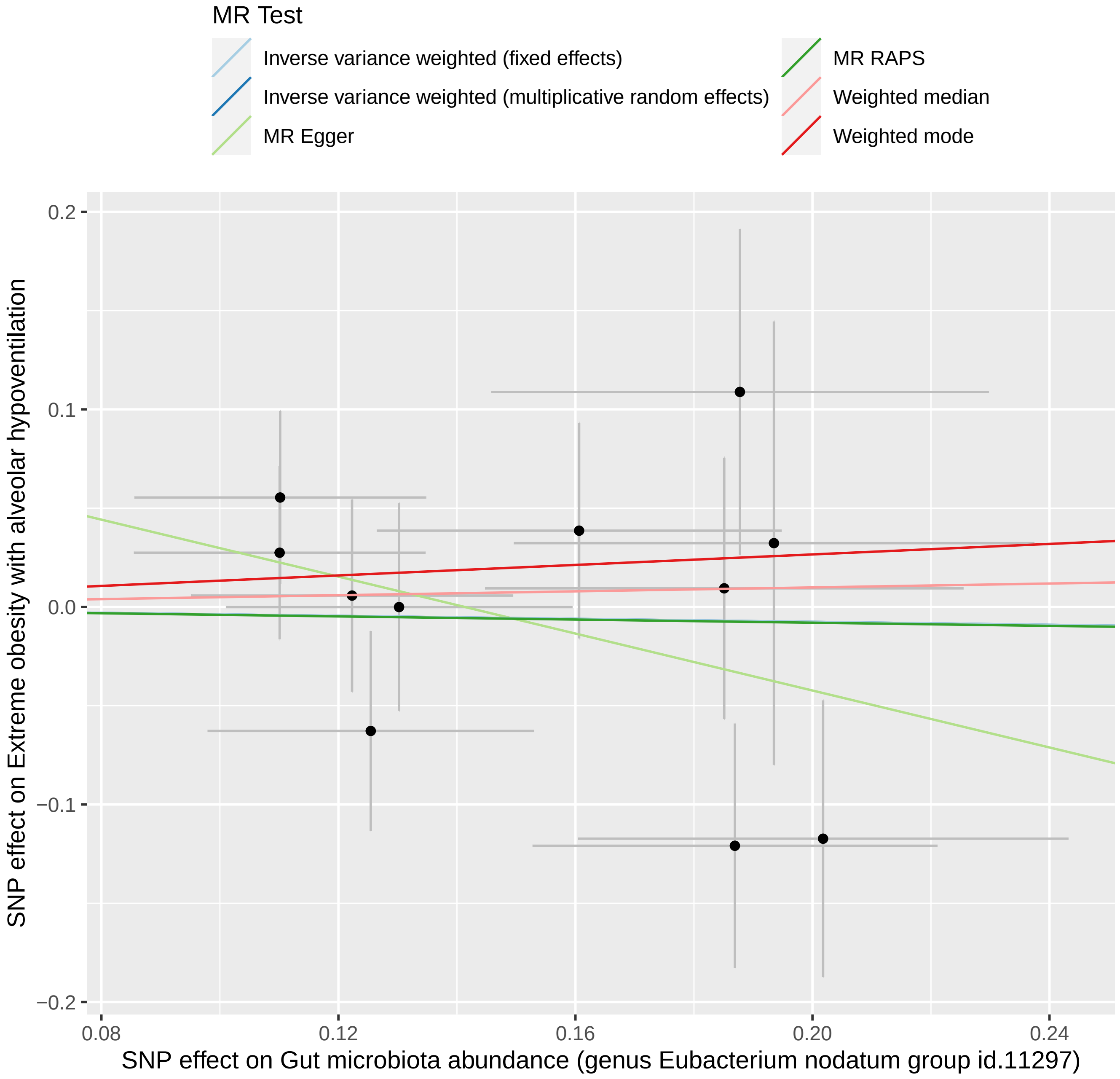

Batch 676 : Gut microbiota abundance (genus Eubacterium oxidoreducens group id.11339) on Extreme obesity with alveolar hypoventilation

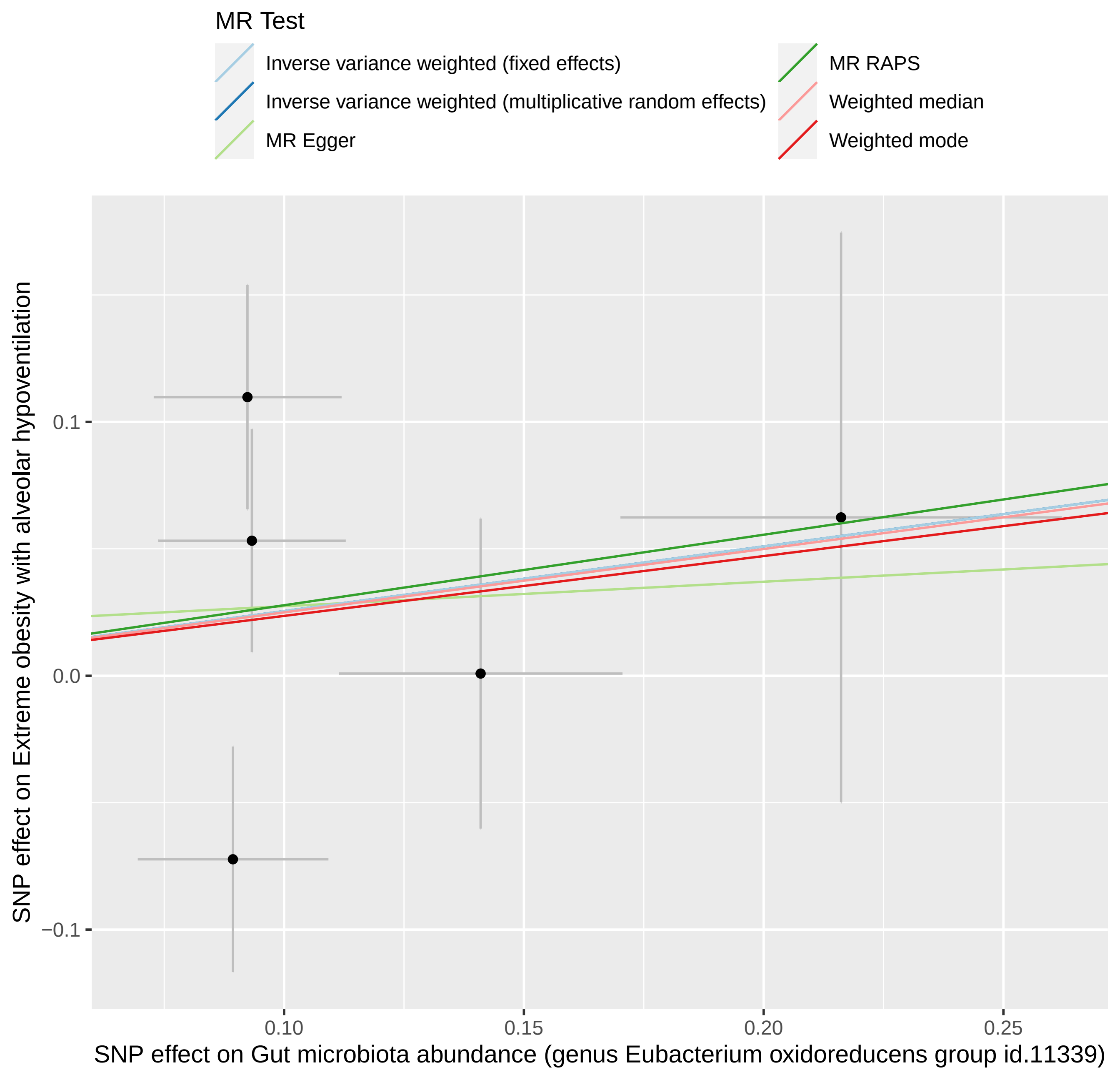

Batch 677 : Gut microbiota abundance (genus Eubacterium rectale group id.14374) on Extreme obesity with alveolar hypoventilation

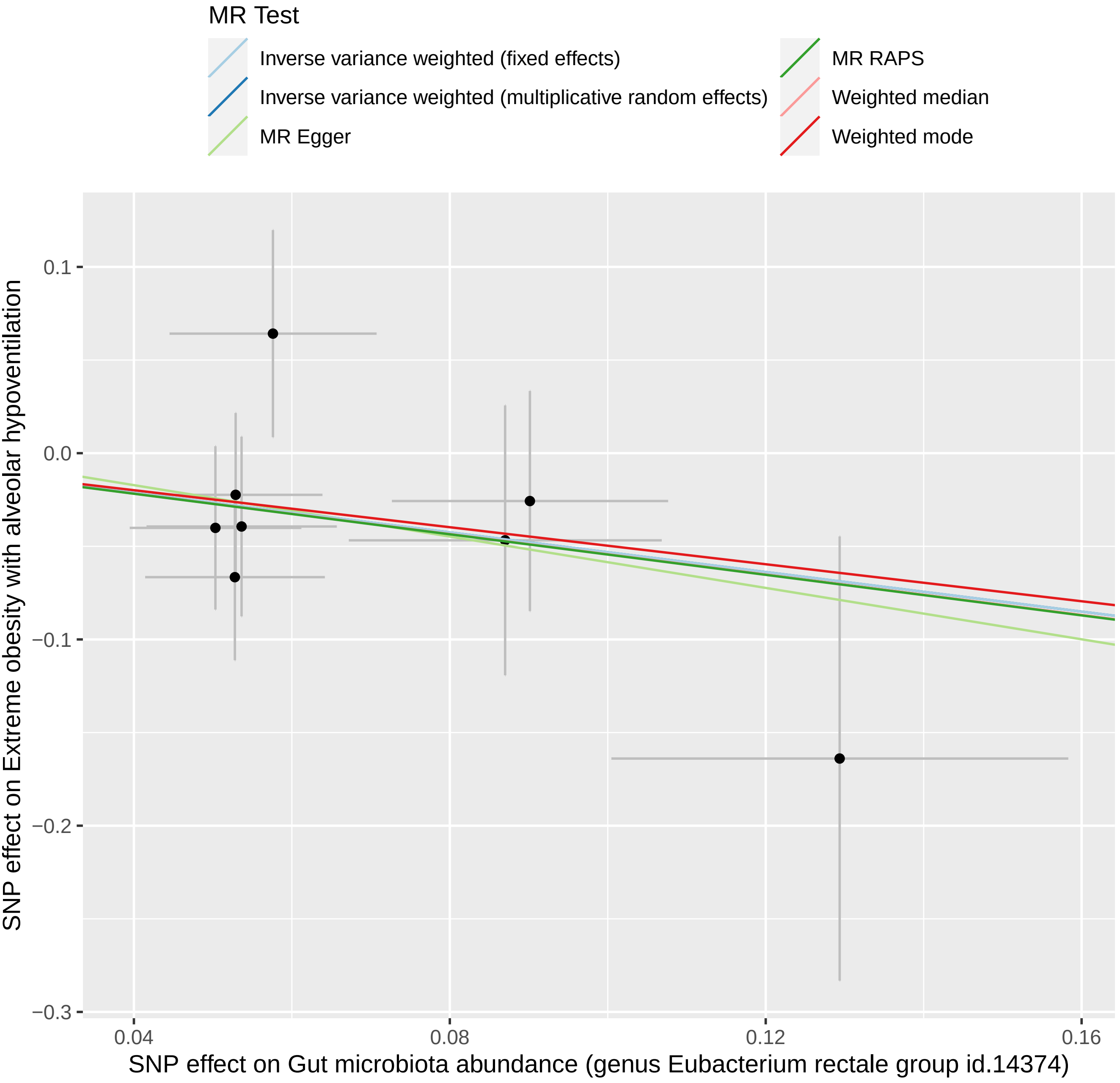

Batch 678 : Gut microbiota abundance (genus Eubacterium ruminantium group id.11340) on Extreme obesity with alveolar hypoventilation

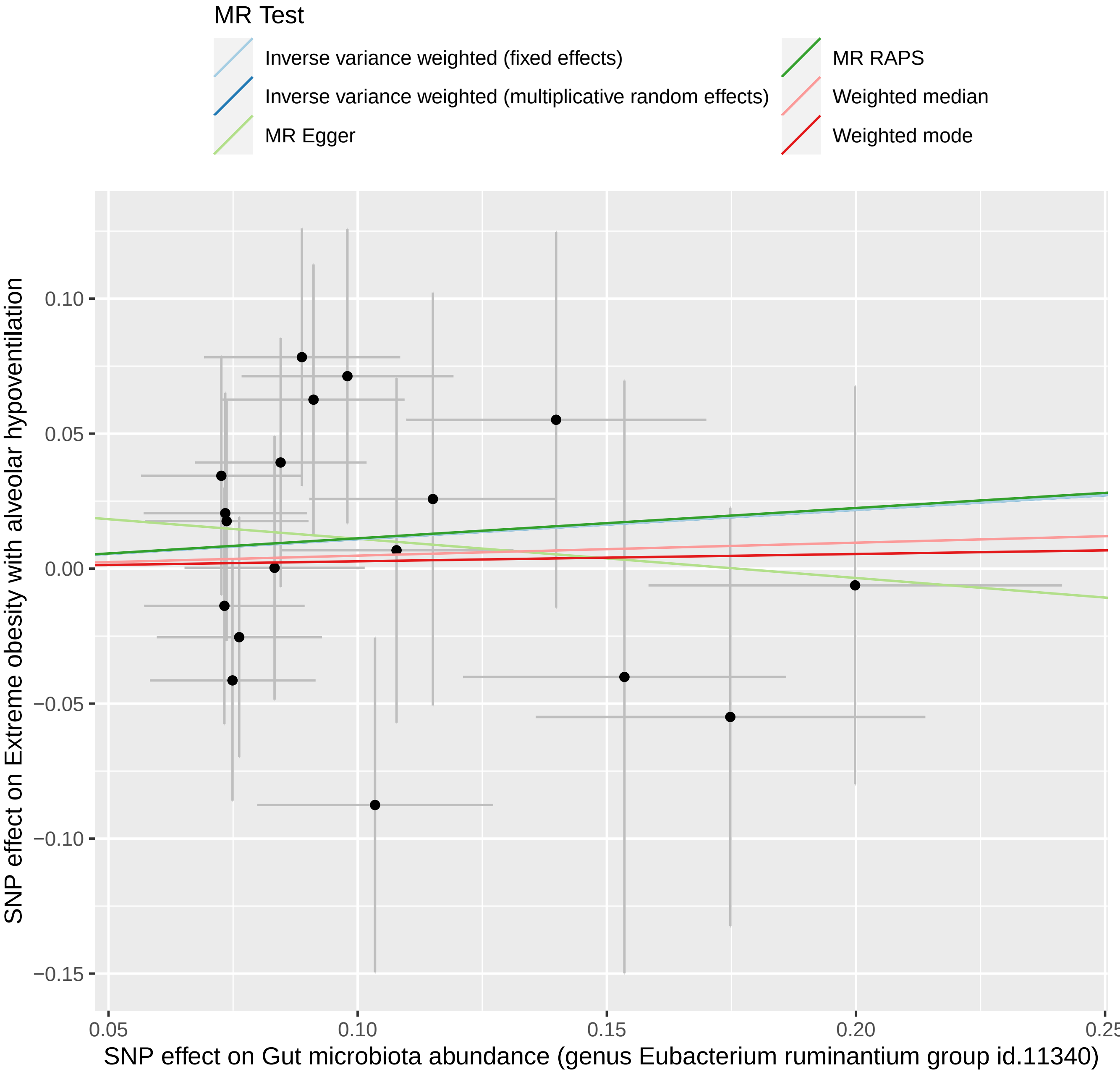

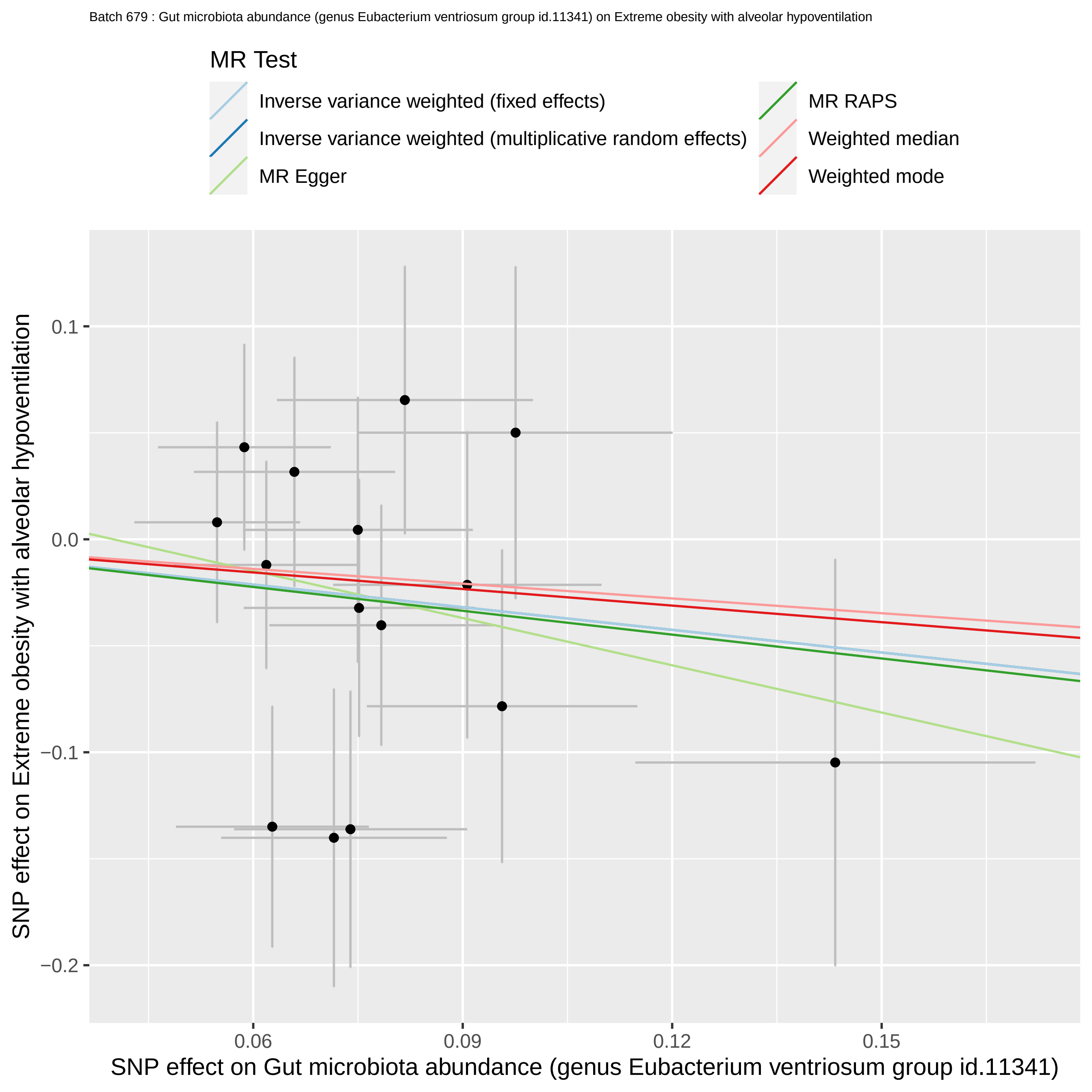

Batch 679 : Gut microbiota abundance (genus Eubacterium ventriosum group id.11341) on Extreme obesity with alveolar hypoventilation
MR Test
Inverse variance weighted (fixed effects)
Inverse variance weighted (multiplicative random effects)
MR Egger
MR RAPS
Weighted median
Weighted mode
0.1
0.0
−0.1
−0.2
0.06
0.09
0.12
0.15
SNP effect on Extreme obesity with alveolar hypoventilation
SNP effect on Gut microbiota abundance (genus Eubacterium ventriosum group id.11341)

Batch 680 : Gut microbiota abundance (genus Eubacterium xylanophilum group id.14375) on Extreme obesity with alveolar hypoventilation

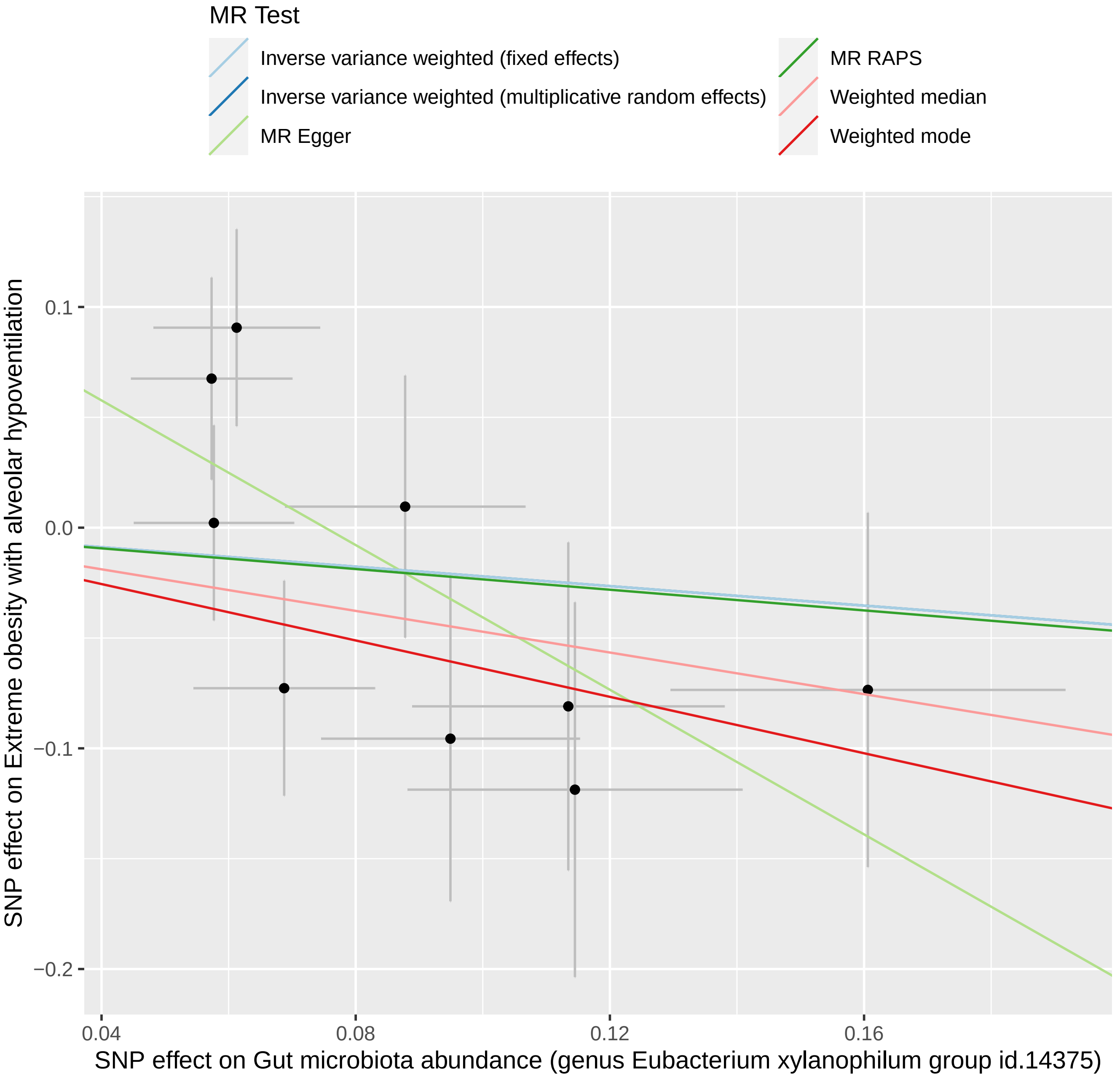

Batch 681 : Gut microbiota abundance (genus Faecalibacterium id.2057) on Extreme obesity with alveolar hypoventilation

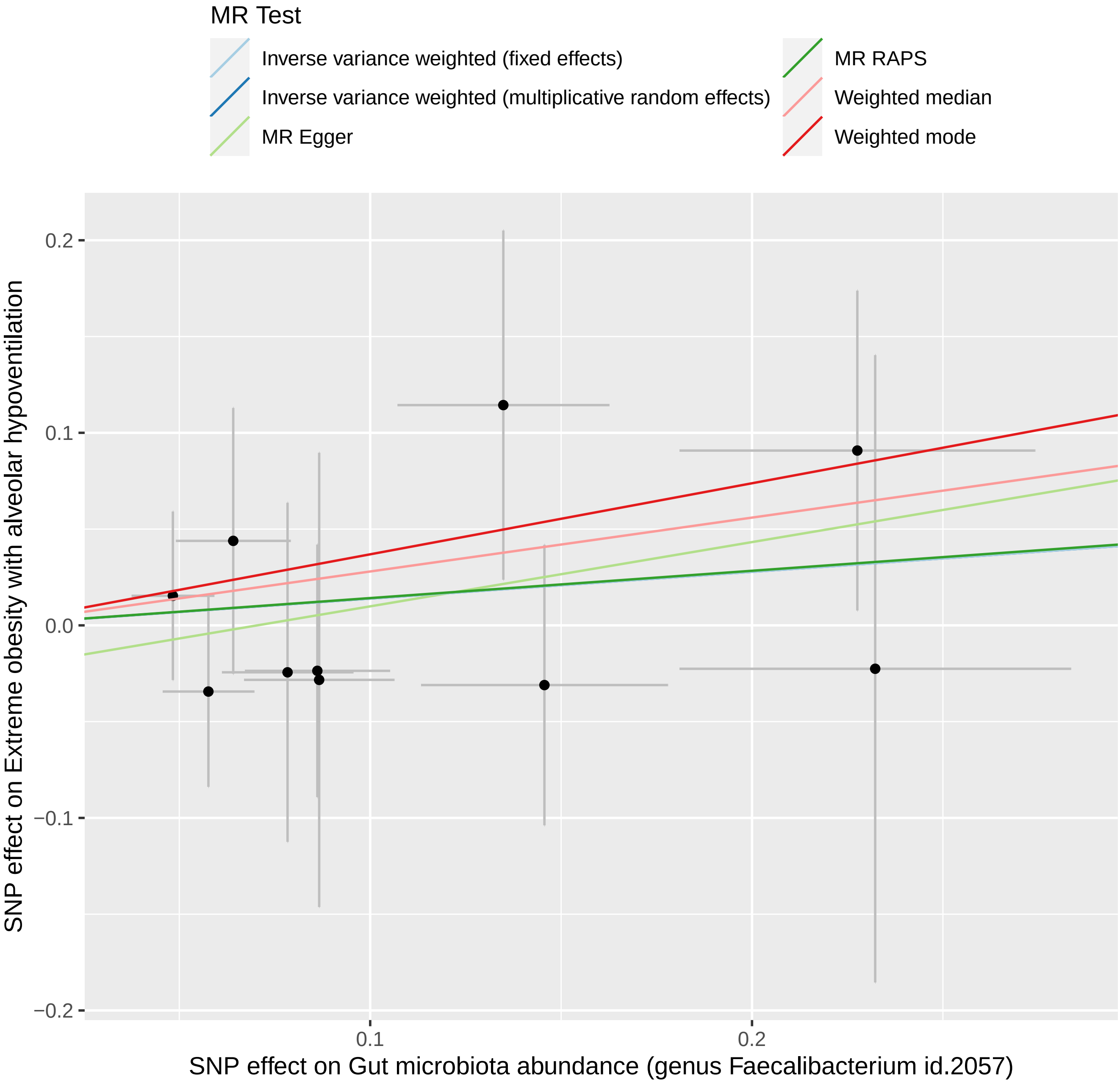

Batch 682 : Gut microbiota abundance (genus Family XIII AD3011 group id.11293) on Extreme obesity with alveolar hypoventilation

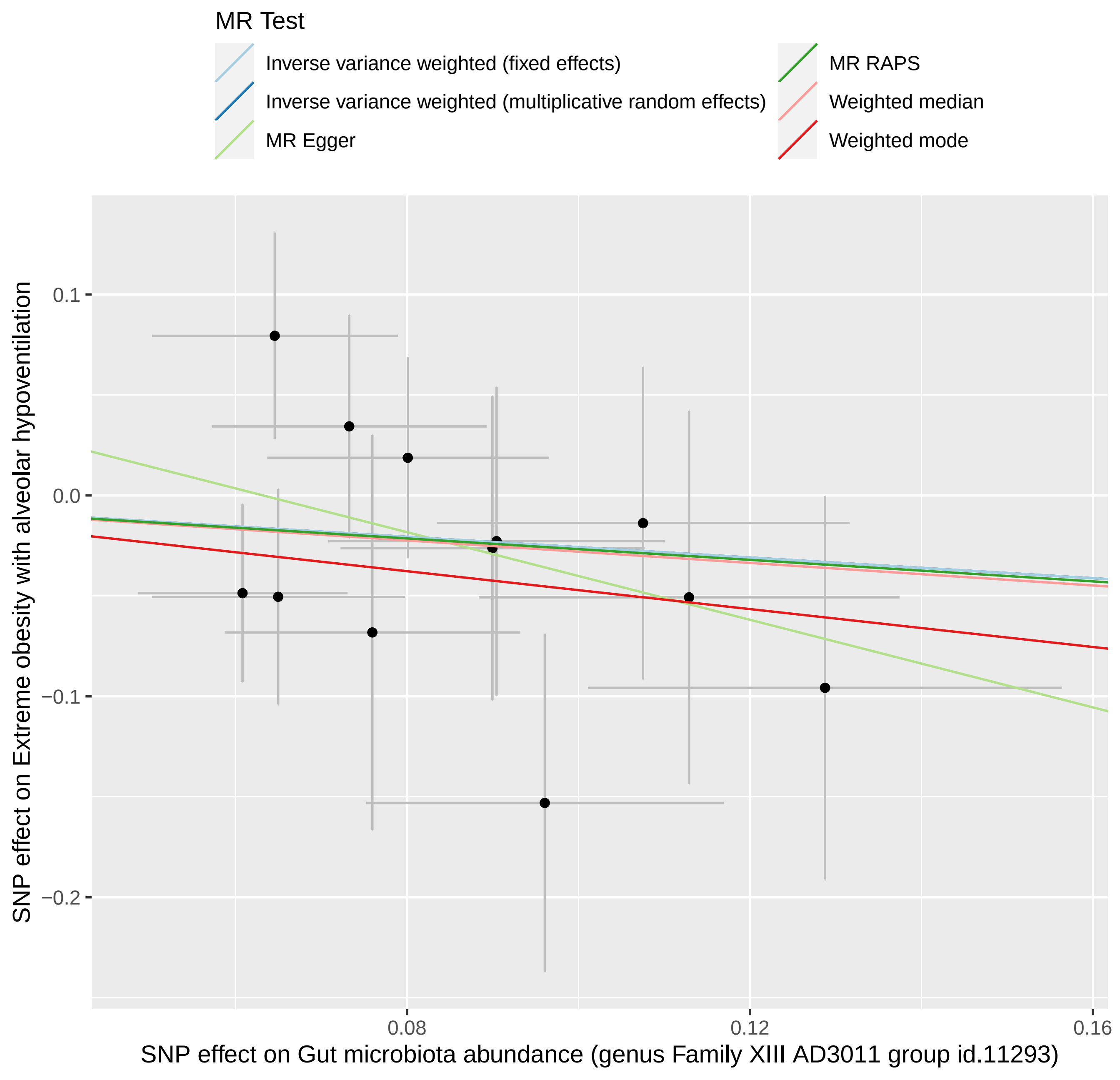

Batch 683 : Gut microbiota abundance (genus Family XIII UCG001 id.11294) on Extreme obesity with alveolar hypoventilation

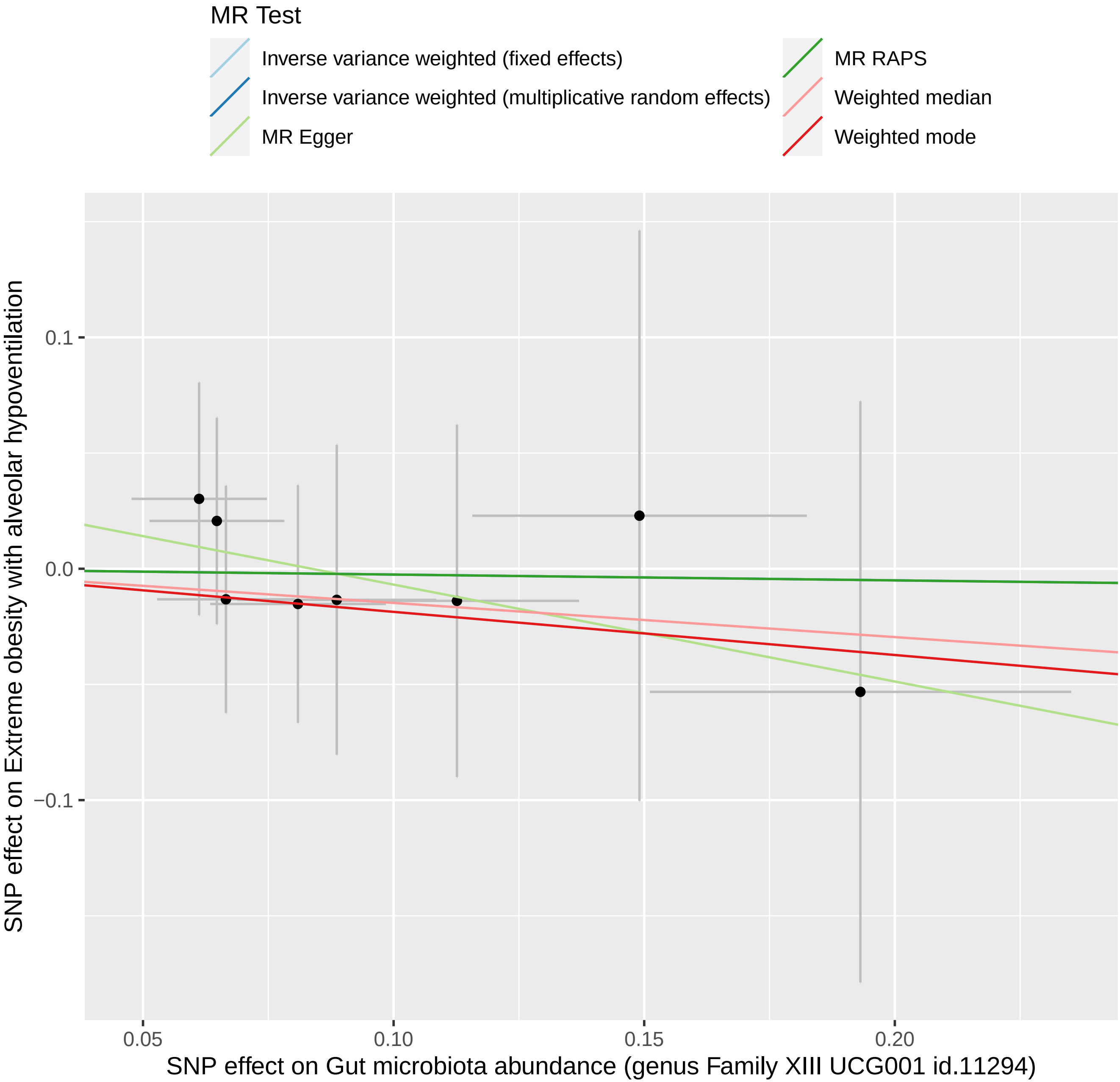

Batch 684 : Gut microbiota abundance (genus Flavonifractor id.2059) on Extreme obesity with alveolar hypoventilation

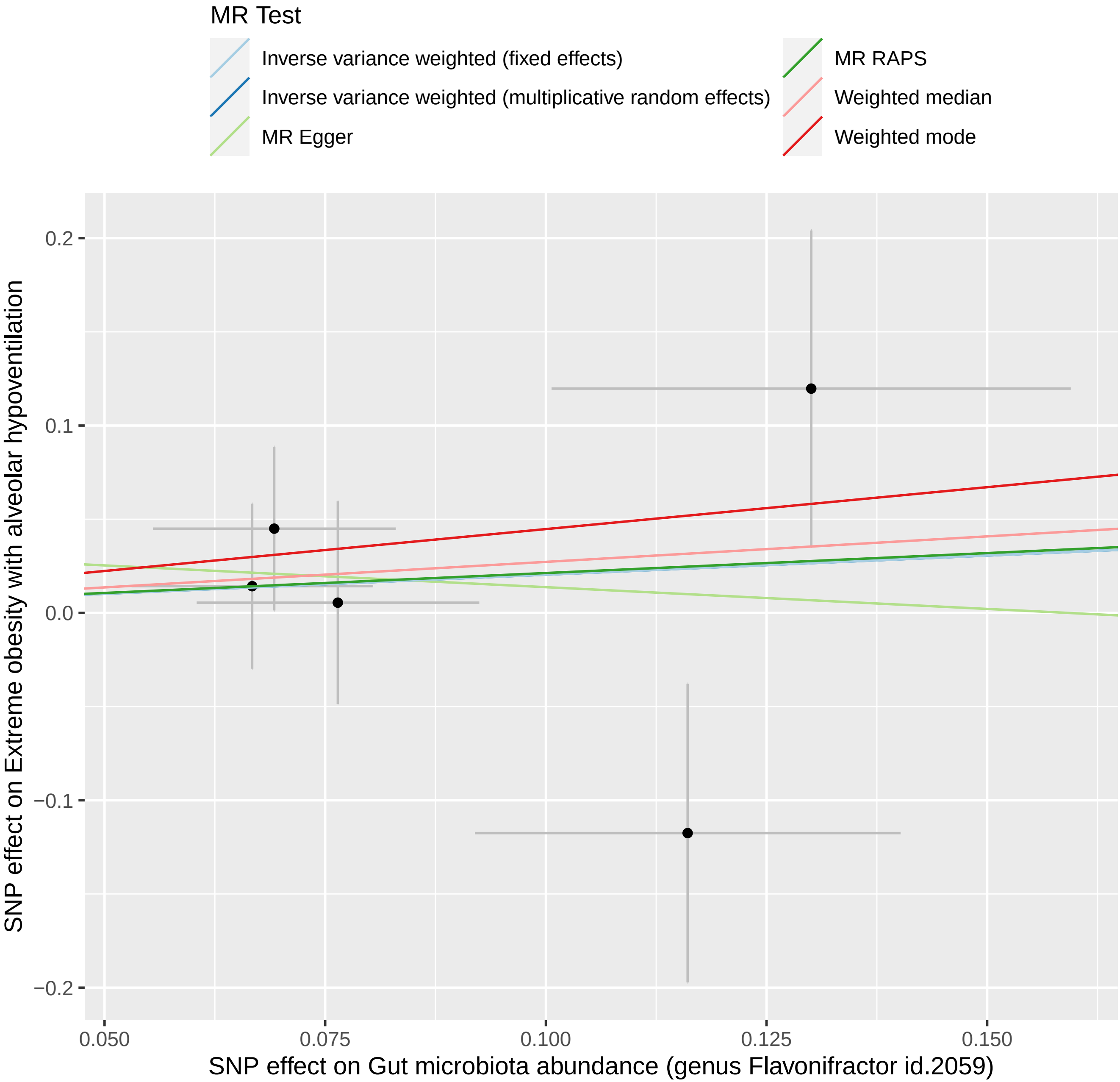

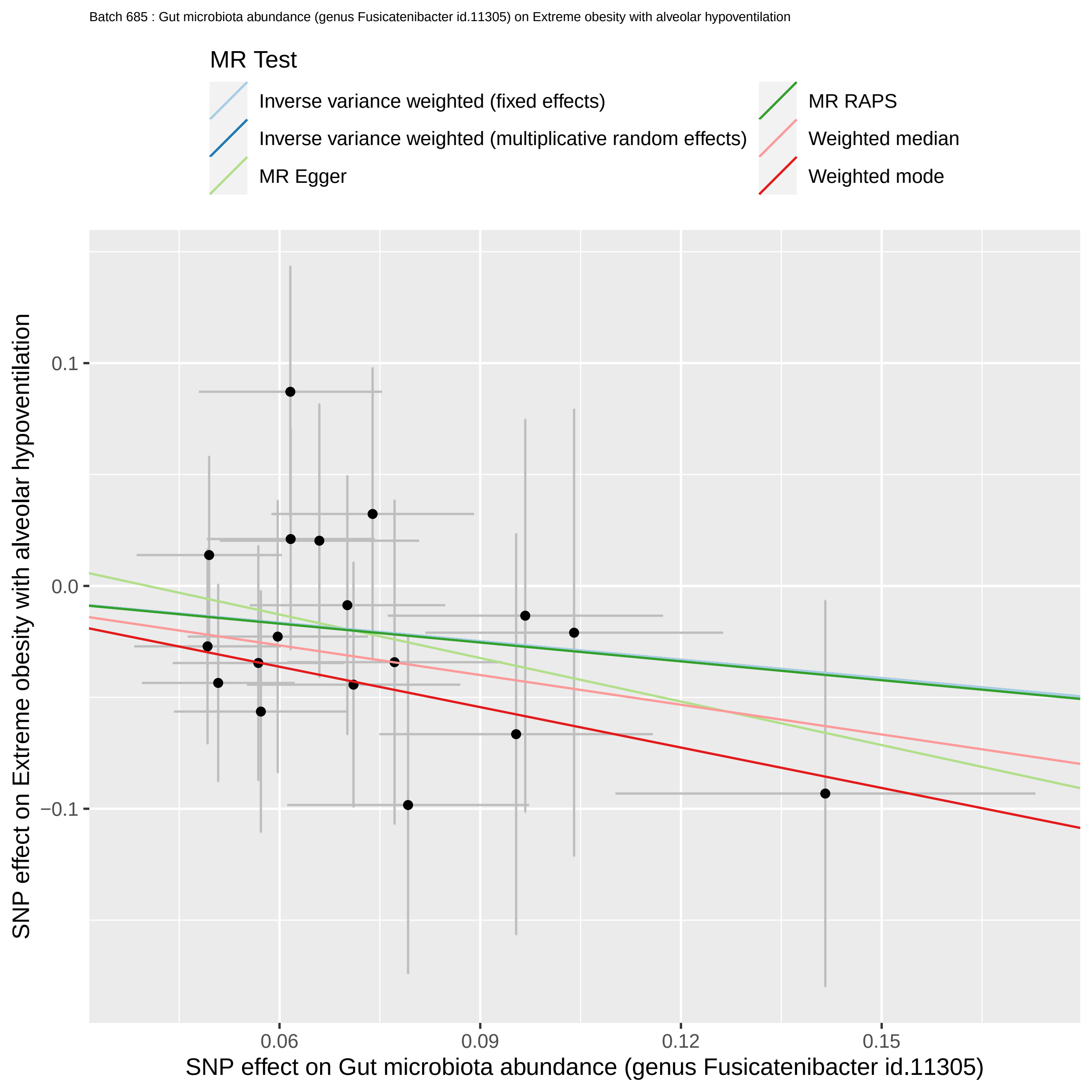
Batch 685 : Gut microbiota abundance (genus Fusicatenibacter id.11305) on Extreme obesity with alveolar hypoventilation
MR Test
Inverse variance weighted (fixed effects)
Inverse variance weighted (multiplicative random effects)
MR Egger
MR RAPS
Weighted median
Weighted mode
0.1
0.0
−0.1
0.06
0.09
0.12
0.15
SNP effect on Extreme obesity with alveolar hypoventilation
SNP effect on Gut microbiota abundance (genus Fusicatenibacter id.11305)

Batch 686 : Gut microbiota abundance (genus Gordonibacter id.821) on Extreme obesity with alveolar hypoventilation

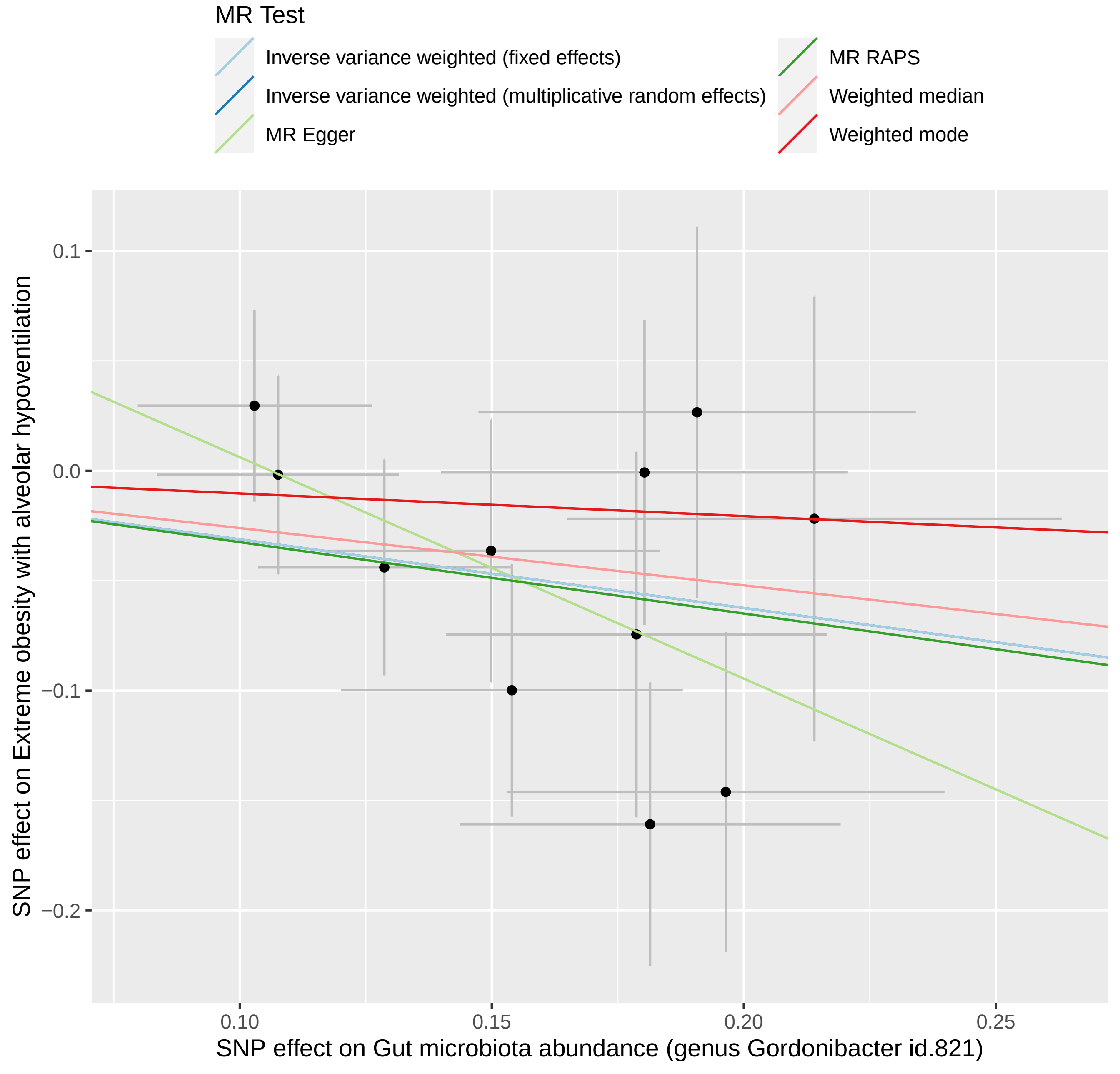

Batch 687 : Gut microbiota abundance (genus Haemophilus id.3698) on Extreme obesity with alveolar hypoventilation

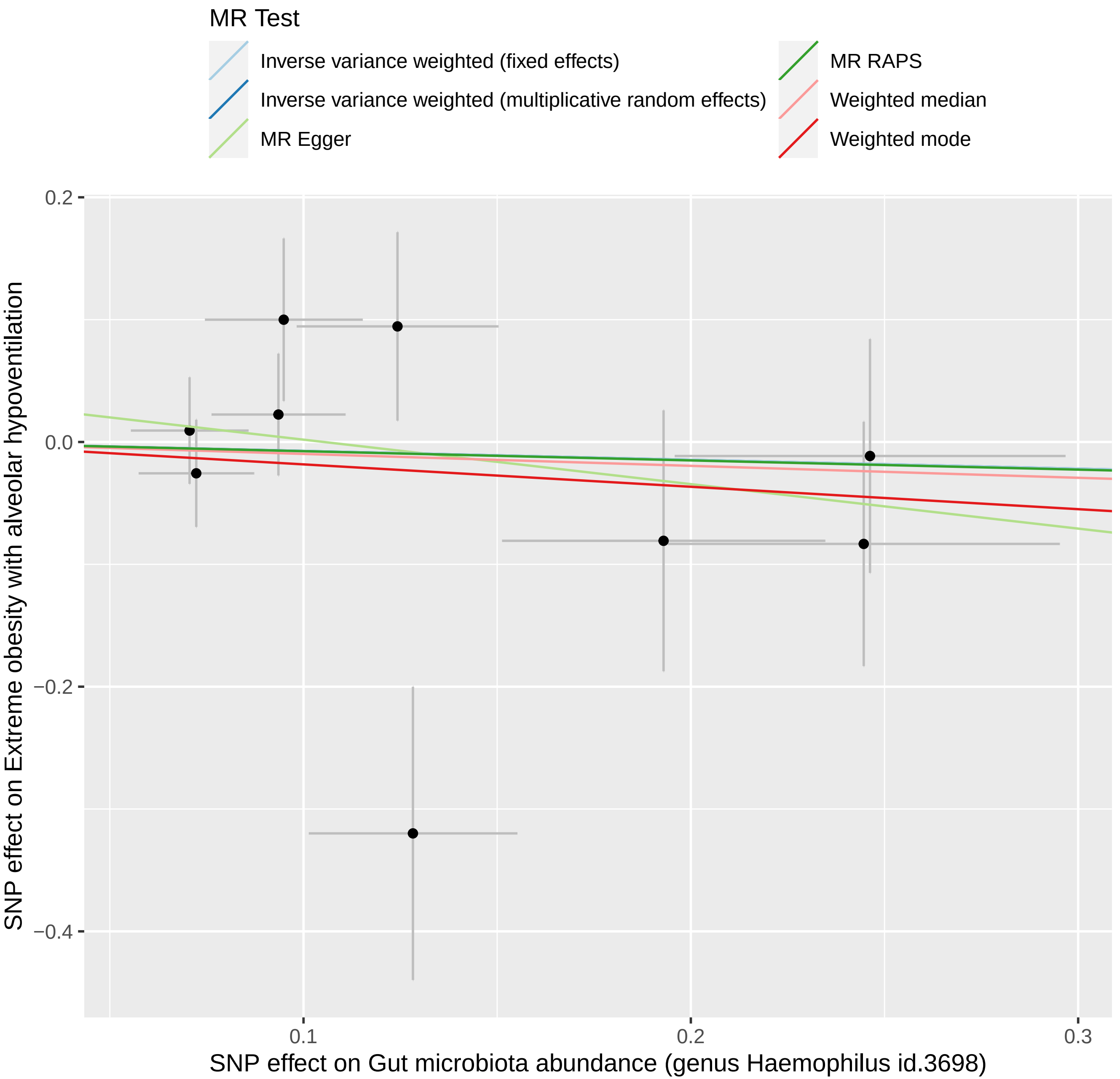

Batch 688 : Gut microbiota abundance (genus Holdemanella id.11393) on Extreme obesity with alveolar hypoventilation

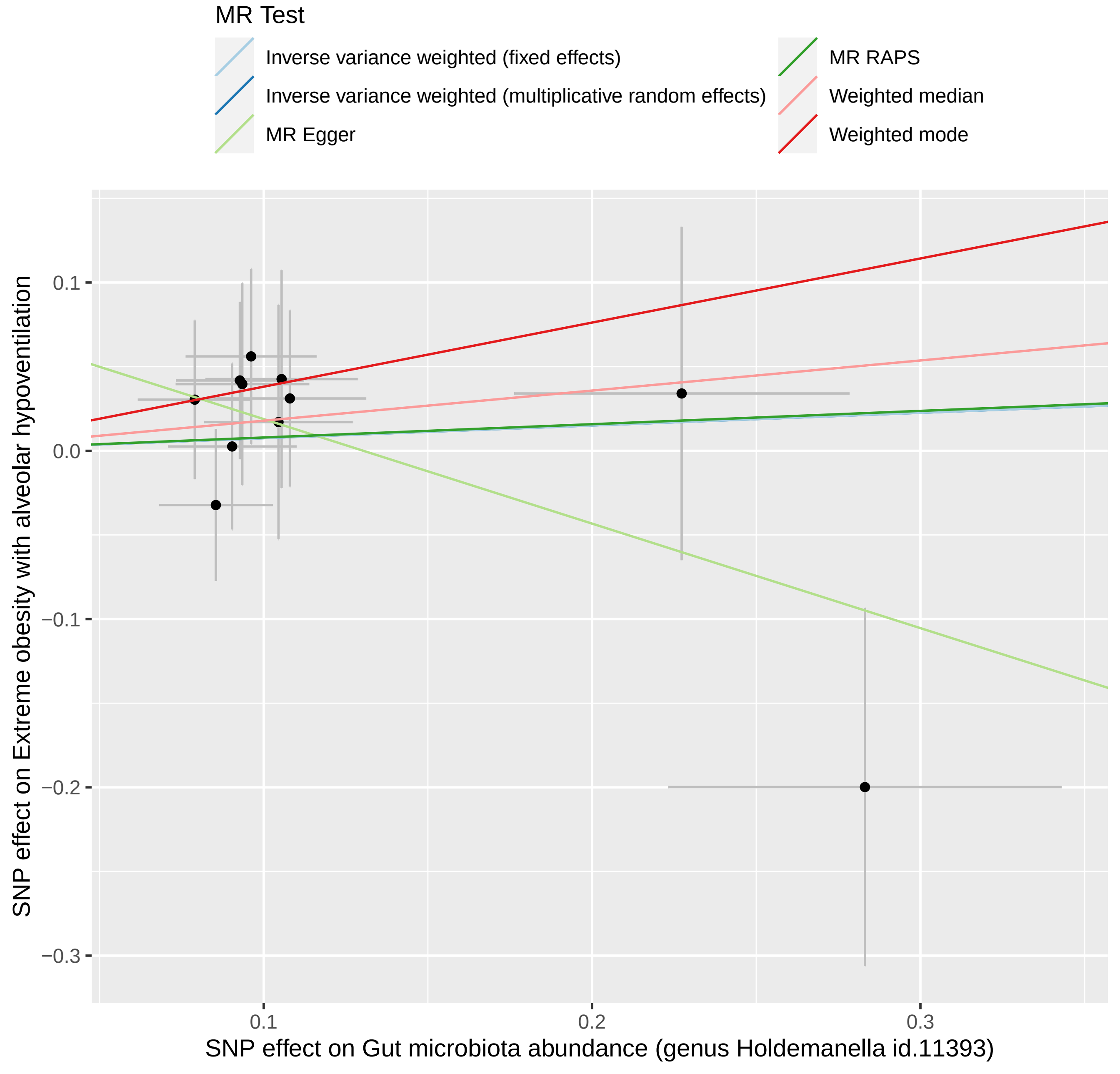

Batch 689 : Gut microbiota abundance (genus Holdemania id.2157) on Extreme obesity with alveolar hypoventilation

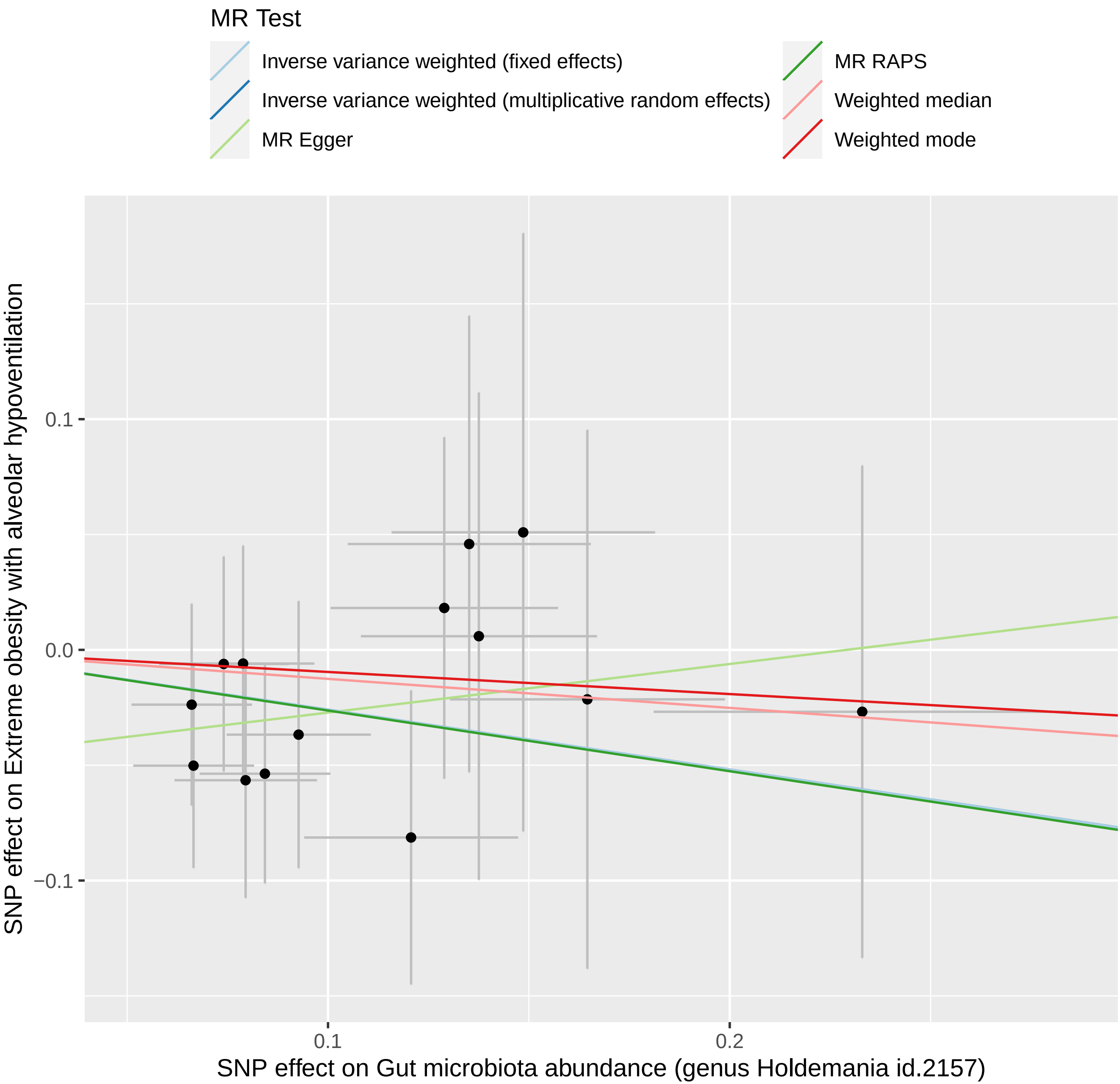

Batch 690 : Gut microbiota abundance (genus Howardella id.2000) on Extreme obesity with alveolar hypoventilation

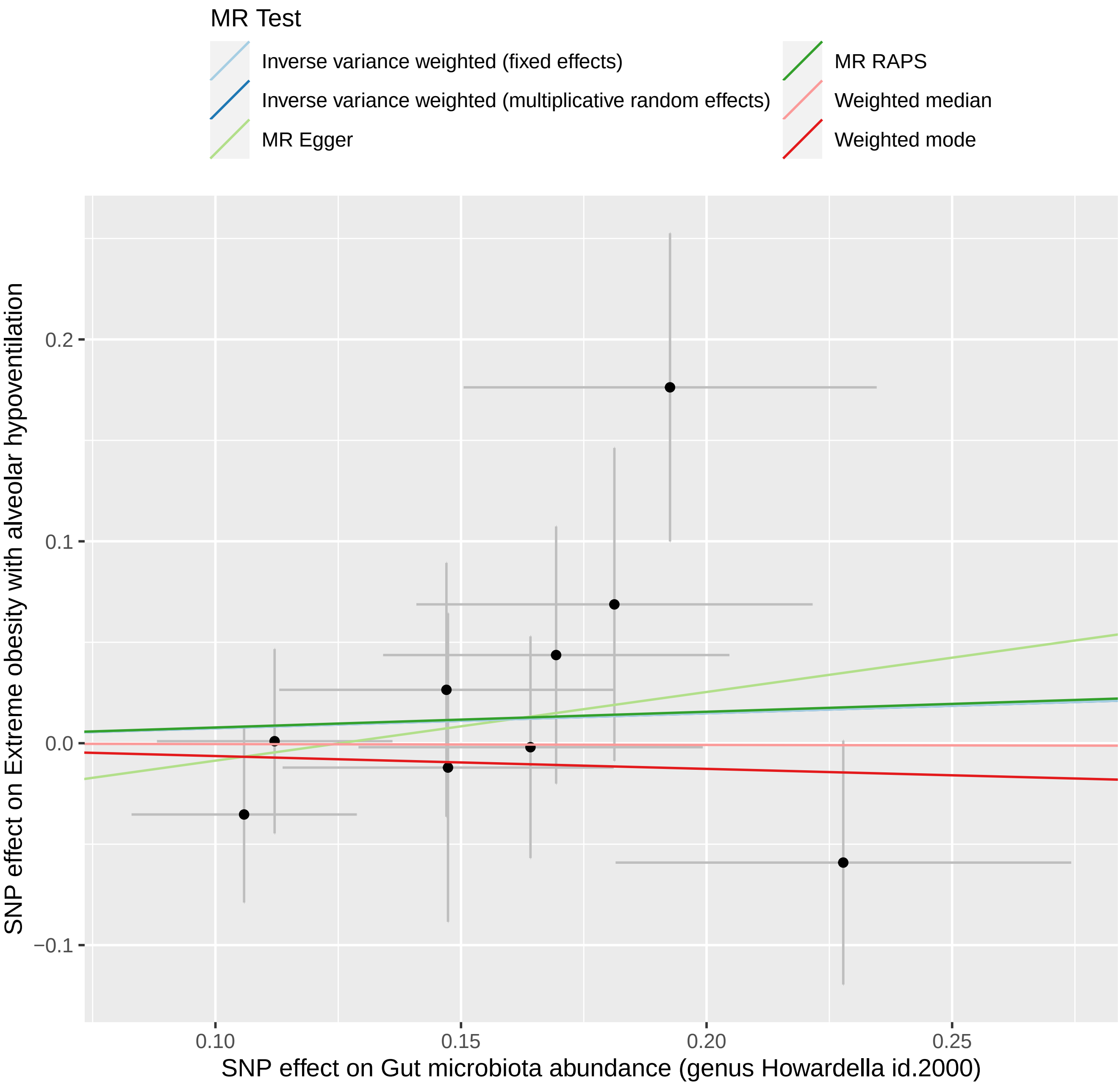

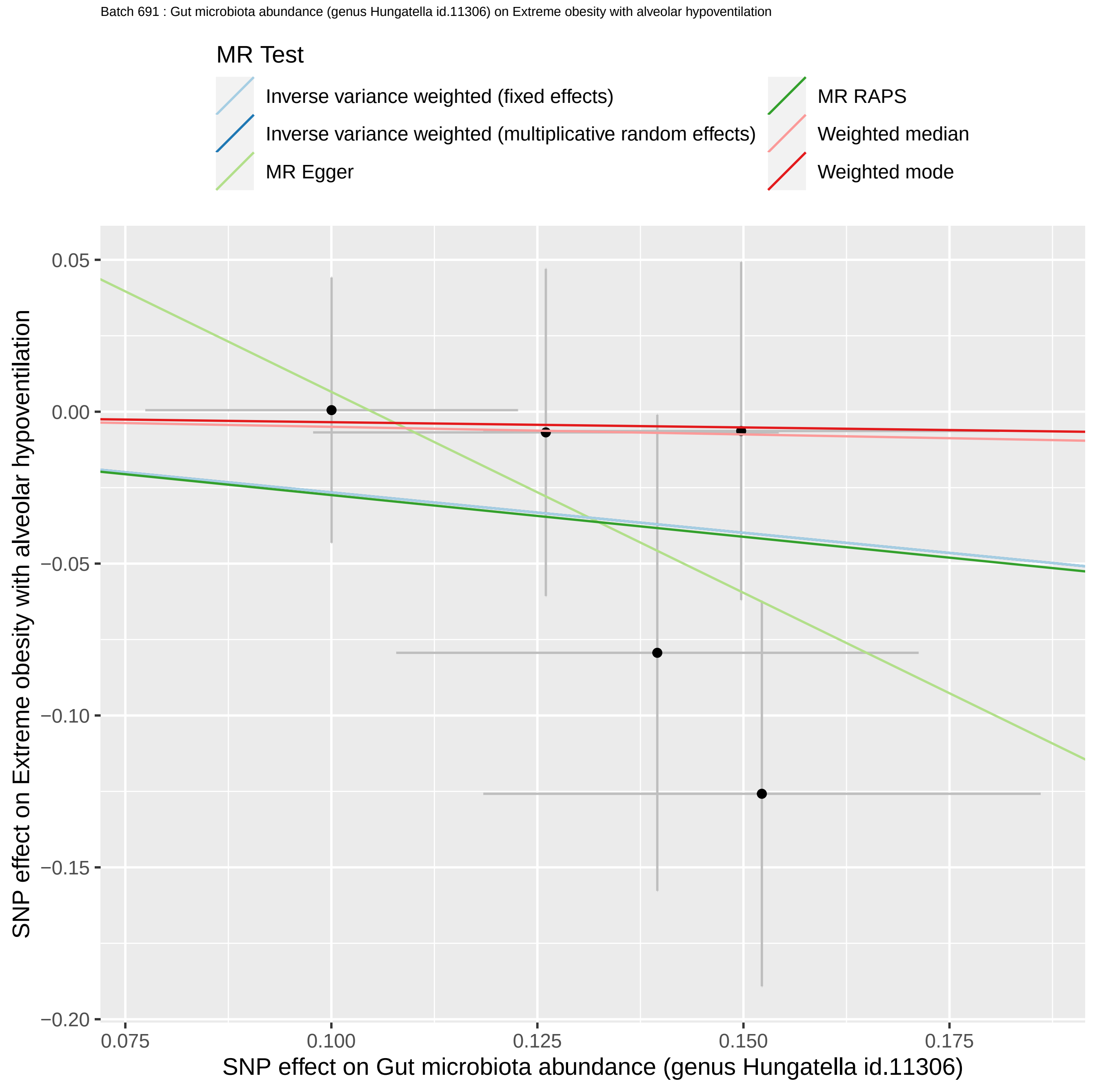

Batch 691 : Gut microbiota abundance (genus Hungatella id.11306) on Extreme obesity with alveolar hypoventilation
MR Test
Inverse variance weighted (fixed effects)
Inverse variance weighted (multiplicative random effects)
MR Egger
MR RAPS
Weighted median
Weighted mode
0.05
0.00
−0.05
−0.10
−0.15
−0.20
0.075
0.100
0.125
0.150
0.175
SNP effect on Extreme obesity with alveolar hypoventilation
SNP effect on Gut microbiota abundance (genus Hungatella id.11306)

Batch 692 : Gut microbiota abundance (genus Intestinibacter id.11345) on Extreme obesity with alveolar hypoventilation

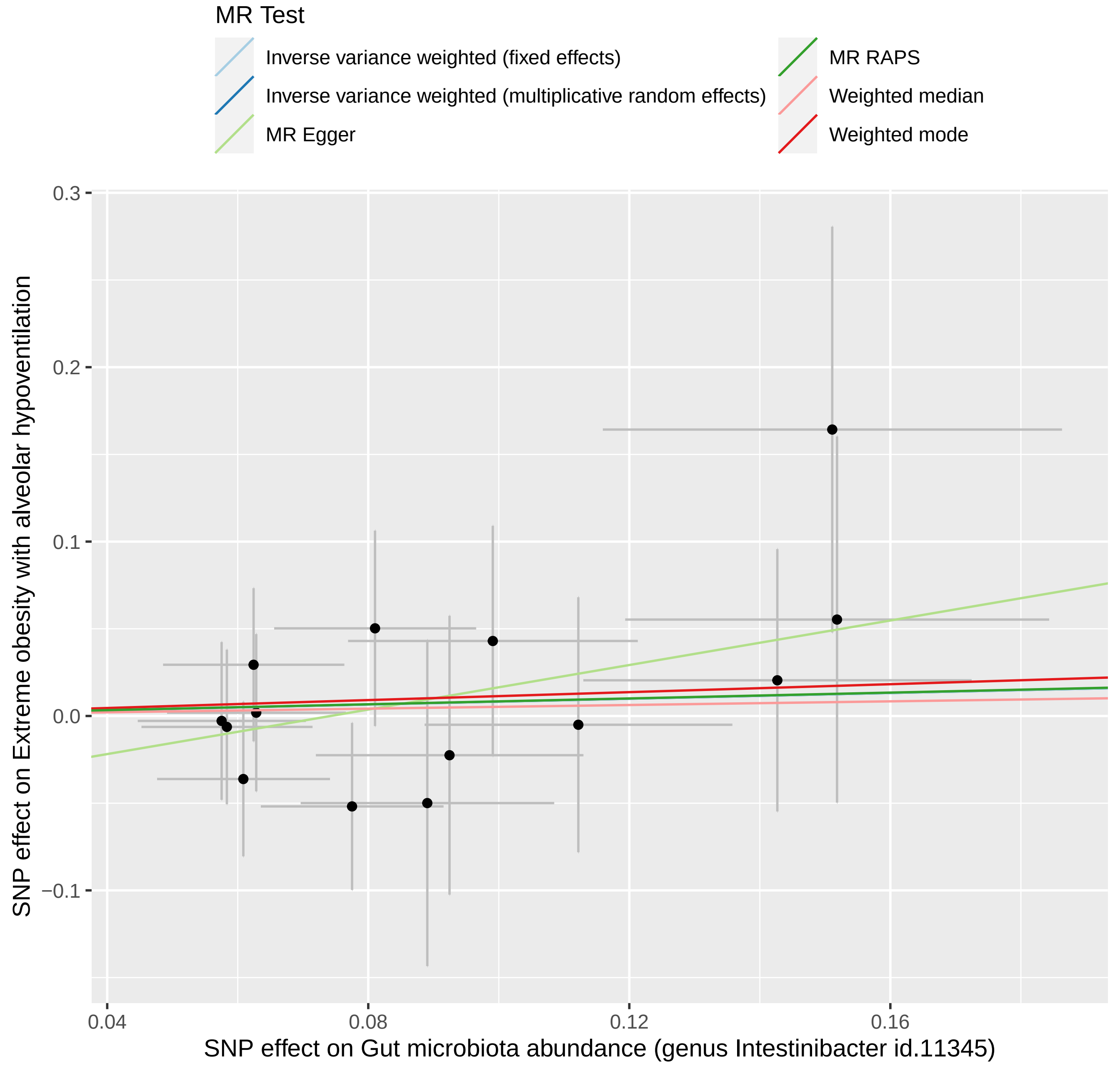

Batch 693 : Gut microbiota abundance (genus Intestinimonas id.2062) on Extreme obesity with alveolar hypoventilation

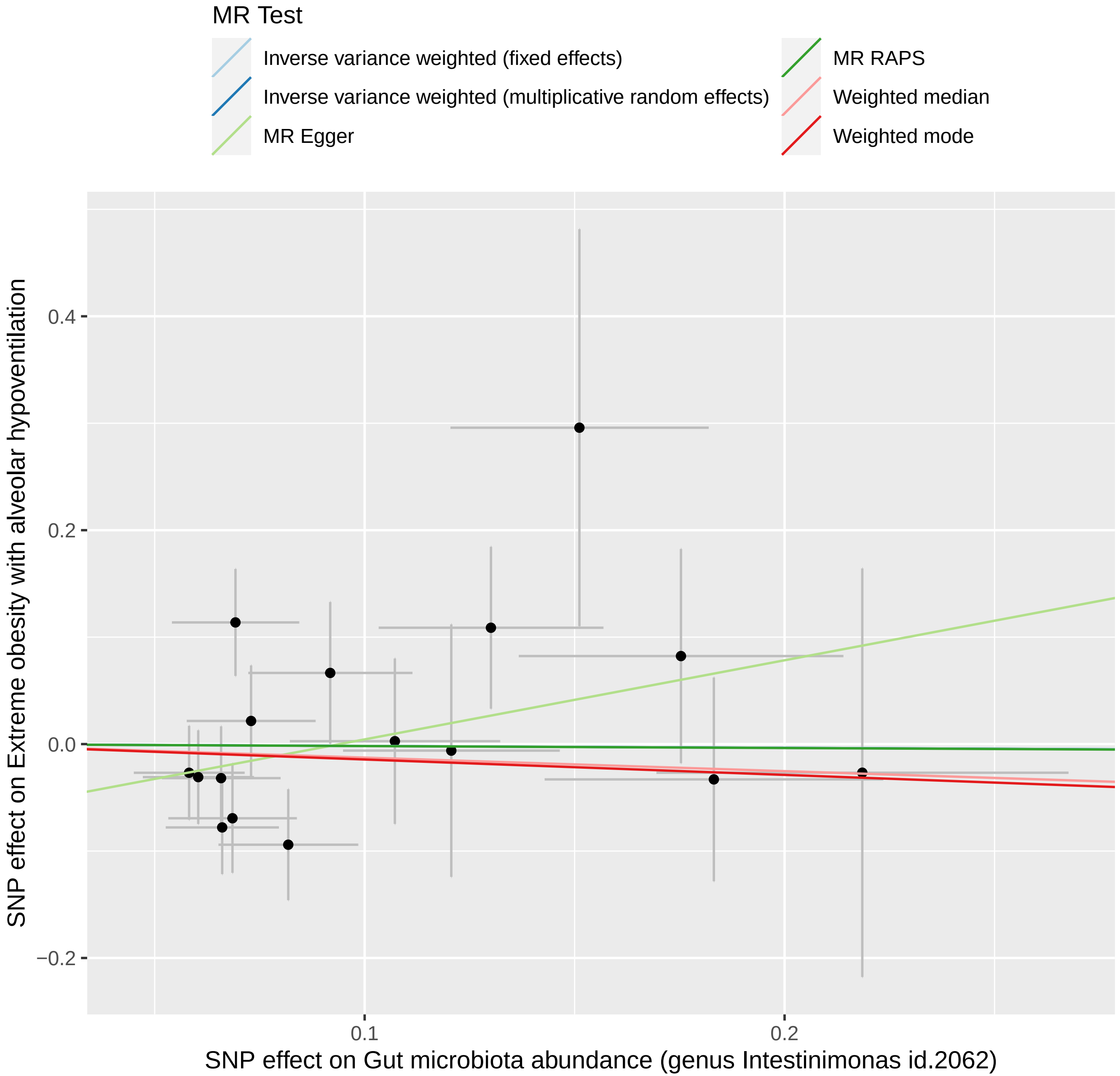

Batch 694 : Gut microbiota abundance (genus Lachnoclostridium id.11308) on Extreme obesity with alveolar hypoventilation

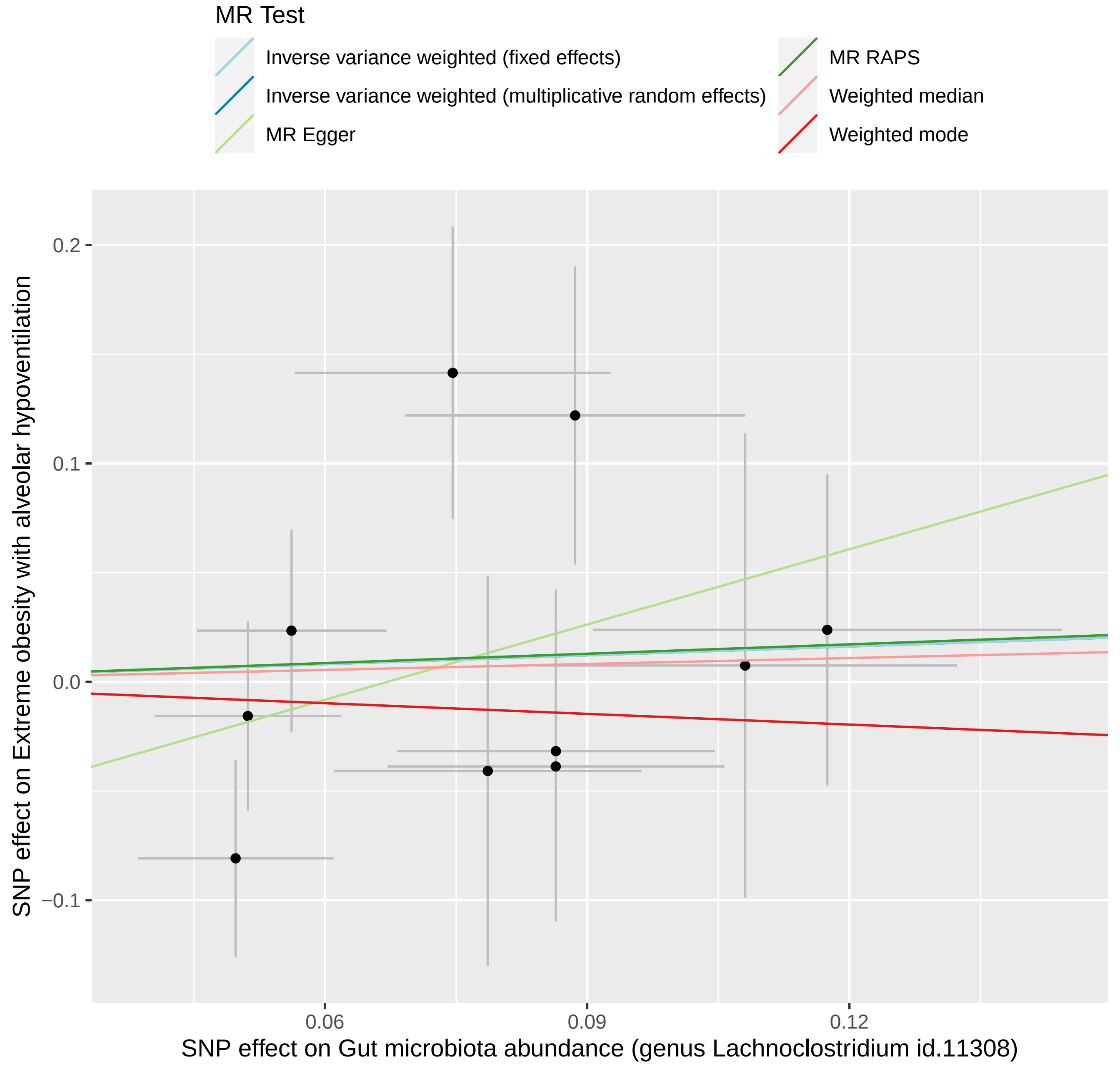

Batch 695 : Gut microbiota abundance (genus Lachnospiraceae FCS020 group id.11314) on Extreme obesity with alveolar hypoventilation

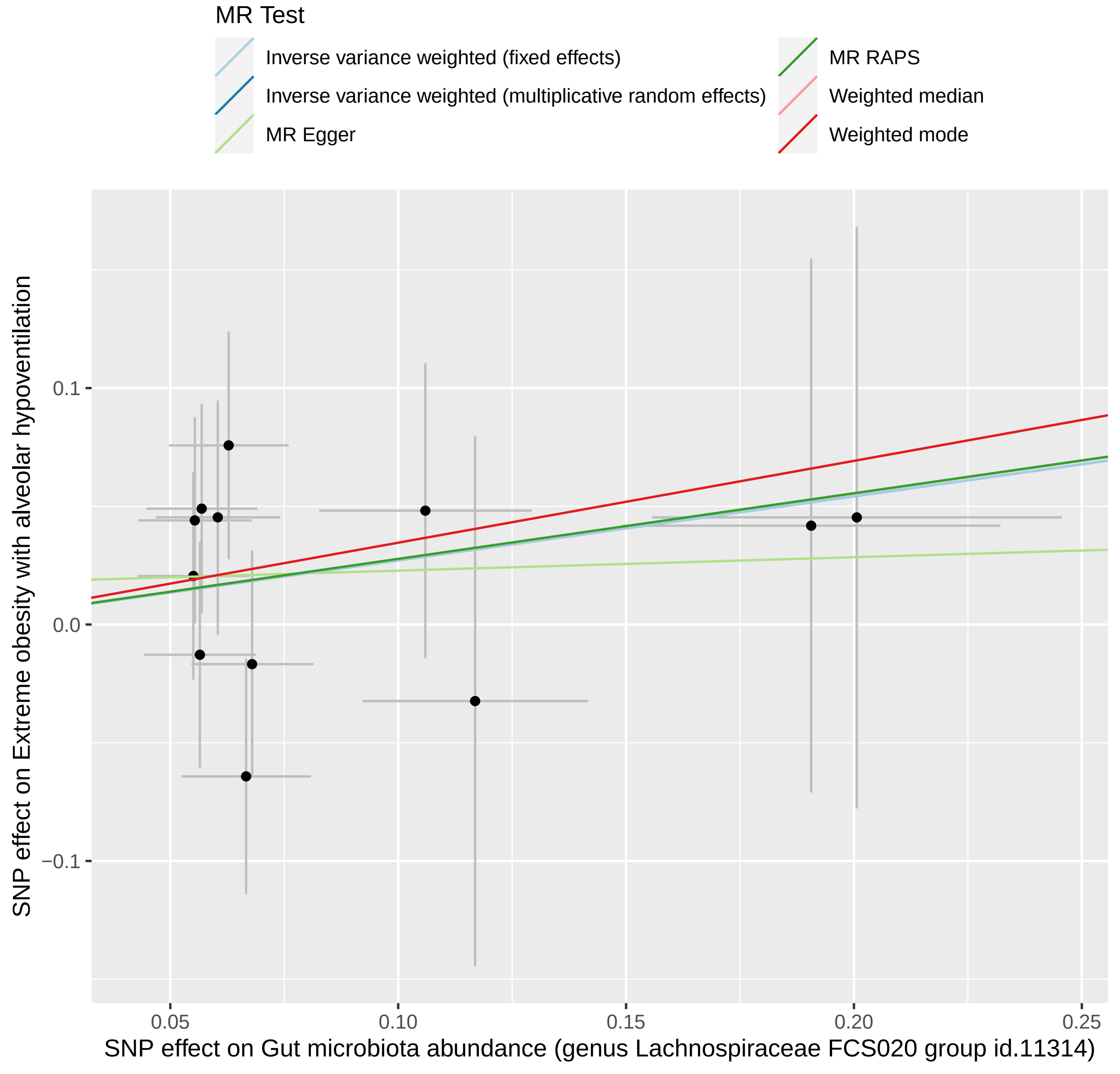

Batch 696 : Gut microbiota abundance (genus Lachnospiraceae NC2004 group id.11316) on Extreme obesity with alveolar hypoventilation

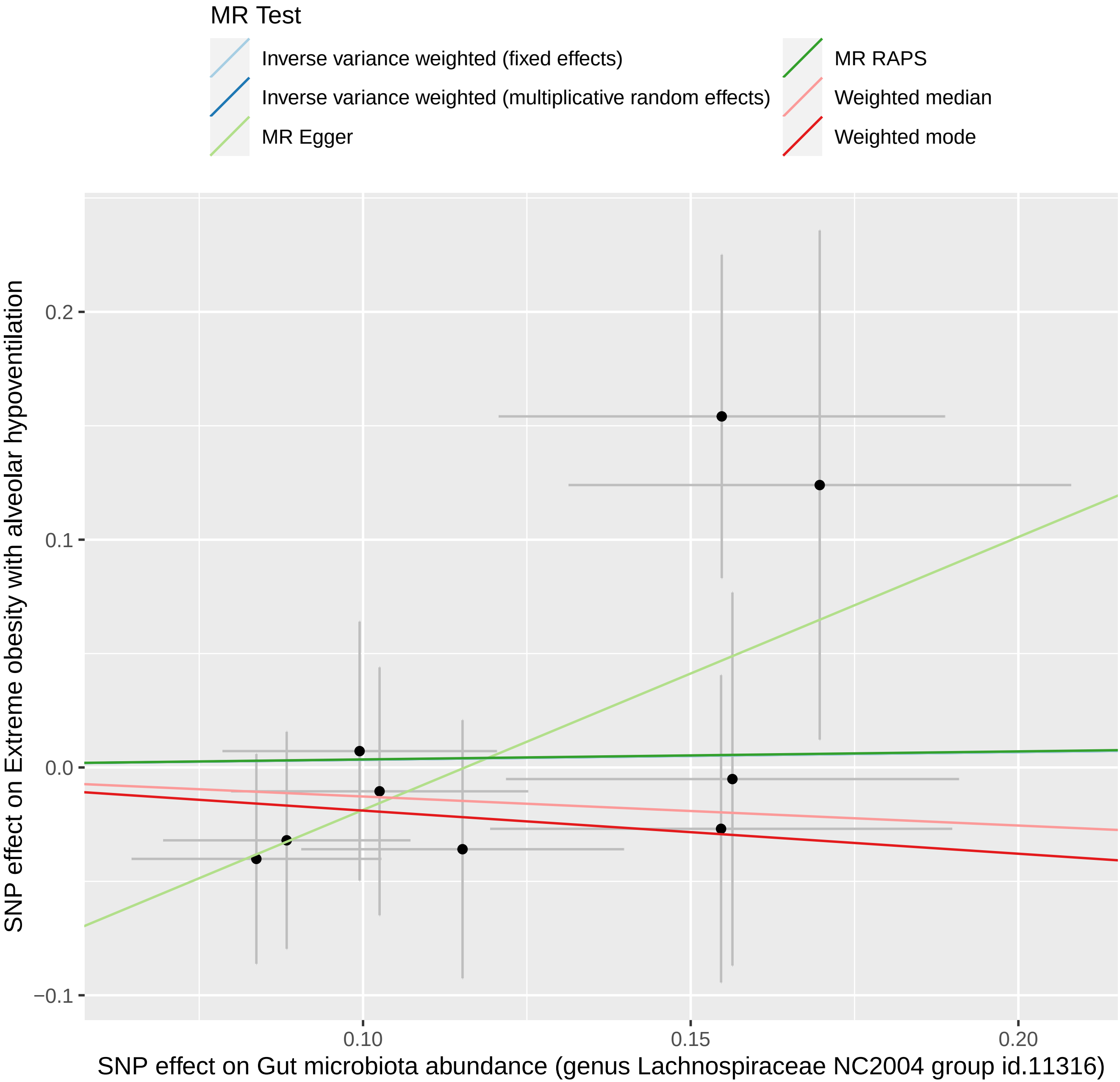

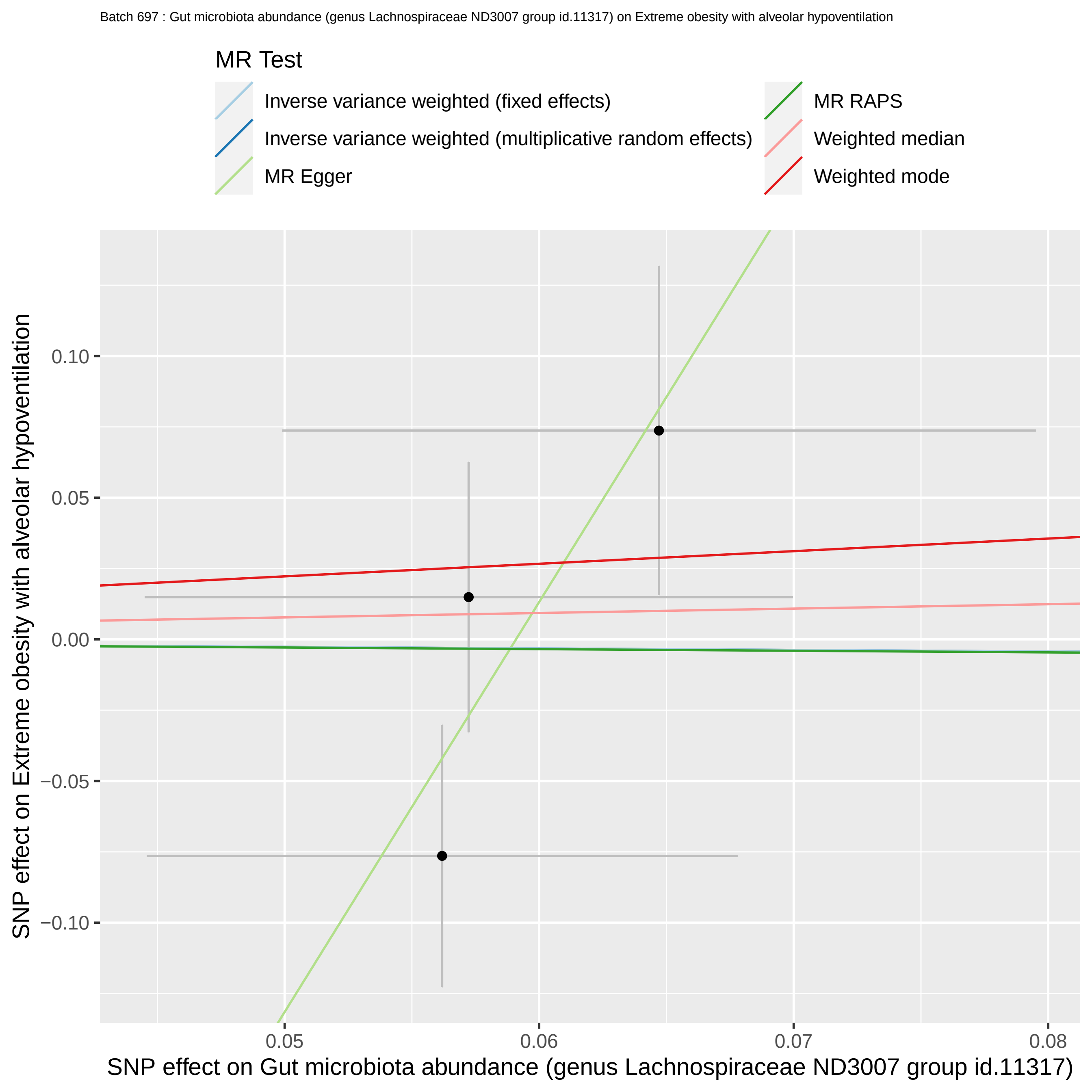

Batch 697 : Gut microbiota abundance (genus Lachnospiraceae ND3007 group id.11317) on Extreme obesity with alveolar hypoventilation
MR Test
Inverse variance weighted (fixed effects)
Inverse variance weighted (multiplicative random effects)
MR Egger
MR RAPS
Weighted median
Weighted mode
0.10
0.05
0.00
−0.05
−0.10
0.05
0.06
0.07
0.08
SNP effect on Extreme obesity with alveolar hypoventilation
SNP effect on Gut microbiota abundance (genus Lachnospiraceae ND3007 group id.11317)

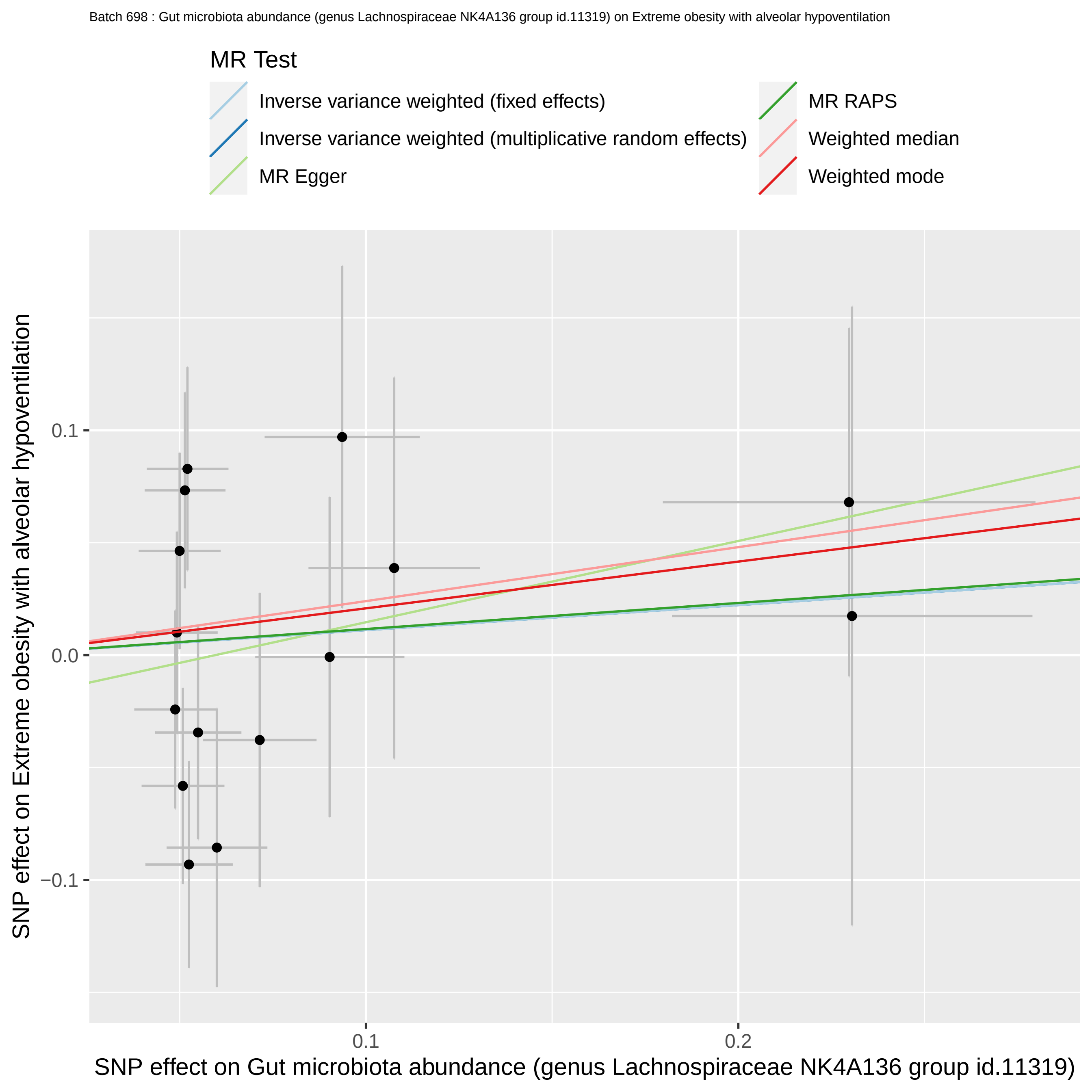

Batch 698 : Gut microbiota abundance (genus Lachnospiraceae NK4A136 group id.11319) on Extreme obesity with alveolar hypoventilation
MR Test
Inverse variance weighted (fixed effects)
Inverse variance weighted (multiplicative random effects)
MR Egger
MR RAPS
Weighted median
Weighted mode
0.1
0.0
−0.1
0.1
0.2
SNP effect on Extreme obesity with alveolar hypoventilation
SNP effect on Gut microbiota abundance (genus Lachnospiraceae NK4A136 group id.11319)

Batch 699 : Gut microbiota abundance (genus Lachnospiraceae UCG001 id.11321) on Extreme obesity with alveolar hypoventilation

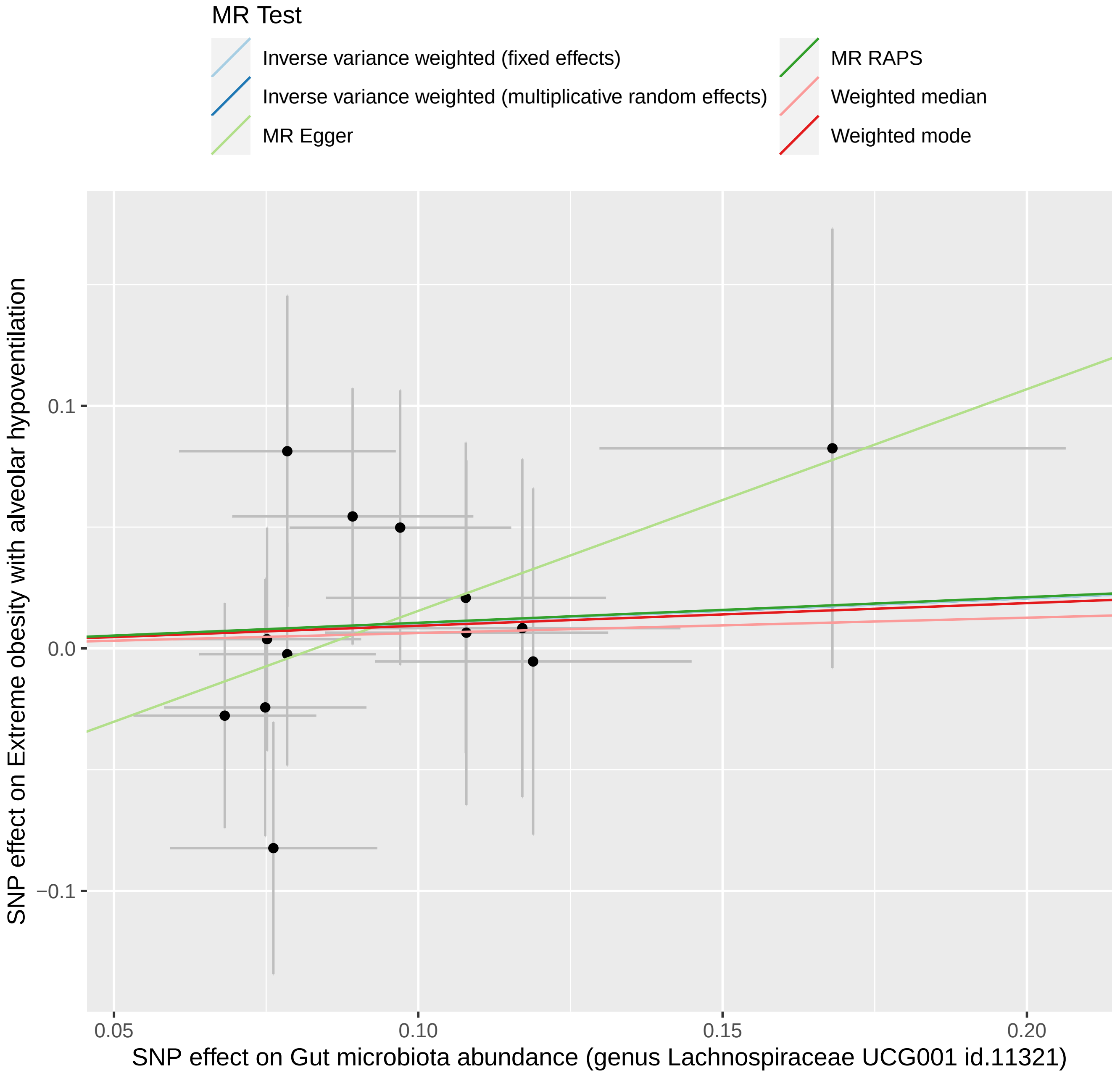

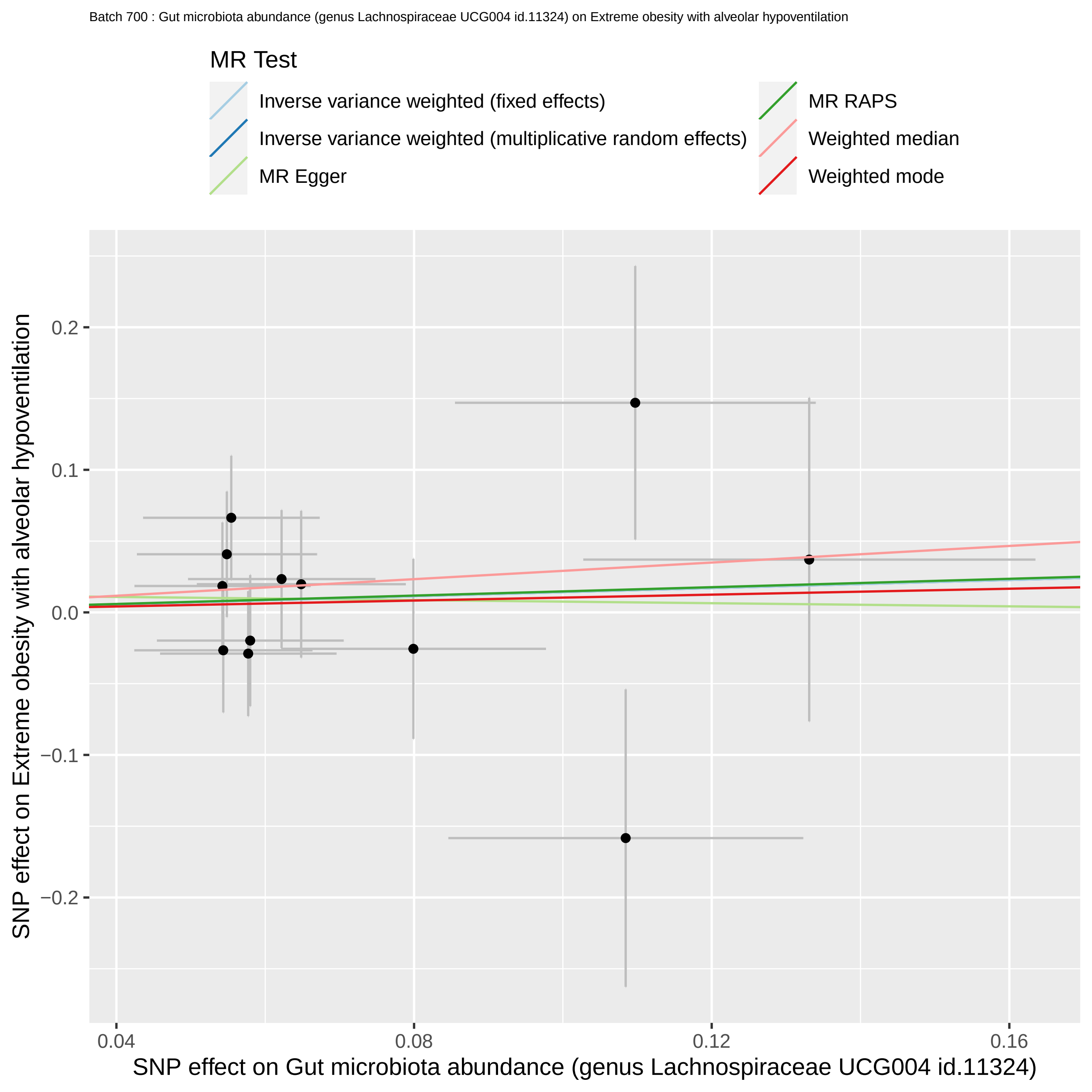

Batch 700 : Gut microbiota abundance (genus Lachnospiraceae UCG004 id.11324) on Extreme obesity with alveolar hypoventilation
MR Test
Inverse variance weighted (fixed effects)
Inverse variance weighted (multiplicative random effects)
MR Egger
MR RAPS
Weighted median
Weighted mode
SNP effect on Extreme obesity with alveolar hypoventilation
0.2
0.1
0.0
−0.1
−0.2
0.04
0.08
0.12
0.16
SNP effect on Gut microbiota abundance (genus Lachnospiraceae UCG004 id.11324)

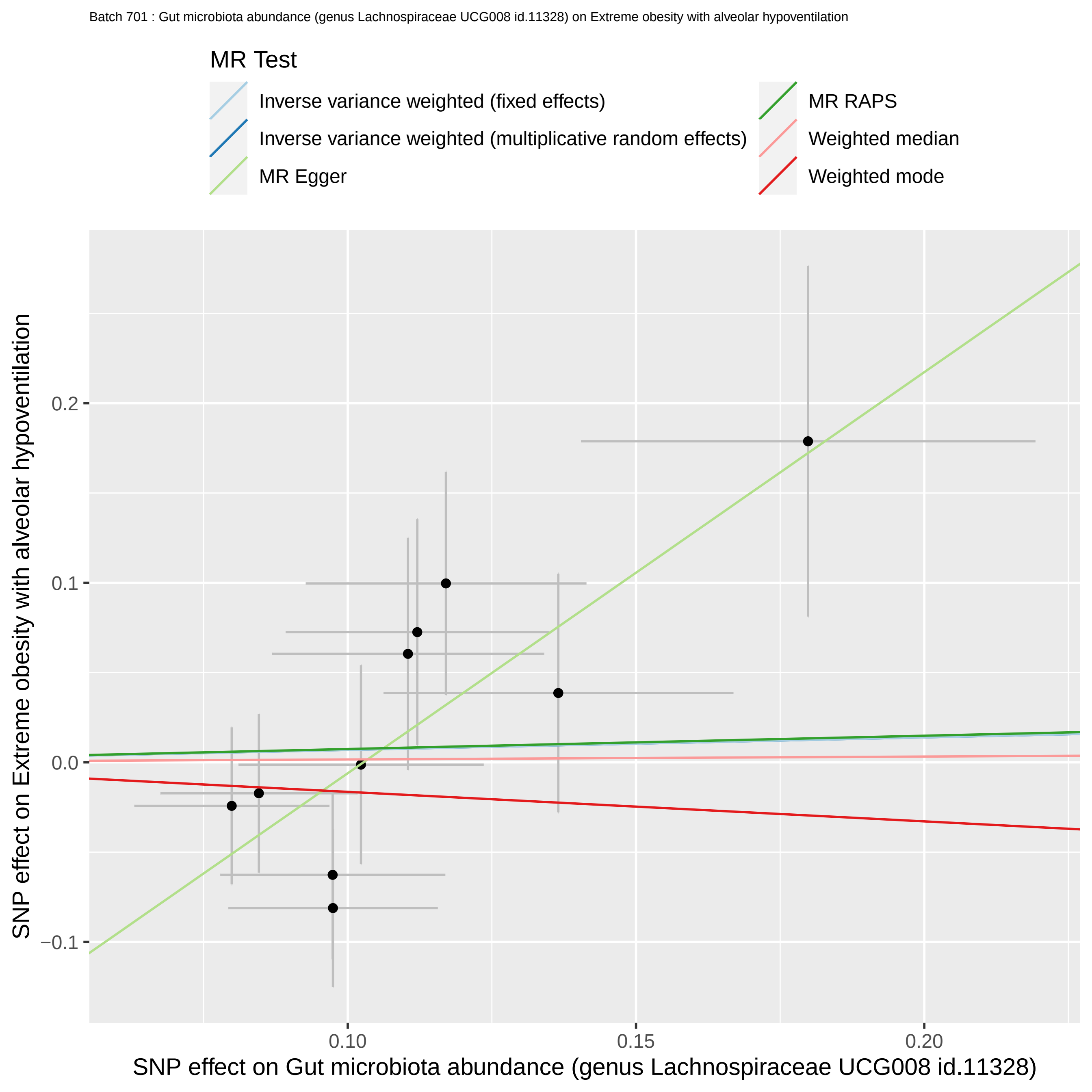

Batch 701 : Gut microbiota abundance (genus Lachnospiraceae UCG008 id.11328) on Extreme obesity with alveolar hypoventilation
MR Test
Inverse variance weighted (fixed effects)
Inverse variance weighted (multiplicative random effects)
MR Egger
MR RAPS
Weighted median
Weighted mode
SNP effect on Extreme obesity with alveolar hypoventilation
0.2
0.1
0.0
−0.1
0.10
0.15
0.20
SNP effect on Gut microbiota abundance (genus Lachnospiraceae UCG008 id.11328)

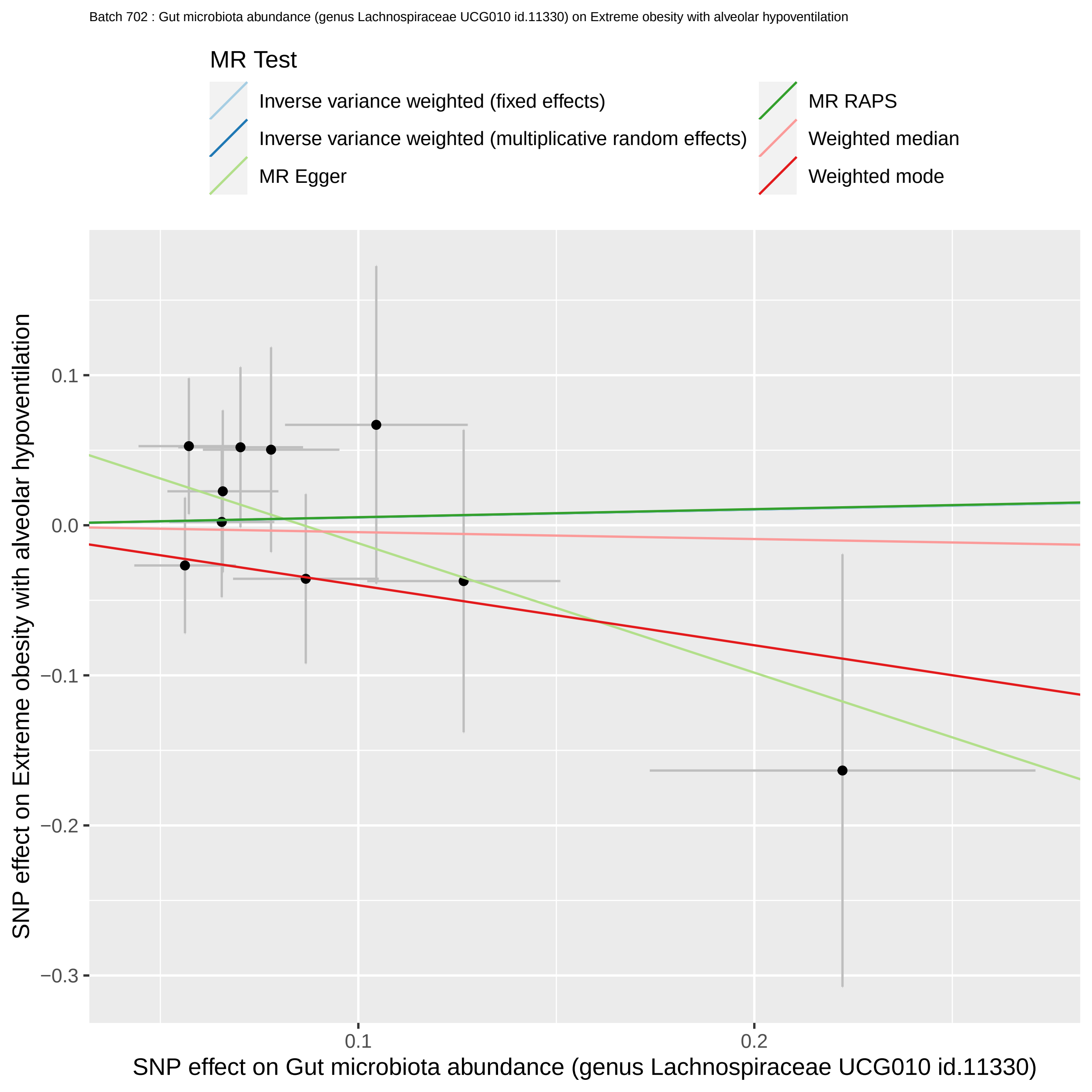

Batch 702 : Gut microbiota abundance (genus Lachnospiraceae UCG010 id.11330) on Extreme obesity with alveolar hypoventilation
MR Test
Inverse variance weighted (fixed effects)
Inverse variance weighted (multiplicative random effects)
MR Egger
MR RAPS
Weighted median
Weighted mode
SNP effect on Extreme obesity with alveolar hypoventilation
0.1
0.0
−0.1
−0.2
−0.3
0.1
0.2
SNP effect on Gut microbiota abundance (genus Lachnospiraceae UCG010 id.11330)

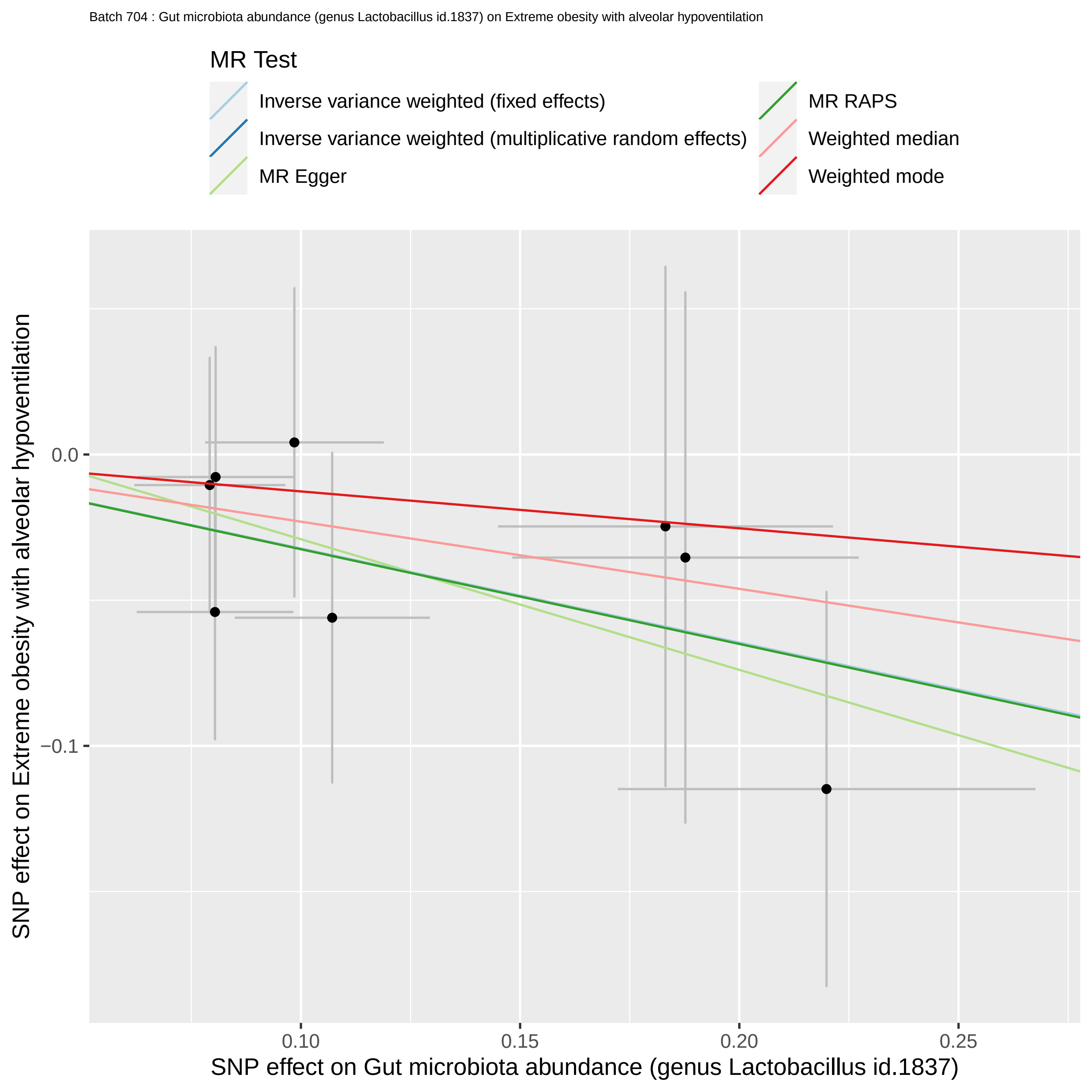
Batch 704 : Gut microbiota abundance (genus Lactobacillus id.1837) on Extreme obesity with alveolar hypoventilation
MR Test
Inverse variance weighted (fixed effects)
Inverse variance weighted (multiplicative random effects)
MR Egger
MR RAPS
Weighted median
Weighted mode
0.0
−0.1
0.10
0.15
0.20
0.25
SNP effect on Gut microbiota abundance (genus Lactobacillus id.1837)
SNP effect on Extreme obesity with alveolar hypoventilation

Batch 705 : Gut microbiota abundance (genus Lactococcus id.1851) on Extreme obesity with alveolar hypoventilation

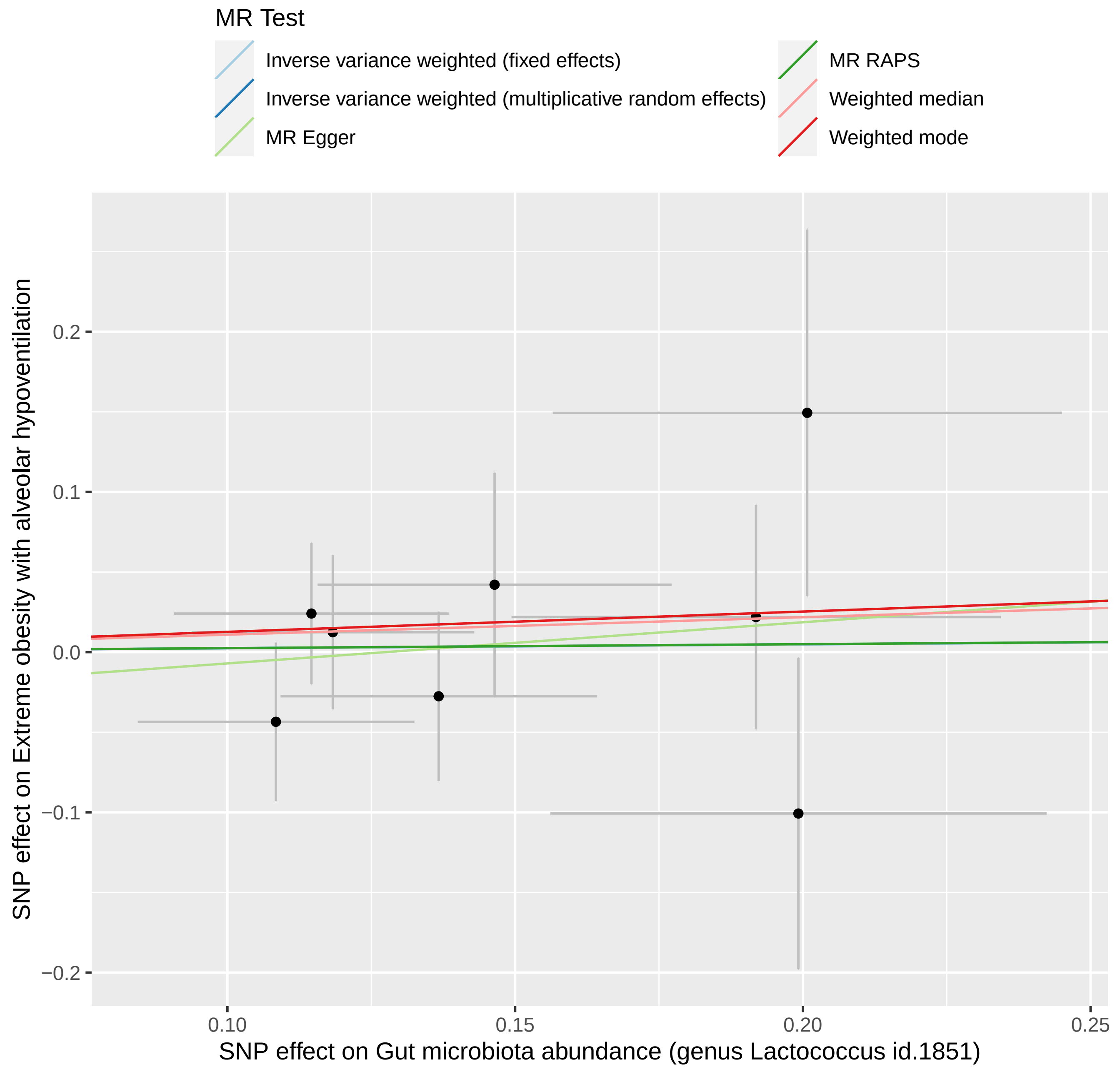

Batch 706 : Gut microbiota abundance (genus Marvinbryantia id.2005) on Extreme obesity with alveolar hypoventilation

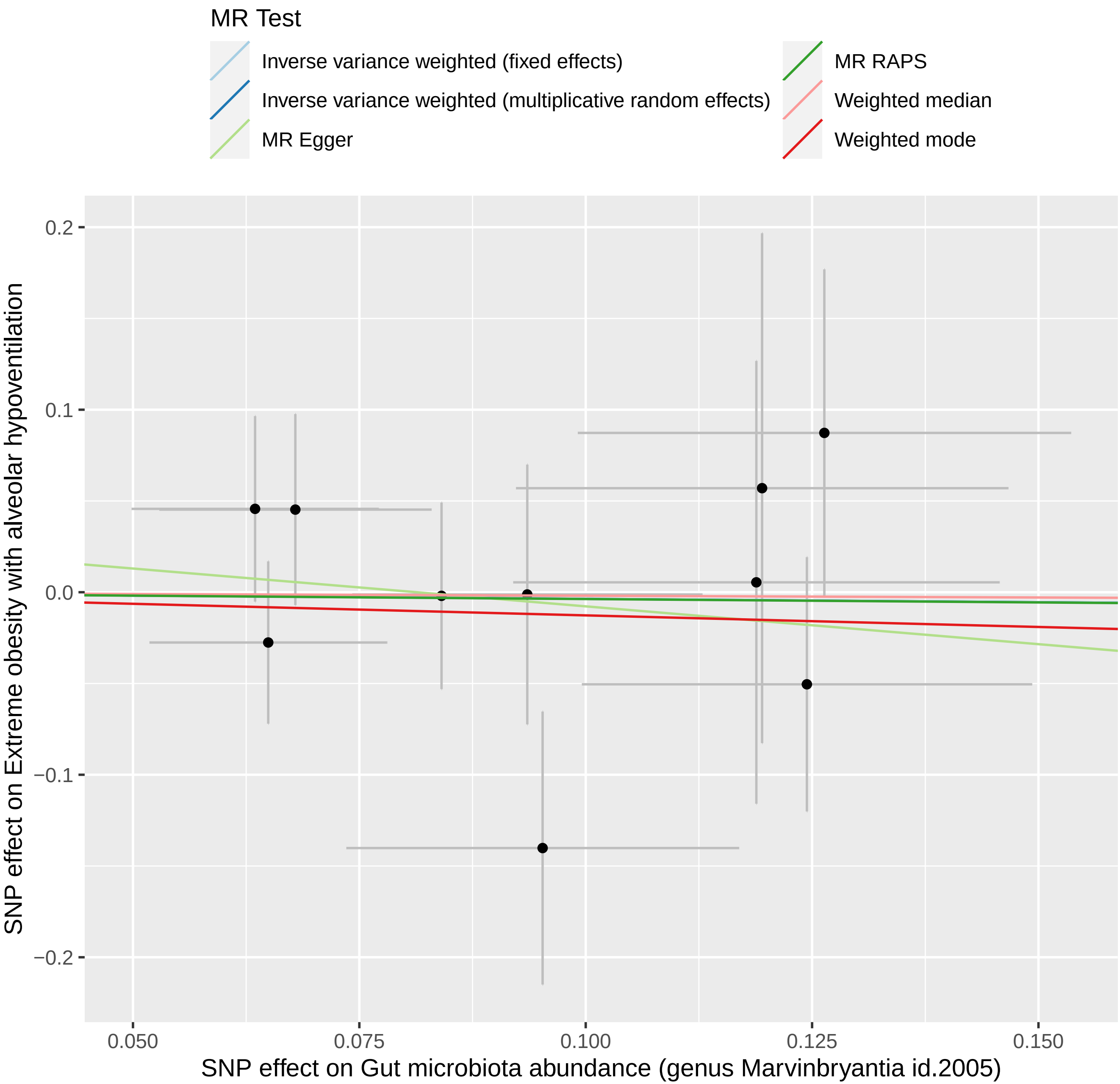

Batch 707 : Gut microbiota abundance (genus Methanobrevibacter id.123) on Extreme obesity with alveolar hypoventilation

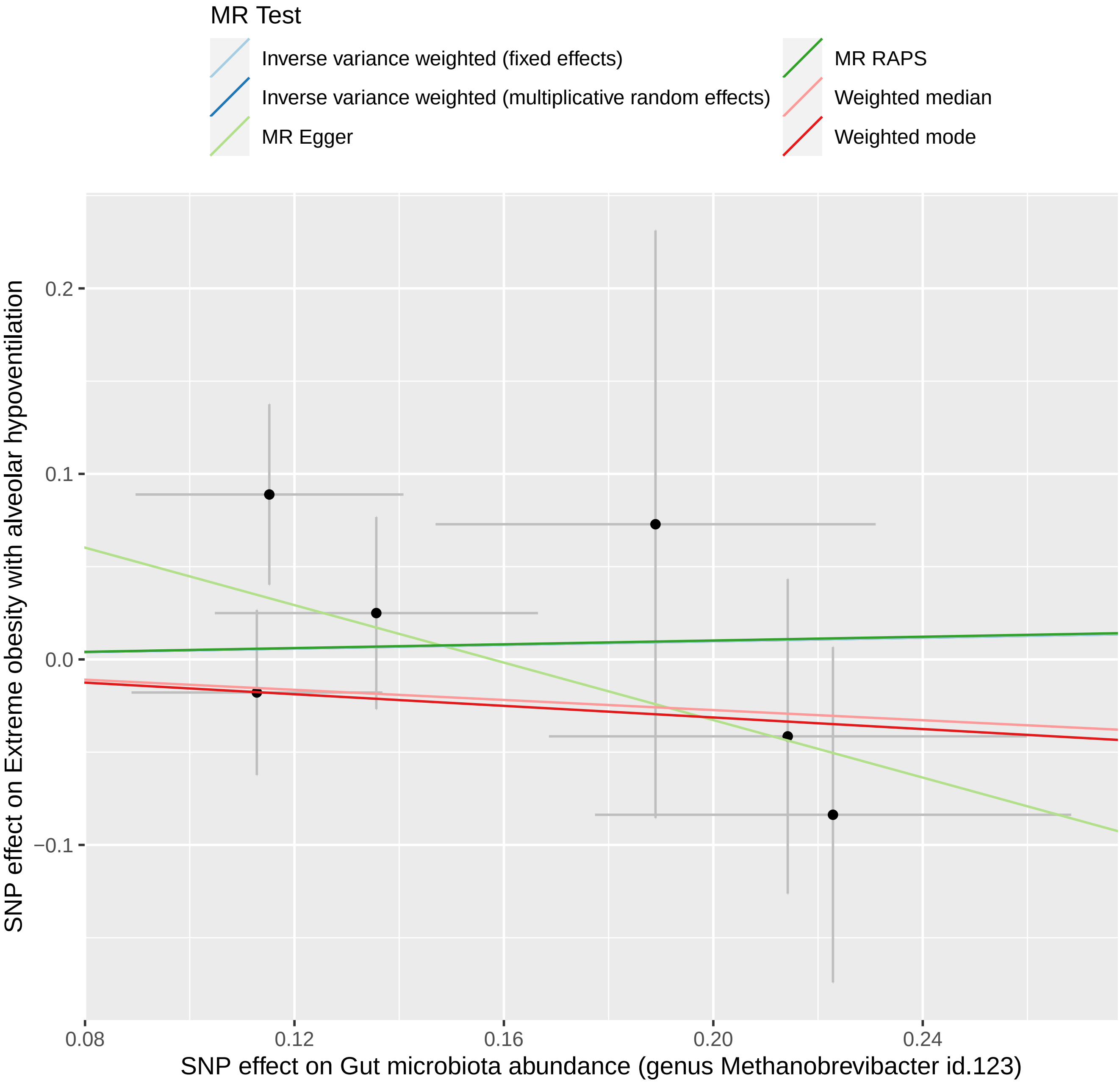

Batch 708 : Gut microbiota abundance (genus Odoribacter id.952) on Extreme obesity with alveolar hypoventilation

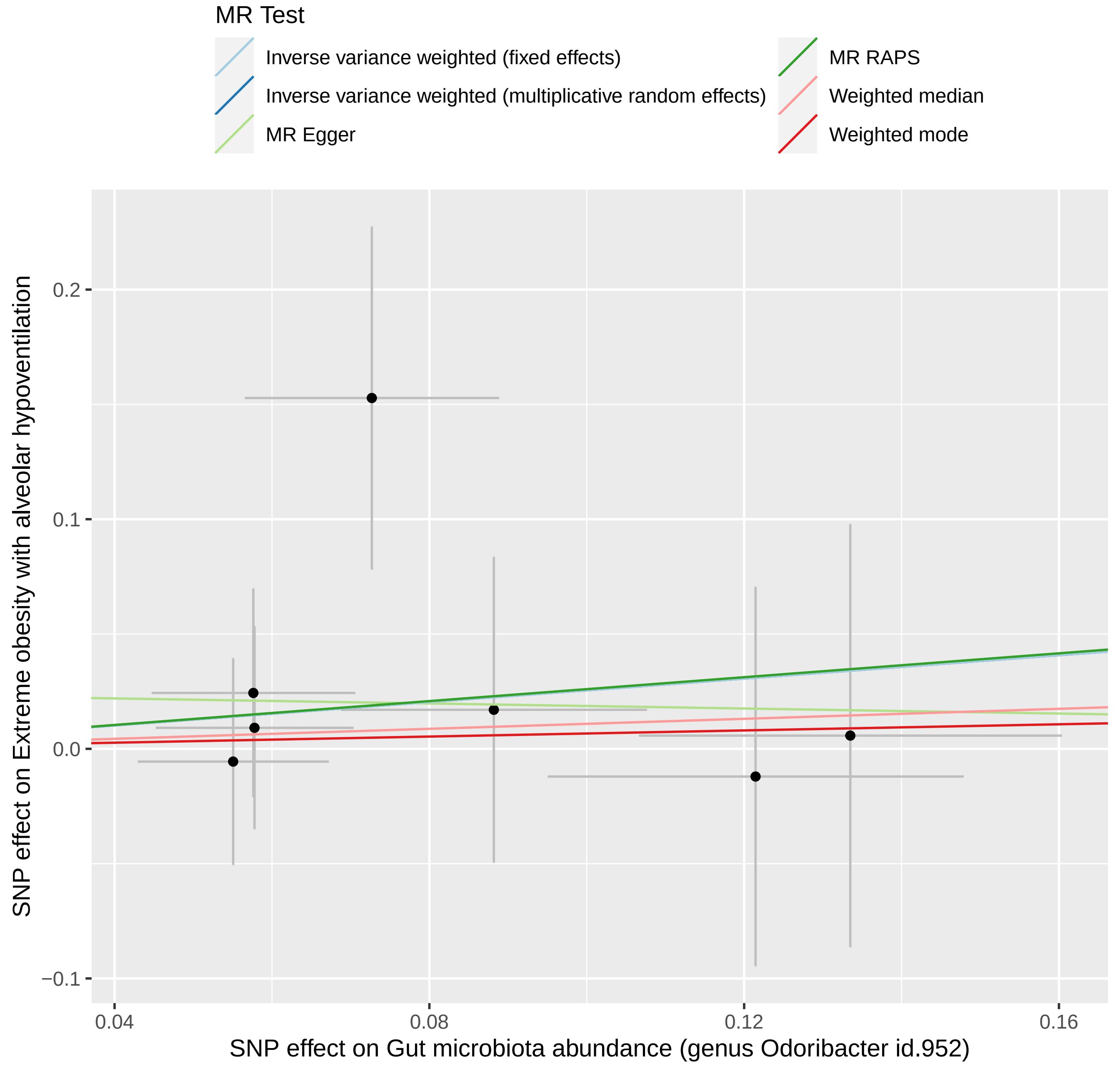

Batch 709 : Gut microbiota abundance (genus Olsenella id.822) on Extreme obesity with alveolar hypoventilation

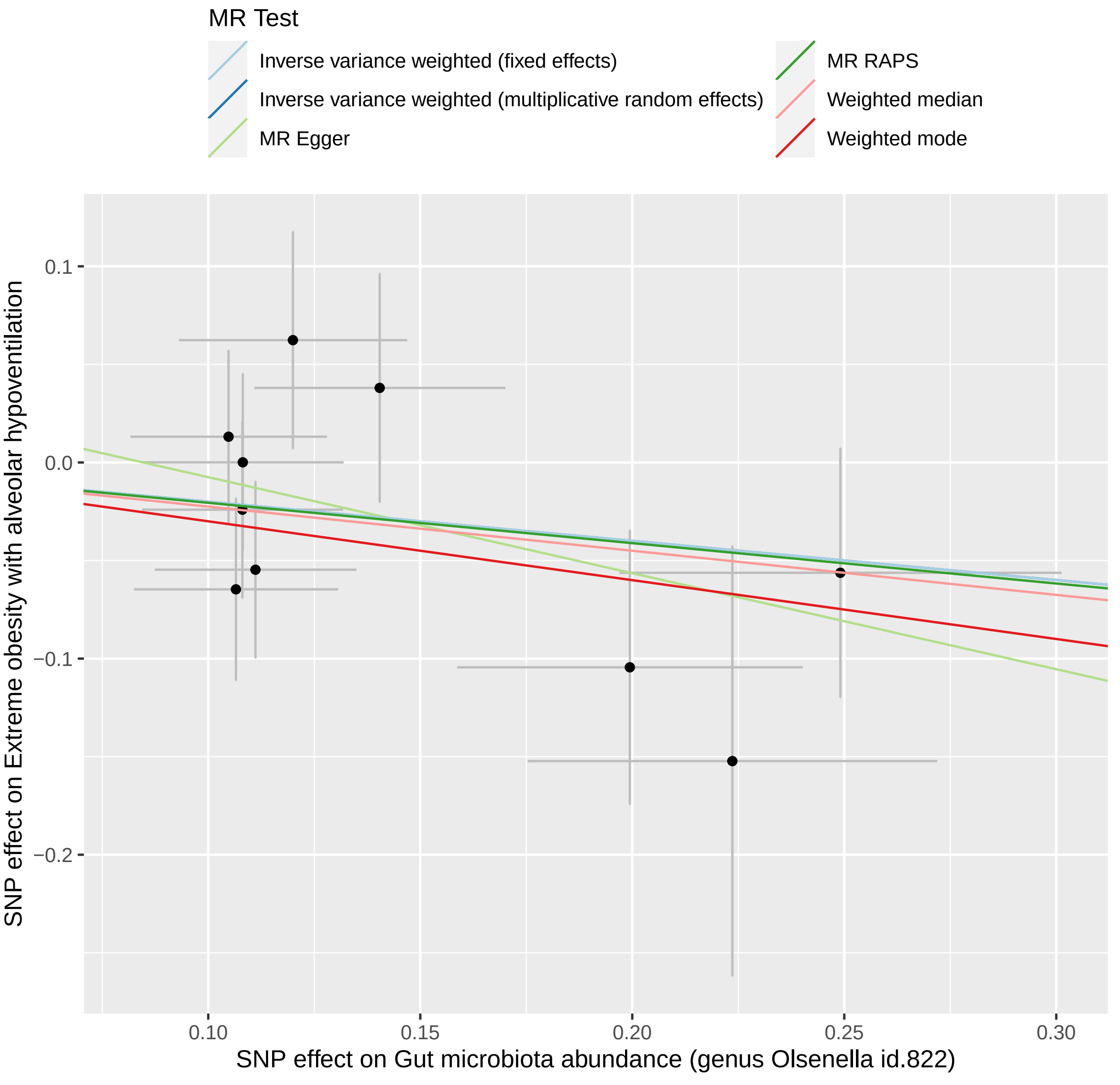

Batch 710 : Gut microbiota abundance (genus Oscillibacter id.2063) on Extreme obesity with alveolar hypoventilation

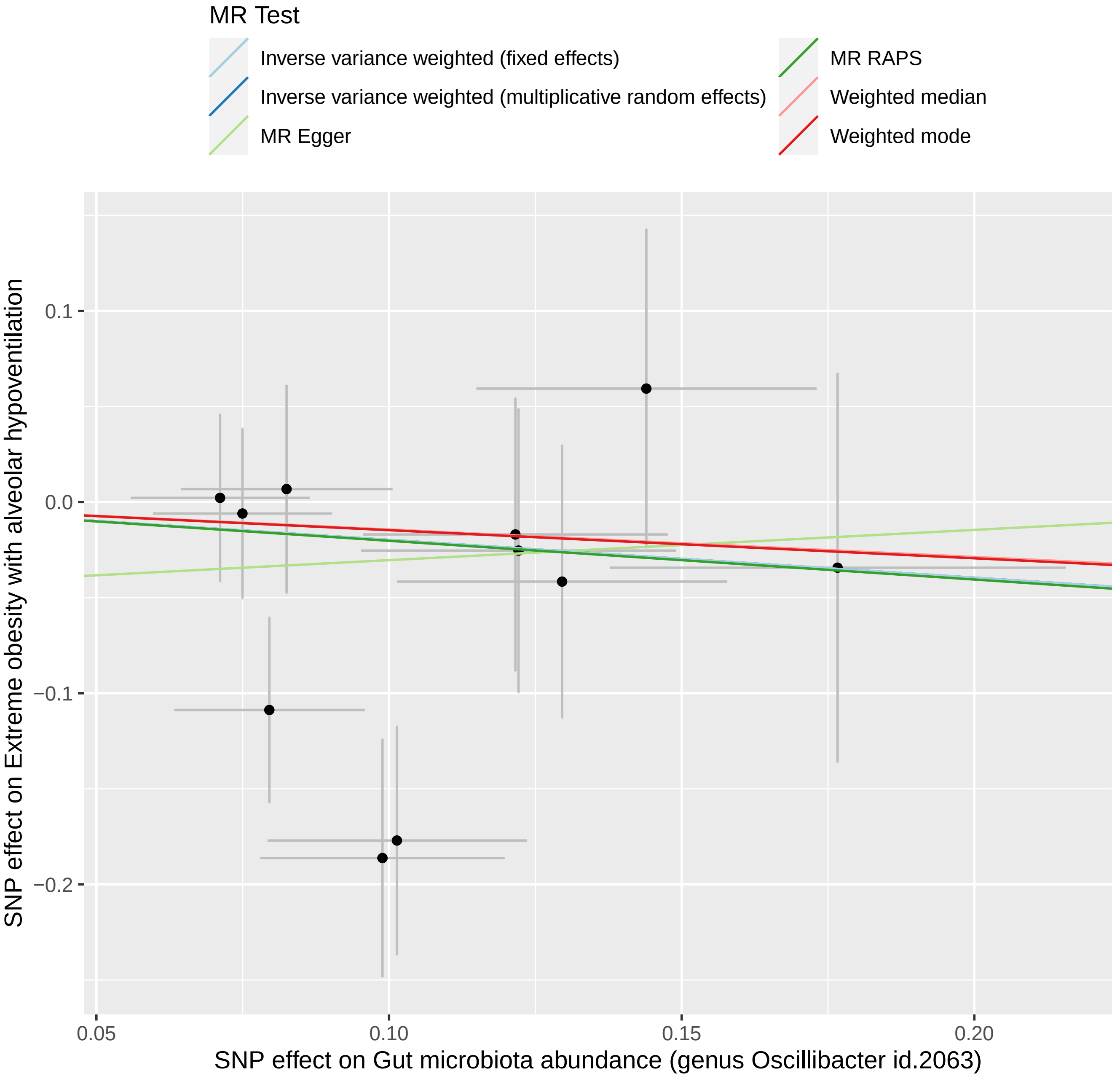

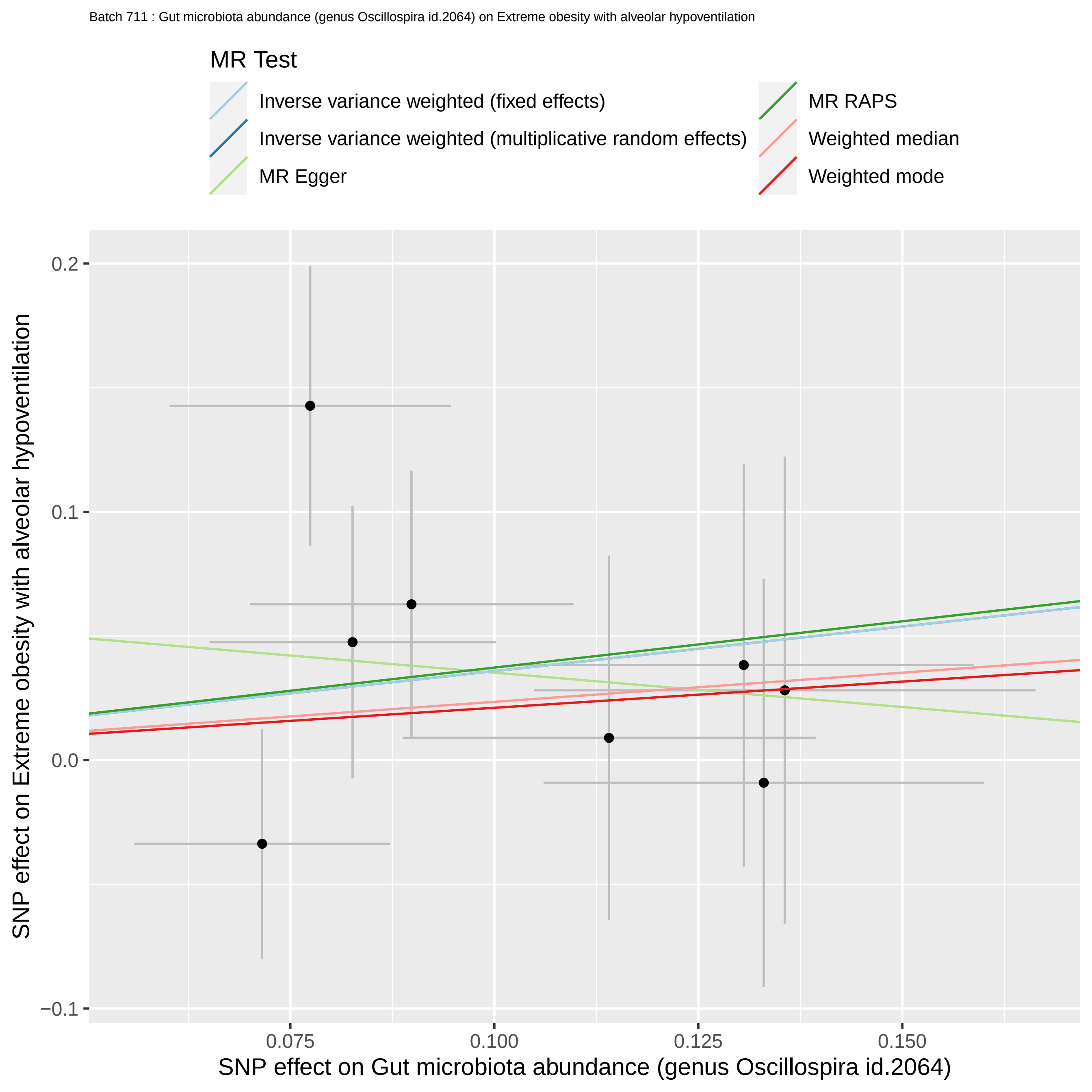
Batch 711 : Gut microbiota abundance (genus Oscillospira id.2064) on Extreme obesity with alveolar hypoventilation
MR Test
Inverse variance weighted (fixed effects)
Inverse variance weighted (multiplicative random effects)
MR Egger
MR RAPS
Weighted median
Weighted mode
0.2
0.1
0.0
−0.1
0.075
0.100
0.125
0.150
SNP effect on Extreme obesity with alveolar hypoventilation
SNP effect on Gut microbiota abundance (genus Oscillospira id.2064)

Batch 712 : Gut microbiota abundance (genus Oxalobacter id.2978) on Extreme obesity with alveolar hypoventilation

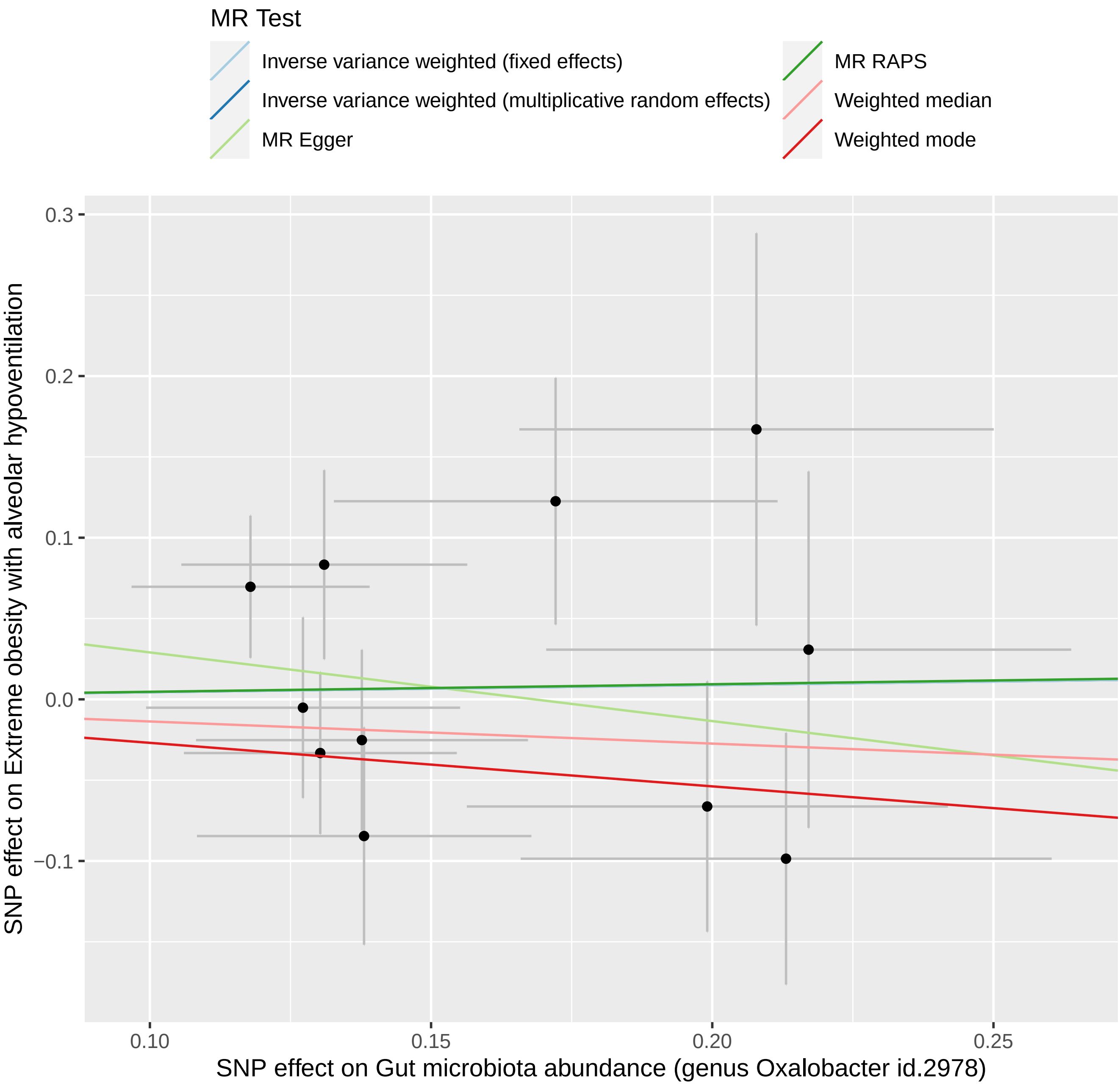

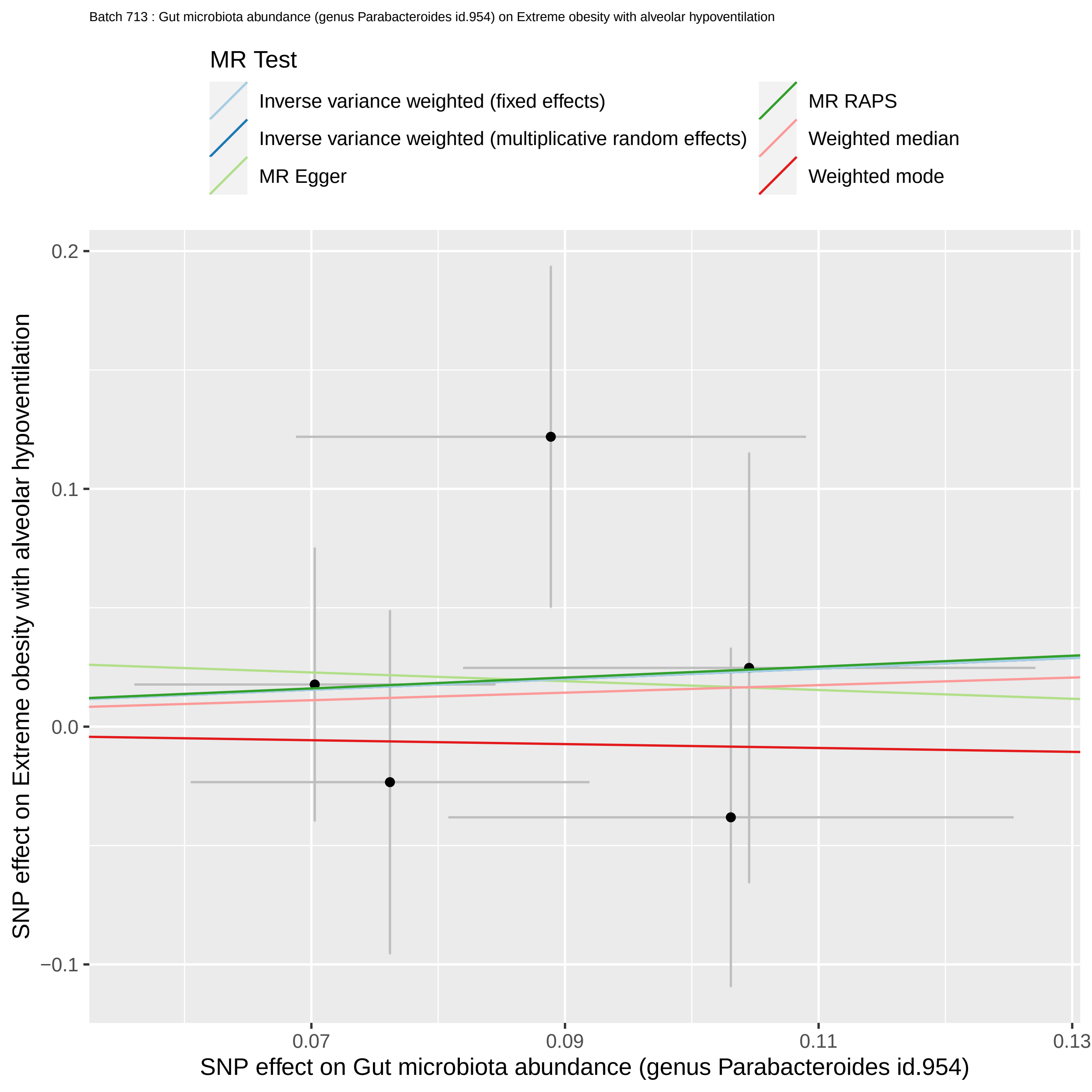

Batch 713 : Gut microbiota abundance (genus Parabacteroides id.954) on Extreme obesity with alveolar hypoventilation
MR Test
Inverse variance weighted (fixed effects)
Inverse variance weighted (multiplicative random effects)
MR Egger
MR RAPS
Weighted median
Weighted mode
0.2
0.1
0.0
−0.1
0.07
0.09
0.11
0.13
SNP effect on Extreme obesity with alveolar hypoventilation
SNP effect on Gut microbiota abundance (genus Parabacteroides id.954)

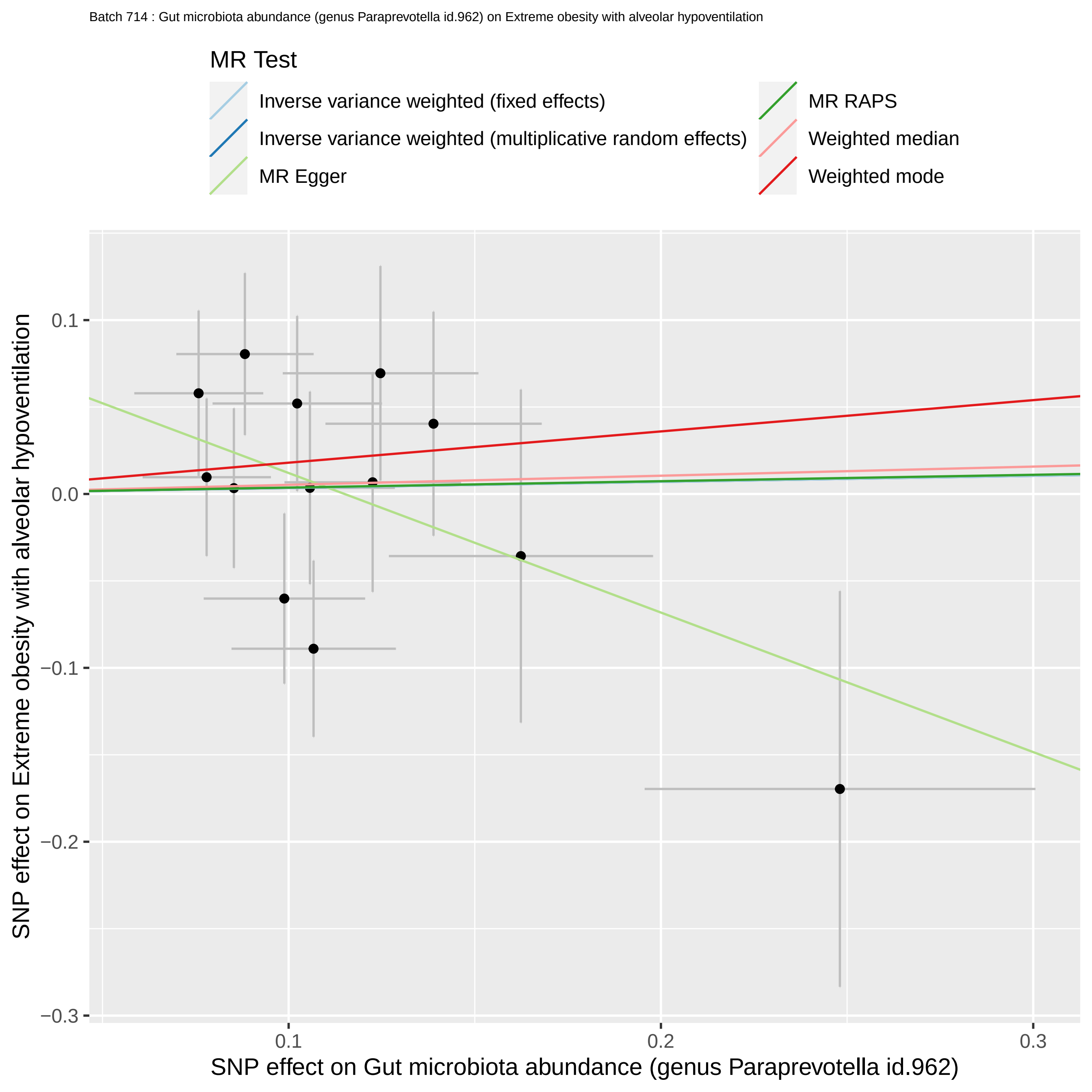

Batch 714 : Gut microbiota abundance (genus Paraprevotella id.962) on Extreme obesity with alveolar hypoventilation
MR Test
Inverse variance weighted (fixed effects)
Inverse variance weighted (multiplicative random effects)
MR Egger
MR RAPS
Weighted median
Weighted mode
0.1
0.0
−0.1
−0.2
−0.3
0.1
0.2
0.3
SNP effect on Extreme obesity with alveolar hypoventilation
SNP effect on Gut microbiota abundance (genus Paraprevotella id.962)

Batch 715 : Gut microbiota abundance (genus Parasutterella id.2892) on Extreme obesity with alveolar hypoventilation

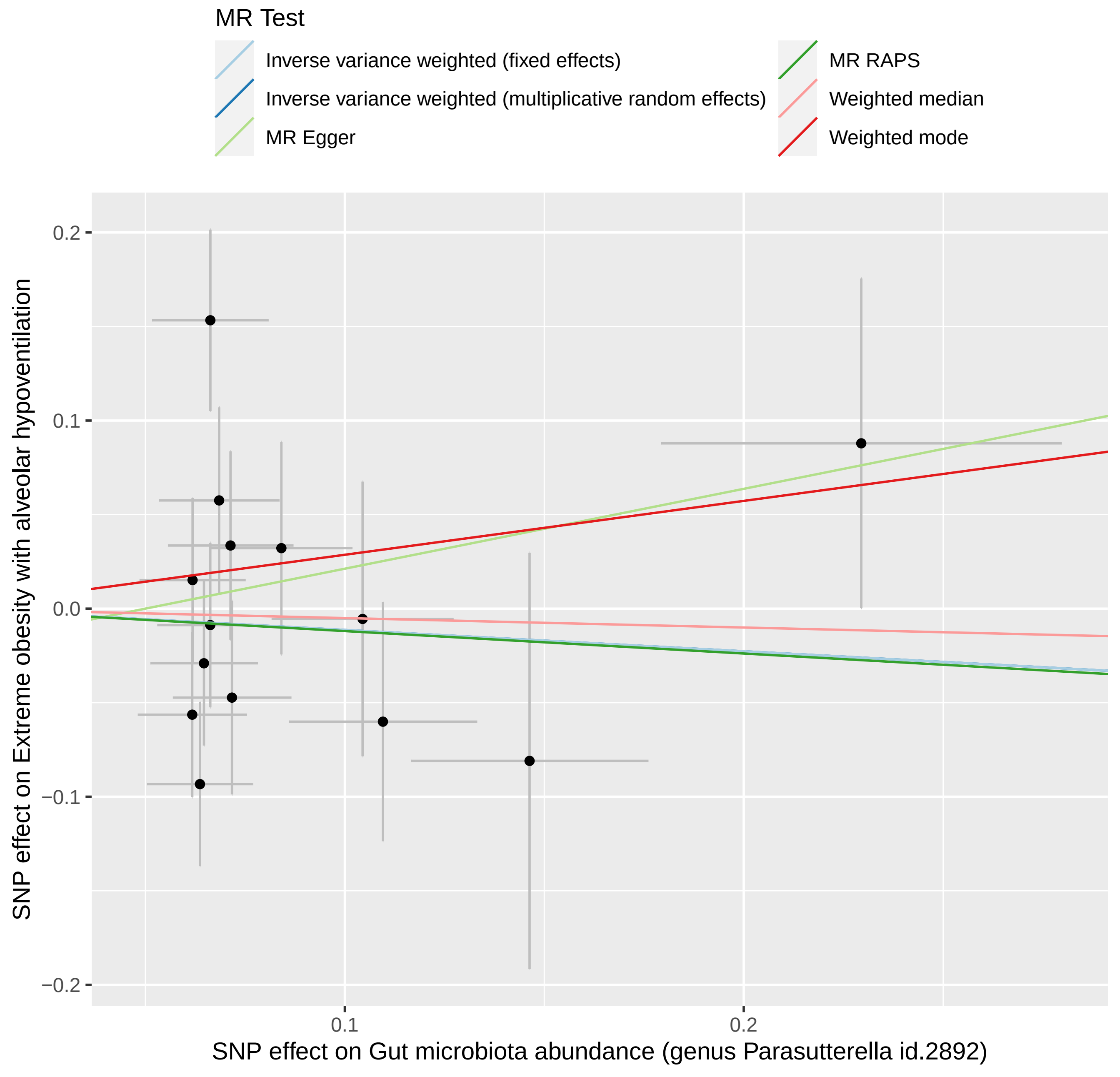

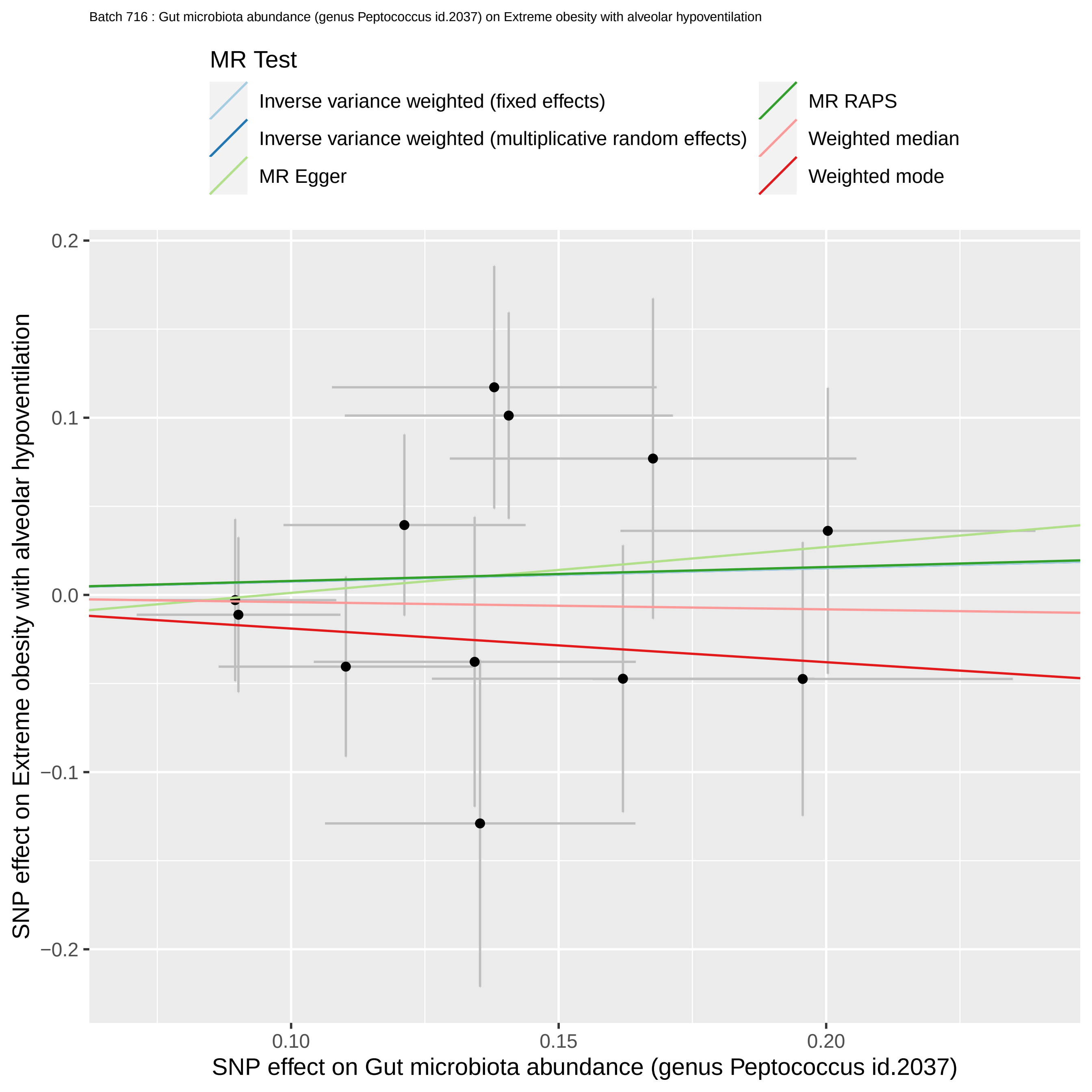

Batch 716 : Gut microbiota abundance (genus Peptococcus id.2037) on Extreme obesity with alveolar hypoventilation
MR Test
Inverse variance weighted (fixed effects)
Inverse variance weighted (multiplicative random effects)
MR Egger
MR RAPS
Weighted median
Weighted mode
0.2
0.1
0.0
−0.1
−0.2
0.10
0.15
0.20
SNP effect on Extreme obesity with alveolar hypoventilation
SNP effect on Gut microbiota abundance (genus Peptococcus id.2037)

Batch 717 : Gut microbiota abundance (genus Phascolarctobacterium id.2168) on Extreme obesity with alveolar hypoventilation

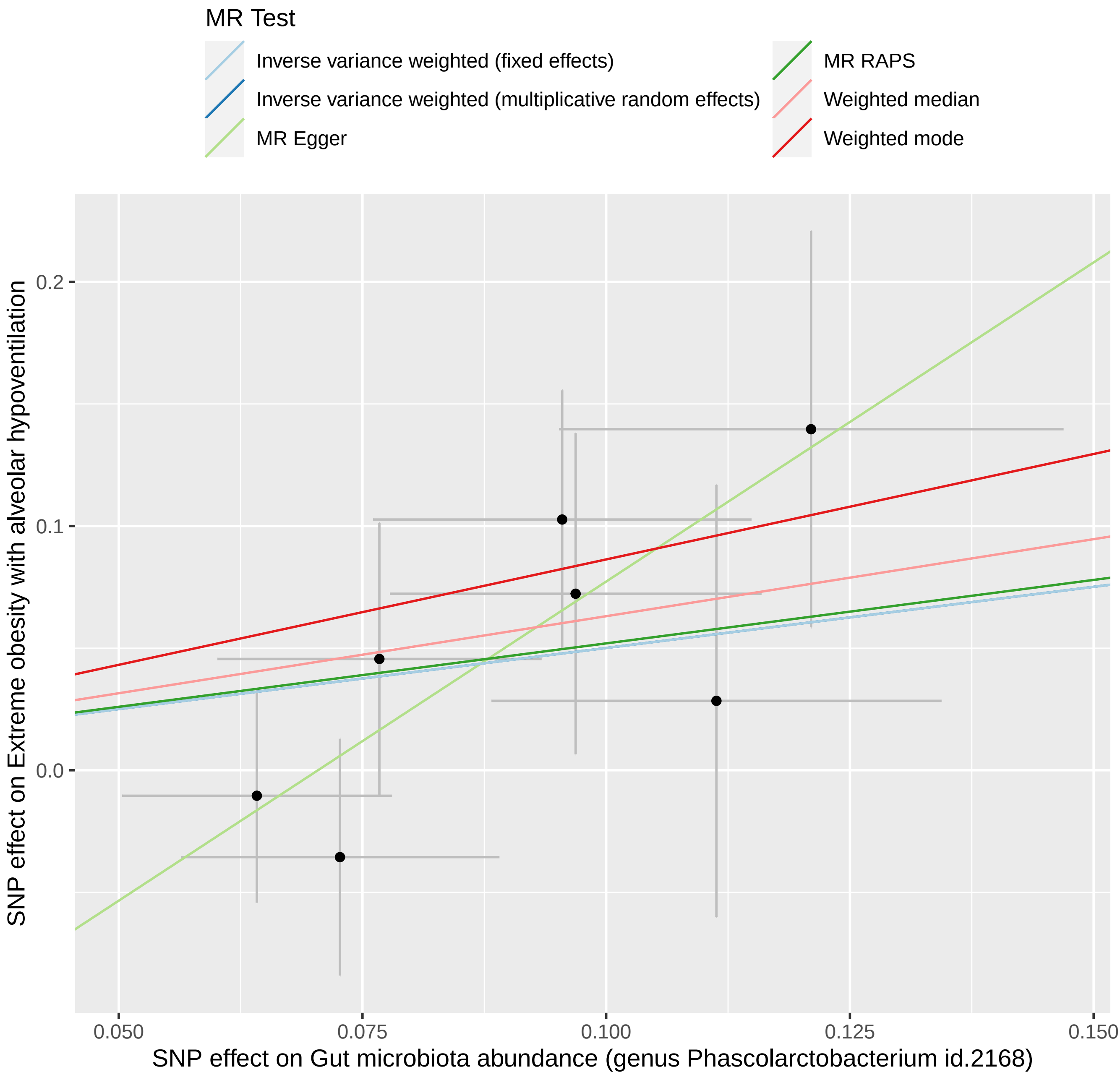

Batch 718 : Gut microbiota abundance (genus Prevotella7 id.11182) on Extreme obesity with alveolar hypoventilation

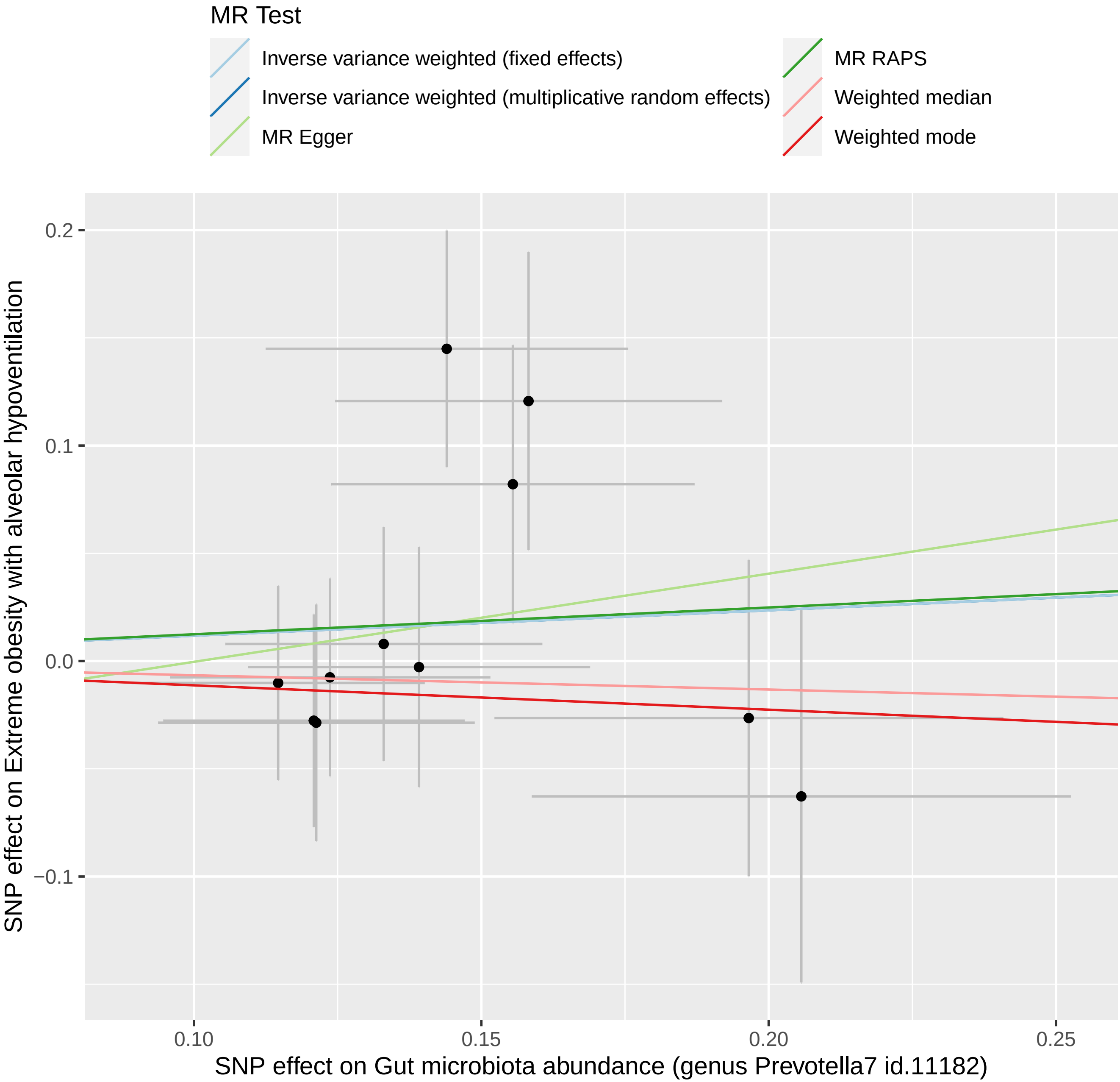

Batch 719 : Gut microbiota abundance (genus Prevotella9 id.11183) on Extreme obesity with alveolar hypoventilation

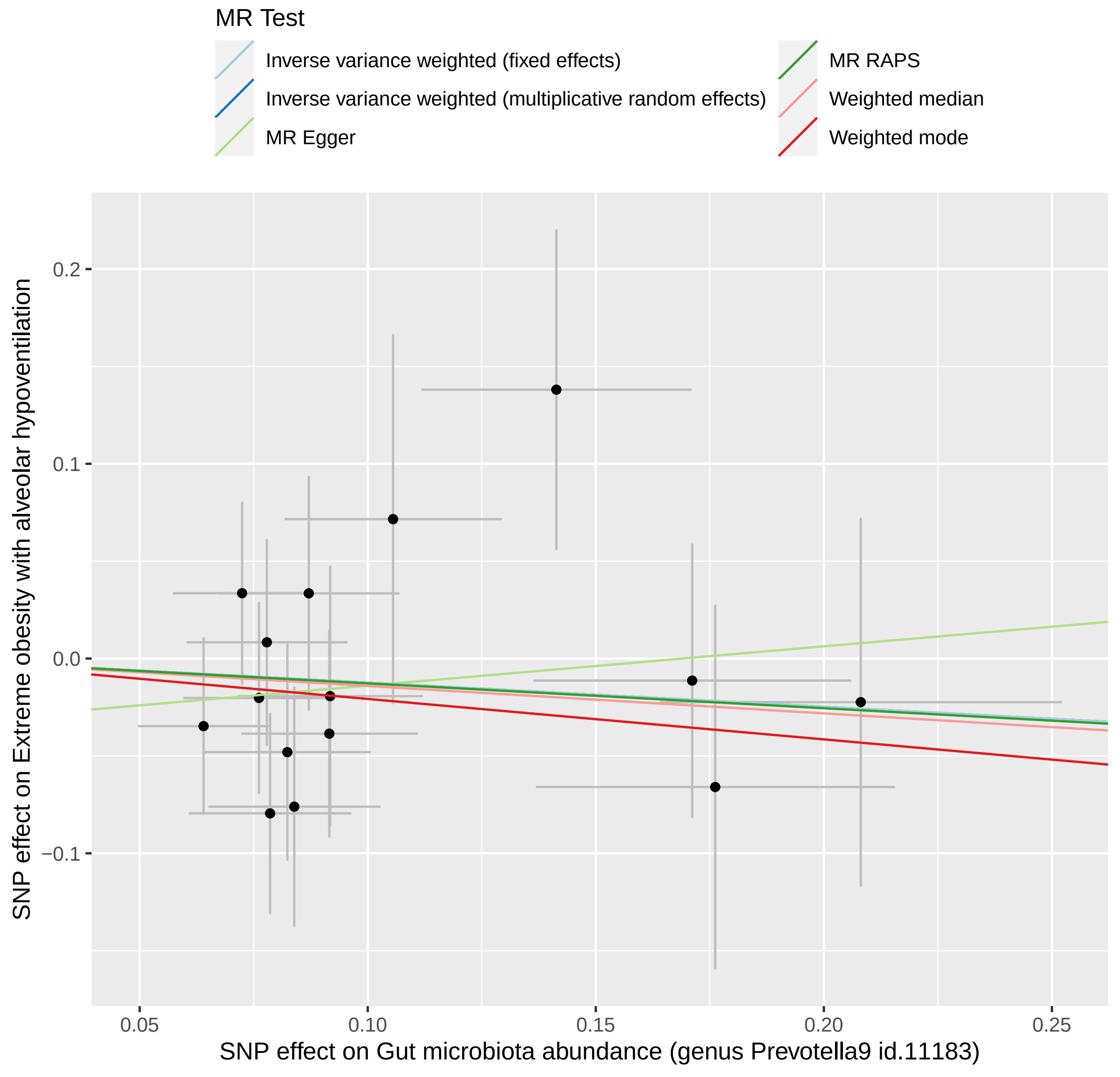

Batch 720 : Gut microbiota abundance (genus Rikenellaceae RC9 gut group id.11191) on Extreme obesity with alveolar hypoventilation

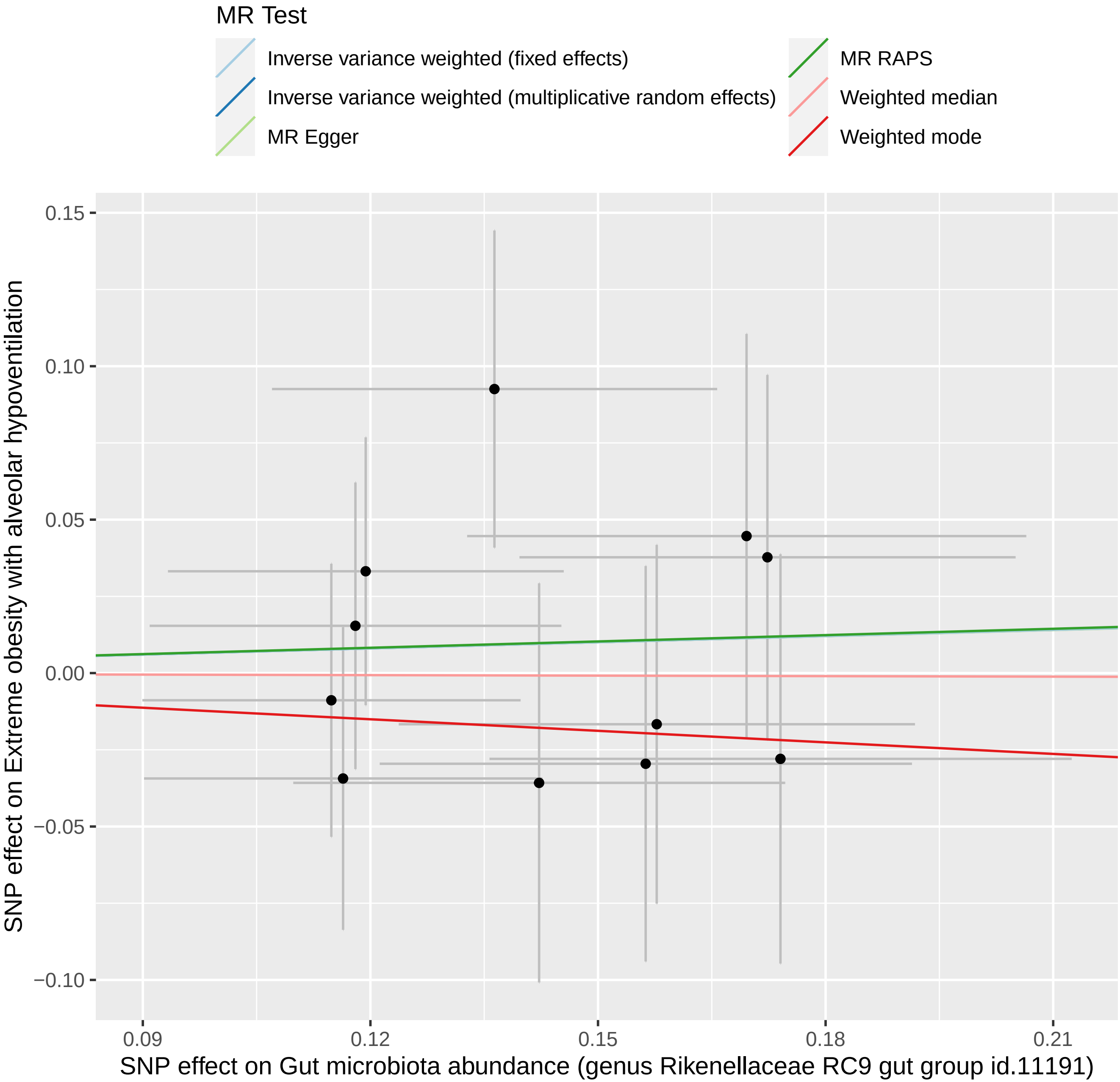

Batch 721 : Gut microbiota abundance (genus Romboutsia id.11347) on Extreme obesity with alveolar hypoventilation

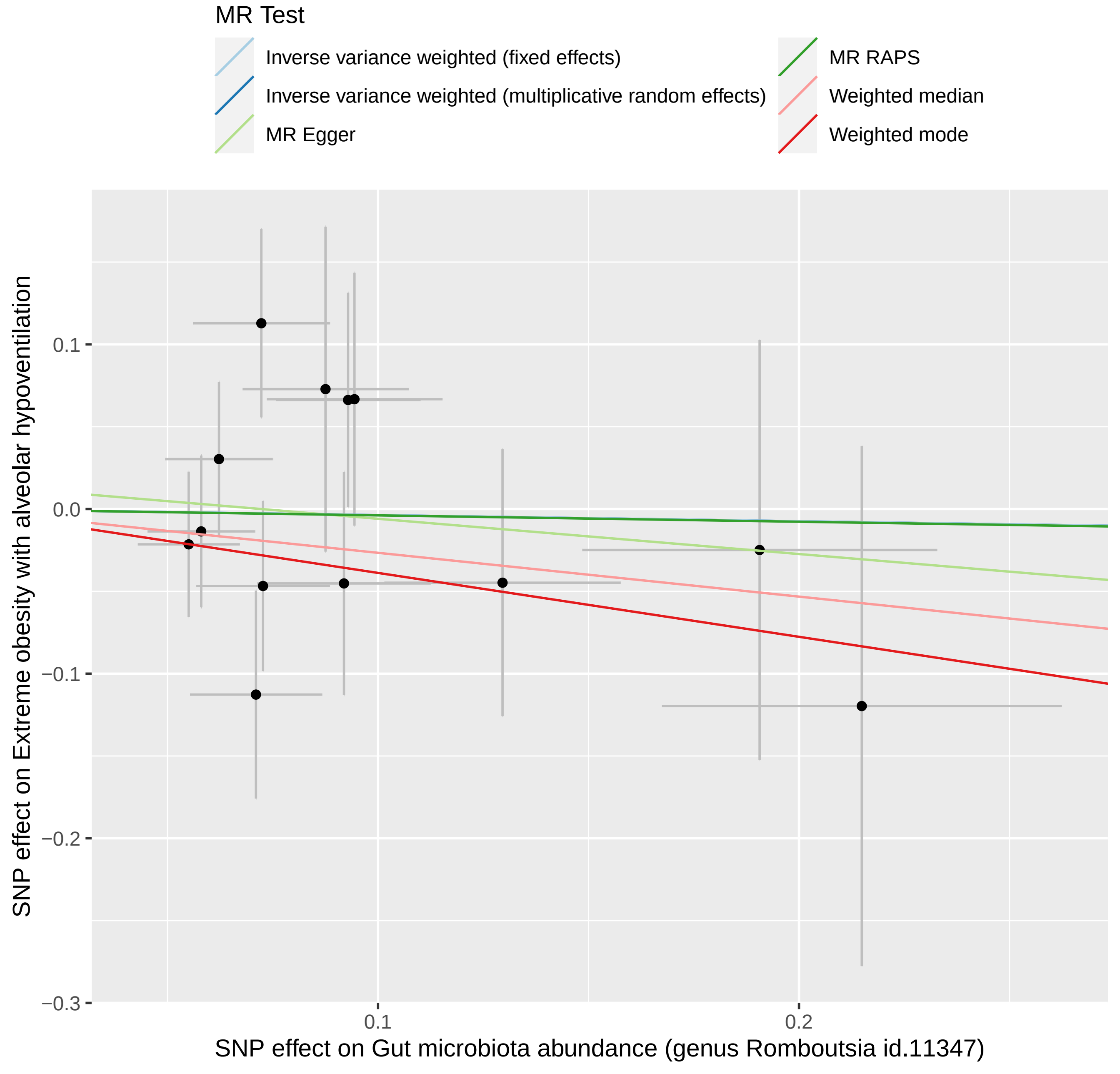

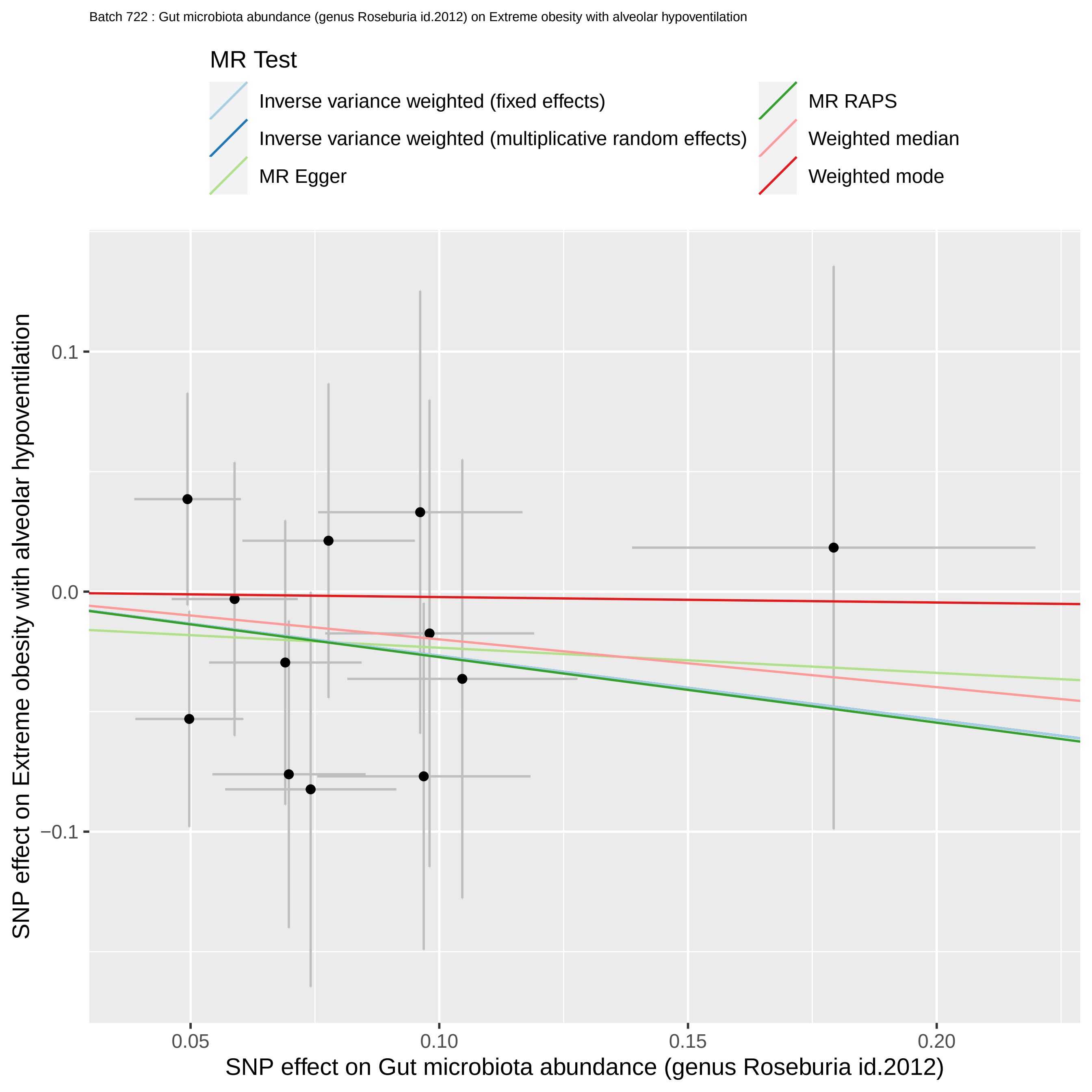

Batch 722 : Gut microbiota abundance (genus Roseburia id.2012) on Extreme obesity with alveolar hypoventilation
MR Test
Inverse variance weighted (fixed effects)
Inverse variance weighted (multiplicative random effects)
MR Egger
MR RAPS
Weighted median
Weighted mode
SNP effect on Extreme obesity with alveolar hypoventilation
0.1
0.0
−0.1
0.05
0.10
0.15
0.20
SNP effect on Gut microbiota abundance (genus Roseburia id.2012)

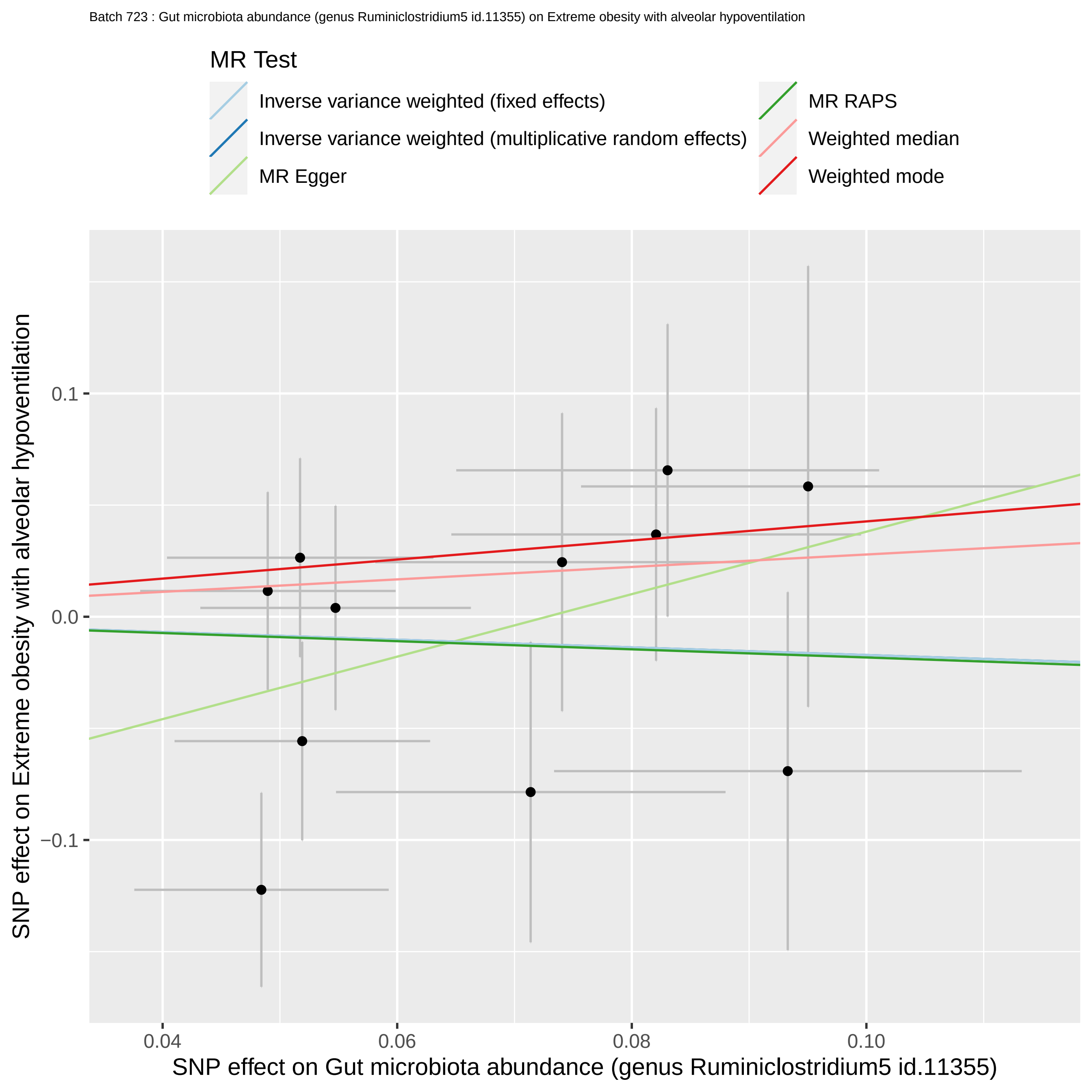

Batch 723 : Gut microbiota abundance (genus Ruminiclostridium5 id.11355) on Extreme obesity with alveolar hypoventilation
MR Test
Inverse variance weighted (fixed effects)
Inverse variance weighted (multiplicative random effects)
MR Egger
MR RAPS
Weighted median
Weighted mode
0.1
0.0
−0.1
0.04
0.06
0.08
0.10
SNP effect on Extreme obesity with alveolar hypoventilation
SNP effect on Gut microbiota abundance (genus Ruminiclostridium5 id.11355)

Batch 724 : Gut microbiota abundance (genus Ruminiclostridium6 id.11356) on Extreme obesity with alveolar hypoventilation

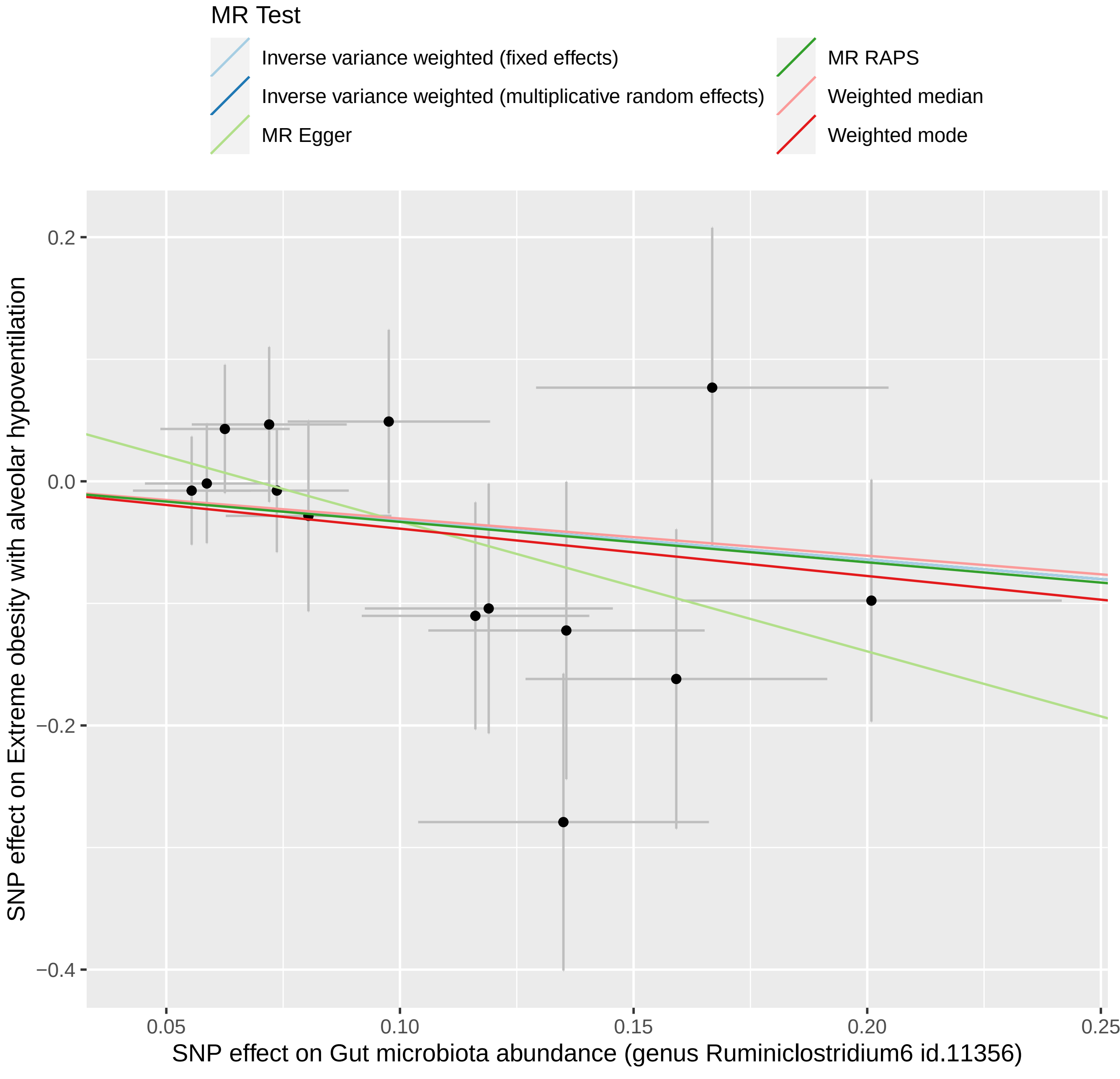

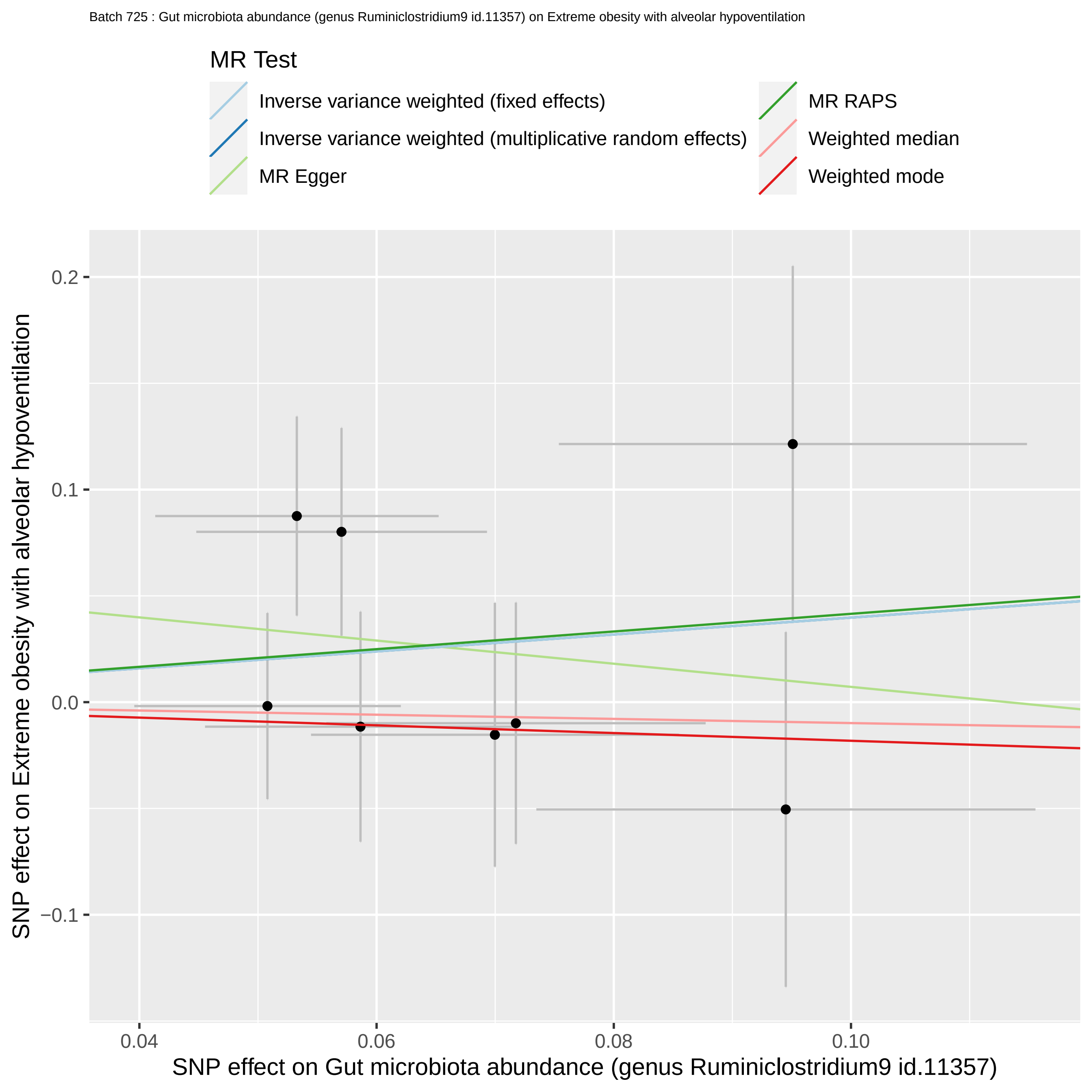
Batch 725 : Gut microbiota abundance (genus Ruminiclostridium9 id.11357) on Extreme obesity with alveolar hypoventilation
MR Test
Inverse variance weighted (fixed effects)
Inverse variance weighted (multiplicative random effects)
MR Egger
MR RAPS
Weighted median
Weighted mode
0.2
0.1
0.0
−0.1
0.04
0.06
0.08
0.10
SNP effect on Extreme obesity with alveolar hypoventilation
SNP effect on Gut microbiota abundance (genus Ruminiclostridium9 id.11357)

Batch 726 : Gut microbiota abundance (genus Ruminococcaceae NK4A214 group id.11358) on Extreme obesity with alveolar hypoventilation

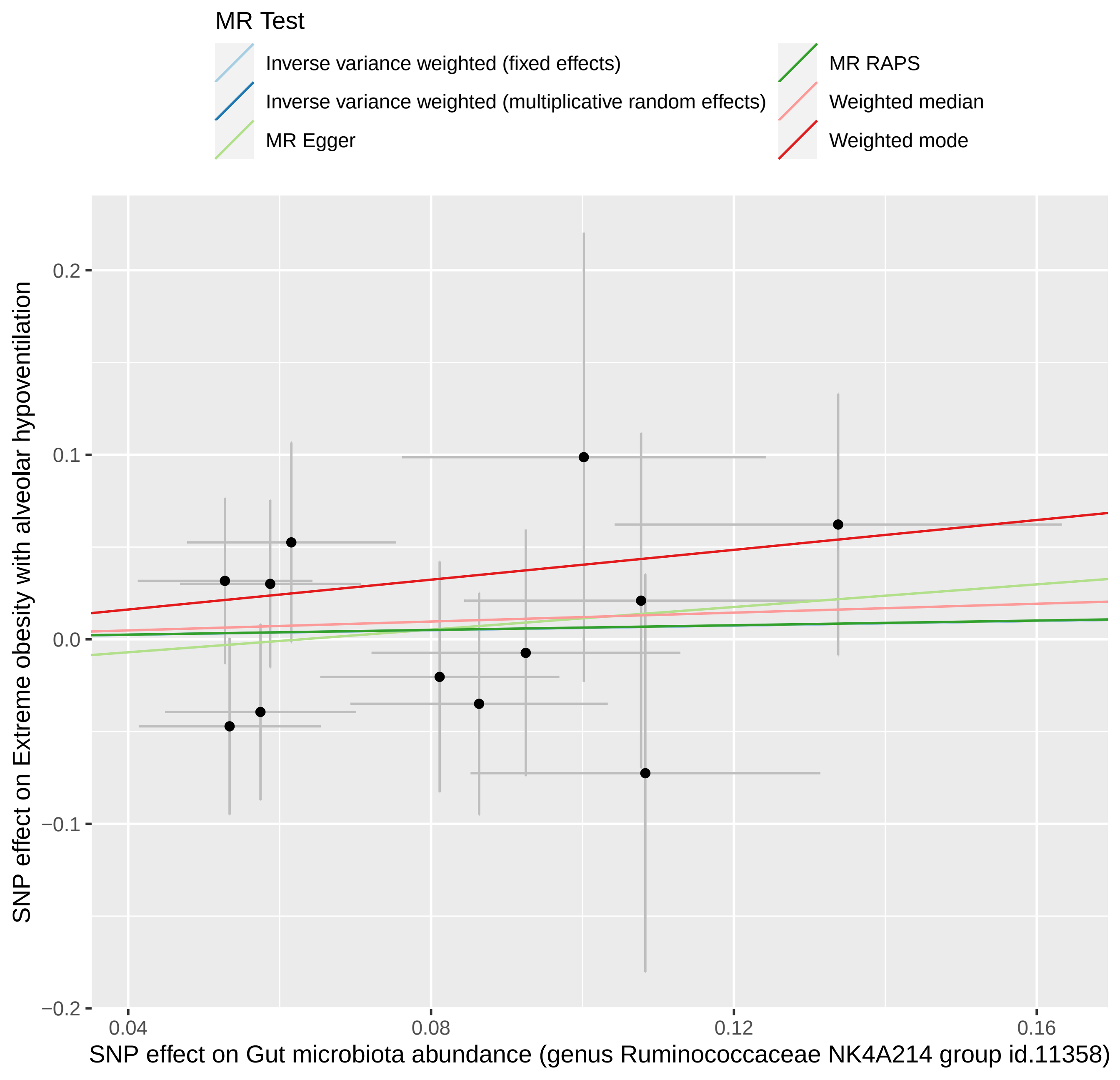

Batch 727 : Gut microbiota abundance (genus Ruminococcaceae UCG002 id.11360) on Extreme obesity with alveolar hypoventilation

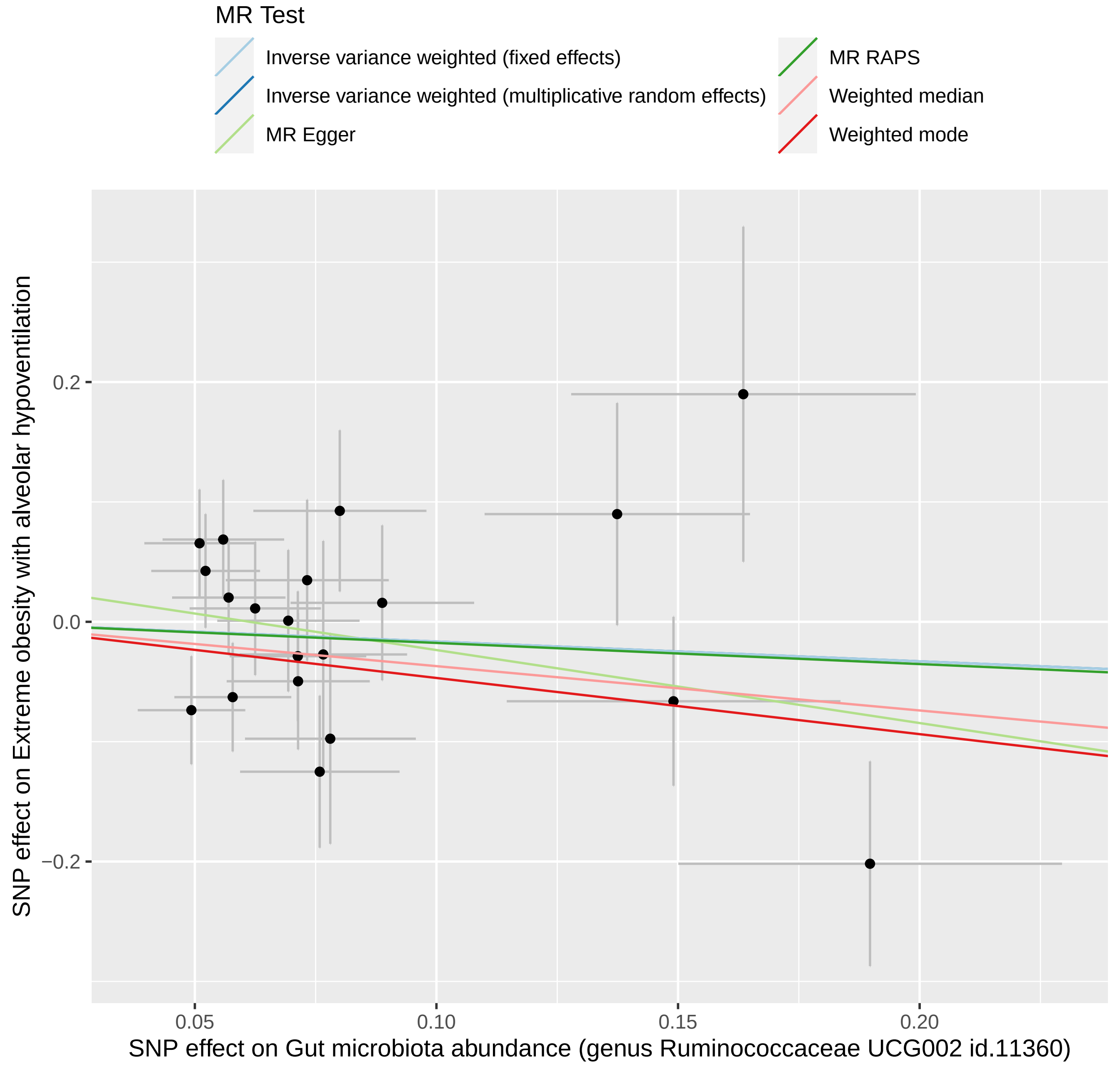

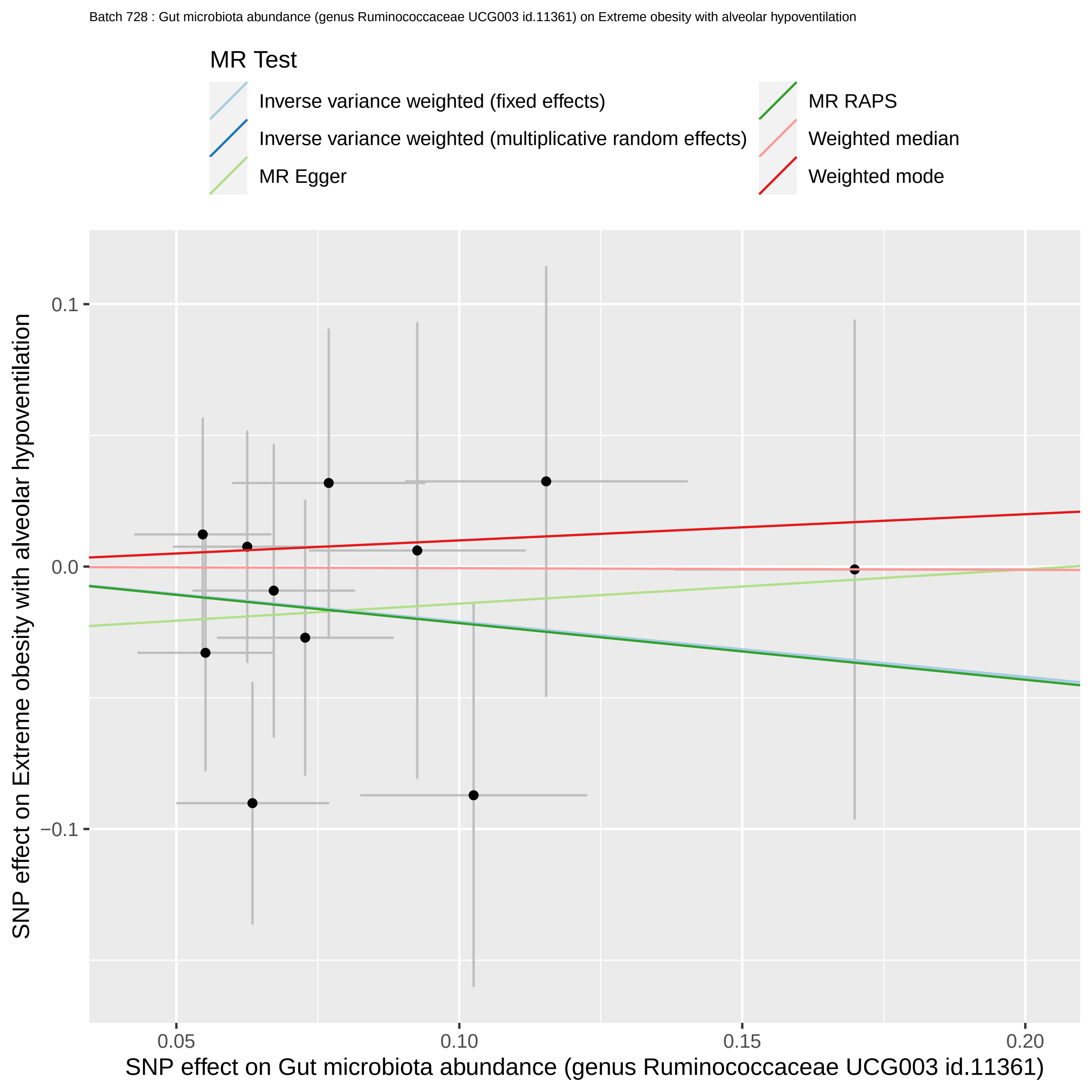

Batch 728 : Gut microbiota abundance (genus Ruminococcaceae UCG003 id.11361) on Extreme obesity with alveolar hypoventilation
MR Test
Inverse variance weighted (fixed effects)
Inverse variance weighted (multiplicative random effects)
MR Egger
MR RAPS
Weighted median
Weighted mode
0.1
0.0
−0.1
0.05
0.10
0.15
0.20
SNP effect on Extreme obesity with alveolar hypoventilation
SNP effect on Gut microbiota abundance (genus Ruminococcaceae UCG003 id.11361)

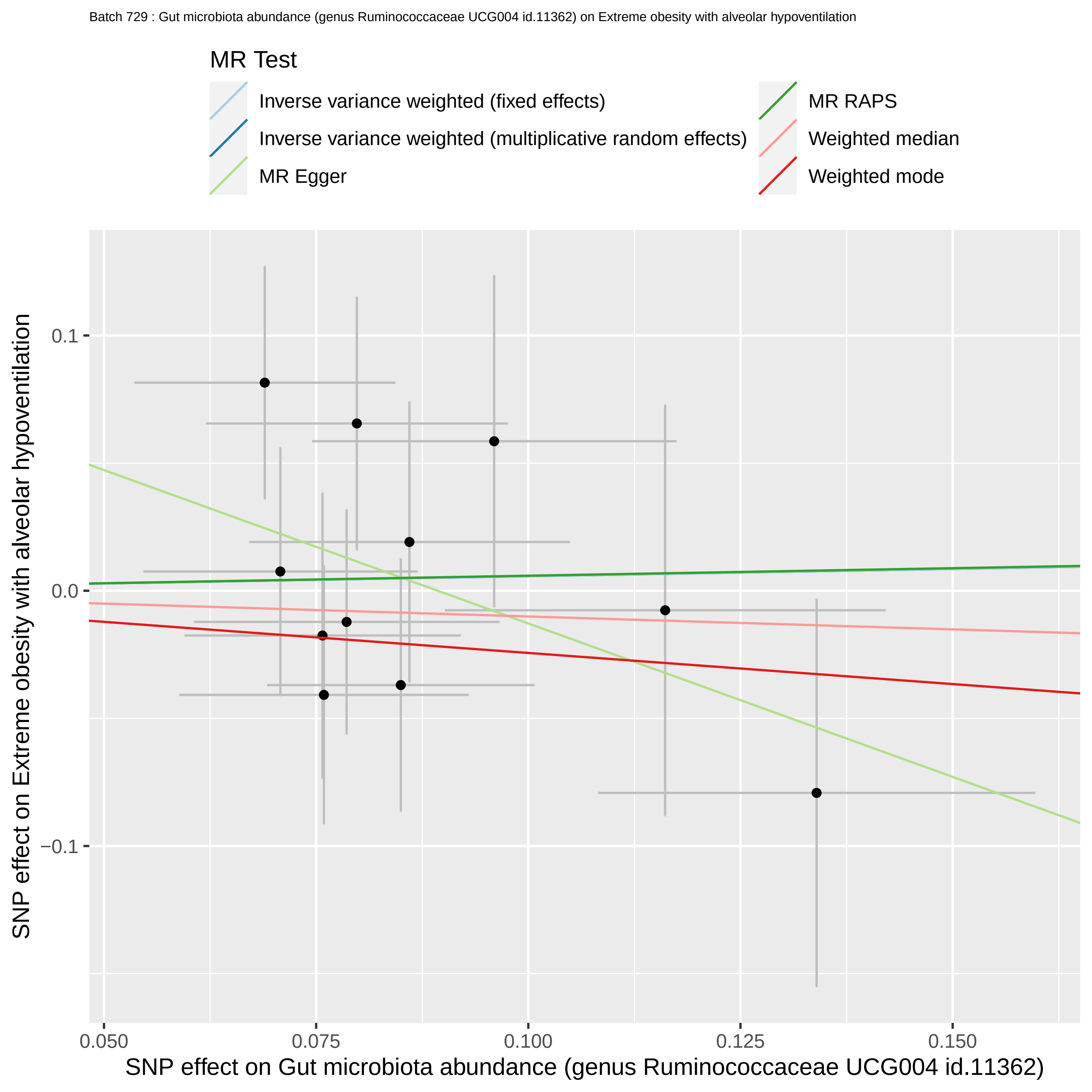
Batch 729 : Gut microbiota abundance (genus Ruminococcaceae UCG004 id.11362) on Extreme obesity with alveolar hypoventilation
MR Test
Inverse variance weighted (fixed effects)
Inverse variance weighted (multiplicative random effects)
MR Egger
MR RAPS
Weighted median
Weighted mode
0.1
0.0
−0.1
0.050
0.075
0.100
0.125
0.150
SNP effect on Extreme obesity with alveolar hypoventilation
SNP effect on Gut microbiota abundance (genus Ruminococcaceae UCG004 id.11362)

Batch 730 : Gut microbiota abundance (genus Ruminococcaceae UCG005 id.11363) on Extreme obesity with alveolar hypoventilation

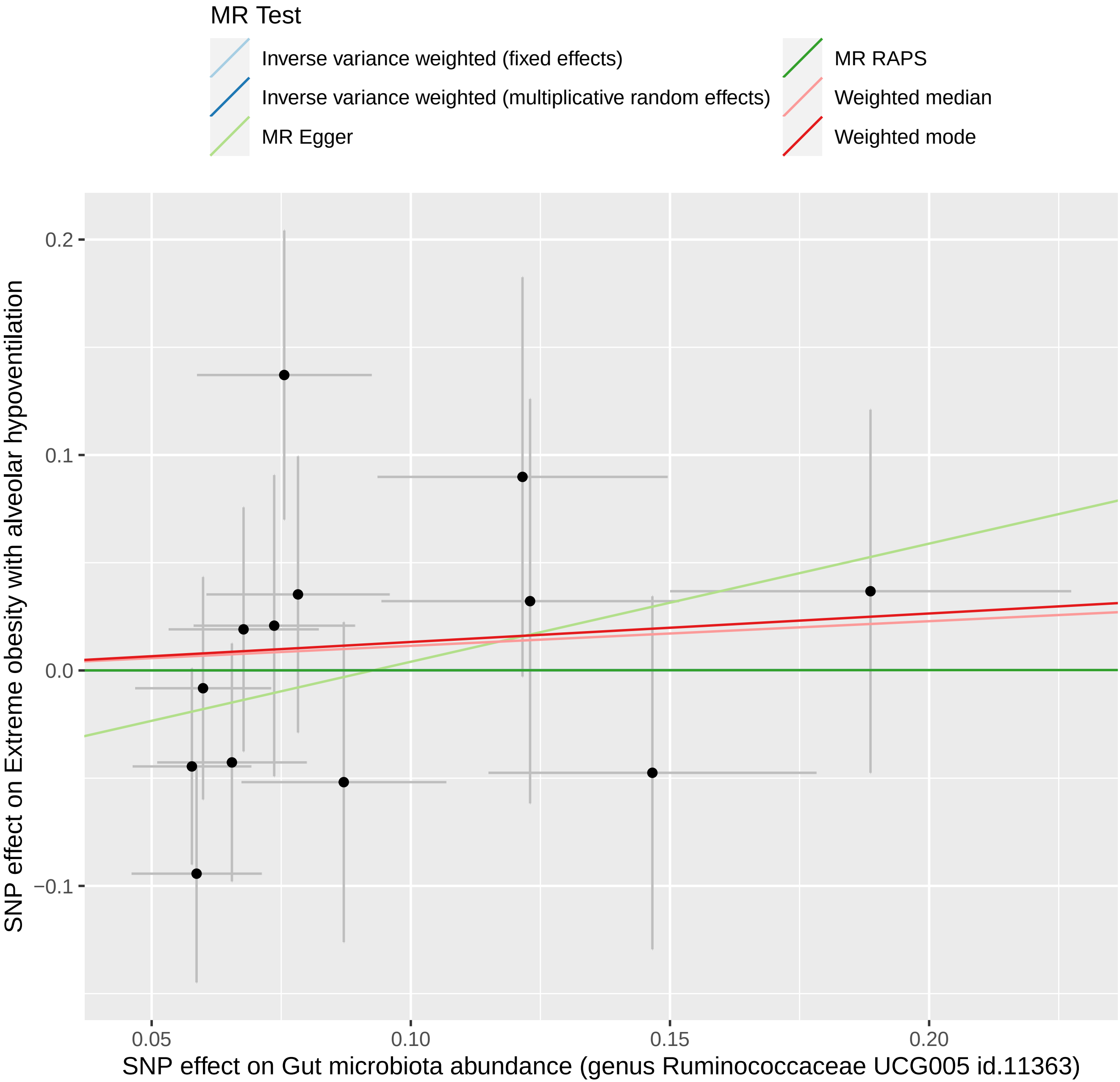

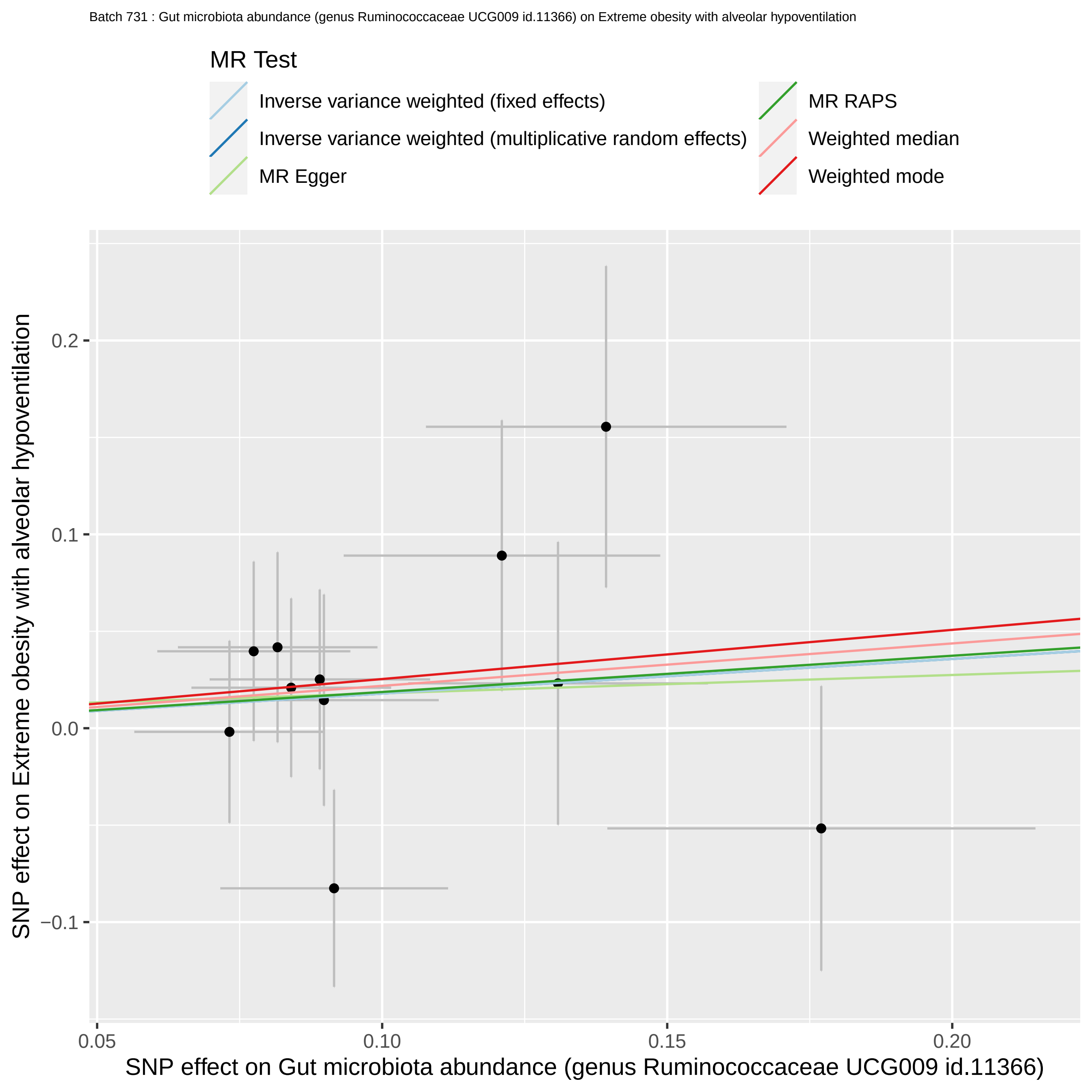

Batch 731 : Gut microbiota abundance (genus Ruminococcaceae UCG009 id.11366) on Extreme obesity with alveolar hypoventilation
MR Test
Inverse variance weighted (fixed effects)
Inverse variance weighted (multiplicative random effects)
MR Egger
MR RAPS
Weighted median
Weighted mode
SNP effect on Extreme obesity with alveolar hypoventilation
0.2
0.1
0.0
−0.1
0.05
0.10
0.15
0.20
SNP effect on Gut microbiota abundance (genus Ruminococcaceae UCG009 id.11366)

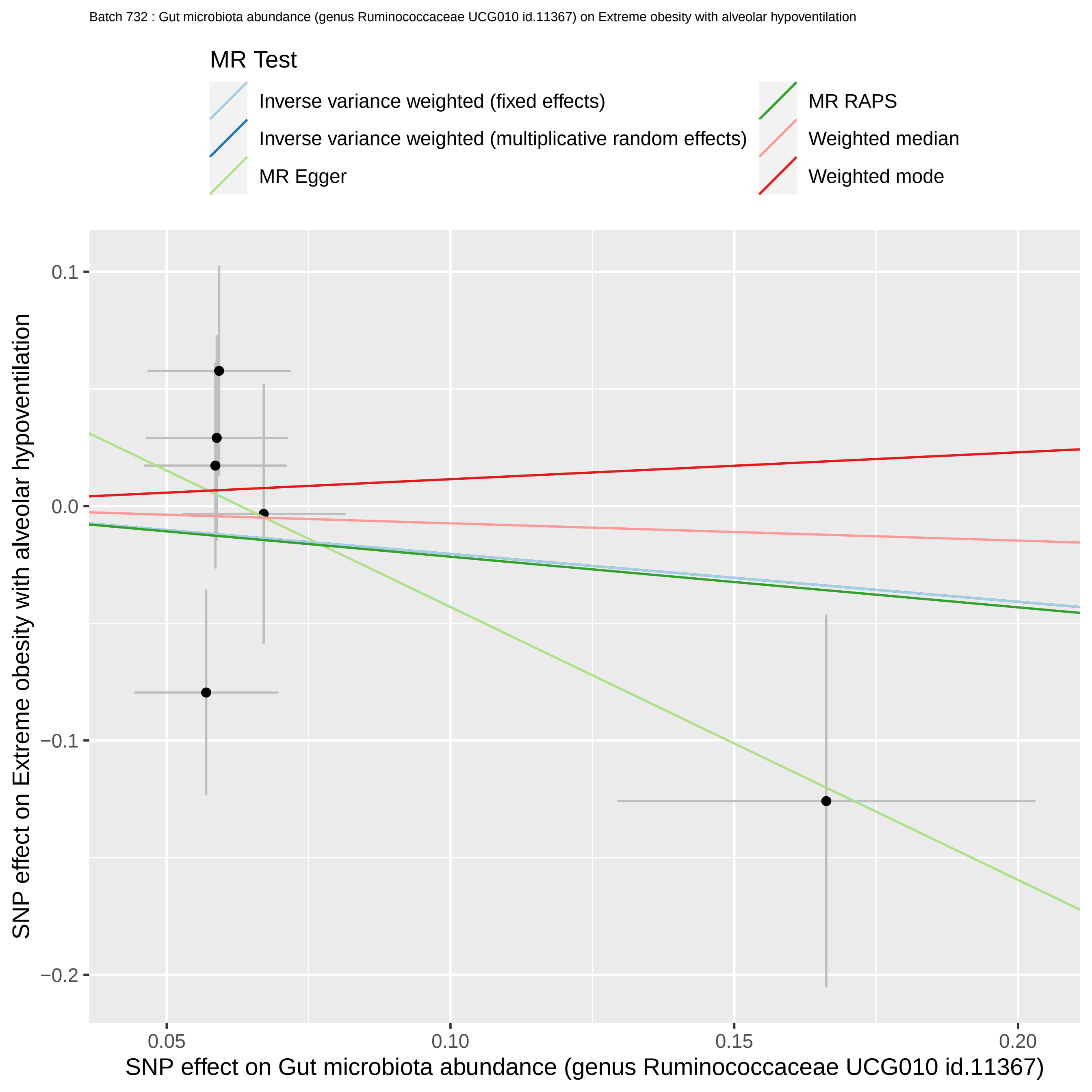
Batch 732 : Gut microbiota abundance (genus Ruminococcaceae UCG010 id.11367) on Extreme obesity with alveolar hypoventilation
MR Test
Inverse variance weighted (fixed effects)
Inverse variance weighted (multiplicative random effects)
MR Egger
MR RAPS
Weighted median
Weighted mode
0.1
0.0
−0.1
−0.2
0.05
0.10
0.15
0.20
SNP effect on Extreme obesity with alveolar hypoventilation
SNP effect on Gut microbiota abundance (genus Ruminococcaceae UCG010 id.11367)

Batch 733 : Gut microbiota abundance (genus Ruminococcaceae UCG011 id.11368) on Extreme obesity with alveolar hypoventilation

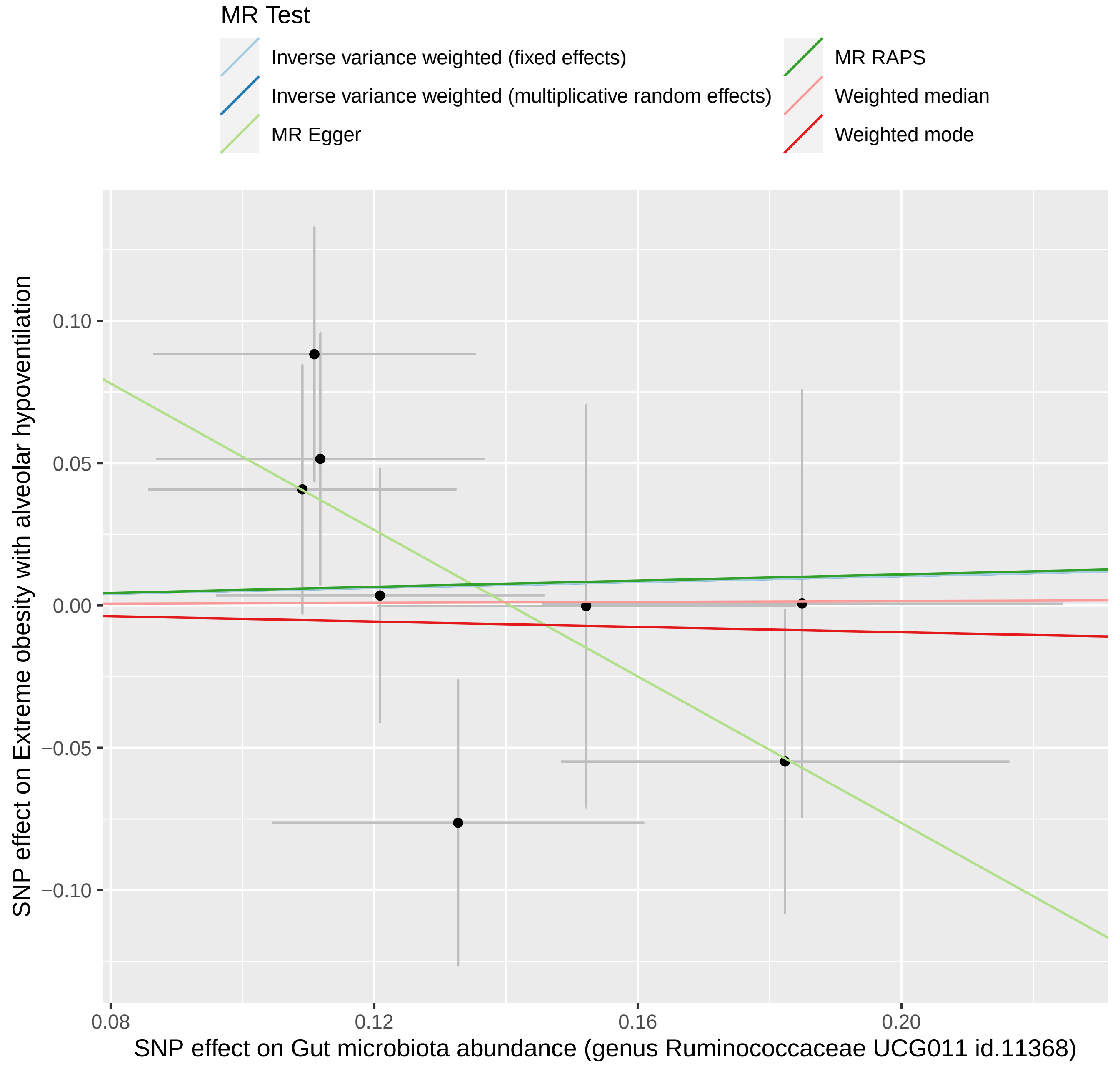

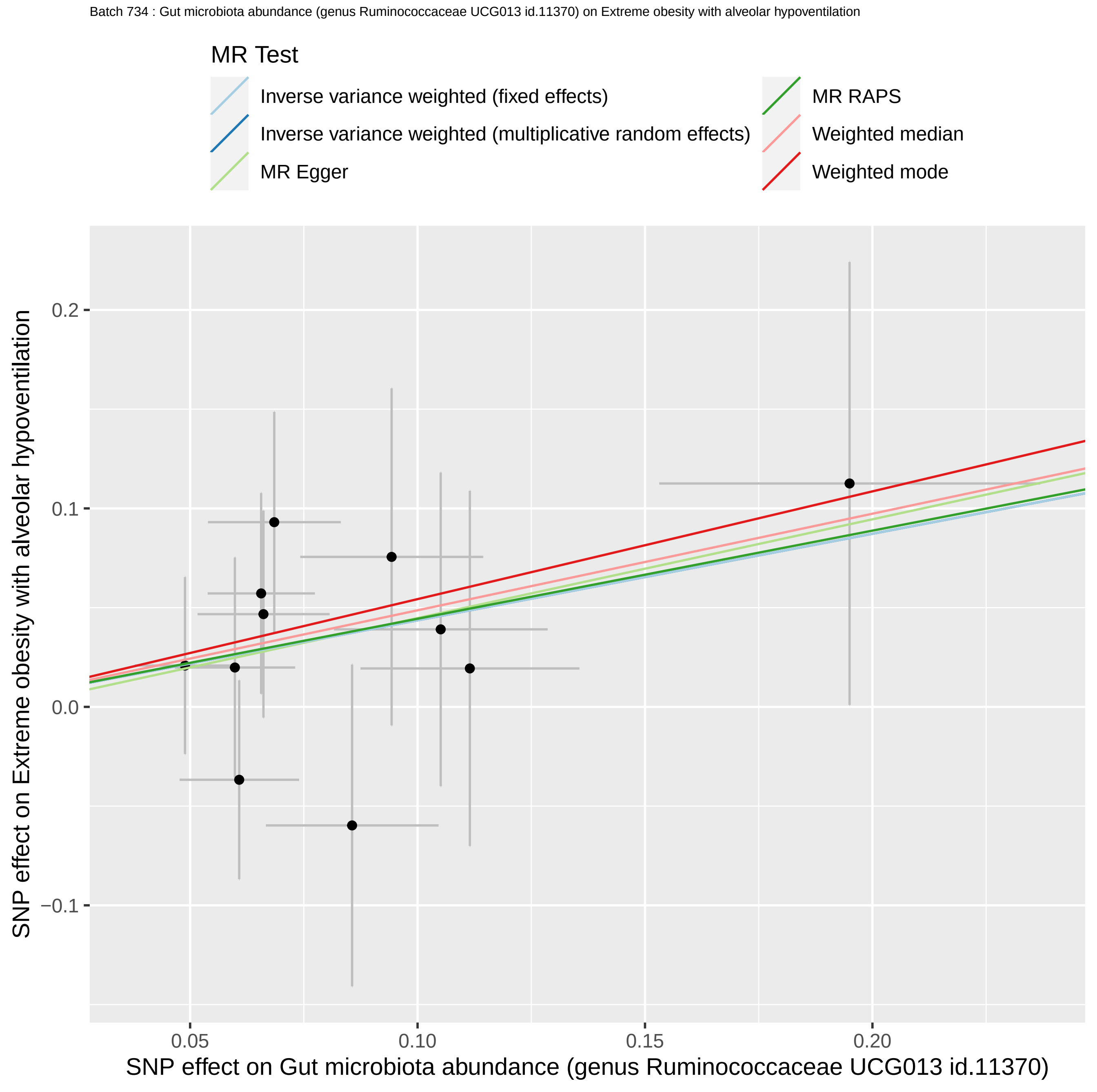
Batch 734 : Gut microbiota abundance (genus Ruminococcaceae UCG013 id.11370) on Extreme obesity with alveolar hypoventilation
MR Test
Inverse variance weighted (fixed effects)
Inverse variance weighted (multiplicative random effects)
MR Egger
MR RAPS
Weighted median
Weighted mode
0.2
0.1
0.0
−0.1
0.05
0.10
0.15
0.20
SNP effect on Extreme obesity with alveolar hypoventilation
SNP effect on Gut microbiota abundance (genus Ruminococcaceae UCG013 id.11370)

Batch 735 : Gut microbiota abundance (genus Ruminococcaceae UCG014 id.11371) on Extreme obesity with alveolar hypoventilation

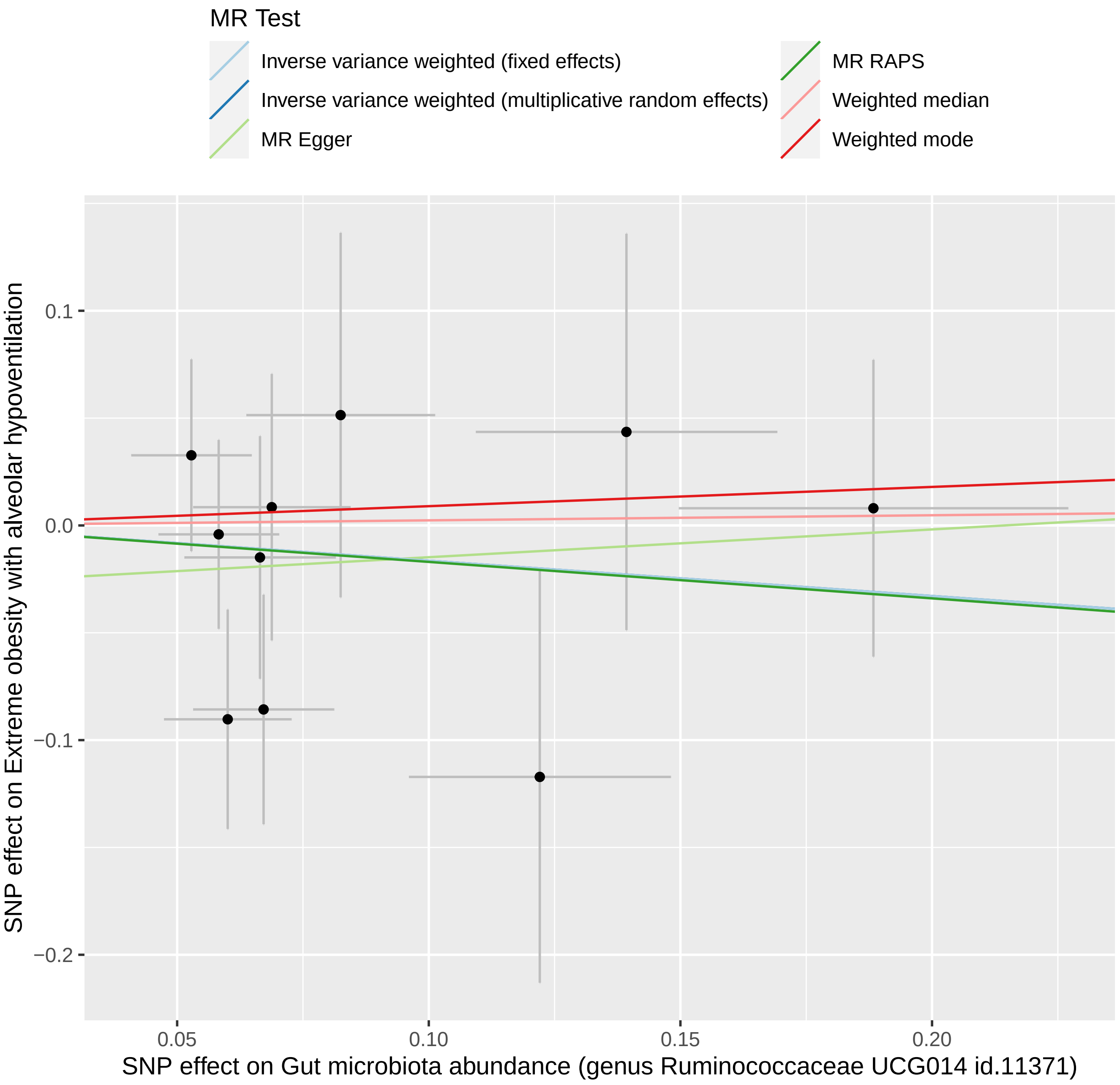

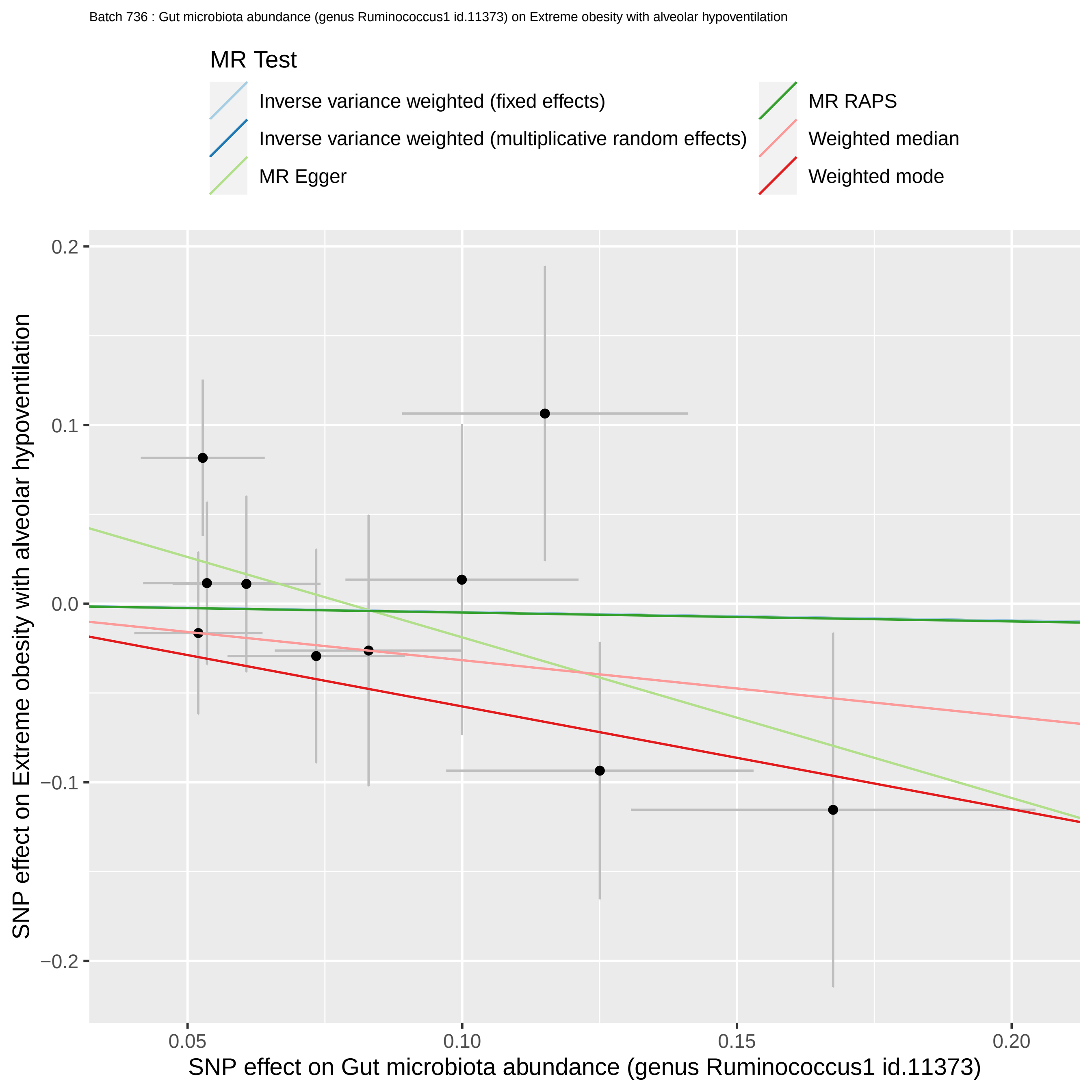
Batch 736 : Gut microbiota abundance (genus Ruminococcus1 id.11373) on Extreme obesity with alveolar hypoventilation
MR Test
Inverse variance weighted (fixed effects)
Inverse variance weighted (multiplicative random effects)
MR Egger
MR RAPS
Weighted median
Weighted mode
0.2
0.1
0.0
−0.1
−0.2
0.05
0.10
0.15
0.20
SNP effect on Extreme obesity with alveolar hypoventilation
SNP effect on Gut microbiota abundance (genus Ruminococcus1 id.11373)

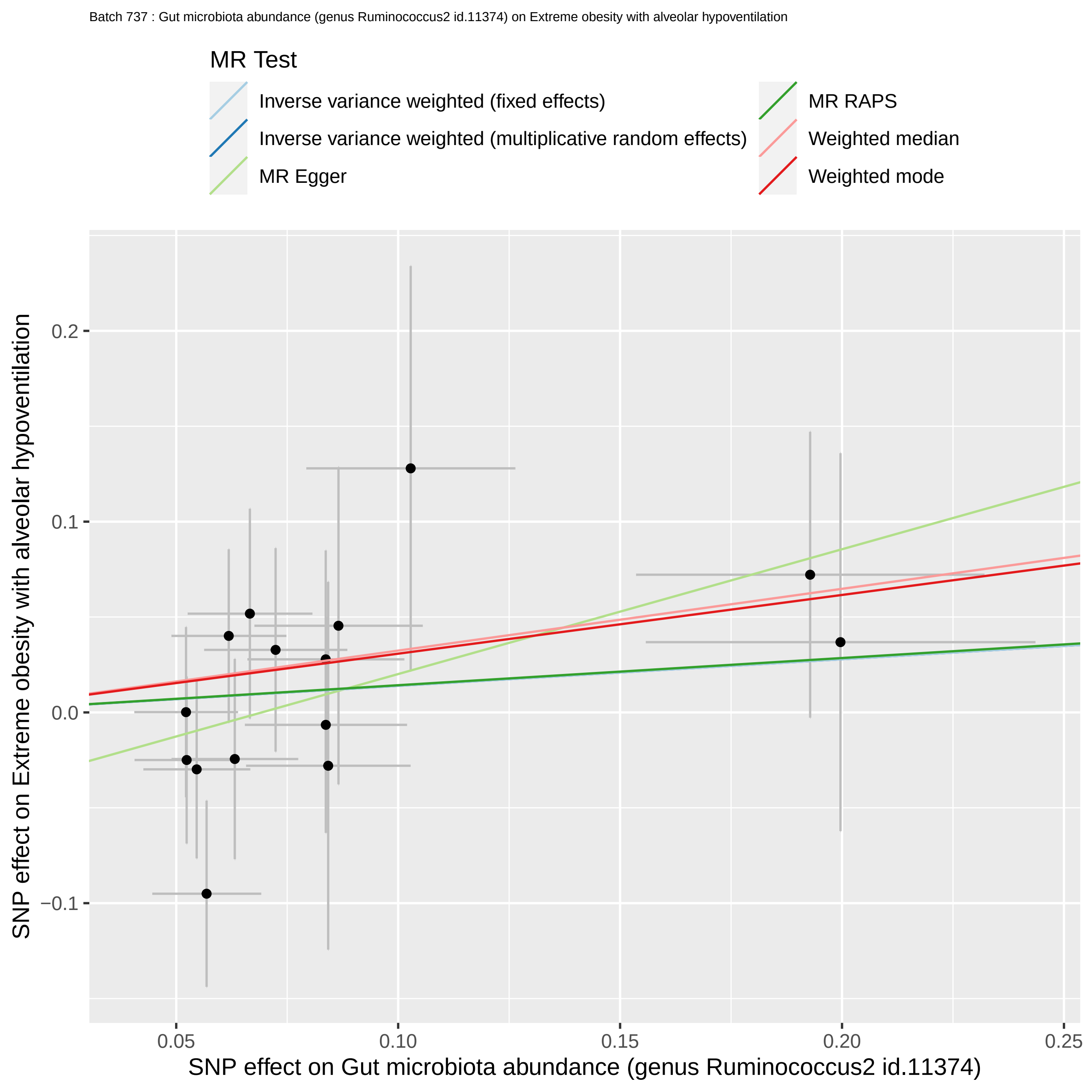
Batch 737 : Gut microbiota abundance (genus Ruminococcus2 id.11374) on Extreme obesity with alveolar hypoventilation
MR Test
Inverse variance weighted (fixed effects)
Inverse variance weighted (multiplicative random effects)
MR Egger
MR RAPS
Weighted median
Weighted mode
SNP effect on Extreme obesity with alveolar hypoventilation
0.2
0.1
0.0
−0.1
0.05
0.10
0.15
0.20
0.25
SNP effect on Gut microbiota abundance (genus Ruminococcus2 id.11374)

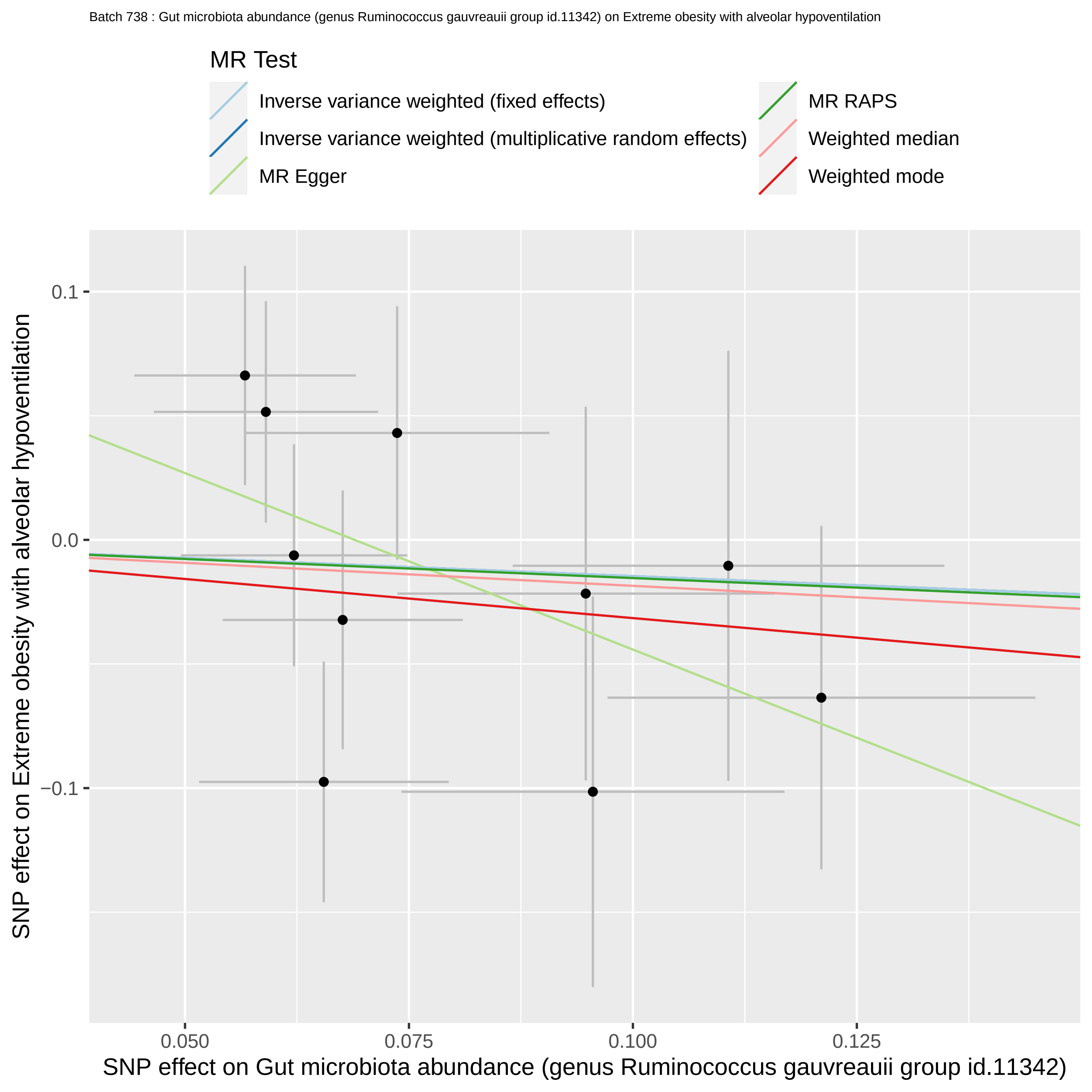

Batch 738 : Gut microbiota abundance (genus Ruminococcus gauvreauii group id.11342) on Extreme obesity with alveolar hypoventilation
MR Test
Inverse variance weighted (fixed effects)
Inverse variance weighted (multiplicative random effects)
MR Egger
MR RAPS
Weighted median
Weighted mode
0.1
0.0
−0.1
0.050
0.075
0.100
0.125
SNP effect on Extreme obesity with alveolar hypoventilation
SNP effect on Gut microbiota abundance (genus Ruminococcus gauvreauii group id.11342)

Batch 739 : Gut microbiota abundance (genus Ruminococcus gnavus group id.14376) on Extreme obesity with alveolar hypoventilation

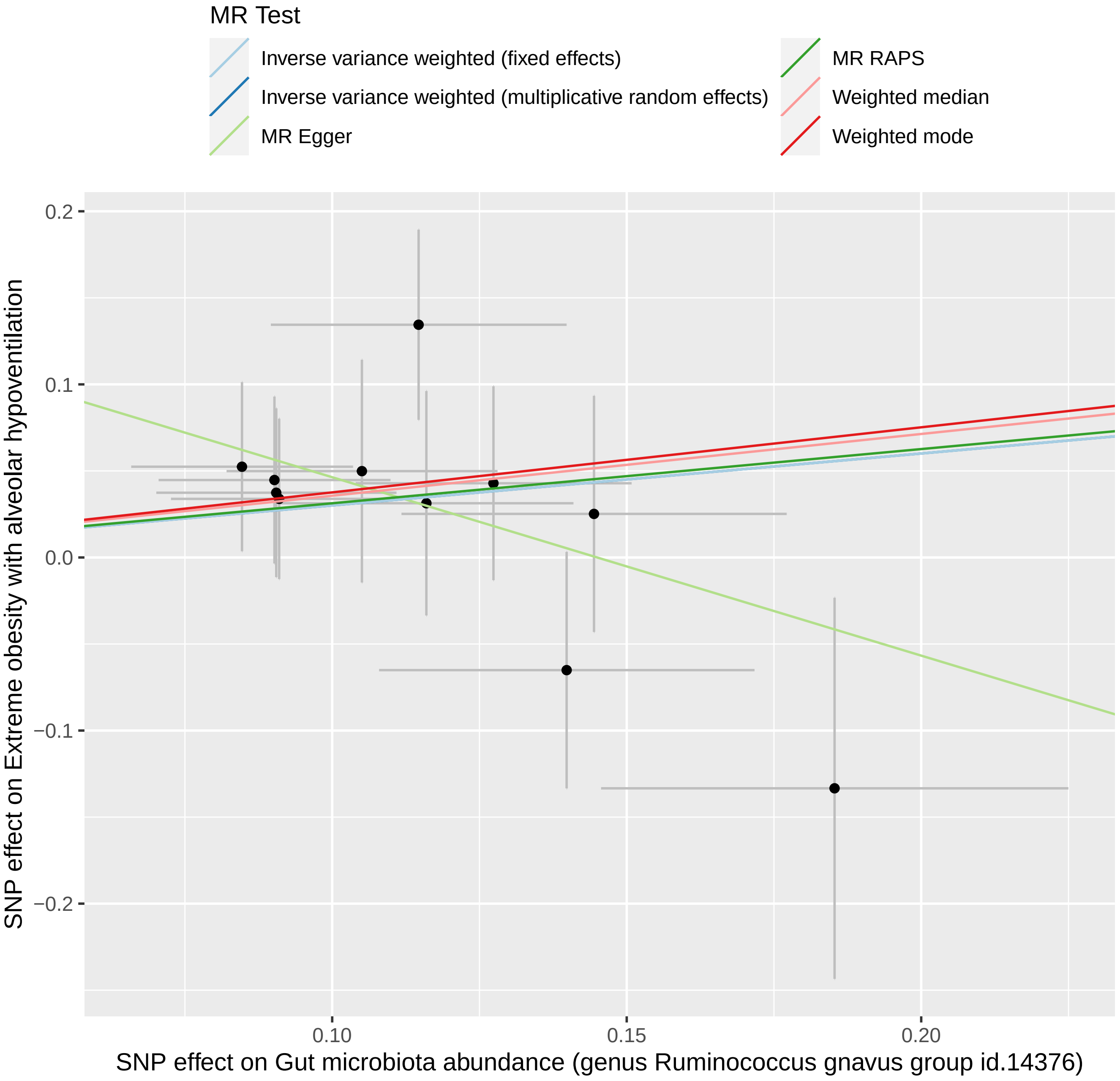

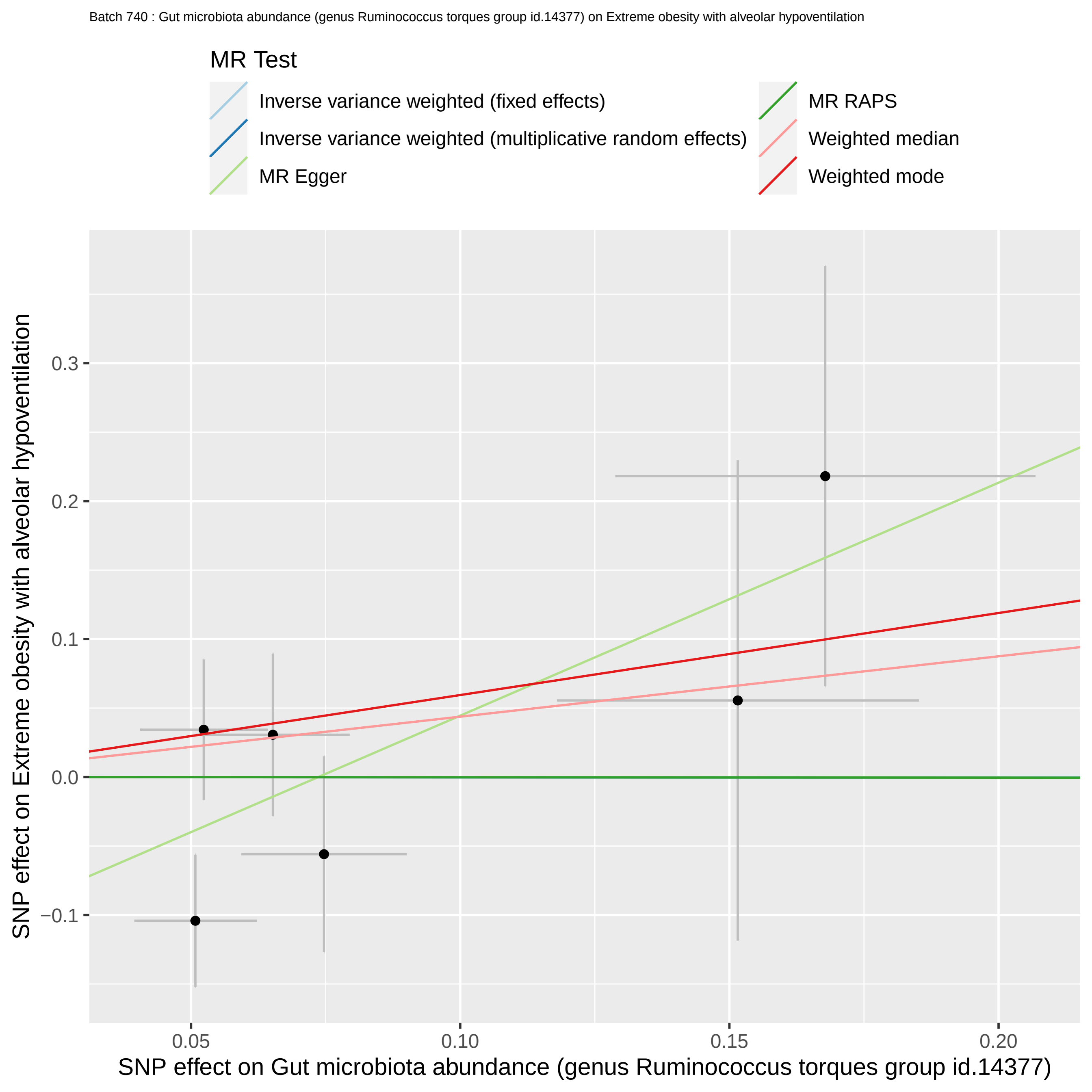
Batch 740 : Gut microbiota abundance (genus Ruminococcus torques group id.14377) on Extreme obesity with alveolar hypoventilation
MR Test
Inverse variance weighted (fixed effects)
Inverse variance weighted (multiplicative random effects)
MR Egger
MR RAPS
Weighted median
Weighted mode
SNP effect on Extreme obesity with alveolar hypoventilation
SNP effect on Gut microbiota abundance (genus Ruminococcus torques group id.14377)
0.3
0.2
0.1
0.0
−0.1
0.05
0.10
0.15
0.20

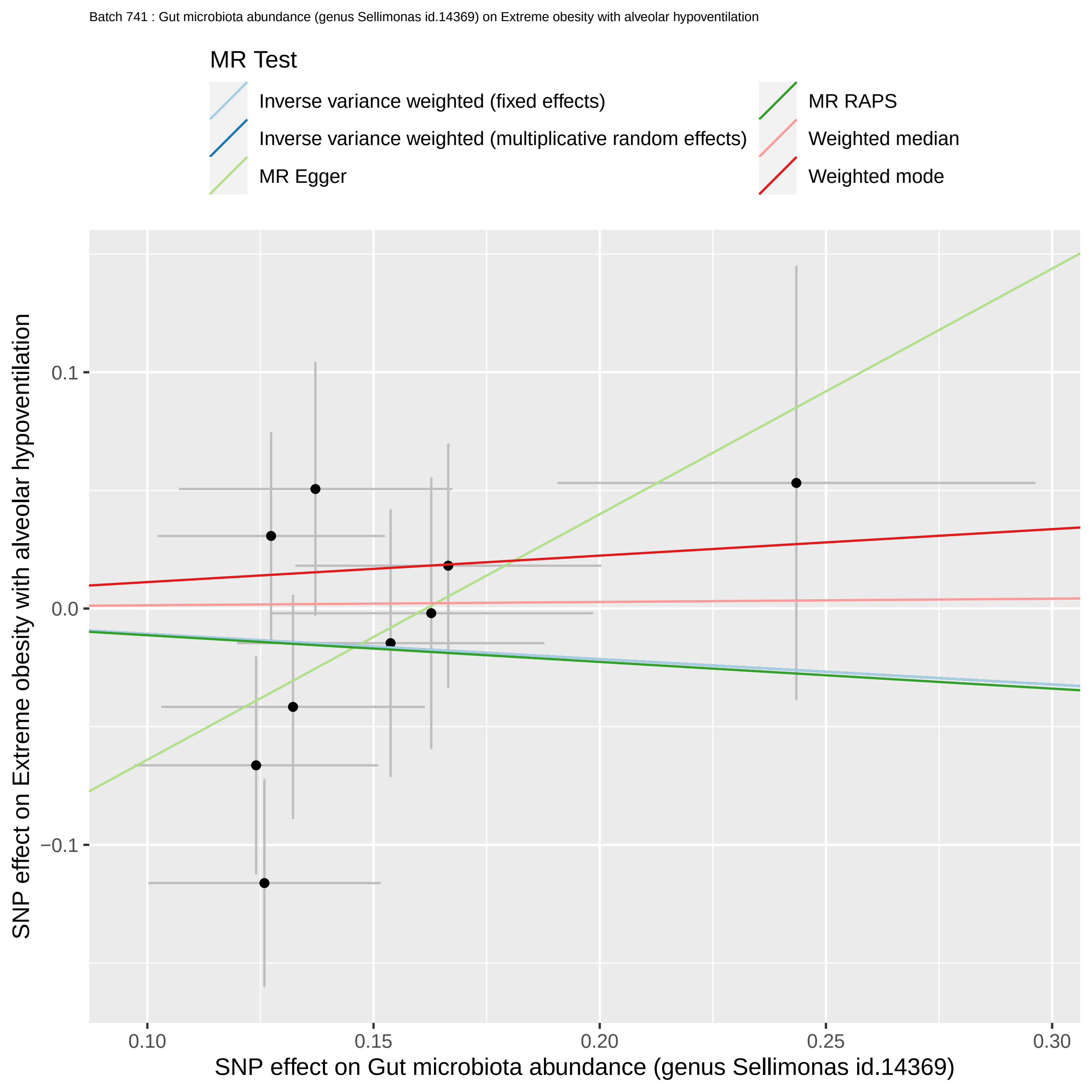
Batch 741 : Gut microbiota abundance (genus Sellimonas id.14369) on Extreme obesity with alveolar hypoventilation
MR Test
Inverse variance weighted (fixed effects)
Inverse variance weighted (multiplicative random effects)
MR Egger
MR RAPS
Weighted median
Weighted mode
0.1
0.0
−0.1
0.10
0.15
0.20
0.25
0.30
SNP effect on Gut microbiota abundance (genus Sellimonas id.14369)
SNP effect on Extreme obesity with alveolar hypoventilation

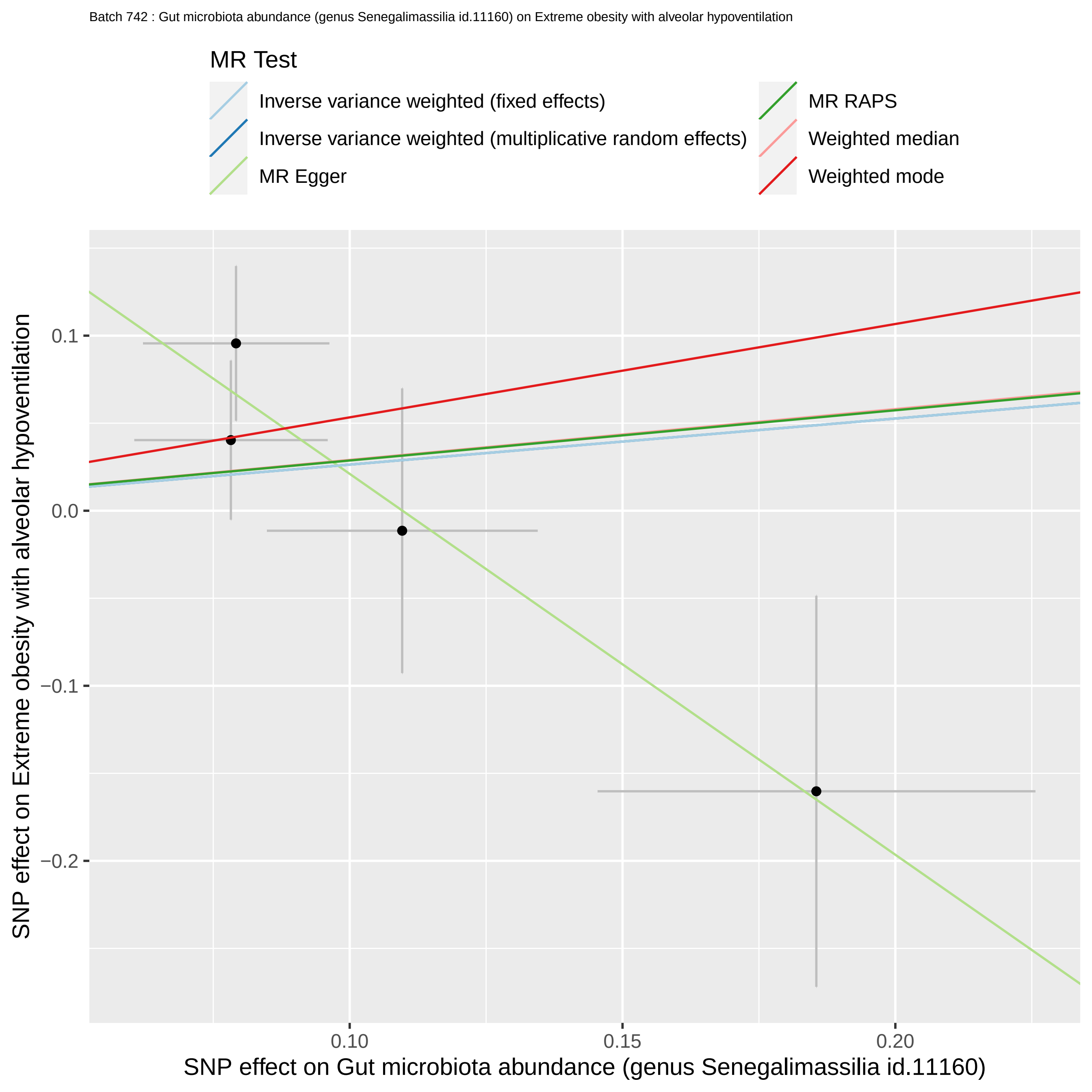
Batch 742 : Gut microbiota abundance (genus Senegalimassilia id.11160) on Extreme obesity with alveolar hypoventilation
MR Test
Inverse variance weighted (fixed effects)
Inverse variance weighted (multiplicative random effects)
MR Egger
MR RAPS
Weighted median
Weighted mode
SNP effect on Extreme obesity with alveolar hypoventilation
0.1
0.0
−0.1
−0.2
0.10
0.15
0.20
SNP effect on Gut microbiota abundance (genus Senegalimassilia id.11160)

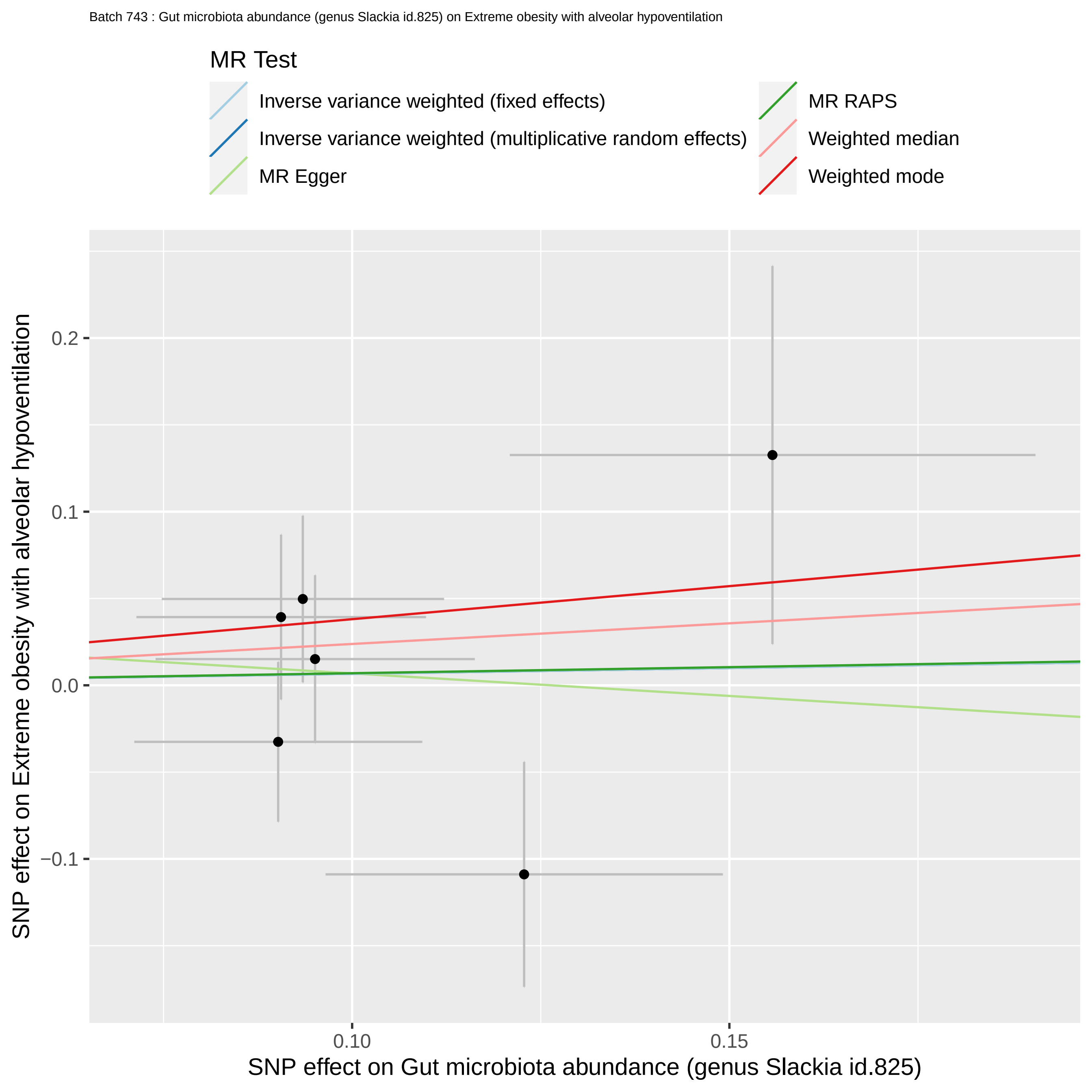
Batch 743 : Gut microbiota abundance (genus Slackia id.825) on Extreme obesity with alveolar hypoventilation
MR Test
Inverse variance weighted (fixed effects)
Inverse variance weighted (multiplicative random effects)
MR Egger
MR RAPS
Weighted median
Weighted mode
0.2
0.1
0.0
−0.1
0.10
0.15
SNP effect on Extreme obesity with alveolar hypoventilation
SNP effect on Gut microbiota abundance (genus Slackia id.825)

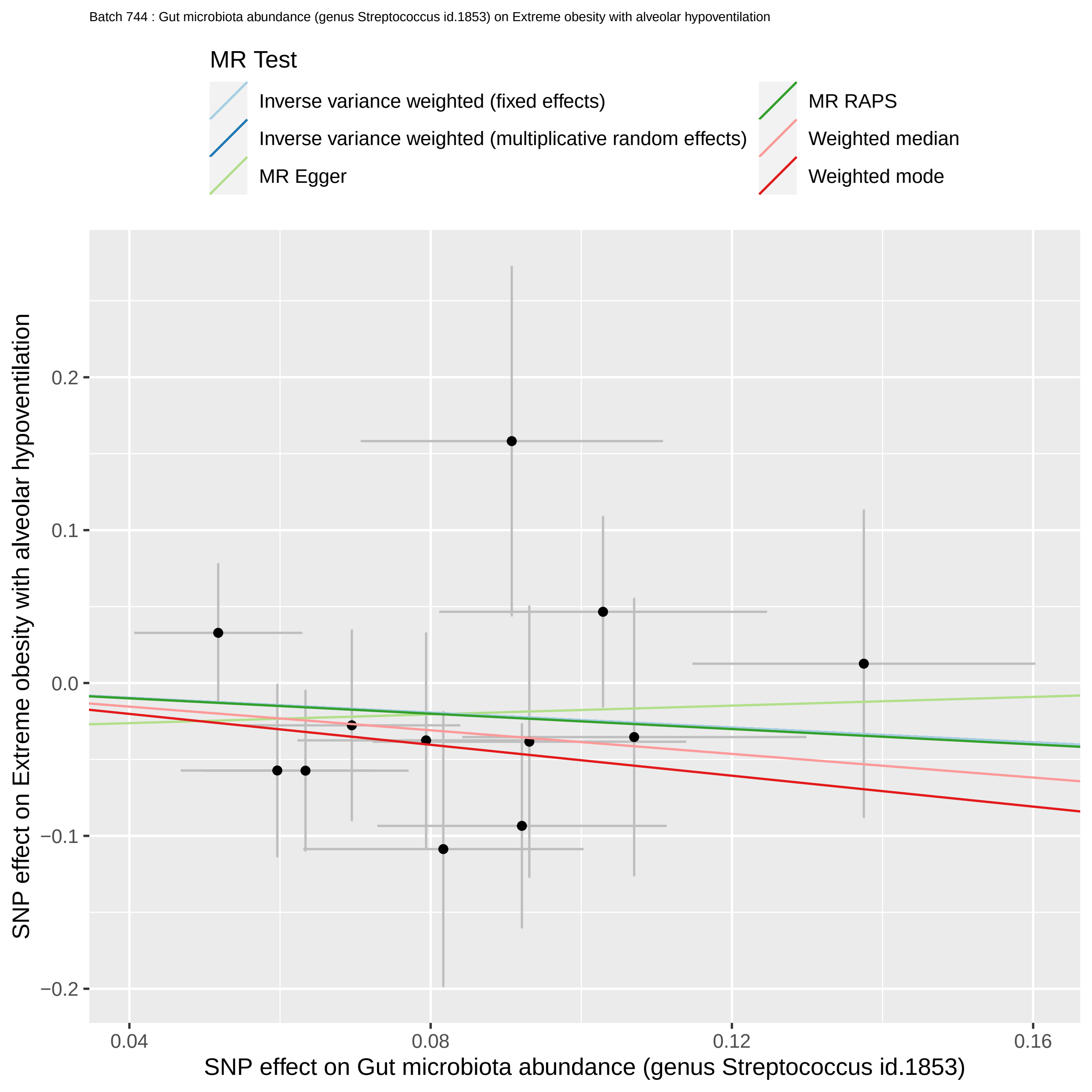
Batch 744 : Gut microbiota abundance (genus Streptococcus id.1853) on Extreme obesity with alveolar hypoventilation
MR Test
Inverse variance weighted (fixed effects)
Inverse variance weighted (multiplicative random effects)
MR Egger
MR RAPS
Weighted median
Weighted mode
SNP effect on Extreme obesity with alveolar hypoventilation
0.2
0.1
0.0
−0.1
−0.2
0.04
0.08
0.12
0.16
SNP effect on Gut microbiota abundance (genus Streptococcus id.1853)

Batch 745 : Gut microbiota abundance (genus Subdoligranulum id.2070) on Extreme obesity with alveolar hypoventilation

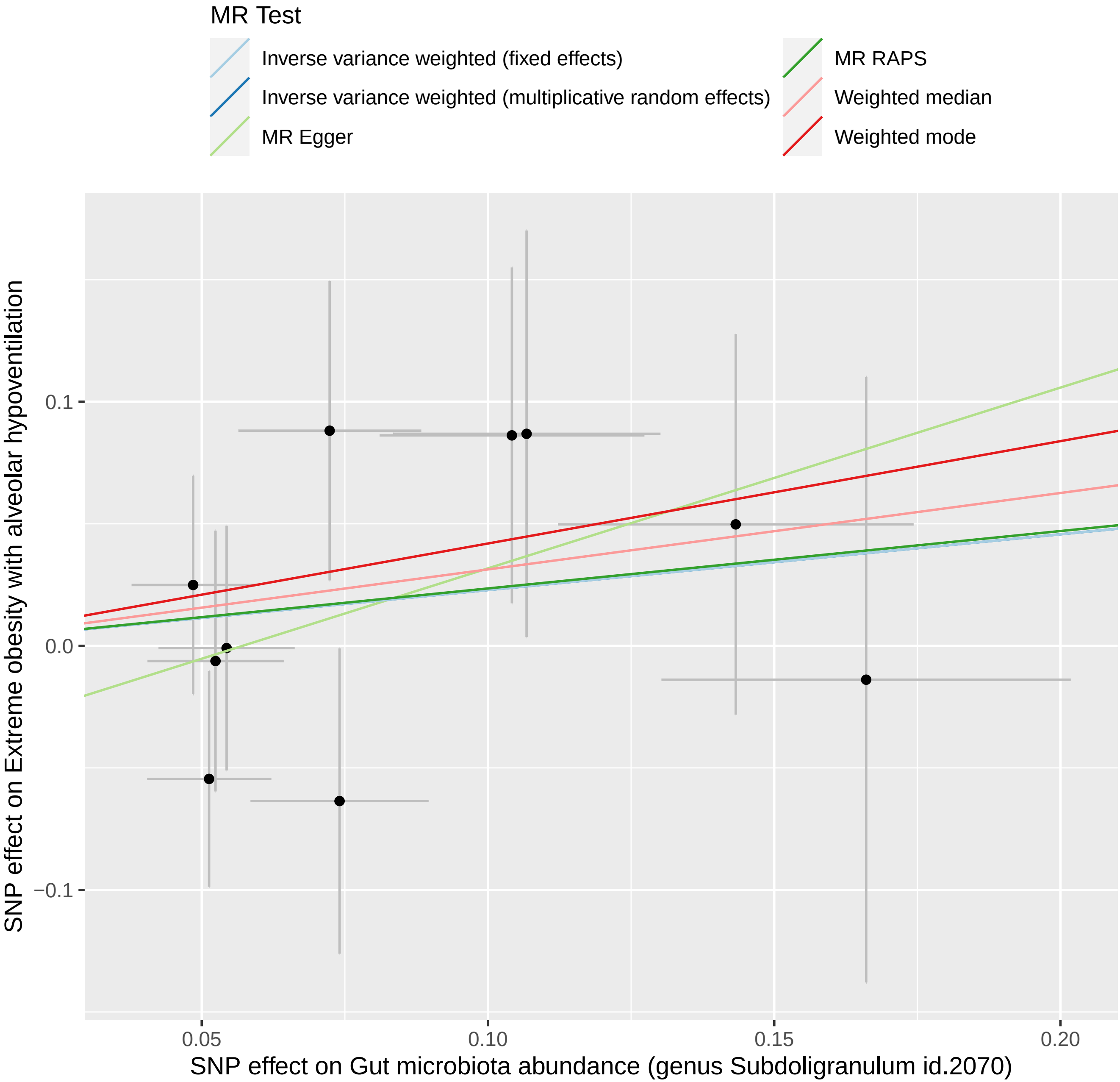

Batch 746 : Gut microbiota abundance (genus Sutterella id.2896) on Extreme obesity with alveolar hypoventilation

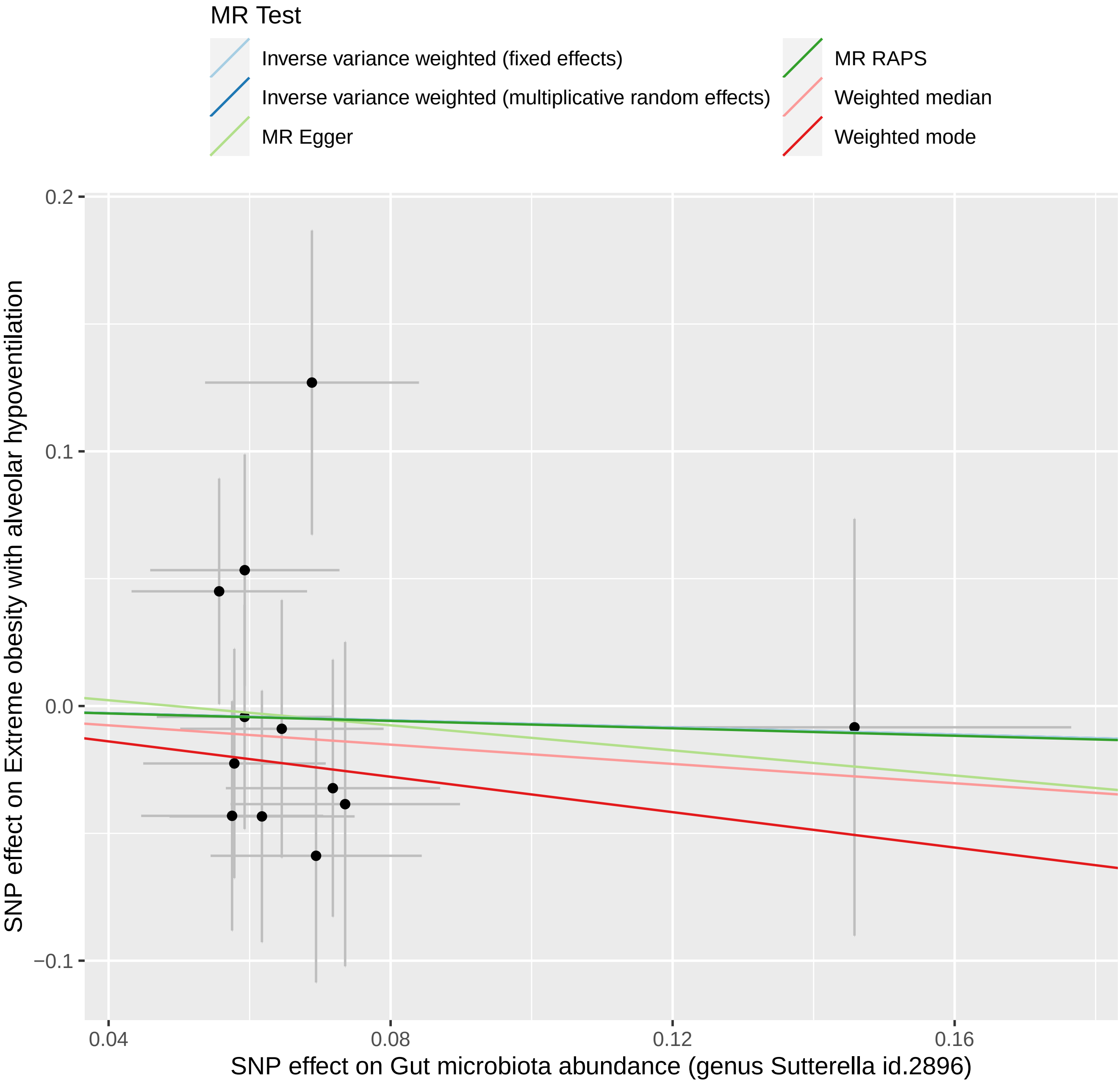

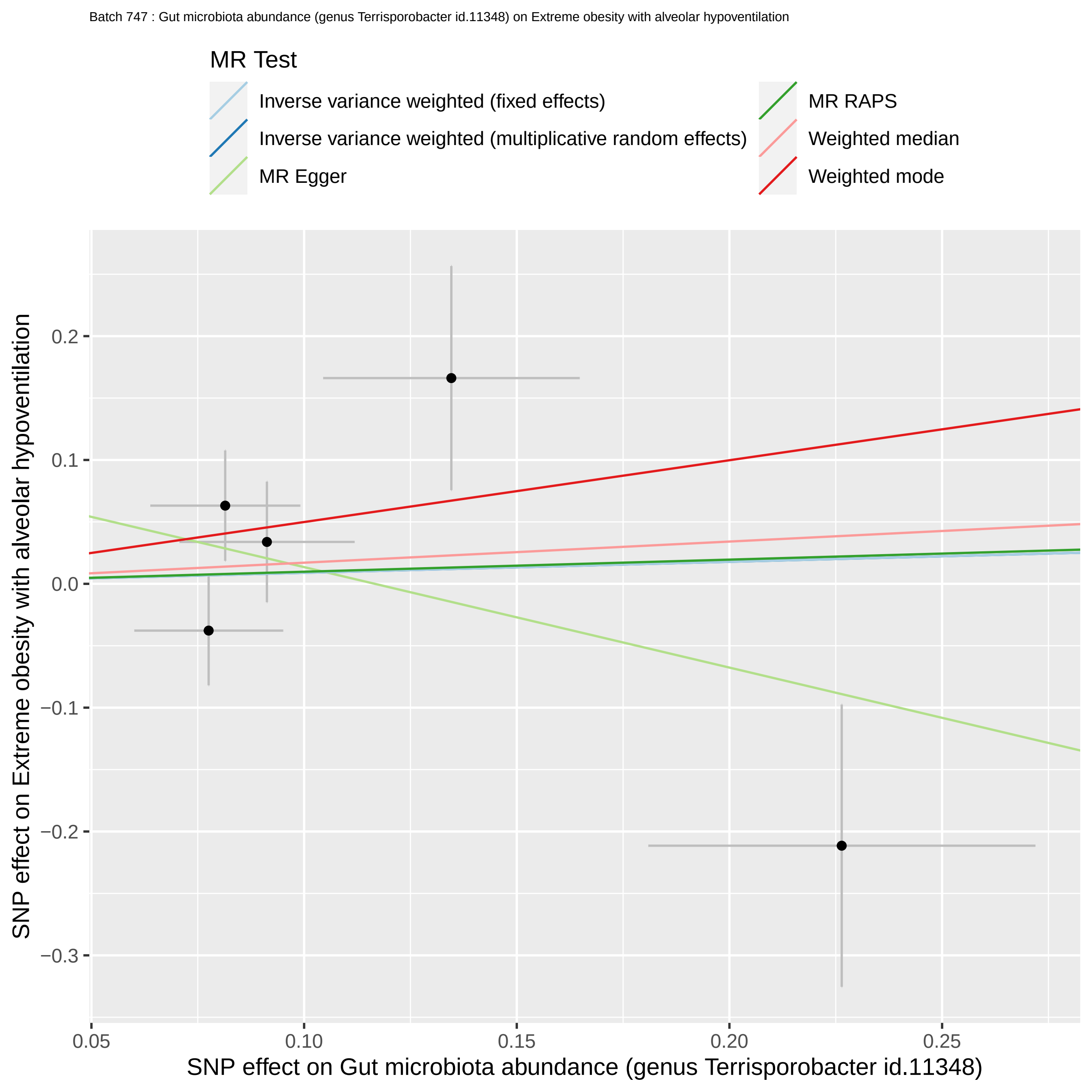
Batch 747 : Gut microbiota abundance (genus Terrisporobacter id.11348) on Extreme obesity with alveolar hypoventilation
MR Test
Inverse variance weighted (fixed effects)
Inverse variance weighted (multiplicative random effects)
MR Egger
MR RAPS
Weighted median
Weighted mode
0.2
0.1
0.0
−0.1
−0.2
−0.3
0.05
0.10
0.15
0.20
0.25
SNP effect on Extreme obesity with alveolar hypoventilation
SNP effect on Gut microbiota abundance (genus Terrisporobacter id.11348)

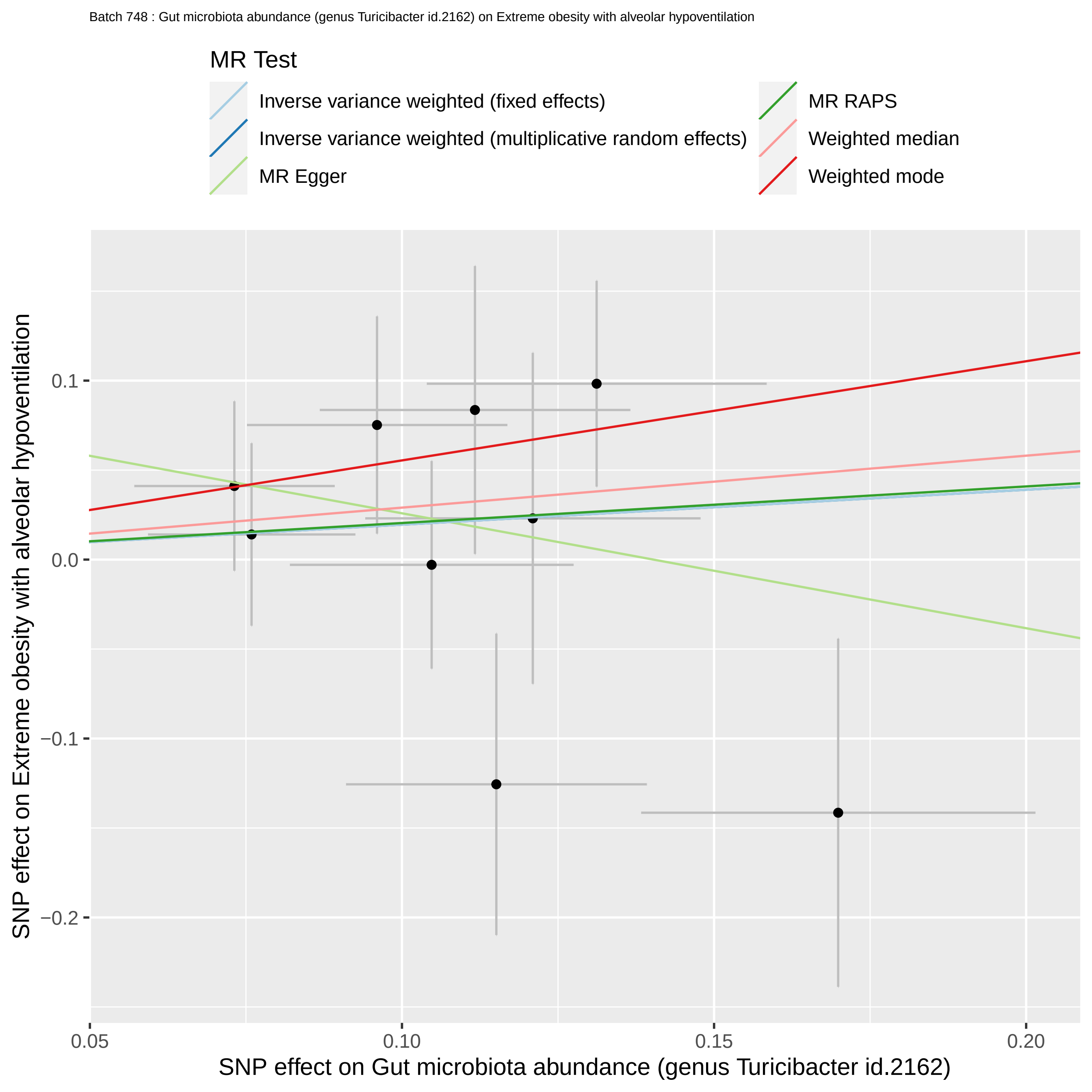

Batch 748 : Gut microbiota abundance (genus Turicibacter id.2162) on Extreme obesity with alveolar hypoventilation
MR Test
Inverse variance weighted (fixed effects)
Inverse variance weighted (multiplicative random effects)
MR Egger
MR RAPS
Weighted median
Weighted mode
0.1
0.0
−0.1
−0.2
0.05
0.10
0.15
0.20
SNP effect on Extreme obesity with alveolar hypoventilation
SNP effect on Gut microbiota abundance (genus Turicibacter id.2162)

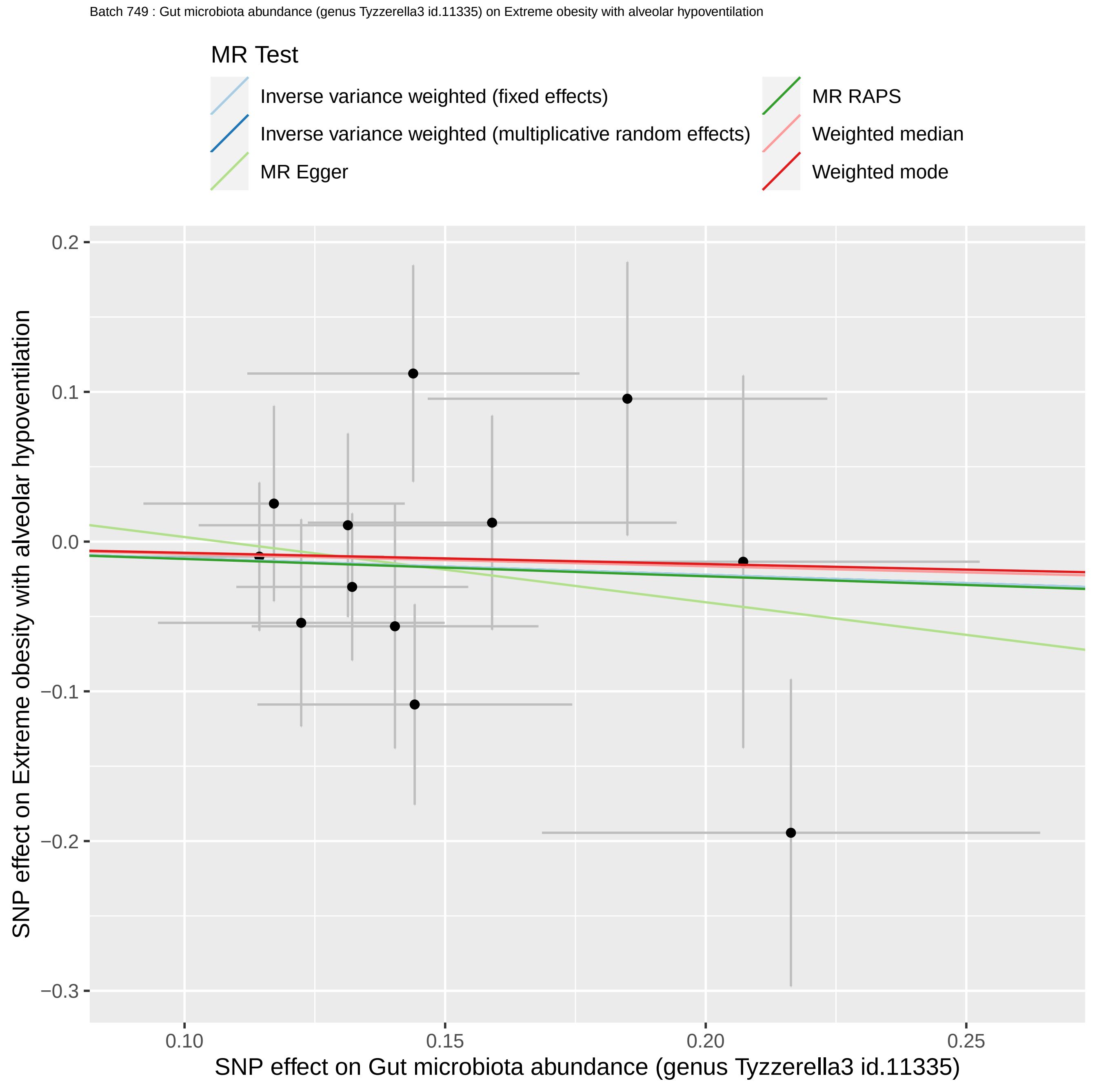
Batch 749 : Gut microbiota abundance (genus Tyzzerella3 id.11335) on Extreme obesity with alveolar hypoventilation
MR Test
Inverse variance weighted (fixed effects)
Inverse variance weighted (multiplicative random effects)
MR Egger
MR RAPS
Weighted median
Weighted mode
0.2
0.1
0.0
−0.1
−0.2
−0.3
0.10
0.15
0.20
0.25
SNP effect on Extreme obesity with alveolar hypoventilation
SNP effect on Gut microbiota abundance (genus Tyzzerella3 id.11335)

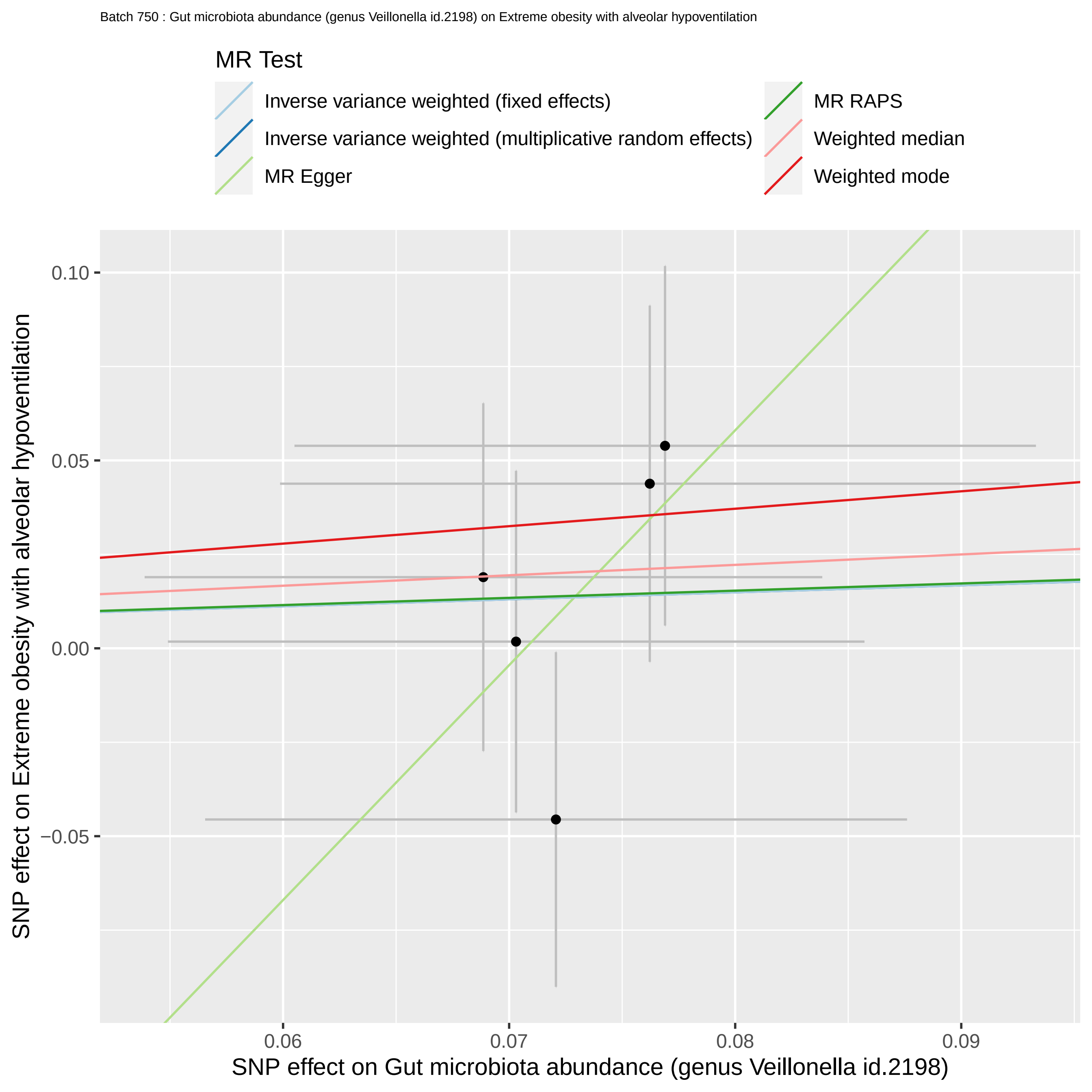
Batch 750 : Gut microbiota abundance (genus Veillonella id.2198) on Extreme obesity with alveolar hypoventilation
MR Test
Inverse variance weighted (fixed effects)
Inverse variance weighted (multiplicative random effects)
MR Egger
MR RAPS
Weighted median
Weighted mode
0.10
0.05
0.00
−0.05
0.06
0.07
0.08
0.09
SNP effect on Extreme obesity with alveolar hypoventilation
SNP effect on Gut microbiota abundance (genus Veillonella id.2198)
