# Supplementary material for: Unraveling the mystery: a Mendelian randomized exploration of gut microbiota and different types of obesity
Source: Front Cell Infect Microbiol. 2024 Feb 5;14:1352109. doi: 10.3389/fcimb.2024.1352109 (PMC10875079; doi:10.3389/fcimb.2024.1352109)
Supplement: Supplementary file 3 [file DataSheet_3.pdf]

Batch 1 : Gut microbiota abundance (family Acidaminococcaceae id.2166) on Localized adiposity

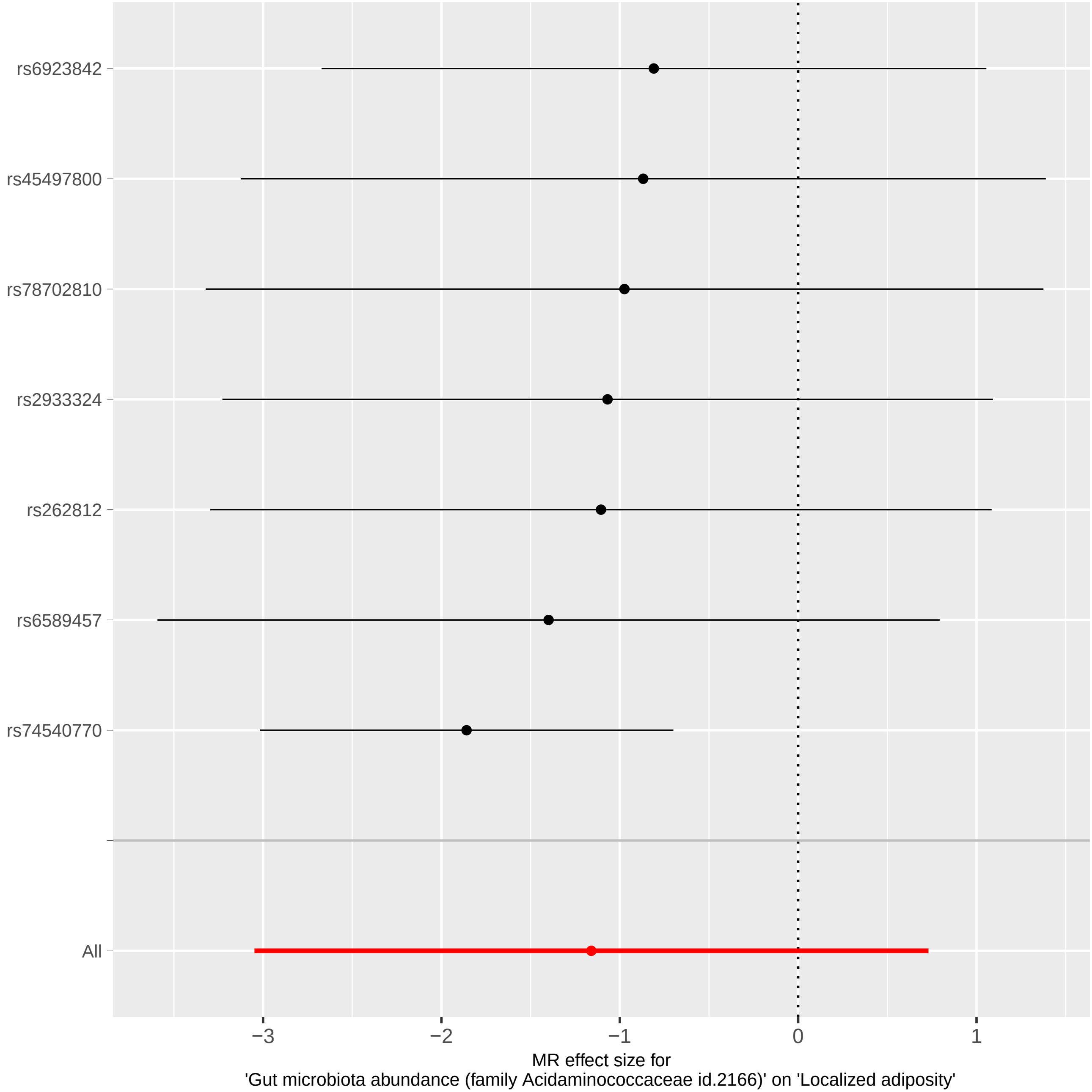

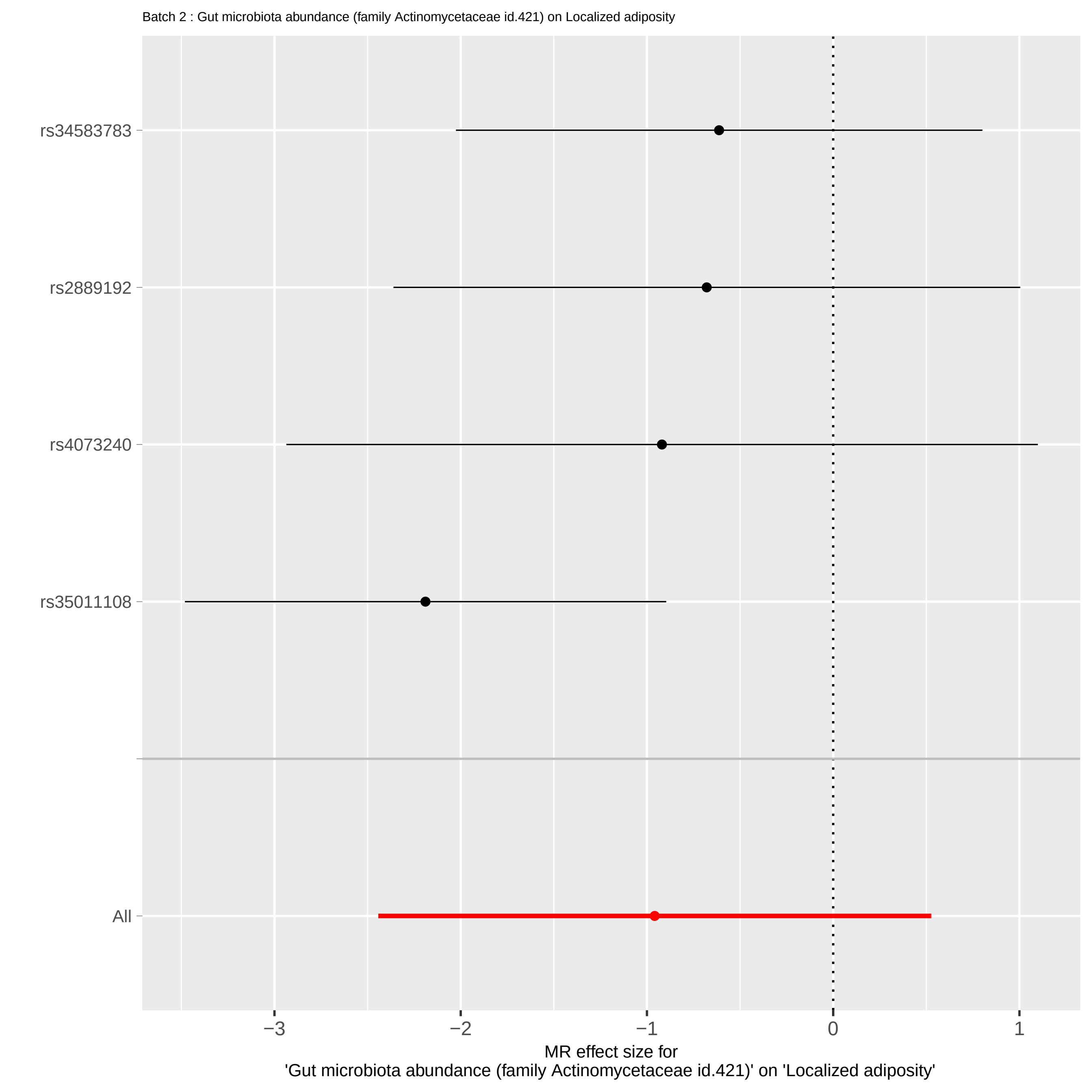
Batch 2 : Gut microbiota abundance (family Actinomycetaceae id.421) on Localized adiposity
rs34583783
rs2889192
rs4073240
rs35011108
All
−3
−2
−1
0
1
MR effect size for
'Gut microbiota abundance (family Actinomycetaceae id.421)' on 'Localized adiposity'

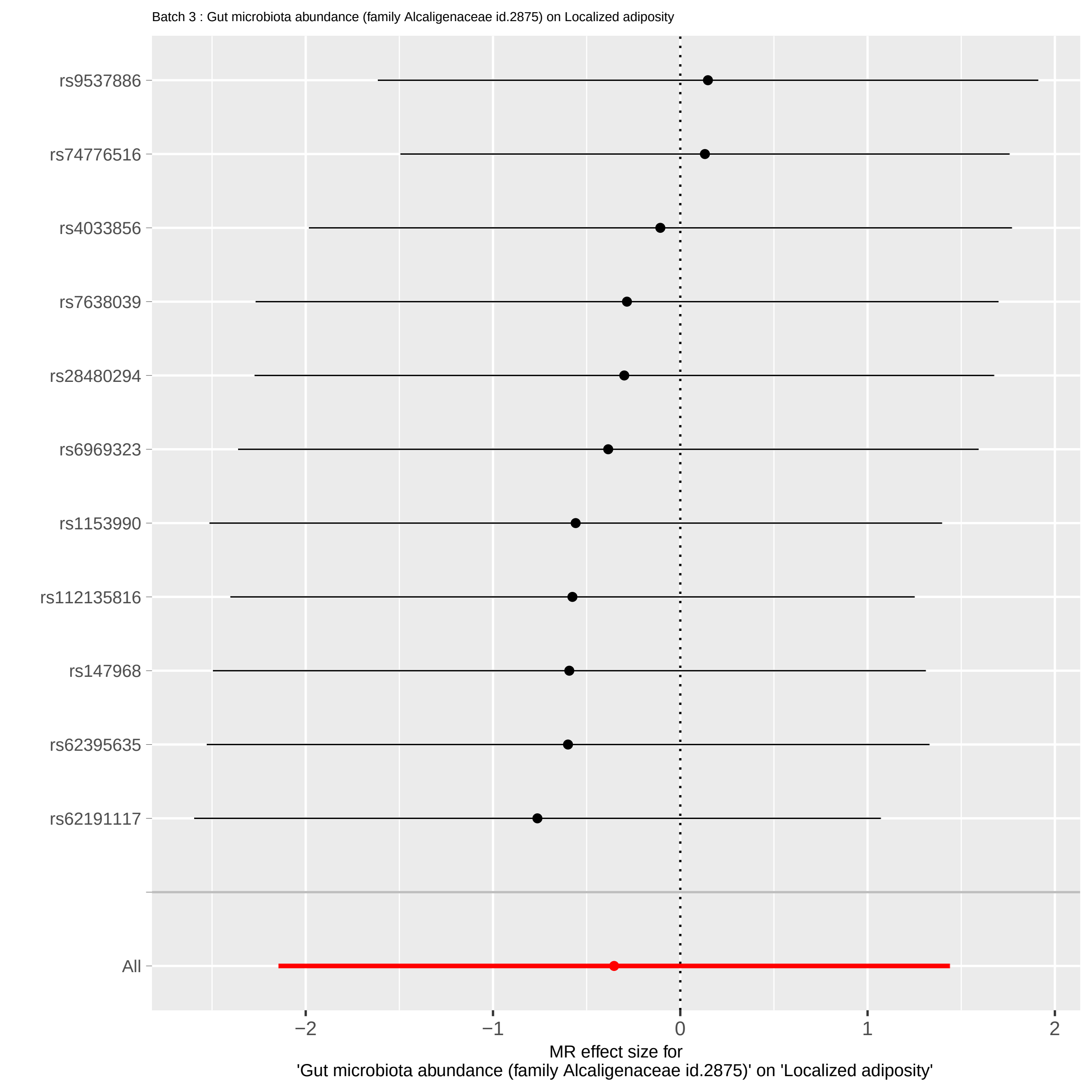
Batch 3 : Gut microbiota abundance (family Alcaligenaceae id.2875) on Localized adiposity
rs9537886
rs74776516
rs4033856
rs7638039
rs28480294
rs6969323
rs1153990
rs112135816
rs147968
rs62395635
rs62191117
All
−2
−1
0
1
2
MR effect size for
'Gut microbiota abundance (family Alcaligenaceae id.2875)' on 'Localized adiposity'

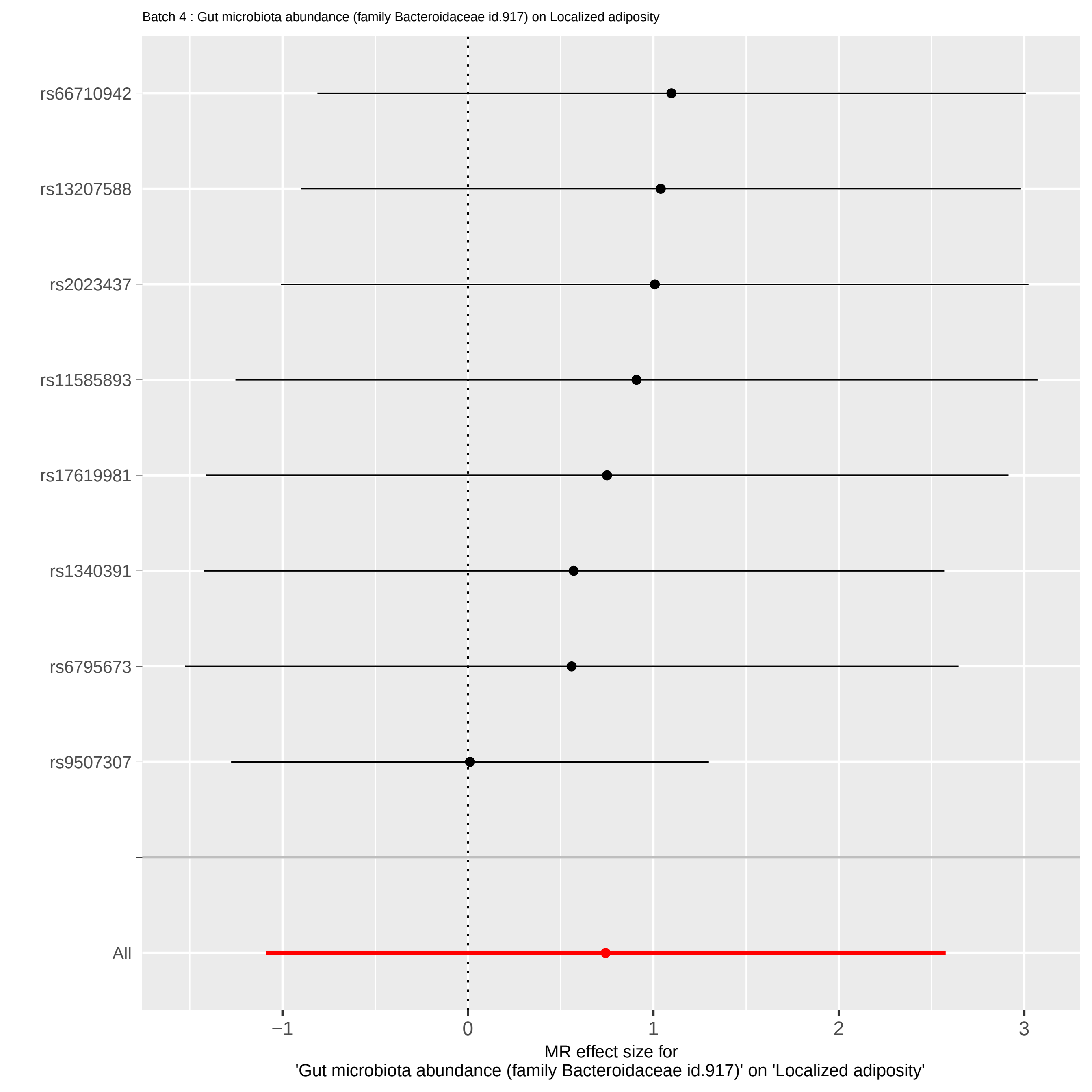

Batch 4 : Gut microbiota abundance (family Bacteroidaceae id.917) on Localized adiposity
rs66710942
rs13207588
rs2023437
rs11585893
rs17619981
rs1340391
rs6795673
rs9507307
All
−1
0
1
2
3
MR effect size for
'Gut microbiota abundance (family Bacteroidaceae id.917)' on 'Localized adiposity'

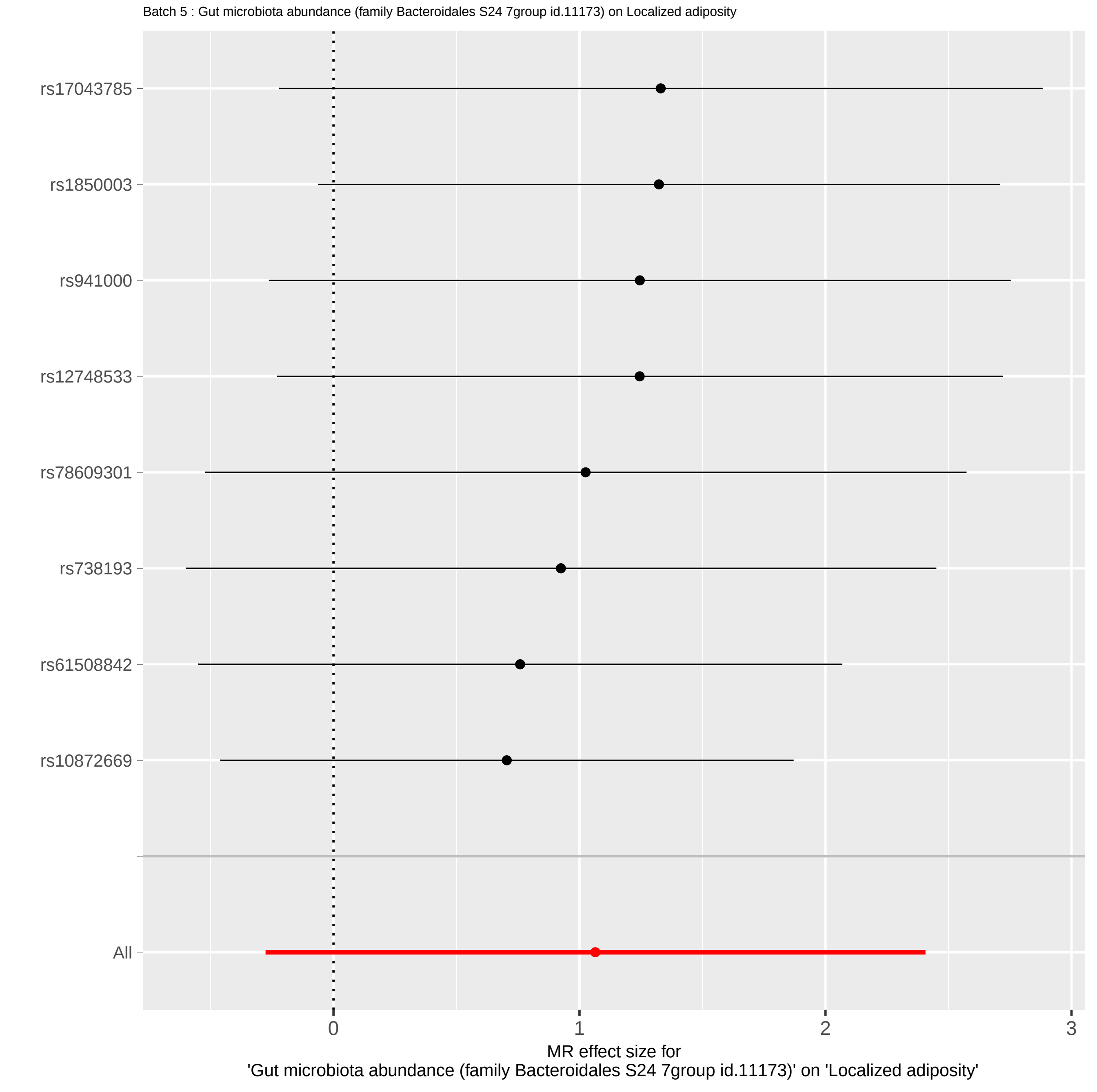
Batch 5 : Gut microbiota abundance (family Bacteroidales S24 7group id.11173) on Localized adiposity
rs17043785
rs1850003
rs941000
rs12748533
rs78609301
rs738193
rs61508842
rs10872669
All
0
1
2
3
MR effect size for
'Gut microbiota abundance (family Bacteroidales S24 7group id.11173)' on 'Localized adiposity'

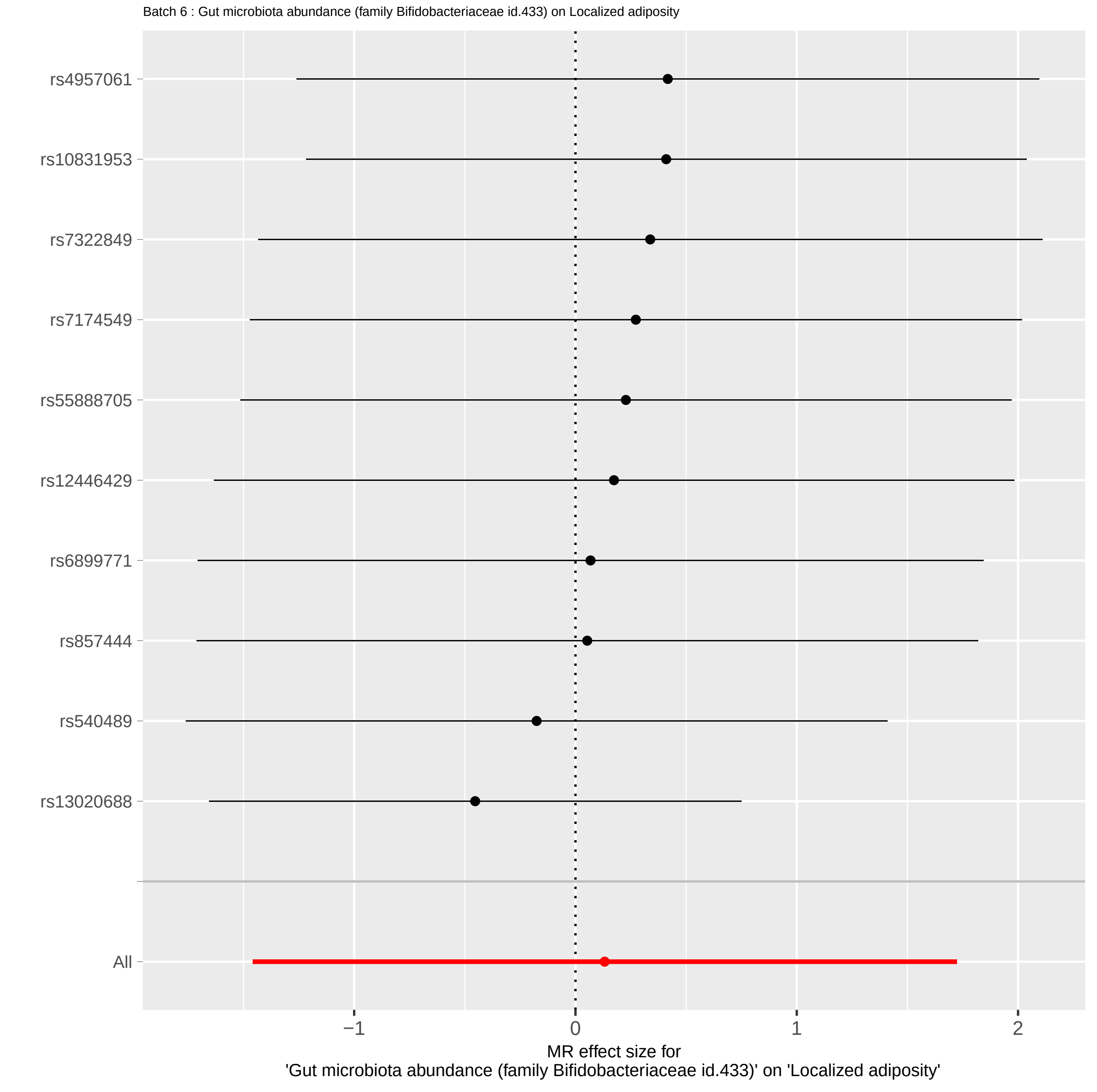

Batch 6 : Gut microbiota abundance (family Bifidobacteriaceae id.433) on Localized adiposity
rs4957061
rs10831953
rs7322849
rs7174549
rs55888705
rs12446429
rs6899771
rs857444
rs540489
rs13020688
All
−1
0
1
2
MR effect size for
'Gut microbiota abundance (family Bifidobacteriaceae id.433)' on 'Localized adiposity'

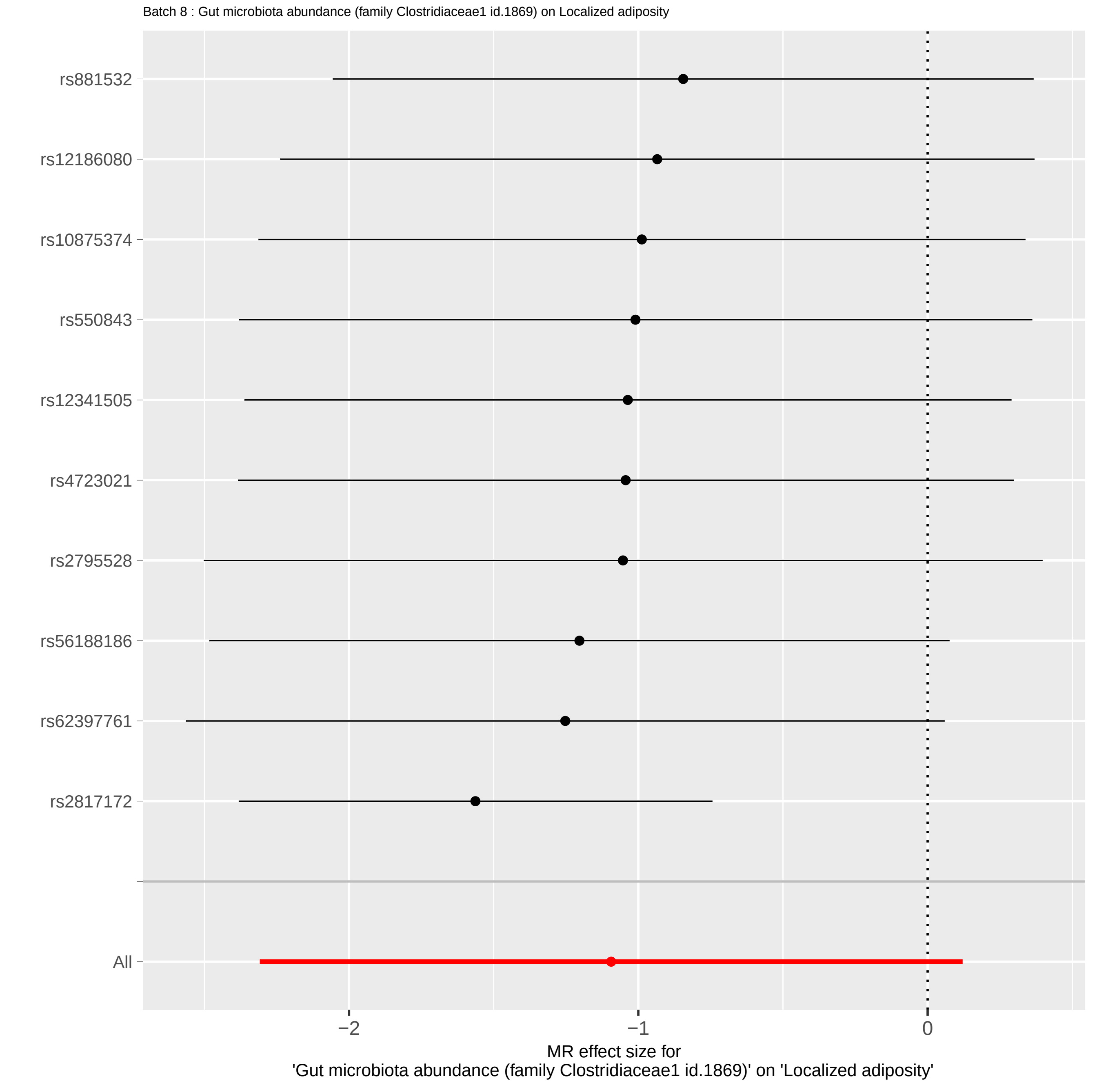

Batch 8 : Gut microbiota abundance (family Clostridiaceae1 id.1869) on Localized adiposity
rs881532
rs12186080
rs10875374
rs550843
rs12341505
rs4723021
rs2795528
rs56188186
rs62397761
rs2817172
All
−2
−1
0
MR effect size for
'Gut microbiota abundance (family Clostridiaceae1 id.1869)' on 'Localized adiposity'

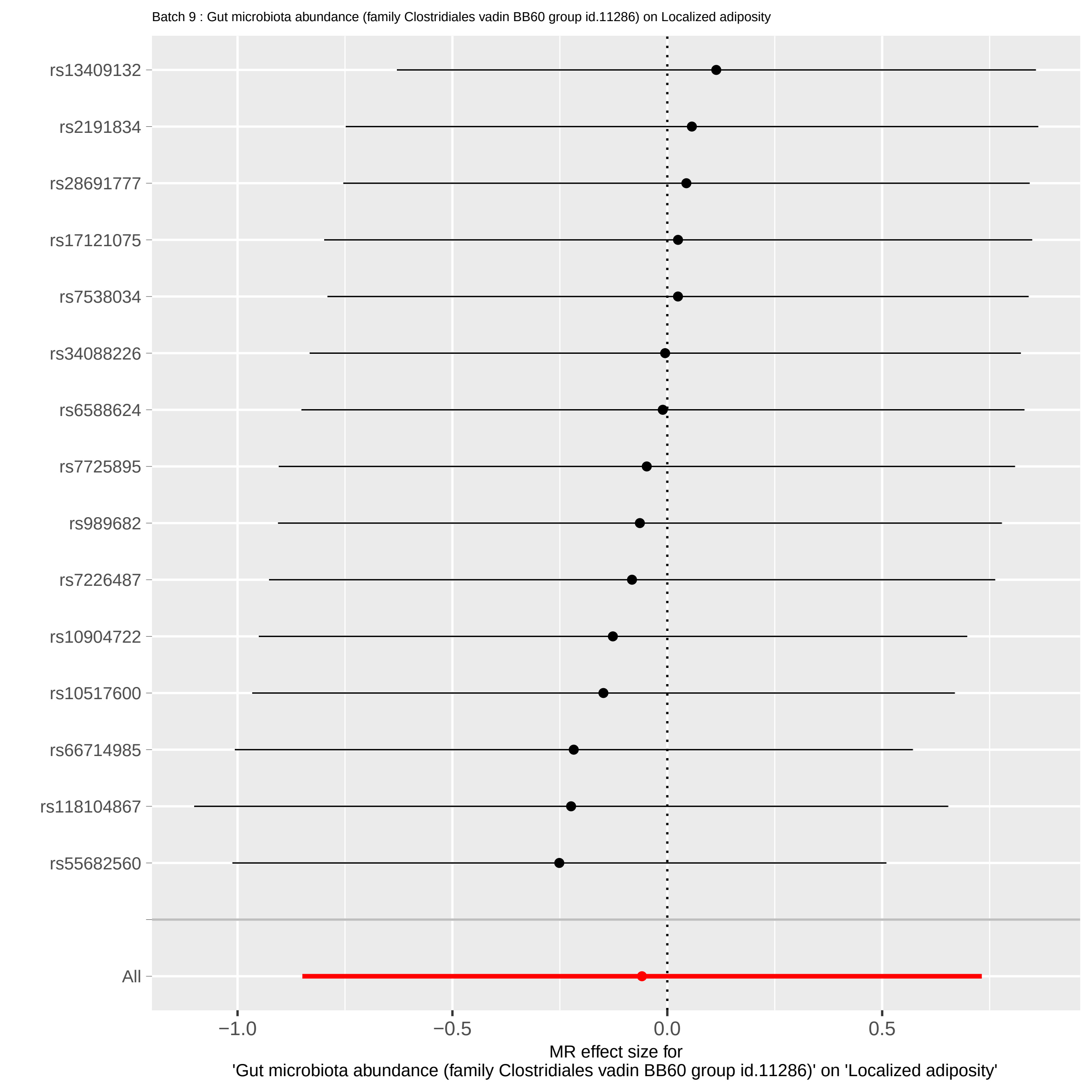
Batch 9 : Gut microbiota abundance (family Clostridiales vadin BB60 group id.11286) on Localized adiposity
rs13409132
rs2191834
rs28691777
rs17121075
rs7538034
rs34088226
rs6588624
rs7725895
rs989682
rs7226487
rs10904722
rs10517600
rs66714985
rs118104867
rs55682560
All
−1.0
−0.5
0.0
0.5
MR effect size for
'Gut microbiota abundance (family Clostridiales vadin BB60 group id.11286)' on 'Localized adiposity'

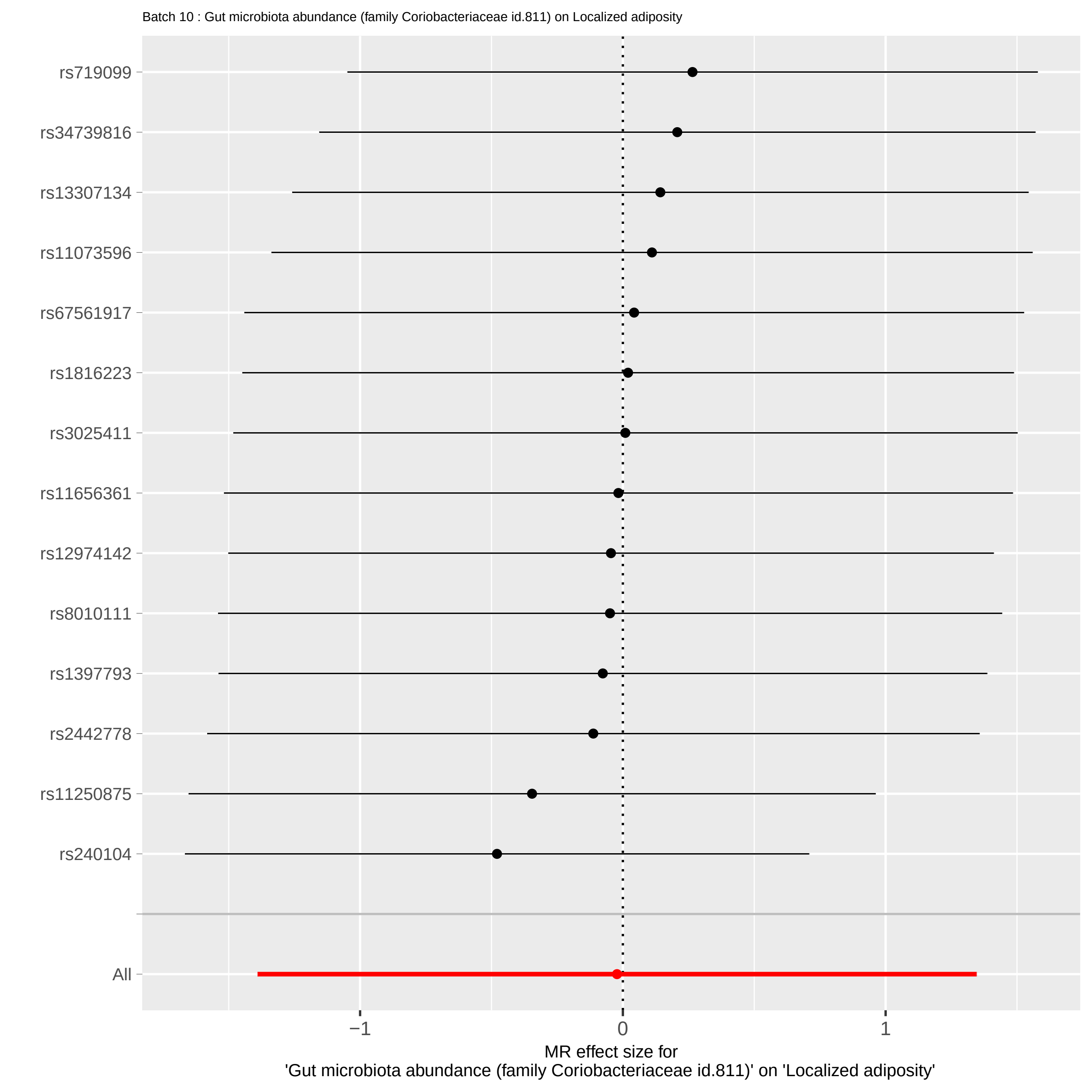

Batch 10 : Gut microbiota abundance (family Coriobacteriaceae id.811) on Localized adiposity
rs719099
rs34739816
rs13307134
rs11073596
rs67561917
rs1816223
rs3025411
rs11656361
rs12974142
rs8010111
rs1397793
rs2442778
rs11250875
rs240104
All
−1
0
1
MR effect size for
'Gut microbiota abundance (family Coriobacteriaceae id.811)' on 'Localized adiposity'

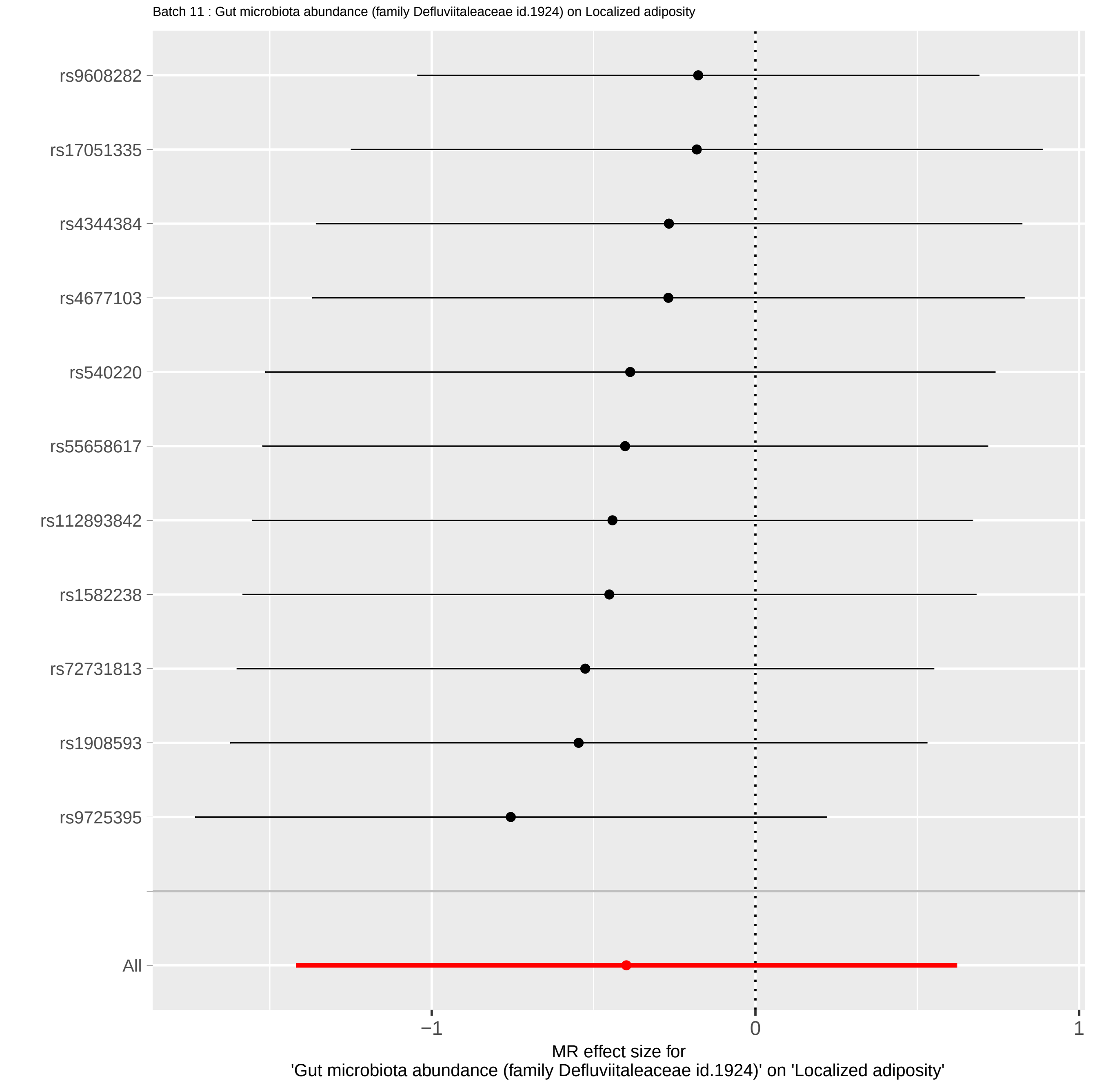
Batch 11 : Gut microbiota abundance (family Defluviitaleaceae id.1924) on Localized adiposity
rs9608282
rs17051335
rs4344384
rs4677103
rs540220
rs55658617
rs112893842
rs1582238
rs72731813
rs1908593
rs9725395
All
−1
0
1
MR effect size for
'Gut microbiota abundance (family Defluviitaleaceae id.1924)' on 'Localized adiposity'

Batch 12 : Gut microbiota abundance (family Desulfovibrionaceae id.3169) on Localized adiposity

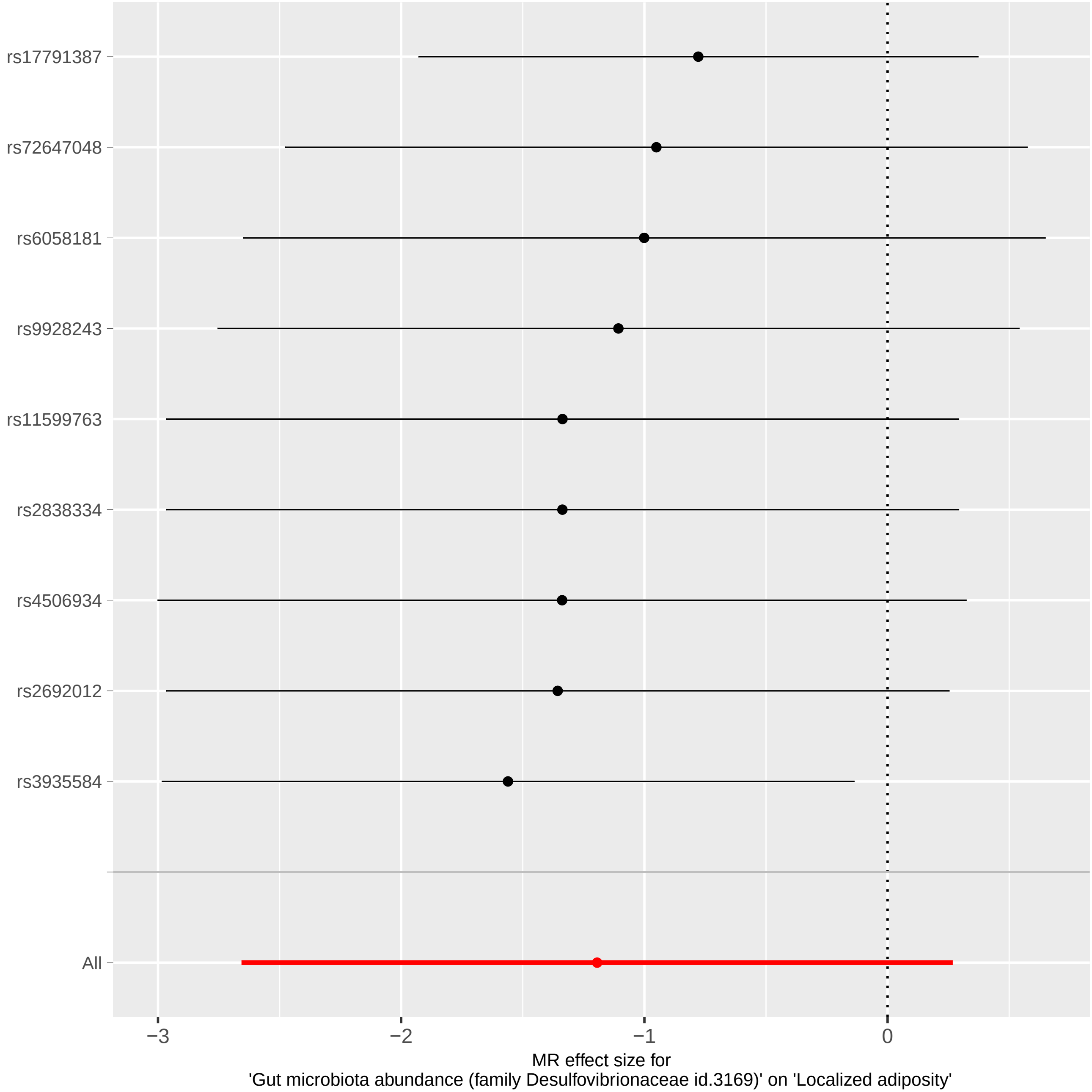

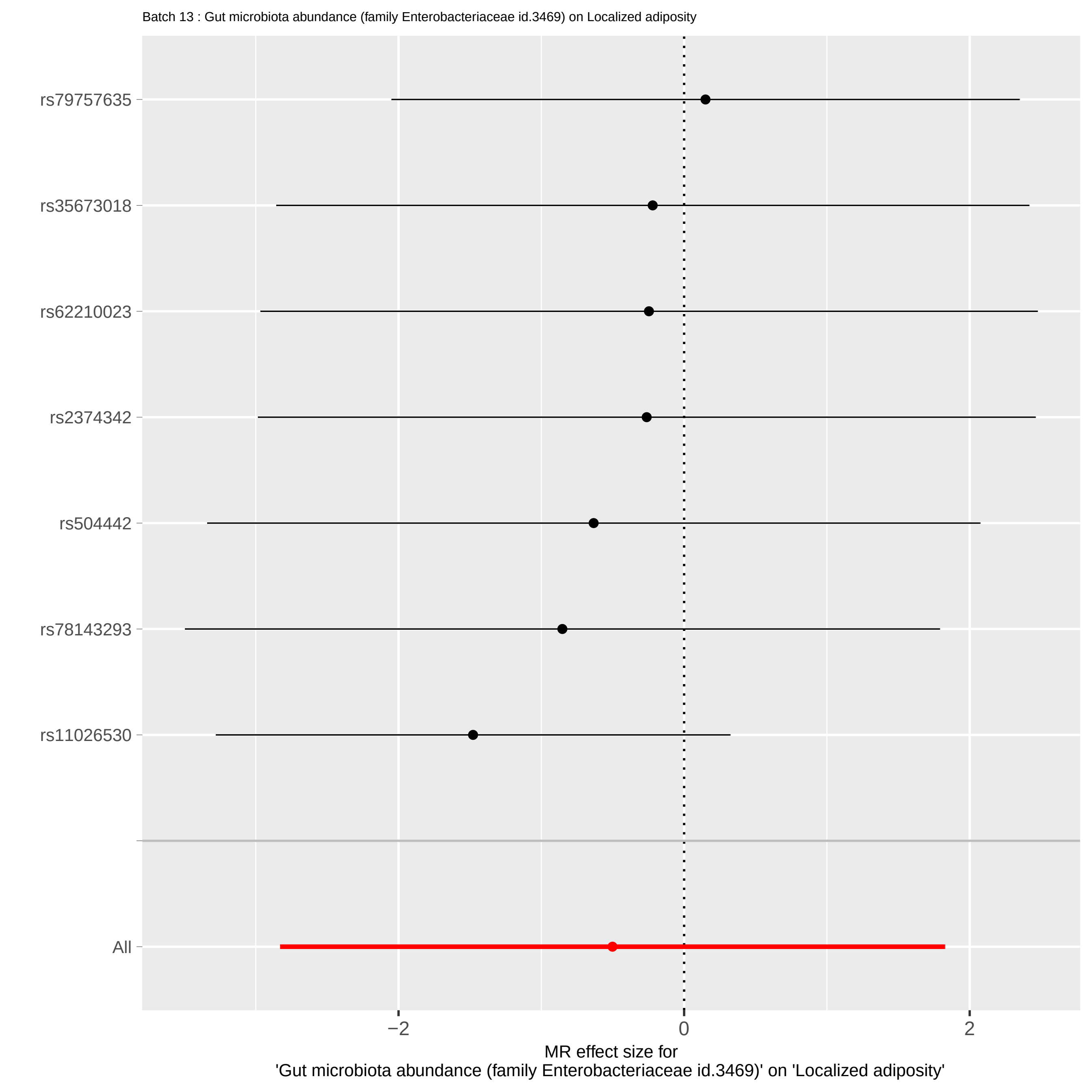

Batch 13 : Gut microbiota abundance (family Enterobacteriaceae id.3469) on Localized adiposity
rs79757635
rs35673018
rs62210023
rs2374342
rs504442
rs78143293
rs11026530
All
−2
0
2
MR effect size for
'Gut microbiota abundance (family Enterobacteriaceae id.3469)' on 'Localized adiposity'

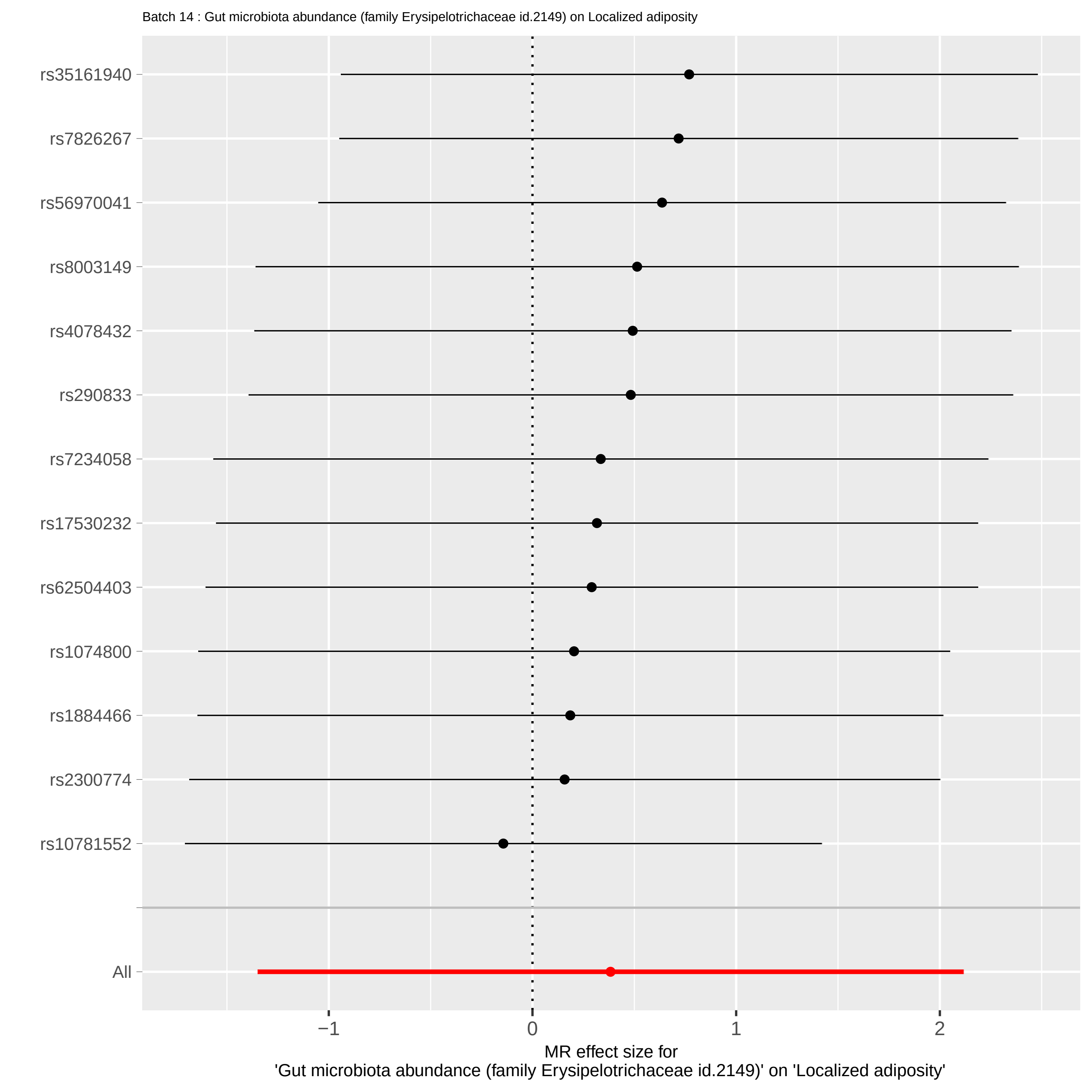
Batch 14 : Gut microbiota abundance (family Erysipelotrichaceae id.2149) on Localized adiposity
rs35161940
rs7826267
rs56970041
rs8003149
rs4078432
rs290833
rs7234058
rs17530232
rs62504403
rs1074800
rs1884466
rs2300774
rs10781552
All
−1
0
1
2
MR effect size for
'Gut microbiota abundance (family Erysipelotrichaceae id.2149)' on 'Localized adiposity'

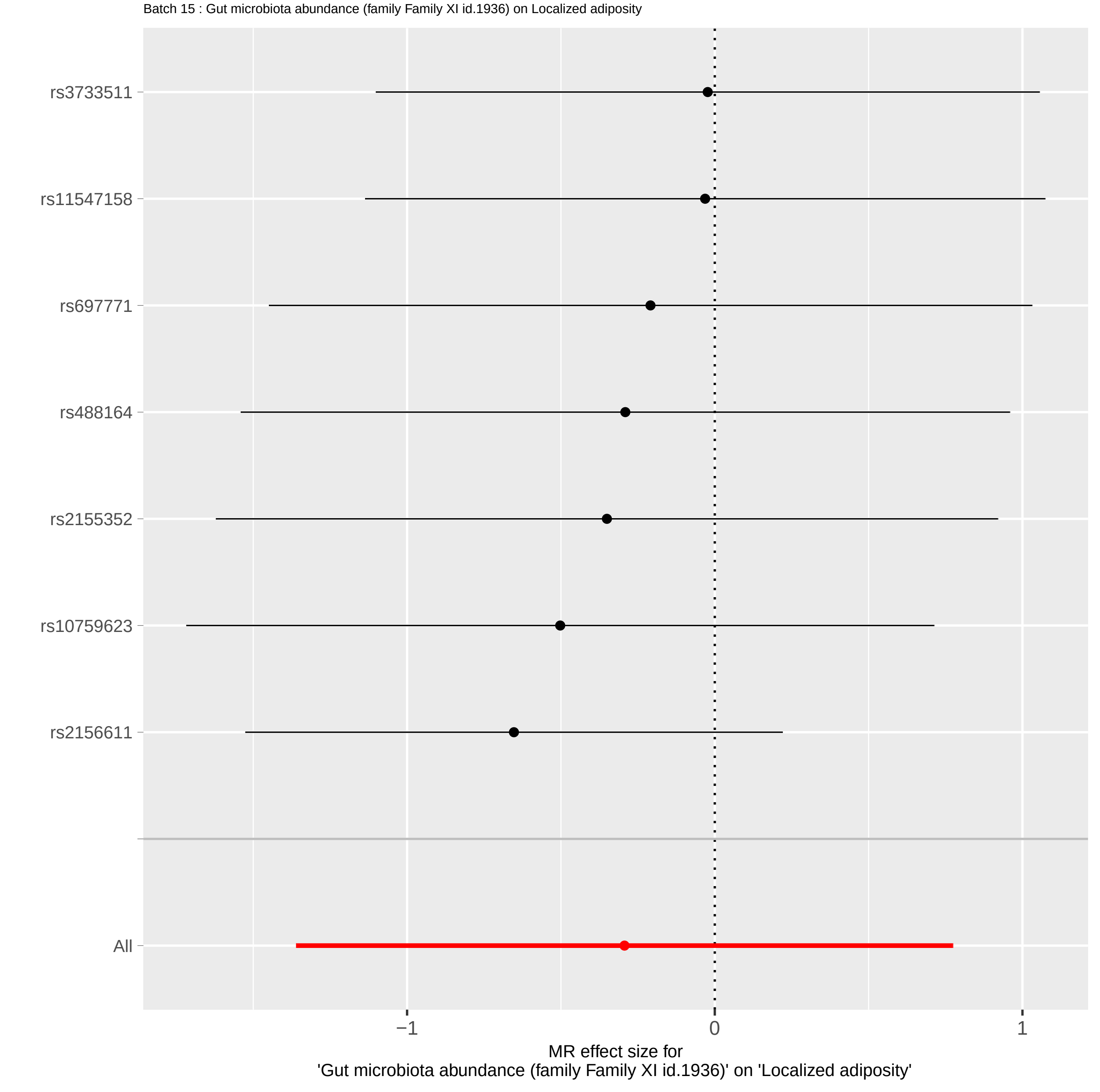

Batch 15 : Gut microbiota abundance (family Family XI id.1936) on Localized adiposity
rs3733511
rs11547158
rs697771
rs488164
rs2155352
rs10759623
rs2156611
All
−1
0
1
MR effect size for
'Gut microbiota abundance (family Family XI id.1936)' on 'Localized adiposity'

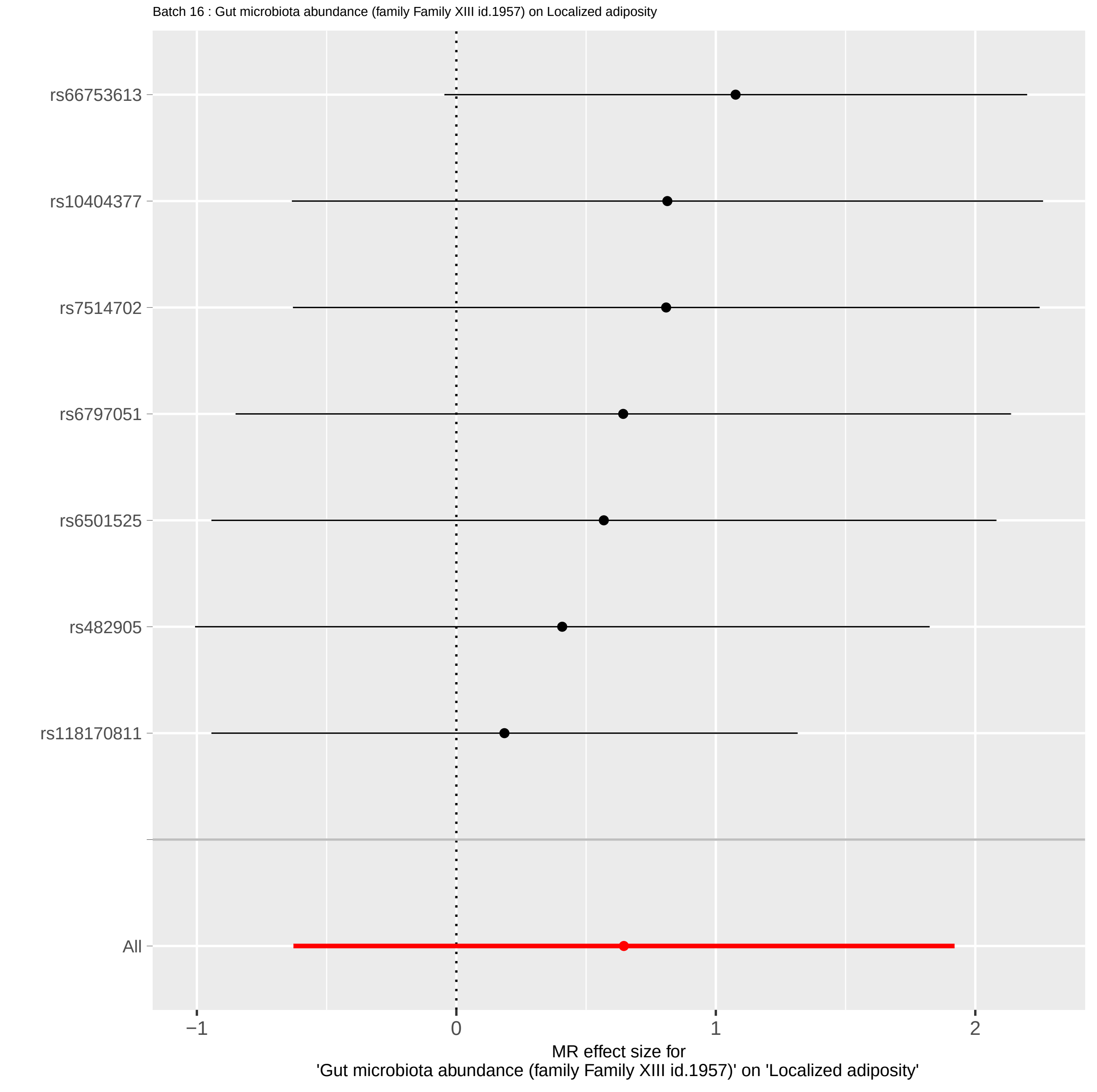

Batch 16 : Gut microbiota abundance (family Family XIII id.1957) on Localized adiposity
rs66753613
rs10404377
rs7514702
rs6797051
rs6501525
rs482905
rs118170811
All
−1
0
1
2
MR effect size for
'Gut microbiota abundance (family Family XIII id.1957)' on 'Localized adiposity'

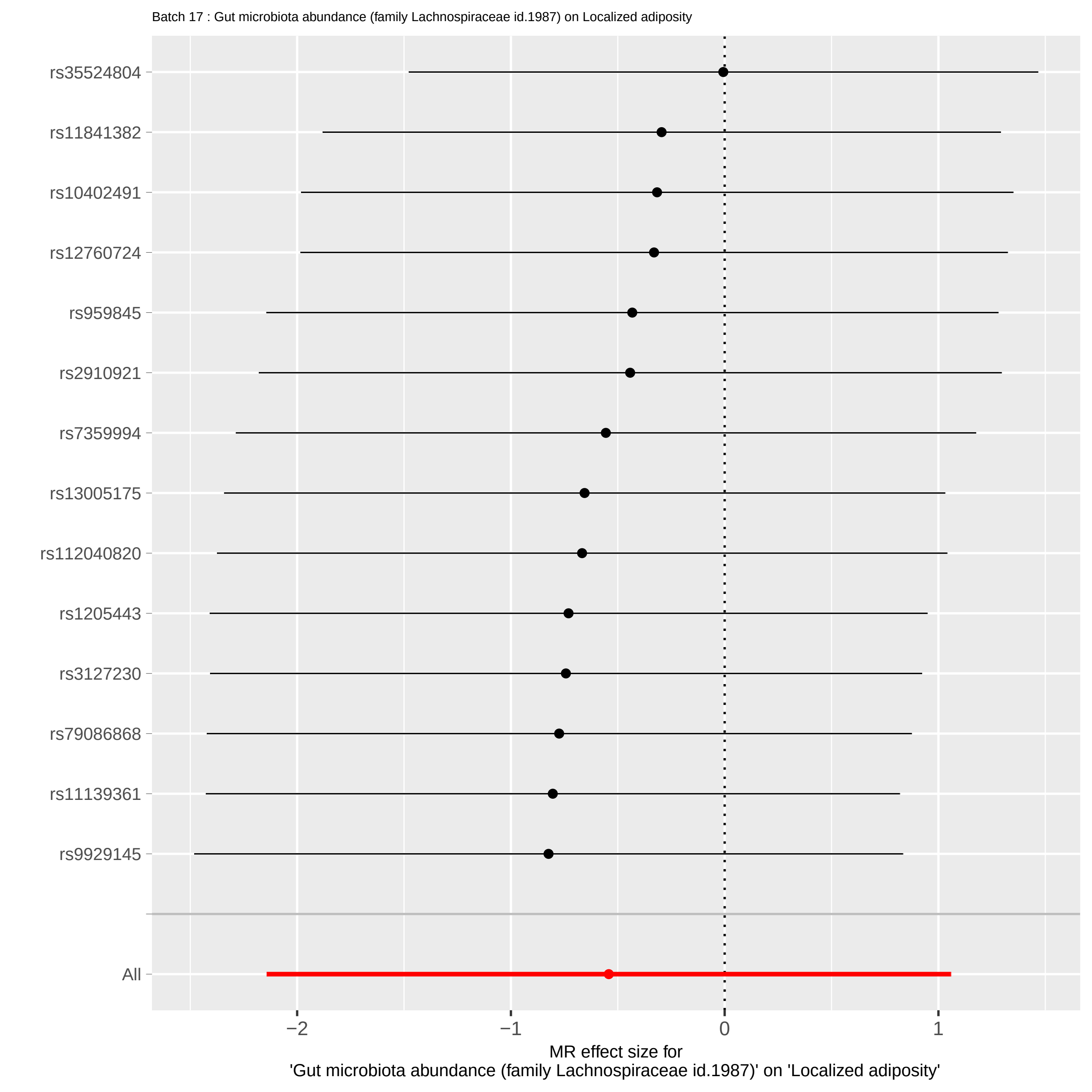
Batch 17 : Gut microbiota abundance (family Lachnospiraceae id.1987) on Localized adiposity
rs35524804
rs11841382
rs10402491
rs12760724
rs959845
rs2910921
rs7359994
rs13005175
rs112040820
rs1205443
rs3127230
rs79086868
rs11139361
rs9929145
All
−2
−1
0
1
MR effect size for
'Gut microbiota abundance (family Lachnospiraceae id.1987)' on 'Localized adiposity'

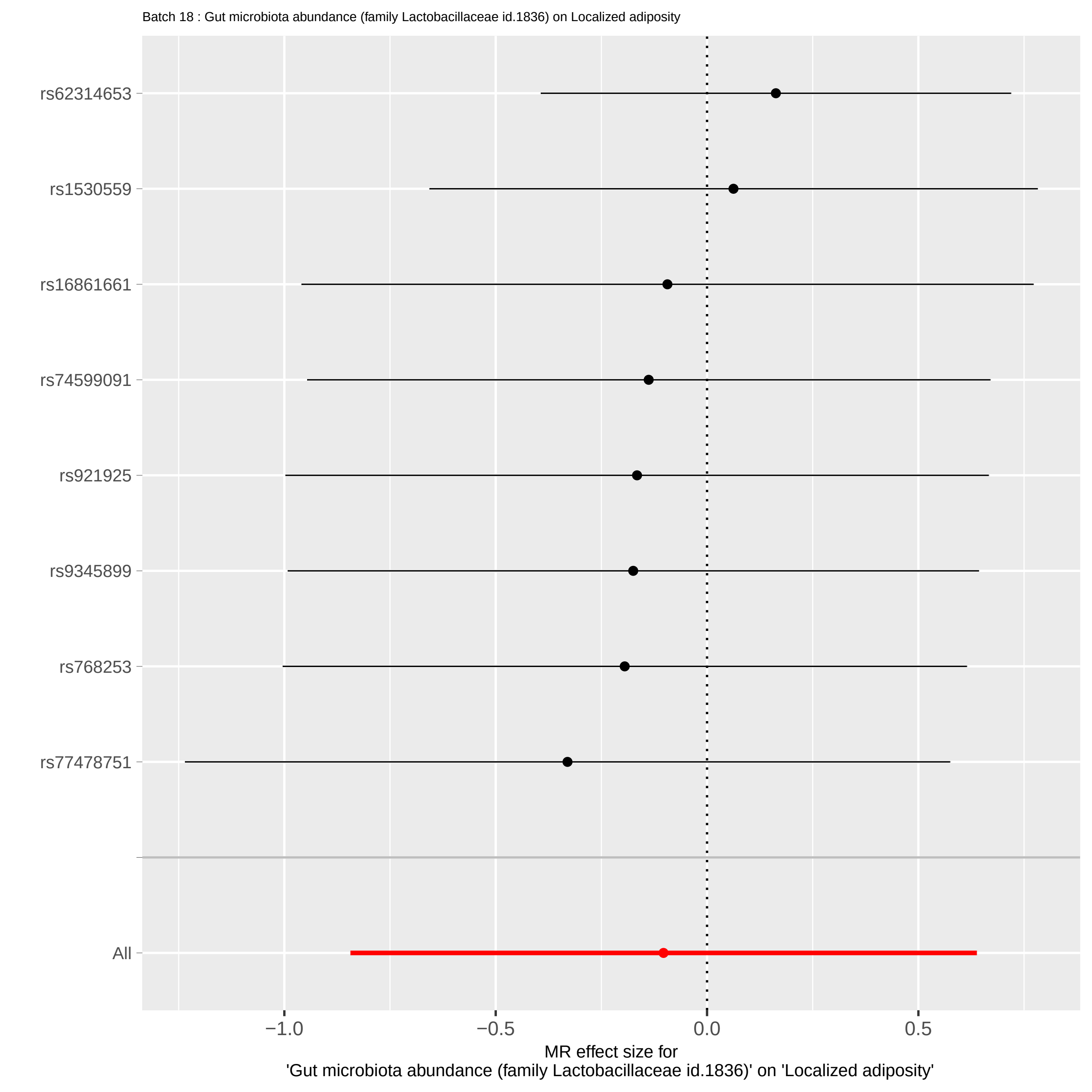

Batch 18 : Gut microbiota abundance (family Lactobacillaceae id.1836) on Localized adiposity
rs62314653
rs1530559
rs16861661
rs74599091
rs921925
rs9345899
rs768253
rs77478751
All
−1.0
−0.5
0.0
0.5
MR effect size for
'Gut microbiota abundance (family Lactobacillaceae id.1836)' on 'Localized adiposity'

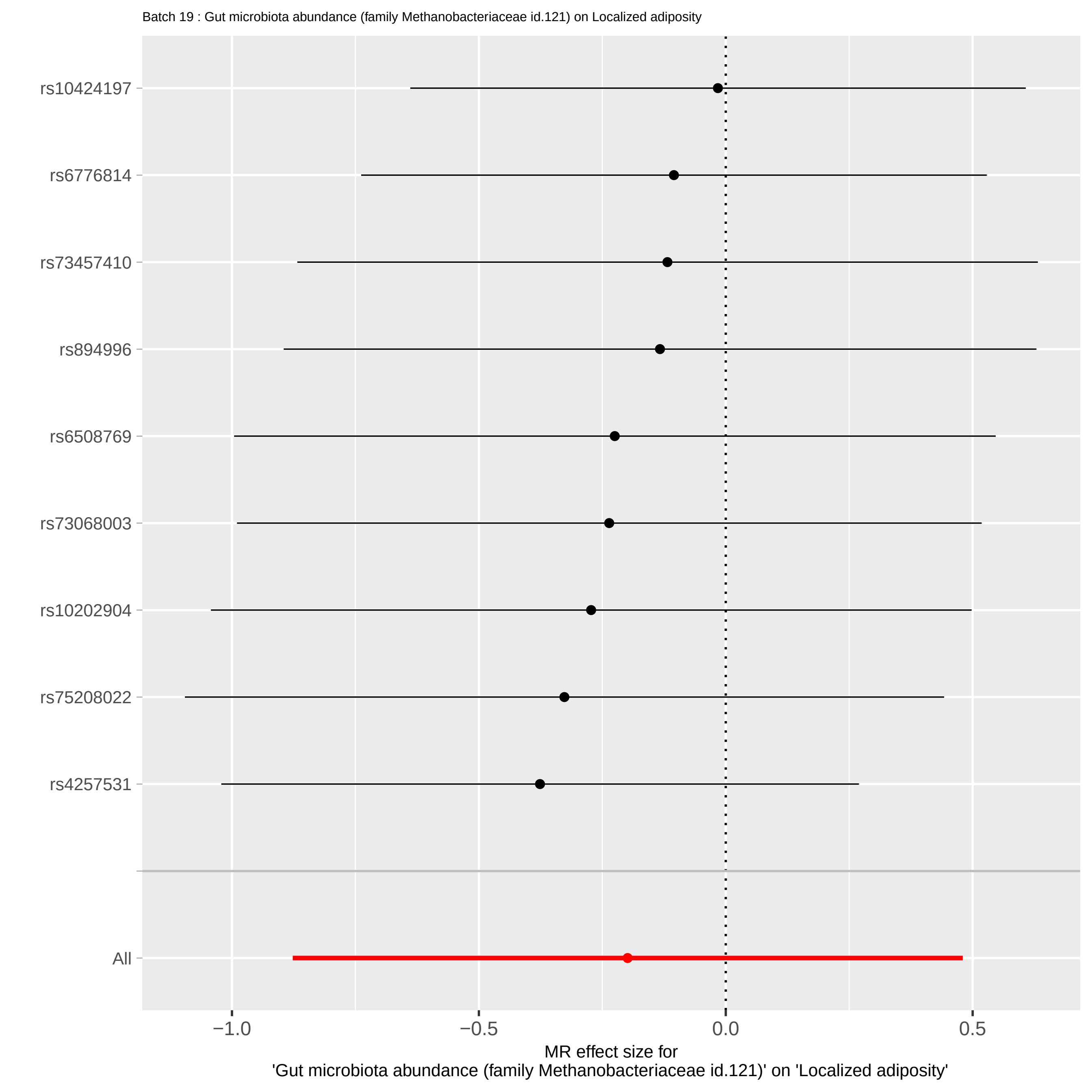
Batch 19 : Gut microbiota abundance (family Methanobacteriaceae id.121) on Localized adiposity
rs10424197
rs6776814
rs73457410
rs894996
rs6508769
rs73068003
rs10202904
rs75208022
rs4257531
All
−1.0
−0.5
0.0
0.5
MR effect size for
'Gut microbiota abundance (family Methanobacteriaceae id.121)' on 'Localized adiposity'

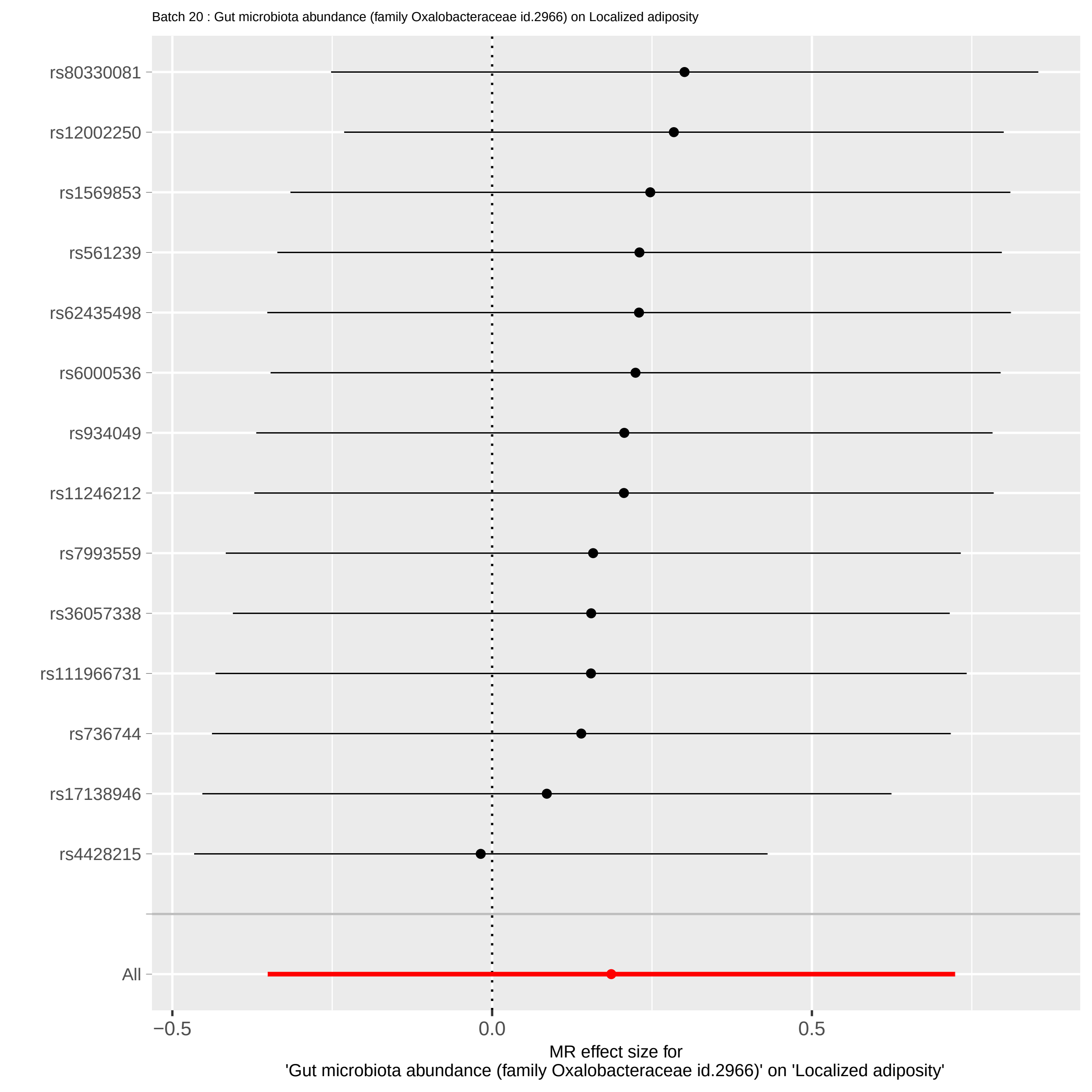

Batch 20 : Gut microbiota abundance (family Oxalobacteraceae id.2966) on Localized adiposity
rs80330081
rs12002250
rs1569853
rs561239
rs62435498
rs6000536
rs934049
rs11246212
rs7993559
rs36057338
rs111966731
rs736744
rs17138946
rs4428215
All
−0.5
0.0
0.5
MR effect size for
'Gut microbiota abundance (family Oxalobacteraceae id.2966)' on 'Localized adiposity'

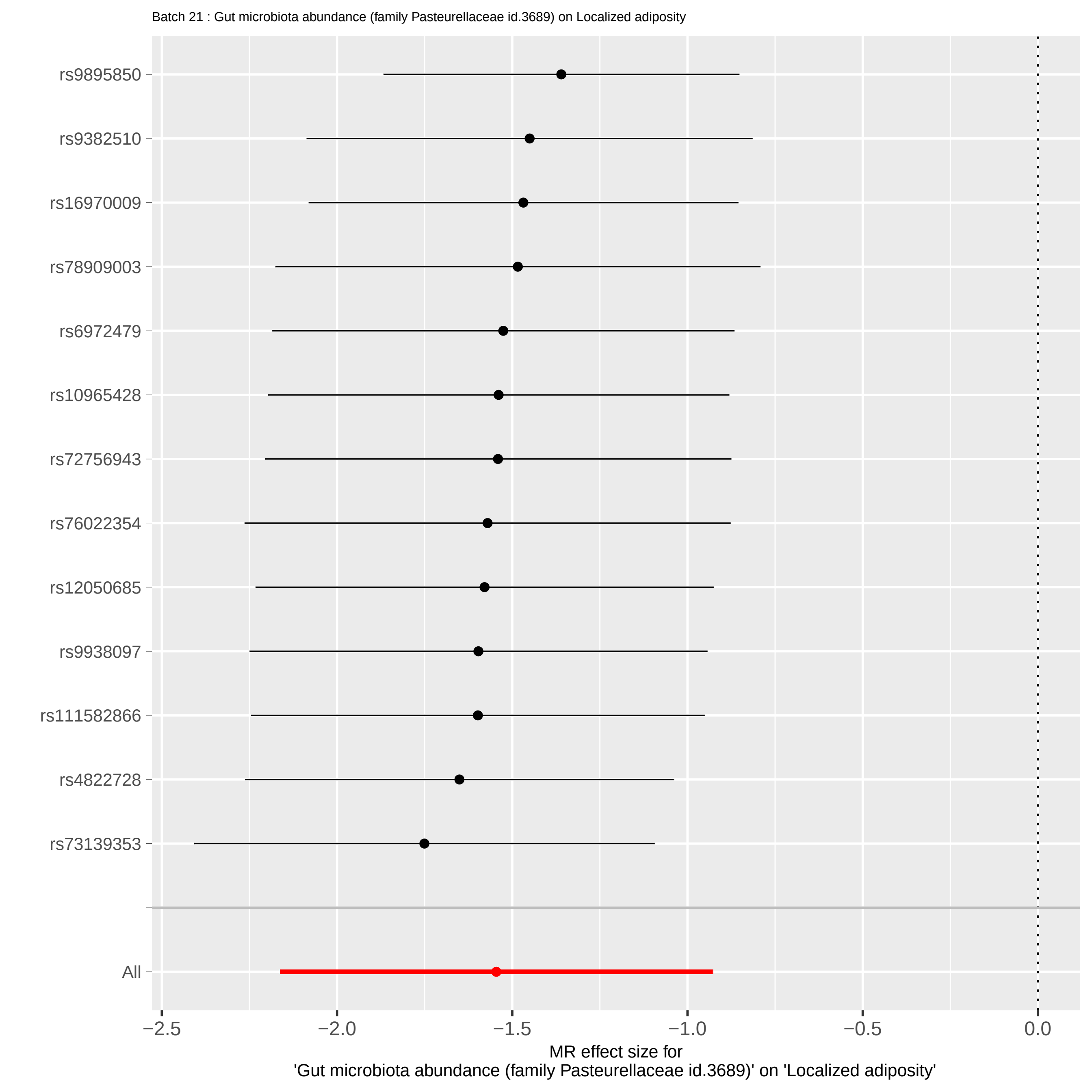
Batch 21 : Gut microbiota abundance (family Pasteurellaceae id.3689) on Localized adiposity
rs9895850
rs9382510
rs16970009
rs78909003
rs6972479
rs10965428
rs72756943
rs76022354
rs12050685
rs9938097
rs111582866
rs4822728
rs73139353
All
−2.5
−2.0
−1.5
−1.0
−0.5
0.0
MR effect size for
'Gut microbiota abundance (family Pasteurellaceae id.3689)' on 'Localized adiposity'

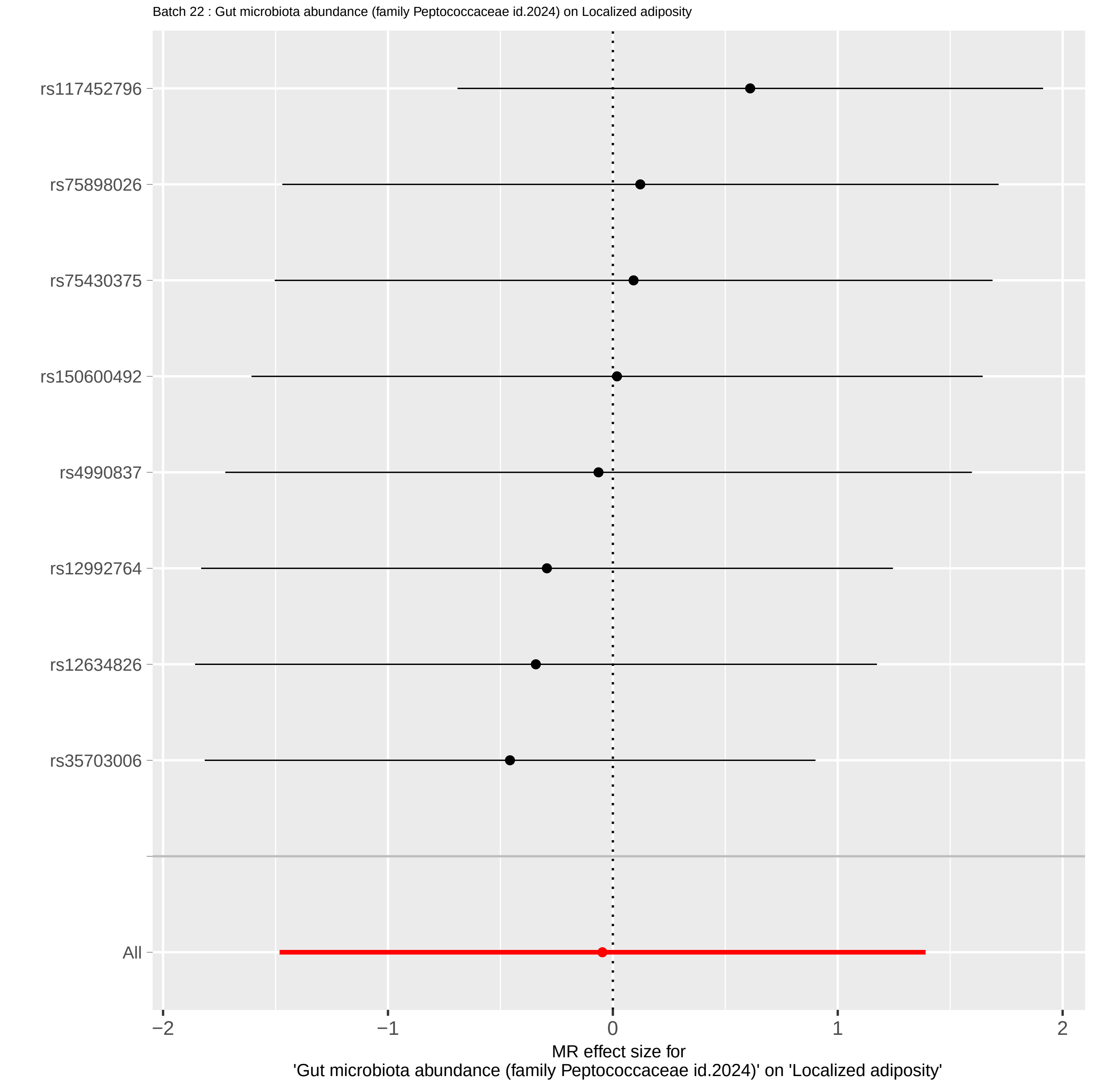

Batch 22 : Gut microbiota abundance (family Peptococcaceae id.2024) on Localized adiposity
rs117452796
rs75898026
rs75430375
rs150600492
rs4990837
rs12992764
rs12634826
rs35703006
All
−2
−1
0
1
2
MR effect size for
'Gut microbiota abundance (family Peptococcaceae id.2024)' on 'Localized adiposity'

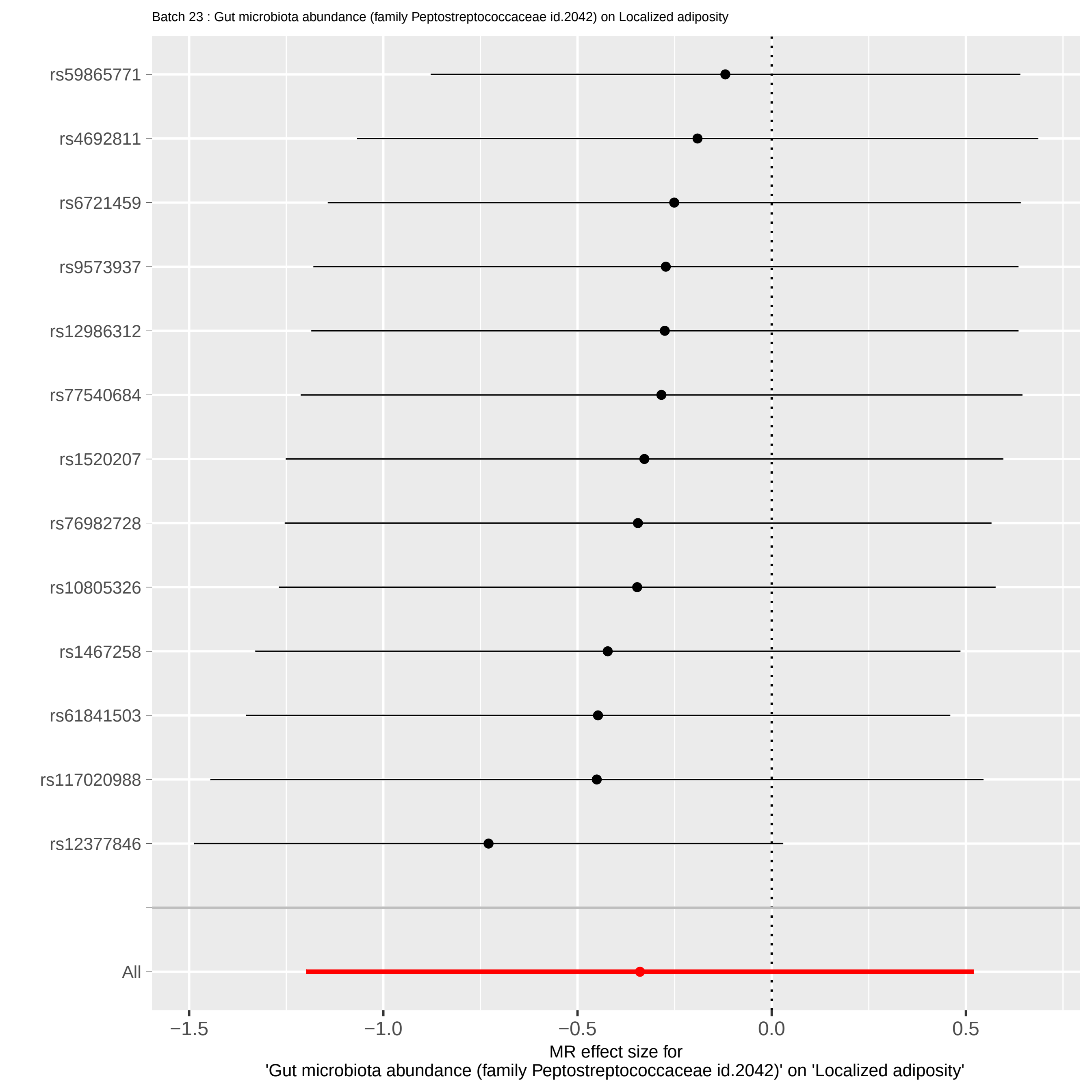
Batch 23 : Gut microbiota abundance (family Peptostreptococcaceae id.2042) on Localized adiposity
rs59865771
rs4692811
rs6721459
rs9573937
rs12986312
rs77540684
rs1520207
rs76982728
rs10805326
rs1467258
rs61841503
rs117020988
rs12377846
All
−1.5
−1.0
−0.5
0.0
0.5
MR effect size for
'Gut microbiota abundance (family Peptostreptococcaceae id.2042)' on 'Localized adiposity'

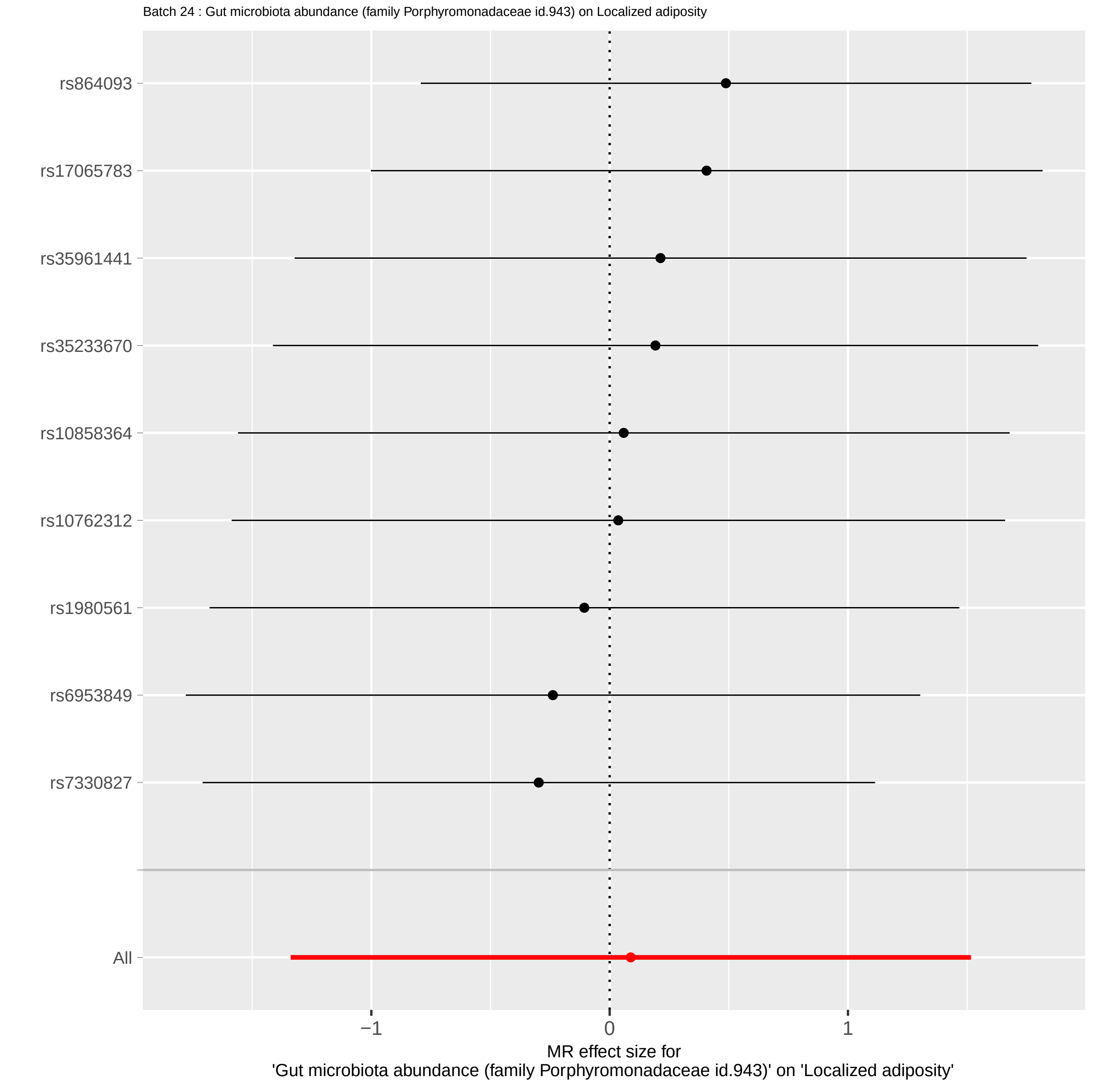
Batch 24 : Gut microbiota abundance (family Porphyromonadaceae id.943) on Localized adiposity
rs864093
rs17065783
rs35961441
rs35233670
rs10858364
rs10762312
rs1980561
rs6953849
rs7330827
All
−1
0
1
MR effect size for
'Gut microbiota abundance (family Porphyromonadaceae id.943)' on 'Localized adiposity'

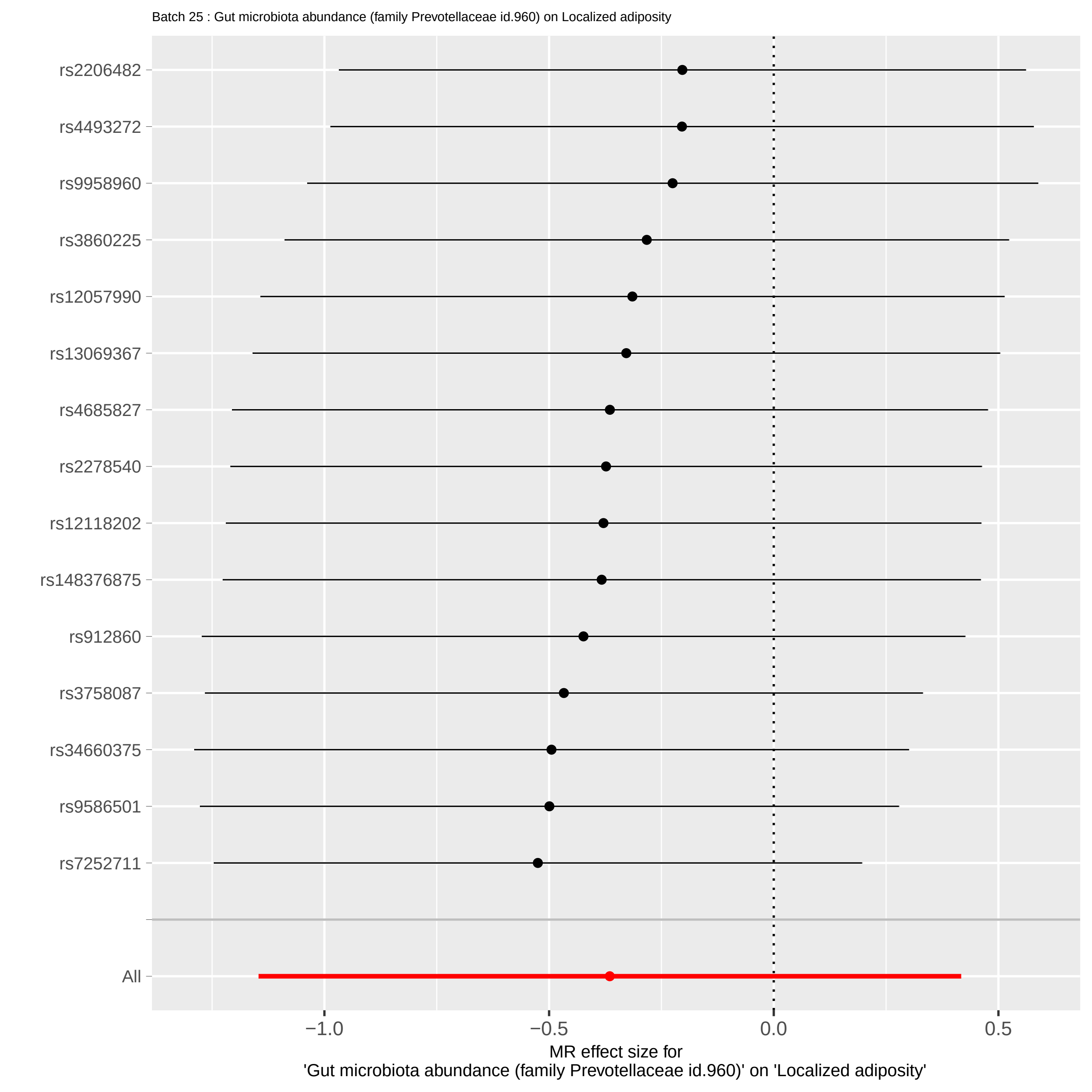
Batch 25 : Gut microbiota abundance (family Prevotellaceae id.960) on Localized adiposity
rs2206482
rs4493272
rs9958960
rs3860225
rs12057990
rs13069367
rs4685827
rs2278540
rs12118202
rs148376875
rs912860
rs3758087
rs34660375
rs9586501
rs7252711
All
−1.0
−0.5
0.0
0.5
MR effect size for
'Gut microbiota abundance (family Prevotellaceae id.960)' on 'Localized adiposity'

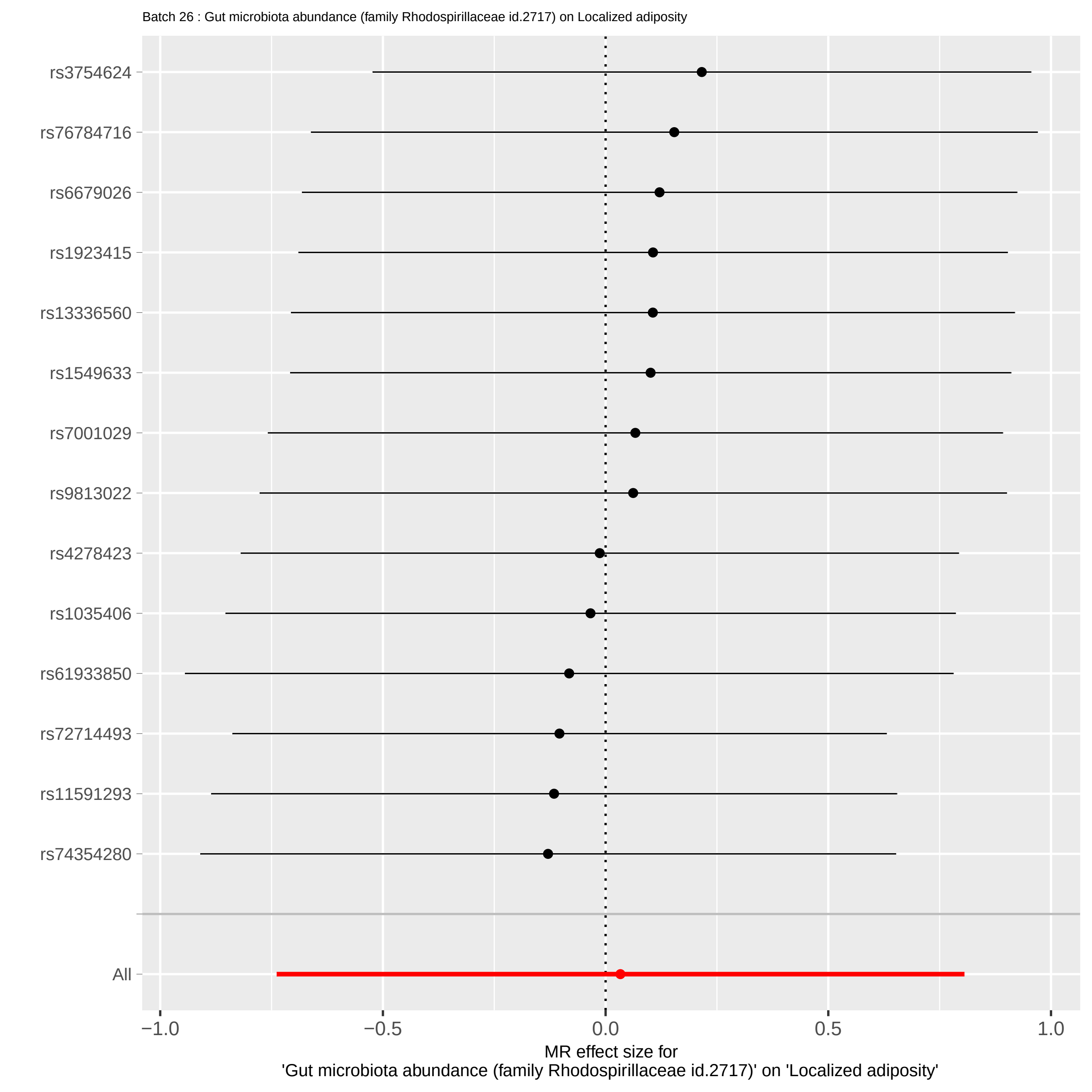
Batch 26 : Gut microbiota abundance (family Rhodospirillaceae id.2717) on Localized adiposity
rs3754624
rs76784716
rs6679026
rs1923415
rs13336560
rs1549633
rs7001029
rs9813022
rs4278423
rs1035406
rs61933850
rs72714493
rs11591293
rs74354280
All
−1.0
−0.5
0.0
0.5
1.0
MR effect size for
'Gut microbiota abundance (family Rhodospirillaceae id.2717)' on 'Localized adiposity'

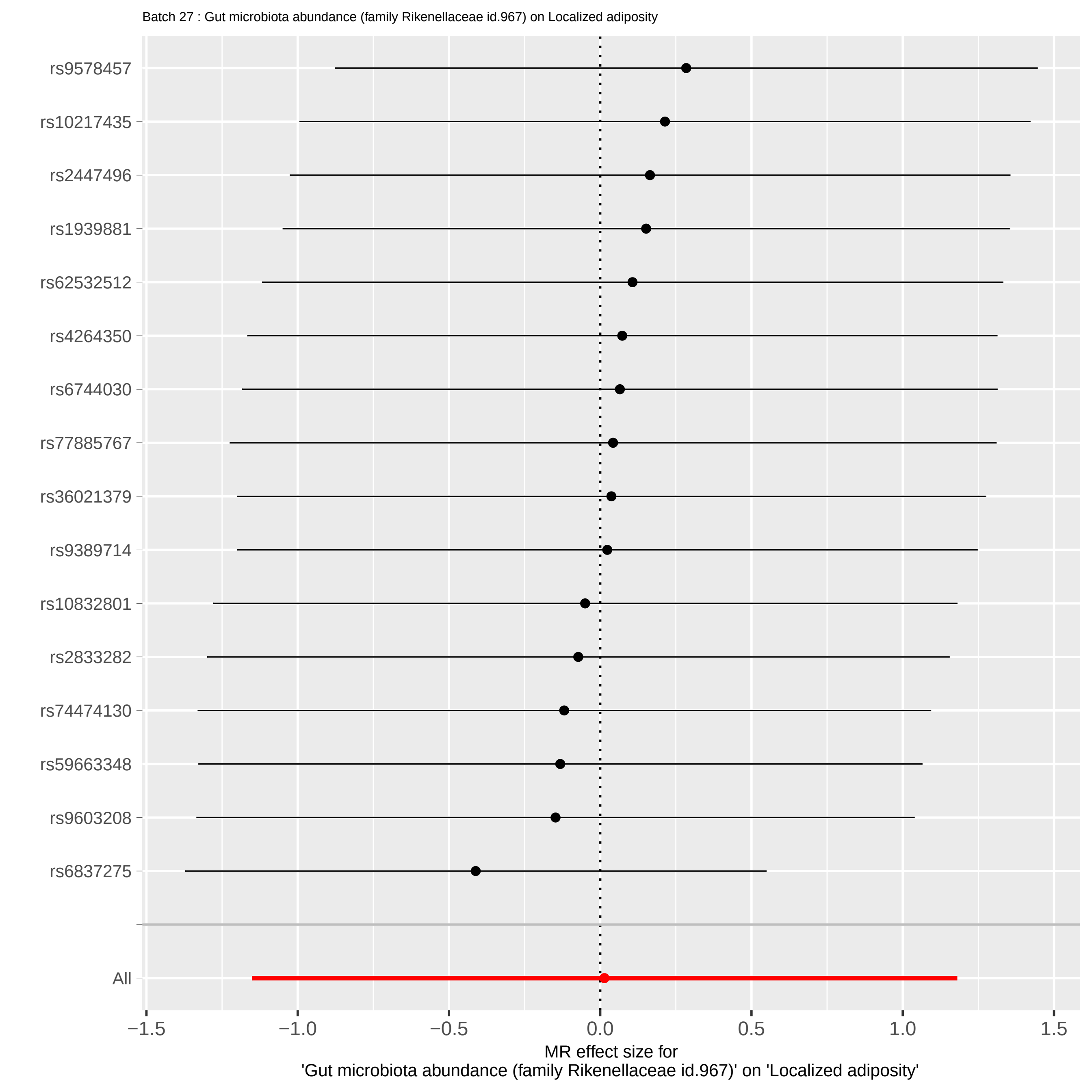

Batch 27 : Gut microbiota abundance (family Rikenellaceae id.967) on Localized adiposity
rs9578457
rs10217435
rs2447496
rs1939881
rs62532512
rs4264350
rs6744030
rs77885767
rs36021379
rs9389714
rs10832801
rs2833282
rs74474130
rs59663348
rs9603208
rs6837275
All
−1.5
−1.0
−0.5
0.0
0.5
1.0
1.5
MR effect size for
'Gut microbiota abundance (family Rikenellaceae id.967)' on 'Localized adiposity'

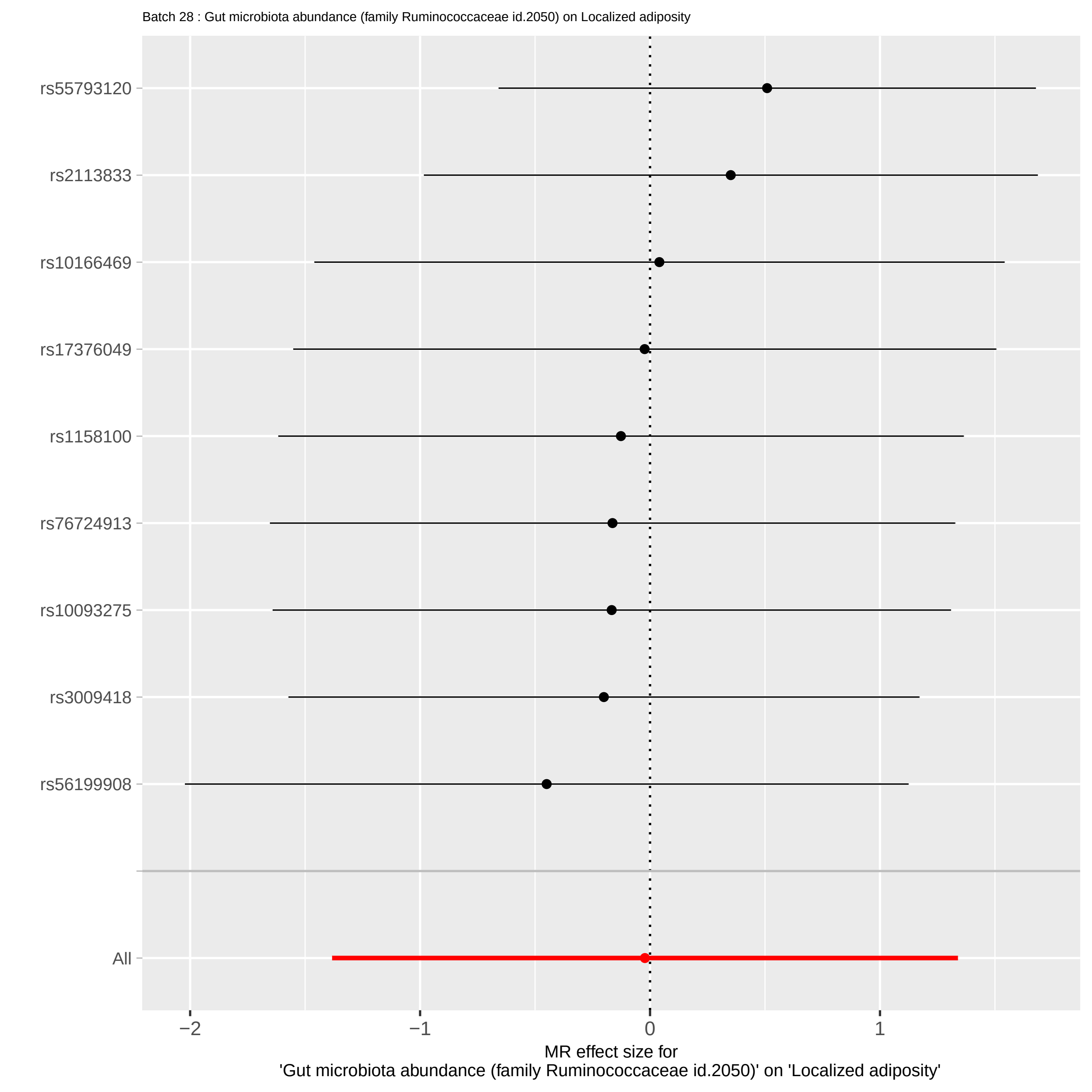

Batch 28 : Gut microbiota abundance (family Ruminococcaceae id.2050) on Localized adiposity
rs55793120
rs2113833
rs10166469
rs17376049
rs1158100
rs76724913
rs10093275
rs3009418
rs56199908
All
−2
−1
0
1
MR effect size for
'Gut microbiota abundance (family Ruminococcaceae id.2050)' on 'Localized adiposity'

Batch 29 : Gut microbiota abundance (family Streptococcaceae id.1850) on Localized adiposity

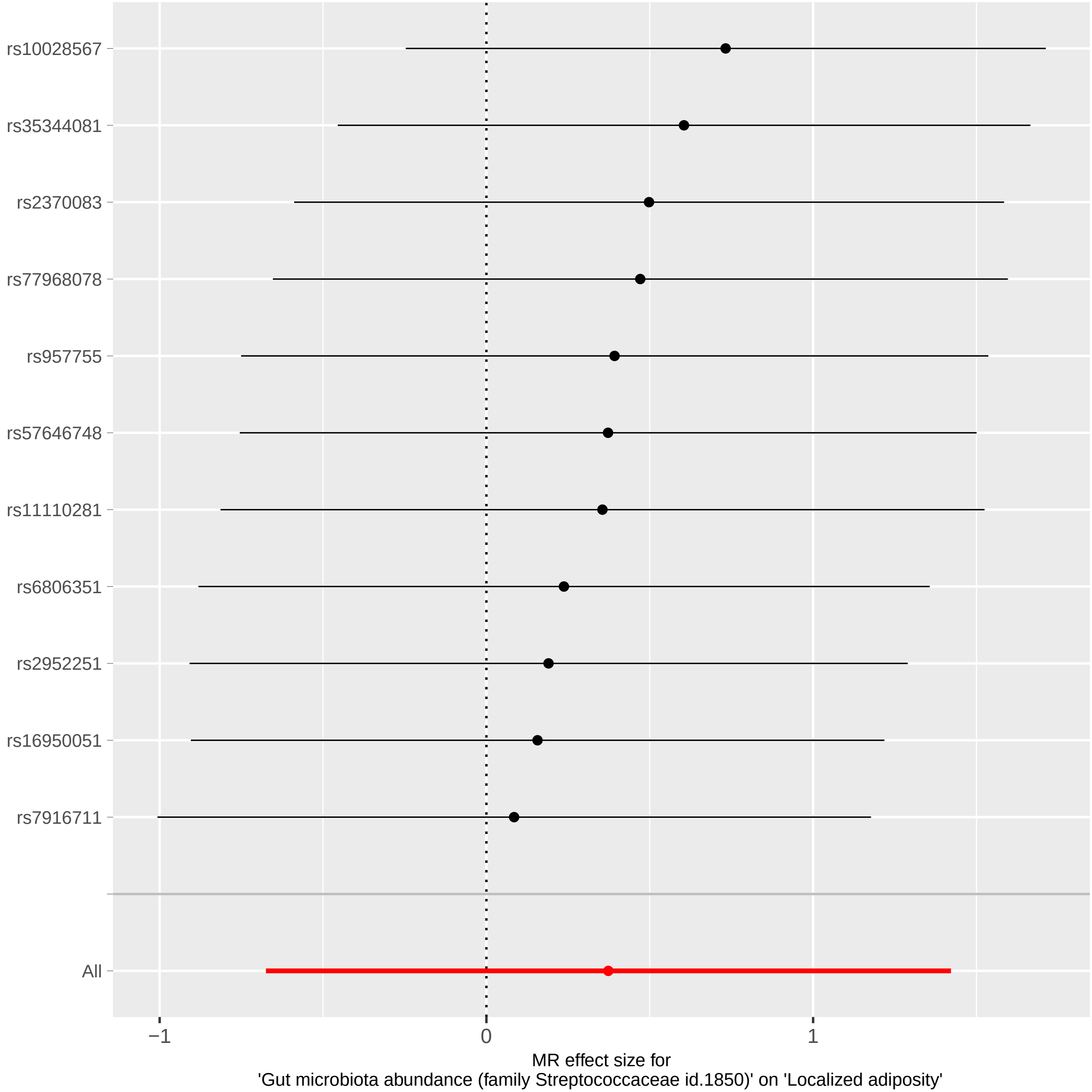

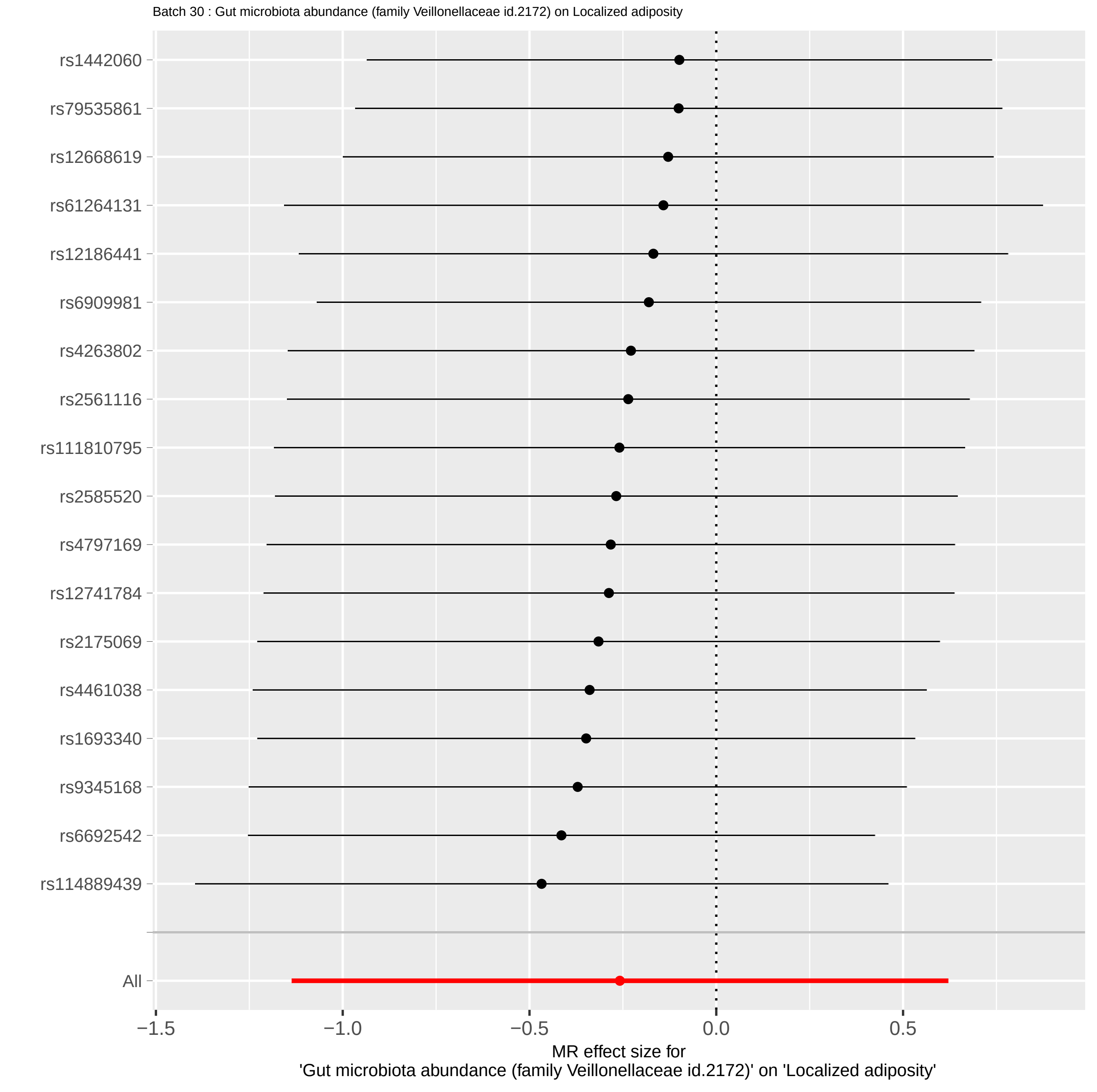

Batch 30 : Gut microbiota abundance (family Veillonellaceae id.2172) on Localized adiposity
rs1442060
rs79535861
rs12668619
rs61264131
rs12186441
rs6909981
rs4263802
rs2561116
rs111810795
rs2585520
rs4797169
rs12741784
rs2175069
rs4461038
rs1693340
rs9345168
rs6692542
rs114889439
All
−1.5
−1.0
−0.5
0.0
0.5
MR effect size for
'Gut microbiota abundance (family Veillonellaceae id.2172)' on 'Localized adiposity'

Batch 31 : Gut microbiota abundance (family Verrucomicrobiaceae id.4036) on Localized adiposity

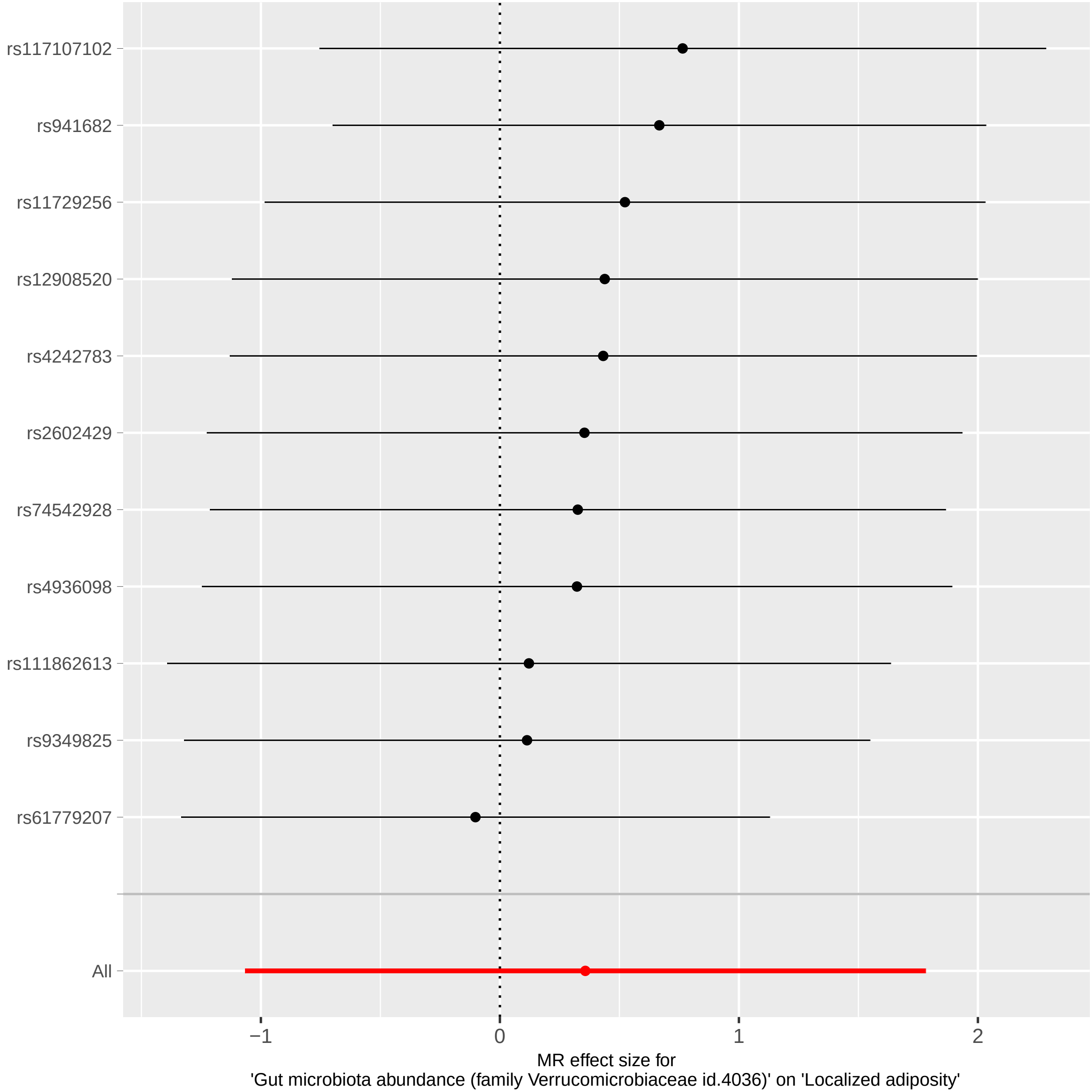

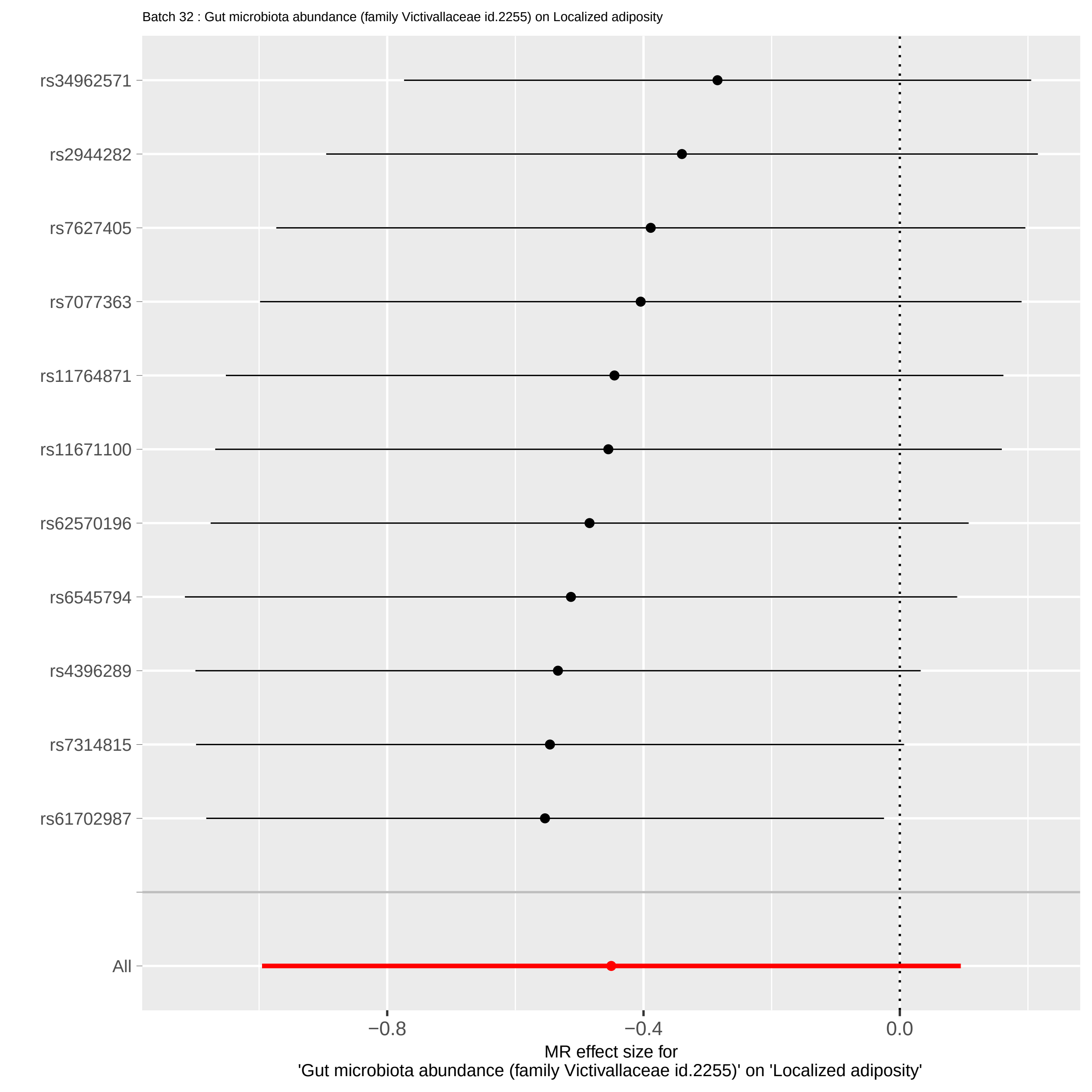

Batch 32 : Gut microbiota abundance (family Victivallaceae id.2255) on Localized adiposity
rs34962571
rs2944282
rs7627405
rs7077363
rs11764871
rs11671100
rs62570196
rs6545794
rs4396289
rs7314815
rs61702987
All
−0.8
−0.4
0.0
MR effect size for
'Gut microbiota abundance (family Victivallaceae id.2255)' on 'Localized adiposity'

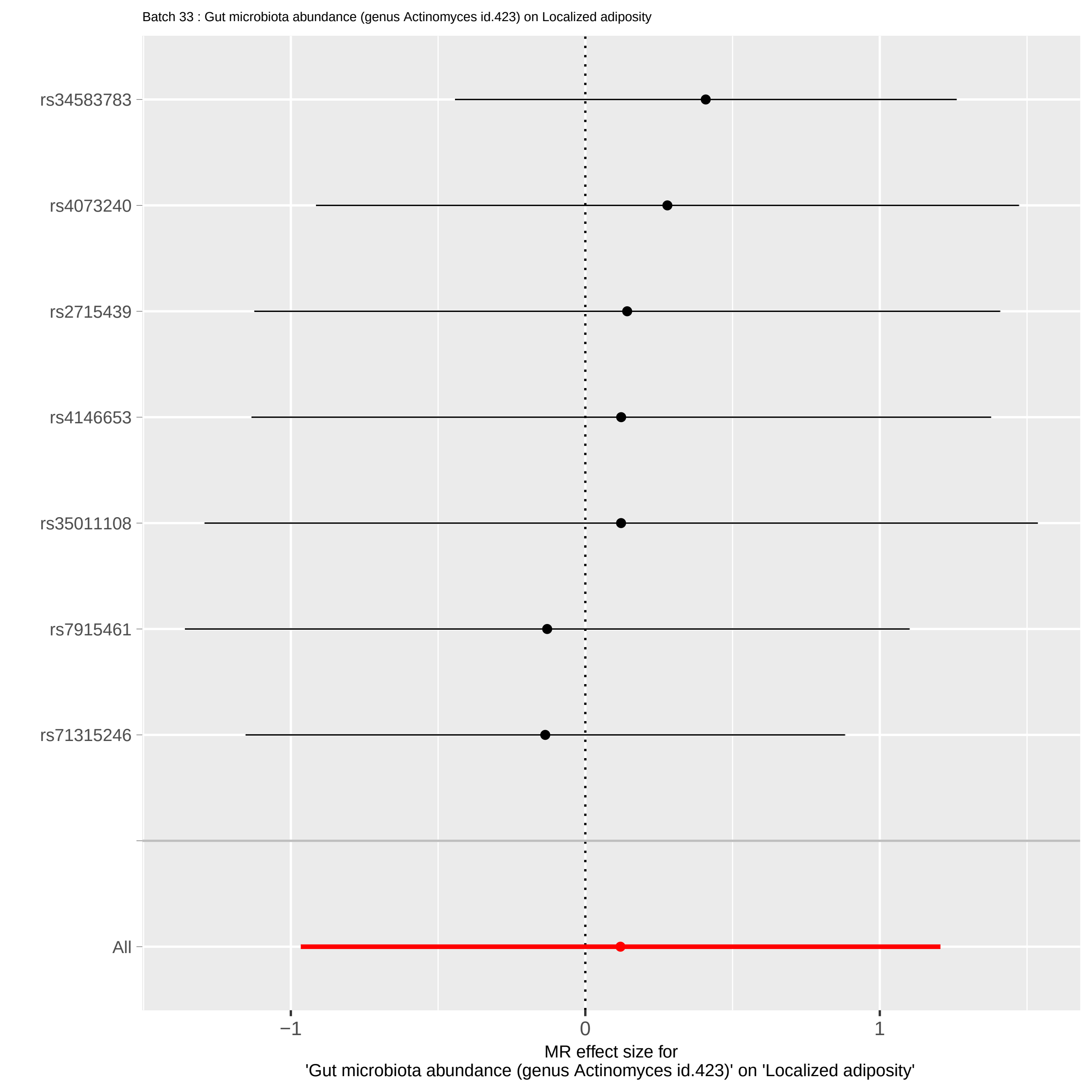
Batch 33 : Gut microbiota abundance (genus Actinomyces id.423) on Localized adiposity
rs34583783
rs4073240
rs2715439
rs4146653
rs35011108
rs7915461
rs71315246
All
−1
0
1
MR effect size for
'Gut microbiota abundance (genus Actinomyces id.423)' on 'Localized adiposity'

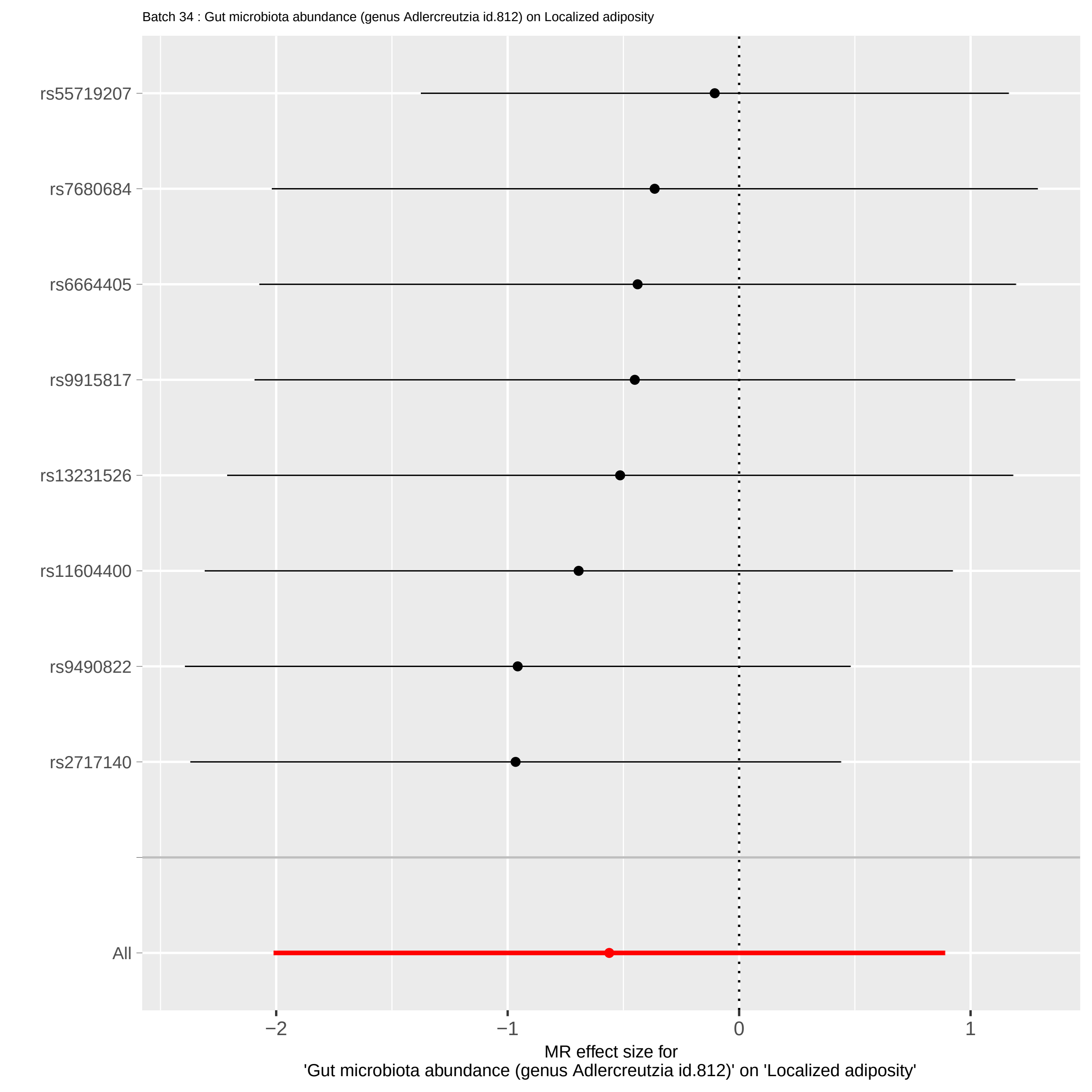
Batch 34 : Gut microbiota abundance (genus Adlercreutzia id.812) on Localized adiposity
rs55719207
rs7680684
rs6664405
rs9915817
rs13231526
rs11604400
rs9490822
rs2717140
All
−2
−1
0
1
MR effect size for
'Gut microbiota abundance (genus Adlercreutzia id.812)' on 'Localized adiposity'

Batch 35 : Gut microbiota abundance (genus Akkermansia id.4037) on Localized adiposity

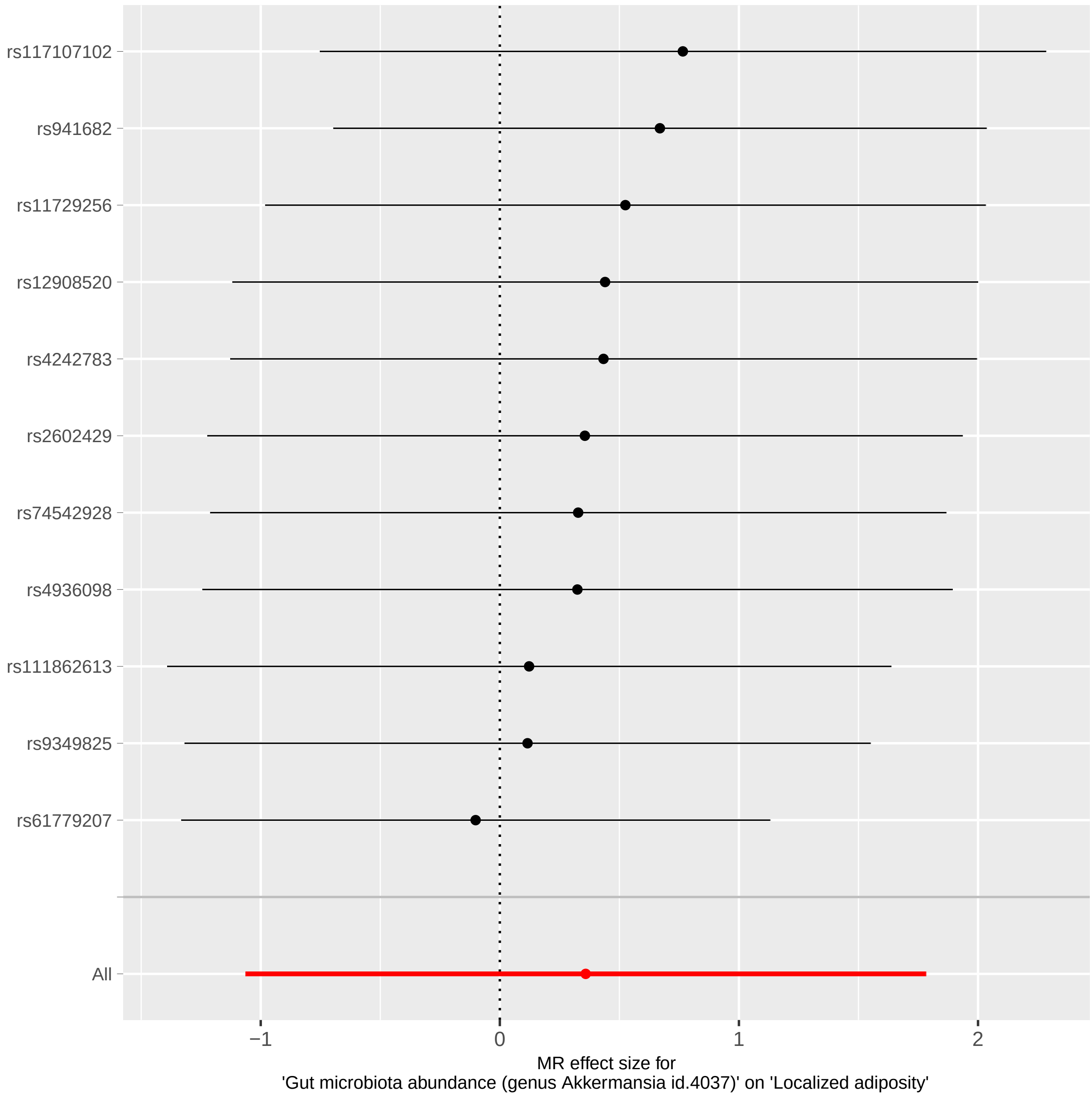

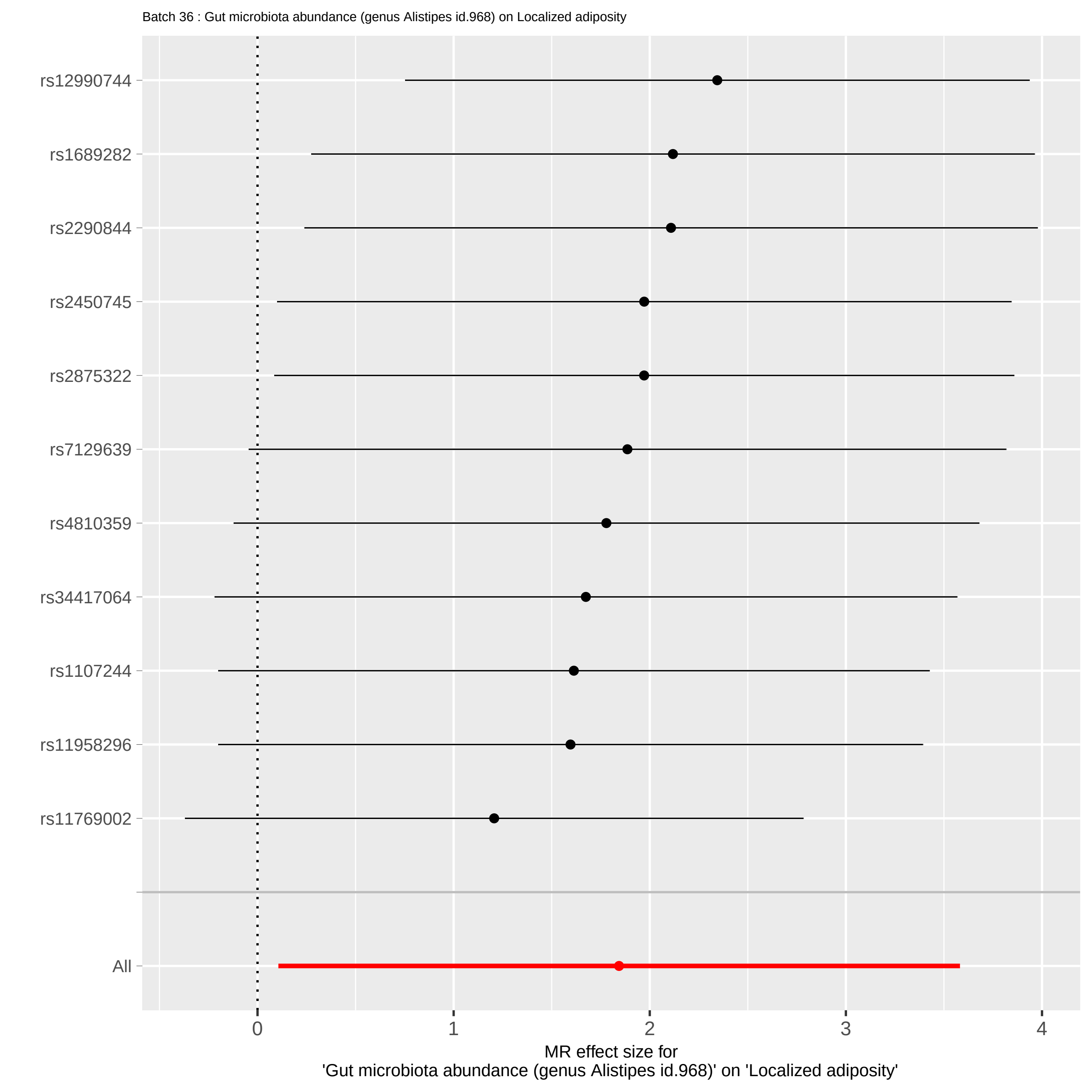

Batch 36 : Gut microbiota abundance (genus Alistipes id.968) on Localized adiposity
rs12990744
rs1689282
rs2290844
rs2450745
rs2875322
rs7129639
rs4810359
rs34417064
rs1107244
rs11958296
rs11769002
All
0
1
2
3
4
MR effect size for
'Gut microbiota abundance (genus Alistipes id.968)' on 'Localized adiposity'

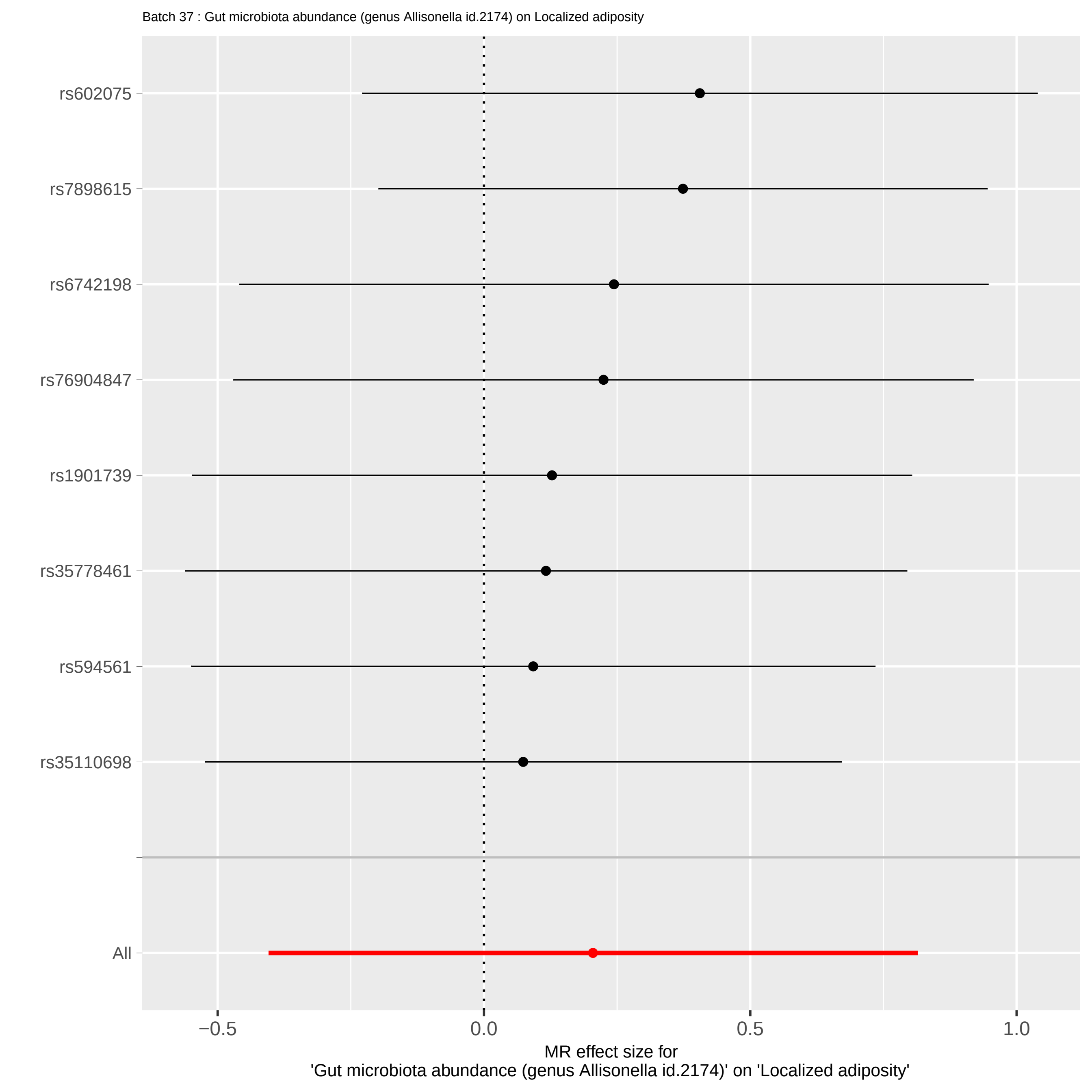

Batch 37 : Gut microbiota abundance (genus Allisonella id.2174) on Localized adiposity
rs602075
rs7898615
rs6742198
rs76904847
rs1901739
rs35778461
rs594561
rs35110698
All
−0.5
0.0
0.5
1.0
MR effect size for
'Gut microbiota abundance (genus Allisonella id.2174)' on 'Localized adiposity'

Batch 38 : Gut microbiota abundance (genus Alloprevotella id.961) on Localized adiposity

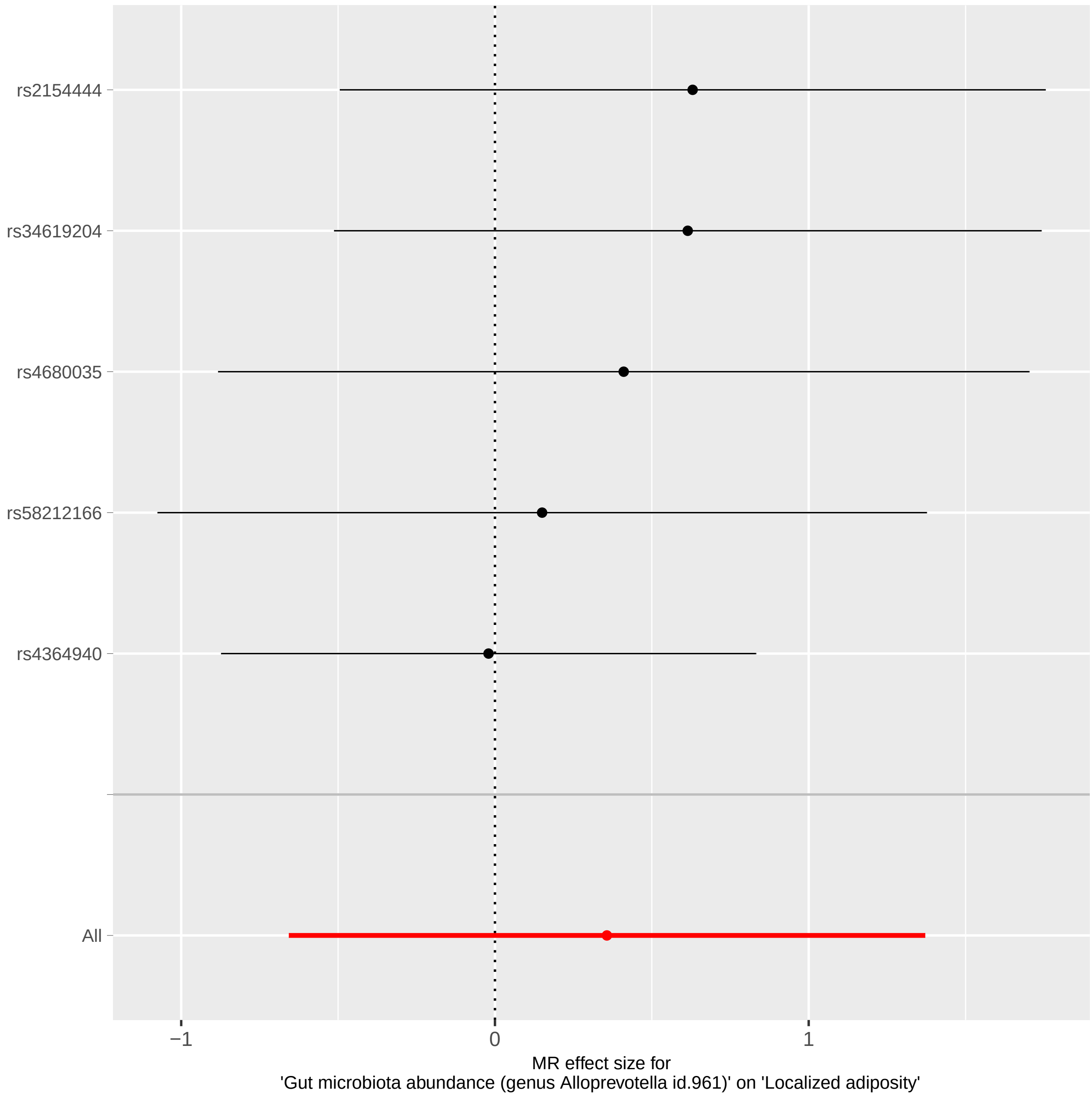

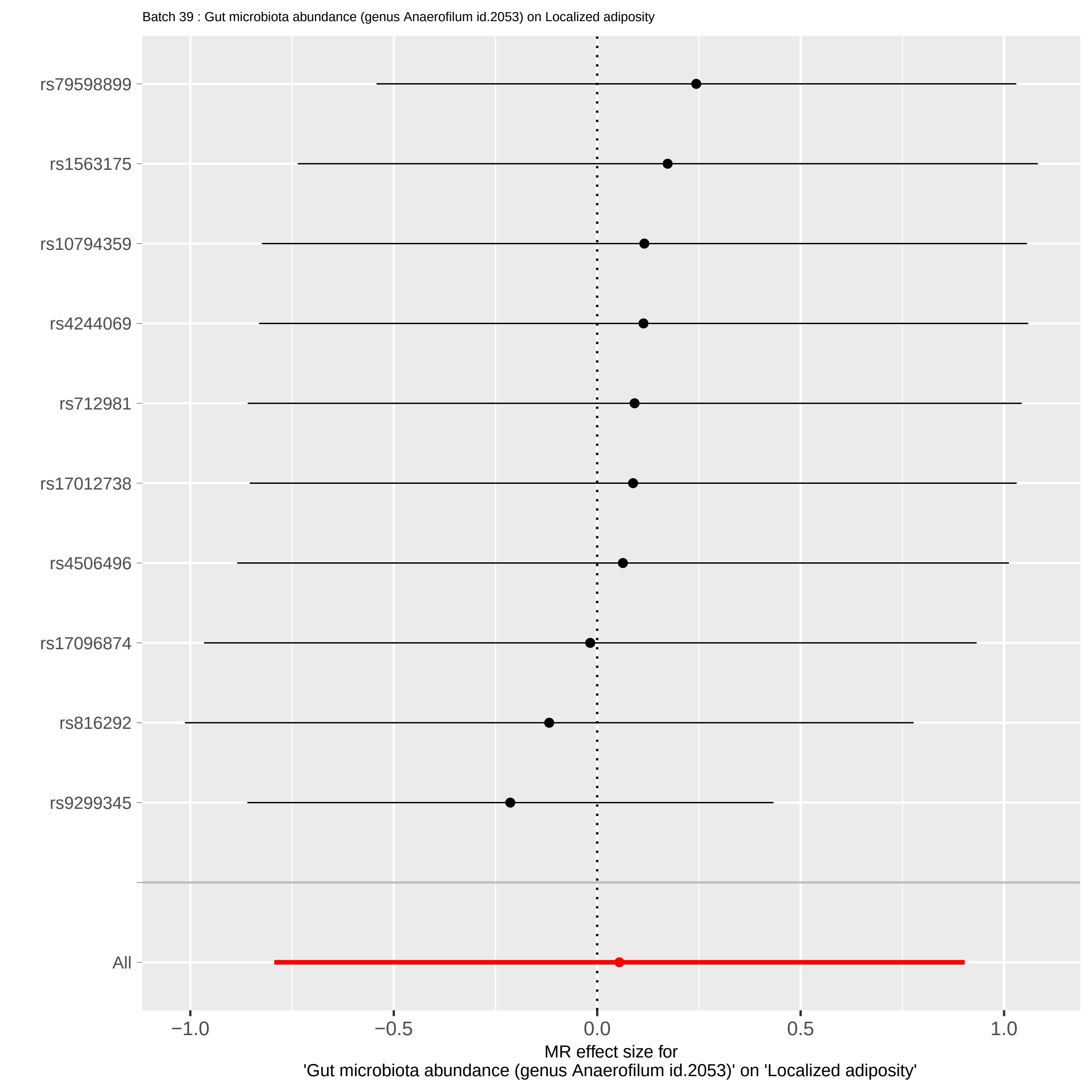

Batch 39 : Gut microbiota abundance (genus Anaerofilum id.2053) on Localized adiposity
rs79598899
rs1563175
rs10794359
rs4244069
rs712981
rs17012738
rs4506496
rs17096874
rs816292
rs9299345
All
−1.0
−0.5
0.0
0.5
1.0
MR effect size for
'Gut microbiota abundance (genus Anaerofilum id.2053)' on 'Localized adiposity'

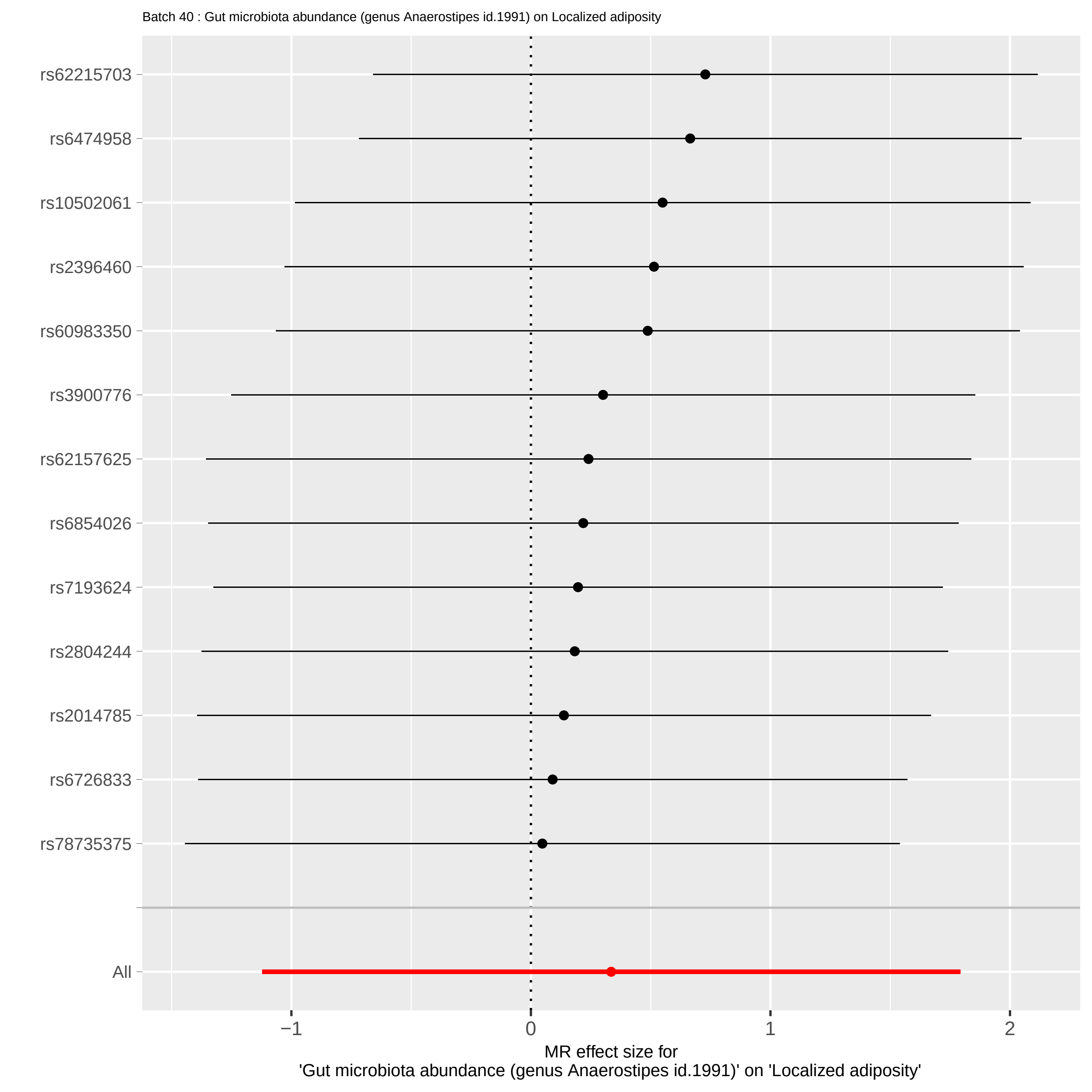

Batch 40 : Gut microbiota abundance (genus Anaerostipes id.1991) on Localized adiposity
rs62215703
rs6474958
rs10502061
rs2396460
rs60983350
rs3900776
rs62157625
rs6854026
rs7193624
rs2804244
rs2014785
rs6726833
rs78735375
All
−1
0
1
2
MR effect size for
'Gut microbiota abundance (genus Anaerostipes id.1991)' on 'Localized adiposity'

Batch 41 : Gut microbiota abundance (genus Anaerotruncus id.2054) on Localized adiposity

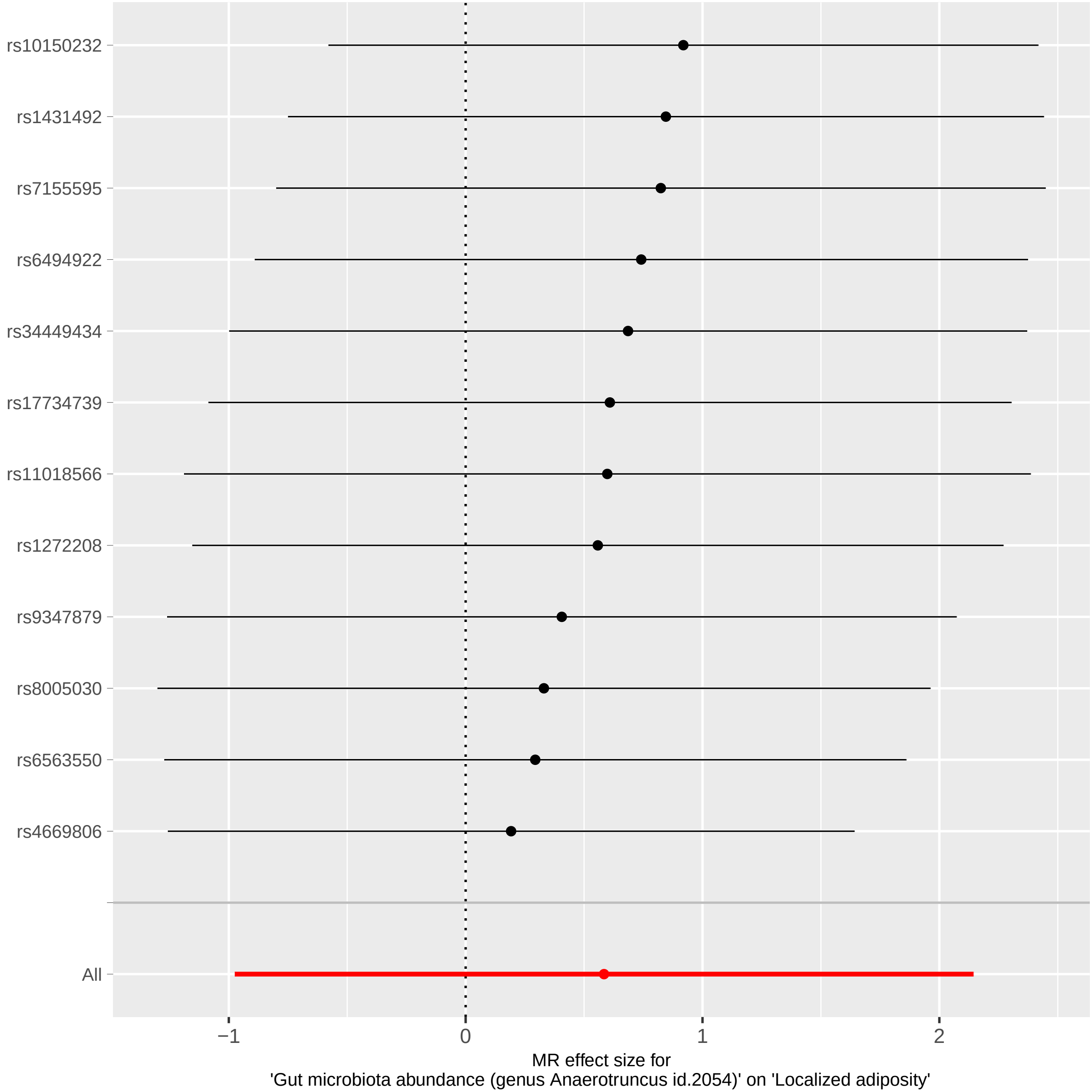

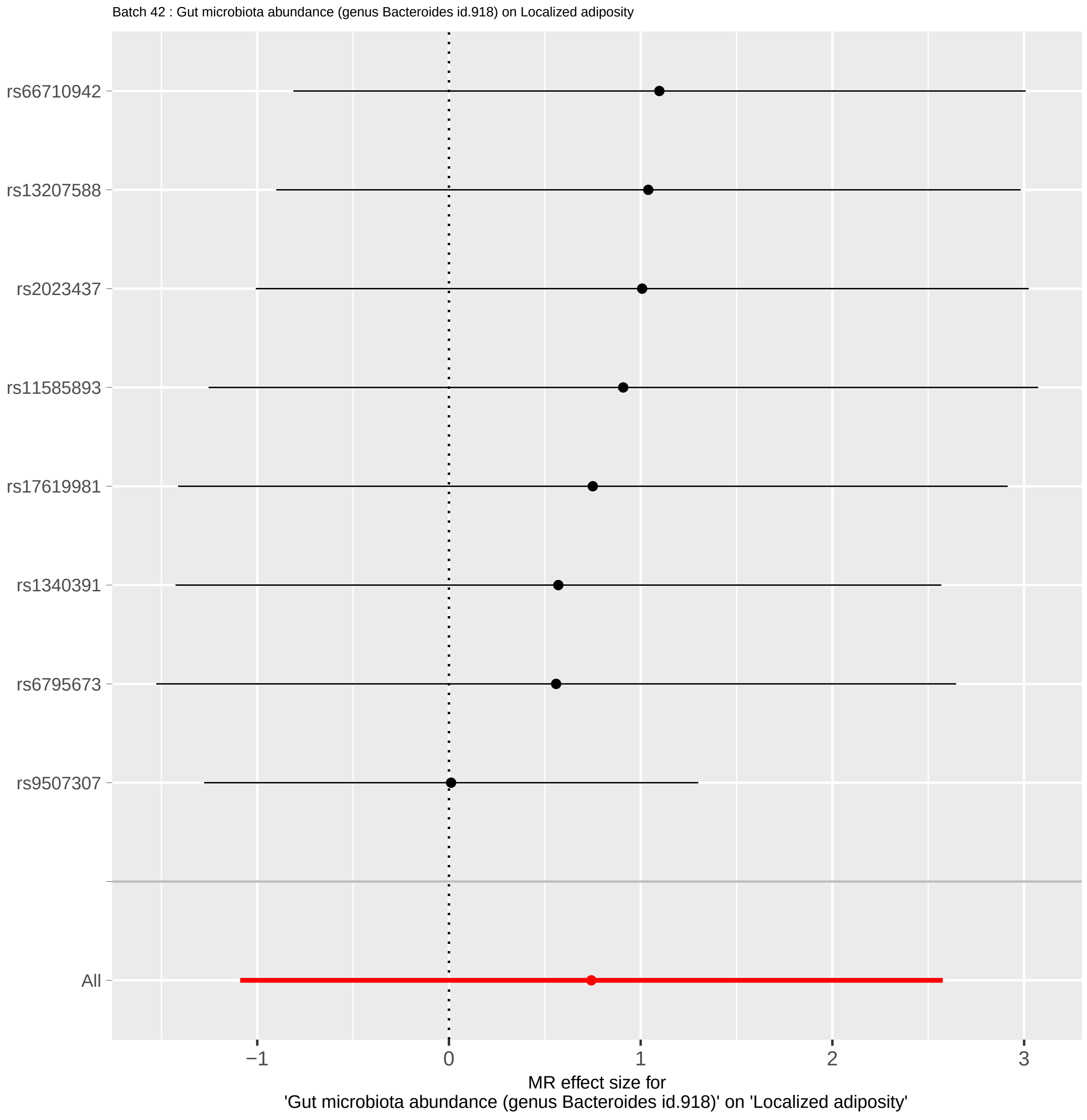
Batch 42 : Gut microbiota abundance (genus Bacteroides id.918) on Localized adiposity
rs66710942
rs13207588
rs2023437
rs11585893
rs17619981
rs1340391
rs6795673
rs9507307
All
−1
0
1
2
3
MR effect size for
'Gut microbiota abundance (genus Bacteroides id.918)' on 'Localized adiposity'

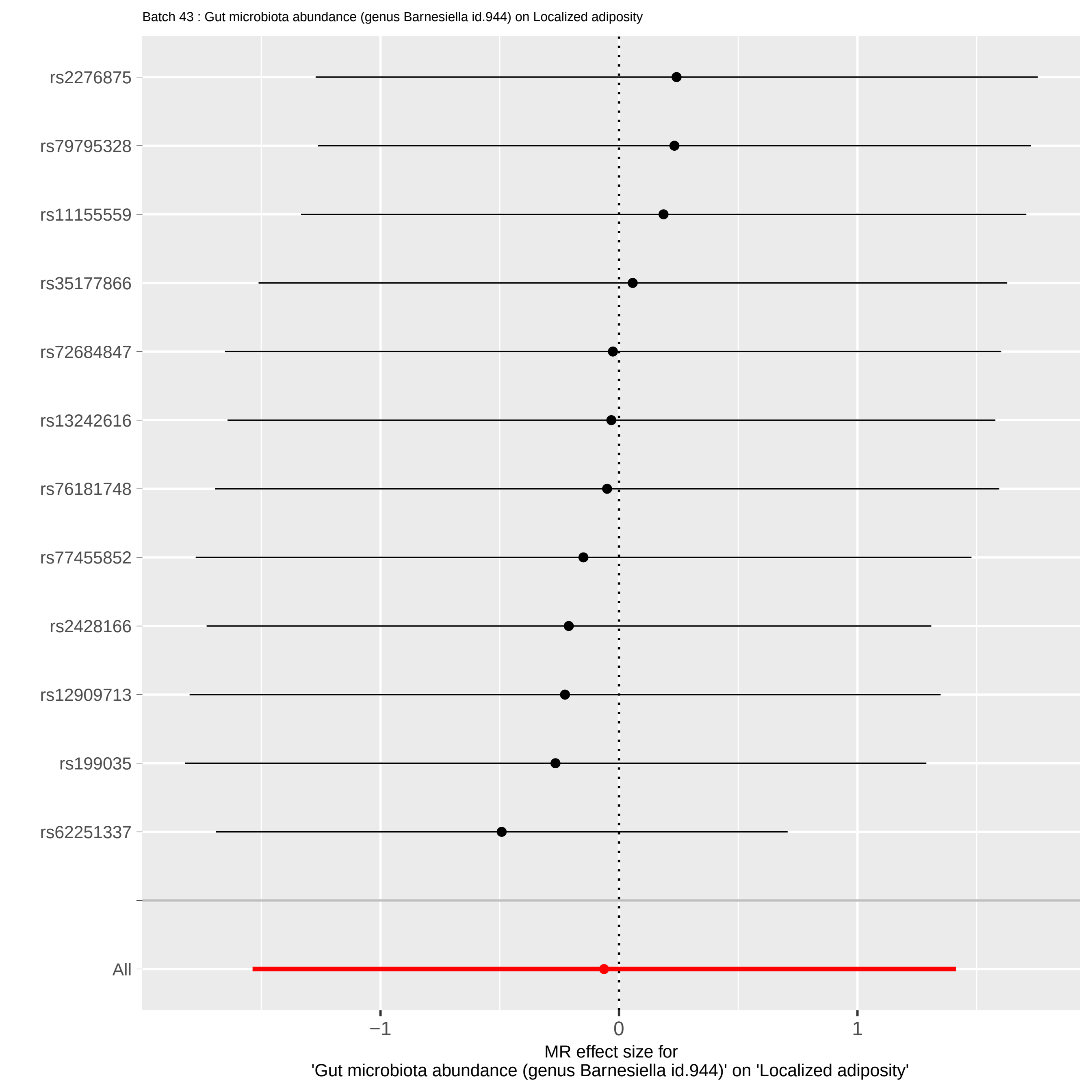
Batch 43 : Gut microbiota abundance (genus Barnesiella id.944) on Localized adiposity
rs2276875
rs79795328
rs11155559
rs35177866
rs72684847
rs13242616
rs76181748
rs77455852
rs2428166
rs12909713
rs199035
rs62251337
All
−1
0
1
MR effect size for
'Gut microbiota abundance (genus Barnesiella id.944)' on 'Localized adiposity'

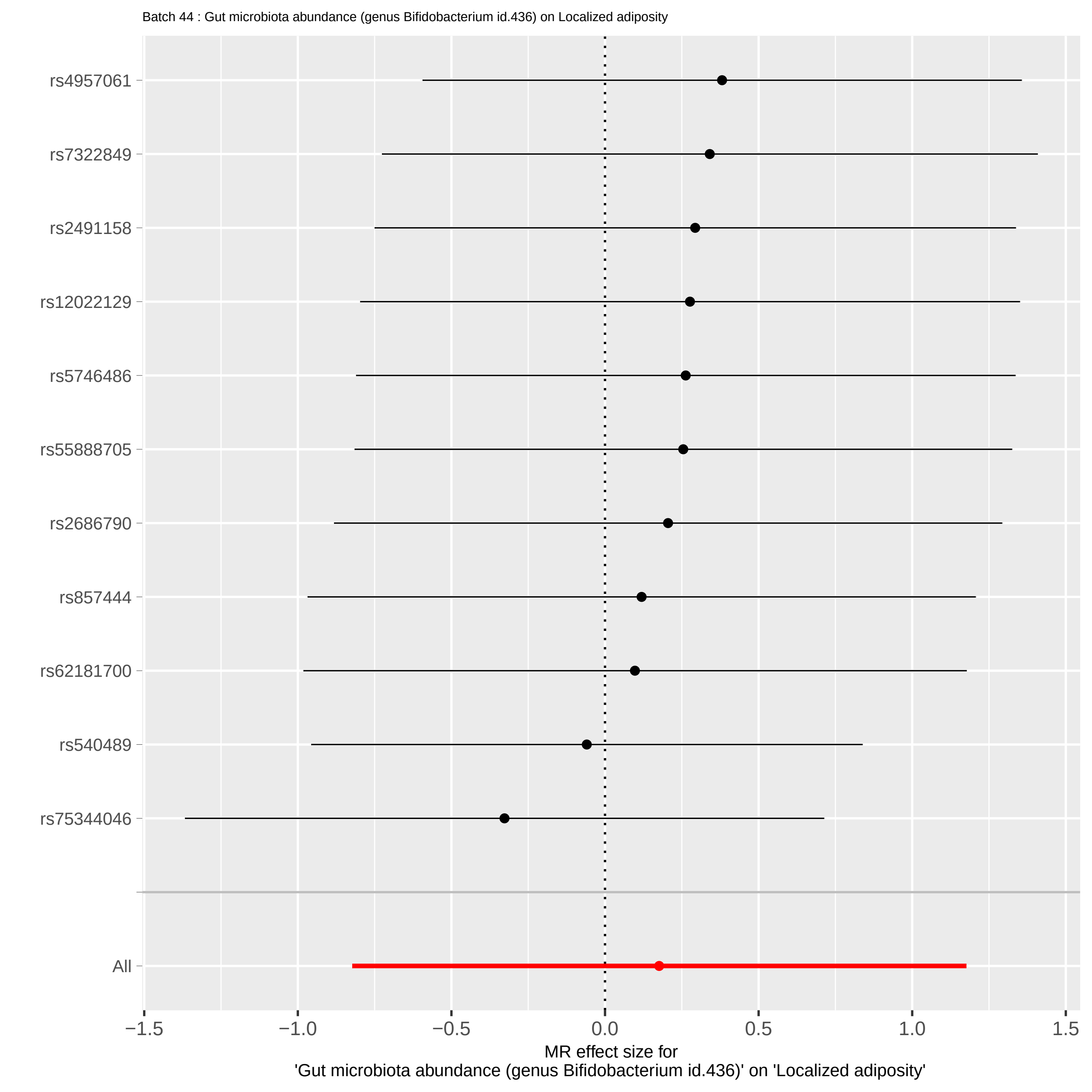

Batch 44 : Gut microbiota abundance (genus Bifidobacterium id.436) on Localized adiposity
rs4957061
rs7322849
rs2491158
rs12022129
rs5746486
rs55888705
rs2686790
rs857444
rs62181700
rs540489
rs75344046
All
−1.5
−1.0
−0.5
0.0
0.5
1.0
1.5
MR effect size for
'Gut microbiota abundance (genus Bifidobacterium id.436)' on 'Localized adiposity'

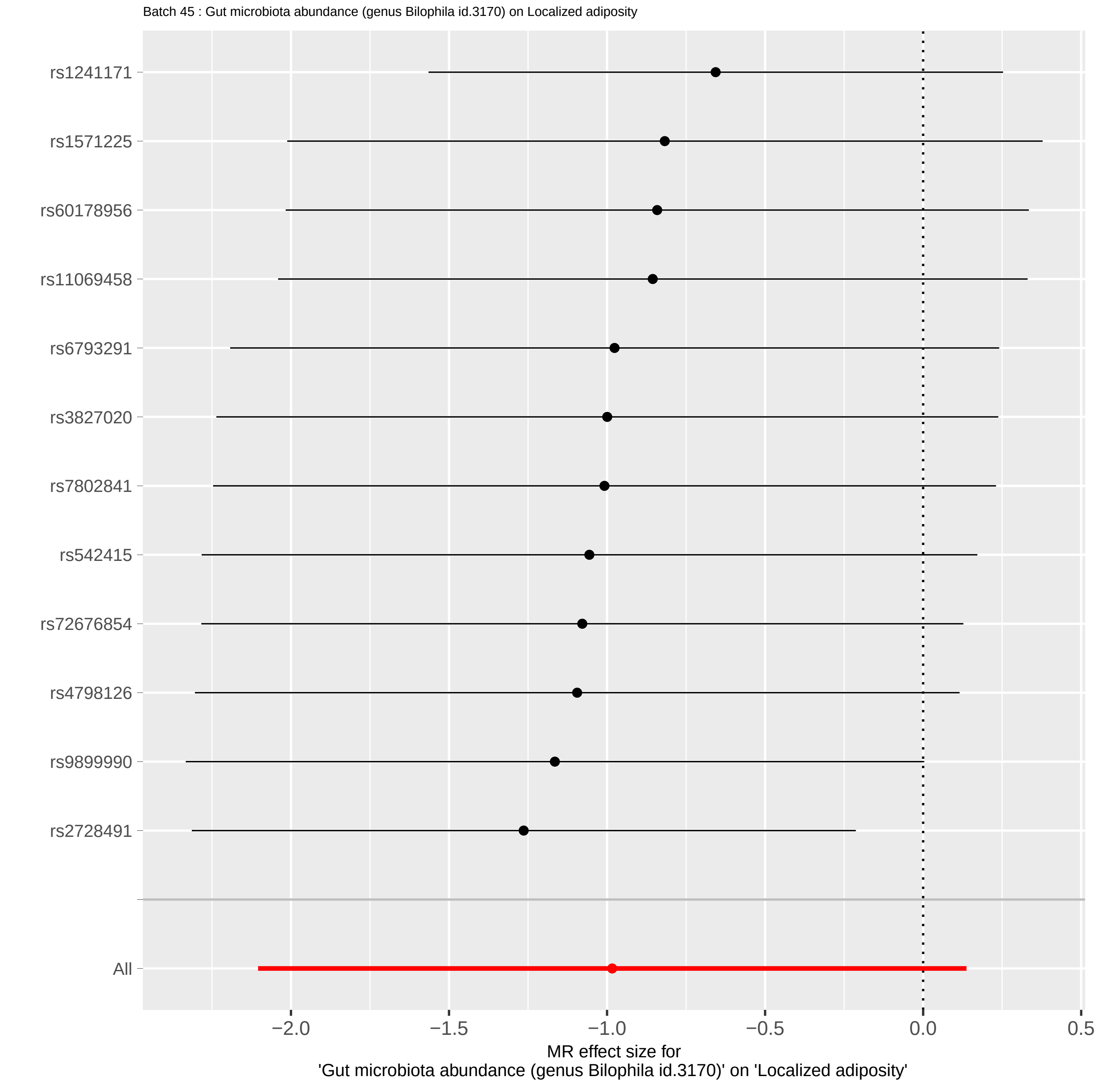
Batch 45 : Gut microbiota abundance (genus Bilophila id.3170) on Localized adiposity
rs1241171
rs1571225
rs60178956
rs11069458
rs6793291
rs3827020
rs7802841
rs542415
rs72676854
rs4798126
rs9899990
rs2728491
All
−2.0
−1.5
−1.0
−0.5
0.0
0.5
MR effect size for
'Gut microbiota abundance (genus Bilophila id.3170)' on 'Localized adiposity'

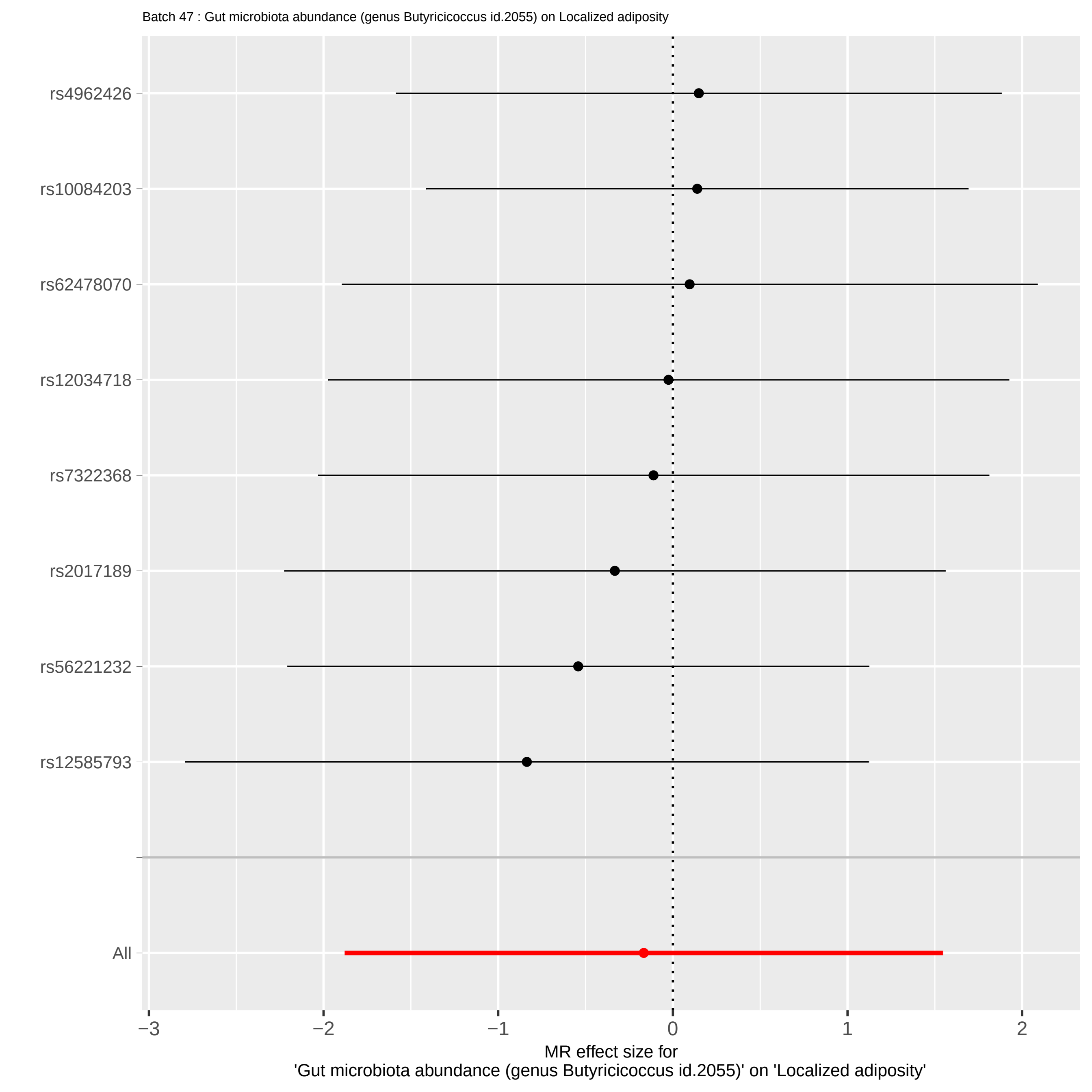

Batch 47 : Gut microbiota abundance (genus Butyricicoccus id.2055) on Localized adiposity
rs4962426
rs10084203
rs62478070
rs12034718
rs7322368
rs2017189
rs56221232
rs12585793
All
−3
−2
−1
0
1
2
MR effect size for
'Gut microbiota abundance (genus Butyricicoccus id.2055)' on 'Localized adiposity'

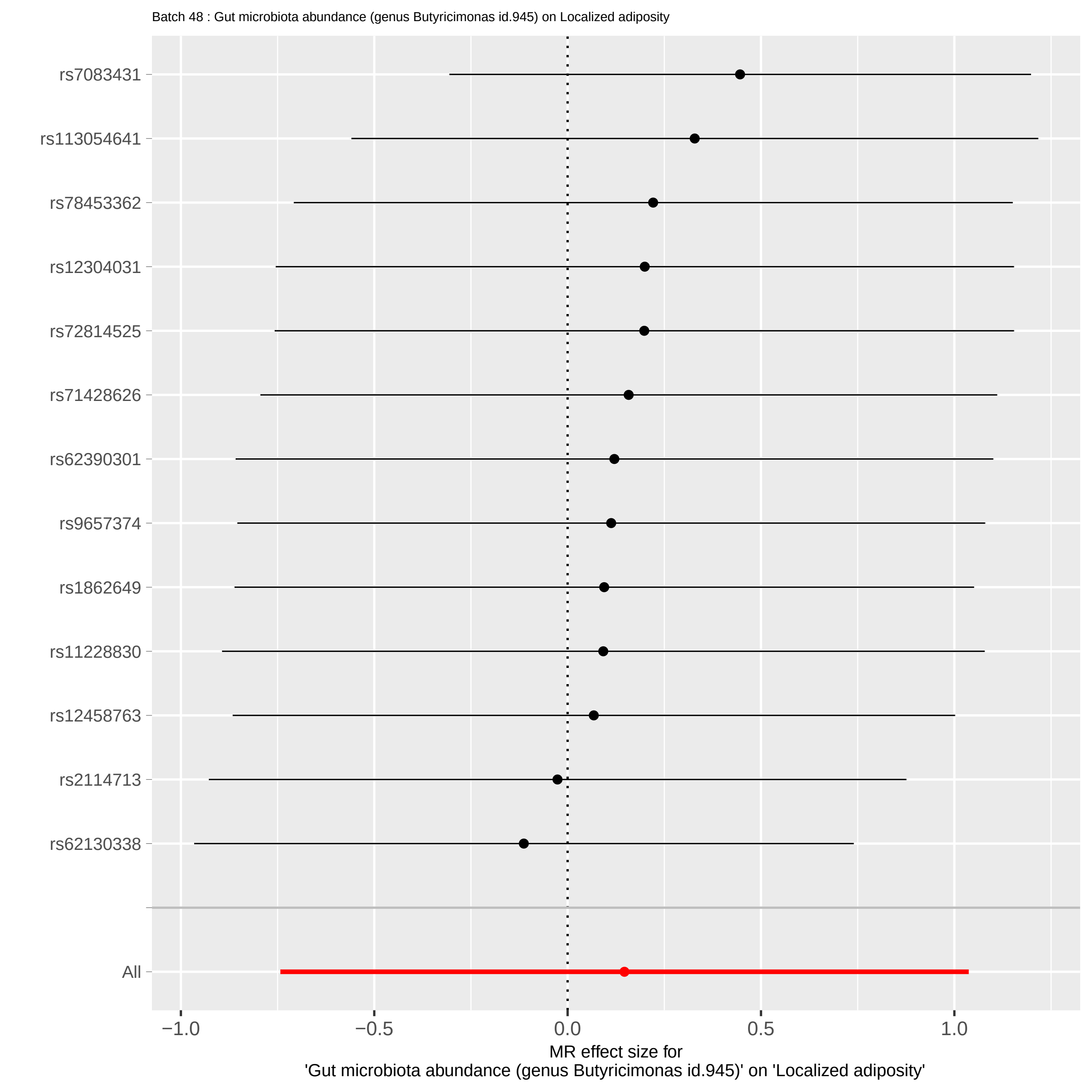

Batch 48 : Gut microbiota abundance (genus Butyricimonas id.945) on Localized adiposity
rs7083431
rs113054641
rs78453362
rs12304031
rs72814525
rs71428626
rs62390301
rs9657374
rs1862649
rs11228830
rs12458763
rs2114713
rs62130338
All
−1.0
−0.5
0.0
0.5
1.0
MR effect size for
'Gut microbiota abundance (genus Butyricimonas id.945)' on 'Localized adiposity'

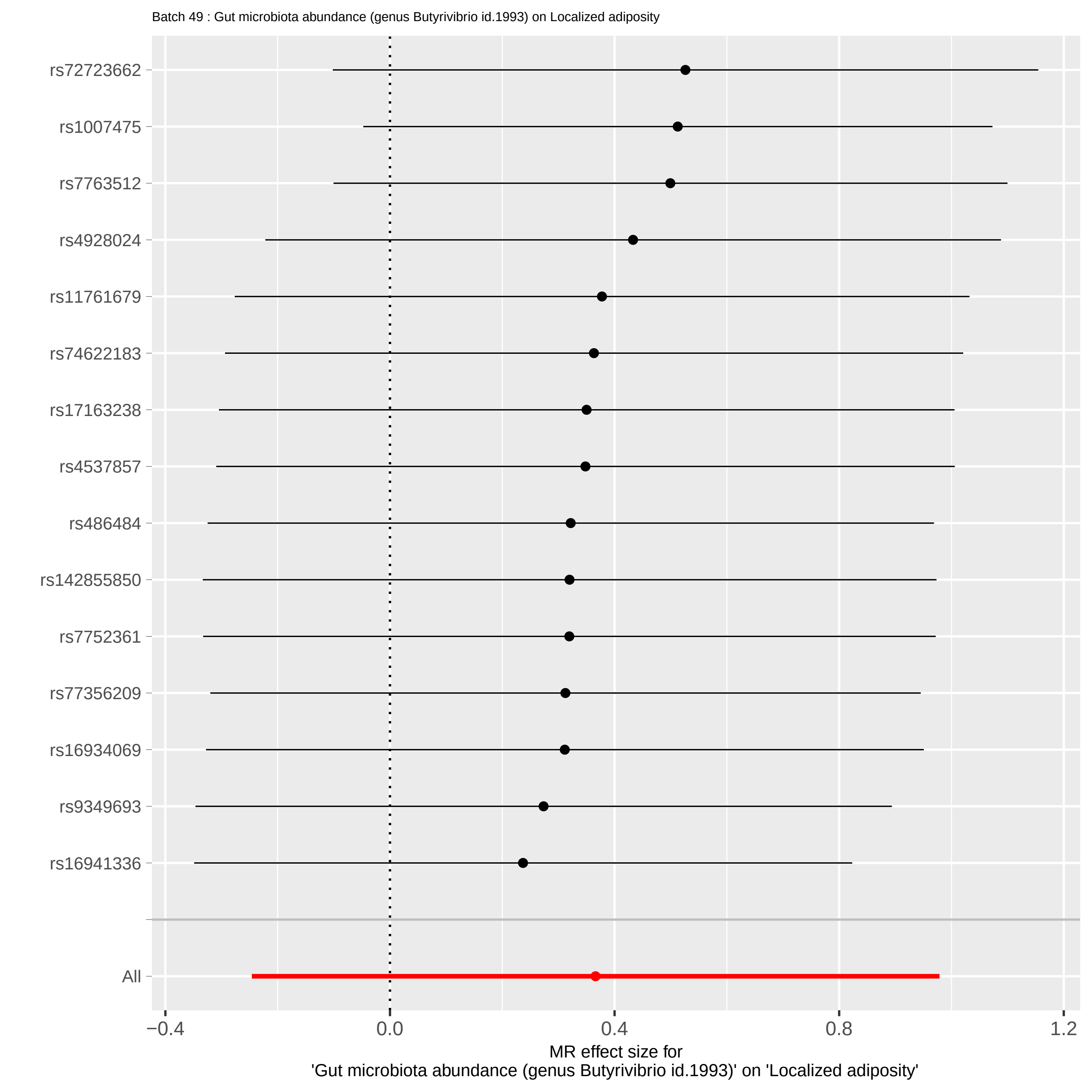

Batch 49 : Gut microbiota abundance (genus Butyrivibrio id.1993) on Localized adiposity
rs72723662
rs1007475
rs7763512
rs4928024
rs11761679
rs74622183
rs17163238
rs4537857
rs486484
rs142855850
rs7752361
rs77356209
rs16934069
rs9349693
rs16941336
All
−0.4
0.0
0.4
0.8
1.2
MR effect size for
'Gut microbiota abundance (genus Butyrivibrio id.1993)' on 'Localized adiposity'

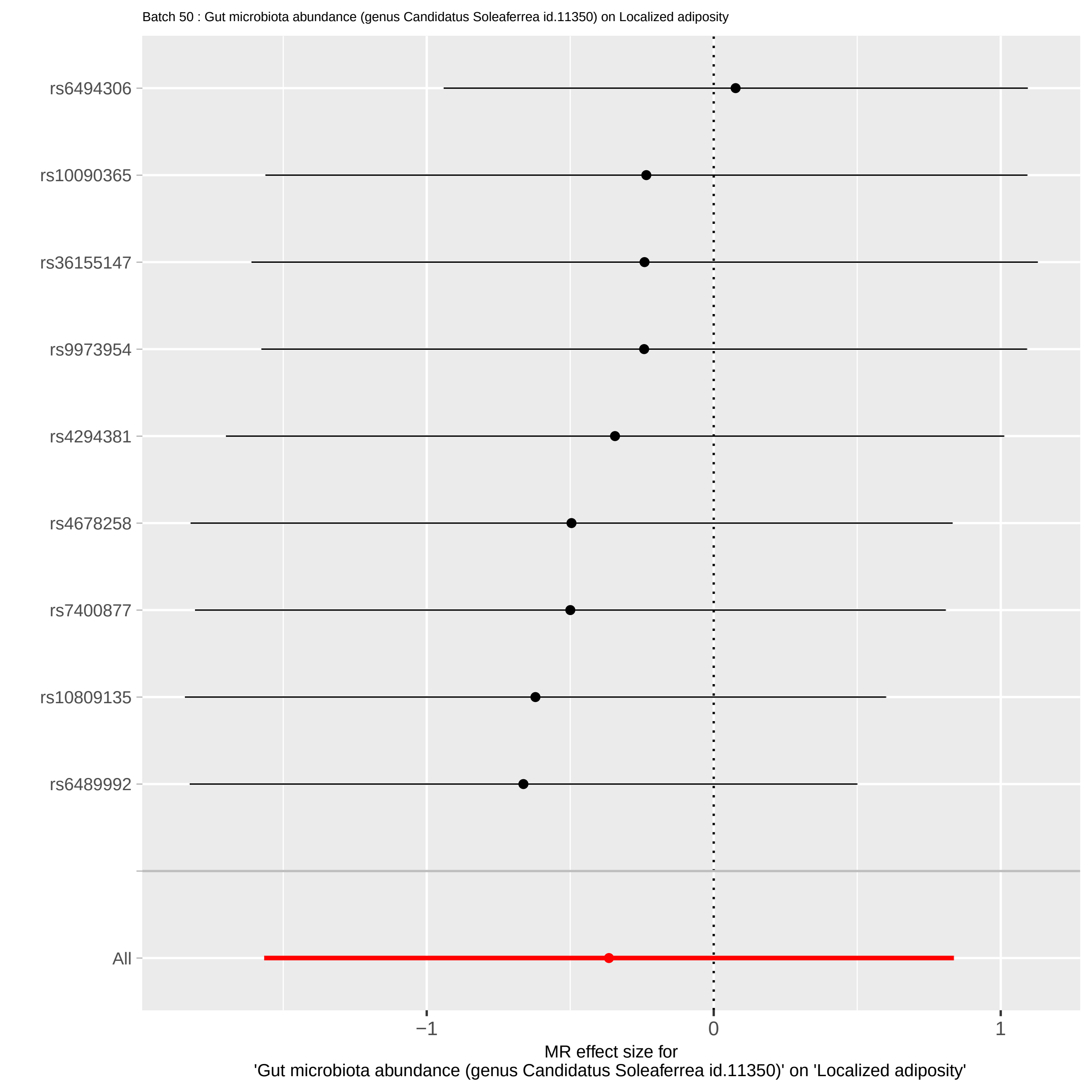
Batch 50 : Gut microbiota abundance (genus Candidatus Soleaferrea id.11350) on Localized adiposity
rs6494306
rs10090365
rs36155147
rs9973954
rs4294381
rs4678258
rs7400877
rs10809135
rs6489992
All
−1
0
1
MR effect size for
'Gut microbiota abundance (genus Candidatus Soleaferrea id.11350)' on 'Localized adiposity'

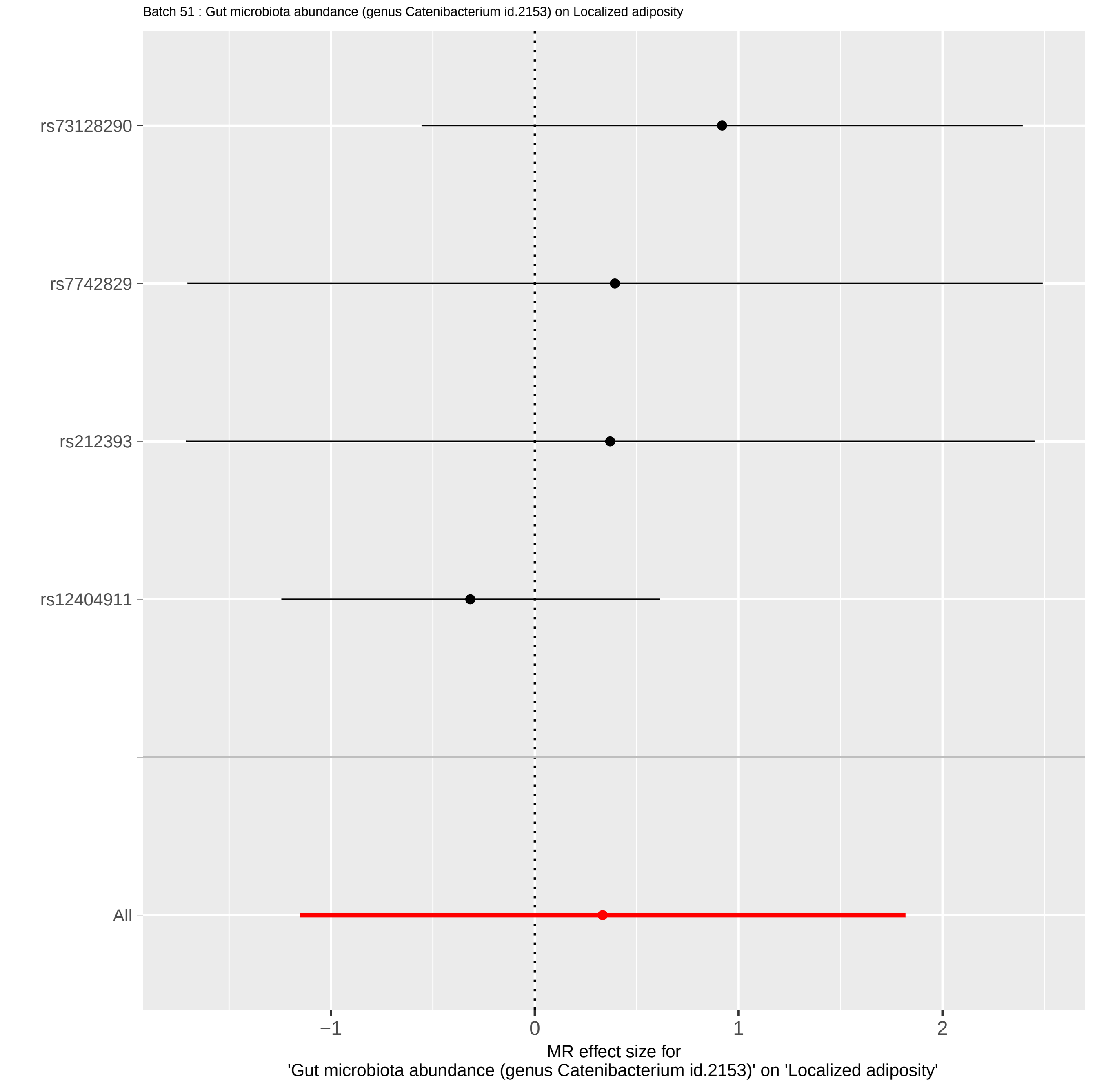
Batch 51 : Gut microbiota abundance (genus Catenibacterium id.2153) on Localized adiposity
rs73128290
rs7742829
rs212393
rs12404911
All
−1
0
1
2
MR effect size for
'Gut microbiota abundance (genus Catenibacterium id.2153)' on 'Localized adiposity'

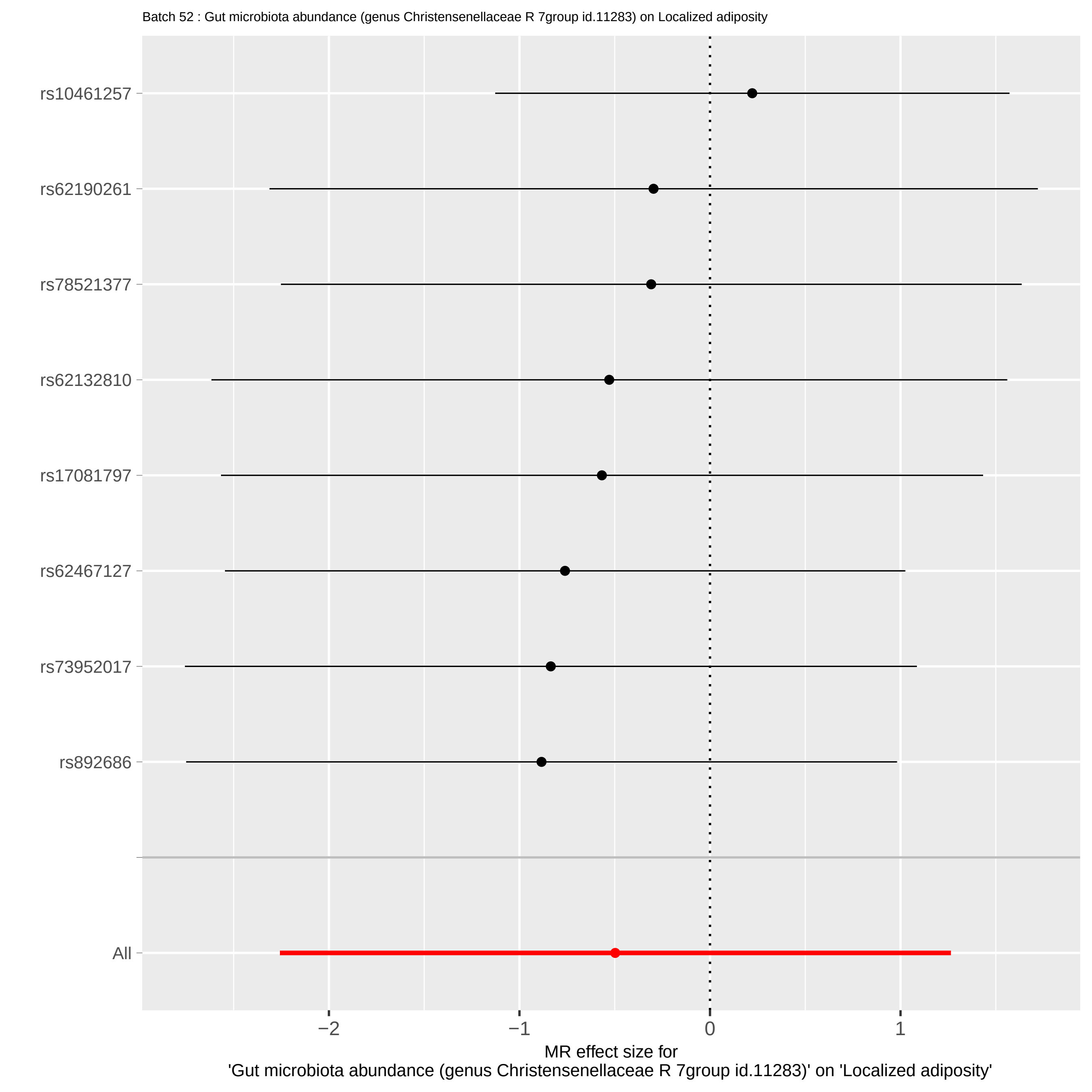

Batch 52 : Gut microbiota abundance (genus Christensenellaceae R 7group id.11283) on Localized adiposity
rs10461257
rs62190261
rs78521377
rs62132810
rs17081797
rs62467127
rs73952017
rs892686
All
−2
−1
0
1
MR effect size for
'Gut microbiota abundance (genus Christensenellaceae R 7group id.11283)' on 'Localized adiposity'

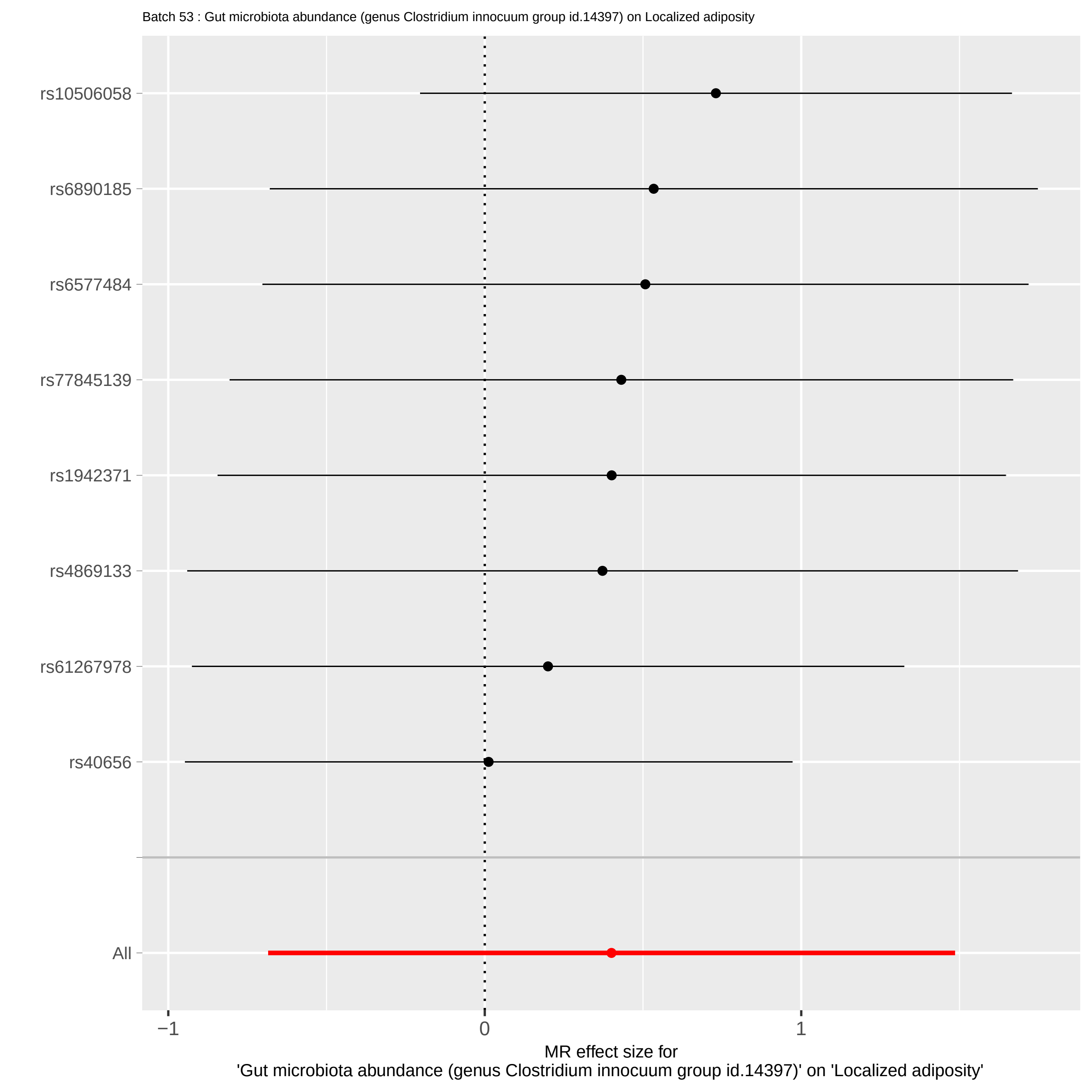

Batch 53 : Gut microbiota abundance (genus Clostridium innocuum group id.14397) on Localized adiposity
rs10506058
rs6890185
rs6577484
rs77845139
rs1942371
rs4869133
rs61267978
rs40656
All
−1
0
1
MR effect size for
'Gut microbiota abundance (genus Clostridium innocuum group id.14397)' on 'Localized adiposity'

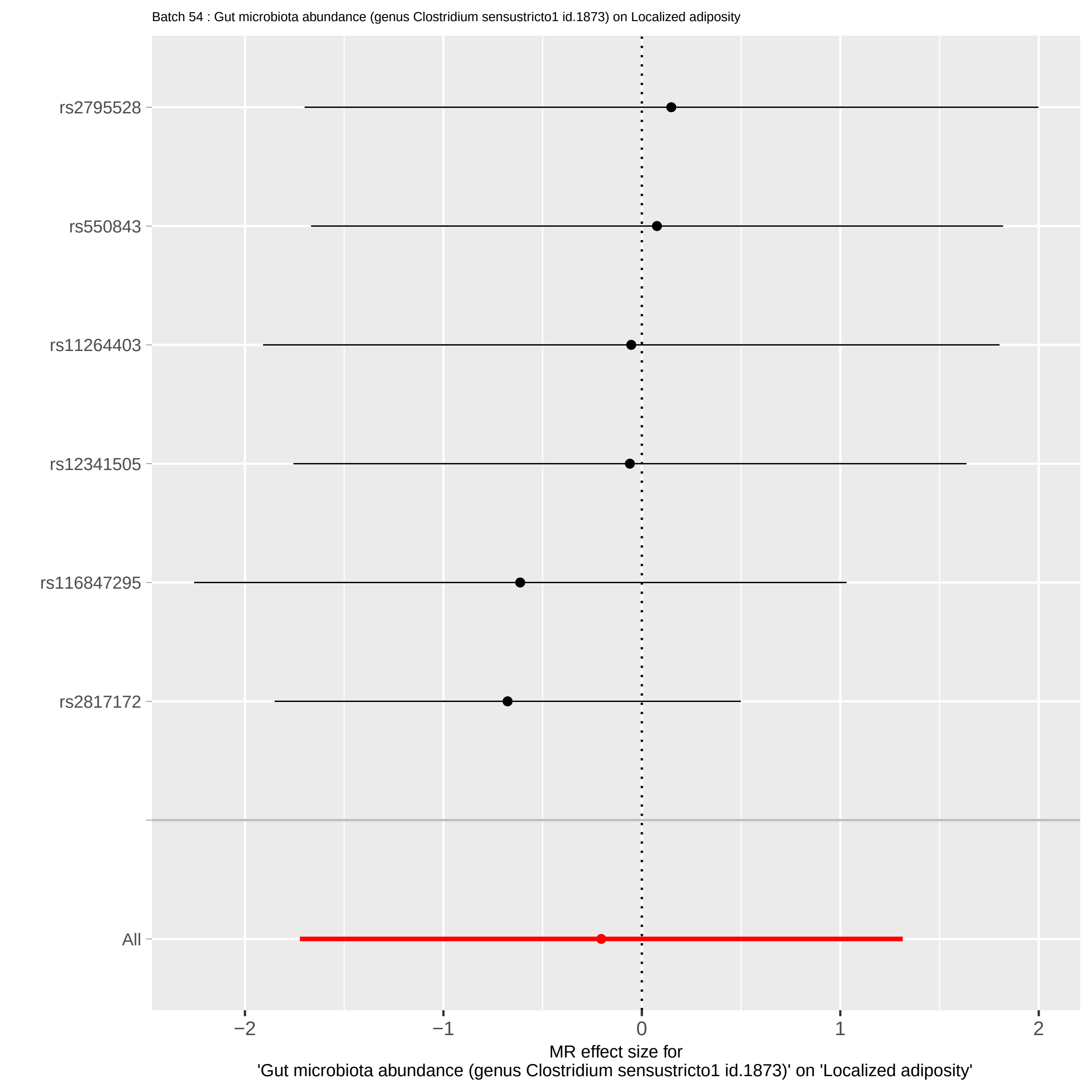

Batch 54 : Gut microbiota abundance (genus Clostridium sensustricto1 id.1873) on Localized adiposity
rs2795528
rs550843
rs11264403
rs12341505
rs116847295
rs2817172
All
−2
−1
0
1
2
MR effect size for
'Gut microbiota abundance (genus Clostridium sensustricto1 id.1873)' on 'Localized adiposity'

Batch 55 : Gut microbiota abundance (genus Collinsella id.815) on Localized adiposity

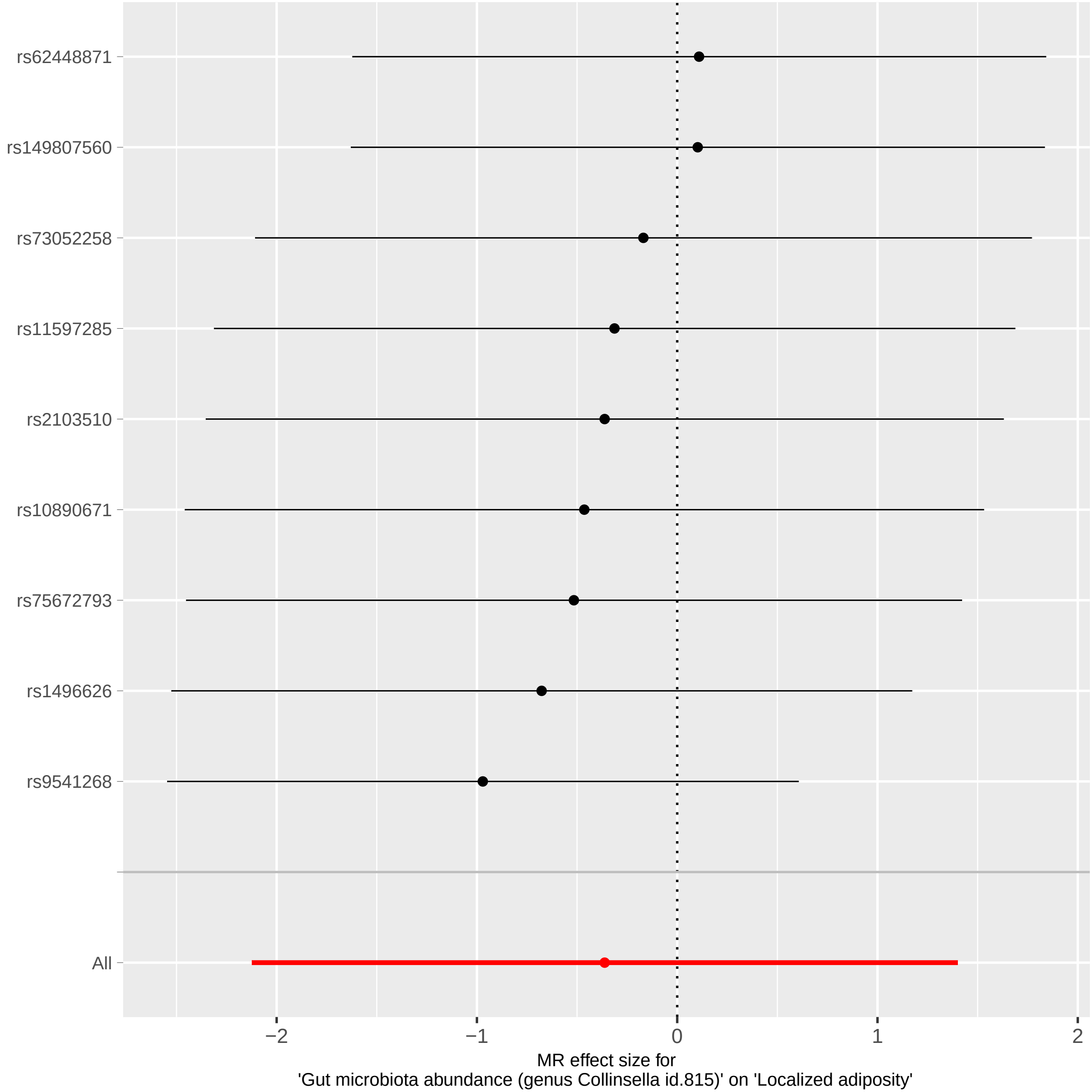

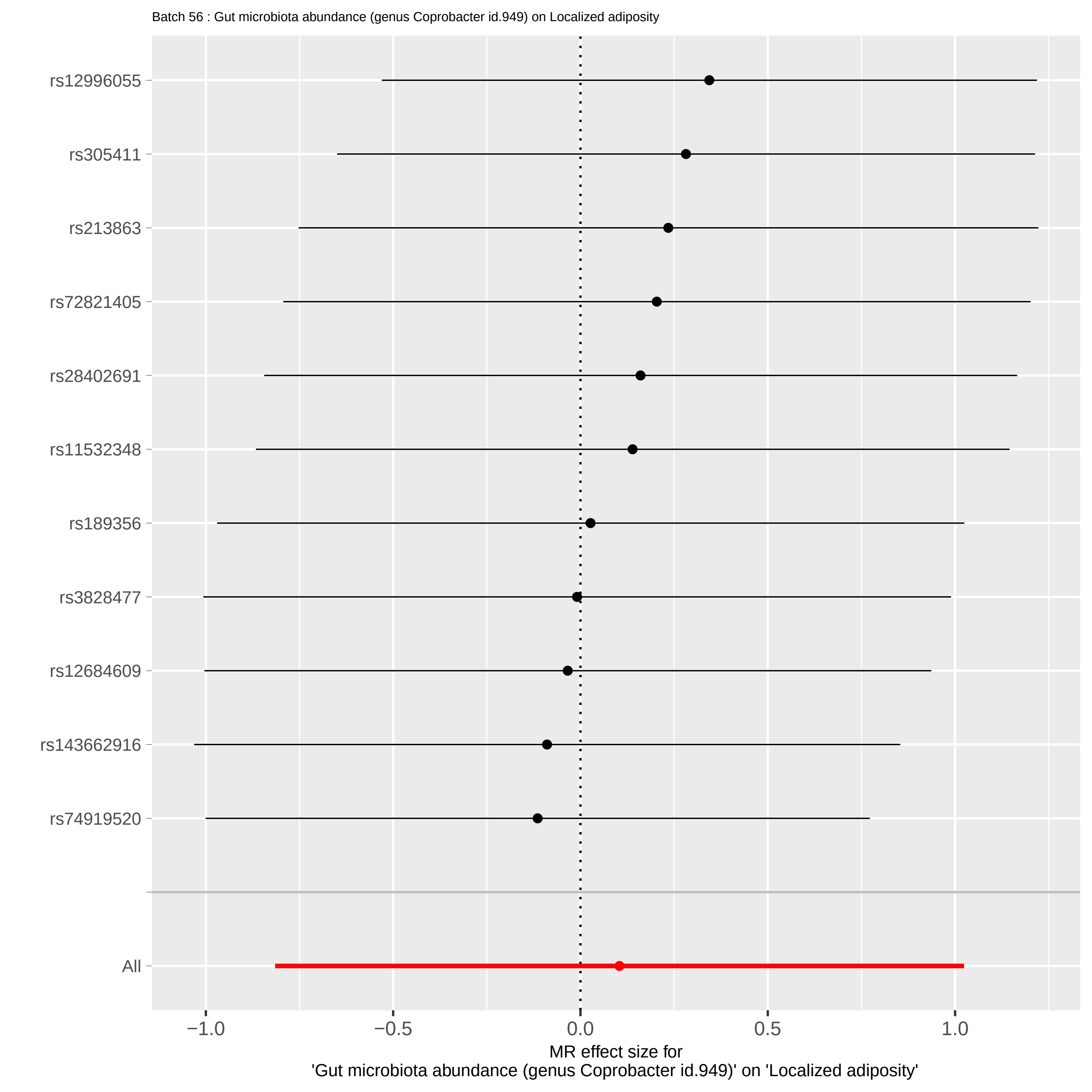

Batch 56 : Gut microbiota abundance (genus Coprobacter id.949) on Localized adiposity
rs12996055
rs305411
rs213863
rs72821405
rs28402691
rs11532348
rs189356
rs3828477
rs12684609
rs143662916
rs74919520
All
−1.0
−0.5
0.0
0.5
1.0
MR effect size for
'Gut microbiota abundance (genus Coprobacter id.949)' on 'Localized adiposity'

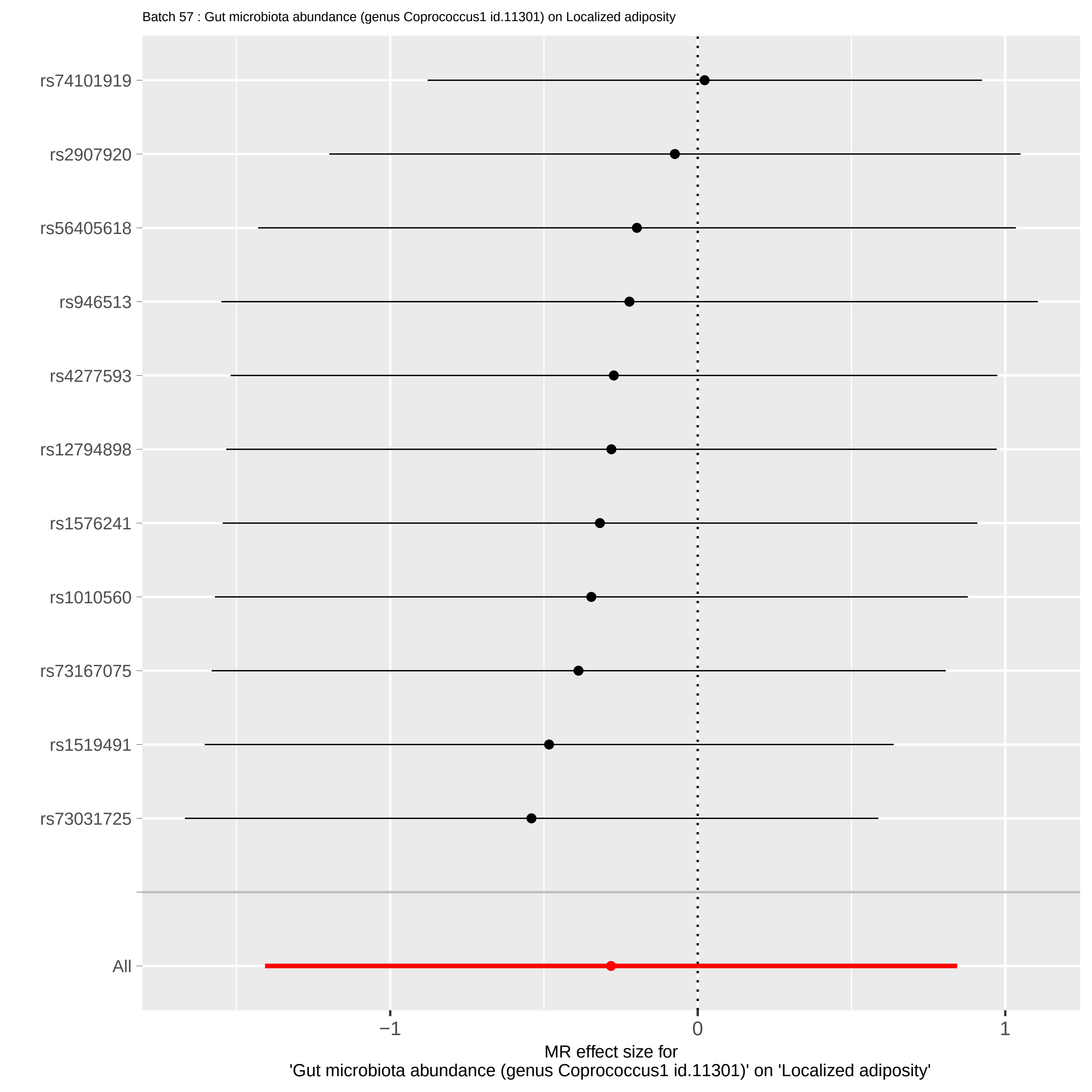
Batch 57 : Gut microbiota abundance (genus Coprococcus1 id.11301) on Localized adiposity
rs74101919
rs2907920
rs56405618
rs946513
rs4277593
rs12794898
rs1576241
rs1010560
rs73167075
rs1519491
rs73031725
All
−1
0
1
MR effect size for
'Gut microbiota abundance (genus Coprococcus1 id.11301)' on 'Localized adiposity'

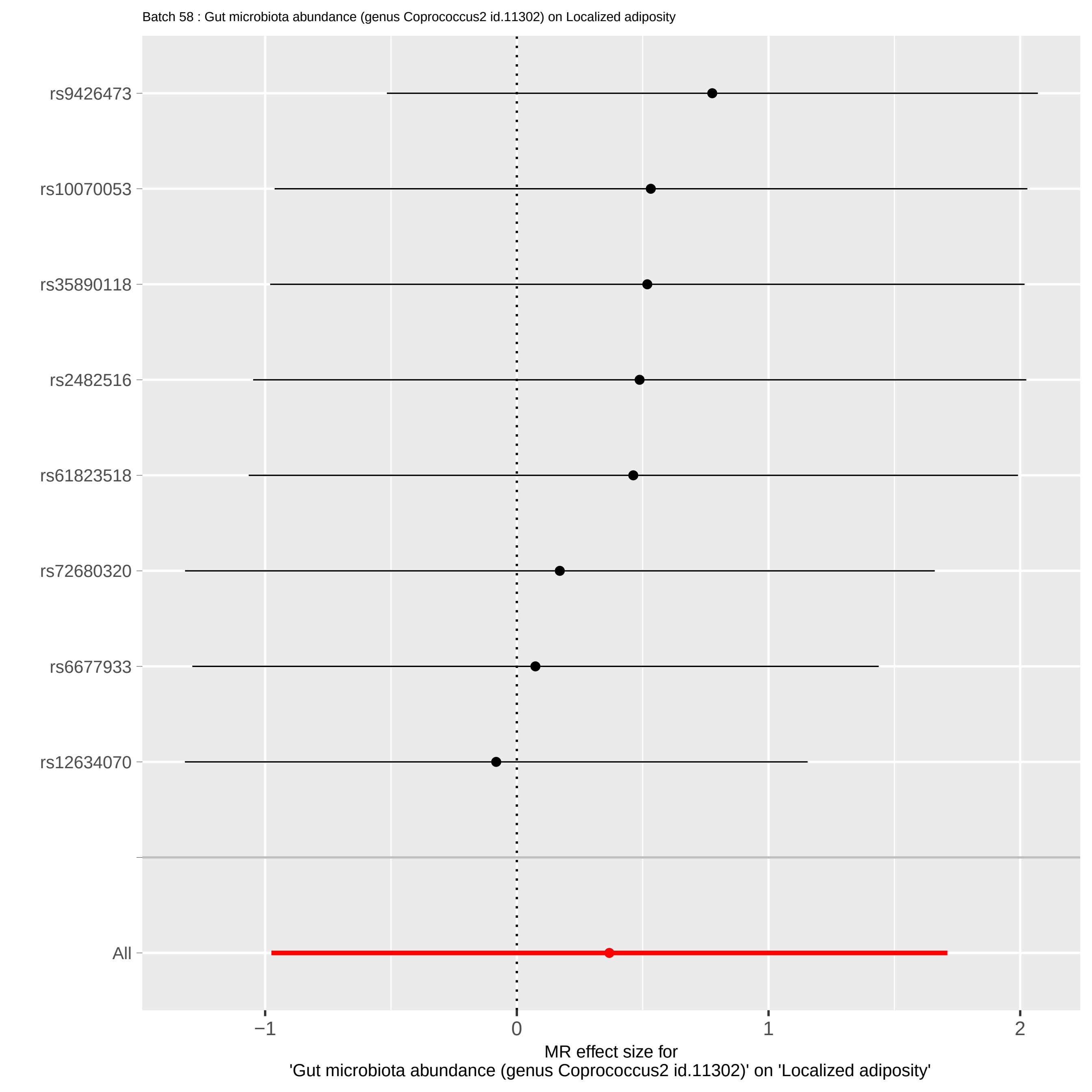

Batch 58 : Gut microbiota abundance (genus Coprococcus2 id.11302) on Localized adiposity
rs9426473
rs10070053
rs35890118
rs2482516
rs61823518
rs72680320
rs6677933
rs12634070
All
−1
0
1
2
MR effect size for
'Gut microbiota abundance (genus Coprococcus2 id.11302)' on 'Localized adiposity'

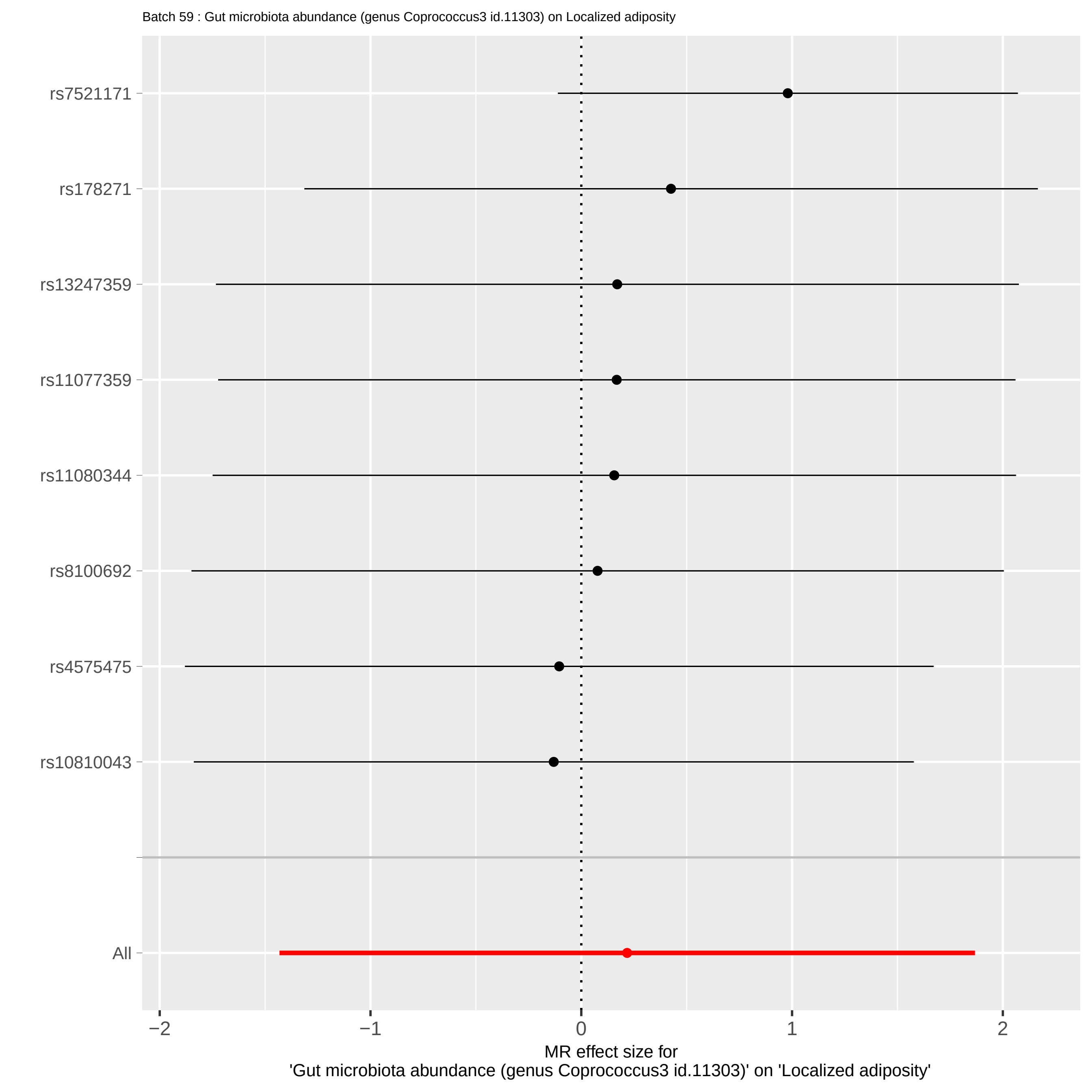

Batch 59 : Gut microbiota abundance (genus Coprococcus3 id.11303) on Localized adiposity
rs7521171
rs178271
rs13247359
rs11077359
rs11080344
rs8100692
rs4575475
rs10810043
All
−2
−1
0
1
2
MR effect size for
'Gut microbiota abundance (genus Coprococcus3 id.11303)' on 'Localized adiposity'

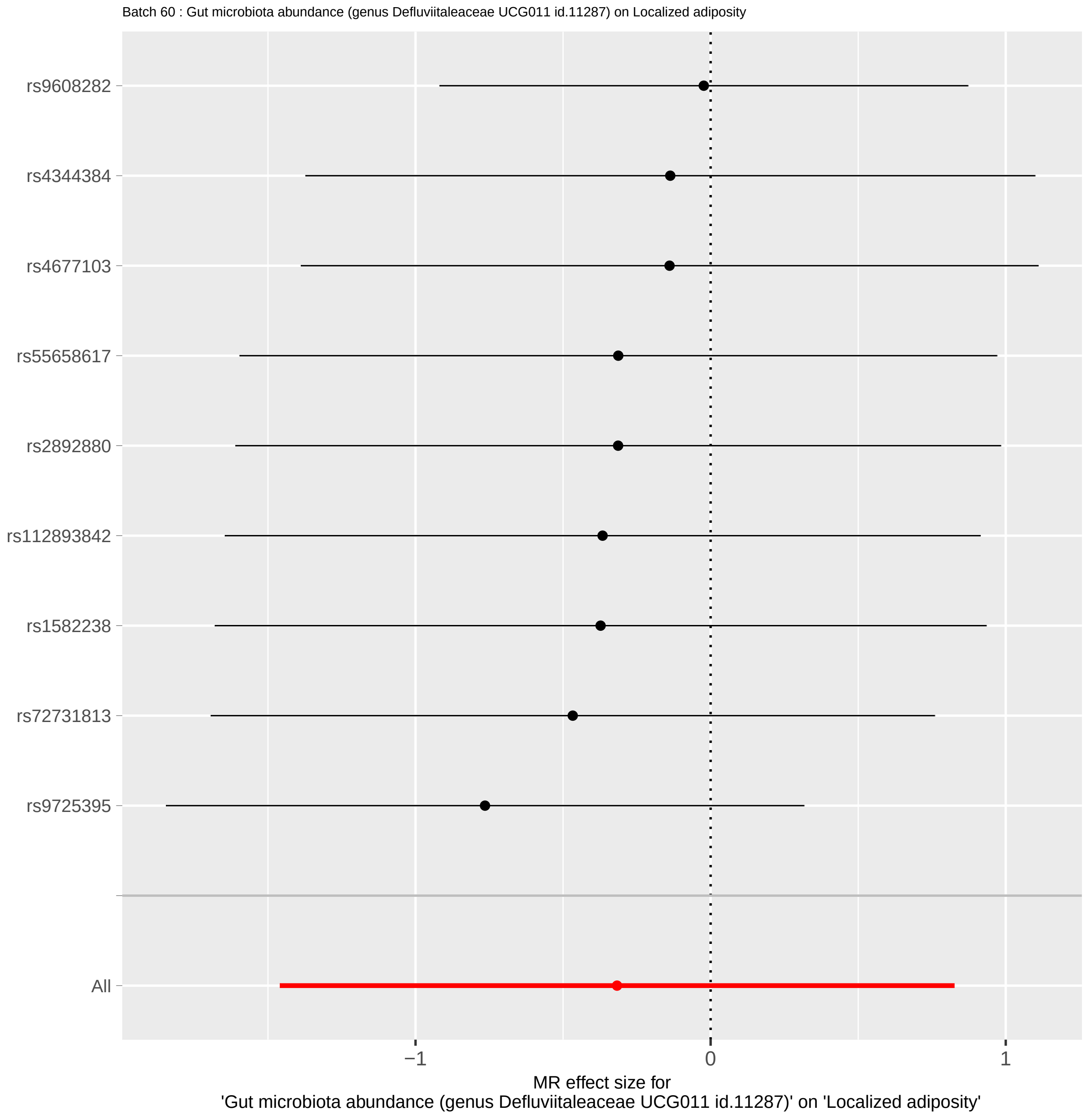

Batch 60 : Gut microbiota abundance (genus Defluviitaleaceae UCG011 id.11287) on Localized adiposity
rs9608282
rs4344384
rs4677103
rs55658617
rs2892880
rs112893842
rs1582238
rs72731813
rs9725395
All
−1
0
1
MR effect size for
'Gut microbiota abundance (genus Defluviitaleaceae UCG011 id.11287)' on 'Localized adiposity'

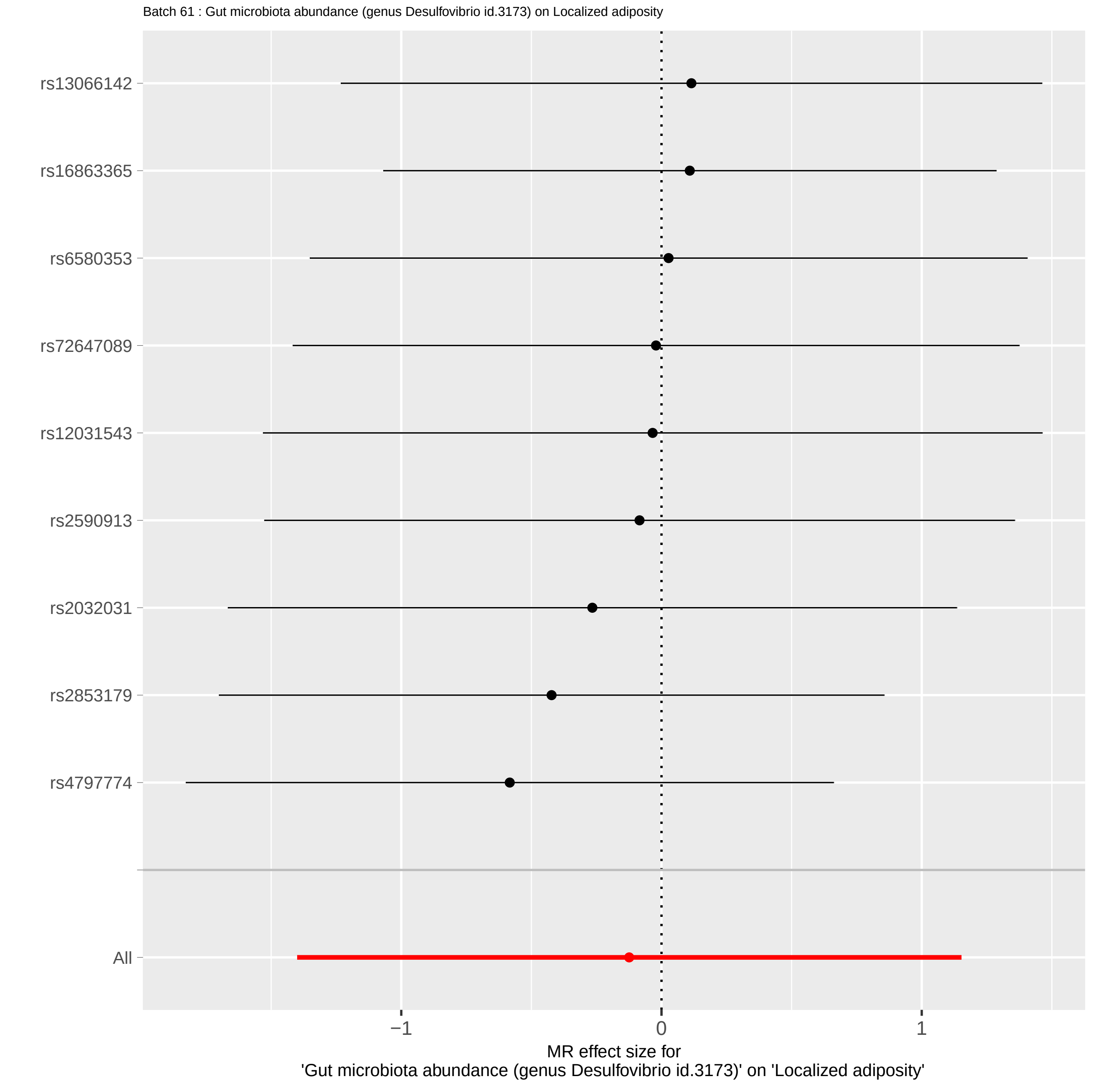
Batch 61 : Gut microbiota abundance (genus Desulfovibrio id.3173) on Localized adiposity
rs13066142
rs16863365
rs6580353
rs72647089
rs12031543
rs2590913
rs2032031
rs2853179
rs4797774
All
−1
0
1
MR effect size for
'Gut microbiota abundance (genus Desulfovibrio id.3173)' on 'Localized adiposity'

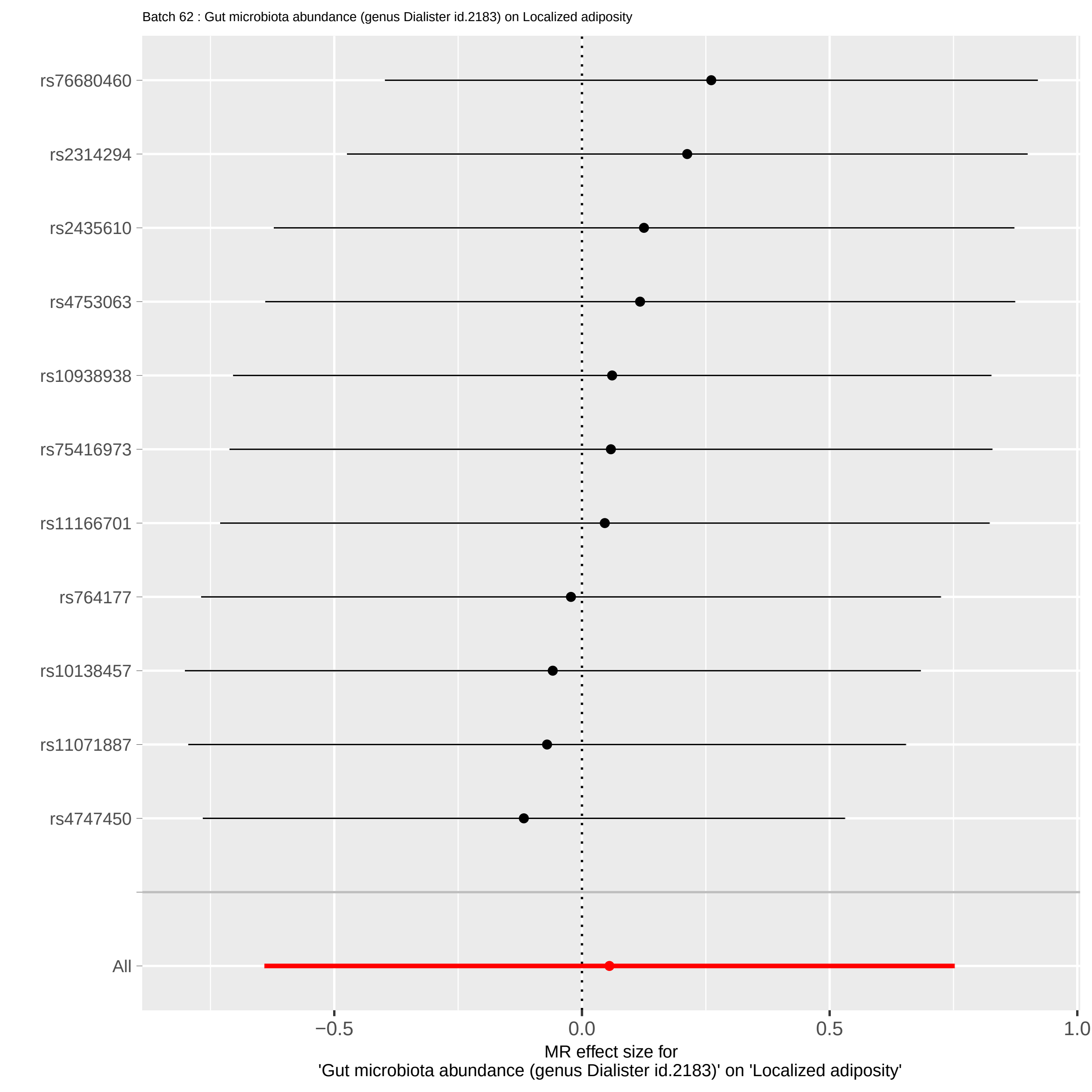
Batch 62 : Gut microbiota abundance (genus Dialister id.2183) on Localized adiposity
rs76680460
rs2314294
rs2435610
rs4753063
rs10938938
rs75416973
rs11166701
rs764177
rs10138457
rs11071887
rs4747450
All
−0.5
0.0
0.5
1.0
MR effect size for
'Gut microbiota abundance (genus Dialister id.2183)' on 'Localized adiposity'

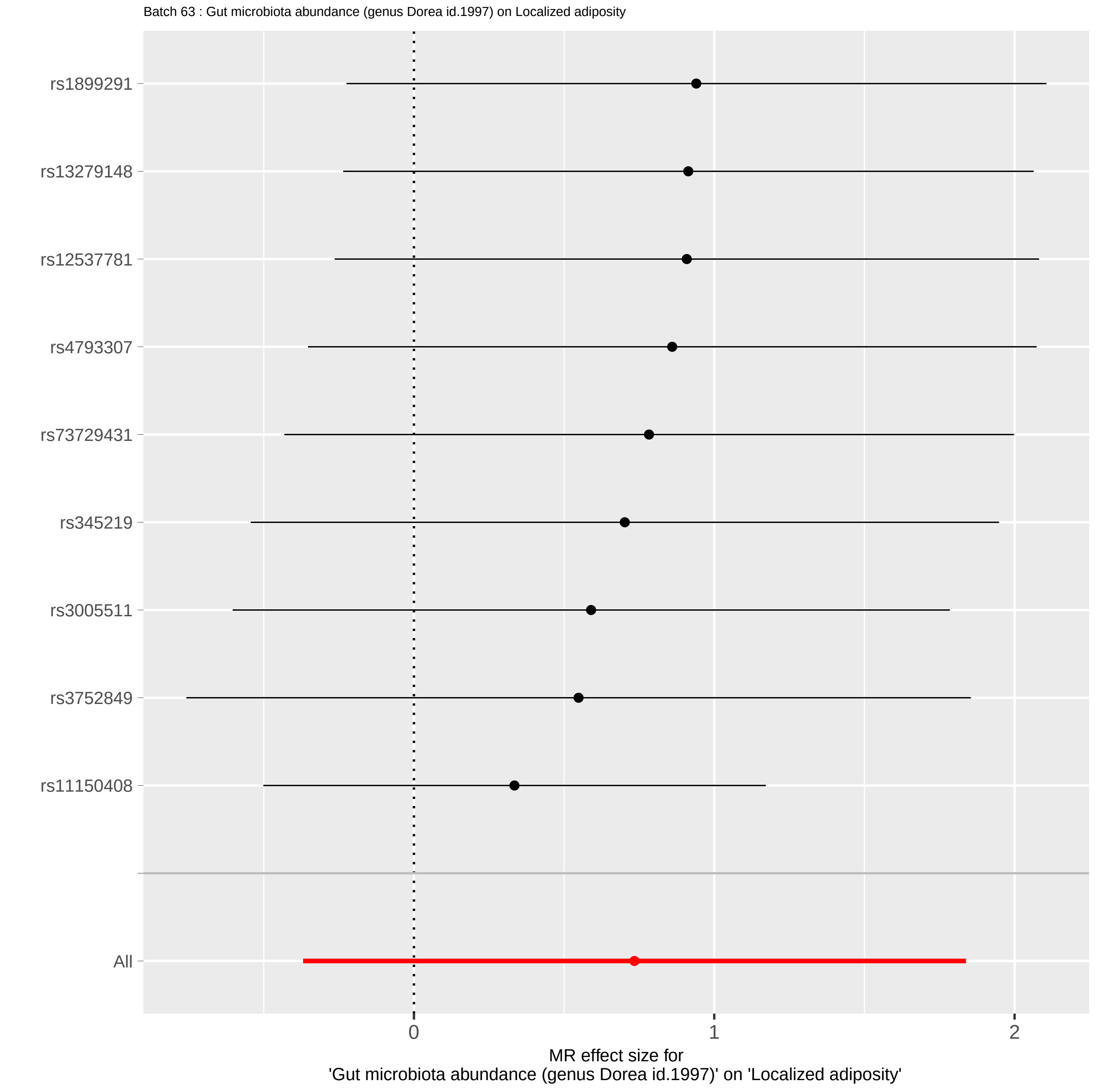
Batch 63 : Gut microbiota abundance (genus Dorea id.1997) on Localized adiposity
rs1899291
rs13279148
rs12537781
rs4793307
rs73729431
rs345219
rs3005511
rs3752849
rs11150408
All
0
1
2
MR effect size for
'Gut microbiota abundance (genus Dorea id.1997)' on 'Localized adiposity'

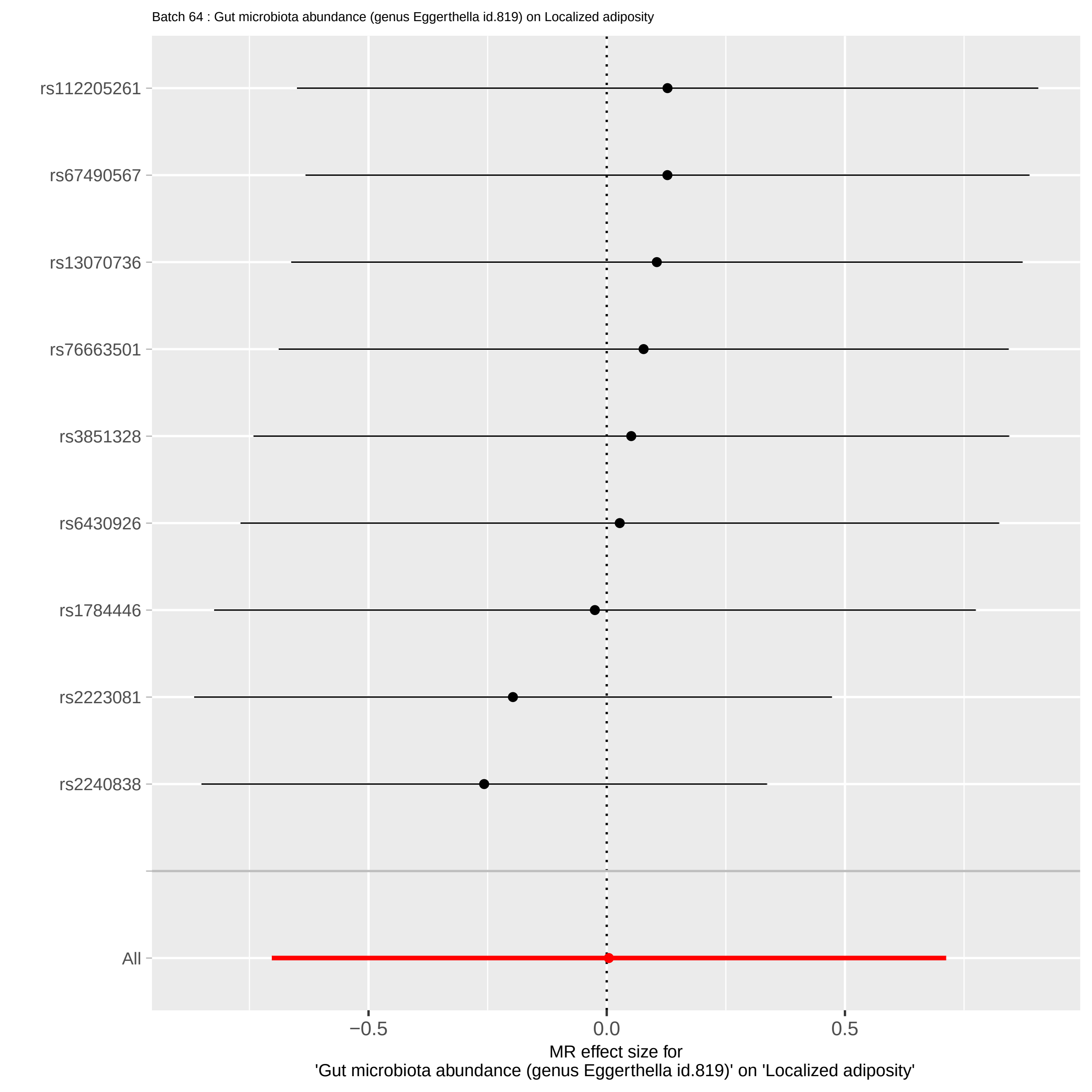

Batch 64 : Gut microbiota abundance (genus Eggerthella id.819) on Localized adiposity
rs112205261
rs67490567
rs13070736
rs76663501
rs3851328
rs6430926
rs1784446
rs2223081
rs2240838
All
−0.5
0.0
0.5
MR effect size for
'Gut microbiota abundance (genus Eggerthella id.819)' on 'Localized adiposity'

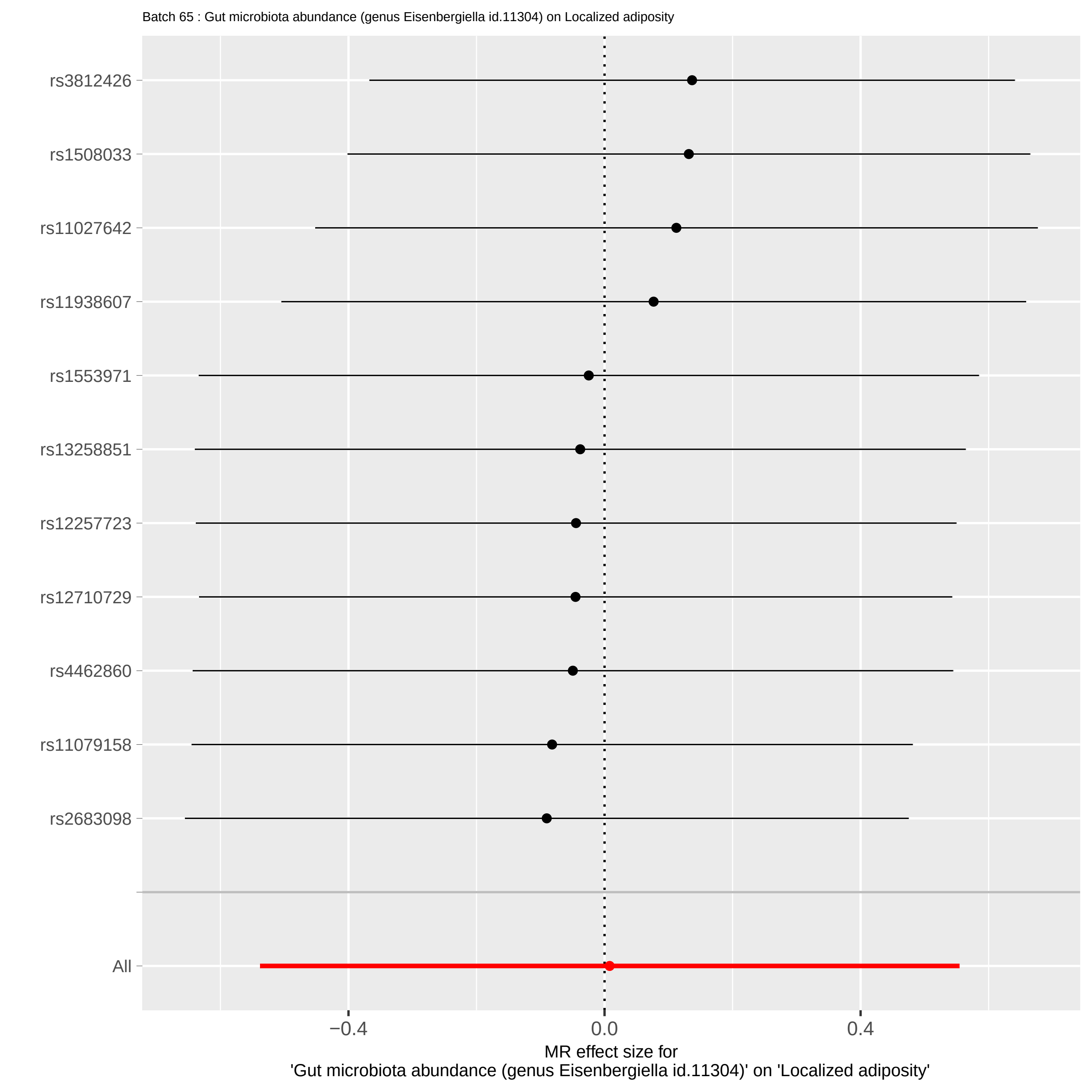
Batch 65 : Gut microbiota abundance (genus Eisenbergiella id.11304) on Localized adiposity
rs3812426
rs1508033
rs11027642
rs11938607
rs1553971
rs13258851
rs12257723
rs12710729
rs4462860
rs11079158
rs2683098
All
−0.4
0.0
0.4
MR effect size for
'Gut microbiota abundance (genus Eisenbergiella id.11304)' on 'Localized adiposity'

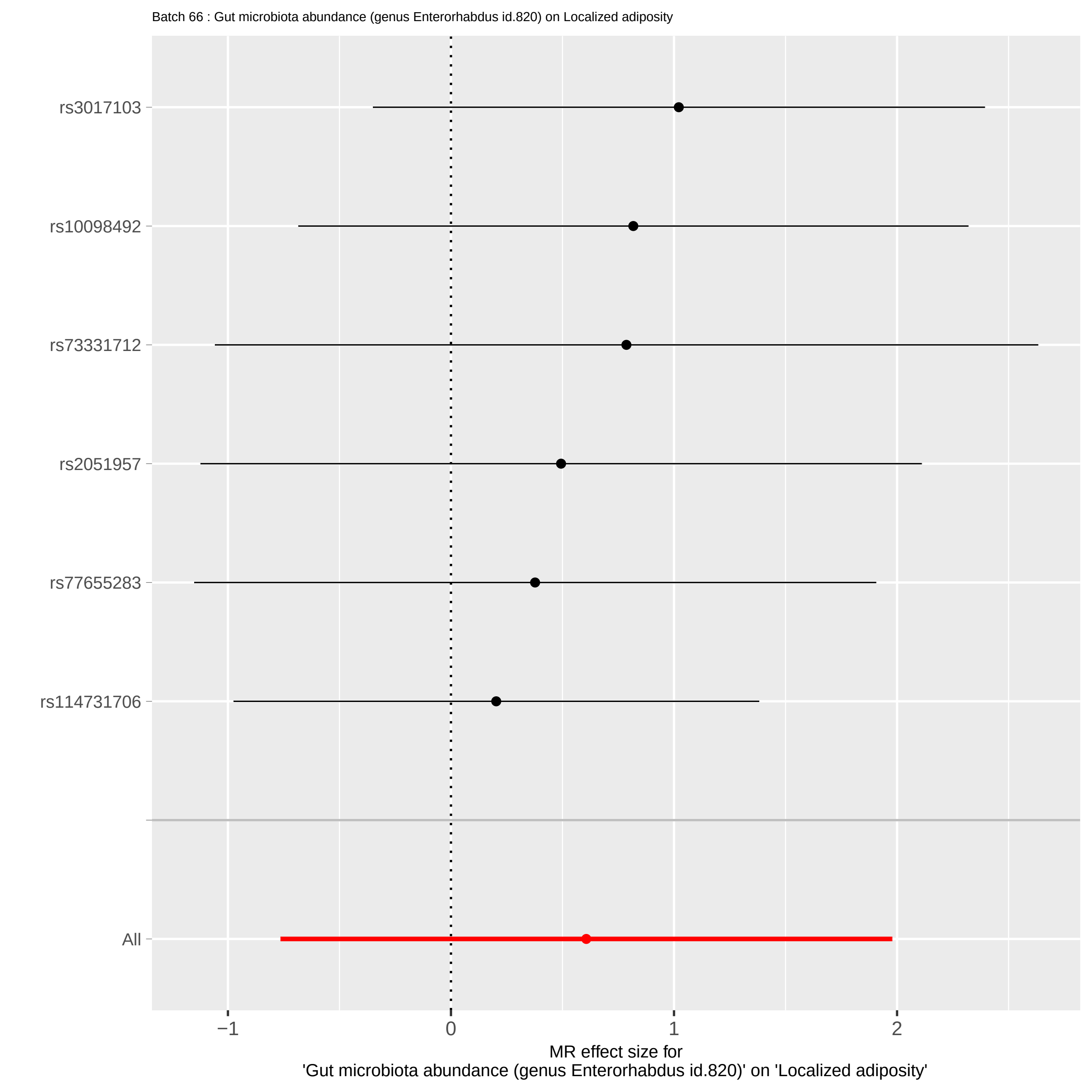

Batch 66 : Gut microbiota abundance (genus Enterorhabdus id.820) on Localized adiposity
rs3017103
rs10098492
rs73331712
rs2051957
rs77655283
rs114731706
All
−1
0
1
2
MR effect size for
'Gut microbiota abundance (genus Enterorhabdus id.820)' on 'Localized adiposity'

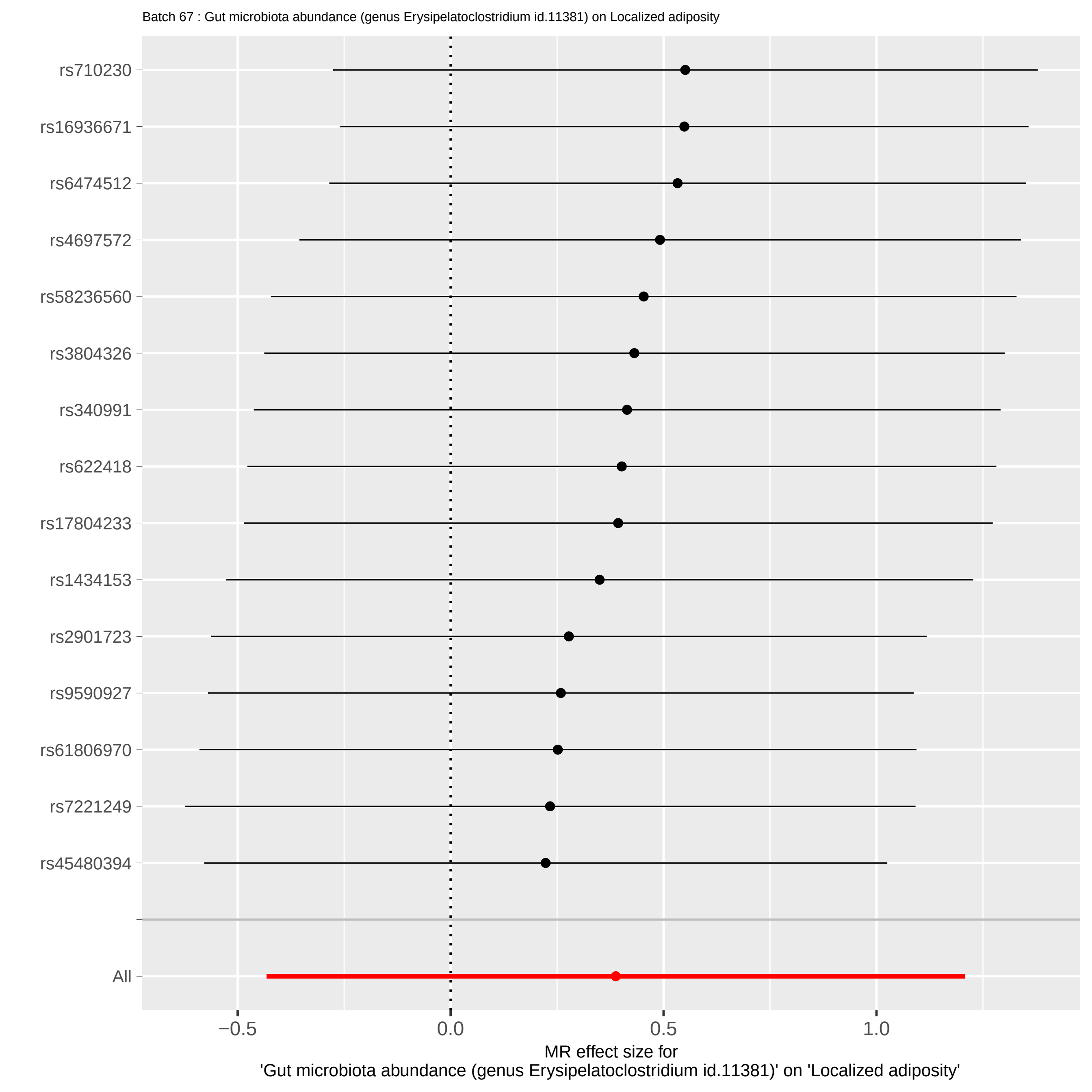

Batch 67 : Gut microbiota abundance (genus Erysipelatoclostridium id.11381) on Localized adiposity
rs710230
rs16936671
rs6474512
rs4697572
rs58236560
rs3804326
rs340991
rs622418
rs17804233
rs1434153
rs2901723
rs9590927
rs61806970
rs7221249
rs45480394
All
−0.5
0.0
0.5
1.0
MR effect size for
'Gut microbiota abundance (genus Erysipelatoclostridium id.11381)' on 'Localized adiposity'

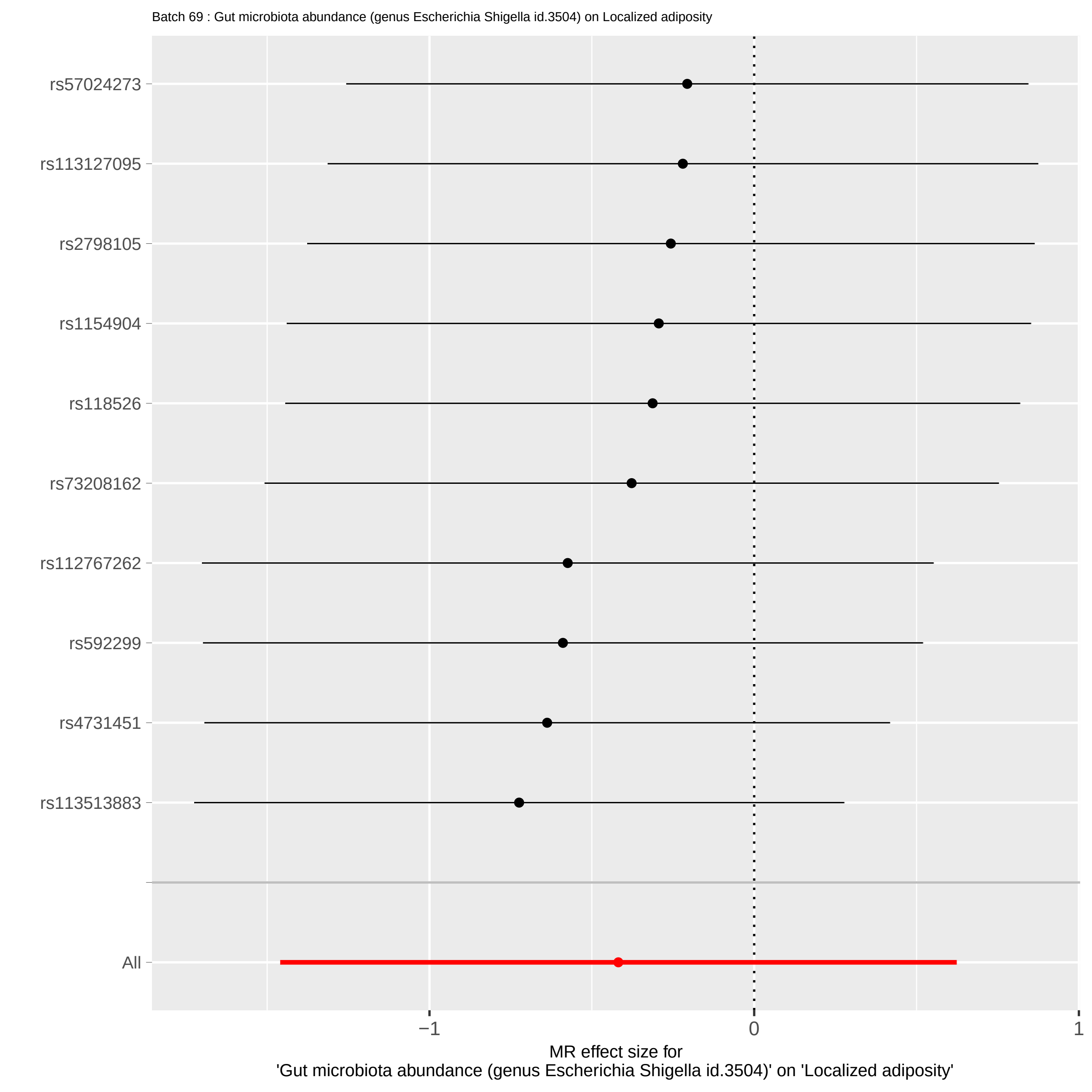

Batch 69 : Gut microbiota abundance (genus Escherichia Shigella id.3504) on Localized adiposity
rs57024273
rs113127095
rs2798105
rs1154904
rs118526
rs73208162
rs112767262
rs592299
rs4731451
rs113513883
All
−1
0
1
MR effect size for
'Gut microbiota abundance (genus Escherichia Shigella id.3504)' on 'Localized adiposity'

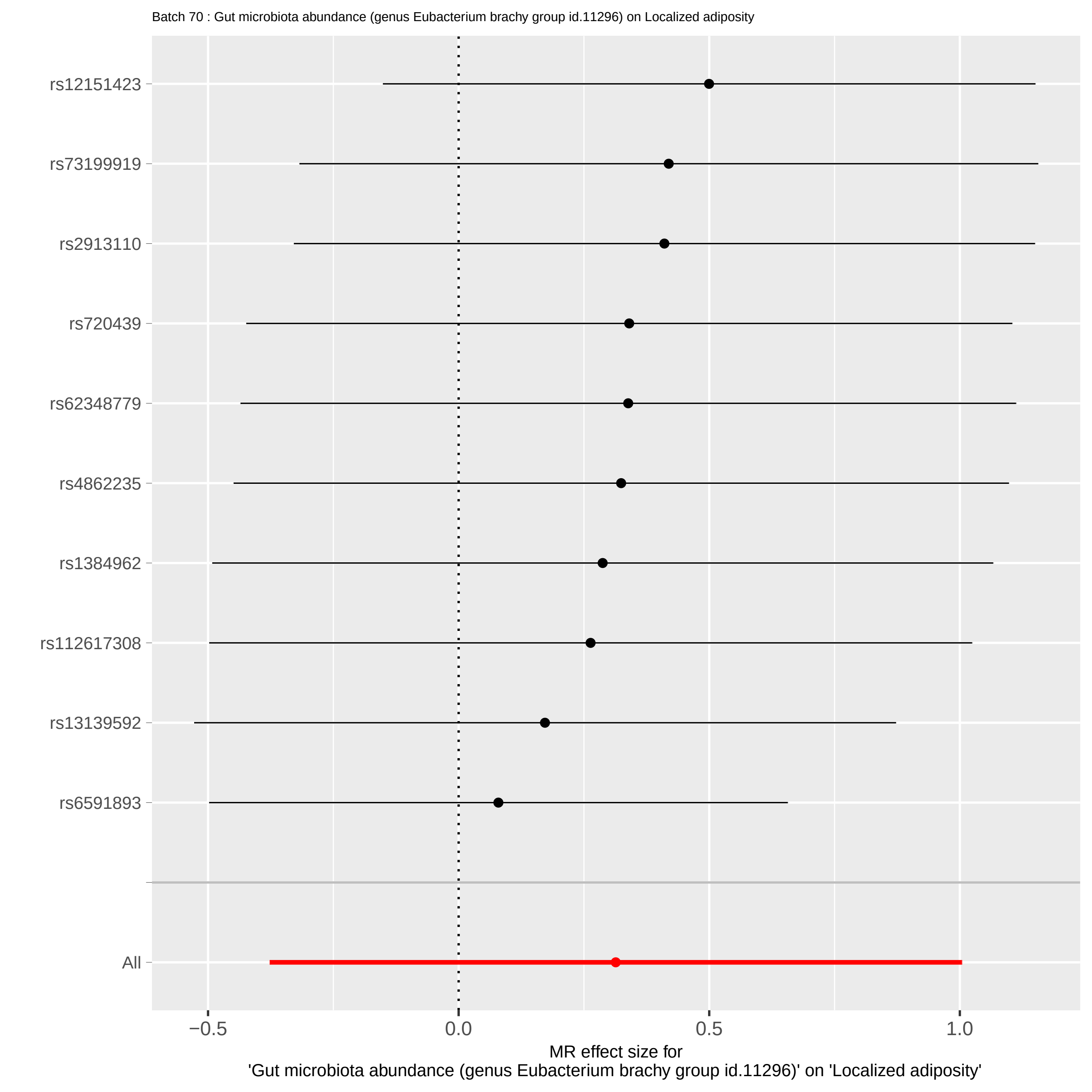

Batch 70 : Gut microbiota abundance (genus Eubacterium brachy group id.11296) on Localized adiposity
rs12151423
rs73199919
rs2913110
rs720439
rs62348779
rs4862235
rs1384962
rs112617308
rs13139592
rs6591893
All
−0.5
0.0
0.5
1.0
MR effect size for
'Gut microbiota abundance (genus Eubacterium brachy group id.11296)' on 'Localized adiposity'

Batch 71 : Gut microbiota abundance (genus Eubacterium coprostanoligenes group id.11375) on Localized adiposity

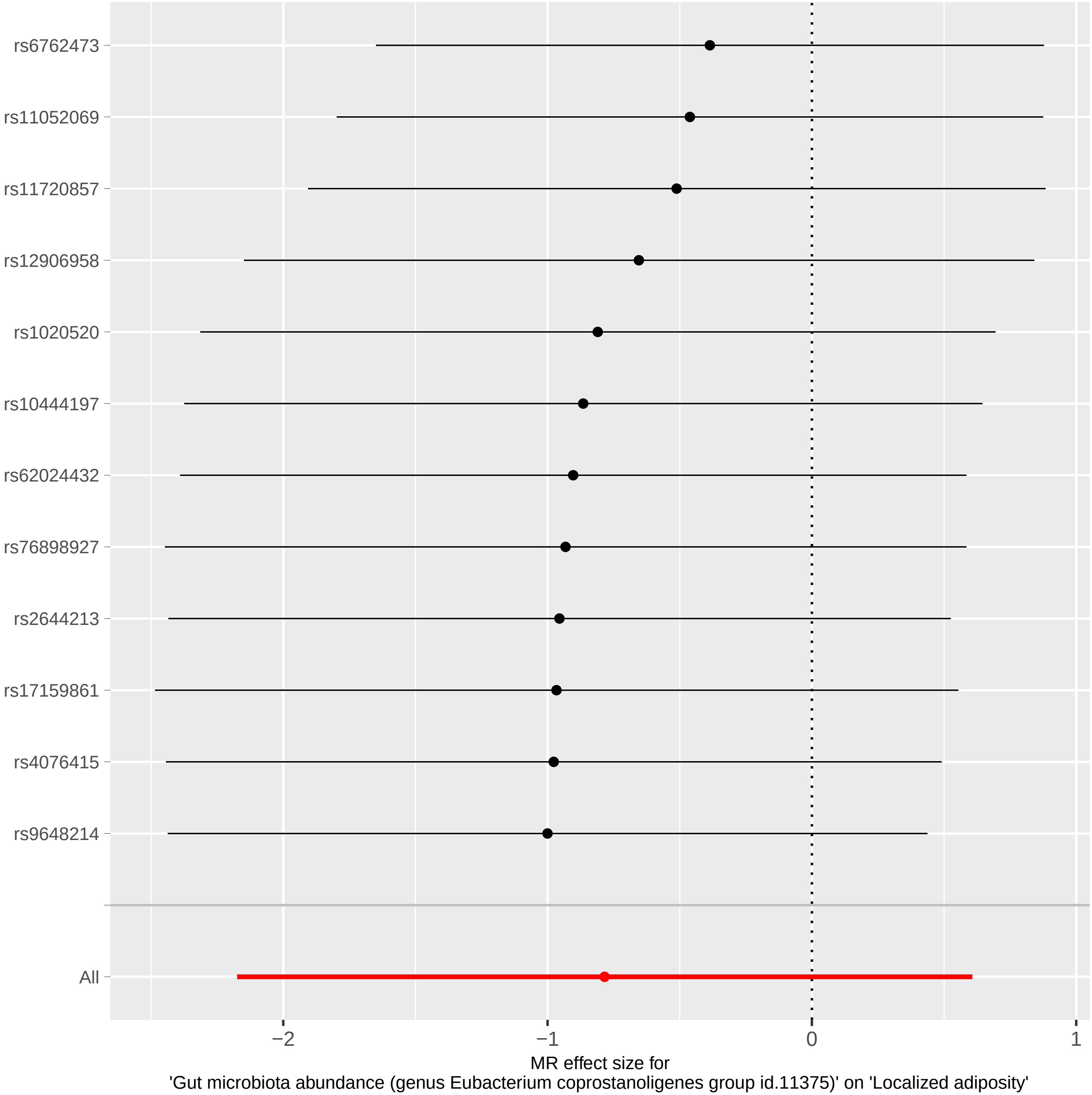

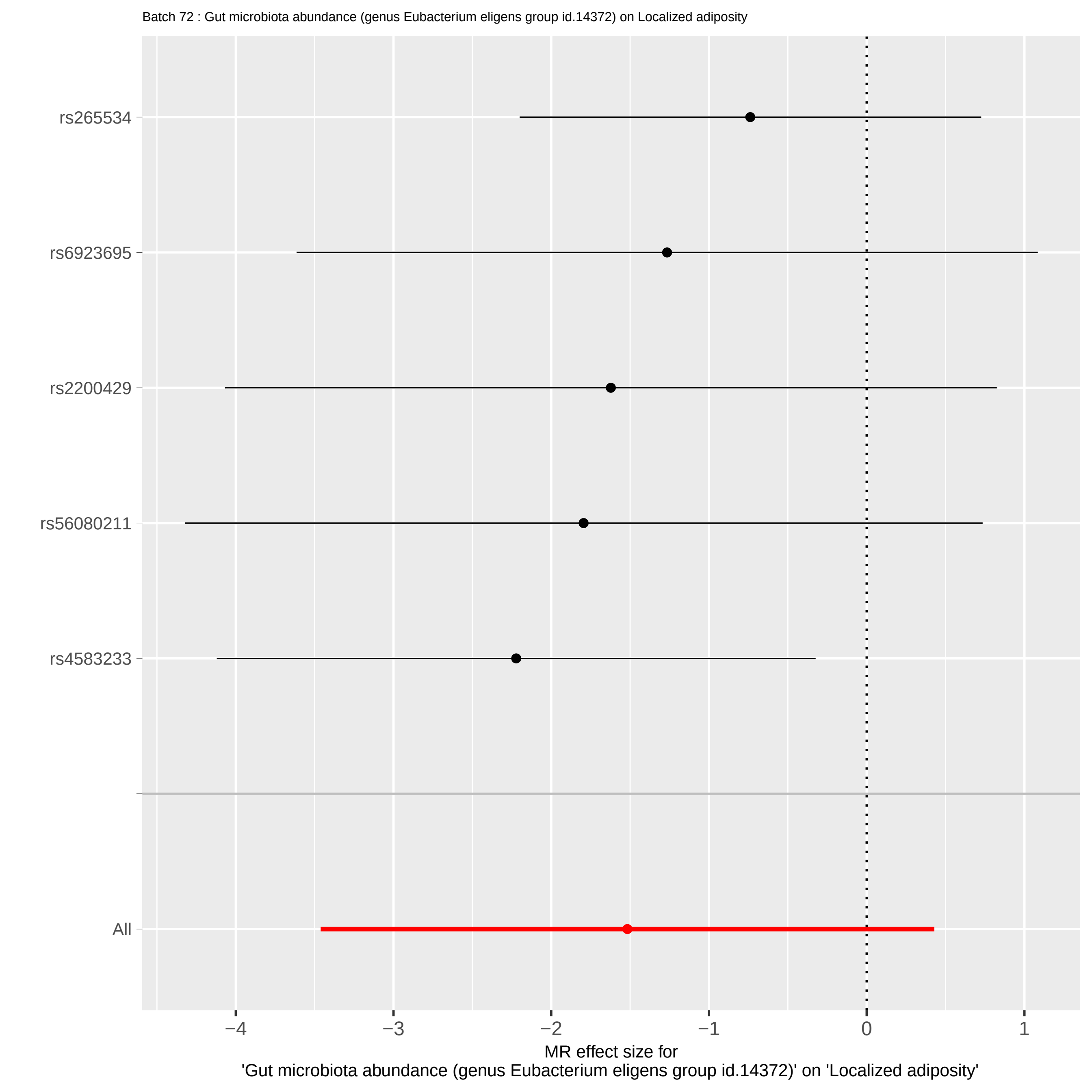
Batch 72 : Gut microbiota abundance (genus Eubacterium eligens group id.14372) on Localized adiposity
rs265534
rs6923695
rs2200429
rs56080211
rs4583233
All
−4
−3
−2
−1
0
1
MR effect size for
'Gut microbiota abundance (genus Eubacterium eligens group id.14372)' on 'Localized adiposity'

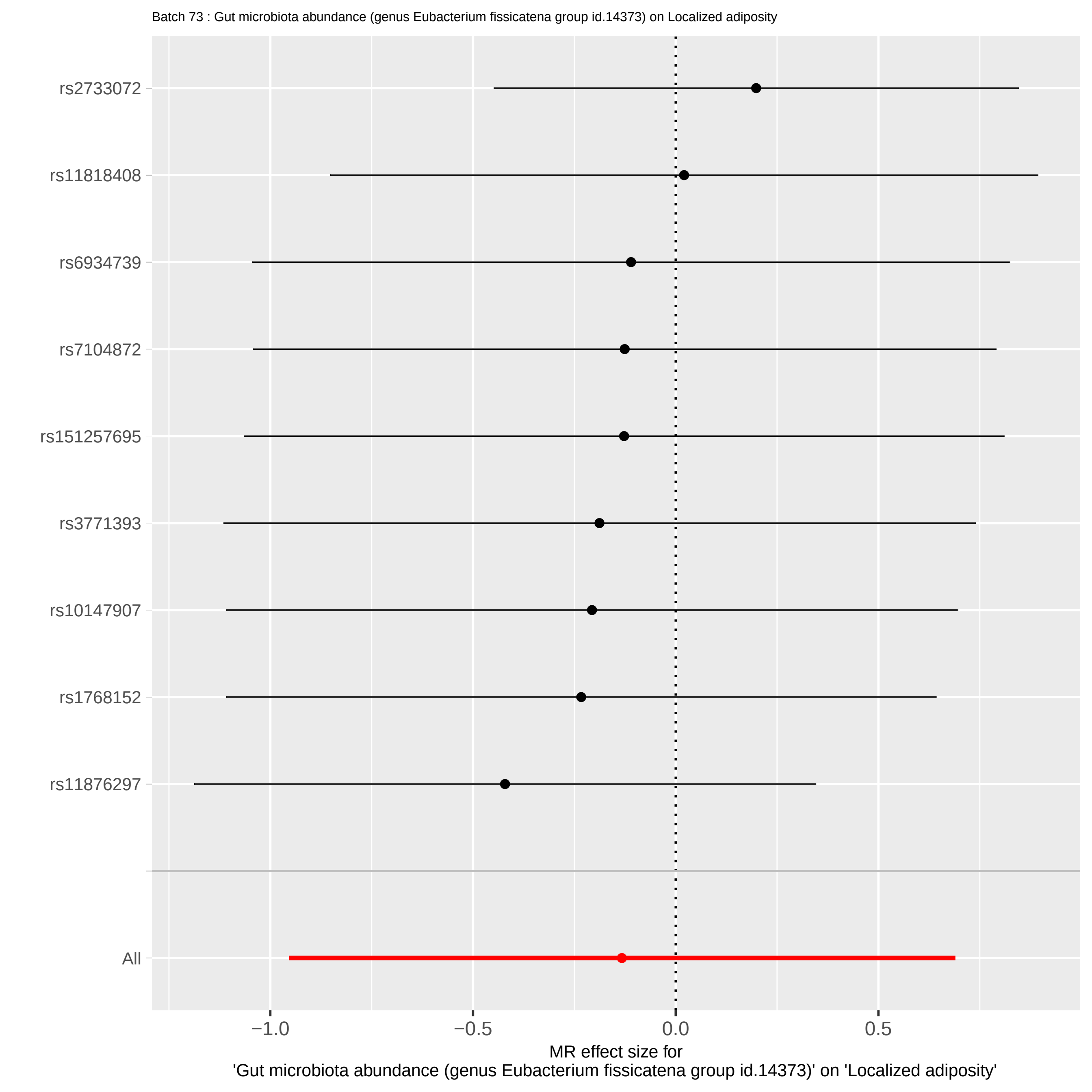

Batch 73 : Gut microbiota abundance (genus Eubacterium fissicatena group id.14373) on Localized adiposity
rs2733072
rs11818408
rs6934739
rs7104872
rs151257695
rs3771393
rs10147907
rs1768152
rs11876297
All
−1.0
−0.5
0.0
0.5
MR effect size for
'Gut microbiota abundance (genus Eubacterium fissicatena group id.14373)' on 'Localized adiposity'

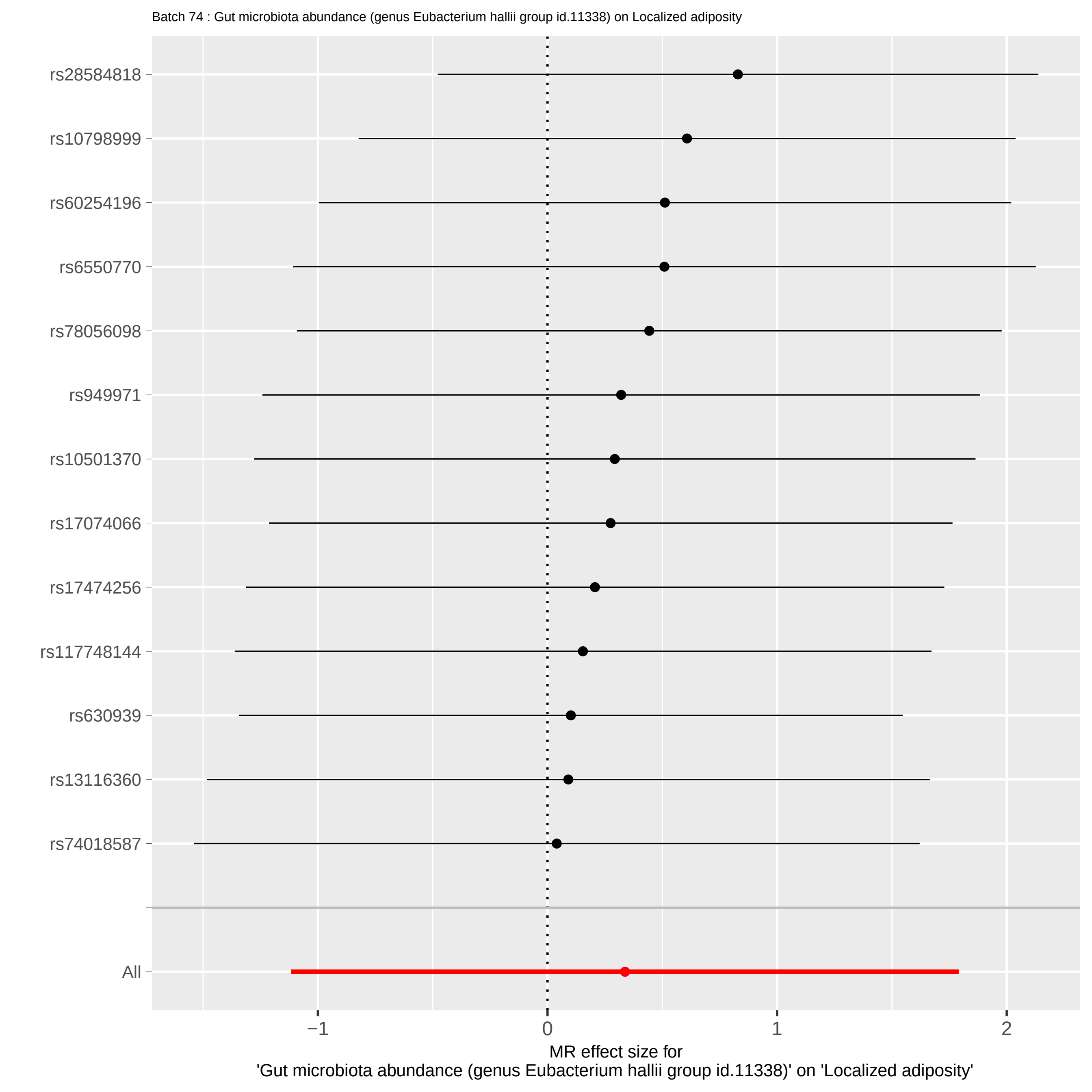

Batch 74 : Gut microbiota abundance (genus Eubacterium hallii group id.11338) on Localized adiposity
rs28584818
rs10798999
rs60254196
rs6550770
rs78056098
rs949971
rs10501370
rs17074066
rs17474256
rs117748144
rs630939
rs13116360
rs74018587
All
−1
0
1
2
MR effect size for
'Gut microbiota abundance (genus Eubacterium hallii group id.11338)' on 'Localized adiposity'

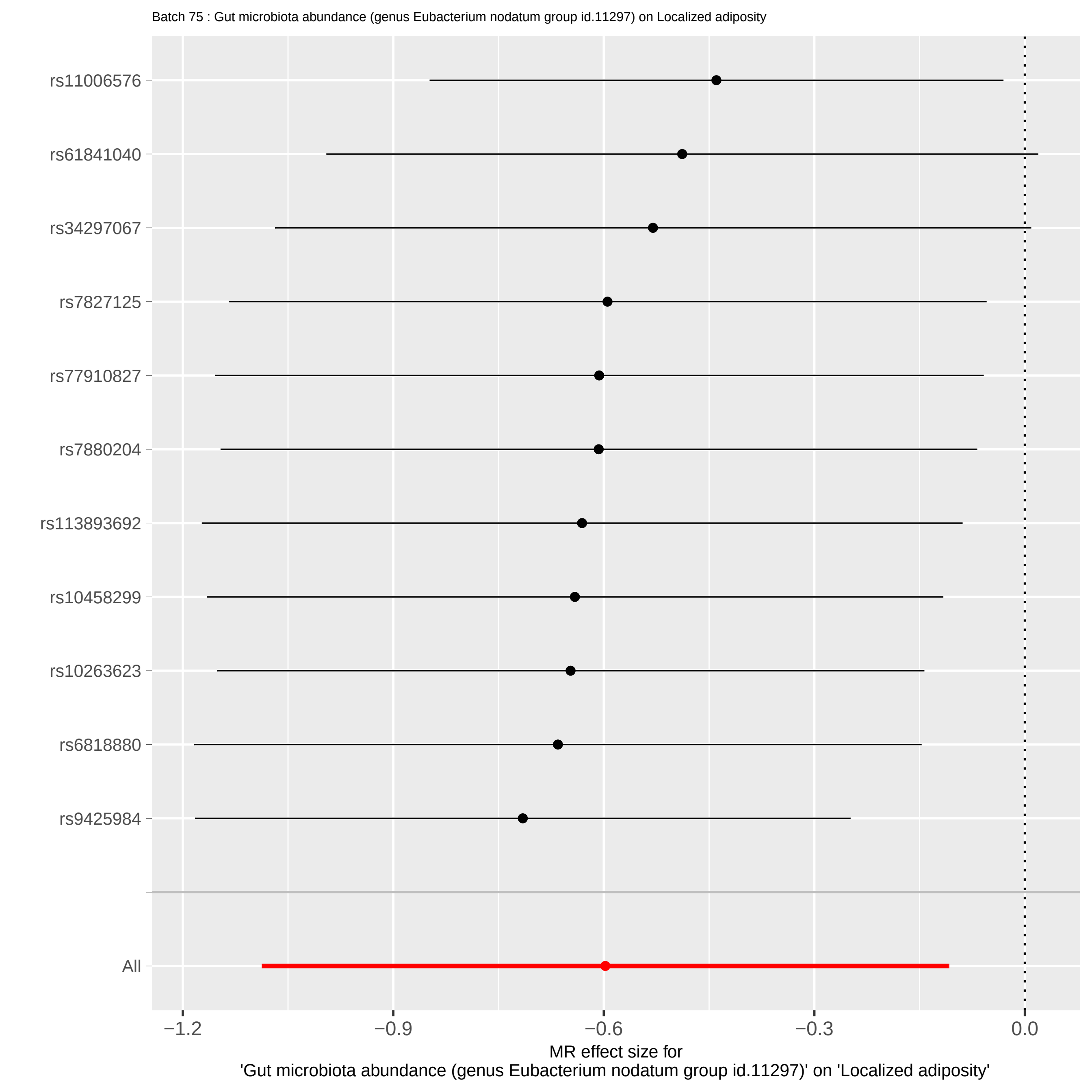

Batch 75 : Gut microbiota abundance (genus Eubacterium nodatum group id.11297) on Localized adiposity
rs11006576
rs61841040
rs34297067
rs7827125
rs77910827
rs7880204
rs113893692
rs10458299
rs10263623
rs6818880
rs9425984
All
−1.2
−0.9
−0.6
−0.3
0.0
MR effect size for
'Gut microbiota abundance (genus Eubacterium nodatum group id.11297)' on 'Localized adiposity'

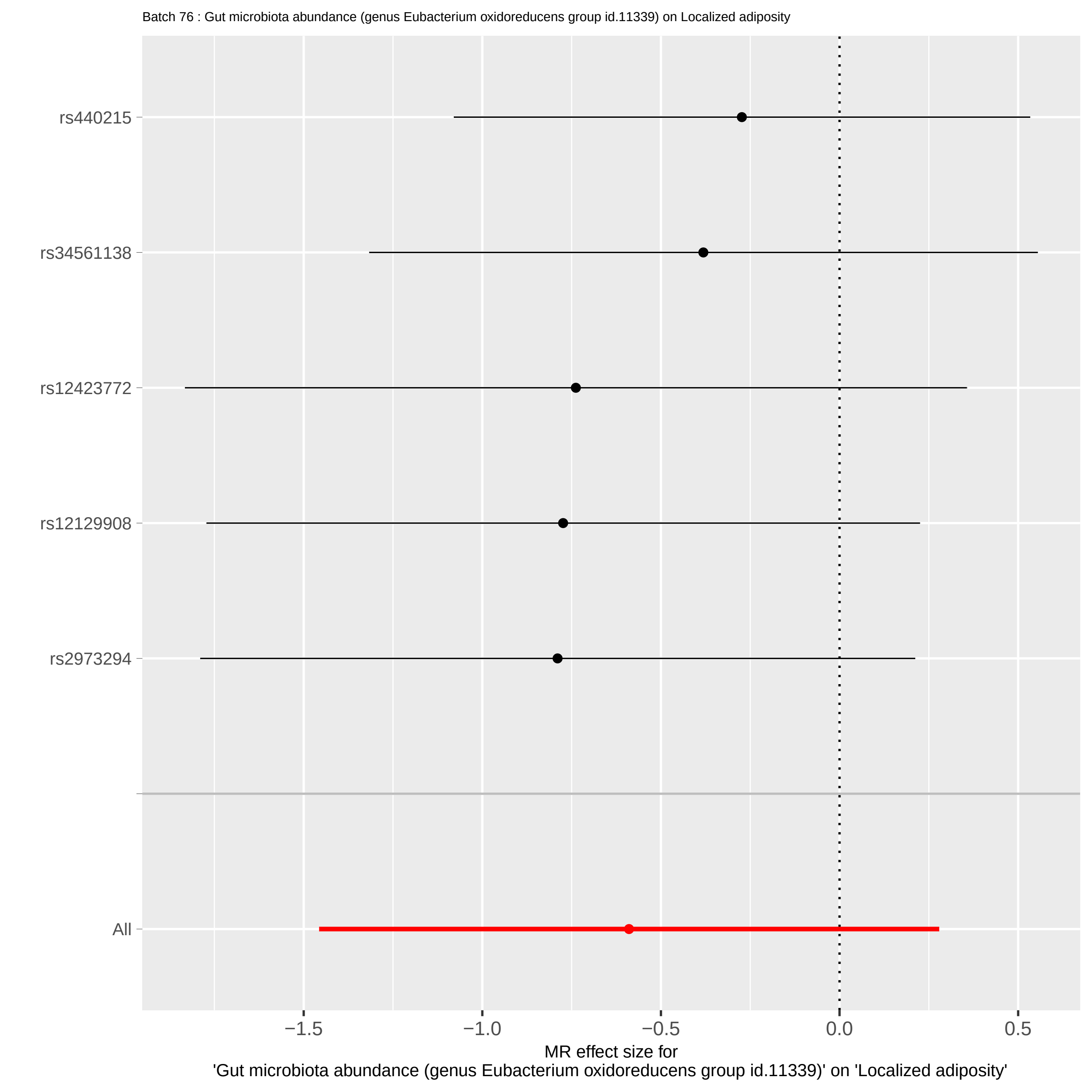

Batch 76 : Gut microbiota abundance (genus Eubacterium oxidoreducens group id.11339) on Localized adiposity
rs440215
rs34561138
rs12423772
rs12129908
rs2973294
All
−1.5
−1.0
−0.5
0.0
0.5
MR effect size for
'Gut microbiota abundance (genus Eubacterium oxidoreducens group id.11339)' on 'Localized adiposity'

Batch 77 : Gut microbiota abundance (genus Eubacterium rectale group id.14374) on Localized adiposity

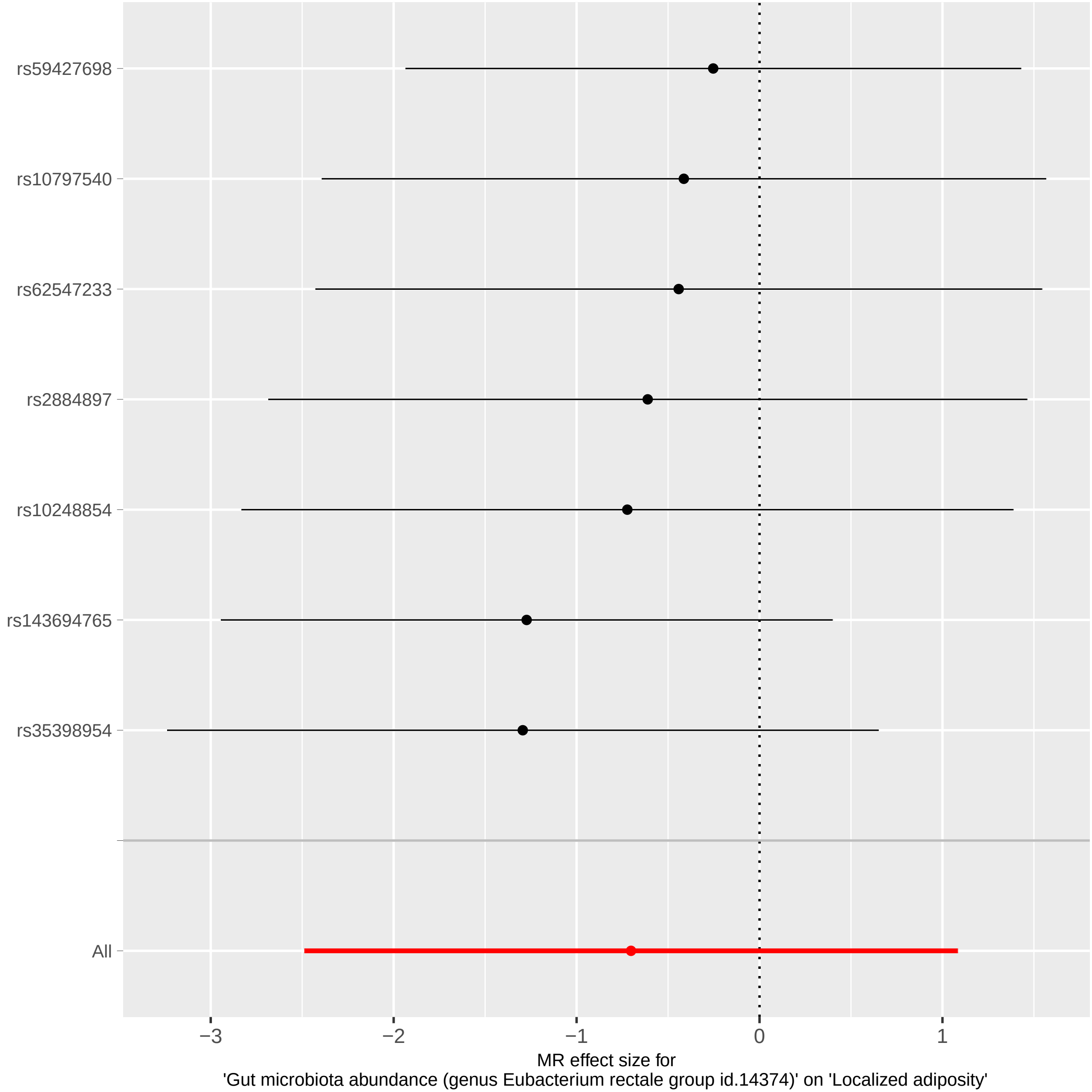

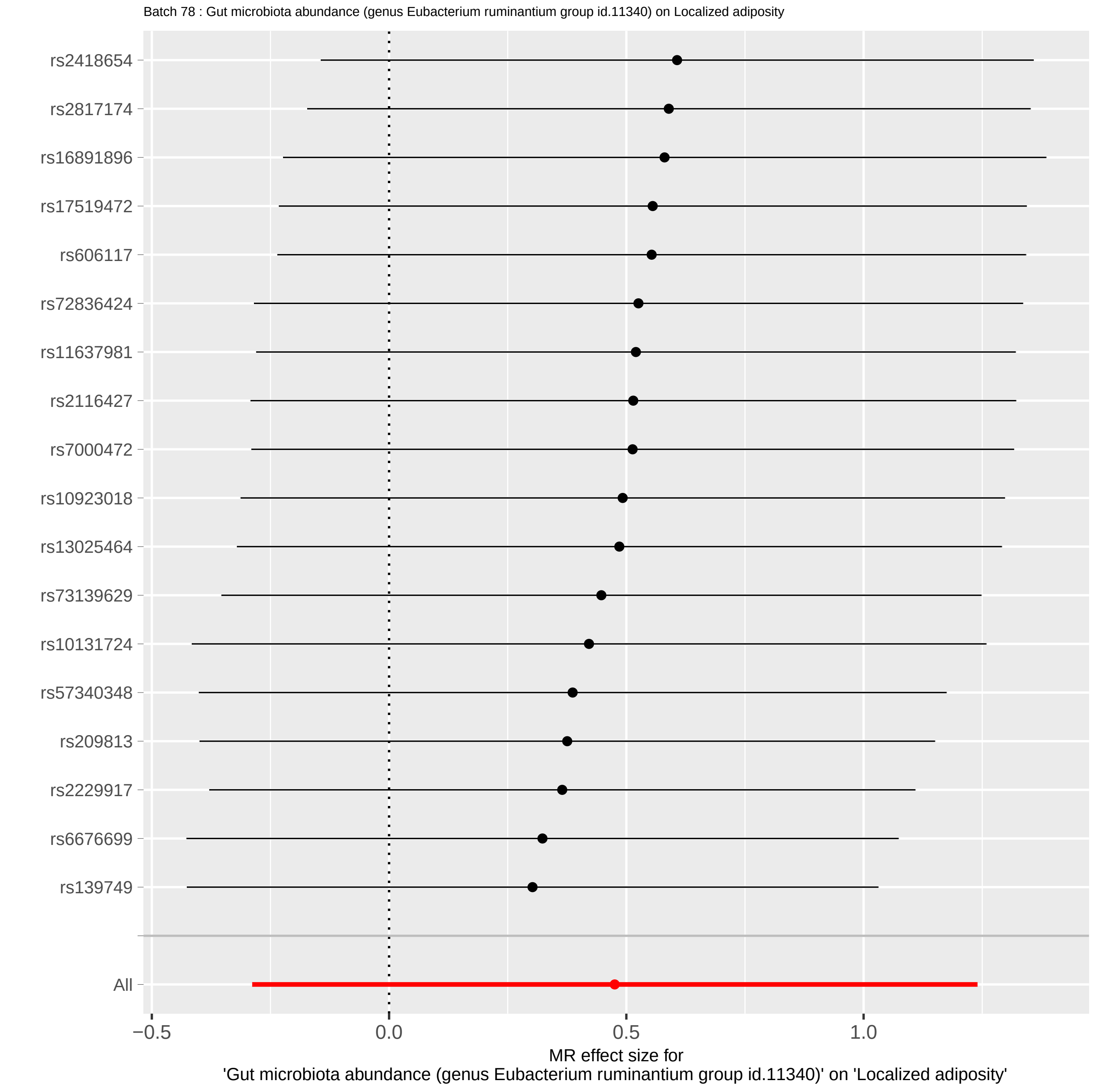
Batch 78 : Gut microbiota abundance (genus Eubacterium ruminantium group id.11340) on Localized adiposity
rs2418654
rs2817174
rs16891896
rs17519472
rs606117
rs72836424
rs11637981
rs2116427
rs7000472
rs10923018
rs13025464
rs73139629
rs10131724
rs57340348
rs209813
rs2229917
rs6676699
rs139749
All
−0.5
0.0
0.5
1.0
MR effect size for
'Gut microbiota abundance (genus Eubacterium ruminantium group id.11340)' on 'Localized adiposity'

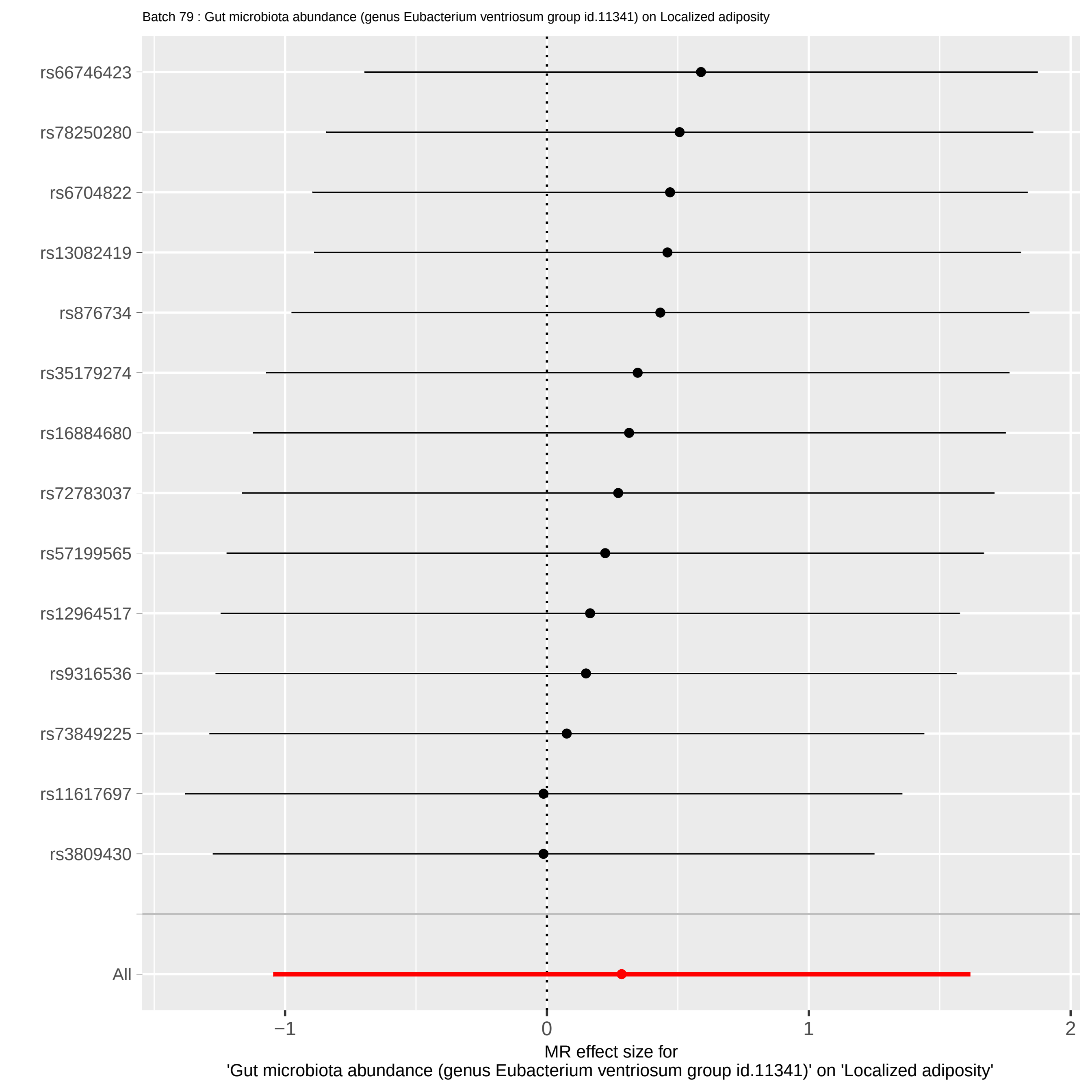

Batch 79 : Gut microbiota abundance (genus Eubacterium ventriosum group id.11341) on Localized adiposity
rs66746423
rs78250280
rs6704822
rs13082419
rs876734
rs35179274
rs16884680
rs72783037
rs57199565
rs12964517
rs9316536
rs73849225
rs11617697
rs3809430
All
−1
0
1
2
MR effect size for
'Gut microbiota abundance (genus Eubacterium ventriosum group id.11341)' on 'Localized adiposity'

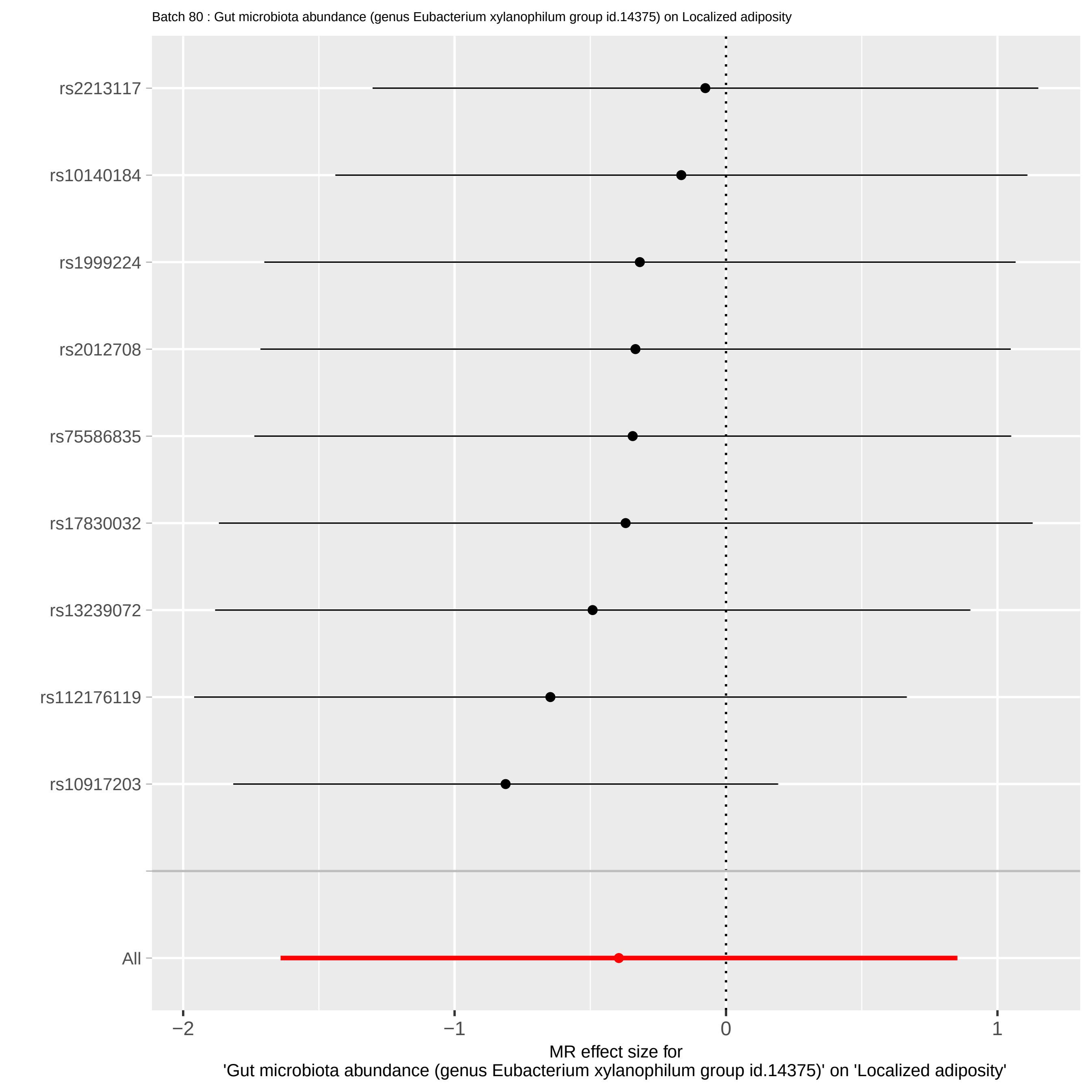
Batch 80 : Gut microbiota abundance (genus Eubacterium xylanophilum group id.14375) on Localized adiposity
rs2213117
rs10140184
rs1999224
rs2012708
rs75586835
rs17830032
rs13239072
rs112176119
rs10917203
All
−2
−1
0
1
MR effect size for
'Gut microbiota abundance (genus Eubacterium xylanophilum group id.14375)' on 'Localized adiposity'

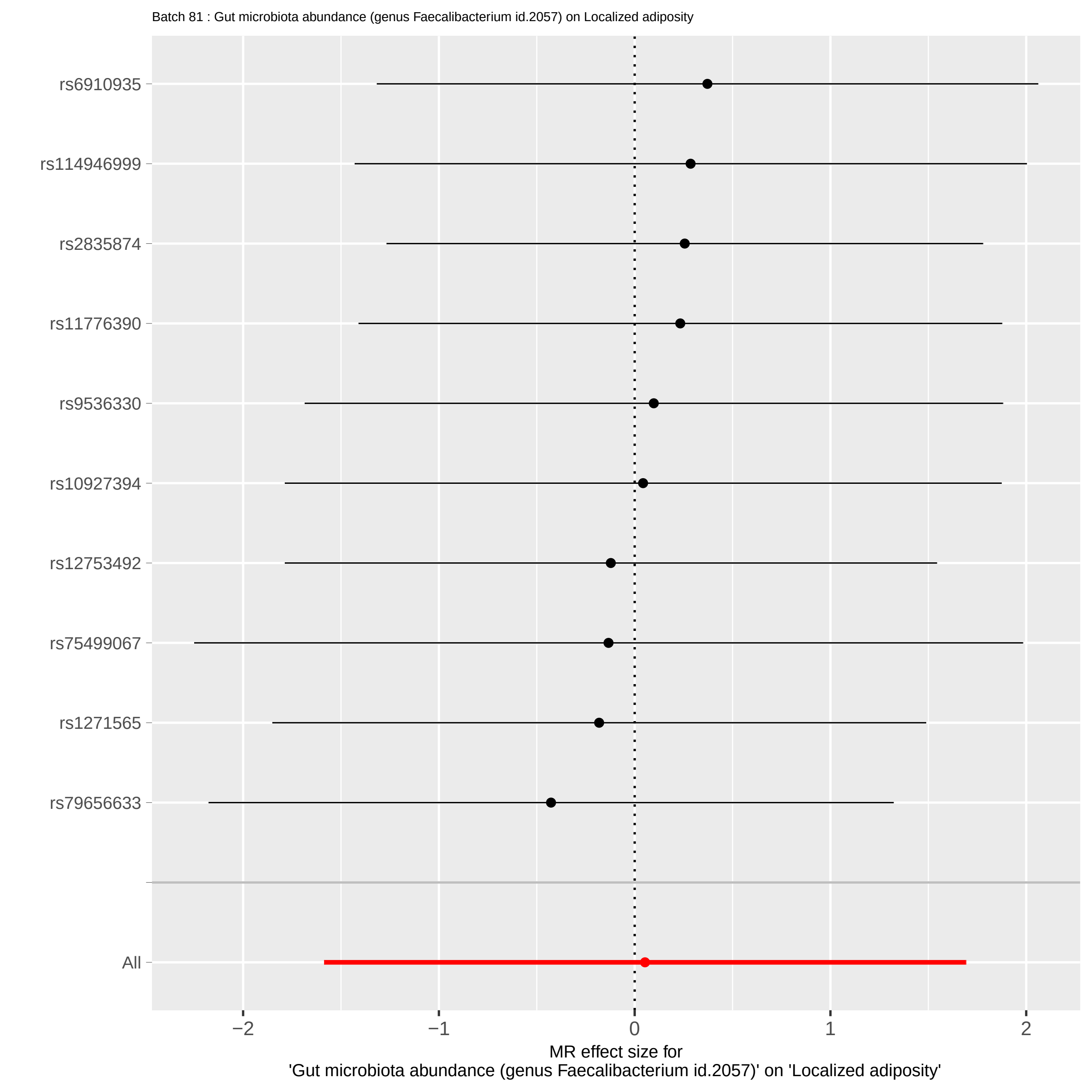
Batch 81 : Gut microbiota abundance (genus Faecalibacterium id.2057) on Localized adiposity
rs6910935
rs114946999
rs2835874
rs11776390
rs9536330
rs10927394
rs12753492
rs75499067
rs1271565
rs79656633
All
−2
−1
0
1
2
MR effect size for
'Gut microbiota abundance (genus Faecalibacterium id.2057)' on 'Localized adiposity'

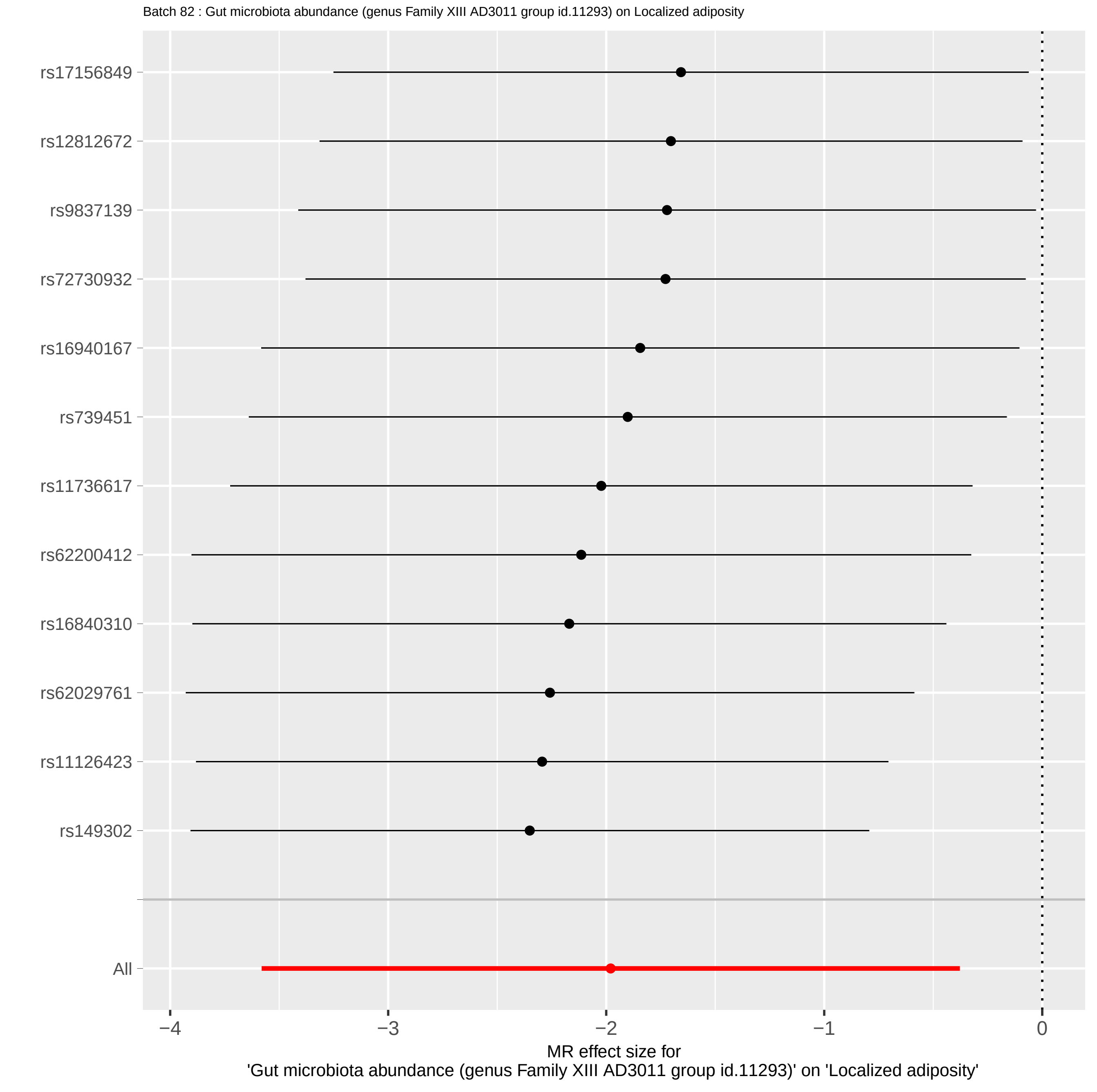

Batch 82 : Gut microbiota abundance (genus Family XIII AD3011 group id.11293) on Localized adiposity
rs17156849
rs12812672
rs9837139
rs72730932
rs16940167
rs739451
rs11736617
rs62200412
rs16840310
rs62029761
rs11126423
rs149302
All
−4
−3
−2
−1
0
MR effect size for
'Gut microbiota abundance (genus Family XIII AD3011 group id.11293)' on 'Localized adiposity'

Batch 83 : Gut microbiota abundance (genus Family XIII UCG001 id.11294) on Localized adiposity

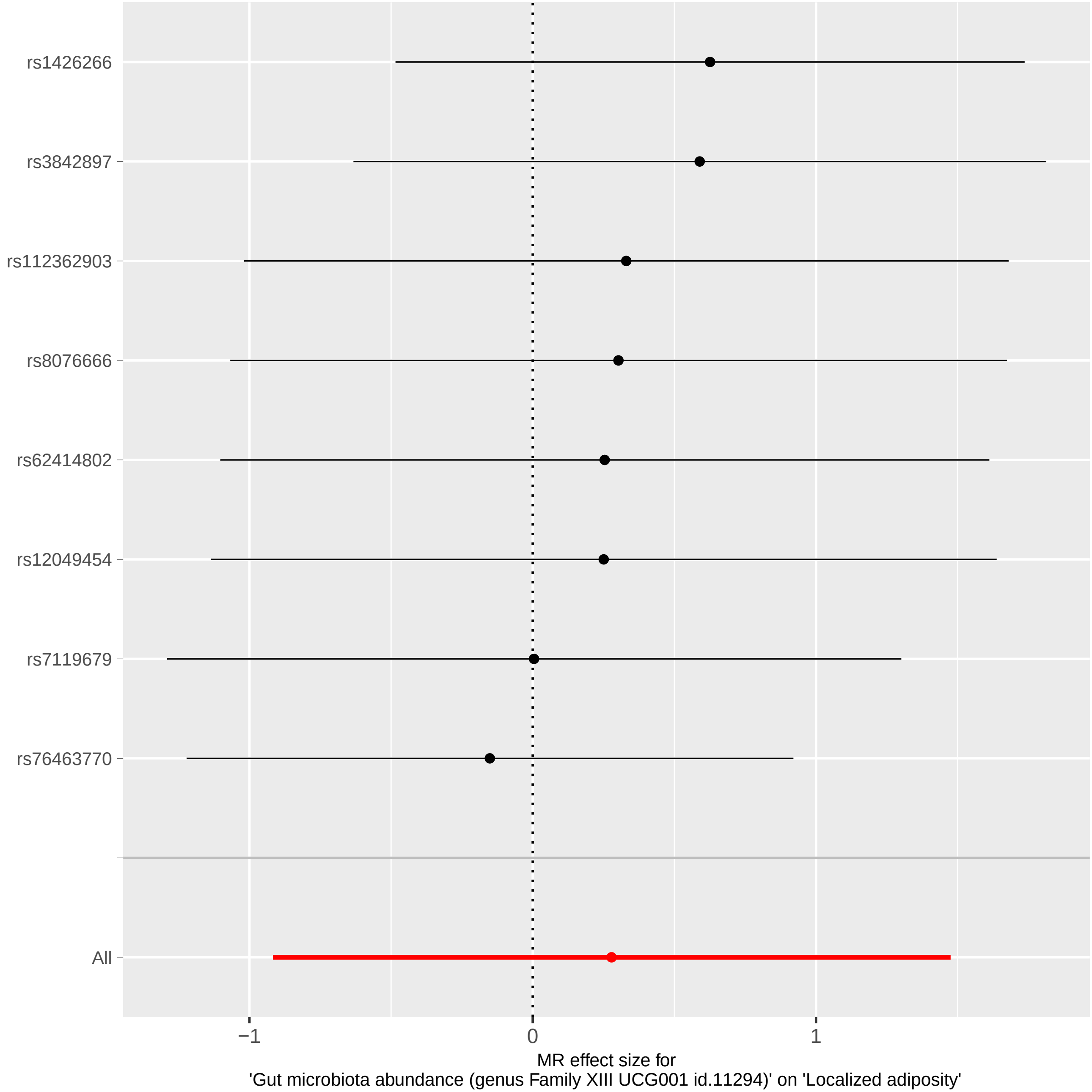

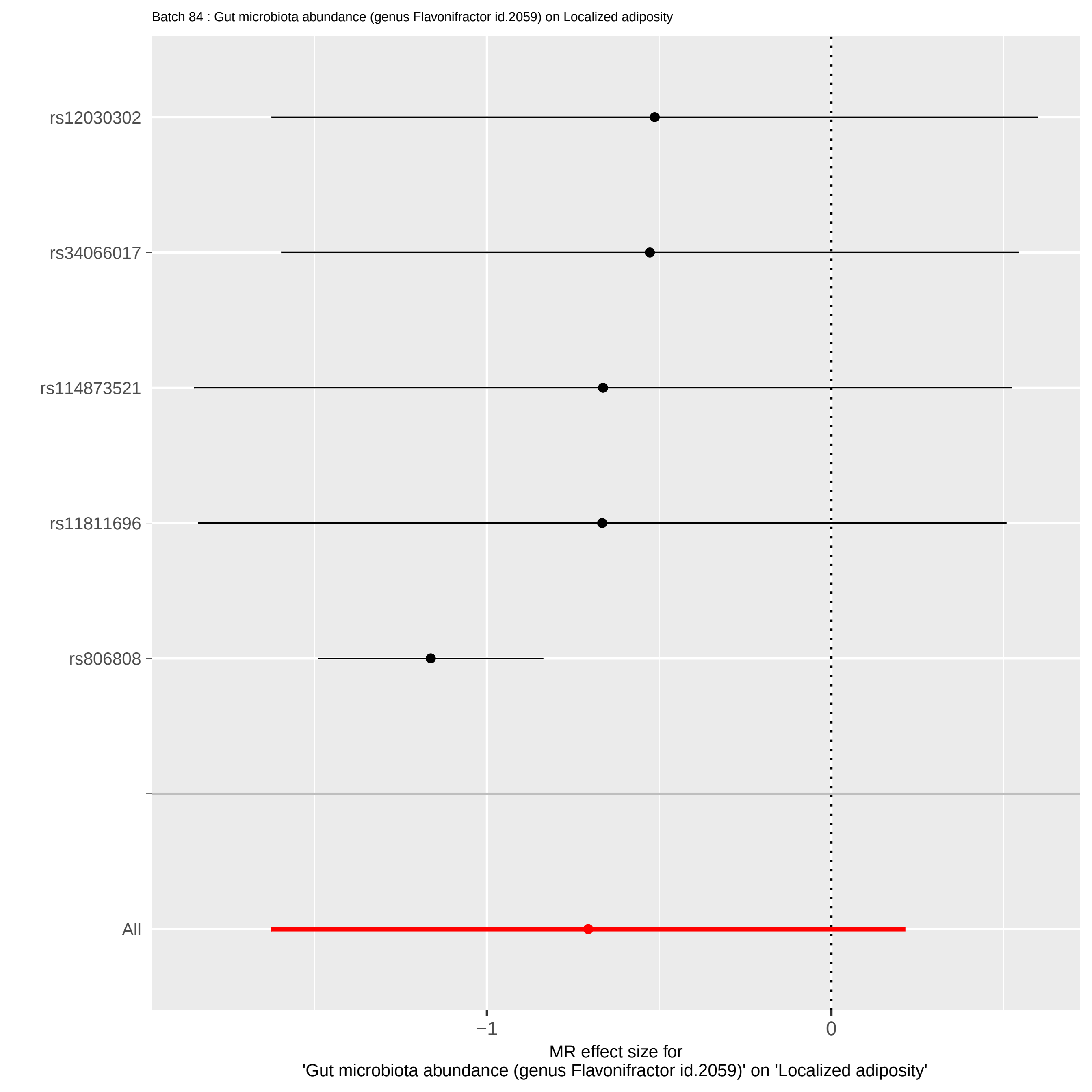

Batch 84 : Gut microbiota abundance (genus Flavonifractor id.2059) on Localized adiposity
rs12030302
rs34066017
rs114873521
rs11811696
rs806808
All
−1
0
MR effect size for
'Gut microbiota abundance (genus Flavonifractor id.2059)' on 'Localized adiposity'

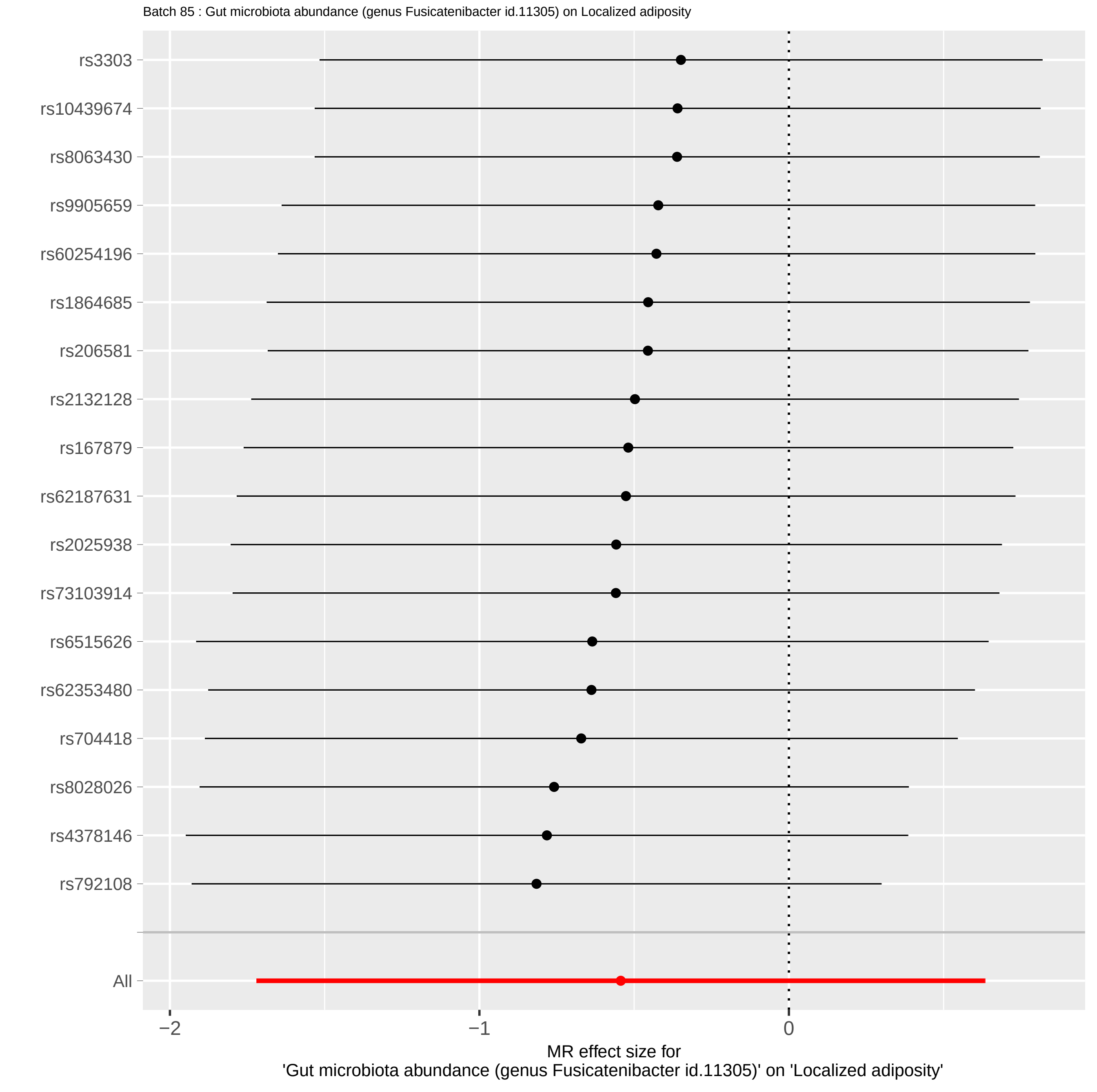

Batch 85 : Gut microbiota abundance (genus Fusicatenibacter id.11305) on Localized adiposity
rs3303
rs10439674
rs8063430
rs9905659
rs60254196
rs1864685
rs206581
rs2132128
rs167879
rs62187631
rs2025938
rs73103914
rs6515626
rs62353480
rs704418
rs8028026
rs4378146
rs792108
All
−2
−1
0
MR effect size for
'Gut microbiota abundance (genus Fusicatenibacter id.11305)' on 'Localized adiposity'

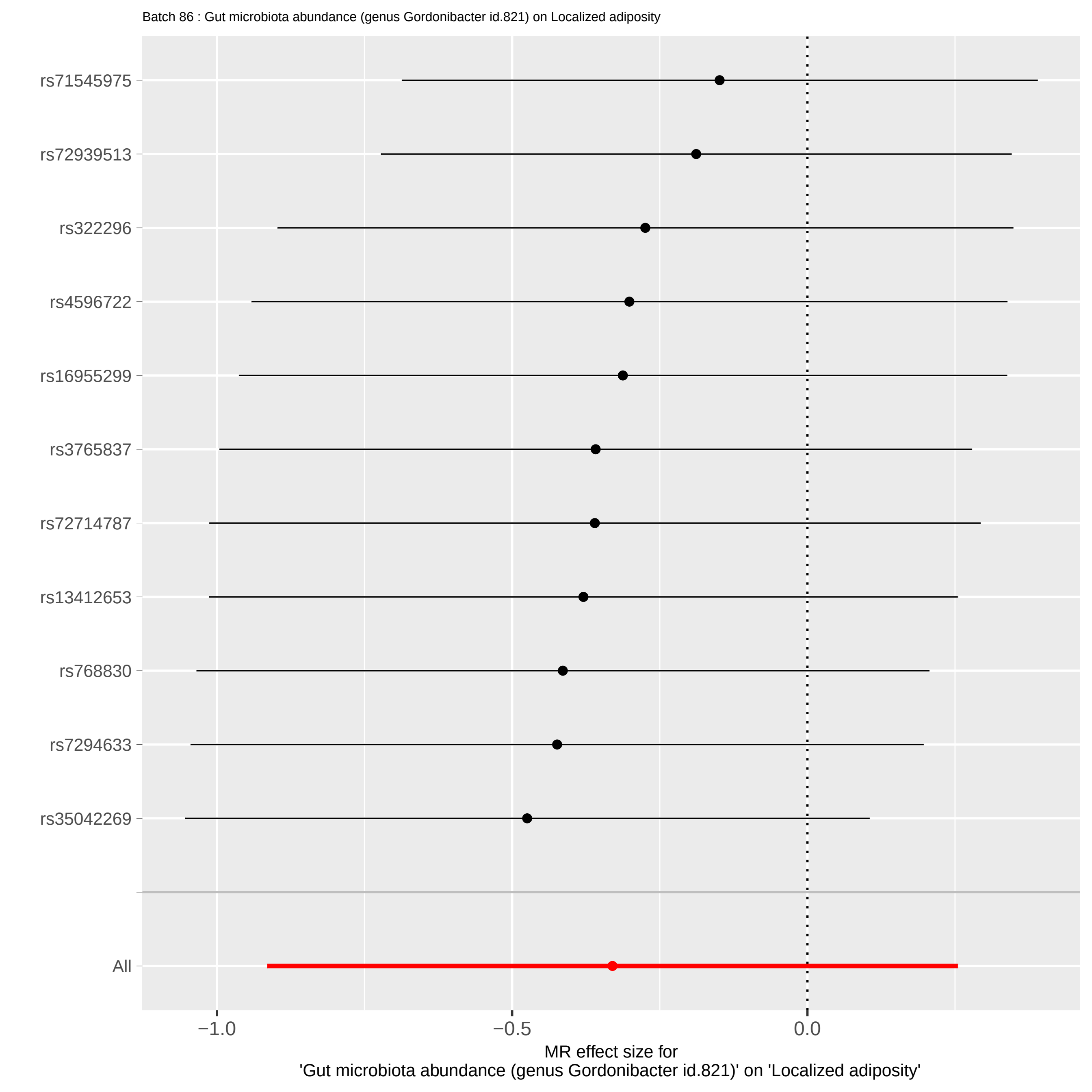

Batch 86 : Gut microbiota abundance (genus Gordonibacter id.821) on Localized adiposity
rs71545975
rs72939513
rs322296
rs4596722
rs16955299
rs3765837
rs72714787
rs13412653
rs768830
rs7294633
rs35042269
All
−1.0
−0.5
0.0
MR effect size for
'Gut microbiota abundance (genus Gordonibacter id.821)' on 'Localized adiposity'

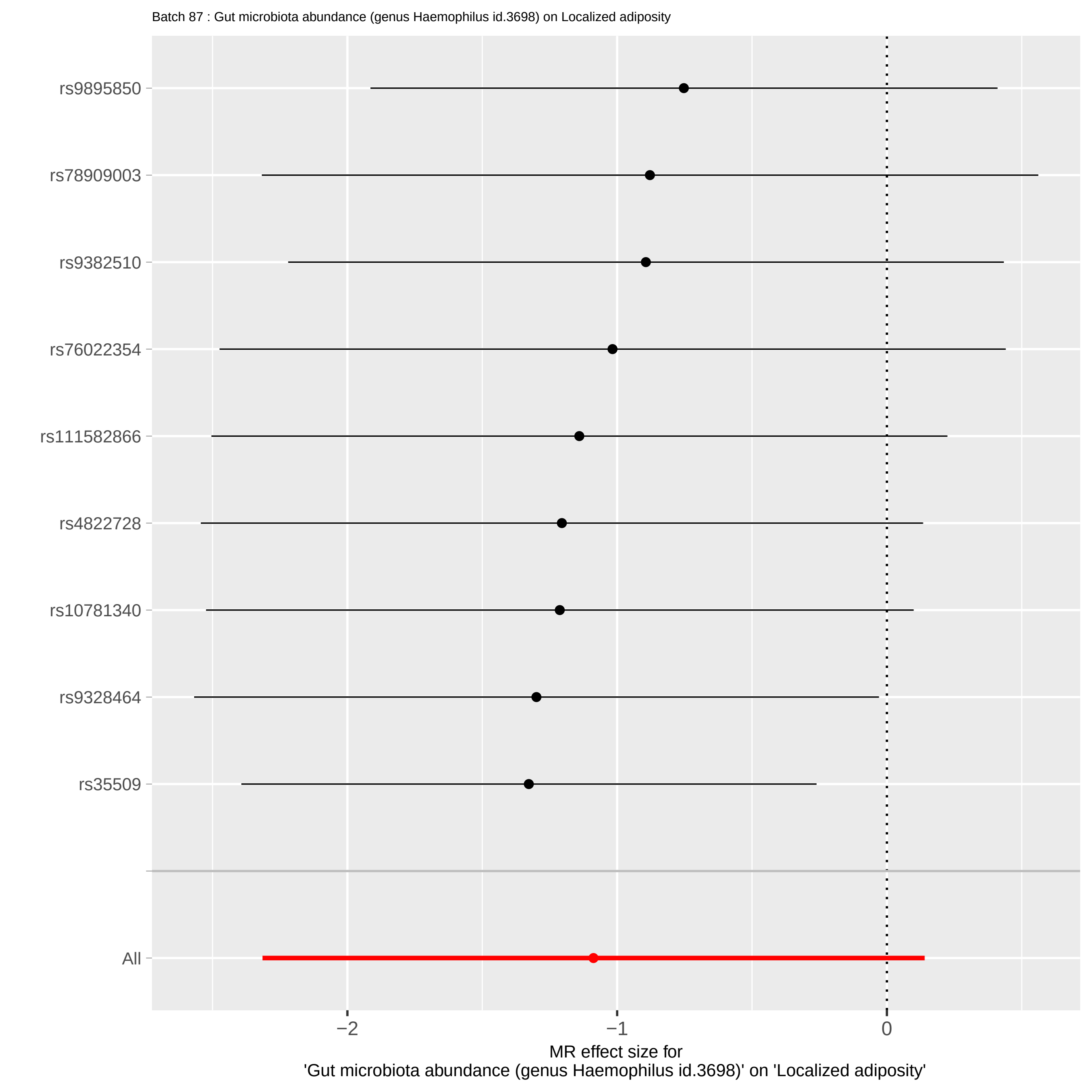

Batch 87 : Gut microbiota abundance (genus Haemophilus id.3698) on Localized adiposity
rs9895850
rs78909003
rs9382510
rs76022354
rs111582866
rs4822728
rs10781340
rs9328464
rs35509
All
−2
−1
0
MR effect size for
'Gut microbiota abundance (genus Haemophilus id.3698)' on 'Localized adiposity'

Batch 88 : Gut microbiota abundance (genus Holdemanella id.11393) on Localized adiposity

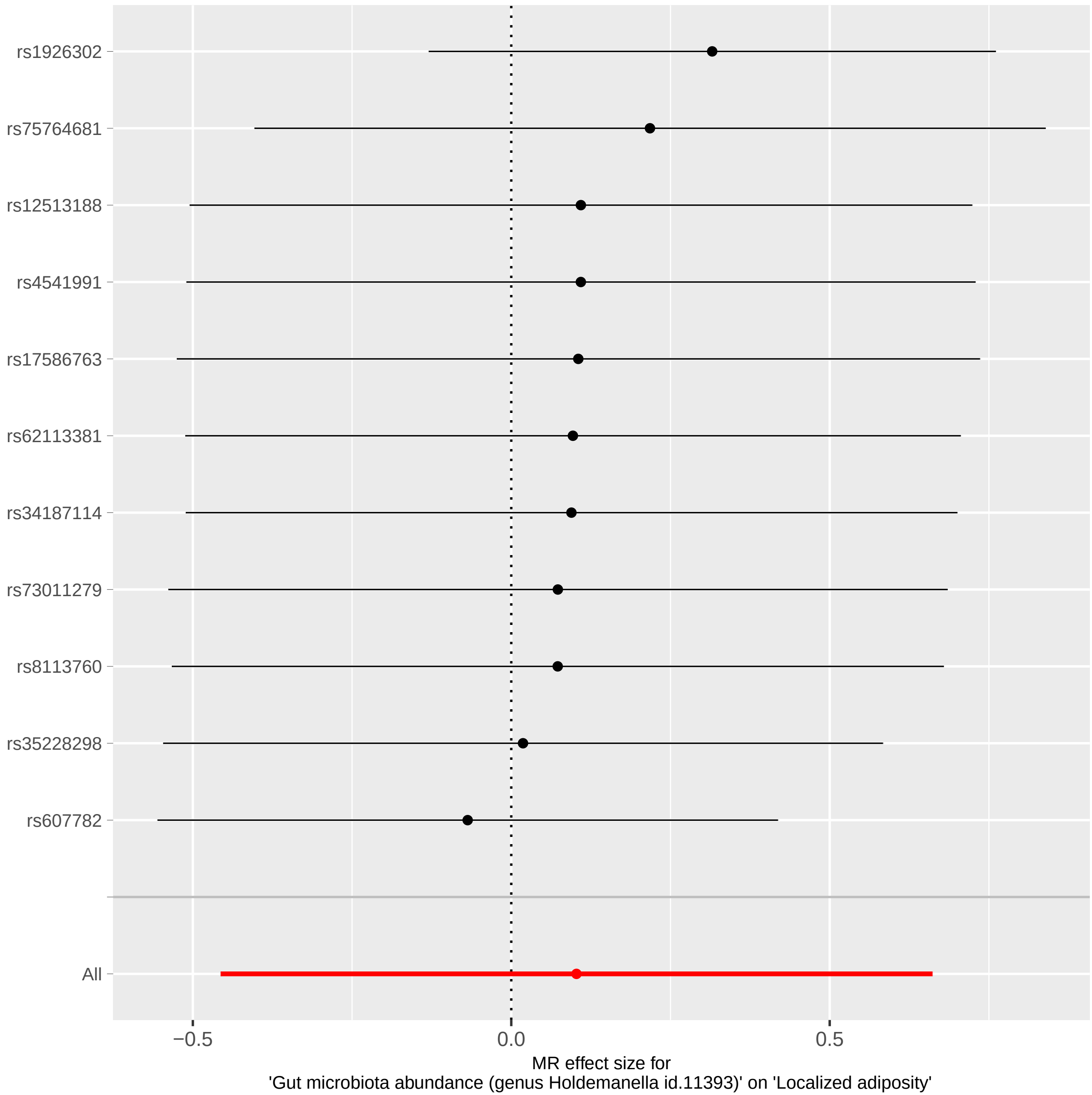

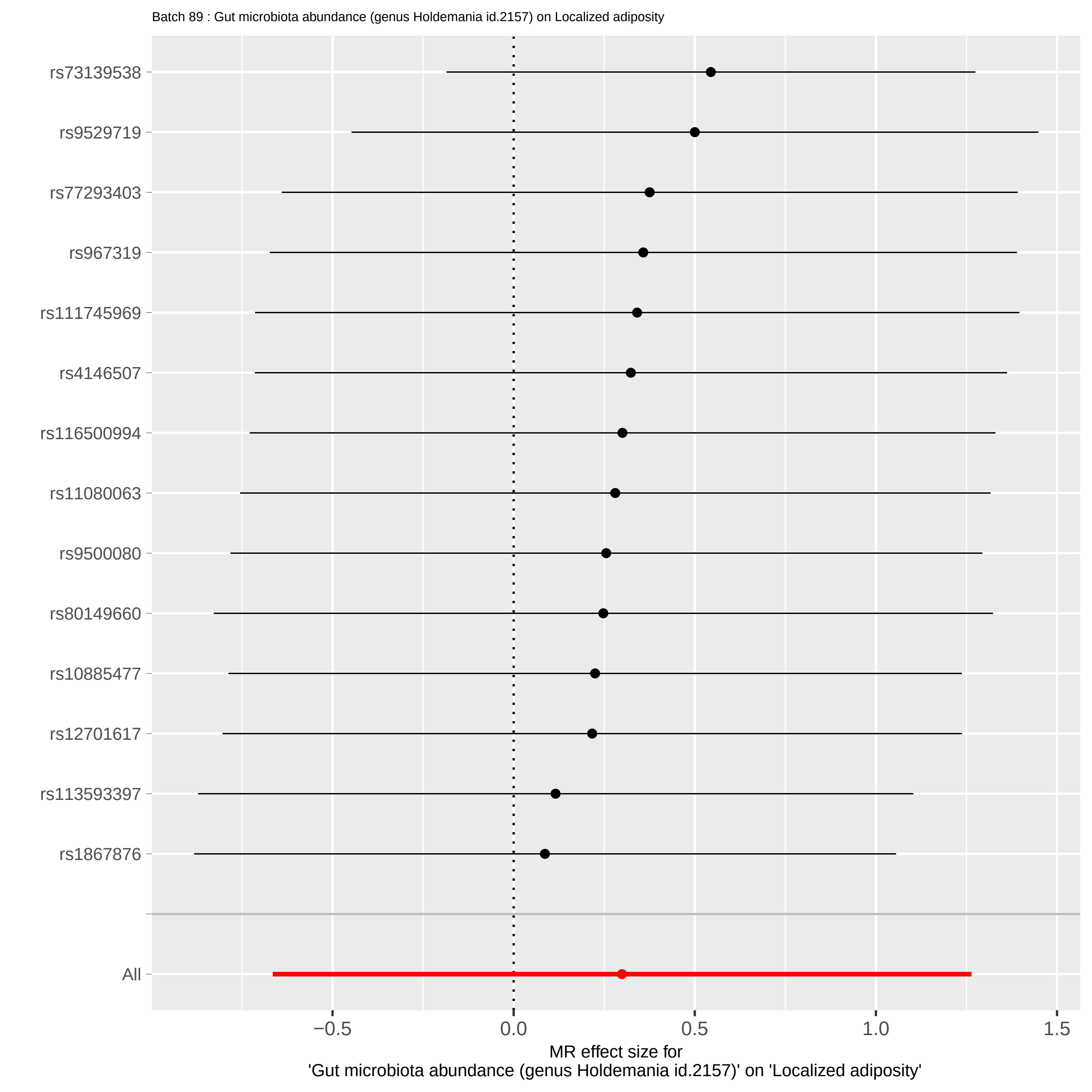

Batch 89 : Gut microbiota abundance (genus Holdemania id.2157) on Localized adiposity
rs73139538
rs9529719
rs77293403
rs967319
rs111745969
rs4146507
rs116500994
rs11080063
rs9500080
rs80149660
rs10885477
rs12701617
rs113593397
rs1867876
All
−0.5
0.0
0.5
1.0
1.5
MR effect size for
'Gut microbiota abundance (genus Holdemania id.2157)' on 'Localized adiposity'

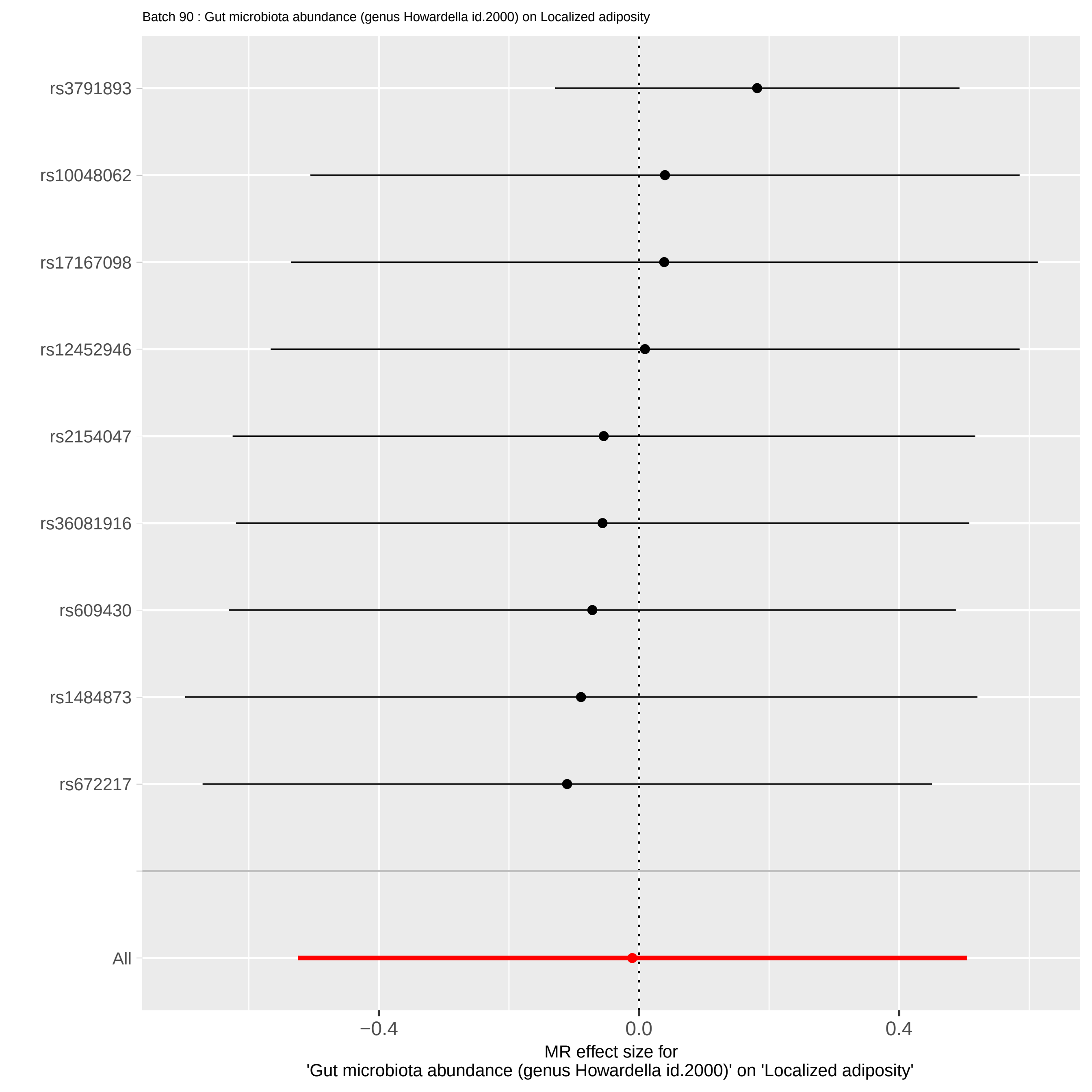

Batch 90 : Gut microbiota abundance (genus Howardella id.2000) on Localized adiposity
rs3791893
rs10048062
rs17167098
rs12452946
rs2154047
rs36081916
rs609430
rs1484873
rs672217
All
−0.4
0.0
0.4
MR effect size for
'Gut microbiota abundance (genus Howardella id.2000)' on 'Localized adiposity'

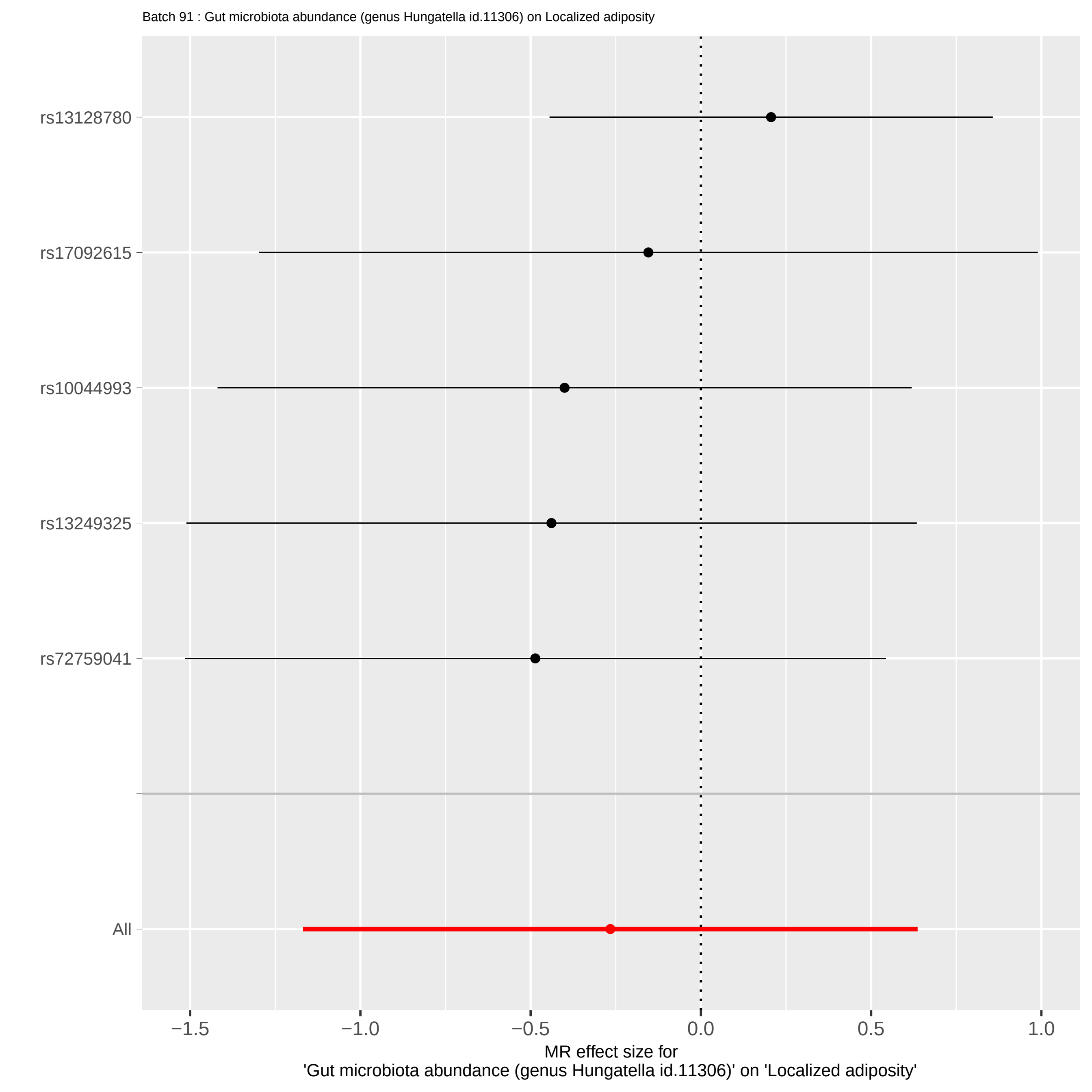

Batch 91 : Gut microbiota abundance (genus Hungatella id.11306) on Localized adiposity
rs13128780
rs17092615
rs10044993
rs13249325
rs72759041
All
−1.5
−1.0
−0.5
0.0
0.5
1.0
MR effect size for
'Gut microbiota abundance (genus Hungatella id.11306)' on 'Localized adiposity'

Batch 92 : Gut microbiota abundance (genus Intestinibacter id.11345) on Localized adiposity

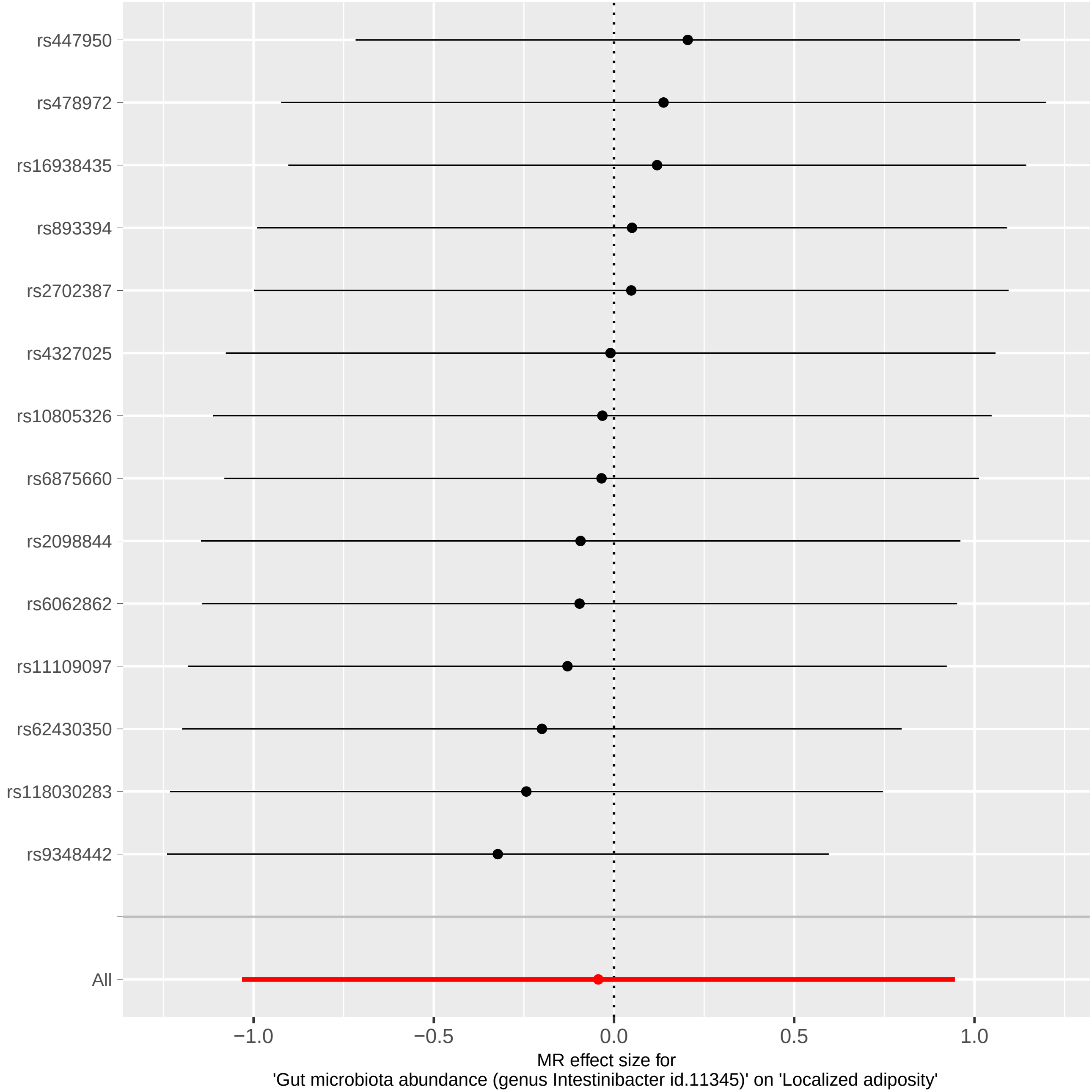

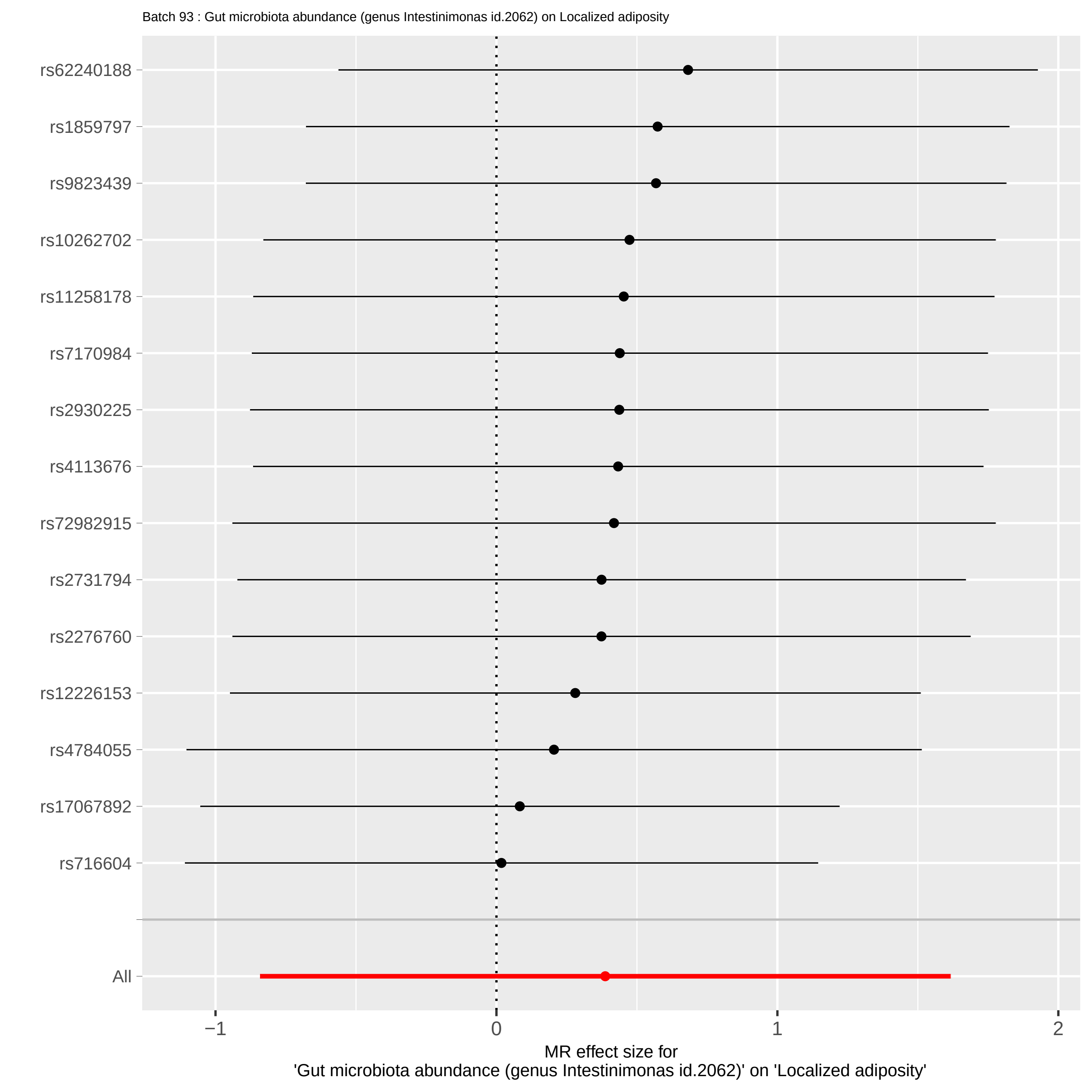

Batch 93 : Gut microbiota abundance (genus Intestinimonas id.2062) on Localized adiposity
rs62240188
rs1859797
rs9823439
rs10262702
rs11258178
rs7170984
rs2930225
rs4113676
rs72982915
rs2731794
rs2276760
rs12226153
rs4784055
rs17067892
rs716604
All
−1
0
1
2
MR effect size for
'Gut microbiota abundance (genus Intestinimonas id.2062)' on 'Localized adiposity'

Batch 94 : Gut microbiota abundance (genus Lachnoclostridium id.11308) on Localized adiposity

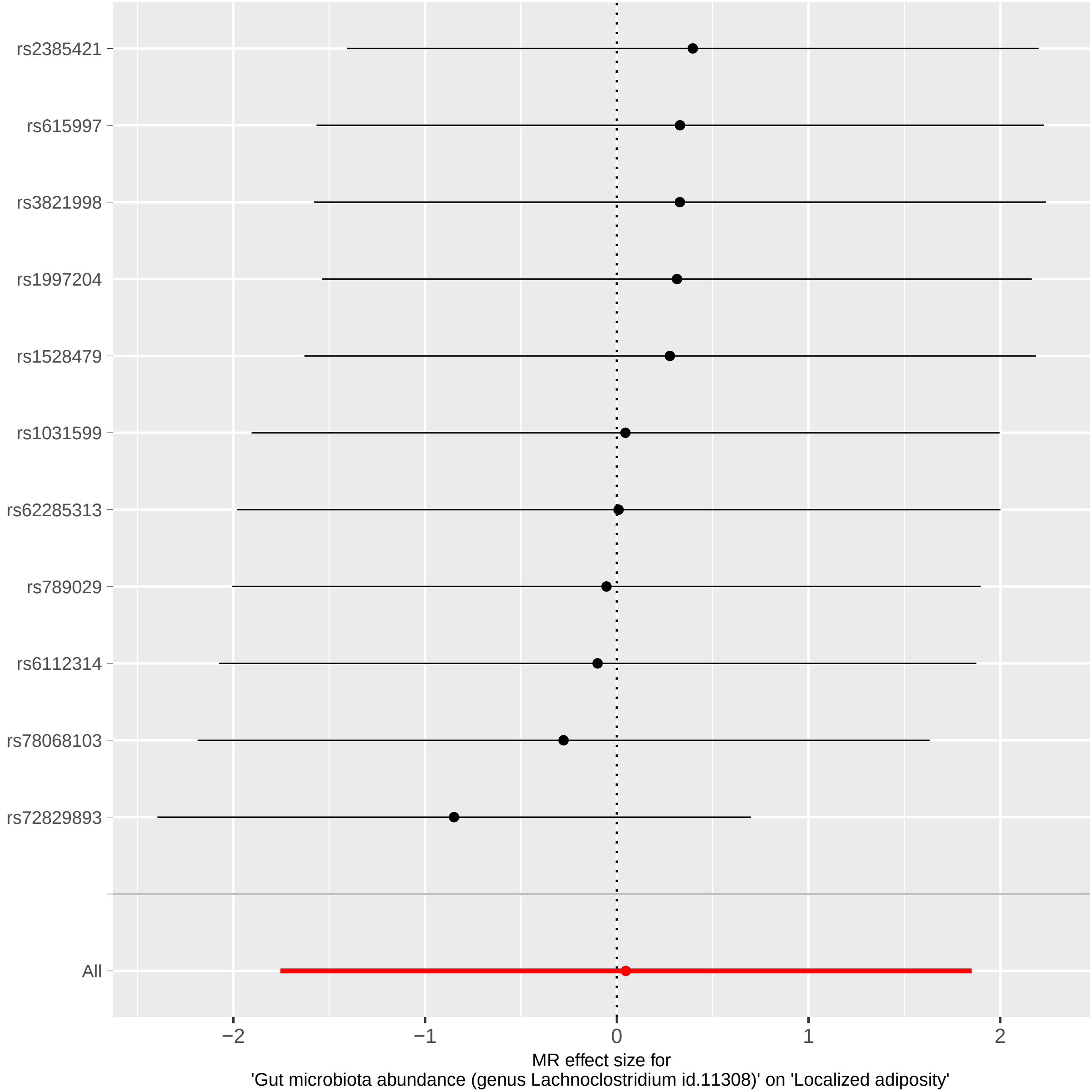

Batch 95 : Gut microbiota abundance (genus Lachnospiraceae FCS020 group id.11314) on Localized adiposity

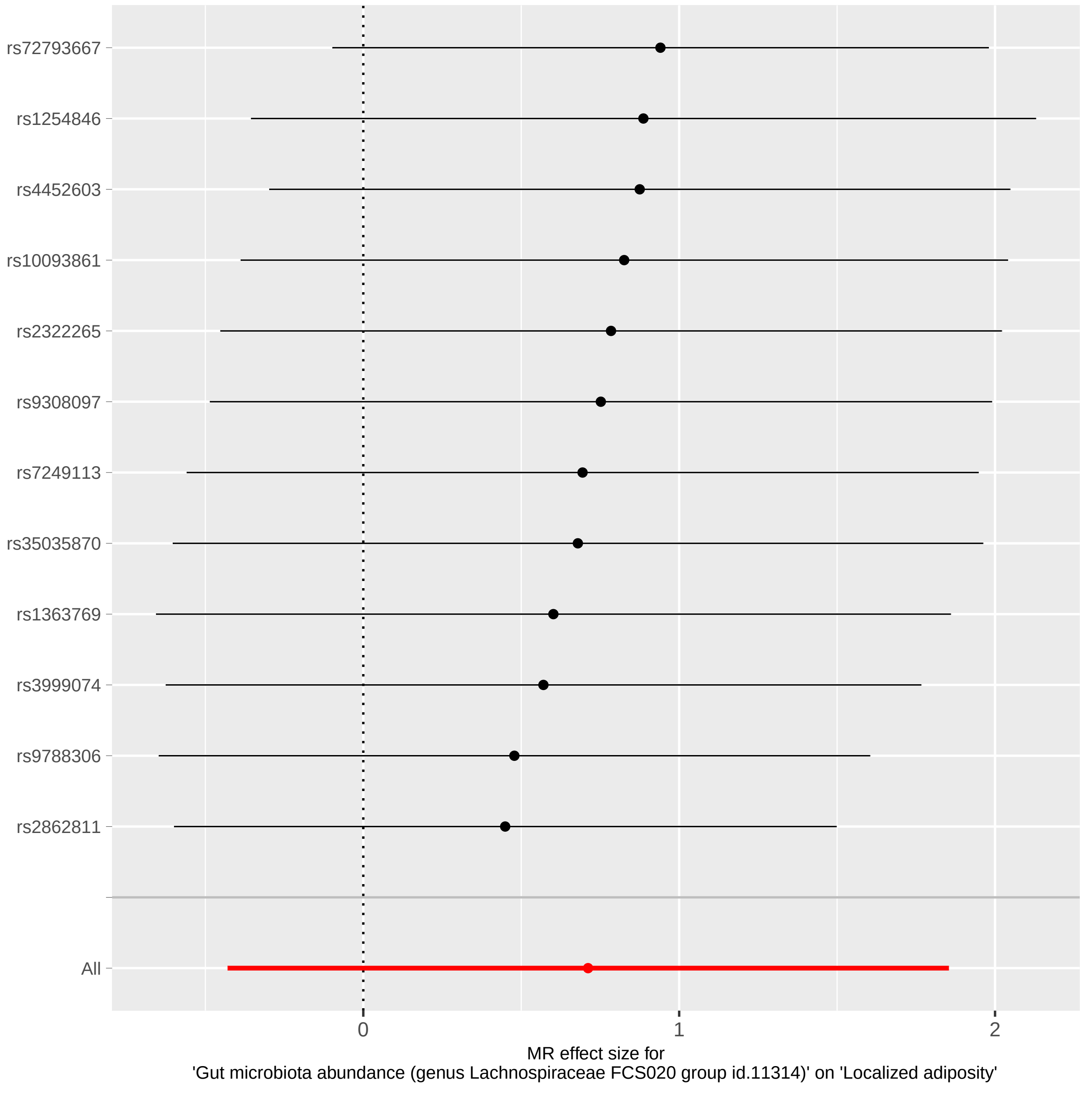

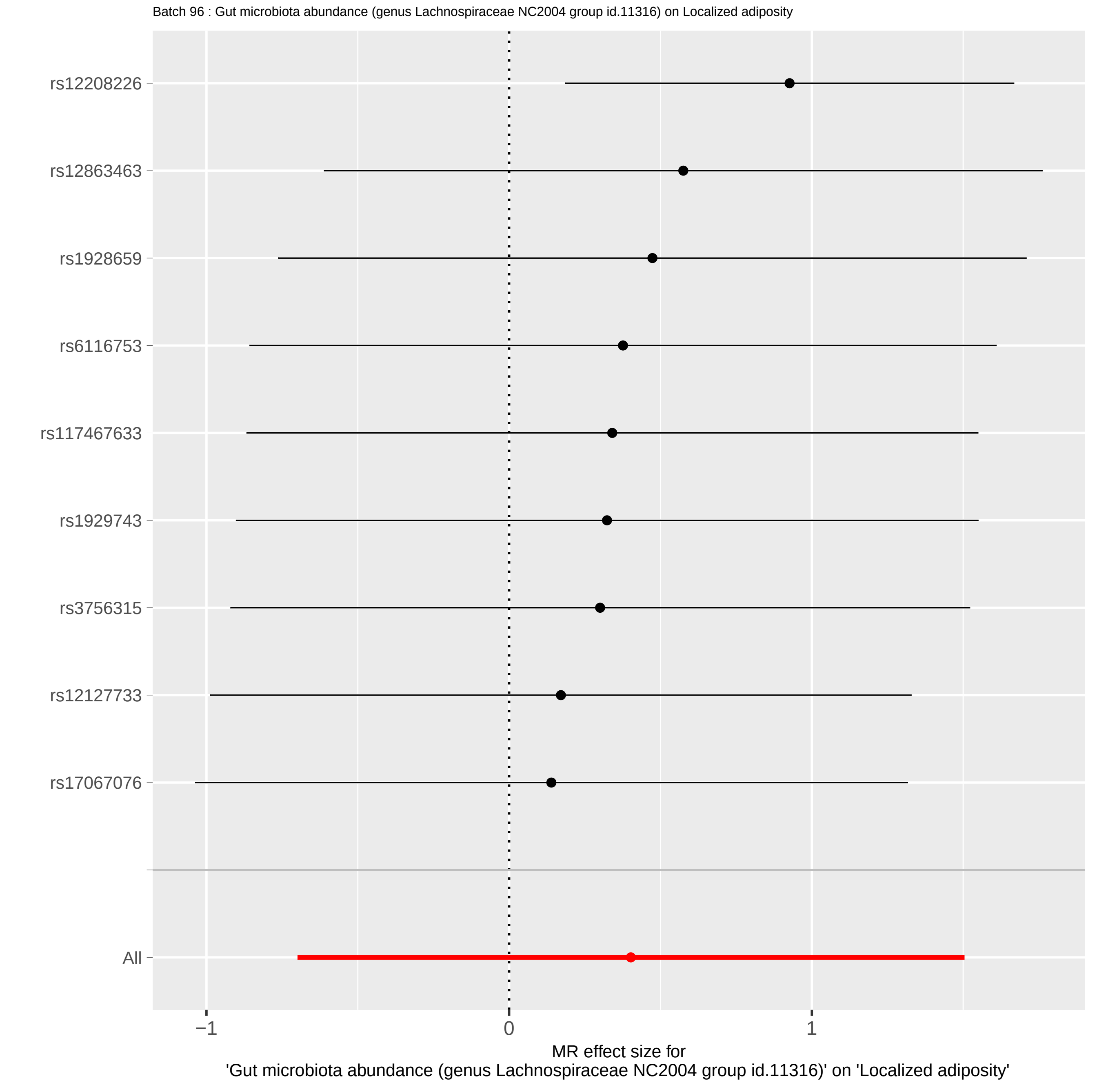
Batch 96 : Gut microbiota abundance (genus Lachnospiraceae NC2004 group id.11316) on Localized adiposity
rs12208226
rs12863463
rs1928659
rs6116753
rs117467633
rs1929743
rs3756315
rs12127733
rs17067076
All
−1
0
1
MR effect size for
'Gut microbiota abundance (genus Lachnospiraceae NC2004 group id.11316)' on 'Localized adiposity'

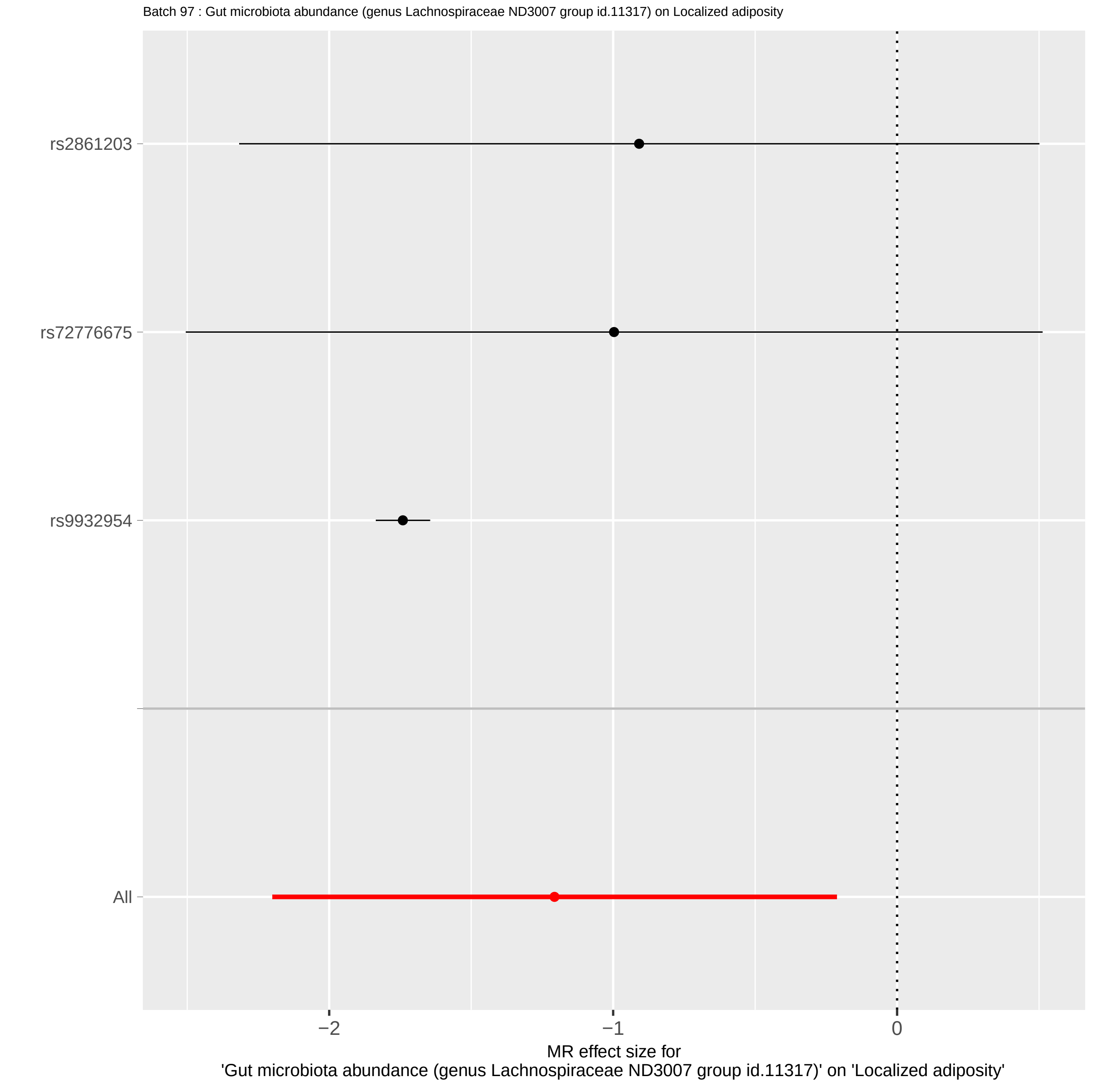
Batch 97 : Gut microbiota abundance (genus Lachnospiraceae ND3007 group id.11317) on Localized adiposity
rs2861203
rs72776675
rs9932954
All
−2
−1
0
MR effect size for
'Gut microbiota abundance (genus Lachnospiraceae ND3007 group id.11317)' on 'Localized adiposity'

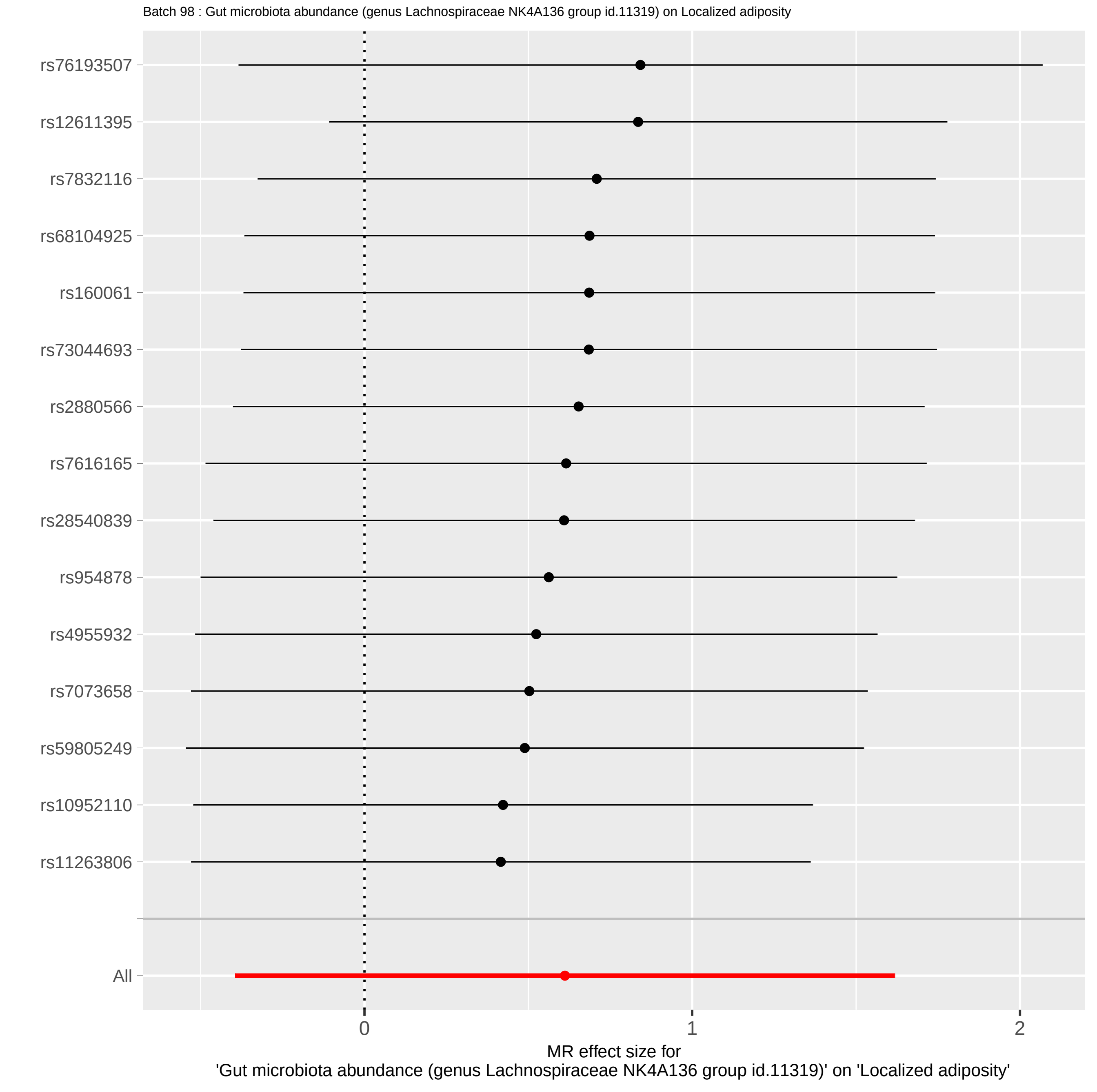

Batch 98 : Gut microbiota abundance (genus Lachnospiraceae NK4A136 group id.11319) on Localized adiposity
rs76193507
rs12611395
rs7832116
rs68104925
rs160061
rs73044693
rs2880566
rs7616165
rs28540839
rs954878
rs4955932
rs7073658
rs59805249
rs10952110
rs11263806
All
0
1
2
MR effect size for
'Gut microbiota abundance (genus Lachnospiraceae NK4A136 group id.11319)' on 'Localized adiposity'

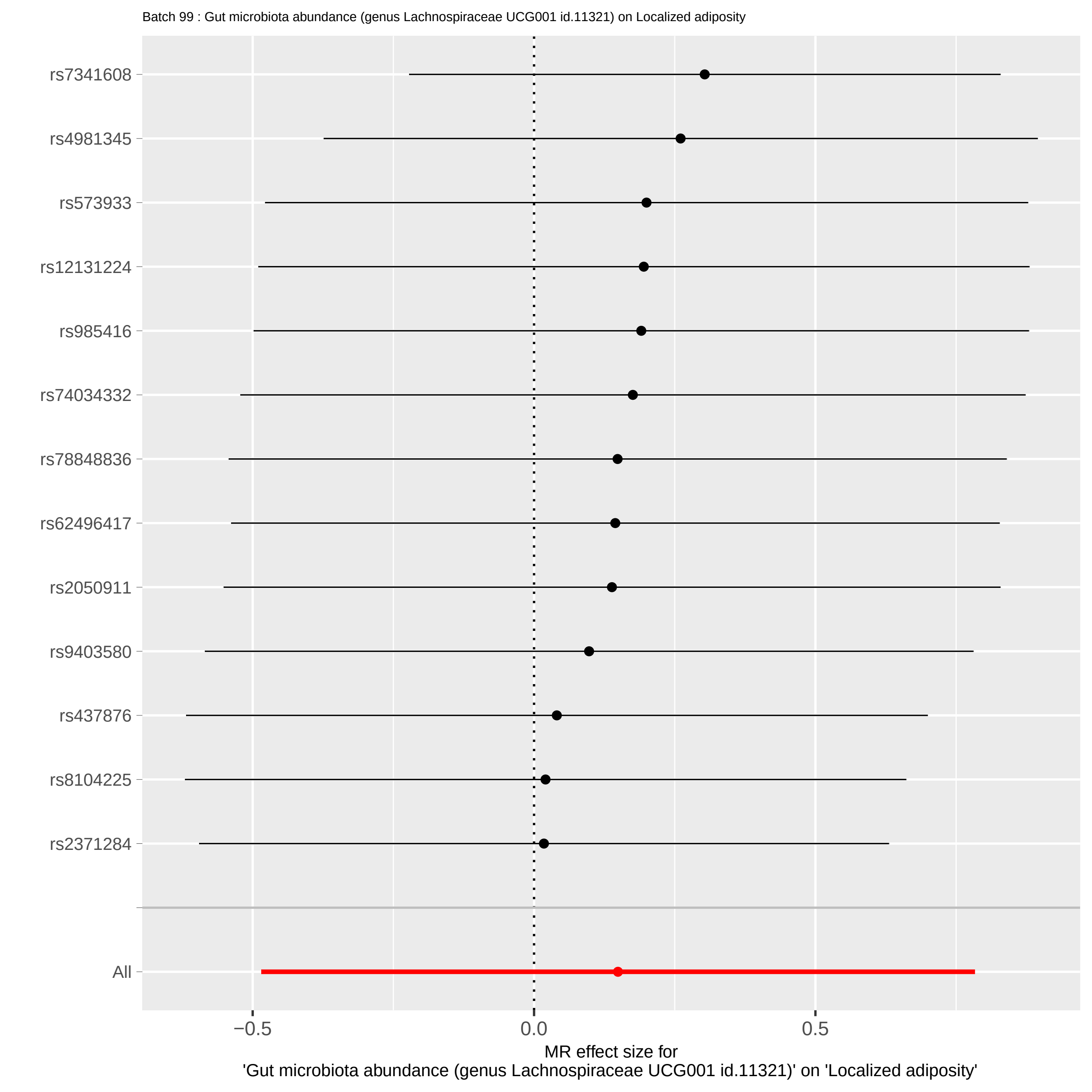

Batch 99 : Gut microbiota abundance (genus Lachnospiraceae UCG001 id.11321) on Localized adiposity
rs7341608
rs4981345
rs573933
rs12131224
rs985416
rs74034332
rs78848836
rs62496417
rs2050911
rs9403580
rs437876
rs8104225
rs2371284
All
−0.5
0.0
0.5
MR effect size for
'Gut microbiota abundance (genus Lachnospiraceae UCG001 id.11321)' on 'Localized adiposity'

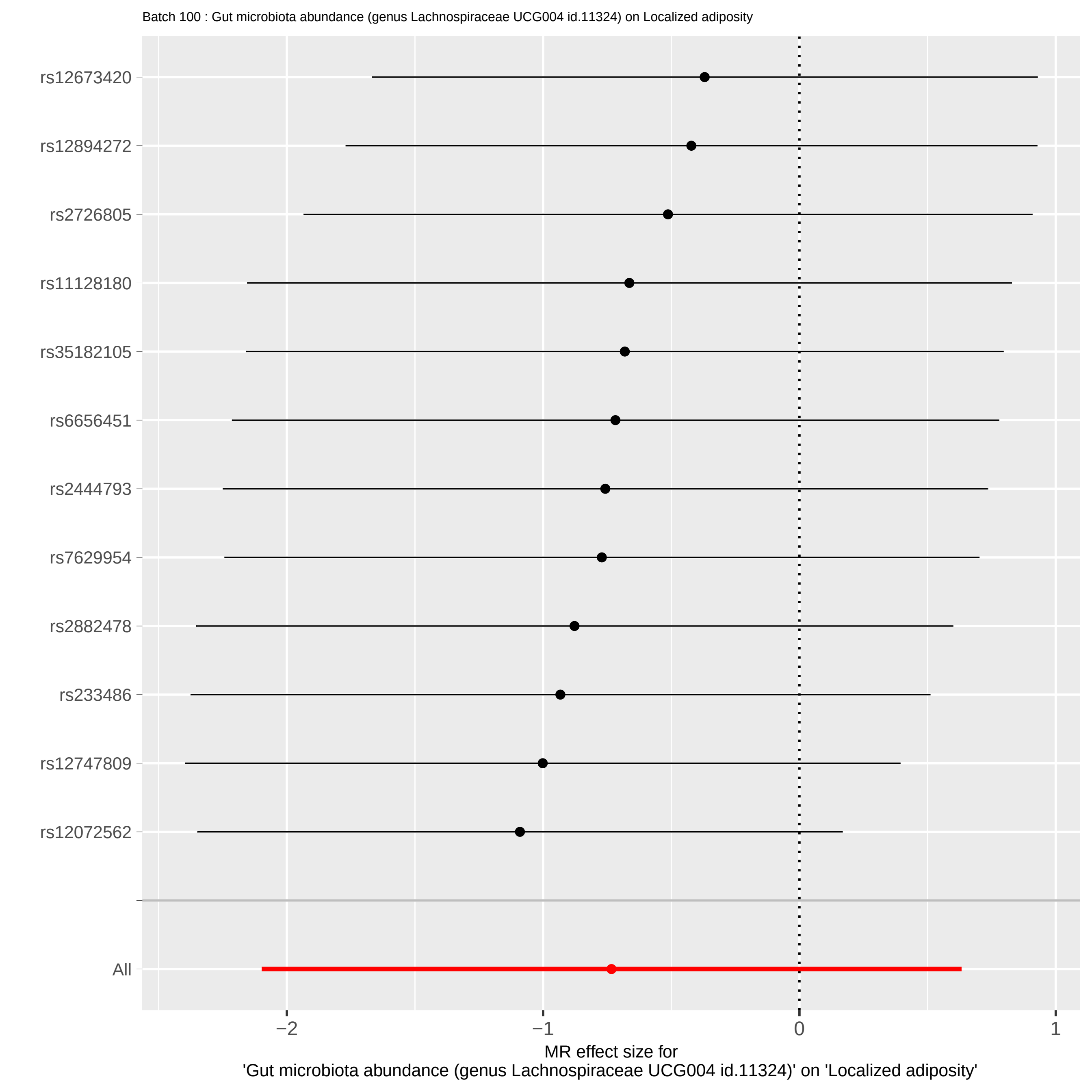
Batch 100 : Gut microbiota abundance (genus Lachnospiraceae UCG004 id.11324) on Localized adiposity
rs12673420
rs12894272
rs2726805
rs11128180
rs35182105
rs6656451
rs2444793
rs7629954
rs2882478
rs233486
rs12747809
rs12072562
All
−2
−1
0
1
MR effect size for
'Gut microbiota abundance (genus Lachnospiraceae UCG004 id.11324)' on 'Localized adiposity'

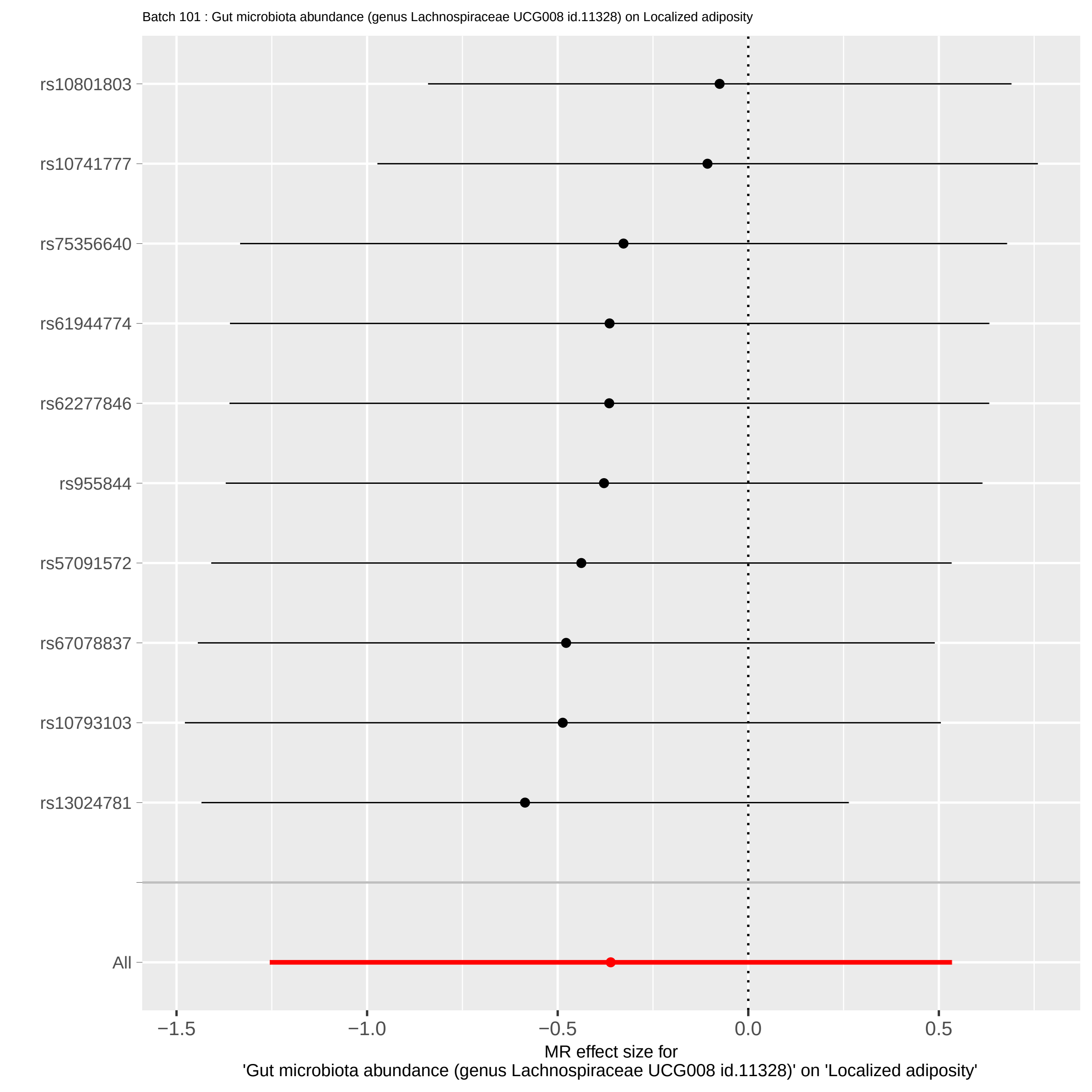

Batch 101 : Gut microbiota abundance (genus Lachnospiraceae UCG008 id.11328) on Localized adiposity
rs10801803
rs10741777
rs75356640
rs61944774
rs62277846
rs955844
rs57091572
rs67078837
rs10793103
rs13024781
All
−1.5
−1.0
−0.5
0.0
0.5
MR effect size for
'Gut microbiota abundance (genus Lachnospiraceae UCG008 id.11328)' on 'Localized adiposity'

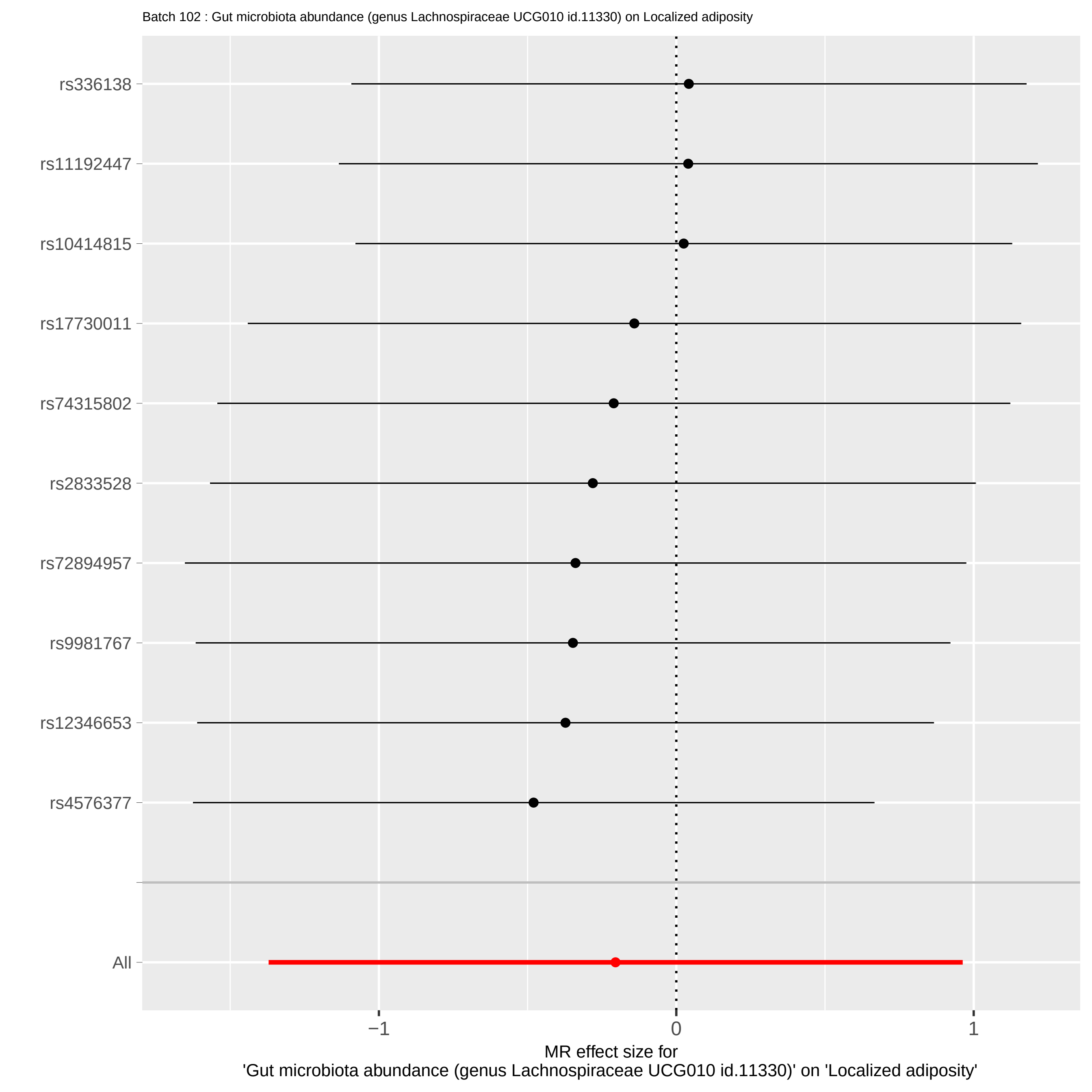
Batch 102 : Gut microbiota abundance (genus Lachnospiraceae UCG010 id.11330) on Localized adiposity
rs336138
rs11192447
rs10414815
rs17730011
rs74315802
rs2833528
rs72894957
rs9981767
rs12346653
rs4576377
All
−1
0
1
MR effect size for
'Gut microbiota abundance (genus Lachnospiraceae UCG010 id.11330)' on 'Localized adiposity'

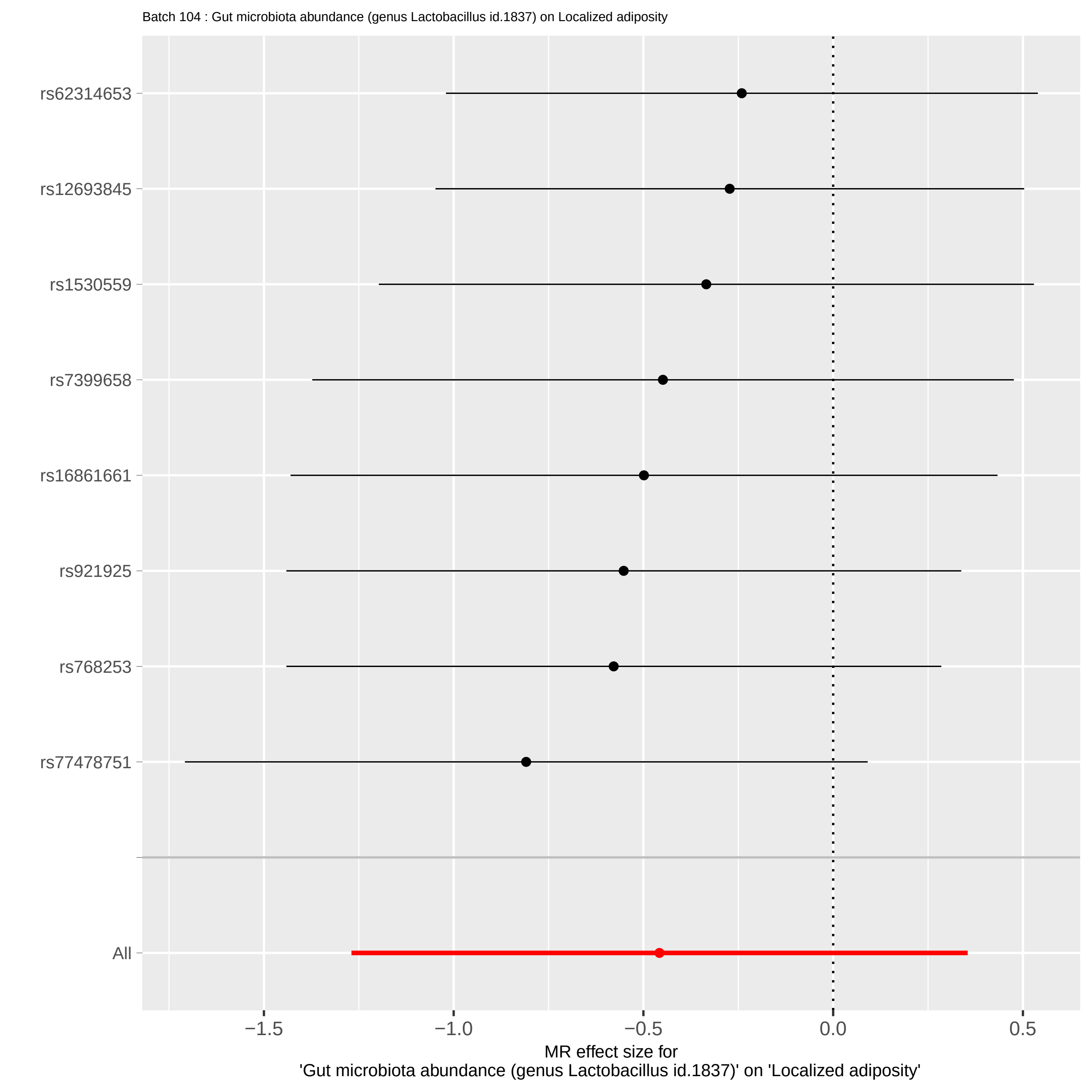
Batch 104 : Gut microbiota abundance (genus Lactobacillus id.1837) on Localized adiposity
rs62314653
rs12693845
rs1530559
rs7399658
rs16861661
rs921925
rs768253
rs77478751
All
−1.5
−1.0
−0.5
0.0
0.5
MR effect size for
'Gut microbiota abundance (genus Lactobacillus id.1837)' on 'Localized adiposity'

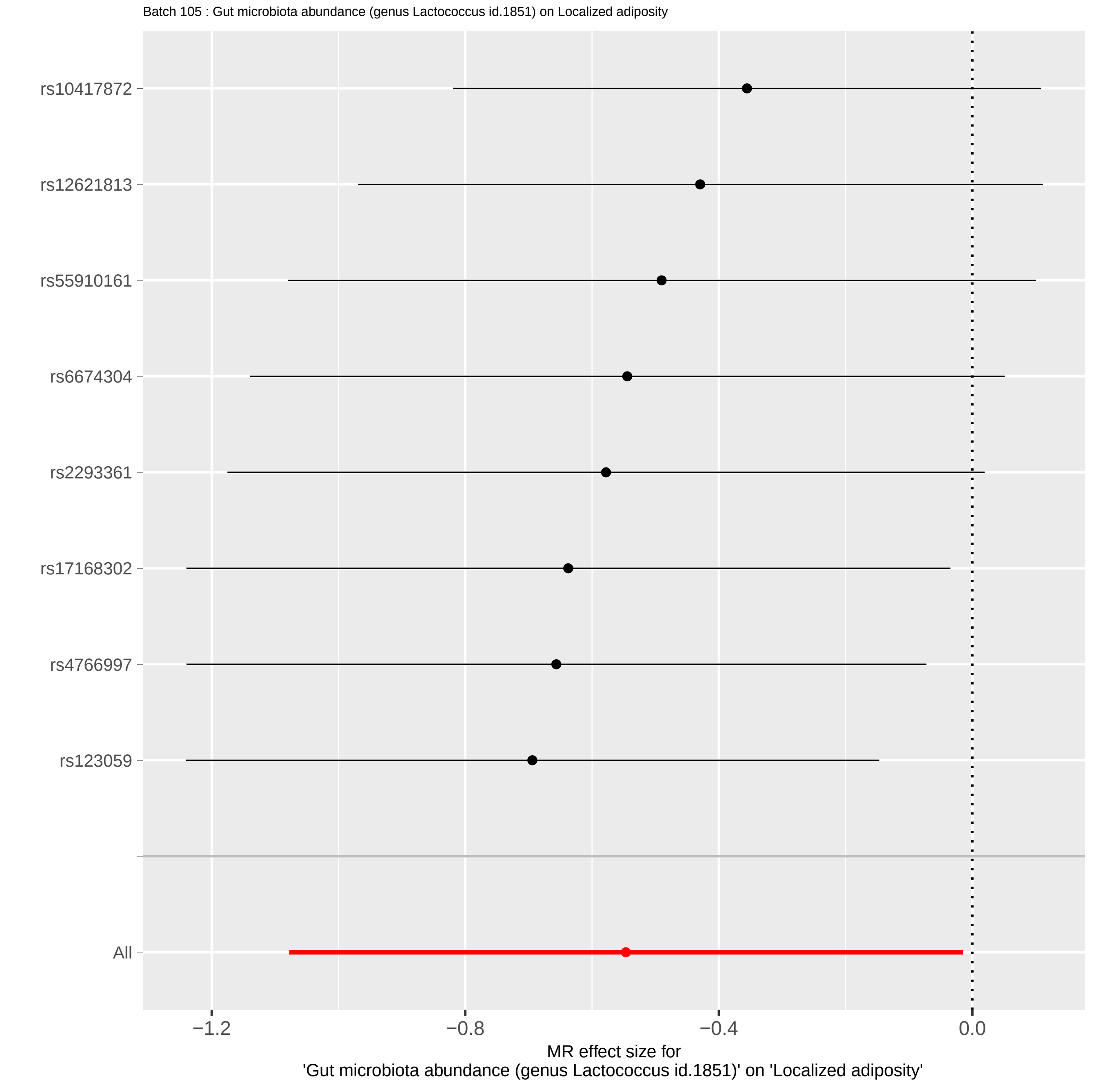

Batch 105 : Gut microbiota abundance (genus Lactococcus id.1851) on Localized adiposity
rs10417872
rs12621813
rs55910161
rs6674304
rs2293361
rs17168302
rs4766997
rs123059
All
−1.2
−0.8
−0.4
0.0
MR effect size for
'Gut microbiota abundance (genus Lactococcus id.1851)' on 'Localized adiposity'

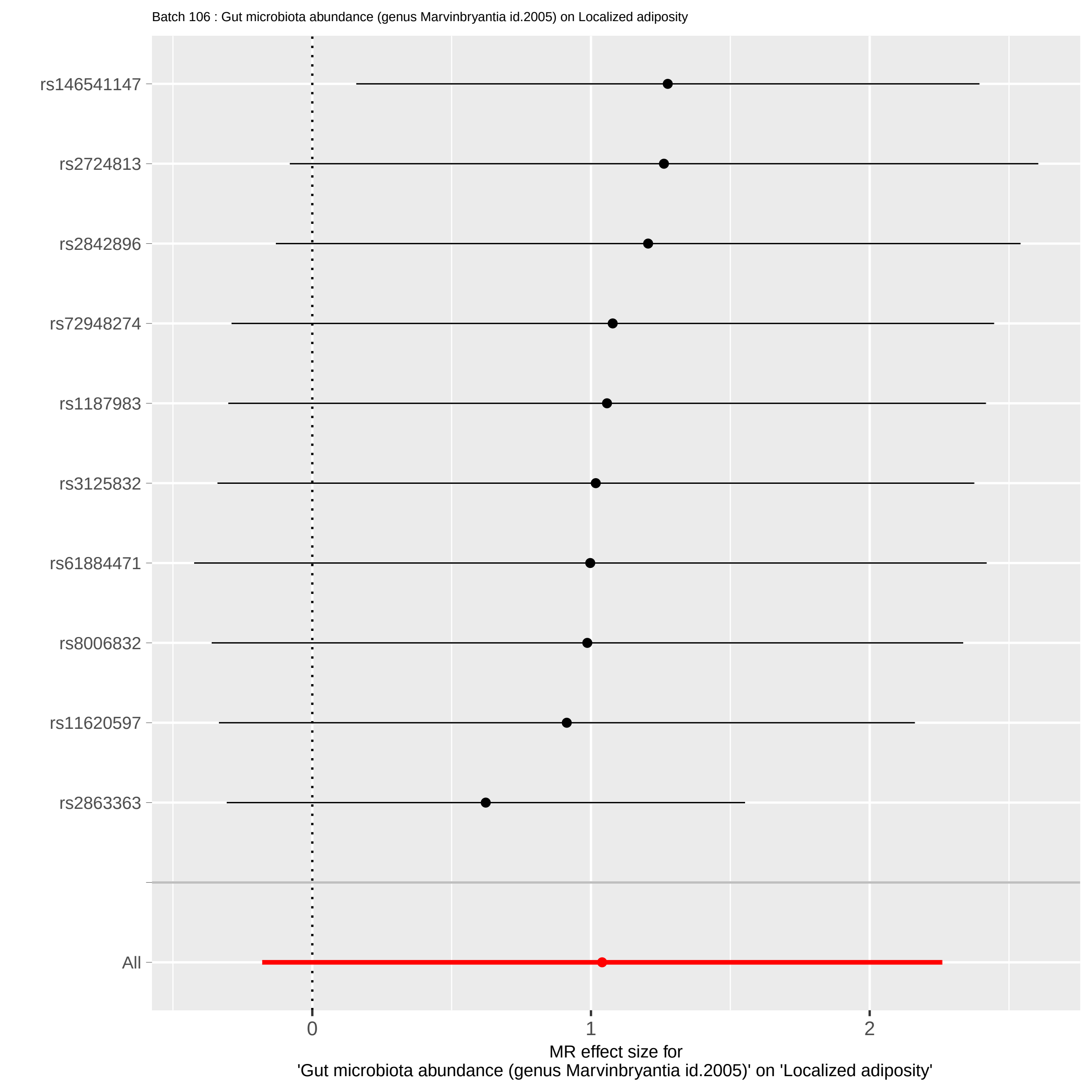

Batch 106 : Gut microbiota abundance (genus Marvinbryantia id.2005) on Localized adiposity
rs146541147
rs2724813
rs2842896
rs72948274
rs1187983
rs3125832
rs61884471
rs8006832
rs11620597
rs2863363
All
0
1
2
MR effect size for
'Gut microbiota abundance (genus Marvinbryantia id.2005)' on 'Localized adiposity'

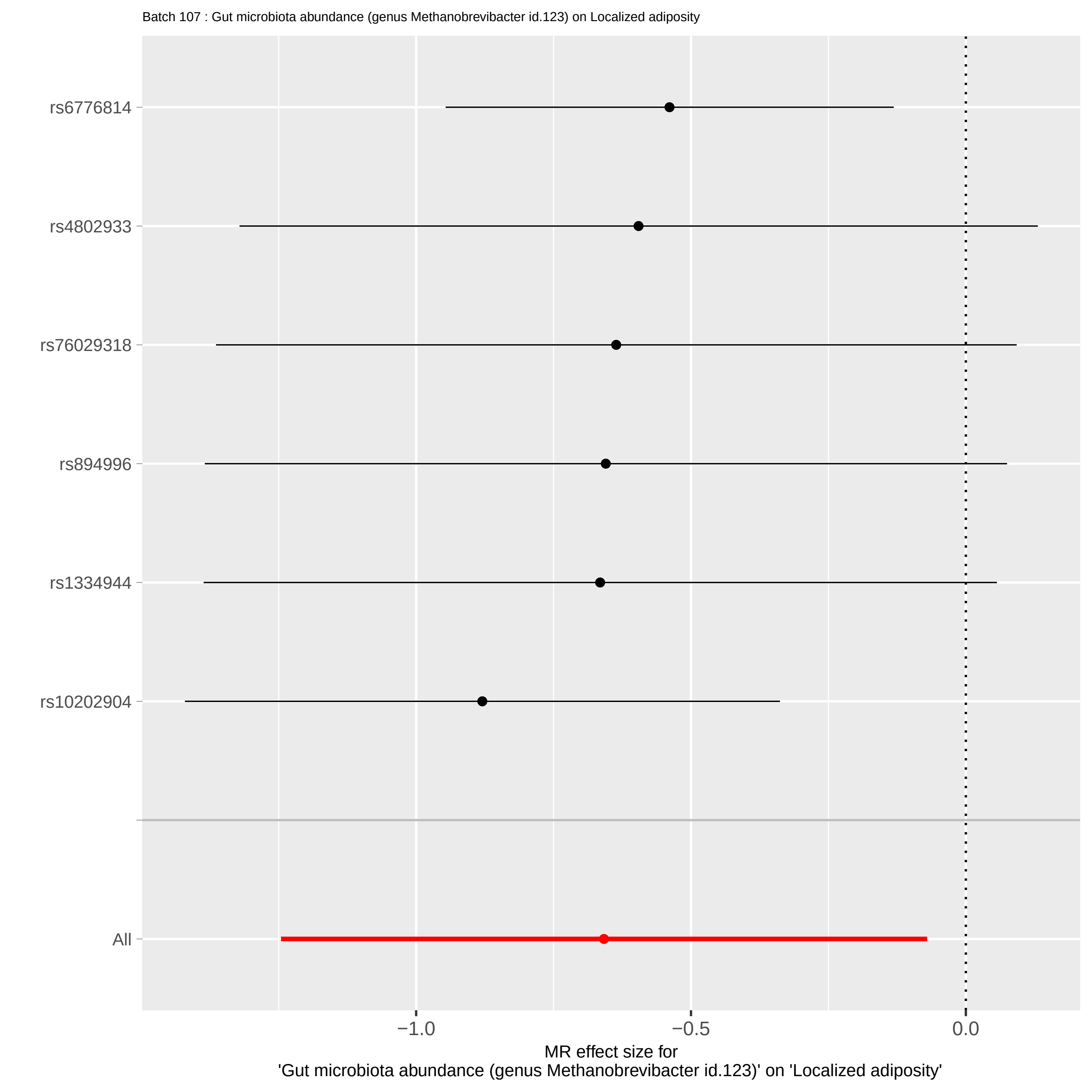

Batch 107 : Gut microbiota abundance (genus Methanobrevibacter id.123) on Localized adiposity
rs6776814
rs4802933
rs76029318
rs894996
rs1334944
rs10202904
All
−1.0
−0.5
0.0
MR effect size for
'Gut microbiota abundance (genus Methanobrevibacter id.123)' on 'Localized adiposity'

Batch 108 : Gut microbiota abundance (genus Odoribacter id.952) on Localized adiposity

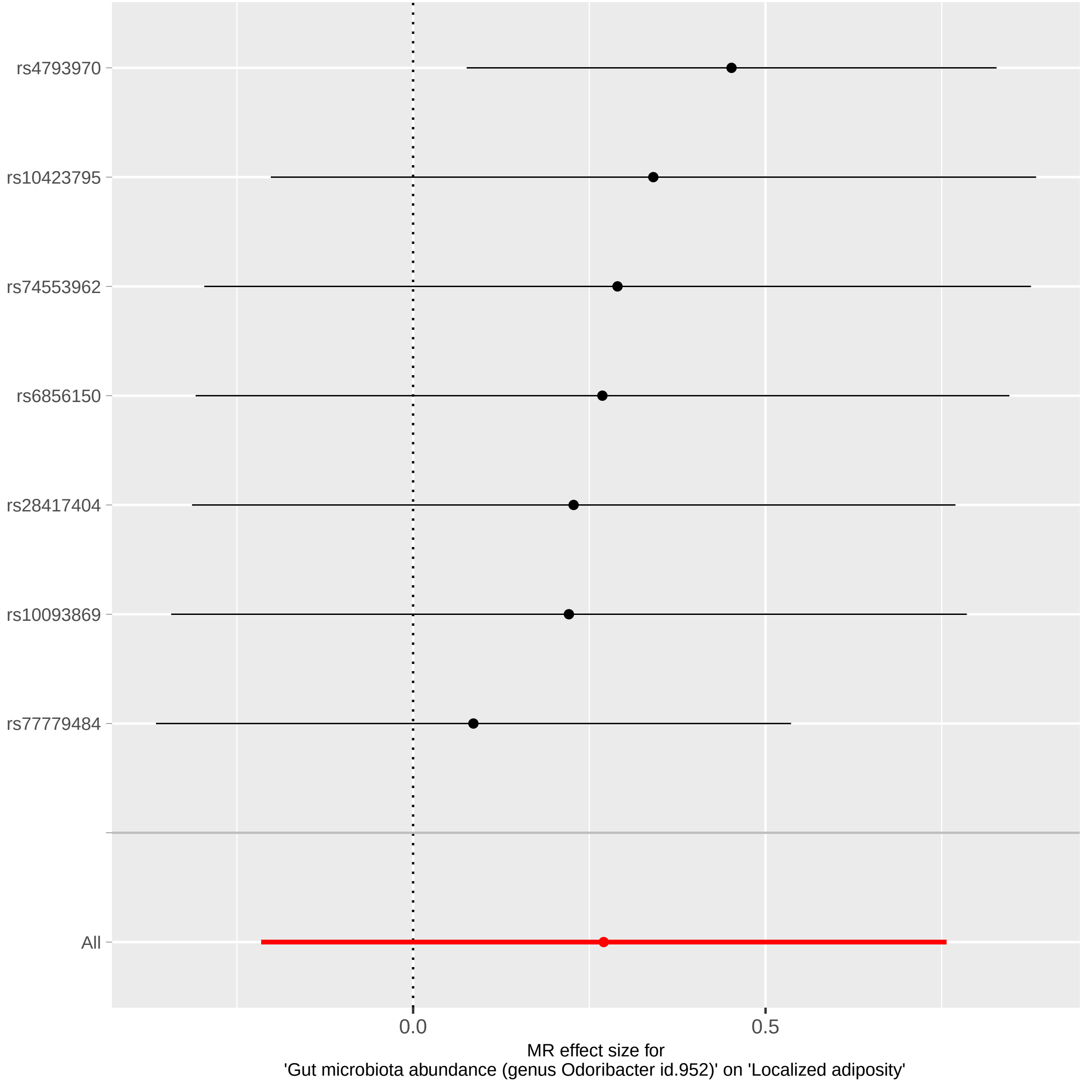

Batch 109 : Gut microbiota abundance (genus Olsenella id.822) on Localized adiposity

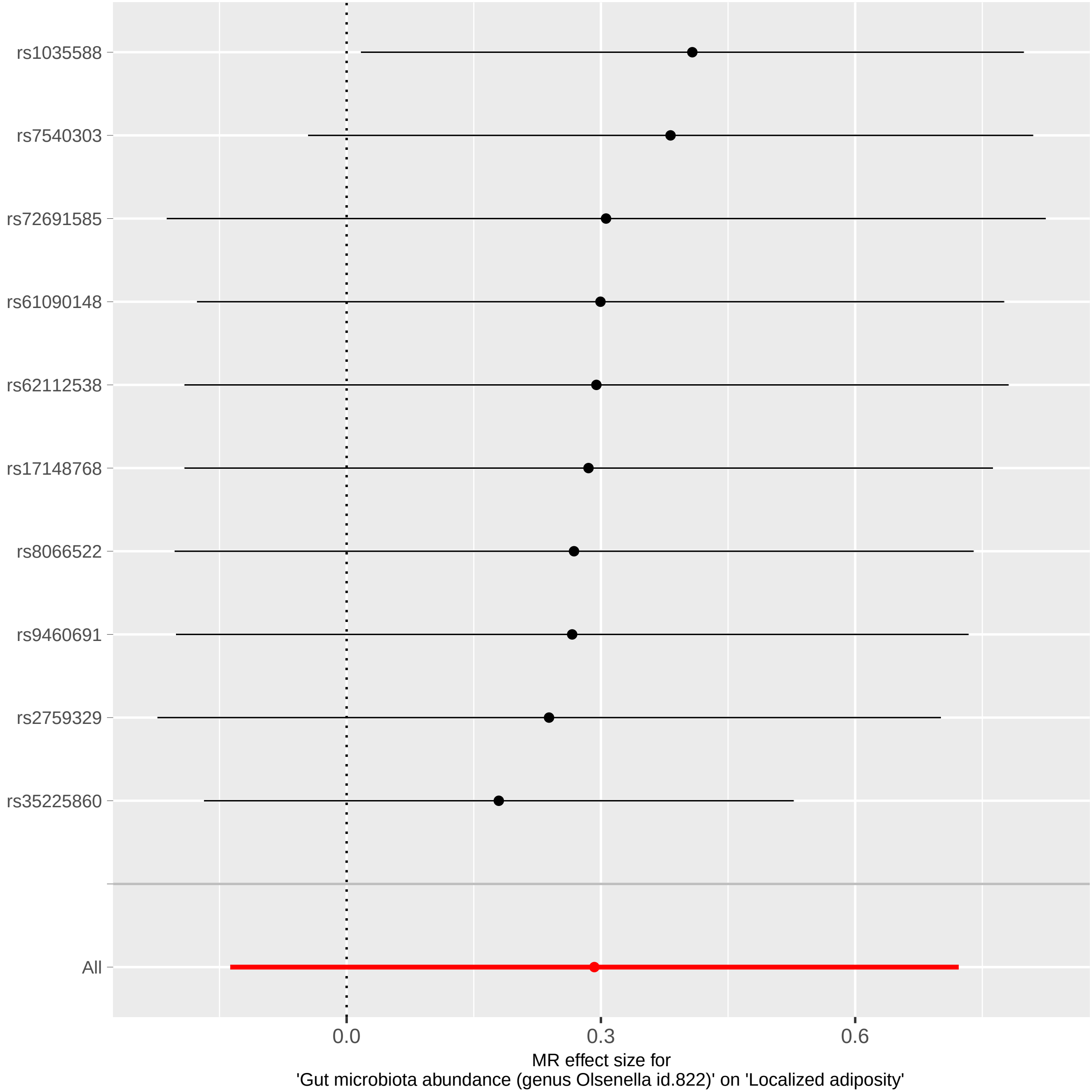

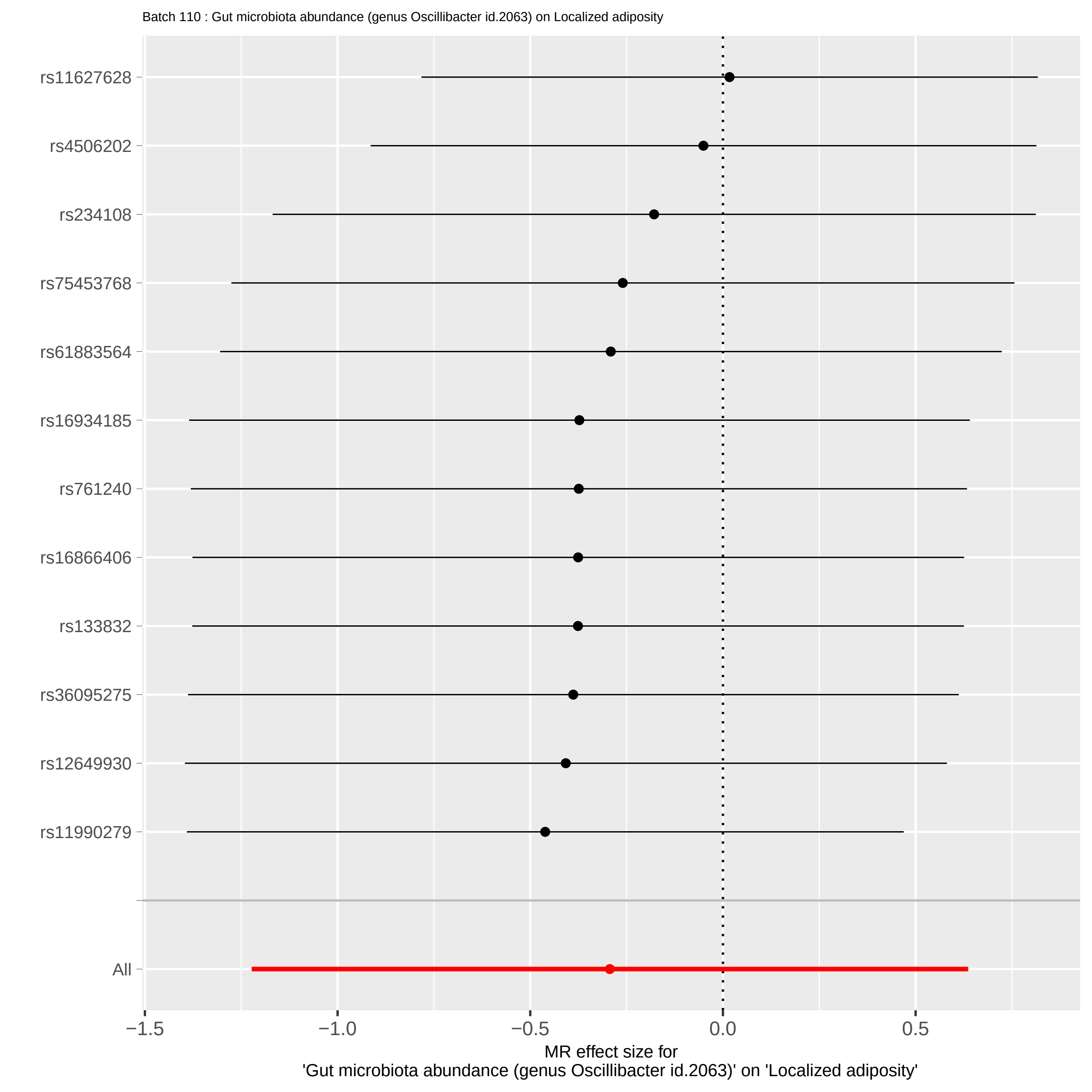

Batch 110 : Gut microbiota abundance (genus Oscillibacter id.2063) on Localized adiposity
rs11627628
rs4506202
rs234108
rs75453768
rs61883564
rs16934185
rs761240
rs16866406
rs133832
rs36095275
rs12649930
rs11990279
All
−1.5
−1.0
−0.5
0.0
0.5
MR effect size for
'Gut microbiota abundance (genus Oscillibacter id.2063)' on 'Localized adiposity'

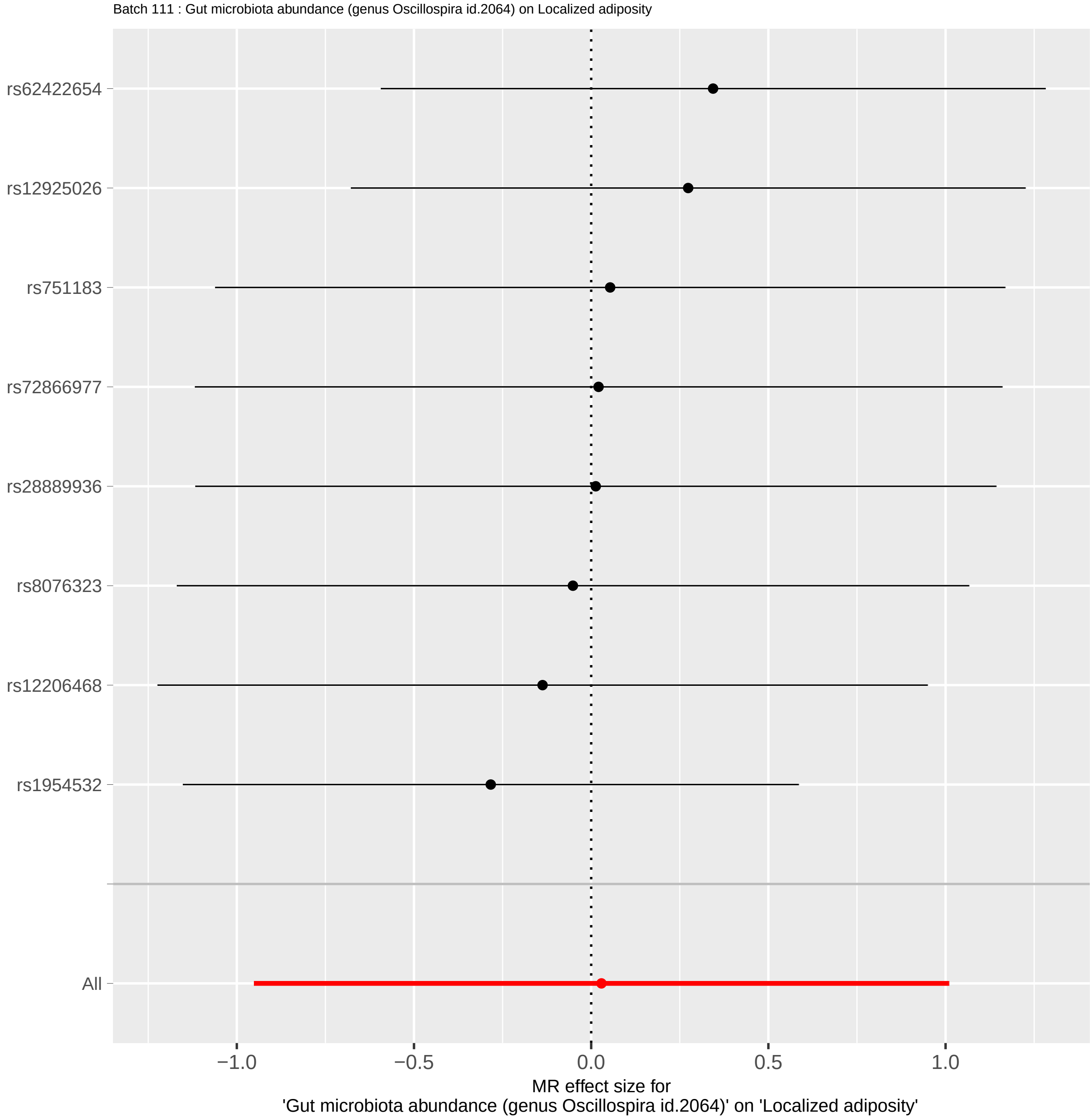
Batch 111 : Gut microbiota abundance (genus Oscillospira id.2064) on Localized adiposity
rs62422654
rs12925026
rs751183
rs72866977
rs28889936
rs8076323
rs12206468
rs1954532
All
−1.0
−0.5
0.0
0.5
1.0
MR effect size for
'Gut microbiota abundance (genus Oscillospira id.2064)' on 'Localized adiposity'

Batch 112 : Gut microbiota abundance (genus Oxalobacter id.2978) on Localized adiposity

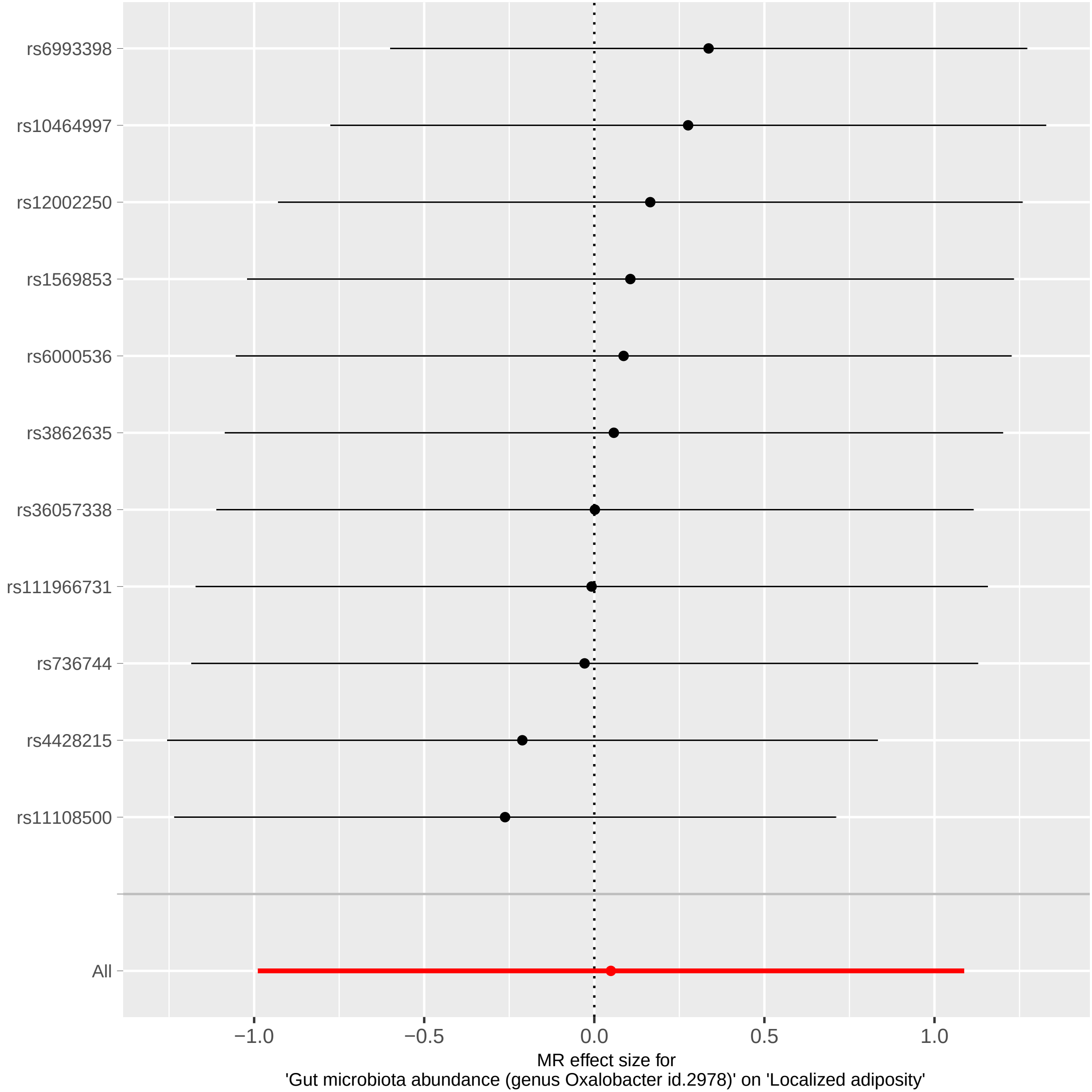

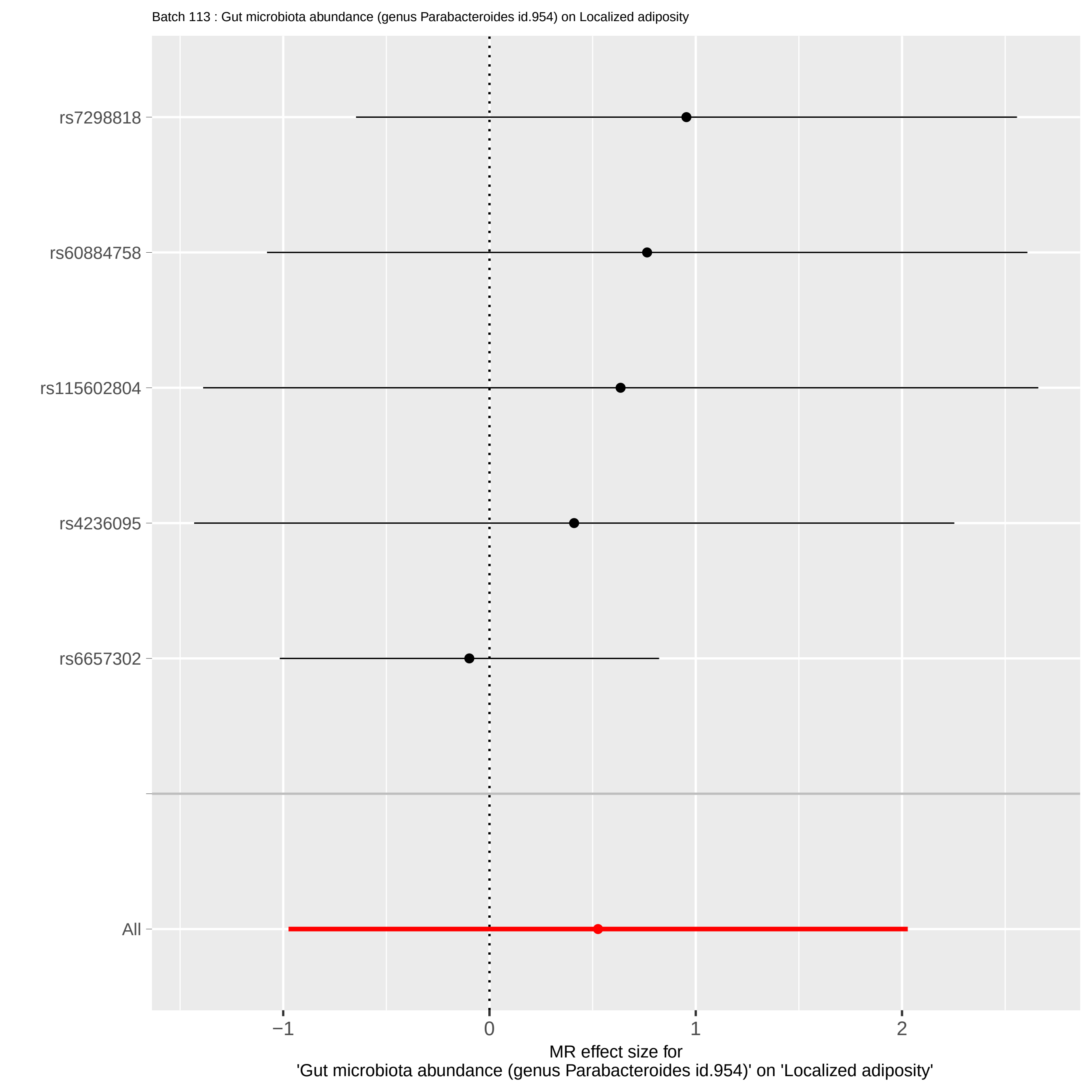

Batch 113 : Gut microbiota abundance (genus Parabacteroides id.954) on Localized adiposity
rs7298818
rs60884758
rs115602804
rs4236095
rs6657302
All
−1
0
1
2
MR effect size for
'Gut microbiota abundance (genus Parabacteroides id.954)' on 'Localized adiposity'

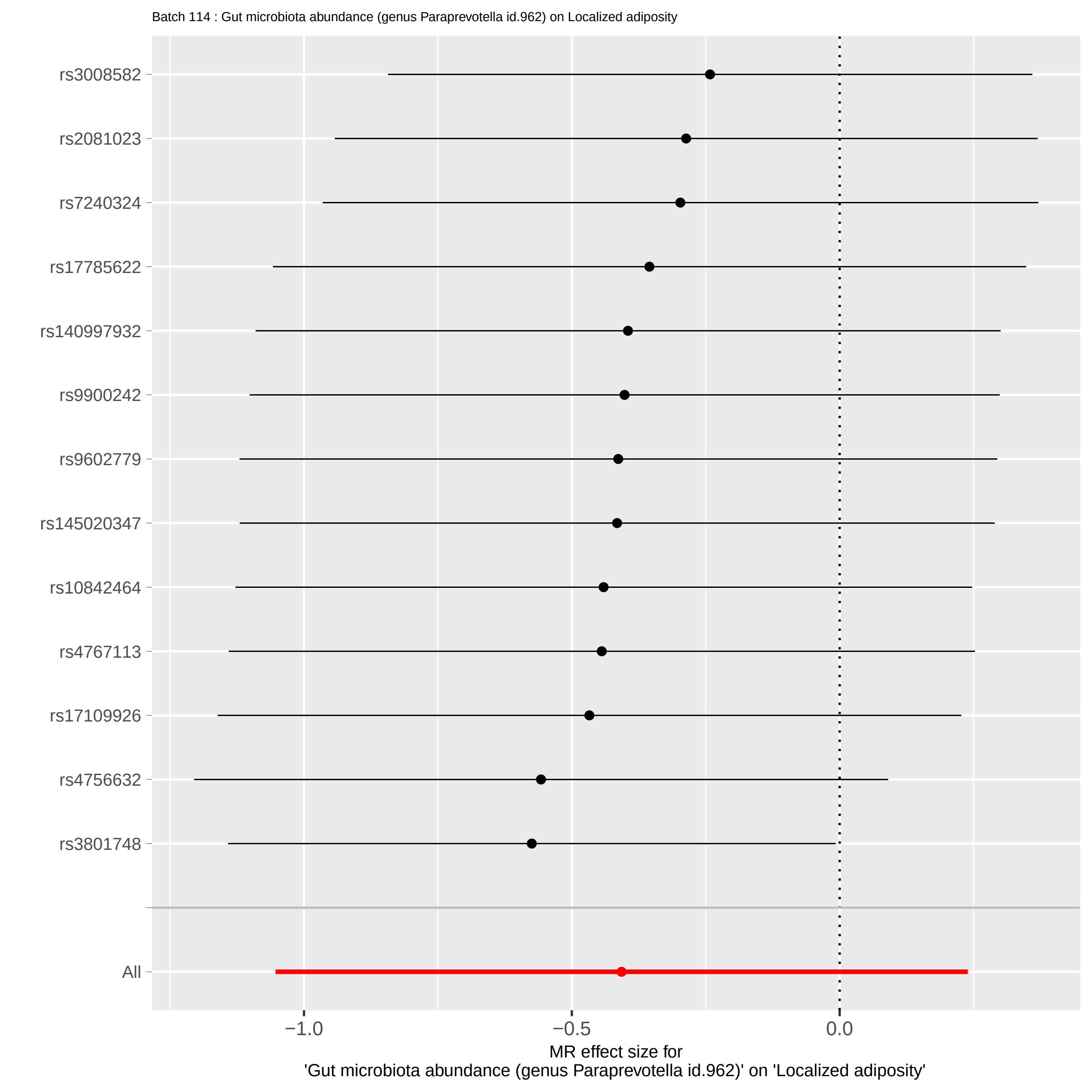

Batch 114 : Gut microbiota abundance (genus Paraprevotella id.962) on Localized adiposity
rs3008582
rs2081023
rs7240324
rs17785622
rs140997932
rs9900242
rs9602779
rs145020347
rs10842464
rs4767113
rs17109926
rs4756632
rs3801748
All
−1.0
−0.5
0.0
MR effect size for
'Gut microbiota abundance (genus Paraprevotella id.962)' on 'Localized adiposity'

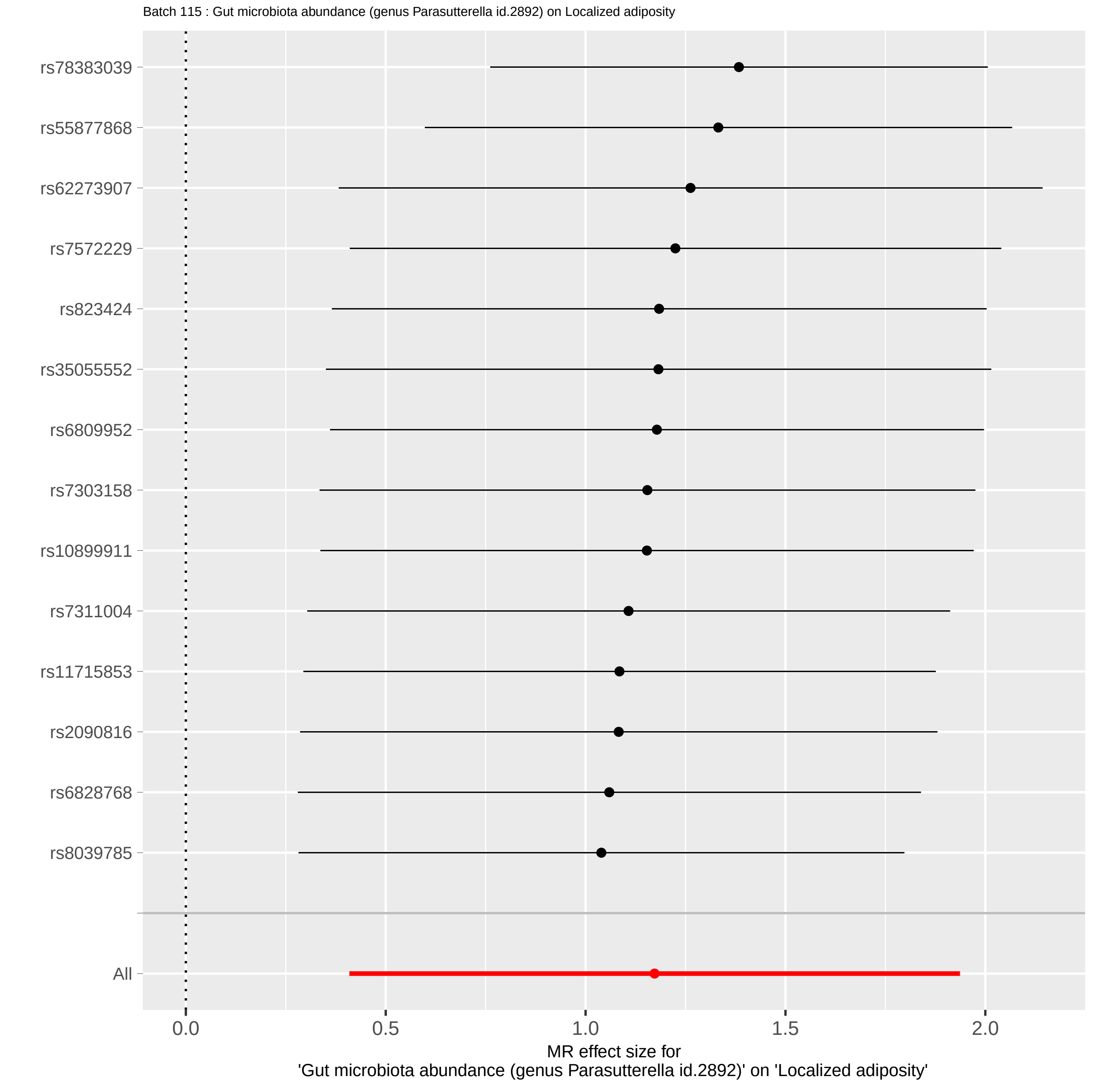
Batch 115 : Gut microbiota abundance (genus Parasutterella id.2892) on Localized adiposity
rs78383039
rs55877868
rs62273907
rs7572229
rs823424
rs35055552
rs6809952
rs7303158
rs10899911
rs7311004
rs11715853
rs2090816
rs6828768
rs8039785
All
0.0
0.5
1.0
1.5
2.0
MR effect size for
'Gut microbiota abundance (genus Parasutterella id.2892)' on 'Localized adiposity'

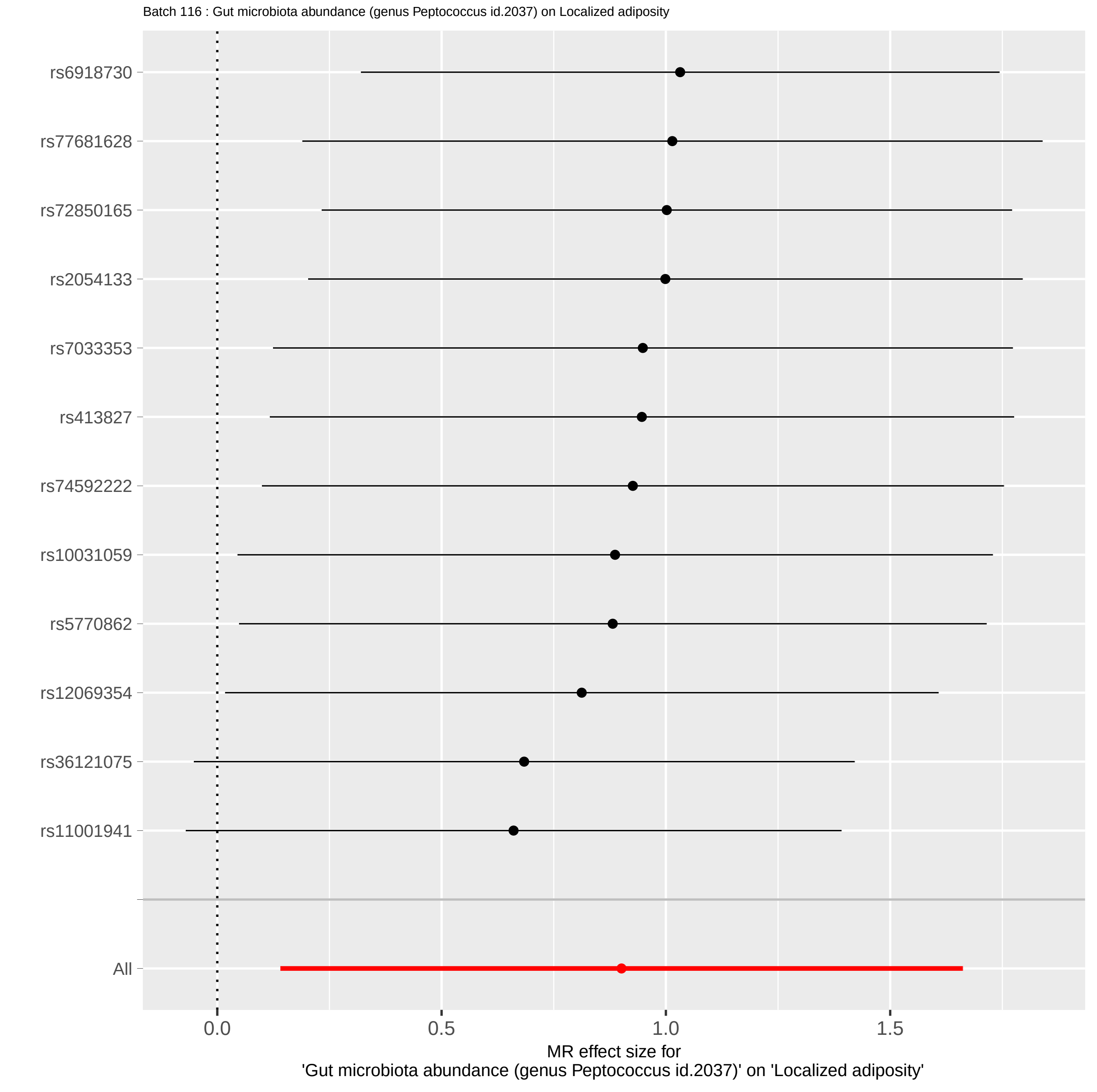

Batch 116 : Gut microbiota abundance (genus Peptococcus id.2037) on Localized adiposity
rs6918730
rs77681628
rs72850165
rs2054133
rs7033353
rs413827
rs74592222
rs10031059
rs5770862
rs12069354
rs36121075
rs11001941
All
0.0
0.5
1.0
1.5
MR effect size for
'Gut microbiota abundance (genus Peptococcus id.2037)' on 'Localized adiposity'

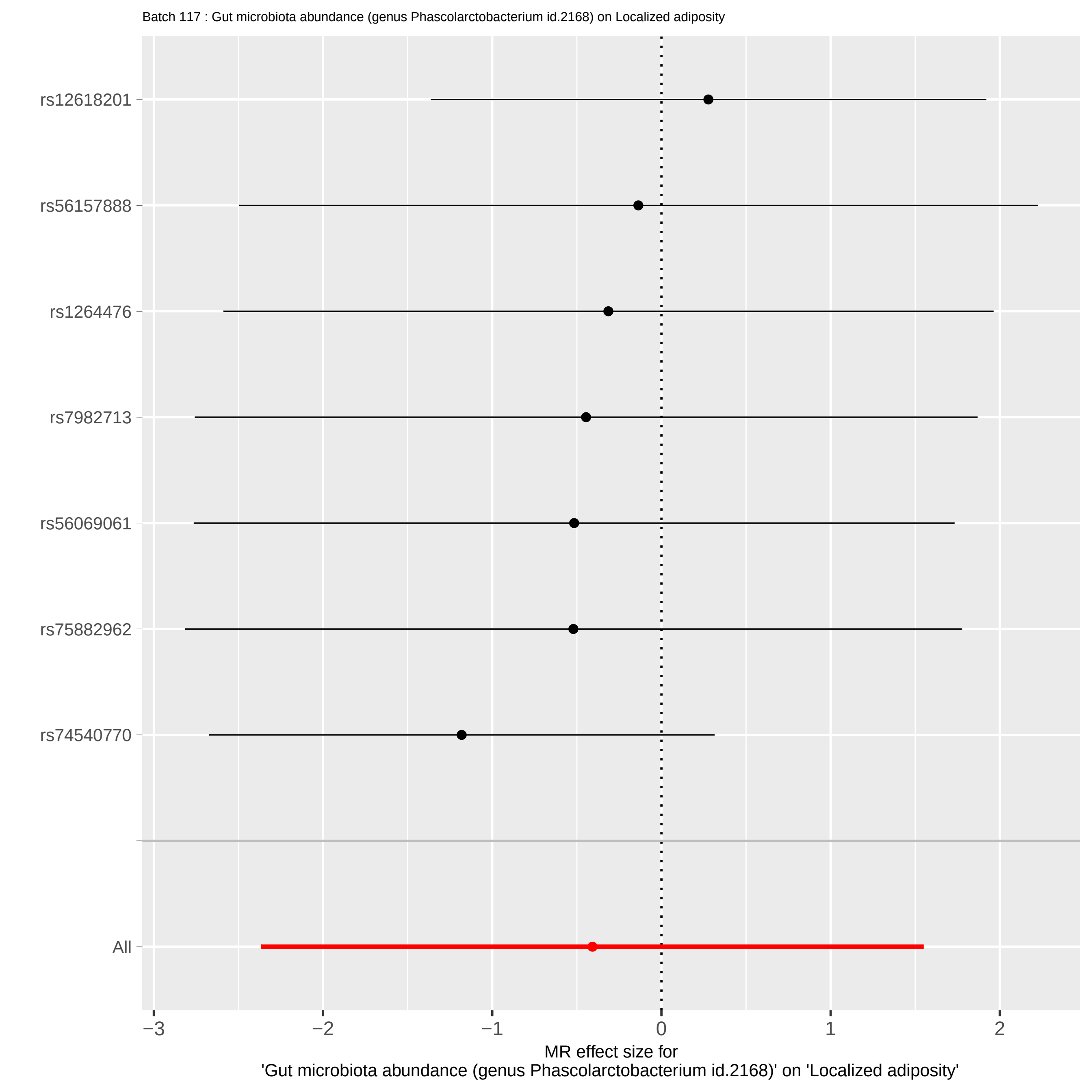

Batch 117 : Gut microbiota abundance (genus Phascolarctobacterium id.2168) on Localized adiposity
rs12618201
rs56157888
rs1264476
rs7982713
rs56069061
rs75882962
rs74540770
All
−3
−2
−1
0
1
2
MR effect size for
'Gut microbiota abundance (genus Phascolarctobacterium id.2168)' on 'Localized adiposity'

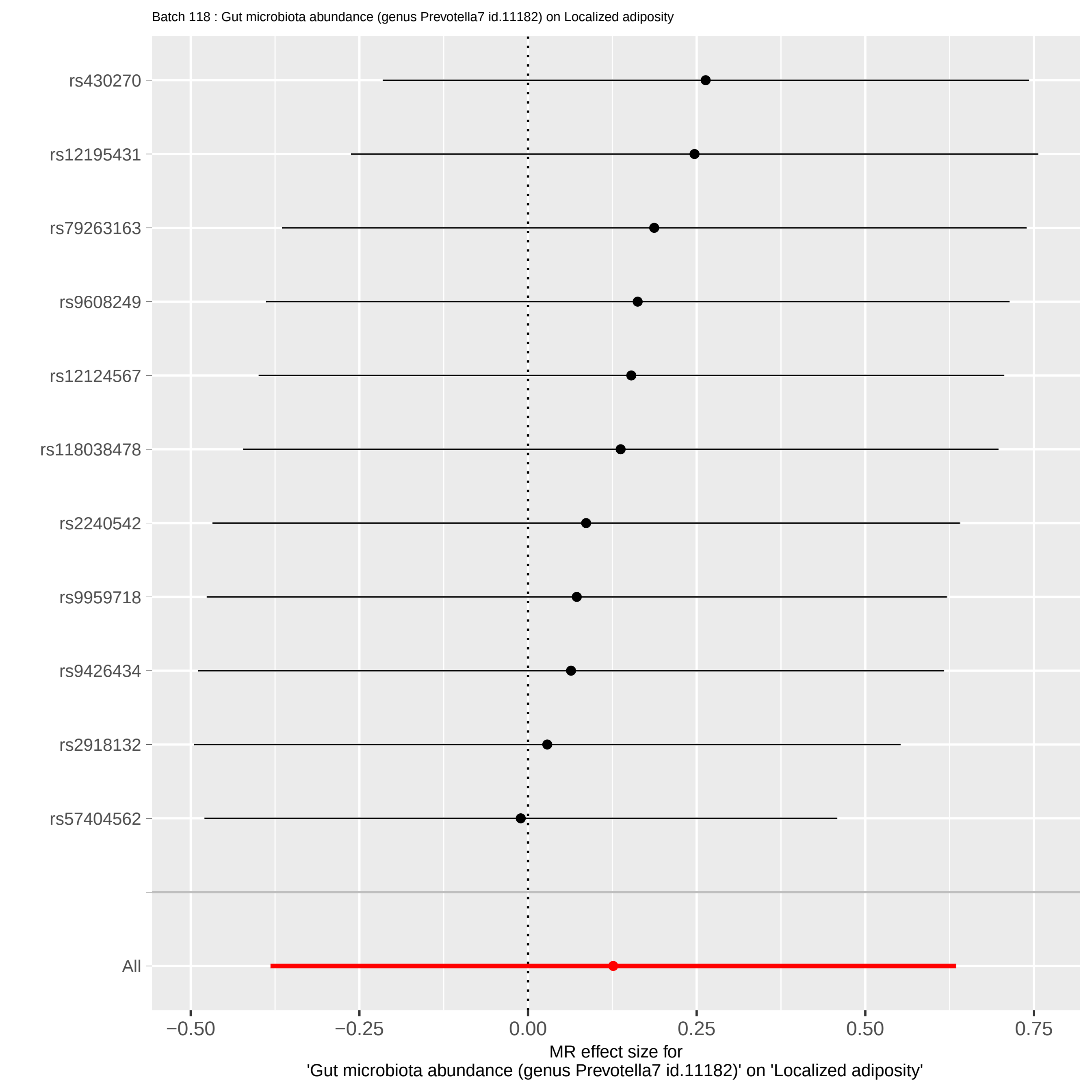

Batch 118 : Gut microbiota abundance (genus Prevotella7 id.11182) on Localized adiposity
rs430270
rs12195431
rs79263163
rs9608249
rs12124567
rs118038478
rs2240542
rs9959718
rs9426434
rs2918132
rs57404562
All
−0.50
−0.25
0.00
0.25
0.50
0.75
MR effect size for
'Gut microbiota abundance (genus Prevotella7 id.11182)' on 'Localized adiposity'

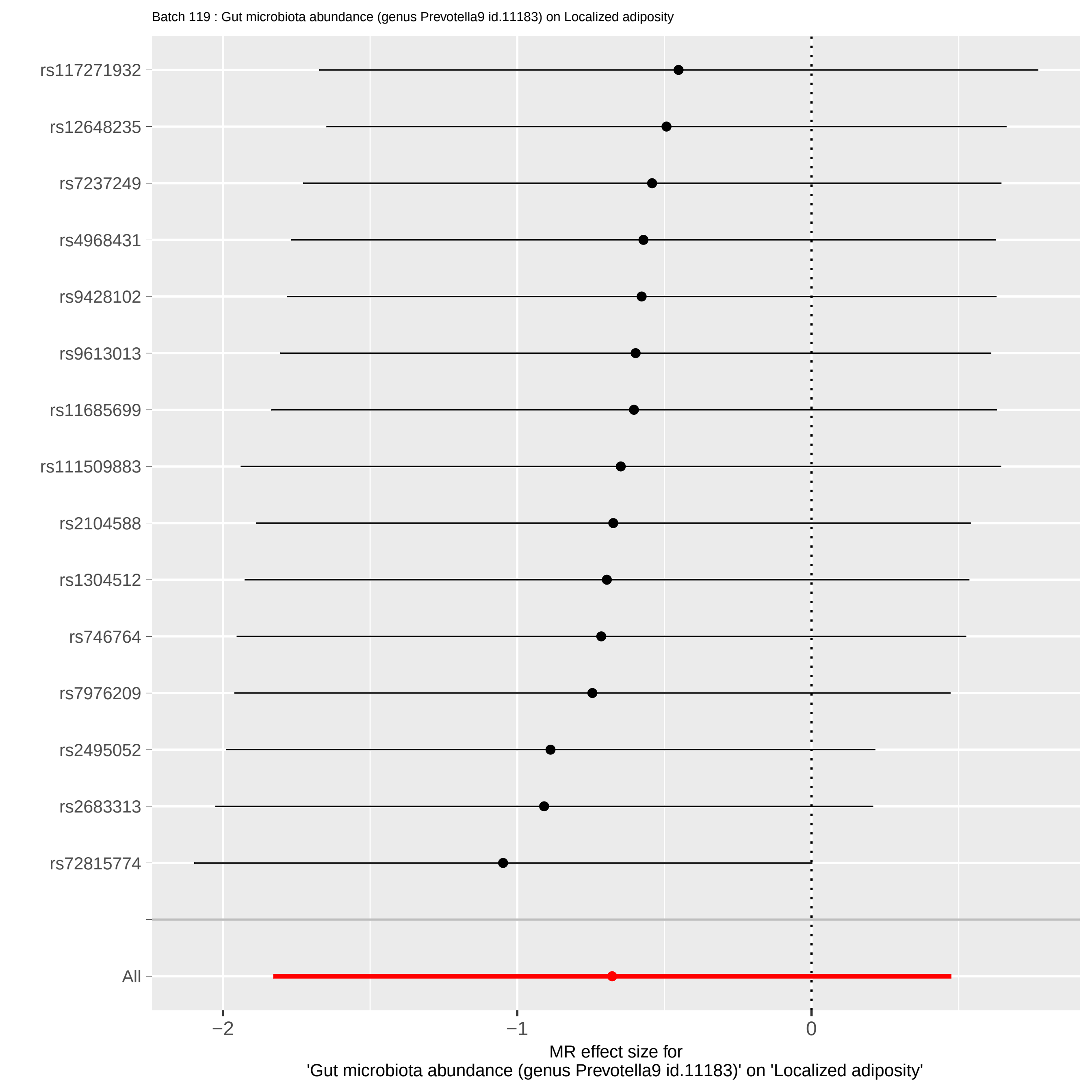
Batch 119 : Gut microbiota abundance (genus Prevotella9 id.11183) on Localized adiposity
rs117271932
rs12648235
rs7237249
rs4968431
rs9428102
rs9613013
rs11685699
rs111509883
rs2104588
rs1304512
rs746764
rs7976209
rs2495052
rs2683313
rs72815774
All
−2
−1
0
MR effect size for
'Gut microbiota abundance (genus Prevotella9 id.11183)' on 'Localized adiposity'

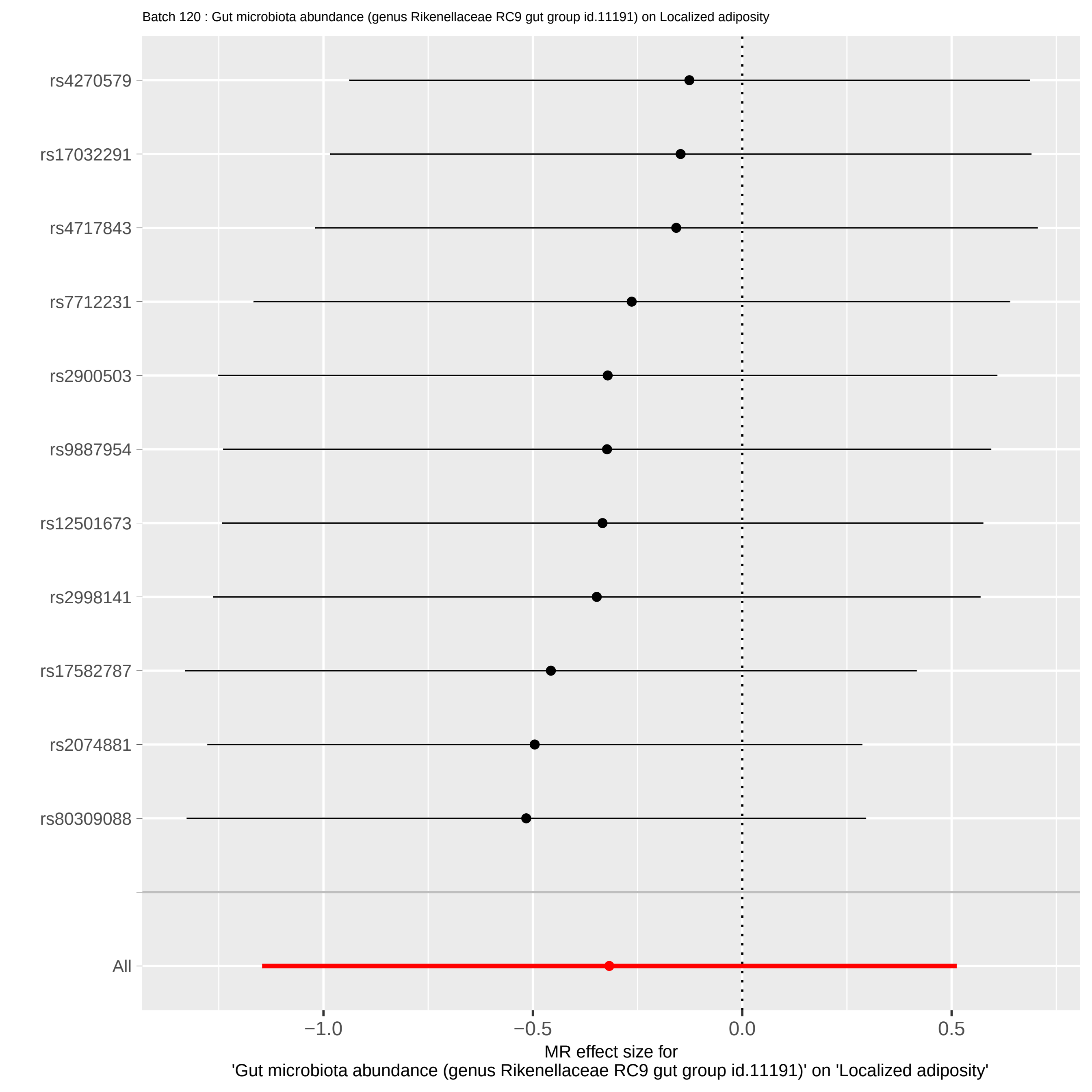

Batch 120 : Gut microbiota abundance (genus Rikenellaceae RC9 gut group id.11191) on Localized adiposity
rs4270579
rs17032291
rs4717843
rs7712231
rs2900503
rs9887954
rs12501673
rs2998141
rs17582787
rs2074881
rs80309088
All
−1.0
−0.5
0.0
0.5
MR effect size for
'Gut microbiota abundance (genus Rikenellaceae RC9 gut group id.11191)' on 'Localized adiposity'

Batch 121 : Gut microbiota abundance (genus Romboutsia id.11347) on Localized adiposity

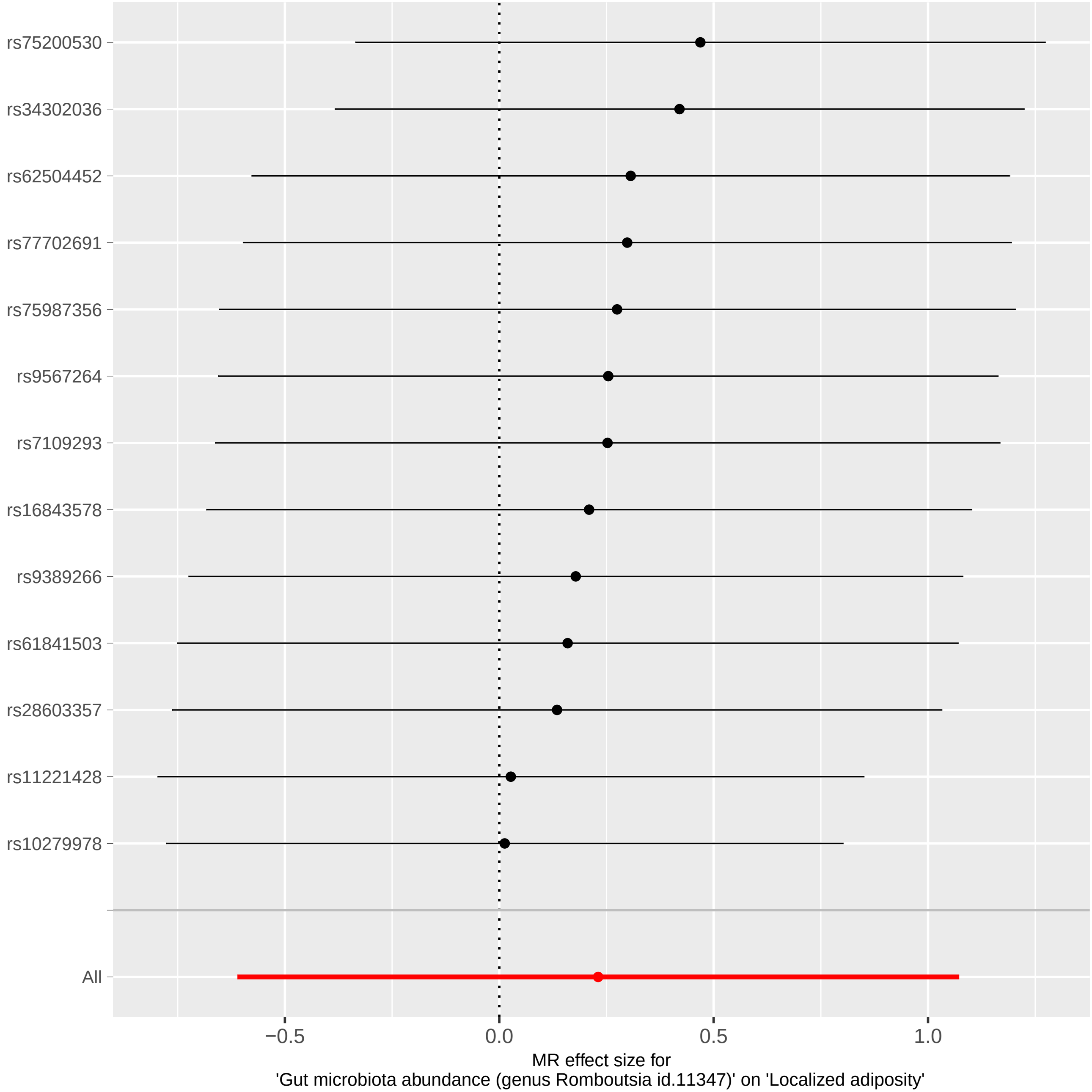

Batch 122 : Gut microbiota abundance (genus Roseburia id.2012) on Localized adiposity

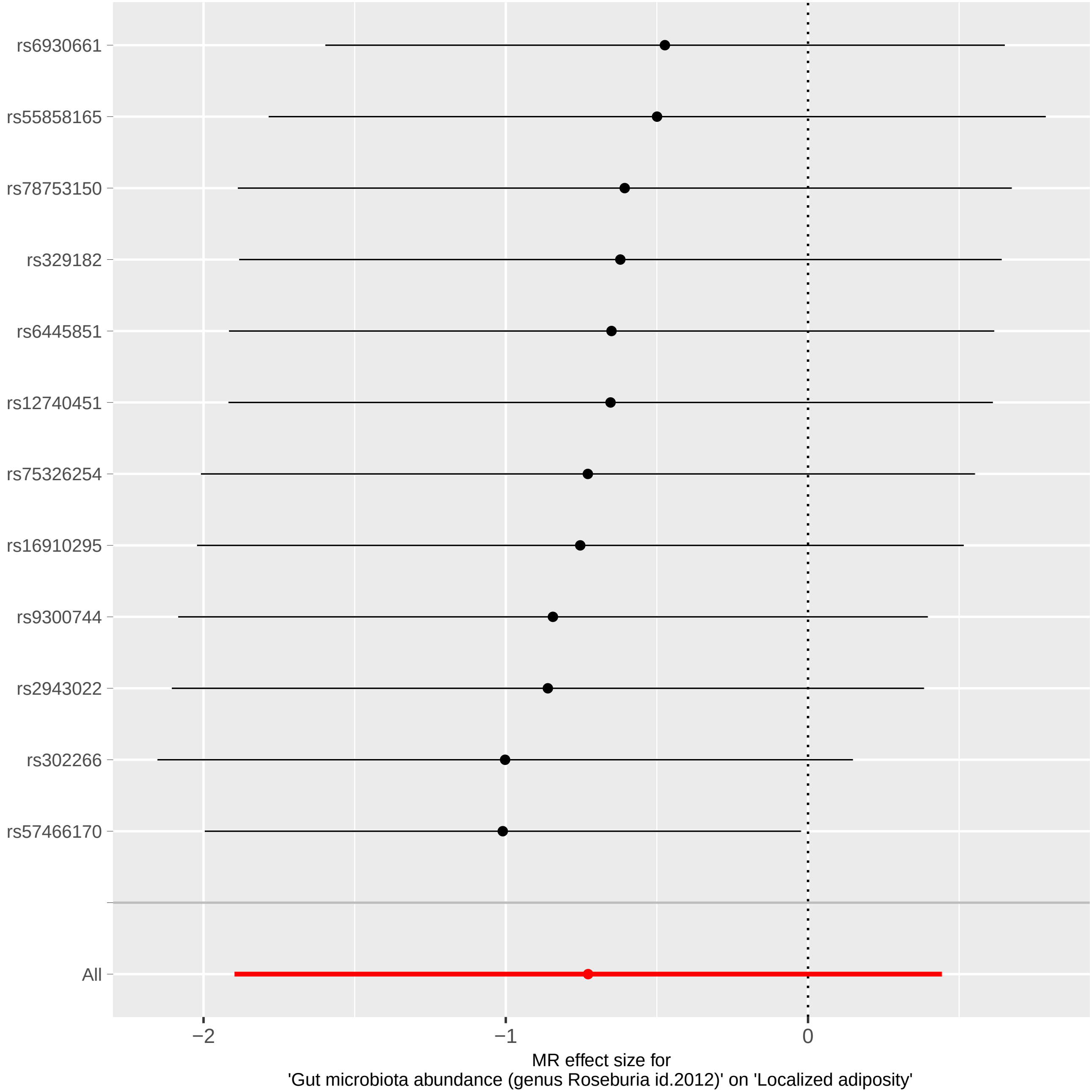

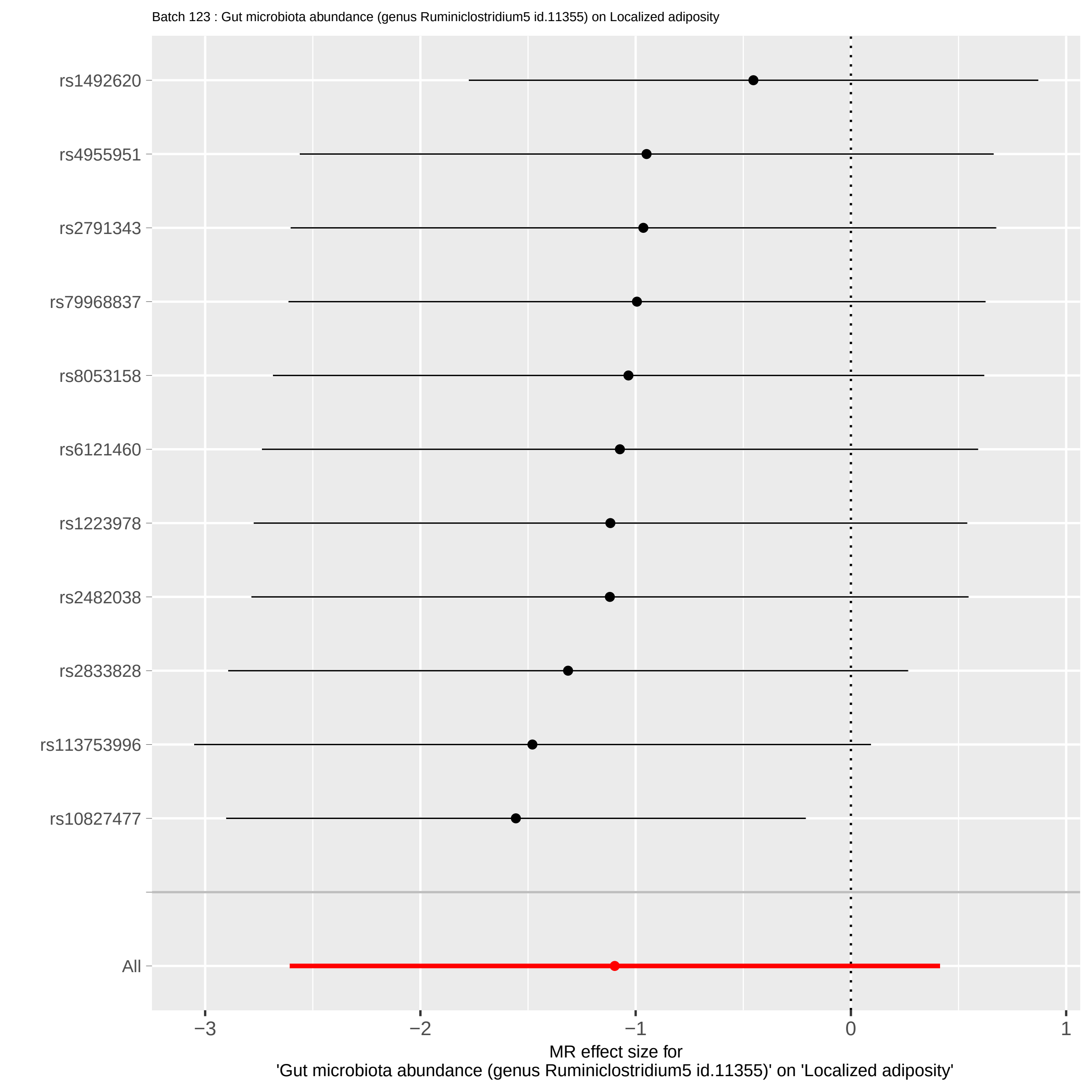
Batch 123 : Gut microbiota abundance (genus Ruminiclostridium5 id.11355) on Localized adiposity
rs1492620
rs4955951
rs2791343
rs79968837
rs8053158
rs6121460
rs1223978
rs2482038
rs2833828
rs113753996
rs10827477
All
−3
−2
−1
0
1
MR effect size for
'Gut microbiota abundance (genus Ruminiclostridium5 id.11355)' on 'Localized adiposity'

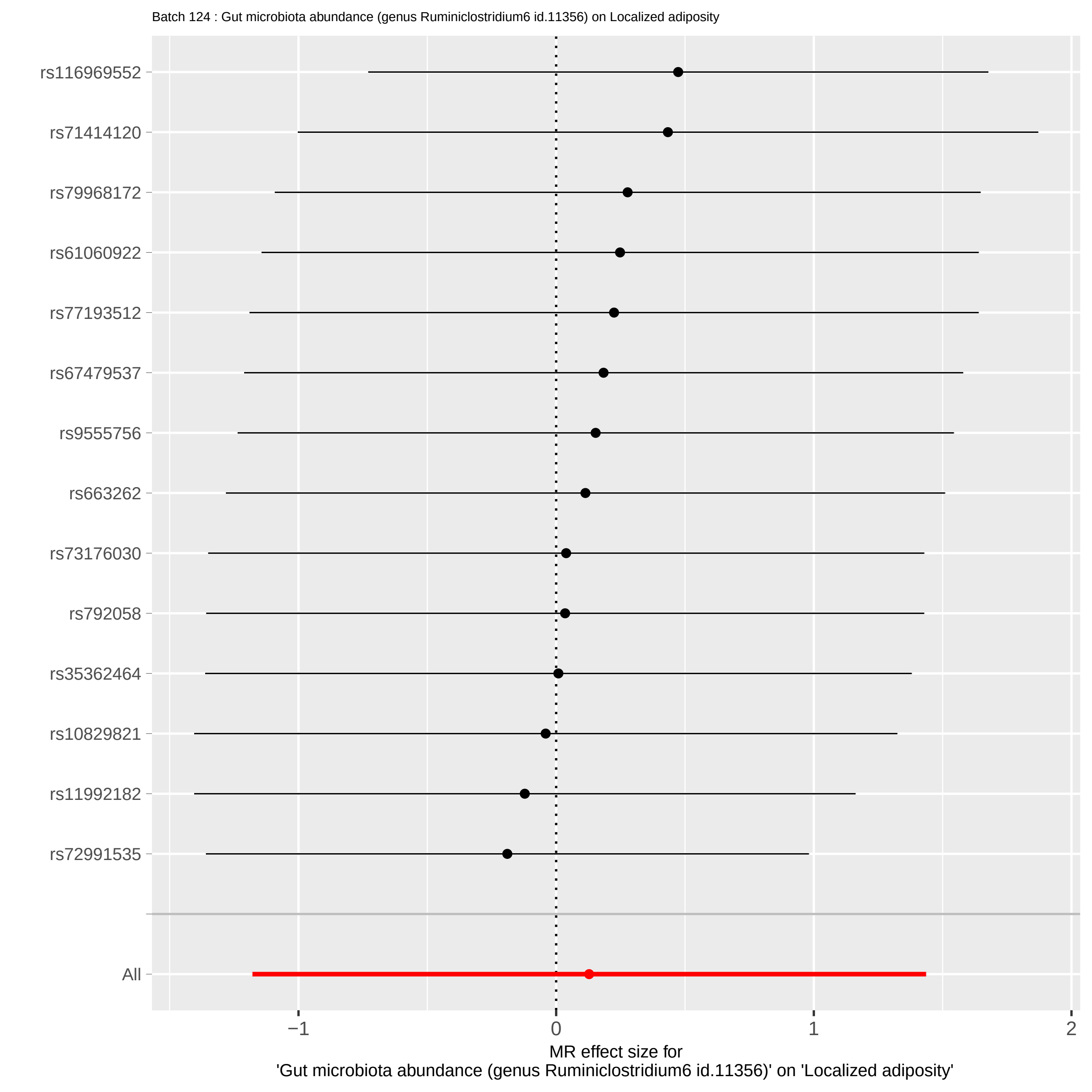

Batch 124 : Gut microbiota abundance (genus Ruminiclostridium6 id.11356) on Localized adiposity
rs116969552
rs71414120
rs79968172
rs61060922
rs77193512
rs67479537
rs9555756
rs663262
rs73176030
rs792058
rs35362464
rs10829821
rs11992182
rs72991535
All
−1
0
1
2
MR effect size for
'Gut microbiota abundance (genus Ruminiclostridium6 id.11356)' on 'Localized adiposity'

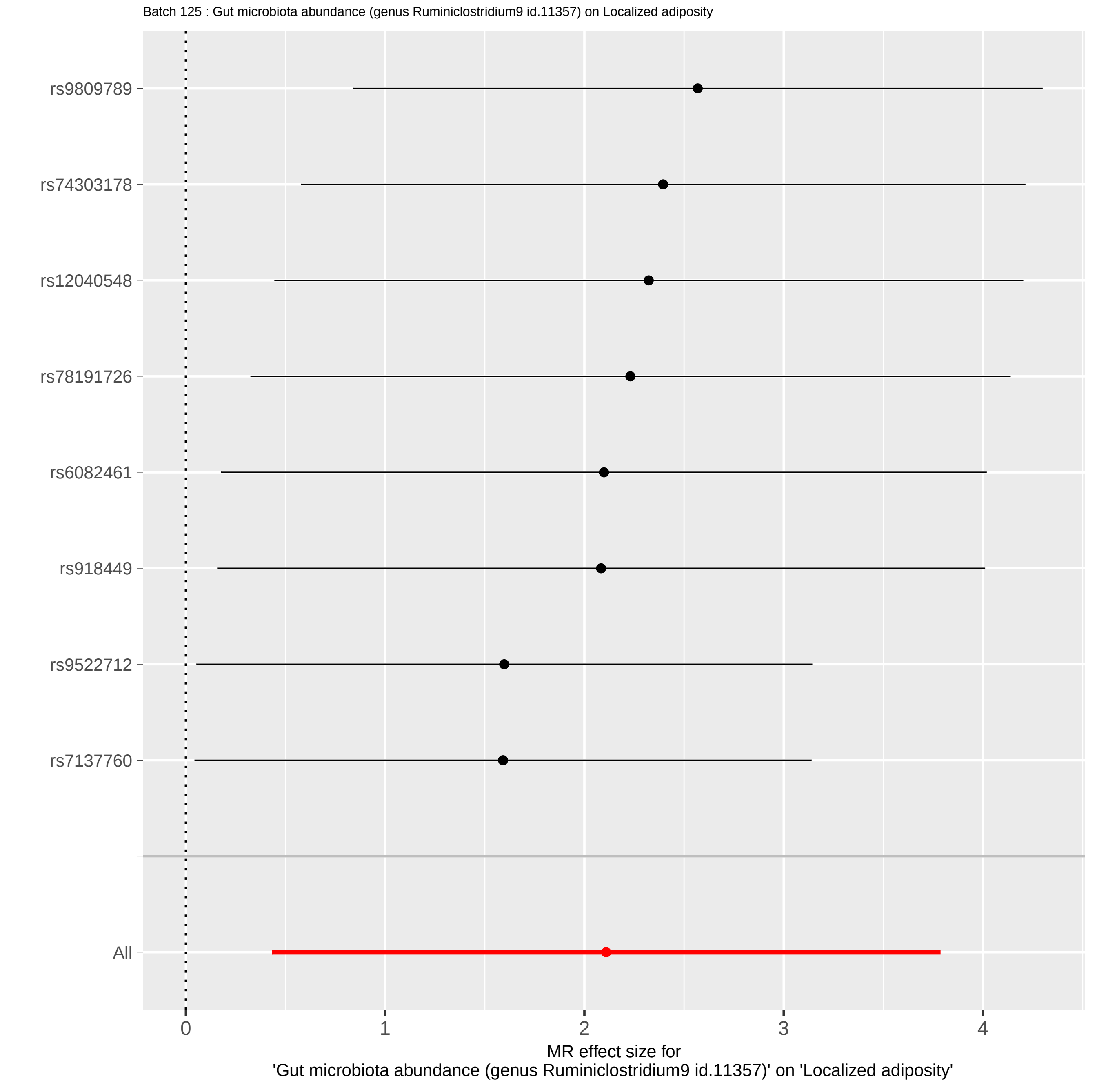

Batch 125 : Gut microbiota abundance (genus Ruminiclostridium9 id.11357) on Localized adiposity
rs9809789
rs74303178
rs12040548
rs78191726
rs6082461
rs918449
rs9522712
rs7137760
All
0
1
2
3
4
MR effect size for
'Gut microbiota abundance (genus Ruminiclostridium9 id.11357)' on 'Localized adiposity'

Batch 126 : Gut microbiota abundance (genus Ruminococcaceae NK4A214 group id.11358) on Localized adiposity

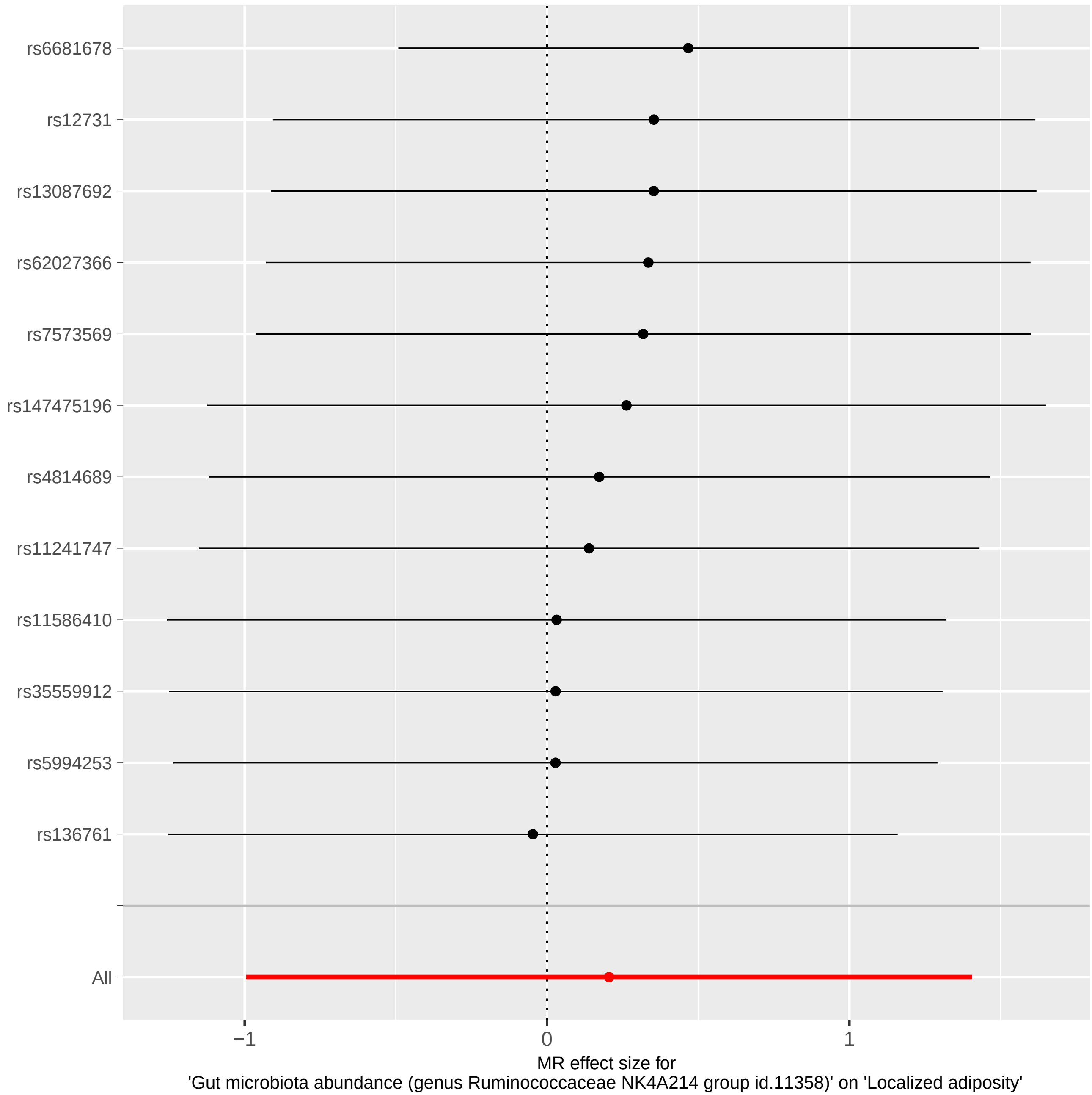

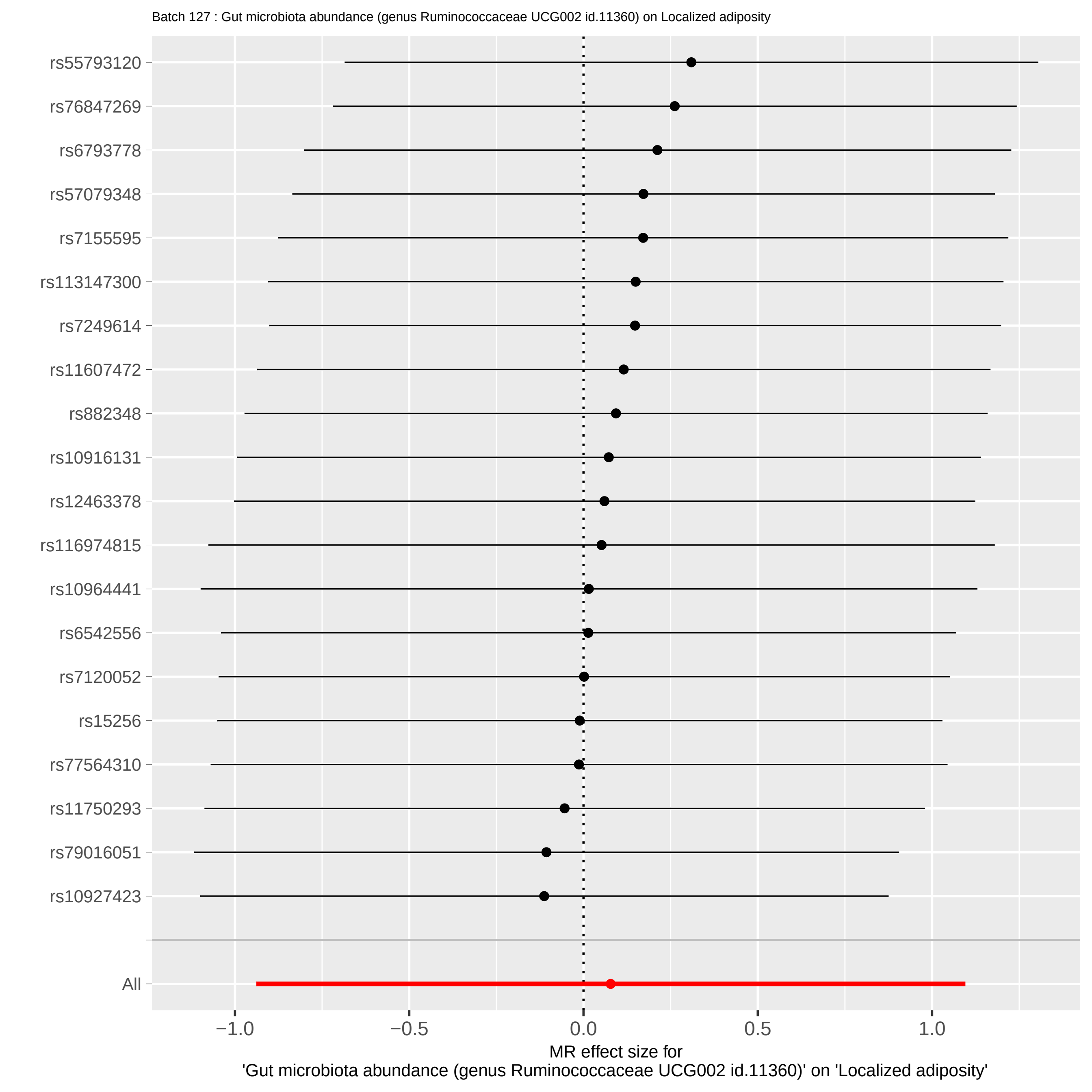
Batch 127 : Gut microbiota abundance (genus Ruminococcaceae UCG002 id.11360) on Localized adiposity
rs55793120
rs76847269
rs6793778
rs57079348
rs7155595
rs113147300
rs7249614
rs11607472
rs882348
rs10916131
rs12463378
rs116974815
rs10964441
rs6542556
rs7120052
rs15256
rs77564310
rs11750293
rs79016051
rs10927423
All
−1.0
−0.5
0.0
0.5
1.0
MR effect size for
'Gut microbiota abundance (genus Ruminococcaceae UCG002 id.11360)' on 'Localized adiposity'

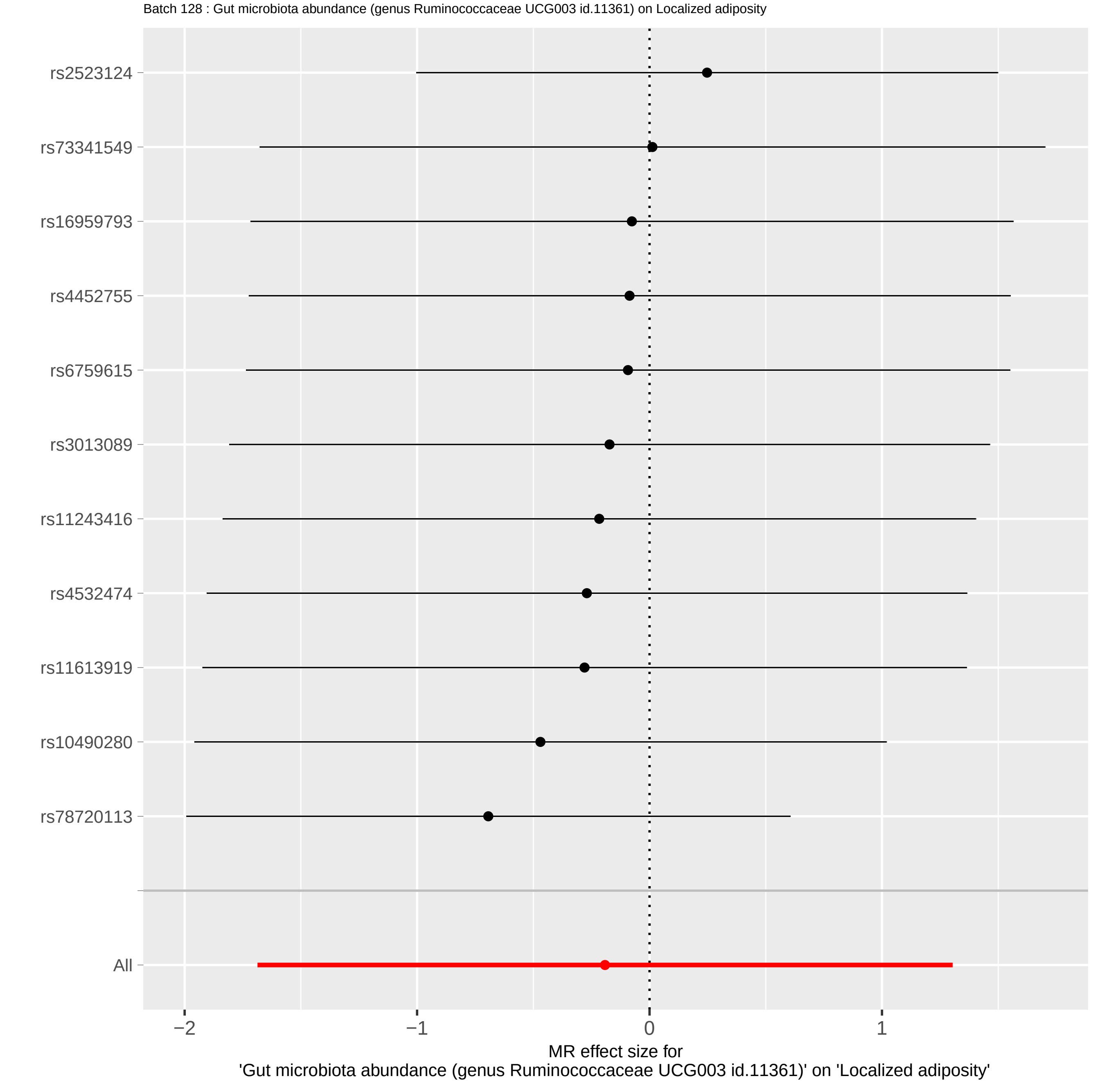

Batch 128 : Gut microbiota abundance (genus Ruminococcaceae UCG003 id.11361) on Localized adiposity
rs2523124
rs73341549
rs16959793
rs4452755
rs6759615
rs3013089
rs11243416
rs4532474
rs11613919
rs10490280
rs78720113
All
−2
−1
0
1
MR effect size for
'Gut microbiota abundance (genus Ruminococcaceae UCG003 id.11361)' on 'Localized adiposity'

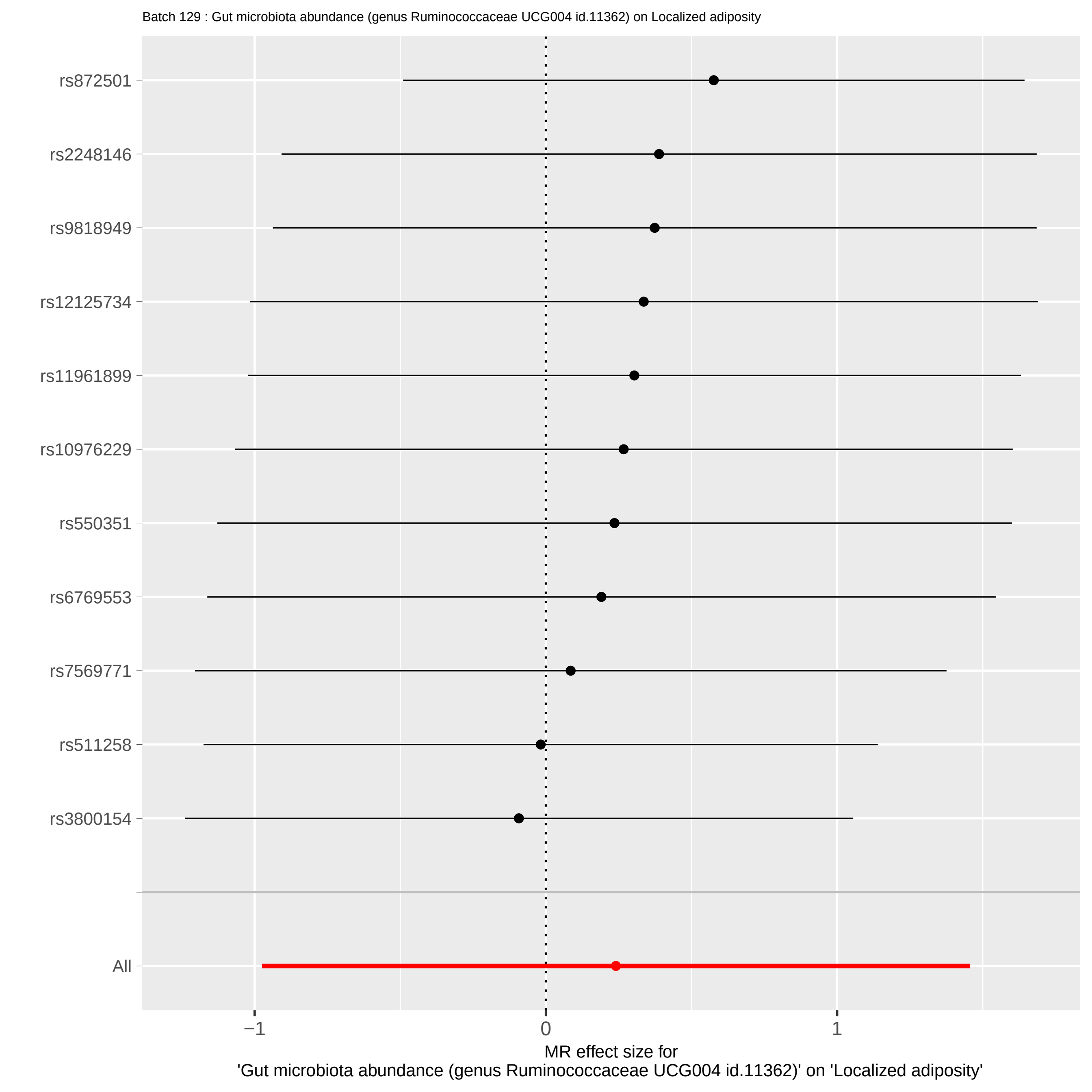

Batch 129 : Gut microbiota abundance (genus Ruminococcaceae UCG004 id.11362) on Localized adiposity
rs872501
rs2248146
rs9818949
rs12125734
rs11961899
rs10976229
rs550351
rs6769553
rs7569771
rs511258
rs3800154
All
−1
0
1
MR effect size for
'Gut microbiota abundance (genus Ruminococcaceae UCG004 id.11362)' on 'Localized adiposity'

Batch 130 : Gut microbiota abundance (genus Ruminococcaceae UCG005 id.11363) on Localized adiposity

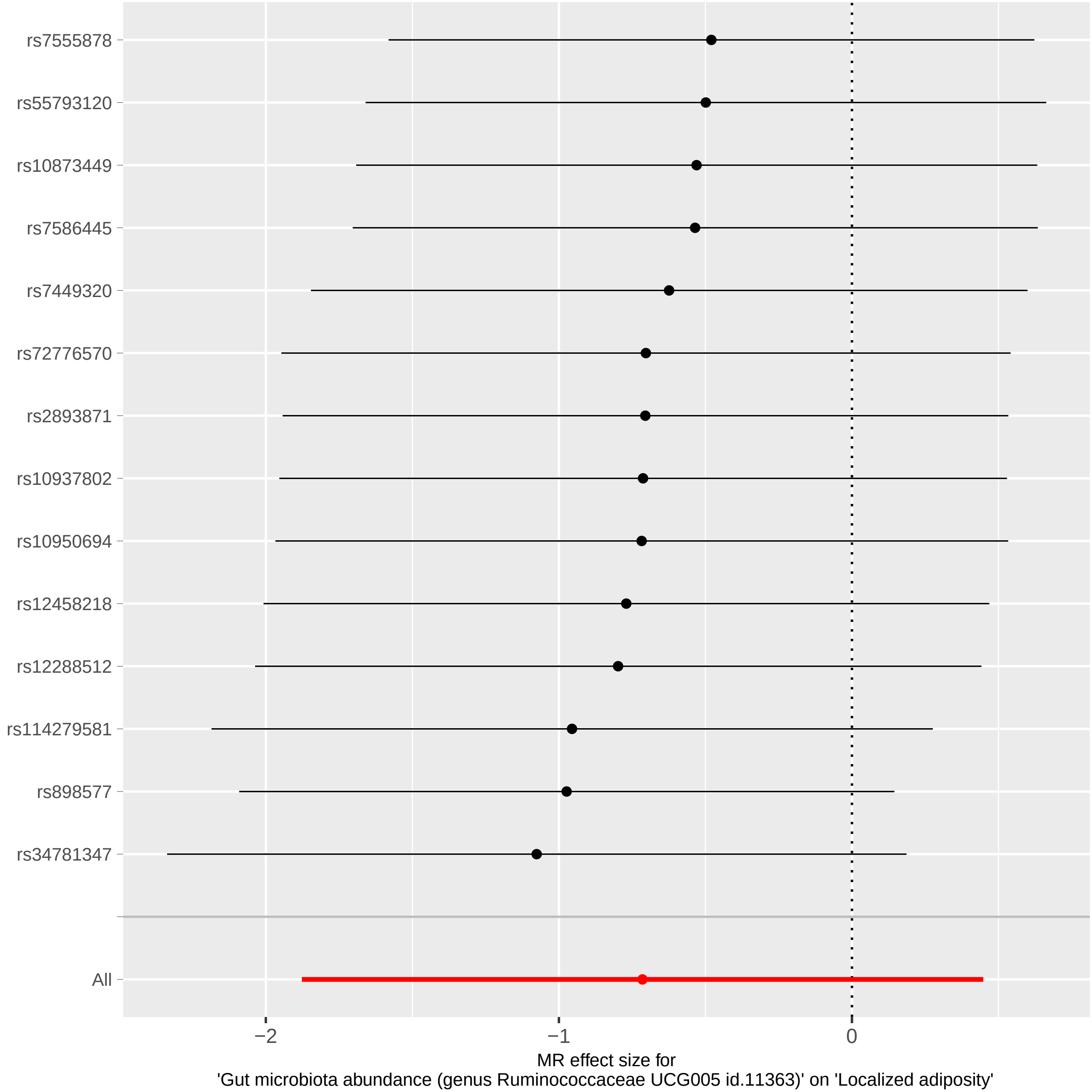

Batch 131 : Gut microbiota abundance (genus Ruminococcaceae UCG009 id.11366) on Localized adiposity

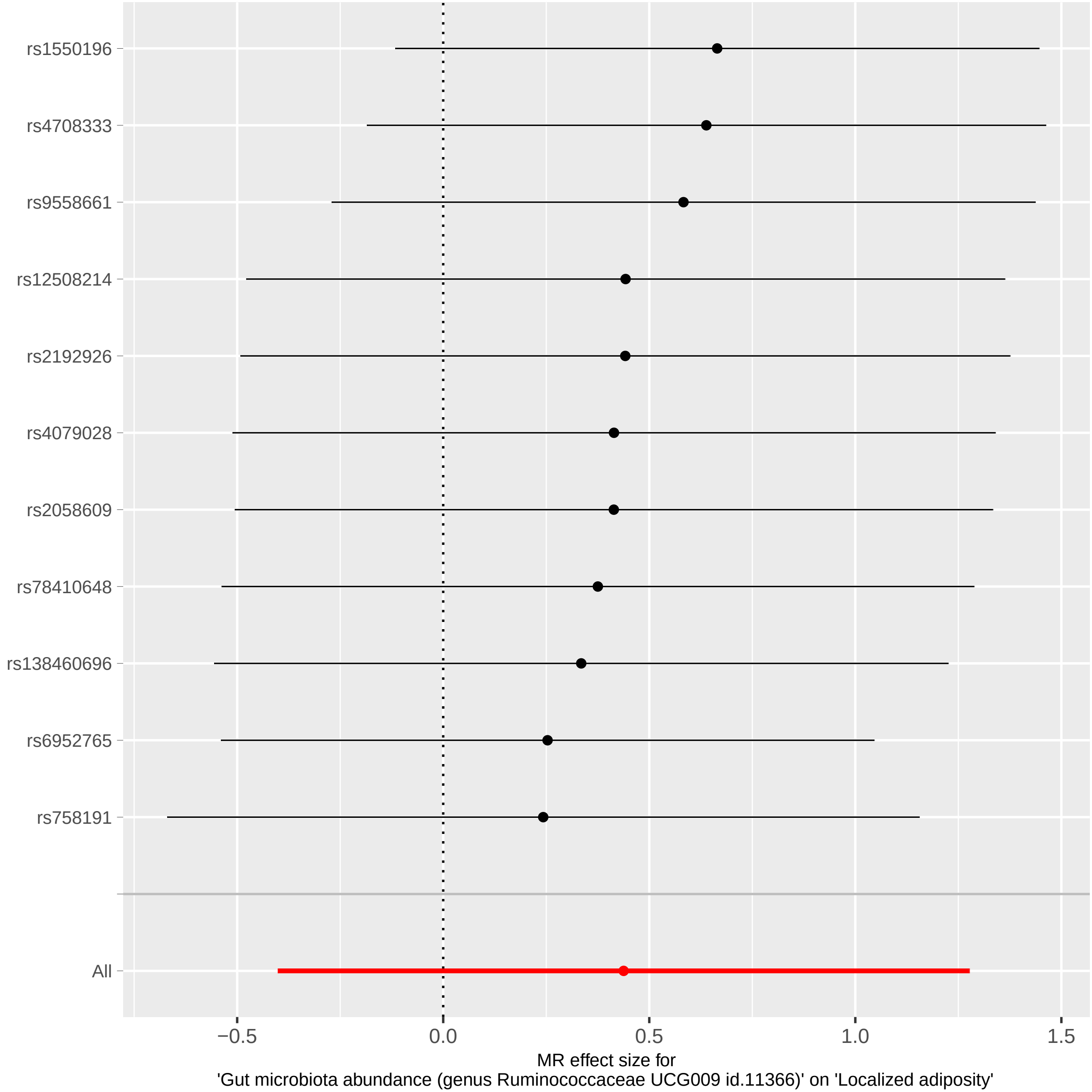

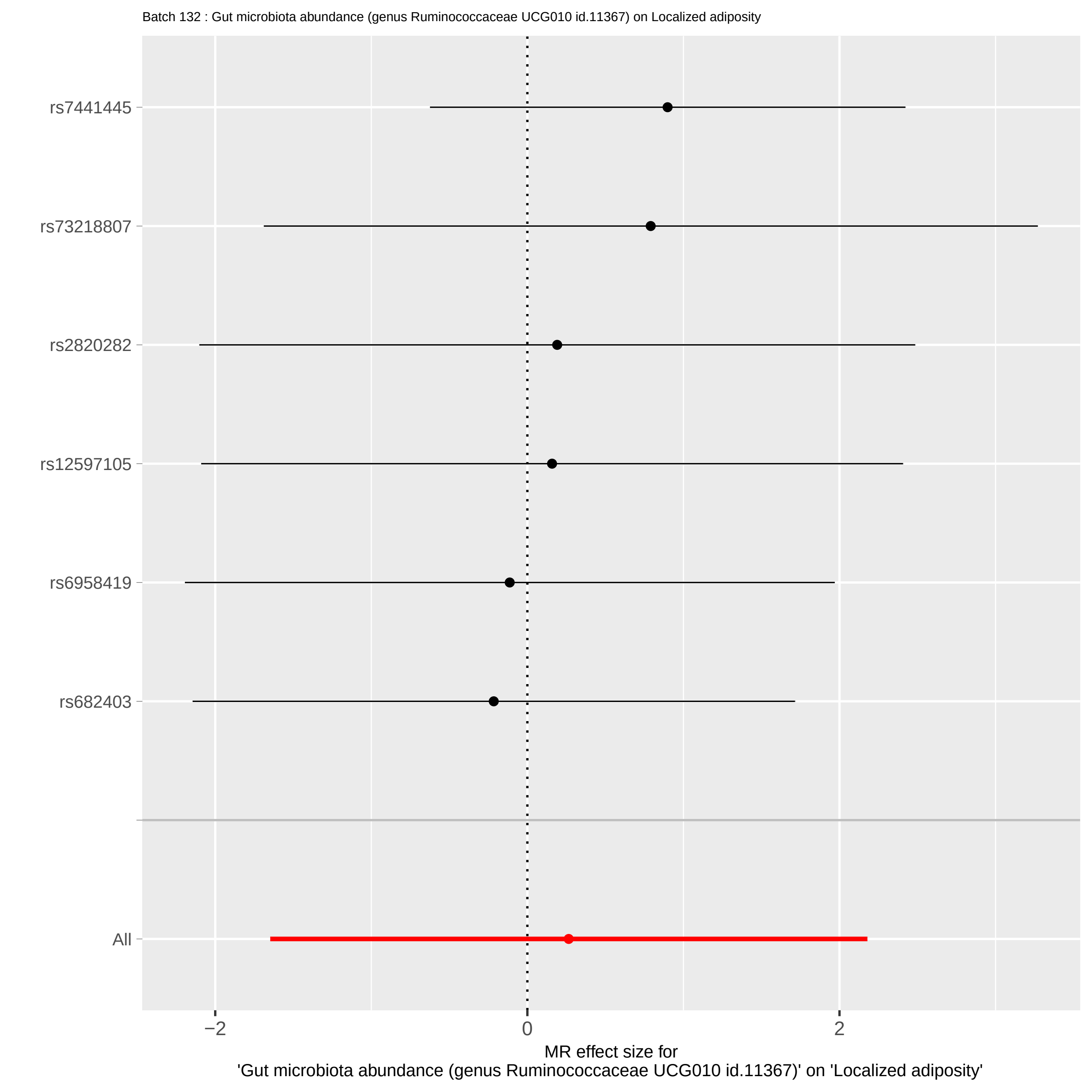

Batch 132 : Gut microbiota abundance (genus Ruminococcaceae UCG010 id.11367) on Localized adiposity
rs7441445
rs73218807
rs2820282
rs12597105
rs6958419
rs682403
All
−2
0
2
MR effect size for
'Gut microbiota abundance (genus Ruminococcaceae UCG010 id.11367)' on 'Localized adiposity'

Batch 133 : Gut microbiota abundance (genus Ruminococcaceae UCG011 id.11368) on Localized adiposity

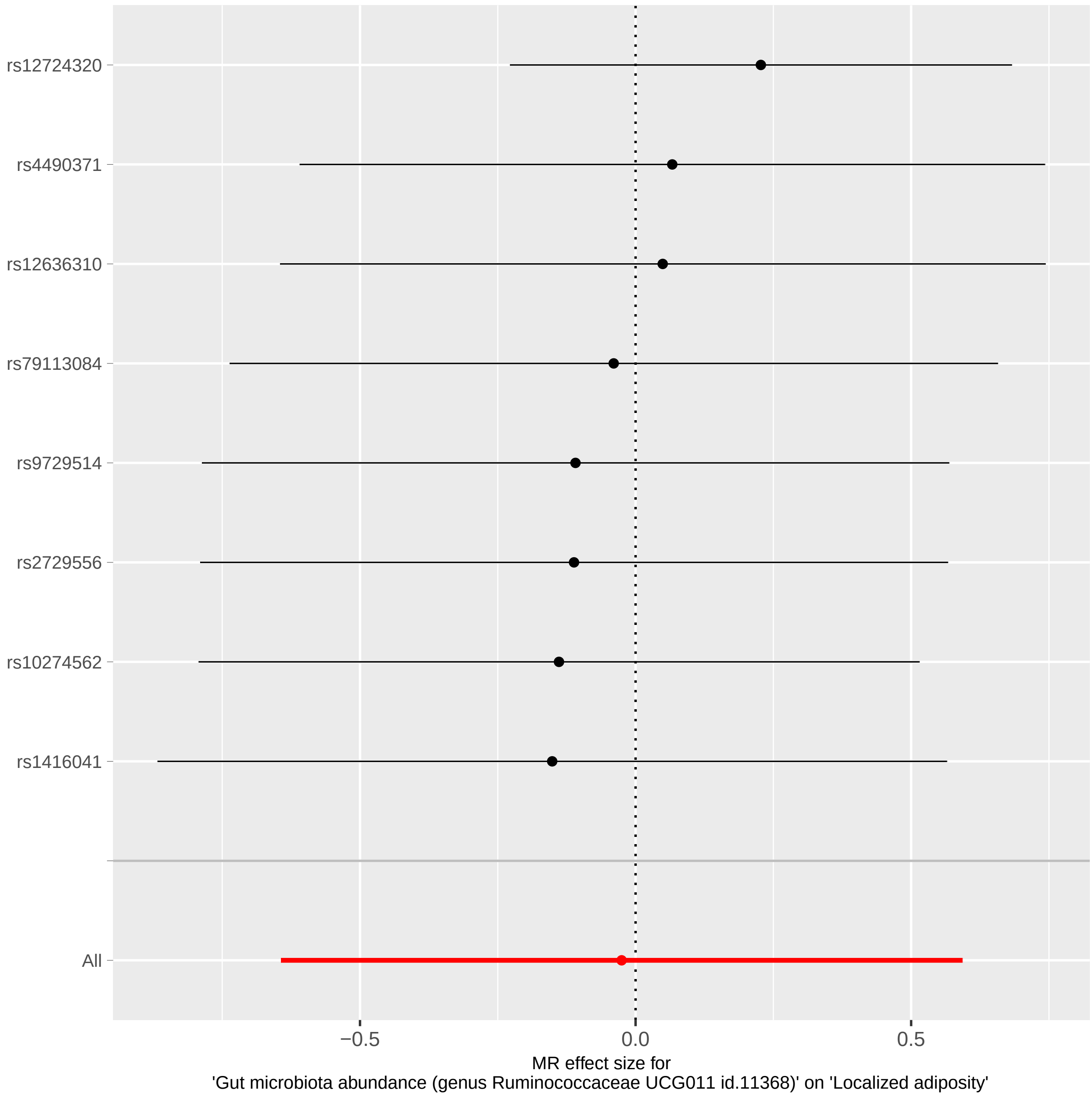

Batch 134 : Gut microbiota abundance (genus Ruminococcaceae UCG013 id.11370) on Localized adiposity

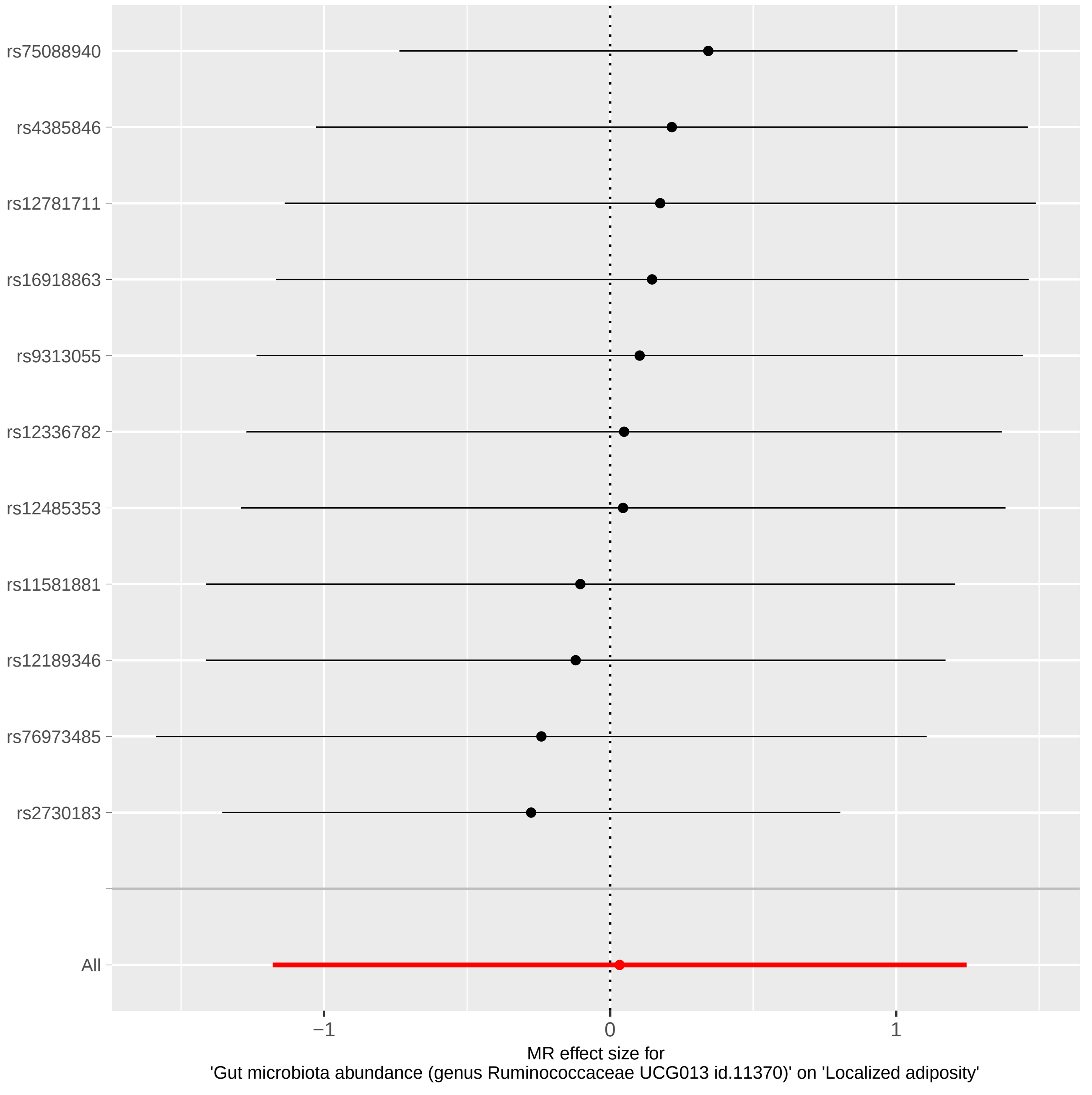

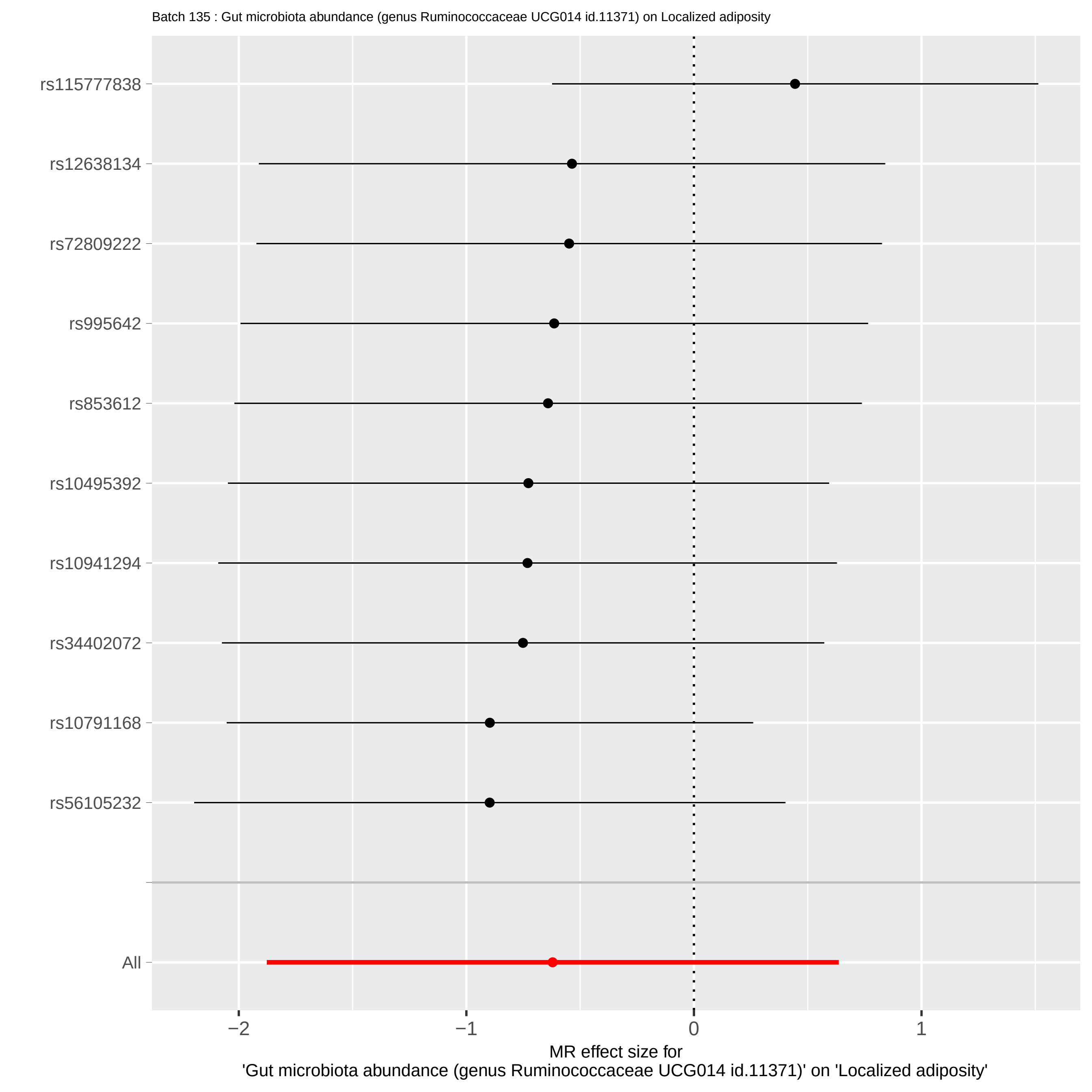
Batch 135 : Gut microbiota abundance (genus Ruminococcaceae UCG014 id.11371) on Localized adiposity
rs115777838
rs12638134
rs72809222
rs995642
rs853612
rs10495392
rs10941294
rs34402072
rs10791168
rs56105232
All
−2
−1
0
1
MR effect size for
'Gut microbiota abundance (genus Ruminococcaceae UCG014 id.11371)' on 'Localized adiposity'

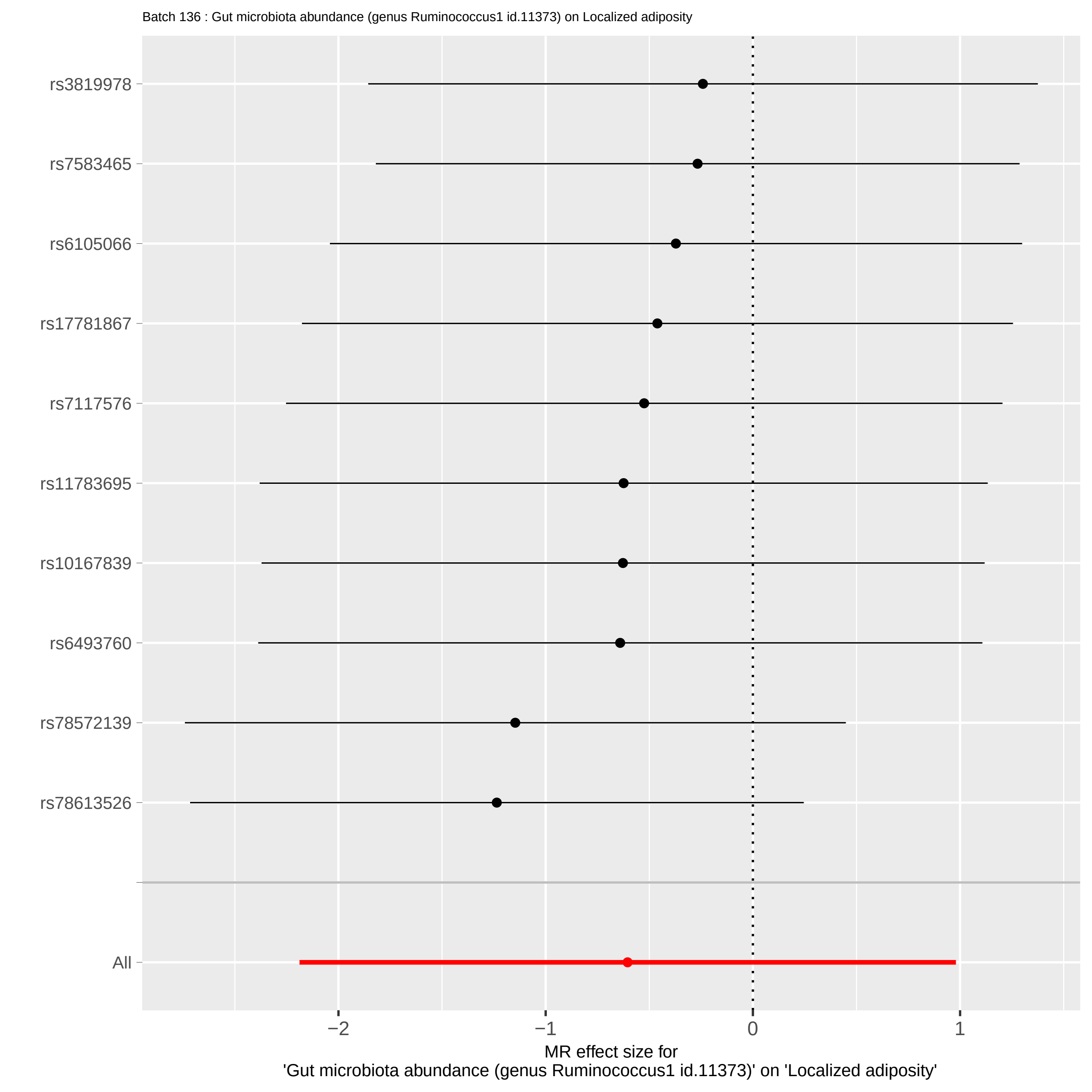
Batch 136 : Gut microbiota abundance (genus Ruminococcus1 id.11373) on Localized adiposity
rs3819978
rs7583465
rs6105066
rs17781867
rs7117576
rs11783695
rs10167839
rs6493760
rs78572139
rs78613526
All
−2
−1
0
1
MR effect size for
'Gut microbiota abundance (genus Ruminococcus1 id.11373)' on 'Localized adiposity'

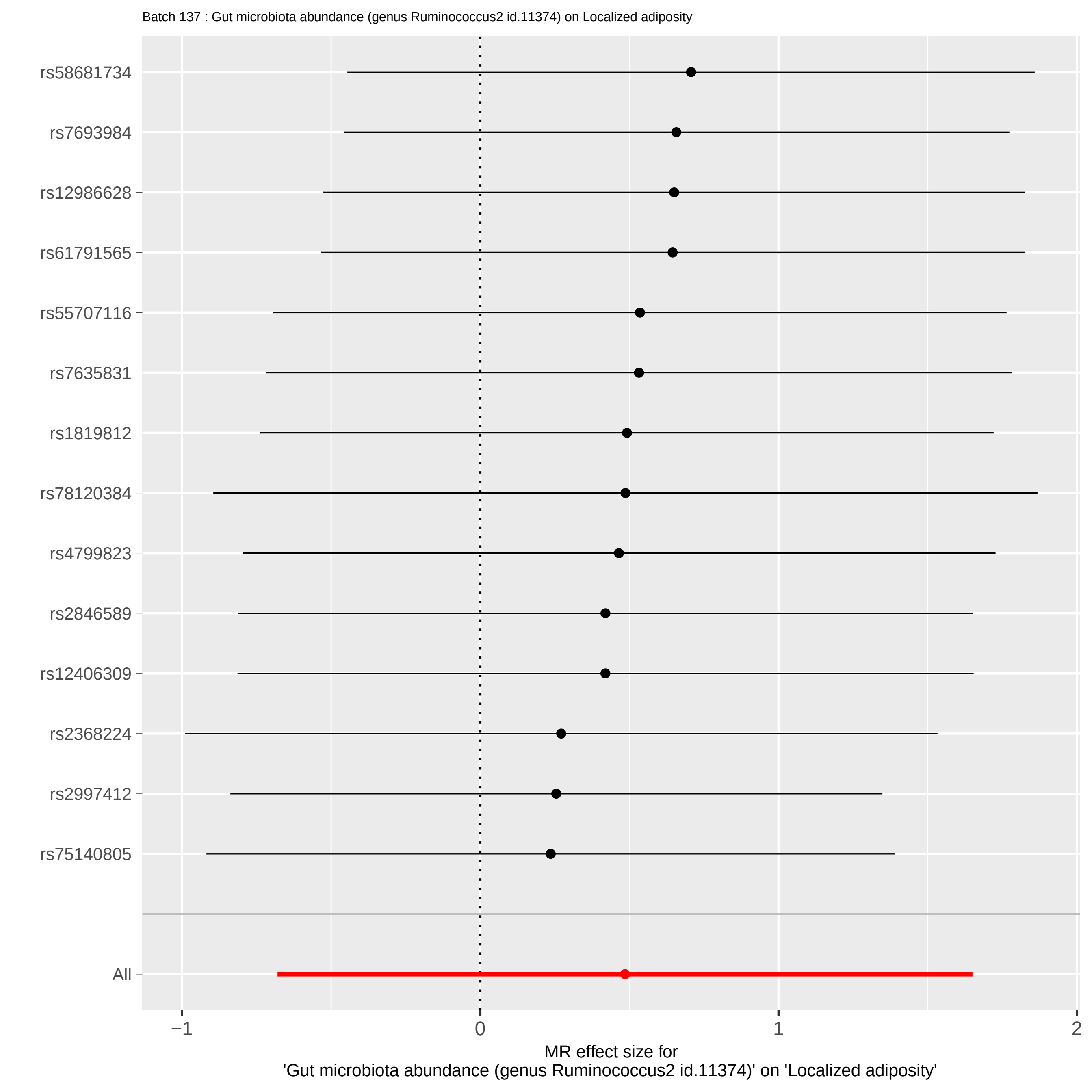

Batch 137 : Gut microbiota abundance (genus Ruminococcus2 id.11374) on Localized adiposity
rs58681734
rs7693984
rs12986628
rs61791565
rs55707116
rs7635831
rs1819812
rs78120384
rs4799823
rs2846589
rs12406309
rs2368224
rs2997412
rs75140805
All
−1
0
1
2
MR effect size for
'Gut microbiota abundance (genus Ruminococcus2 id.11374)' on 'Localized adiposity'

Batch 138 : Gut microbiota abundance (genus Ruminococcus gauvreauii group id.11342) on Localized adiposity

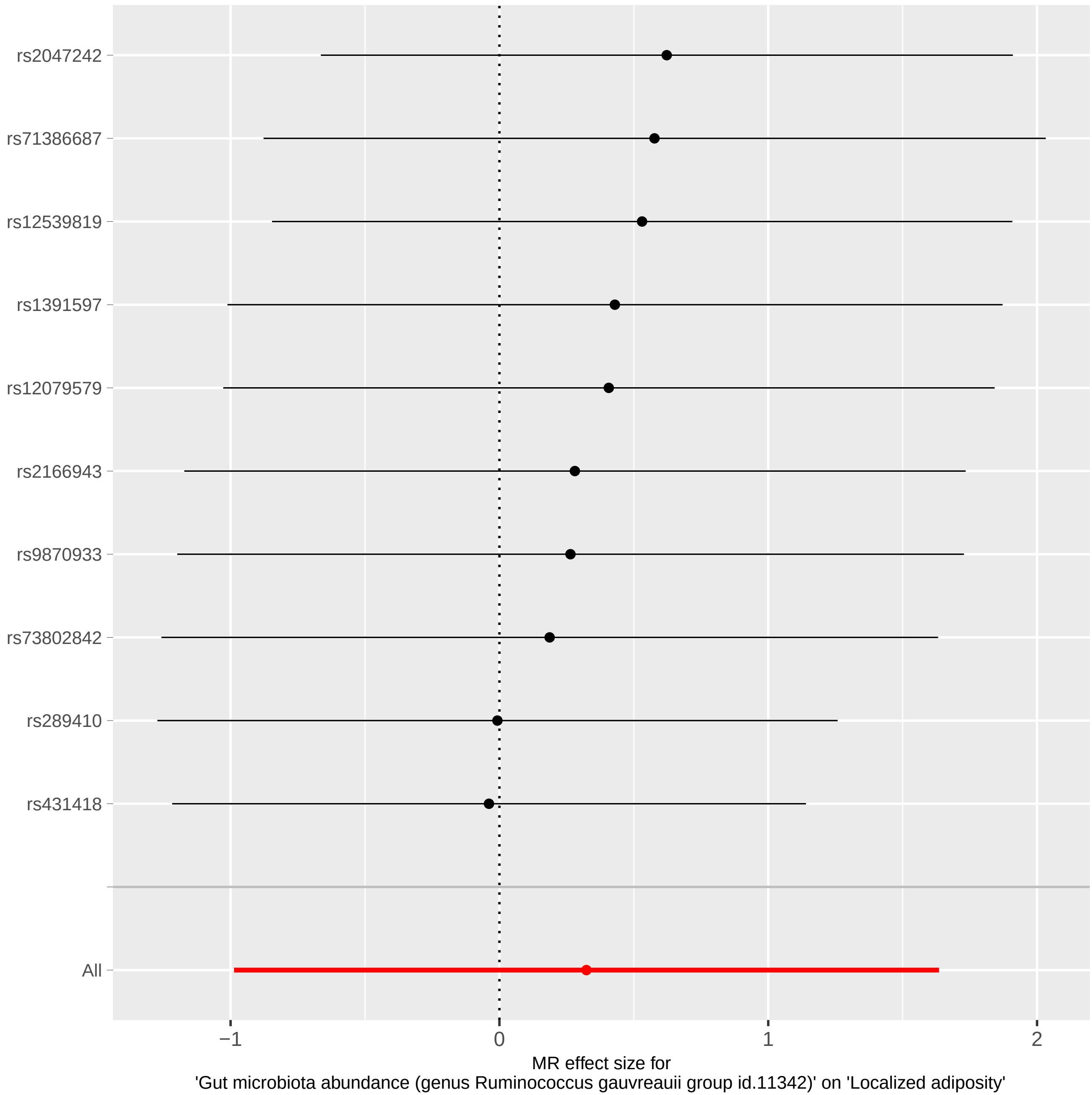

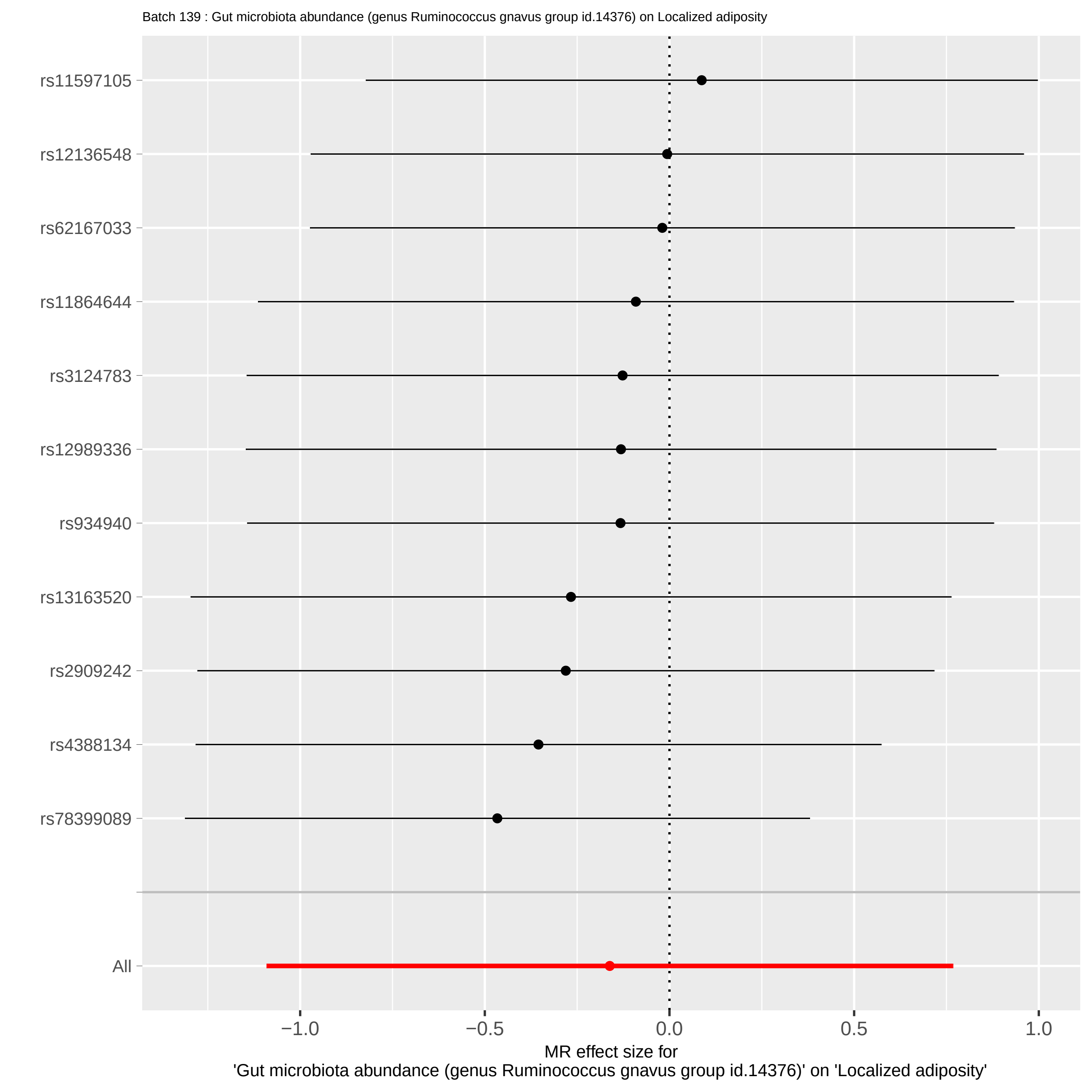

Batch 139 : Gut microbiota abundance (genus Ruminococcus gnavus group id.14376) on Localized adiposity
rs11597105
rs12136548
rs62167033
rs11864644
rs3124783
rs12989336
rs934940
rs13163520
rs2909242
rs4388134
rs78399089
All
−1.0
−0.5
0.0
0.5
1.0
MR effect size for
'Gut microbiota abundance (genus Ruminococcus gnavus group id.14376)' on 'Localized adiposity'

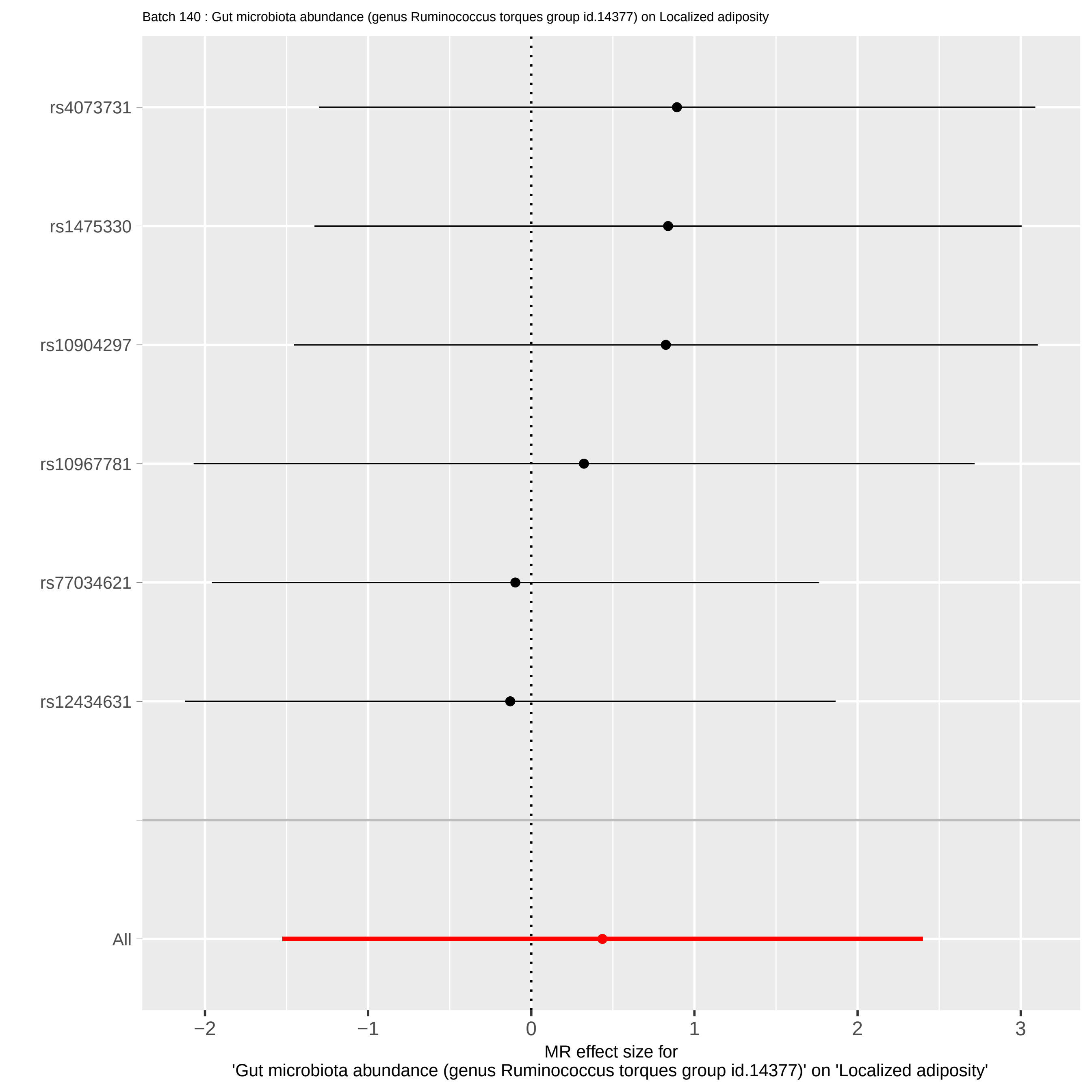

Batch 140 : Gut microbiota abundance (genus Ruminococcus torques group id.14377) on Localized adiposity
rs4073731
rs1475330
rs10904297
rs10967781
rs77034621
rs12434631
All
−2
−1
0
1
2
3
MR effect size for
'Gut microbiota abundance (genus Ruminococcus torques group id.14377)' on 'Localized adiposity'

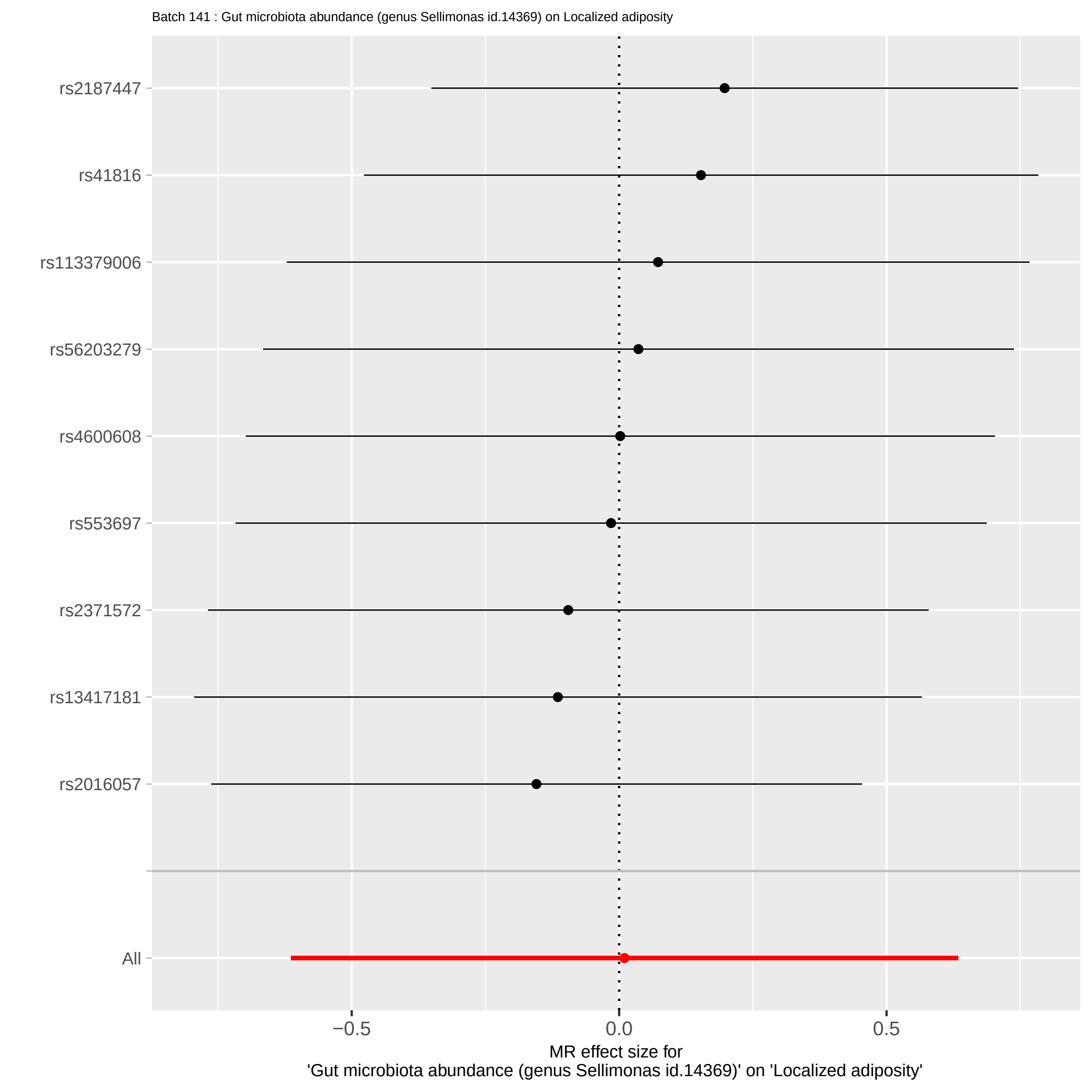
Batch 141 : Gut microbiota abundance (genus Sellimonas id.14369) on Localized adiposity
rs2187447
rs41816
rs113379006
rs56203279
rs4600608
rs553697
rs2371572
rs13417181
rs2016057
All
−0.5
0.0
0.5
MR effect size for
'Gut microbiota abundance (genus Sellimonas id.14369)' on 'Localized adiposity'

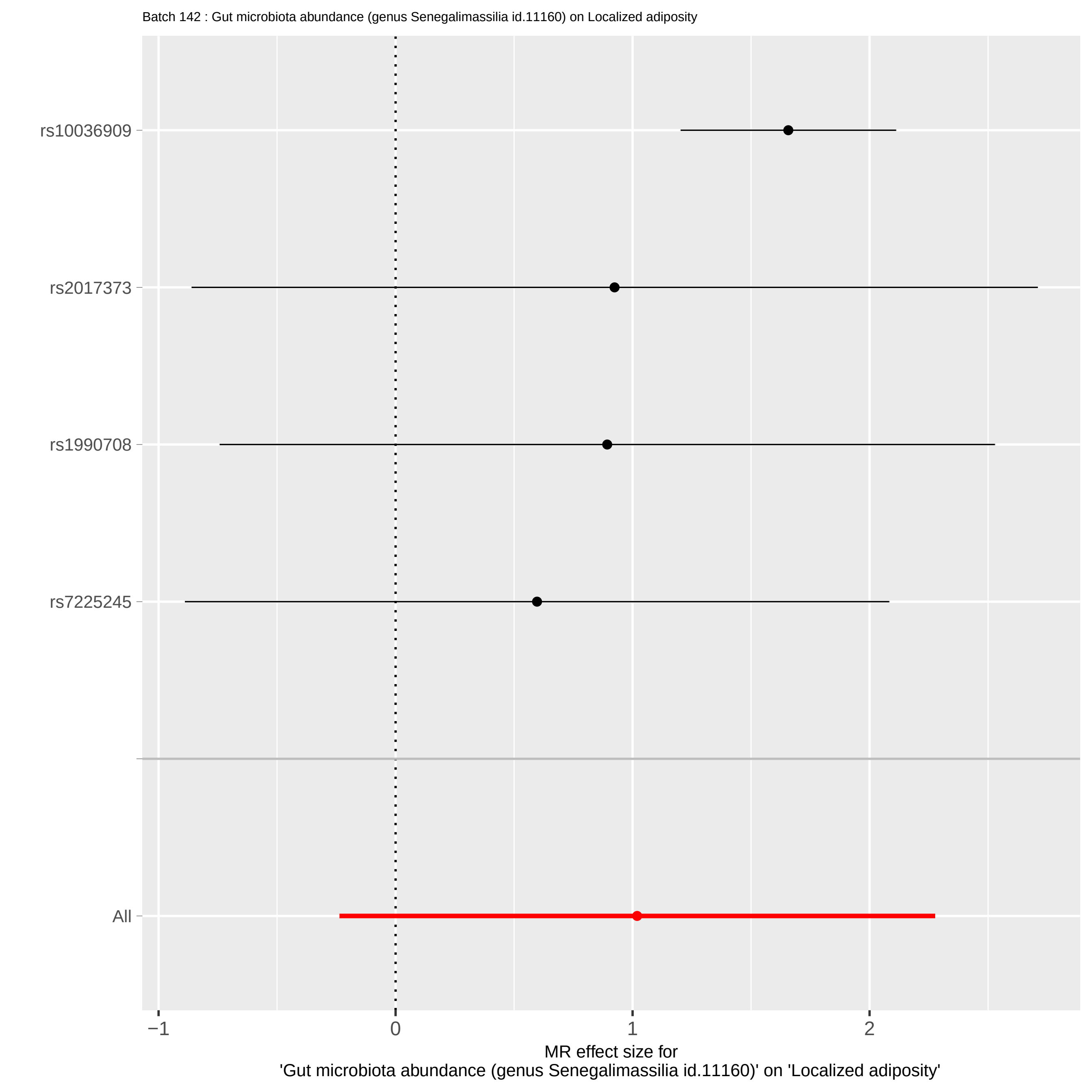

Batch 142 : Gut microbiota abundance (genus Senegalimassilia id.11160) on Localized adiposity
rs10036909
rs2017373
rs1990708
rs7225245
All
−1
0
1
2
MR effect size for
'Gut microbiota abundance (genus Senegalimassilia id.11160)' on 'Localized adiposity'

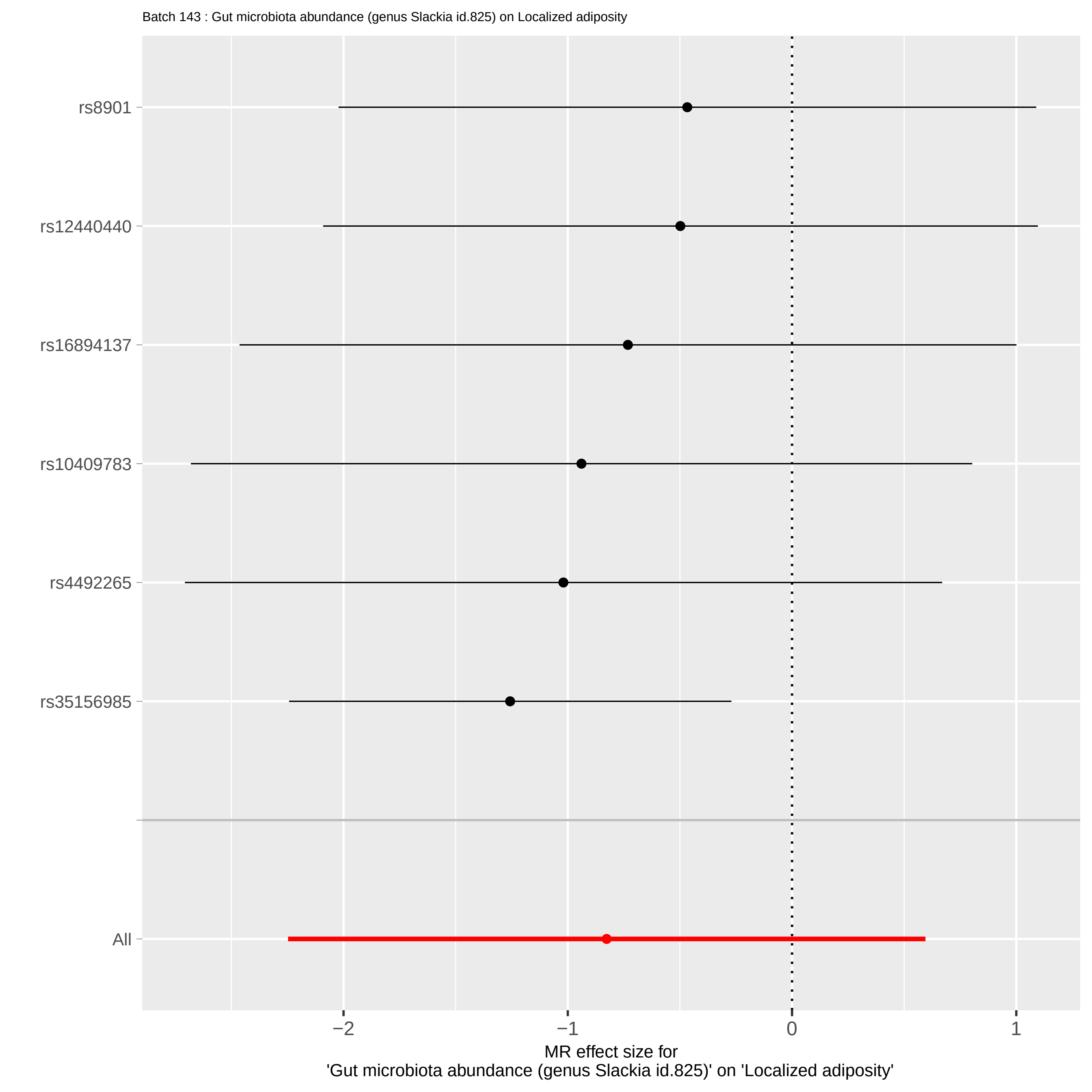

Batch 143 : Gut microbiota abundance (genus Slackia id.825) on Localized adiposity
rs8901
rs12440440
rs16894137
rs10409783
rs4492265
rs35156985
All
−2
−1
0
1
MR effect size for
'Gut microbiota abundance (genus Slackia id.825)' on 'Localized adiposity'

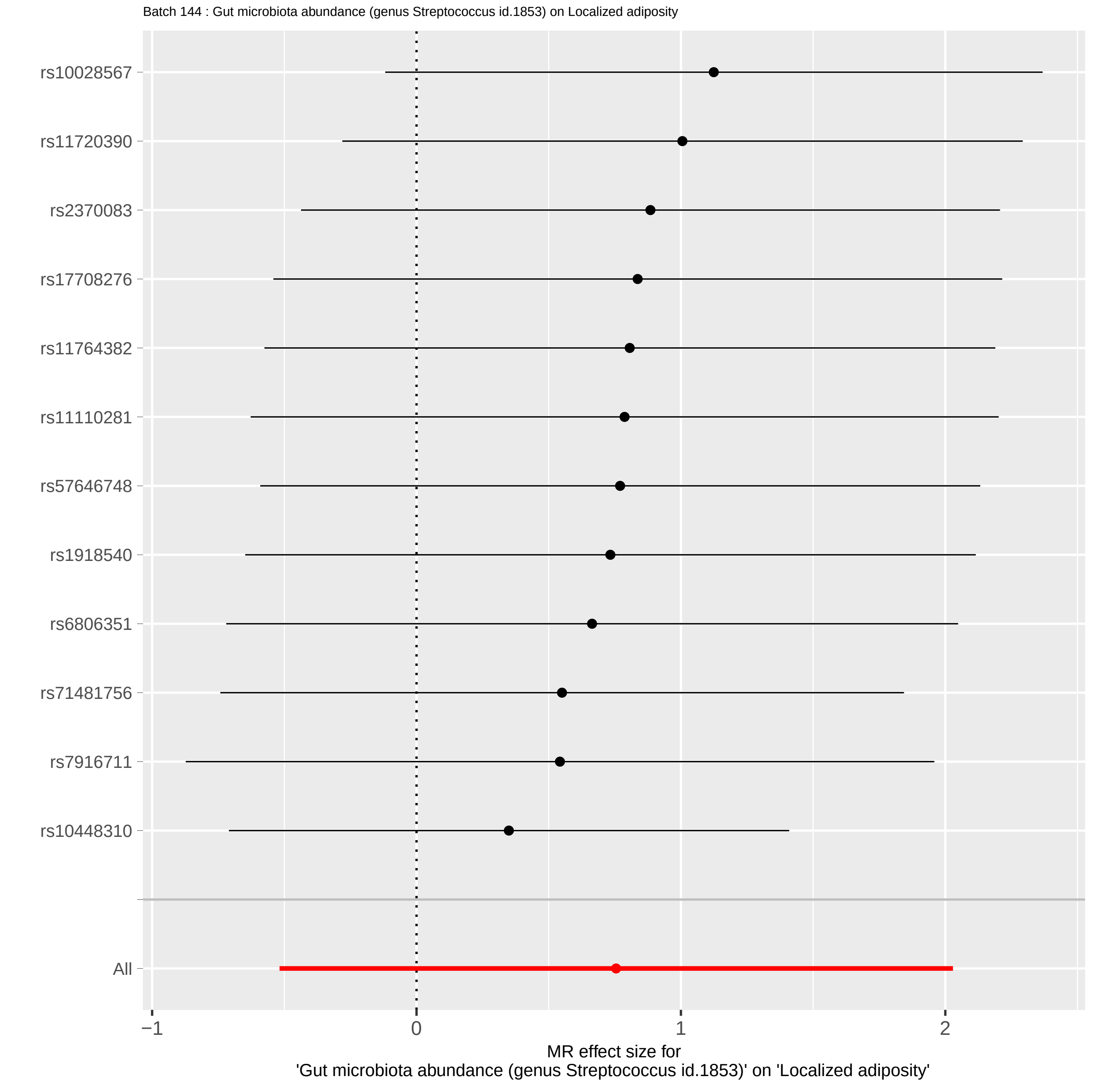

Batch 144 : Gut microbiota abundance (genus Streptococcus id.1853) on Localized adiposity
rs10028567
rs11720390
rs2370083
rs17708276
rs11764382
rs11110281
rs57646748
rs1918540
rs6806351
rs71481756
rs7916711
rs10448310
All
−1
0
1
2
MR effect size for
'Gut microbiota abundance (genus Streptococcus id.1853)' on 'Localized adiposity'

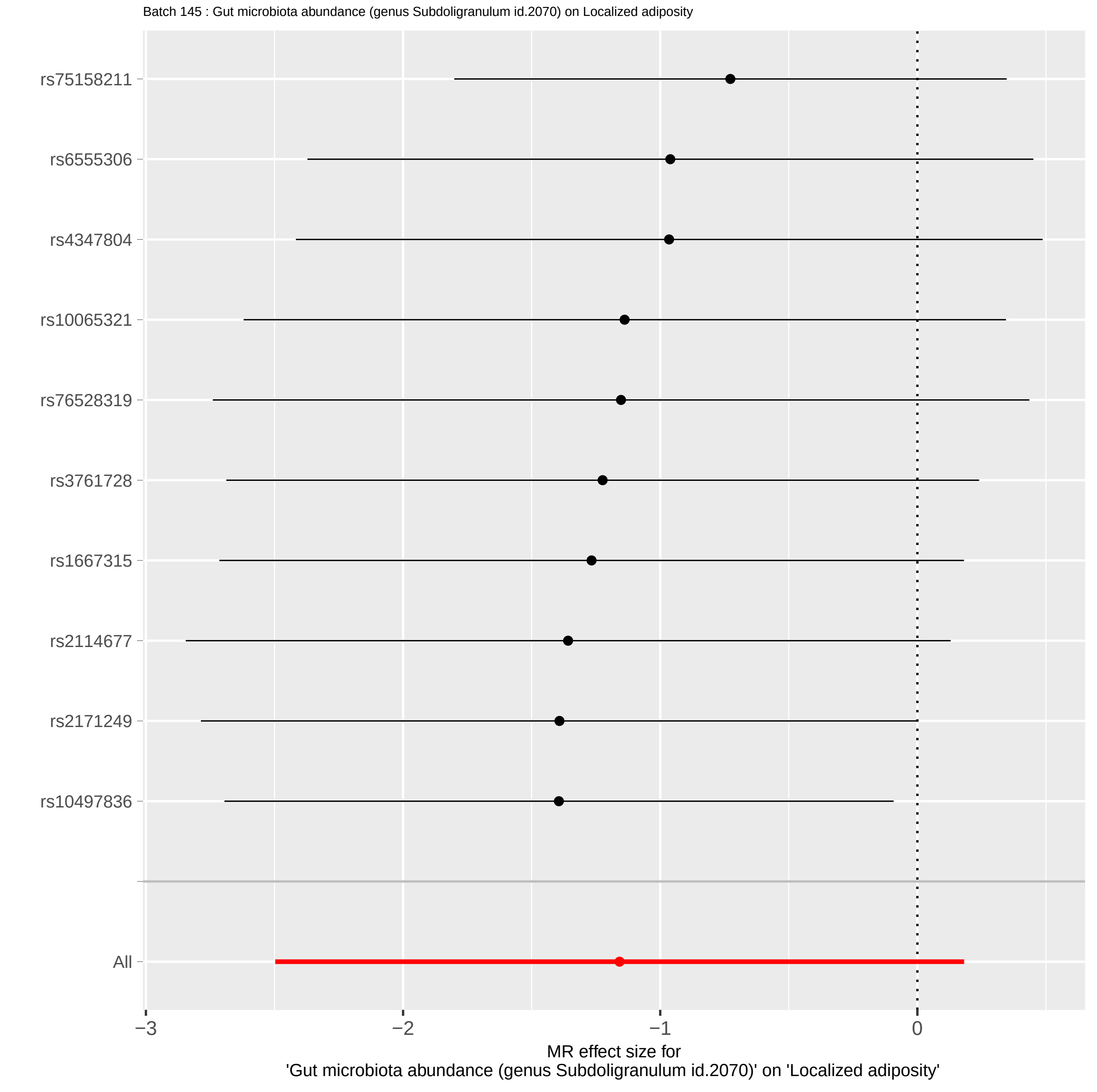

Batch 145 : Gut microbiota abundance (genus Subdoligranulum id.2070) on Localized adiposity
rs75158211
rs6555306
rs4347804
rs10065321
rs76528319
rs3761728
rs1667315
rs2114677
rs2171249
rs10497836
All
−3
−2
−1
0
MR effect size for
'Gut microbiota abundance (genus Subdoligranulum id.2070)' on 'Localized adiposity'

Batch 146 : Gut microbiota abundance (genus Sutterella id.2896) on Localized adiposity

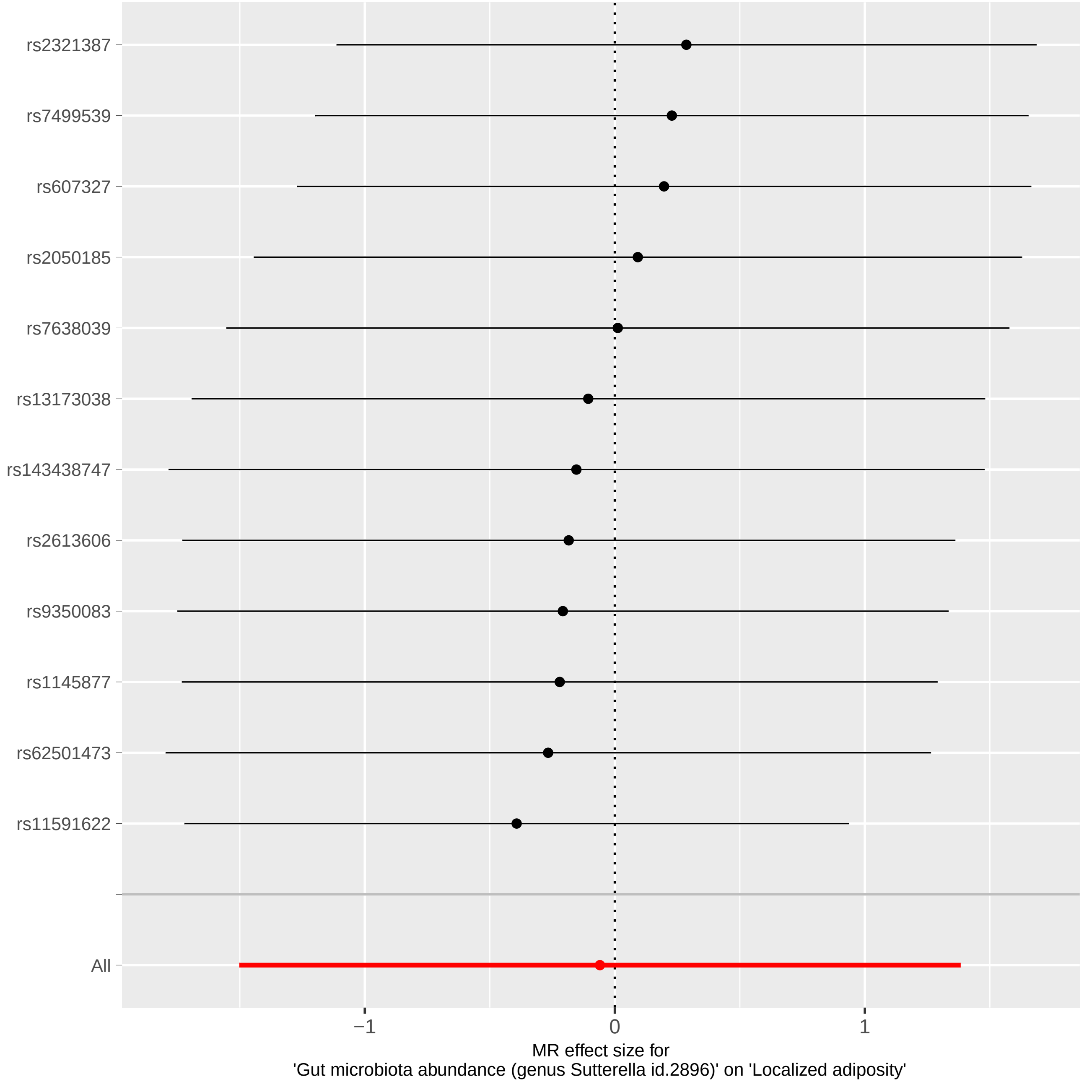

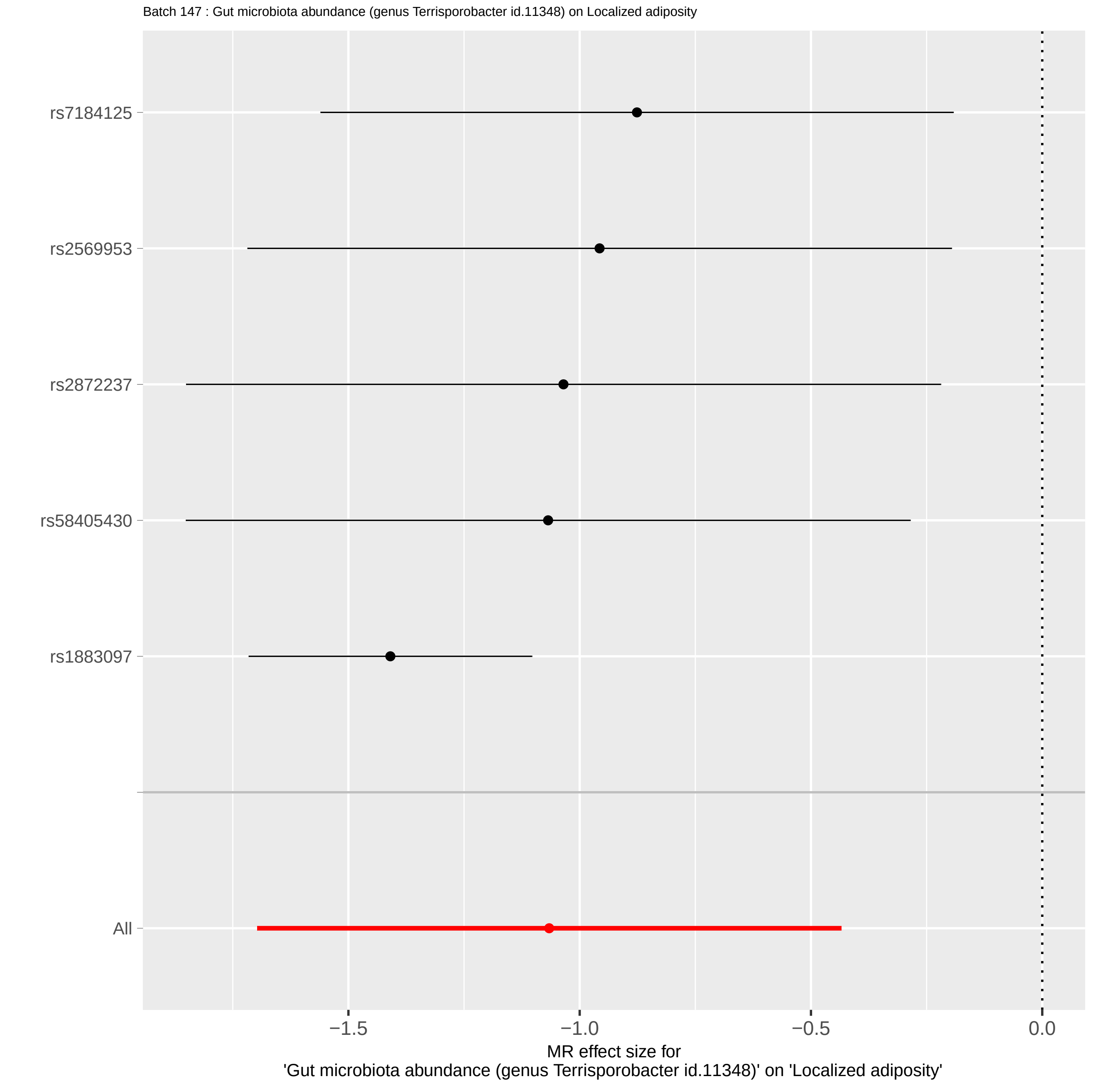
Batch 147 : Gut microbiota abundance (genus Terrisporobacter id.11348) on Localized adiposity
rs7184125
rs2569953
rs2872237
rs58405430
rs1883097
All
−1.5
−1.0
−0.5
0.0
MR effect size for
'Gut microbiota abundance (genus Terrisporobacter id.11348)' on 'Localized adiposity'

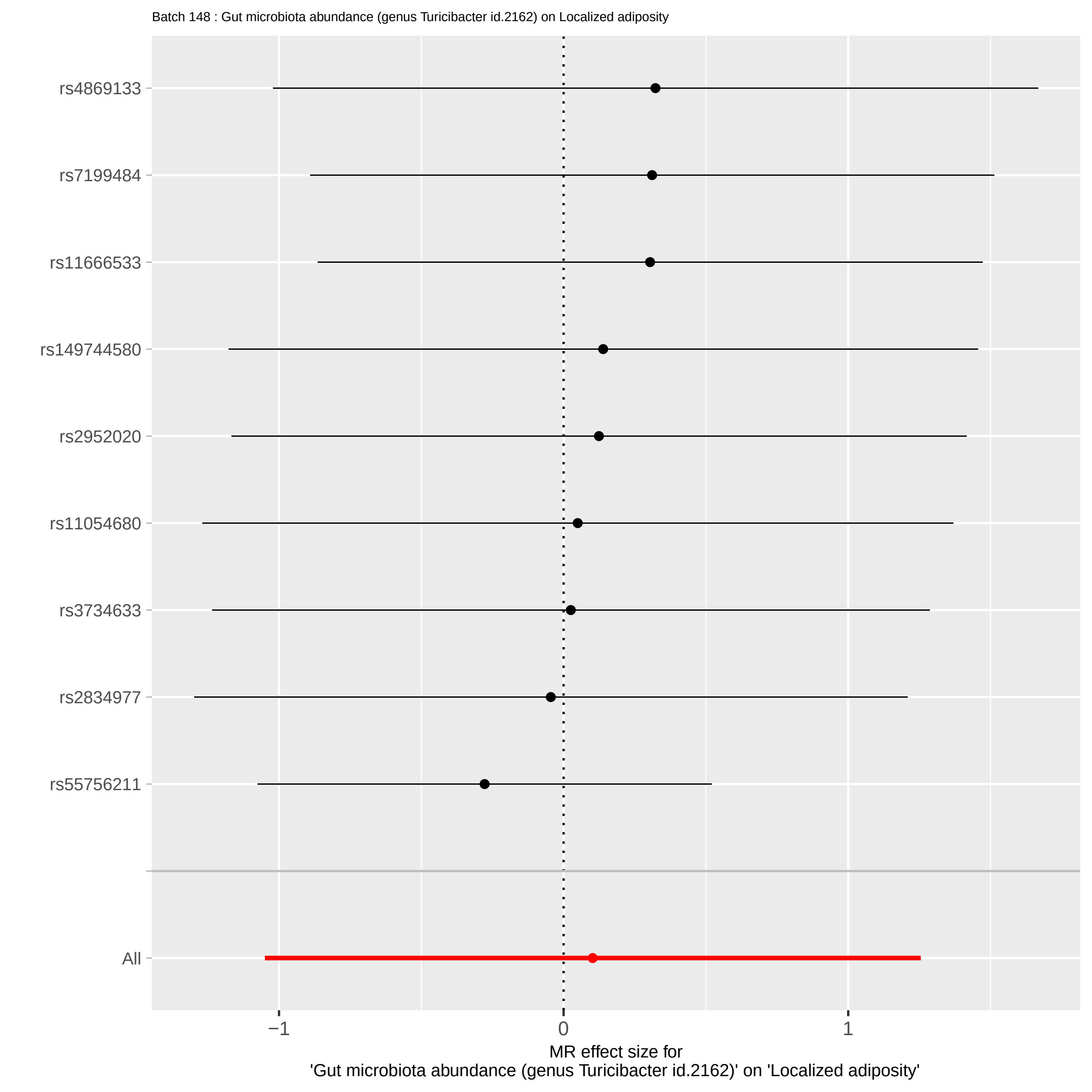

Batch 148 : Gut microbiota abundance (genus Turicibacter id.2162) on Localized adiposity
rs4869133
rs7199484
rs11666533
rs149744580
rs2952020
rs11054680
rs3734633
rs2834977
rs55756211
All
−1
0
1
MR effect size for
'Gut microbiota abundance (genus Turicibacter id.2162)' on 'Localized adiposity'

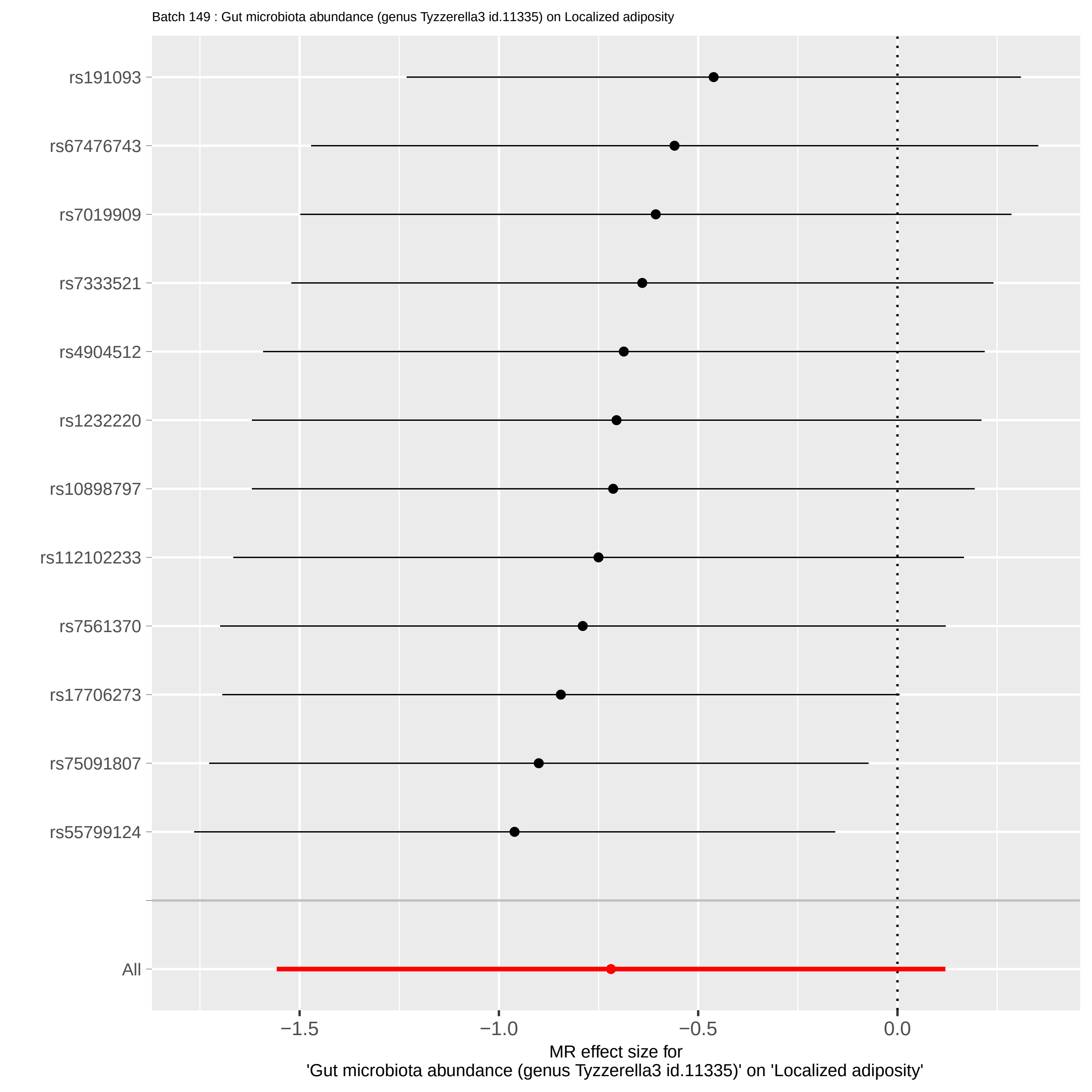

Batch 149 : Gut microbiota abundance (genus Tyzzerella3 id.11335) on Localized adiposity
rs191093
rs67476743
rs7019909
rs7333521
rs4904512
rs1232220
rs10898797
rs112102233
rs7561370
rs17706273
rs75091807
rs55799124
All
−1.5
−1.0
−0.5
0.0
MR effect size for
'Gut microbiota abundance (genus Tyzzerella3 id.11335)' on 'Localized adiposity'

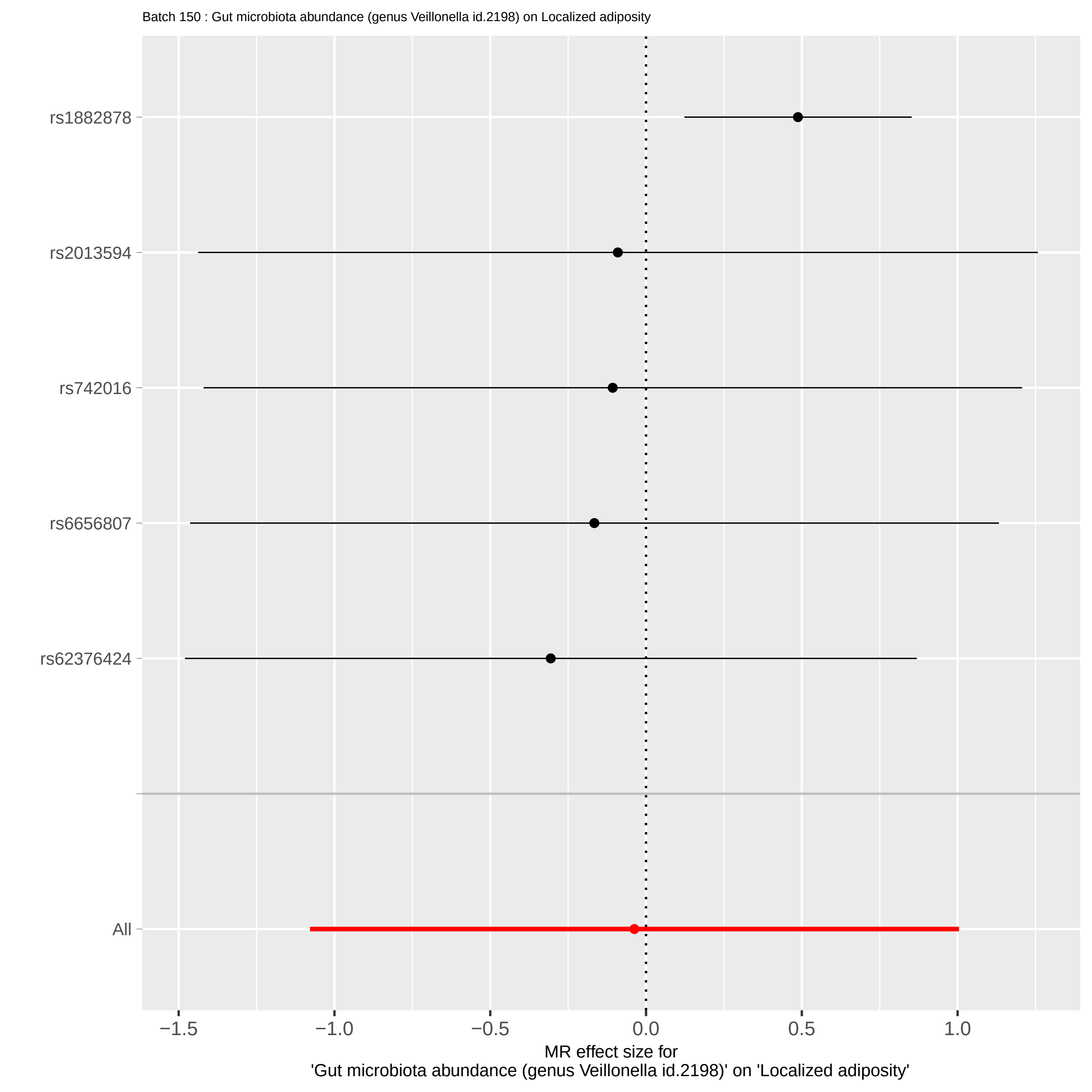

Batch 150 : Gut microbiota abundance (genus Veillonella id.2198) on Localized adiposity
rs1882878
rs2013594
rs742016
rs6656807
rs62376424
All
−1.5
−1.0
−0.5
0.0
0.5
1.0
MR effect size for
'Gut microbiota abundance (genus Veillonella id.2198)' on 'Localized adiposity'

Batch 301 : Gut microbiota abundance (family Acidaminococcaceae id.2166) on Obesity due to excess calories

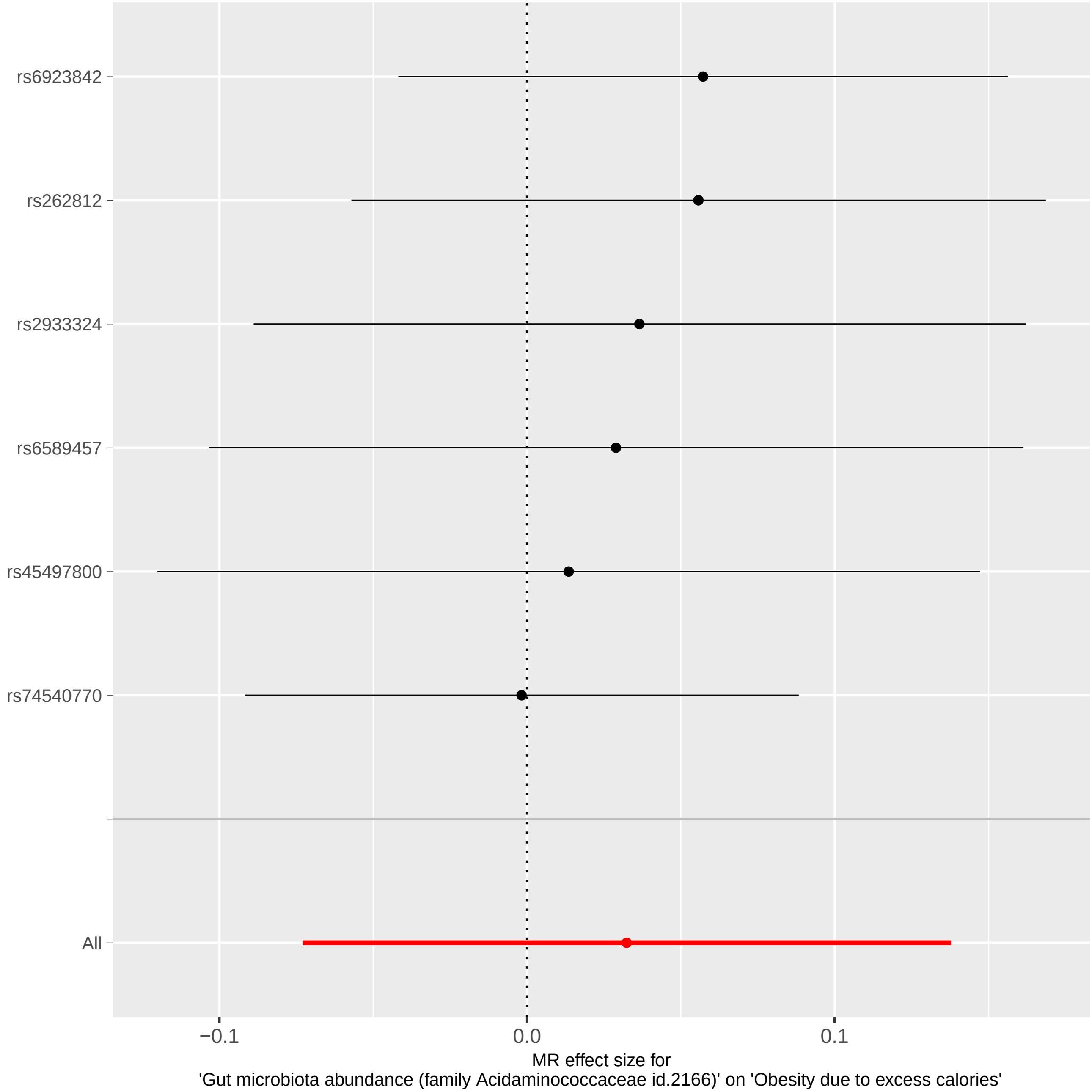

Batch 302 : Gut microbiota abundance (family Actinomycetaceae id.421) on Obesity due to excess calories

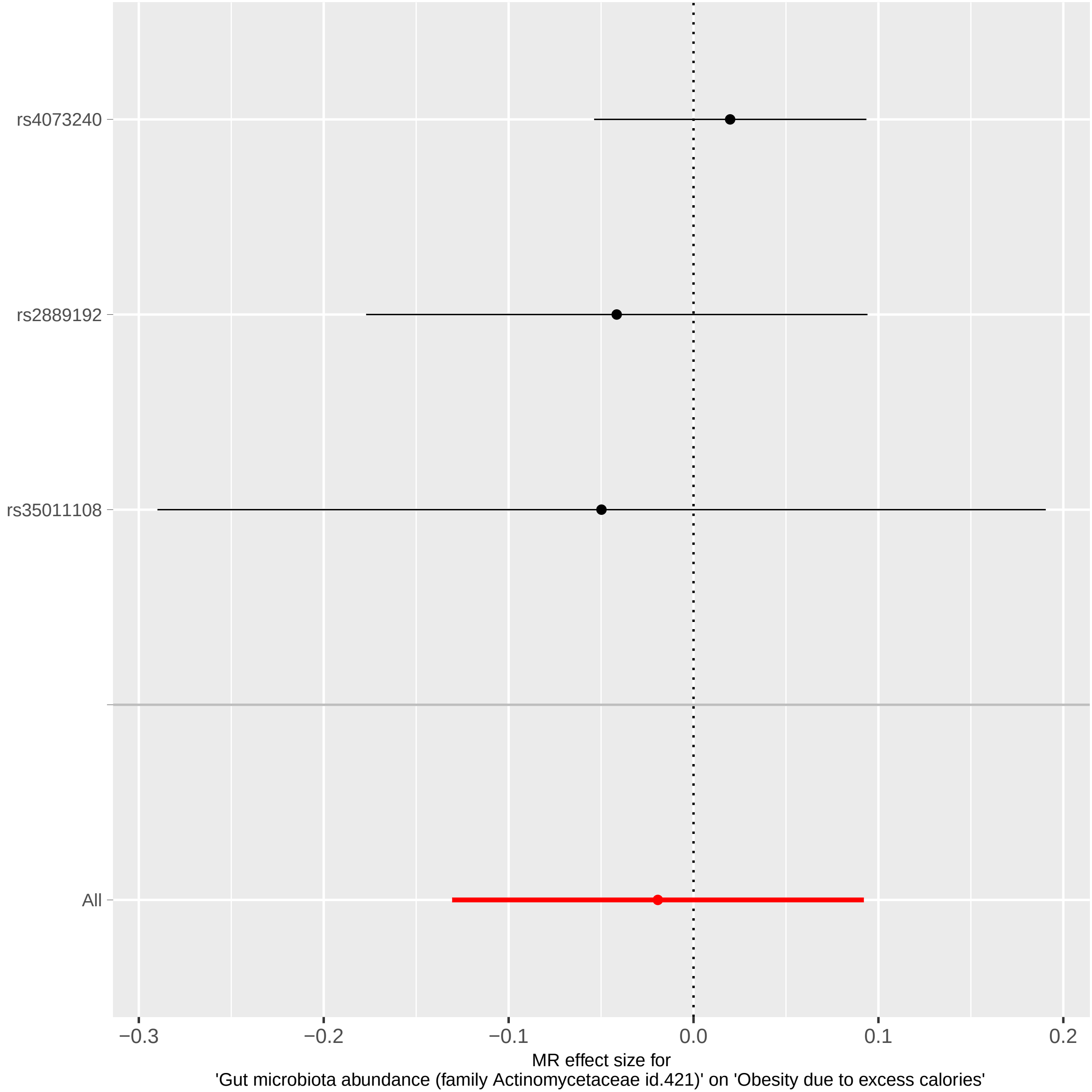

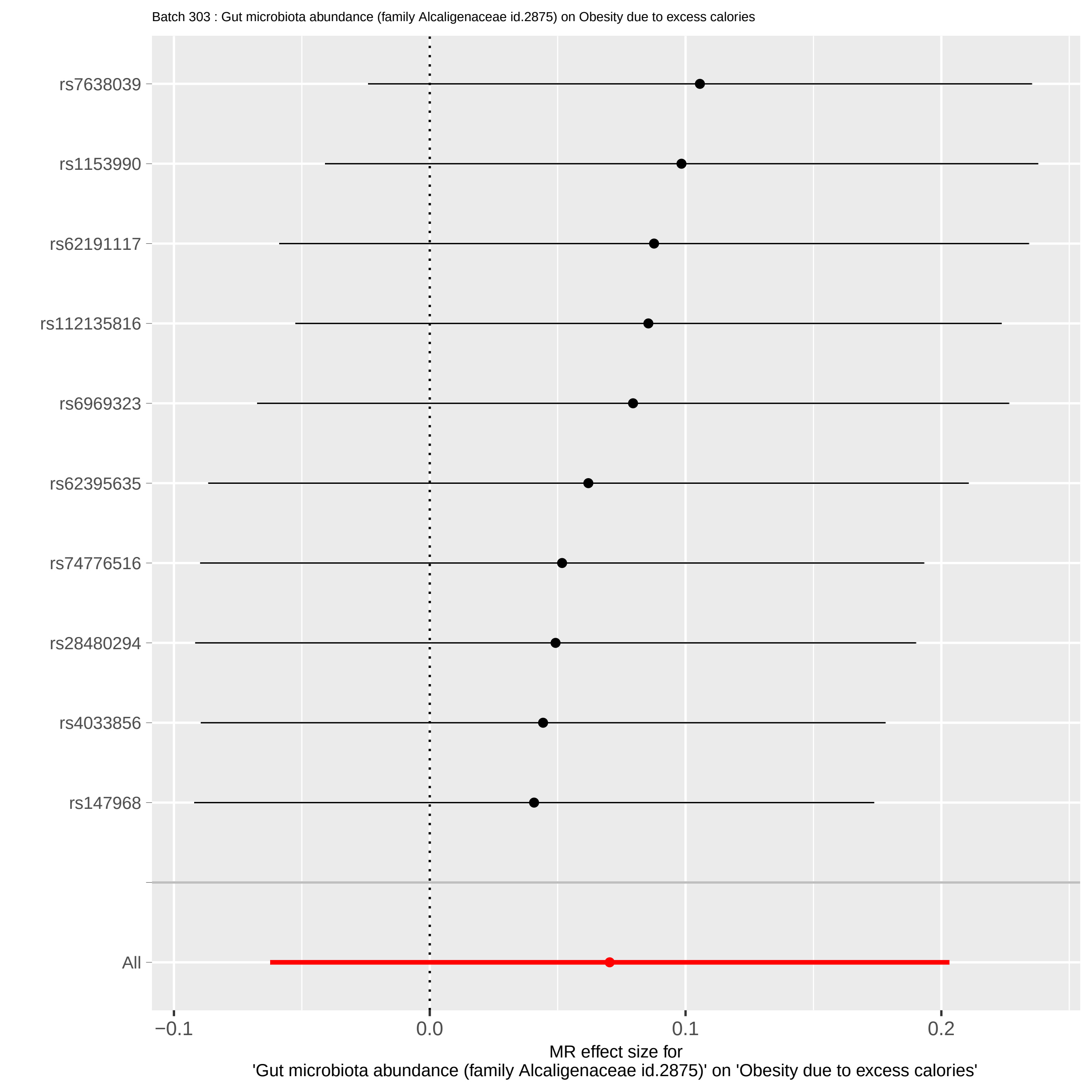
Batch 303 : Gut microbiota abundance (family Alcaligenaceae id.2875) on Obesity due to excess calories
rs7638039
rs1153990
rs62191117
rs112135816
rs6969323
rs62395635
rs74776516
rs28480294
rs4033856
rs147968
All
−0.1
0.0
0.1
0.2
MR effect size for
'Gut microbiota abundance (family Alcaligenaceae id.2875)' on 'Obesity due to excess calories'

Batch 304 : Gut microbiota abundance (family Bacteroidaceae id.917) on Obesity due to excess calories

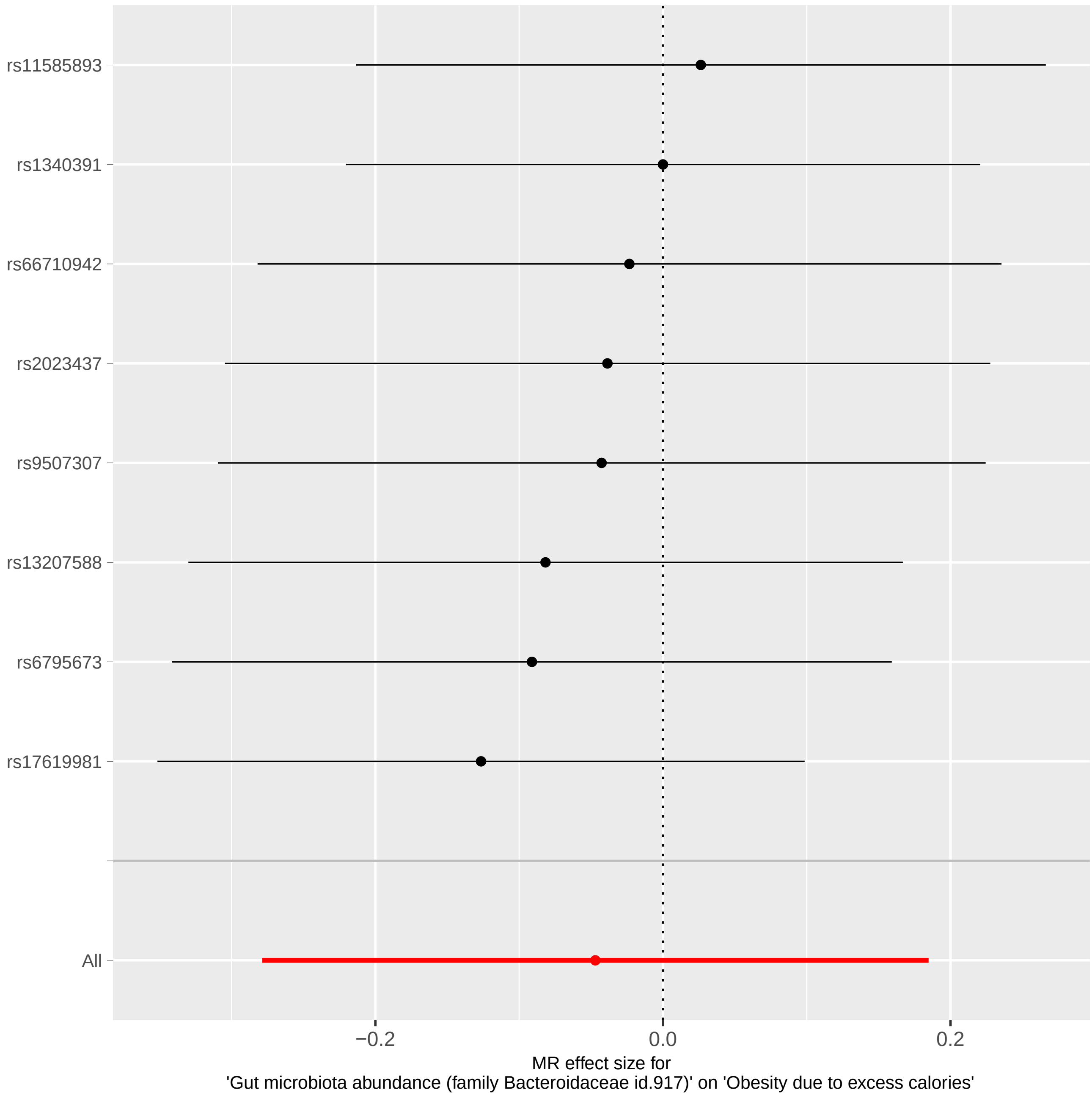

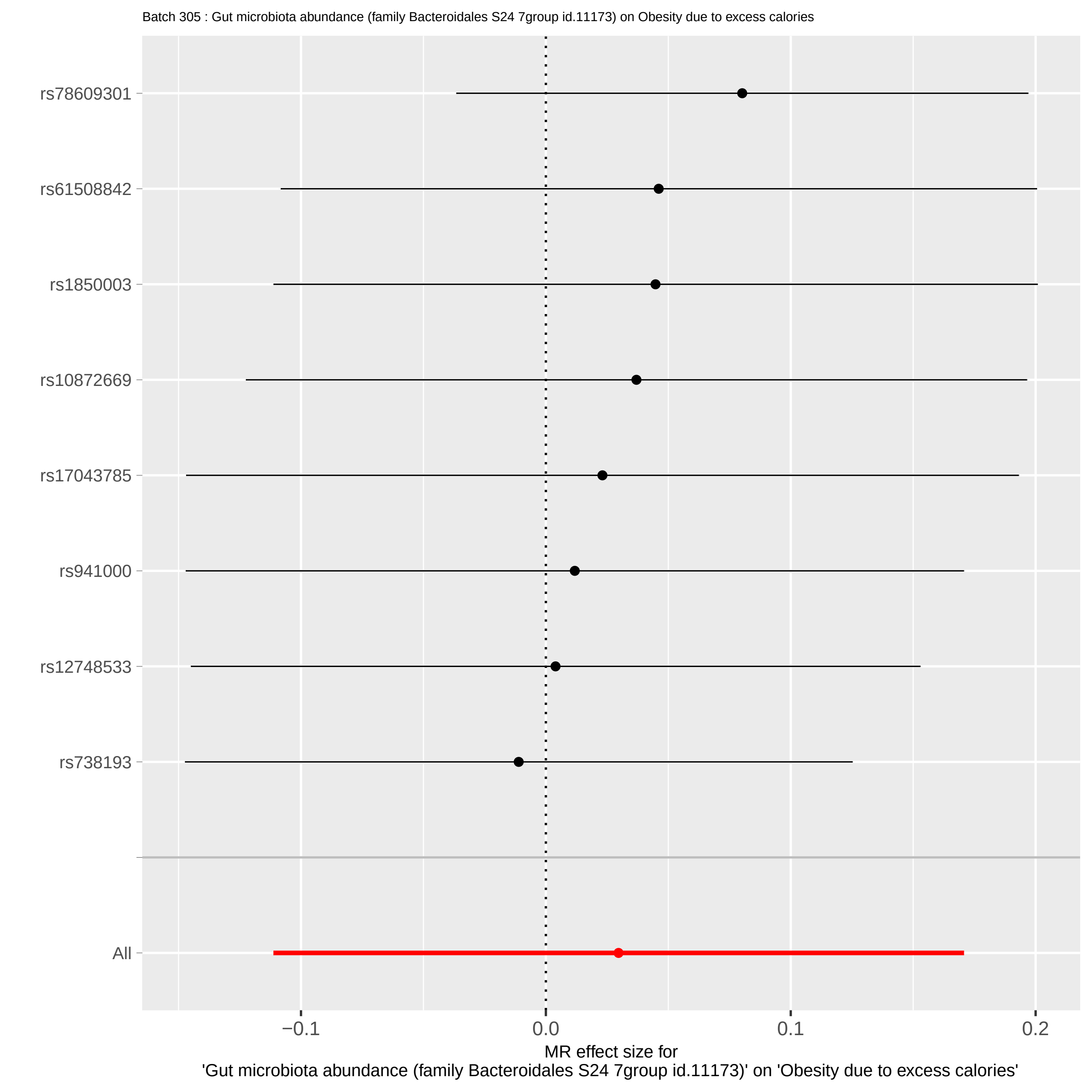

Batch 305 : Gut microbiota abundance (family Bacteroidales S24 7group id.11173) on Obesity due to excess calories
rs78609301
rs61508842
rs1850003
rs10872669
rs17043785
rs941000
rs12748533
rs738193
All
−0.1
0.0
0.1
0.2
MR effect size for
'Gut microbiota abundance (family Bacteroidales S24 7group id.11173)' on 'Obesity due to excess calories'

Batch 306 : Gut microbiota abundance (family Bifidobacteriaceae id.433) on Obesity due to excess calories

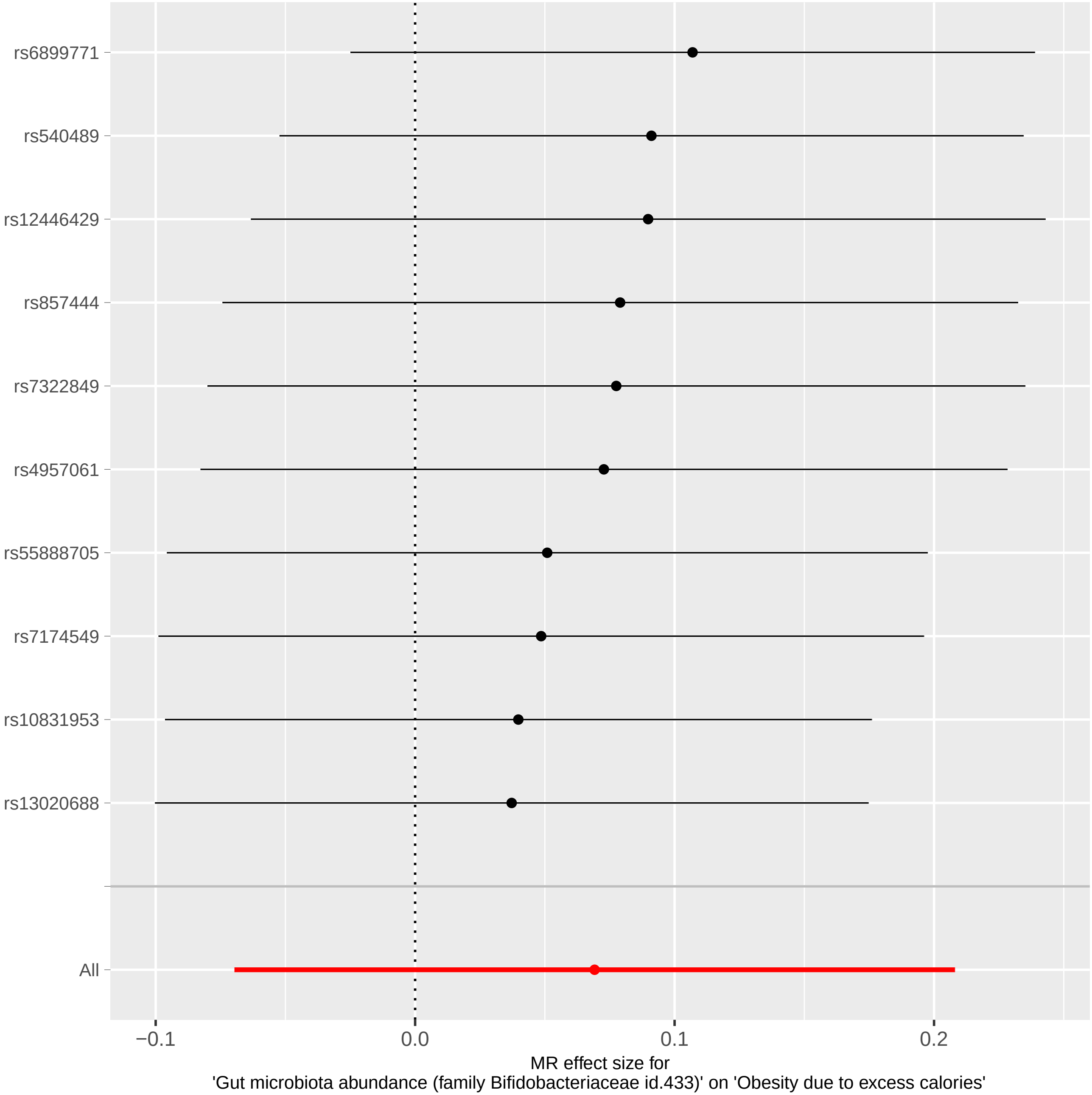

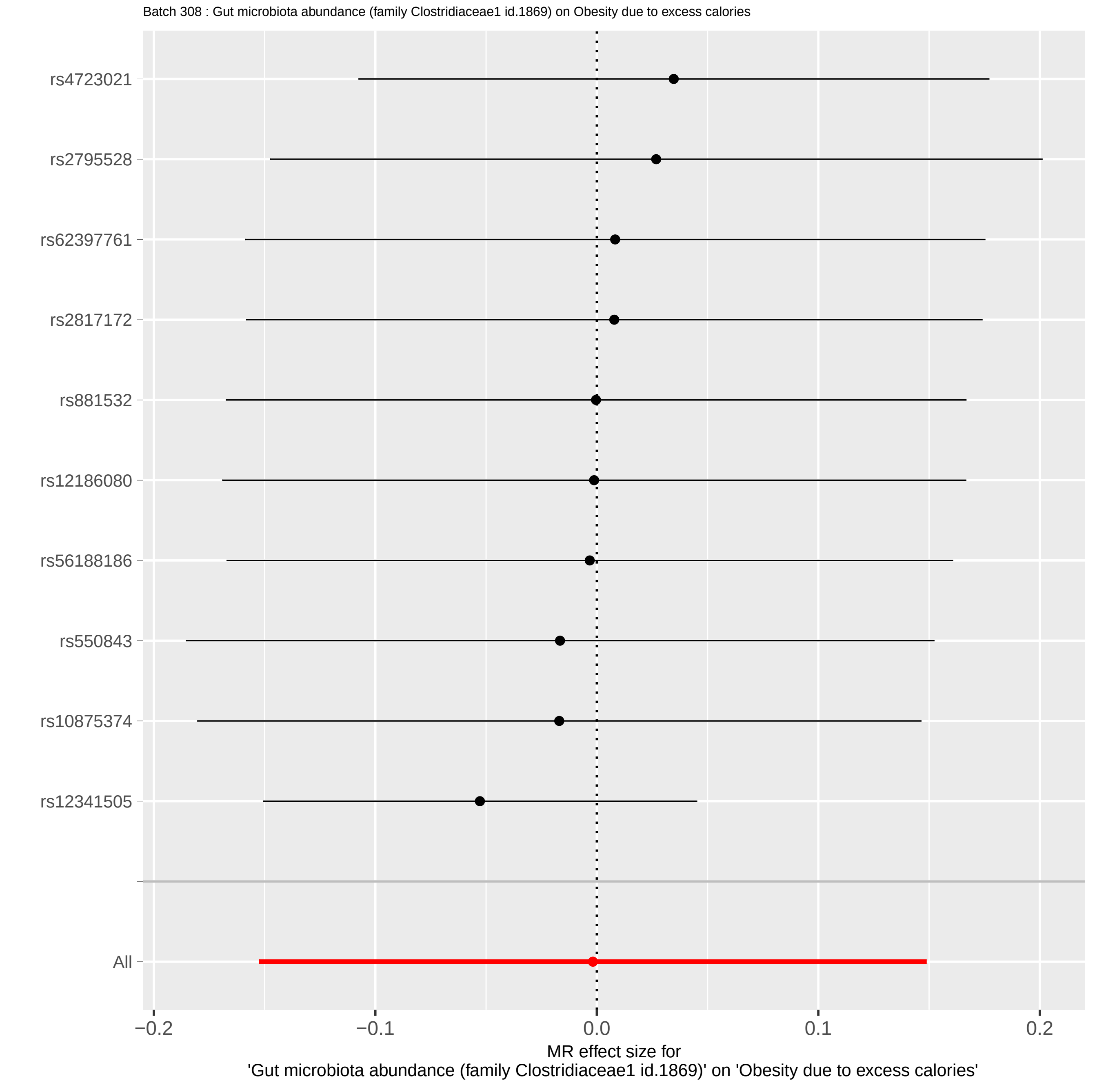

Batch 308 : Gut microbiota abundance (family Clostridiaceae1 id.1869) on Obesity due to excess calories
rs4723021
rs2795528
rs62397761
rs2817172
rs881532
rs12186080
rs56188186
rs550843
rs10875374
rs12341505
All
−0.2
−0.1
0.0
0.1
0.2
MR effect size for
'Gut microbiota abundance (family Clostridiaceae1 id.1869)' on 'Obesity due to excess calories'

Batch 309 : Gut microbiota abundance (family Clostridiales vadin BB60 group id.11286) on Obesity due to excess calories

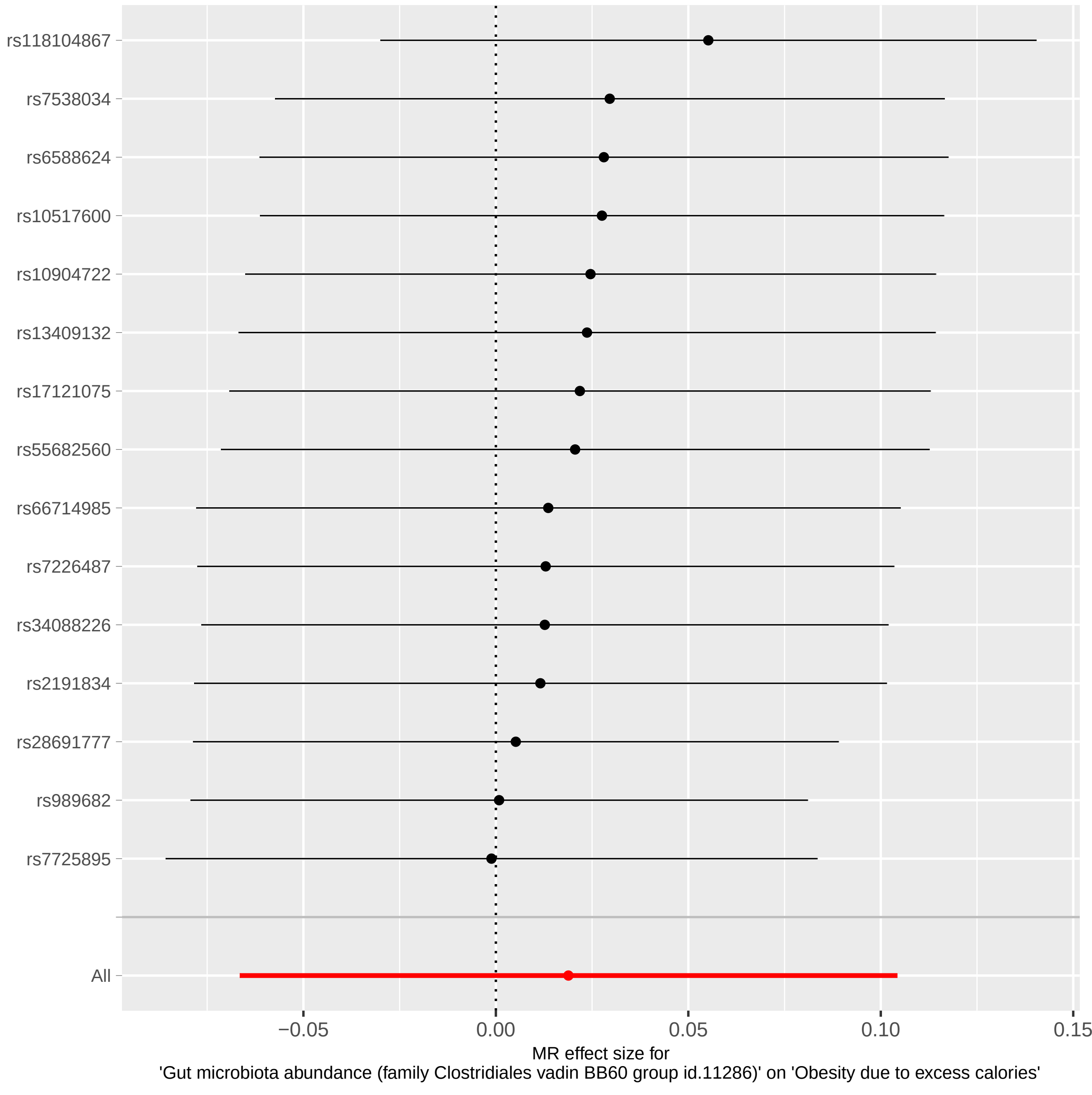

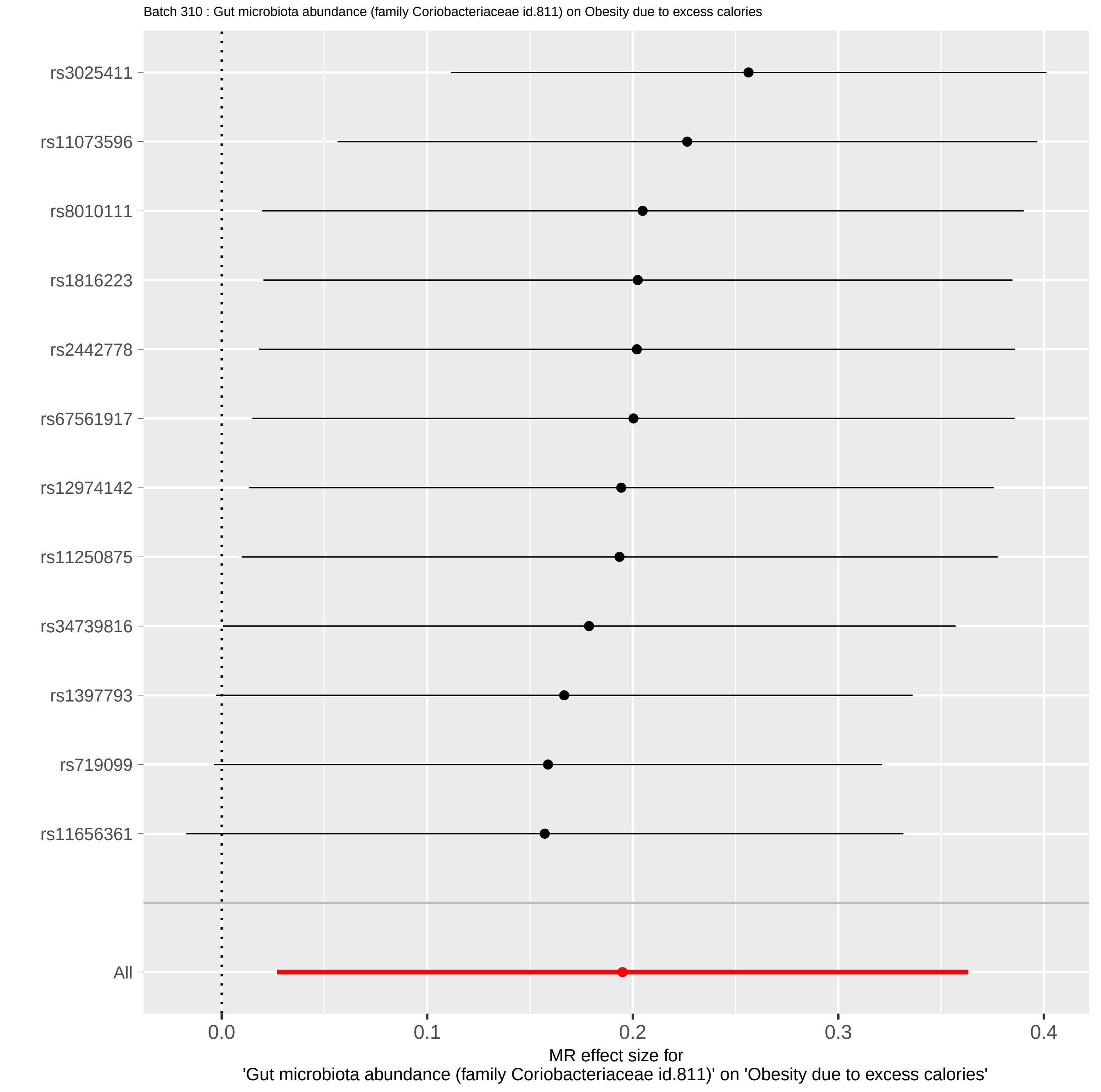
Batch 310 : Gut microbiota abundance (family Coriobacteriaceae id.811) on Obesity due to excess calories
rs3025411
rs11073596
rs8010111
rs1816223
rs2442778
rs67561917
rs12974142
rs11250875
rs34739816
rs1397793
rs719099
rs11656361
All
0.0
0.1
0.2
0.3
0.4
MR effect size for
'Gut microbiota abundance (family Coriobacteriaceae id.811)' on 'Obesity due to excess calories'

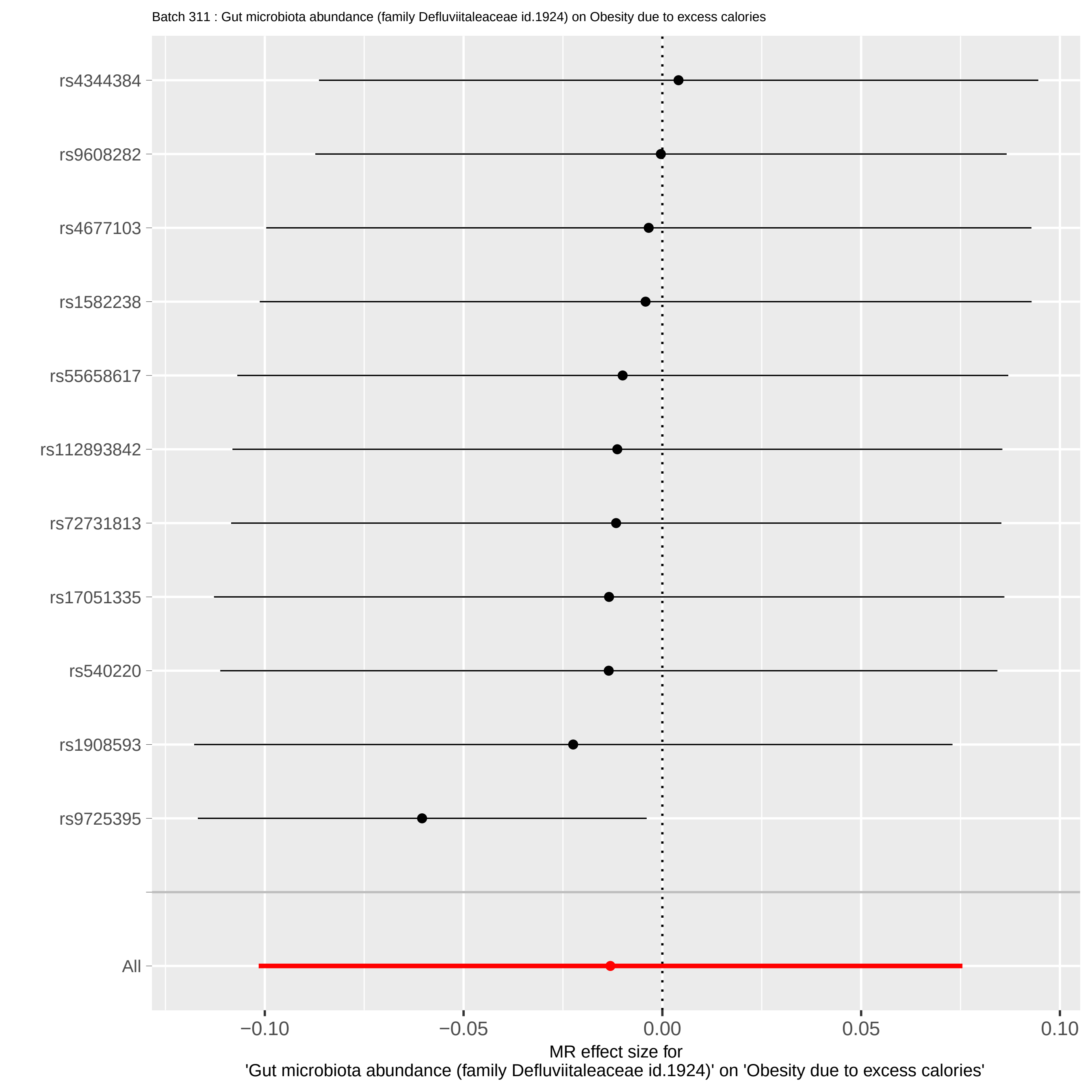
Batch 311 : Gut microbiota abundance (family Defluviitaleaceae id.1924) on Obesity due to excess calories
rs4344384
rs9608282
rs4677103
rs1582238
rs55658617
rs112893842
rs72731813
rs17051335
rs540220
rs1908593
rs9725395
All
−0.10
−0.05
0.00
0.05
0.10
MR effect size for
'Gut microbiota abundance (family Defluviitaleaceae id.1924)' on 'Obesity due to excess calories'

Batch 312 : Gut microbiota abundance (family Desulfovibrionaceae id.3169) on Obesity due to excess calories

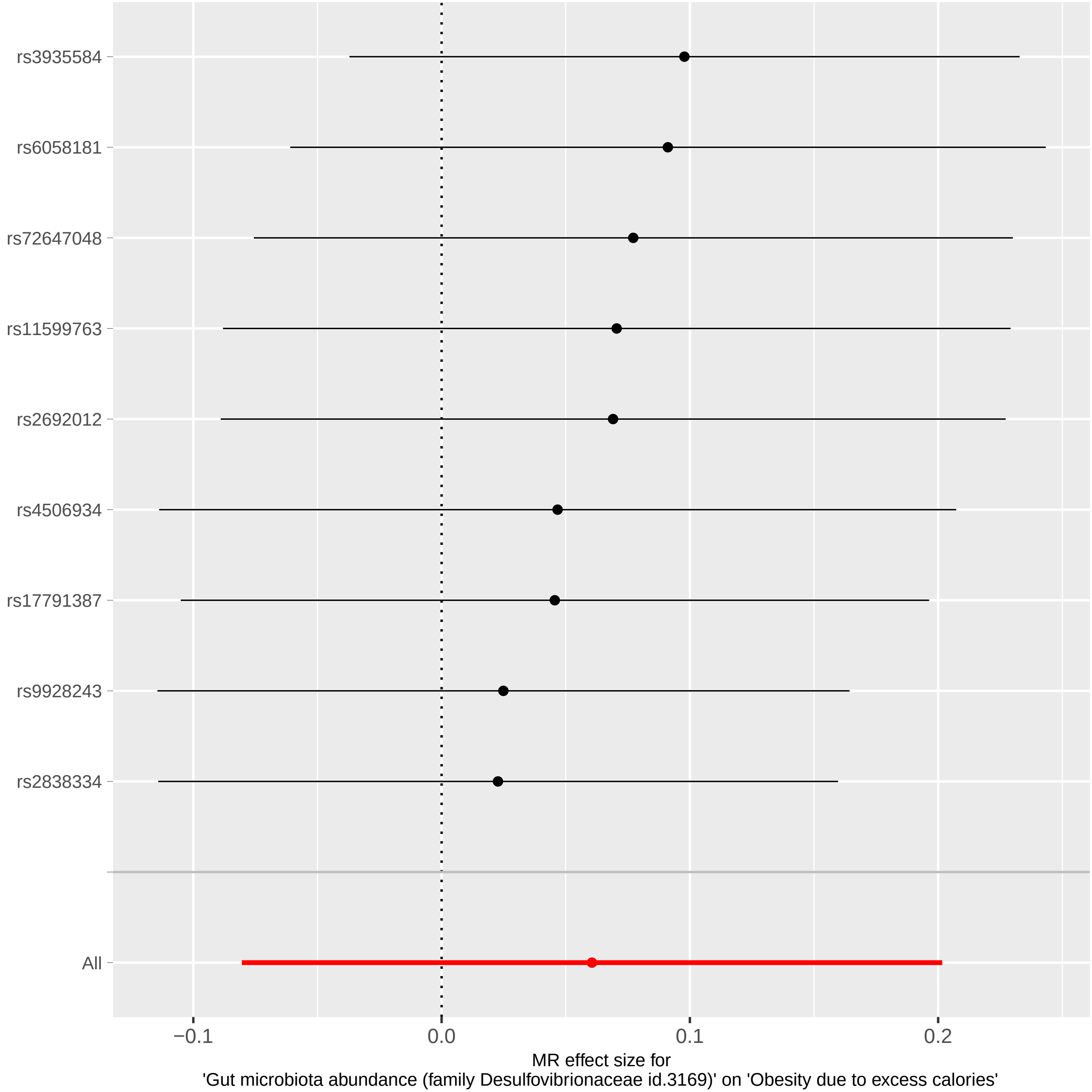

Batch 313 : Gut microbiota abundance (family Enterobacteriaceae id.3469) on Obesity due to excess calories

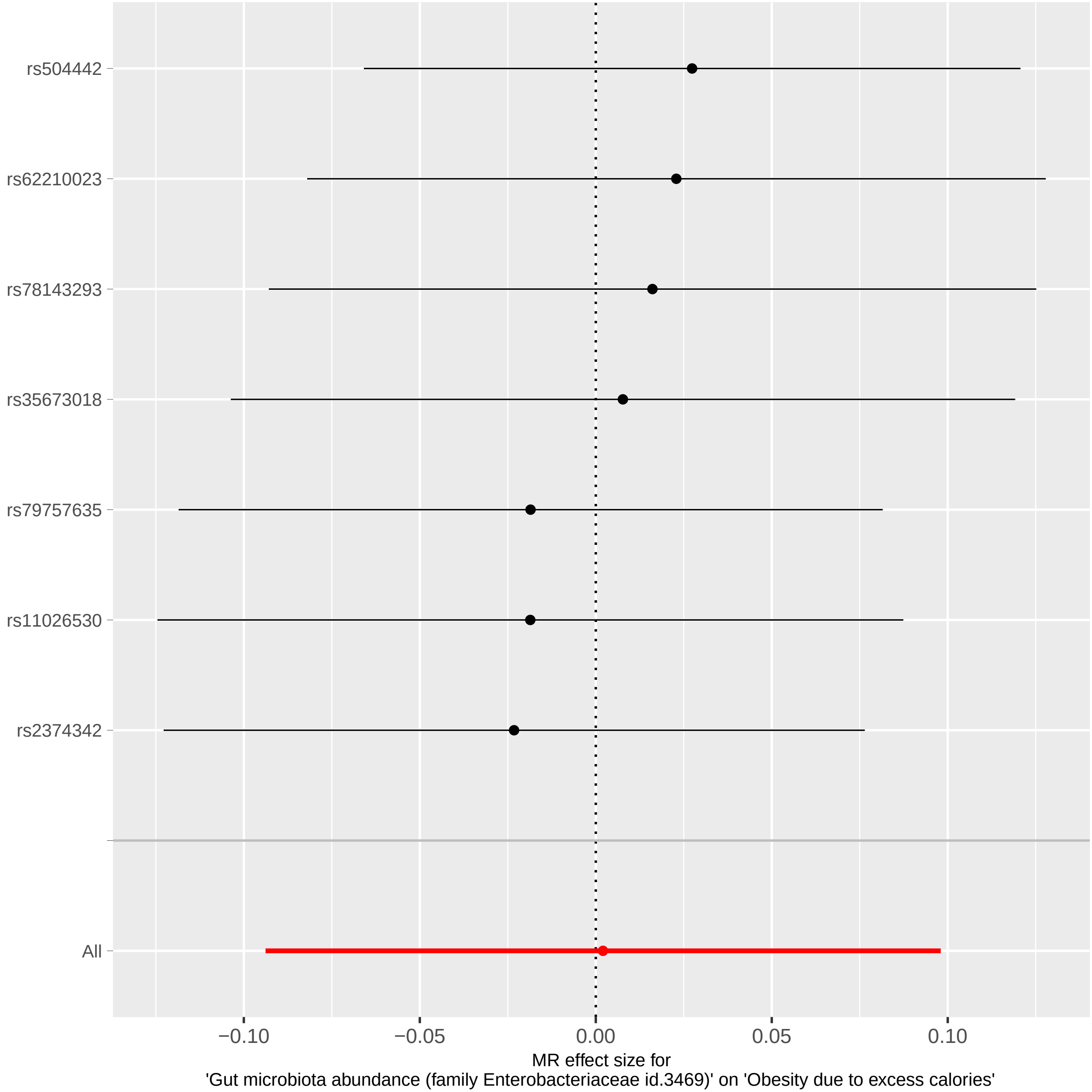

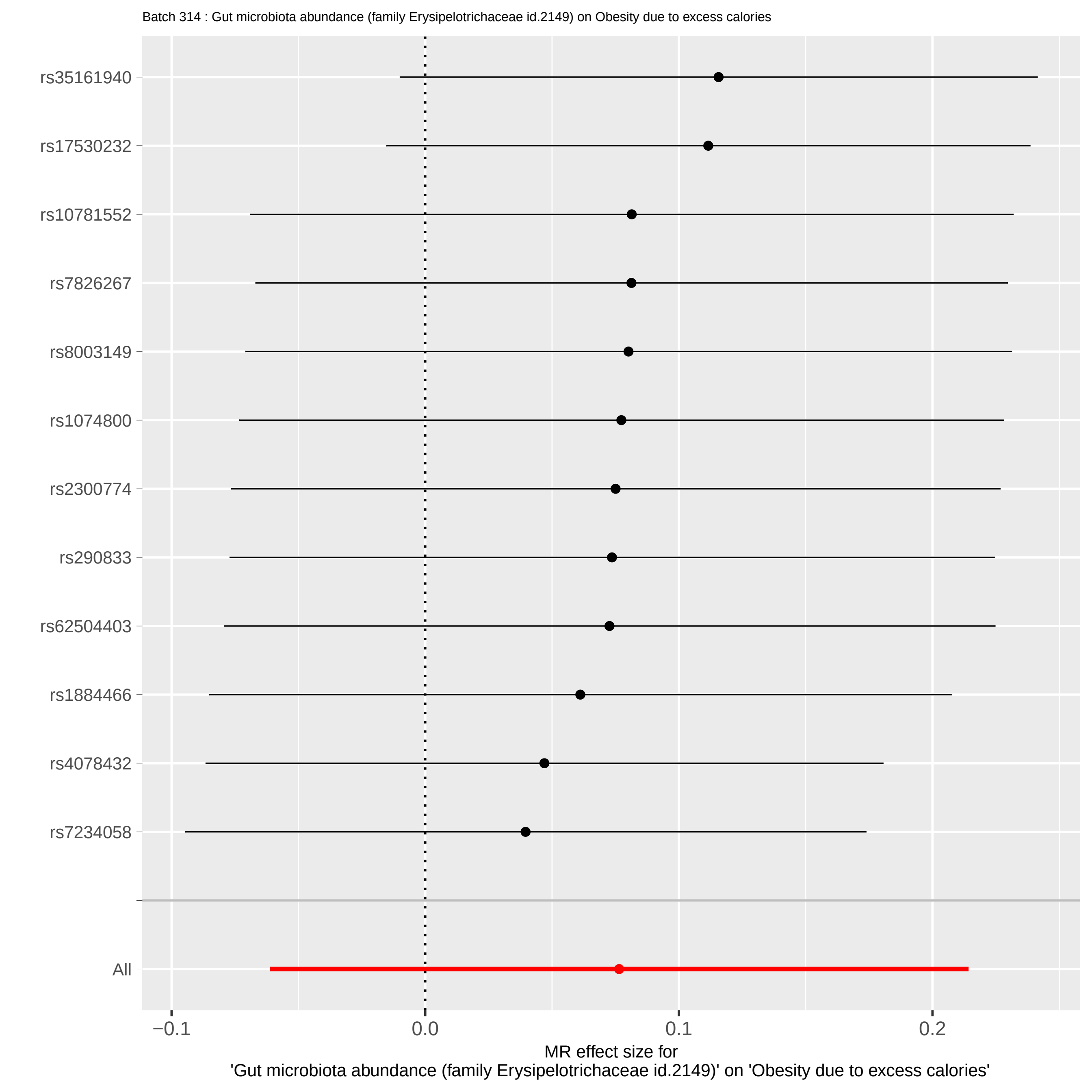

Batch 314 : Gut microbiota abundance (family Erysipelotrichaceae id.2149) on Obesity due to excess calories
rs35161940
rs17530232
rs10781552
rs7826267
rs8003149
rs1074800
rs2300774
rs290833
rs62504403
rs1884466
rs4078432
rs7234058
All
−0.1
0.0
0.1
0.2
MR effect size for
'Gut microbiota abundance (family Erysipelotrichaceae id.2149)' on 'Obesity due to excess calories'

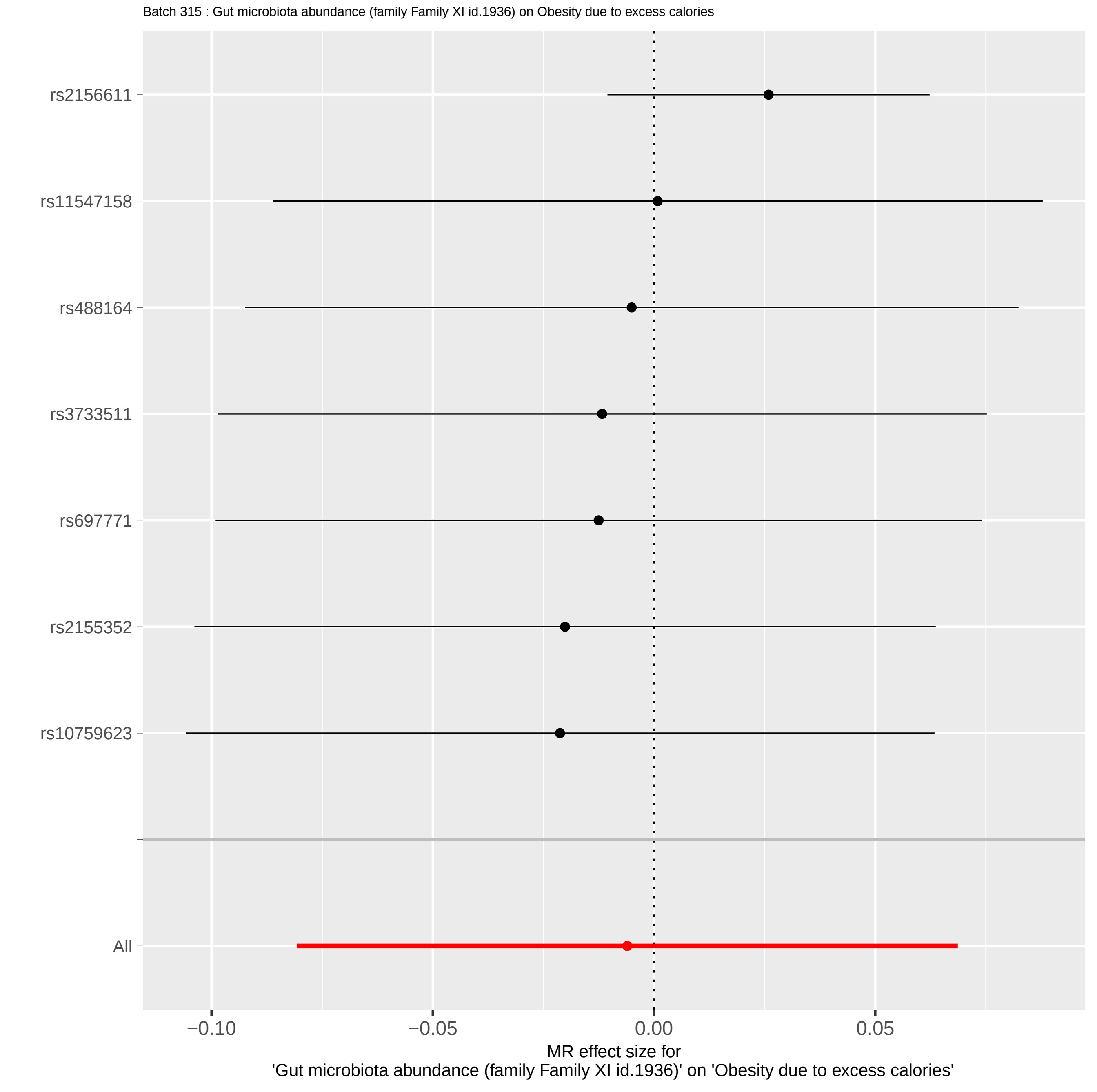

Batch 315 : Gut microbiota abundance (family Family XI id.1936) on Obesity due to excess calories
rs2156611
rs11547158
rs488164
rs3733511
rs697771
rs2155352
rs10759623
All
−0.10
−0.05
0.00
0.05
MR effect size for
'Gut microbiota abundance (family Family XI id.1936)' on 'Obesity due to excess calories'

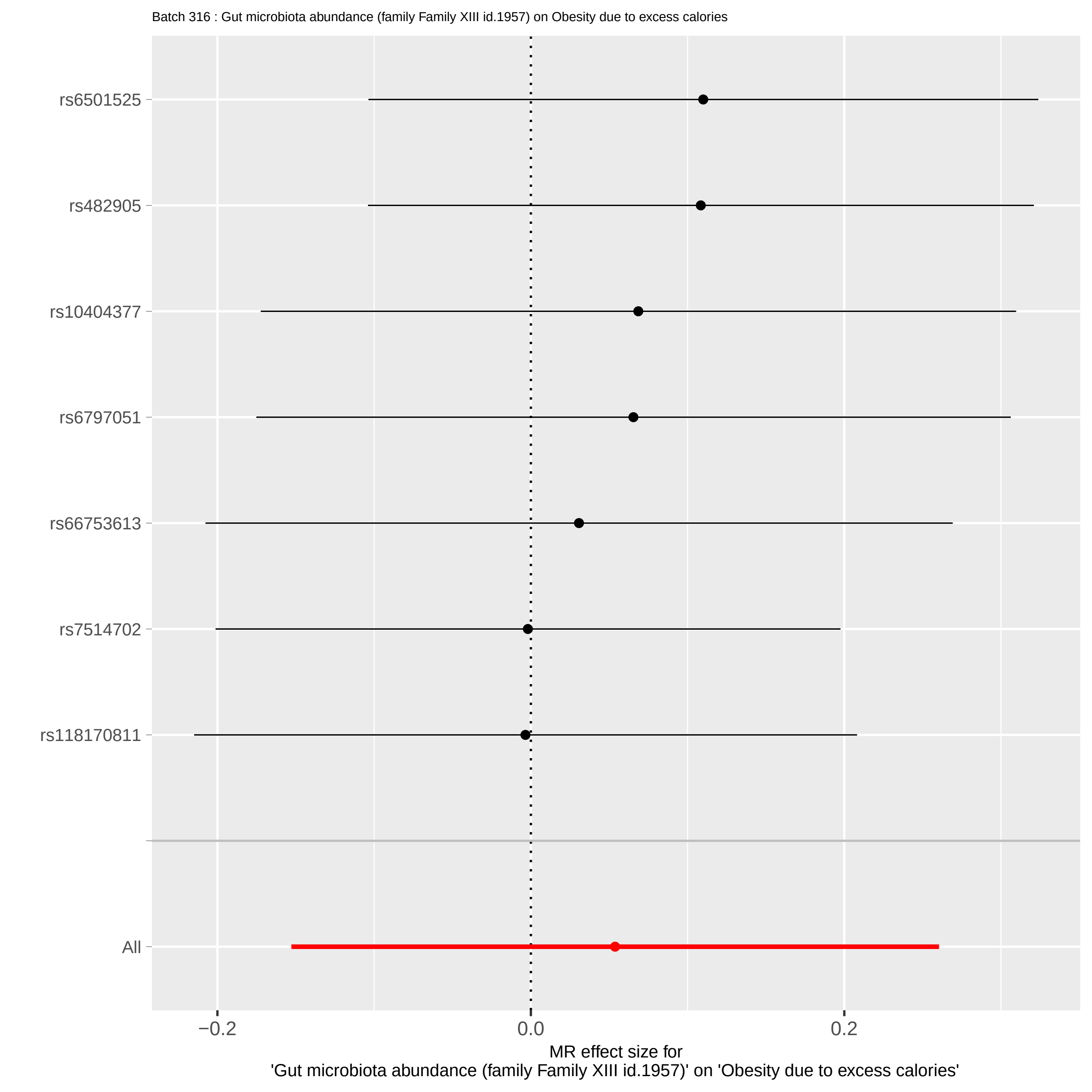
Batch 316 : Gut microbiota abundance (family Family XIII id.1957) on Obesity due to excess calories
rs6501525
rs482905
rs10404377
rs6797051
rs66753613
rs7514702
rs118170811
All
−0.2
0.0
0.2
MR effect size for
'Gut microbiota abundance (family Family XIII id.1957)' on 'Obesity due to excess calories'

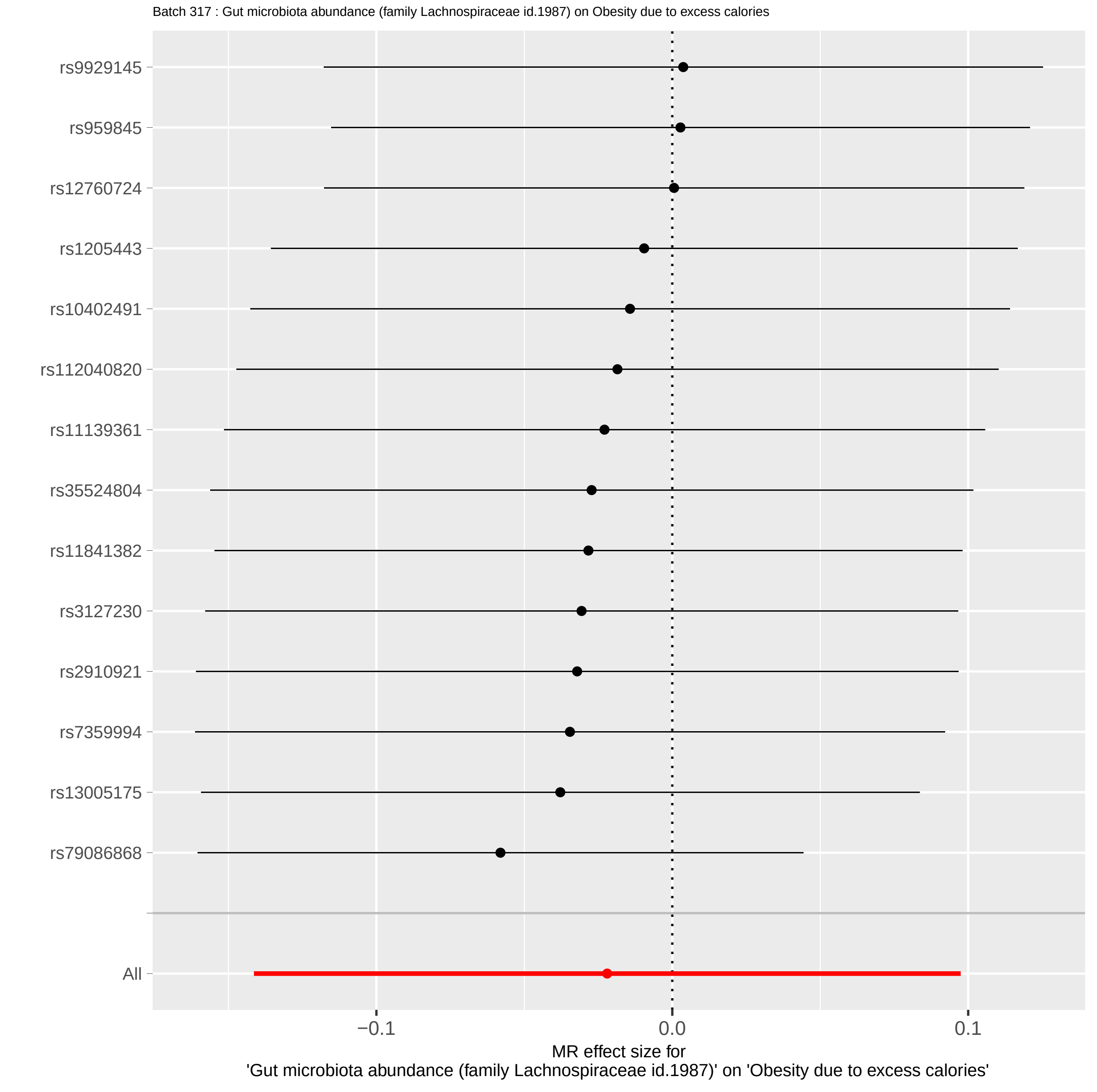
Batch 317 : Gut microbiota abundance (family Lachnospiraceae id.1987) on Obesity due to excess calories
rs9929145
rs959845
rs12760724
rs1205443
rs10402491
rs112040820
rs11139361
rs35524804
rs11841382
rs3127230
rs2910921
rs7359994
rs13005175
rs79086868
All
−0.1
0.0
0.1
MR effect size for
'Gut microbiota abundance (family Lachnospiraceae id.1987)' on 'Obesity due to excess calories'

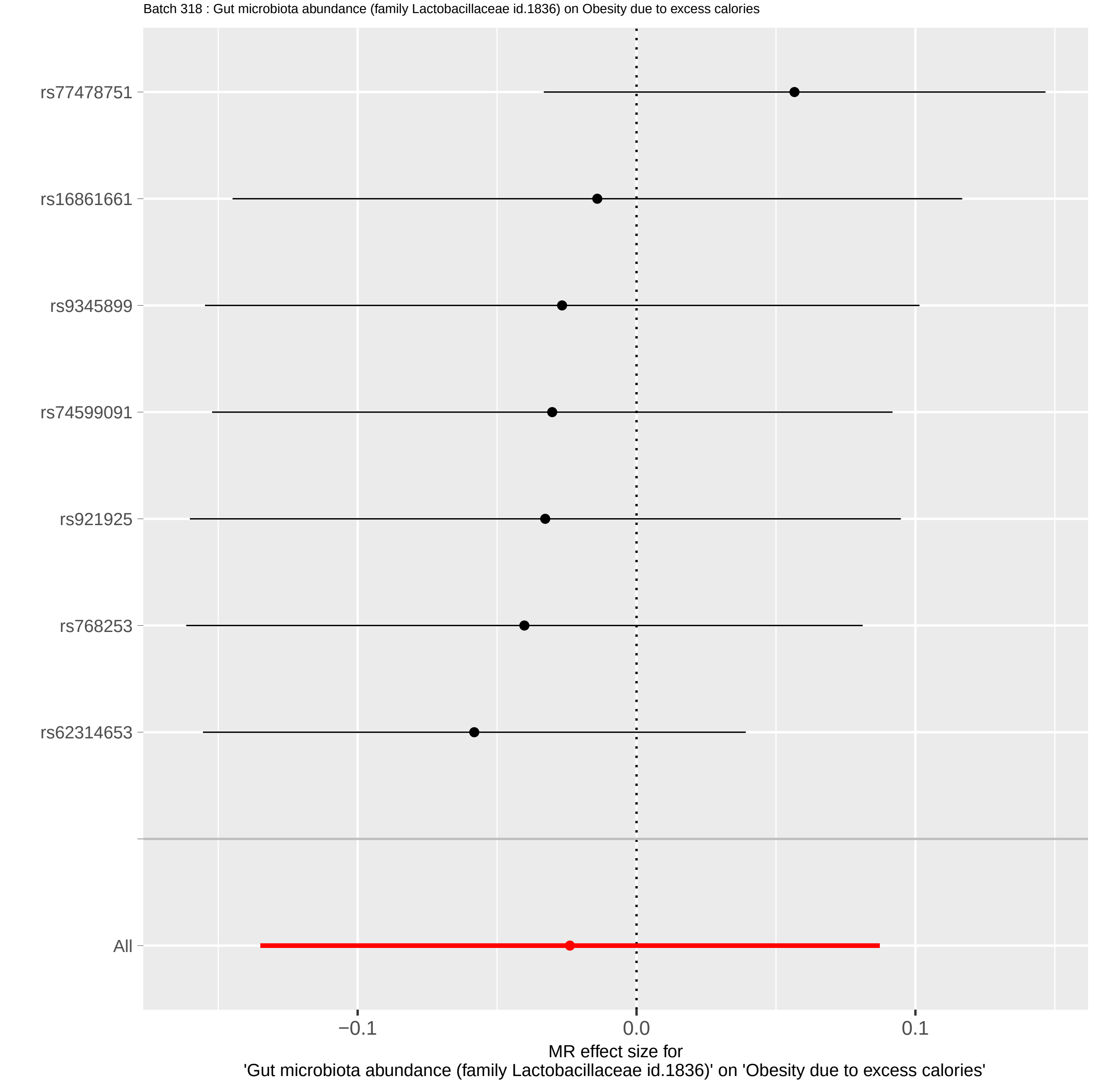

Batch 318 : Gut microbiota abundance (family Lactobacillaceae id.1836) on Obesity due to excess calories
rs77478751
rs16861661
rs9345899
rs74599091
rs921925
rs768253
rs62314653
All
−0.1
0.0
0.1
MR effect size for
'Gut microbiota abundance (family Lactobacillaceae id.1836)' on 'Obesity due to excess calories'

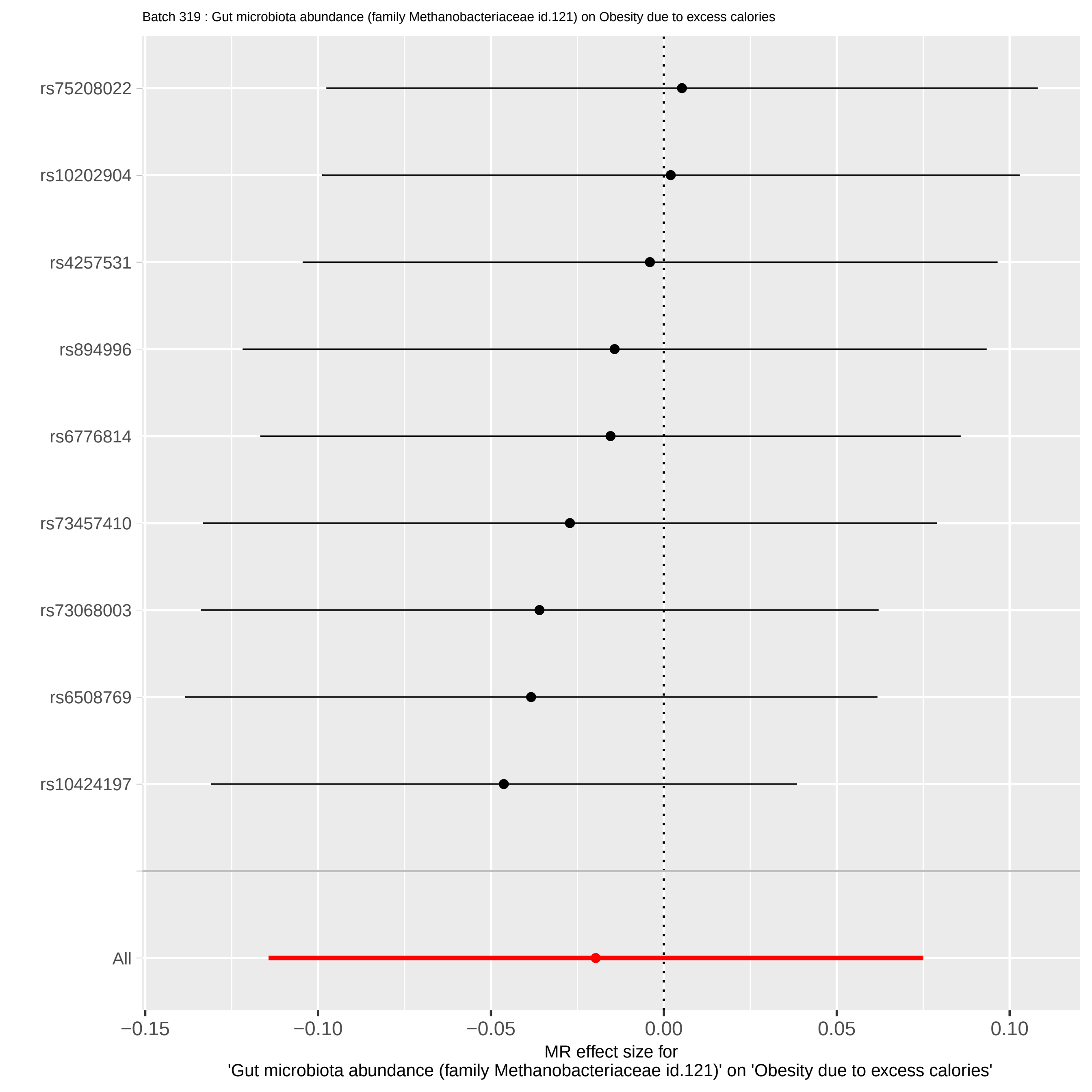
Batch 319 : Gut microbiota abundance (family Methanobacteriaceae id.121) on Obesity due to excess calories
rs75208022
rs10202904
rs4257531
rs894996
rs6776814
rs73457410
rs73068003
rs6508769
rs10424197
All
−0.15
−0.10
−0.05
0.00
0.05
0.10
MR effect size for
'Gut microbiota abundance (family Methanobacteriaceae id.121)' on 'Obesity due to excess calories'

Batch 320 : Gut microbiota abundance (family Oxalobacteraceae id.2966) on Obesity due to excess calories

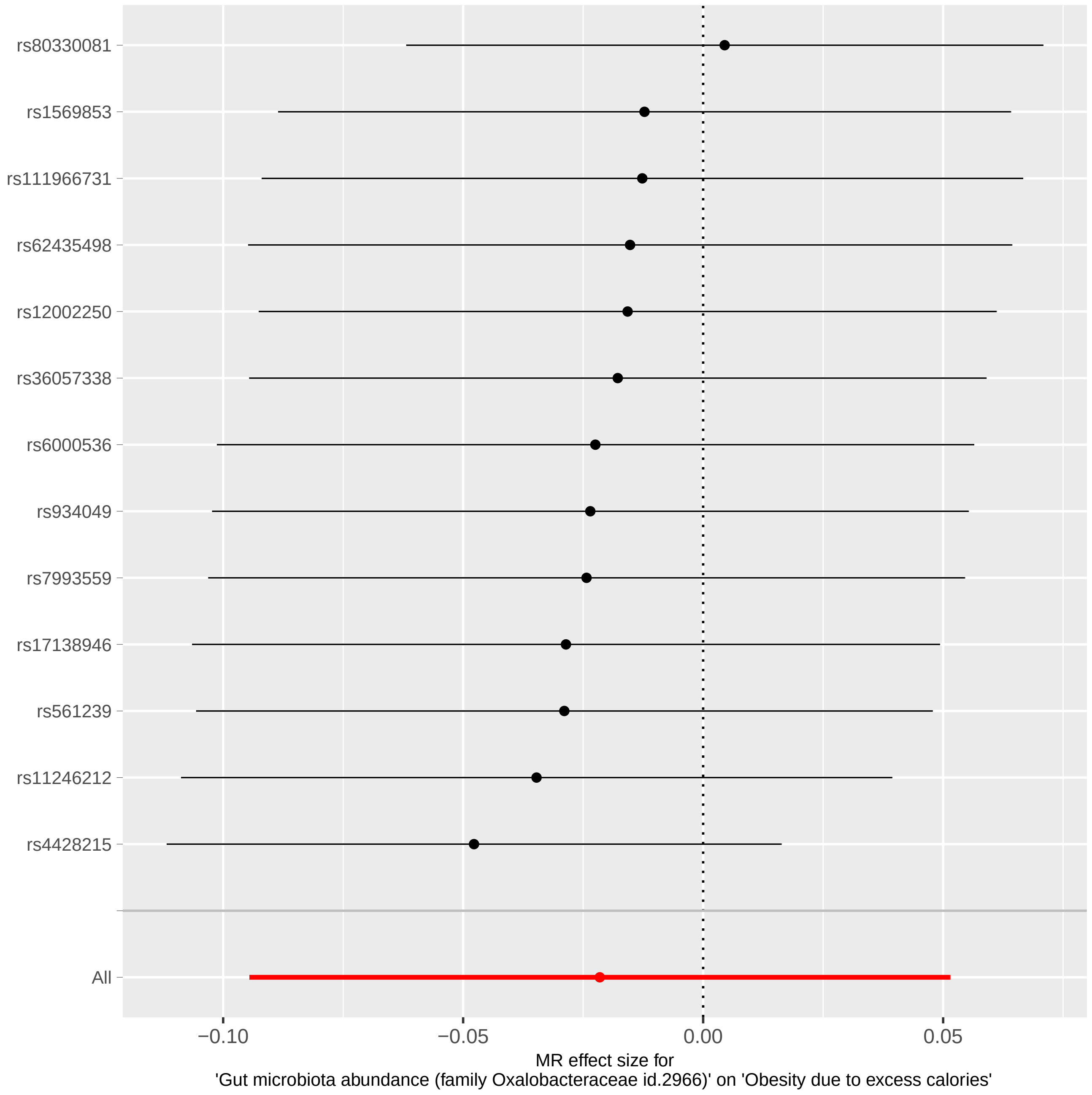

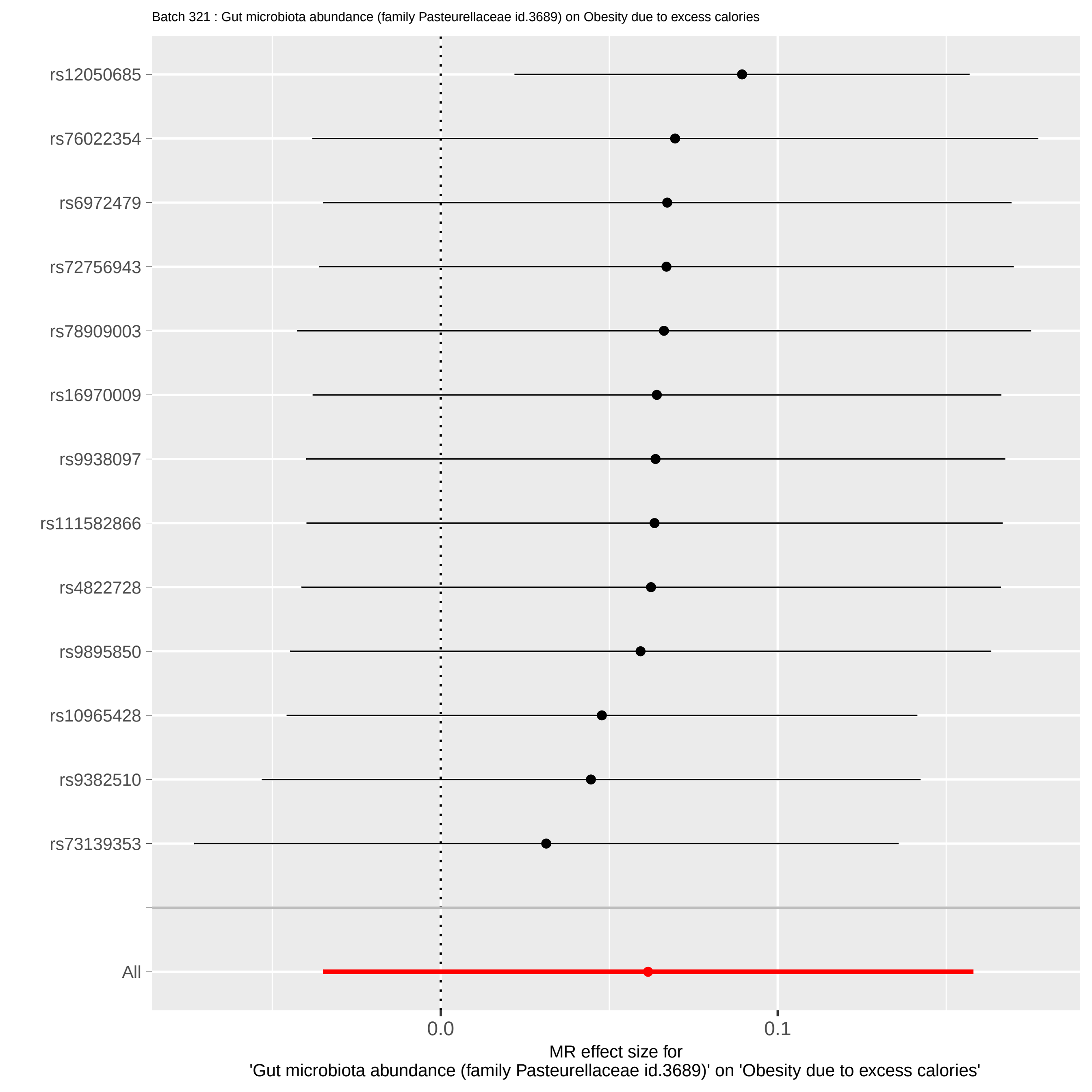

Batch 321 : Gut microbiota abundance (family Pasteurellaceae id.3689) on Obesity due to excess calories
rs12050685
rs76022354
rs6972479
rs72756943
rs78909003
rs16970009
rs9938097
rs111582866
rs4822728
rs9895850
rs10965428
rs9382510
rs73139353
All
0.0
0.1
MR effect size for
'Gut microbiota abundance (family Pasteurellaceae id.3689)' on 'Obesity due to excess calories'

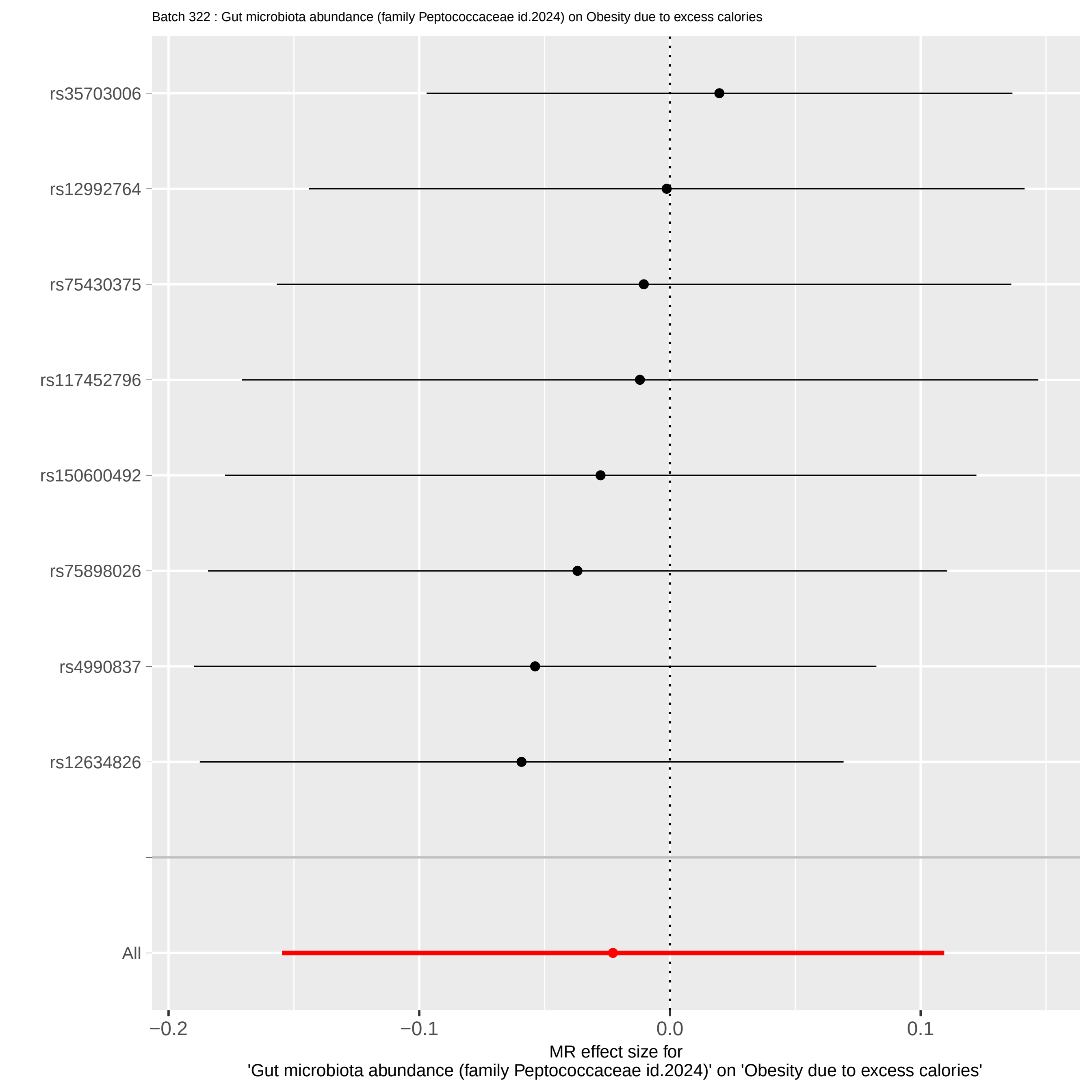
Batch 322 : Gut microbiota abundance (family Peptococcaceae id.2024) on Obesity due to excess calories
rs35703006
rs12992764
rs75430375
rs117452796
rs150600492
rs75898026
rs4990837
rs12634826
All
−0.2
−0.1
0.0
0.1
MR effect size for
'Gut microbiota abundance (family Peptococcaceae id.2024)' on 'Obesity due to excess calories'

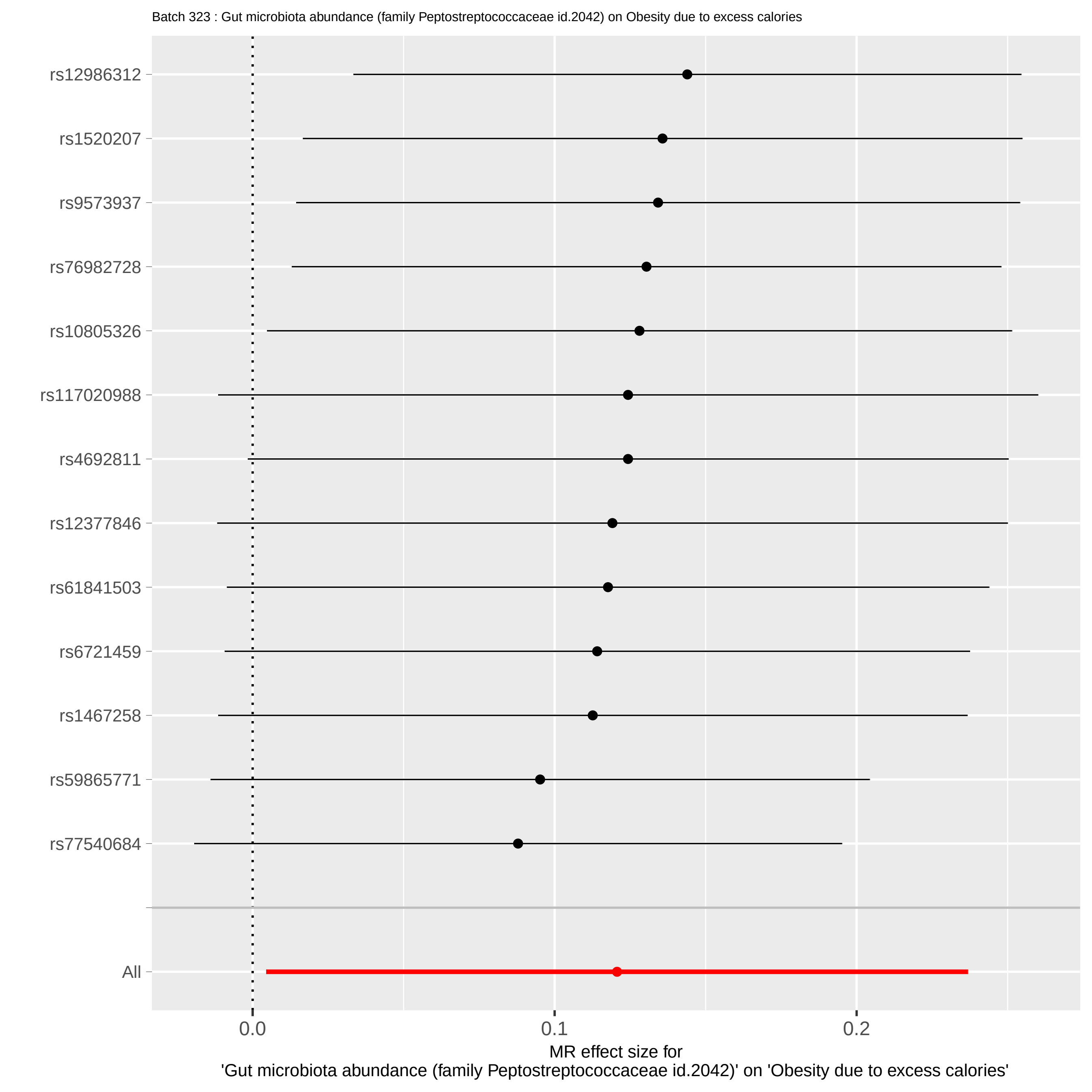

Batch 323 : Gut microbiota abundance (family Peptostreptococcaceae id.2042) on Obesity due to excess calories
rs12986312
rs1520207
rs9573937
rs76982728
rs10805326
rs117020988
rs4692811
rs12377846
rs61841503
rs6721459
rs1467258
rs59865771
rs77540684
All
0.0
0.1
0.2
MR effect size for
'Gut microbiota abundance (family Peptostreptococcaceae id.2042)' on 'Obesity due to excess calories'

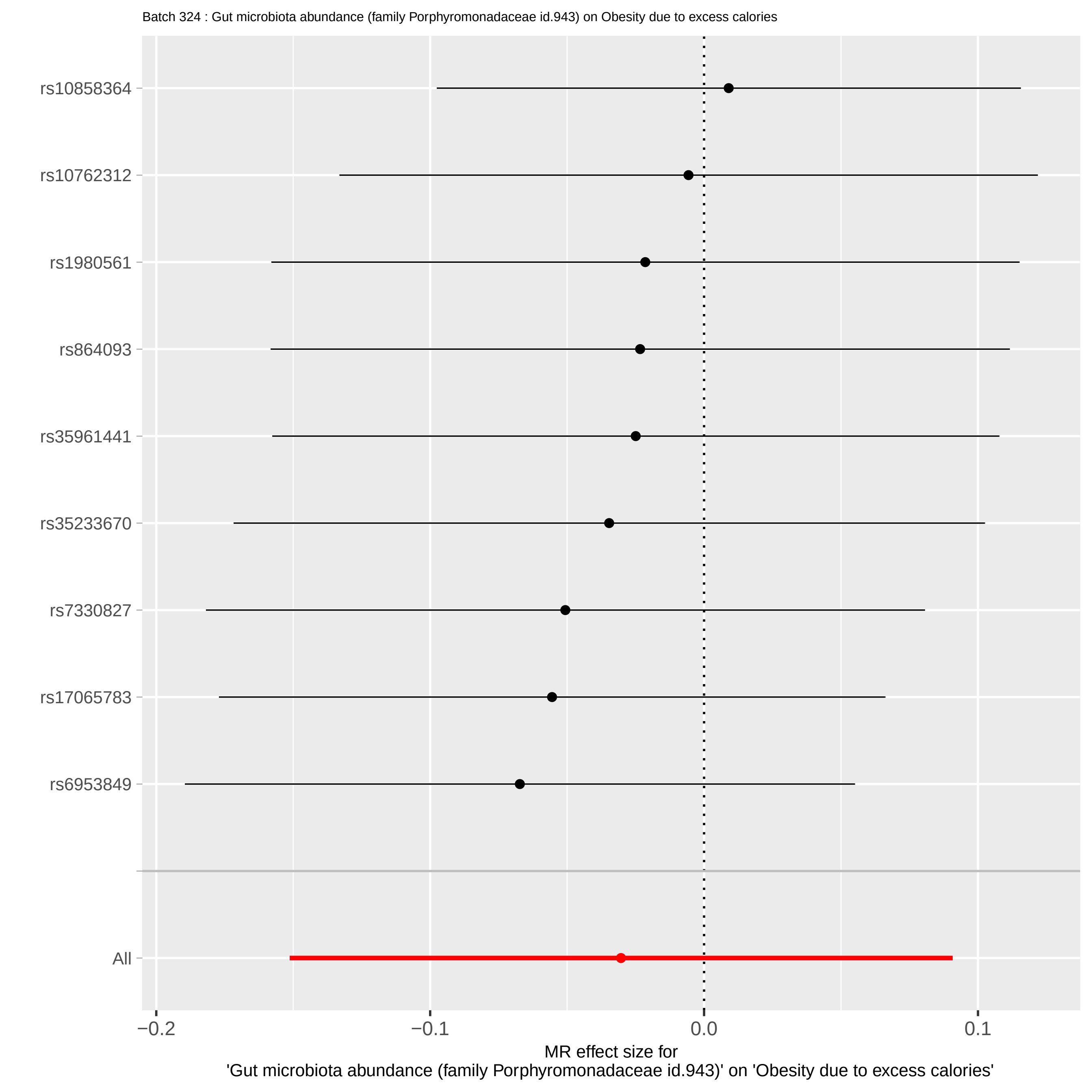
Batch 324 : Gut microbiota abundance (family Porphyromonadaceae id.943) on Obesity due to excess calories
rs10858364
rs10762312
rs1980561
rs864093
rs35961441
rs35233670
rs7330827
rs17065783
rs6953849
All
−0.2
−0.1
0.0
0.1
MR effect size for
'Gut microbiota abundance (family Porphyromonadaceae id.943)' on 'Obesity due to excess calories'

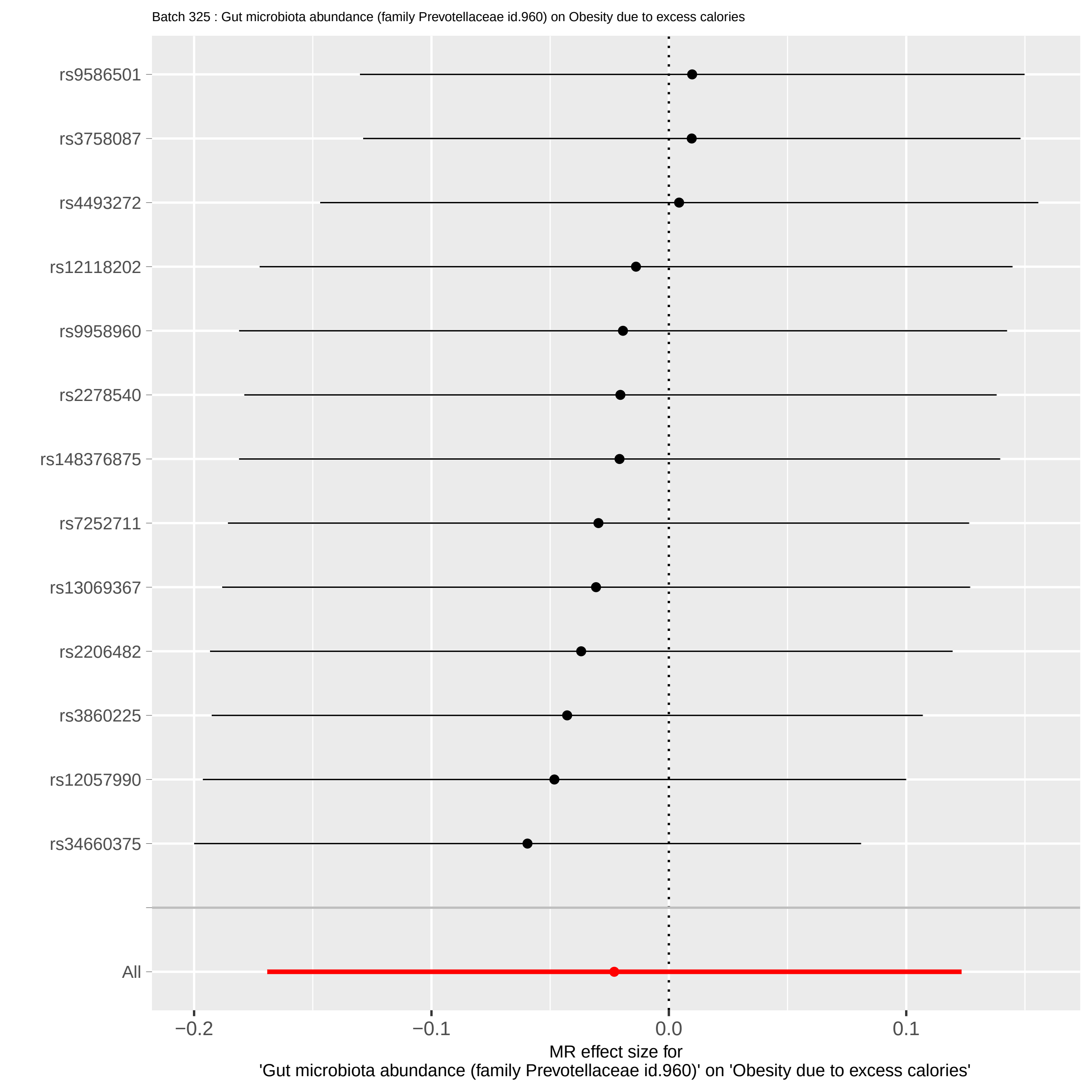
Batch 325 : Gut microbiota abundance (family Prevotellaceae id.960) on Obesity due to excess calories
rs9586501
rs3758087
rs4493272
rs12118202
rs9958960
rs2278540
rs148376875
rs7252711
rs13069367
rs2206482
rs3860225
rs12057990
rs34660375
All
−0.2
−0.1
0.0
0.1
MR effect size for
'Gut microbiota abundance (family Prevotellaceae id.960)' on 'Obesity due to excess calories'

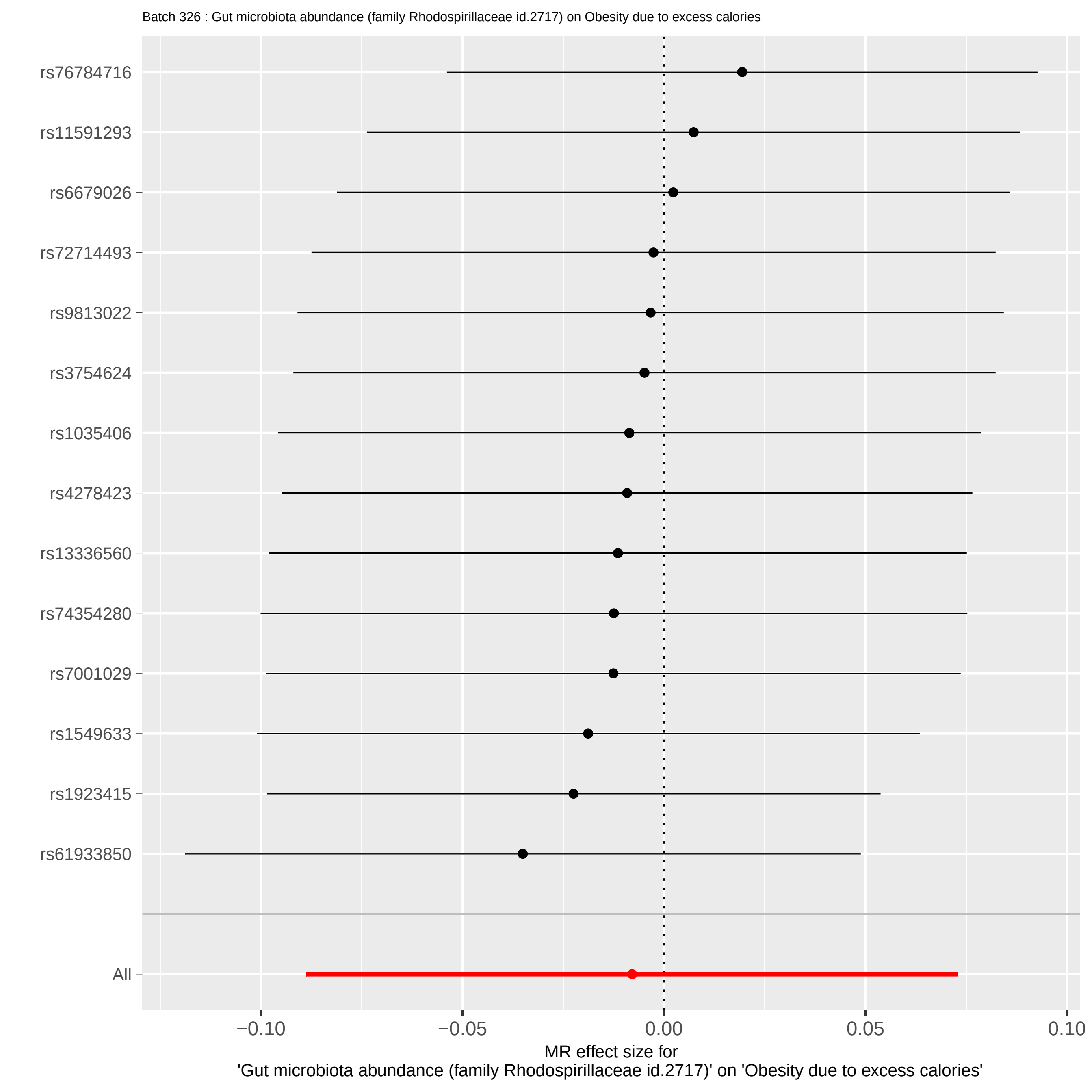

Batch 326 : Gut microbiota abundance (family Rhodospirillaceae id.2717) on Obesity due to excess calories
rs76784716
rs11591293
rs6679026
rs72714493
rs9813022
rs3754624
rs1035406
rs4278423
rs13336560
rs74354280
rs7001029
rs1549633
rs1923415
rs61933850
All
−0.10
−0.05
0.00
0.05
0.10
MR effect size for
'Gut microbiota abundance (family Rhodospirillaceae id.2717)' on 'Obesity due to excess calories'

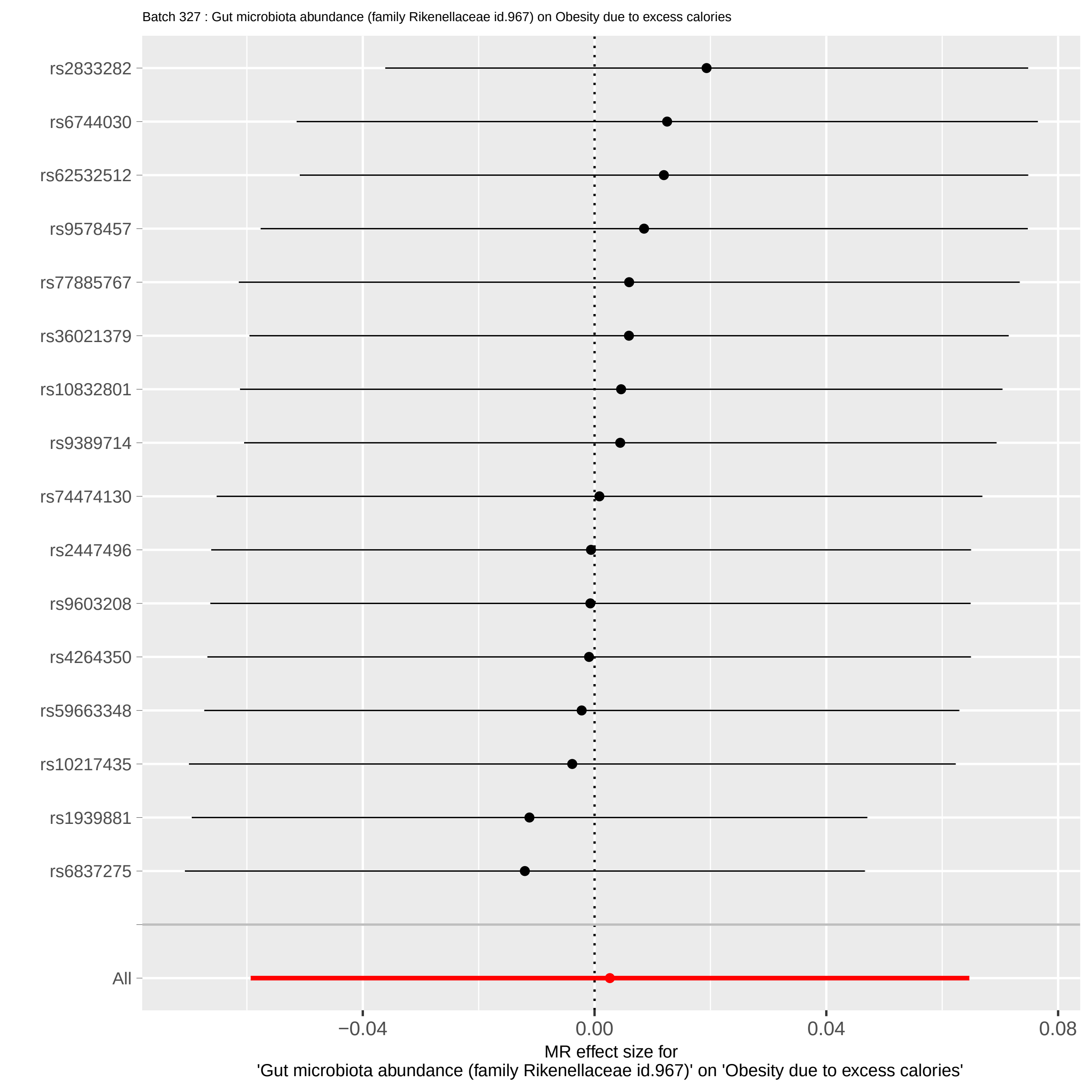

Batch 327 : Gut microbiota abundance (family Rikenellaceae id.967) on Obesity due to excess calories
rs2833282
rs6744030
rs62532512
rs9578457
rs77885767
rs36021379
rs10832801
rs9389714
rs74474130
rs2447496
rs9603208
rs4264350
rs59663348
rs10217435
rs1939881
rs6837275
All
−0.04
0.00
0.04
0.08
MR effect size for
'Gut microbiota abundance (family Rikenellaceae id.967)' on 'Obesity due to excess calories'

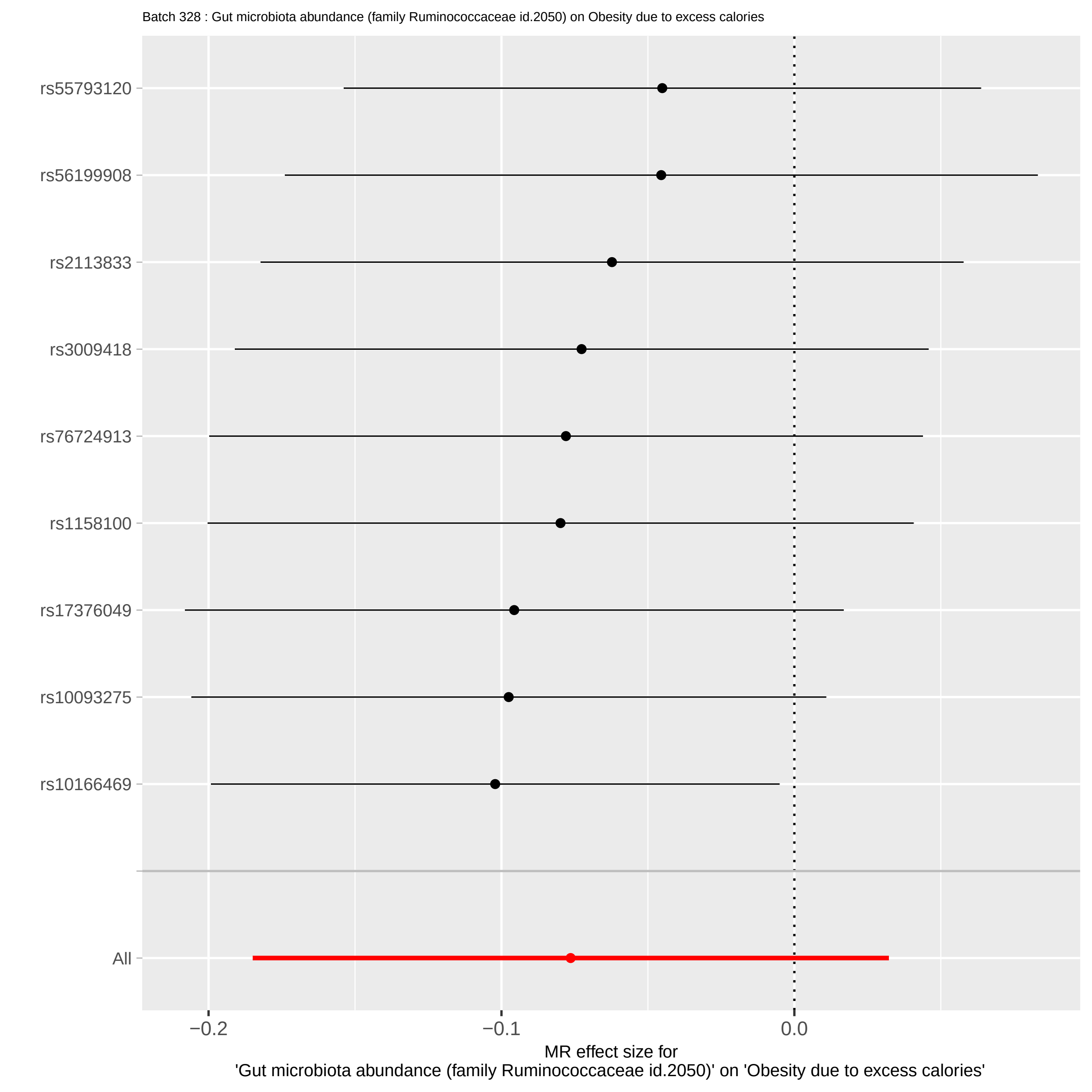
Batch 328 : Gut microbiota abundance (family Ruminococcaceae id.2050) on Obesity due to excess calories
rs55793120
rs56199908
rs2113833
rs3009418
rs76724913
rs1158100
rs17376049
rs10093275
rs10166469
All
−0.2
−0.1
0.0
MR effect size for
'Gut microbiota abundance (family Ruminococcaceae id.2050)' on 'Obesity due to excess calories'

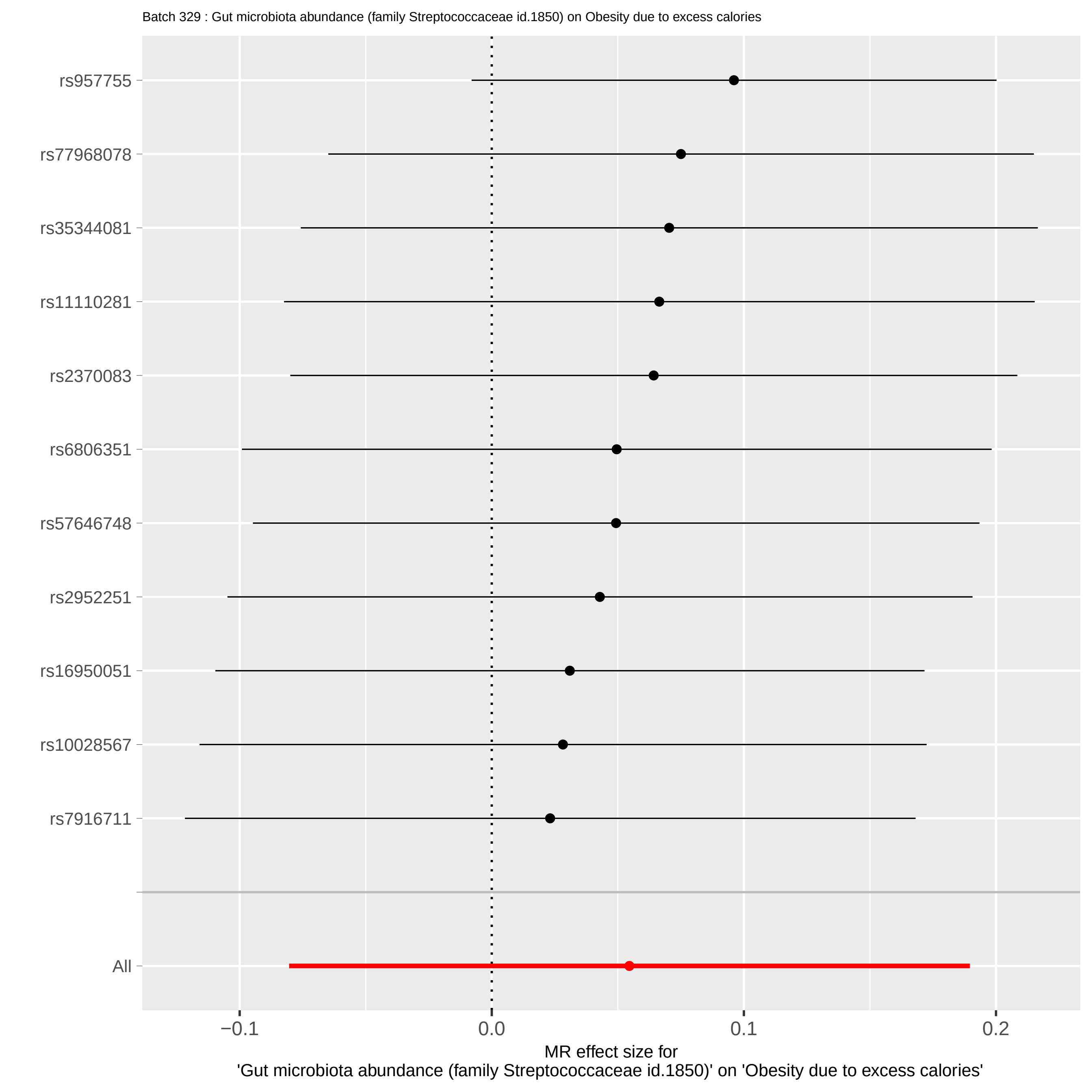

Batch 329 : Gut microbiota abundance (family Streptococcaceae id.1850) on Obesity due to excess calories
rs957755
rs77968078
rs35344081
rs11110281
rs2370083
rs6806351
rs57646748
rs2952251
rs16950051
rs10028567
rs7916711
All
−0.1
0.0
0.1
0.2
MR effect size for
'Gut microbiota abundance (family Streptococcaceae id.1850)' on 'Obesity due to excess calories'

Batch 330 : Gut microbiota abundance (family Veillonellaceae id.2172) on Obesity due to excess calories

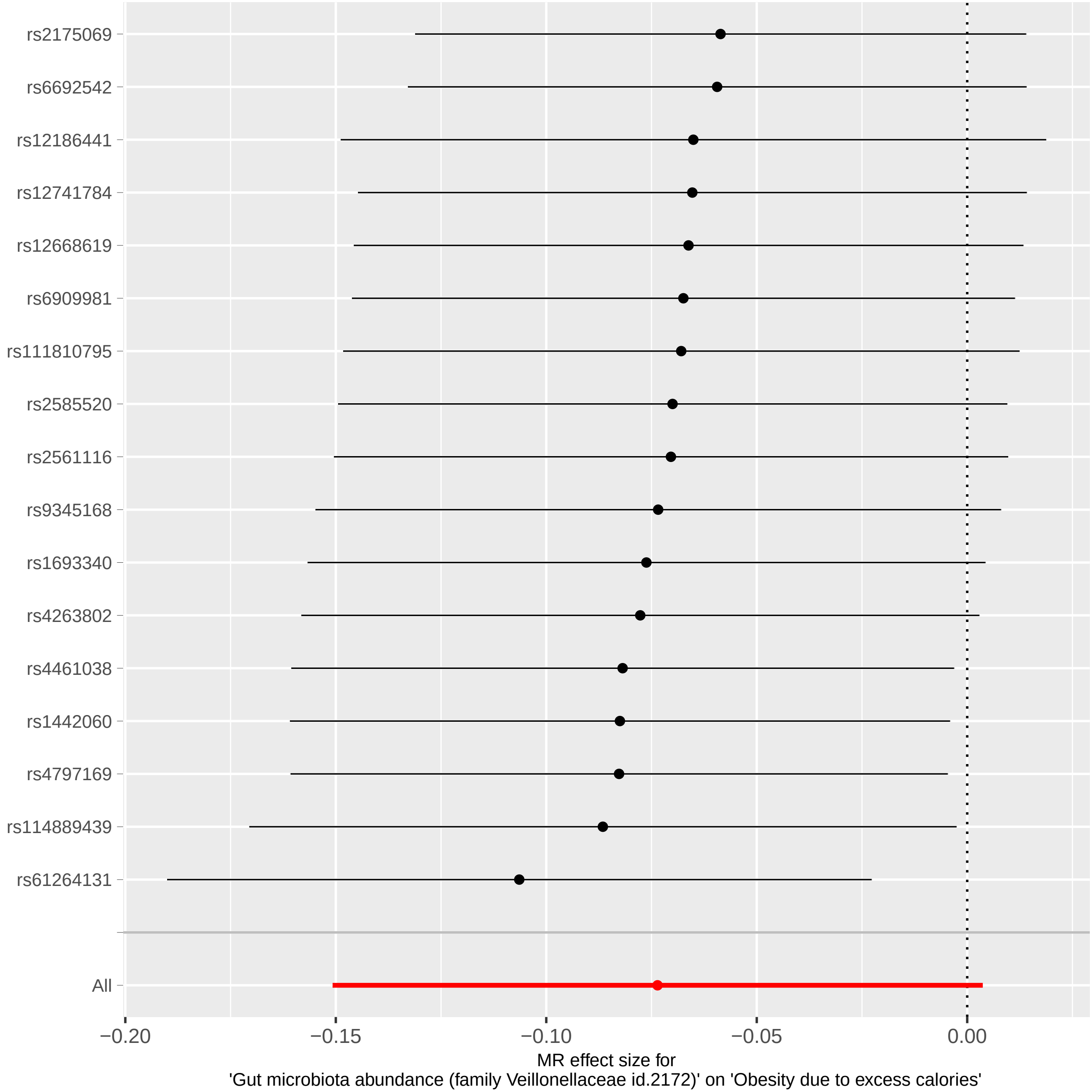

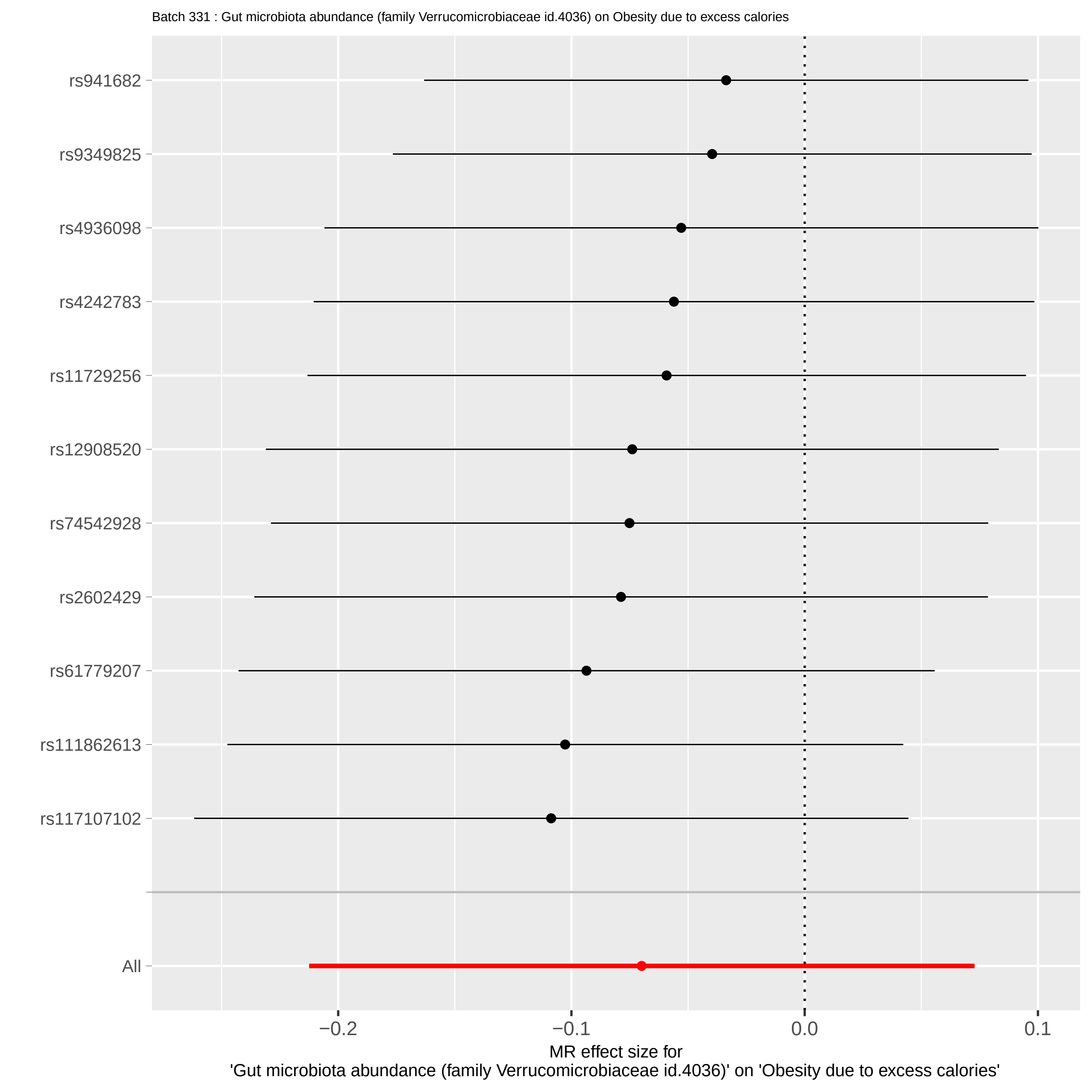

Batch 331 : Gut microbiota abundance (family Verrucomicrobiaceae id.4036) on Obesity due to excess calories
rs941682
rs9349825
rs4936098
rs4242783
rs11729256
rs12908520
rs74542928
rs2602429
rs61779207
rs111862613
rs117107102
All
−0.2
−0.1
0.0
0.1
MR effect size for
'Gut microbiota abundance (family Verrucomicrobiaceae id.4036)' on 'Obesity due to excess calories'

Batch 332 : Gut microbiota abundance (family Victivallaceae id.2255) on Obesity due to excess calories

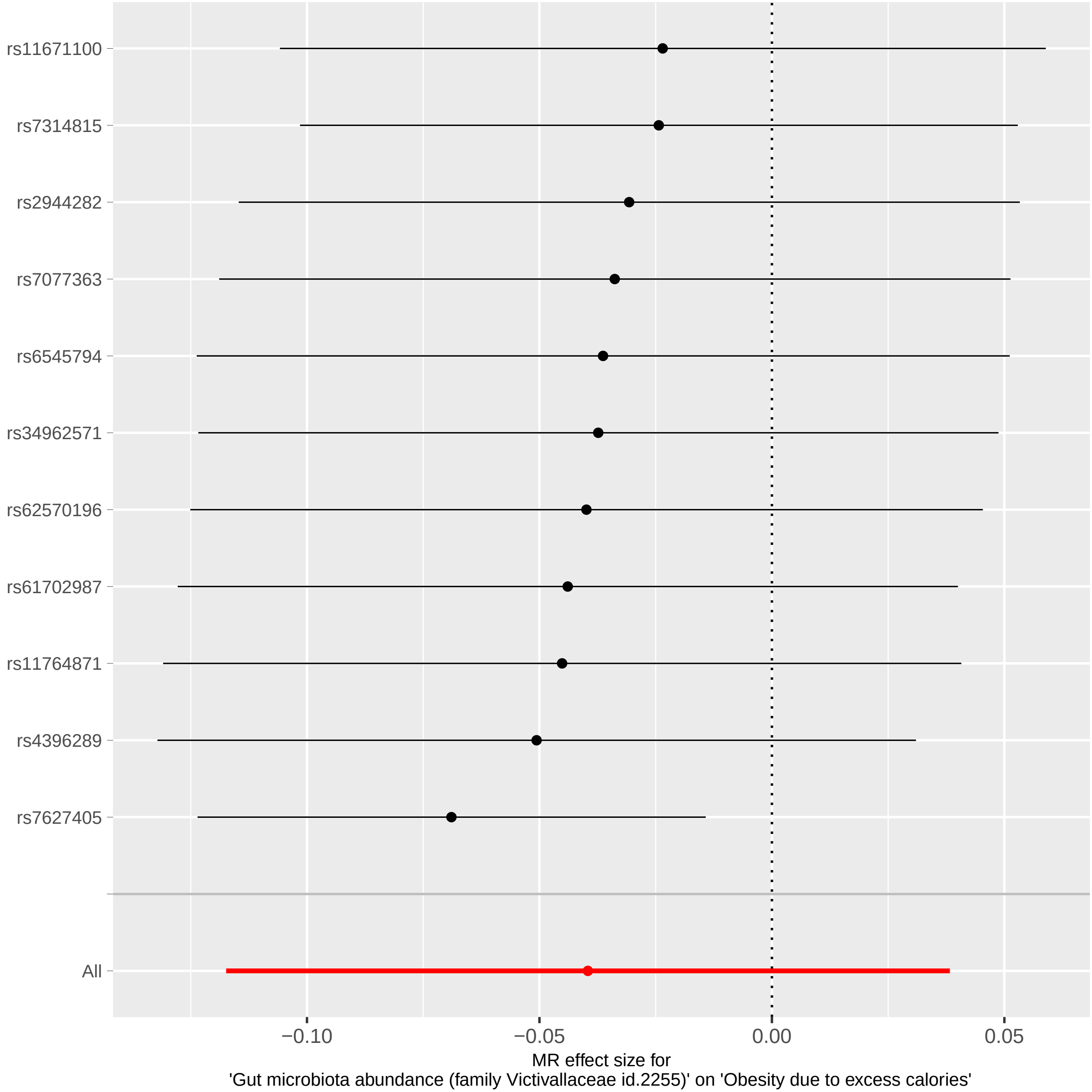

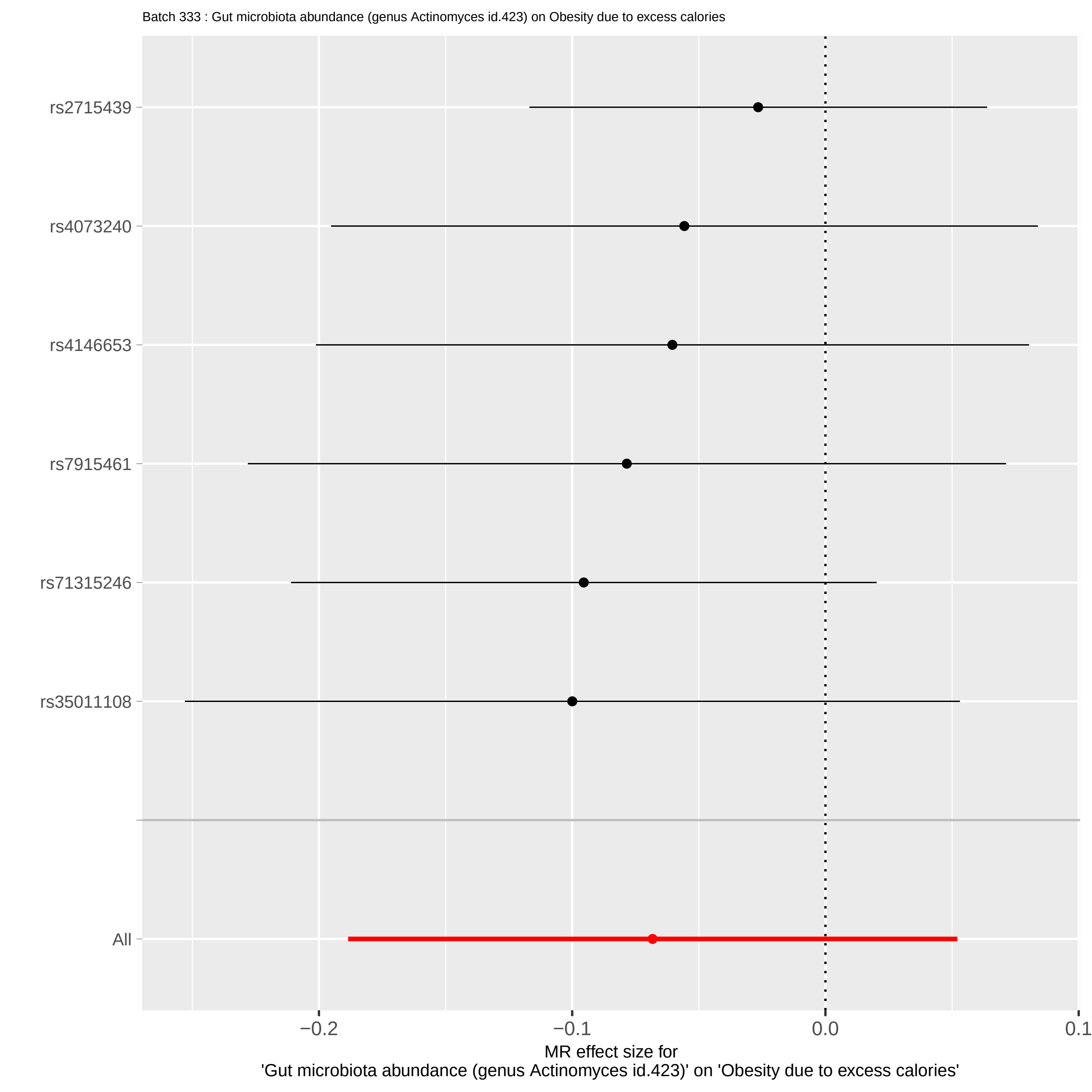

Batch 333 : Gut microbiota abundance (genus Actinomyces id.423) on Obesity due to excess calories
rs2715439
rs4073240
rs4146653
rs7915461
rs71315246
rs35011108
All
−0.2
−0.1
0.0
0.1
MR effect size for
'Gut microbiota abundance (genus Actinomyces id.423)' on 'Obesity due to excess calories'

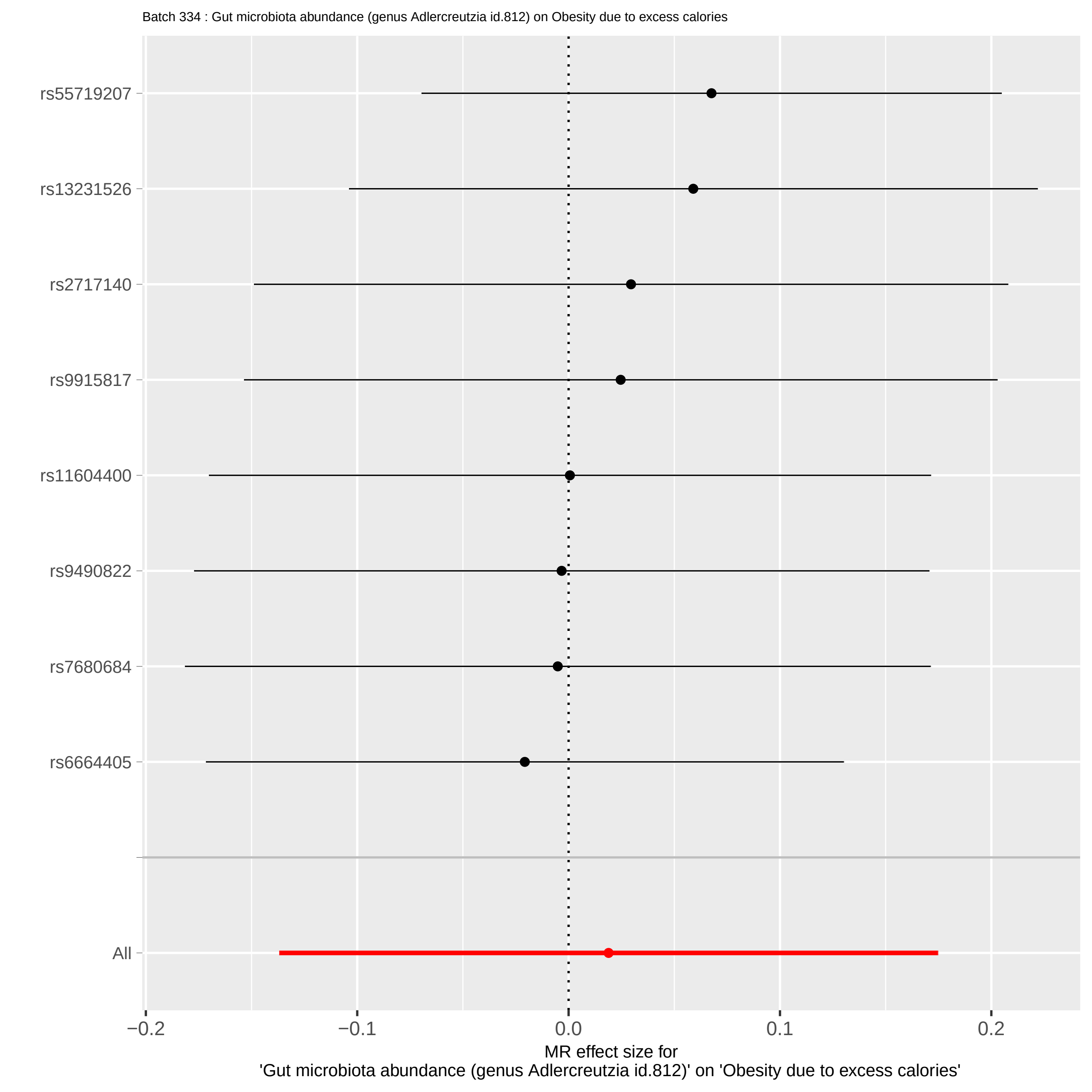
Batch 334 : Gut microbiota abundance (genus Adlercreutzia id.812) on Obesity due to excess calories
rs55719207
rs13231526
rs2717140
rs9915817
rs11604400
rs9490822
rs7680684
rs6664405
All
−0.2
−0.1
0.0
0.1
0.2
MR effect size for
'Gut microbiota abundance (genus Adlercreutzia id.812)' on 'Obesity due to excess calories'

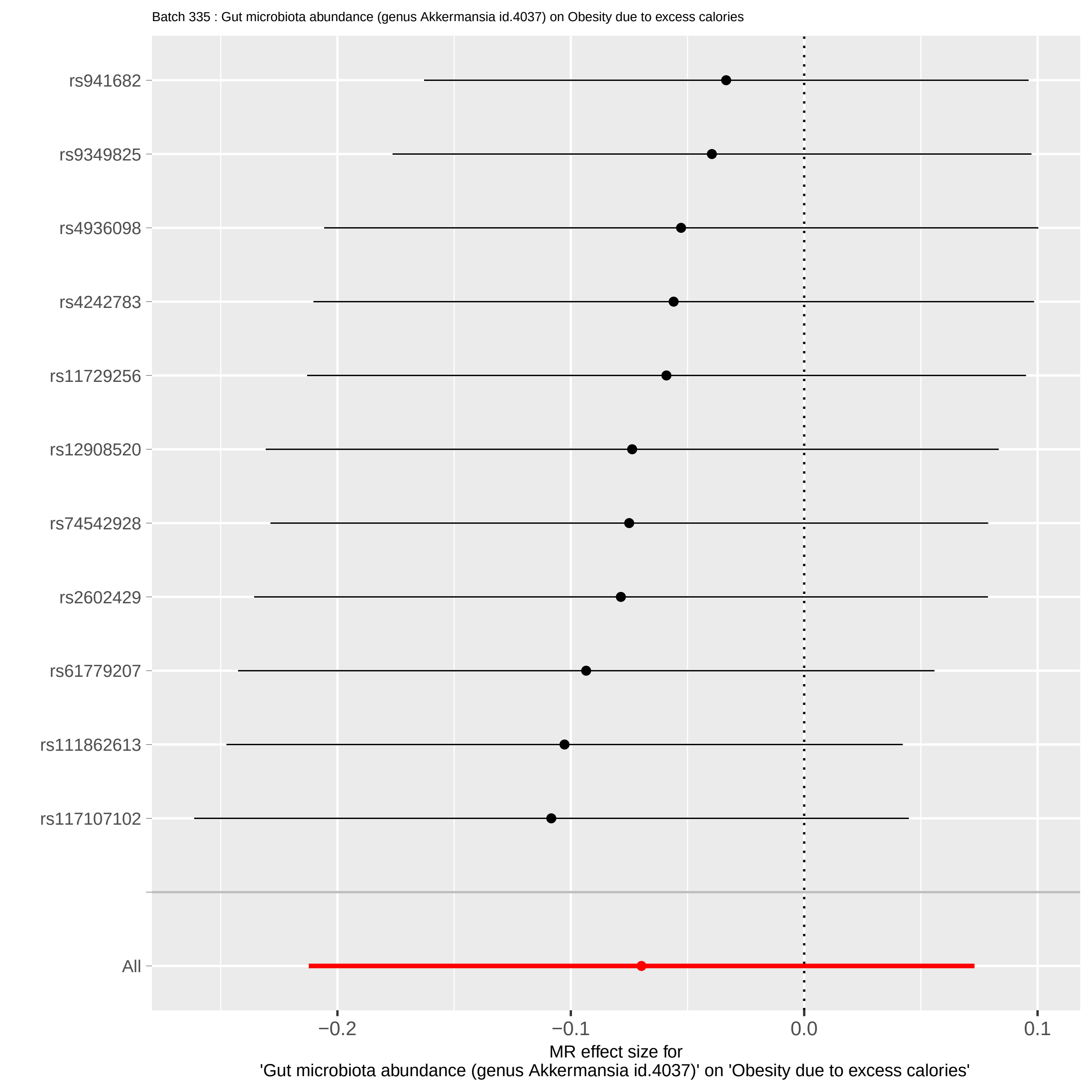

Batch 335 : Gut microbiota abundance (genus Akkermansia id.4037) on Obesity due to excess calories
rs941682
rs9349825
rs4936098
rs4242783
rs11729256
rs12908520
rs74542928
rs2602429
rs61779207
rs111862613
rs117107102
All
−0.2
−0.1
0.0
0.1
MR effect size for
'Gut microbiota abundance (genus Akkermansia id.4037)' on 'Obesity due to excess calories'

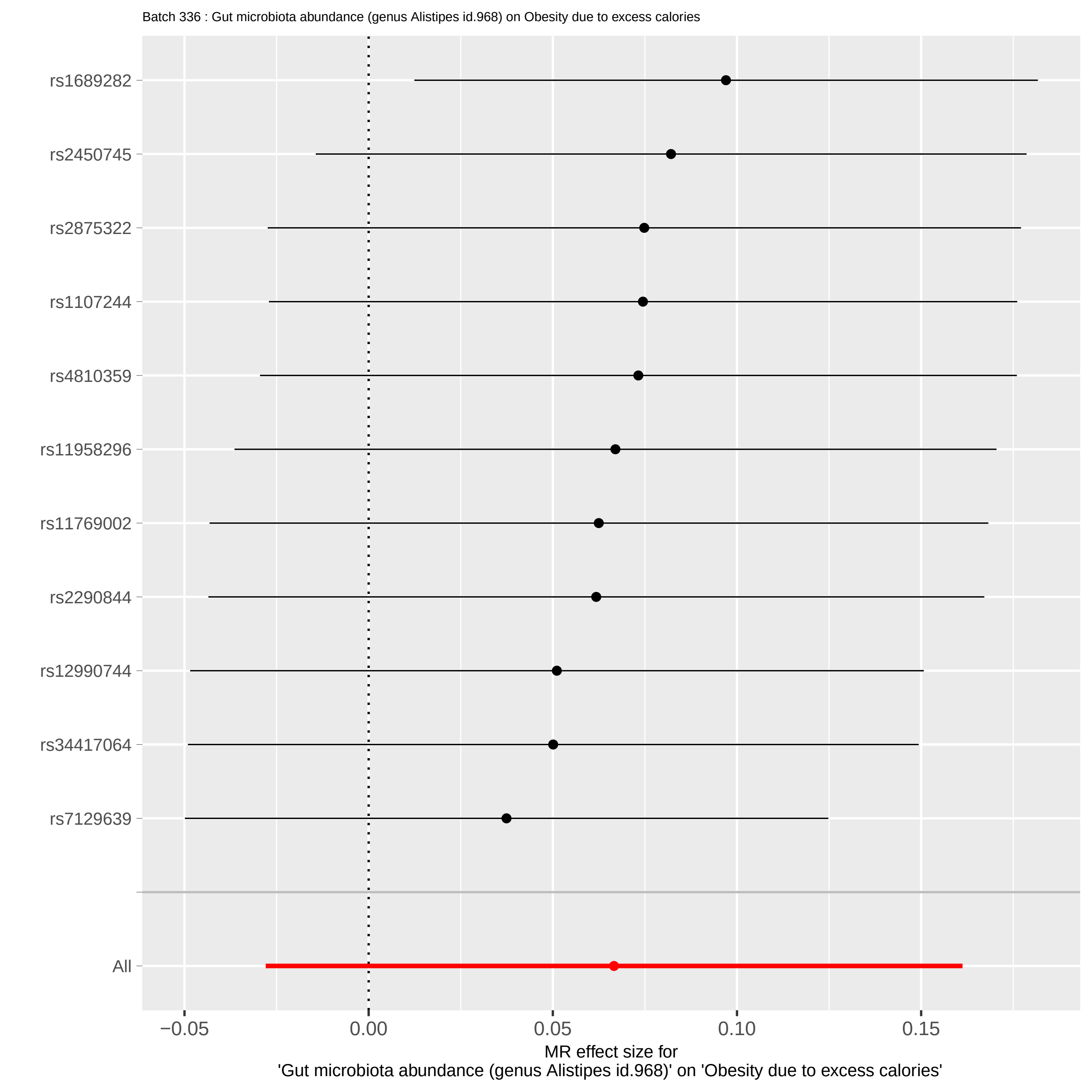
Batch 336 : Gut microbiota abundance (genus Alistipes id.968) on Obesity due to excess calories
rs1689282
rs2450745
rs2875322
rs1107244
rs4810359
rs11958296
rs11769002
rs2290844
rs12990744
rs34417064
rs7129639
All
−0.05
0.00
0.05
0.10
0.15
MR effect size for
'Gut microbiota abundance (genus Alistipes id.968)' on 'Obesity due to excess calories'

Batch 337 : Gut microbiota abundance (genus Allisonella id.2174) on Obesity due to excess calories

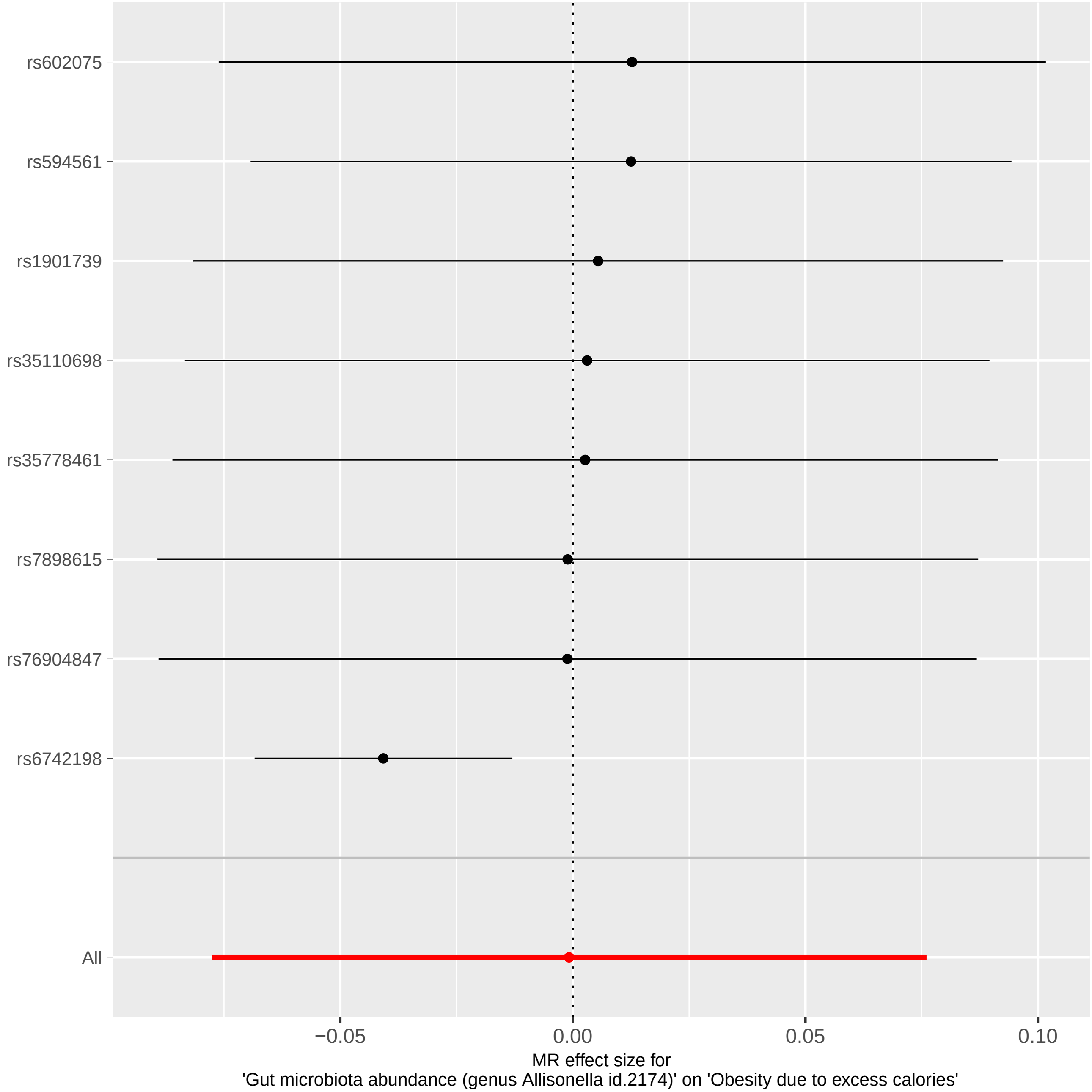

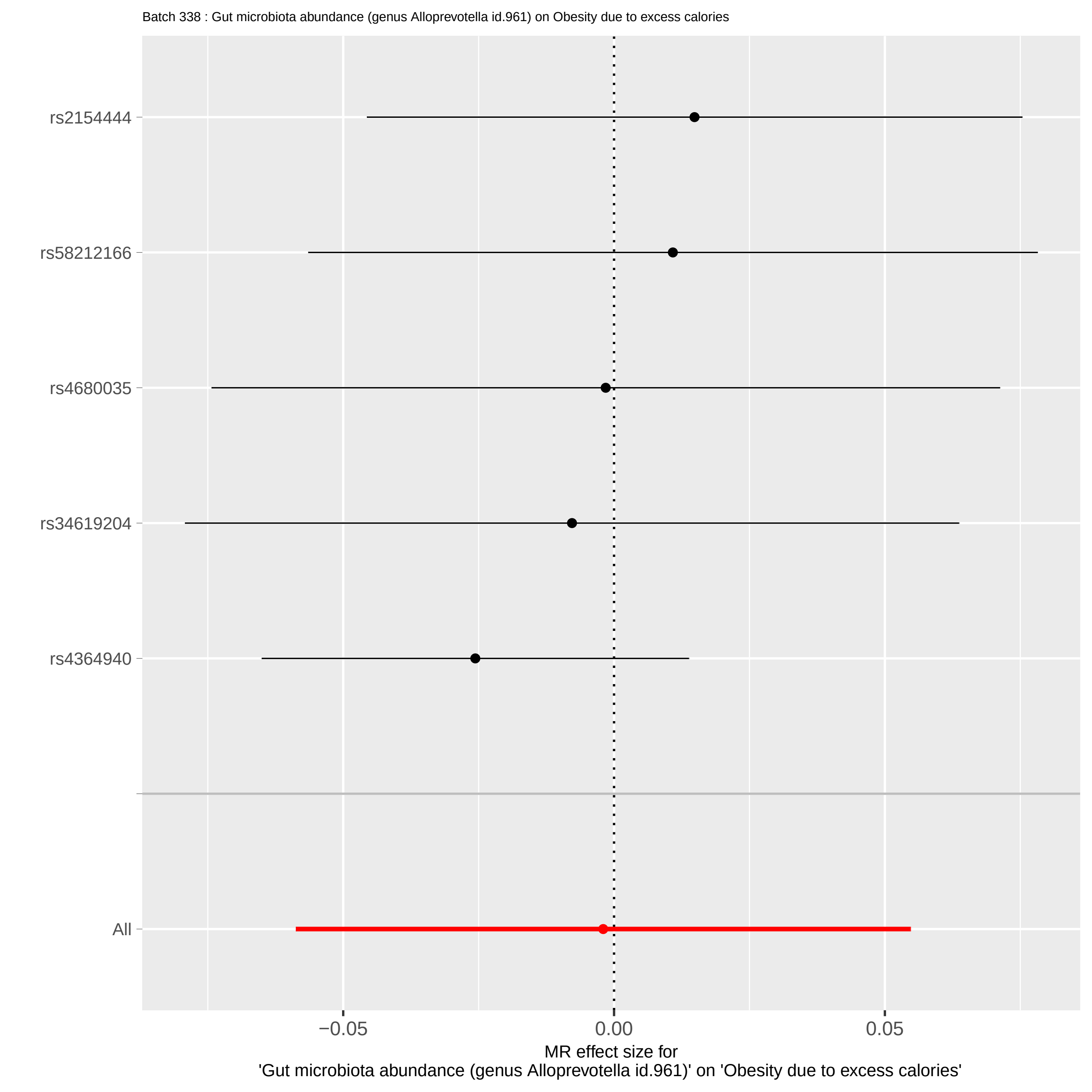

Batch 338 : Gut microbiota abundance (genus Alloprevotella id.961) on Obesity due to excess calories
rs2154444
rs58212166
rs4680035
rs34619204
rs4364940
All
−0.05
0.00
0.05
MR effect size for
'Gut microbiota abundance (genus Alloprevotella id.961)' on 'Obesity due to excess calories'

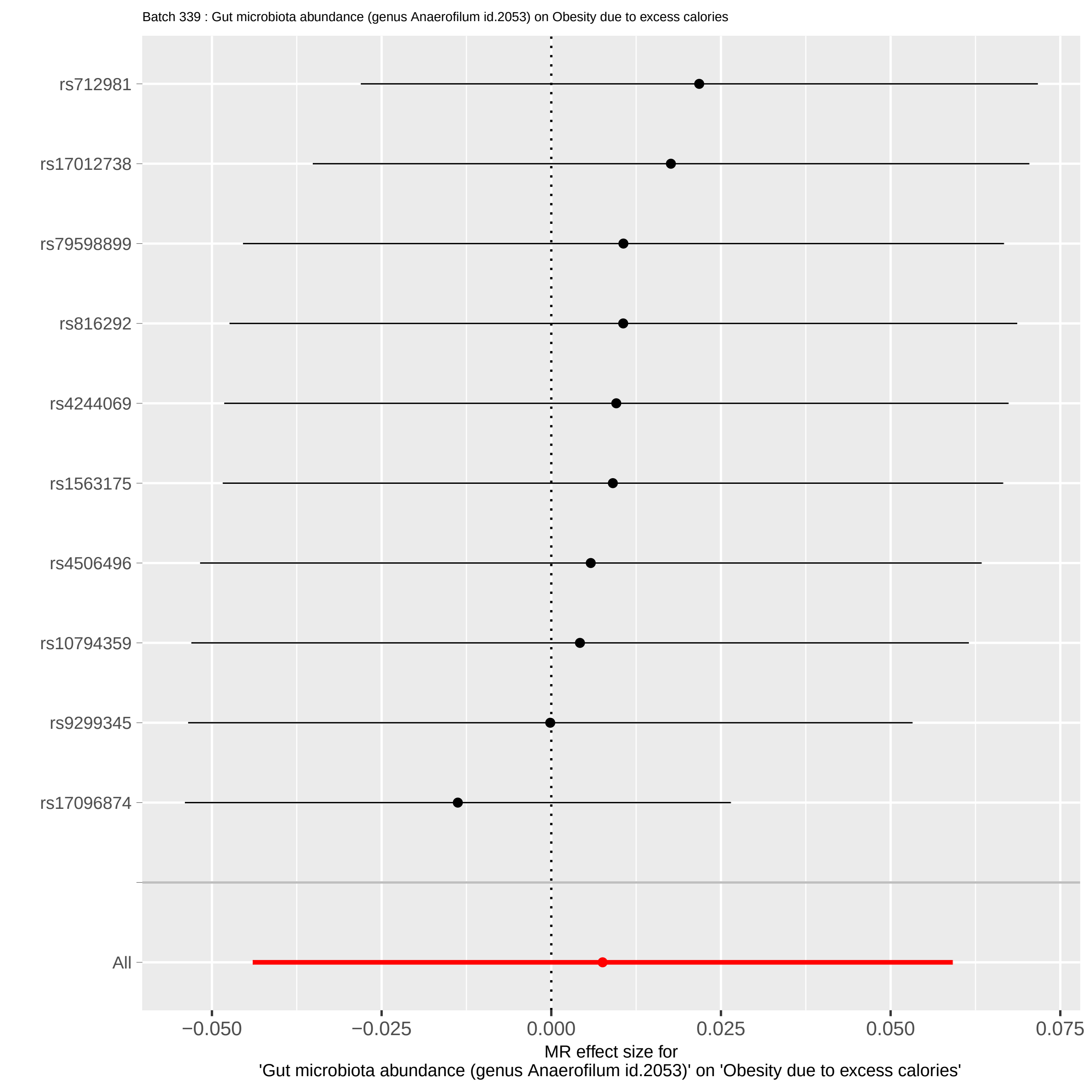
Batch 339 : Gut microbiota abundance (genus Anaerofilum id.2053) on Obesity due to excess calories
rs712981
rs17012738
rs79598899
rs816292
rs4244069
rs1563175
rs4506496
rs10794359
rs9299345
rs17096874
All
−0.050
−0.025
0.000
0.025
0.050
0.075
MR effect size for
'Gut microbiota abundance (genus Anaerofilum id.2053)' on 'Obesity due to excess calories'

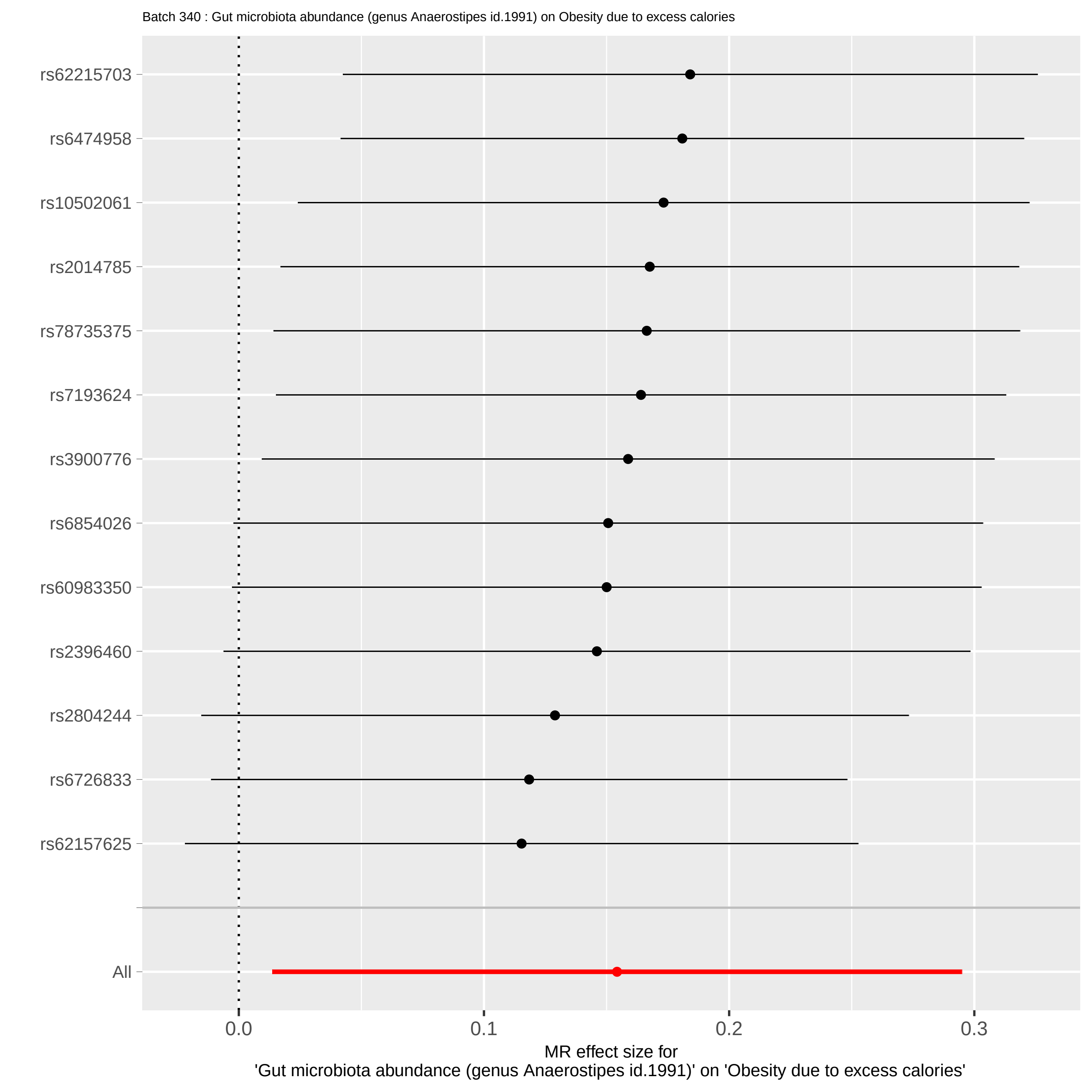

Batch 340 : Gut microbiota abundance (genus Anaerostipes id.1991) on Obesity due to excess calories
rs62215703
rs6474958
rs10502061
rs2014785
rs78735375
rs7193624
rs3900776
rs6854026
rs60983350
rs2396460
rs2804244
rs6726833
rs62157625
All
0.0
0.1
0.2
0.3
MR effect size for
'Gut microbiota abundance (genus Anaerostipes id.1991)' on 'Obesity due to excess calories'

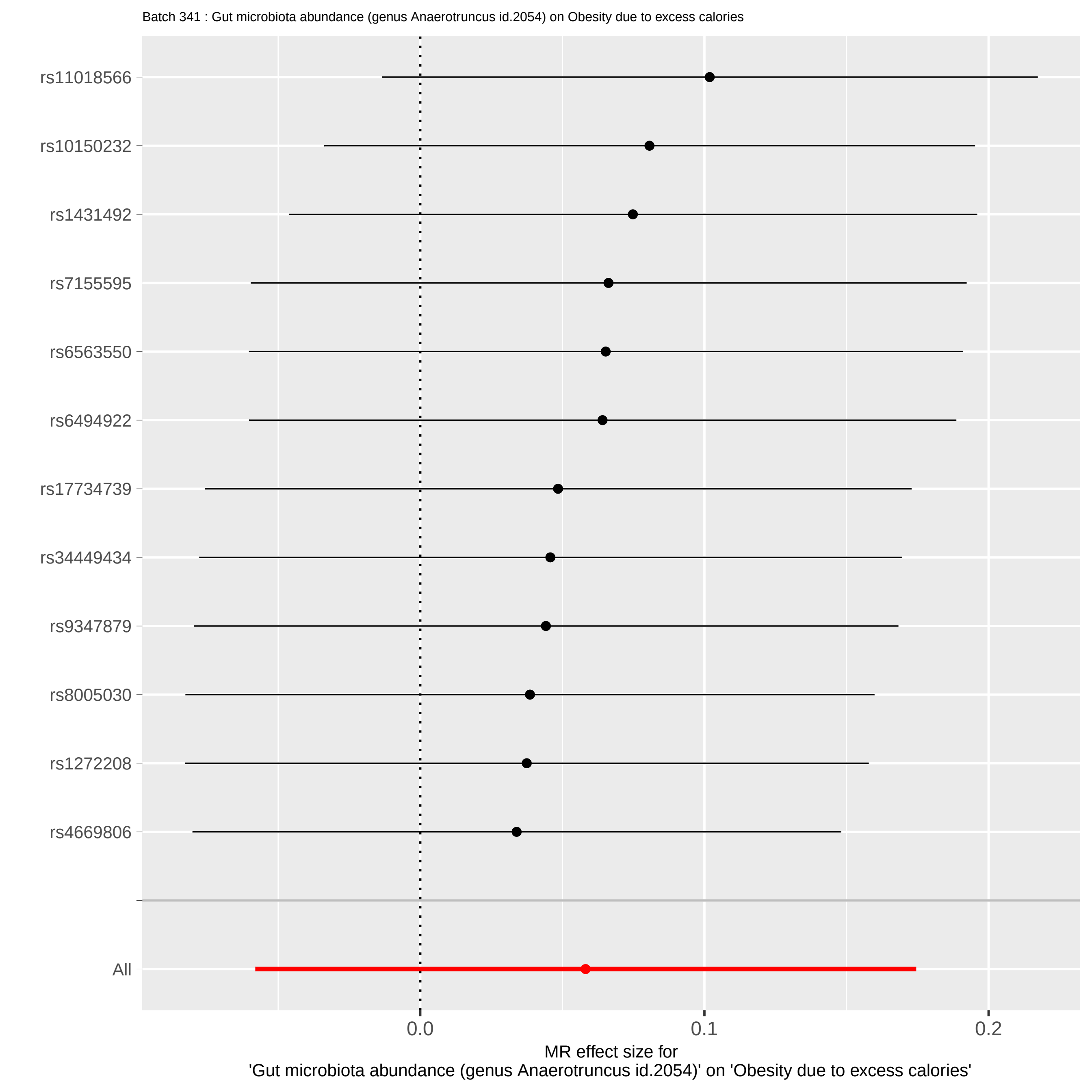

Batch 341 : Gut microbiota abundance (genus Anaerotruncus id.2054) on Obesity due to excess calories
rs11018566
rs10150232
rs1431492
rs7155595
rs6563550
rs6494922
rs17734739
rs34449434
rs9347879
rs8005030
rs1272208
rs4669806
All
0.0
0.1
0.2
MR effect size for
'Gut microbiota abundance (genus Anaerotruncus id.2054)' on 'Obesity due to excess calories'

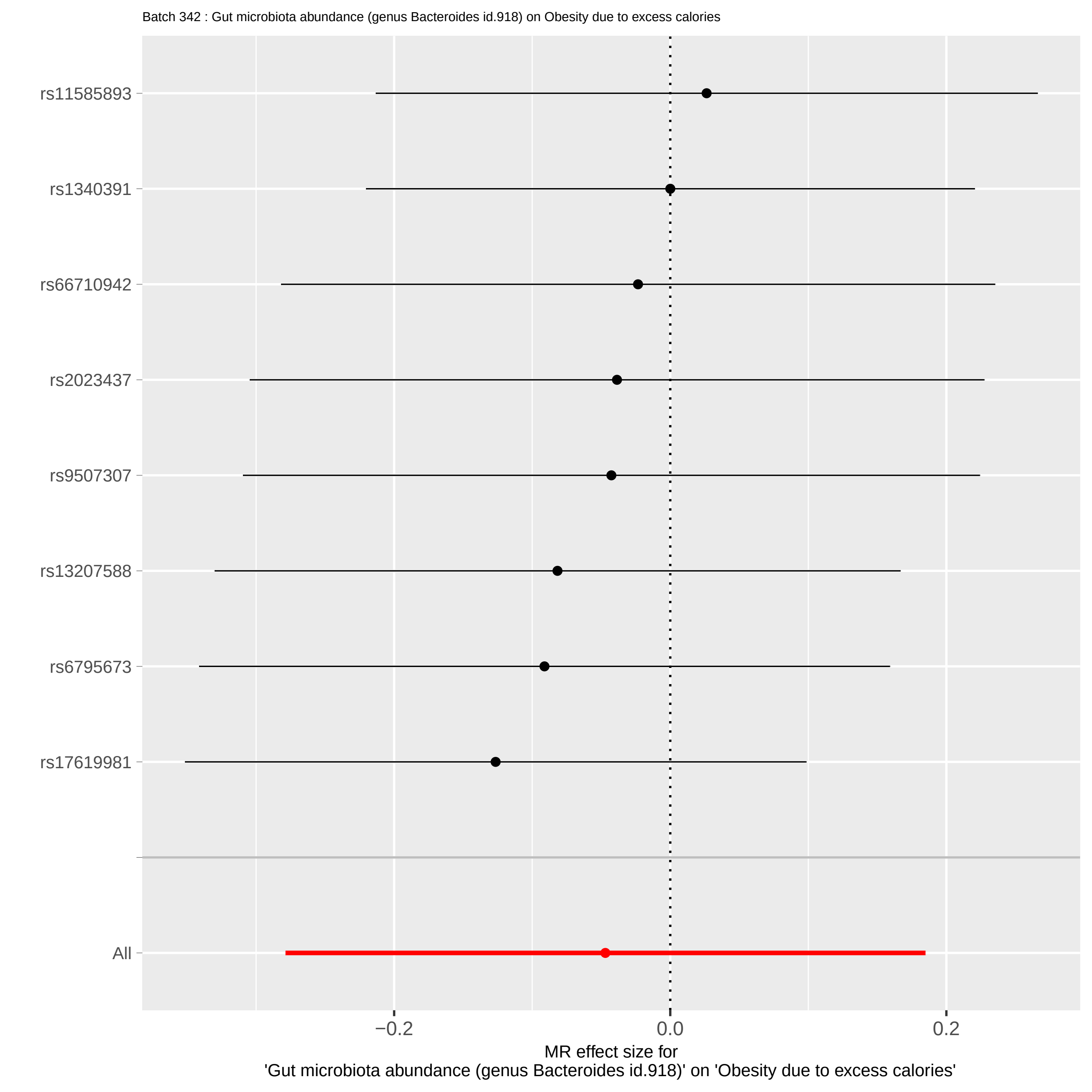
Batch 342 : Gut microbiota abundance (genus Bacteroides id.918) on Obesity due to excess calories
rs11585893
rs1340391
rs66710942
rs2023437
rs9507307
rs13207588
rs6795673
rs17619981
All
−0.2
0.0
0.2
MR effect size for
'Gut microbiota abundance (genus Bacteroides id.918)' on 'Obesity due to excess calories'

Batch 343 : Gut microbiota abundance (genus Barnesiella id.944) on Obesity due to excess calories

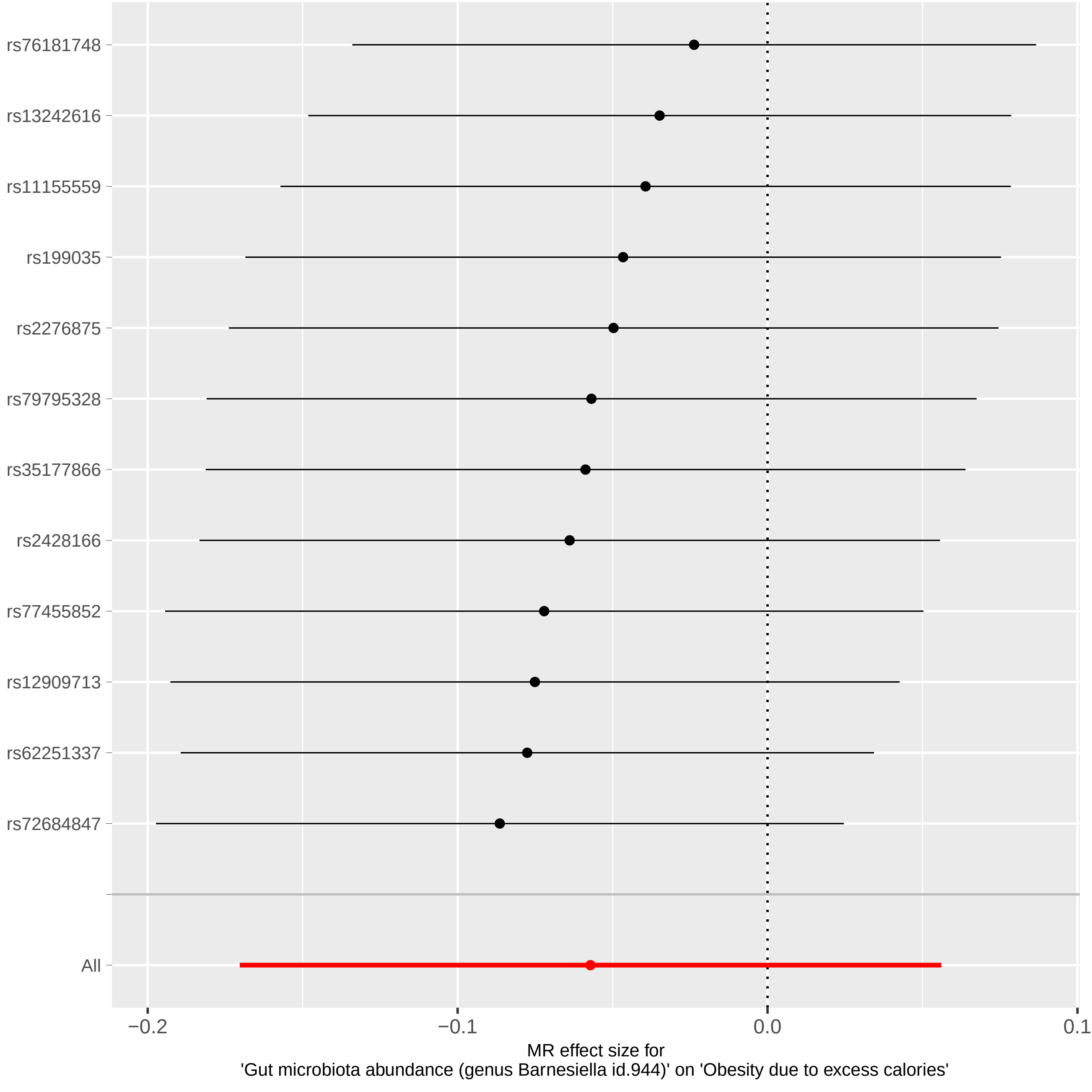

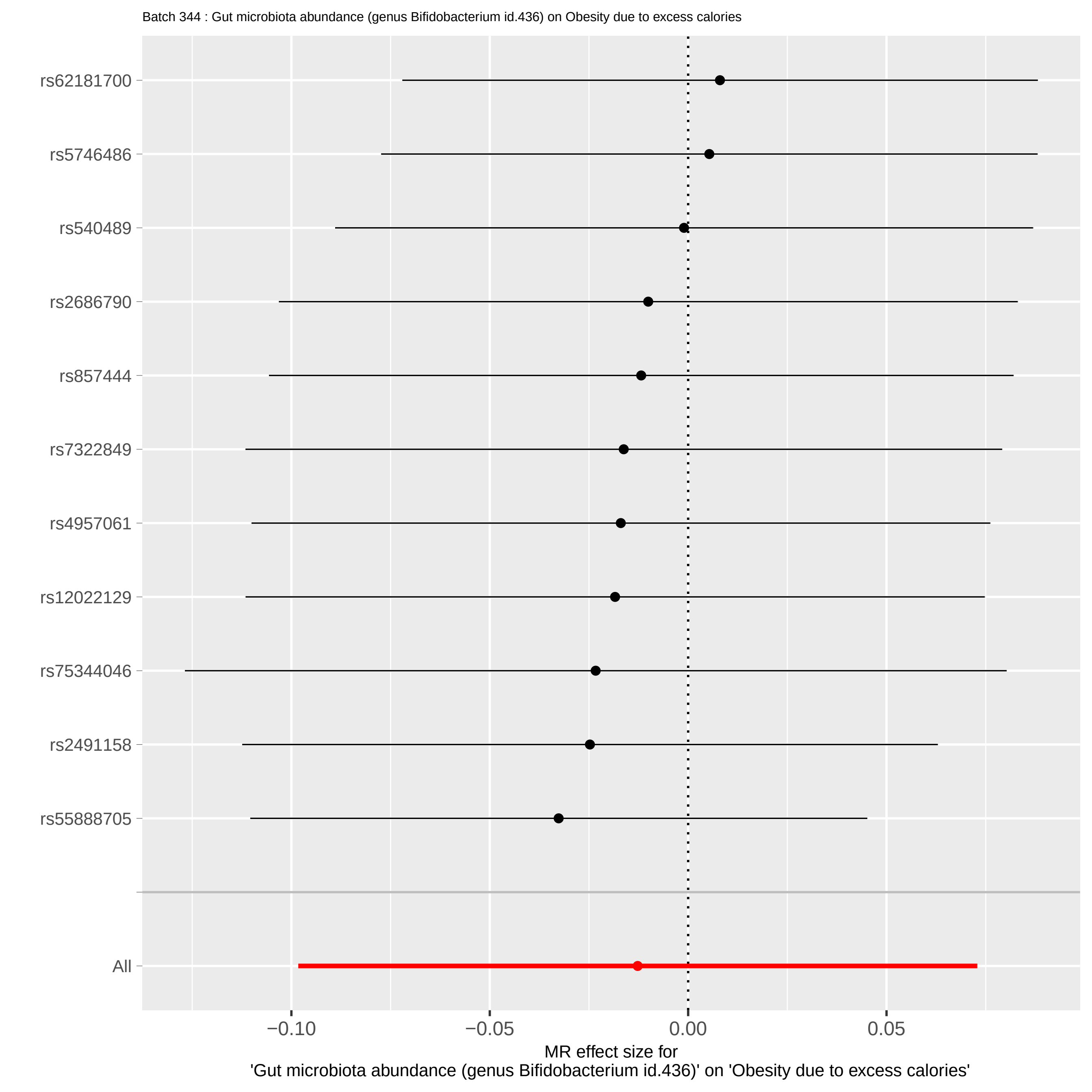
Batch 344 : Gut microbiota abundance (genus Bifidobacterium id.436) on Obesity due to excess calories
rs62181700
rs5746486
rs540489
rs2686790
rs857444
rs7322849
rs4957061
rs12022129
rs75344046
rs2491158
rs55888705
All
−0.10
−0.05
0.00
0.05
MR effect size for
'Gut microbiota abundance (genus Bifidobacterium id.436)' on 'Obesity due to excess calories'

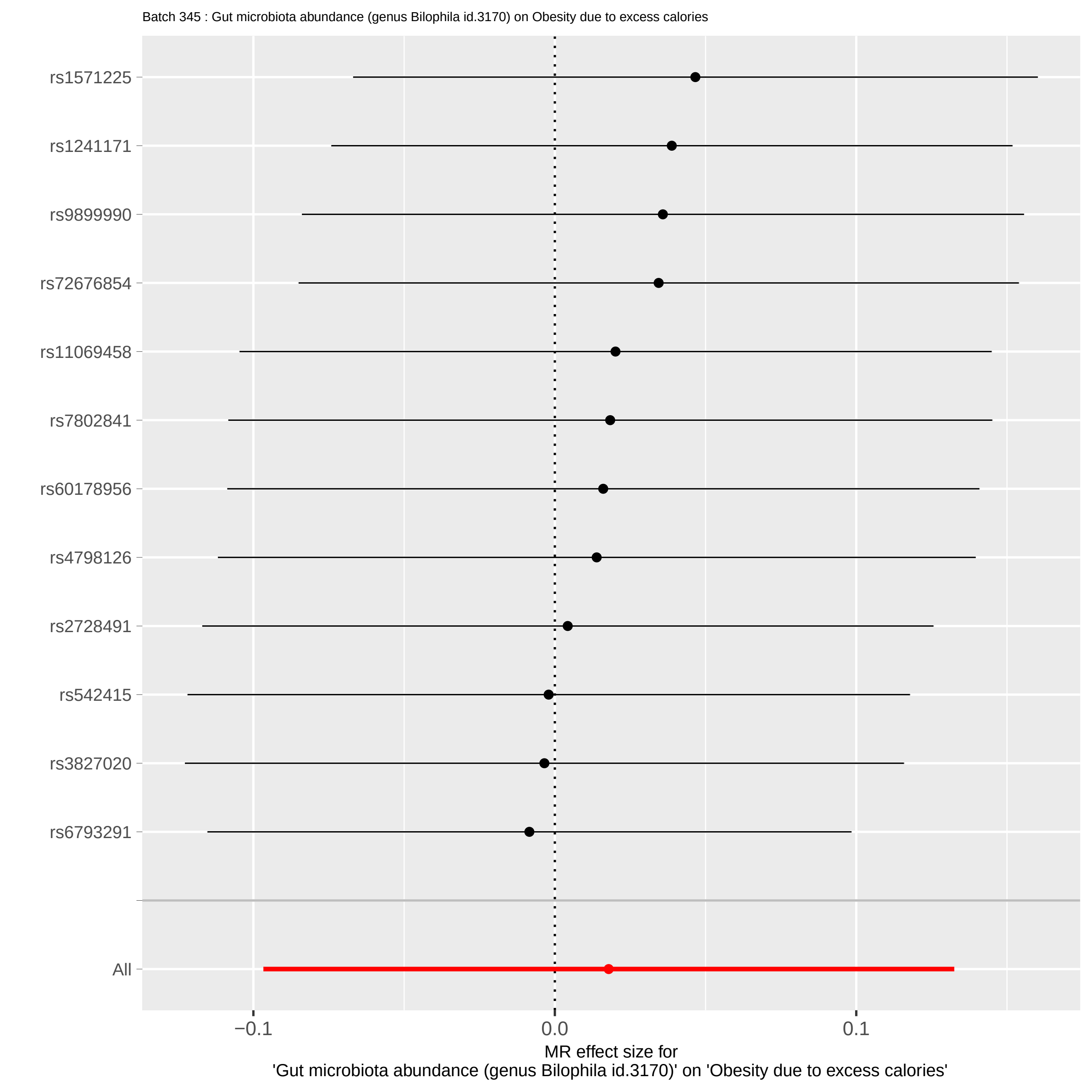
Batch 345 : Gut microbiota abundance (genus Bilophila id.3170) on Obesity due to excess calories
rs1571225
rs1241171
rs9899990
rs72676854
rs11069458
rs7802841
rs60178956
rs4798126
rs2728491
rs542415
rs3827020
rs6793291
All
−0.1
0.0
0.1
MR effect size for
'Gut microbiota abundance (genus Bilophila id.3170)' on 'Obesity due to excess calories'

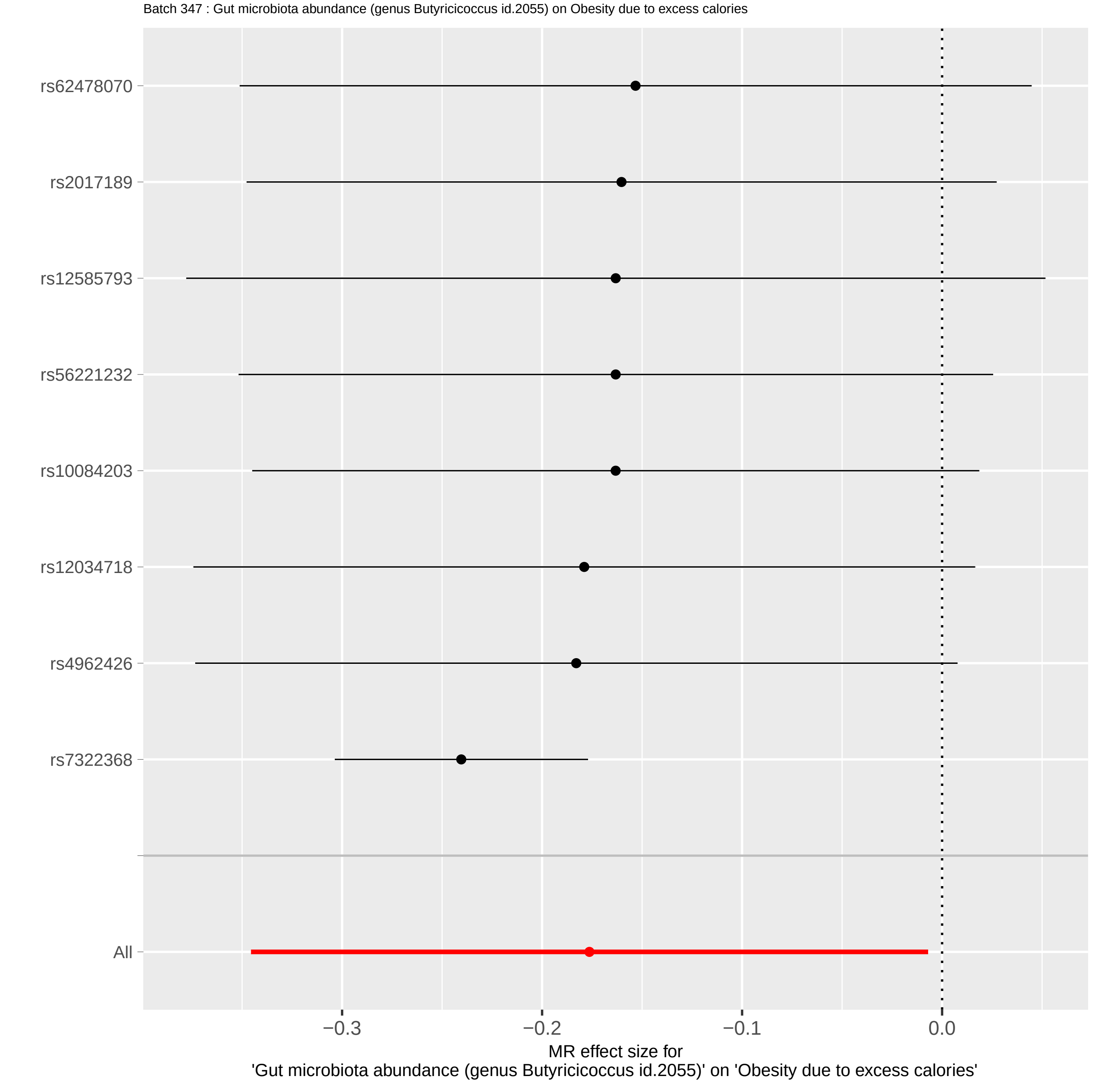
Batch 347 : Gut microbiota abundance (genus Butyricicoccus id.2055) on Obesity due to excess calories
rs62478070
rs2017189
rs12585793
rs56221232
rs10084203
rs12034718
rs4962426
rs7322368
All
−0.3
−0.2
−0.1
0.0
MR effect size for
'Gut microbiota abundance (genus Butyricicoccus id.2055)' on 'Obesity due to excess calories'

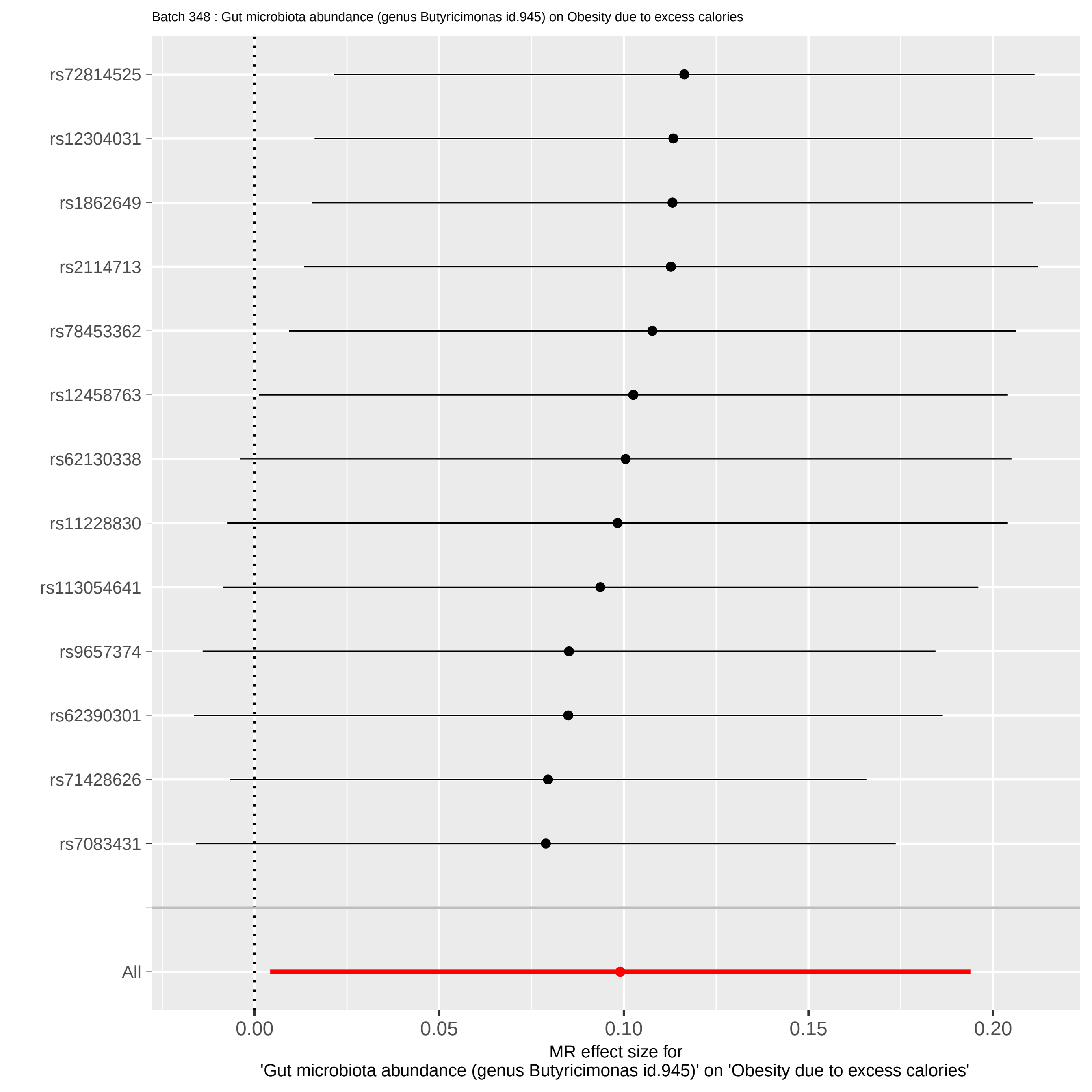

Batch 348 : Gut microbiota abundance (genus Butyricimonas id.945) on Obesity due to excess calories
rs72814525
rs12304031
rs1862649
rs2114713
rs78453362
rs12458763
rs62130338
rs11228830
rs113054641
rs9657374
rs62390301
rs71428626
rs7083431
All
0.00
0.05
0.10
0.15
0.20
MR effect size for
'Gut microbiota abundance (genus Butyricimonas id.945)' on 'Obesity due to excess calories'

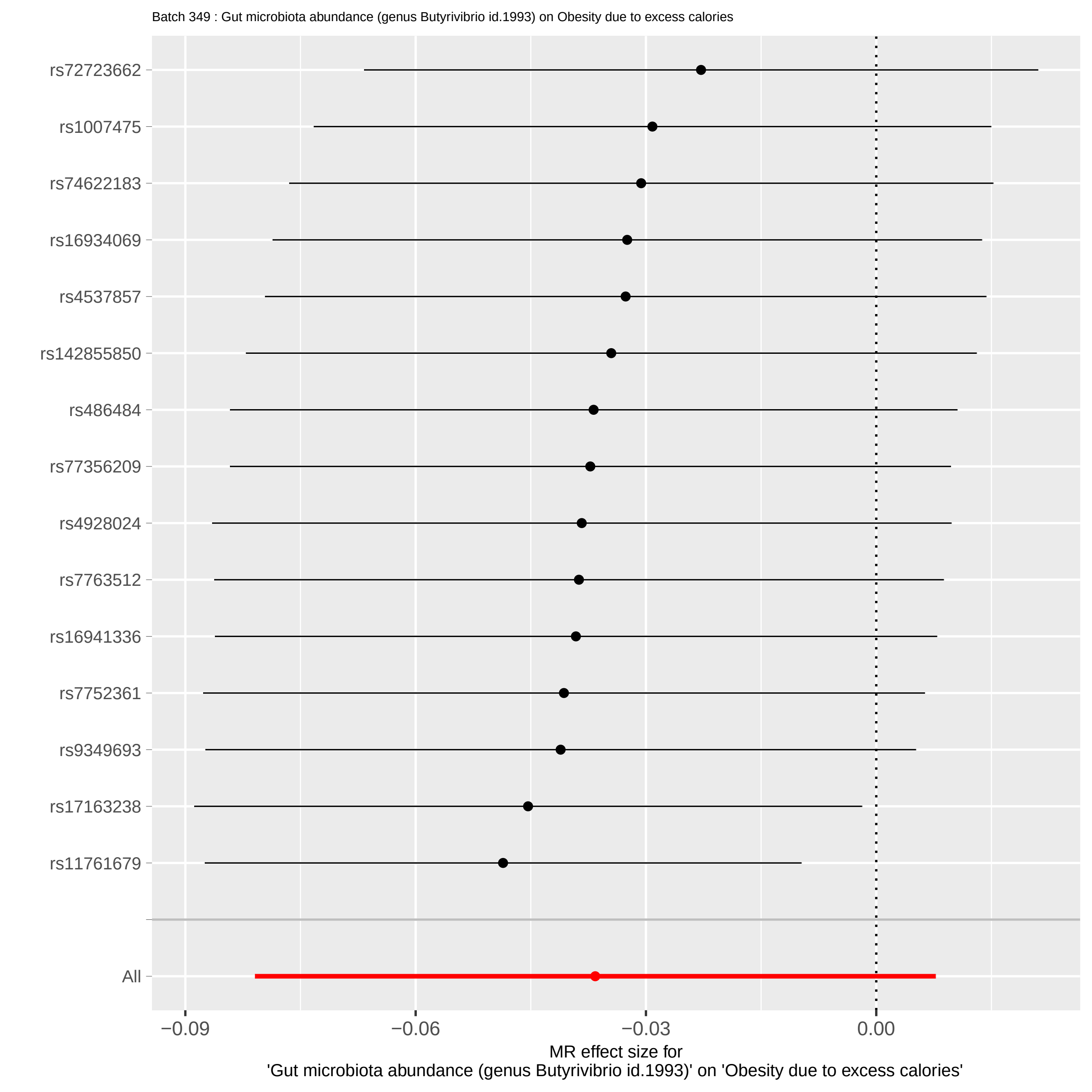

Batch 349 : Gut microbiota abundance (genus Butyrivibrio id.1993) on Obesity due to excess calories
rs72723662
rs1007475
rs74622183
rs16934069
rs4537857
rs142855850
rs486484
rs77356209
rs4928024
rs7763512
rs16941336
rs7752361
rs9349693
rs17163238
rs11761679
All
−0.09
−0.06
−0.03
0.00
MR effect size for
'Gut microbiota abundance (genus Butyrivibrio id.1993)' on 'Obesity due to excess calories'

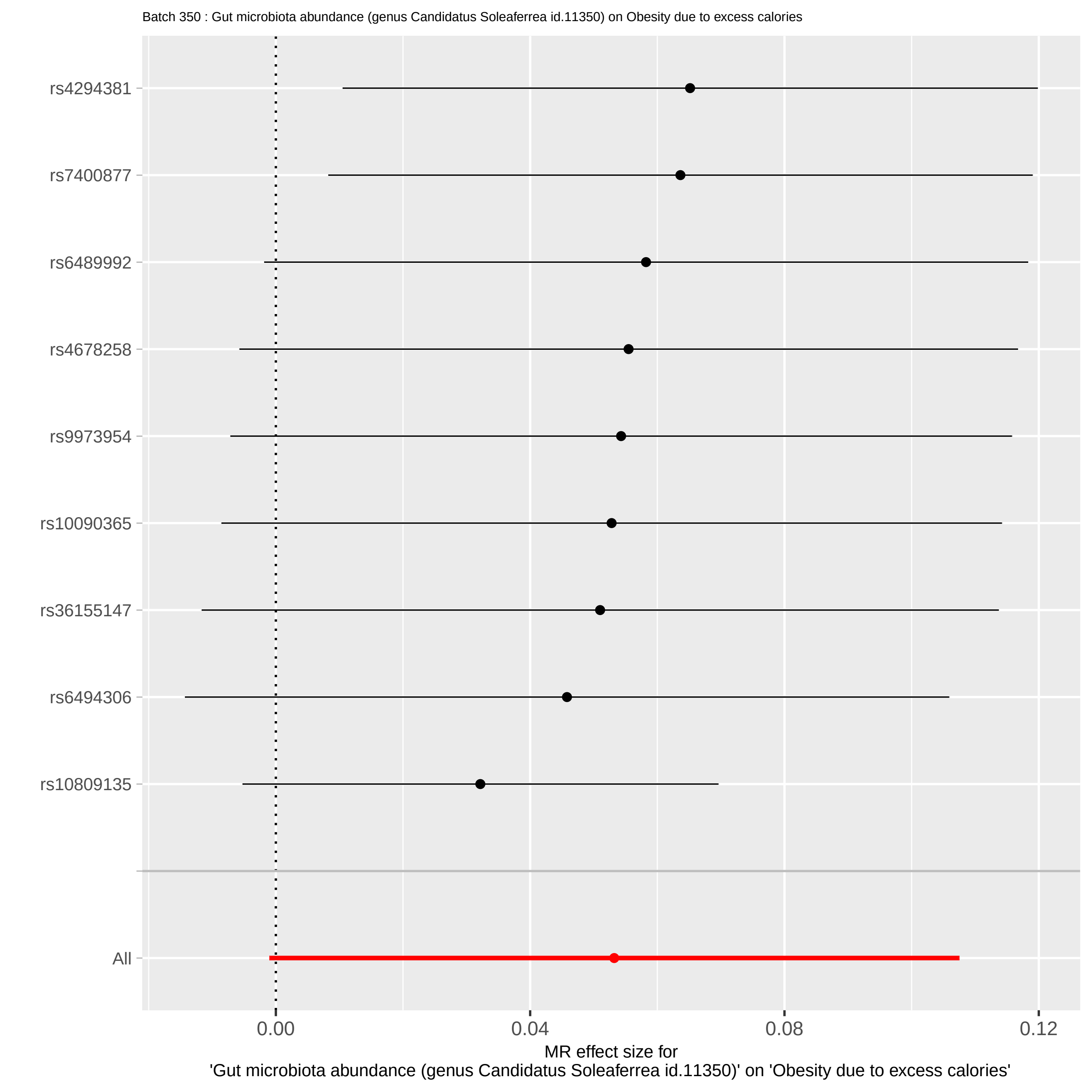
Batch 350 : Gut microbiota abundance (genus Candidatus Soleaferrea id.11350) on Obesity due to excess calories
rs4294381
rs7400877
rs6489992
rs4678258
rs9973954
rs10090365
rs36155147
rs6494306
rs10809135
All
0.00
0.04
0.08
0.12
MR effect size for
'Gut microbiota abundance (genus Candidatus Soleaferrea id.11350)' on 'Obesity due to excess calories'

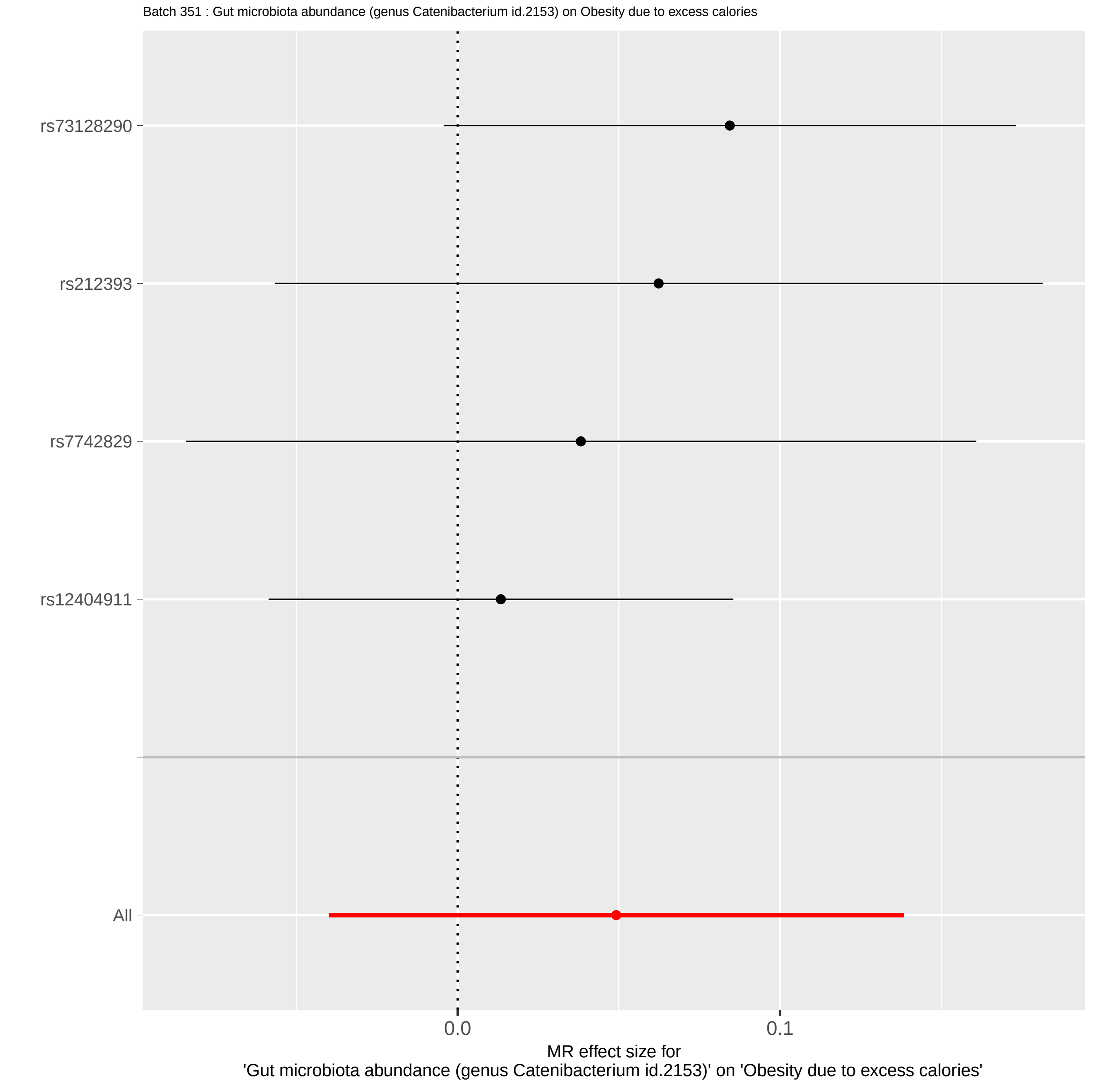

Batch 351 : Gut microbiota abundance (genus Catenibacterium id.2153) on Obesity due to excess calories
rs73128290
rs212393
rs7742829
rs12404911
All
0.0
0.1
MR effect size for
'Gut microbiota abundance (genus Catenibacterium id.2153)' on 'Obesity due to excess calories'

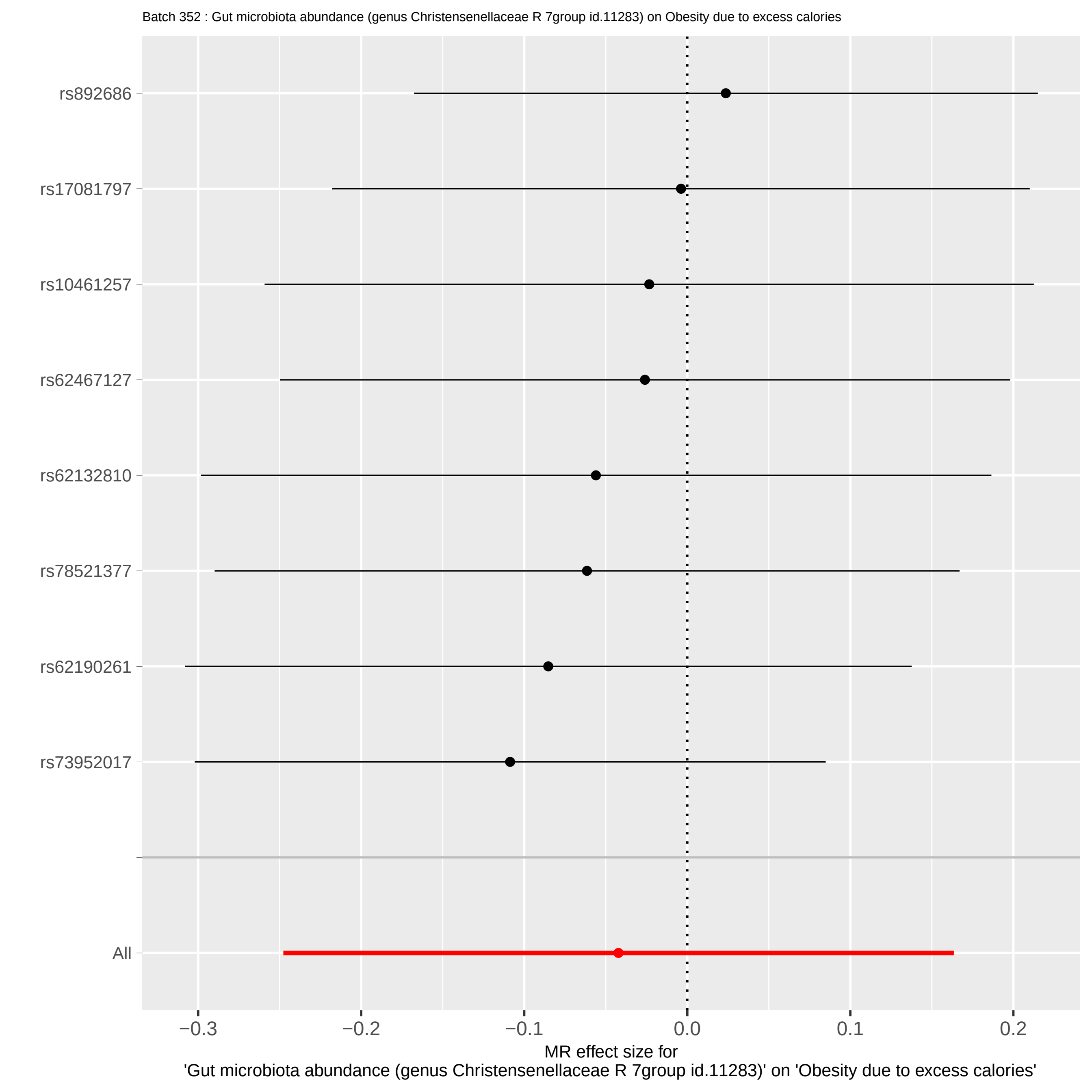
Batch 352 : Gut microbiota abundance (genus Christensenellaceae R 7group id.11283) on Obesity due to excess calories
rs892686
rs17081797
rs10461257
rs62467127
rs62132810
rs78521377
rs62190261
rs73952017
All
−0.3
−0.2
−0.1
0.0
0.1
0.2
MR effect size for
'Gut microbiota abundance (genus Christensenellaceae R 7group id.11283)' on 'Obesity due to excess calories'

Batch 353 : Gut microbiota abundance (genus Clostridium innocuum group id.14397) on Obesity due to excess calories

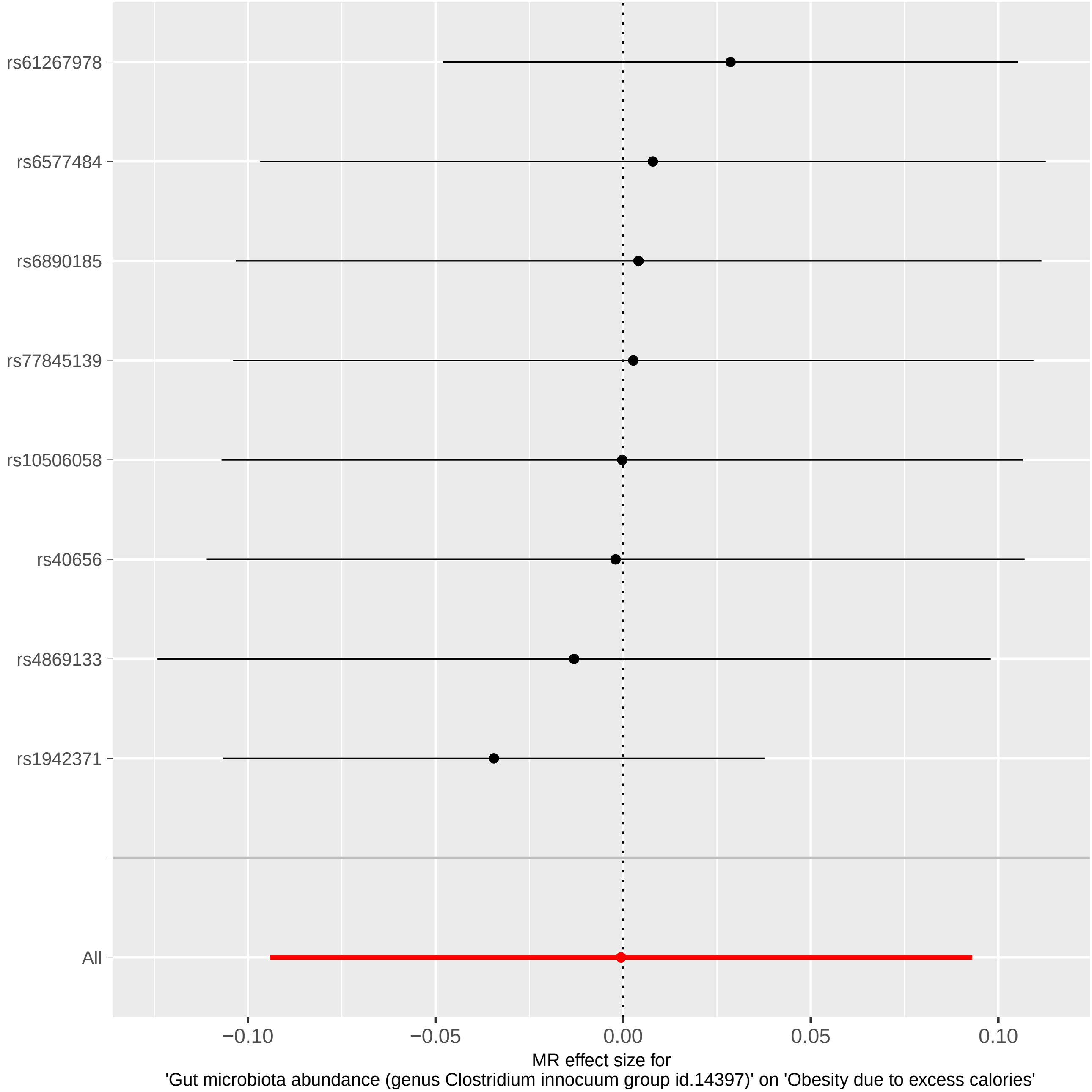

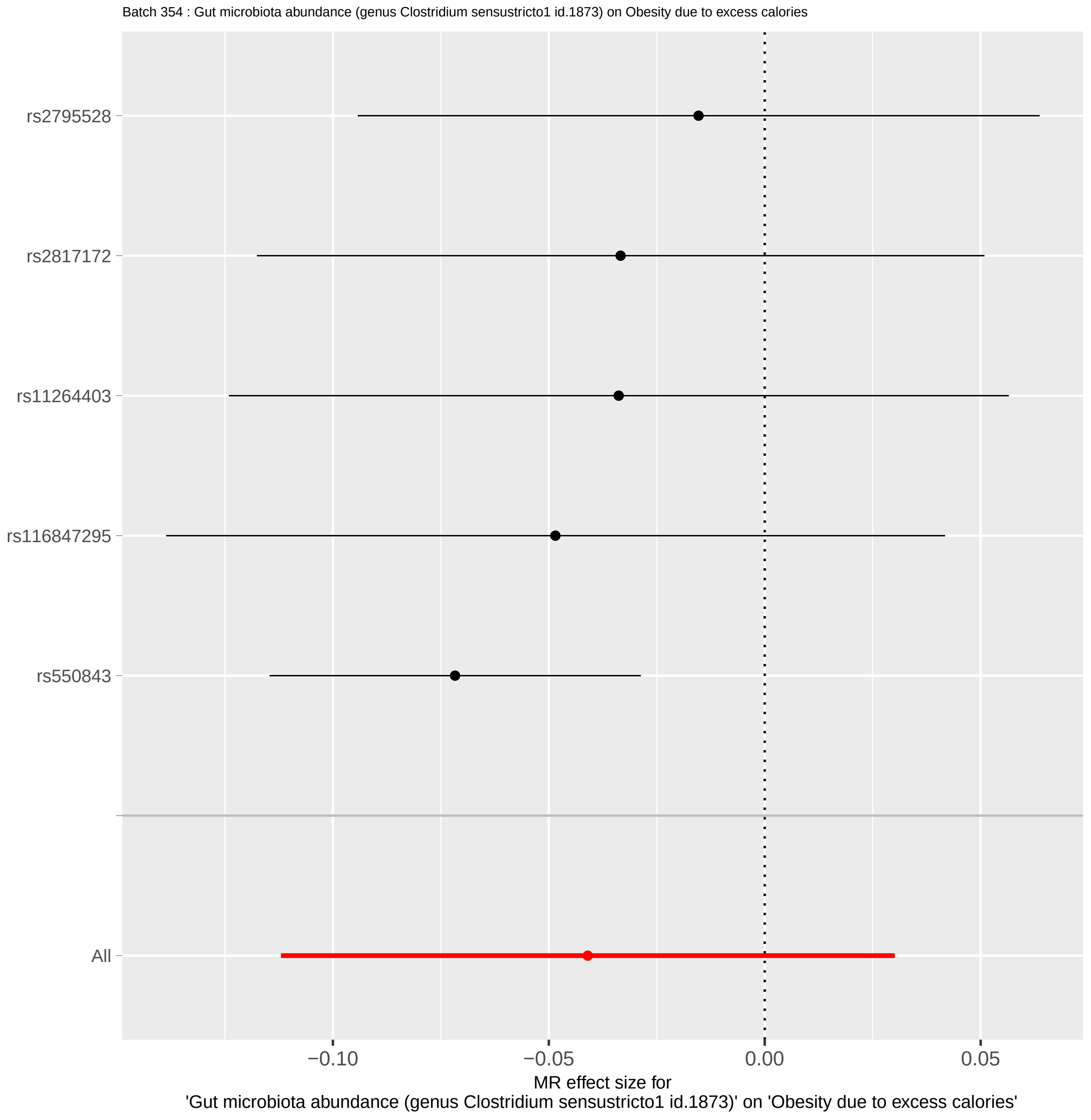

Batch 354 : Gut microbiota abundance (genus Clostridium sensustricto1 id.1873) on Obesity due to excess calories
rs2795528
rs2817172
rs11264403
rs116847295
rs550843
All
−0.10
−0.05
0.00
0.05
MR effect size for
'Gut microbiota abundance (genus Clostridium sensustricto1 id.1873)' on 'Obesity due to excess calories'

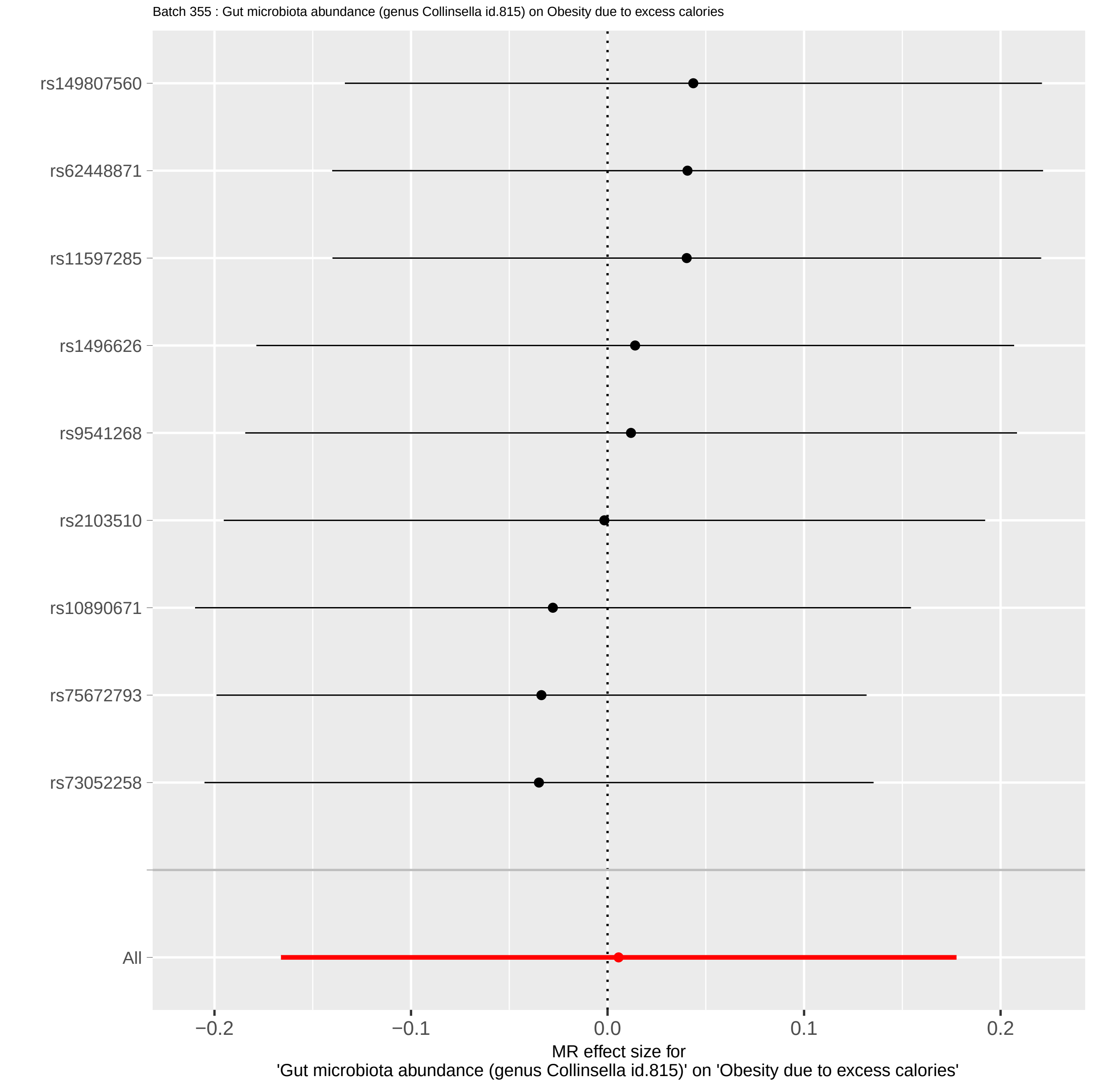
Batch 355 : Gut microbiota abundance (genus Collinsella id.815) on Obesity due to excess calories
rs149807560
rs62448871
rs11597285
rs1496626
rs9541268
rs2103510
rs10890671
rs75672793
rs73052258
All
−0.2
−0.1
0.0
0.1
0.2
MR effect size for
'Gut microbiota abundance (genus Collinsella id.815)' on 'Obesity due to excess calories'

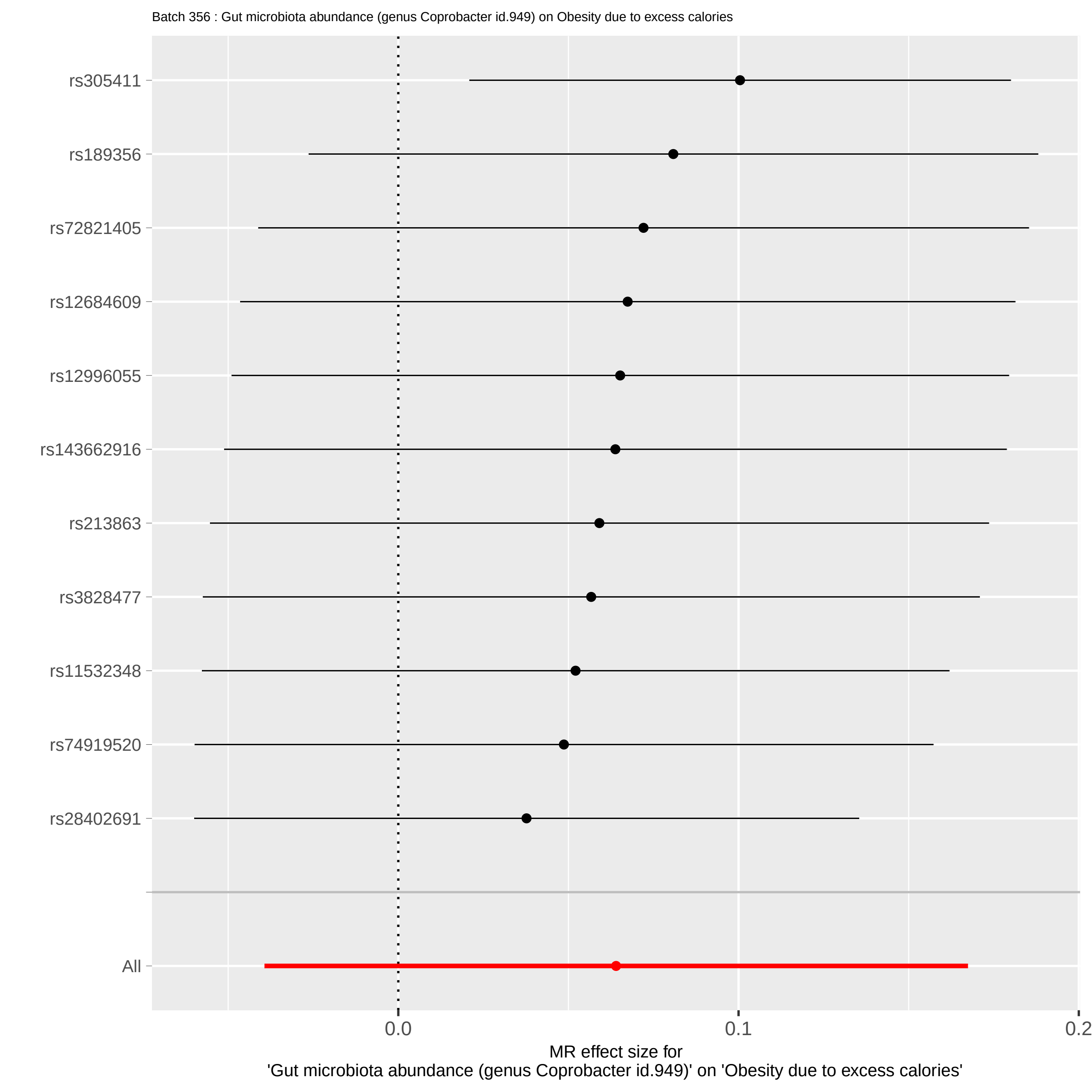

Batch 356 : Gut microbiota abundance (genus Coprobacter id.949) on Obesity due to excess calories
rs305411
rs189356
rs72821405
rs12684609
rs12996055
rs143662916
rs213863
rs3828477
rs11532348
rs74919520
rs28402691
All
0.0
0.1
0.2
MR effect size for
'Gut microbiota abundance (genus Coprobacter id.949)' on 'Obesity due to excess calories'

Batch 357 : Gut microbiota abundance (genus Coprococcus1 id.11301) on Obesity due to excess calories

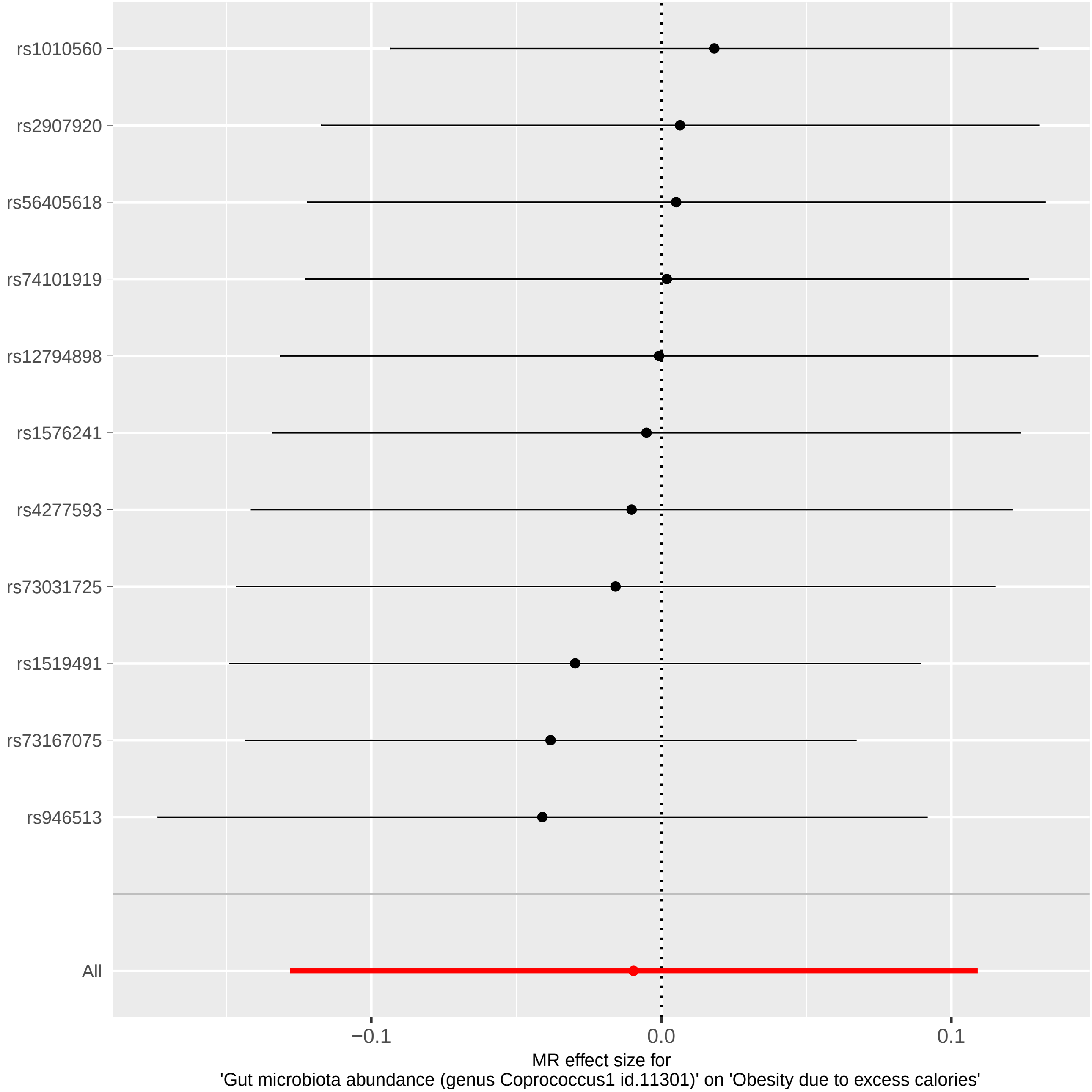

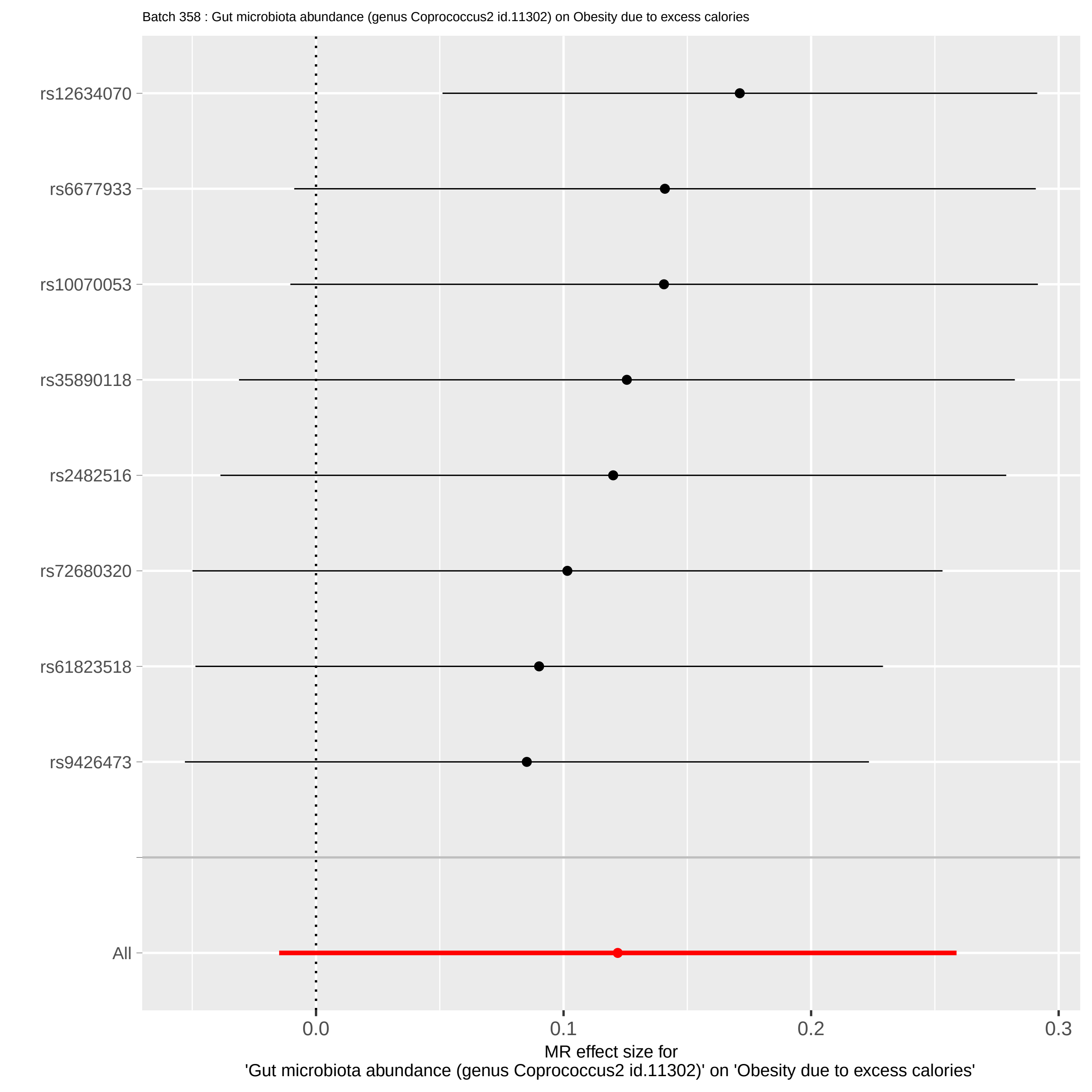

Batch 358 : Gut microbiota abundance (genus Coprococcus2 id.11302) on Obesity due to excess calories
rs12634070
rs6677933
rs10070053
rs35890118
rs2482516
rs72680320
rs61823518
rs9426473
All
0.0
0.1
0.2
0.3
MR effect size for
'Gut microbiota abundance (genus Coprococcus2 id.11302)' on 'Obesity due to excess calories'

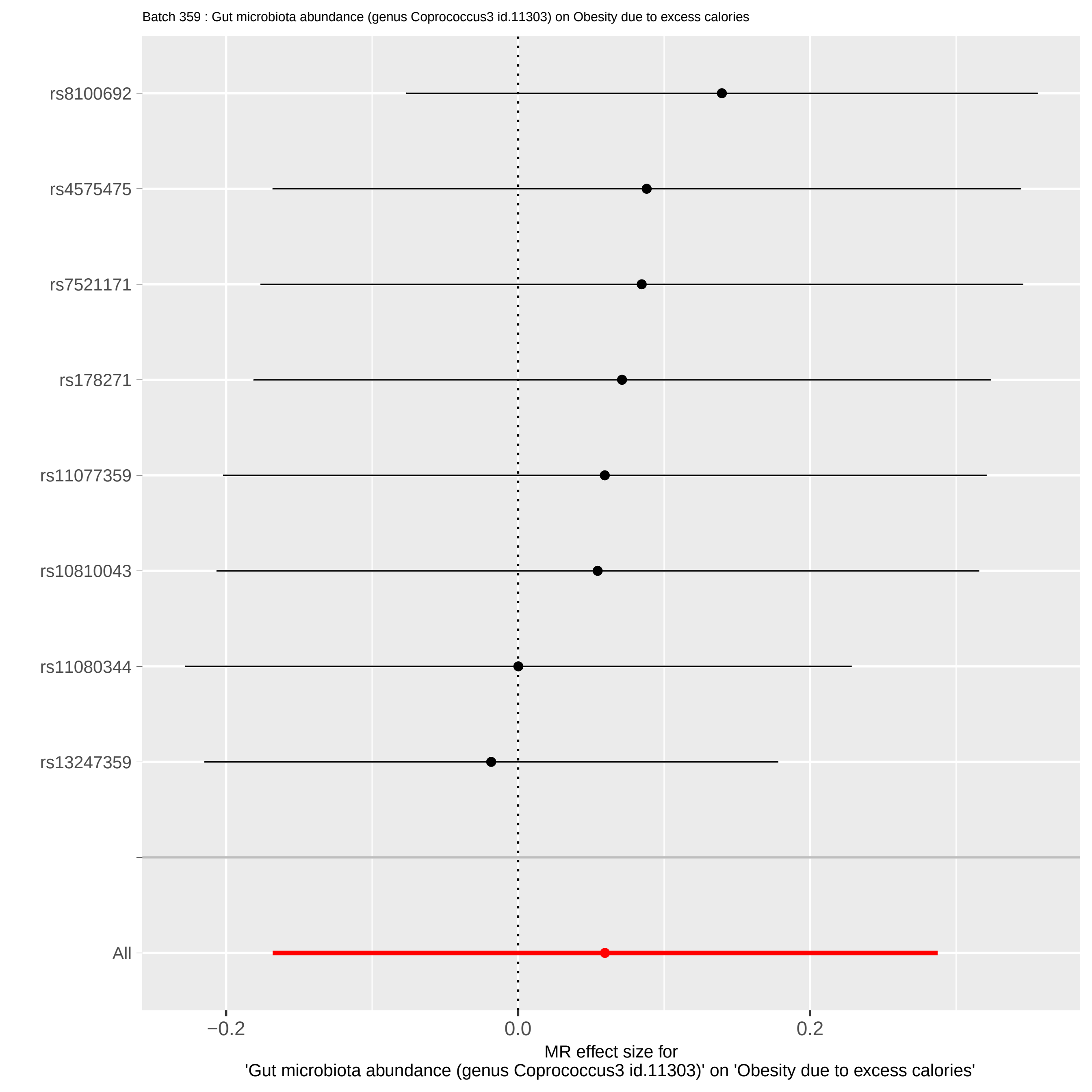
Batch 359 : Gut microbiota abundance (genus Coprococcus3 id.11303) on Obesity due to excess calories
rs8100692
rs4575475
rs7521171
rs178271
rs11077359
rs10810043
rs11080344
rs13247359
All
−0.2
0.0
0.2
MR effect size for
'Gut microbiota abundance (genus Coprococcus3 id.11303)' on 'Obesity due to excess calories'

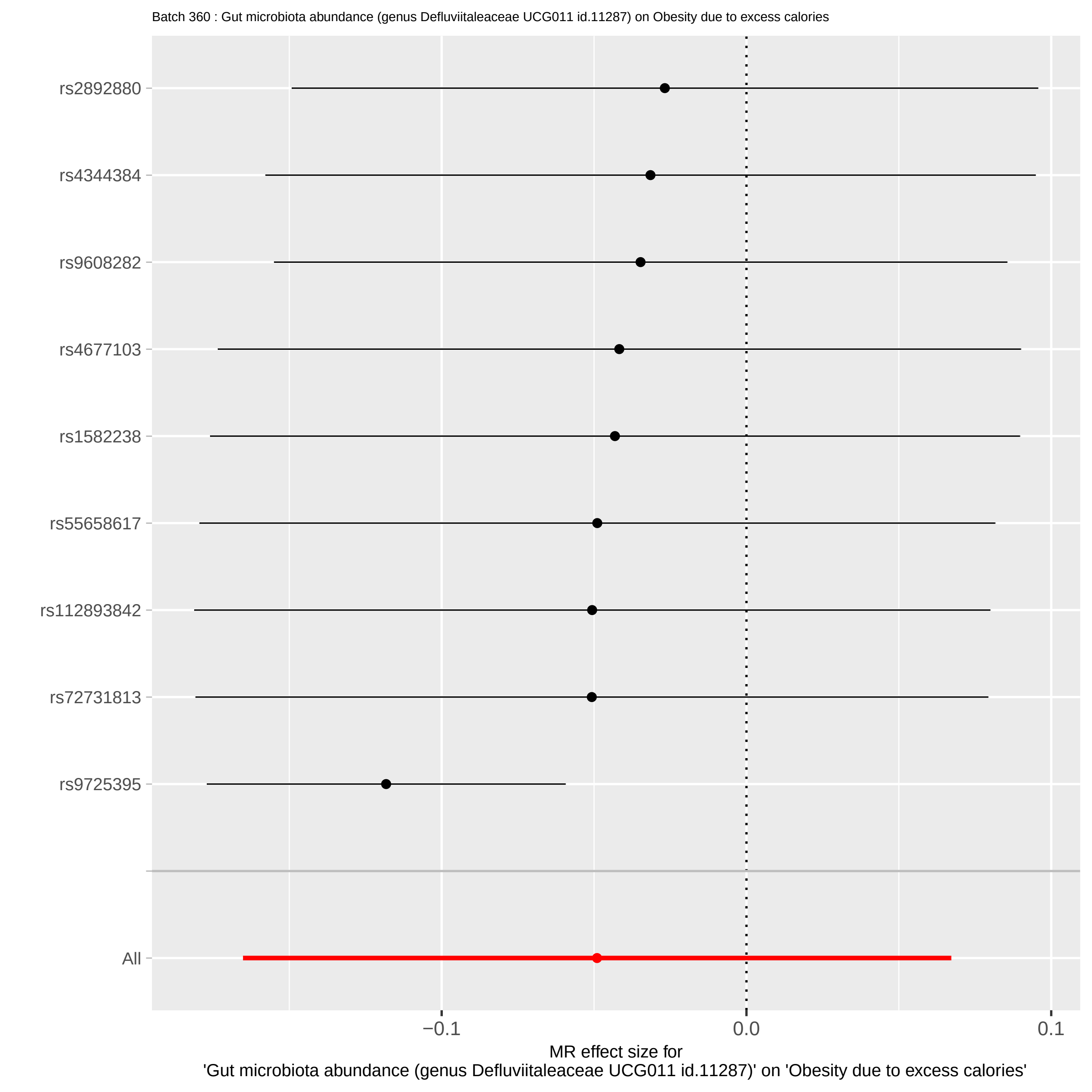
Batch 360 : Gut microbiota abundance (genus Defluviitaleaceae UCG011 id.11287) on Obesity due to excess calories
rs2892880
rs4344384
rs9608282
rs4677103
rs1582238
rs55658617
rs112893842
rs72731813
rs9725395
All
−0.1
0.0
0.1
MR effect size for
'Gut microbiota abundance (genus Defluviitaleaceae UCG011 id.11287)' on 'Obesity due to excess calories'

Batch 361 : Gut microbiota abundance (genus Desulfovibrio id.3173) on Obesity due to excess calories

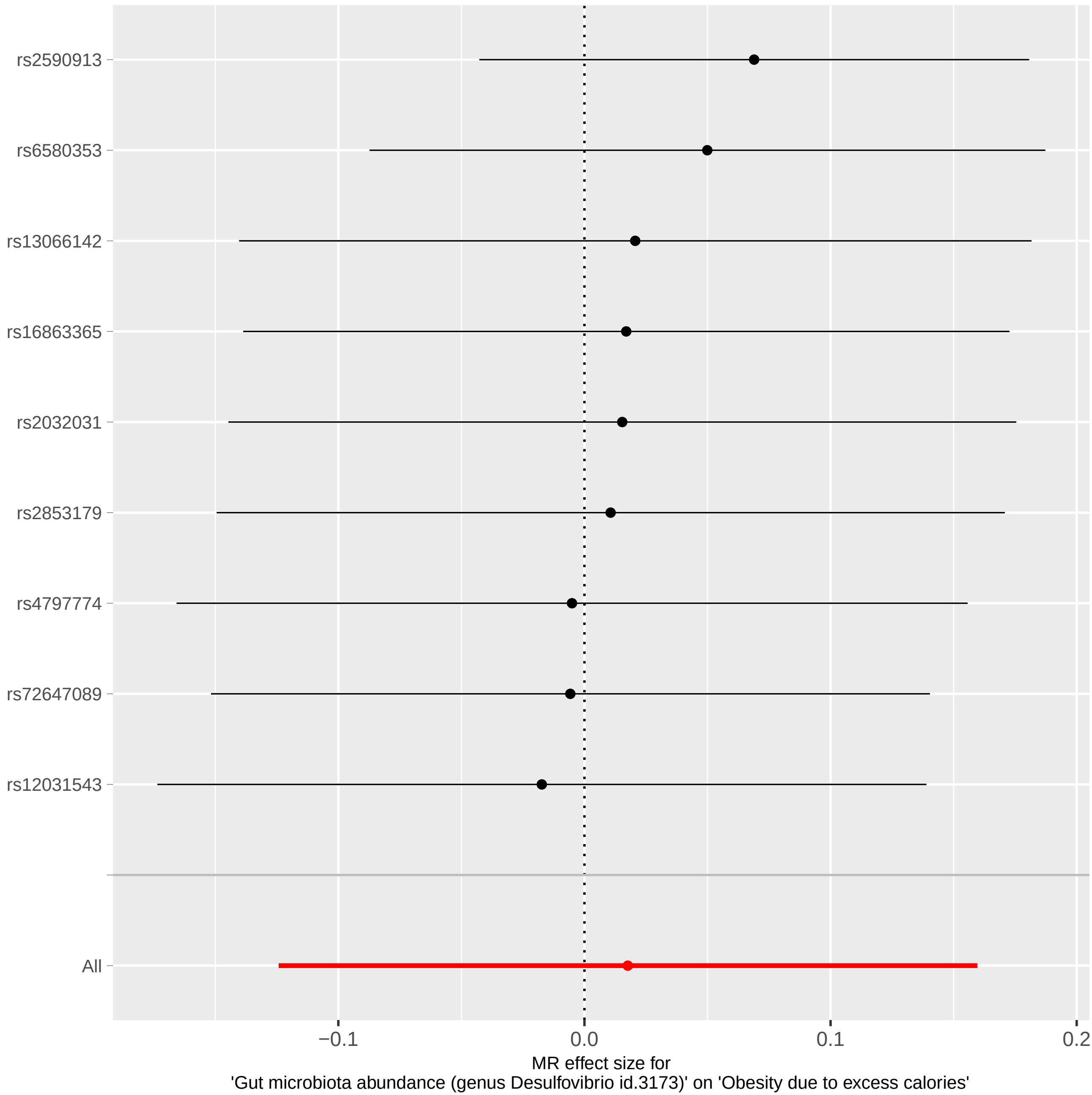

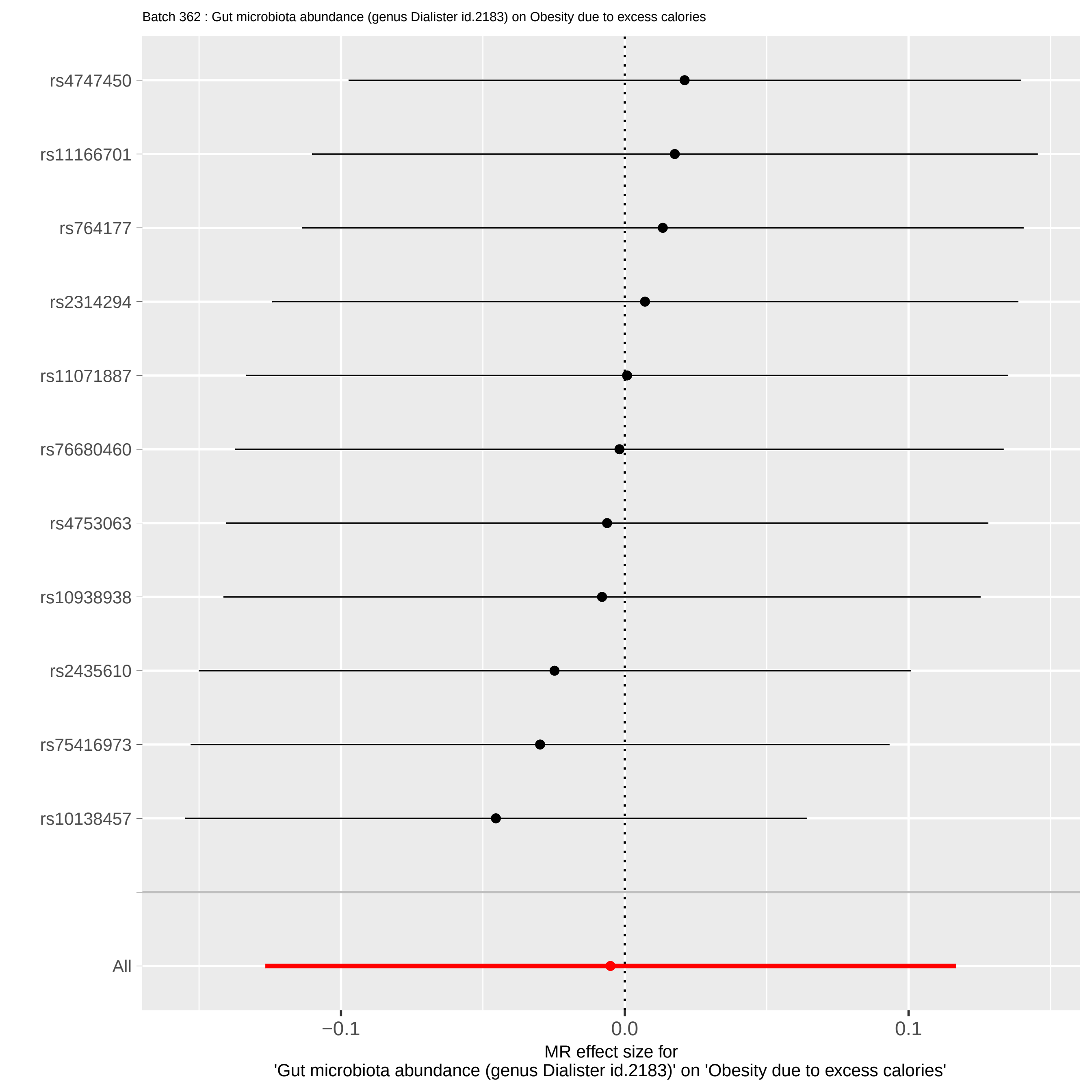
Batch 362 : Gut microbiota abundance (genus Dialister id.2183) on Obesity due to excess calories
rs4747450
rs11166701
rs764177
rs2314294
rs11071887
rs76680460
rs4753063
rs10938938
rs2435610
rs75416973
rs10138457
All
−0.1
0.0
0.1
MR effect size for
'Gut microbiota abundance (genus Dialister id.2183)' on 'Obesity due to excess calories'

Batch 363 : Gut microbiota abundance (genus Dorea id.1997) on Obesity due to excess calories

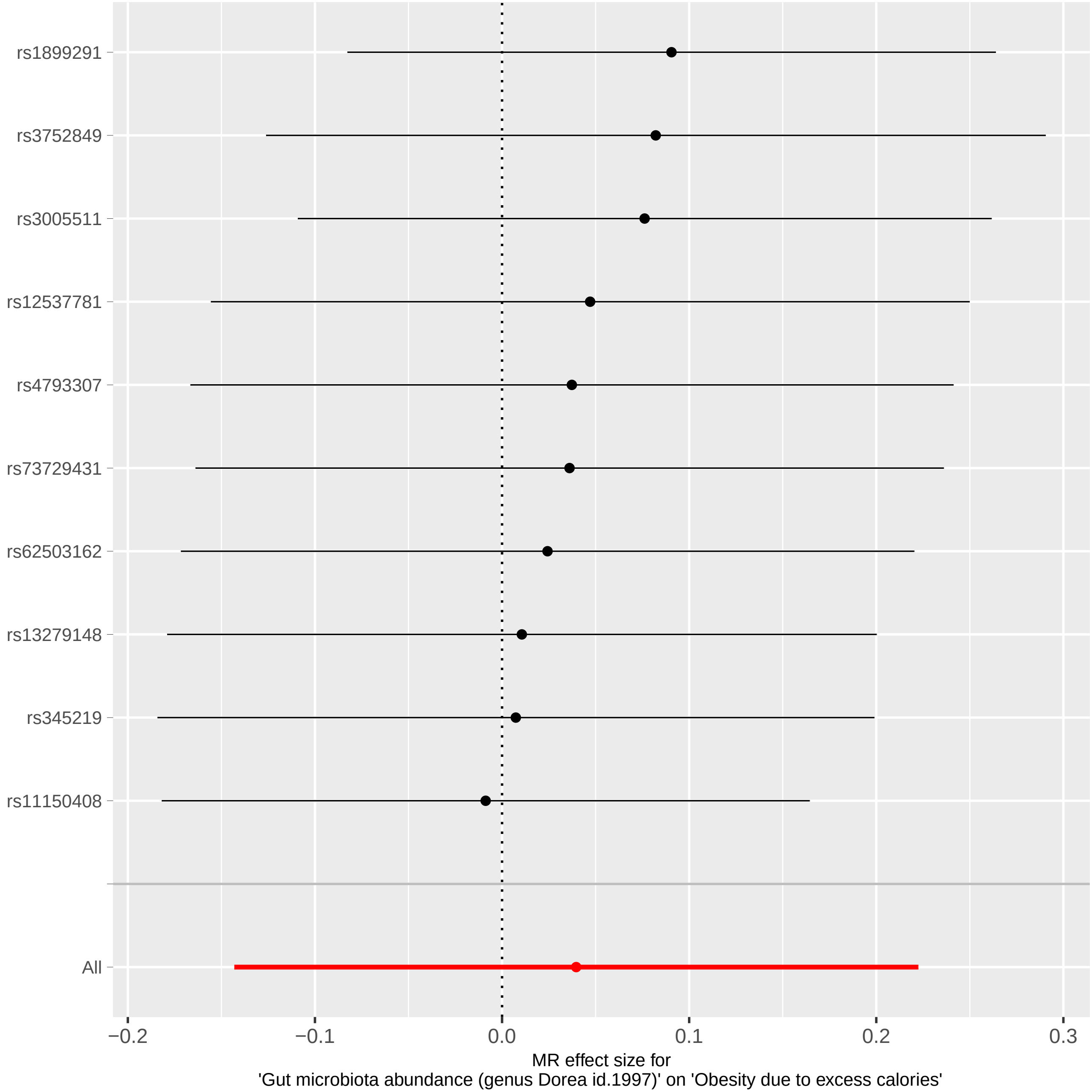

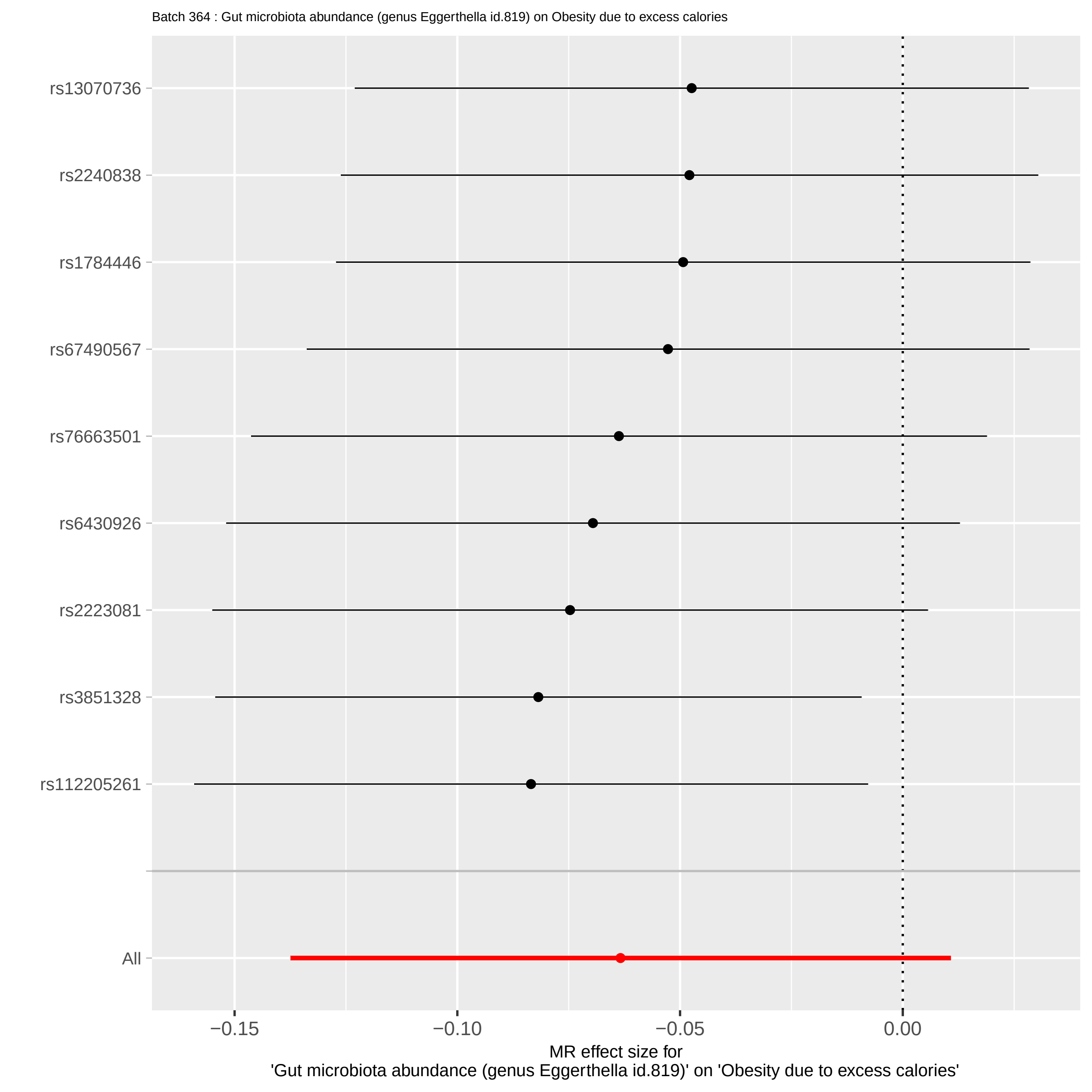
Batch 364 : Gut microbiota abundance (genus Eggerthella id.819) on Obesity due to excess calories
rs13070736
rs2240838
rs1784446
rs67490567
rs76663501
rs6430926
rs2223081
rs3851328
rs112205261
All
−0.15
−0.10
−0.05
0.00
MR effect size for
'Gut microbiota abundance (genus Eggerthella id.819)' on 'Obesity due to excess calories'

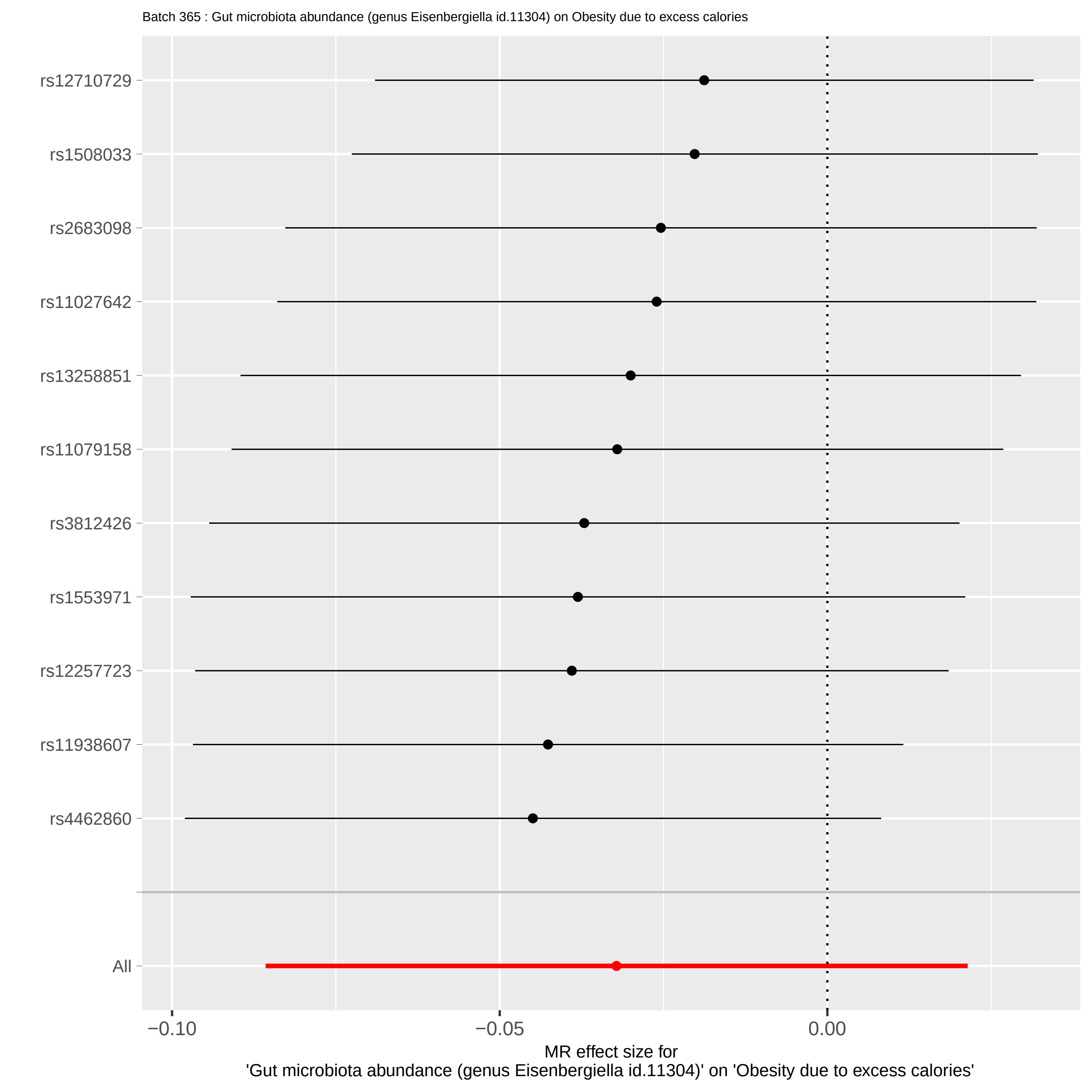

Batch 365 : Gut microbiota abundance (genus Eisenbergiella id.11304) on Obesity due to excess calories
rs12710729
rs1508033
rs2683098
rs11027642
rs13258851
rs11079158
rs3812426
rs1553971
rs12257723
rs11938607
rs4462860
All
−0.10
−0.05
0.00
MR effect size for
'Gut microbiota abundance (genus Eisenbergiella id.11304)' on 'Obesity due to excess calories'

Batch 366 : Gut microbiota abundance (genus Enterorhabdus id.820) on Obesity due to excess calories

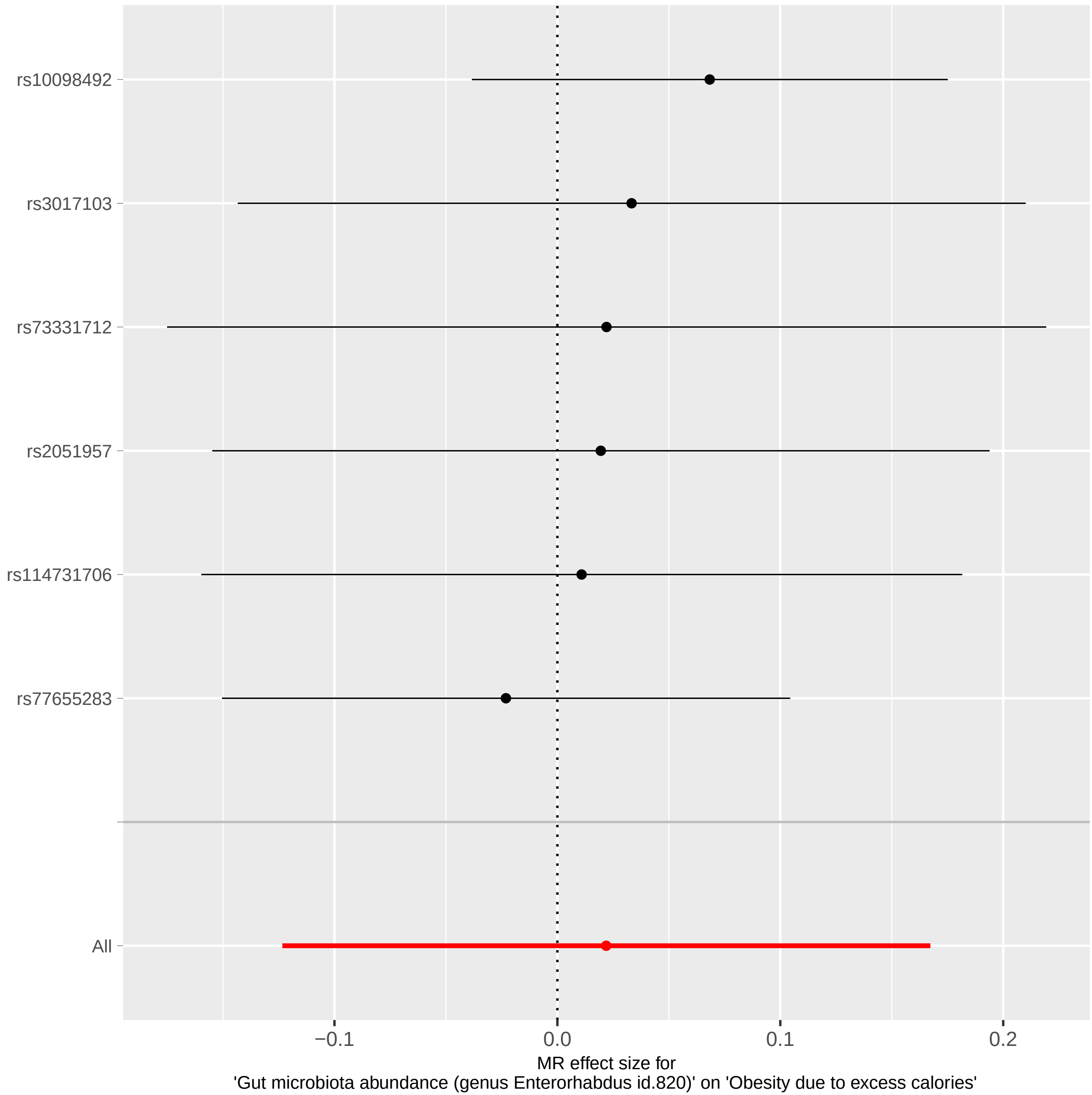

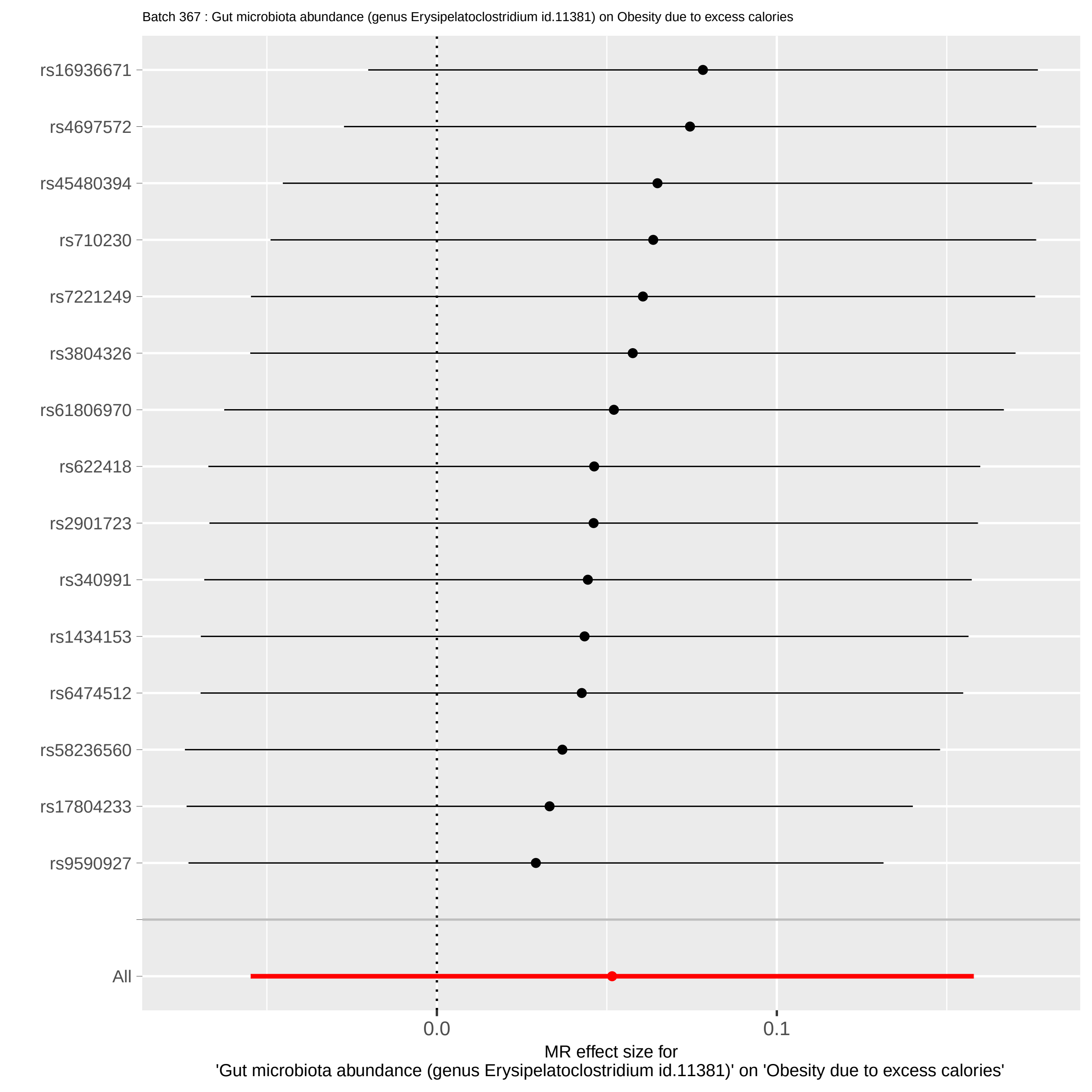

Batch 367 : Gut microbiota abundance (genus Erysipelatoclostridium id.11381) on Obesity due to excess calories
rs16936671
rs4697572
rs45480394
rs710230
rs7221249
rs3804326
rs61806970
rs622418
rs2901723
rs340991
rs1434153
rs6474512
rs58236560
rs17804233
rs9590927
All
0.0
0.1
MR effect size for
'Gut microbiota abundance (genus Erysipelatoclostridium id.11381)' on 'Obesity due to excess calories'

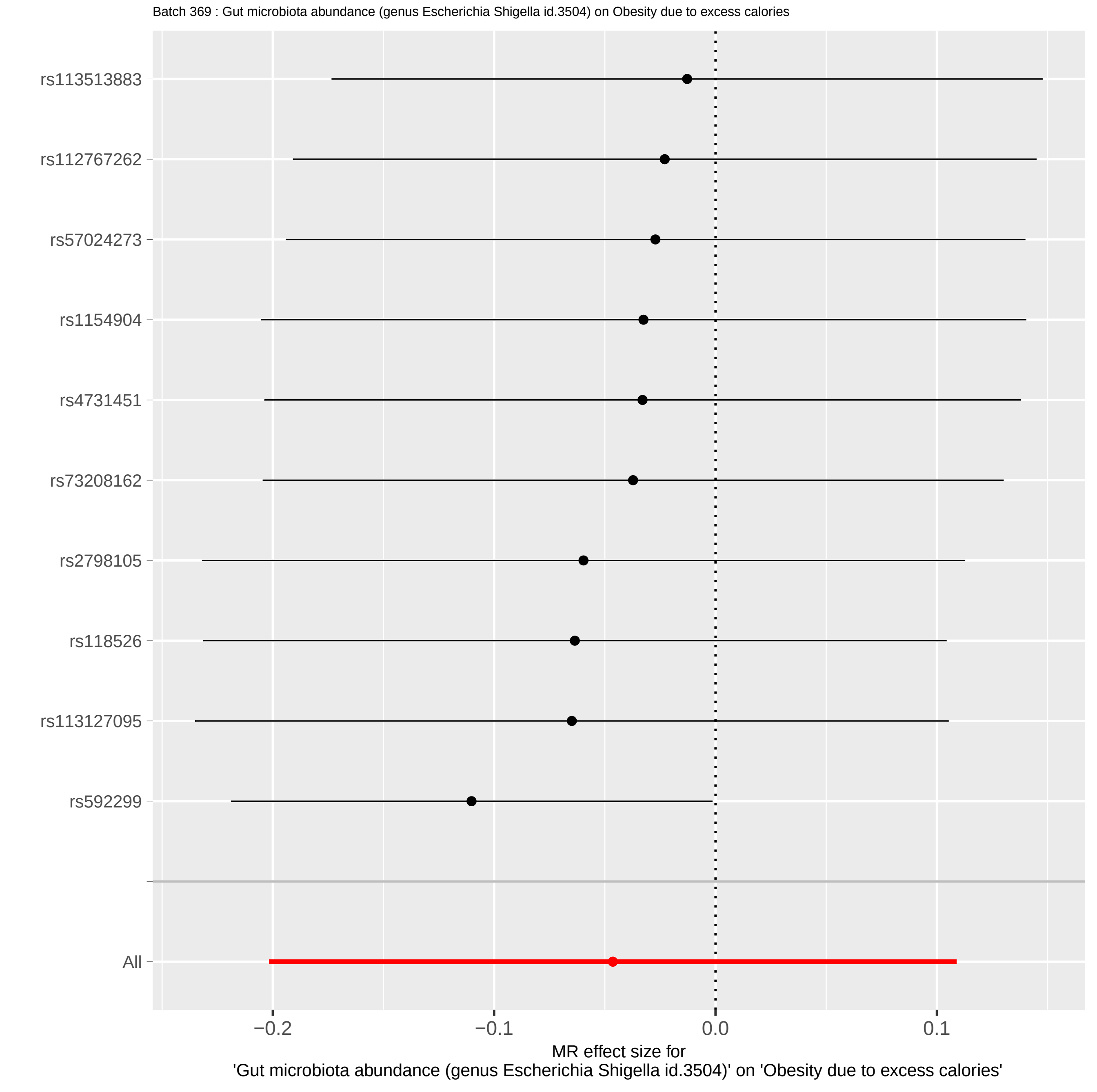
Batch 369 : Gut microbiota abundance (genus Escherichia Shigella id.3504) on Obesity due to excess calories
rs113513883
rs112767262
rs57024273
rs1154904
rs4731451
rs73208162
rs2798105
rs118526
rs113127095
rs592299
All
−0.2
−0.1
0.0
0.1
MR effect size for
'Gut microbiota abundance (genus Escherichia Shigella id.3504)' on 'Obesity due to excess calories'

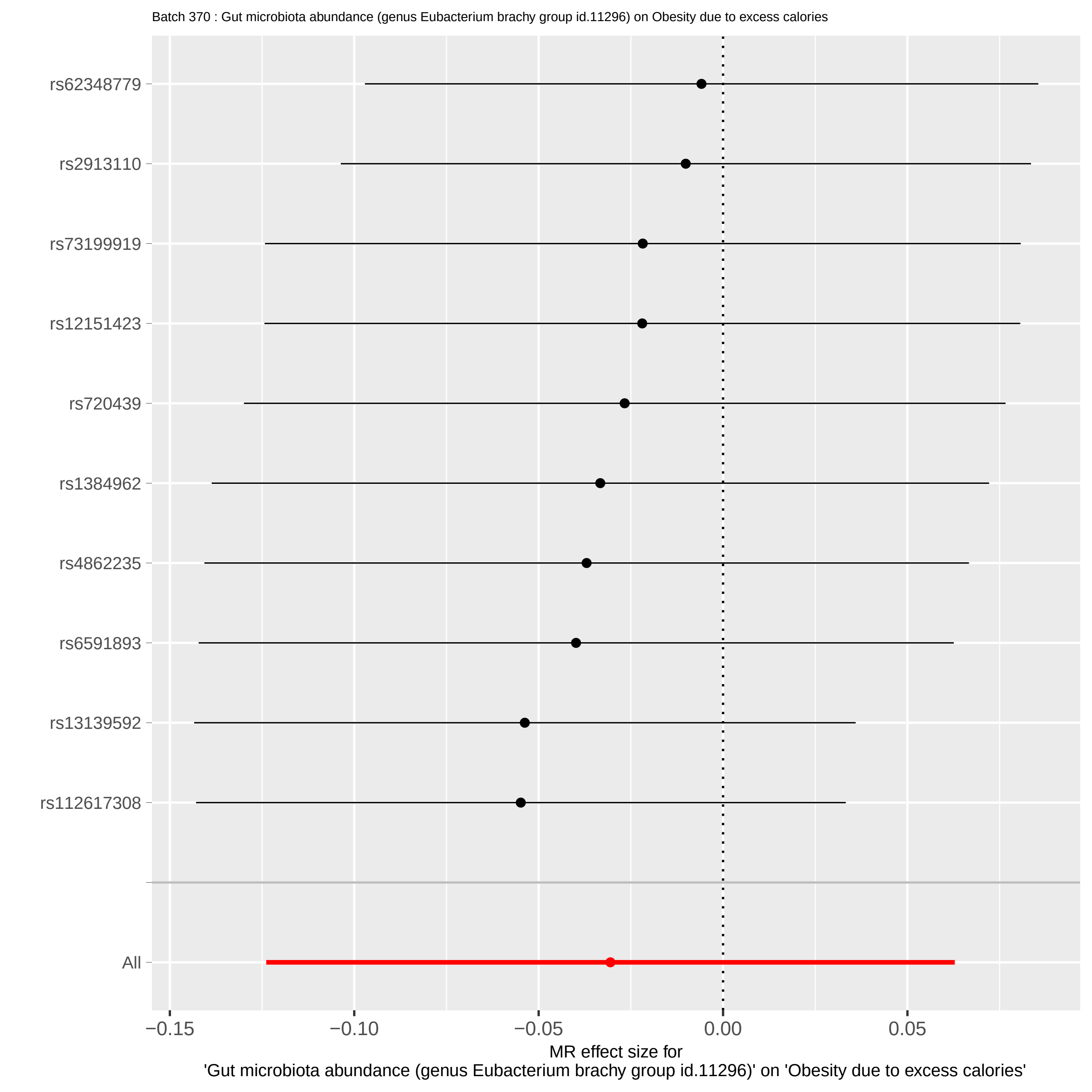
Batch 370 : Gut microbiota abundance (genus Eubacterium brachy group id.11296) on Obesity due to excess calories
rs62348779
rs2913110
rs73199919
rs12151423
rs720439
rs1384962
rs4862235
rs6591893
rs13139592
rs112617308
All
−0.15
−0.10
−0.05
0.00
0.05
MR effect size for
'Gut microbiota abundance (genus Eubacterium brachy group id.11296)' on 'Obesity due to excess calories'

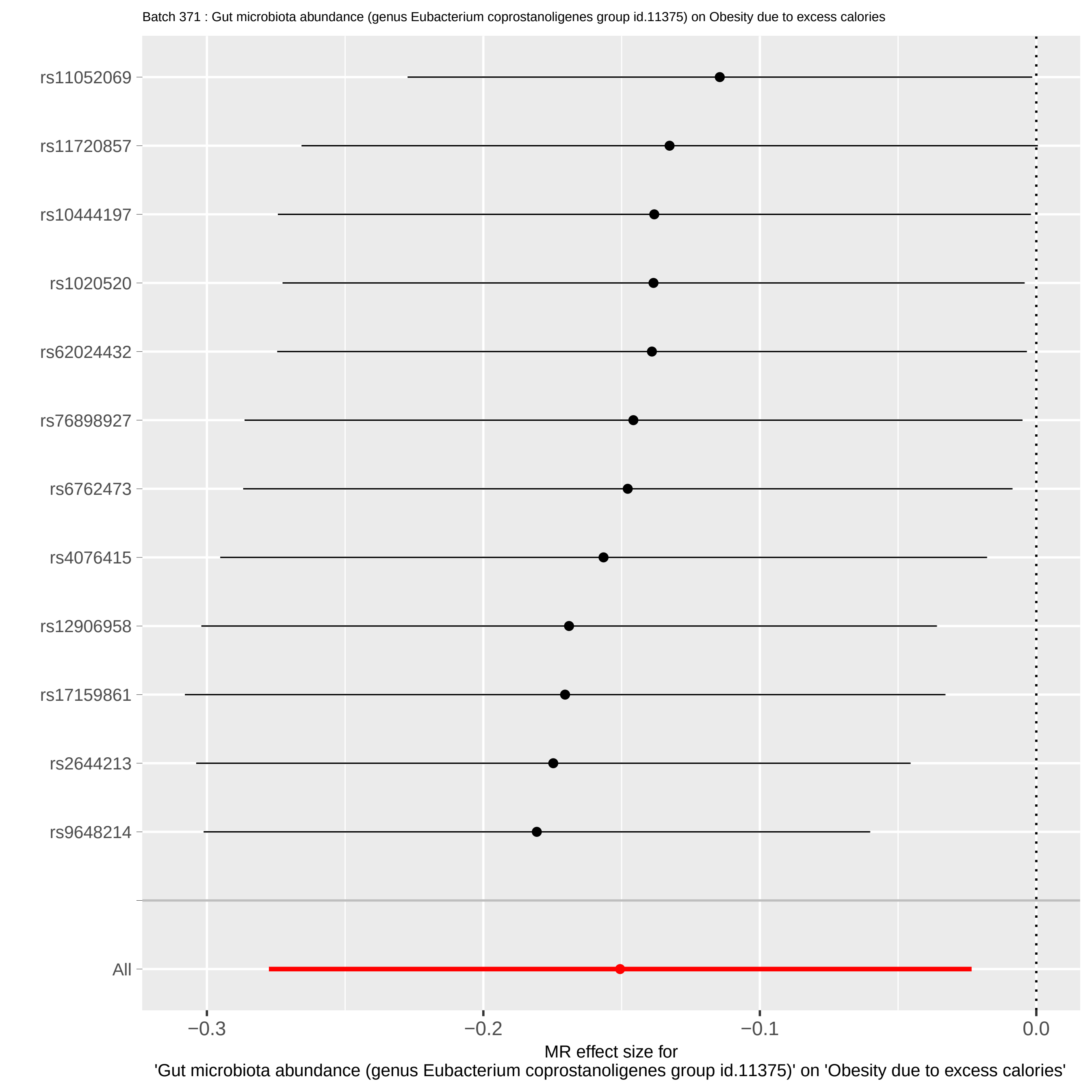

Batch 371 : Gut microbiota abundance (genus Eubacterium coprostanoligenes group id.11375) on Obesity due to excess calories
rs11052069
rs11720857
rs10444197
rs1020520
rs62024432
rs76898927
rs6762473
rs4076415
rs12906958
rs17159861
rs2644213
rs9648214
All
−0.3
−0.2
−0.1
0.0
MR effect size for
'Gut microbiota abundance (genus Eubacterium coprostanoligenes group id.11375)' on 'Obesity due to excess calories'

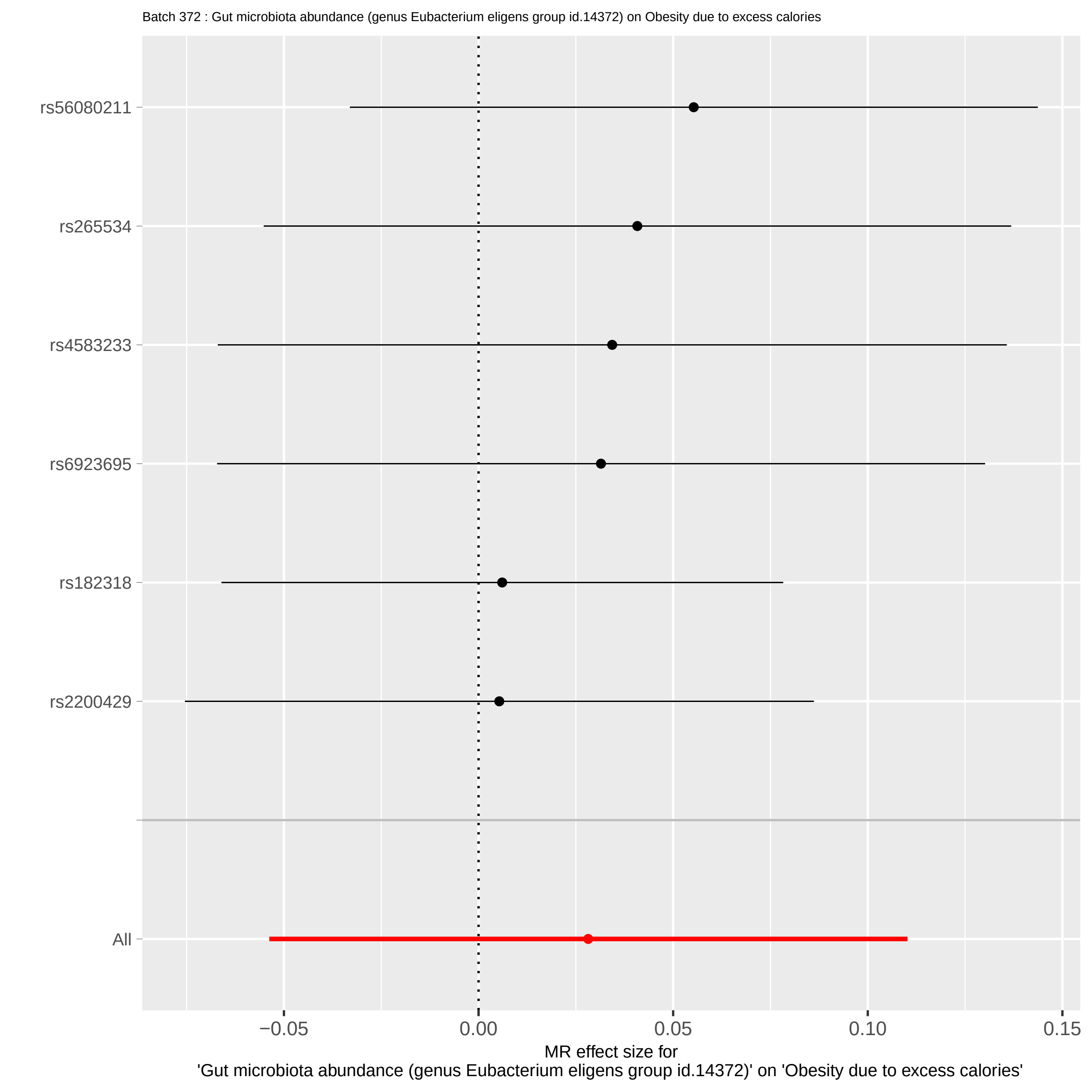

Batch 372 : Gut microbiota abundance (genus Eubacterium eligens group id.14372) on Obesity due to excess calories
rs56080211
rs265534
rs4583233
rs6923695
rs182318
rs2200429
All
−0.05
0.00
0.05
0.10
0.15
MR effect size for
'Gut microbiota abundance (genus Eubacterium eligens group id.14372)' on 'Obesity due to excess calories'

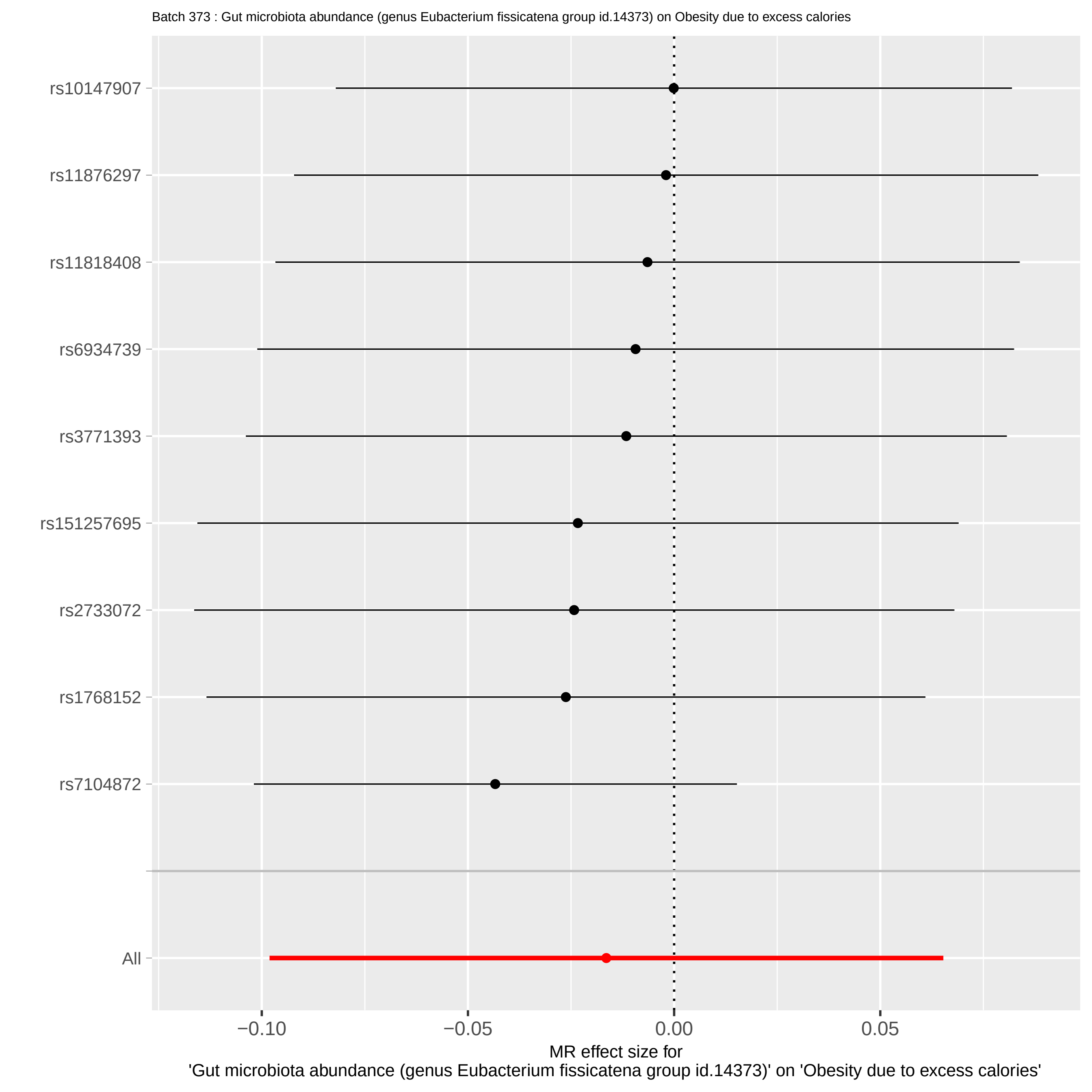

Batch 373 : Gut microbiota abundance (genus Eubacterium fissicatena group id.14373) on Obesity due to excess calories
rs10147907
rs11876297
rs11818408
rs6934739
rs3771393
rs151257695
rs2733072
rs1768152
rs7104872
All
−0.10
−0.05
0.00
0.05
MR effect size for
'Gut microbiota abundance (genus Eubacterium fissicatena group id.14373)' on 'Obesity due to excess calories'

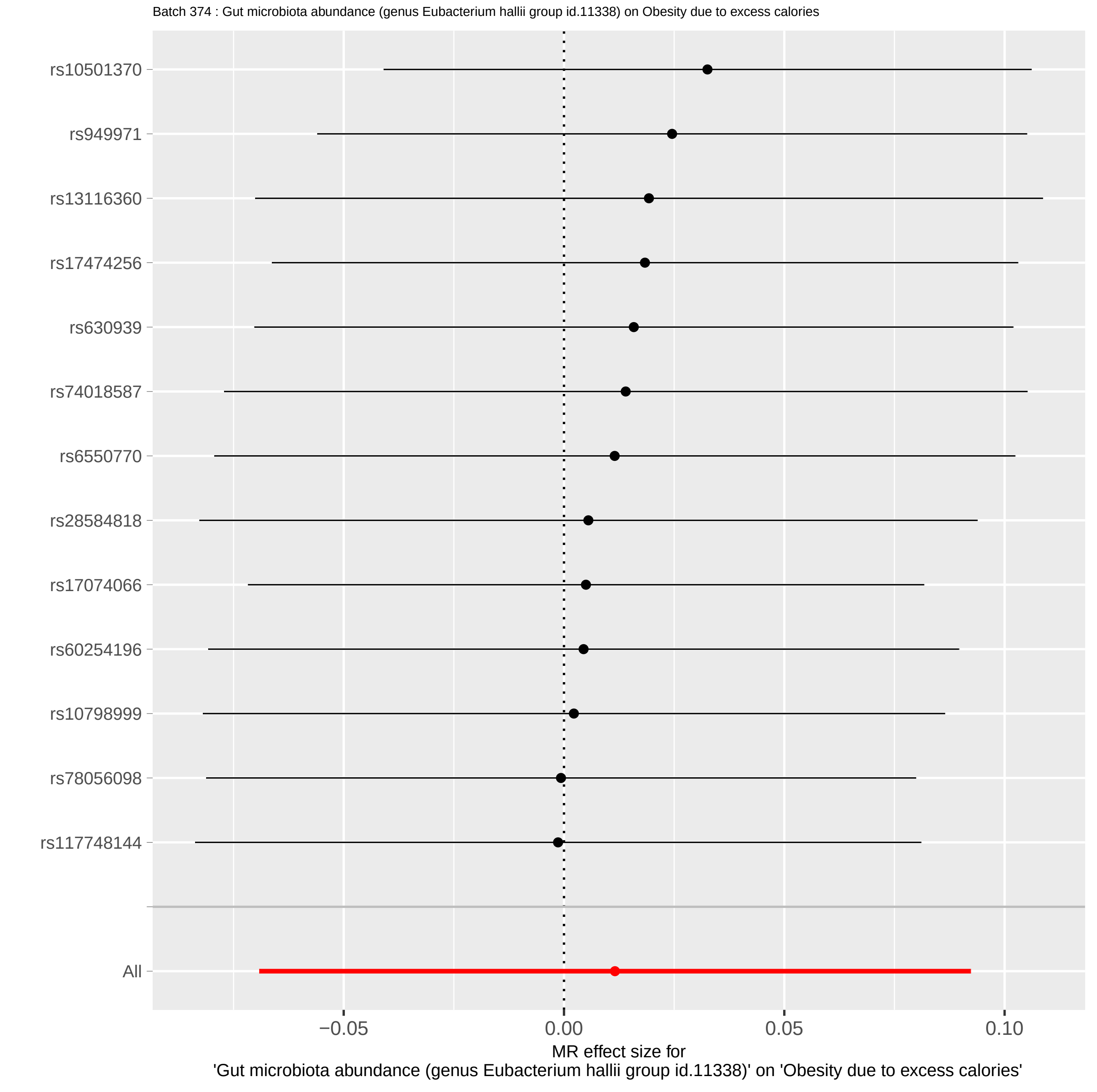

Batch 374 : Gut microbiota abundance (genus Eubacterium hallii group id.11338) on Obesity due to excess calories
rs10501370
rs949971
rs13116360
rs17474256
rs630939
rs74018587
rs6550770
rs28584818
rs17074066
rs60254196
rs10798999
rs78056098
rs117748144
All
−0.05
0.00
0.05
0.10
MR effect size for
'Gut microbiota abundance (genus Eubacterium hallii group id.11338)' on 'Obesity due to excess calories'

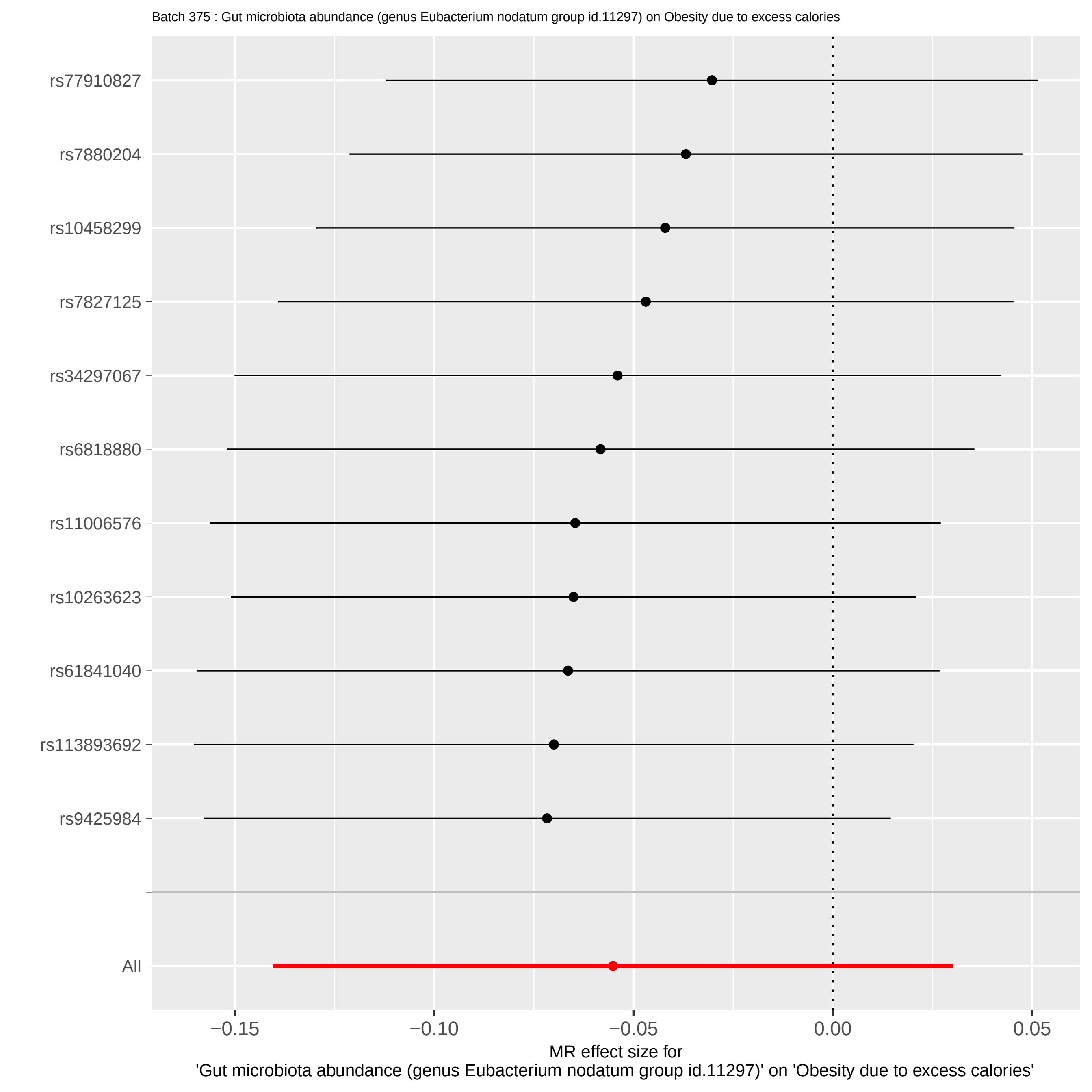

Batch 375 : Gut microbiota abundance (genus Eubacterium nodatum group id.11297) on Obesity due to excess calories
rs77910827
rs7880204
rs10458299
rs7827125
rs34297067
rs6818880
rs11006576
rs10263623
rs61841040
rs113893692
rs9425984
All
−0.15
−0.10
−0.05
0.00
0.05
MR effect size for
'Gut microbiota abundance (genus Eubacterium nodatum group id.11297)' on 'Obesity due to excess calories'

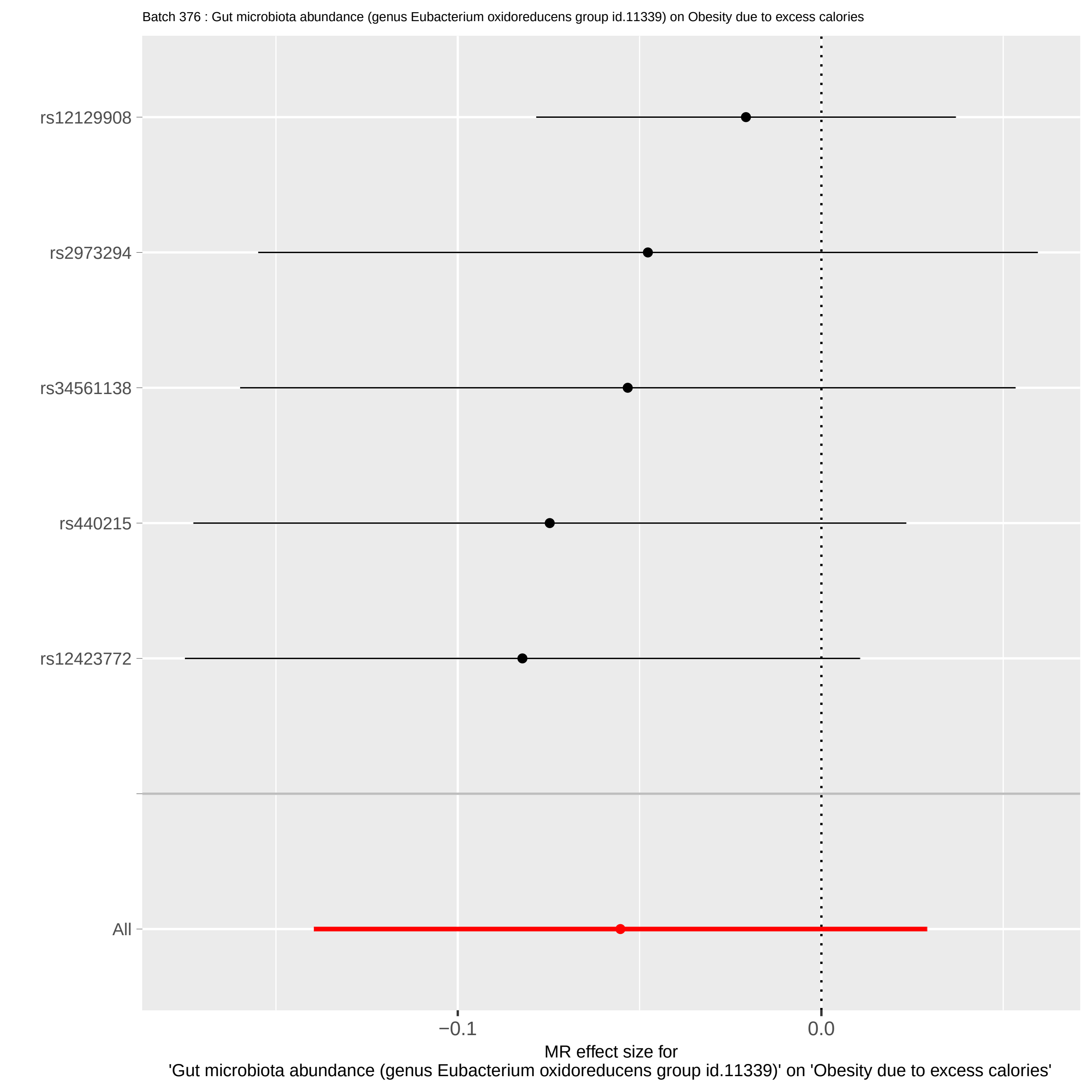
Batch 376 : Gut microbiota abundance (genus Eubacterium oxidoreducens group id.11339) on Obesity due to excess calories
rs12129908
rs2973294
rs34561138
rs440215
rs12423772
All
−0.1
0.0
MR effect size for
'Gut microbiota abundance (genus Eubacterium oxidoreducens group id.11339)' on 'Obesity due to excess calories'

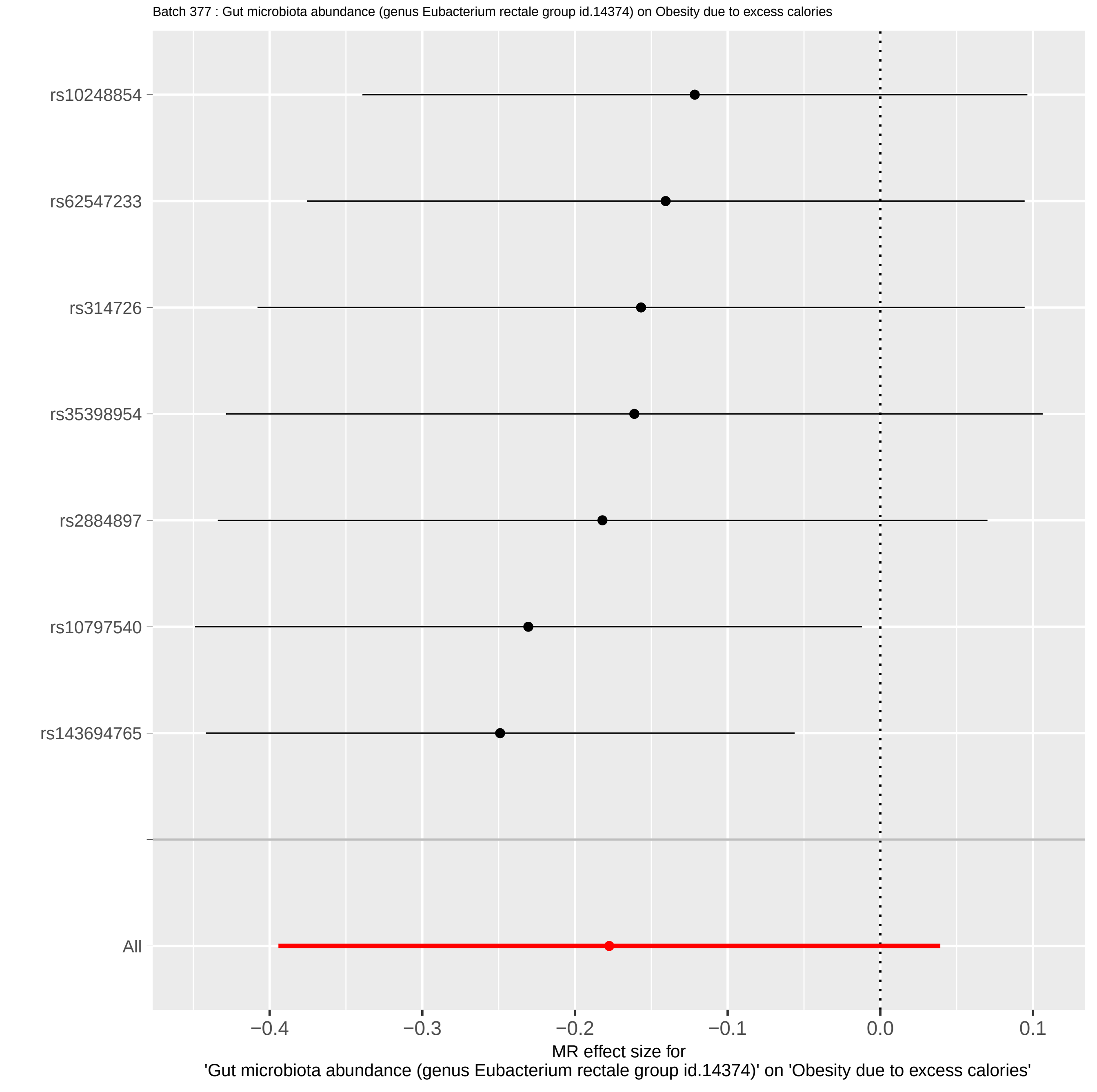
Batch 377 : Gut microbiota abundance (genus Eubacterium rectale group id.14374) on Obesity due to excess calories
rs10248854
rs62547233
rs314726
rs35398954
rs2884897
rs10797540
rs143694765
All
−0.4
−0.3
−0.2
−0.1
0.0
0.1
MR effect size for
'Gut microbiota abundance (genus Eubacterium rectale group id.14374)' on 'Obesity due to excess calories'

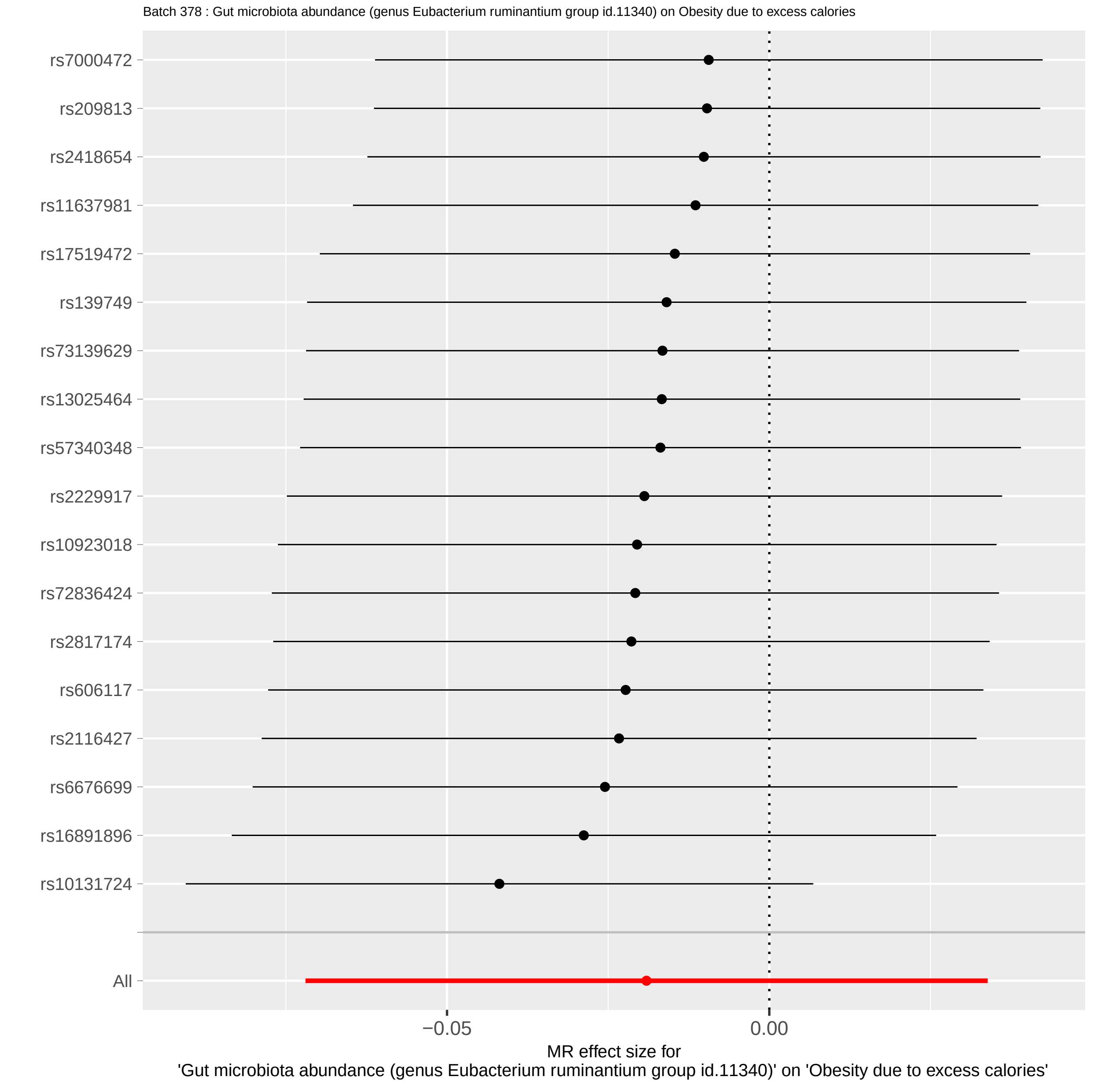

Batch 378 : Gut microbiota abundance (genus Eubacterium ruminantium group id.11340) on Obesity due to excess calories
rs7000472
rs209813
rs2418654
rs11637981
rs17519472
rs139749
rs73139629
rs13025464
rs57340348
rs2229917
rs10923018
rs72836424
rs2817174
rs606117
rs2116427
rs6676699
rs16891896
rs10131724
All
−0.05
0.00
MR effect size for
'Gut microbiota abundance (genus Eubacterium ruminantium group id.11340)' on 'Obesity due to excess calories'

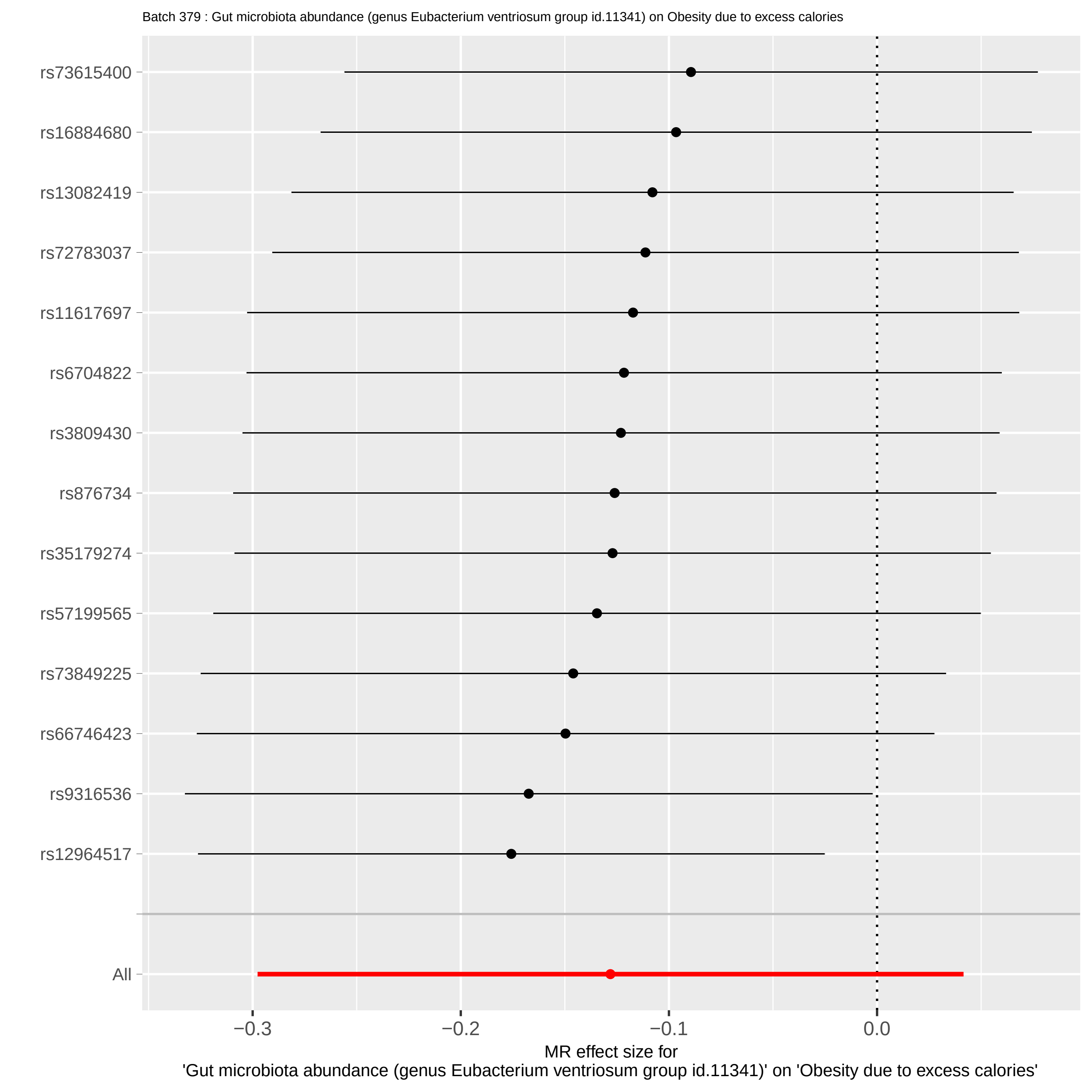

Batch 379 : Gut microbiota abundance (genus Eubacterium ventriosum group id.11341) on Obesity due to excess calories
rs73615400
rs16884680
rs13082419
rs72783037
rs11617697
rs6704822
rs3809430
rs876734
rs35179274
rs57199565
rs73849225
rs66746423
rs9316536
rs12964517
All
−0.3
−0.2
−0.1
0.0
MR effect size for
'Gut microbiota abundance (genus Eubacterium ventriosum group id.11341)' on 'Obesity due to excess calories'

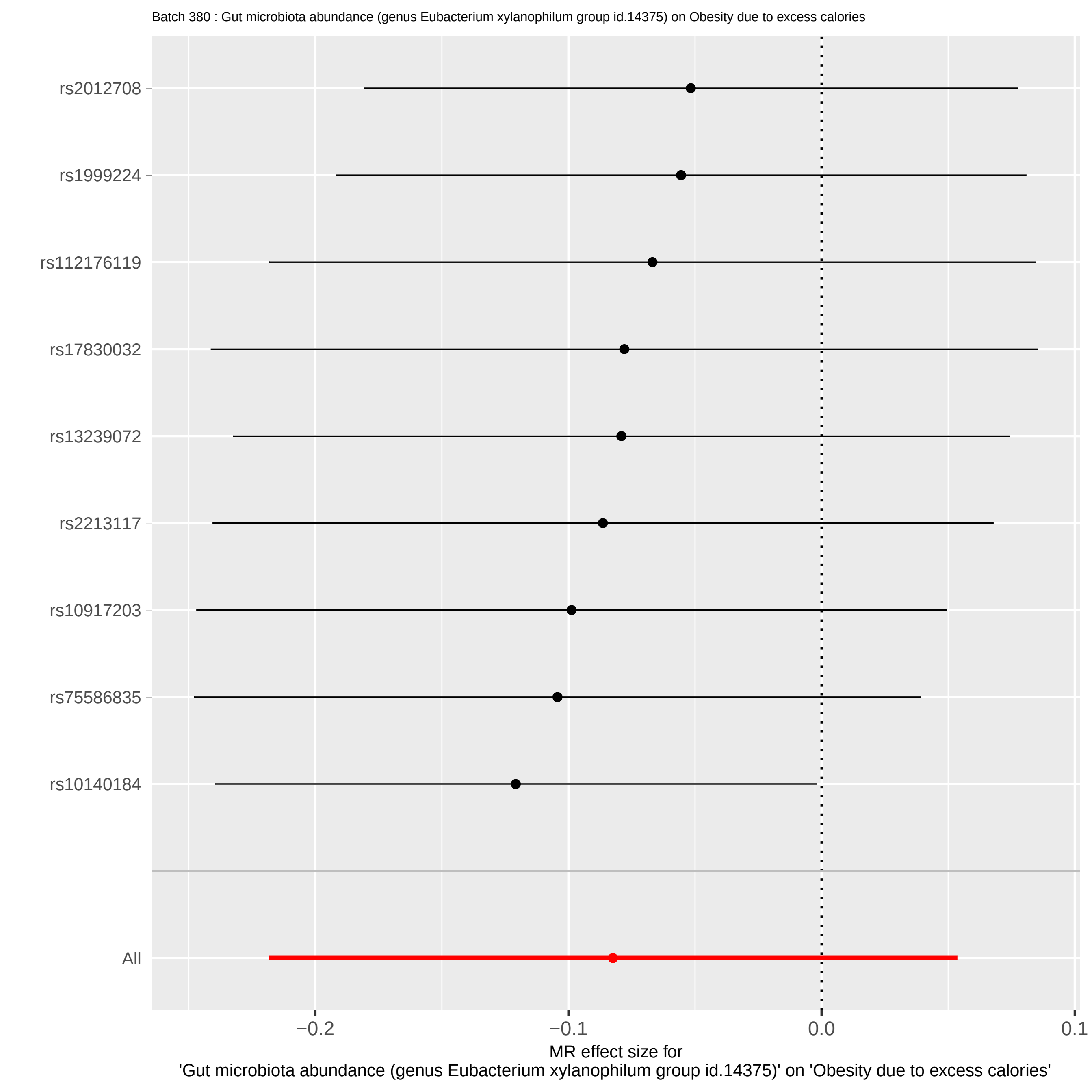

Batch 380 : Gut microbiota abundance (genus Eubacterium xylanophilum group id.14375) on Obesity due to excess calories
rs2012708
rs1999224
rs112176119
rs17830032
rs13239072
rs2213117
rs10917203
rs75586835
rs10140184
All
−0.2
−0.1
0.0
0.1
MR effect size for
'Gut microbiota abundance (genus Eubacterium xylanophilum group id.14375)' on 'Obesity due to excess calories'

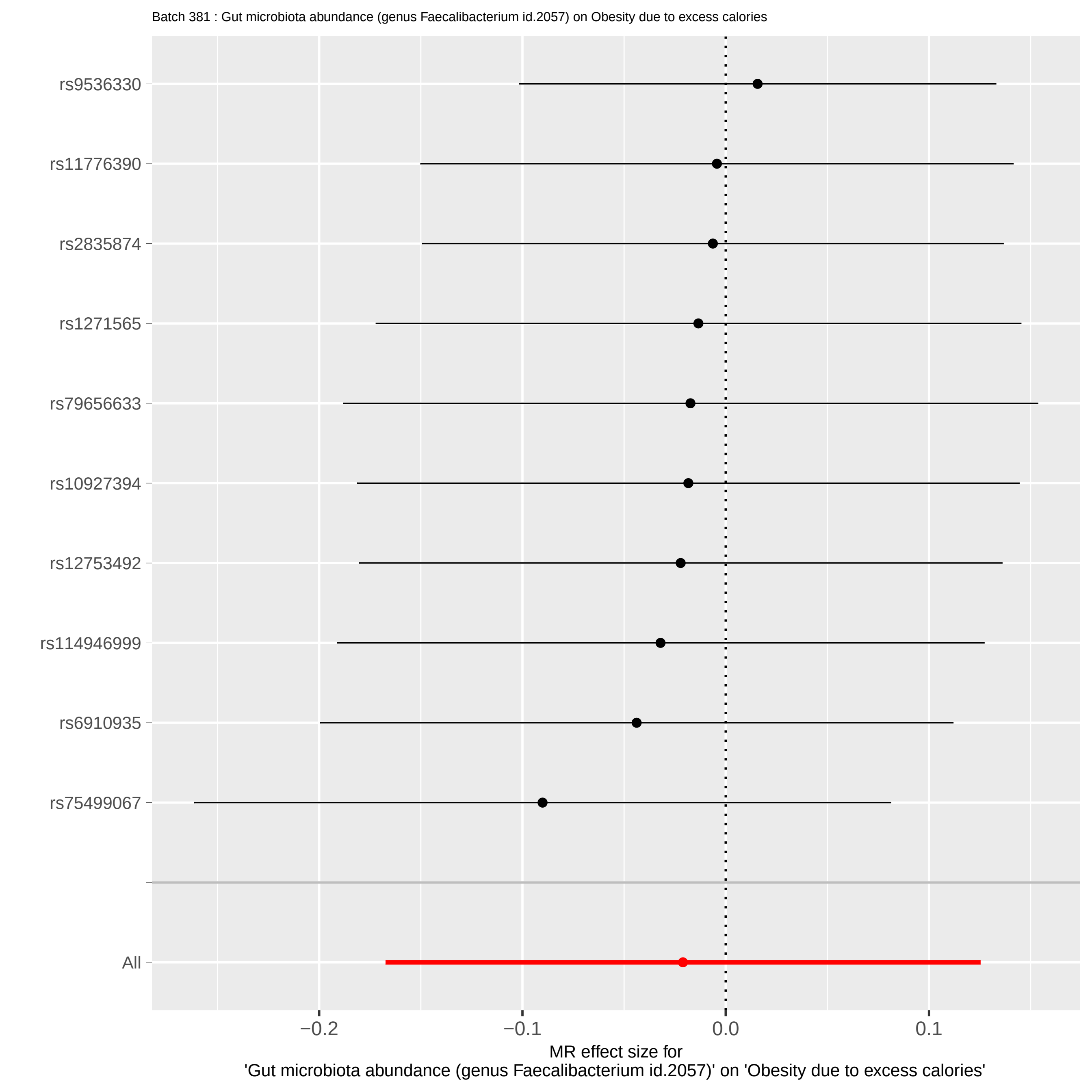

Batch 381 : Gut microbiota abundance (genus Faecalibacterium id.2057) on Obesity due to excess calories
rs9536330
rs11776390
rs2835874
rs1271565
rs79656633
rs10927394
rs12753492
rs114946999
rs6910935
rs75499067
All
−0.2
−0.1
0.0
0.1
MR effect size for
'Gut microbiota abundance (genus Faecalibacterium id.2057)' on 'Obesity due to excess calories'

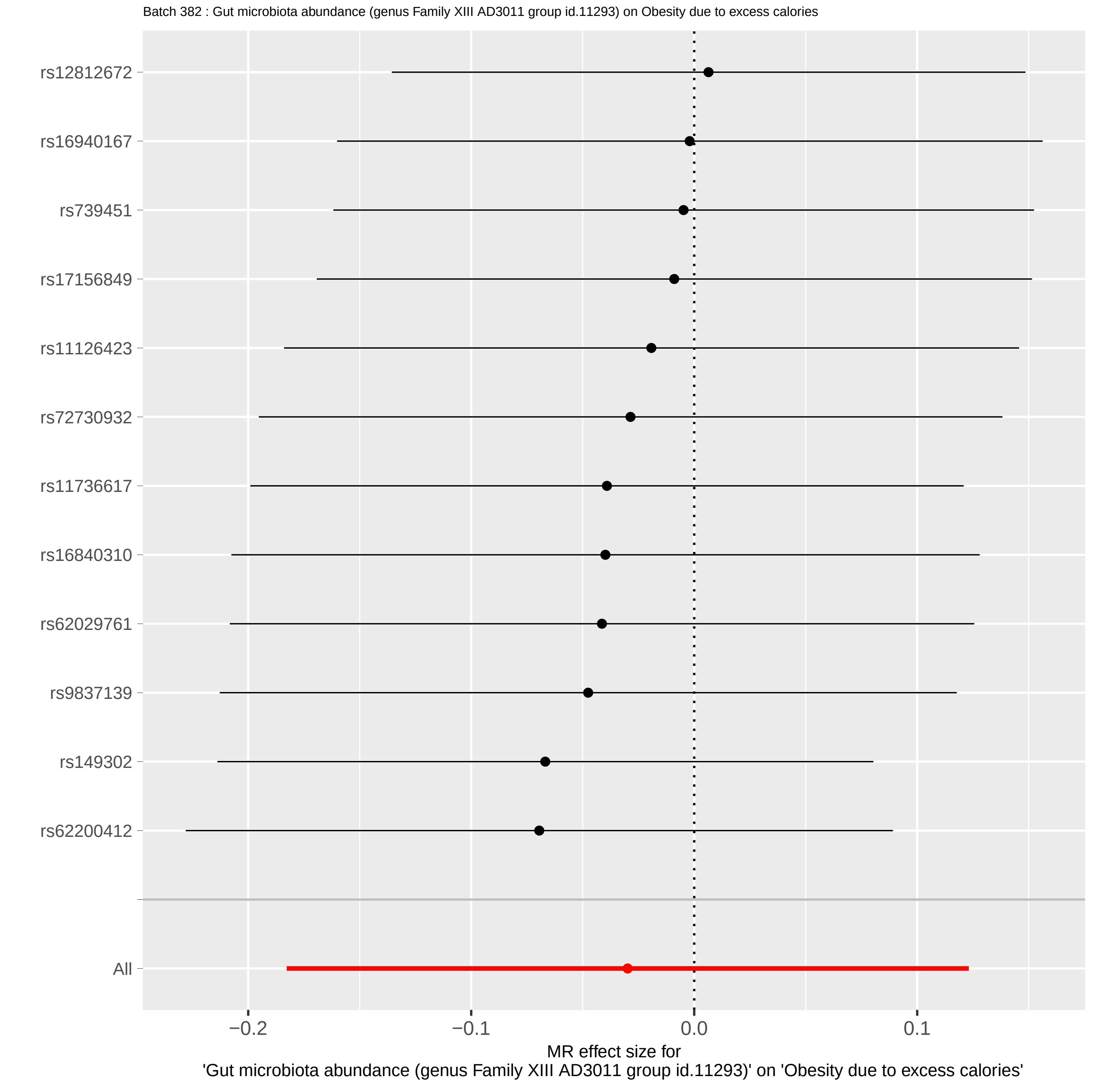

Batch 382 : Gut microbiota abundance (genus Family XIII AD3011 group id.11293) on Obesity due to excess calories
rs12812672
rs16940167
rs739451
rs17156849
rs11126423
rs72730932
rs11736617
rs16840310
rs62029761
rs9837139
rs149302
rs62200412
All
−0.2
−0.1
0.0
0.1
MR effect size for
'Gut microbiota abundance (genus Family XIII AD3011 group id.11293)' on 'Obesity due to excess calories'

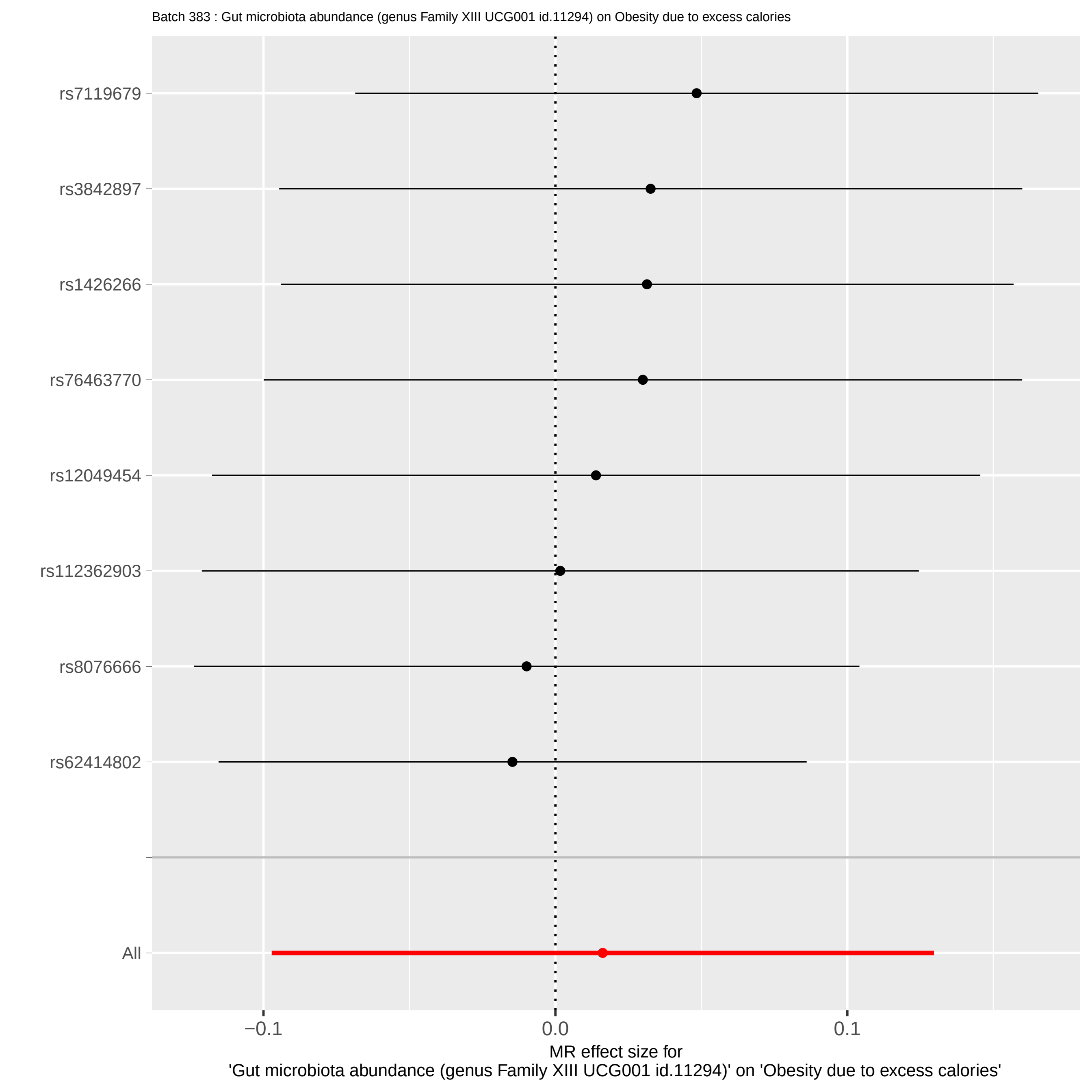
Batch 383 : Gut microbiota abundance (genus Family XIII UCG001 id.11294) on Obesity due to excess calories
rs7119679
rs3842897
rs1426266
rs76463770
rs12049454
rs112362903
rs8076666
rs62414802
All
−0.1
0.0
0.1
MR effect size for
'Gut microbiota abundance (genus Family XIII UCG001 id.11294)' on 'Obesity due to excess calories'

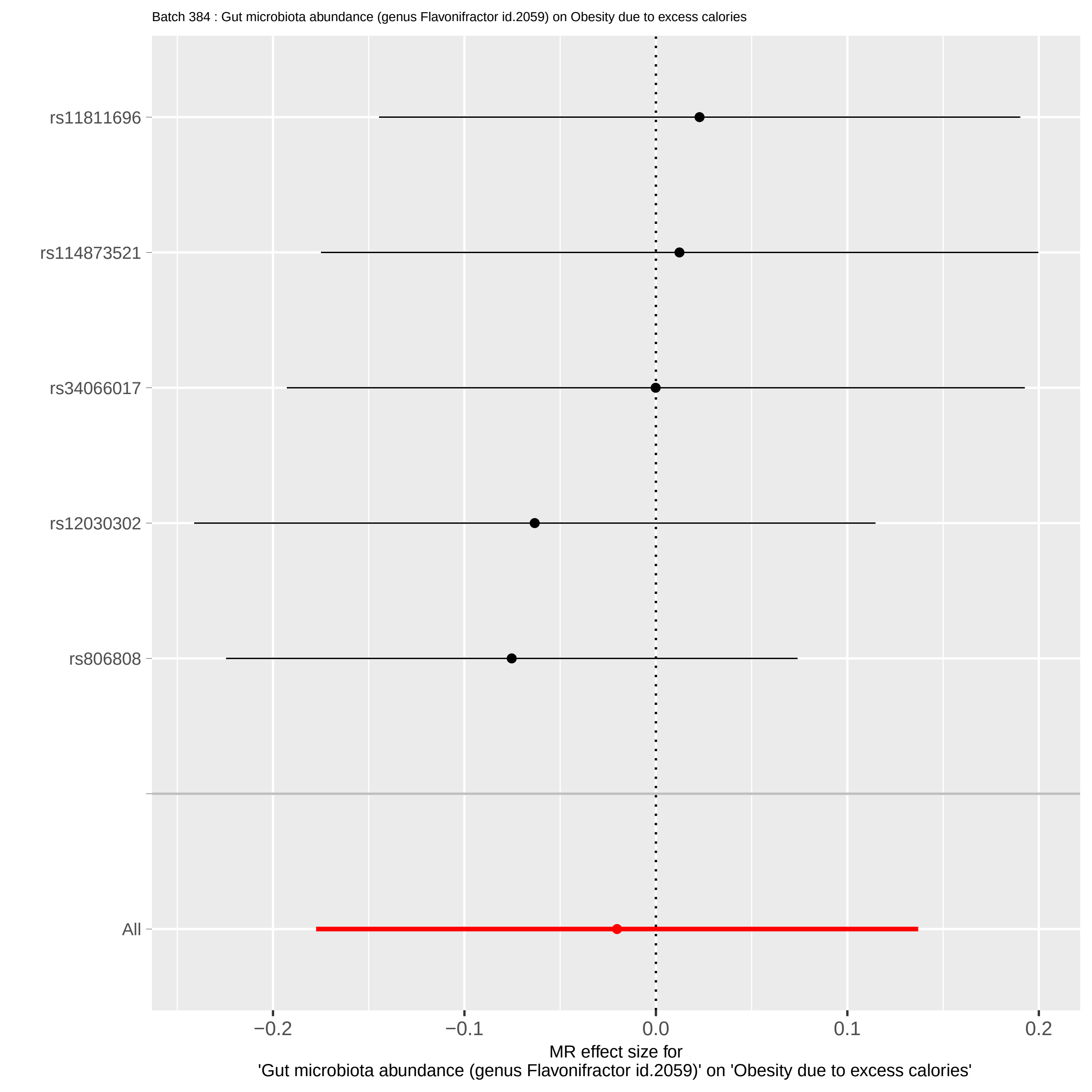

Batch 384 : Gut microbiota abundance (genus Flavonifractor id.2059) on Obesity due to excess calories
rs11811696
rs114873521
rs34066017
rs12030302
rs806808
All
−0.2
−0.1
0.0
0.1
0.2
MR effect size for
'Gut microbiota abundance (genus Flavonifractor id.2059)' on 'Obesity due to excess calories'

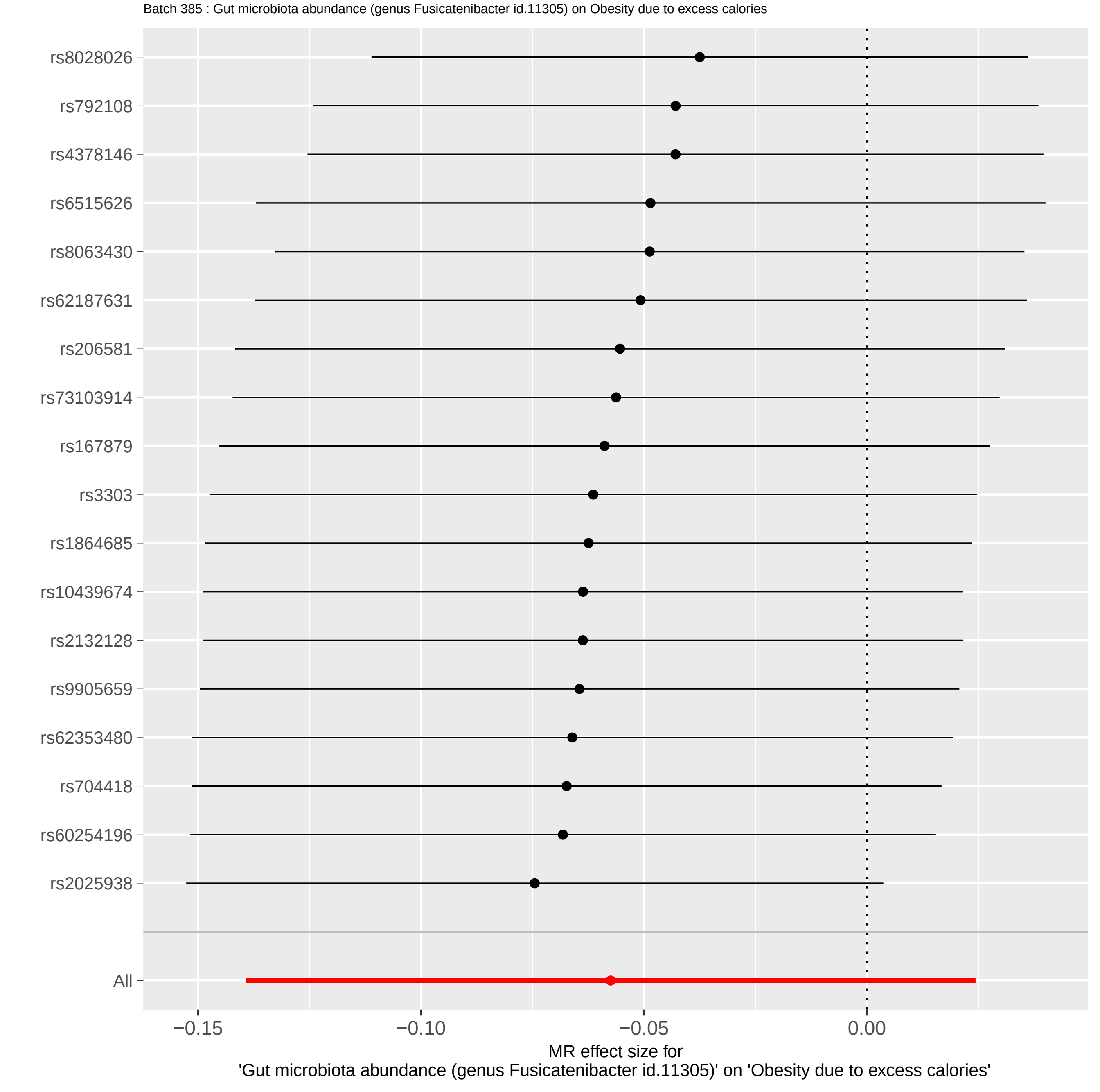

Batch 385 : Gut microbiota abundance (genus Fusicatenibacter id.11305) on Obesity due to excess calories
rs8028026
rs792108
rs4378146
rs6515626
rs8063430
rs62187631
rs206581
rs73103914
rs167879
rs3303
rs1864685
rs10439674
rs2132128
rs9905659
rs62353480
rs704418
rs60254196
rs2025938
All
−0.15
−0.10
−0.05
0.00
MR effect size for
'Gut microbiota abundance (genus Fusicatenibacter id.11305)' on 'Obesity due to excess calories'

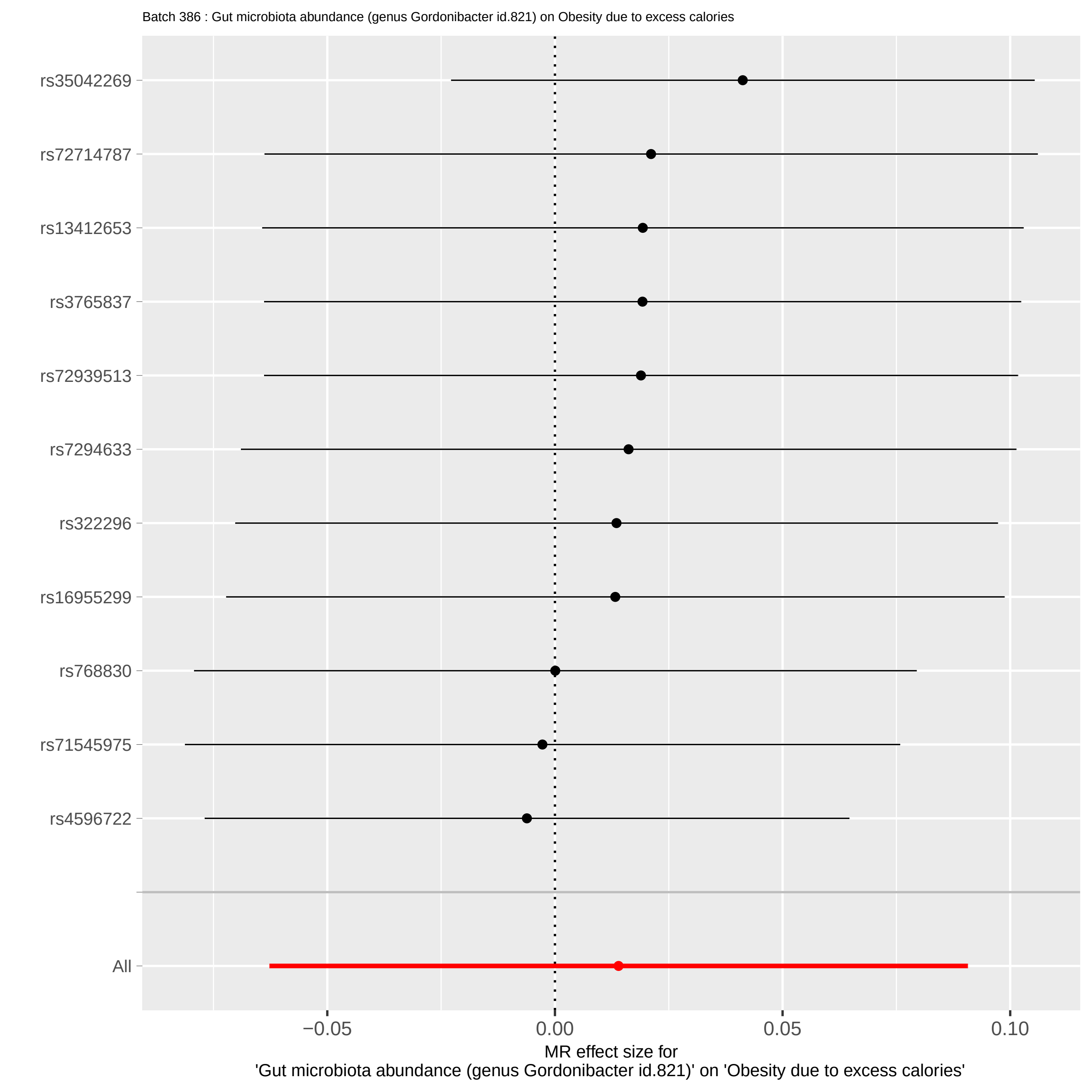

Batch 386 : Gut microbiota abundance (genus Gordonibacter id.821) on Obesity due to excess calories
rs35042269
rs72714787
rs13412653
rs3765837
rs72939513
rs7294633
rs322296
rs16955299
rs768830
rs71545975
rs4596722
All
−0.05
0.00
0.05
0.10
MR effect size for
'Gut microbiota abundance (genus Gordonibacter id.821)' on 'Obesity due to excess calories'

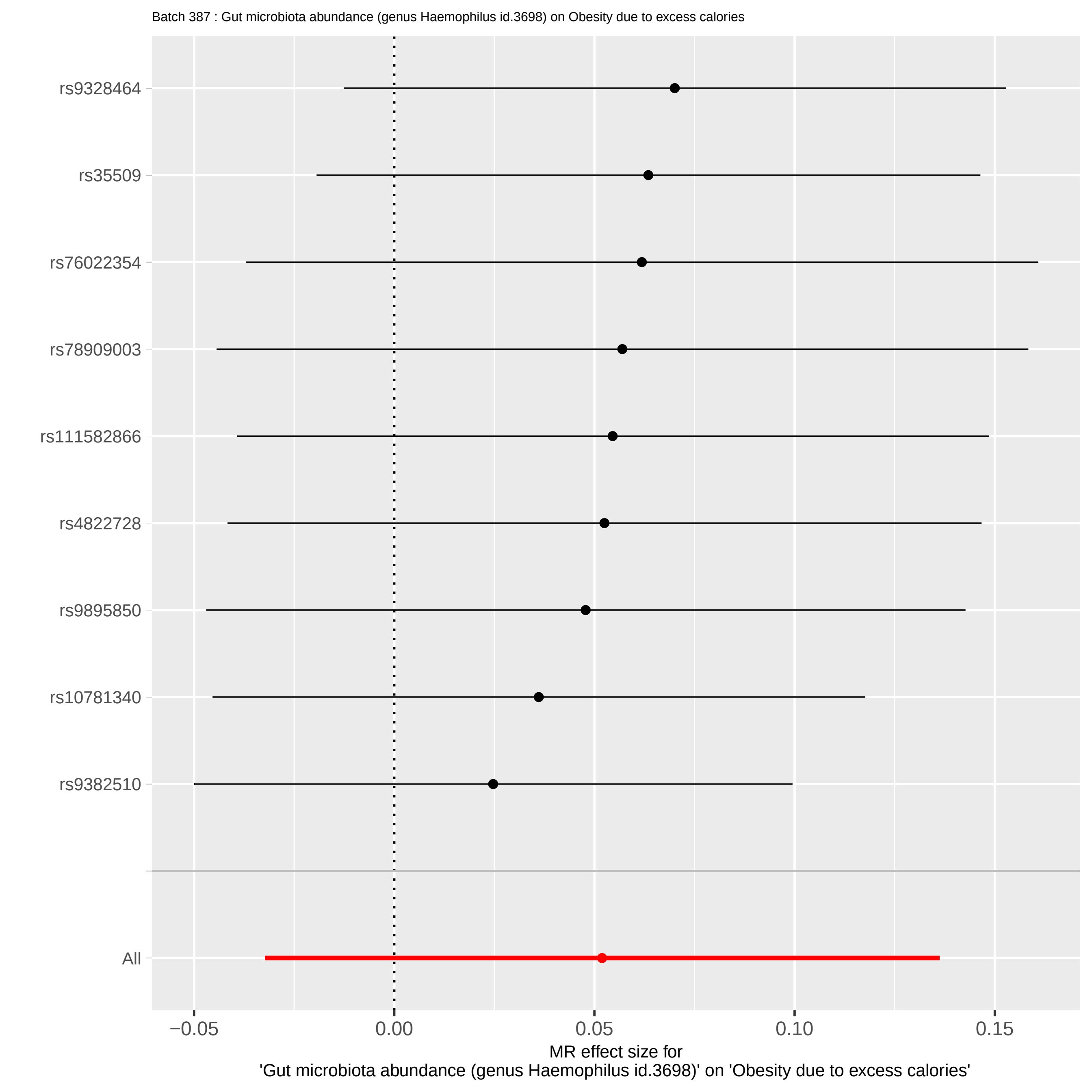

Batch 387 : Gut microbiota abundance (genus Haemophilus id.3698) on Obesity due to excess calories
rs9328464
rs35509
rs76022354
rs78909003
rs111582866
rs4822728
rs9895850
rs10781340
rs9382510
All
−0.05
0.00
0.05
0.10
0.15
MR effect size for
'Gut microbiota abundance (genus Haemophilus id.3698)' on 'Obesity due to excess calories'

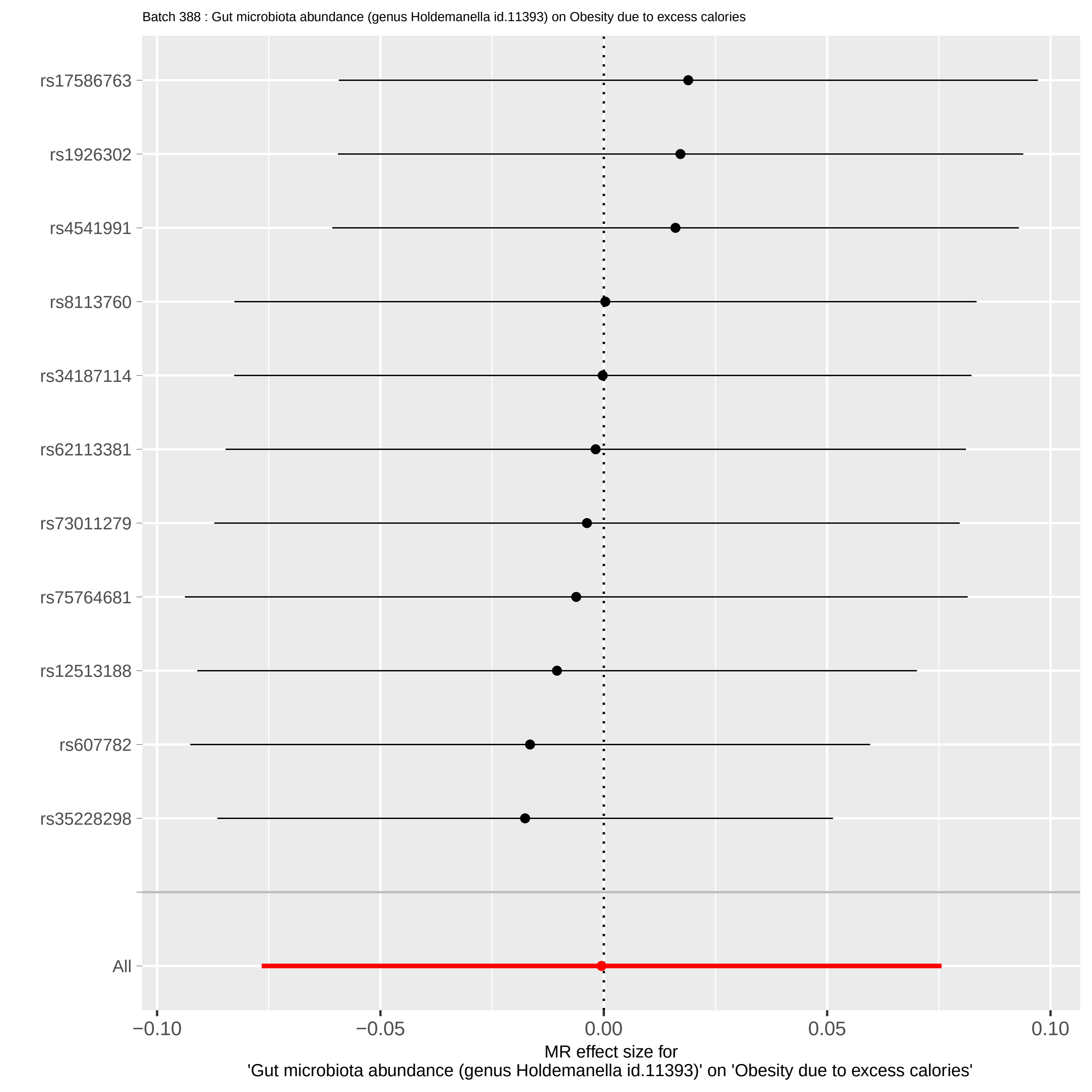

Batch 388 : Gut microbiota abundance (genus Holdemanella id.11393) on Obesity due to excess calories
rs17586763
rs1926302
rs4541991
rs8113760
rs34187114
rs62113381
rs73011279
rs75764681
rs12513188
rs607782
rs35228298
All
−0.10
−0.05
0.00
0.05
0.10
MR effect size for
'Gut microbiota abundance (genus Holdemanella id.11393)' on 'Obesity due to excess calories'

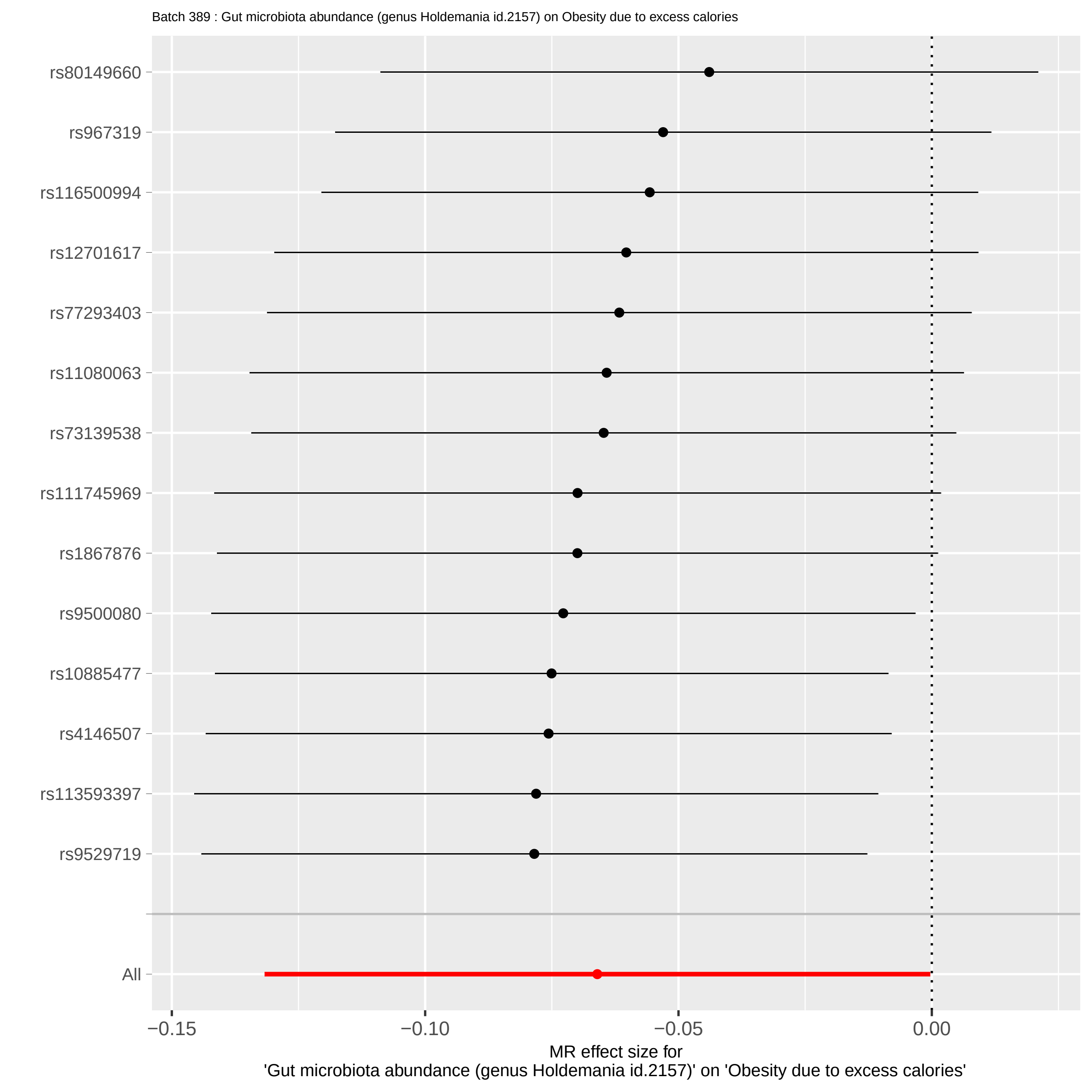

Batch 389 : Gut microbiota abundance (genus Holdemania id.2157) on Obesity due to excess calories
rs80149660
rs967319
rs116500994
rs12701617
rs77293403
rs11080063
rs73139538
rs111745969
rs1867876
rs9500080
rs10885477
rs4146507
rs113593397
rs9529719
All
−0.15
−0.10
−0.05
0.00
MR effect size for
'Gut microbiota abundance (genus Holdemania id.2157)' on 'Obesity due to excess calories'

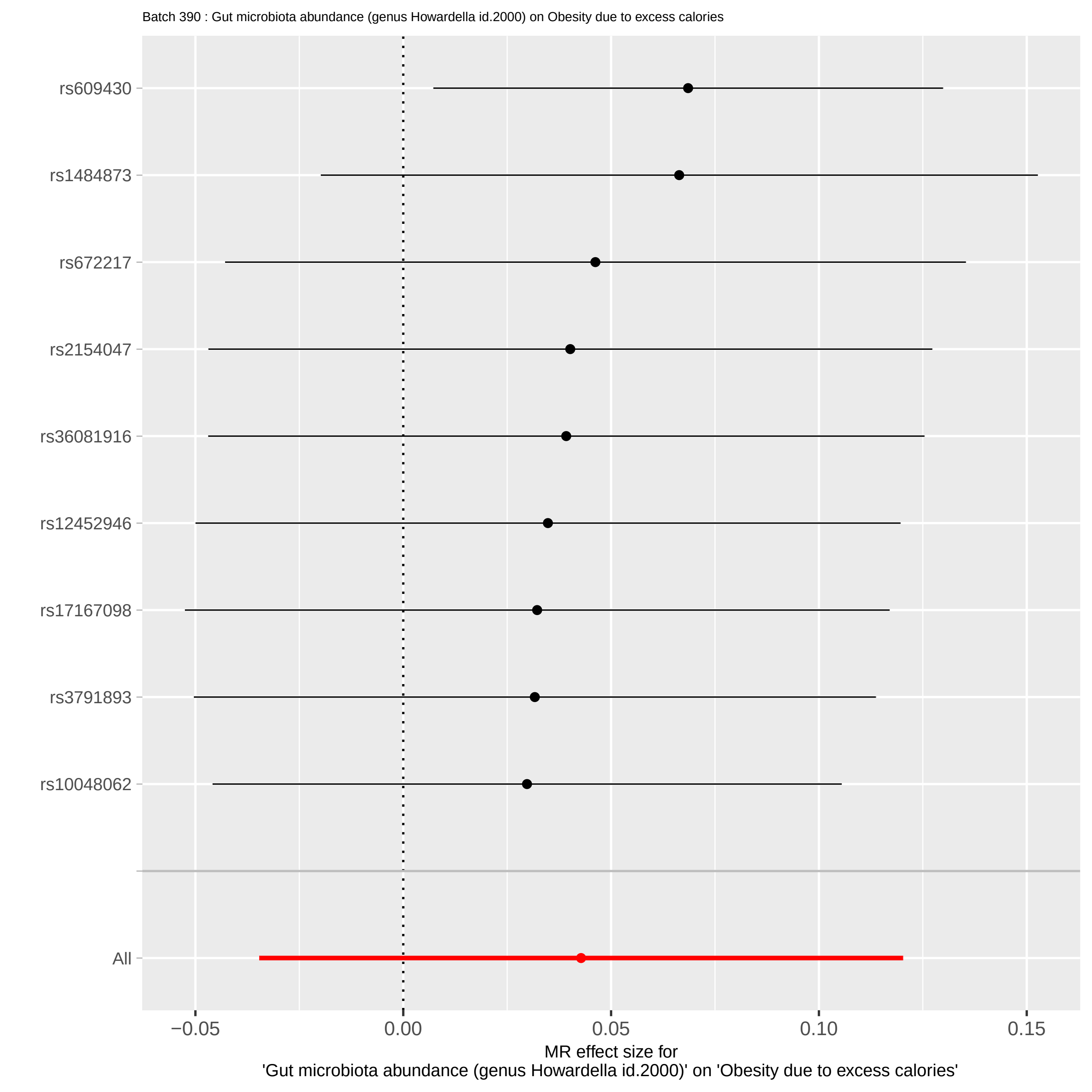
Batch 390 : Gut microbiota abundance (genus Howardella id.2000) on Obesity due to excess calories
rs609430
rs1484873
rs672217
rs2154047
rs36081916
rs12452946
rs17167098
rs3791893
rs10048062
All
−0.05
0.00
0.05
0.10
0.15
MR effect size for
'Gut microbiota abundance (genus Howardella id.2000)' on 'Obesity due to excess calories'

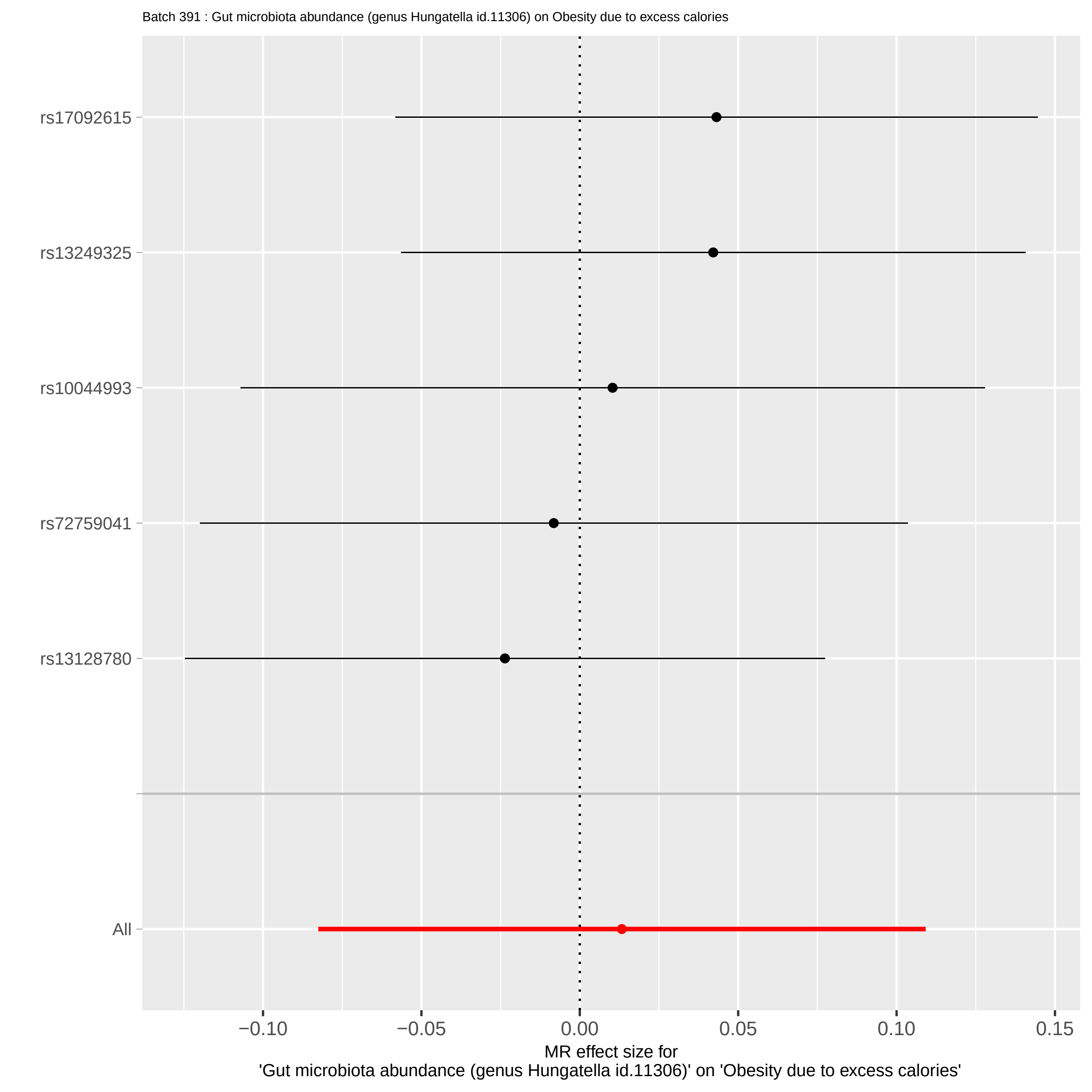

Batch 391 : Gut microbiota abundance (genus Hungatella id.11306) on Obesity due to excess calories
rs17092615
rs13249325
rs10044993
rs72759041
rs13128780
All
−0.10
−0.05
0.00
0.05
0.10
0.15
MR effect size for
'Gut microbiota abundance (genus Hungatella id.11306)' on 'Obesity due to excess calories'

Batch 392 : Gut microbiota abundance (genus Intestinibacter id.11345) on Obesity due to excess calories

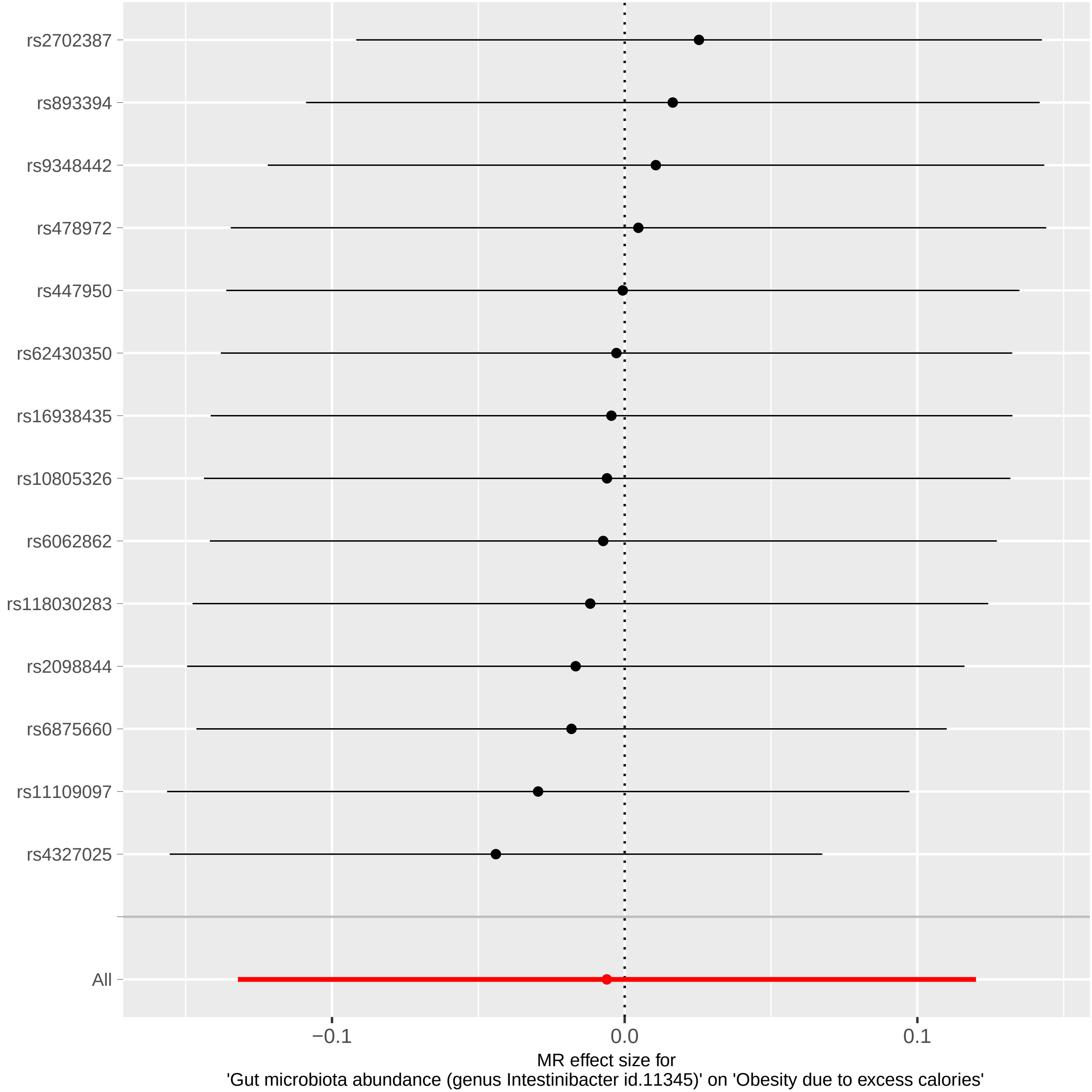

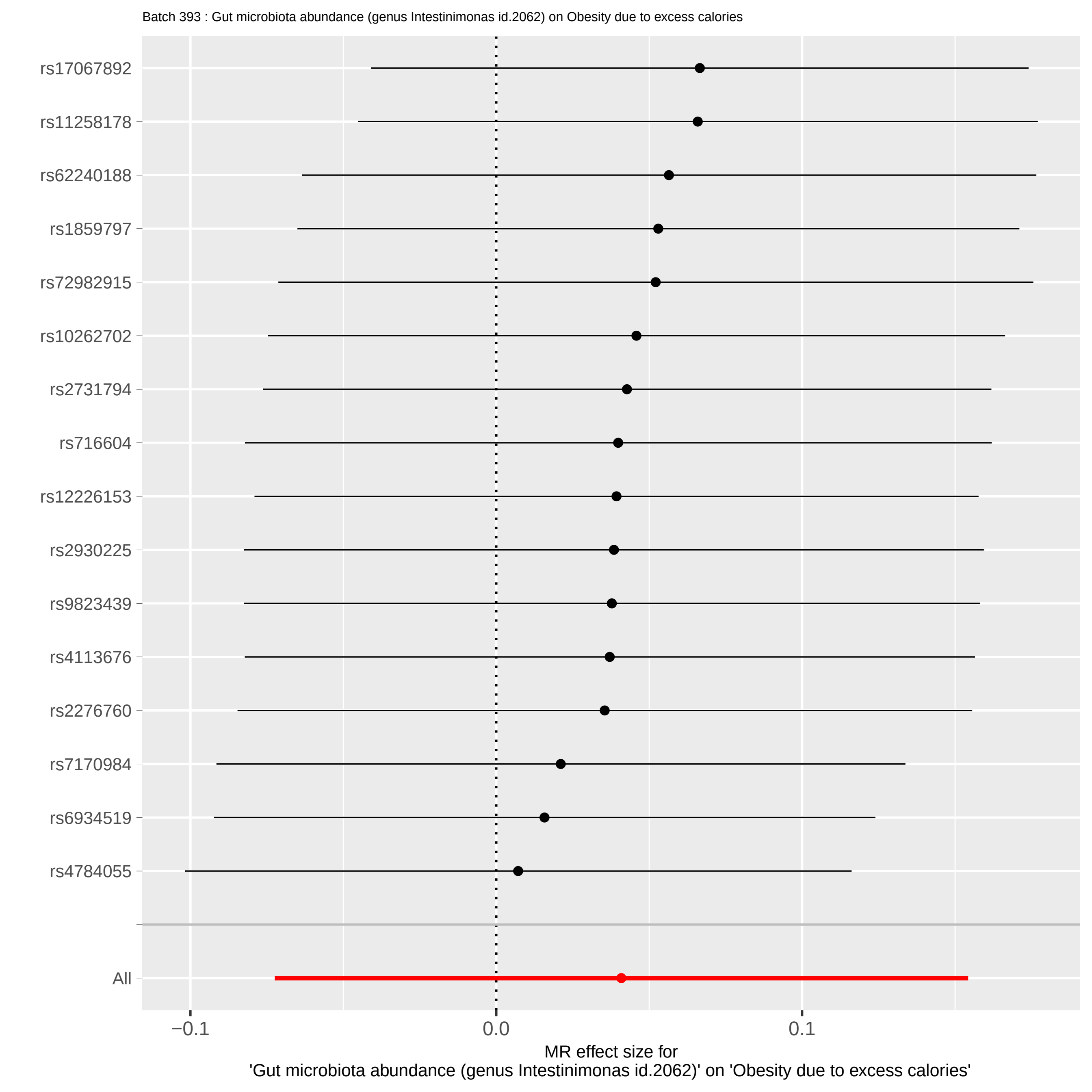

Batch 393 : Gut microbiota abundance (genus Intestinimonas id.2062) on Obesity due to excess calories
rs17067892
rs11258178
rs62240188
rs1859797
rs72982915
rs10262702
rs2731794
rs716604
rs12226153
rs2930225
rs9823439
rs4113676
rs2276760
rs7170984
rs6934519
rs4784055
All
−0.1
0.0
0.1
MR effect size for
'Gut microbiota abundance (genus Intestinimonas id.2062)' on 'Obesity due to excess calories'

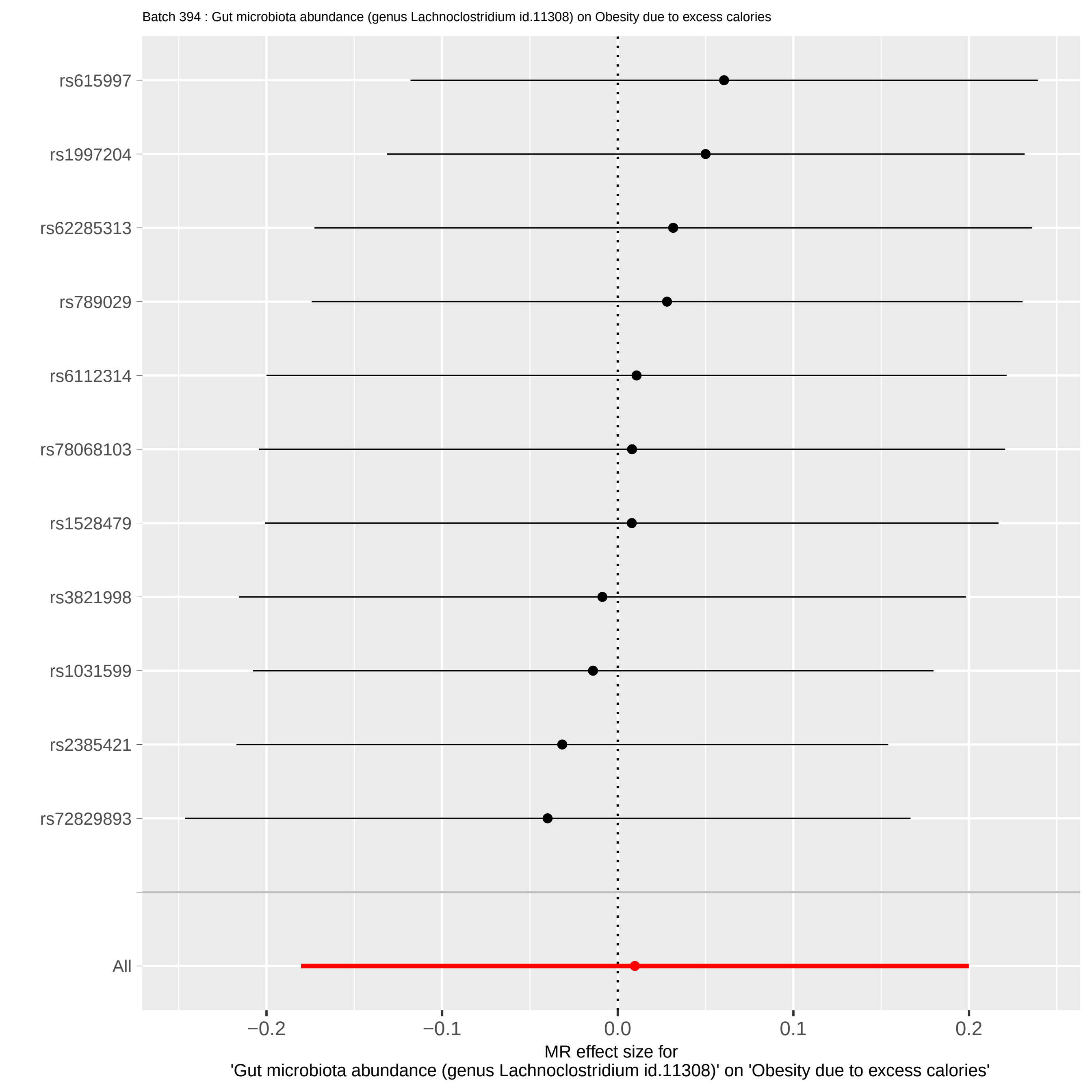

Batch 394 : Gut microbiota abundance (genus Lachnoclostridium id.11308) on Obesity due to excess calories
rs615997
rs1997204
rs62285313
rs789029
rs6112314
rs78068103
rs1528479
rs3821998
rs1031599
rs2385421
rs72829893
All
−0.2
−0.1
0.0
0.1
0.2
MR effect size for
'Gut microbiota abundance (genus Lachnoclostridium id.11308)' on 'Obesity due to excess calories'

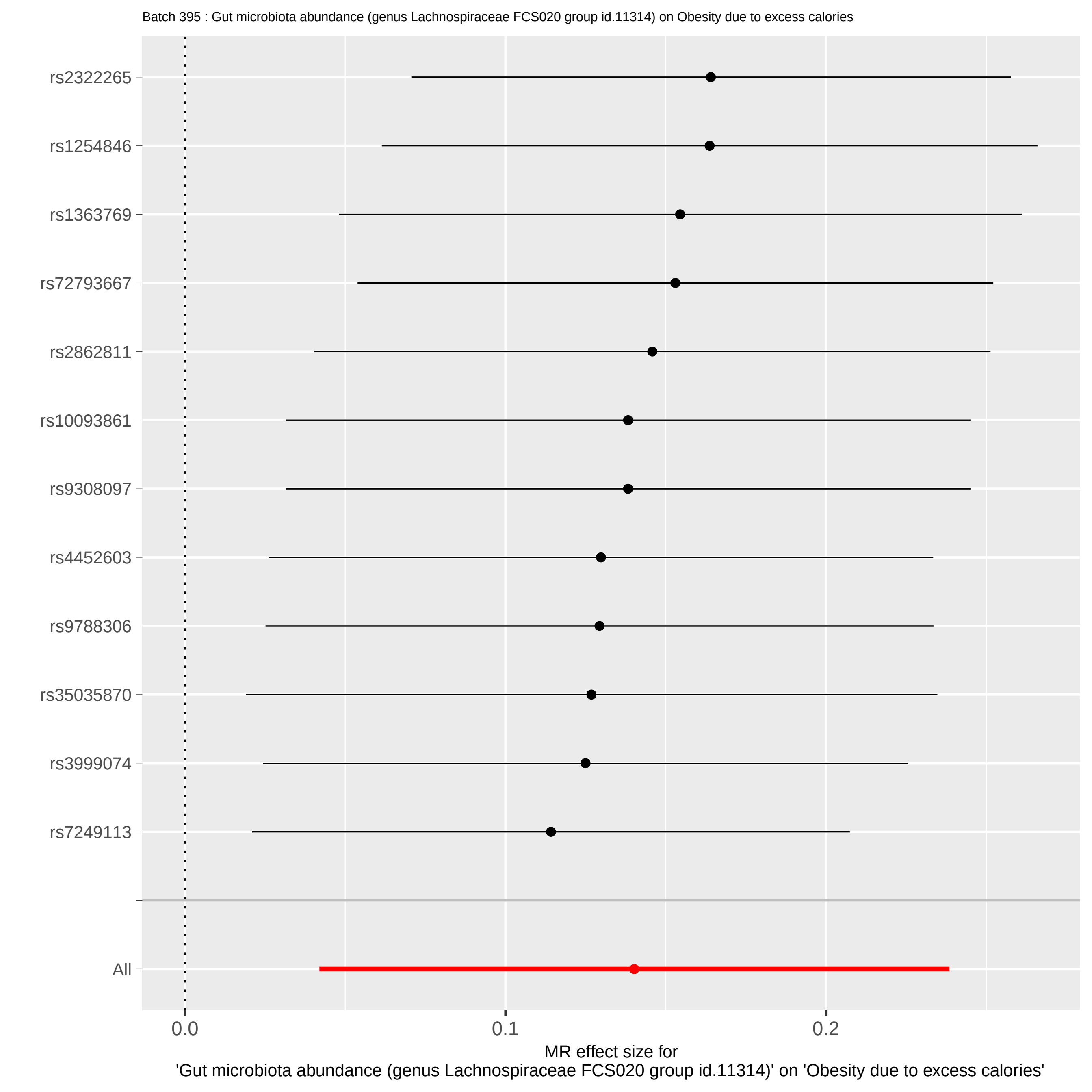
Batch 395 : Gut microbiota abundance (genus Lachnospiraceae FCS020 group id.11314) on Obesity due to excess calories
rs2322265
rs1254846
rs1363769
rs72793667
rs2862811
rs10093861
rs9308097
rs4452603
rs9788306
rs35035870
rs3999074
rs7249113
All
0.0
0.1
0.2
MR effect size for
'Gut microbiota abundance (genus Lachnospiraceae FCS020 group id.11314)' on 'Obesity due to excess calories'

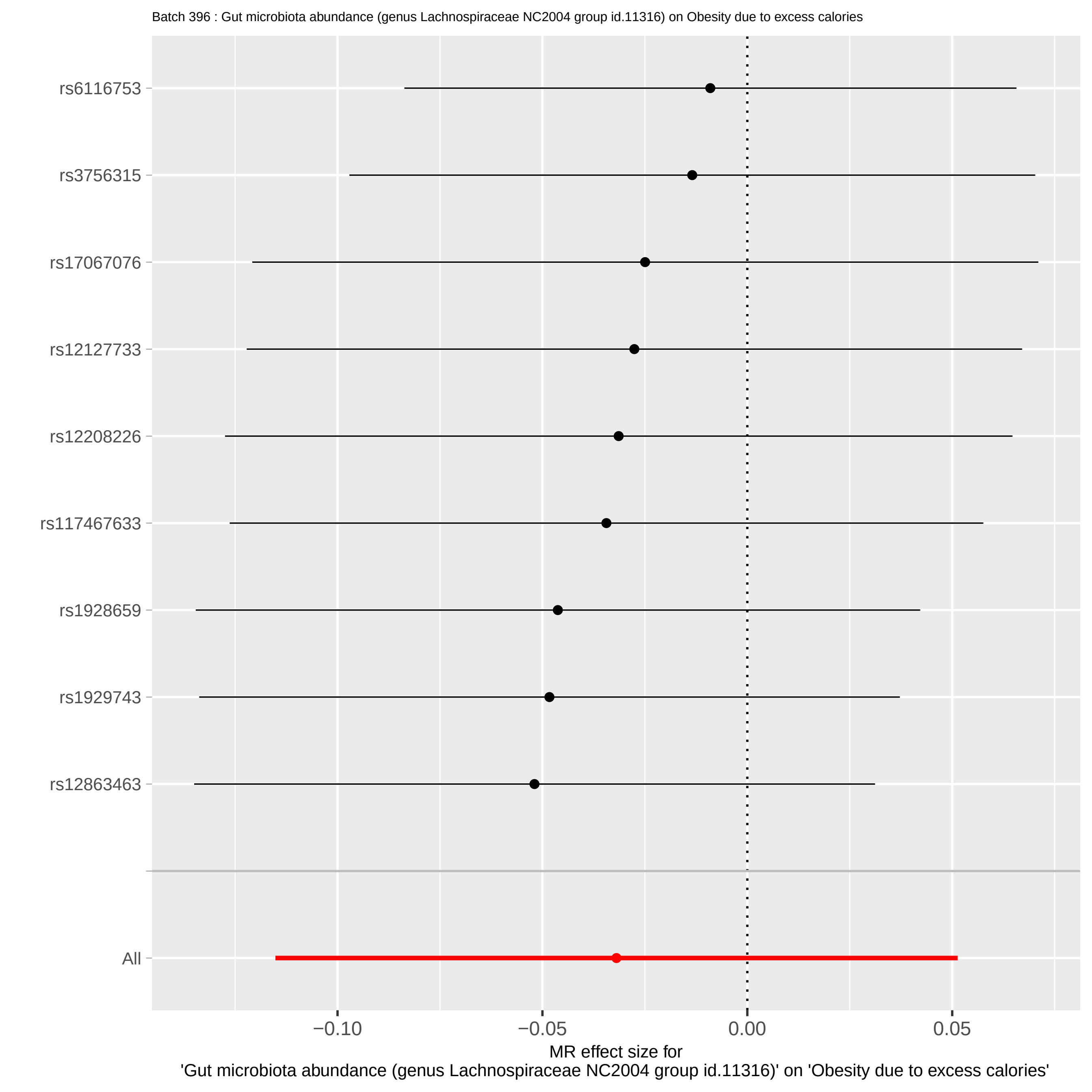
Batch 396 : Gut microbiota abundance (genus Lachnospiraceae NC2004 group id.11316) on Obesity due to excess calories
rs6116753
rs3756315
rs17067076
rs12127733
rs12208226
rs117467633
rs1928659
rs1929743
rs12863463
All
−0.10
−0.05
0.00
0.05
MR effect size for
'Gut microbiota abundance (genus Lachnospiraceae NC2004 group id.11316)' on 'Obesity due to excess calories'

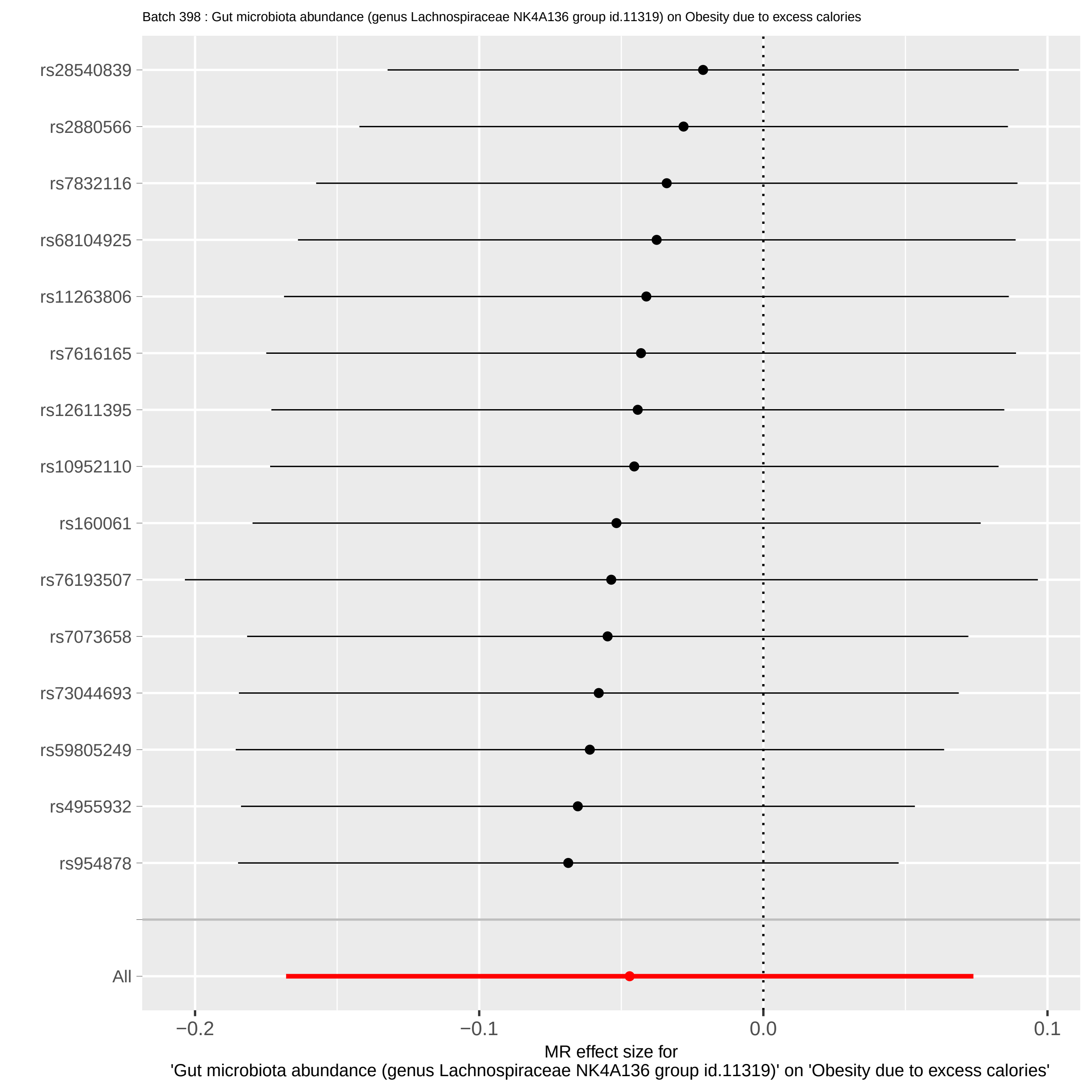

Batch 398 : Gut microbiota abundance (genus Lachnospiraceae NK4A136 group id.11319) on Obesity due to excess calories
rs28540839
rs2880566
rs7832116
rs68104925
rs11263806
rs7616165
rs12611395
rs10952110
rs160061
rs76193507
rs7073658
rs73044693
rs59805249
rs4955932
rs954878
All
−0.2
−0.1
0.0
0.1
MR effect size for
'Gut microbiota abundance (genus Lachnospiraceae NK4A136 group id.11319)' on 'Obesity due to excess calories'

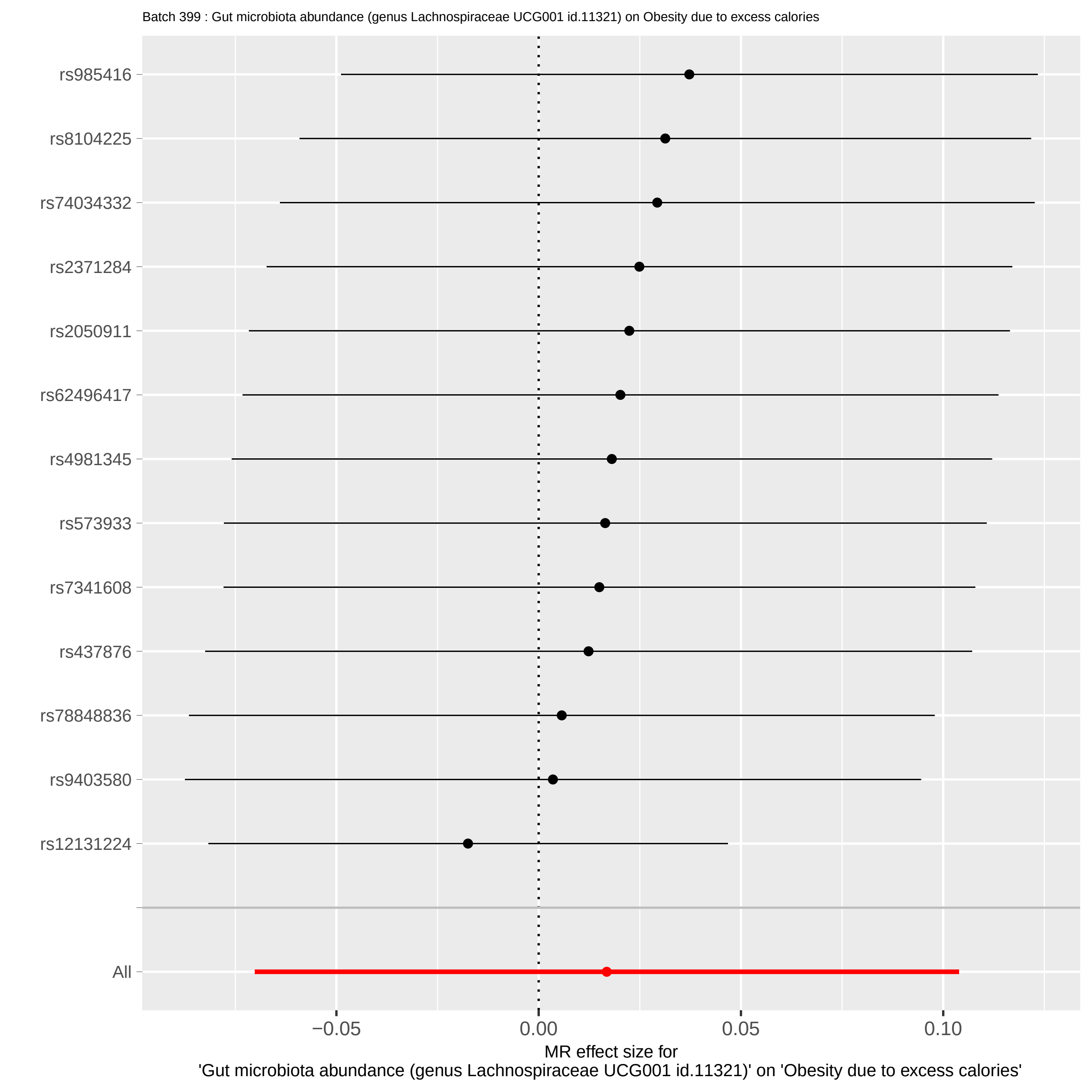
Batch 399 : Gut microbiota abundance (genus Lachnospiraceae UCG001 id.11321) on Obesity due to excess calories
rs985416
rs8104225
rs74034332
rs2371284
rs2050911
rs62496417
rs4981345
rs573933
rs7341608
rs437876
rs78848836
rs9403580
rs12131224
All
−0.05
0.00
0.05
0.10
MR effect size for
'Gut microbiota abundance (genus Lachnospiraceae UCG001 id.11321)' on 'Obesity due to excess calories'

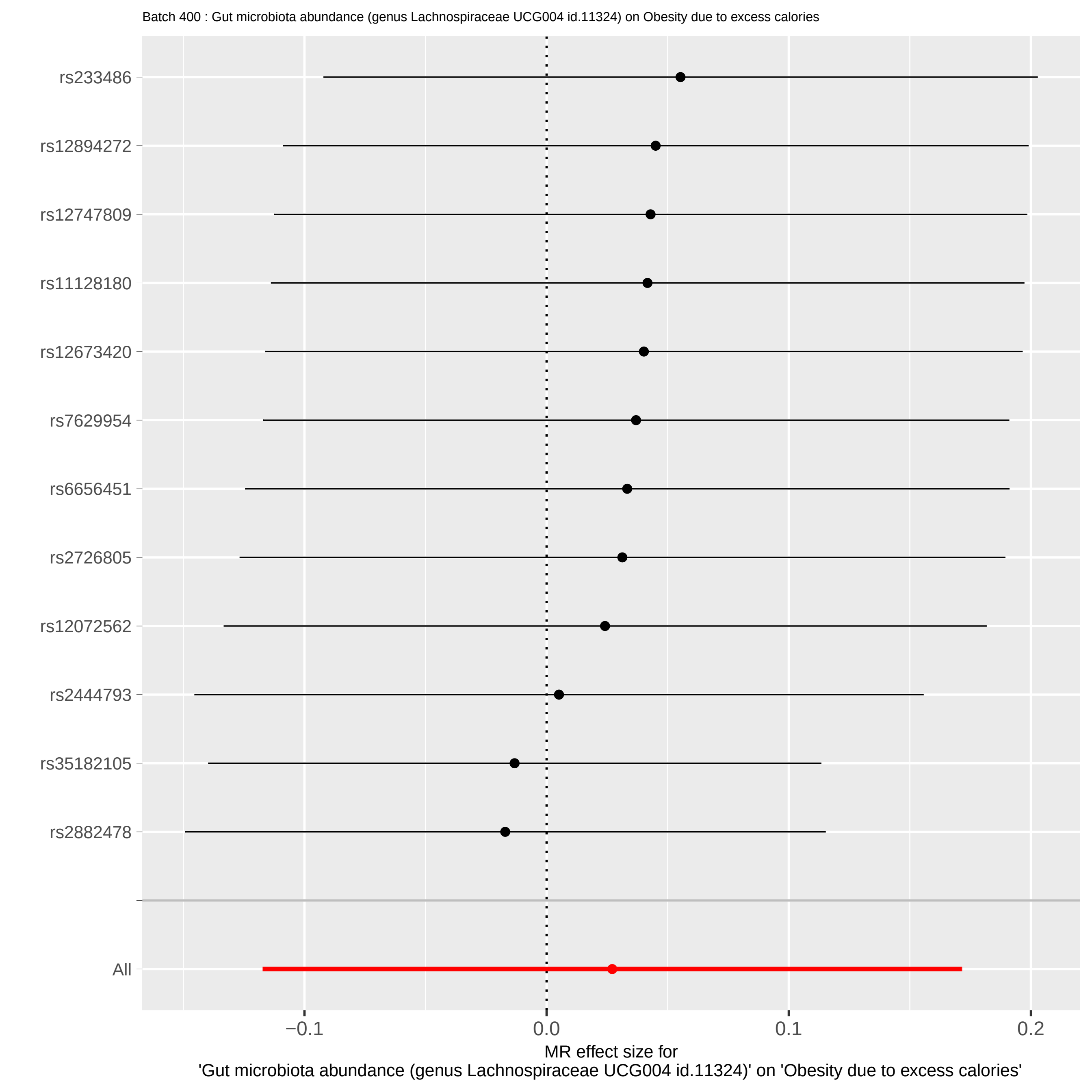

Batch 400 : Gut microbiota abundance (genus Lachnospiraceae UCG004 id.11324) on Obesity due to excess calories
rs233486
rs12894272
rs12747809
rs11128180
rs12673420
rs7629954
rs6656451
rs2726805
rs12072562
rs2444793
rs35182105
rs2882478
All
−0.1
0.0
0.1
0.2
MR effect size for
'Gut microbiota abundance (genus Lachnospiraceae UCG004 id.11324)' on 'Obesity due to excess calories'

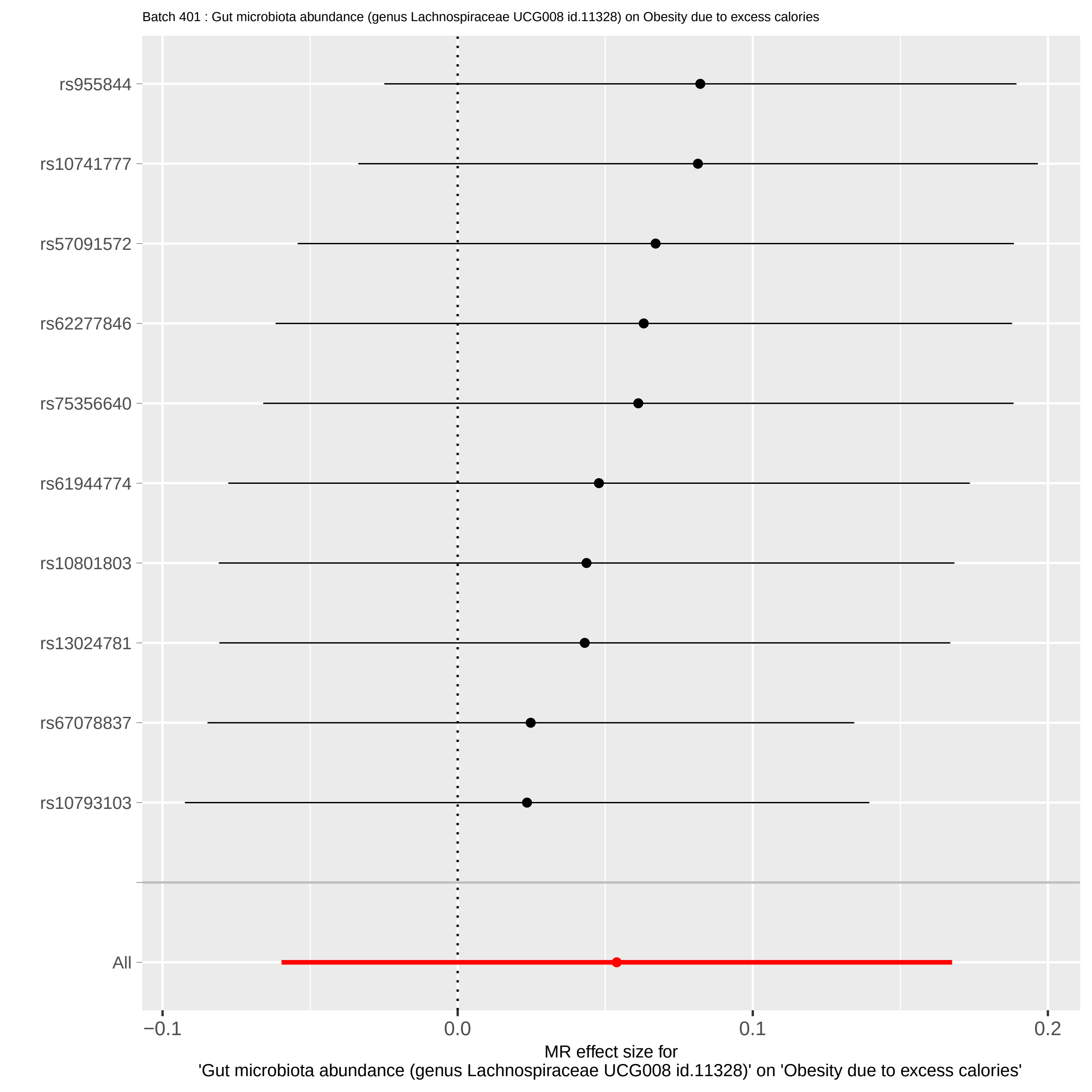

Batch 401 : Gut microbiota abundance (genus Lachnospiraceae UCG008 id.11328) on Obesity due to excess calories
rs955844
rs10741777
rs57091572
rs62277846
rs75356640
rs61944774
rs10801803
rs13024781
rs67078837
rs10793103
All
−0.1
0.0
0.1
0.2
MR effect size for
'Gut microbiota abundance (genus Lachnospiraceae UCG008 id.11328)' on 'Obesity due to excess calories'

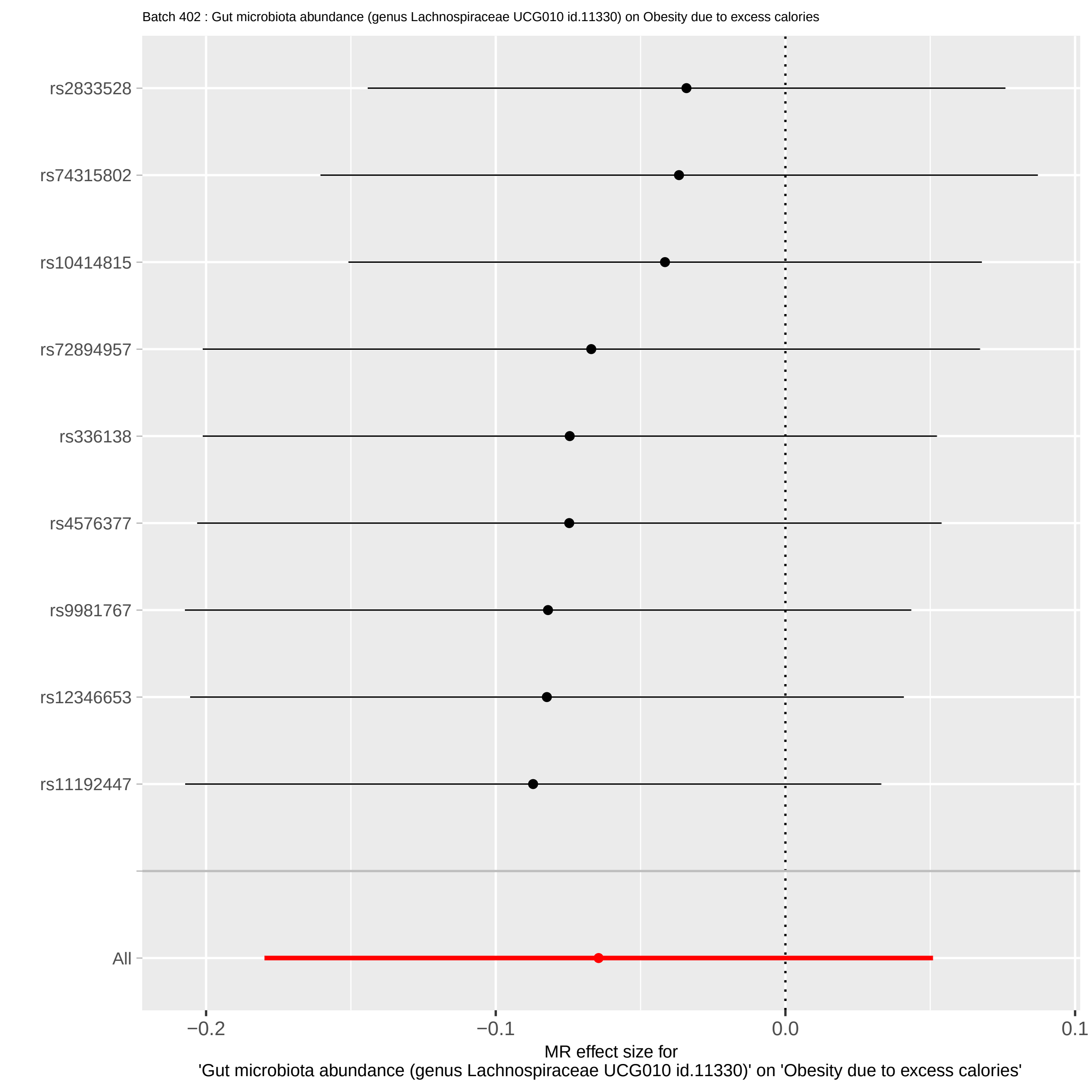

Batch 402 : Gut microbiota abundance (genus Lachnospiraceae UCG010 id.11330) on Obesity due to excess calories
rs2833528
rs74315802
rs10414815
rs72894957
rs336138
rs4576377
rs9981767
rs12346653
rs11192447
All
−0.2
−0.1
0.0
0.1
MR effect size for
'Gut microbiota abundance (genus Lachnospiraceae UCG010 id.11330)' on 'Obesity due to excess calories'

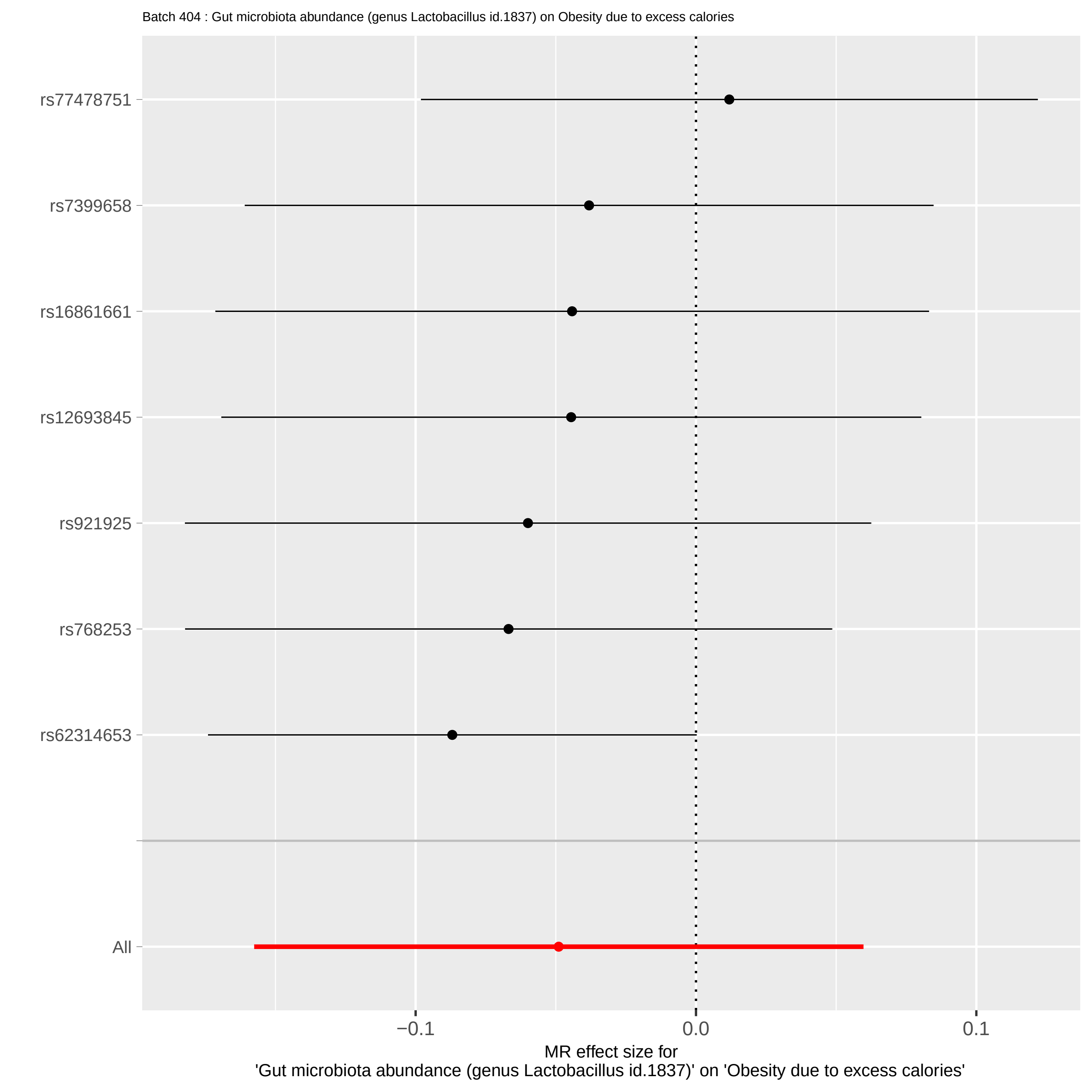

Batch 404 : Gut microbiota abundance (genus Lactobacillus id.1837) on Obesity due to excess calories
rs77478751
rs7399658
rs16861661
rs12693845
rs921925
rs768253
rs62314653
All
−0.1
0.0
0.1
MR effect size for
'Gut microbiota abundance (genus Lactobacillus id.1837)' on 'Obesity due to excess calories'

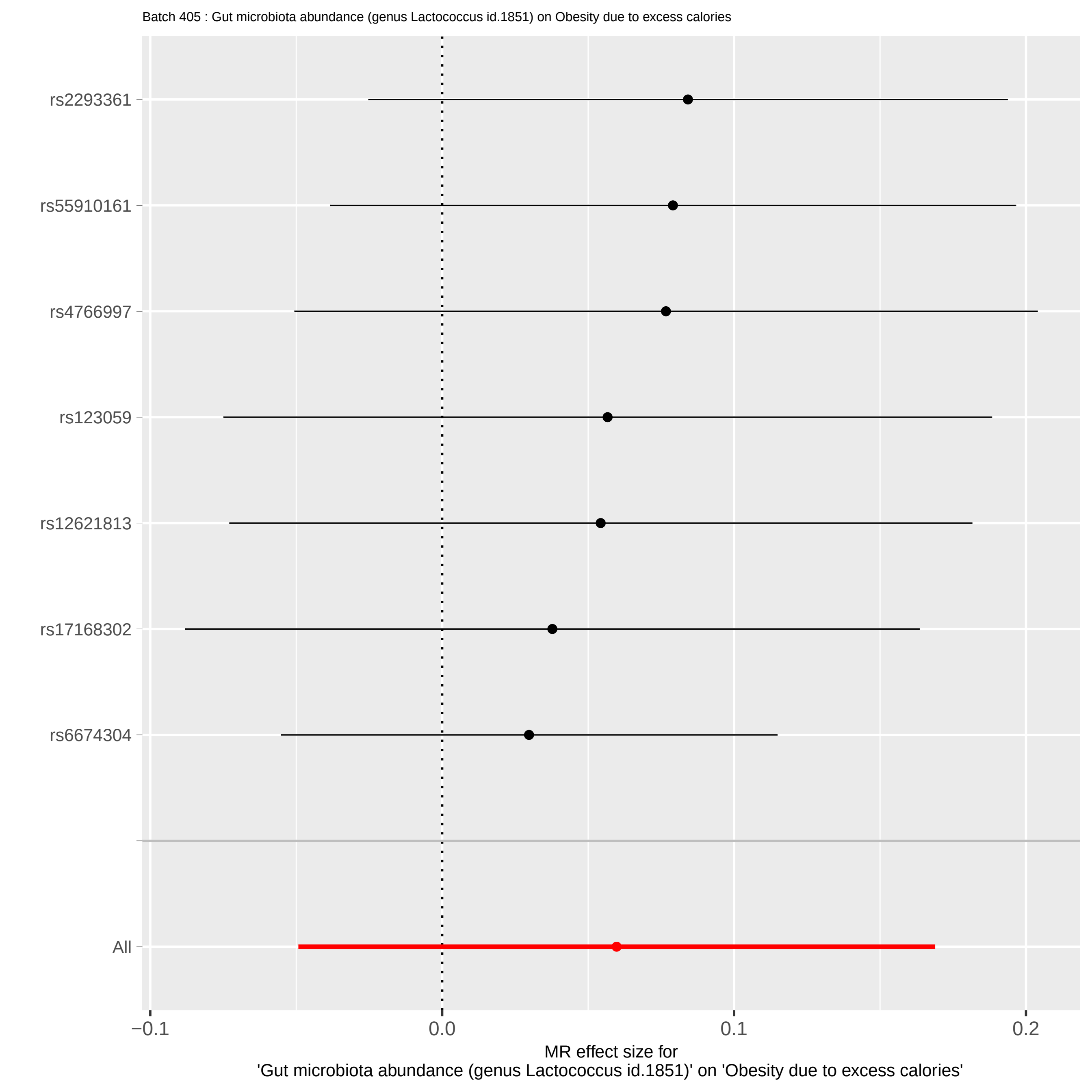

Batch 405 : Gut microbiota abundance (genus Lactococcus id.1851) on Obesity due to excess calories
rs2293361
rs55910161
rs4766997
rs123059
rs12621813
rs17168302
rs6674304
All
−0.1
0.0
0.1
0.2
MR effect size for
'Gut microbiota abundance (genus Lactococcus id.1851)' on 'Obesity due to excess calories'

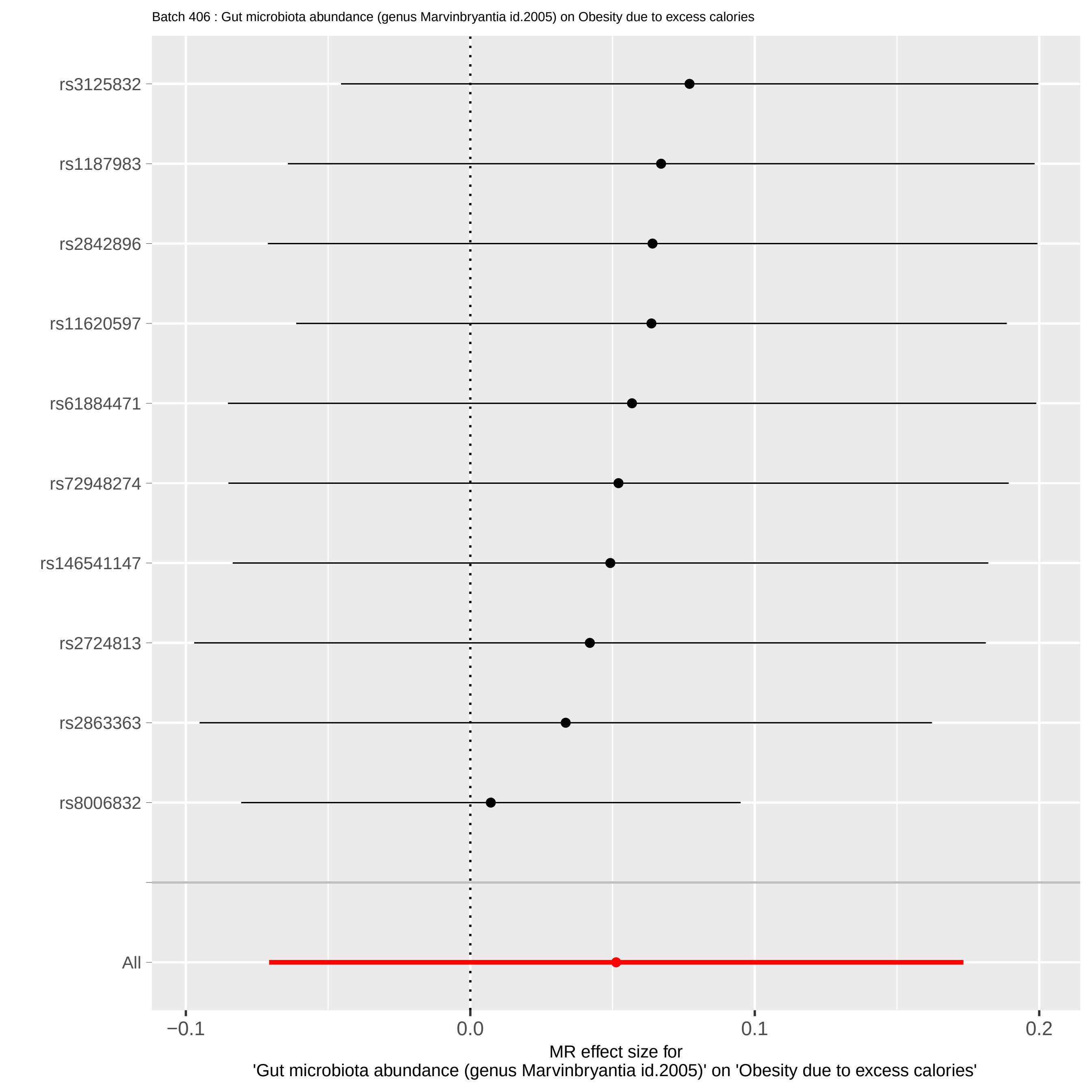
Batch 406 : Gut microbiota abundance (genus Marvinbryantia id.2005) on Obesity due to excess calories
rs3125832
rs1187983
rs2842896
rs11620597
rs61884471
rs72948274
rs146541147
rs2724813
rs2863363
rs8006832
All
−0.1
0.0
0.1
0.2
MR effect size for
'Gut microbiota abundance (genus Marvinbryantia id.2005)' on 'Obesity due to excess calories'

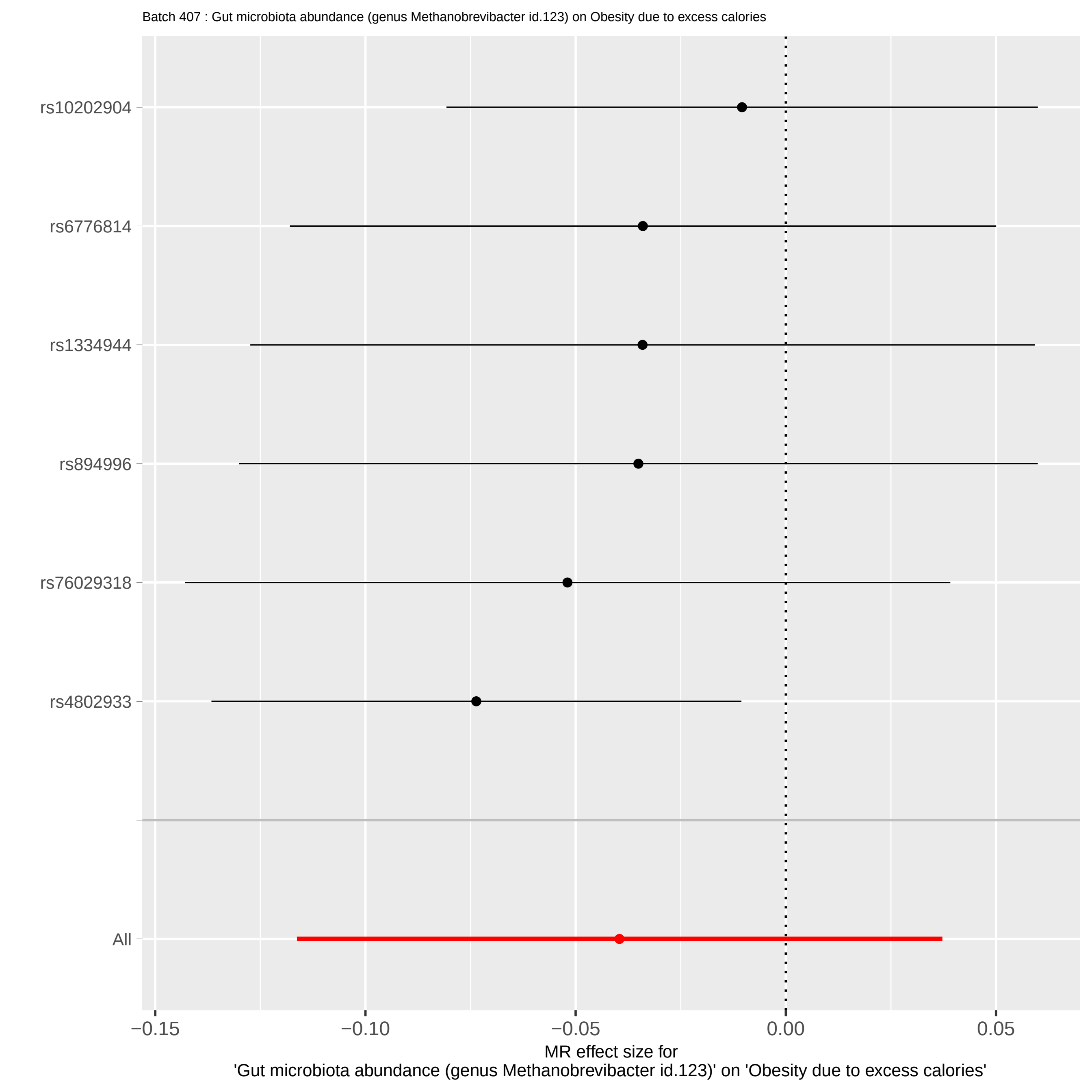

Batch 407 : Gut microbiota abundance (genus Methanobrevibacter id.123) on Obesity due to excess calories
rs10202904
rs6776814
rs1334944
rs894996
rs76029318
rs4802933
All
−0.15
−0.10
−0.05
0.00
0.05
MR effect size for
'Gut microbiota abundance (genus Methanobrevibacter id.123)' on 'Obesity due to excess calories'

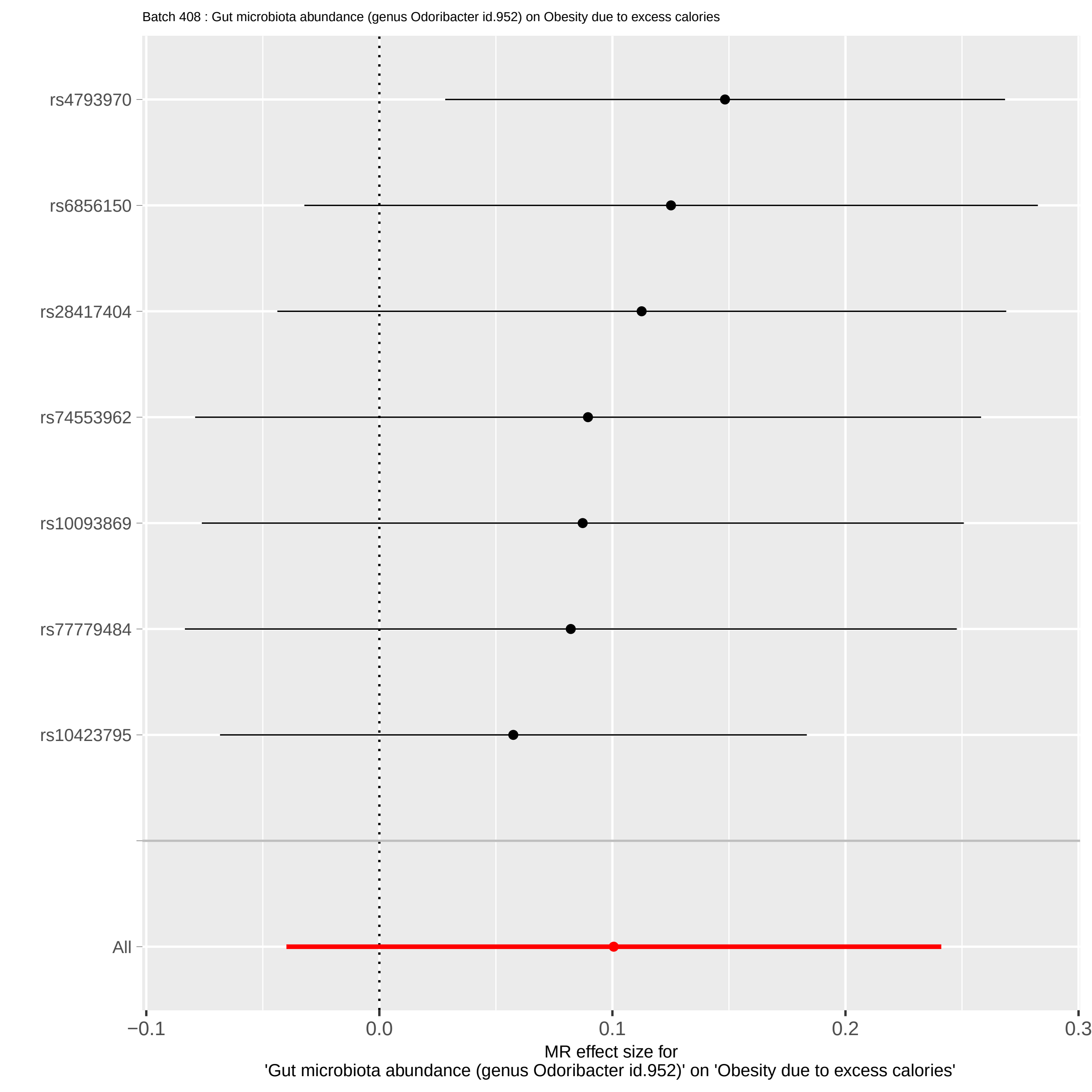

Batch 408 : Gut microbiota abundance (genus Odoribacter id.952) on Obesity due to excess calories
rs4793970
rs6856150
rs28417404
rs74553962
rs10093869
rs77779484
rs10423795
All
−0.1
0.0
0.1
0.2
0.3
MR effect size for
'Gut microbiota abundance (genus Odoribacter id.952)' on 'Obesity due to excess calories'

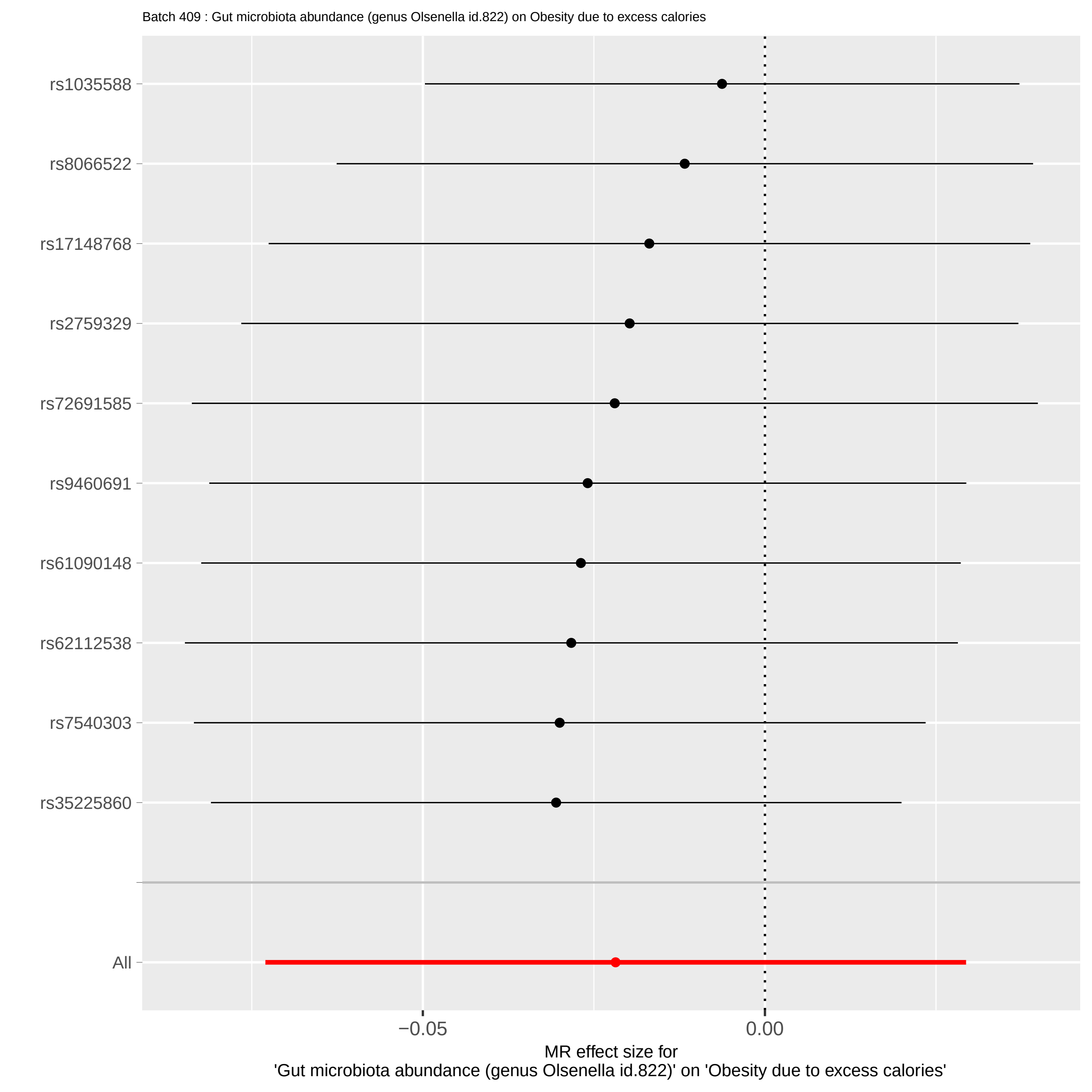
Batch 409 : Gut microbiota abundance (genus Olsenella id.822) on Obesity due to excess calories
rs1035588
rs8066522
rs17148768
rs2759329
rs72691585
rs9460691
rs61090148
rs62112538
rs7540303
rs35225860
All
−0.05
0.00
MR effect size for
'Gut microbiota abundance (genus Olsenella id.822)' on 'Obesity due to excess calories'

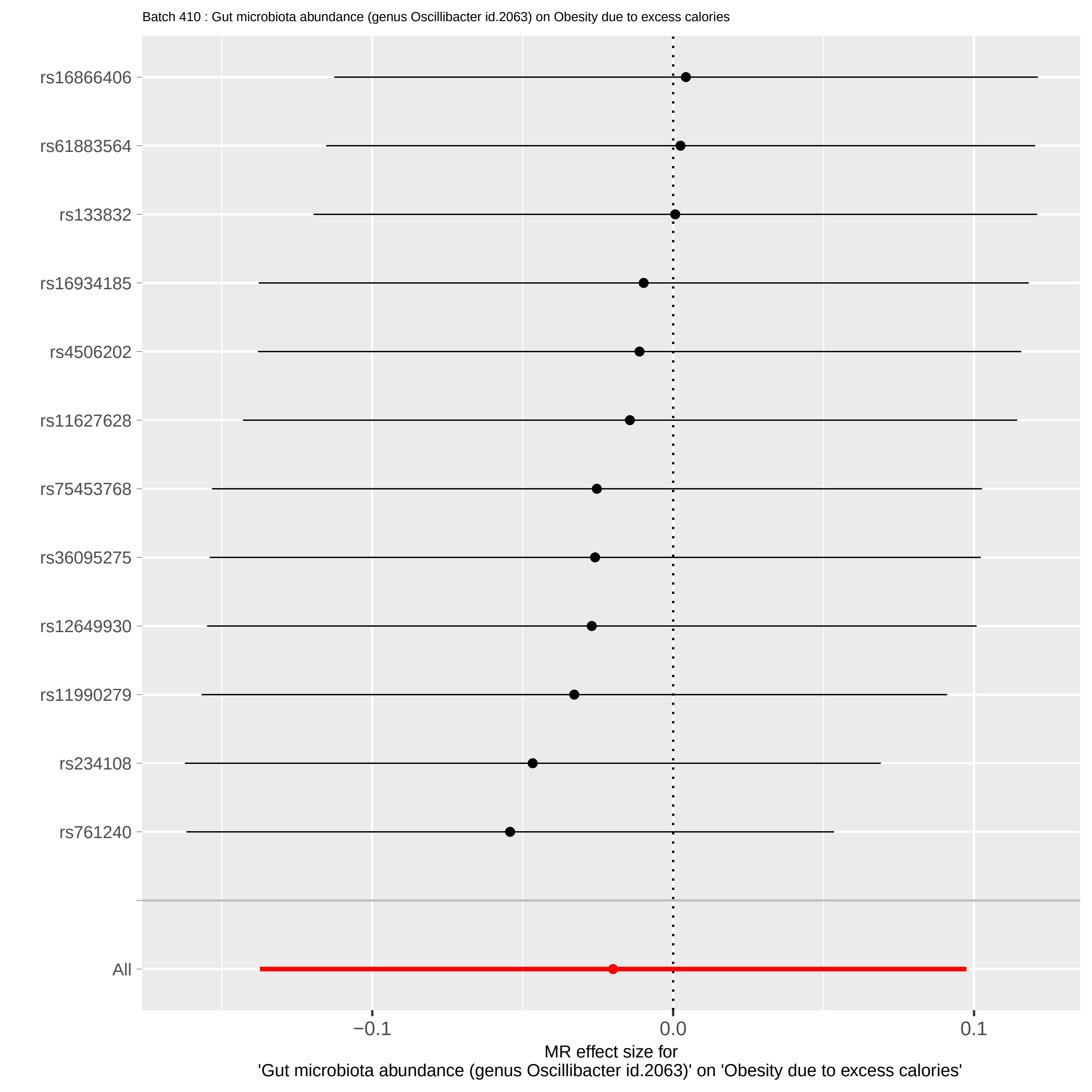

Batch 410 : Gut microbiota abundance (genus Oscillibacter id.2063) on Obesity due to excess calories
rs16866406
rs61883564
rs133832
rs16934185
rs4506202
rs11627628
rs75453768
rs36095275
rs12649930
rs11990279
rs234108
rs761240
All
−0.1
0.0
0.1
MR effect size for
'Gut microbiota abundance (genus Oscillibacter id.2063)' on 'Obesity due to excess calories'

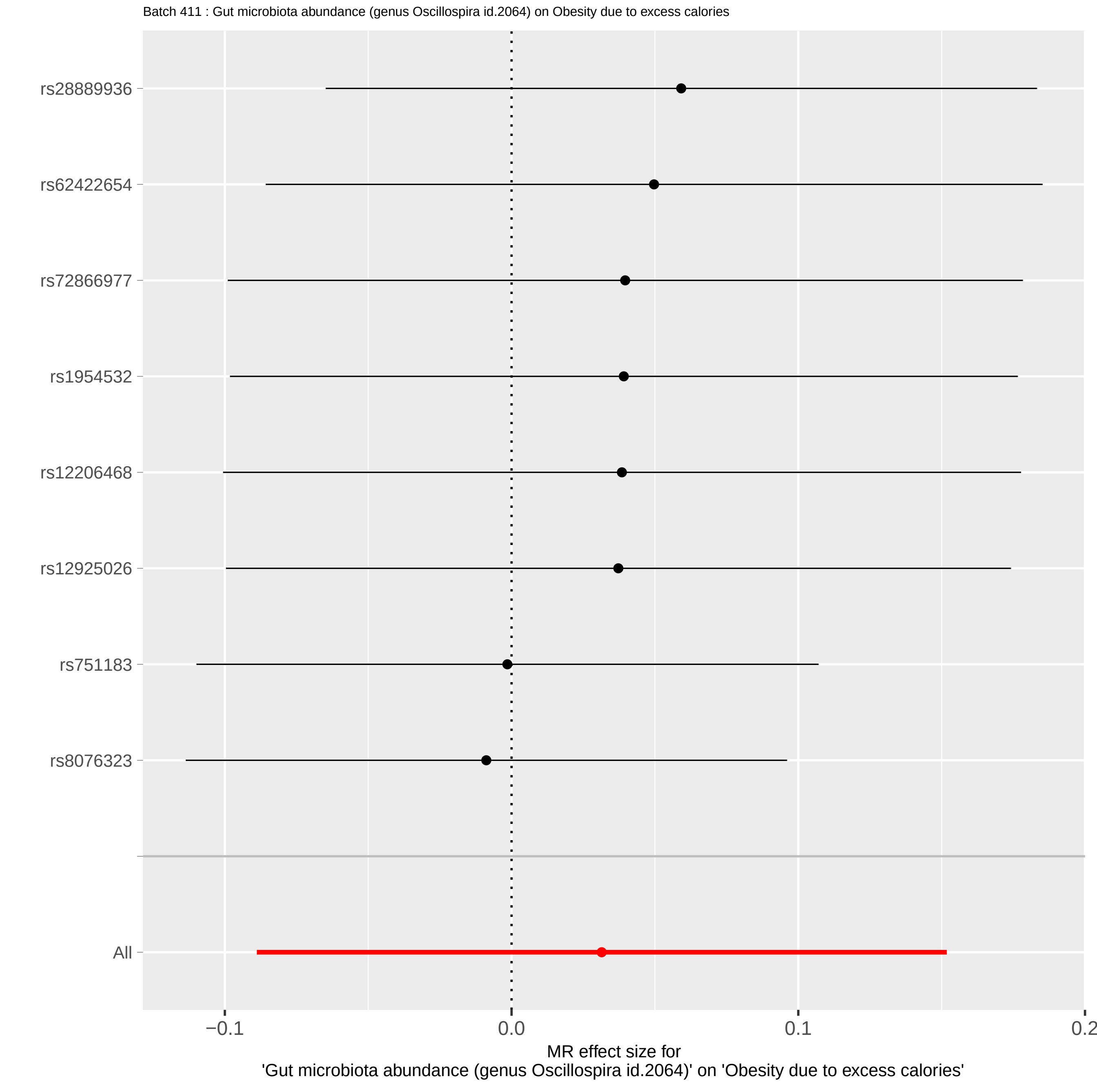

Batch 411 : Gut microbiota abundance (genus Oscillospira id.2064) on Obesity due to excess calories
rs28889936
rs62422654
rs72866977
rs1954532
rs12206468
rs12925026
rs751183
rs8076323
All
−0.1
0.0
0.1
0.2
MR effect size for
'Gut microbiota abundance (genus Oscillospira id.2064)' on 'Obesity due to excess calories'

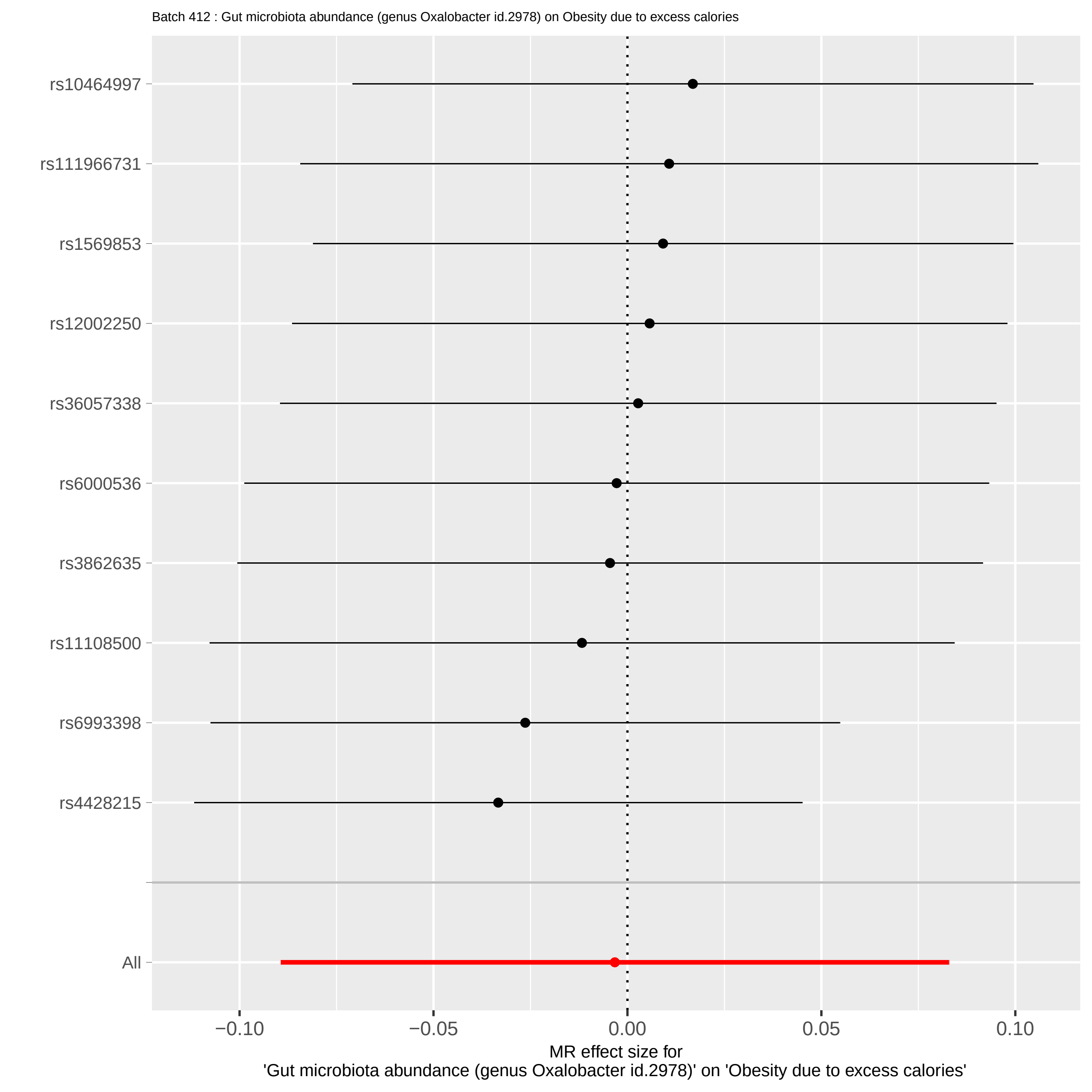
Batch 412 : Gut microbiota abundance (genus Oxalobacter id.2978) on Obesity due to excess calories
rs10464997
rs111966731
rs1569853
rs12002250
rs36057338
rs6000536
rs3862635
rs11108500
rs6993398
rs4428215
All
−0.10
−0.05
0.00
0.05
0.10
MR effect size for
'Gut microbiota abundance (genus Oxalobacter id.2978)' on 'Obesity due to excess calories'

Batch 413 : Gut microbiota abundance (genus Parabacteroides id.954) on Obesity due to excess calories

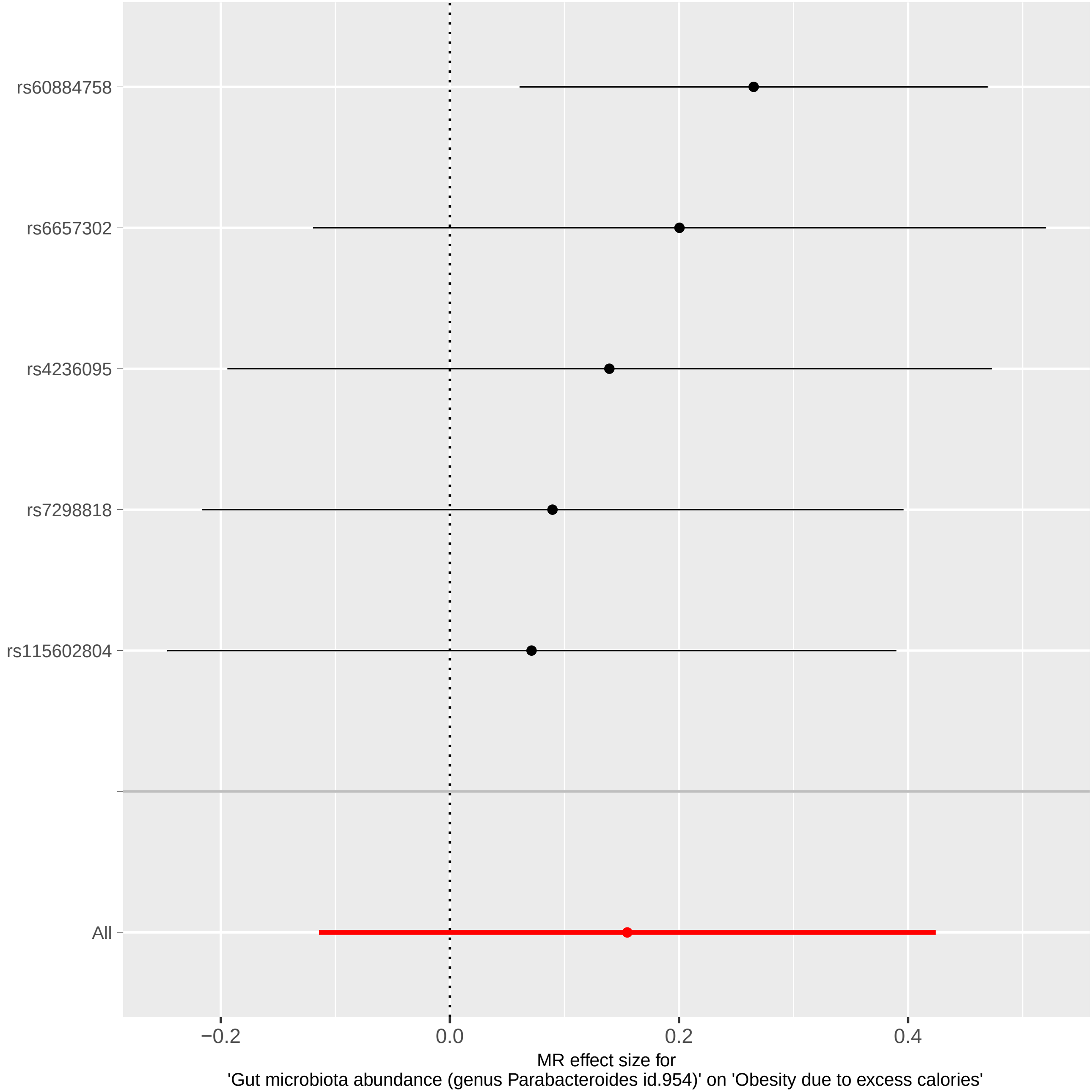

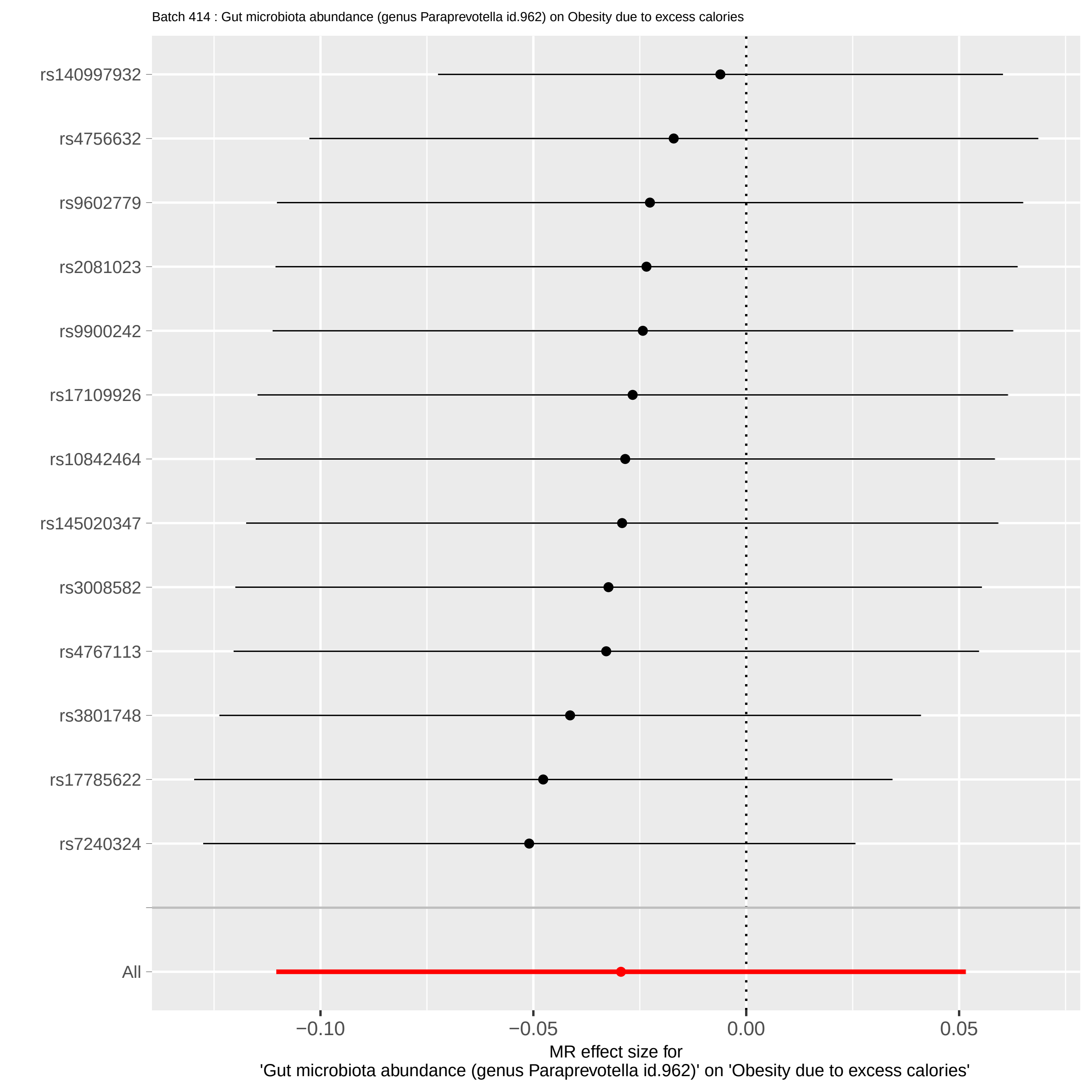
Batch 414 : Gut microbiota abundance (genus Paraprevotella id.962) on Obesity due to excess calories
rs140997932
rs4756632
rs9602779
rs2081023
rs9900242
rs17109926
rs10842464
rs145020347
rs3008582
rs4767113
rs3801748
rs17785622
rs7240324
All
−0.10
−0.05
0.00
0.05
MR effect size for
'Gut microbiota abundance (genus Paraprevotella id.962)' on 'Obesity due to excess calories'

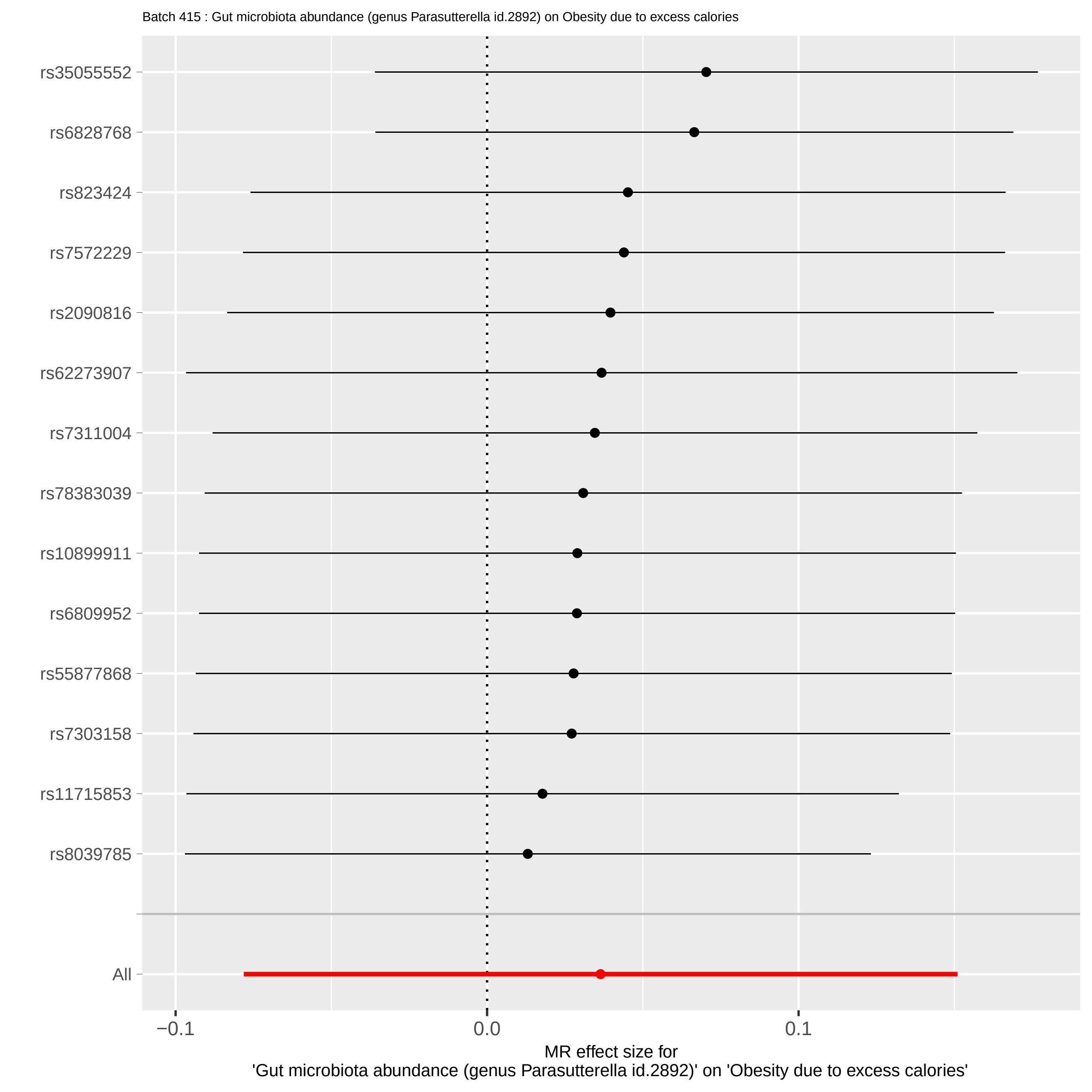
Batch 415 : Gut microbiota abundance (genus Parasutterella id.2892) on Obesity due to excess calories
rs35055552
rs6828768
rs823424
rs7572229
rs2090816
rs62273907
rs7311004
rs78383039
rs10899911
rs6809952
rs55877868
rs7303158
rs11715853
rs8039785
All
−0.1
0.0
0.1
MR effect size for
'Gut microbiota abundance (genus Parasutterella id.2892)' on 'Obesity due to excess calories'

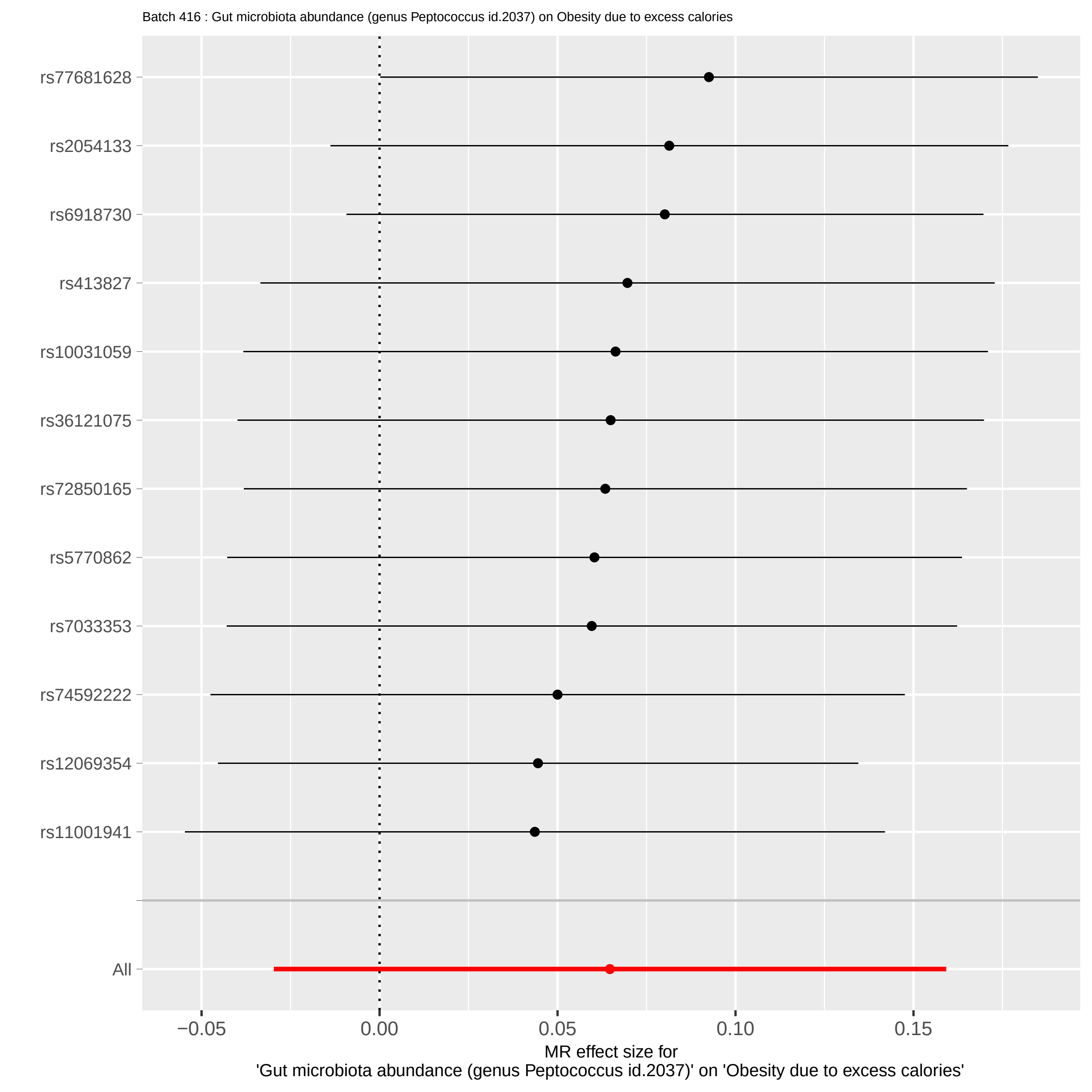

Batch 416 : Gut microbiota abundance (genus Peptococcus id.2037) on Obesity due to excess calories
rs77681628
rs2054133
rs6918730
rs413827
rs10031059
rs36121075
rs72850165
rs5770862
rs7033353
rs74592222
rs12069354
rs11001941
All
−0.05
0.00
0.05
0.10
0.15
MR effect size for
'Gut microbiota abundance (genus Peptococcus id.2037)' on 'Obesity due to excess calories'

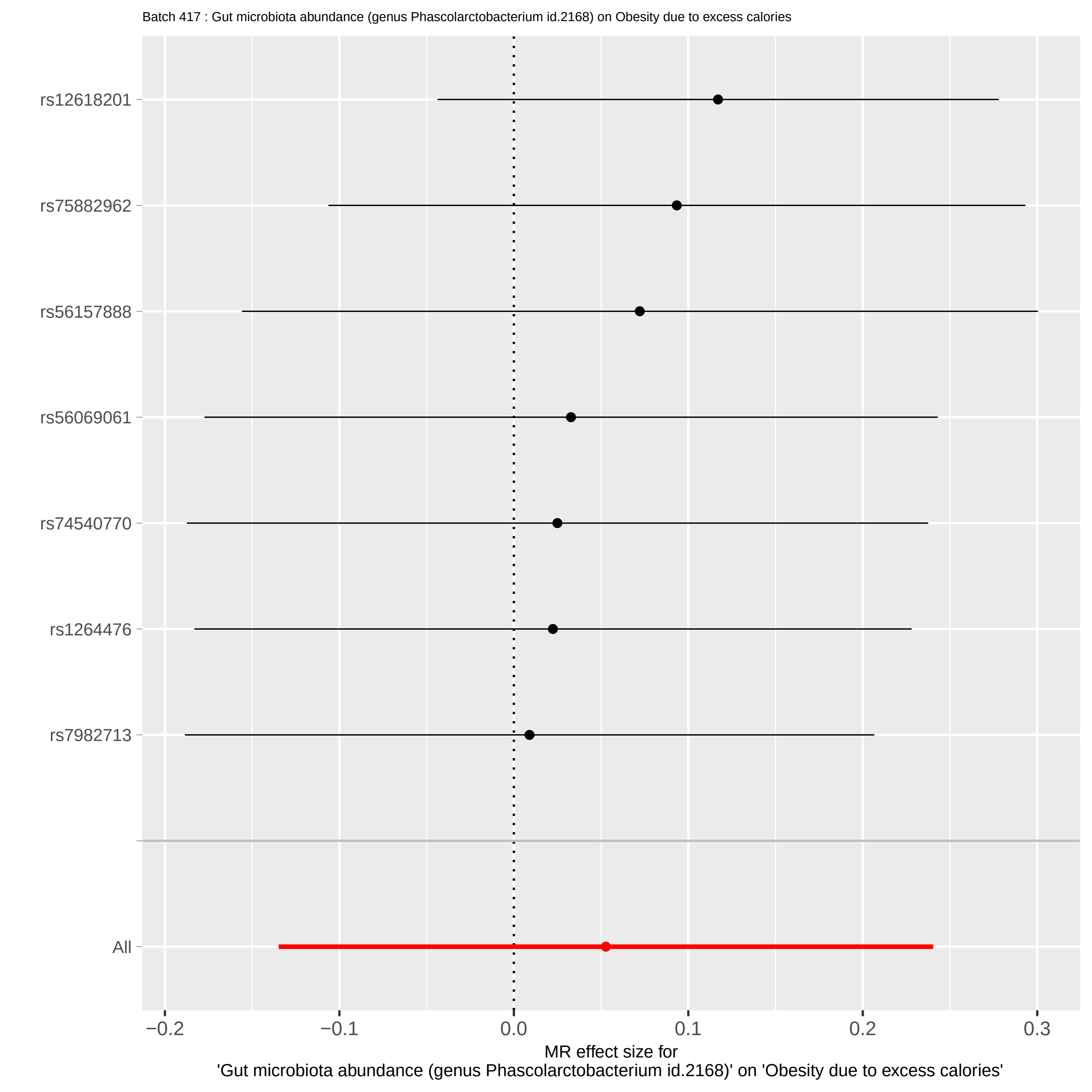

Batch 417 : Gut microbiota abundance (genus Phascolarctobacterium id.2168) on Obesity due to excess calories
rs12618201
rs75882962
rs56157888
rs56069061
rs74540770
rs1264476
rs7982713
All
−0.2
−0.1
0.0
0.1
0.2
0.3
MR effect size for
'Gut microbiota abundance (genus Phascolarctobacterium id.2168)' on 'Obesity due to excess calories'

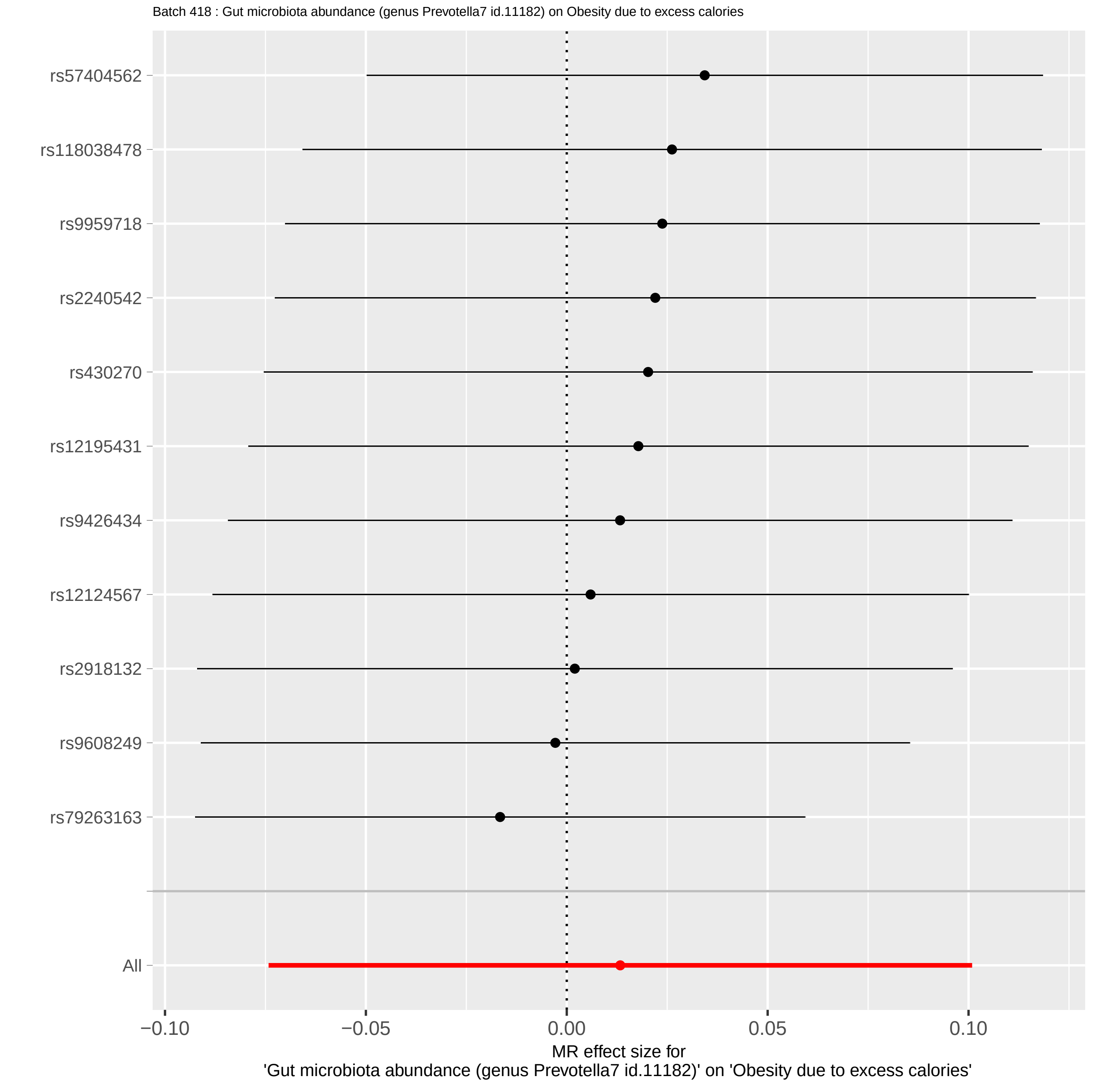

Batch 418 : Gut microbiota abundance (genus Prevotella7 id.11182) on Obesity due to excess calories
rs57404562
rs118038478
rs9959718
rs2240542
rs430270
rs12195431
rs9426434
rs12124567
rs2918132
rs9608249
rs79263163
All
−0.10
−0.05
0.00
0.05
0.10
MR effect size for
'Gut microbiota abundance (genus Prevotella7 id.11182)' on 'Obesity due to excess calories'

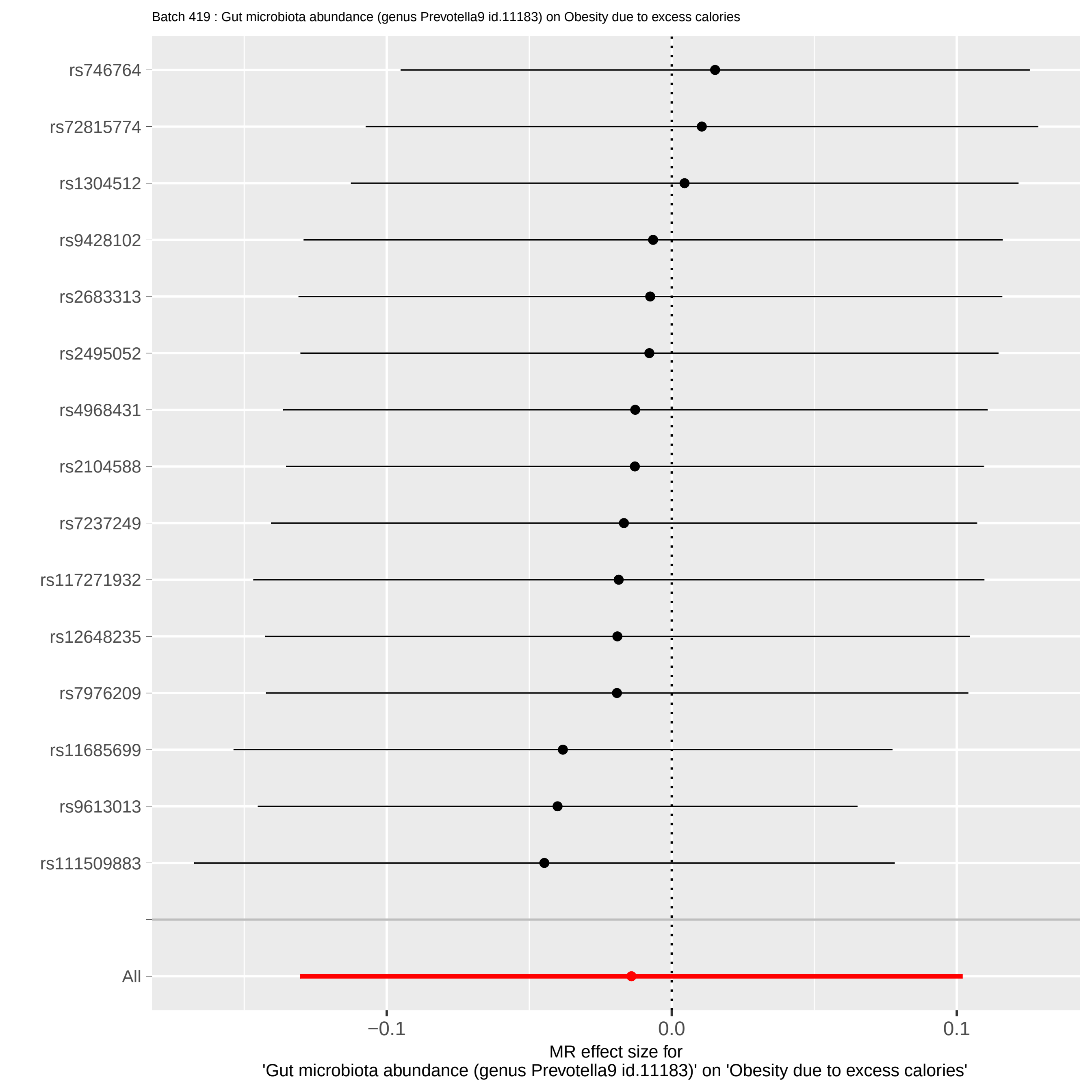

Batch 419 : Gut microbiota abundance (genus Prevotella9 id.11183) on Obesity due to excess calories
rs746764
rs72815774
rs1304512
rs9428102
rs2683313
rs2495052
rs4968431
rs2104588
rs7237249
rs117271932
rs12648235
rs7976209
rs11685699
rs9613013
rs111509883
All
−0.1
0.0
0.1
MR effect size for
'Gut microbiota abundance (genus Prevotella9 id.11183)' on 'Obesity due to excess calories'

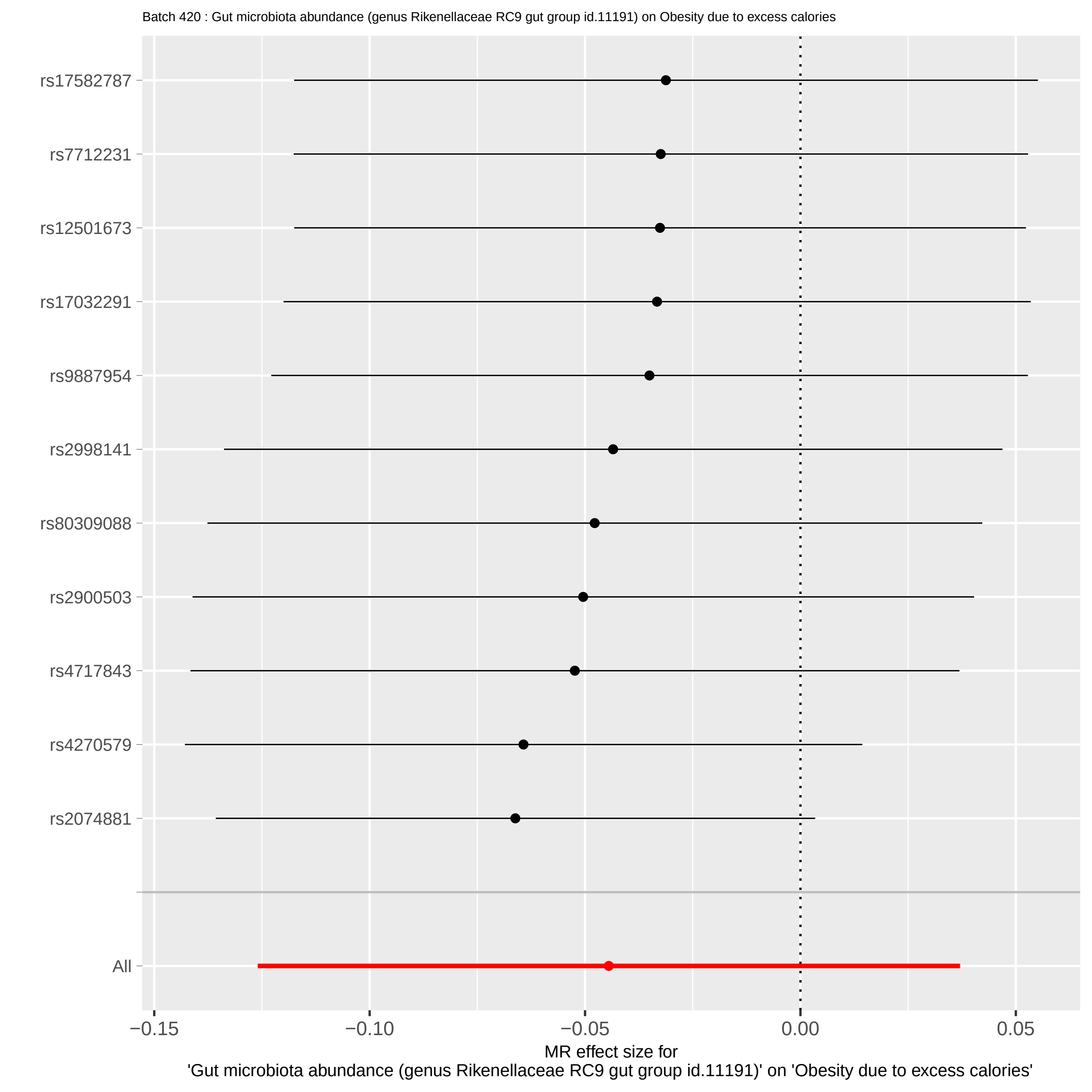
Batch 420 : Gut microbiota abundance (genus Rikenellaceae RC9 gut group id.11191) on Obesity due to excess calories
rs17582787
rs7712231
rs12501673
rs17032291
rs9887954
rs2998141
rs80309088
rs2900503
rs4717843
rs4270579
rs2074881
All
−0.15
−0.10
−0.05
0.00
0.05
MR effect size for
'Gut microbiota abundance (genus Rikenellaceae RC9 gut group id.11191)' on 'Obesity due to excess calories'

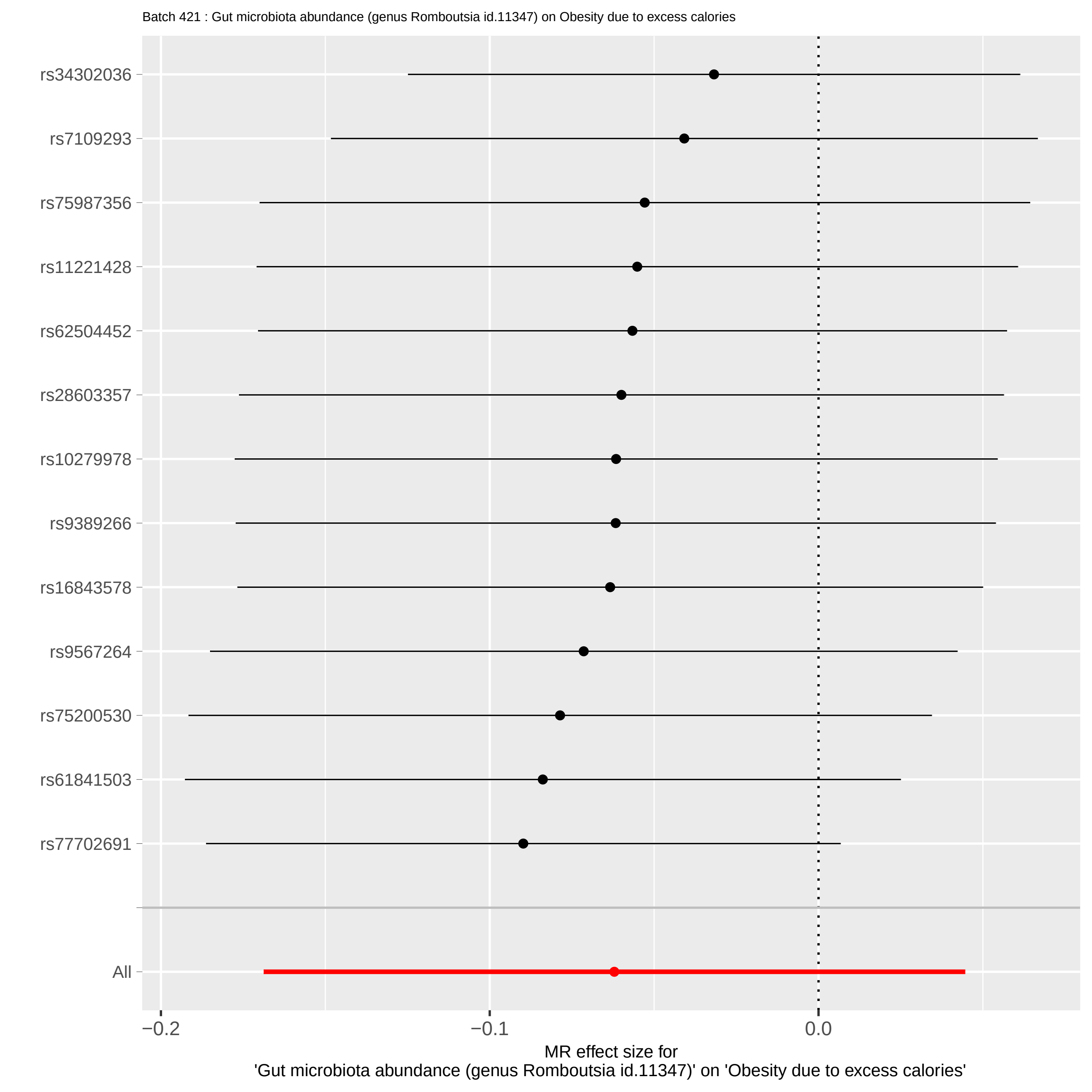

Batch 421 : Gut microbiota abundance (genus Romboutsia id.11347) on Obesity due to excess calories
rs34302036
rs7109293
rs75987356
rs11221428
rs62504452
rs28603357
rs10279978
rs9389266
rs16843578
rs9567264
rs75200530
rs61841503
rs77702691
All
−0.2
−0.1
0.0
MR effect size for
'Gut microbiota abundance (genus Romboutsia id.11347)' on 'Obesity due to excess calories'

Batch 422 : Gut microbiota abundance (genus Roseburia id.2012) on Obesity due to excess calories

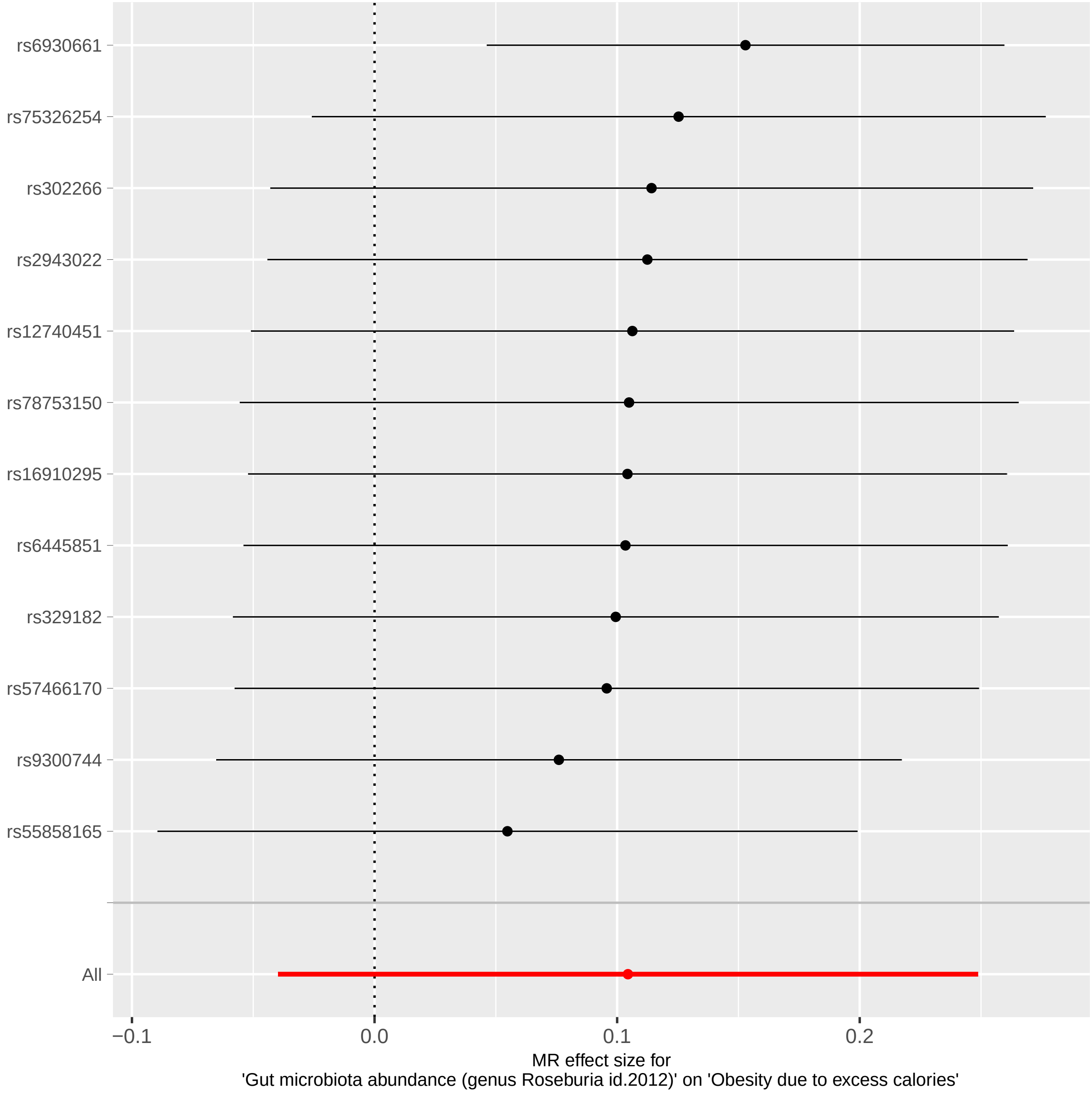

Batch 423 : Gut microbiota abundance (genus Ruminiclostridium5 id.11355) on Obesity due to excess calories

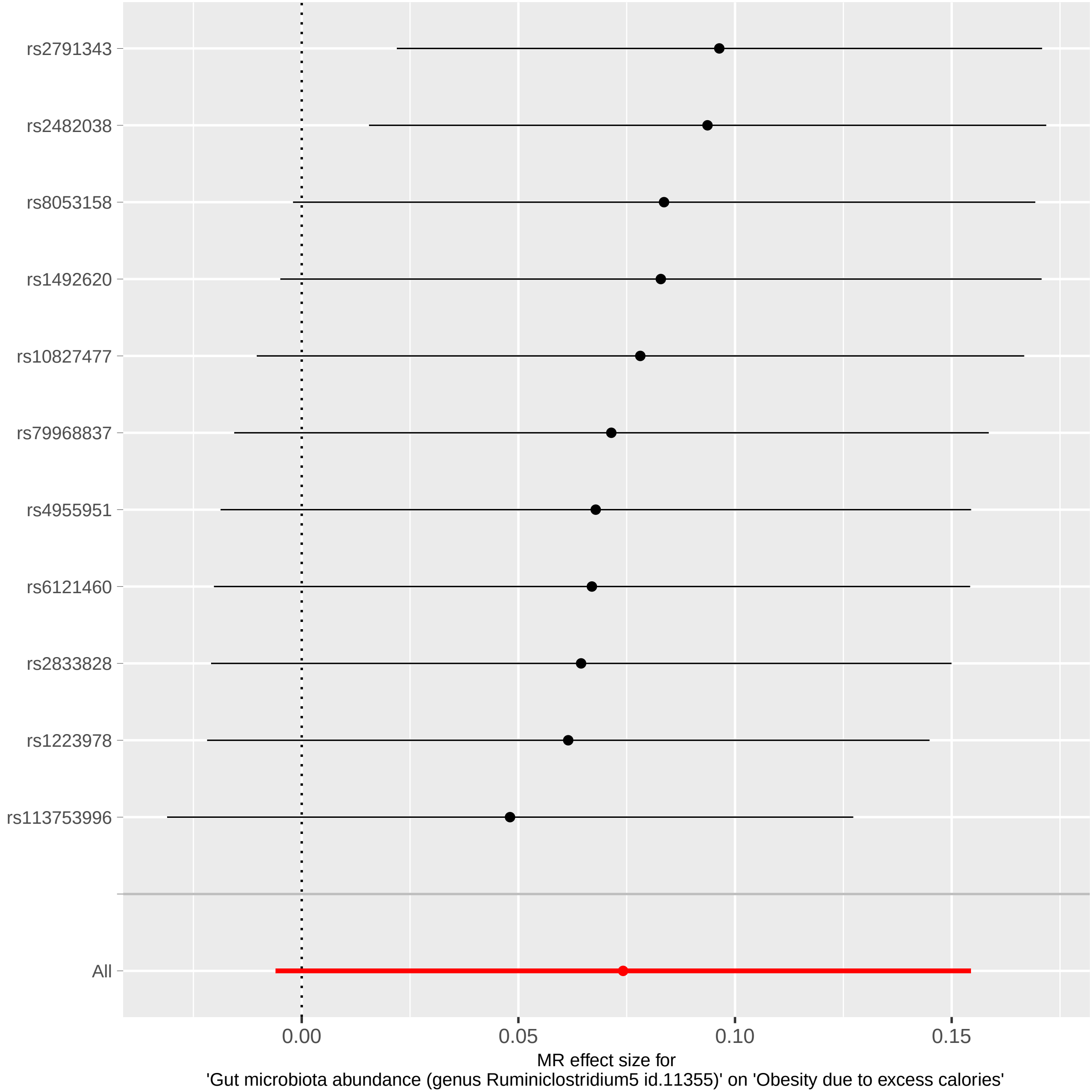

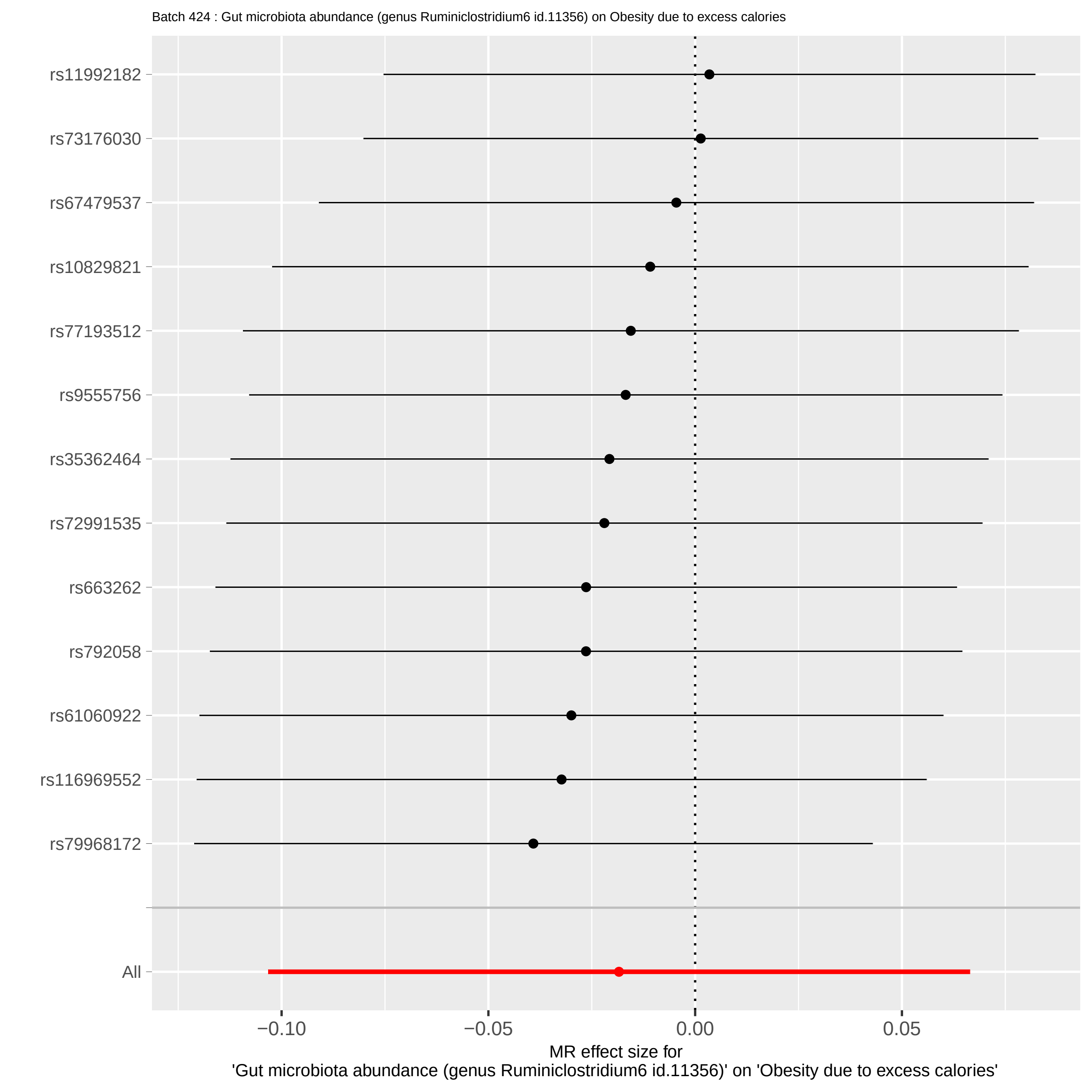

Batch 424 : Gut microbiota abundance (genus Ruminiclostridium6 id.11356) on Obesity due to excess calories
rs11992182
rs73176030
rs67479537
rs10829821
rs77193512
rs9555756
rs35362464
rs72991535
rs663262
rs792058
rs61060922
rs116969552
rs79968172
All
−0.10
−0.05
0.00
0.05
MR effect size for
'Gut microbiota abundance (genus Ruminiclostridium6 id.11356)' on 'Obesity due to excess calories'

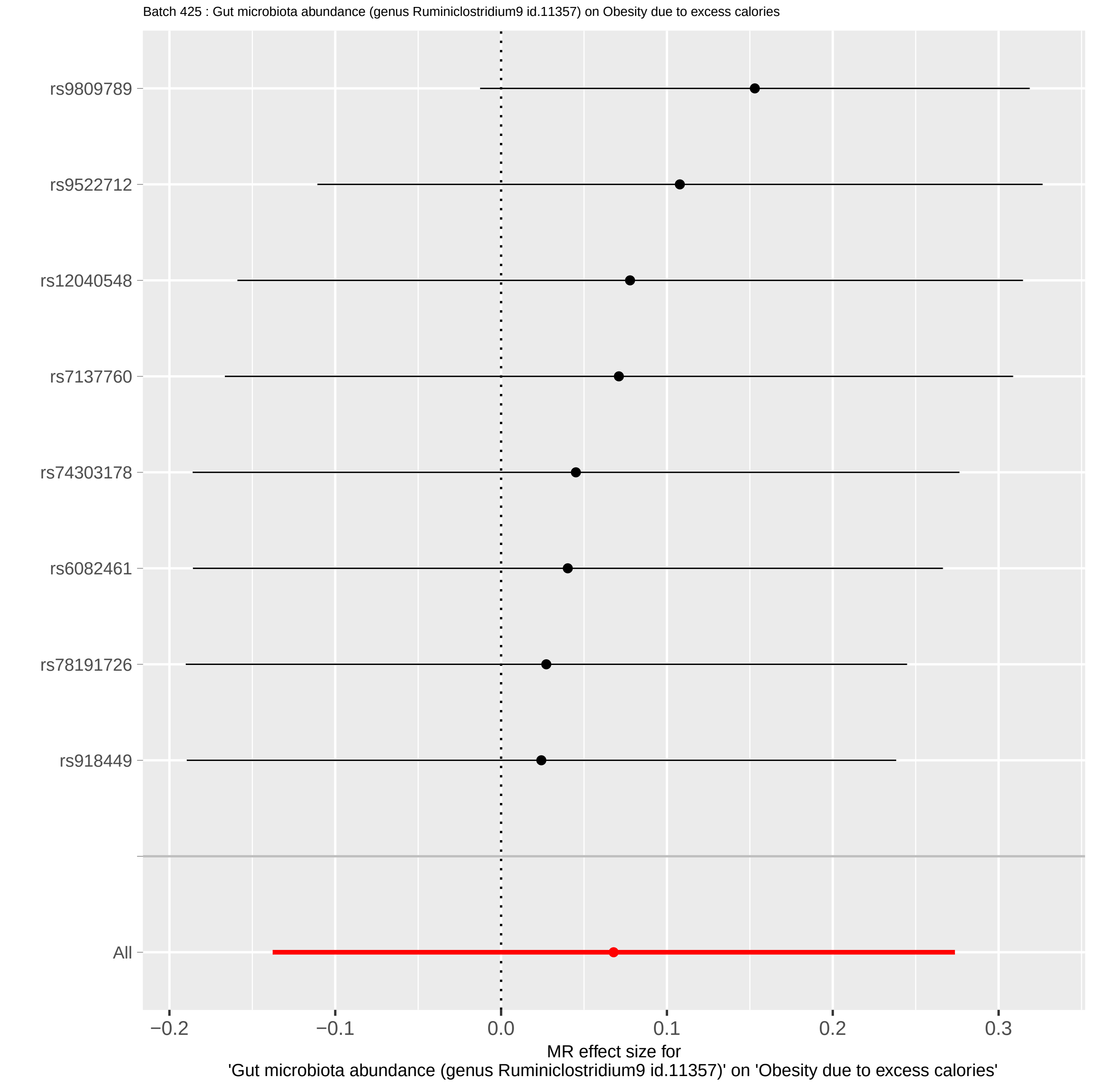

Batch 425 : Gut microbiota abundance (genus Ruminiclostridium9 id.11357) on Obesity due to excess calories
rs9809789
rs9522712
rs12040548
rs7137760
rs74303178
rs6082461
rs78191726
rs918449
All
−0.2
−0.1
0.0
0.1
0.2
0.3
MR effect size for
'Gut microbiota abundance (genus Ruminiclostridium9 id.11357)' on 'Obesity due to excess calories'

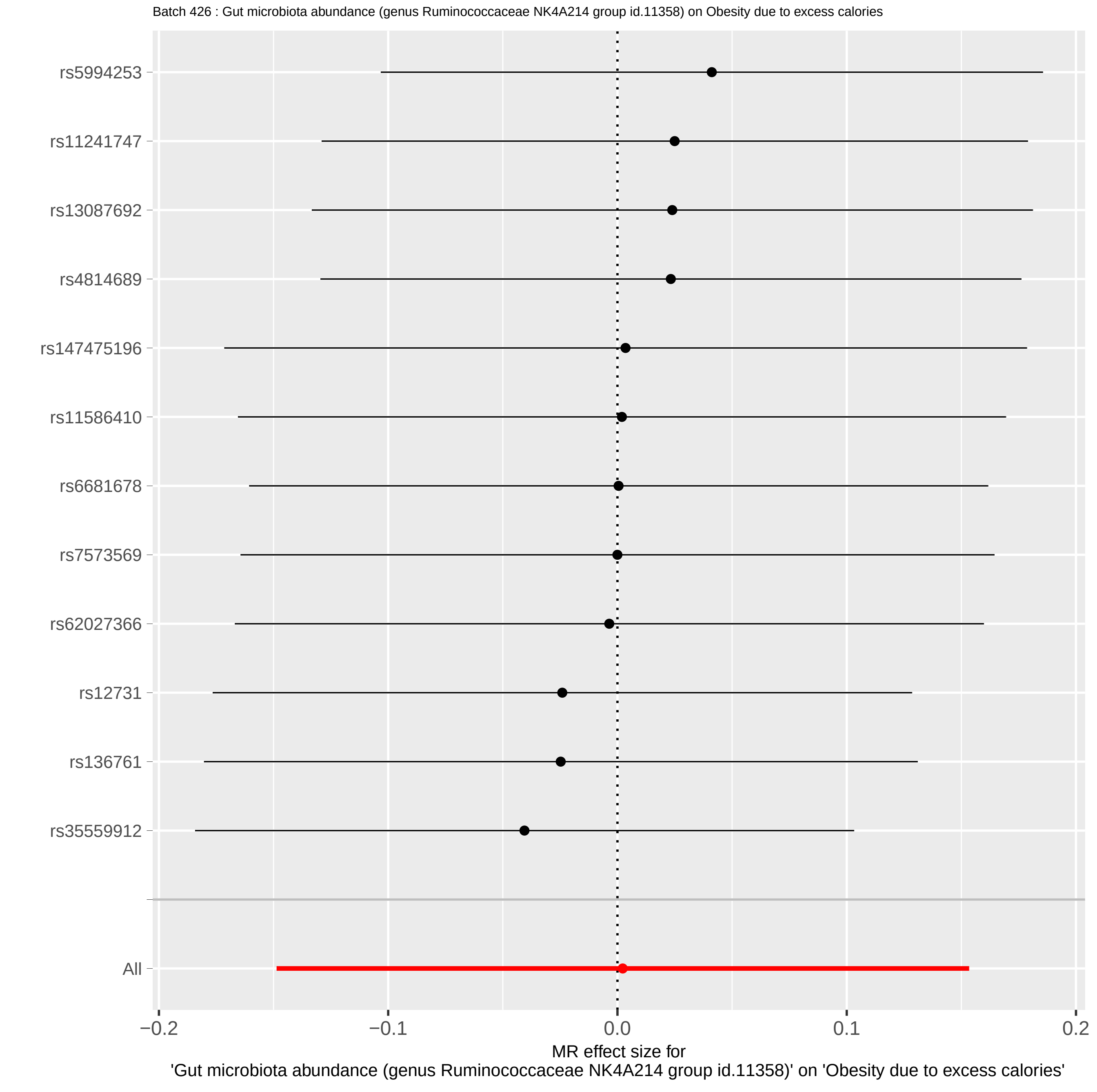

Batch 426 : Gut microbiota abundance (genus Ruminococcaceae NK4A214 group id.11358) on Obesity due to excess calories
rs5994253
rs11241747
rs13087692
rs4814689
rs147475196
rs11586410
rs6681678
rs7573569
rs62027366
rs12731
rs136761
rs35559912
All
−0.2
−0.1
0.0
0.1
0.2
MR effect size for
'Gut microbiota abundance (genus Ruminococcaceae NK4A214 group id.11358)' on 'Obesity due to excess calories'

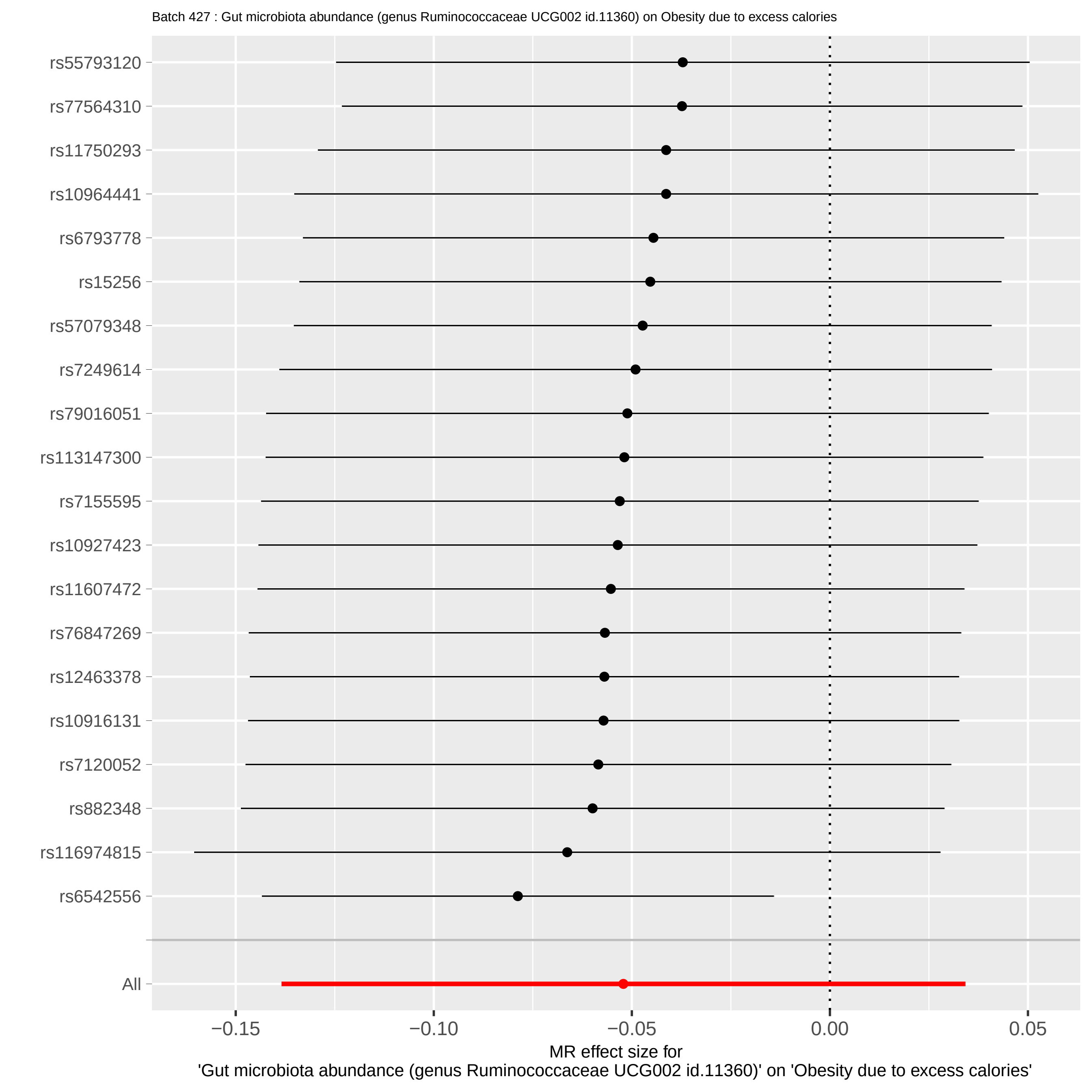

Batch 427 : Gut microbiota abundance (genus Ruminococcaceae UCG002 id.11360) on Obesity due to excess calories
rs55793120
rs77564310
rs11750293
rs10964441
rs6793778
rs15256
rs57079348
rs7249614
rs79016051
rs113147300
rs7155595
rs10927423
rs11607472
rs76847269
rs12463378
rs10916131
rs7120052
rs882348
rs116974815
rs6542556
All
−0.15
−0.10
−0.05
0.00
0.05
MR effect size for
'Gut microbiota abundance (genus Ruminococcaceae UCG002 id.11360)' on 'Obesity due to excess calories'

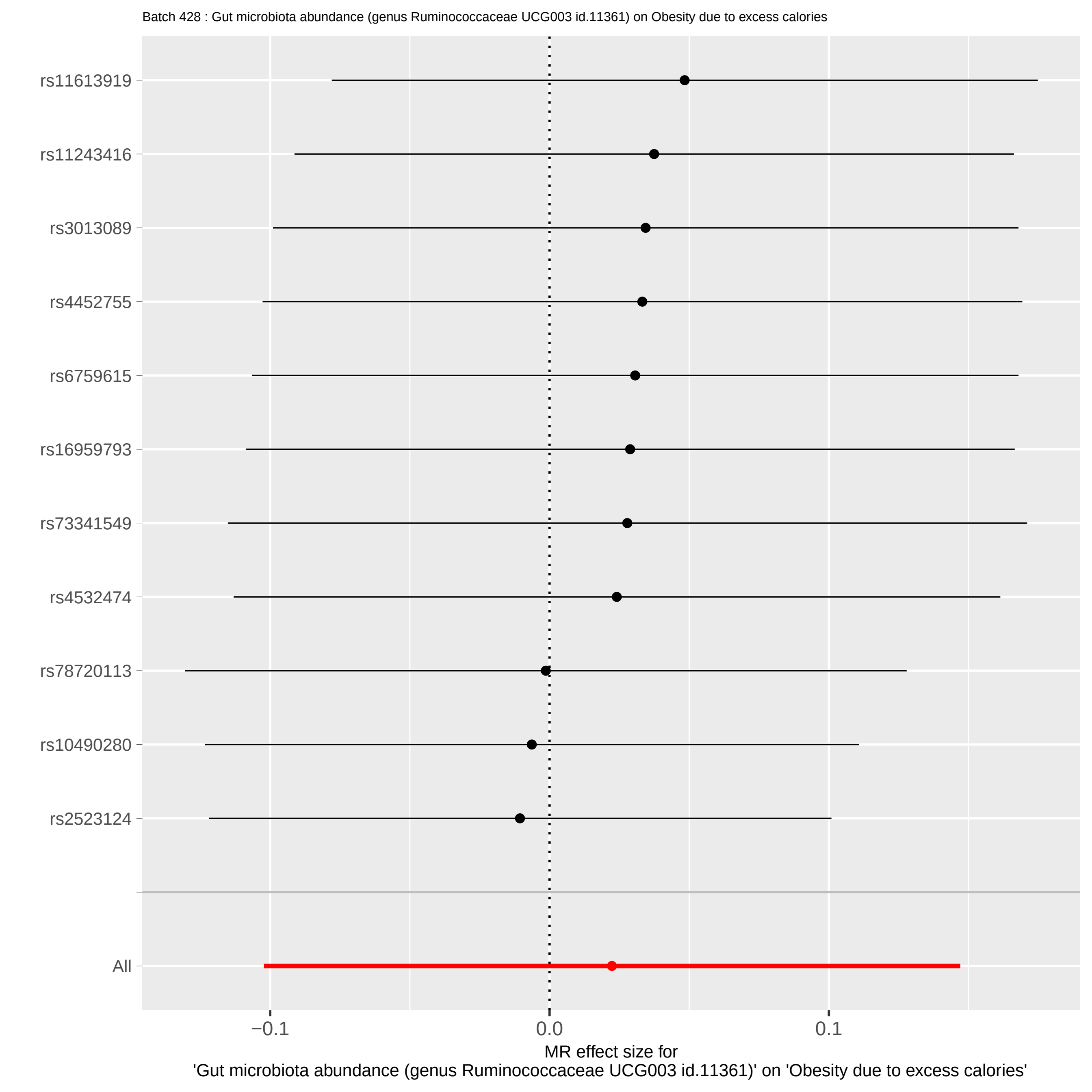

Batch 428 : Gut microbiota abundance (genus Ruminococcaceae UCG003 id.11361) on Obesity due to excess calories
rs11613919
rs11243416
rs3013089
rs4452755
rs6759615
rs16959793
rs73341549
rs4532474
rs78720113
rs10490280
rs2523124
All
−0.1
0.0
0.1
MR effect size for
'Gut microbiota abundance (genus Ruminococcaceae UCG003 id.11361)' on 'Obesity due to excess calories'

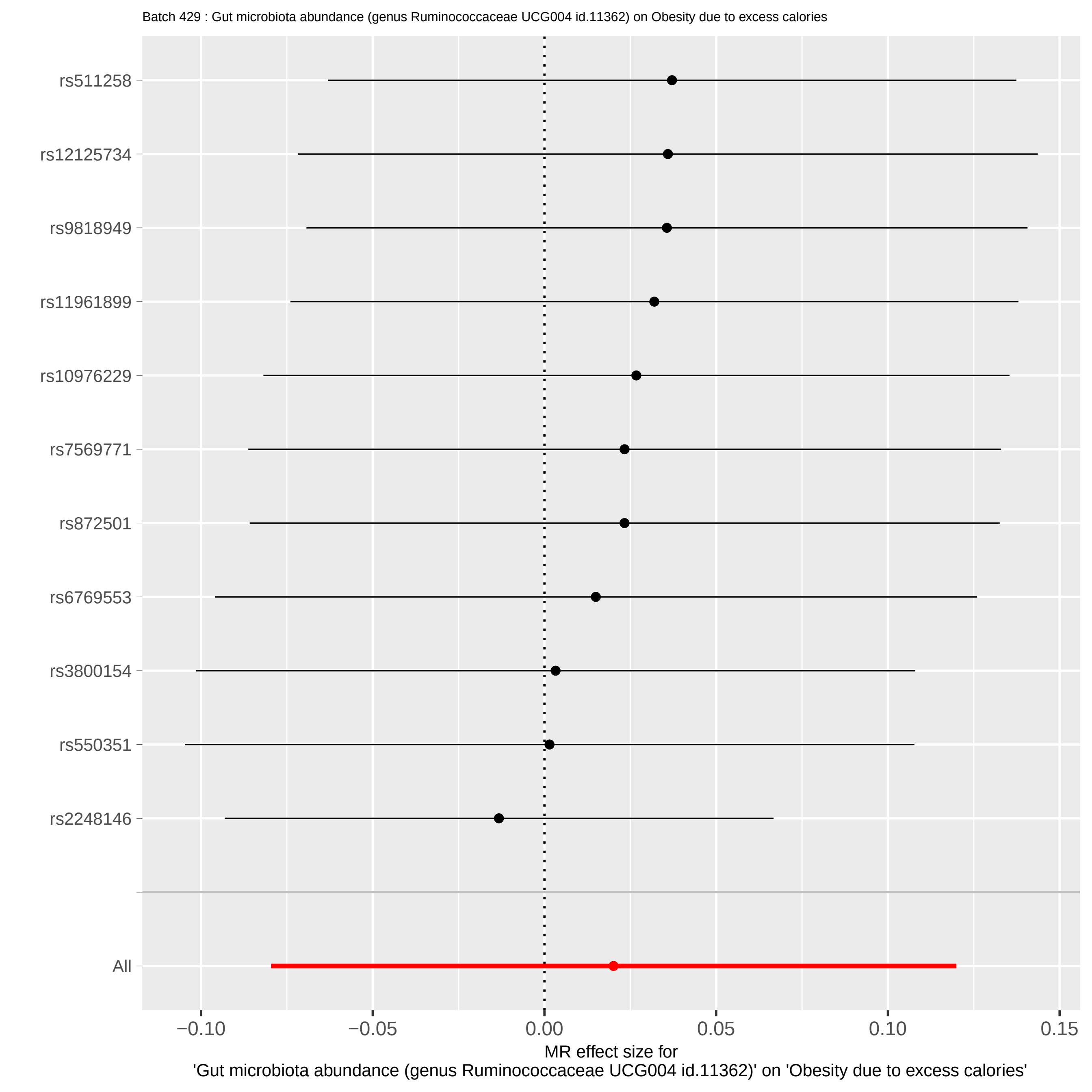
Batch 429 : Gut microbiota abundance (genus Ruminococcaceae UCG004 id.11362) on Obesity due to excess calories
rs511258
rs12125734
rs9818949
rs11961899
rs10976229
rs7569771
rs872501
rs6769553
rs3800154
rs550351
rs2248146
All
−0.10
−0.05
0.00
0.05
0.10
0.15
MR effect size for
'Gut microbiota abundance (genus Ruminococcaceae UCG004 id.11362)' on 'Obesity due to excess calories'

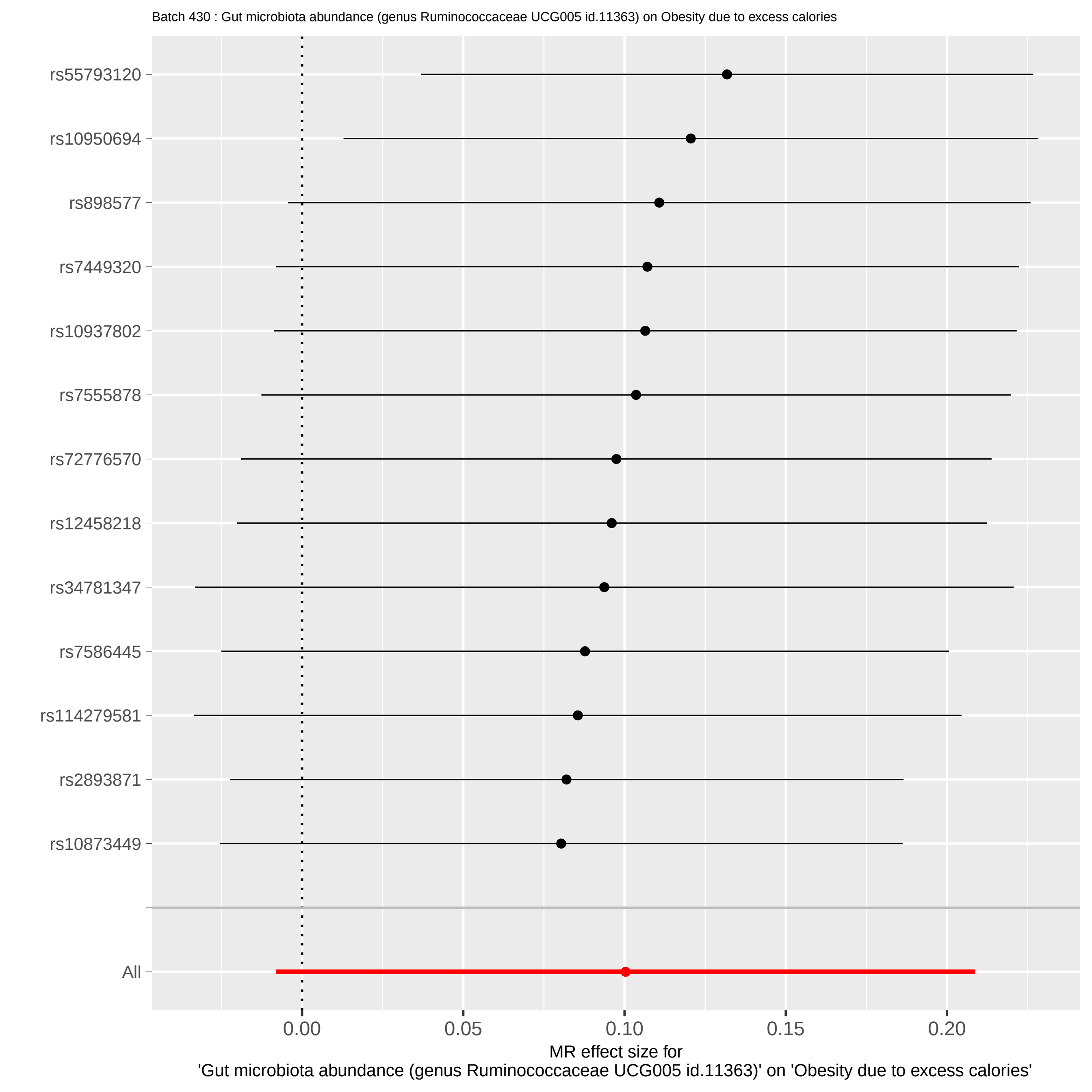

Batch 430 : Gut microbiota abundance (genus Ruminococcaceae UCG005 id.11363) on Obesity due to excess calories
rs55793120
rs10950694
rs898577
rs7449320
rs10937802
rs7555878
rs72776570
rs12458218
rs34781347
rs7586445
rs114279581
rs2893871
rs10873449
All
0.00
0.05
0.10
0.15
0.20
MR effect size for
'Gut microbiota abundance (genus Ruminococcaceae UCG005 id.11363)' on 'Obesity due to excess calories'

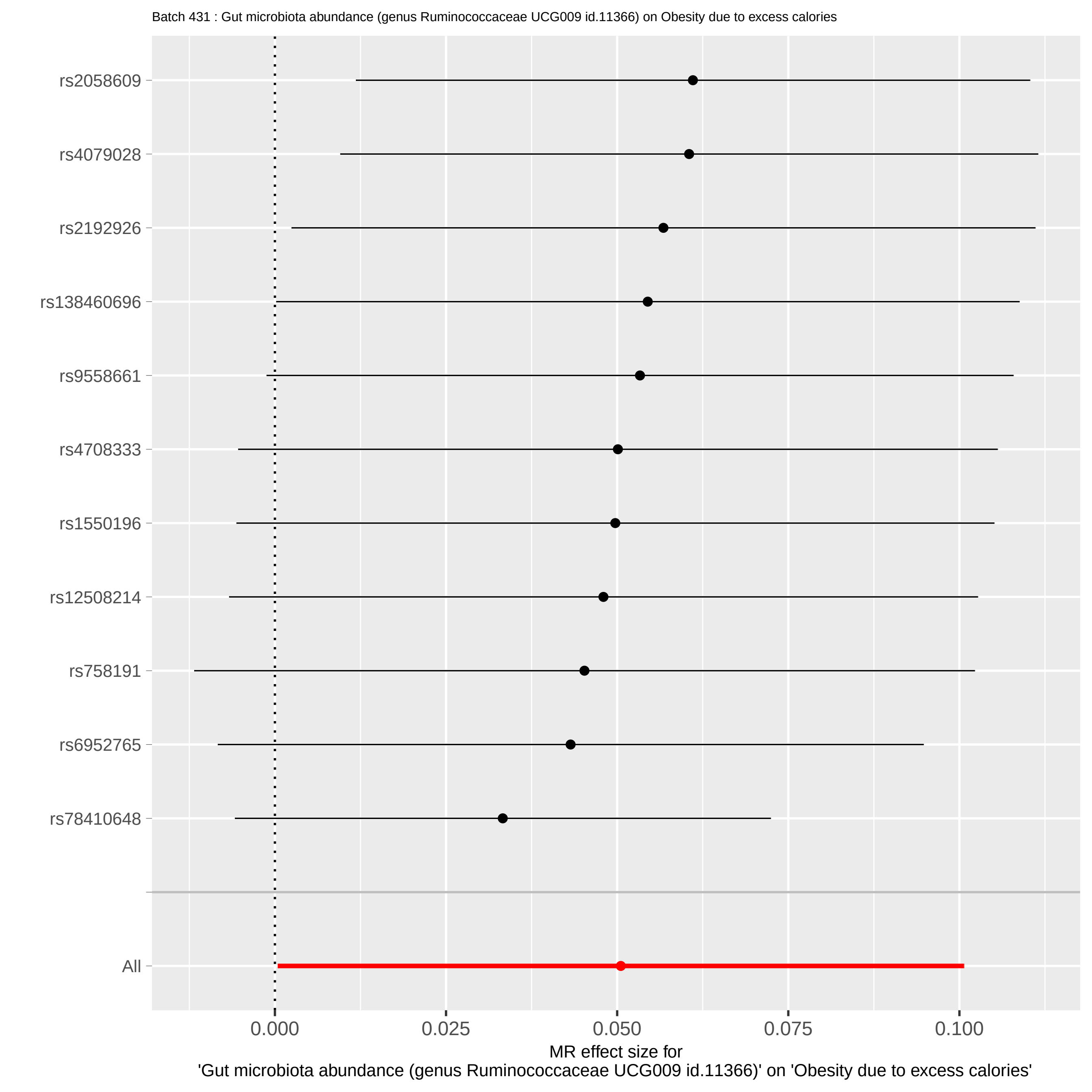

Batch 431 : Gut microbiota abundance (genus Ruminococcaceae UCG009 id.11366) on Obesity due to excess calories
rs2058609
rs4079028
rs2192926
rs138460696
rs9558661
rs4708333
rs1550196
rs12508214
rs758191
rs6952765
rs78410648
All
0.000
0.025
0.050
0.075
0.100
MR effect size for
'Gut microbiota abundance (genus Ruminococcaceae UCG009 id.11366)' on 'Obesity due to excess calories'

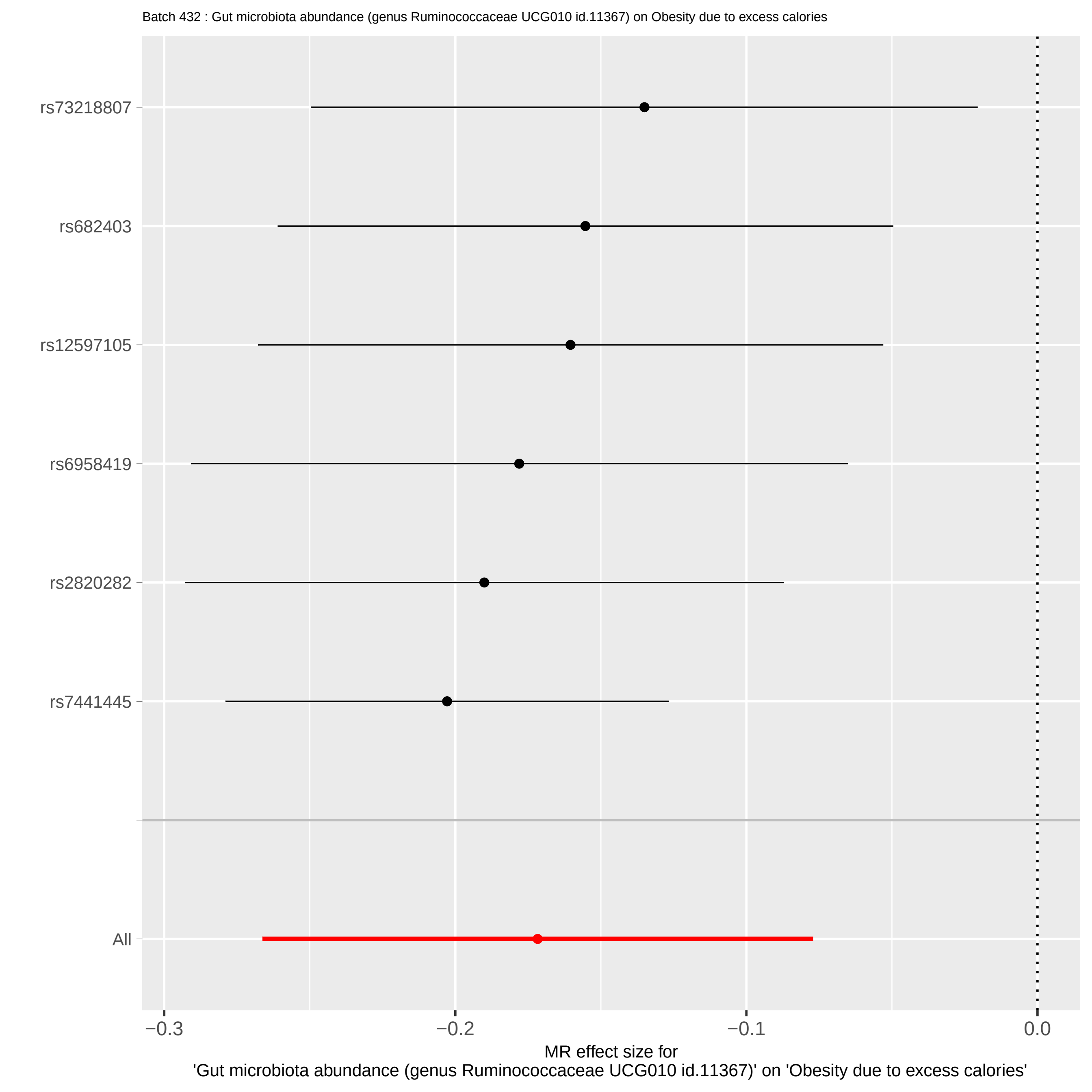

Batch 432 : Gut microbiota abundance (genus Ruminococcaceae UCG010 id.11367) on Obesity due to excess calories
rs73218807
rs682403
rs12597105
rs6958419
rs2820282
rs7441445
All
−0.3
−0.2
−0.1
0.0
MR effect size for
'Gut microbiota abundance (genus Ruminococcaceae UCG010 id.11367)' on 'Obesity due to excess calories'

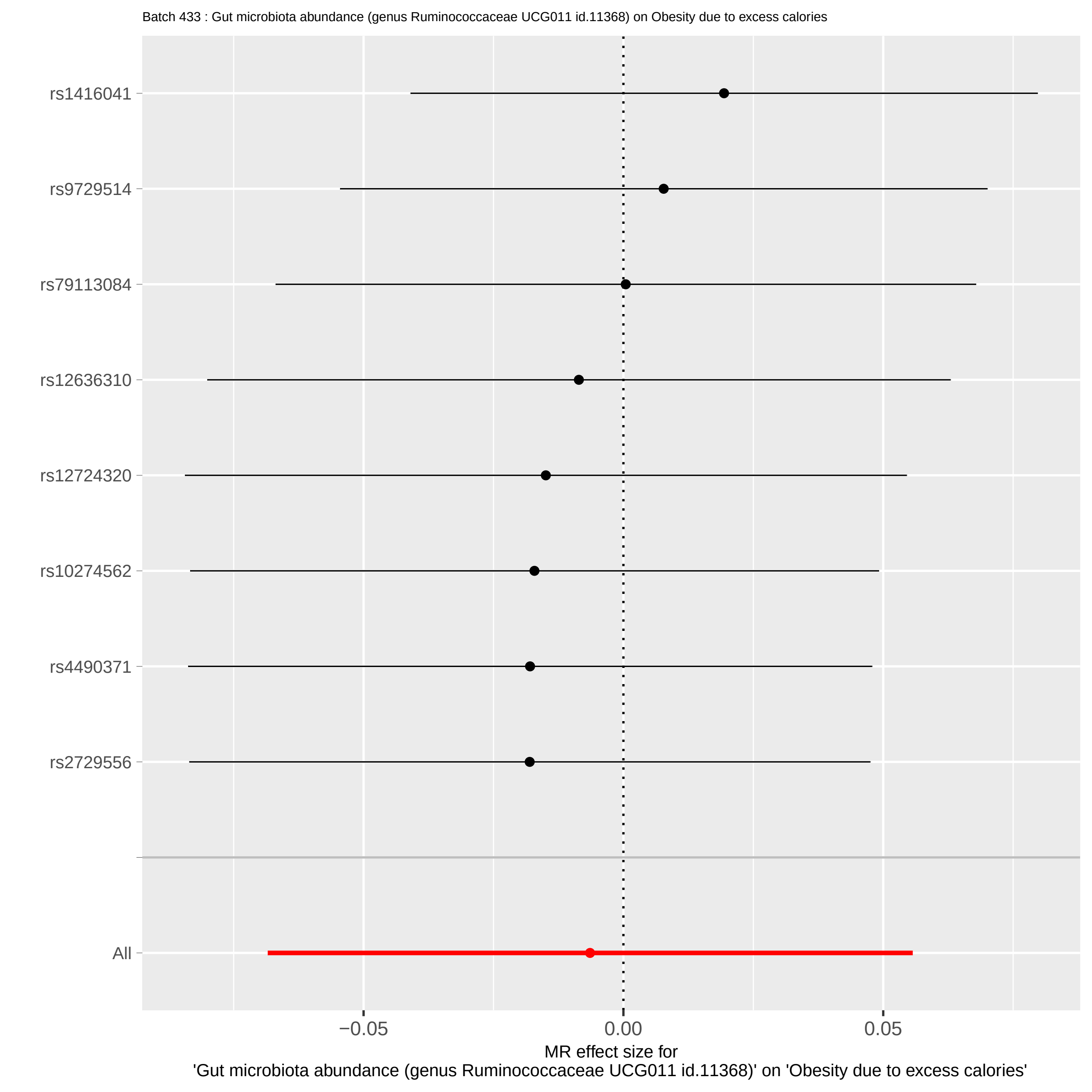

Batch 433 : Gut microbiota abundance (genus Ruminococcaceae UCG011 id.11368) on Obesity due to excess calories
rs1416041
rs9729514
rs79113084
rs12636310
rs12724320
rs10274562
rs4490371
rs2729556
All
−0.05
0.00
0.05
MR effect size for
'Gut microbiota abundance (genus Ruminococcaceae UCG011 id.11368)' on 'Obesity due to excess calories'

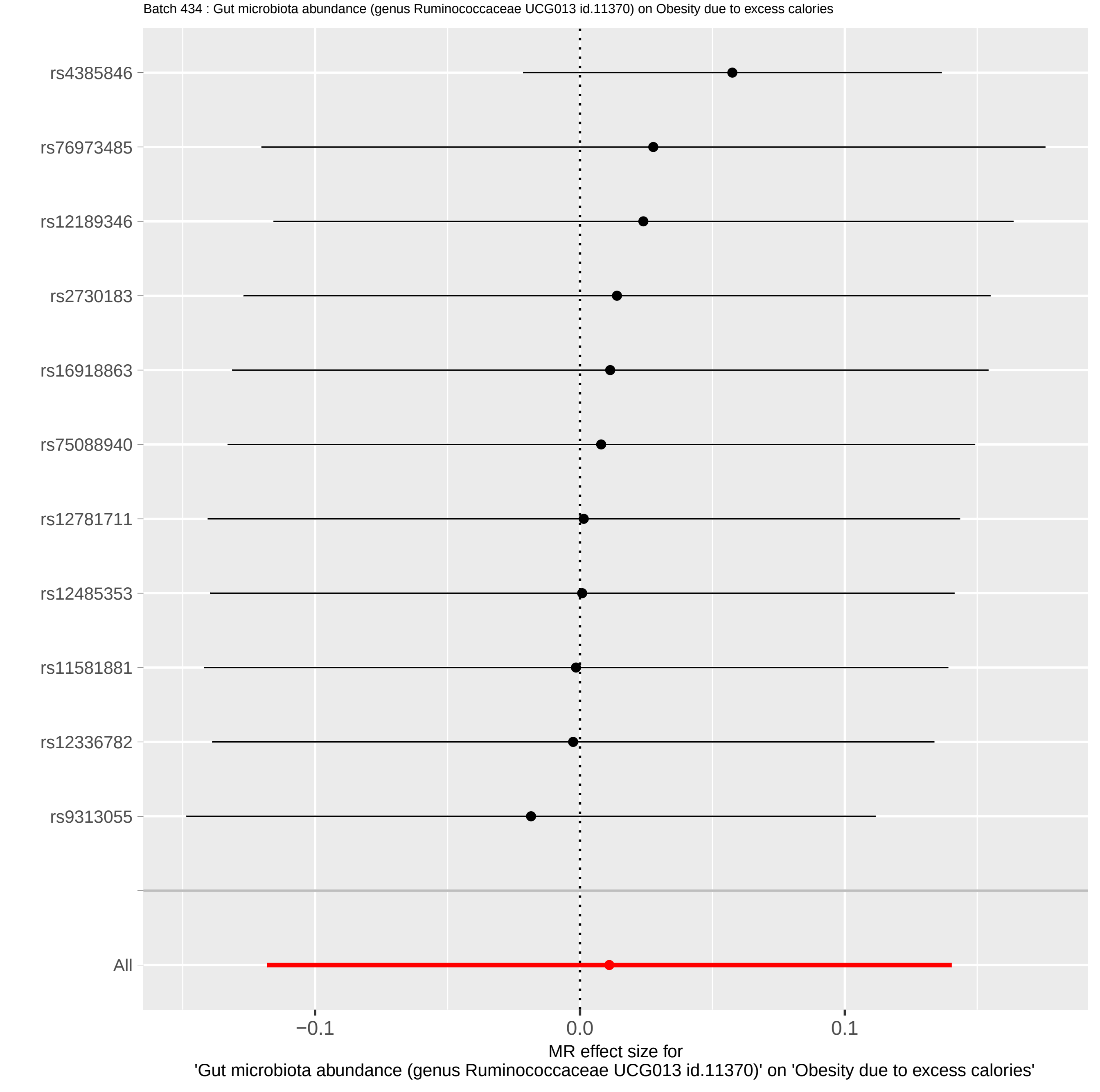

Batch 434 : Gut microbiota abundance (genus Ruminococcaceae UCG013 id.11370) on Obesity due to excess calories
rs4385846
rs76973485
rs12189346
rs2730183
rs16918863
rs75088940
rs12781711
rs12485353
rs11581881
rs12336782
rs9313055
All
−0.1
0.0
0.1
MR effect size for
'Gut microbiota abundance (genus Ruminococcaceae UCG013 id.11370)' on 'Obesity due to excess calories'

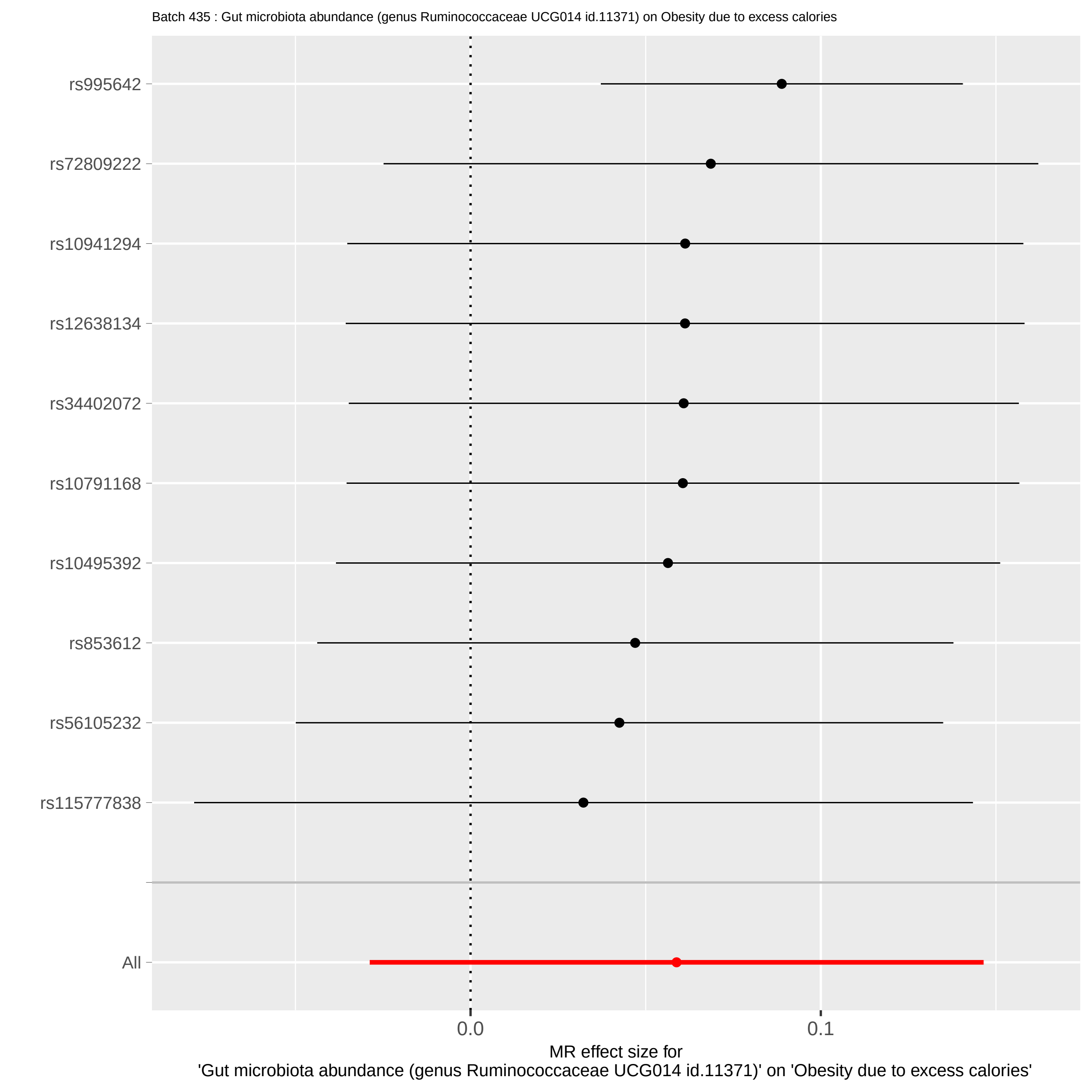

Batch 435 : Gut microbiota abundance (genus Ruminococcaceae UCG014 id.11371) on Obesity due to excess calories
rs995642
rs72809222
rs10941294
rs12638134
rs34402072
rs10791168
rs10495392
rs853612
rs56105232
rs115777838
All
0.0
0.1
MR effect size for
'Gut microbiota abundance (genus Ruminococcaceae UCG014 id.11371)' on 'Obesity due to excess calories'

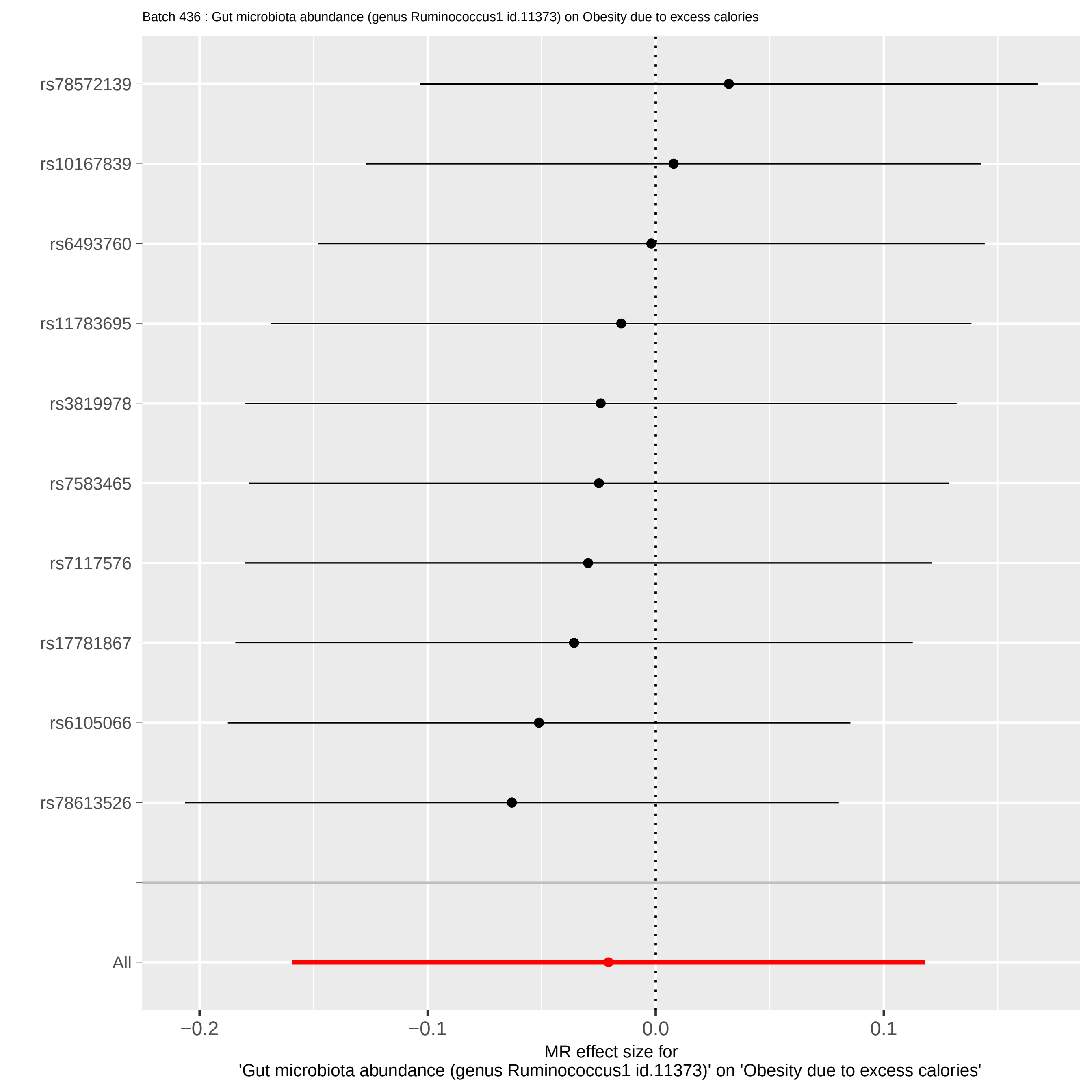

Batch 436 : Gut microbiota abundance (genus Ruminococcus1 id.11373) on Obesity due to excess calories
rs78572139
rs10167839
rs6493760
rs11783695
rs3819978
rs7583465
rs7117576
rs17781867
rs6105066
rs78613526
All
−0.2
−0.1
0.0
0.1
MR effect size for
'Gut microbiota abundance (genus Ruminococcus1 id.11373)' on 'Obesity due to excess calories'

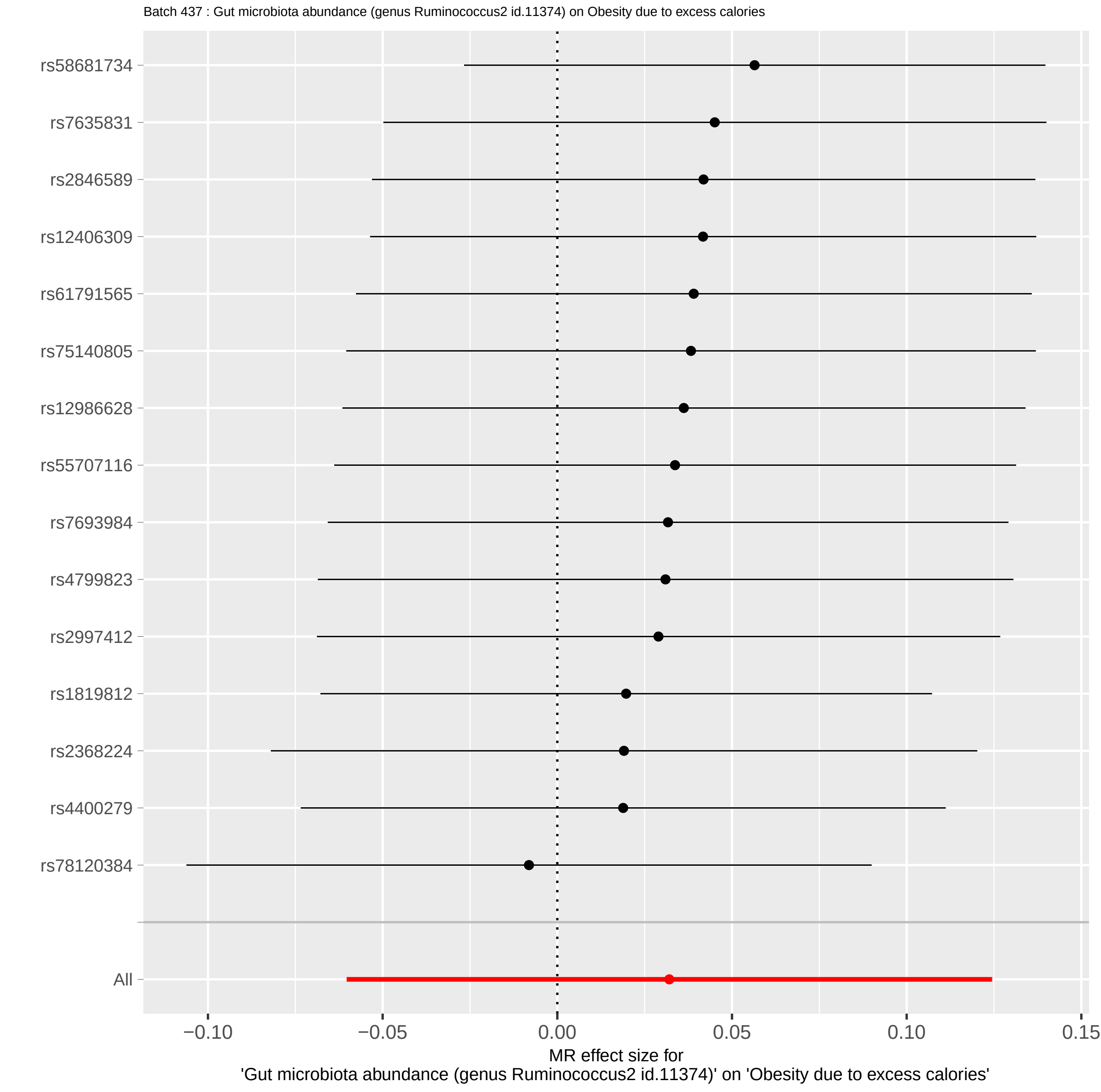

Batch 437 : Gut microbiota abundance (genus Ruminococcus2 id.11374) on Obesity due to excess calories
rs58681734
rs7635831
rs2846589
rs12406309
rs61791565
rs75140805
rs12986628
rs55707116
rs7693984
rs4799823
rs2997412
rs1819812
rs2368224
rs4400279
rs78120384
All
−0.10
−0.05
0.00
0.05
0.10
0.15
MR effect size for
'Gut microbiota abundance (genus Ruminococcus2 id.11374)' on 'Obesity due to excess calories'

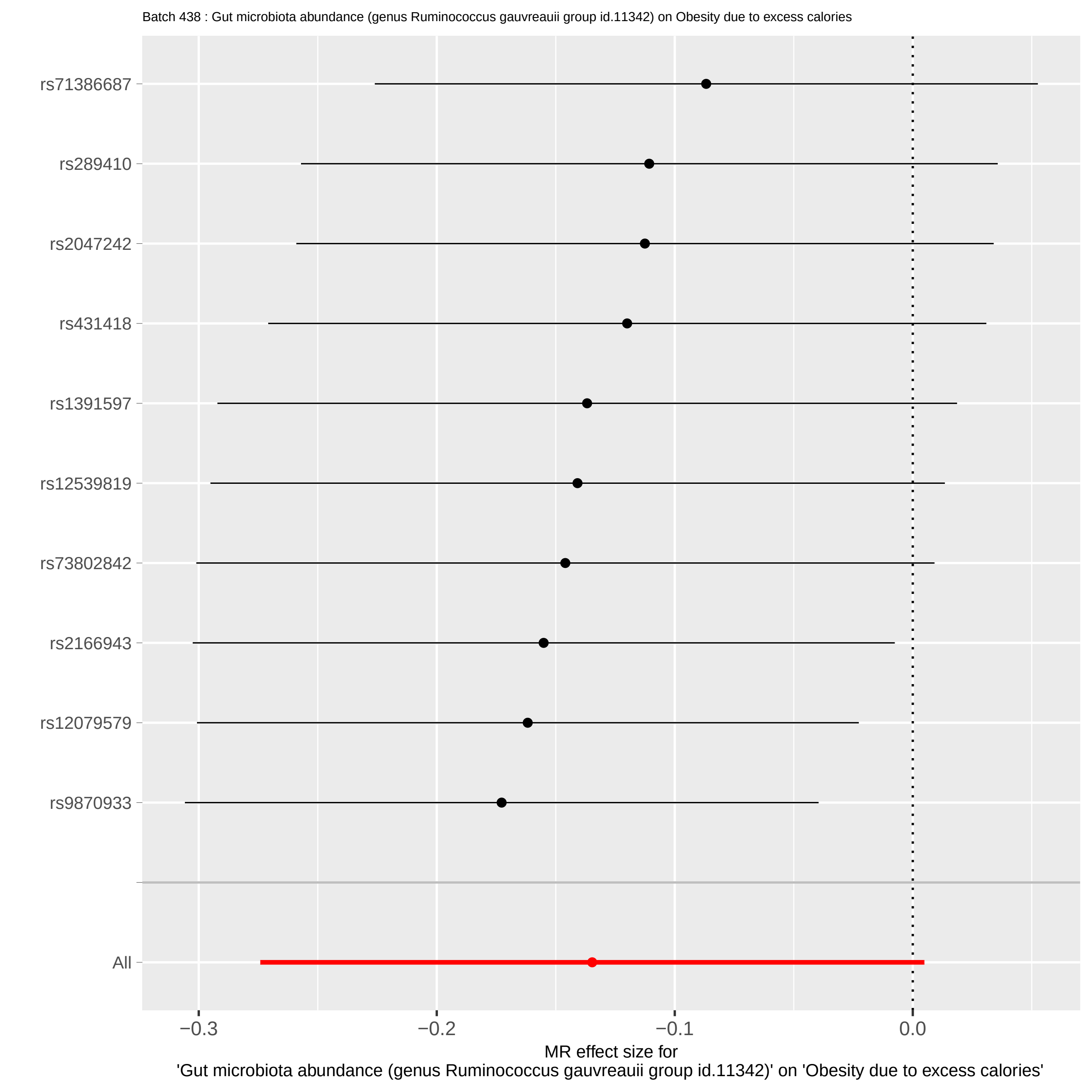
Batch 438 : Gut microbiota abundance (genus Ruminococcus gauvreauii group id.11342) on Obesity due to excess calories
rs71386687
rs289410
rs2047242
rs431418
rs1391597
rs12539819
rs73802842
rs2166943
rs12079579
rs9870933
All
−0.3
−0.2
−0.1
0.0
MR effect size for
'Gut microbiota abundance (genus Ruminococcus gauvreauii group id.11342)' on 'Obesity due to excess calories'

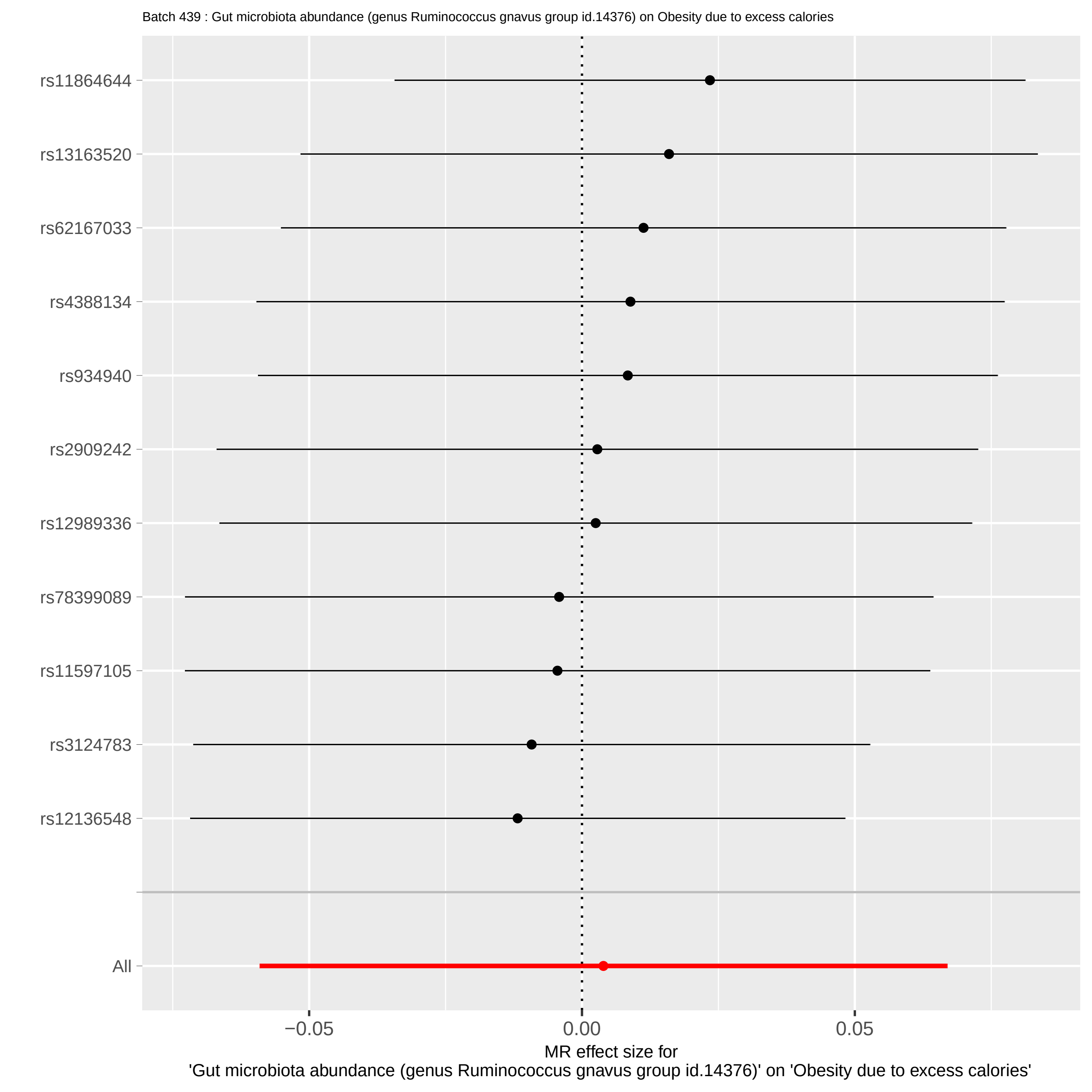

Batch 439 : Gut microbiota abundance (genus Ruminococcus gnavus group id.14376) on Obesity due to excess calories
rs11864644
rs13163520
rs62167033
rs4388134
rs934940
rs2909242
rs12989336
rs78399089
rs11597105
rs3124783
rs12136548
All
−0.05
0.00
0.05
MR effect size for
'Gut microbiota abundance (genus Ruminococcus gnavus group id.14376)' on 'Obesity due to excess calories'

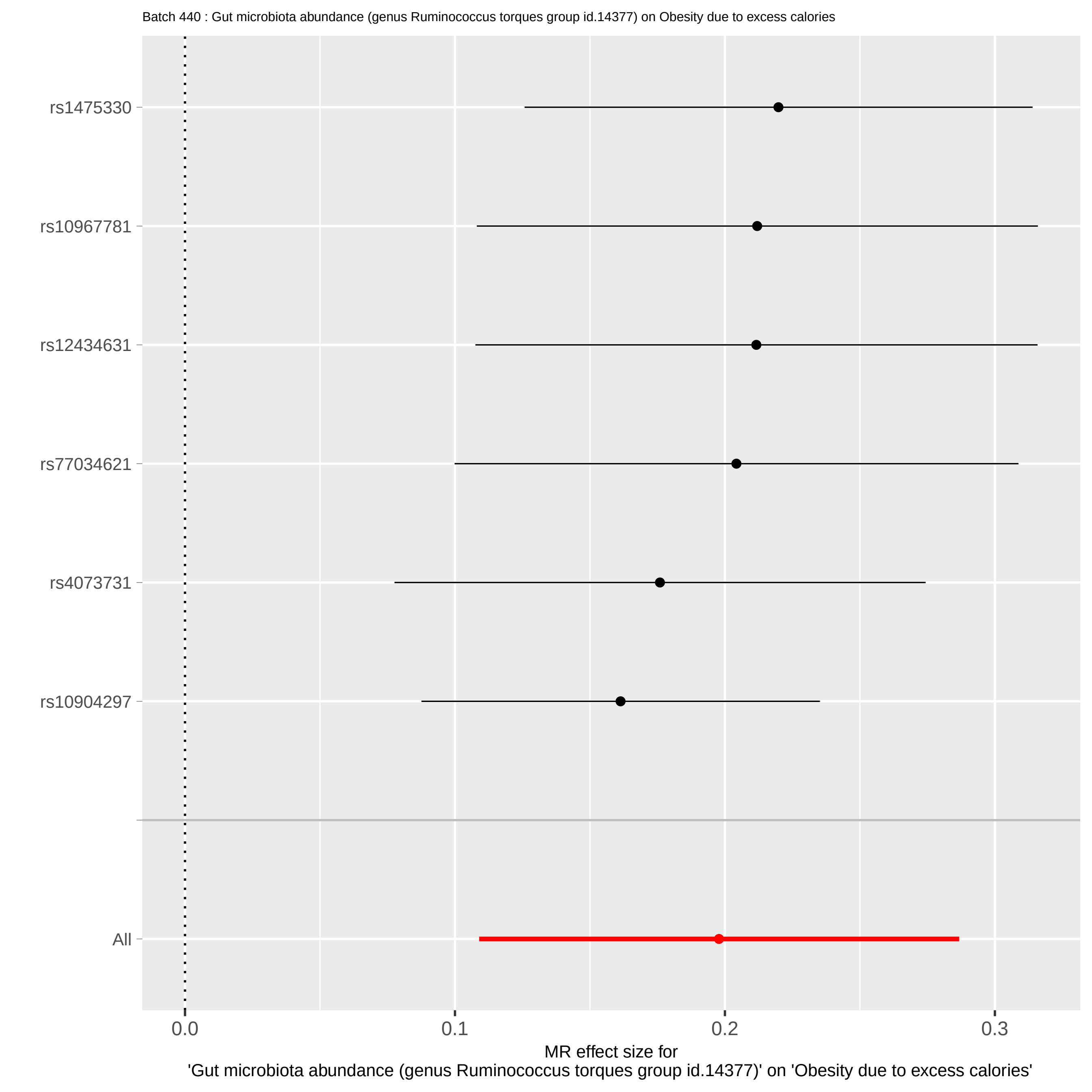
Batch 440 : Gut microbiota abundance (genus Ruminococcus torques group id.14377) on Obesity due to excess calories
rs1475330
rs10967781
rs12434631
rs77034621
rs4073731
rs10904297
All
0.0
0.1
0.2
0.3
MR effect size for
'Gut microbiota abundance (genus Ruminococcus torques group id.14377)' on 'Obesity due to excess calories'

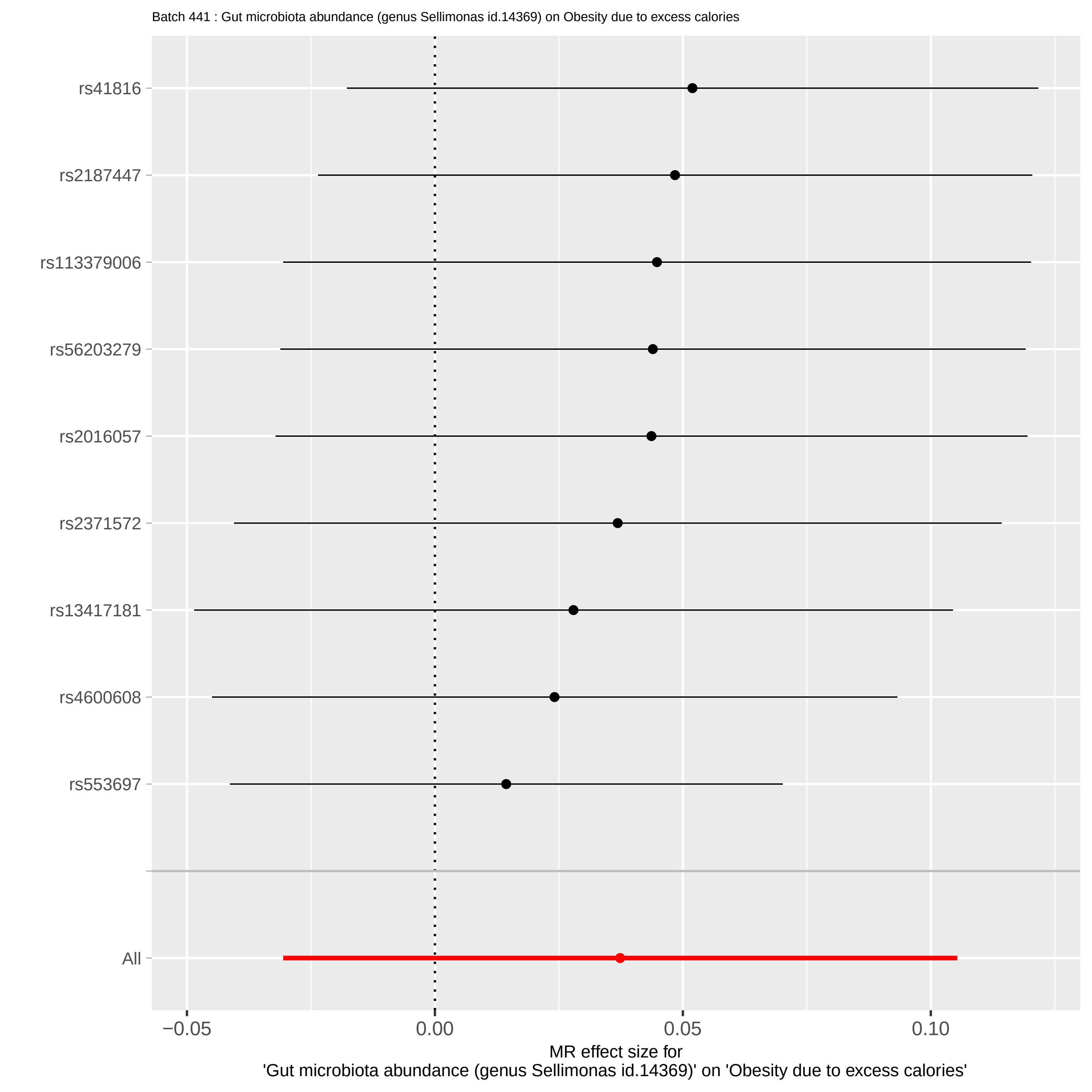

Batch 441 : Gut microbiota abundance (genus Sellimonas id.14369) on Obesity due to excess calories
rs41816
rs2187447
rs113379006
rs56203279
rs2016057
rs2371572
rs13417181
rs4600608
rs553697
All
−0.05
0.00
0.05
0.10
MR effect size for
'Gut microbiota abundance (genus Sellimonas id.14369)' on 'Obesity due to excess calories'

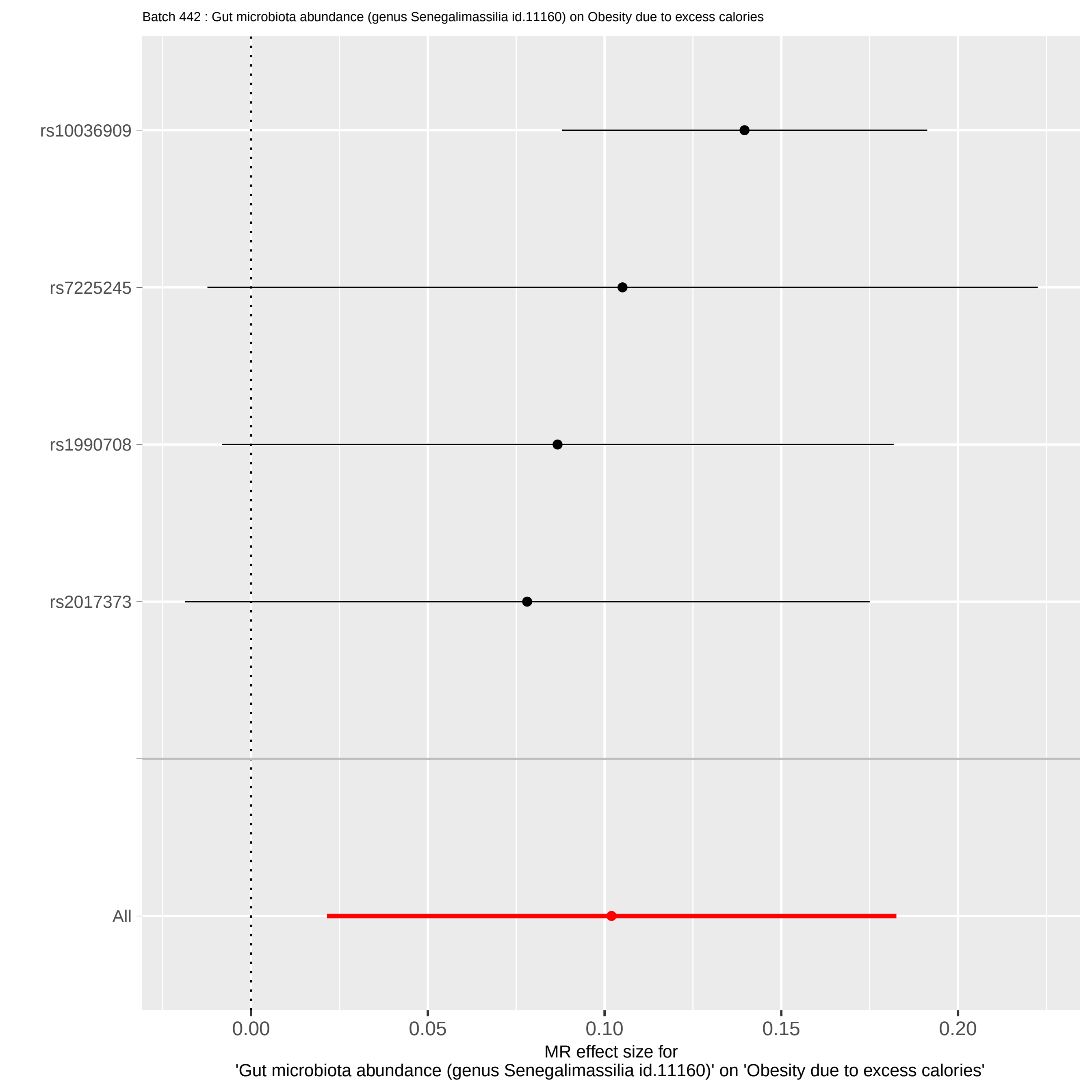
Batch 442 : Gut microbiota abundance (genus Senegalimassilia id.11160) on Obesity due to excess calories
rs10036909
rs7225245
rs1990708
rs2017373
All
0.00
0.05
0.10
0.15
0.20
MR effect size for
'Gut microbiota abundance (genus Senegalimassilia id.11160)' on 'Obesity due to excess calories'

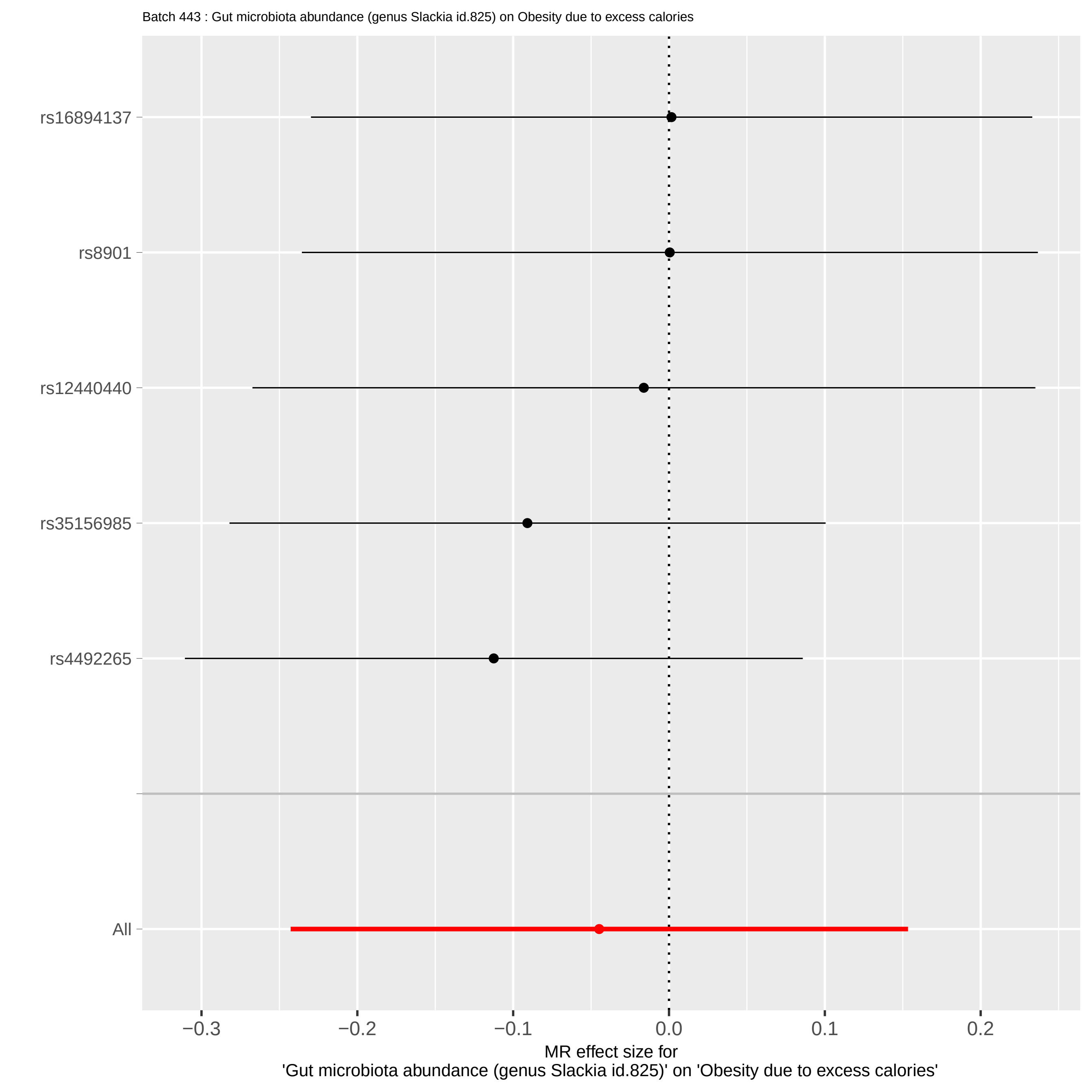

Batch 443 : Gut microbiota abundance (genus Slackia id.825) on Obesity due to excess calories
rs16894137
rs8901
rs12440440
rs35156985
rs4492265
All
−0.3
−0.2
−0.1
0.0
0.1
0.2
MR effect size for
'Gut microbiota abundance (genus Slackia id.825)' on 'Obesity due to excess calories'

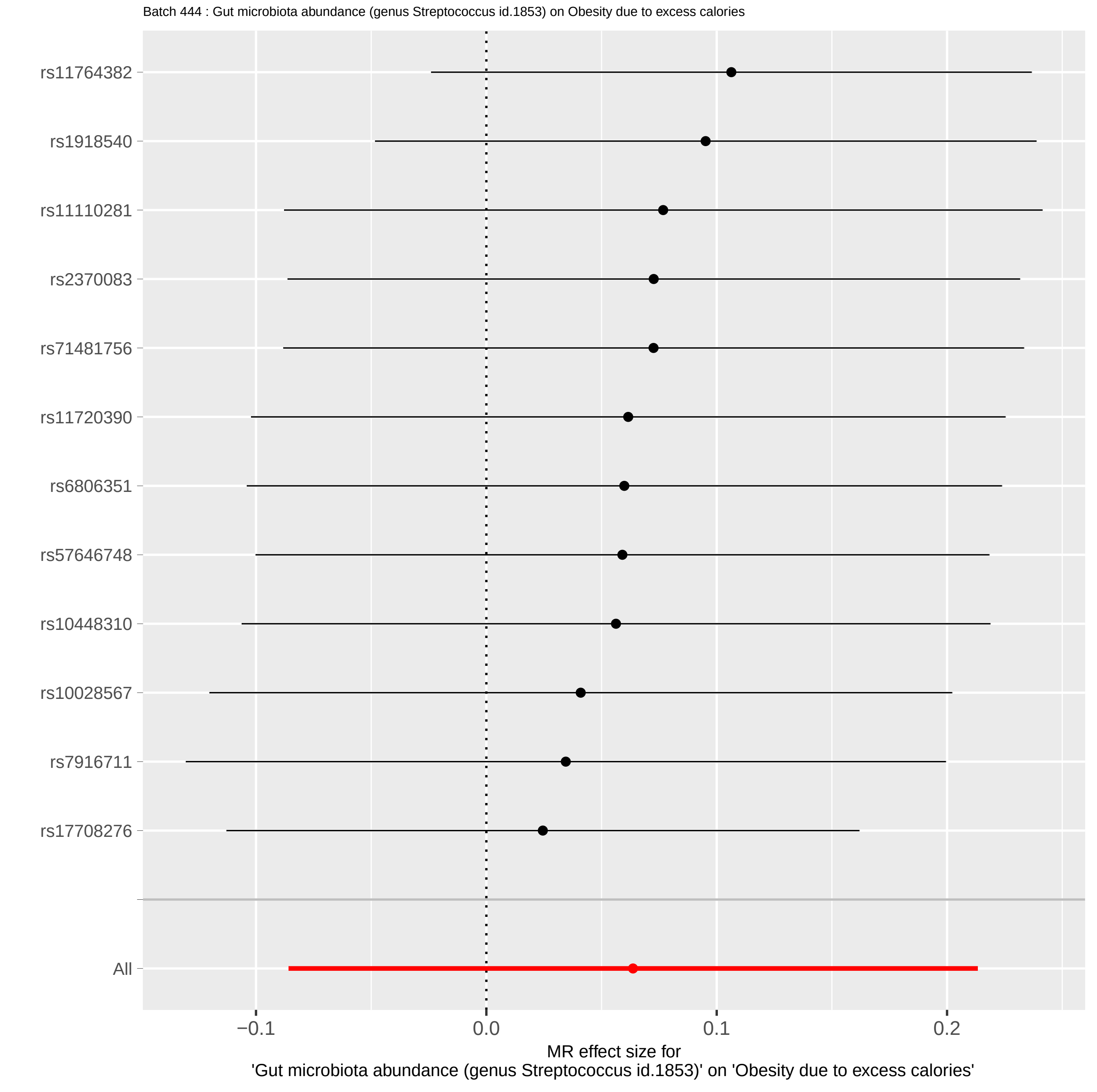
Batch 444 : Gut microbiota abundance (genus Streptococcus id.1853) on Obesity due to excess calories
rs11764382
rs1918540
rs11110281
rs2370083
rs71481756
rs11720390
rs6806351
rs57646748
rs10448310
rs10028567
rs7916711
rs17708276
All
−0.1
0.0
0.1
0.2
MR effect size for
'Gut microbiota abundance (genus Streptococcus id.1853)' on 'Obesity due to excess calories'

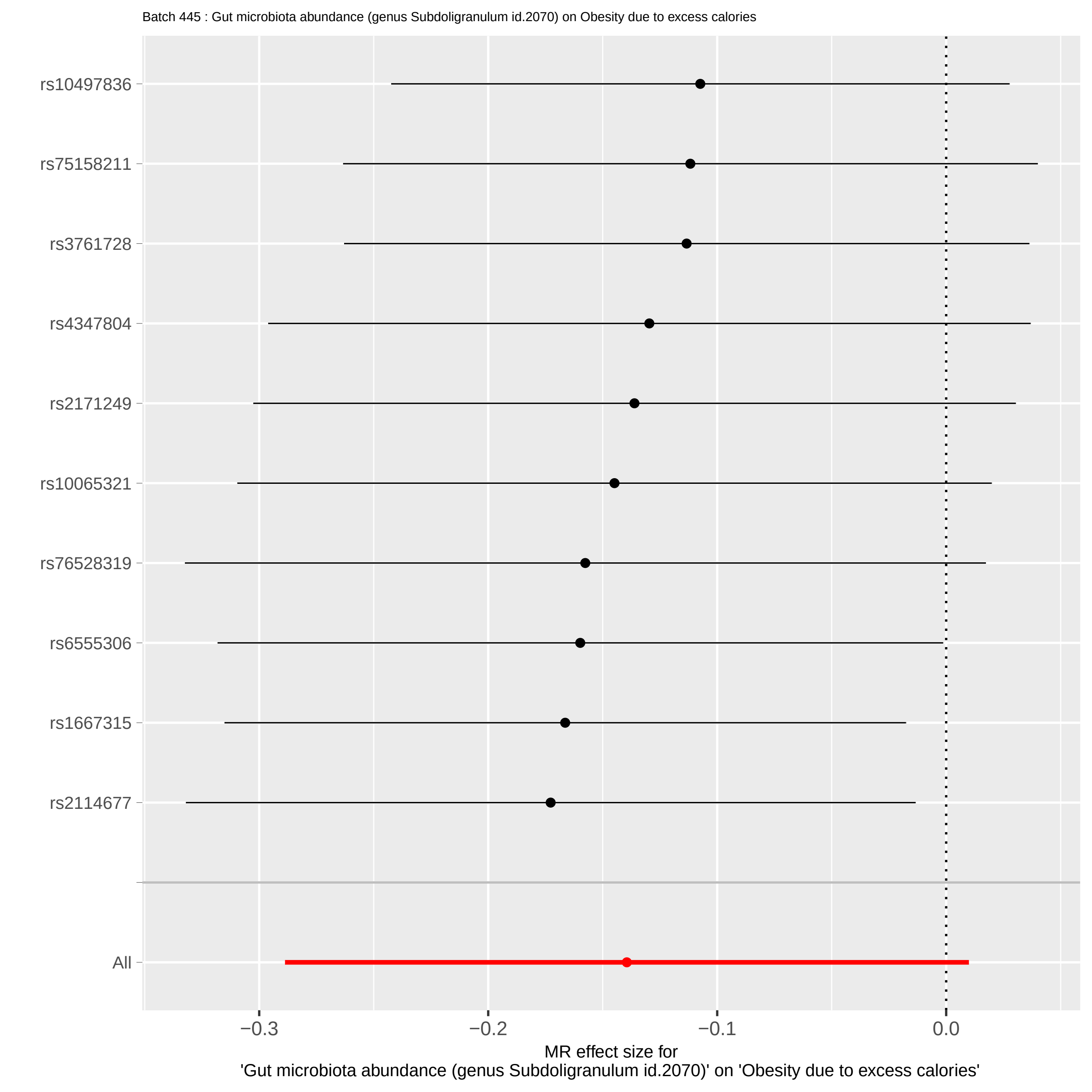

Batch 445 : Gut microbiota abundance (genus Subdoligranulum id.2070) on Obesity due to excess calories
rs10497836
rs75158211
rs3761728
rs4347804
rs2171249
rs10065321
rs76528319
rs6555306
rs1667315
rs2114677
All
−0.3
−0.2
−0.1
0.0
MR effect size for
'Gut microbiota abundance (genus Subdoligranulum id.2070)' on 'Obesity due to excess calories'

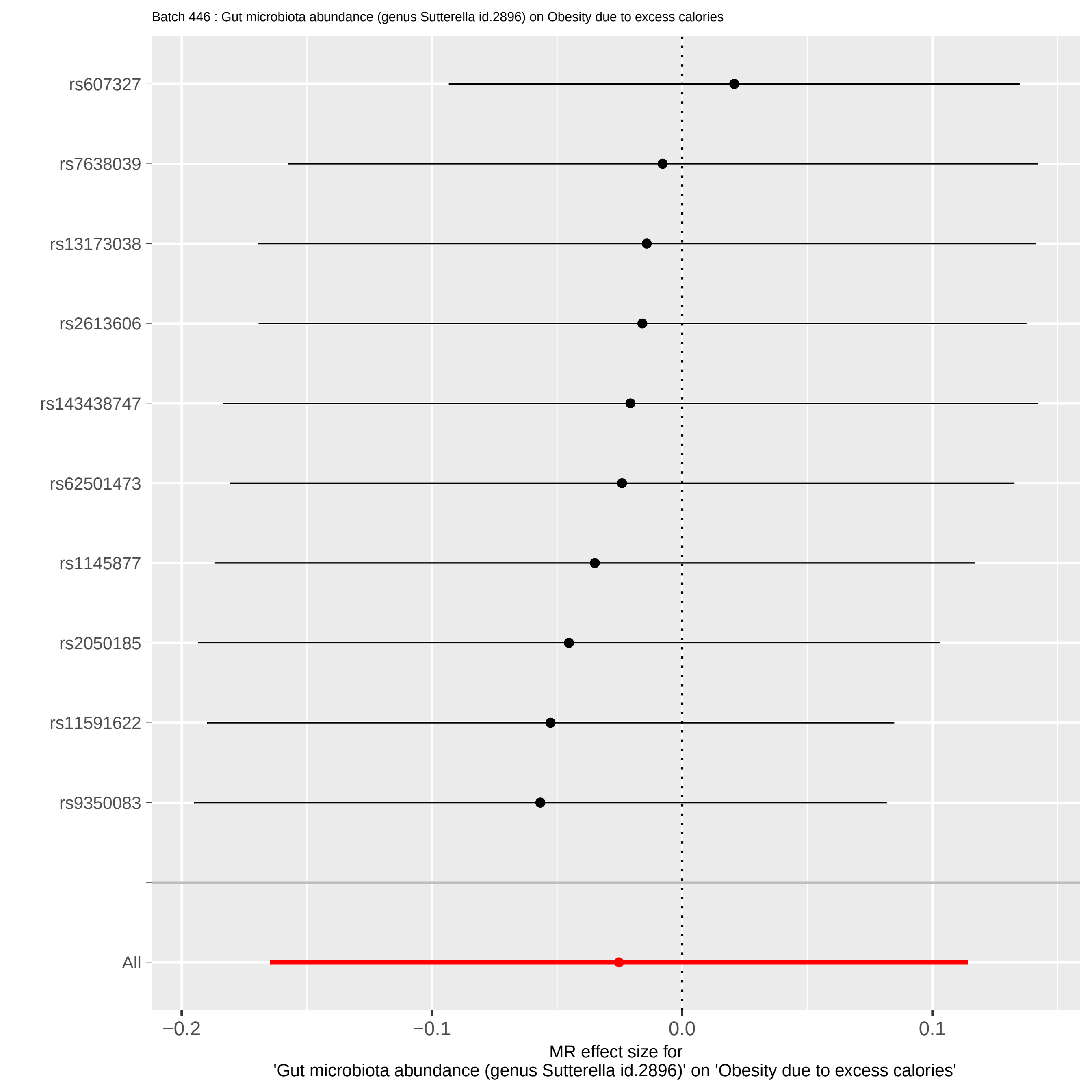

Batch 446 : Gut microbiota abundance (genus Sutterella id.2896) on Obesity due to excess calories
rs607327
rs7638039
rs13173038
rs2613606
rs143438747
rs62501473
rs1145877
rs2050185
rs11591622
rs9350083
All
−0.2
−0.1
0.0
0.1
MR effect size for
'Gut microbiota abundance (genus Sutterella id.2896)' on 'Obesity due to excess calories'

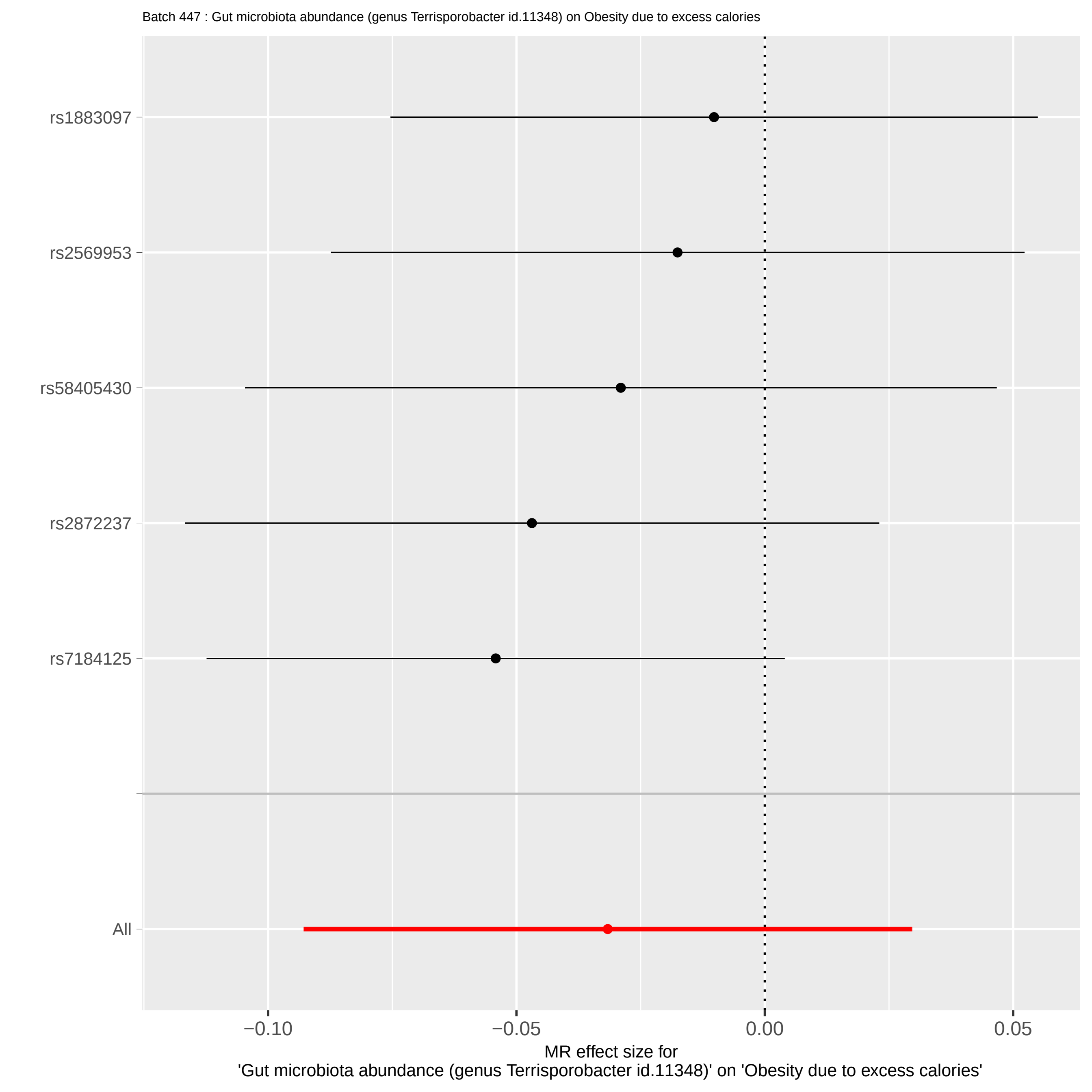

Batch 447 : Gut microbiota abundance (genus Terrisporobacter id.11348) on Obesity due to excess calories
rs1883097
rs2569953
rs58405430
rs2872237
rs7184125
All
−0.10
−0.05
0.00
0.05
MR effect size for
'Gut microbiota abundance (genus Terrisporobacter id.11348)' on 'Obesity due to excess calories'

Batch 448 : Gut microbiota abundance (genus Turicibacter id.2162) on Obesity due to excess calories

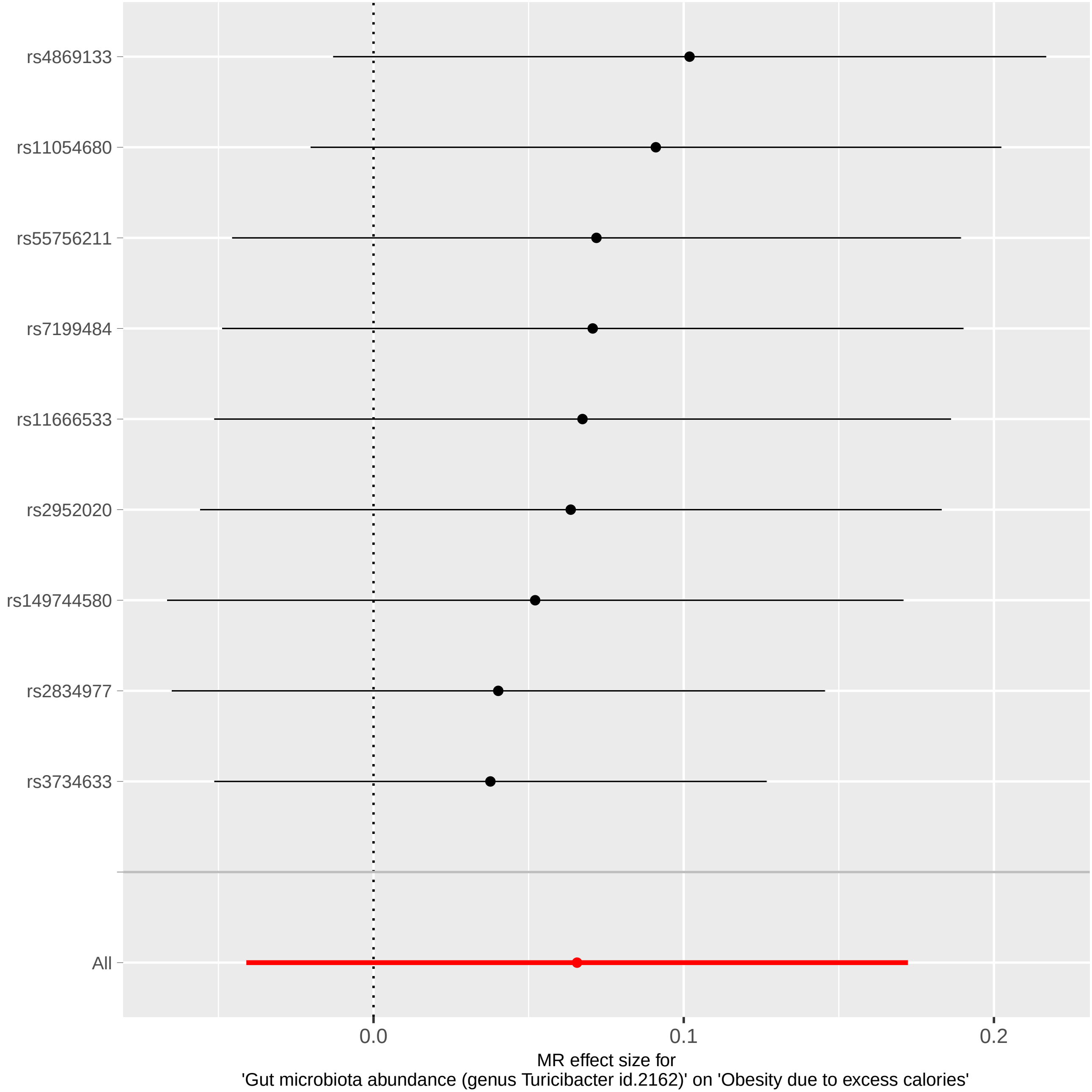

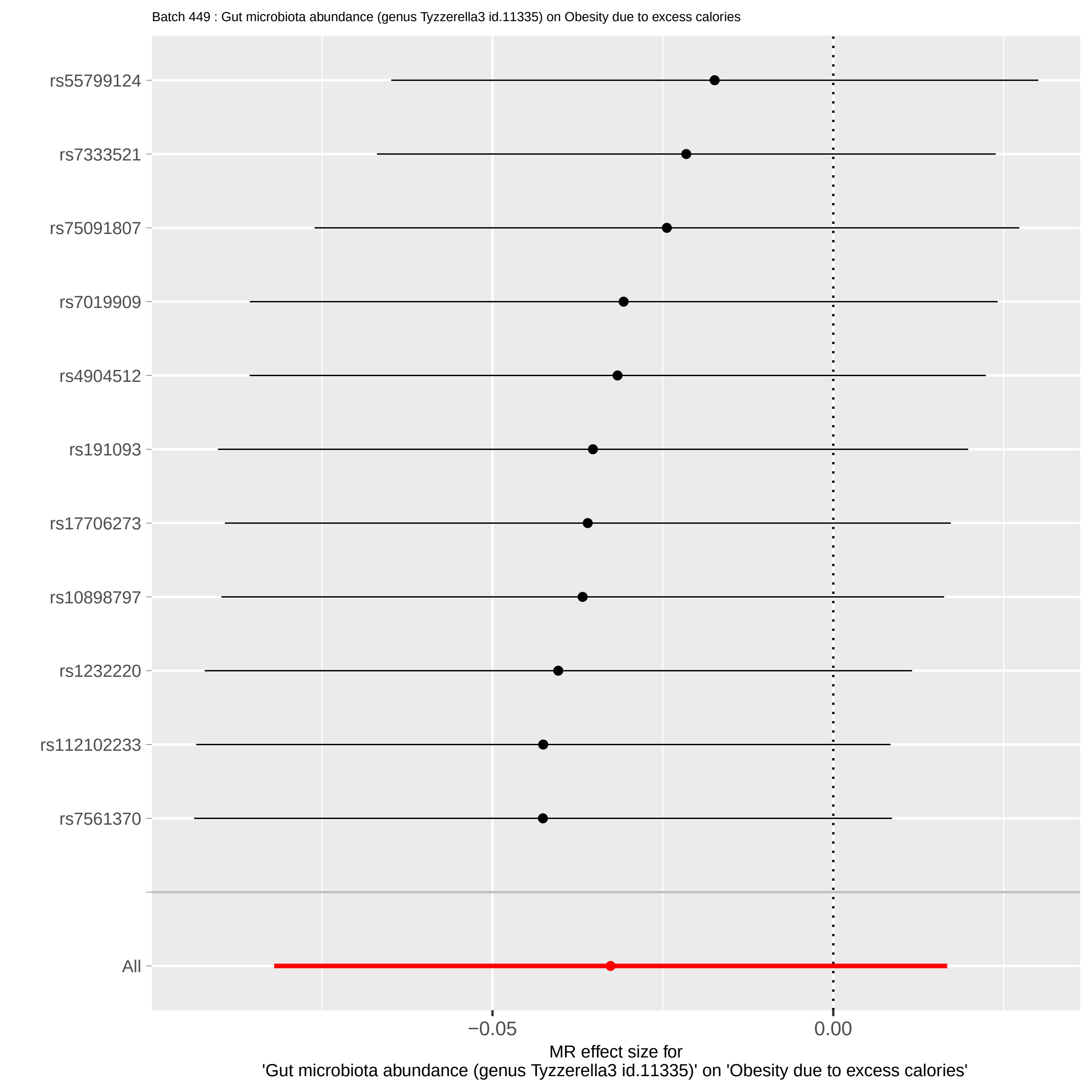

Batch 449 : Gut microbiota abundance (genus Tyzzerella3 id.11335) on Obesity due to excess calories
rs55799124
rs7333521
rs75091807
rs7019909
rs4904512
rs191093
rs17706273
rs10898797
rs1232220
rs112102233
rs7561370
All
−0.05
0.00
MR effect size for
'Gut microbiota abundance (genus Tyzzerella3 id.11335)' on 'Obesity due to excess calories'

Batch 450 : Gut microbiota abundance (genus Veillonella id.2198) on Obesity due to excess calories

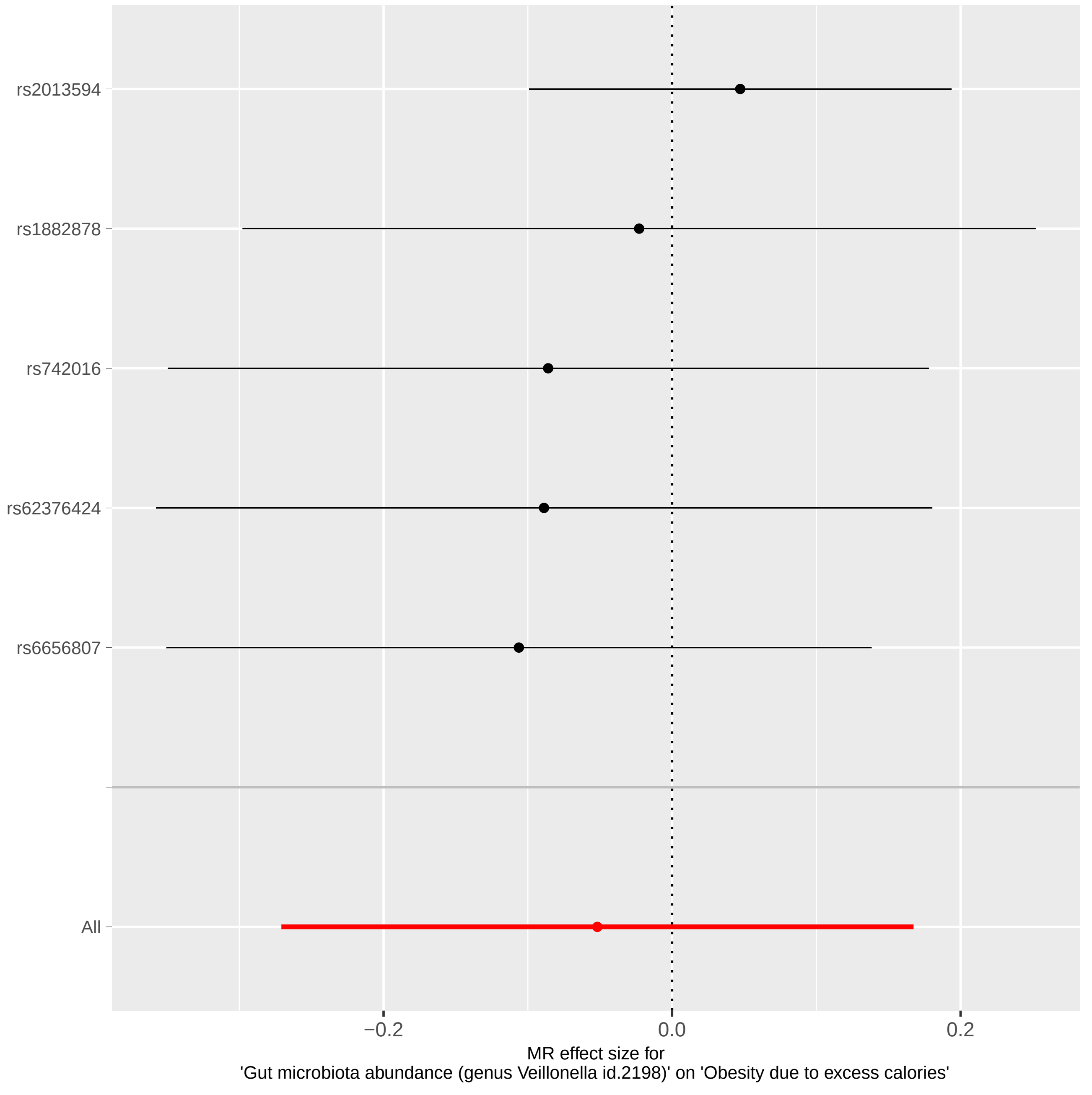

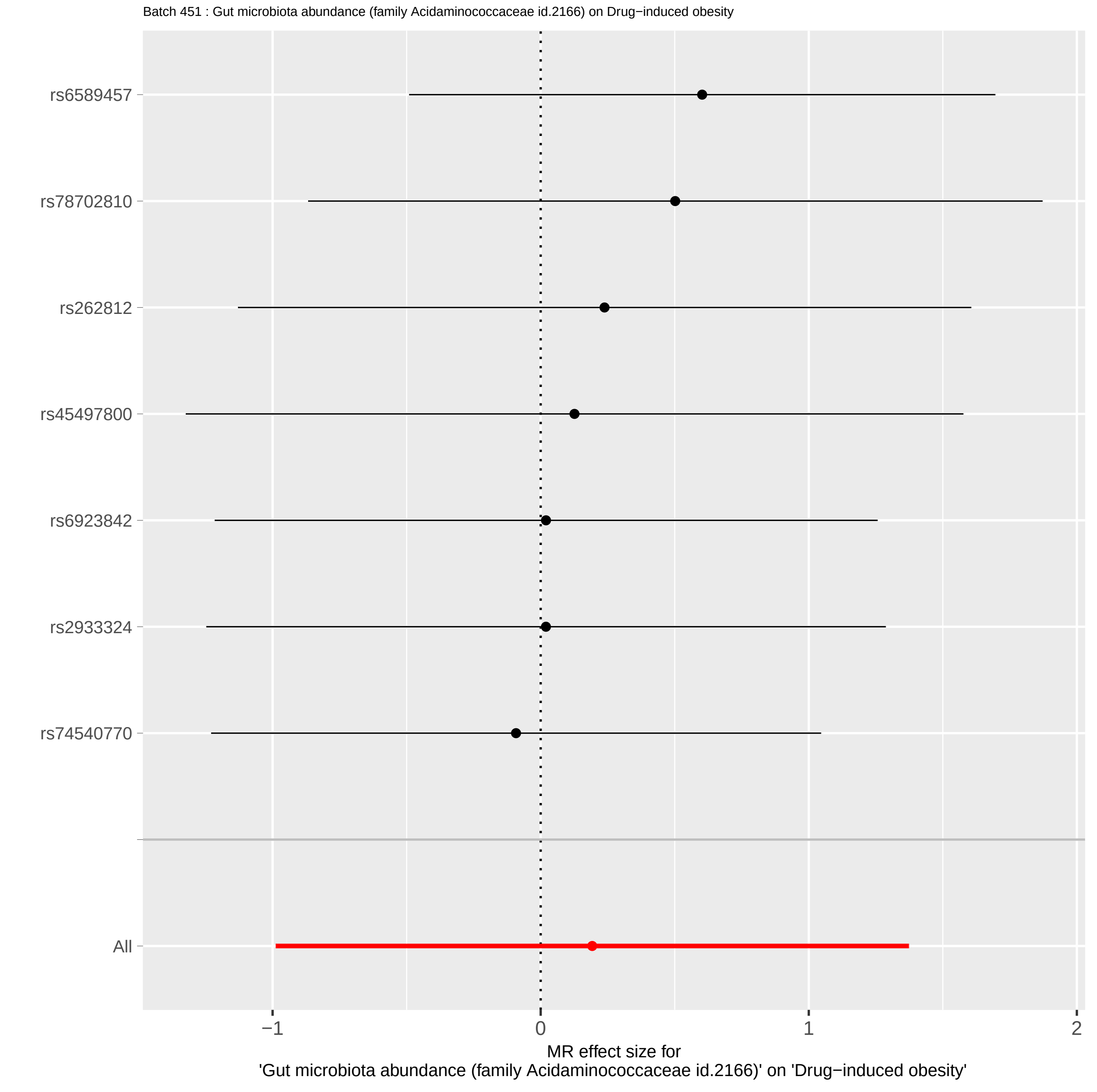
Batch 451 : Gut microbiota abundance (family Acidaminococcaceae id.2166) on Drug−induced obesity
rs6589457
rs78702810
rs262812
rs45497800
rs6923842
rs2933324
rs74540770
All
−1
0
1
2
MR effect size for
'Gut microbiota abundance (family Acidaminococcaceae id.2166)' on 'Drug−induced obesity'

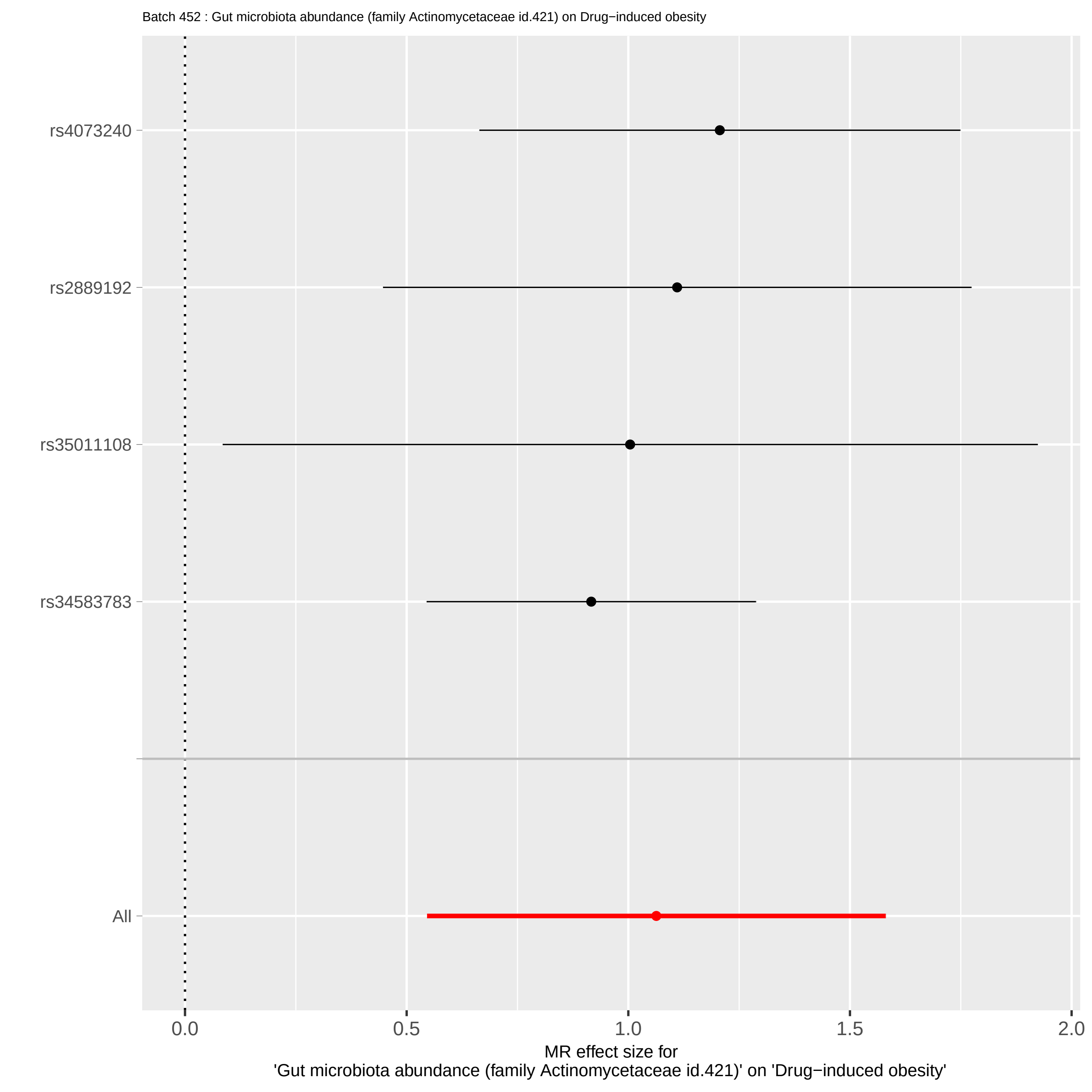
Batch 452 : Gut microbiota abundance (family Actinomycetaceae id.421) on Drug−induced obesity
rs4073240
rs2889192
rs35011108
rs34583783
All
0.0
0.5
1.0
1.5
2.0
MR effect size for
'Gut microbiota abundance (family Actinomycetaceae id.421)' on 'Drug−induced obesity'

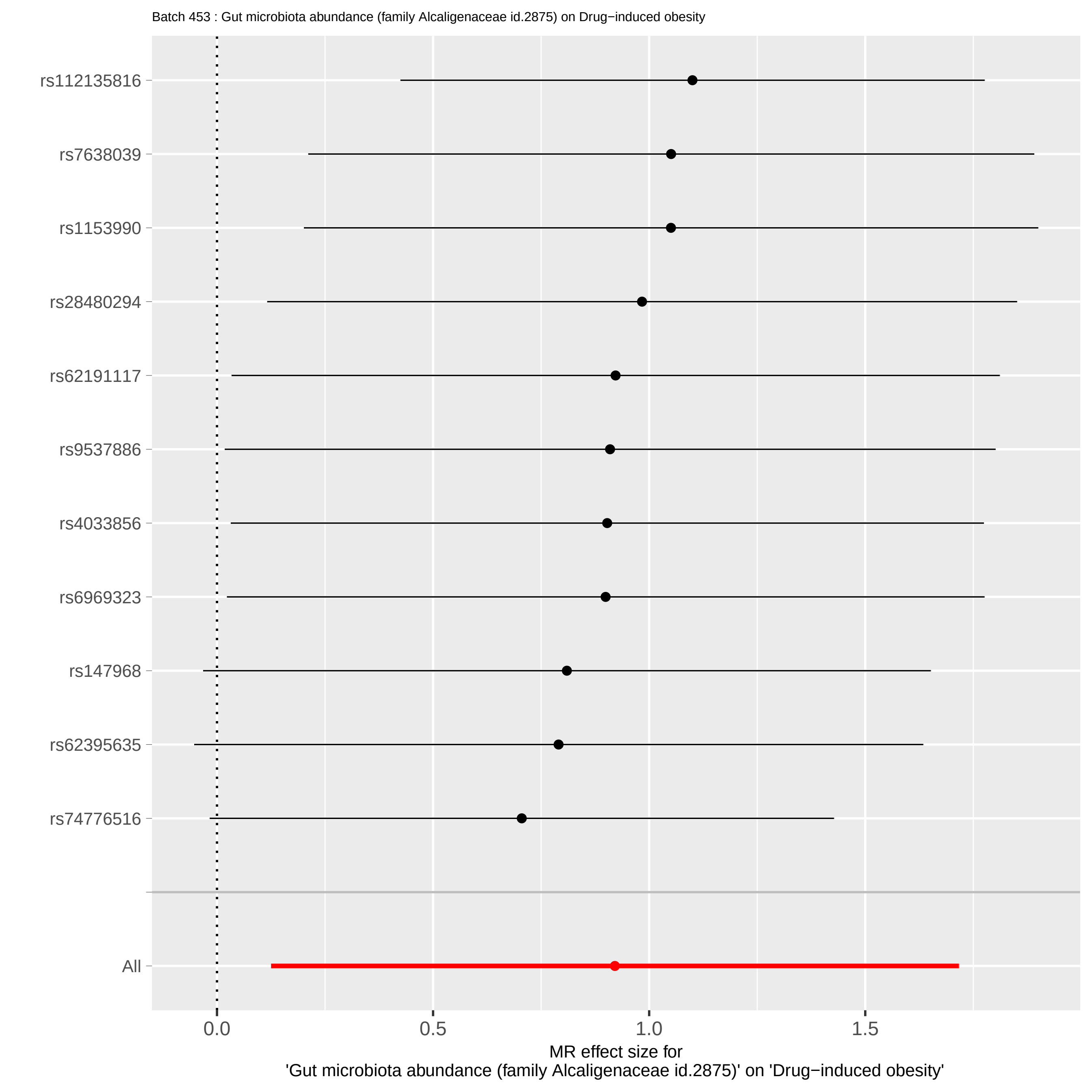
Batch 453 : Gut microbiota abundance (family Alcaligenaceae id.2875) on Drug−induced obesity
rs112135816
rs7638039
rs1153990
rs28480294
rs62191117
rs9537886
rs4033856
rs6969323
rs147968
rs62395635
rs74776516
All
0.0
0.5
1.0
1.5
MR effect size for
'Gut microbiota abundance (family Alcaligenaceae id.2875)' on 'Drug−induced obesity'

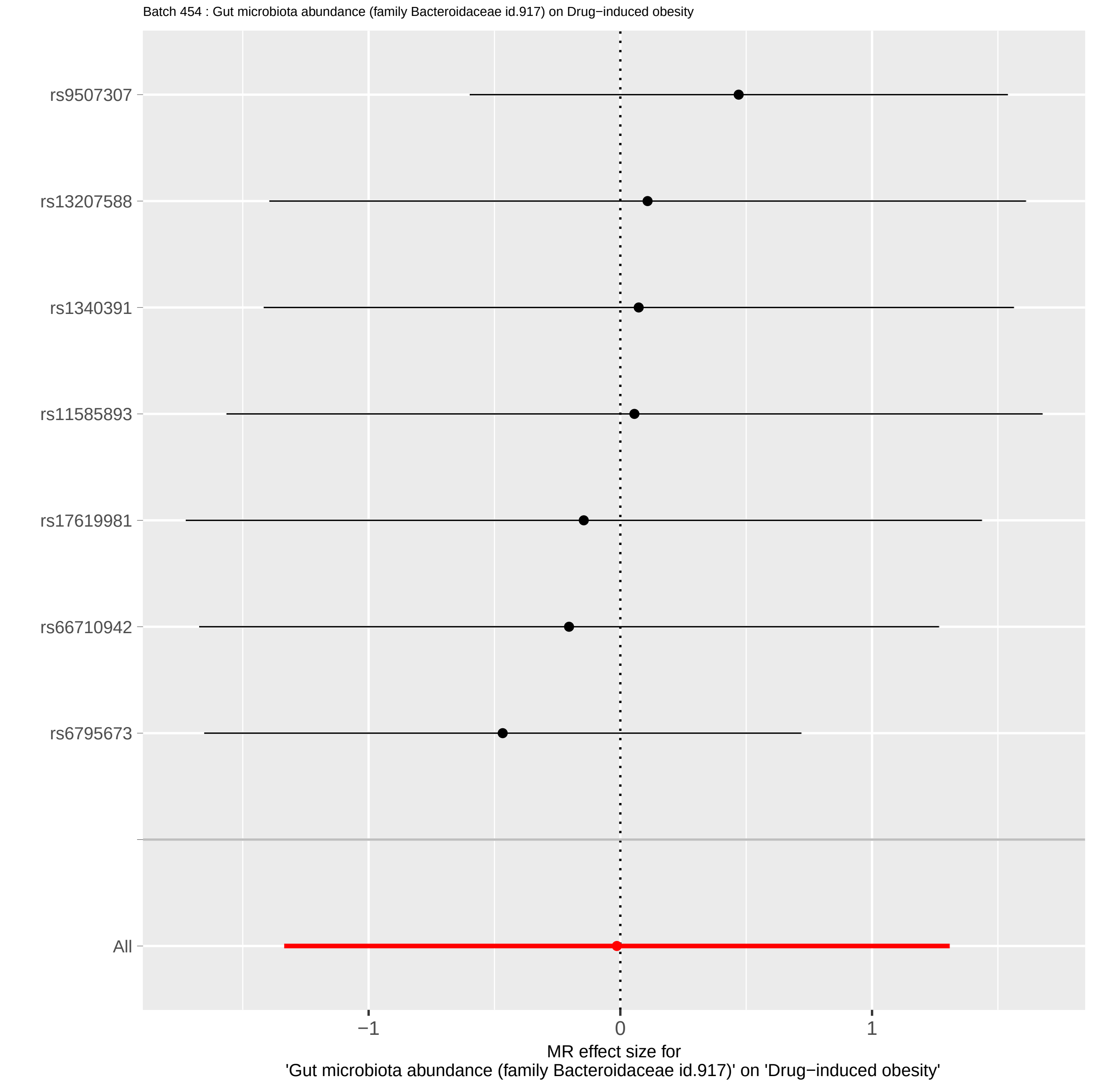

Batch 454 : Gut microbiota abundance (family Bacteroidaceae id.917) on Drug−induced obesity
rs9507307
rs13207588
rs1340391
rs11585893
rs17619981
rs66710942
rs6795673
All
−1
0
1
MR effect size for
'Gut microbiota abundance (family Bacteroidaceae id.917)' on 'Drug−induced obesity'

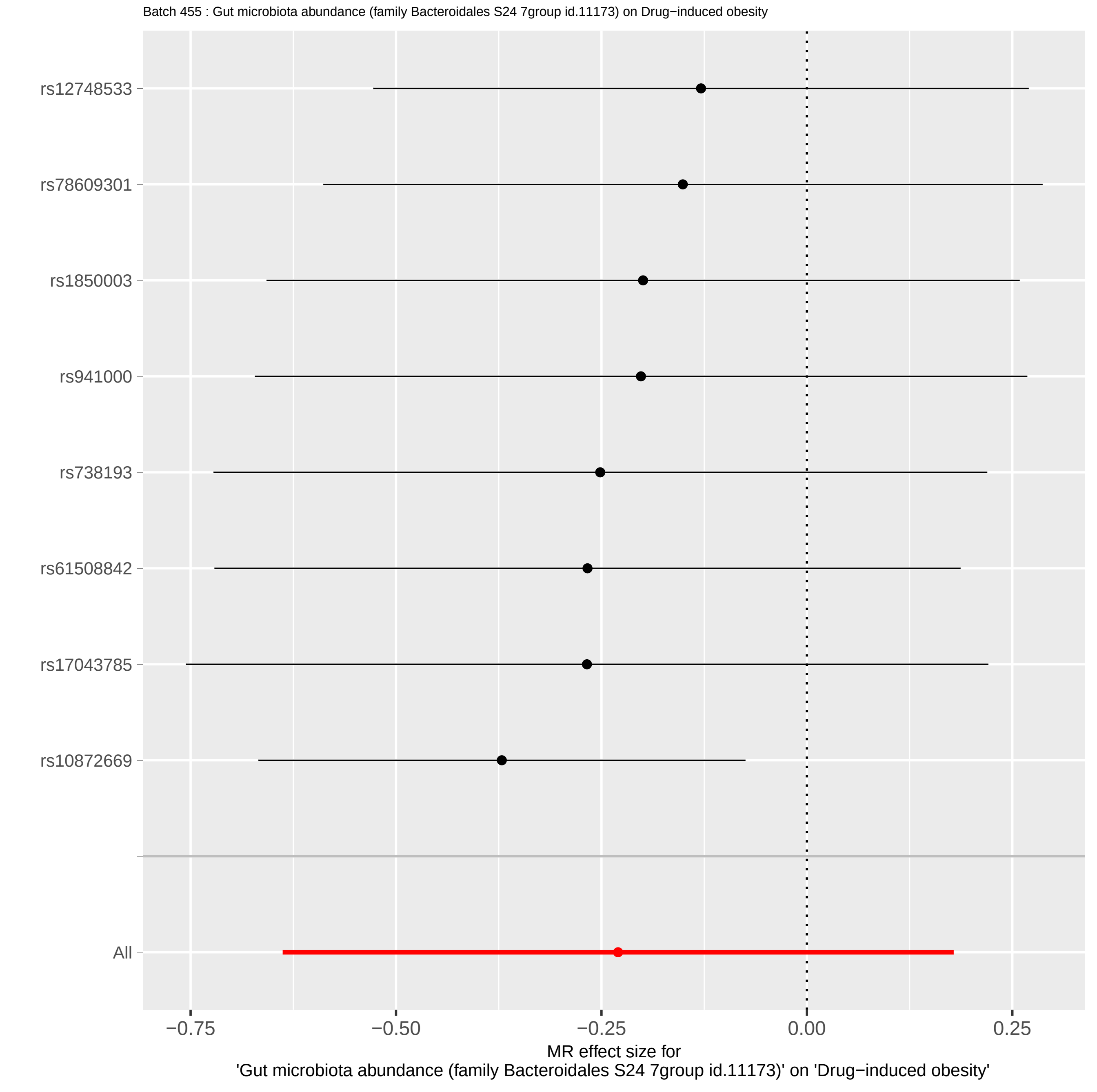
Batch 455 : Gut microbiota abundance (family Bacteroidales S24 7group id.11173) on Drug−induced obesity
rs12748533
rs78609301
rs1850003
rs941000
rs738193
rs61508842
rs17043785
rs10872669
All
−0.75
−0.50
−0.25
0.00
0.25
MR effect size for
'Gut microbiota abundance (family Bacteroidales S24 7group id.11173)' on 'Drug−induced obesity'

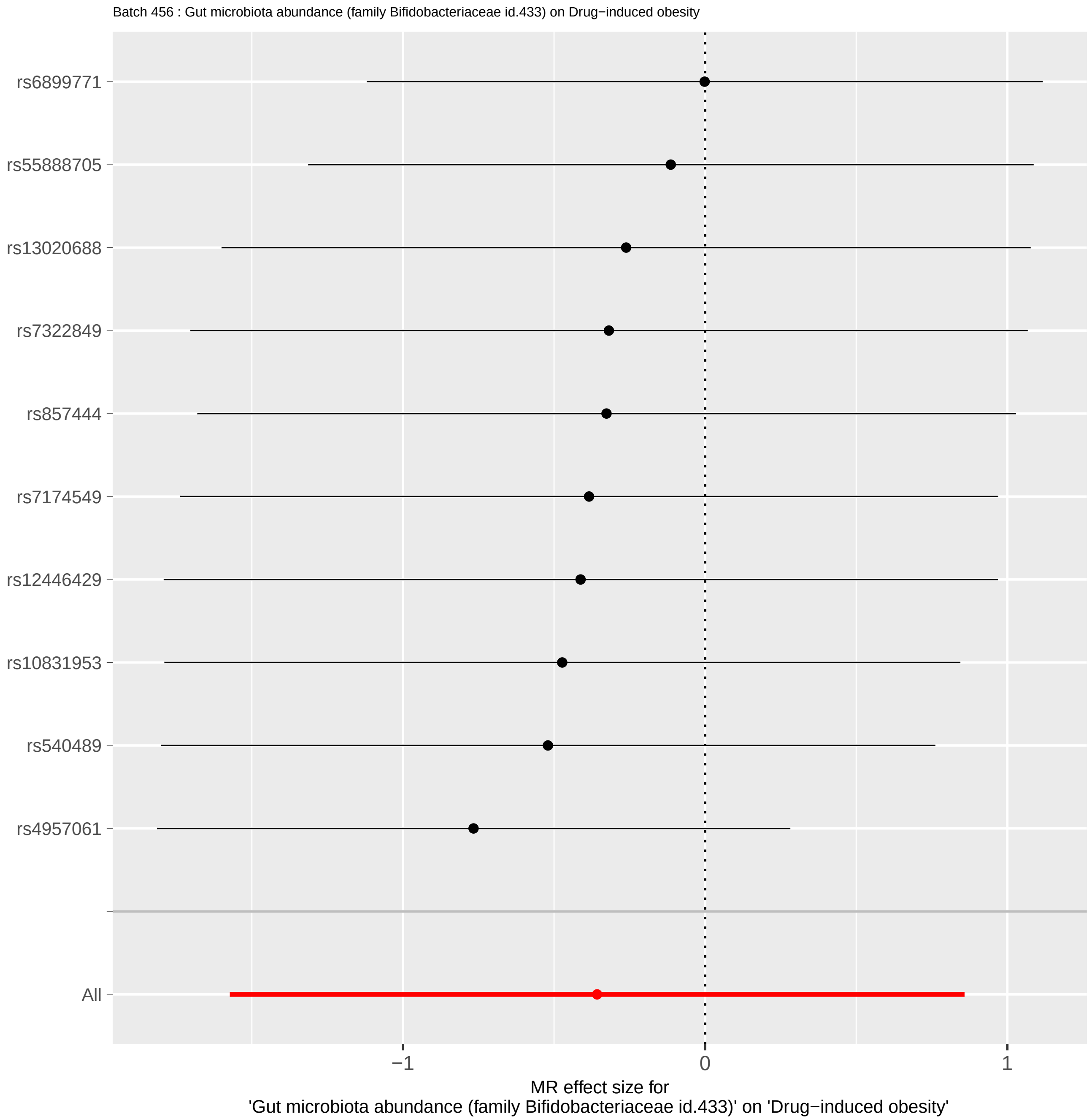
Batch 456 : Gut microbiota abundance (family Bifidobacteriaceae id.433) on Drug−induced obesity
rs6899771
rs55888705
rs13020688
rs7322849
rs857444
rs7174549
rs12446429
rs10831953
rs540489
rs4957061
All
−1
0
1
MR effect size for
'Gut microbiota abundance (family Bifidobacteriaceae id.433)' on 'Drug−induced obesity'

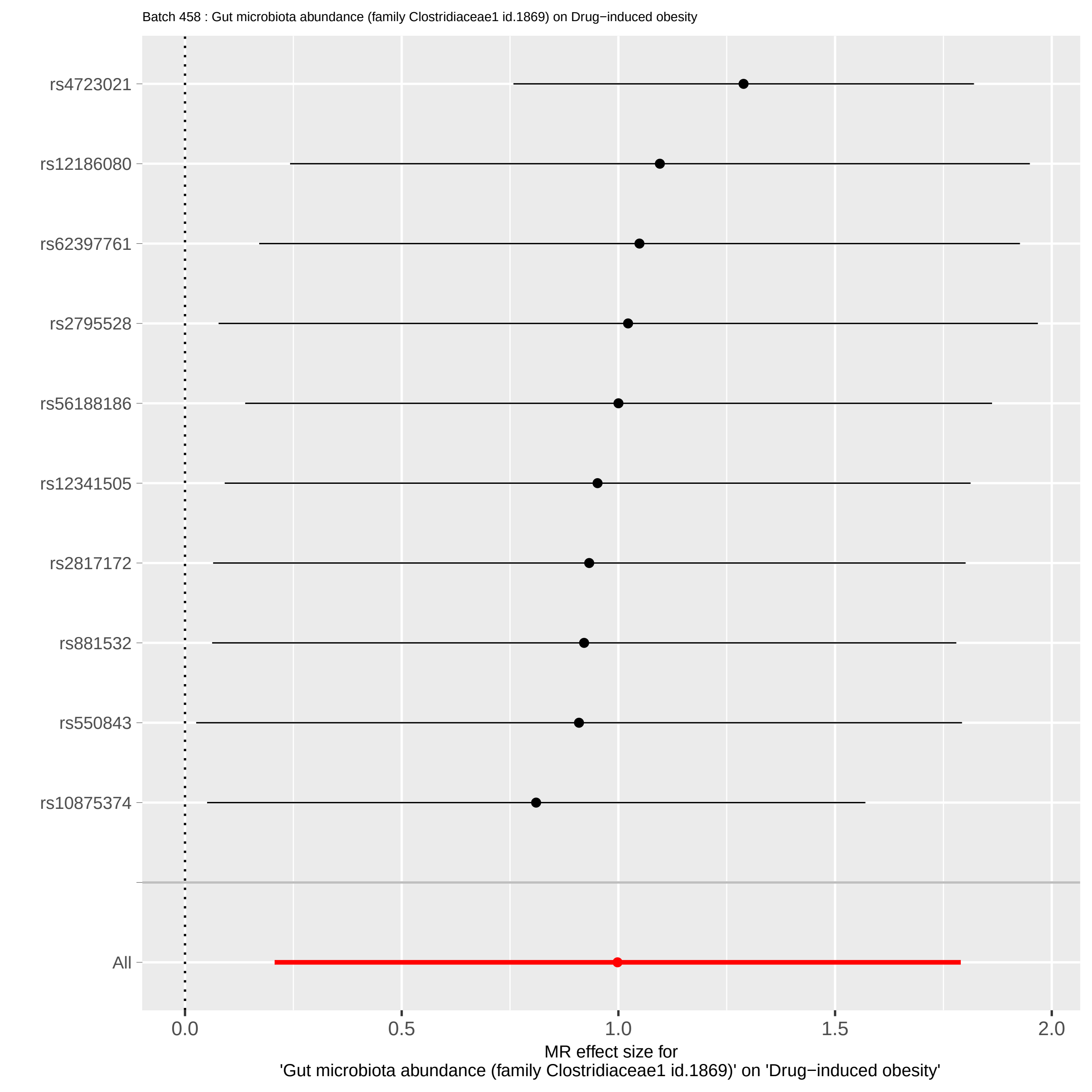
Batch 458 : Gut microbiota abundance (family Clostridiaceae1 id.1869) on Drug−induced obesity
rs4723021
rs12186080
rs62397761
rs2795528
rs56188186
rs12341505
rs2817172
rs881532
rs550843
rs10875374
All
0.0
0.5
1.0
1.5
2.0
MR effect size for
'Gut microbiota abundance (family Clostridiaceae1 id.1869)' on 'Drug−induced obesity'

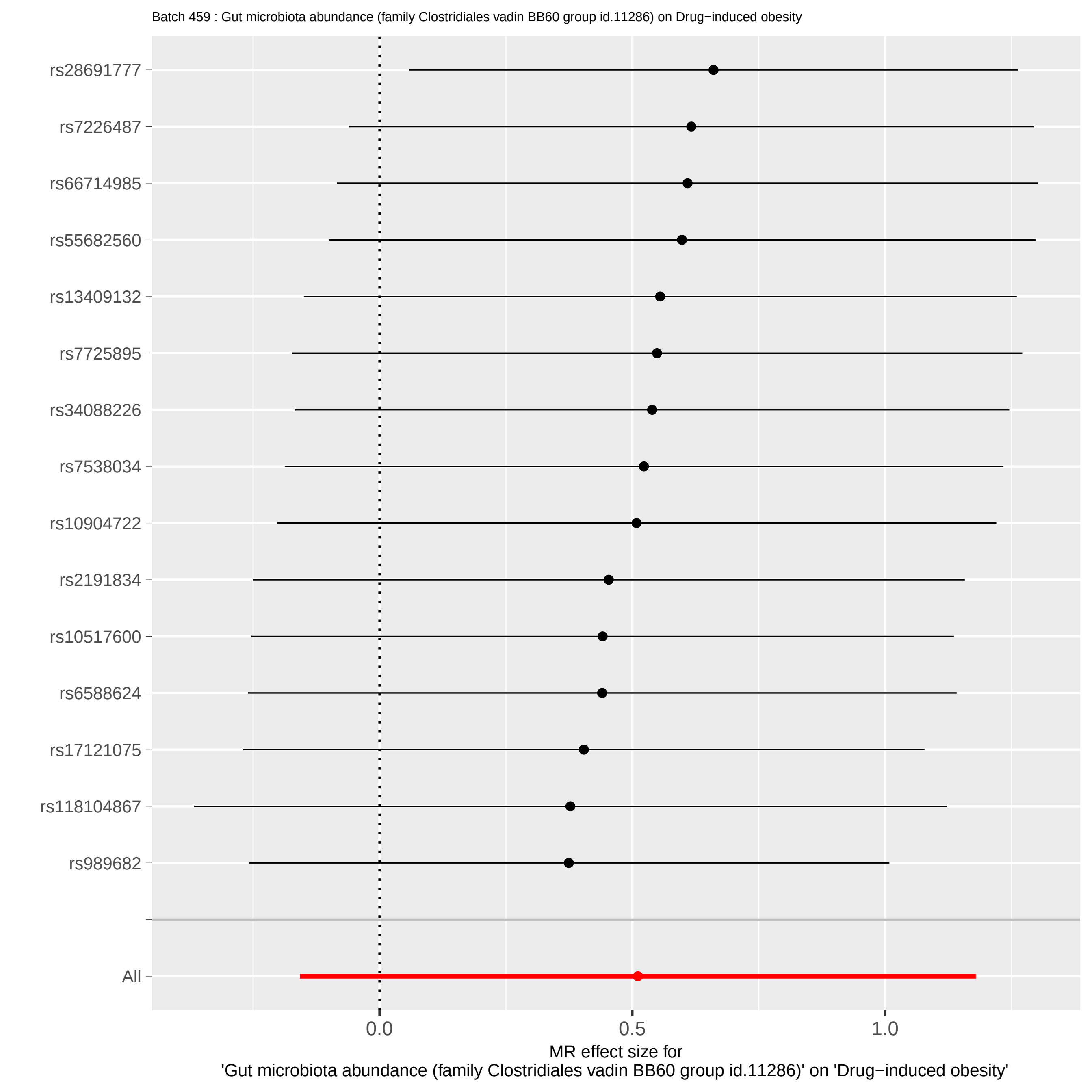

Batch 459 : Gut microbiota abundance (family Clostridiales vadin BB60 group id.11286) on Drug−induced obesity
rs28691777
rs7226487
rs66714985
rs55682560
rs13409132
rs7725895
rs34088226
rs7538034
rs10904722
rs2191834
rs10517600
rs6588624
rs17121075
rs118104867
rs989682
All
0.0
0.5
1.0
MR effect size for
'Gut microbiota abundance (family Clostridiales vadin BB60 group id.11286)' on 'Drug−induced obesity'

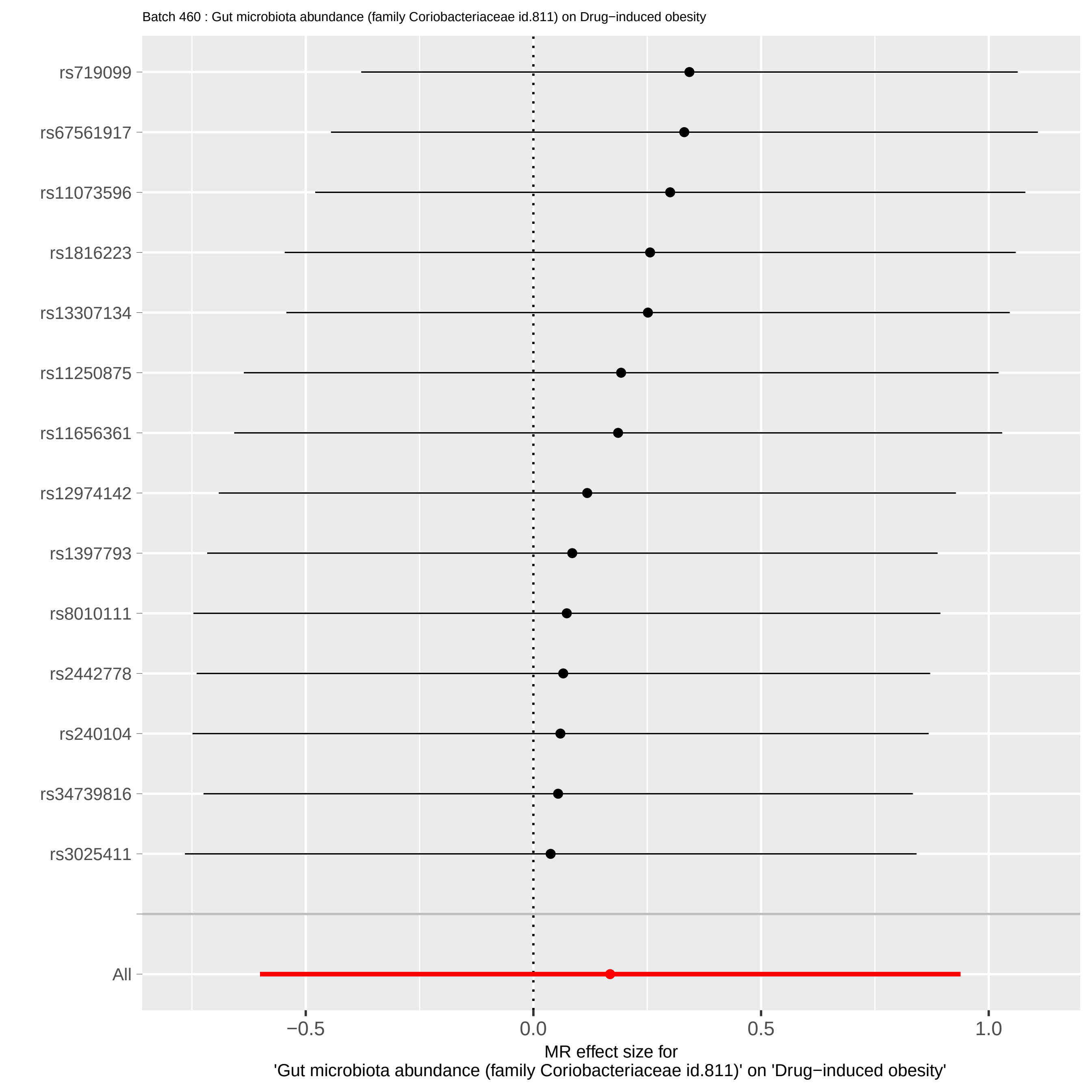
Batch 460 : Gut microbiota abundance (family Coriobacteriaceae id.811) on Drug−induced obesity
rs719099
rs67561917
rs11073596
rs1816223
rs13307134
rs11250875
rs11656361
rs12974142
rs1397793
rs8010111
rs2442778
rs240104
rs34739816
rs3025411
All
−0.5
0.0
0.5
1.0
MR effect size for
'Gut microbiota abundance (family Coriobacteriaceae id.811)' on 'Drug−induced obesity'

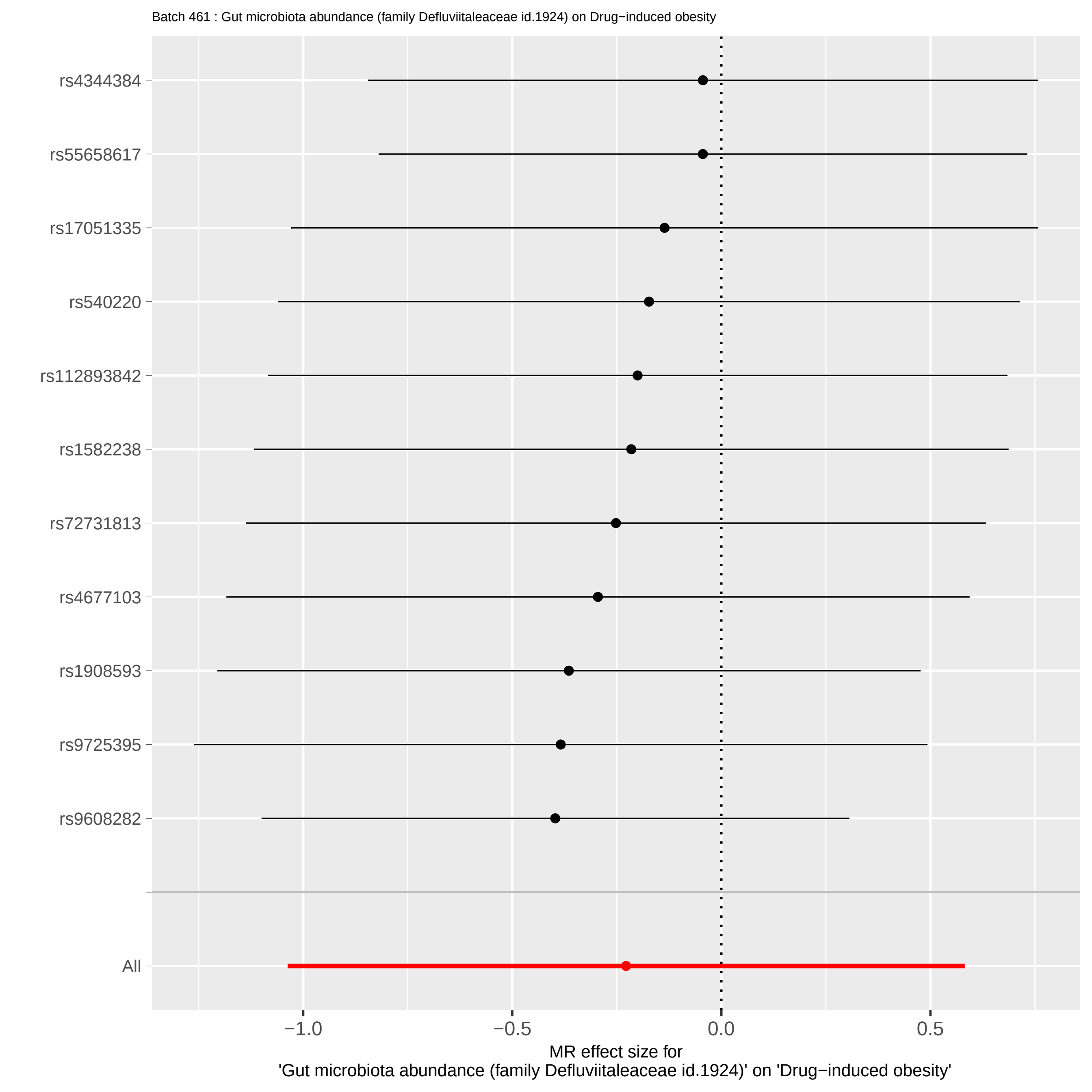

Batch 461 : Gut microbiota abundance (family Defluviitaleaceae id.1924) on Drug−induced obesity
rs4344384
rs55658617
rs17051335
rs540220
rs112893842
rs1582238
rs72731813
rs4677103
rs1908593
rs9725395
rs9608282
All
−1.0
−0.5
0.0
0.5
MR effect size for
'Gut microbiota abundance (family Defluviitaleaceae id.1924)' on 'Drug−induced obesity'

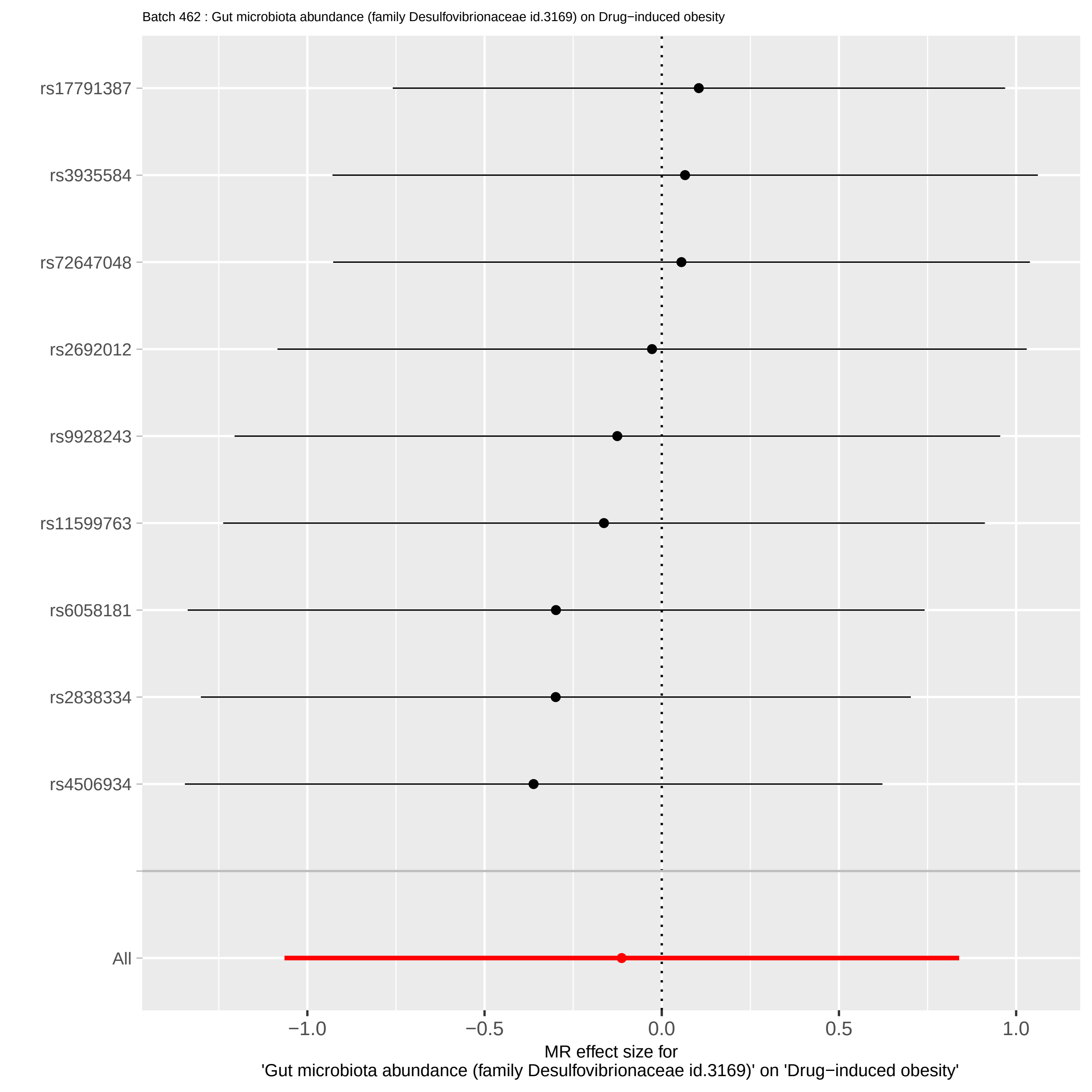

Batch 462 : Gut microbiota abundance (family Desulfovibrionaceae id.3169) on Drug−induced obesity
rs17791387
rs3935584
rs72647048
rs2692012
rs9928243
rs11599763
rs6058181
rs2838334
rs4506934
All
−1.0
−0.5
0.0
0.5
1.0
MR effect size for
'Gut microbiota abundance (family Desulfovibrionaceae id.3169)' on 'Drug−induced obesity'

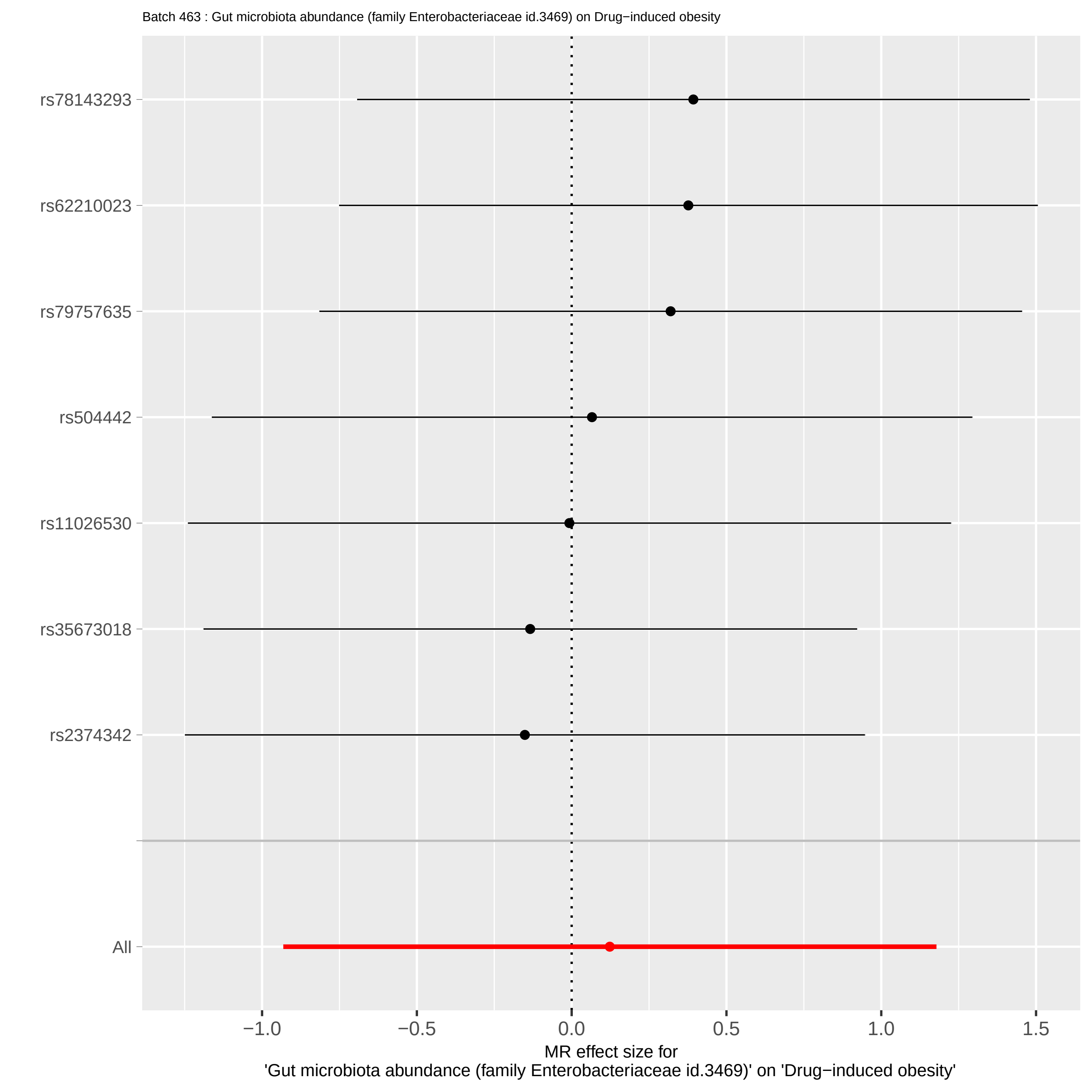

Batch 463 : Gut microbiota abundance (family Enterobacteriaceae id.3469) on Drug−induced obesity
rs78143293
rs62210023
rs79757635
rs504442
rs11026530
rs35673018
rs2374342
All
−1.0
−0.5
0.0
0.5
1.0
1.5
MR effect size for
'Gut microbiota abundance (family Enterobacteriaceae id.3469)' on 'Drug−induced obesity'

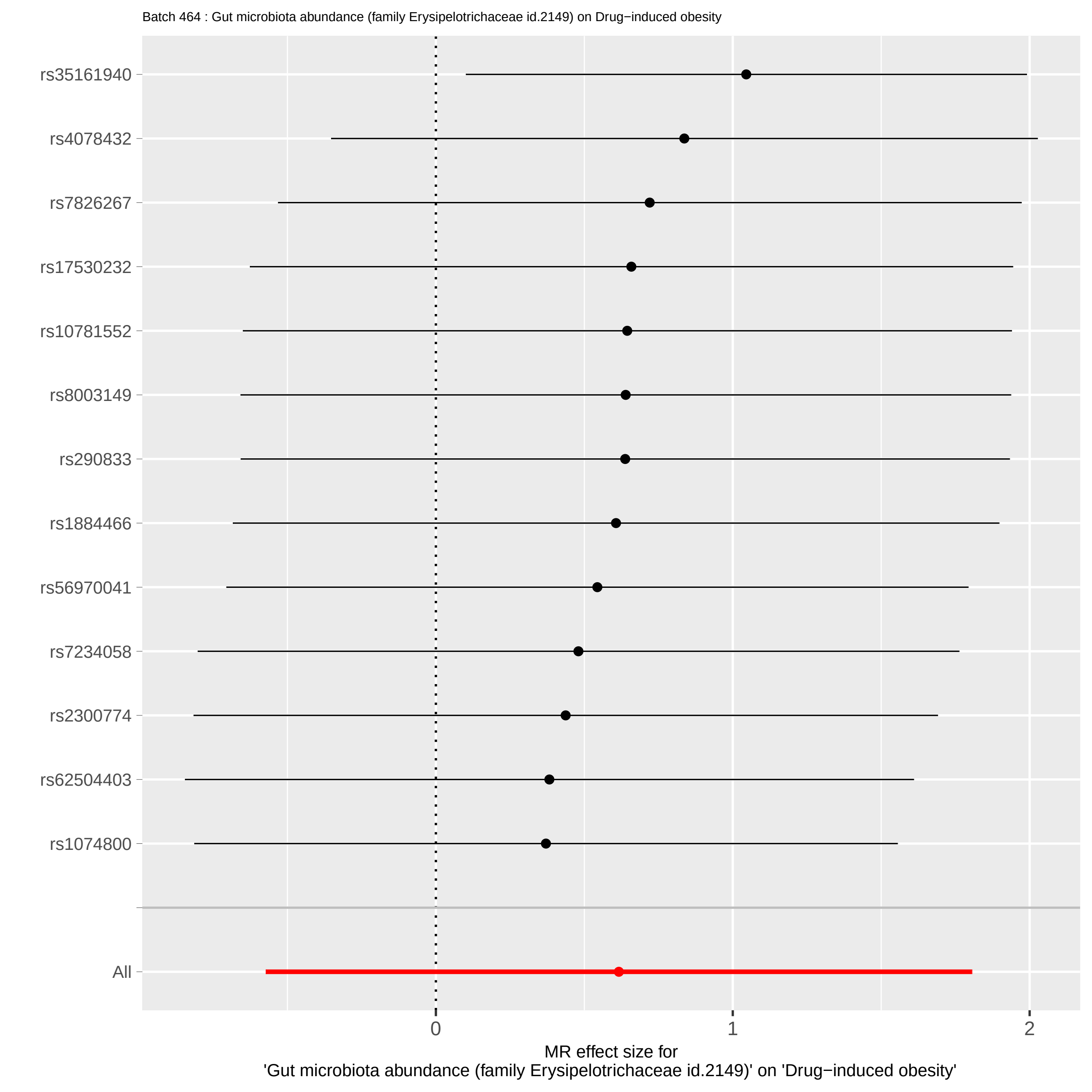

Batch 464 : Gut microbiota abundance (family Erysipelotrichaceae id.2149) on Drug−induced obesity
rs35161940
rs4078432
rs7826267
rs17530232
rs10781552
rs8003149
rs290833
rs1884466
rs56970041
rs7234058
rs2300774
rs62504403
rs1074800
All
0
1
2
MR effect size for
'Gut microbiota abundance (family Erysipelotrichaceae id.2149)' on 'Drug−induced obesity'

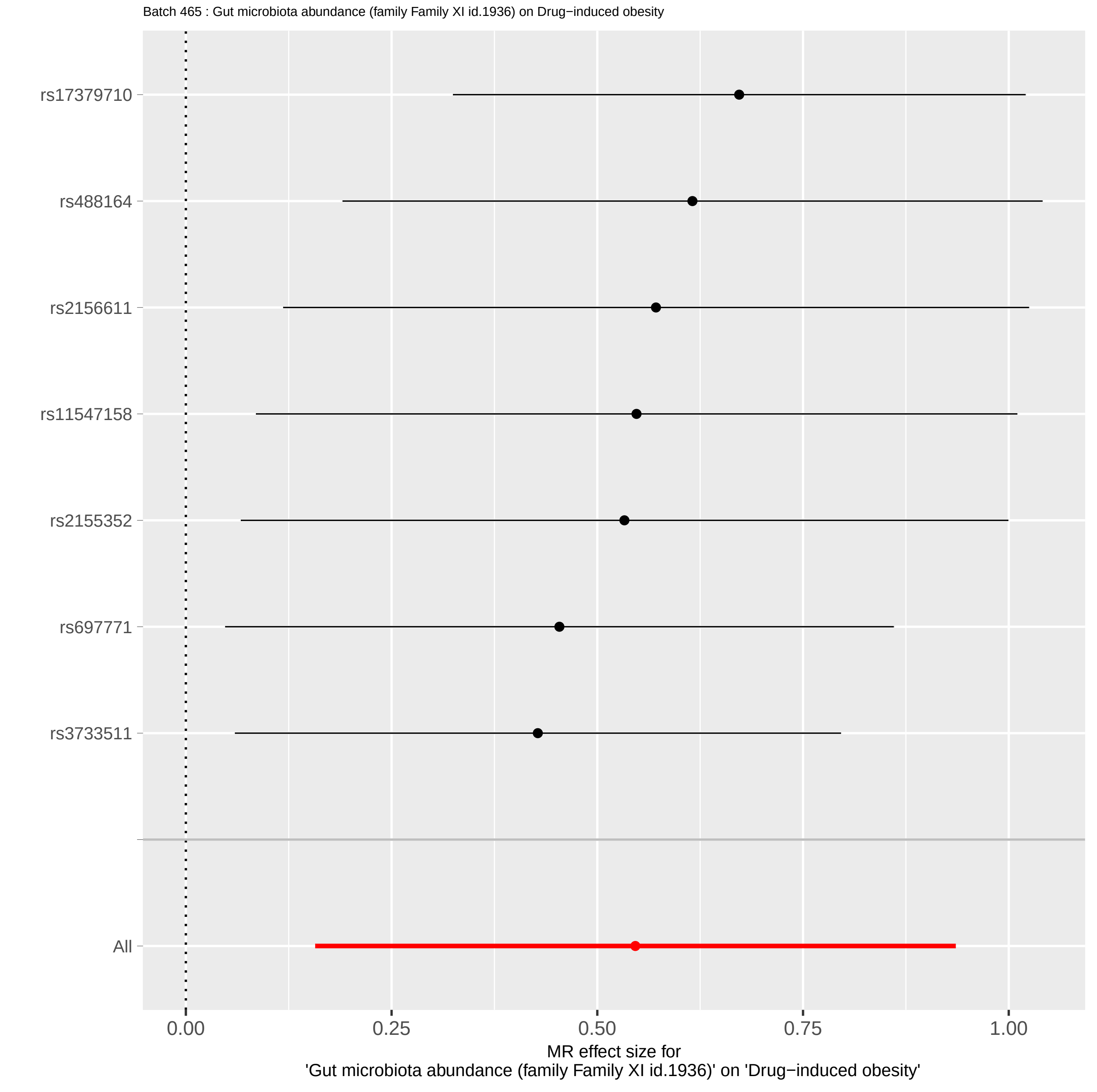

Batch 465 : Gut microbiota abundance (family Family XI id.1936) on Drug−induced obesity
rs17379710
rs488164
rs2156611
rs11547158
rs2155352
rs697771
rs3733511
All
0.00
0.25
0.50
0.75
1.00
MR effect size for
'Gut microbiota abundance (family Family XI id.1936)' on 'Drug−induced obesity'

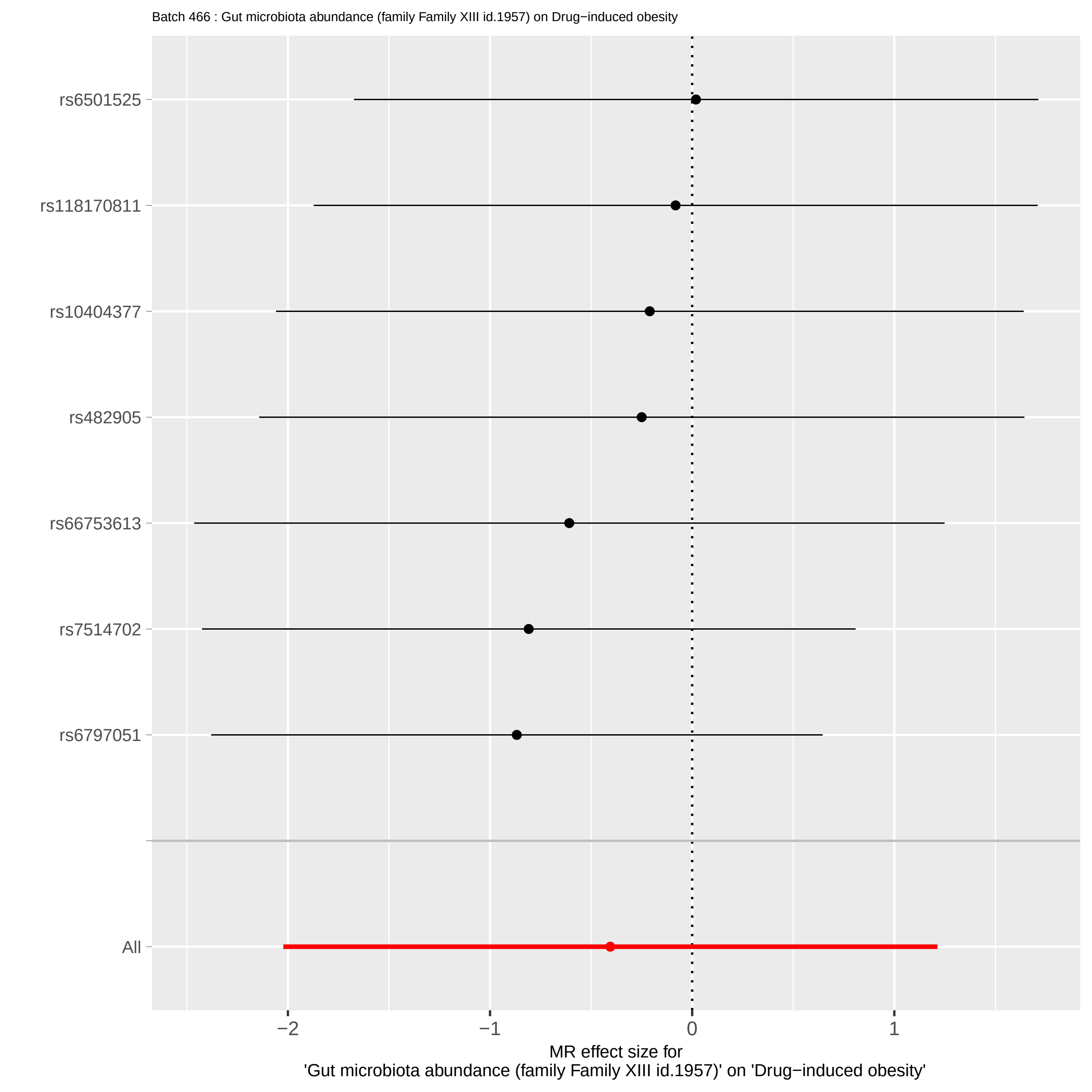

Batch 466 : Gut microbiota abundance (family Family XIII id.1957) on Drug−induced obesity
rs6501525
rs118170811
rs10404377
rs482905
rs66753613
rs7514702
rs6797051
All
−2
−1
0
1
MR effect size for
'Gut microbiota abundance (family Family XIII id.1957)' on 'Drug−induced obesity'

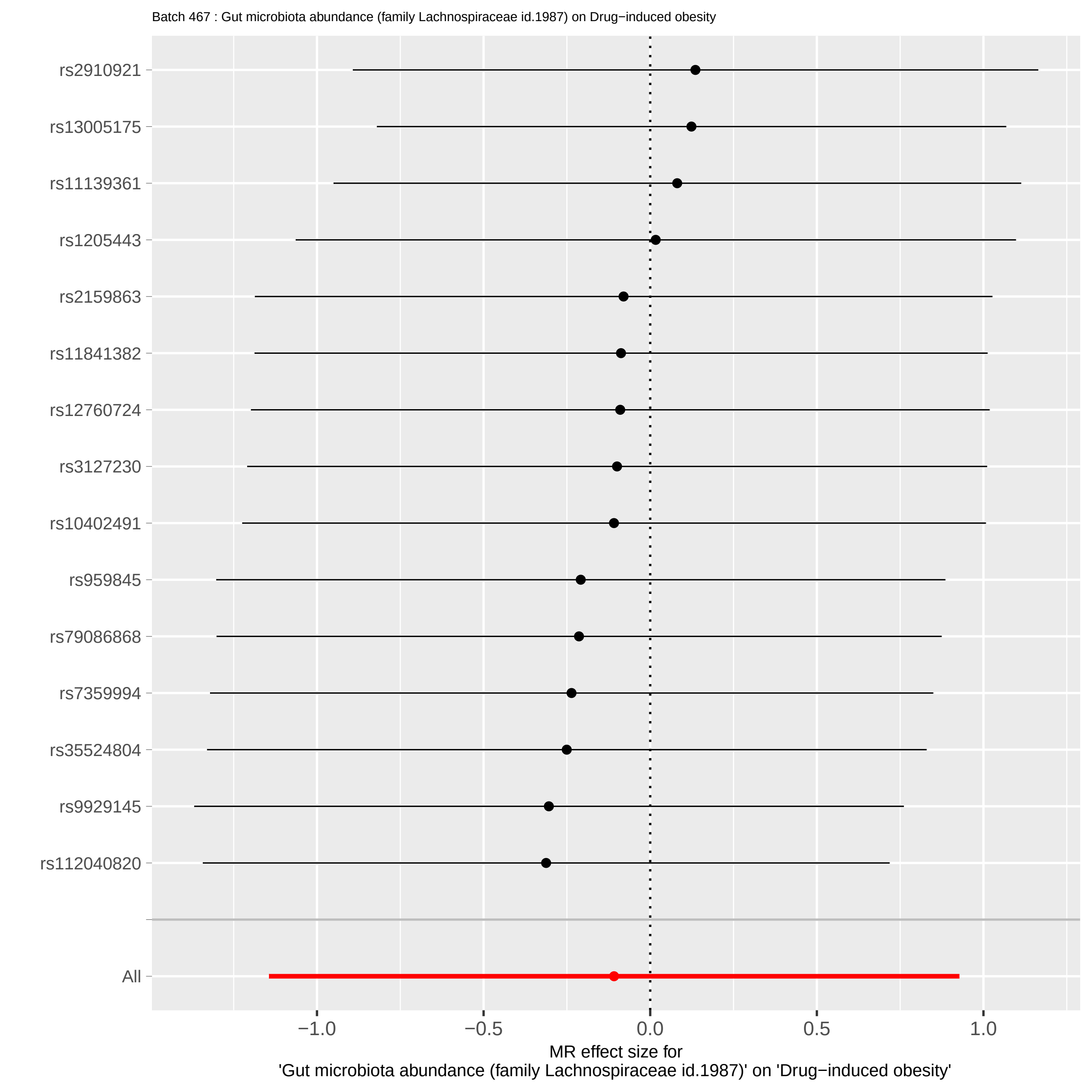

Batch 467 : Gut microbiota abundance (family Lachnospiraceae id.1987) on Drug−induced obesity
rs2910921
rs13005175
rs11139361
rs1205443
rs2159863
rs11841382
rs12760724
rs3127230
rs10402491
rs959845
rs79086868
rs7359994
rs35524804
rs9929145
rs112040820
All
−1.0
−0.5
0.0
0.5
1.0
MR effect size for
'Gut microbiota abundance (family Lachnospiraceae id.1987)' on 'Drug−induced obesity'

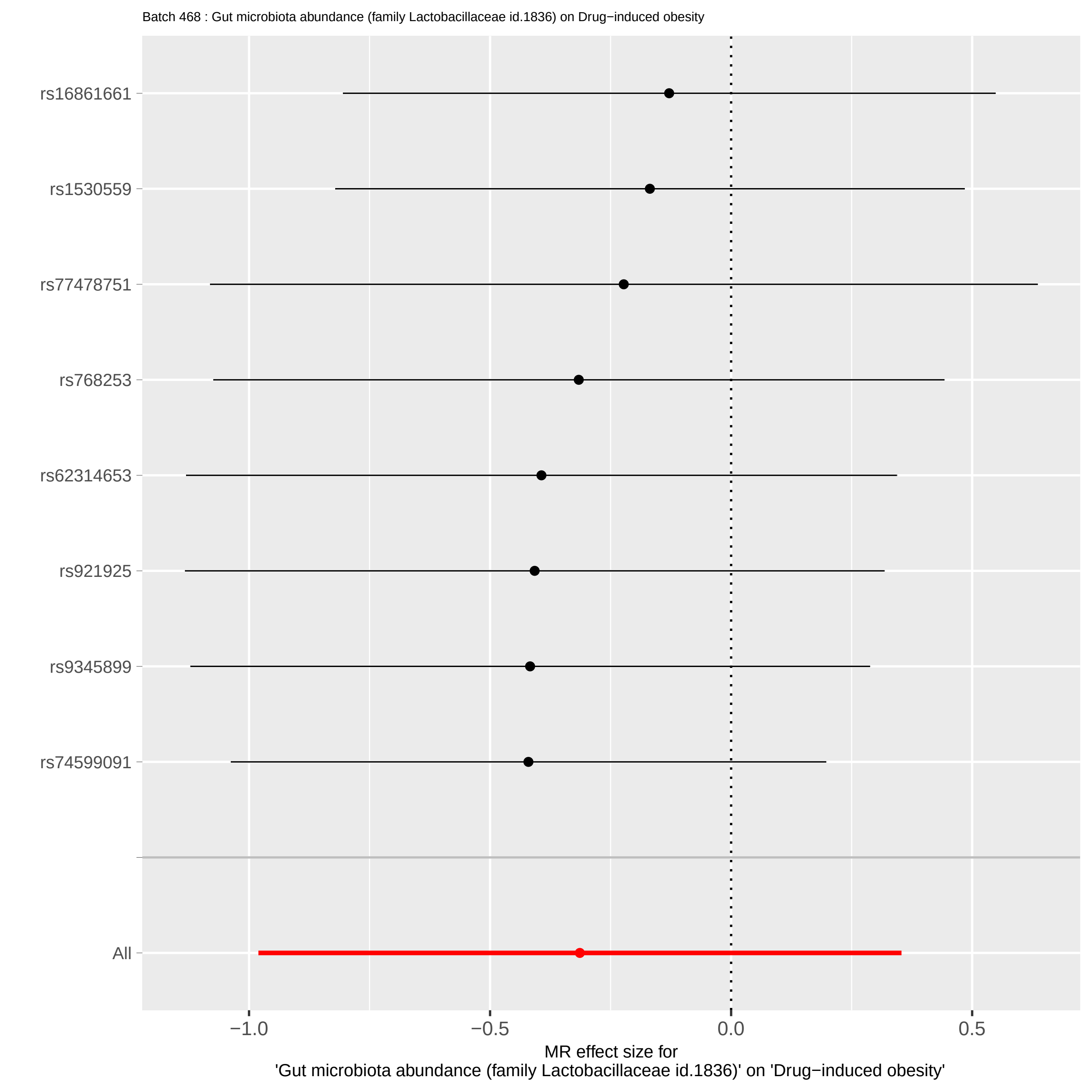

Batch 468 : Gut microbiota abundance (family Lactobacillaceae id.1836) on Drug−induced obesity
rs16861661
rs1530559
rs77478751
rs768253
rs62314653
rs921925
rs9345899
rs74599091
All
−1.0
−0.5
0.0
0.5
MR effect size for
'Gut microbiota abundance (family Lactobacillaceae id.1836)' on 'Drug−induced obesity'

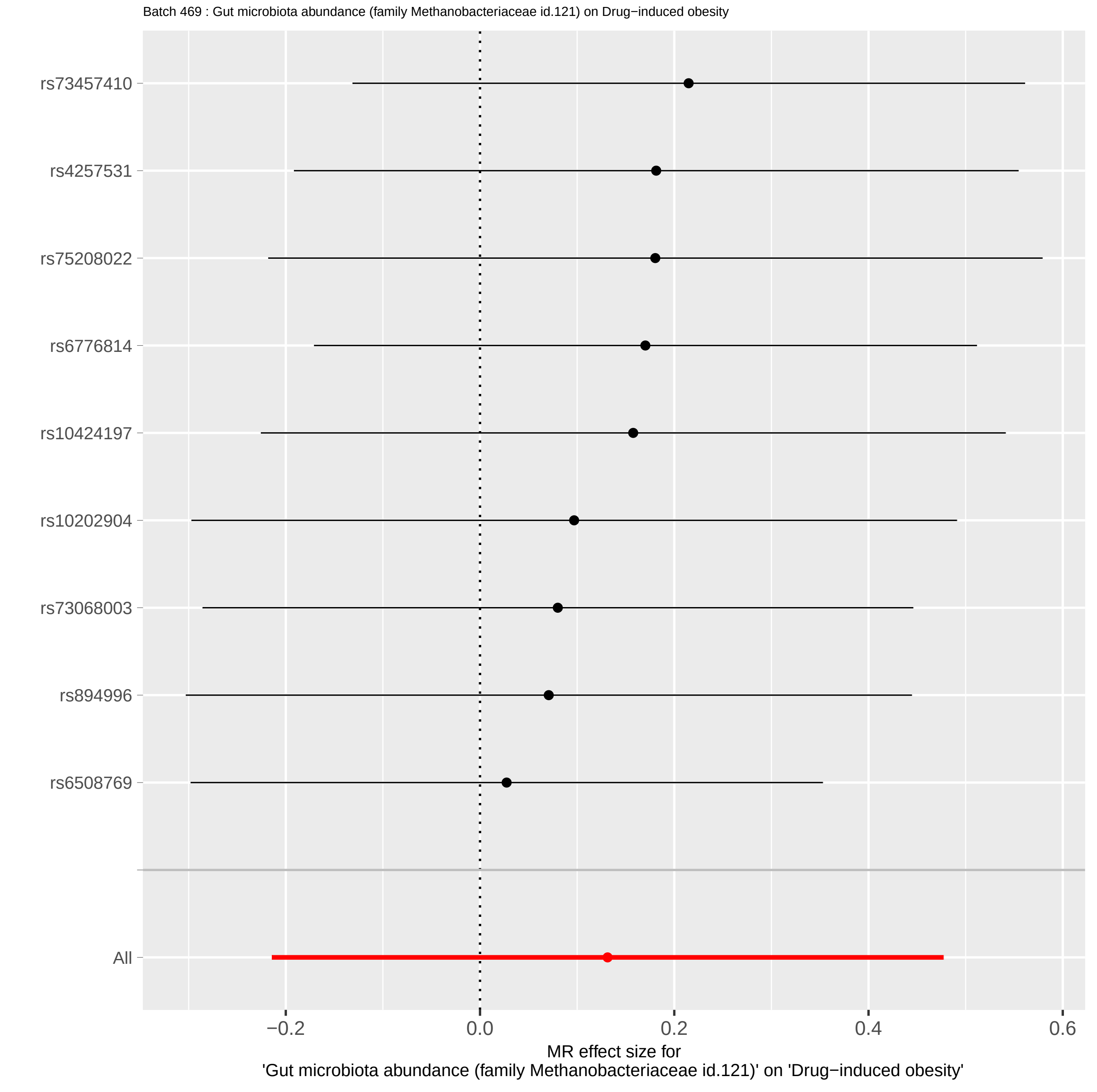

Batch 469 : Gut microbiota abundance (family Methanobacteriaceae id.121) on Drug−induced obesity
rs73457410
rs4257531
rs75208022
rs6776814
rs10424197
rs10202904
rs73068003
rs894996
rs6508769
All
−0.2
0.0
0.2
0.4
0.6
MR effect size for
'Gut microbiota abundance (family Methanobacteriaceae id.121)' on 'Drug−induced obesity'

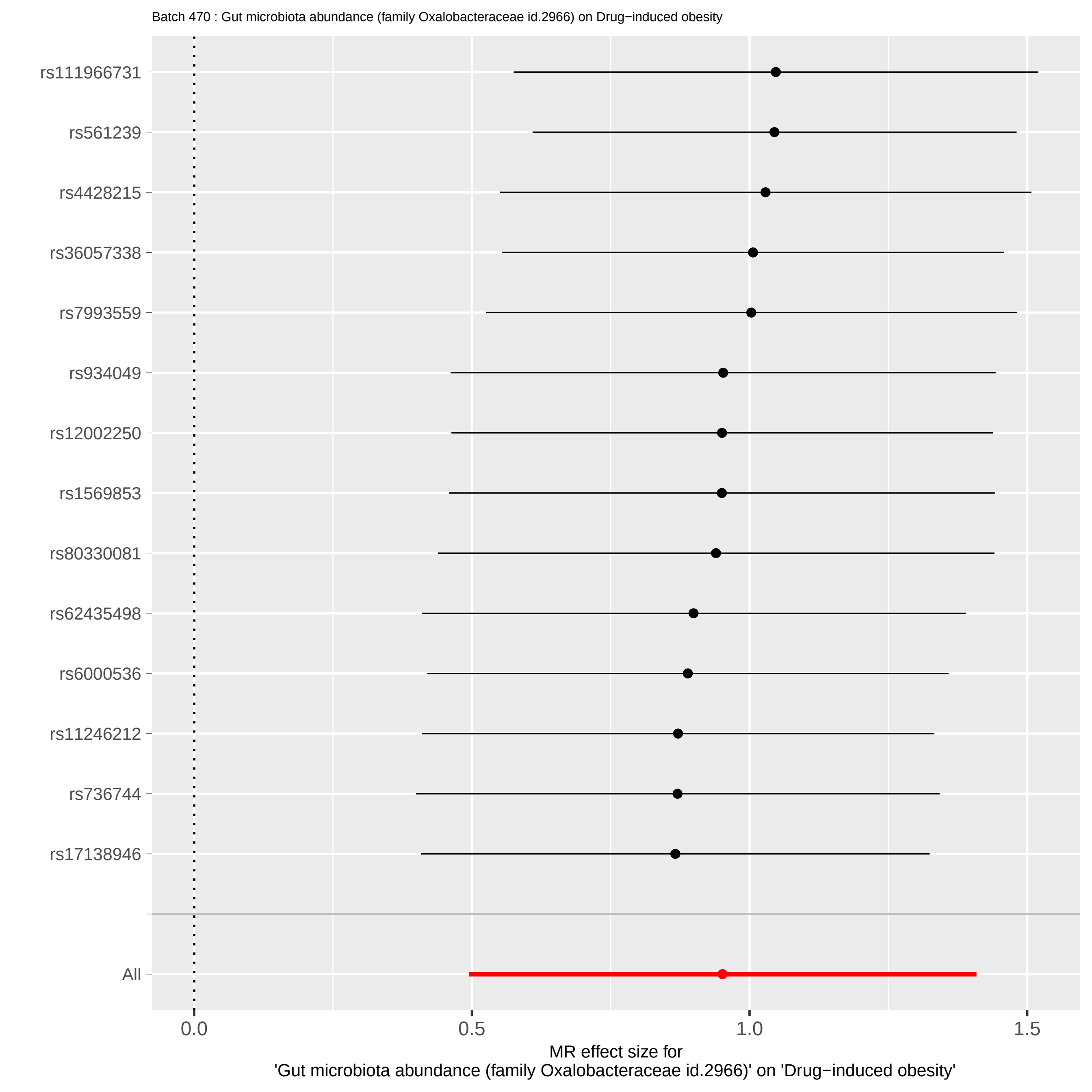

Batch 470 : Gut microbiota abundance (family Oxalobacteraceae id.2966) on Drug−induced obesity
rs111966731
rs561239
rs4428215
rs36057338
rs7993559
rs934049
rs12002250
rs1569853
rs80330081
rs62435498
rs6000536
rs11246212
rs736744
rs17138946
All
0.0
0.5
1.0
1.5
MR effect size for
'Gut microbiota abundance (family Oxalobacteraceae id.2966)' on 'Drug−induced obesity'

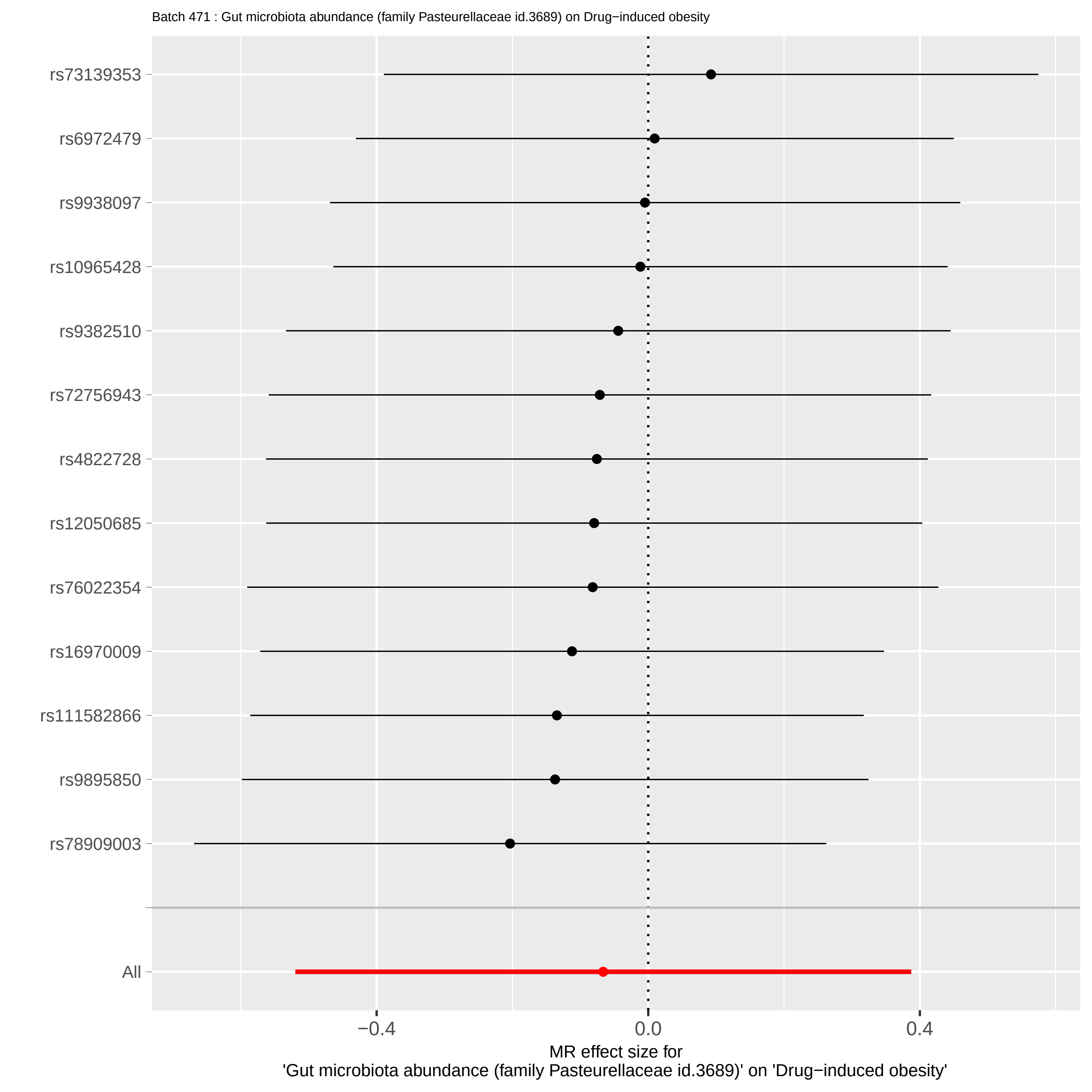

Batch 471 : Gut microbiota abundance (family Pasteurellaceae id.3689) on Drug−induced obesity
rs73139353
rs6972479
rs9938097
rs10965428
rs9382510
rs72756943
rs4822728
rs12050685
rs76022354
rs16970009
rs111582866
rs9895850
rs78909003
All
−0.4
0.0
0.4
MR effect size for
'Gut microbiota abundance (family Pasteurellaceae id.3689)' on 'Drug−induced obesity'

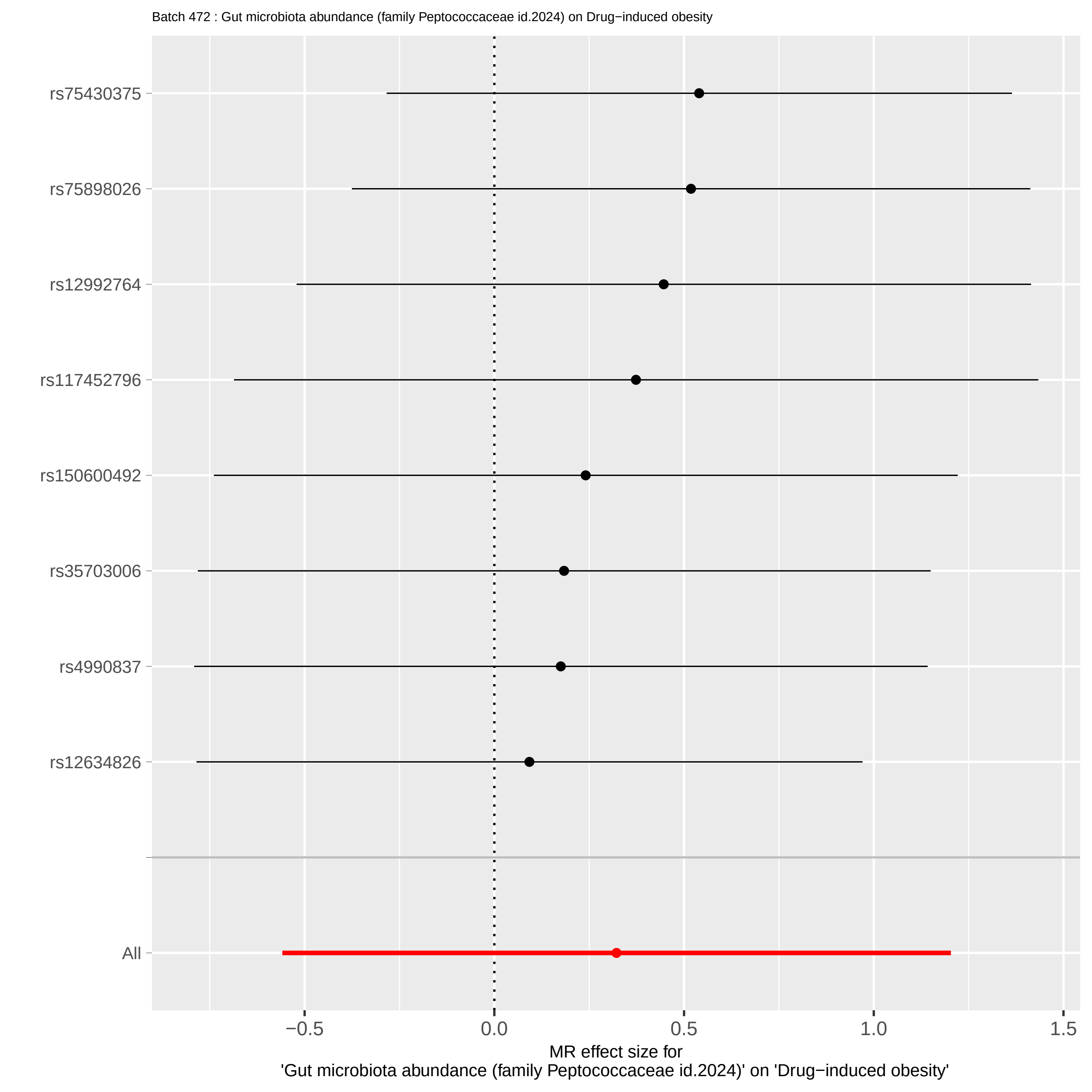
Batch 472 : Gut microbiota abundance (family Peptococcaceae id.2024) on Drug−induced obesity
rs75430375
rs75898026
rs12992764
rs117452796
rs150600492
rs35703006
rs4990837
rs12634826
All
−0.5
0.0
0.5
1.0
1.5
MR effect size for
'Gut microbiota abundance (family Peptococcaceae id.2024)' on 'Drug−induced obesity'

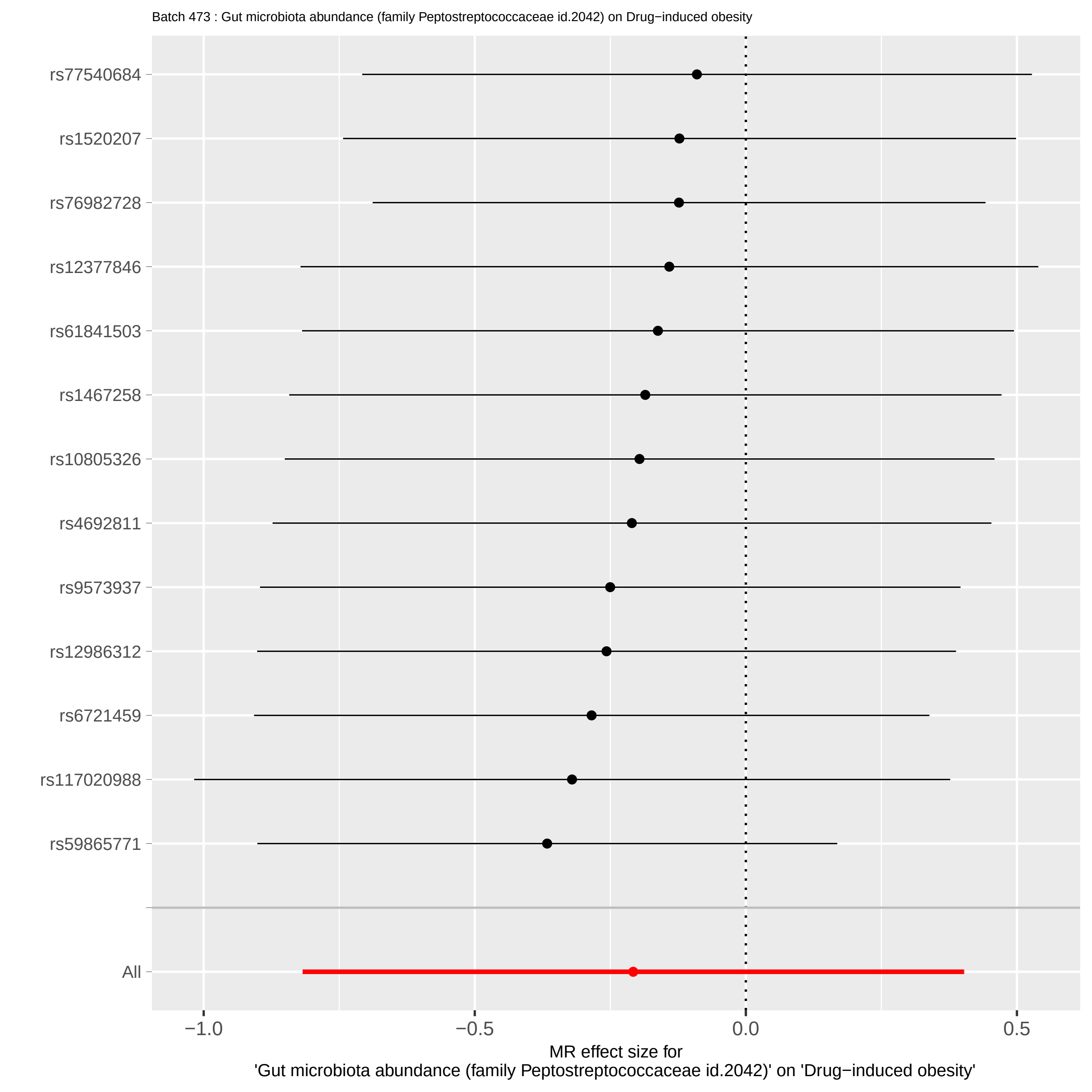

Batch 473 : Gut microbiota abundance (family Peptostreptococcaceae id.2042) on Drug−induced obesity
rs77540684
rs1520207
rs76982728
rs12377846
rs61841503
rs1467258
rs10805326
rs4692811
rs9573937
rs12986312
rs6721459
rs117020988
rs59865771
All
−1.0
−0.5
0.0
0.5
MR effect size for
'Gut microbiota abundance (family Peptostreptococcaceae id.2042)' on 'Drug−induced obesity'

Batch 474 : Gut microbiota abundance (family Porphyromonadaceae id.943) on Drug−induced obesity

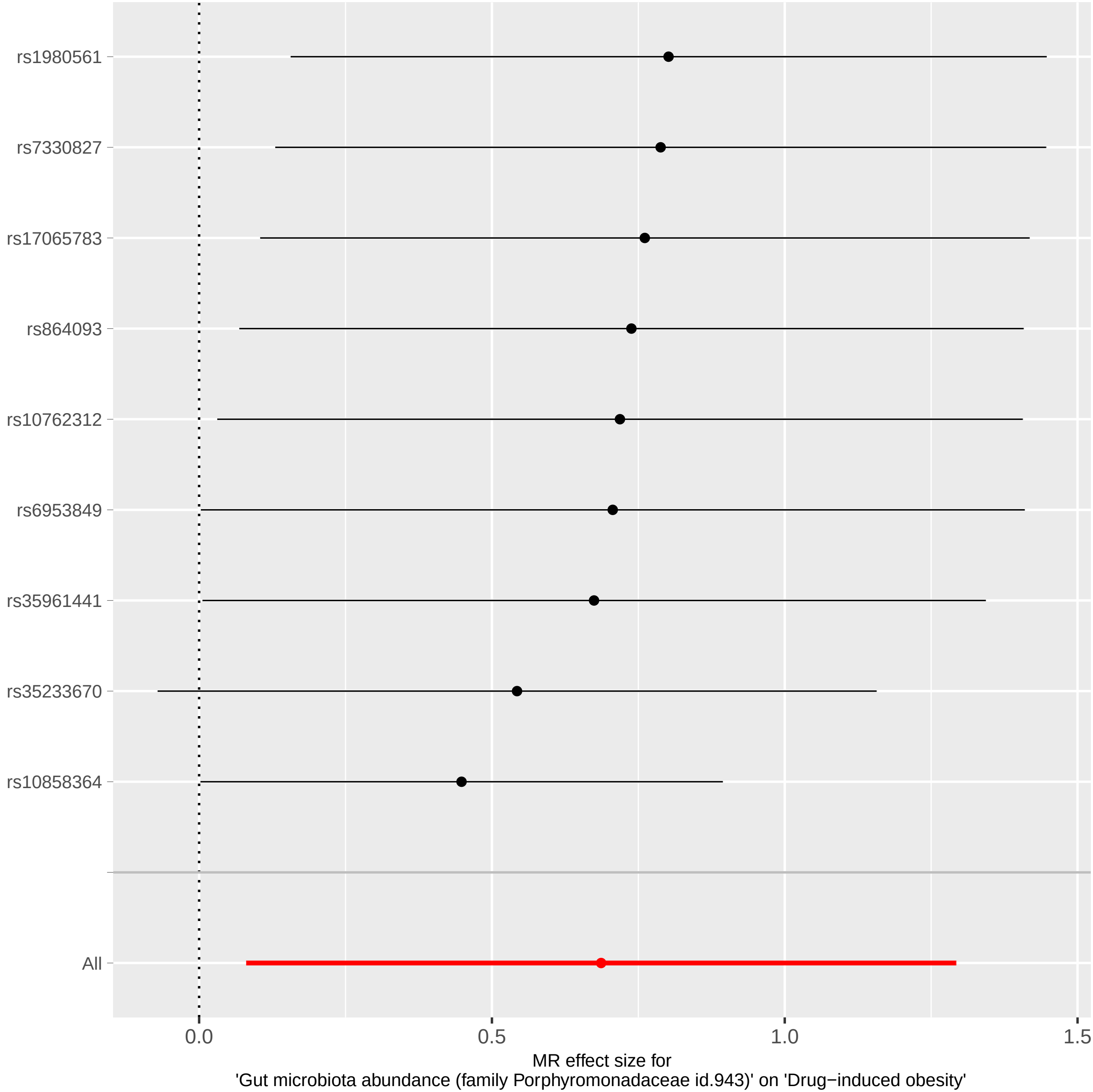

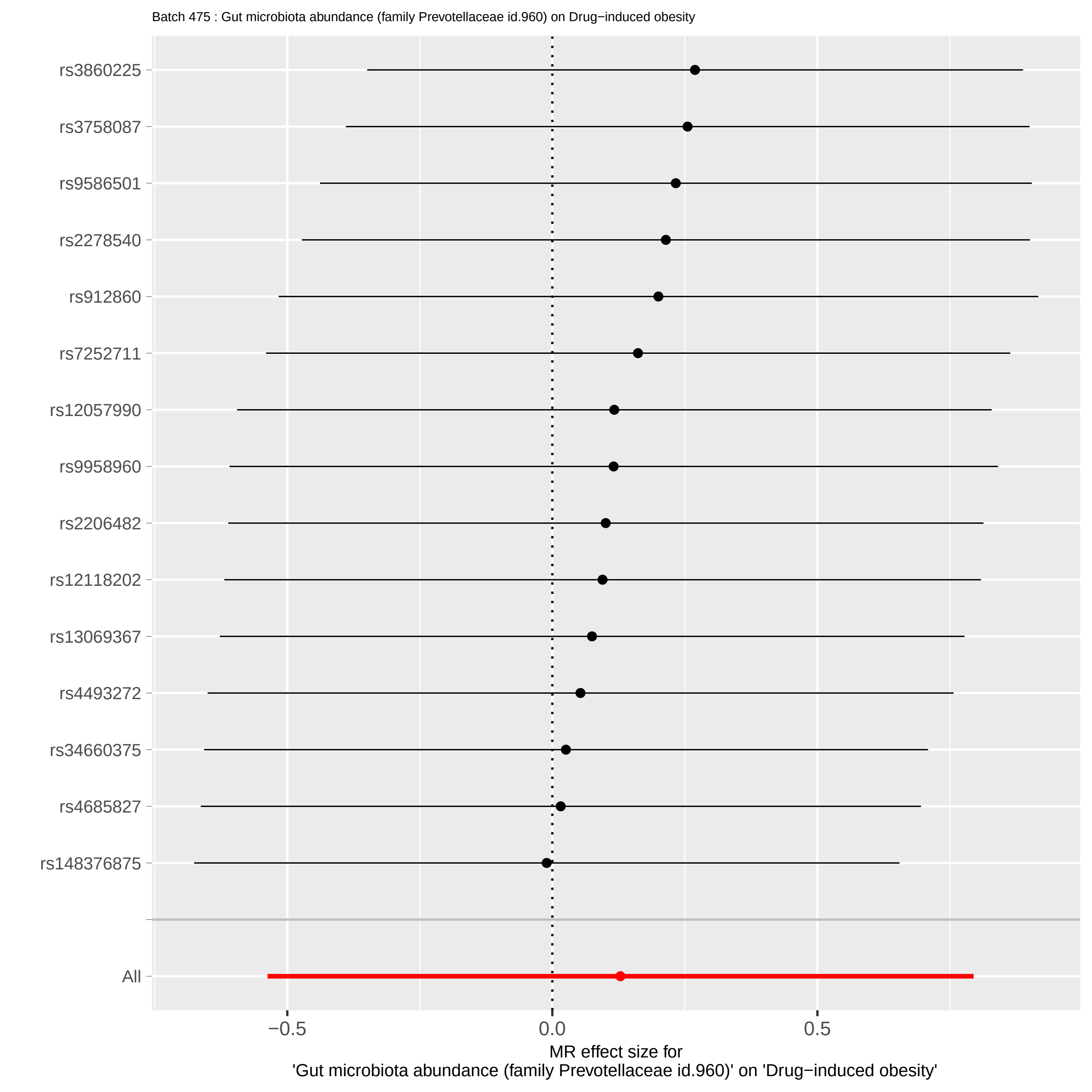

Batch 475 : Gut microbiota abundance (family Prevotellaceae id.960) on Drug−induced obesity
rs3860225
rs3758087
rs9586501
rs2278540
rs912860
rs7252711
rs12057990
rs9958960
rs2206482
rs12118202
rs13069367
rs4493272
rs34660375
rs4685827
rs148376875
All
−0.5
0.0
0.5
MR effect size for
'Gut microbiota abundance (family Prevotellaceae id.960)' on 'Drug−induced obesity'

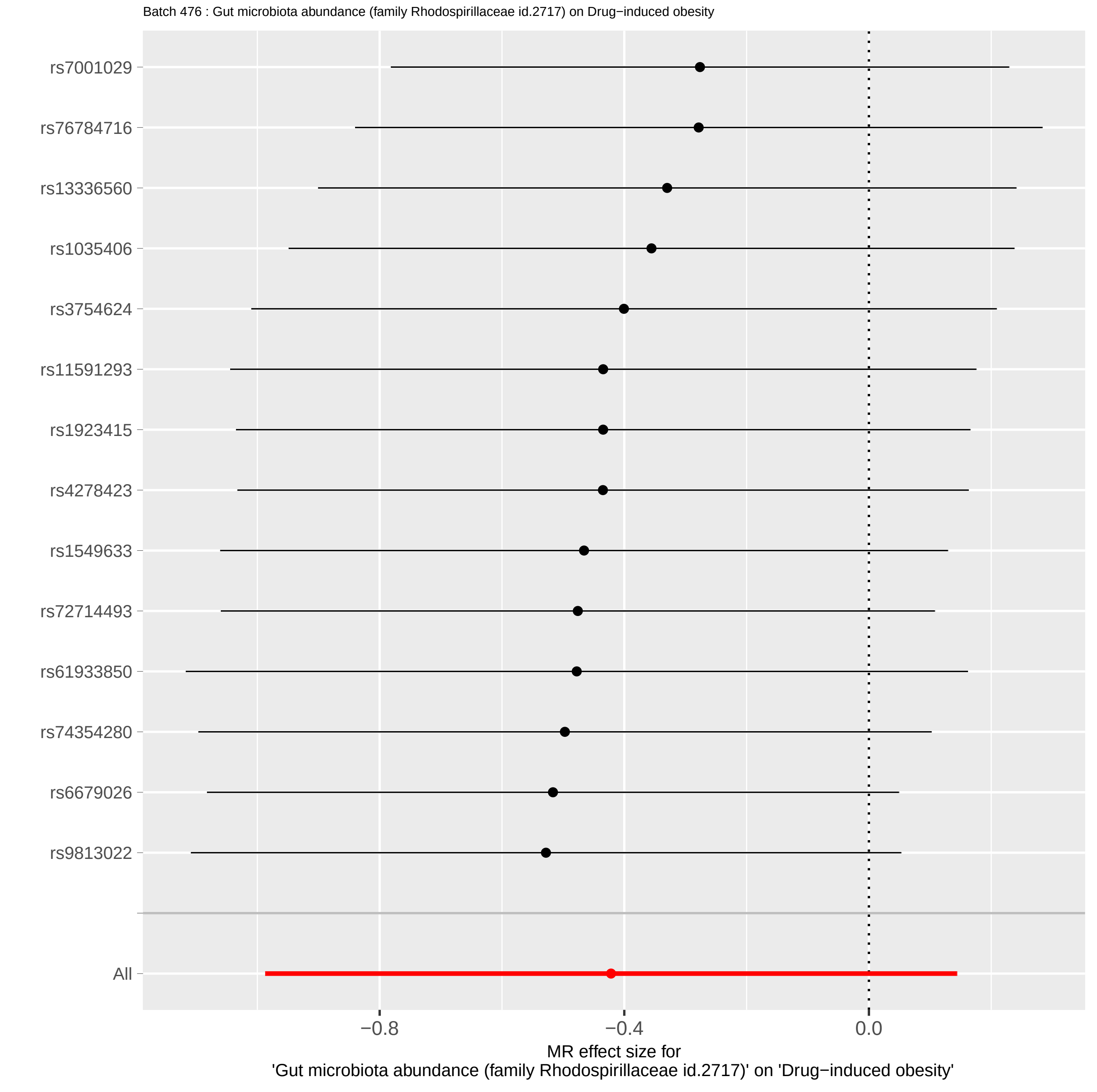

Batch 476 : Gut microbiota abundance (family Rhodospirillaceae id.2717) on Drug−induced obesity
rs7001029
rs76784716
rs13336560
rs1035406
rs3754624
rs11591293
rs1923415
rs4278423
rs1549633
rs72714493
rs61933850
rs74354280
rs6679026
rs9813022
All
−0.8
−0.4
0.0
MR effect size for
'Gut microbiota abundance (family Rhodospirillaceae id.2717)' on 'Drug−induced obesity'

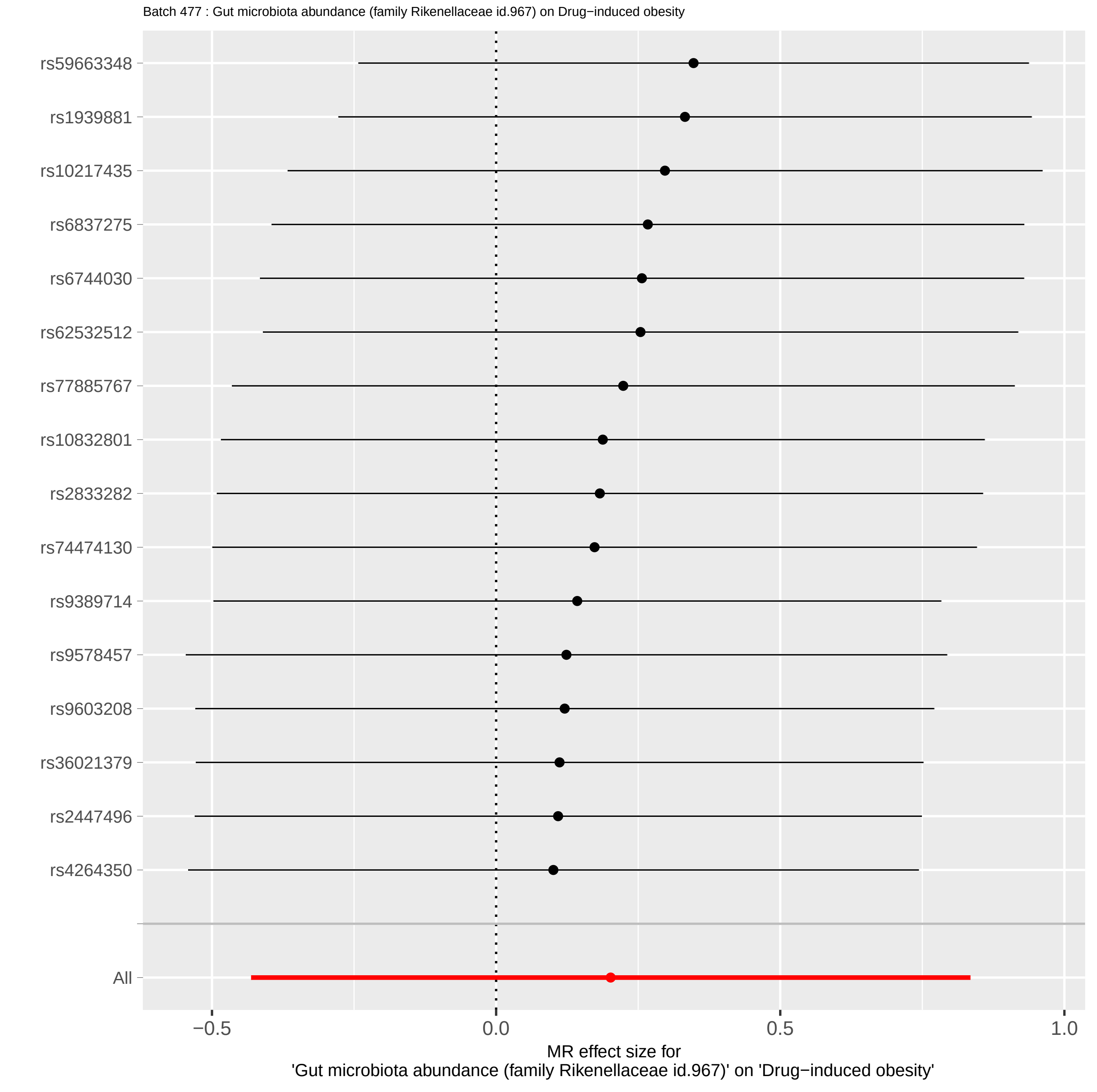

Batch 477 : Gut microbiota abundance (family Rikenellaceae id.967) on Drug−induced obesity
rs59663348
rs1939881
rs10217435
rs6837275
rs6744030
rs62532512
rs77885767
rs10832801
rs2833282
rs74474130
rs9389714
rs9578457
rs9603208
rs36021379
rs2447496
rs4264350
All
−0.5
0.0
0.5
1.0
MR effect size for
'Gut microbiota abundance (family Rikenellaceae id.967)' on 'Drug−induced obesity'

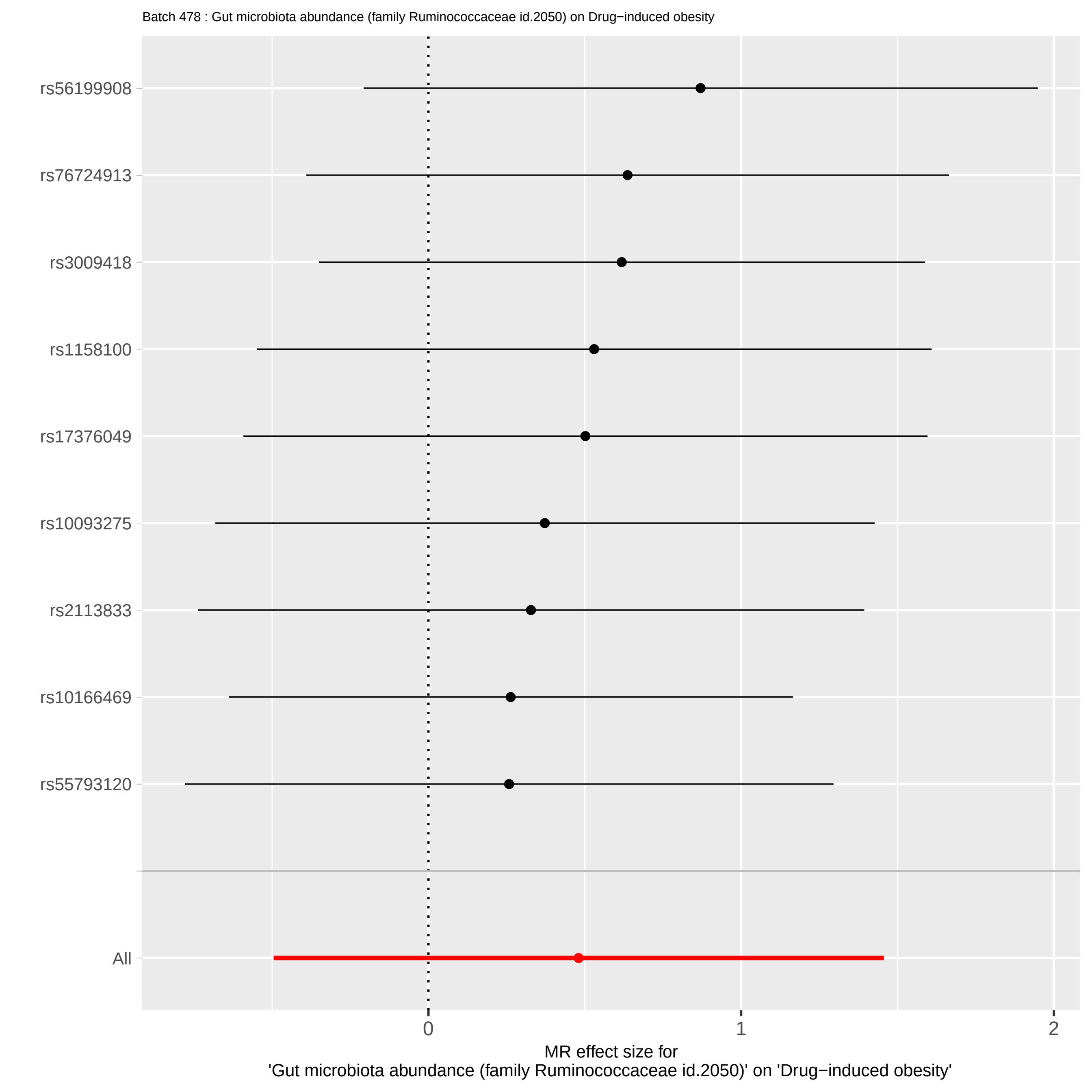

Batch 478 : Gut microbiota abundance (family Ruminococcaceae id.2050) on Drug−induced obesity
rs56199908
rs76724913
rs3009418
rs1158100
rs17376049
rs10093275
rs2113833
rs10166469
rs55793120
All
0
1
2
MR effect size for
'Gut microbiota abundance (family Ruminococcaceae id.2050)' on 'Drug−induced obesity'

Batch 479 : Gut microbiota abundance (family Streptococcaceae id.1850) on Drug−induced obesity

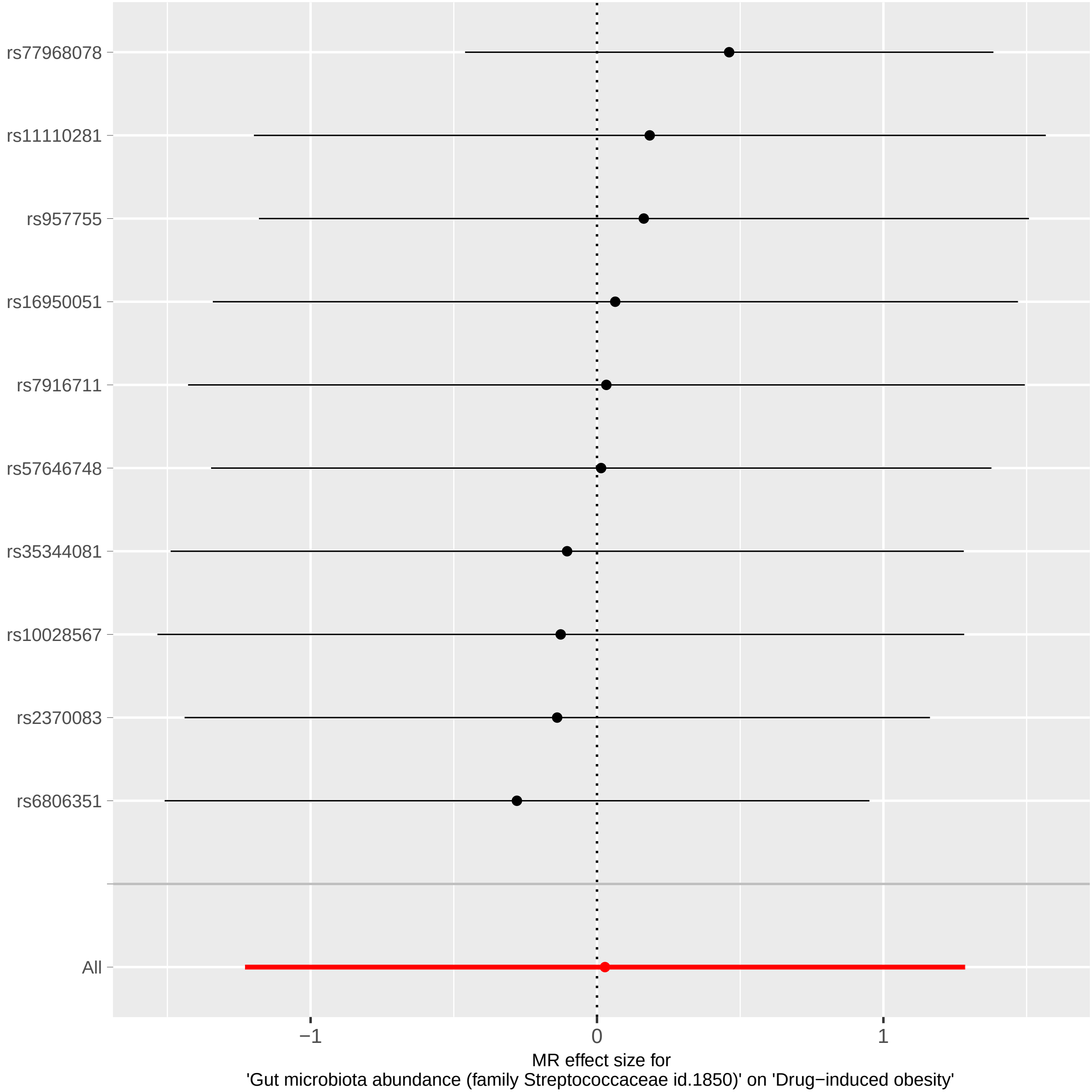

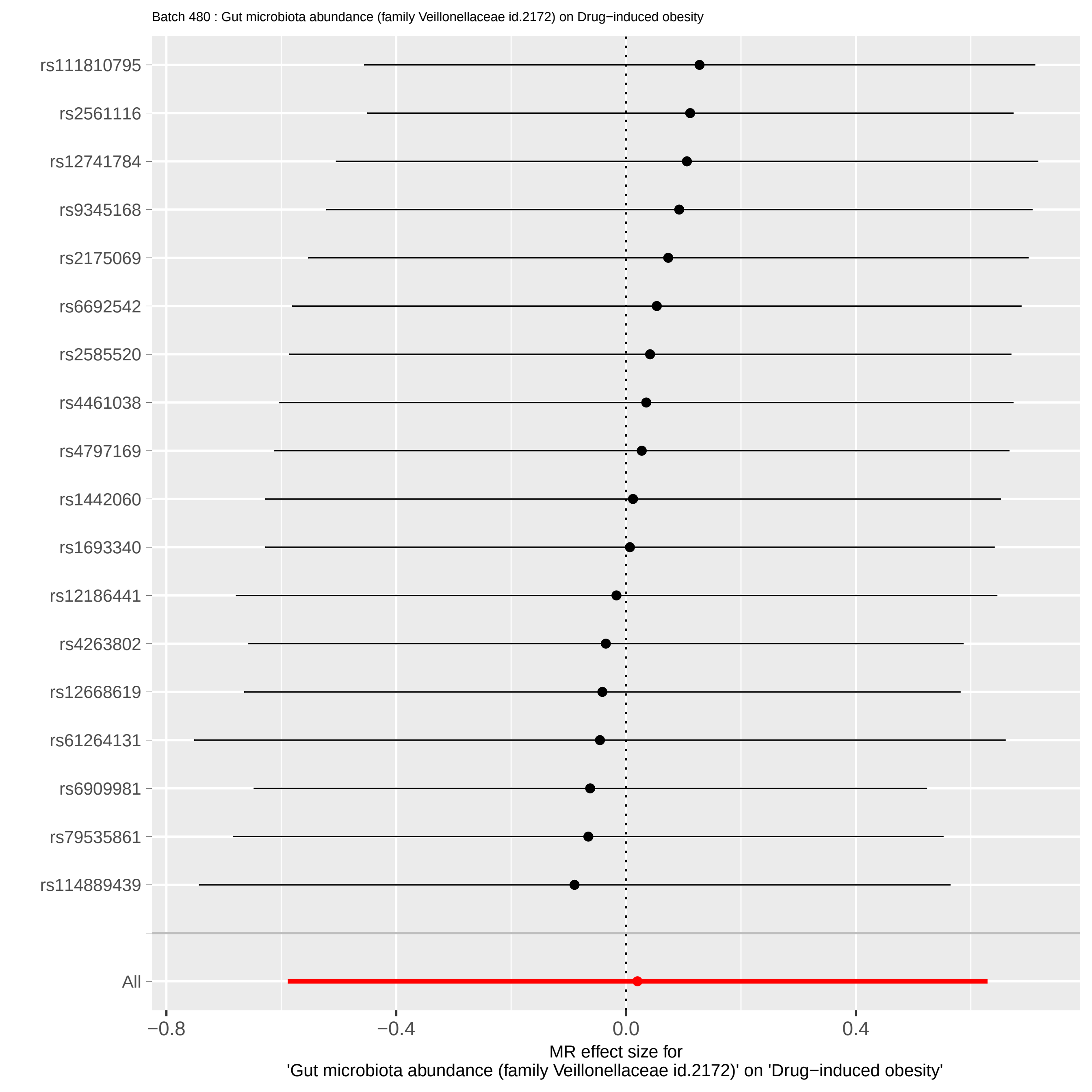
Batch 480 : Gut microbiota abundance (family Veillonellaceae id.2172) on Drug−induced obesity
rs111810795
rs2561116
rs12741784
rs9345168
rs2175069
rs6692542
rs2585520
rs4461038
rs4797169
rs1442060
rs1693340
rs12186441
rs4263802
rs12668619
rs61264131
rs6909981
rs79535861
rs114889439
All
−0.8
−0.4
0.0
0.4
MR effect size for
'Gut microbiota abundance (family Veillonellaceae id.2172)' on 'Drug−induced obesity'

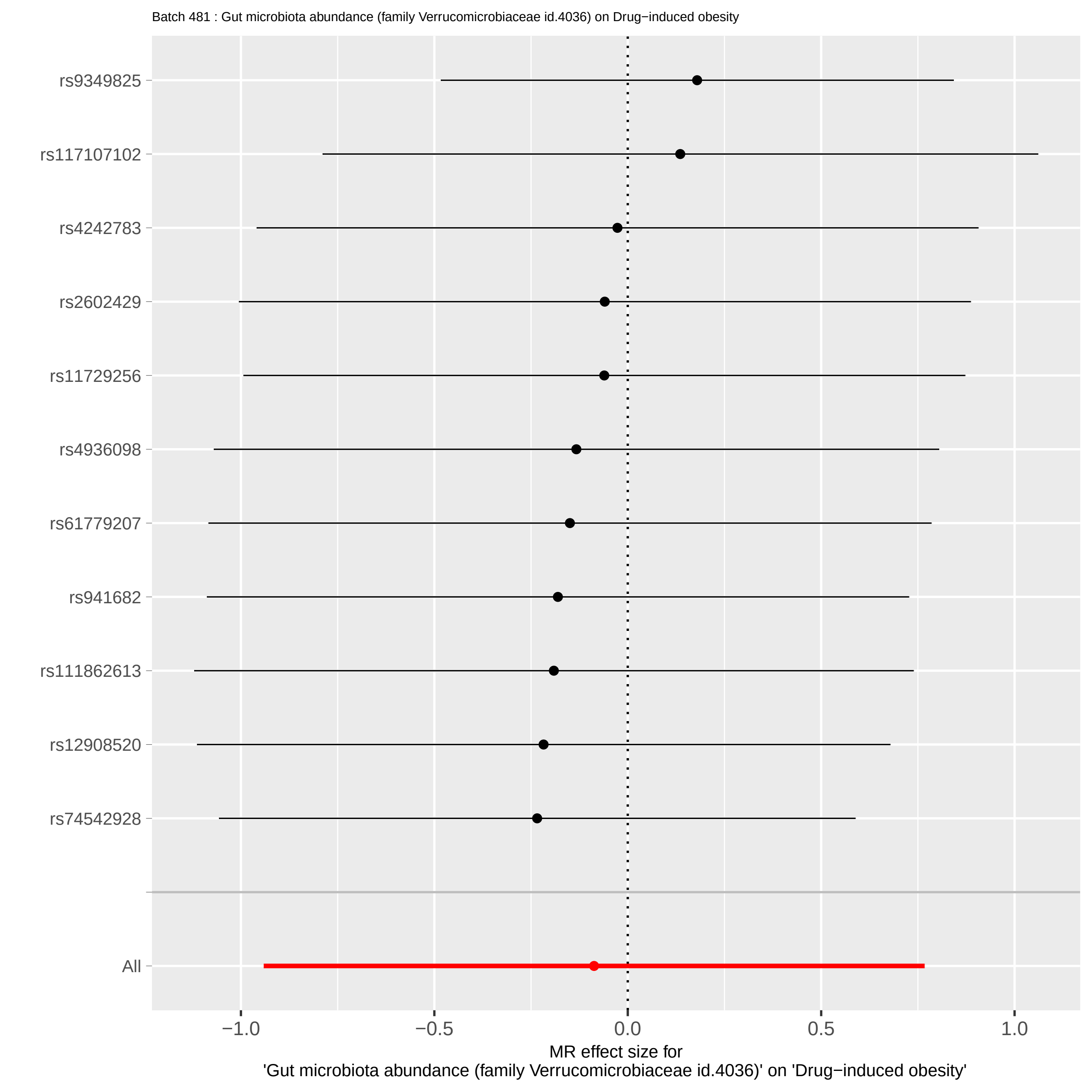
Batch 481 : Gut microbiota abundance (family Verrucomicrobiaceae id.4036) on Drug−induced obesity
rs9349825
rs117107102
rs4242783
rs2602429
rs11729256
rs4936098
rs61779207
rs941682
rs111862613
rs12908520
rs74542928
All
−1.0
−0.5
0.0
0.5
1.0
MR effect size for
'Gut microbiota abundance (family Verrucomicrobiaceae id.4036)' on 'Drug−induced obesity'

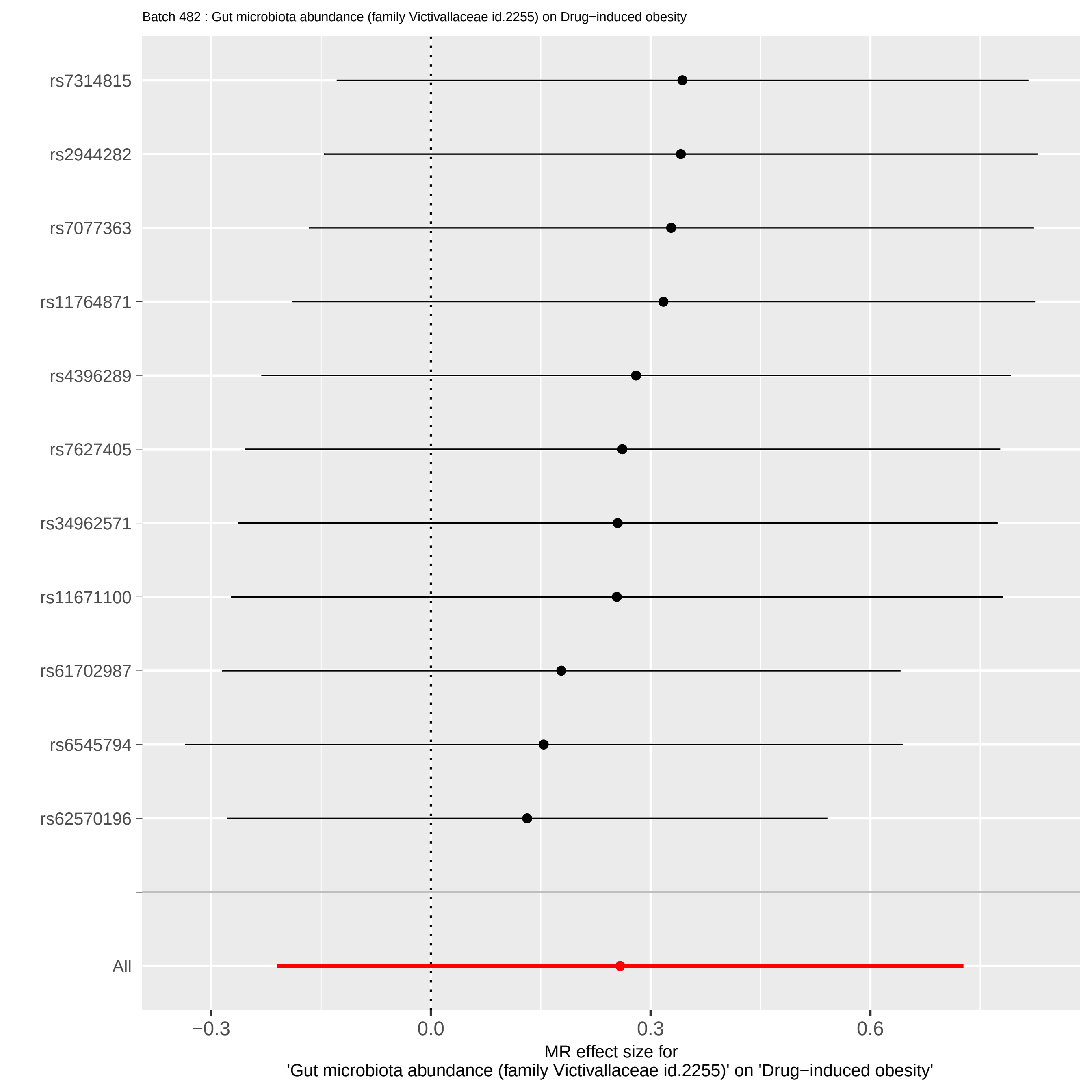
Batch 482 : Gut microbiota abundance (family Victivallaceae id.2255) on Drug−induced obesity
rs7314815
rs2944282
rs7077363
rs11764871
rs4396289
rs7627405
rs34962571
rs11671100
rs61702987
rs6545794
rs62570196
All
−0.3
0.0
0.3
0.6
MR effect size for
'Gut microbiota abundance (family Victivallaceae id.2255)' on 'Drug−induced obesity'

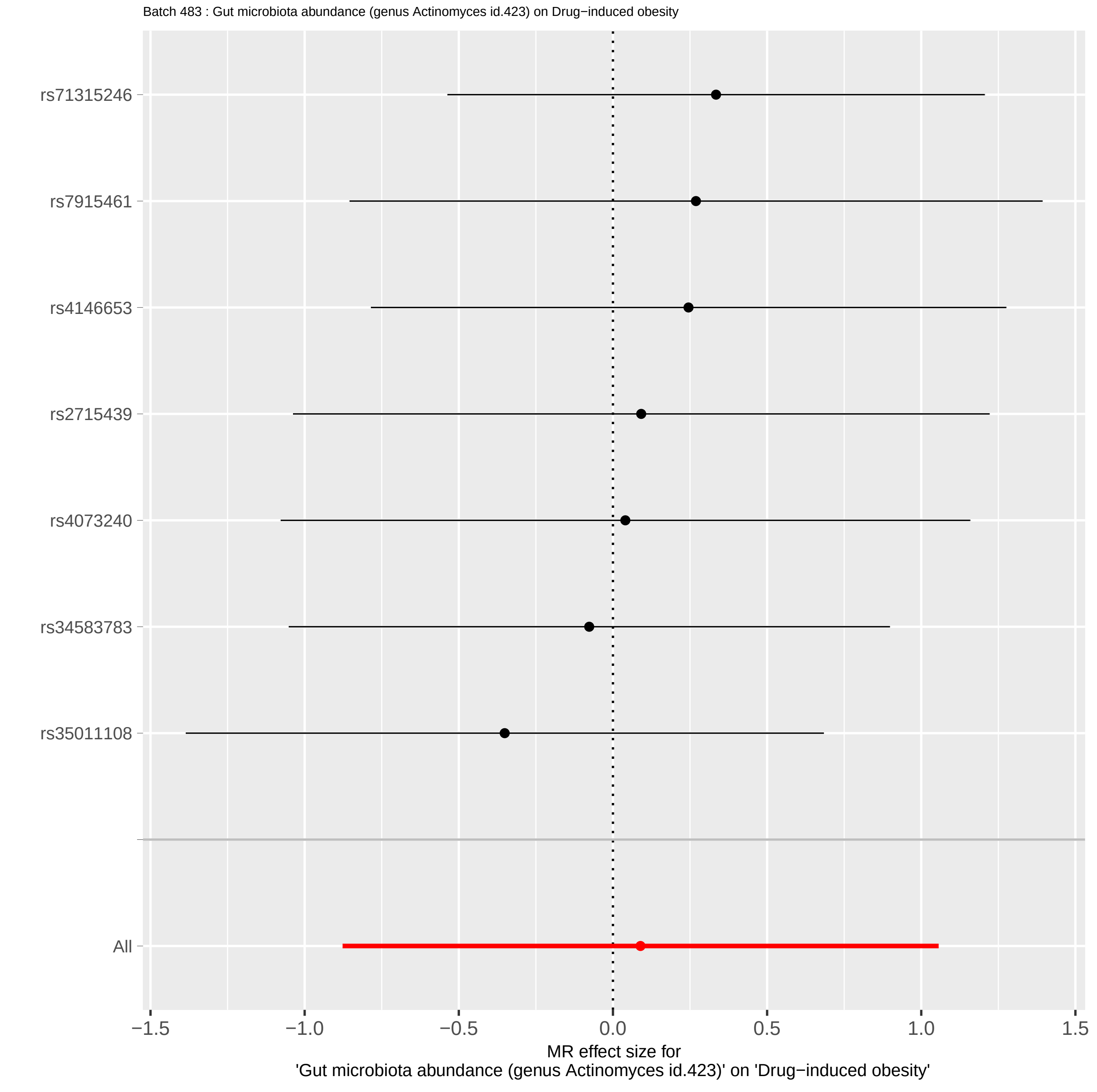

Batch 483 : Gut microbiota abundance (genus Actinomyces id.423) on Drug−induced obesity
rs71315246
rs7915461
rs4146653
rs2715439
rs4073240
rs34583783
rs35011108
All
−1.5
−1.0
−0.5
0.0
0.5
1.0
1.5
MR effect size for
'Gut microbiota abundance (genus Actinomyces id.423)' on 'Drug−induced obesity'

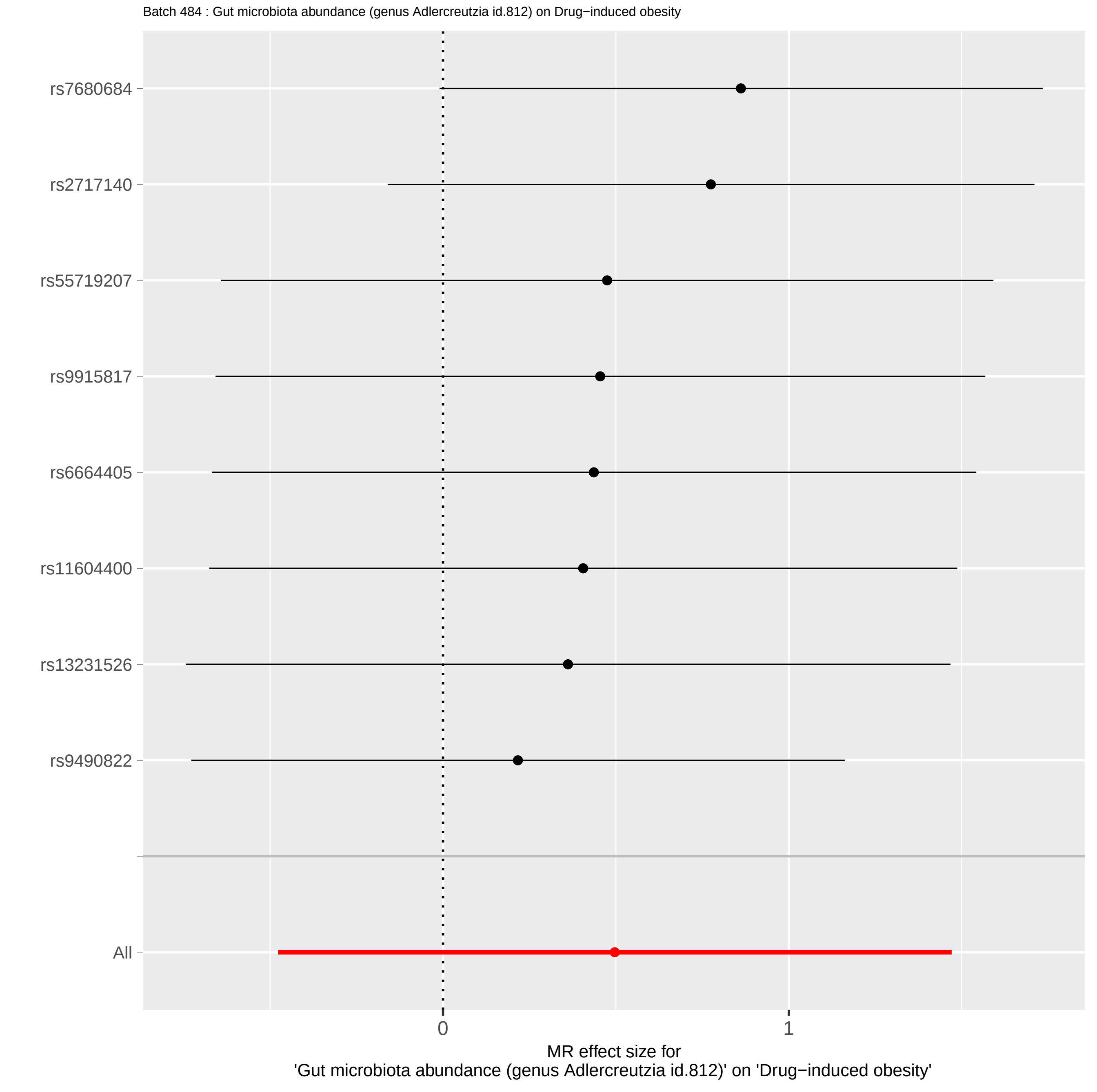

Batch 484 : Gut microbiota abundance (genus Adlercreutzia id.812) on Drug−induced obesity
rs7680684
rs2717140
rs55719207
rs9915817
rs6664405
rs11604400
rs13231526
rs9490822
All
0
1
MR effect size for
'Gut microbiota abundance (genus Adlercreutzia id.812)' on 'Drug−induced obesity'

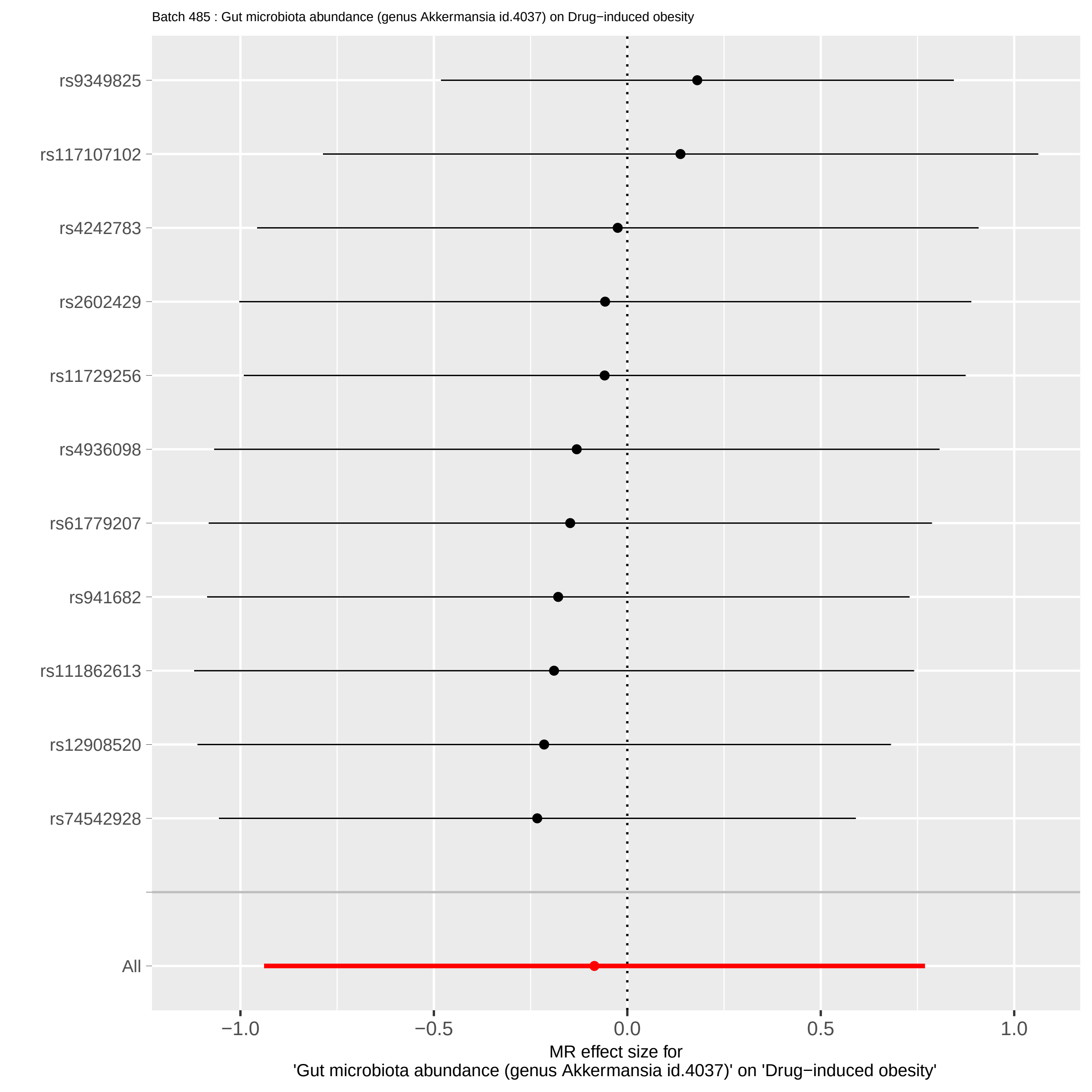
Batch 485 : Gut microbiota abundance (genus Akkermansia id.4037) on Drug−induced obesity
rs9349825
rs117107102
rs4242783
rs2602429
rs11729256
rs4936098
rs61779207
rs941682
rs111862613
rs12908520
rs74542928
All
−1.0
−0.5
0.0
0.5
1.0
MR effect size for
'Gut microbiota abundance (genus Akkermansia id.4037)' on 'Drug−induced obesity'

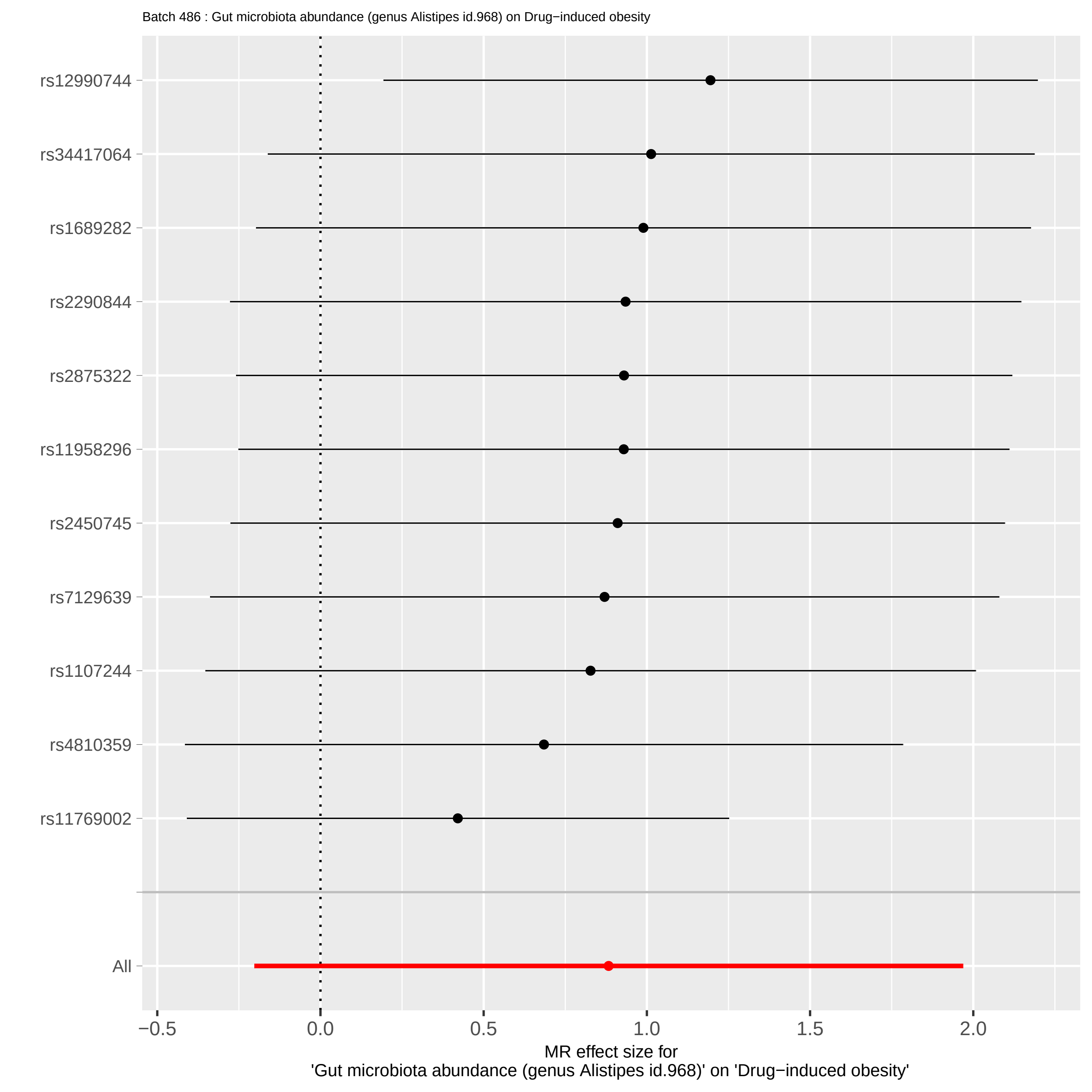
Batch 486 : Gut microbiota abundance (genus Alistipes id.968) on Drug−induced obesity
rs12990744
rs34417064
rs1689282
rs2290844
rs2875322
rs11958296
rs2450745
rs7129639
rs1107244
rs4810359
rs11769002
All
−0.5
0.0
0.5
1.0
1.5
2.0
MR effect size for
'Gut microbiota abundance (genus Alistipes id.968)' on 'Drug−induced obesity'

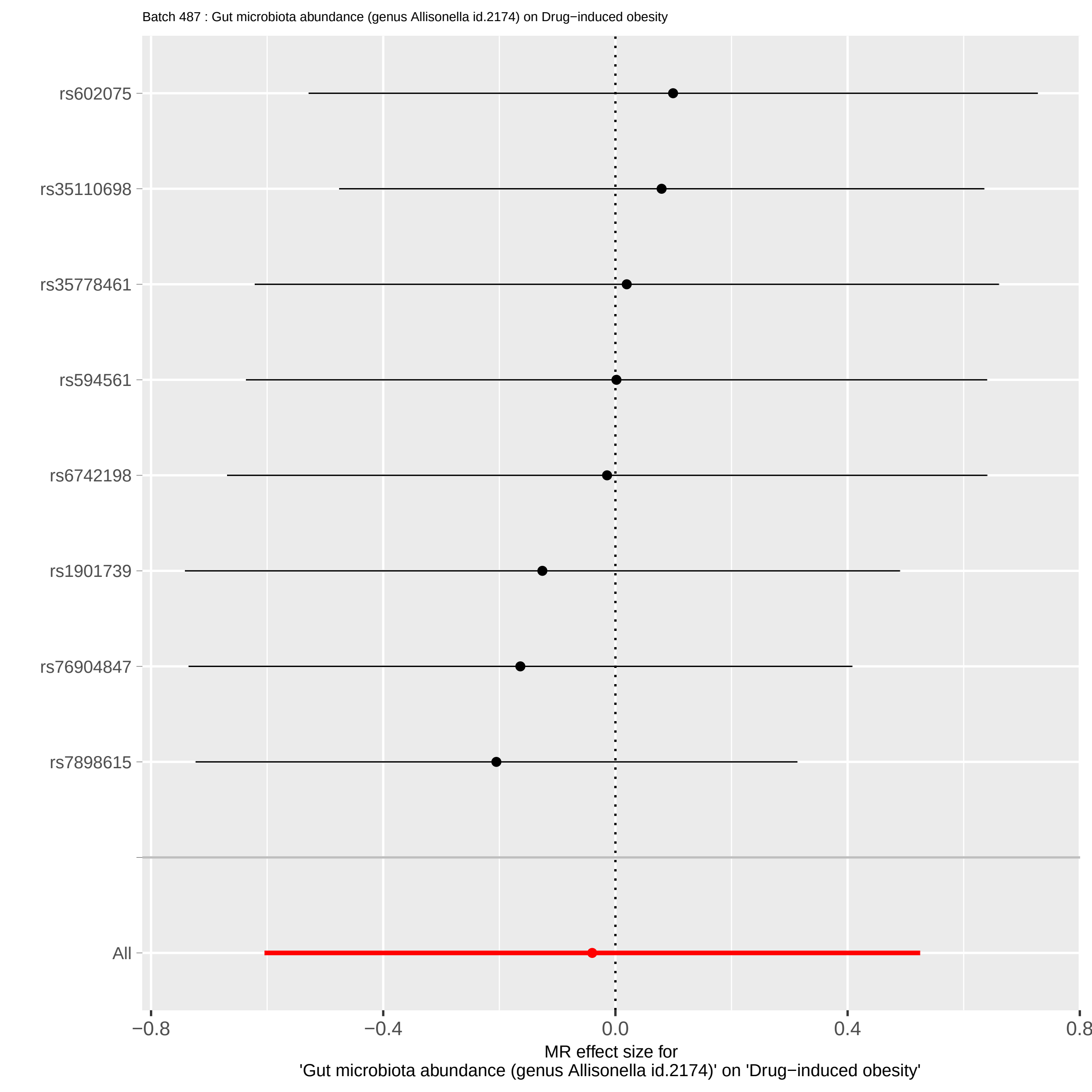
Batch 487 : Gut microbiota abundance (genus Allisonella id.2174) on Drug−induced obesity
rs602075
rs35110698
rs35778461
rs594561
rs6742198
rs1901739
rs76904847
rs7898615
All
−0.8
−0.4
0.0
0.4
0.8
MR effect size for
'Gut microbiota abundance (genus Allisonella id.2174)' on 'Drug−induced obesity'

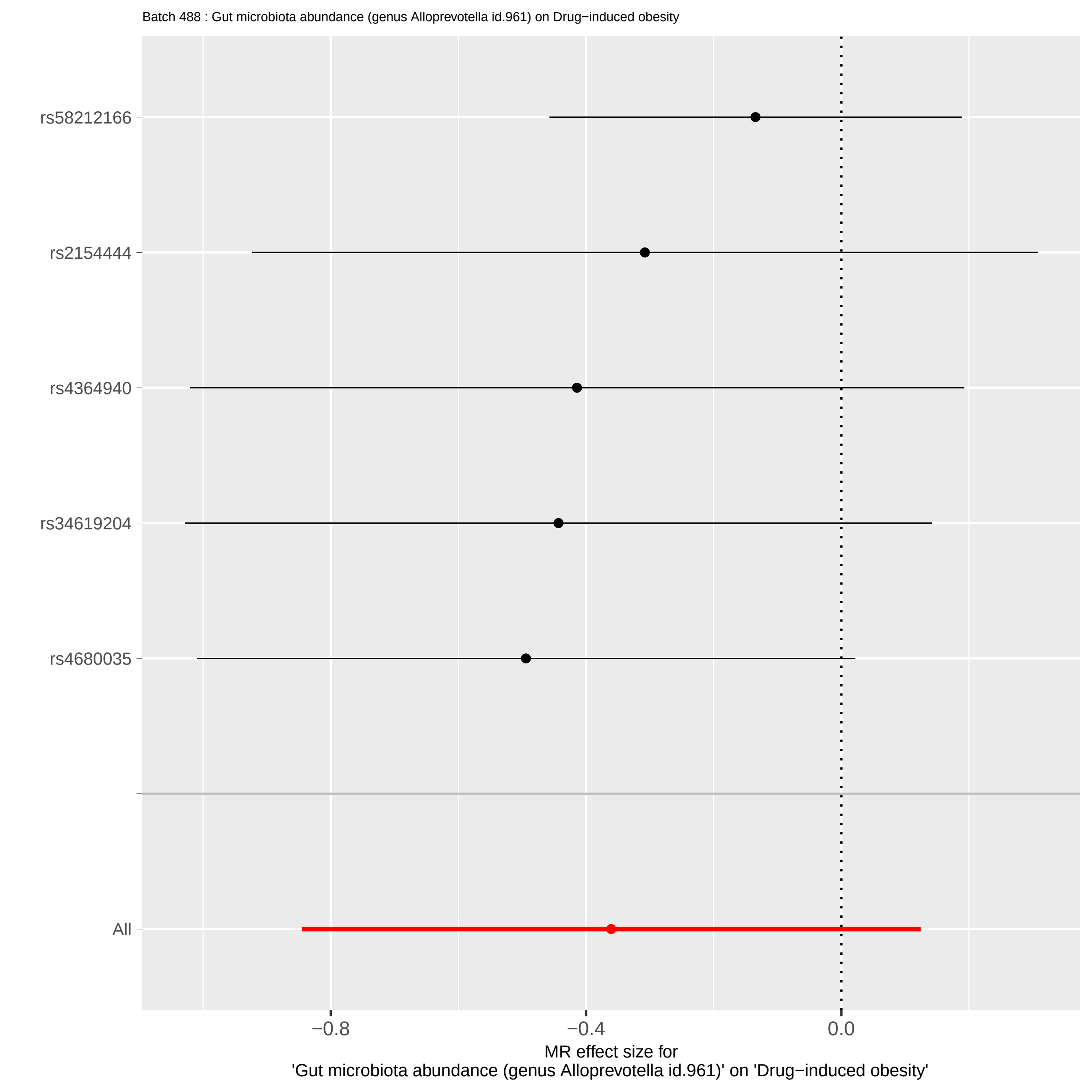

Batch 488 : Gut microbiota abundance (genus Alloprevotella id.961) on Drug−induced obesity
rs58212166
rs2154444
rs4364940
rs34619204
rs4680035
All
−0.8
−0.4
0.0
MR effect size for
'Gut microbiota abundance (genus Alloprevotella id.961)' on 'Drug−induced obesity'

Batch 489 : Gut microbiota abundance (genus Anaerofilum id.2053) on Drug−induced obesity

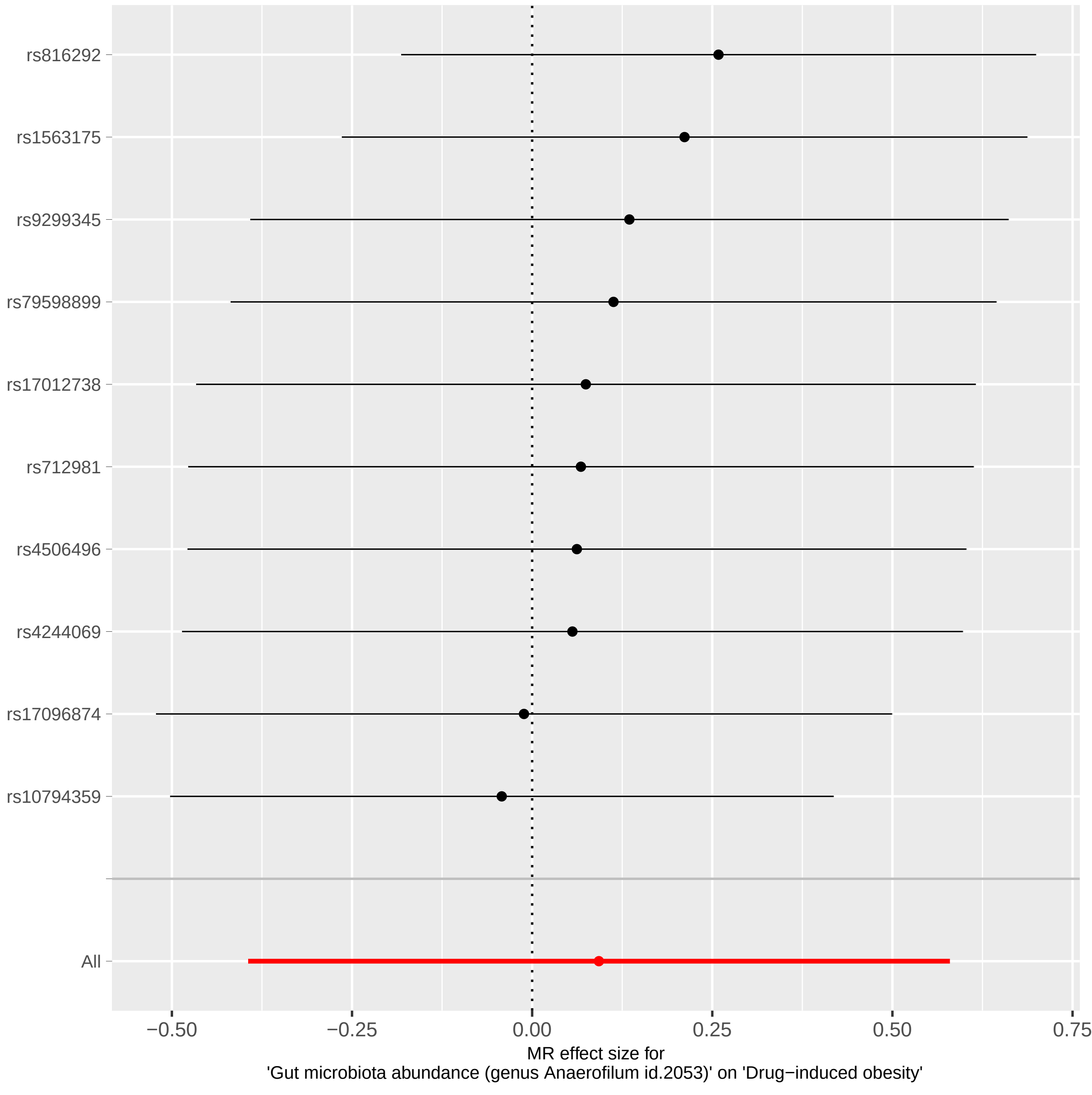

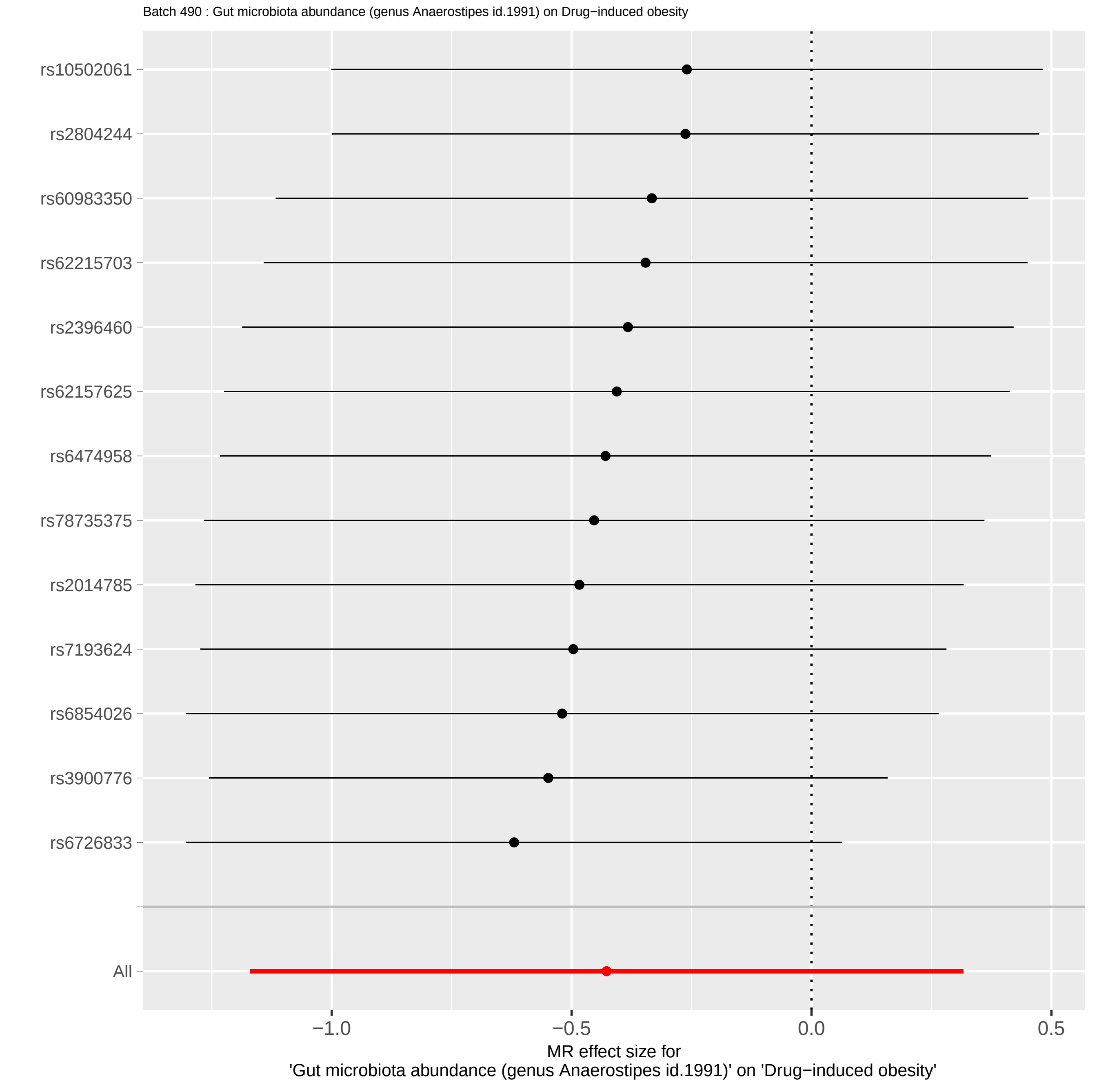
Batch 490 : Gut microbiota abundance (genus Anaerostipes id.1991) on Drug−induced obesity
rs10502061
rs2804244
rs60983350
rs62215703
rs2396460
rs62157625
rs6474958
rs78735375
rs2014785
rs7193624
rs6854026
rs3900776
rs6726833
All
−1.0
−0.5
0.0
0.5
MR effect size for
'Gut microbiota abundance (genus Anaerostipes id.1991)' on 'Drug−induced obesity'

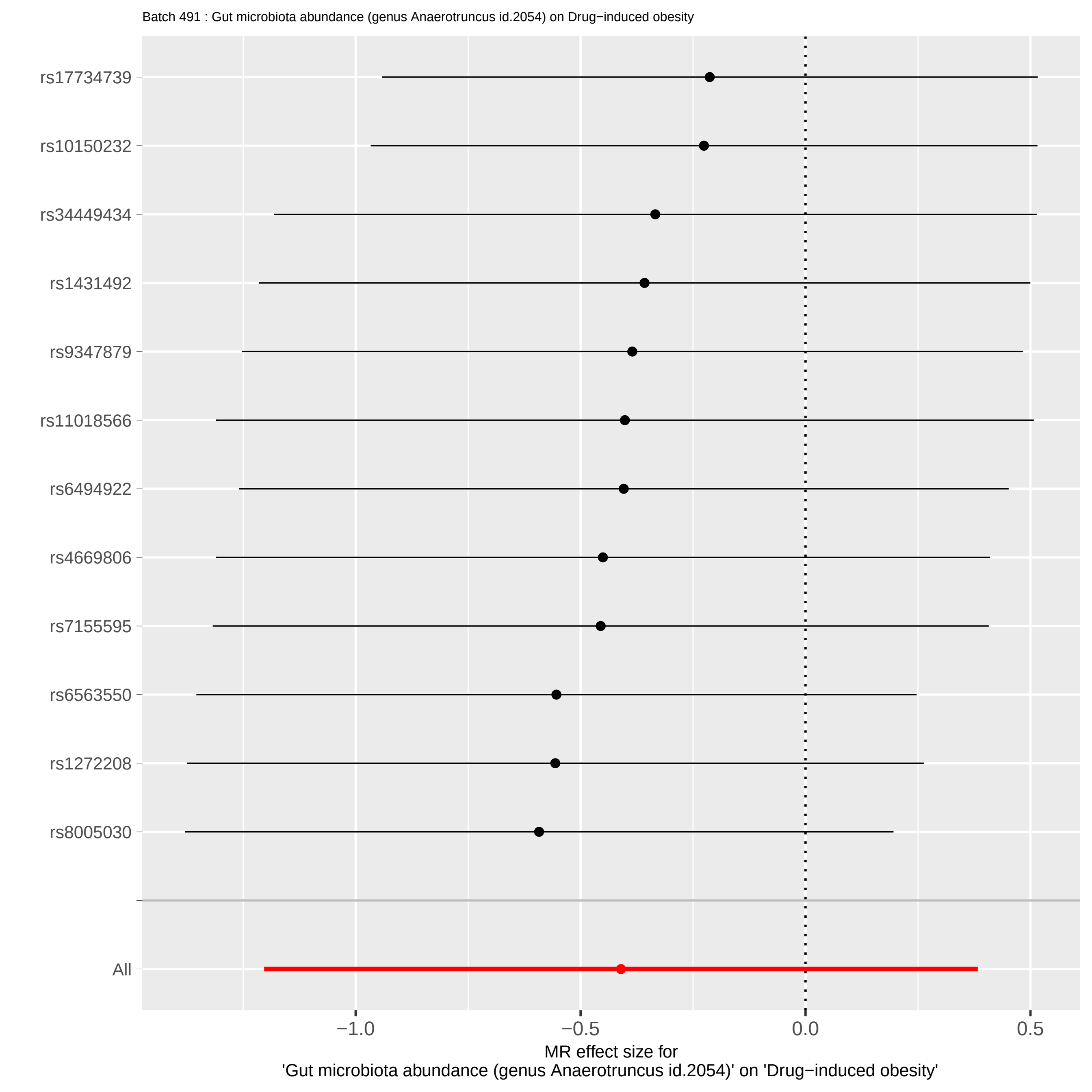
Batch 491 : Gut microbiota abundance (genus Anaerotruncus id.2054) on Drug−induced obesity
rs17734739
rs10150232
rs34449434
rs1431492
rs9347879
rs11018566
rs6494922
rs4669806
rs7155595
rs6563550
rs1272208
rs8005030
All
−1.0
−0.5
0.0
0.5
MR effect size for
'Gut microbiota abundance (genus Anaerotruncus id.2054)' on 'Drug−induced obesity'

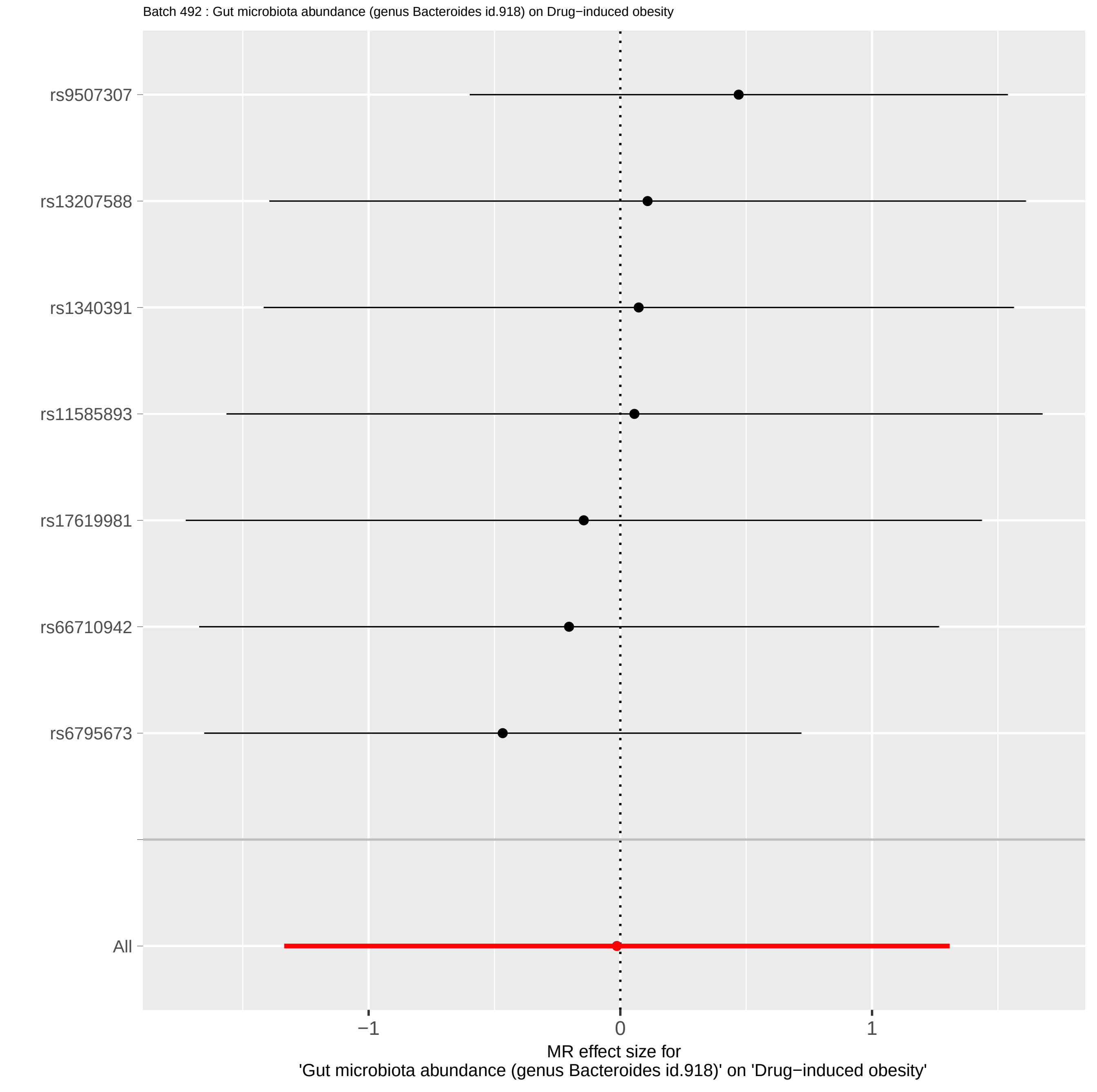

Batch 492 : Gut microbiota abundance (genus Bacteroides id.918) on Drug−induced obesity
rs9507307
rs13207588
rs1340391
rs11585893
rs17619981
rs66710942
rs6795673
All
−1
0
1
MR effect size for
'Gut microbiota abundance (genus Bacteroides id.918)' on 'Drug−induced obesity'

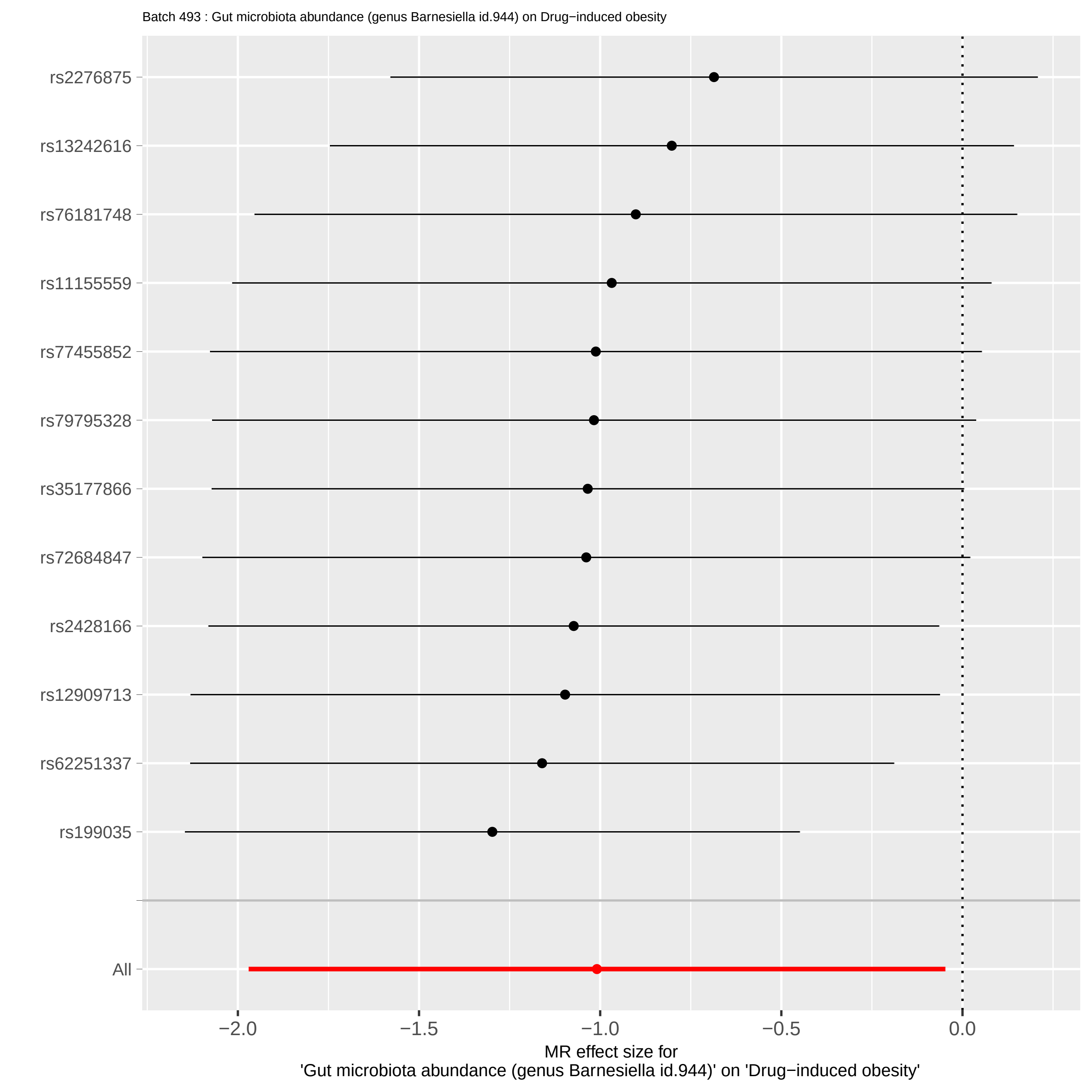

Batch 493 : Gut microbiota abundance (genus Barnesiella id.944) on Drug−induced obesity
rs2276875
rs13242616
rs76181748
rs11155559
rs77455852
rs79795328
rs35177866
rs72684847
rs2428166
rs12909713
rs62251337
rs199035
All
−2.0
−1.5
−1.0
−0.5
0.0
MR effect size for
'Gut microbiota abundance (genus Barnesiella id.944)' on 'Drug−induced obesity'

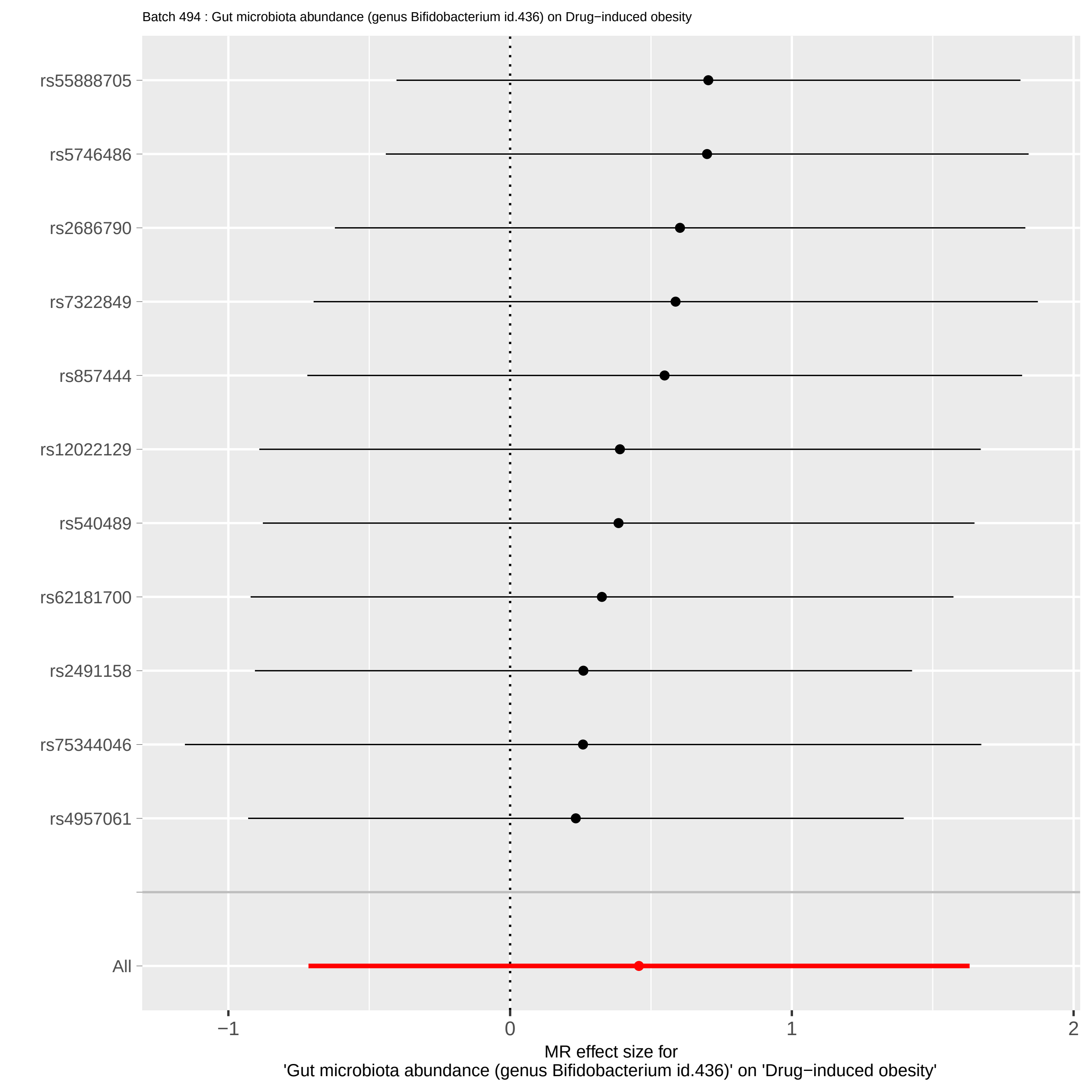

Batch 494 : Gut microbiota abundance (genus Bifidobacterium id.436) on Drug−induced obesity
rs55888705
rs5746486
rs2686790
rs7322849
rs857444
rs12022129
rs540489
rs62181700
rs2491158
rs75344046
rs4957061
All
−1
0
1
2
MR effect size for
'Gut microbiota abundance (genus Bifidobacterium id.436)' on 'Drug−induced obesity'

Batch 495 : Gut microbiota abundance (genus Bilophila id.3170) on Drug−induced obesity

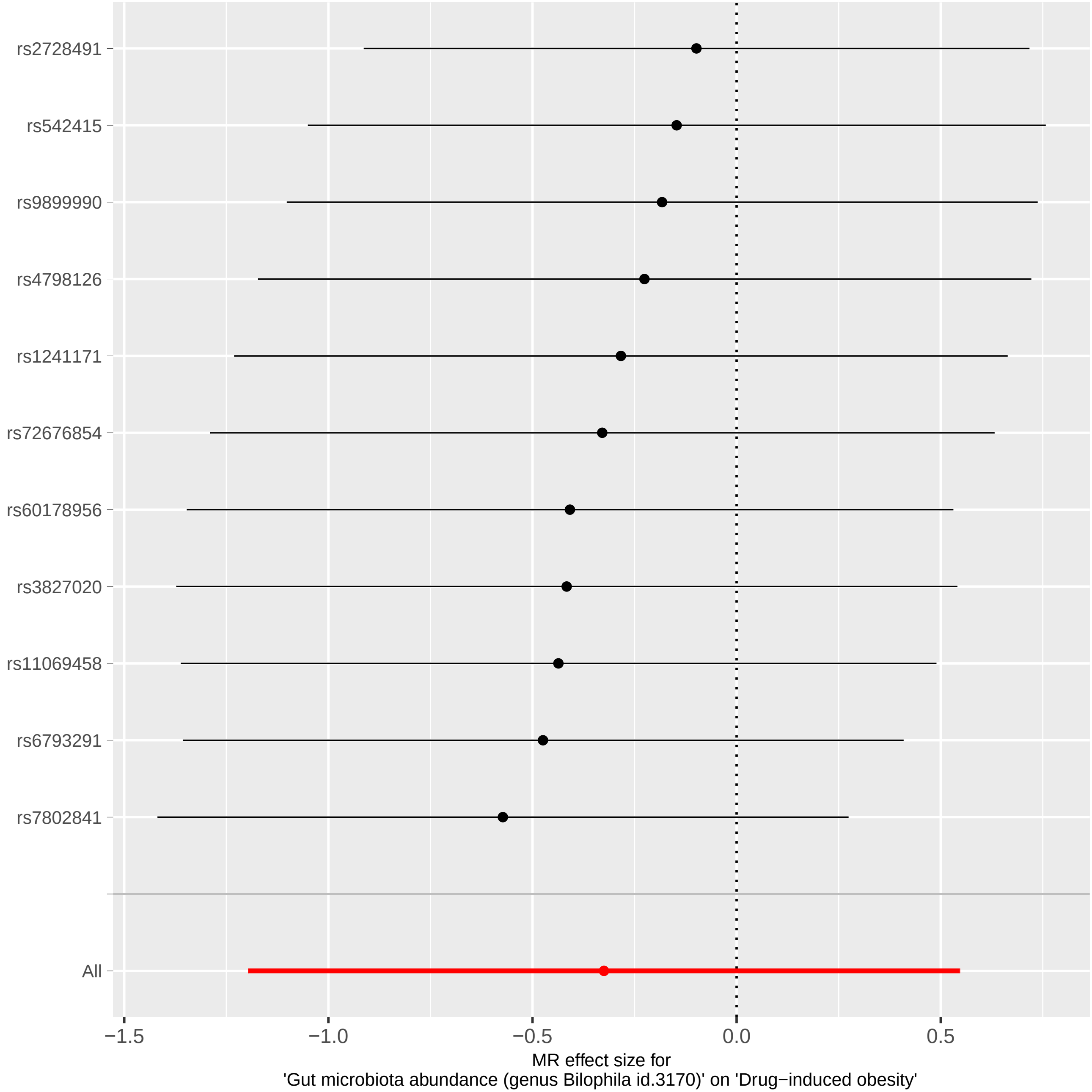

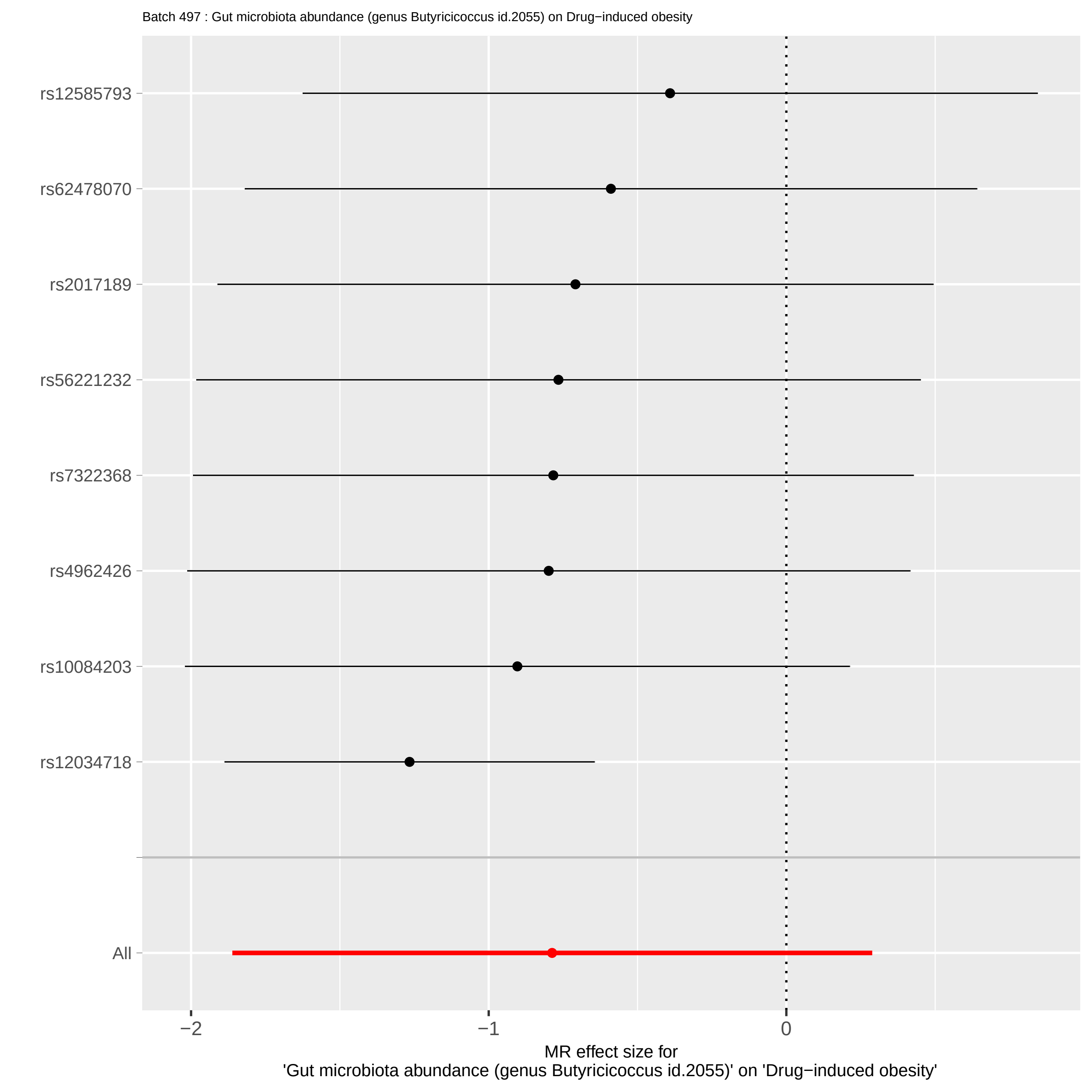

Batch 497 : Gut microbiota abundance (genus Butyricicoccus id.2055) on Drug−induced obesity
rs12585793
rs62478070
rs2017189
rs56221232
rs7322368
rs4962426
rs10084203
rs12034718
All
−2
−1
0
MR effect size for
'Gut microbiota abundance (genus Butyricicoccus id.2055)' on 'Drug−induced obesity'

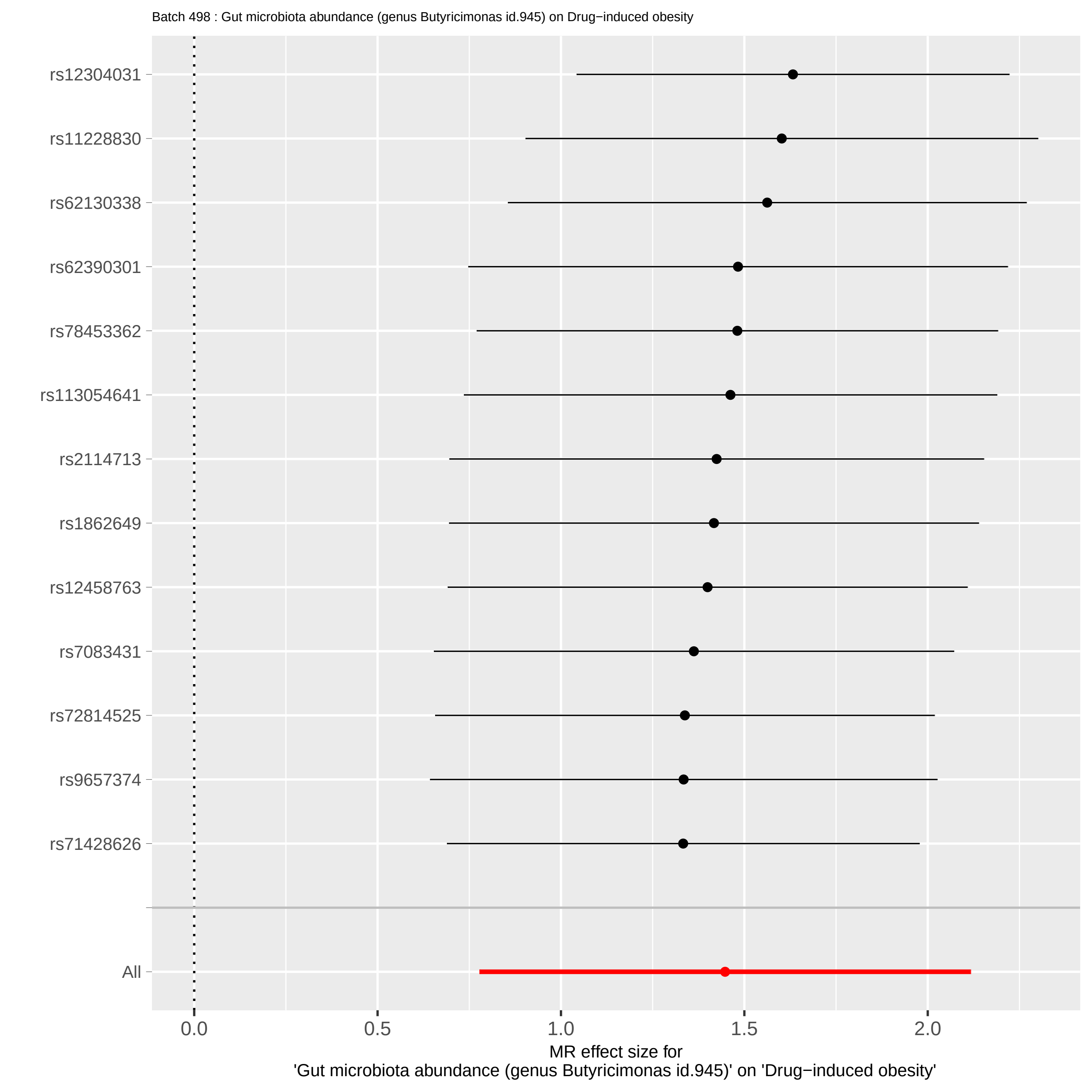
Batch 498 : Gut microbiota abundance (genus Butyricimonas id.945) on Drug−induced obesity
rs12304031
rs11228830
rs62130338
rs62390301
rs78453362
rs113054641
rs2114713
rs1862649
rs12458763
rs7083431
rs72814525
rs9657374
rs71428626
All
0.0
0.5
1.0
1.5
2.0
MR effect size for
'Gut microbiota abundance (genus Butyricimonas id.945)' on 'Drug−induced obesity'

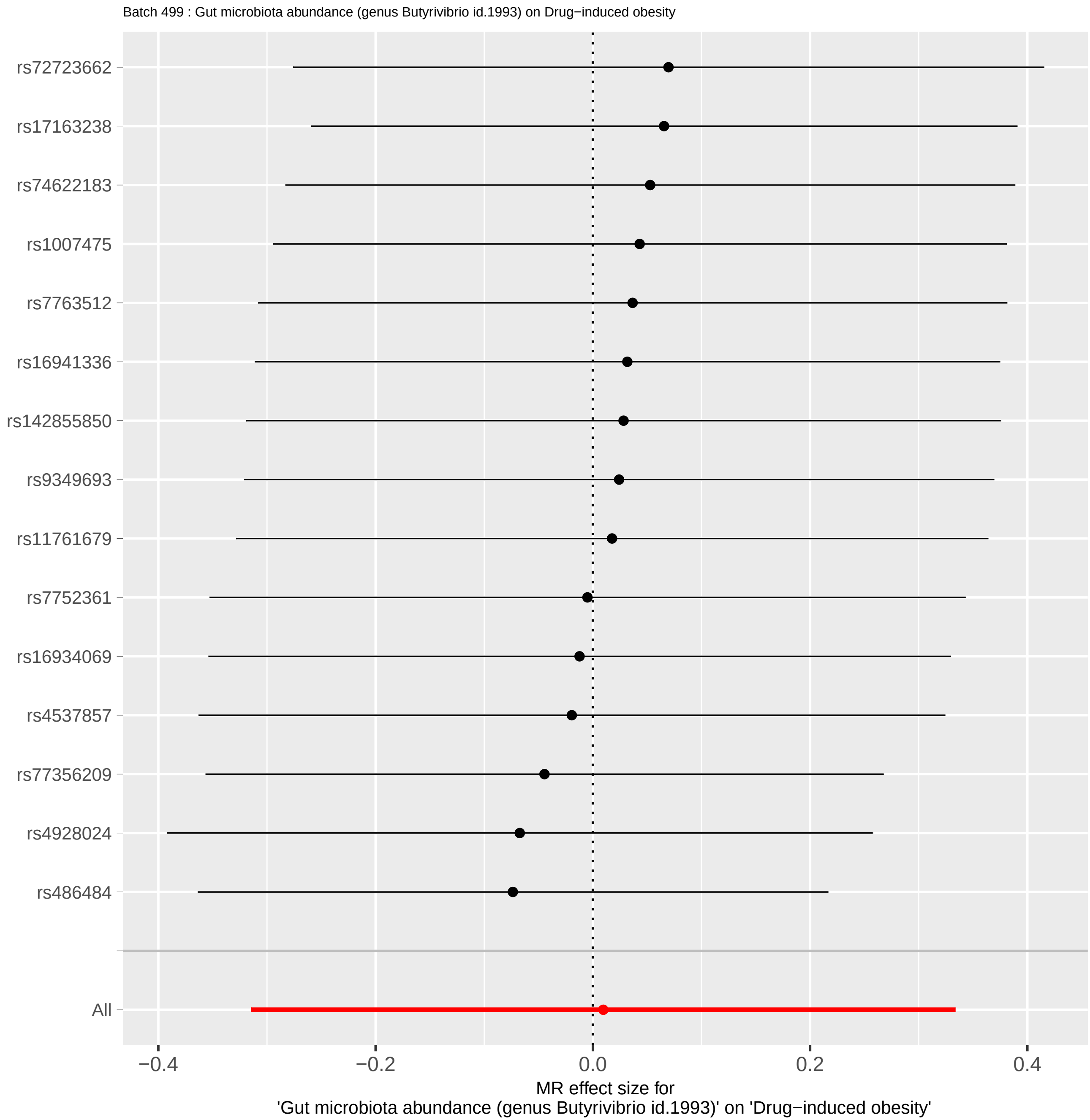
Batch 499 : Gut microbiota abundance (genus Butyrivibrio id.1993) on Drug−induced obesity
rs72723662
rs17163238
rs74622183
rs1007475
rs7763512
rs16941336
rs142855850
rs9349693
rs11761679
rs7752361
rs16934069
rs4537857
rs77356209
rs4928024
rs486484
All
−0.4
−0.2
0.0
0.2
0.4
MR effect size for
'Gut microbiota abundance (genus Butyrivibrio id.1993)' on 'Drug−induced obesity'

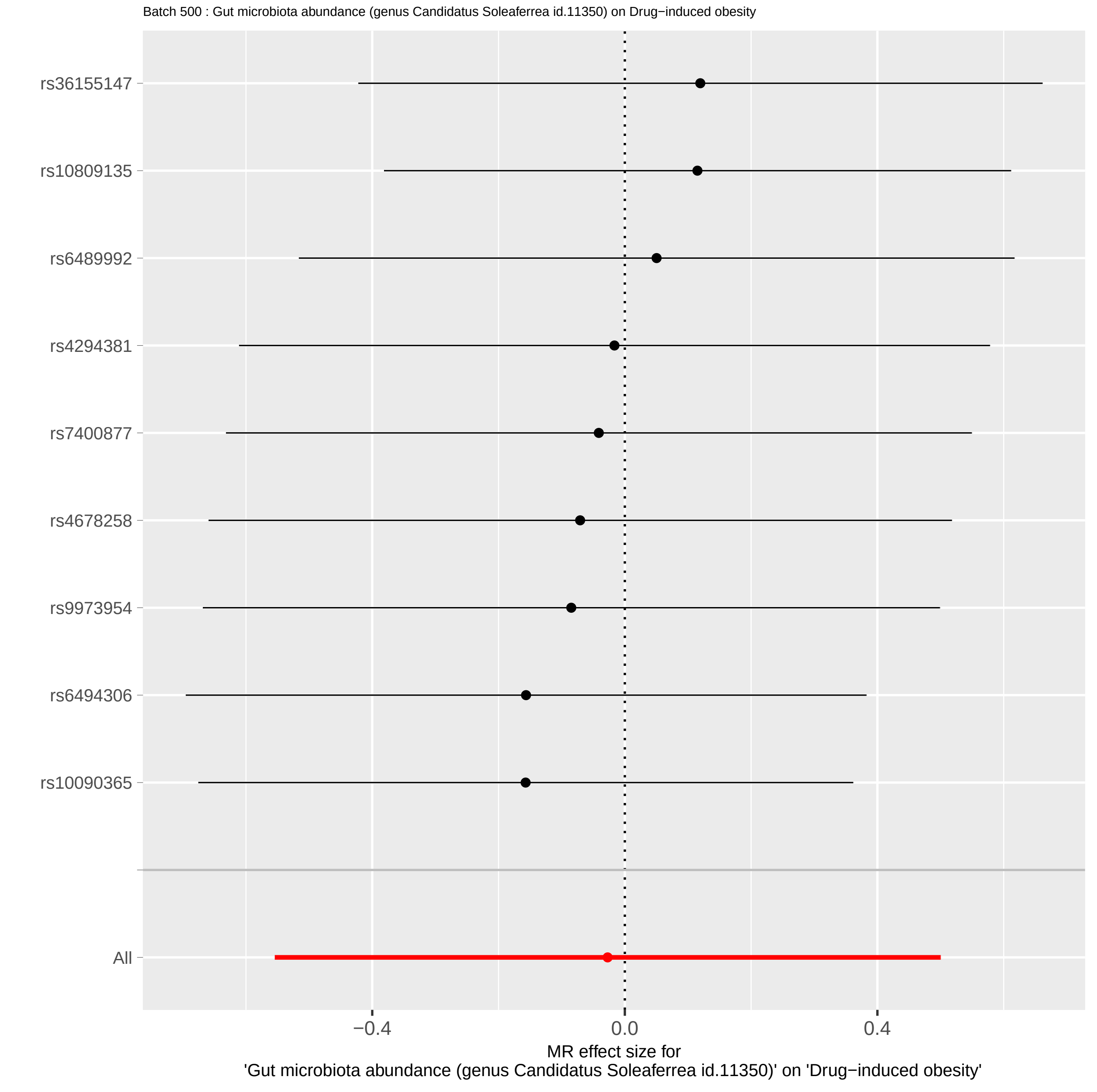
Batch 500 : Gut microbiota abundance (genus Candidatus Soleaferrea id.11350) on Drug−induced obesity
rs36155147
rs10809135
rs6489992
rs4294381
rs7400877
rs4678258
rs9973954
rs6494306
rs10090365
All
−0.4
0.0
0.4
MR effect size for
'Gut microbiota abundance (genus Candidatus Soleaferrea id.11350)' on 'Drug−induced obesity'

Batch 501 : Gut microbiota abundance (genus Catenibacterium id.2153) on Drug−induced obesity

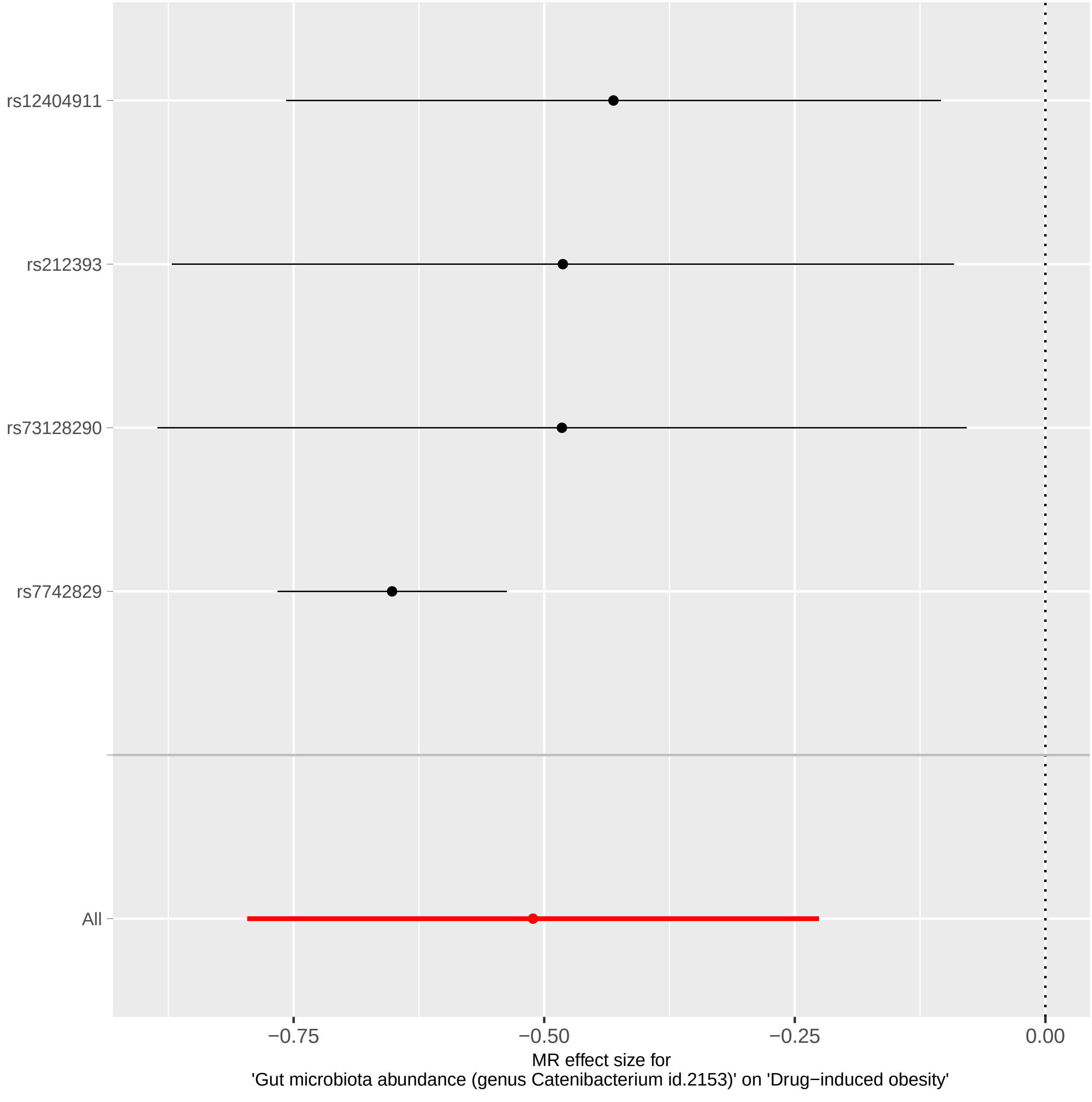

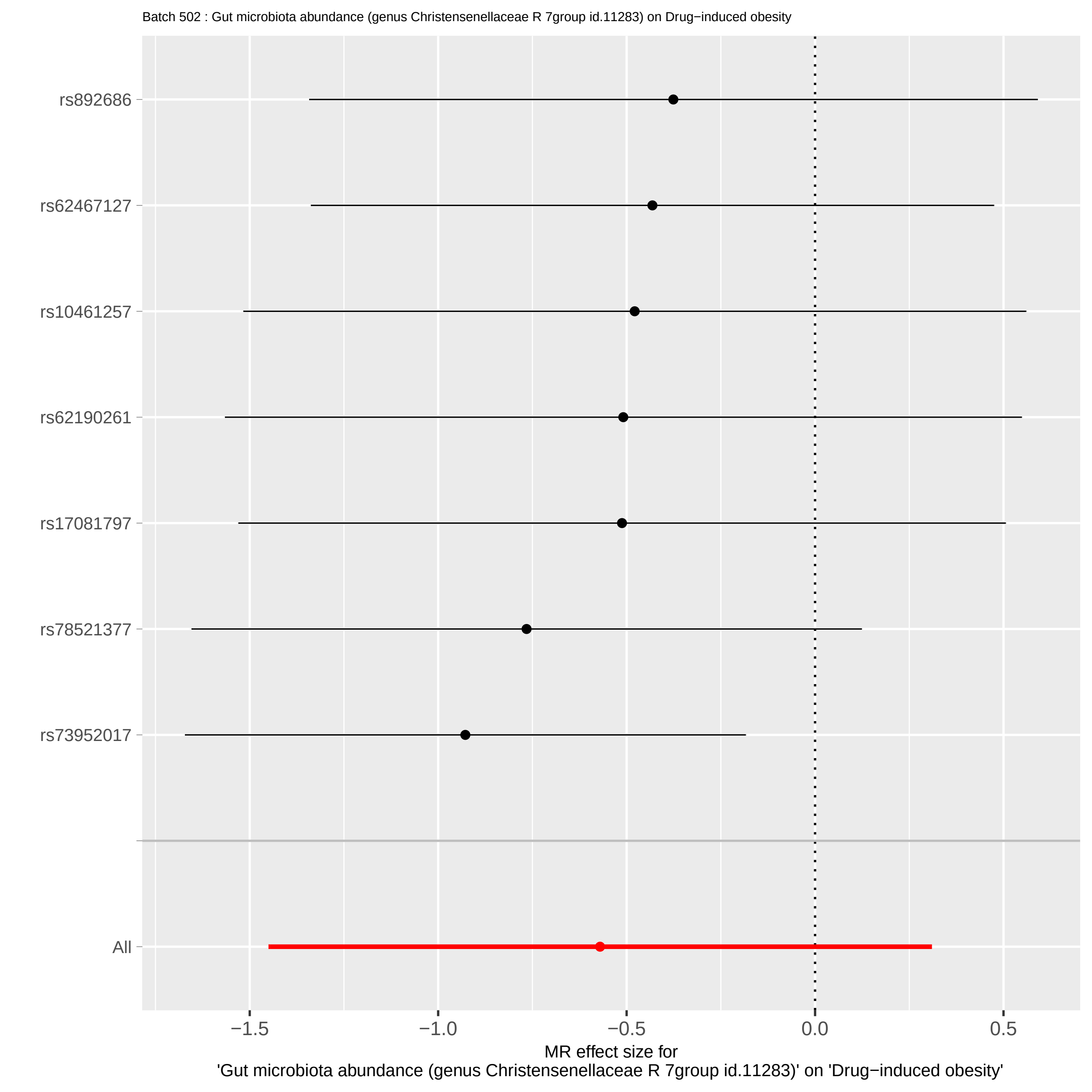
Batch 502 : Gut microbiota abundance (genus Christensenellaceae R 7group id.11283) on Drug−induced obesity
rs892686
rs62467127
rs10461257
rs62190261
rs17081797
rs78521377
rs73952017
All
−1.5
−1.0
−0.5
0.0
0.5
MR effect size for
'Gut microbiota abundance (genus Christensenellaceae R 7group id.11283)' on 'Drug−induced obesity'

Batch 503 : Gut microbiota abundance (genus Clostridium innocuum group id.14397) on Drug−induced obesity

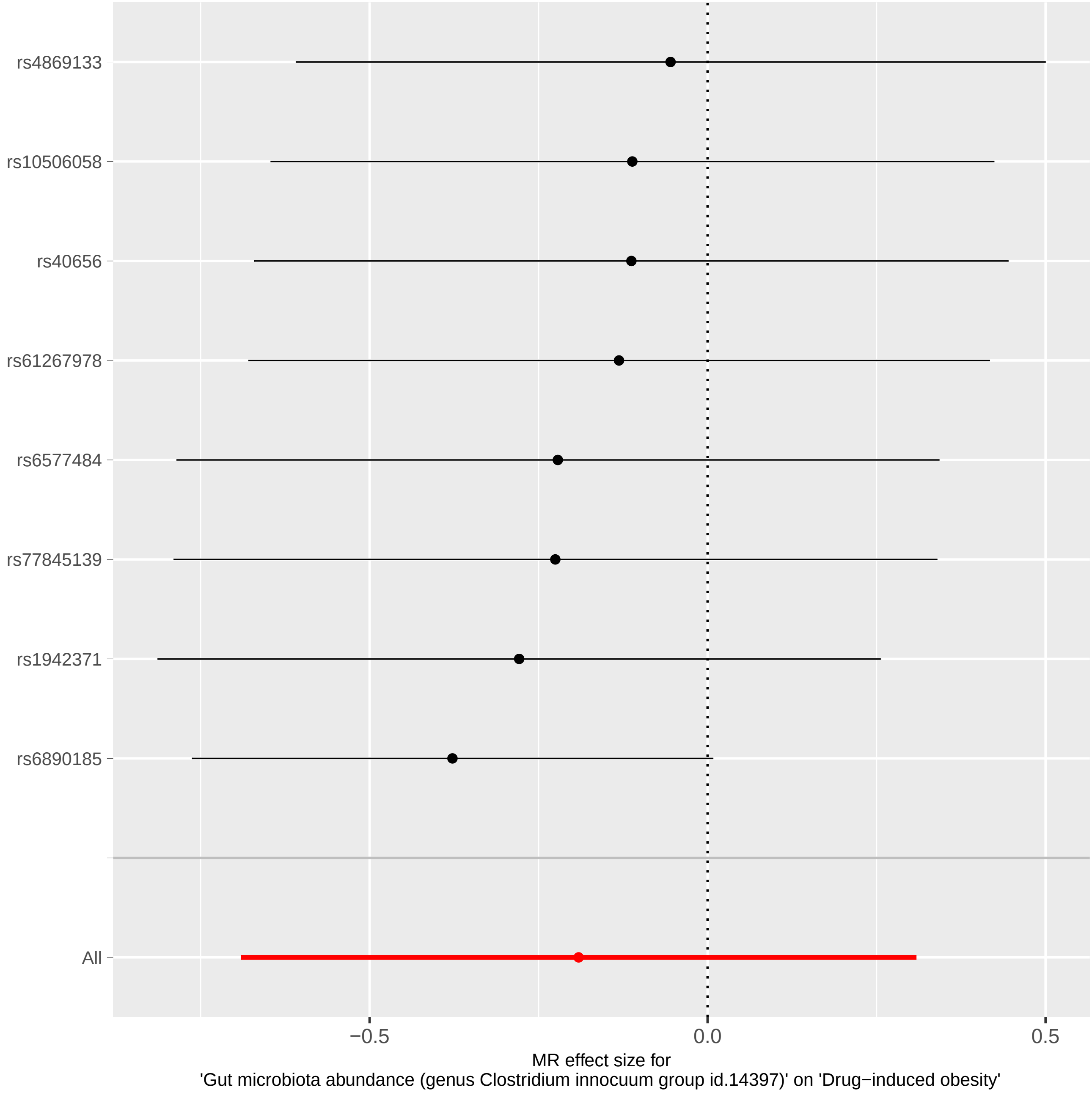

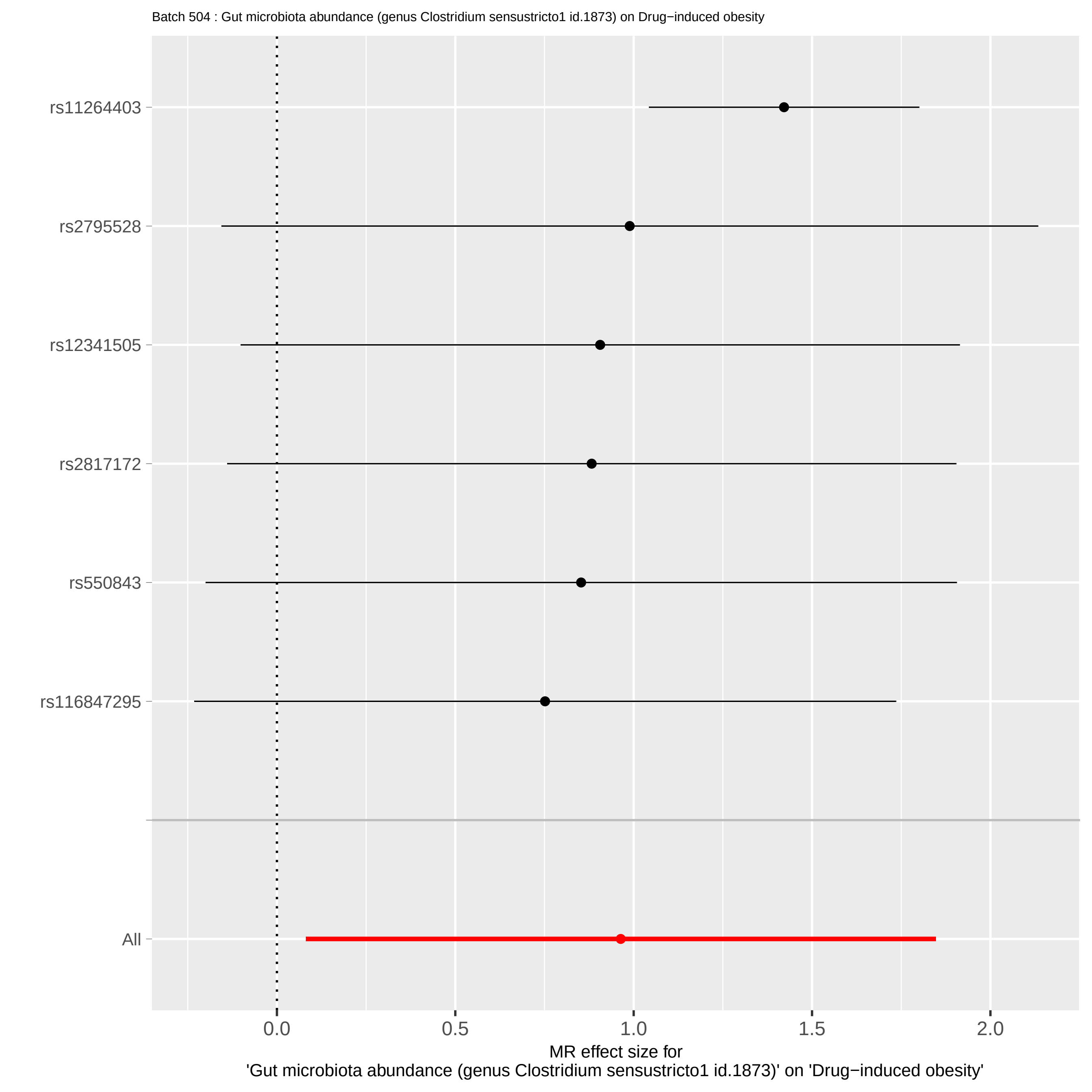
Batch 504 : Gut microbiota abundance (genus Clostridium sensustricto1 id.1873) on Drug−induced obesity
rs11264403
rs2795528
rs12341505
rs2817172
rs550843
rs116847295
All
0.0
0.5
1.0
1.5
2.0
MR effect size for
'Gut microbiota abundance (genus Clostridium sensustricto1 id.1873)' on 'Drug−induced obesity'

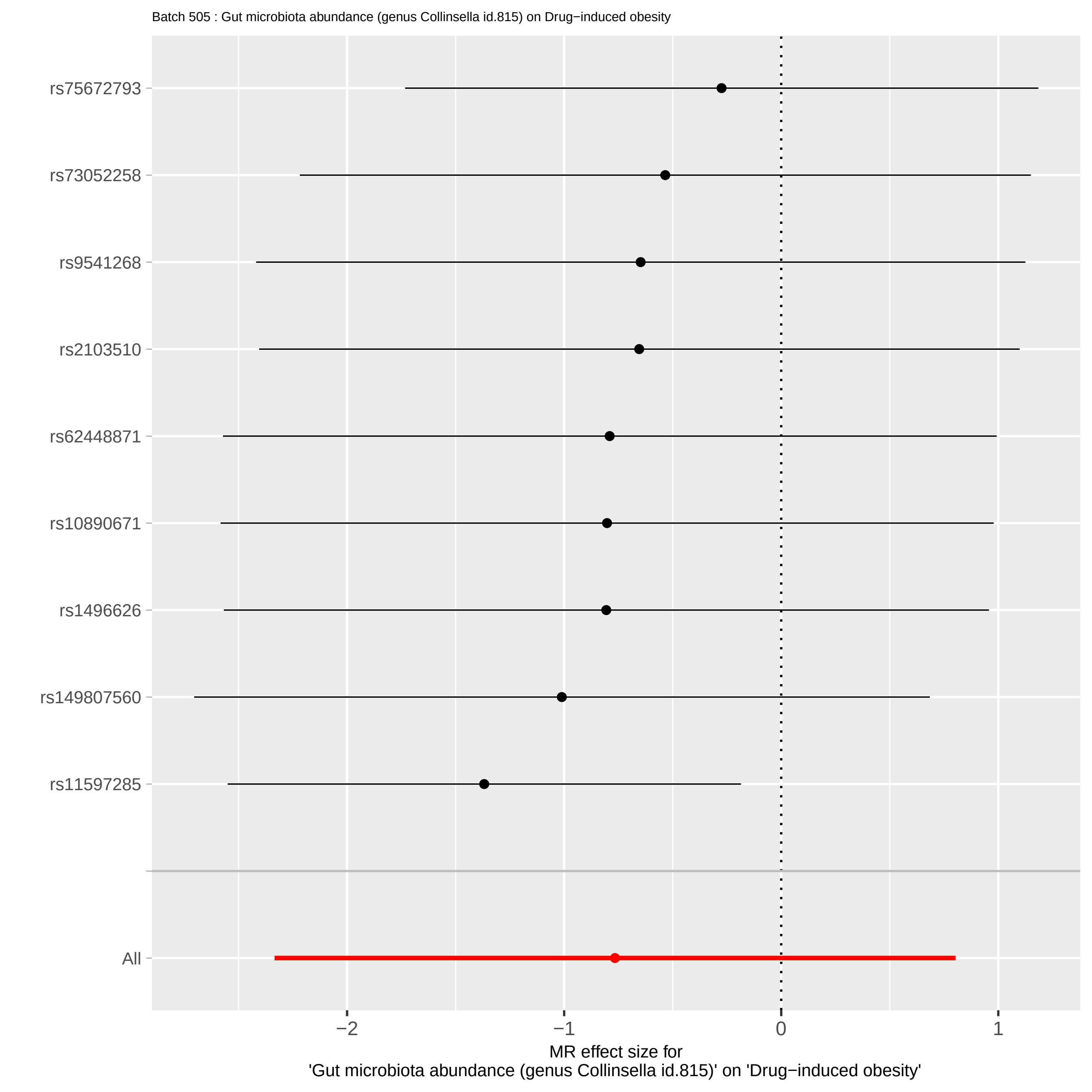

Batch 505 : Gut microbiota abundance (genus Collinsella id.815) on Drug−induced obesity
rs75672793
rs73052258
rs9541268
rs2103510
rs62448871
rs10890671
rs1496626
rs149807560
rs11597285
All
−2
−1
0
1
MR effect size for
'Gut microbiota abundance (genus Collinsella id.815)' on 'Drug−induced obesity'

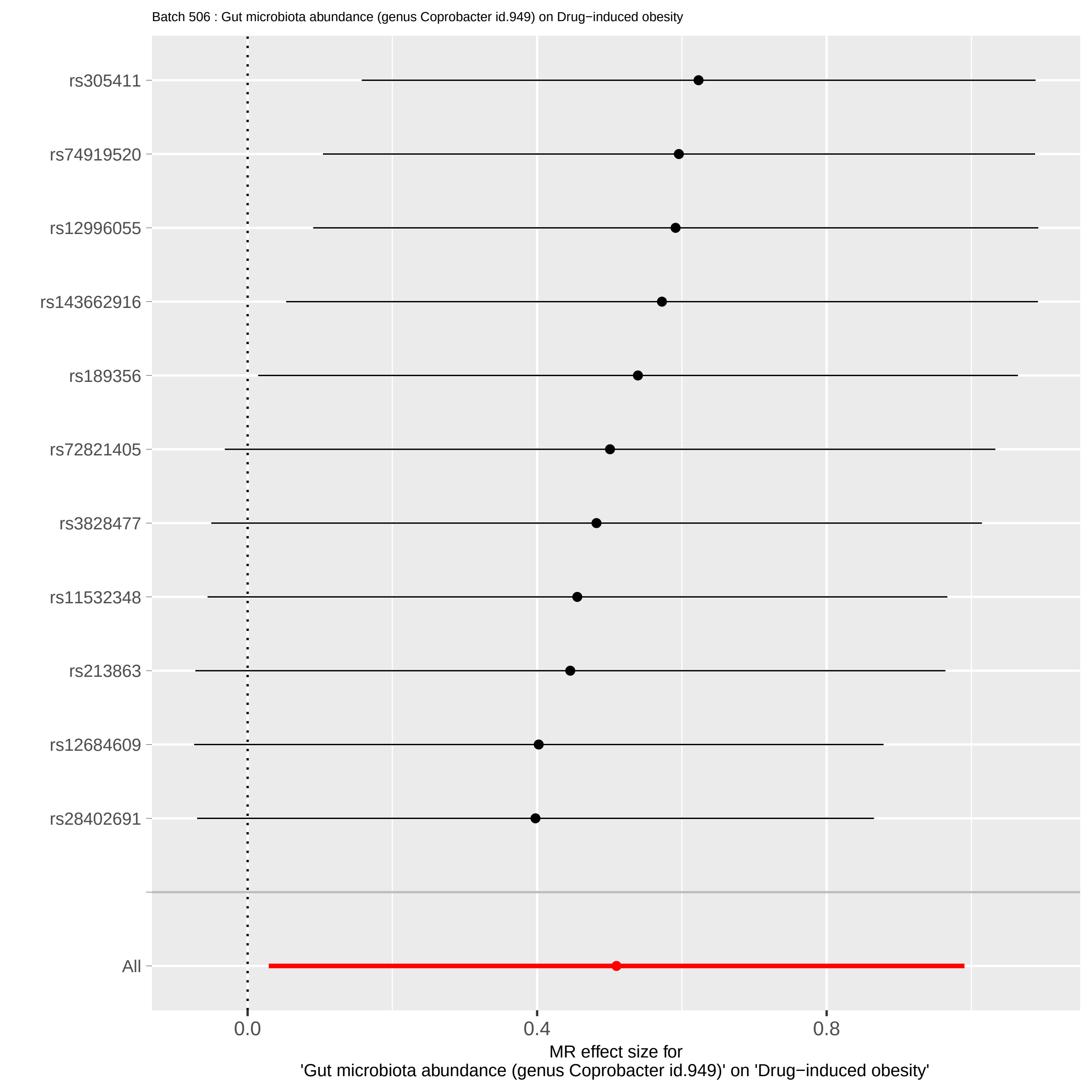

Batch 506 : Gut microbiota abundance (genus Coprobacter id.949) on Drug−induced obesity
rs305411
rs74919520
rs12996055
rs143662916
rs189356
rs72821405
rs3828477
rs11532348
rs213863
rs12684609
rs28402691
All
0.0
0.4
0.8
MR effect size for
'Gut microbiota abundance (genus Coprobacter id.949)' on 'Drug−induced obesity'

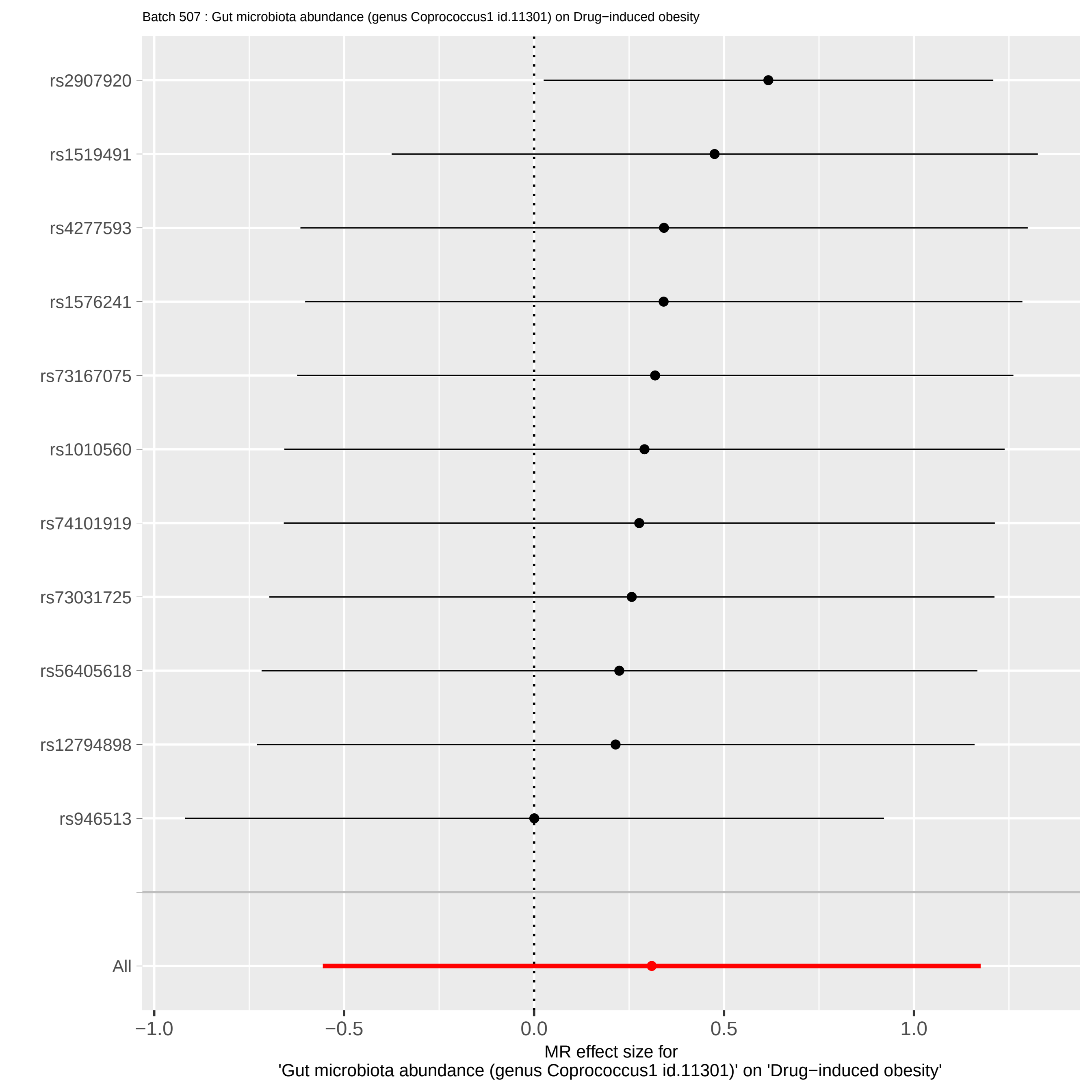

Batch 507 : Gut microbiota abundance (genus Coprococcus1 id.11301) on Drug−induced obesity
rs2907920
rs1519491
rs4277593
rs1576241
rs73167075
rs1010560
rs74101919
rs73031725
rs56405618
rs12794898
rs946513
All
−1.0
−0.5
0.0
0.5
1.0
MR effect size for
'Gut microbiota abundance (genus Coprococcus1 id.11301)' on 'Drug−induced obesity'

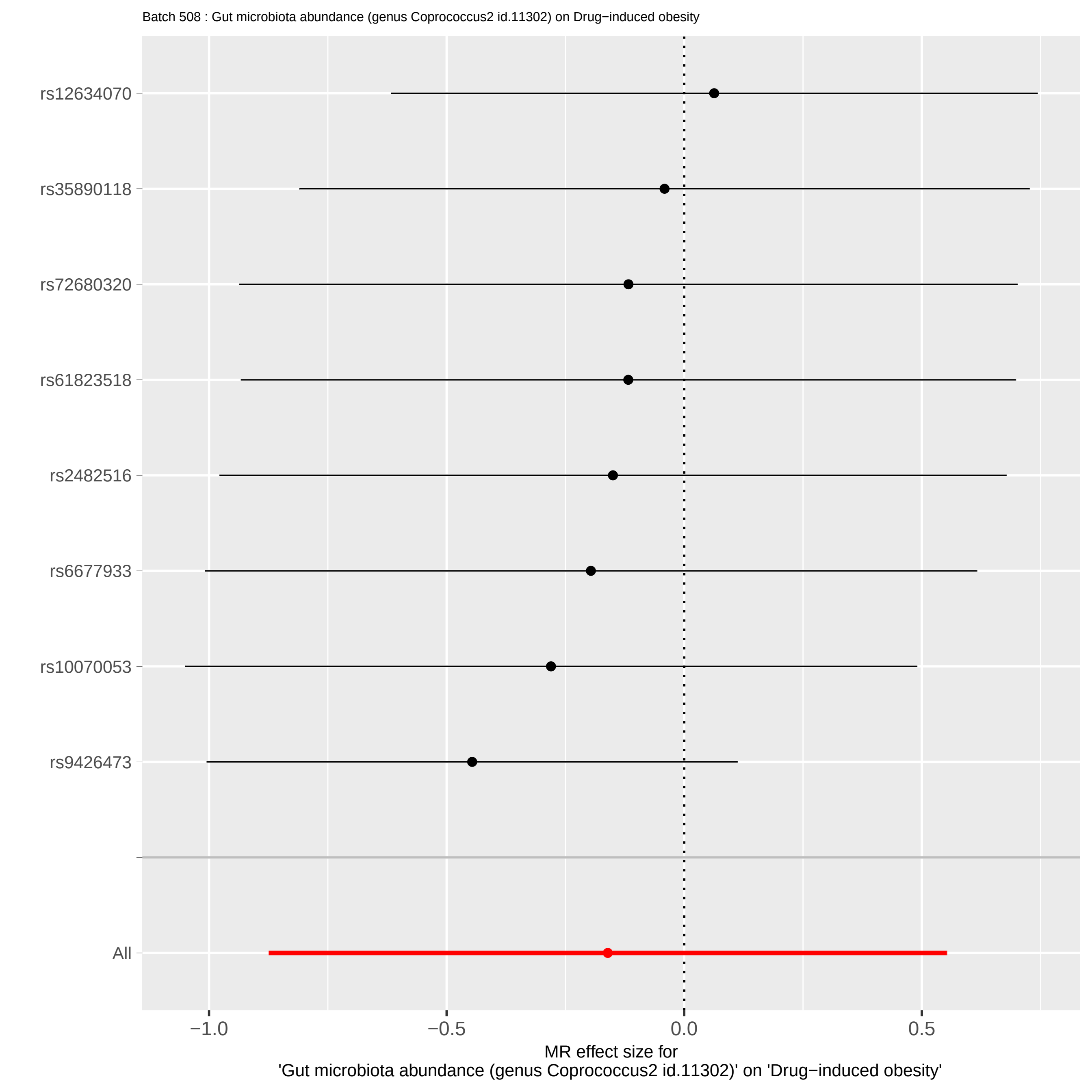
Batch 508 : Gut microbiota abundance (genus Coprococcus2 id.11302) on Drug−induced obesity
rs12634070
rs35890118
rs72680320
rs61823518
rs2482516
rs6677933
rs10070053
rs9426473
All
−1.0
−0.5
0.0
0.5
MR effect size for
'Gut microbiota abundance (genus Coprococcus2 id.11302)' on 'Drug−induced obesity'

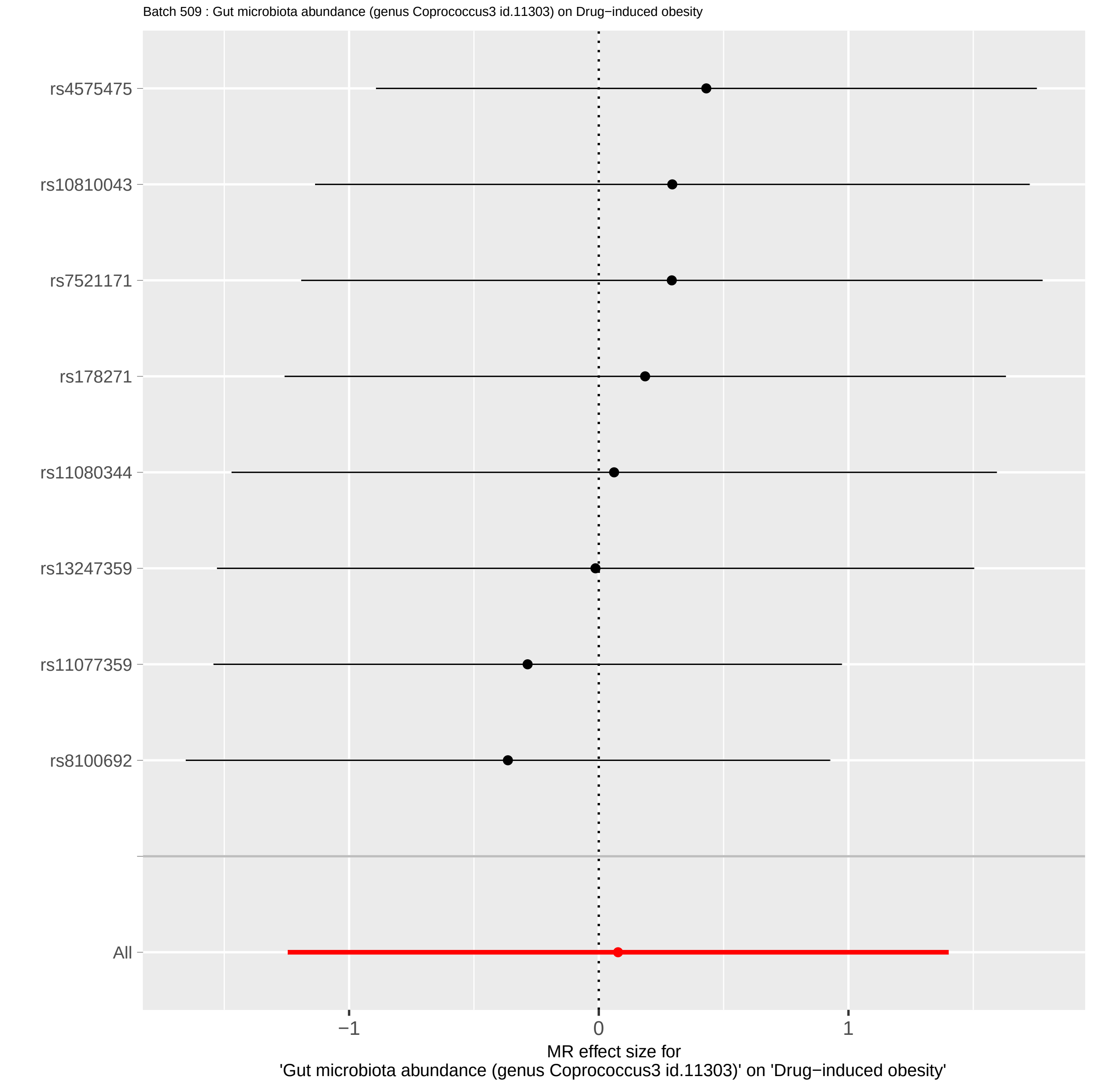
Batch 509 : Gut microbiota abundance (genus Coprococcus3 id.11303) on Drug−induced obesity
rs4575475
rs10810043
rs7521171
rs178271
rs11080344
rs13247359
rs11077359
rs8100692
All
−1
0
1
MR effect size for
'Gut microbiota abundance (genus Coprococcus3 id.11303)' on 'Drug−induced obesity'

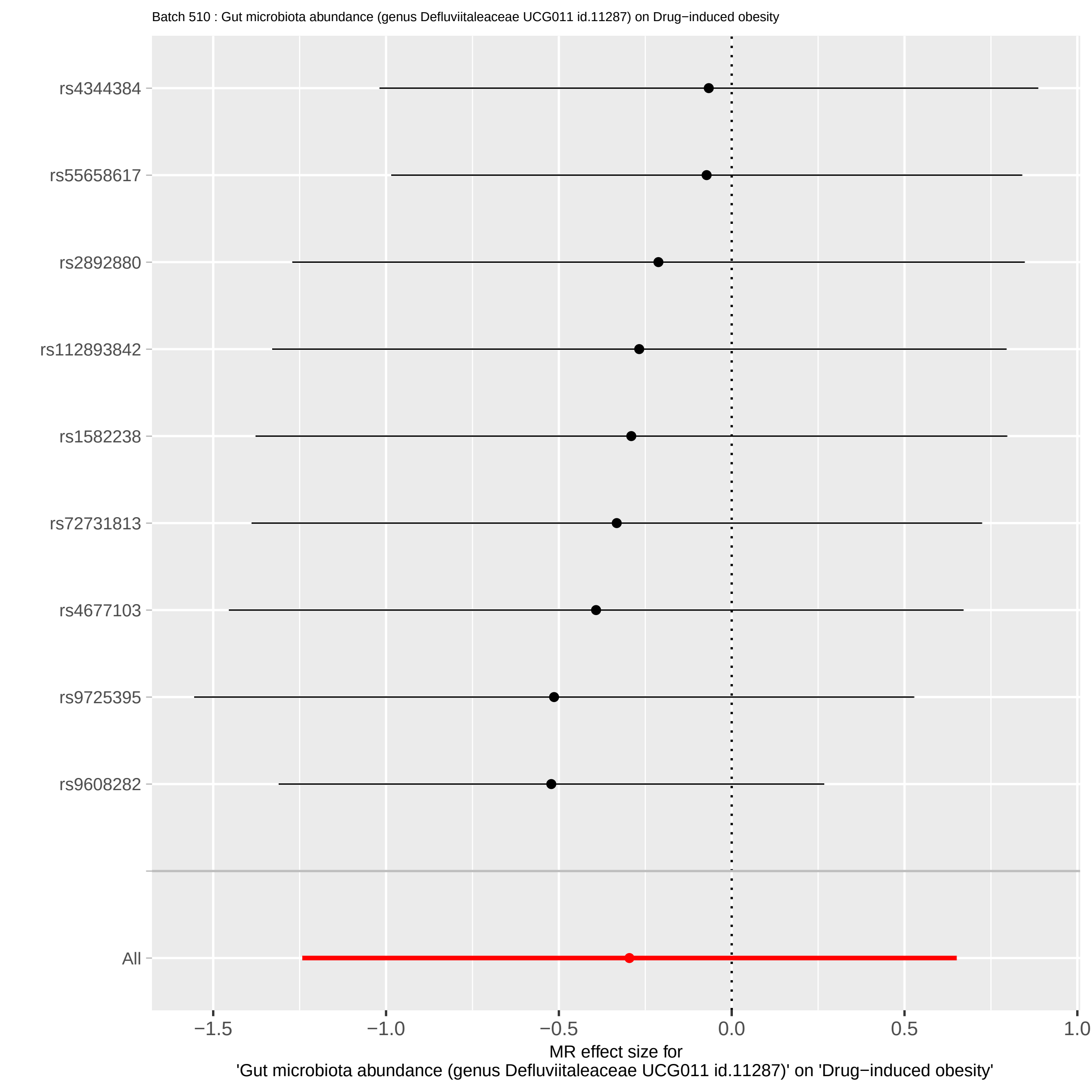

Batch 510 : Gut microbiota abundance (genus Defluviitaleaceae UCG011 id.11287) on Drug−induced obesity
rs4344384
rs55658617
rs2892880
rs112893842
rs1582238
rs72731813
rs4677103
rs9725395
rs9608282
All
−1.5
−1.0
−0.5
0.0
0.5
1.0
MR effect size for
'Gut microbiota abundance (genus Defluviitaleaceae UCG011 id.11287)' on 'Drug−induced obesity'

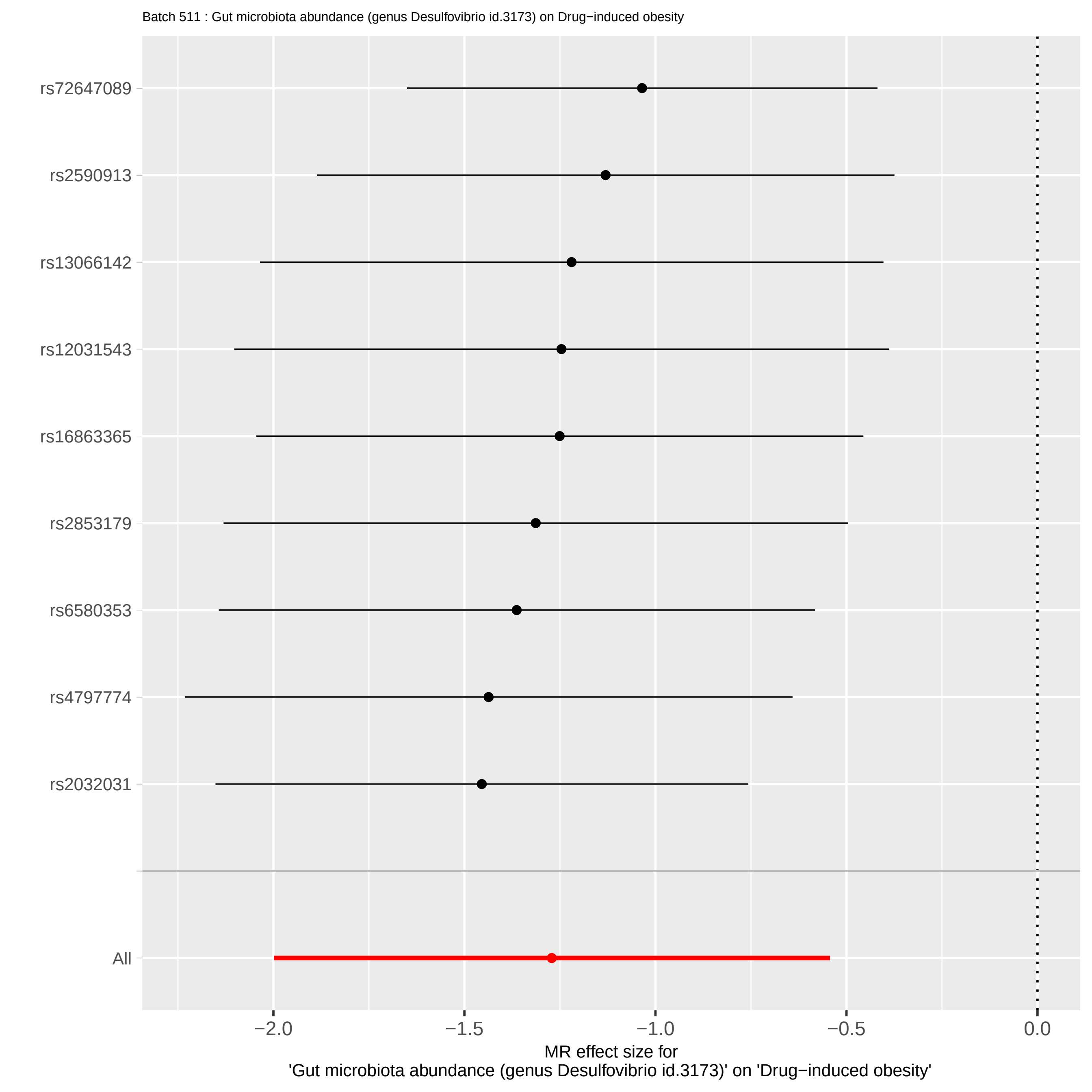

Batch 511 : Gut microbiota abundance (genus Desulfovibrio id.3173) on Drug−induced obesity
rs72647089
rs2590913
rs13066142
rs12031543
rs16863365
rs2853179
rs6580353
rs4797774
rs2032031
All
−2.0
−1.5
−1.0
−0.5
0.0
MR effect size for
'Gut microbiota abundance (genus Desulfovibrio id.3173)' on 'Drug−induced obesity'

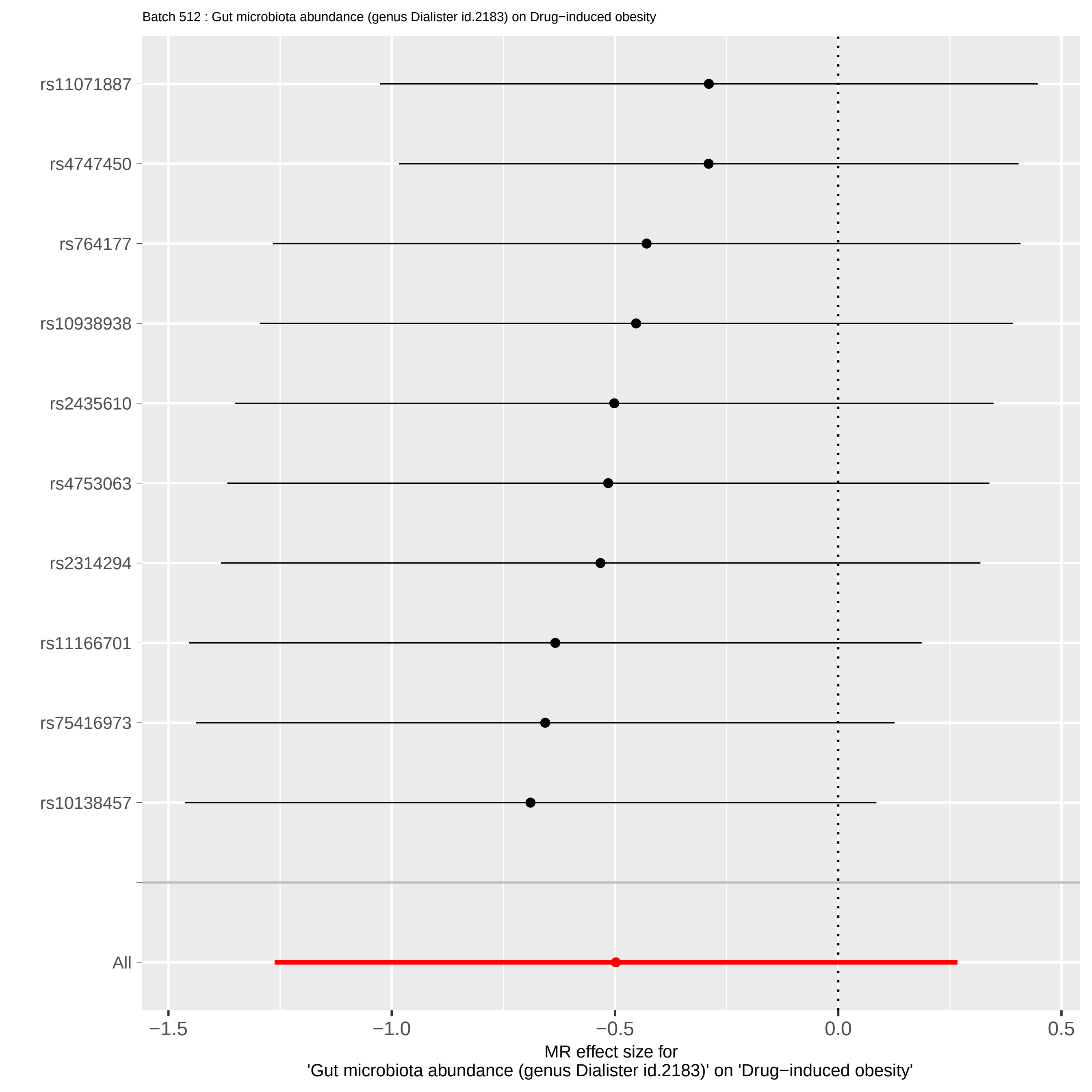

Batch 512 : Gut microbiota abundance (genus Dialister id.2183) on Drug−induced obesity
rs11071887
rs4747450
rs764177
rs10938938
rs2435610
rs4753063
rs2314294
rs11166701
rs75416973
rs10138457
All
−1.5
−1.0
−0.5
0.0
0.5
MR effect size for
'Gut microbiota abundance (genus Dialister id.2183)' on 'Drug−induced obesity'

Batch 513 : Gut microbiota abundance (genus Dorea id.1997) on Drug−induced obesity

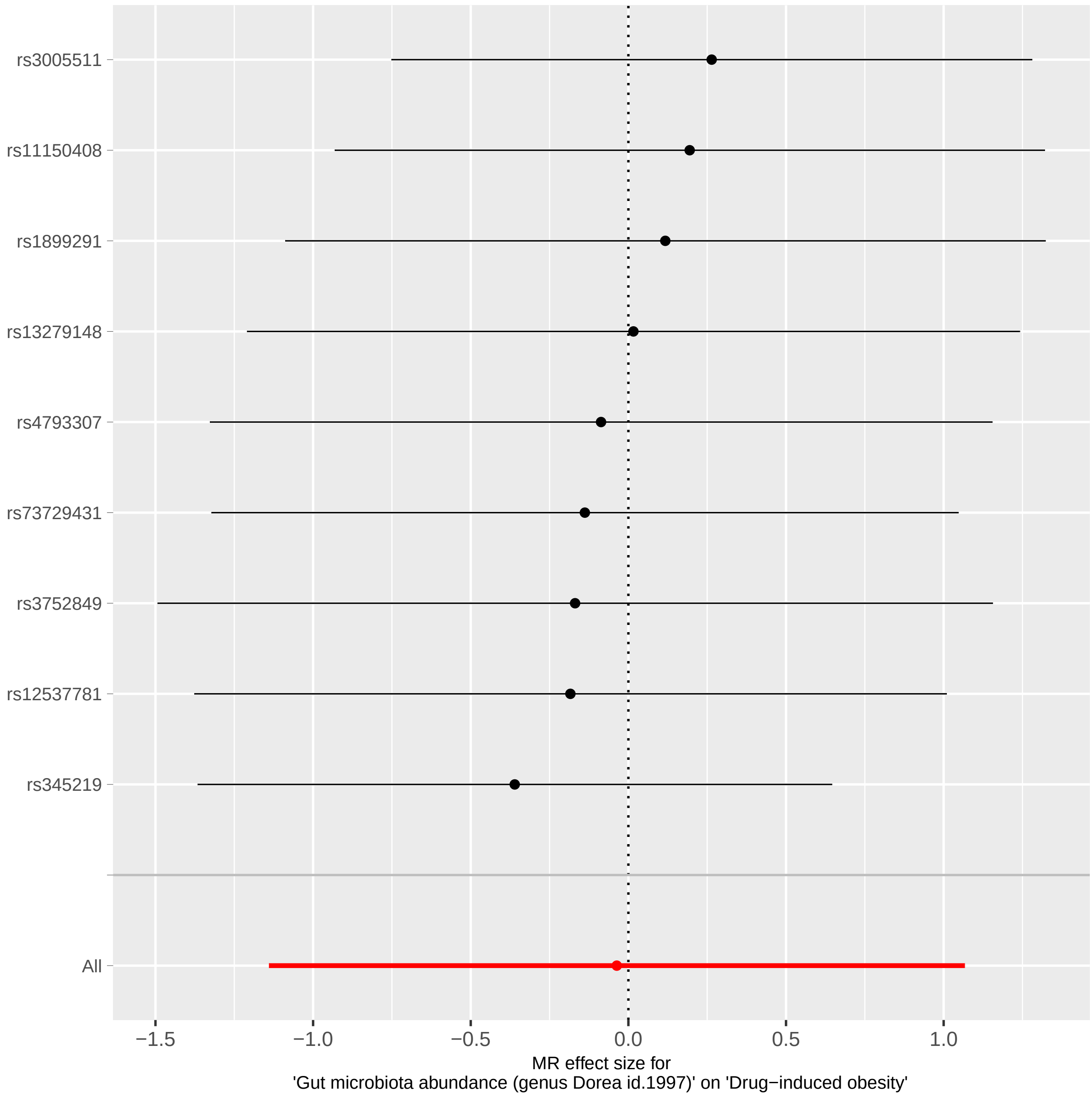

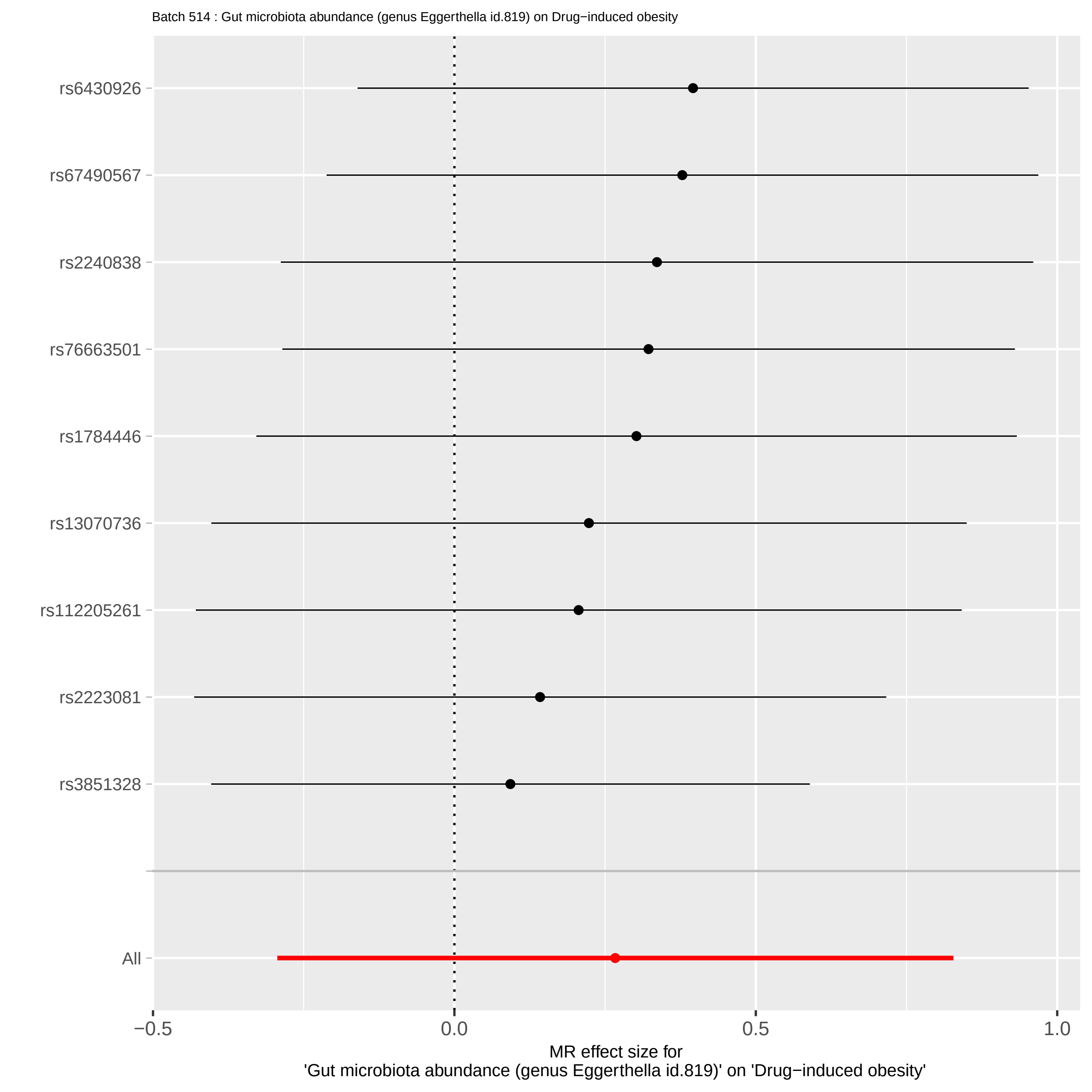

Batch 514 : Gut microbiota abundance (genus Eggerthella id.819) on Drug−induced obesity
rs6430926
rs67490567
rs2240838
rs76663501
rs1784446
rs13070736
rs112205261
rs2223081
rs3851328
All
−0.5
0.0
0.5
1.0
MR effect size for
'Gut microbiota abundance (genus Eggerthella id.819)' on 'Drug−induced obesity'

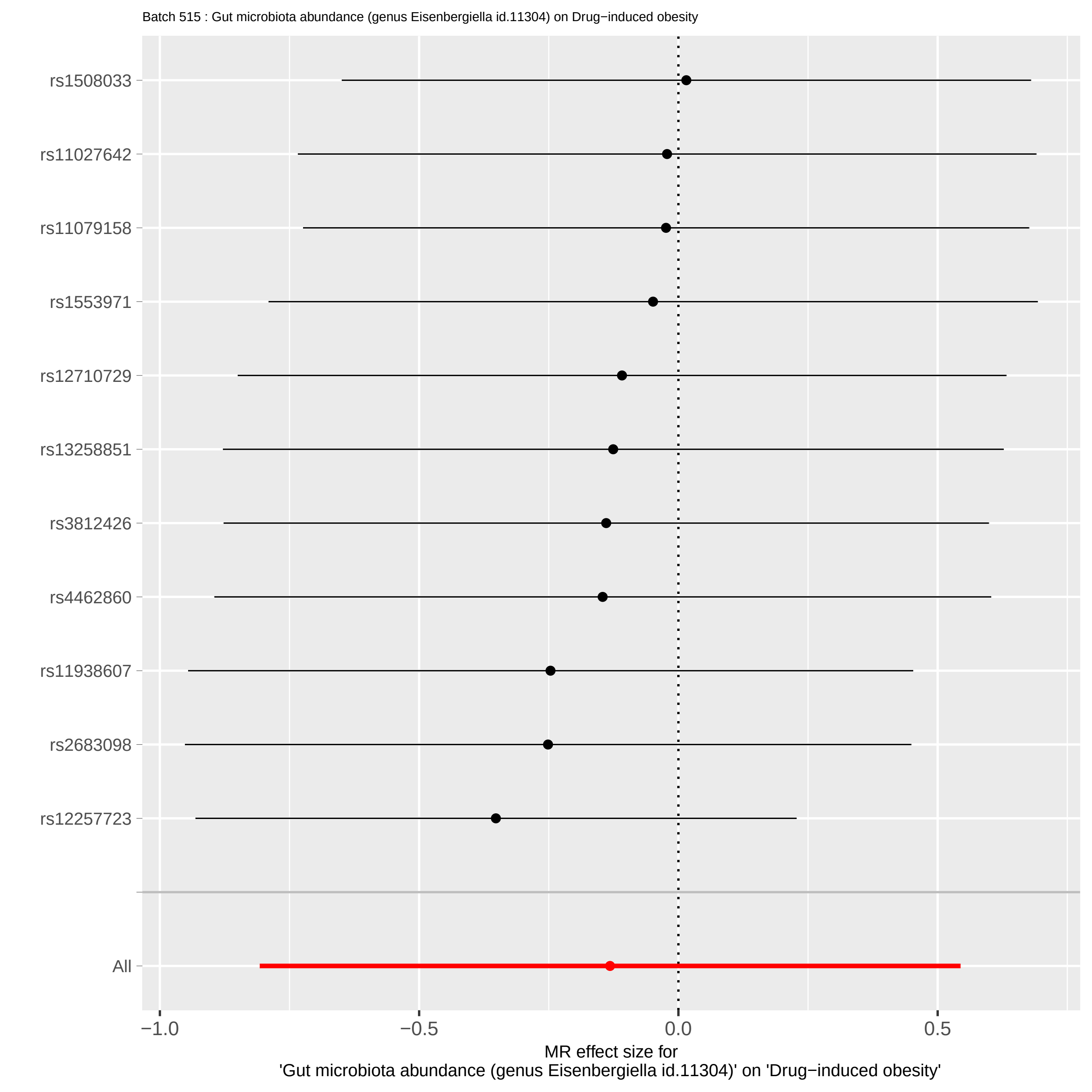
Batch 515 : Gut microbiota abundance (genus Eisenbergiella id.11304) on Drug−induced obesity
rs1508033
rs11027642
rs11079158
rs1553971
rs12710729
rs13258851
rs3812426
rs4462860
rs11938607
rs2683098
rs12257723
All
−1.0
−0.5
0.0
0.5
MR effect size for
'Gut microbiota abundance (genus Eisenbergiella id.11304)' on 'Drug−induced obesity'

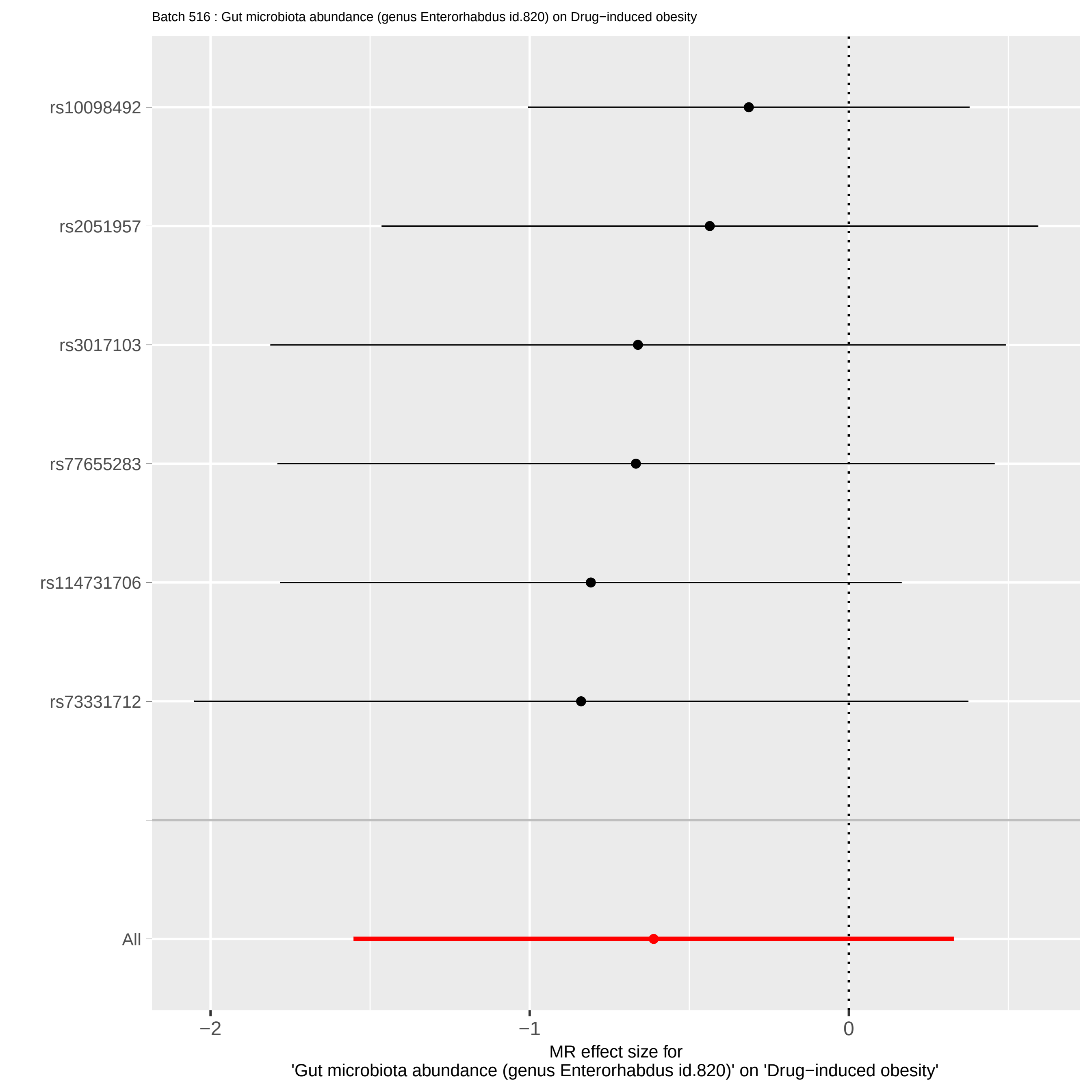
Batch 516 : Gut microbiota abundance (genus Enterorhabdus id.820) on Drug−induced obesity
rs10098492
rs2051957
rs3017103
rs77655283
rs114731706
rs73331712
All
−2
−1
0
MR effect size for
'Gut microbiota abundance (genus Enterorhabdus id.820)' on 'Drug−induced obesity'

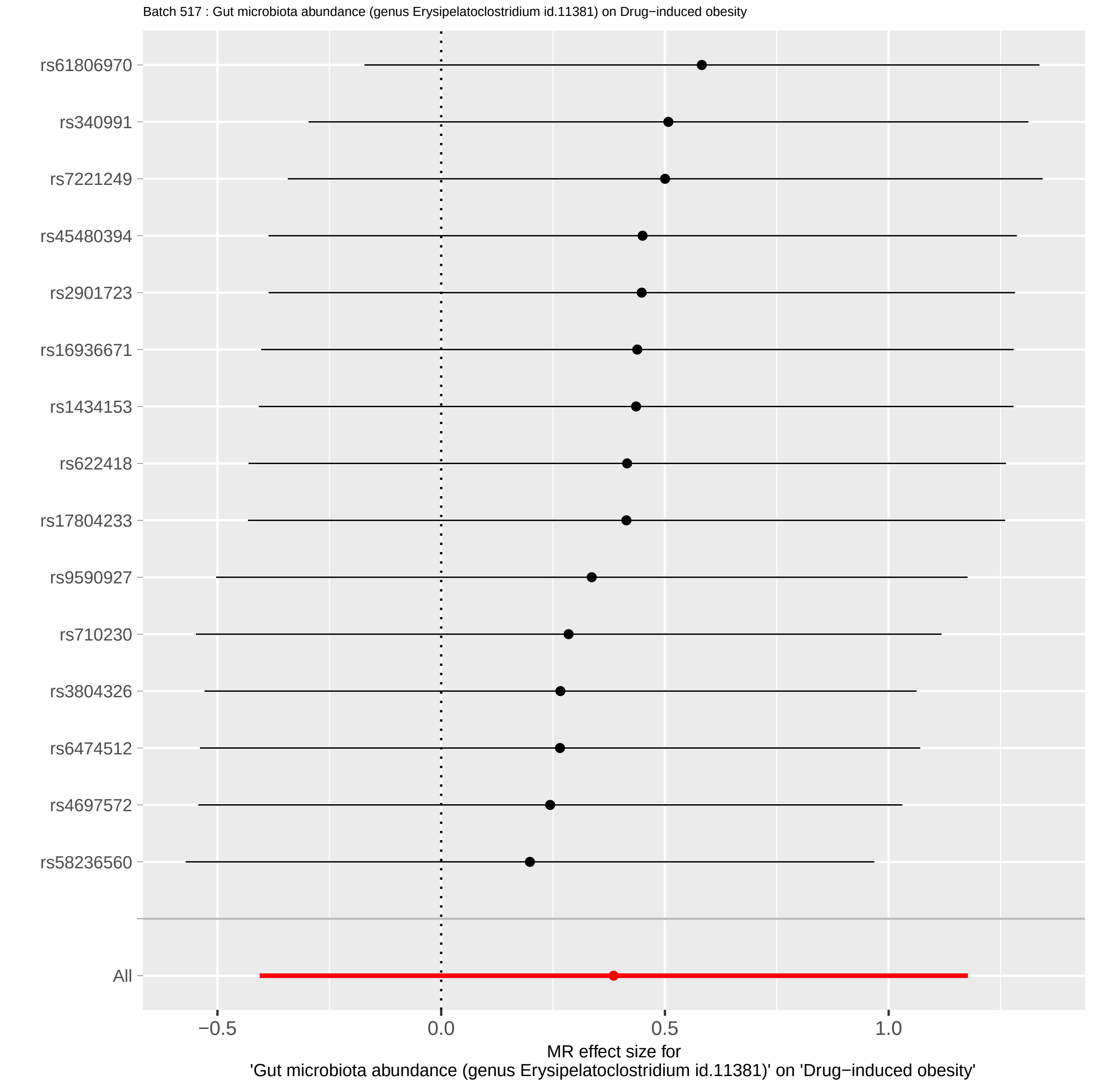

Batch 517 : Gut microbiota abundance (genus Erysipelatoclostridium id.11381) on Drug−induced obesity
rs61806970
rs340991
rs7221249
rs45480394
rs2901723
rs16936671
rs1434153
rs622418
rs17804233
rs9590927
rs710230
rs3804326
rs6474512
rs4697572
rs58236560
All
−0.5
0.0
0.5
1.0
MR effect size for
'Gut microbiota abundance (genus Erysipelatoclostridium id.11381)' on 'Drug−induced obesity'

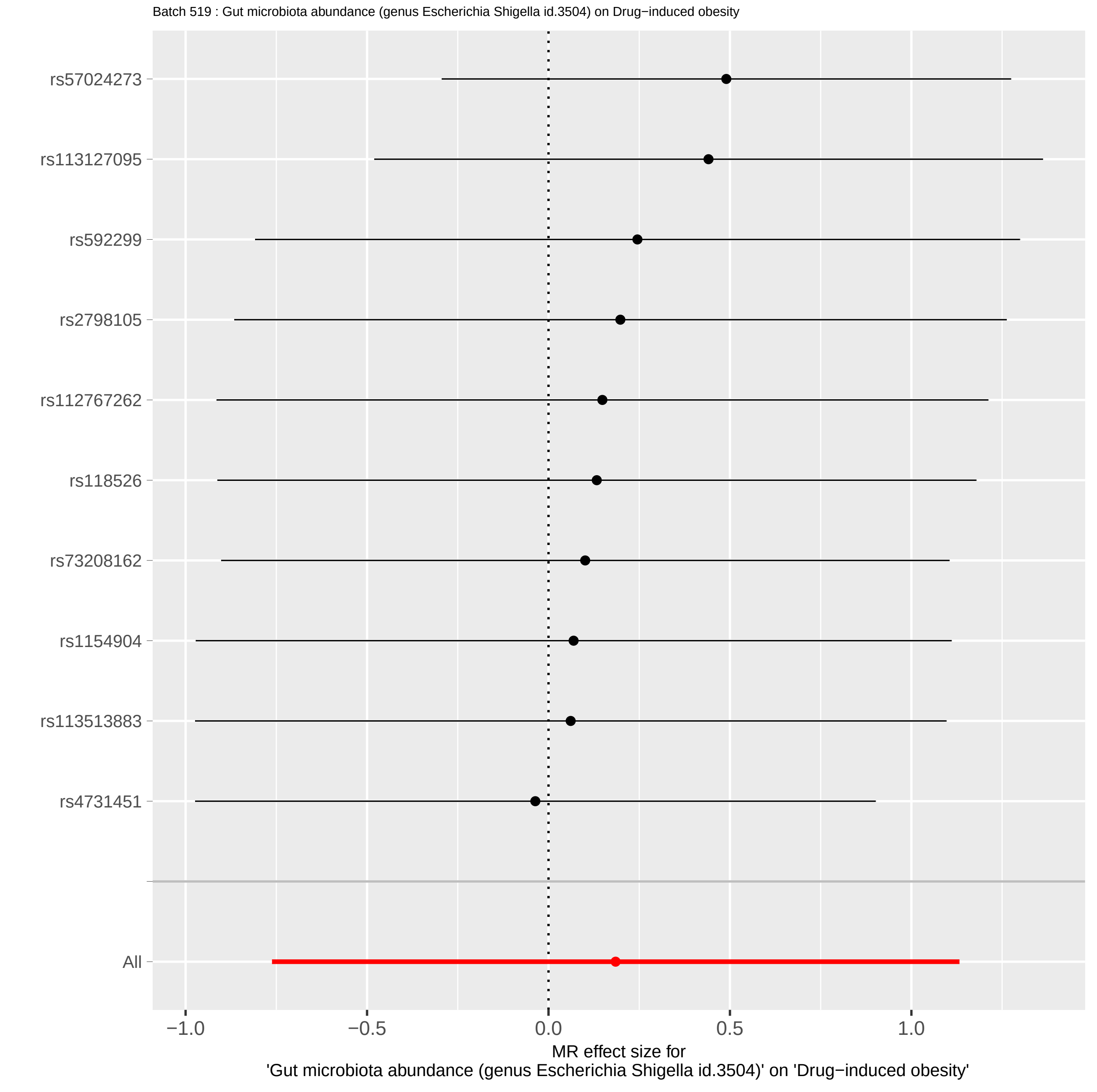

Batch 519 : Gut microbiota abundance (genus Escherichia Shigella id.3504) on Drug−induced obesity
rs57024273
rs113127095
rs592299
rs2798105
rs112767262
rs118526
rs73208162
rs1154904
rs113513883
rs4731451
All
−1.0
−0.5
0.0
0.5
1.0
MR effect size for
'Gut microbiota abundance (genus Escherichia Shigella id.3504)' on 'Drug−induced obesity'

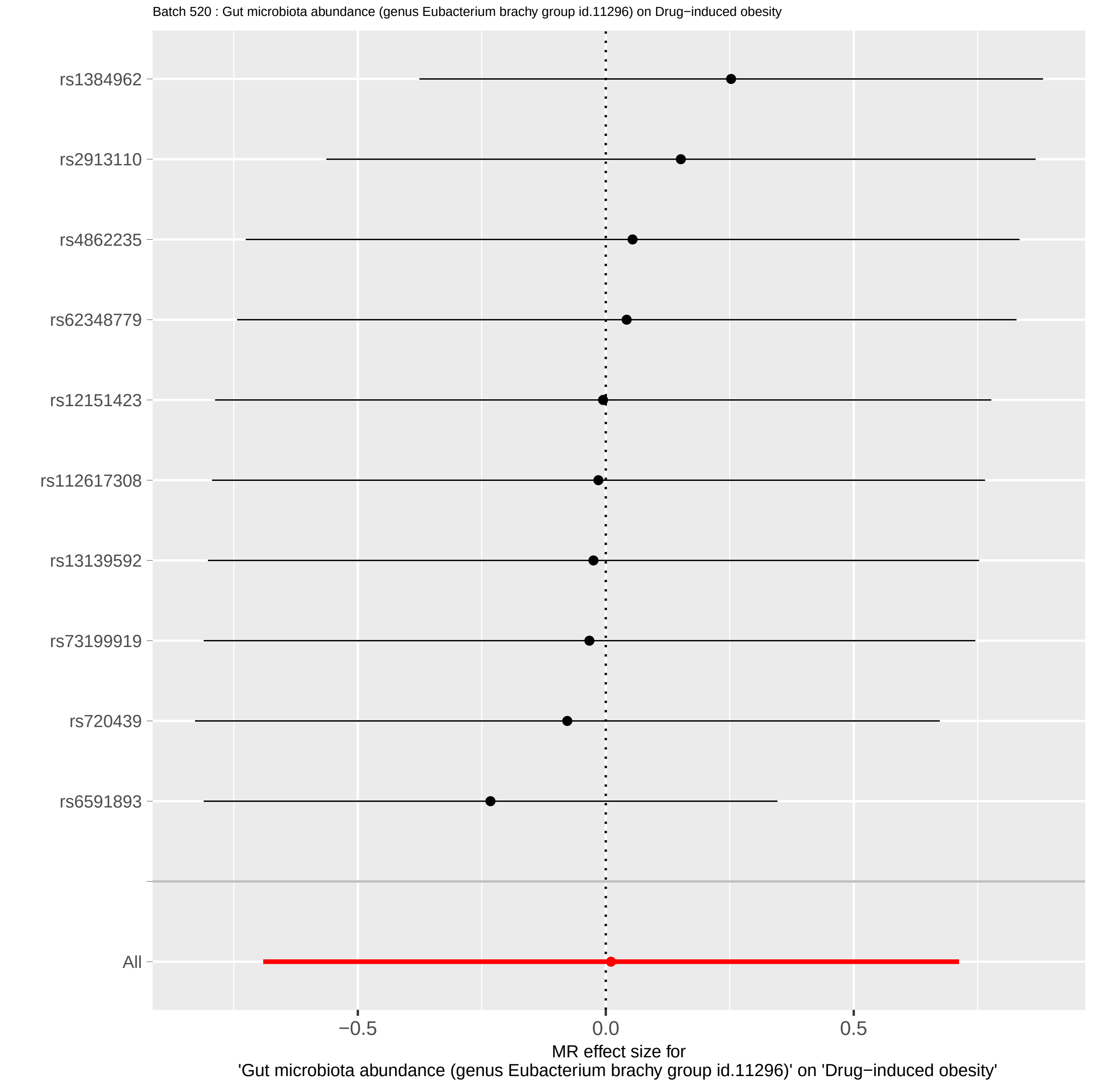
Batch 520 : Gut microbiota abundance (genus Eubacterium brachy group id.11296) on Drug−induced obesity
rs1384962
rs2913110
rs4862235
rs62348779
rs12151423
rs112617308
rs13139592
rs73199919
rs720439
rs6591893
All
−0.5
0.0
0.5
MR effect size for
'Gut microbiota abundance (genus Eubacterium brachy group id.11296)' on 'Drug−induced obesity'

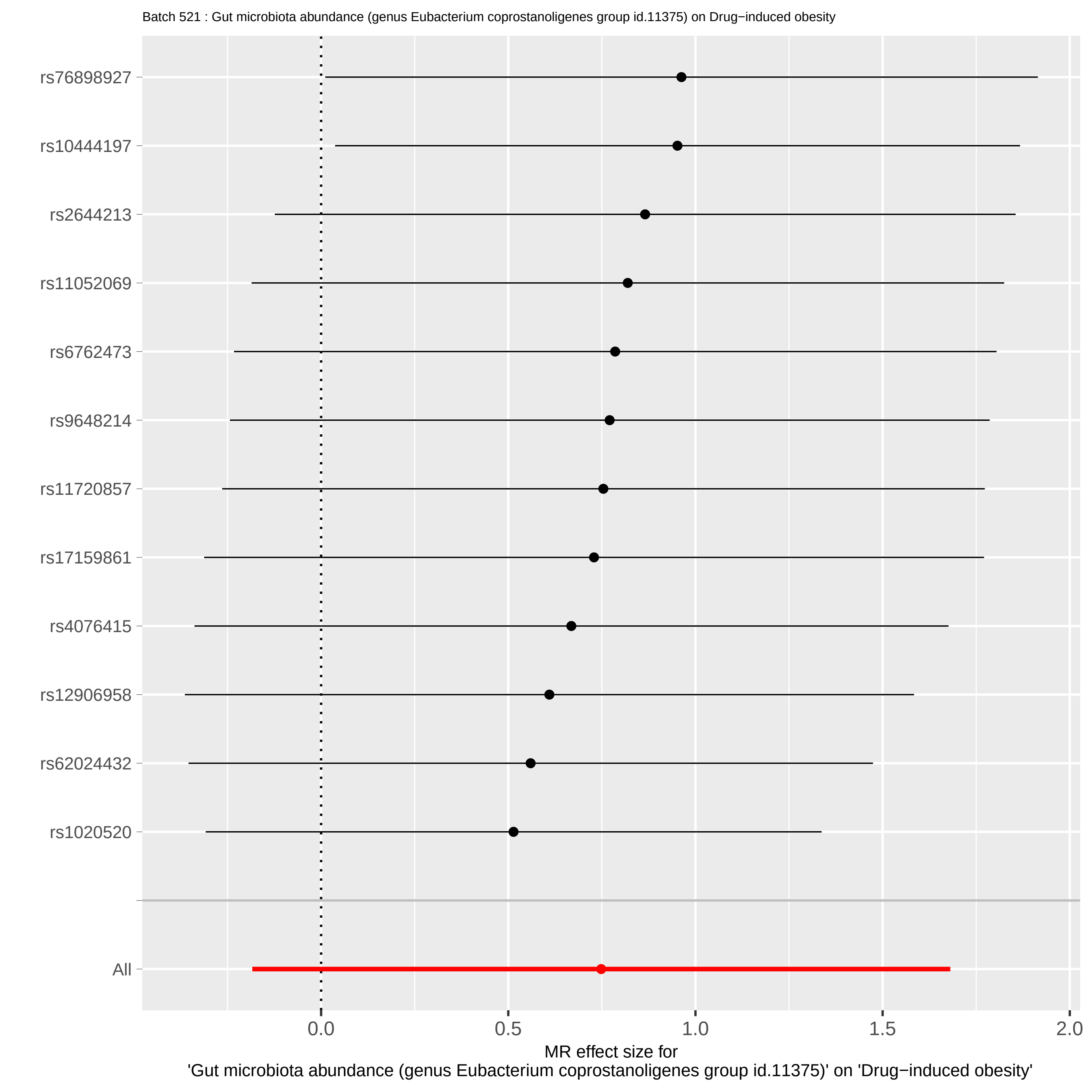

Batch 521 : Gut microbiota abundance (genus Eubacterium coprostanoligenes group id.11375) on Drug−induced obesity
rs76898927
rs10444197
rs2644213
rs11052069
rs6762473
rs9648214
rs11720857
rs17159861
rs4076415
rs12906958
rs62024432
rs1020520
All
0.0
0.5
1.0
1.5
2.0
MR effect size for
'Gut microbiota abundance (genus Eubacterium coprostanoligenes group id.11375)' on 'Drug−induced obesity'

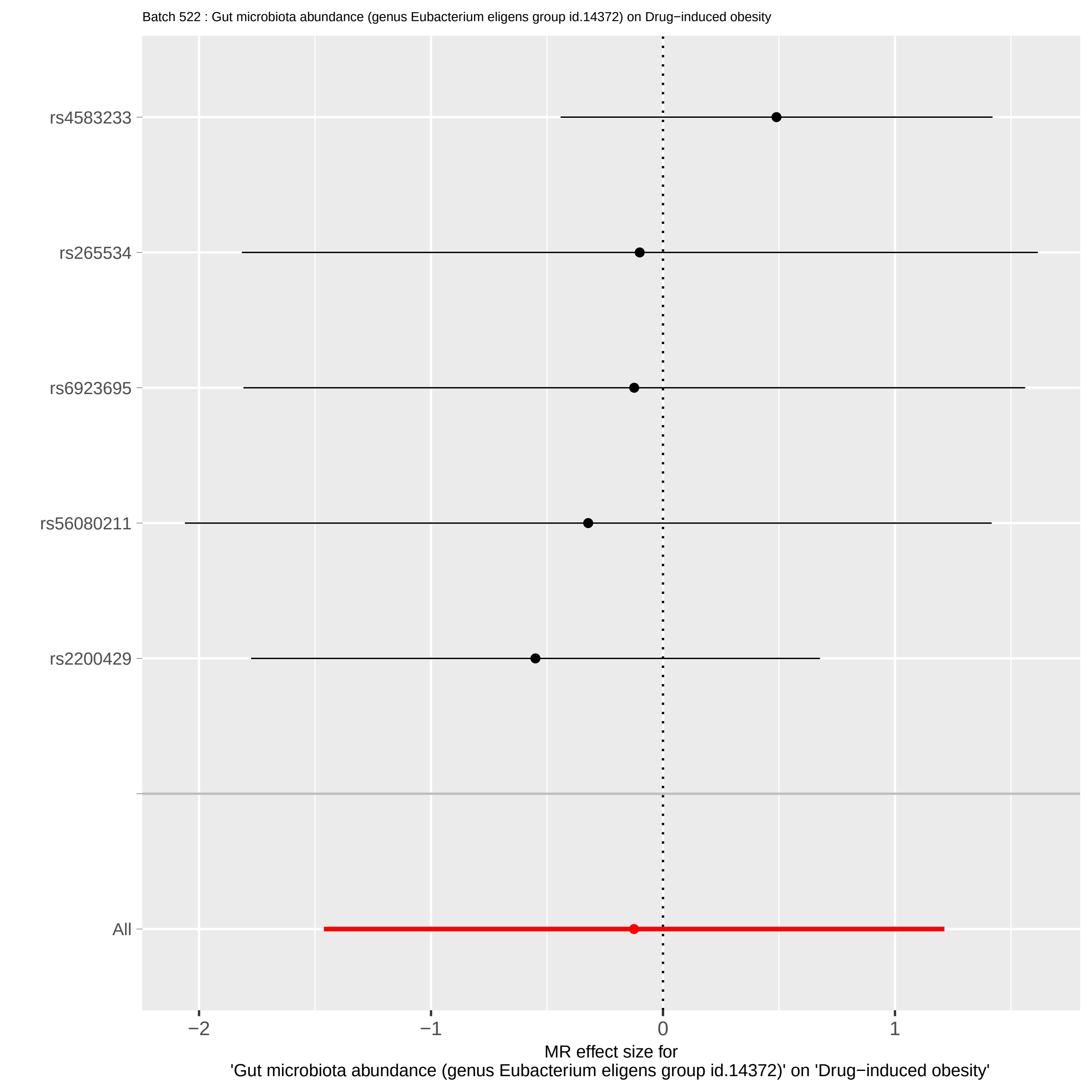

Batch 522 : Gut microbiota abundance (genus Eubacterium eligens group id.14372) on Drug−induced obesity
rs4583233
rs265534
rs6923695
rs56080211
rs2200429
All
−2
−1
0
1
MR effect size for
'Gut microbiota abundance (genus Eubacterium eligens group id.14372)' on 'Drug−induced obesity'

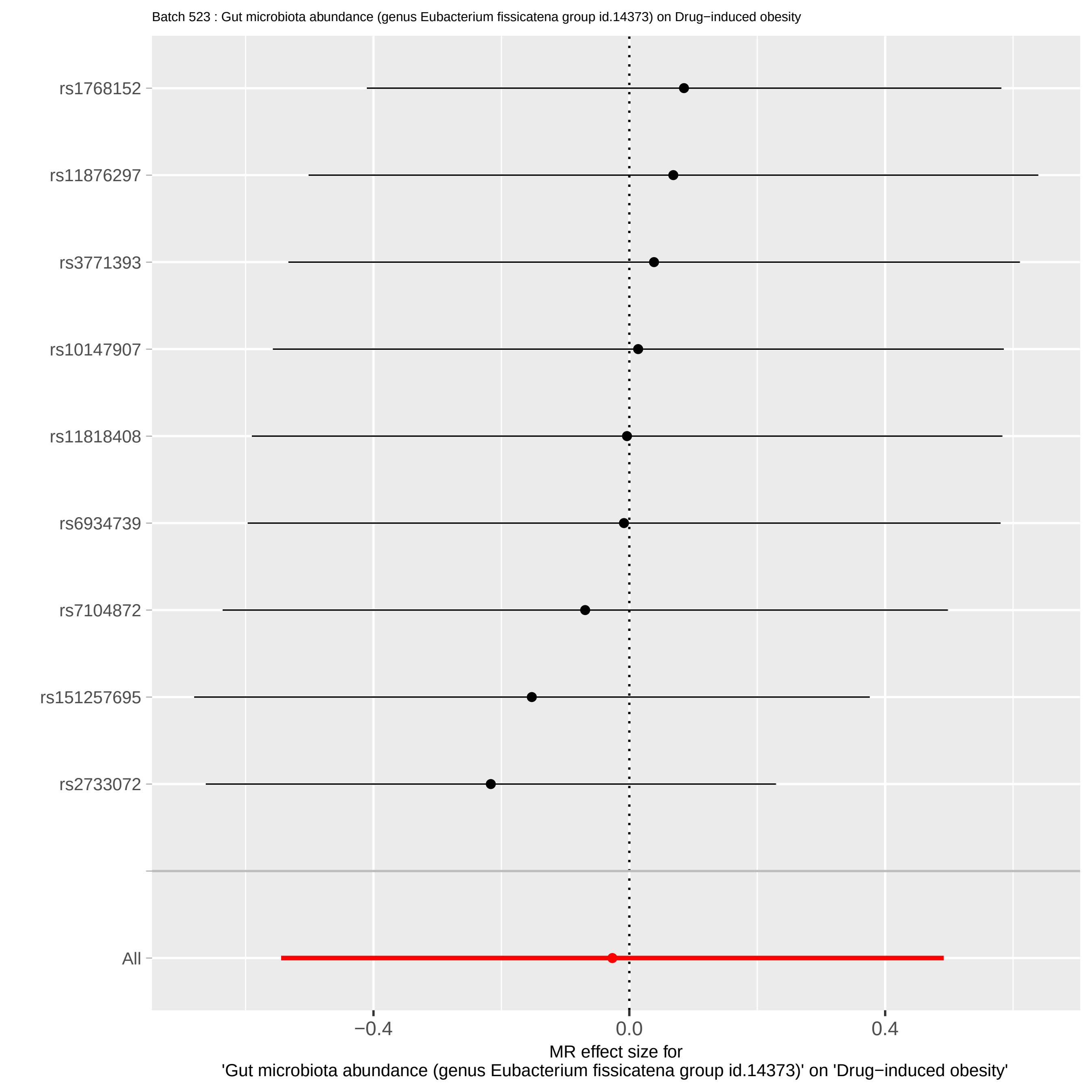

Batch 523 : Gut microbiota abundance (genus Eubacterium fissicatena group id.14373) on Drug−induced obesity
rs1768152
rs11876297
rs3771393
rs10147907
rs11818408
rs6934739
rs7104872
rs151257695
rs2733072
All
−0.4
0.0
0.4
MR effect size for
'Gut microbiota abundance (genus Eubacterium fissicatena group id.14373)' on 'Drug−induced obesity'

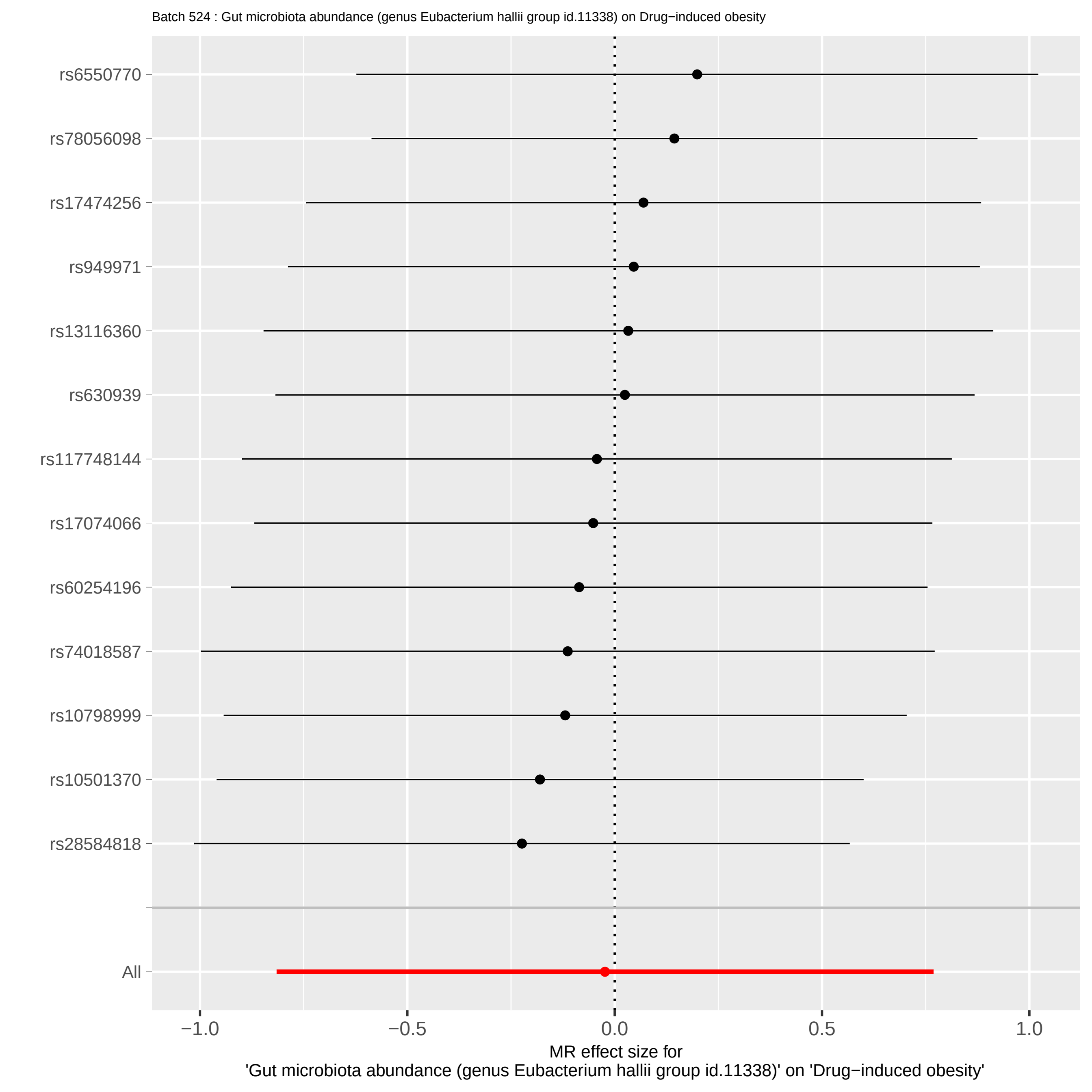

Batch 524 : Gut microbiota abundance (genus Eubacterium hallii group id.11338) on Drug−induced obesity
rs6550770
rs78056098
rs17474256
rs949971
rs13116360
rs630939
rs117748144
rs17074066
rs60254196
rs74018587
rs10798999
rs10501370
rs28584818
All
−1.0
−0.5
0.0
0.5
1.0
MR effect size for
'Gut microbiota abundance (genus Eubacterium hallii group id.11338)' on 'Drug−induced obesity'

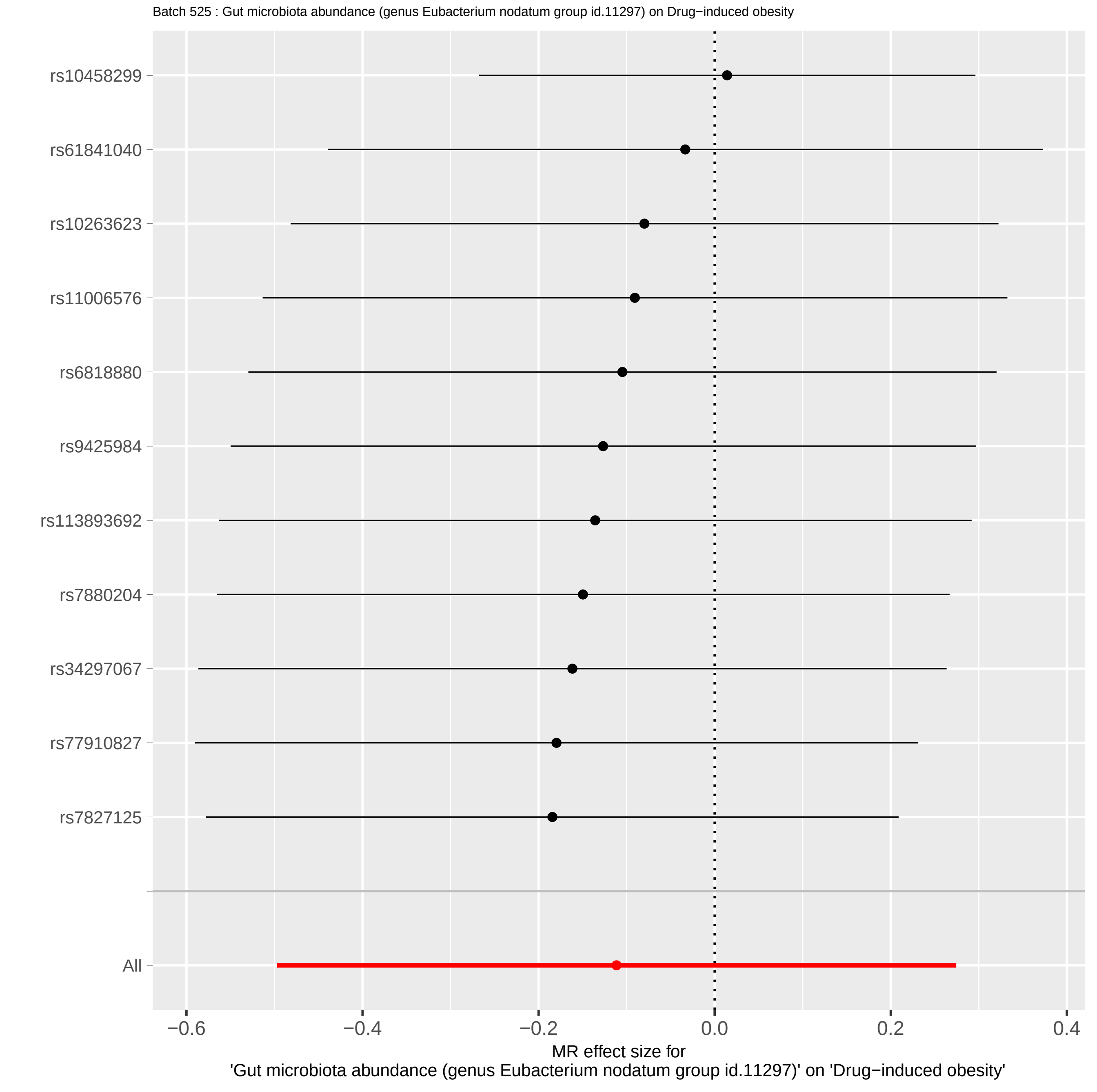
Batch 525 : Gut microbiota abundance (genus Eubacterium nodatum group id.11297) on Drug−induced obesity
rs10458299
rs61841040
rs10263623
rs11006576
rs6818880
rs9425984
rs113893692
rs7880204
rs34297067
rs77910827
rs7827125
All
−0.6
−0.4
−0.2
0.0
0.2
0.4
MR effect size for
'Gut microbiota abundance (genus Eubacterium nodatum group id.11297)' on 'Drug−induced obesity'

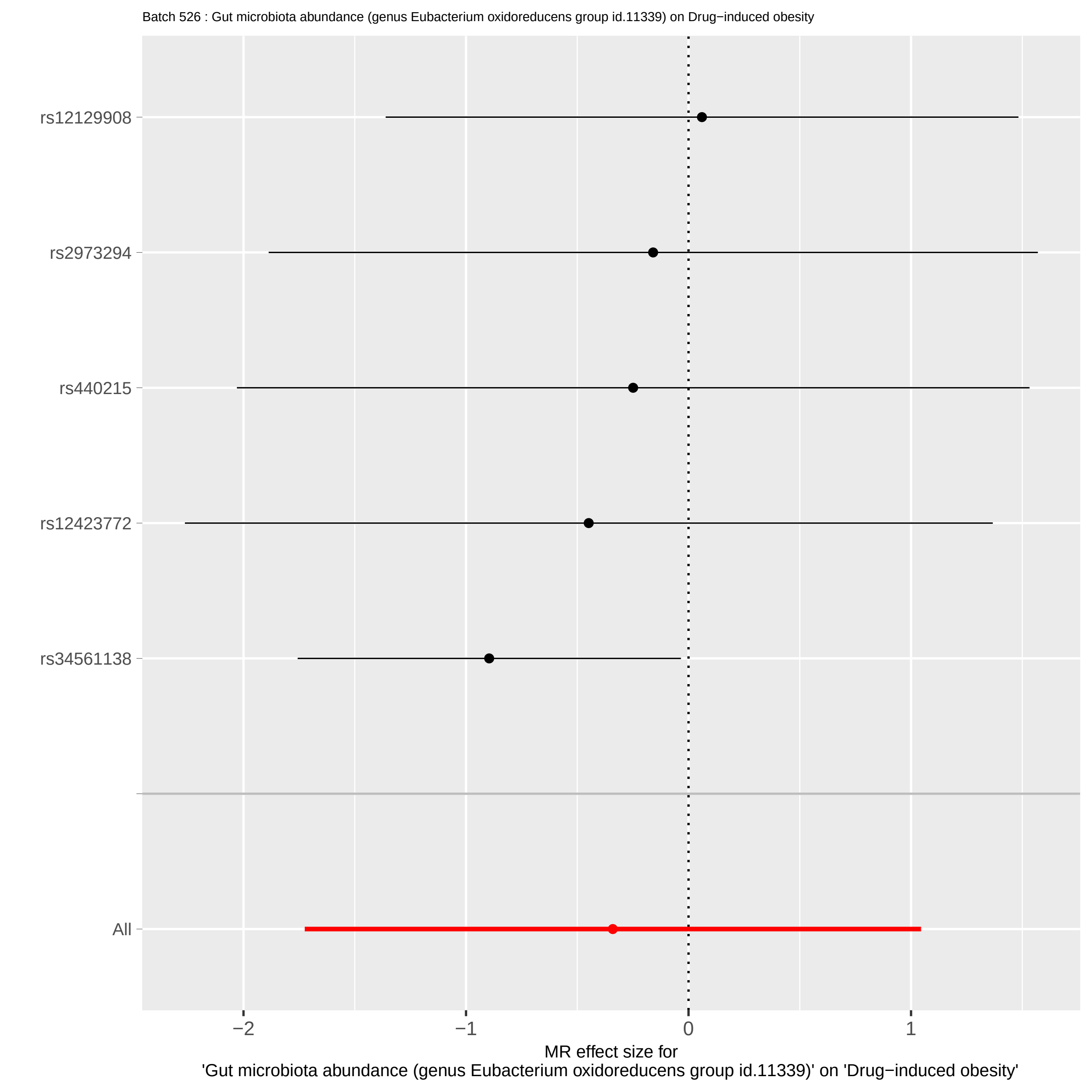
Batch 526 : Gut microbiota abundance (genus Eubacterium oxidoreducens group id.11339) on Drug−induced obesity
rs12129908
rs2973294
rs440215
rs12423772
rs34561138
All
−2
−1
0
1
MR effect size for
'Gut microbiota abundance (genus Eubacterium oxidoreducens group id.11339)' on 'Drug−induced obesity'

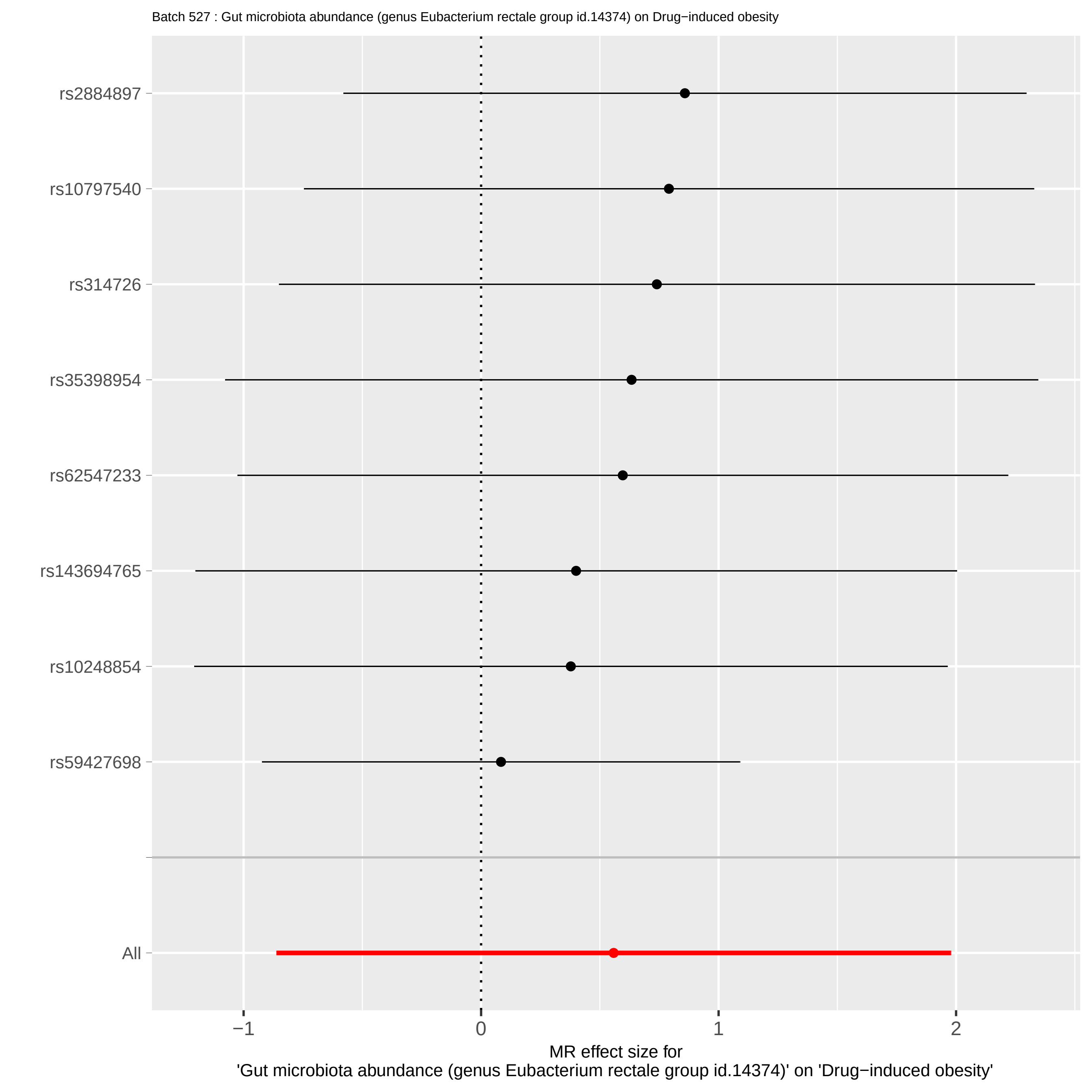
Batch 527 : Gut microbiota abundance (genus Eubacterium rectale group id.14374) on Drug−induced obesity
rs2884897
rs10797540
rs314726
rs35398954
rs62547233
rs143694765
rs10248854
rs59427698
All
−1
0
1
2
MR effect size for
'Gut microbiota abundance (genus Eubacterium rectale group id.14374)' on 'Drug−induced obesity'

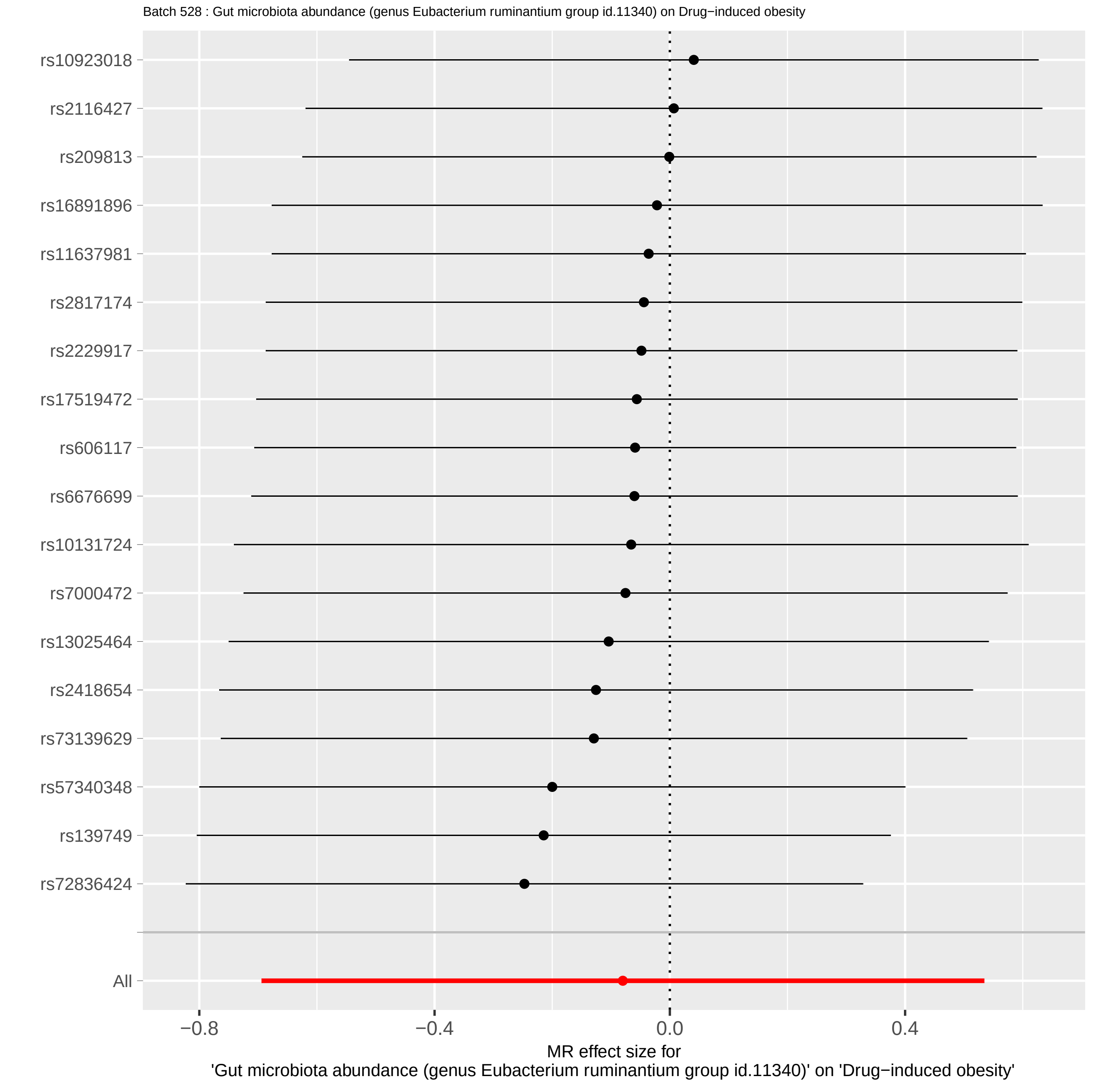

Batch 528 : Gut microbiota abundance (genus Eubacterium ruminantium group id.11340) on Drug−induced obesity
rs10923018
rs2116427
rs209813
rs16891896
rs11637981
rs2817174
rs2229917
rs17519472
rs606117
rs6676699
rs10131724
rs7000472
rs13025464
rs2418654
rs73139629
rs57340348
rs139749
rs72836424
All
−0.8
−0.4
0.0
0.4
MR effect size for
'Gut microbiota abundance (genus Eubacterium ruminantium group id.11340)' on 'Drug−induced obesity'

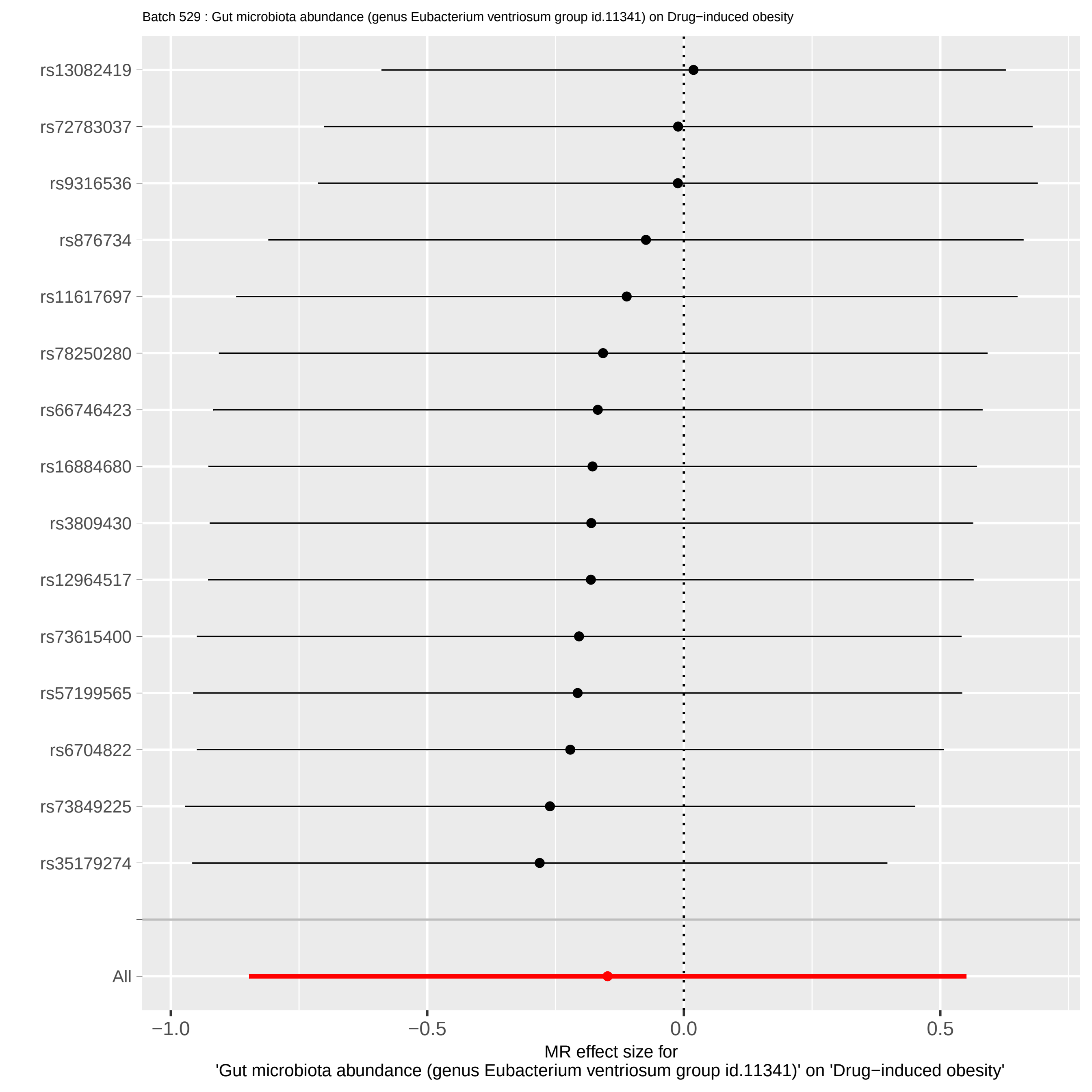
Batch 529 : Gut microbiota abundance (genus Eubacterium ventriosum group id.11341) on Drug−induced obesity
rs13082419
rs72783037
rs9316536
rs876734
rs11617697
rs78250280
rs66746423
rs16884680
rs3809430
rs12964517
rs73615400
rs57199565
rs6704822
rs73849225
rs35179274
All
−1.0
−0.5
0.0
0.5
MR effect size for
'Gut microbiota abundance (genus Eubacterium ventriosum group id.11341)' on 'Drug−induced obesity'

Batch 530 : Gut microbiota abundance (genus Eubacterium xylanophilum group id.14375) on Drug−induced obesity

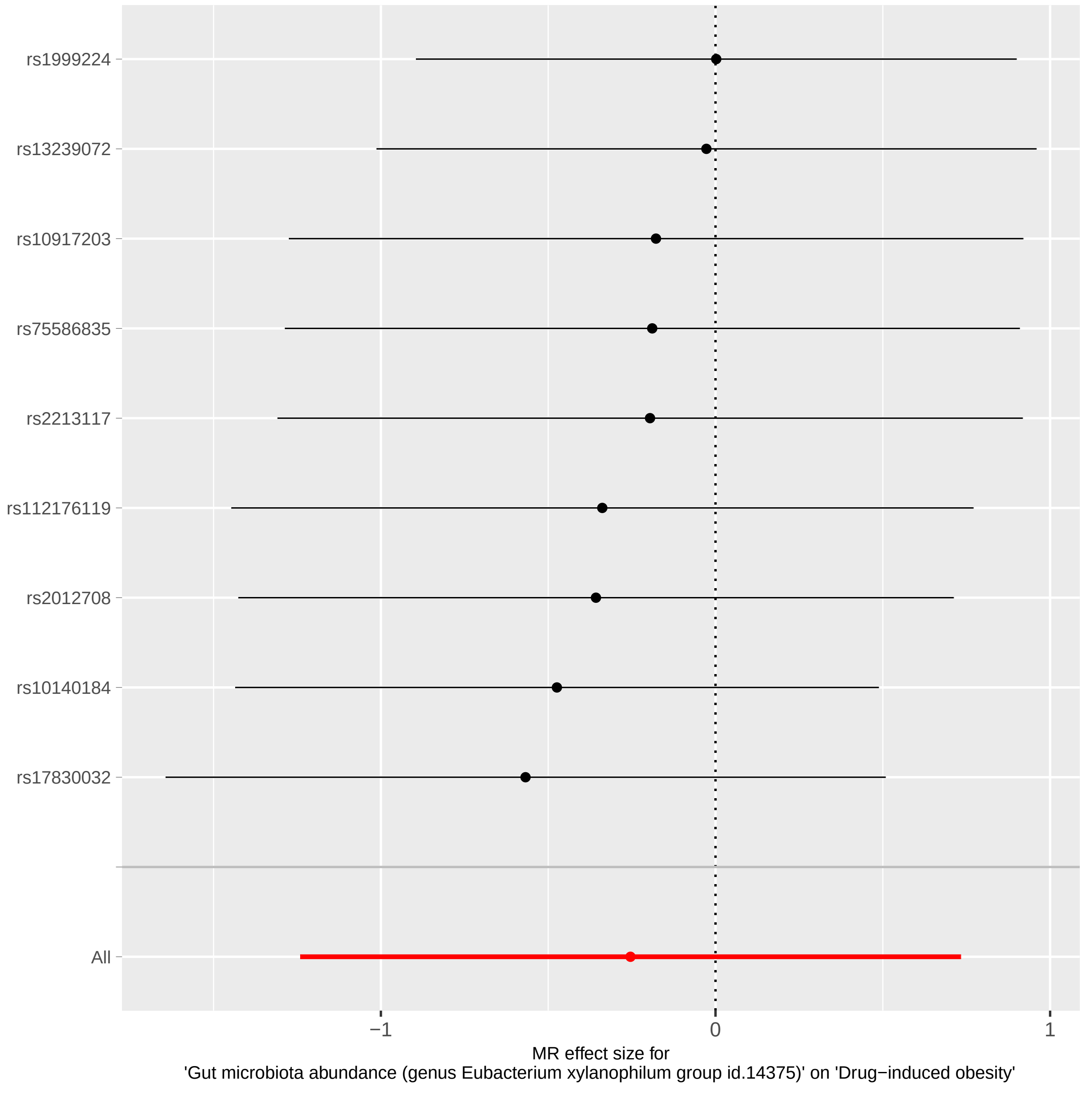

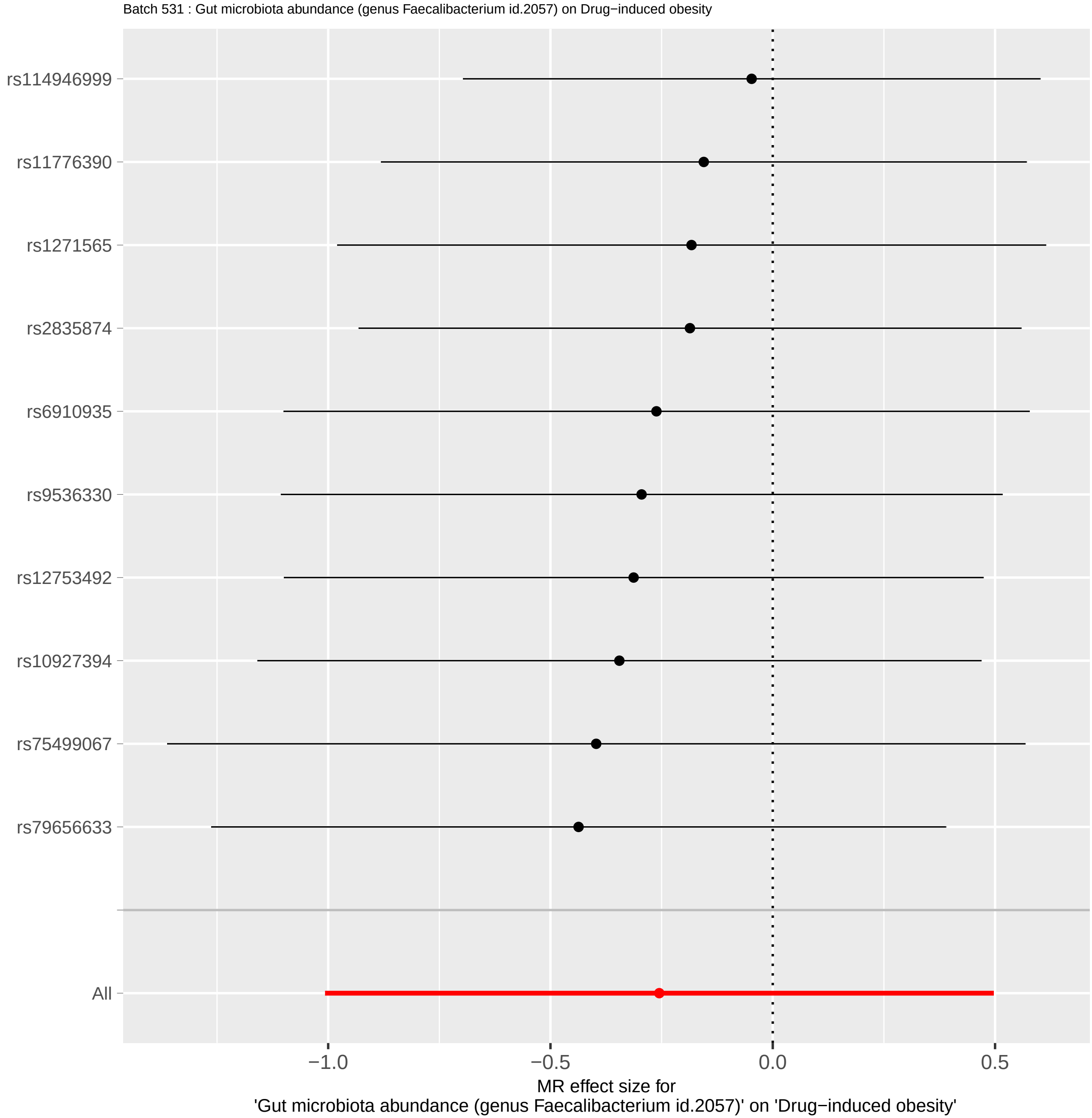
Batch 531 : Gut microbiota abundance (genus Faecalibacterium id.2057) on Drug−induced obesity
rs114946999
rs11776390
rs1271565
rs2835874
rs6910935
rs9536330
rs12753492
rs10927394
rs75499067
rs79656633
All
−1.0
−0.5
0.0
0.5
MR effect size for
'Gut microbiota abundance (genus Faecalibacterium id.2057)' on 'Drug−induced obesity'

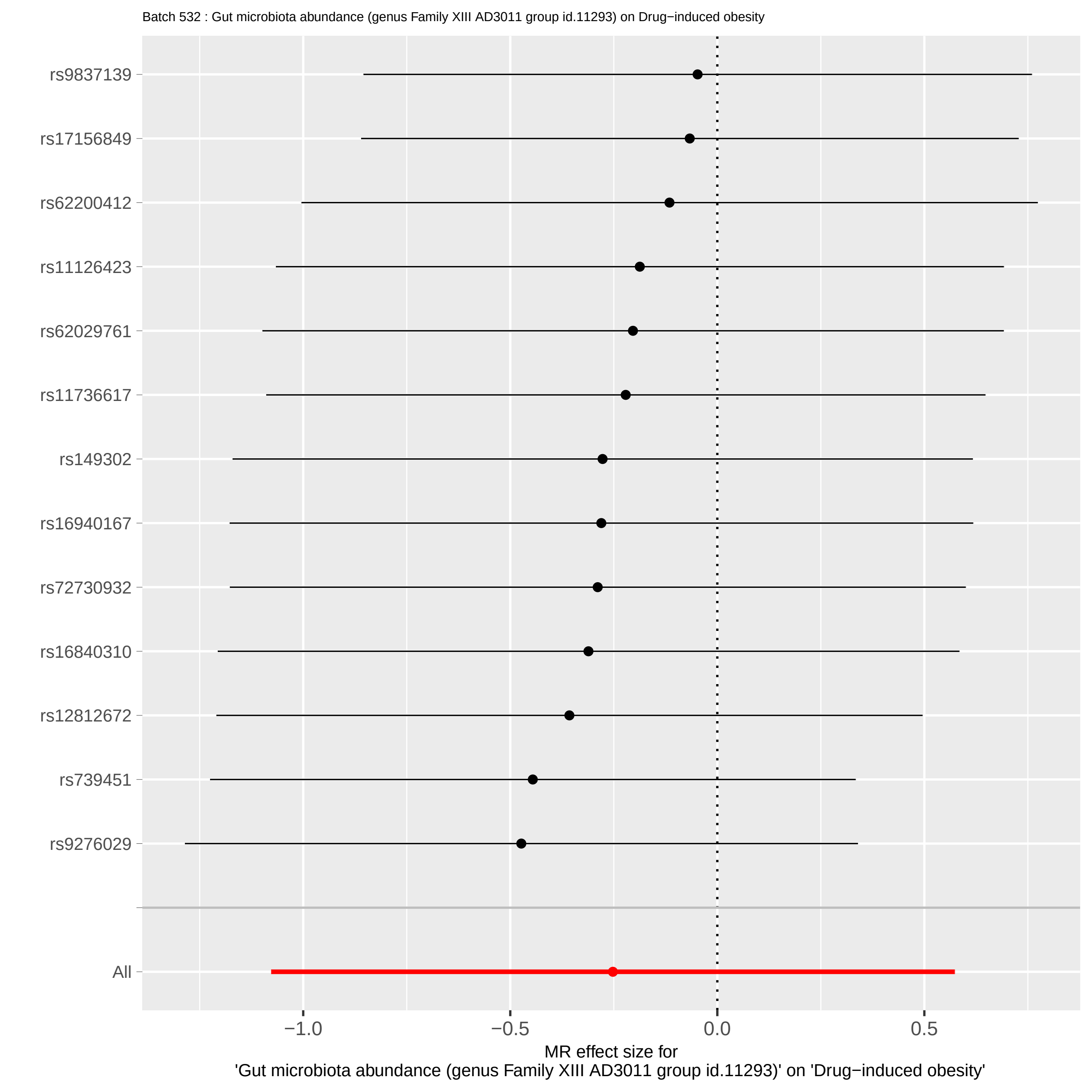

Batch 532 : Gut microbiota abundance (genus Family XIII AD3011 group id.11293) on Drug−induced obesity
rs9837139
rs17156849
rs62200412
rs11126423
rs62029761
rs11736617
rs149302
rs16940167
rs72730932
rs16840310
rs12812672
rs739451
rs9276029
All
−1.0
−0.5
0.0
0.5
MR effect size for
'Gut microbiota abundance (genus Family XIII AD3011 group id.11293)' on 'Drug−induced obesity'

Batch 533 : Gut microbiota abundance (genus Family XIII UCG001 id.11294) on Drug−induced obesity

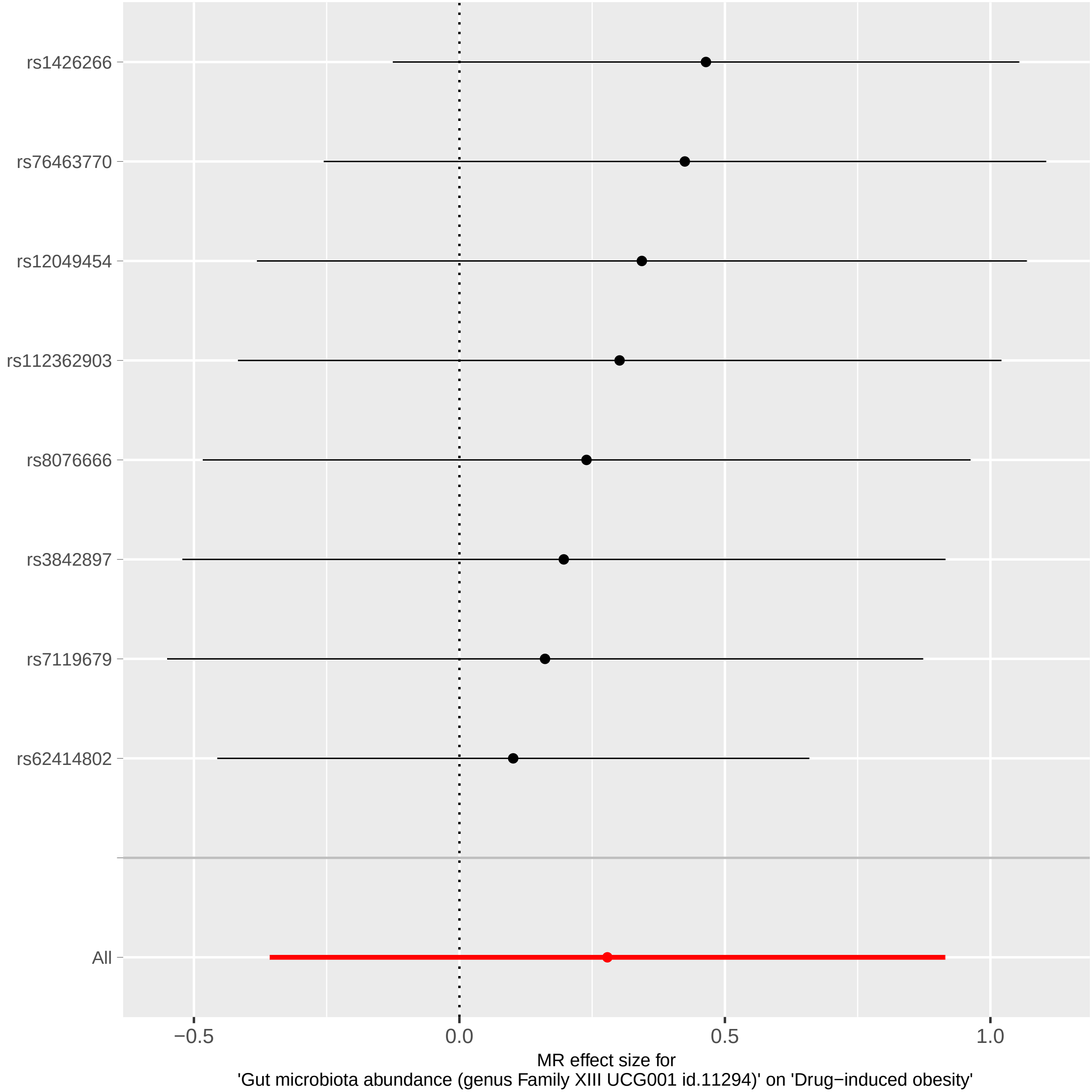

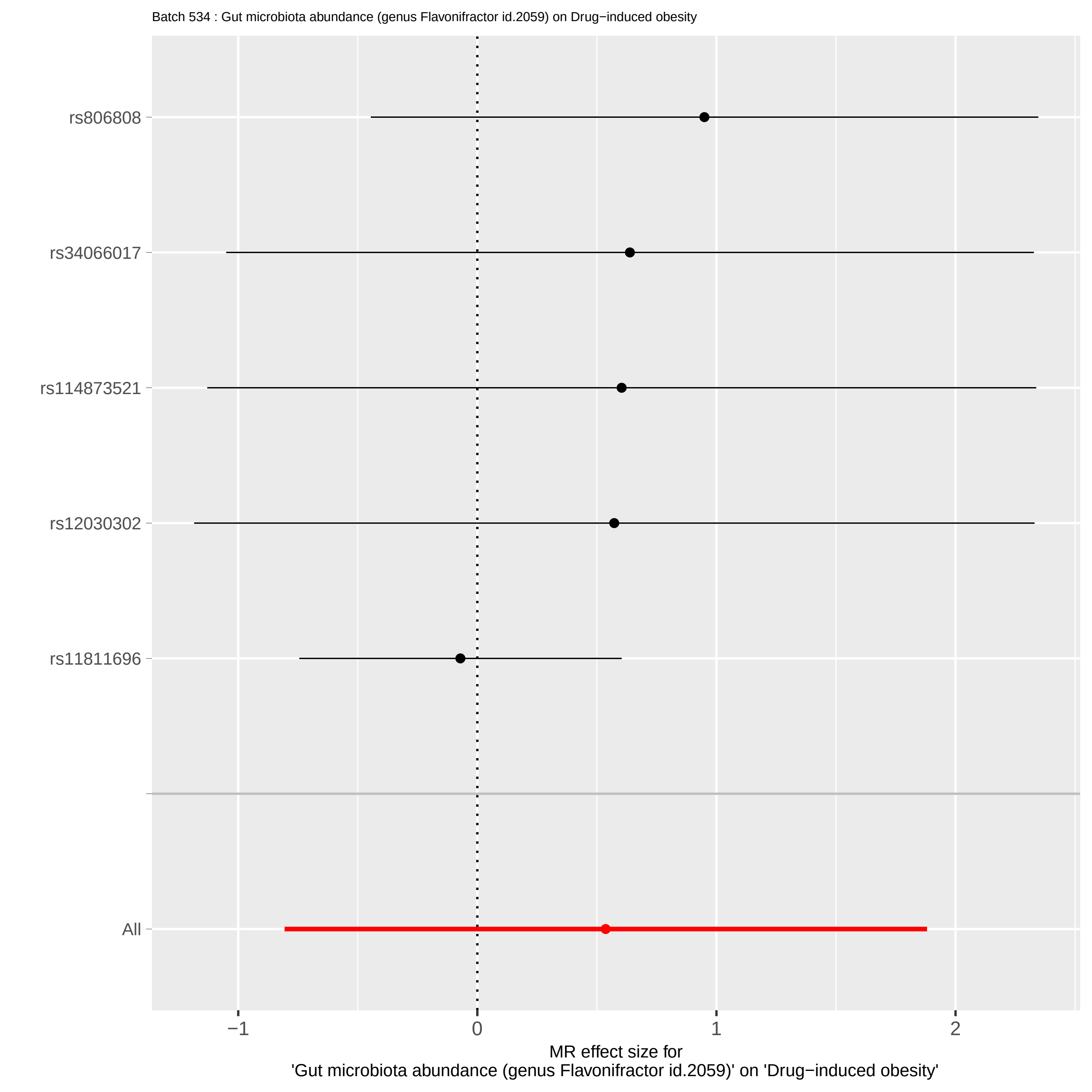

Batch 534 : Gut microbiota abundance (genus Flavonifractor id.2059) on Drug−induced obesity
rs806808
rs34066017
rs114873521
rs12030302
rs11811696
All
−1
0
1
2
MR effect size for
'Gut microbiota abundance (genus Flavonifractor id.2059)' on 'Drug−induced obesity'

Batch 535 : Gut microbiota abundance (genus Fusicatenibacter id.11305) on Drug−induced obesity

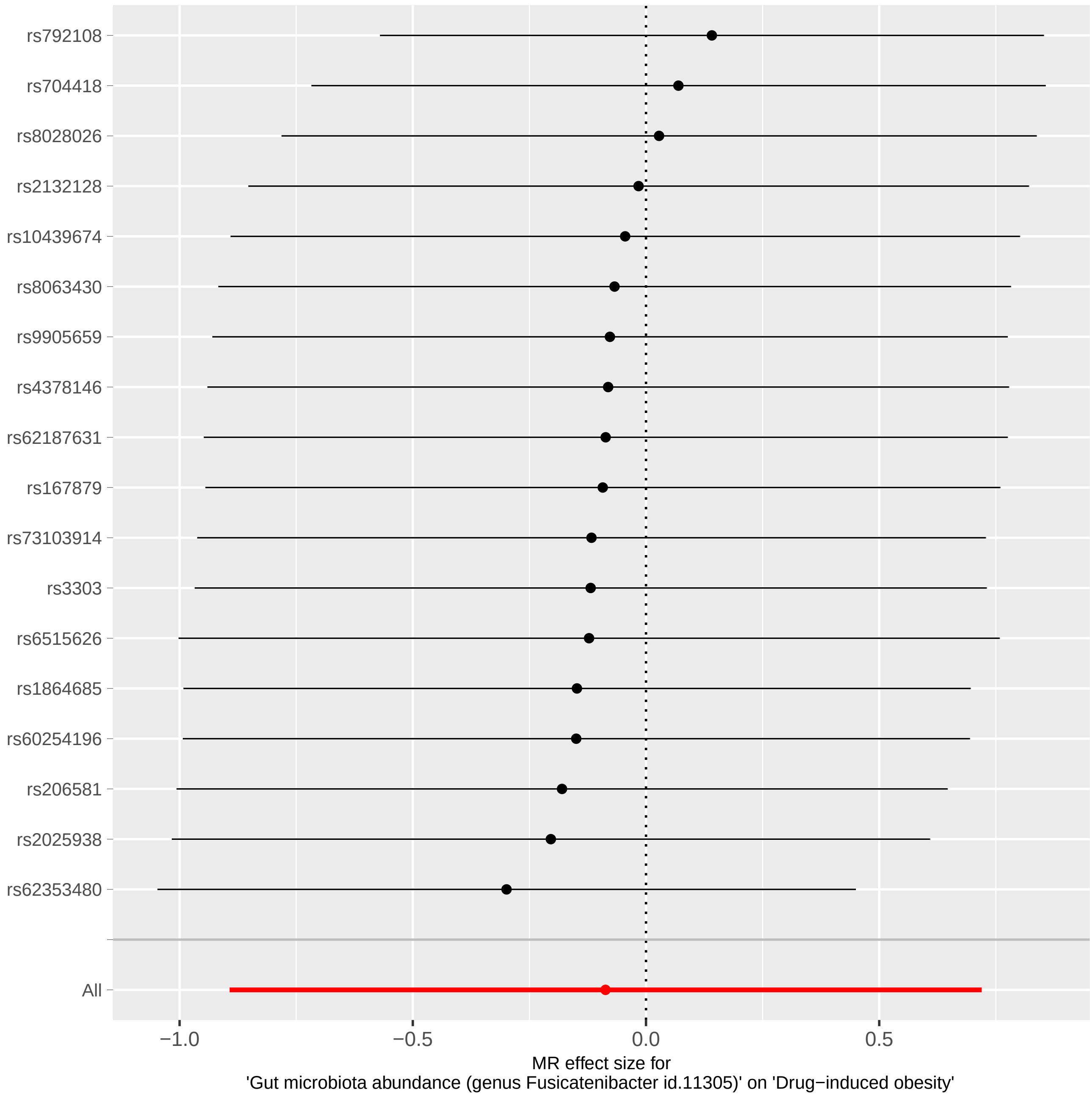

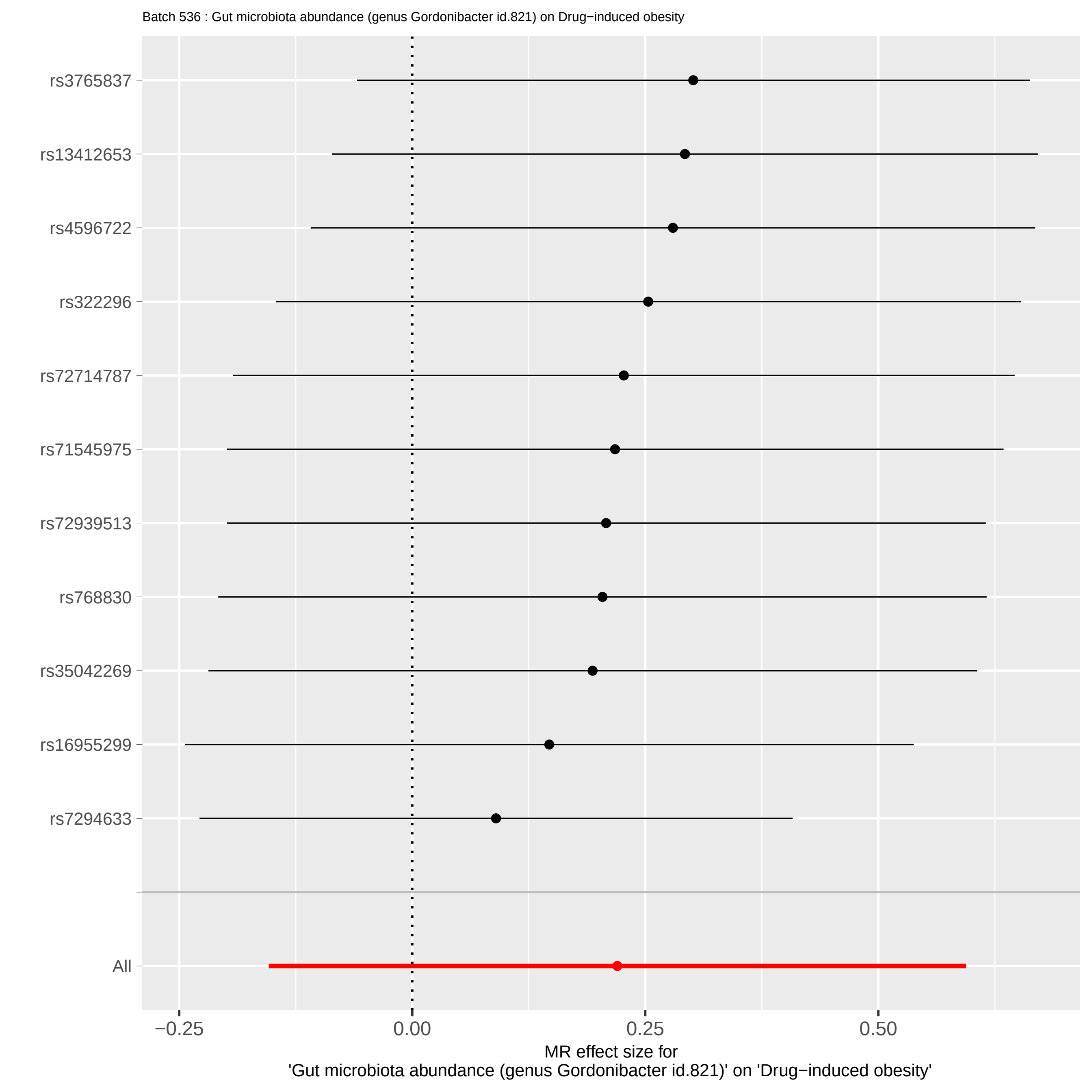

Batch 536 : Gut microbiota abundance (genus Gordonibacter id.821) on Drug−induced obesity
rs3765837
rs13412653
rs4596722
rs322296
rs72714787
rs71545975
rs72939513
rs768830
rs35042269
rs16955299
rs7294633
All
−0.25
0.00
0.25
0.50
MR effect size for
'Gut microbiota abundance (genus Gordonibacter id.821)' on 'Drug−induced obesity'

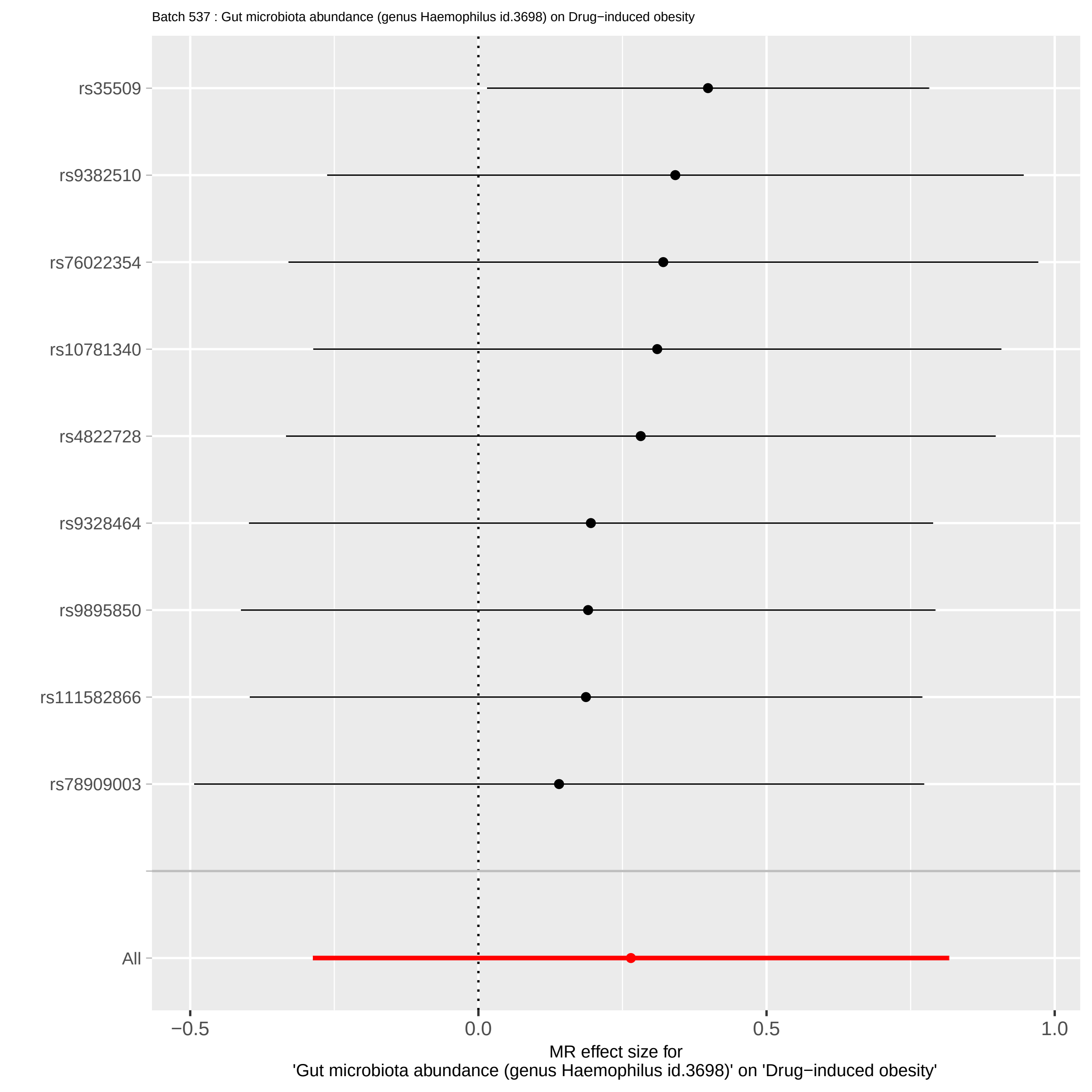

Batch 537 : Gut microbiota abundance (genus Haemophilus id.3698) on Drug−induced obesity
rs35509
rs9382510
rs76022354
rs10781340
rs4822728
rs9328464
rs9895850
rs111582866
rs78909003
All
−0.5
0.0
0.5
1.0
MR effect size for
'Gut microbiota abundance (genus Haemophilus id.3698)' on 'Drug−induced obesity'

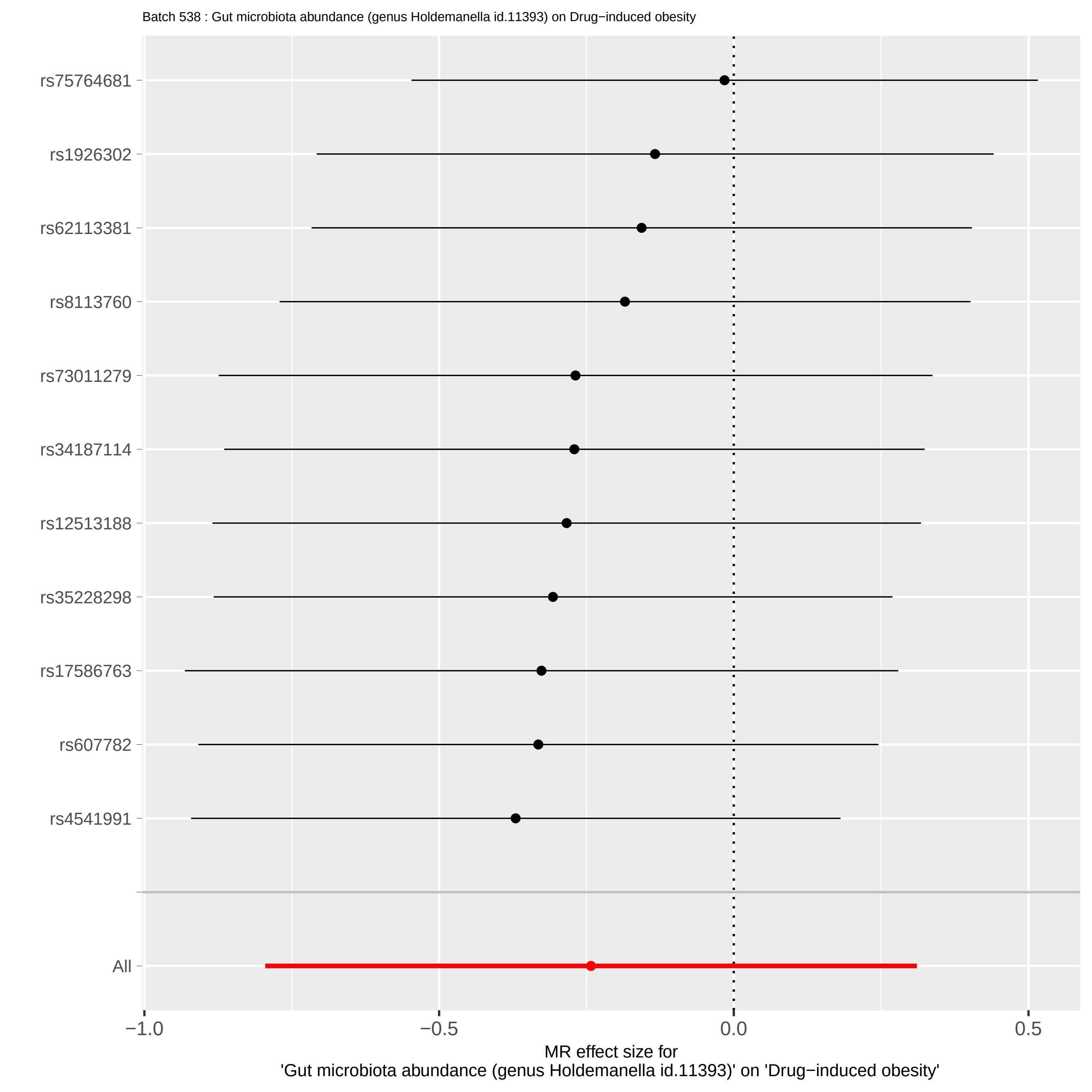

Batch 538 : Gut microbiota abundance (genus Holdemanella id.11393) on Drug−induced obesity
rs75764681
rs1926302
rs62113381
rs8113760
rs73011279
rs34187114
rs12513188
rs35228298
rs17586763
rs607782
rs4541991
All
−1.0
−0.5
0.0
0.5
MR effect size for
'Gut microbiota abundance (genus Holdemanella id.11393)' on 'Drug−induced obesity'

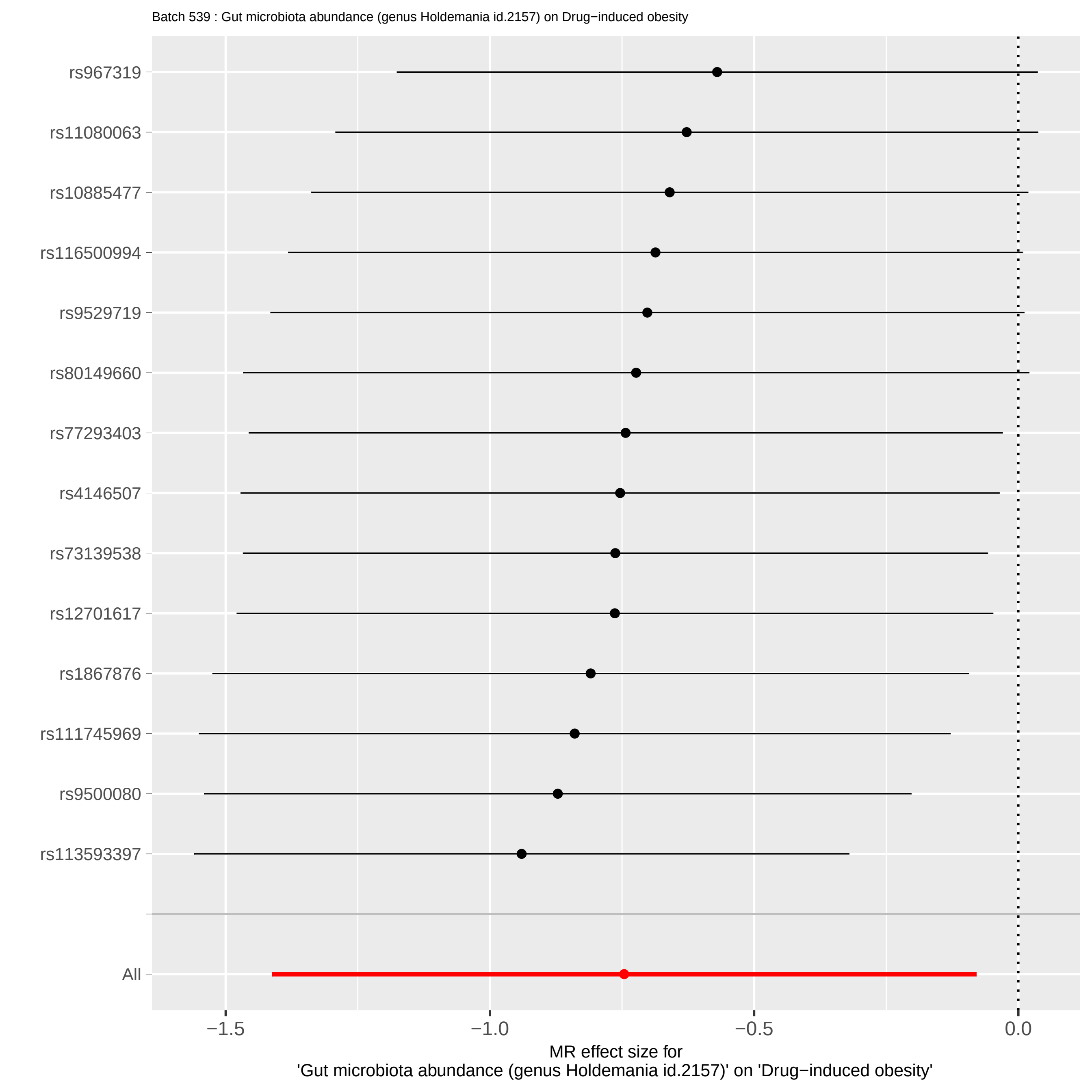

Batch 539 : Gut microbiota abundance (genus Holdemania id.2157) on Drug−induced obesity
rs967319
rs11080063
rs10885477
rs116500994
rs9529719
rs80149660
rs77293403
rs4146507
rs73139538
rs12701617
rs1867876
rs111745969
rs9500080
rs113593397
All
−1.5
−1.0
−0.5
0.0
MR effect size for
'Gut microbiota abundance (genus Holdemania id.2157)' on 'Drug−induced obesity'

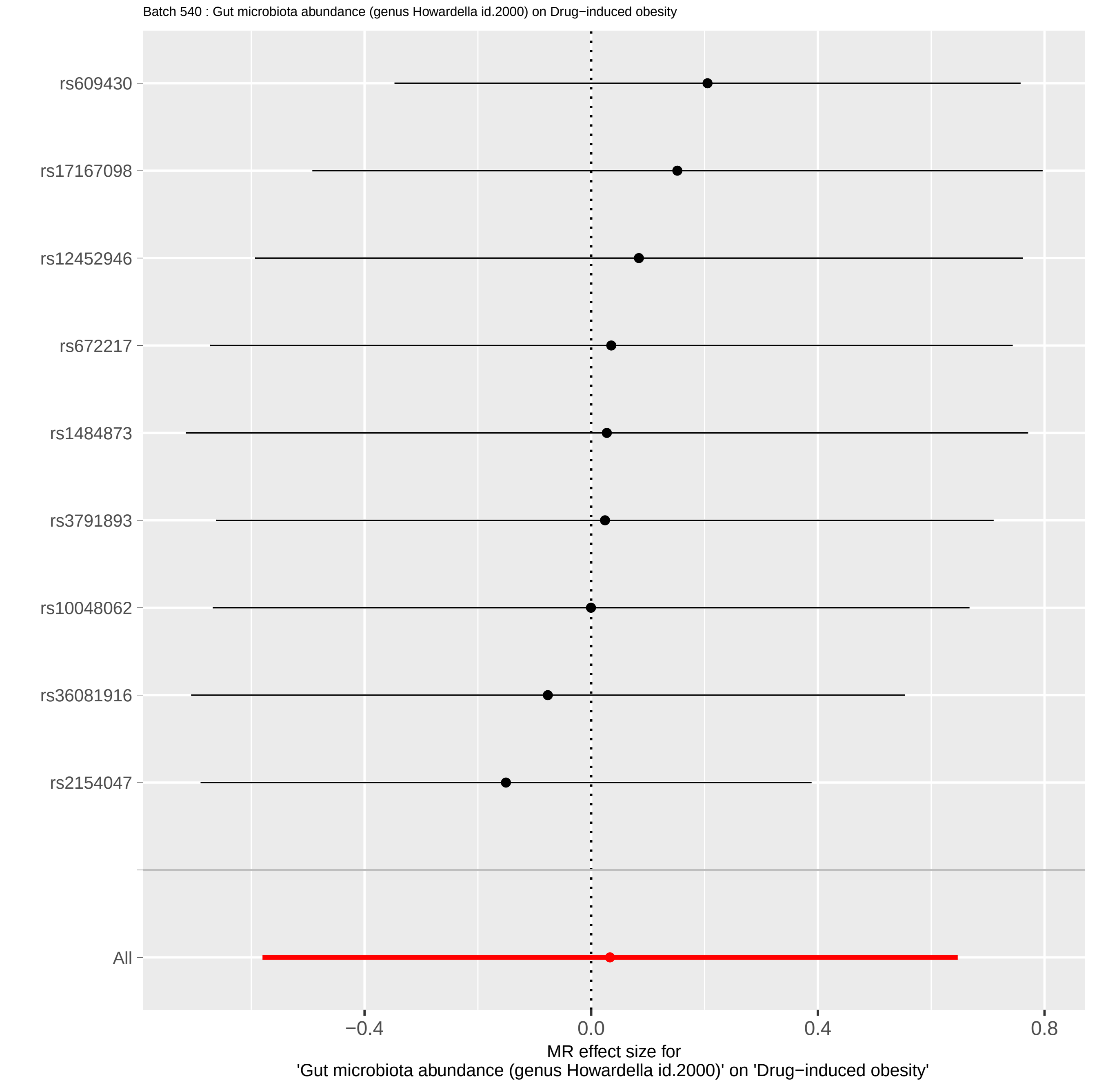
Batch 540 : Gut microbiota abundance (genus Howardella id.2000) on Drug−induced obesity
rs609430
rs17167098
rs12452946
rs672217
rs1484873
rs3791893
rs10048062
rs36081916
rs2154047
All
−0.4
0.0
0.4
0.8
MR effect size for
'Gut microbiota abundance (genus Howardella id.2000)' on 'Drug−induced obesity'

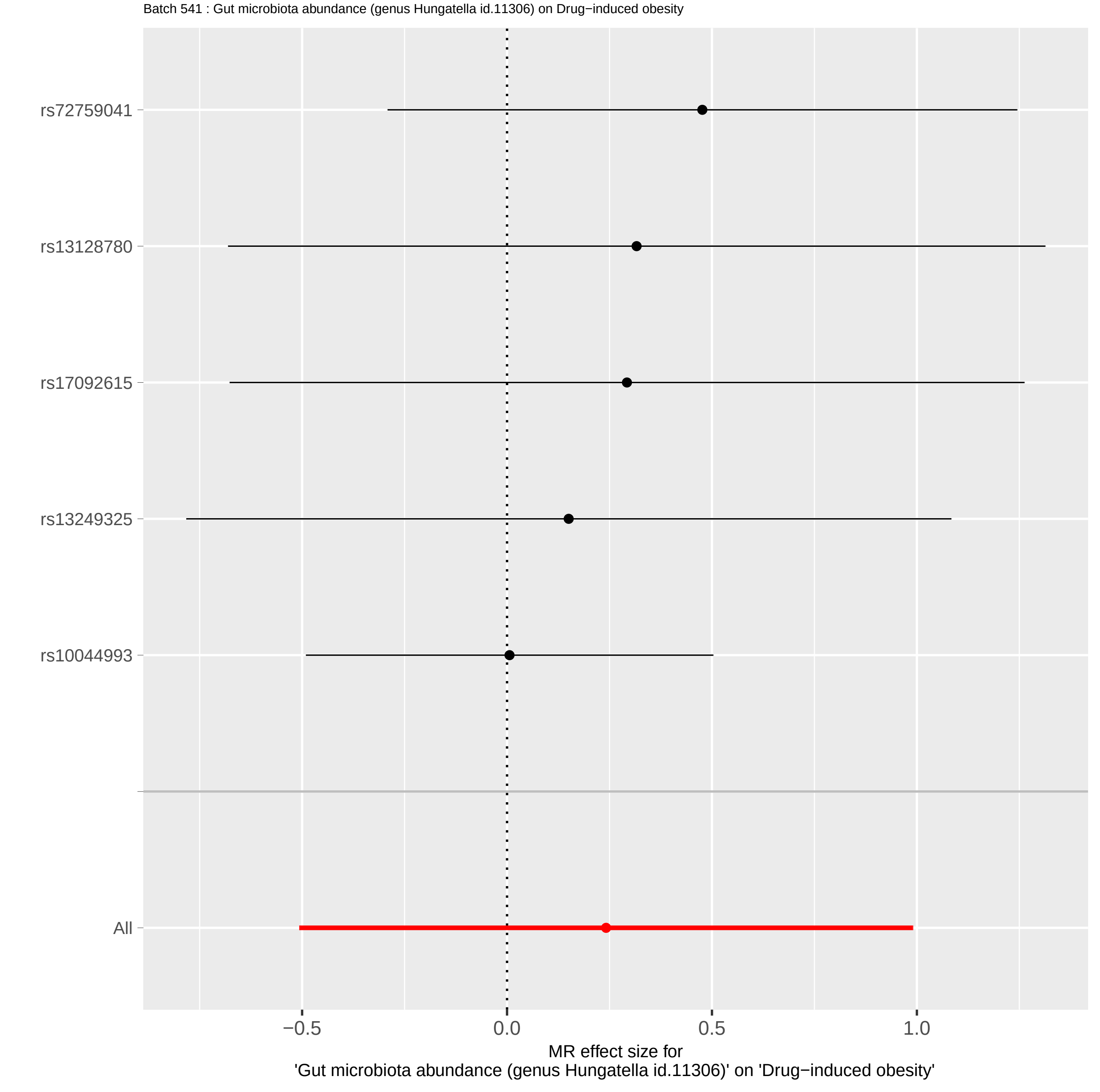

Batch 541 : Gut microbiota abundance (genus Hungatella id.11306) on Drug−induced obesity
rs72759041
rs13128780
rs17092615
rs13249325
rs10044993
All
−0.5
0.0
0.5
1.0
MR effect size for
'Gut microbiota abundance (genus Hungatella id.11306)' on 'Drug−induced obesity'

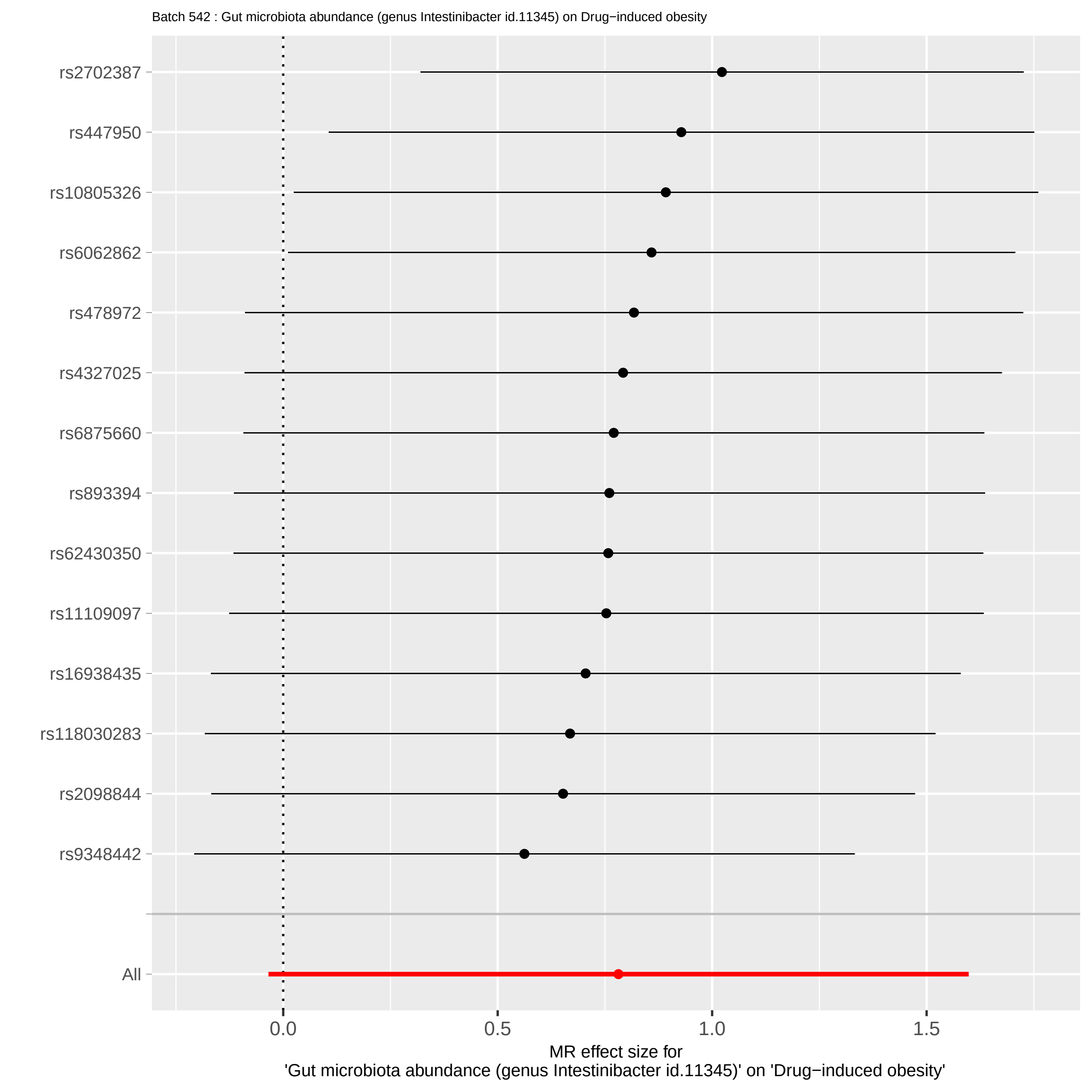
Batch 542 : Gut microbiota abundance (genus Intestinibacter id.11345) on Drug−induced obesity
rs2702387
rs447950
rs10805326
rs6062862
rs478972
rs4327025
rs6875660
rs893394
rs62430350
rs11109097
rs16938435
rs118030283
rs2098844
rs9348442
All
0.0
0.5
1.0
1.5
MR effect size for
'Gut microbiota abundance (genus Intestinibacter id.11345)' on 'Drug−induced obesity'

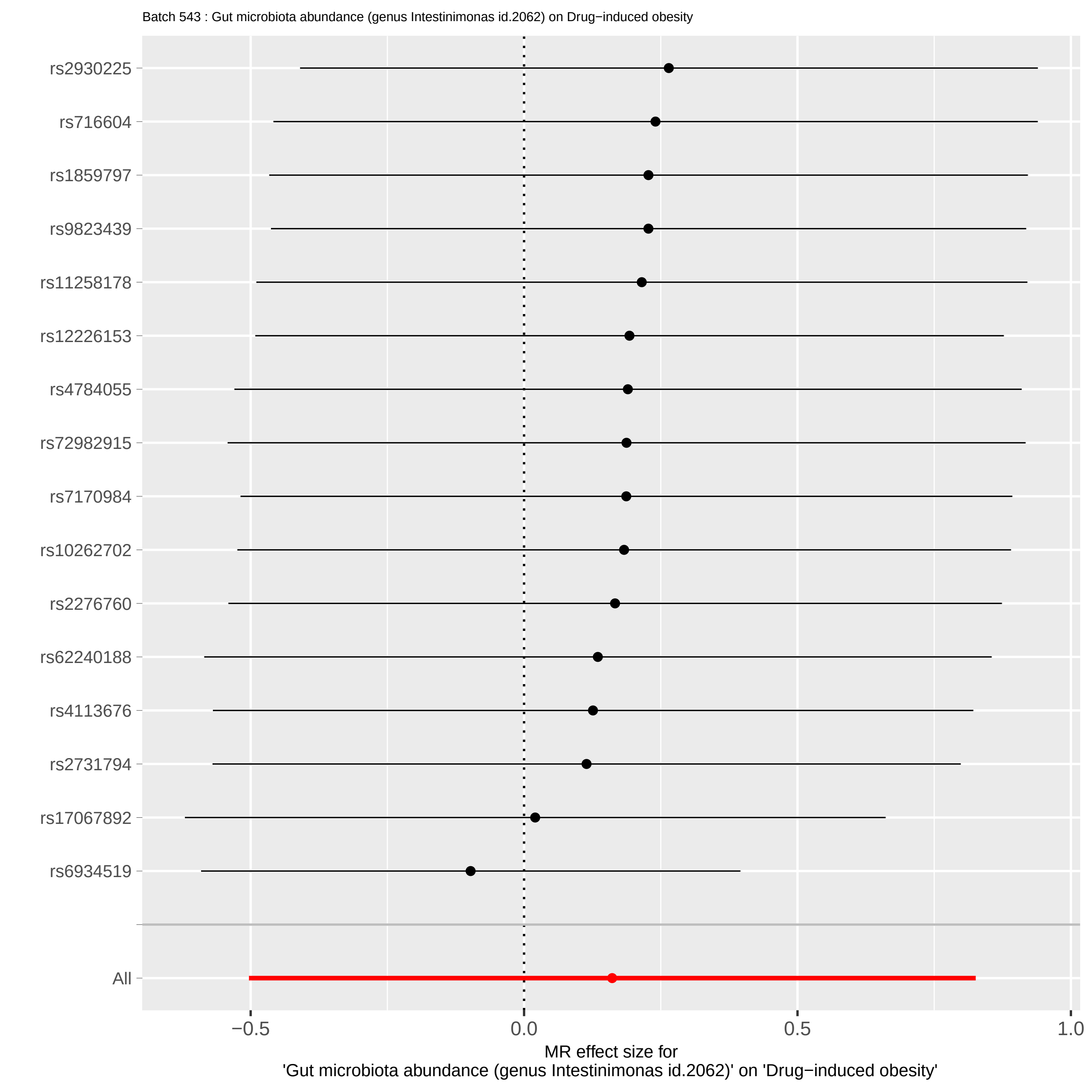

Batch 543 : Gut microbiota abundance (genus Intestinimonas id.2062) on Drug−induced obesity
rs2930225
rs716604
rs1859797
rs9823439
rs11258178
rs12226153
rs4784055
rs72982915
rs7170984
rs10262702
rs2276760
rs62240188
rs4113676
rs2731794
rs17067892
rs6934519
All
−0.5
0.0
0.5
1.0
MR effect size for
'Gut microbiota abundance (genus Intestinimonas id.2062)' on 'Drug−induced obesity'

Batch 544 : Gut microbiota abundance (genus Lachnoclostridium id.11308) on Drug−induced obesity

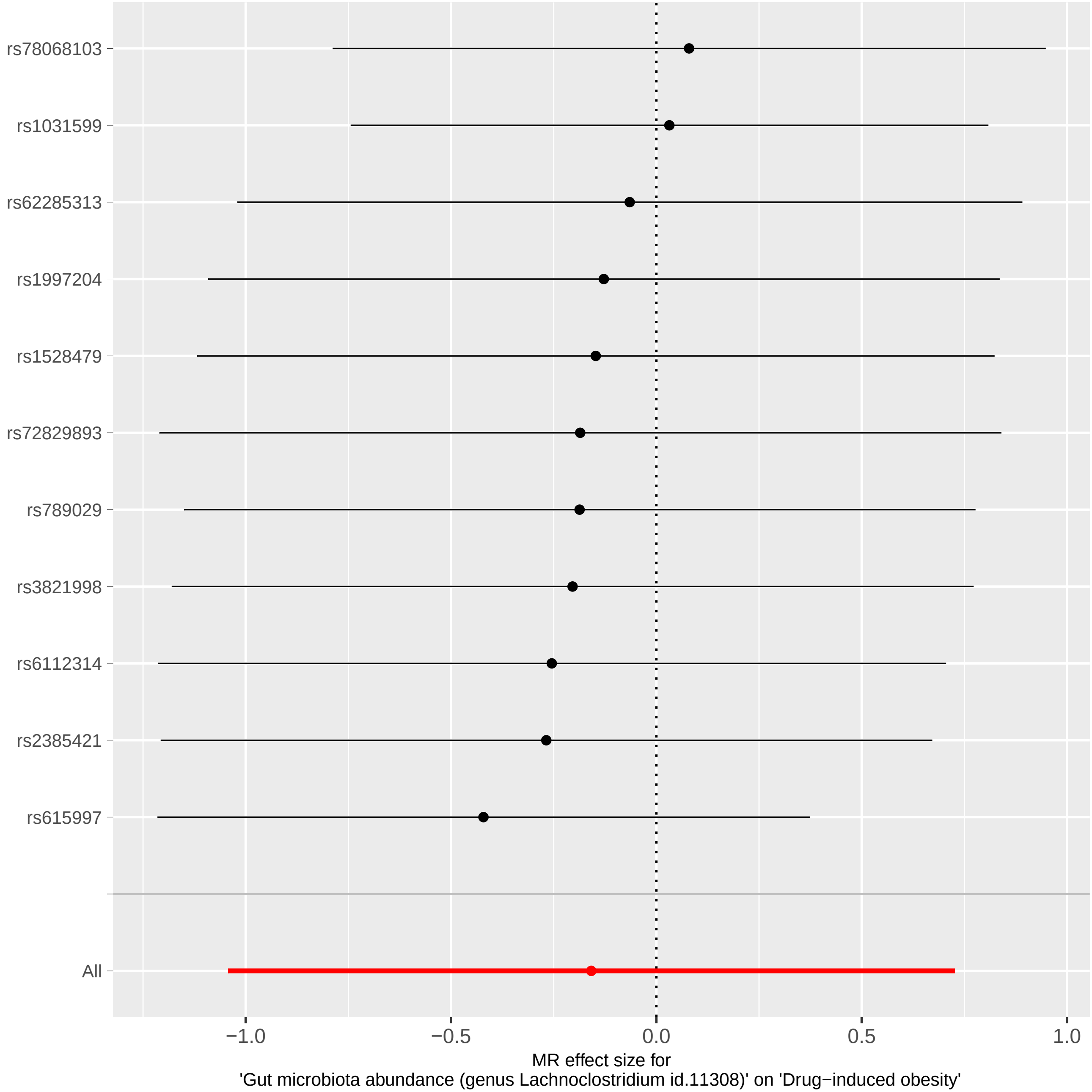

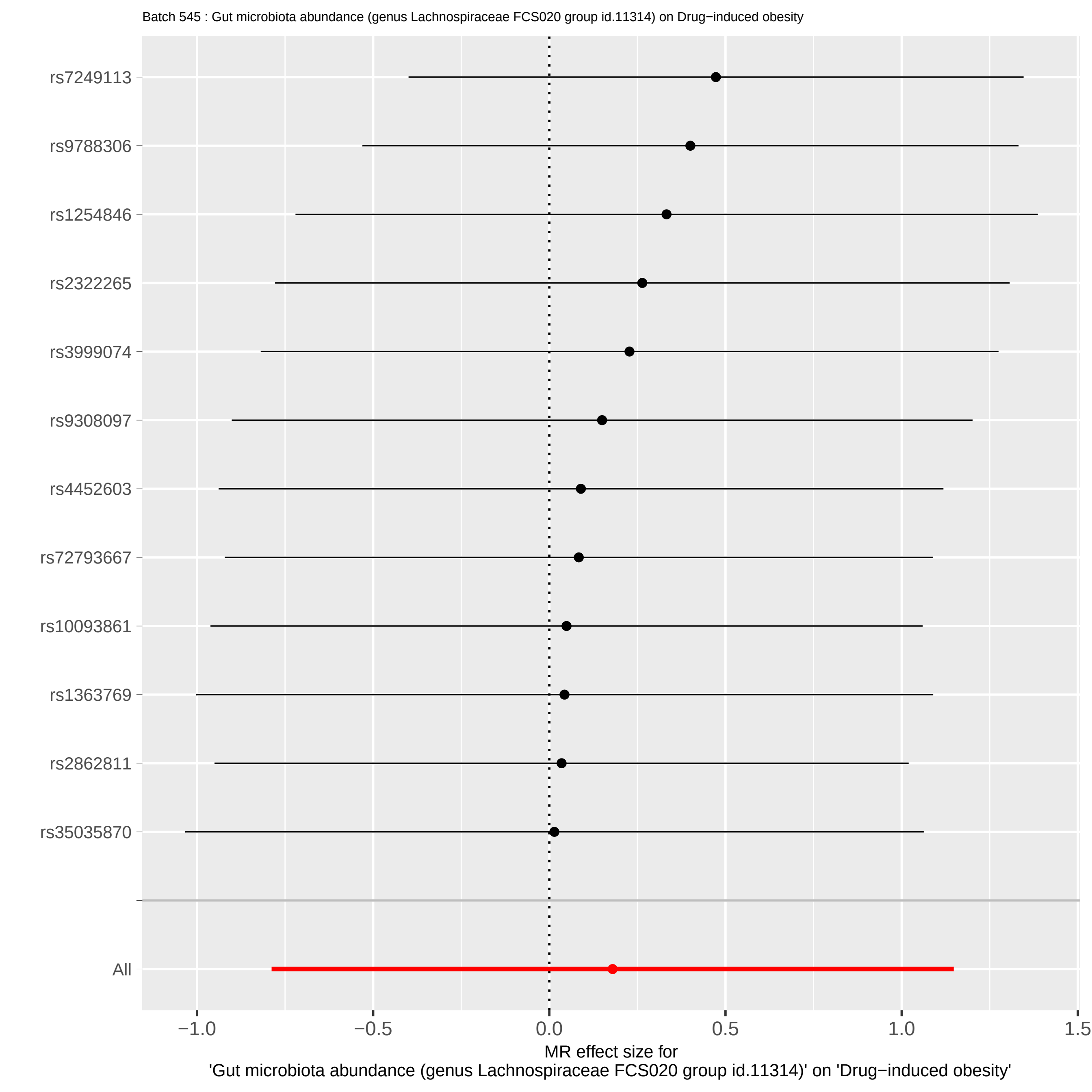

Batch 545 : Gut microbiota abundance (genus Lachnospiraceae FCS020 group id.11314) on Drug−induced obesity
rs7249113
rs9788306
rs1254846
rs2322265
rs3999074
rs9308097
rs4452603
rs72793667
rs10093861
rs1363769
rs2862811
rs35035870
All
−1.0
−0.5
0.0
0.5
1.0
1.5
MR effect size for
'Gut microbiota abundance (genus Lachnospiraceae FCS020 group id.11314)' on 'Drug−induced obesity'

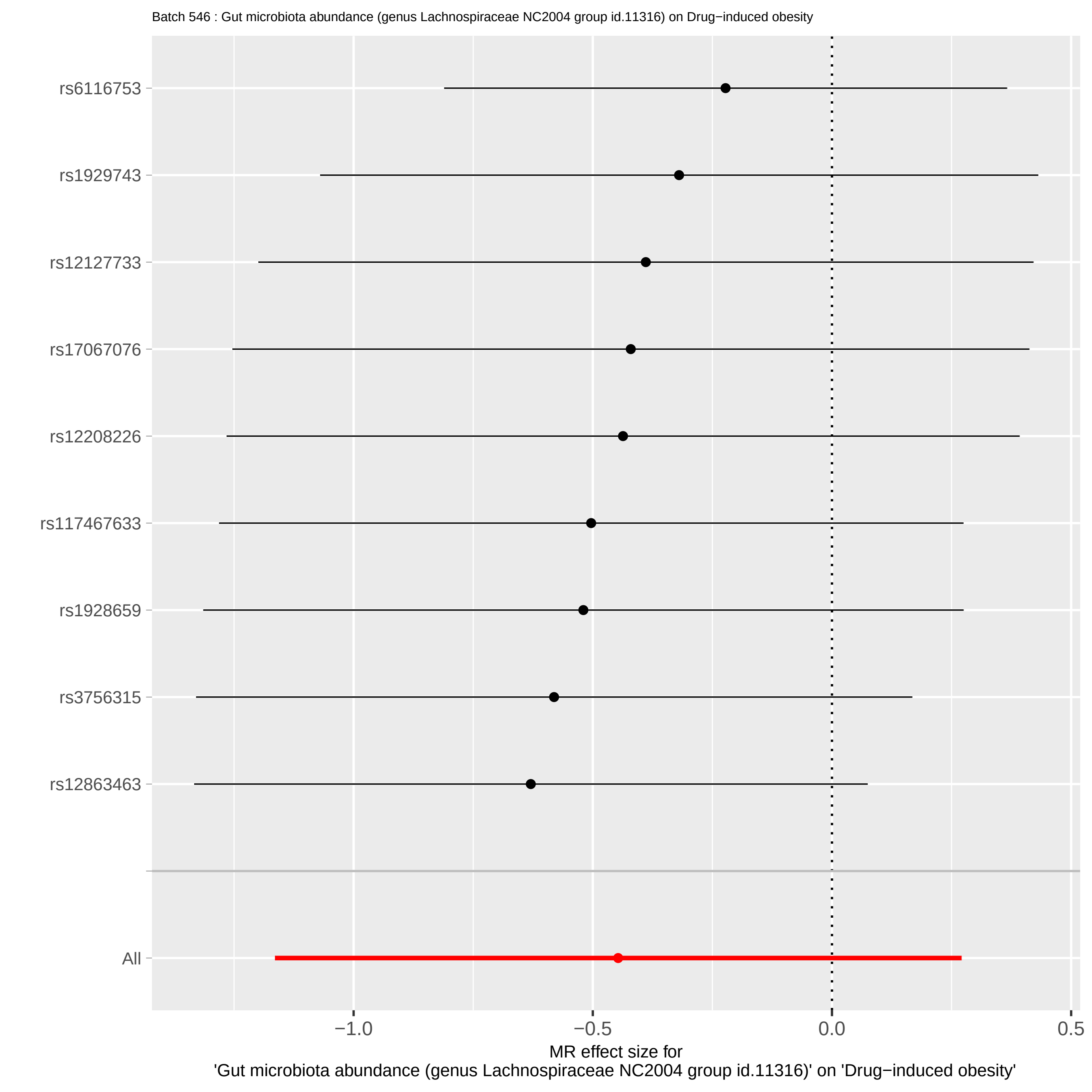

Batch 546 : Gut microbiota abundance (genus Lachnospiraceae NC2004 group id.11316) on Drug−induced obesity
rs6116753
rs1929743
rs12127733
rs17067076
rs12208226
rs117467633
rs1928659
rs3756315
rs12863463
All
−1.0
−0.5
0.0
0.5
MR effect size for
'Gut microbiota abundance (genus Lachnospiraceae NC2004 group id.11316)' on 'Drug−induced obesity'

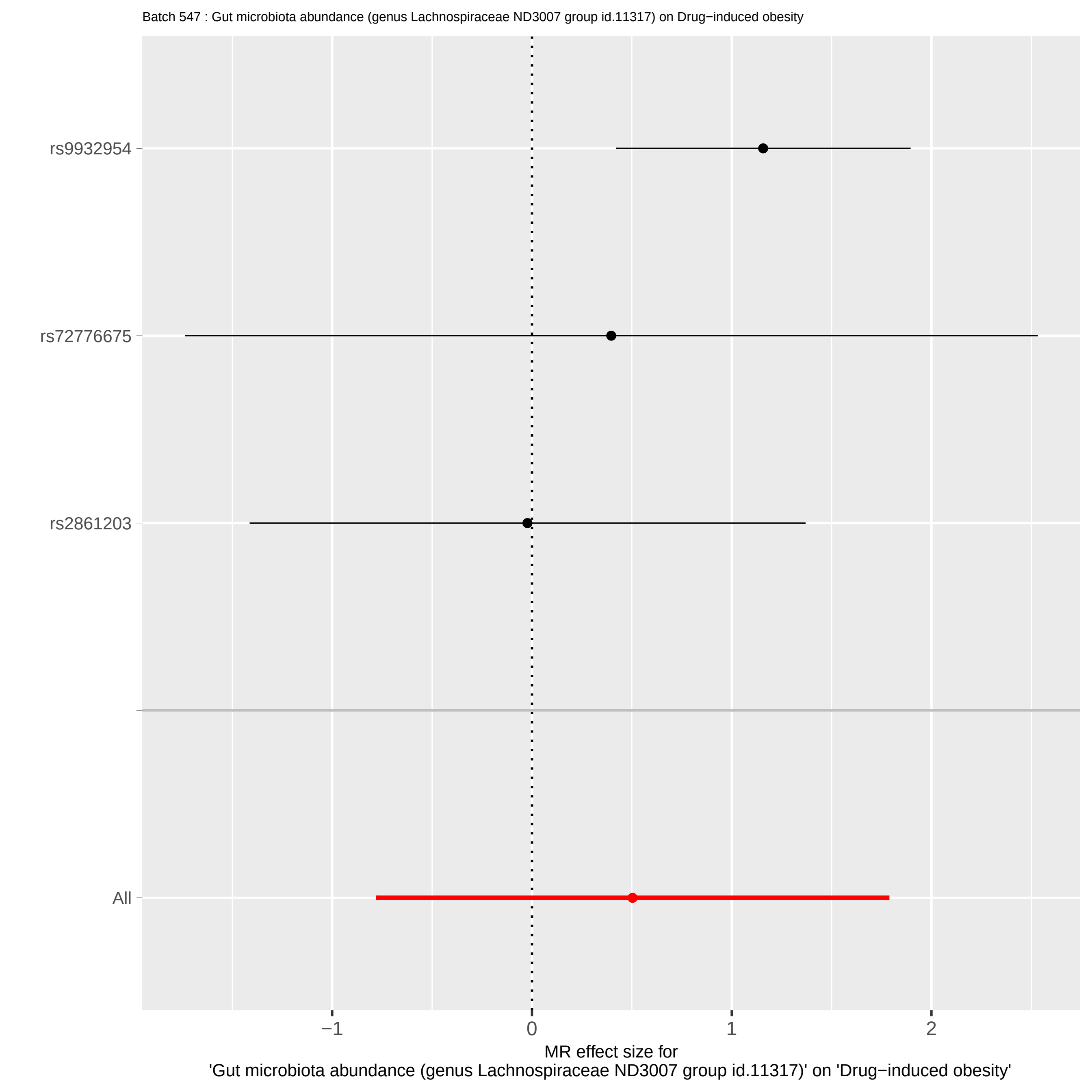

Batch 547 : Gut microbiota abundance (genus Lachnospiraceae ND3007 group id.11317) on Drug−induced obesity
rs9932954
rs72776675
rs2861203
All
−1
0
1
2
MR effect size for
'Gut microbiota abundance (genus Lachnospiraceae ND3007 group id.11317)' on 'Drug−induced obesity'

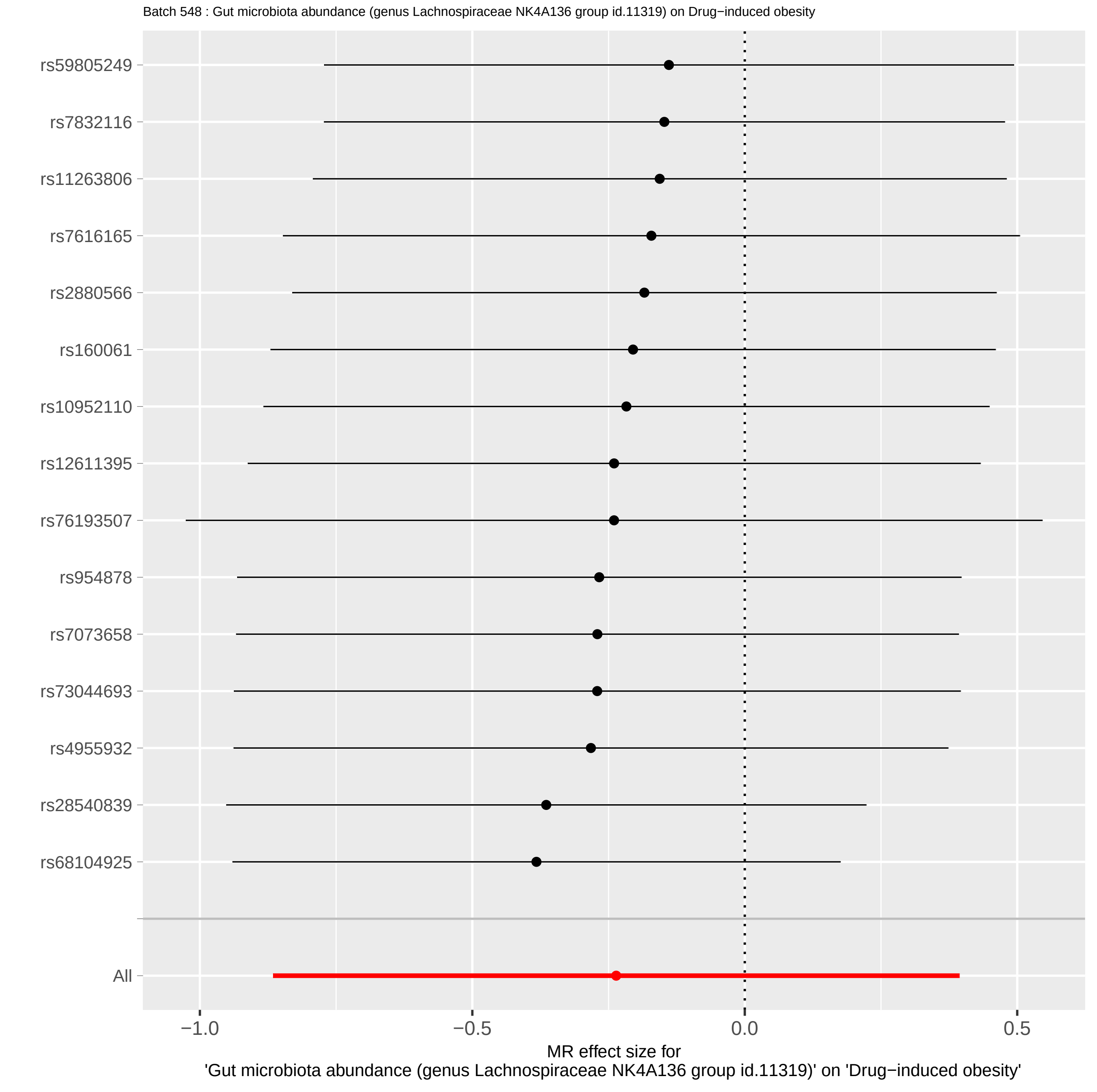

Batch 548 : Gut microbiota abundance (genus Lachnospiraceae NK4A136 group id.11319) on Drug−induced obesity
rs59805249
rs7832116
rs11263806
rs7616165
rs2880566
rs160061
rs10952110
rs12611395
rs76193507
rs954878
rs7073658
rs73044693
rs4955932
rs28540839
rs68104925
All
−1.0
−0.5
0.0
0.5
MR effect size for
'Gut microbiota abundance (genus Lachnospiraceae NK4A136 group id.11319)' on 'Drug−induced obesity'

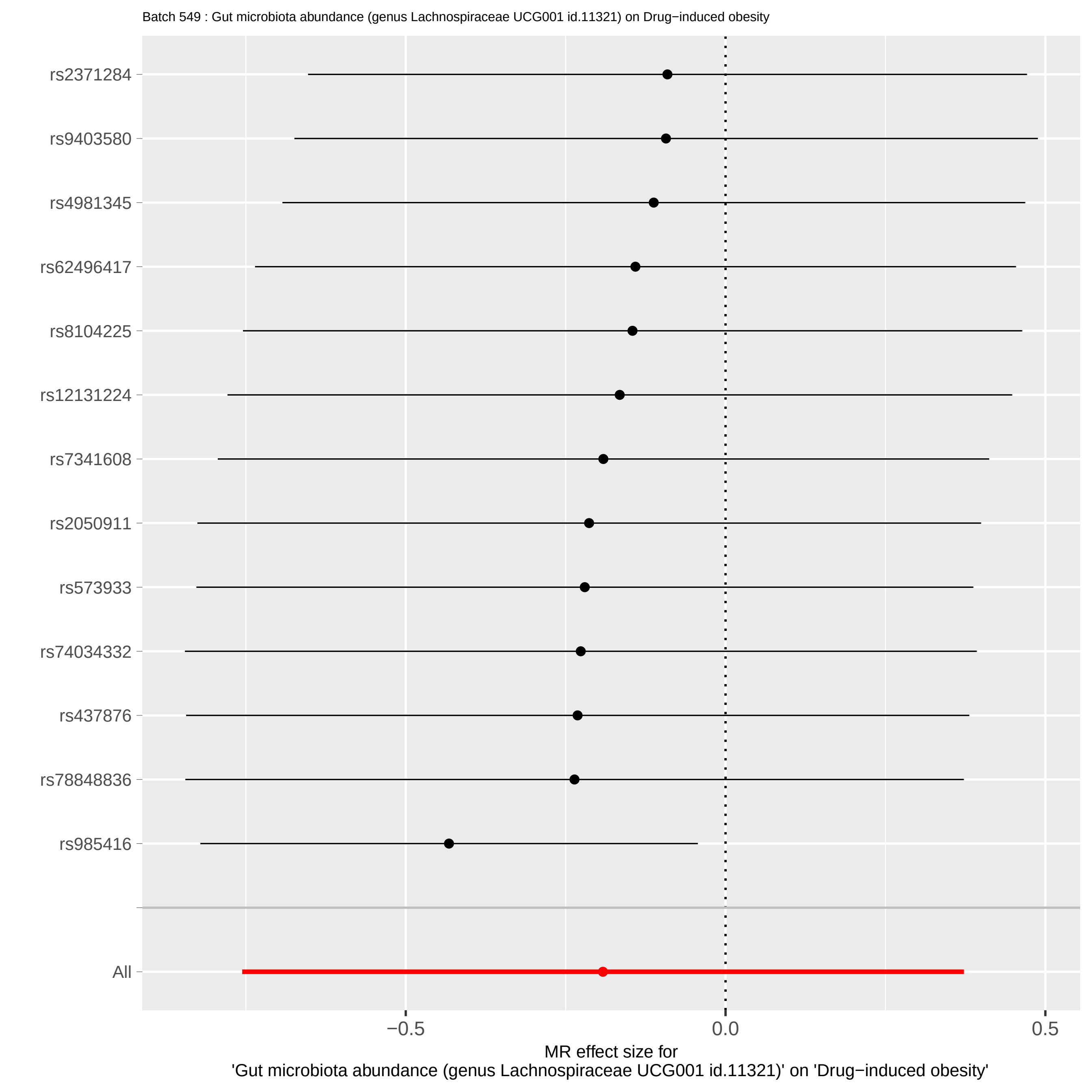
Batch 549 : Gut microbiota abundance (genus Lachnospiraceae UCG001 id.11321) on Drug−induced obesity
rs2371284
rs9403580
rs4981345
rs62496417
rs8104225
rs12131224
rs7341608
rs2050911
rs573933
rs74034332
rs437876
rs78848836
rs985416
All
−0.5
0.0
0.5
MR effect size for
'Gut microbiota abundance (genus Lachnospiraceae UCG001 id.11321)' on 'Drug−induced obesity'

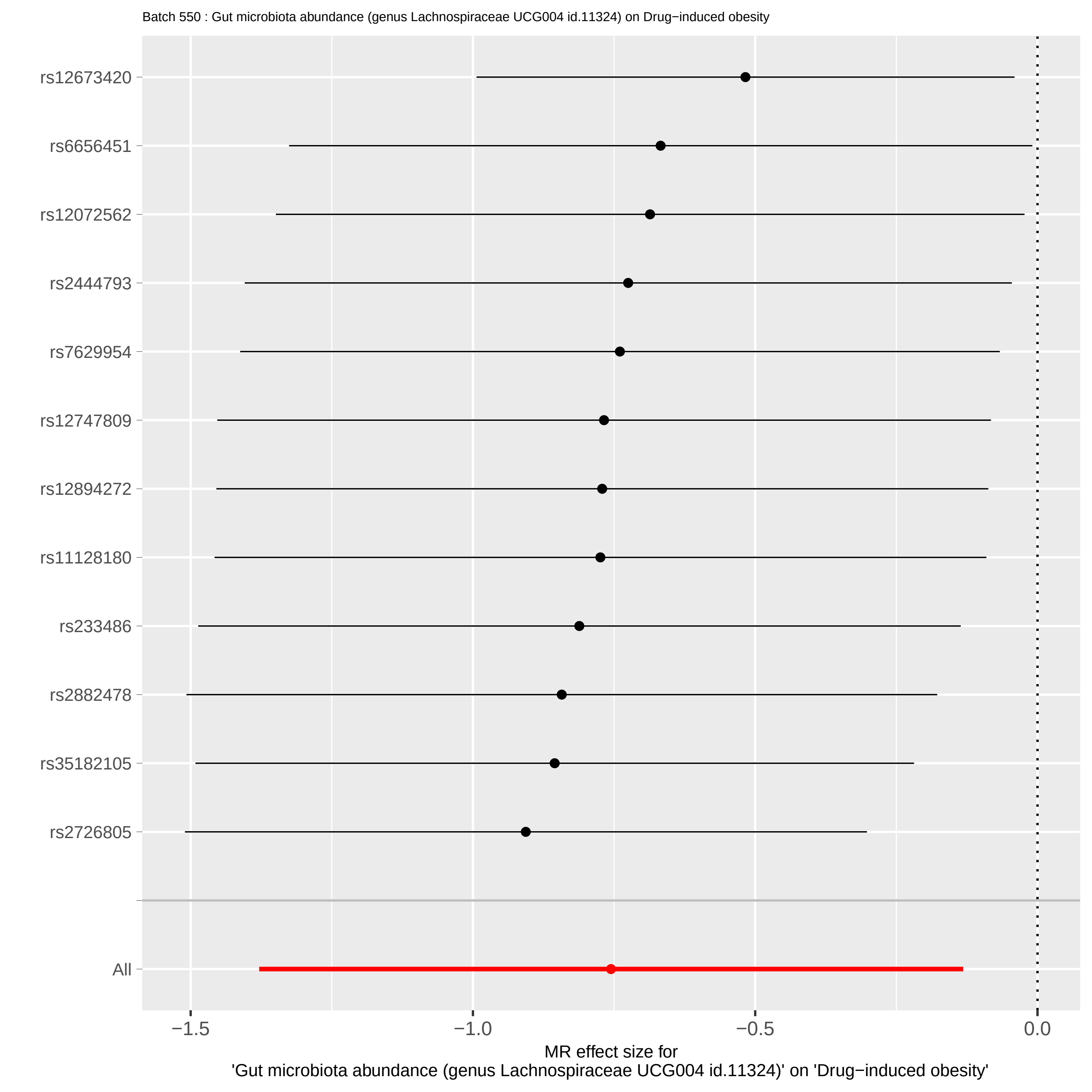

Batch 550 : Gut microbiota abundance (genus Lachnospiraceae UCG004 id.11324) on Drug−induced obesity
rs12673420
rs6656451
rs12072562
rs2444793
rs7629954
rs12747809
rs12894272
rs11128180
rs233486
rs2882478
rs35182105
rs2726805
All
−1.5
−1.0
−0.5
0.0
MR effect size for
'Gut microbiota abundance (genus Lachnospiraceae UCG004 id.11324)' on 'Drug−induced obesity'

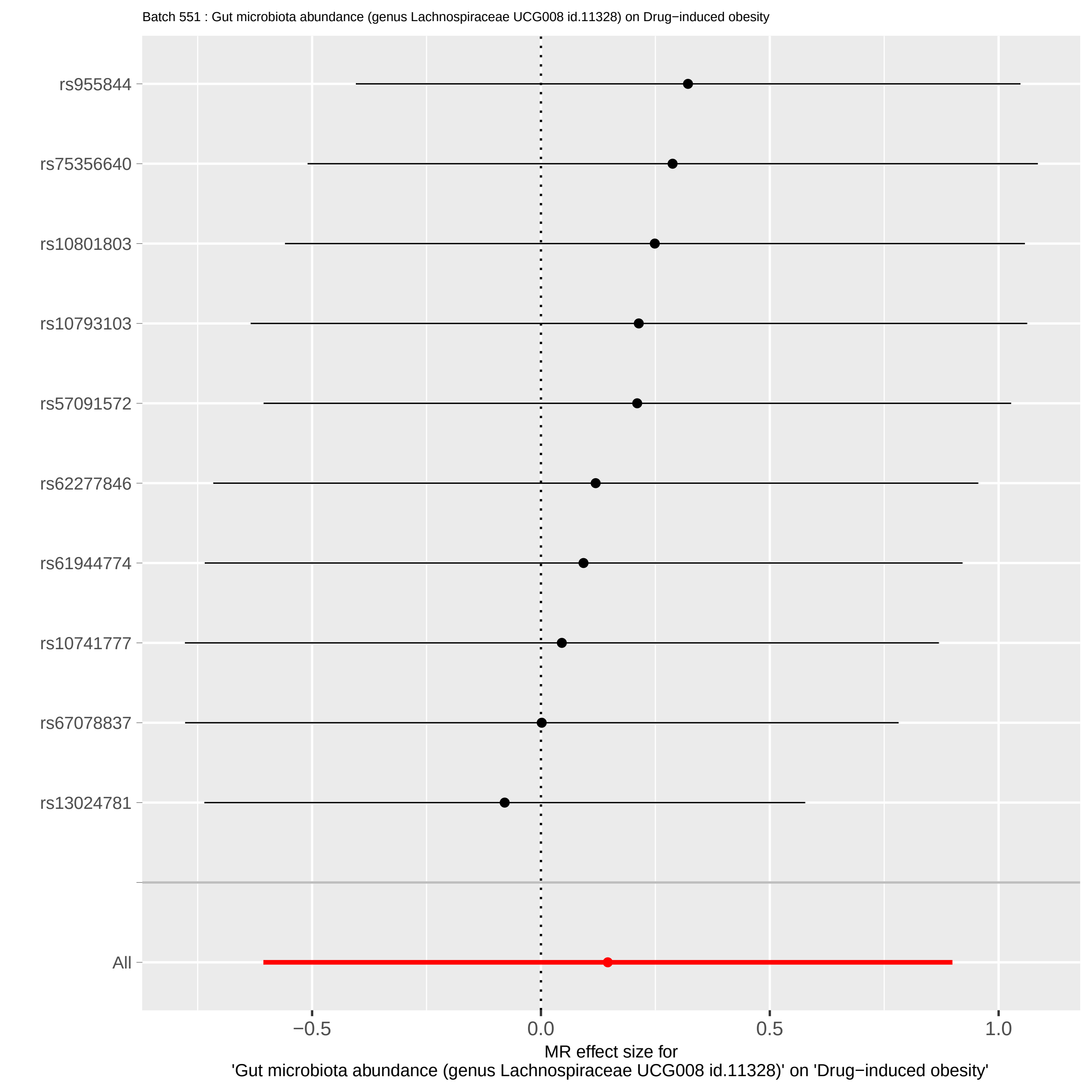

Batch 551 : Gut microbiota abundance (genus Lachnospiraceae UCG008 id.11328) on Drug−induced obesity
rs955844
rs75356640
rs10801803
rs10793103
rs57091572
rs62277846
rs61944774
rs10741777
rs67078837
rs13024781
All
−0.5
0.0
0.5
1.0
MR effect size for
'Gut microbiota abundance (genus Lachnospiraceae UCG008 id.11328)' on 'Drug−induced obesity'

Batch 552 : Gut microbiota abundance (genus Lachnospiraceae UCG010 id.11330) on Drug−induced obesity

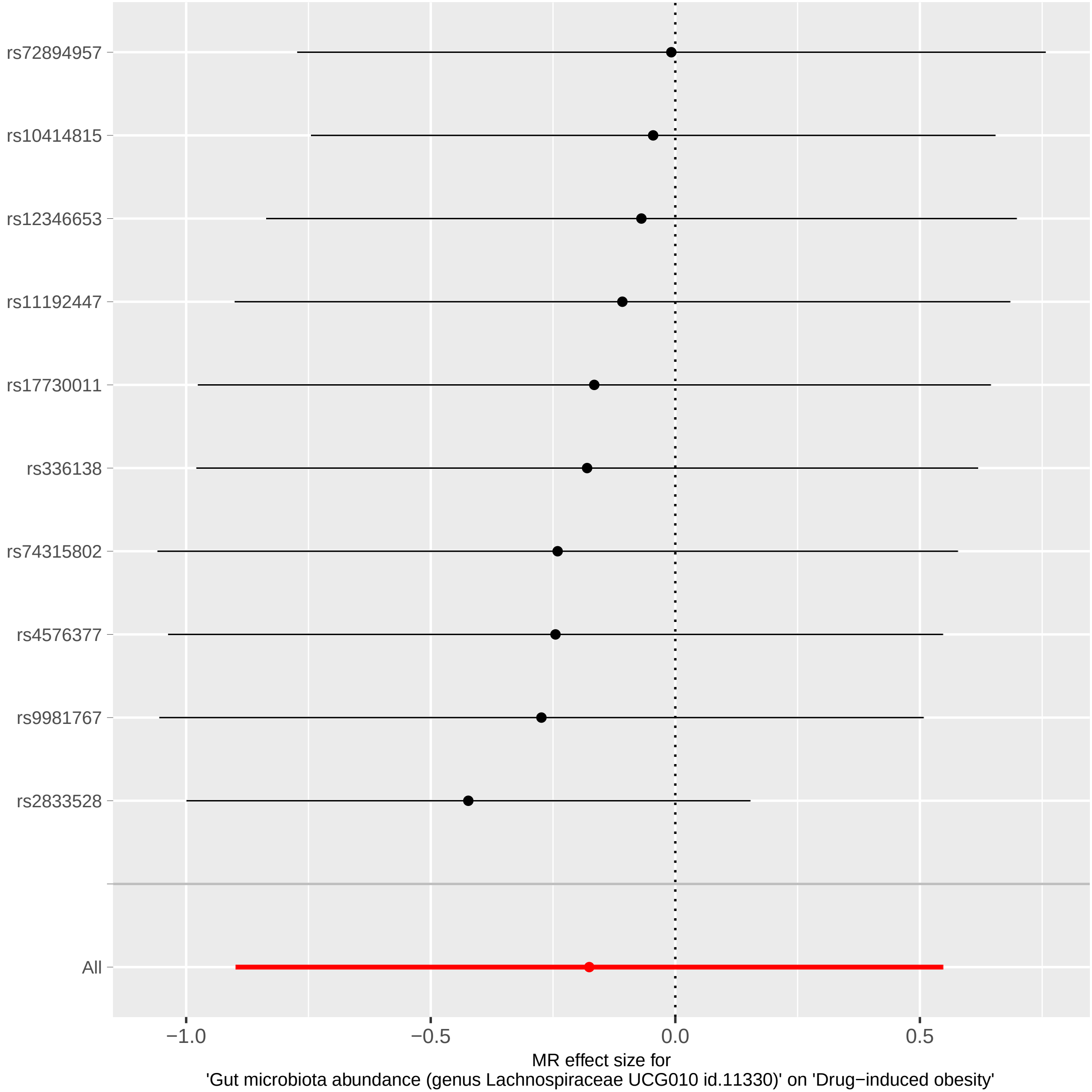

Batch 554 : Gut microbiota abundance (genus Lactobacillus id.1837) on Drug−induced obesity

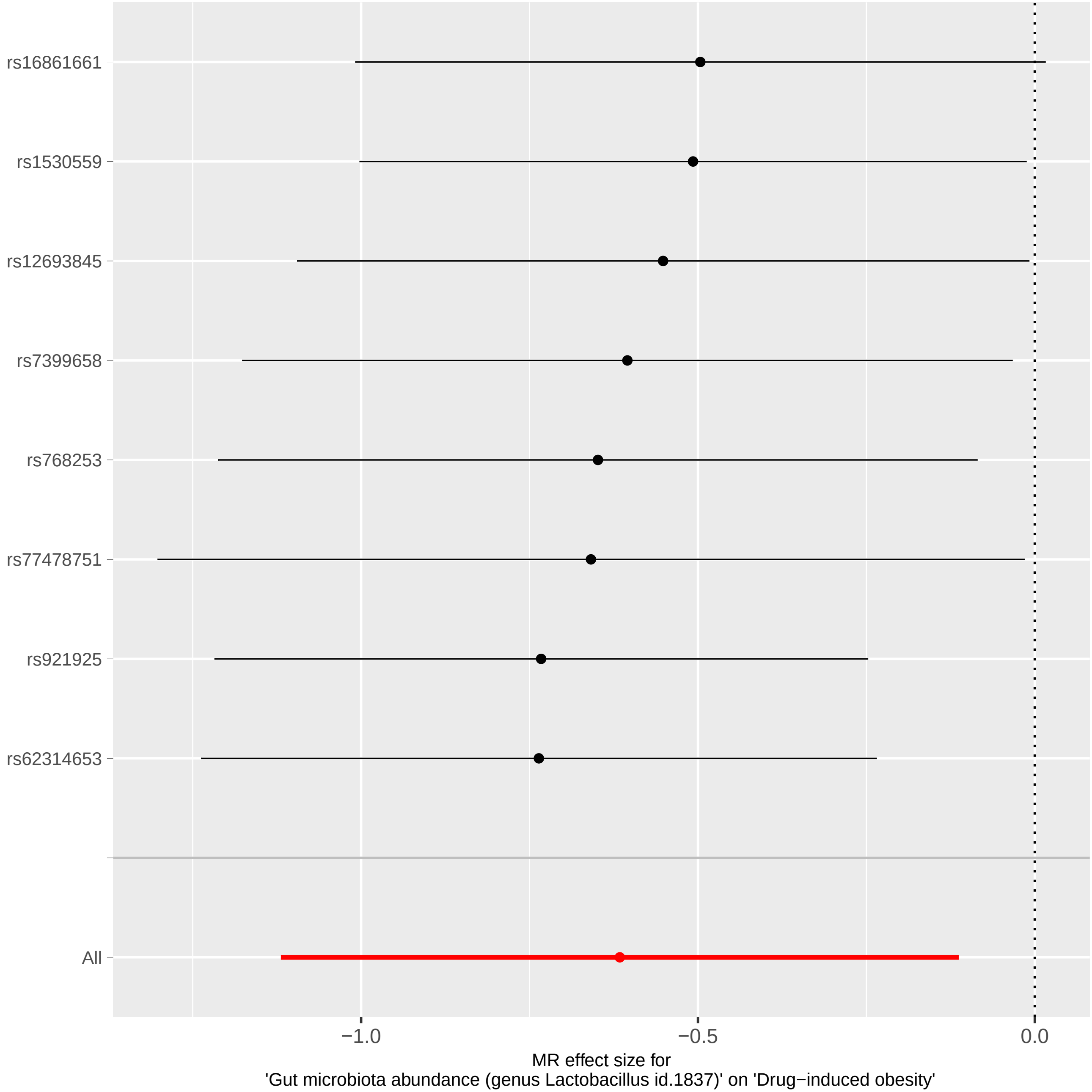

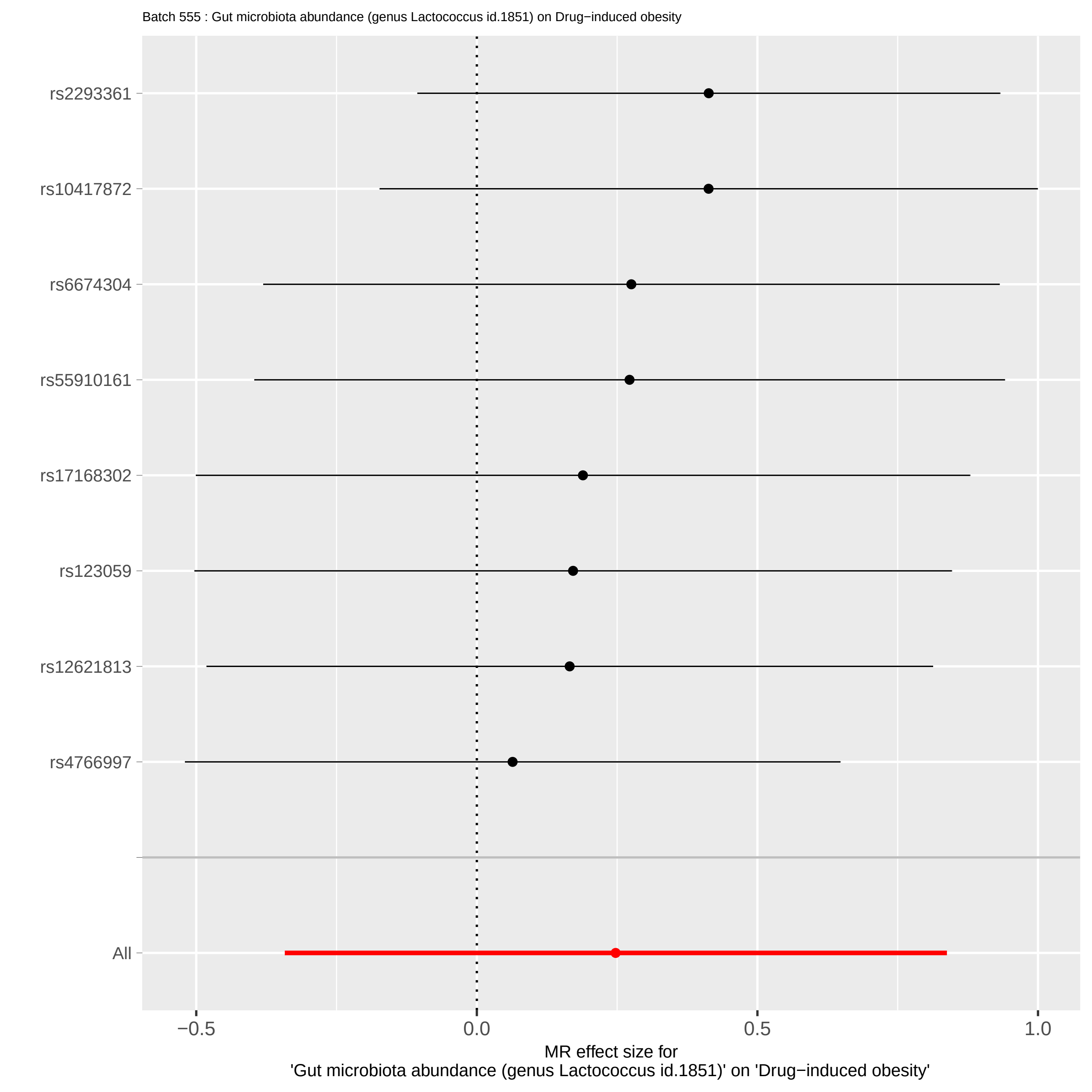
Batch 555 : Gut microbiota abundance (genus Lactococcus id.1851) on Drug−induced obesity
rs2293361
rs10417872
rs6674304
rs55910161
rs17168302
rs123059
rs12621813
rs4766997
All
−0.5
0.0
0.5
1.0
MR effect size for
'Gut microbiota abundance (genus Lactococcus id.1851)' on 'Drug−induced obesity'

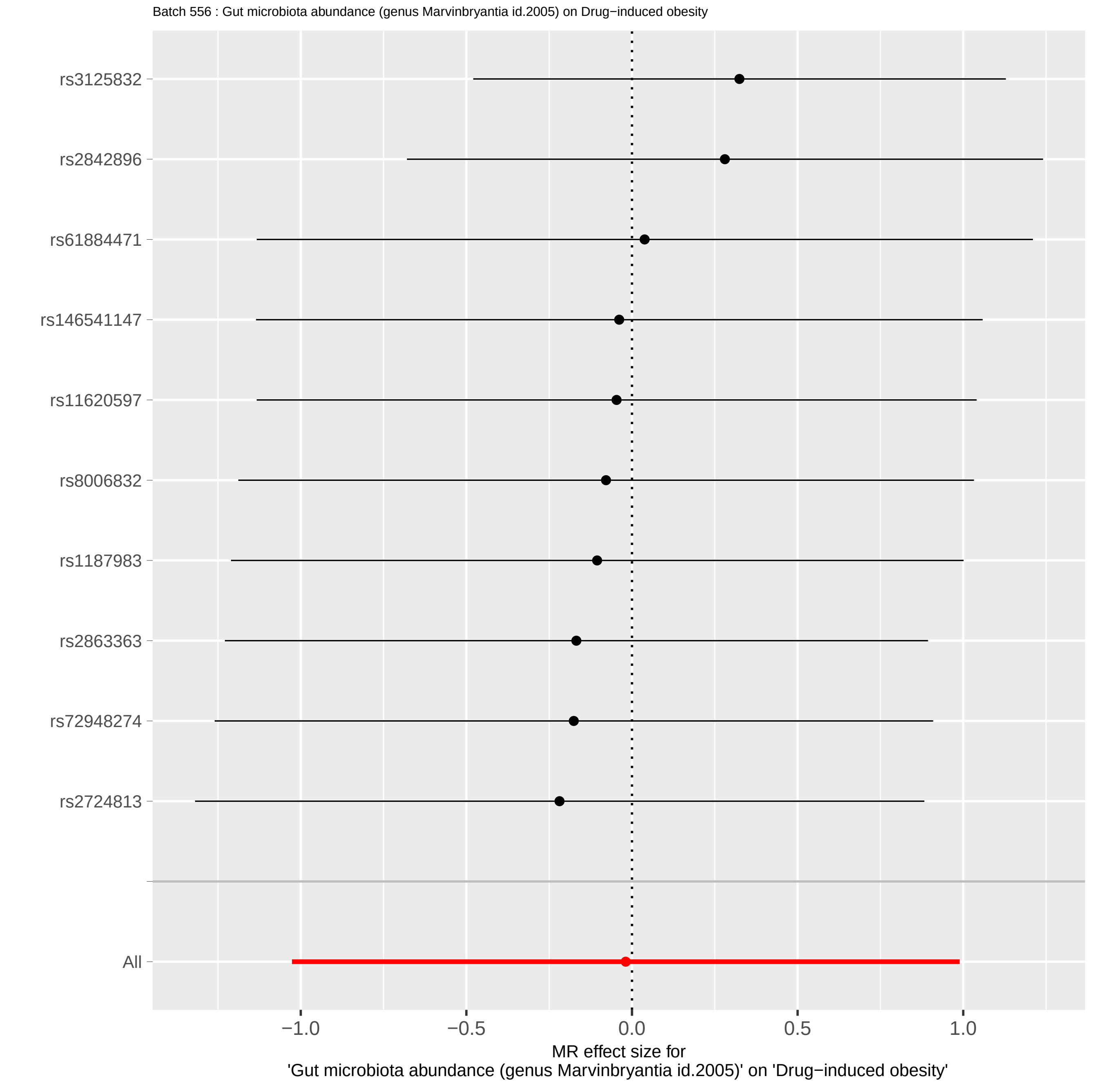

Batch 556 : Gut microbiota abundance (genus Marvinbryantia id.2005) on Drug−induced obesity
rs3125832
rs2842896
rs61884471
rs146541147
rs11620597
rs8006832
rs1187983
rs2863363
rs72948274
rs2724813
All
−1.0
−0.5
0.0
0.5
1.0
MR effect size for
'Gut microbiota abundance (genus Marvinbryantia id.2005)' on 'Drug−induced obesity'

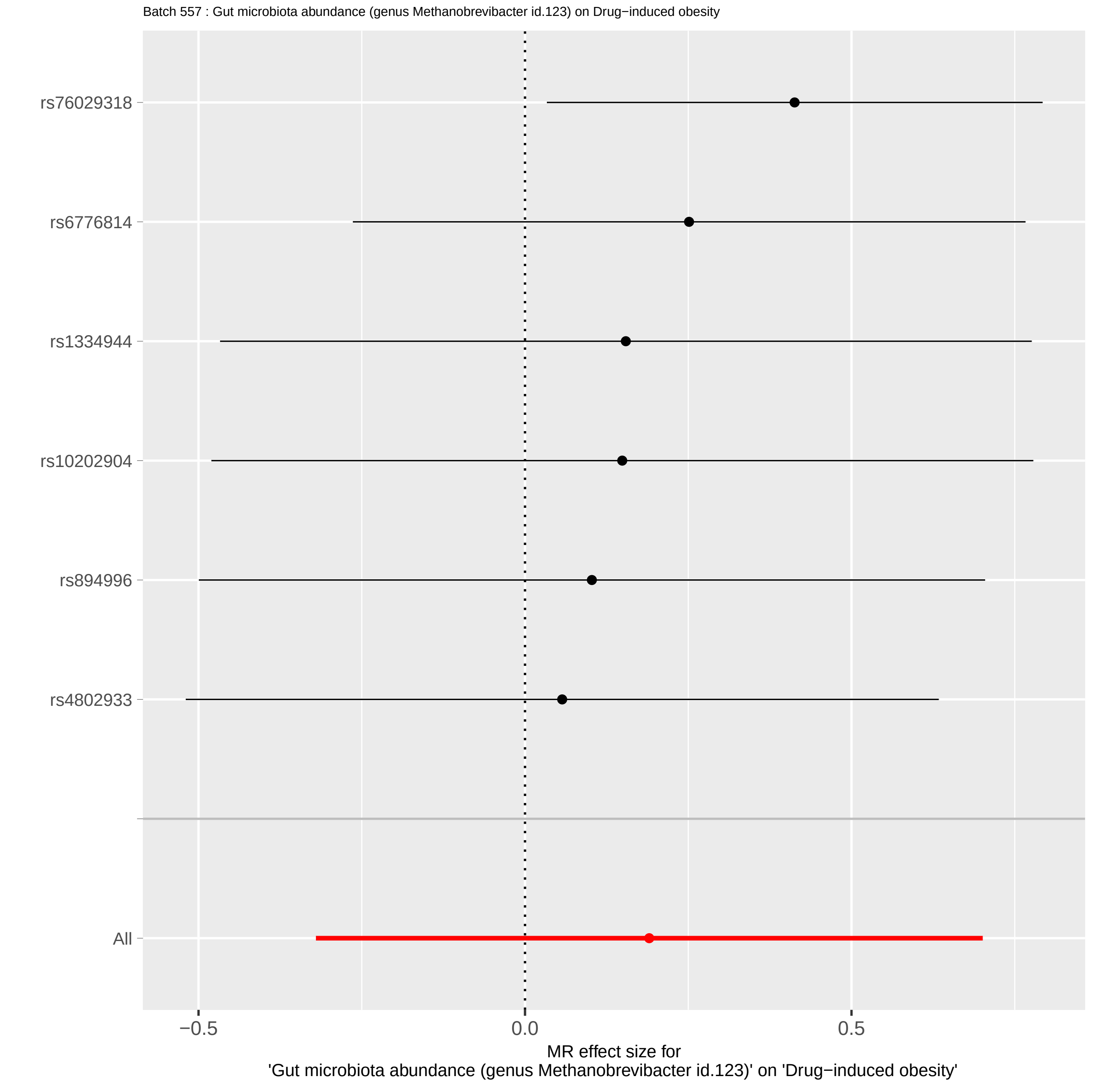

Batch 557 : Gut microbiota abundance (genus Methanobrevibacter id.123) on Drug−induced obesity
rs76029318
rs6776814
rs1334944
rs10202904
rs894996
rs4802933
All
−0.5
0.0
0.5
MR effect size for
'Gut microbiota abundance (genus Methanobrevibacter id.123)' on 'Drug−induced obesity'

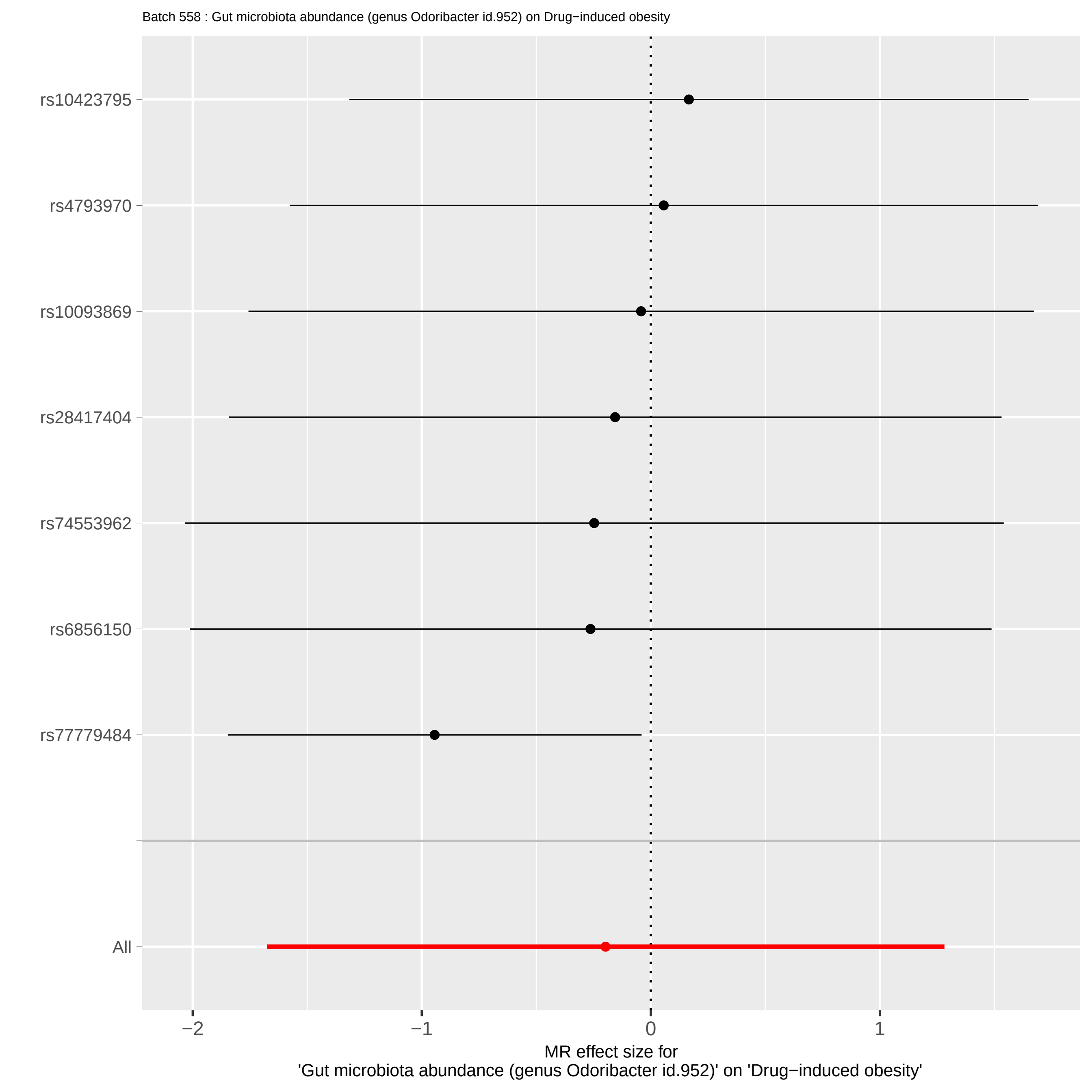

Batch 558 : Gut microbiota abundance (genus Odoribacter id.952) on Drug−induced obesity
rs10423795
rs4793970
rs10093869
rs28417404
rs74553962
rs6856150
rs77779484
All
−2
−1
0
1
MR effect size for
'Gut microbiota abundance (genus Odoribacter id.952)' on 'Drug−induced obesity'

Batch 559 : Gut microbiota abundance (genus Olsenella id.822) on Drug−induced obesity

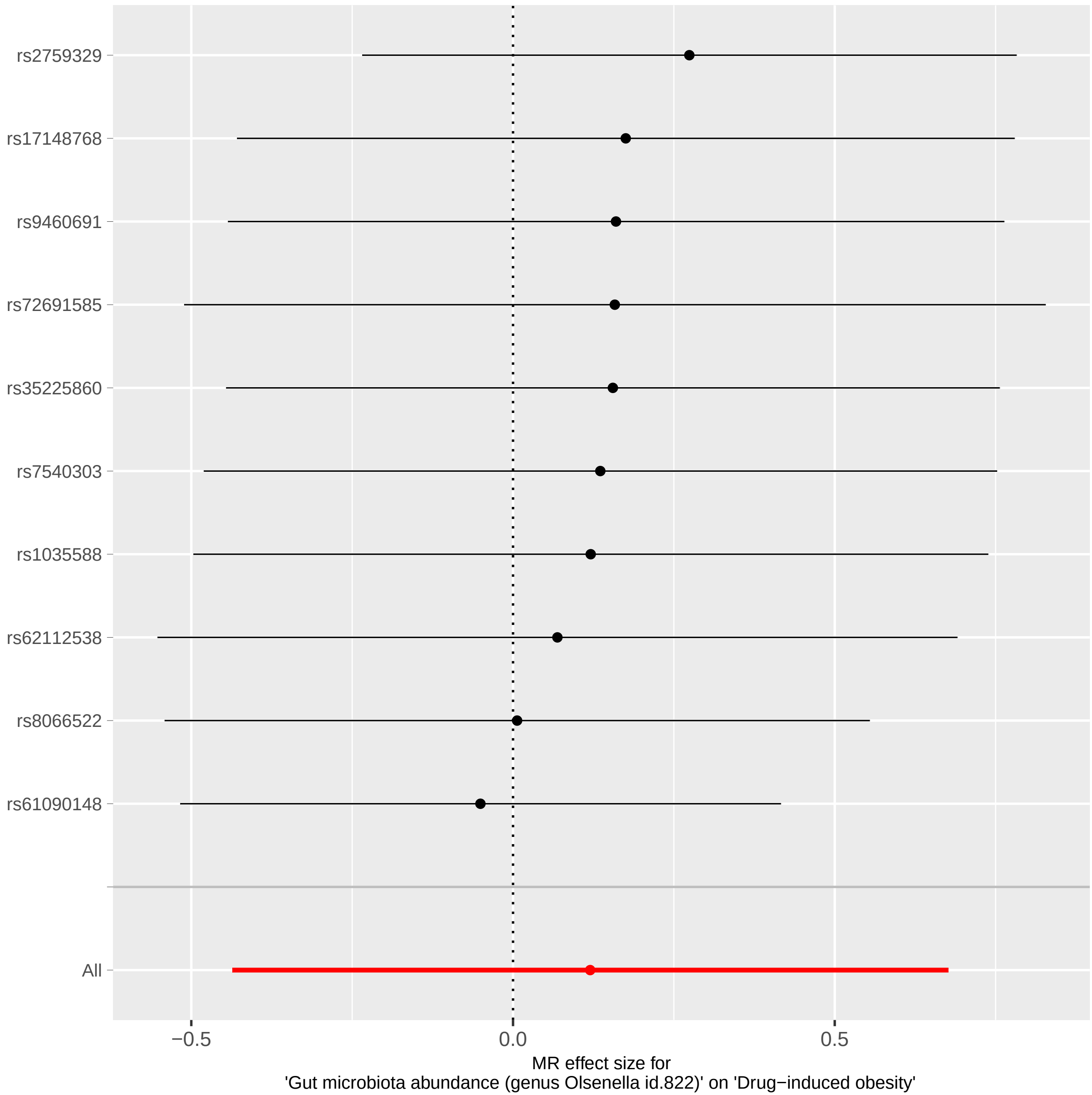

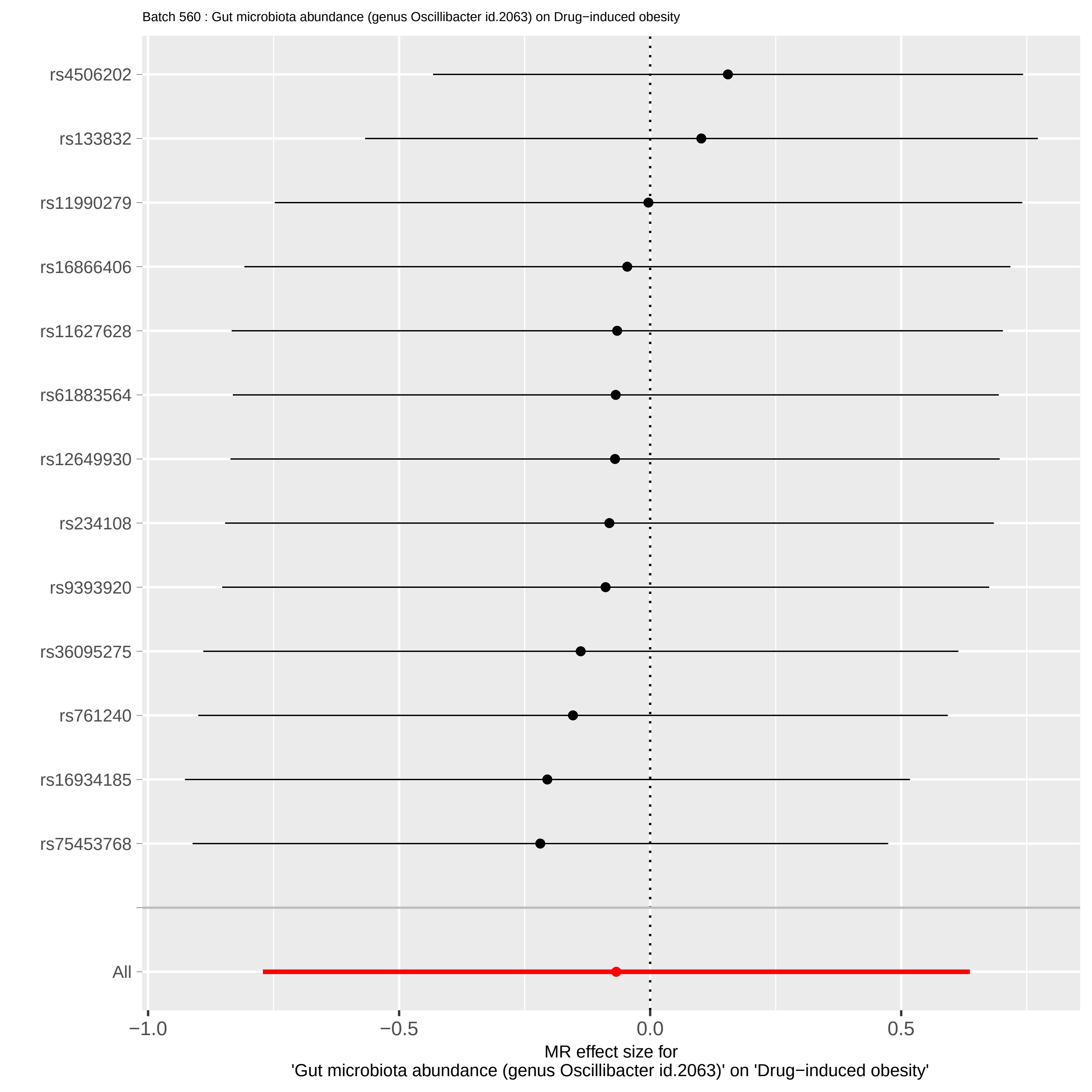
Batch 560 : Gut microbiota abundance (genus Oscillibacter id.2063) on Drug−induced obesity
rs4506202
rs133832
rs11990279
rs16866406
rs11627628
rs61883564
rs12649930
rs234108
rs9393920
rs36095275
rs761240
rs16934185
rs75453768
All
−1.0
−0.5
0.0
0.5
MR effect size for
'Gut microbiota abundance (genus Oscillibacter id.2063)' on 'Drug−induced obesity'

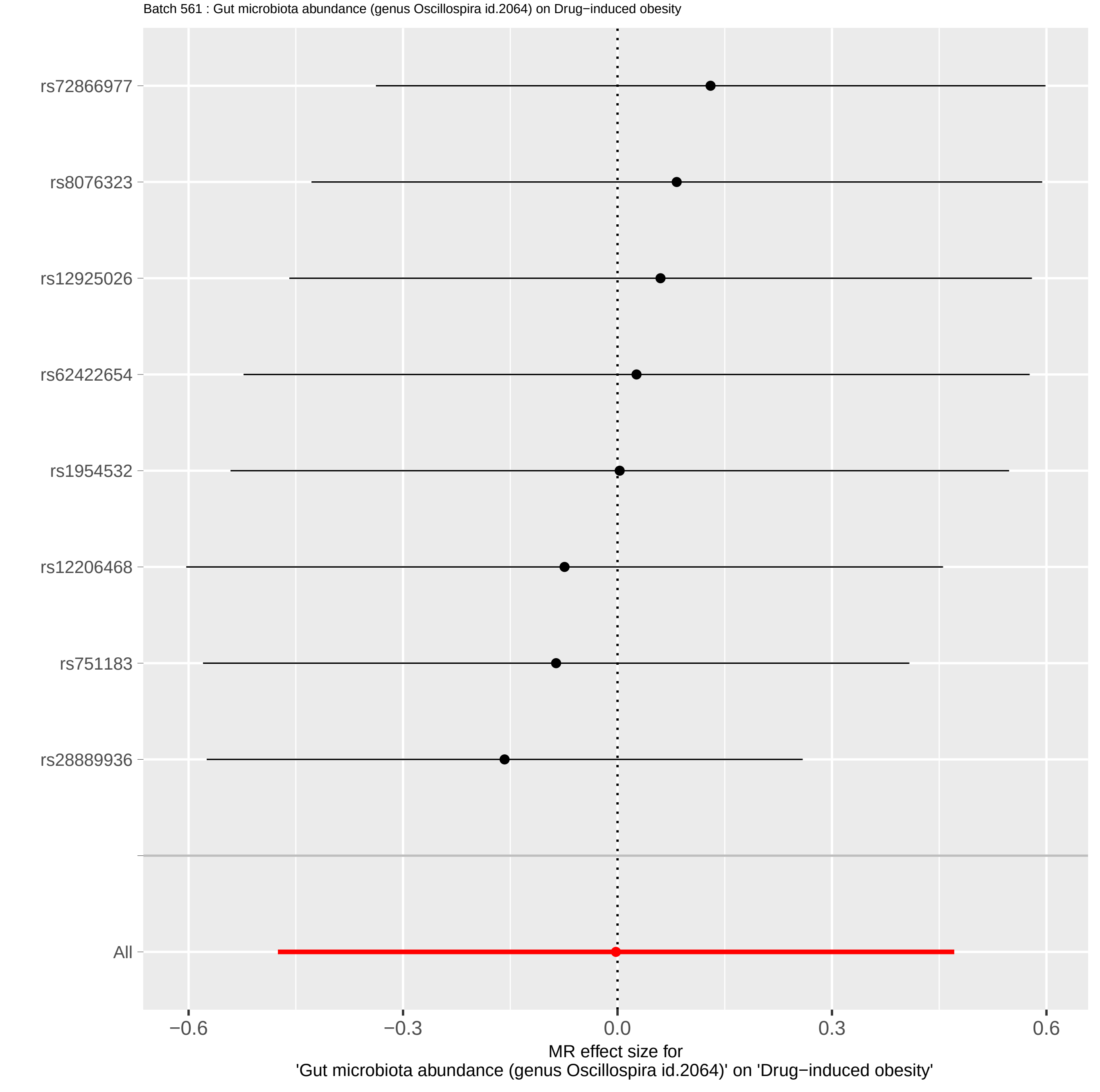

Batch 561 : Gut microbiota abundance (genus Oscillospira id.2064) on Drug−induced obesity
rs72866977
rs8076323
rs12925026
rs62422654
rs1954532
rs12206468
rs751183
rs28889936
All
−0.6
−0.3
0.0
0.3
0.6
MR effect size for
'Gut microbiota abundance (genus Oscillospira id.2064)' on 'Drug−induced obesity'

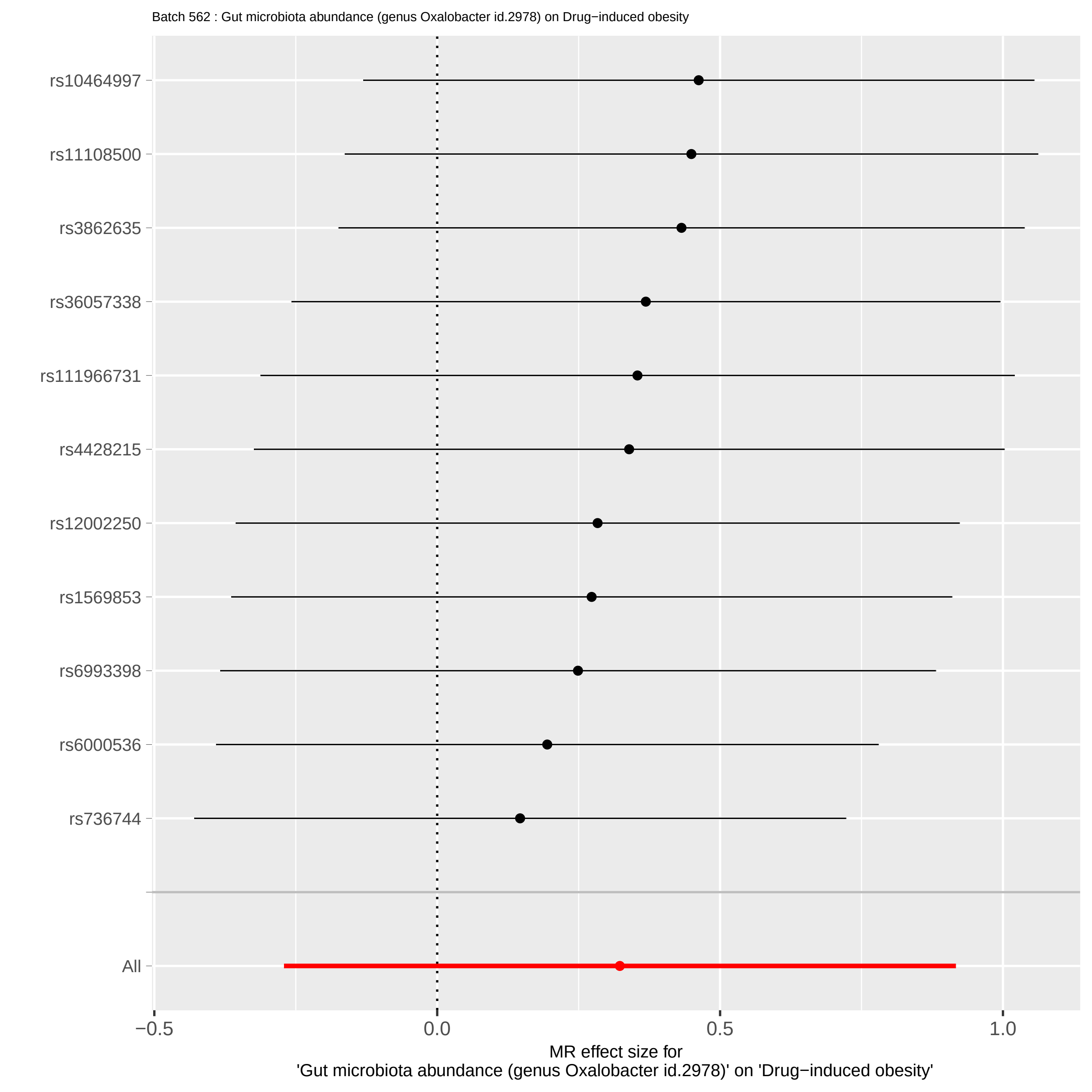
Batch 562 : Gut microbiota abundance (genus Oxalobacter id.2978) on Drug−induced obesity
rs10464997
rs11108500
rs3862635
rs36057338
rs111966731
rs4428215
rs12002250
rs1569853
rs6993398
rs6000536
rs736744
All
−0.5
0.0
0.5
1.0
MR effect size for
'Gut microbiota abundance (genus Oxalobacter id.2978)' on 'Drug−induced obesity'

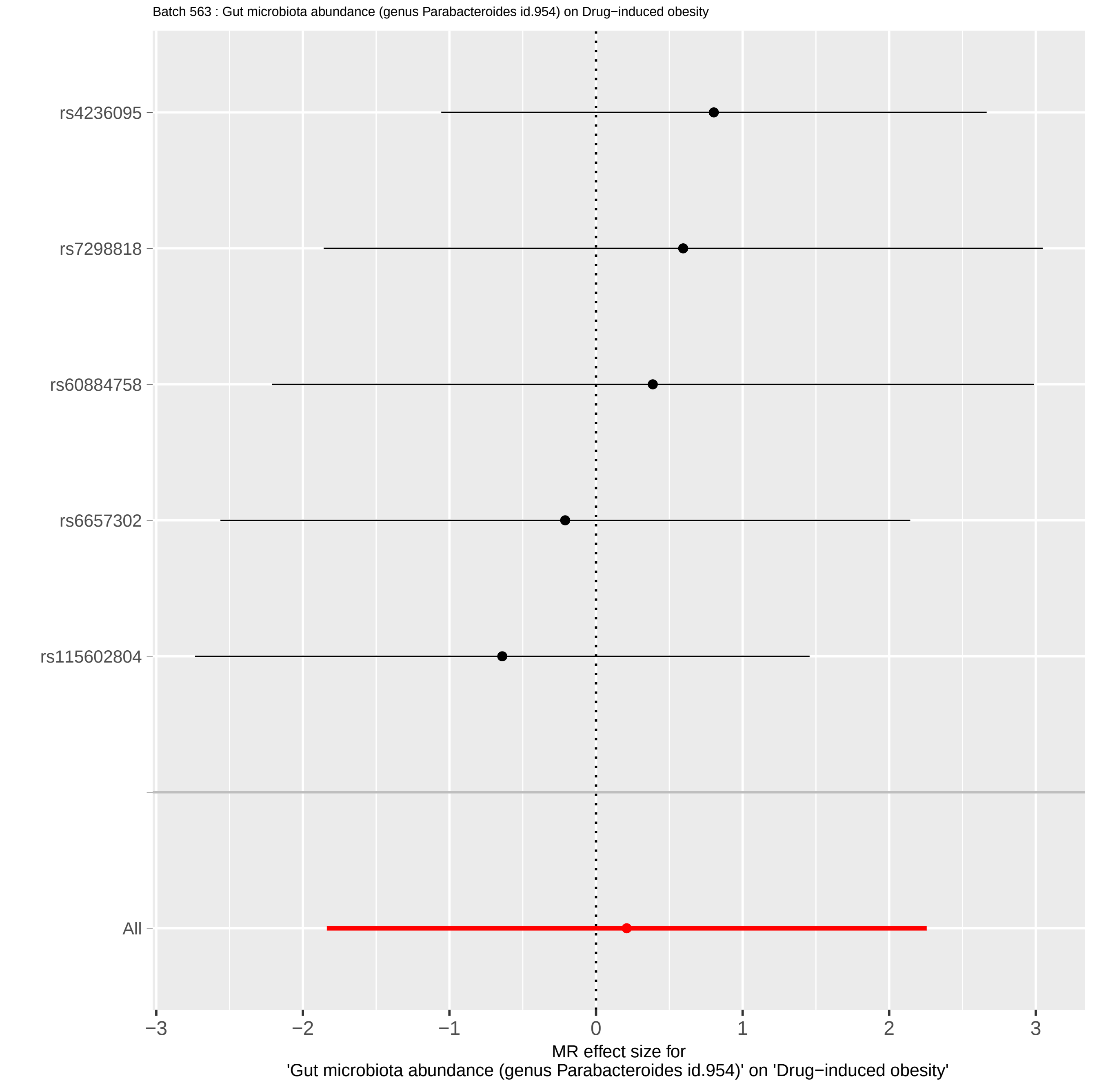

Batch 563 : Gut microbiota abundance (genus Parabacteroides id.954) on Drug−induced obesity
rs4236095
rs7298818
rs60884758
rs6657302
rs115602804
All
−3
−2
−1
0
1
2
3
MR effect size for
'Gut microbiota abundance (genus Parabacteroides id.954)' on 'Drug−induced obesity'

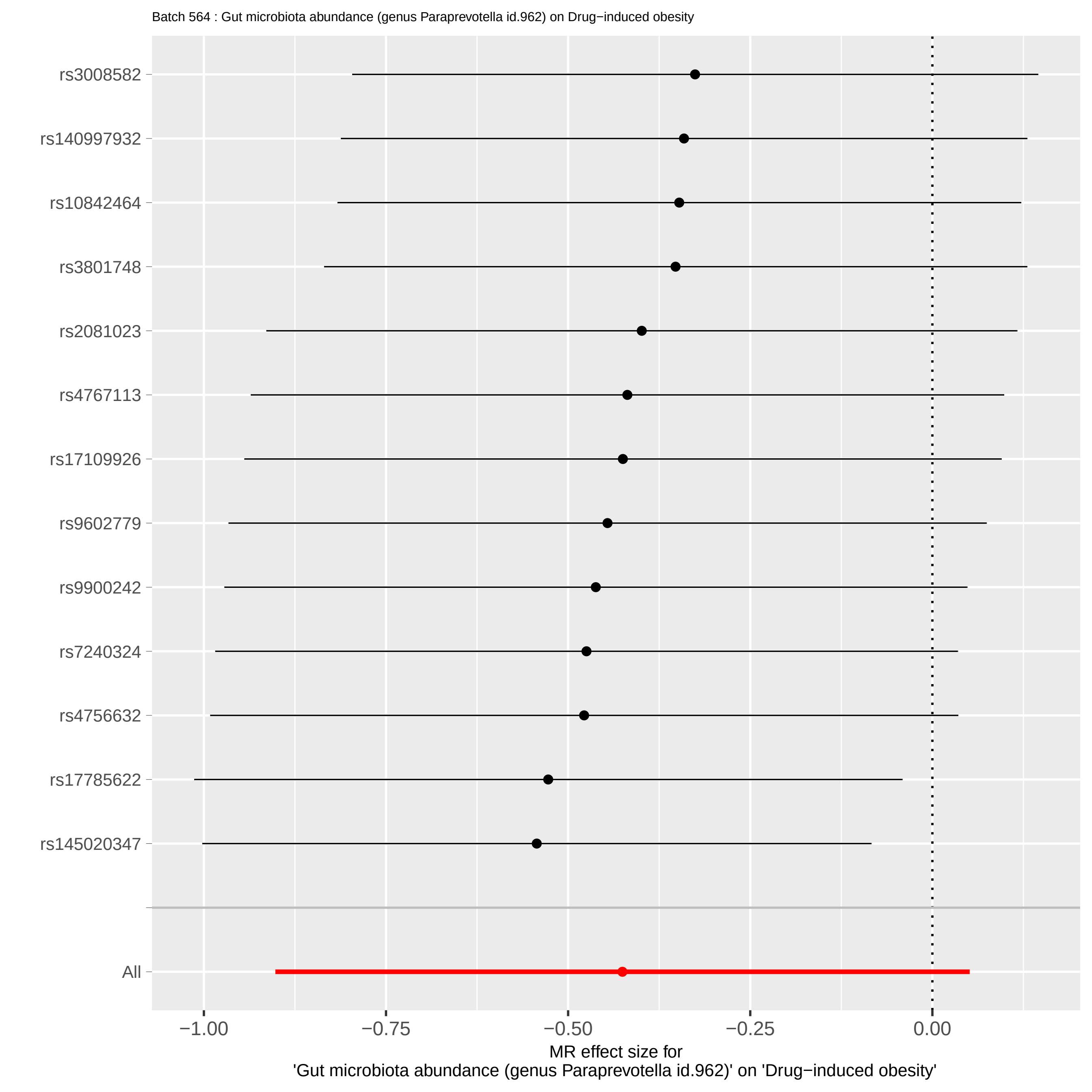

Batch 564 : Gut microbiota abundance (genus Paraprevotella id.962) on Drug−induced obesity
rs3008582
rs140997932
rs10842464
rs3801748
rs2081023
rs4767113
rs17109926
rs9602779
rs9900242
rs7240324
rs4756632
rs17785622
rs145020347
All
−1.00
−0.75
−0.50
−0.25
0.00
MR effect size for
'Gut microbiota abundance (genus Paraprevotella id.962)' on 'Drug−induced obesity'

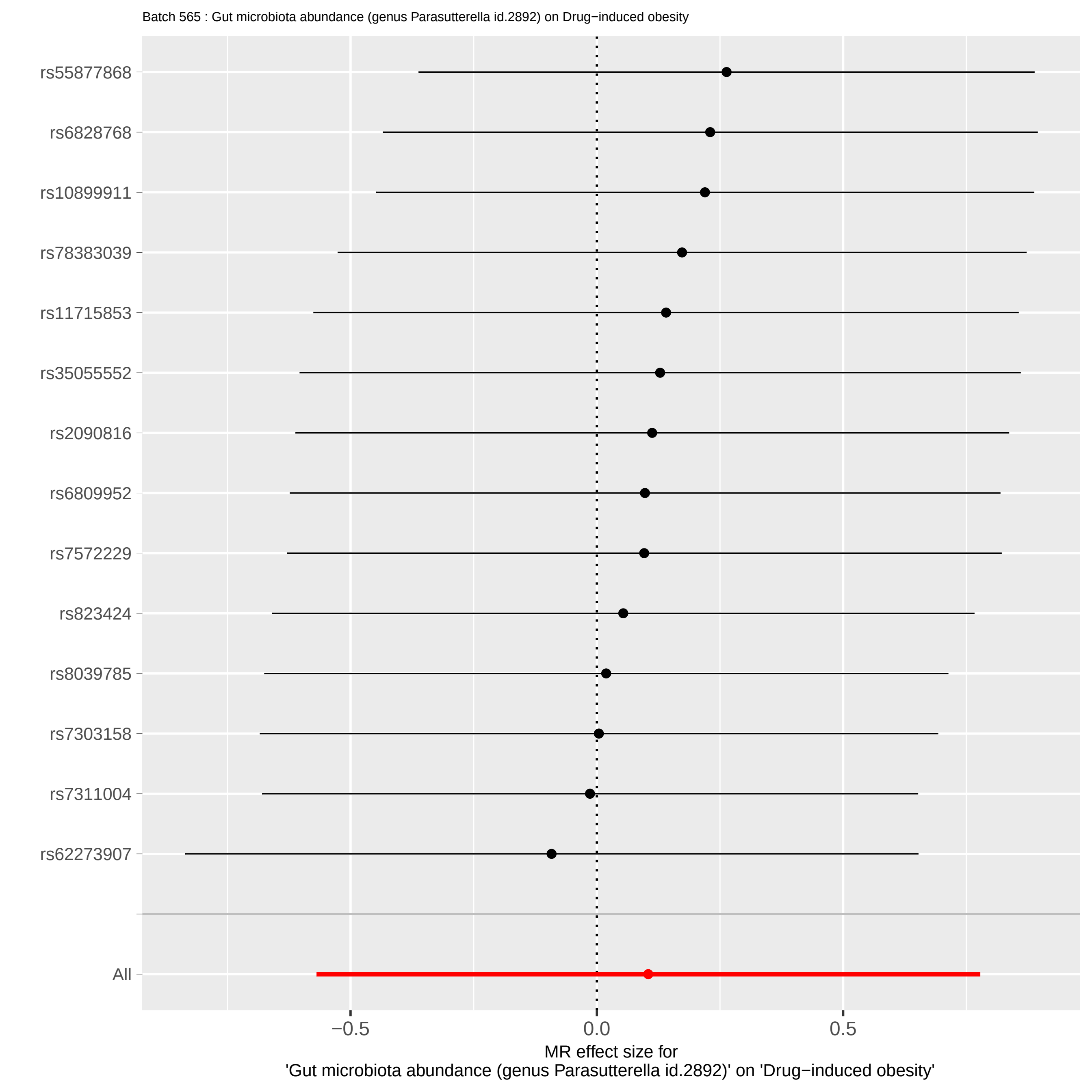

Batch 565 : Gut microbiota abundance (genus Parasutterella id.2892) on Drug−induced obesity
rs55877868
rs6828768
rs10899911
rs78383039
rs11715853
rs35055552
rs2090816
rs6809952
rs7572229
rs823424
rs8039785
rs7303158
rs7311004
rs62273907
All
−0.5
0.0
0.5
MR effect size for
'Gut microbiota abundance (genus Parasutterella id.2892)' on 'Drug−induced obesity'

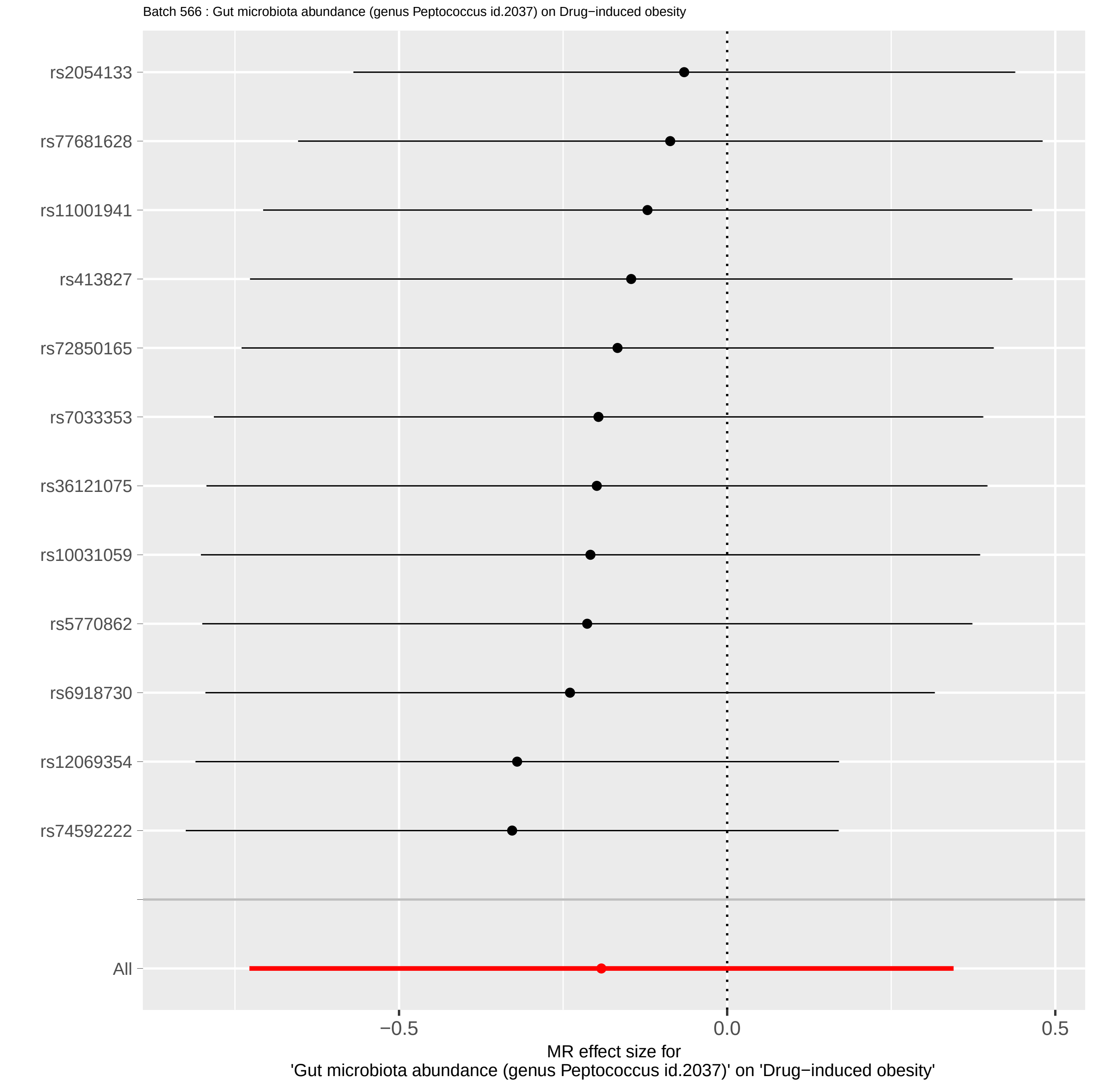
Batch 566 : Gut microbiota abundance (genus Peptococcus id.2037) on Drug−induced obesity
rs2054133
rs77681628
rs11001941
rs413827
rs72850165
rs7033353
rs36121075
rs10031059
rs5770862
rs6918730
rs12069354
rs74592222
All
−0.5
0.0
0.5
MR effect size for
'Gut microbiota abundance (genus Peptococcus id.2037)' on 'Drug−induced obesity'

Batch 567 : Gut microbiota abundance (genus Phascolarctobacterium id.2168) on Drug−induced obesity

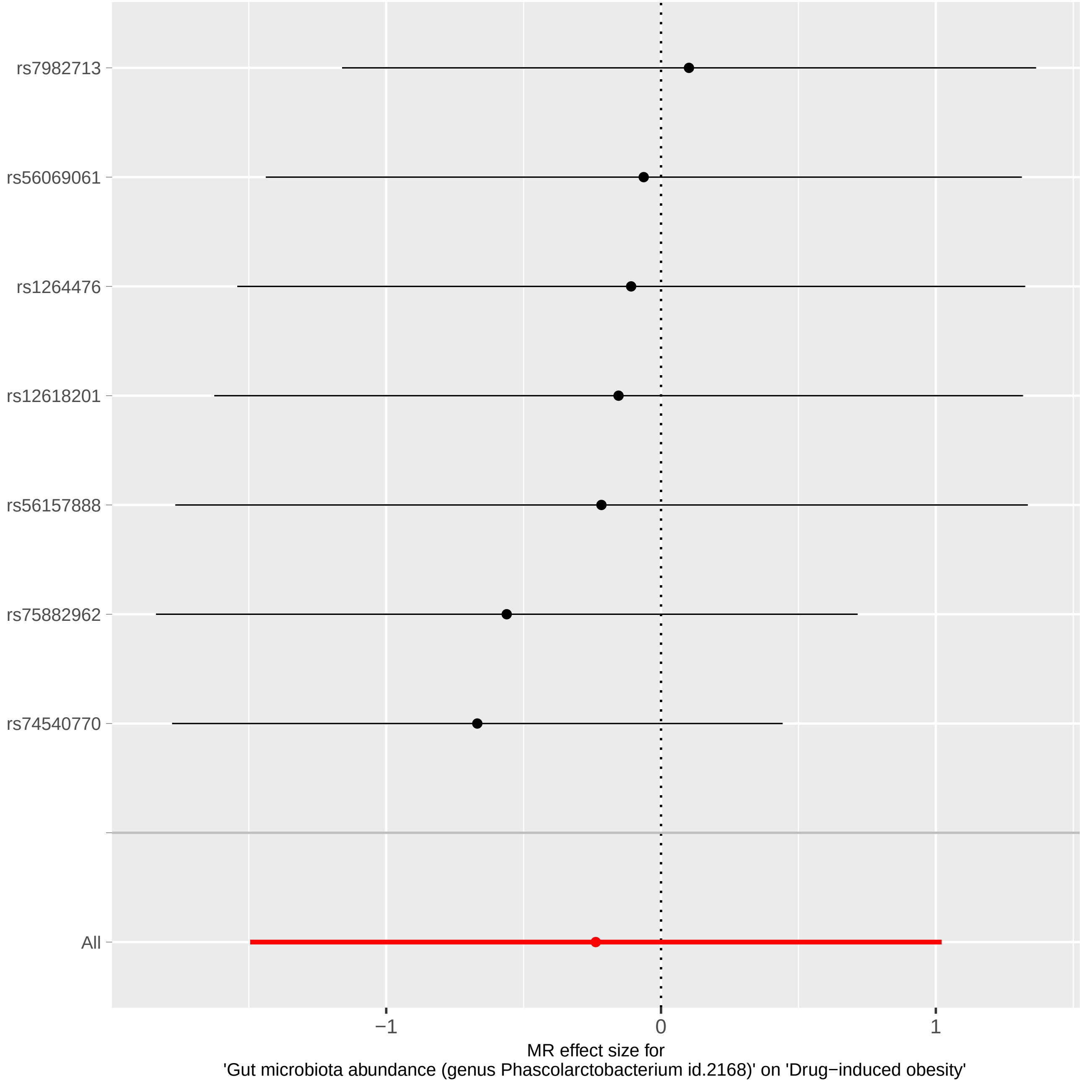

Batch 568 : Gut microbiota abundance (genus Prevotella7 id.11182) on Drug−induced obesity

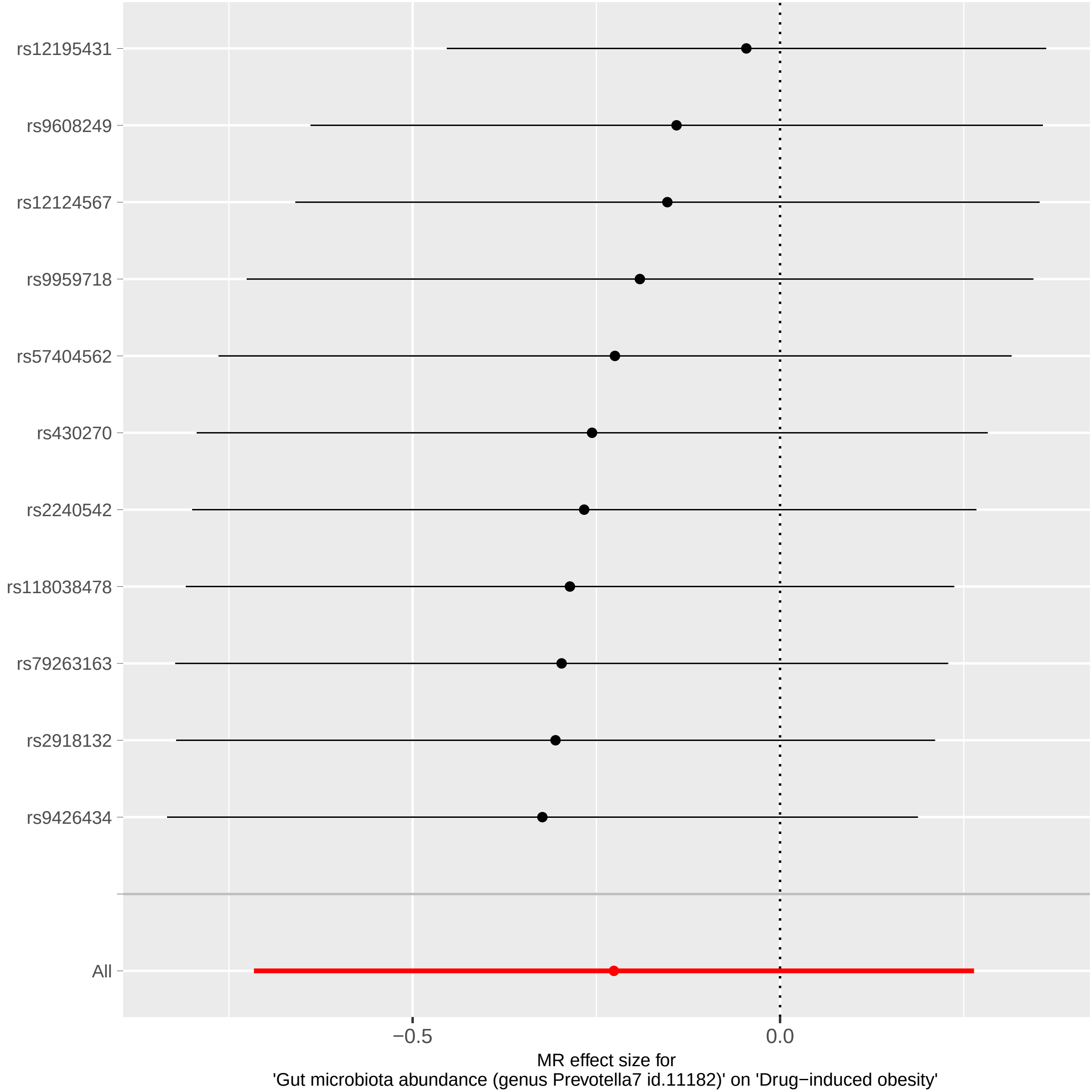

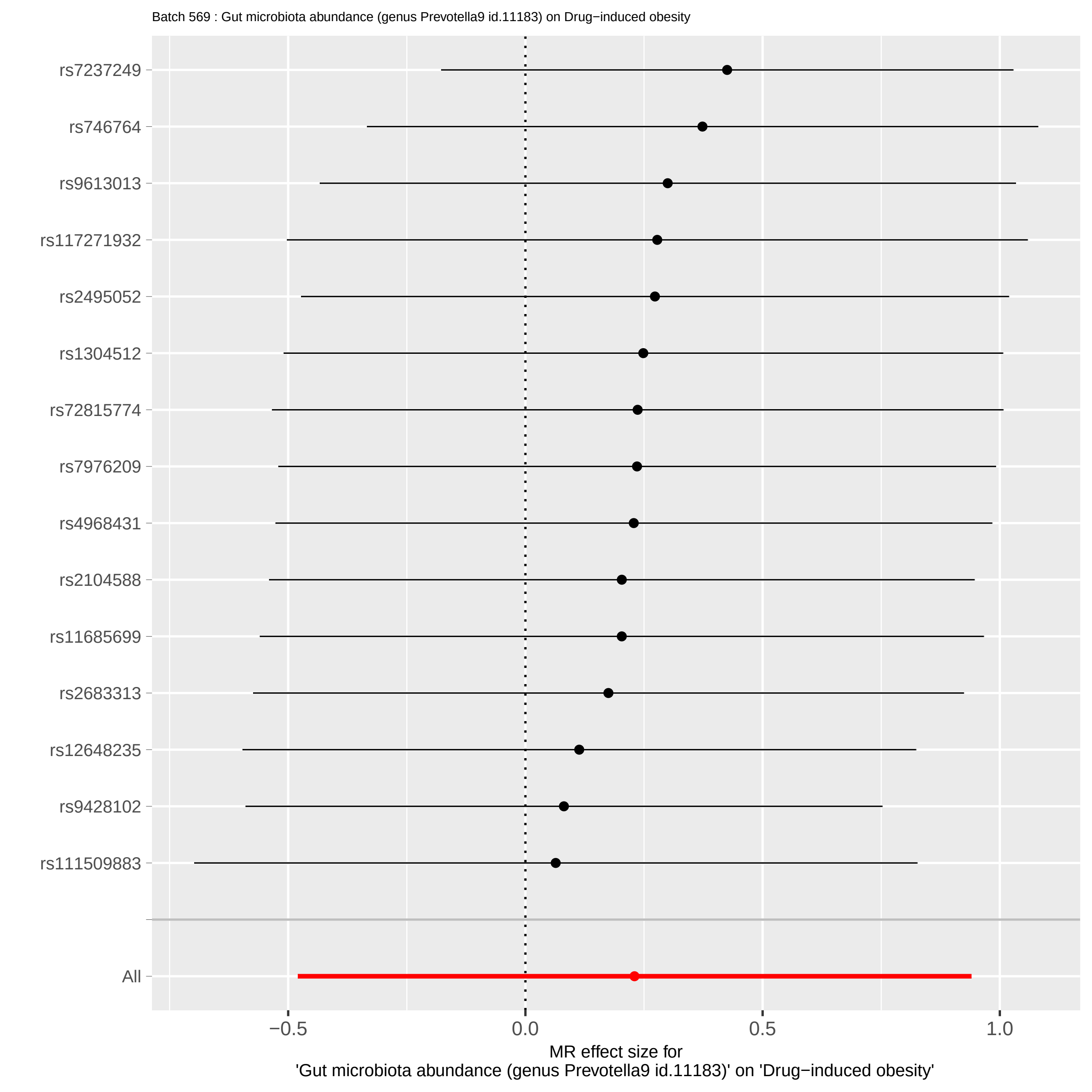
Batch 569 : Gut microbiota abundance (genus Prevotella9 id.11183) on Drug−induced obesity
rs7237249
rs746764
rs9613013
rs117271932
rs2495052
rs1304512
rs72815774
rs7976209
rs4968431
rs2104588
rs11685699
rs2683313
rs12648235
rs9428102
rs111509883
All
−0.5
0.0
0.5
1.0
MR effect size for
'Gut microbiota abundance (genus Prevotella9 id.11183)' on 'Drug−induced obesity'

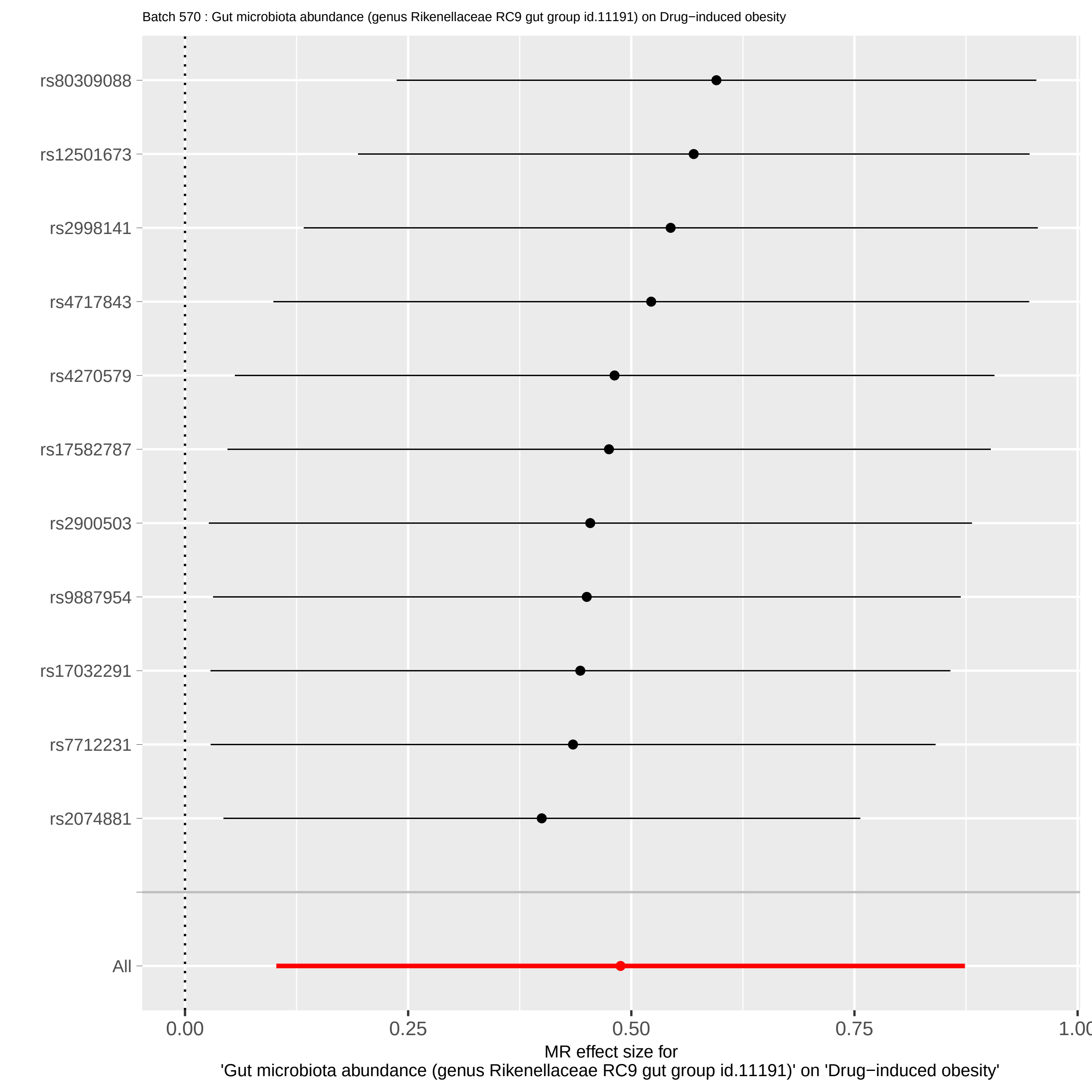
Batch 570 : Gut microbiota abundance (genus Rikenellaceae RC9 gut group id.11191) on Drug−induced obesity
rs80309088
rs12501673
rs2998141
rs4717843
rs4270579
rs17582787
rs2900503
rs9887954
rs17032291
rs7712231
rs2074881
All
0.00
0.25
0.50
0.75
1.00
MR effect size for
'Gut microbiota abundance (genus Rikenellaceae RC9 gut group id.11191)' on 'Drug−induced obesity'

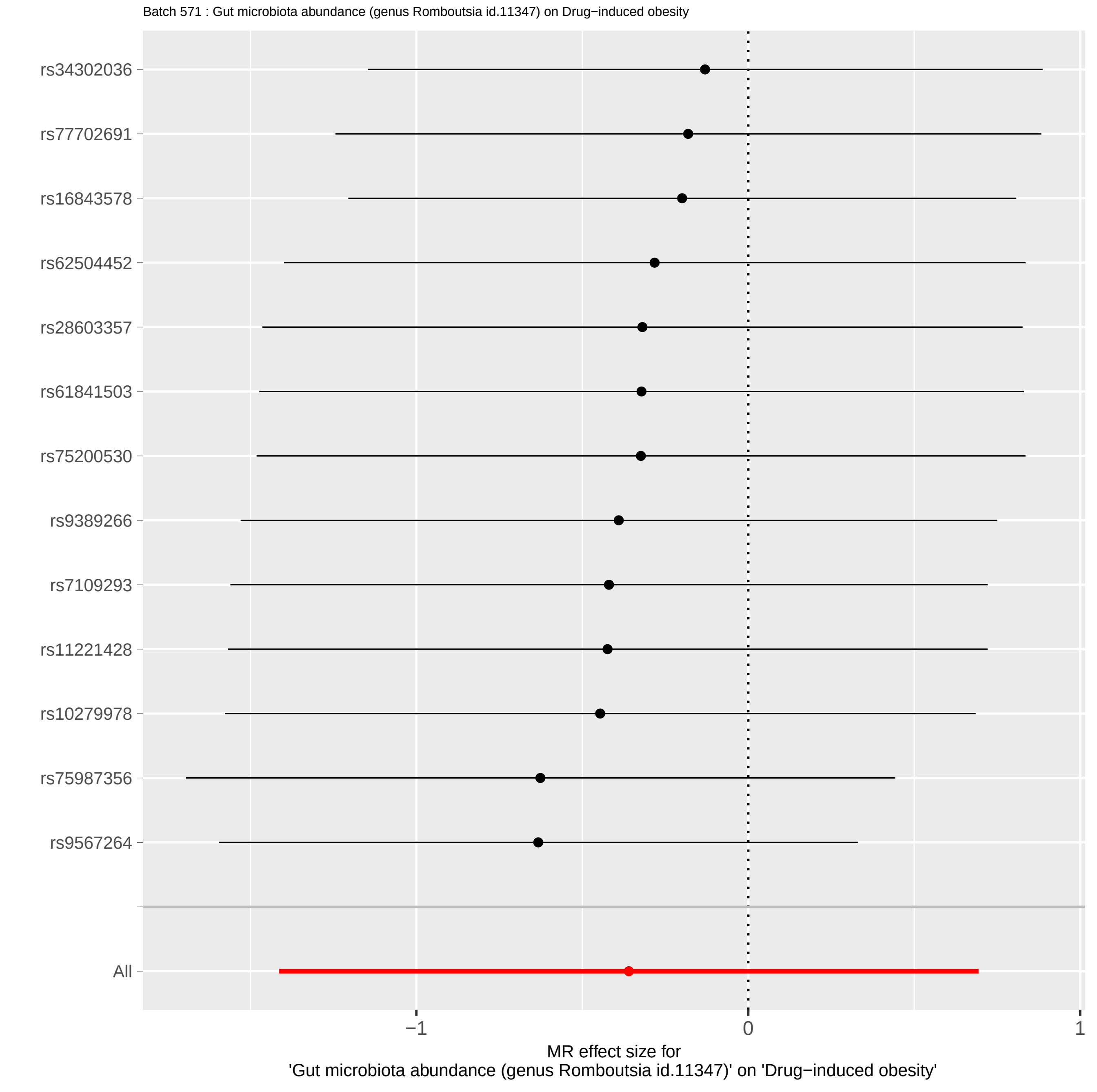

Batch 571 : Gut microbiota abundance (genus Romboutsia id.11347) on Drug−induced obesity
rs34302036
rs77702691
rs16843578
rs62504452
rs28603357
rs61841503
rs75200530
rs9389266
rs7109293
rs11221428
rs10279978
rs75987356
rs9567264
All
−1
0
1
MR effect size for
'Gut microbiota abundance (genus Romboutsia id.11347)' on 'Drug−induced obesity'

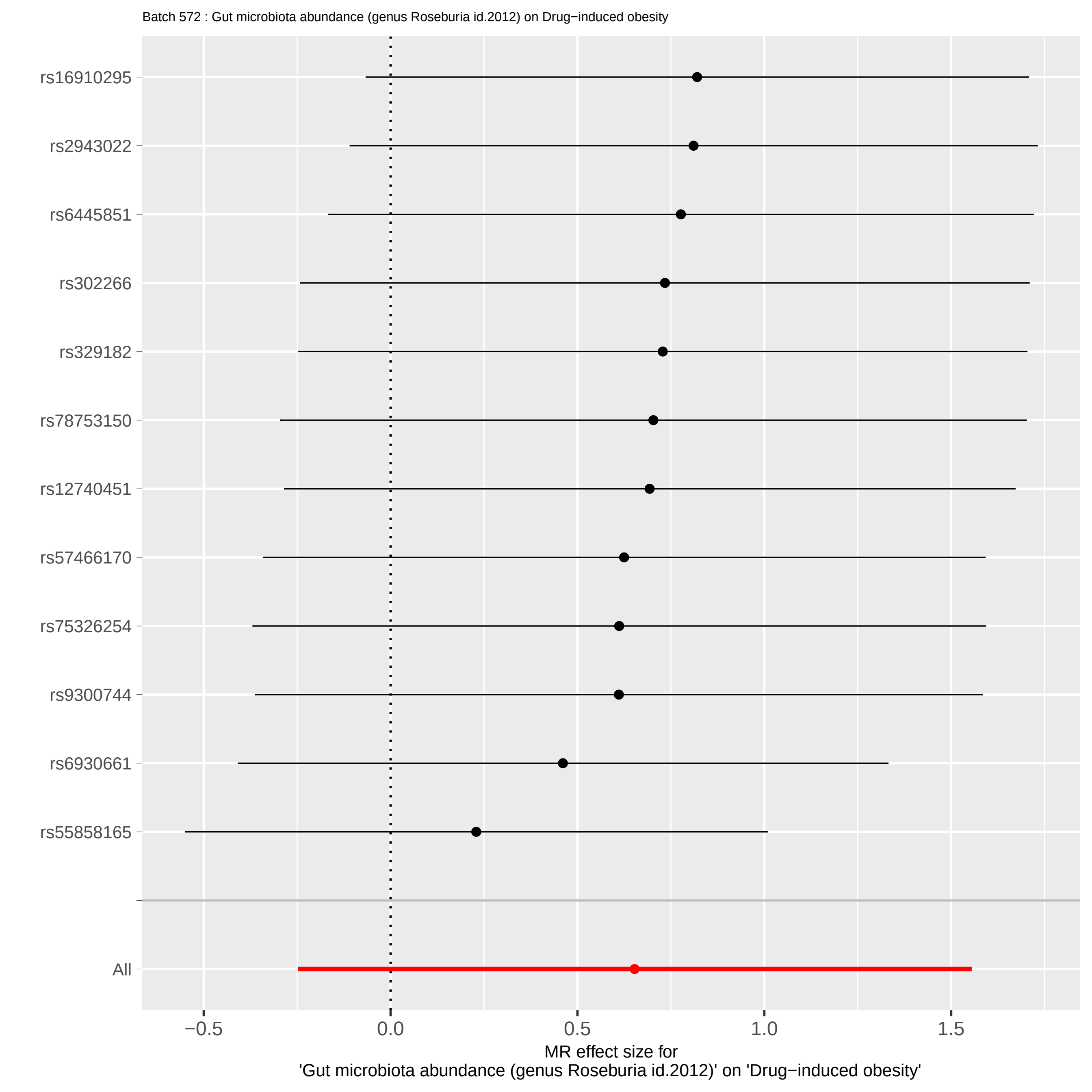

Batch 572 : Gut microbiota abundance (genus Roseburia id.2012) on Drug−induced obesity
rs16910295
rs2943022
rs6445851
rs302266
rs329182
rs78753150
rs12740451
rs57466170
rs75326254
rs9300744
rs6930661
rs55858165
All
−0.5
0.0
0.5
1.0
1.5
MR effect size for
'Gut microbiota abundance (genus Roseburia id.2012)' on 'Drug−induced obesity'

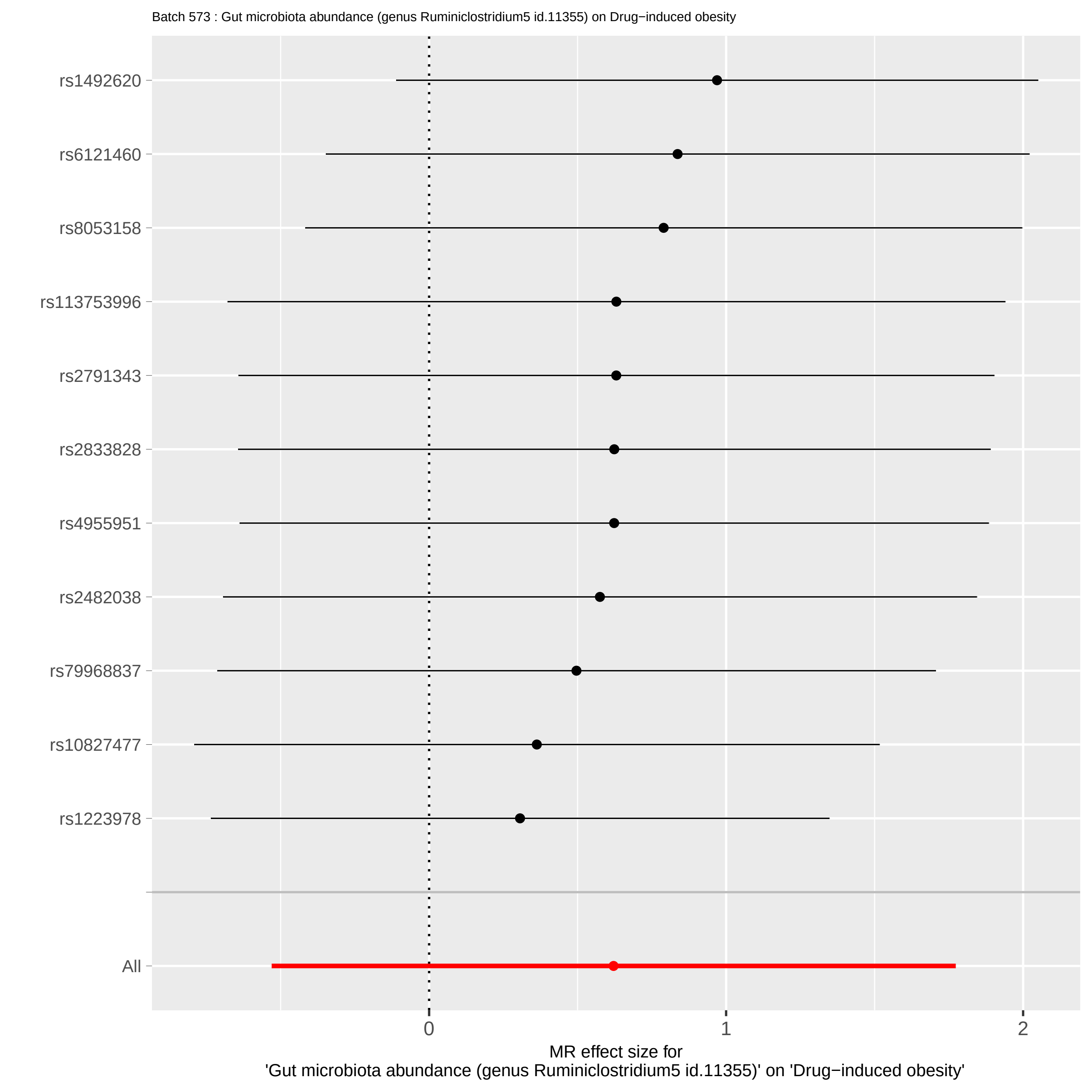
Batch 573 : Gut microbiota abundance (genus Ruminiclostridium5 id.11355) on Drug−induced obesity
rs1492620
rs6121460
rs8053158
rs113753996
rs2791343
rs2833828
rs4955951
rs2482038
rs79968837
rs10827477
rs1223978
All
0
1
2
MR effect size for
'Gut microbiota abundance (genus Ruminiclostridium5 id.11355)' on 'Drug−induced obesity'

Batch 574 : Gut microbiota abundance (genus Ruminiclostridium6 id.11356) on Drug−induced obesity

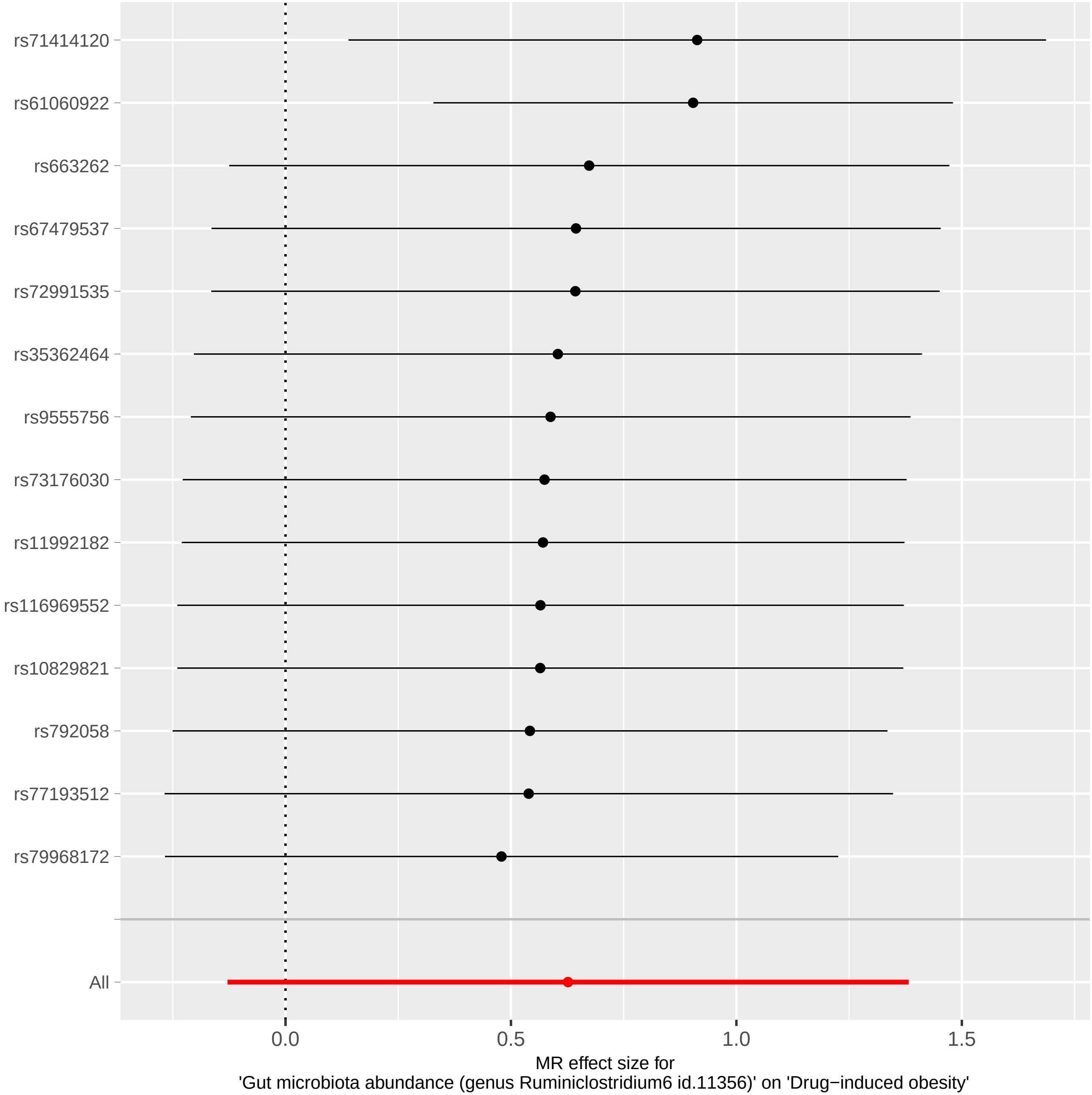

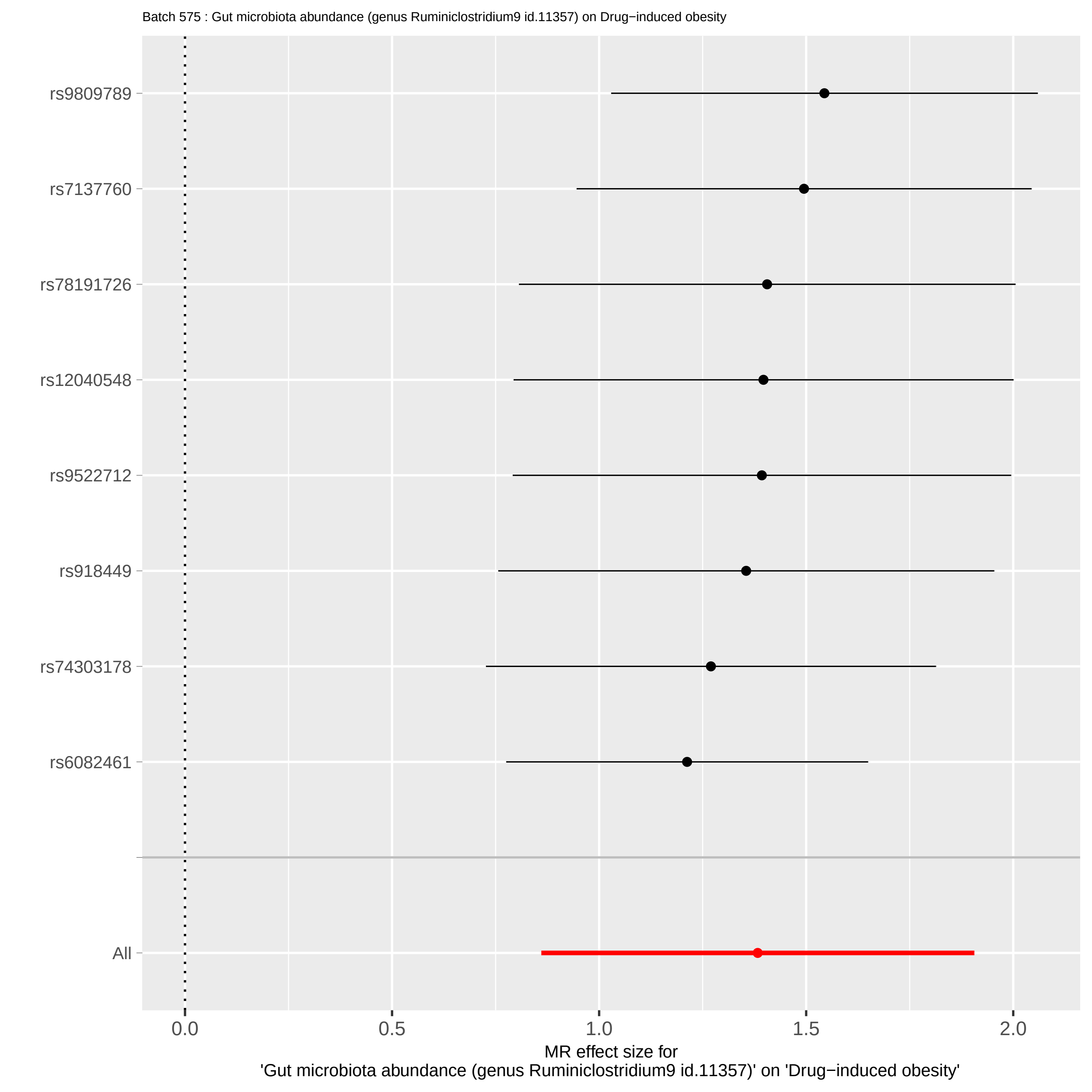

Batch 575 : Gut microbiota abundance (genus Ruminiclostridium9 id.11357) on Drug−induced obesity
rs9809789
rs7137760
rs78191726
rs12040548
rs9522712
rs918449
rs74303178
rs6082461
All
0.0
0.5
1.0
1.5
2.0
MR effect size for
'Gut microbiota abundance (genus Ruminiclostridium9 id.11357)' on 'Drug−induced obesity'

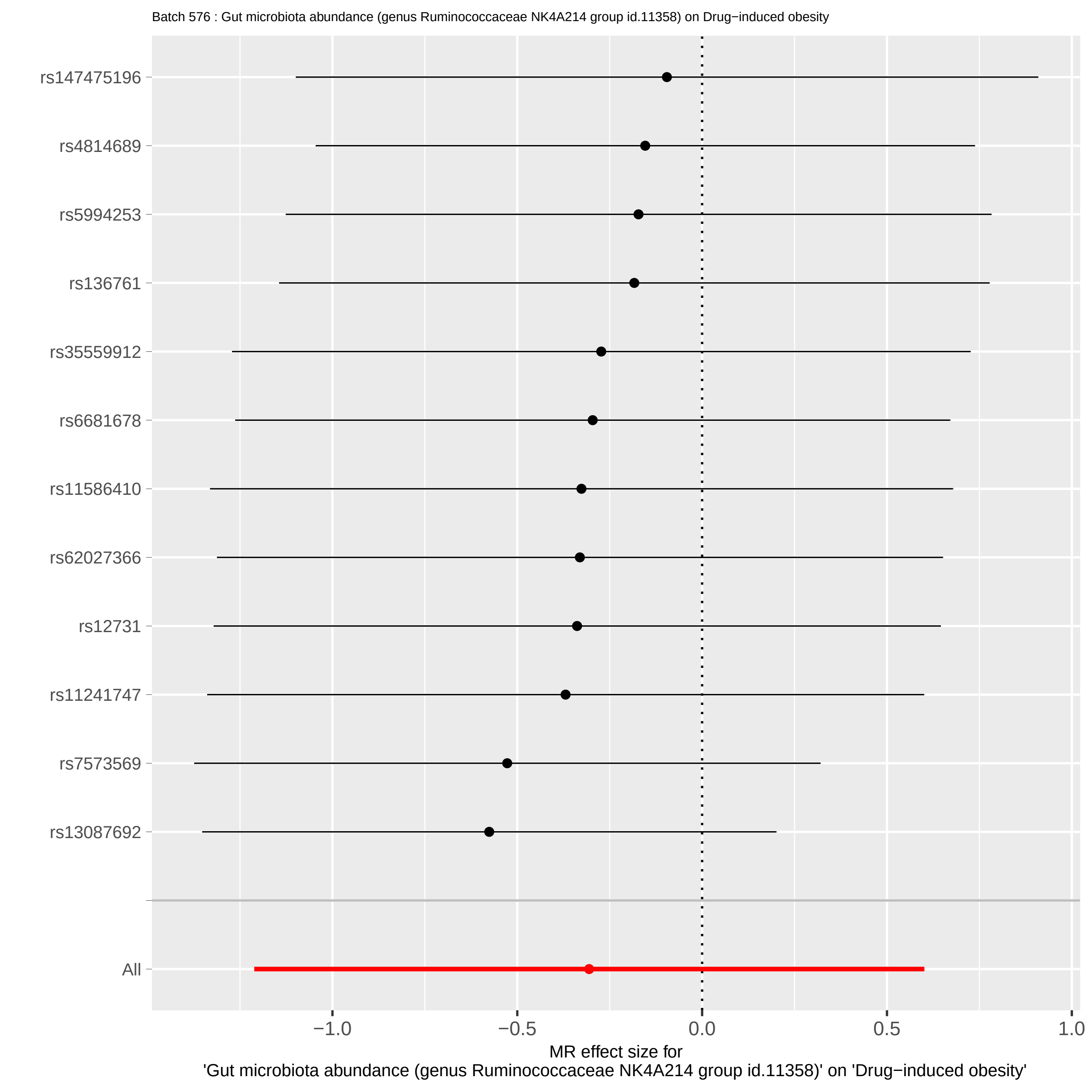
Batch 576 : Gut microbiota abundance (genus Ruminococcaceae NK4A214 group id.11358) on Drug−induced obesity
rs147475196
rs4814689
rs5994253
rs136761
rs35559912
rs6681678
rs11586410
rs62027366
rs12731
rs11241747
rs7573569
rs13087692
All
−1.0
−0.5
0.0
0.5
1.0
MR effect size for
'Gut microbiota abundance (genus Ruminococcaceae NK4A214 group id.11358)' on 'Drug−induced obesity'

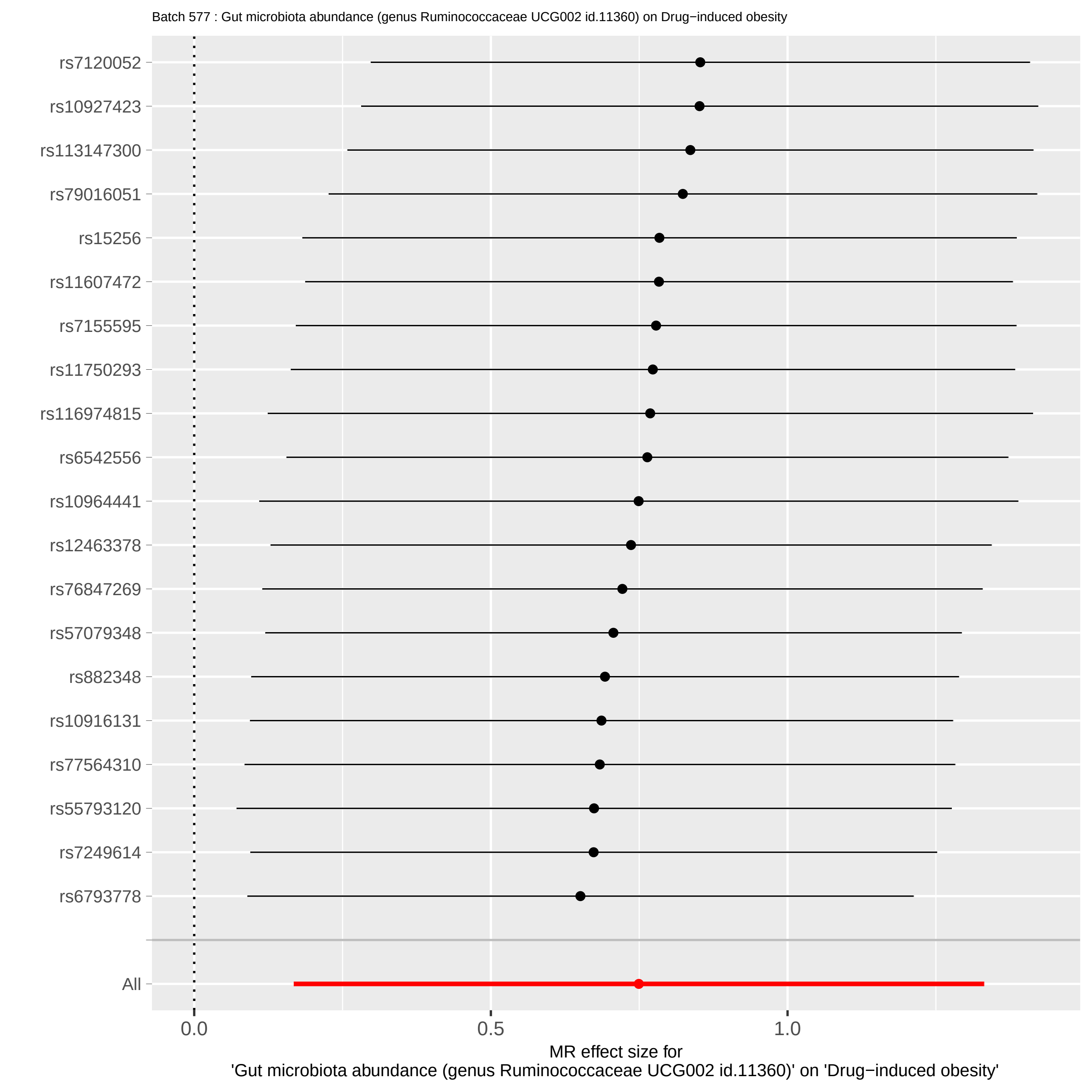

Batch 577 : Gut microbiota abundance (genus Ruminococcaceae UCG002 id.11360) on Drug−induced obesity
rs7120052
rs10927423
rs113147300
rs79016051
rs15256
rs11607472
rs7155595
rs11750293
rs116974815
rs6542556
rs10964441
rs12463378
rs76847269
rs57079348
rs882348
rs10916131
rs77564310
rs55793120
rs7249614
rs6793778
All
0.0
0.5
1.0
MR effect size for
'Gut microbiota abundance (genus Ruminococcaceae UCG002 id.11360)' on 'Drug−induced obesity'

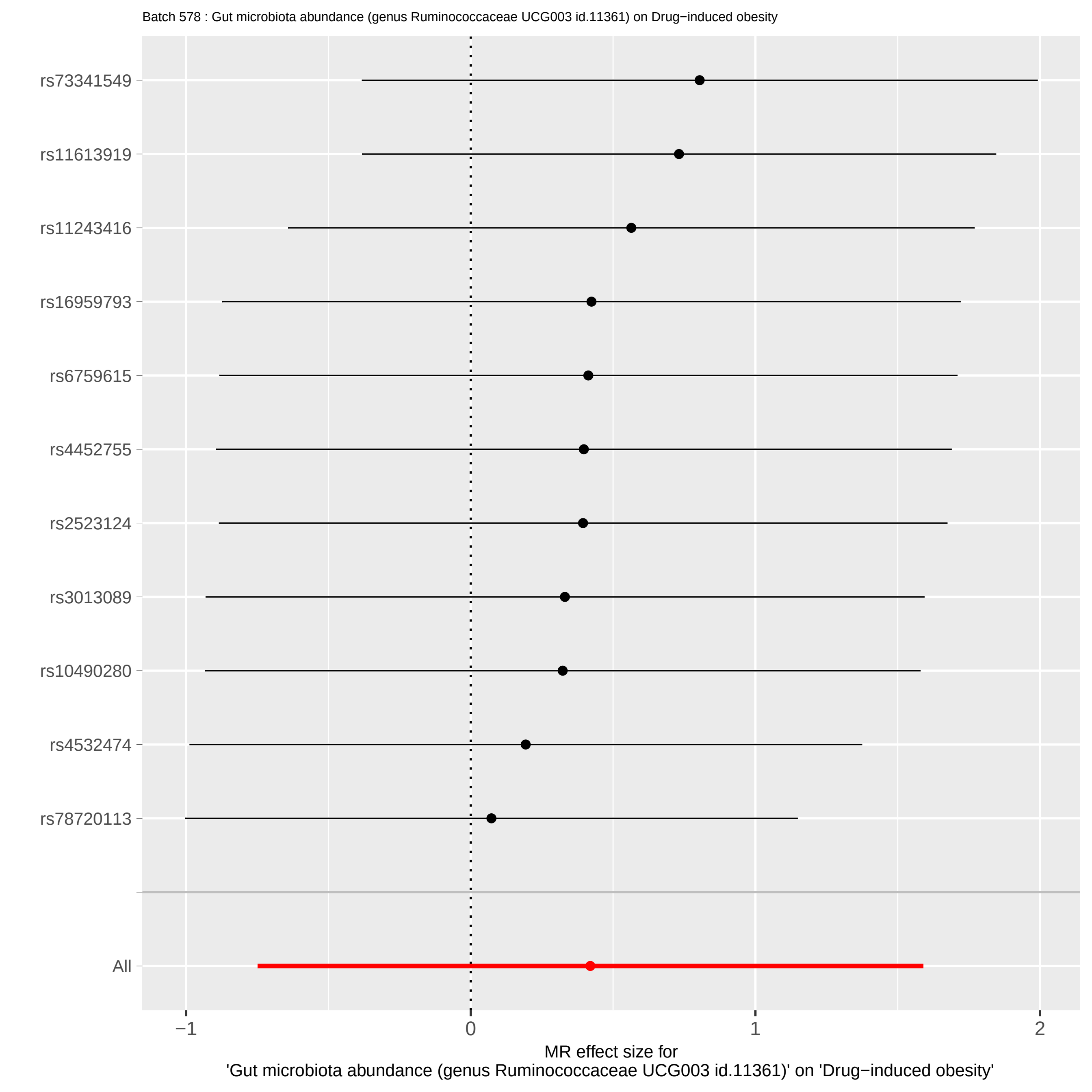

Batch 578 : Gut microbiota abundance (genus Ruminococcaceae UCG003 id.11361) on Drug−induced obesity
rs73341549
rs11613919
rs11243416
rs16959793
rs6759615
rs4452755
rs2523124
rs3013089
rs10490280
rs4532474
rs78720113
All
−1
0
1
2
MR effect size for
'Gut microbiota abundance (genus Ruminococcaceae UCG003 id.11361)' on 'Drug−induced obesity'

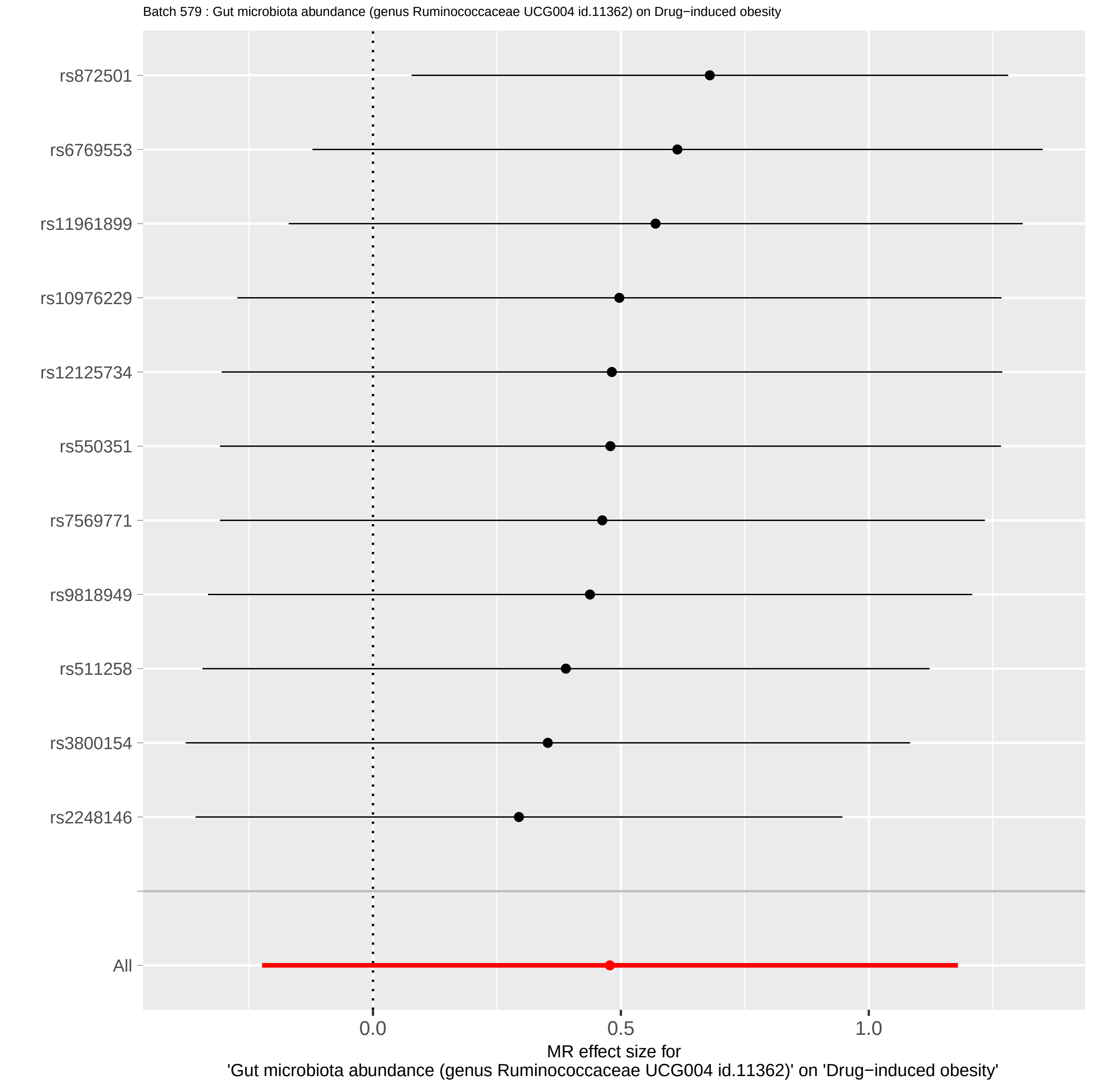
Batch 579 : Gut microbiota abundance (genus Ruminococcaceae UCG004 id.11362) on Drug−induced obesity
rs872501
rs6769553
rs11961899
rs10976229
rs12125734
rs550351
rs7569771
rs9818949
rs511258
rs3800154
rs2248146
All
0.0
0.5
1.0
MR effect size for
'Gut microbiota abundance (genus Ruminococcaceae UCG004 id.11362)' on 'Drug−induced obesity'

Batch 580 : Gut microbiota abundance (genus Ruminococcaceae UCG005 id.11363) on Drug−induced obesity

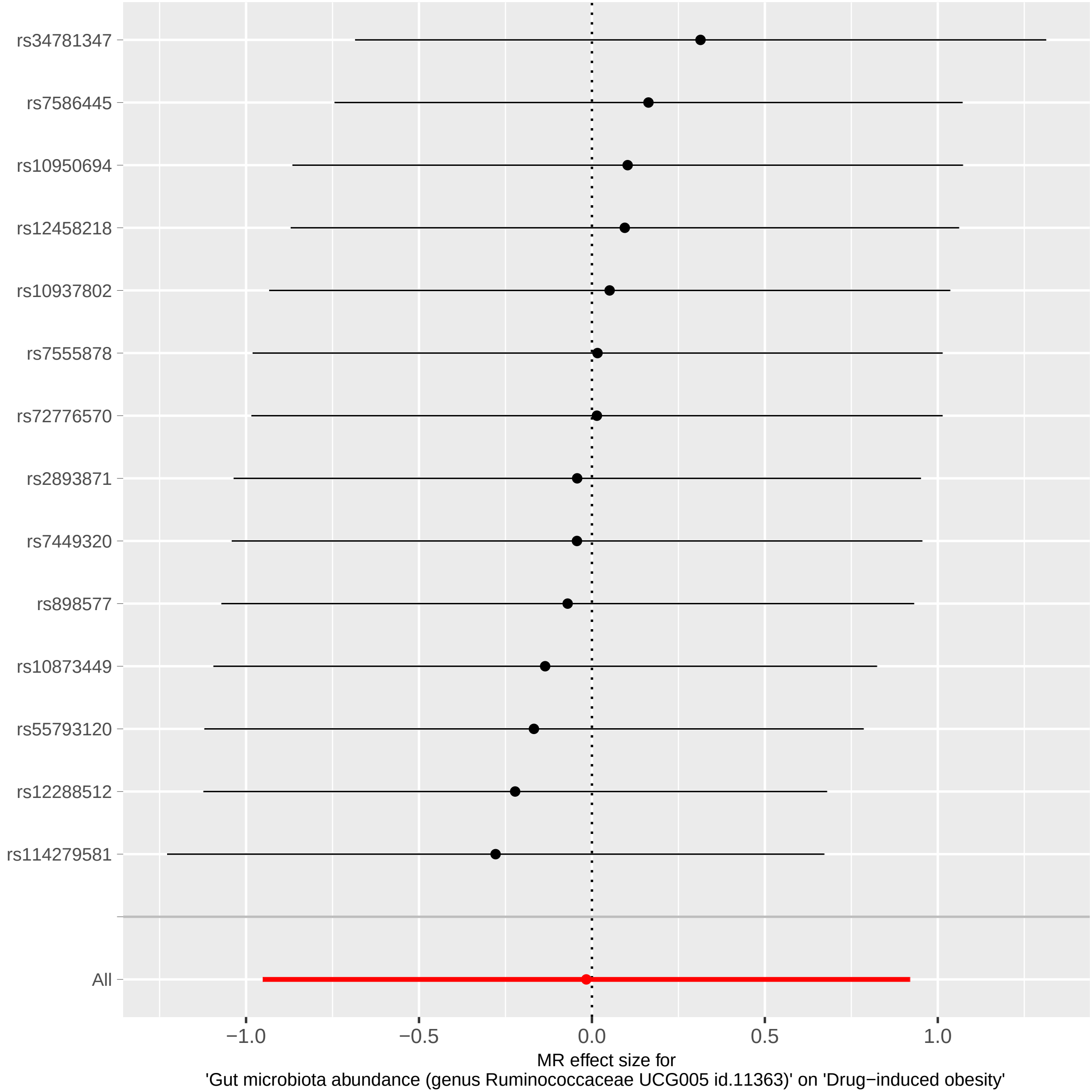

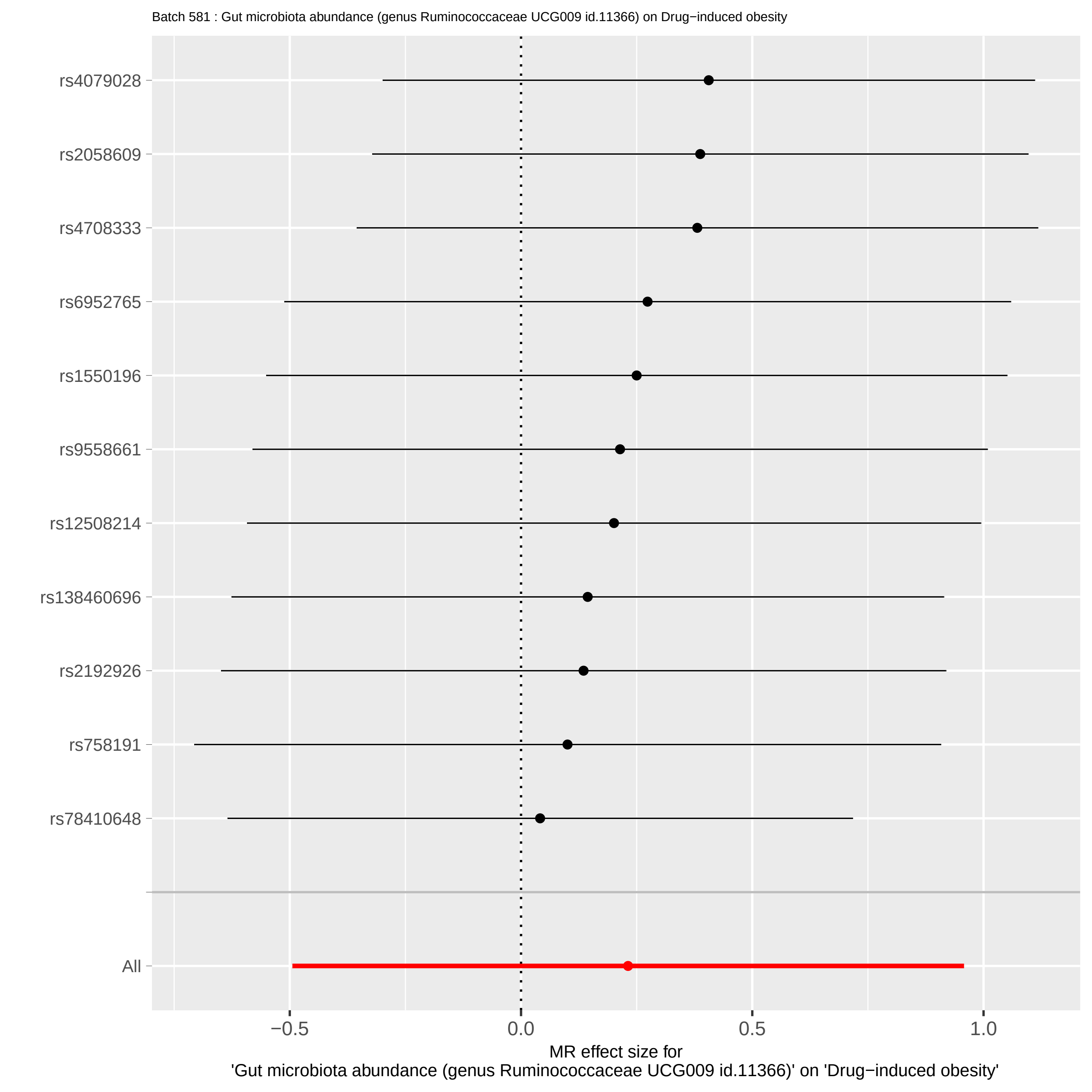

Batch 581 : Gut microbiota abundance (genus Ruminococcaceae UCG009 id.11366) on Drug−induced obesity
rs4079028
rs2058609
rs4708333
rs6952765
rs1550196
rs9558661
rs12508214
rs138460696
rs2192926
rs758191
rs78410648
All
−0.5
0.0
0.5
1.0
MR effect size for
'Gut microbiota abundance (genus Ruminococcaceae UCG009 id.11366)' on 'Drug−induced obesity'

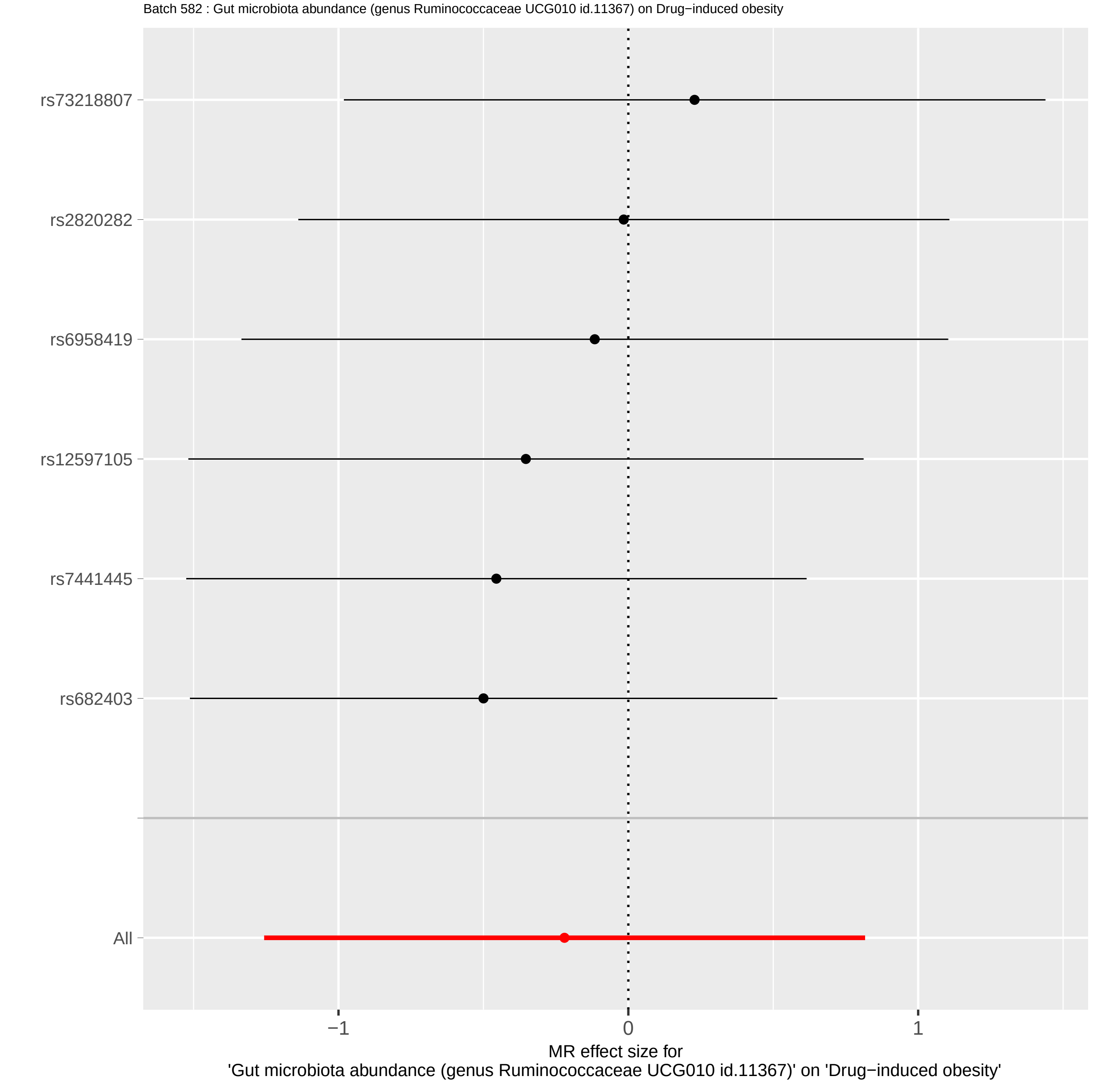

Batch 582 : Gut microbiota abundance (genus Ruminococcaceae UCG010 id.11367) on Drug−induced obesity
rs73218807
rs2820282
rs6958419
rs12597105
rs7441445
rs682403
All
−1
0
1
MR effect size for
'Gut microbiota abundance (genus Ruminococcaceae UCG010 id.11367)' on 'Drug−induced obesity'

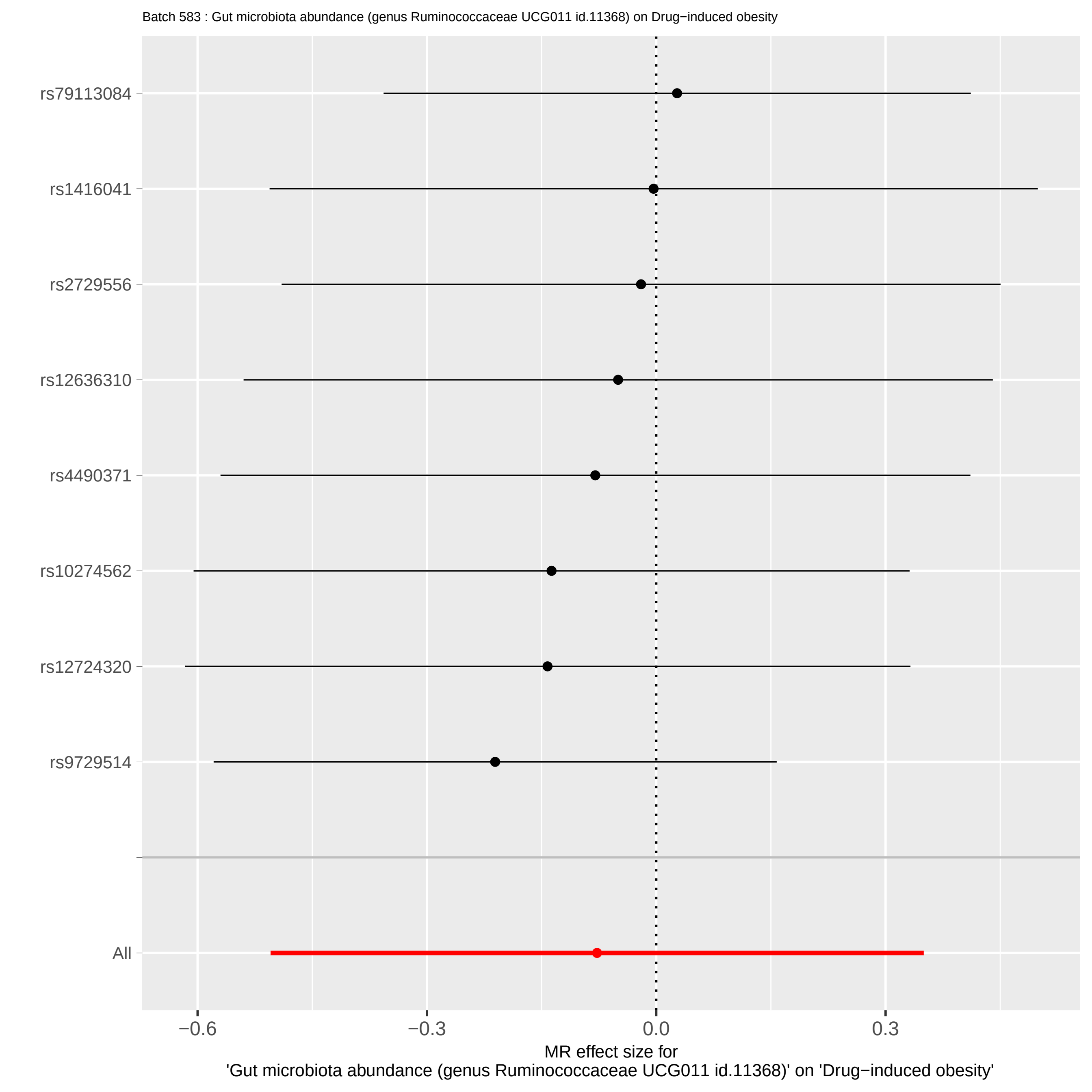

Batch 583 : Gut microbiota abundance (genus Ruminococcaceae UCG011 id.11368) on Drug−induced obesity
rs79113084
rs1416041
rs2729556
rs12636310
rs4490371
rs10274562
rs12724320
rs9729514
All
−0.6
−0.3
0.0
0.3
MR effect size for
'Gut microbiota abundance (genus Ruminococcaceae UCG011 id.11368)' on 'Drug−induced obesity'

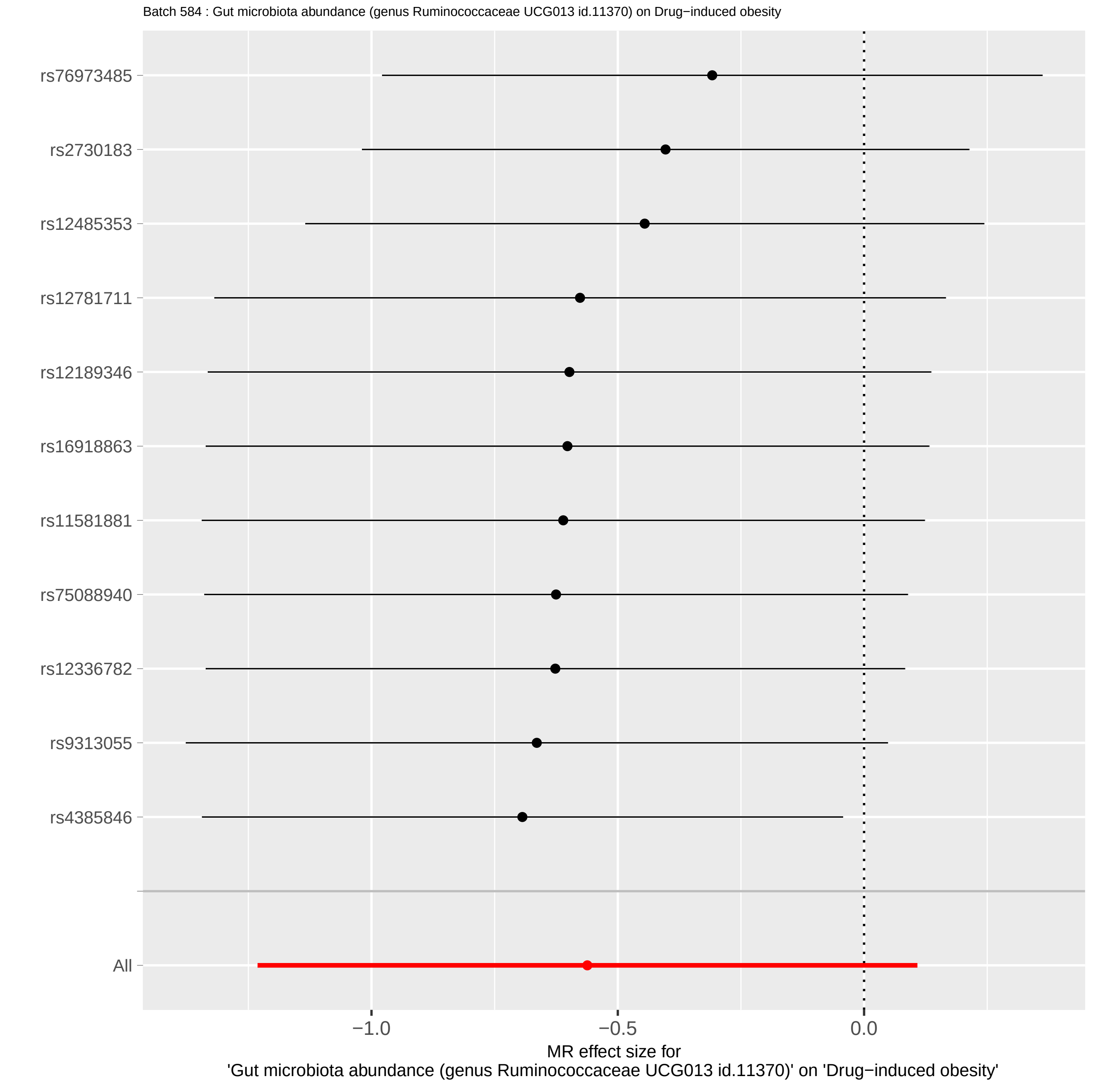
Batch 584 : Gut microbiota abundance (genus Ruminococcaceae UCG013 id.11370) on Drug−induced obesity
rs76973485
rs2730183
rs12485353
rs12781711
rs12189346
rs16918863
rs11581881
rs75088940
rs12336782
rs9313055
rs4385846
All
−1.0
−0.5
0.0
MR effect size for
'Gut microbiota abundance (genus Ruminococcaceae UCG013 id.11370)' on 'Drug−induced obesity'

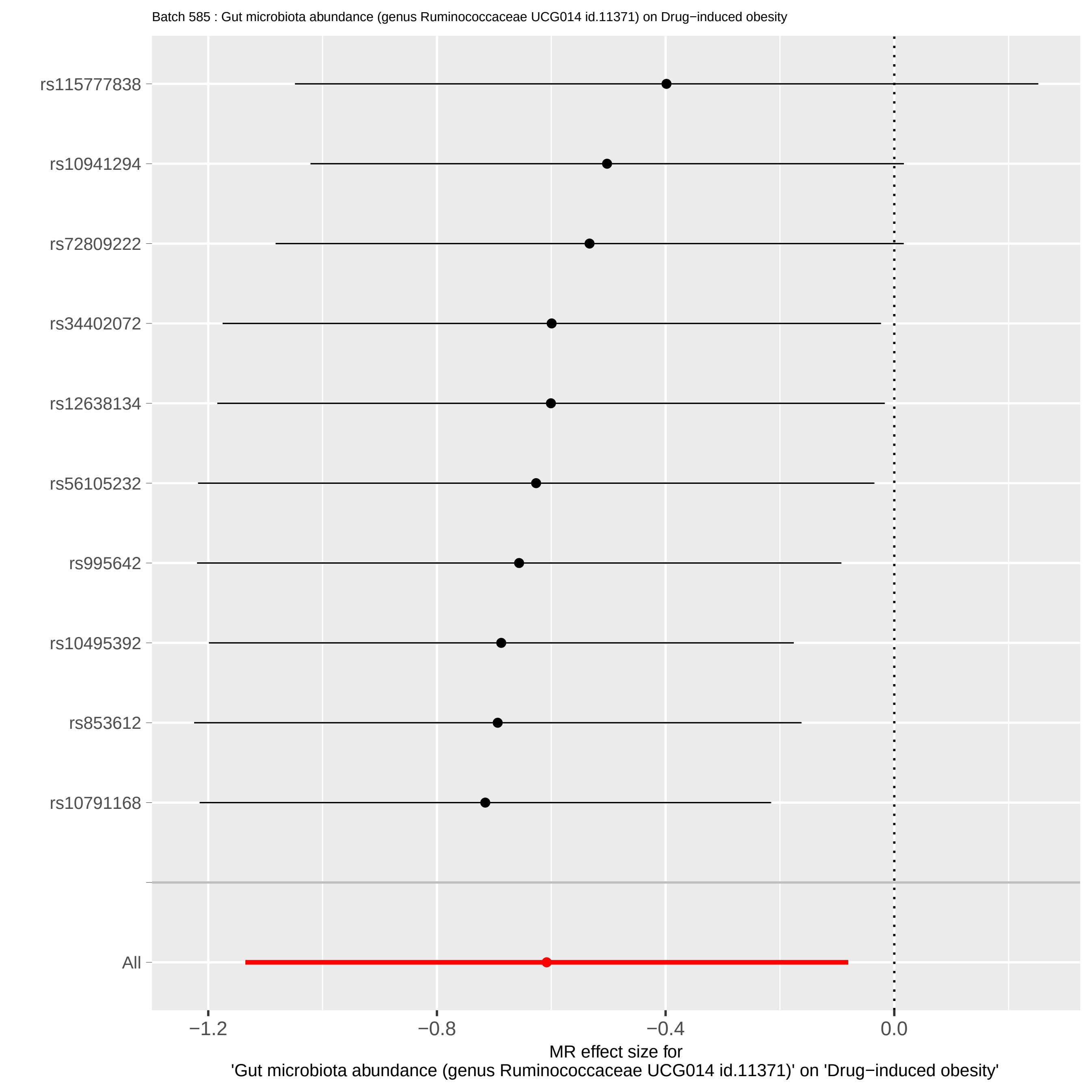

Batch 585 : Gut microbiota abundance (genus Ruminococcaceae UCG014 id.11371) on Drug−induced obesity
rs115777838
rs10941294
rs72809222
rs34402072
rs12638134
rs56105232
rs995642
rs10495392
rs853612
rs10791168
All
−1.2
−0.8
−0.4
0.0
MR effect size for
'Gut microbiota abundance (genus Ruminococcaceae UCG014 id.11371)' on 'Drug−induced obesity'

Batch 586 : Gut microbiota abundance (genus Ruminococcus1 id.11373) on Drug−induced obesity

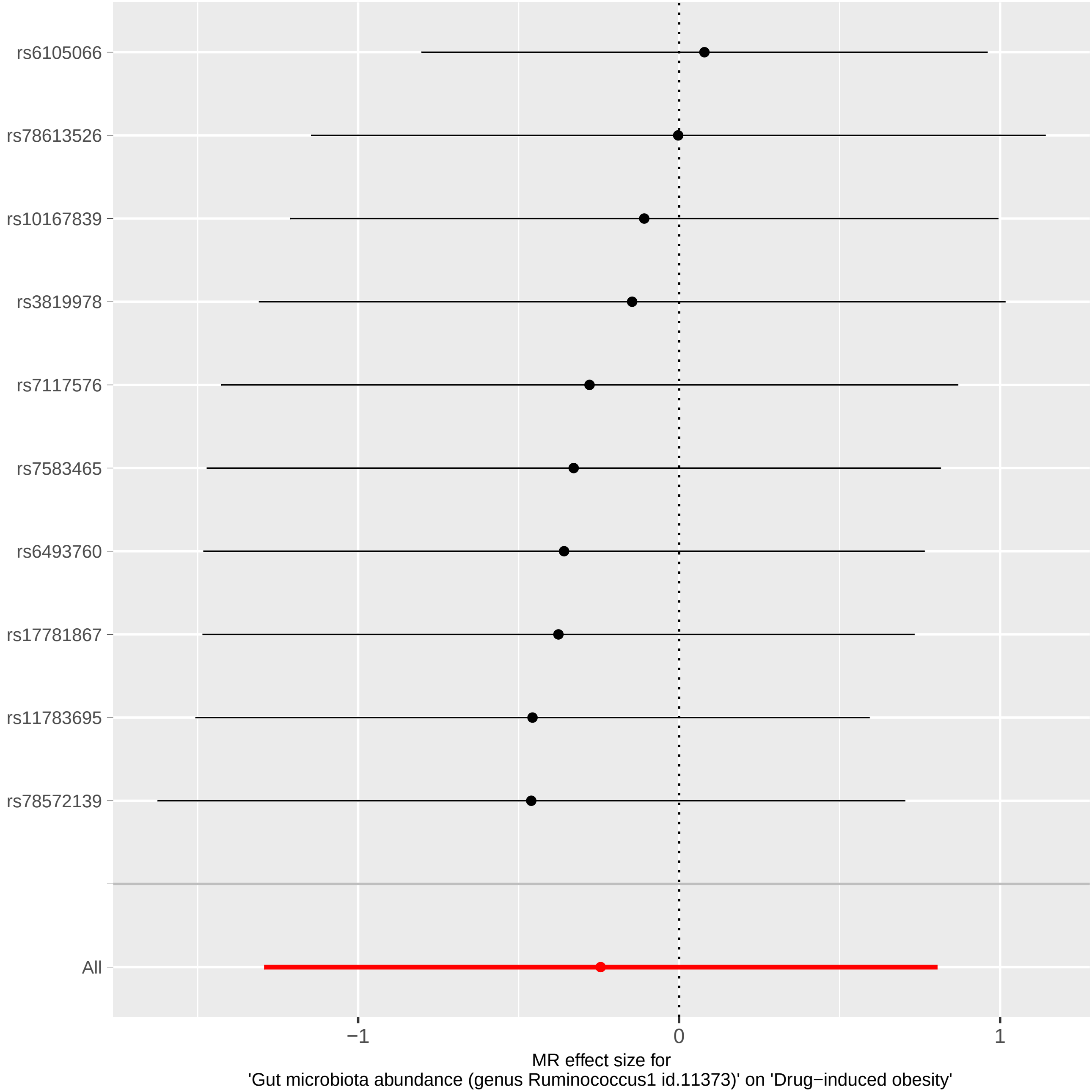

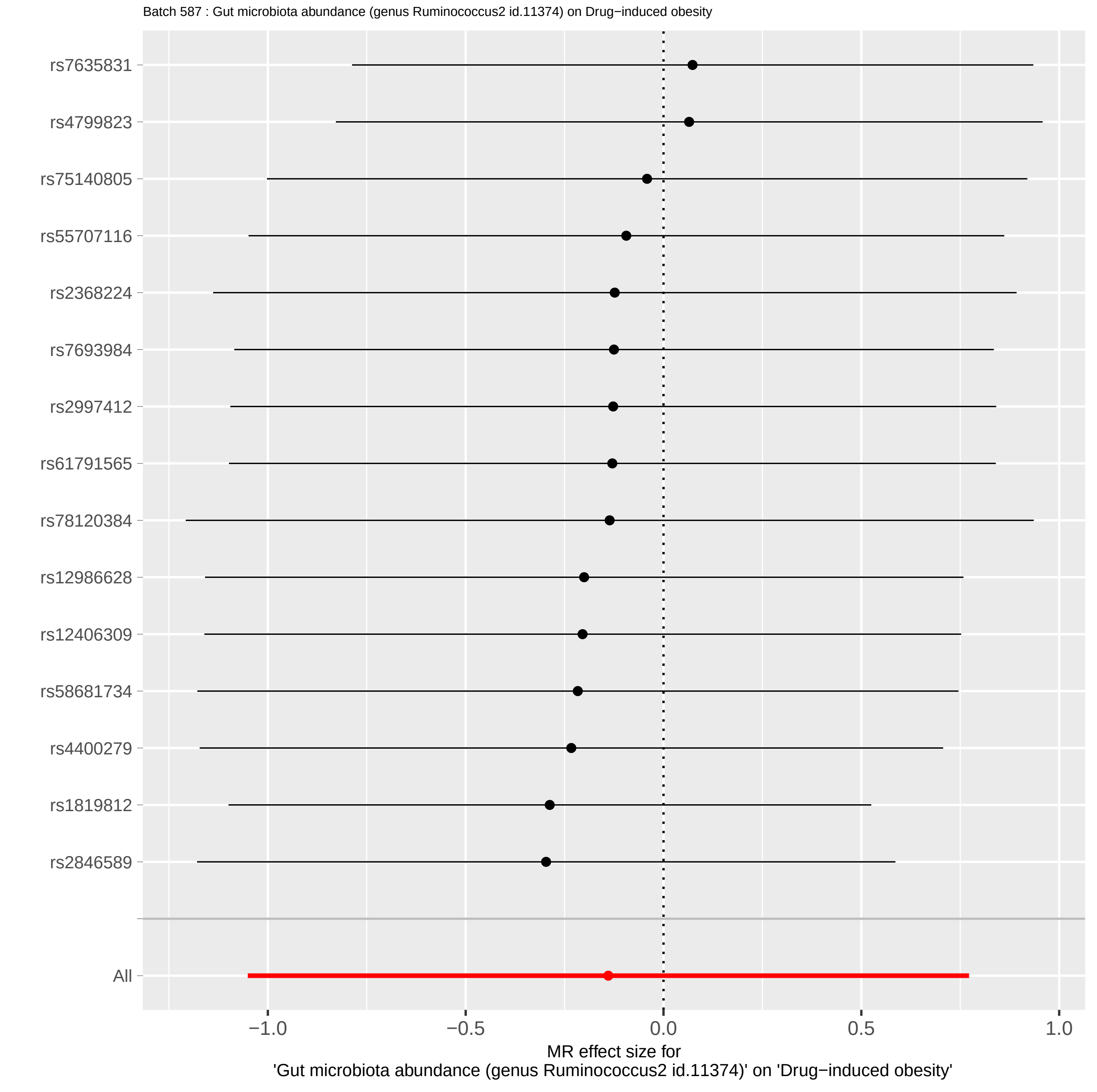

Batch 587 : Gut microbiota abundance (genus Ruminococcus2 id.11374) on Drug−induced obesity
rs7635831
rs4799823
rs75140805
rs55707116
rs2368224
rs7693984
rs2997412
rs61791565
rs78120384
rs12986628
rs12406309
rs58681734
rs4400279
rs1819812
rs2846589
All
−1.0
−0.5
0.0
0.5
1.0
MR effect size for
'Gut microbiota abundance (genus Ruminococcus2 id.11374)' on 'Drug−induced obesity'

Batch 588 : Gut microbiota abundance (genus Ruminococcus gauvreauii group id.11342) on Drug−induced obesity

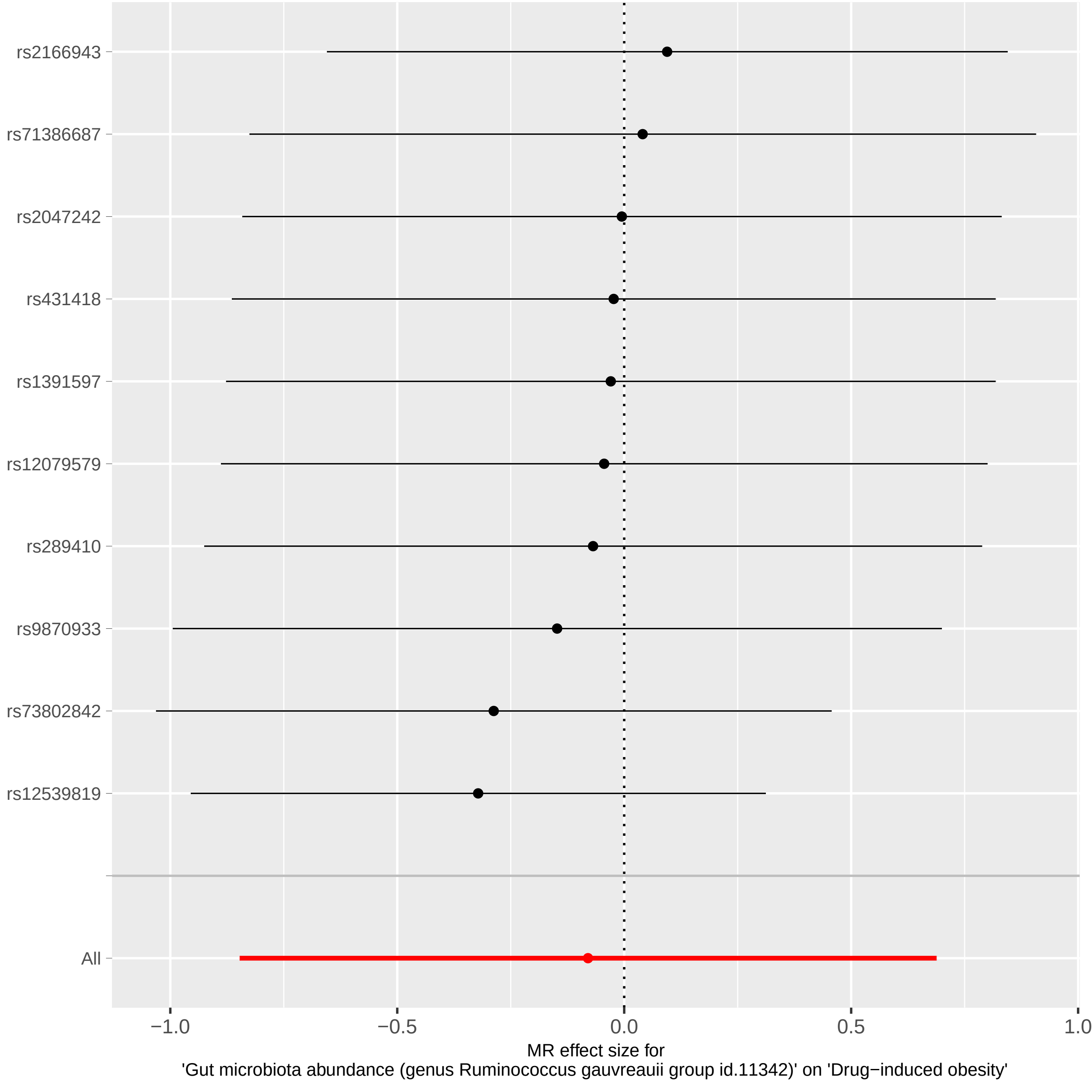

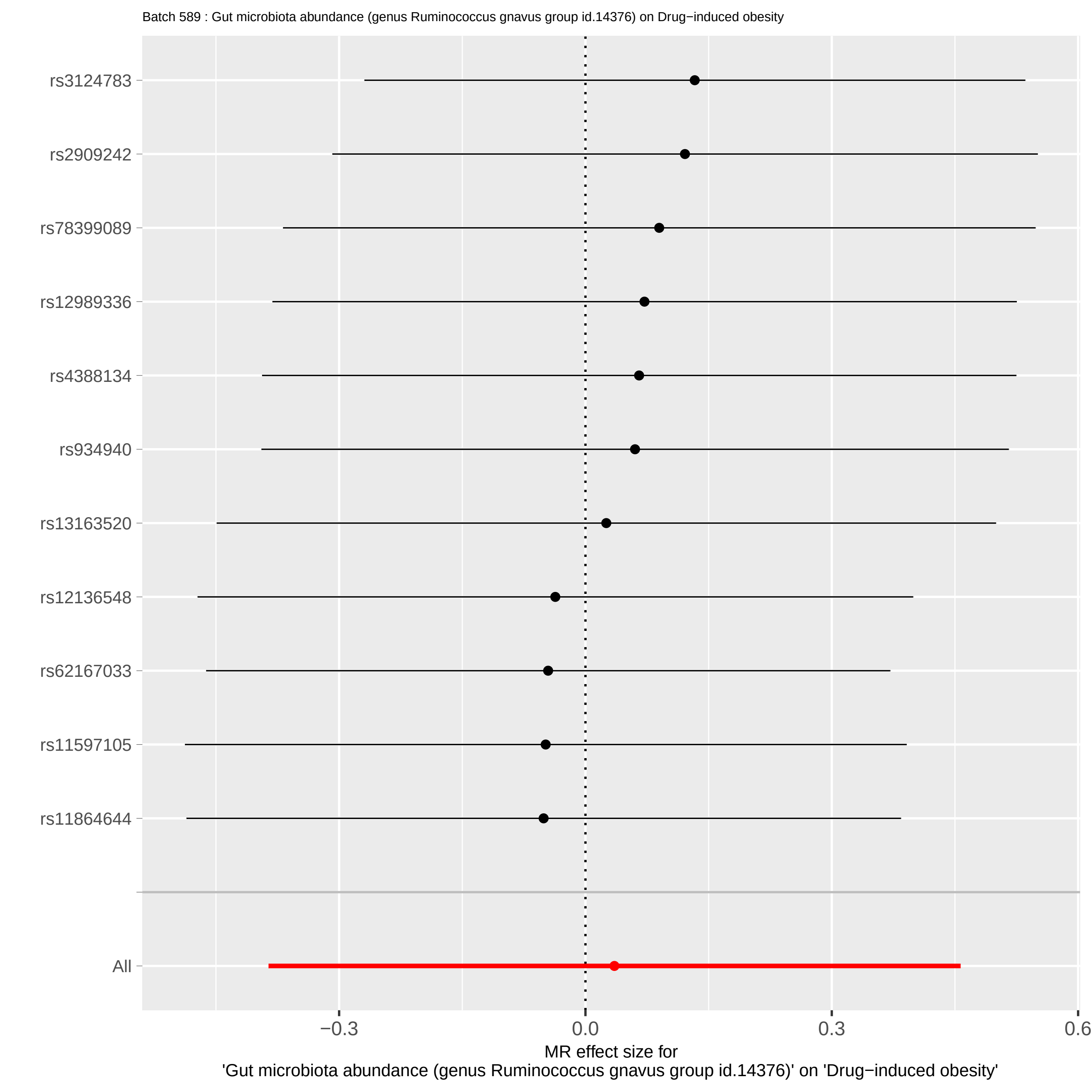

Batch 589 : Gut microbiota abundance (genus Ruminococcus gnavus group id.14376) on Drug−induced obesity
rs3124783
rs2909242
rs78399089
rs12989336
rs4388134
rs934940
rs13163520
rs12136548
rs62167033
rs11597105
rs11864644
All
−0.3
0.0
0.3
0.6
MR effect size for
'Gut microbiota abundance (genus Ruminococcus gnavus group id.14376)' on 'Drug−induced obesity'

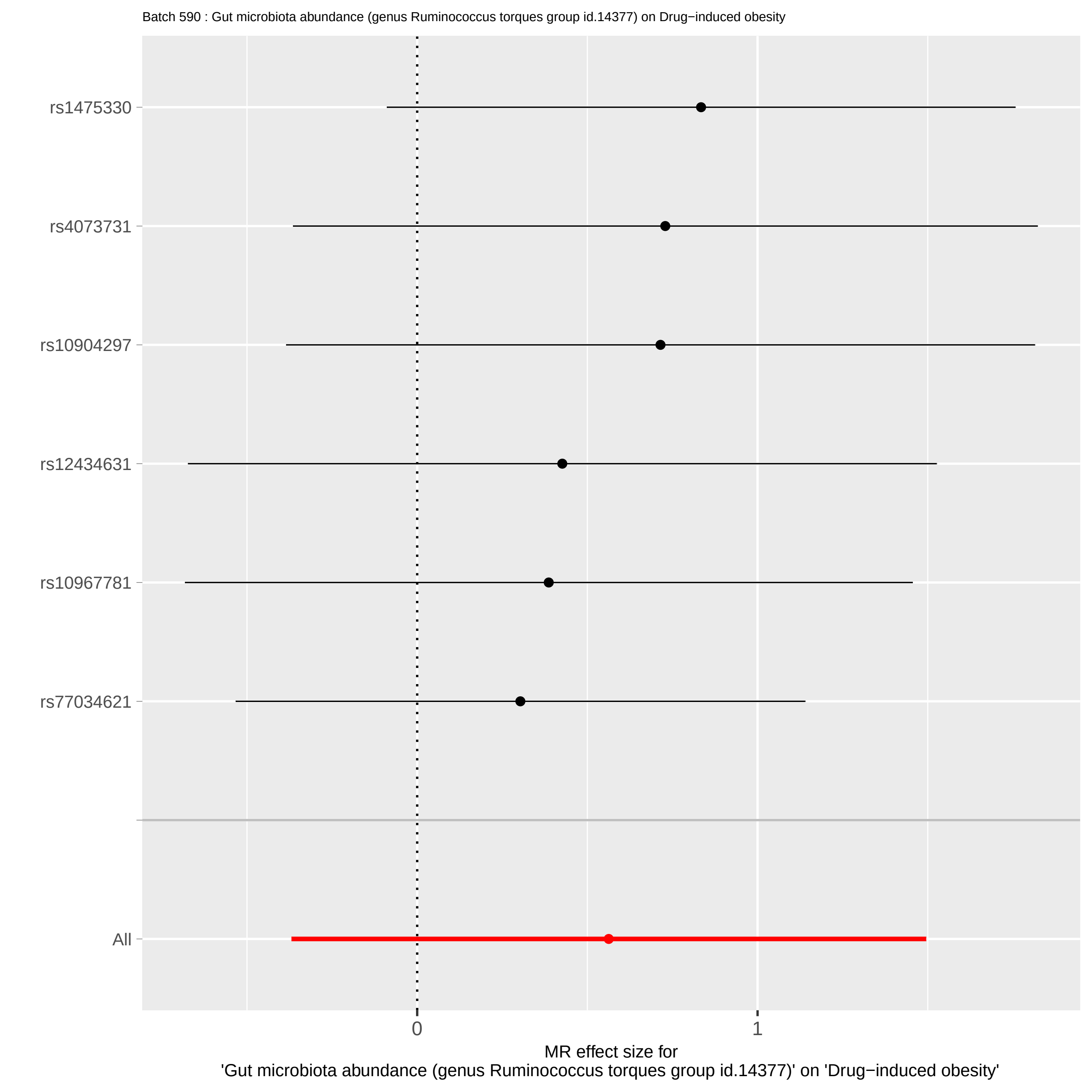

Batch 590 : Gut microbiota abundance (genus Ruminococcus torques group id.14377) on Drug−induced obesity
rs1475330
rs4073731
rs10904297
rs12434631
rs10967781
rs77034621
All
0
1
MR effect size for
'Gut microbiota abundance (genus Ruminococcus torques group id.14377)' on 'Drug−induced obesity'

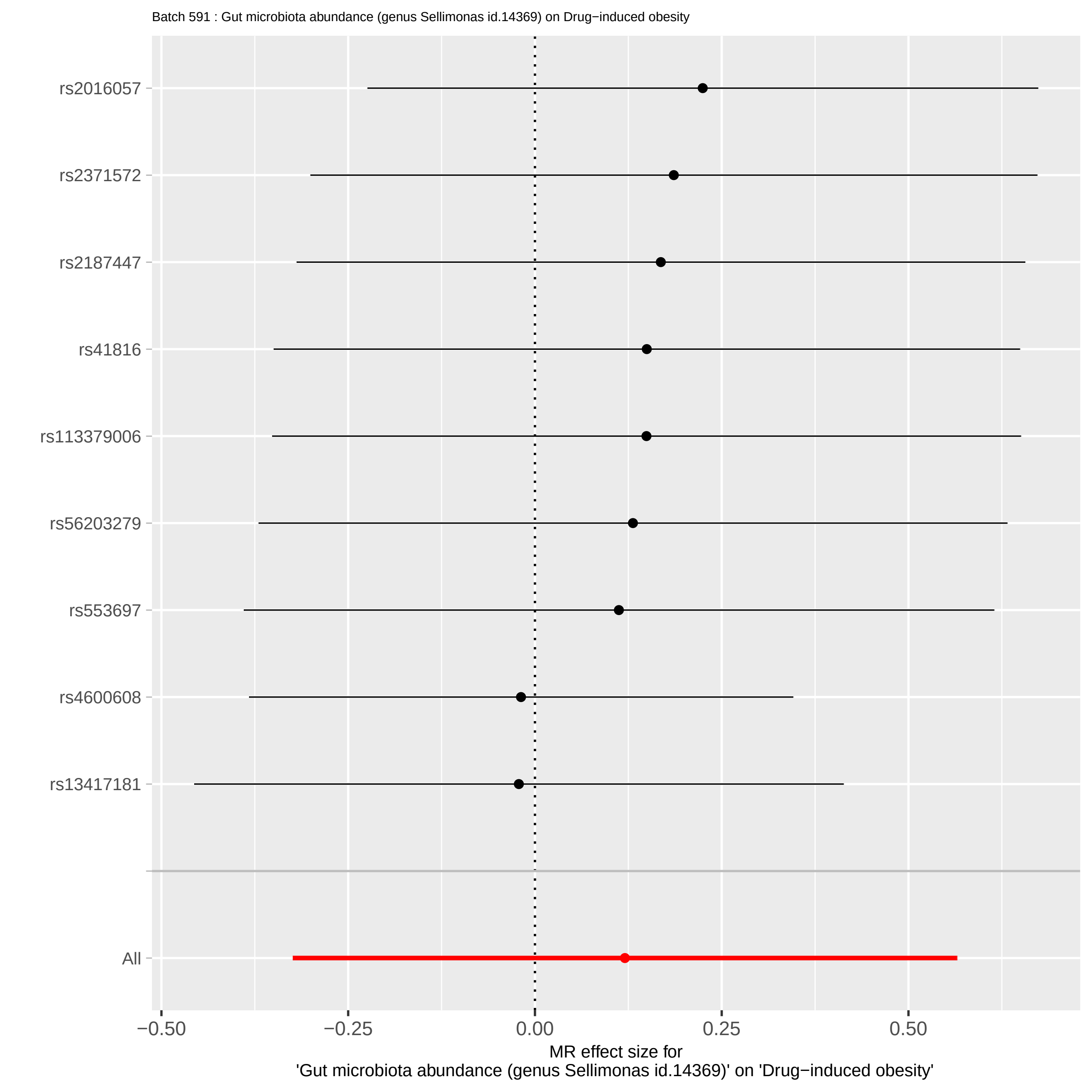
Batch 591 : Gut microbiota abundance (genus Sellimonas id.14369) on Drug−induced obesity
rs2016057
rs2371572
rs2187447
rs41816
rs113379006
rs56203279
rs553697
rs4600608
rs13417181
All
−0.50
−0.25
0.00
0.25
0.50
MR effect size for
'Gut microbiota abundance (genus Sellimonas id.14369)' on 'Drug−induced obesity'

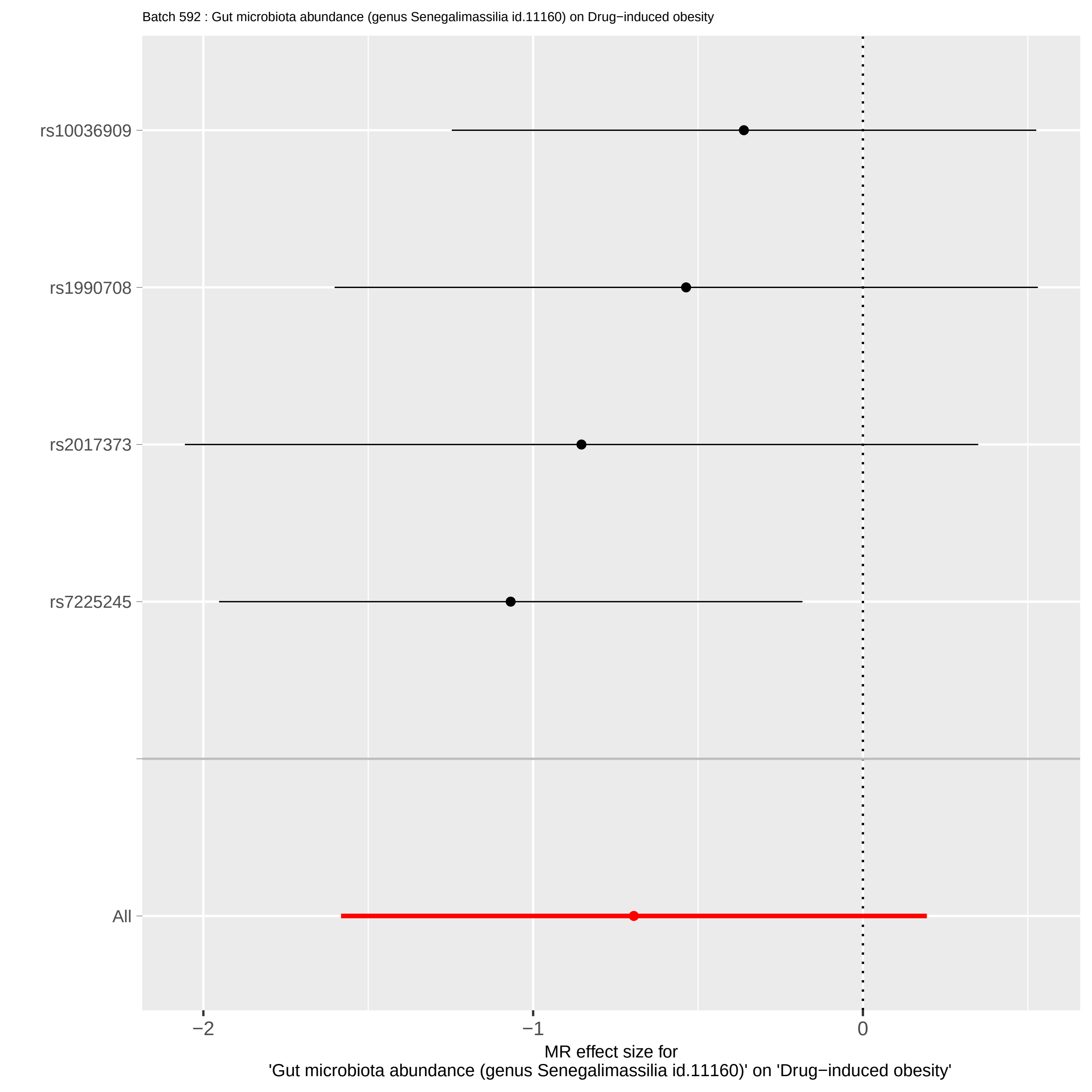
Batch 592 : Gut microbiota abundance (genus Senegalimassilia id.11160) on Drug−induced obesity
rs10036909
rs1990708
rs2017373
rs7225245
All
−2
−1
0
MR effect size for
'Gut microbiota abundance (genus Senegalimassilia id.11160)' on 'Drug−induced obesity'

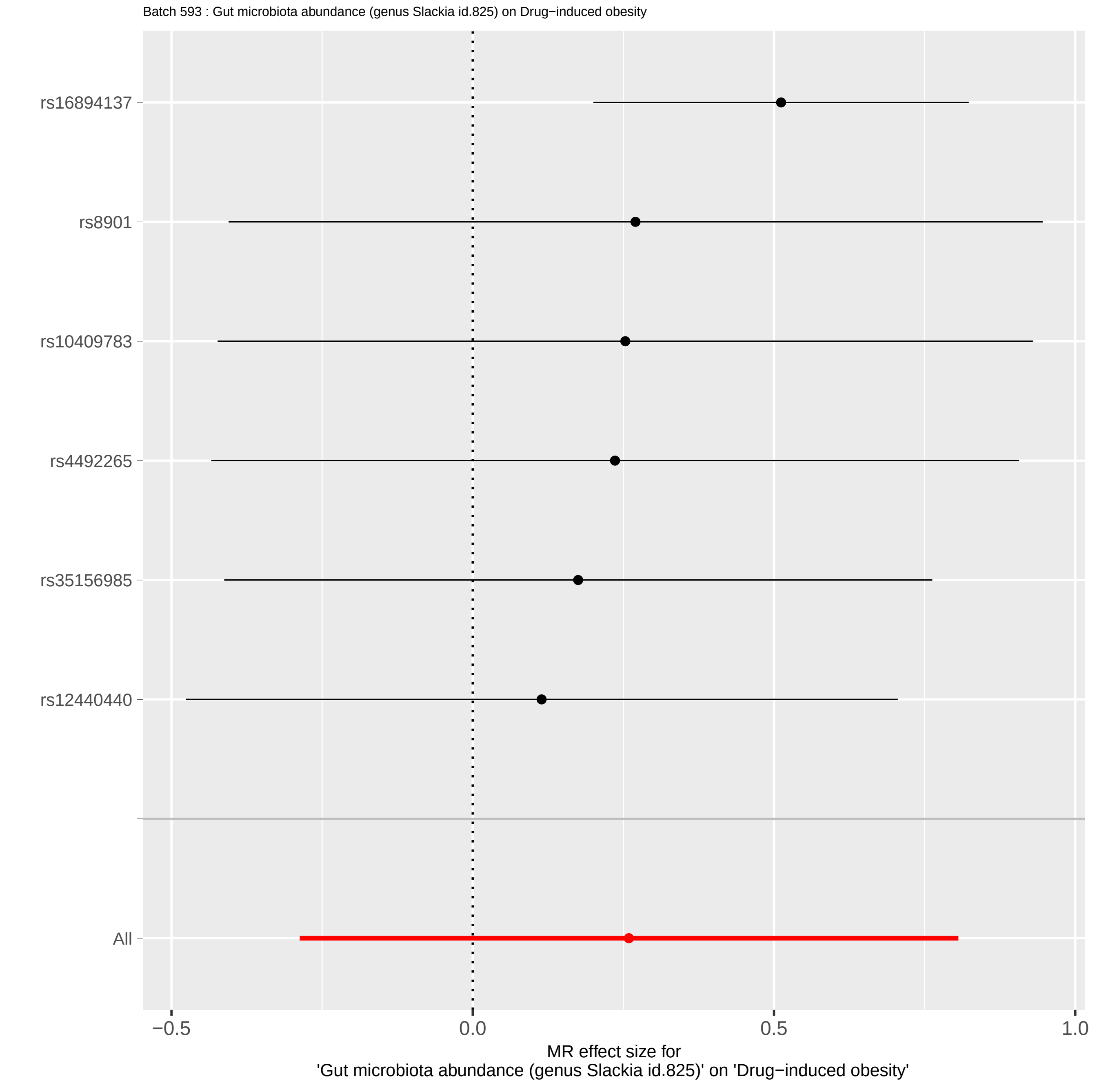
Batch 593 : Gut microbiota abundance (genus Slackia id.825) on Drug−induced obesity
rs16894137
rs8901
rs10409783
rs4492265
rs35156985
rs12440440
All
−0.5
0.0
0.5
1.0
MR effect size for
'Gut microbiota abundance (genus Slackia id.825)' on 'Drug−induced obesity'

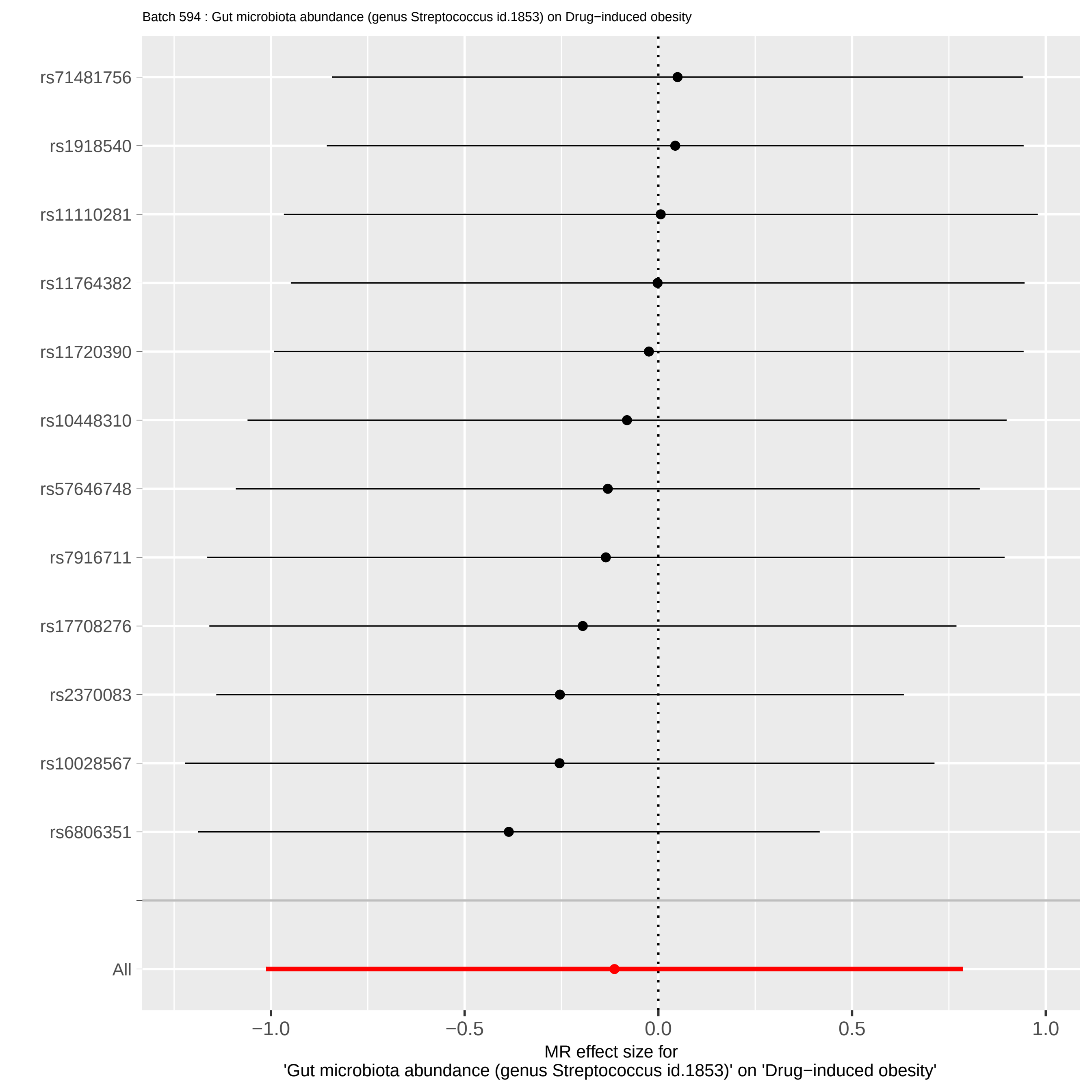

Batch 594 : Gut microbiota abundance (genus Streptococcus id.1853) on Drug−induced obesity
rs71481756
rs1918540
rs11110281
rs11764382
rs11720390
rs10448310
rs57646748
rs7916711
rs17708276
rs2370083
rs10028567
rs6806351
All
−1.0
−0.5
0.0
0.5
1.0
MR effect size for
'Gut microbiota abundance (genus Streptococcus id.1853)' on 'Drug−induced obesity'

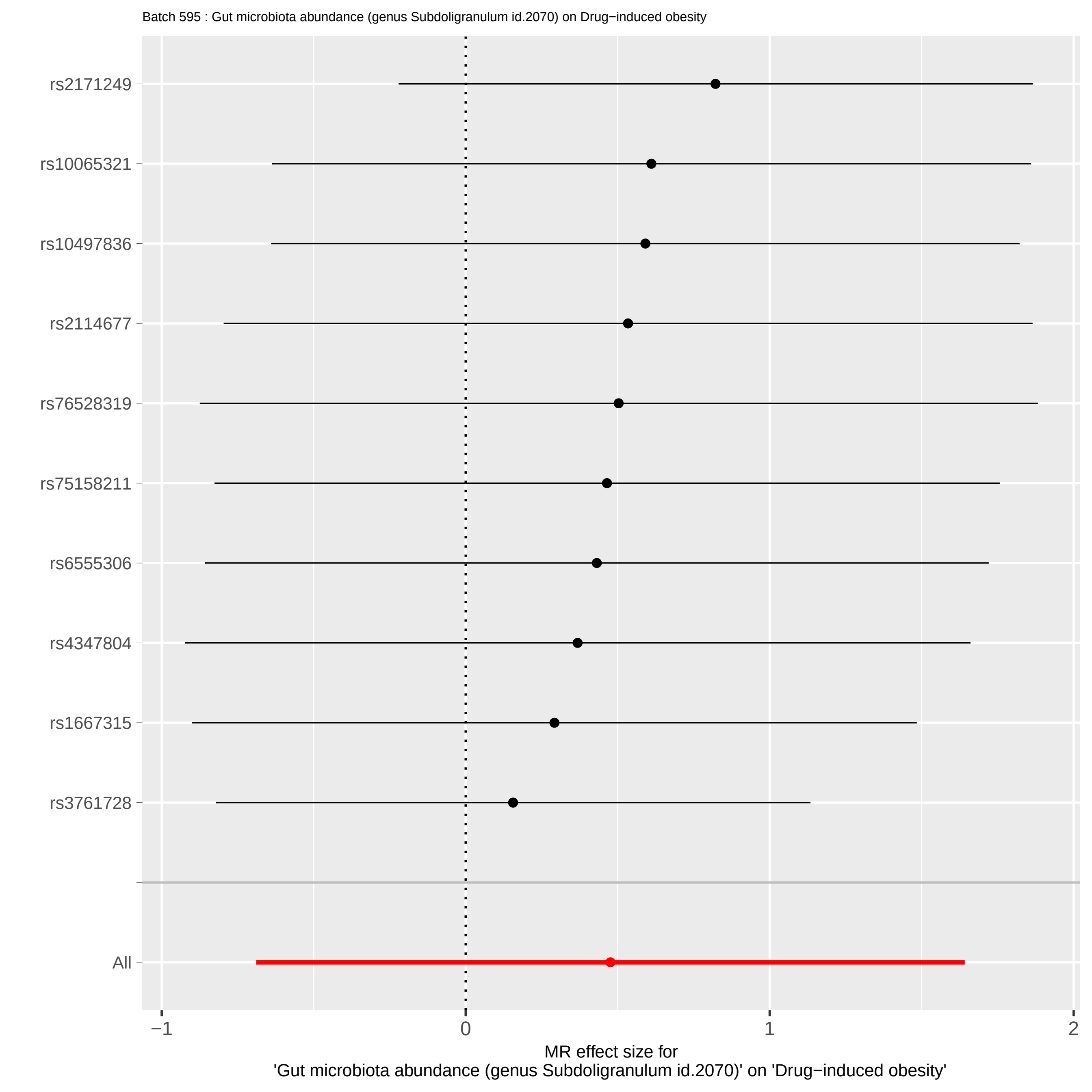

Batch 595 : Gut microbiota abundance (genus Subdoligranulum id.2070) on Drug−induced obesity
rs2171249
rs10065321
rs10497836
rs2114677
rs76528319
rs75158211
rs6555306
rs4347804
rs1667315
rs3761728
All
−1
0
1
2
MR effect size for
'Gut microbiota abundance (genus Subdoligranulum id.2070)' on 'Drug−induced obesity'

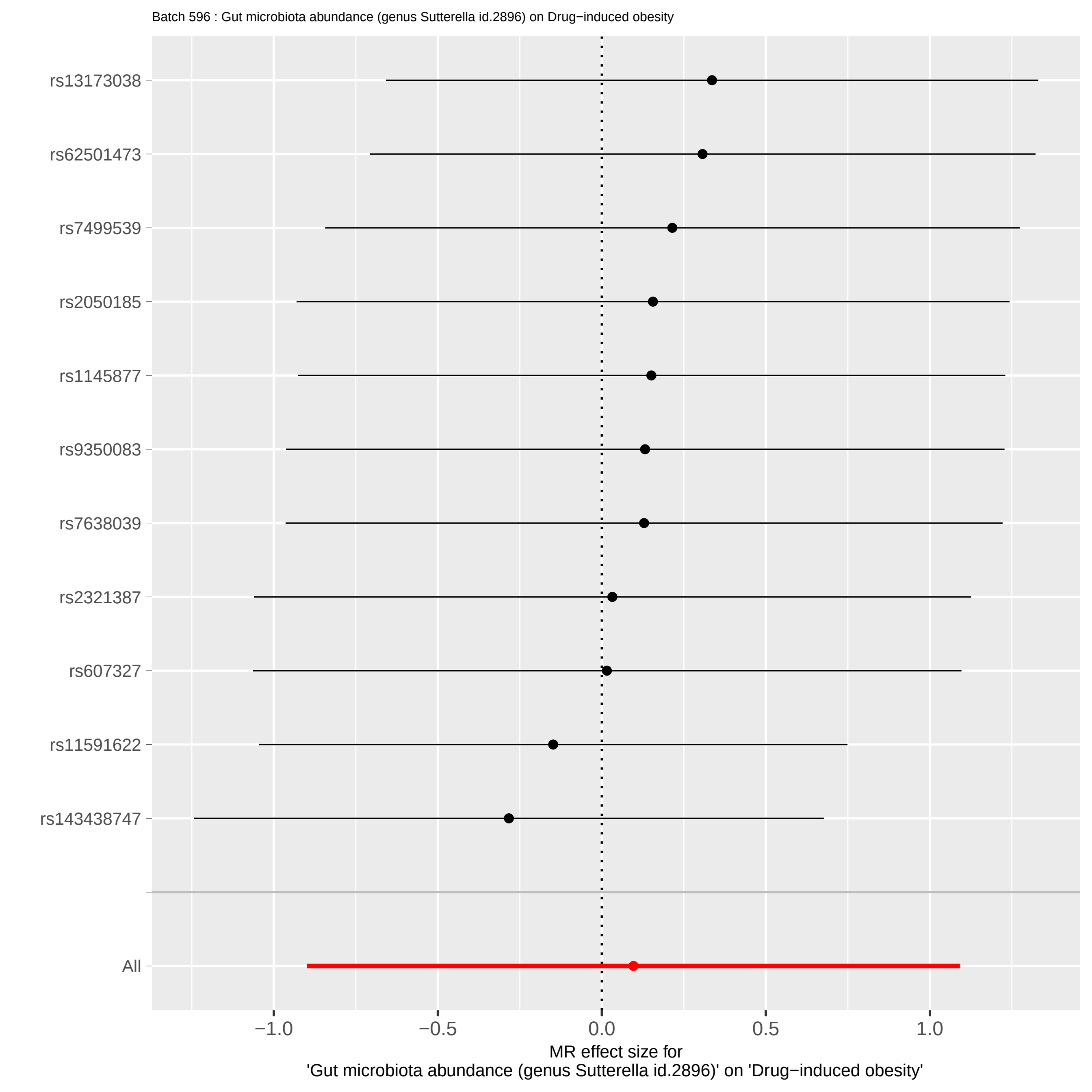

Batch 596 : Gut microbiota abundance (genus Sutterella id.2896) on Drug−induced obesity
rs13173038
rs62501473
rs7499539
rs2050185
rs1145877
rs9350083
rs7638039
rs2321387
rs607327
rs11591622
rs143438747
All
−1.0
−0.5
0.0
0.5
1.0
MR effect size for
'Gut microbiota abundance (genus Sutterella id.2896)' on 'Drug−induced obesity'

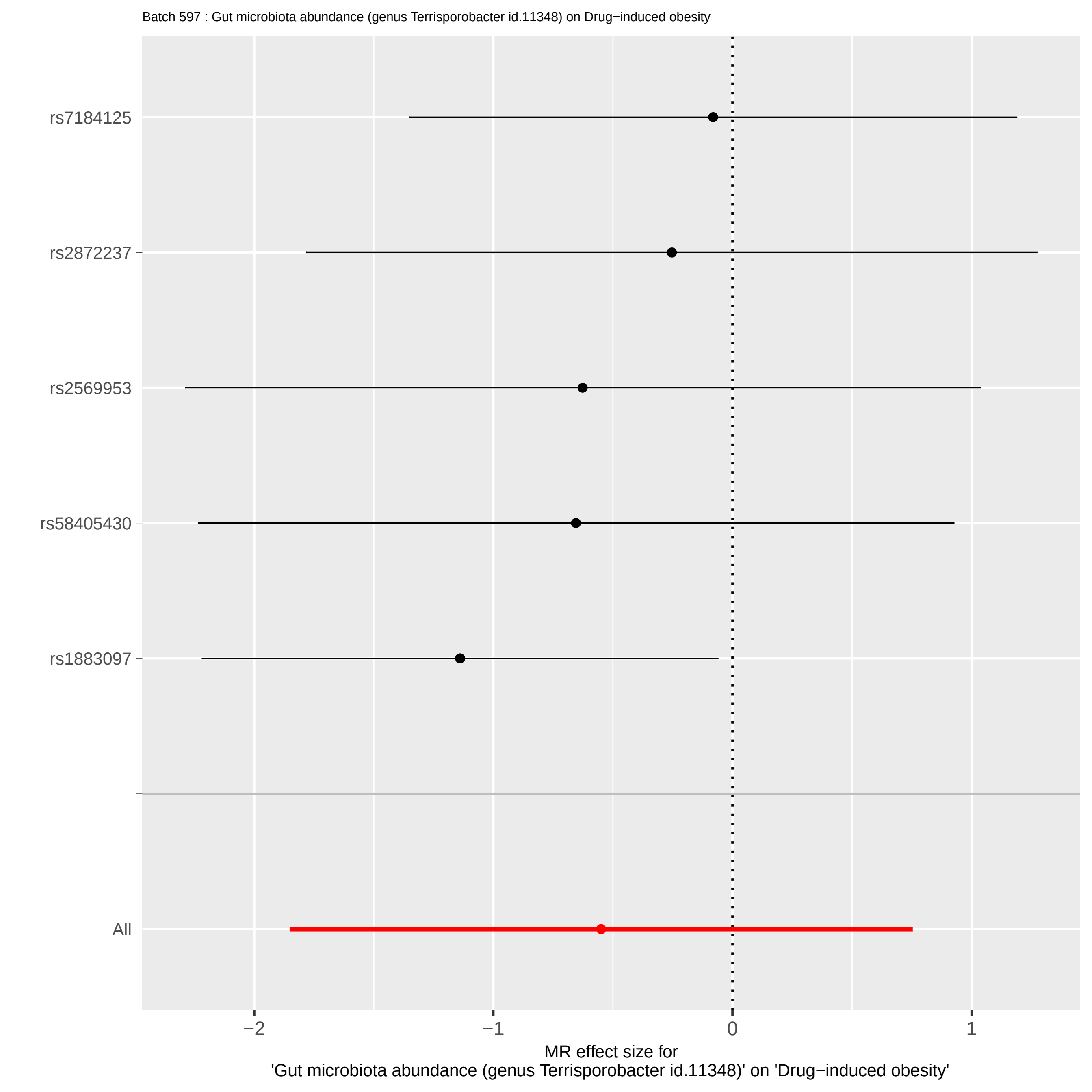

Batch 597 : Gut microbiota abundance (genus Terrisporobacter id.11348) on Drug−induced obesity
rs7184125
rs2872237
rs2569953
rs58405430
rs1883097
All
−2
−1
0
1
MR effect size for
'Gut microbiota abundance (genus Terrisporobacter id.11348)' on 'Drug−induced obesity'

Batch 598 : Gut microbiota abundance (genus Turicibacter id.2162) on Drug−induced obesity

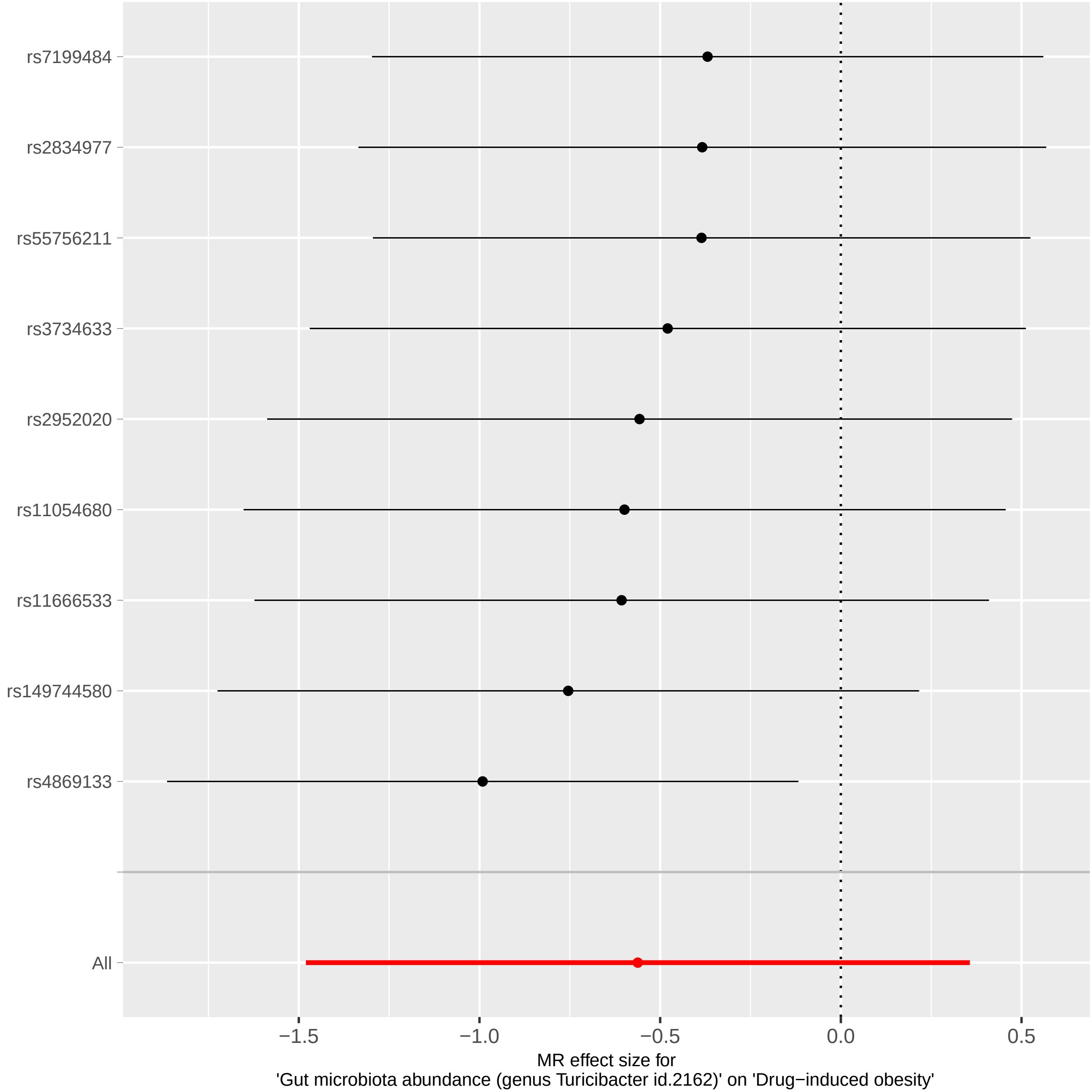

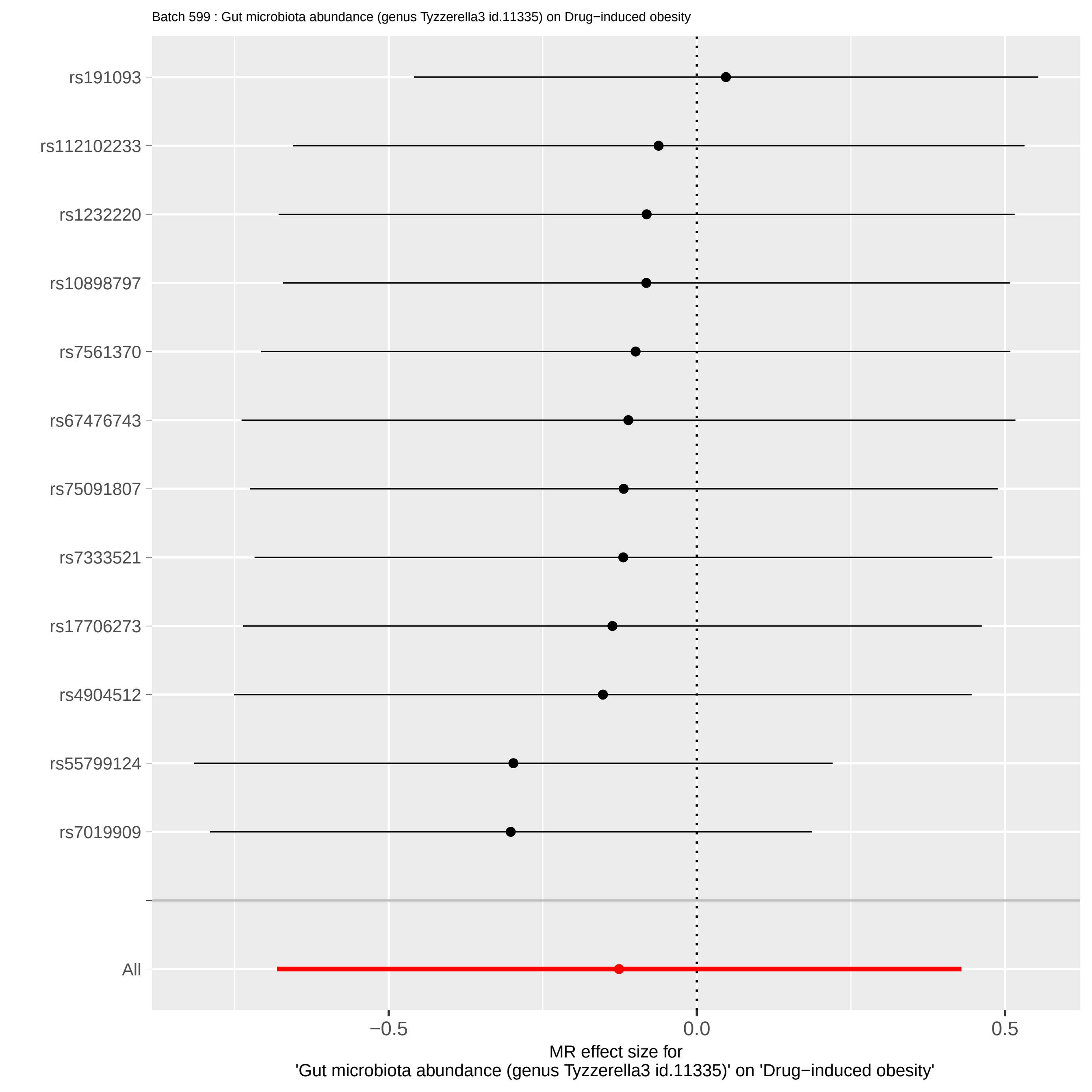
Batch 599 : Gut microbiota abundance (genus Tyzzerella3 id.11335) on Drug−induced obesity
rs191093
rs112102233
rs1232220
rs10898797
rs7561370
rs67476743
rs75091807
rs7333521
rs17706273
rs4904512
rs55799124
rs7019909
All
−0.5
0.0
0.5
MR effect size for
'Gut microbiota abundance (genus Tyzzerella3 id.11335)' on 'Drug−induced obesity'

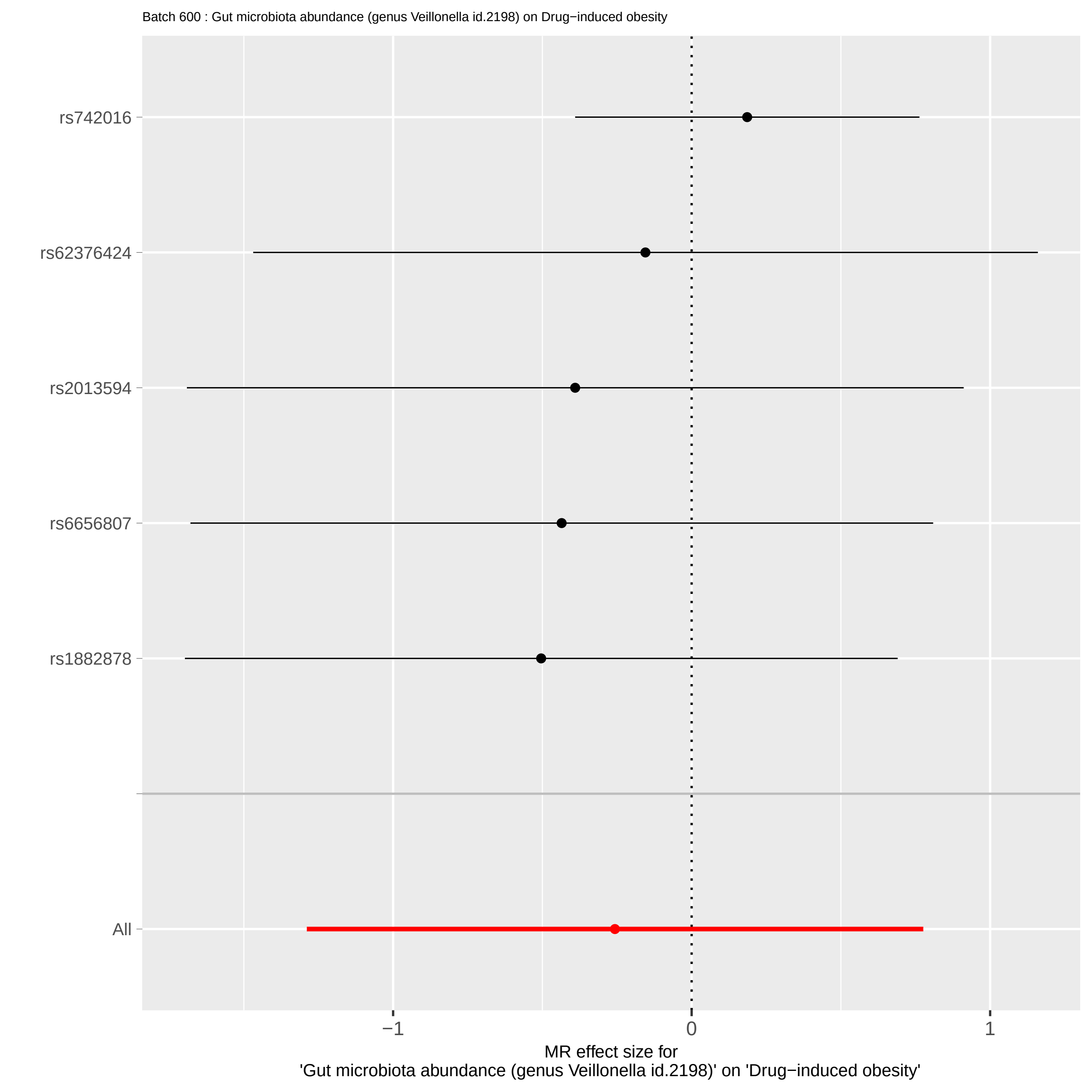

Batch 600 : Gut microbiota abundance (genus Veillonella id.2198) on Drug−induced obesity
rs742016
rs62376424
rs2013594
rs6656807
rs1882878
All
−1
0
1
MR effect size for
'Gut microbiota abundance (genus Veillonella id.2198)' on 'Drug−induced obesity'

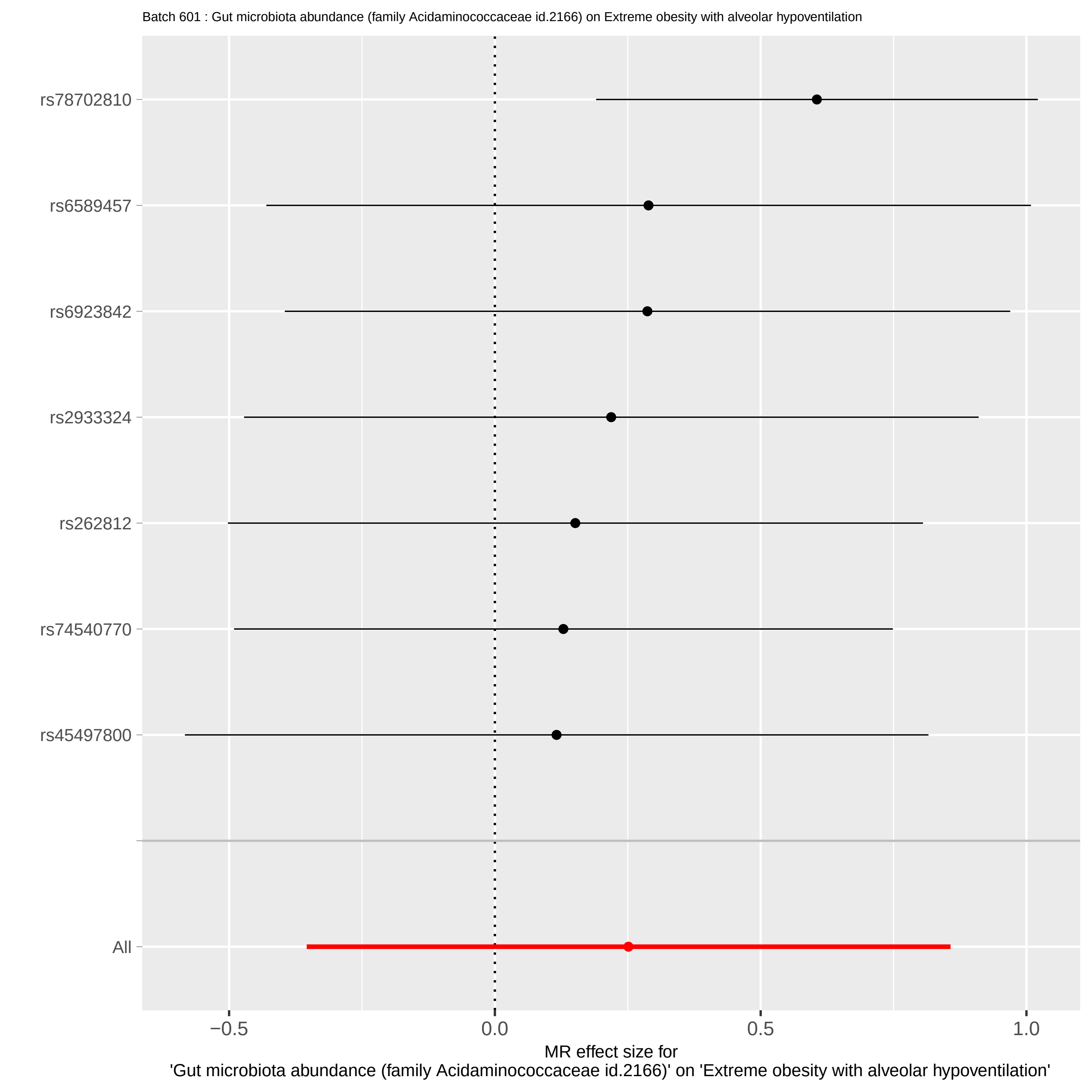
Batch 601 : Gut microbiota abundance (family Acidaminococcaceae id.2166) on Extreme obesity with alveolar hypoventilation
rs78702810
rs6589457
rs6923842
rs2933324
rs262812
rs74540770
rs45497800
All
−0.5
0.0
0.5
1.0
MR effect size for
'Gut microbiota abundance (family Acidaminococcaceae id.2166)' on 'Extreme obesity with alveolar hypoventilation'

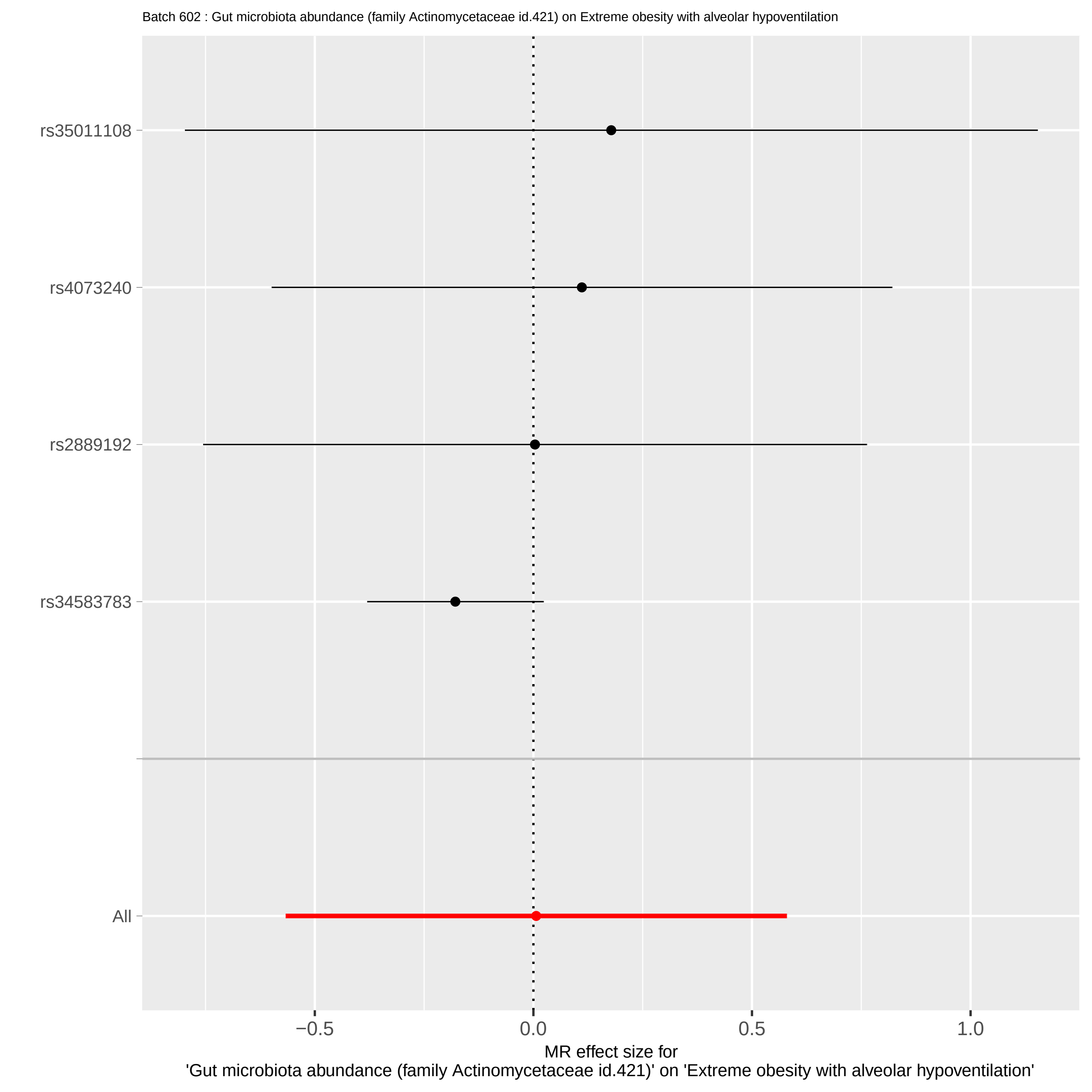
Batch 602 : Gut microbiota abundance (family Actinomycetaceae id.421) on Extreme obesity with alveolar hypoventilation
rs35011108
rs4073240
rs2889192
rs34583783
All
−0.5
0.0
0.5
1.0
MR effect size for
'Gut microbiota abundance (family Actinomycetaceae id.421)' on 'Extreme obesity with alveolar hypoventilation'

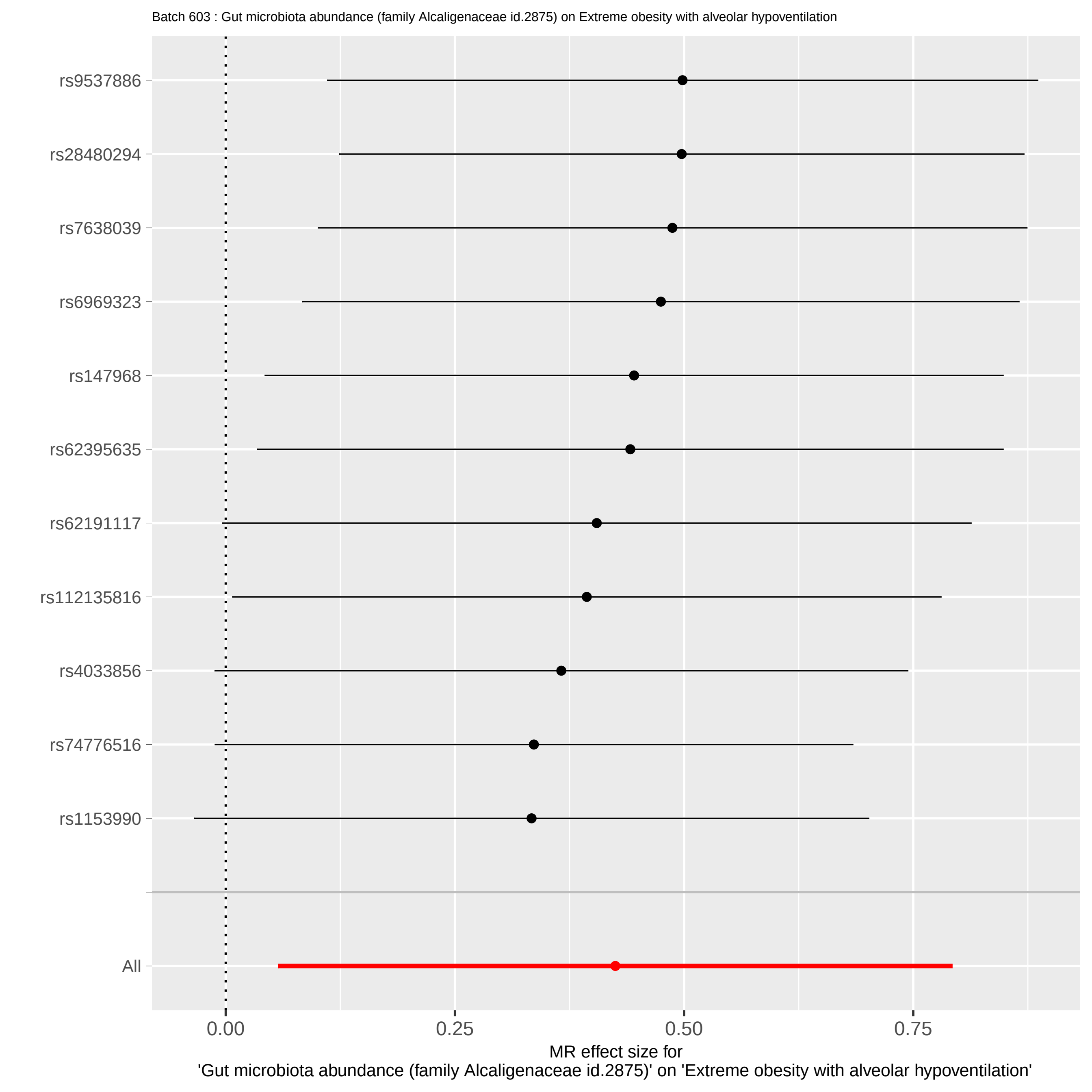

Batch 603 : Gut microbiota abundance (family Alcaligenaceae id.2875) on Extreme obesity with alveolar hypoventilation
rs9537886
rs28480294
rs7638039
rs6969323
rs147968
rs62395635
rs62191117
rs112135816
rs4033856
rs74776516
rs1153990
All
0.00
0.25
0.50
0.75
MR effect size for
'Gut microbiota abundance (family Alcaligenaceae id.2875)' on 'Extreme obesity with alveolar hypoventilation'

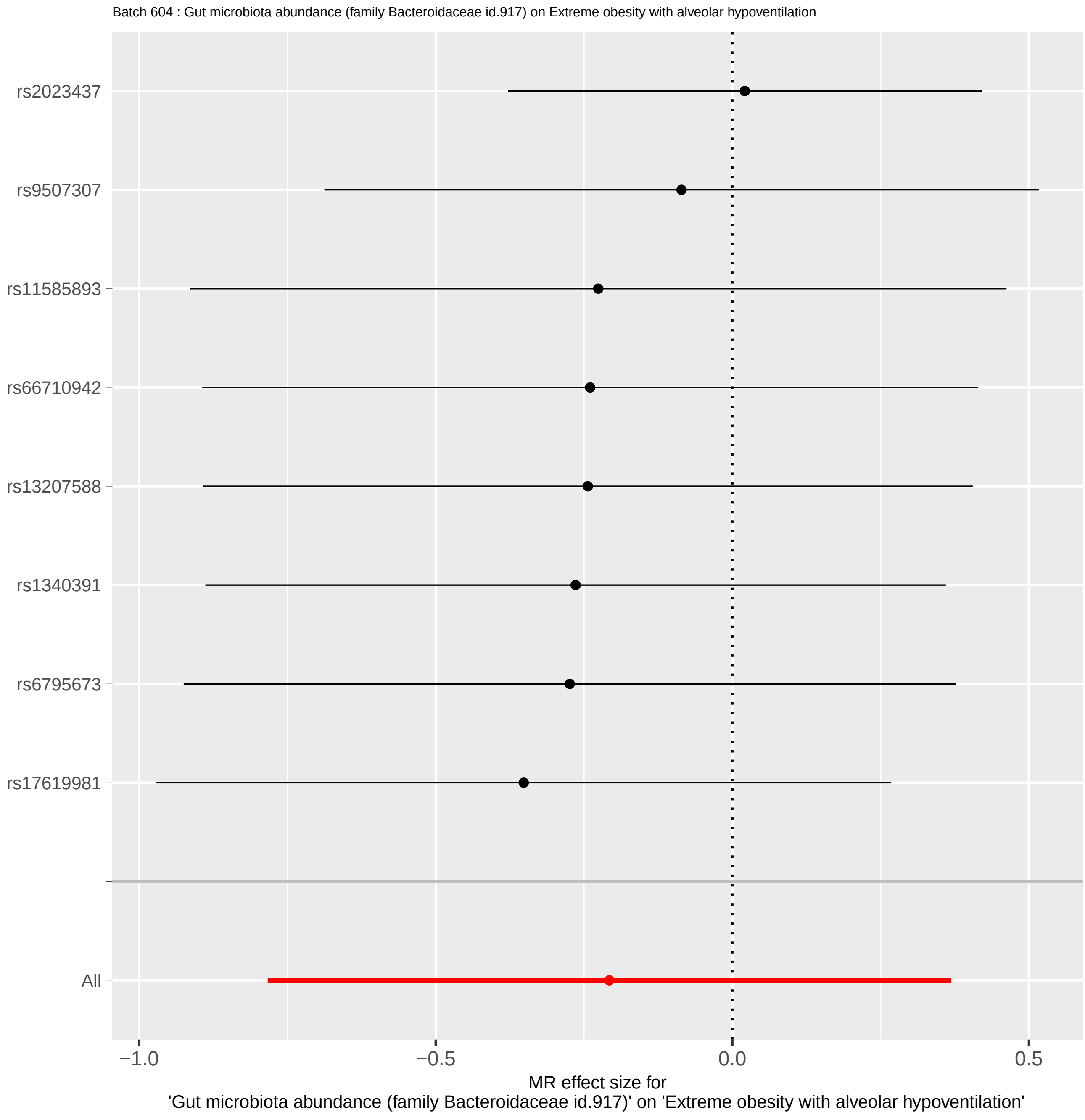
Batch 604 : Gut microbiota abundance (family Bacteroidaceae id.917) on Extreme obesity with alveolar hypoventilation
rs2023437
rs9507307
rs11585893
rs66710942
rs13207588
rs1340391
rs6795673
rs17619981
All
−1.0
−0.5
0.0
0.5
MR effect size for
'Gut microbiota abundance (family Bacteroidaceae id.917)' on 'Extreme obesity with alveolar hypoventilation'

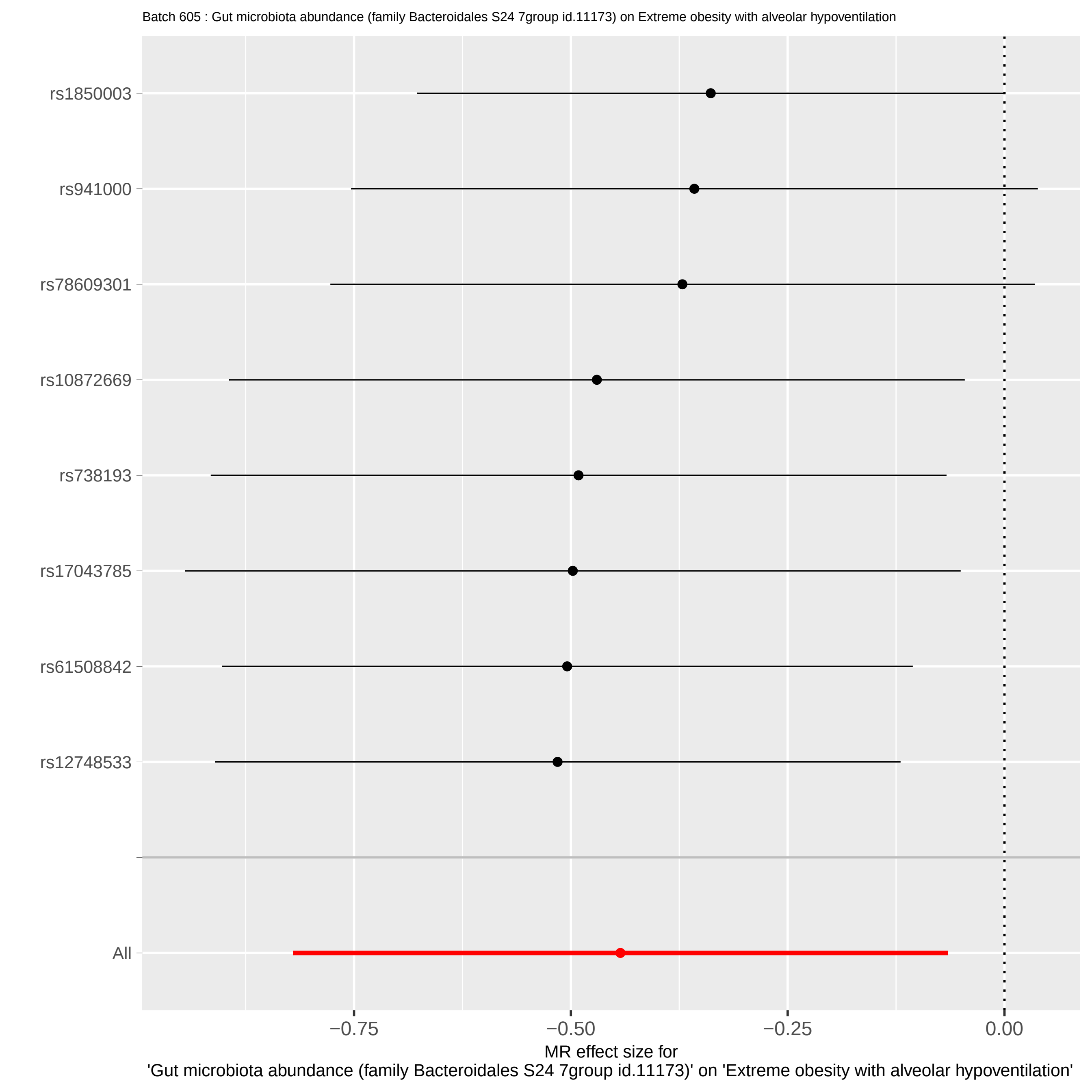

Batch 605 : Gut microbiota abundance (family Bacteroidales S24 7group id.11173) on Extreme obesity with alveolar hypoventilation
rs1850003
rs941000
rs78609301
rs10872669
rs738193
rs17043785
rs61508842
rs12748533
All
−0.75
−0.50
−0.25
0.00
MR effect size for
'Gut microbiota abundance (family Bacteroidales S24 7group id.11173)' on 'Extreme obesity with alveolar hypoventilation'

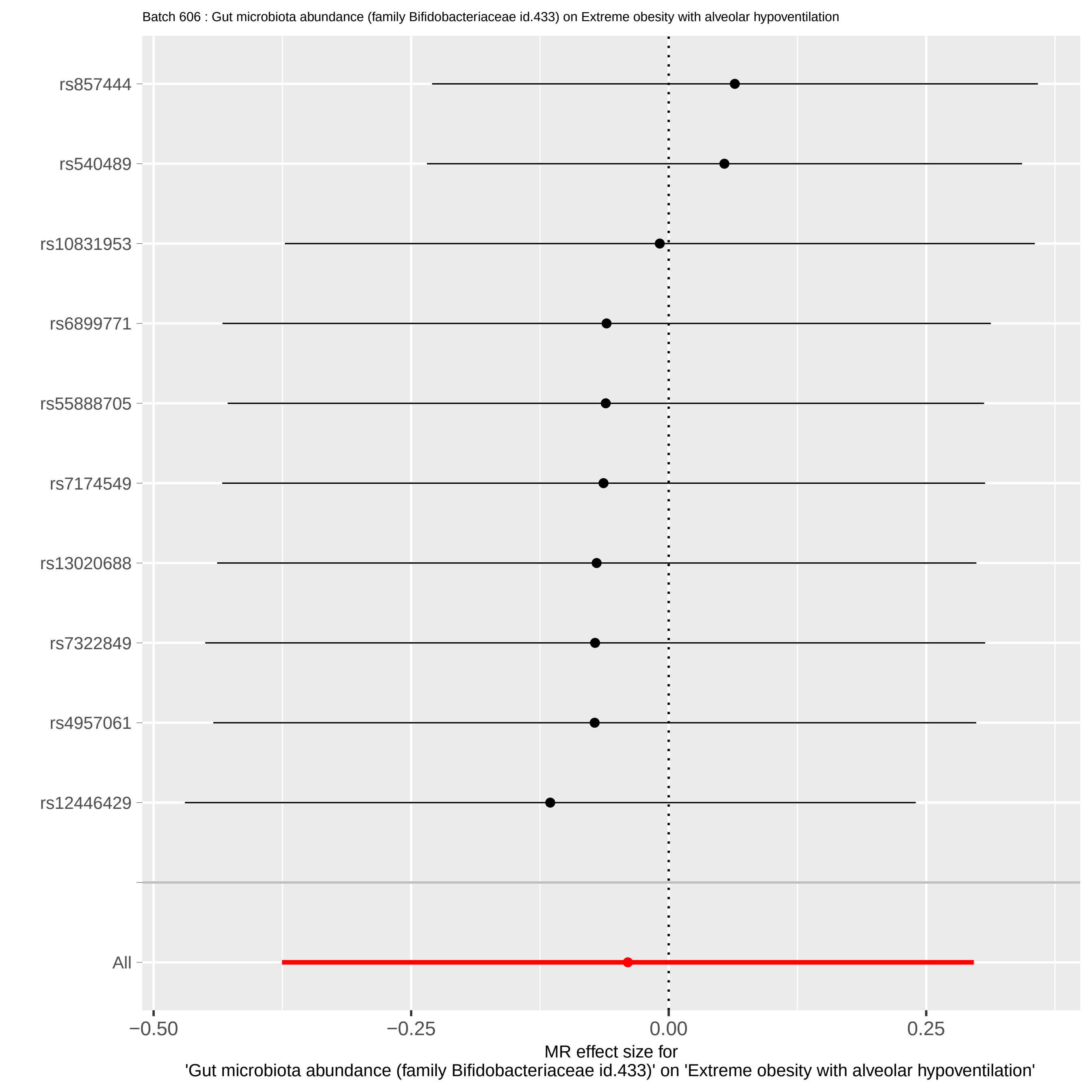

Batch 606 : Gut microbiota abundance (family Bifidobacteriaceae id.433) on Extreme obesity with alveolar hypoventilation
rs857444
rs540489
rs10831953
rs6899771
rs55888705
rs7174549
rs13020688
rs7322849
rs4957061
rs12446429
All
−0.50
−0.25
0.00
0.25
MR effect size for
'Gut microbiota abundance (family Bifidobacteriaceae id.433)' on 'Extreme obesity with alveolar hypoventilation'

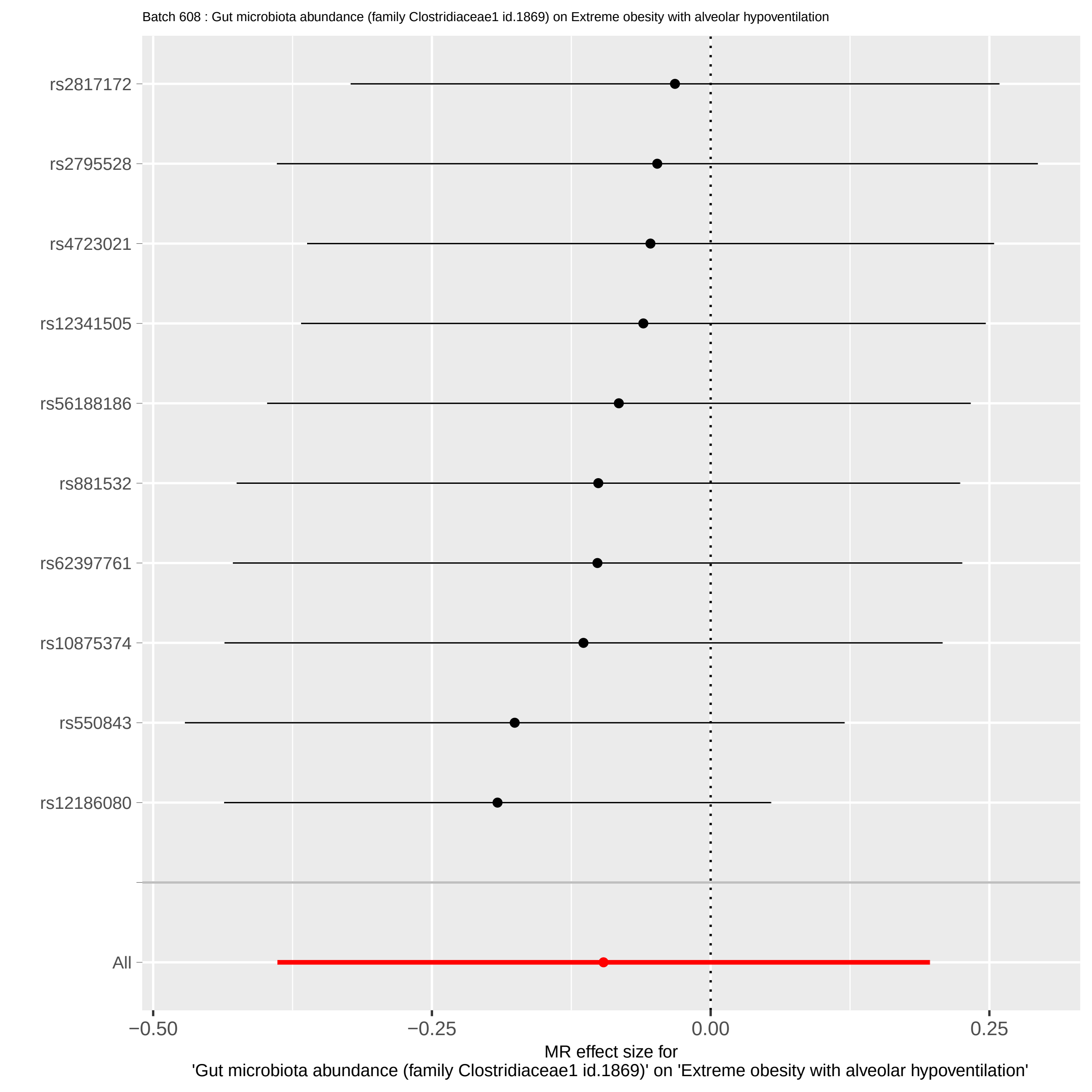

Batch 608 : Gut microbiota abundance (family Clostridiaceae1 id.1869) on Extreme obesity with alveolar hypoventilation
rs2817172
rs2795528
rs4723021
rs12341505
rs56188186
rs881532
rs62397761
rs10875374
rs550843
rs12186080
All
−0.50
−0.25
0.00
0.25
MR effect size for
'Gut microbiota abundance (family Clostridiaceae1 id.1869)' on 'Extreme obesity with alveolar hypoventilation'

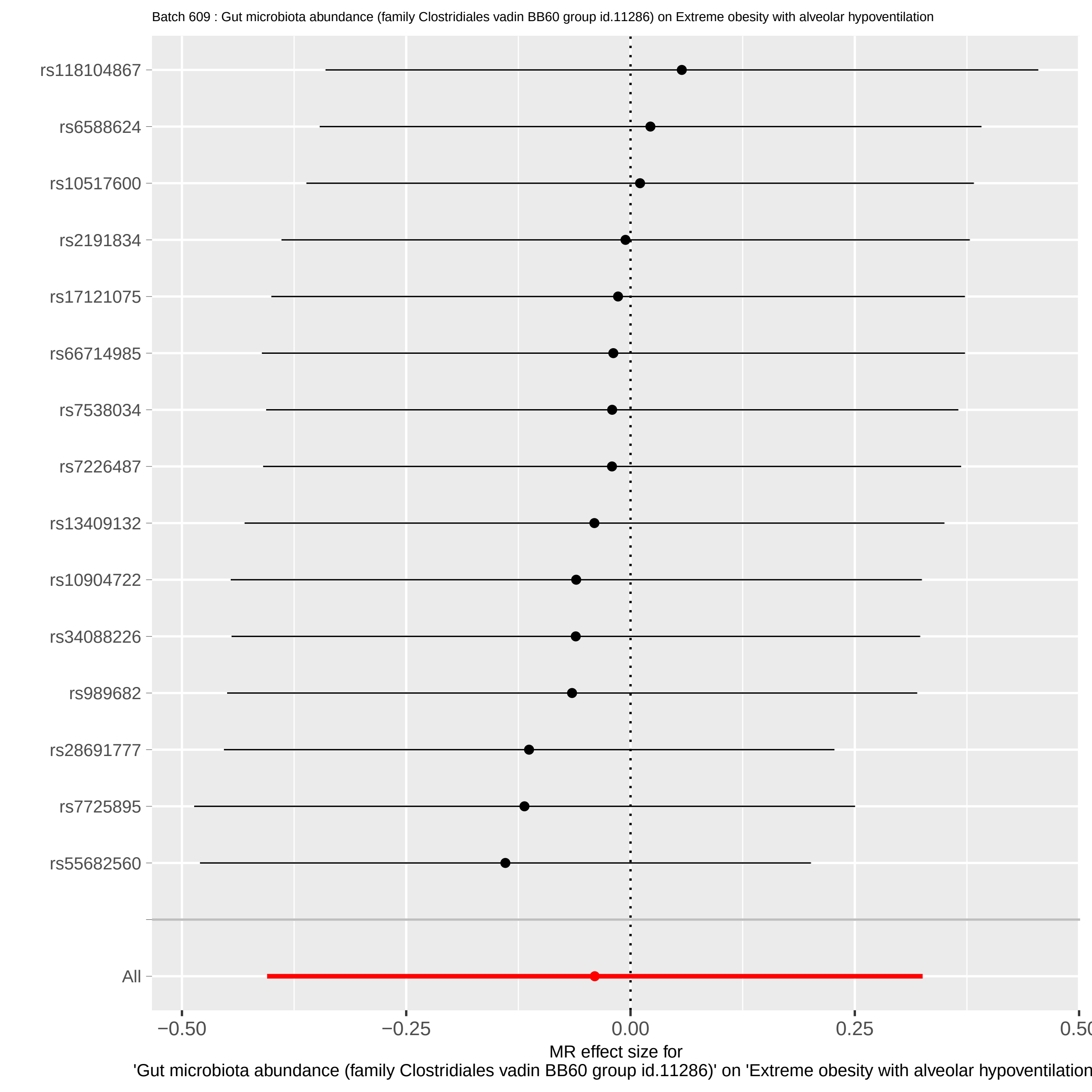

Batch 609 : Gut microbiota abundance (family Clostridiales vadin BB60 group id.11286) on Extreme obesity with alveolar hypoventilation
rs118104867
rs6588624
rs10517600
rs2191834
rs17121075
rs66714985
rs7538034
rs7226487
rs13409132
rs10904722
rs34088226
rs989682
rs28691777
rs7725895
rs55682560
All
−0.50
−0.25
0.00
0.25
0.50
MR effect size for
'Gut microbiota abundance (family Clostridiales vadin BB60 group id.11286)' on 'Extreme obesity with alveolar hypoventilation'

Batch 610 : Gut microbiota abundance (family Coriobacteriaceae id.811) on Extreme obesity with alveolar hypoventilation

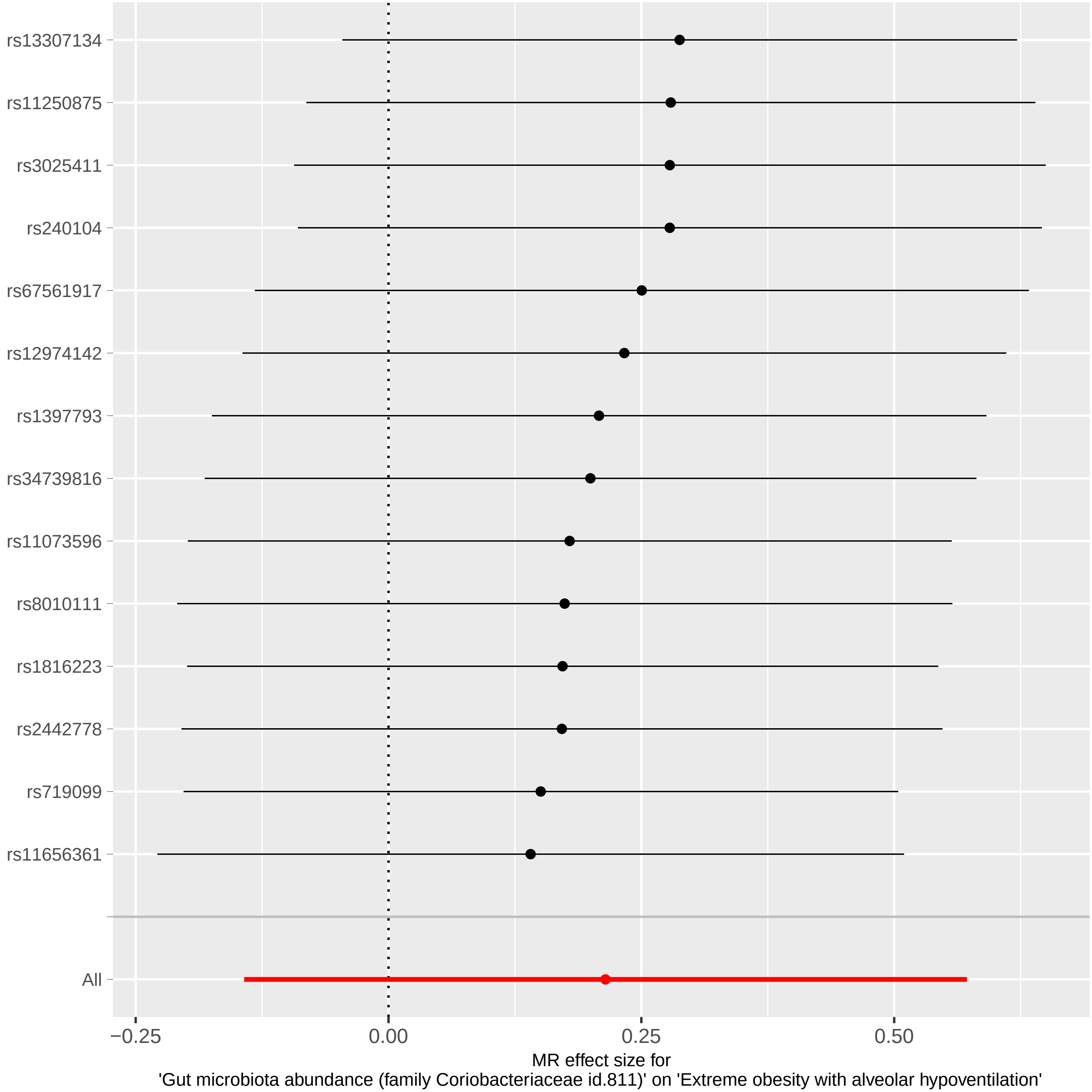

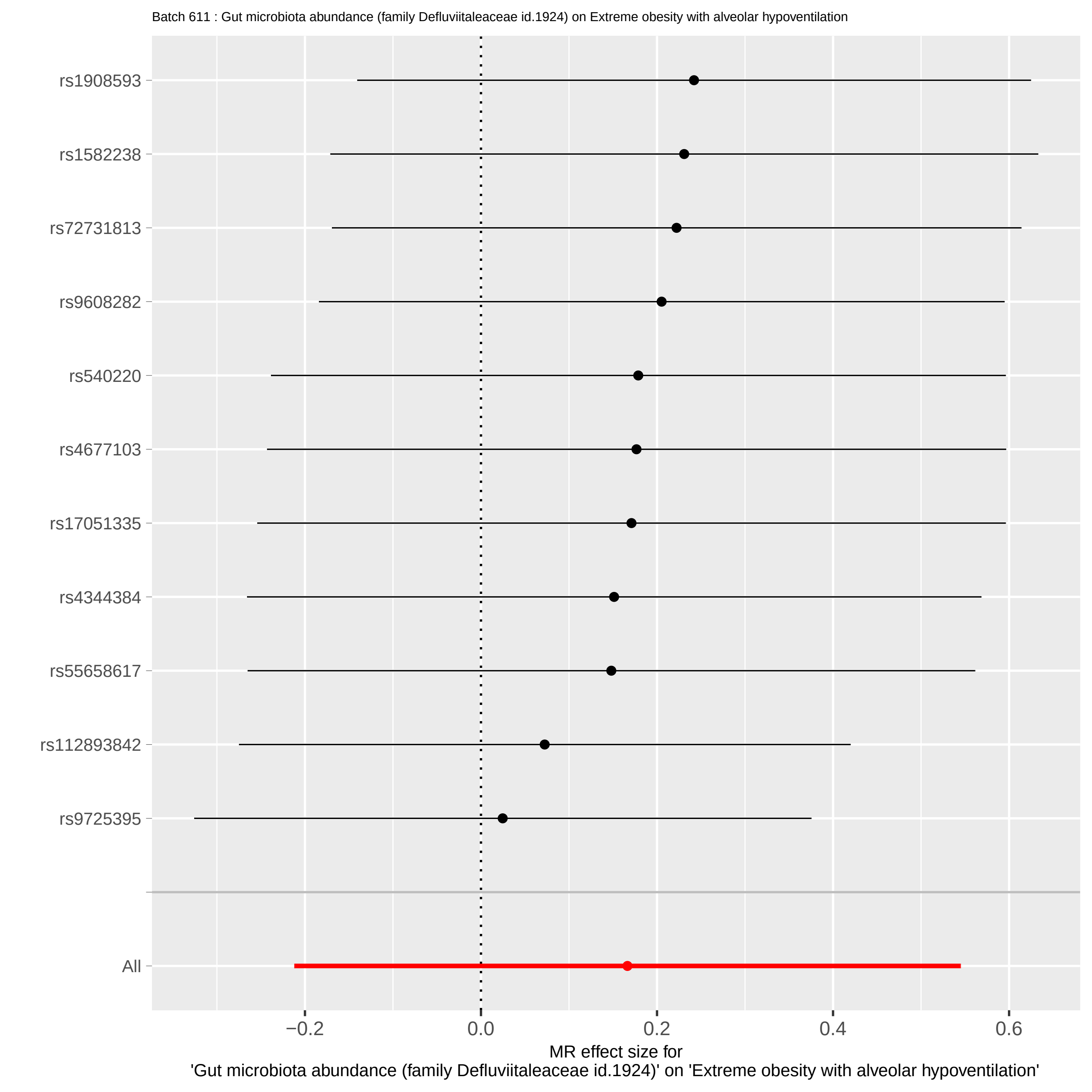

Batch 611 : Gut microbiota abundance (family Defluviitaleaceae id.1924) on Extreme obesity with alveolar hypoventilation
rs1908593
rs1582238
rs72731813
rs9608282
rs540220
rs4677103
rs17051335
rs4344384
rs55658617
rs112893842
rs9725395
All
−0.2
0.0
0.2
0.4
0.6
MR effect size for
'Gut microbiota abundance (family Defluviitaleaceae id.1924)' on 'Extreme obesity with alveolar hypoventilation'

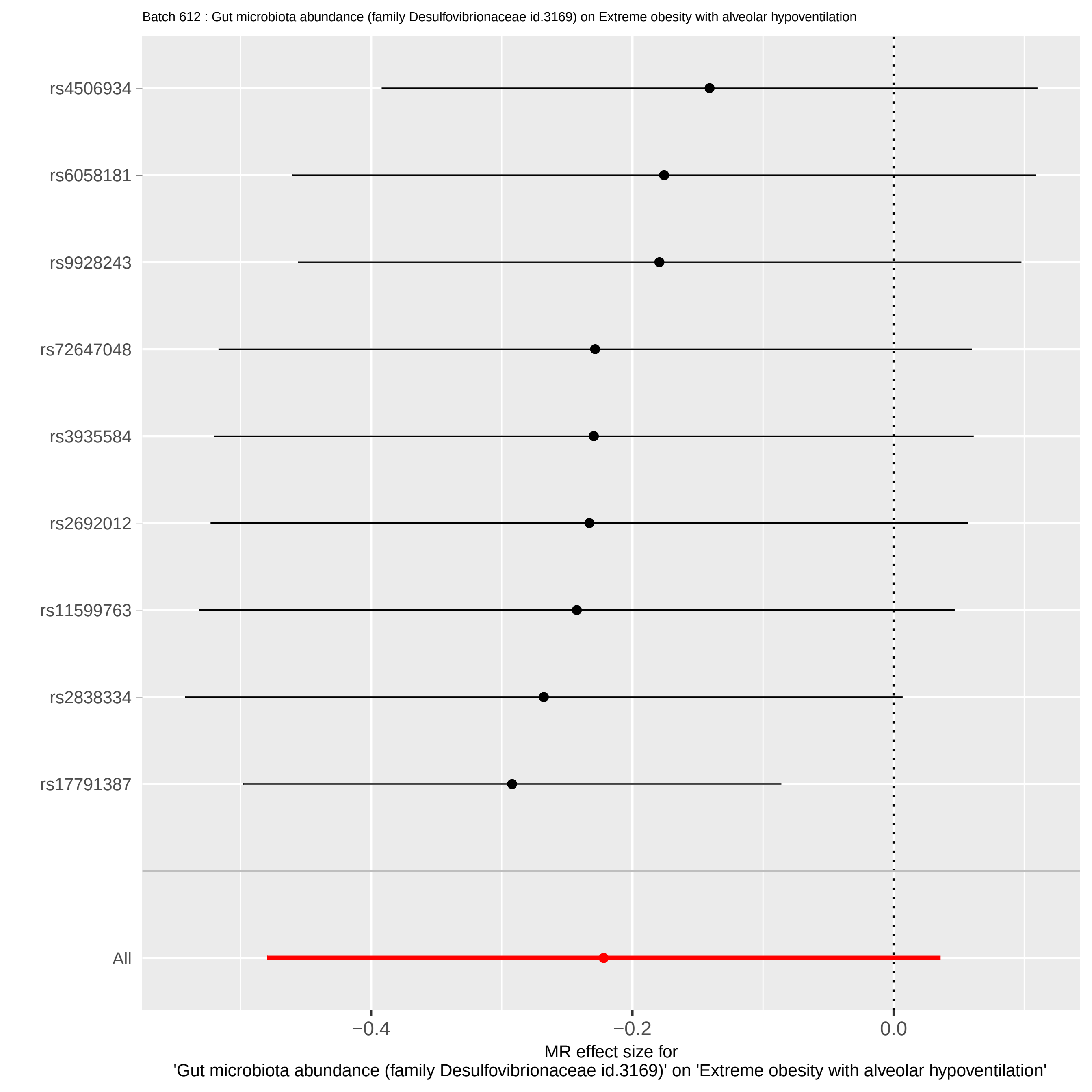
Batch 612 : Gut microbiota abundance (family Desulfovibrionaceae id.3169) on Extreme obesity with alveolar hypoventilation
rs4506934
rs6058181
rs9928243
rs72647048
rs3935584
rs2692012
rs11599763
rs2838334
rs17791387
All
−0.4
−0.2
0.0
MR effect size for
'Gut microbiota abundance (family Desulfovibrionaceae id.3169)' on 'Extreme obesity with alveolar hypoventilation'

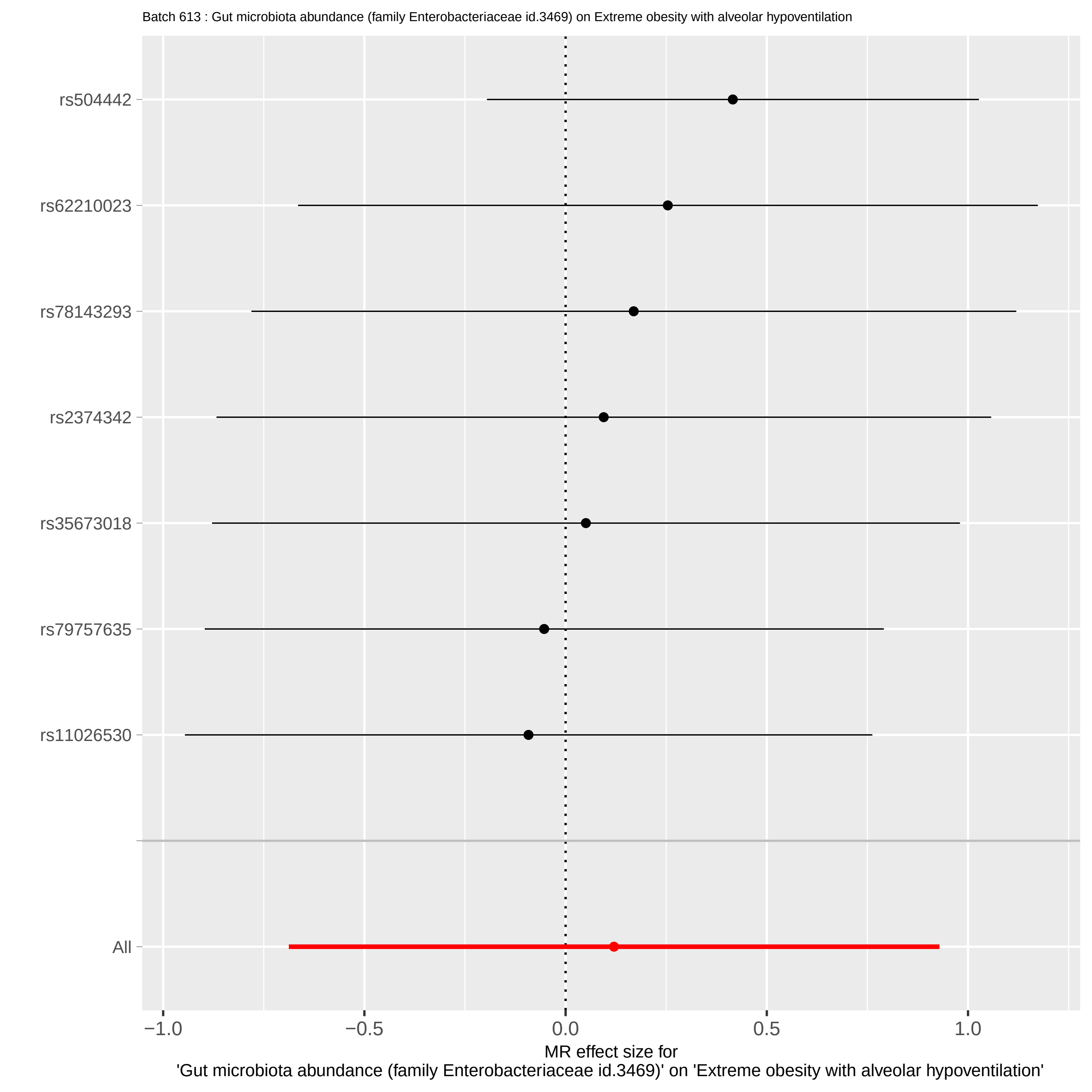
Batch 613 : Gut microbiota abundance (family Enterobacteriaceae id.3469) on Extreme obesity with alveolar hypoventilation
rs504442
rs62210023
rs78143293
rs2374342
rs35673018
rs79757635
rs11026530
All
−1.0
−0.5
0.0
0.5
1.0
MR effect size for
'Gut microbiota abundance (family Enterobacteriaceae id.3469)' on 'Extreme obesity with alveolar hypoventilation'

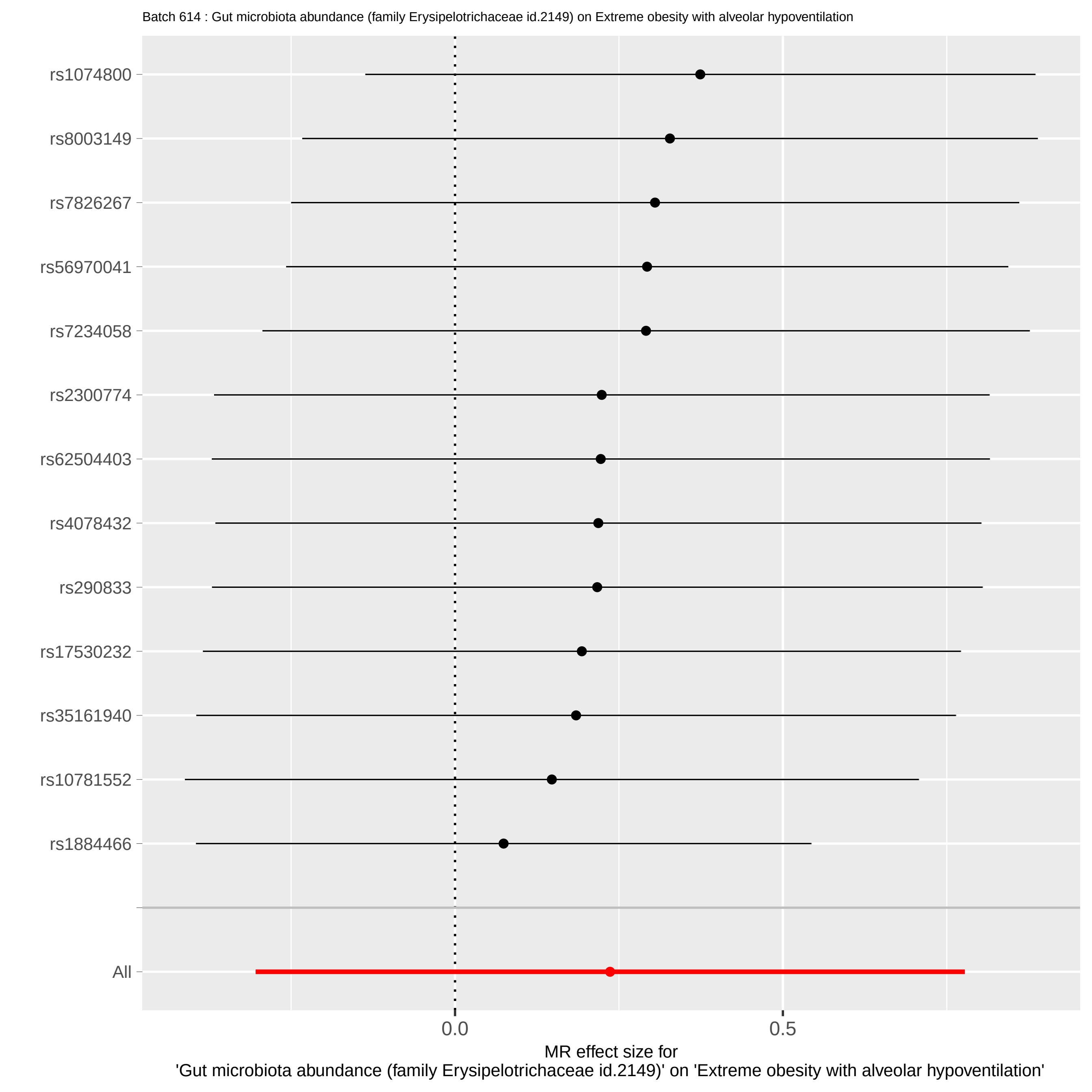

Batch 614 : Gut microbiota abundance (family Erysipelotrichaceae id.2149) on Extreme obesity with alveolar hypoventilation
rs1074800
rs8003149
rs7826267
rs56970041
rs7234058
rs2300774
rs62504403
rs4078432
rs290833
rs17530232
rs35161940
rs10781552
rs1884466
All
0.0
0.5
MR effect size for
'Gut microbiota abundance (family Erysipelotrichaceae id.2149)' on 'Extreme obesity with alveolar hypoventilation'

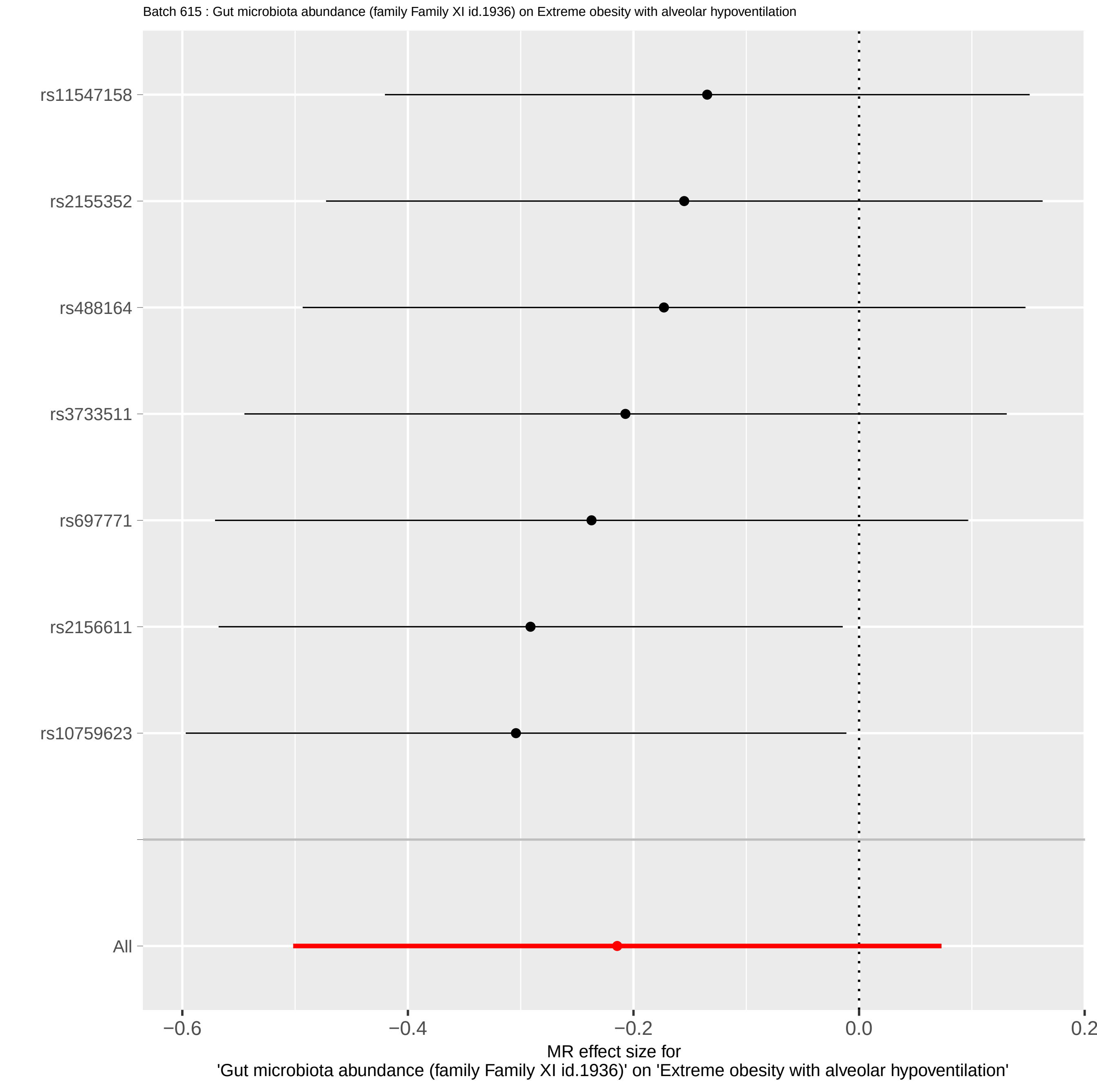

Batch 615 : Gut microbiota abundance (family Family XI id.1936) on Extreme obesity with alveolar hypoventilation
rs11547158
rs2155352
rs488164
rs3733511
rs697771
rs2156611
rs10759623
All
−0.6
−0.4
−0.2
0.0
0.2
MR effect size for
'Gut microbiota abundance (family Family XI id.1936)' on 'Extreme obesity with alveolar hypoventilation'

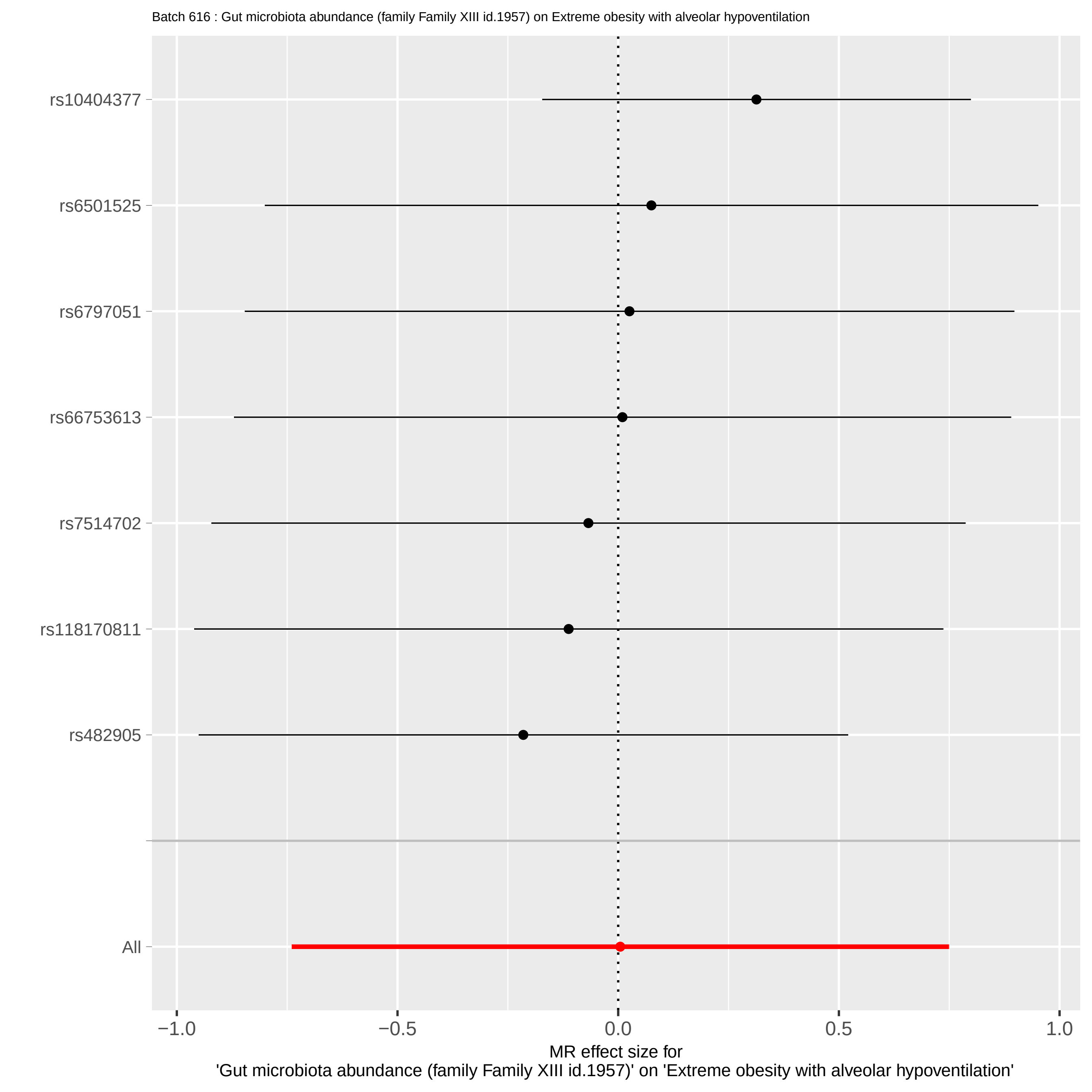
Batch 616 : Gut microbiota abundance (family Family XIII id.1957) on Extreme obesity with alveolar hypoventilation
rs10404377
rs6501525
rs6797051
rs66753613
rs7514702
rs118170811
rs482905
All
−1.0
−0.5
0.0
0.5
1.0
MR effect size for
'Gut microbiota abundance (family Family XIII id.1957)' on 'Extreme obesity with alveolar hypoventilation'

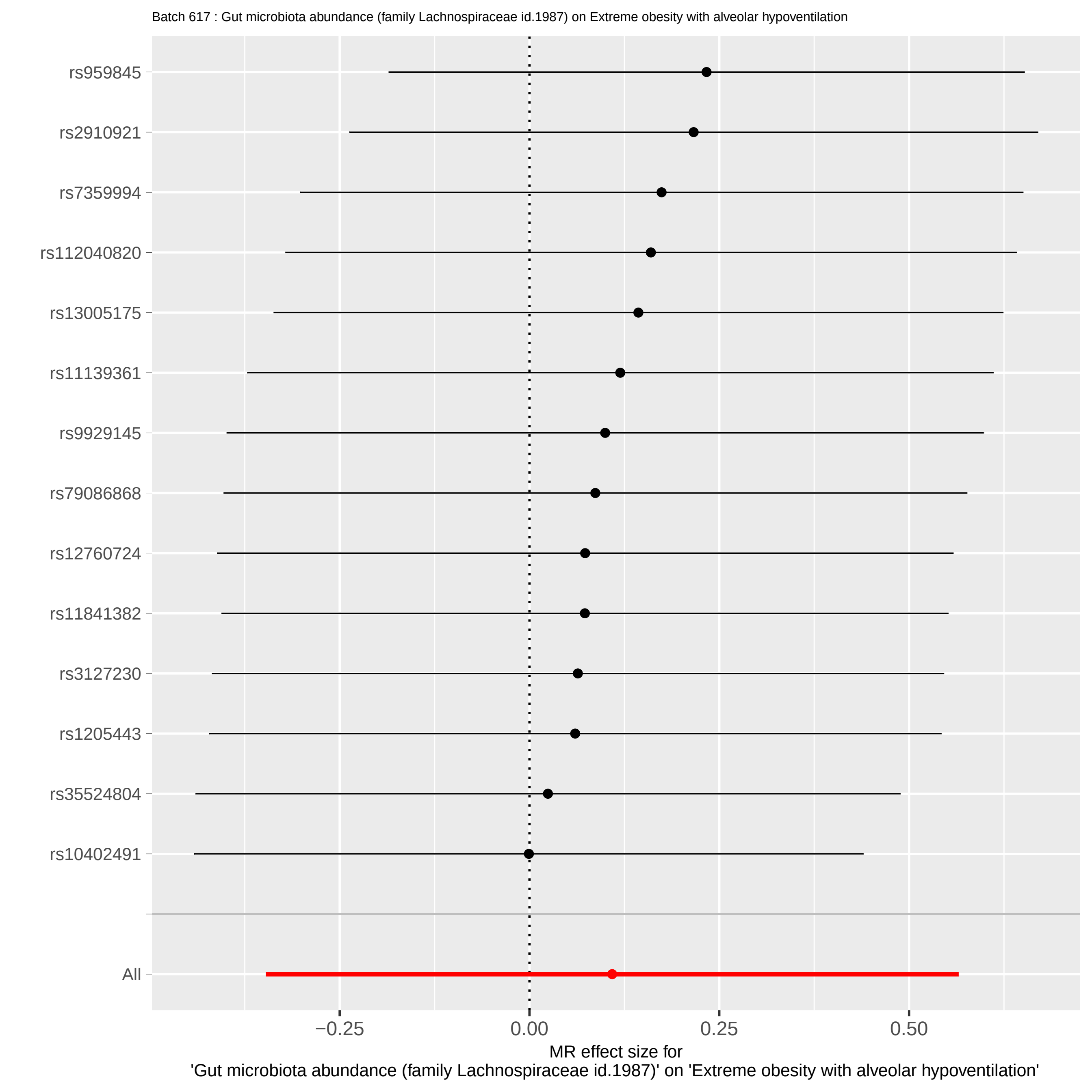

Batch 617 : Gut microbiota abundance (family Lachnospiraceae id.1987) on Extreme obesity with alveolar hypoventilation
rs959845
rs2910921
rs7359994
rs112040820
rs13005175
rs11139361
rs9929145
rs79086868
rs12760724
rs11841382
rs3127230
rs1205443
rs35524804
rs10402491
All
−0.25
0.00
0.25
0.50
MR effect size for
'Gut microbiota abundance (family Lachnospiraceae id.1987)' on 'Extreme obesity with alveolar hypoventilation'

Batch 618 : Gut microbiota abundance (family Lactobacillaceae id.1836) on Extreme obesity with alveolar hypoventilation

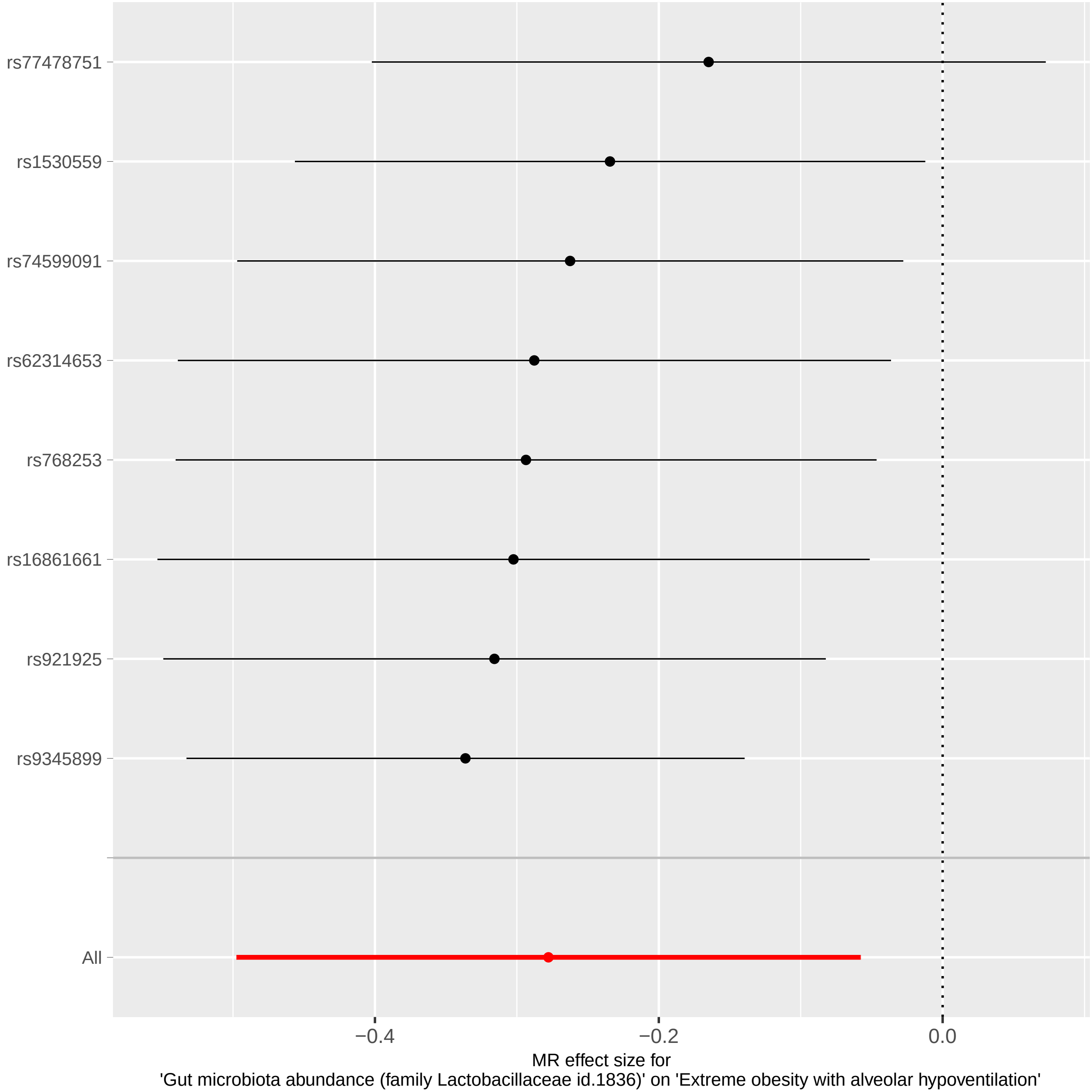

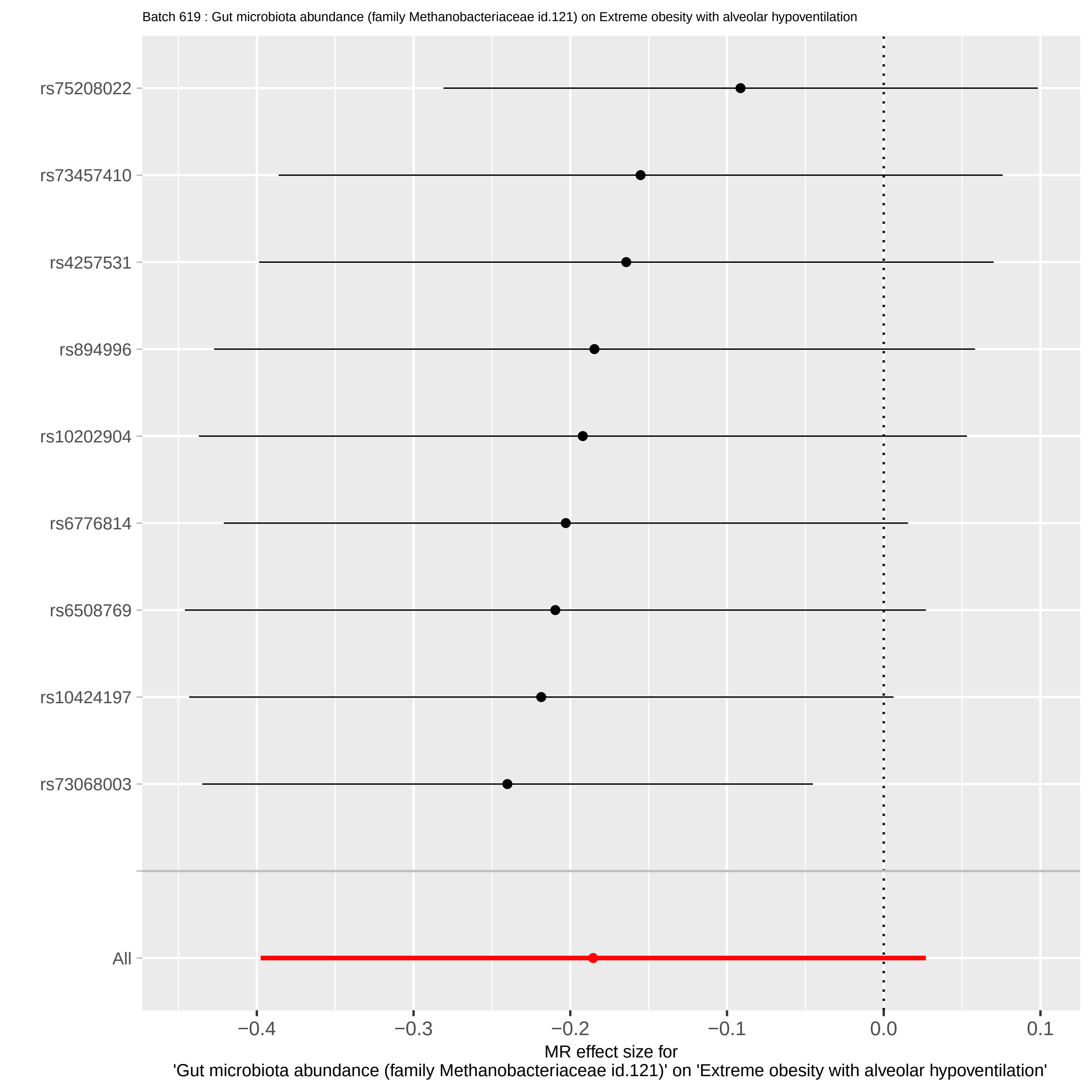

Batch 619 : Gut microbiota abundance (family Methanobacteriaceae id.121) on Extreme obesity with alveolar hypoventilation
rs75208022
rs73457410
rs4257531
rs894996
rs10202904
rs6776814
rs6508769
rs10424197
rs73068003
All
−0.4
−0.3
−0.2
−0.1
0.0
0.1
MR effect size for
'Gut microbiota abundance (family Methanobacteriaceae id.121)' on 'Extreme obesity with alveolar hypoventilation'

Batch 620 : Gut microbiota abundance (family Oxalobacteraceae id.2966) on Extreme obesity with alveolar hypoventilation

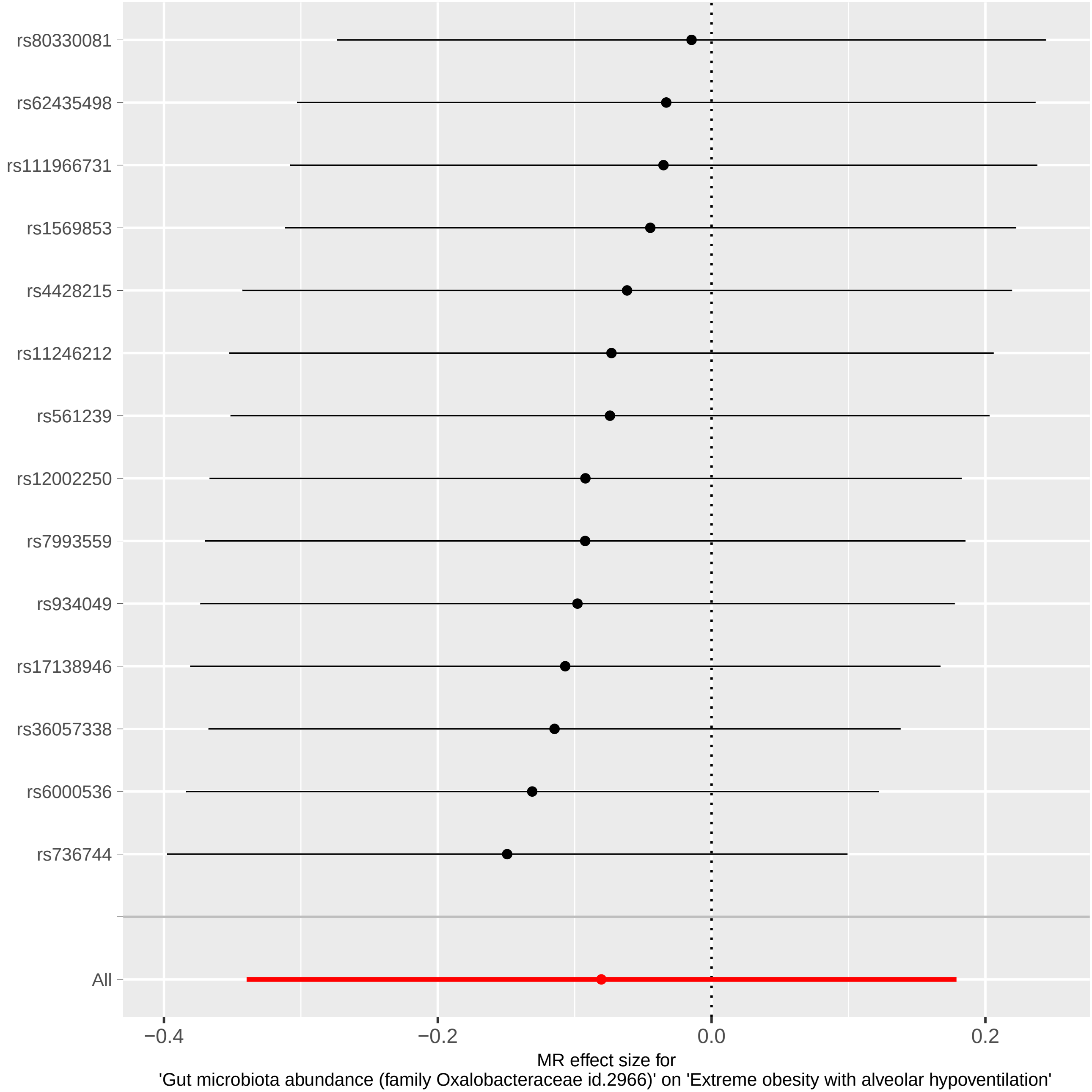

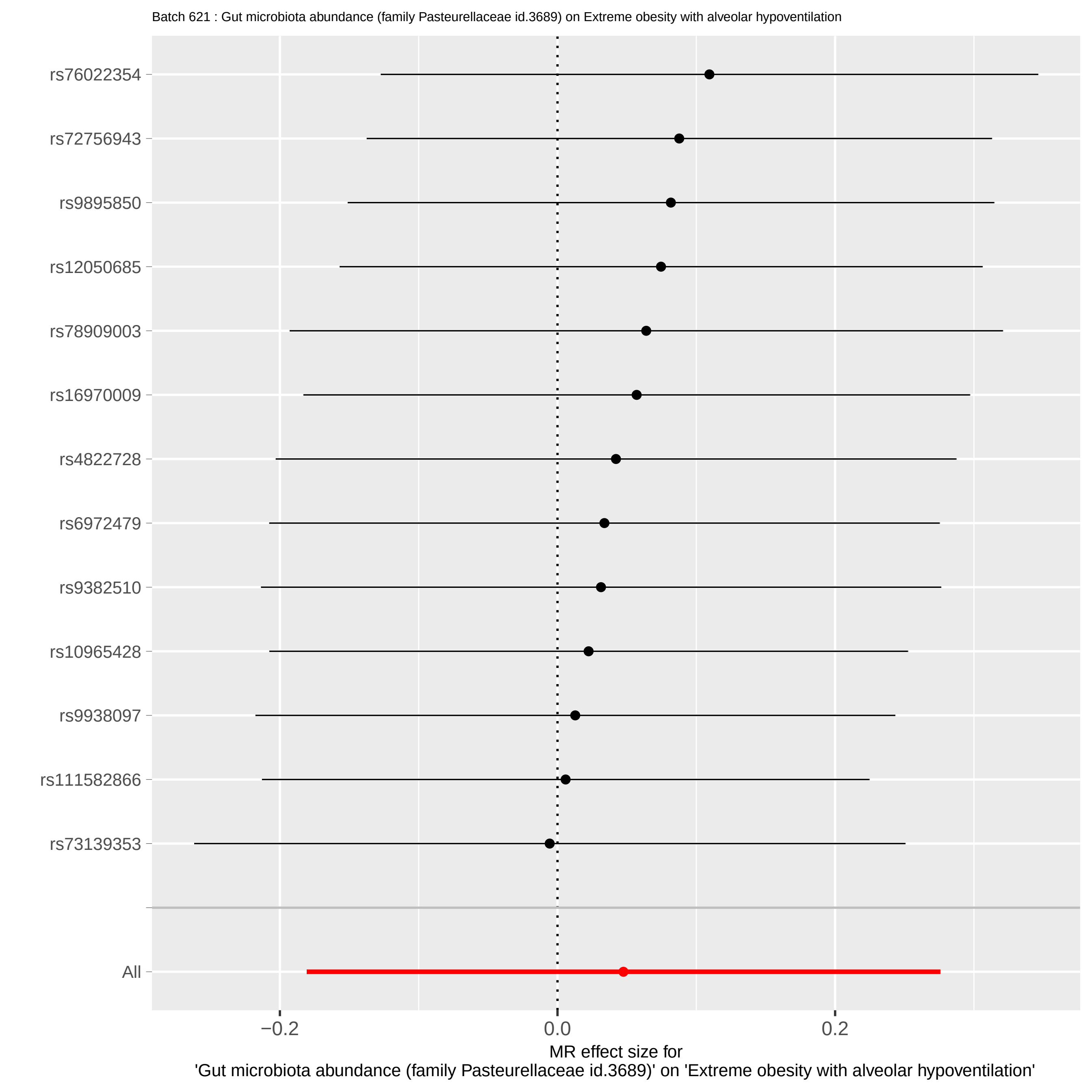

Batch 621 : Gut microbiota abundance (family Pasteurellaceae id.3689) on Extreme obesity with alveolar hypoventilation
rs76022354
rs72756943
rs9895850
rs12050685
rs78909003
rs16970009
rs4822728
rs6972479
rs9382510
rs10965428
rs9938097
rs111582866
rs73139353
All
−0.2
0.0
0.2
MR effect size for
'Gut microbiota abundance (family Pasteurellaceae id.3689)' on 'Extreme obesity with alveolar hypoventilation'

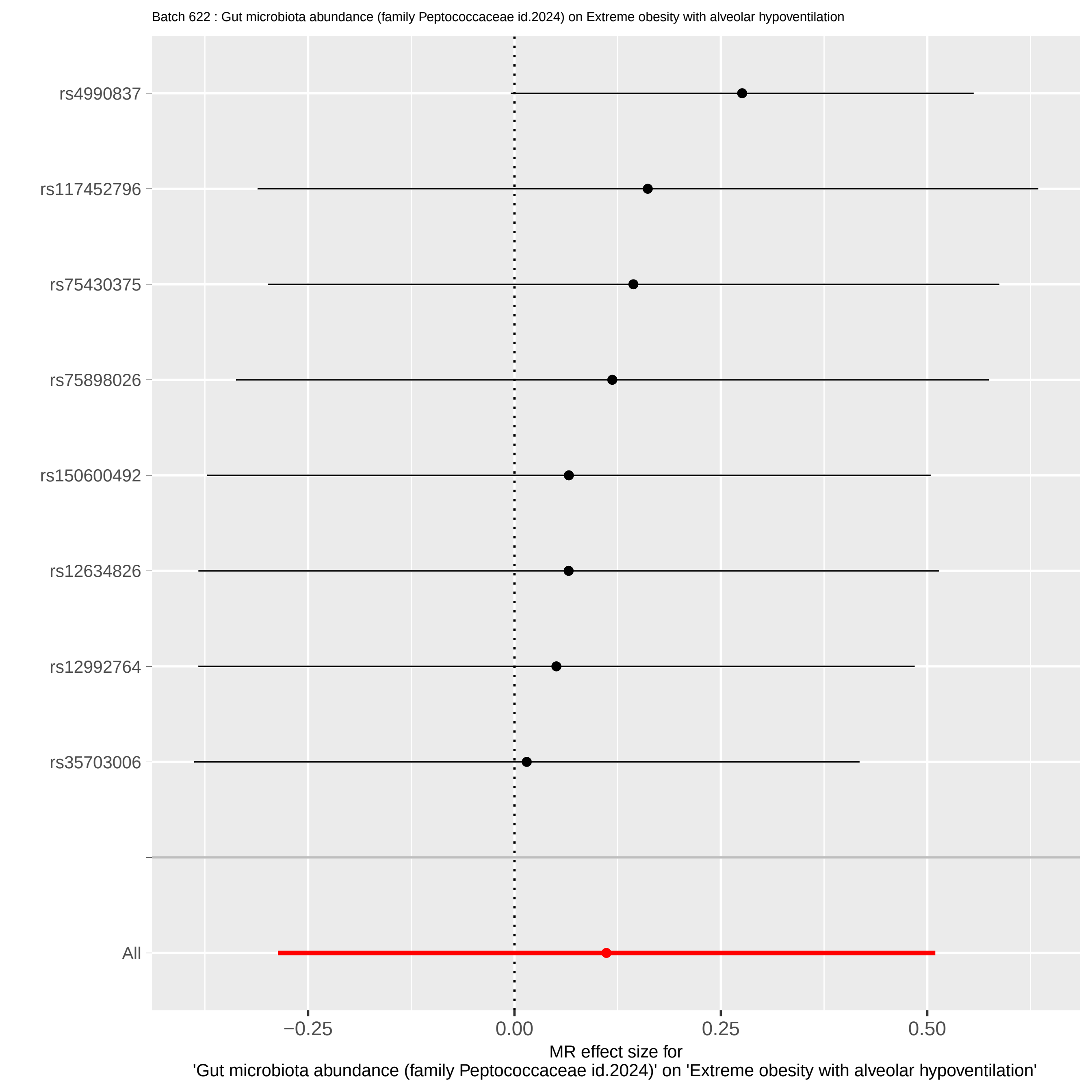
Batch 622 : Gut microbiota abundance (family Peptococcaceae id.2024) on Extreme obesity with alveolar hypoventilation
rs4990837
rs117452796
rs75430375
rs75898026
rs150600492
rs12634826
rs12992764
rs35703006
All
−0.25
0.00
0.25
0.50
MR effect size for
'Gut microbiota abundance (family Peptococcaceae id.2024)' on 'Extreme obesity with alveolar hypoventilation'

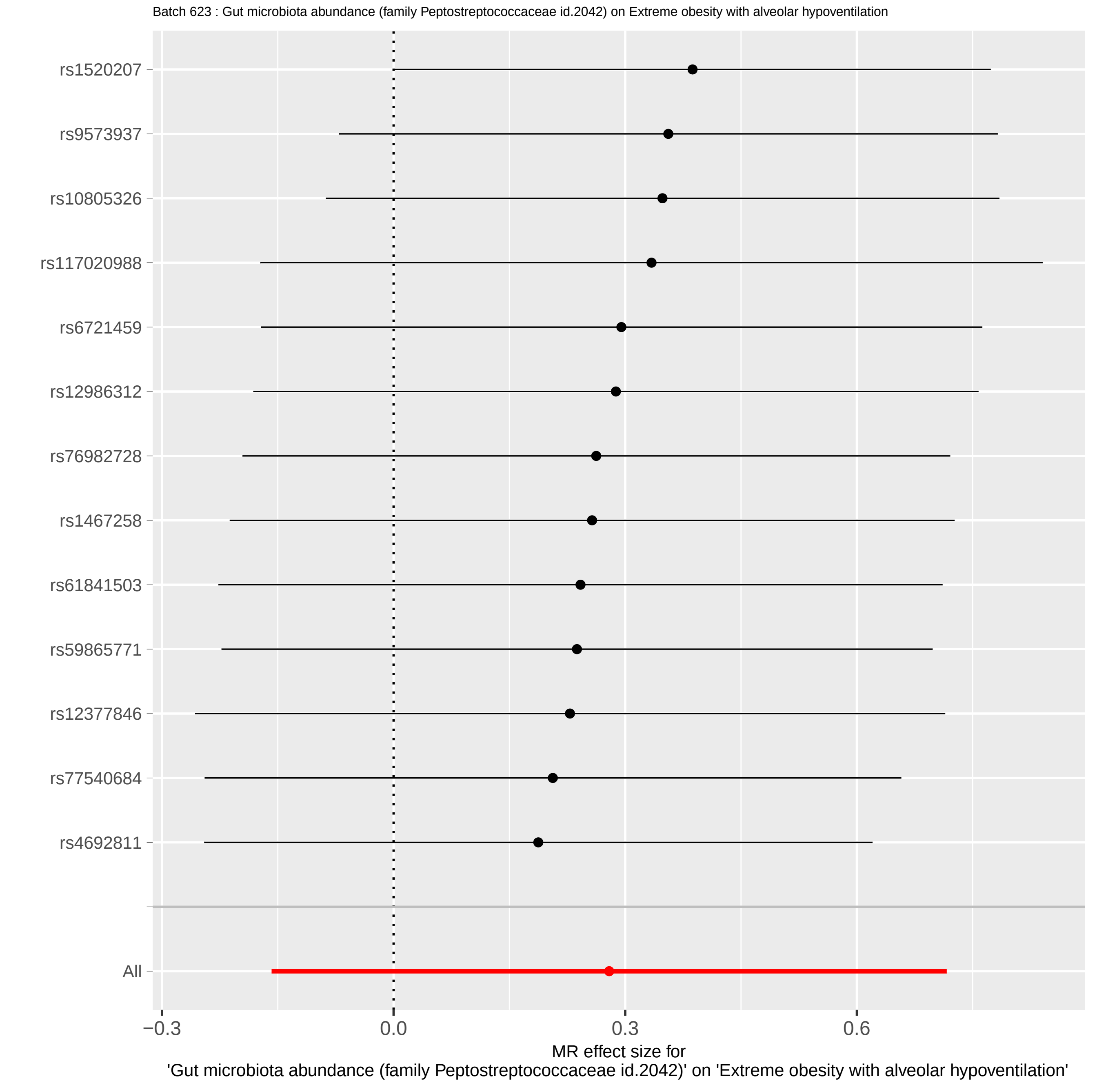

Batch 623 : Gut microbiota abundance (family Peptostreptococcaceae id.2042) on Extreme obesity with alveolar hypoventilation
rs1520207
rs9573937
rs10805326
rs117020988
rs6721459
rs12986312
rs76982728
rs1467258
rs61841503
rs59865771
rs12377846
rs77540684
rs4692811
All
−0.3
0.0
0.3
0.6
MR effect size for
'Gut microbiota abundance (family Peptostreptococcaceae id.2042)' on 'Extreme obesity with alveolar hypoventilation'

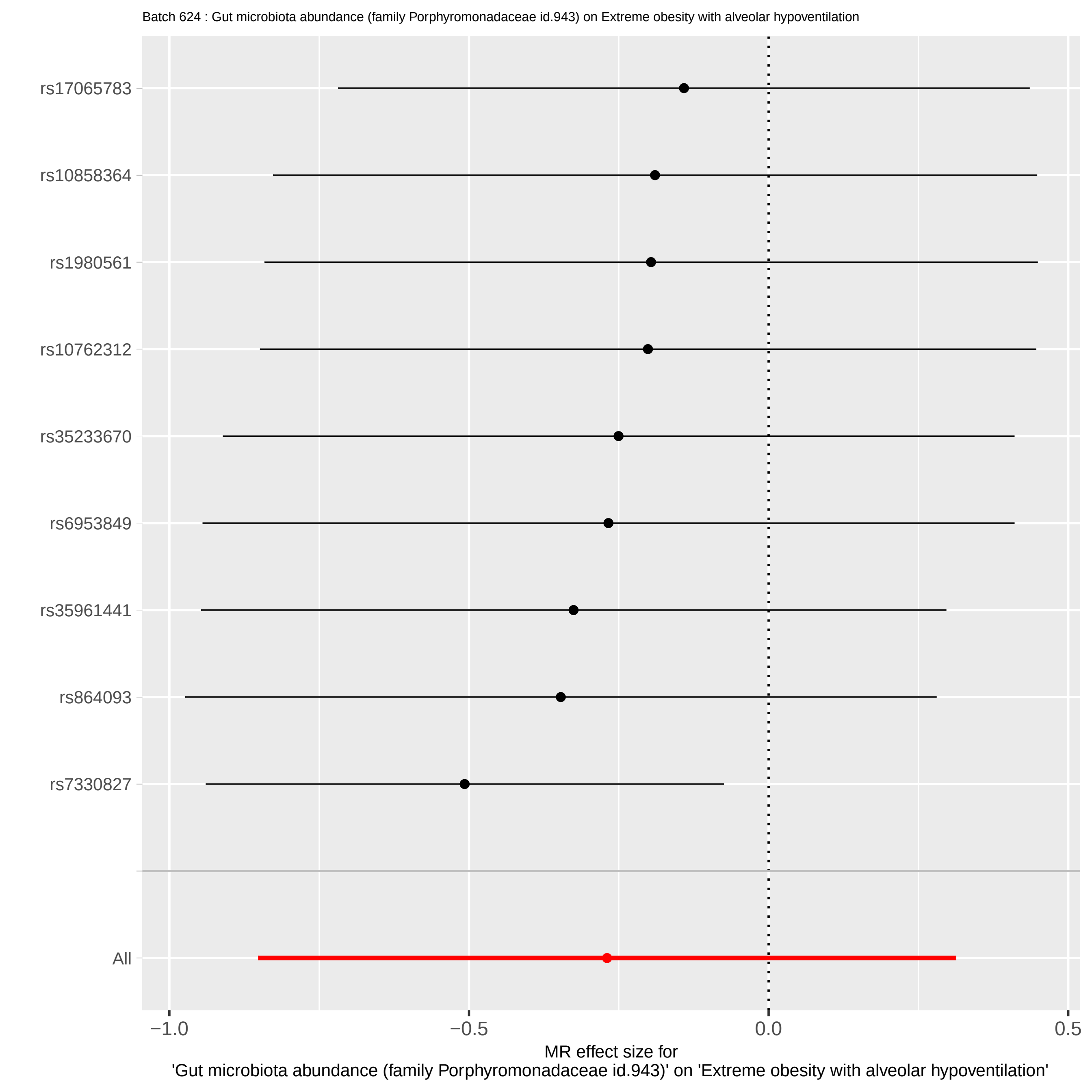

Batch 624 : Gut microbiota abundance (family Porphyromonadaceae id.943) on Extreme obesity with alveolar hypoventilation
rs17065783
rs10858364
rs1980561
rs10762312
rs35233670
rs6953849
rs35961441
rs864093
rs7330827
All
−1.0
−0.5
0.0
0.5
MR effect size for
'Gut microbiota abundance (family Porphyromonadaceae id.943)' on 'Extreme obesity with alveolar hypoventilation'

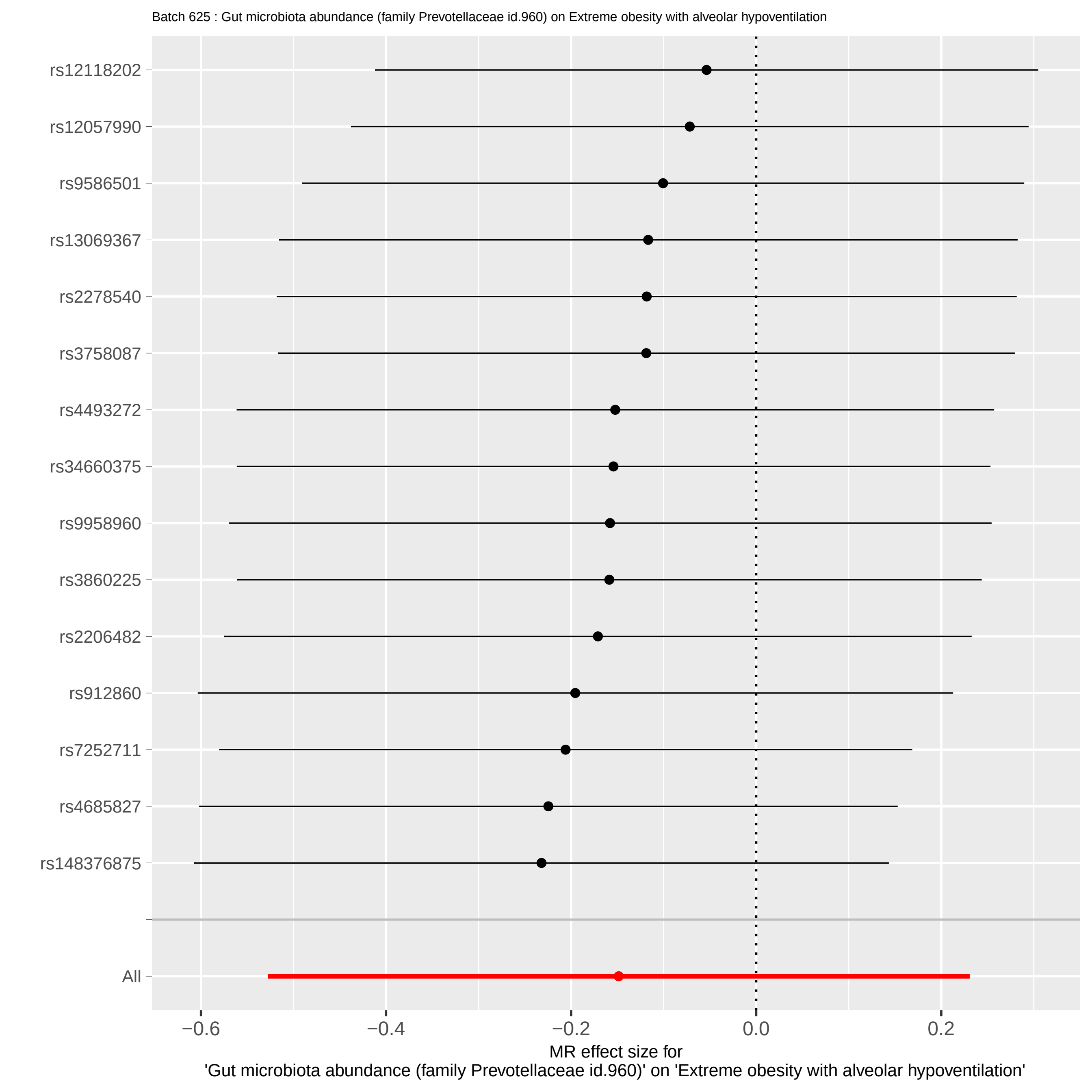

Batch 625 : Gut microbiota abundance (family Prevotellaceae id.960) on Extreme obesity with alveolar hypoventilation
rs12118202
rs12057990
rs9586501
rs13069367
rs2278540
rs3758087
rs4493272
rs34660375
rs9958960
rs3860225
rs2206482
rs912860
rs7252711
rs4685827
rs148376875
All
−0.6
−0.4
−0.2
0.0
0.2
MR effect size for
'Gut microbiota abundance (family Prevotellaceae id.960)' on 'Extreme obesity with alveolar hypoventilation'

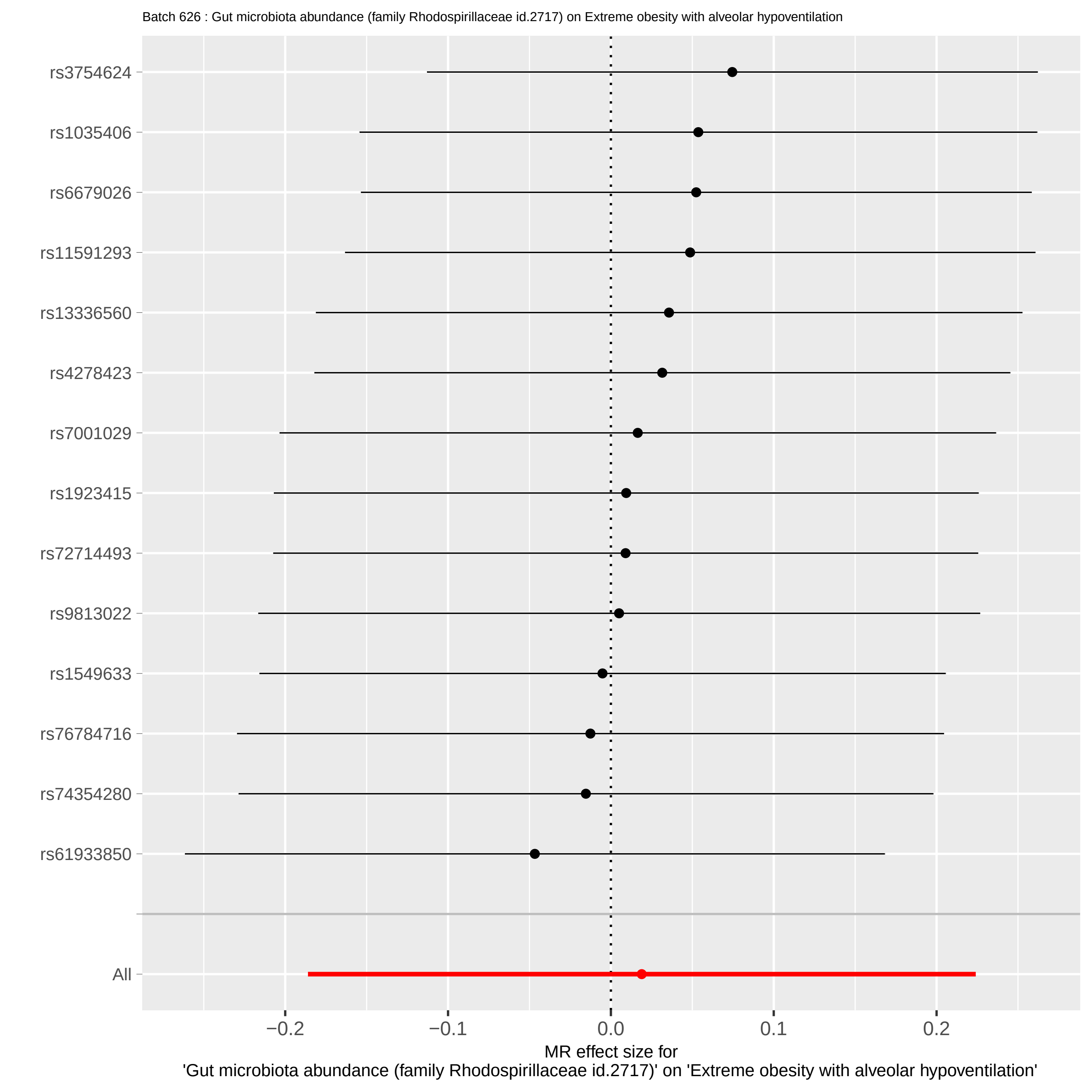
Batch 626 : Gut microbiota abundance (family Rhodospirillaceae id.2717) on Extreme obesity with alveolar hypoventilation
rs3754624
rs1035406
rs6679026
rs11591293
rs13336560
rs4278423
rs7001029
rs1923415
rs72714493
rs9813022
rs1549633
rs76784716
rs74354280
rs61933850
All
−0.2
−0.1
0.0
0.1
0.2
MR effect size for
'Gut microbiota abundance (family Rhodospirillaceae id.2717)' on 'Extreme obesity with alveolar hypoventilation'

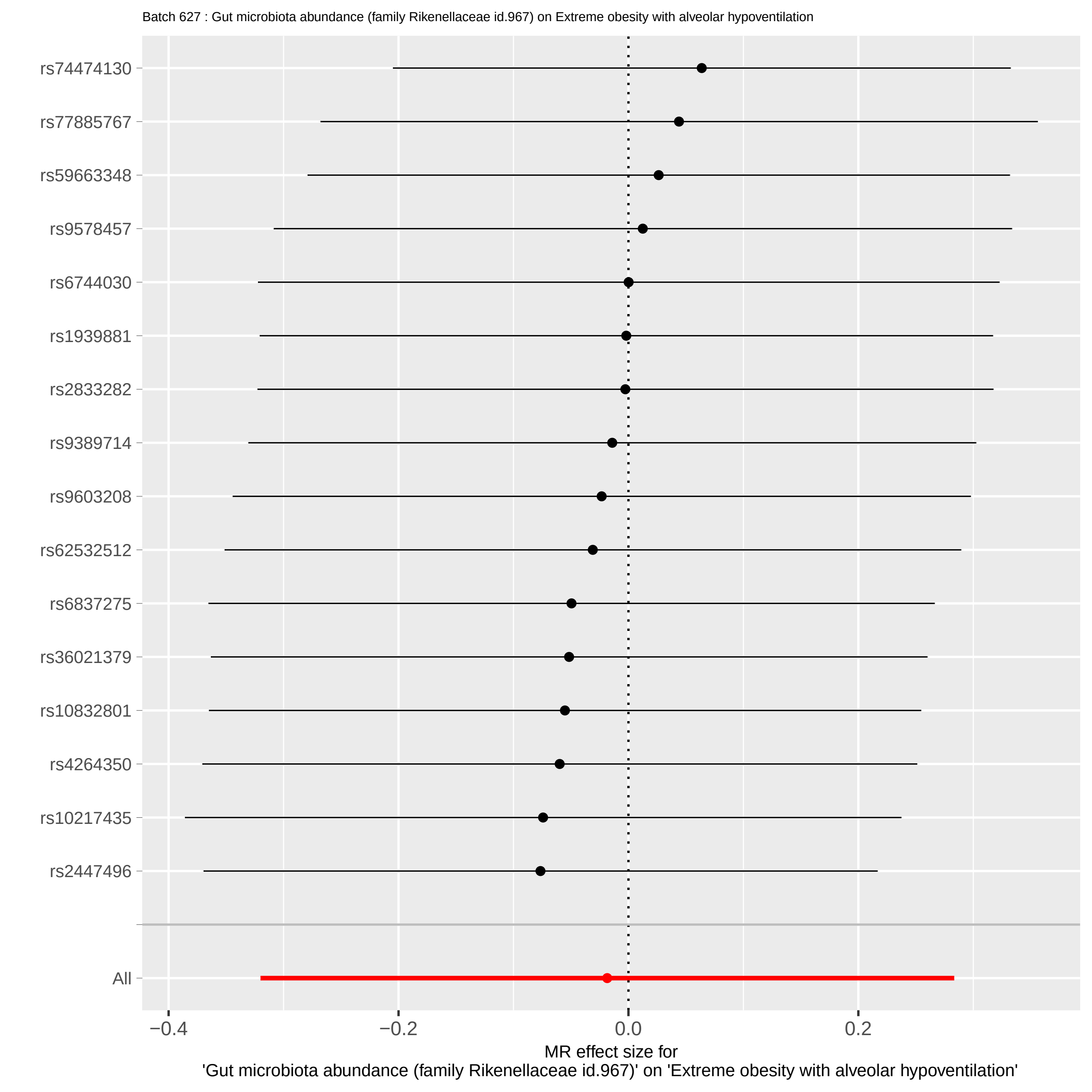
Batch 627 : Gut microbiota abundance (family Rikenellaceae id.967) on Extreme obesity with alveolar hypoventilation
rs74474130
rs77885767
rs59663348
rs9578457
rs6744030
rs1939881
rs2833282
rs9389714
rs9603208
rs62532512
rs6837275
rs36021379
rs10832801
rs4264350
rs10217435
rs2447496
All
−0.4
−0.2
0.0
0.2
MR effect size for
'Gut microbiota abundance (family Rikenellaceae id.967)' on 'Extreme obesity with alveolar hypoventilation'

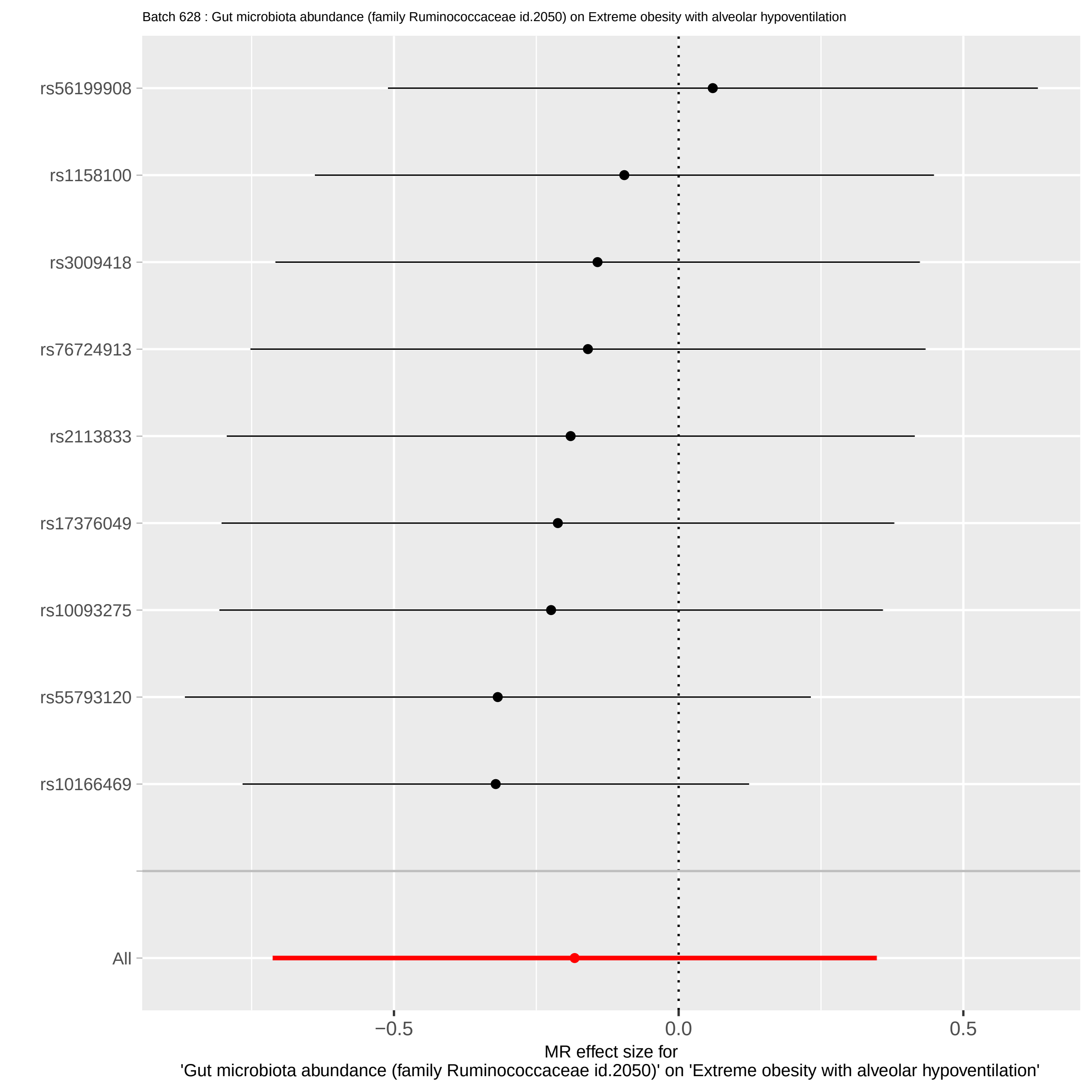

Batch 628 : Gut microbiota abundance (family Ruminococcaceae id.2050) on Extreme obesity with alveolar hypoventilation
rs56199908
rs1158100
rs3009418
rs76724913
rs2113833
rs17376049
rs10093275
rs55793120
rs10166469
All
−0.5
0.0
0.5
MR effect size for
'Gut microbiota abundance (family Ruminococcaceae id.2050)' on 'Extreme obesity with alveolar hypoventilation'

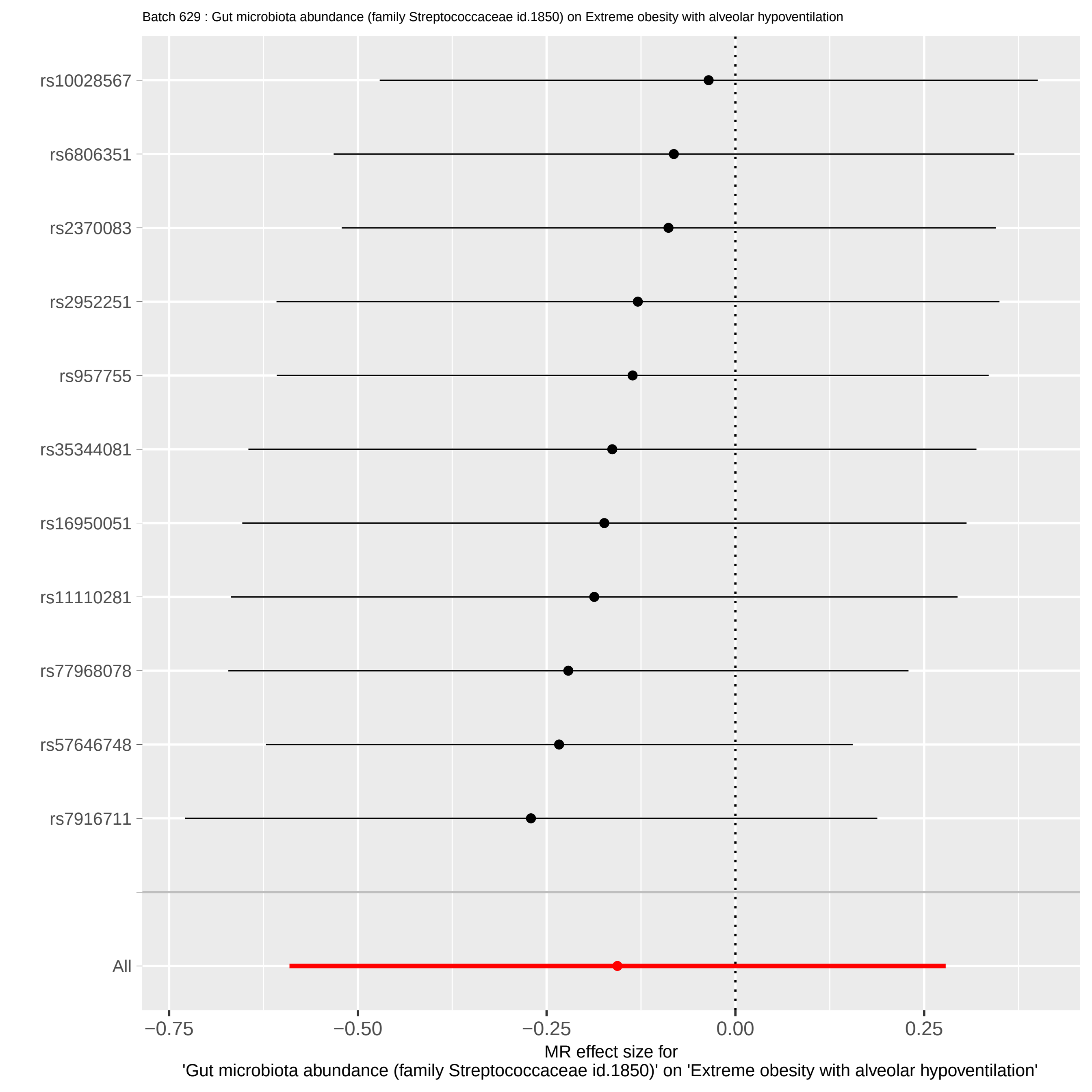

Batch 629 : Gut microbiota abundance (family Streptococcaceae id.1850) on Extreme obesity with alveolar hypoventilation
rs10028567
rs6806351
rs2370083
rs2952251
rs957755
rs35344081
rs16950051
rs11110281
rs77968078
rs57646748
rs7916711
All
−0.75
−0.50
−0.25
0.00
0.25
MR effect size for
'Gut microbiota abundance (family Streptococcaceae id.1850)' on 'Extreme obesity with alveolar hypoventilation'

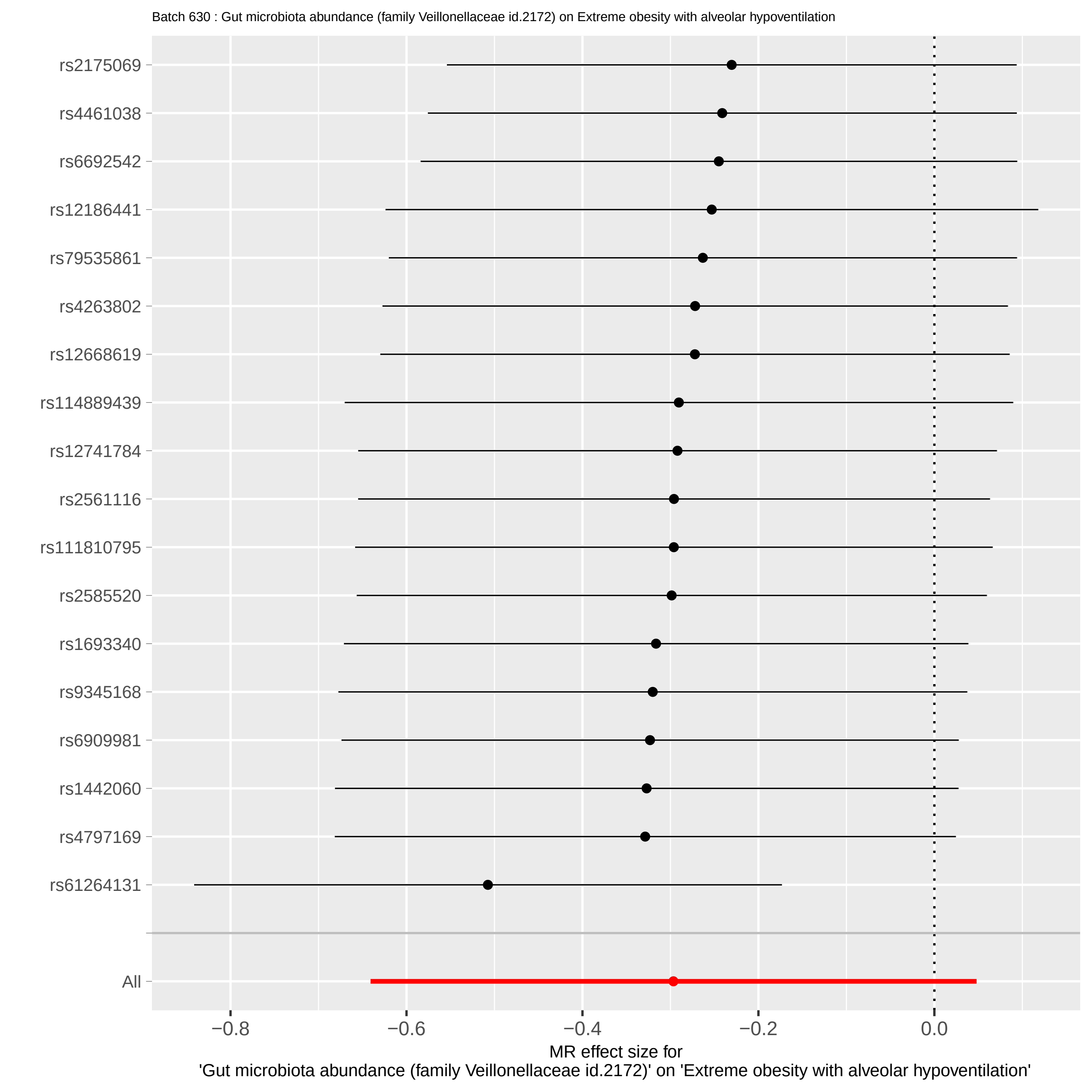
Batch 630 : Gut microbiota abundance (family Veillonellaceae id.2172) on Extreme obesity with alveolar hypoventilation
rs2175069
rs4461038
rs6692542
rs12186441
rs79535861
rs4263802
rs12668619
rs114889439
rs12741784
rs2561116
rs111810795
rs2585520
rs1693340
rs9345168
rs6909981
rs1442060
rs4797169
rs61264131
All
−0.8
−0.6
−0.4
−0.2
0.0
MR effect size for
'Gut microbiota abundance (family Veillonellaceae id.2172)' on 'Extreme obesity with alveolar hypoventilation'

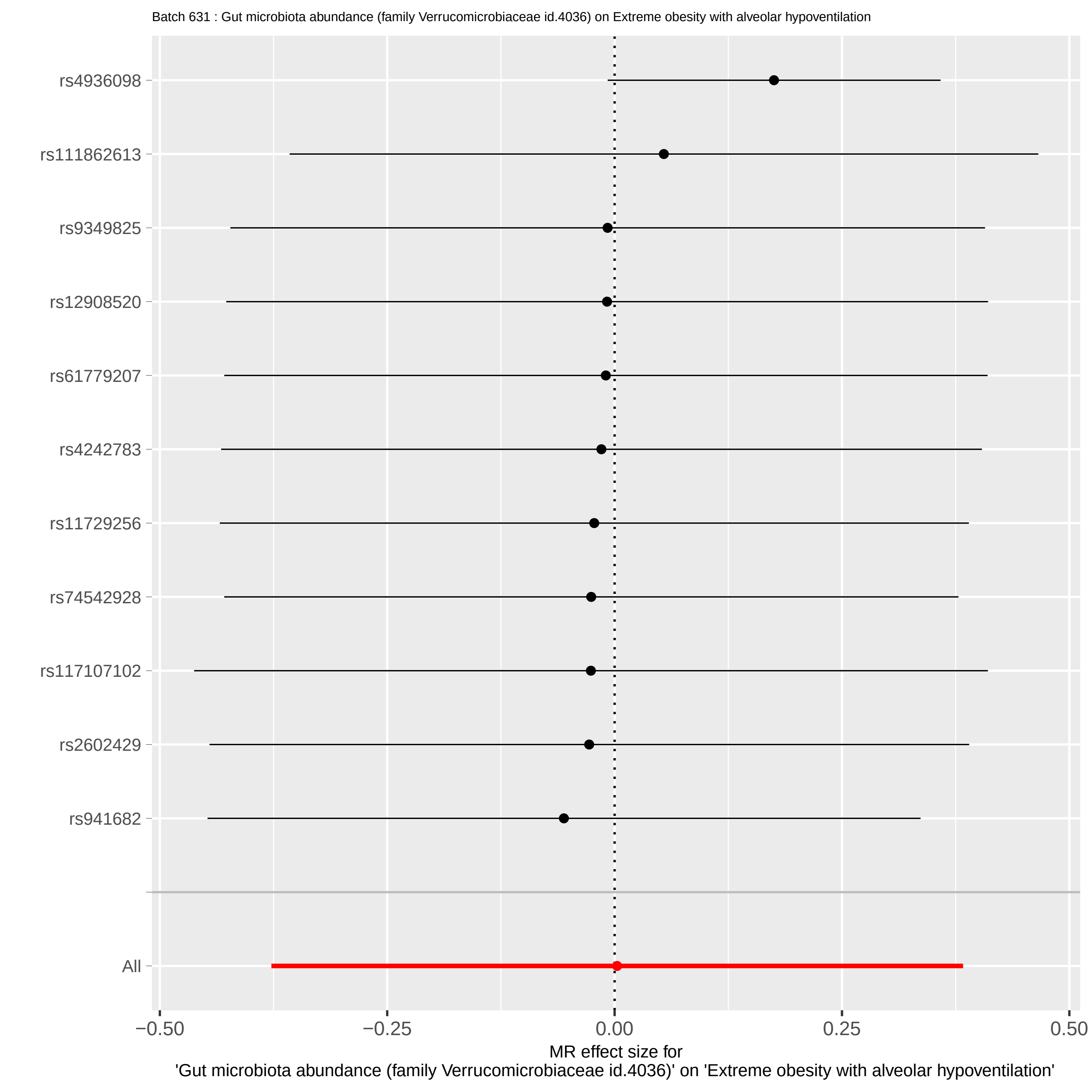
Batch 631 : Gut microbiota abundance (family Verrucomicrobiaceae id.4036) on Extreme obesity with alveolar hypoventilation
rs4936098
rs111862613
rs9349825
rs12908520
rs61779207
rs4242783
rs11729256
rs74542928
rs117107102
rs2602429
rs941682
All
−0.50
−0.25
0.00
0.25
0.50
MR effect size for
'Gut microbiota abundance (family Verrucomicrobiaceae id.4036)' on 'Extreme obesity with alveolar hypoventilation'

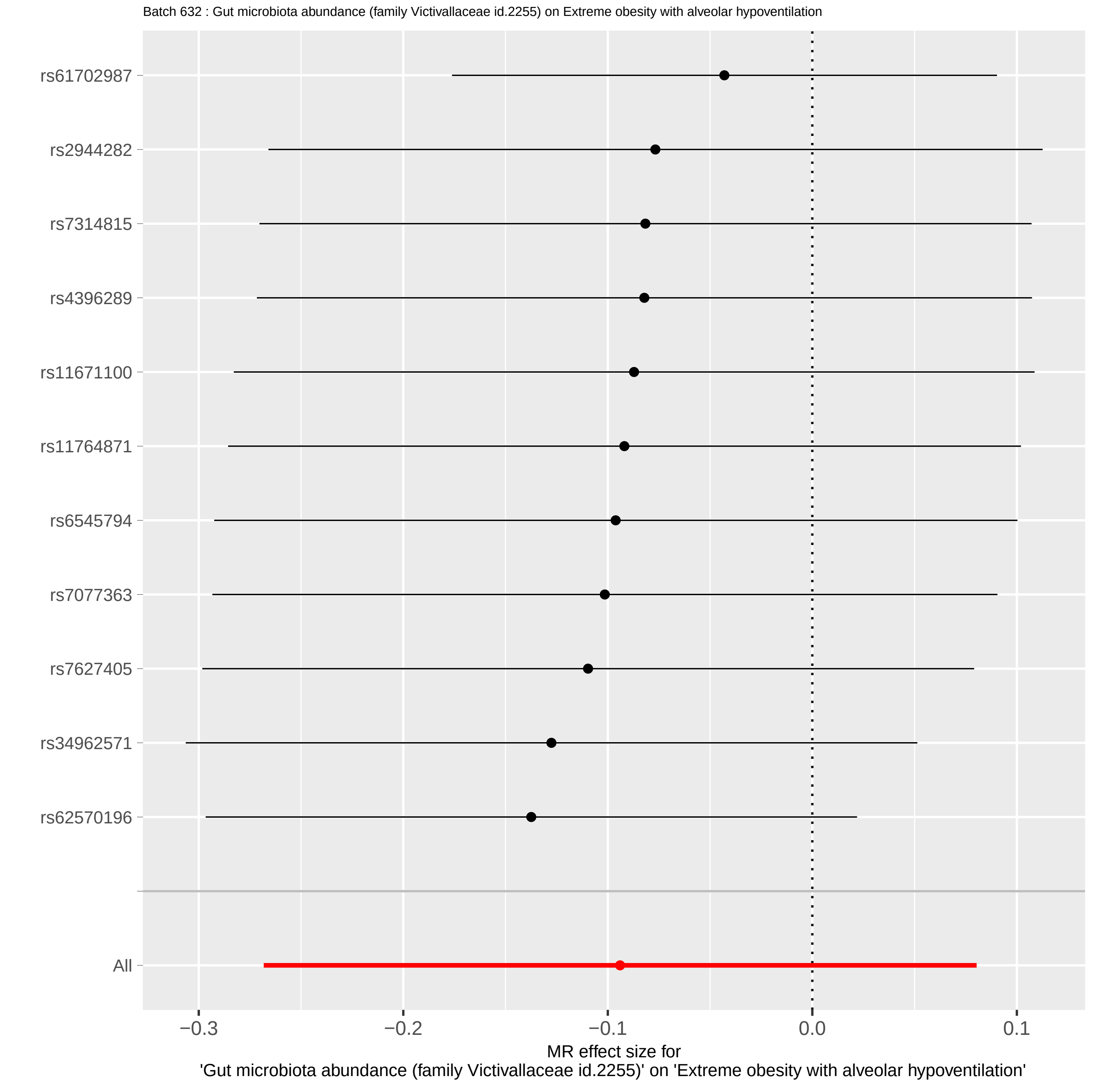

Batch 632 : Gut microbiota abundance (family Victivallaceae id.2255) on Extreme obesity with alveolar hypoventilation
rs61702987
rs2944282
rs7314815
rs4396289
rs11671100
rs11764871
rs6545794
rs7077363
rs7627405
rs34962571
rs62570196
All
−0.3
−0.2
−0.1
0.0
0.1
MR effect size for
'Gut microbiota abundance (family Victivallaceae id.2255)' on 'Extreme obesity with alveolar hypoventilation'

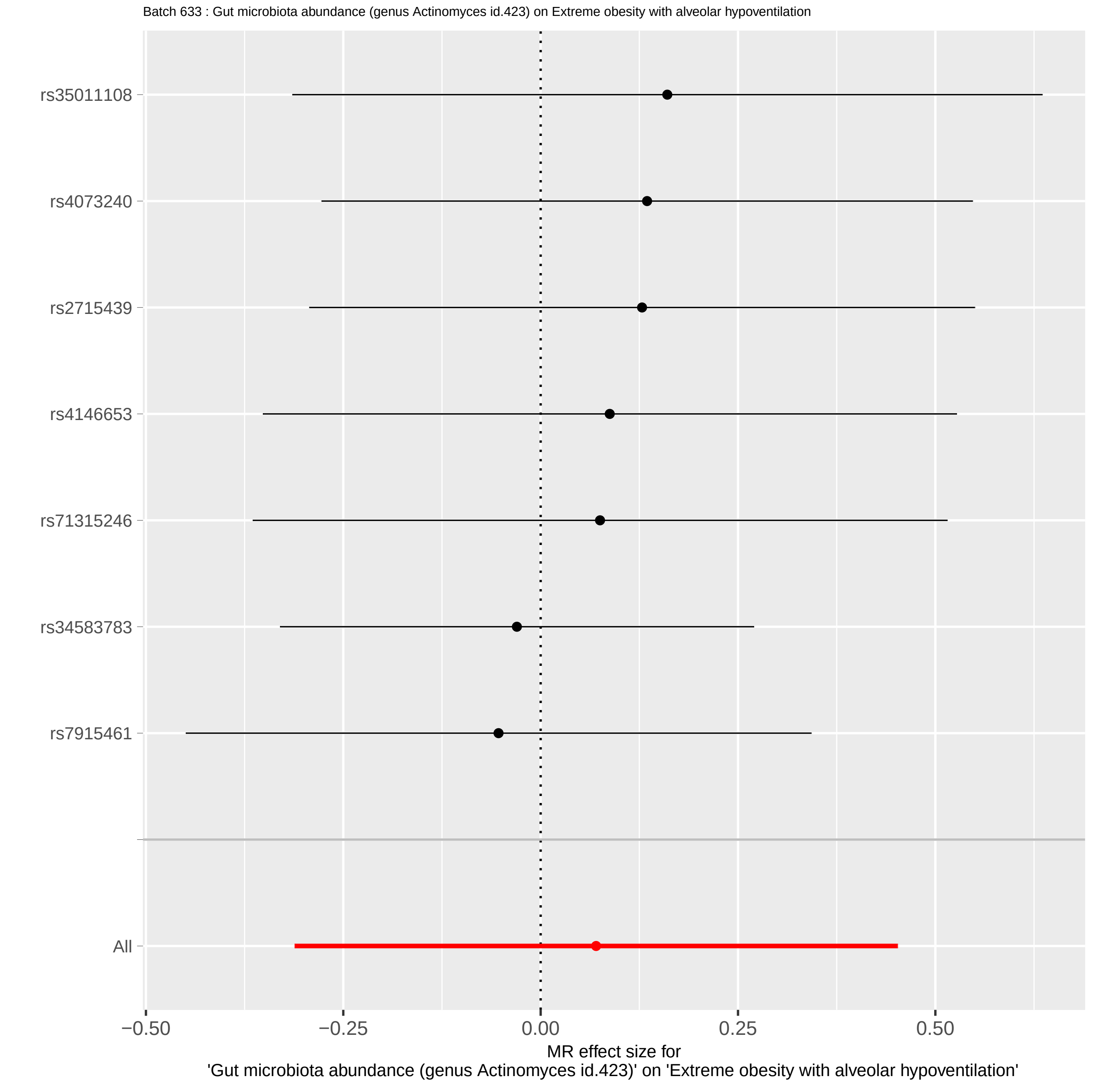
Batch 633 : Gut microbiota abundance (genus Actinomyces id.423) on Extreme obesity with alveolar hypoventilation
rs35011108
rs4073240
rs2715439
rs4146653
rs71315246
rs34583783
rs7915461
All
−0.50
−0.25
0.00
0.25
0.50
MR effect size for
'Gut microbiota abundance (genus Actinomyces id.423)' on 'Extreme obesity with alveolar hypoventilation'

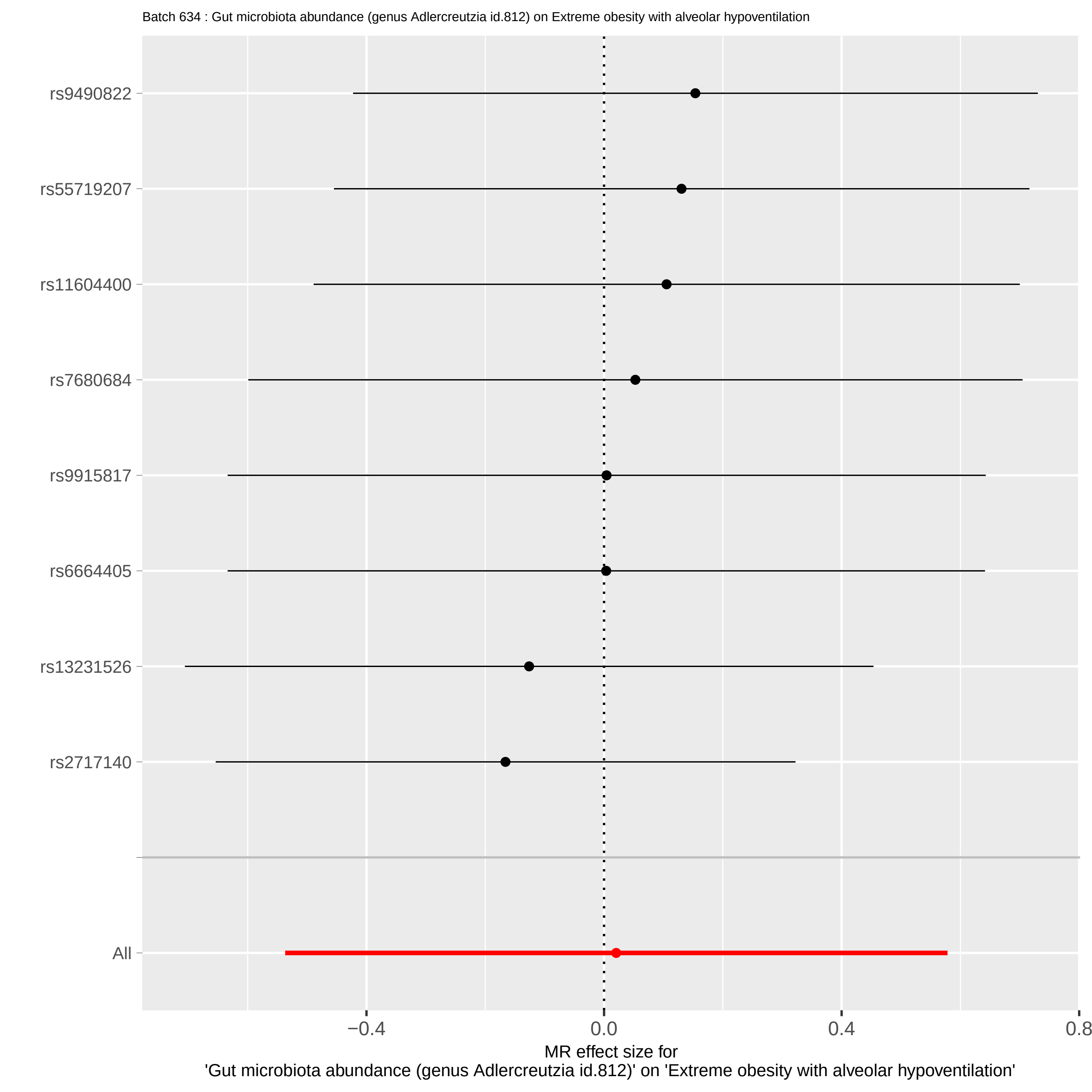
Batch 634 : Gut microbiota abundance (genus Adlercreutzia id.812) on Extreme obesity with alveolar hypoventilation
rs9490822
rs55719207
rs11604400
rs7680684
rs9915817
rs6664405
rs13231526
rs2717140
All
−0.4
0.0
0.4
0.8
MR effect size for
'Gut microbiota abundance (genus Adlercreutzia id.812)' on 'Extreme obesity with alveolar hypoventilation'

Batch 635 : Gut microbiota abundance (genus Akkermansia id.4037) on Extreme obesity with alveolar hypoventilation

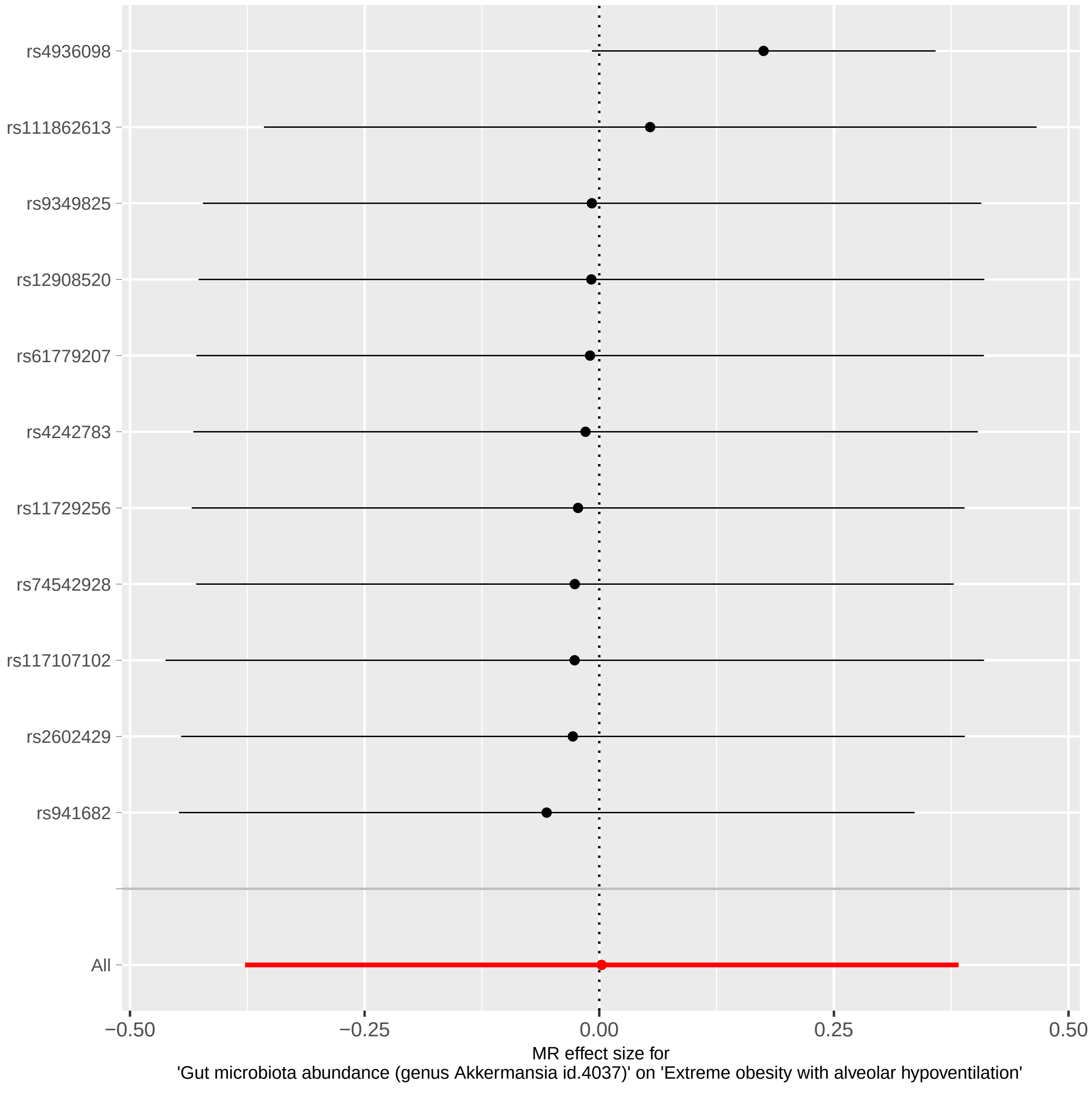

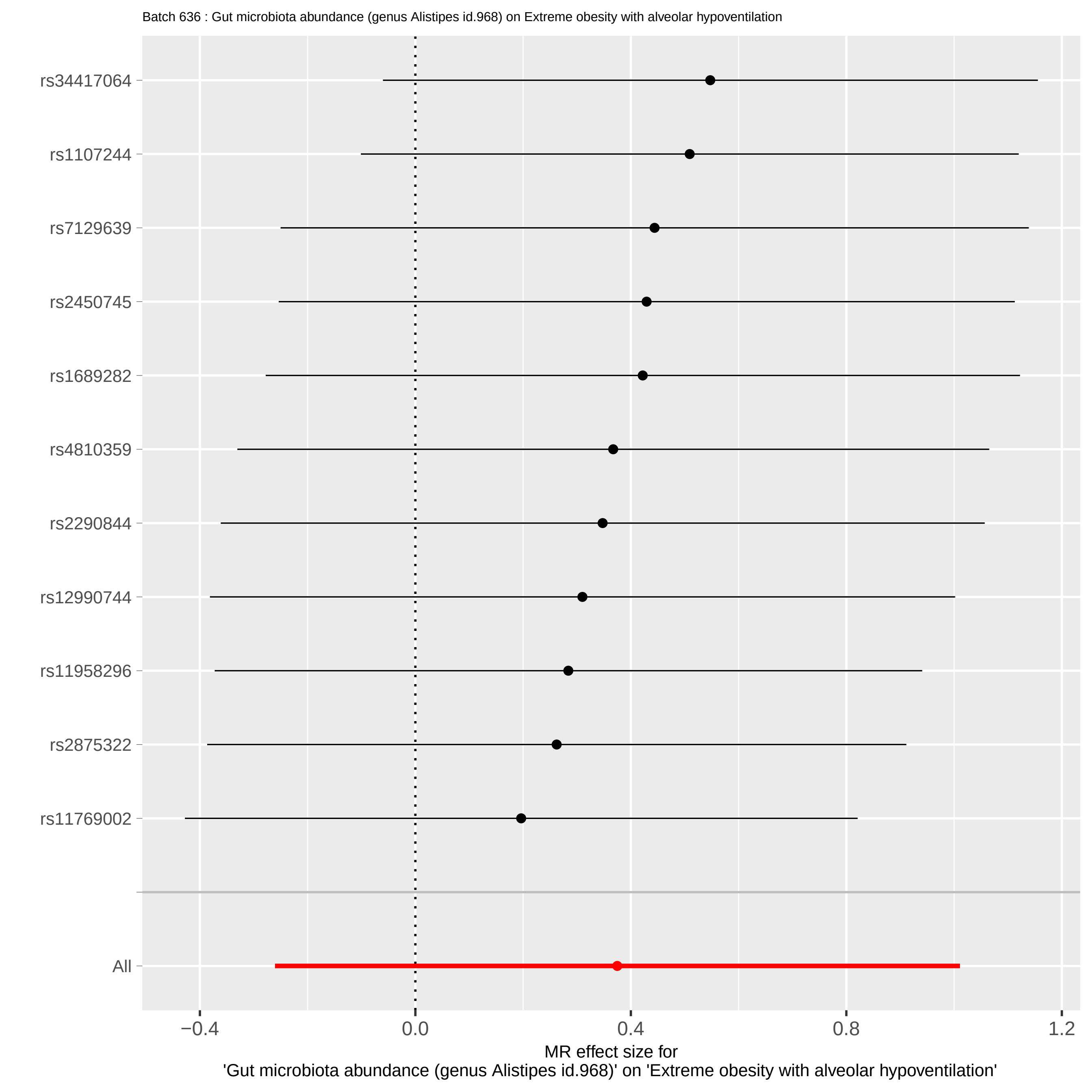
Batch 636 : Gut microbiota abundance (genus Alistipes id.968) on Extreme obesity with alveolar hypoventilation
rs34417064
rs1107244
rs7129639
rs2450745
rs1689282
rs4810359
rs2290844
rs12990744
rs11958296
rs2875322
rs11769002
All
−0.4
0.0
0.4
0.8
1.2
MR effect size for
'Gut microbiota abundance (genus Alistipes id.968)' on 'Extreme obesity with alveolar hypoventilation'

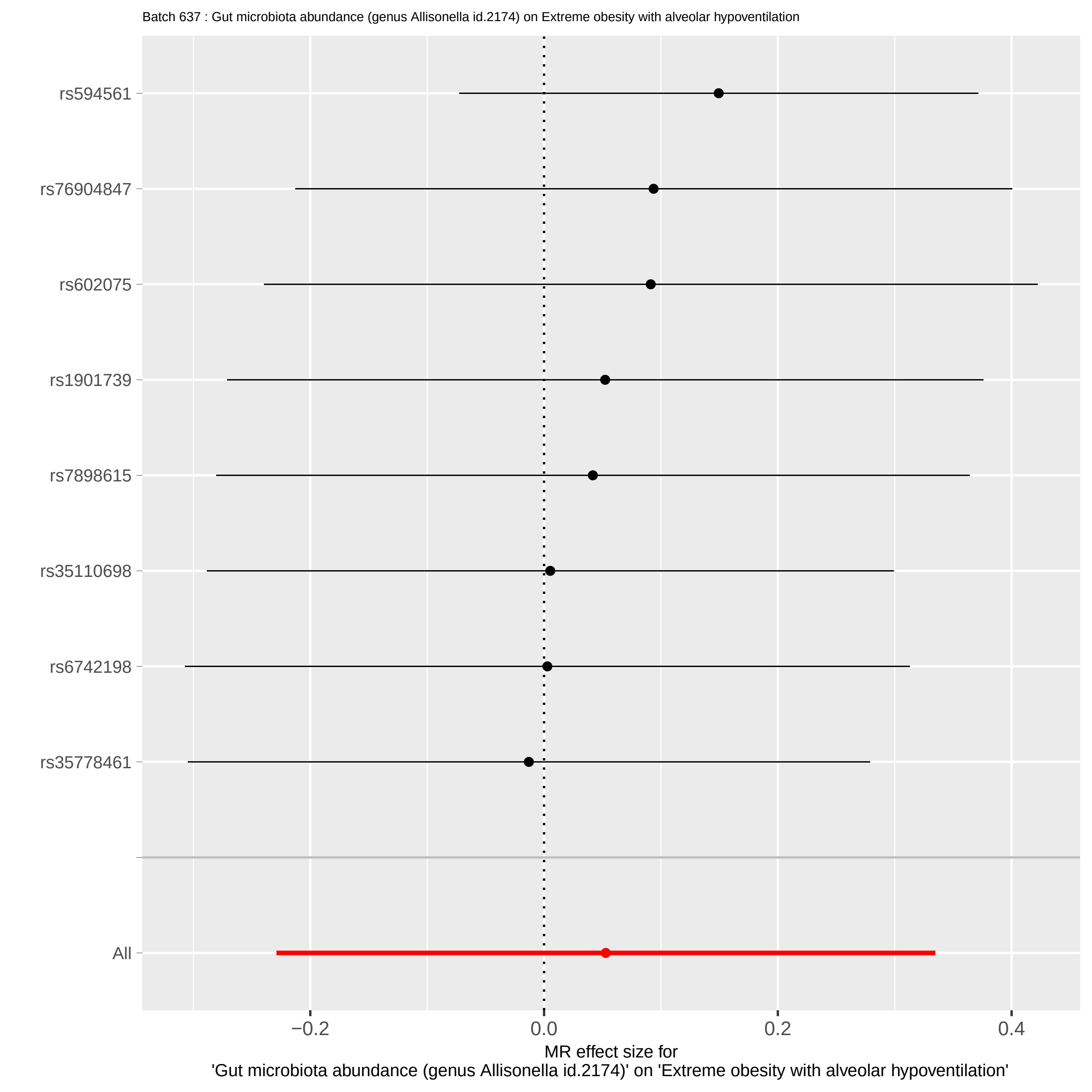
Batch 637 : Gut microbiota abundance (genus Allisonella id.2174) on Extreme obesity with alveolar hypoventilation
rs594561
rs76904847
rs602075
rs1901739
rs7898615
rs35110698
rs6742198
rs35778461
All
−0.2
0.0
0.2
0.4
MR effect size for
'Gut microbiota abundance (genus Allisonella id.2174)' on 'Extreme obesity with alveolar hypoventilation'

Batch 638 : Gut microbiota abundance (genus Alloprevotella id.961) on Extreme obesity with alveolar hypoventilation

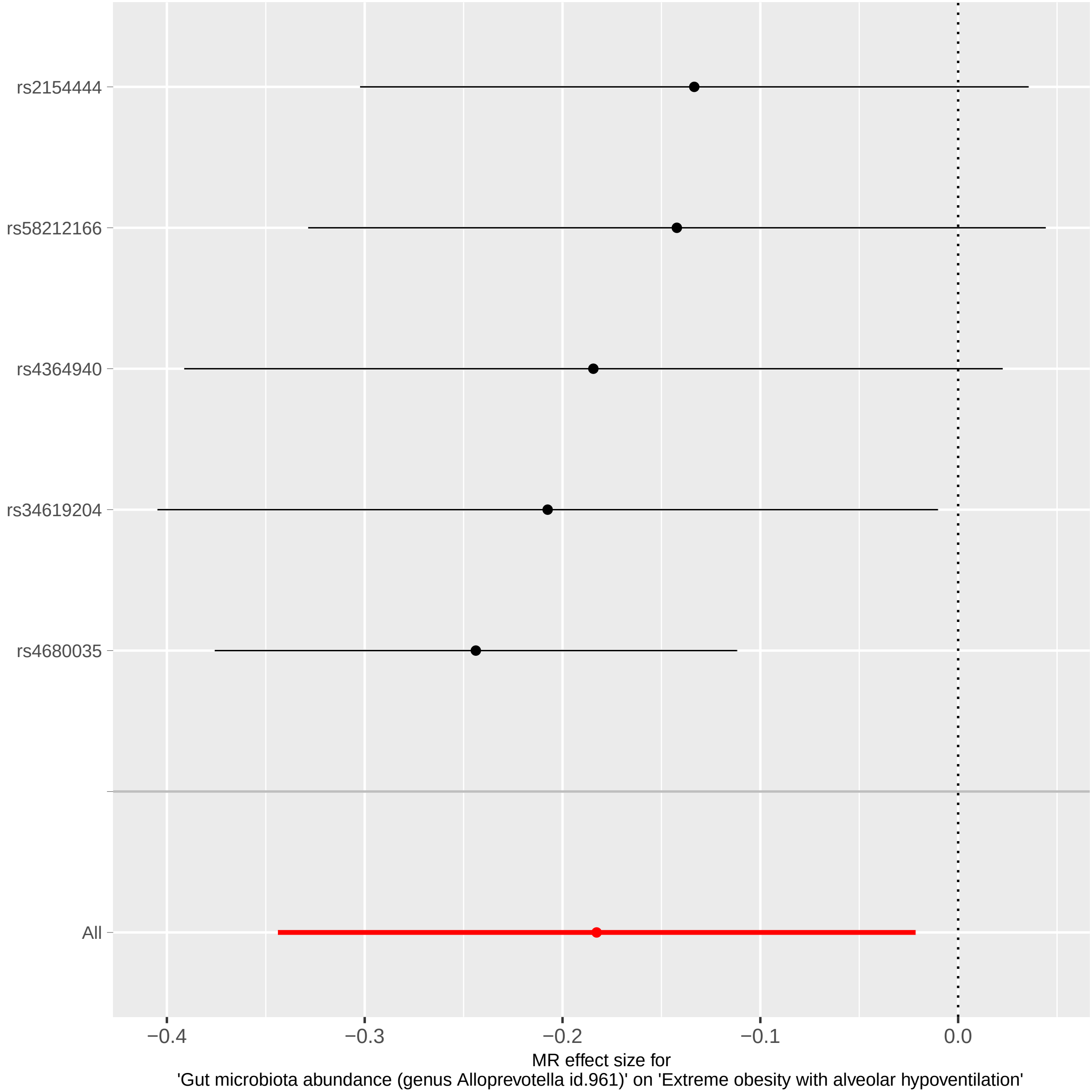

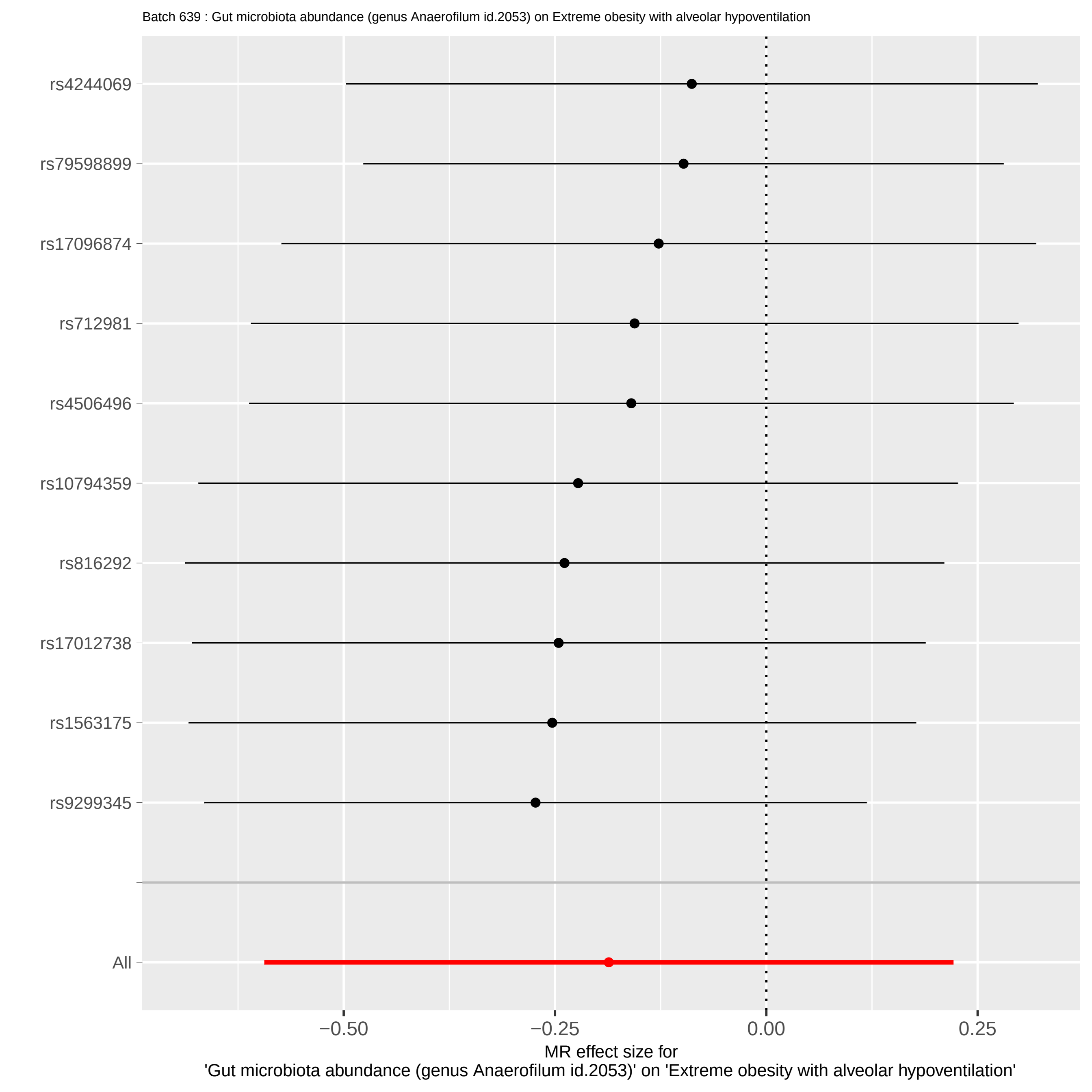

Batch 639 : Gut microbiota abundance (genus Anaerofilum id.2053) on Extreme obesity with alveolar hypoventilation
rs4244069
rs79598899
rs17096874
rs712981
rs4506496
rs10794359
rs816292
rs17012738
rs1563175
rs9299345
All
−0.50
−0.25
0.00
0.25
MR effect size for
'Gut microbiota abundance (genus Anaerofilum id.2053)' on 'Extreme obesity with alveolar hypoventilation'

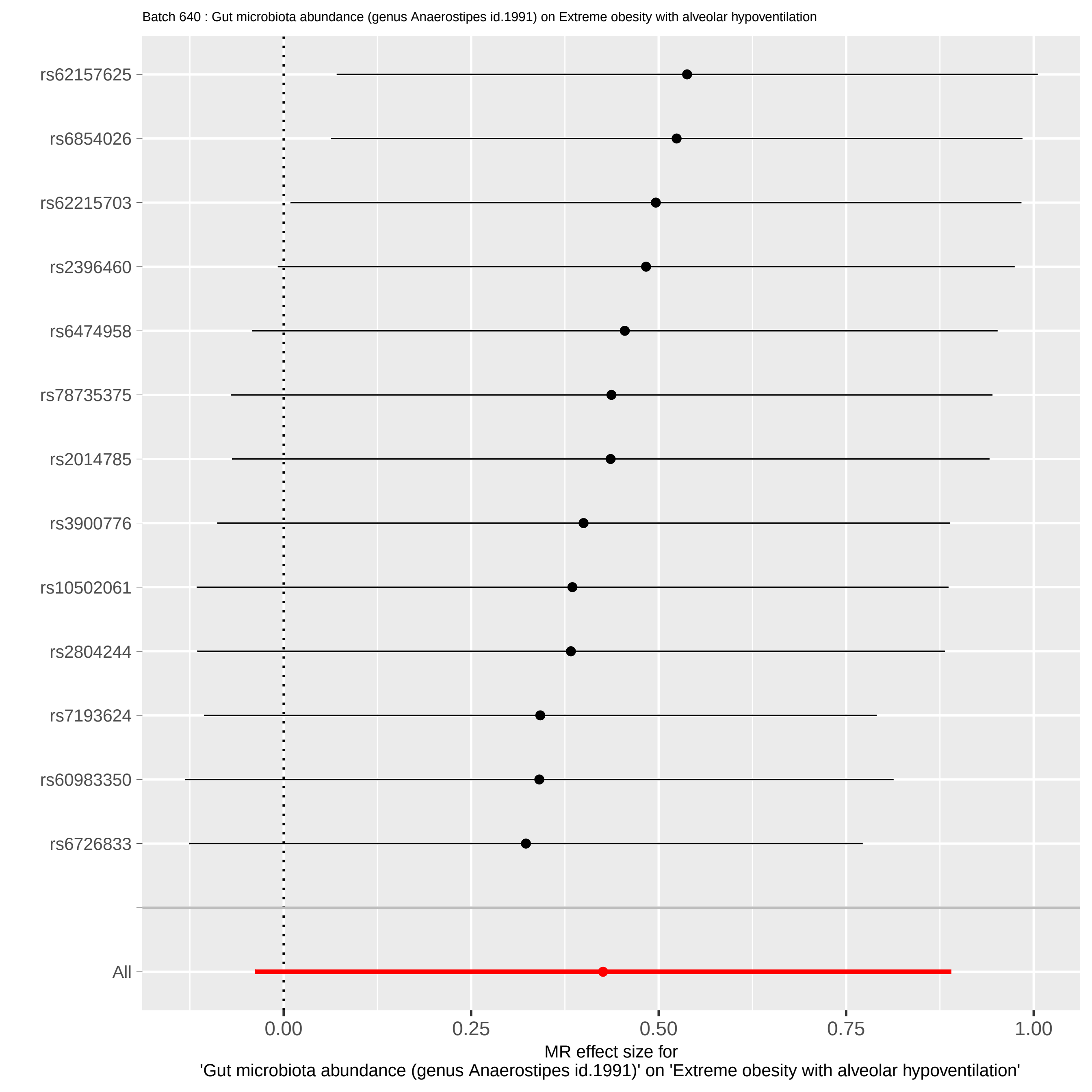

Batch 640 : Gut microbiota abundance (genus Anaerostipes id.1991) on Extreme obesity with alveolar hypoventilation
rs62157625
rs6854026
rs62215703
rs2396460
rs6474958
rs78735375
rs2014785
rs3900776
rs10502061
rs2804244
rs7193624
rs60983350
rs6726833
All
0.00
0.25
0.50
0.75
1.00
MR effect size for
'Gut microbiota abundance (genus Anaerostipes id.1991)' on 'Extreme obesity with alveolar hypoventilation'

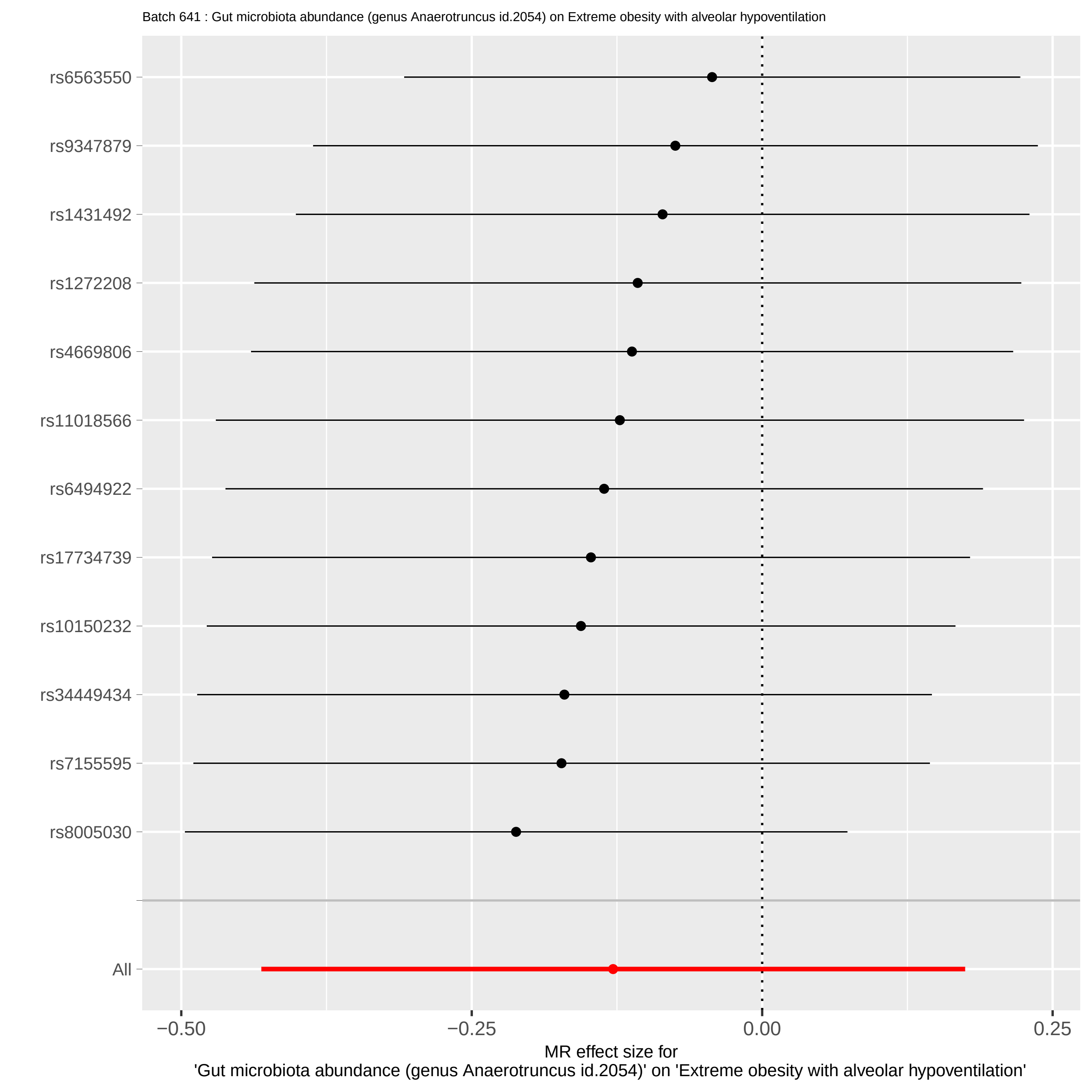

Batch 641 : Gut microbiota abundance (genus Anaerotruncus id.2054) on Extreme obesity with alveolar hypoventilation
rs6563550
rs9347879
rs1431492
rs1272208
rs4669806
rs11018566
rs6494922
rs17734739
rs10150232
rs34449434
rs7155595
rs8005030
All
−0.50
−0.25
0.00
0.25
MR effect size for
'Gut microbiota abundance (genus Anaerotruncus id.2054)' on 'Extreme obesity with alveolar hypoventilation'

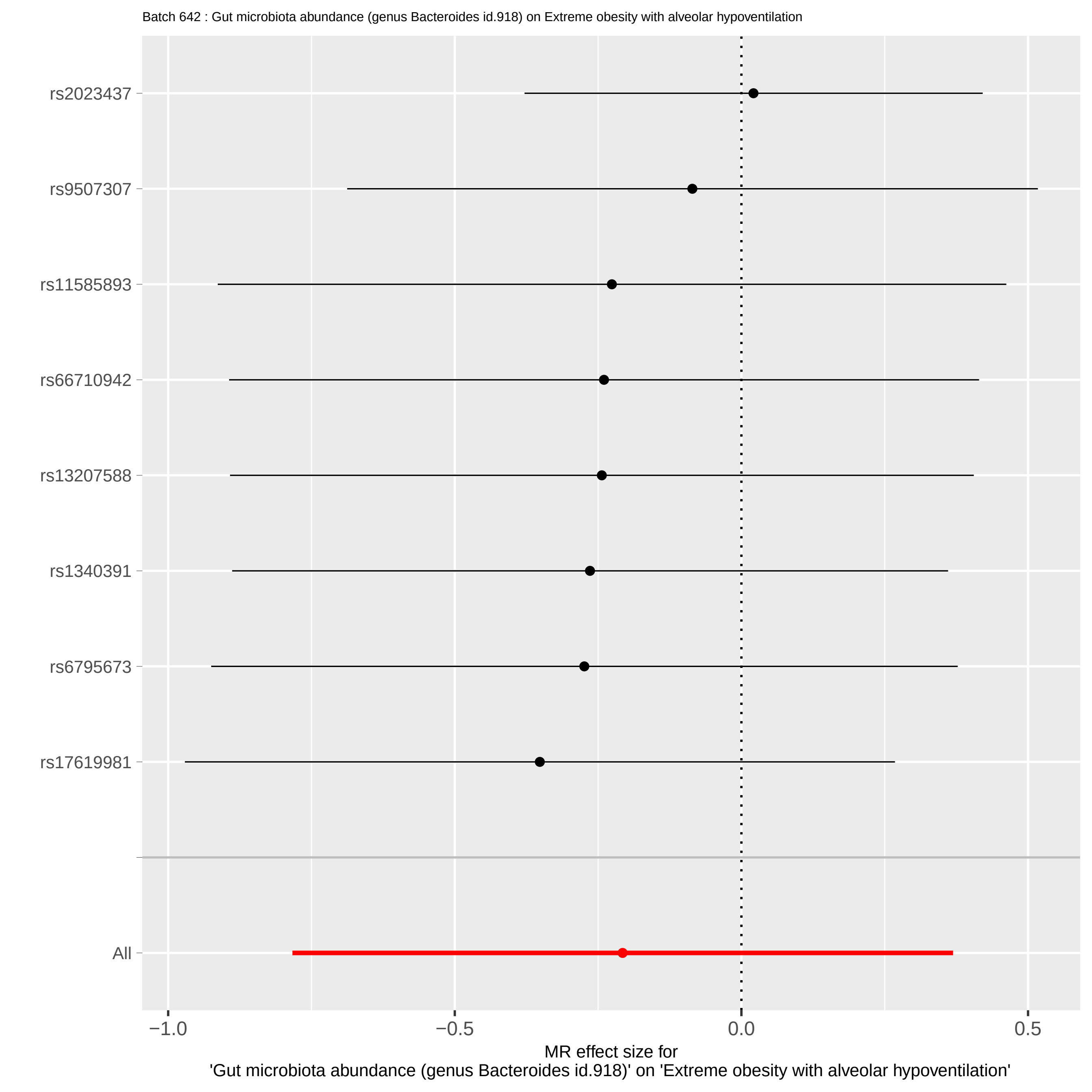
Batch 642 : Gut microbiota abundance (genus Bacteroides id.918) on Extreme obesity with alveolar hypoventilation
rs2023437
rs9507307
rs11585893
rs66710942
rs13207588
rs1340391
rs6795673
rs17619981
All
−1.0
−0.5
0.0
0.5
MR effect size for
'Gut microbiota abundance (genus Bacteroides id.918)' on 'Extreme obesity with alveolar hypoventilation'

Batch 643 : Gut microbiota abundance (genus Barnesiella id.944) on Extreme obesity with alveolar hypoventilation

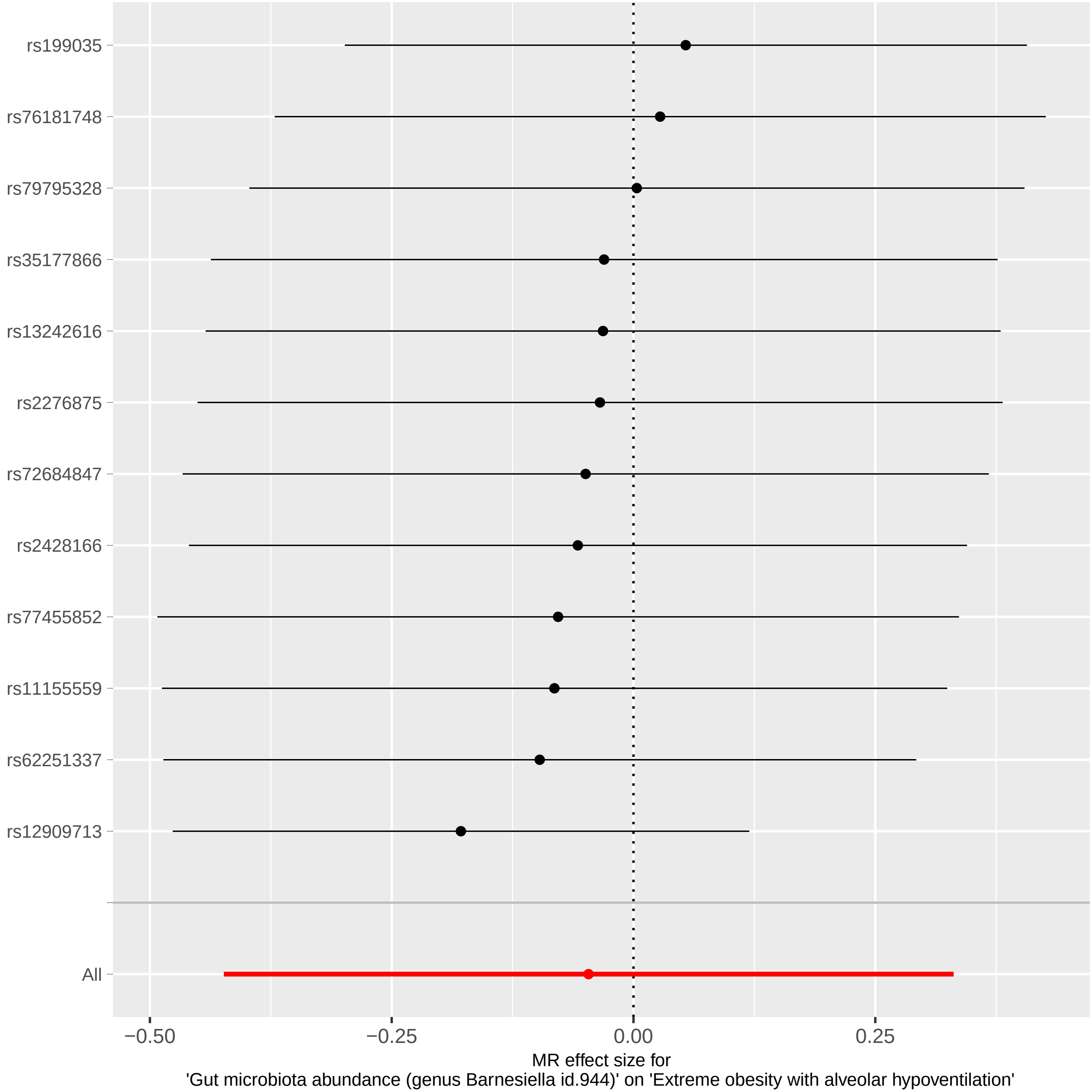

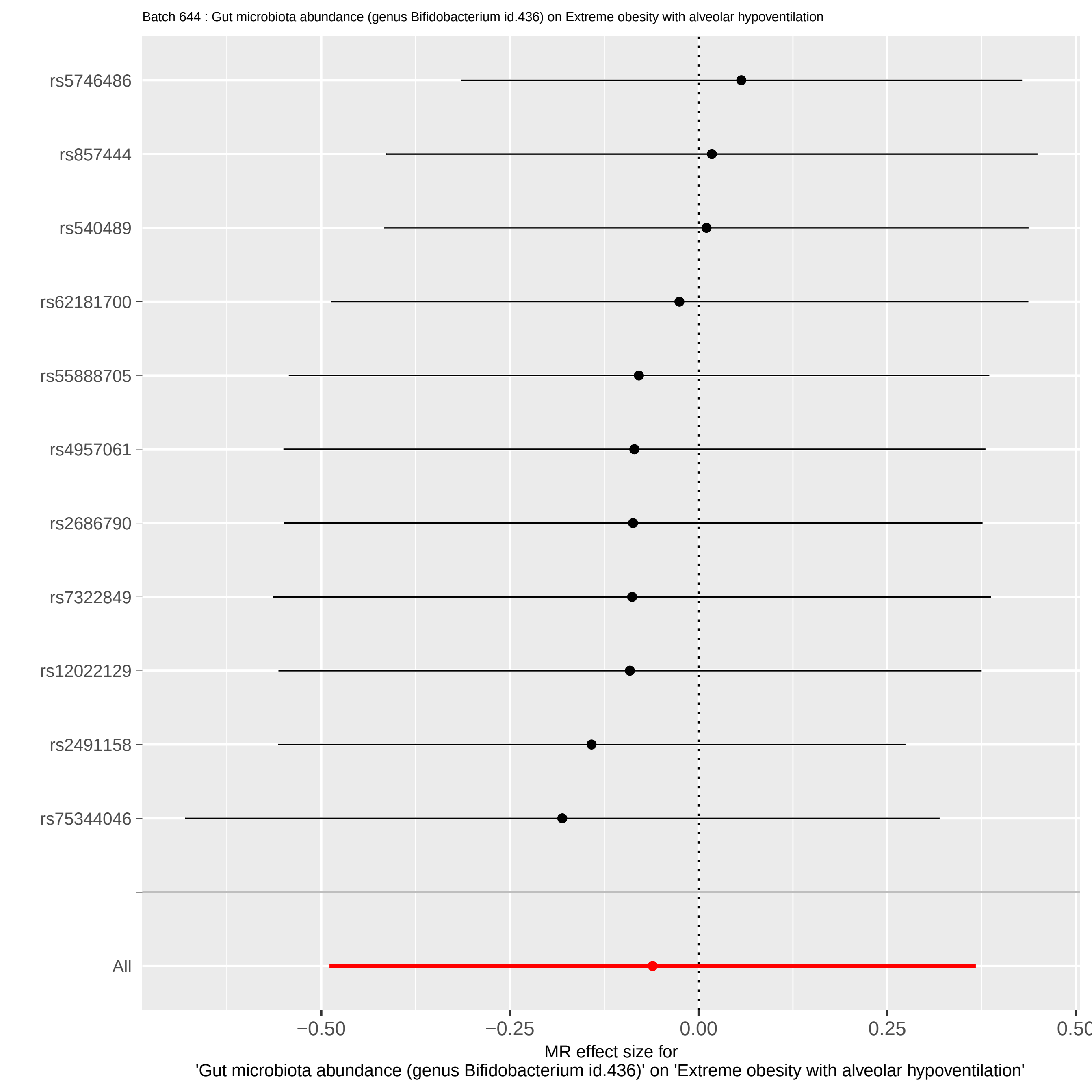

Batch 644 : Gut microbiota abundance (genus Bifidobacterium id.436) on Extreme obesity with alveolar hypoventilation
rs5746486
rs857444
rs540489
rs62181700
rs55888705
rs4957061
rs2686790
rs7322849
rs12022129
rs2491158
rs75344046
All
−0.50
−0.25
0.00
0.25
0.50
MR effect size for
'Gut microbiota abundance (genus Bifidobacterium id.436)' on 'Extreme obesity with alveolar hypoventilation'

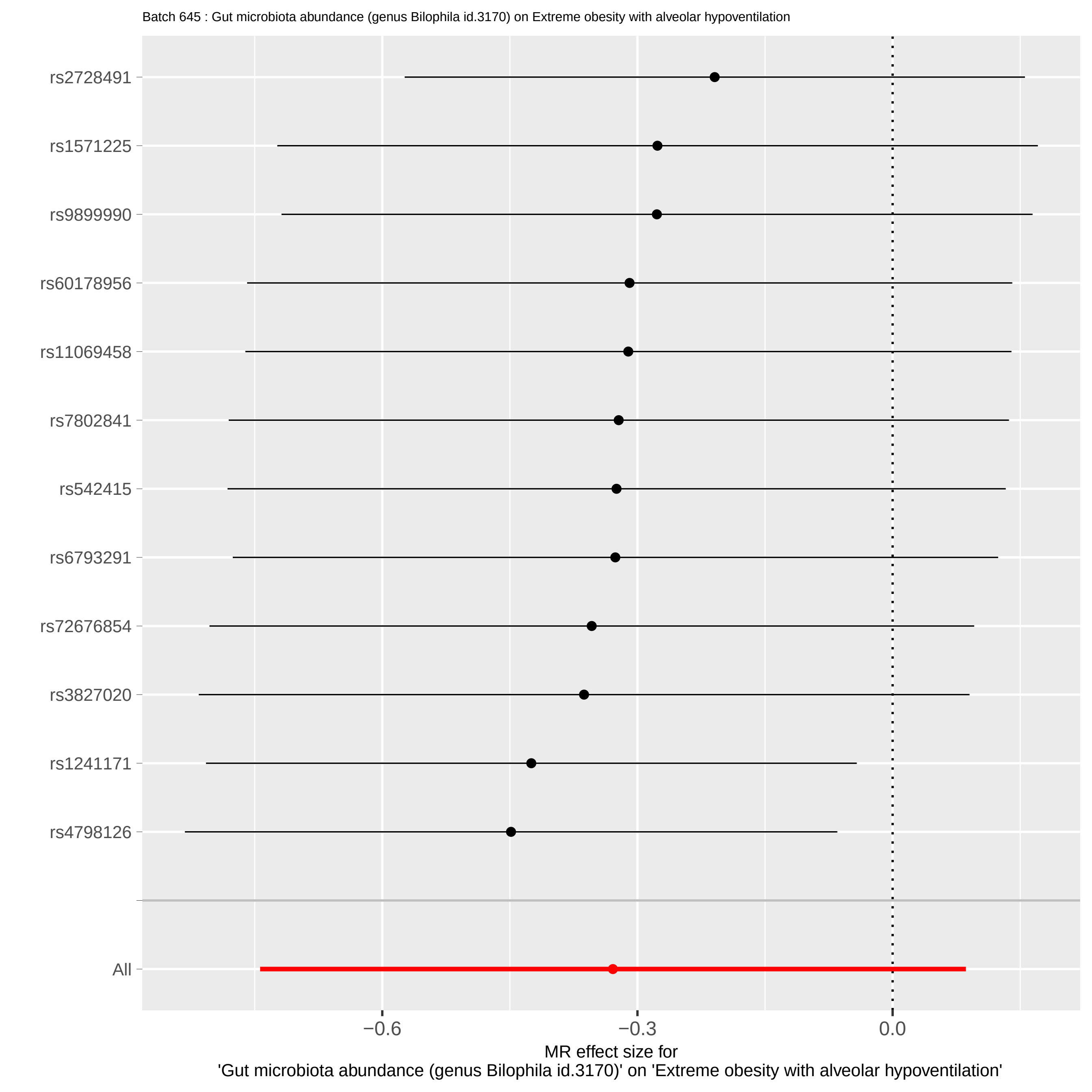
Batch 645 : Gut microbiota abundance (genus Bilophila id.3170) on Extreme obesity with alveolar hypoventilation
rs2728491
rs1571225
rs9899990
rs60178956
rs11069458
rs7802841
rs542415
rs6793291
rs72676854
rs3827020
rs1241171
rs4798126
All
−0.6
−0.3
0.0
MR effect size for
'Gut microbiota abundance (genus Bilophila id.3170)' on 'Extreme obesity with alveolar hypoventilation'

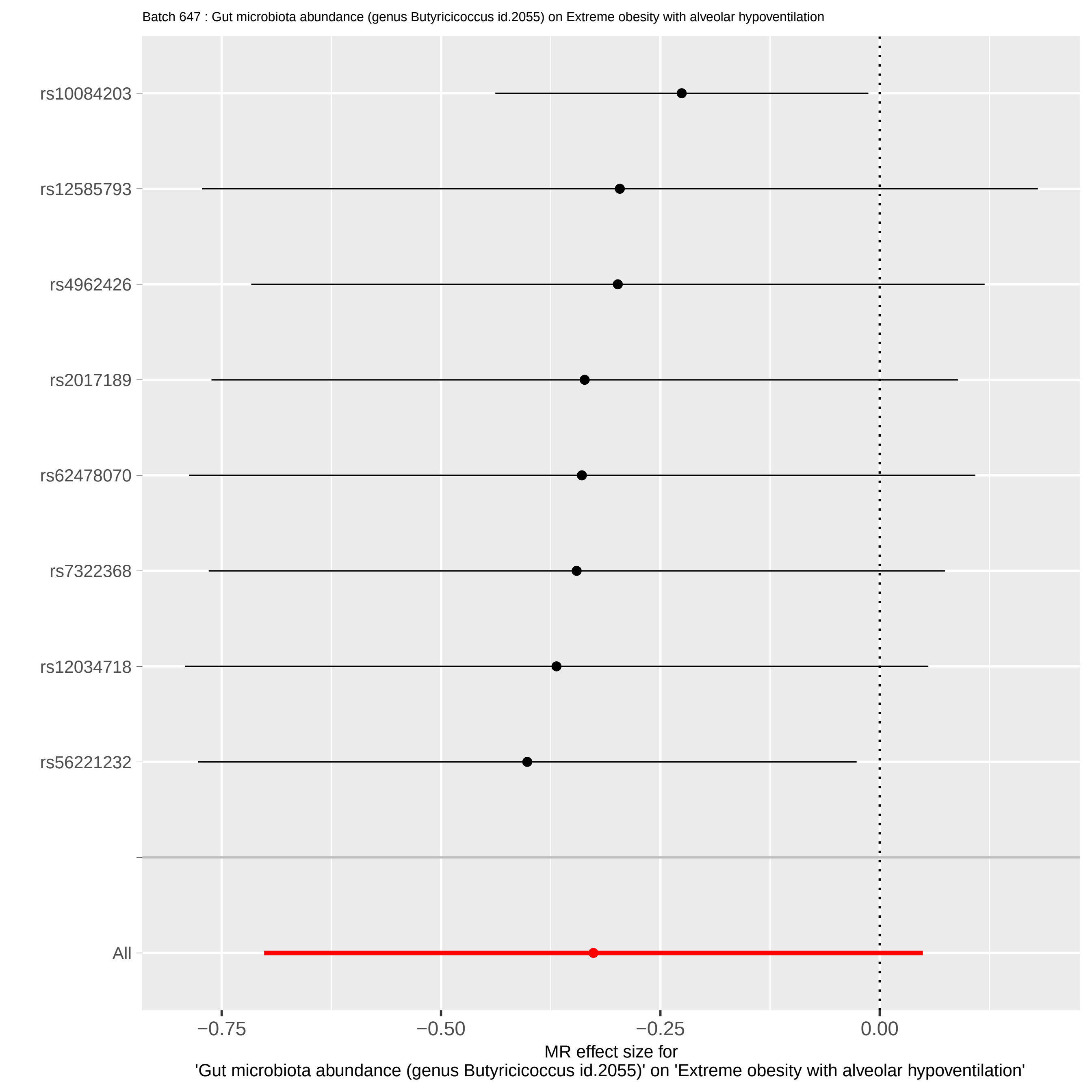
Batch 647 : Gut microbiota abundance (genus Butyricicoccus id.2055) on Extreme obesity with alveolar hypoventilation
rs10084203
rs12585793
rs4962426
rs2017189
rs62478070
rs7322368
rs12034718
rs56221232
All
−0.75
−0.50
−0.25
0.00
MR effect size for
'Gut microbiota abundance (genus Butyricicoccus id.2055)' on 'Extreme obesity with alveolar hypoventilation'

Batch 648 : Gut microbiota abundance (genus Butyricimonas id.945) on Extreme obesity with alveolar hypoventilation

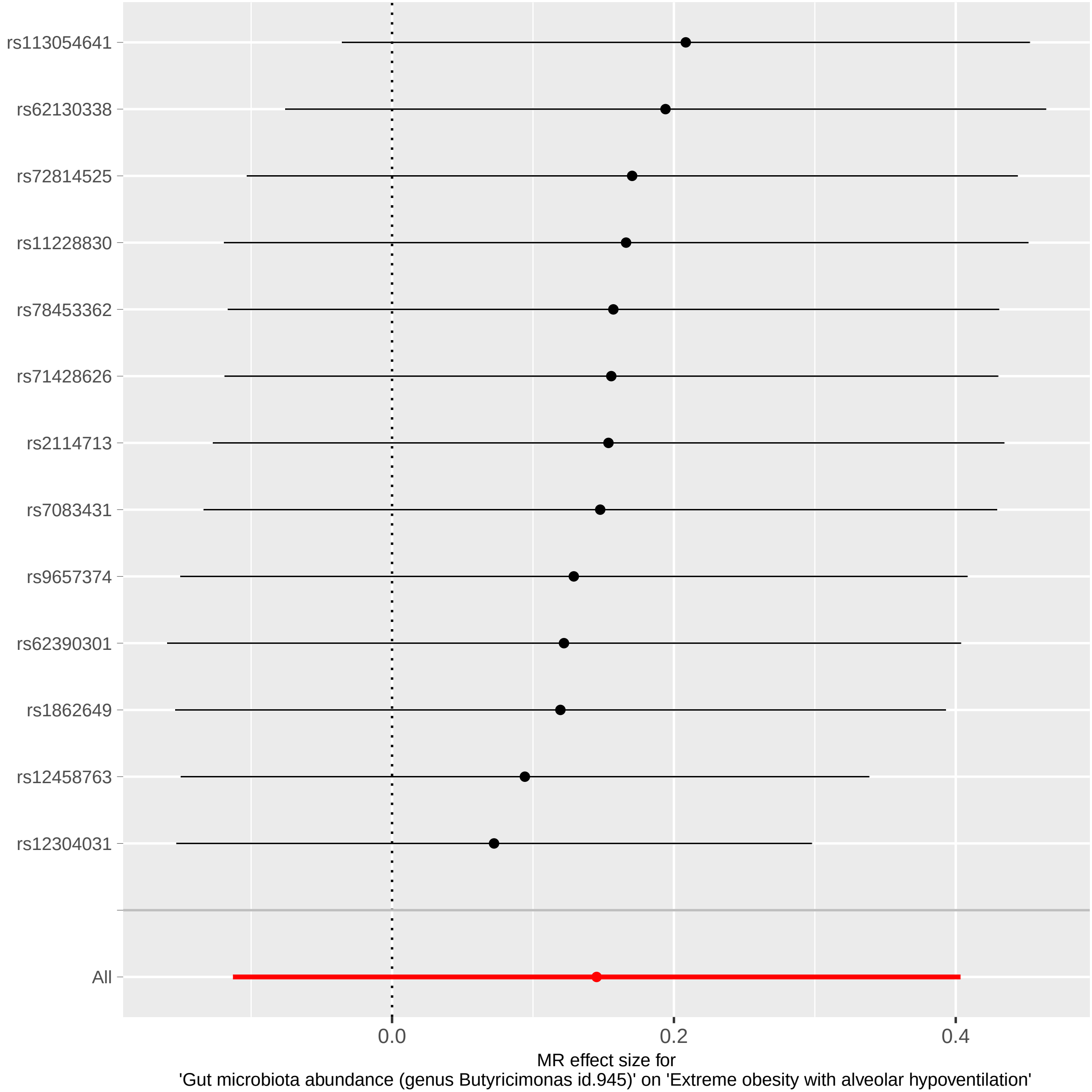

Batch 649 : Gut microbiota abundance (genus Butyrivibrio id.1993) on Extreme obesity with alveolar hypoventilation

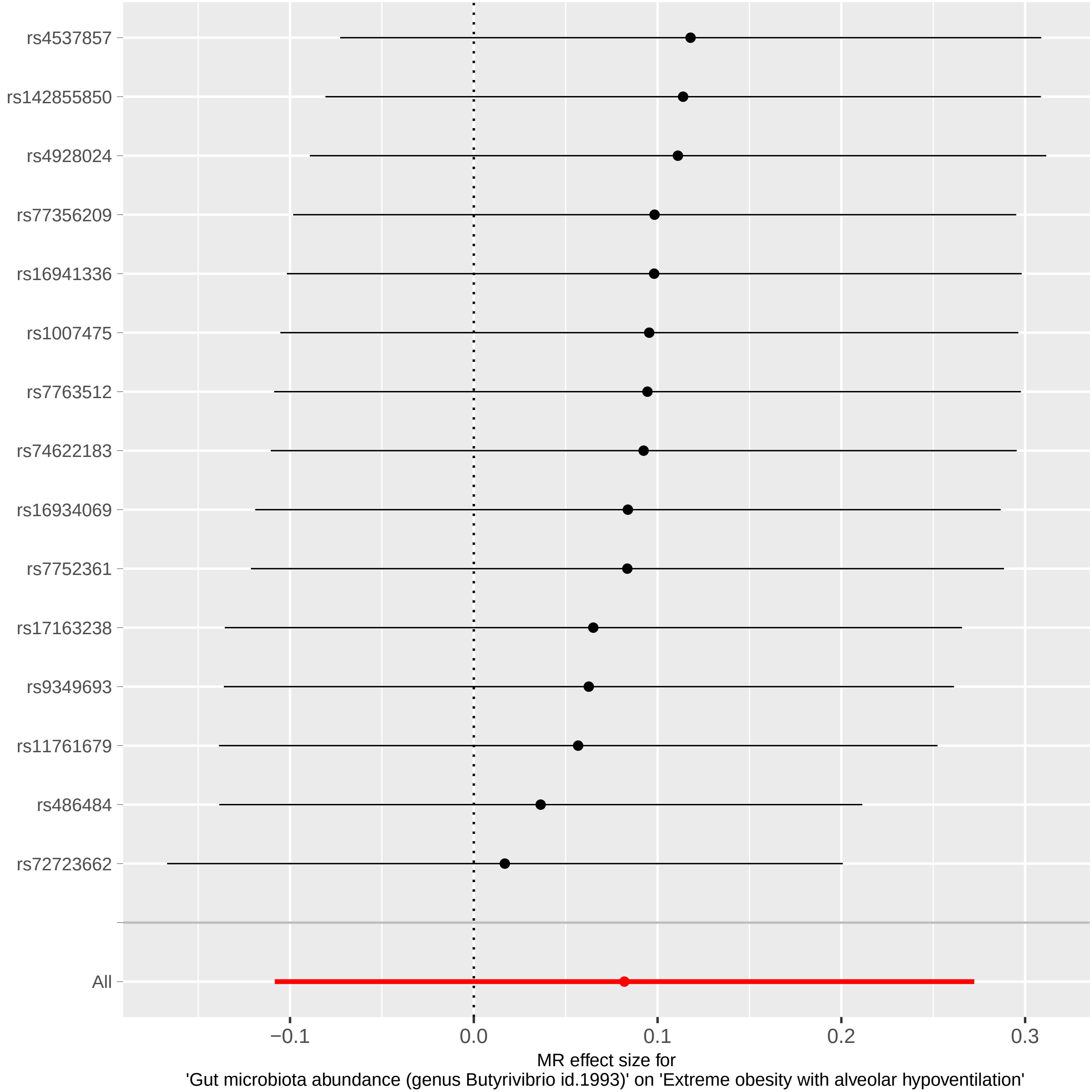

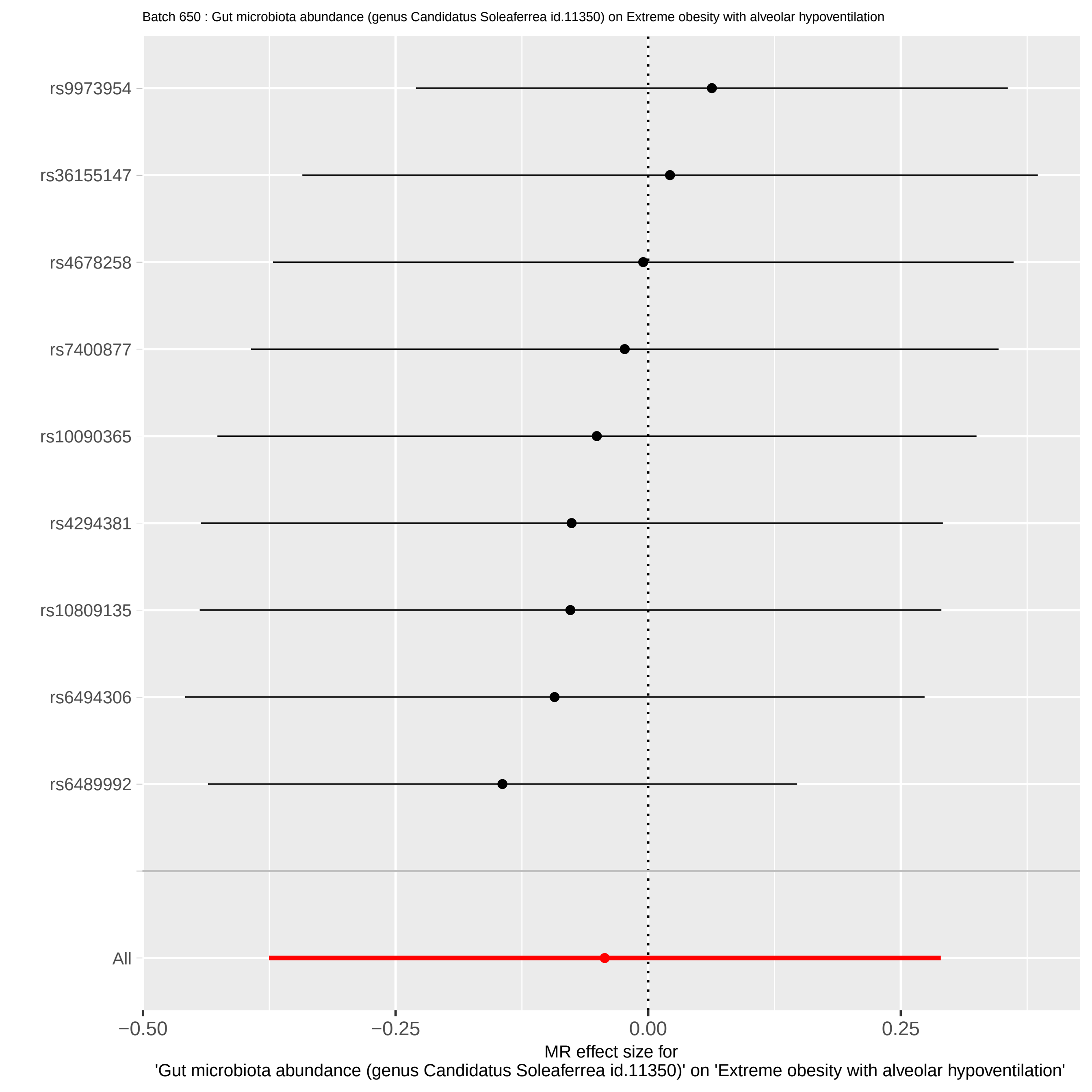

Batch 650 : Gut microbiota abundance (genus Candidatus Soleaferrea id.11350) on Extreme obesity with alveolar hypoventilation
rs9973954
rs36155147
rs4678258
rs7400877
rs10090365
rs4294381
rs10809135
rs6494306
rs6489992
All
−0.50
−0.25
0.00
0.25
MR effect size for
'Gut microbiota abundance (genus Candidatus Soleaferrea id.11350)' on 'Extreme obesity with alveolar hypoventilation'

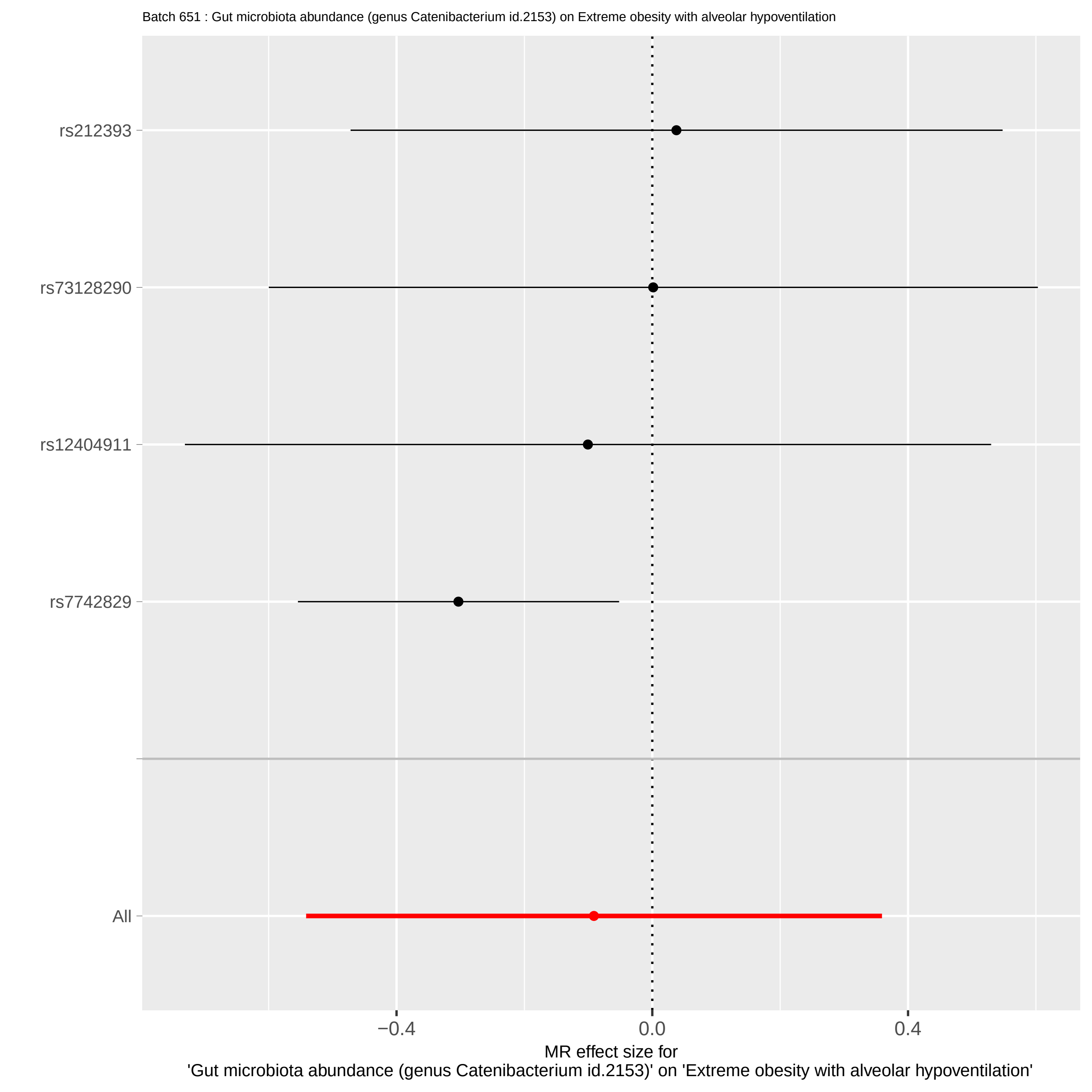
Batch 651 : Gut microbiota abundance (genus Catenibacterium id.2153) on Extreme obesity with alveolar hypoventilation
rs212393
rs73128290
rs12404911
rs7742829
All
−0.4
0.0
0.4
MR effect size for
'Gut microbiota abundance (genus Catenibacterium id.2153)' on 'Extreme obesity with alveolar hypoventilation'

Batch 652 : Gut microbiota abundance (genus Christensenellaceae R 7group id.11283) on Extreme obesity with alveolar hypoventilation

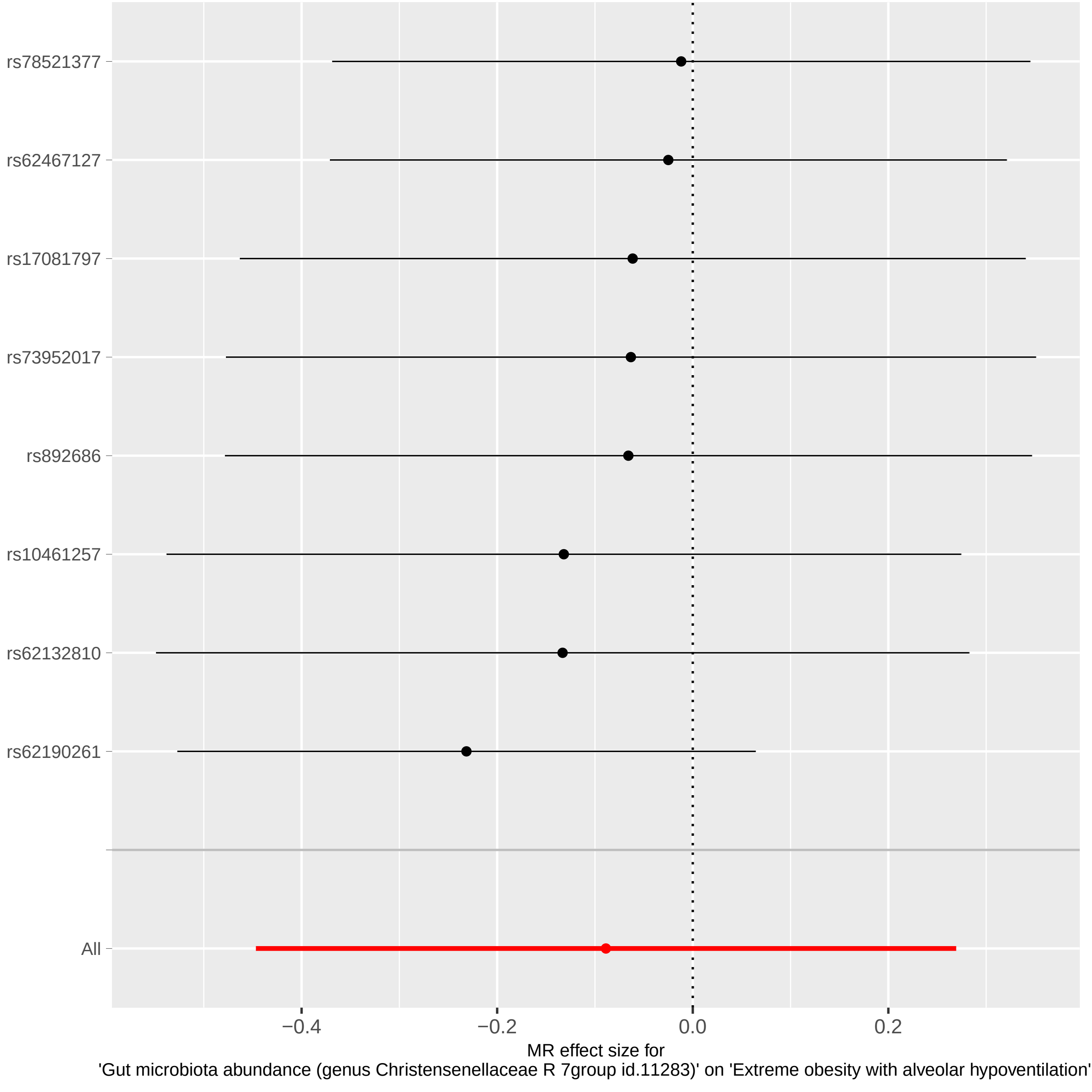

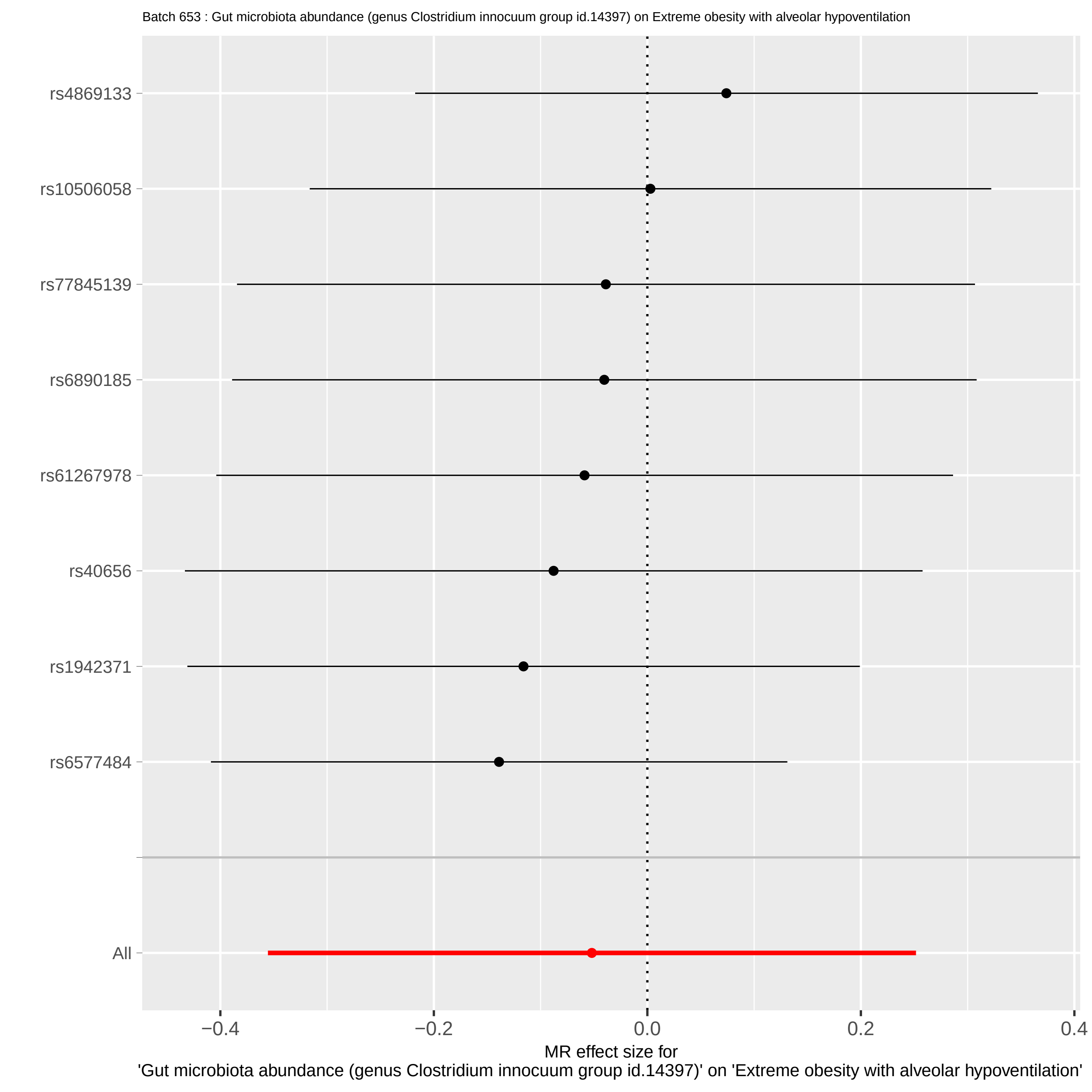

Batch 653 : Gut microbiota abundance (genus Clostridium innocuum group id.14397) on Extreme obesity with alveolar hypoventilation
rs4869133
rs10506058
rs77845139
rs6890185
rs61267978
rs40656
rs1942371
rs6577484
All
−0.4
−0.2
0.0
0.2
0.4
MR effect size for
'Gut microbiota abundance (genus Clostridium innocuum group id.14397)' on 'Extreme obesity with alveolar hypoventilation'

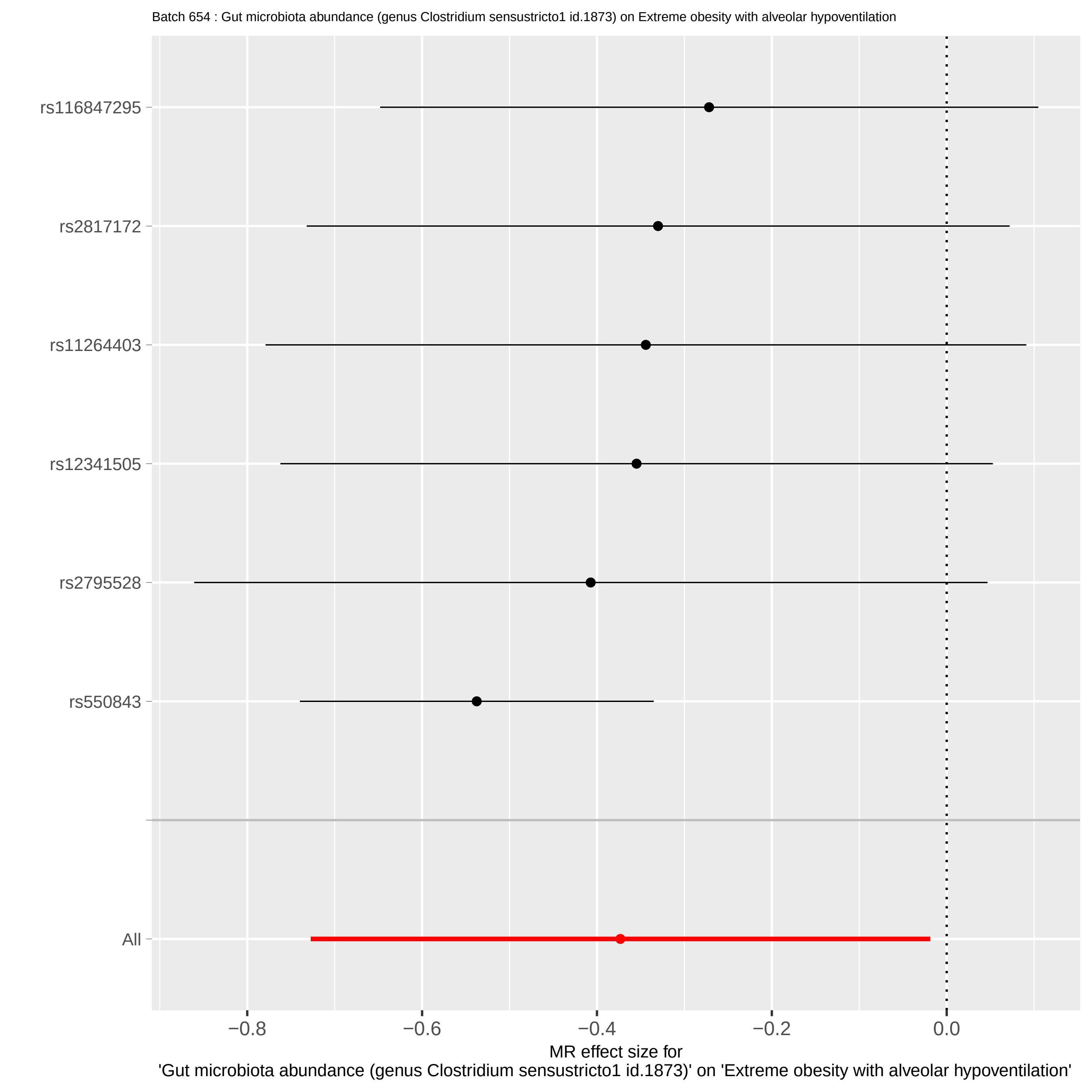

Batch 654 : Gut microbiota abundance (genus Clostridium sensustricto1 id.1873) on Extreme obesity with alveolar hypoventilation
rs116847295
rs2817172
rs11264403
rs12341505
rs2795528
rs550843
All
−0.8
−0.6
−0.4
−0.2
0.0
MR effect size for
'Gut microbiota abundance (genus Clostridium sensustricto1 id.1873)' on 'Extreme obesity with alveolar hypoventilation'

Batch 655 : Gut microbiota abundance (genus Collinsella id.815) on Extreme obesity with alveolar hypoventilation

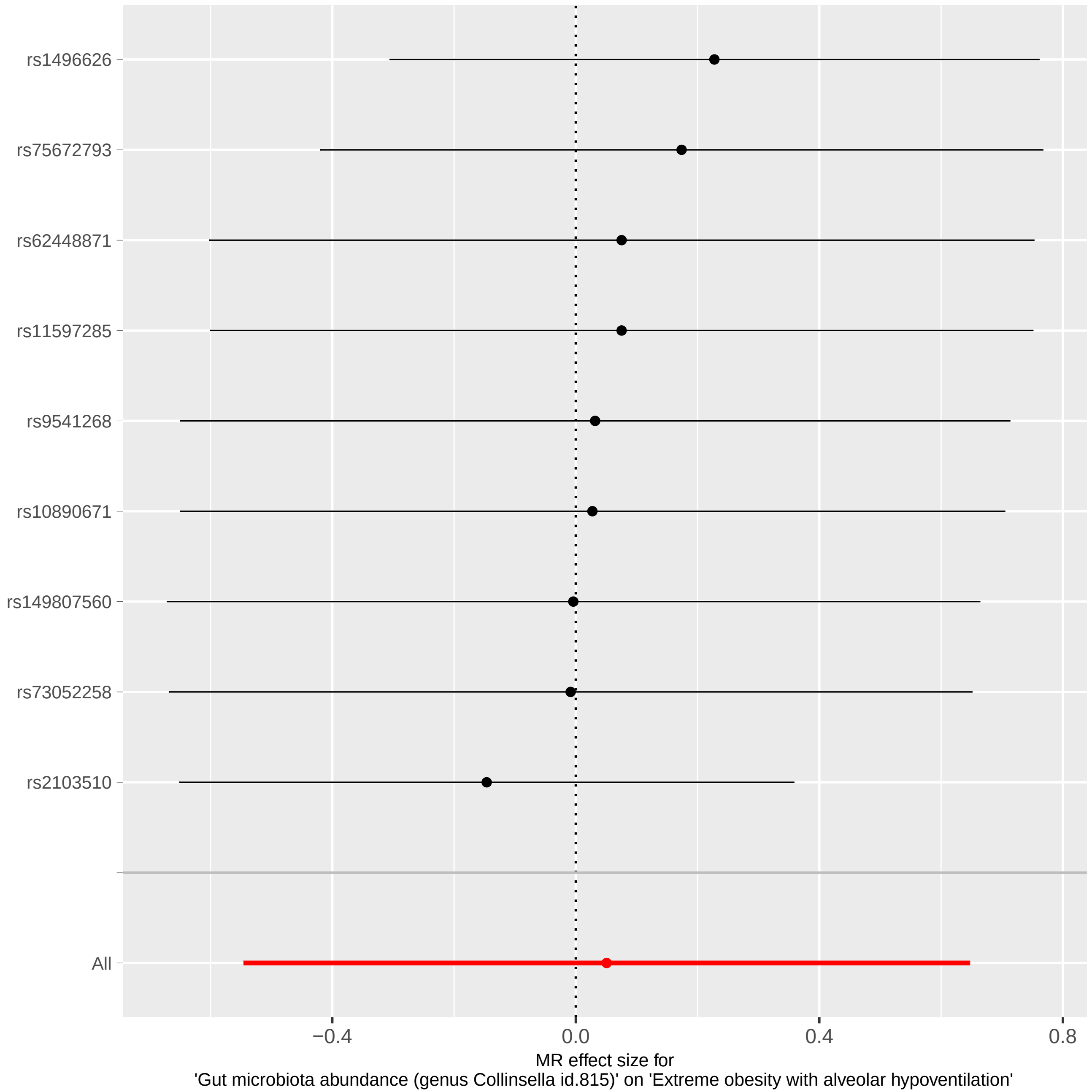

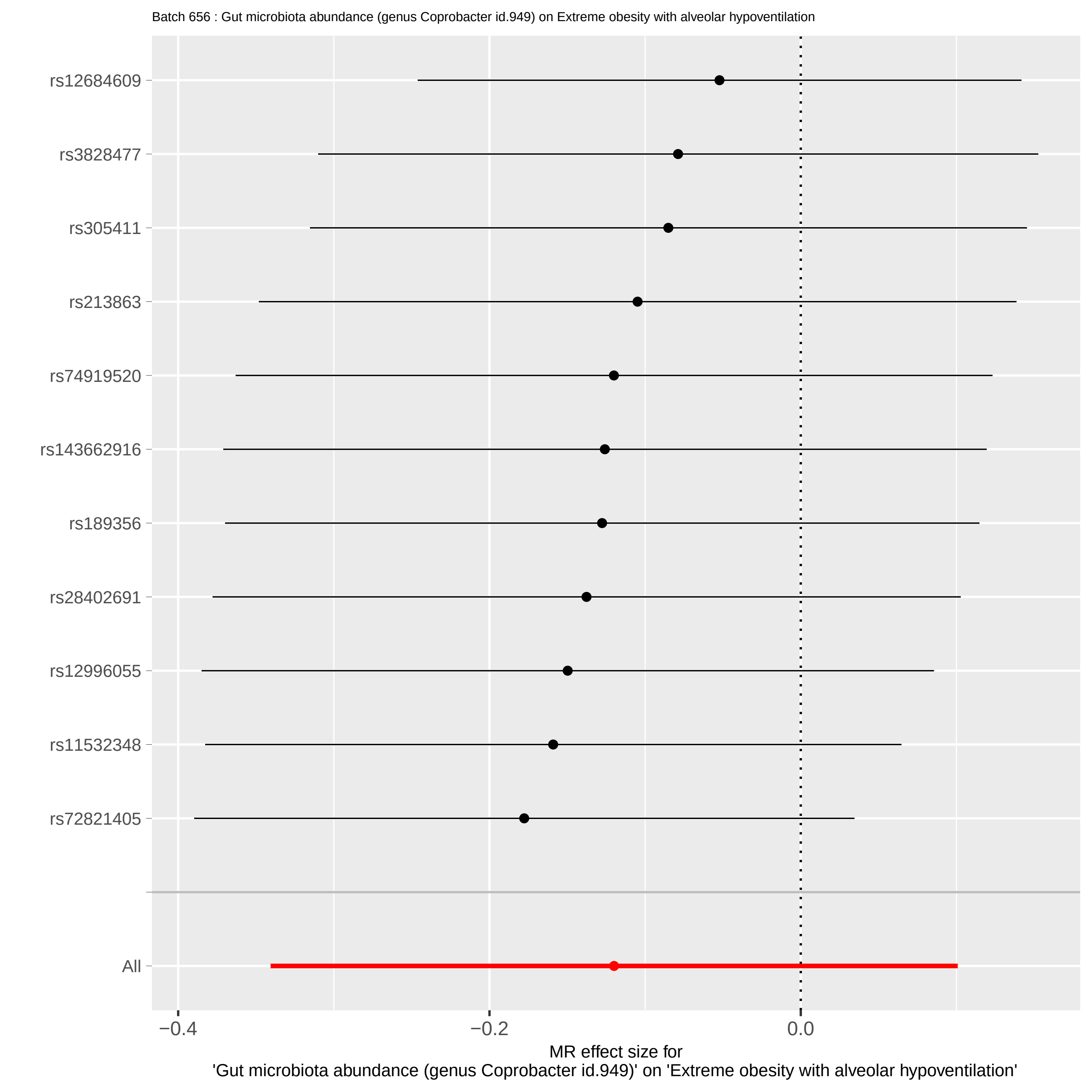

Batch 656 : Gut microbiota abundance (genus Coprobacter id.949) on Extreme obesity with alveolar hypoventilation
rs12684609
rs3828477
rs305411
rs213863
rs74919520
rs143662916
rs189356
rs28402691
rs12996055
rs11532348
rs72821405
All
−0.4
−0.2
0.0
MR effect size for
'Gut microbiota abundance (genus Coprobacter id.949)' on 'Extreme obesity with alveolar hypoventilation'

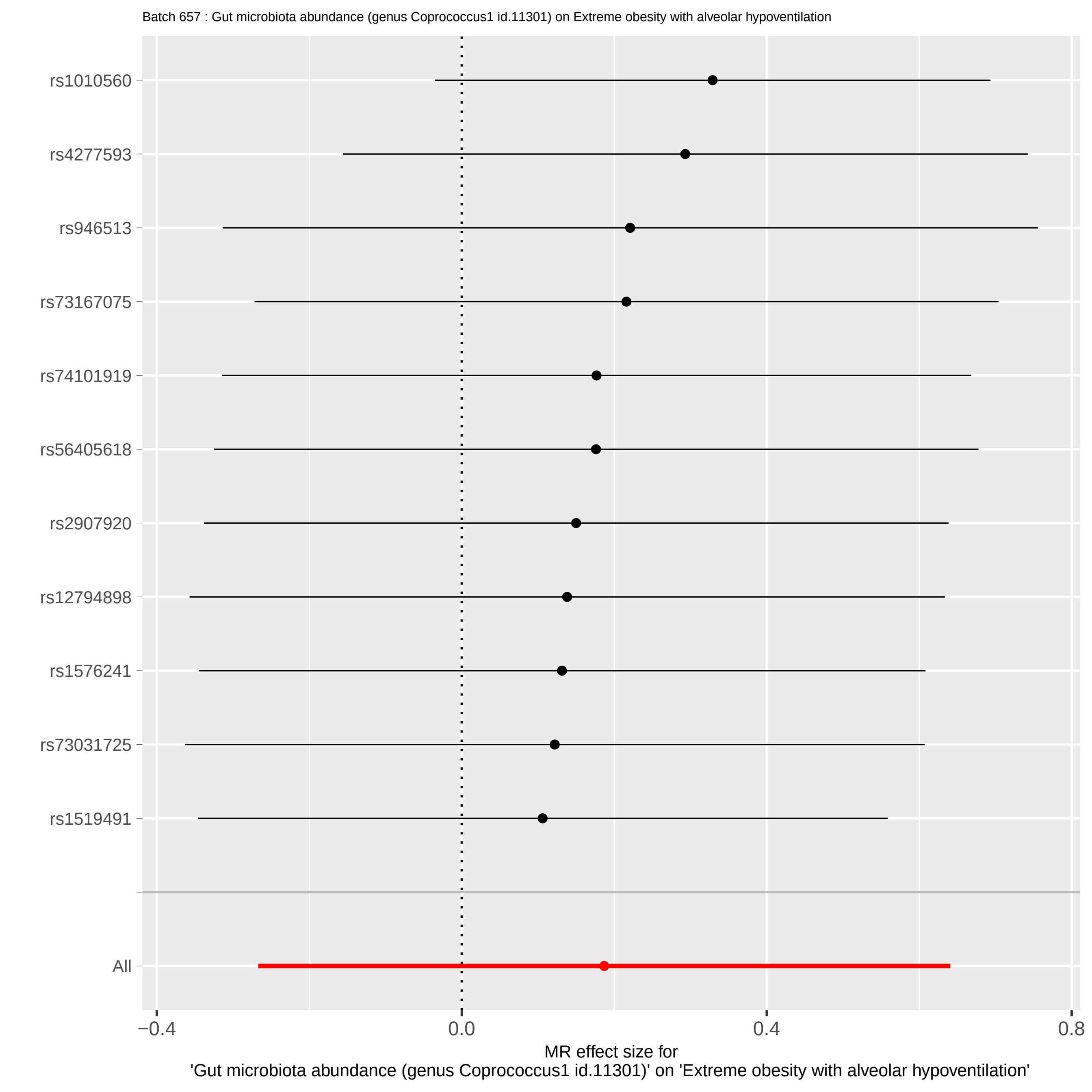
Batch 657 : Gut microbiota abundance (genus Coprococcus1 id.11301) on Extreme obesity with alveolar hypoventilation
rs1010560
rs4277593
rs946513
rs73167075
rs74101919
rs56405618
rs2907920
rs12794898
rs1576241
rs73031725
rs1519491
All
−0.4
0.0
0.4
0.8
MR effect size for
'Gut microbiota abundance (genus Coprococcus1 id.11301)' on 'Extreme obesity with alveolar hypoventilation'

Batch 658 : Gut microbiota abundance (genus Coprococcus2 id.11302) on Extreme obesity with alveolar hypoventilation

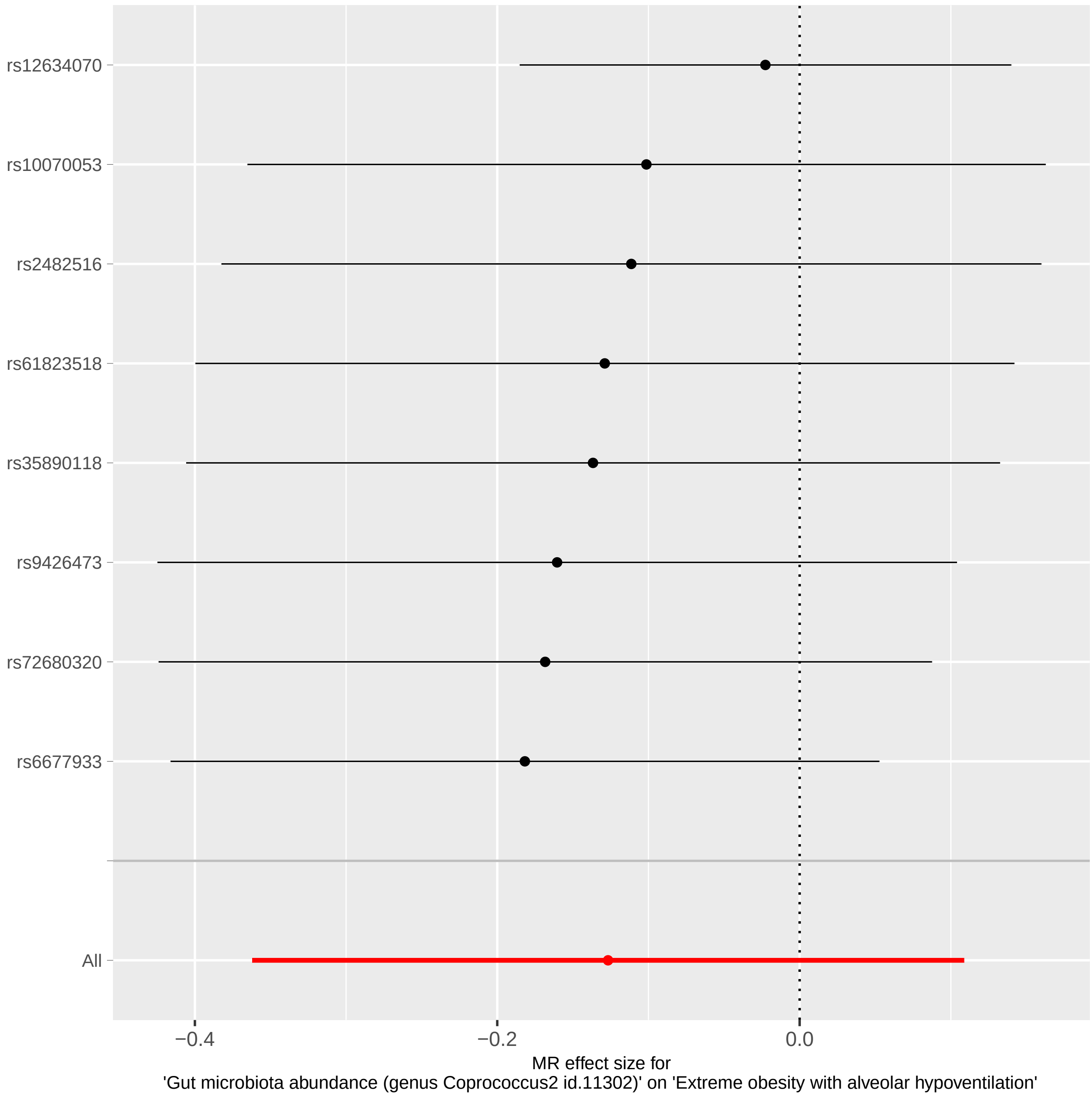

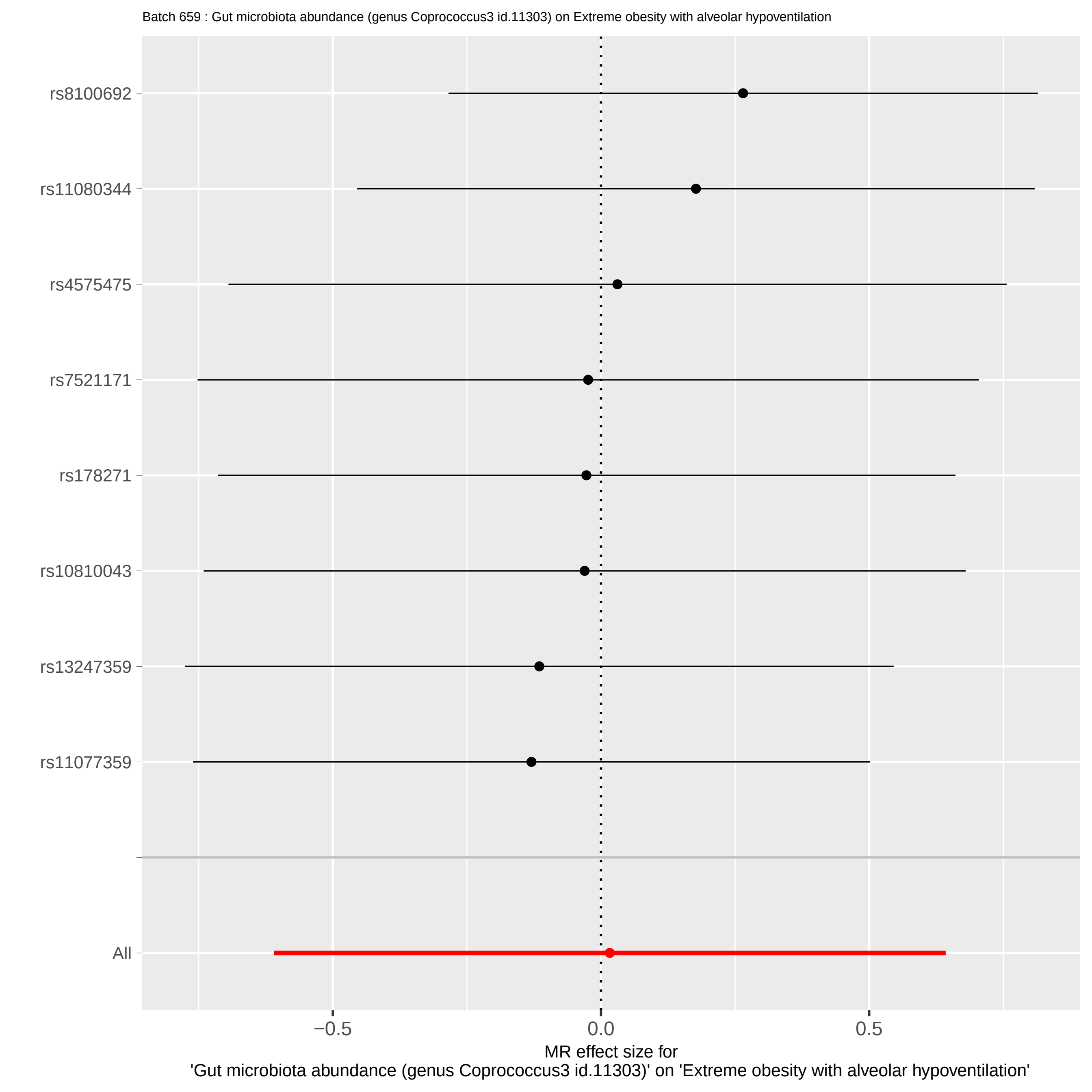

Batch 659 : Gut microbiota abundance (genus Coprococcus3 id.11303) on Extreme obesity with alveolar hypoventilation
rs8100692
rs11080344
rs4575475
rs7521171
rs178271
rs10810043
rs13247359
rs11077359
All
−0.5
0.0
0.5
MR effect size for
'Gut microbiota abundance (genus Coprococcus3 id.11303)' on 'Extreme obesity with alveolar hypoventilation'

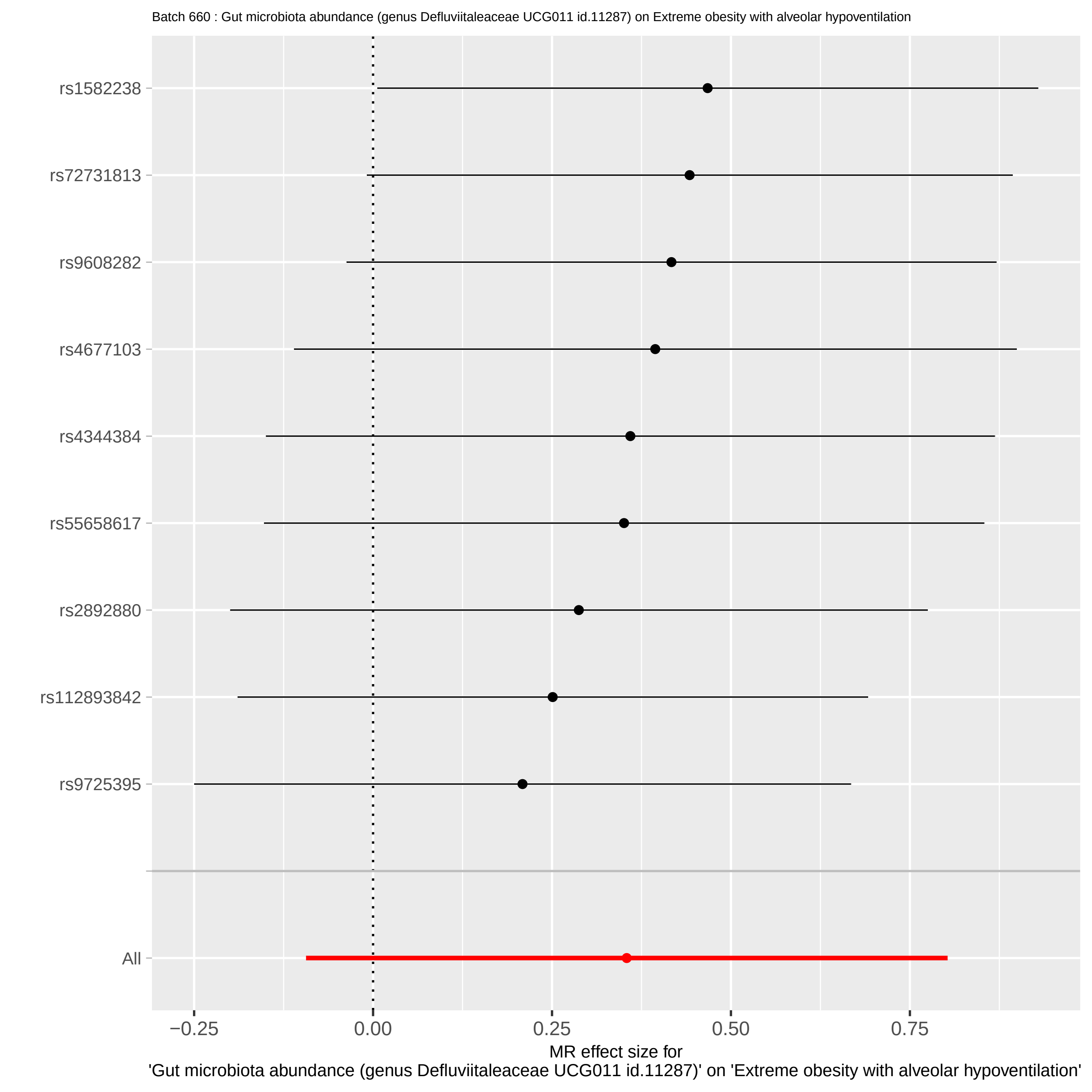

Batch 660 : Gut microbiota abundance (genus Defluviitaleaceae UCG011 id.11287) on Extreme obesity with alveolar hypoventilation
rs1582238
rs72731813
rs9608282
rs4677103
rs4344384
rs55658617
rs2892880
rs112893842
rs9725395
All
−0.25
0.00
0.25
0.50
0.75
MR effect size for
'Gut microbiota abundance (genus Defluviitaleaceae UCG011 id.11287)' on 'Extreme obesity with alveolar hypoventilation'

Batch 661 : Gut microbiota abundance (genus Desulfovibrio id.3173) on Extreme obesity with alveolar hypoventilation

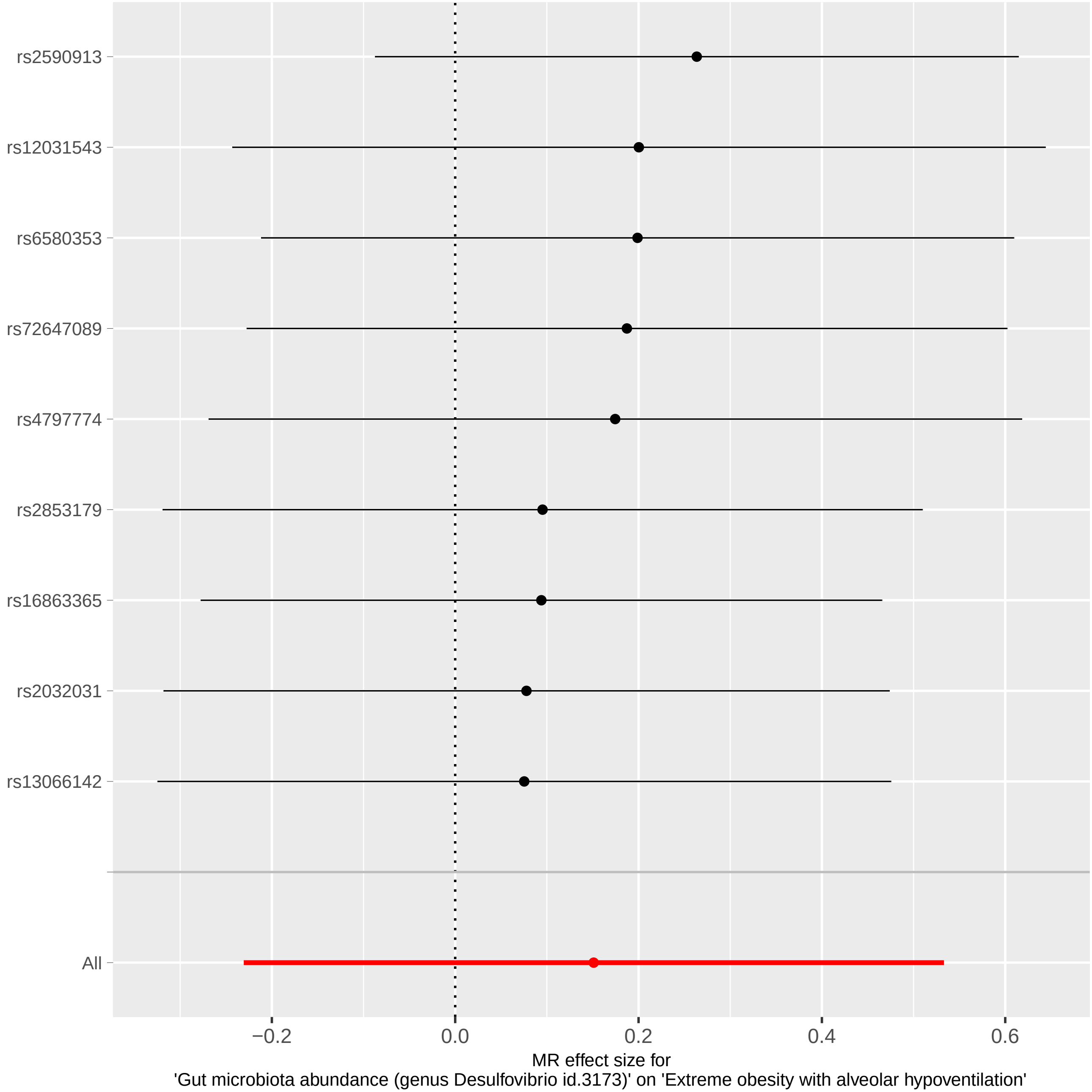

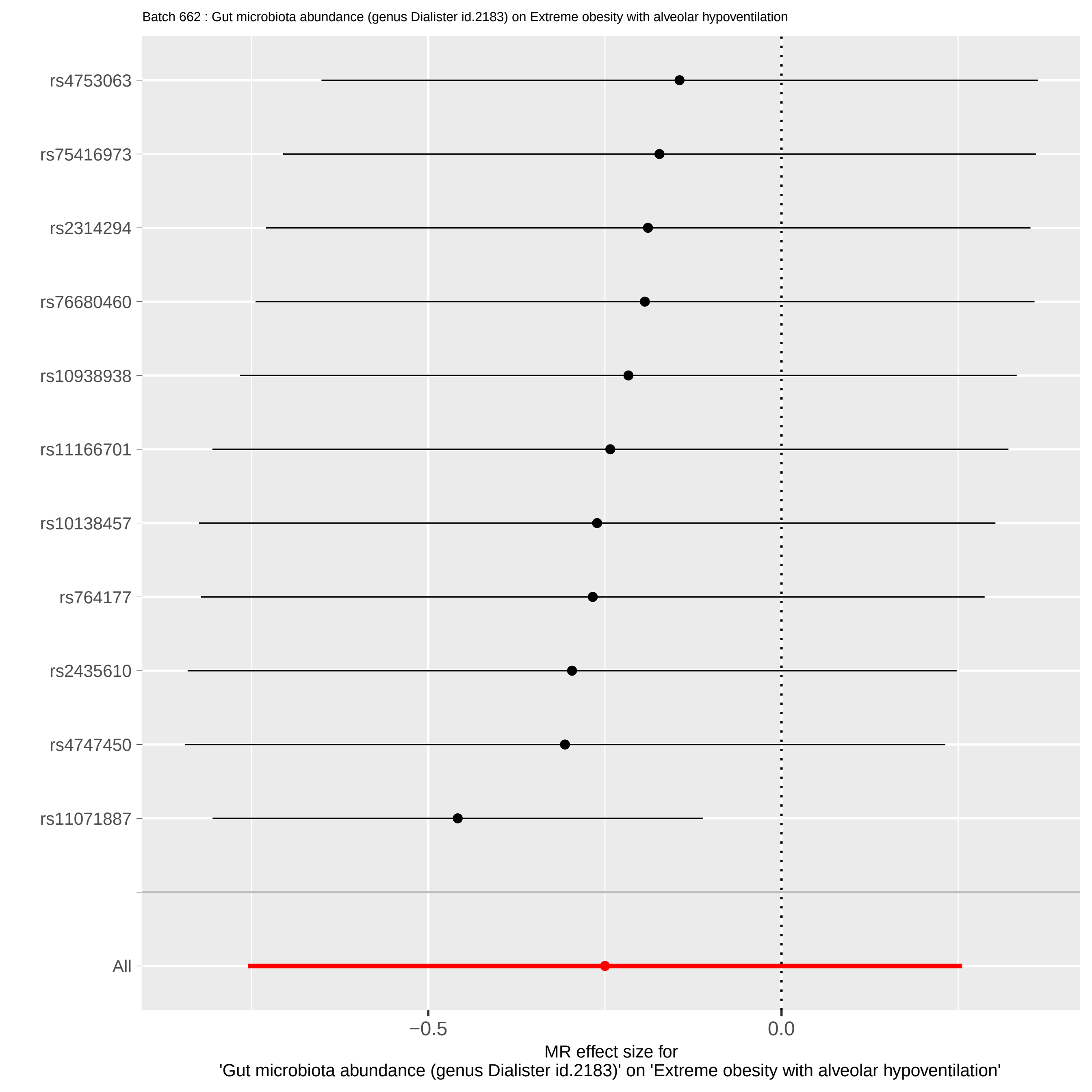

Batch 662 : Gut microbiota abundance (genus Dialister id.2183) on Extreme obesity with alveolar hypoventilation
rs4753063
rs75416973
rs2314294
rs76680460
rs10938938
rs11166701
rs10138457
rs764177
rs2435610
rs4747450
rs11071887
All
−0.5
0.0
MR effect size for
'Gut microbiota abundance (genus Dialister id.2183)' on 'Extreme obesity with alveolar hypoventilation'

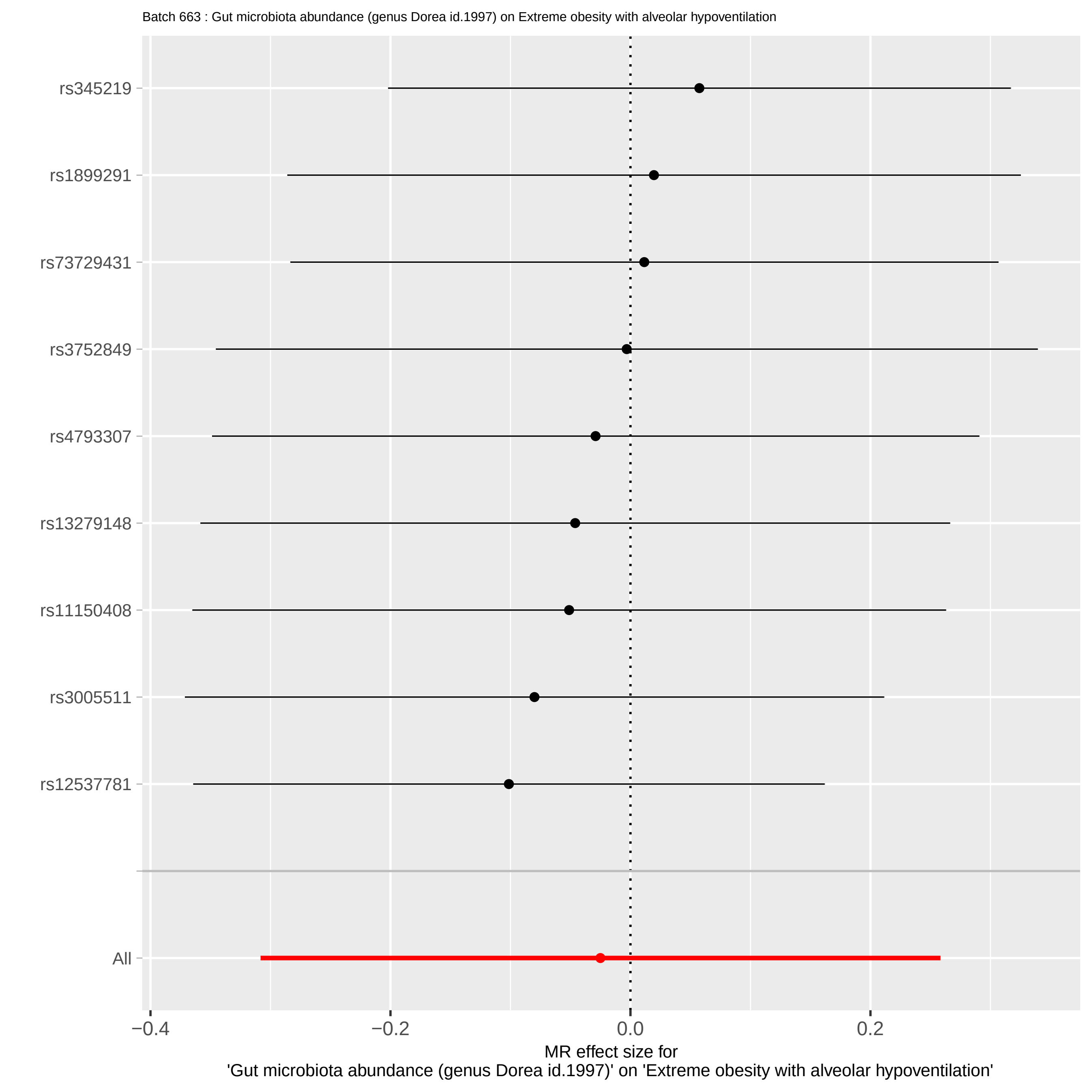
Batch 663 : Gut microbiota abundance (genus Dorea id.1997) on Extreme obesity with alveolar hypoventilation
rs345219
rs1899291
rs73729431
rs3752849
rs4793307
rs13279148
rs11150408
rs3005511
rs12537781
All
−0.4
−0.2
0.0
0.2
MR effect size for
'Gut microbiota abundance (genus Dorea id.1997)' on 'Extreme obesity with alveolar hypoventilation'

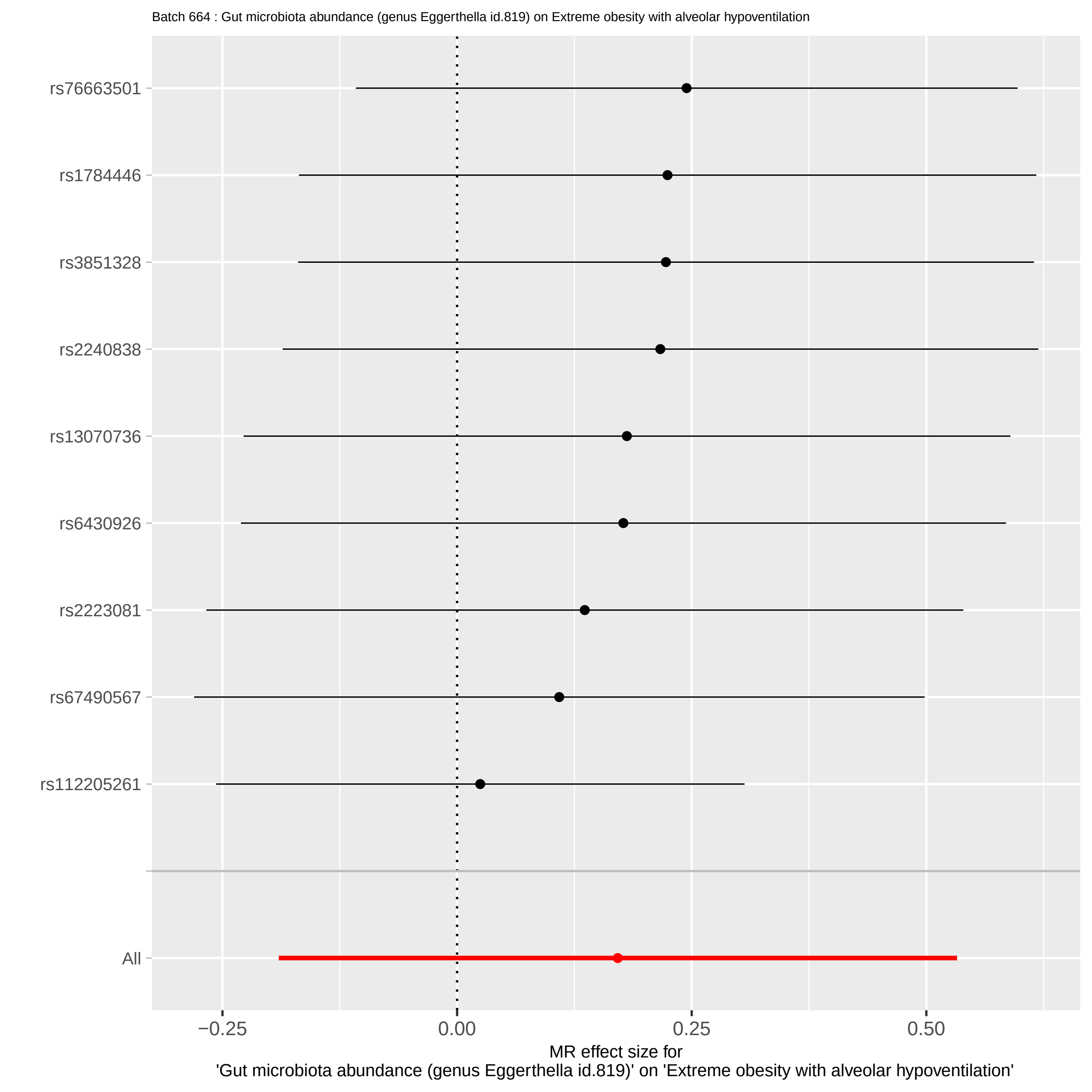

Batch 664 : Gut microbiota abundance (genus Eggerthella id.819) on Extreme obesity with alveolar hypoventilation
rs76663501
rs1784446
rs3851328
rs2240838
rs13070736
rs6430926
rs2223081
rs67490567
rs112205261
All
−0.25
0.00
0.25
0.50
MR effect size for
'Gut microbiota abundance (genus Eggerthella id.819)' on 'Extreme obesity with alveolar hypoventilation'

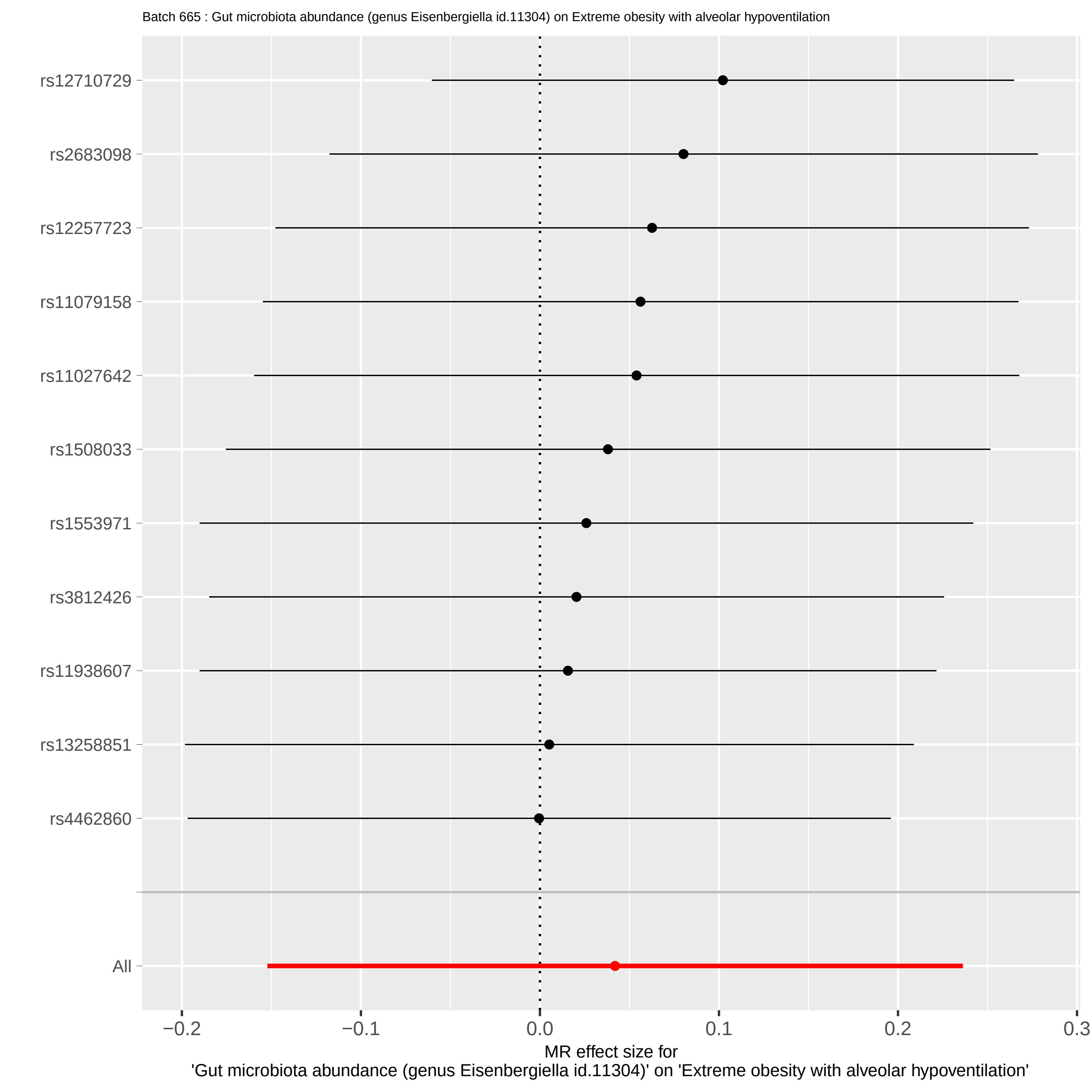
Batch 665 : Gut microbiota abundance (genus Eisenbergiella id.11304) on Extreme obesity with alveolar hypoventilation
rs12710729
rs2683098
rs12257723
rs11079158
rs11027642
rs1508033
rs1553971
rs3812426
rs11938607
rs13258851
rs4462860
All
−0.2
−0.1
0.0
0.1
0.2
0.3
MR effect size for
'Gut microbiota abundance (genus Eisenbergiella id.11304)' on 'Extreme obesity with alveolar hypoventilation'

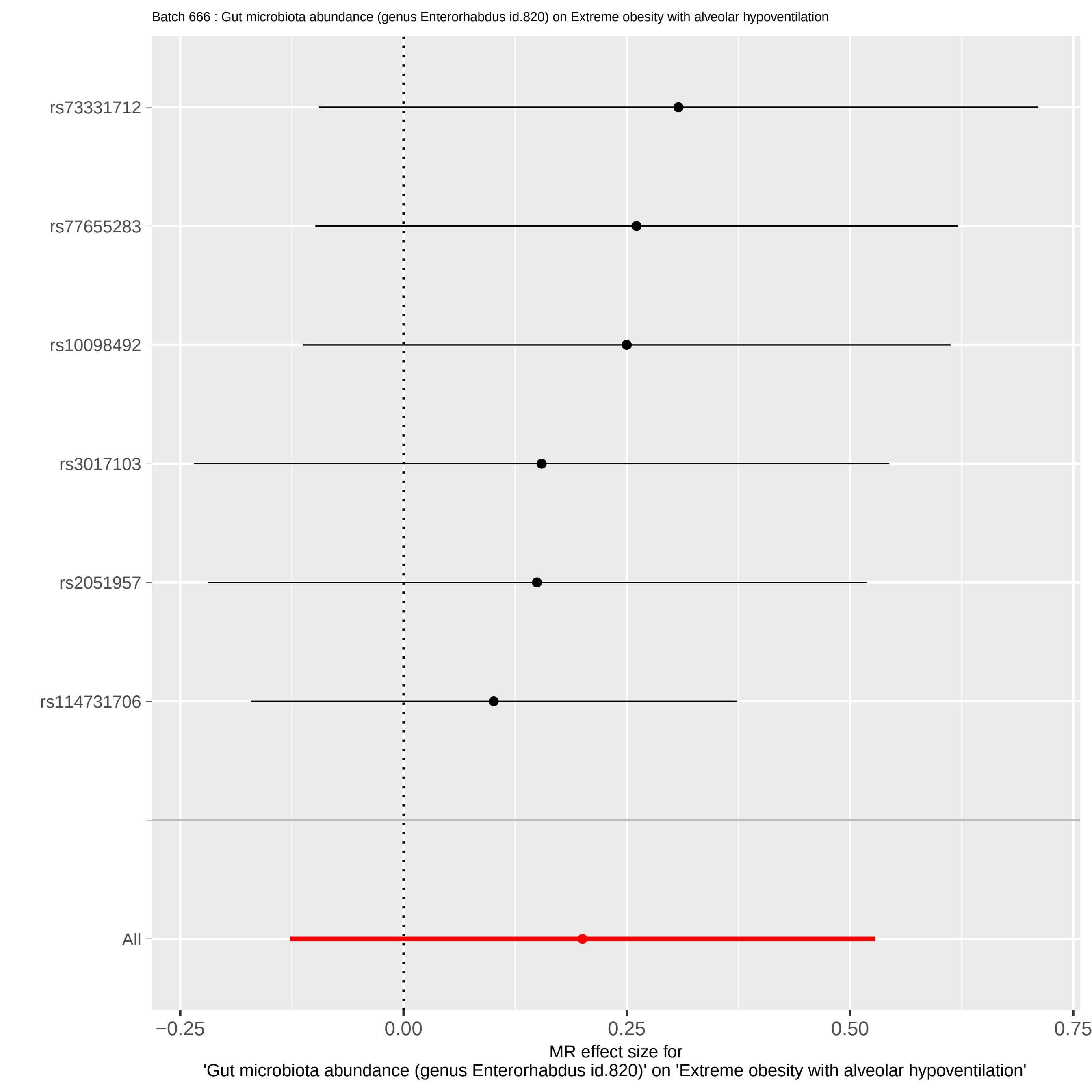

Batch 666 : Gut microbiota abundance (genus Enterorhabdus id.820) on Extreme obesity with alveolar hypoventilation
rs73331712
rs77655283
rs10098492
rs3017103
rs2051957
rs114731706
All
−0.25
0.00
0.25
0.50
0.75
MR effect size for
'Gut microbiota abundance (genus Enterorhabdus id.820)' on 'Extreme obesity with alveolar hypoventilation'

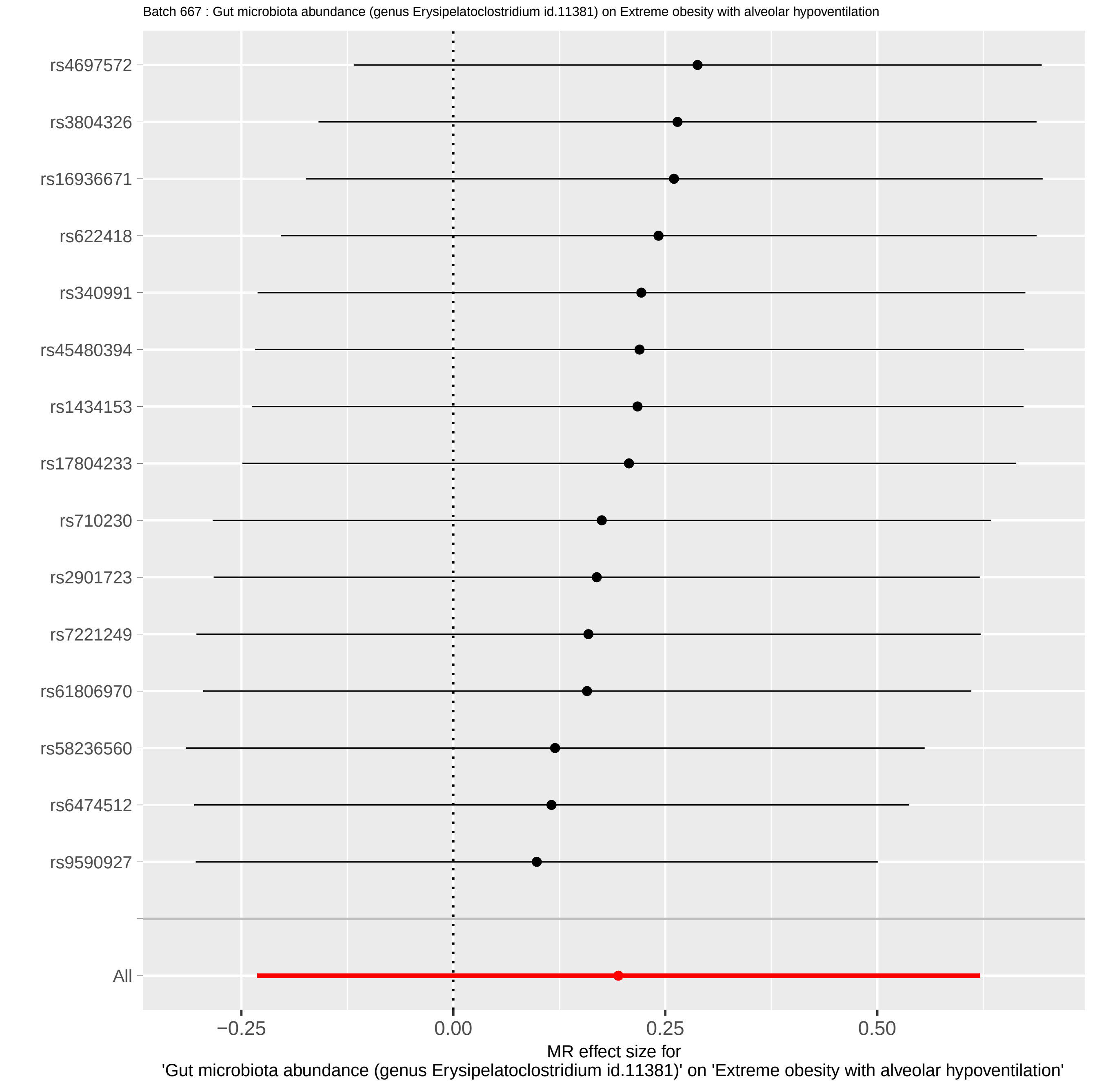

Batch 667 : Gut microbiota abundance (genus Erysipelatoclostridium id.11381) on Extreme obesity with alveolar hypoventilation
rs4697572
rs3804326
rs16936671
rs622418
rs340991
rs45480394
rs1434153
rs17804233
rs710230
rs2901723
rs7221249
rs61806970
rs58236560
rs6474512
rs9590927
All
−0.25
0.00
0.25
0.50
MR effect size for
'Gut microbiota abundance (genus Erysipelatoclostridium id.11381)' on 'Extreme obesity with alveolar hypoventilation'

Batch 669 : Gut microbiota abundance (genus Escherichia Shigella id.3504) on Extreme obesity with alveolar hypoventilation

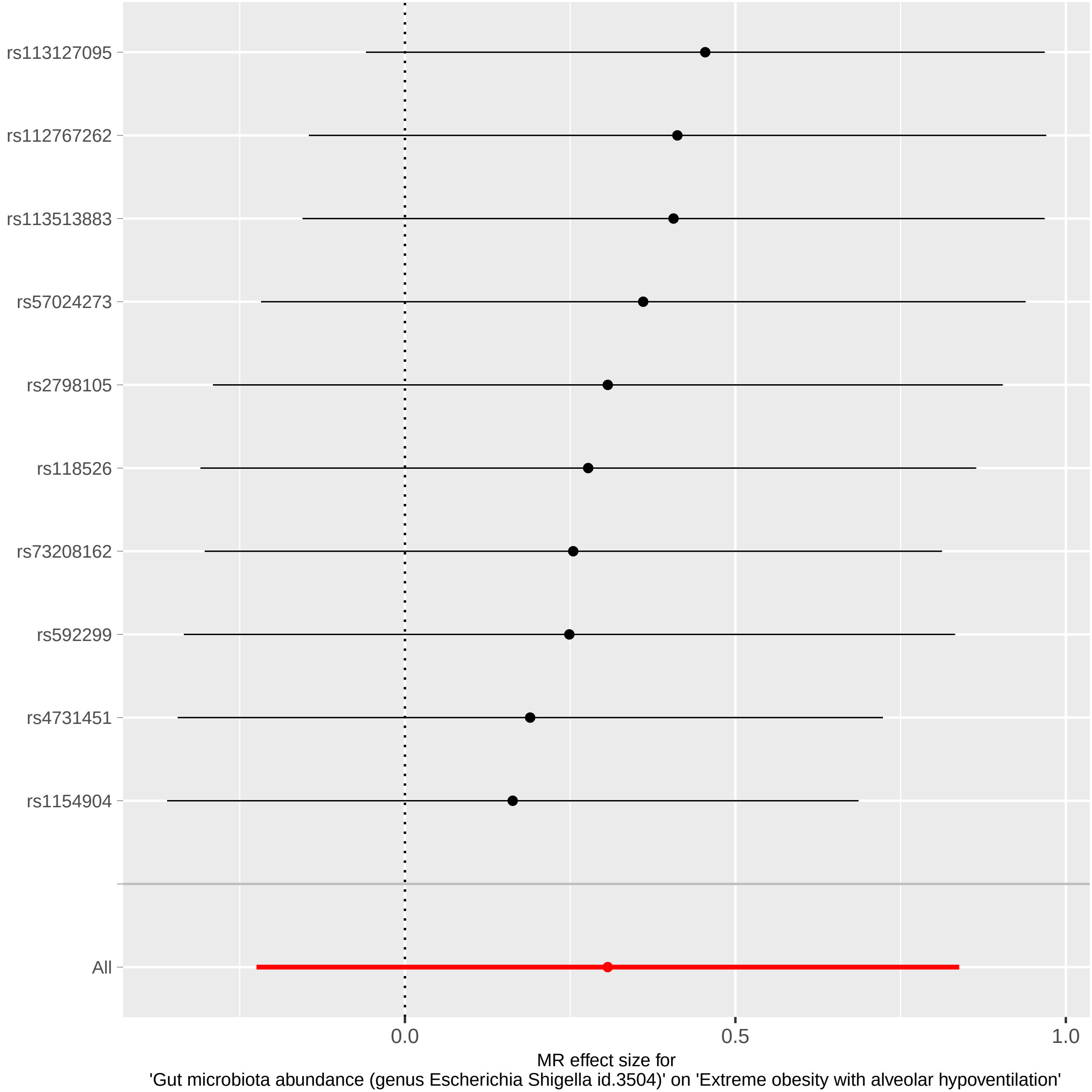

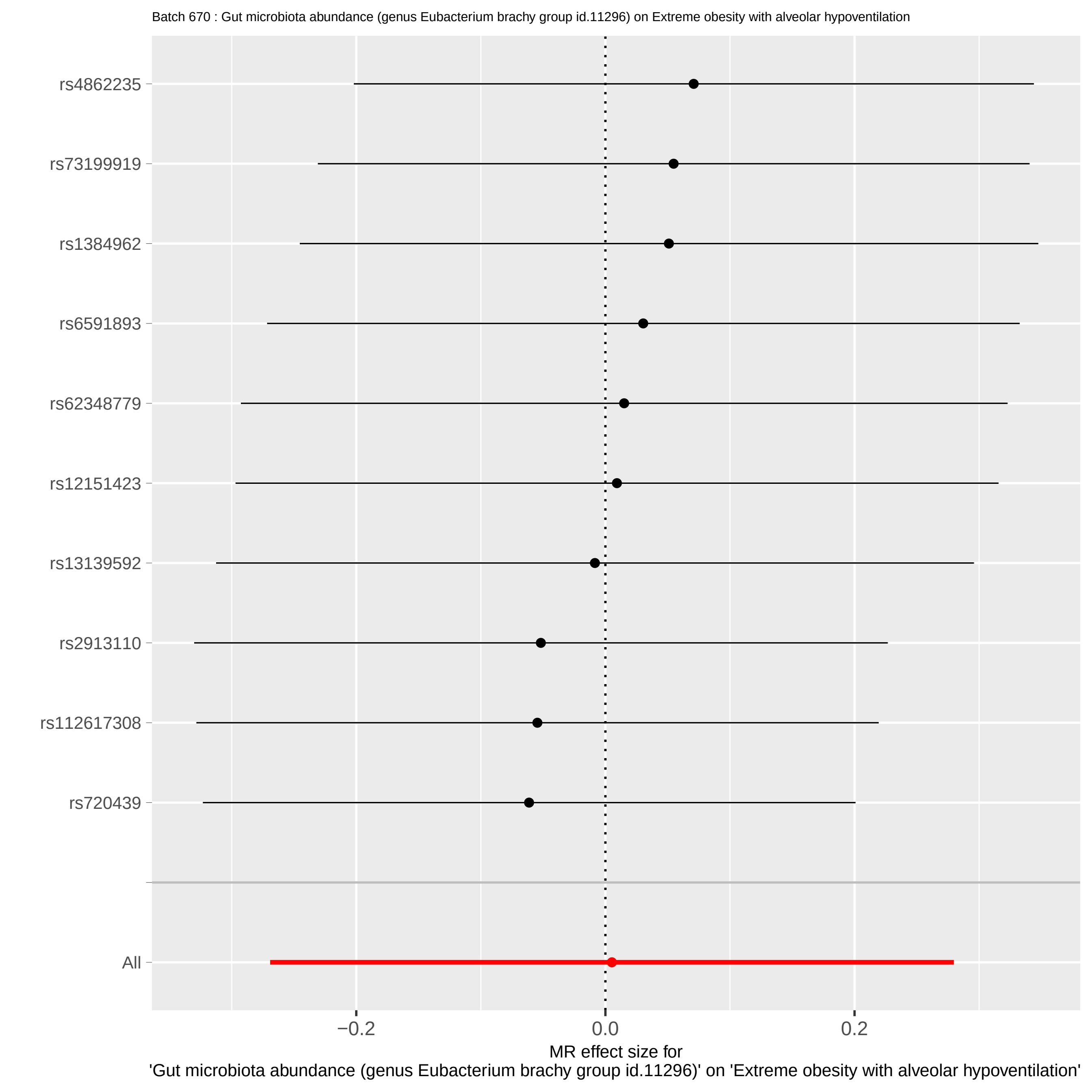

Batch 670 : Gut microbiota abundance (genus Eubacterium brachy group id.11296) on Extreme obesity with alveolar hypoventilation
rs4862235
rs73199919
rs1384962
rs6591893
rs62348779
rs12151423
rs13139592
rs2913110
rs112617308
rs720439
All
−0.2
0.0
0.2
MR effect size for
'Gut microbiota abundance (genus Eubacterium brachy group id.11296)' on 'Extreme obesity with alveolar hypoventilation'

Batch 671 : Gut microbiota abundance (genus Eubacterium coprostanoligenes group id.11375) on Extreme obesity with alveolar hypoventilation

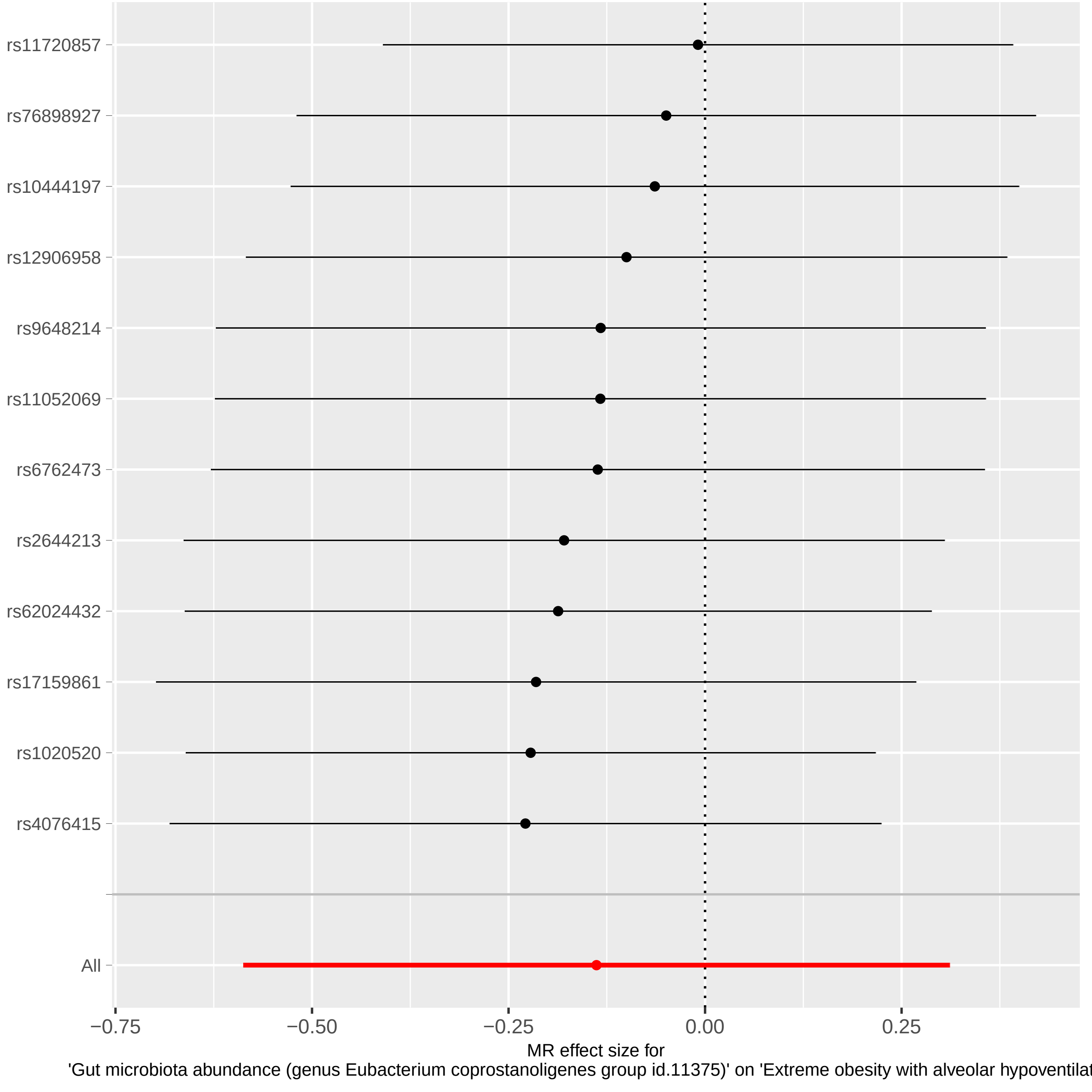

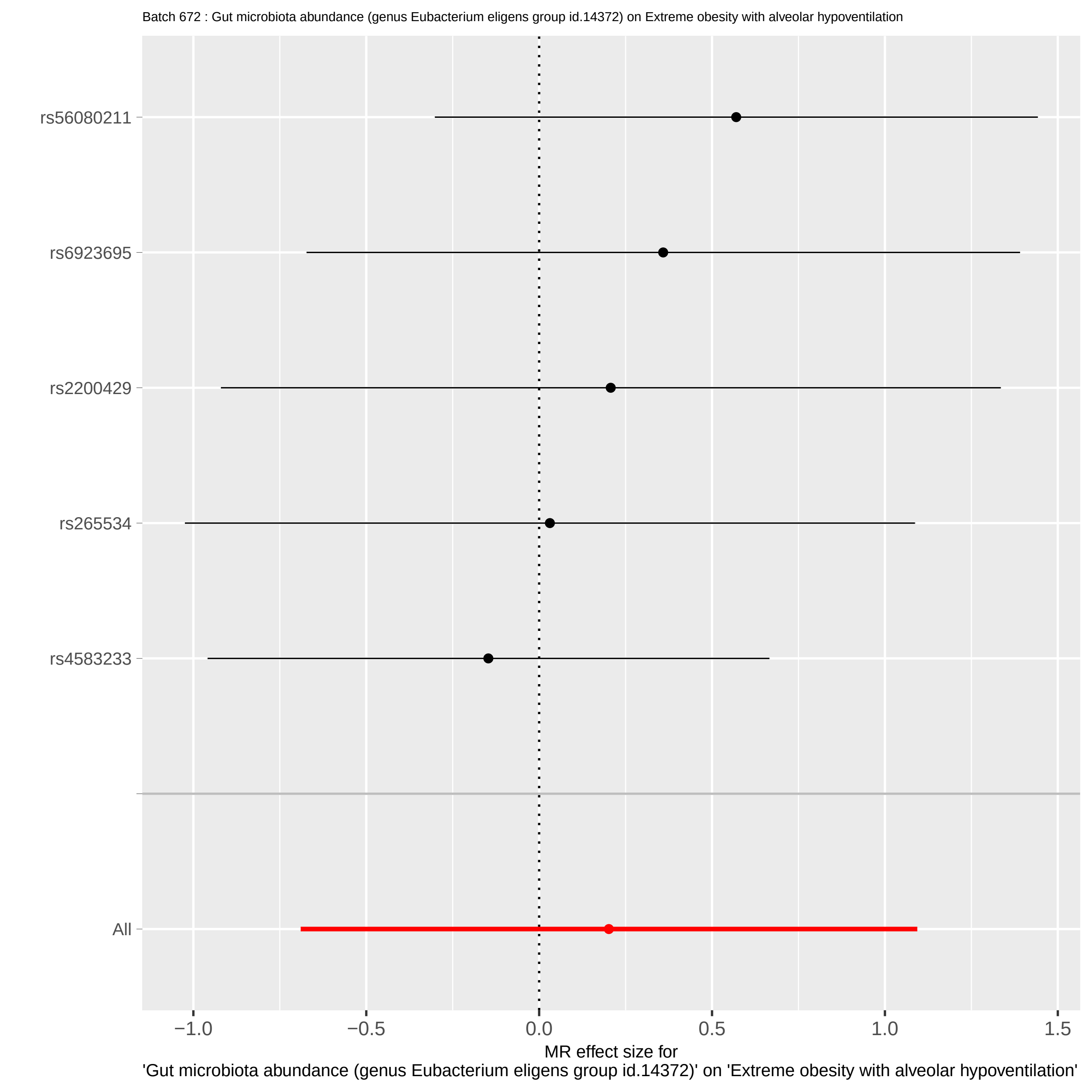

Batch 672 : Gut microbiota abundance (genus Eubacterium eligens group id.14372) on Extreme obesity with alveolar hypoventilation
rs56080211
rs6923695
rs2200429
rs265534
rs4583233
All
−1.0
−0.5
0.0
0.5
1.0
1.5
MR effect size for
'Gut microbiota abundance (genus Eubacterium eligens group id.14372)' on 'Extreme obesity with alveolar hypoventilation'

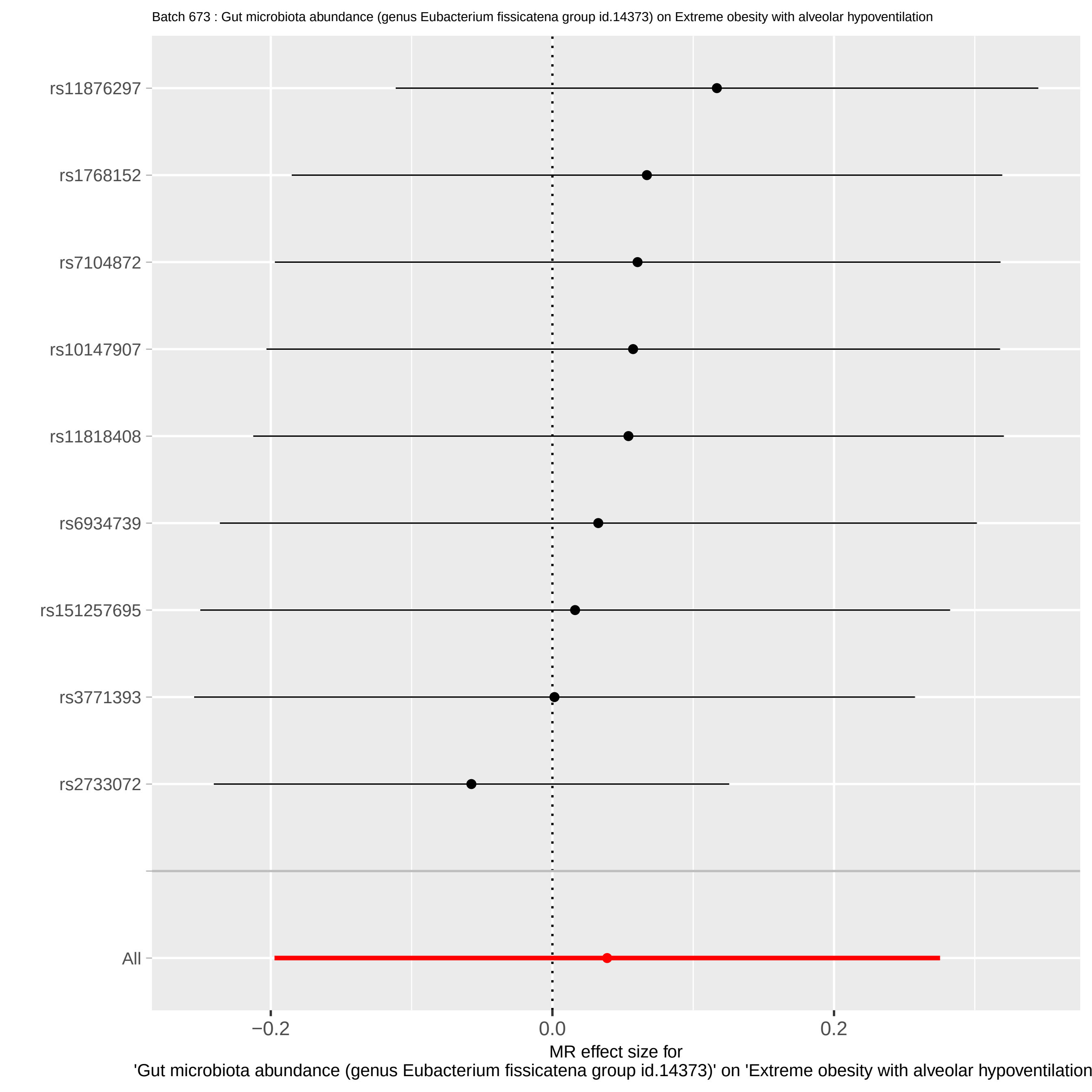
Batch 673 : Gut microbiota abundance (genus Eubacterium fissicatena group id.14373) on Extreme obesity with alveolar hypoventilation
rs11876297
rs1768152
rs7104872
rs10147907
rs11818408
rs6934739
rs151257695
rs3771393
rs2733072
All
−0.2
0.0
0.2
MR effect size for
'Gut microbiota abundance (genus Eubacterium fissicatena group id.14373)' on 'Extreme obesity with alveolar hypoventilation

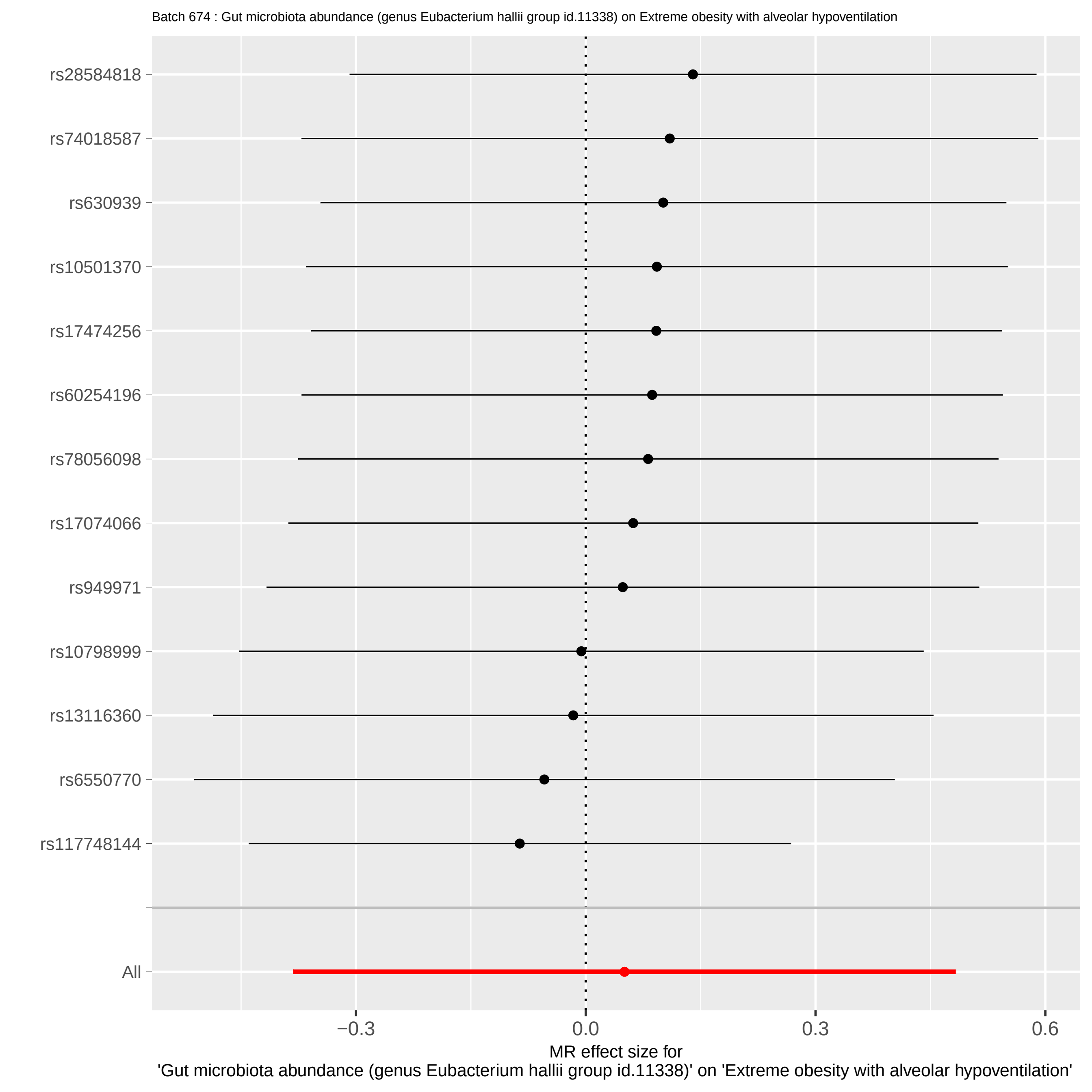

Batch 674 : Gut microbiota abundance (genus Eubacterium hallii group id.11338) on Extreme obesity with alveolar hypoventilation
rs28584818
rs74018587
rs630939
rs10501370
rs17474256
rs60254196
rs78056098
rs17074066
rs949971
rs10798999
rs13116360
rs6550770
rs117748144
All
−0.3
0.0
0.3
0.6
MR effect size for
'Gut microbiota abundance (genus Eubacterium hallii group id.11338)' on 'Extreme obesity with alveolar hypoventilation'

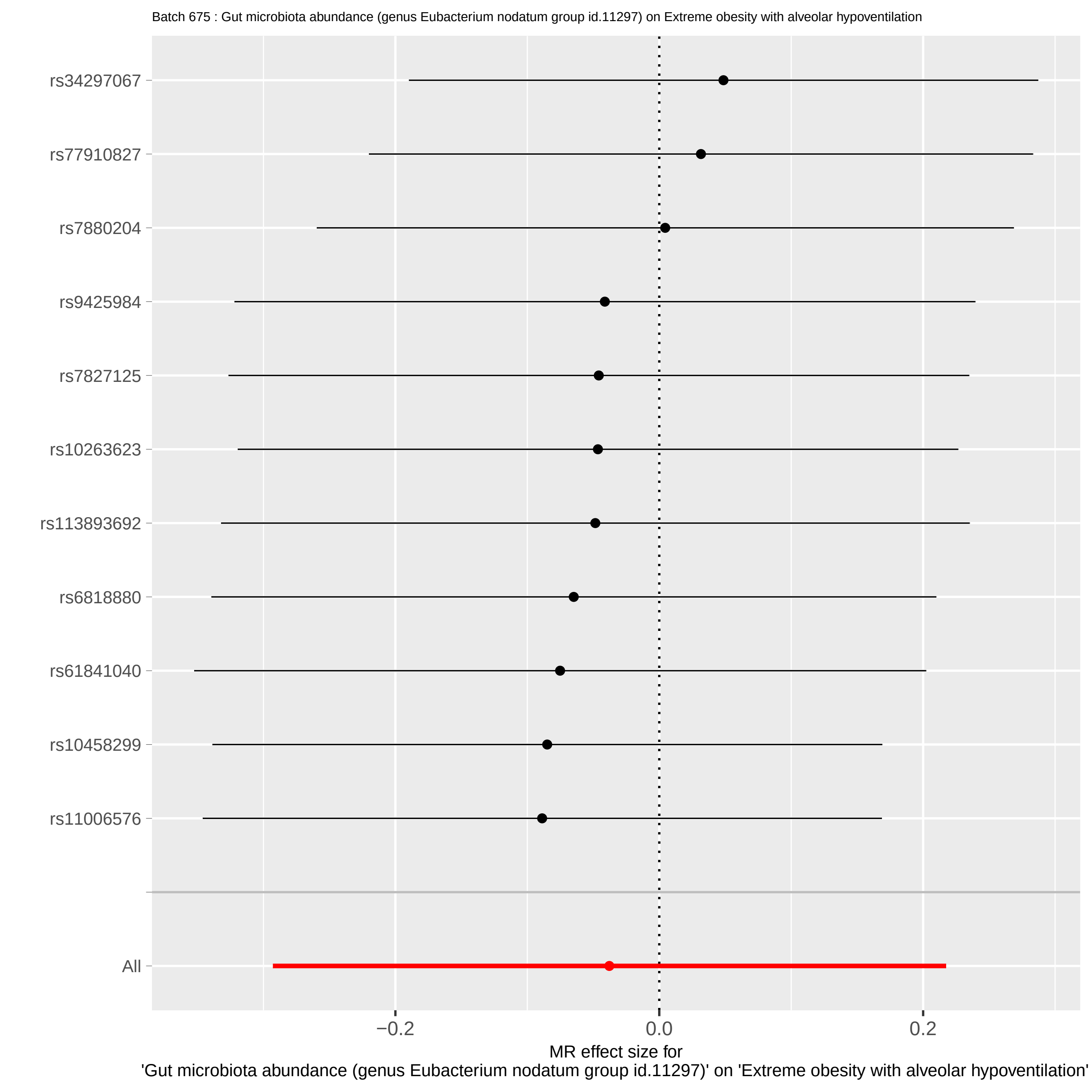

Batch 675 : Gut microbiota abundance (genus Eubacterium nodatum group id.11297) on Extreme obesity with alveolar hypoventilation
rs34297067
rs77910827
rs7880204
rs9425984
rs7827125
rs10263623
rs113893692
rs6818880
rs61841040
rs10458299
rs11006576
All
−0.2
0.0
0.2
MR effect size for
'Gut microbiota abundance (genus Eubacterium nodatum group id.11297)' on 'Extreme obesity with alveolar hypoventilation'

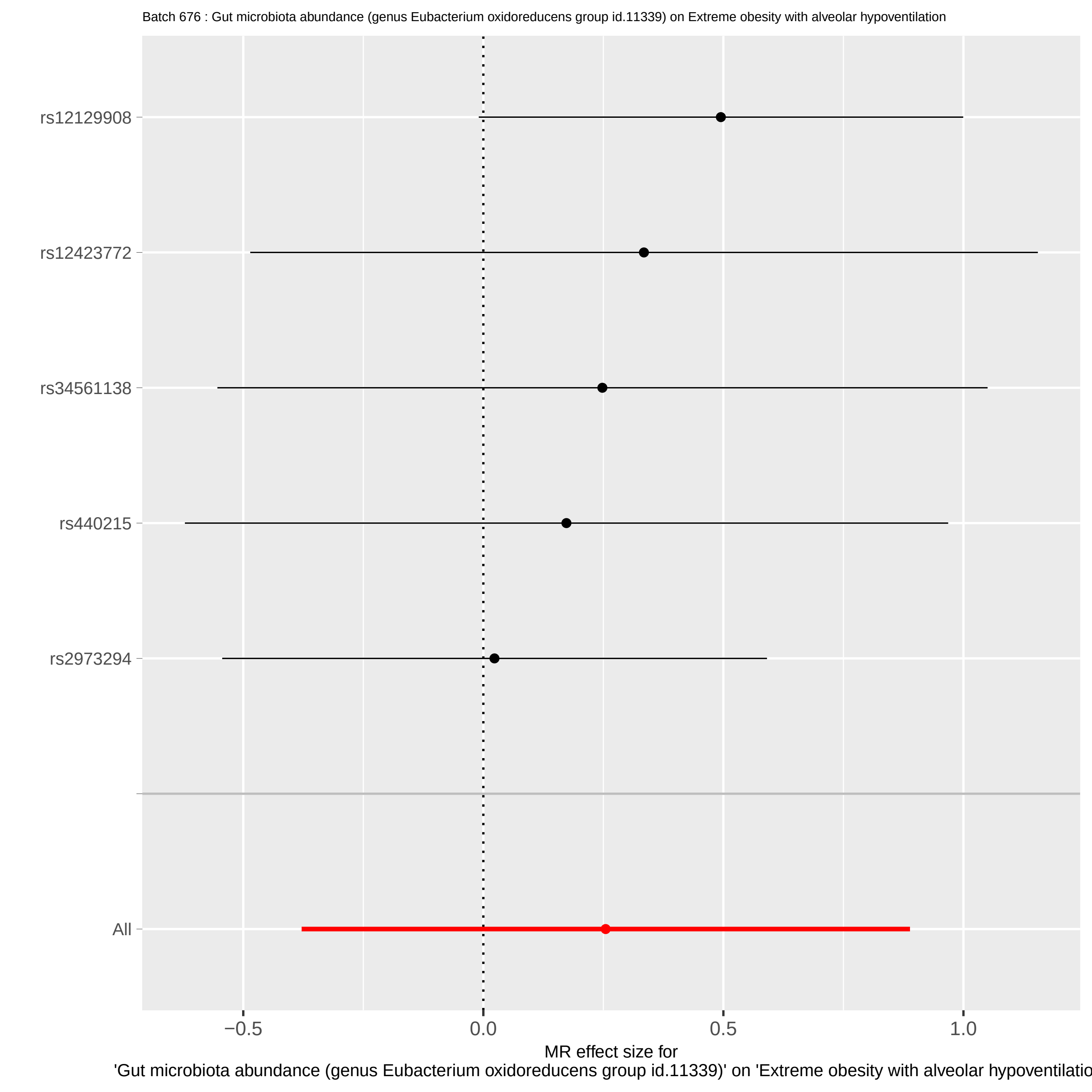

Batch 676 : Gut microbiota abundance (genus Eubacterium oxidoreducens group id.11339) on Extreme obesity with alveolar hypoventilation
rs12129908
rs12423772
rs34561138
rs440215
rs2973294
All
−0.5
0.0
0.5
1.0
MR effect size for
'Gut microbiota abundance (genus Eubacterium oxidoreducens group id.11339)' on 'Extreme obesity with alveolar hypoventilatio

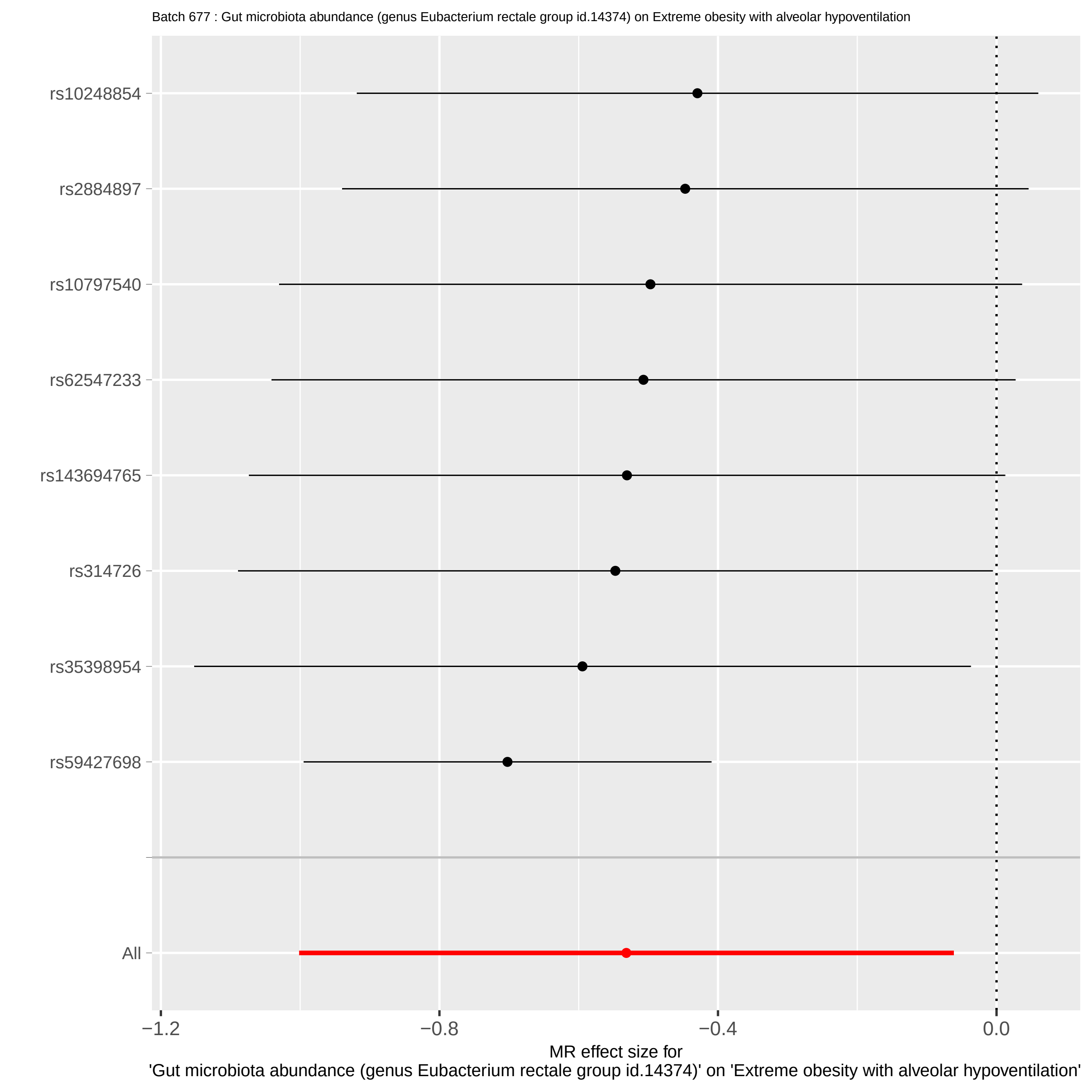
Batch 677 : Gut microbiota abundance (genus Eubacterium rectale group id.14374) on Extreme obesity with alveolar hypoventilation
rs10248854
rs2884897
rs10797540
rs62547233
rs143694765
rs314726
rs35398954
rs59427698
All
−1.2
−0.8
−0.4
0.0
MR effect size for
'Gut microbiota abundance (genus Eubacterium rectale group id.14374)' on 'Extreme obesity with alveolar hypoventilation'

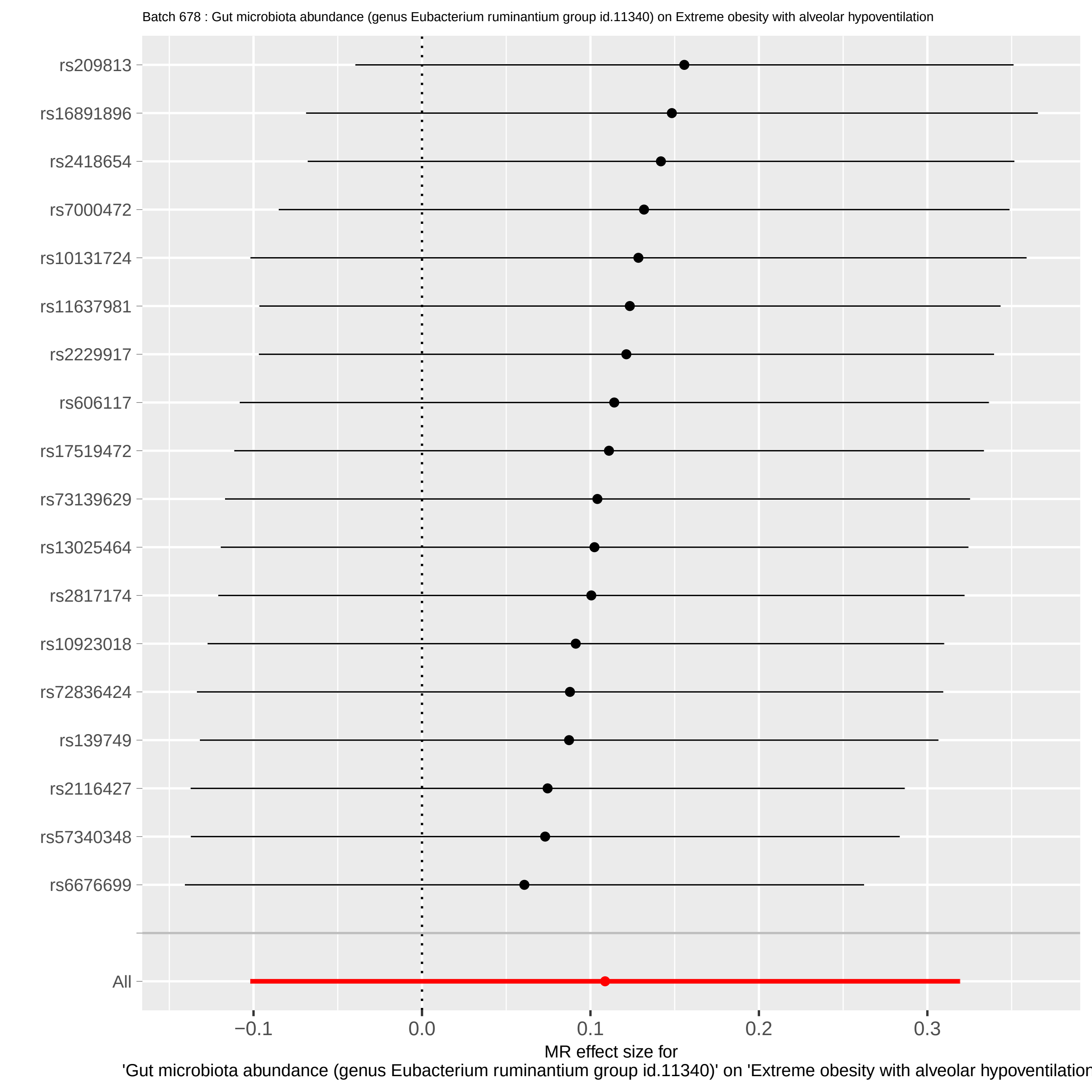

Batch 678 : Gut microbiota abundance (genus Eubacterium ruminantium group id.11340) on Extreme obesity with alveolar hypoventilation
rs209813
rs16891896
rs2418654
rs7000472
rs10131724
rs11637981
rs2229917
rs606117
rs17519472
rs73139629
rs13025464
rs2817174
rs10923018
rs72836424
rs139749
rs2116427
rs57340348
rs6676699
All
−0.1
0.0
0.1
0.2
0.3
MR effect size for
'Gut microbiota abundance (genus Eubacterium ruminantium group id.11340)' on 'Extreme obesity with alveolar hypoventilation

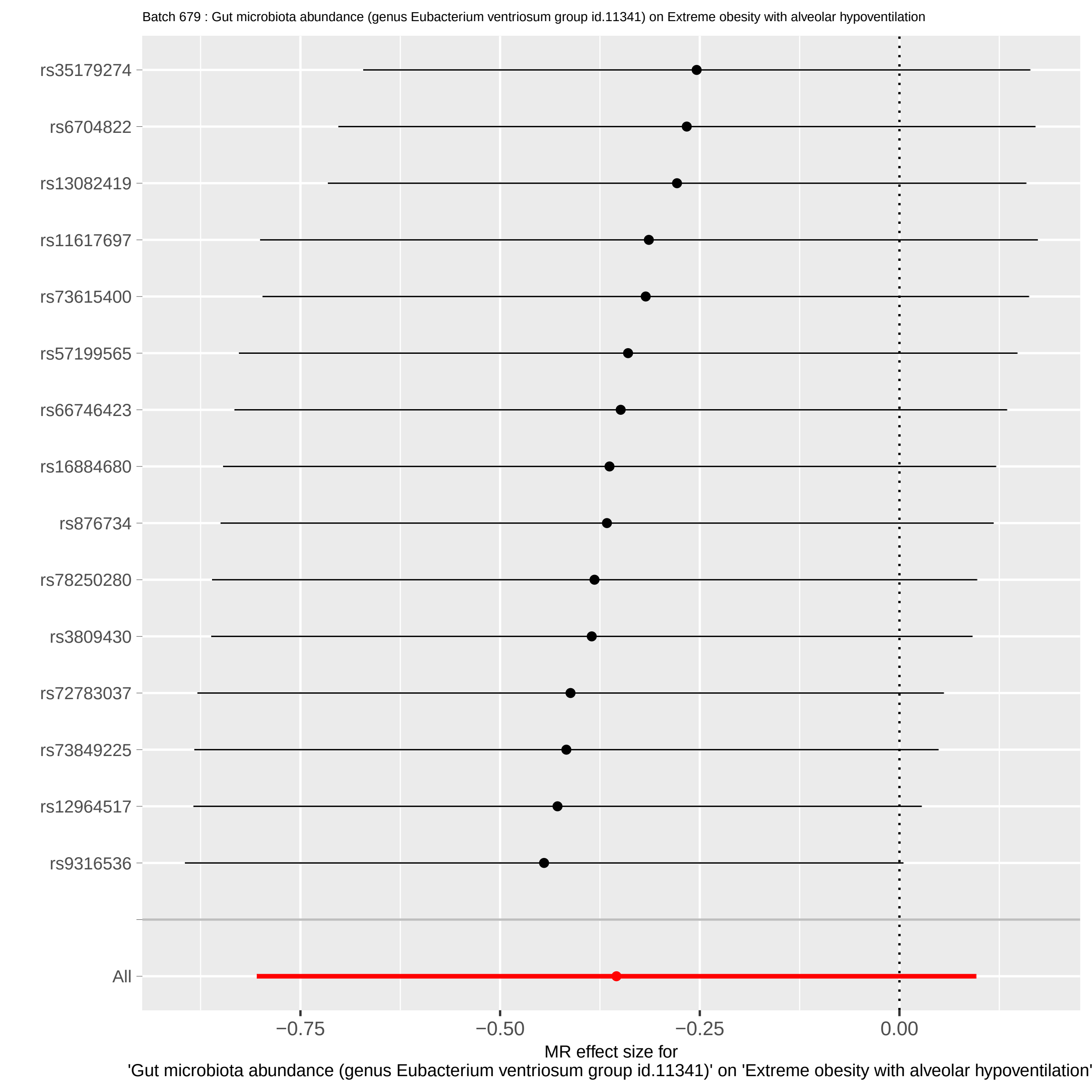

Batch 679 : Gut microbiota abundance (genus Eubacterium ventriosum group id.11341) on Extreme obesity with alveolar hypoventilation
rs35179274
rs6704822
rs13082419
rs11617697
rs73615400
rs57199565
rs66746423
rs16884680
rs876734
rs78250280
rs3809430
rs72783037
rs73849225
rs12964517
rs9316536
All
−0.75
−0.50
−0.25
0.00
MR effect size for
'Gut microbiota abundance (genus Eubacterium ventriosum group id.11341)' on 'Extreme obesity with alveolar hypoventilation'

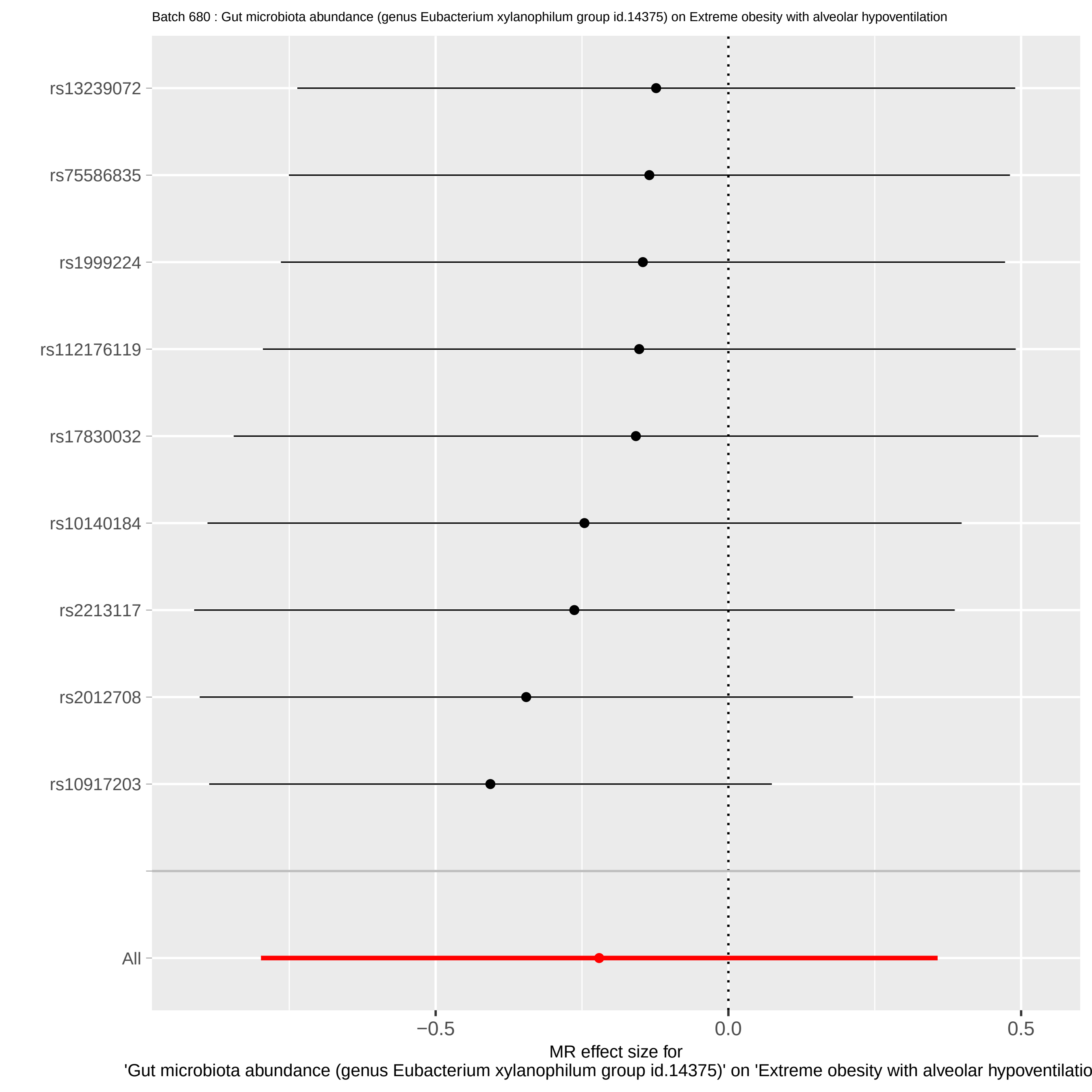

Batch 680 : Gut microbiota abundance (genus Eubacterium xylanophilum group id.14375) on Extreme obesity with alveolar hypoventilation
rs13239072
rs75586835
rs1999224
rs112176119
rs17830032
rs10140184
rs2213117
rs2012708
rs10917203
All
−0.5
0.0
0.5
MR effect size for
'Gut microbiota abundance (genus Eubacterium xylanophilum group id.14375)' on 'Extreme obesity with alveolar hypoventilatio

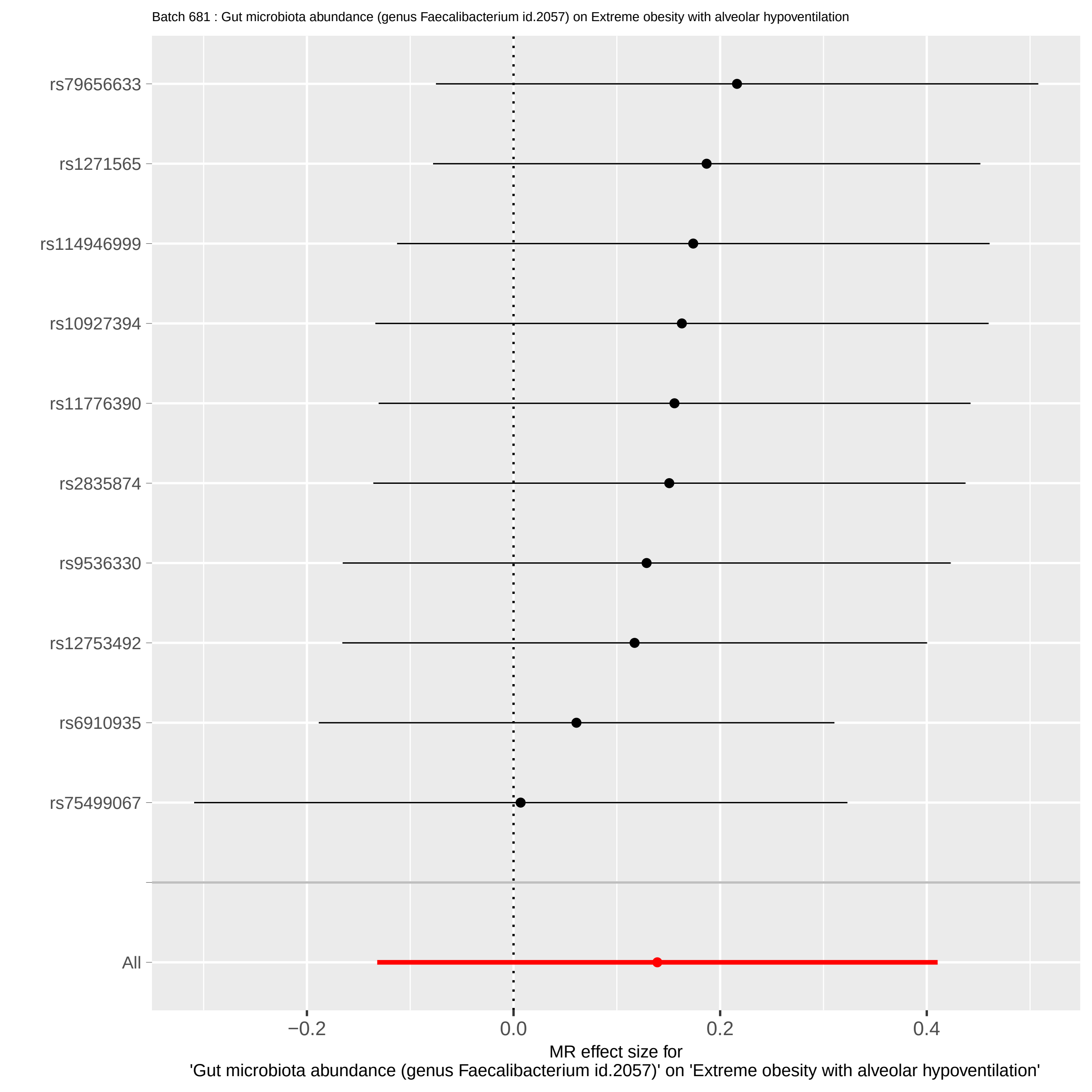

Batch 681 : Gut microbiota abundance (genus Faecalibacterium id.2057) on Extreme obesity with alveolar hypoventilation
rs79656633
rs1271565
rs114946999
rs10927394
rs11776390
rs2835874
rs9536330
rs12753492
rs6910935
rs75499067
All
−0.2
0.0
0.2
0.4
MR effect size for
'Gut microbiota abundance (genus Faecalibacterium id.2057)' on 'Extreme obesity with alveolar hypoventilation'

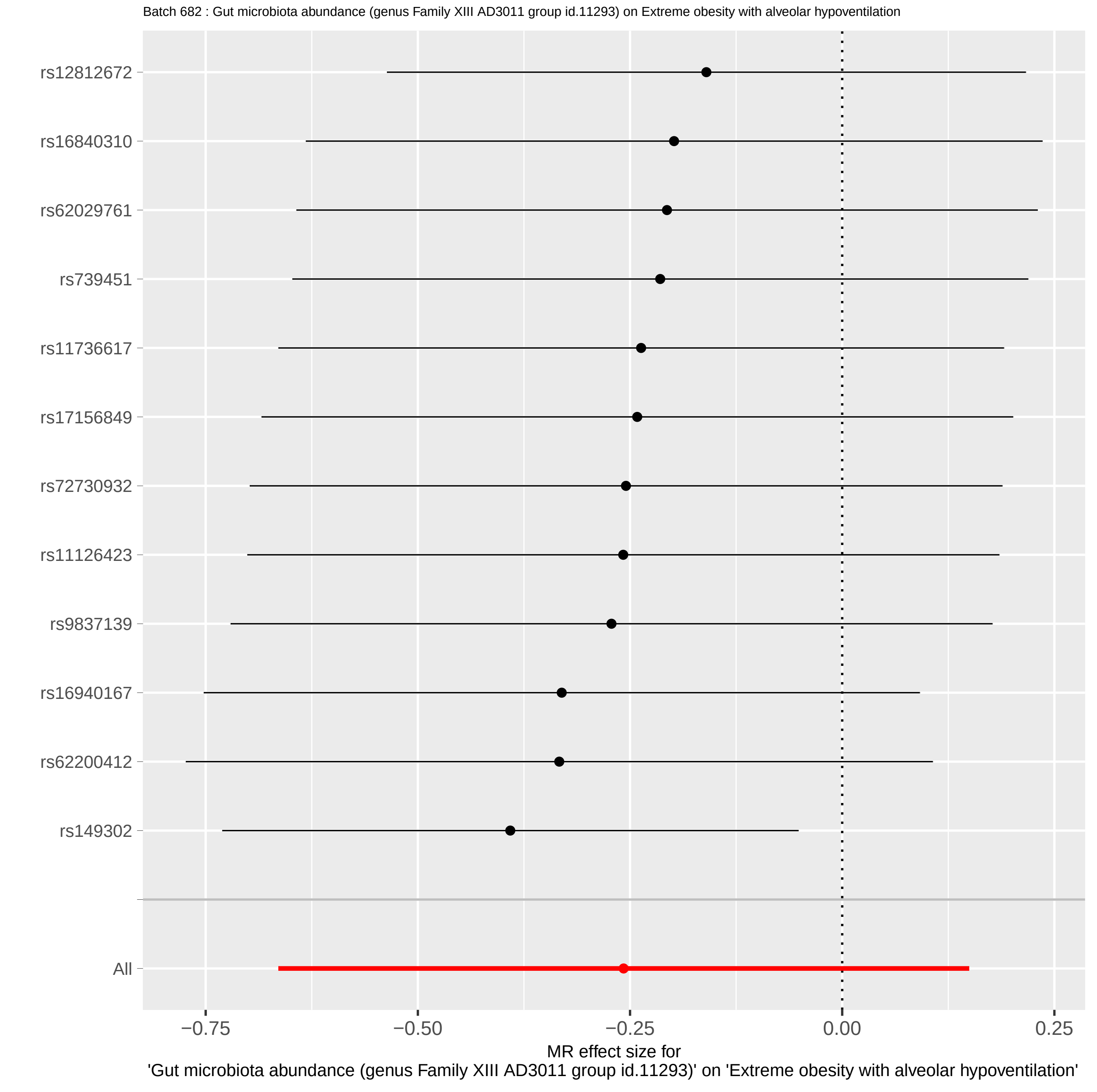

Batch 682 : Gut microbiota abundance (genus Family XIII AD3011 group id.11293) on Extreme obesity with alveolar hypoventilation
rs12812672
rs16840310
rs62029761
rs739451
rs11736617
rs17156849
rs72730932
rs11126423
rs9837139
rs16940167
rs62200412
rs149302
All
−0.75
−0.50
−0.25
0.00
0.25
MR effect size for
'Gut microbiota abundance (genus Family XIII AD3011 group id.11293)' on 'Extreme obesity with alveolar hypoventilation'

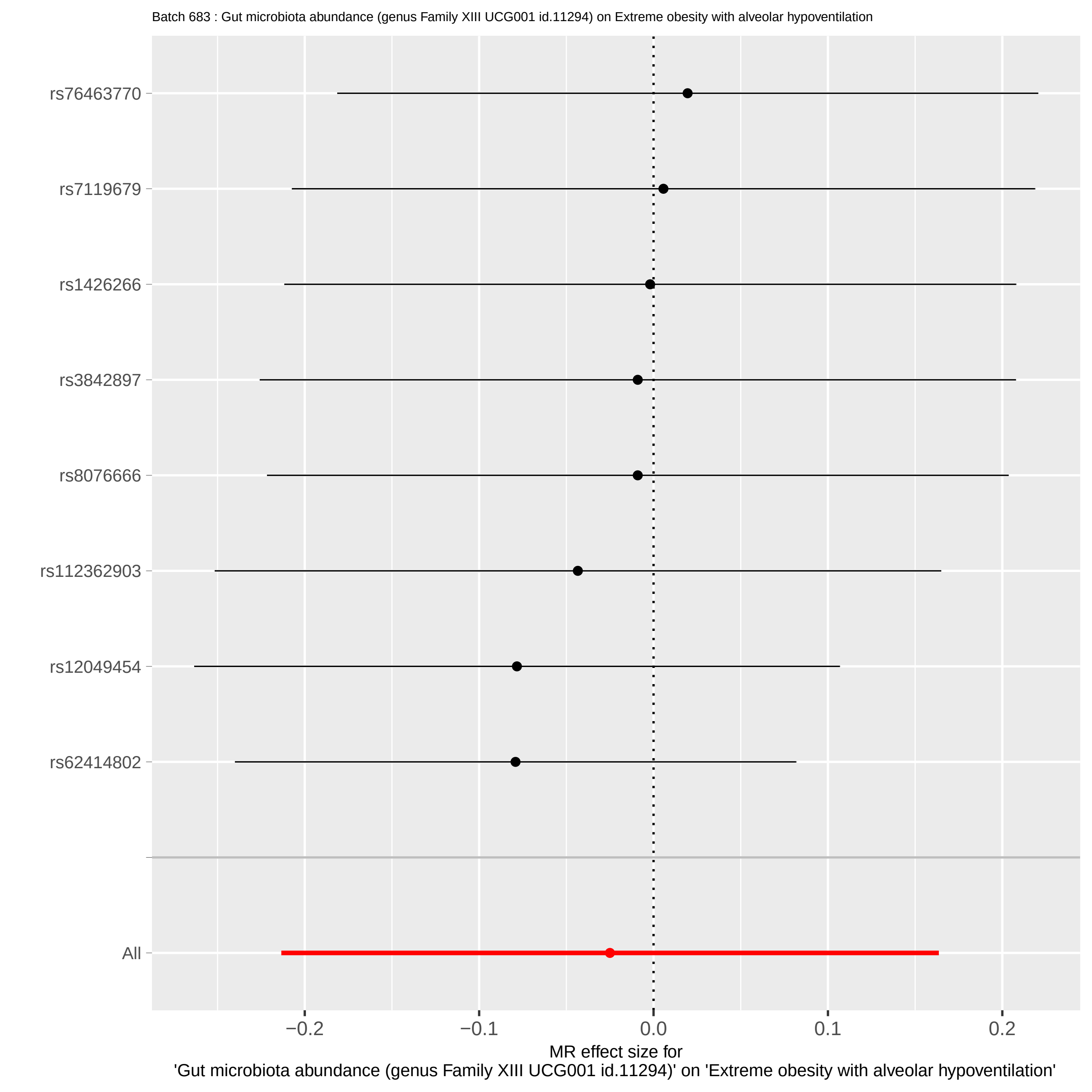
Batch 683 : Gut microbiota abundance (genus Family XIII UCG001 id.11294) on Extreme obesity with alveolar hypoventilation
rs76463770
rs7119679
rs1426266
rs3842897
rs8076666
rs112362903
rs12049454
rs62414802
All
−0.2
−0.1
0.0
0.1
0.2
MR effect size for
'Gut microbiota abundance (genus Family XIII UCG001 id.11294)' on 'Extreme obesity with alveolar hypoventilation'

Batch 684 : Gut microbiota abundance (genus Flavonifractor id.2059) on Extreme obesity with alveolar hypoventilation

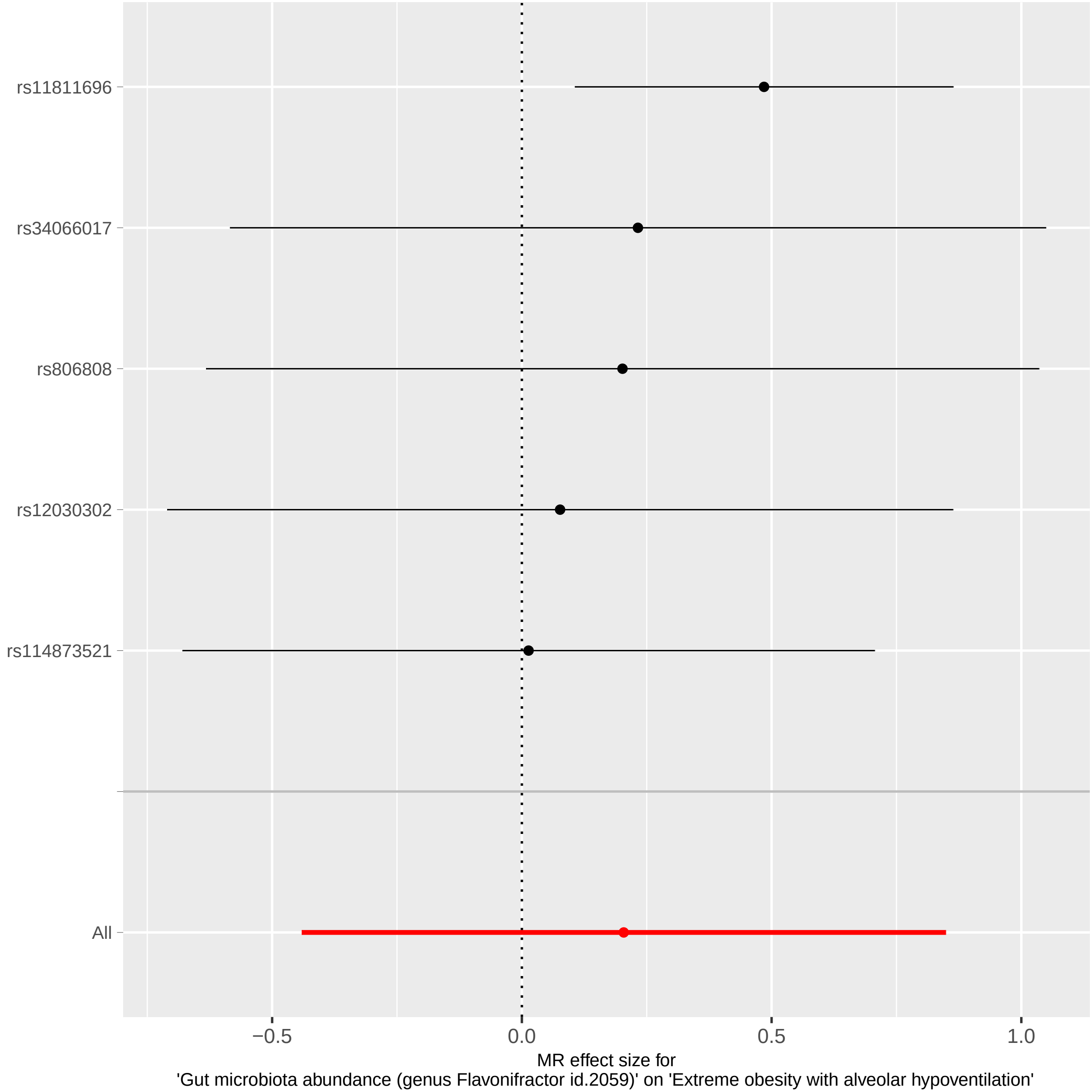

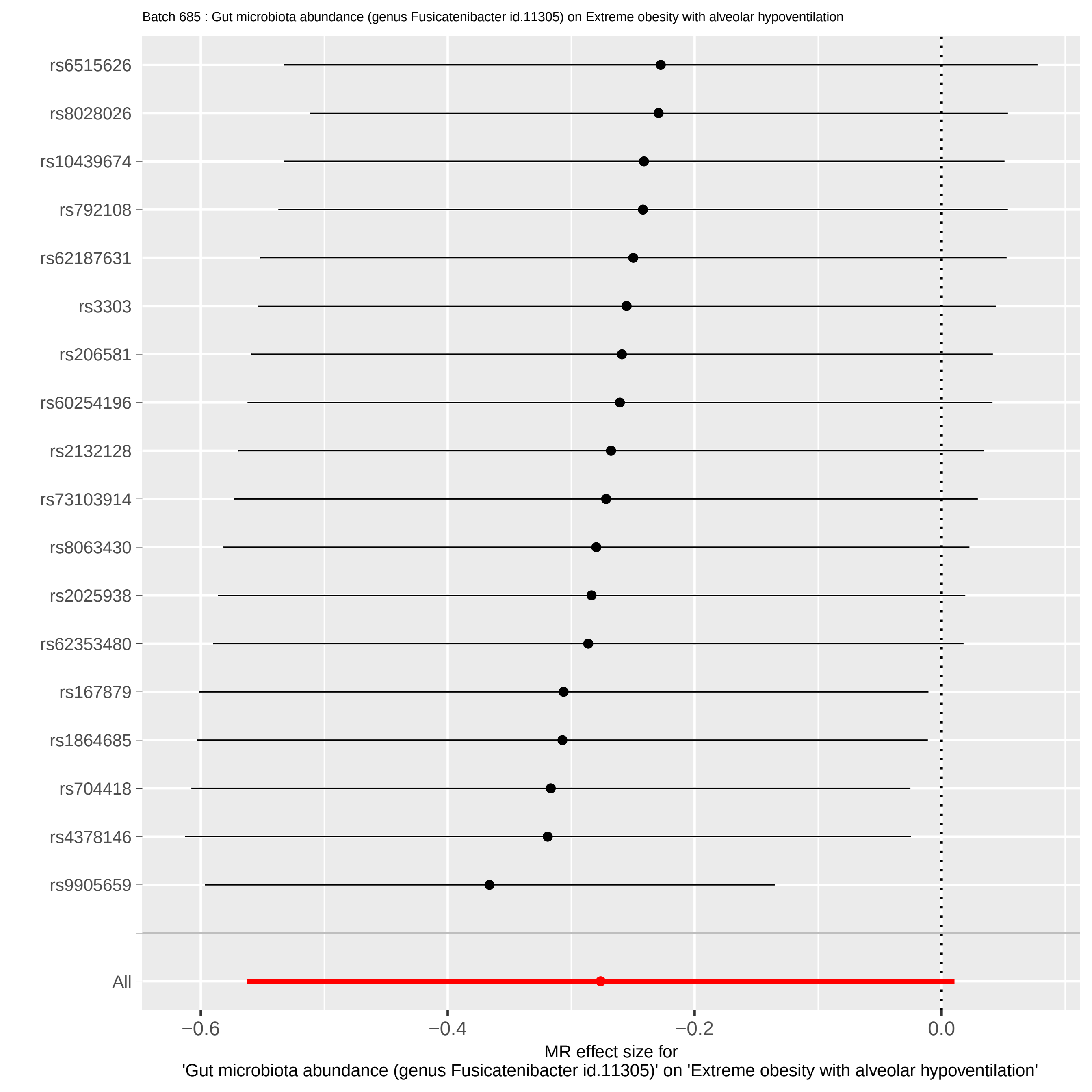

Batch 685 : Gut microbiota abundance (genus Fusicatenibacter id.11305) on Extreme obesity with alveolar hypoventilation
rs6515626
rs8028026
rs10439674
rs792108
rs62187631
rs3303
rs206581
rs60254196
rs2132128
rs73103914
rs8063430
rs2025938
rs62353480
rs167879
rs1864685
rs704418
rs4378146
rs9905659
All
−0.6
−0.4
−0.2
0.0
MR effect size for
'Gut microbiota abundance (genus Fusicatenibacter id.11305)' on 'Extreme obesity with alveolar hypoventilation'

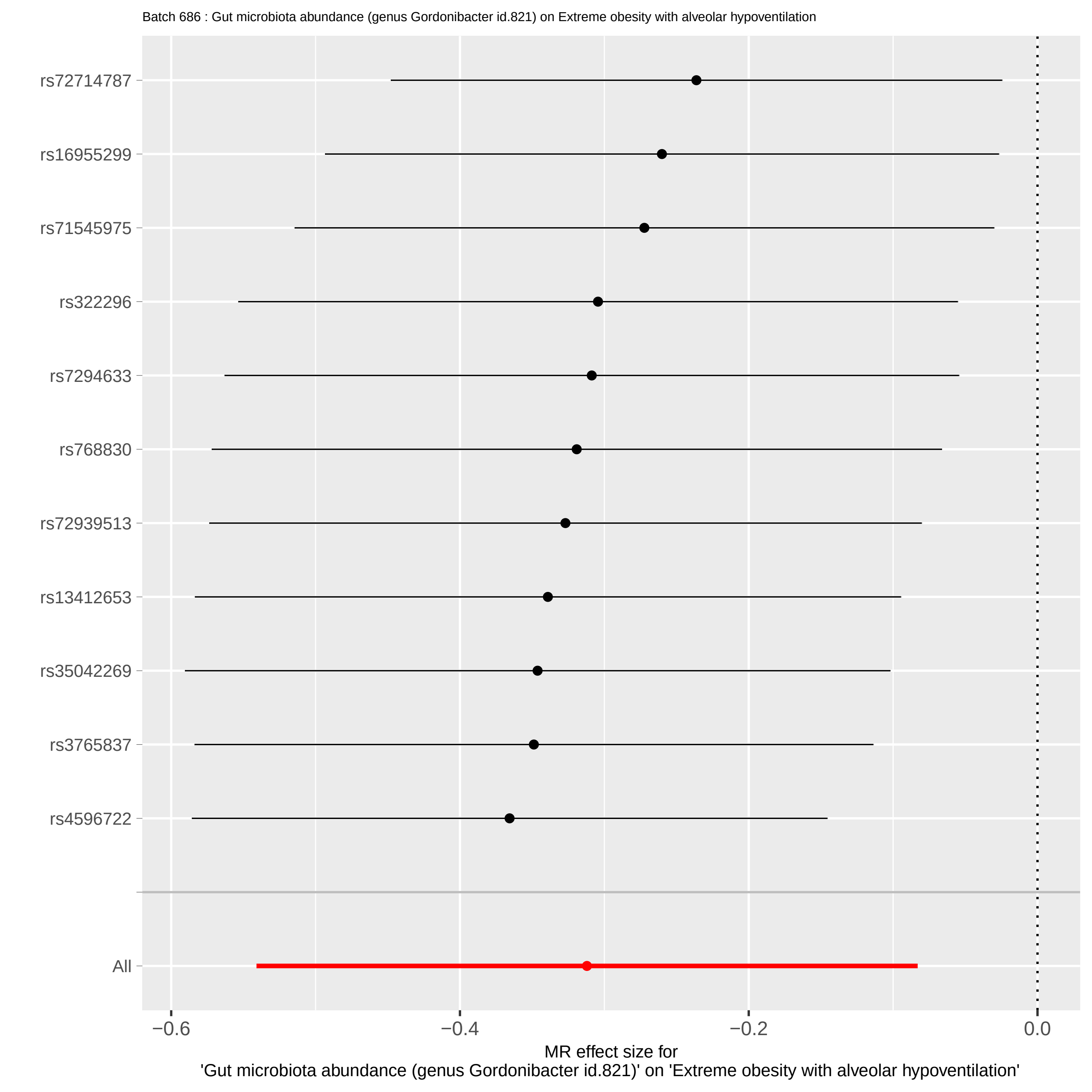

Batch 686 : Gut microbiota abundance (genus Gordonibacter id.821) on Extreme obesity with alveolar hypoventilation
rs72714787
rs16955299
rs71545975
rs322296
rs7294633
rs768830
rs72939513
rs13412653
rs35042269
rs3765837
rs4596722
All
−0.6
−0.4
−0.2
0.0
MR effect size for
'Gut microbiota abundance (genus Gordonibacter id.821)' on 'Extreme obesity with alveolar hypoventilation'

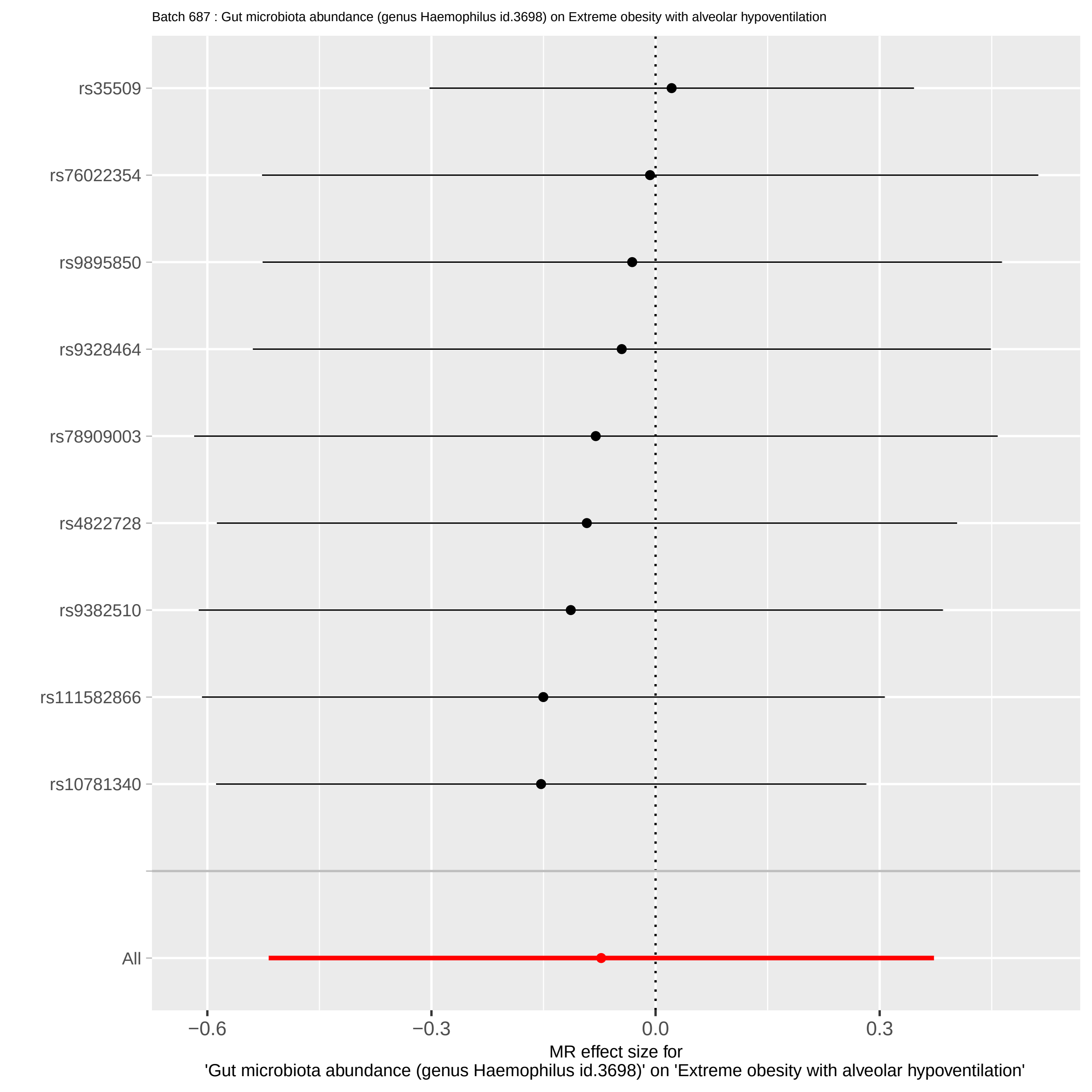

Batch 687 : Gut microbiota abundance (genus Haemophilus id.3698) on Extreme obesity with alveolar hypoventilation
rs35509
rs76022354
rs9895850
rs9328464
rs78909003
rs4822728
rs9382510
rs111582866
rs10781340
All
−0.6
−0.3
0.0
0.3
MR effect size for
'Gut microbiota abundance (genus Haemophilus id.3698)' on 'Extreme obesity with alveolar hypoventilation'

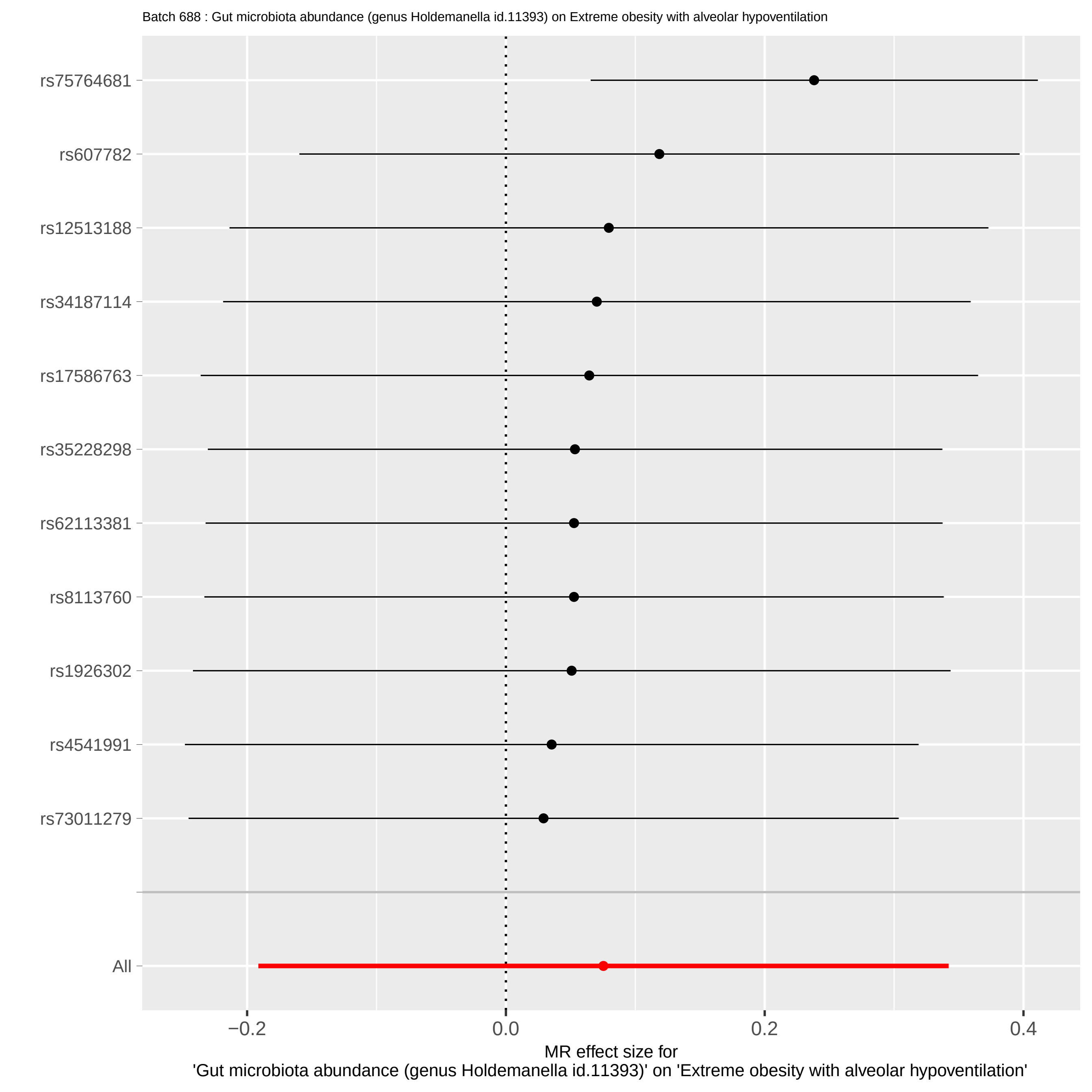

Batch 688 : Gut microbiota abundance (genus Holdemanella id.11393) on Extreme obesity with alveolar hypoventilation
rs75764681
rs607782
rs12513188
rs34187114
rs17586763
rs35228298
rs62113381
rs8113760
rs1926302
rs4541991
rs73011279
All
−0.2
0.0
0.2
0.4
MR effect size for
'Gut microbiota abundance (genus Holdemanella id.11393)' on 'Extreme obesity with alveolar hypoventilation'

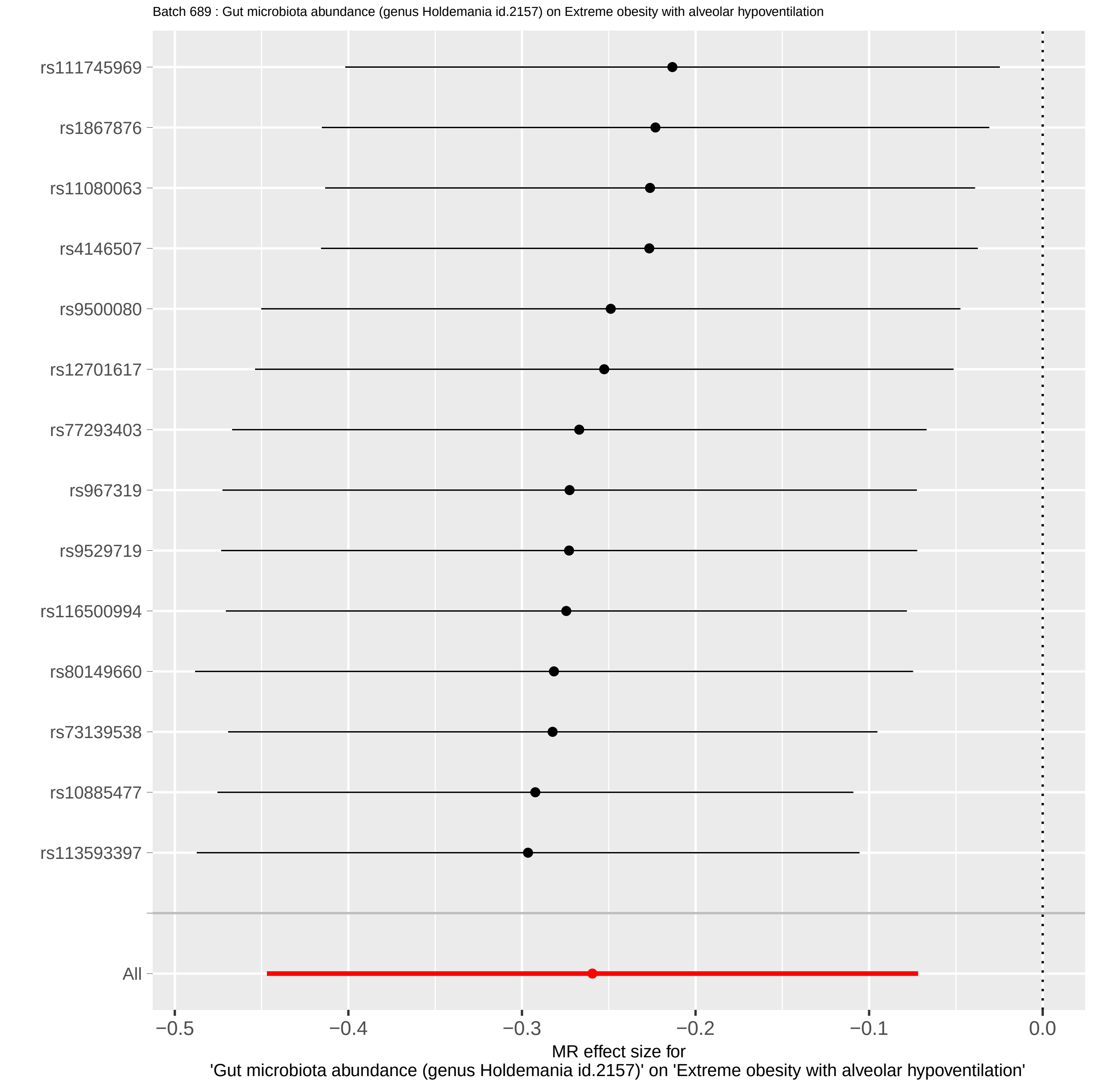

Batch 689 : Gut microbiota abundance (genus Holdemania id.2157) on Extreme obesity with alveolar hypoventilation
rs111745969
rs1867876
rs11080063
rs4146507
rs9500080
rs12701617
rs77293403
rs967319
rs9529719
rs116500994
rs80149660
rs73139538
rs10885477
rs113593397
All
−0.5
−0.4
−0.3
−0.2
−0.1
0.0
MR effect size for
'Gut microbiota abundance (genus Holdemania id.2157)' on 'Extreme obesity with alveolar hypoventilation'

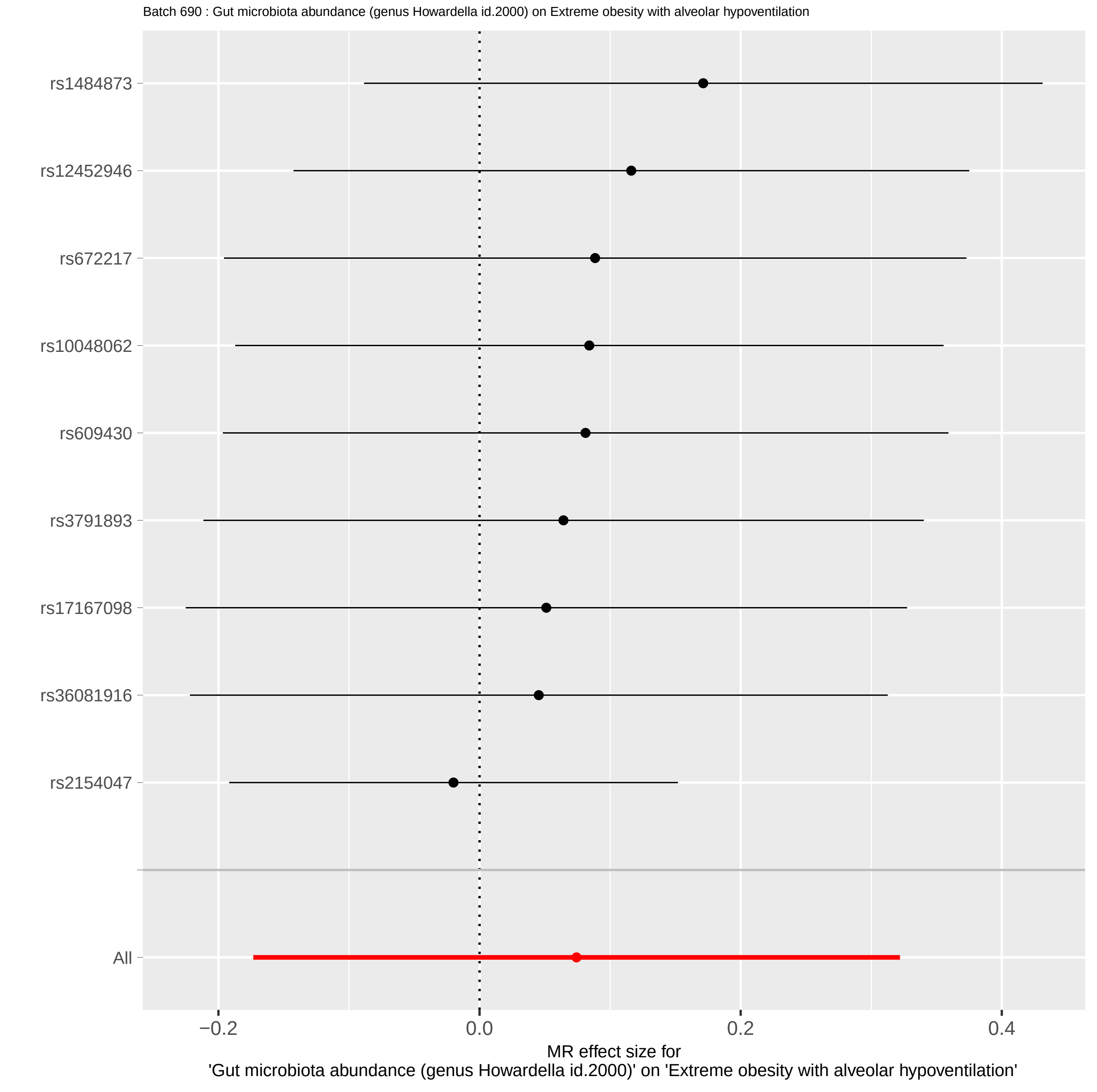
Batch 690 : Gut microbiota abundance (genus Howardella id.2000) on Extreme obesity with alveolar hypoventilation
rs1484873
rs12452946
rs672217
rs10048062
rs609430
rs3791893
rs17167098
rs36081916
rs2154047
All
−0.2
0.0
0.2
0.4
MR effect size for
'Gut microbiota abundance (genus Howardella id.2000)' on 'Extreme obesity with alveolar hypoventilation'

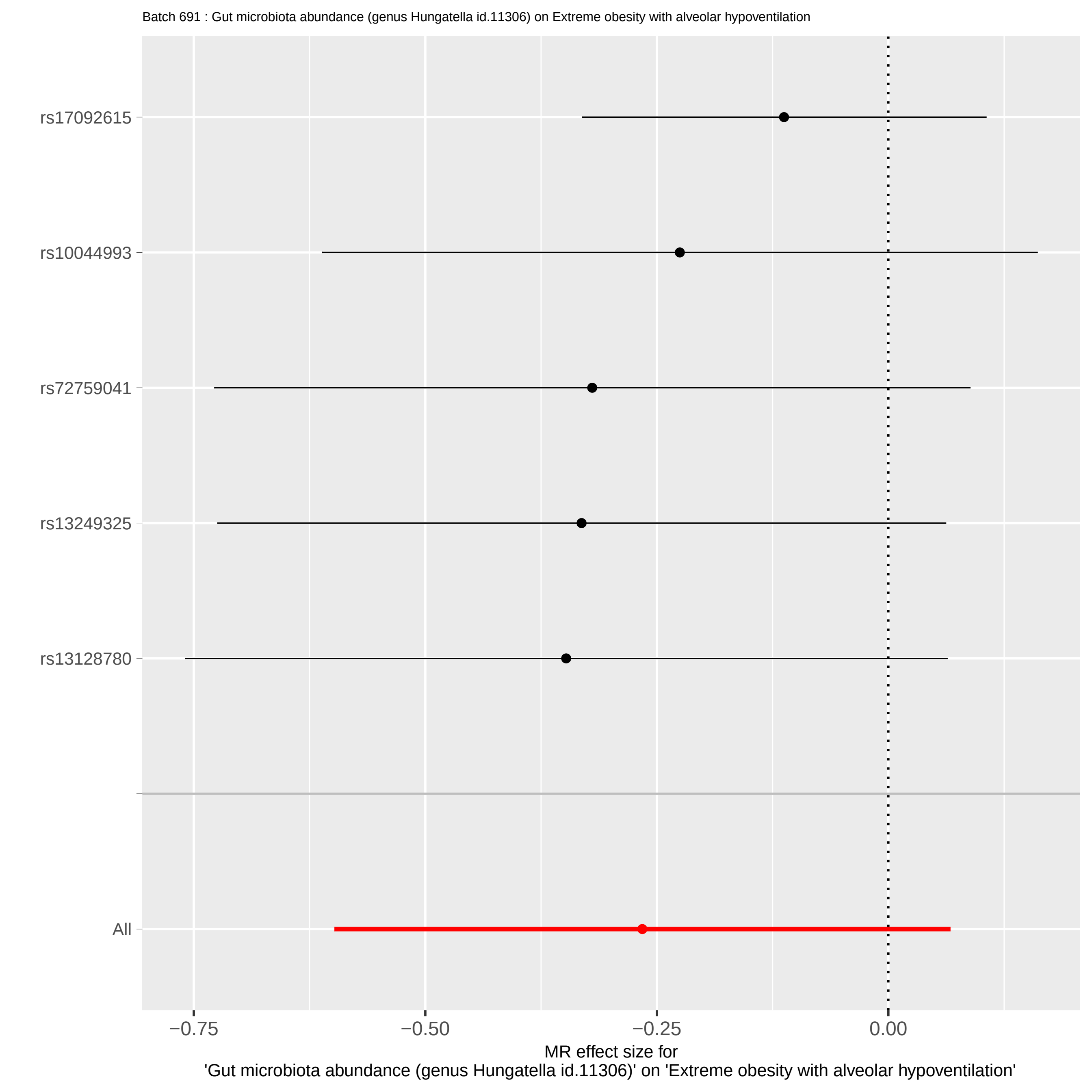

Batch 691 : Gut microbiota abundance (genus Hungatella id.11306) on Extreme obesity with alveolar hypoventilation
rs17092615
rs10044993
rs72759041
rs13249325
rs13128780
All
−0.75
−0.50
−0.25
0.00
MR effect size for
'Gut microbiota abundance (genus Hungatella id.11306)' on 'Extreme obesity with alveolar hypoventilation'

Batch 692 : Gut microbiota abundance (genus Intestinibacter id.11345) on Extreme obesity with alveolar hypoventilation

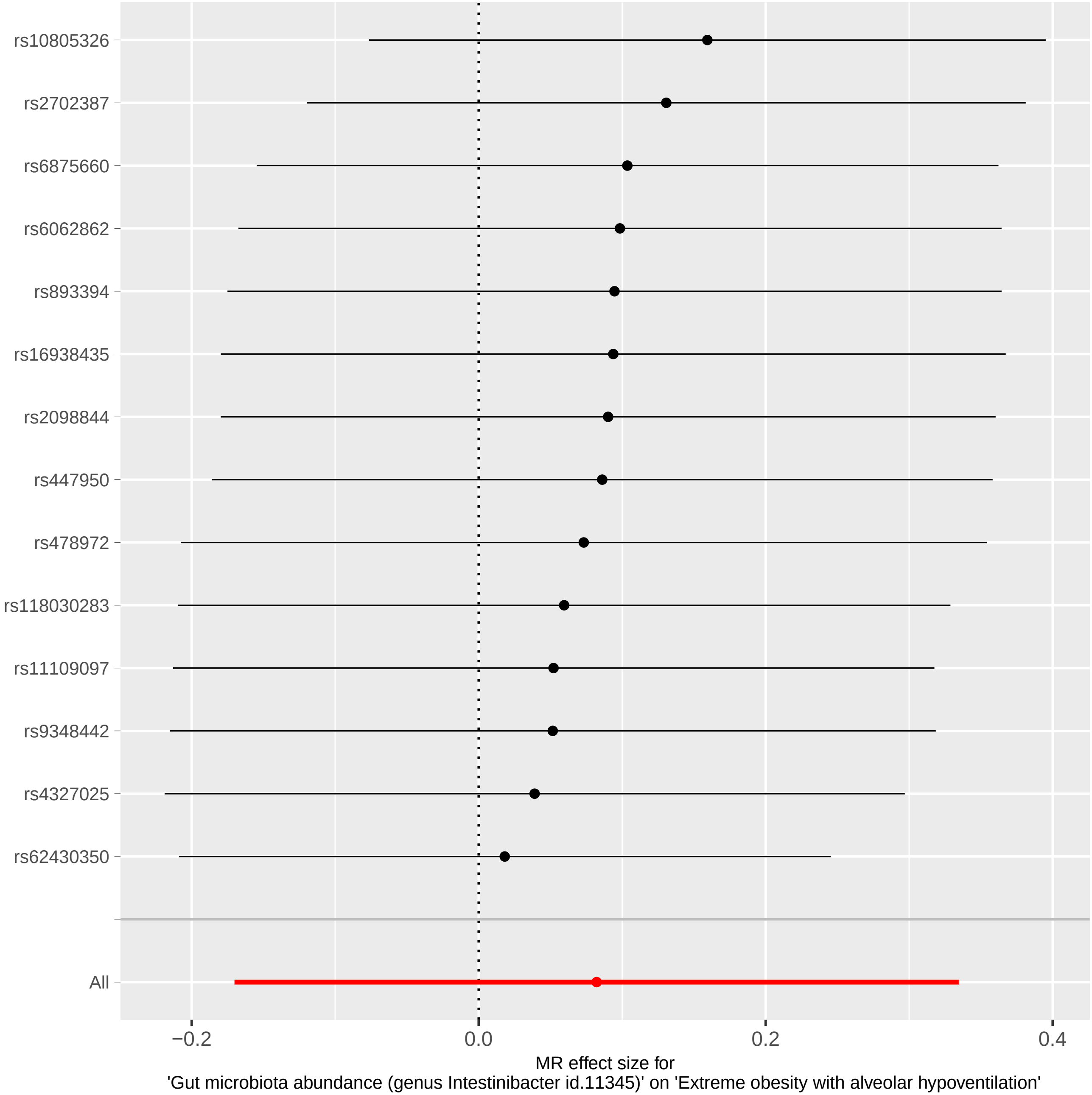

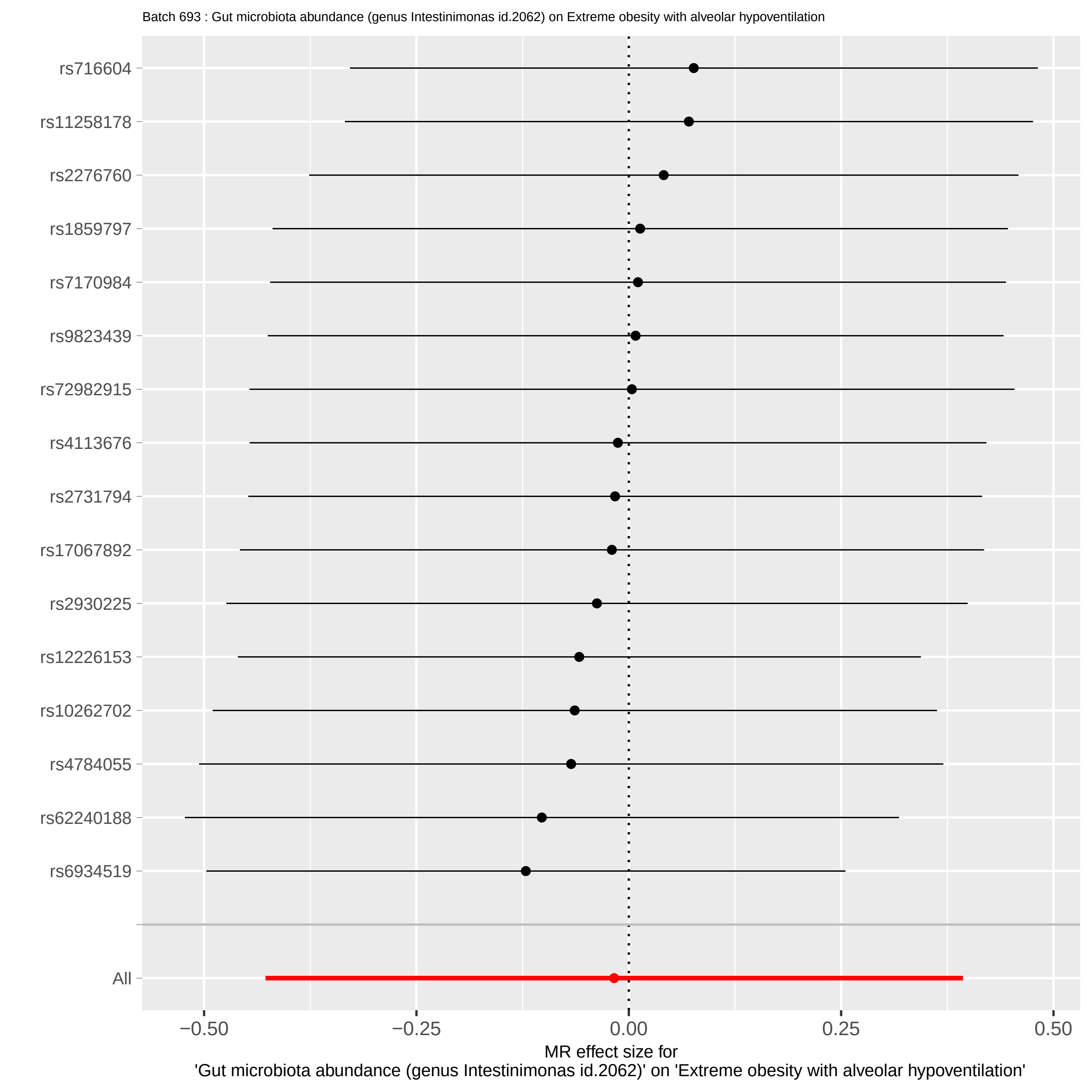

Batch 693 : Gut microbiota abundance (genus Intestinimonas id.2062) on Extreme obesity with alveolar hypoventilation
rs716604
rs11258178
rs2276760
rs1859797
rs7170984
rs9823439
rs72982915
rs4113676
rs2731794
rs17067892
rs2930225
rs12226153
rs10262702
rs4784055
rs62240188
rs6934519
All
−0.50
−0.25
0.00
0.25
0.50
MR effect size for
'Gut microbiota abundance (genus Intestinimonas id.2062)' on 'Extreme obesity with alveolar hypoventilation'

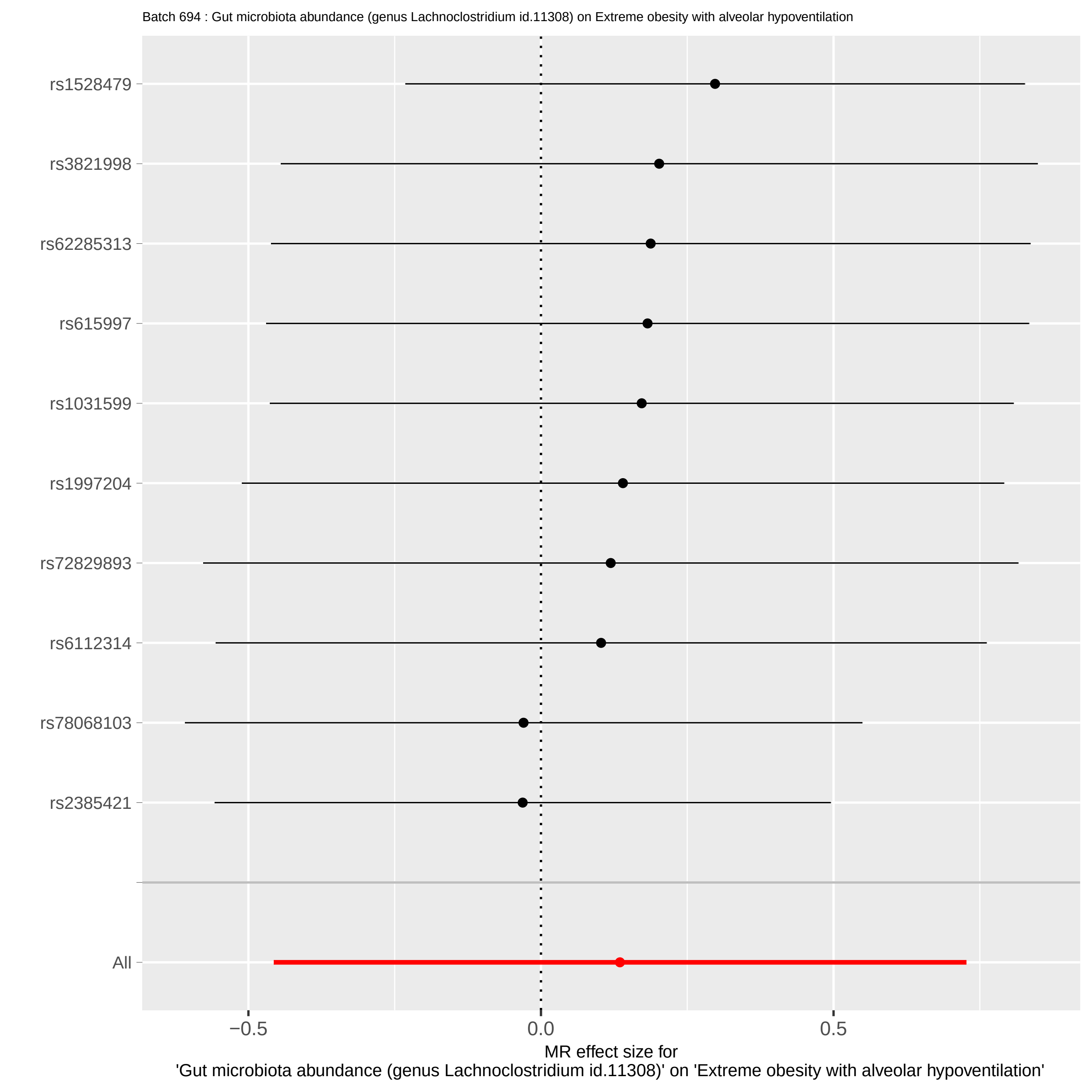

Batch 694 : Gut microbiota abundance (genus Lachnoclostridium id.11308) on Extreme obesity with alveolar hypoventilation
rs1528479
rs3821998
rs62285313
rs615997
rs1031599
rs1997204
rs72829893
rs6112314
rs78068103
rs2385421
All
−0.5
0.0
0.5
MR effect size for
'Gut microbiota abundance (genus Lachnoclostridium id.11308)' on 'Extreme obesity with alveolar hypoventilation'

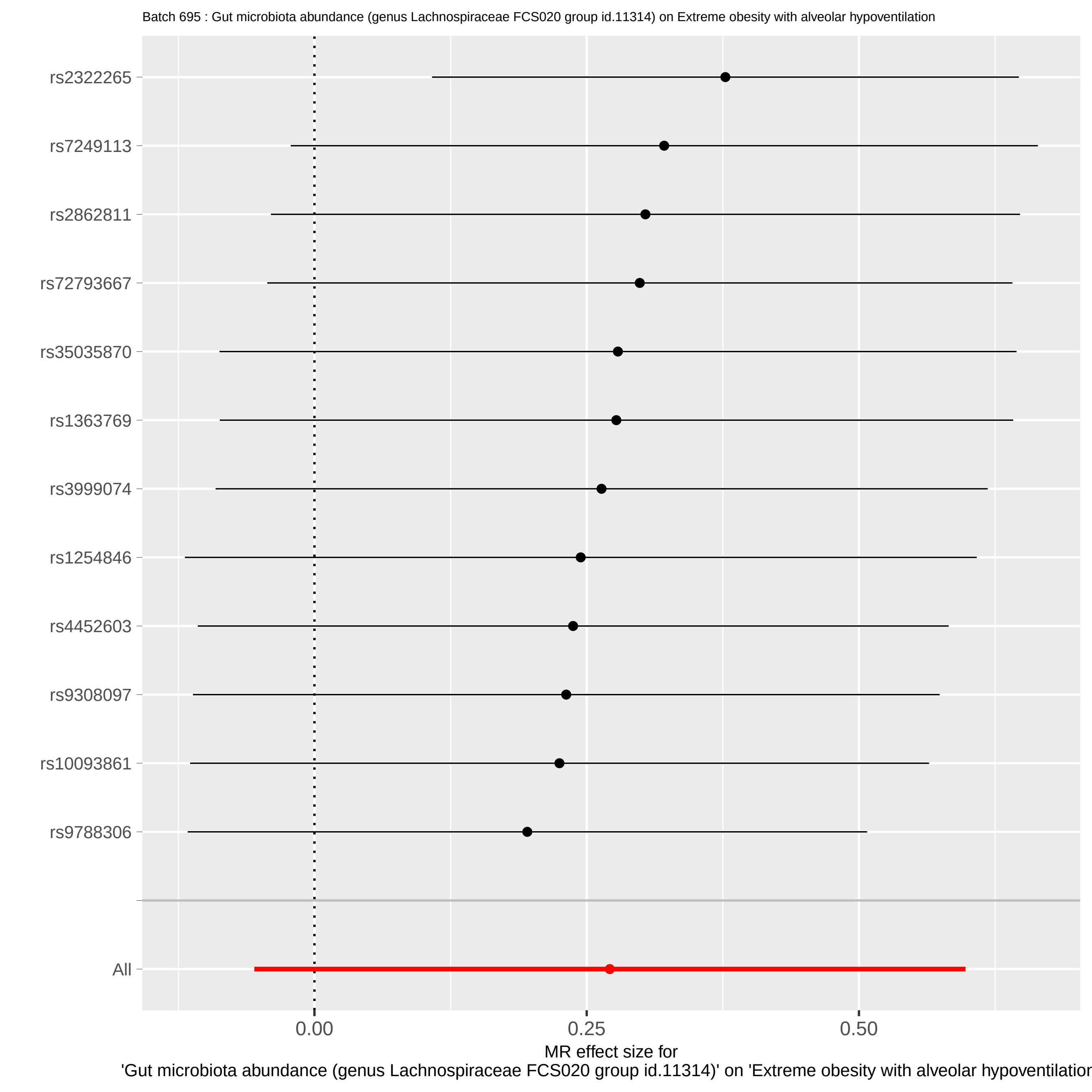
Batch 695 : Gut microbiota abundance (genus Lachnospiraceae FCS020 group id.11314) on Extreme obesity with alveolar hypoventilation
rs2322265
rs7249113
rs2862811
rs72793667
rs35035870
rs1363769
rs3999074
rs1254846
rs4452603
rs9308097
rs10093861
rs9788306
All
0.00
0.25
0.50
MR effect size for
'Gut microbiota abundance (genus Lachnospiraceae FCS020 group id.11314)' on 'Extreme obesity with alveolar hypoventilatio

Batch 696 : Gut microbiota abundance (genus Lachnospiraceae NC2004 group id.11316) on Extreme obesity with alveolar hypoventilation

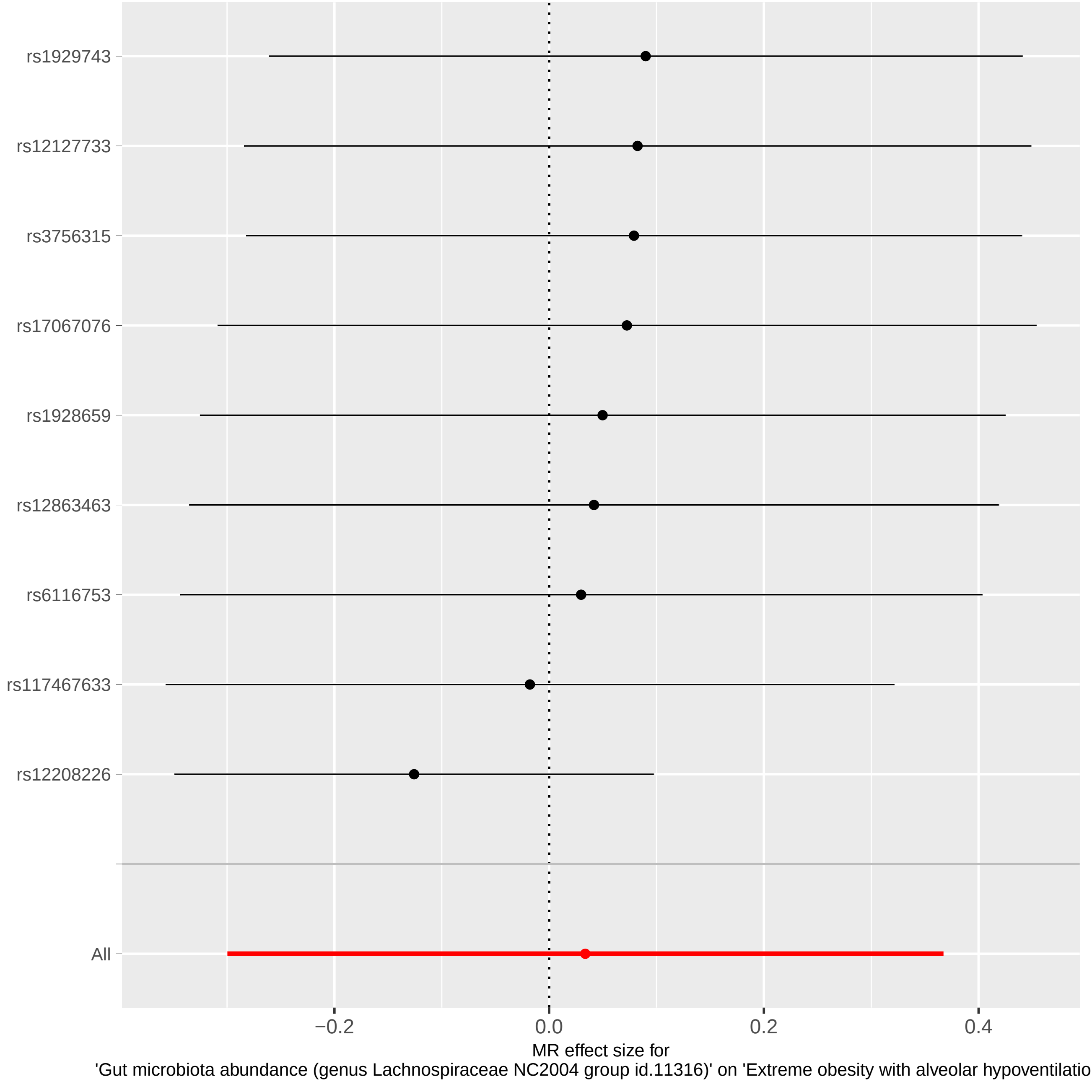

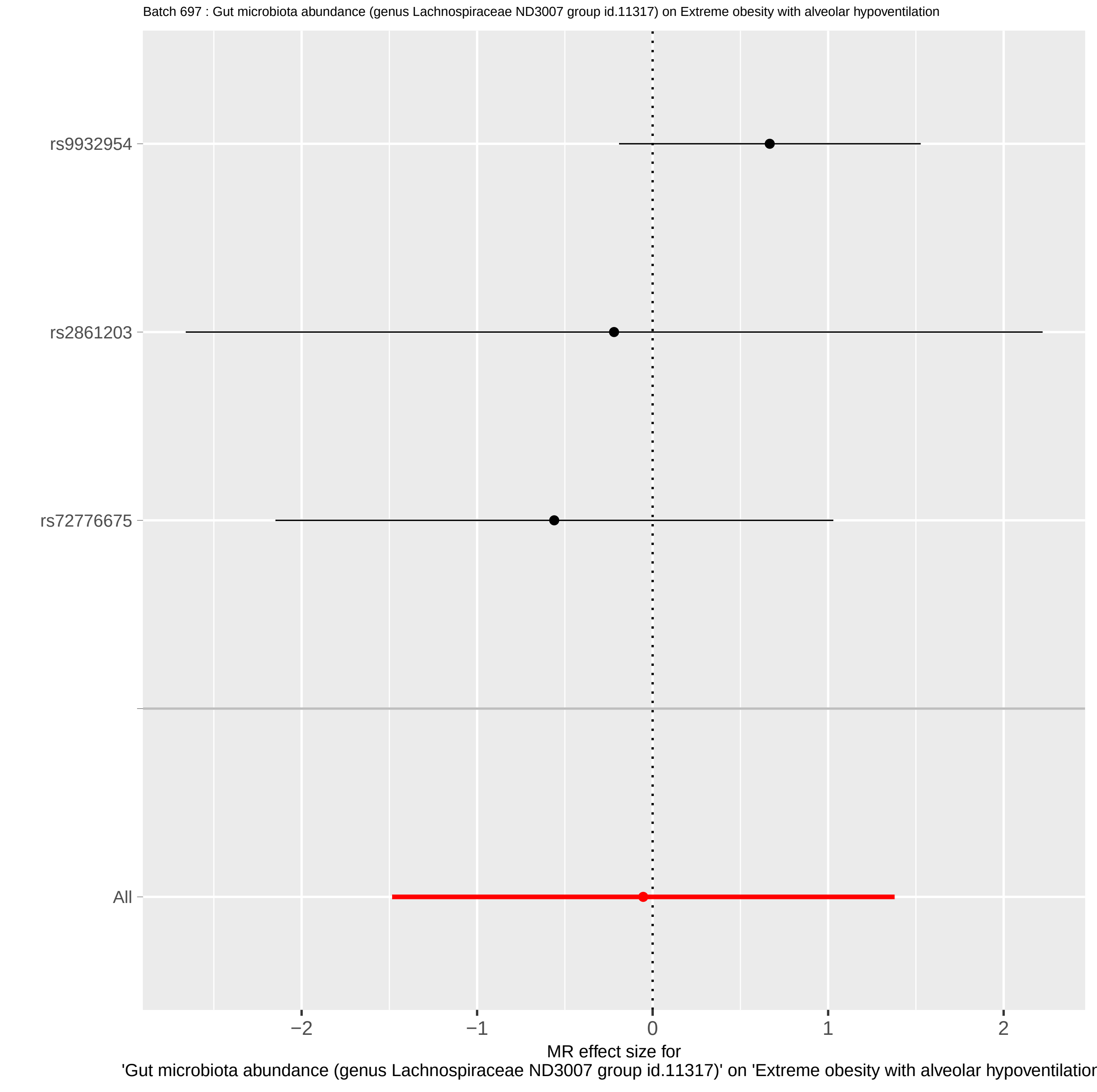

Batch 697 : Gut microbiota abundance (genus Lachnospiraceae ND3007 group id.11317) on Extreme obesity with alveolar hypoventilation
rs9932954
rs2861203
rs72776675
All
−2
−1
0
1
2
MR effect size for
'Gut microbiota abundance (genus Lachnospiraceae ND3007 group id.11317)' on 'Extreme obesity with alveolar hypoventilatio

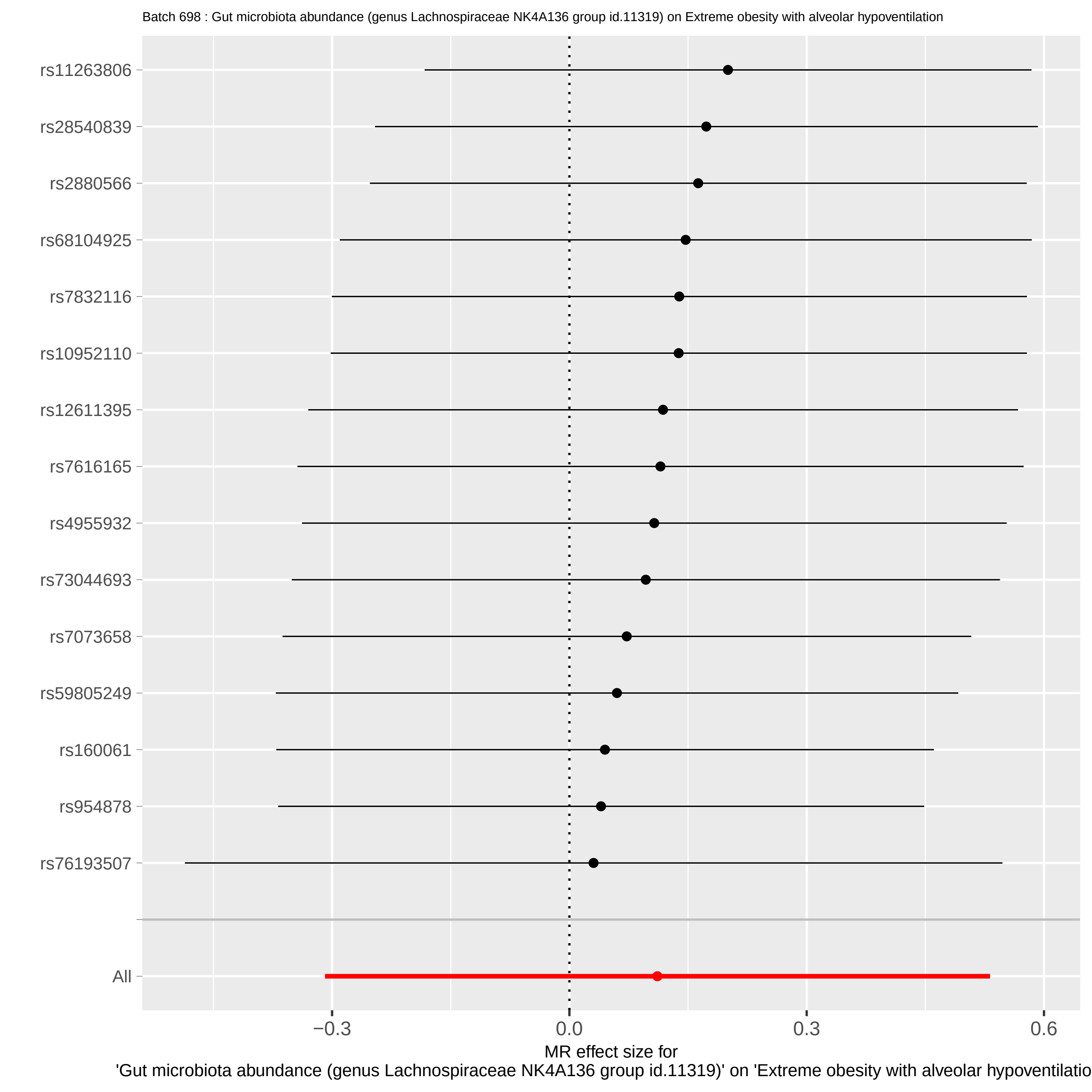

Batch 698 : Gut microbiota abundance (genus Lachnospiraceae NK4A136 group id.11319) on Extreme obesity with alveolar hypoventilation
rs11263806
rs28540839
rs2880566
rs68104925
rs7832116
rs10952110
rs12611395
rs7616165
rs4955932
rs73044693
rs7073658
rs59805249
rs160061
rs954878
rs76193507
All
−0.3
0.0
0.3
0.6
MR effect size for
'Gut microbiota abundance (genus Lachnospiraceae NK4A136 group id.11319)' on 'Extreme obesity with alveolar hypoventilatio

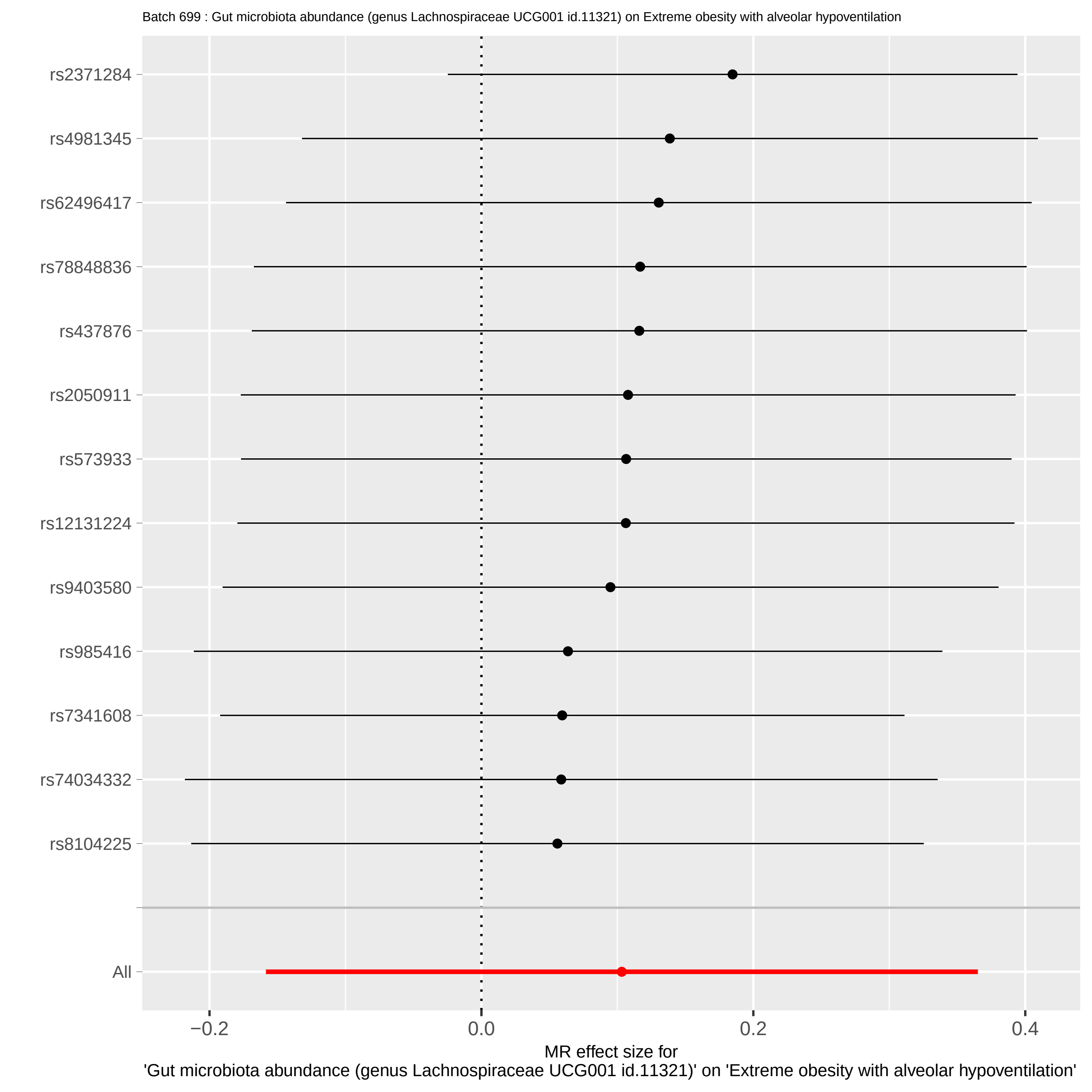

Batch 699 : Gut microbiota abundance (genus Lachnospiraceae UCG001 id.11321) on Extreme obesity with alveolar hypoventilation
rs2371284
rs4981345
rs62496417
rs78848836
rs437876
rs2050911
rs573933
rs12131224
rs9403580
rs985416
rs7341608
rs74034332
rs8104225
All
−0.2
0.0
0.2
0.4
MR effect size for
'Gut microbiota abundance (genus Lachnospiraceae UCG001 id.11321)' on 'Extreme obesity with alveolar hypoventilation'

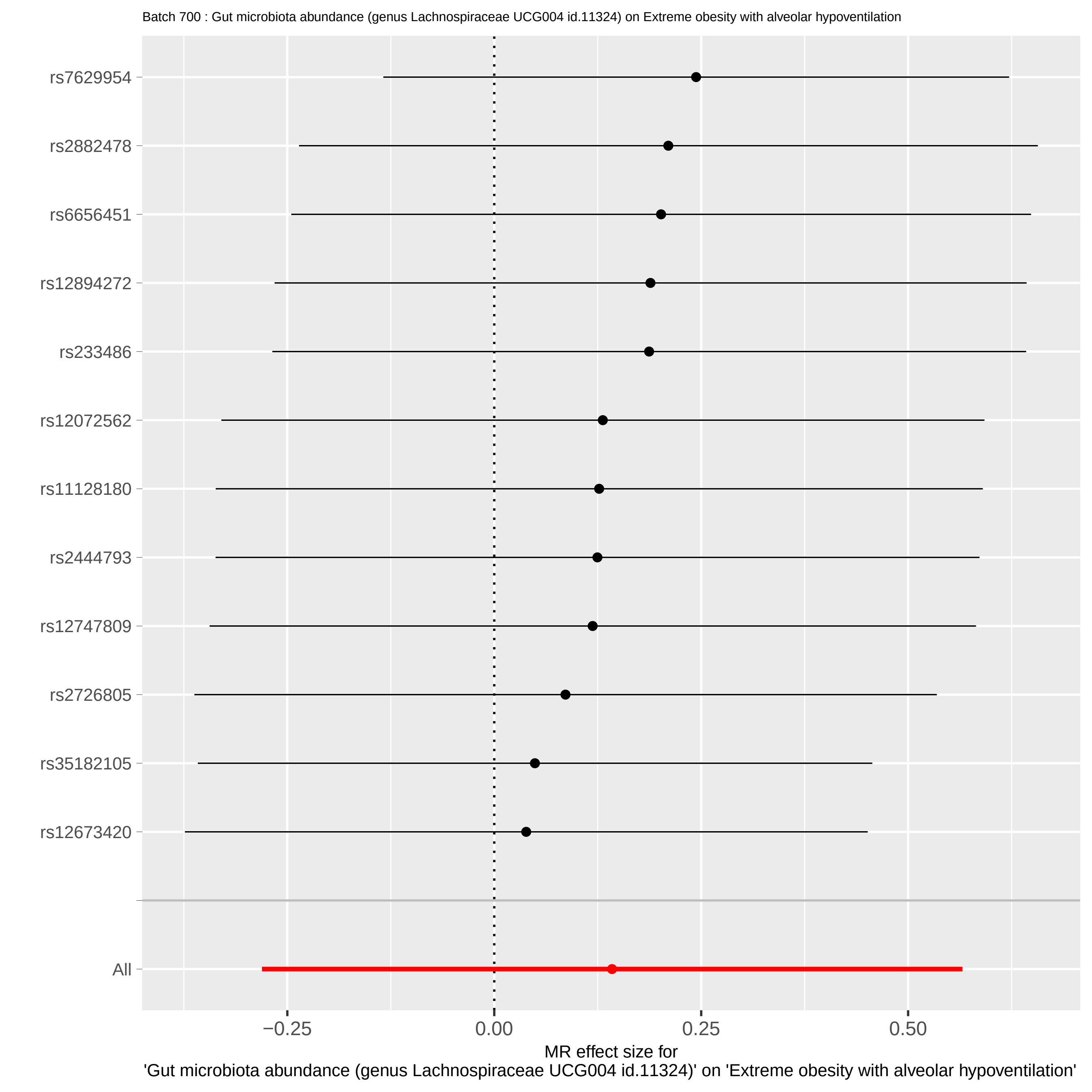

Batch 700 : Gut microbiota abundance (genus Lachnospiraceae UCG004 id.11324) on Extreme obesity with alveolar hypoventilation
rs7629954
rs2882478
rs6656451
rs12894272
rs233486
rs12072562
rs11128180
rs2444793
rs12747809
rs2726805
rs35182105
rs12673420
All
−0.25
0.00
0.25
0.50
MR effect size for
'Gut microbiota abundance (genus Lachnospiraceae UCG004 id.11324)' on 'Extreme obesity with alveolar hypoventilation'

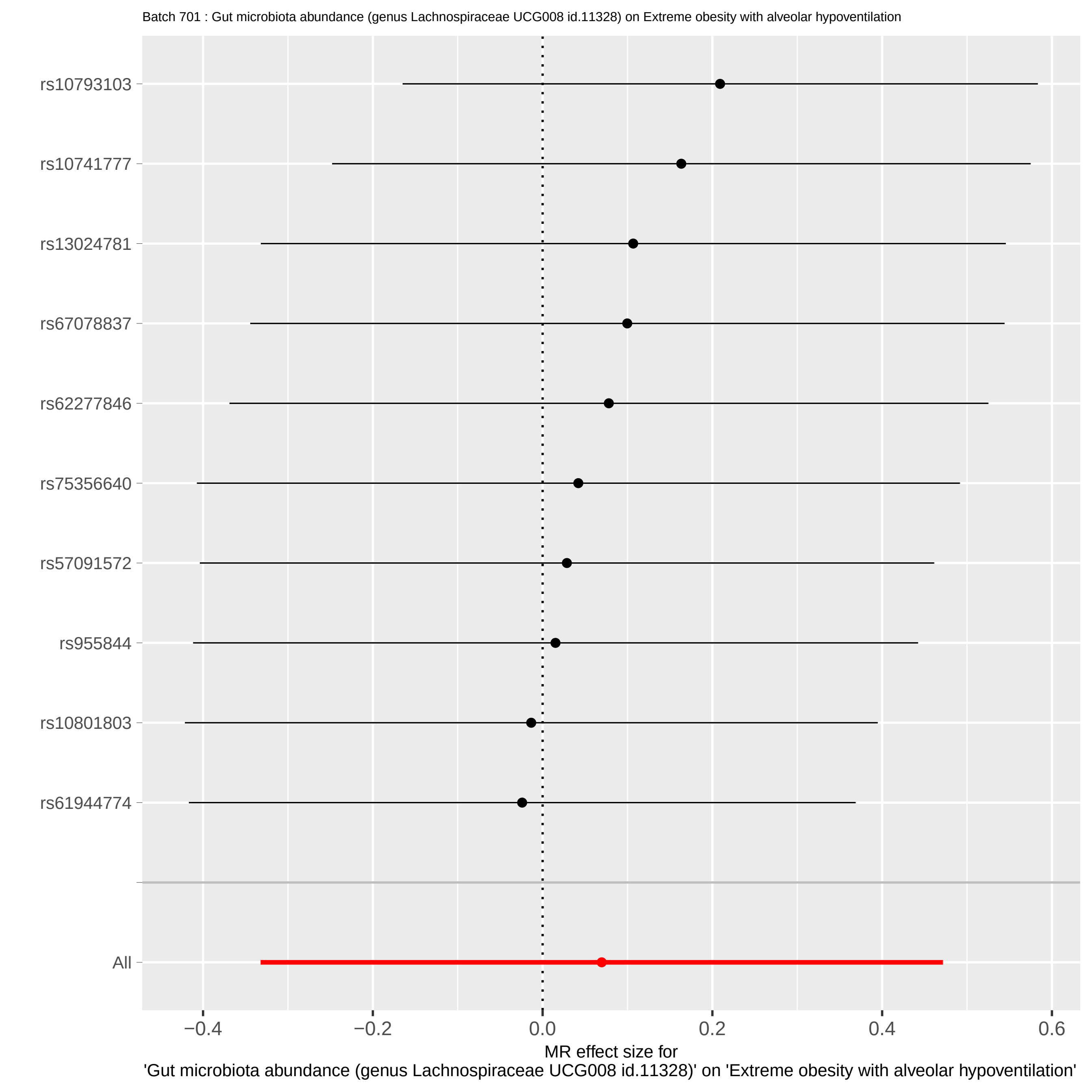

Batch 701 : Gut microbiota abundance (genus Lachnospiraceae UCG008 id.11328) on Extreme obesity with alveolar hypoventilation
rs10793103
rs10741777
rs13024781
rs67078837
rs62277846
rs75356640
rs57091572
rs955844
rs10801803
rs61944774
All
−0.4
−0.2
0.0
0.2
0.4
0.6
MR effect size for
'Gut microbiota abundance (genus Lachnospiraceae UCG008 id.11328)' on 'Extreme obesity with alveolar hypoventilation'

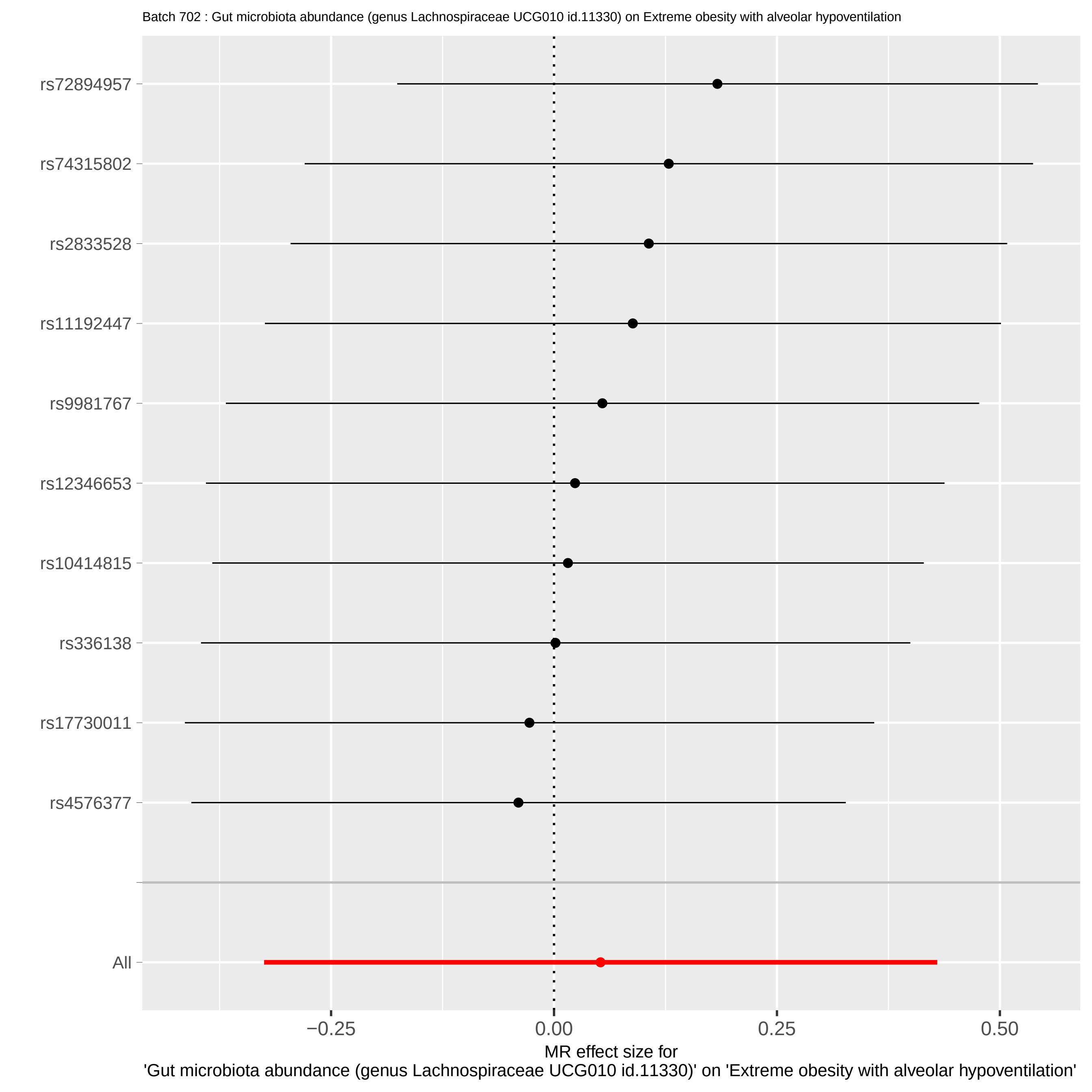
Batch 702 : Gut microbiota abundance (genus Lachnospiraceae UCG010 id.11330) on Extreme obesity with alveolar hypoventilation
rs72894957
rs74315802
rs2833528
rs11192447
rs9981767
rs12346653
rs10414815
rs336138
rs17730011
rs4576377
All
−0.25
0.00
0.25
0.50
MR effect size for
'Gut microbiota abundance (genus Lachnospiraceae UCG010 id.11330)' on 'Extreme obesity with alveolar hypoventilation'

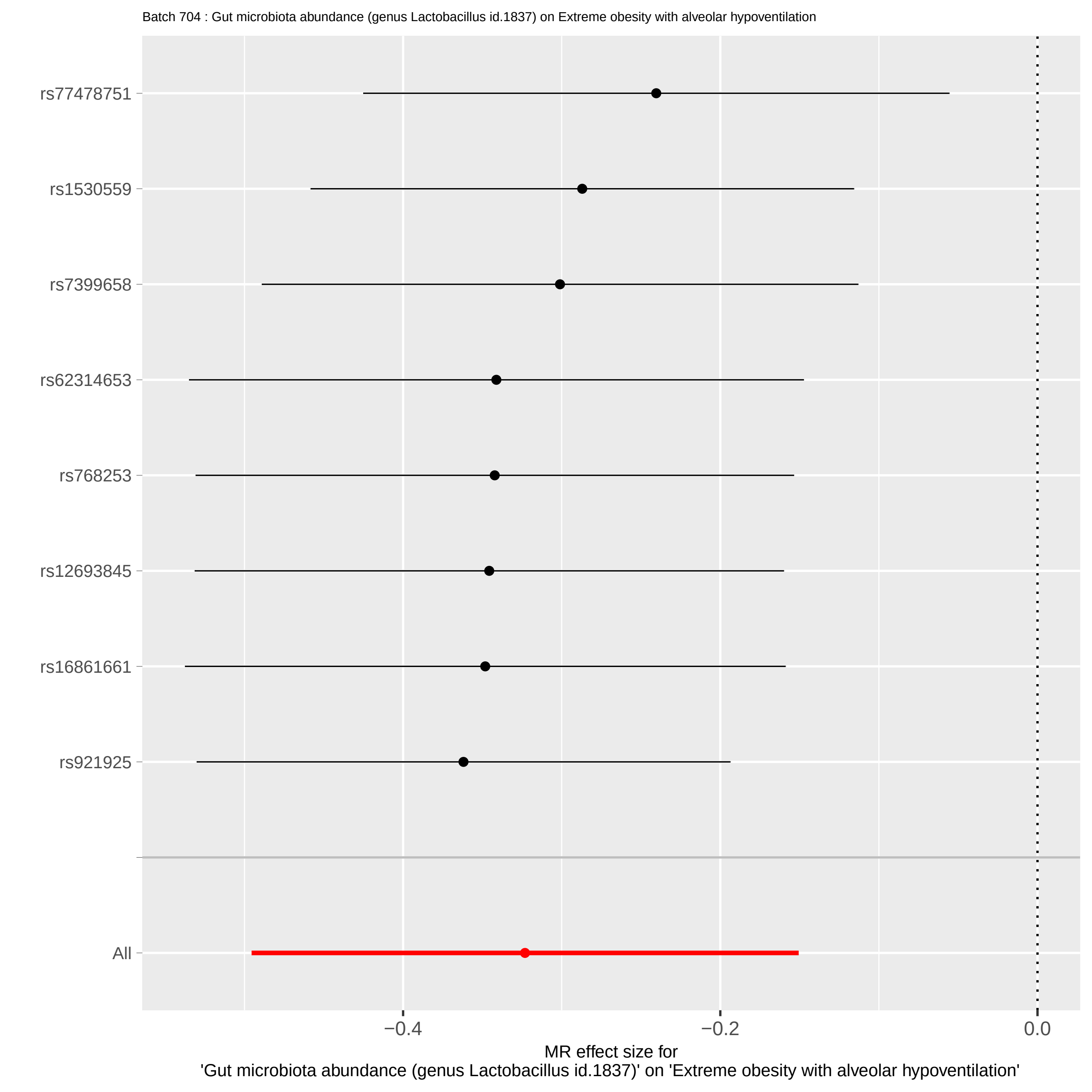

Batch 704 : Gut microbiota abundance (genus Lactobacillus id.1837) on Extreme obesity with alveolar hypoventilation
rs77478751
rs1530559
rs7399658
rs62314653
rs768253
rs12693845
rs16861661
rs921925
All
−0.4
−0.2
0.0
MR effect size for
'Gut microbiota abundance (genus Lactobacillus id.1837)' on 'Extreme obesity with alveolar hypoventilation'

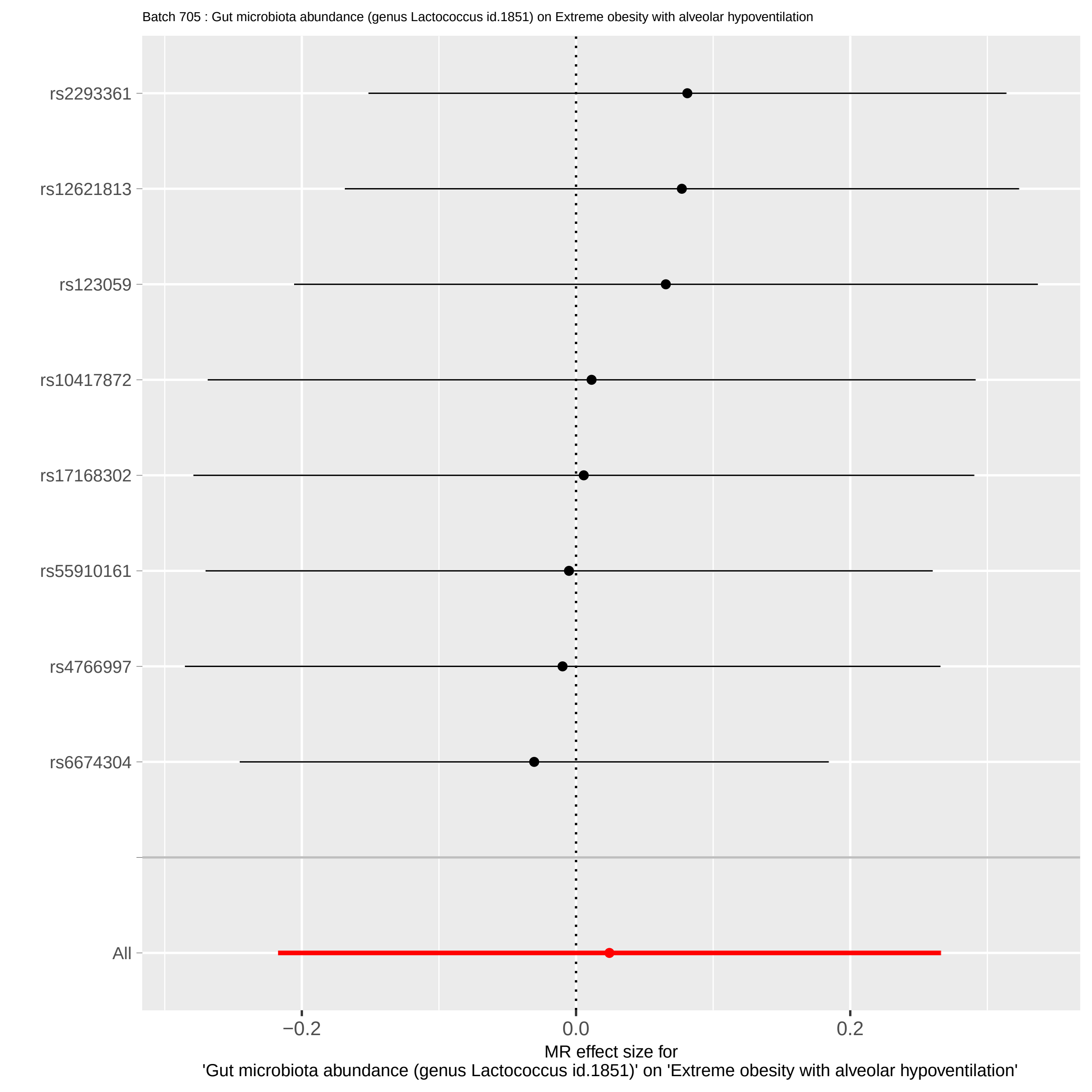

Batch 705 : Gut microbiota abundance (genus Lactococcus id.1851) on Extreme obesity with alveolar hypoventilation
rs2293361
rs12621813
rs123059
rs10417872
rs17168302
rs55910161
rs4766997
rs6674304
All
−0.2
0.0
0.2
MR effect size for
'Gut microbiota abundance (genus Lactococcus id.1851)' on 'Extreme obesity with alveolar hypoventilation'

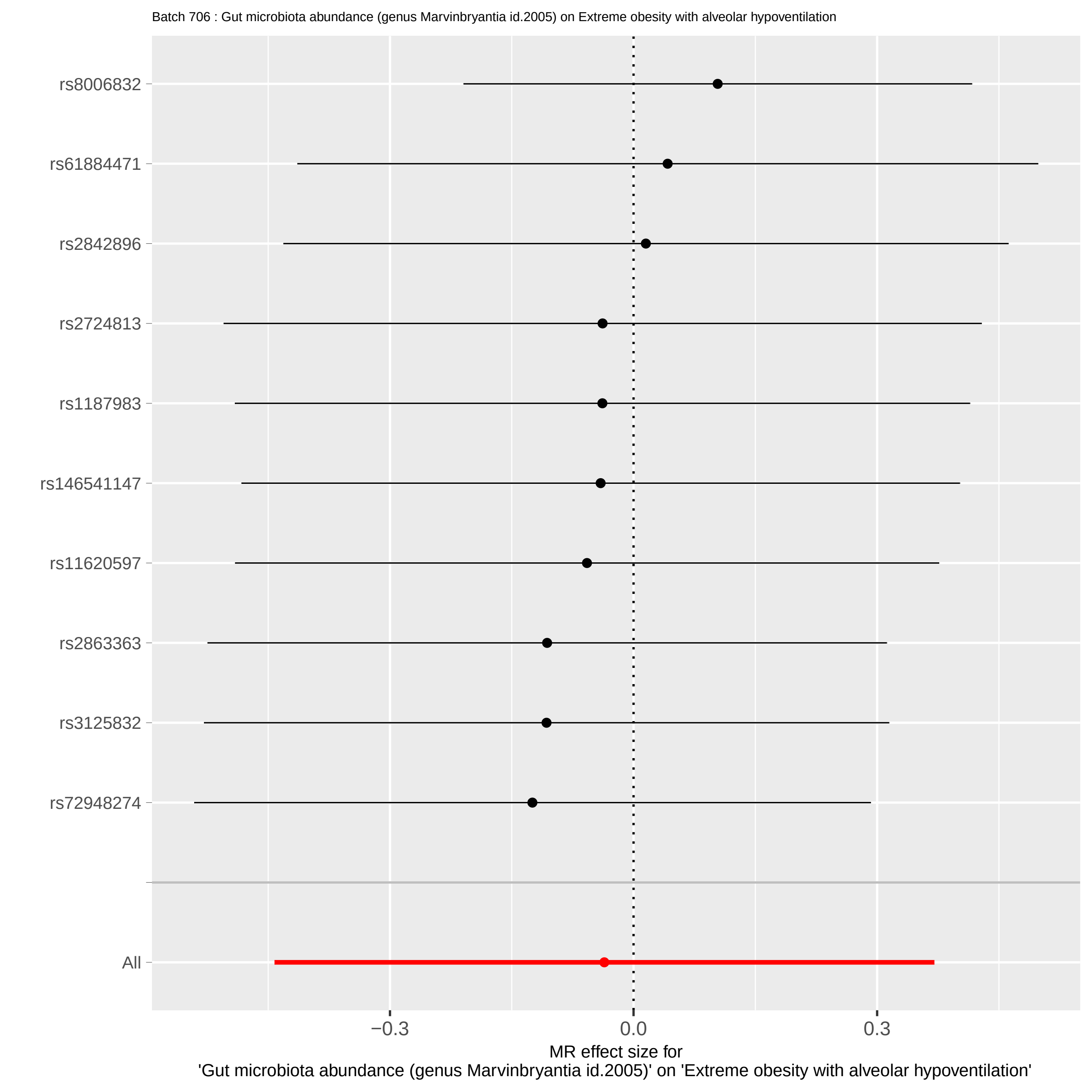

Batch 706 : Gut microbiota abundance (genus Marvinbryantia id.2005) on Extreme obesity with alveolar hypoventilation
rs8006832
rs61884471
rs2842896
rs2724813
rs1187983
rs146541147
rs11620597
rs2863363
rs3125832
rs72948274
All
−0.3
0.0
0.3
MR effect size for
'Gut microbiota abundance (genus Marvinbryantia id.2005)' on 'Extreme obesity with alveolar hypoventilation'

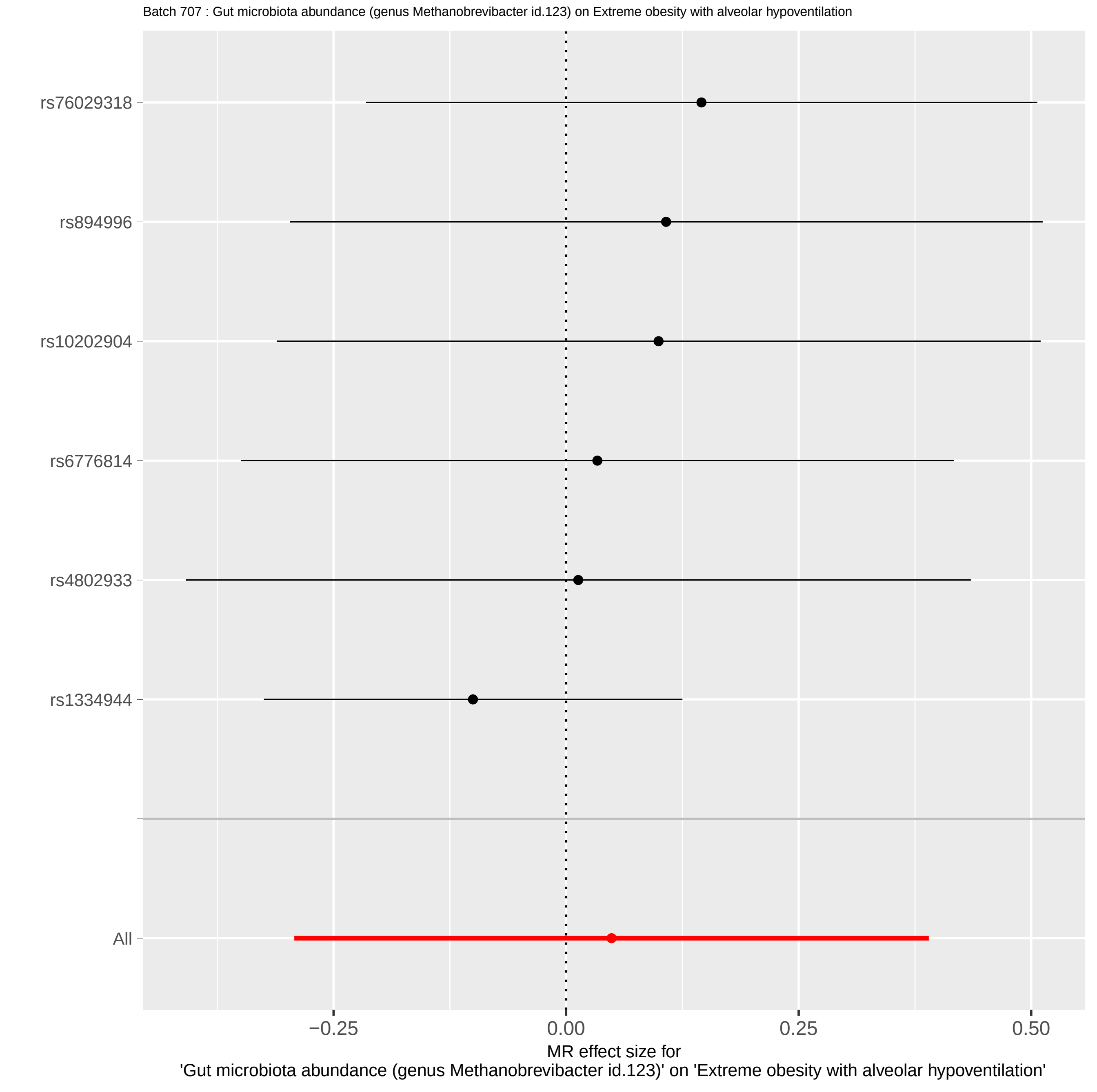

Batch 707 : Gut microbiota abundance (genus Methanobrevibacter id.123) on Extreme obesity with alveolar hypoventilation
rs76029318
rs894996
rs10202904
rs6776814
rs4802933
rs1334944
All
−0.25
0.00
0.25
0.50
MR effect size for
'Gut microbiota abundance (genus Methanobrevibacter id.123)' on 'Extreme obesity with alveolar hypoventilation'

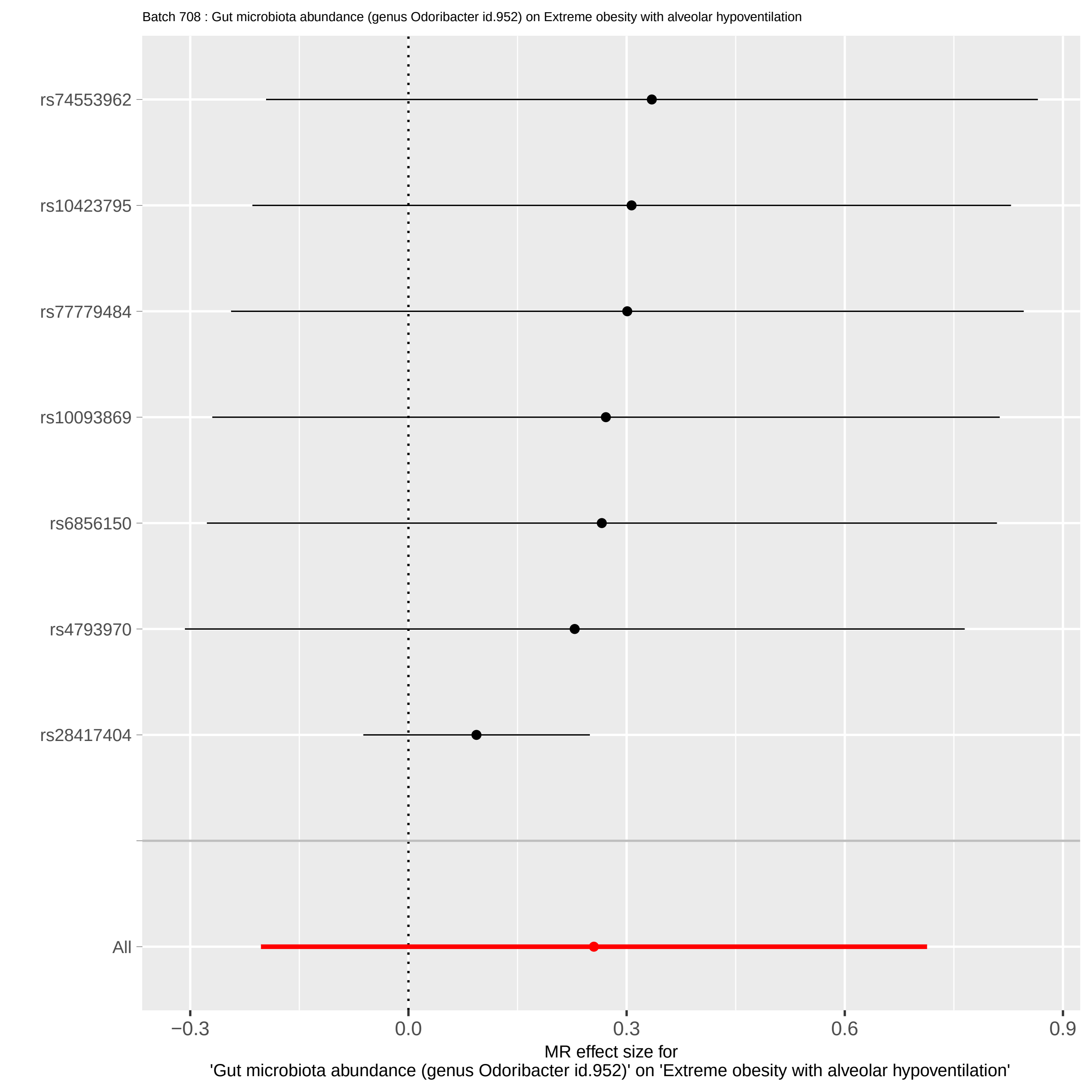
Batch 708 : Gut microbiota abundance (genus Odoribacter id.952) on Extreme obesity with alveolar hypoventilation
rs74553962
rs10423795
rs77779484
rs10093869
rs6856150
rs4793970
rs28417404
All
−0.3
0.0
0.3
0.6
0.9
MR effect size for
'Gut microbiota abundance (genus Odoribacter id.952)' on 'Extreme obesity with alveolar hypoventilation'

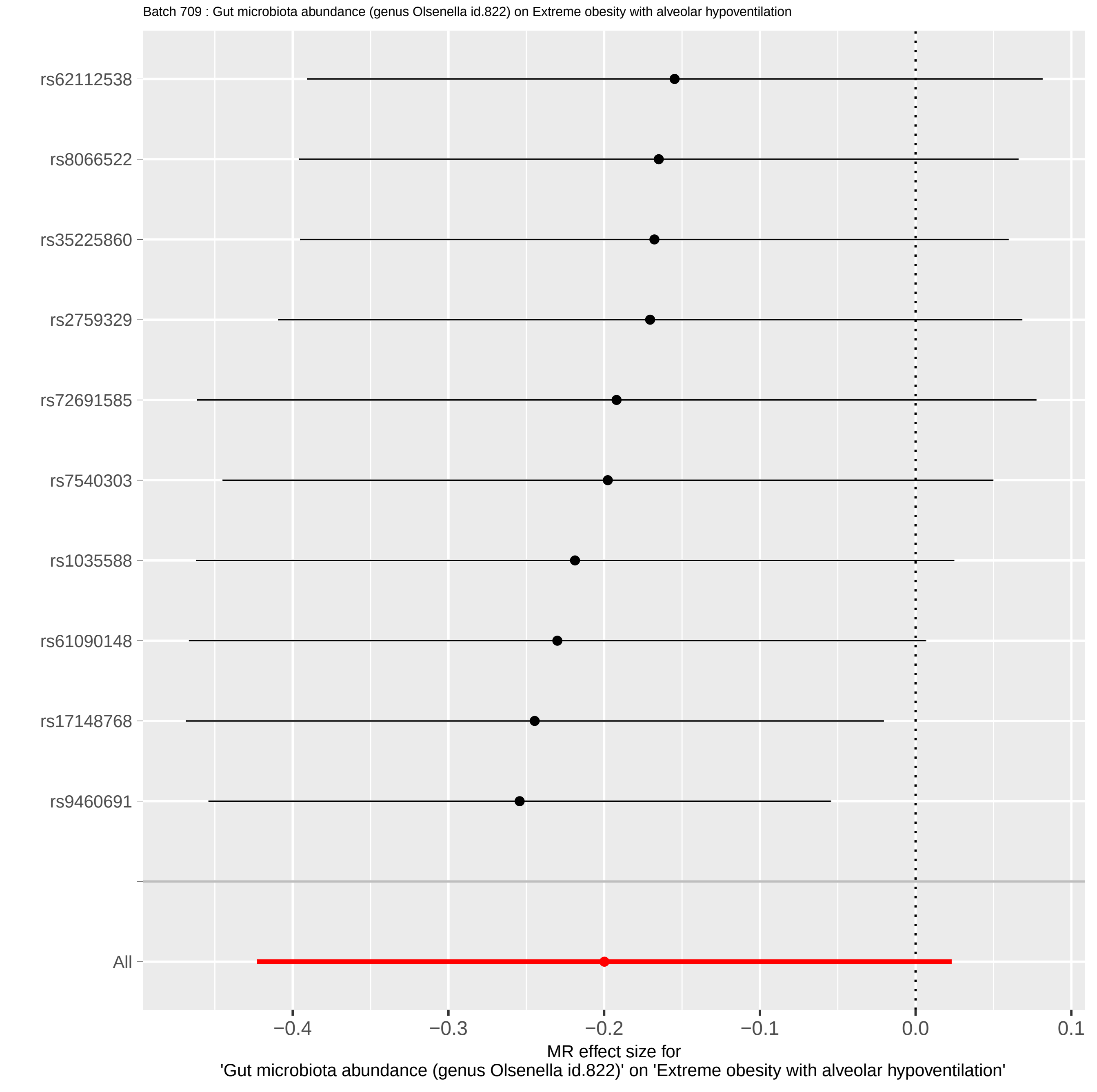
Batch 709 : Gut microbiota abundance (genus Olsenella id.822) on Extreme obesity with alveolar hypoventilation
rs62112538
rs8066522
rs35225860
rs2759329
rs72691585
rs7540303
rs1035588
rs61090148
rs17148768
rs9460691
All
−0.4
−0.3
−0.2
−0.1
0.0
0.1
MR effect size for
'Gut microbiota abundance (genus Olsenella id.822)' on 'Extreme obesity with alveolar hypoventilation'

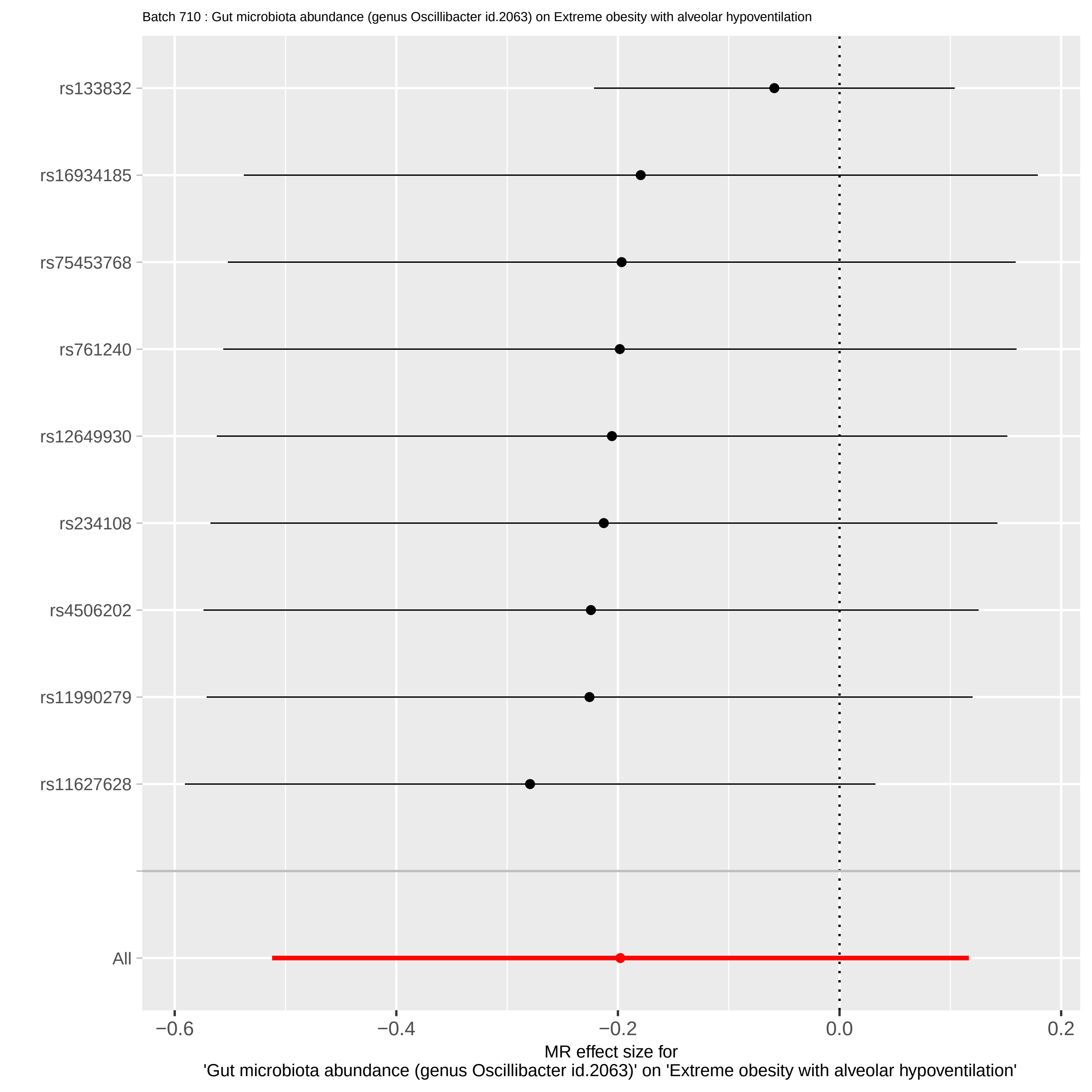

Batch 710 : Gut microbiota abundance (genus Oscillibacter id.2063) on Extreme obesity with alveolar hypoventilation
rs133832
rs16934185
rs75453768
rs761240
rs12649930
rs234108
rs4506202
rs11990279
rs11627628
All
−0.6
−0.4
−0.2
0.0
0.2
MR effect size for
'Gut microbiota abundance (genus Oscillibacter id.2063)' on 'Extreme obesity with alveolar hypoventilation'

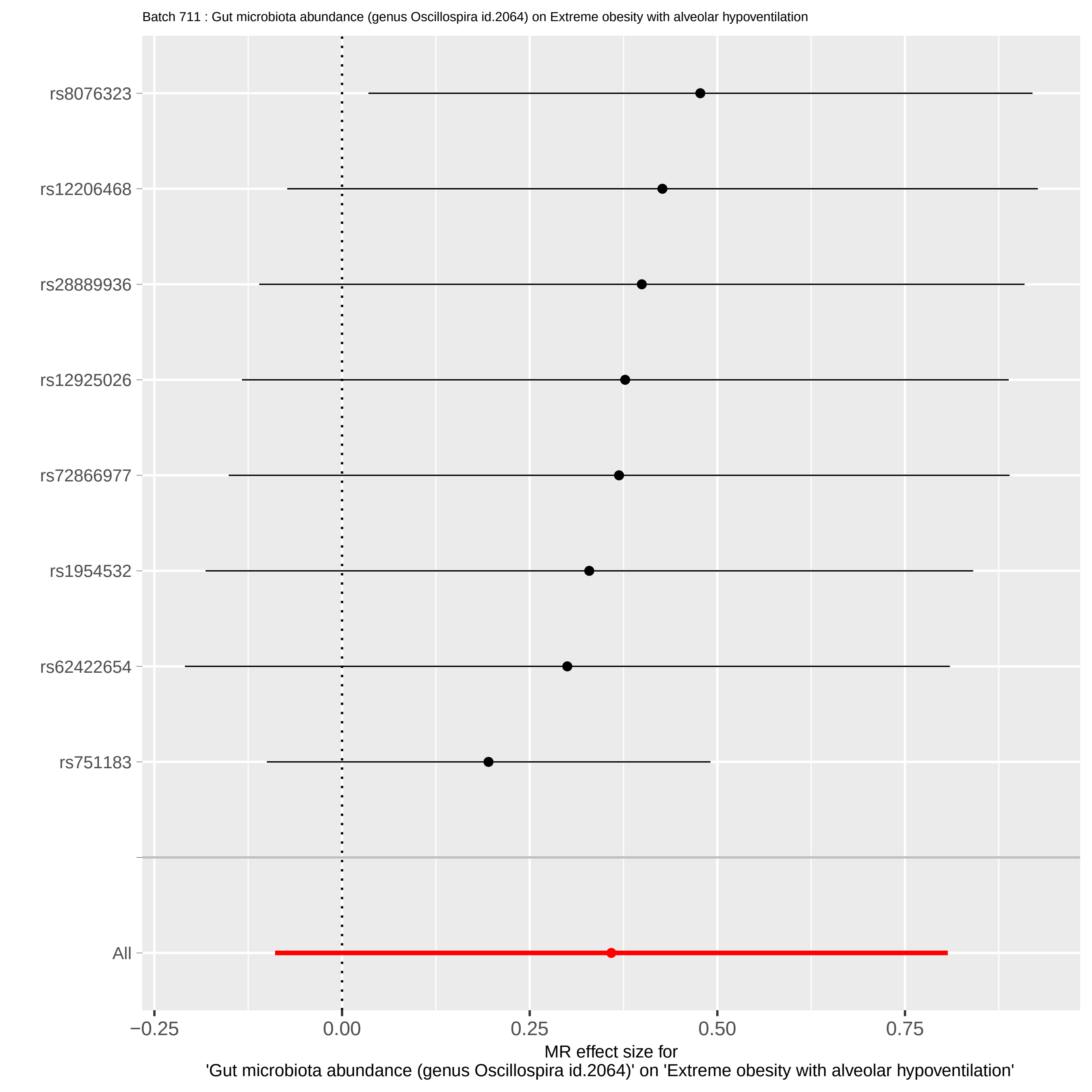
Batch 711 : Gut microbiota abundance (genus Oscillospira id.2064) on Extreme obesity with alveolar hypoventilation
rs8076323
rs12206468
rs28889936
rs12925026
rs72866977
rs1954532
rs62422654
rs751183
All
−0.25
0.00
0.25
0.50
0.75
MR effect size for
'Gut microbiota abundance (genus Oscillospira id.2064)' on 'Extreme obesity with alveolar hypoventilation'

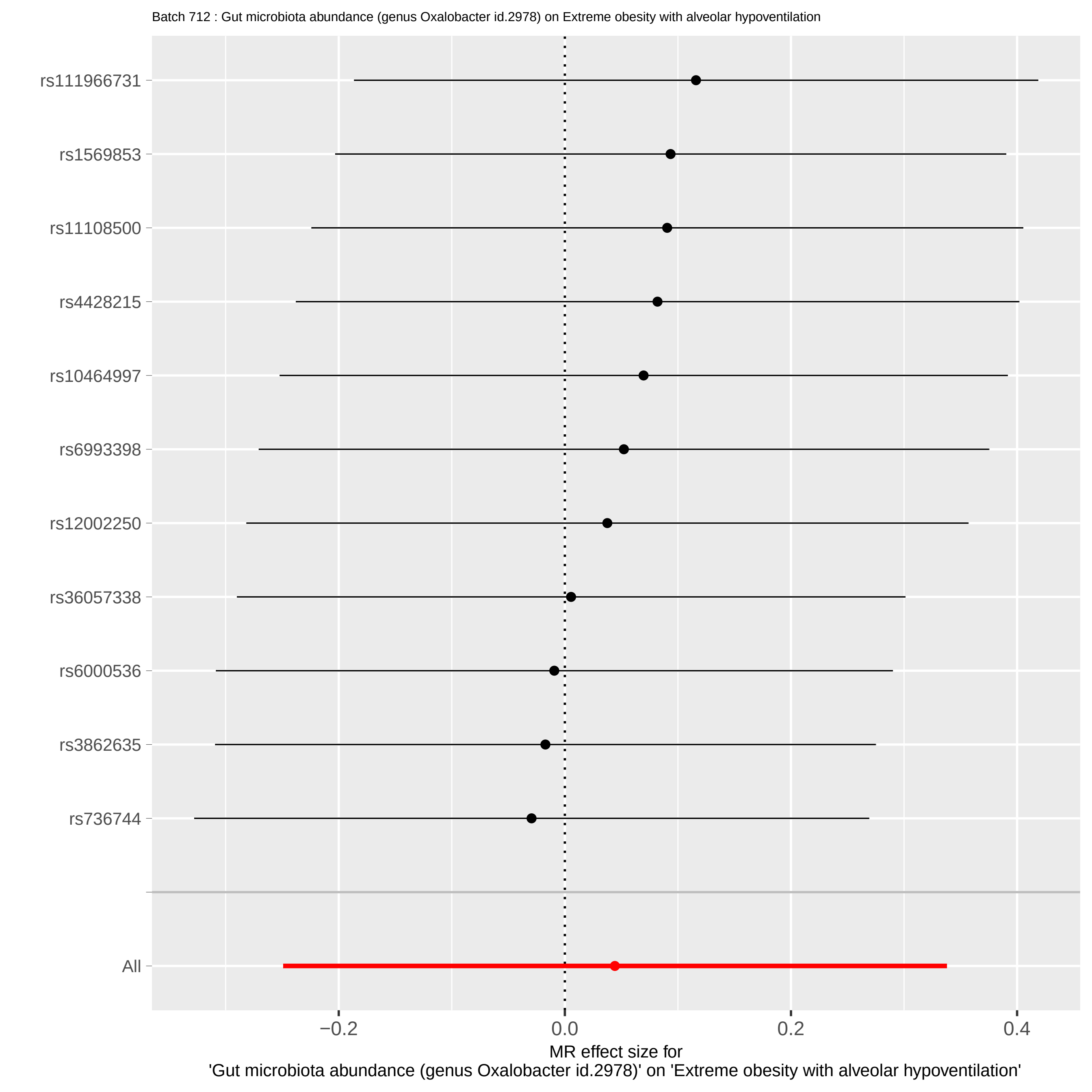

Batch 712 : Gut microbiota abundance (genus Oxalobacter id.2978) on Extreme obesity with alveolar hypoventilation
rs111966731
rs1569853
rs11108500
rs4428215
rs10464997
rs6993398
rs12002250
rs36057338
rs6000536
rs3862635
rs736744
All
−0.2
0.0
0.2
0.4
MR effect size for
'Gut microbiota abundance (genus Oxalobacter id.2978)' on 'Extreme obesity with alveolar hypoventilation'

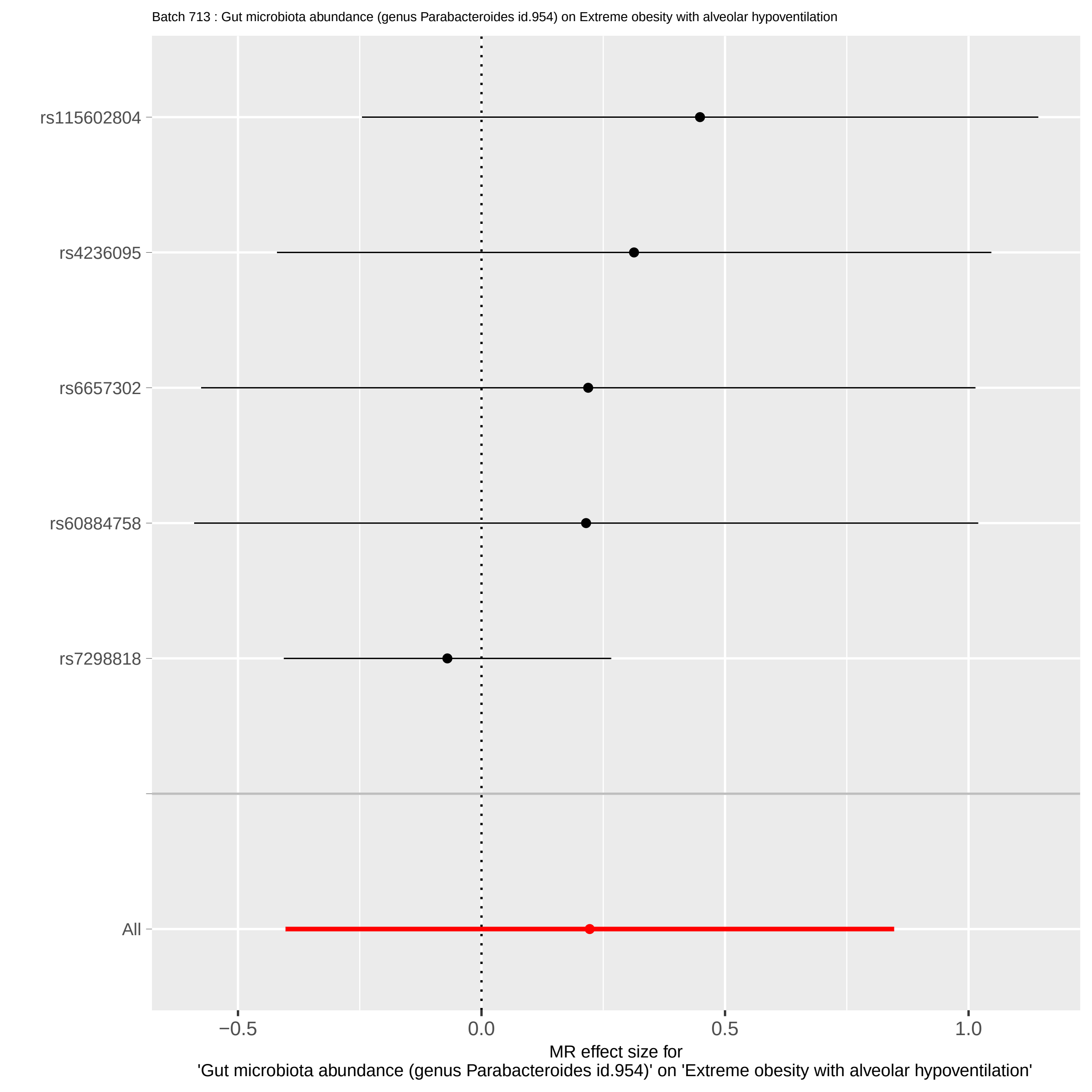

Batch 713 : Gut microbiota abundance (genus Parabacteroides id.954) on Extreme obesity with alveolar hypoventilation
rs115602804
rs4236095
rs6657302
rs60884758
rs7298818
All
−0.5
0.0
0.5
1.0
MR effect size for
'Gut microbiota abundance (genus Parabacteroides id.954)' on 'Extreme obesity with alveolar hypoventilation'

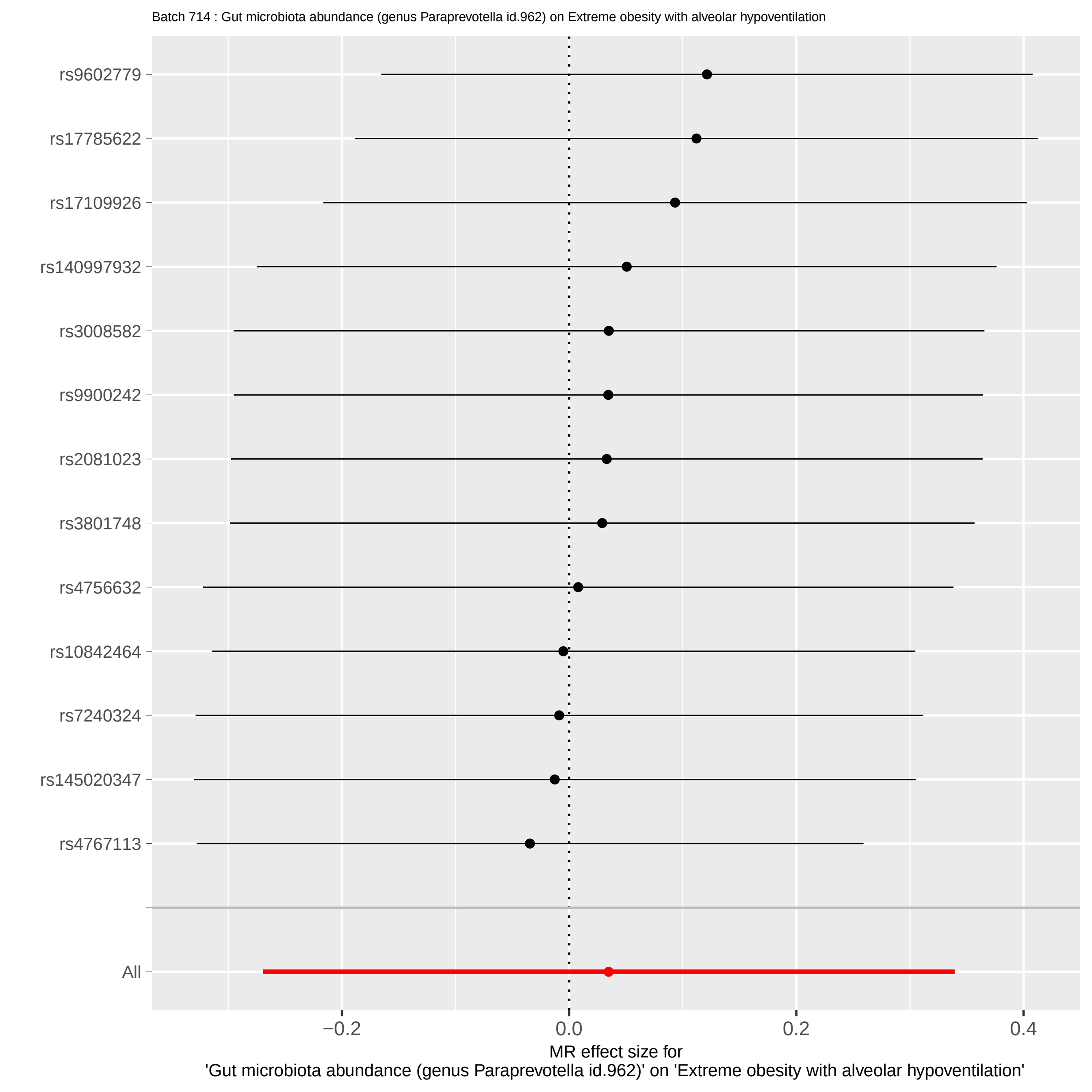

Batch 714 : Gut microbiota abundance (genus Paraprevotella id.962) on Extreme obesity with alveolar hypoventilation
rs9602779
rs17785622
rs17109926
rs140997932
rs3008582
rs9900242
rs2081023
rs3801748
rs4756632
rs10842464
rs7240324
rs145020347
rs4767113
All
−0.2
0.0
0.2
0.4
MR effect size for
'Gut microbiota abundance (genus Paraprevotella id.962)' on 'Extreme obesity with alveolar hypoventilation'

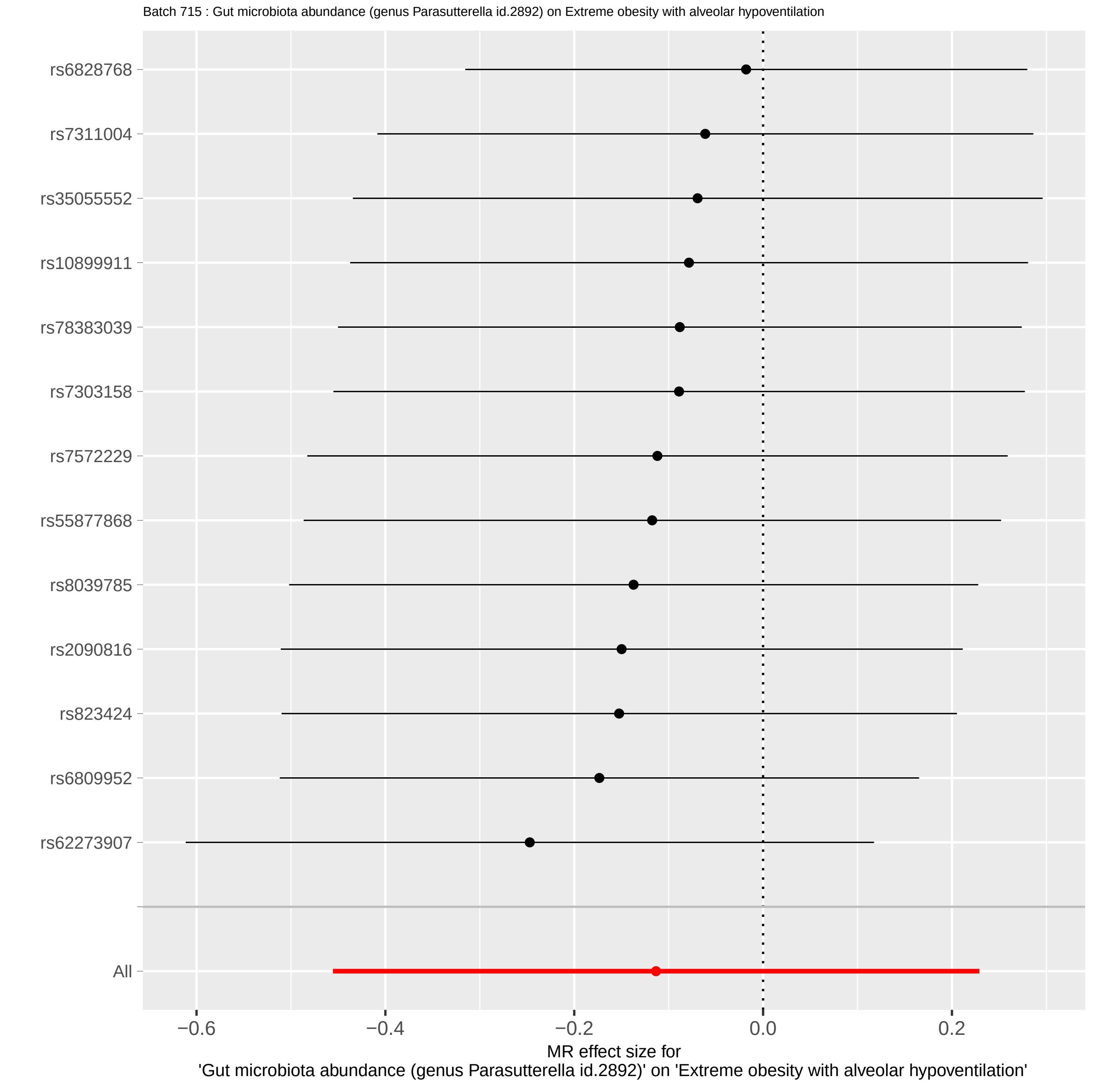

Batch 715 : Gut microbiota abundance (genus Parasutterella id.2892) on Extreme obesity with alveolar hypoventilation
rs6828768
rs7311004
rs35055552
rs10899911
rs78383039
rs7303158
rs7572229
rs55877868
rs8039785
rs2090816
rs823424
rs6809952
rs62273907
All
−0.6
−0.4
−0.2
0.0
0.2
MR effect size for
'Gut microbiota abundance (genus Parasutterella id.2892)' on 'Extreme obesity with alveolar hypoventilation'

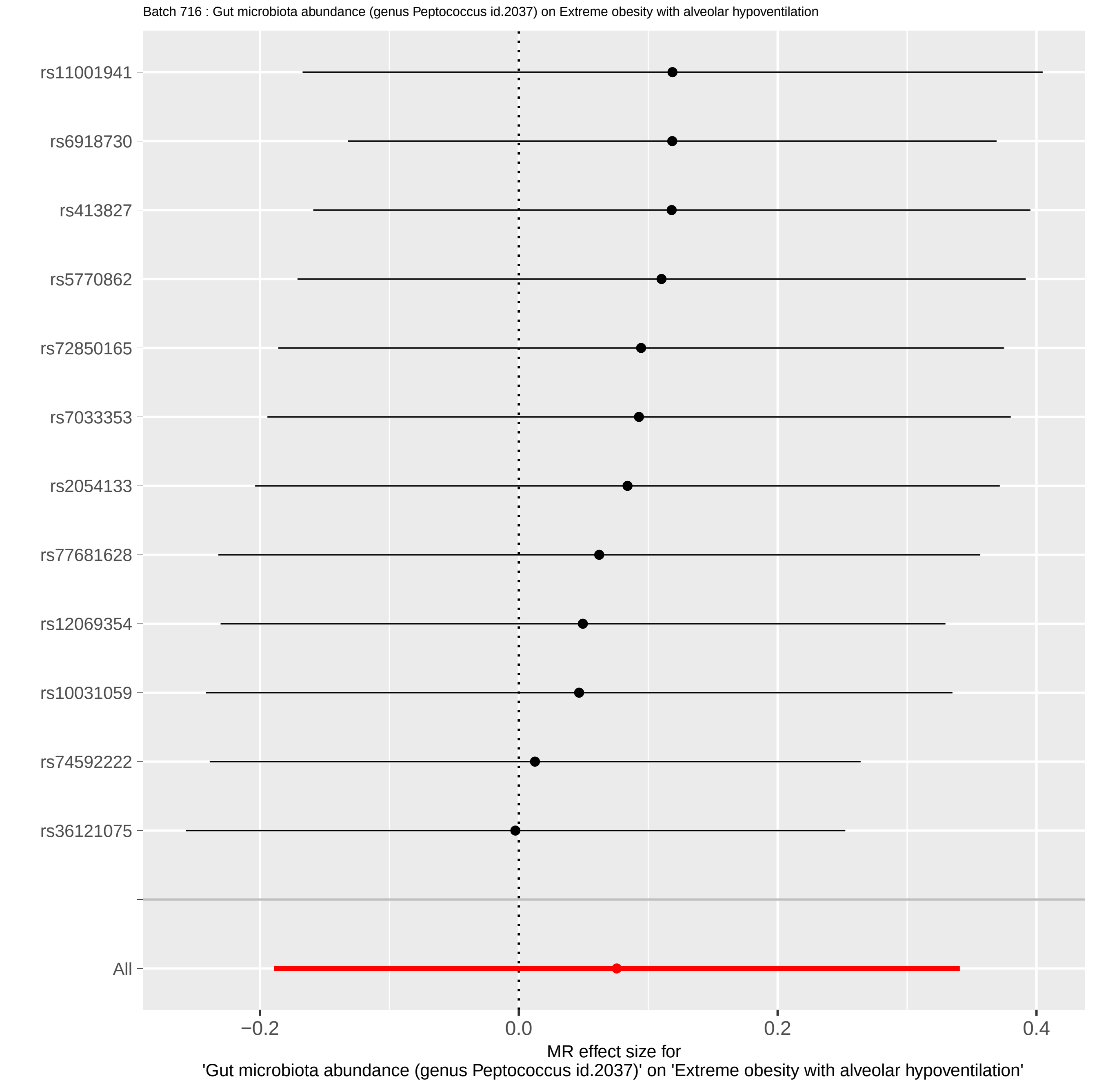

Batch 716 : Gut microbiota abundance (genus Peptococcus id.2037) on Extreme obesity with alveolar hypoventilation
rs11001941
rs6918730
rs413827
rs5770862
rs72850165
rs7033353
rs2054133
rs77681628
rs12069354
rs10031059
rs74592222
rs36121075
All
−0.2
0.0
0.2
0.4
MR effect size for
'Gut microbiota abundance (genus Peptococcus id.2037)' on 'Extreme obesity with alveolar hypoventilation'

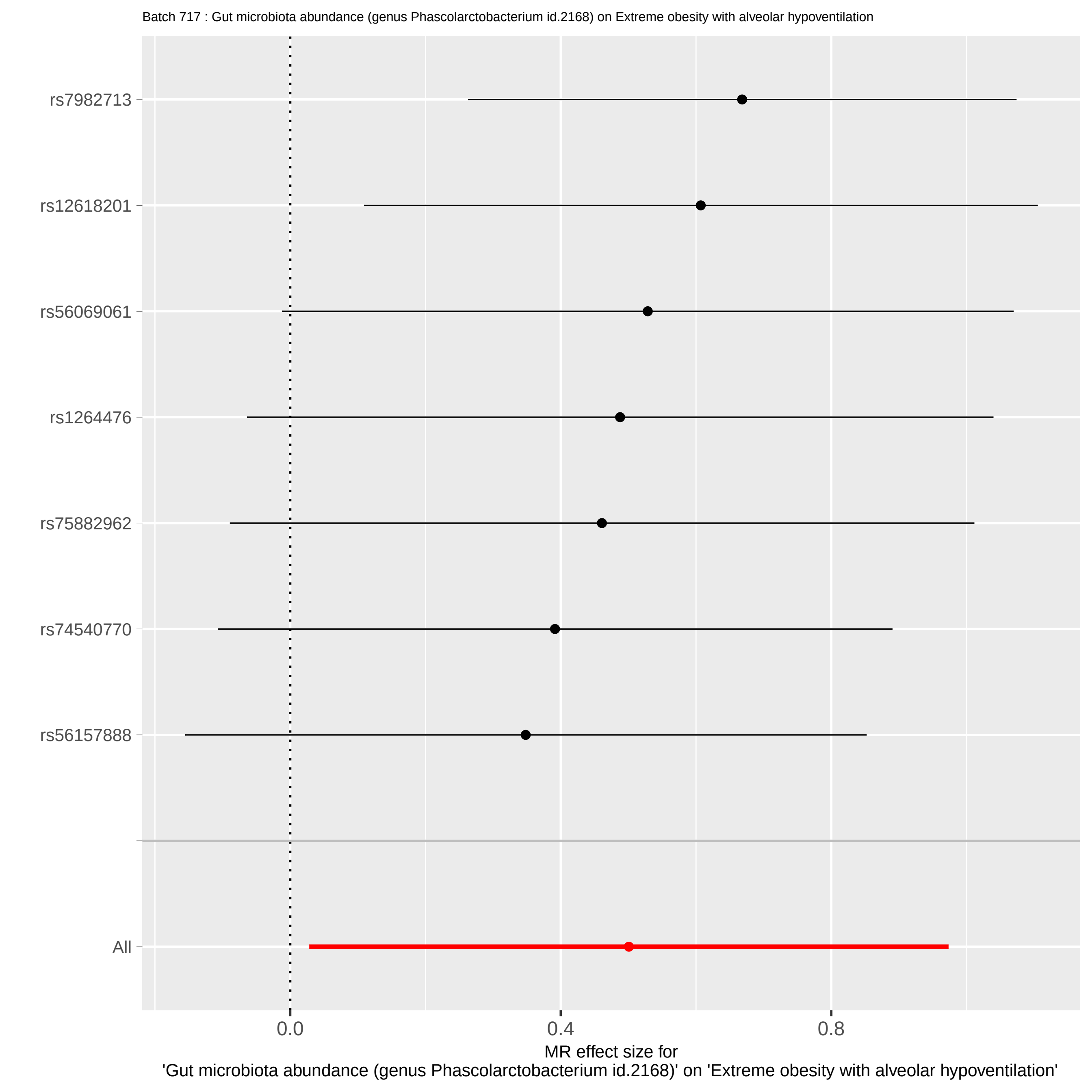
Batch 717 : Gut microbiota abundance (genus Phascolarctobacterium id.2168) on Extreme obesity with alveolar hypoventilation
rs7982713
rs12618201
rs56069061
rs1264476
rs75882962
rs74540770
rs56157888
All
0.0
0.4
0.8
MR effect size for
'Gut microbiota abundance (genus Phascolarctobacterium id.2168)' on 'Extreme obesity with alveolar hypoventilation'

Batch 718 : Gut microbiota abundance (genus Prevotella7 id.11182) on Extreme obesity with alveolar hypoventilation

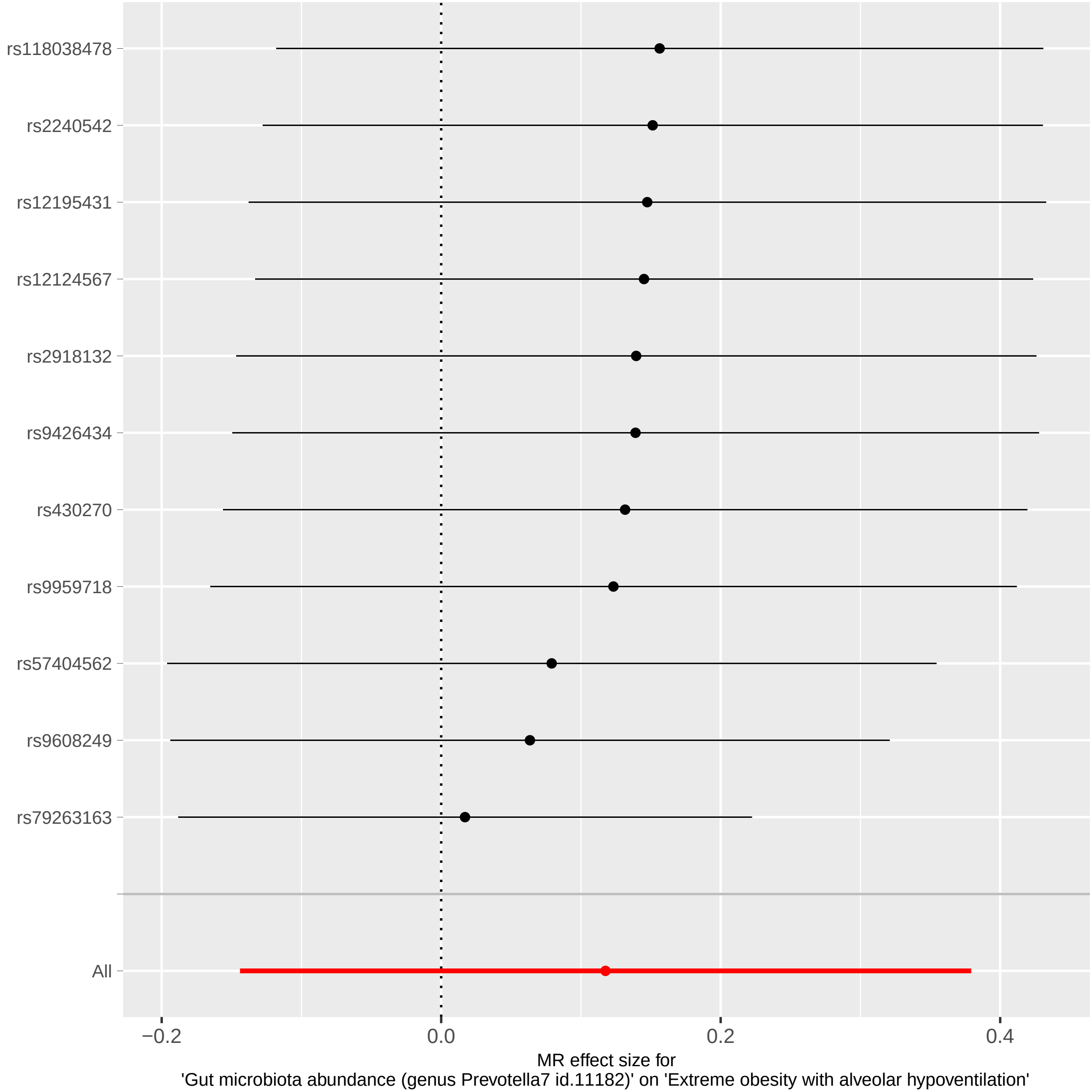

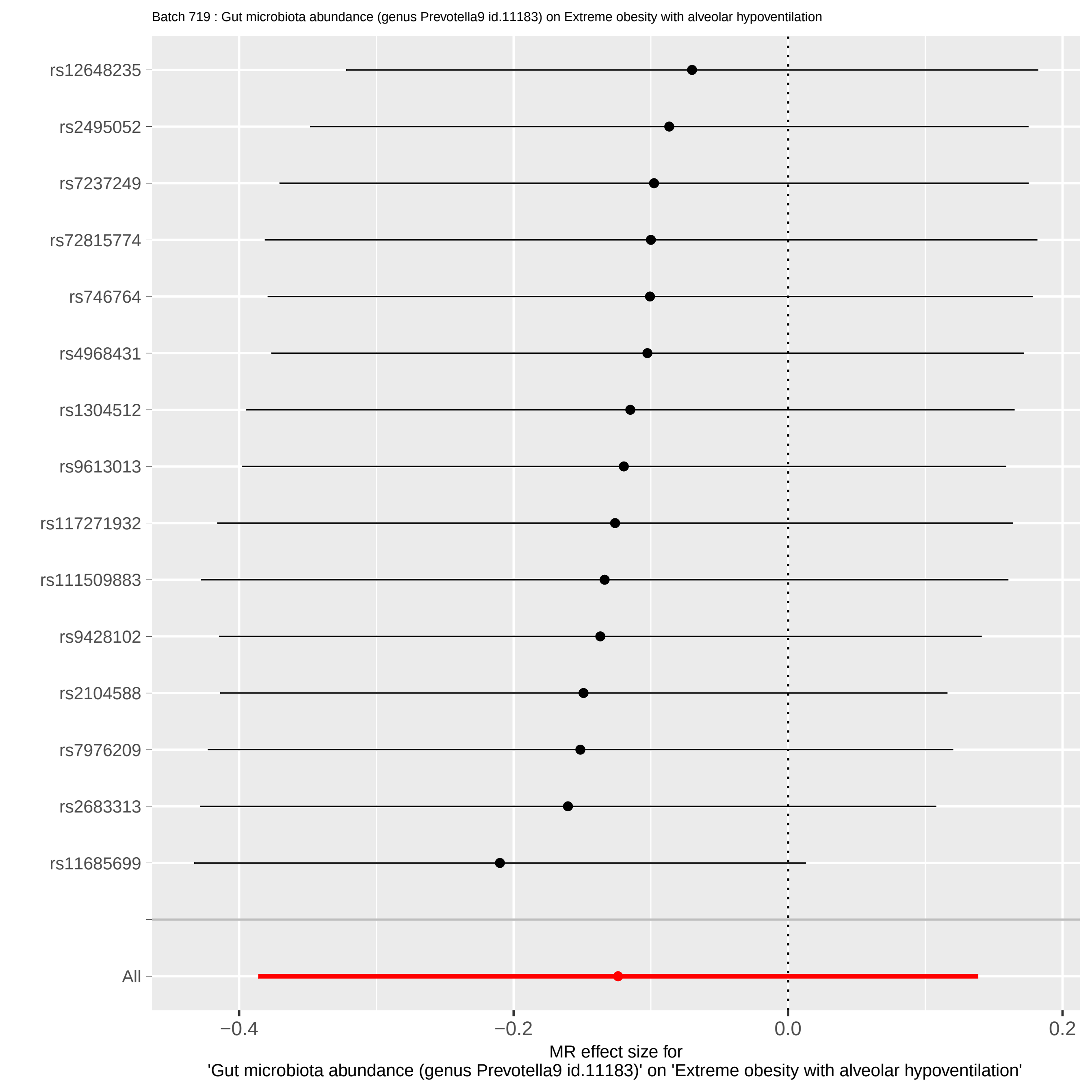

Batch 719 : Gut microbiota abundance (genus Prevotella9 id.11183) on Extreme obesity with alveolar hypoventilation
rs12648235
rs2495052
rs7237249
rs72815774
rs746764
rs4968431
rs1304512
rs9613013
rs117271932
rs111509883
rs9428102
rs2104588
rs7976209
rs2683313
rs11685699
All
−0.4
−0.2
0.0
0.2
MR effect size for
'Gut microbiota abundance (genus Prevotella9 id.11183)' on 'Extreme obesity with alveolar hypoventilation'

Batch 720 : Gut microbiota abundance (genus Rikenellaceae RC9 gut group id.11191) on Extreme obesity with alveolar hypoventilation

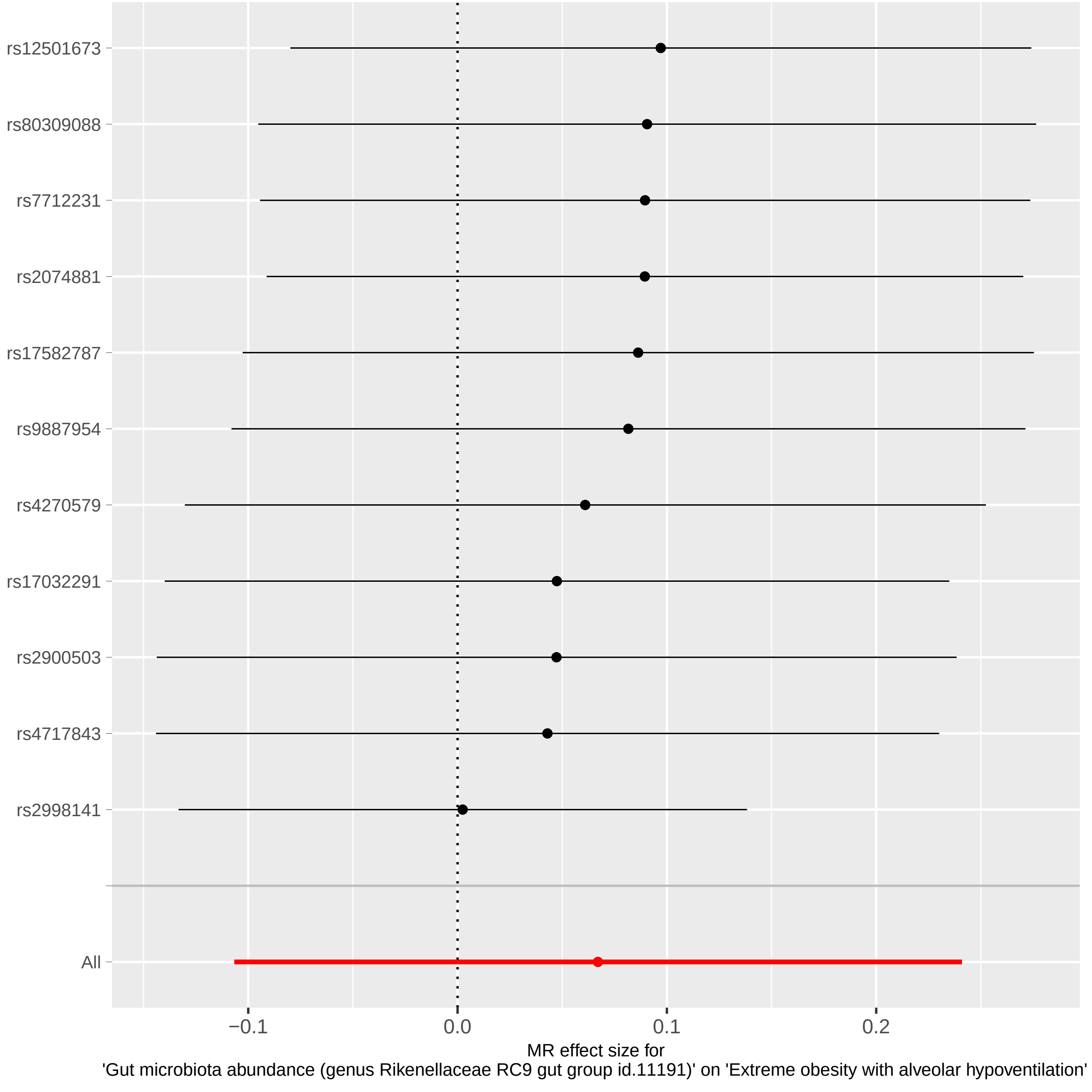

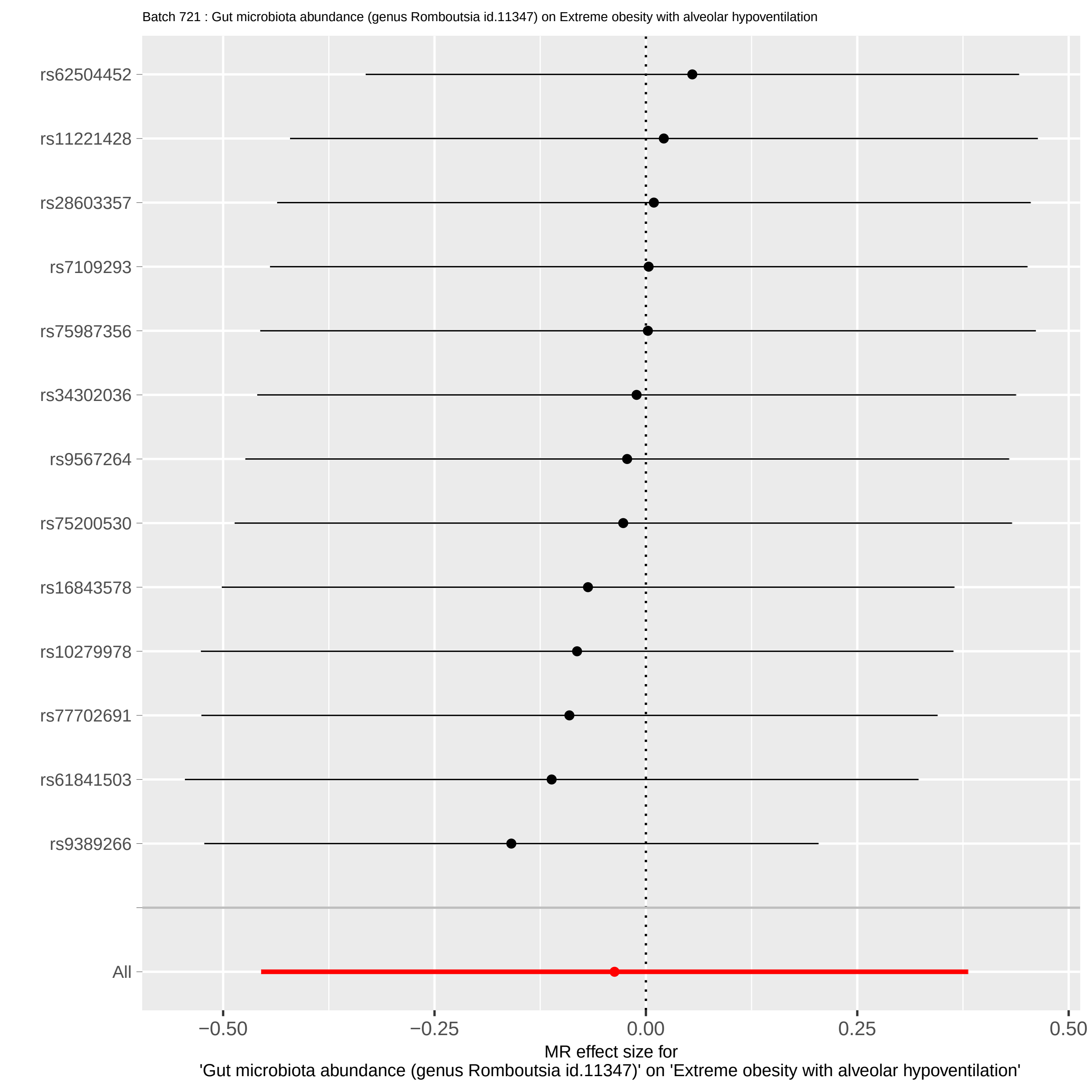

Batch 721 : Gut microbiota abundance (genus Romboutsia id.11347) on Extreme obesity with alveolar hypoventilation
rs62504452
rs11221428
rs28603357
rs7109293
rs75987356
rs34302036
rs9567264
rs75200530
rs16843578
rs10279978
rs77702691
rs61841503
rs9389266
All
−0.50
−0.25
0.00
0.25
0.50
MR effect size for
'Gut microbiota abundance (genus Romboutsia id.11347)' on 'Extreme obesity with alveolar hypoventilation'

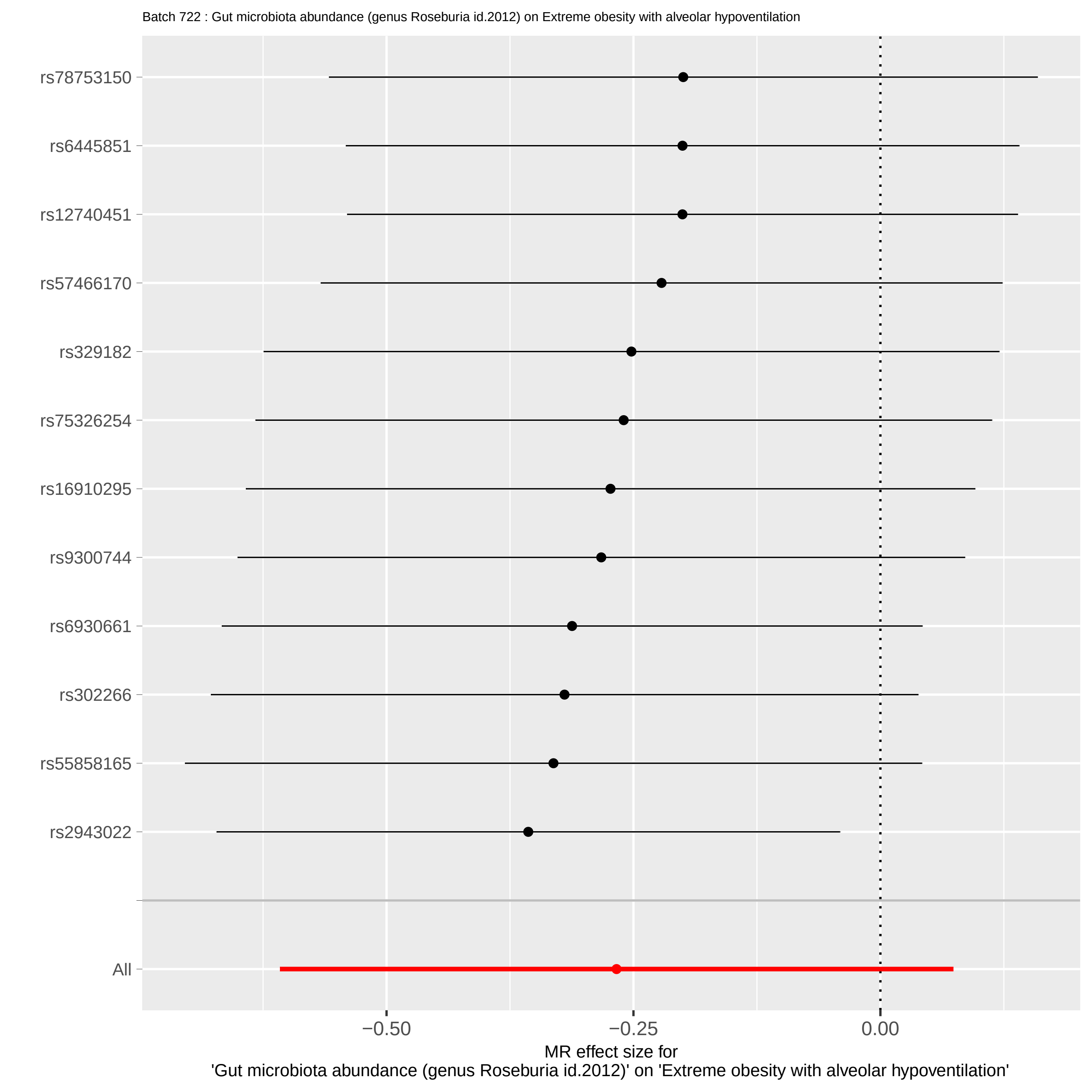

Batch 722 : Gut microbiota abundance (genus Roseburia id.2012) on Extreme obesity with alveolar hypoventilation
rs78753150
rs6445851
rs12740451
rs57466170
rs329182
rs75326254
rs16910295
rs9300744
rs6930661
rs302266
rs55858165
rs2943022
All
−0.50
−0.25
0.00
MR effect size for
'Gut microbiota abundance (genus Roseburia id.2012)' on 'Extreme obesity with alveolar hypoventilation'

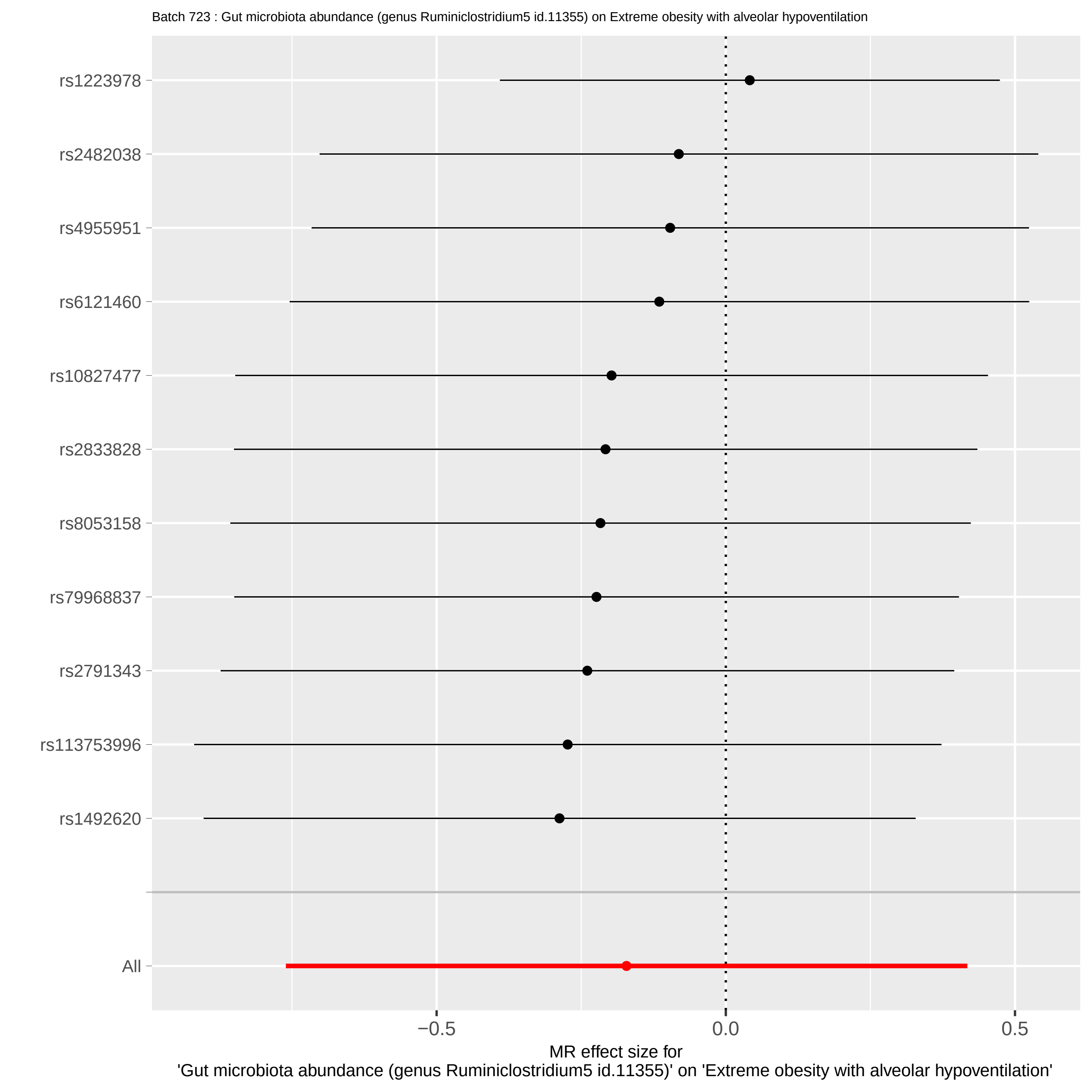

Batch 723 : Gut microbiota abundance (genus Ruminiclostridium5 id.11355) on Extreme obesity with alveolar hypoventilation
rs1223978
rs2482038
rs4955951
rs6121460
rs10827477
rs2833828
rs8053158
rs79968837
rs2791343
rs113753996
rs1492620
All
−0.5
0.0
0.5
MR effect size for
'Gut microbiota abundance (genus Ruminiclostridium5 id.11355)' on 'Extreme obesity with alveolar hypoventilation'

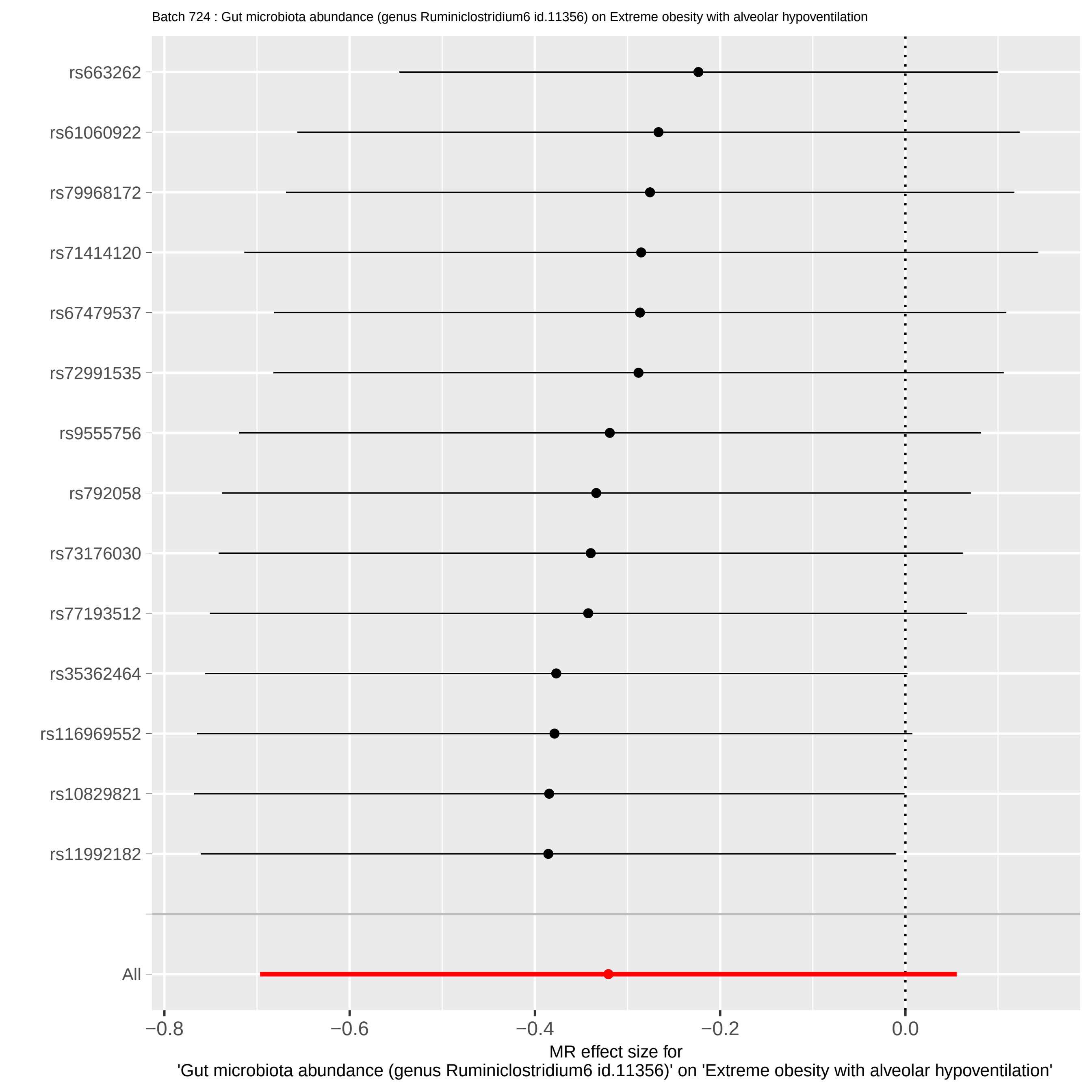

Batch 724 : Gut microbiota abundance (genus Ruminiclostridium6 id.11356) on Extreme obesity with alveolar hypoventilation
rs663262
rs61060922
rs79968172
rs71414120
rs67479537
rs72991535
rs9555756
rs792058
rs73176030
rs77193512
rs35362464
rs116969552
rs10829821
rs11992182
All
−0.8
−0.6
−0.4
−0.2
0.0
MR effect size for
'Gut microbiota abundance (genus Ruminiclostridium6 id.11356)' on 'Extreme obesity with alveolar hypoventilation'

Batch 725 : Gut microbiota abundance (genus Ruminiclostridium9 id.11357) on Extreme obesity with alveolar hypoventilation

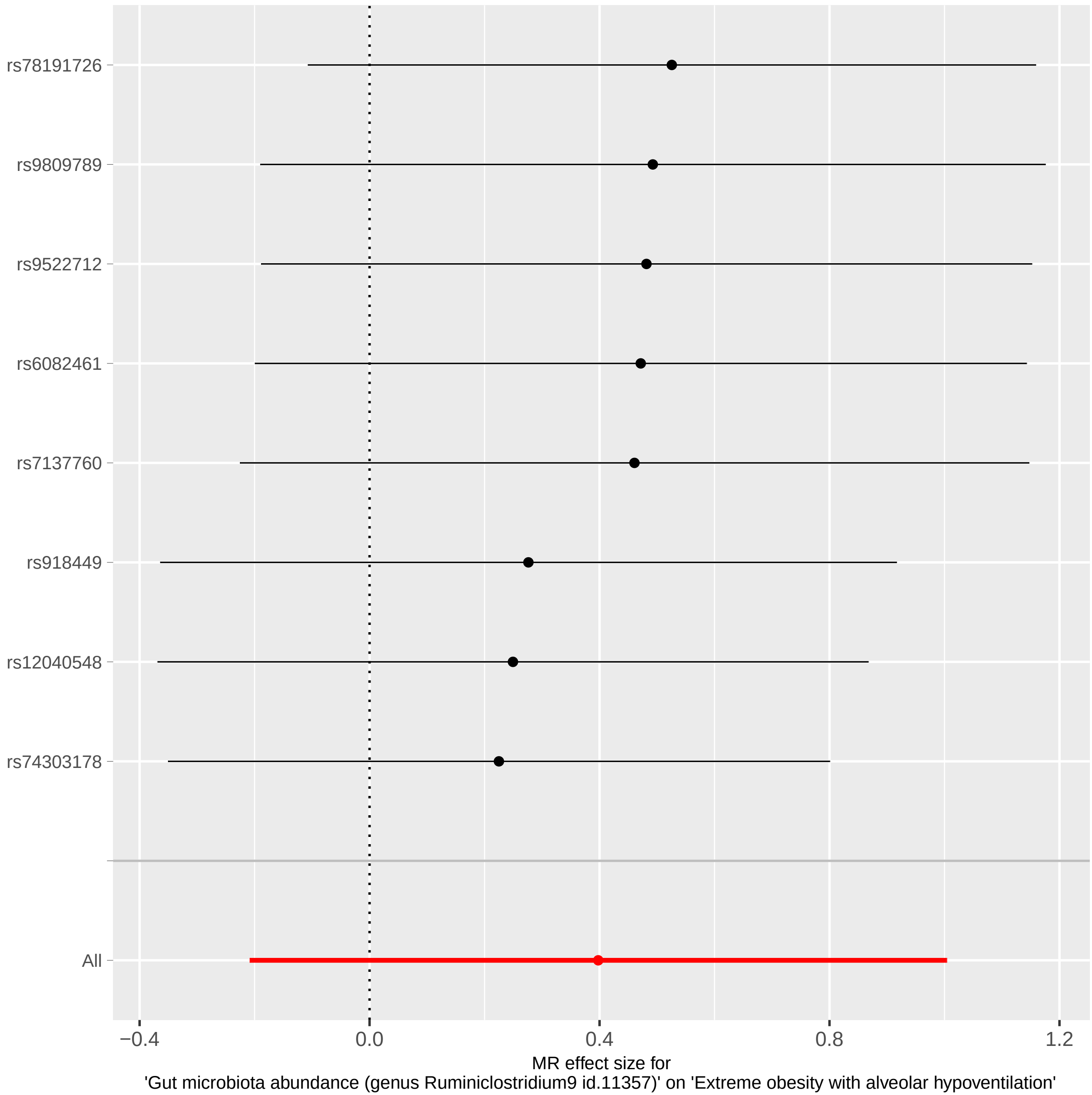

Batch 726 : Gut microbiota abundance (genus Ruminococcaceae NK4A214 group id.11358) on Extreme obesity with alveolar hypoventilation

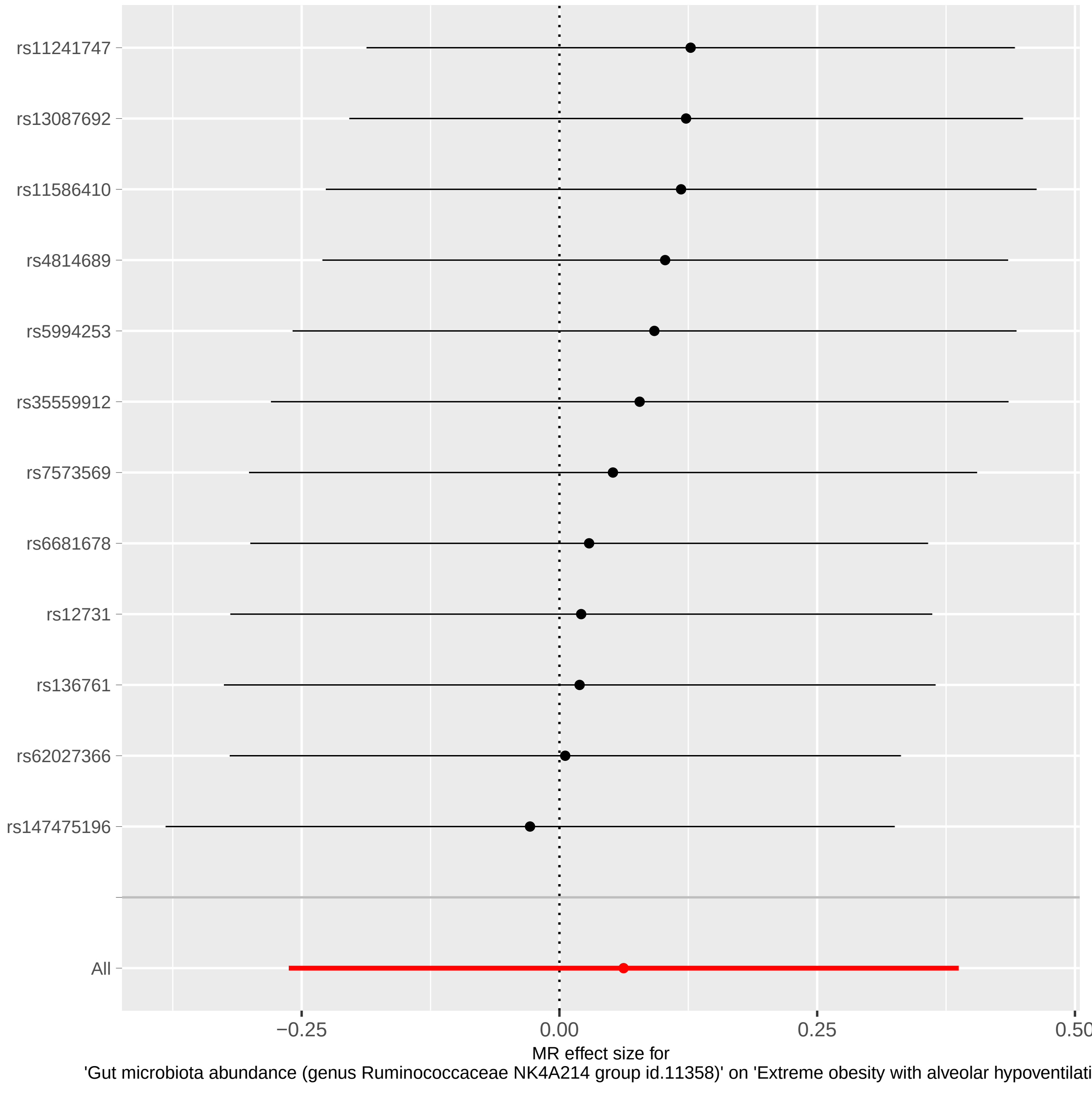

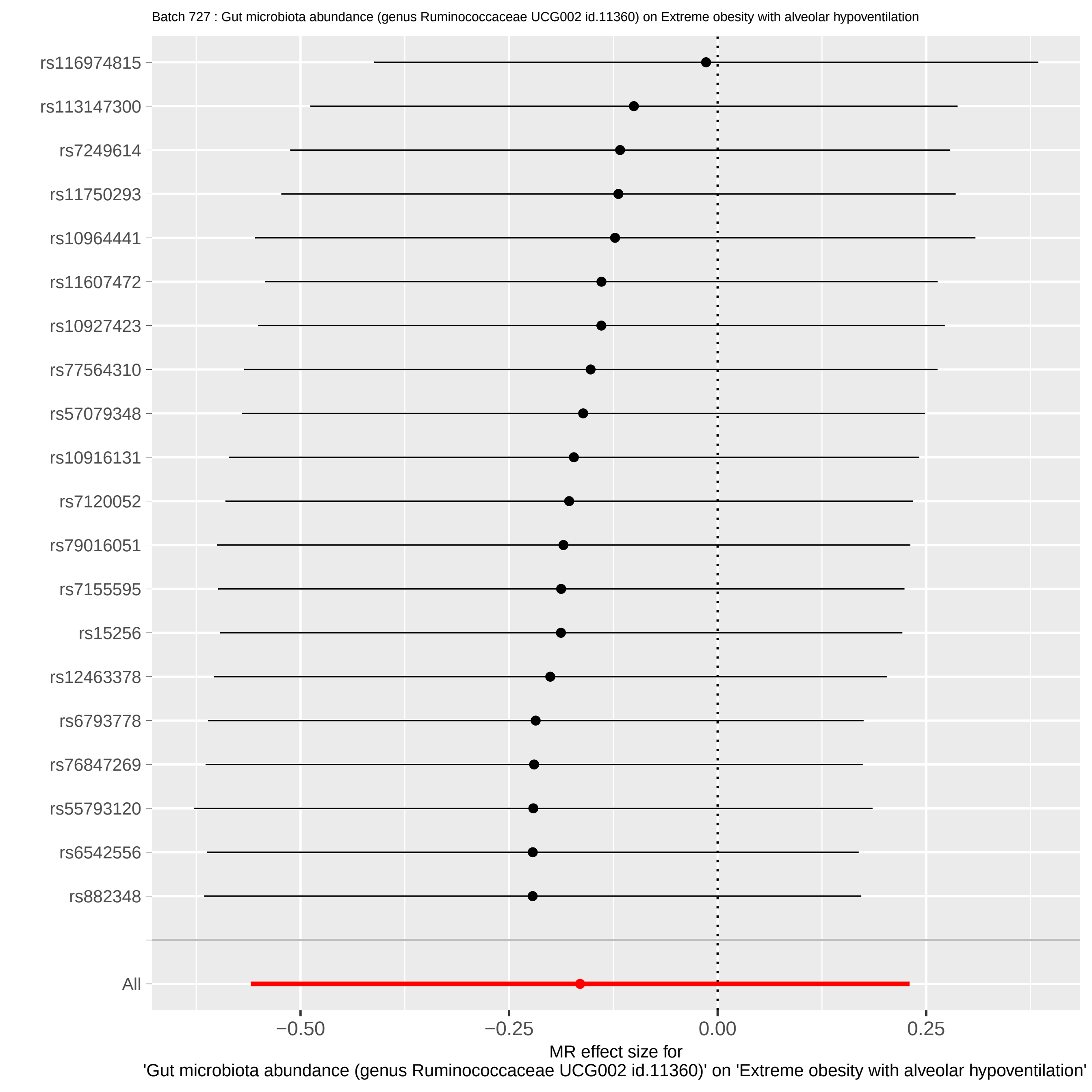

Batch 727 : Gut microbiota abundance (genus Ruminococcaceae UCG002 id.11360) on Extreme obesity with alveolar hypoventilation
rs116974815
rs113147300
rs7249614
rs11750293
rs10964441
rs11607472
rs10927423
rs77564310
rs57079348
rs10916131
rs7120052
rs79016051
rs7155595
rs15256
rs12463378
rs6793778
rs76847269
rs55793120
rs6542556
rs882348
All
−0.50
−0.25
0.00
0.25
MR effect size for
'Gut microbiota abundance (genus Ruminococcaceae UCG002 id.11360)' on 'Extreme obesity with alveolar hypoventilation'

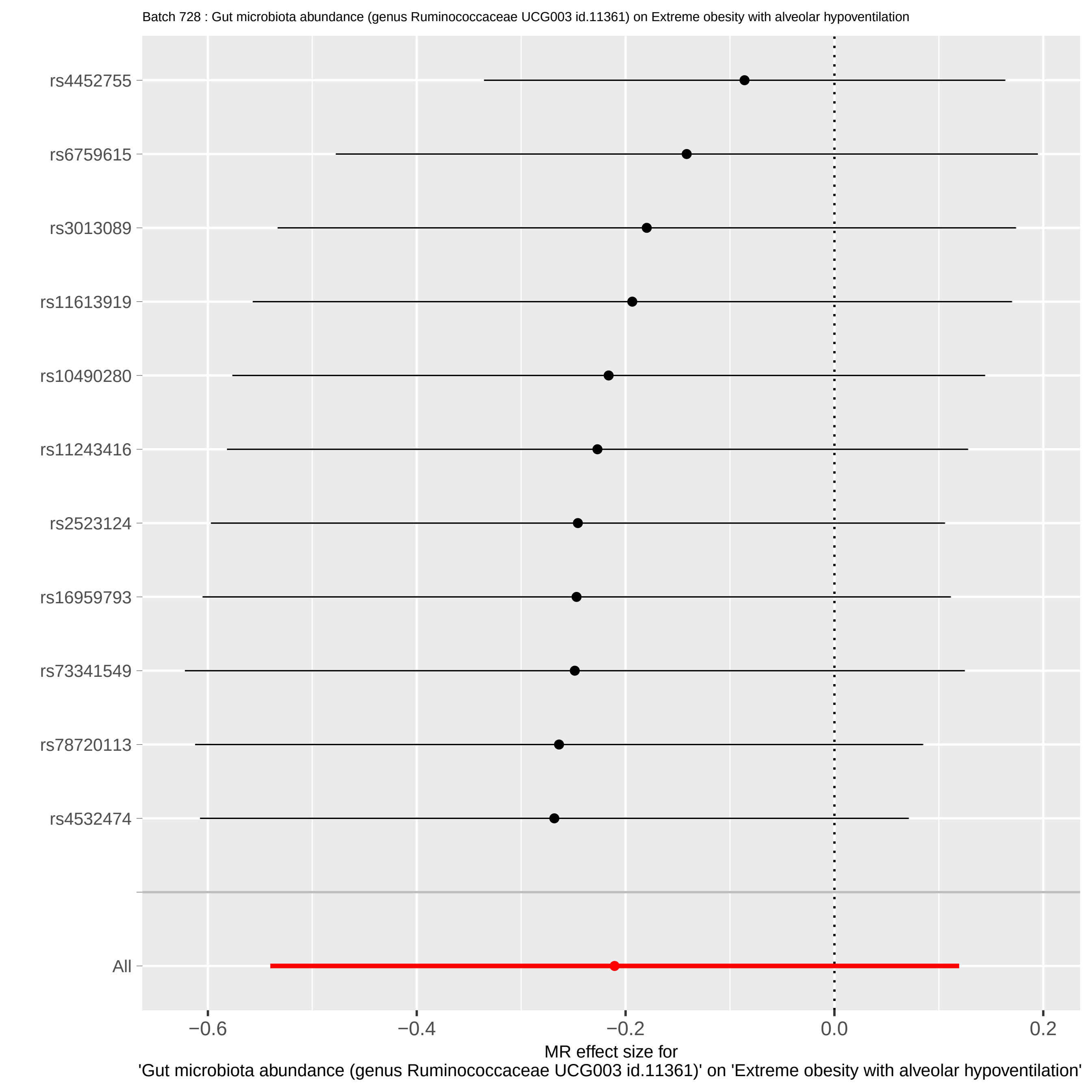

Batch 728 : Gut microbiota abundance (genus Ruminococcaceae UCG003 id.11361) on Extreme obesity with alveolar hypoventilation
rs4452755
rs6759615
rs3013089
rs11613919
rs10490280
rs11243416
rs2523124
rs16959793
rs73341549
rs78720113
rs4532474
All
−0.6
−0.4
−0.2
0.0
0.2
MR effect size for
'Gut microbiota abundance (genus Ruminococcaceae UCG003 id.11361)' on 'Extreme obesity with alveolar hypoventilation'

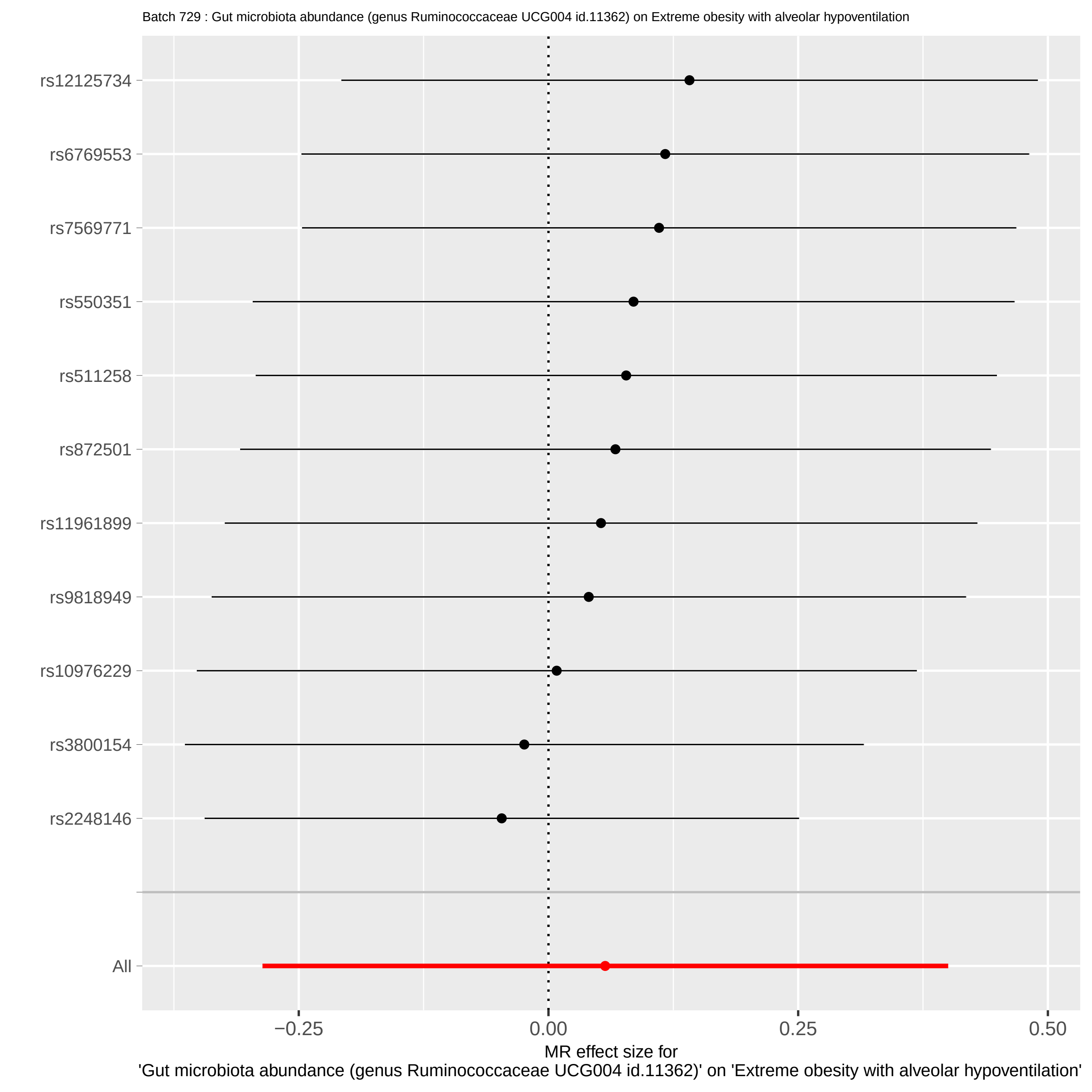

Batch 729 : Gut microbiota abundance (genus Ruminococcaceae UCG004 id.11362) on Extreme obesity with alveolar hypoventilation
rs12125734
rs6769553
rs7569771
rs550351
rs511258
rs872501
rs11961899
rs9818949
rs10976229
rs3800154
rs2248146
All
−0.25
0.00
0.25
0.50
MR effect size for
'Gut microbiota abundance (genus Ruminococcaceae UCG004 id.11362)' on 'Extreme obesity with alveolar hypoventilation'

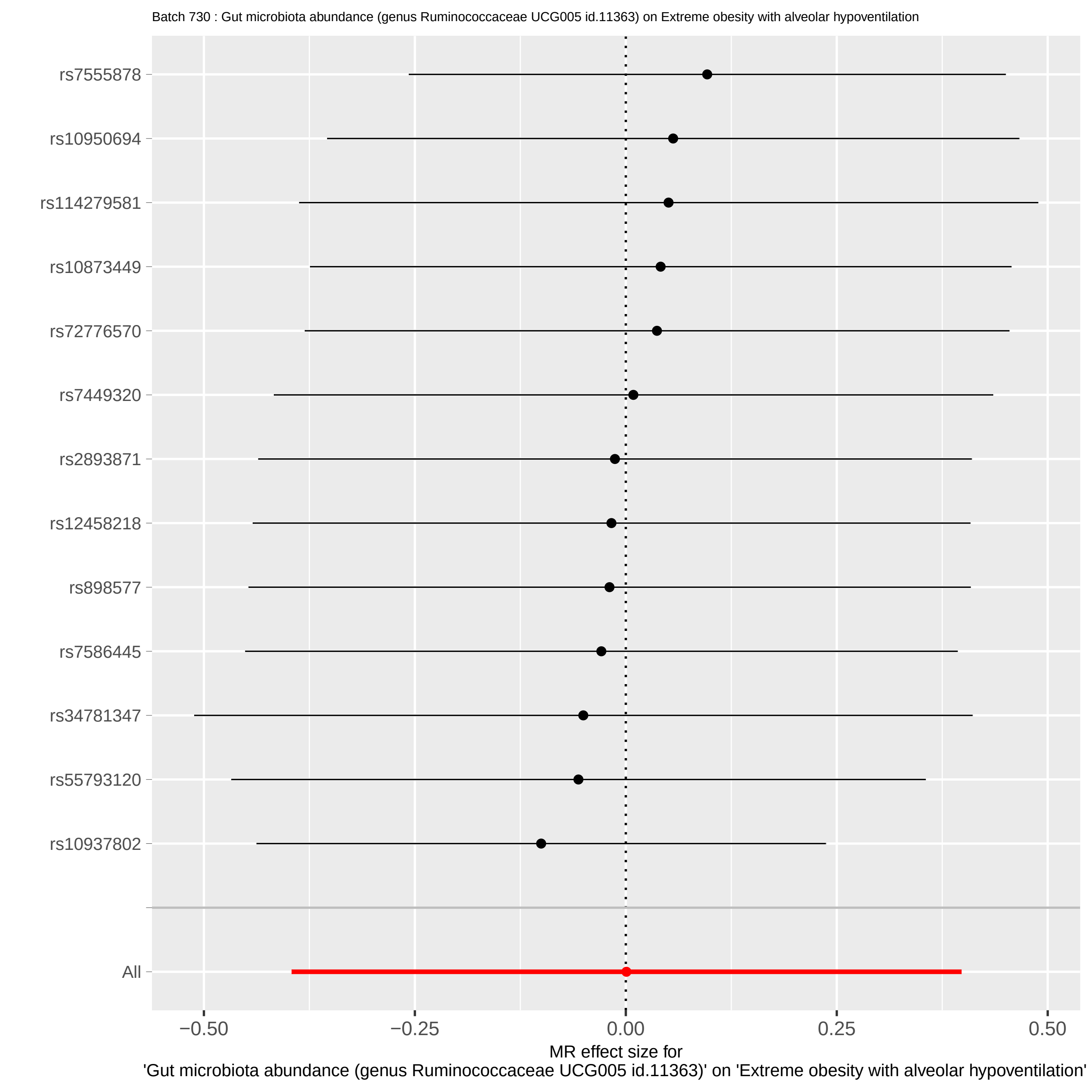
Batch 730 : Gut microbiota abundance (genus Ruminococcaceae UCG005 id.11363) on Extreme obesity with alveolar hypoventilation
rs7555878
rs10950694
rs114279581
rs10873449
rs72776570
rs7449320
rs2893871
rs12458218
rs898577
rs7586445
rs34781347
rs55793120
rs10937802
All
−0.50
−0.25
0.00
0.25
0.50
MR effect size for
'Gut microbiota abundance (genus Ruminococcaceae UCG005 id.11363)' on 'Extreme obesity with alveolar hypoventilation'

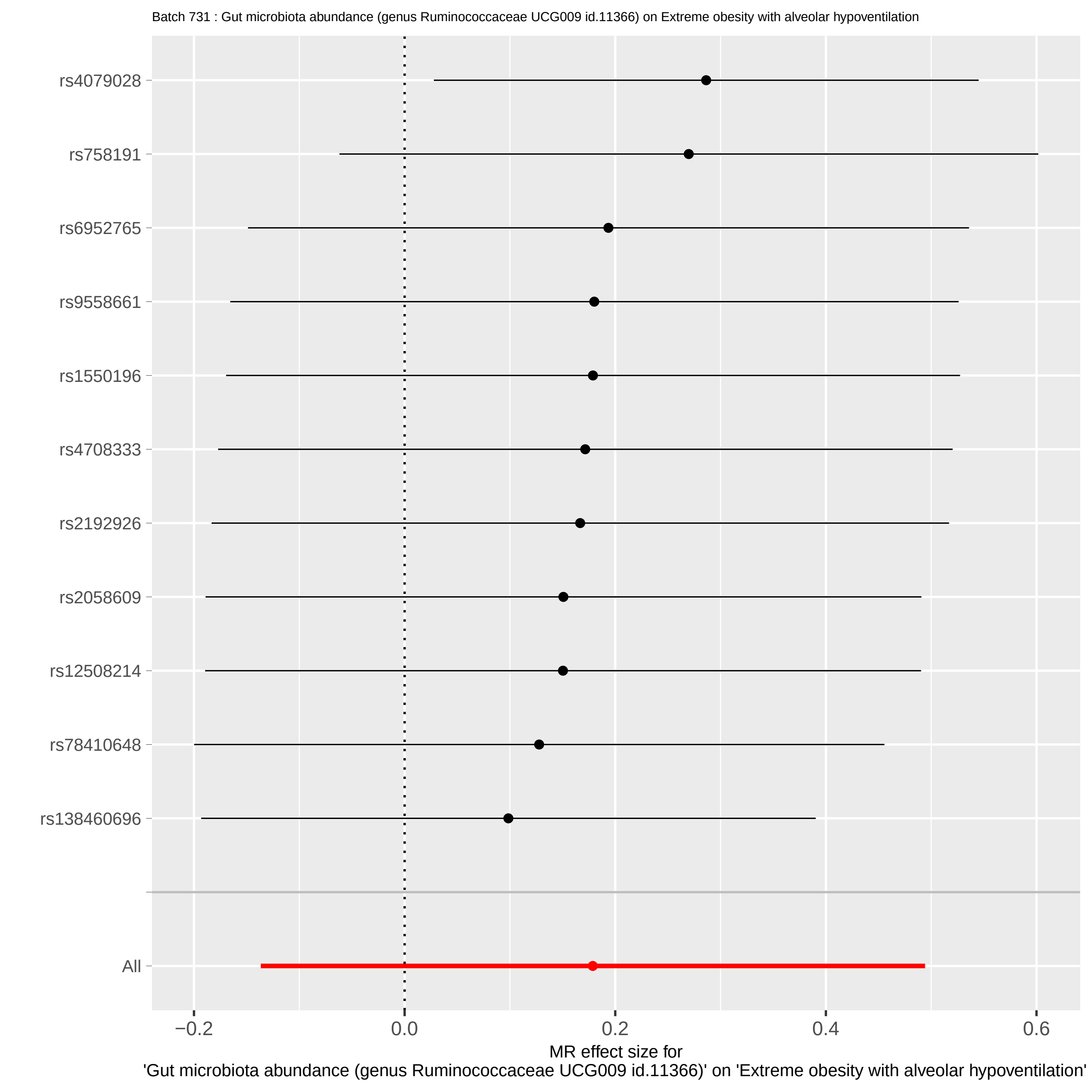

Batch 731 : Gut microbiota abundance (genus Ruminococcaceae UCG009 id.11366) on Extreme obesity with alveolar hypoventilation
rs4079028
rs758191
rs6952765
rs9558661
rs1550196
rs4708333
rs2192926
rs2058609
rs12508214
rs78410648
rs138460696
All
−0.2
0.0
0.2
0.4
0.6
MR effect size for
'Gut microbiota abundance (genus Ruminococcaceae UCG009 id.11366)' on 'Extreme obesity with alveolar hypoventilation'

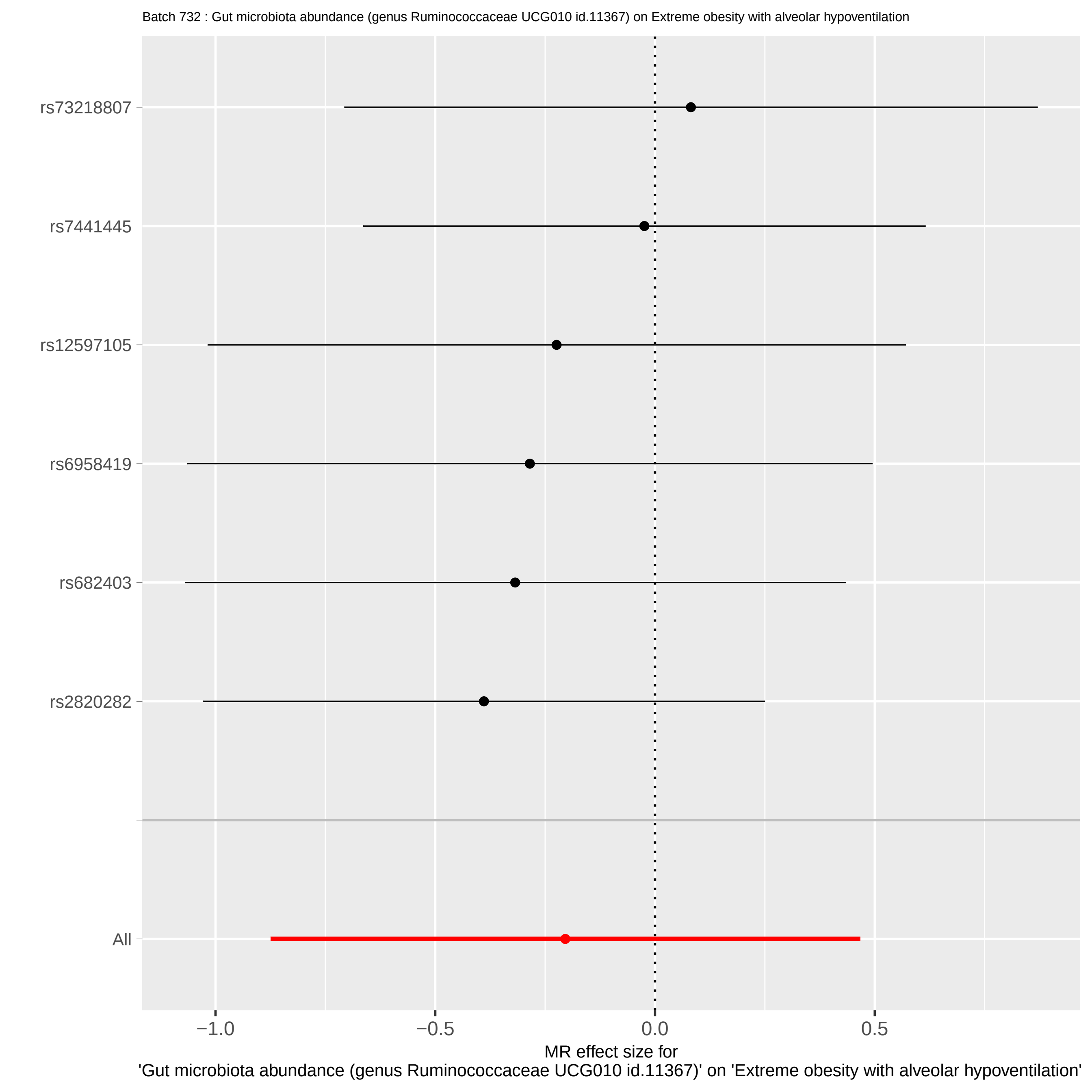

Batch 732 : Gut microbiota abundance (genus Ruminococcaceae UCG010 id.11367) on Extreme obesity with alveolar hypoventilation
rs73218807
rs7441445
rs12597105
rs6958419
rs682403
rs2820282
All
−1.0
−0.5
0.0
0.5
MR effect size for
'Gut microbiota abundance (genus Ruminococcaceae UCG010 id.11367)' on 'Extreme obesity with alveolar hypoventilation'

Batch 733 : Gut microbiota abundance (genus Ruminococcaceae UCG011 id.11368) on Extreme obesity with alveolar hypoventilation

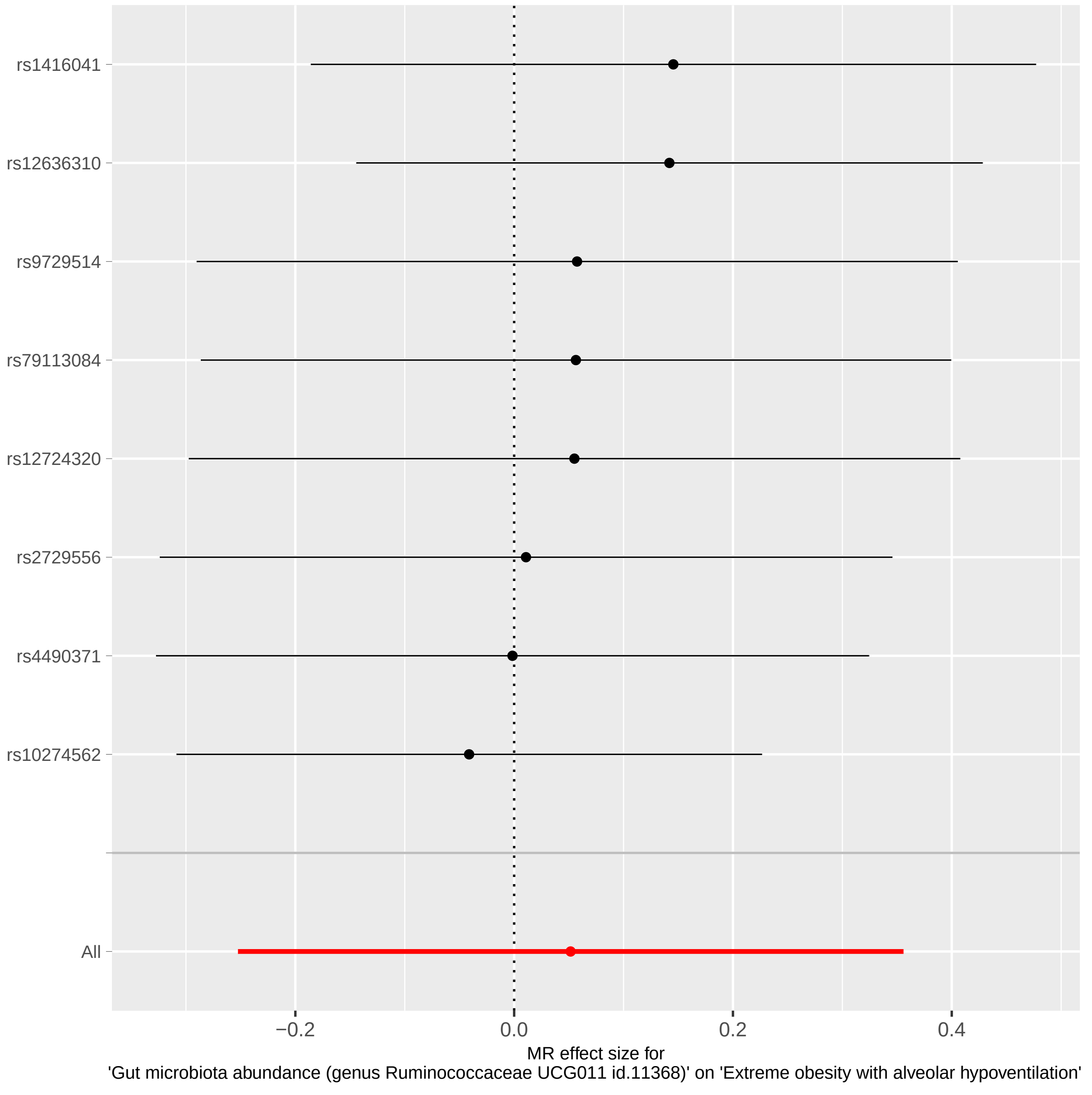

Batch 734 : Gut microbiota abundance (genus Ruminococcaceae UCG013 id.11370) on Extreme obesity with alveolar hypoventilation

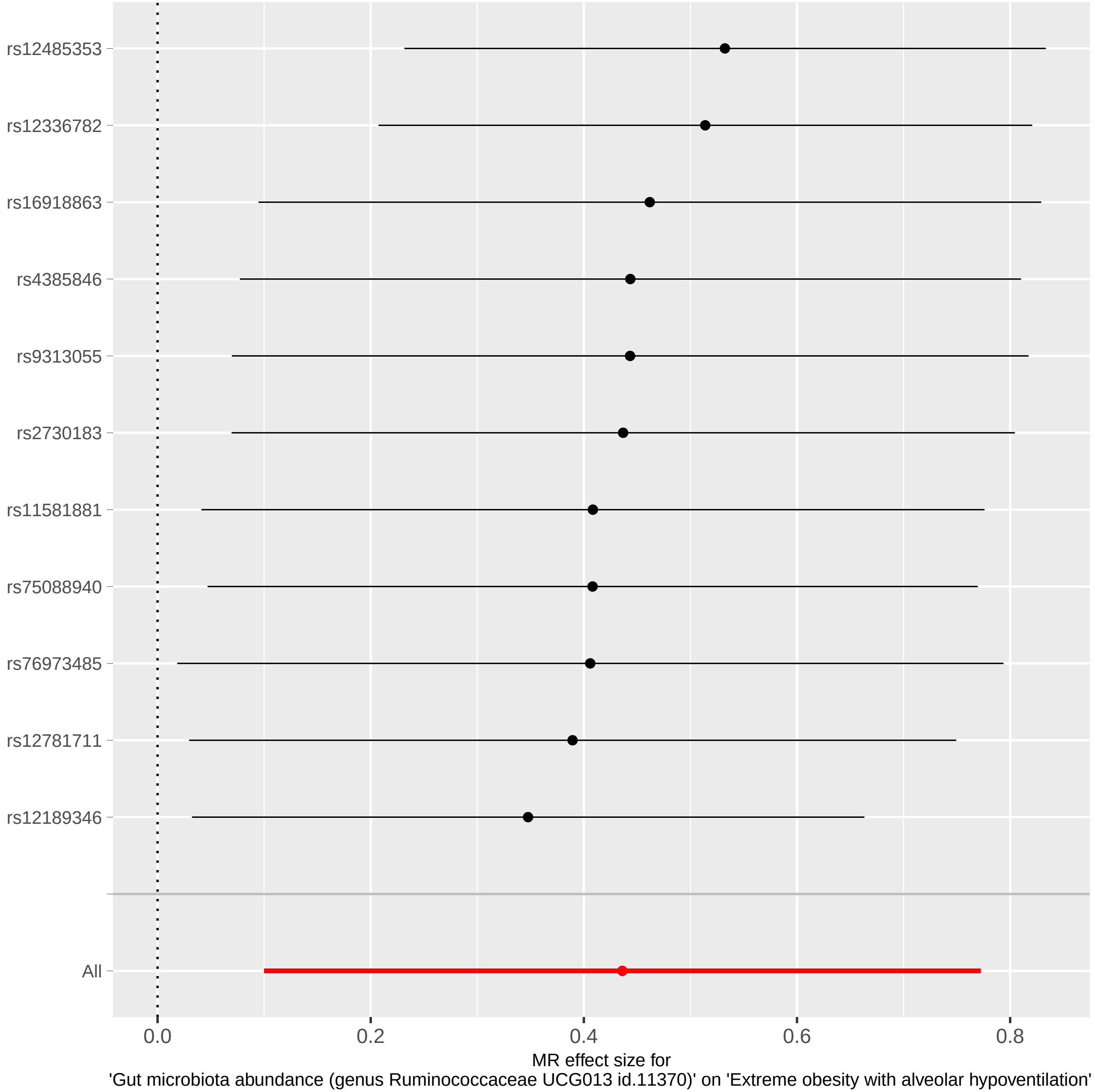

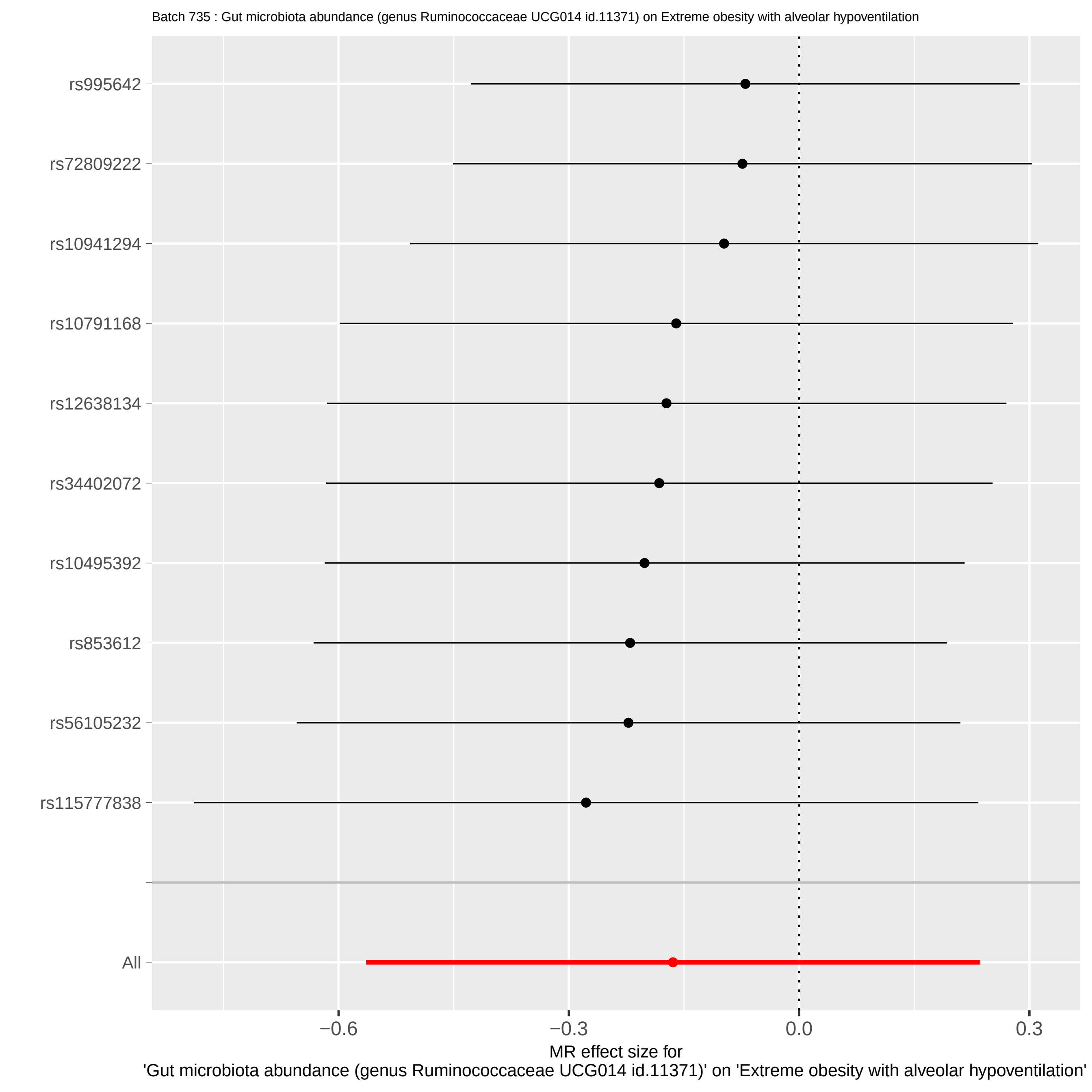

Batch 735 : Gut microbiota abundance (genus Ruminococcaceae UCG014 id.11371) on Extreme obesity with alveolar hypoventilation
rs995642
rs72809222
rs10941294
rs10791168
rs12638134
rs34402072
rs10495392
rs853612
rs56105232
rs115777838
All
−0.6
−0.3
0.0
0.3
MR effect size for
'Gut microbiota abundance (genus Ruminococcaceae UCG014 id.11371)' on 'Extreme obesity with alveolar hypoventilation'

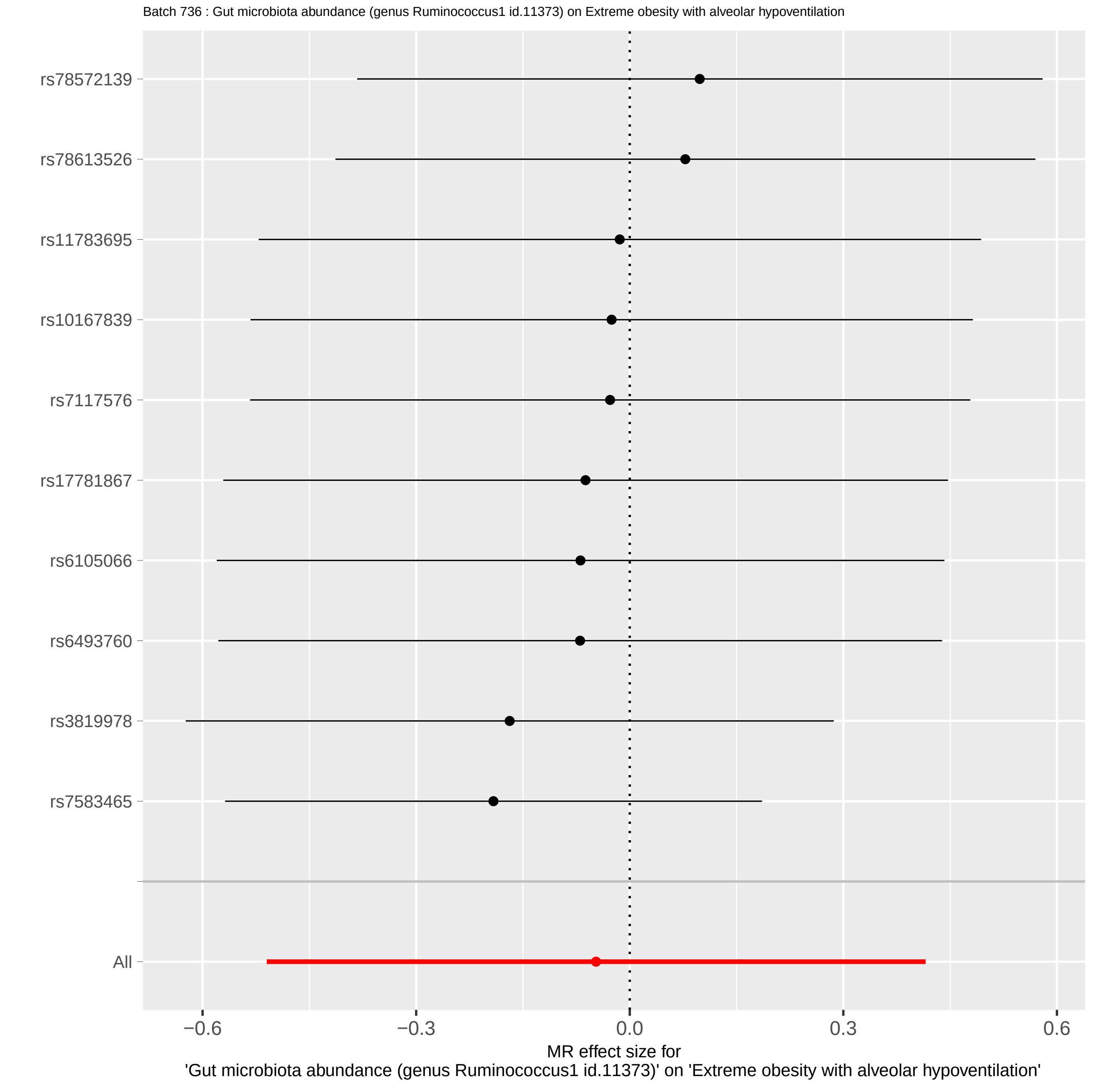

Batch 736 : Gut microbiota abundance (genus Ruminococcus1 id.11373) on Extreme obesity with alveolar hypoventilation
rs78572139
rs78613526
rs11783695
rs10167839
rs7117576
rs17781867
rs6105066
rs6493760
rs3819978
rs7583465
All
−0.6
−0.3
0.0
0.3
0.6
MR effect size for
'Gut microbiota abundance (genus Ruminococcus1 id.11373)' on 'Extreme obesity with alveolar hypoventilation'

Batch 737 : Gut microbiota abundance (genus Ruminococcus2 id.11374) on Extreme obesity with alveolar hypoventilation

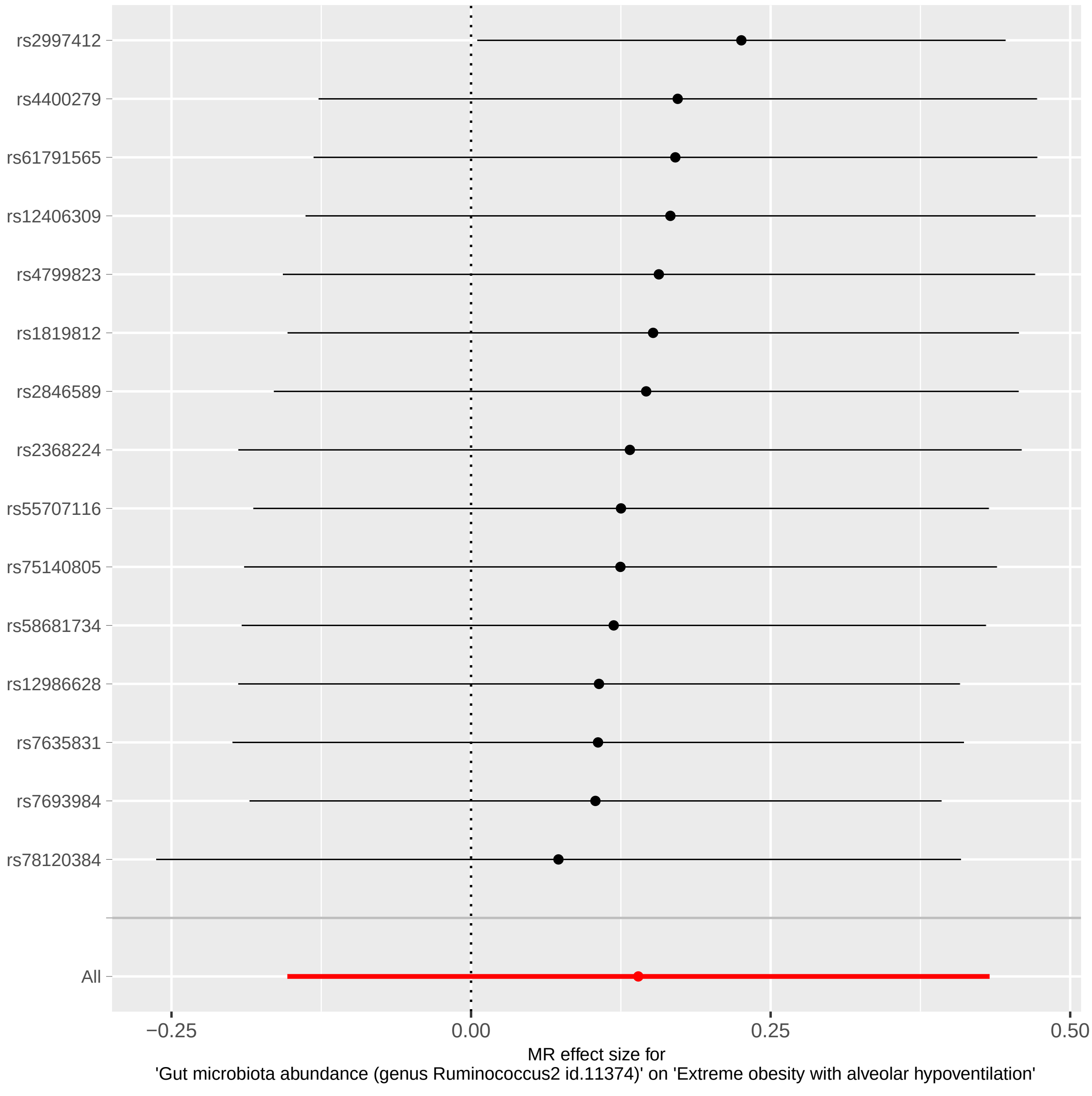

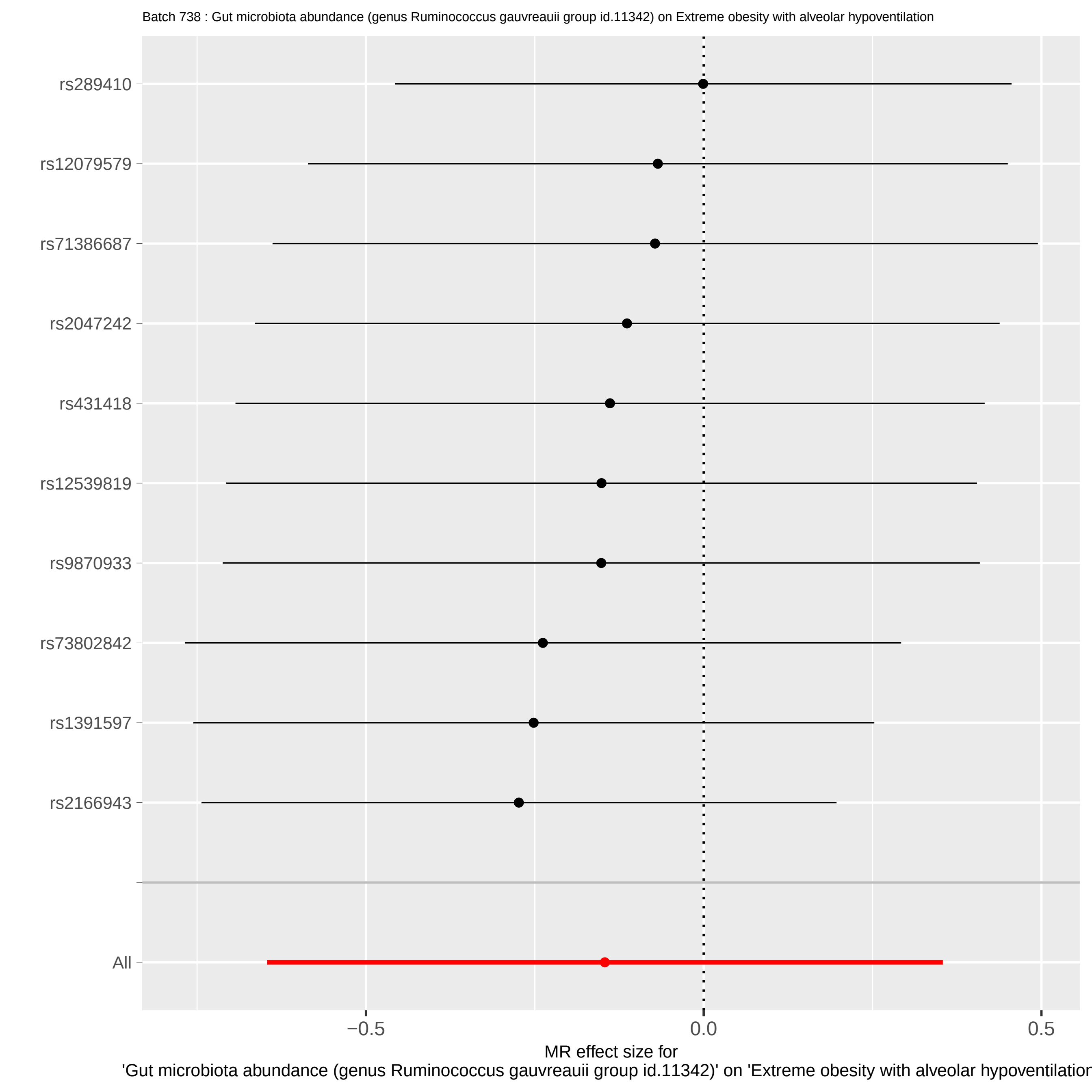
Batch 738 : Gut microbiota abundance (genus Ruminococcus gauvreauii group id.11342) on Extreme obesity with alveolar hypoventilation
rs289410
rs12079579
rs71386687
rs2047242
rs431418
rs12539819
rs9870933
rs73802842
rs1391597
rs2166943
All
−0.5
0.0
0.5
MR effect size for
'Gut microbiota abundance (genus Ruminococcus gauvreauii group id.11342)' on 'Extreme obesity with alveolar hypoventilation

Batch 739 : Gut microbiota abundance (genus Ruminococcus gnavus group id.14376) on Extreme obesity with alveolar hypoventilation

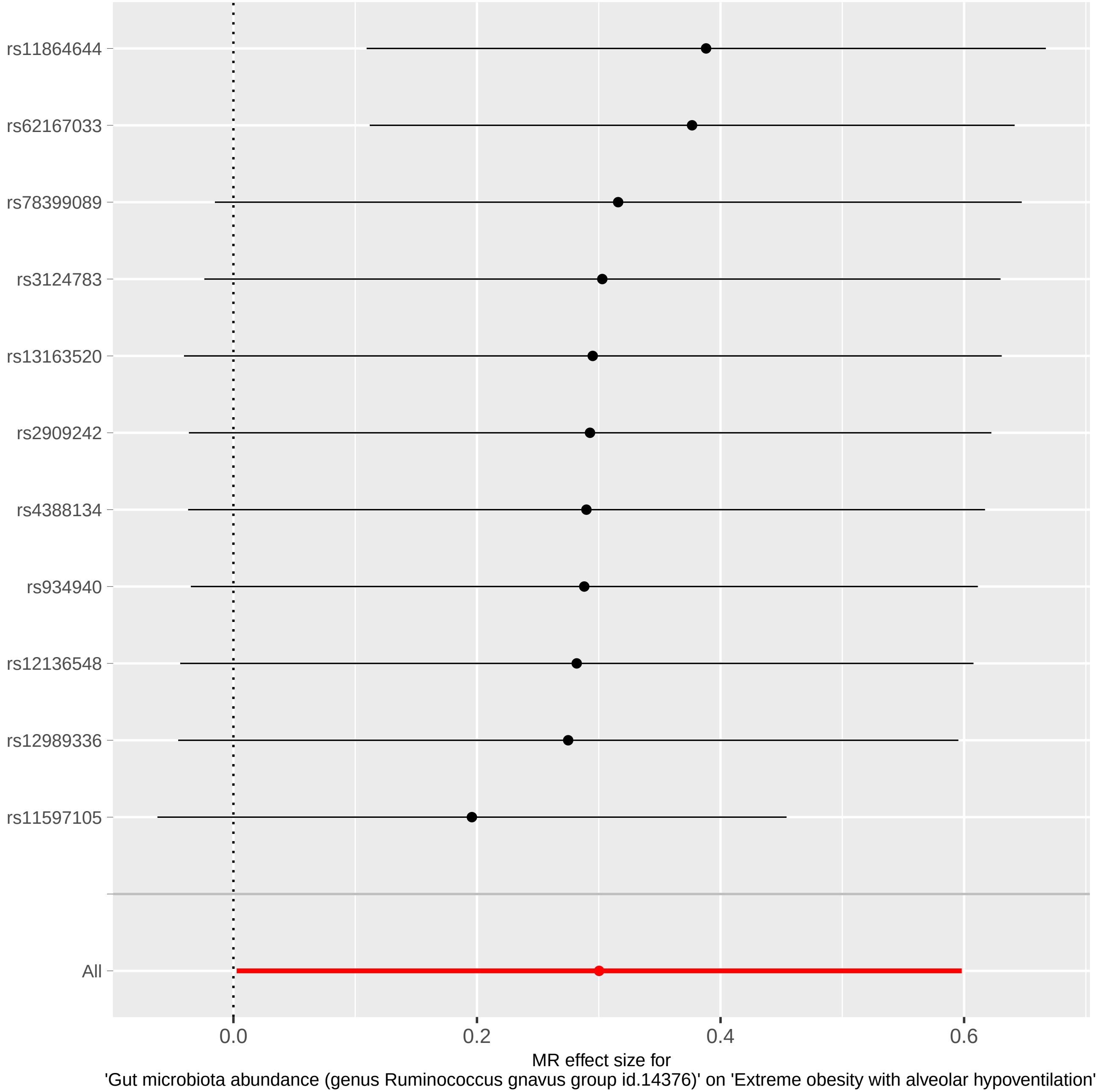

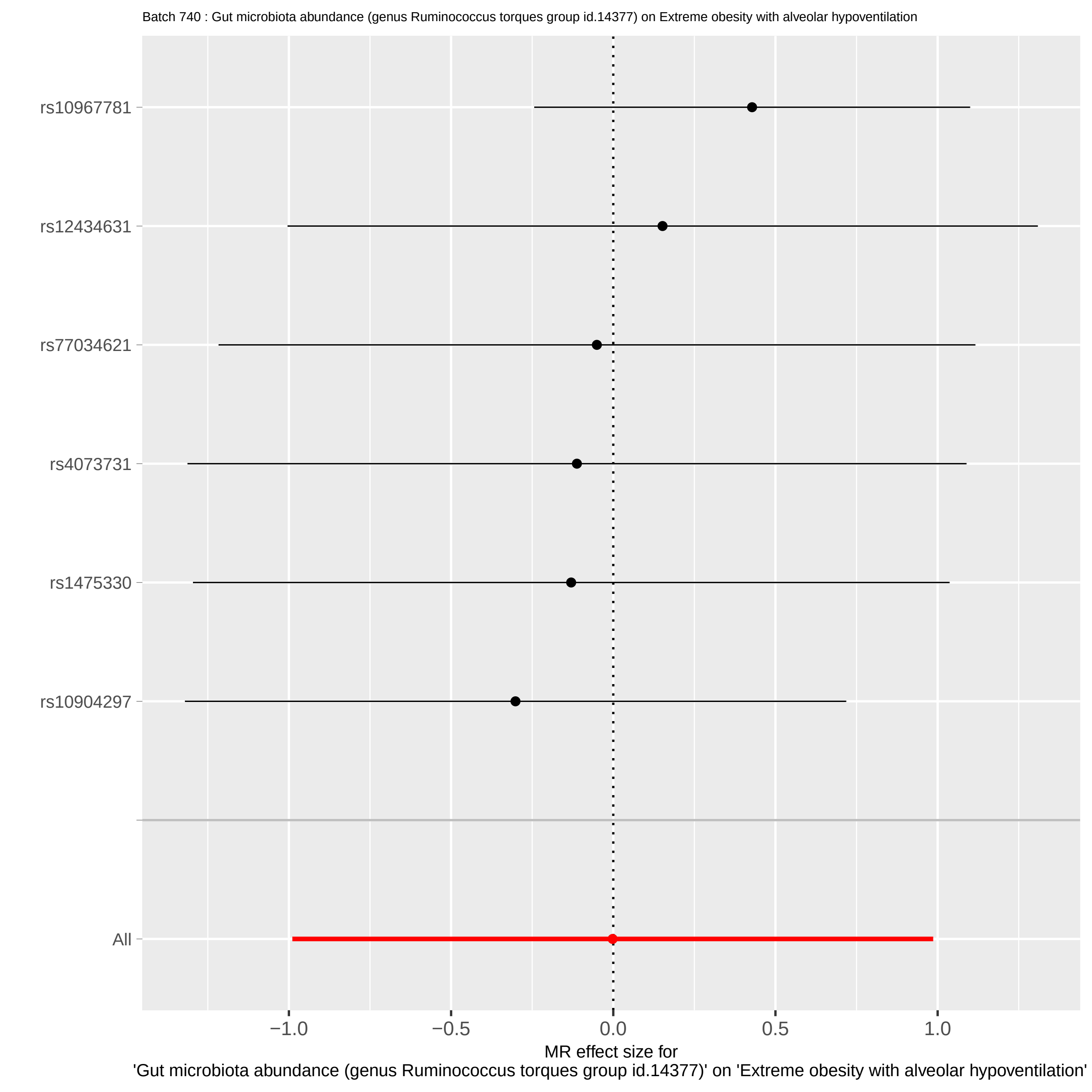

Batch 740 : Gut microbiota abundance (genus Ruminococcus torques group id.14377) on Extreme obesity with alveolar hypoventilation
rs10967781
rs12434631
rs77034621
rs4073731
rs1475330
rs10904297
All
−1.0
−0.5
0.0
0.5
1.0
MR effect size for
'Gut microbiota abundance (genus Ruminococcus torques group id.14377)' on 'Extreme obesity with alveolar hypoventilation'

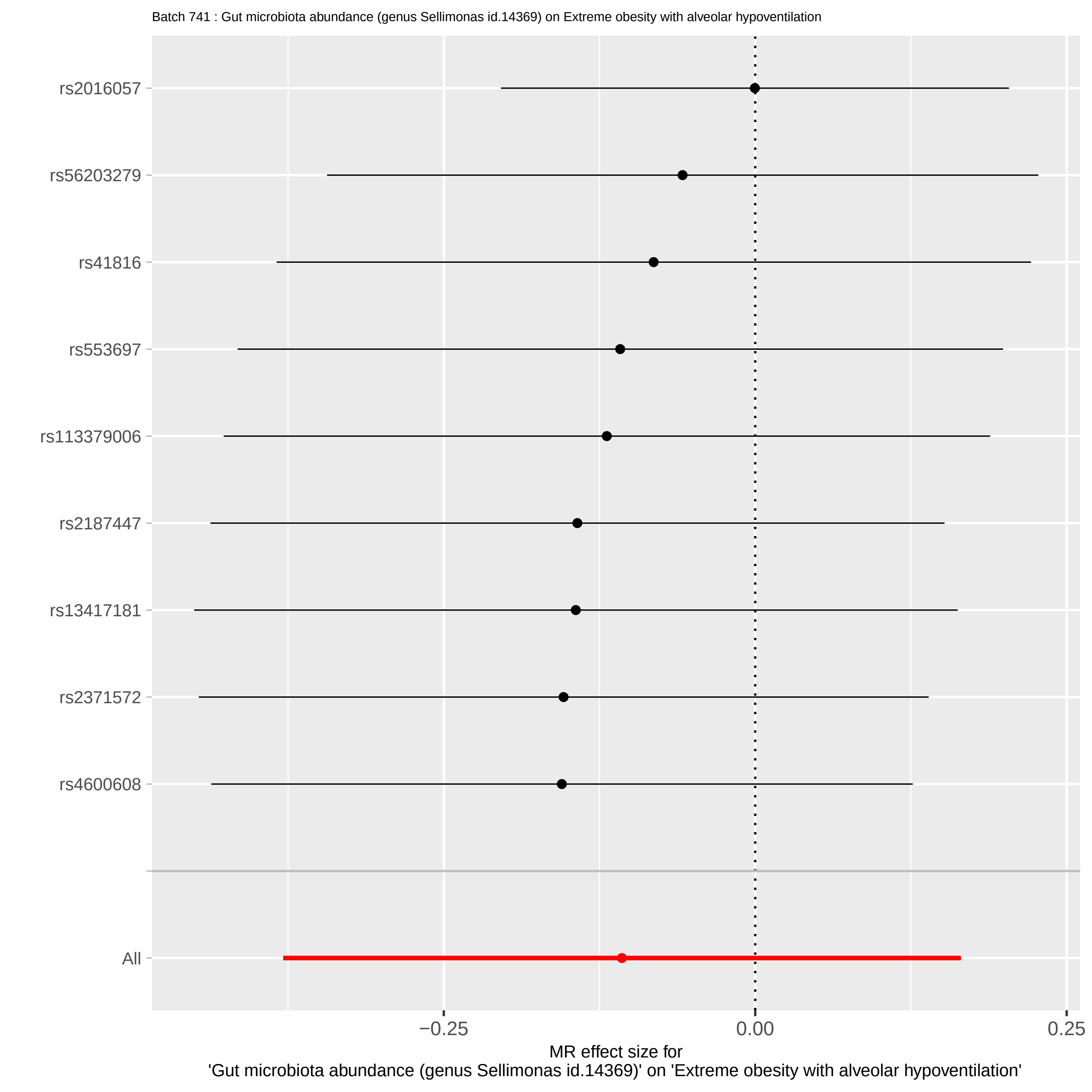
Batch 741 : Gut microbiota abundance (genus Sellimonas id.14369) on Extreme obesity with alveolar hypoventilation
rs2016057
rs56203279
rs41816
rs553697
rs113379006
rs2187447
rs13417181
rs2371572
rs4600608
All
−0.25
0.00
0.25
MR effect size for
'Gut microbiota abundance (genus Sellimonas id.14369)' on 'Extreme obesity with alveolar hypoventilation'

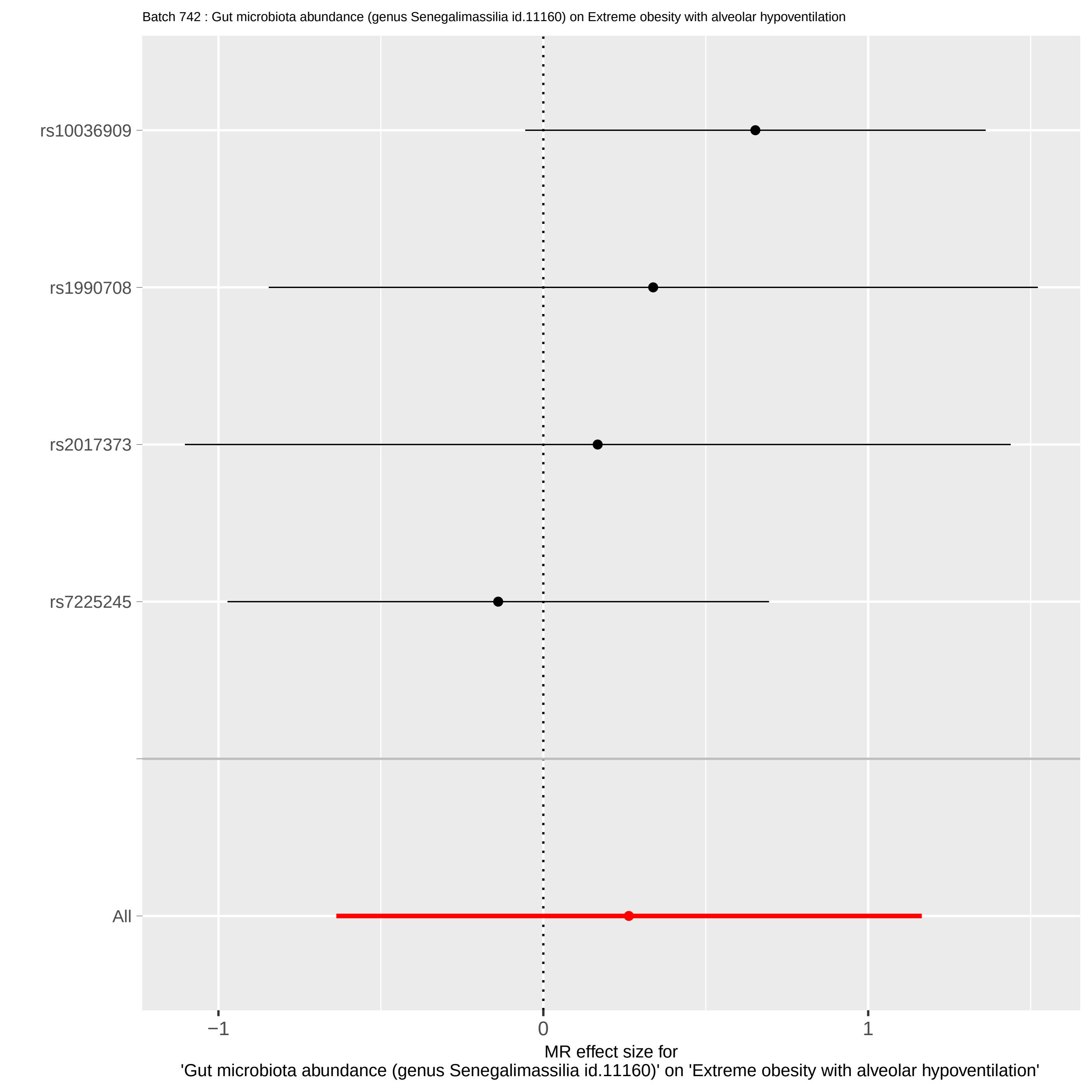
Batch 742 : Gut microbiota abundance (genus Senegalimassilia id.11160) on Extreme obesity with alveolar hypoventilation
rs10036909
rs1990708
rs2017373
rs7225245
All
−1
0
1
MR effect size for
'Gut microbiota abundance (genus Senegalimassilia id.11160)' on 'Extreme obesity with alveolar hypoventilation'

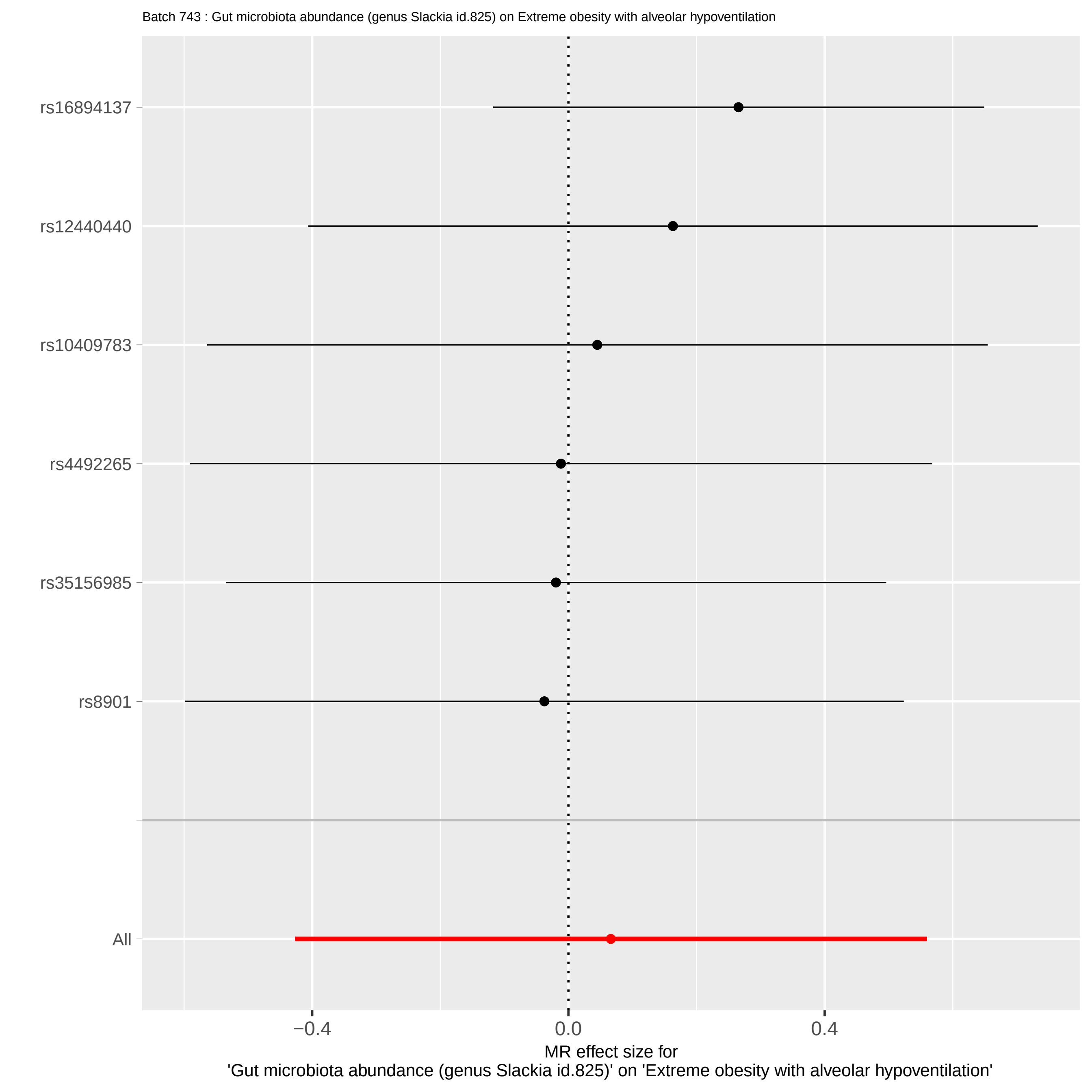
Batch 743 : Gut microbiota abundance (genus Slackia id.825) on Extreme obesity with alveolar hypoventilation
rs16894137
rs12440440
rs10409783
rs4492265
rs35156985
rs8901
All
−0.4
0.0
0.4
MR effect size for
'Gut microbiota abundance (genus Slackia id.825)' on 'Extreme obesity with alveolar hypoventilation'

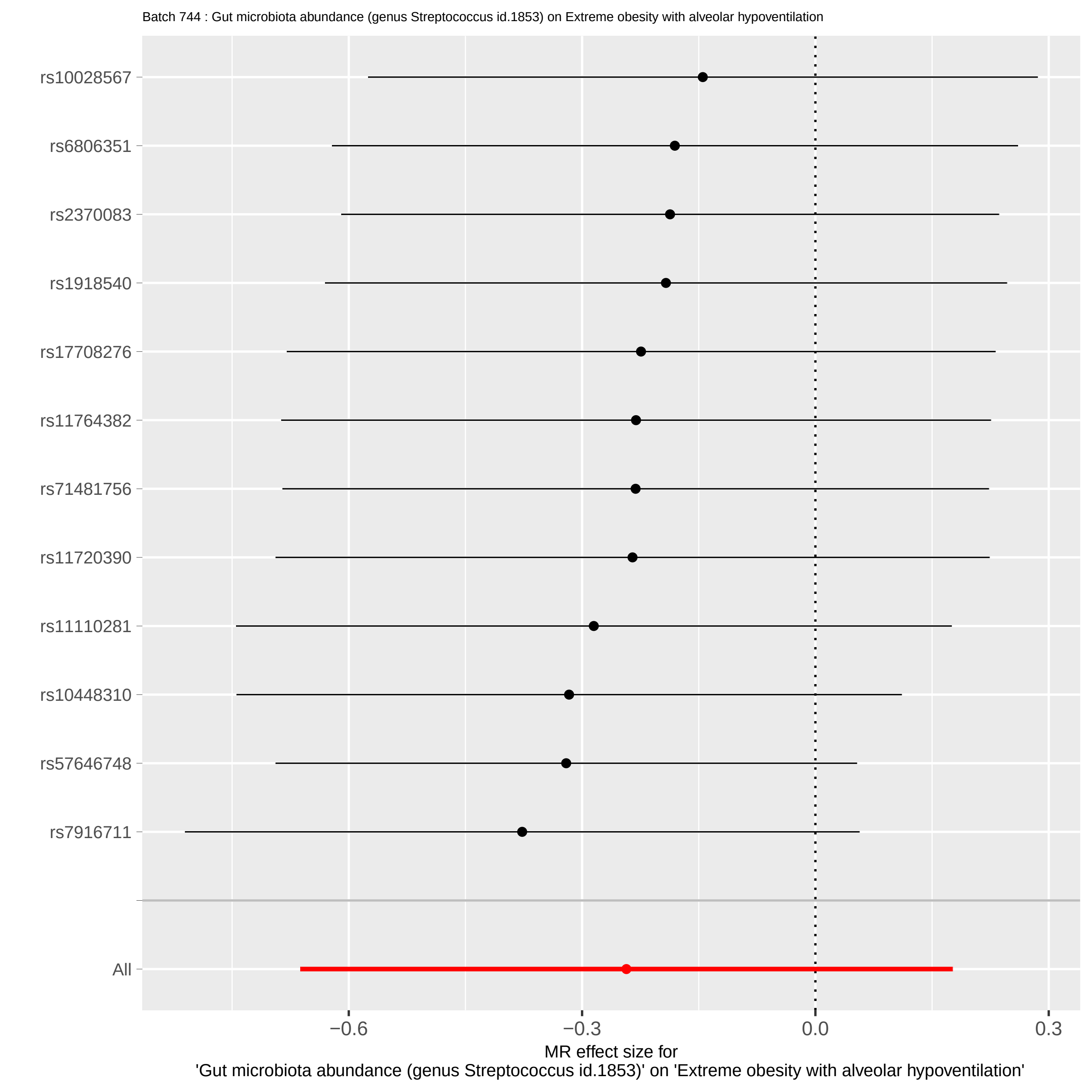
Batch 744 : Gut microbiota abundance (genus Streptococcus id.1853) on Extreme obesity with alveolar hypoventilation
rs10028567
rs6806351
rs2370083
rs1918540
rs17708276
rs11764382
rs71481756
rs11720390
rs11110281
rs10448310
rs57646748
rs7916711
All
−0.6
−0.3
0.0
0.3
MR effect size for
'Gut microbiota abundance (genus Streptococcus id.1853)' on 'Extreme obesity with alveolar hypoventilation'

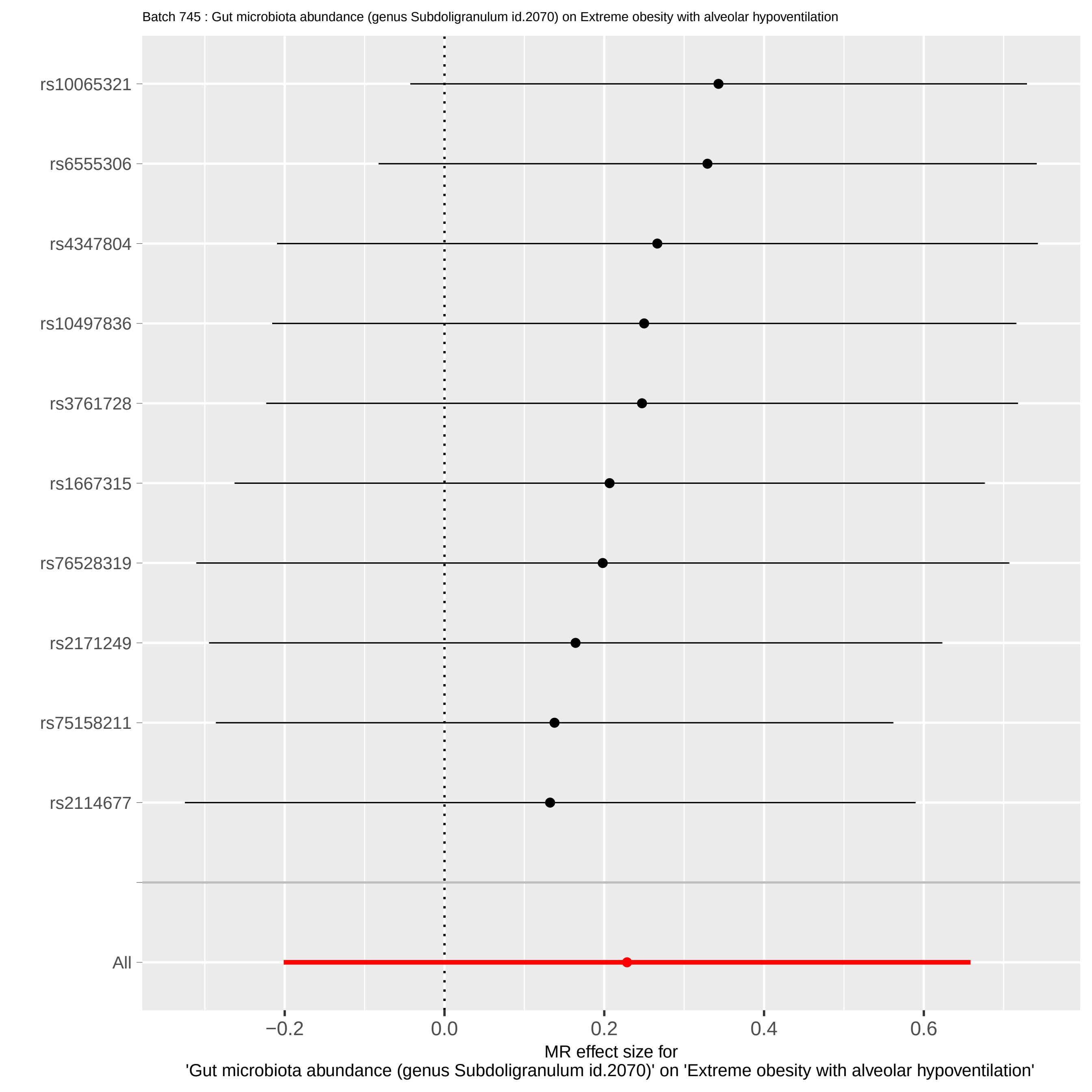

Batch 745 : Gut microbiota abundance (genus Subdoligranulum id.2070) on Extreme obesity with alveolar hypoventilation
rs10065321
rs6555306
rs4347804
rs10497836
rs3761728
rs1667315
rs76528319
rs2171249
rs75158211
rs2114677
All
−0.2
0.0
0.2
0.4
0.6
MR effect size for
'Gut microbiota abundance (genus Subdoligranulum id.2070)' on 'Extreme obesity with alveolar hypoventilation'

Batch 746 : Gut microbiota abundance (genus Sutterella id.2896) on Extreme obesity with alveolar hypoventilation

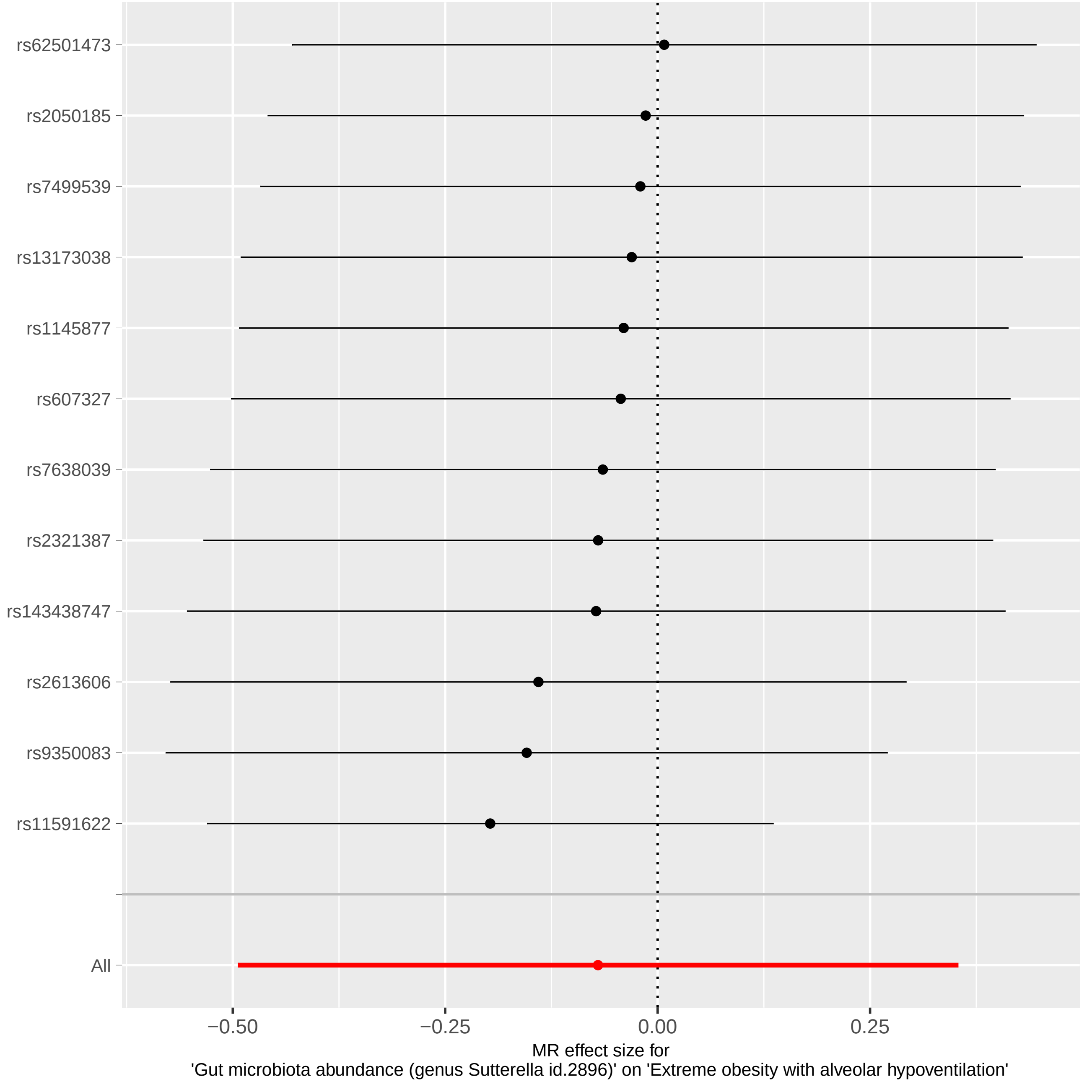

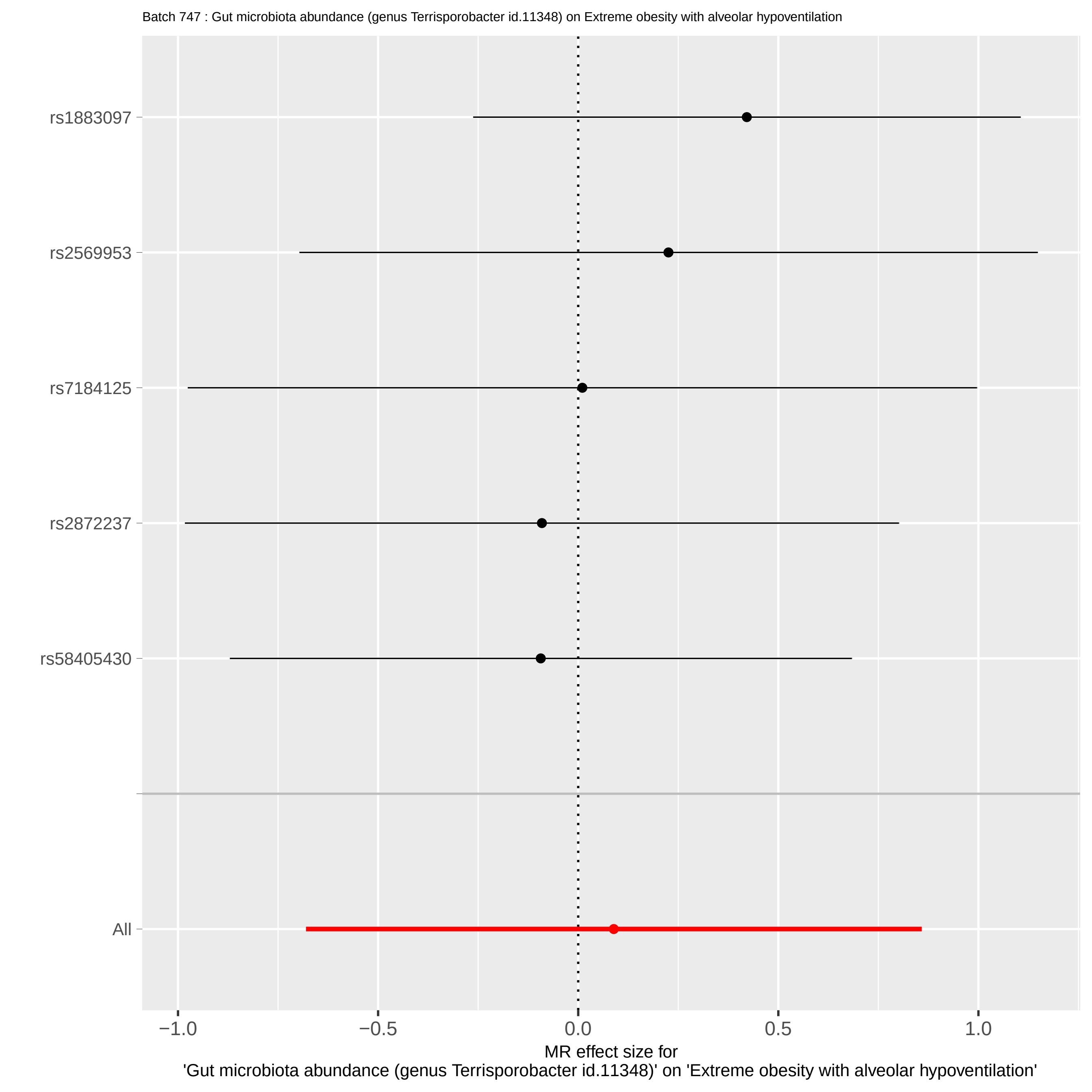
Batch 747 : Gut microbiota abundance (genus Terrisporobacter id.11348) on Extreme obesity with alveolar hypoventilation
rs1883097
rs2569953
rs7184125
rs2872237
rs58405430
All
−1.0
−0.5
0.0
0.5
1.0
MR effect size for
'Gut microbiota abundance (genus Terrisporobacter id.11348)' on 'Extreme obesity with alveolar hypoventilation'

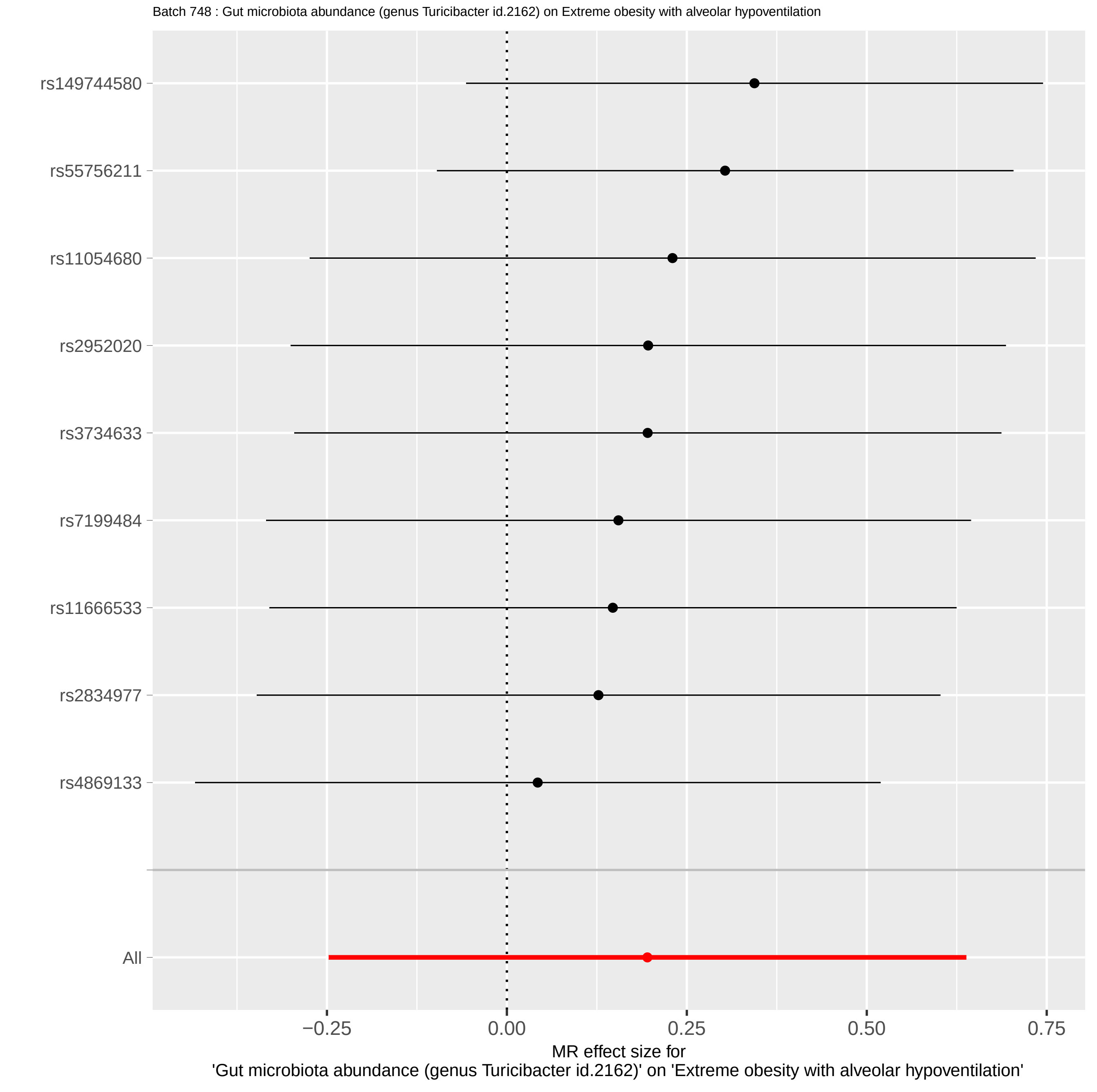
Batch 748 : Gut microbiota abundance (genus Turicibacter id.2162) on Extreme obesity with alveolar hypoventilation
rs149744580
rs55756211
rs11054680
rs2952020
rs3734633
rs7199484
rs11666533
rs2834977
rs4869133
All
−0.25
0.00
0.25
0.50
0.75
MR effect size for
'Gut microbiota abundance (genus Turicibacter id.2162)' on 'Extreme obesity with alveolar hypoventilation'

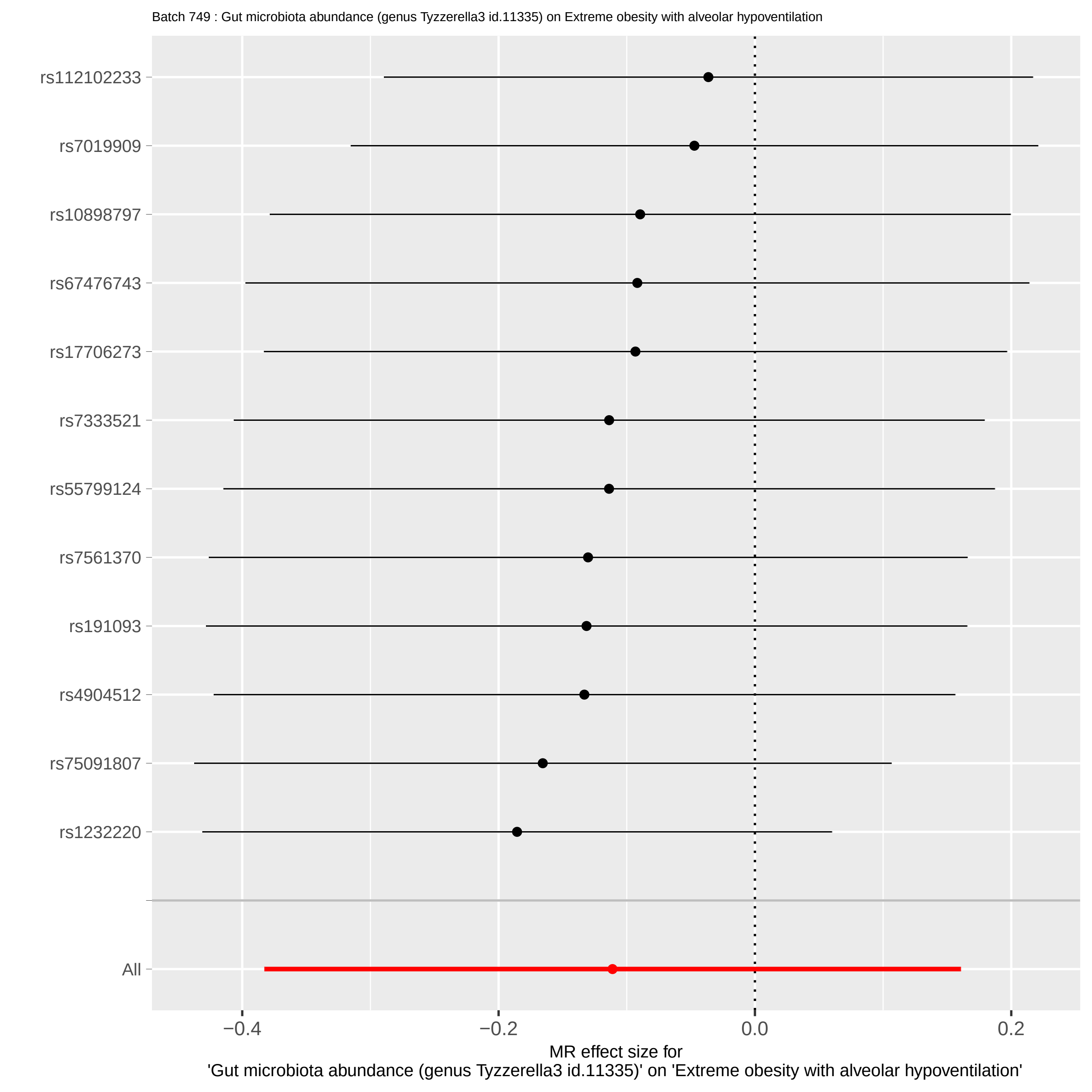

Batch 749 : Gut microbiota abundance (genus Tyzzerella3 id.11335) on Extreme obesity with alveolar hypoventilation
rs112102233
rs7019909
rs10898797
rs67476743
rs17706273
rs7333521
rs55799124
rs7561370
rs191093
rs4904512
rs75091807
rs1232220
All
−0.4
−0.2
0.0
0.2
MR effect size for
'Gut microbiota abundance (genus Tyzzerella3 id.11335)' on 'Extreme obesity with alveolar hypoventilation'

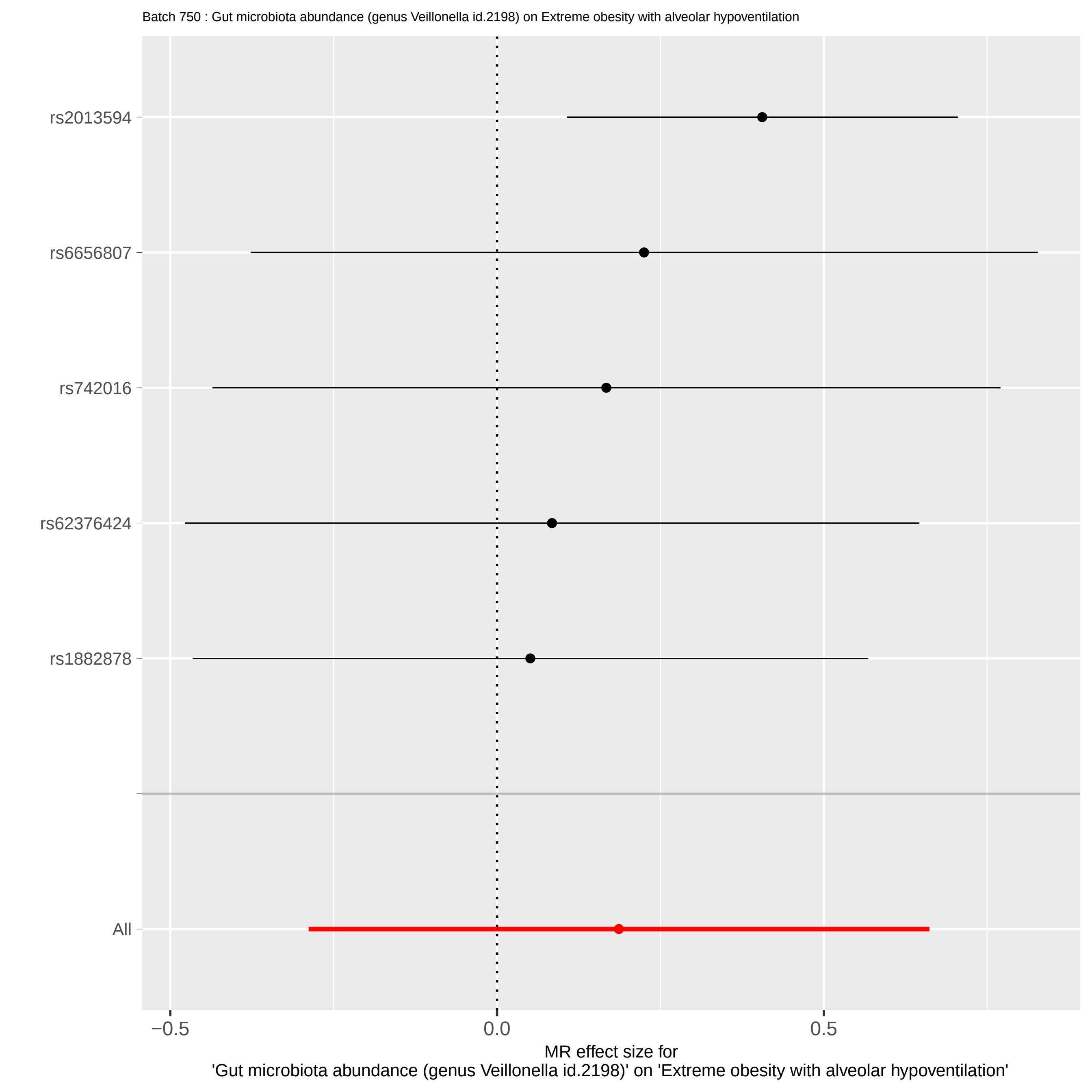
Batch 750 : Gut microbiota abundance (genus Veillonella id.2198) on Extreme obesity with alveolar hypoventilation
rs2013594
rs6656807
rs742016
rs62376424
rs1882878
All
−0.5
0.0
0.5
MR effect size for
'Gut microbiota abundance (genus Veillonella id.2198)' on 'Extreme obesity with alveolar hypoventilation'
