# Supplementary material for: Unraveling the mystery: a Mendelian randomized exploration of gut microbiota and different types of obesity
Source: Front Cell Infect Microbiol. 2024 Feb 5;14:1352109. doi: 10.3389/fcimb.2024.1352109 (PMC10875079; doi:10.3389/fcimb.2024.1352109)

Batch 1 : Gut microbiota abundance (family Acidaminococcaceae id.2166) on Localized adiposity

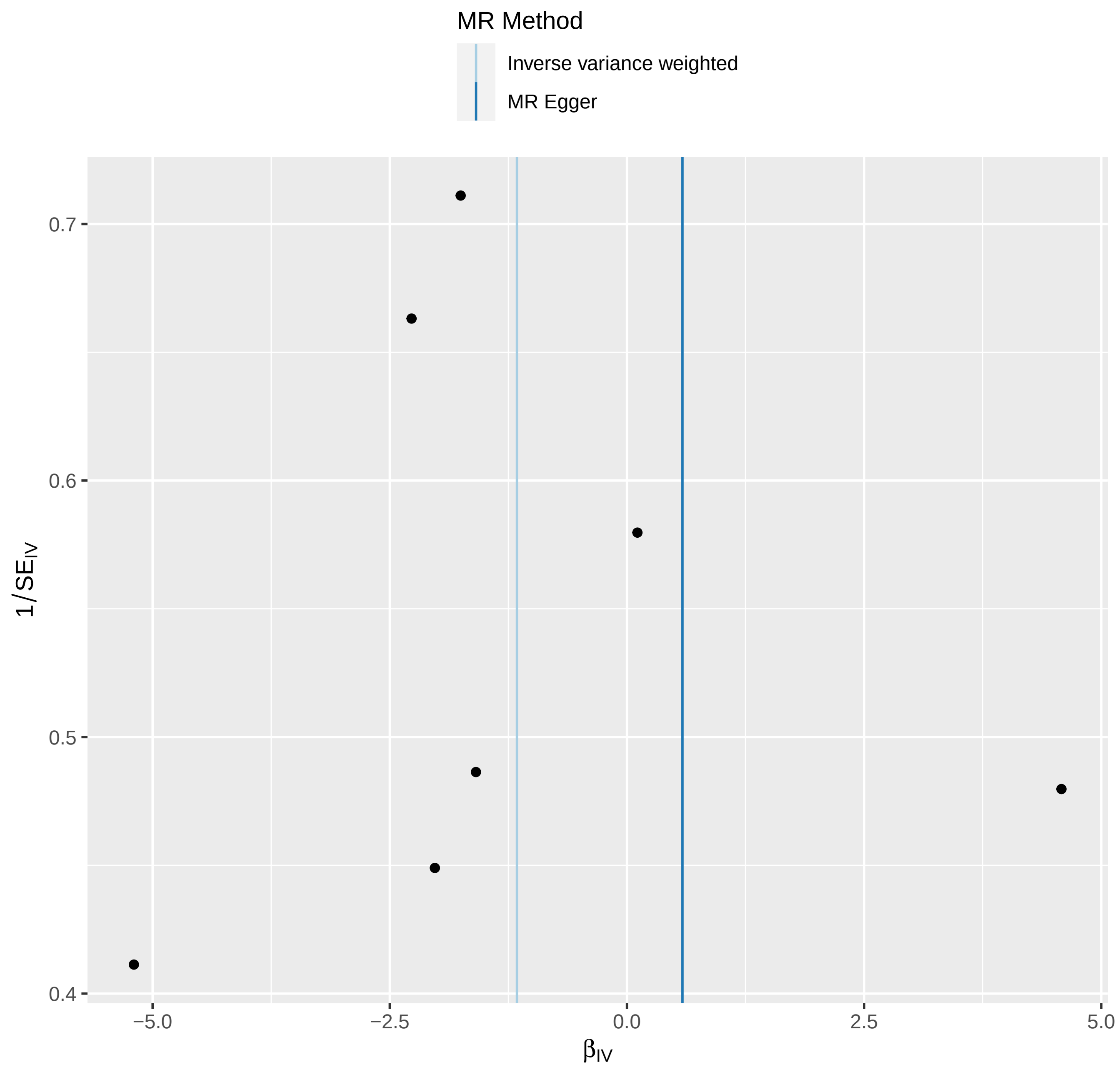

Batch 2 : Gut microbiota abundance (family Actinomycetaceae id.421) on Localized adiposity

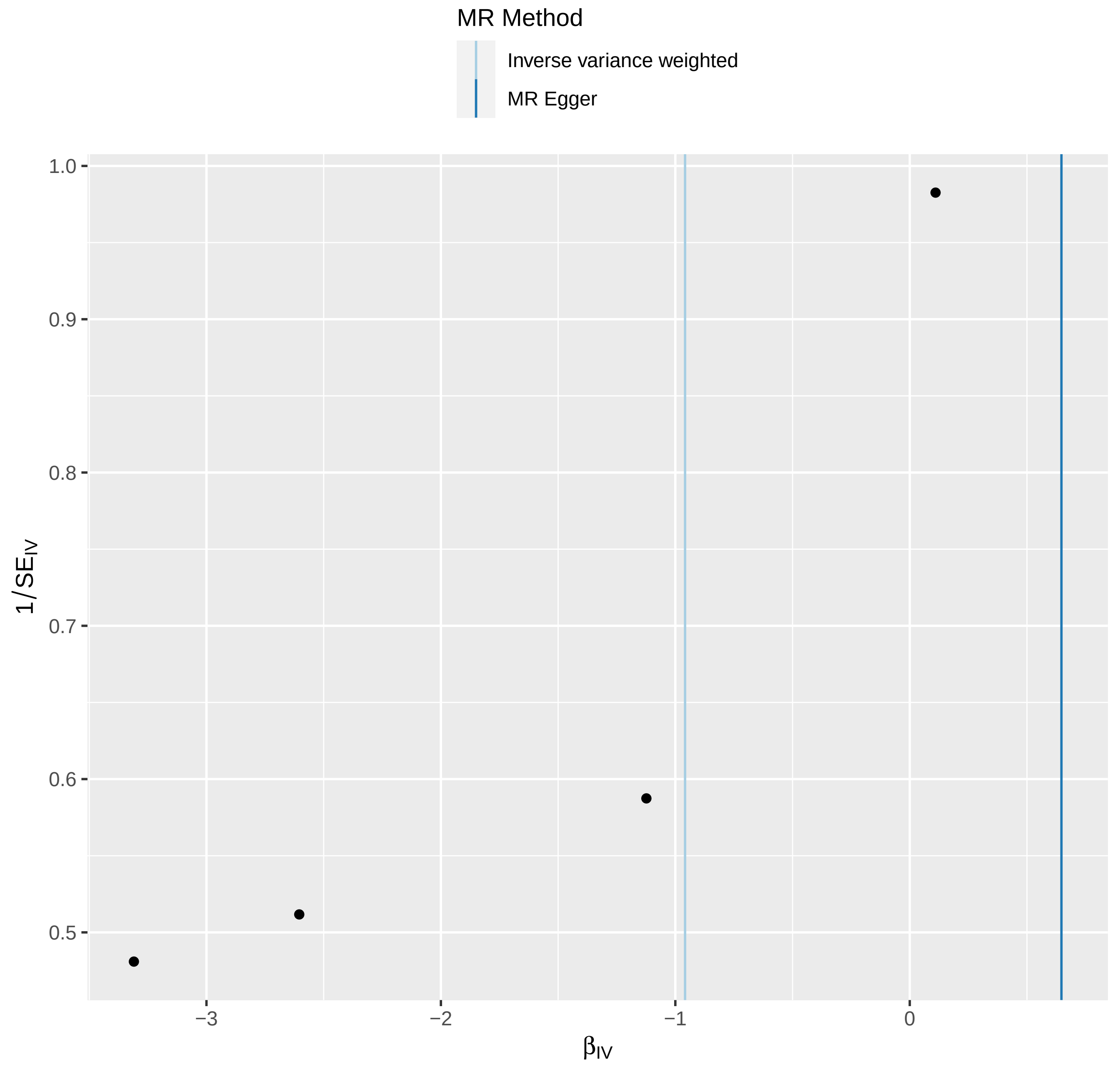

Batch 3 : Gut microbiota abundance (family Alcaligenaceae id.2875) on Localized adiposity

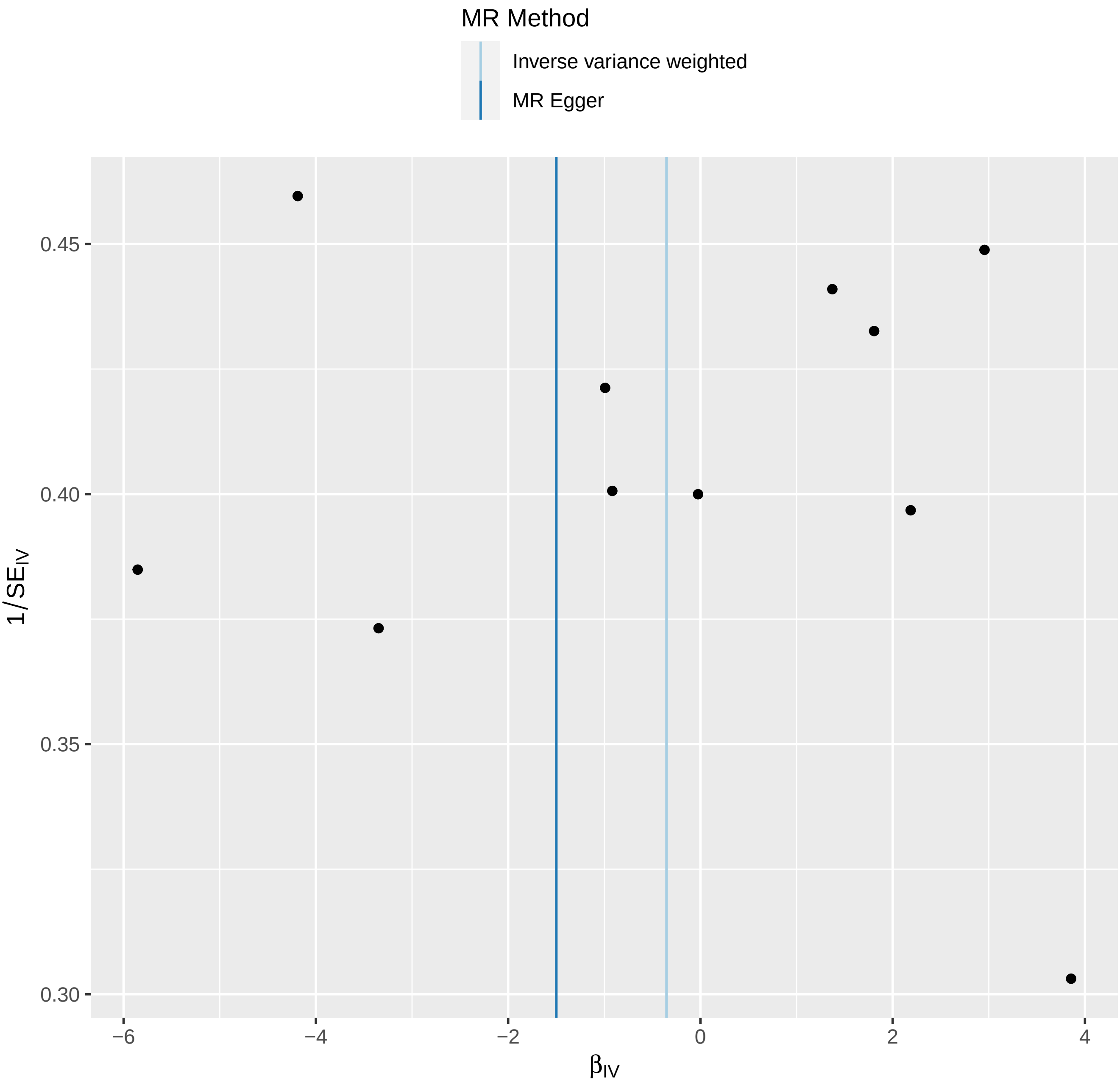

Batch 4 : Gut microbiota abundance (family Bacteroidaceae id.917) on Localized adiposity

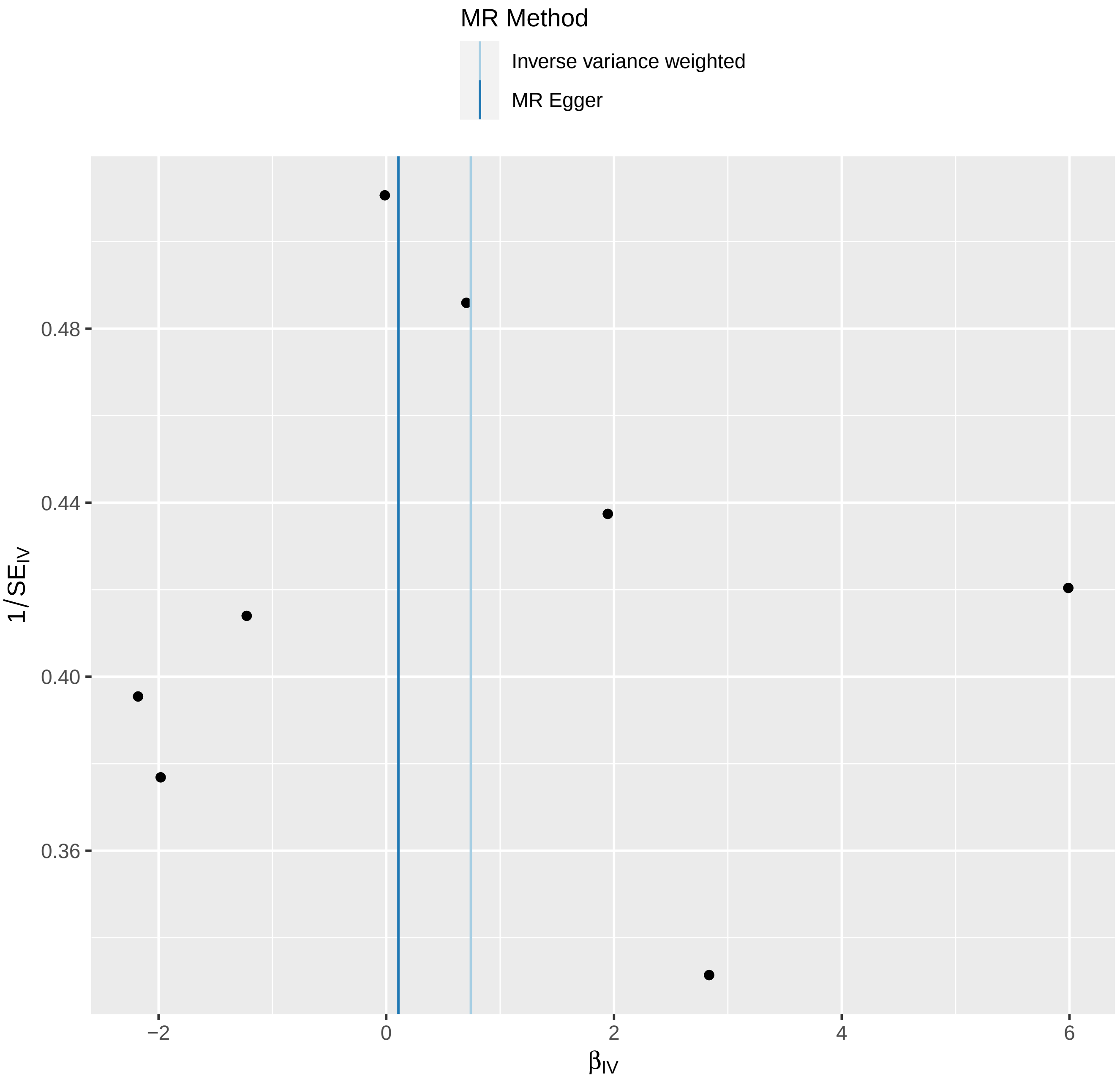

Batch 5 : Gut microbiota abundance (family Bacteroidales S24 7group id.11173) on Localized adiposity

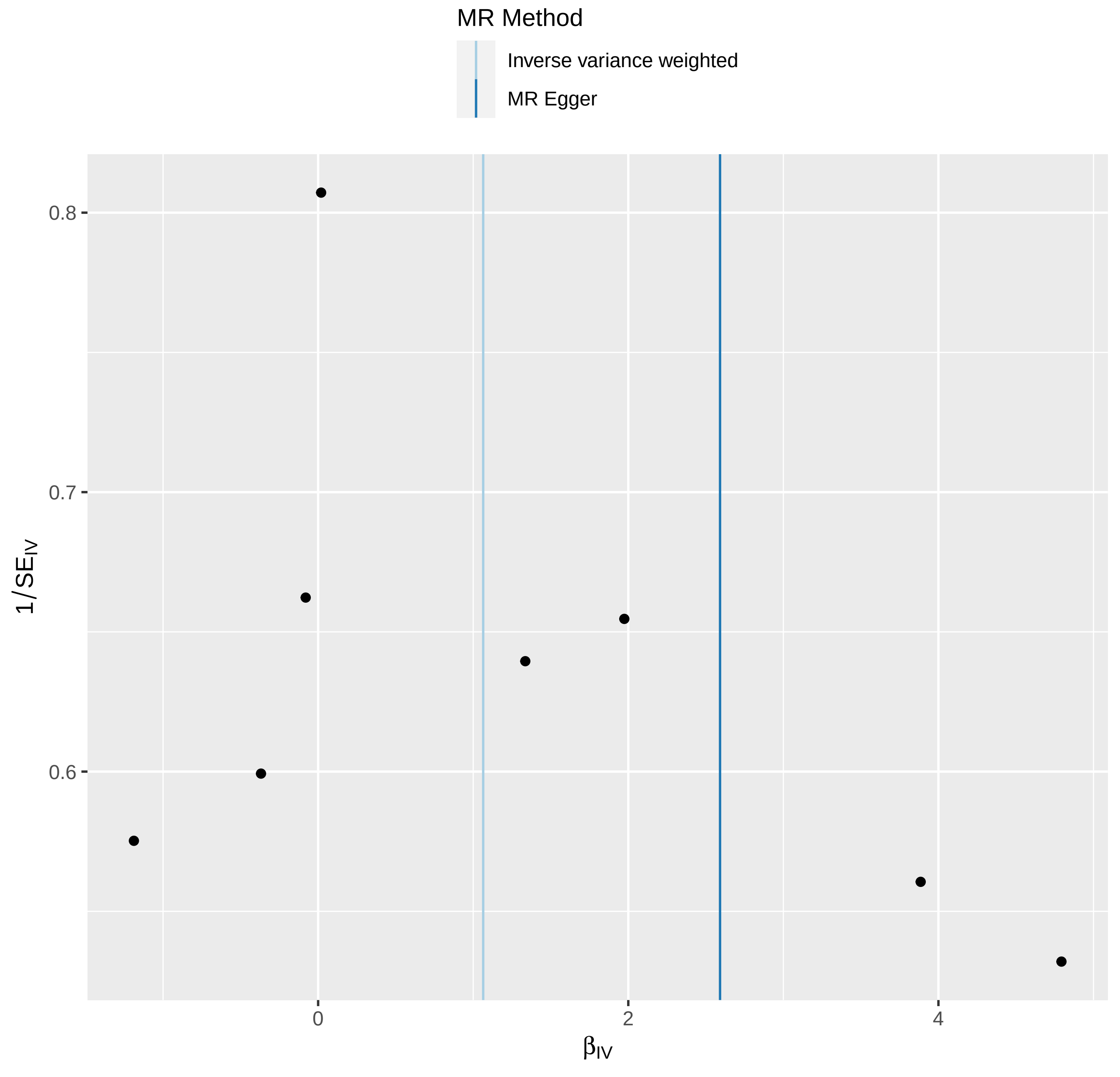

Batch 6 : Gut microbiota abundance (family Bifidobacteriaceae id.433) on Localized adiposity

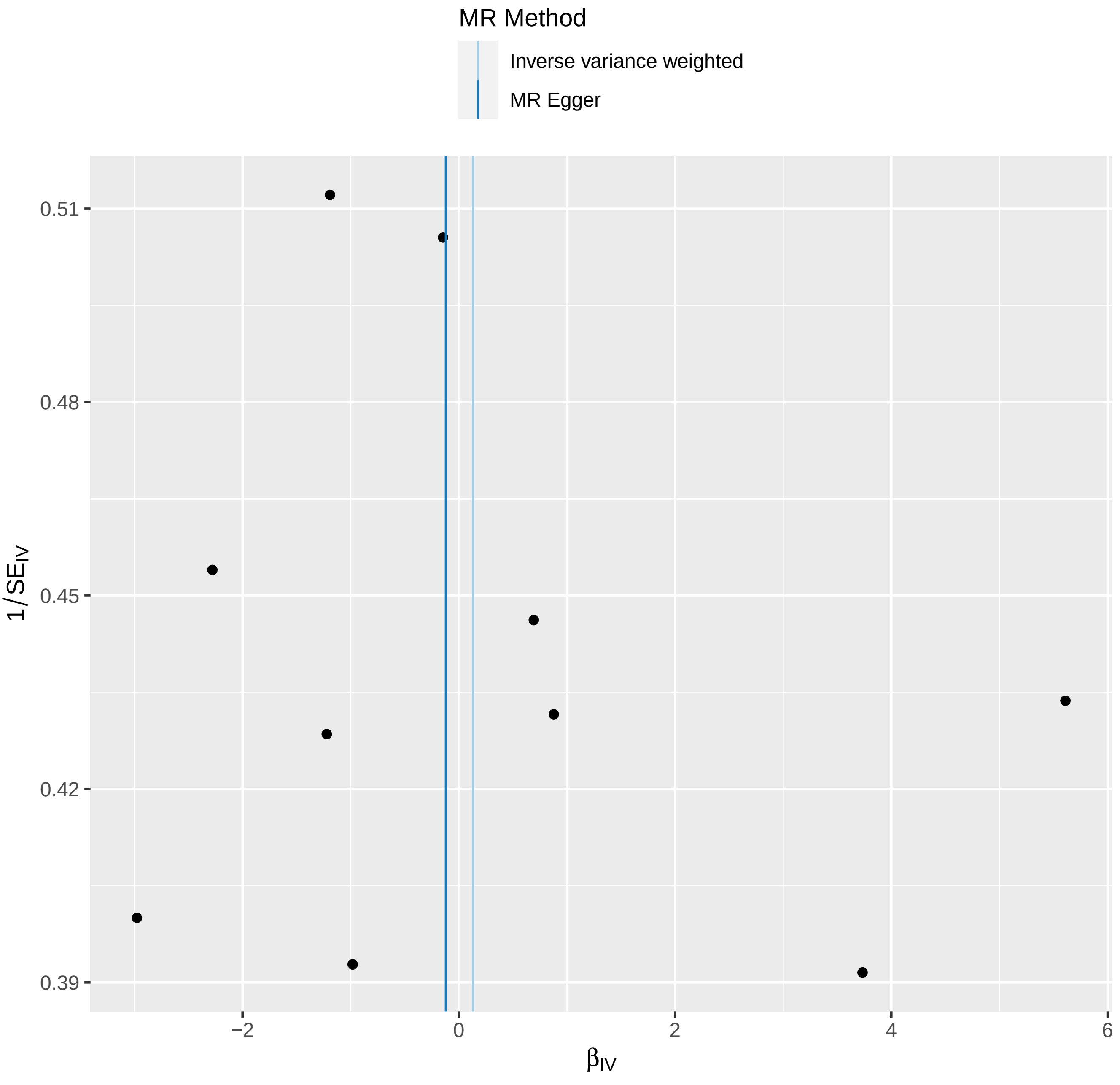

Batch 8 : Gut microbiota abundance (family Clostridiaceae1 id.1869) on Localized adiposity

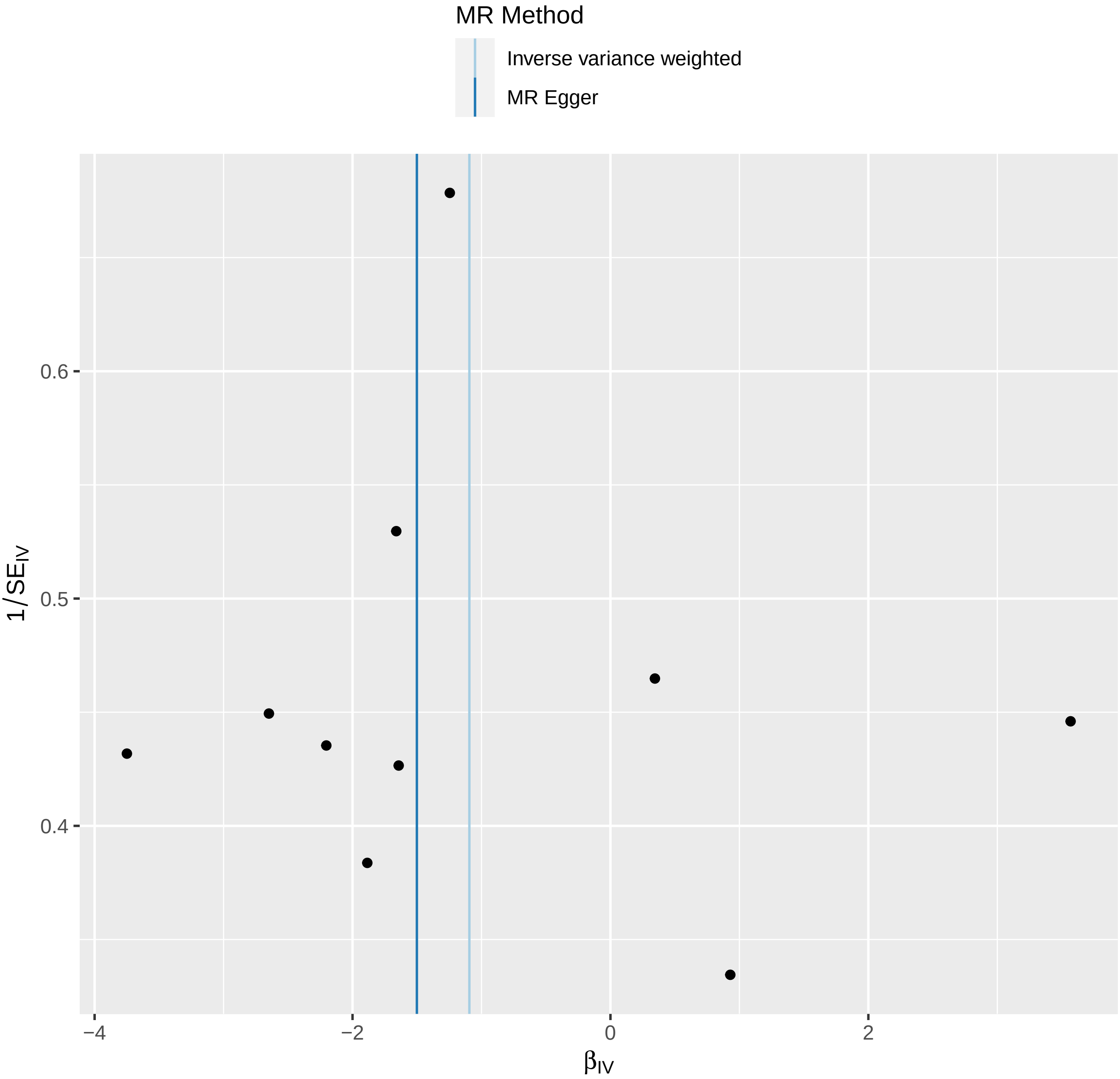

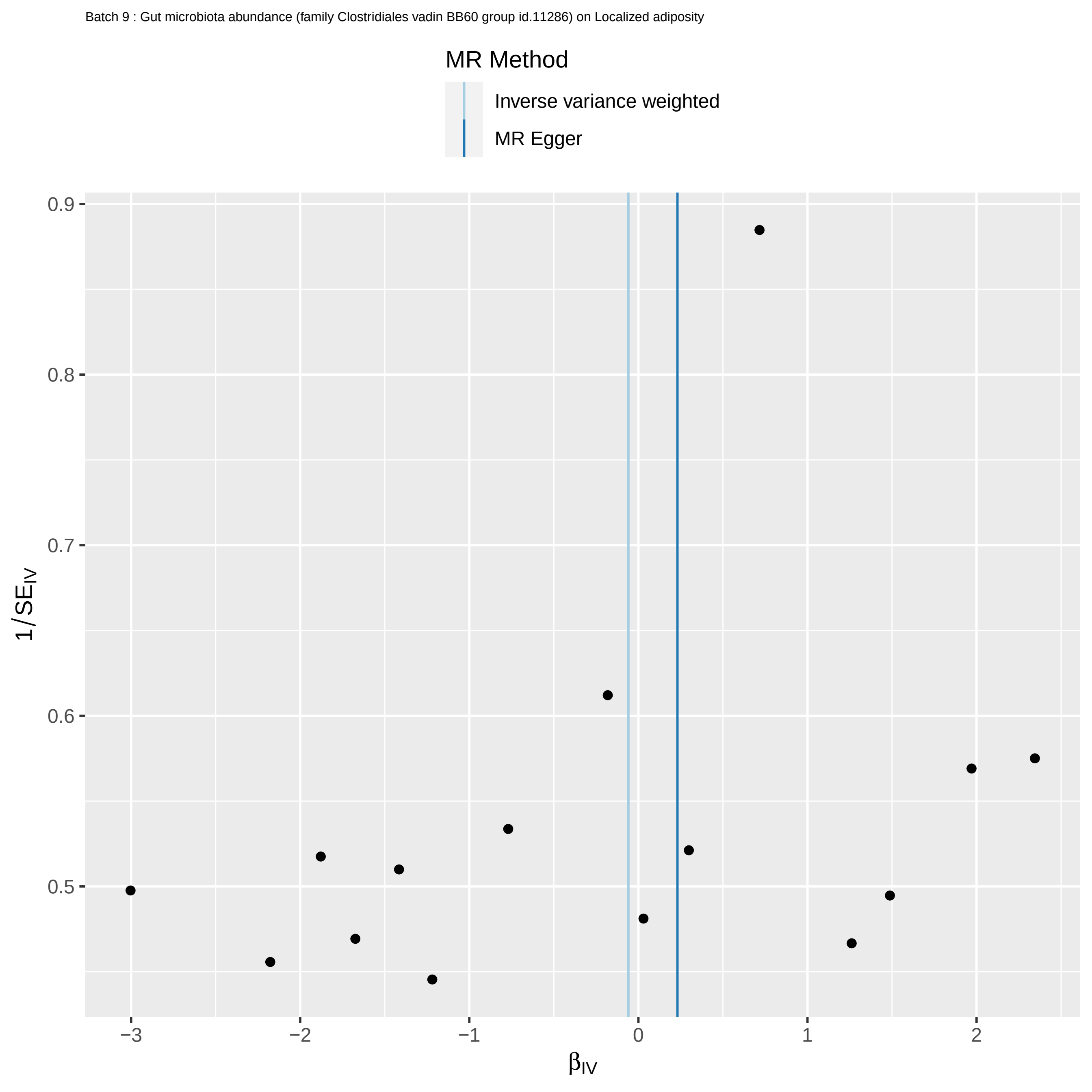

Batch 9 : Gut microbiota abundance (family Clostridiales vadin BB60 group id.11286) on Localized adiposity
MR Method
Inverse variance weighted
MR Egger
0.9
0.8
0.7
0.6
0.5
$1/SE_{IV}$
−3
−2
−1
0
1
2
$\beta_{IV}$

Batch 10 : Gut microbiota abundance (family Coriobacteriaceae id.811) on Localized adiposity

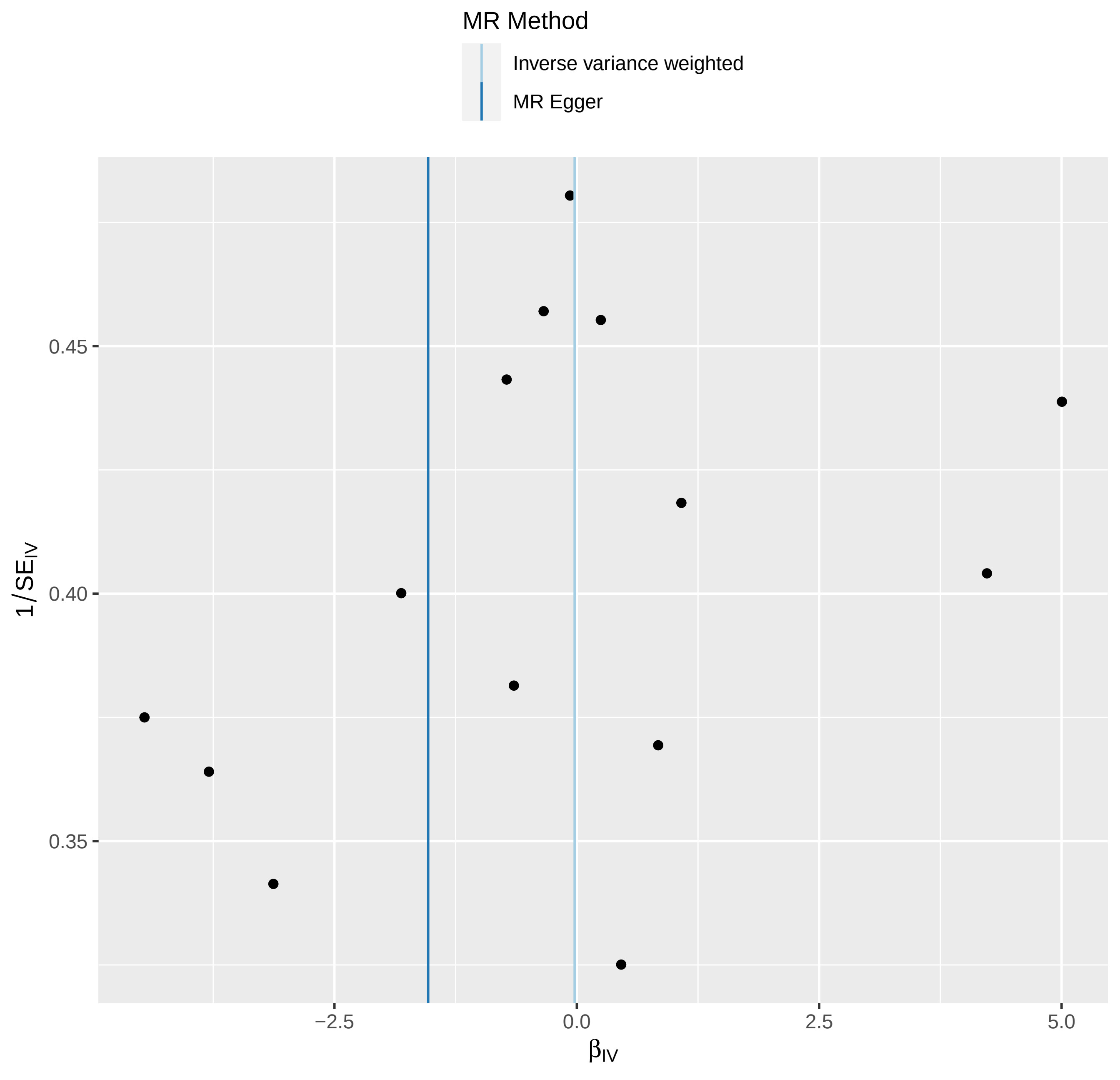

Batch 11 : Gut microbiota abundance (family Defluviitaleaceae id.1924) on Localized adiposity

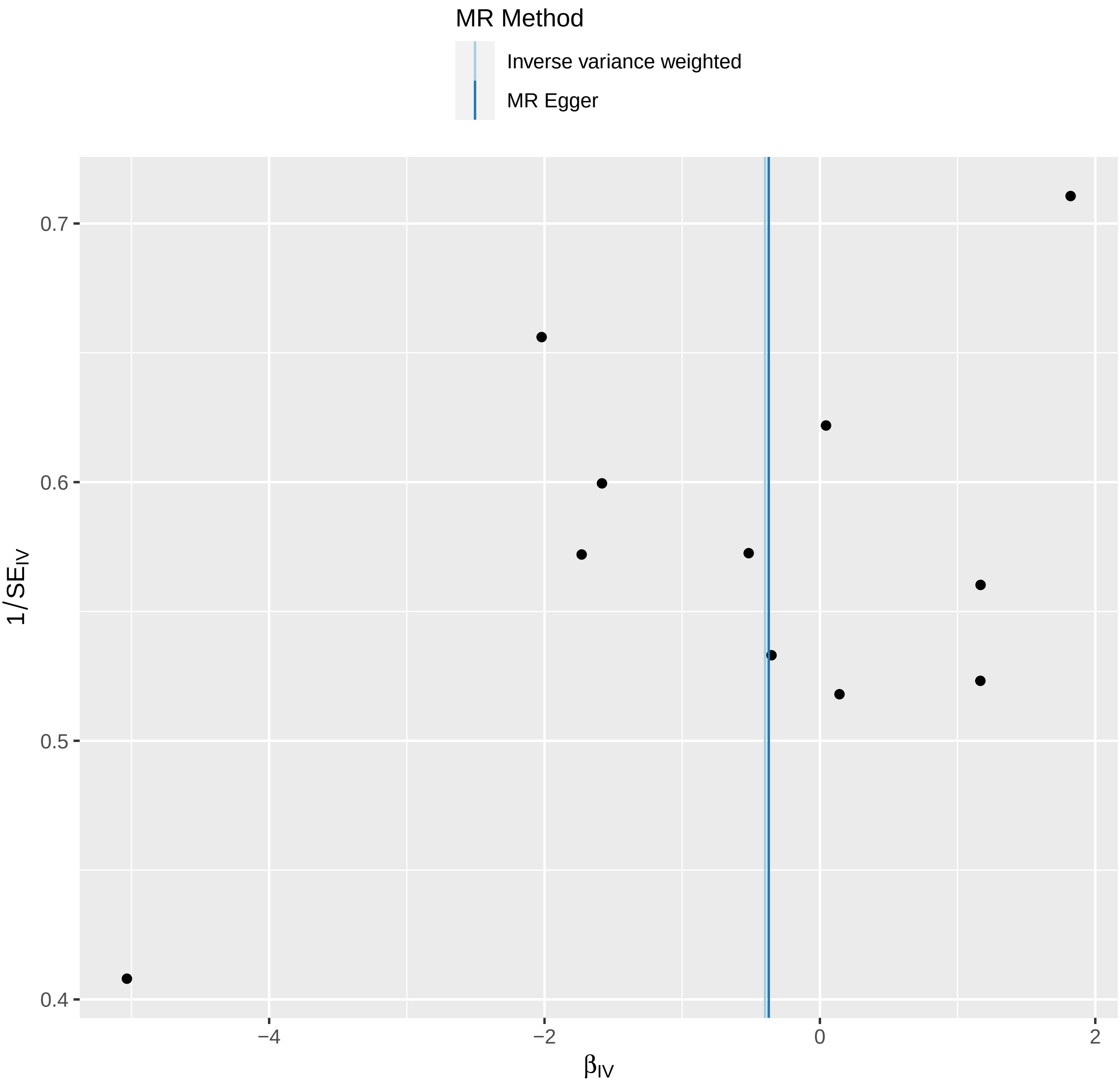

Batch 12 : Gut microbiota abundance (family Desulfovibrionaceae id.3169) on Localized adiposity

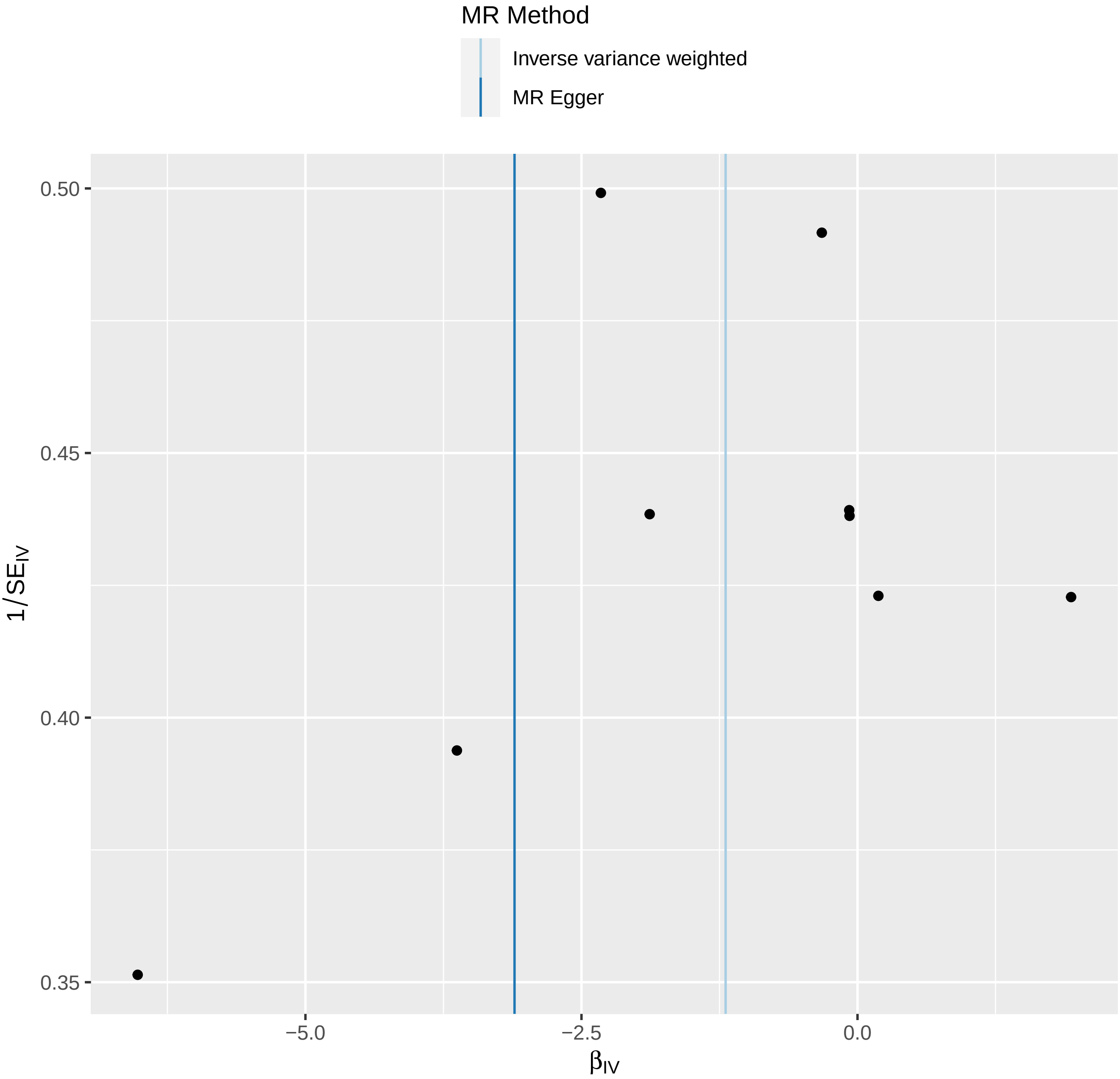

Batch 13 : Gut microbiota abundance (family Enterobacteriaceae id.3469) on Localized adiposity

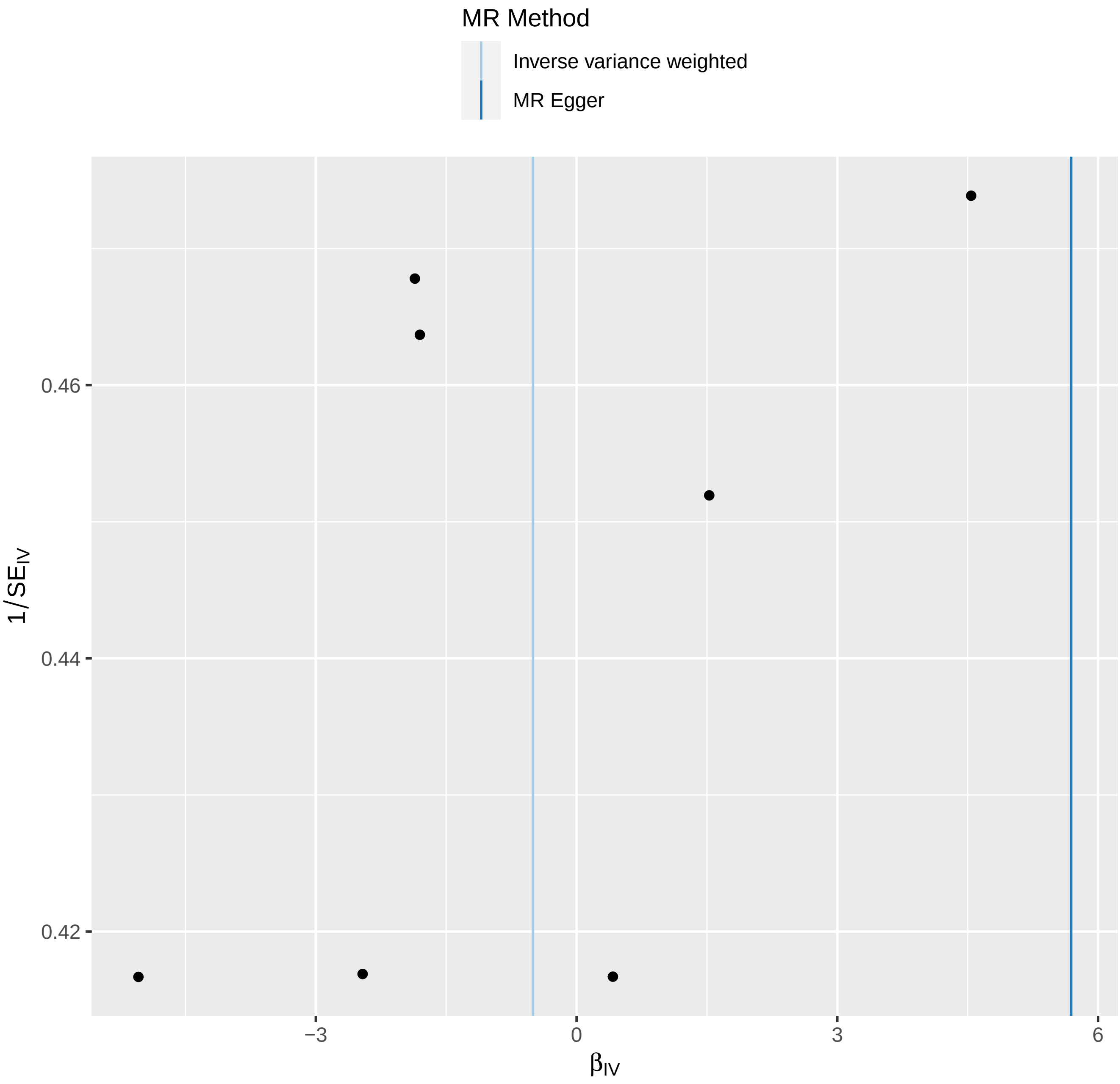

Batch 14 : Gut microbiota abundance (family Erysipelotrichaceae id.2149) on Localized adiposity

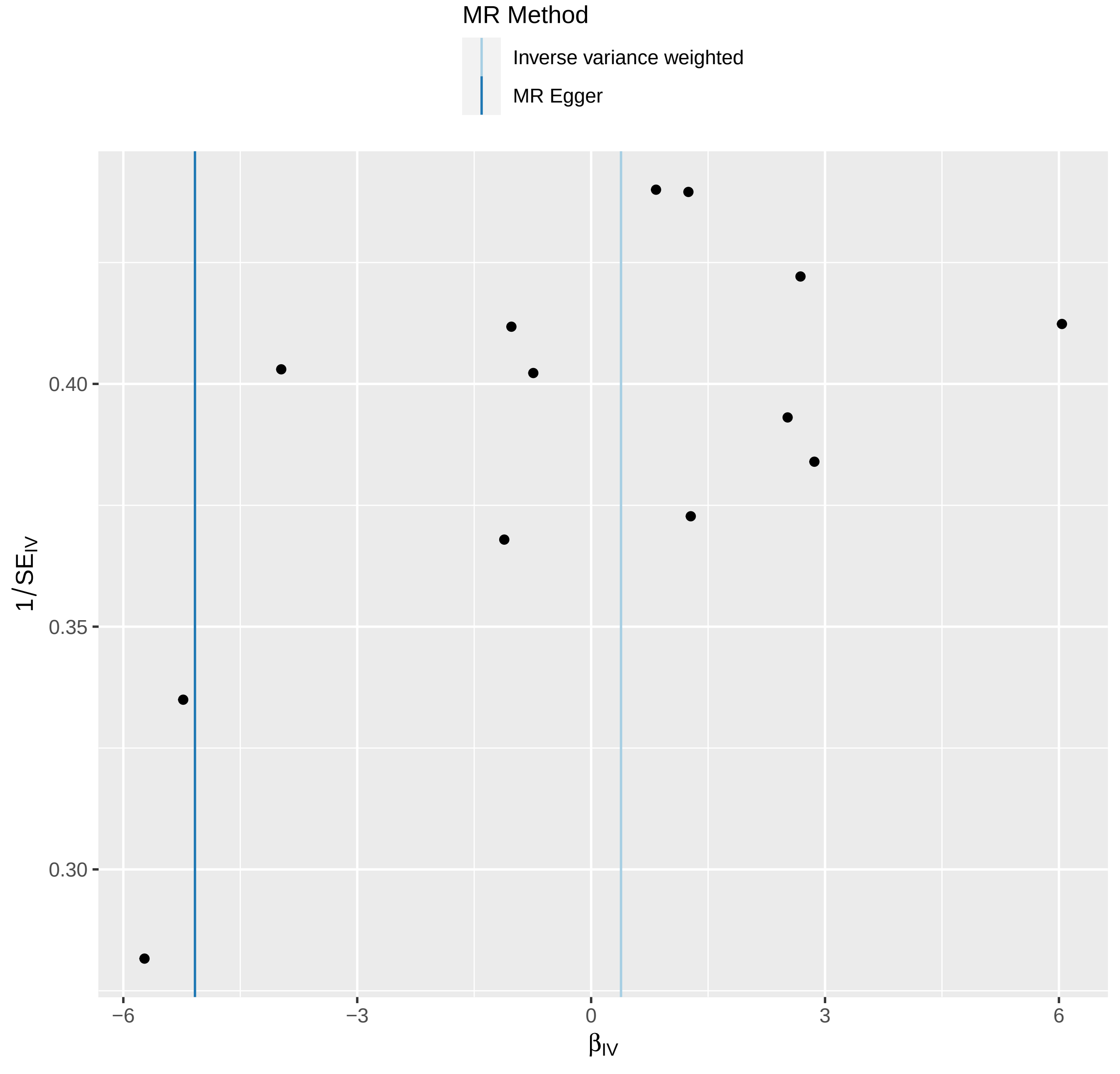

Batch 15 : Gut microbiota abundance (family Family XI id.1936) on Localized adiposity

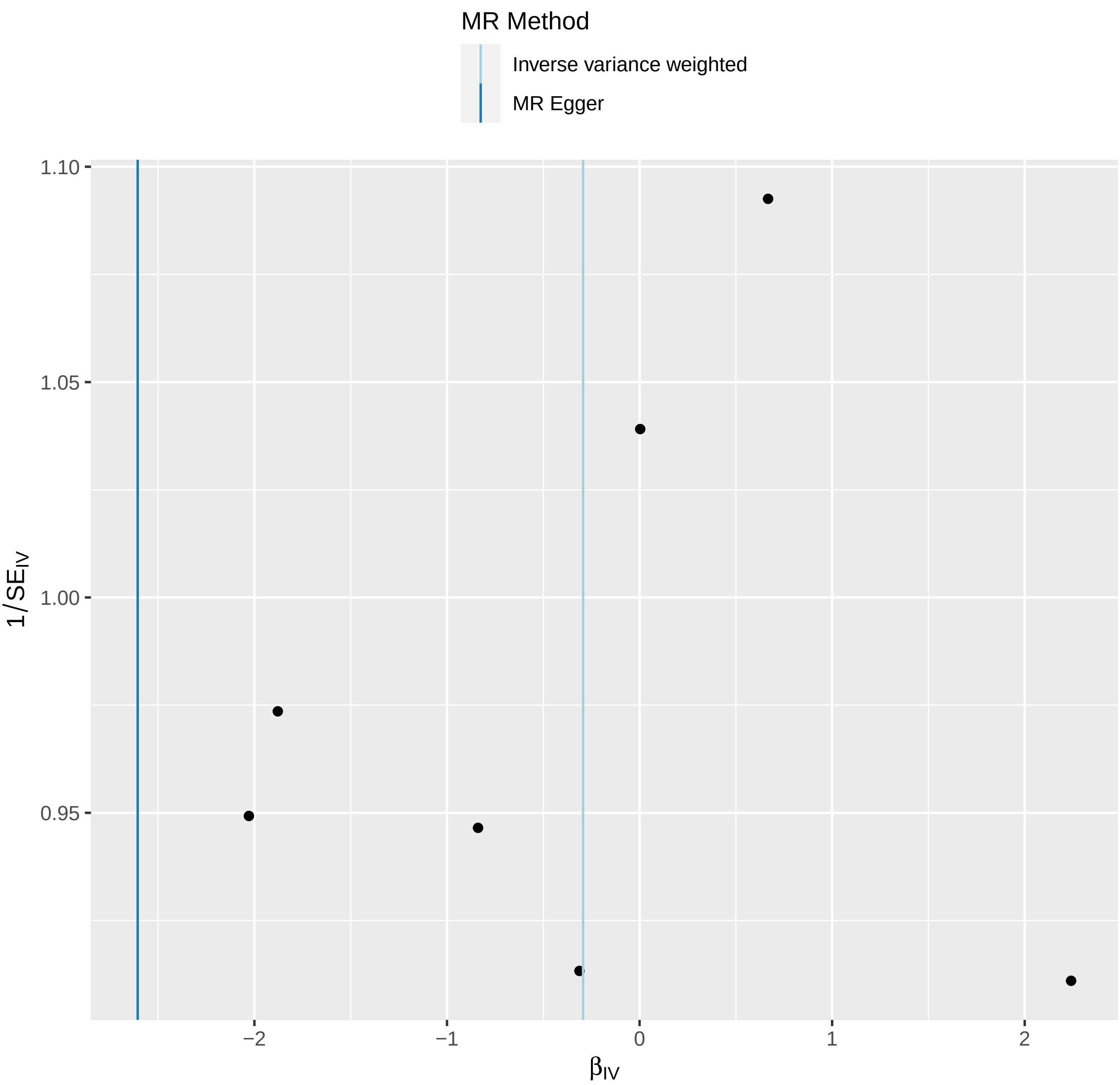

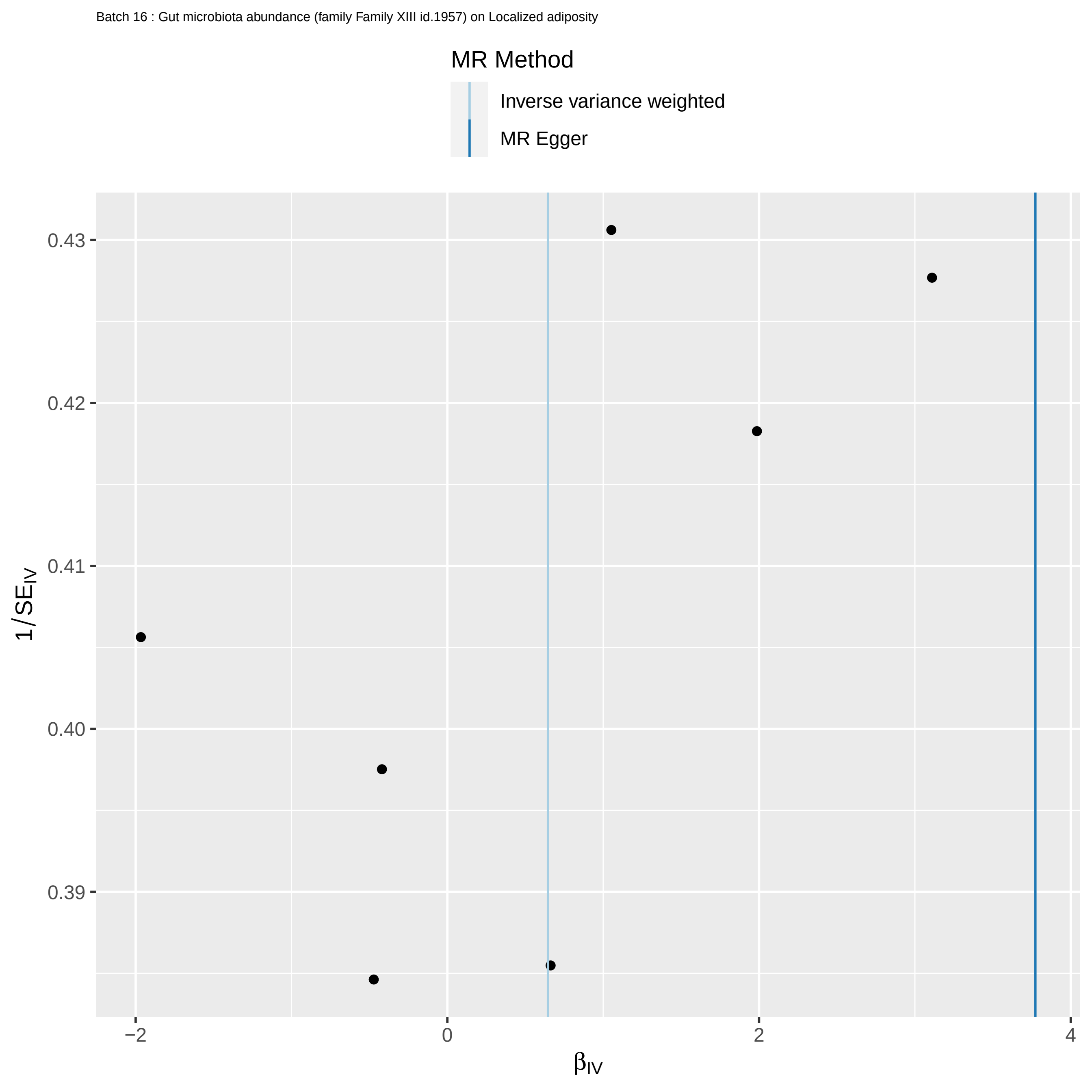

Batch 16 : Gut microbiota abundance (family Family XIII id.1957) on Localized adiposity
MR Method
Inverse variance weighted
MR Egger
0.43
0.42
0.41
0.40
0.39
1/SE_IV
−2
0
2
4
β_IV

Batch 17 : Gut microbiota abundance (family Lachnospiraceae id.1987) on Localized adiposity

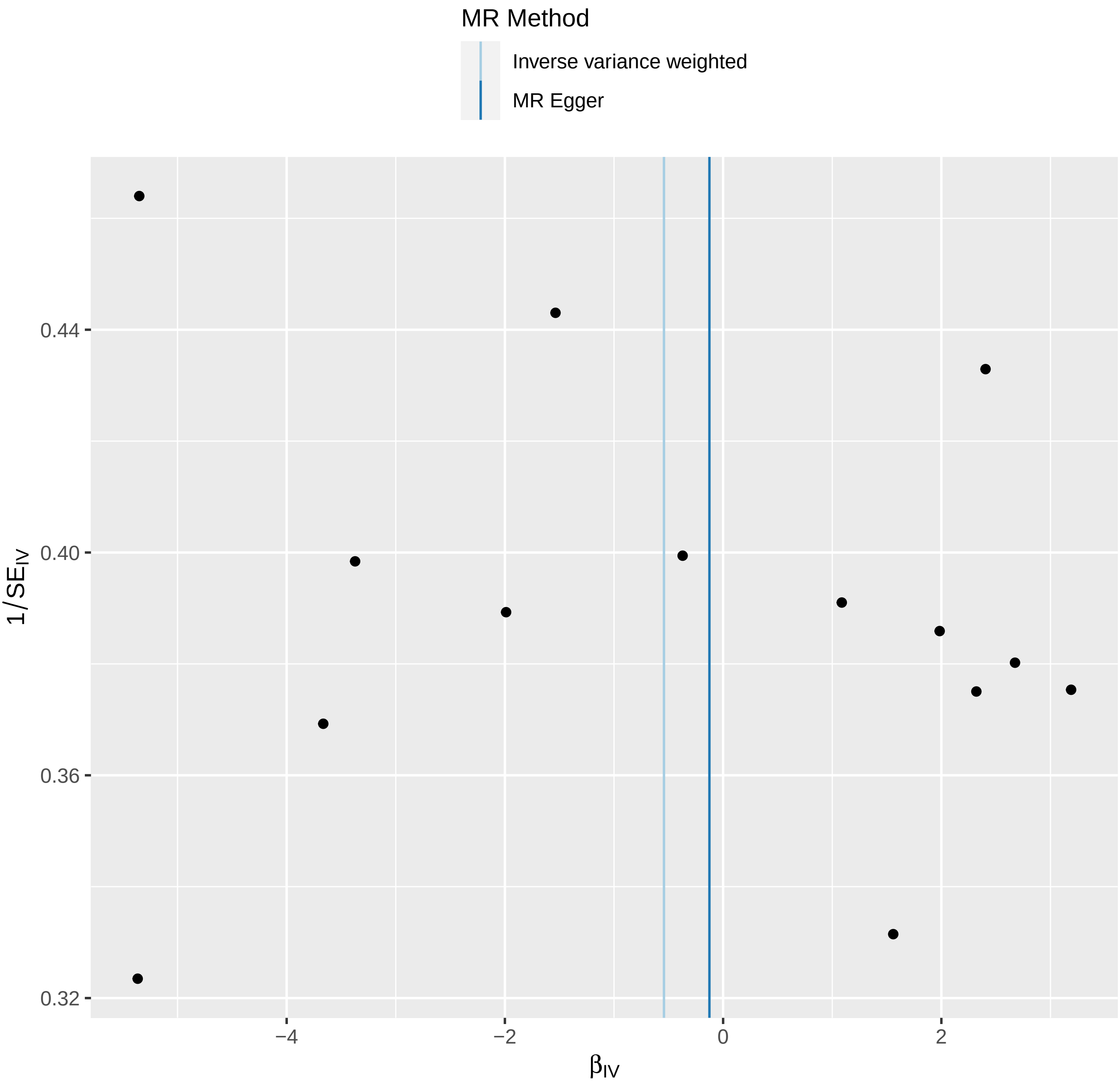

Batch 18 : Gut microbiota abundance (family Lactobacillaceae id.1836) on Localized adiposity

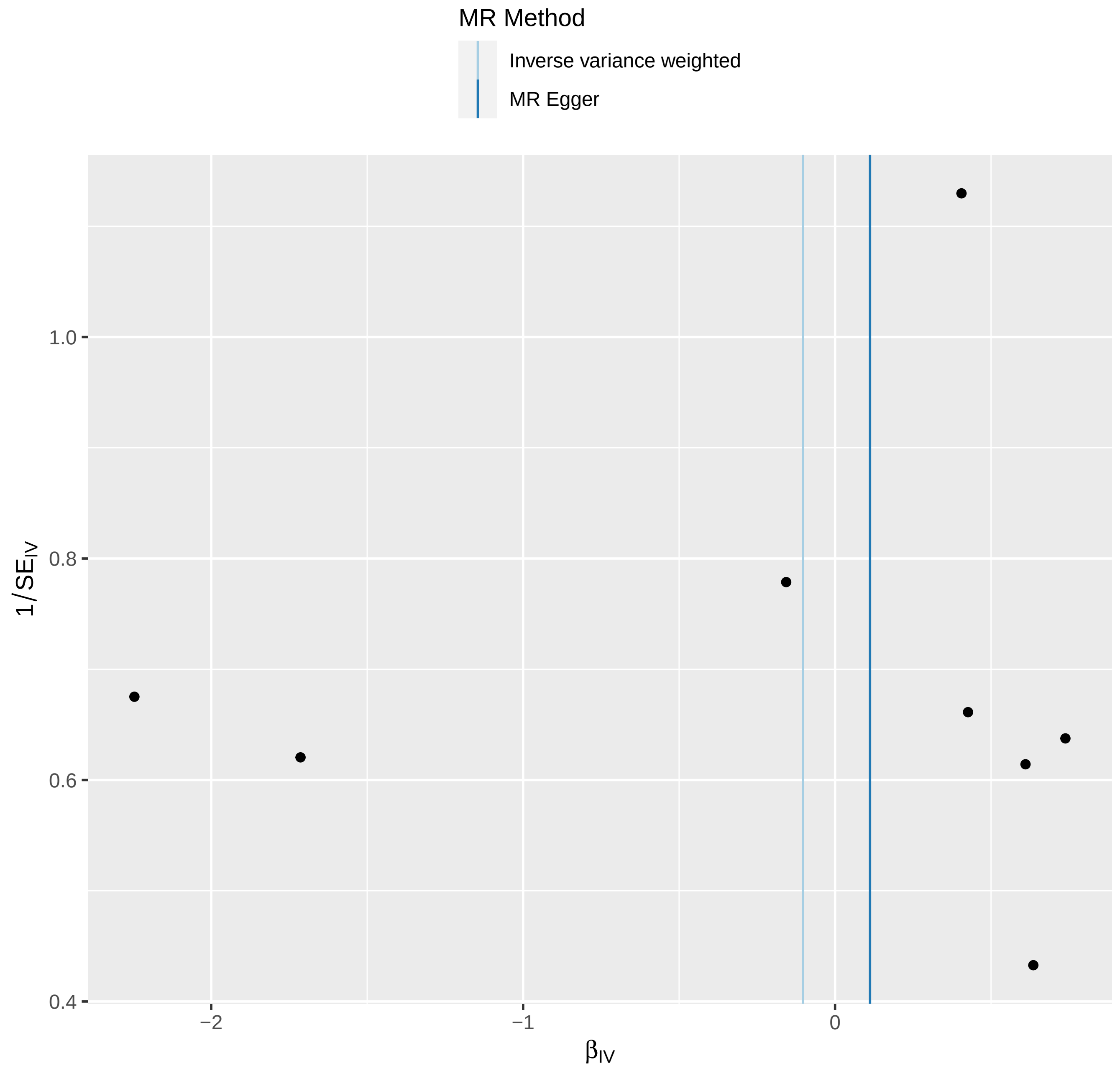

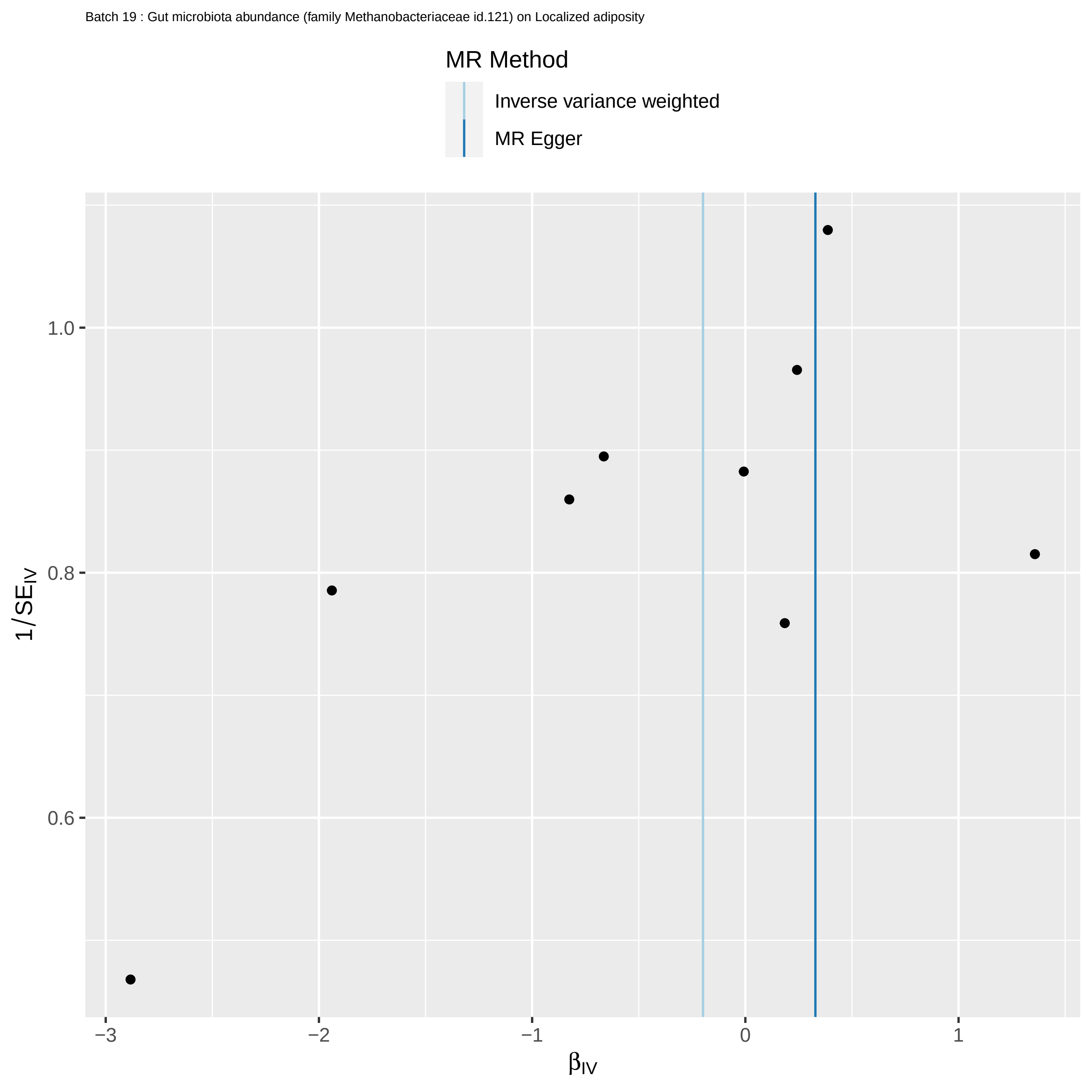
Batch 19 : Gut microbiota abundance (family Methanobacteriaceae id.121) on Localized adiposity
MR Method
Inverse variance weighted
MR Egger
1.0
0.8
0.6
1/SE_IV
−3
−2
−1
0
1
β_IV

Batch 20 : Gut microbiota abundance (family Oxalobacteraceae id.2966) on Localized adiposity

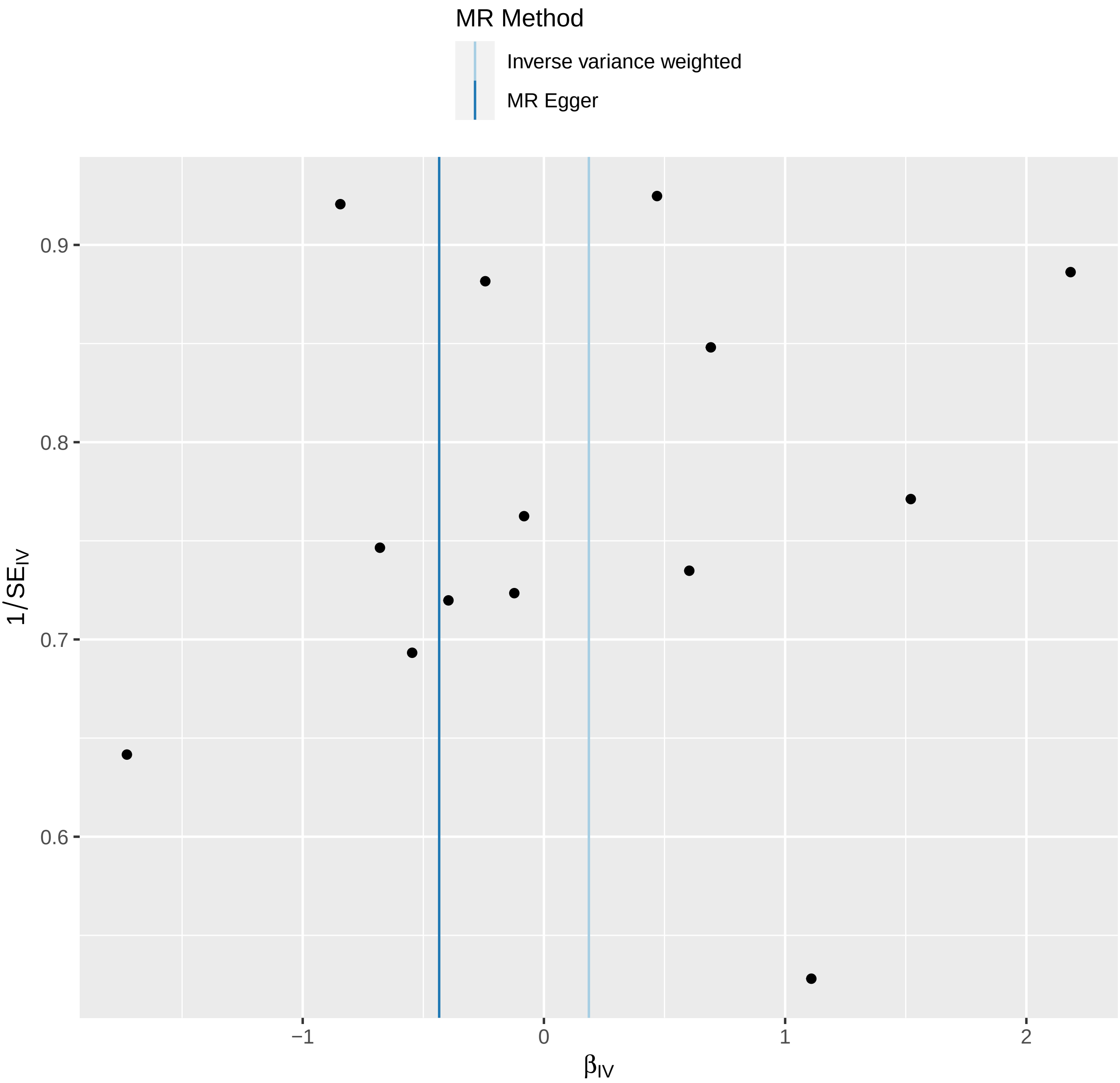

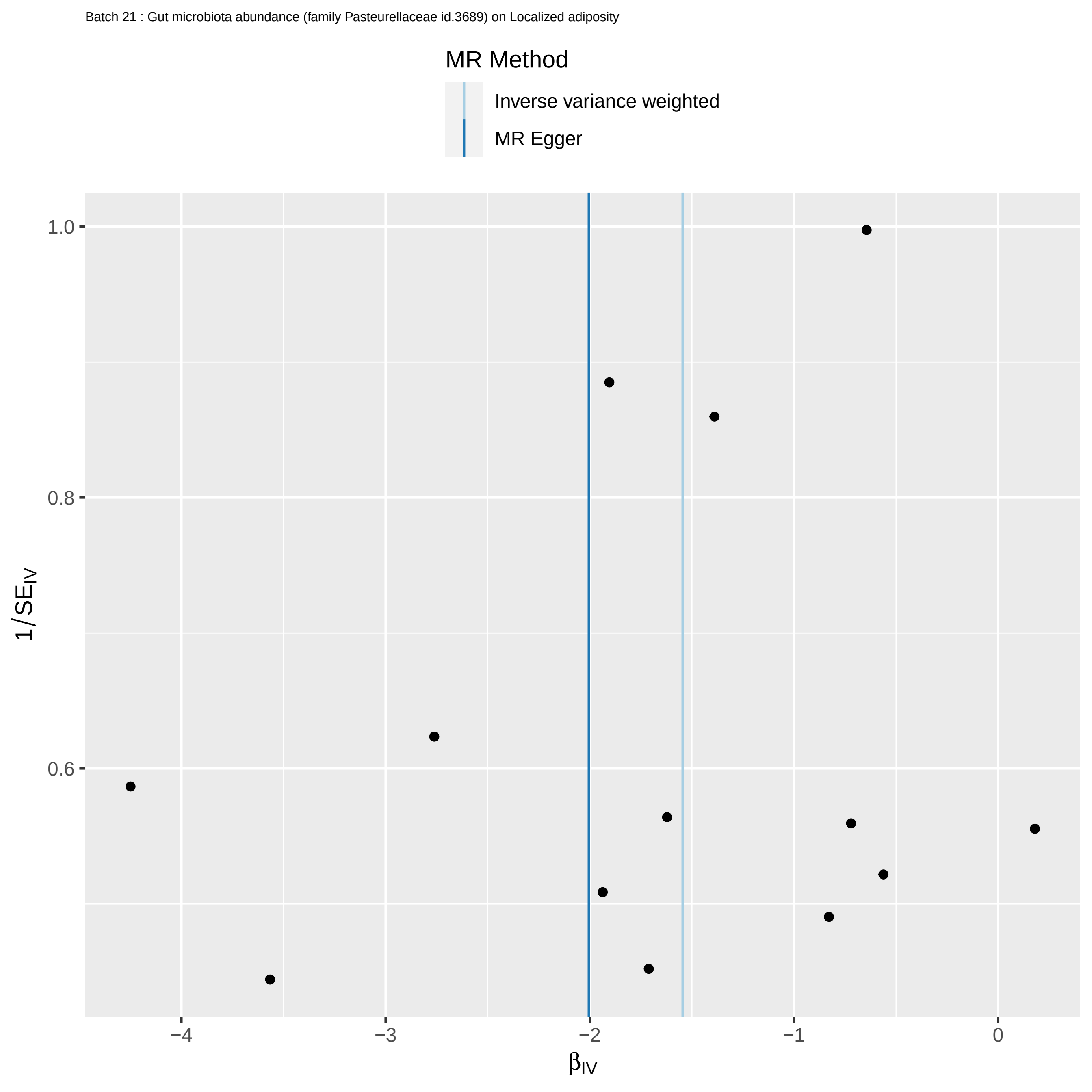

Batch 21 : Gut microbiota abundance (family Pasteurellaceae id.3689) on Localized adiposity
MR Method
Inverse variance weighted
MR Egger
1.0
0.8
0.6
1/SE$_{IV}$
−4
−3
−2
−1
0
β$_{IV}$

Batch 22 : Gut microbiota abundance (family Peptococcaceae id.2024) on Localized adiposity

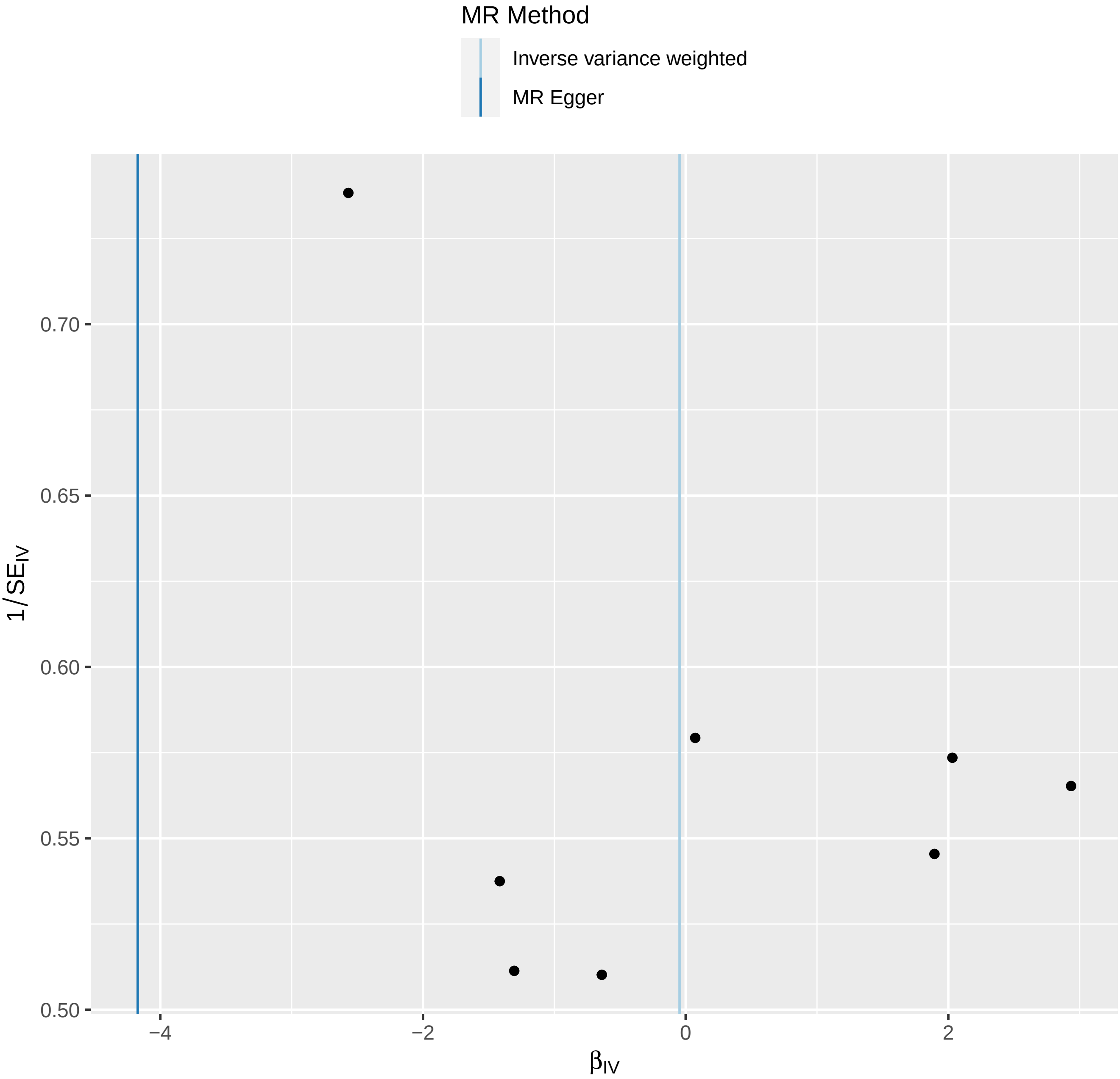

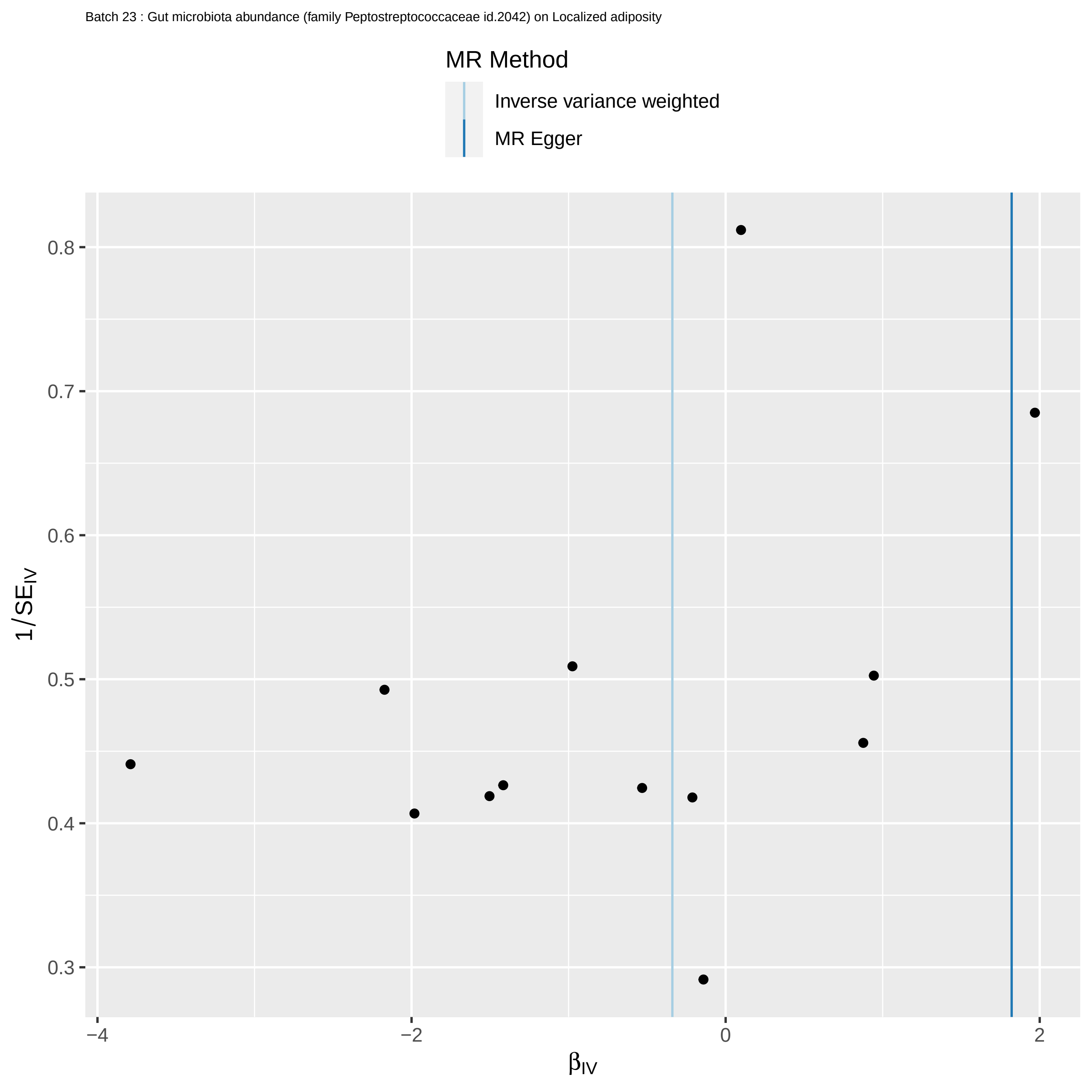
Batch 23 : Gut microbiota abundance (family Peptostreptococcaceae id.2042) on Localized adiposity
MR Method
Inverse variance weighted
MR Egger
0.8
0.7
0.6
0.5
0.4
0.3
1/SE_IV
−4
−2
0
2
β_IV

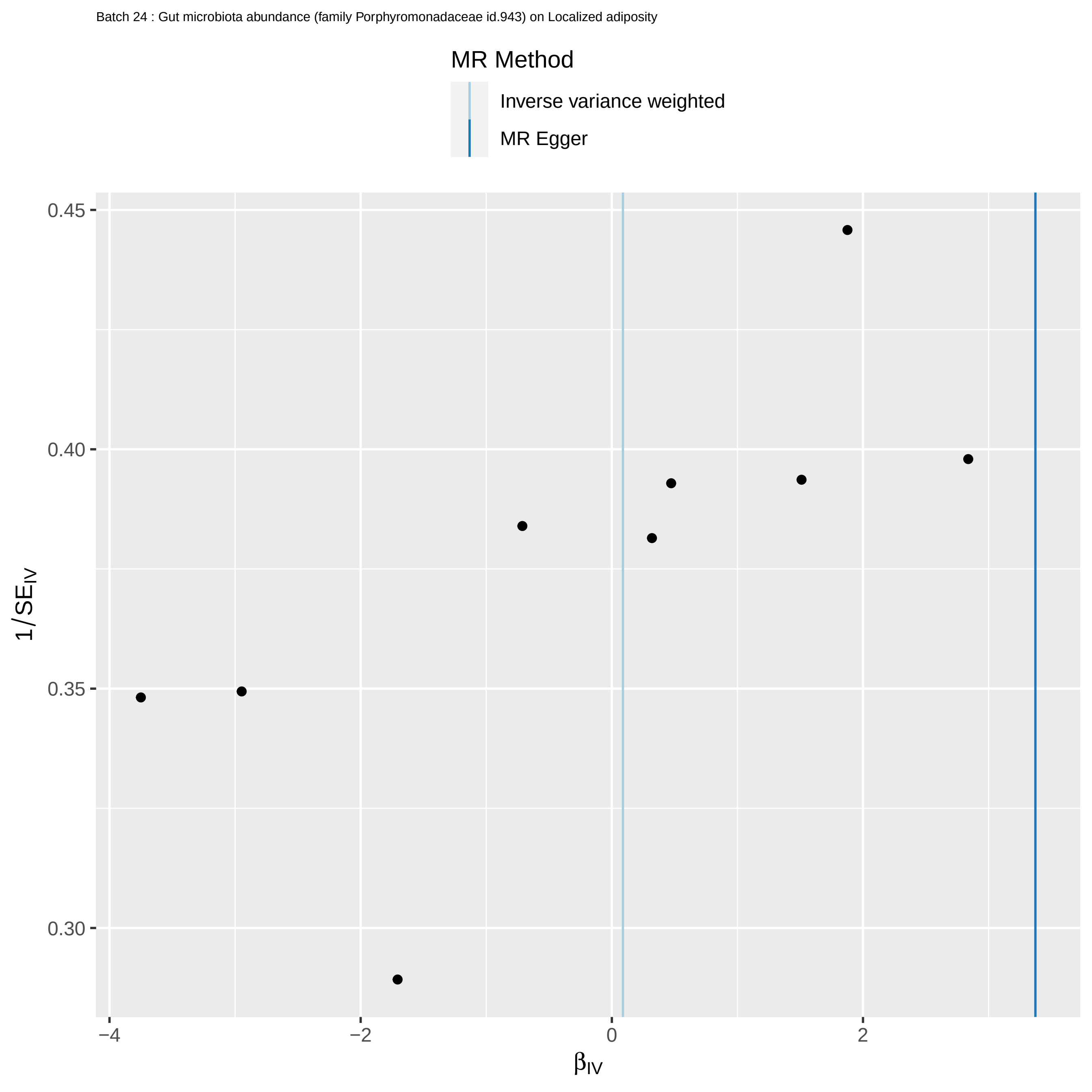

Batch 24 : Gut microbiota abundance (family Porphyromonadaceae id.943) on Localized adiposity
MR Method
Inverse variance weighted
MR Egger
0.45
0.40
0.35
0.30
1/SE$_{IV}$
−4
−2
0
2
β$_{IV}$

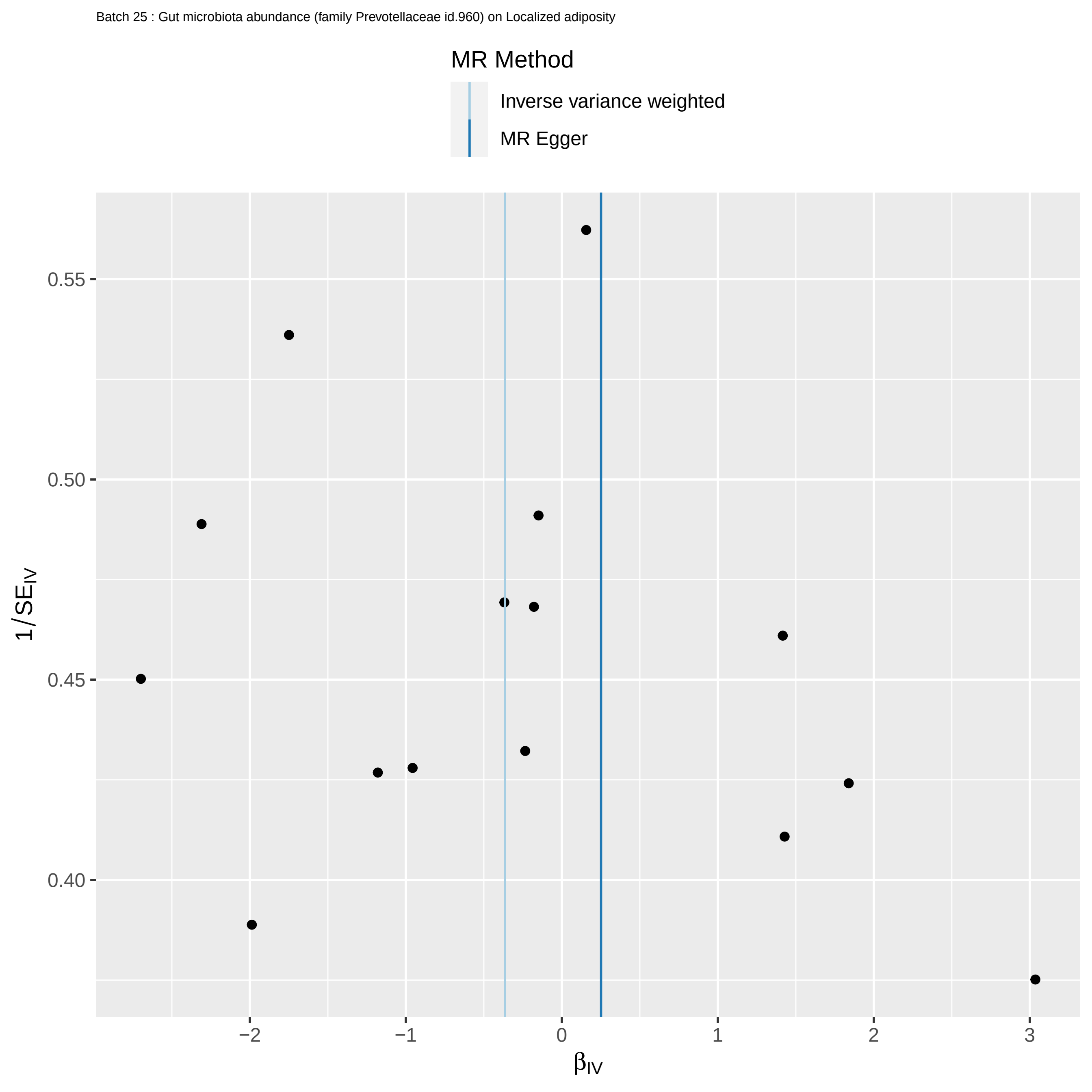

Batch 25 : Gut microbiota abundance (family Prevotellaceae id.960) on Localized adiposity
MR Method
Inverse variance weighted
MR Egger
0.55
0.50
0.45
0.40
1/SE$_{IV}$
−2
−1
0
1
2
3
$\beta_{IV}$

Batch 26 : Gut microbiota abundance (family Rhodospirillaceae id.2717) on Localized adiposity

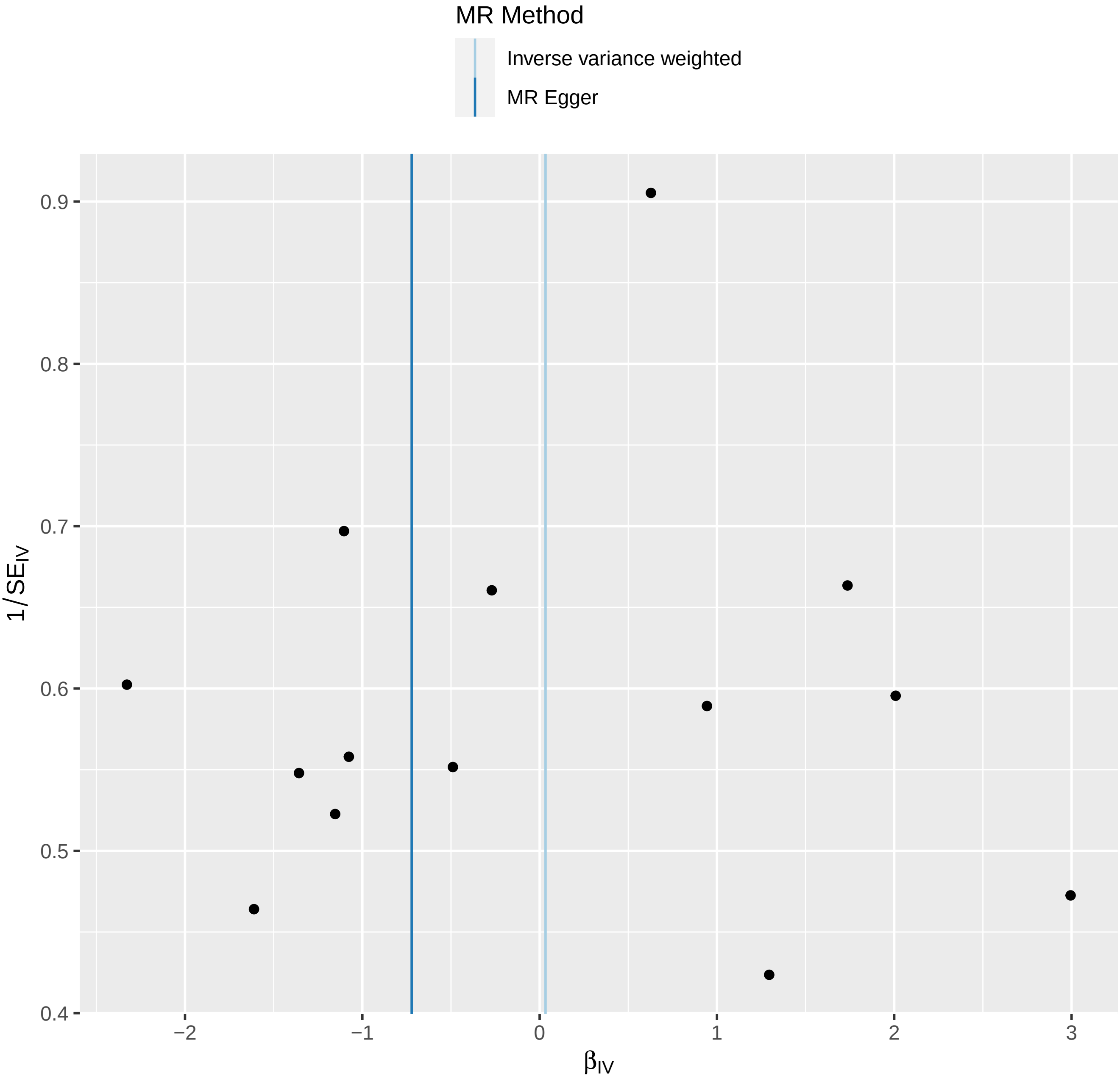

Batch 27 : Gut microbiota abundance (family Rikenellaceae id.967) on Localized adiposity

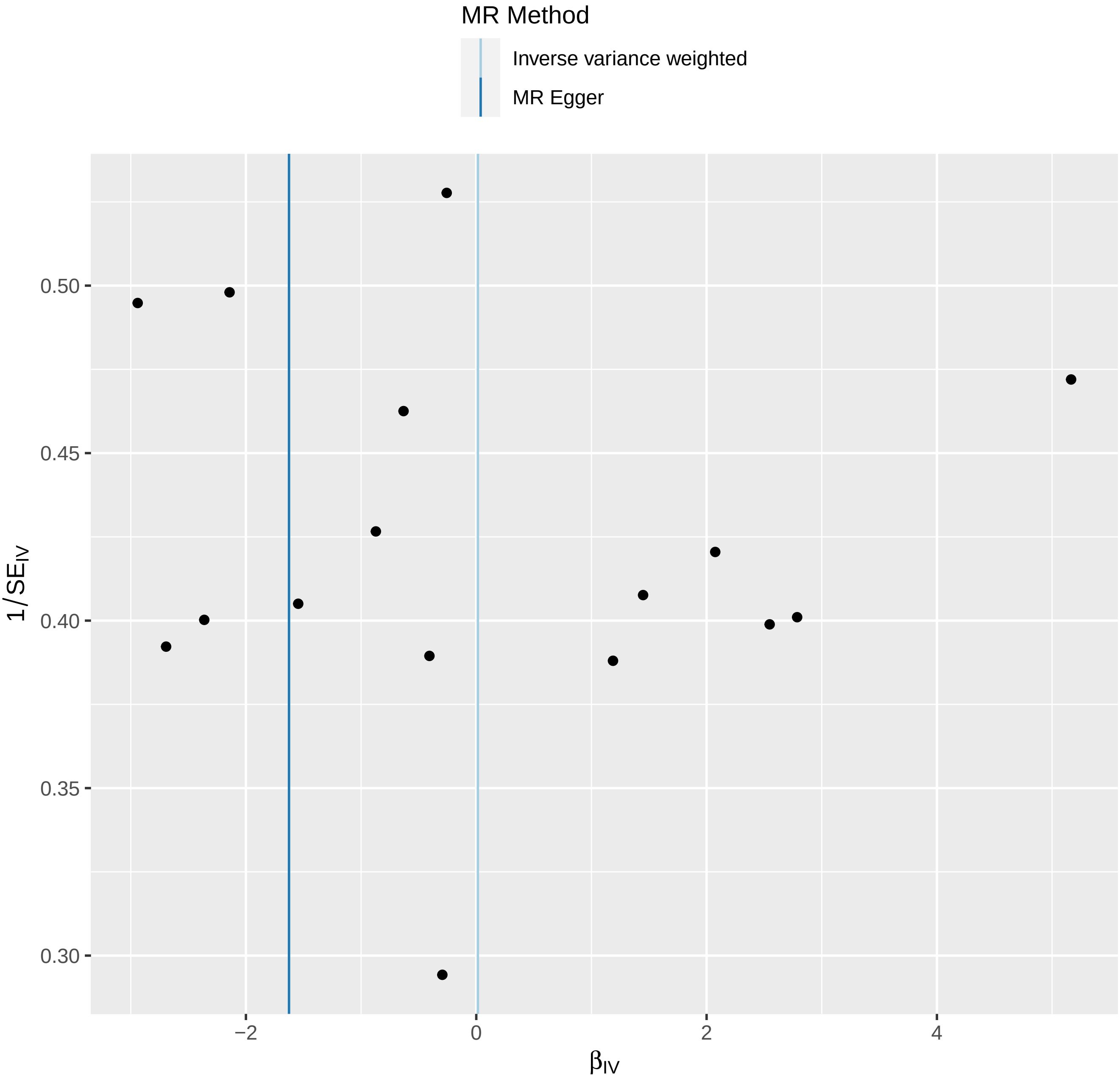

Batch 28 : Gut microbiota abundance (family Ruminococcaceae id.2050) on Localized adiposity

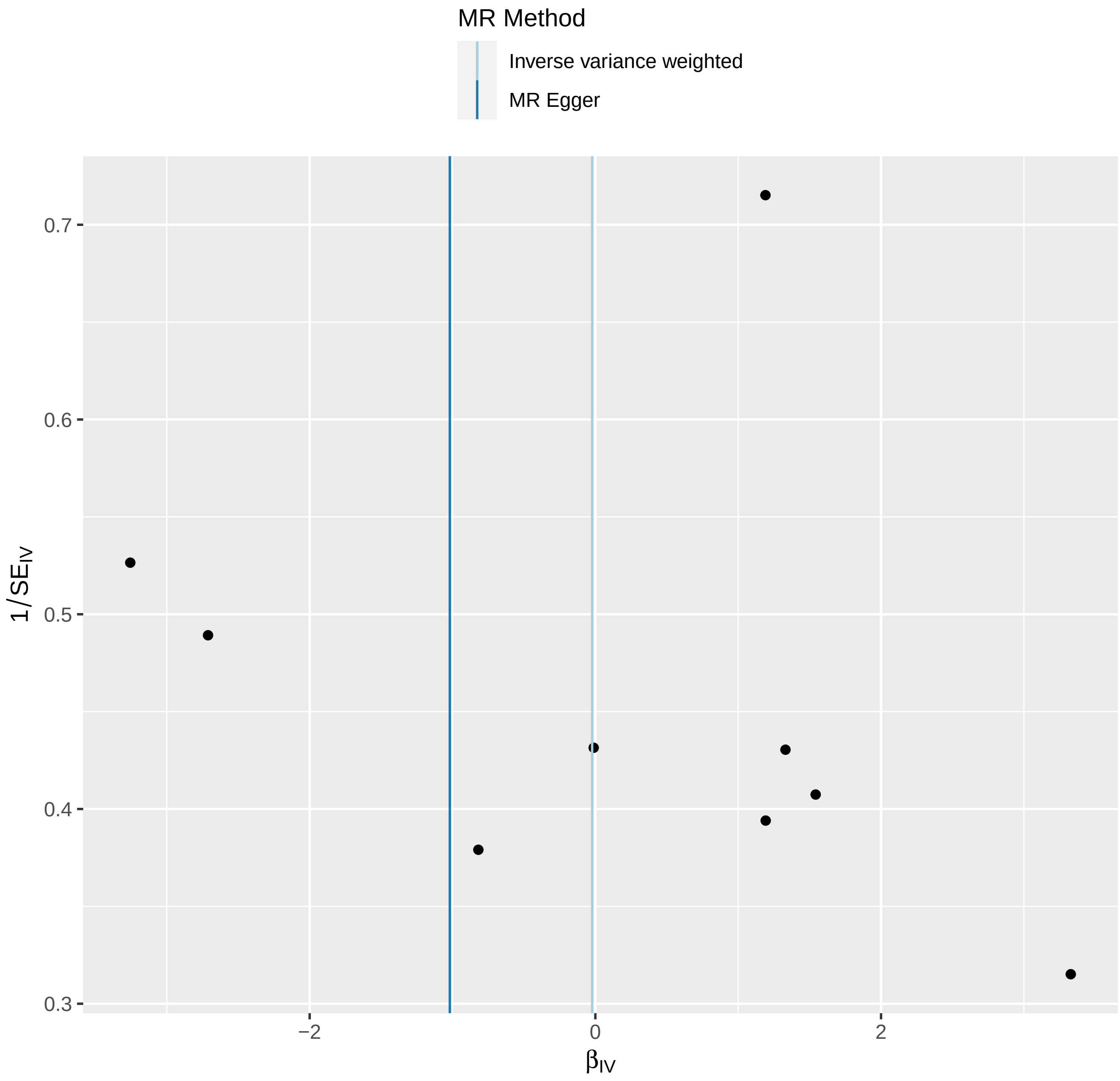

Batch 29 : Gut microbiota abundance (family Streptococcaceae id.1850) on Localized adiposity

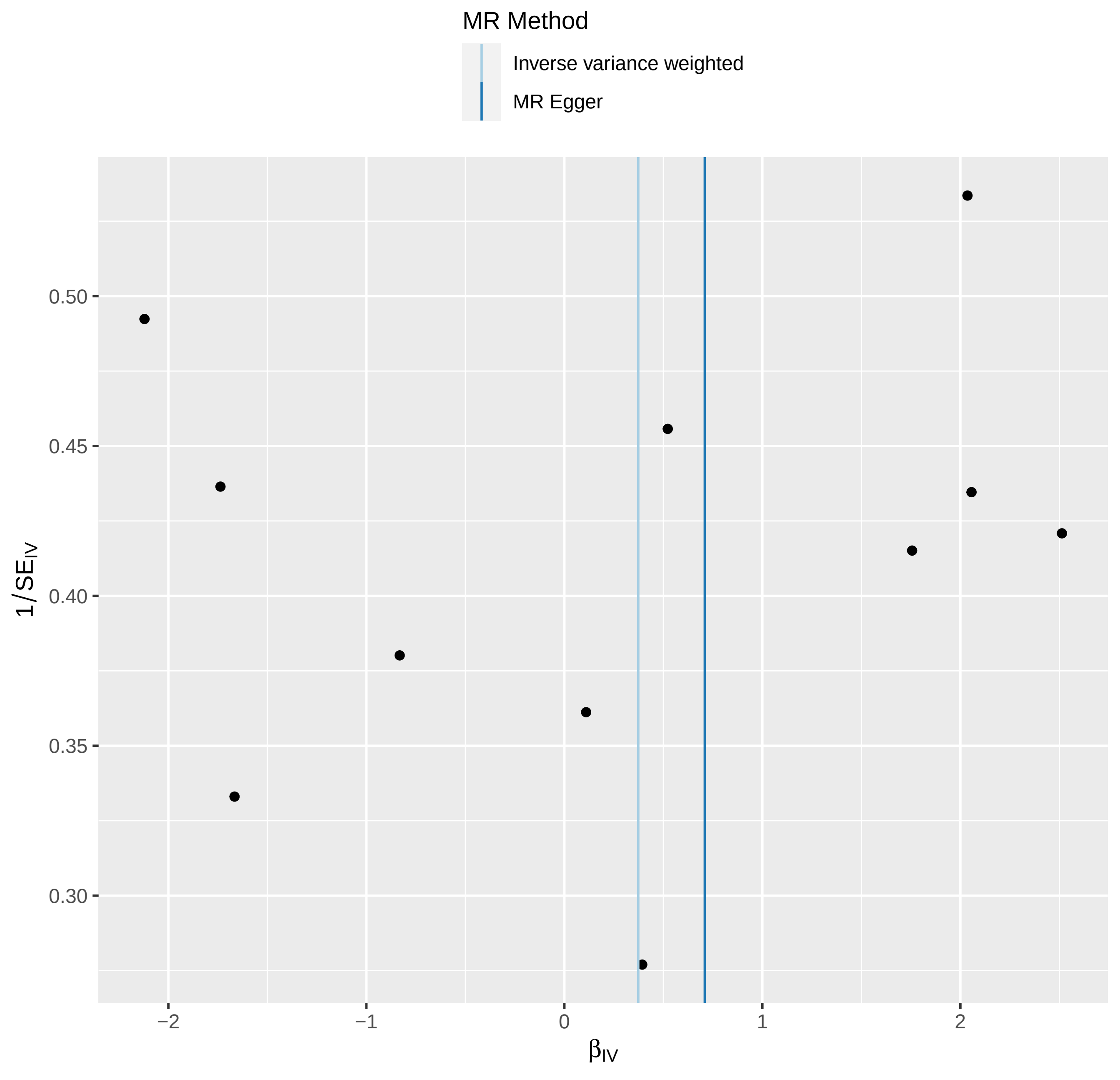

Batch 30 : Gut microbiota abundance (family Veillonellaceae id.2172) on Localized adiposity

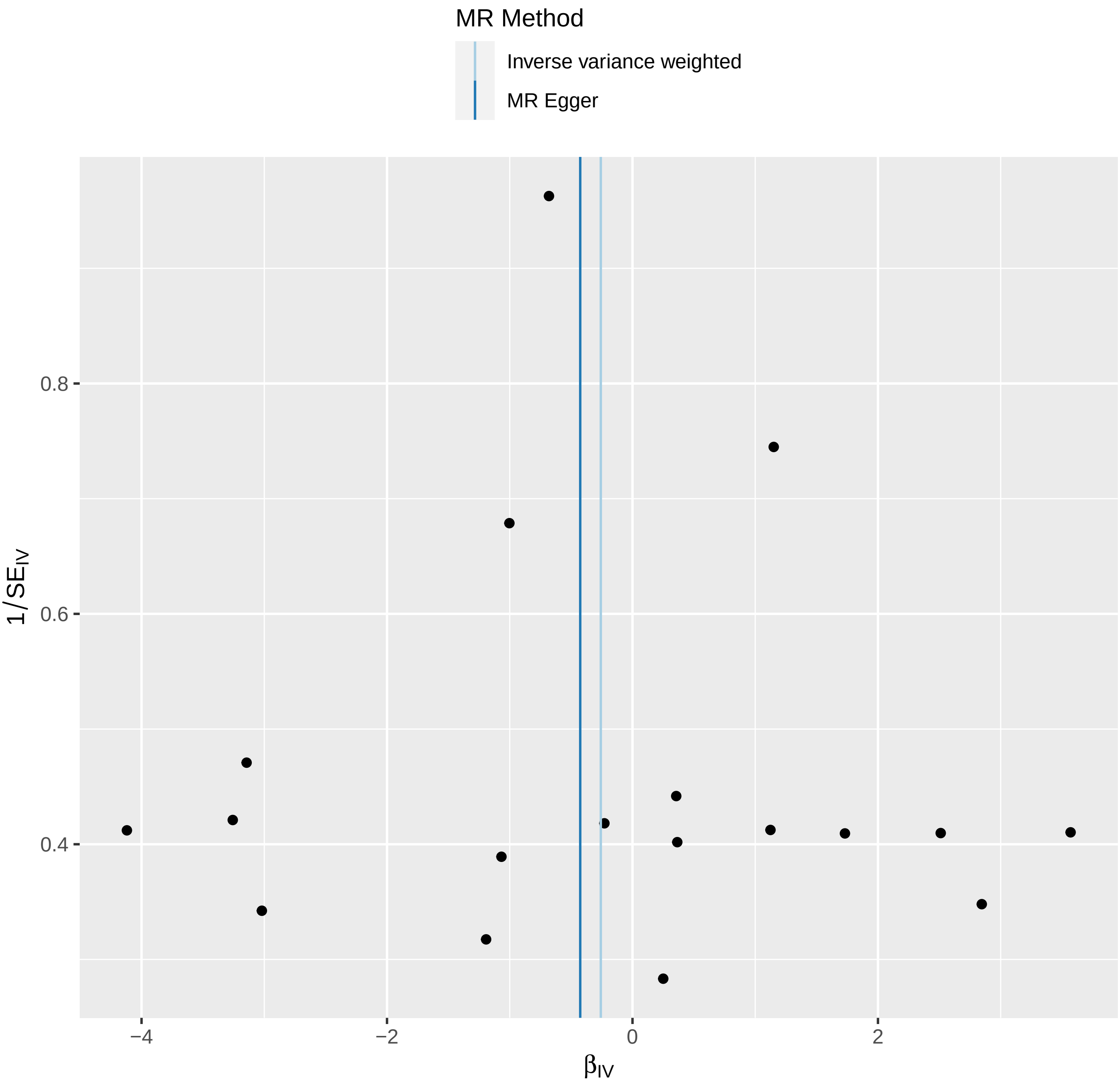

Batch 31 : Gut microbiota abundance (family Verrucomicrobiaceae id.4036) on Localized adiposity

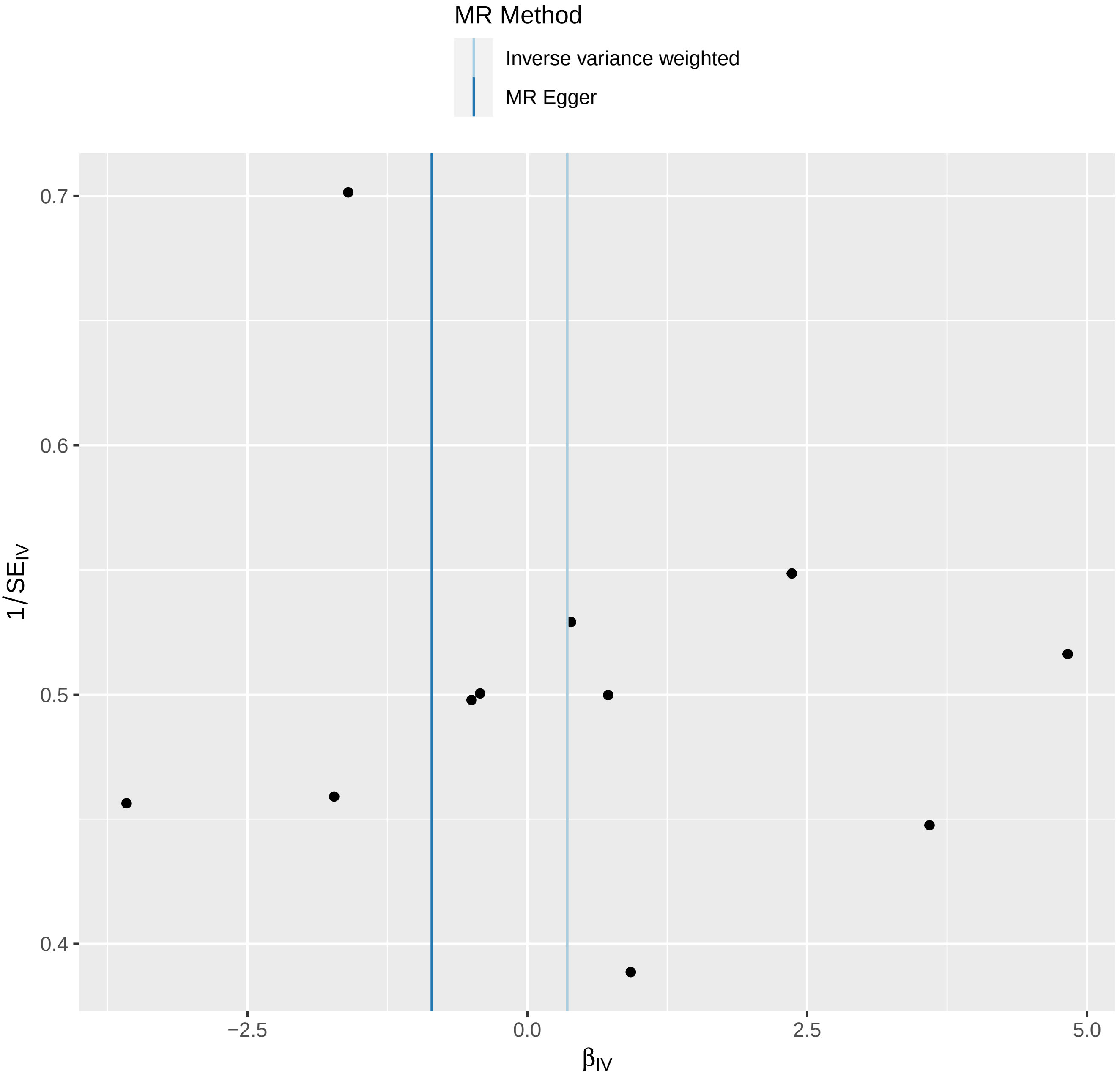

Batch 32 : Gut microbiota abundance (family Victivallaceae id.2255) on Localized adiposity

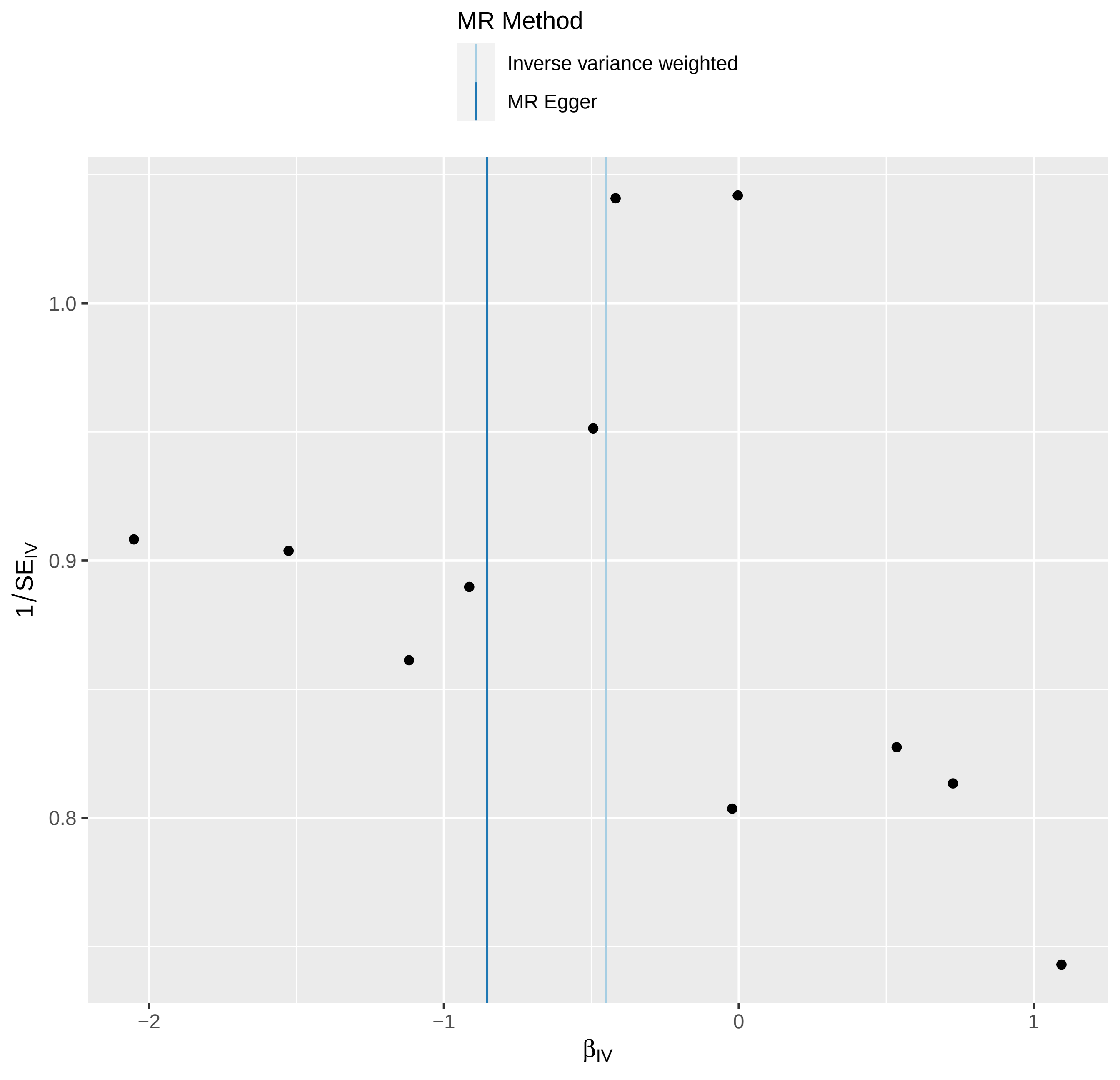

Batch 33 : Gut microbiota abundance (genus Actinomyces id.423) on Localized adiposity

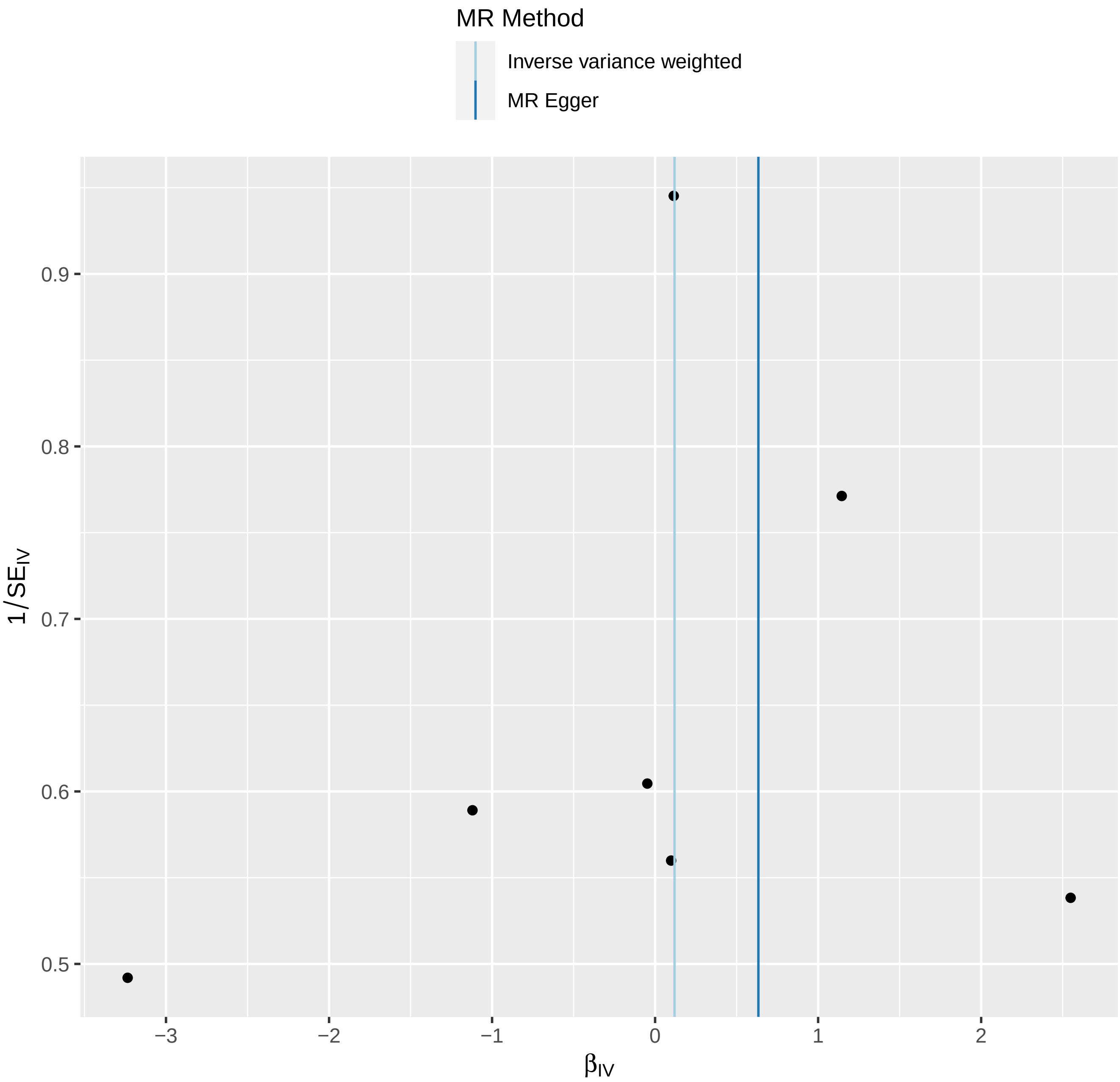

Batch 34 : Gut microbiota abundance (genus Adlercreutzia id.812) on Localized adiposity

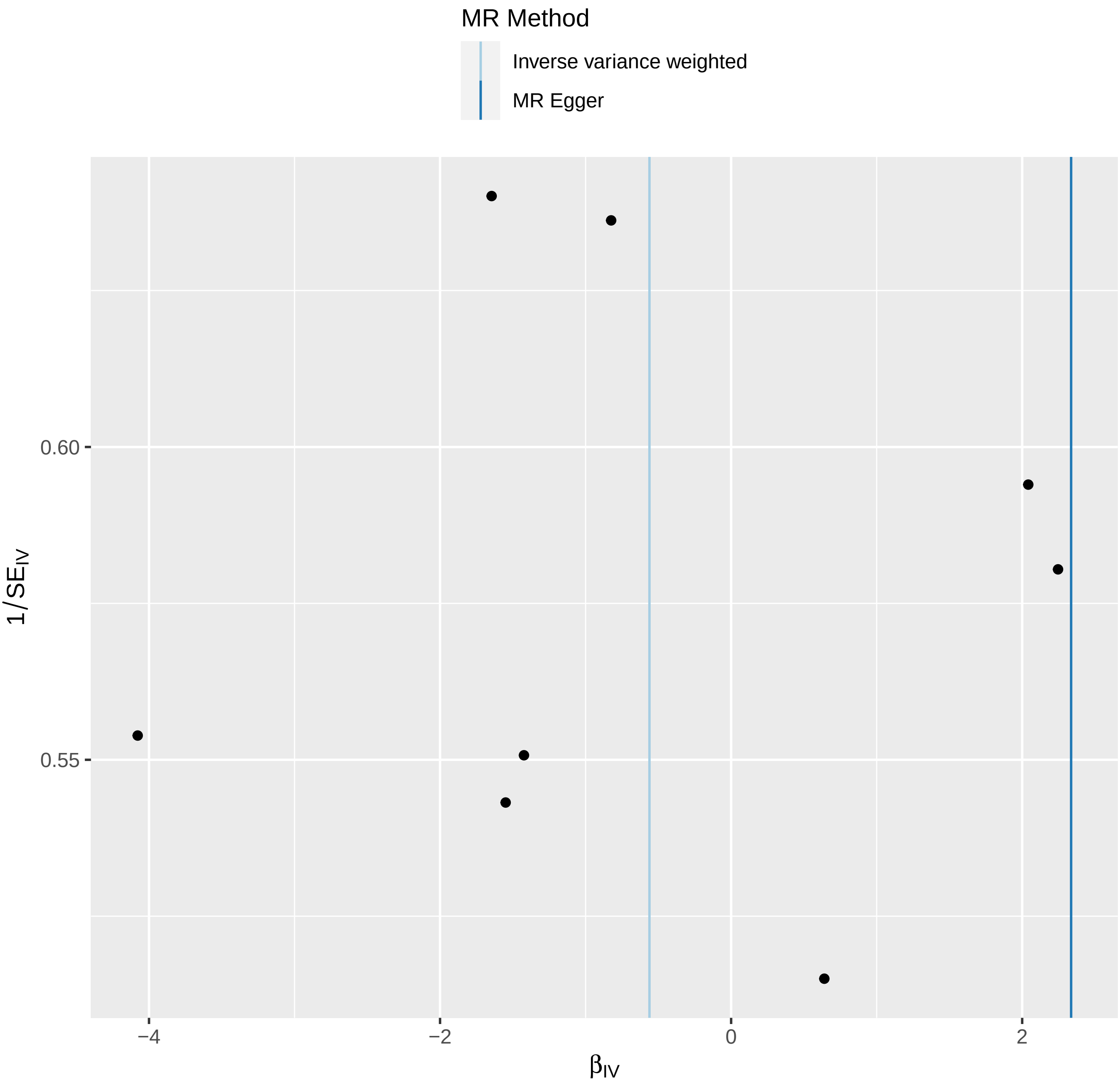

Batch 35 : Gut microbiota abundance (genus Akkermansia id.4037) on Localized adiposity

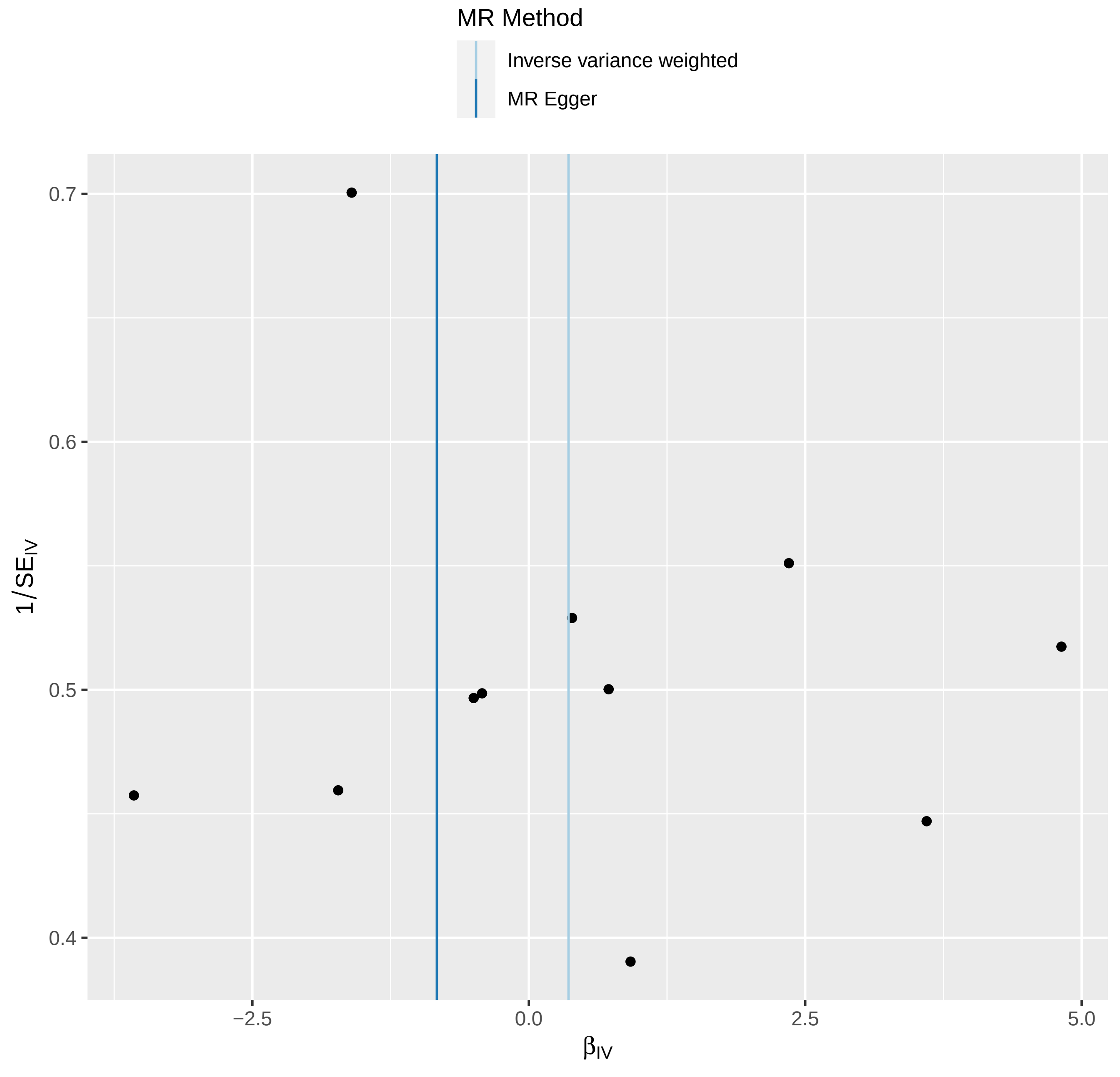

Batch 36 : Gut microbiota abundance (genus Alistipes id.968) on Localized adiposity

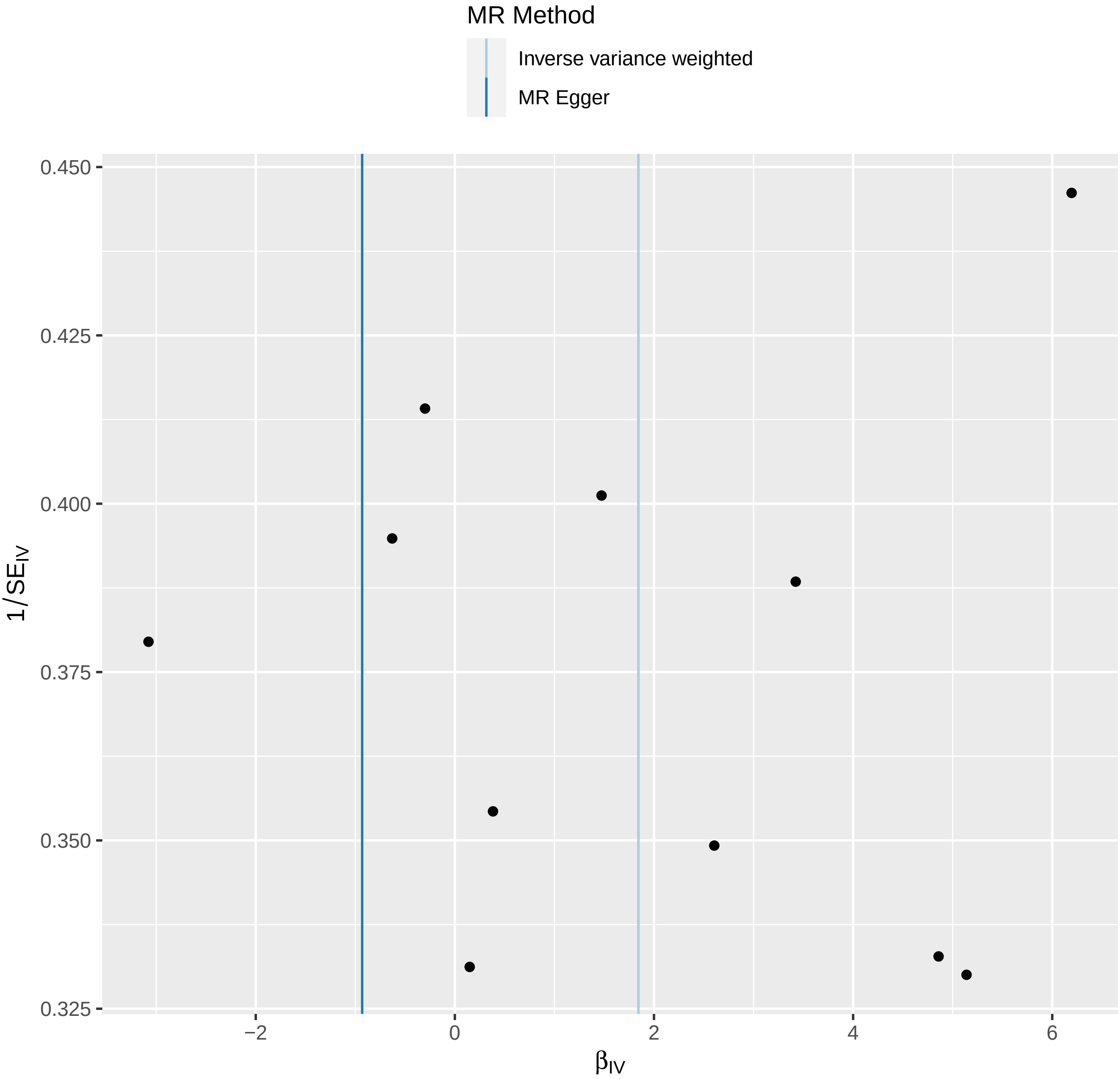

Batch 37 : Gut microbiota abundance (genus Allisonella id.2174) on Localized adiposity

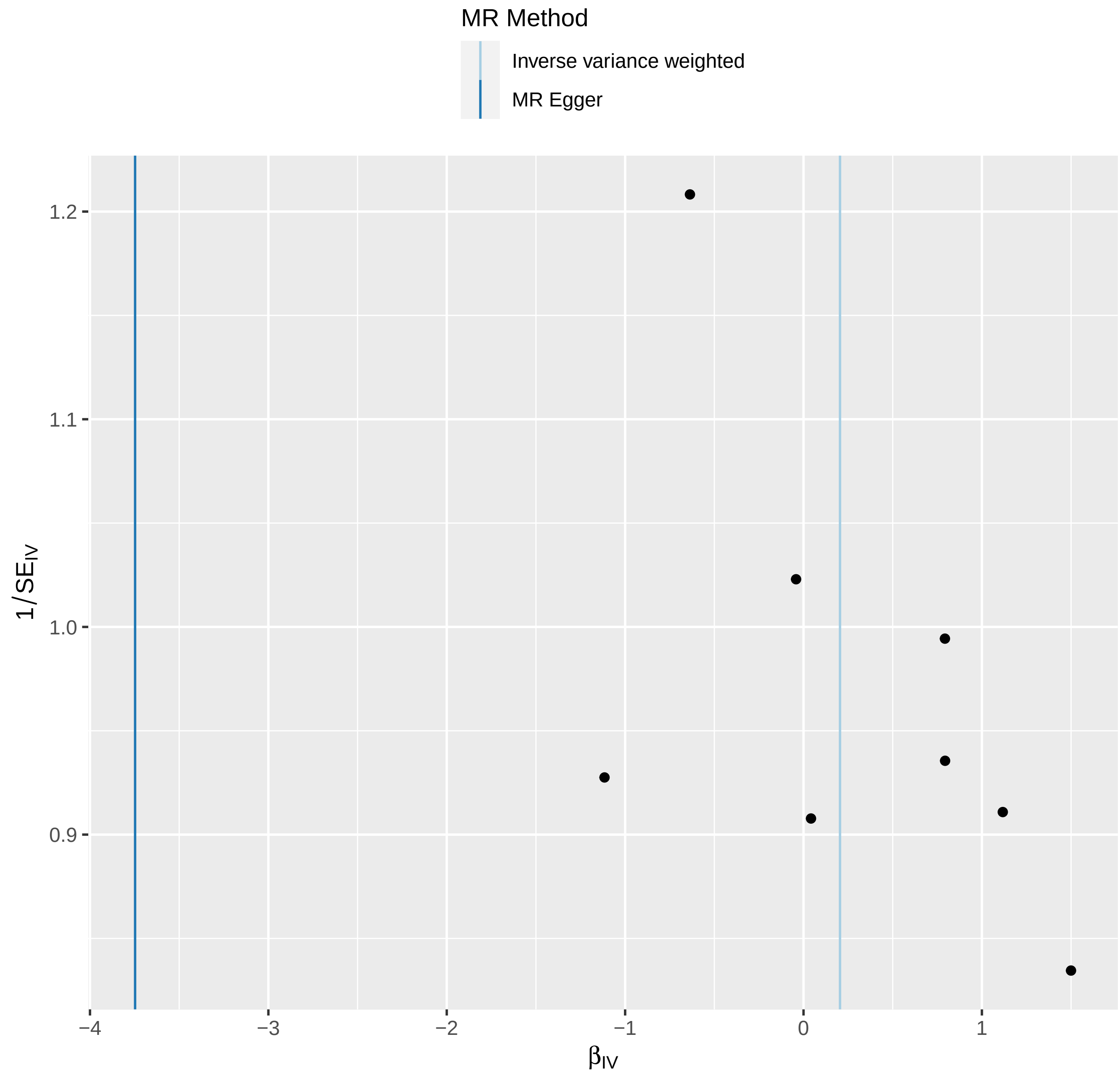

Batch 38 : Gut microbiota abundance (genus Alloprevotella id.961) on Localized adiposity

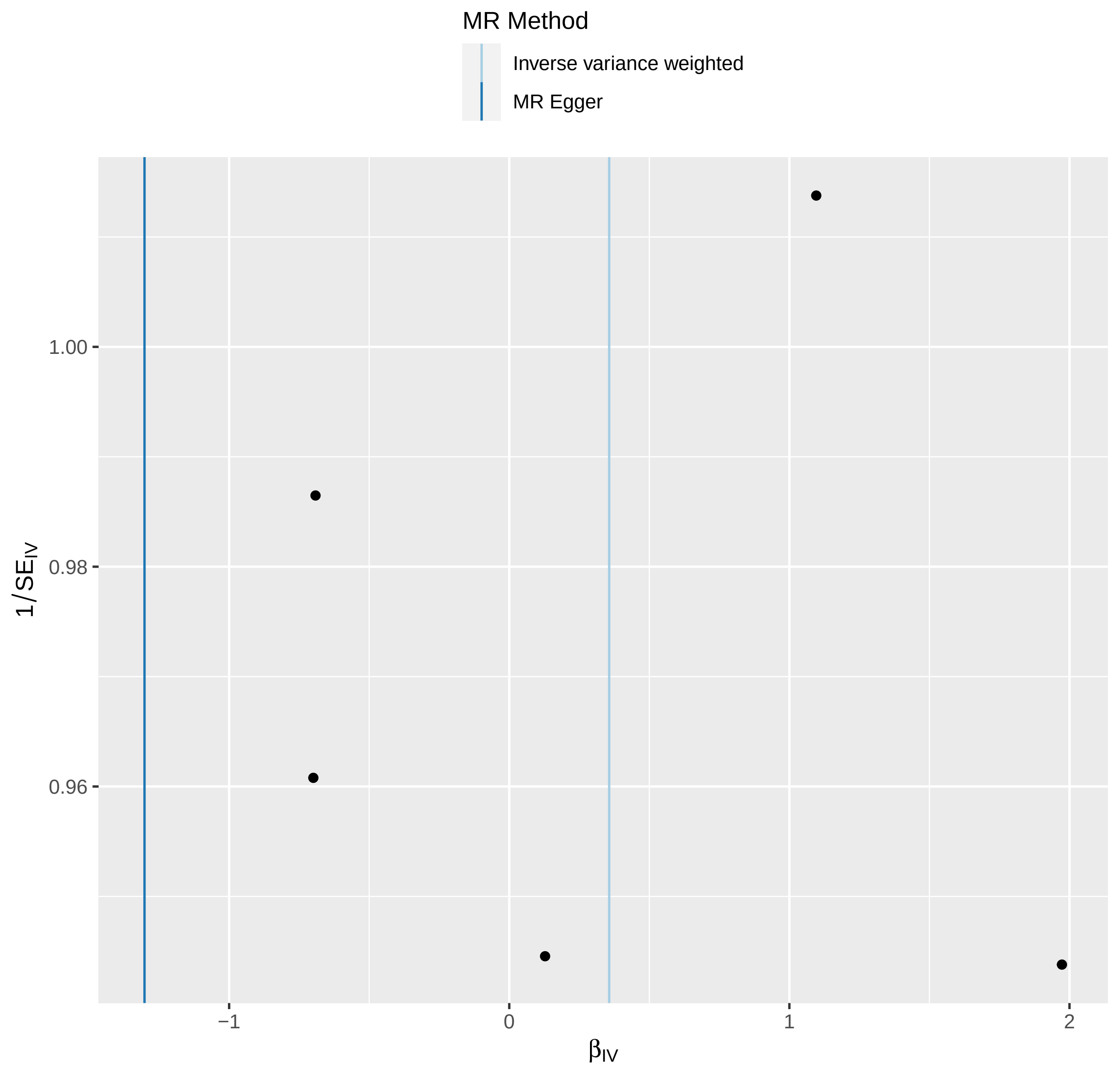

Batch 39 : Gut microbiota abundance (genus Anaerofilum id.2053) on Localized adiposity

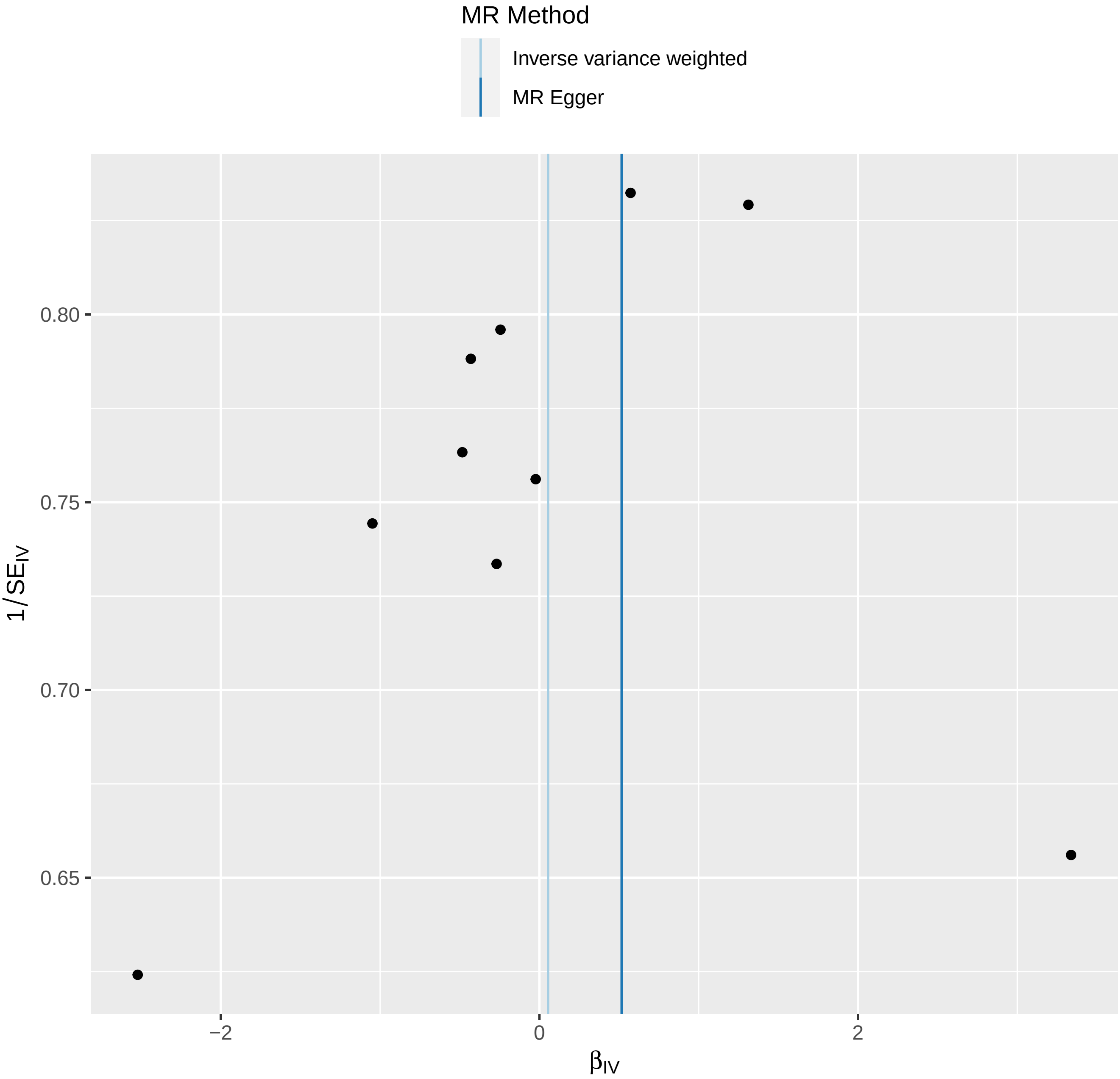

Batch 40 : Gut microbiota abundance (genus Anaerostipes id.1991) on Localized adiposity

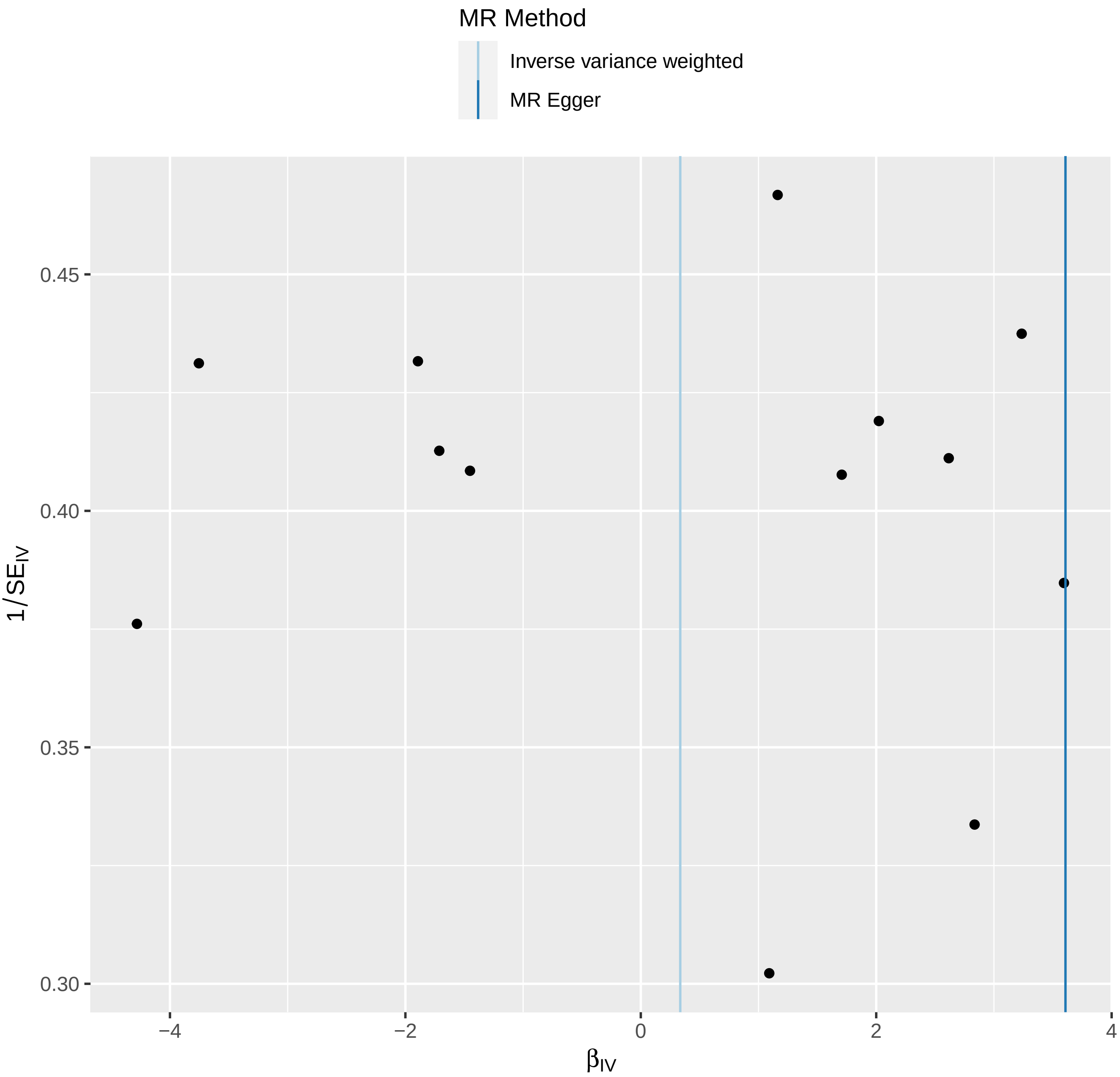

Batch 41 : Gut microbiota abundance (genus Anaerotruncus id.2054) on Localized adiposity

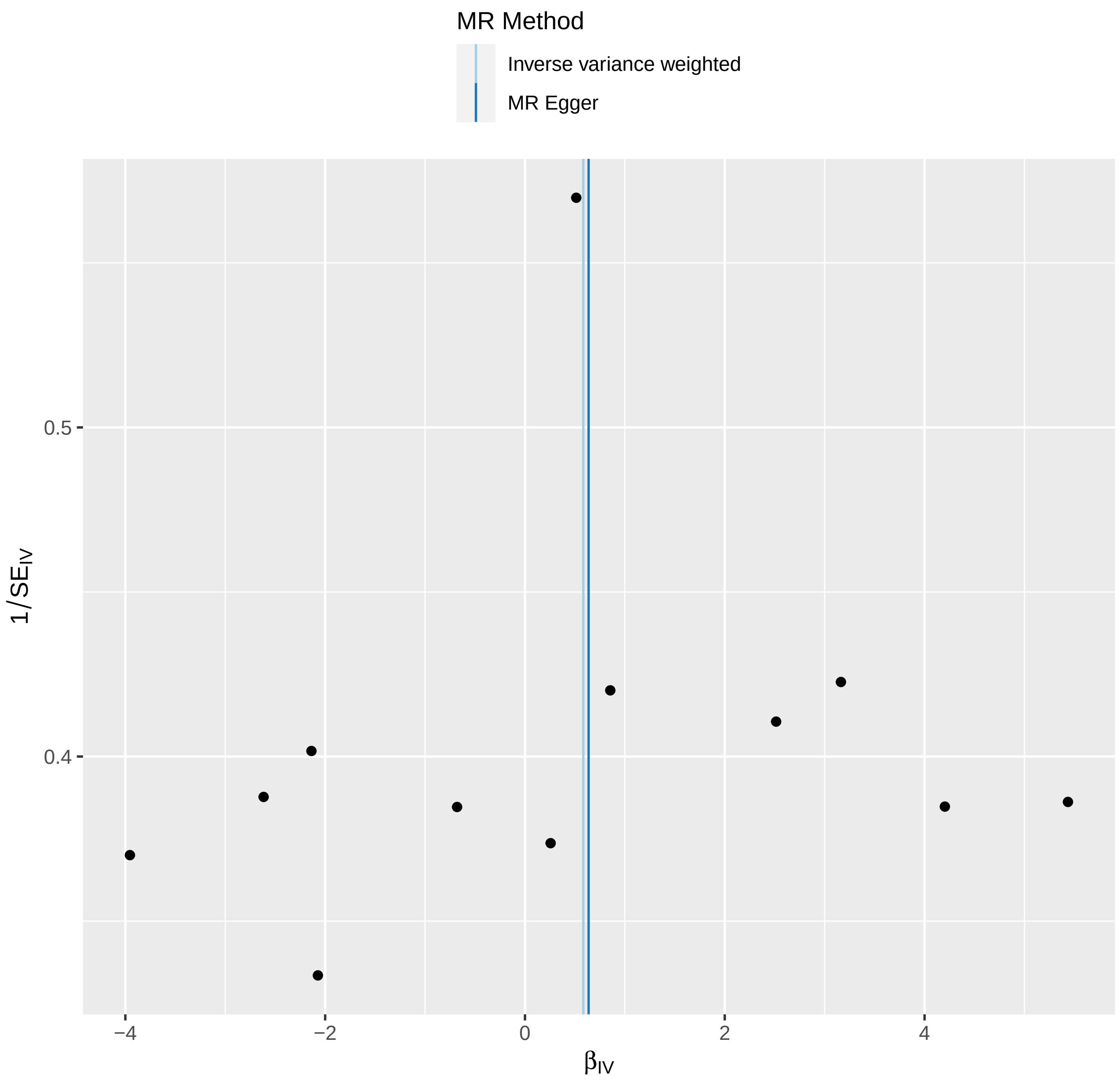

Batch 42 : Gut microbiota abundance (genus Bacteroides id.918) on Localized adiposity

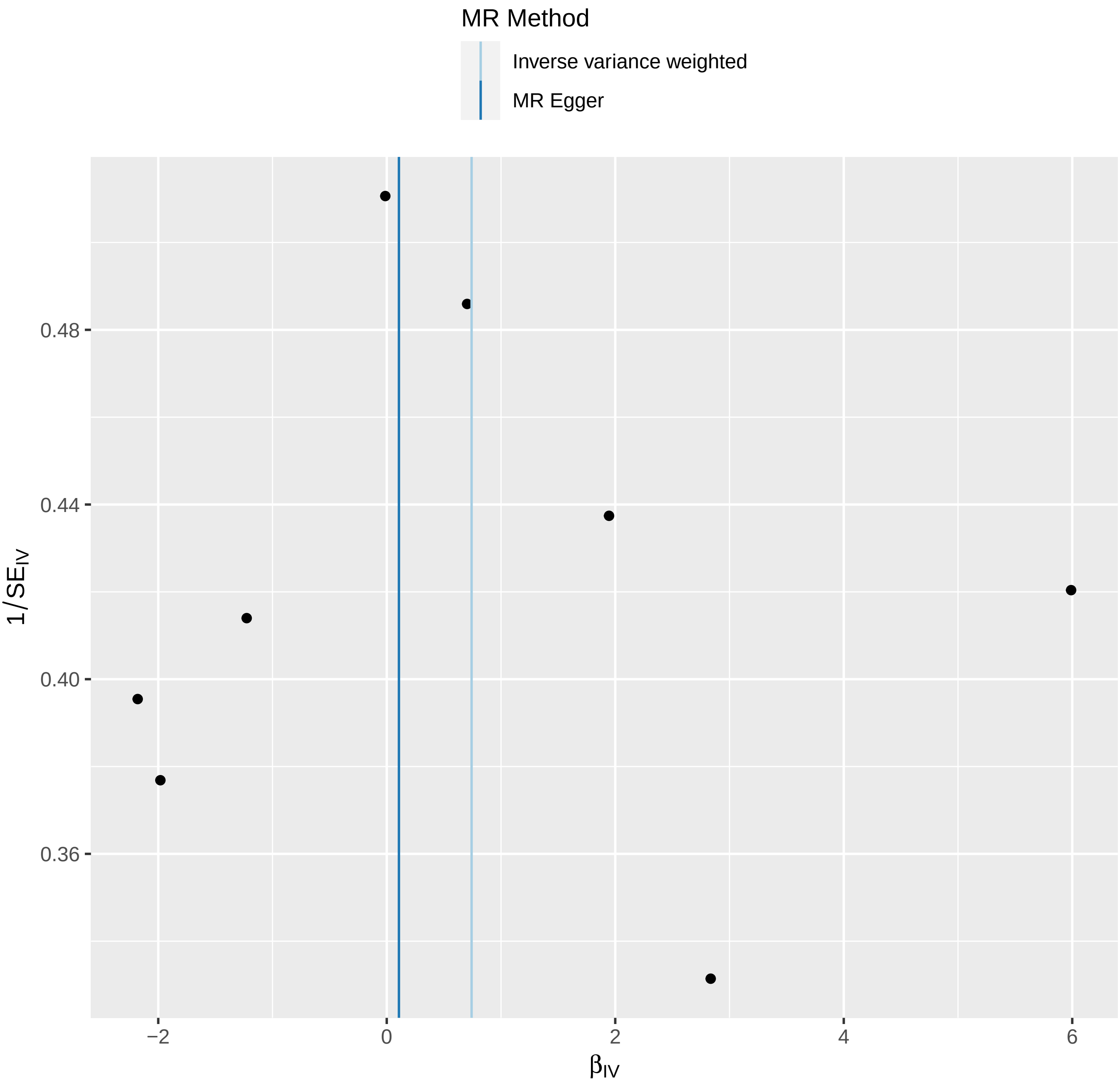

Batch 43 : Gut microbiota abundance (genus Barnesiella id.944) on Localized adiposity

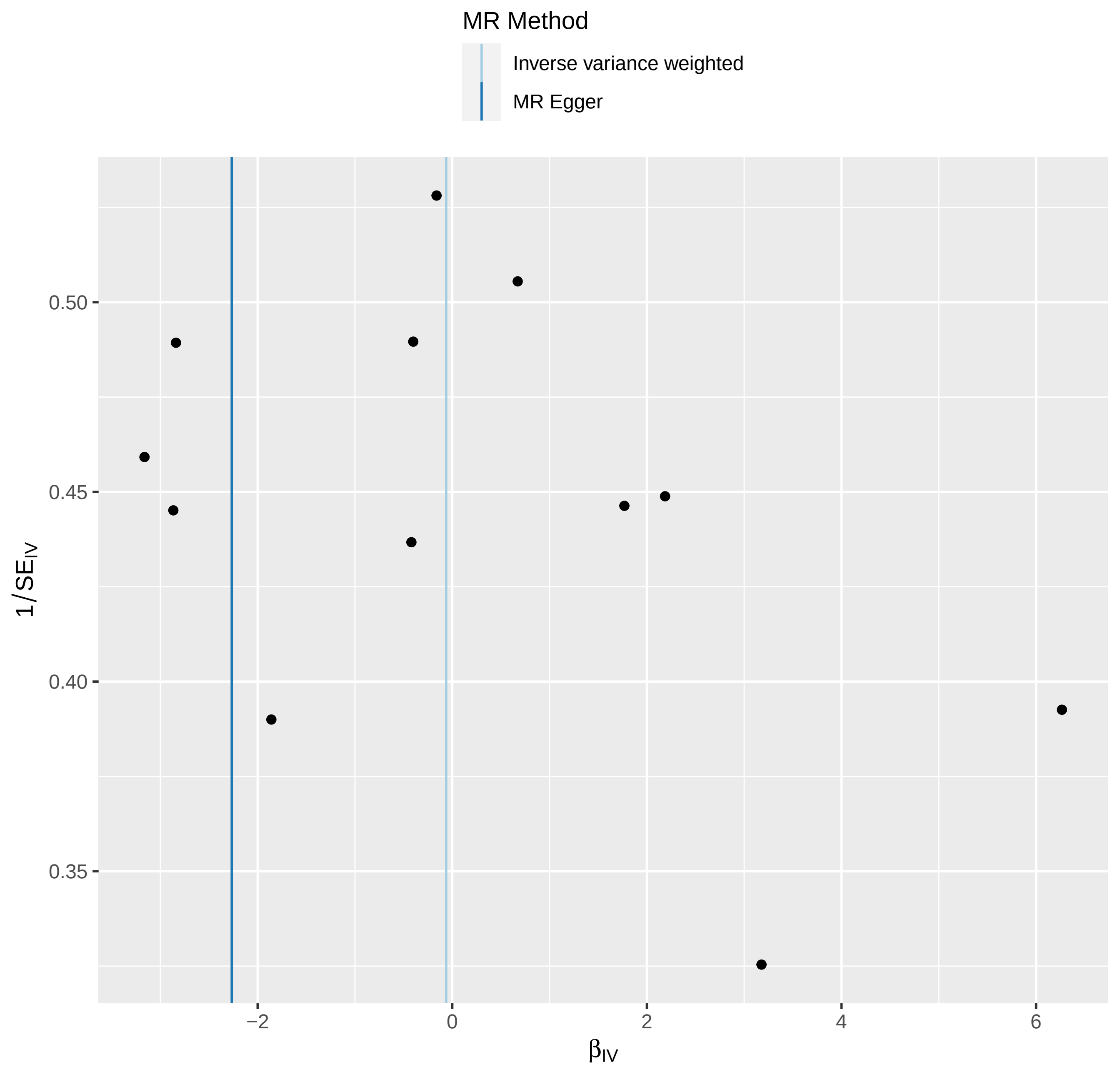

Batch 44 : Gut microbiota abundance (genus Bifidobacterium id.436) on Localized adiposity

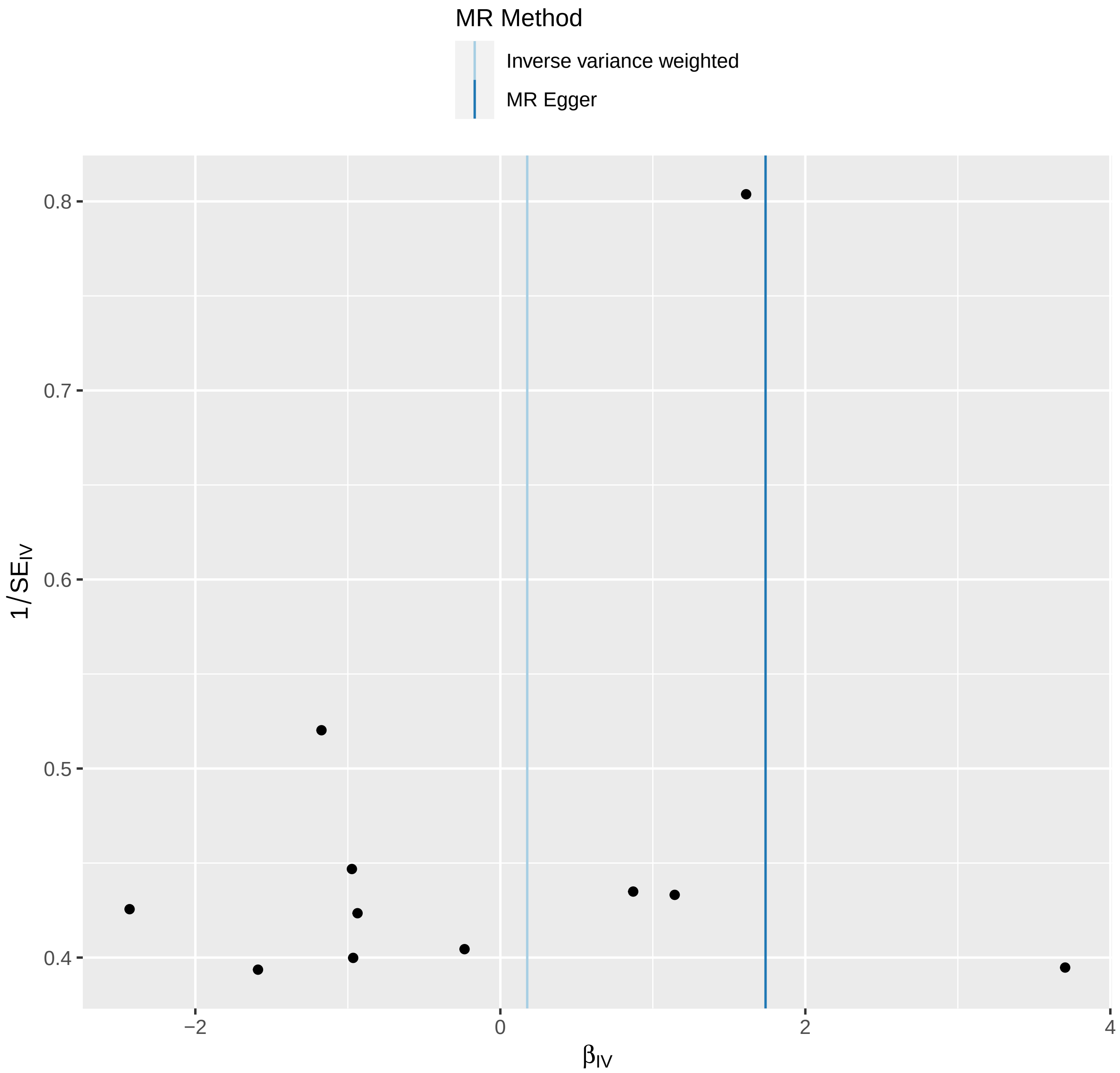

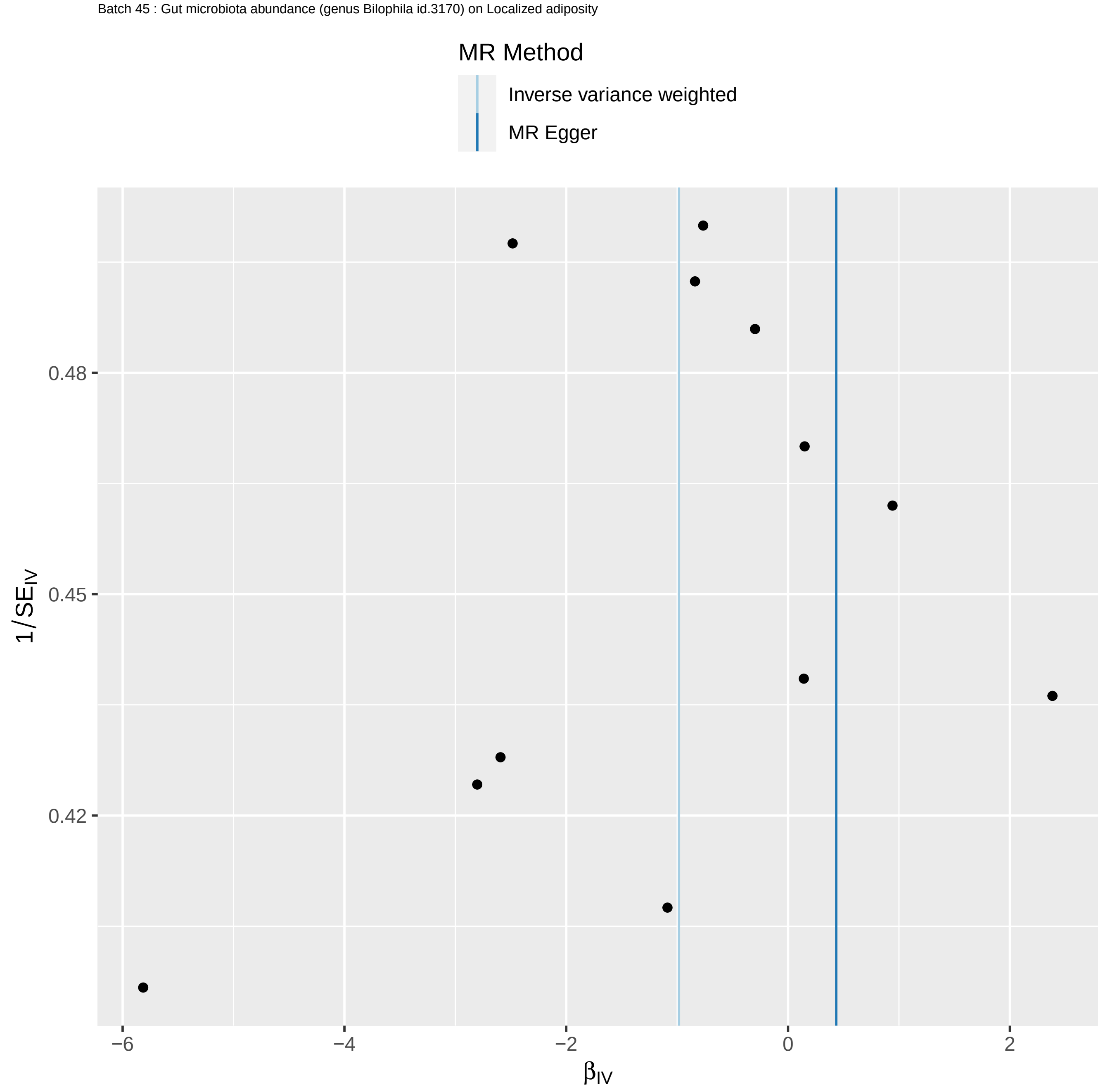

Batch 45 : Gut microbiota abundance (genus Bilophila id.3170) on Localized adiposity
MR Method
Inverse variance weighted
MR Egger
0.48
0.45
0.42
$1/SE_{IV}$
−6
−4
−2
0
2
$\beta_{IV}$

Batch 46 : Gut microbiota abundance (genus Blautia id.1992) on Localized adiposity

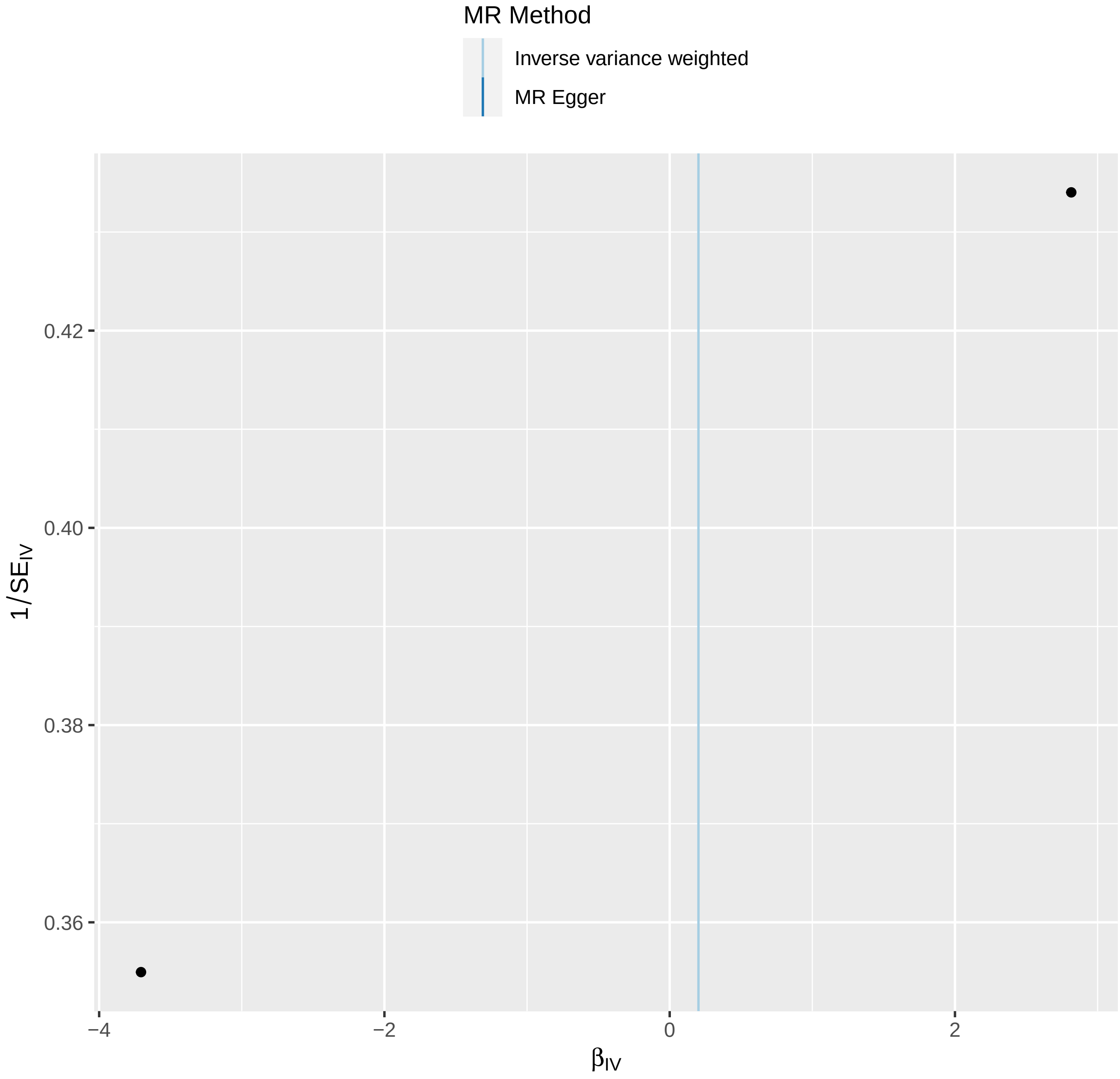

Batch 47 : Gut microbiota abundance (genus Butyricicoccus id.2055) on Localized adiposity

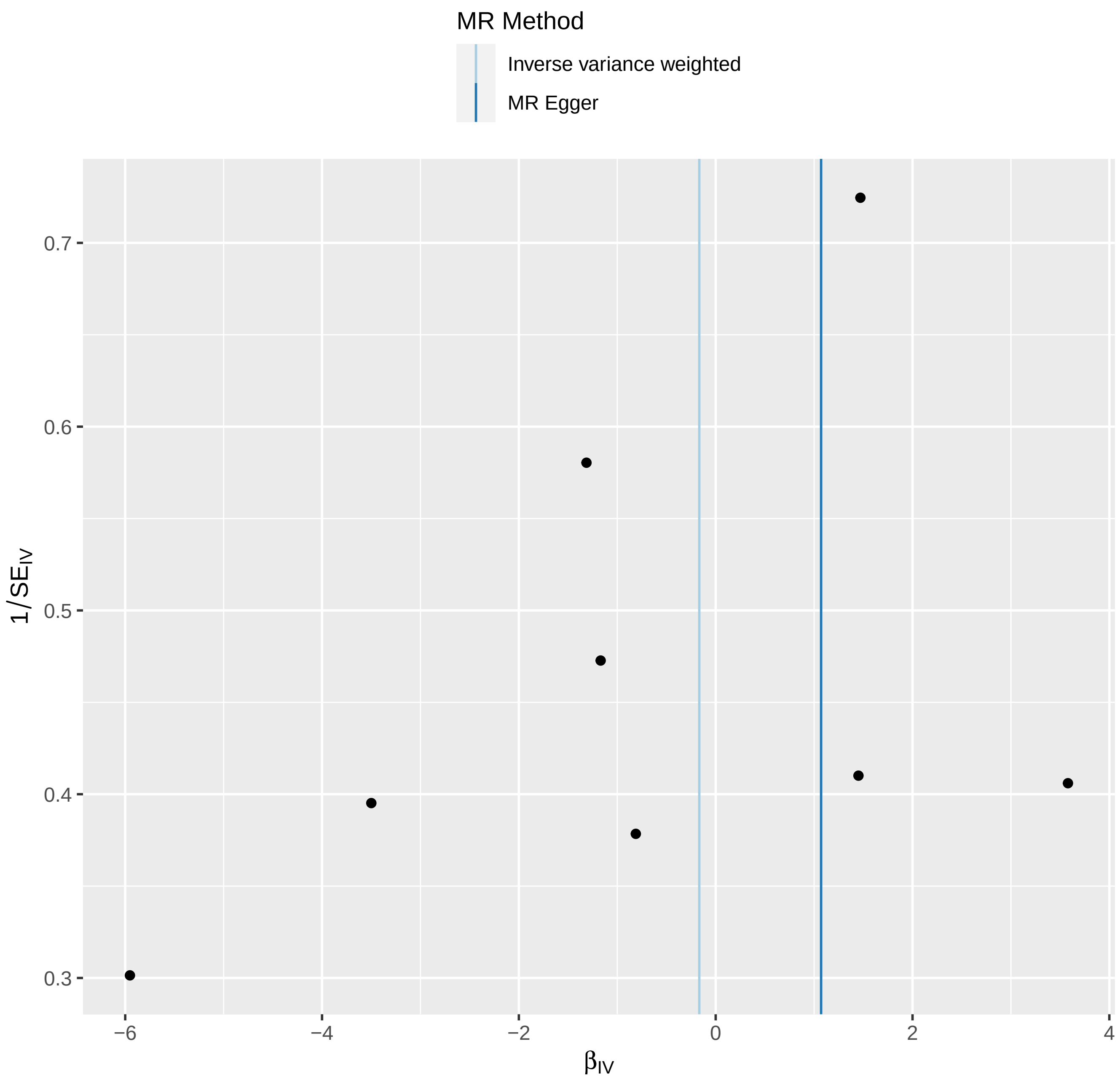

Batch 48 : Gut microbiota abundance (genus Butyricimonas id.945) on Localized adiposity

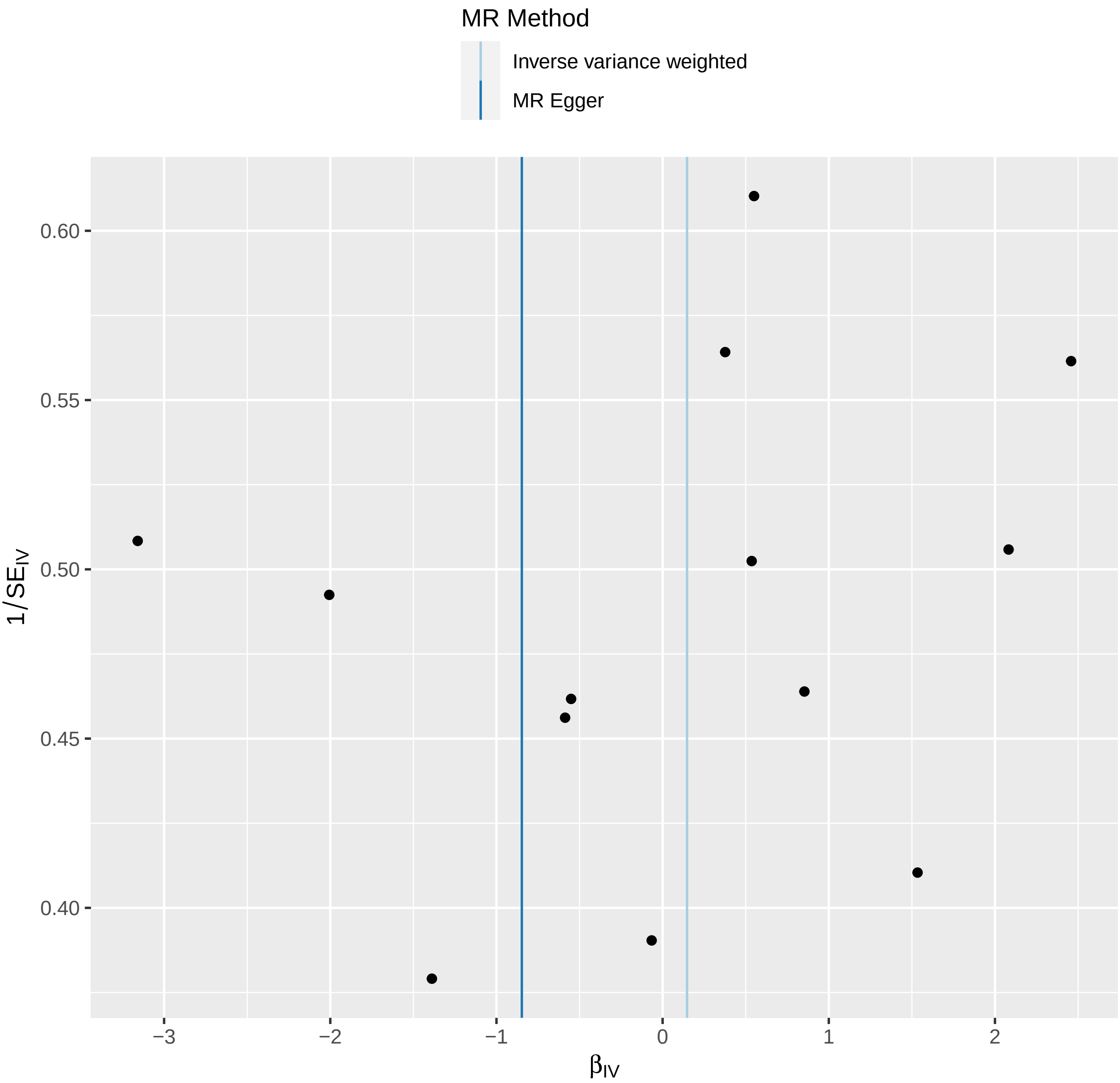

Batch 49 : Gut microbiota abundance (genus Butyrivibrio id.1993) on Localized adiposity

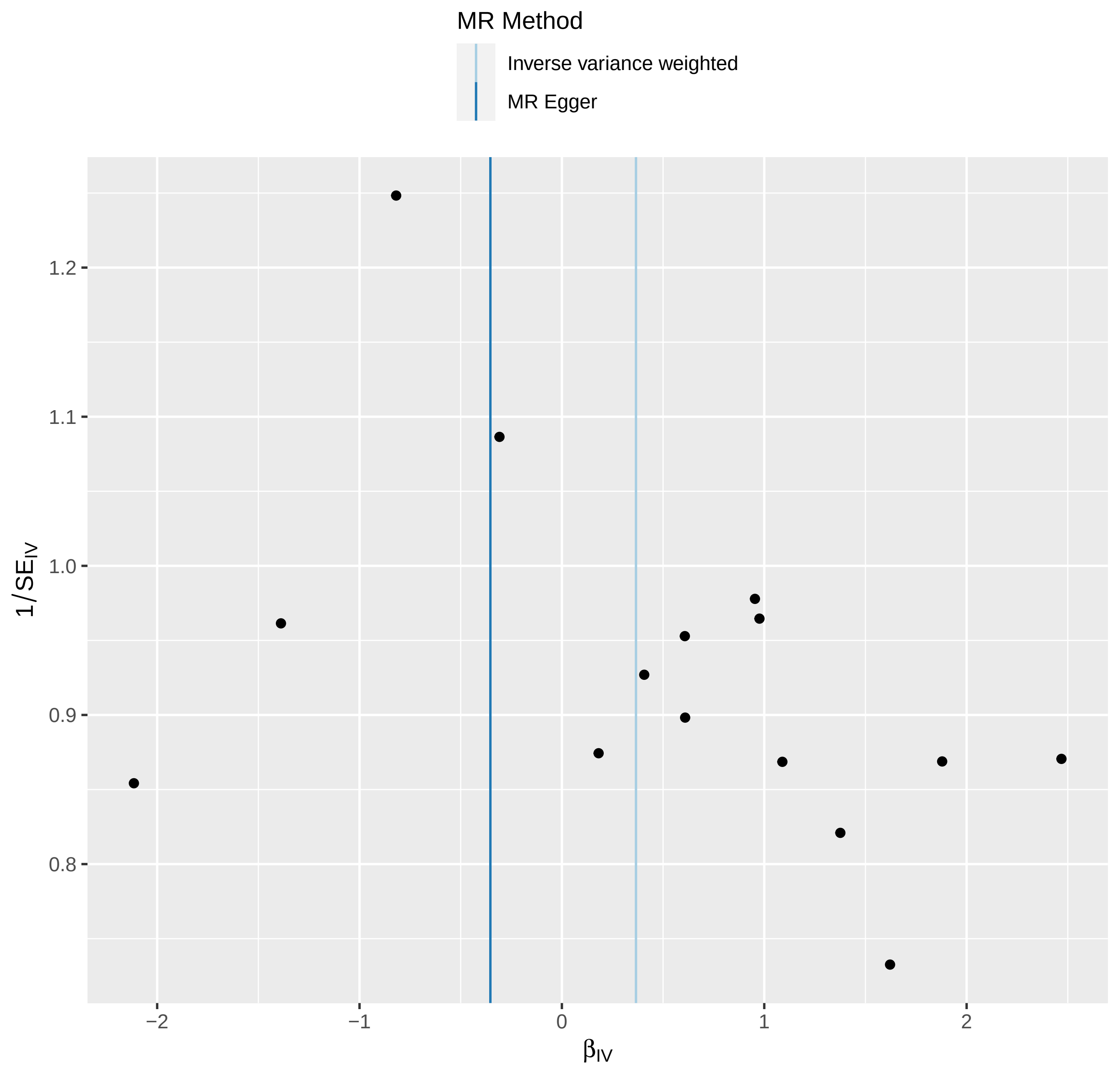

Batch 50 : Gut microbiota abundance (genus Candidatus Soleaferrea id.11350) on Localized adiposity

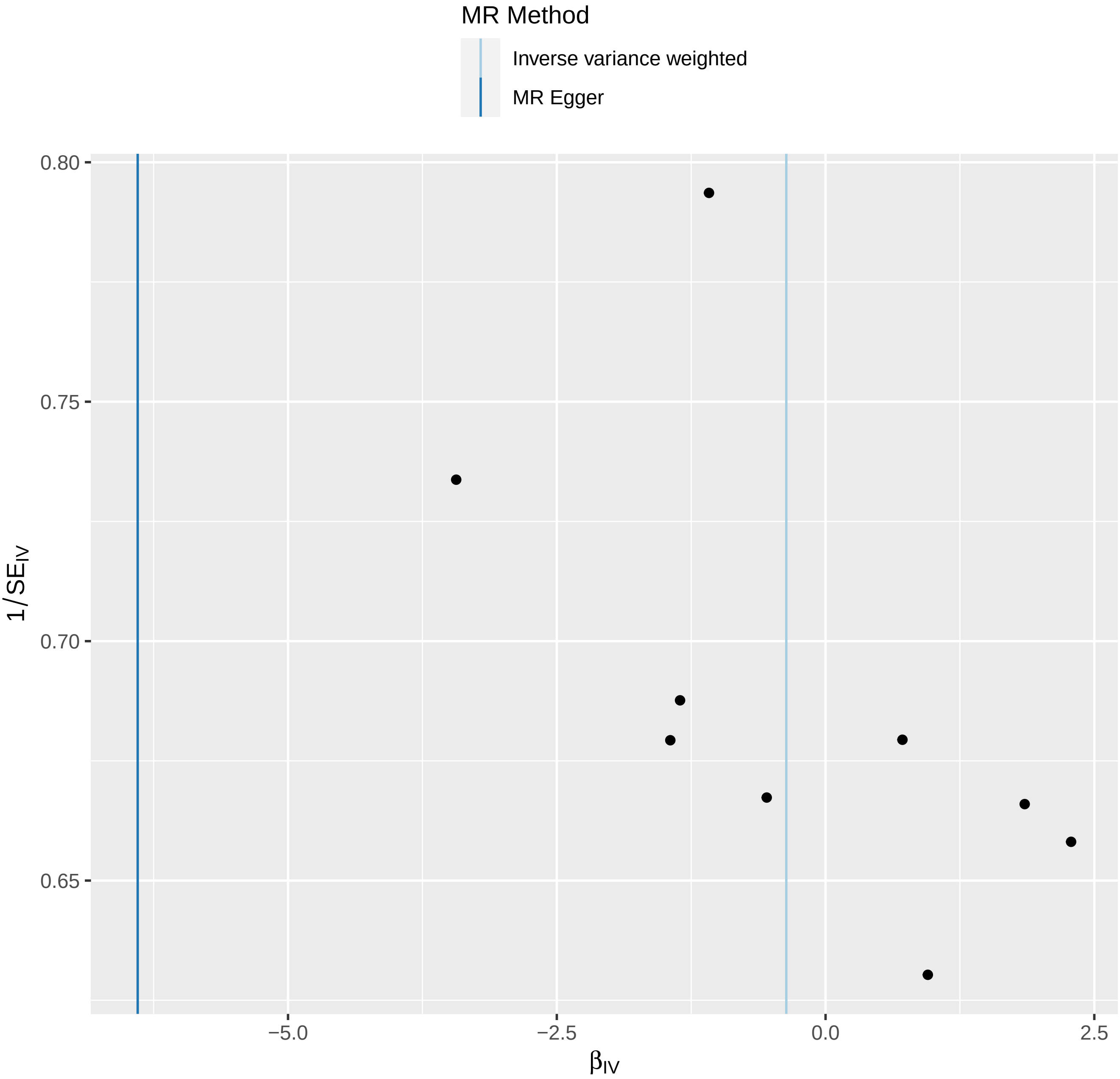

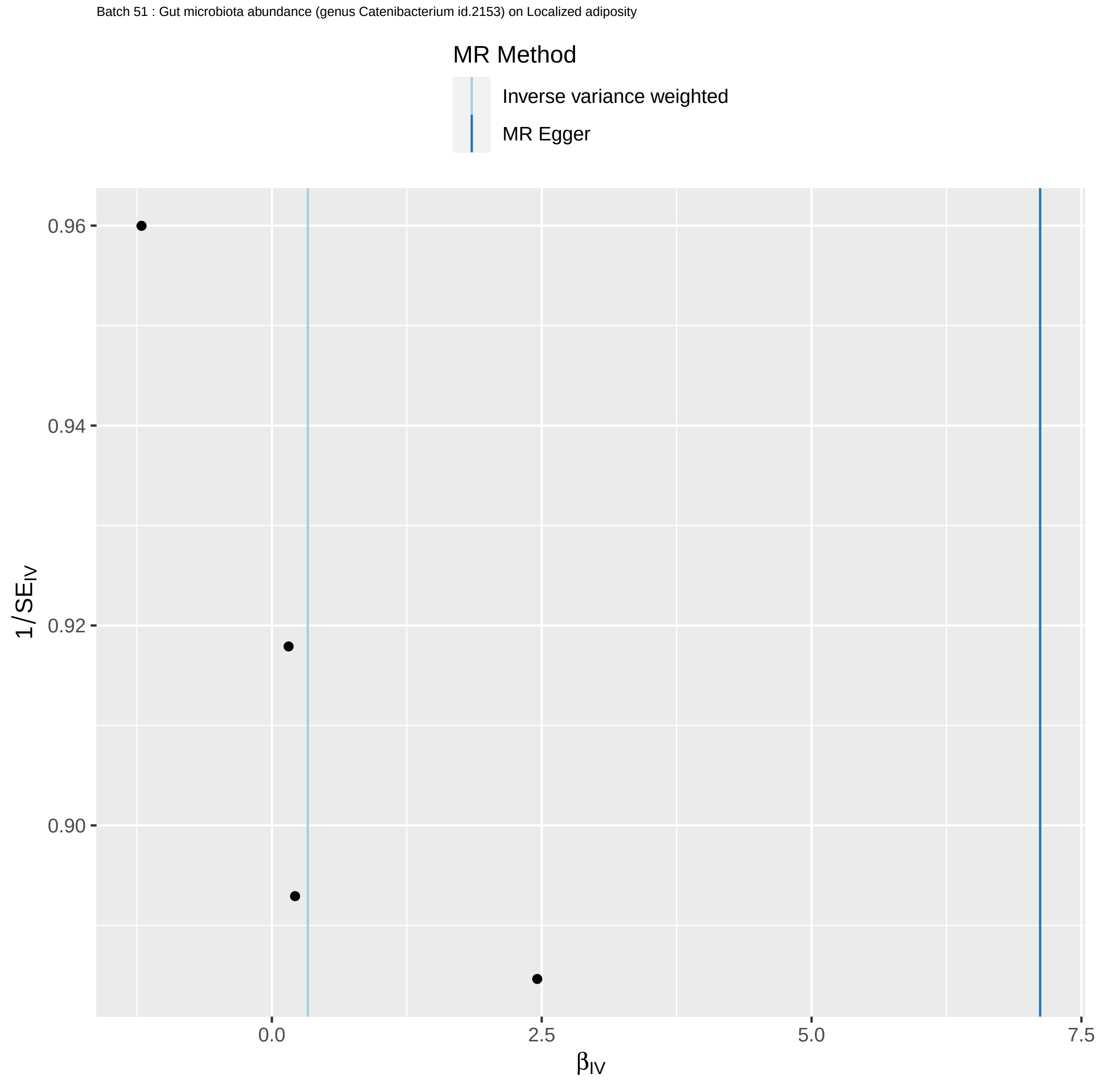

Batch 51 : Gut microbiota abundance (genus Catenibacterium id.2153) on Localized adiposity
MR Method
Inverse variance weighted
MR Egger
0.96
0.94
0.92
0.90
1/SE_IV
0.0
2.5
5.0
7.5
β_IV

Batch 52 : Gut microbiota abundance (genus Christensenellaceae R 7group id.11283) on Localized adiposity

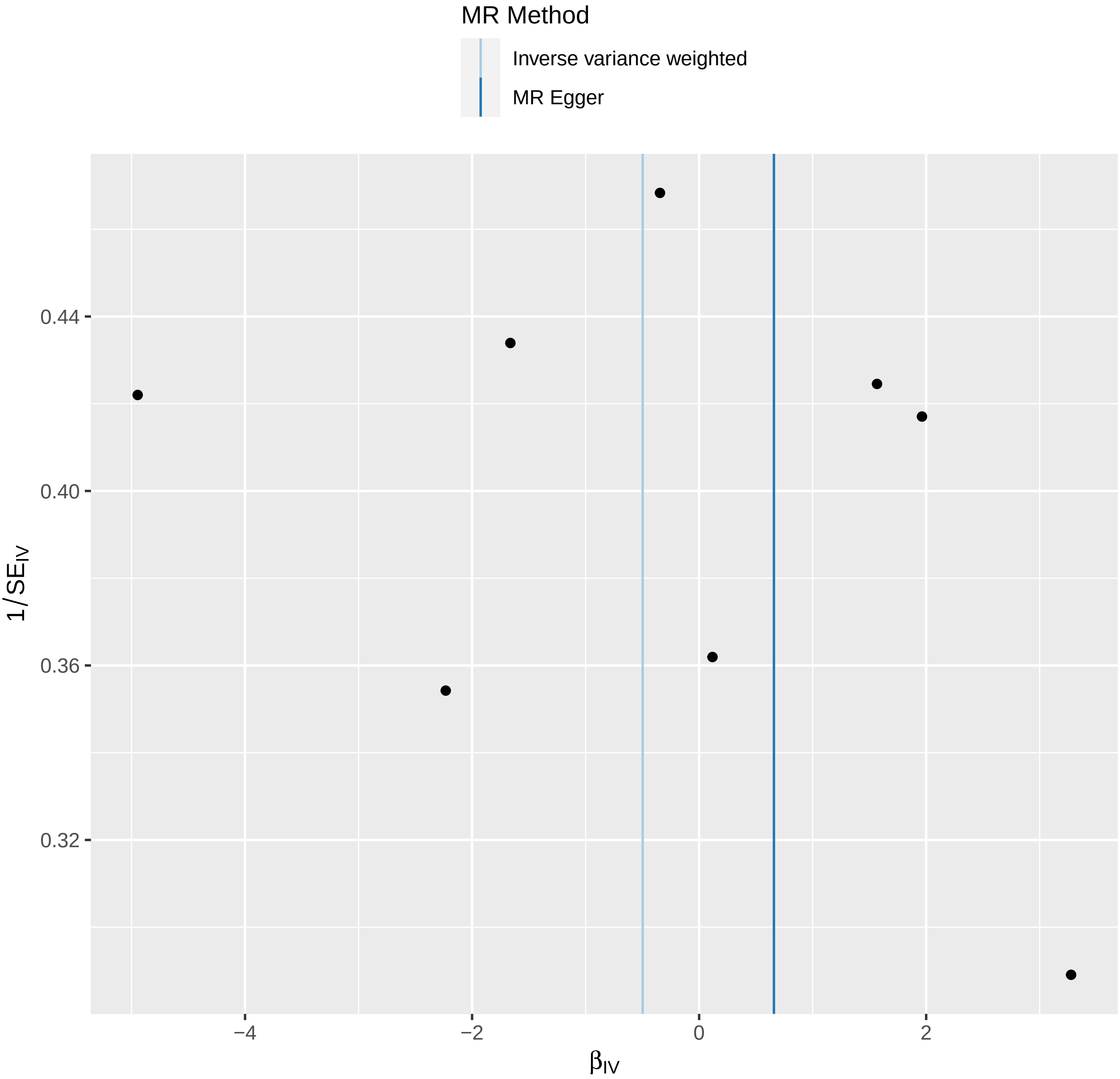

Batch 53 : Gut microbiota abundance (genus Clostridium innocuum group id.14397) on Localized adiposity

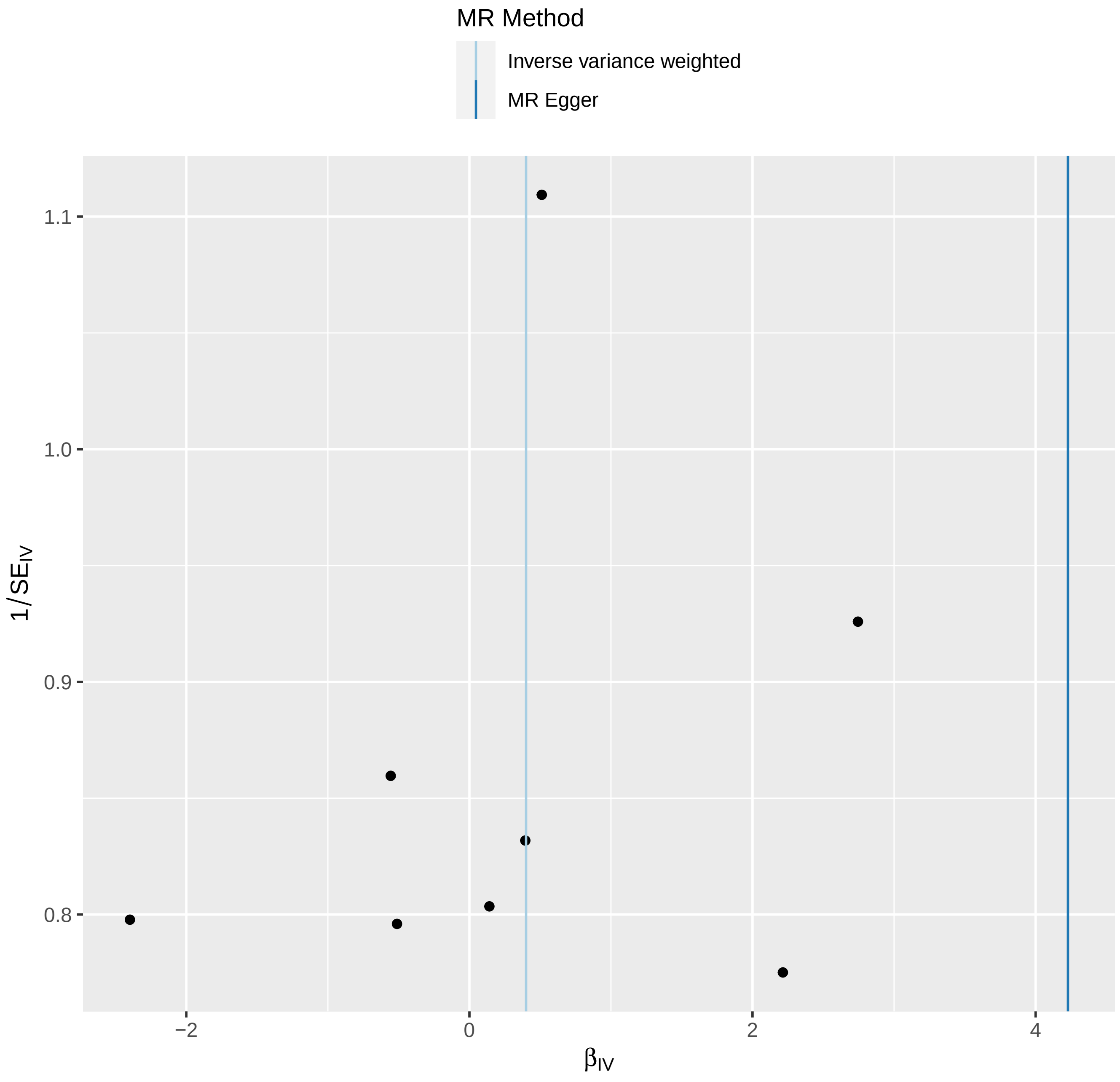

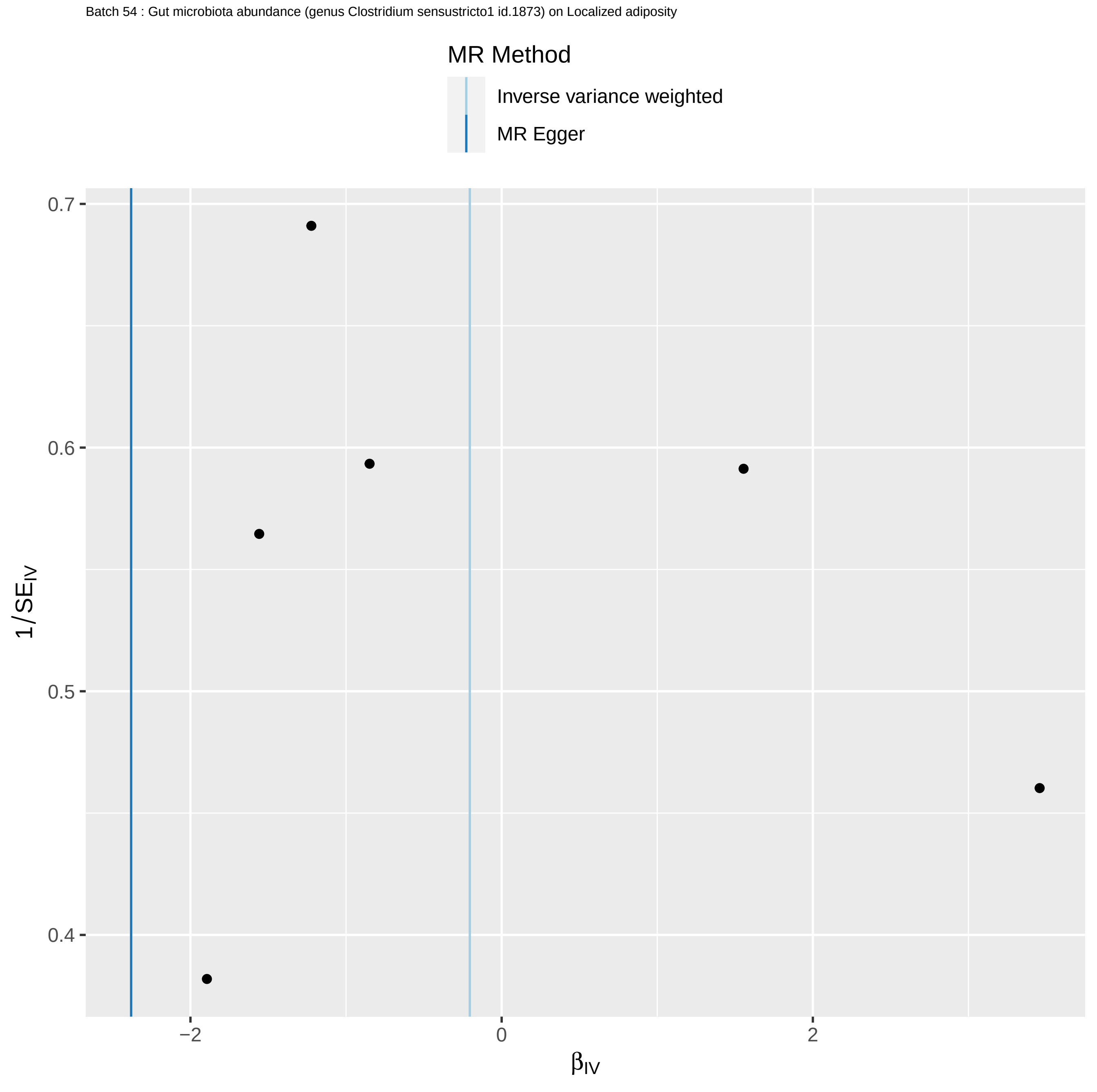

Batch 54 : Gut microbiota abundance (genus Clostridium sensustricto1 id.1873) on Localized adiposity
MR Method
Inverse variance weighted
MR Egger
0.7
0.6
0.5
0.4
1/SE$_{IV}$
−2
0
2
$\beta_{IV}$

Batch 55 : Gut microbiota abundance (genus Collinsella id.815) on Localized adiposity

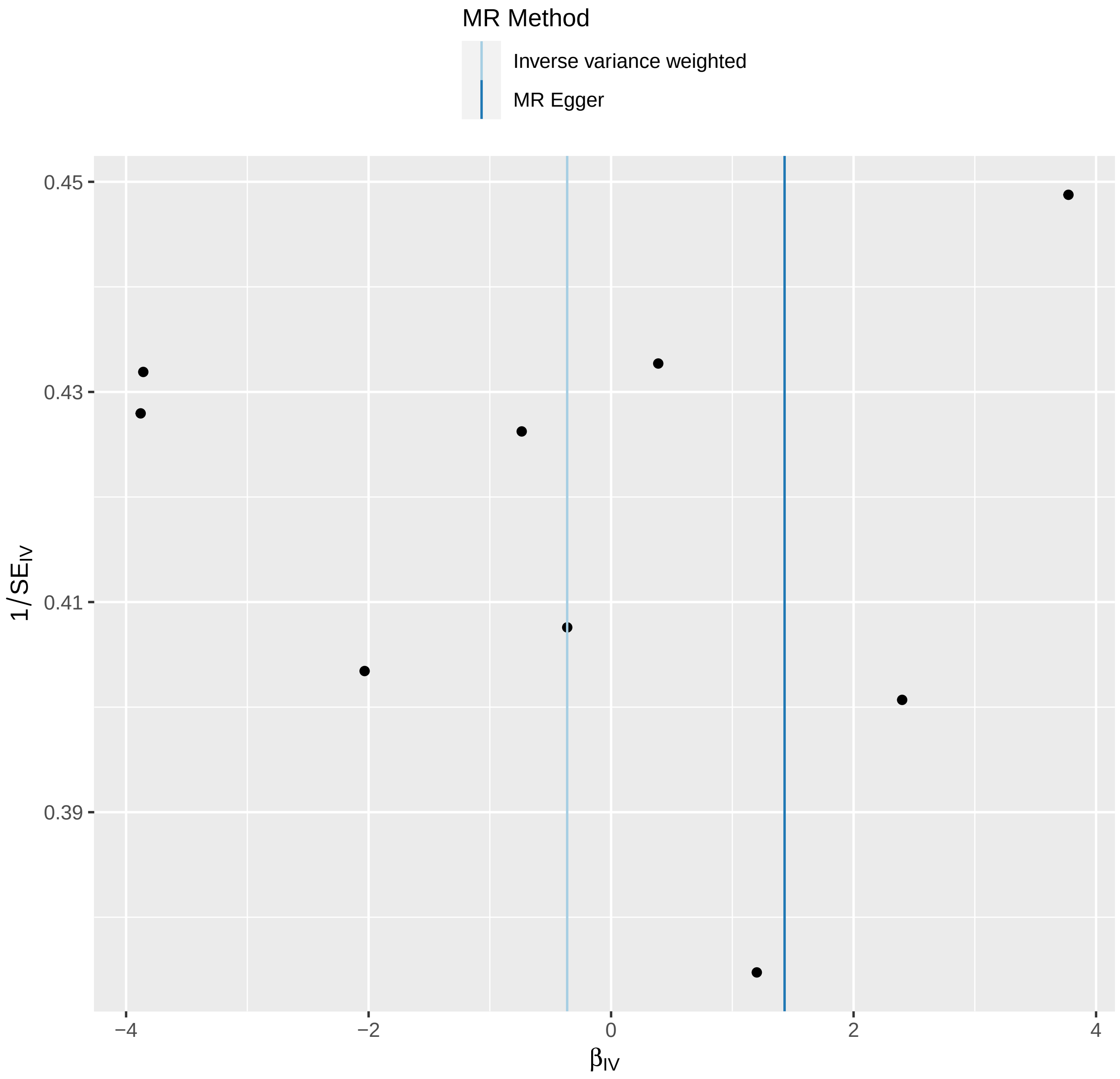

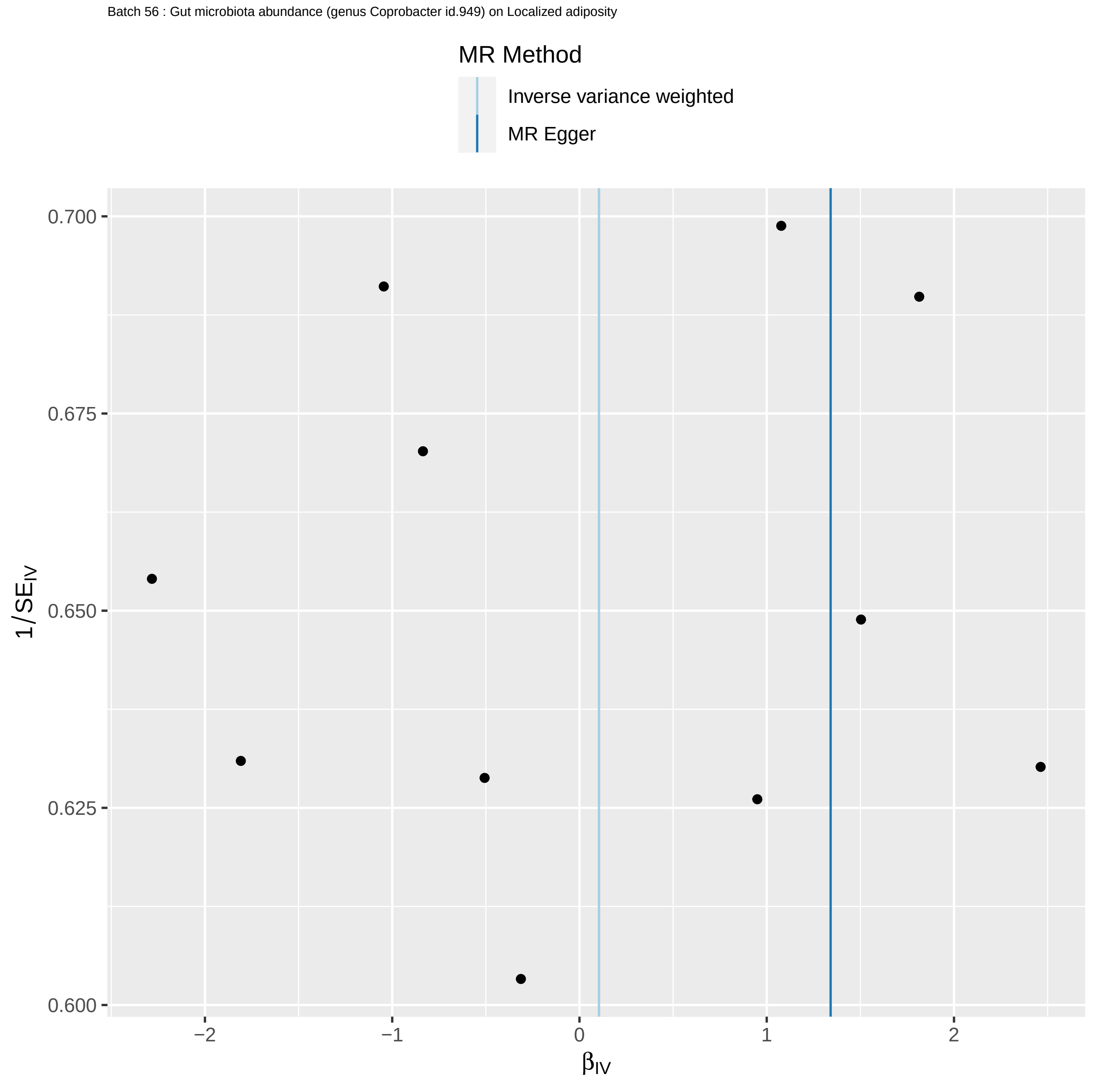

Batch 56 : Gut microbiota abundance (genus Coprobacter id.949) on Localized adiposity
MR Method
Inverse variance weighted
MR Egger
0.700
0.675
0.650
0.625
0.600
1/SE_IV
−2
−1
0
1
2
β_IV

Batch 57 : Gut microbiota abundance (genus Coprococcus1 id.11301) on Localized adiposity

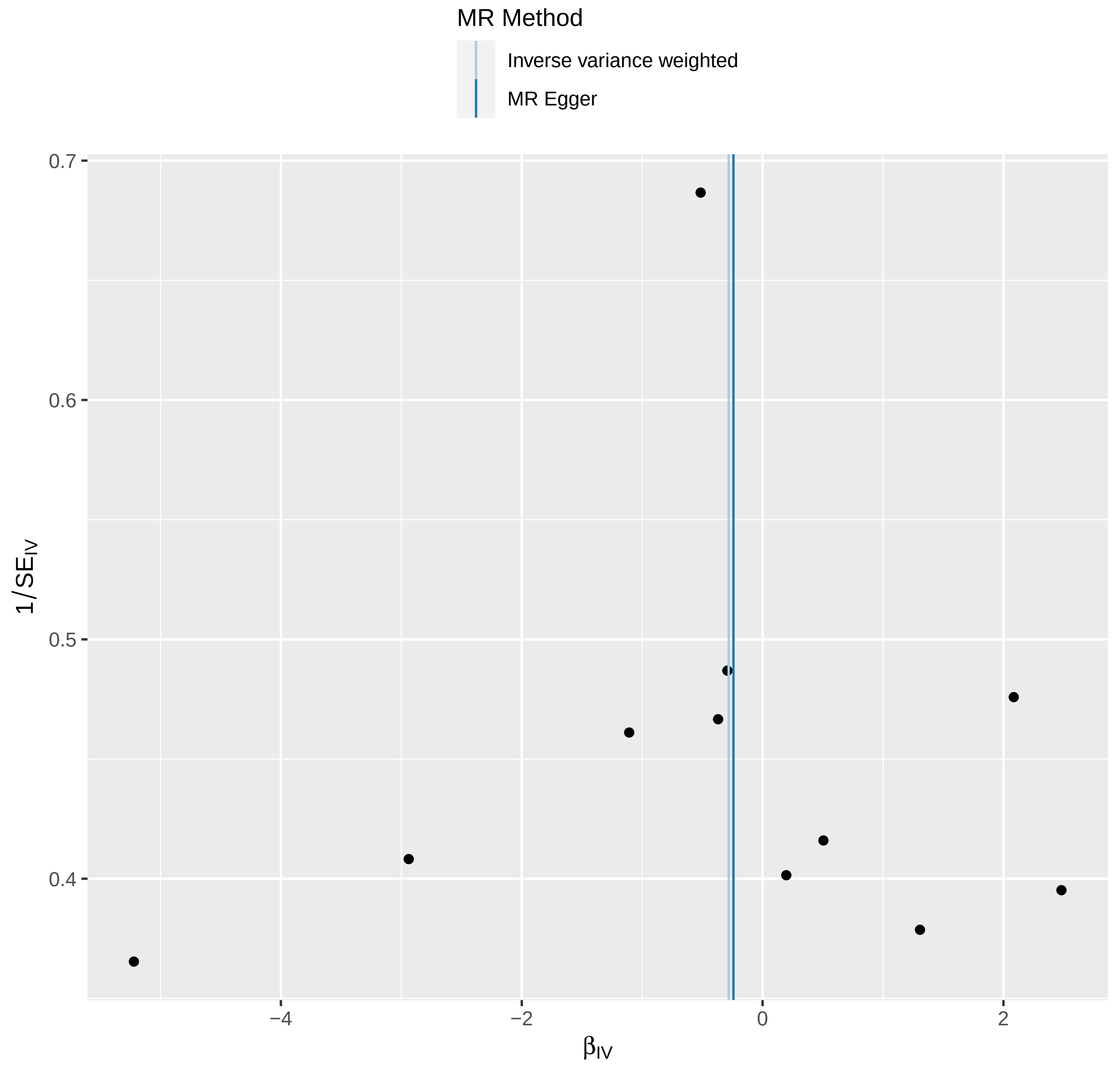

Batch 58 : Gut microbiota abundance (genus Coprococcus2 id.11302) on Localized adiposity

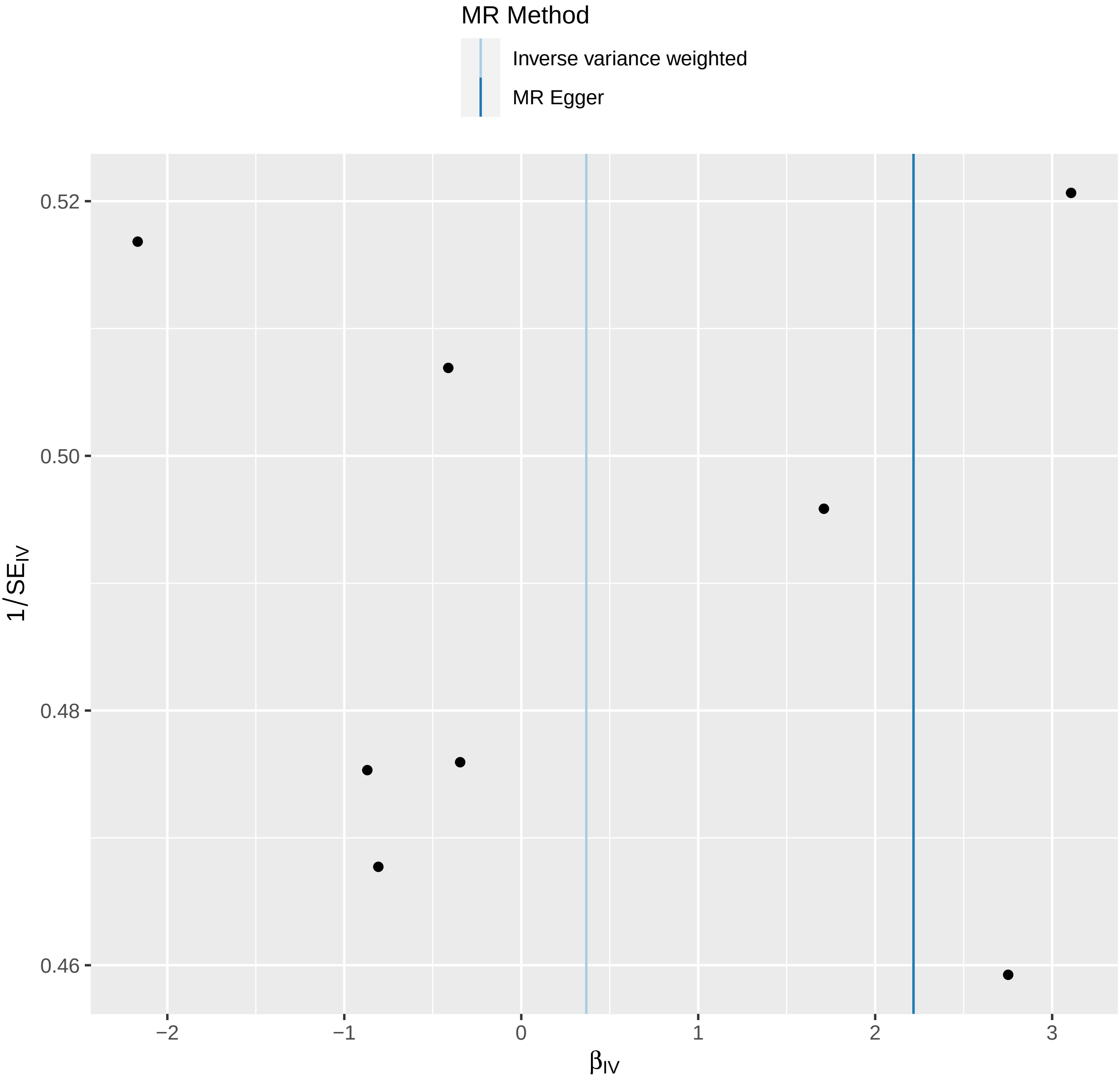

Batch 59 : Gut microbiota abundance (genus Coprococcus3 id.11303) on Localized adiposity

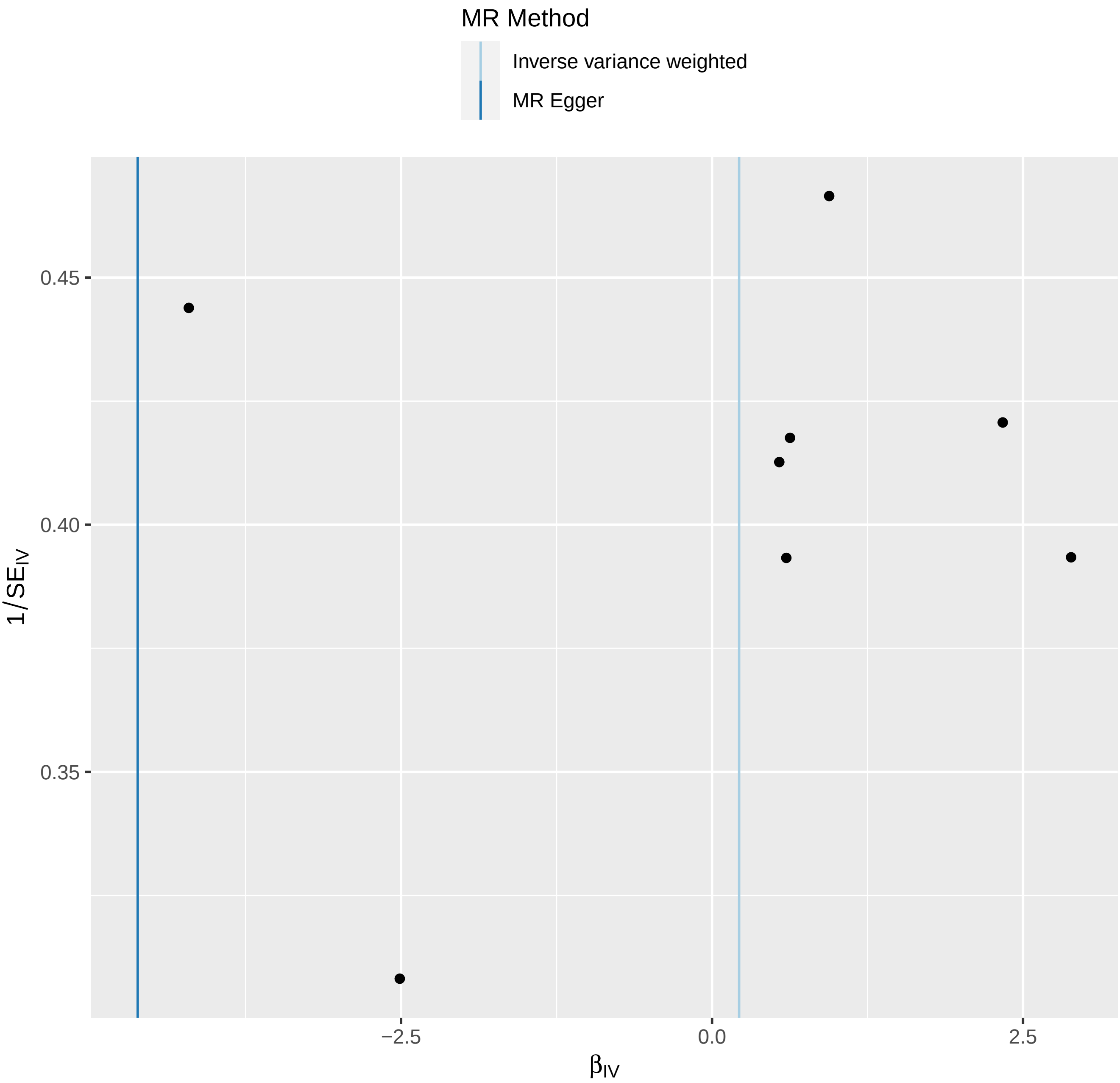

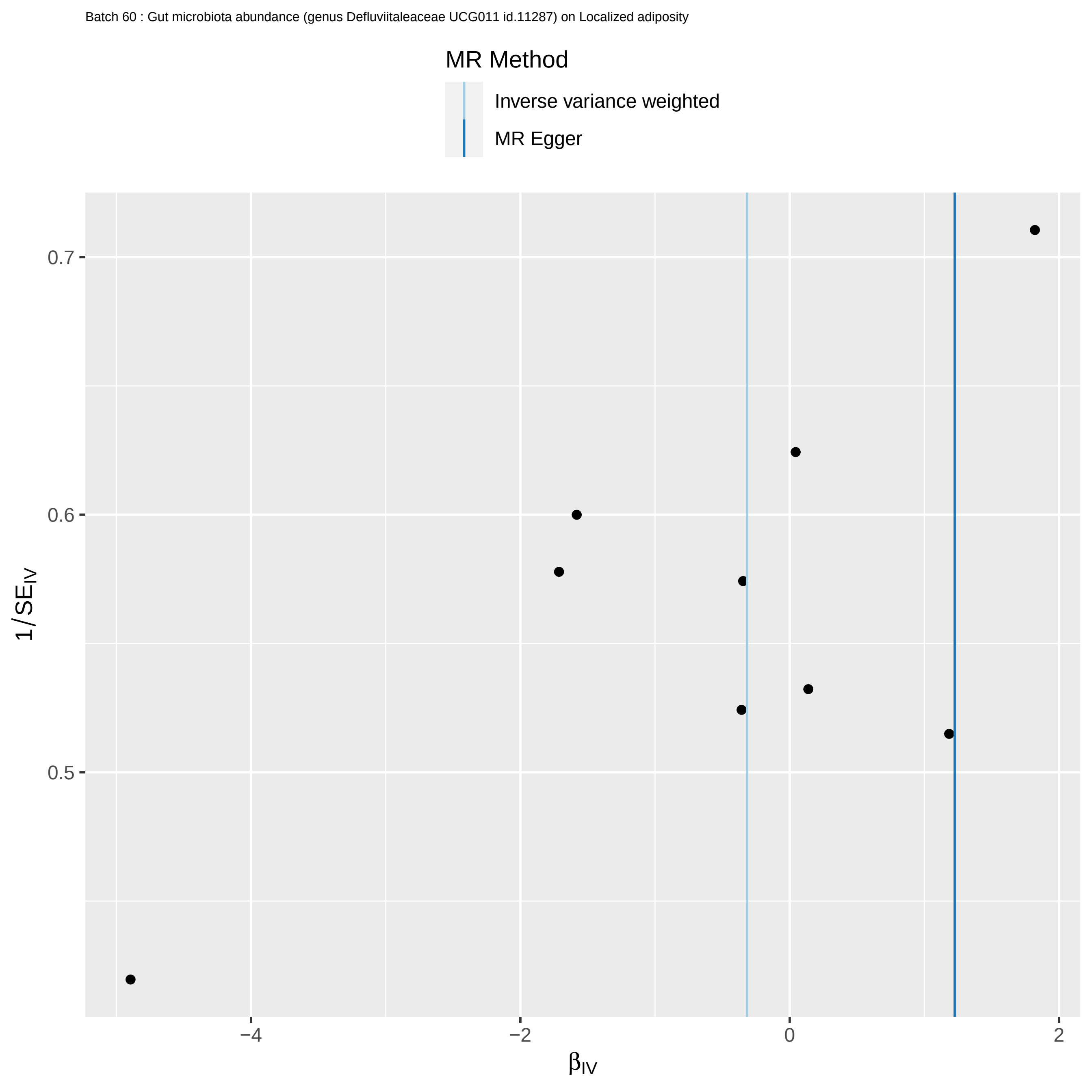

Batch 60 : Gut microbiota abundance (genus Defluviitaleaceae UCG011 id.11287) on Localized adiposity
MR Method
Inverse variance weighted
MR Egger
0.7
0.6
0.5
1/SE_IV
−4
−2
0
2
β_IV

Batch 61 : Gut microbiota abundance (genus Desulfovibrio id.3173) on Localized adiposity

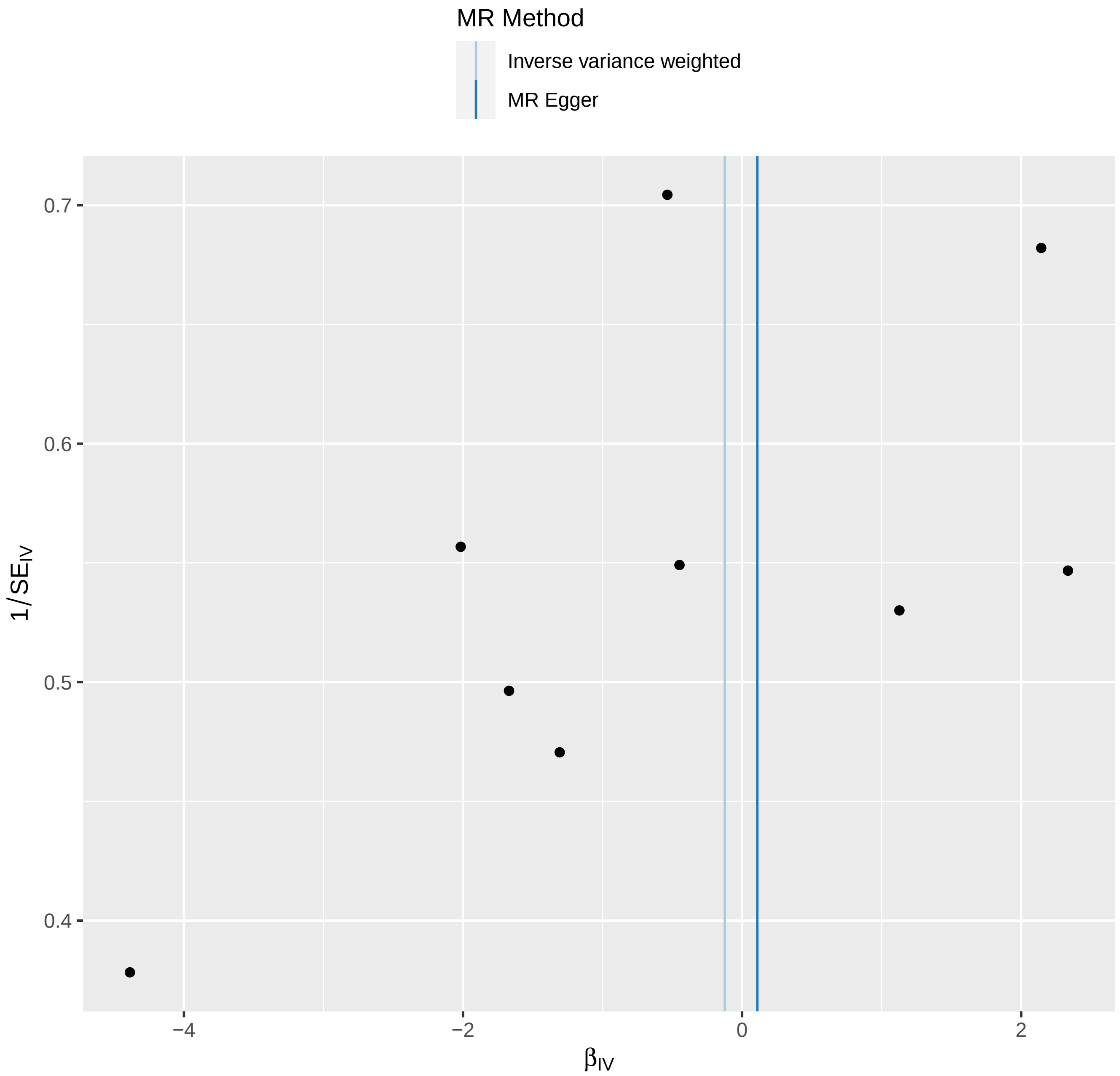

Batch 62 : Gut microbiota abundance (genus Dialister id.2183) on Localized adiposity

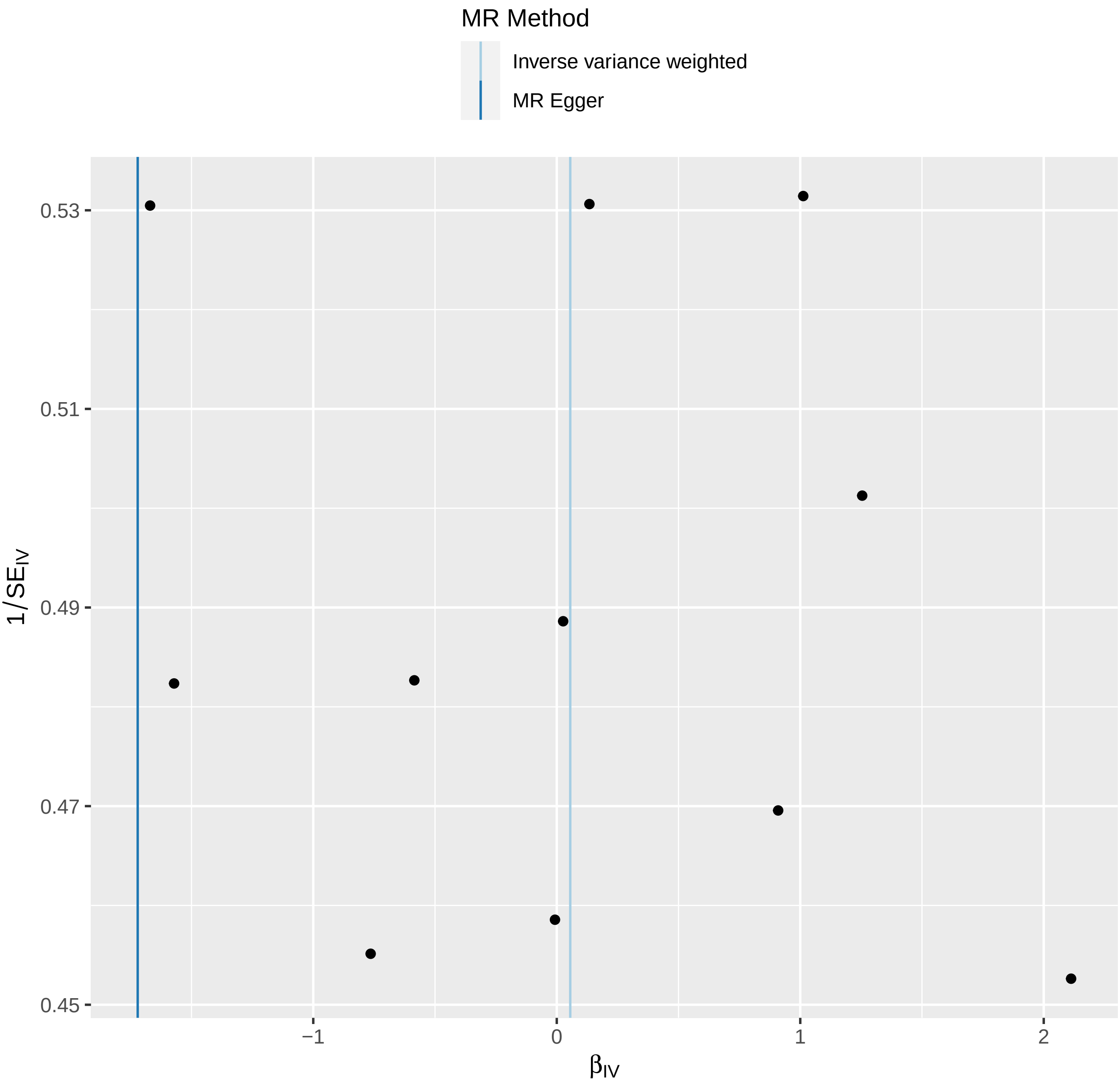

Batch 63 : Gut microbiota abundance (genus Dorea id.1997) on Localized adiposity

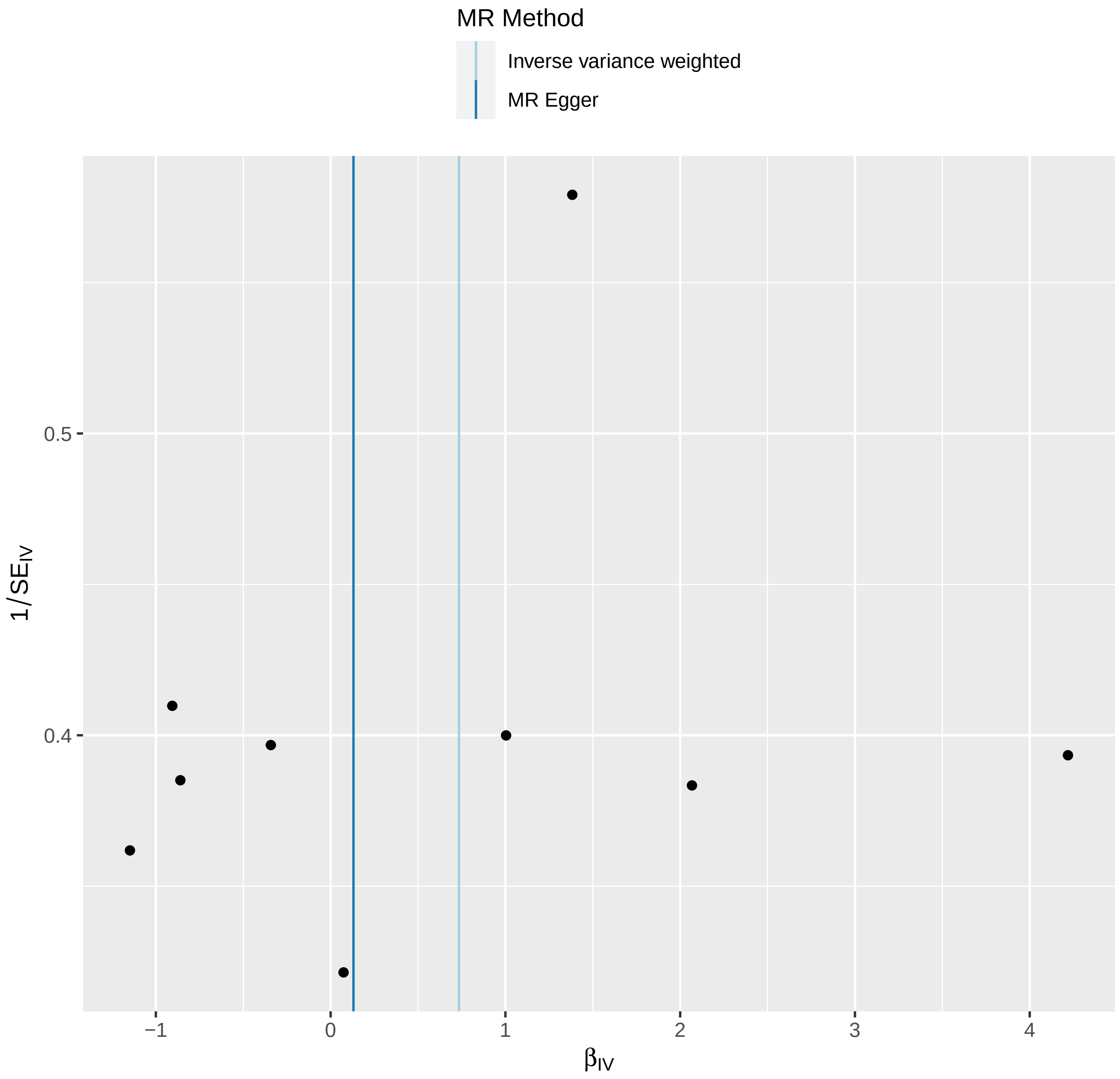

Batch 64 : Gut microbiota abundance (genus Eggerthella id.819) on Localized adiposity

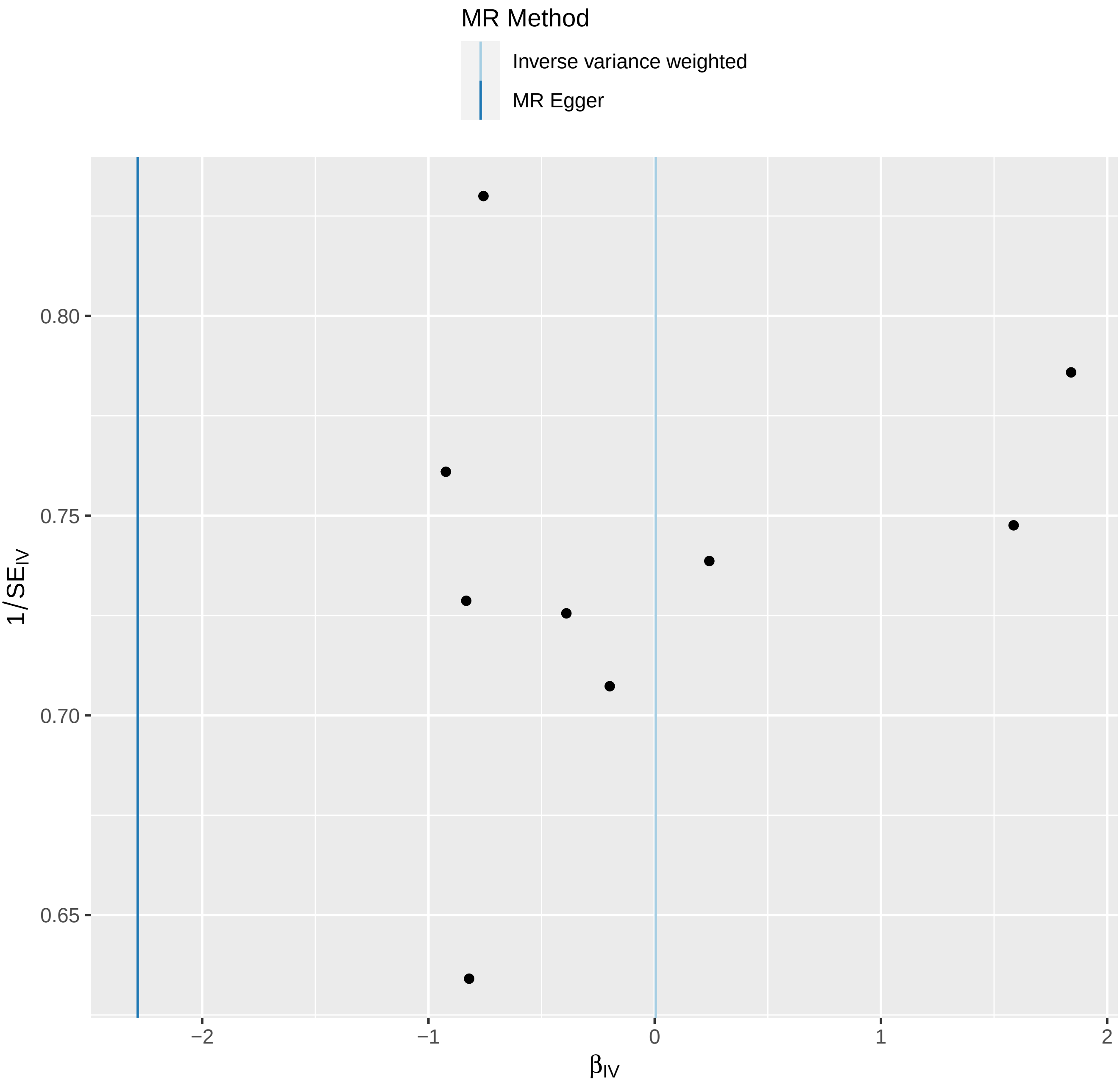

Batch 65 : Gut microbiota abundance (genus Eisenbergiella id.11304) on Localized adiposity

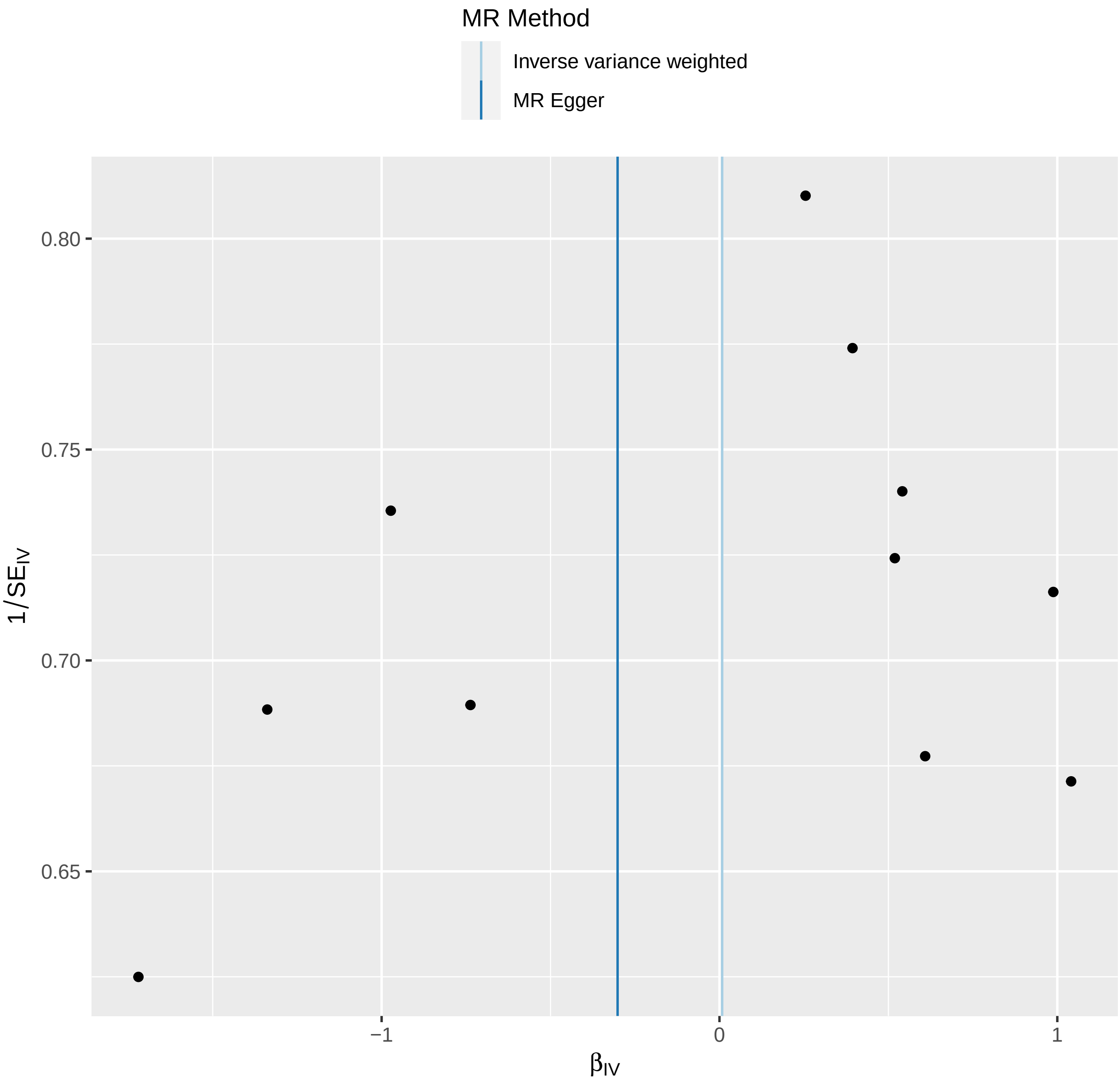

Batch 66 : Gut microbiota abundance (genus Enterorhabdus id.820) on Localized adiposity

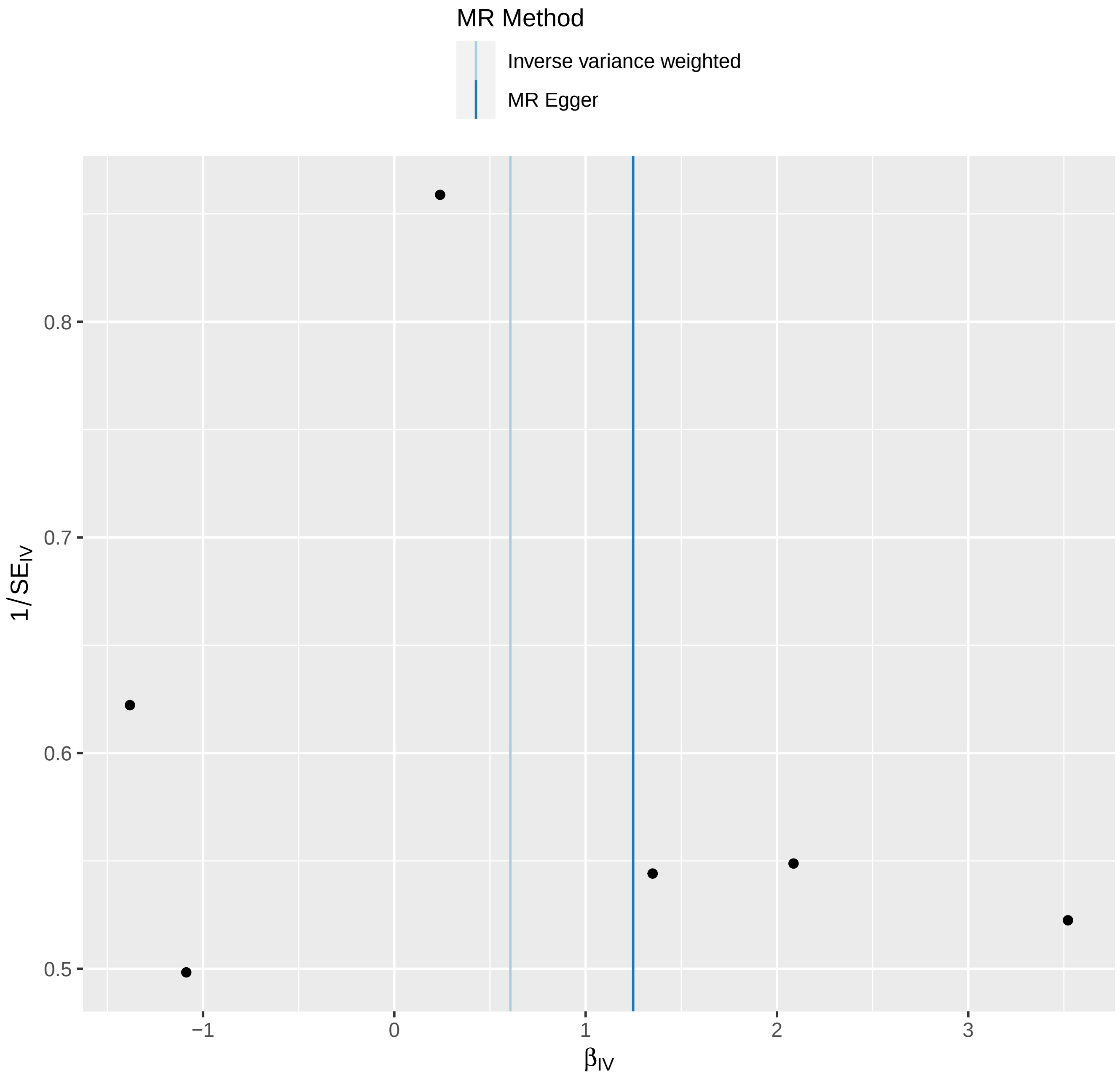

Batch 67 : Gut microbiota abundance (genus Erysipelatoclostridium id.11381) on Localized adiposity

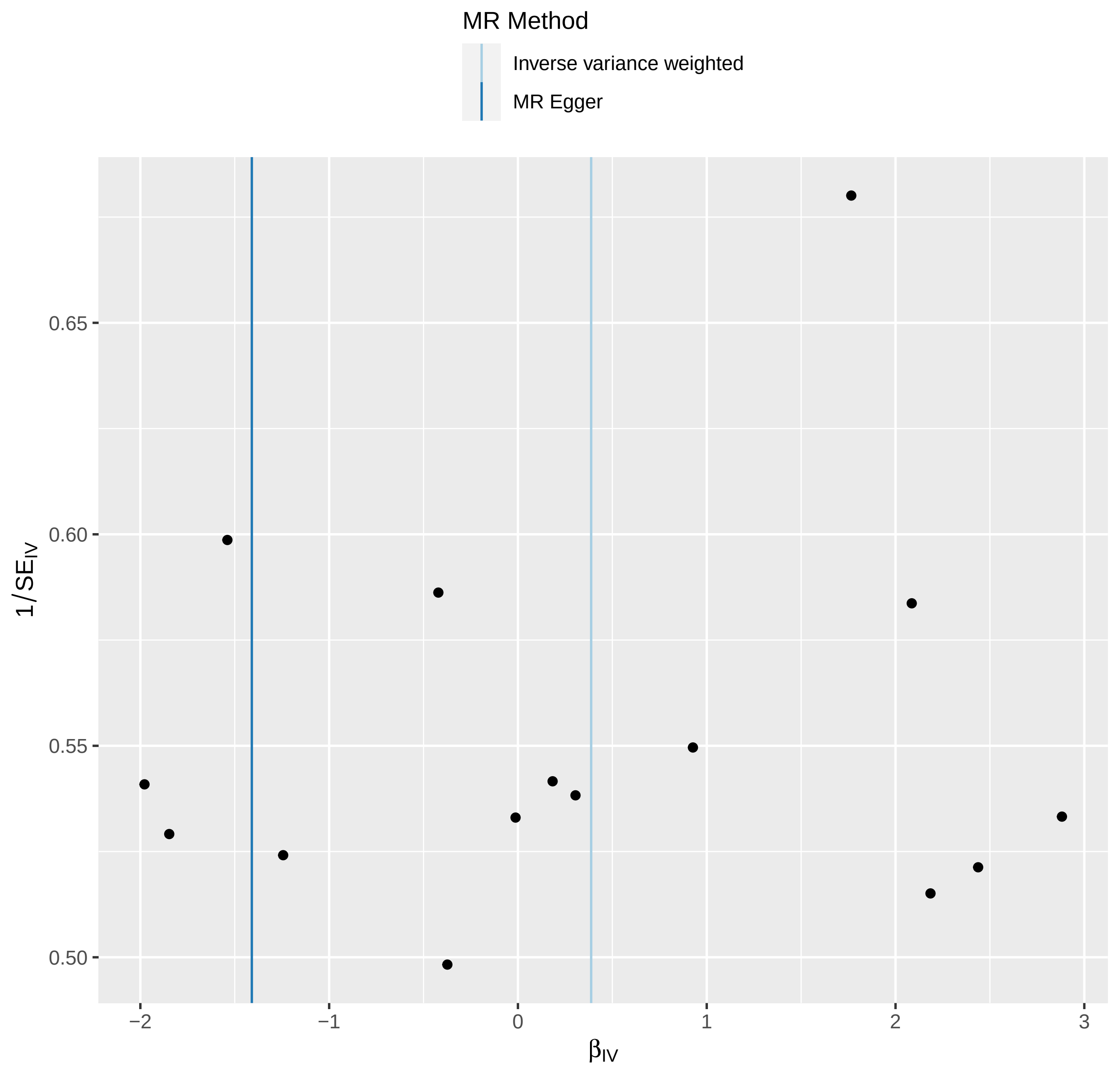

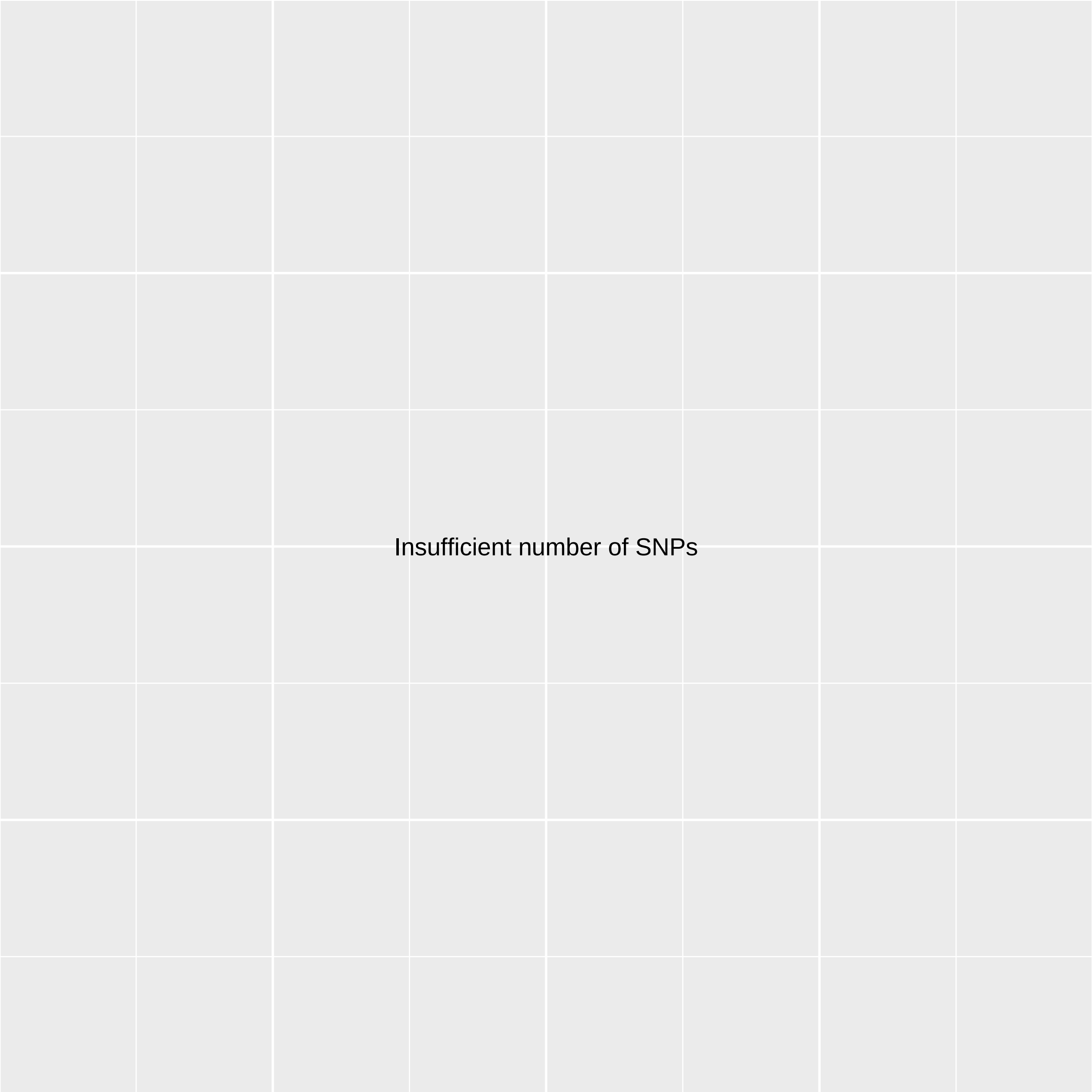
Insufficient number of SNPs

Batch 69 : Gut microbiota abundance (genus Escherichia Shigella id.3504) on Localized adiposity

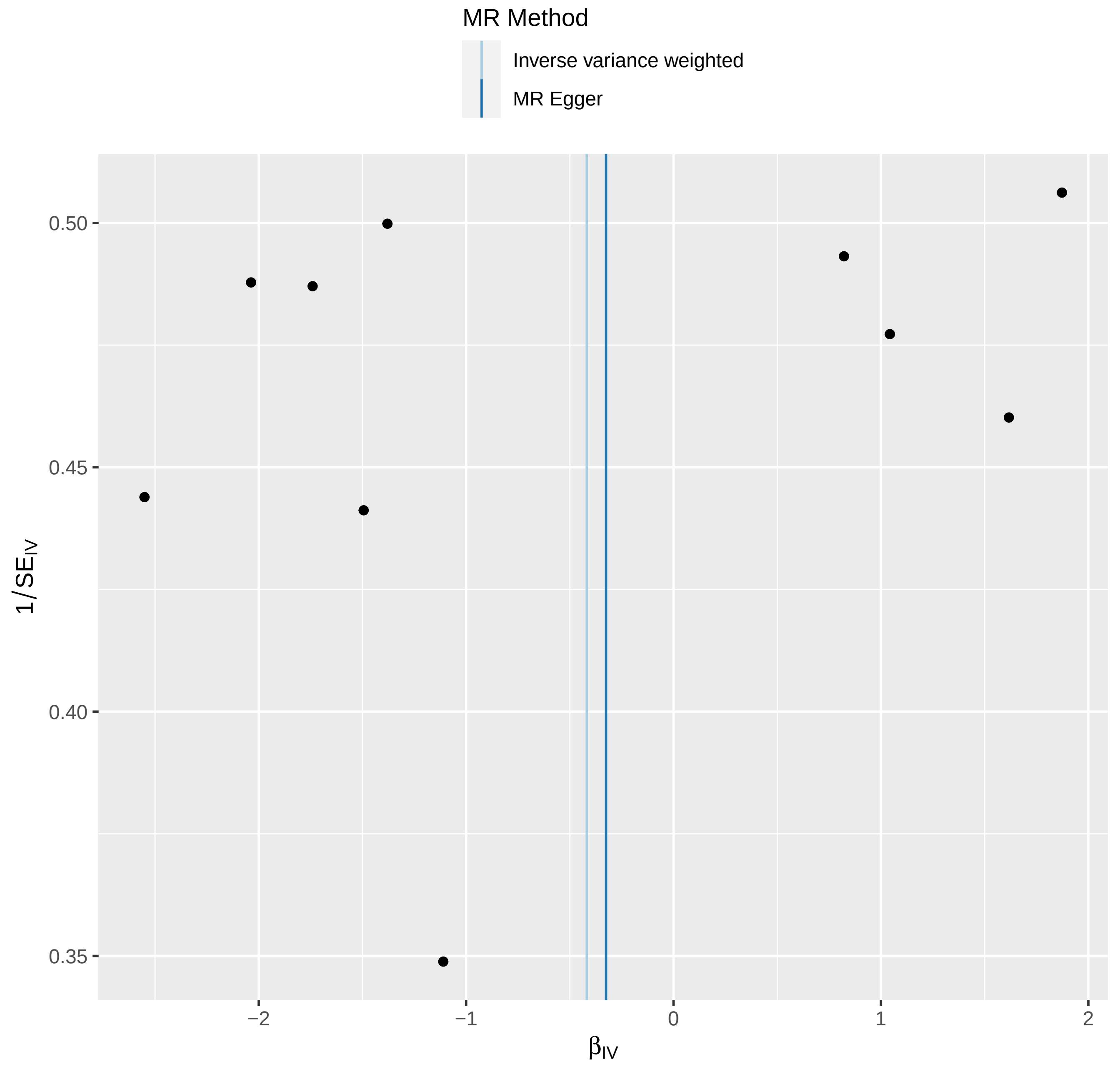

Batch 70 : Gut microbiota abundance (genus Eubacterium brachy group id.11296) on Localized adiposity

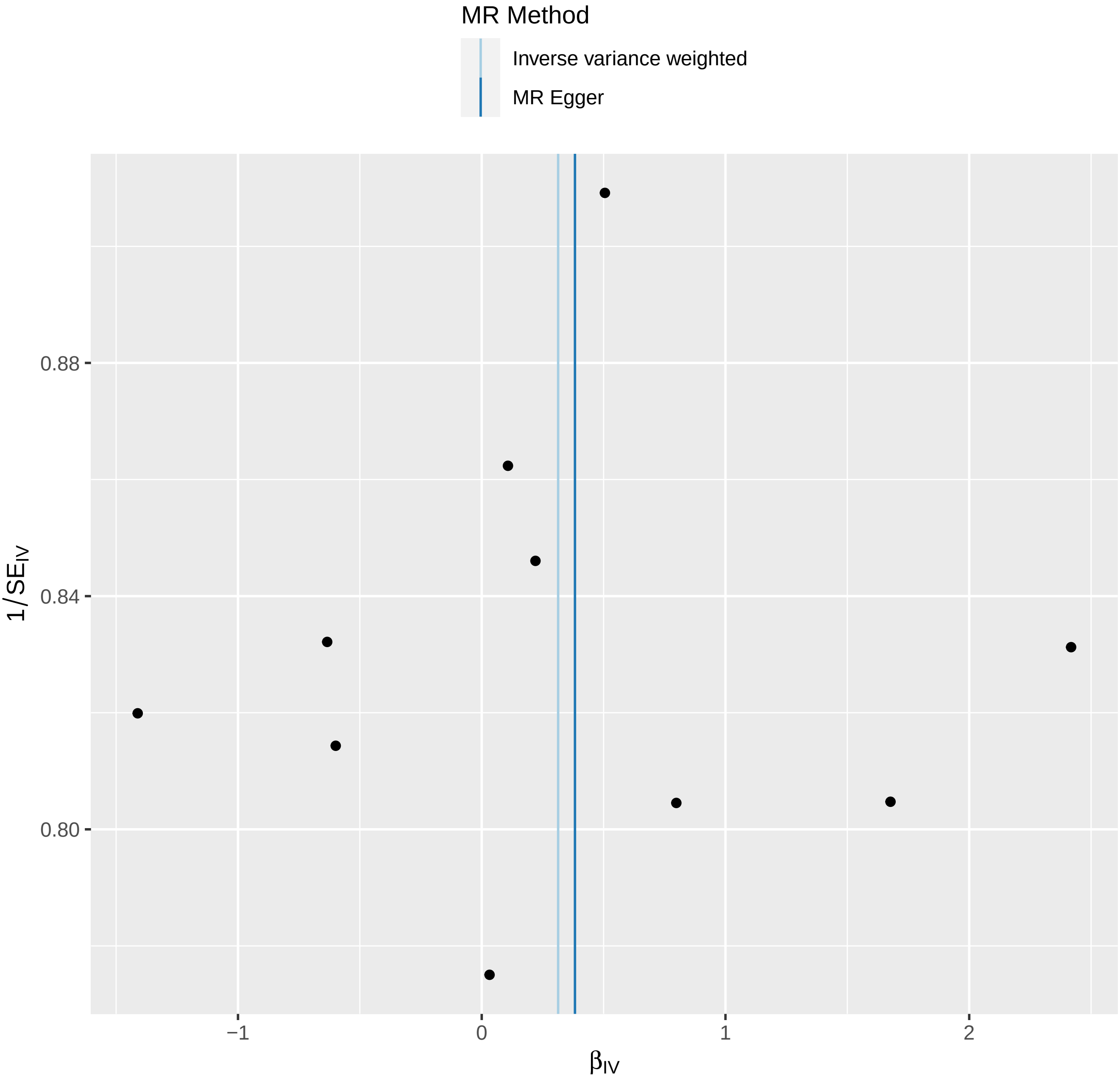

Batch 71 : Gut microbiota abundance (genus Eubacterium coprostanoligenes group id.11375) on Localized adiposity

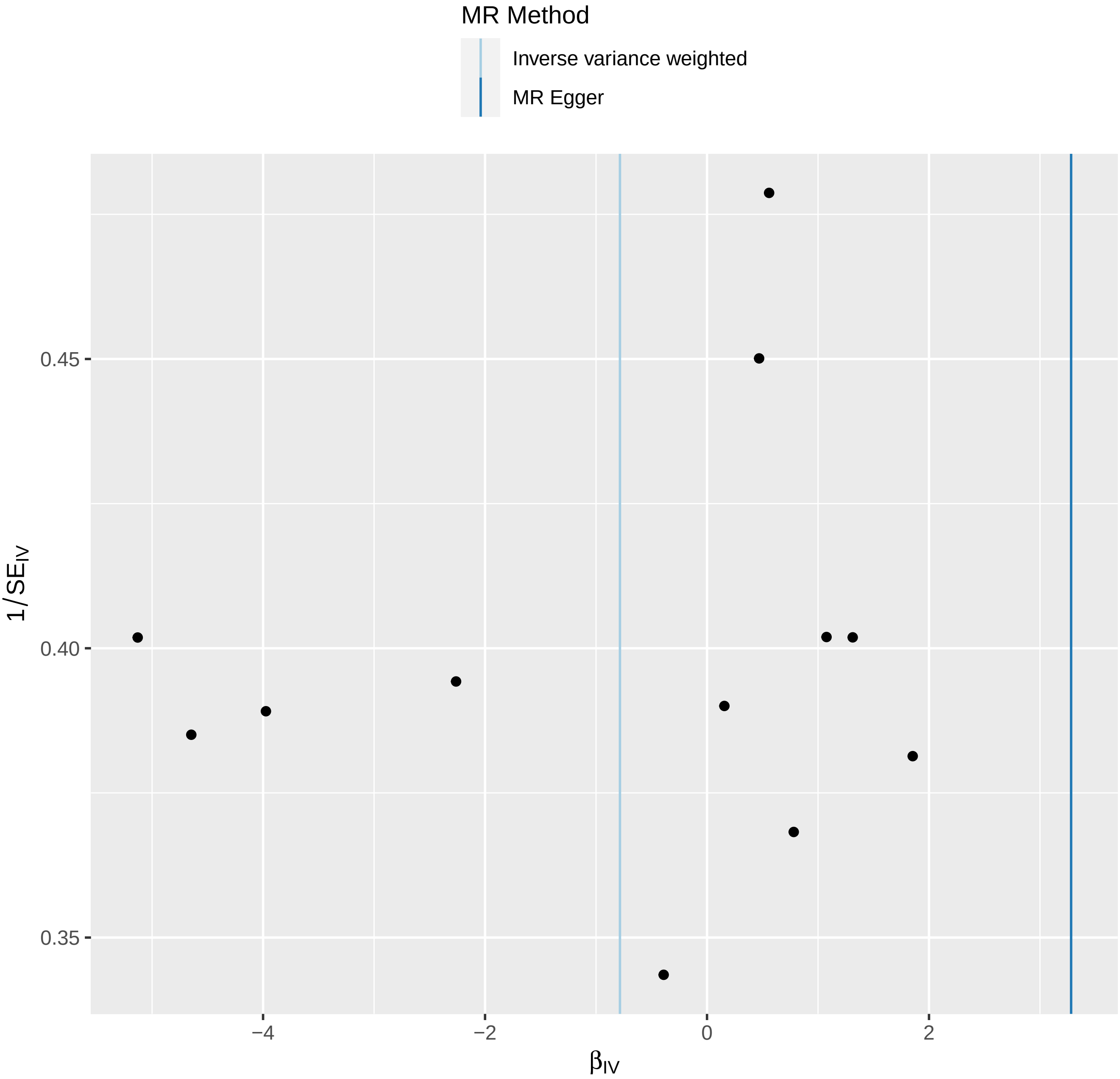

Batch 72 : Gut microbiota abundance (genus Eubacterium eligens group id.14372) on Localized adiposity

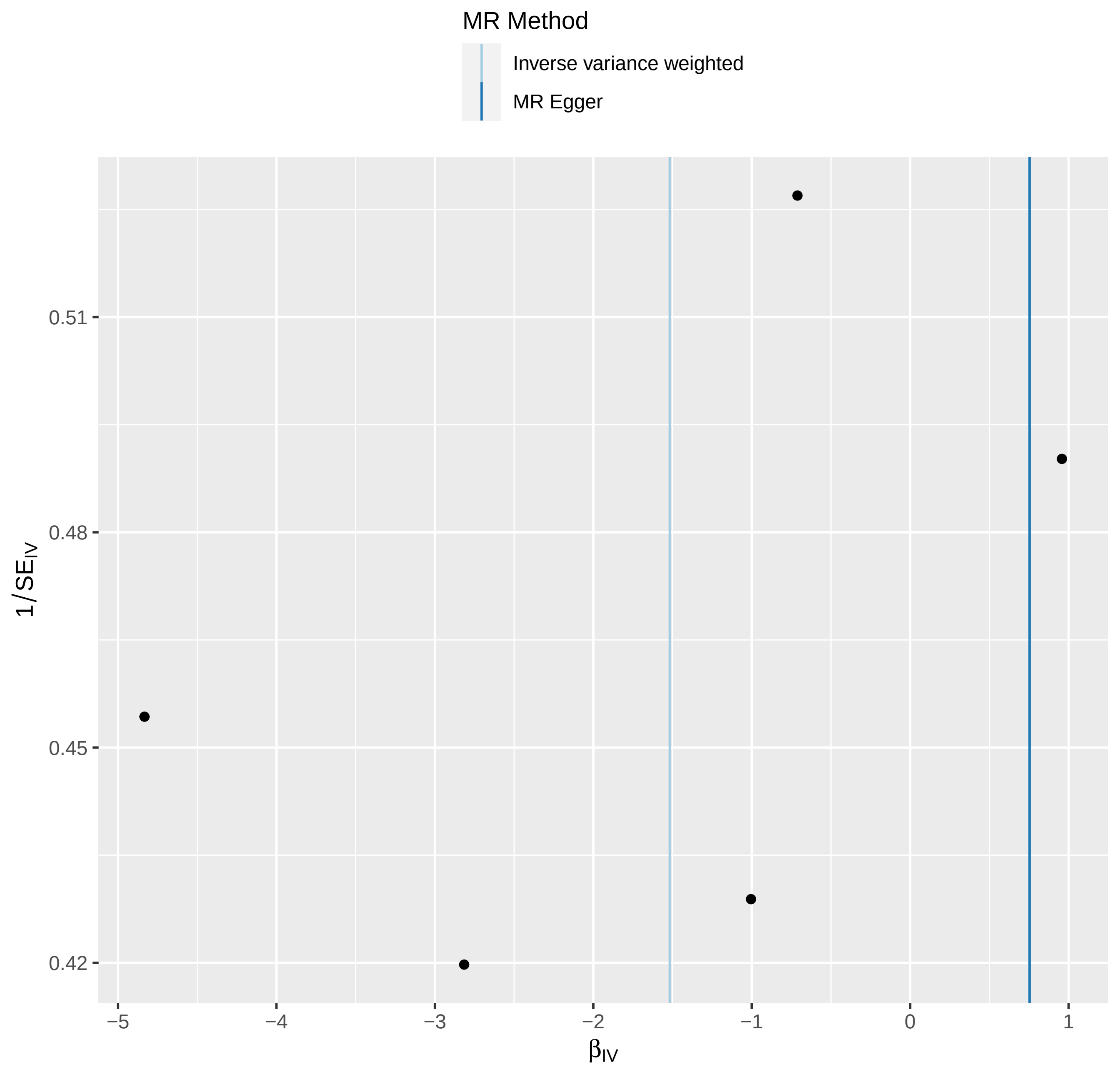

Batch 73 : Gut microbiota abundance (genus Eubacterium fissicatena group id.14373) on Localized adiposity

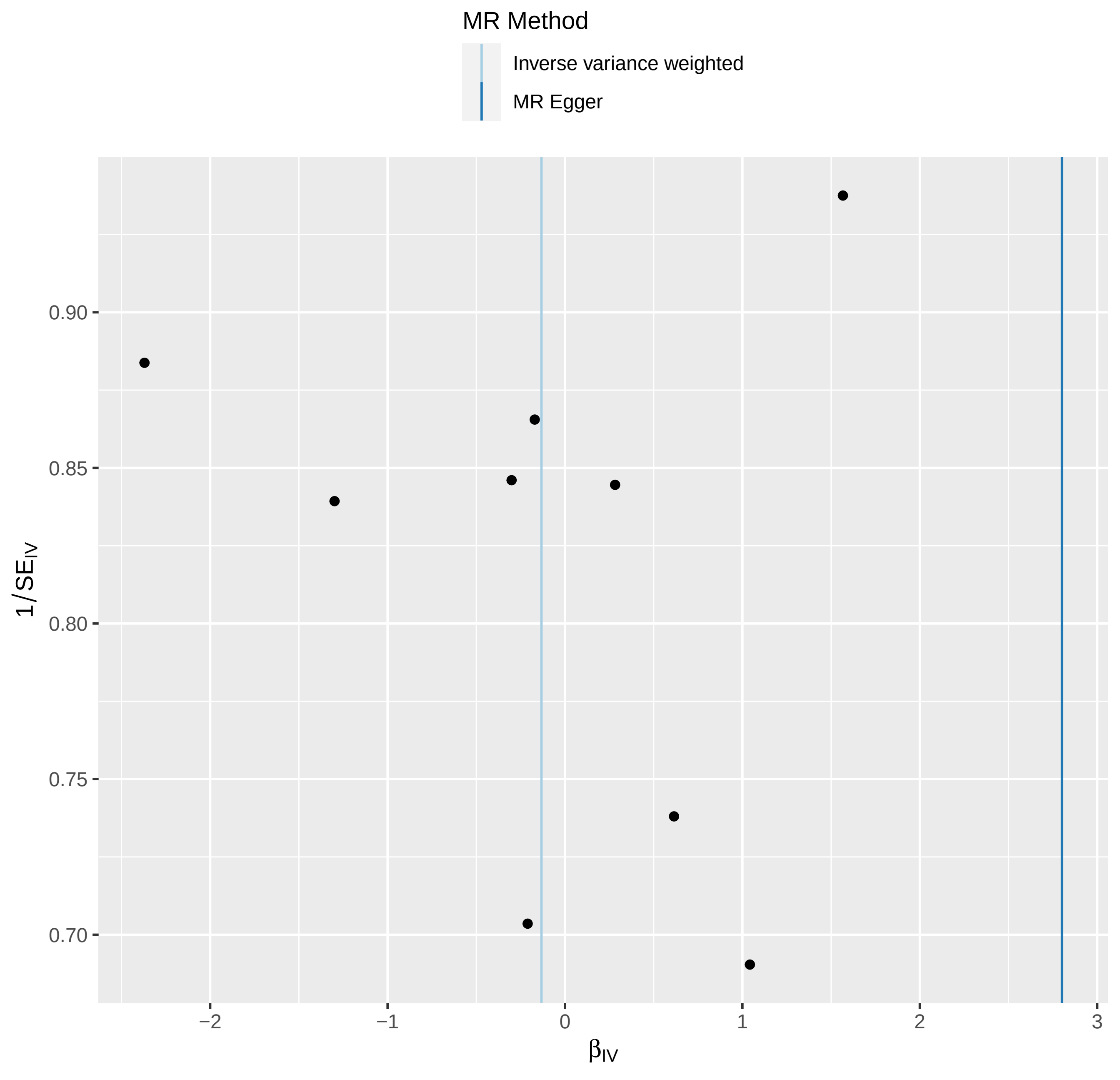

Batch 74 : Gut microbiota abundance (genus Eubacterium hallii group id.11338) on Localized adiposity

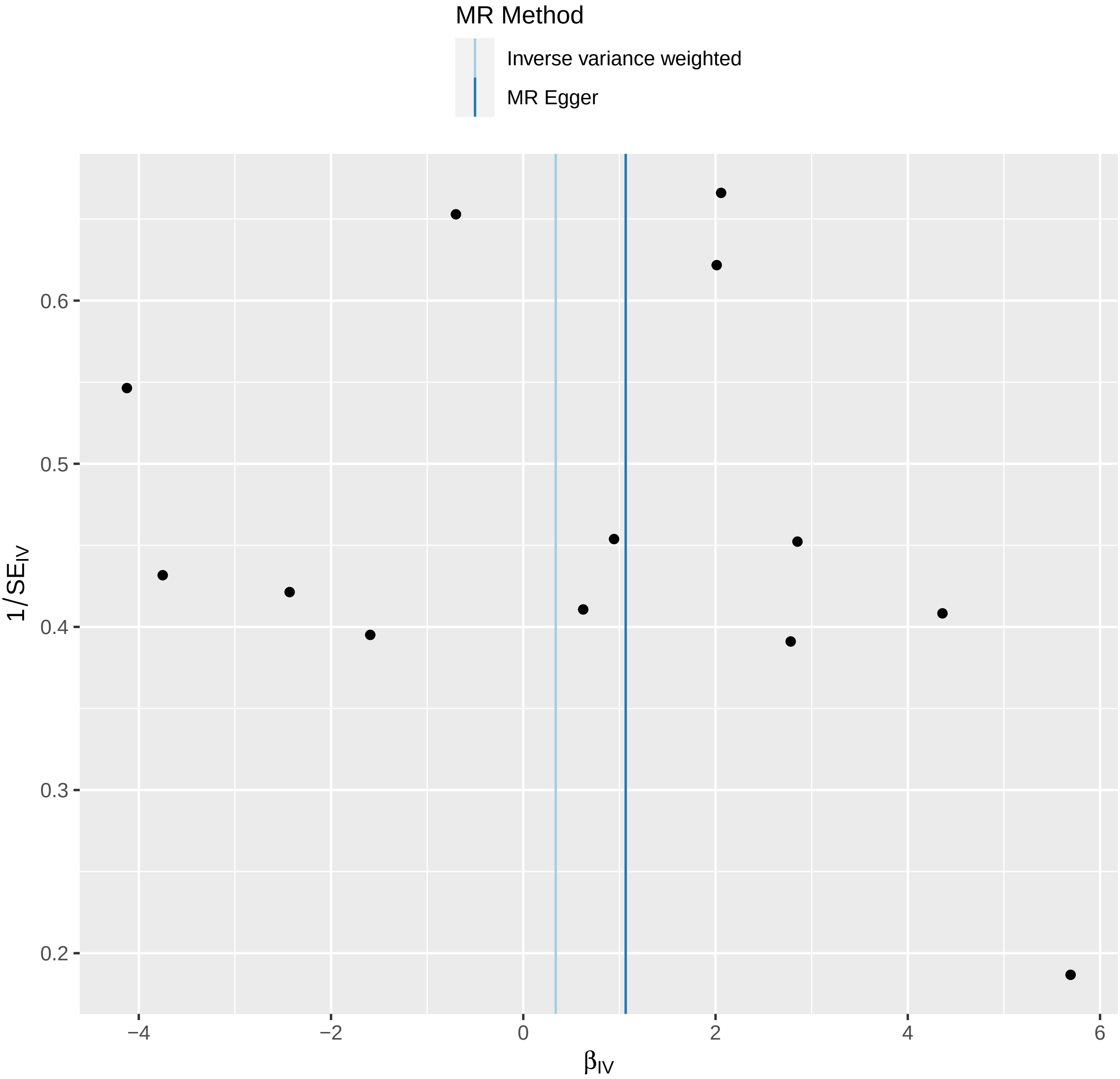

Batch 75 : Gut microbiota abundance (genus Eubacterium nodatum group id.11297) on Localized adiposity

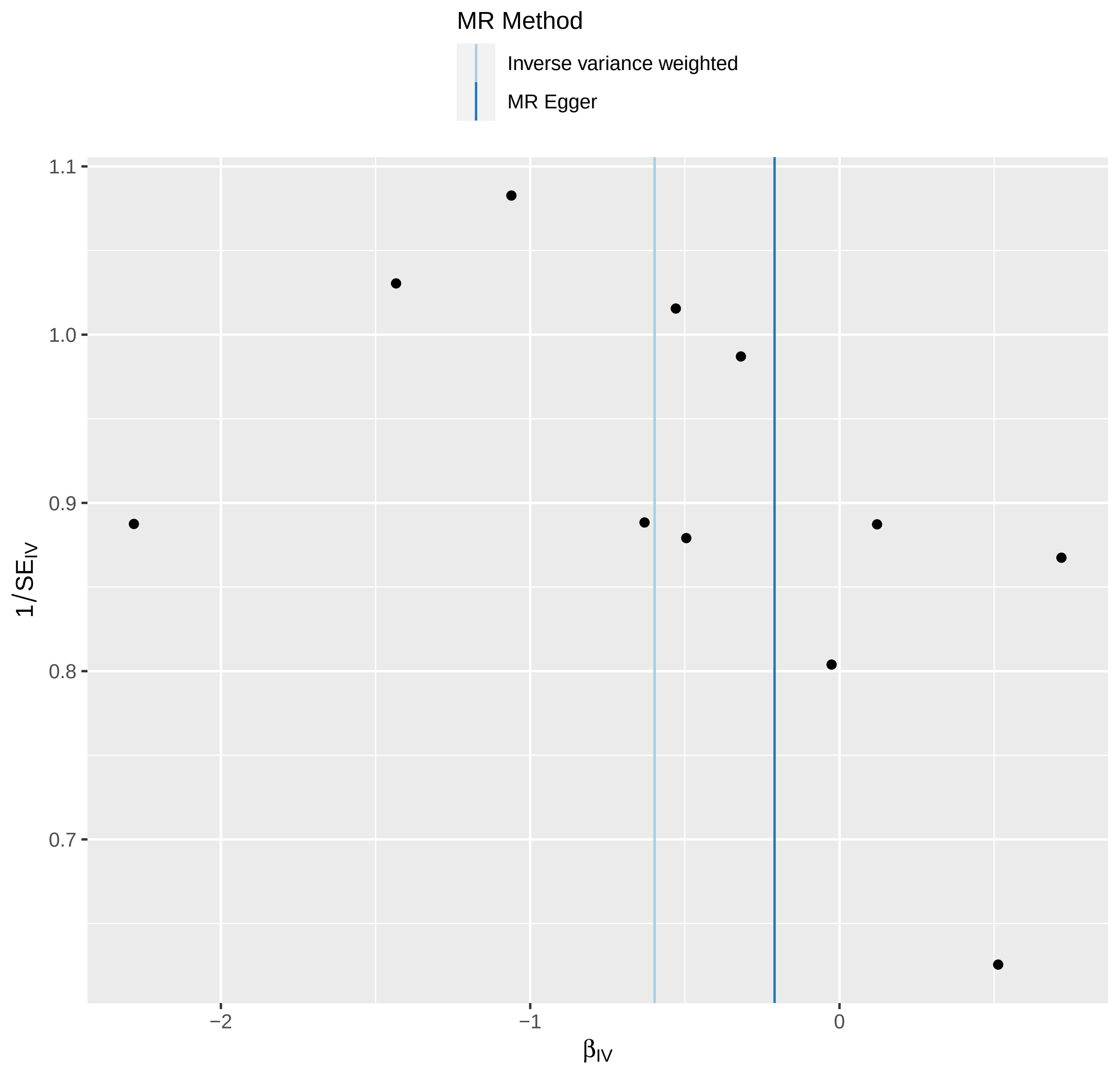

Batch 76 : Gut microbiota abundance (genus Eubacterium oxidoreducens group id.11339) on Localized adiposity

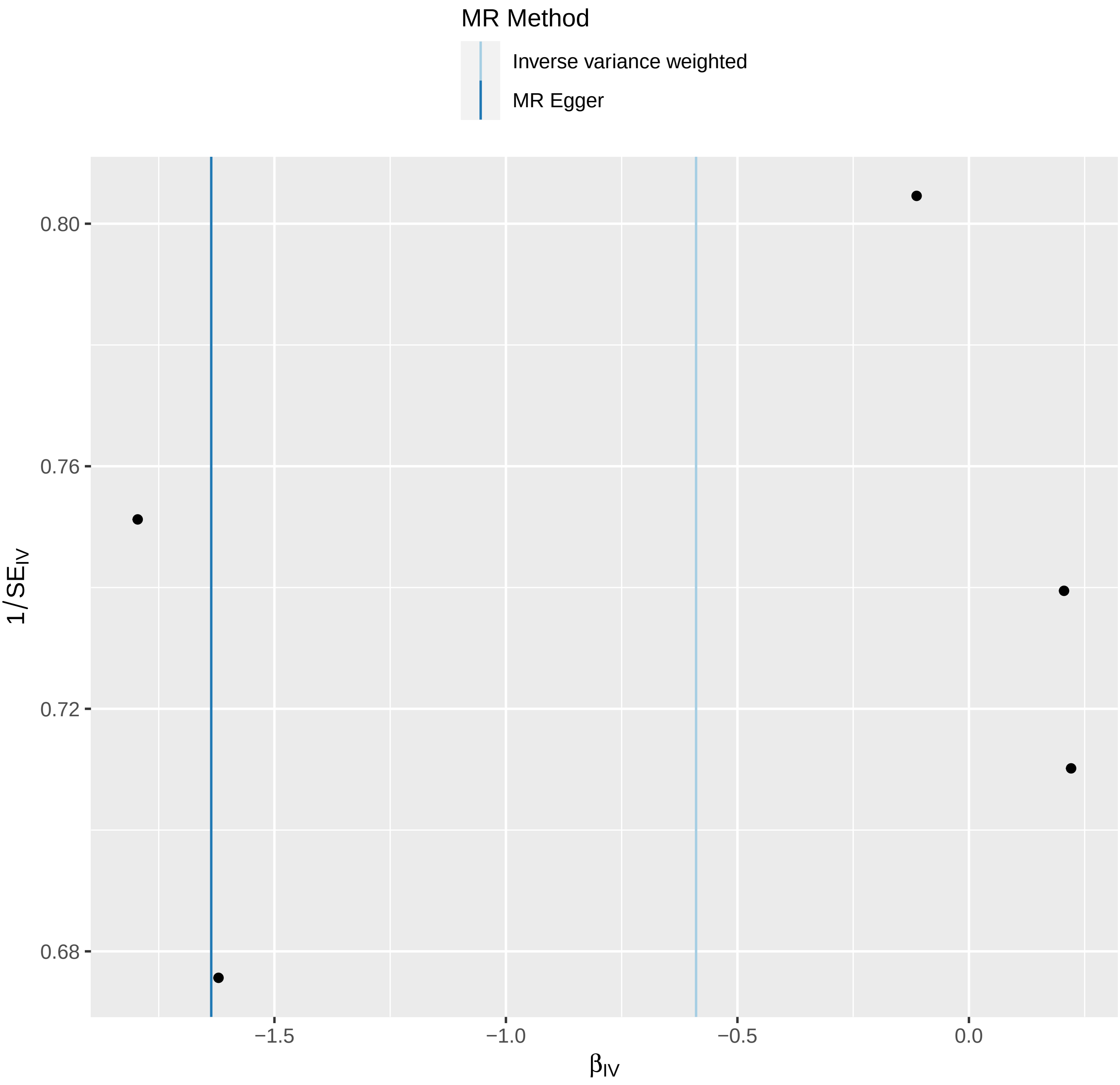

Batch 77 : Gut microbiota abundance (genus Eubacterium rectale group id.14374) on Localized adiposity

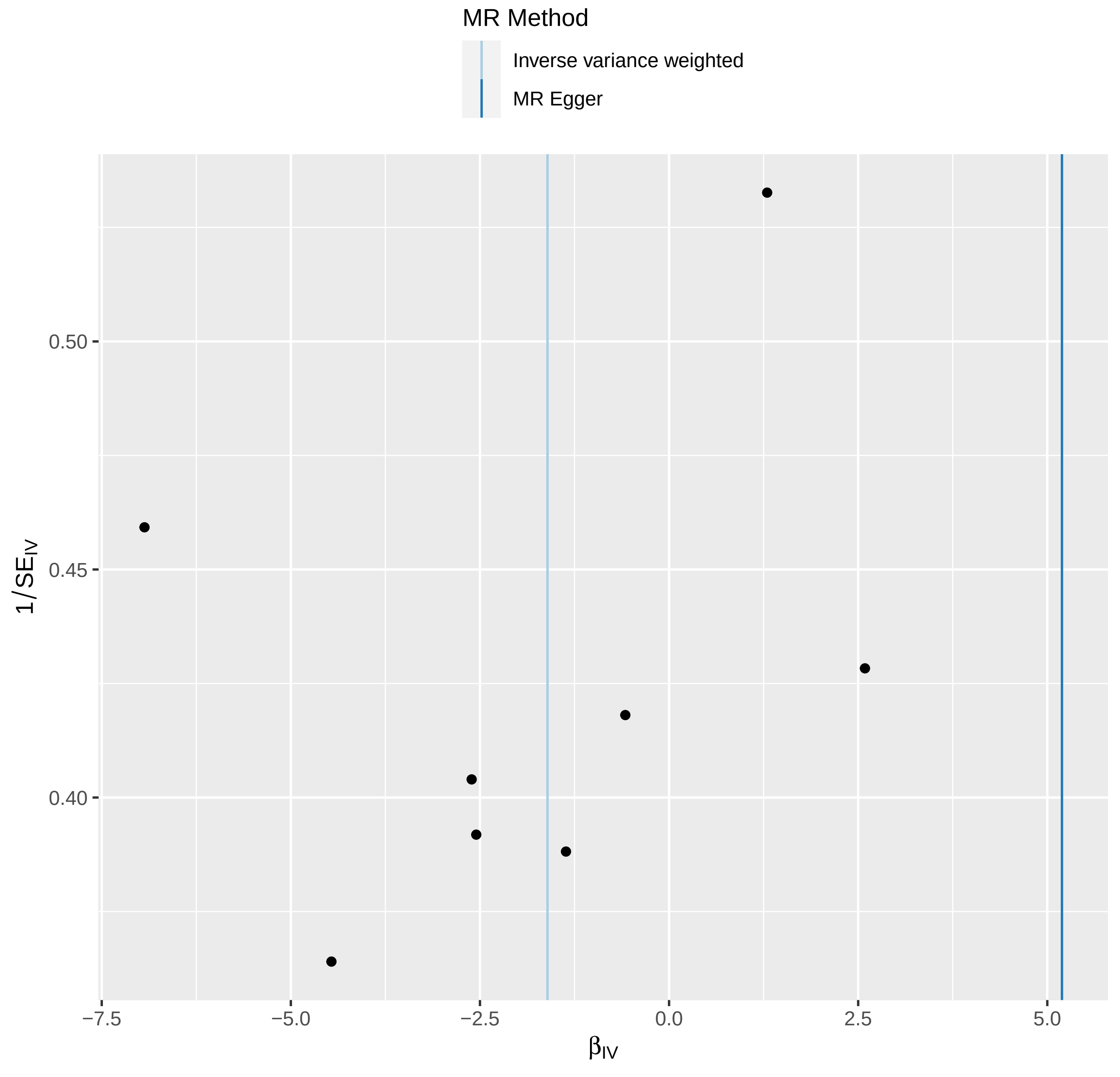

Batch 78 : Gut microbiota abundance (genus Eubacterium ruminantium group id.11340) on Localized adiposity

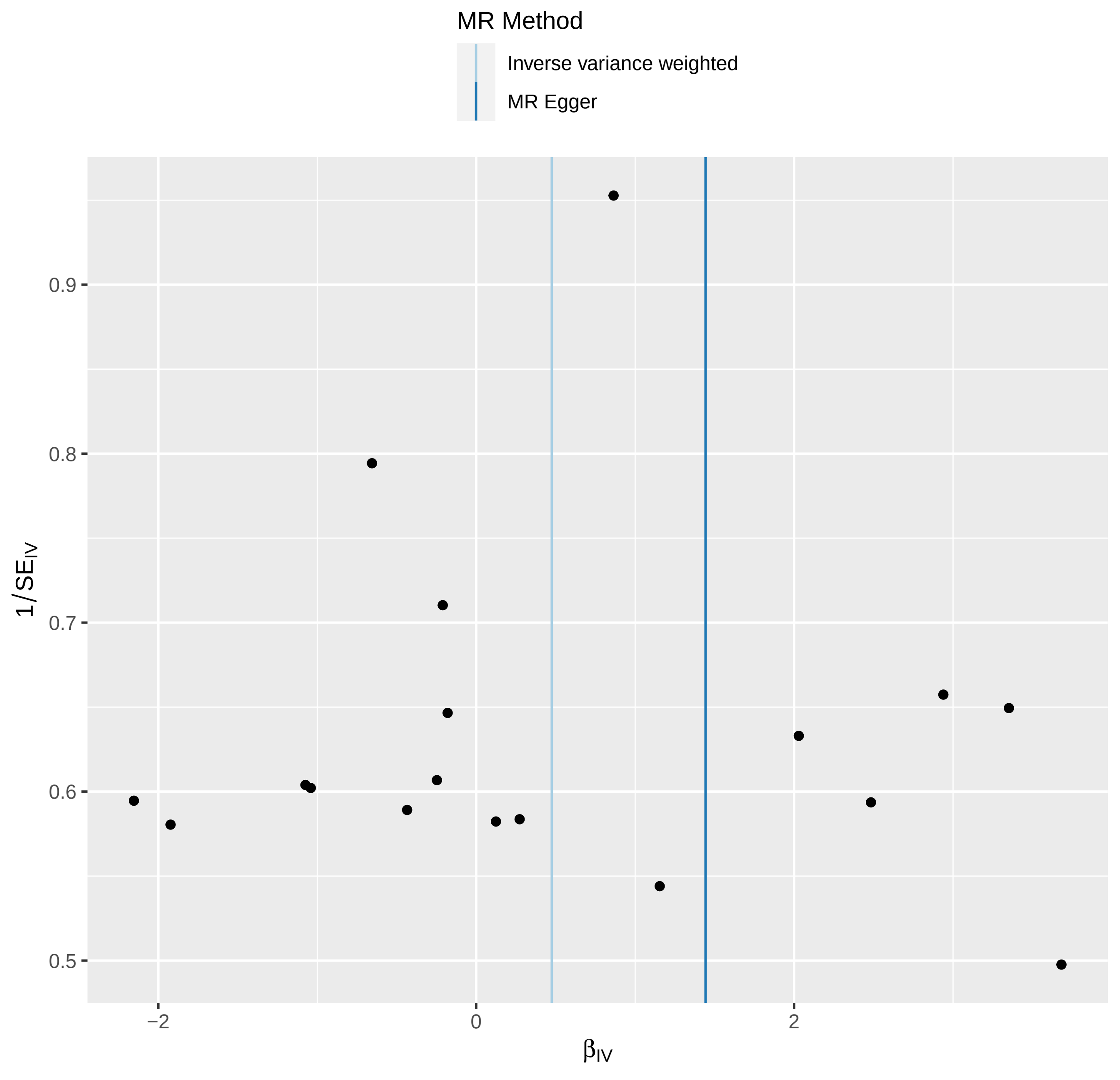

Batch 79 : Gut microbiota abundance (genus Eubacterium ventriosum group id.11341) on Localized adiposity

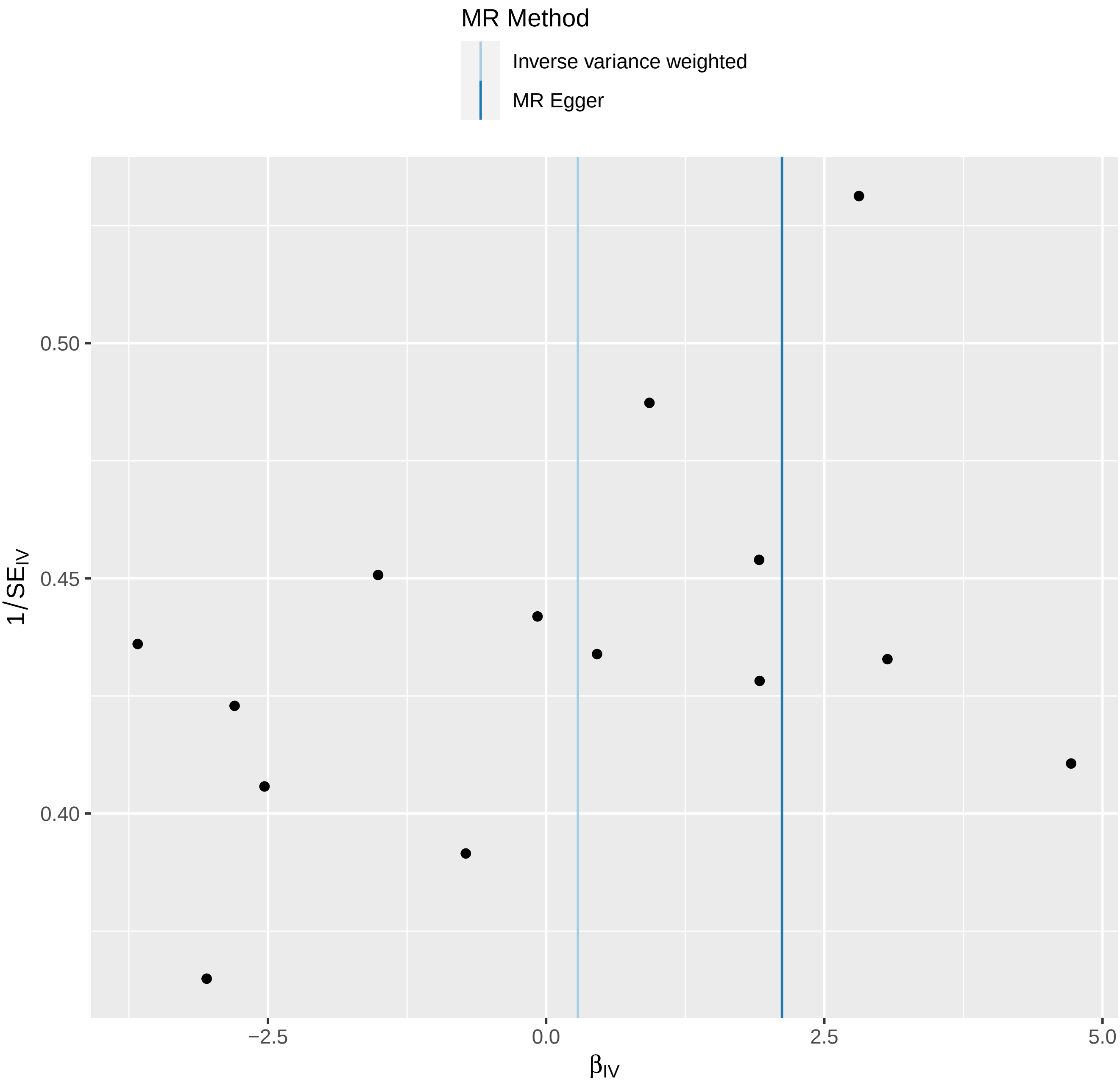

Batch 80 : Gut microbiota abundance (genus Eubacterium xylanophilum group id.14375) on Localized adiposity

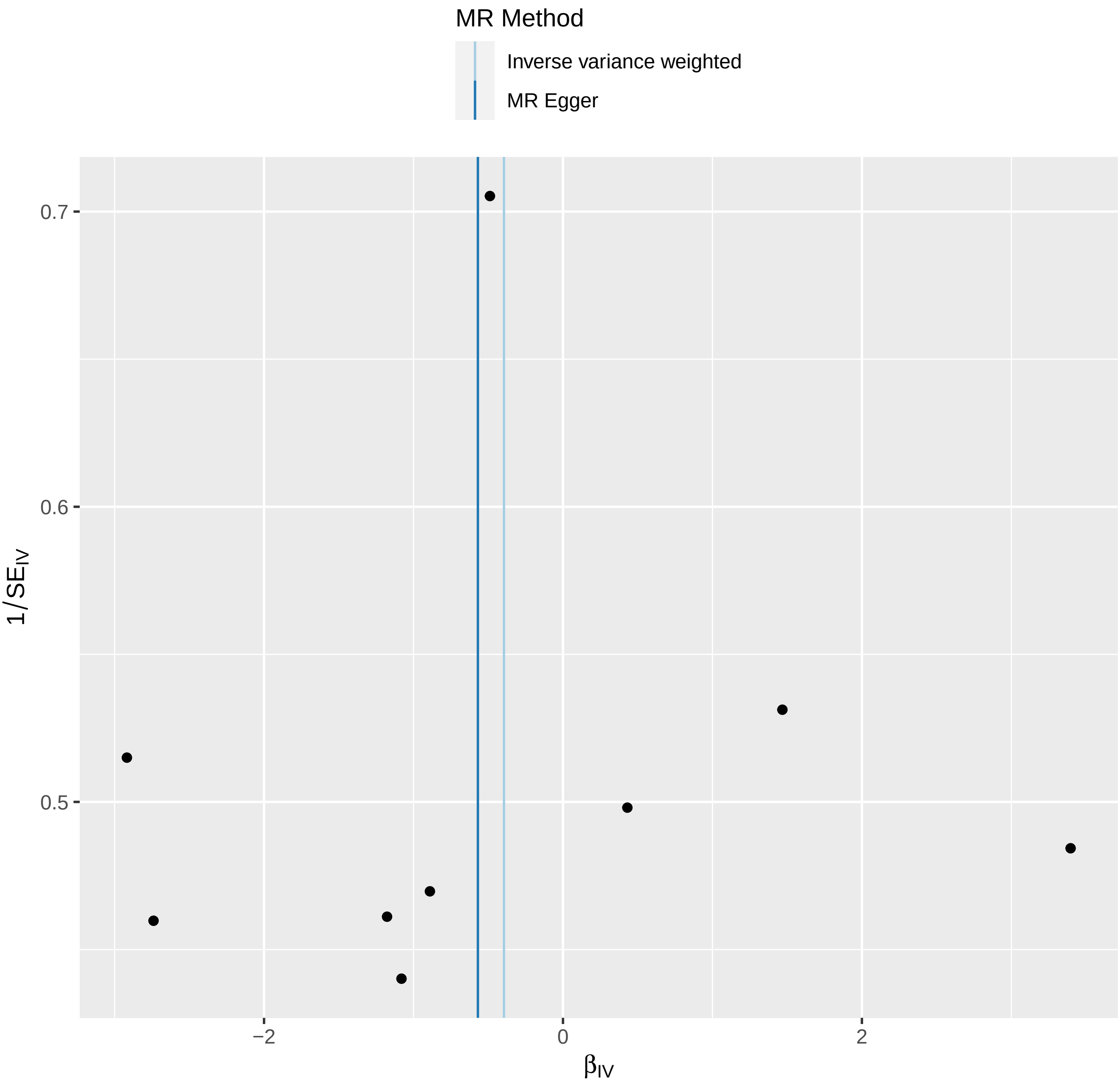

Batch 81 : Gut microbiota abundance (genus Faecalibacterium id.2057) on Localized adiposity

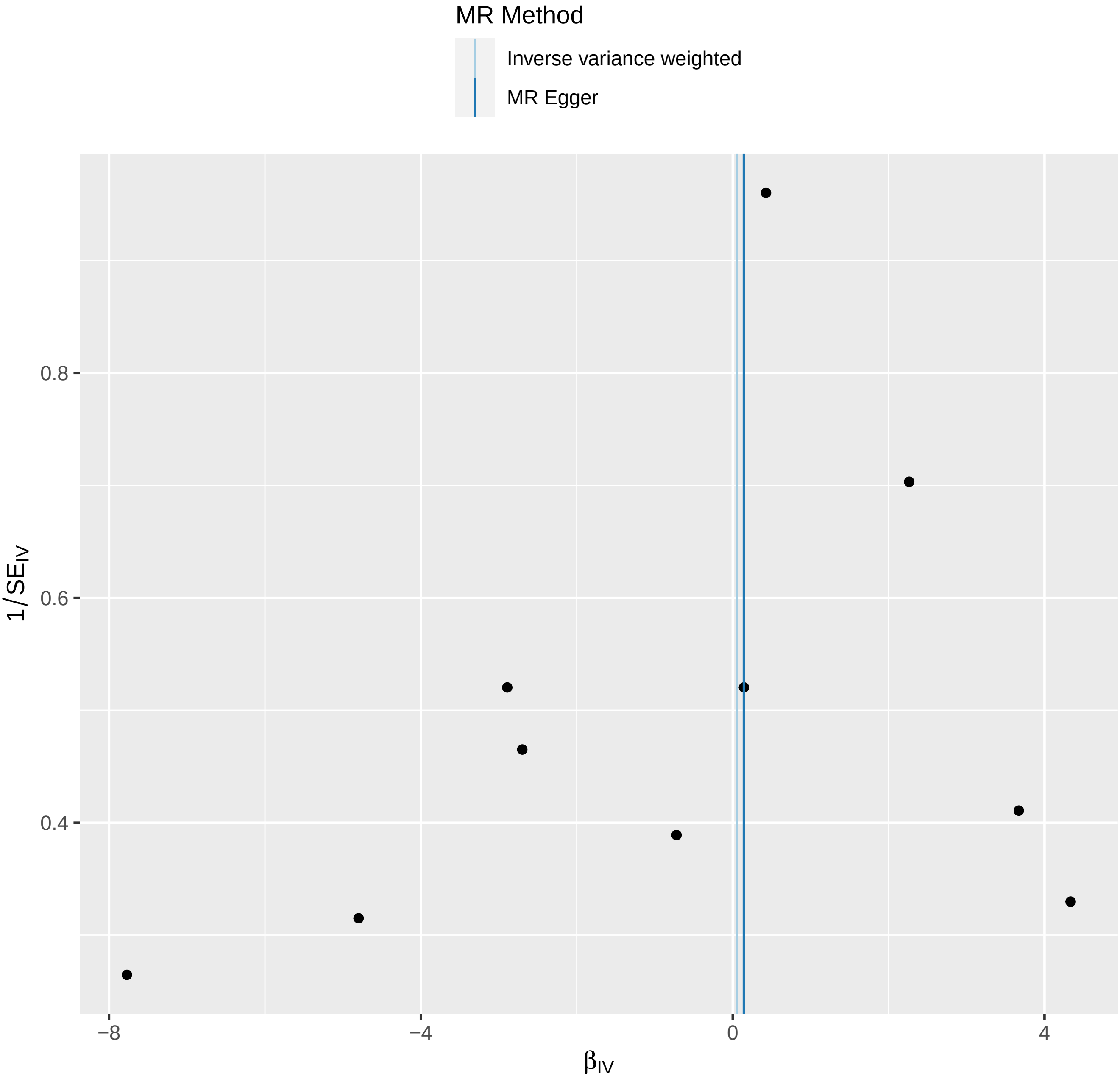

Batch 82 : Gut microbiota abundance (genus Family XIII AD3011 group id.11293) on Localized adiposity

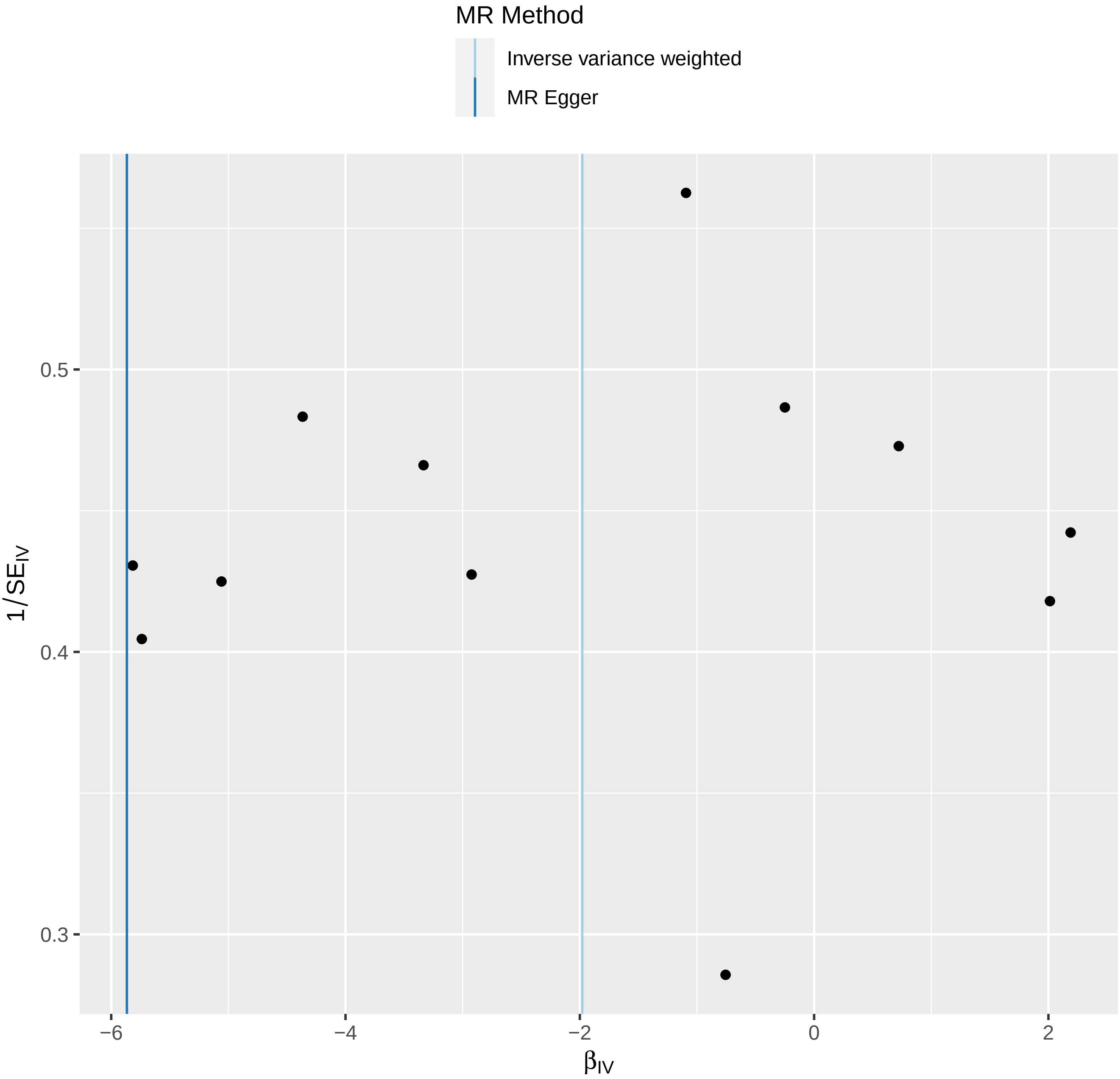

Batch 83 : Gut microbiota abundance (genus Family XIII UCG001 id.11294) on Localized adiposity

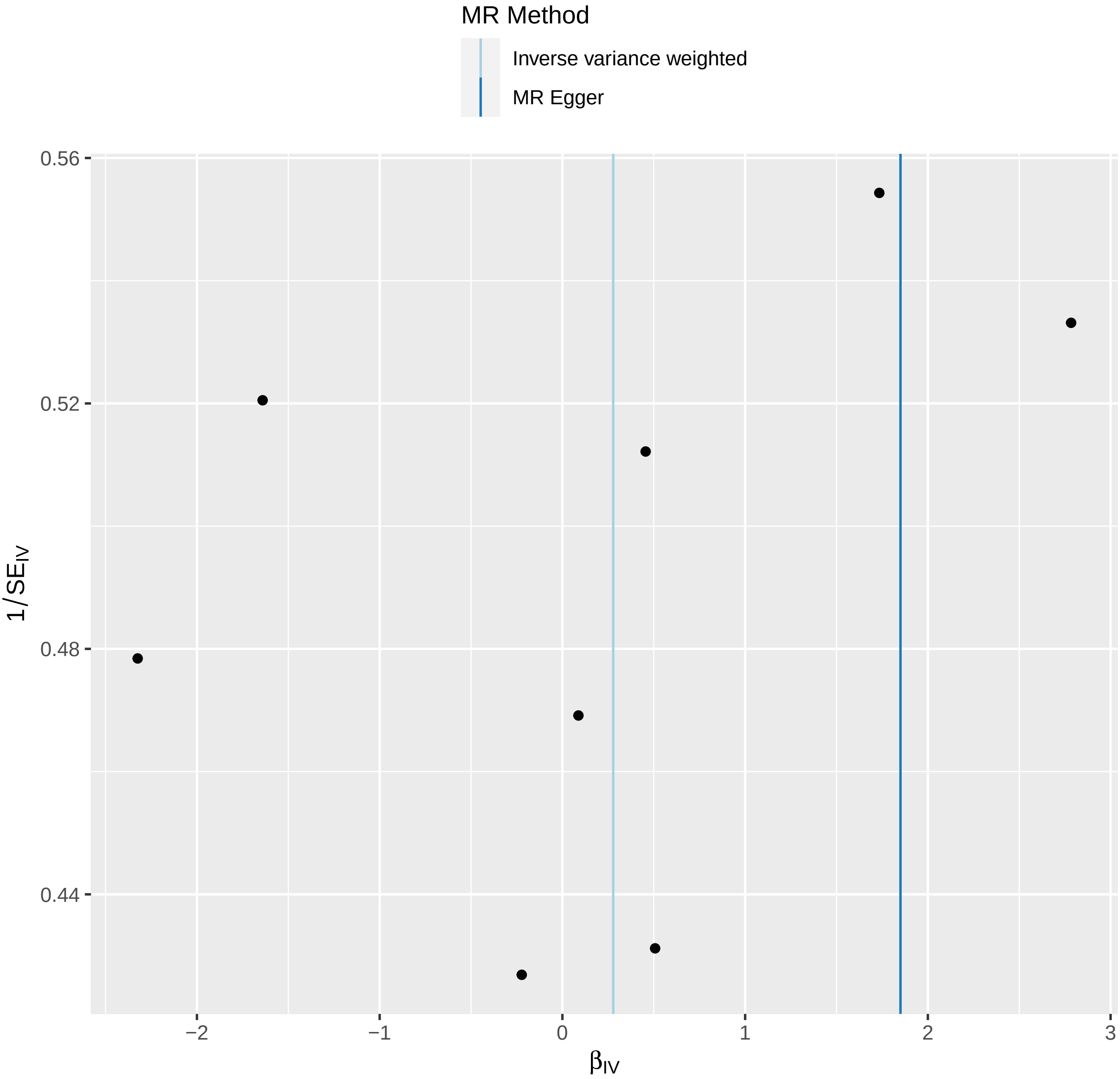

Batch 84 : Gut microbiota abundance (genus Flavonifractor id.2059) on Localized adiposity

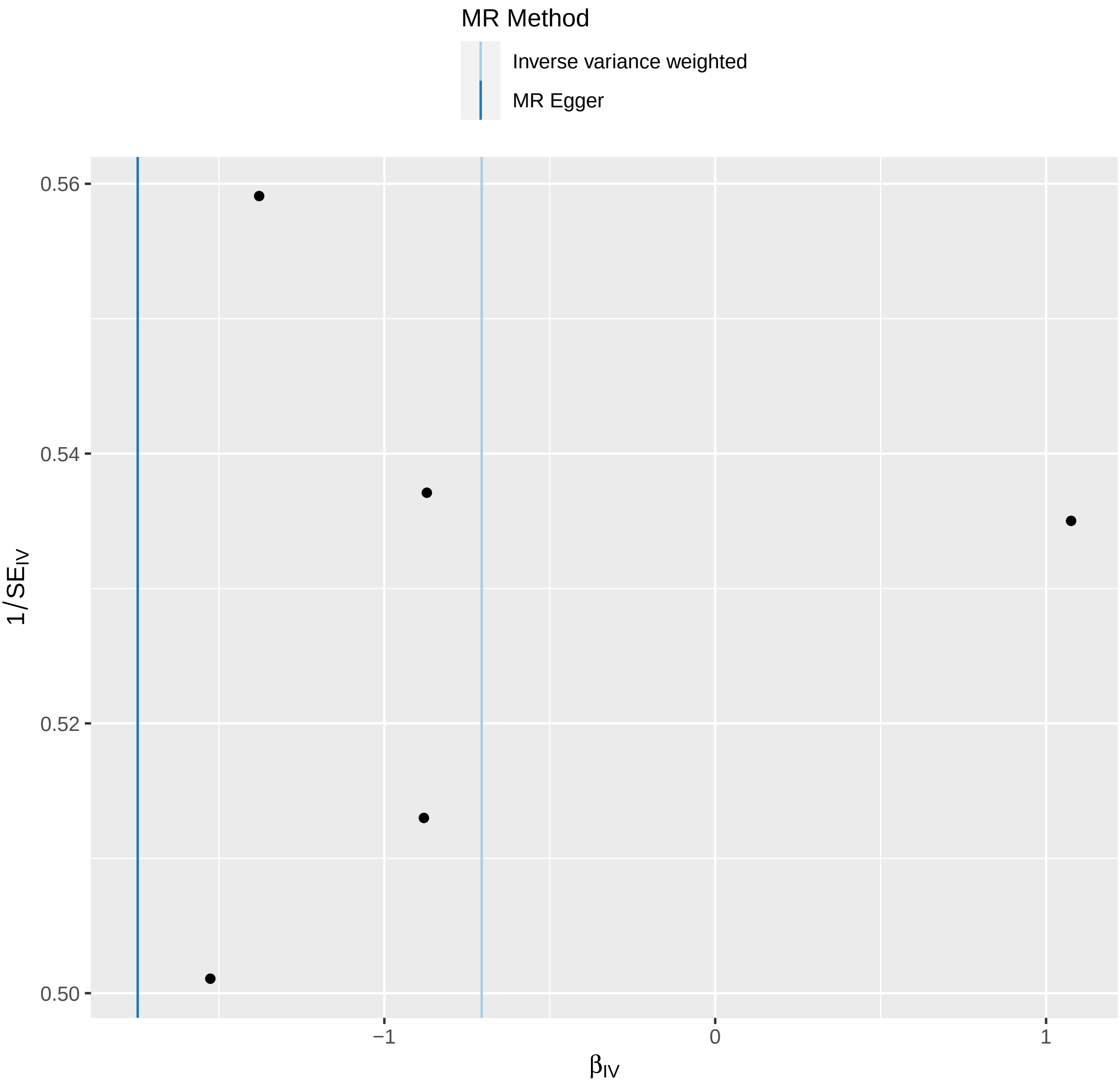

Batch 85 : Gut microbiota abundance (genus Fusicatenibacter id.11305) on Localized adiposity

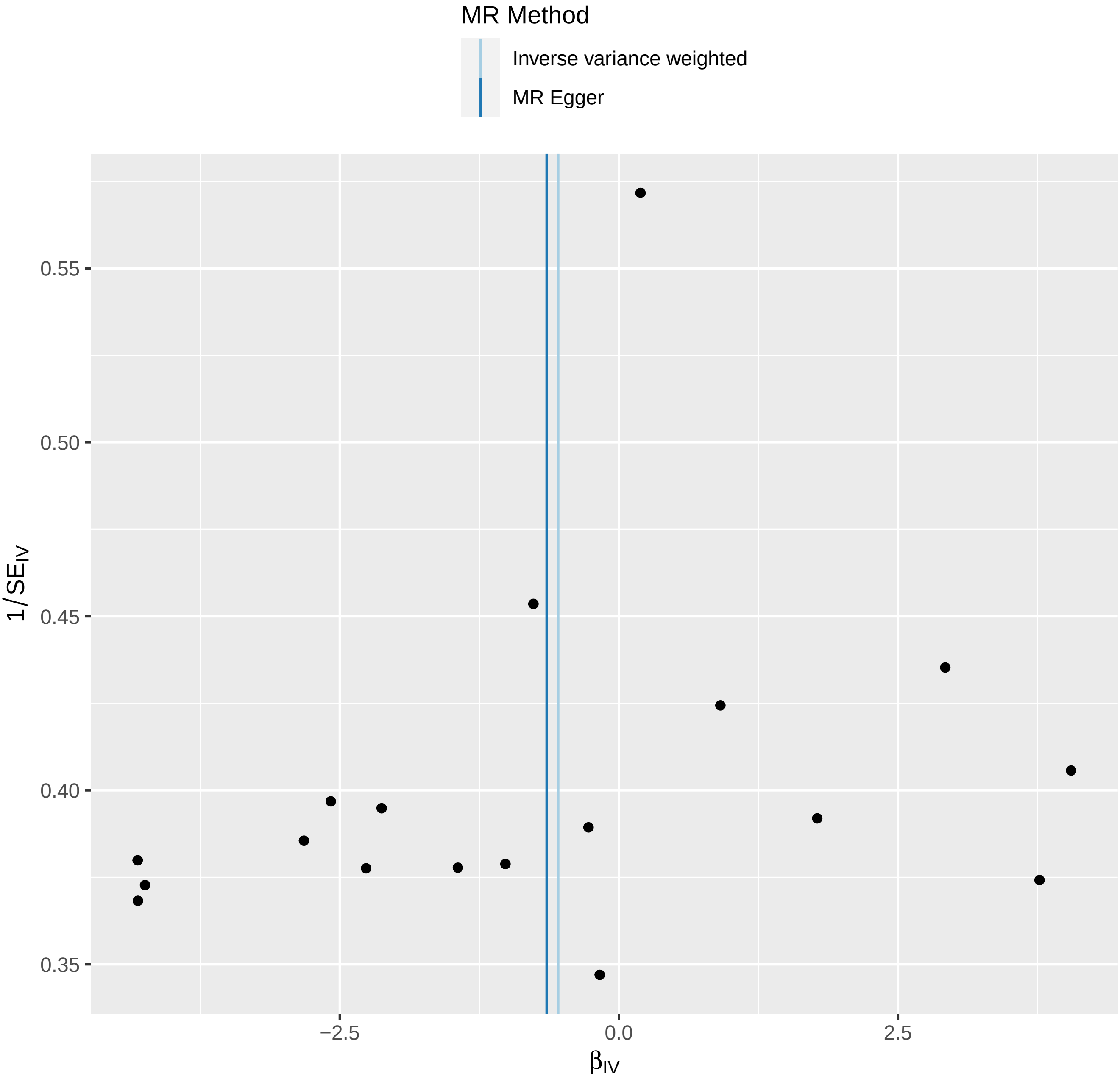

Batch 86 : Gut microbiota abundance (genus Gordonibacter id.821) on Localized adiposity

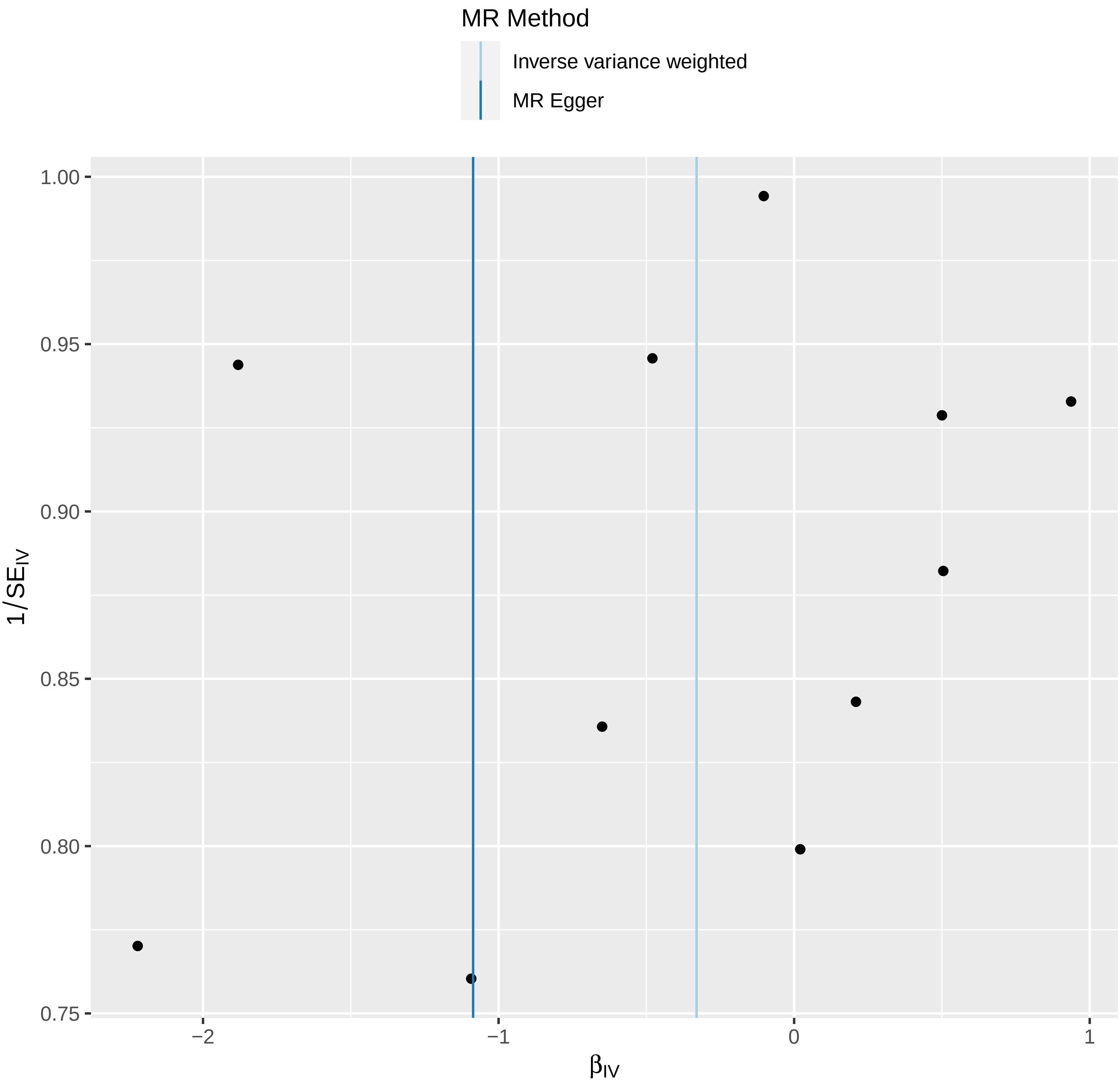

Batch 87 : Gut microbiota abundance (genus Haemophilus id.3698) on Localized adiposity

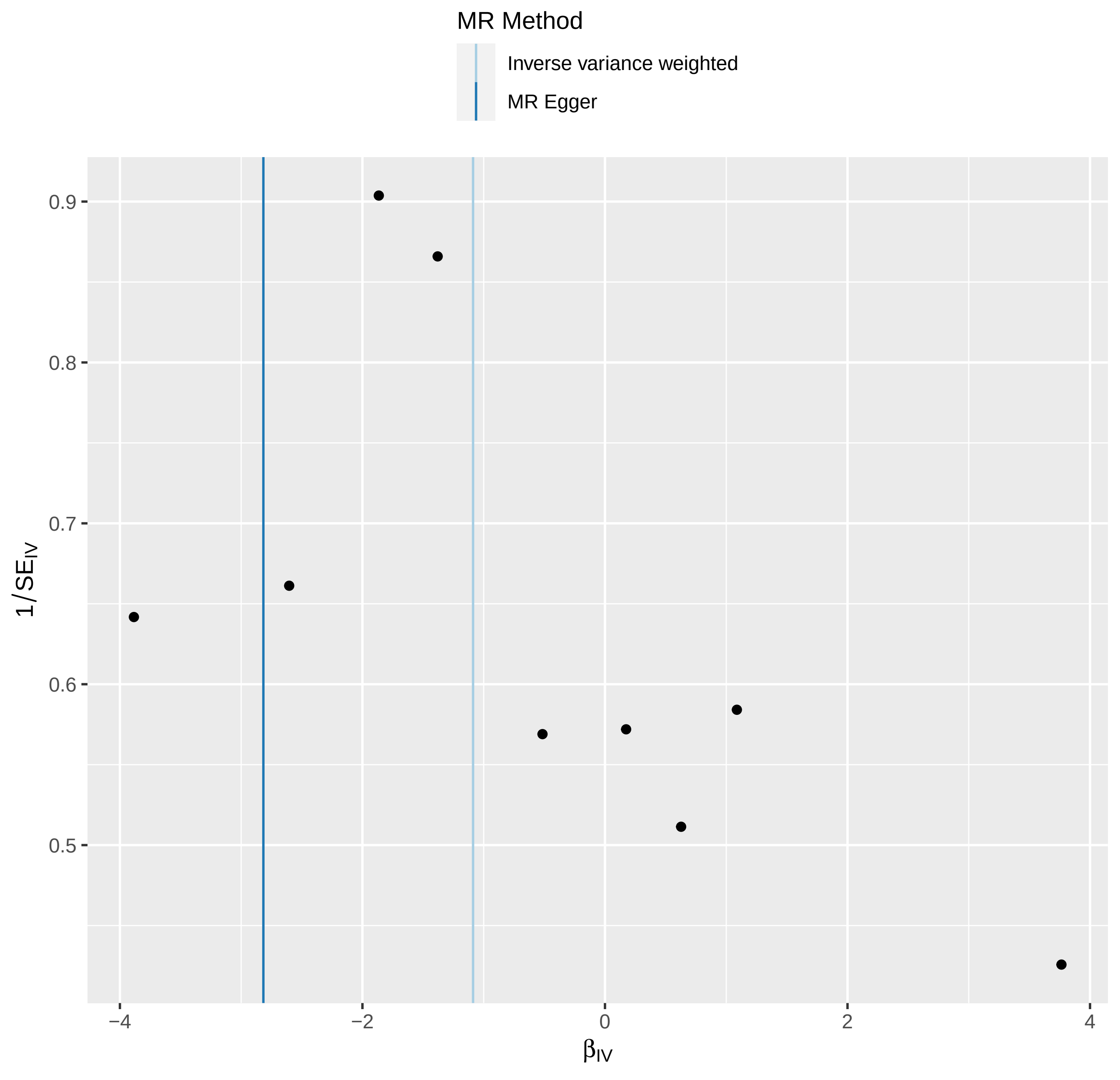

Batch 88 : Gut microbiota abundance (genus Holdemanella id.11393) on Localized adiposity

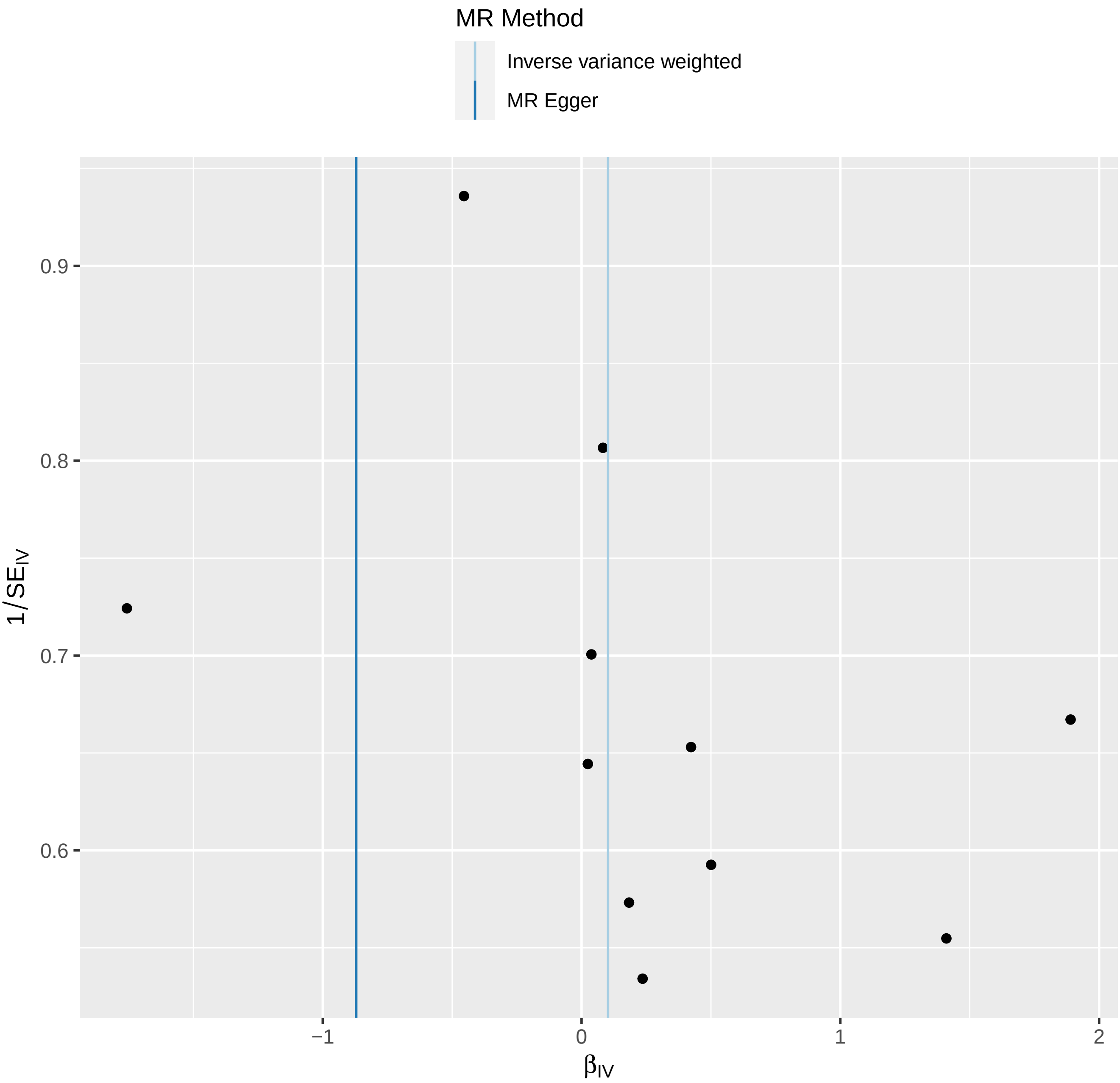

Batch 89 : Gut microbiota abundance (genus Holdemania id.2157) on Localized adiposity

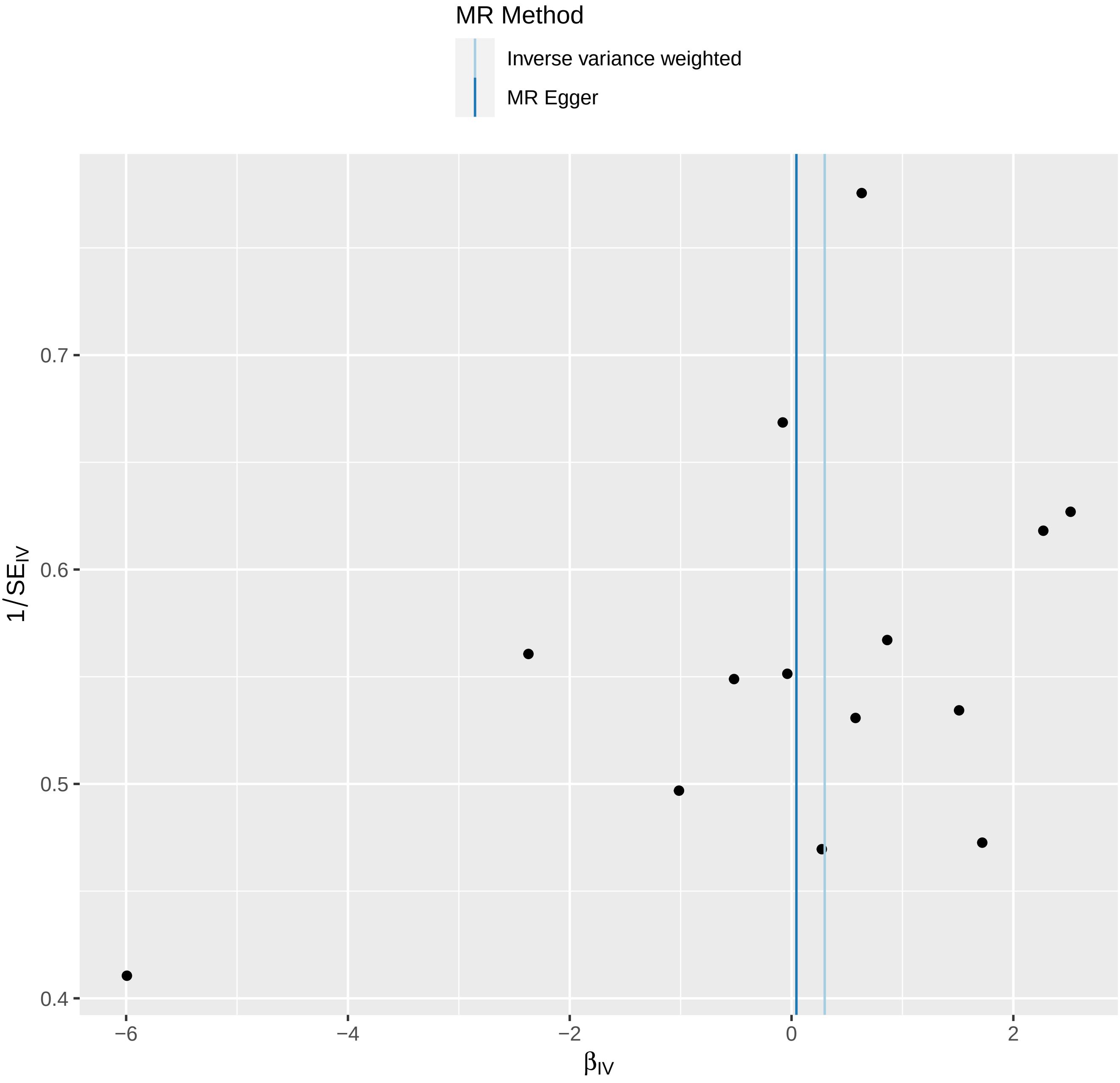

Batch 90 : Gut microbiota abundance (genus Howardella id.2000) on Localized adiposity

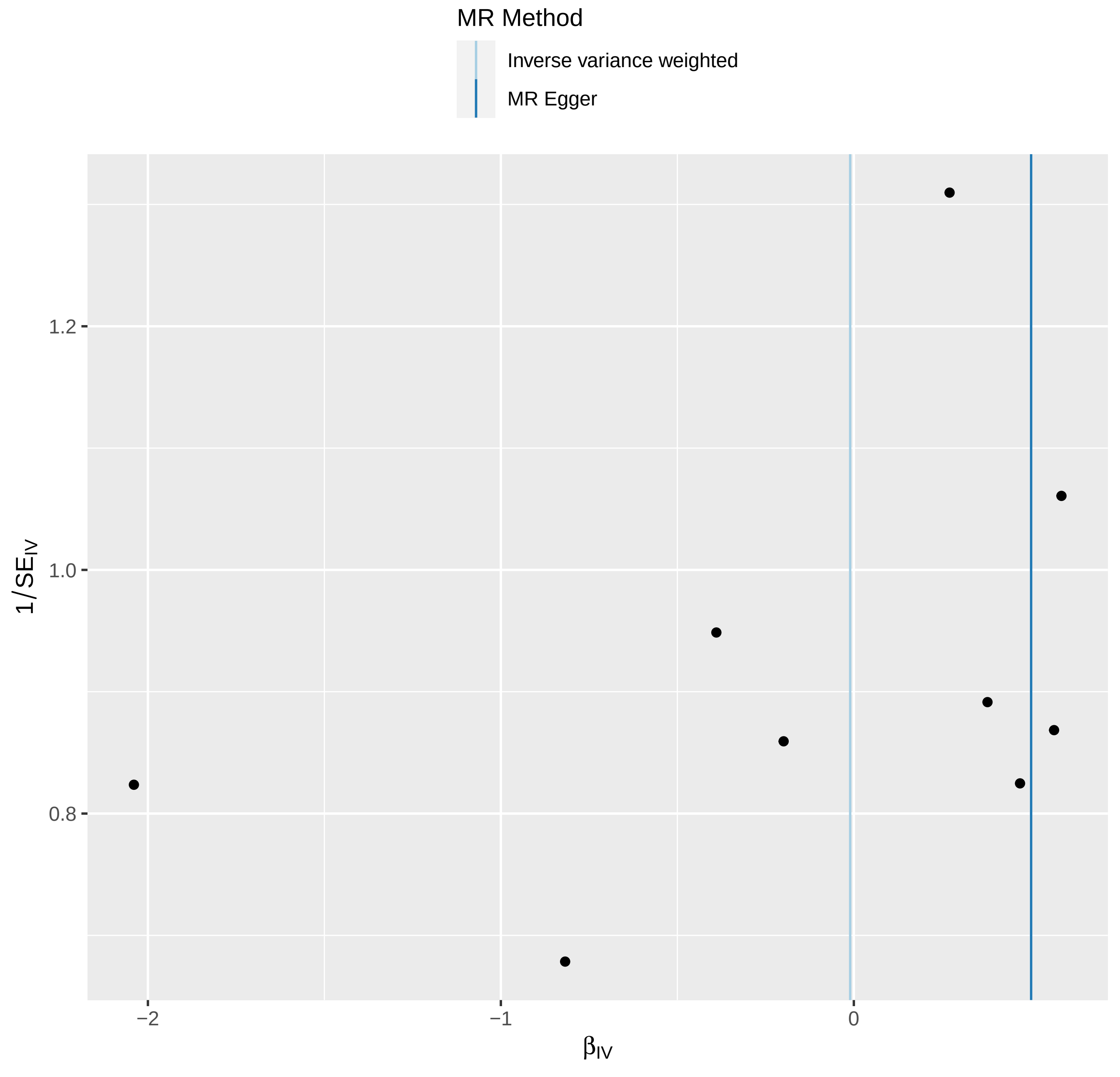

Batch 91 : Gut microbiota abundance (genus Hungatella id.11306) on Localized adiposity

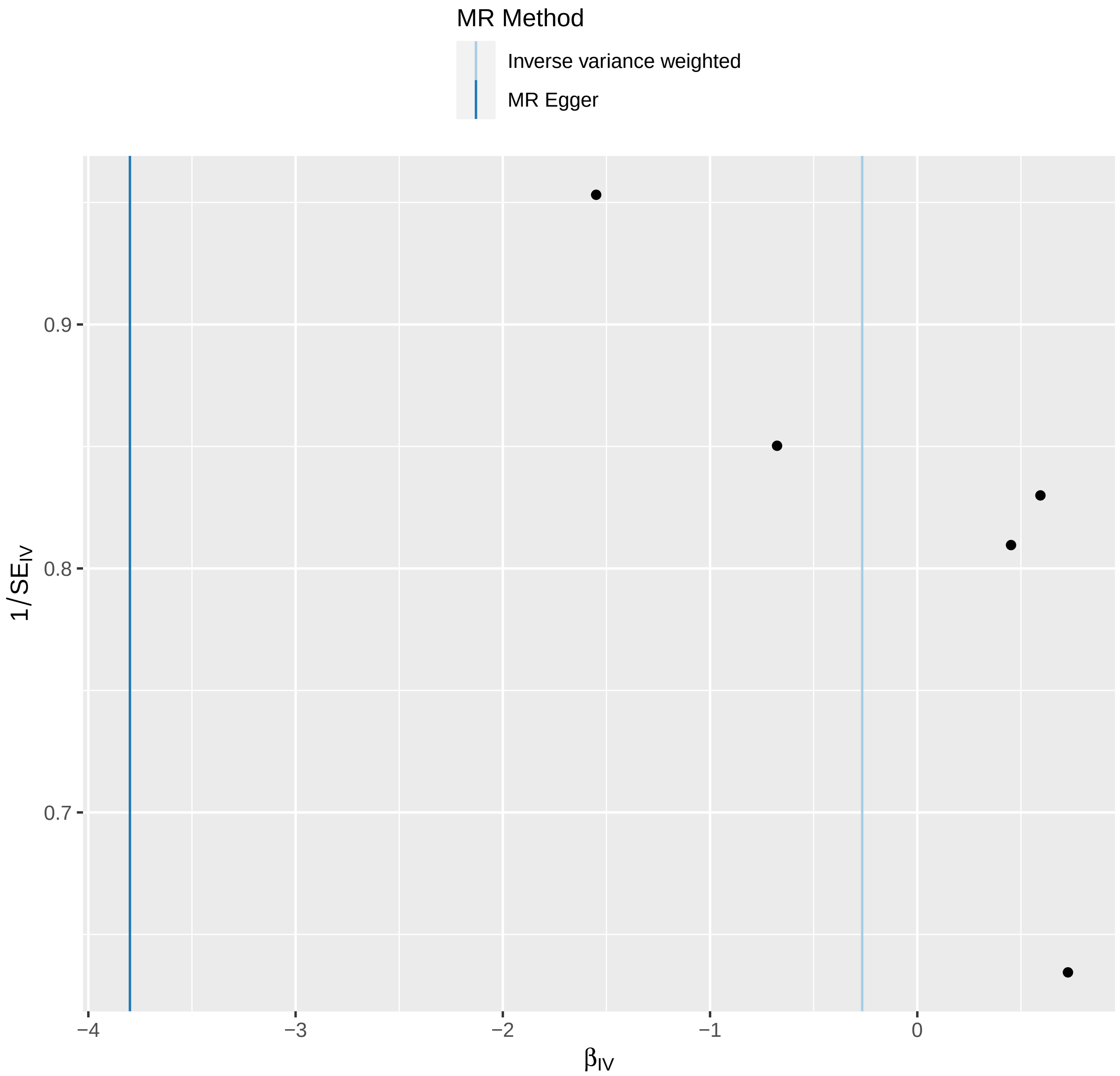

Batch 92 : Gut microbiota abundance (genus Intestinibacter id.11345) on Localized adiposity

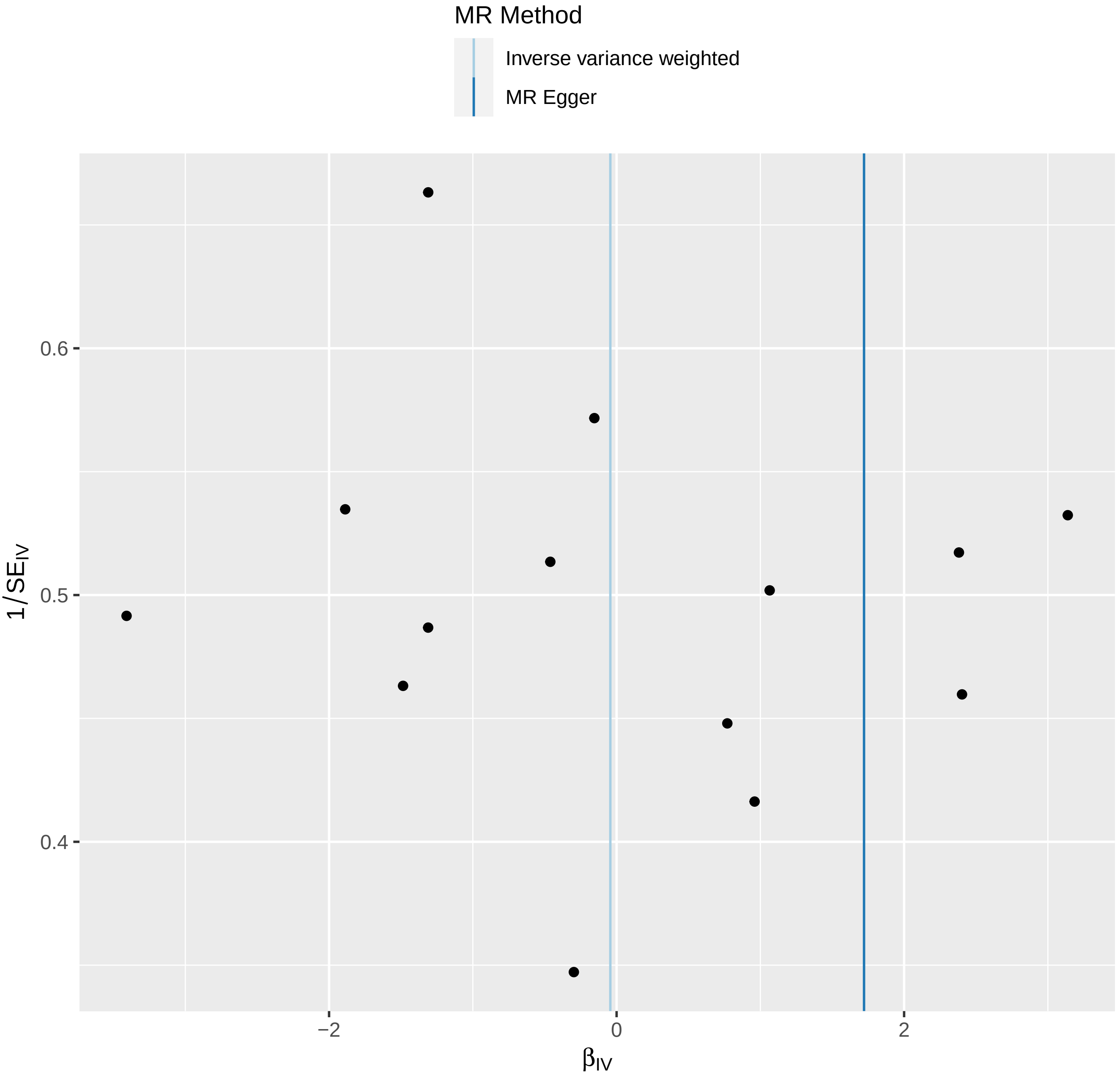

Batch 93 : Gut microbiota abundance (genus Intestinimonas id.2062) on Localized adiposity

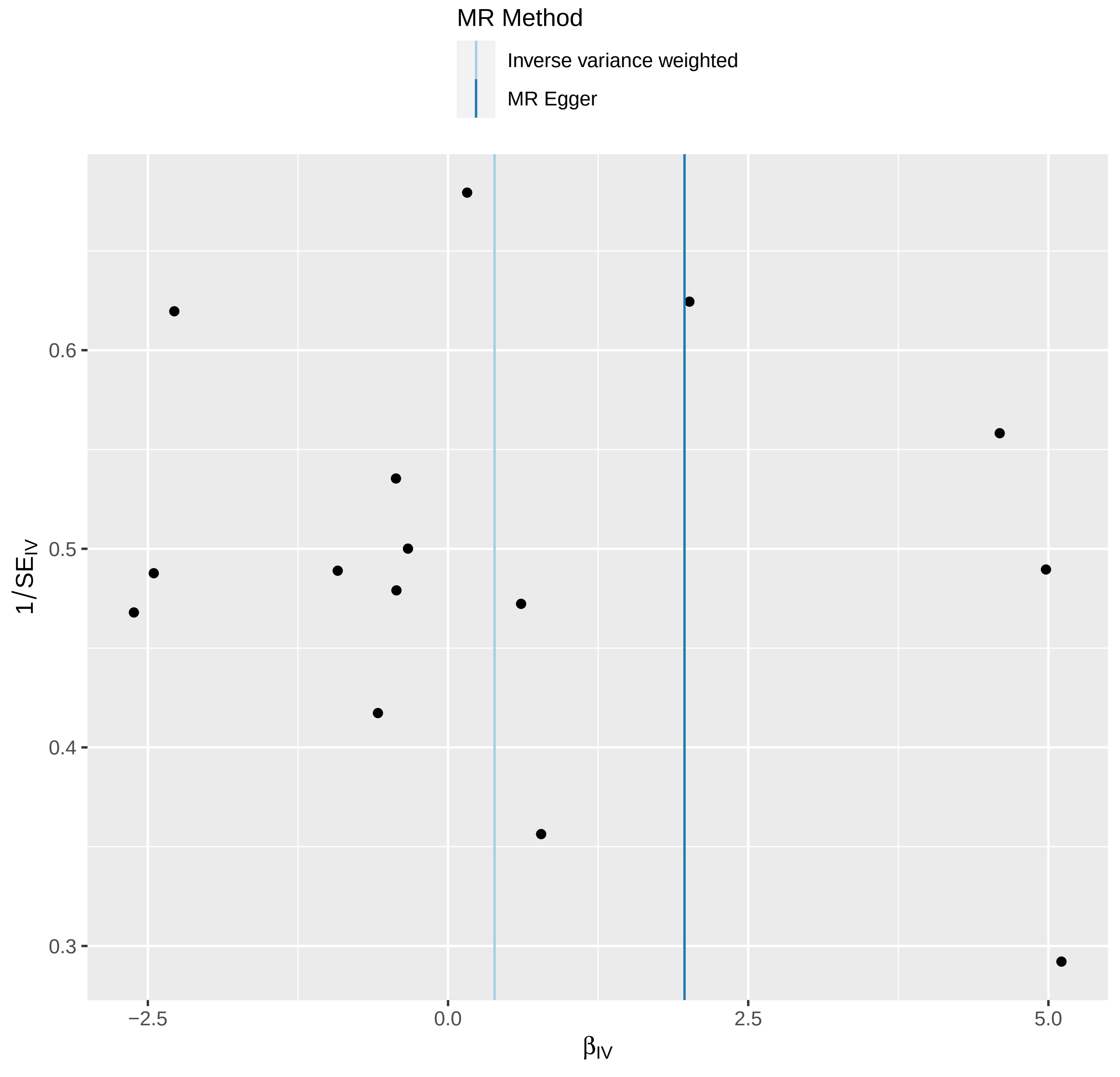

Batch 94 : Gut microbiota abundance (genus Lachnoclostridium id.11308) on Localized adiposity

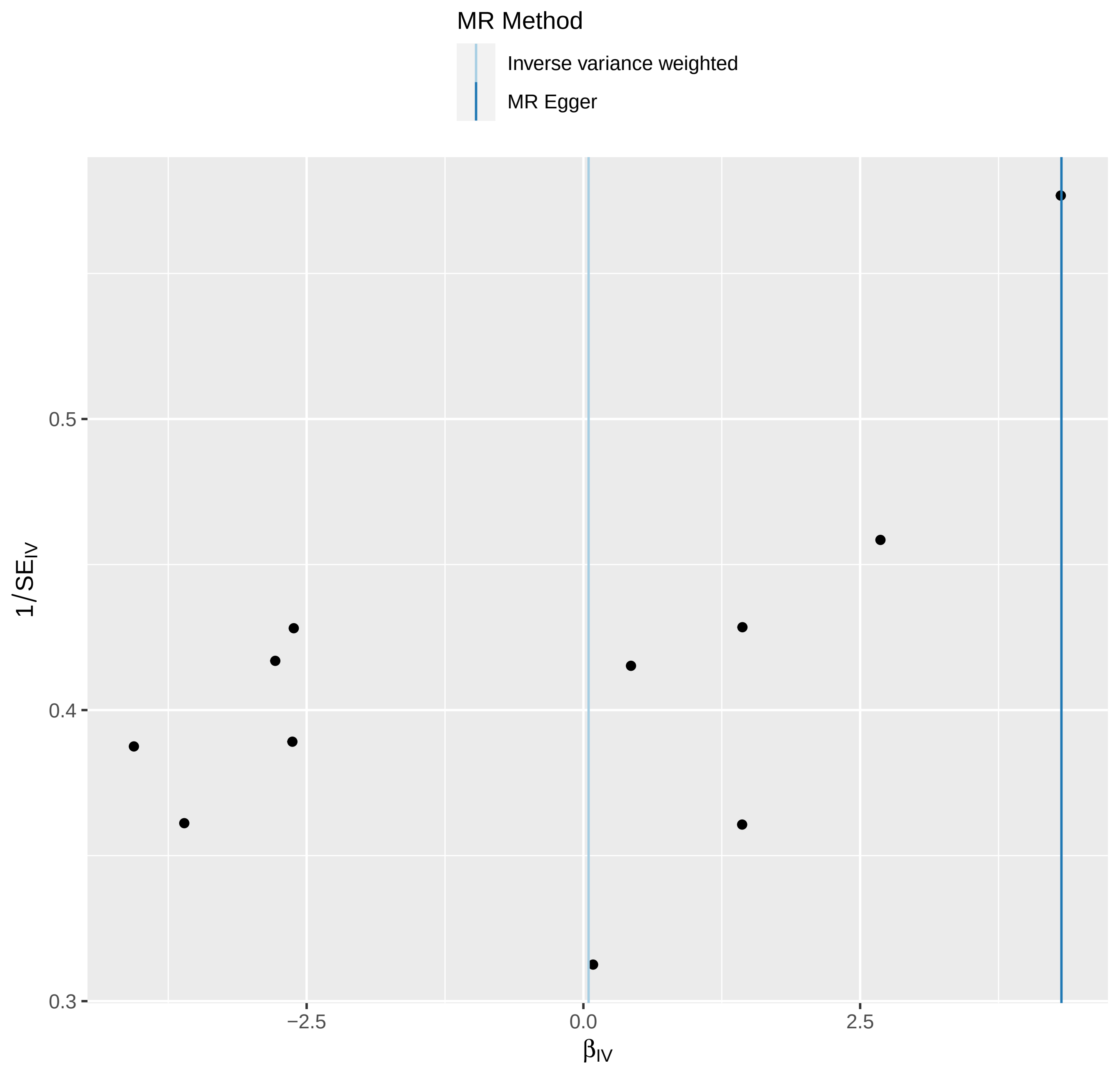

Batch 95 : Gut microbiota abundance (genus Lachnospiraceae FCS020 group id.11314) on Localized adiposity

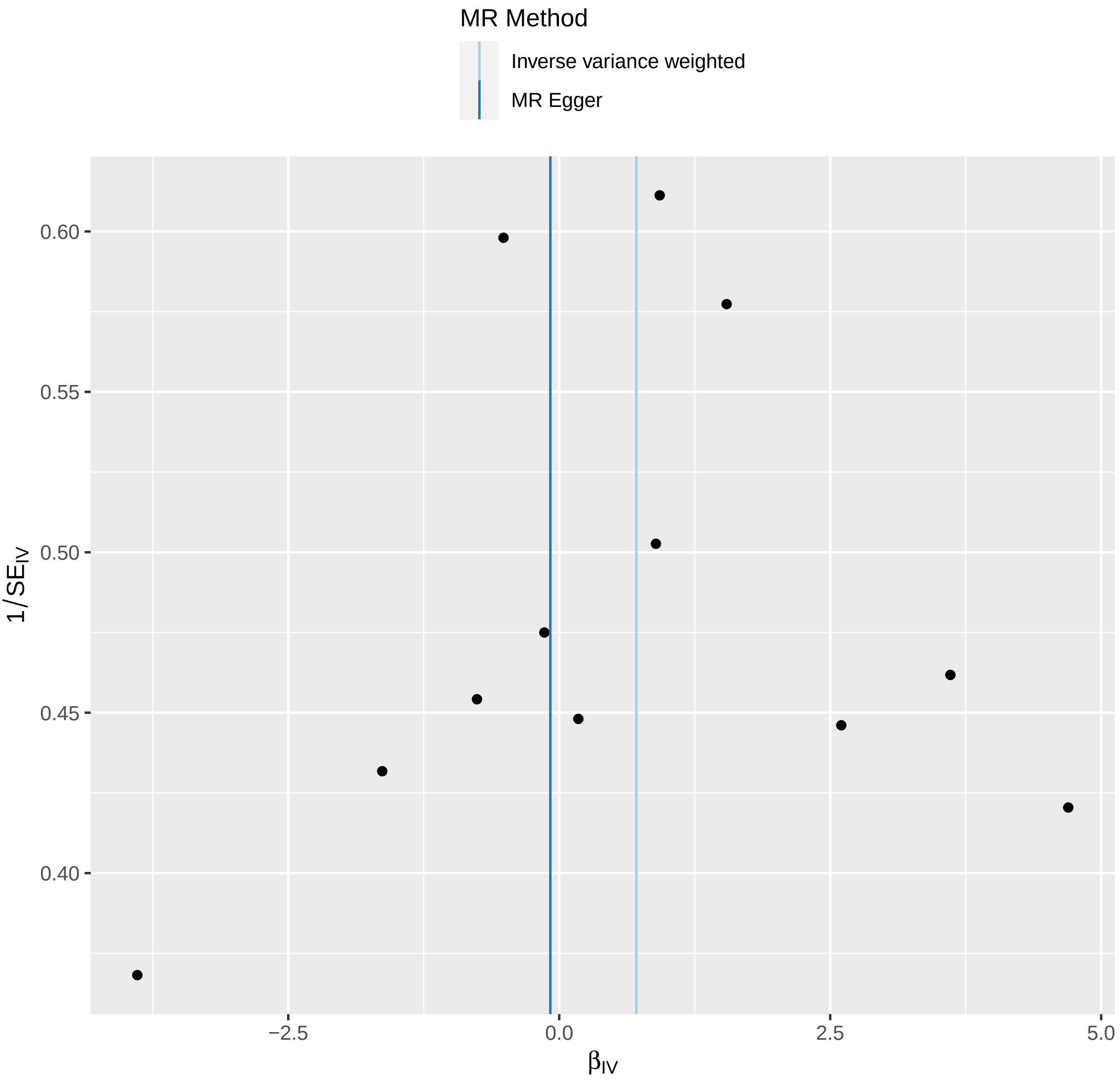

Batch 96 : Gut microbiota abundance (genus Lachnospiraceae NC2004 group id.11316) on Localized adiposity

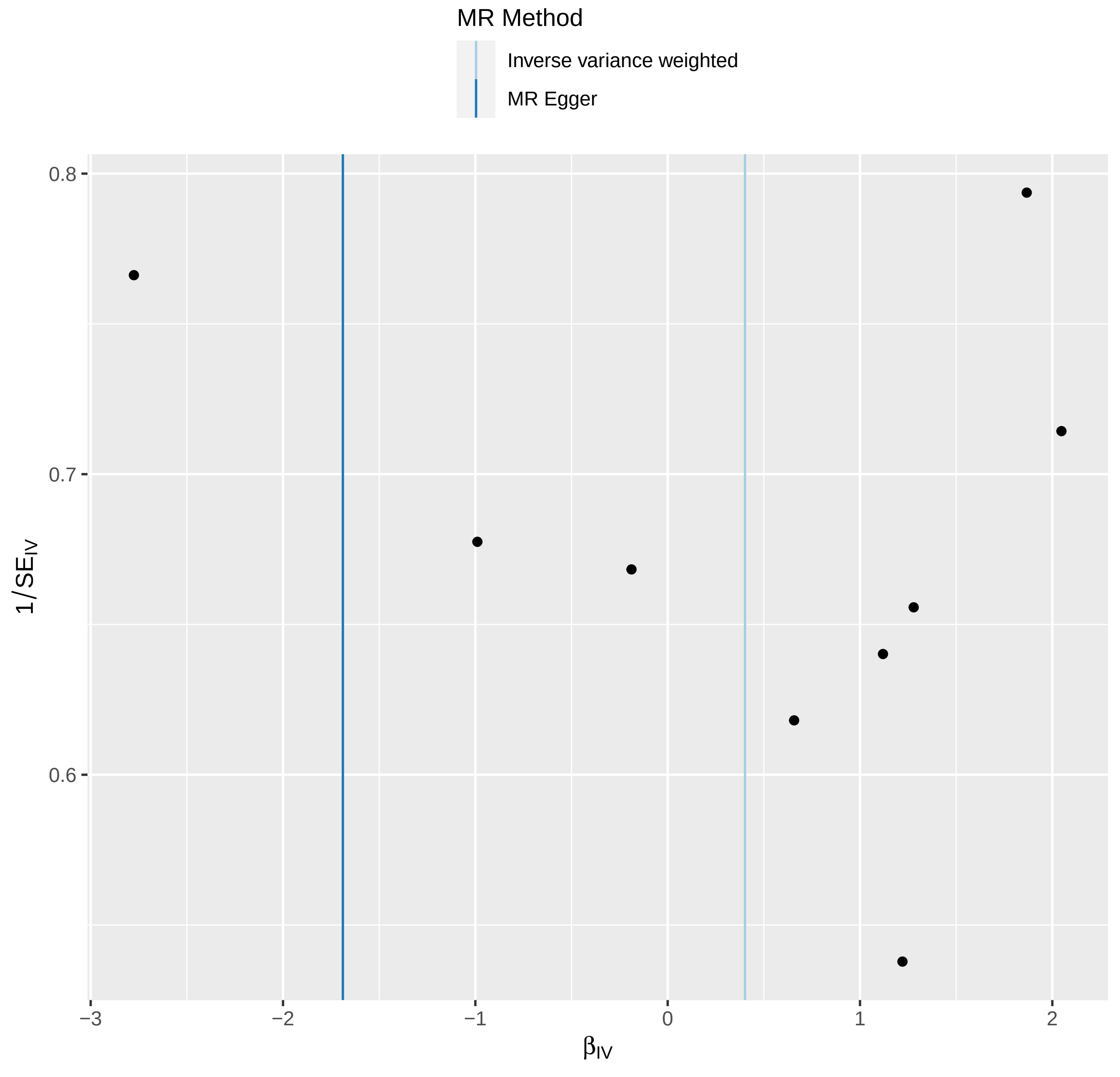

Batch 97 : Gut microbiota abundance (genus Lachnospiraceae ND3007 group id.11317) on Localized adiposity

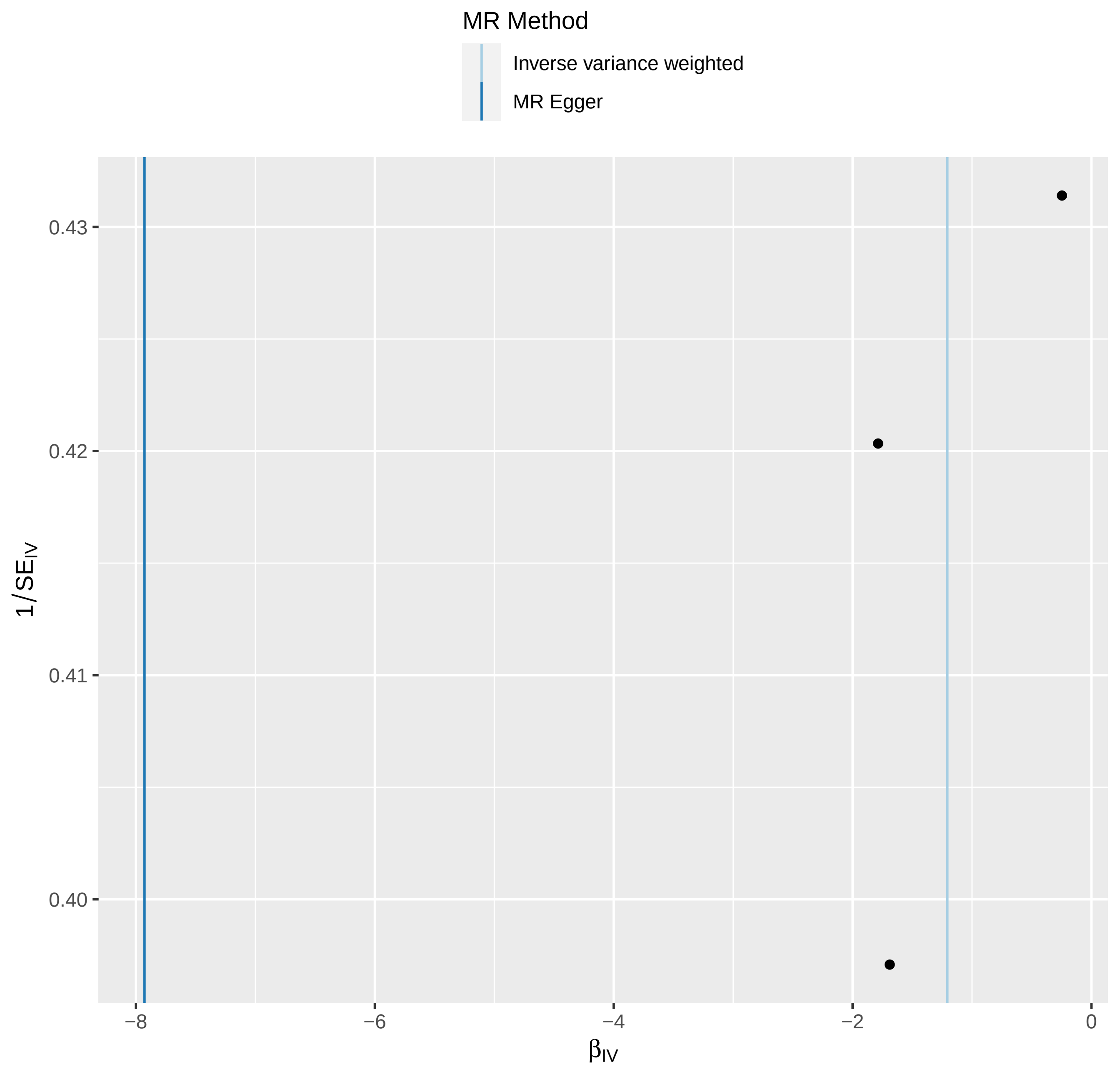

Batch 98 : Gut microbiota abundance (genus Lachnospiraceae NK4A136 group id.11319) on Localized adiposity

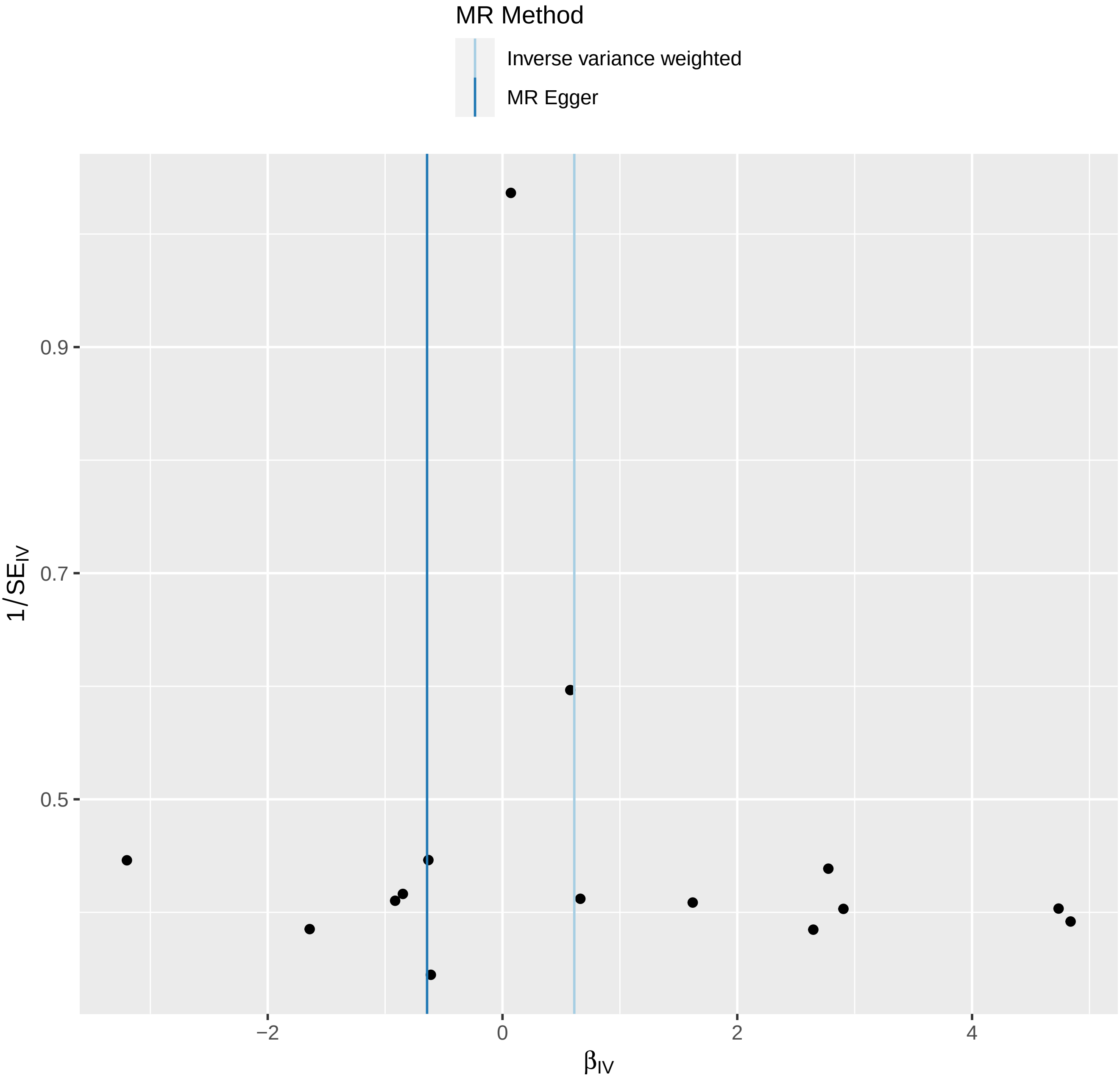

Batch 99 : Gut microbiota abundance (genus Lachnospiraceae UCG001 id.11321) on Localized adiposity

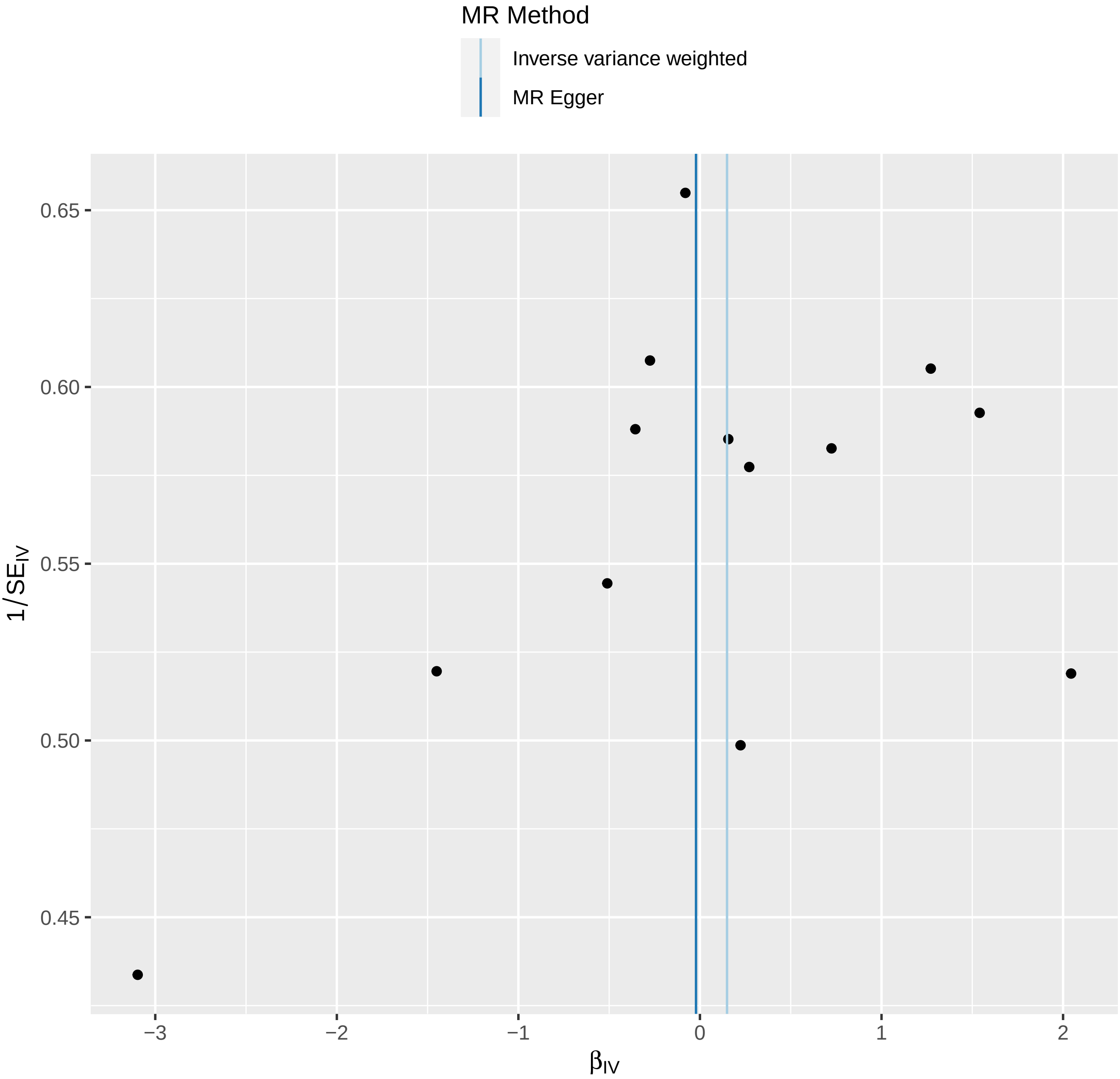

Batch 100 : Gut microbiota abundance (genus Lachnospiraceae UCG004 id.11324) on Localized adiposity

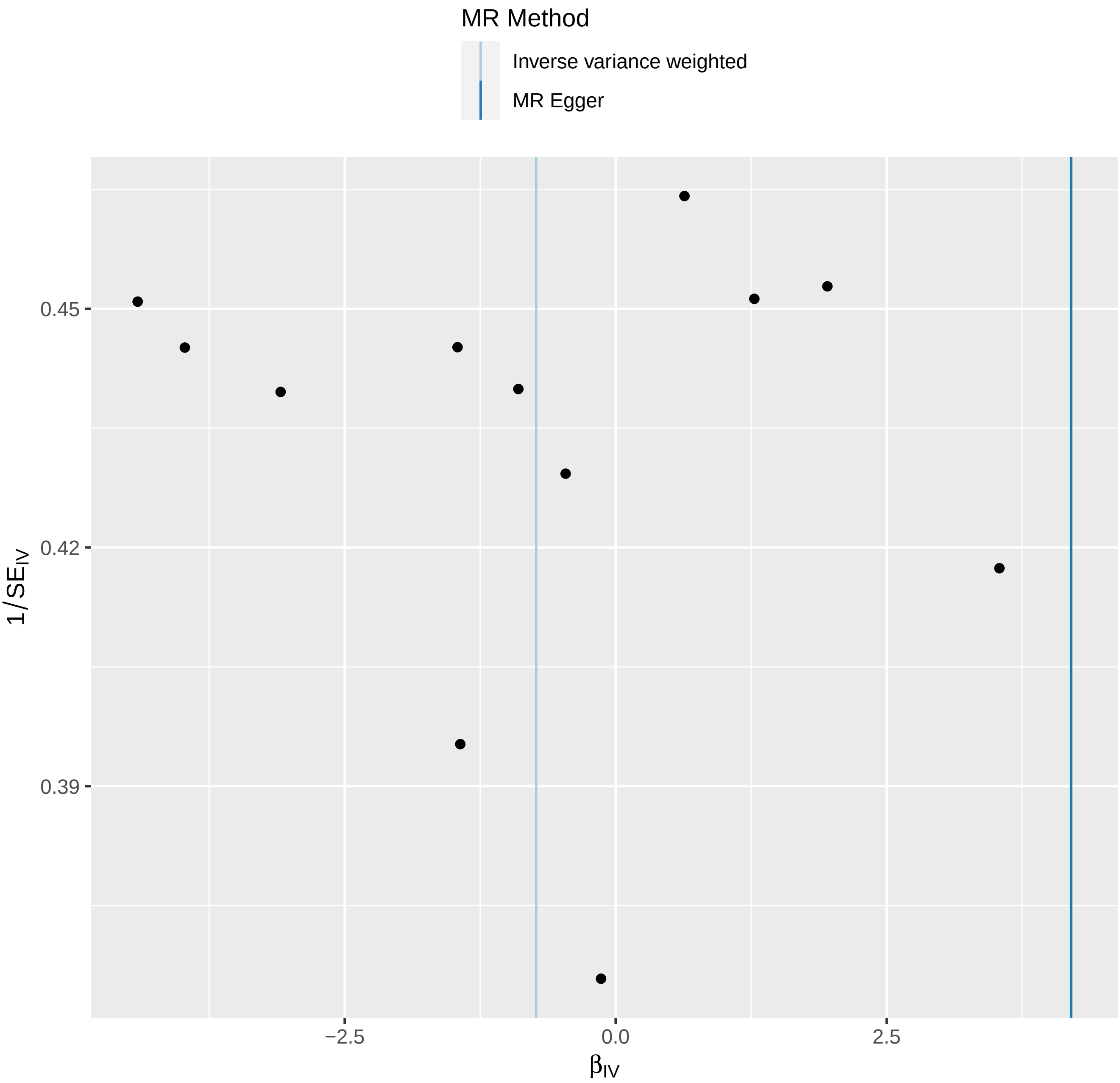

Batch 101 : Gut microbiota abundance (genus Lachnospiraceae UCG008 id.11328) on Localized adiposity

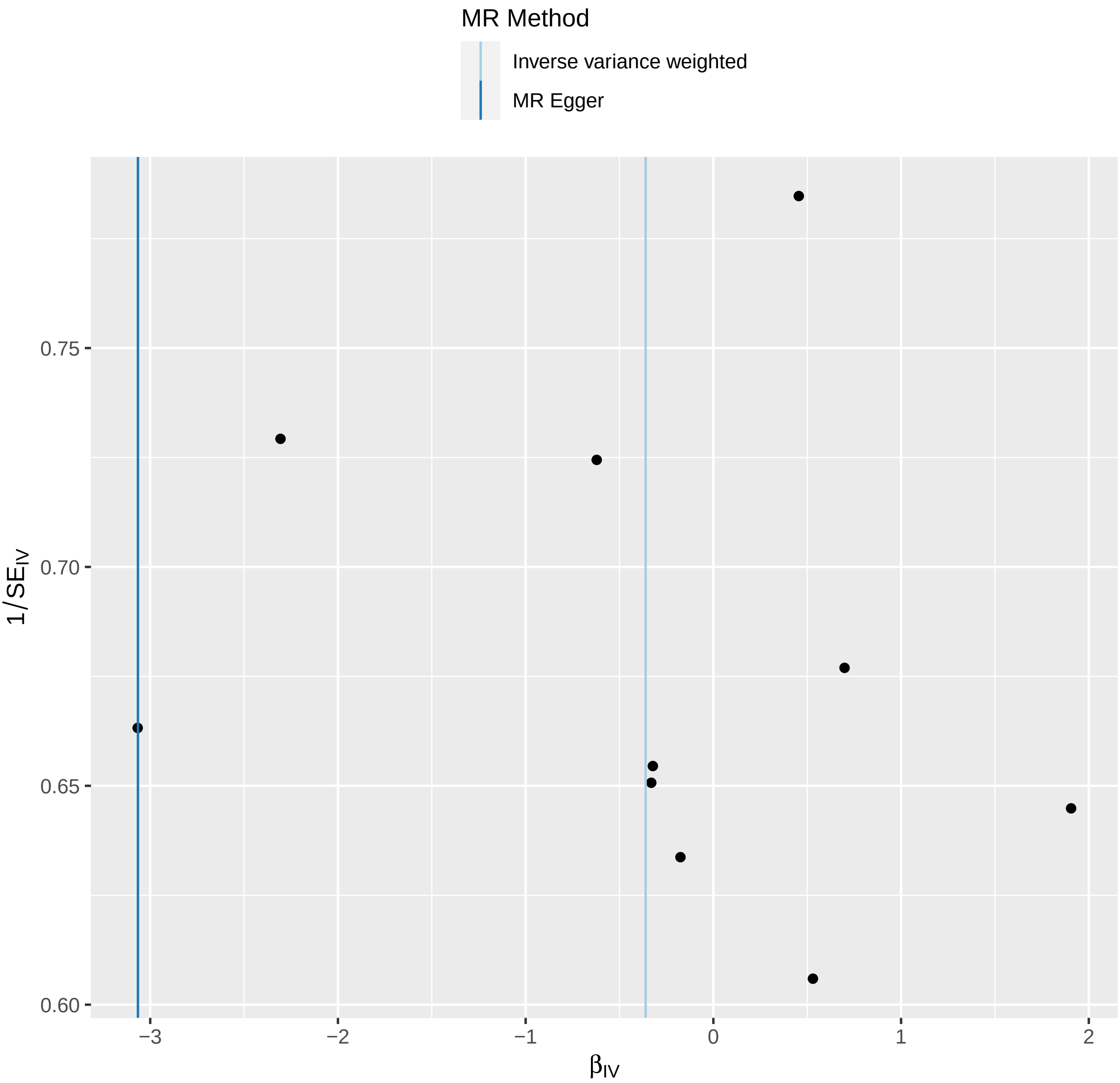

Batch 102 : Gut microbiota abundance (genus Lachnospiraceae UCG010 id.11330) on Localized adiposity

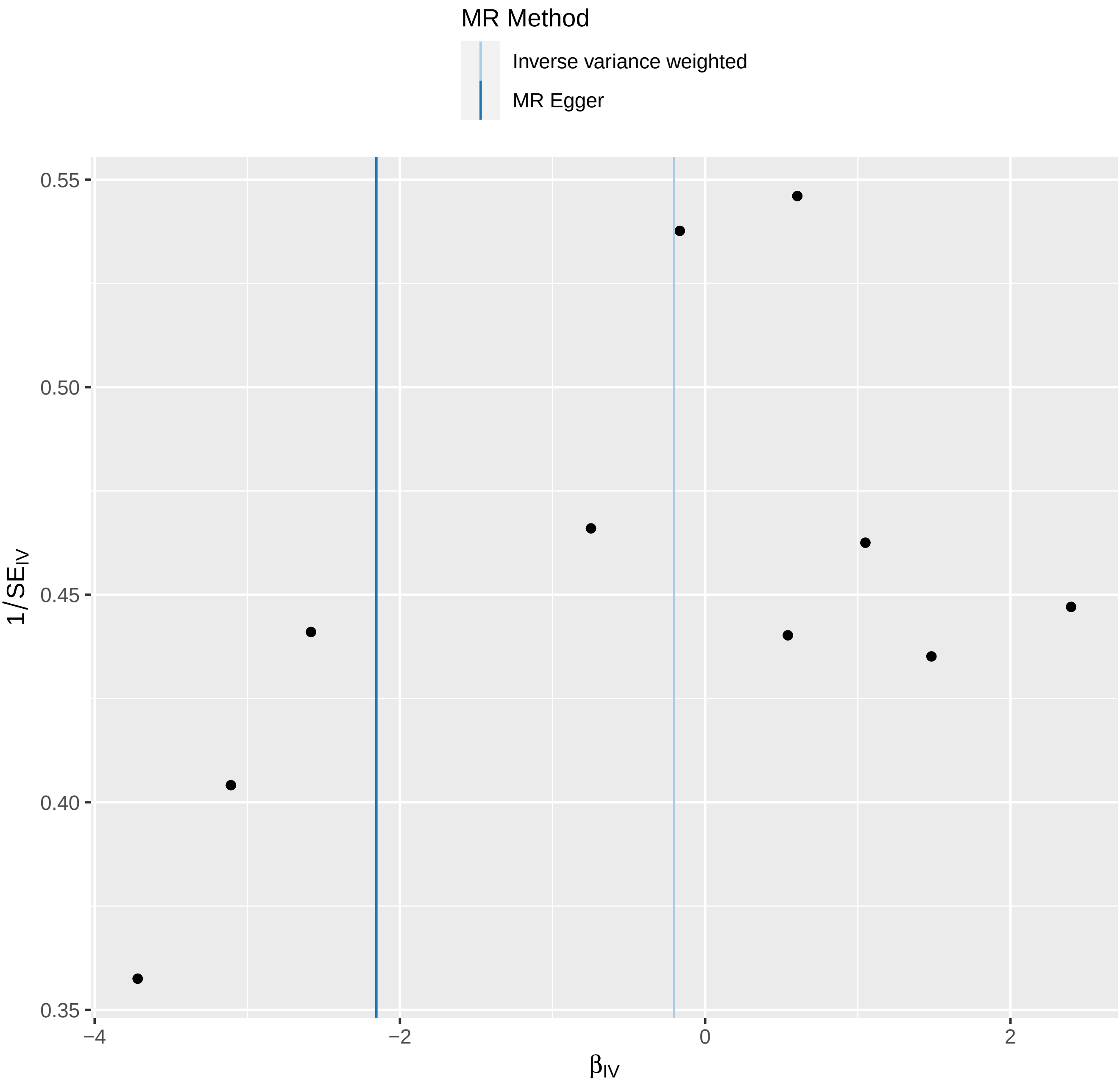

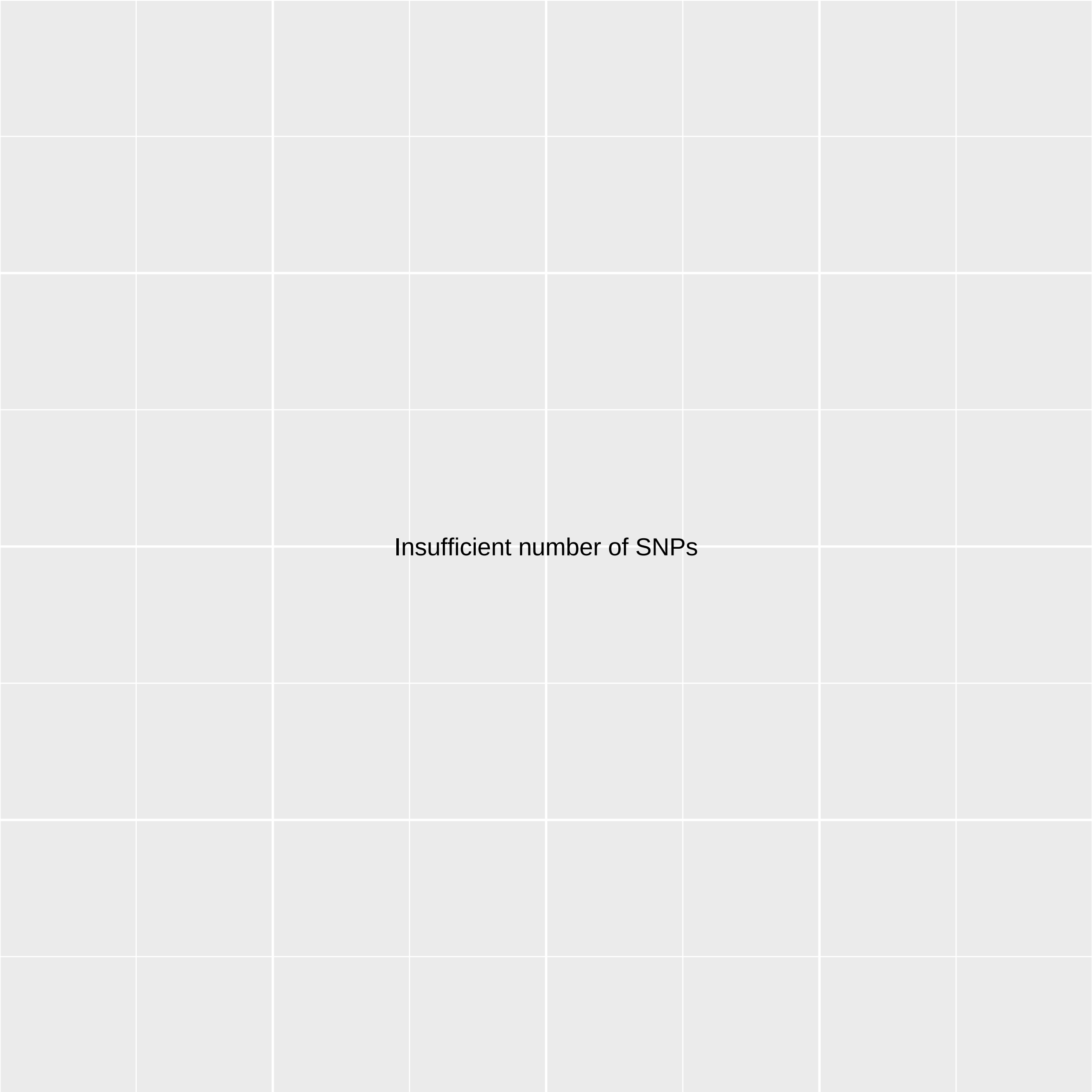
Insufficient number of SNPs

Batch 104 : Gut microbiota abundance (genus Lactobacillus id.1837) on Localized adiposity

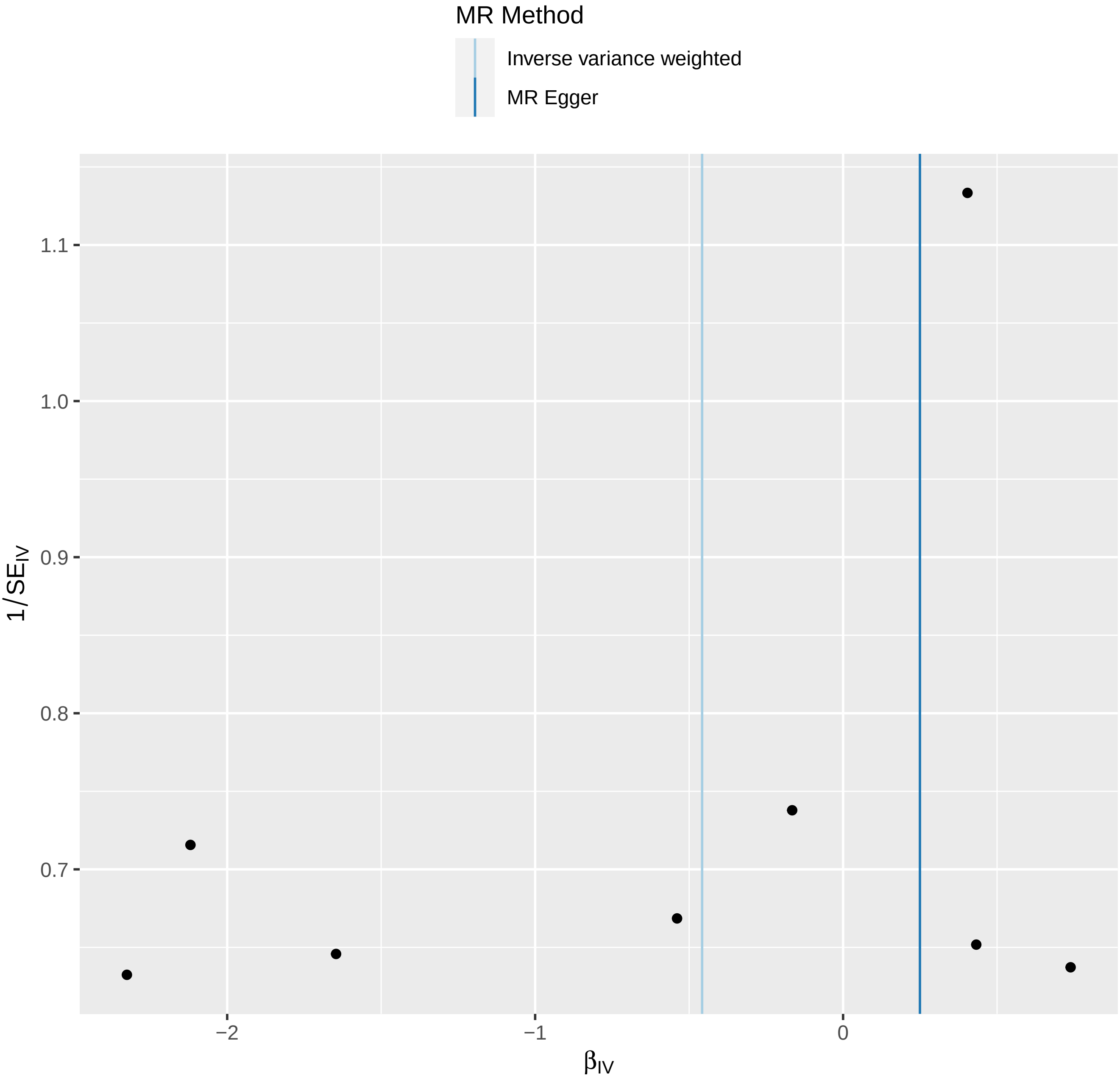

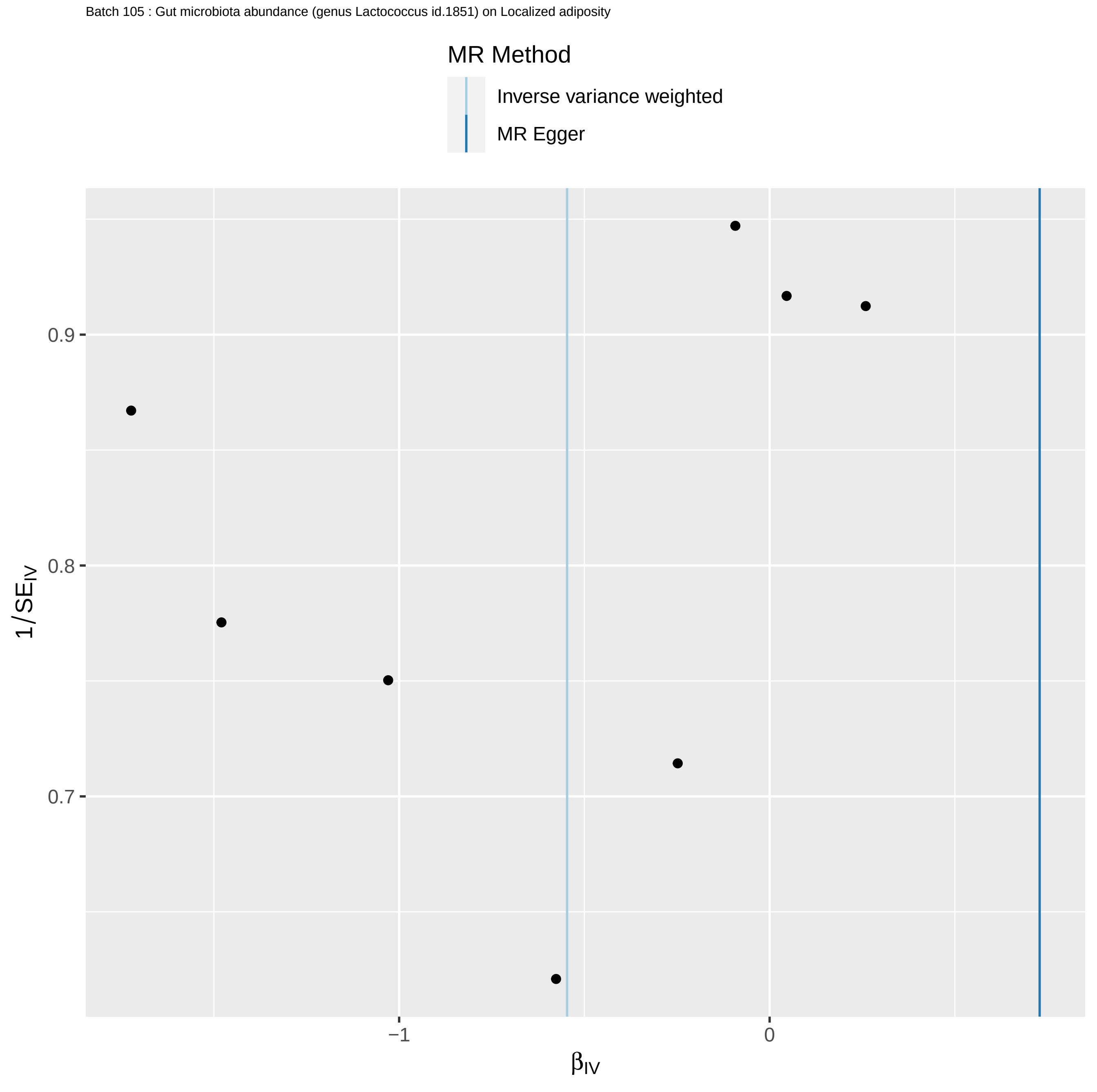

Batch 105 : Gut microbiota abundance (genus Lactococcus id.1851) on Localized adiposity
MR Method
Inverse variance weighted
MR Egger
0.9
0.8
0.7
−1
0
$1/SE_{IV}$
$\beta_{IV}$

Batch 106 : Gut microbiota abundance (genus Marvinbryantia id.2005) on Localized adiposity

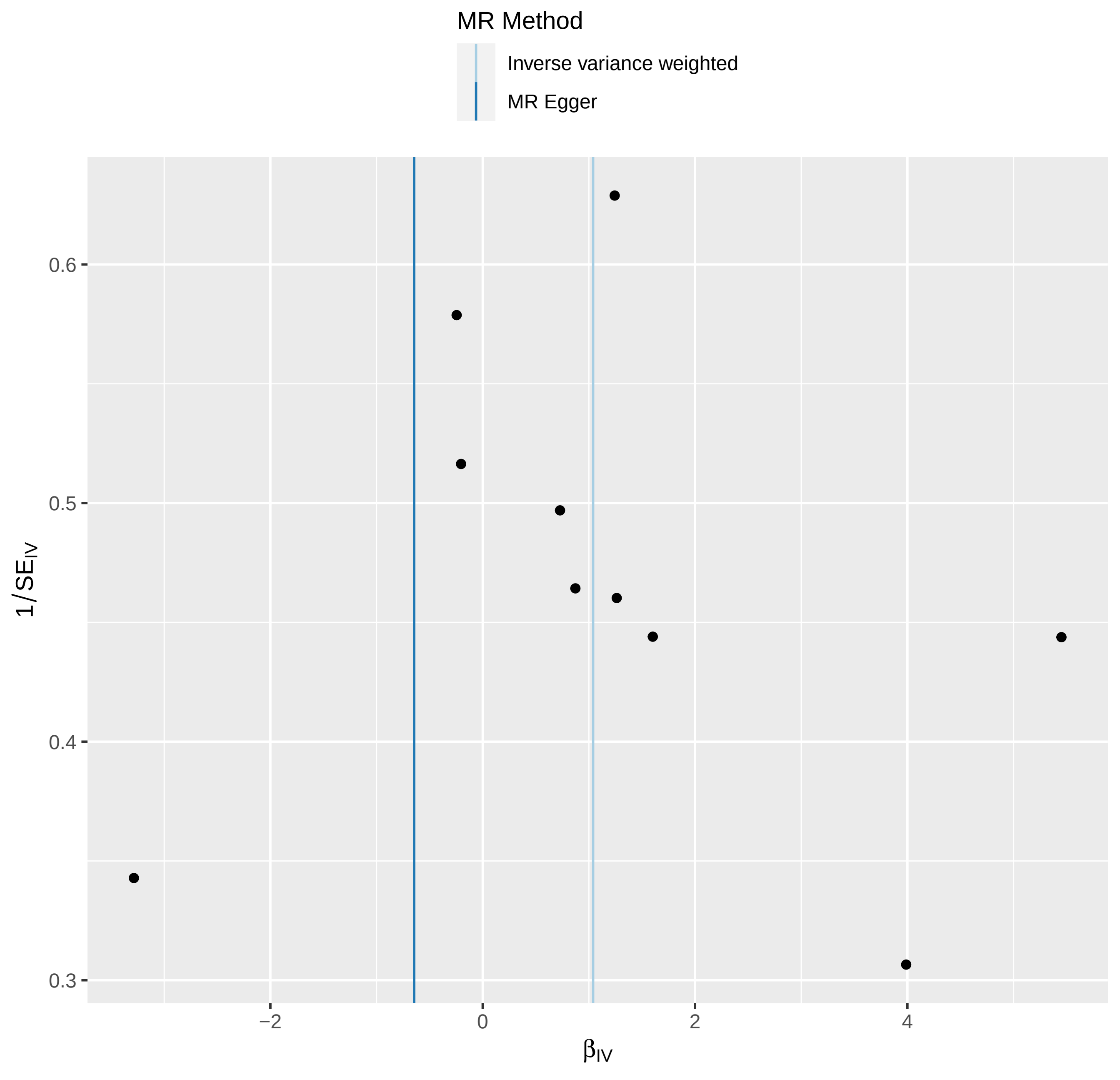

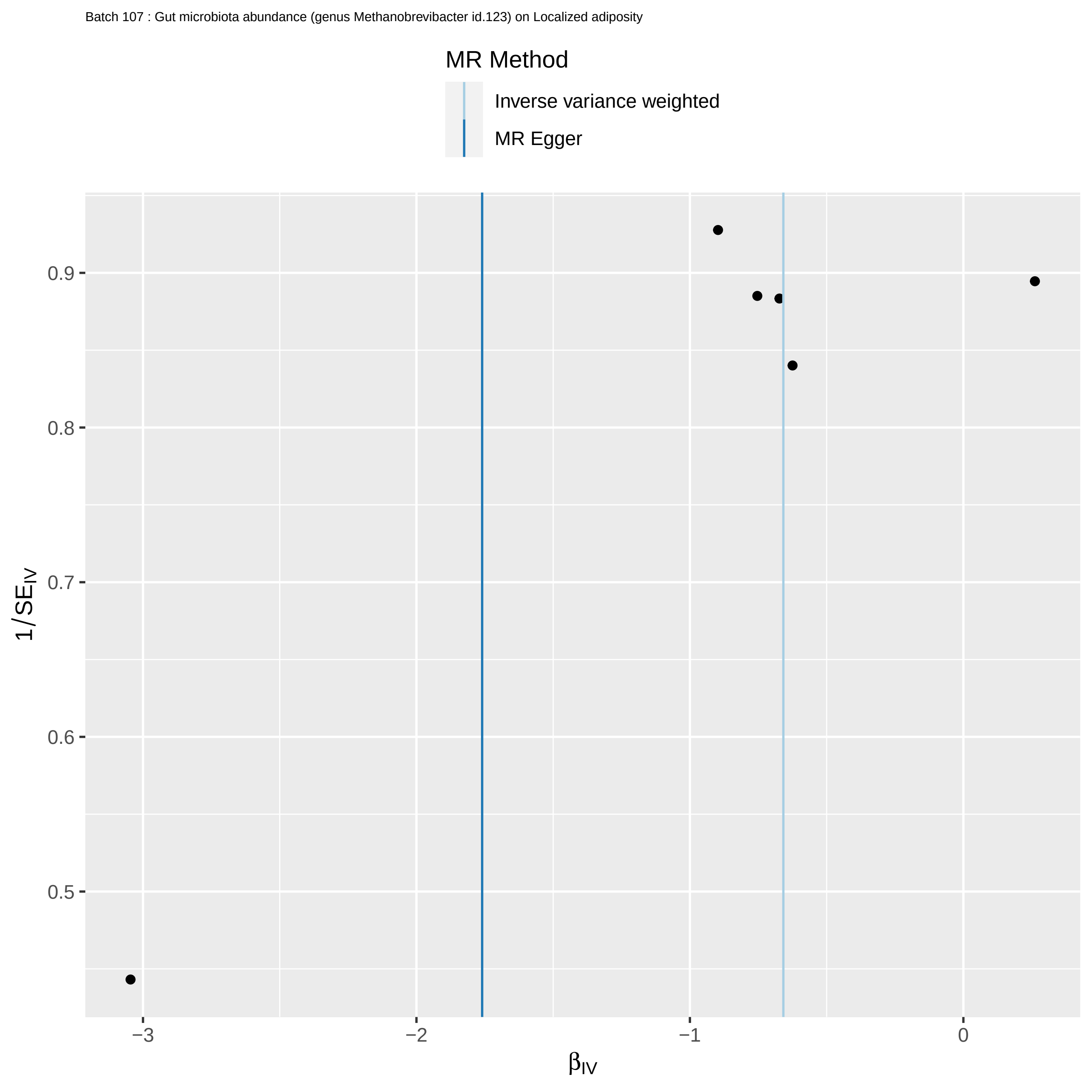

Batch 107 : Gut microbiota abundance (genus Methanobrevibacter id.123) on Localized adiposity
MR Method
Inverse variance weighted
MR Egger
0.9
0.8
0.7
0.6
0.5
1/SE_IV
−3
−2
−1
0
β_IV

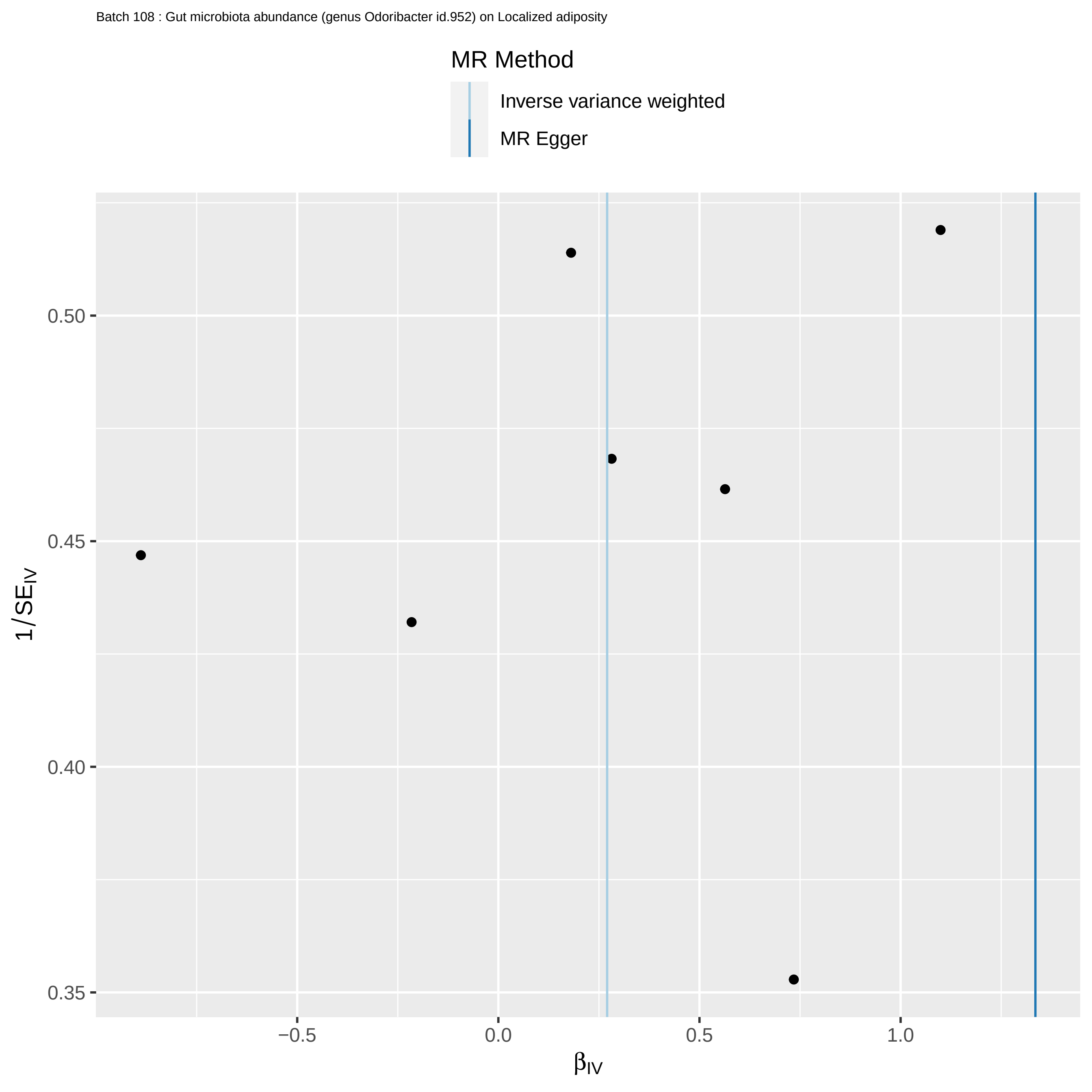
Batch 108 : Gut microbiota abundance (genus Odoribacter id.952) on Localized adiposity
MR Method
Inverse variance weighted
MR Egger
0.50
0.45
0.40
0.35
1/SE_IV
−0.5
0.0
0.5
1.0
β_IV

Batch 109 : Gut microbiota abundance (genus Olsenella id.822) on Localized adiposity

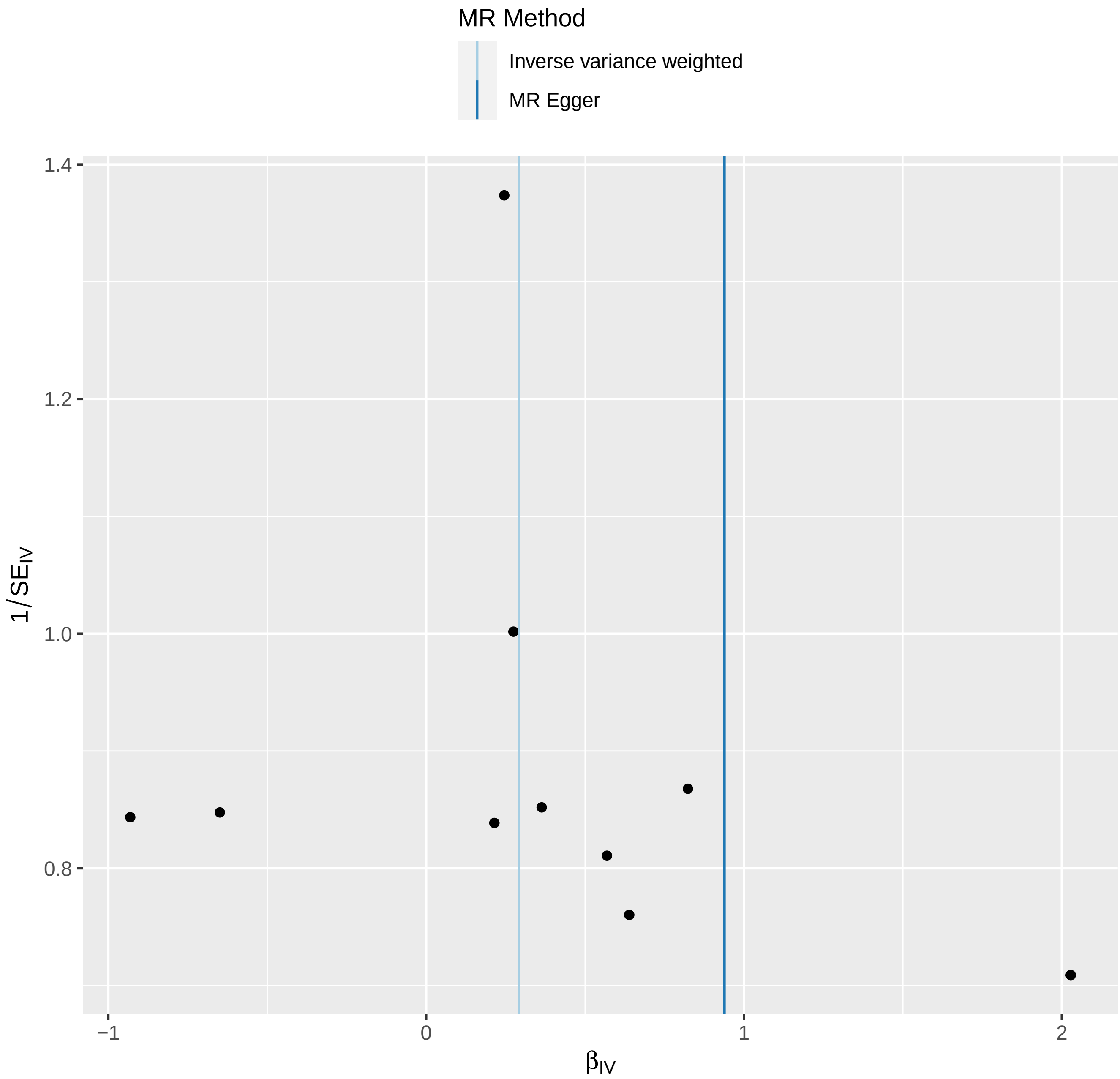

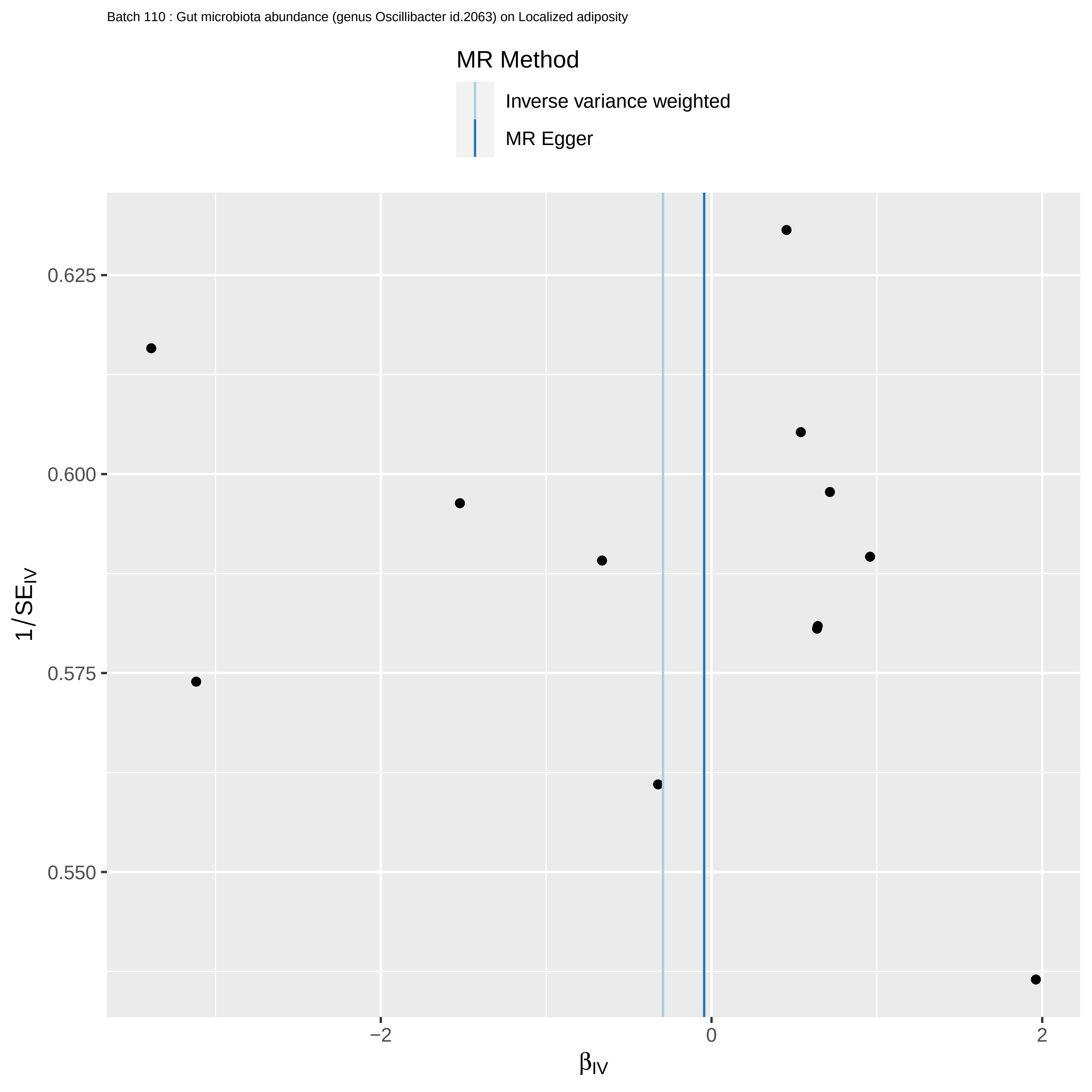

Batch 110 : Gut microbiota abundance (genus Oscillibacter id.2063) on Localized adiposity
MR Method
Inverse variance weighted
MR Egger
0.625
0.600
0.575
0.550
1/SE_IV
−2
0
2
β_IV

Batch 111 : Gut microbiota abundance (genus Oscillospira id.2064) on Localized adiposity

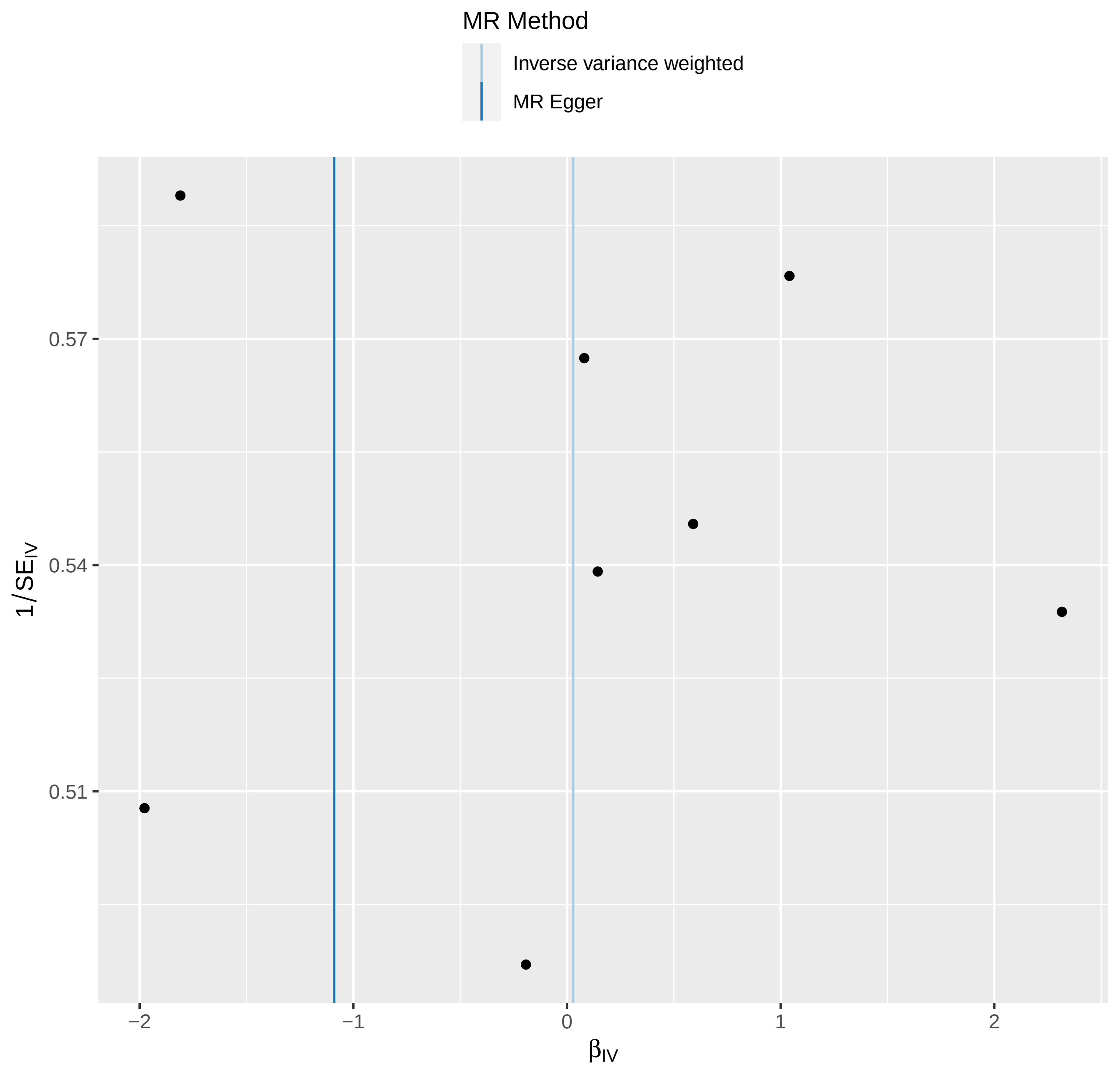

Batch 112 : Gut microbiota abundance (genus Oxalobacter id.2978) on Localized adiposity

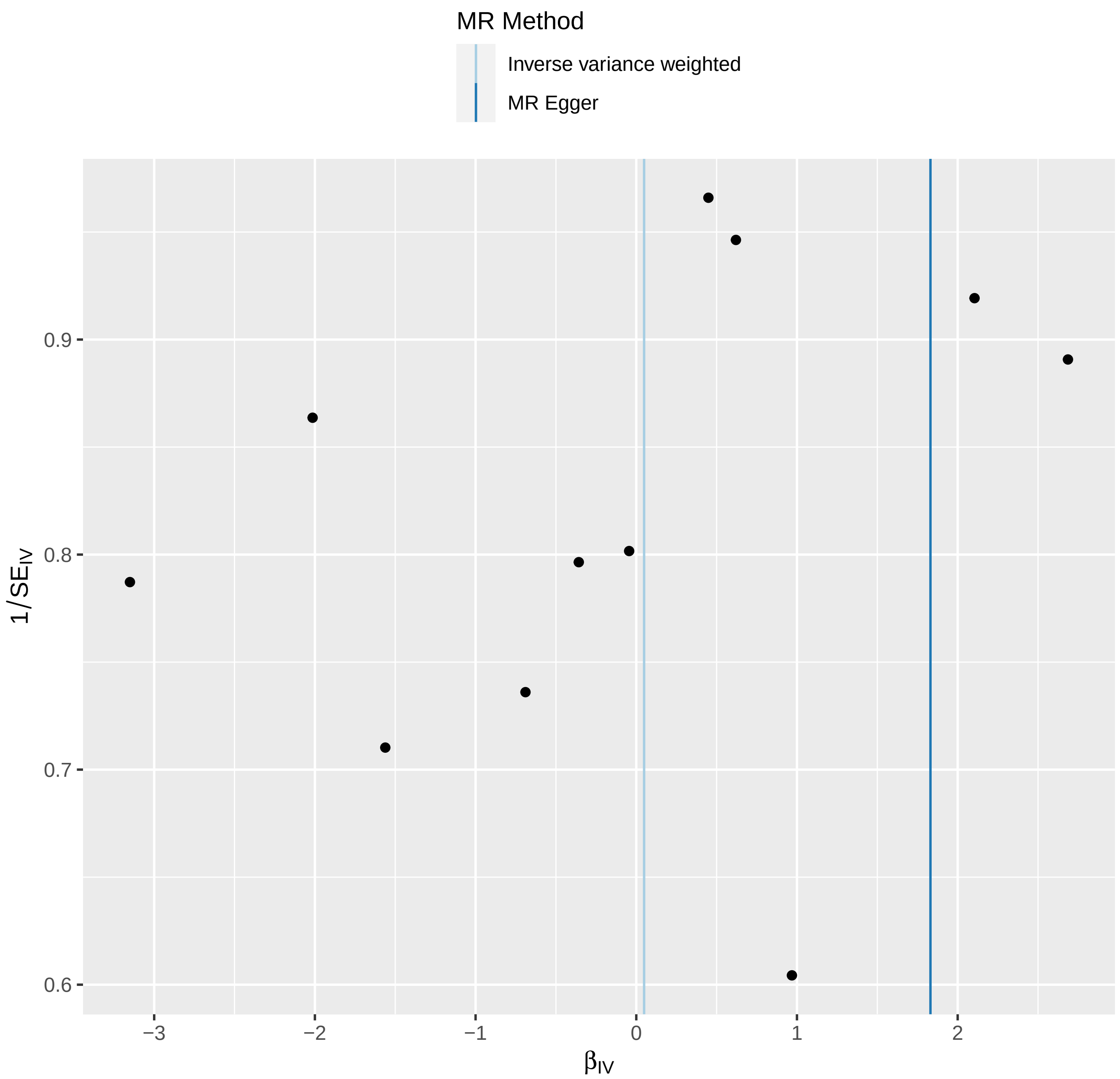

Batch 113 : Gut microbiota abundance (genus Parabacteroides id.954) on Localized adiposity

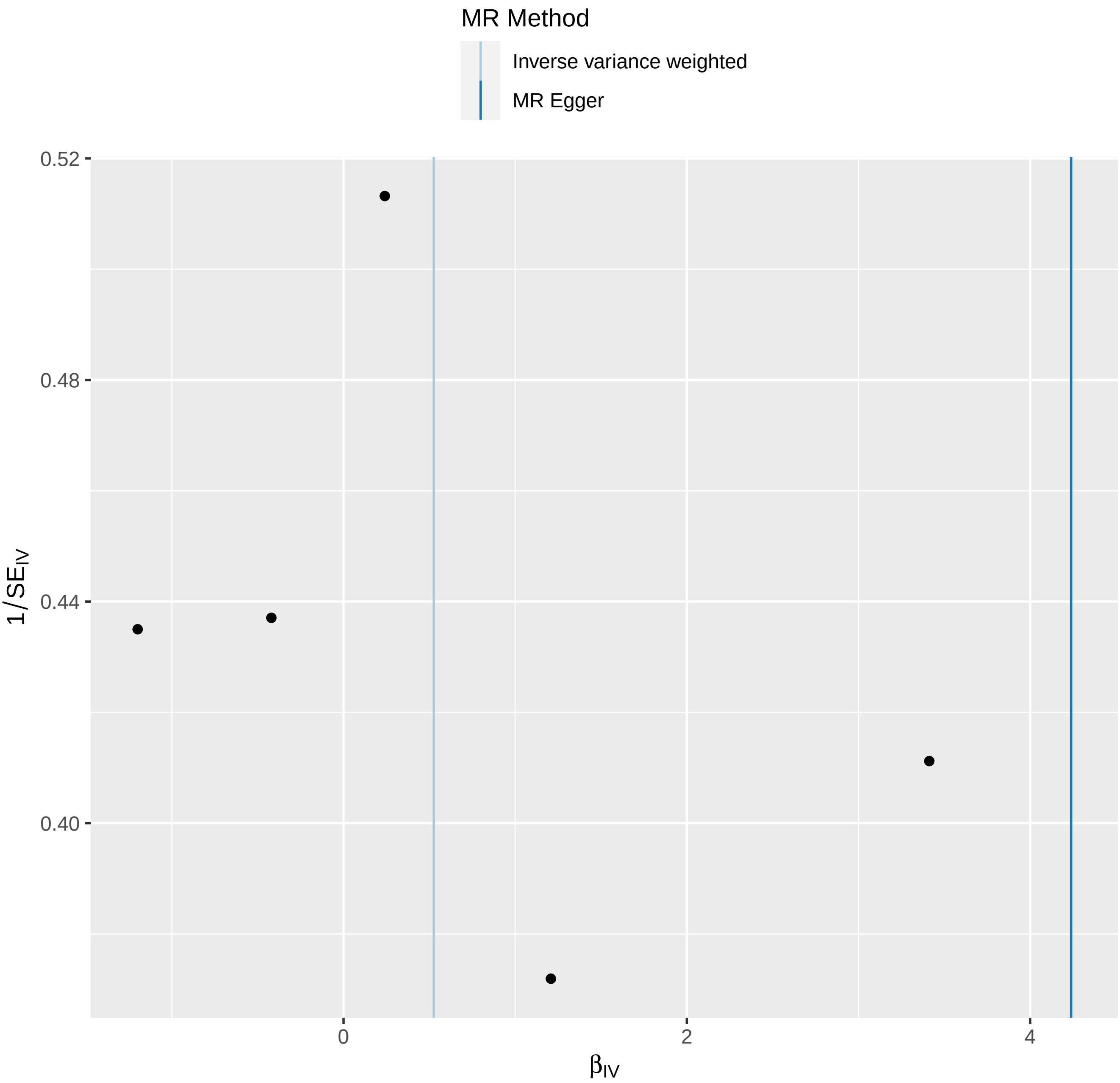

Batch 114 : Gut microbiota abundance (genus Paraprevotella id.962) on Localized adiposity

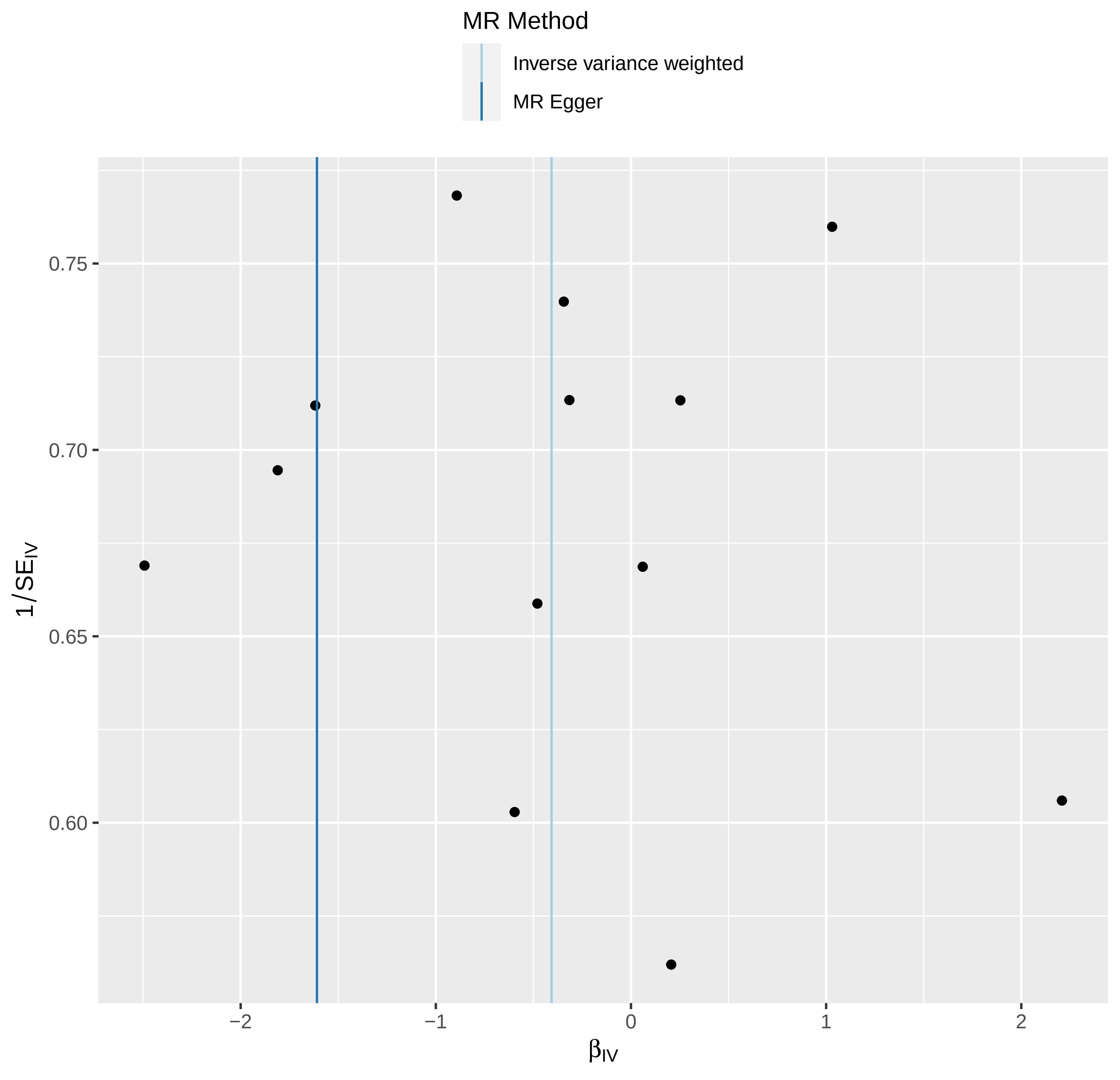

Batch 115 : Gut microbiota abundance (genus Parasutterella id.2892) on Localized adiposity

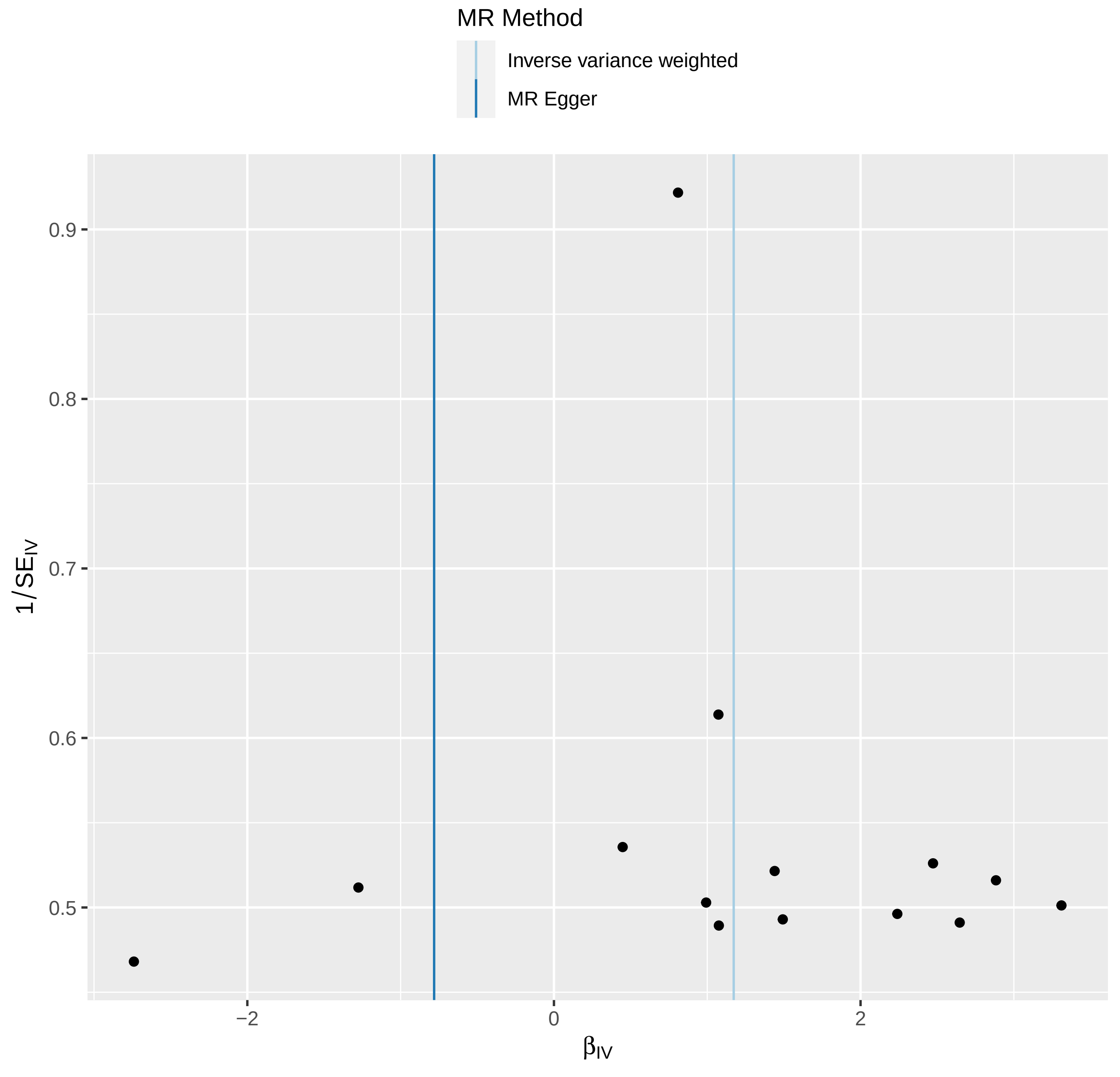

Batch 116 : Gut microbiota abundance (genus Peptococcus id.2037) on Localized adiposity

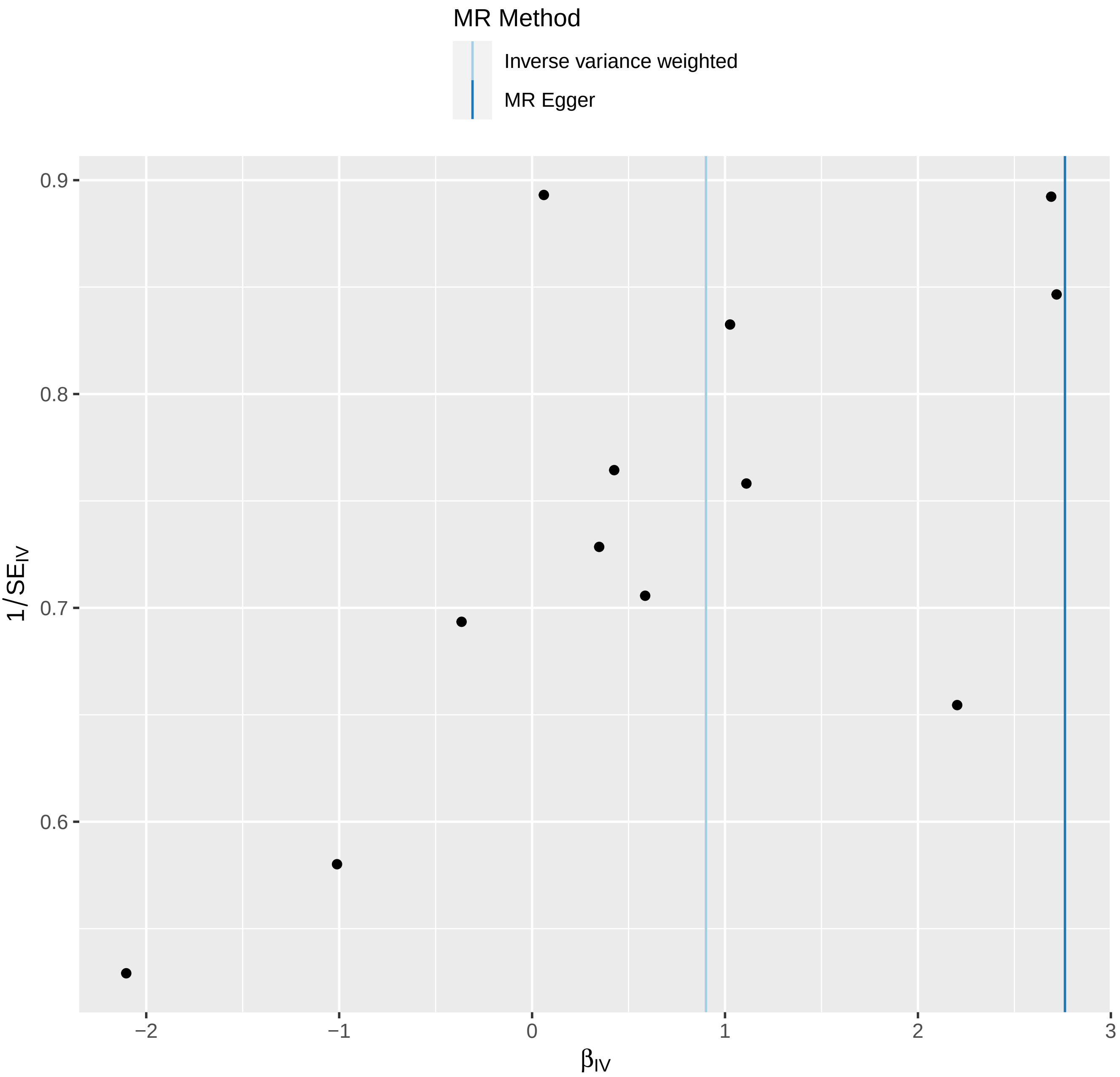

Batch 117 : Gut microbiota abundance (genus Phascolarctobacterium id.2168) on Localized adiposity

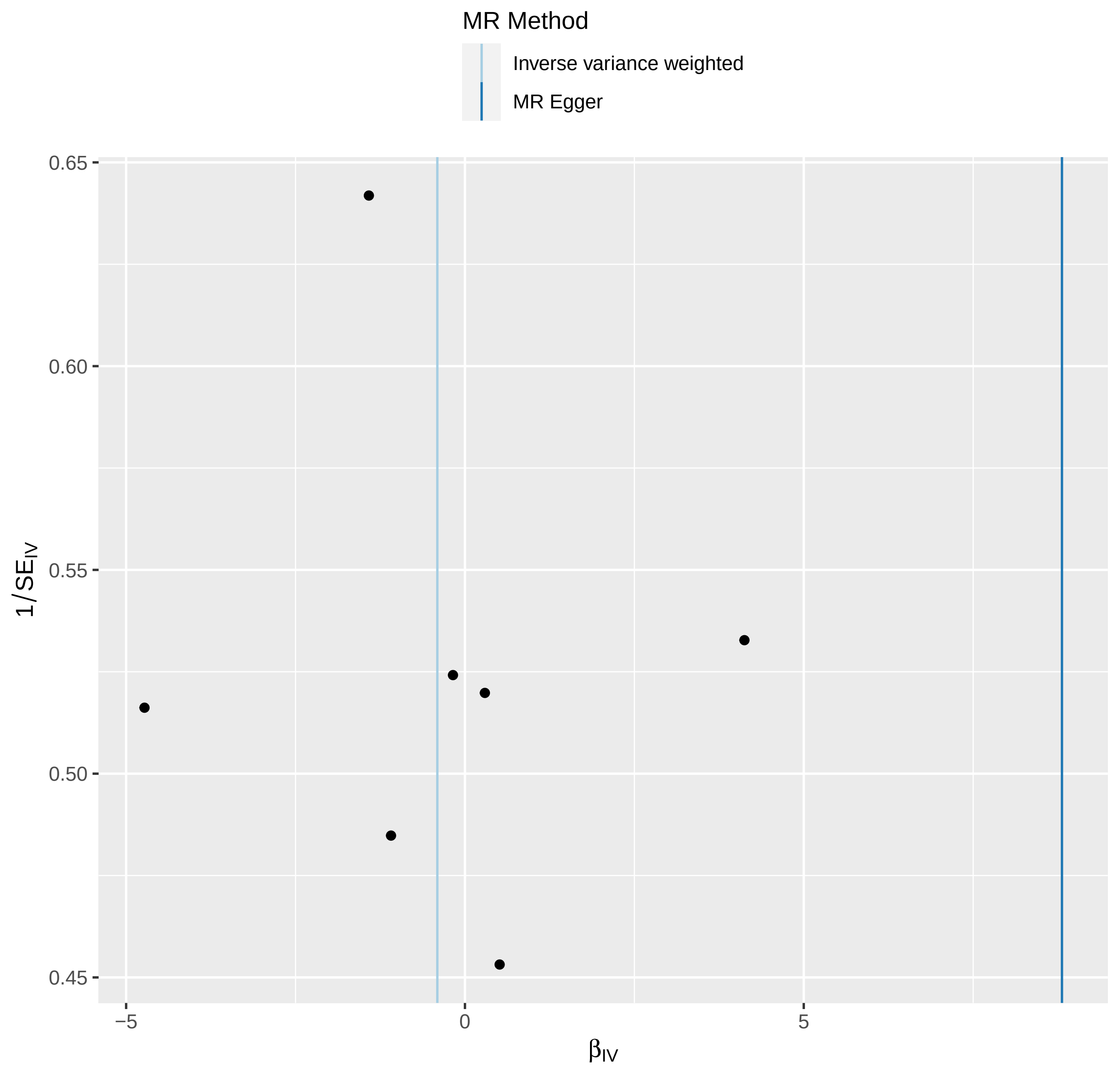

Batch 118 : Gut microbiota abundance (genus Prevotella7 id.11182) on Localized adiposity

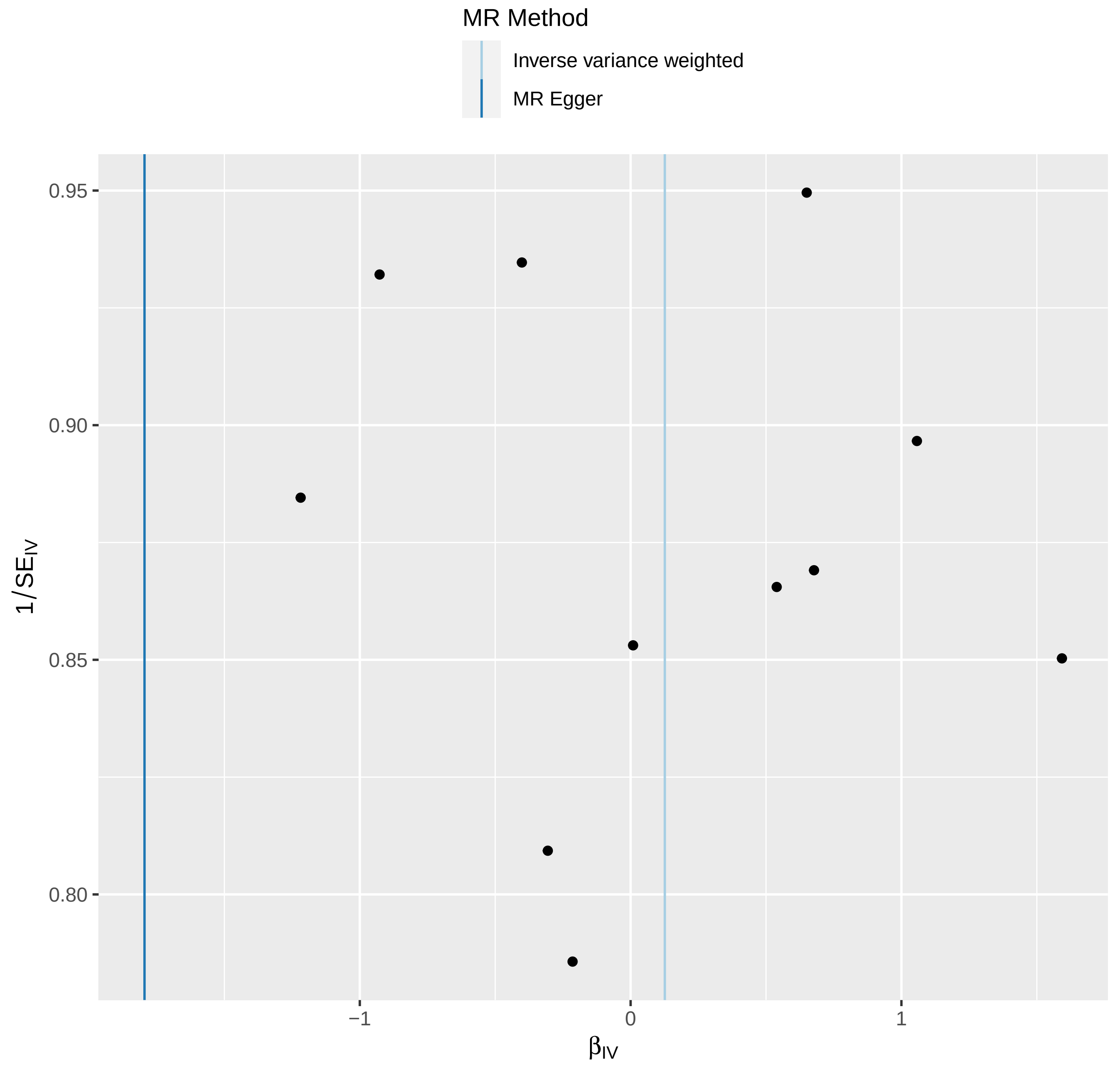

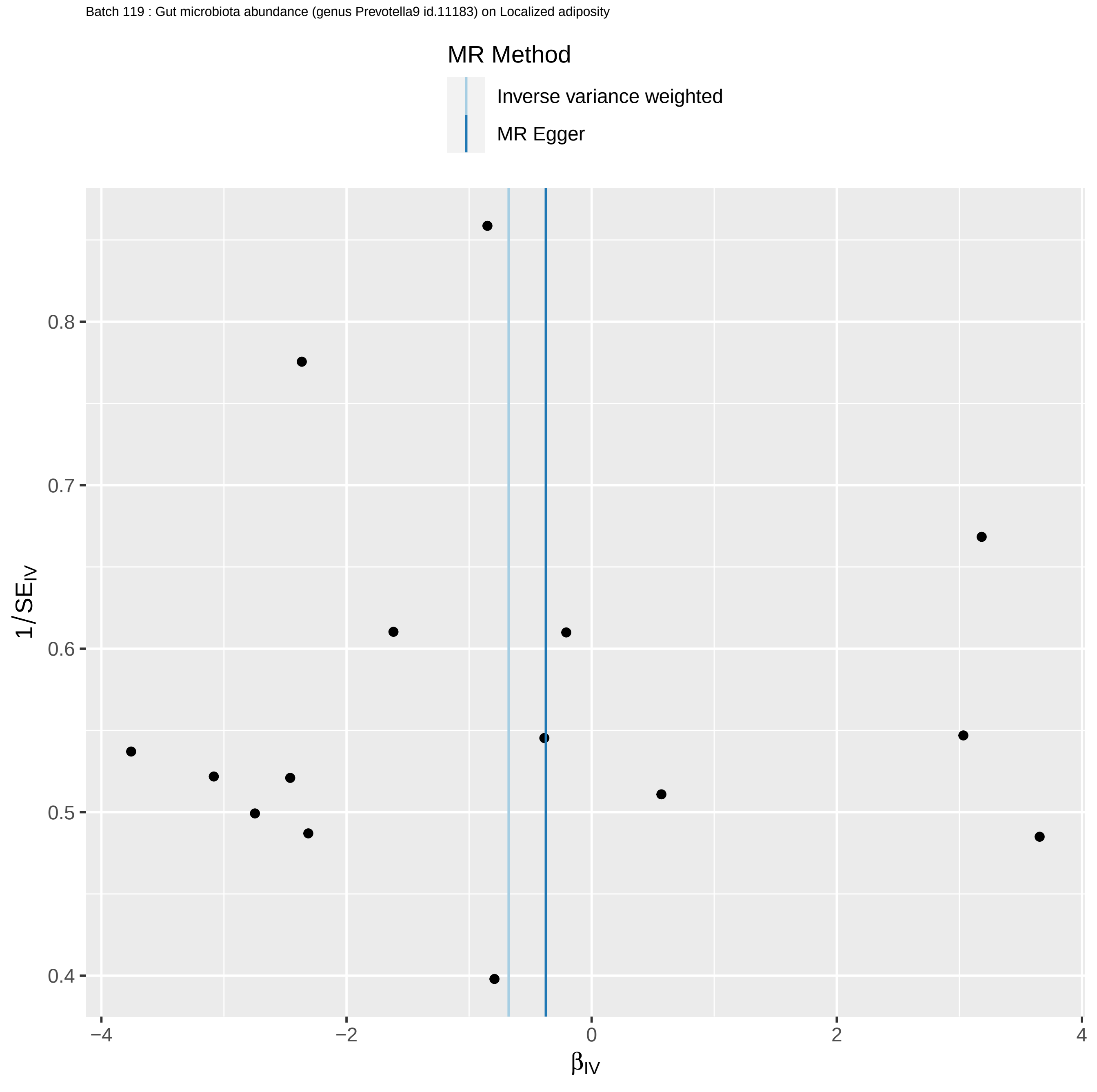
Batch 119 : Gut microbiota abundance (genus Prevotella9 id.11183) on Localized adiposity
MR Method
Inverse variance weighted
MR Egger
0.8
0.7
0.6
0.5
0.4
1/SE_IV
−4
−2
0
2
4
β_IV

Batch 120 : Gut microbiota abundance (genus Rikenellaceae RC9 gut group id.11191) on Localized adiposity

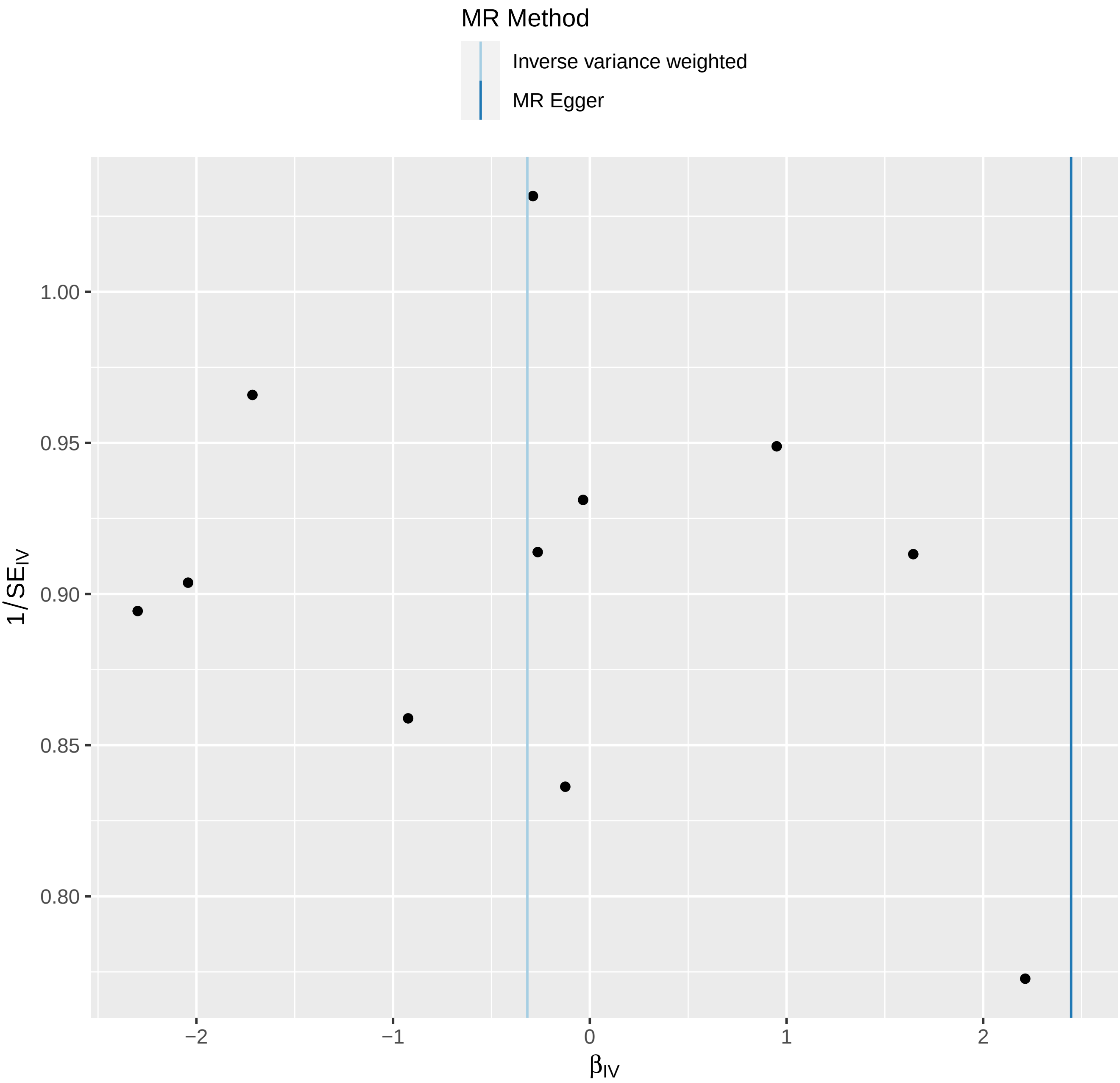

Batch 121 : Gut microbiota abundance (genus Romboutsia id.11347) on Localized adiposity

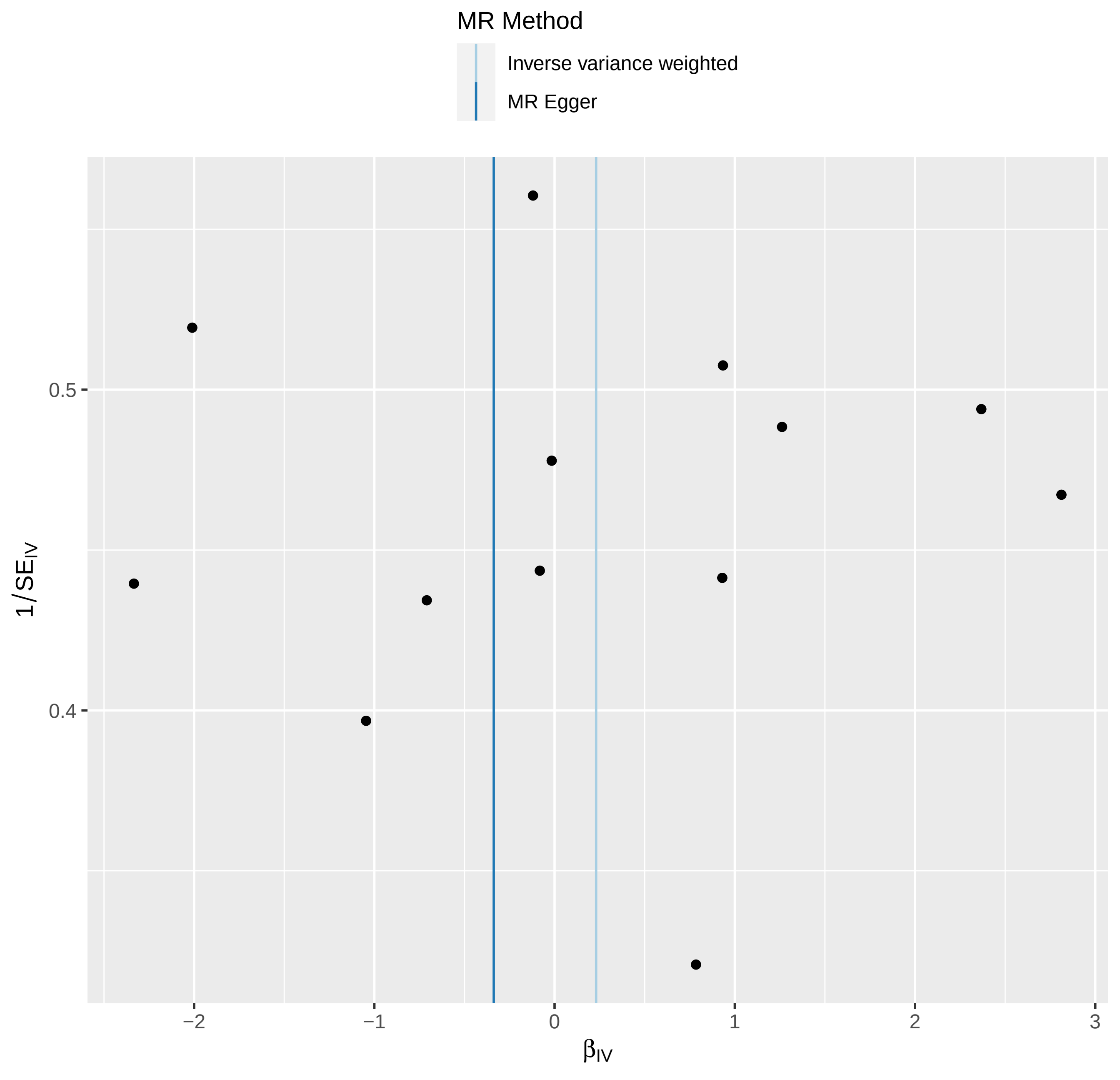

Batch 122 : Gut microbiota abundance (genus Roseburia id.2012) on Localized adiposity

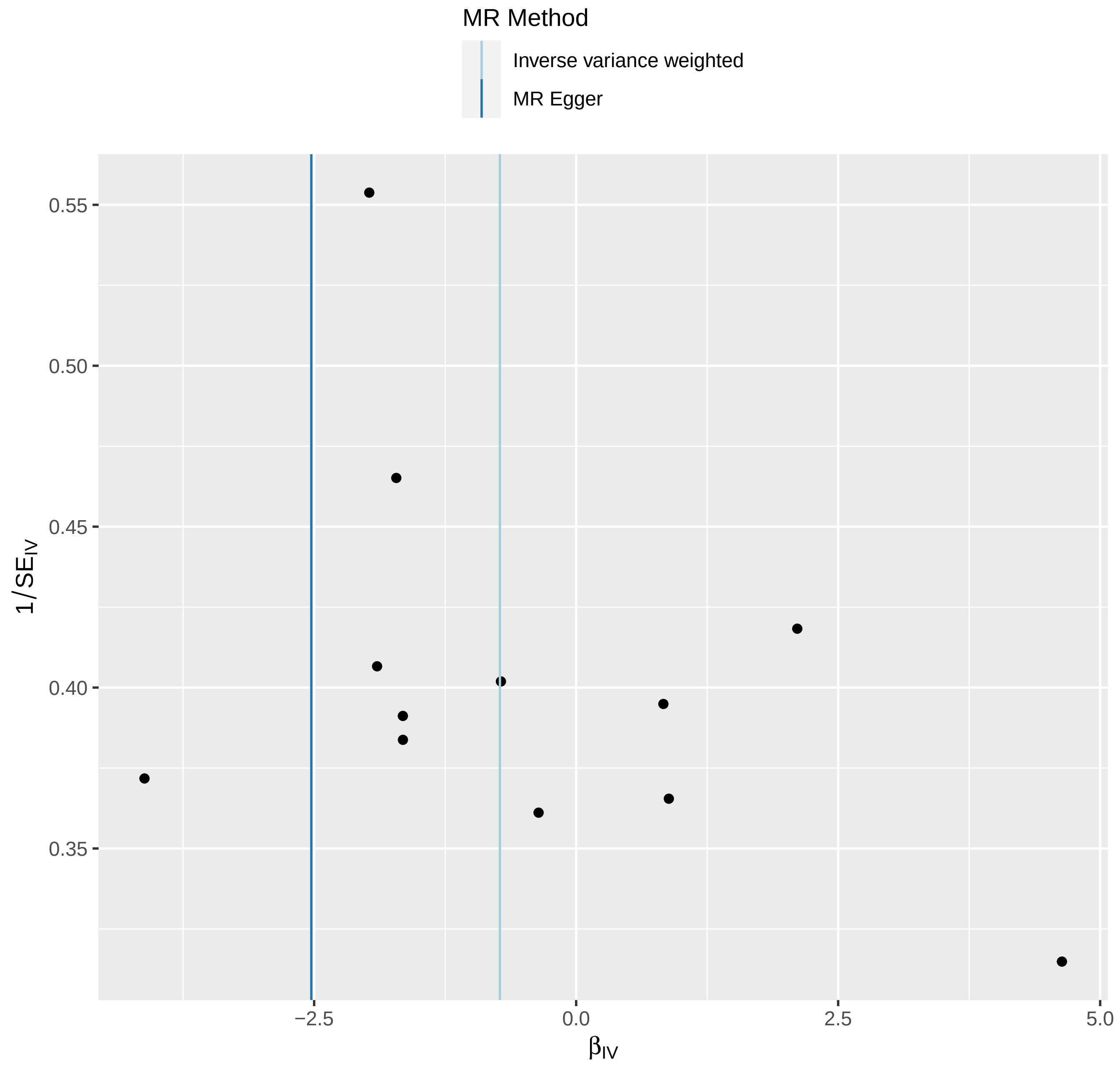

Batch 123 : Gut microbiota abundance (genus Ruminiclostridium5 id.11355) on Localized adiposity

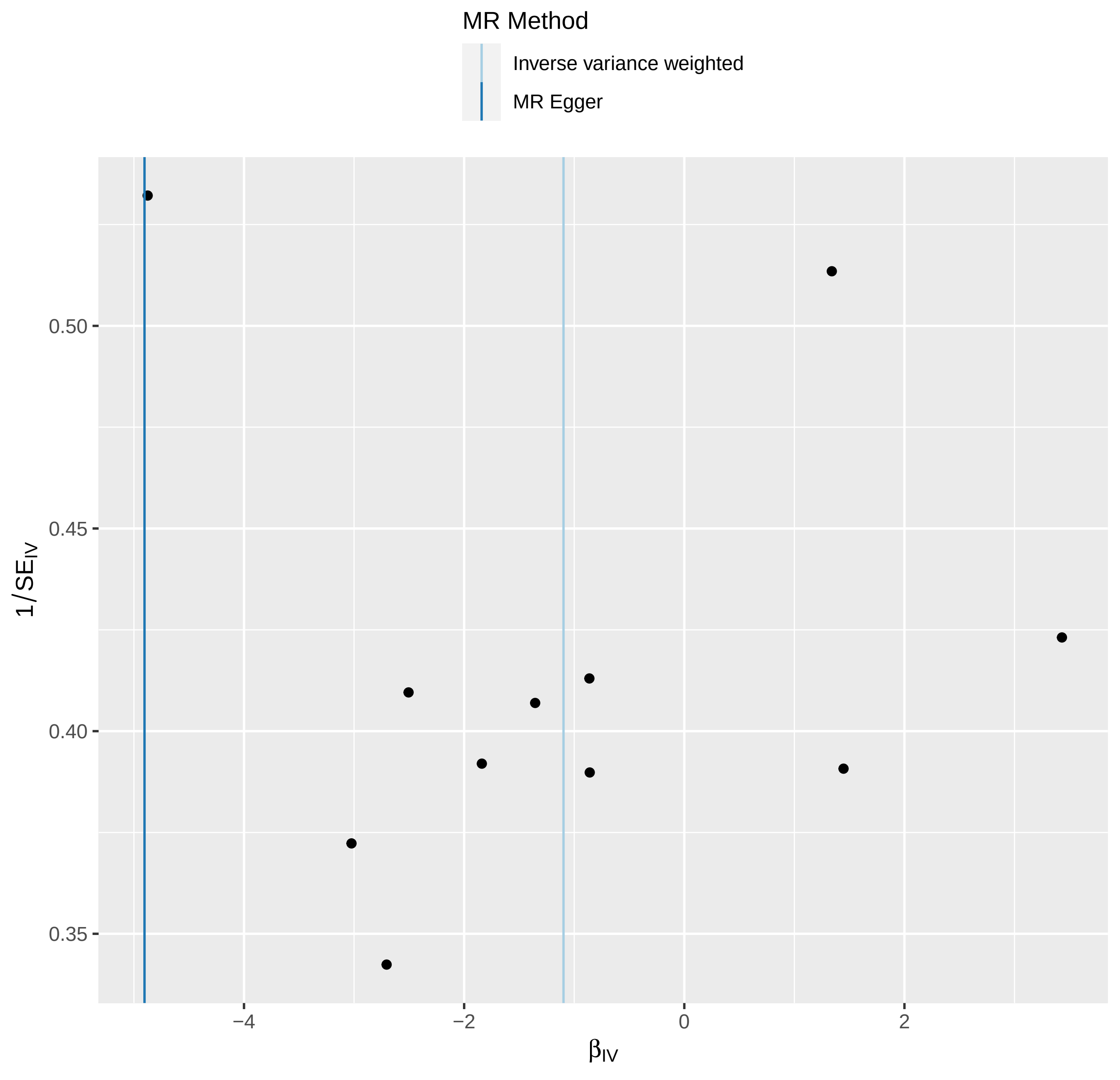

Batch 124 : Gut microbiota abundance (genus Ruminiclostridium6 id.11356) on Localized adiposity

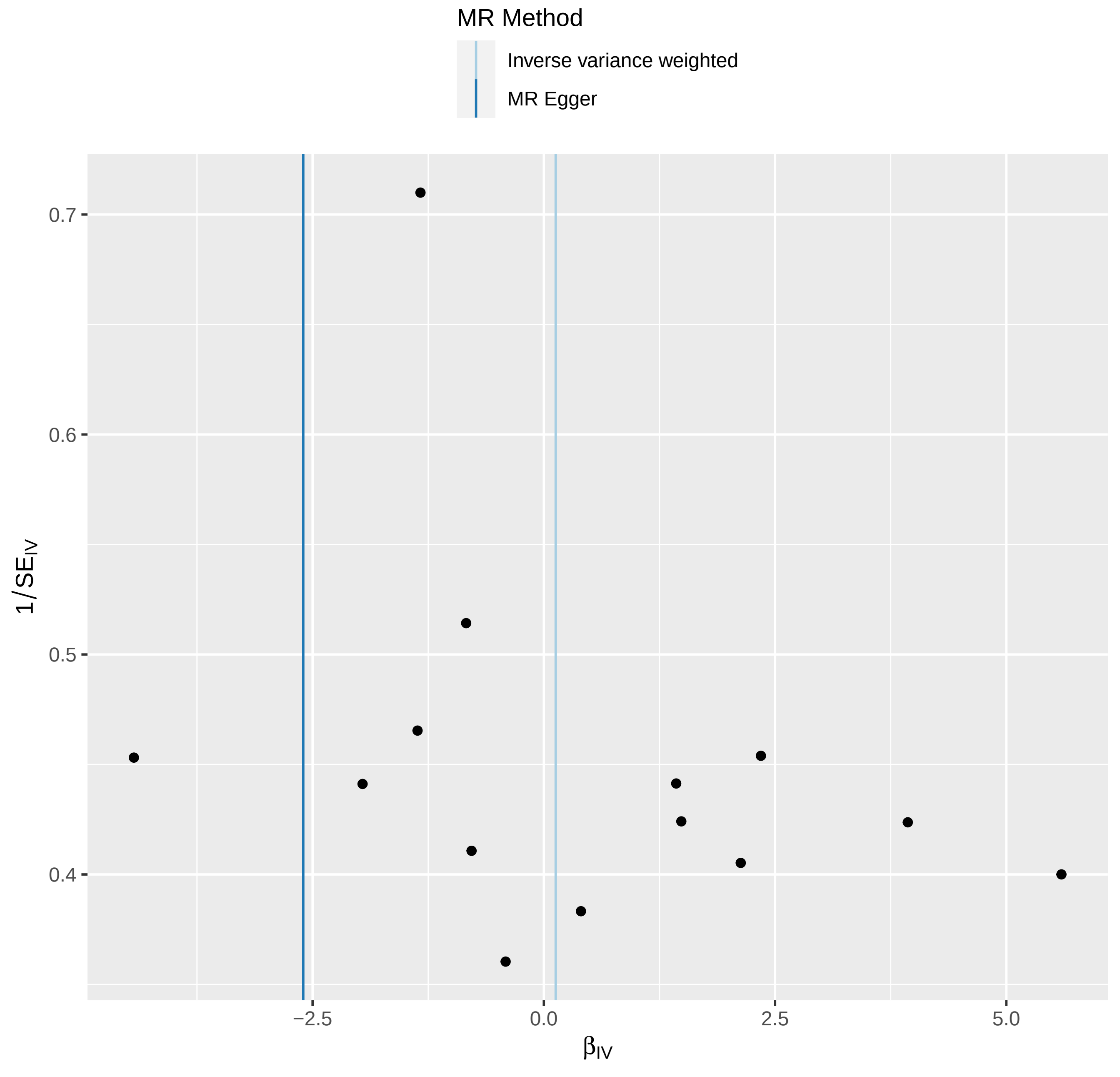

Batch 125 : Gut microbiota abundance (genus Ruminiclostridium9 id.11357) on Localized adiposity

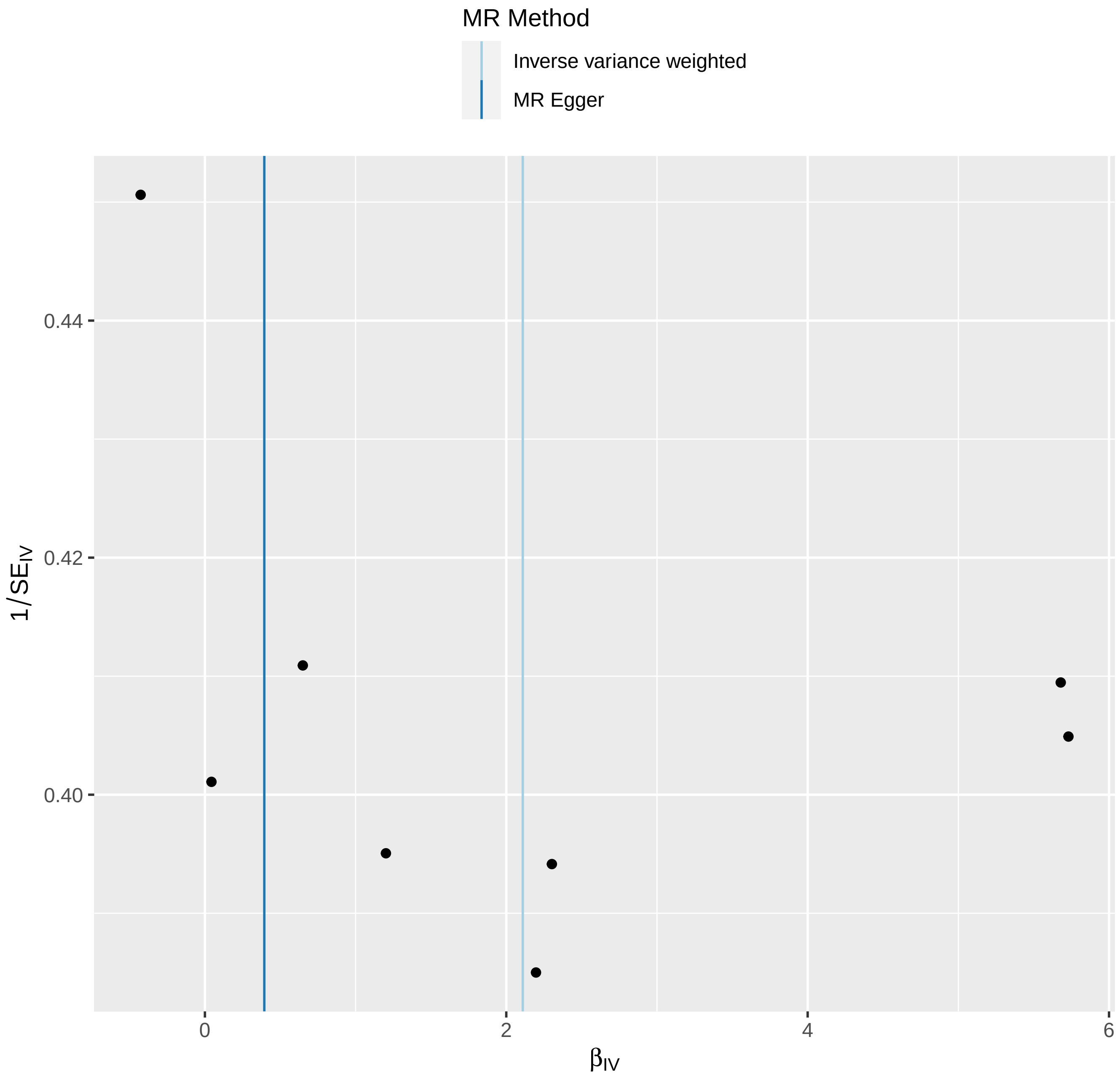

Batch 126 : Gut microbiota abundance (genus Ruminococcaceae NK4A214 group id.11358) on Localized adiposity

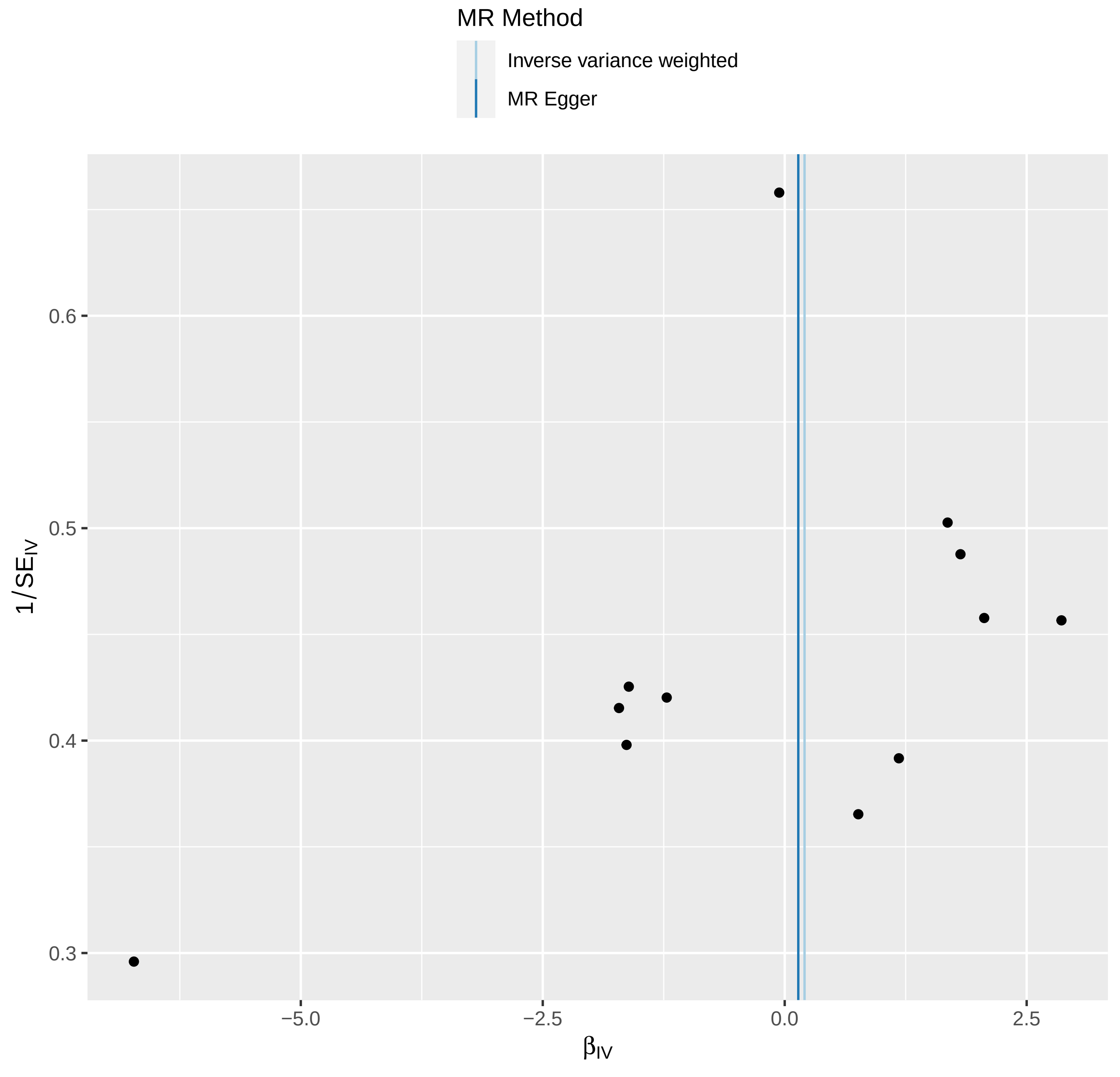

Batch 127 : Gut microbiota abundance (genus Ruminococcaceae UCG002 id.11360) on Localized adiposity

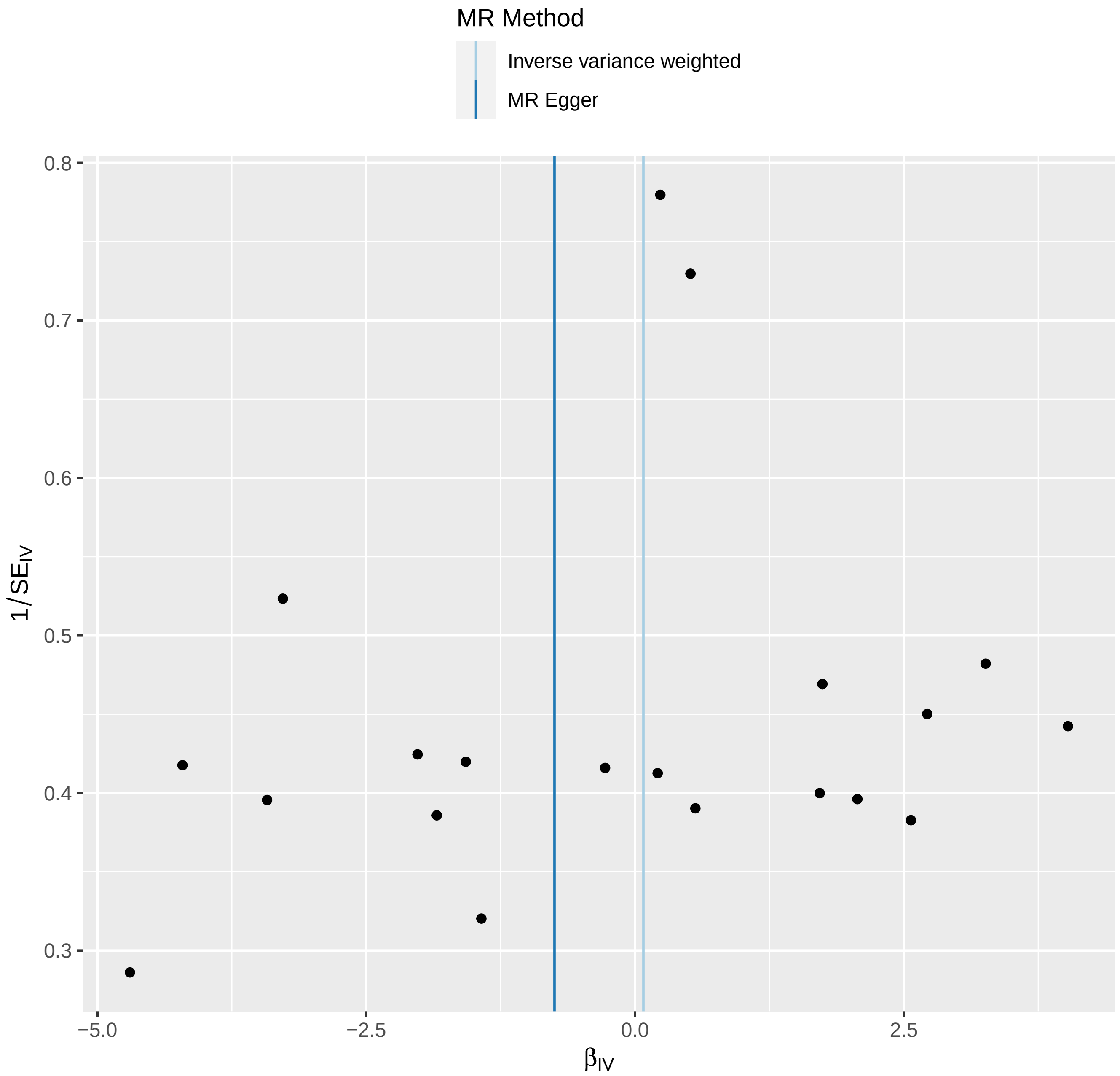

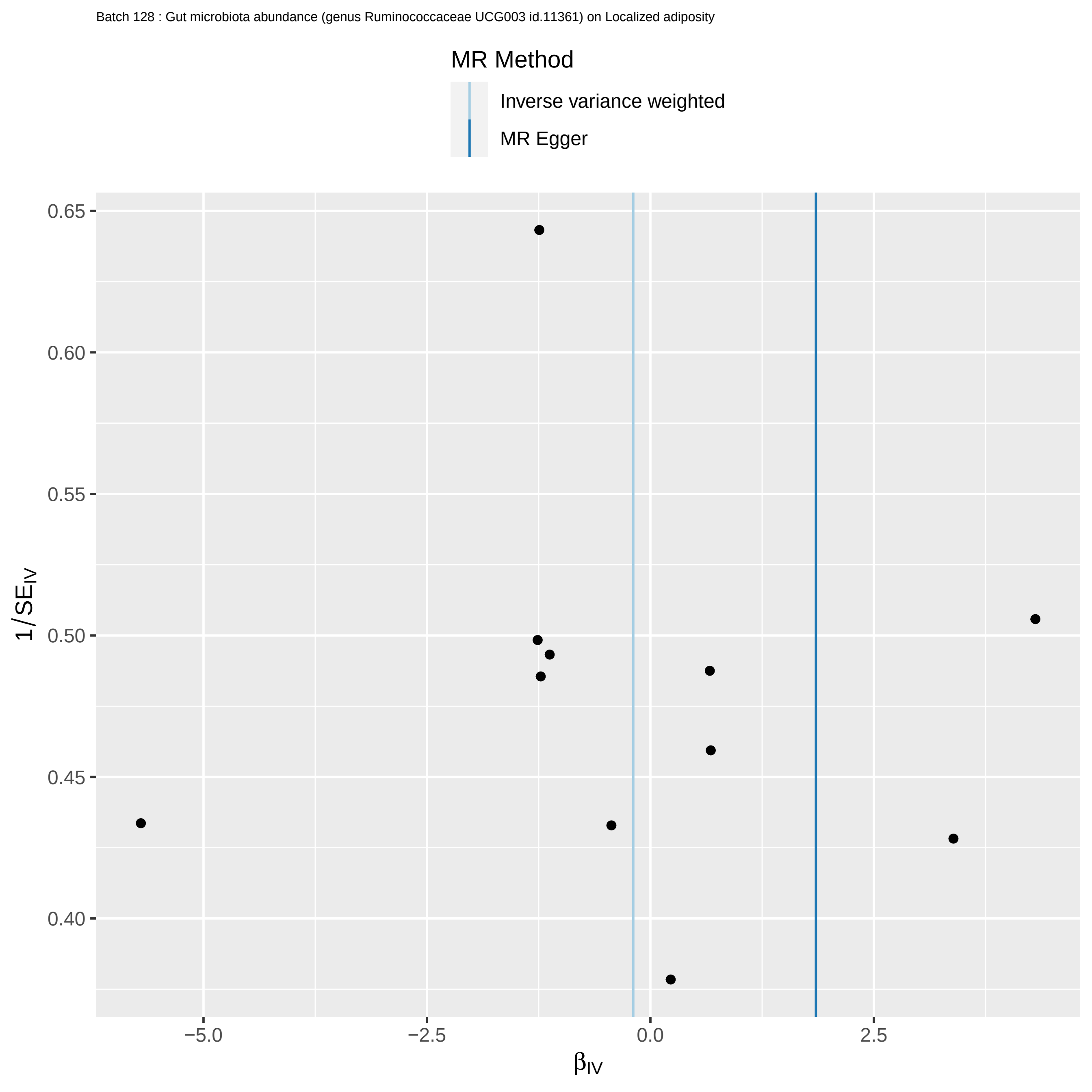

Batch 128 : Gut microbiota abundance (genus Ruminococcaceae UCG003 id.11361) on Localized adiposity
MR Method
Inverse variance weighted
MR Egger
0.65
0.60
0.55
0.50
0.45
0.40
1/SE_IV
−5.0
−2.5
0.0
2.5
β_IV

Batch 129 : Gut microbiota abundance (genus Ruminococcaceae UCG004 id.11362) on Localized adiposity

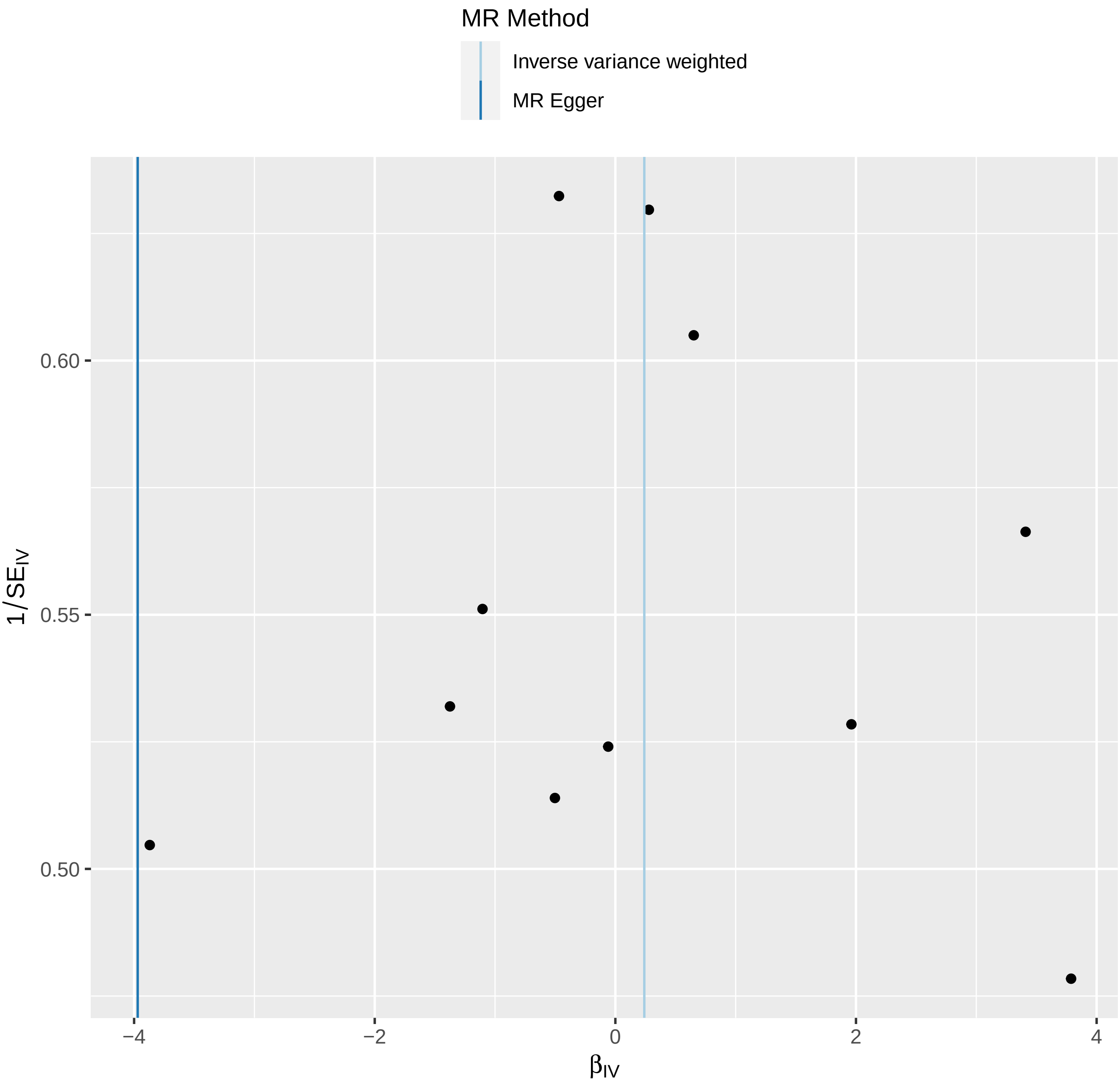

Batch 130 : Gut microbiota abundance (genus Ruminococcaceae UCG005 id.11363) on Localized adiposity

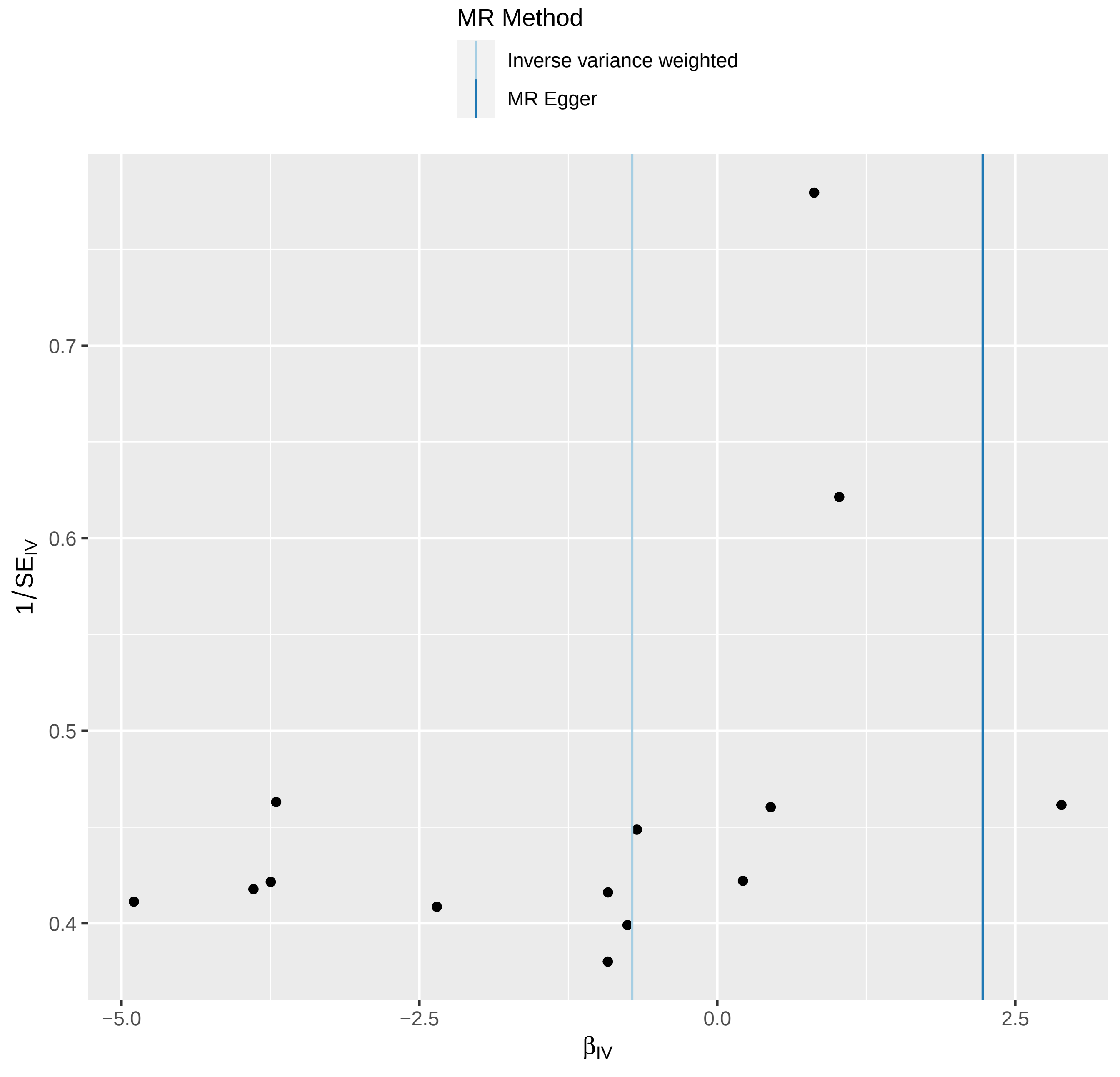

Batch 131 : Gut microbiota abundance (genus Ruminococcaceae UCG009 id.11366) on Localized adiposity

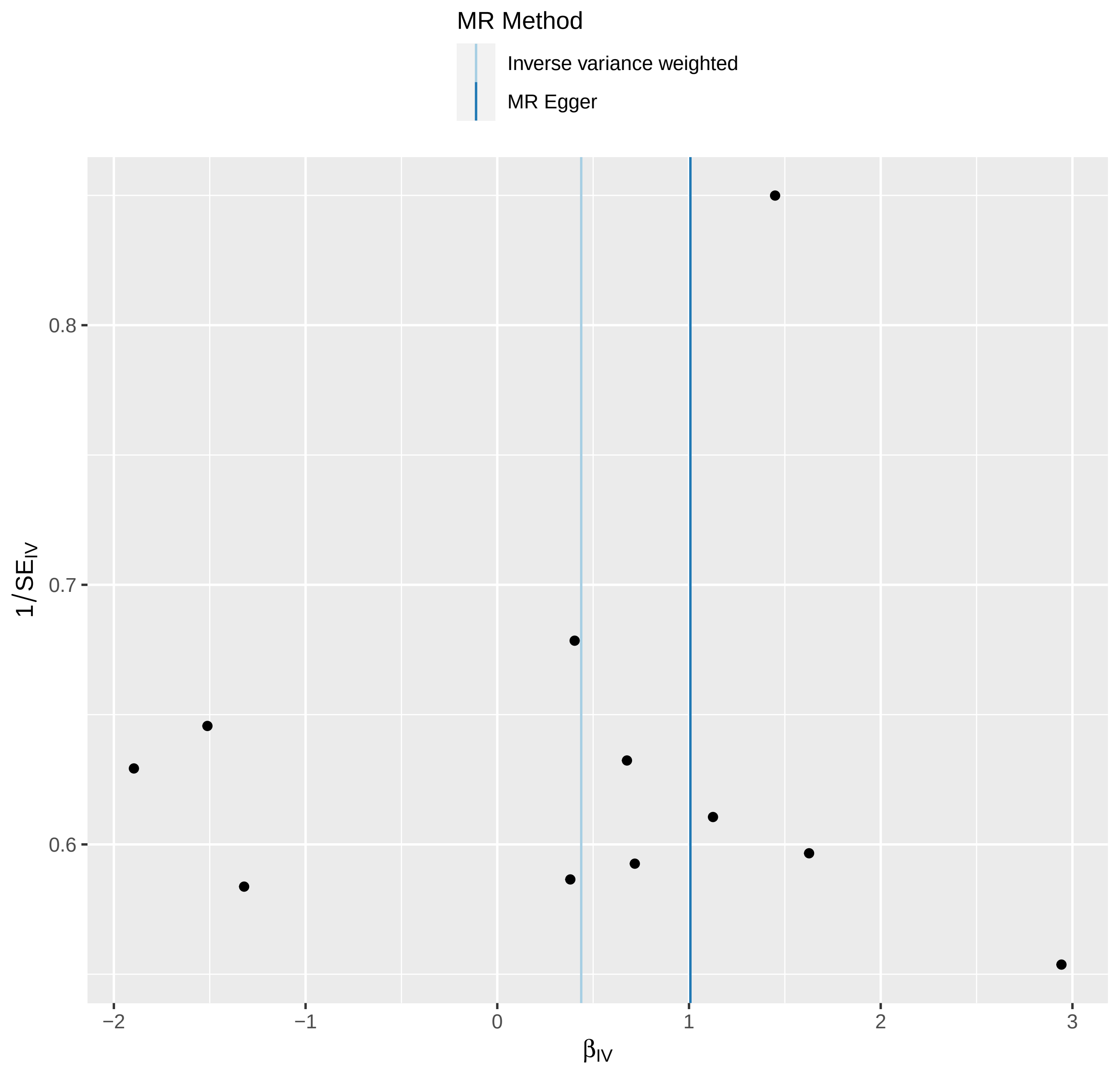

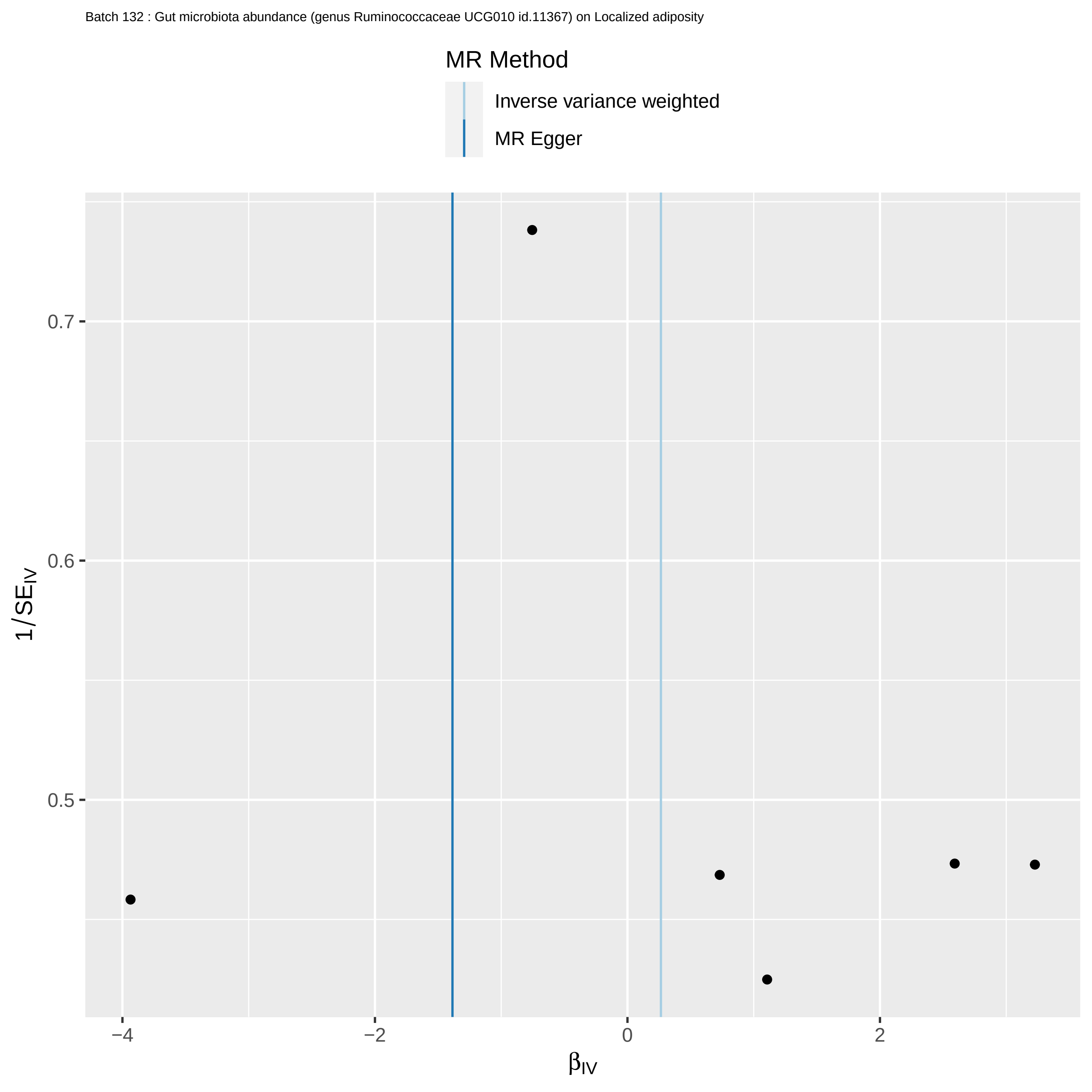

Batch 132 : Gut microbiota abundance (genus Ruminococcaceae UCG010 id.11367) on Localized adiposity
MR Method
Inverse variance weighted
MR Egger
0.7
0.6
0.5
1/SE$_{IV}$
−4
−2
0
2
$\beta_{IV}$

Batch 133 : Gut microbiota abundance (genus Ruminococcaceae UCG011 id.11368) on Localized adiposity

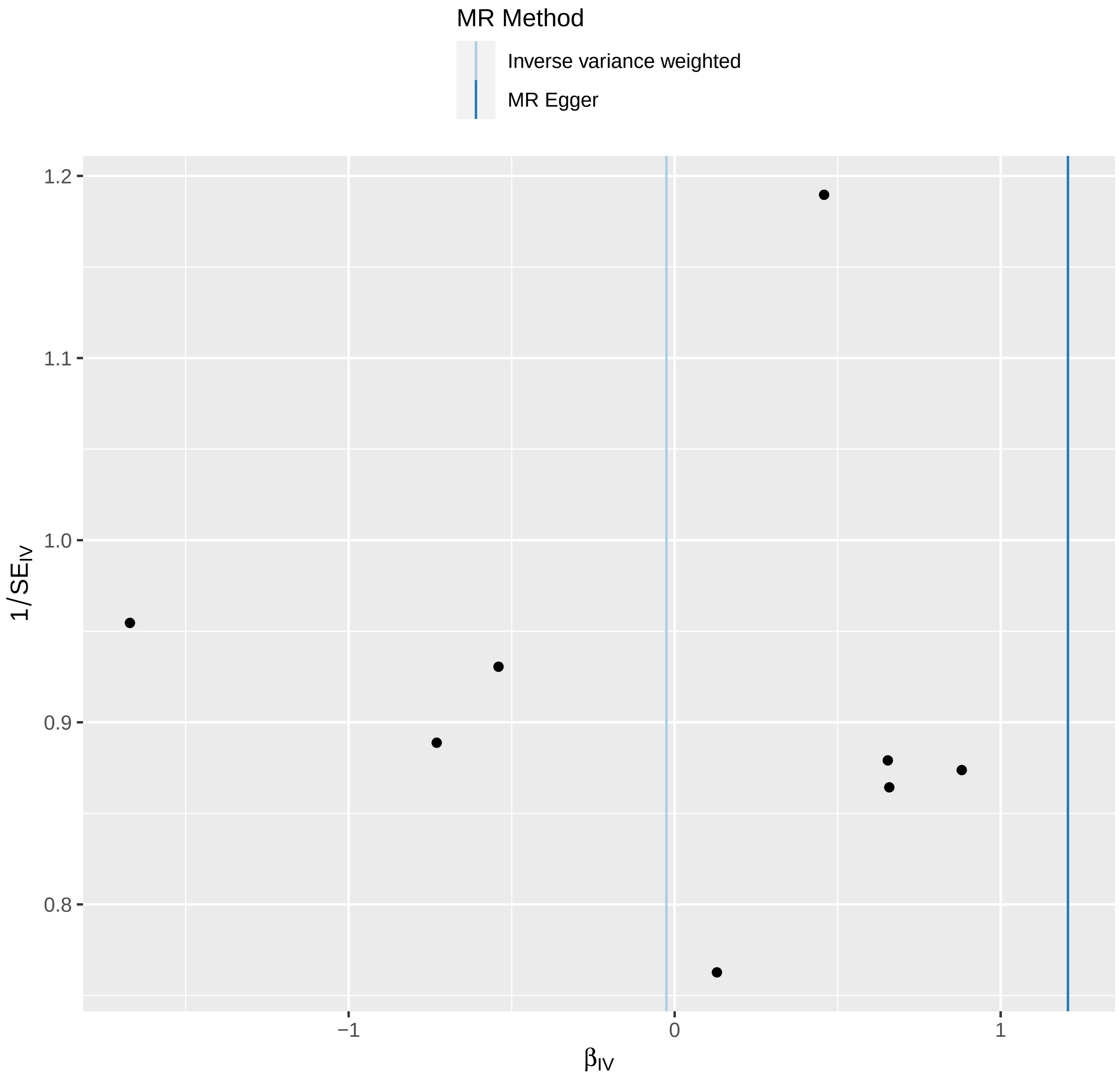

Batch 134 : Gut microbiota abundance (genus Ruminococcaceae UCG013 id.11370) on Localized adiposity

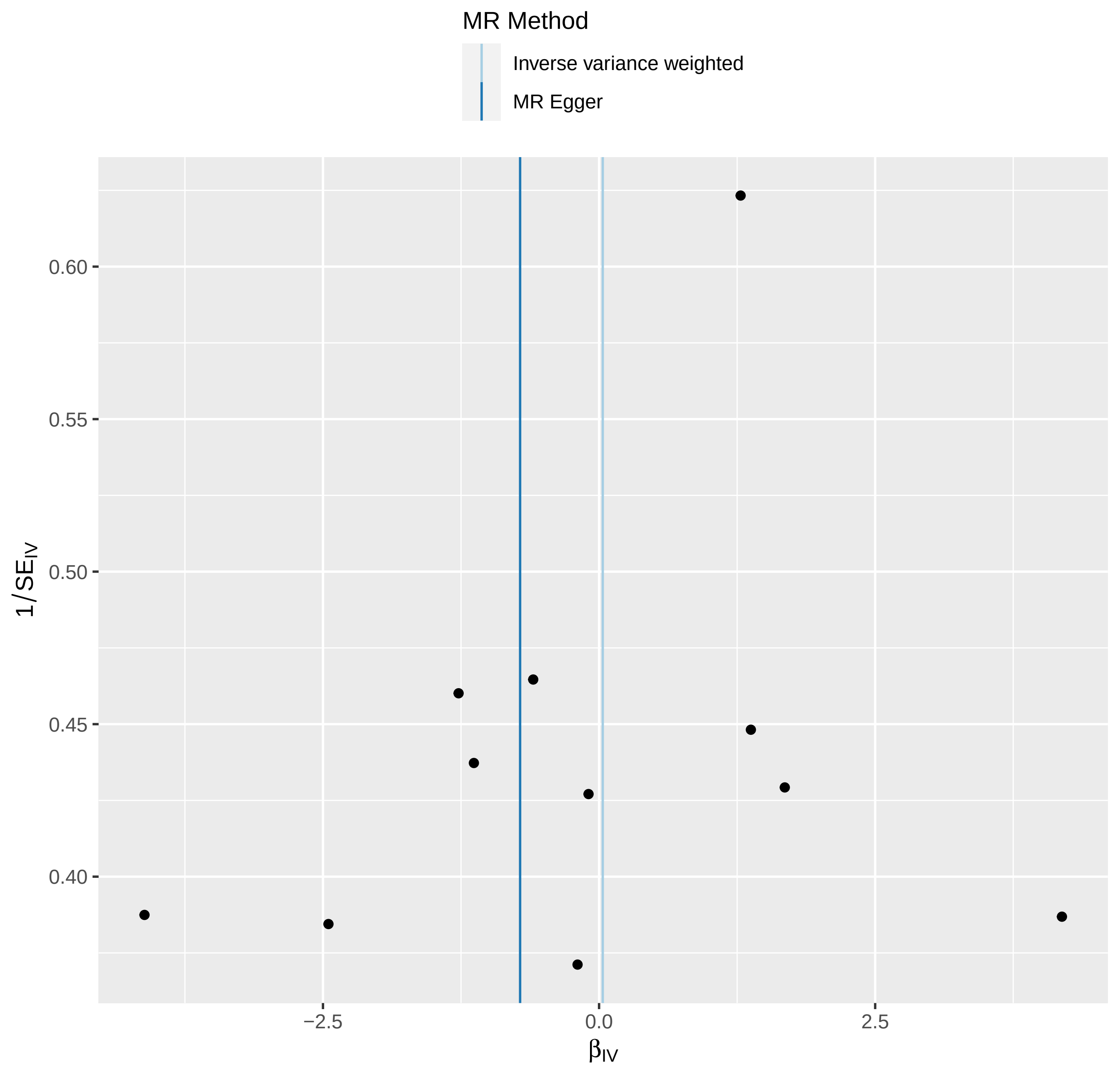

Batch 135 : Gut microbiota abundance (genus Ruminococcaceae UCG014 id.11371) on Localized adiposity

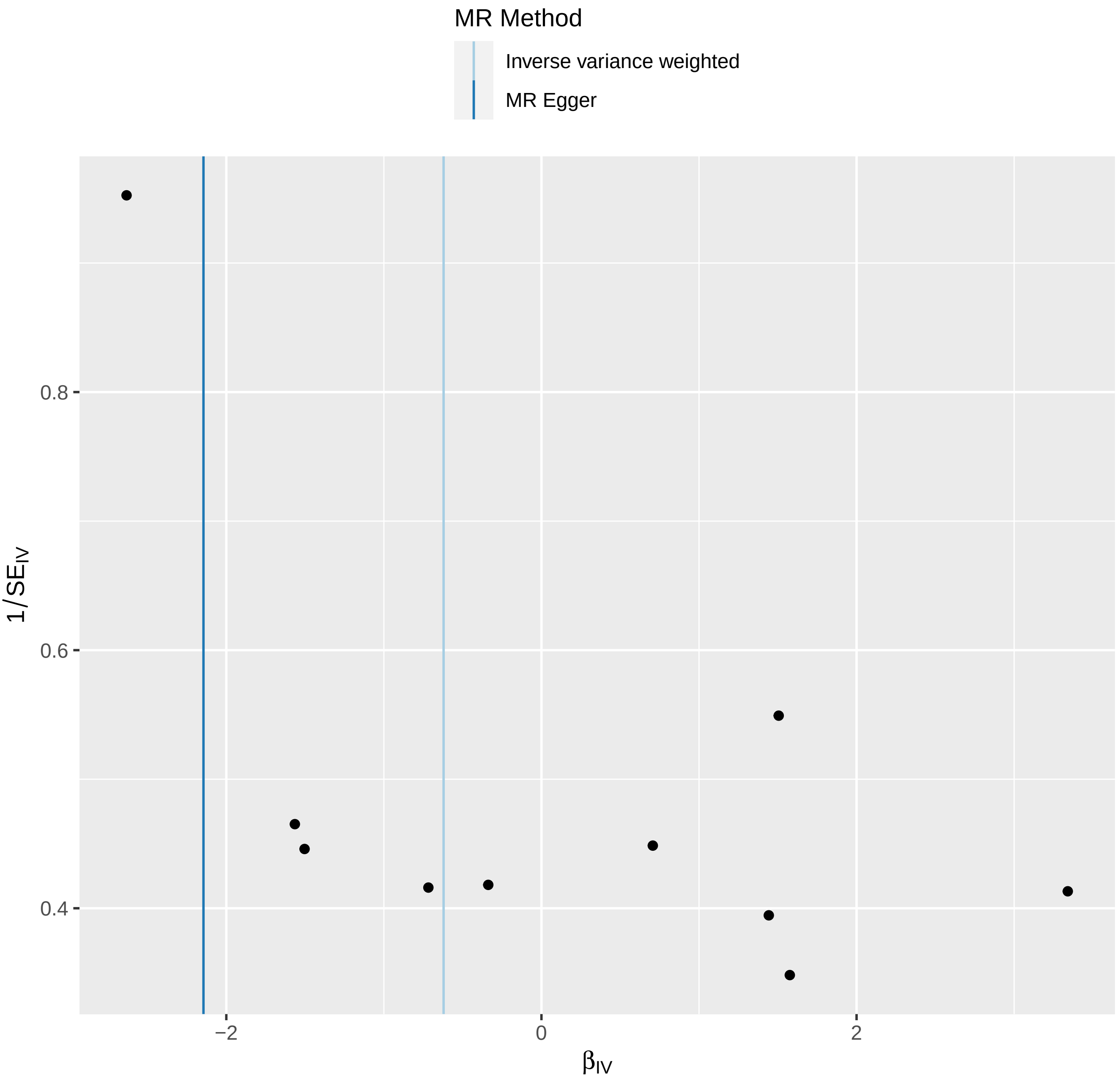

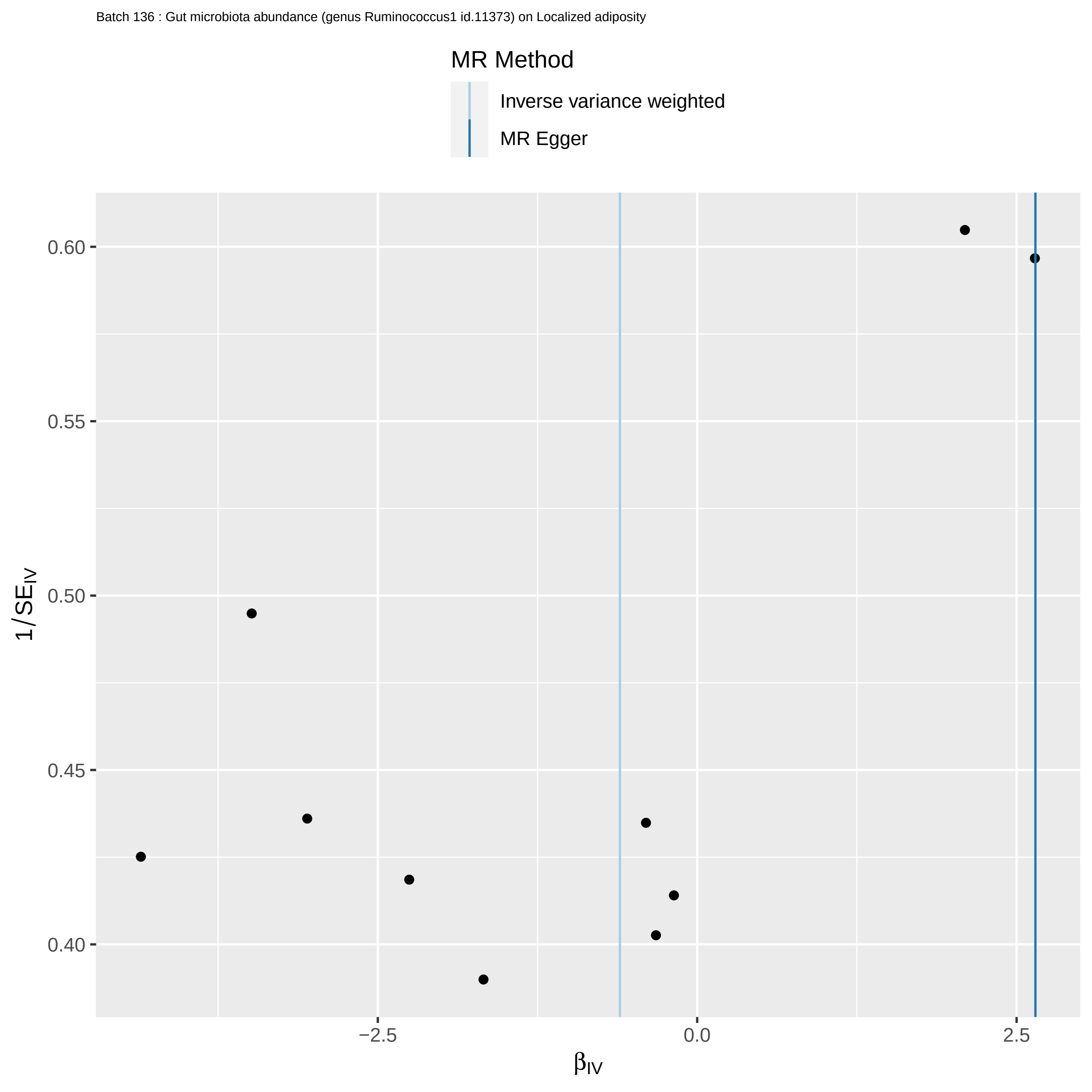

Batch 136 : Gut microbiota abundance (genus Ruminococcus1 id.11373) on Localized adiposity
MR Method
Inverse variance weighted
MR Egger
0.60
0.55
0.50
0.45
0.40
1/SE$_{IV}$
−2.5
0.0
2.5
β$_{IV}$

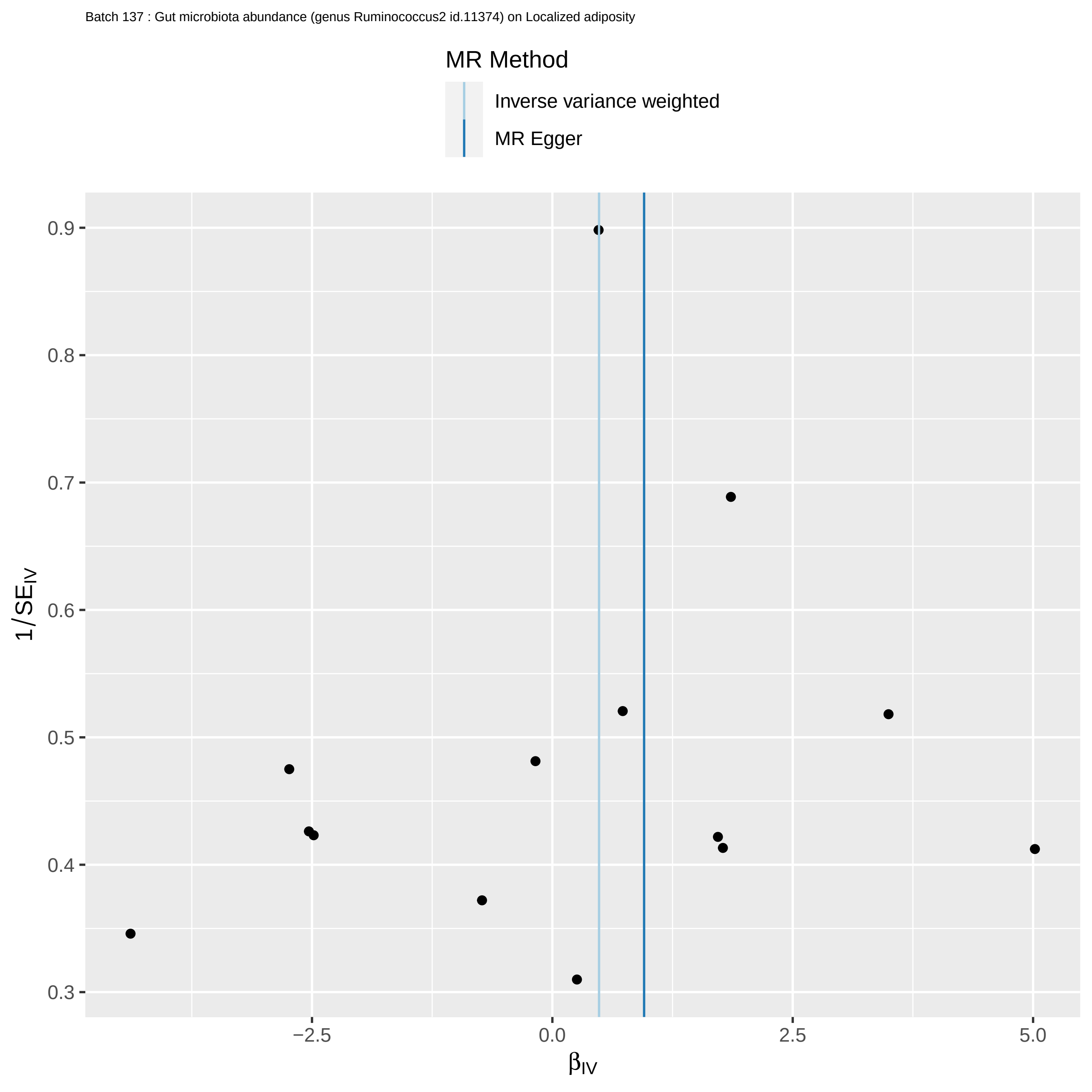
Batch 137 : Gut microbiota abundance (genus Ruminococcus2 id.11374) on Localized adiposity
MR Method
Inverse variance weighted
MR Egger
0.9
0.8
0.7
0.6
0.5
0.4
0.3
1/SE_IV
−2.5
0.0
2.5
5.0
β_IV

Batch 138 : Gut microbiota abundance (genus Ruminococcus gauvreauii group id.11342) on Localized adiposity

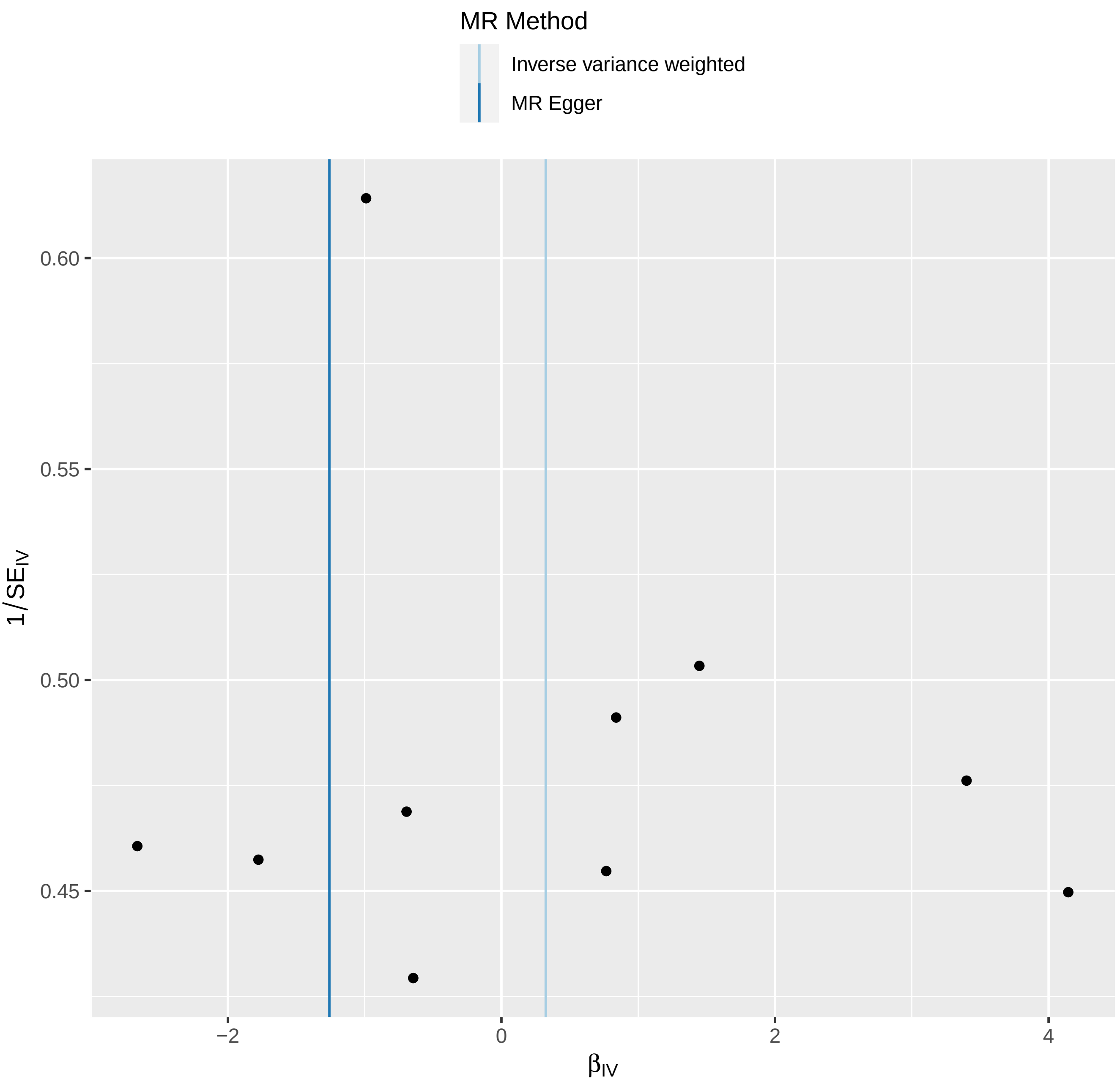

Batch 139 : Gut microbiota abundance (genus Ruminococcus gnavus group id.14376) on Localized adiposity

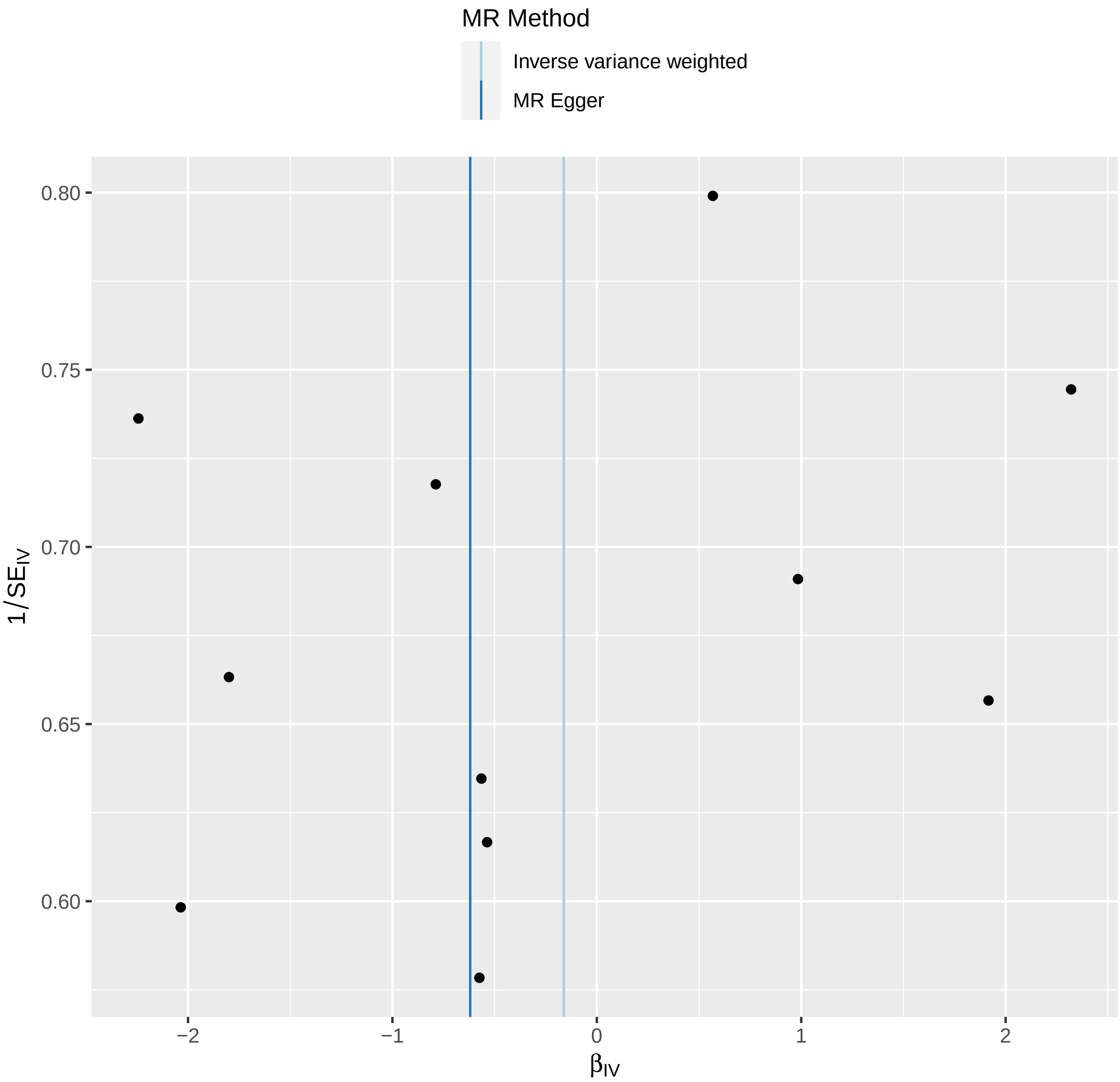

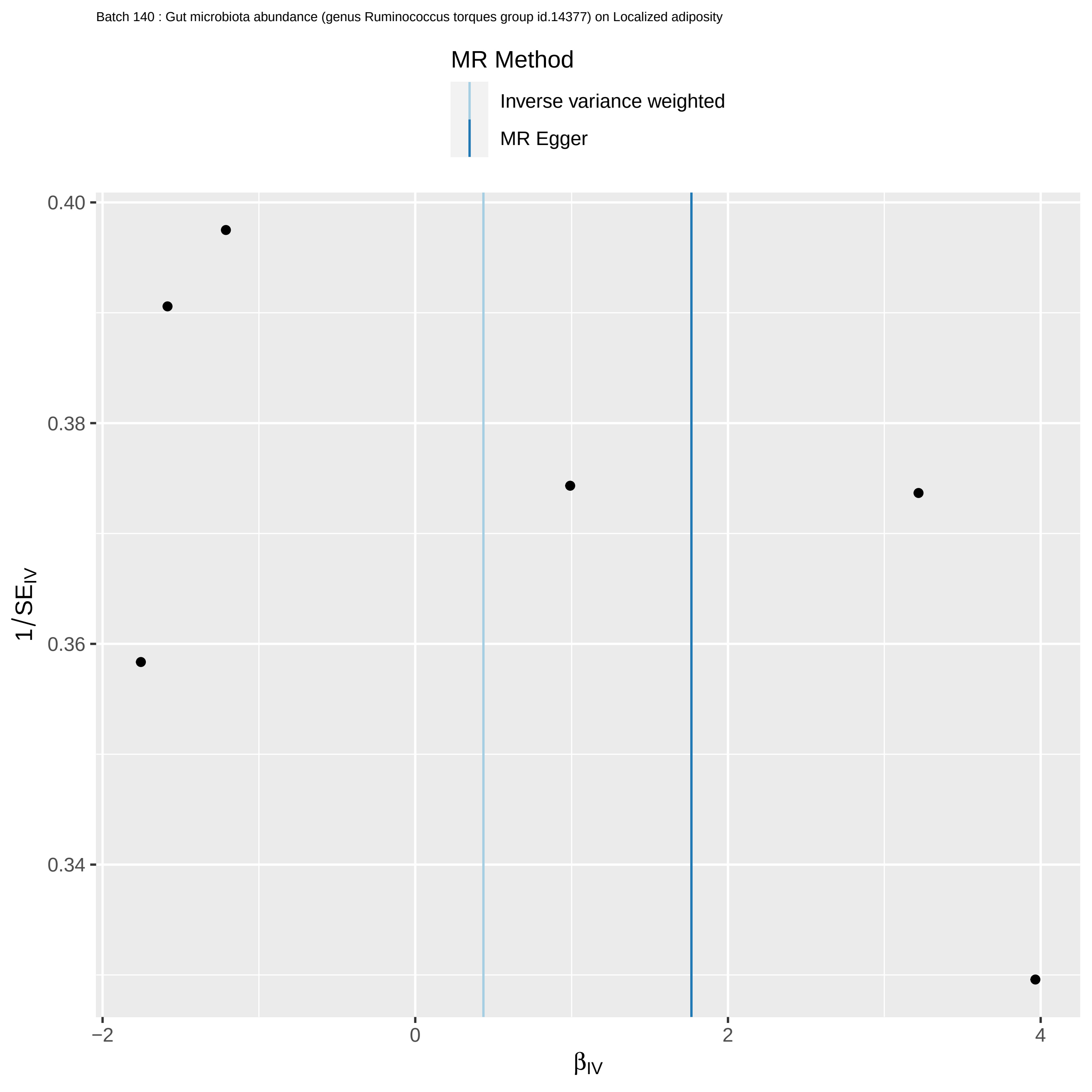
Batch 140 : Gut microbiota abundance (genus Ruminococcus torques group id.14377) on Localized adiposity
MR Method
Inverse variance weighted
MR Egger
0.40
0.38
0.36
0.34
1/SE_IV
−2
0
2
4
β_IV

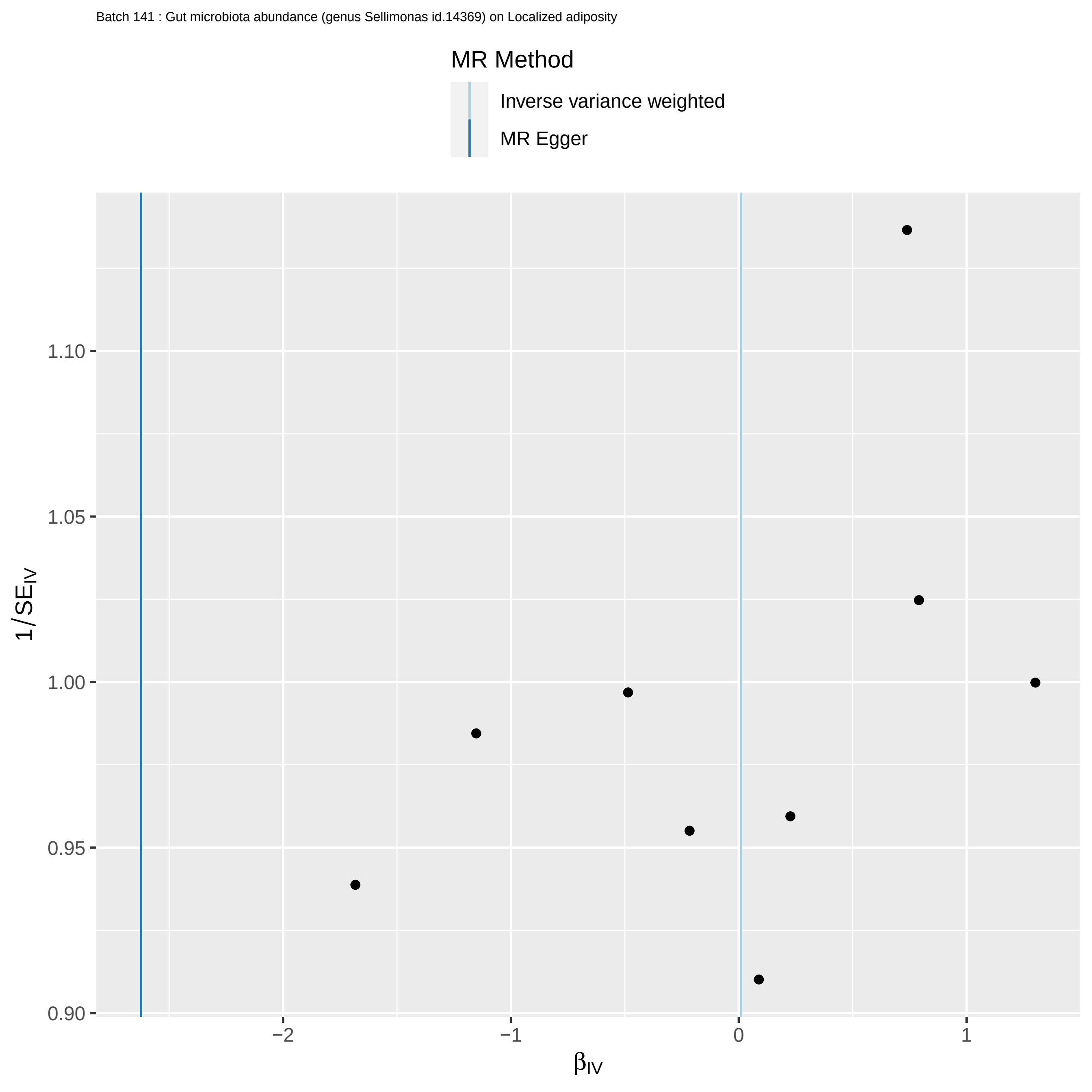

Batch 141 : Gut microbiota abundance (genus Sellimonas id.14369) on Localized adiposity
MR Method
Inverse variance weighted
MR Egger
1.10
1.05
1.00
0.95
0.90
1/SE_IV
−2
−1
0
1
β_IV

Batch 142 : Gut microbiota abundance (genus Senegalimassilia id.11160) on Localized adiposity

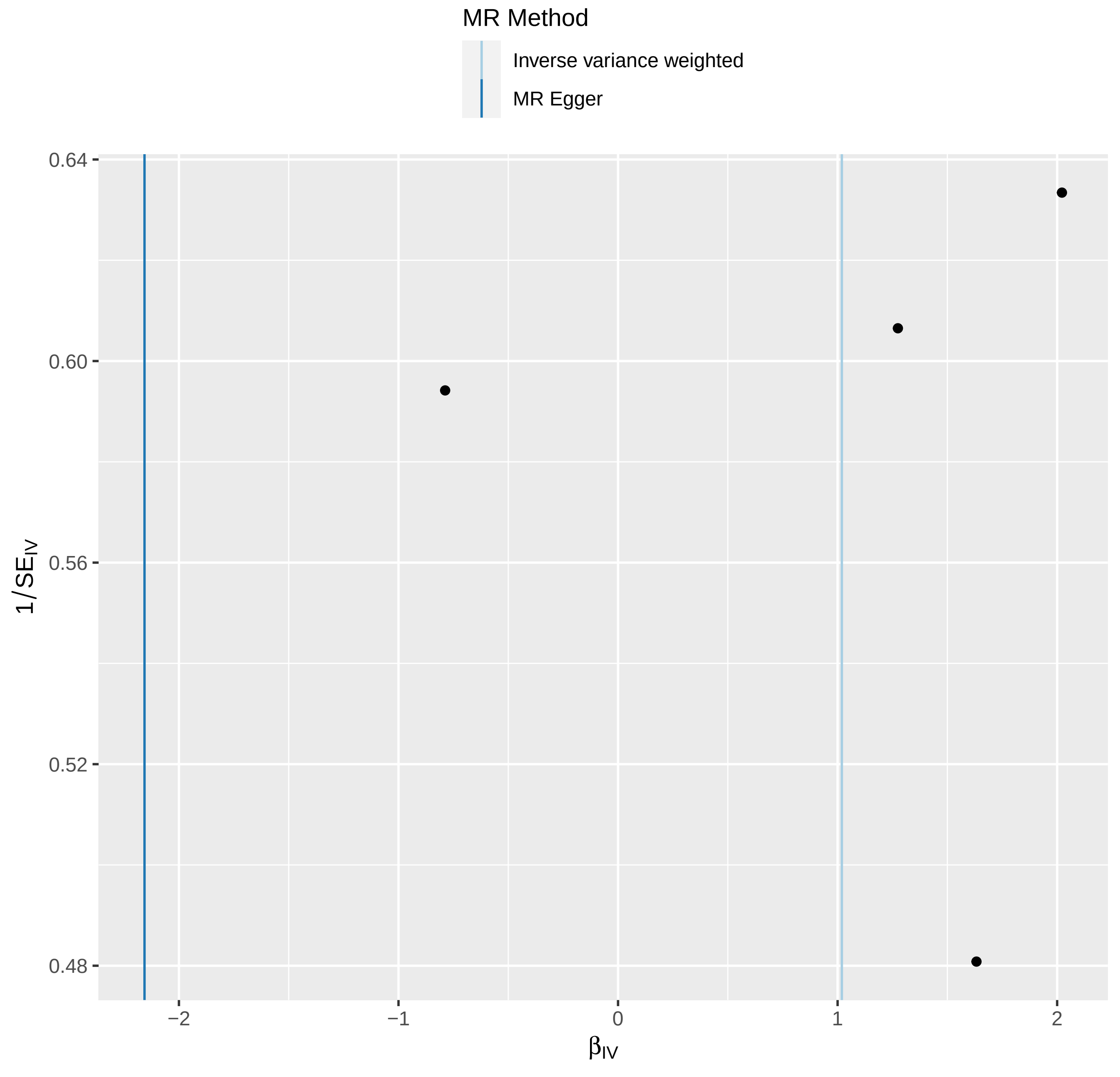

Batch 143 : Gut microbiota abundance (genus Slackia id.825) on Localized adiposity

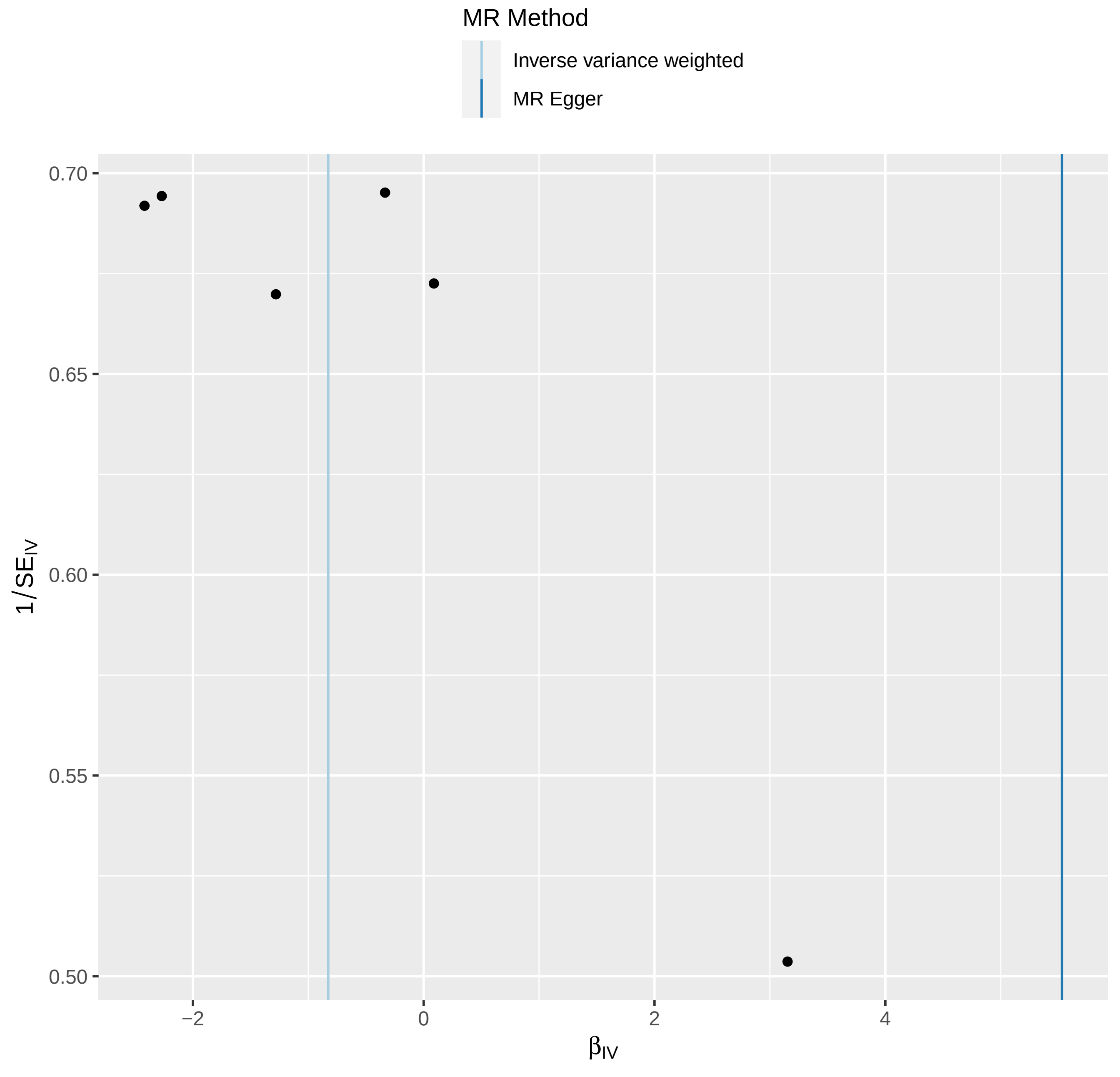

Batch 144 : Gut microbiota abundance (genus Streptococcus id.1853) on Localized adiposity

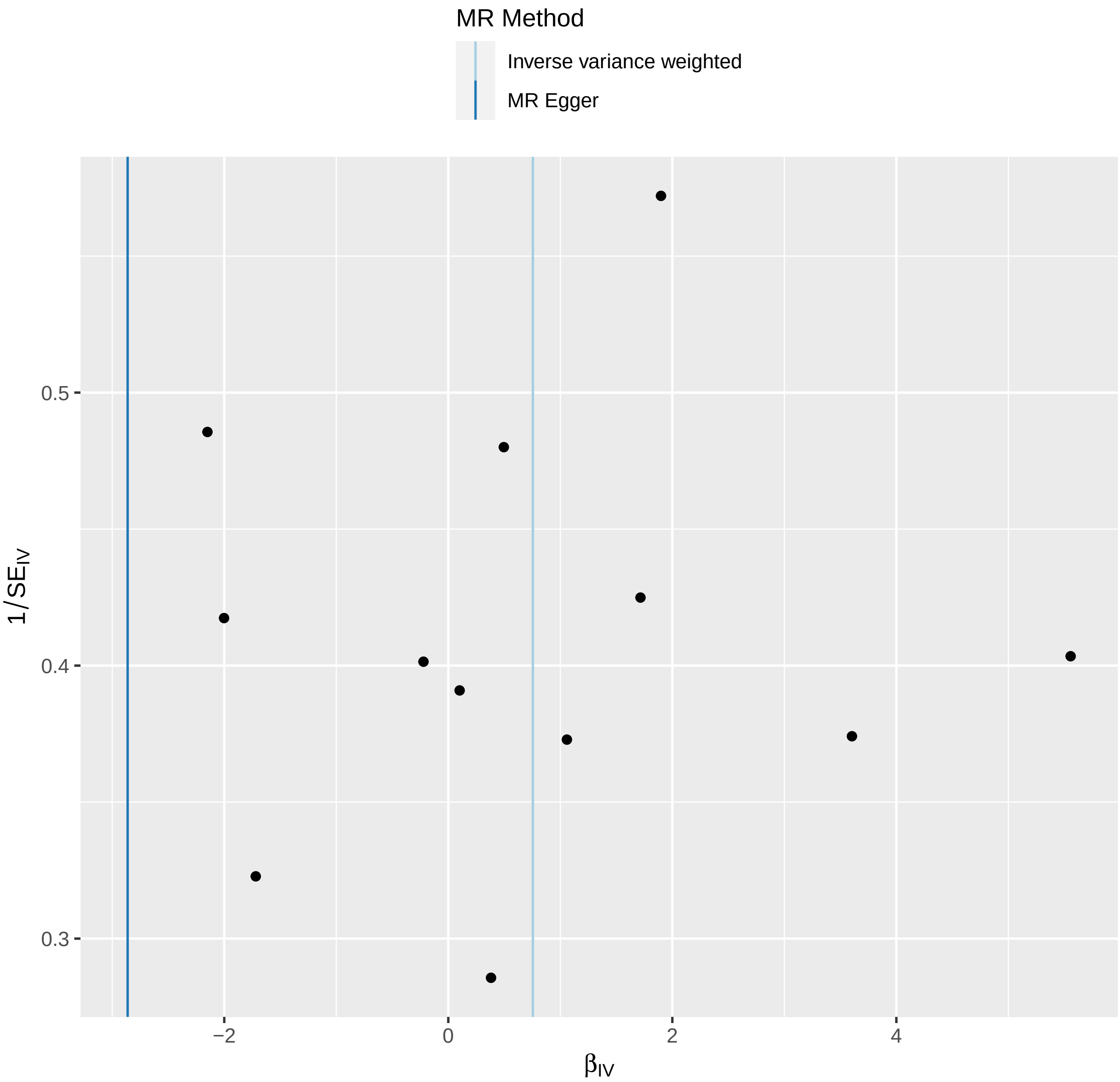

Batch 145 : Gut microbiota abundance (genus Subdoligranulum id.2070) on Localized adiposity

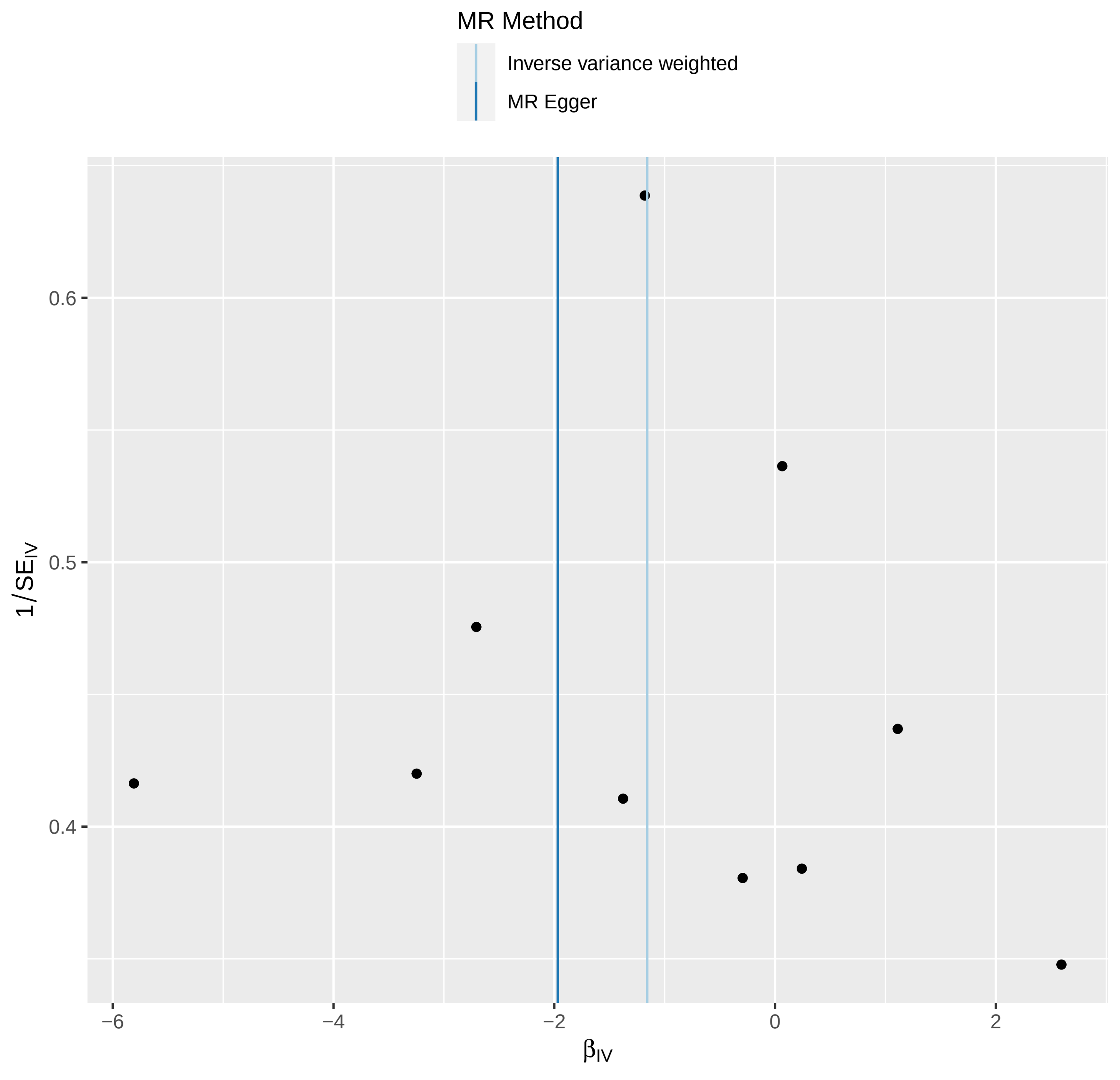

Batch 146 : Gut microbiota abundance (genus Sutterella id.2896) on Localized adiposity

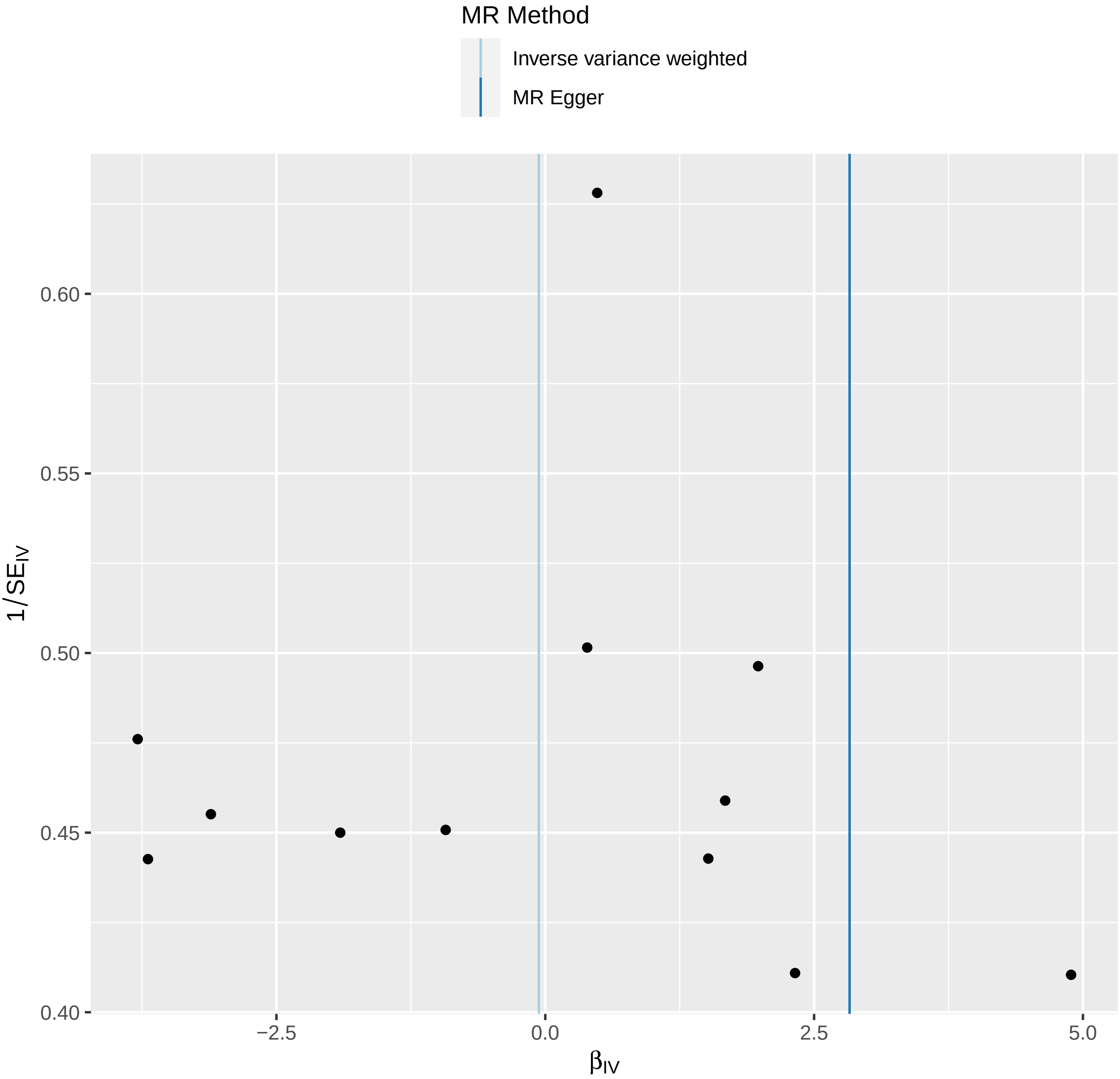

Batch 147 : Gut microbiota abundance (genus Terrisporobacter id.11348) on Localized adiposity

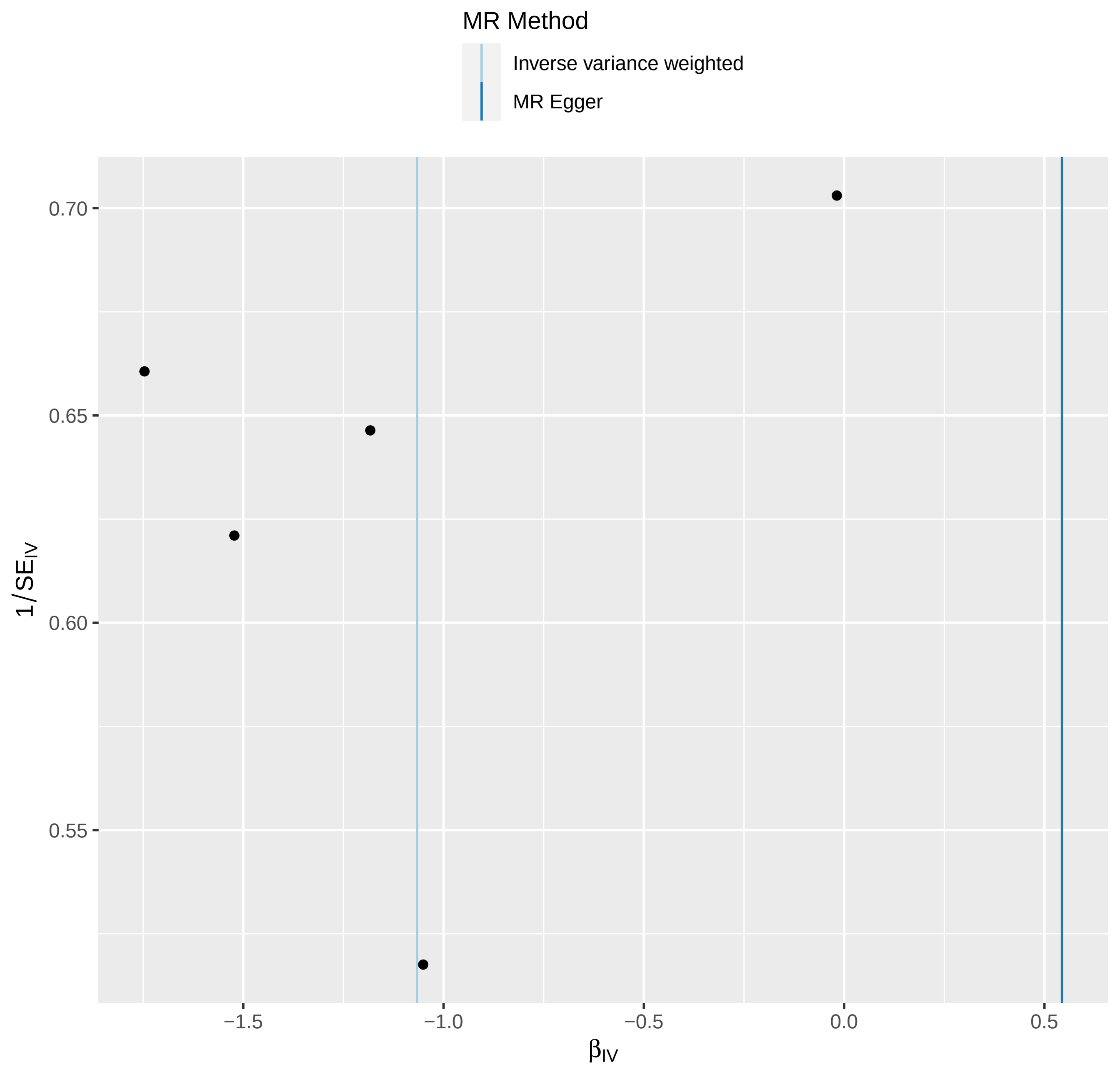

Batch 148 : Gut microbiota abundance (genus Turicibacter id.2162) on Localized adiposity

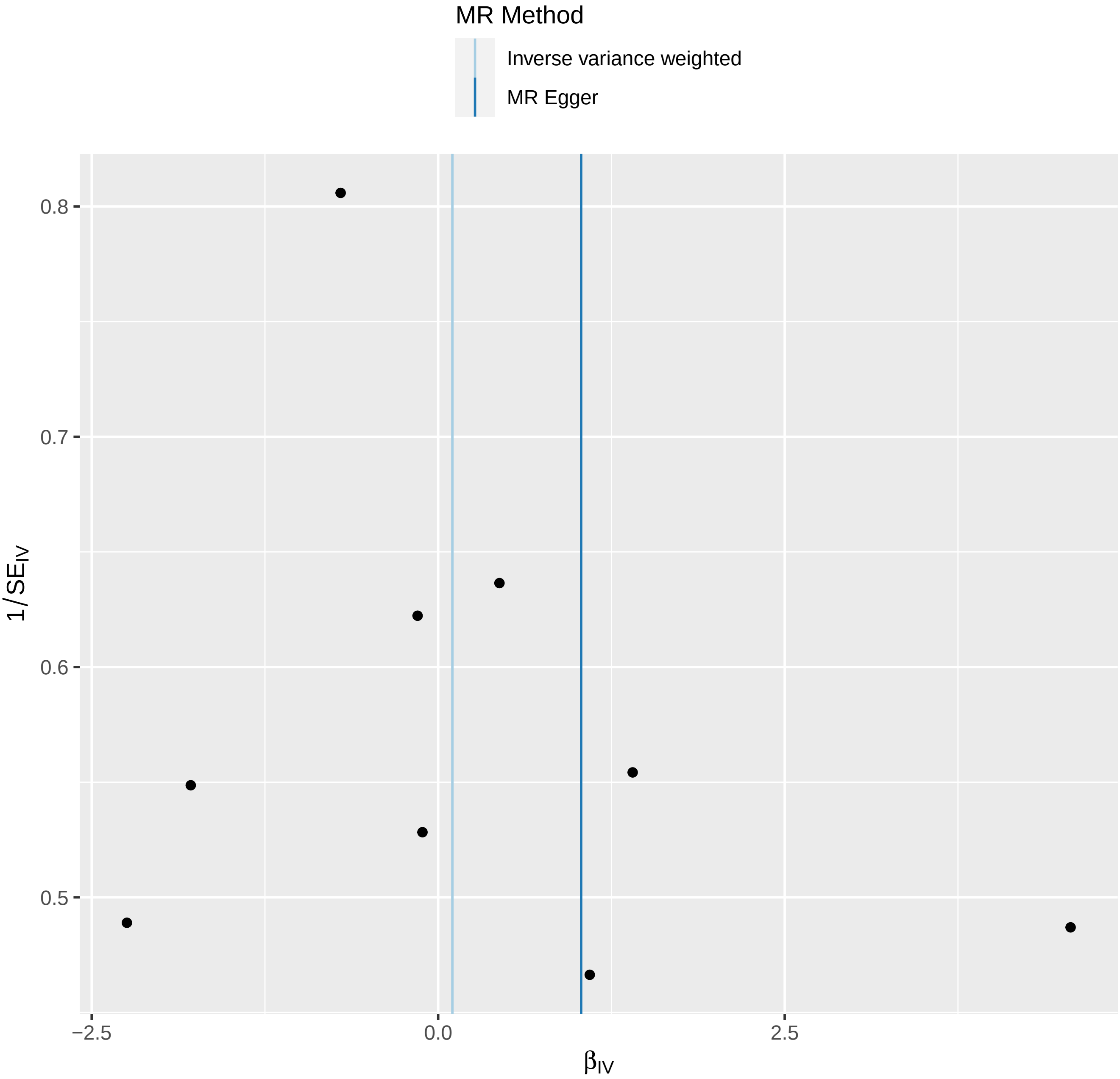

Batch 149 : Gut microbiota abundance (genus Tyzzerella3 id.11335) on Localized adiposity

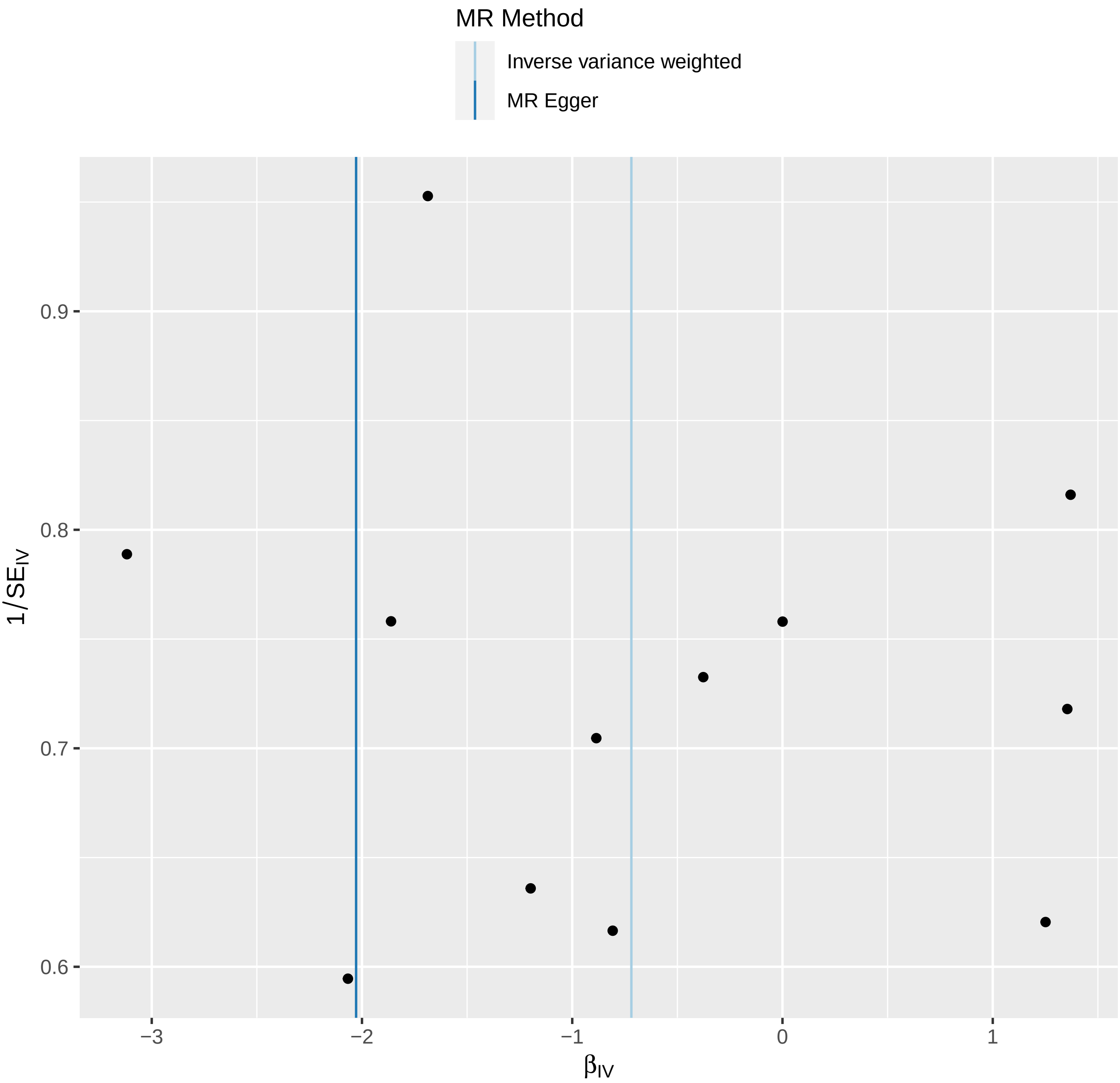

Batch 150 : Gut microbiota abundance (genus Veillonella id.2198) on Localized adiposity

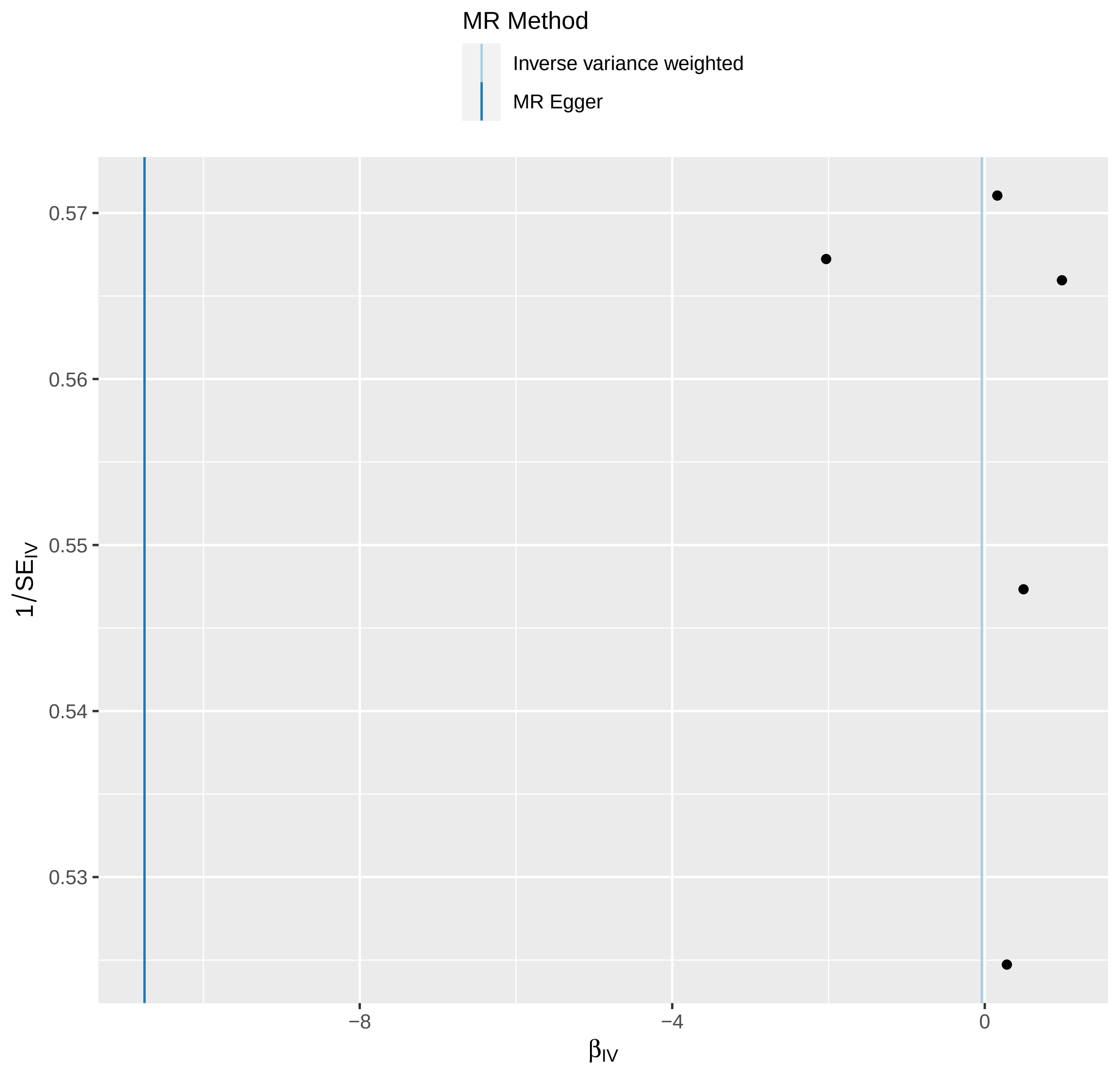

Batch 301 : Gut microbiota abundance (family Acidaminococcaceae id.2166) on Obesity due to excess calories

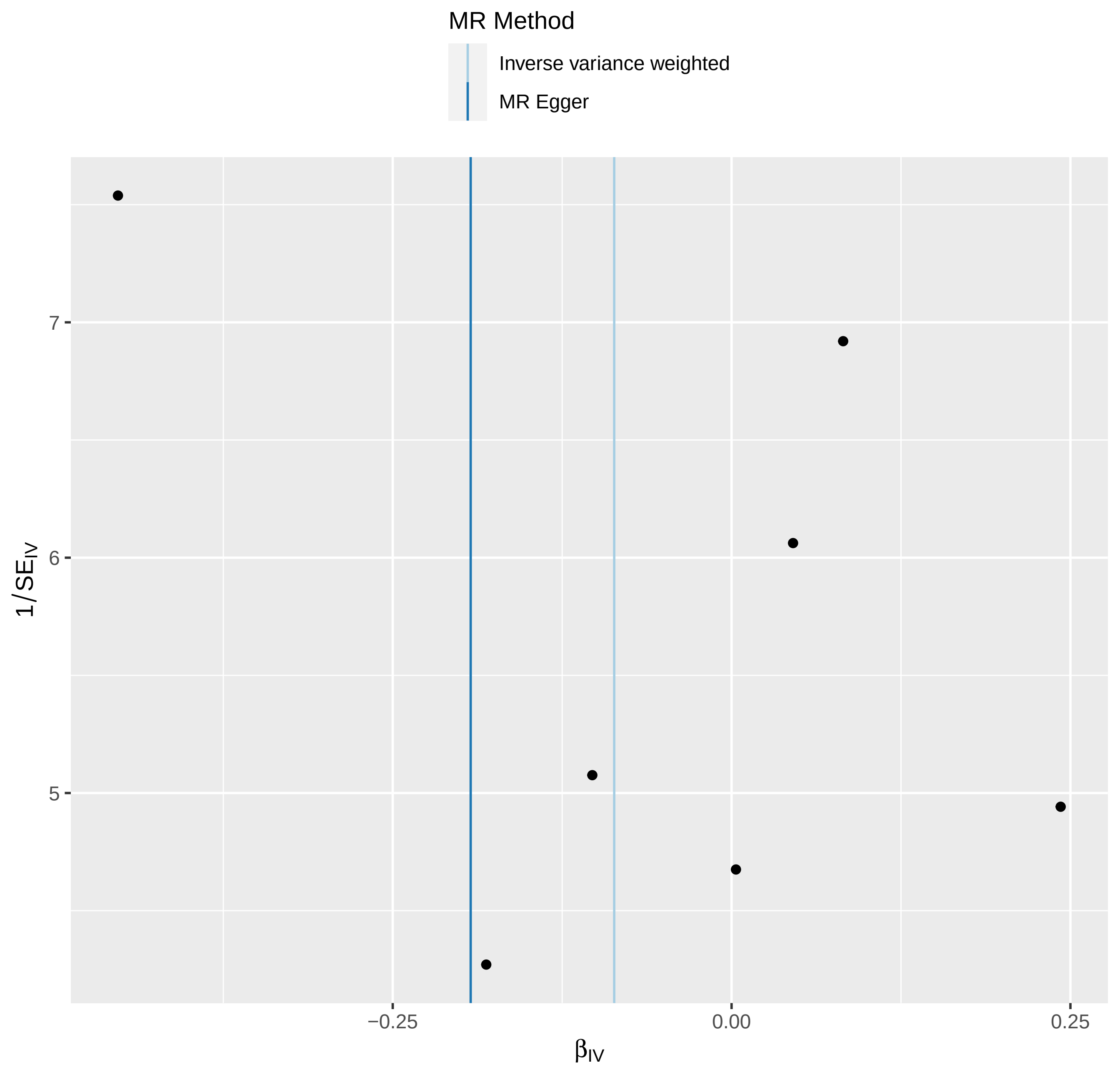

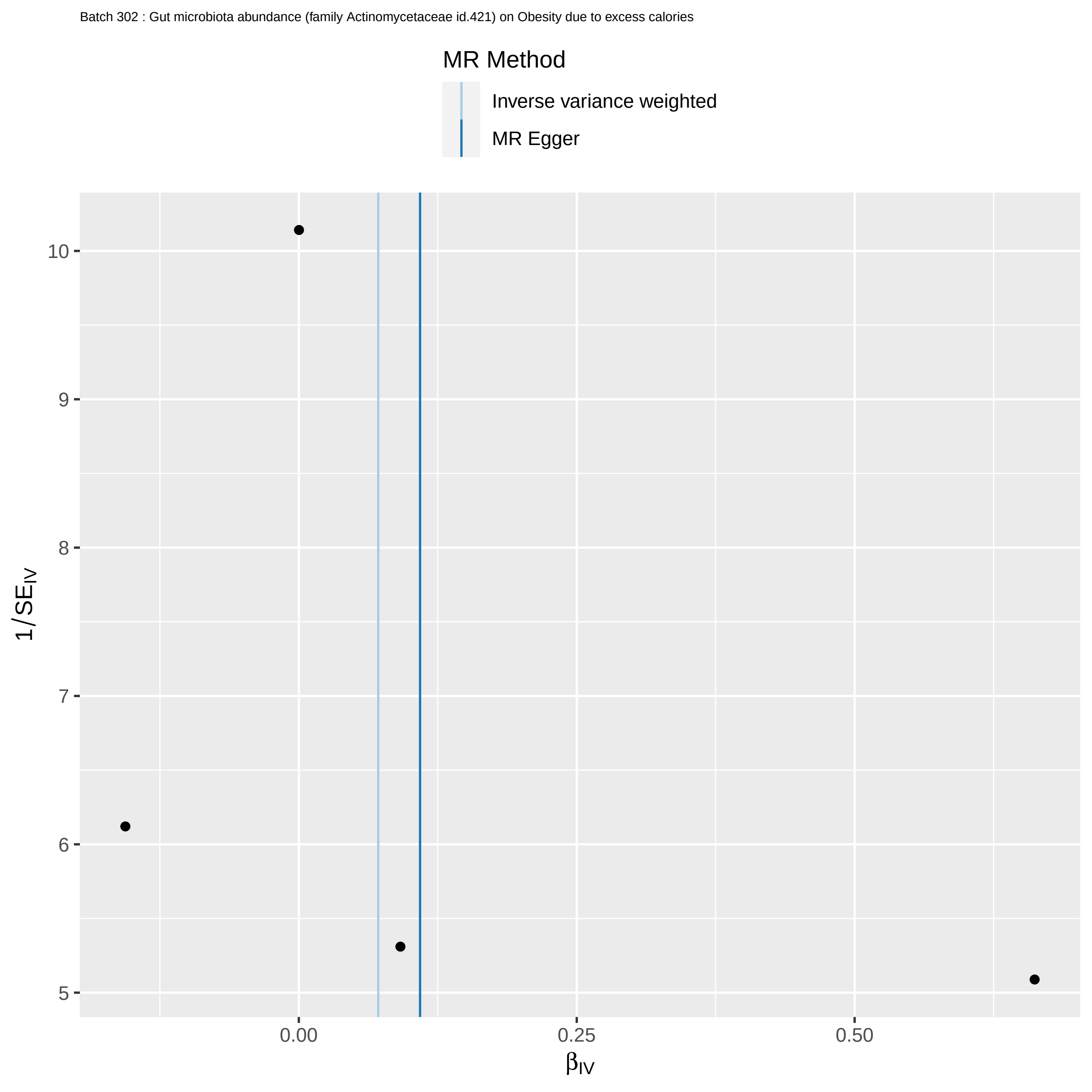
Batch 302 : Gut microbiota abundance (family Actinomycetaceae id.421) on Obesity due to excess calories
MR Method
Inverse variance weighted
MR Egger
1/SE_IV
β_IV
10
9
8
7
6
5
0.00
0.25
0.50

Batch 303 : Gut microbiota abundance (family Alcaligenaceae id.2875) on Obesity due to excess calories

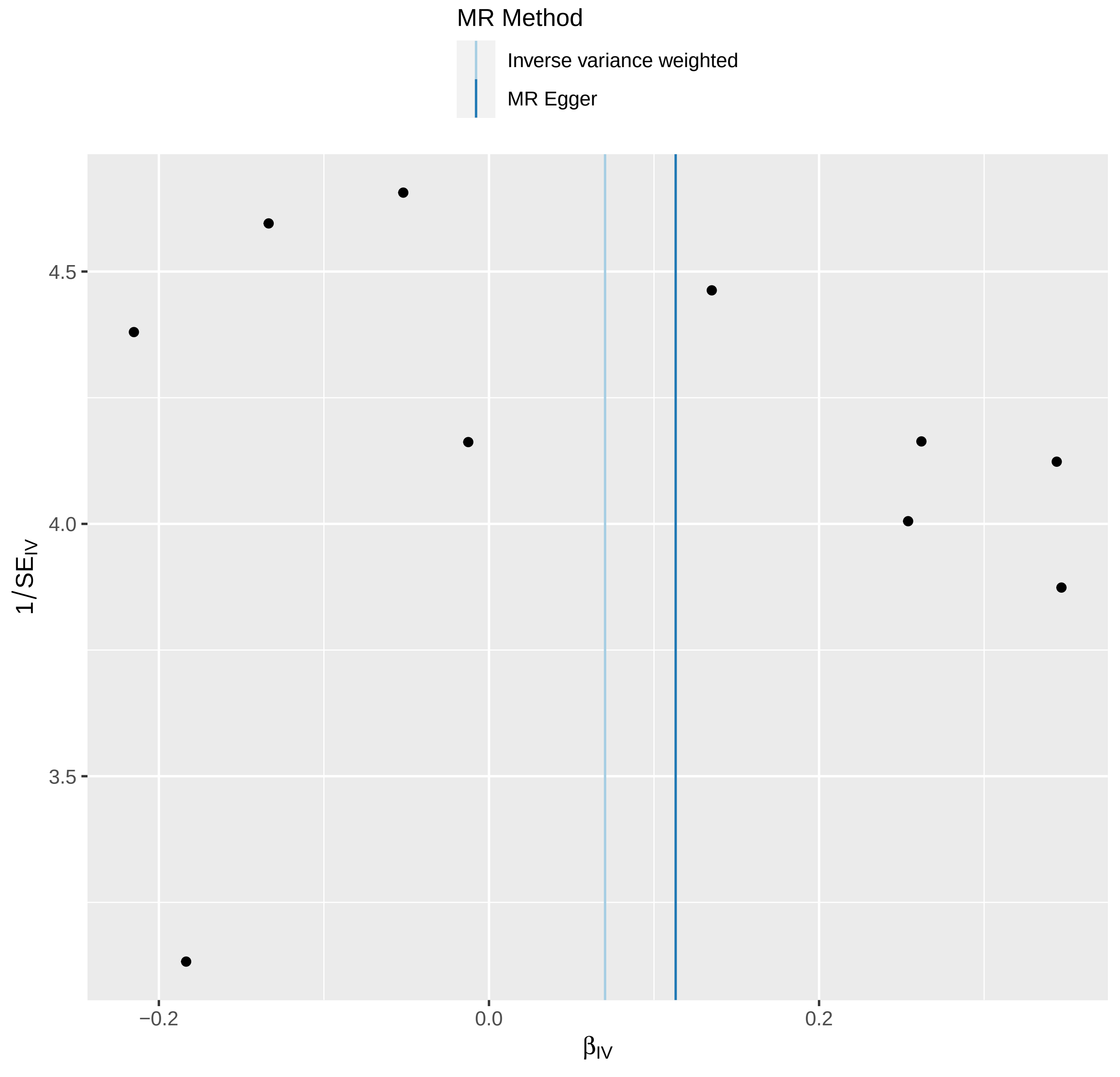

Batch 304 : Gut microbiota abundance (family Bacteroidaceae id.917) on Obesity due to excess calories

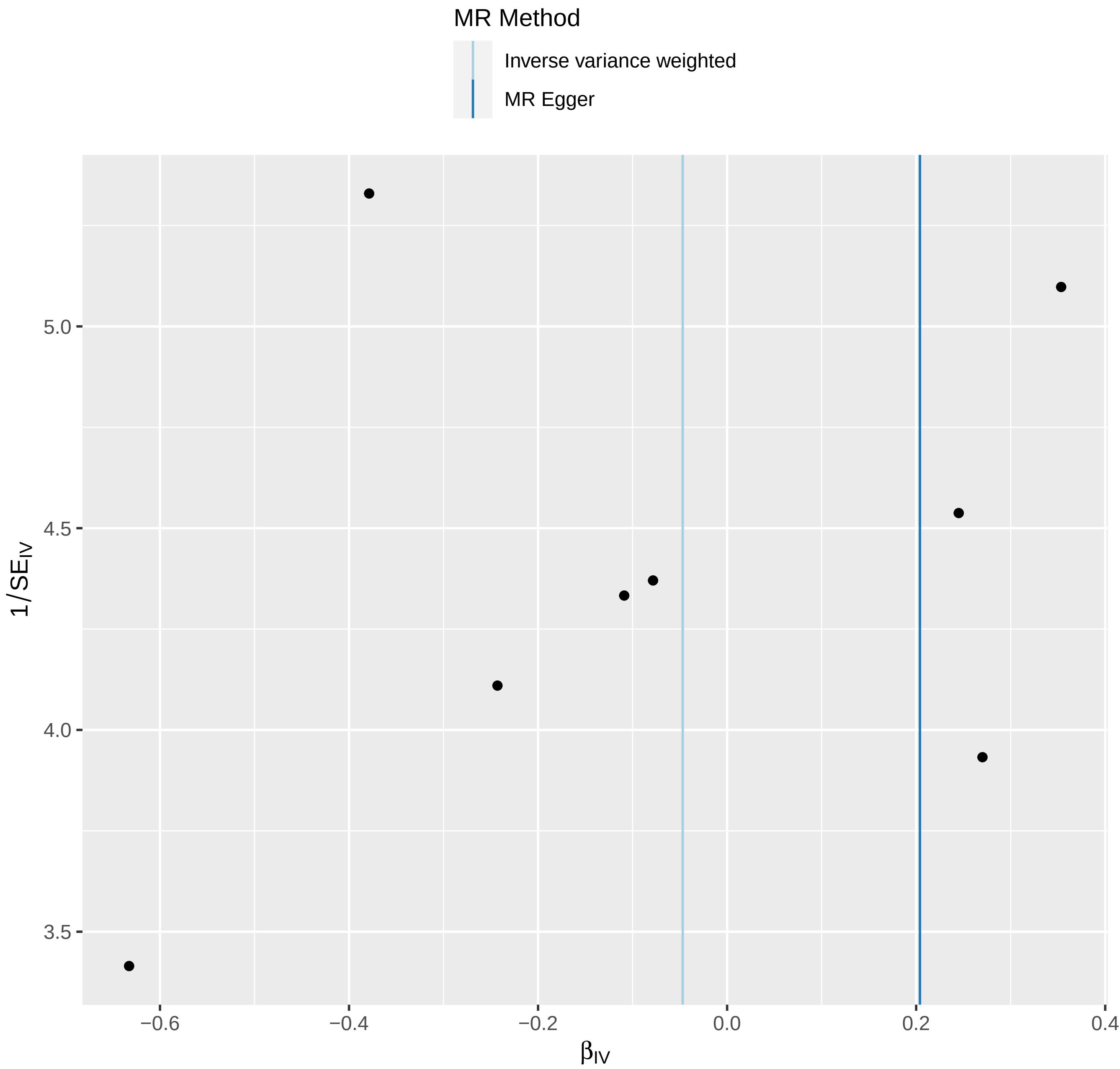

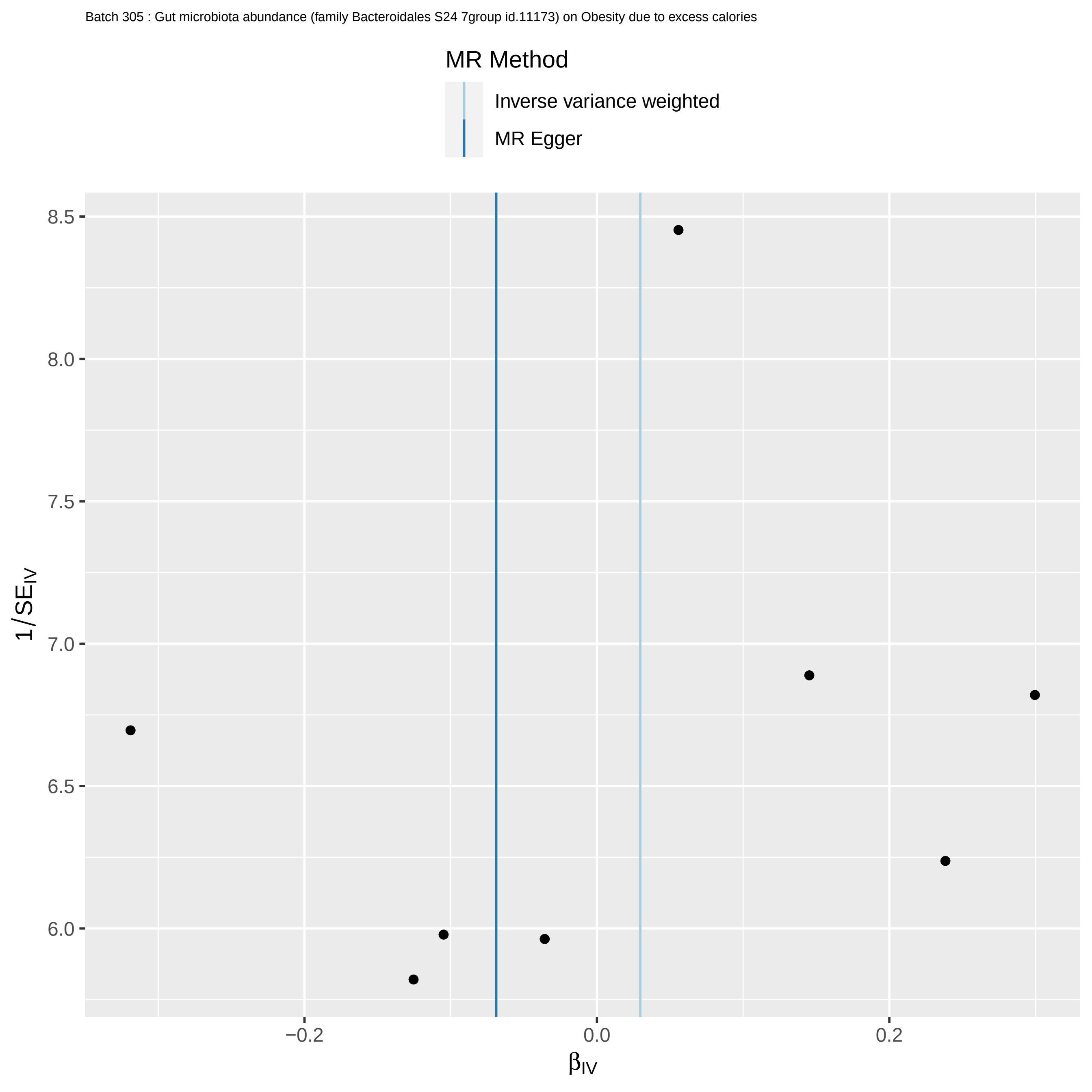

Batch 305 : Gut microbiota abundance (family Bacteroidales S24 7group id.11173) on Obesity due to excess calories
MR Method
Inverse variance weighted
MR Egger
8.5
8.0
7.5
7.0
6.5
6.0
1/SE_IV
−0.2
0.0
0.2
β_IV

Batch 306 : Gut microbiota abundance (family Bifidobacteriaceae id.433) on Obesity due to excess calories

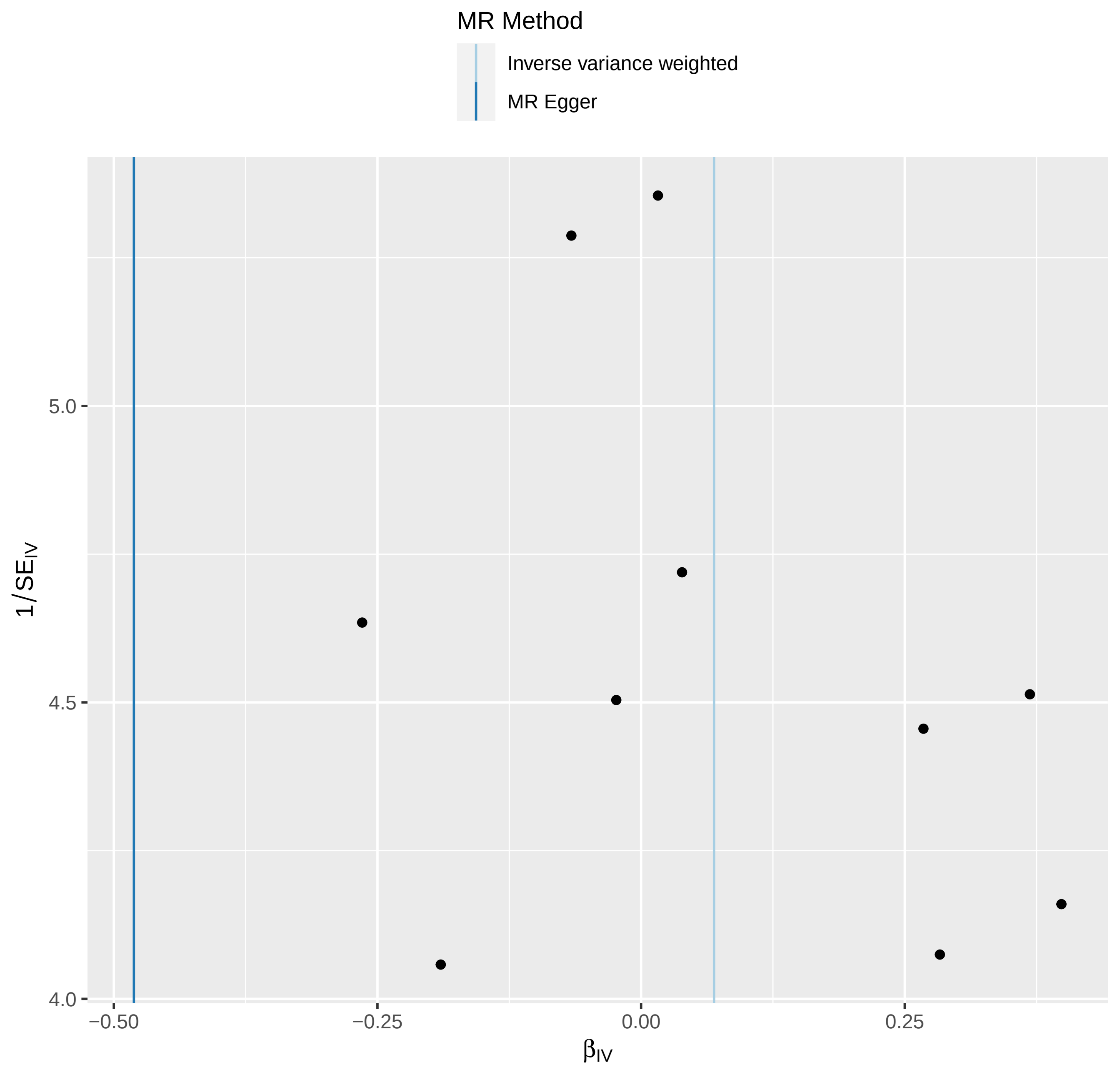

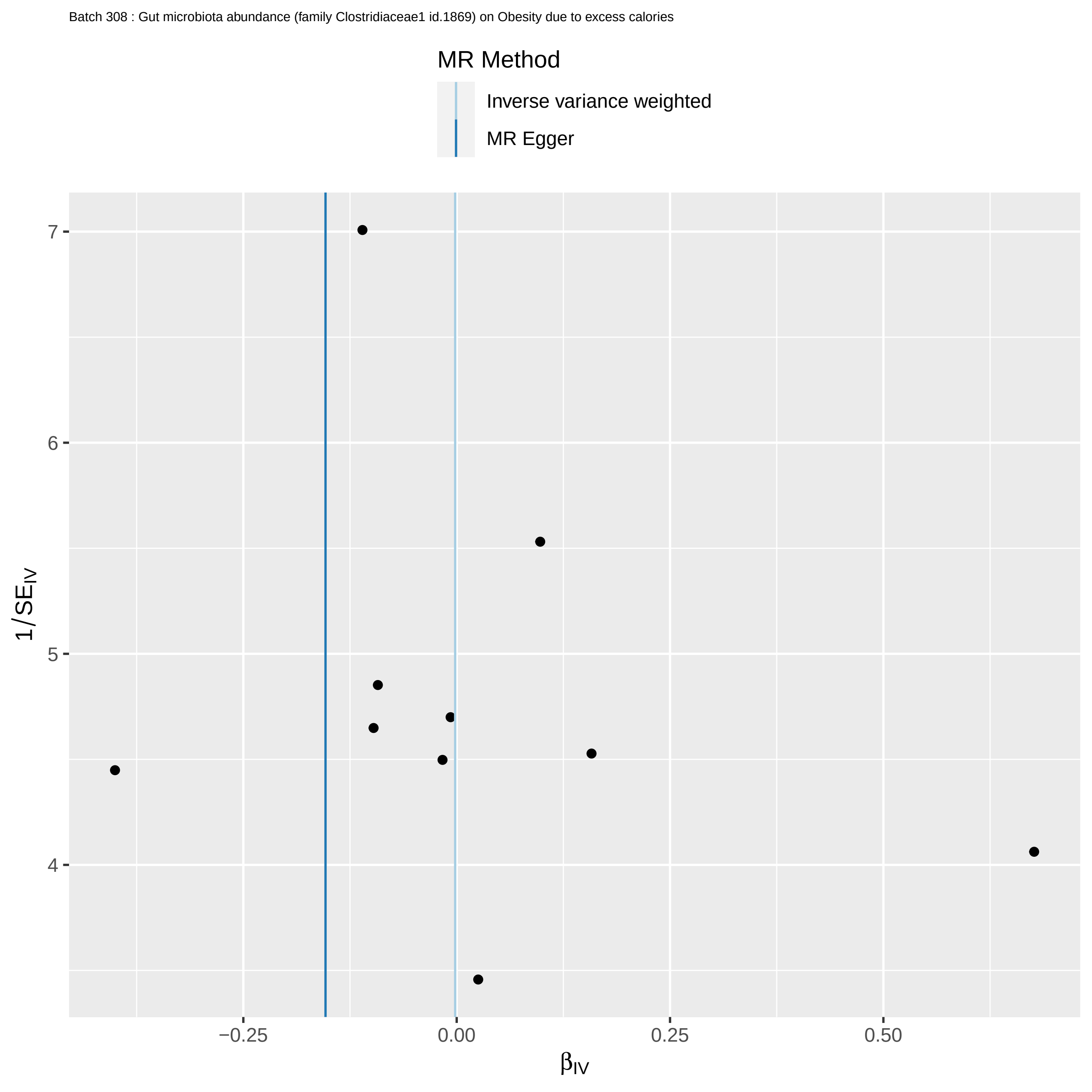

Batch 308 : Gut microbiota abundance (family Clostridiaceae1 id.1869) on Obesity due to excess calories
MR Method
Inverse variance weighted
MR Egger
7
6
5
4
1/SE_IV
−0.25
0.00
0.25
0.50
β_IV

Batch 309 : Gut microbiota abundance (family Clostridiales vadin BB60 group id.11286) on Obesity due to excess calories

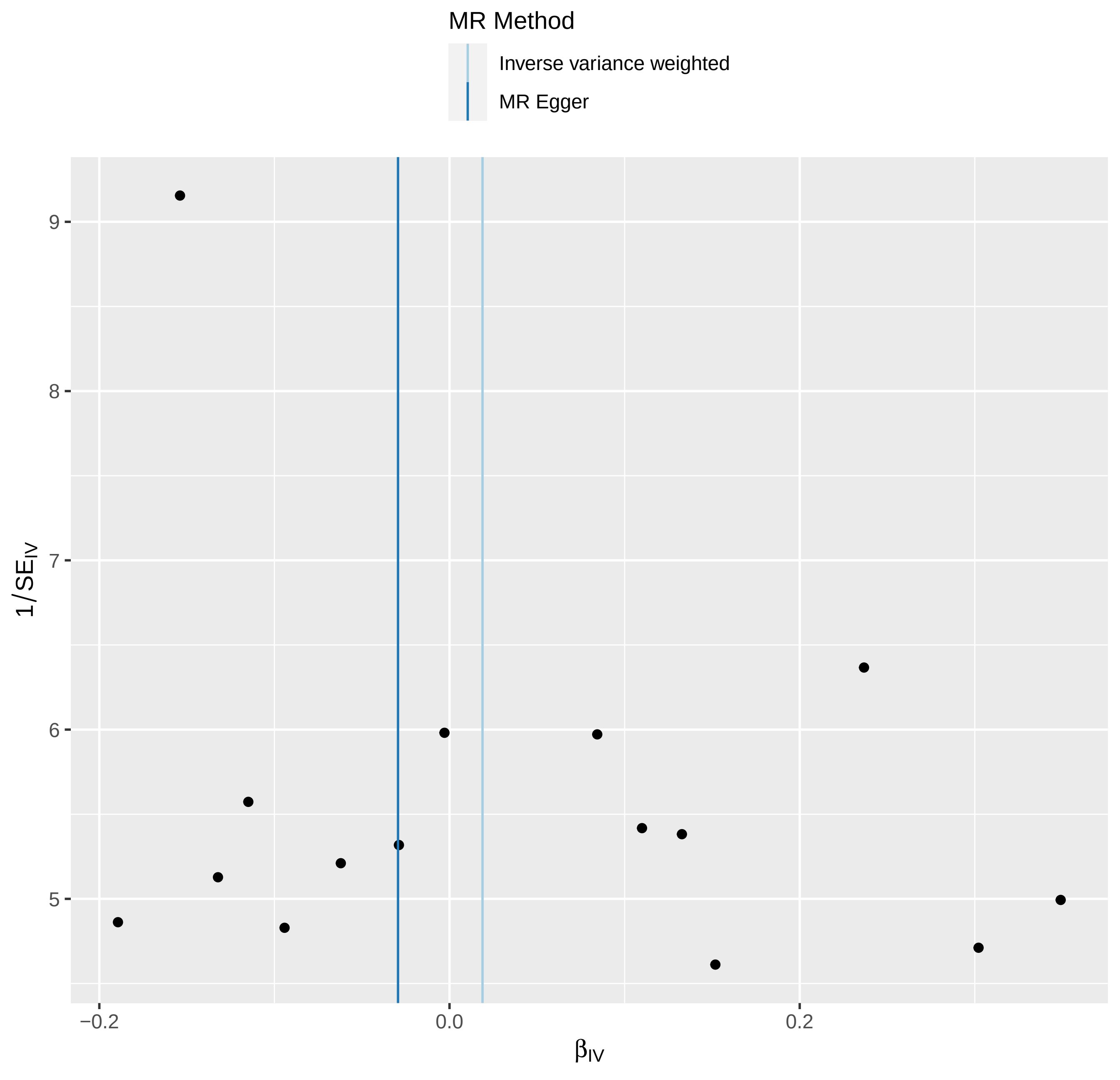

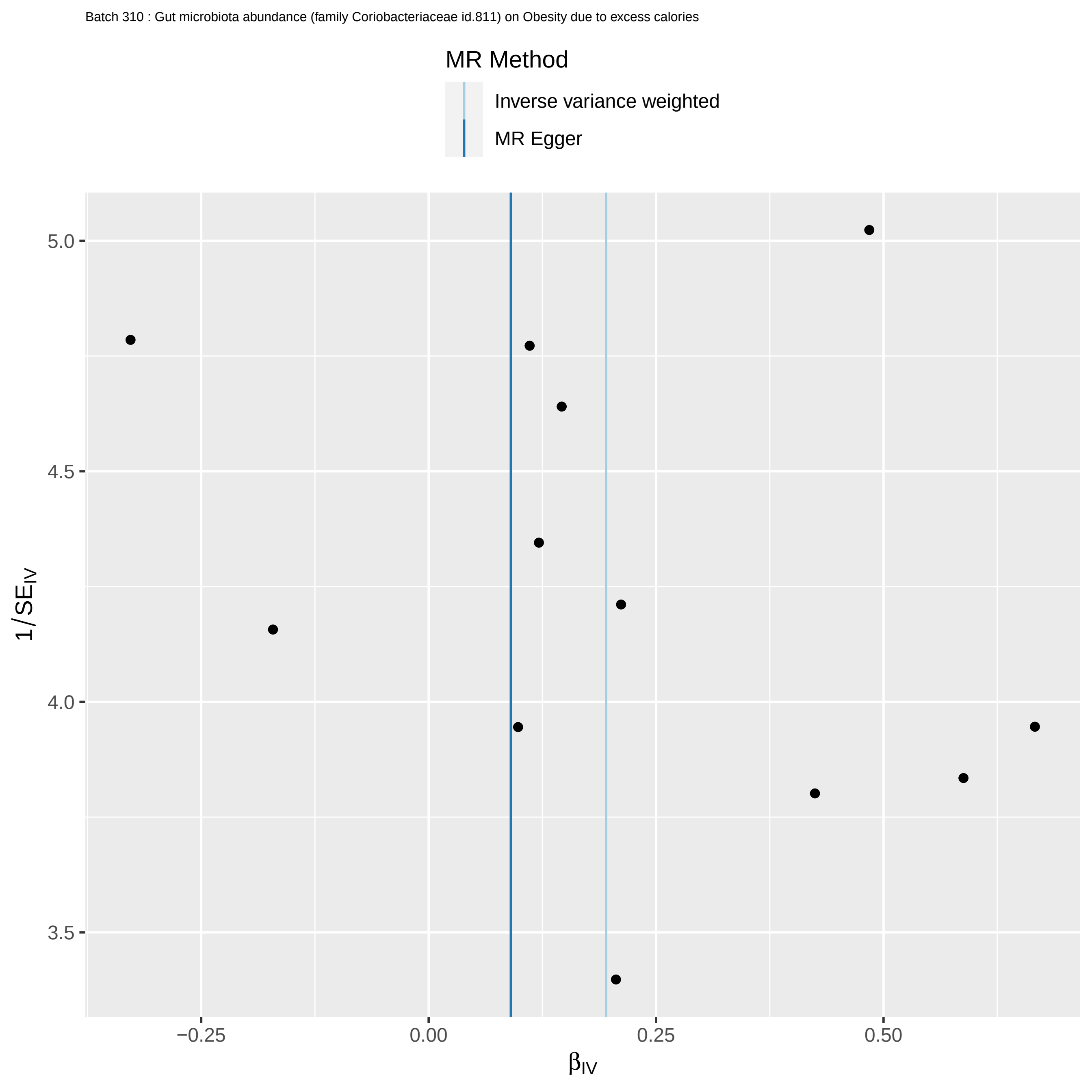

Batch 310 : Gut microbiota abundance (family Coriobacteriaceae id.811) on Obesity due to excess calories
MR Method
Inverse variance weighted
MR Egger
5.0
4.5
4.0
3.5
1/SE$_{IV}$
−0.25
0.00
0.25
0.50
β$_{IV}$

Batch 311 : Gut microbiota abundance (family Defluviitaleaceae id.1924) on Obesity due to excess calories

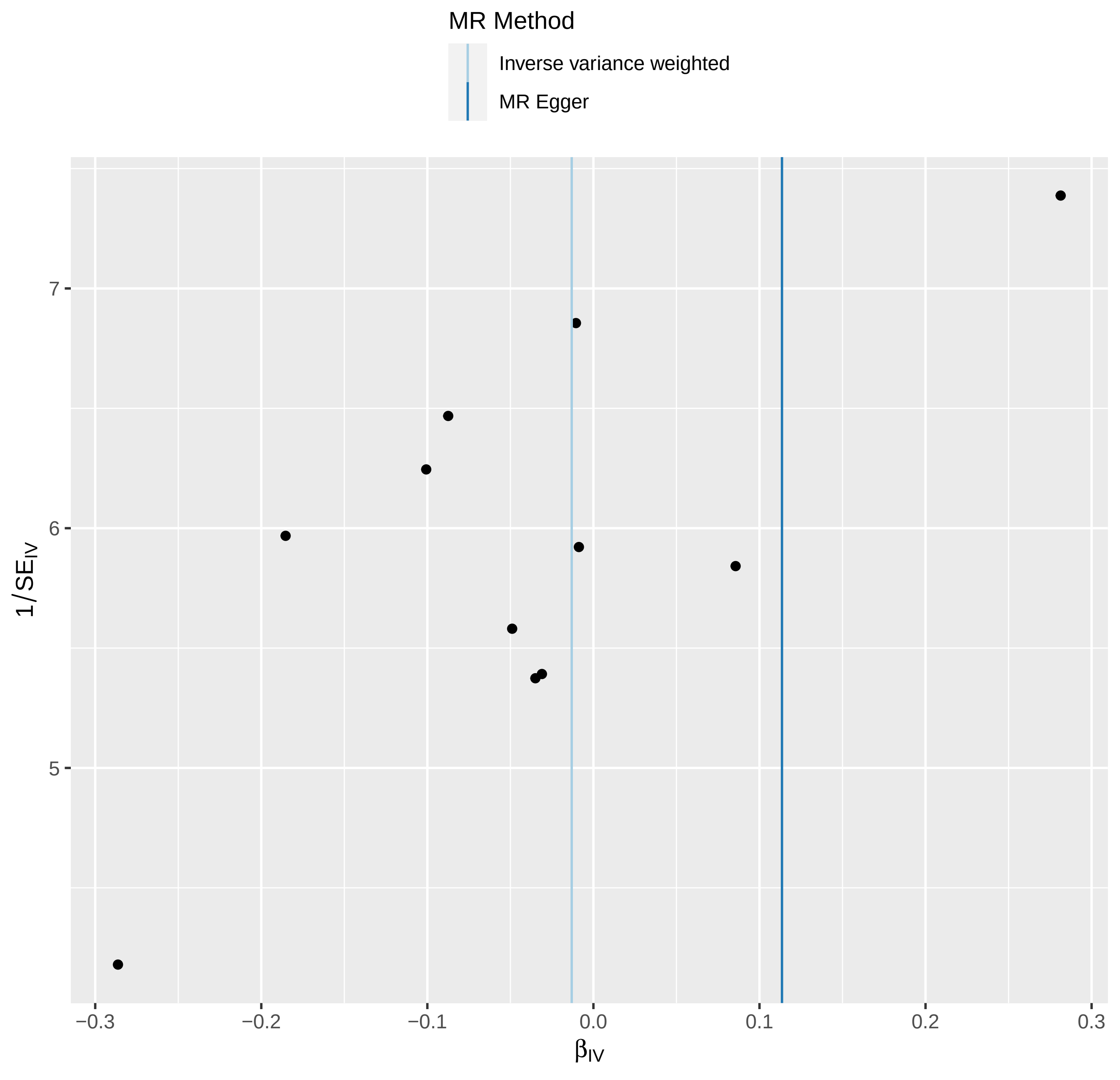

Batch 312 : Gut microbiota abundance (family Desulfovibrionaceae id.3169) on Obesity due to excess calories

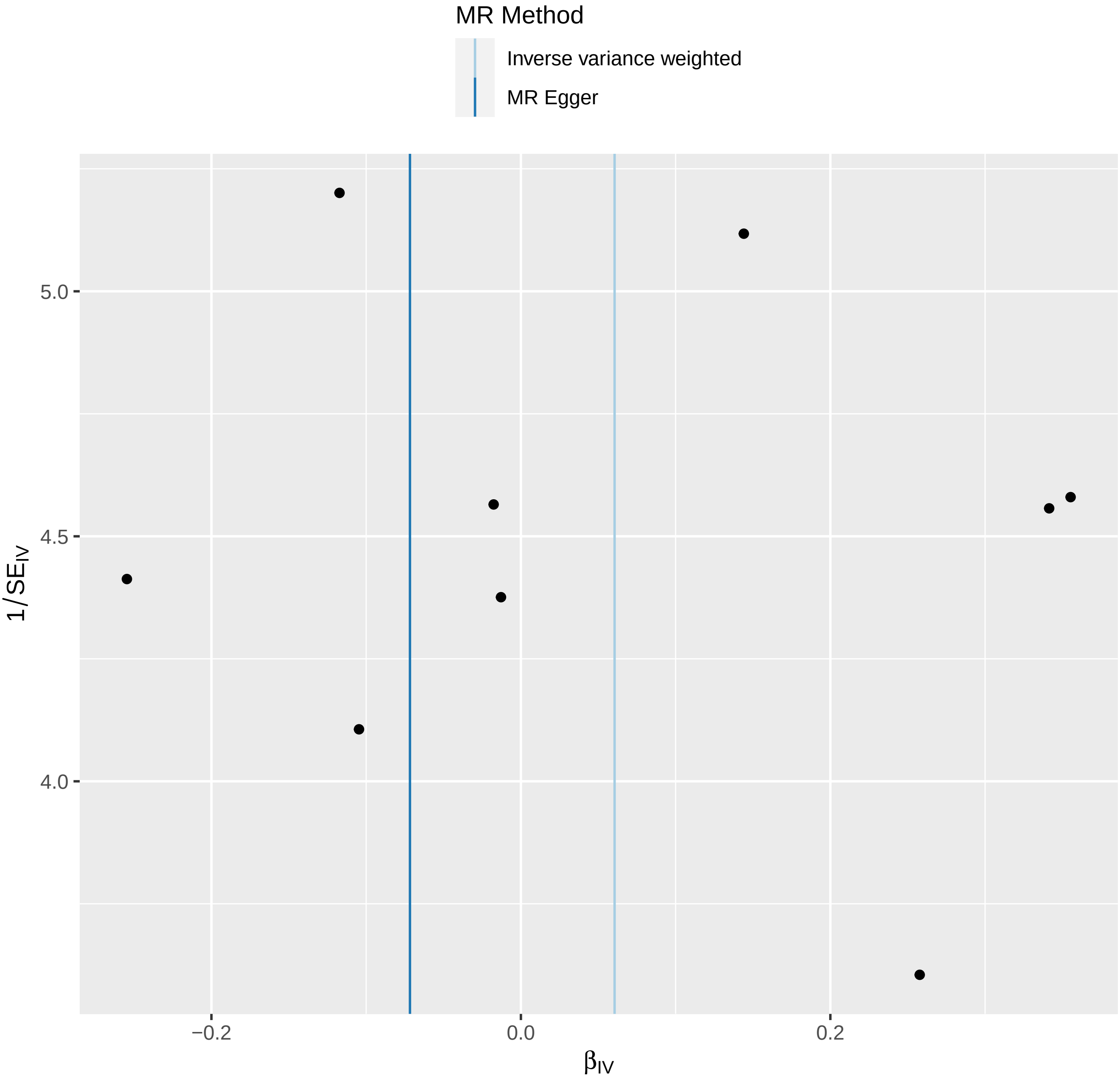

Batch 313 : Gut microbiota abundance (family Enterobacteriaceae id.3469) on Obesity due to excess calories

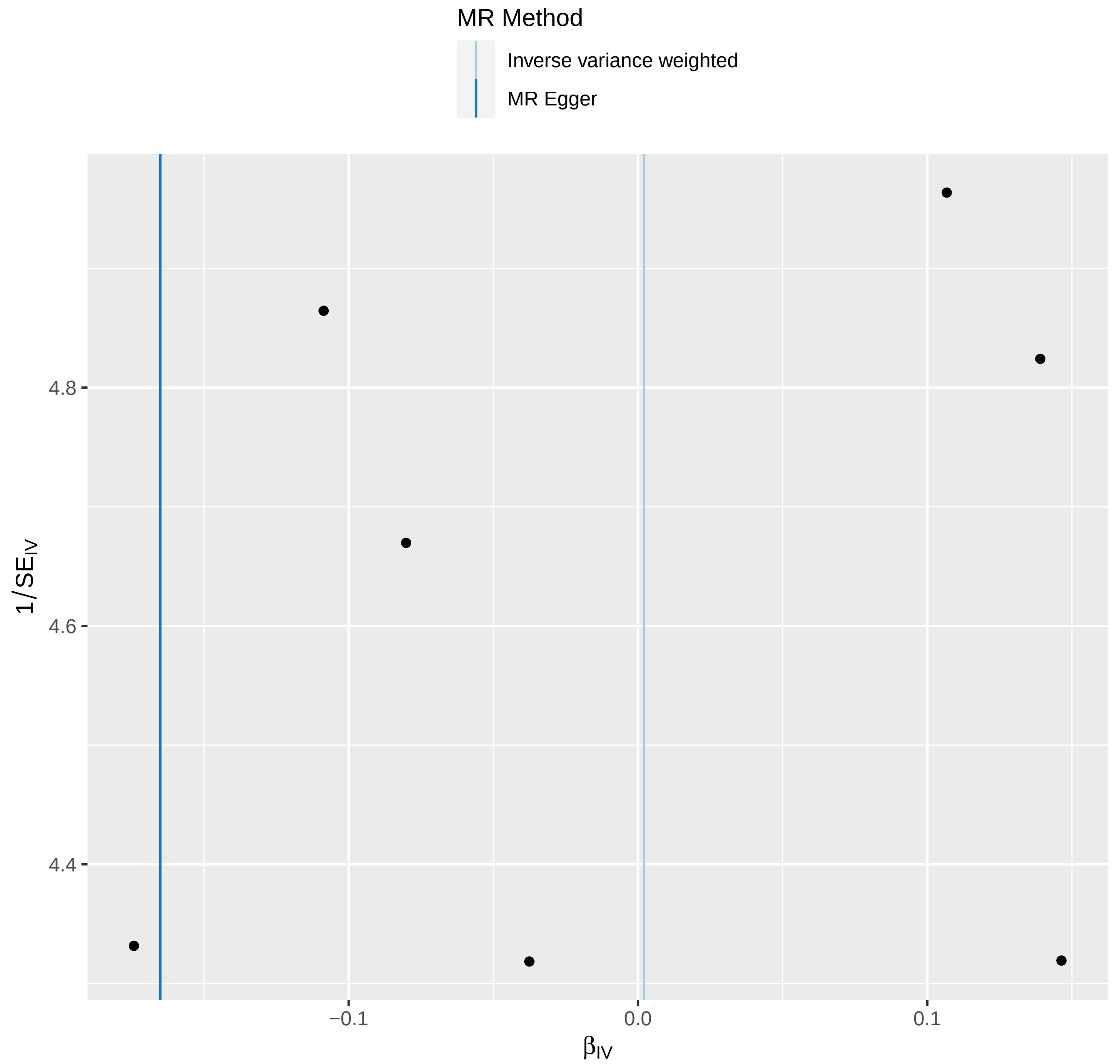

Batch 314 : Gut microbiota abundance (family Erysipelotrichaceae id.2149) on Obesity due to excess calories

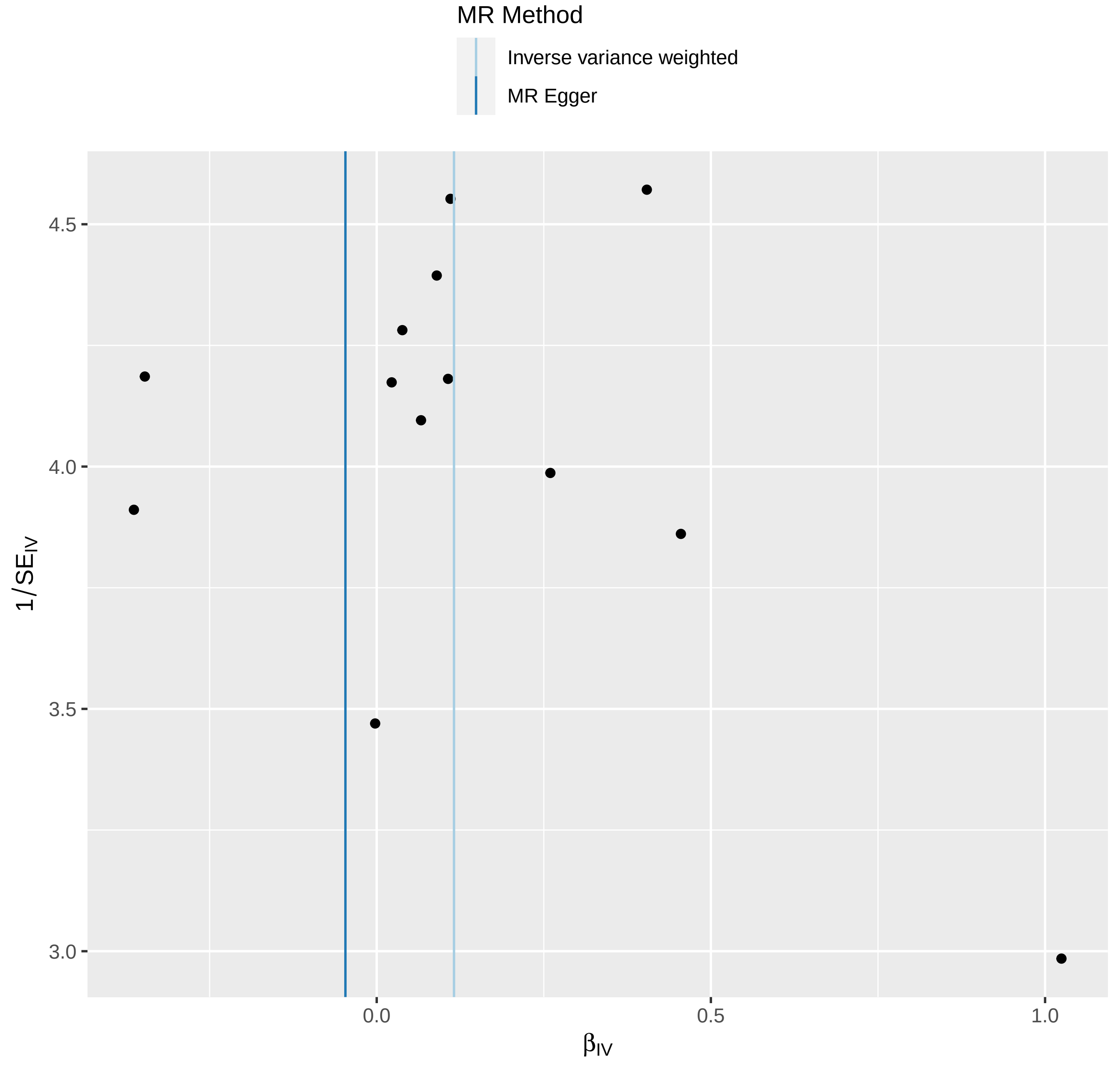

Batch 315 : Gut microbiota abundance (family Family XI id.1936) on Obesity due to excess calories

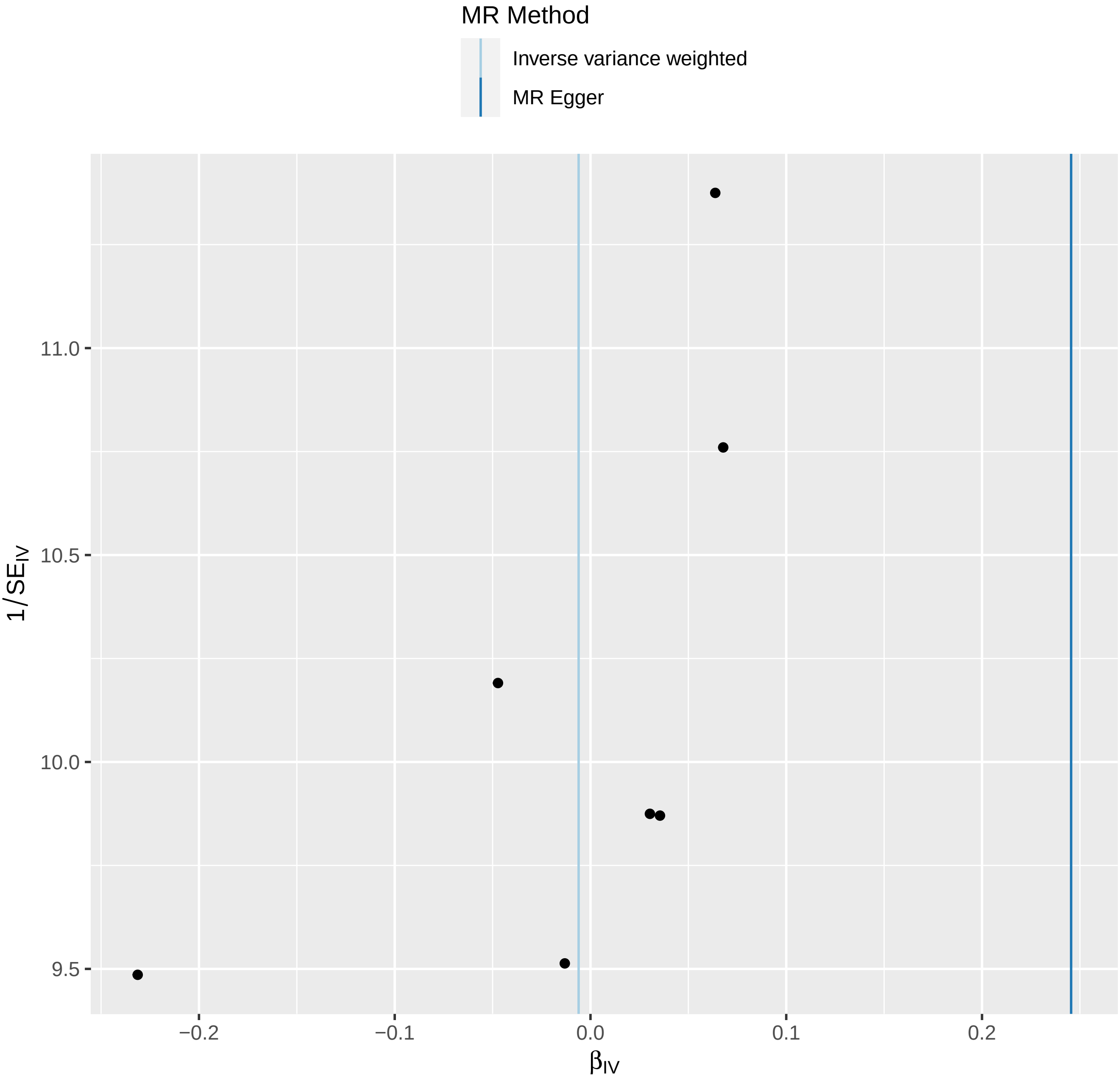

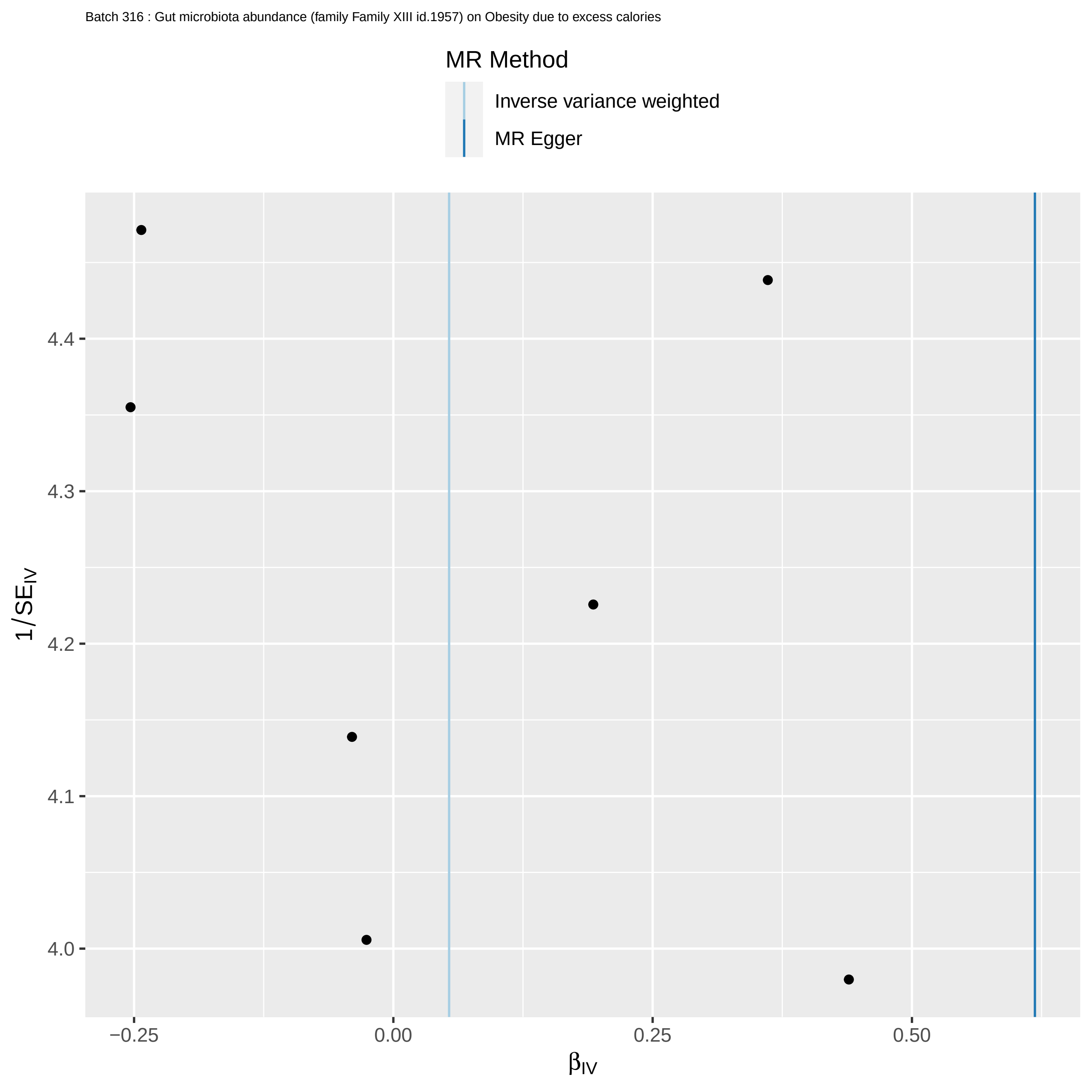

Batch 316 : Gut microbiota abundance (family Family XIII id.1957) on Obesity due to excess calories
MR Method
Inverse variance weighted
MR Egger
4.4
4.3
4.2
4.1
4.0
1/SE_IV
−0.25
0.00
0.25
0.50
β_IV

Batch 317 : Gut microbiota abundance (family Lachnospiraceae id.1987) on Obesity due to excess calories

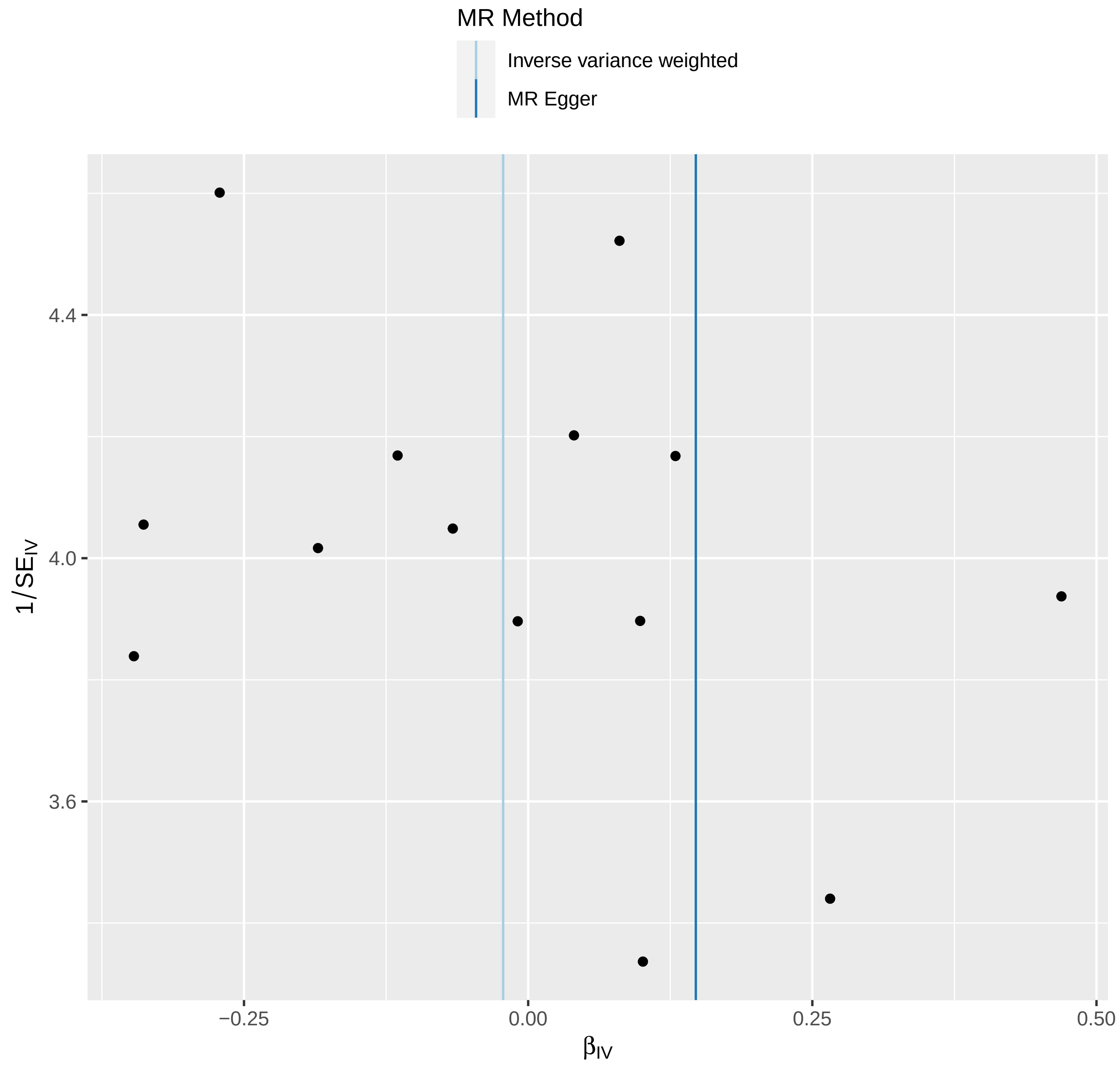

Batch 318 : Gut microbiota abundance (family Lactobacillaceae id.1836) on Obesity due to excess calories

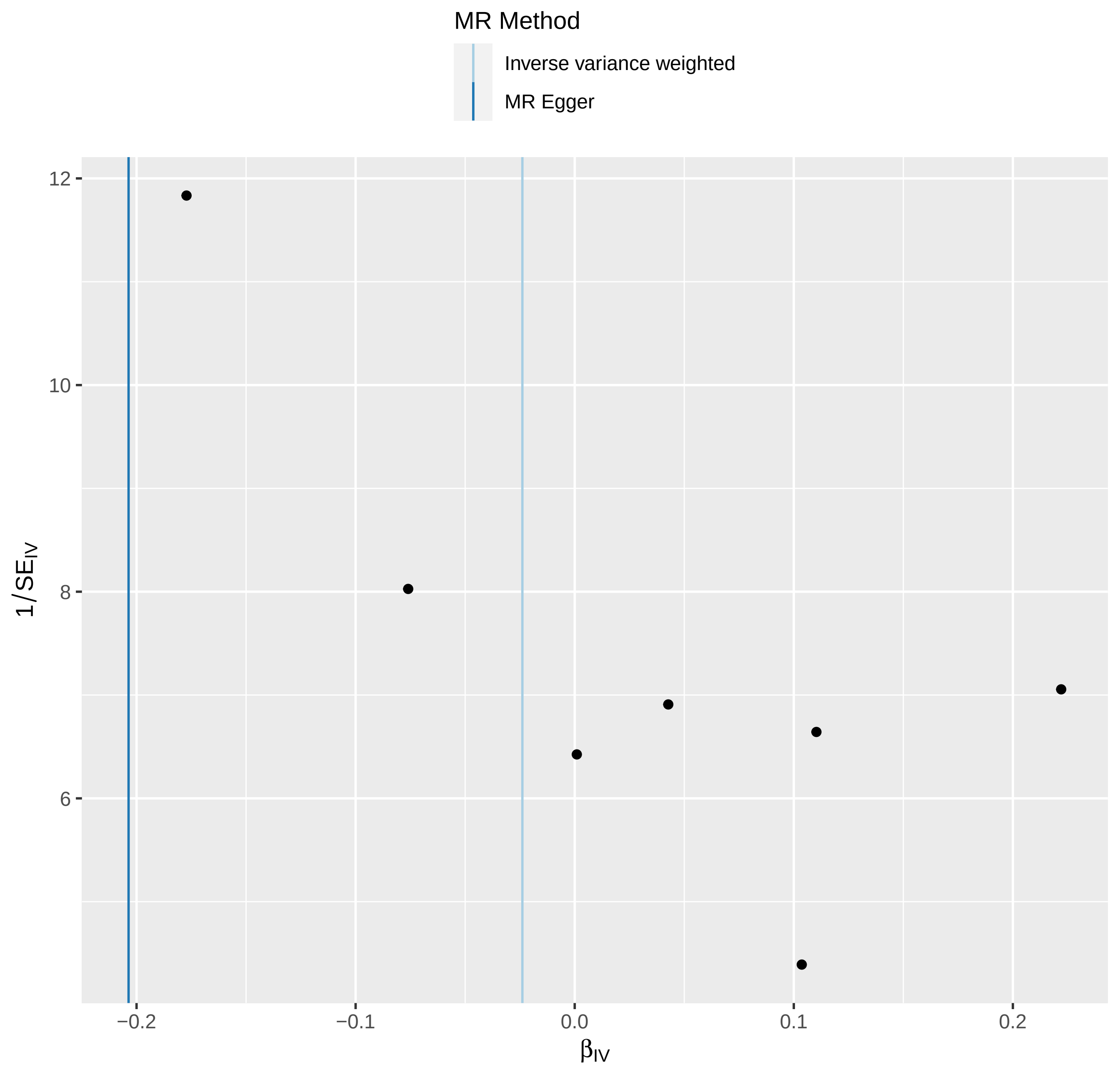

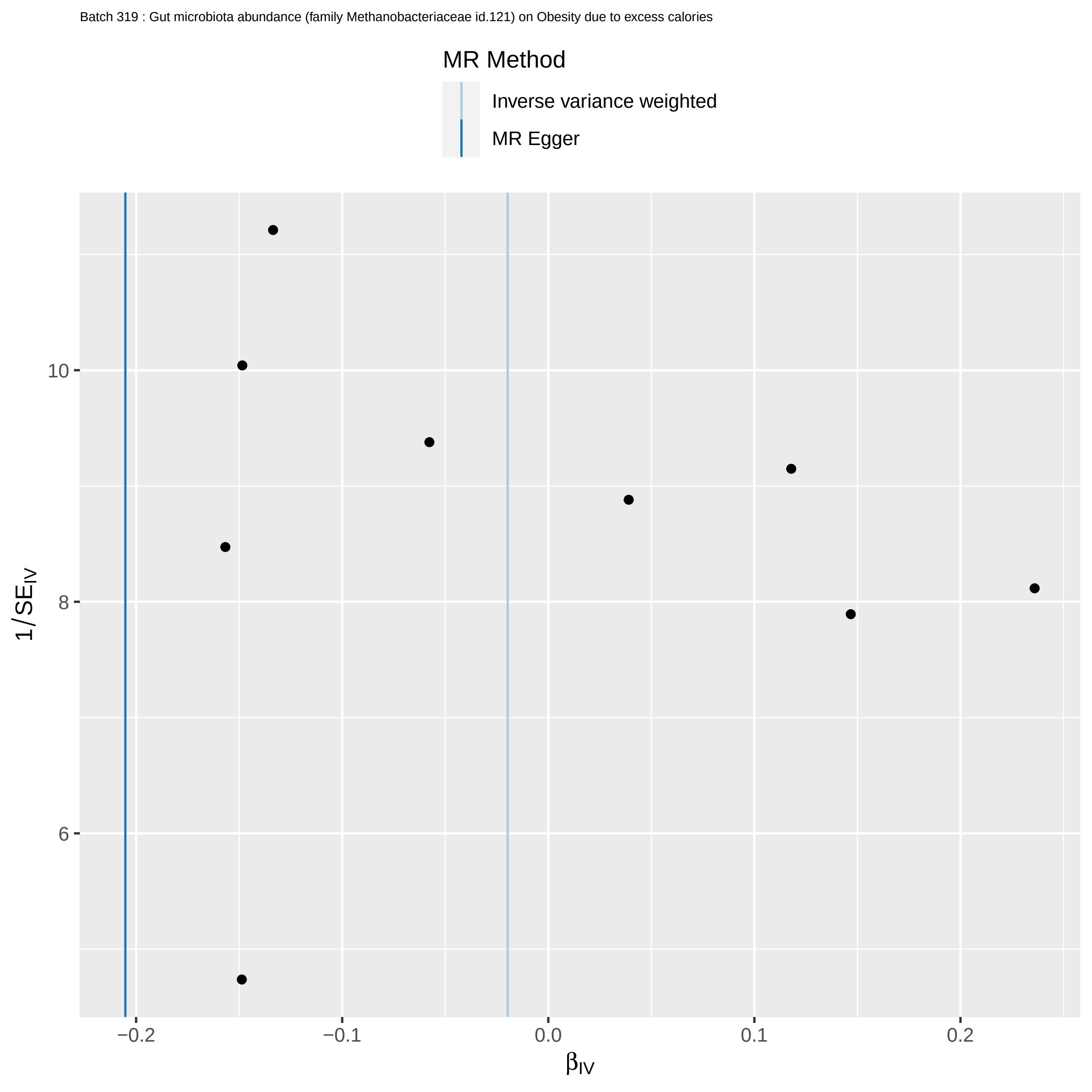
Batch 319 : Gut microbiota abundance (family Methanobacteriaceae id.121) on Obesity due to excess calories
MR Method
Inverse variance weighted
MR Egger
10
8
6
−0.2
−0.1
0.0
0.1
0.2
$\beta_{IV}$
$1/SE_{IV}$

Batch 320 : Gut microbiota abundance (family Oxalobacteraceae id.2966) on Obesity due to excess calories

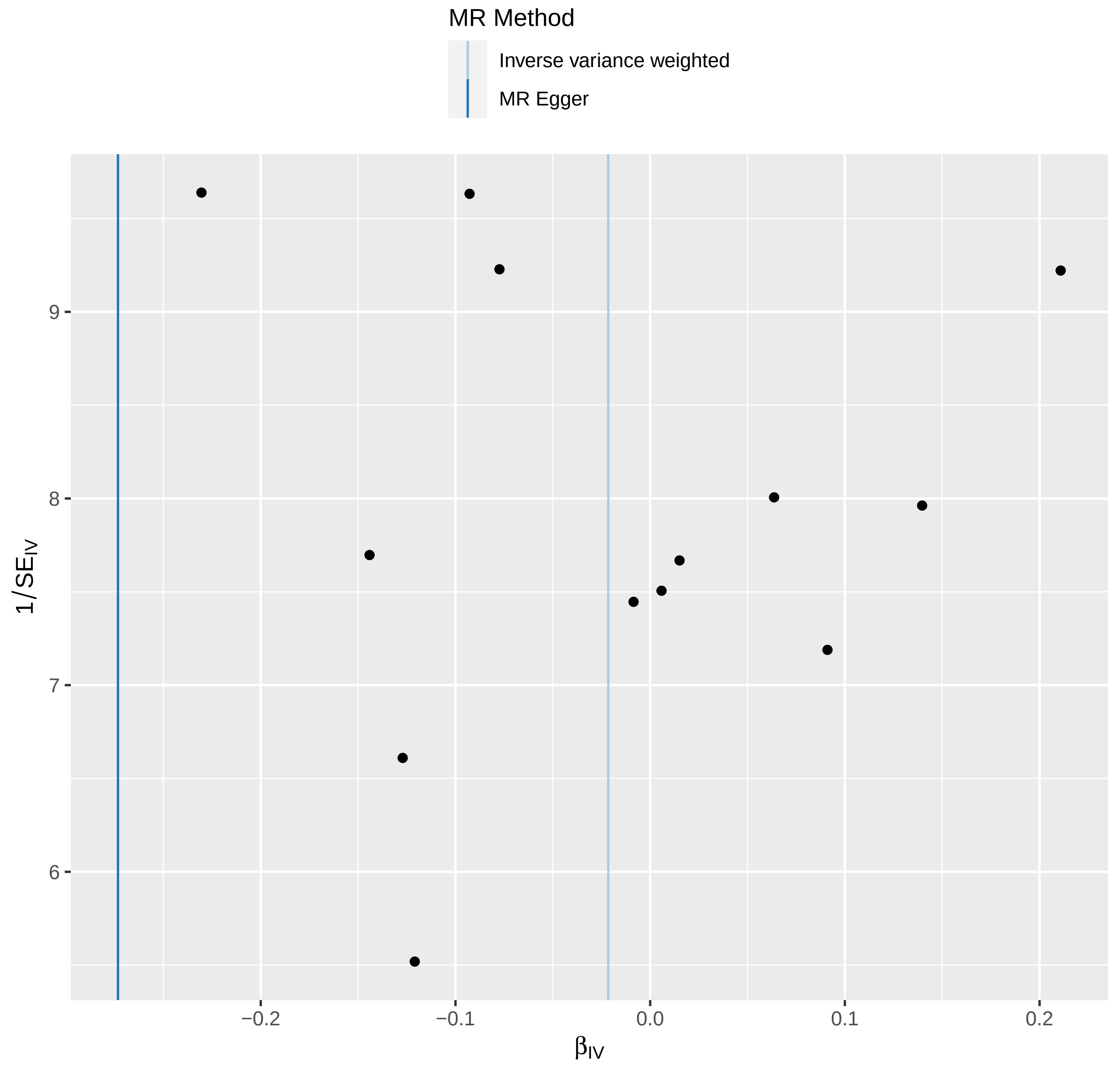

Batch 321 : Gut microbiota abundance (family Pasteurellaceae id.3689) on Obesity due to excess calories

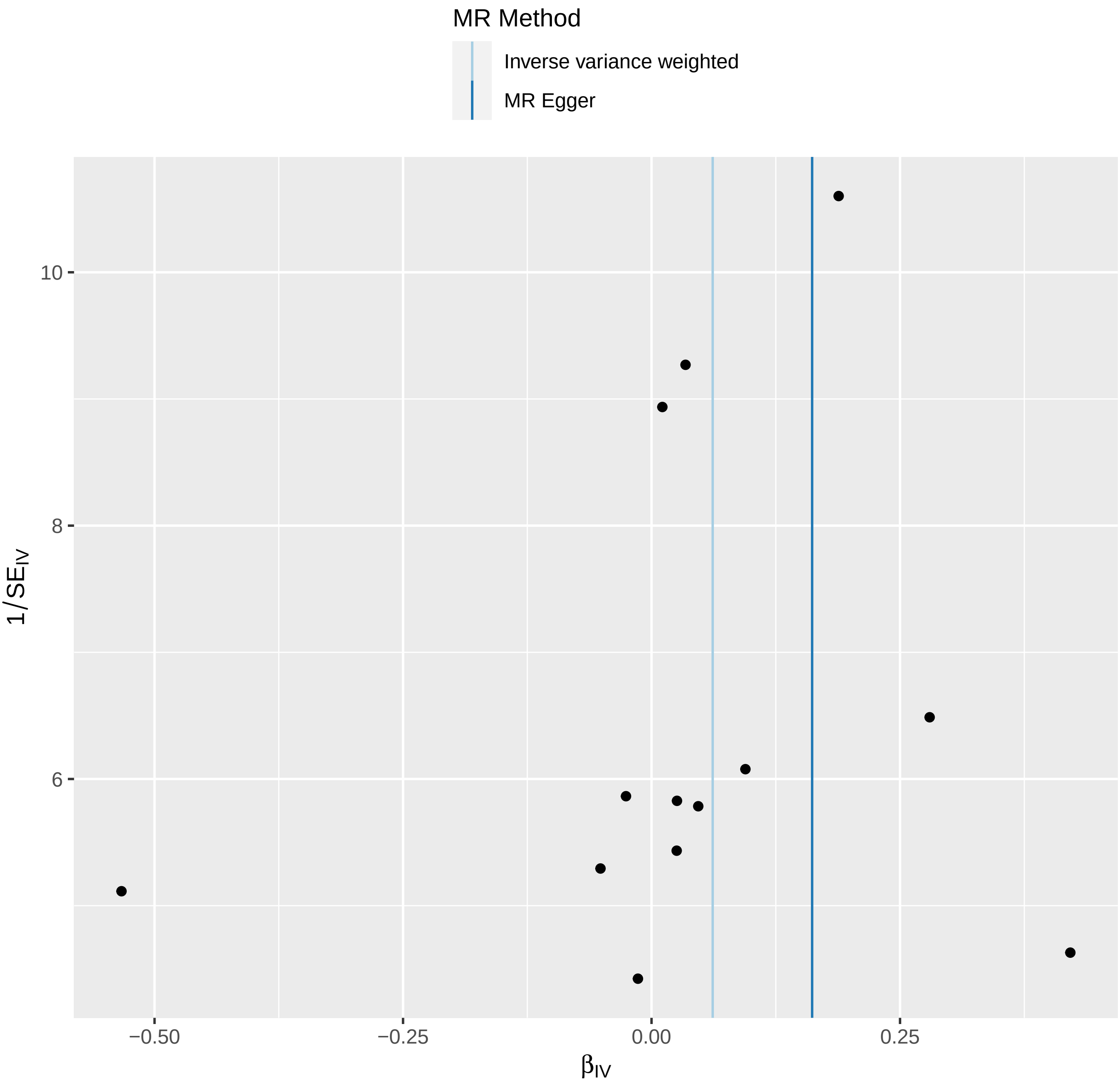

Batch 322 : Gut microbiota abundance (family Peptococcaceae id.2024) on Obesity due to excess calories

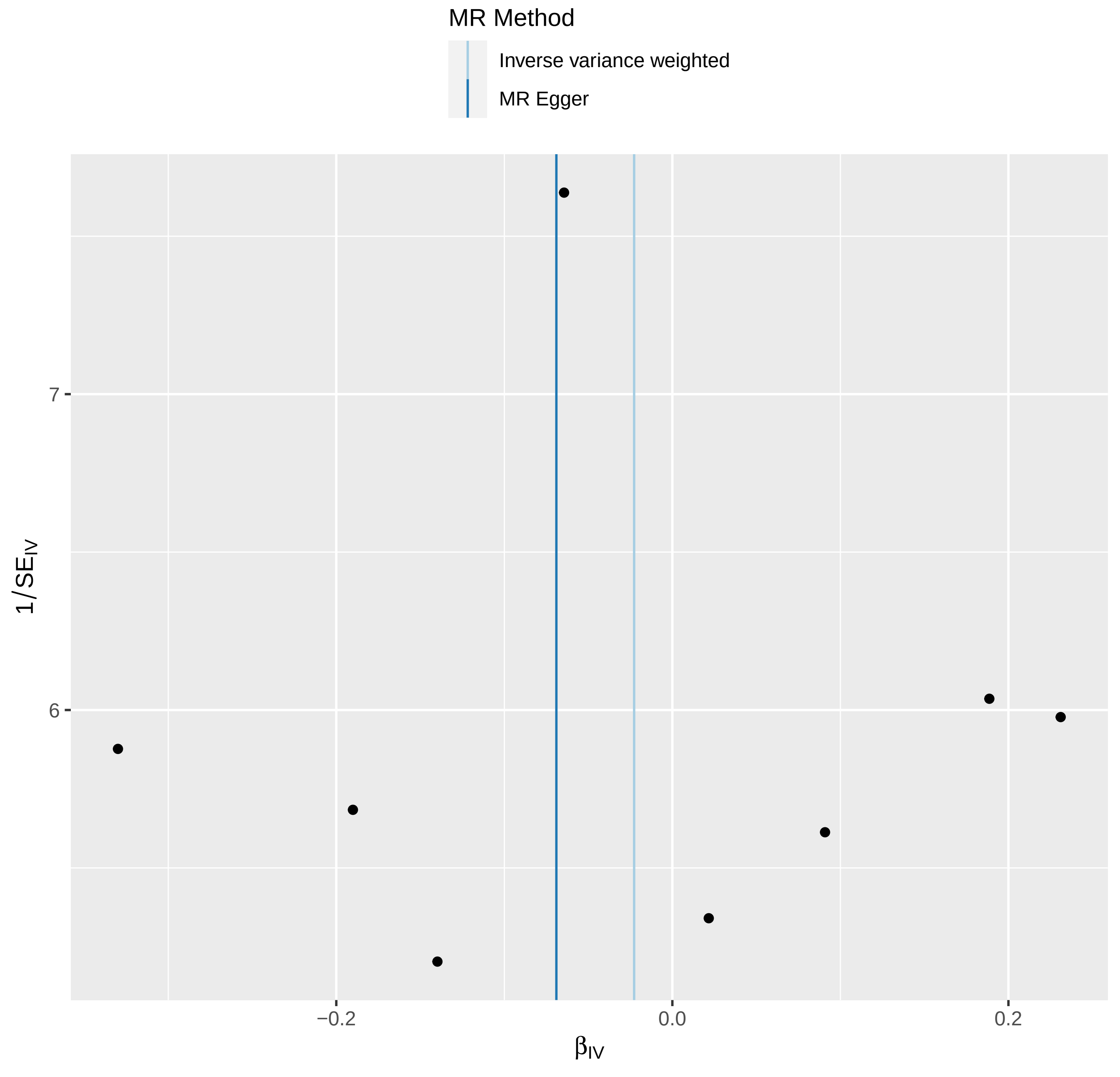

Batch 323 : Gut microbiota abundance (family Peptostreptococcaceae id.2042) on Obesity due to excess calories

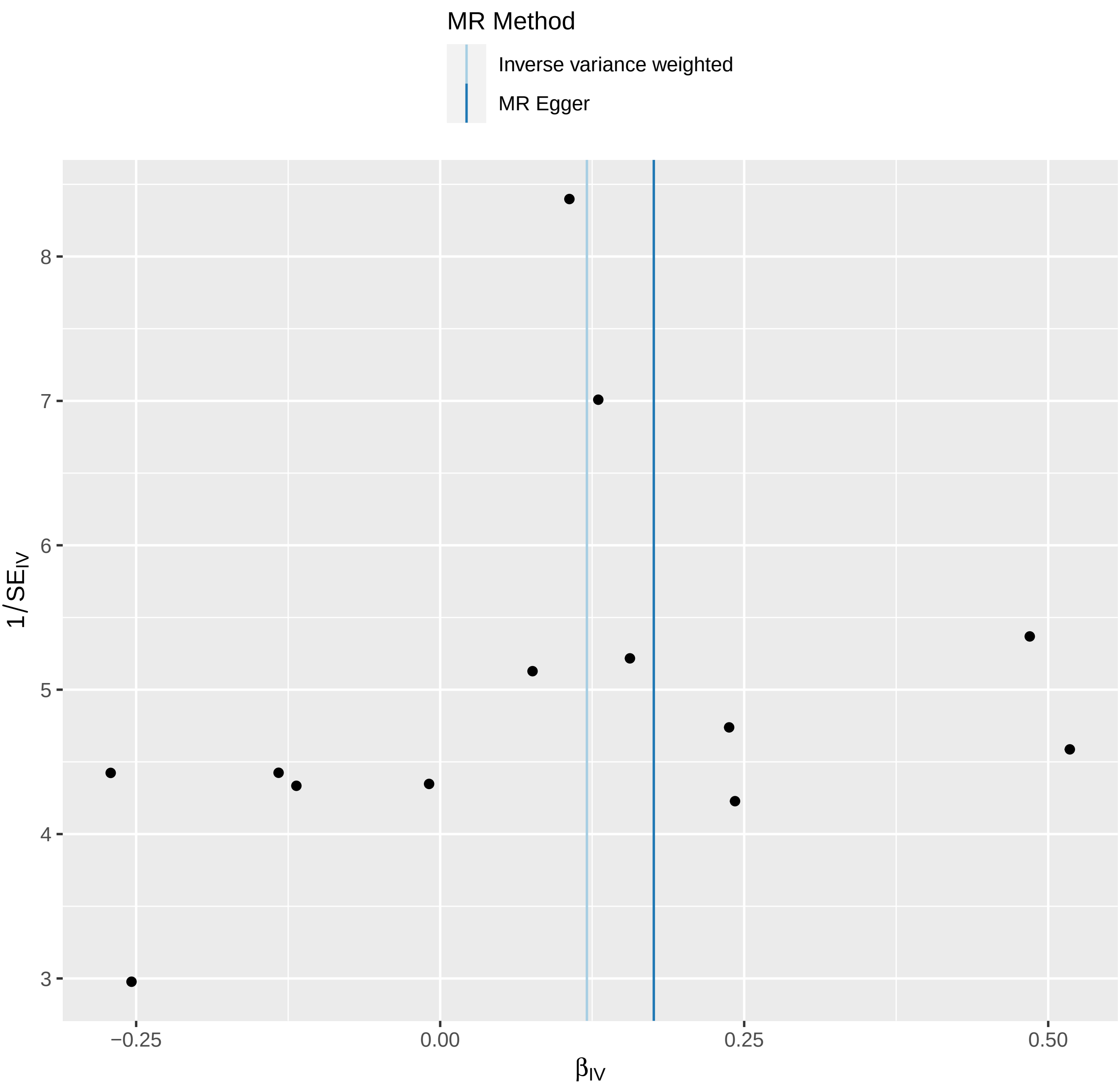

Batch 324 : Gut microbiota abundance (family Porphyromonadaceae id.943) on Obesity due to excess calories

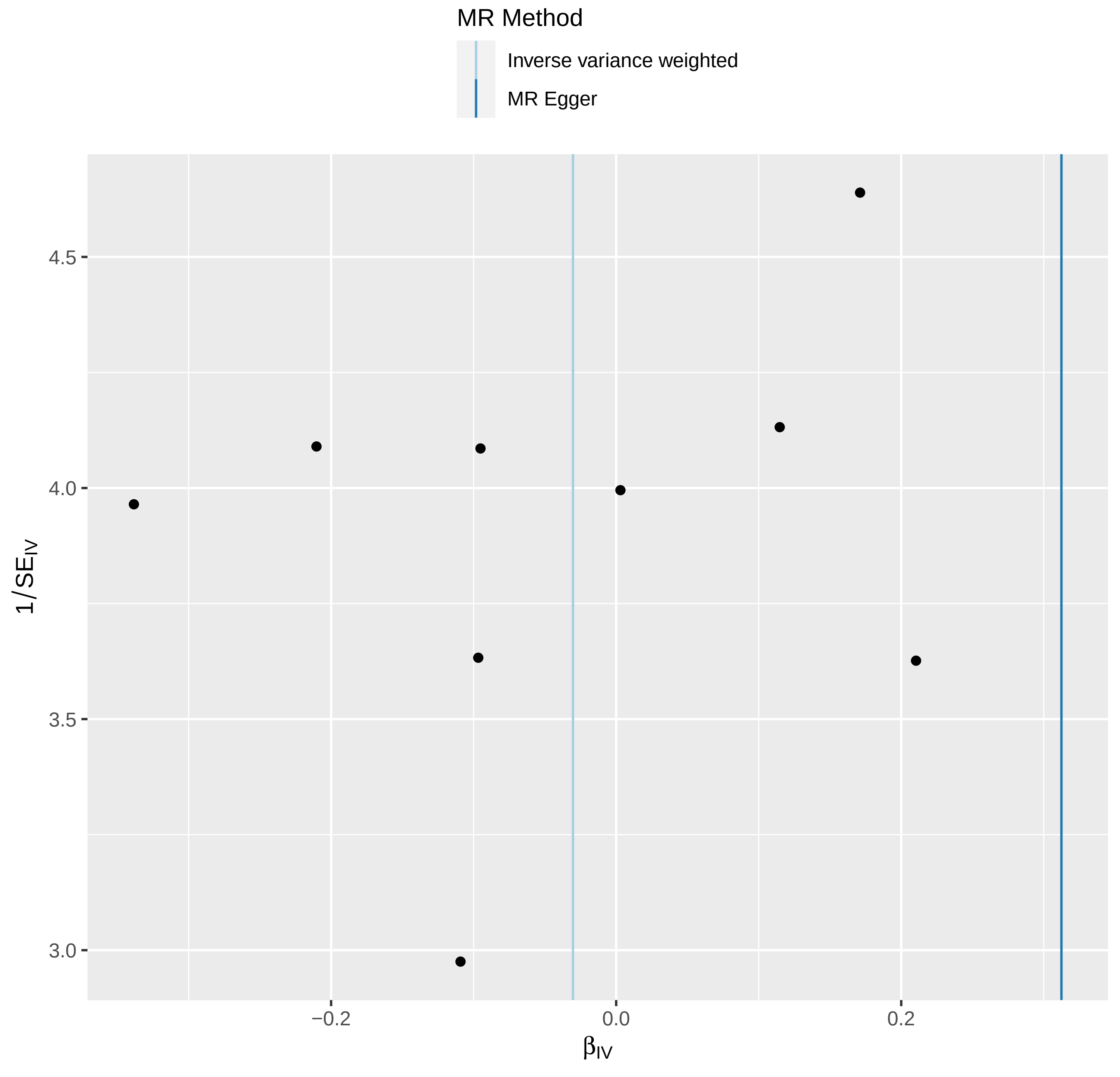

Batch 325 : Gut microbiota abundance (family Prevotellaceae id.960) on Obesity due to excess calories

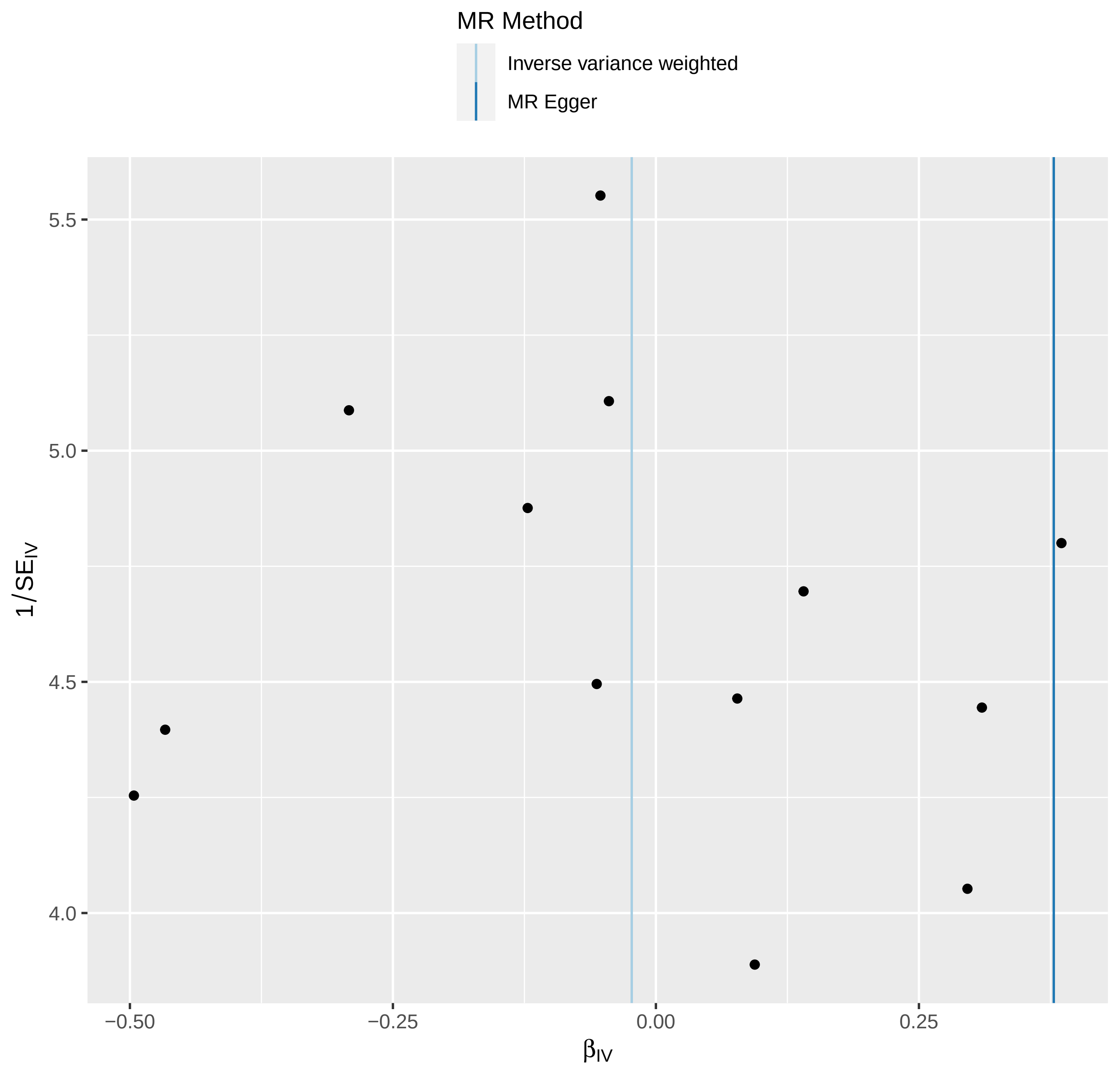

Batch 326 : Gut microbiota abundance (family Rhodospirillaceae id.2717) on Obesity due to excess calories

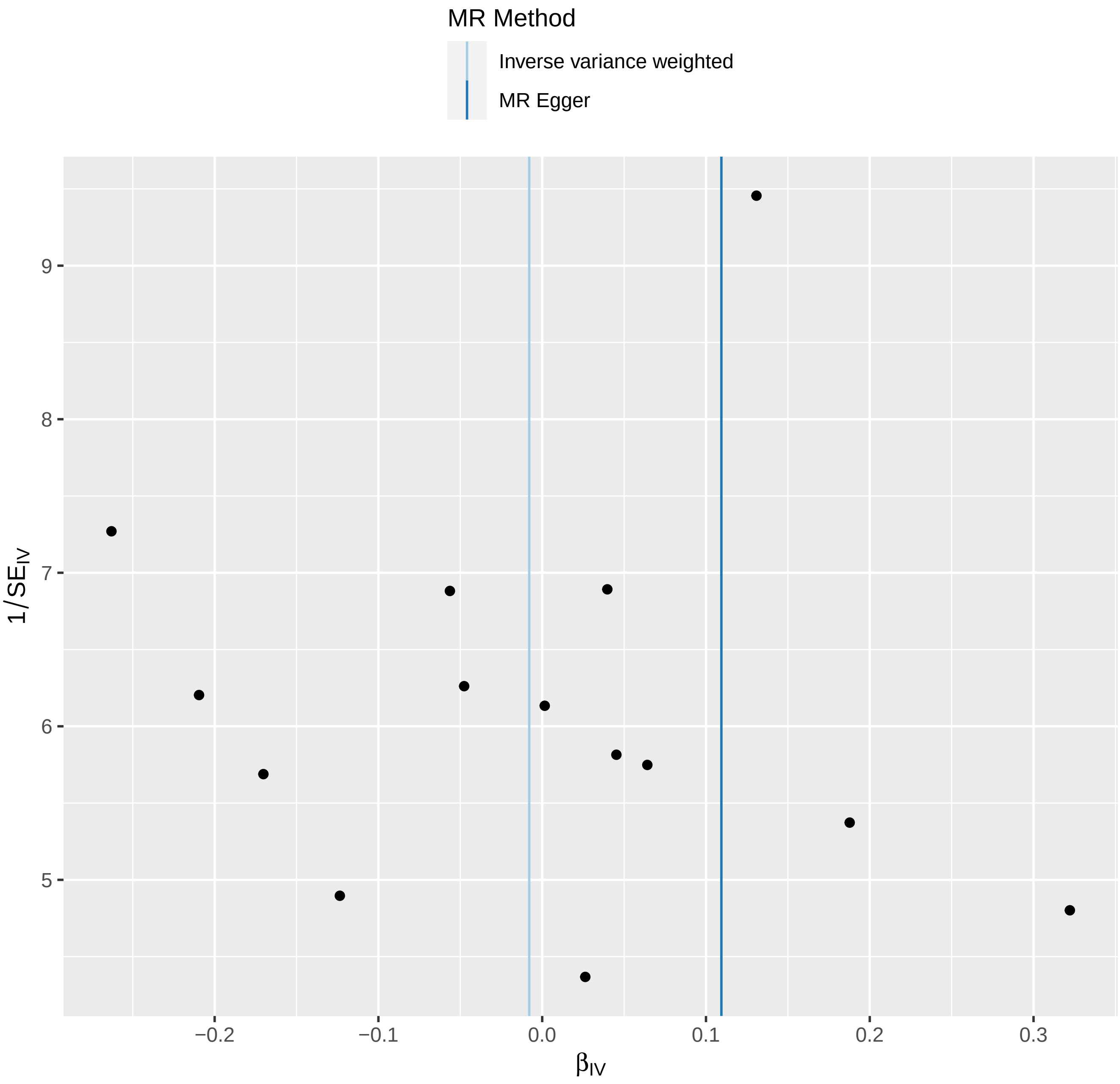

Batch 327 : Gut microbiota abundance (family Rikenellaceae id.967) on Obesity due to excess calories

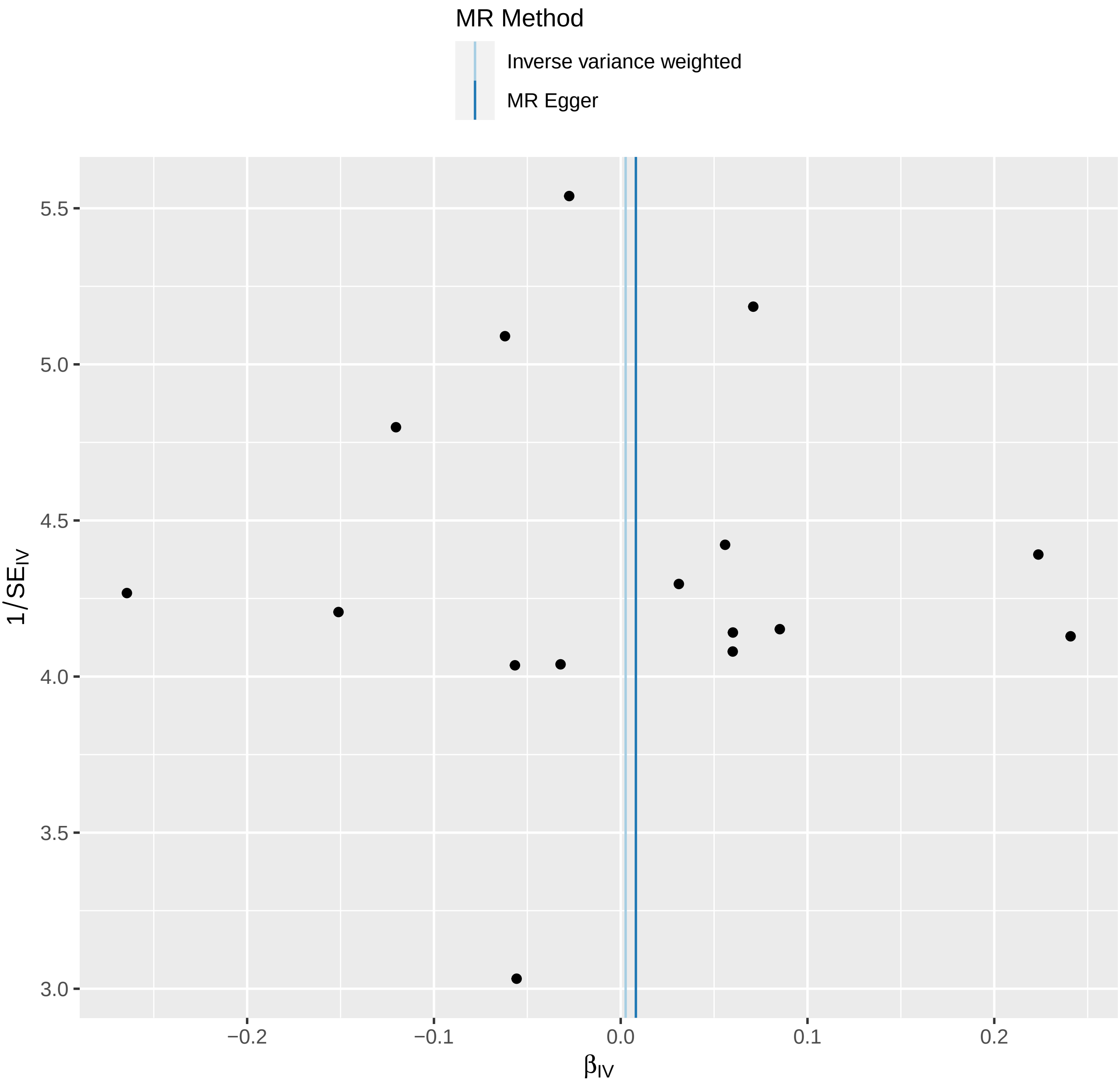

Batch 328 : Gut microbiota abundance (family Ruminococcaceae id.2050) on Obesity due to excess calories

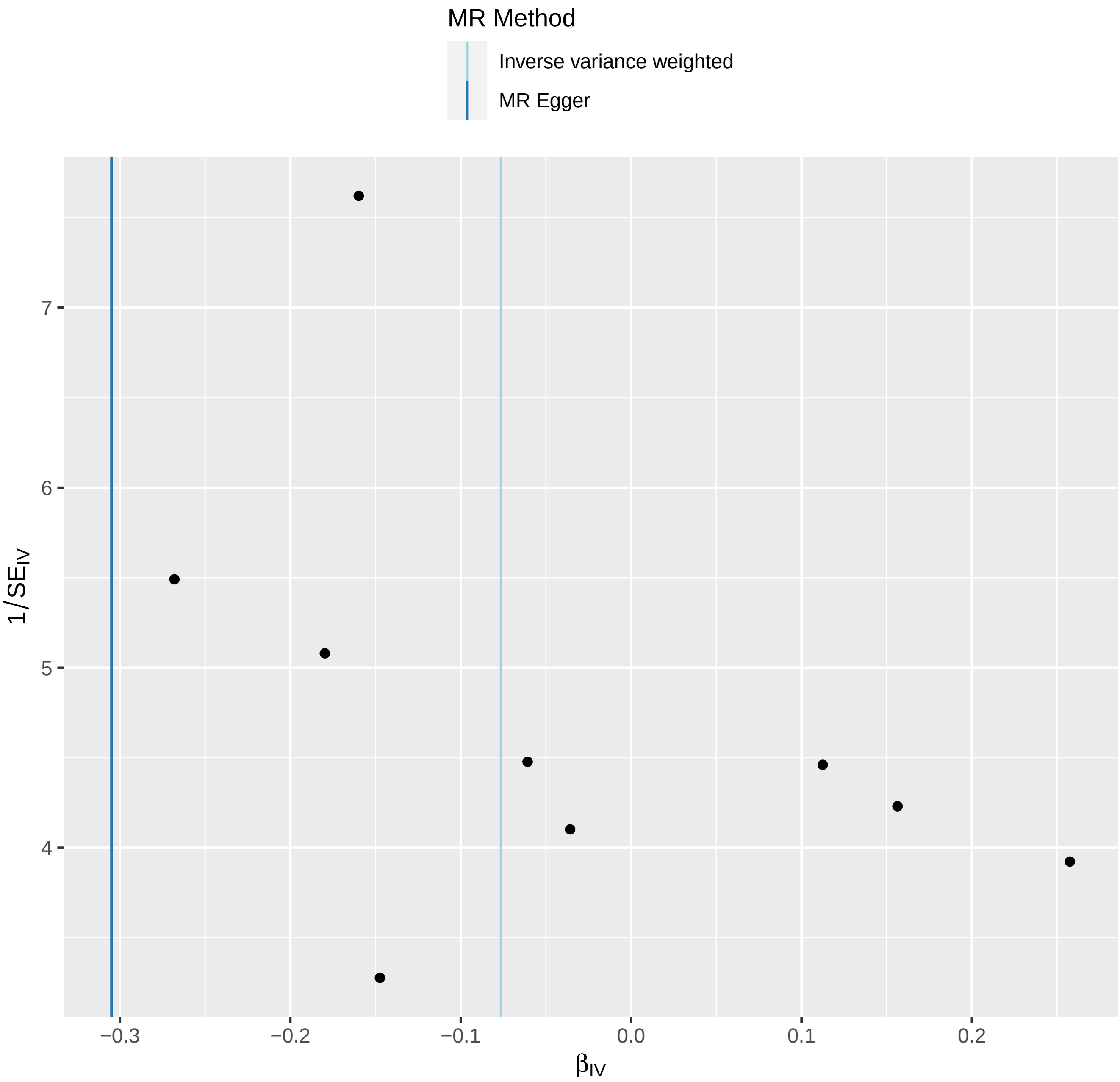

Batch 329 : Gut microbiota abundance (family Streptococcaceae id.1850) on Obesity due to excess calories

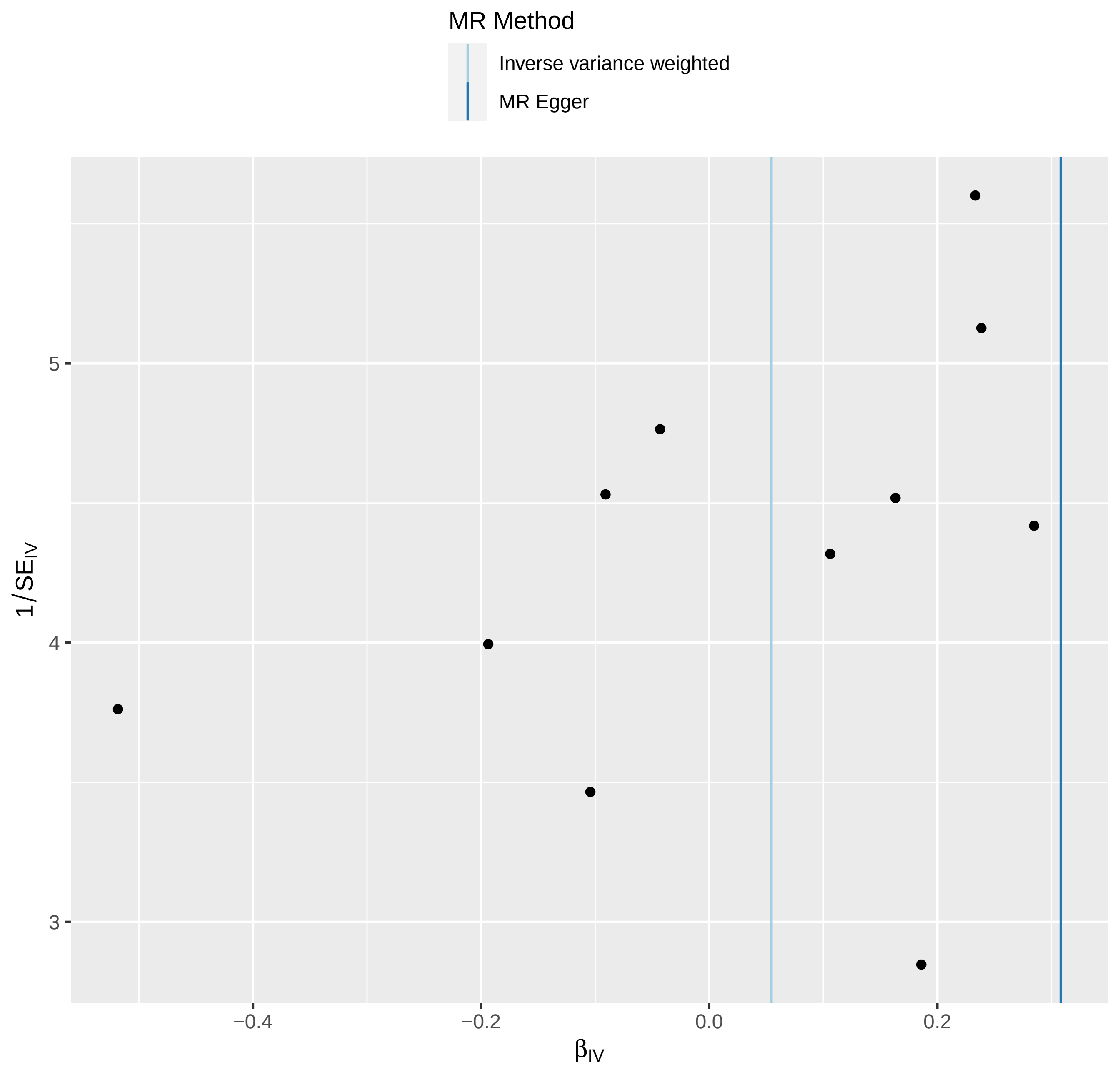

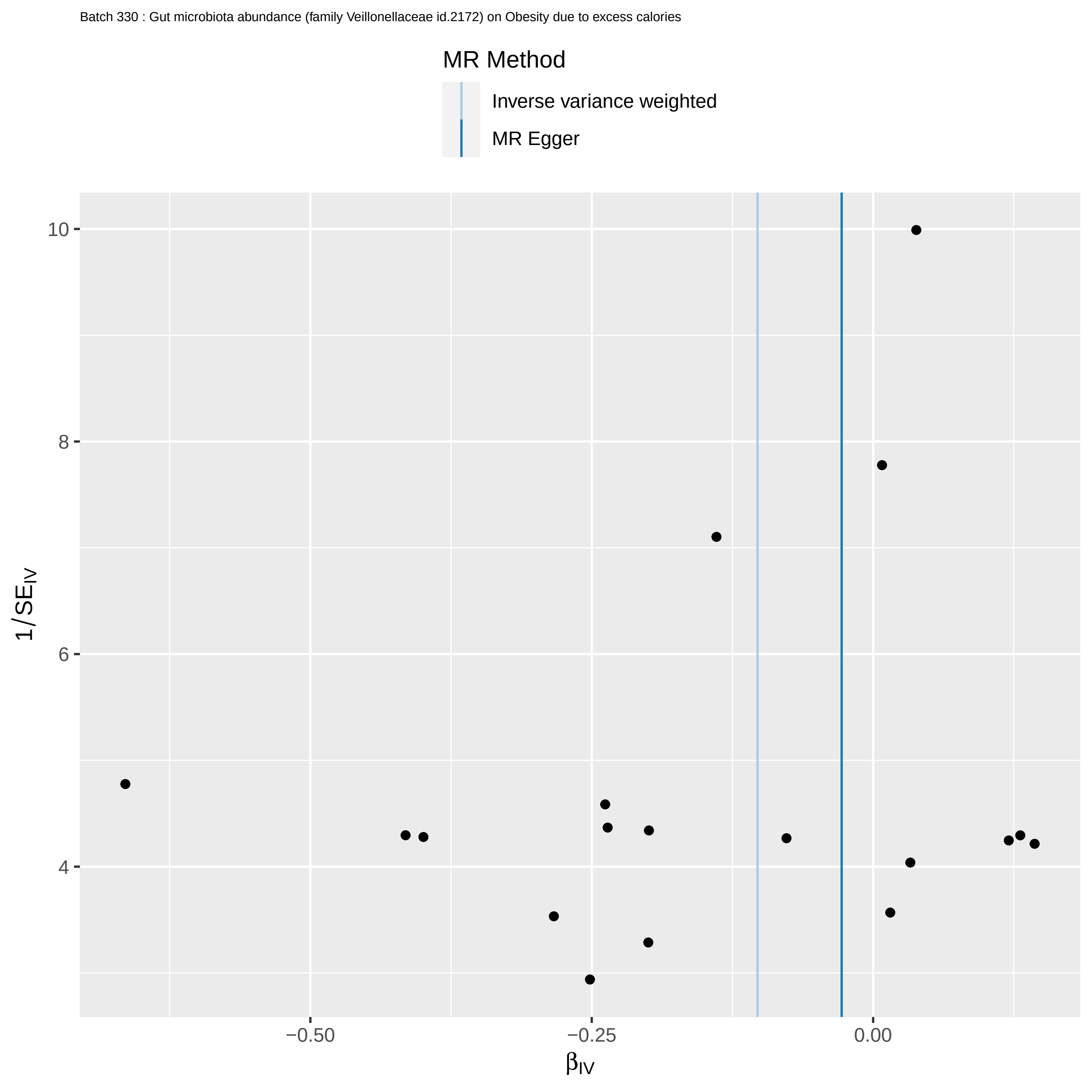
Batch 330 : Gut microbiota abundance (family Veillonellaceae id.2172) on Obesity due to excess calories
MR Method
Inverse variance weighted
MR Egger
10
8
6
4
1/SE_IV
−0.50
−0.25
0.00
β_IV

Batch 331 : Gut microbiota abundance (family Verrucomicrobiaceae id.4036) on Obesity due to excess calories

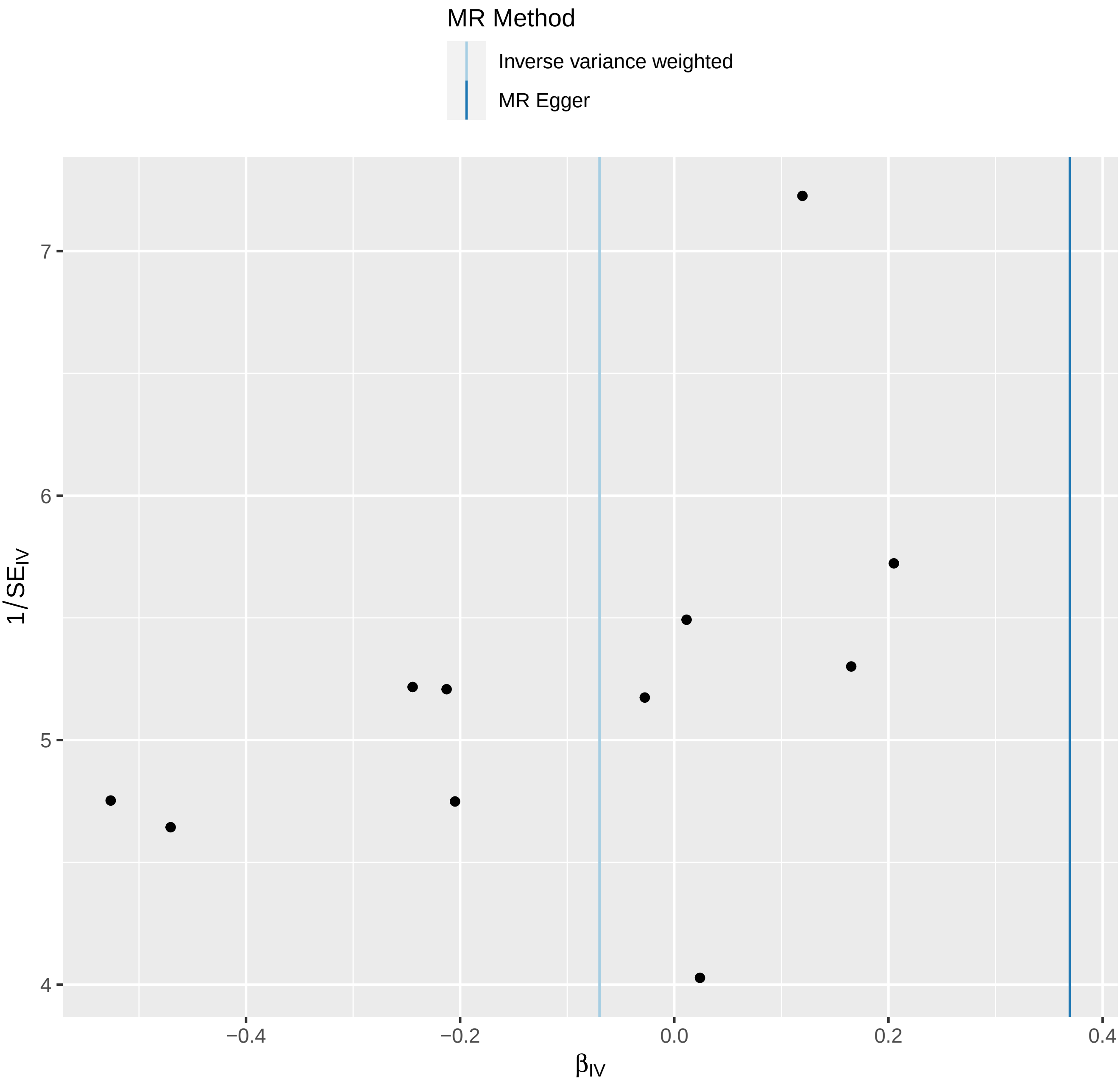

Batch 332 : Gut microbiota abundance (family Victivallaceae id.2255) on Obesity due to excess calories

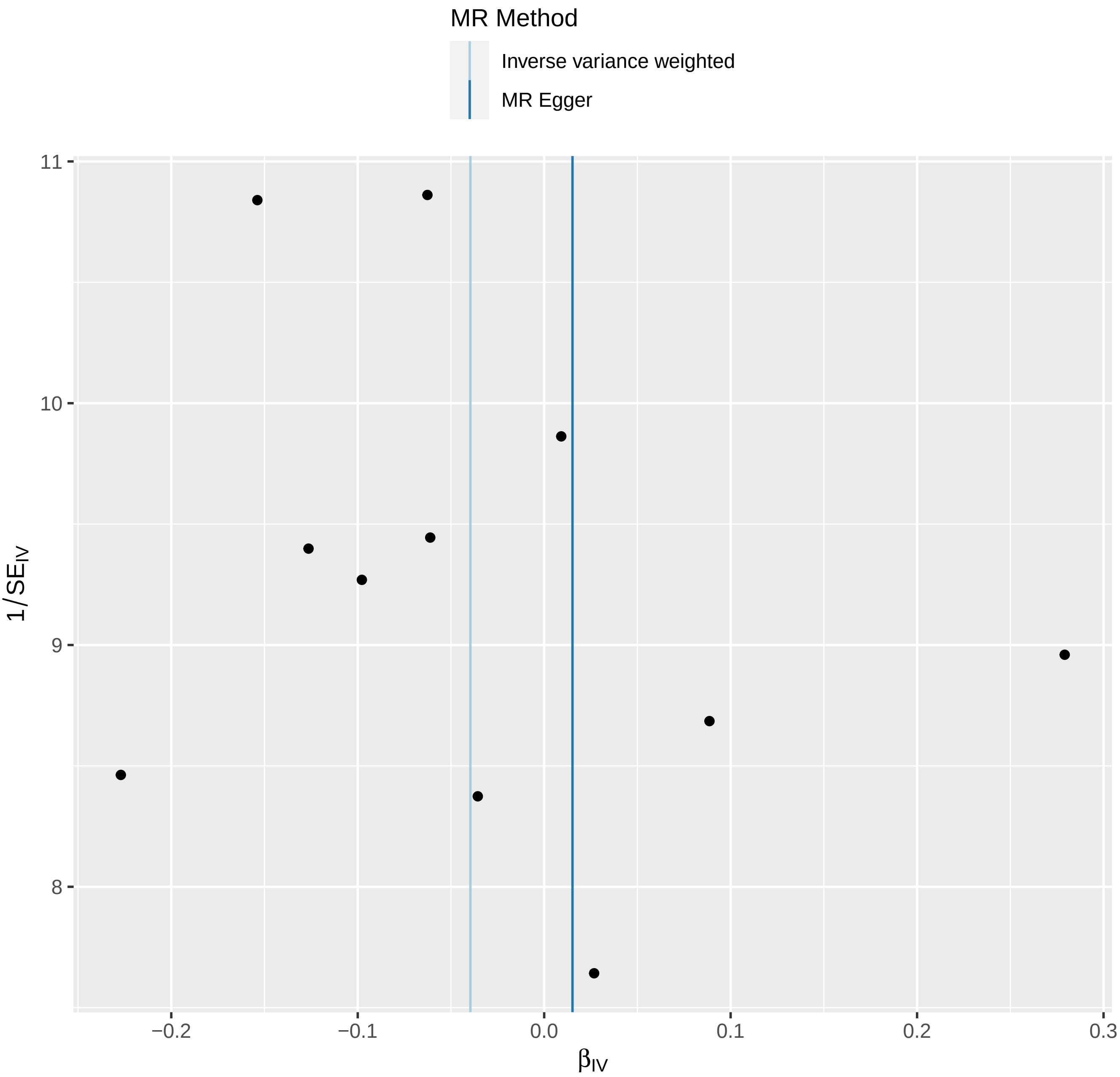

Batch 333 : Gut microbiota abundance (genus Actinomyces id.423) on Obesity due to excess calories

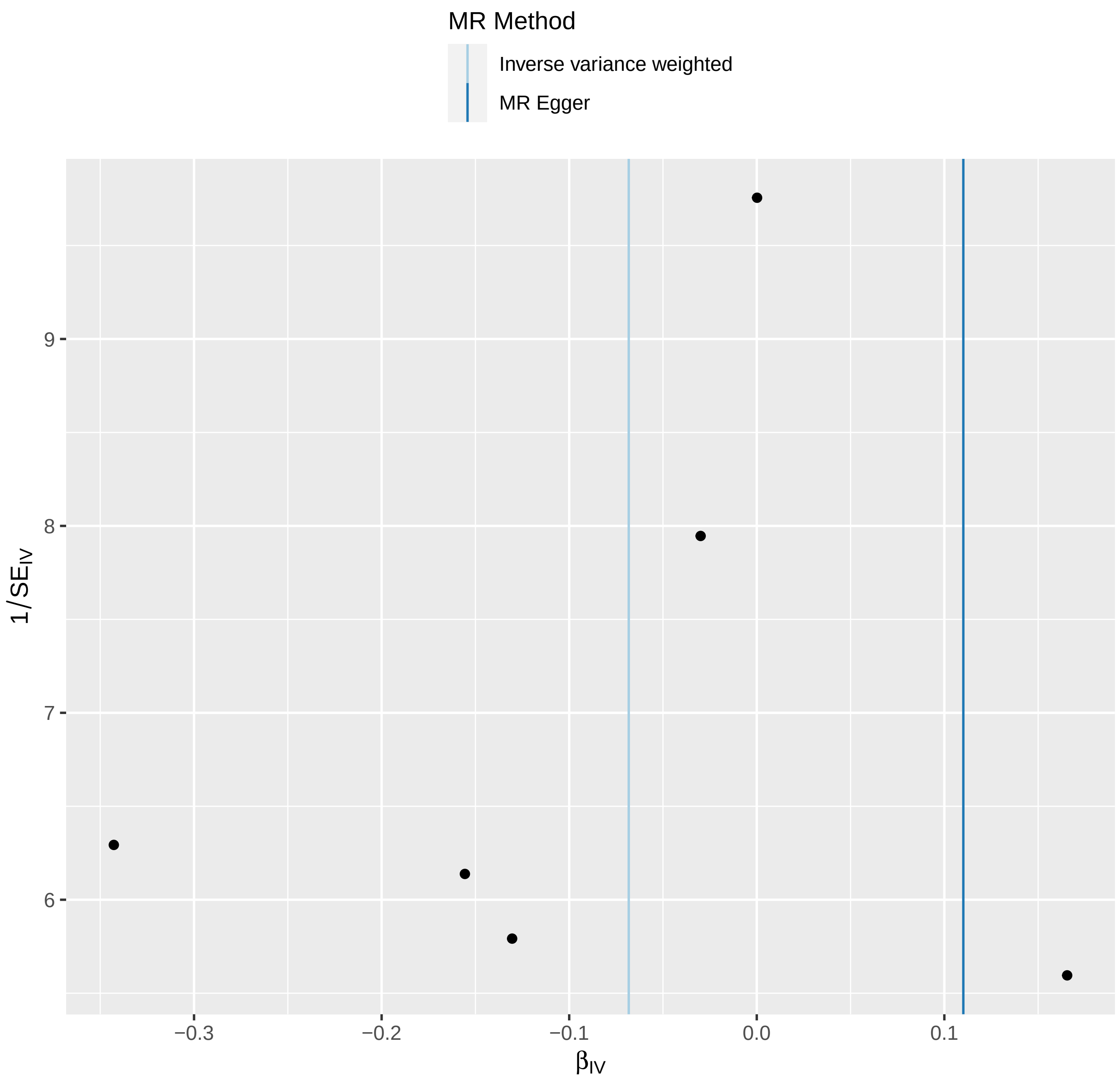

Batch 334 : Gut microbiota abundance (genus Adlercreutzia id.812) on Obesity due to excess calories

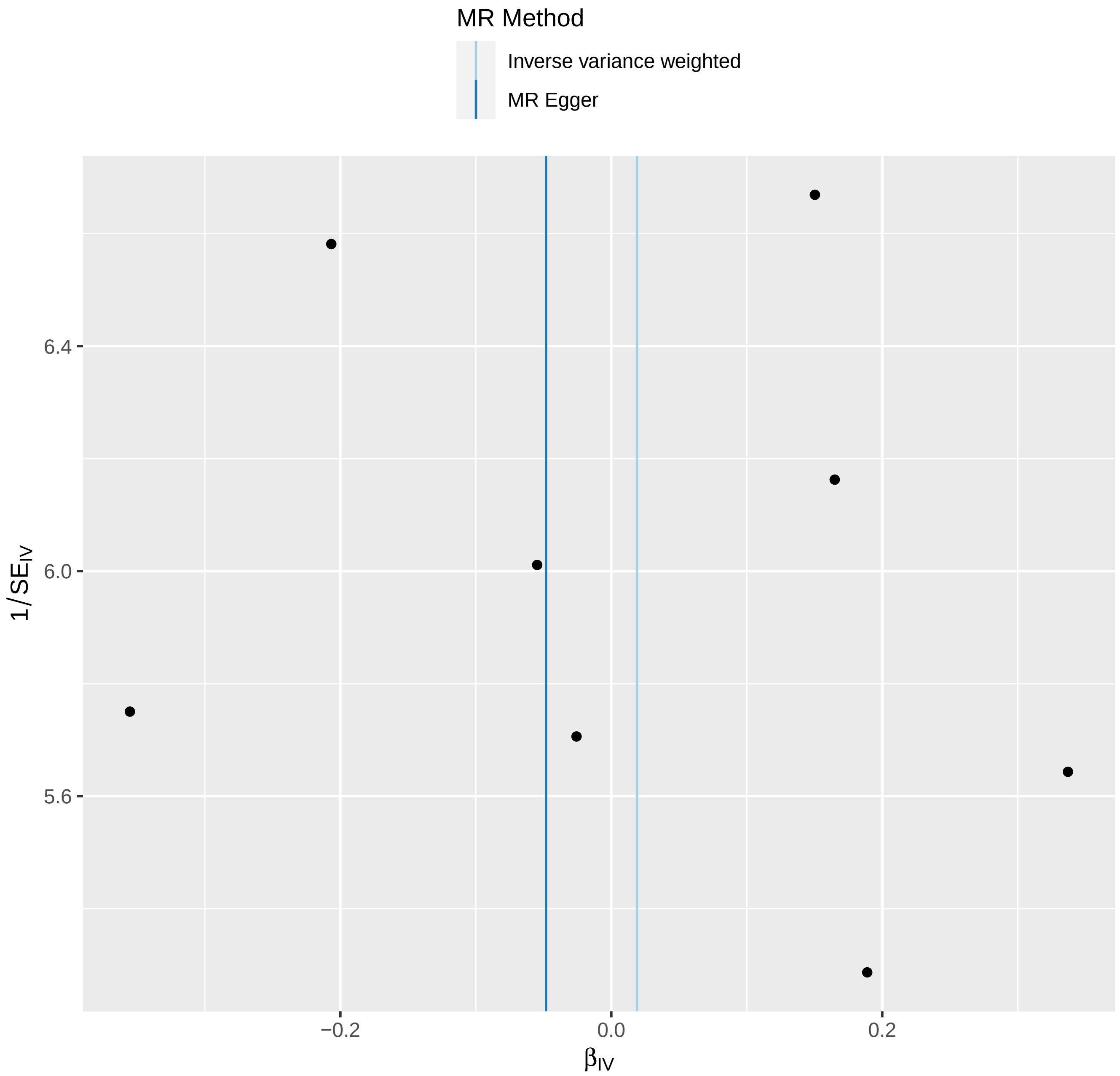

Batch 335 : Gut microbiota abundance (genus Akkermansia id.4037) on Obesity due to excess calories

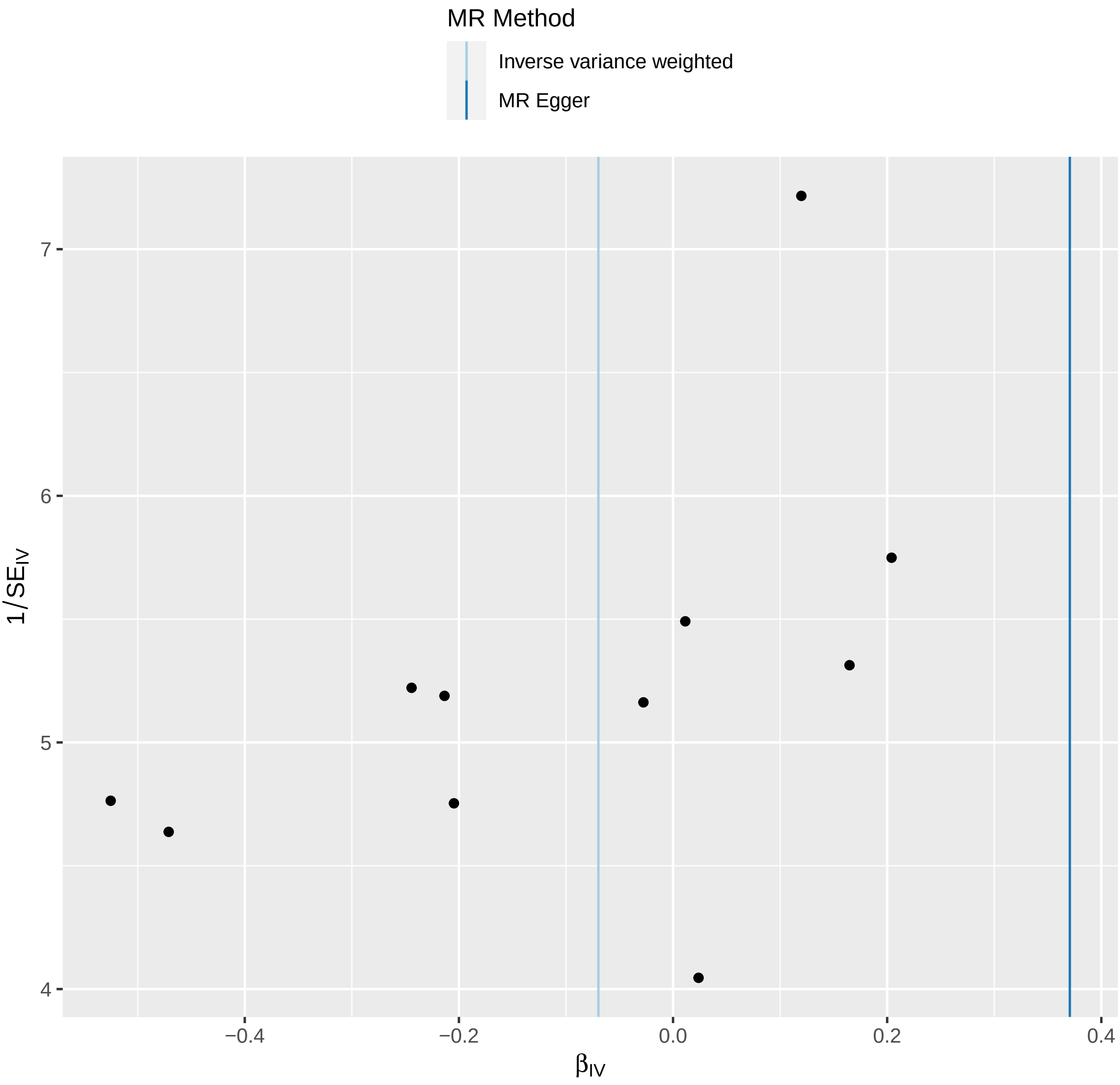

Batch 336 : Gut microbiota abundance (genus Alistipes id.968) on Obesity due to excess calories

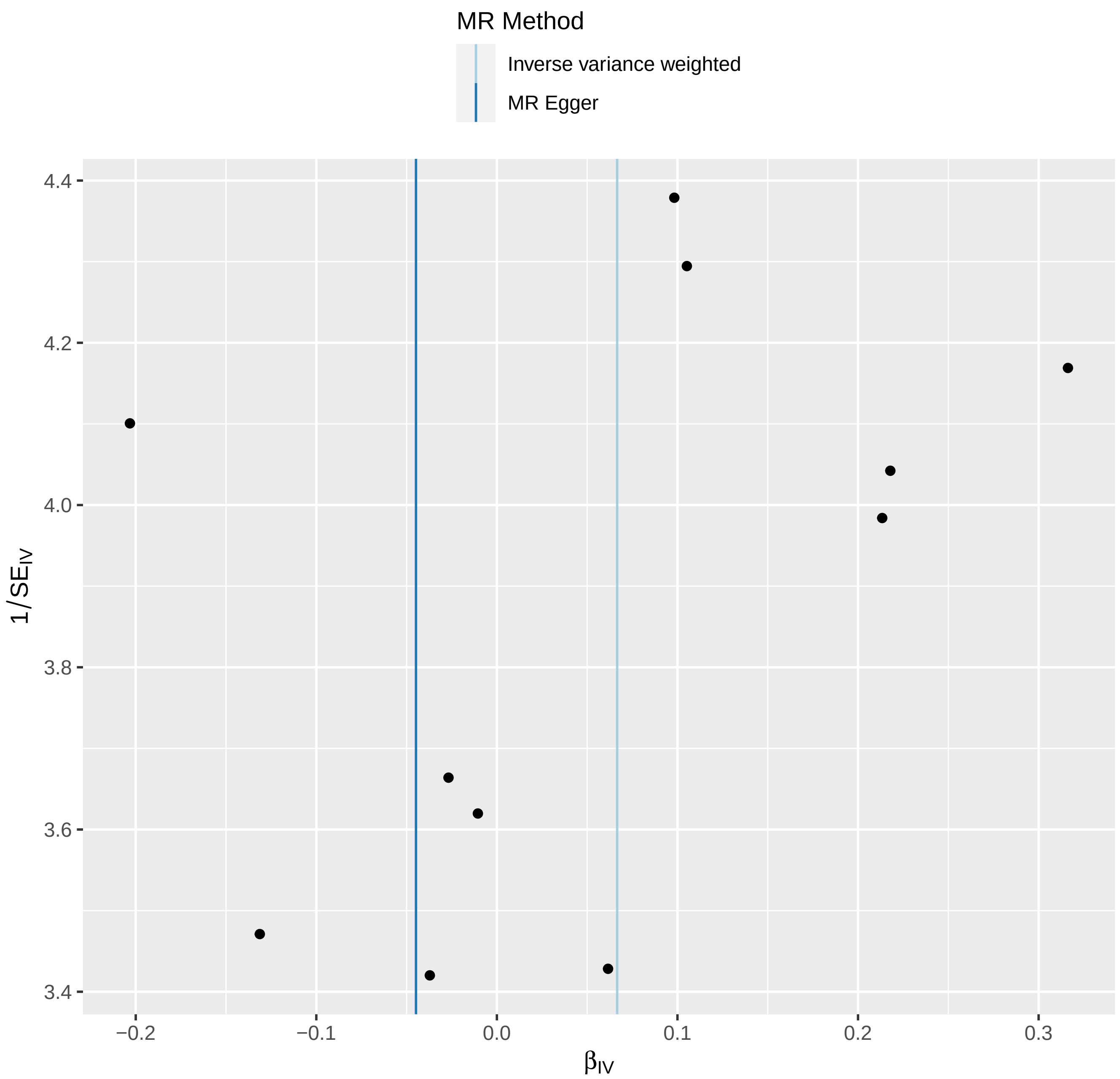

Batch 337 : Gut microbiota abundance (genus Allisonella id.2174) on Obesity due to excess calories

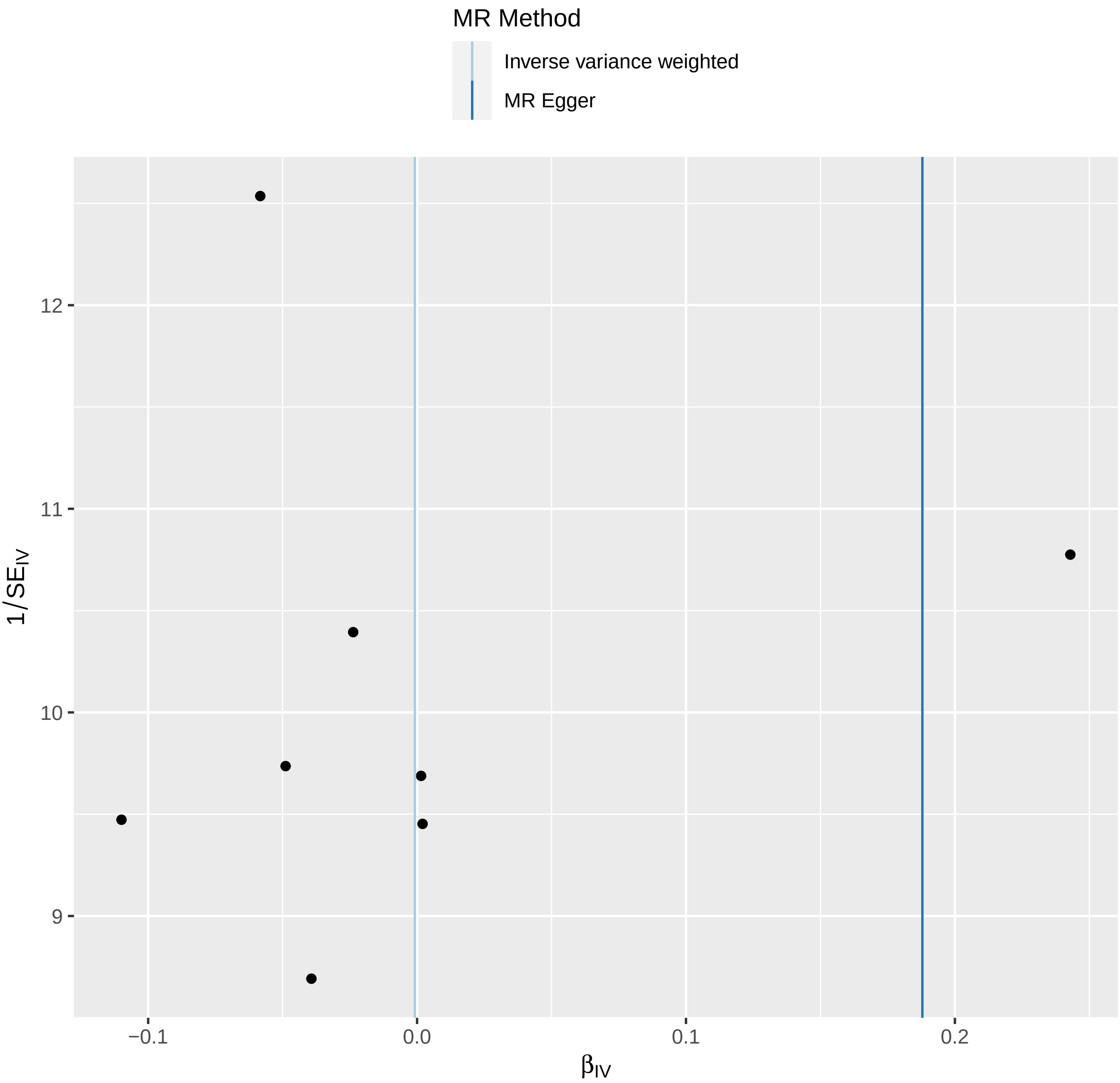

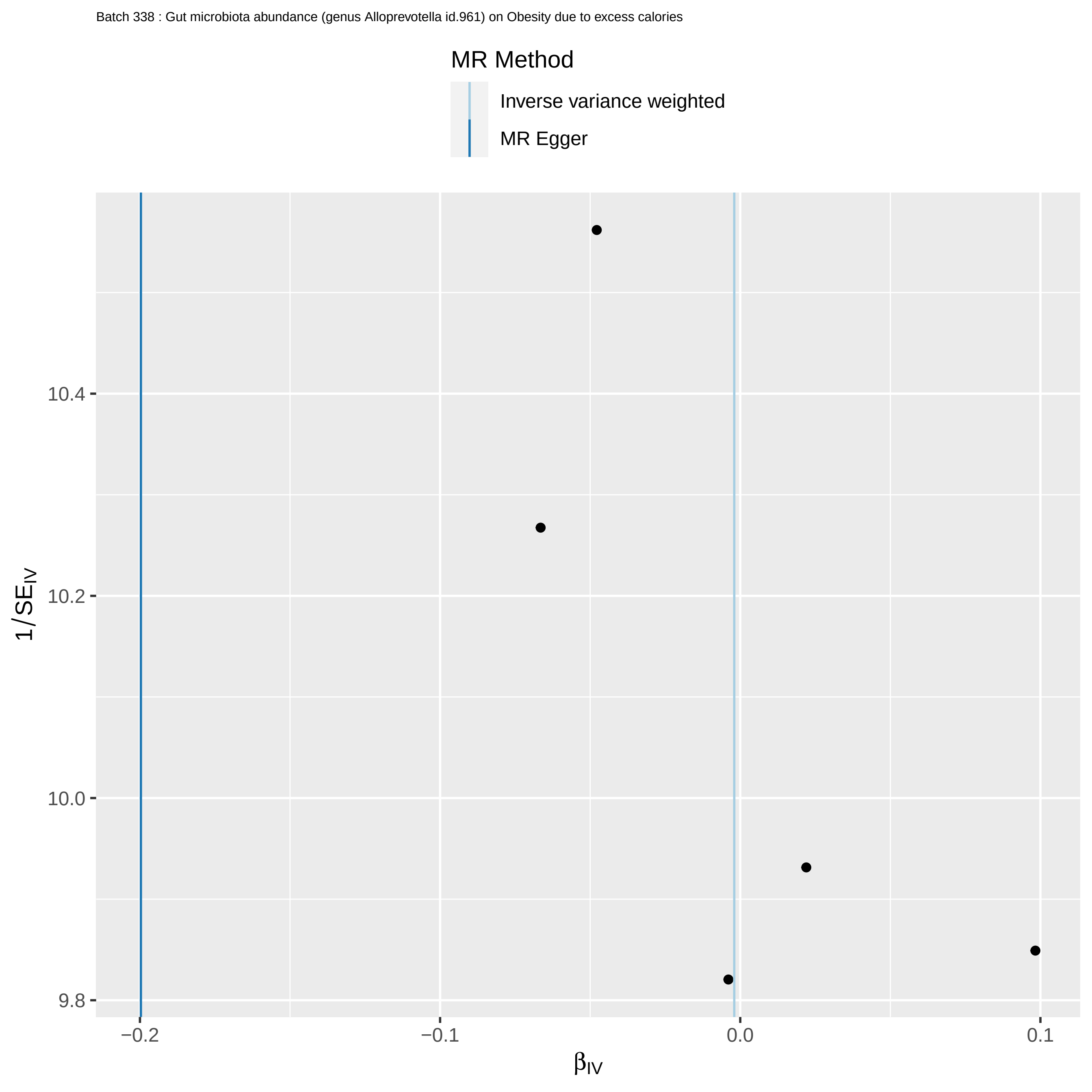

Batch 338 : Gut microbiota abundance (genus Alloprevotella id.961) on Obesity due to excess calories
MR Method
Inverse variance weighted
MR Egger
10.4
10.2
10.0
9.8
1/SE$_{IV}$
−0.2
−0.1
0.0
0.1
β$_{IV}$

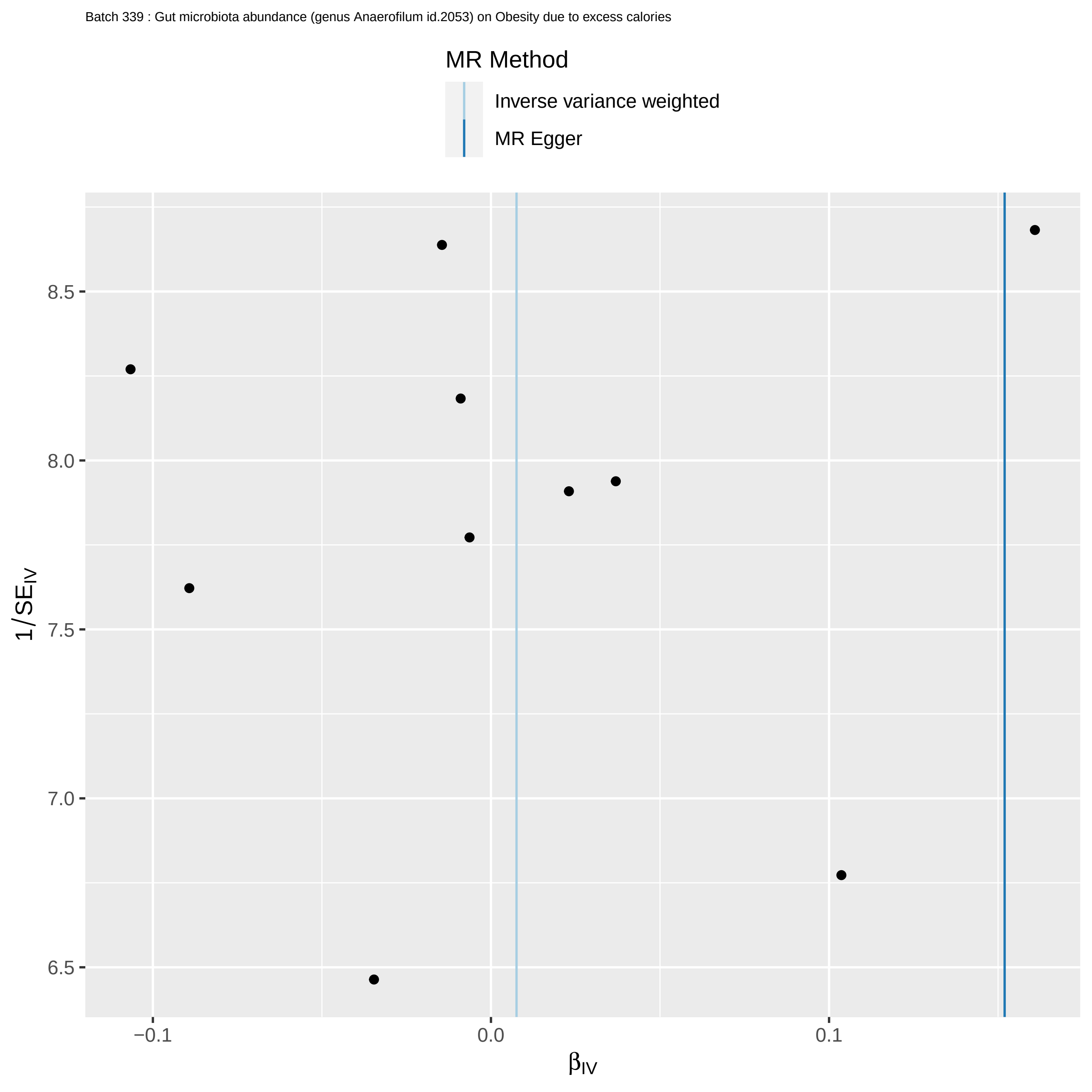

Batch 339 : Gut microbiota abundance (genus Anaerofilum id.2053) on Obesity due to excess calories
MR Method
Inverse variance weighted
MR Egger
8.5
8.0
7.5
7.0
6.5
1/SE$_{IV}$
−0.1
0.0
0.1
β$_{IV}$

Batch 340 : Gut microbiota abundance (genus Anaerostipes id.1991) on Obesity due to excess calories

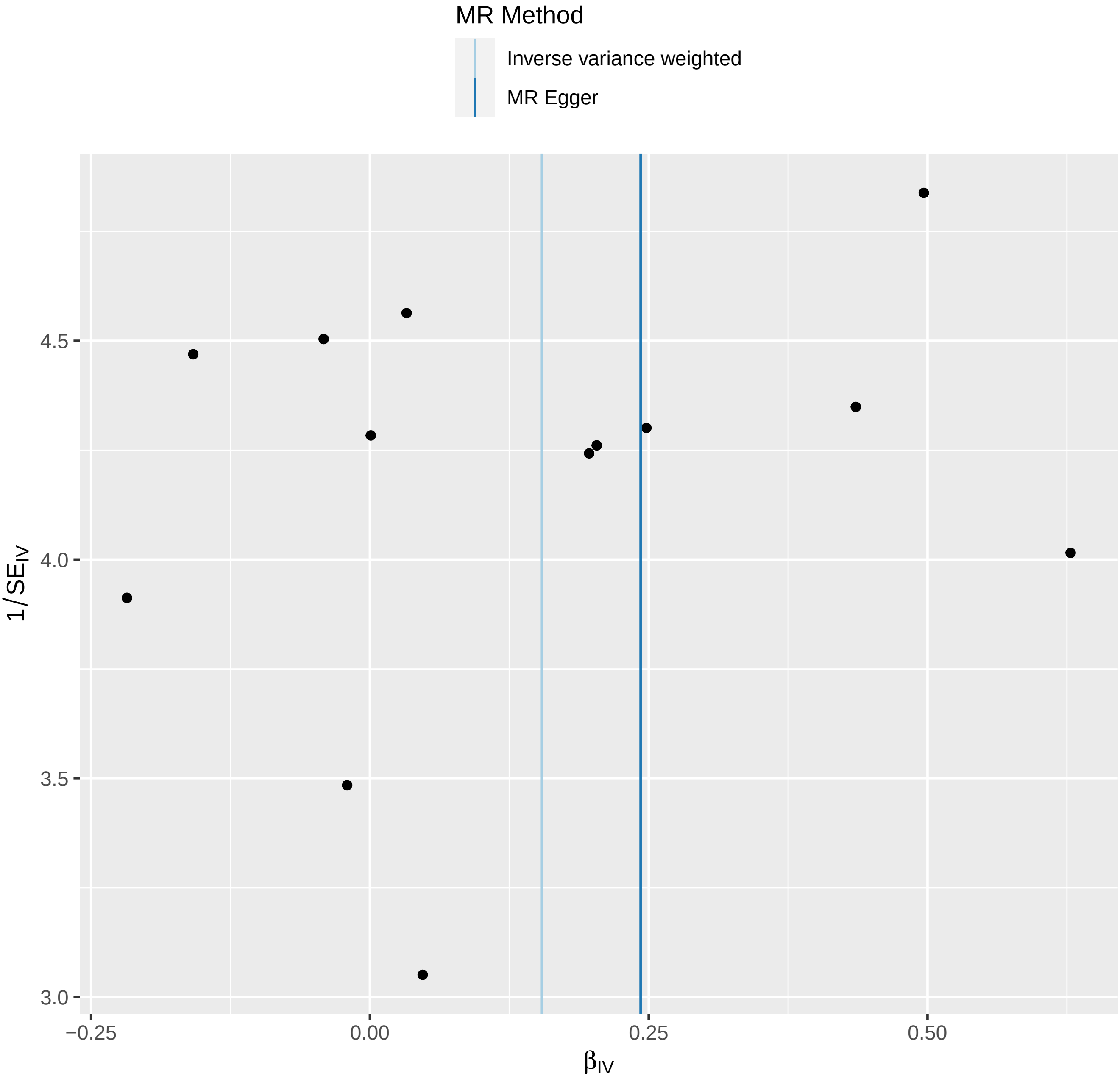

Batch 341 : Gut microbiota abundance (genus Anaerotruncus id.2054) on Obesity due to excess calories

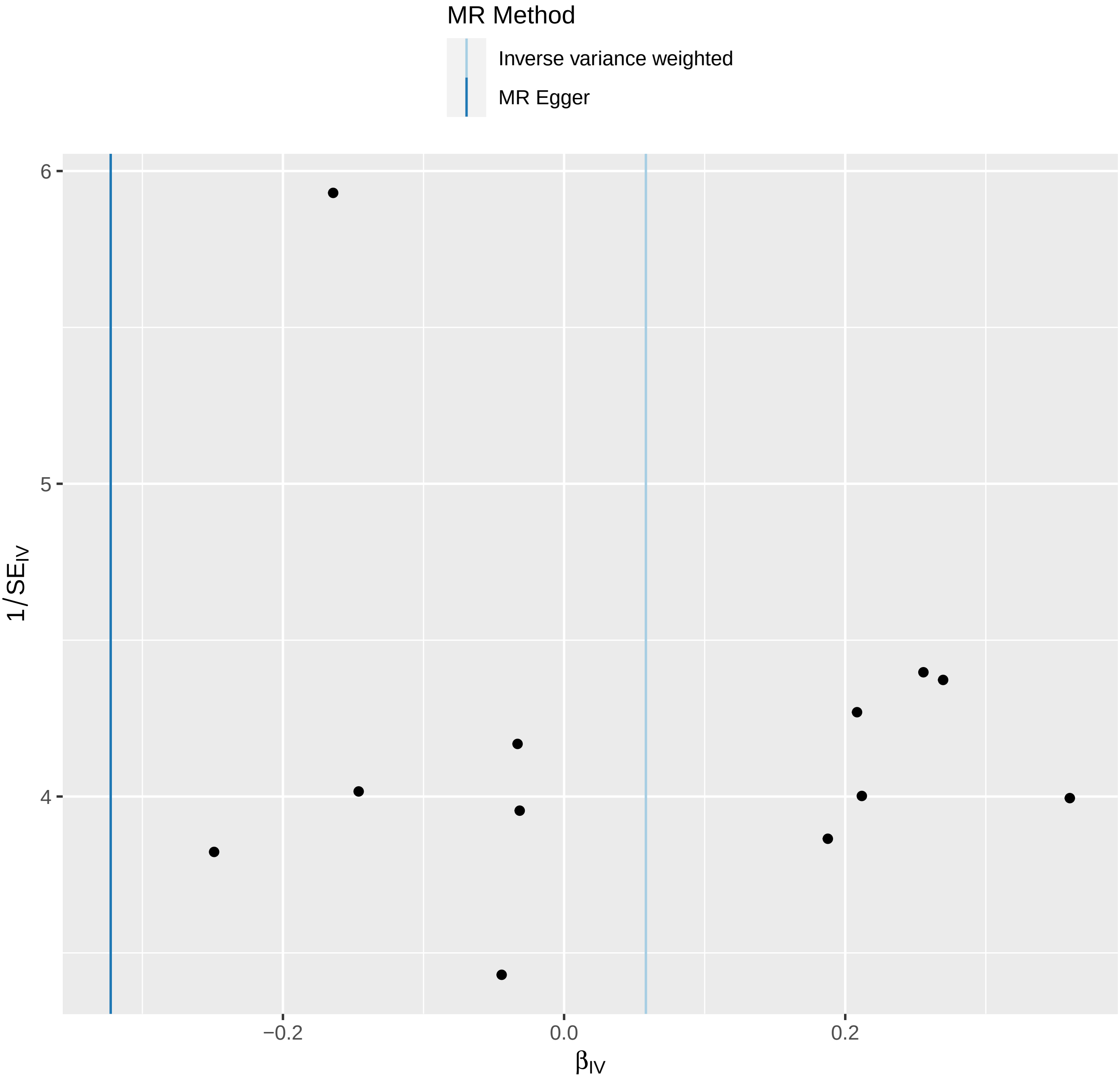

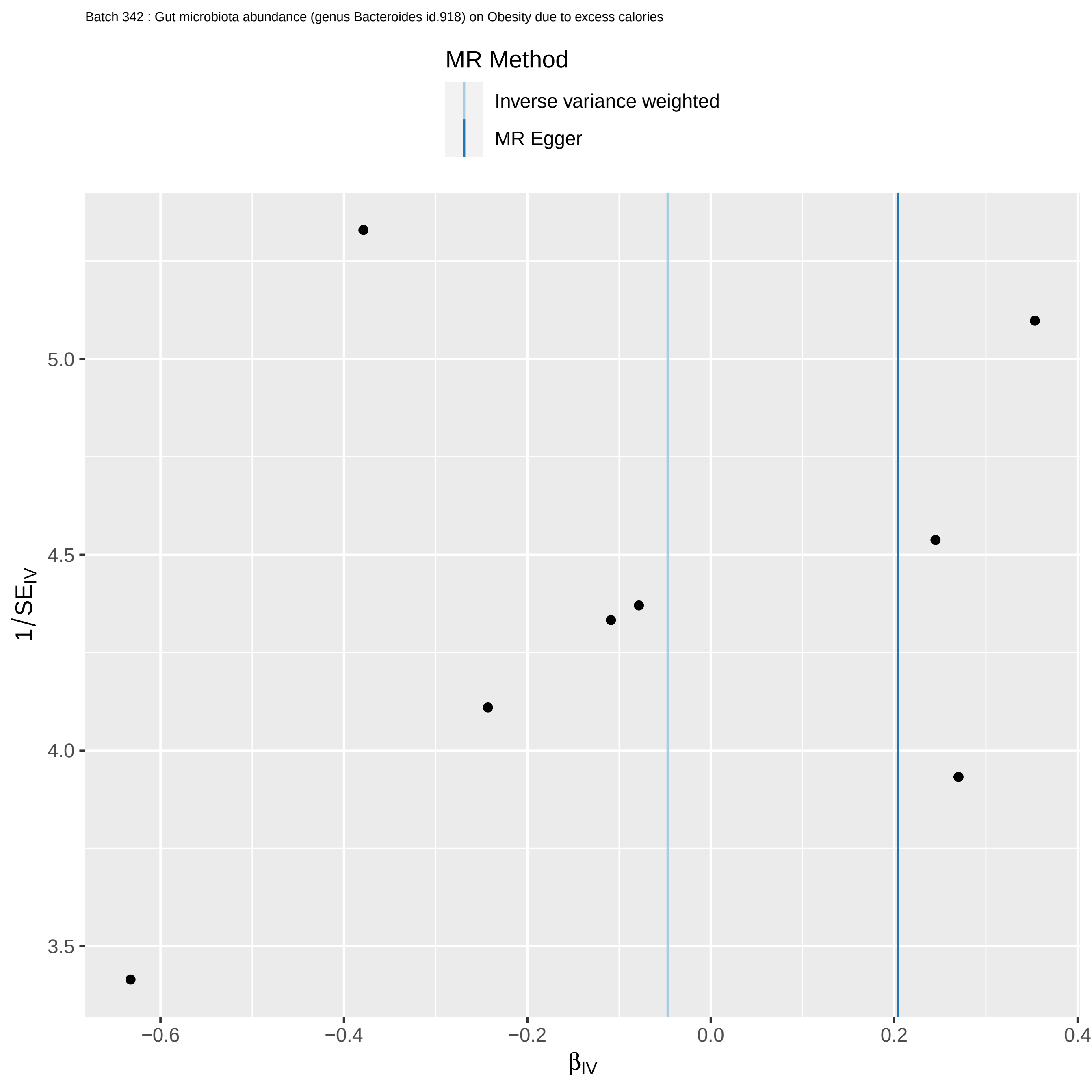
Batch 342 : Gut microbiota abundance (genus Bacteroides id.918) on Obesity due to excess calories
MR Method
Inverse variance weighted
MR Egger
5.0
4.5
4.0
3.5
1/SE_IV
−0.6
−0.4
−0.2
0.0
0.2
0.4
β_IV

Batch 343 : Gut microbiota abundance (genus Barnesiella id.944) on Obesity due to excess calories

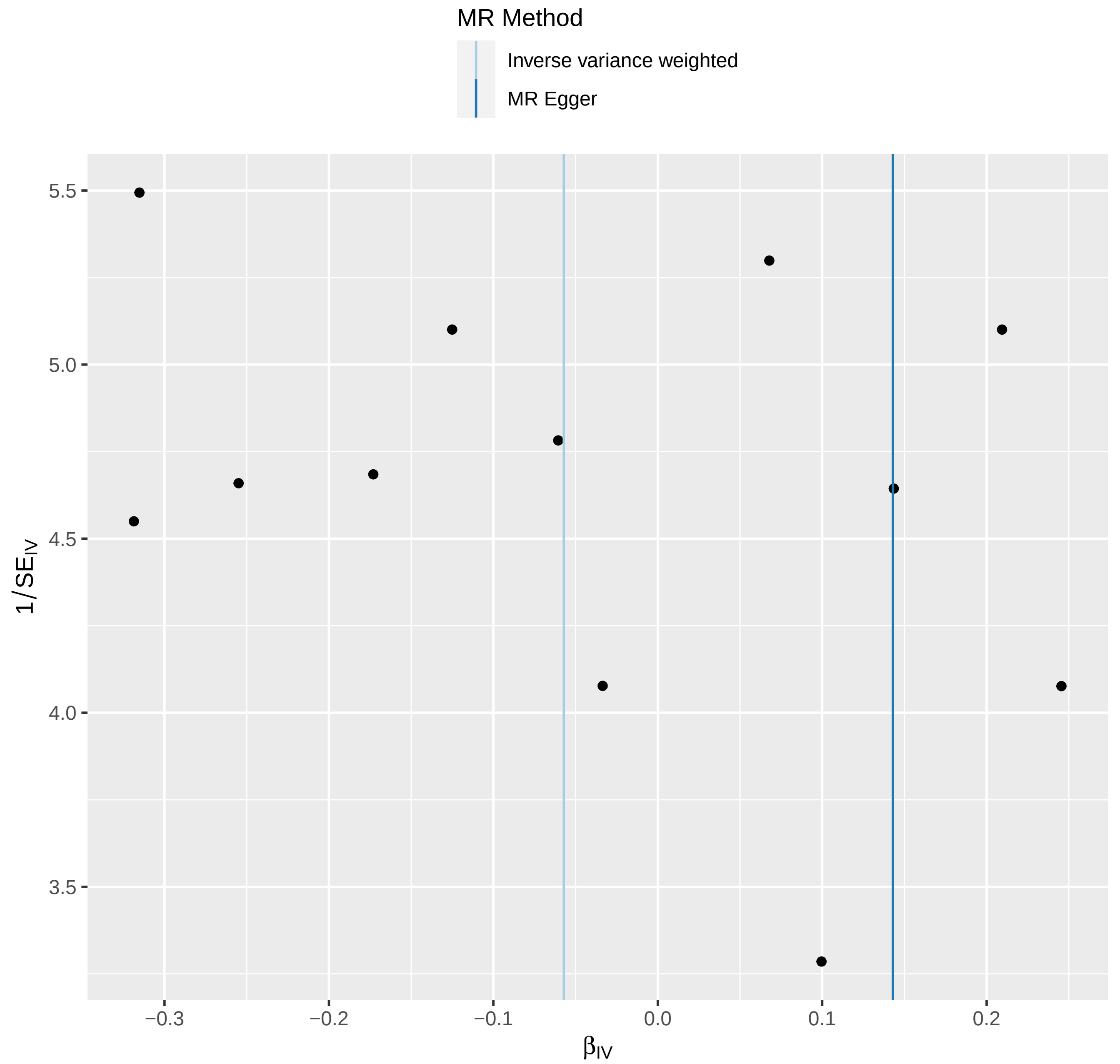

Batch 344 : Gut microbiota abundance (genus Bifidobacterium id.436) on Obesity due to excess calories

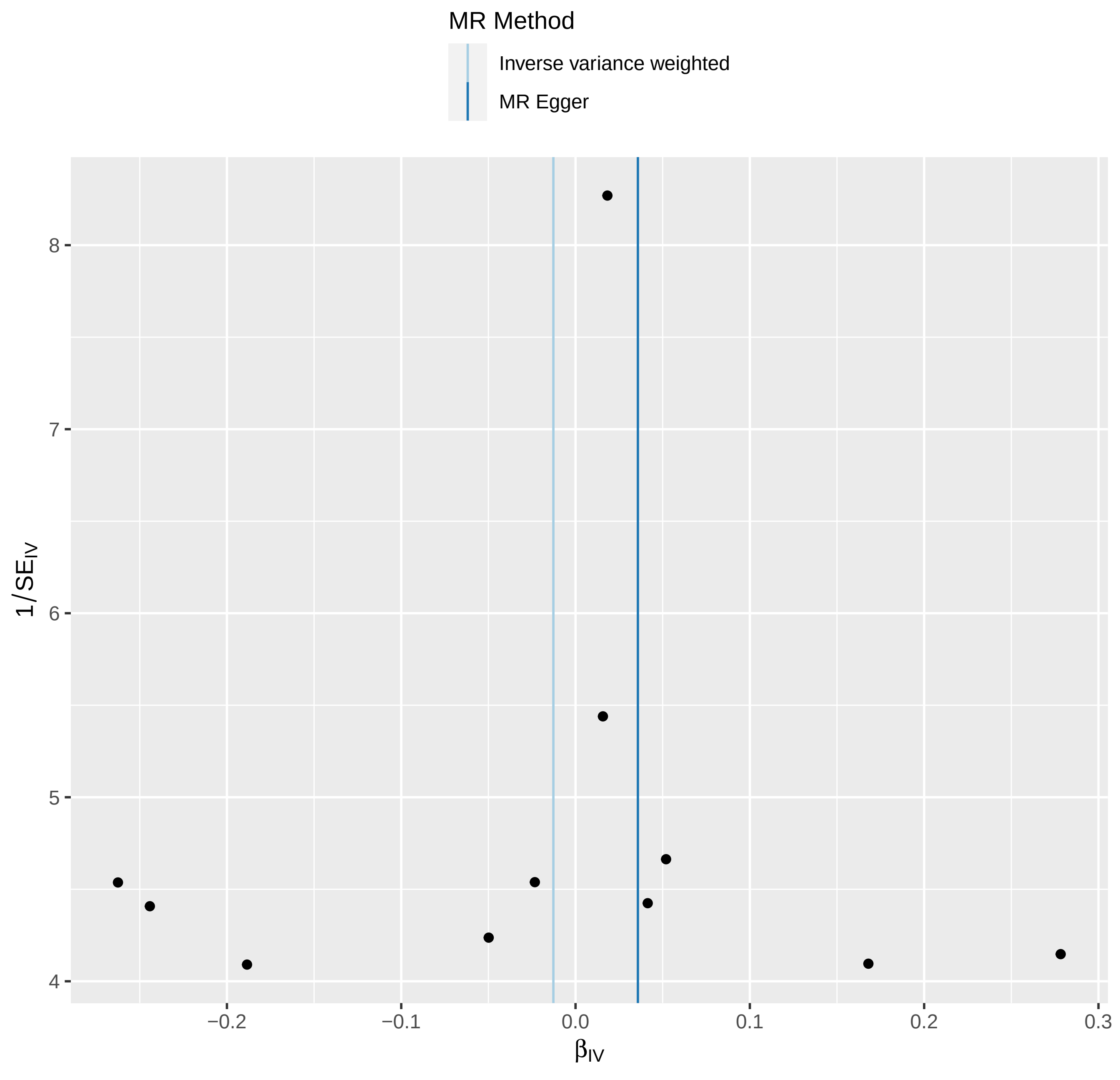

Batch 345 : Gut microbiota abundance (genus Bilophila id.3170) on Obesity due to excess calories

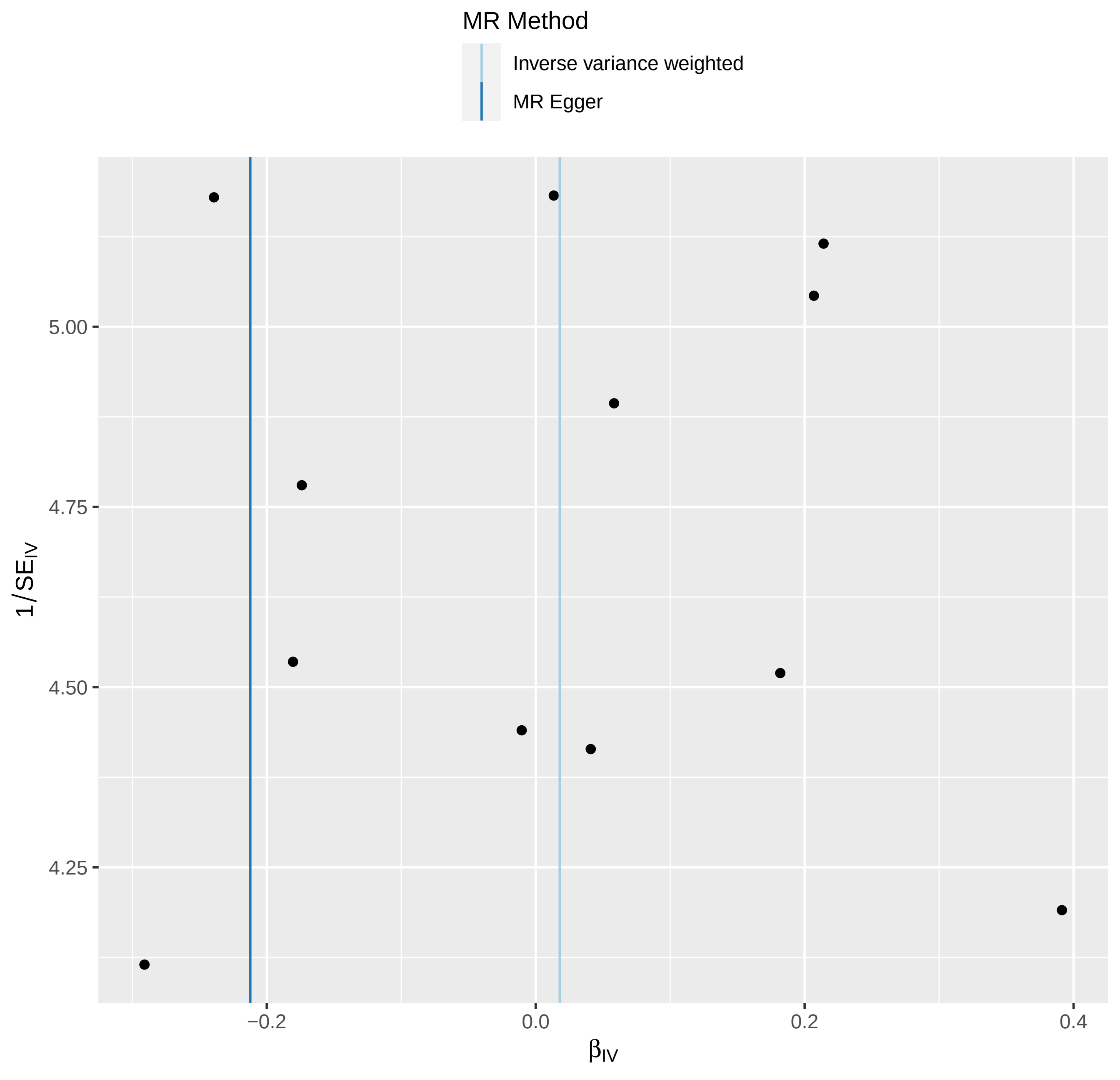

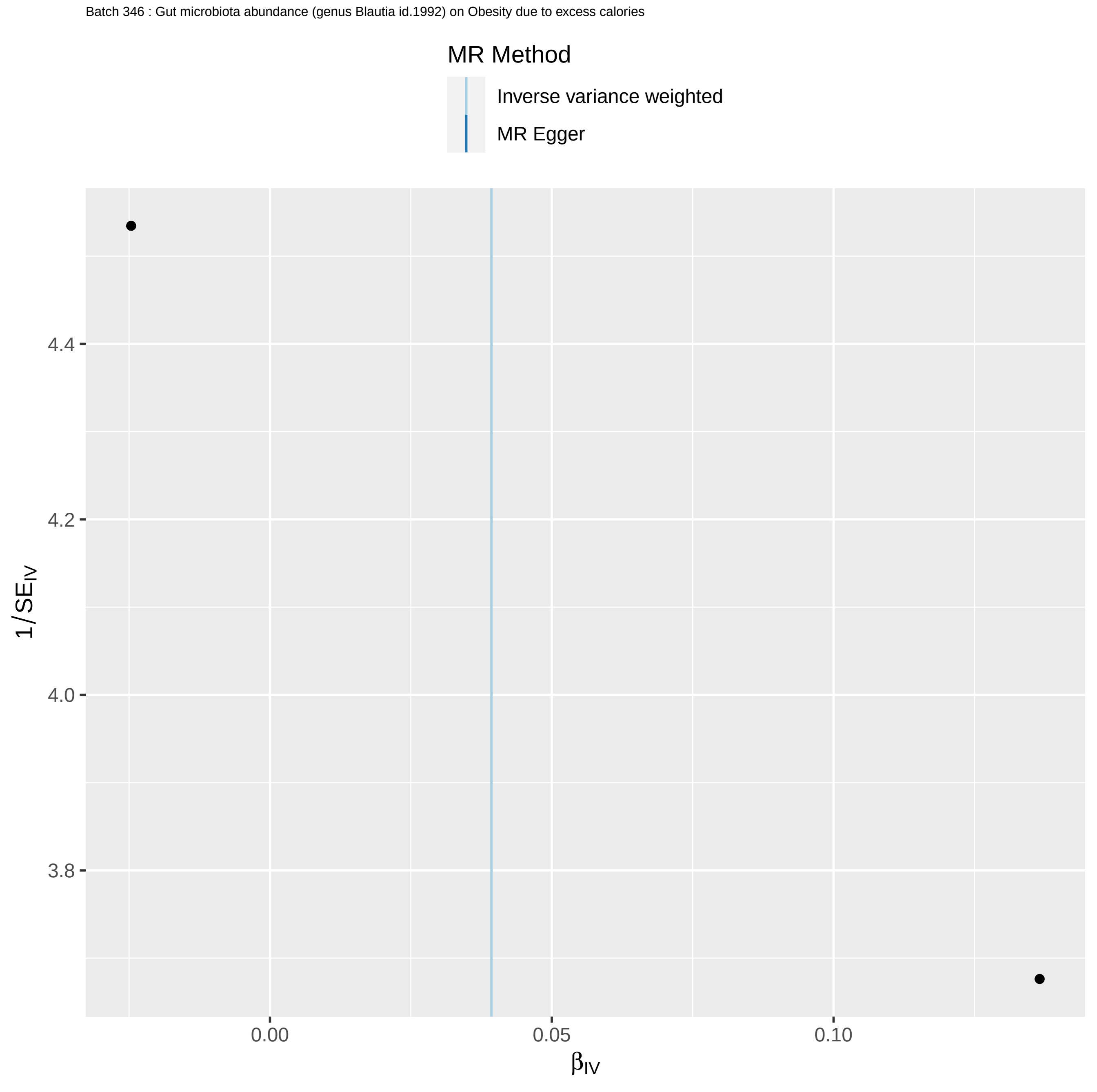

Batch 346 : Gut microbiota abundance (genus Blautia id.1992) on Obesity due to excess calories
MR Method
Inverse variance weighted
MR Egger
4.4
4.2
4.0
3.8
$1/SE_{IV}$
0.00
0.05
0.10
$\beta_{IV}$

Batch 347 : Gut microbiota abundance (genus Butyricicoccus id.2055) on Obesity due to excess calories

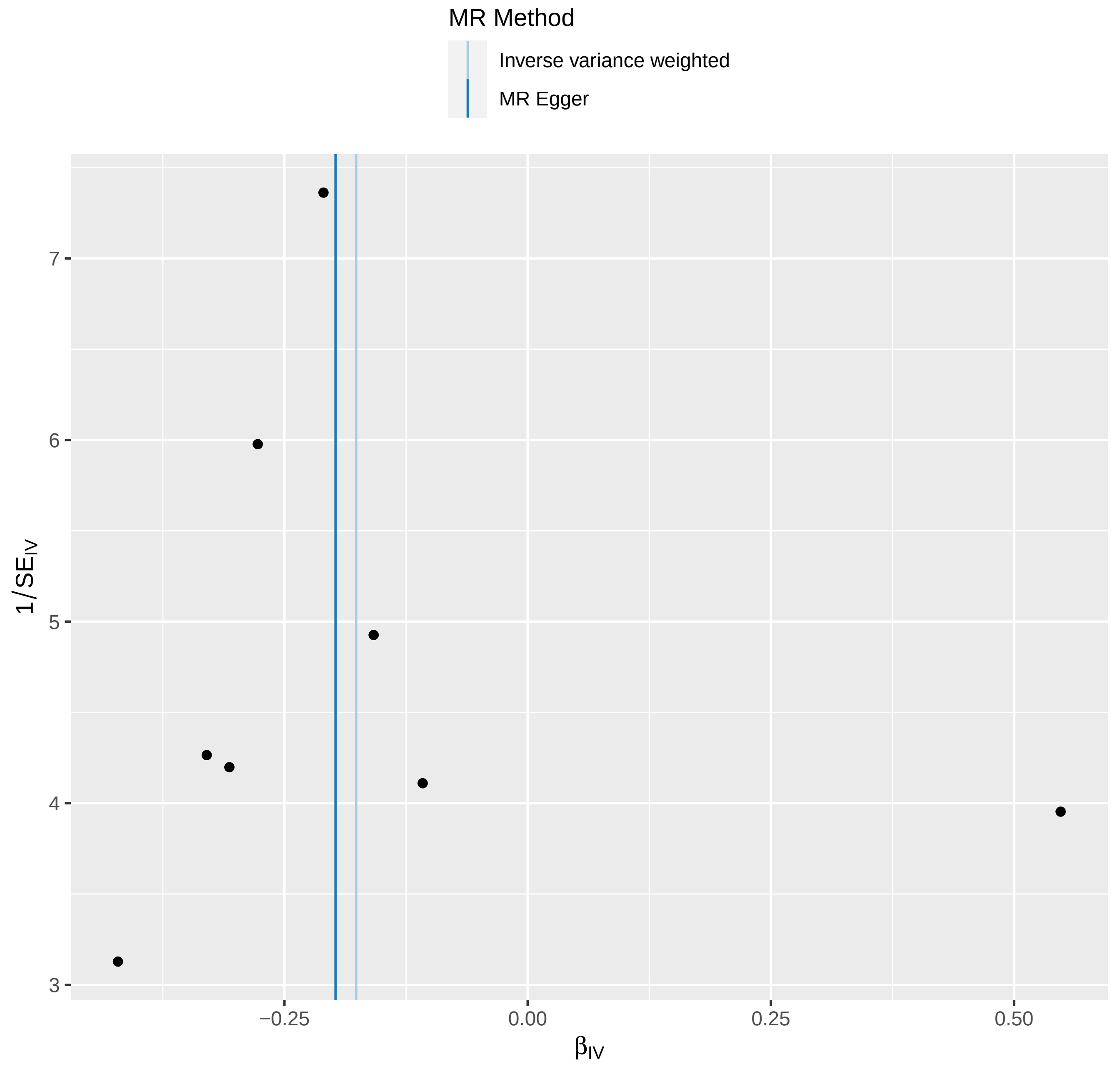

Batch 348 : Gut microbiota abundance (genus Butyricimonas id.945) on Obesity due to excess calories

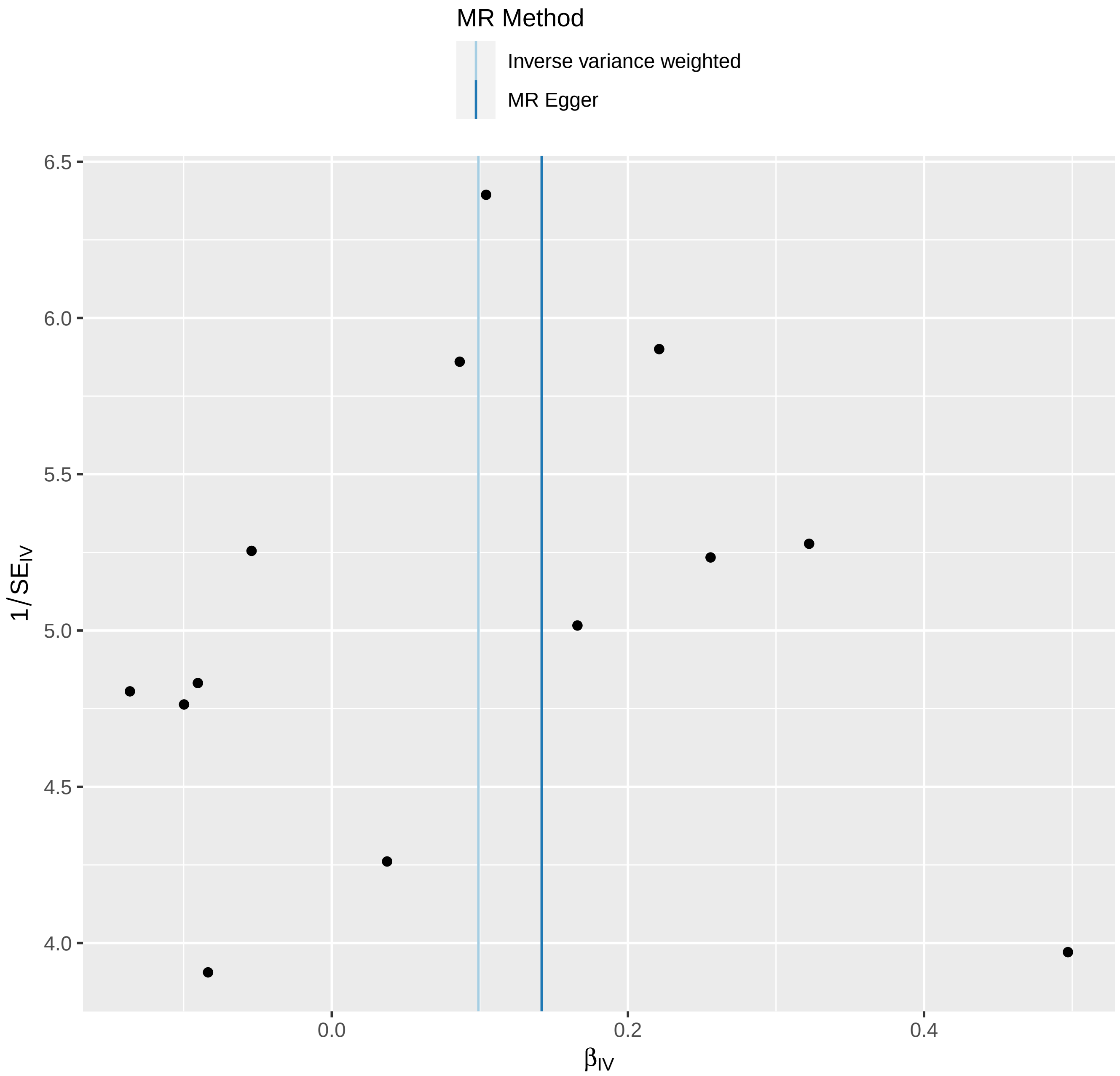

Batch 349 : Gut microbiota abundance (genus Butyrivibrio id.1993) on Obesity due to excess calories

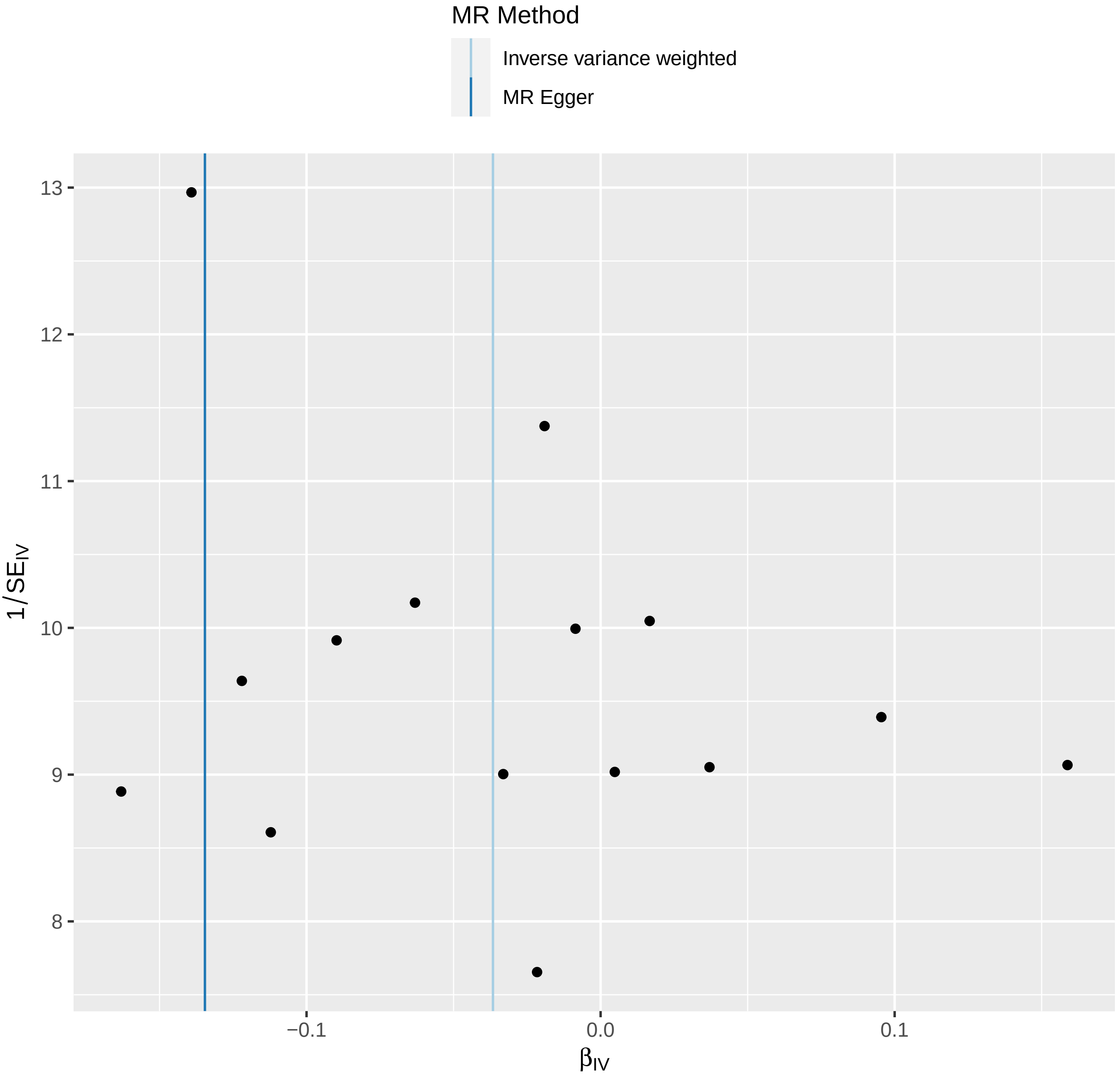

Batch 350 : Gut microbiota abundance (genus Candidatus Soleaferrea id.11350) on Obesity due to excess calories

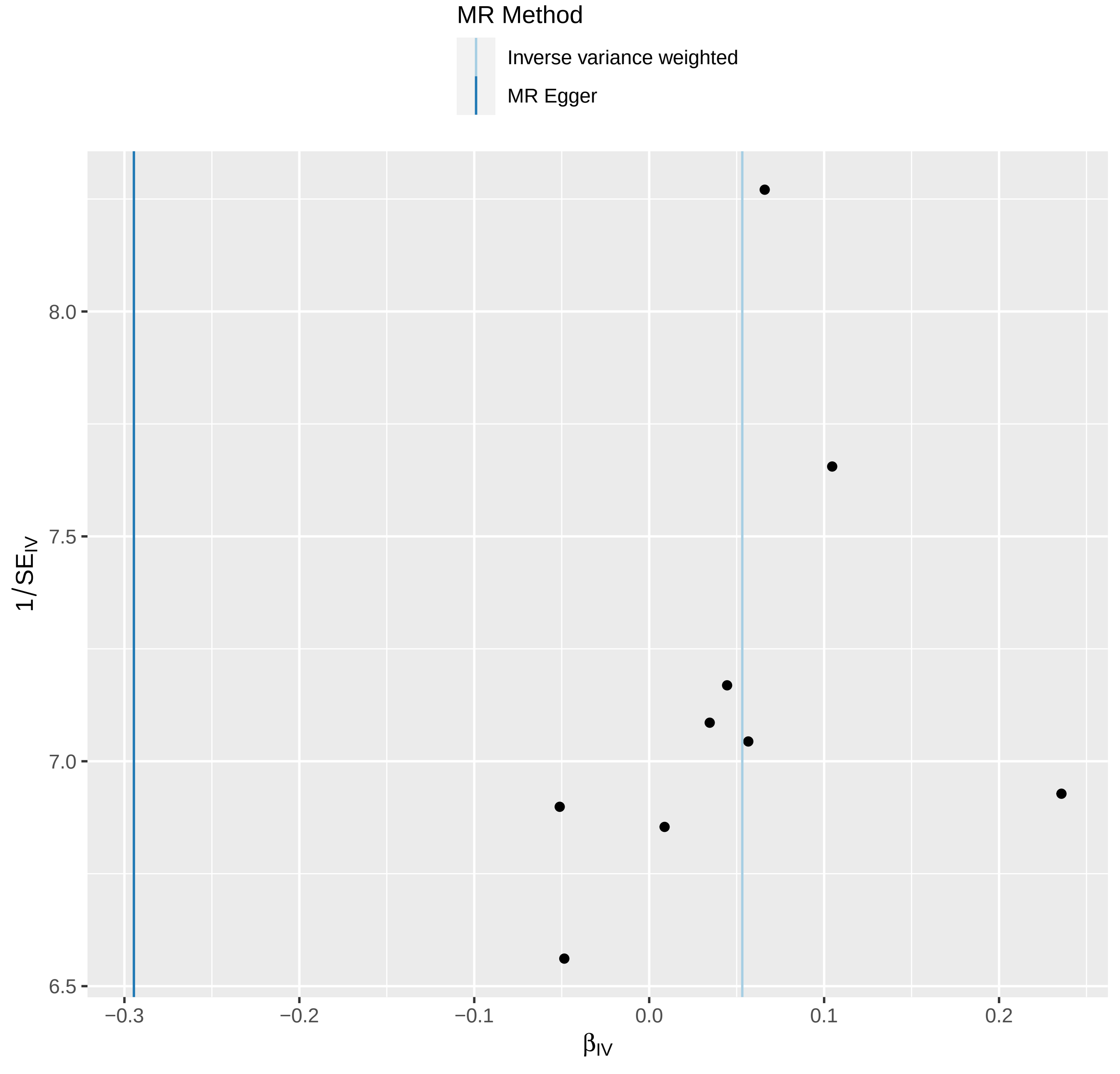

Batch 351 : Gut microbiota abundance (genus Catenibacterium id.2153) on Obesity due to excess calories

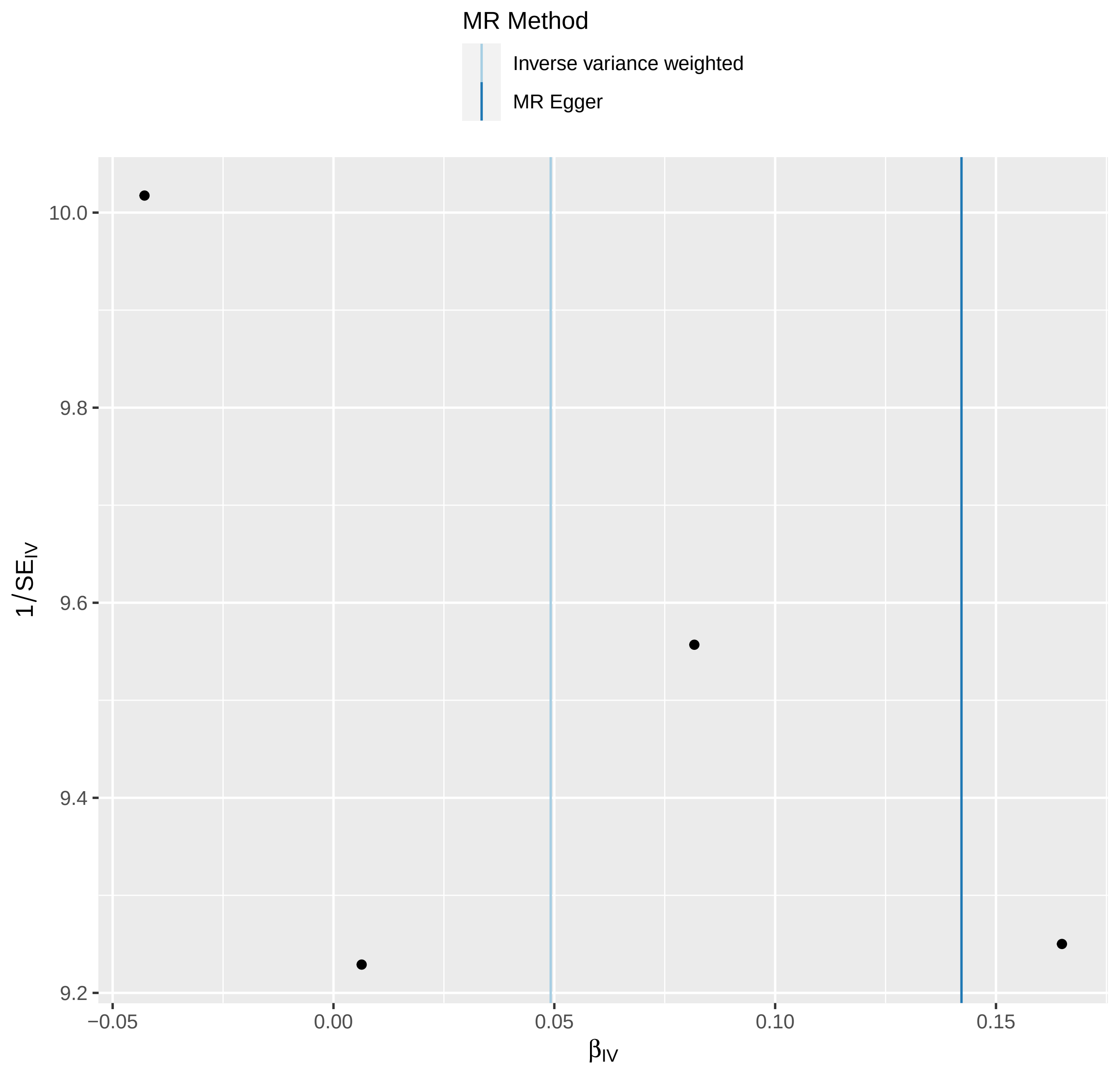

Batch 352 : Gut microbiota abundance (genus Christensenellaceae R 7group id.11283) on Obesity due to excess calories

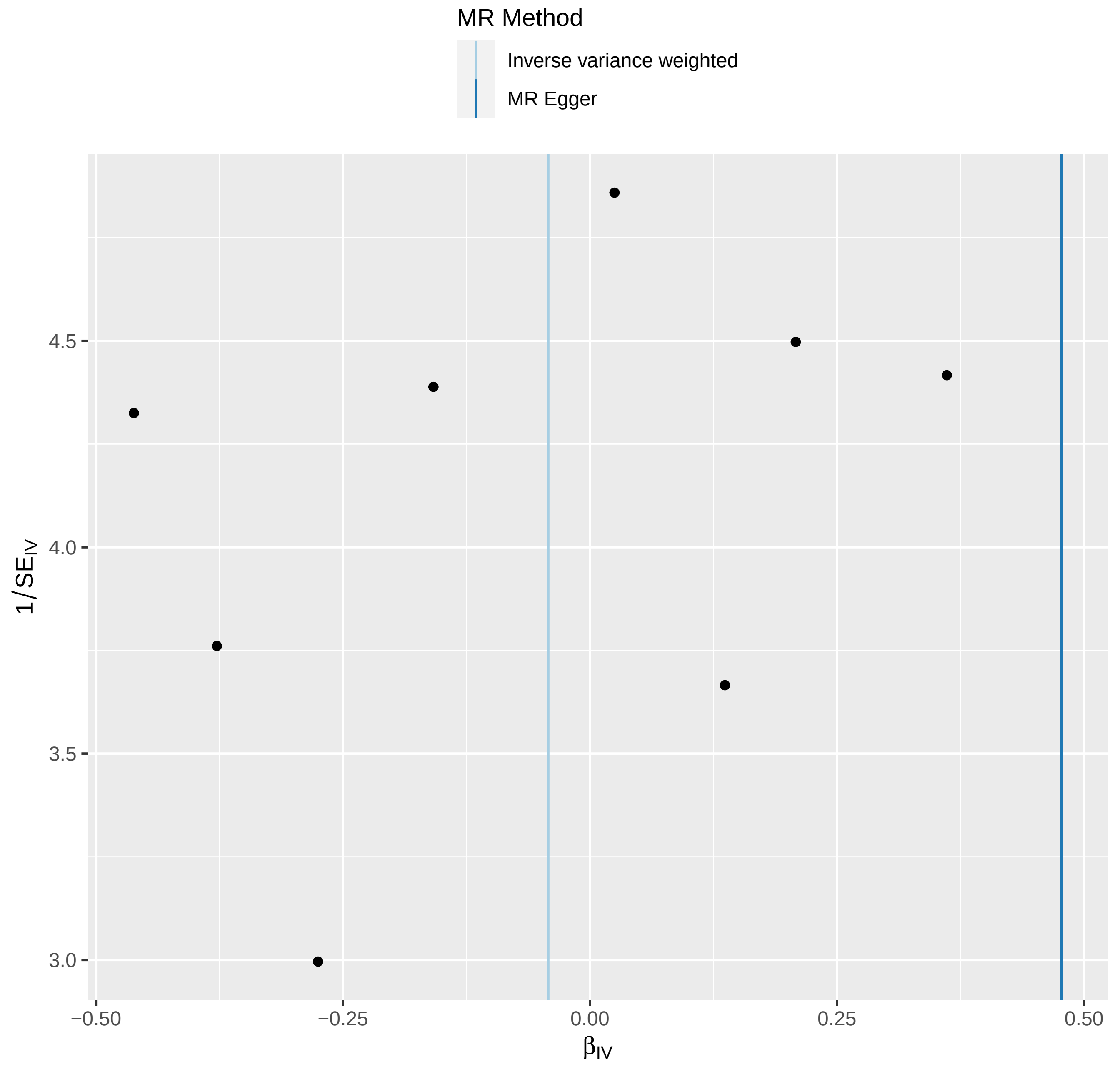

Batch 353 : Gut microbiota abundance (genus Clostridium innocuum group id.14397) on Obesity due to excess calories

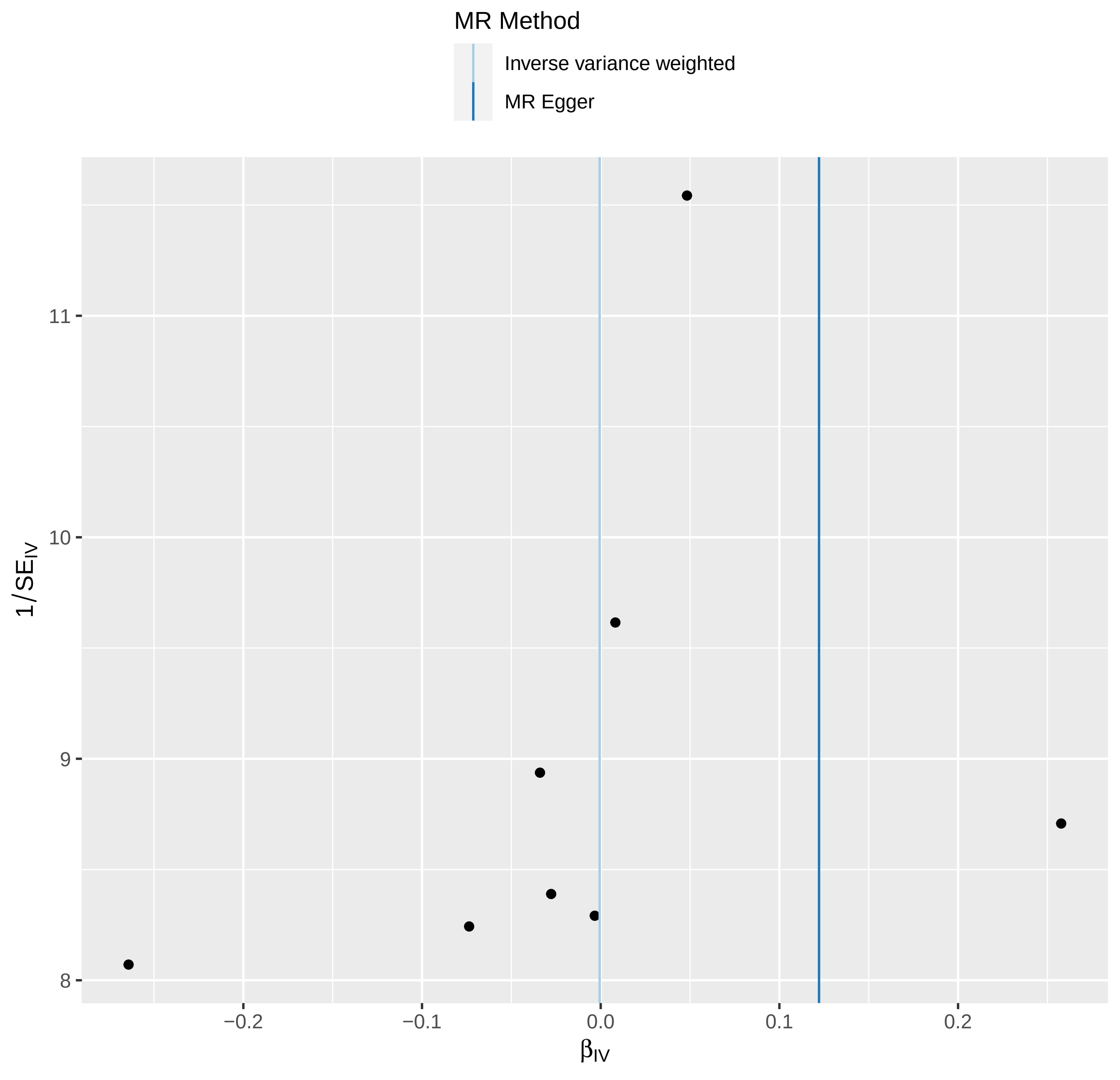

Batch 354 : Gut microbiota abundance (genus Clostridium sensustricto1 id.1873) on Obesity due to excess calories

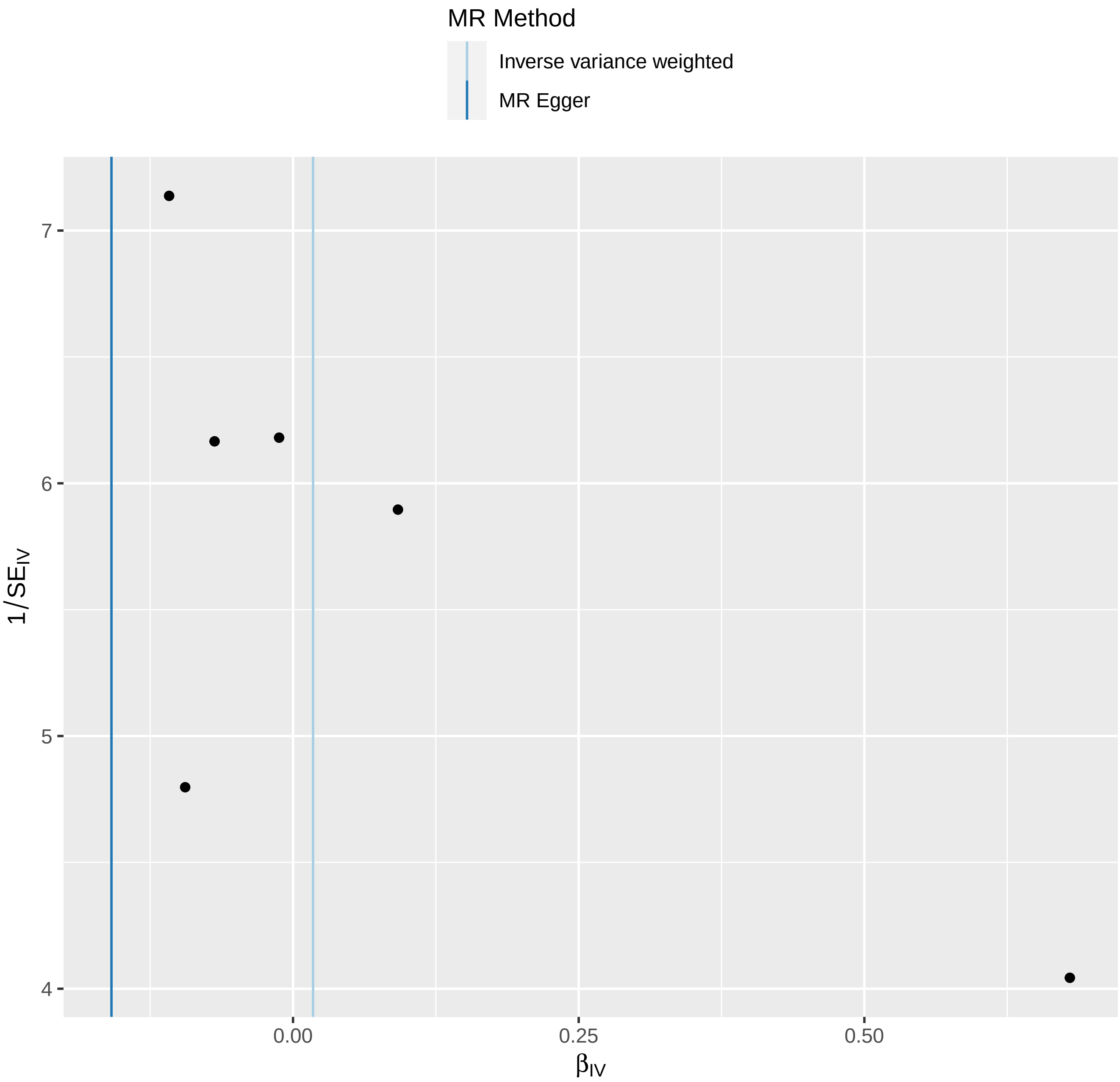

Batch 355 : Gut microbiota abundance (genus Collinsella id.815) on Obesity due to excess calories

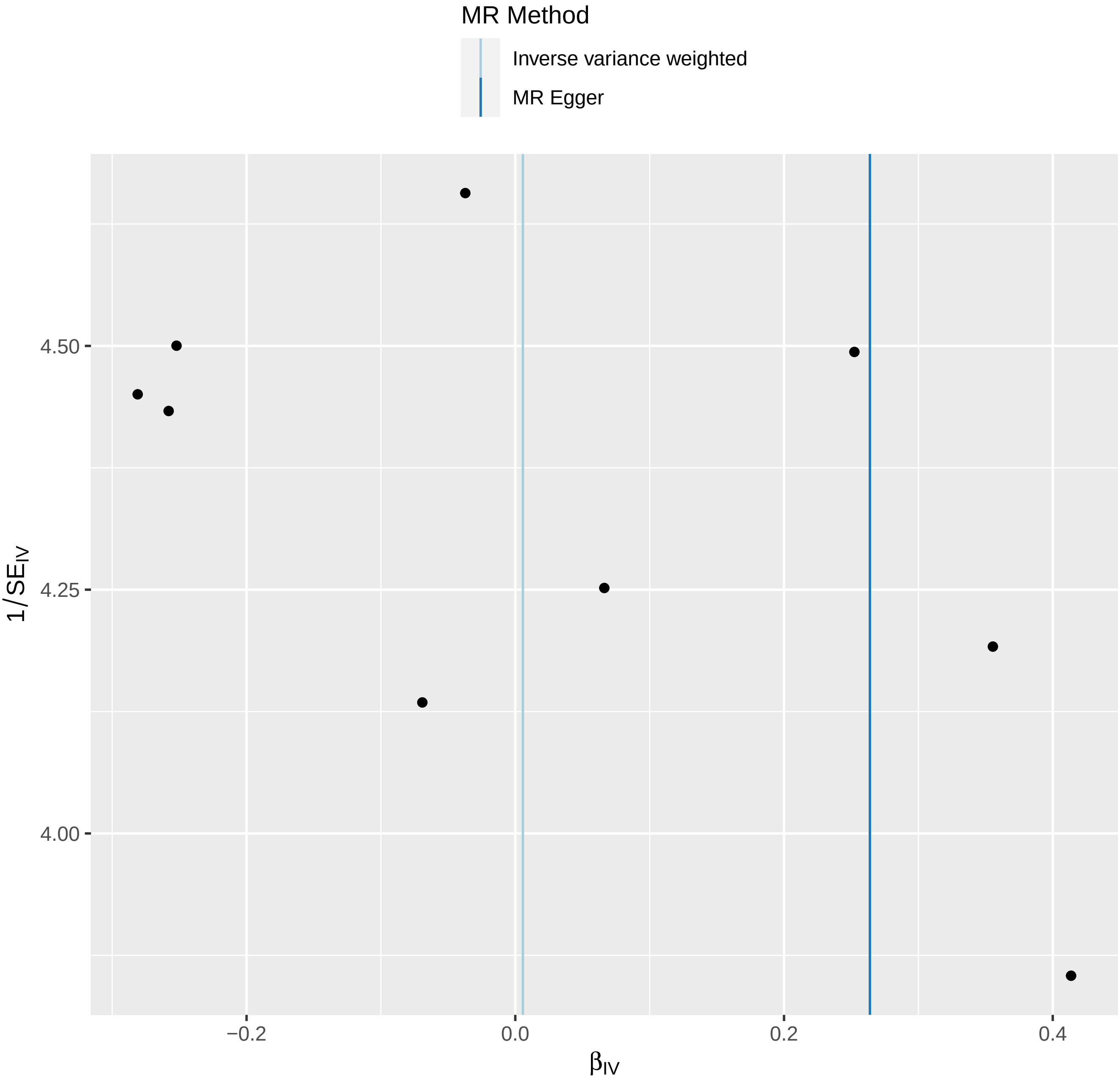

Batch 356 : Gut microbiota abundance (genus Coprobacter id.949) on Obesity due to excess calories

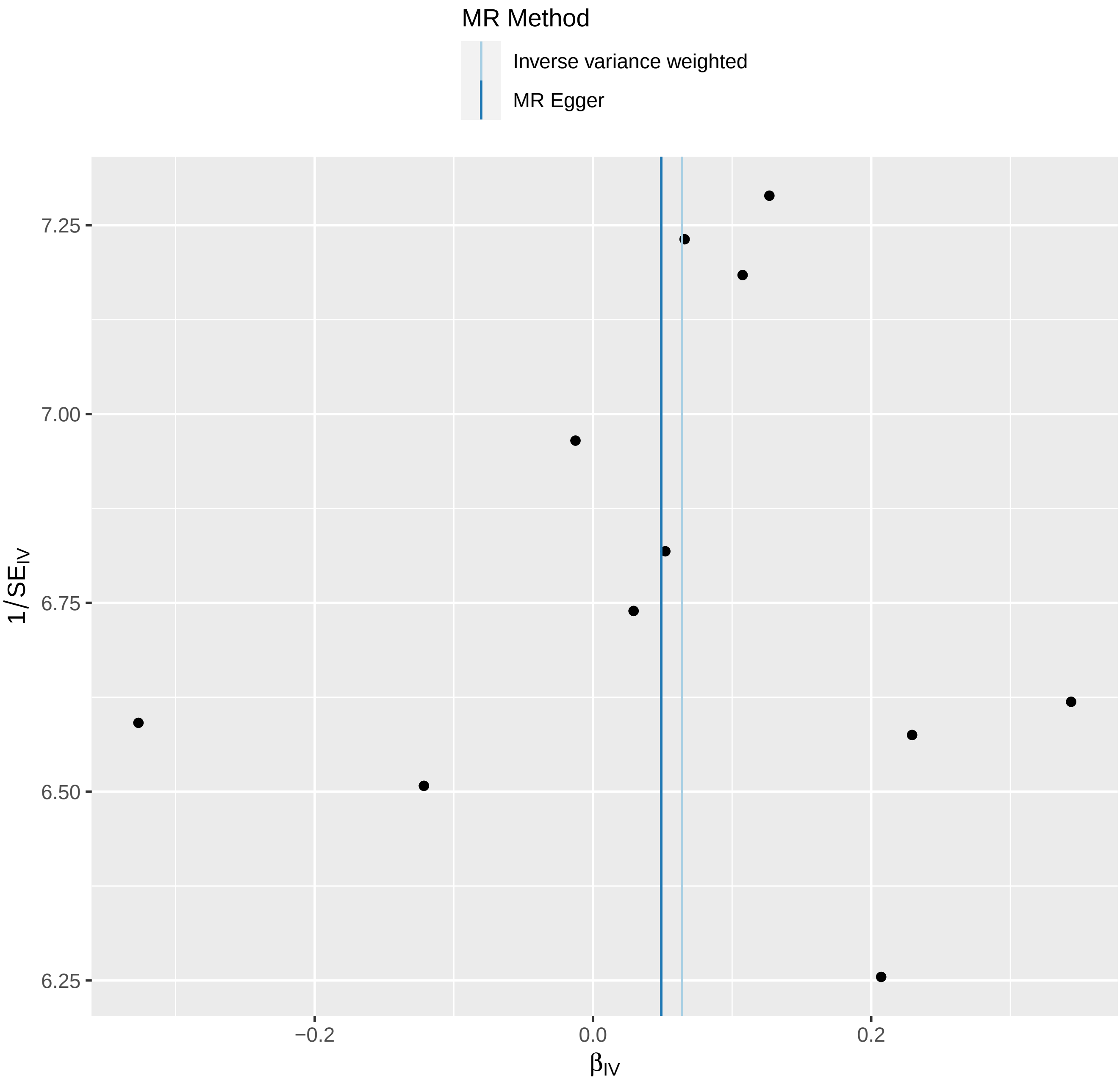

Batch 357 : Gut microbiota abundance (genus Coprococcus1 id.11301) on Obesity due to excess calories

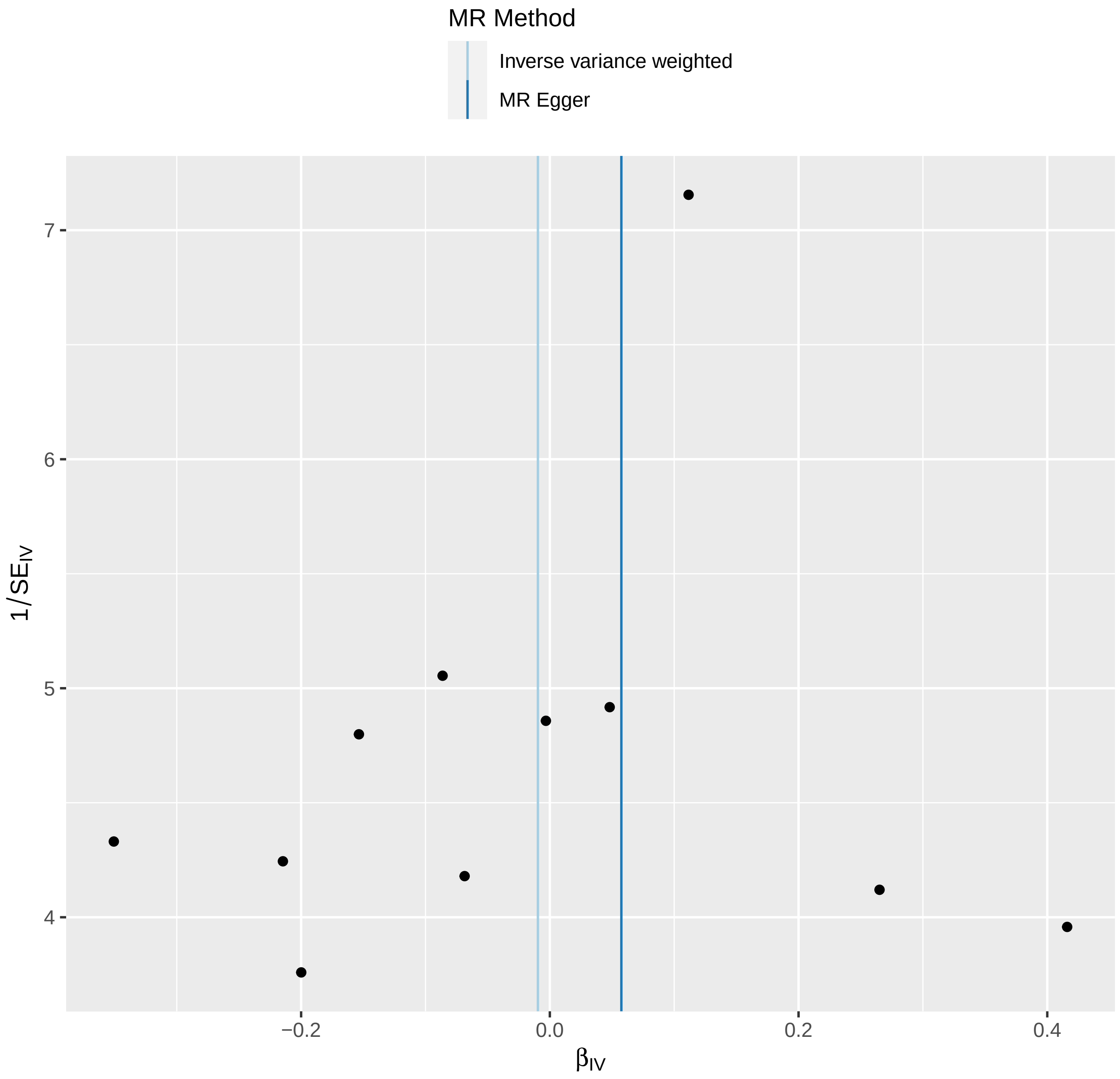

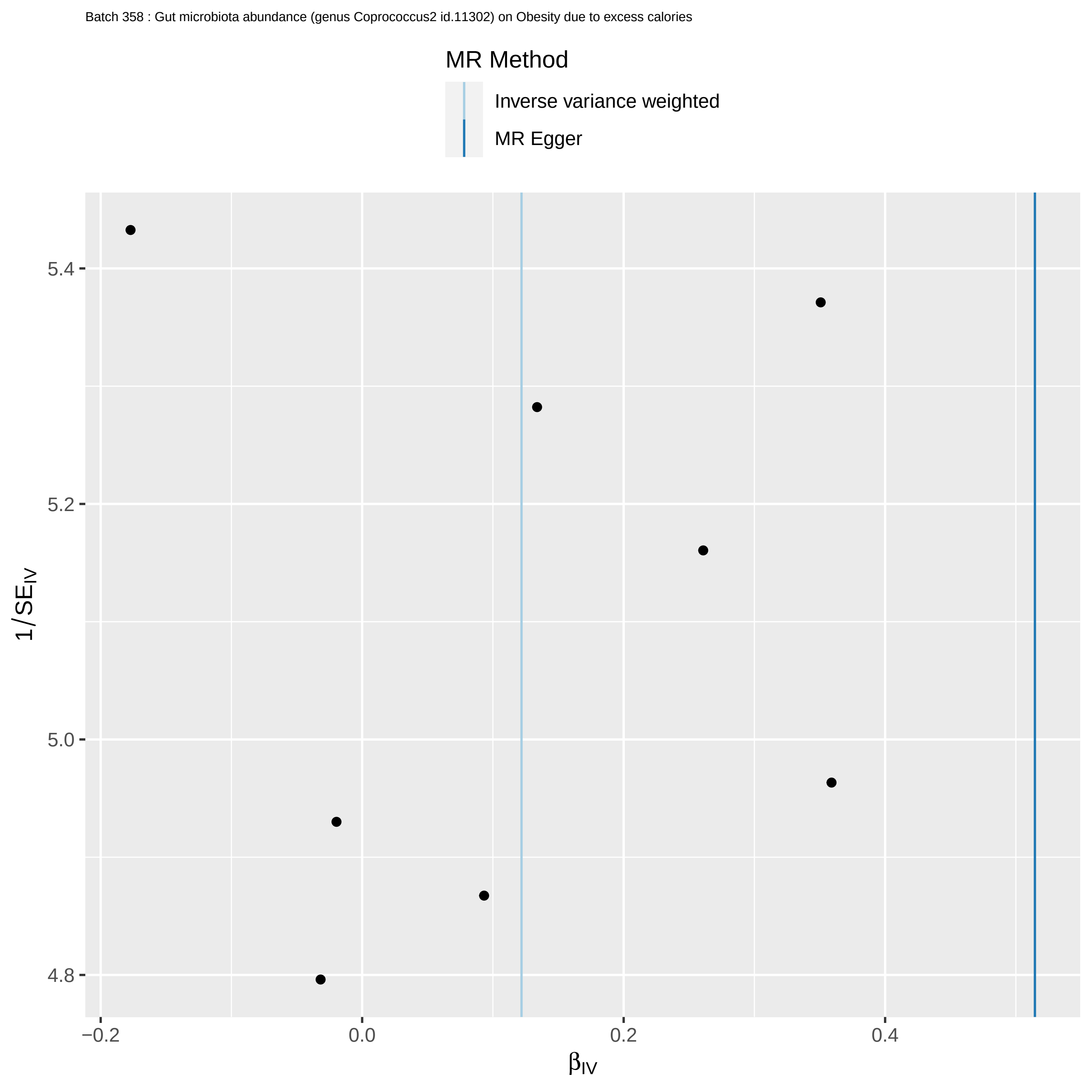

Batch 358 : Gut microbiota abundance (genus Coprococcus2 id.11302) on Obesity due to excess calories
MR Method
Inverse variance weighted
MR Egger
5.4
5.2
5.0
4.8
1/SE_IV
−0.2
0.0
0.2
0.4
β_IV

Batch 359 : Gut microbiota abundance (genus Coprococcus3 id.11303) on Obesity due to excess calories

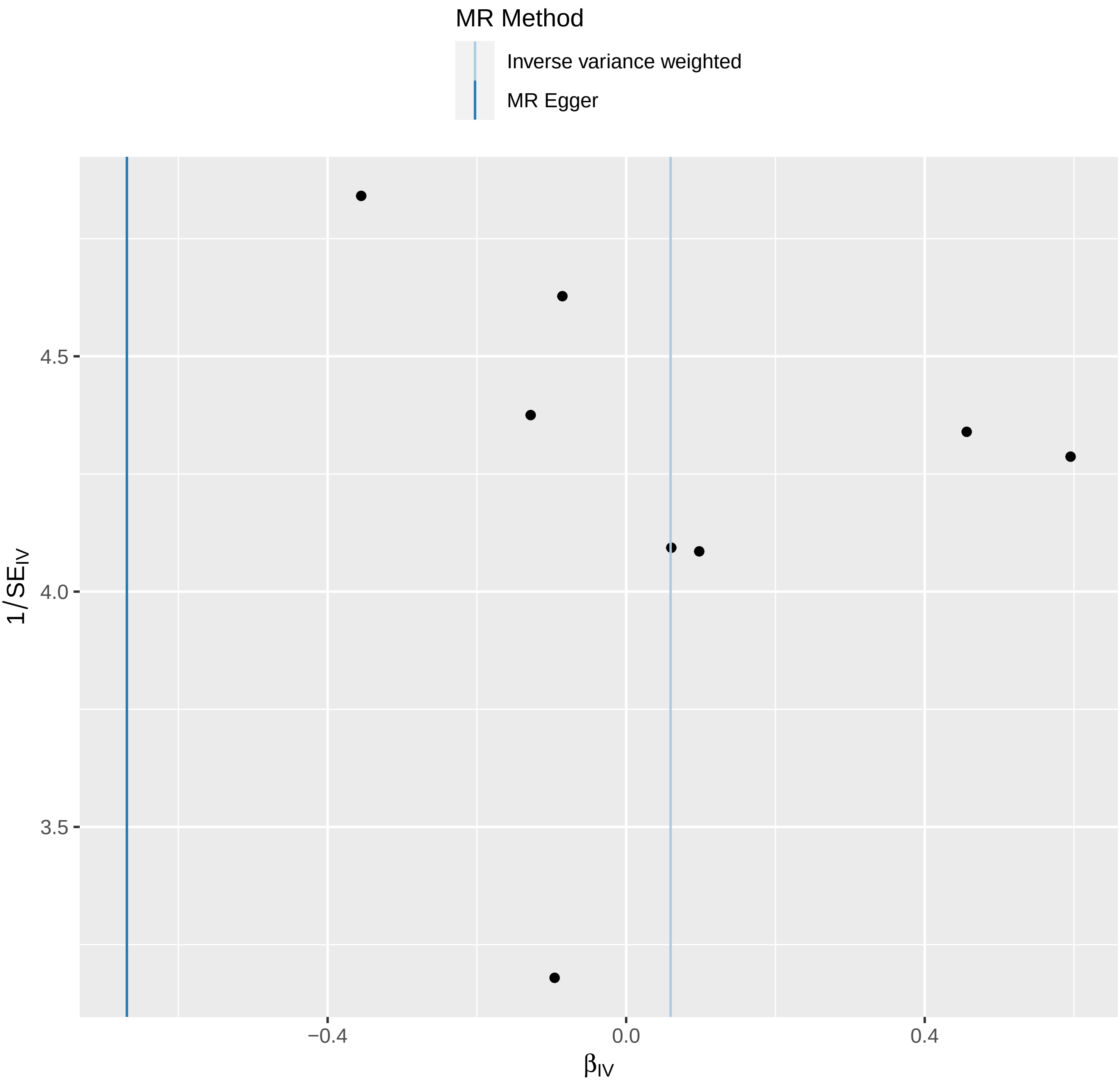

Batch 360 : Gut microbiota abundance (genus Defluviitaleaceae UCG011 id.11287) on Obesity due to excess calories

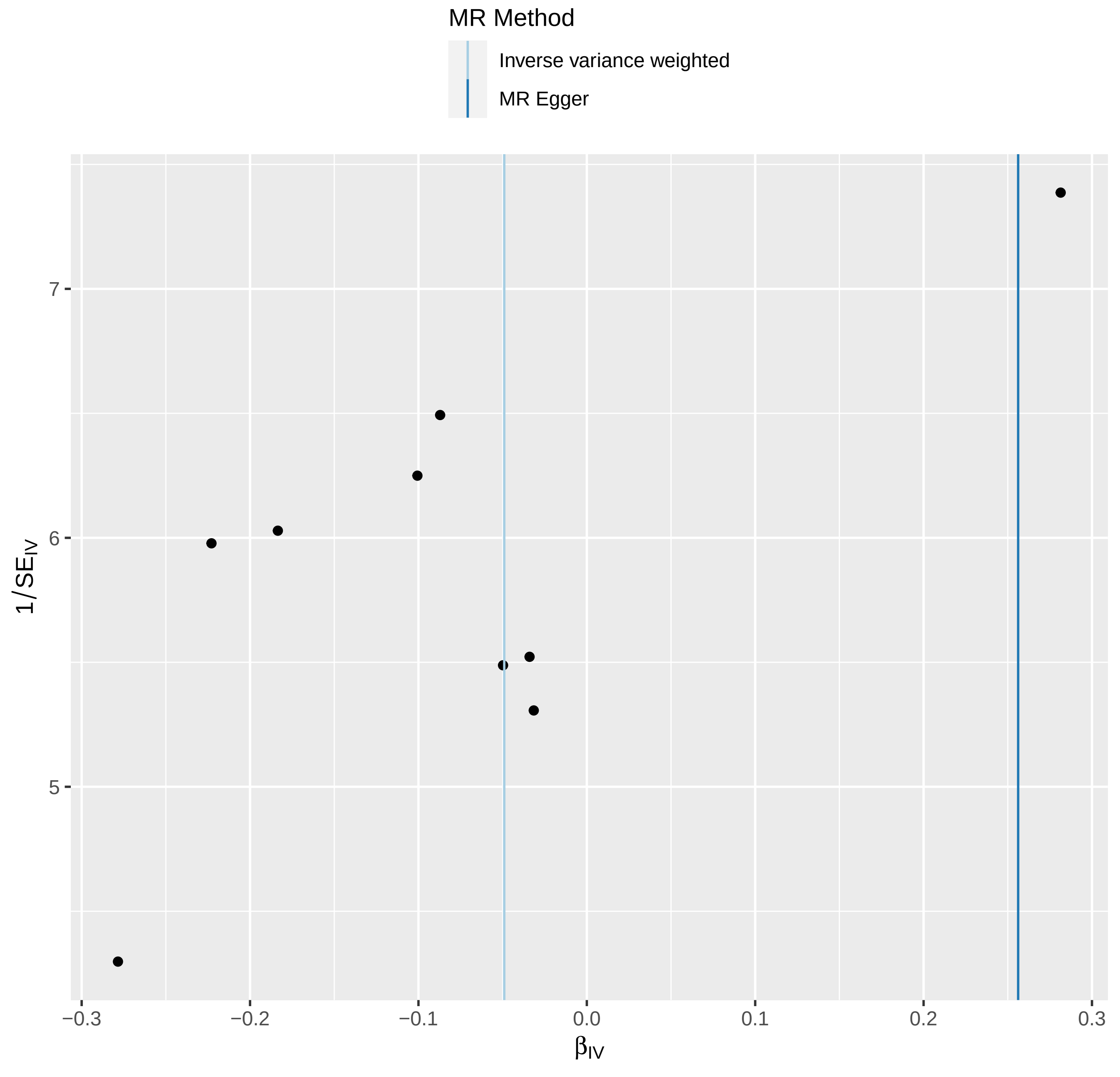

Batch 361 : Gut microbiota abundance (genus Desulfovibrio id.3173) on Obesity due to excess calories

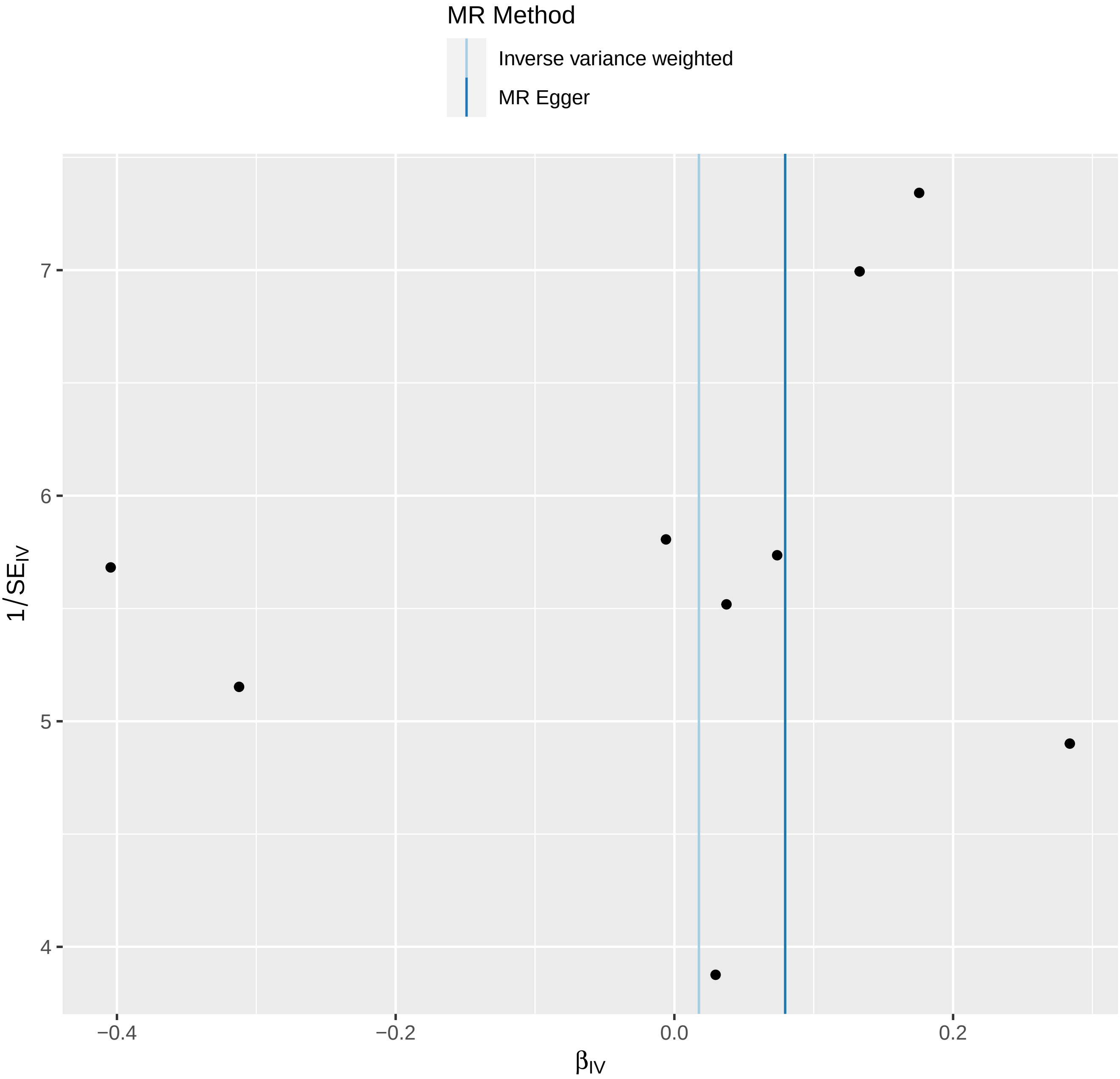

Batch 362 : Gut microbiota abundance (genus Dialister id.2183) on Obesity due to excess calories

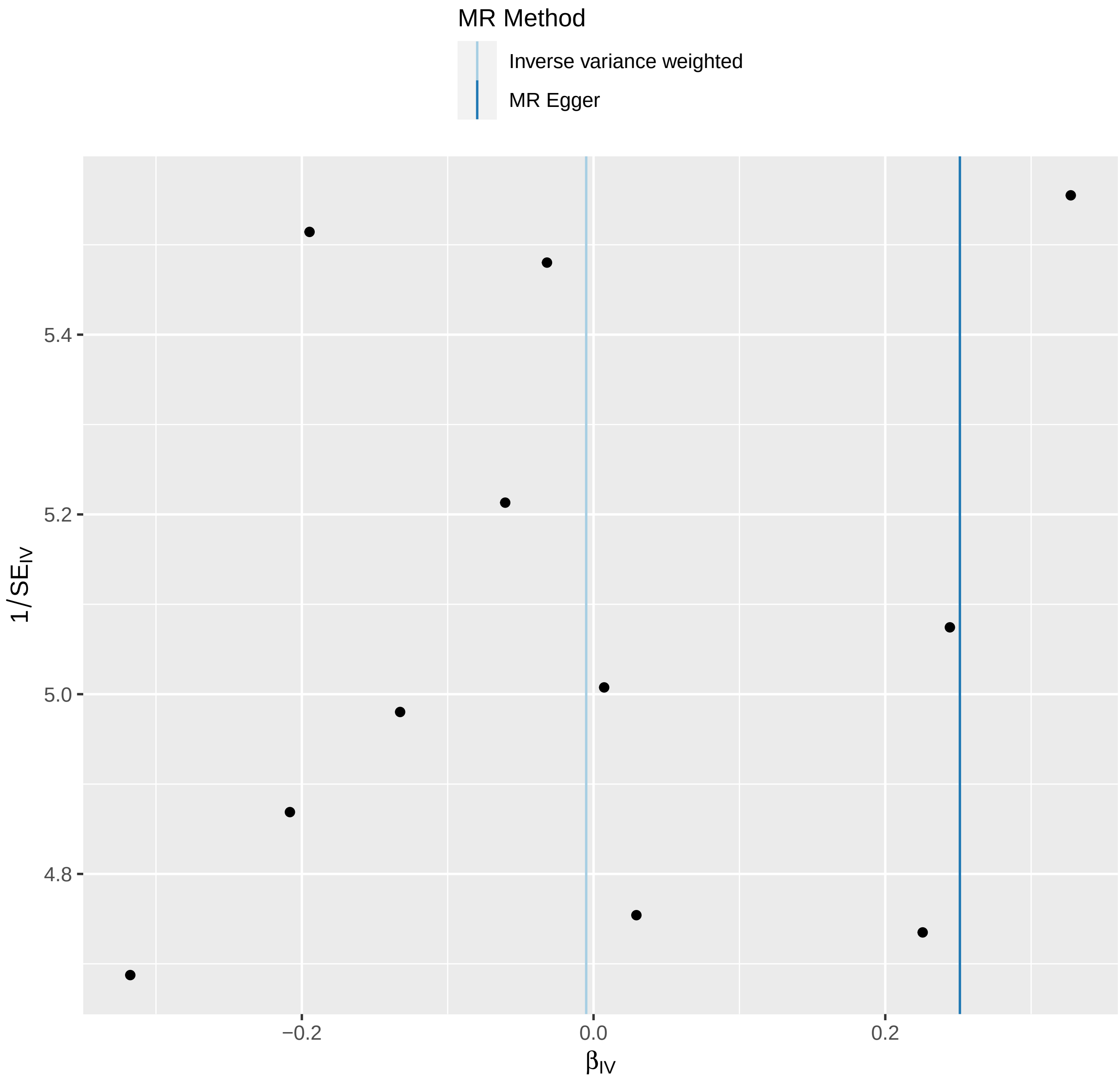

Batch 363 : Gut microbiota abundance (genus Dorea id.1997) on Obesity due to excess calories

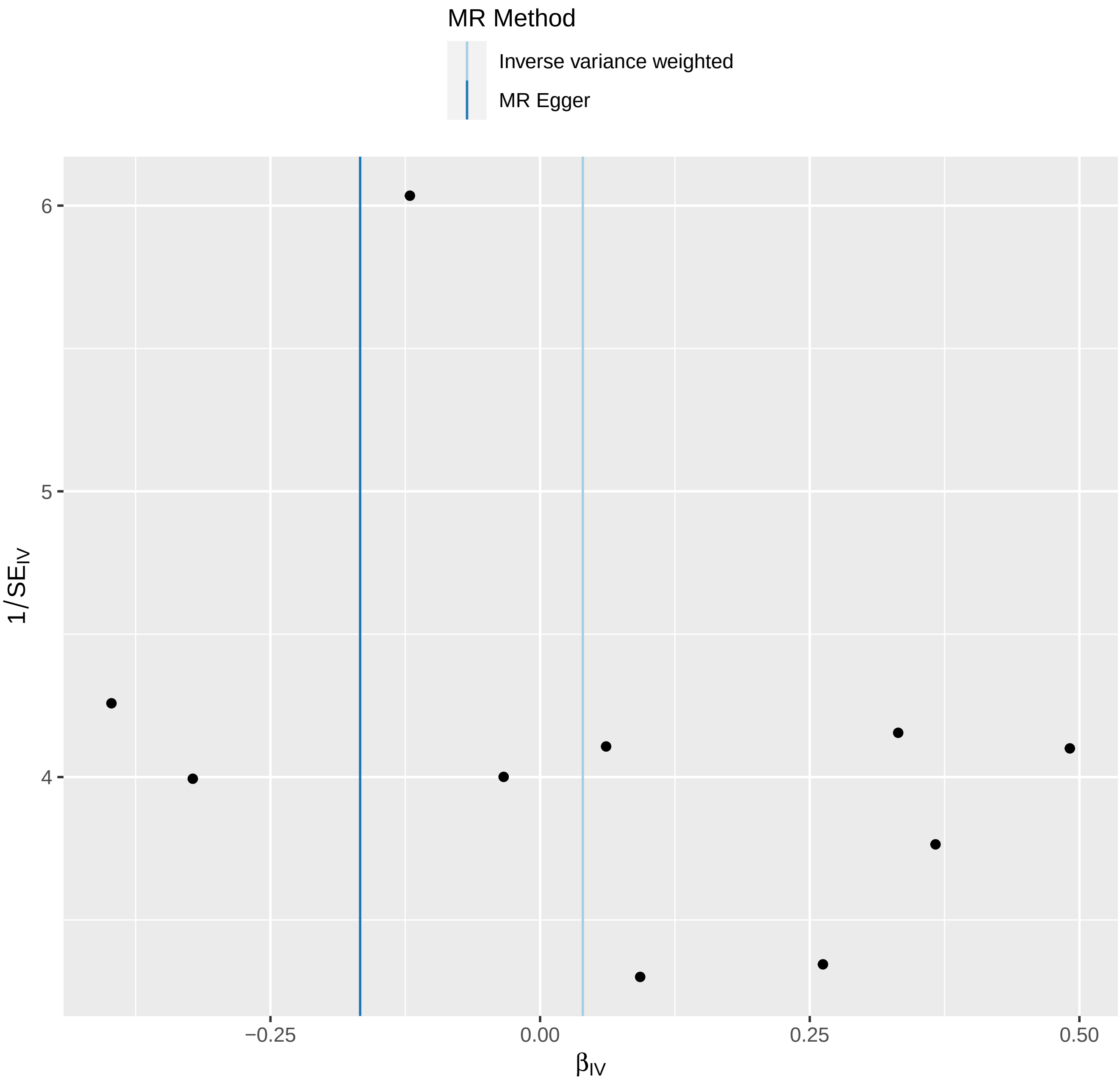

Batch 364 : Gut microbiota abundance (genus Eggerthella id.819) on Obesity due to excess calories

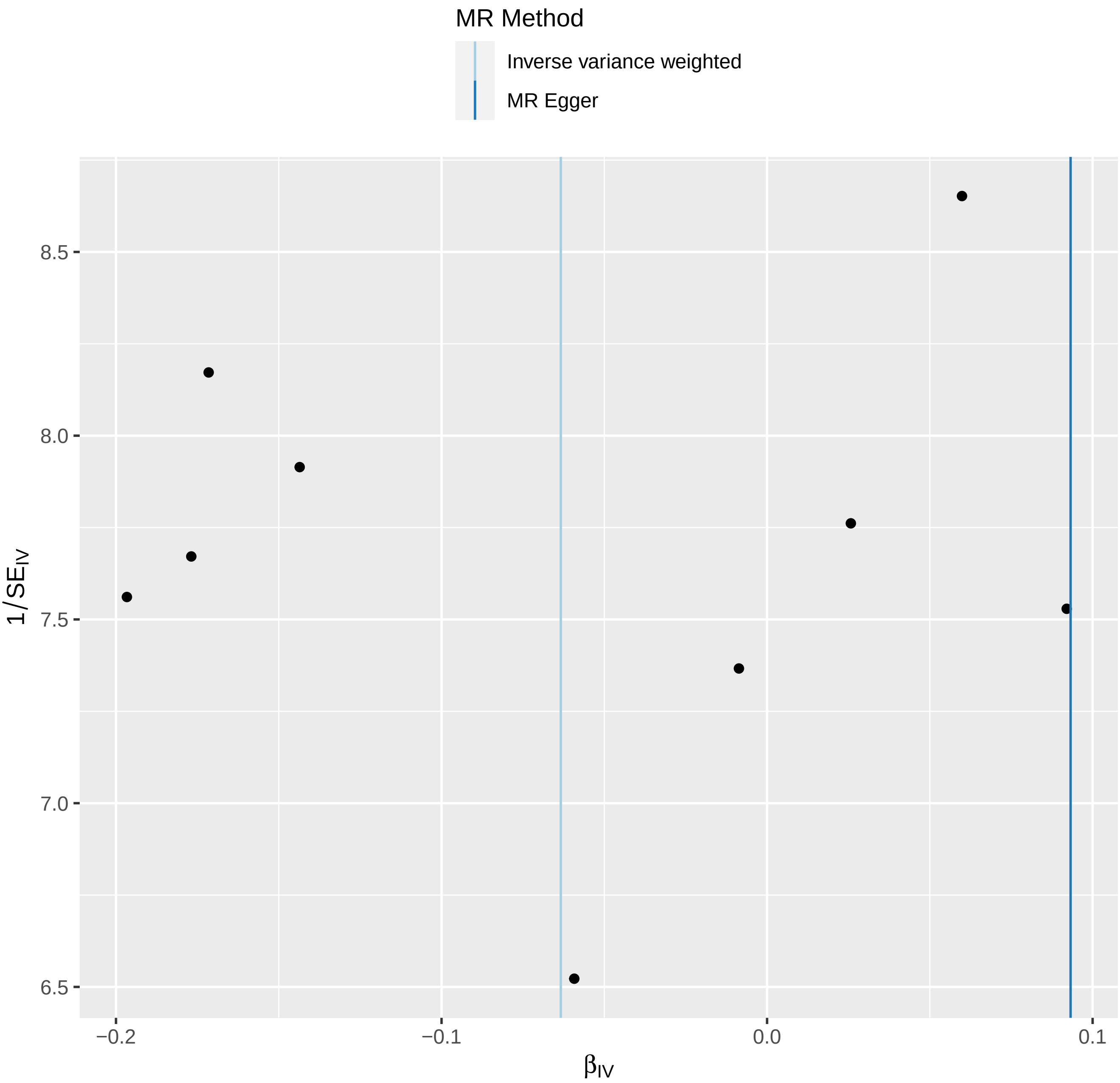

Batch 365 : Gut microbiota abundance (genus Eisenbergiella id.11304) on Obesity due to excess calories

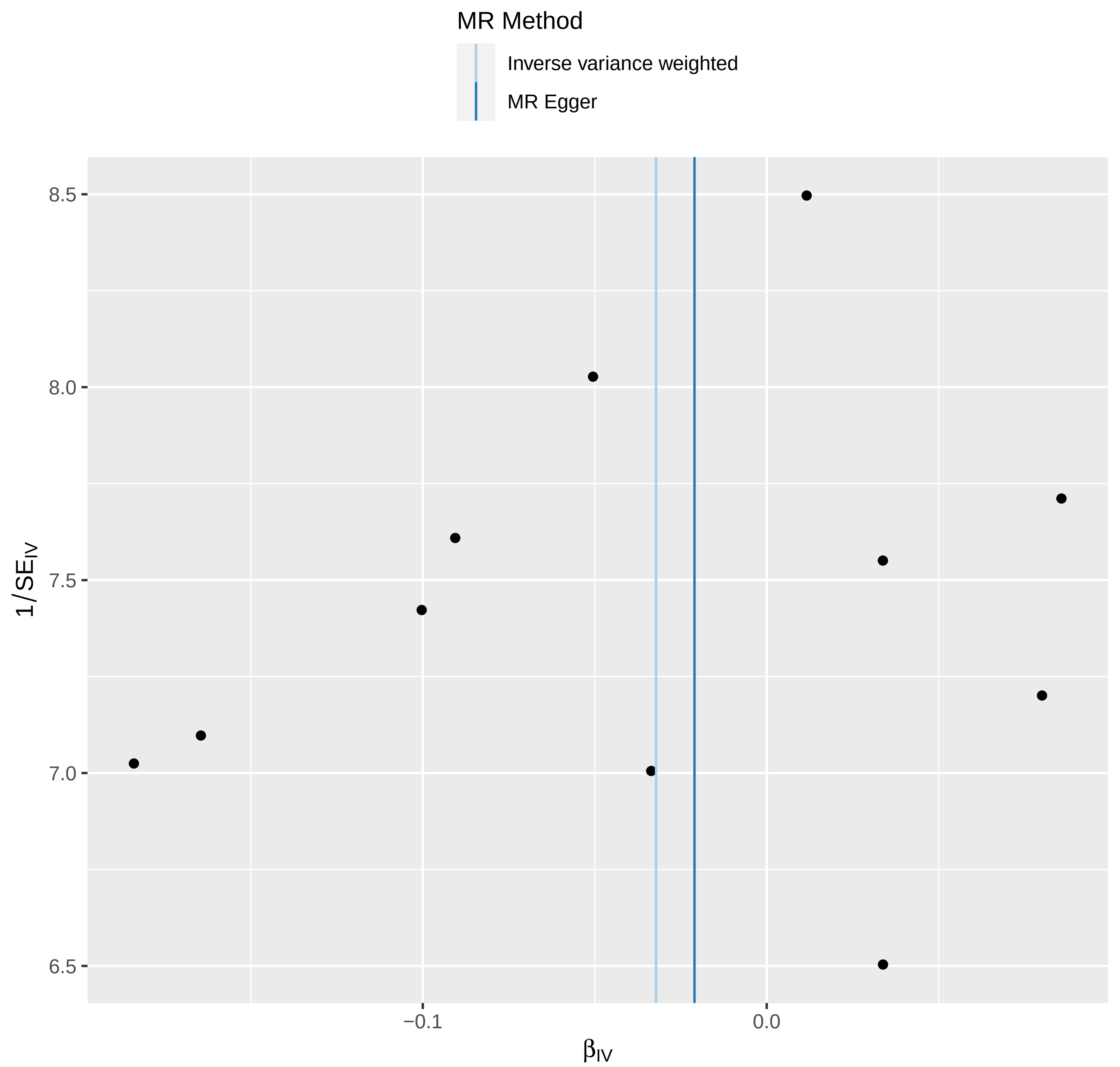

Batch 366 : Gut microbiota abundance (genus Enterorhabdus id.820) on Obesity due to excess calories

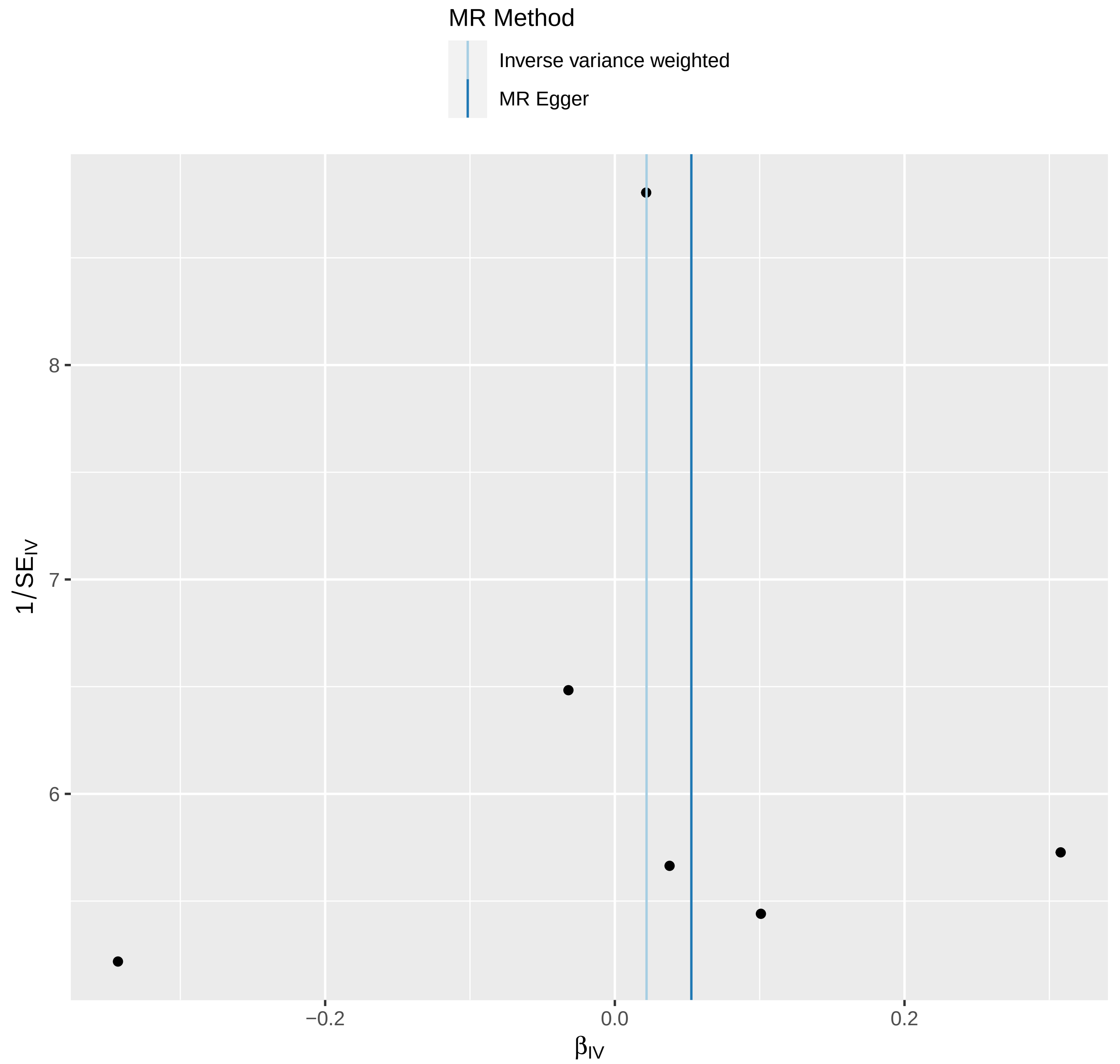

Batch 367 : Gut microbiota abundance (genus Erysipelatoclostridium id.11381) on Obesity due to excess calories

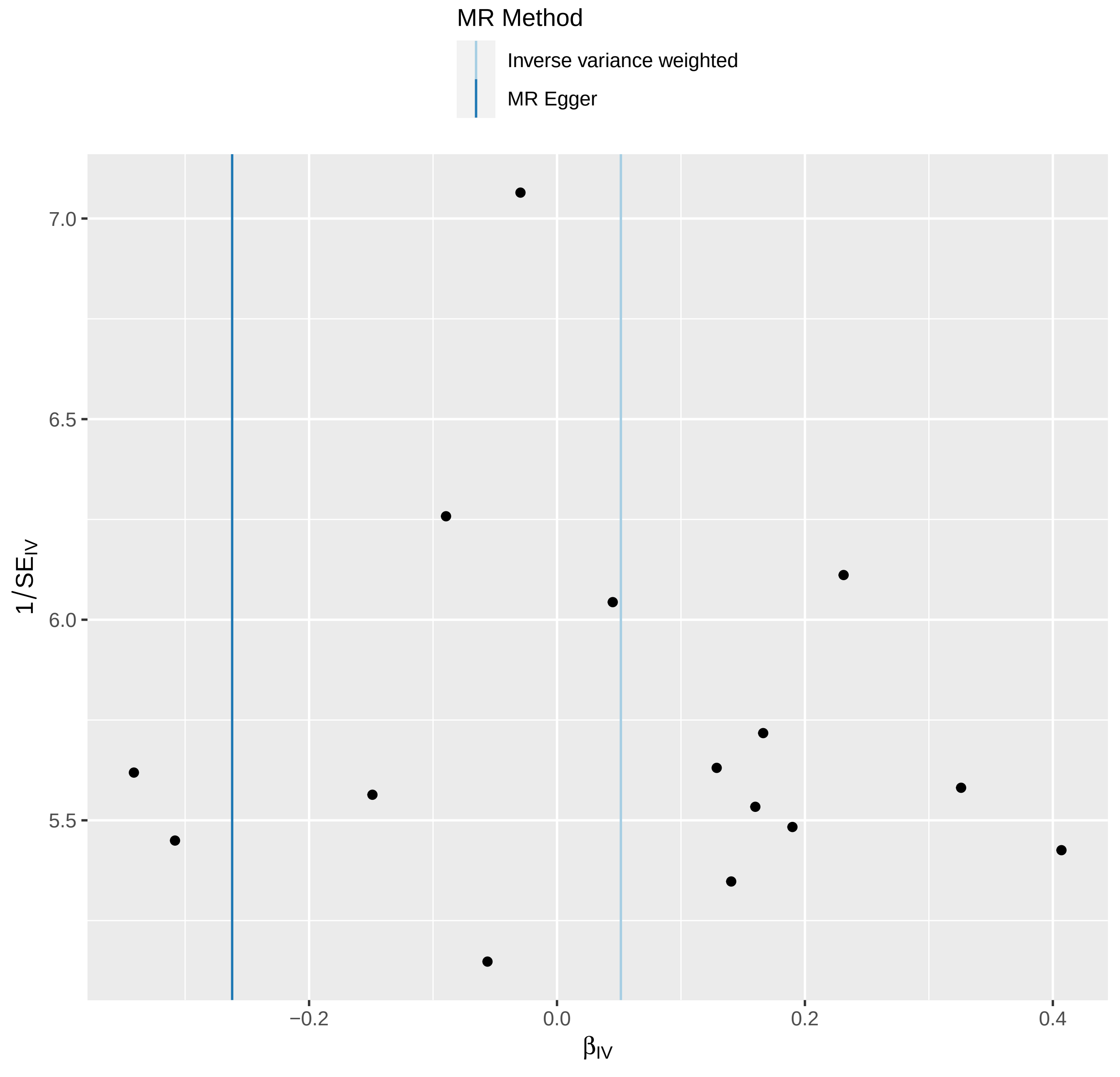

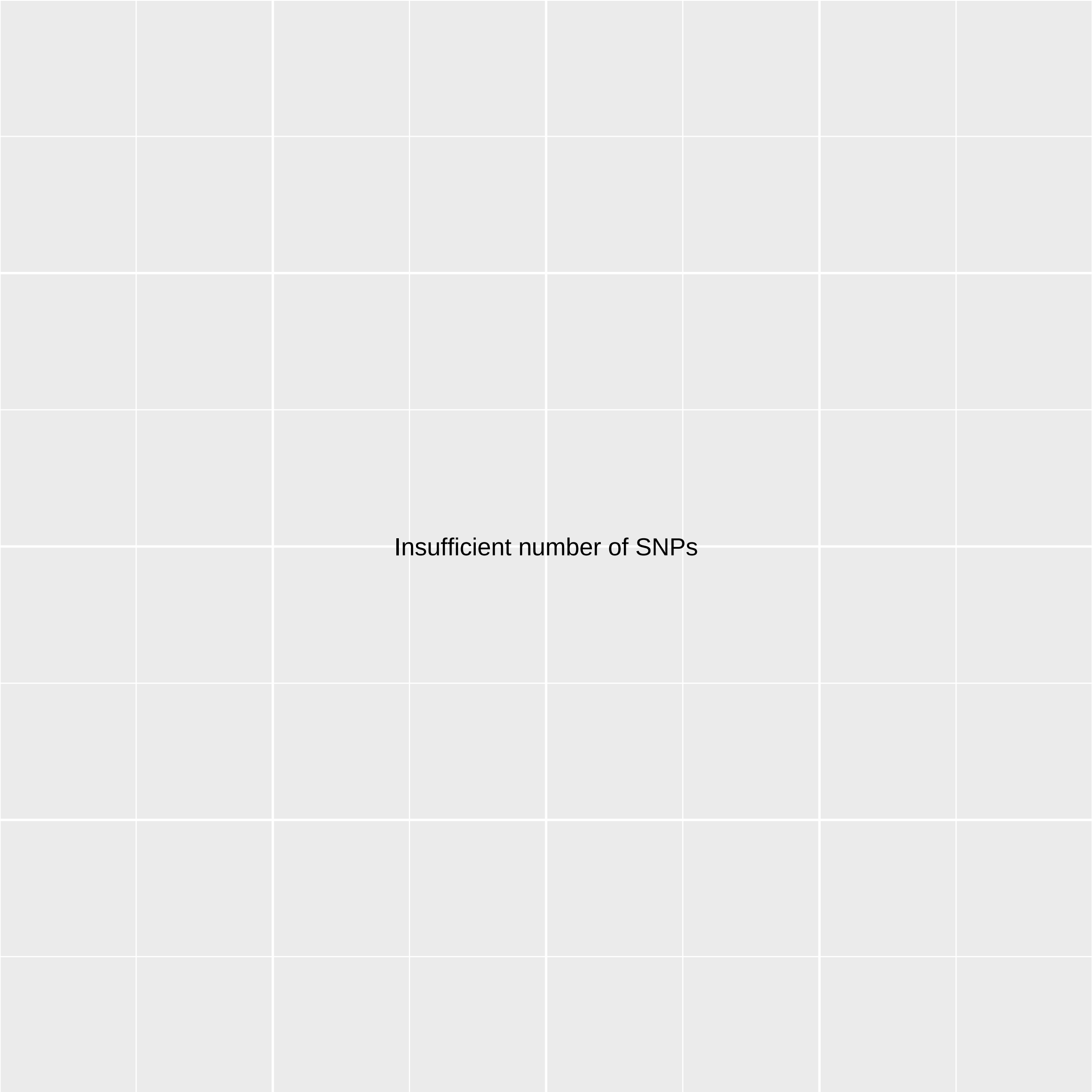
Insufficient number of SNPs

Batch 369 : Gut microbiota abundance (genus Escherichia Shigella id.3504) on Obesity due to excess calories

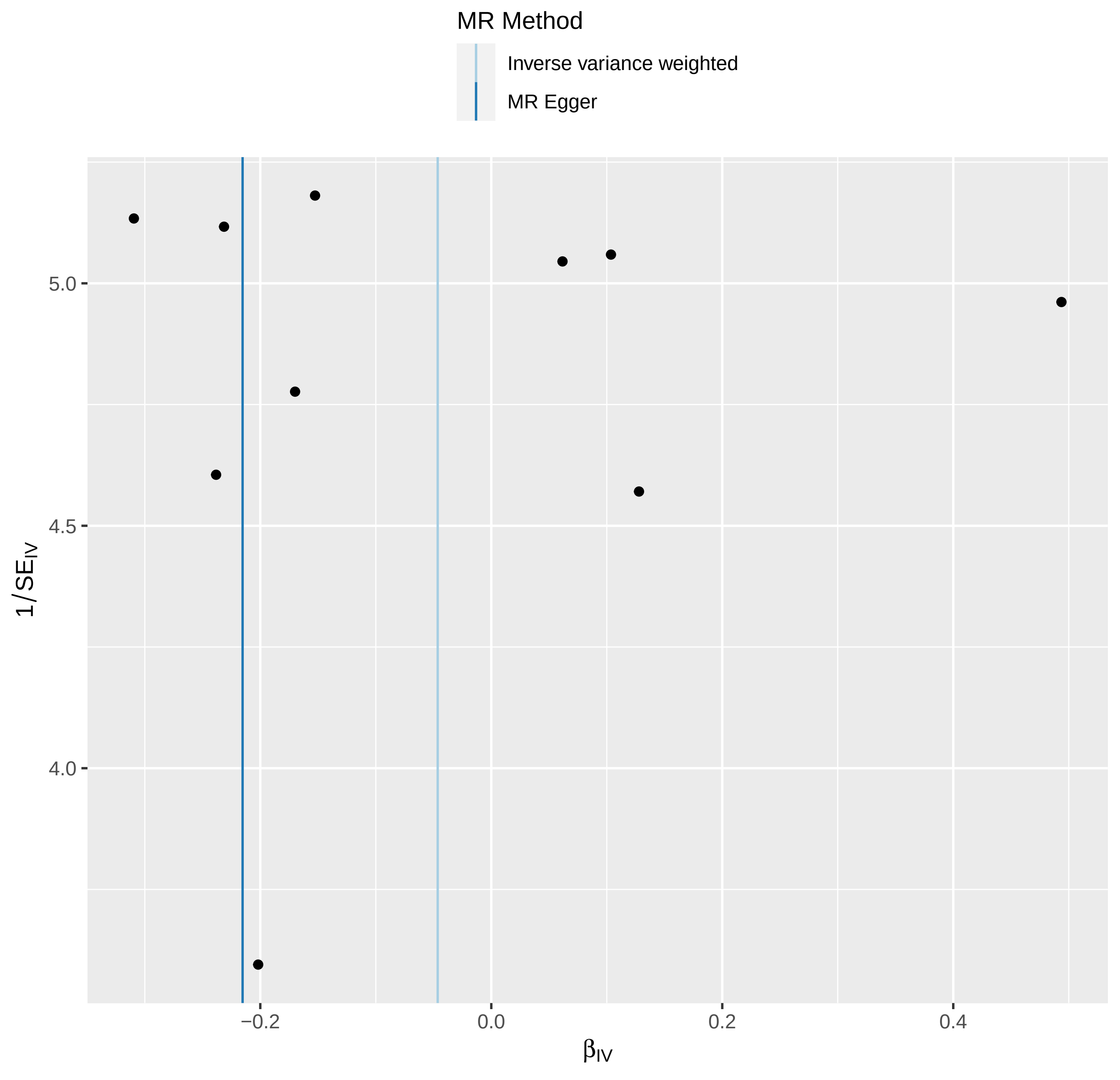

Batch 370 : Gut microbiota abundance (genus Eubacterium brachy group id.11296) on Obesity due to excess calories

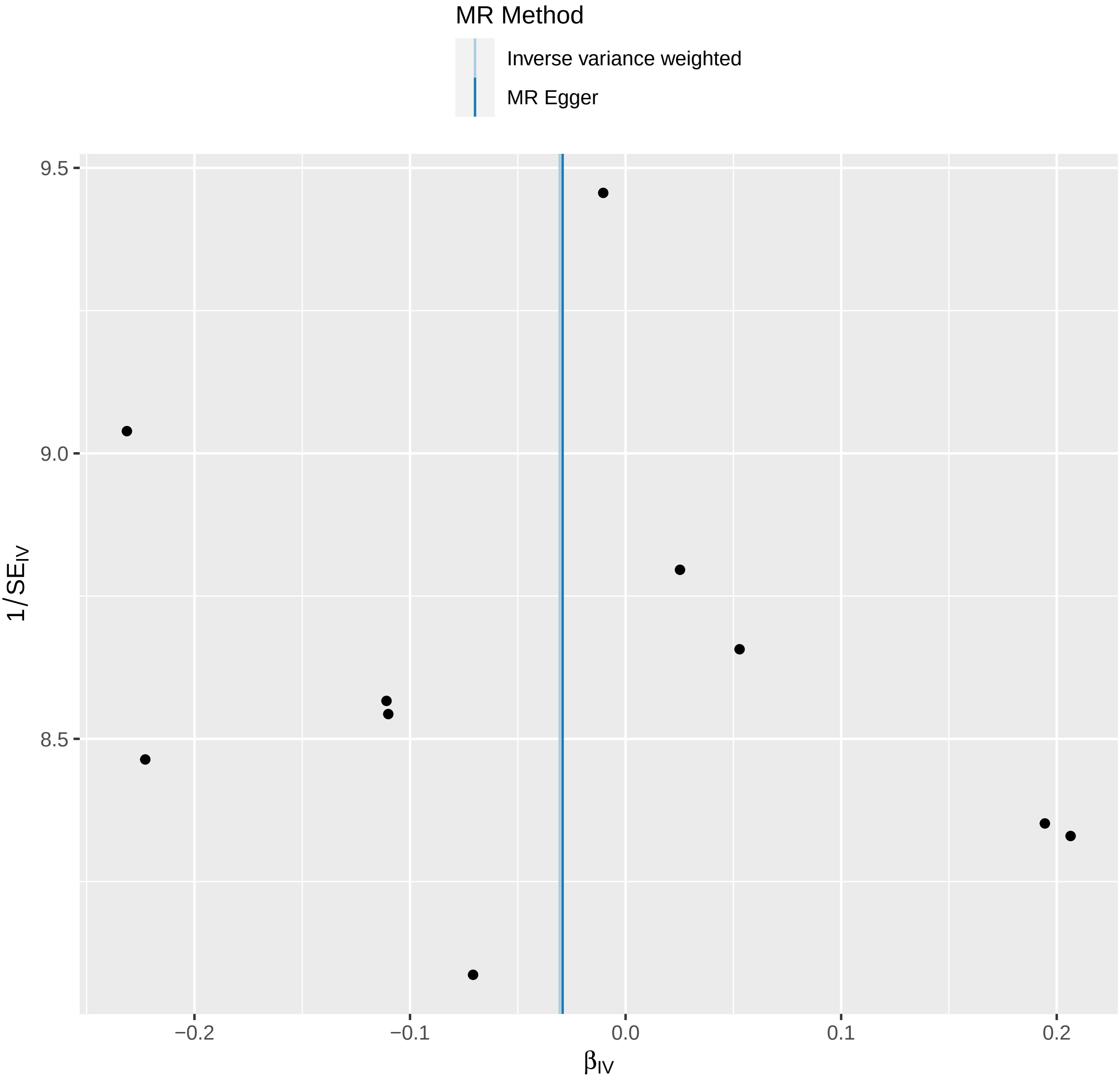

Batch 371 : Gut microbiota abundance (genus Eubacterium coprostanoligenes group id.11375) on Obesity due to excess calories

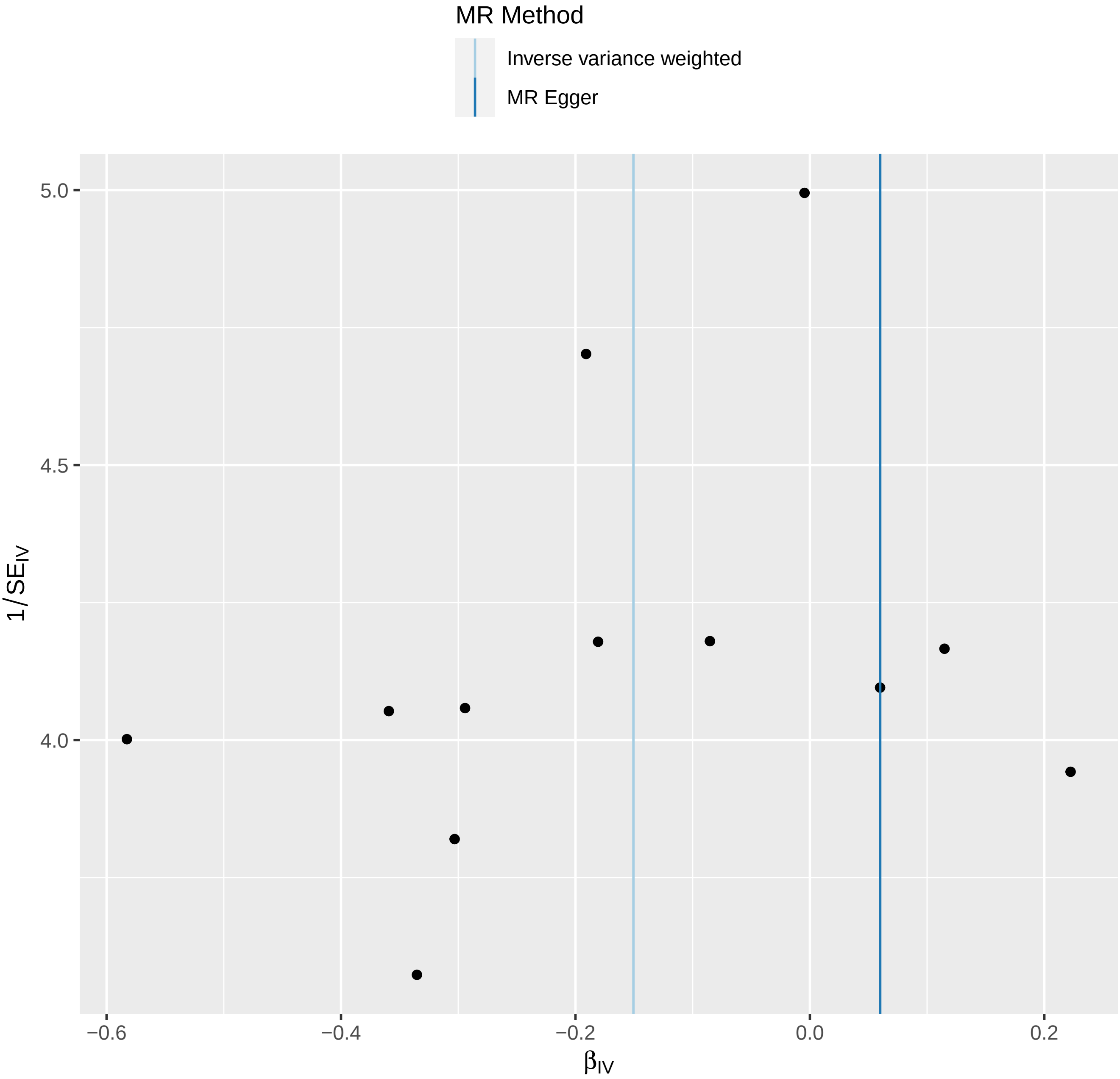

Batch 372 : Gut microbiota abundance (genus Eubacterium eligens group id.14372) on Obesity due to excess calories

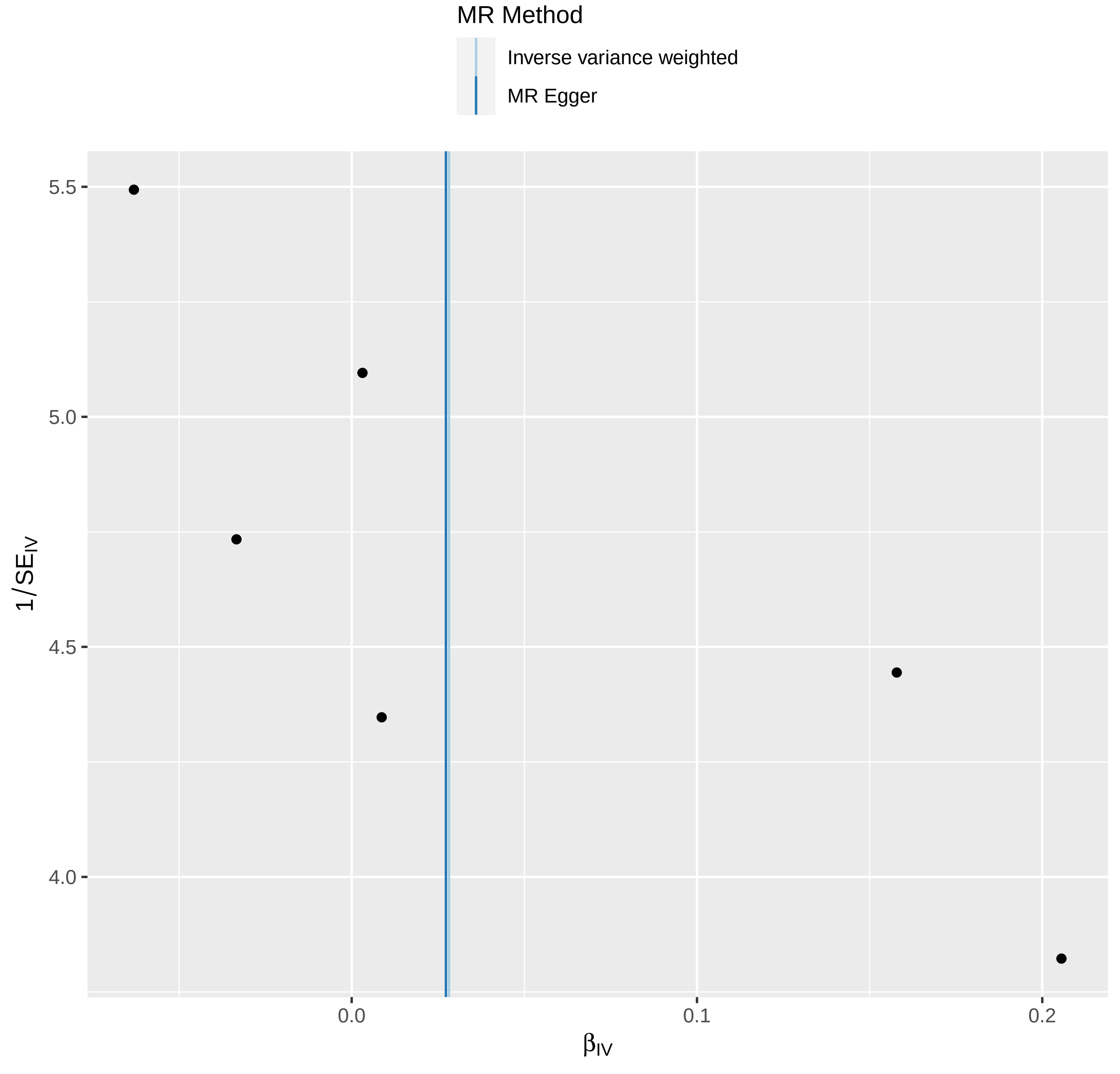

Batch 373 : Gut microbiota abundance (genus Eubacterium fissicatena group id.14373) on Obesity due to excess calories

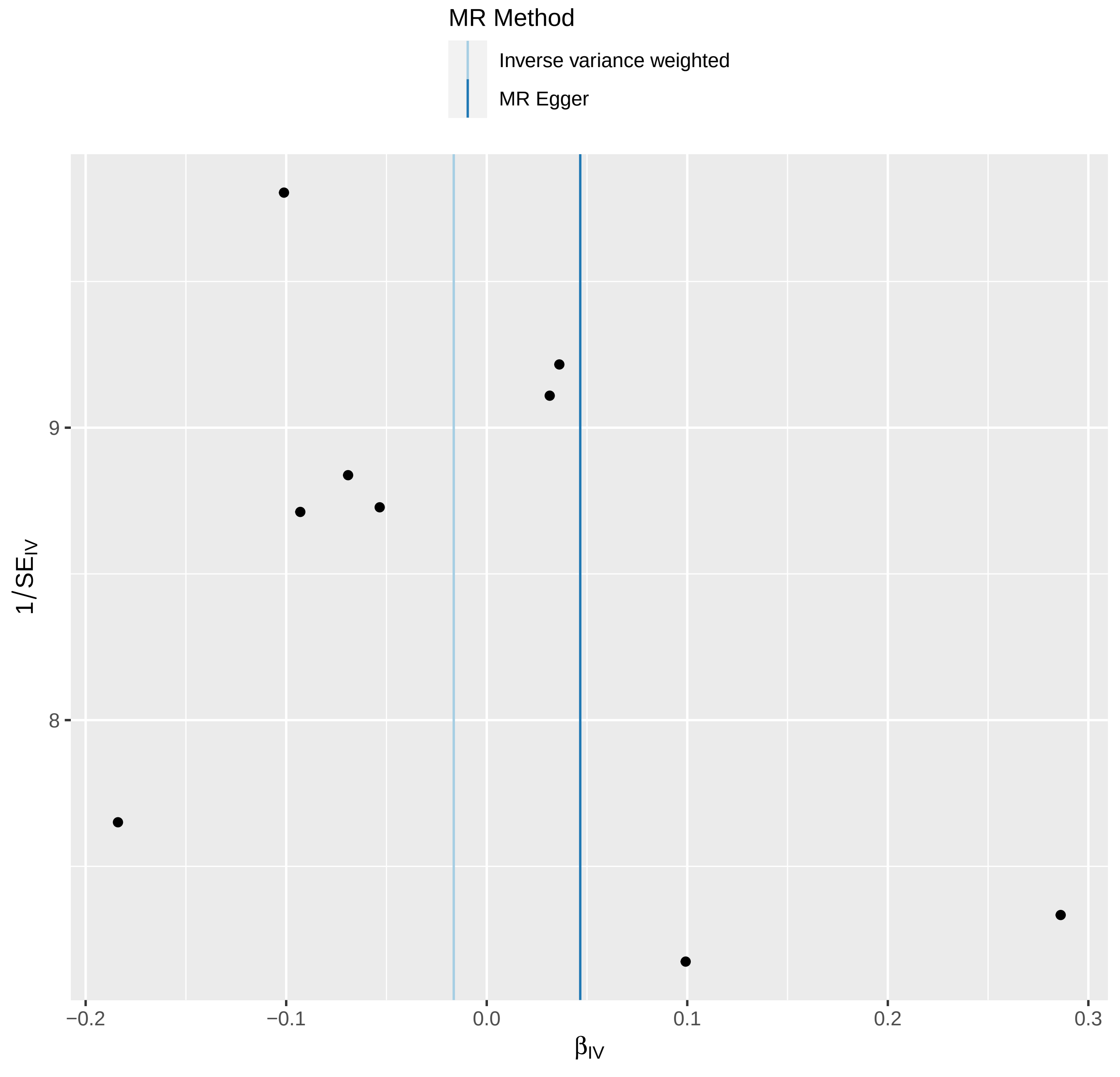

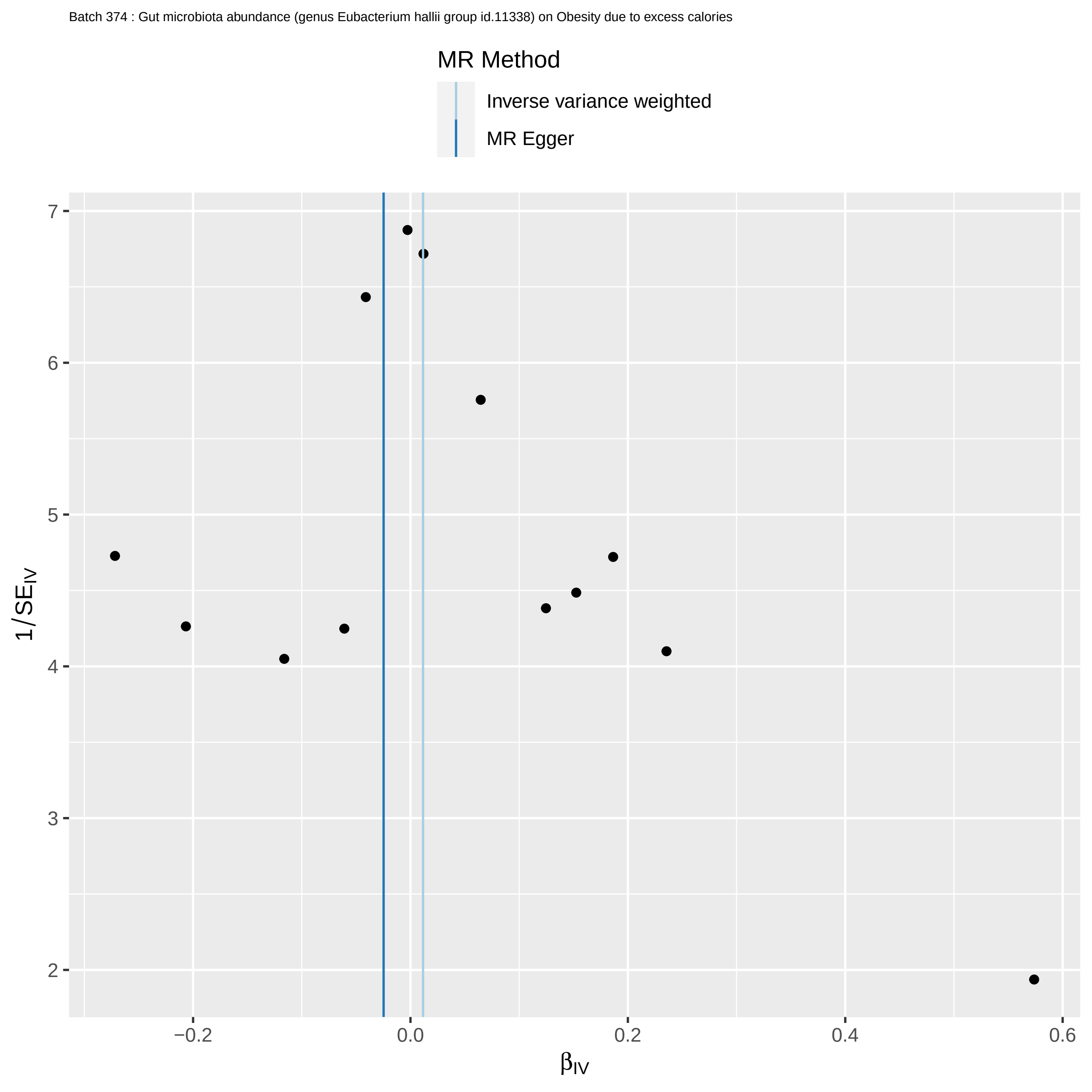

Batch 374 : Gut microbiota abundance (genus Eubacterium hallii group id.11338) on Obesity due to excess calories
MR Method
Inverse variance weighted
MR Egger
7
6
5
4
3
2
1/SE_IV
−0.2
0.0
0.2
0.4
0.6
β_IV

Batch 375 : Gut microbiota abundance (genus Eubacterium nodatum group id.11297) on Obesity due to excess calories

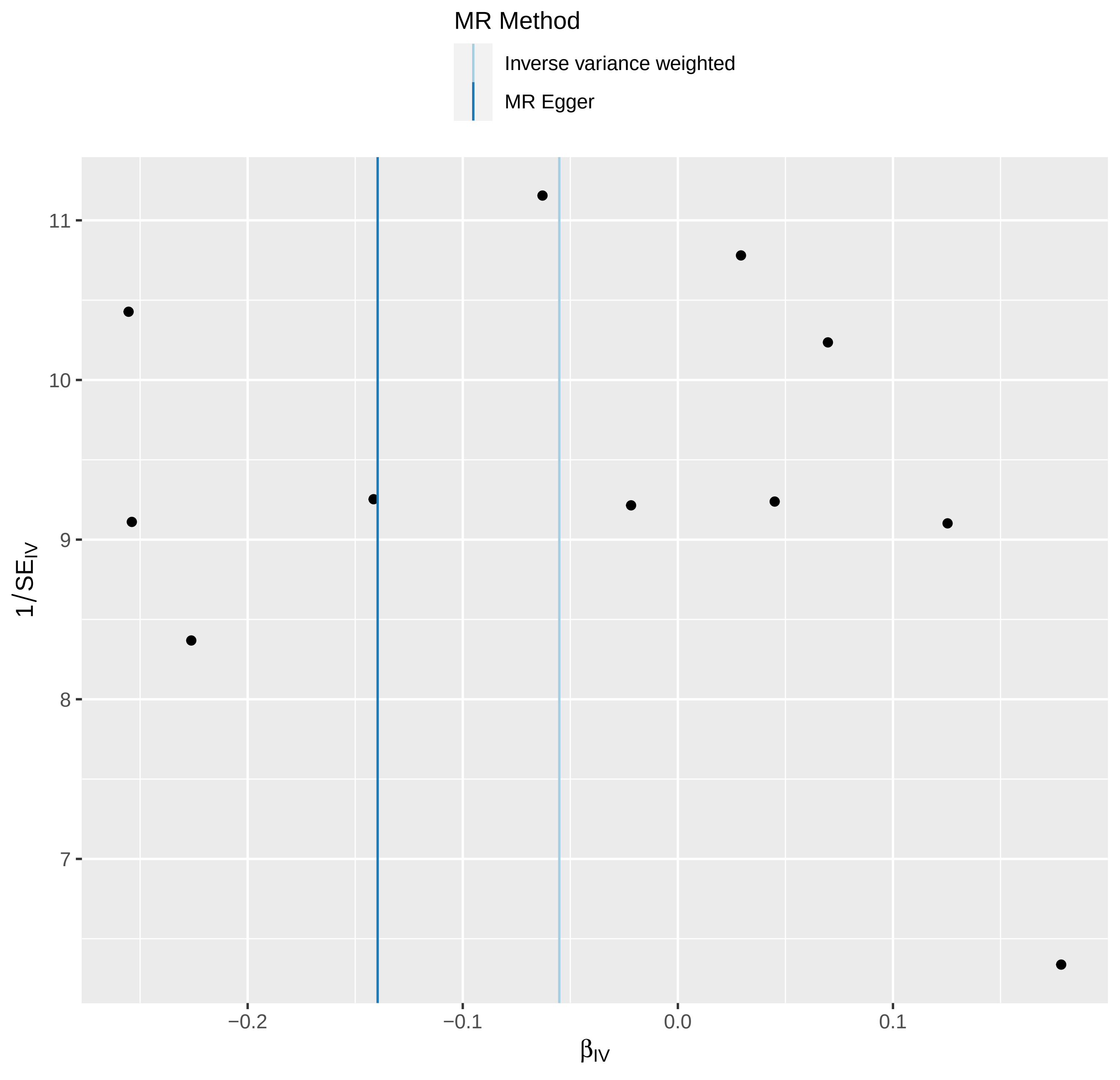

Batch 376 : Gut microbiota abundance (genus Eubacterium oxidoreducens group id.11339) on Obesity due to excess calories

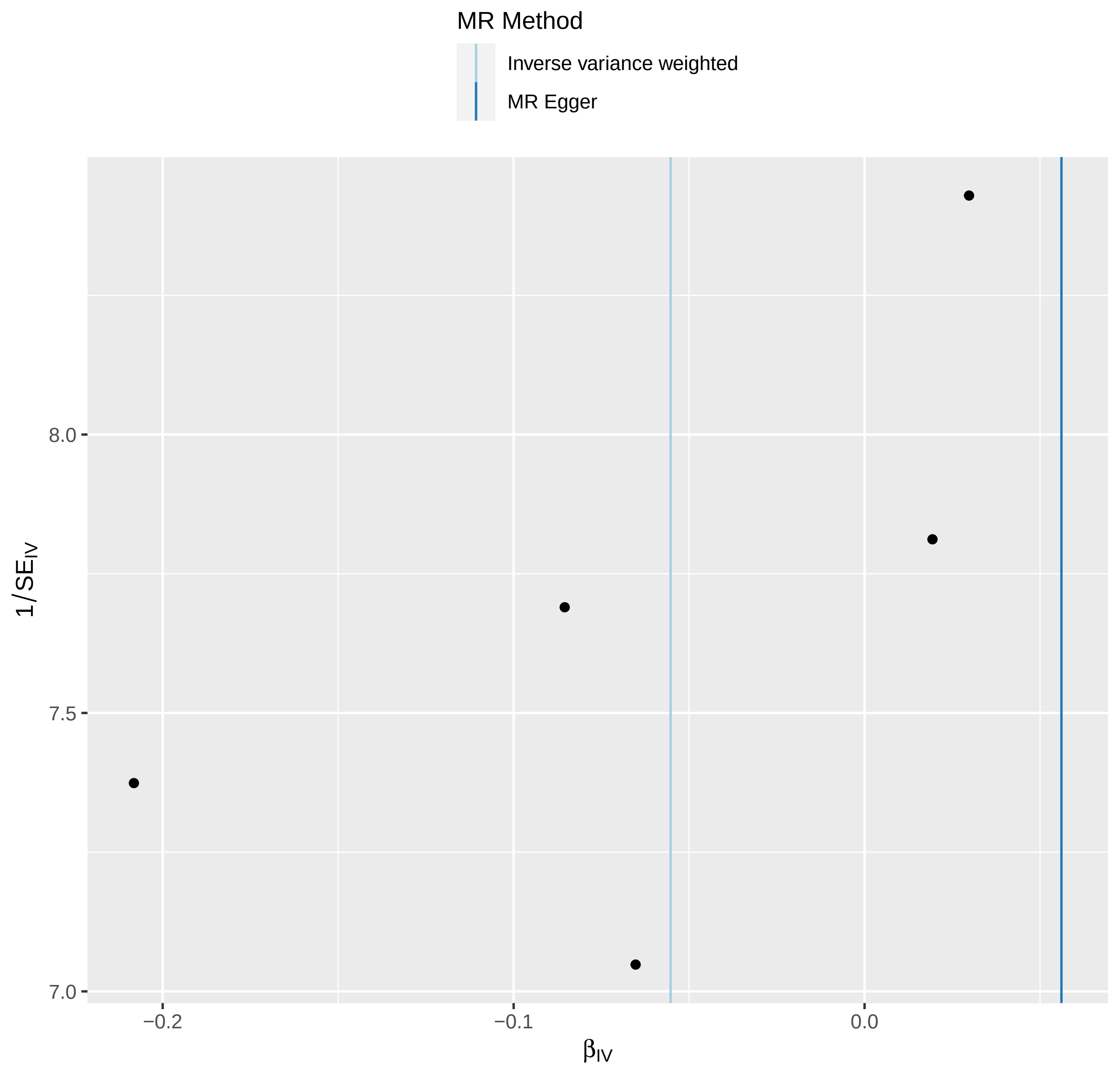

Batch 377 : Gut microbiota abundance (genus Eubacterium rectale group id.14374) on Obesity due to excess calories

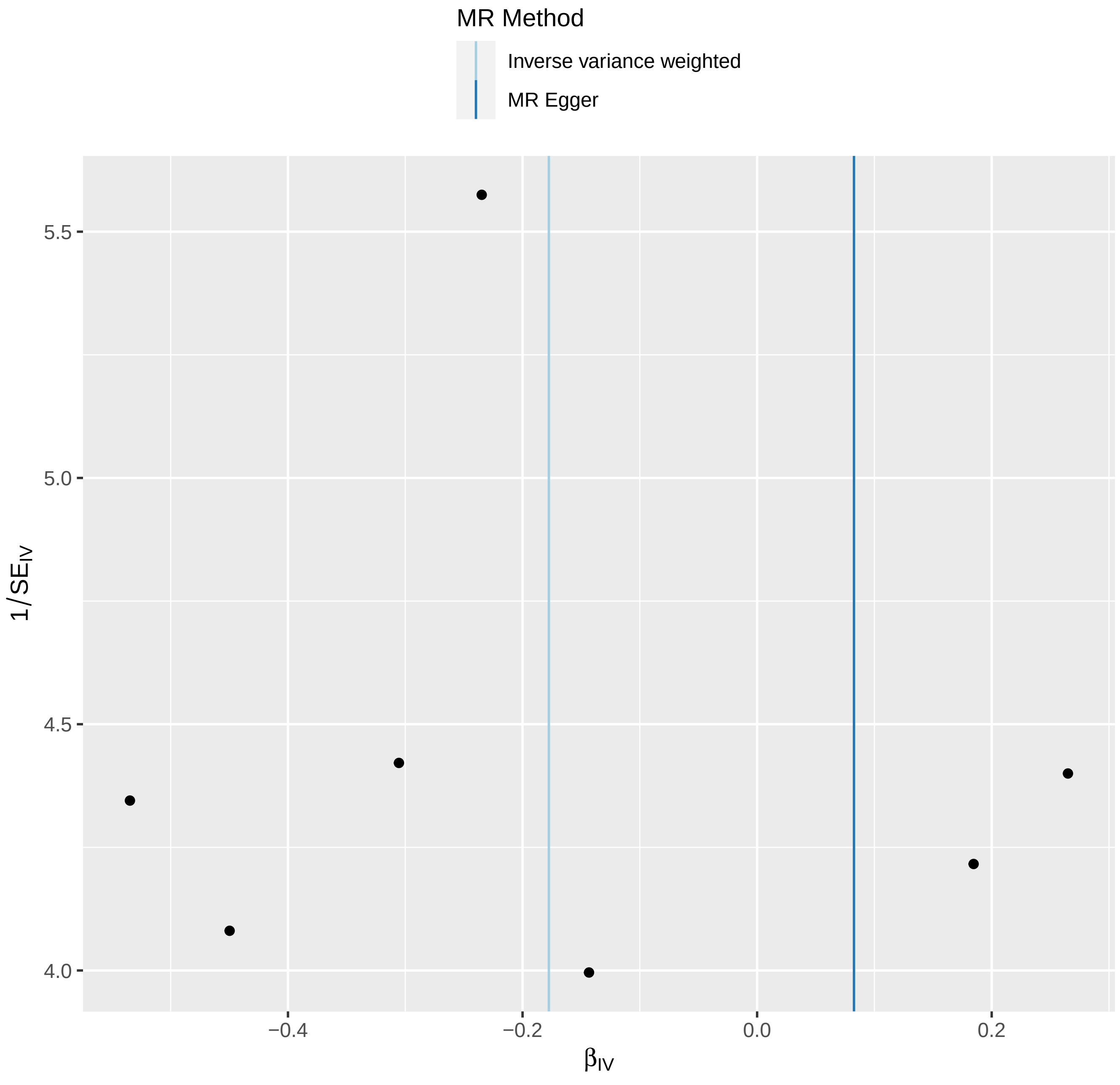

Batch 378 : Gut microbiota abundance (genus Eubacterium ruminantium group id.11340) on Obesity due to excess calories

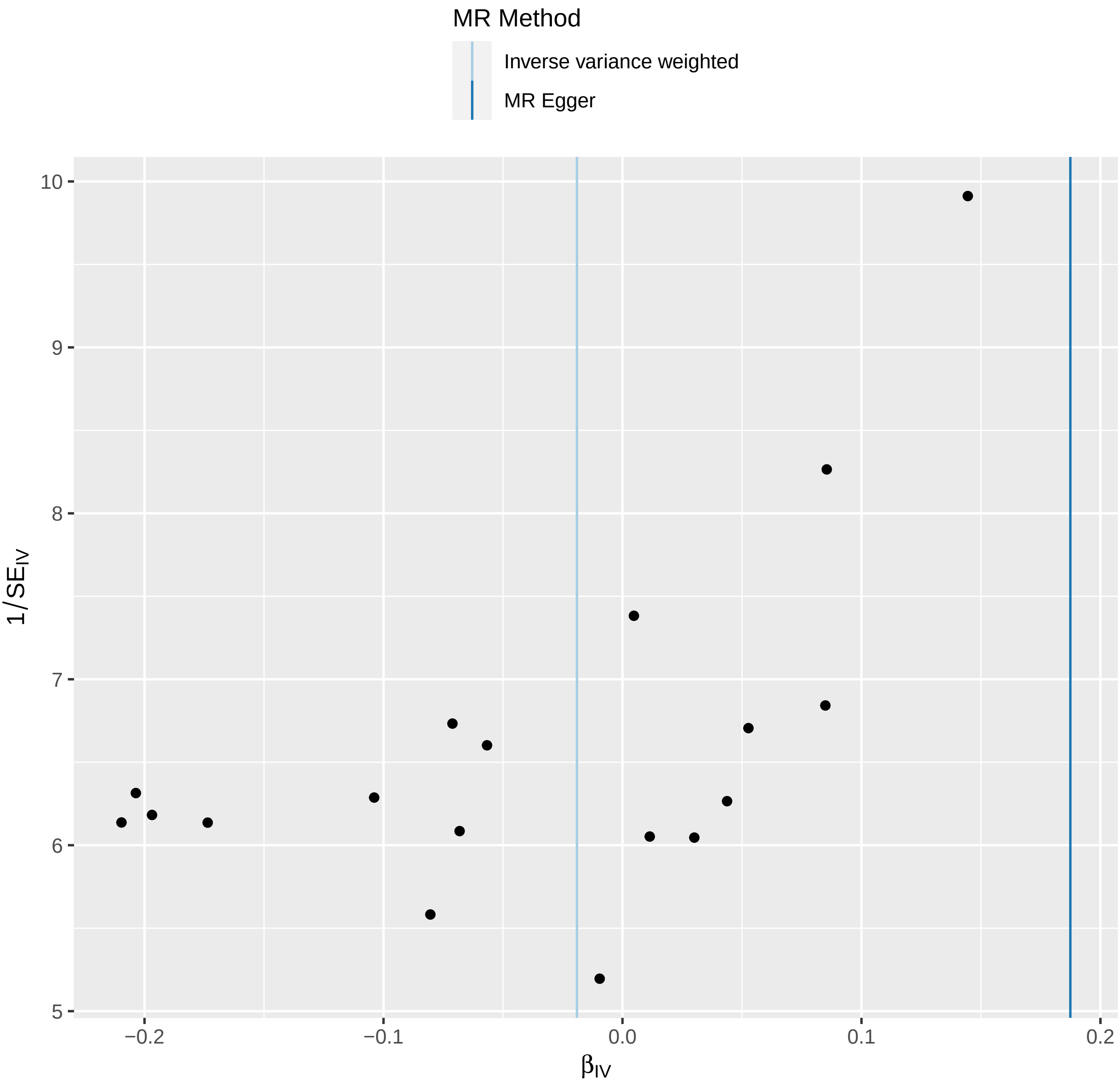

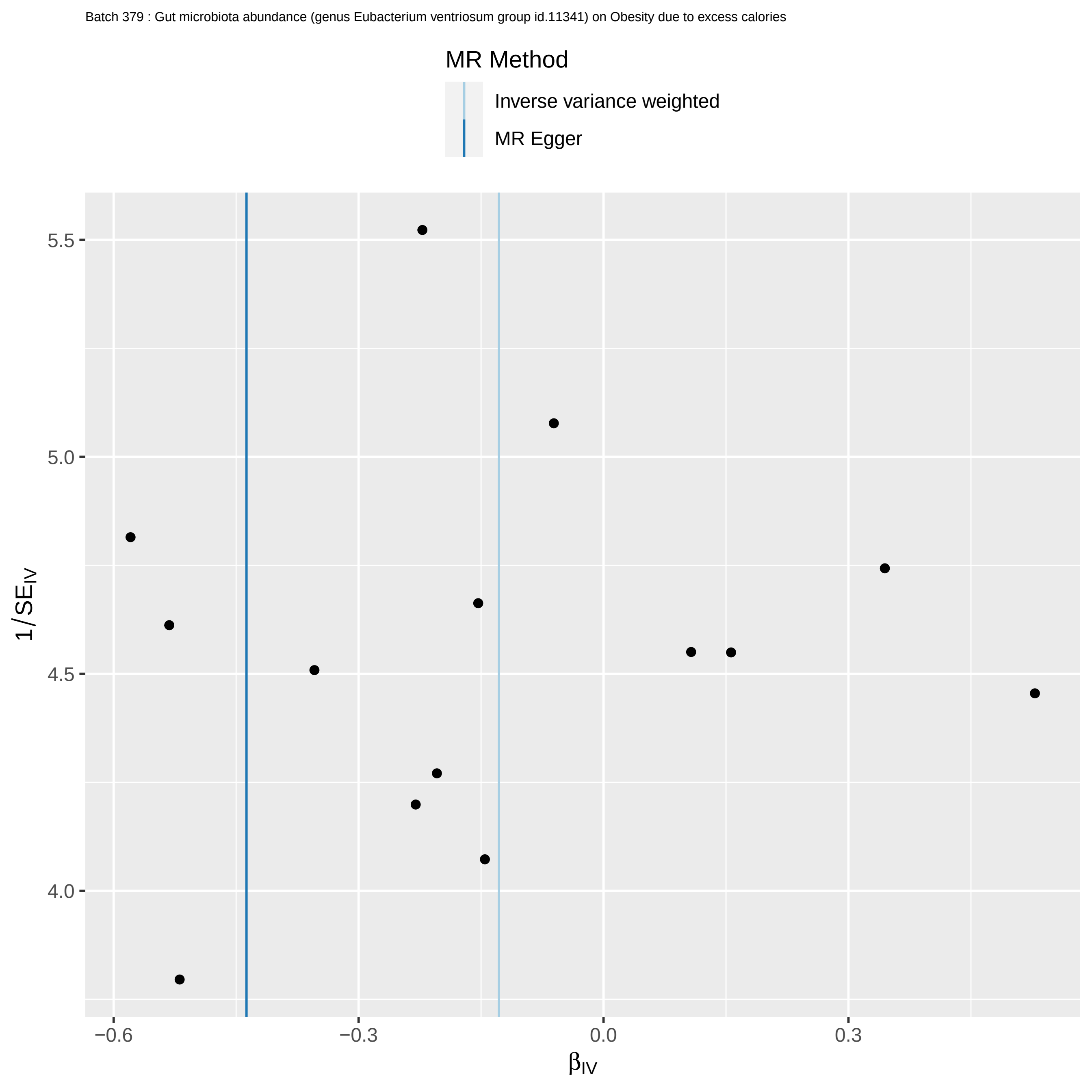

Batch 379 : Gut microbiota abundance (genus Eubacterium ventriosum group id.11341) on Obesity due to excess calories
MR Method
Inverse variance weighted
MR Egger
5.5
5.0
4.5
4.0
1/SE_IV
−0.6
−0.3
0.0
0.3
β_IV

Batch 380 : Gut microbiota abundance (genus Eubacterium xylanophilum group id.14375) on Obesity due to excess calories

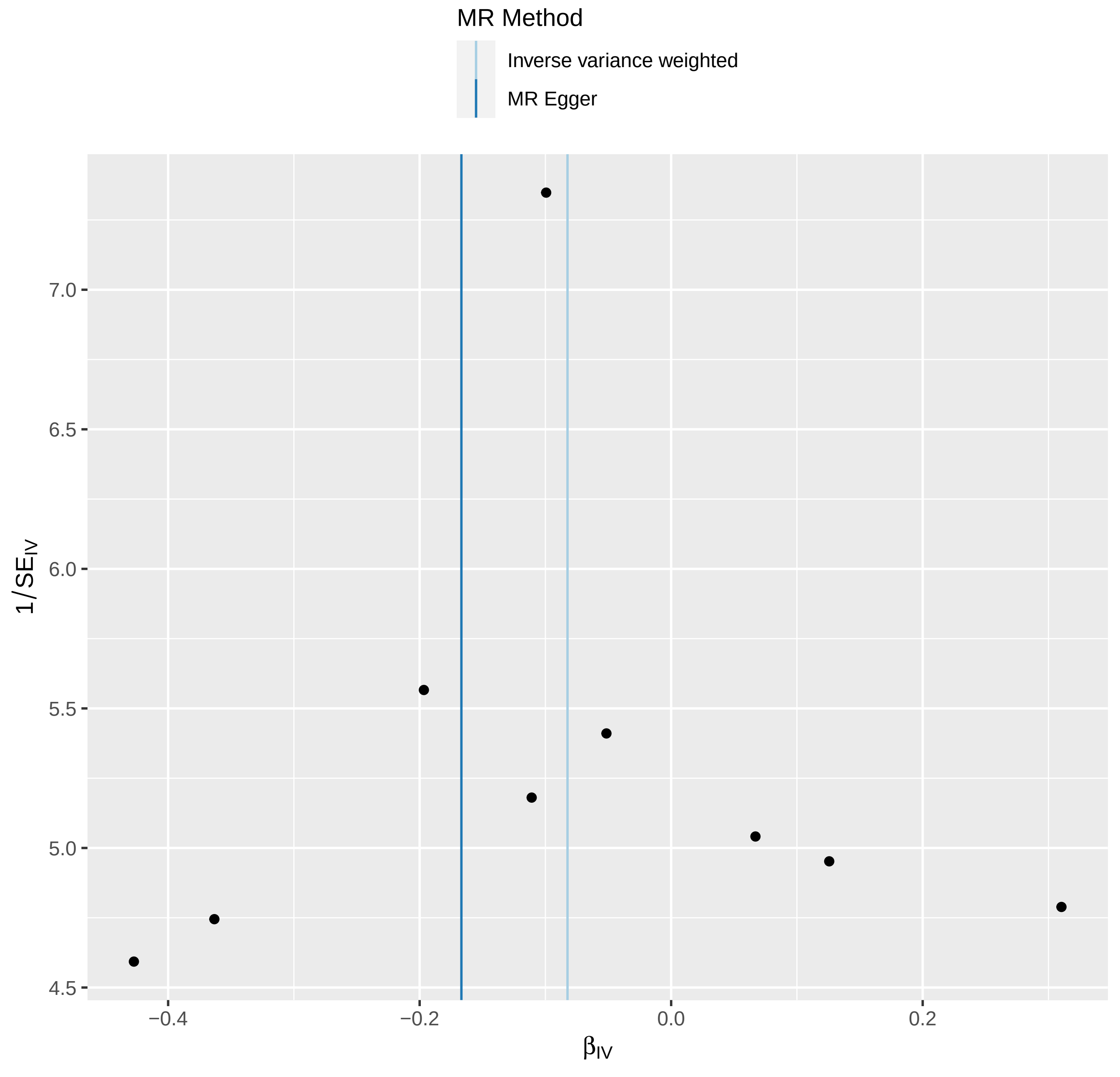

Batch 381 : Gut microbiota abundance (genus Faecalibacterium id.2057) on Obesity due to excess calories

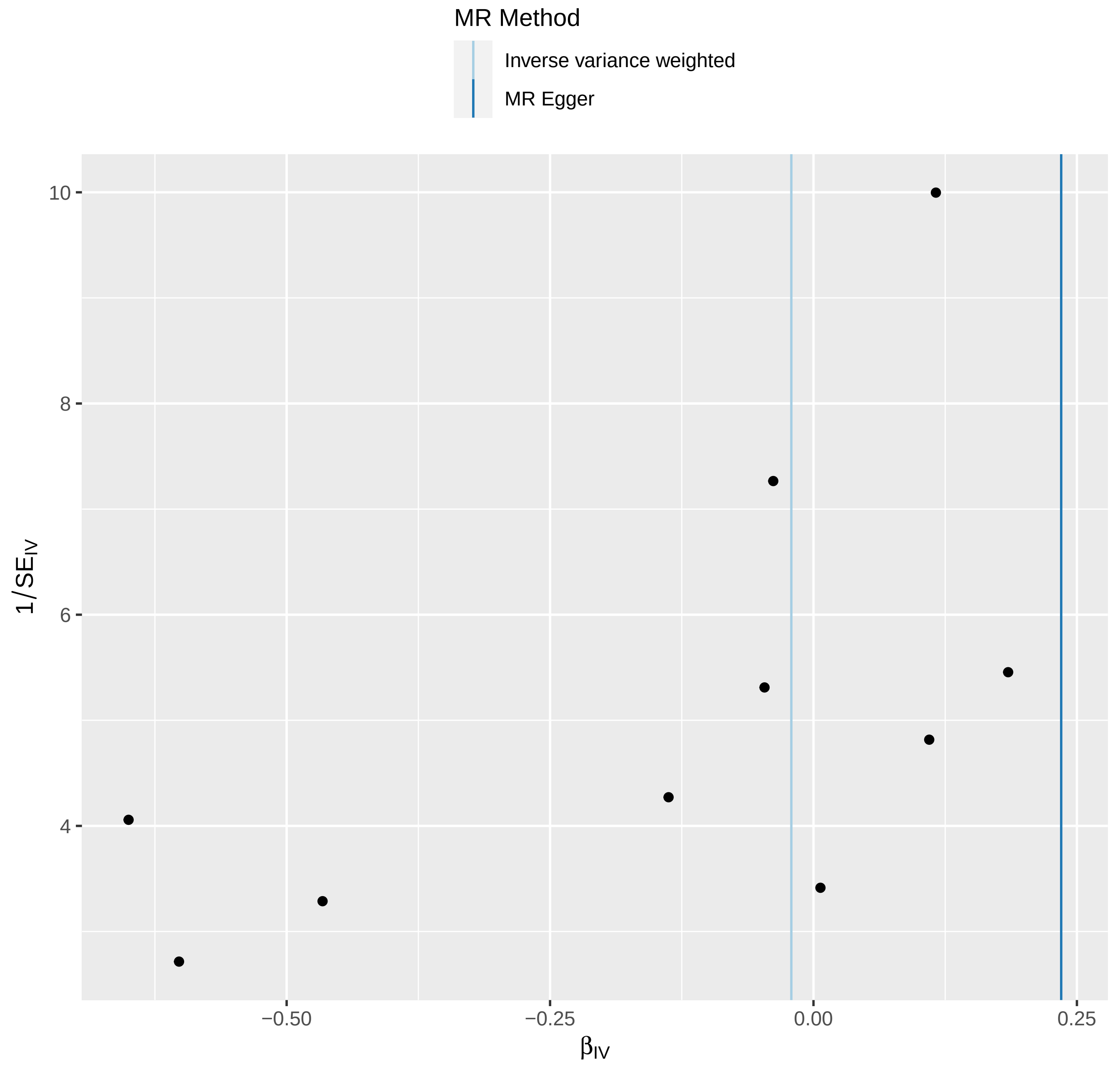

Batch 382 : Gut microbiota abundance (genus Family XIII AD3011 group id.11293) on Obesity due to excess calories

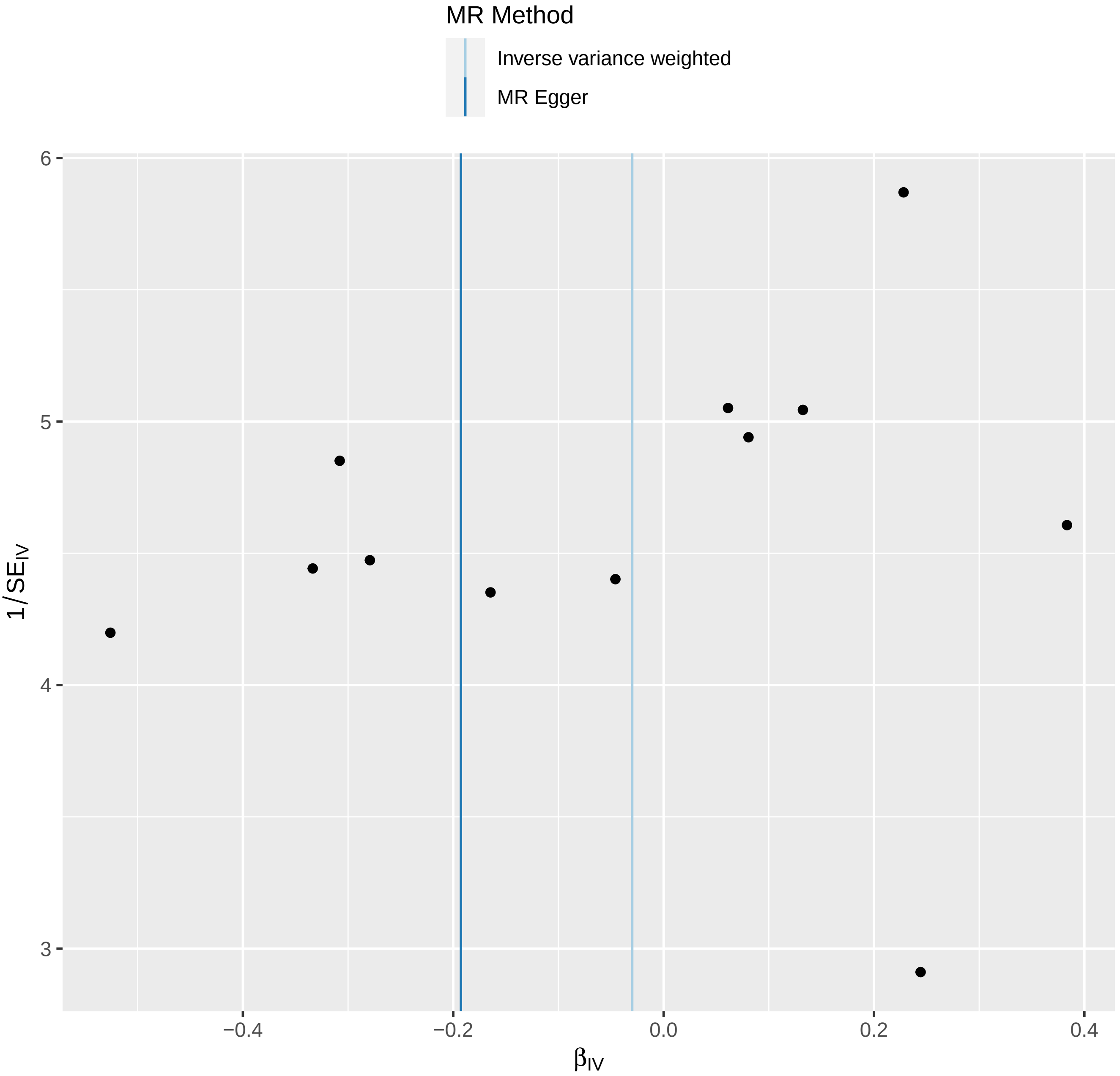

Batch 383 : Gut microbiota abundance (genus Family XIII UCG001 id.11294) on Obesity due to excess calories

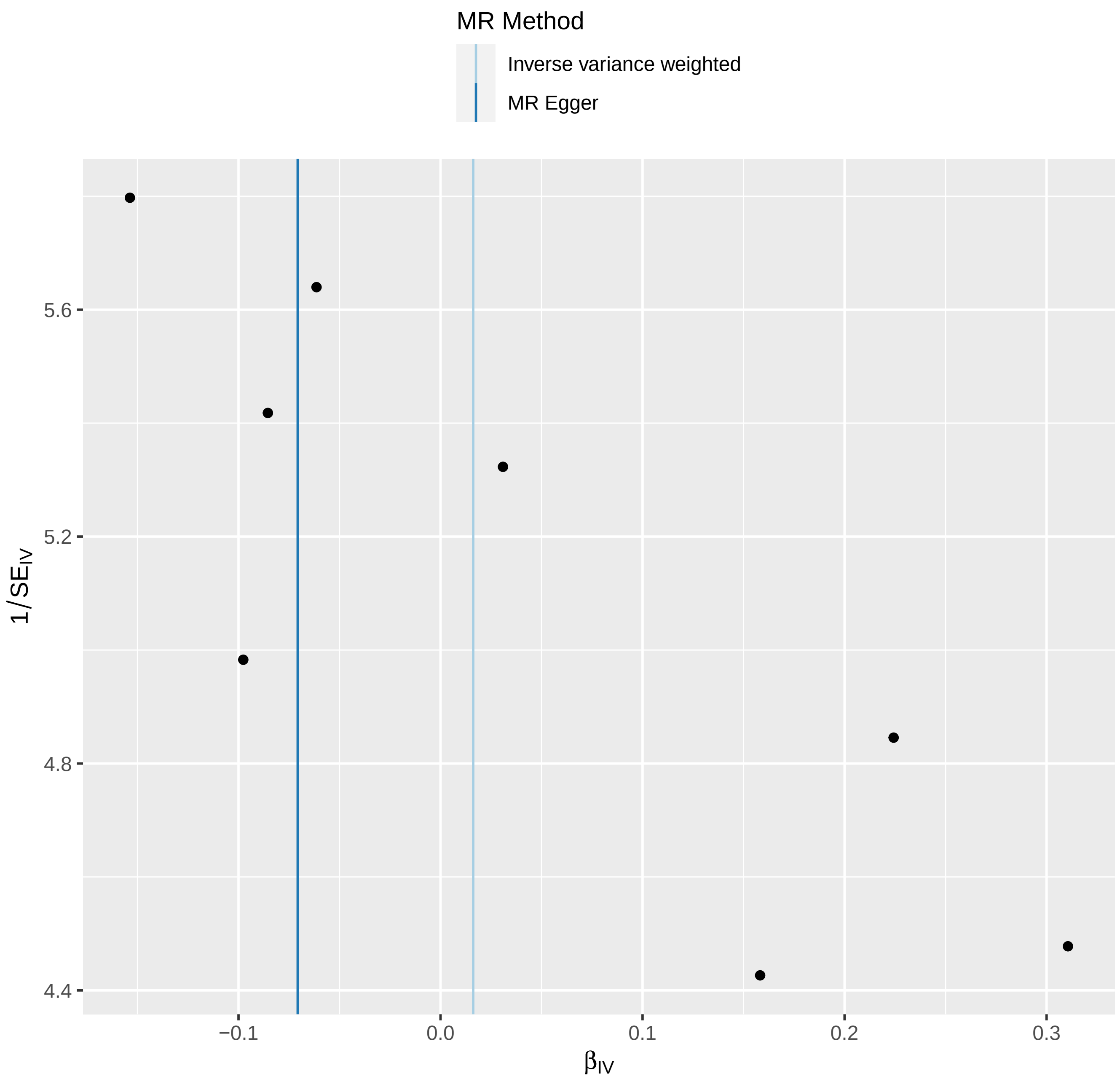

Batch 384 : Gut microbiota abundance (genus Flavonifractor id.2059) on Obesity due to excess calories

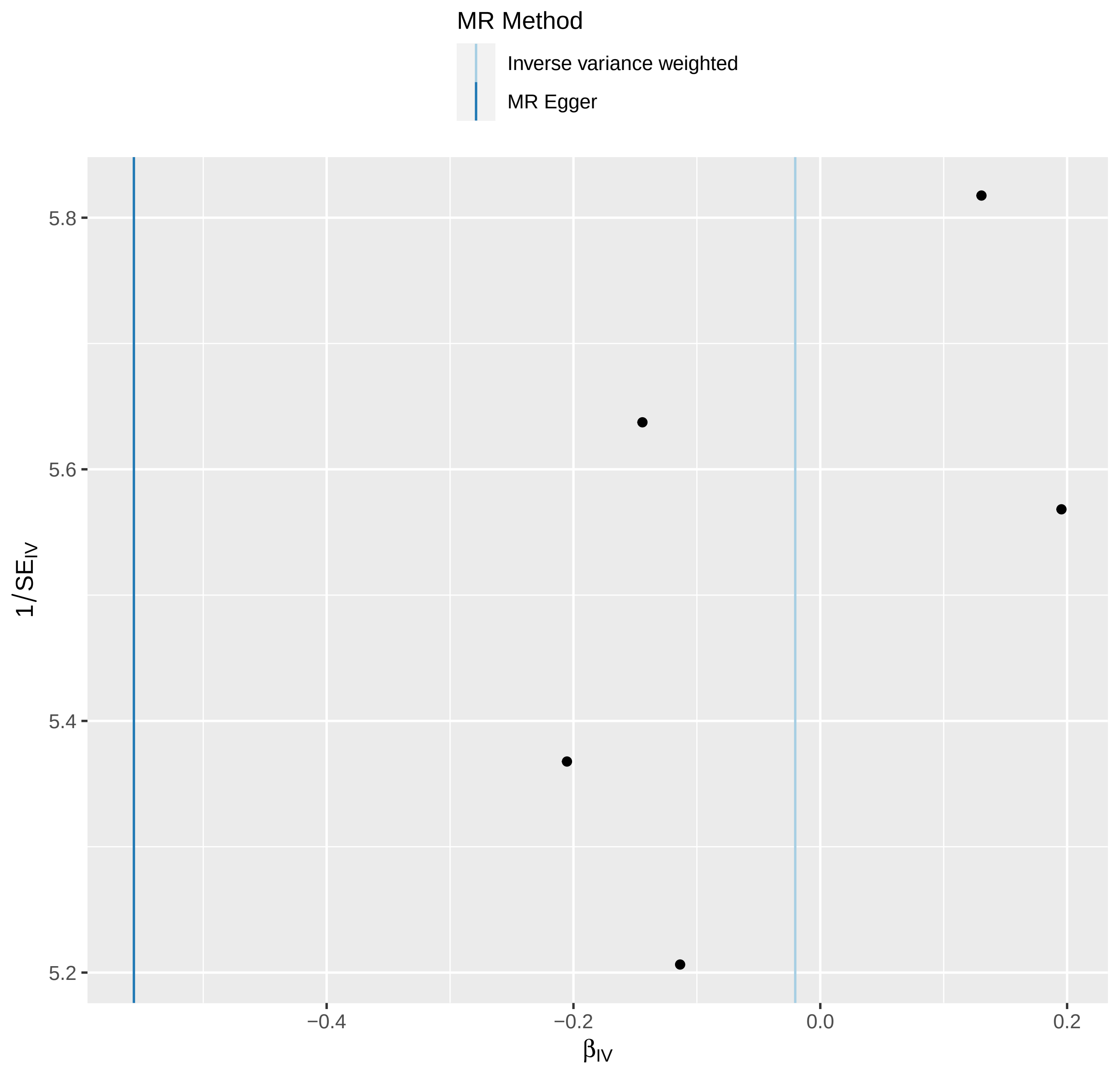

Batch 385 : Gut microbiota abundance (genus Fusicatenibacter id.11305) on Obesity due to excess calories

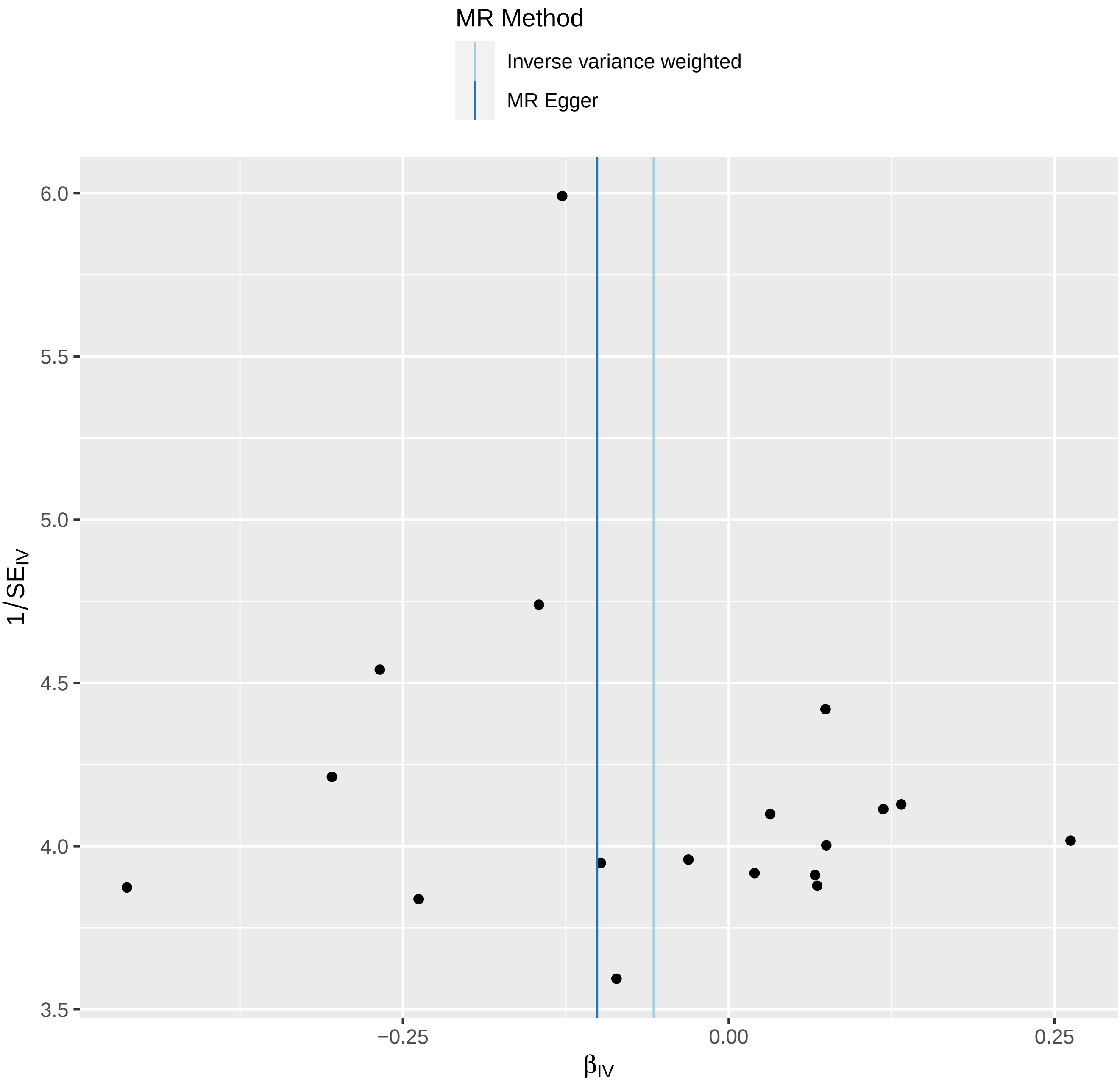

Batch 386 : Gut microbiota abundance (genus Gordonibacter id.821) on Obesity due to excess calories

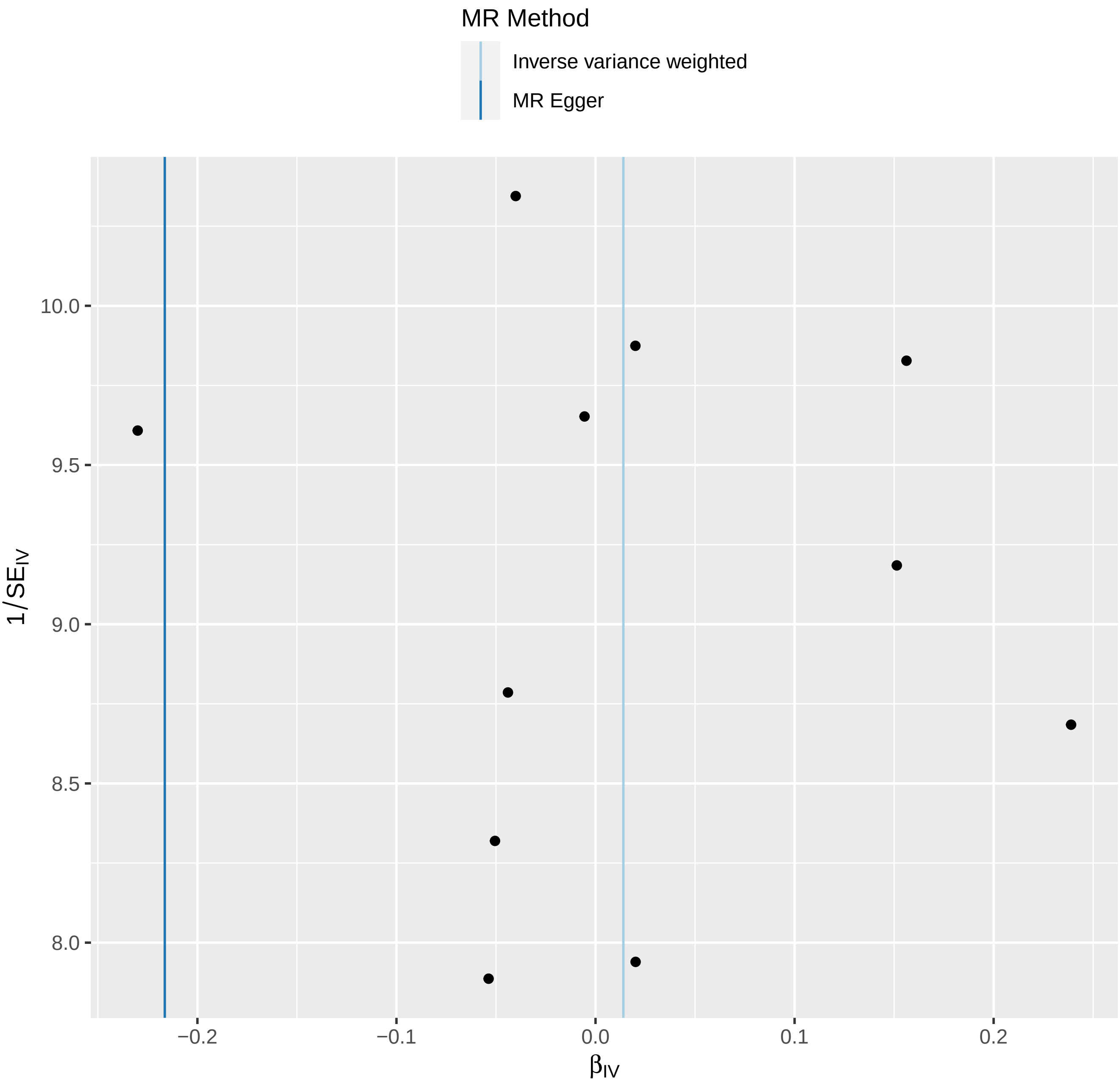

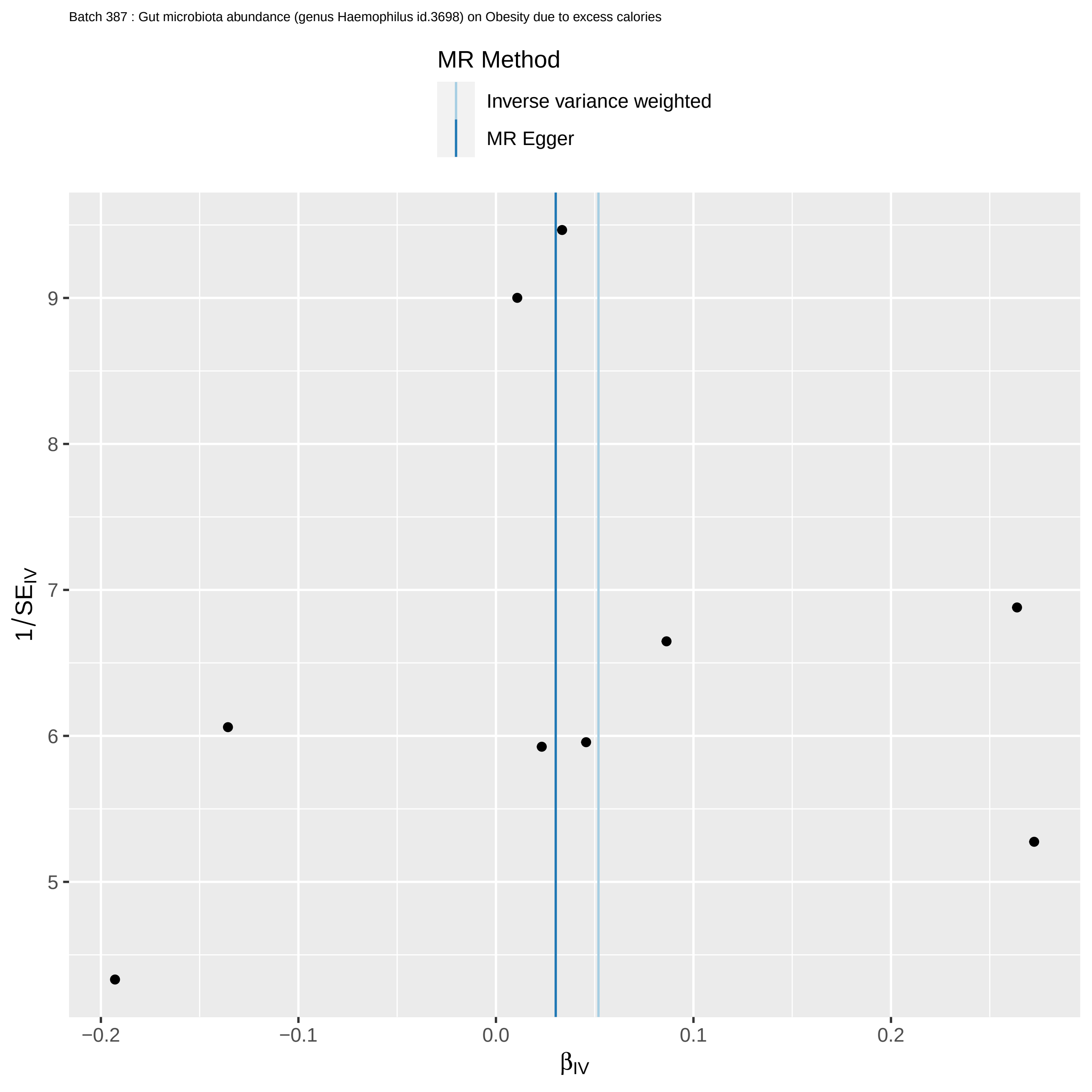
Batch 387 : Gut microbiota abundance (genus Haemophilus id.3698) on Obesity due to excess calories
MR Method
Inverse variance weighted
MR Egger
9
8
7
6
5
1/SE_IV
−0.2
−0.1
0.0
0.1
0.2
β_IV

Batch 388 : Gut microbiota abundance (genus Holdemanella id.11393) on Obesity due to excess calories

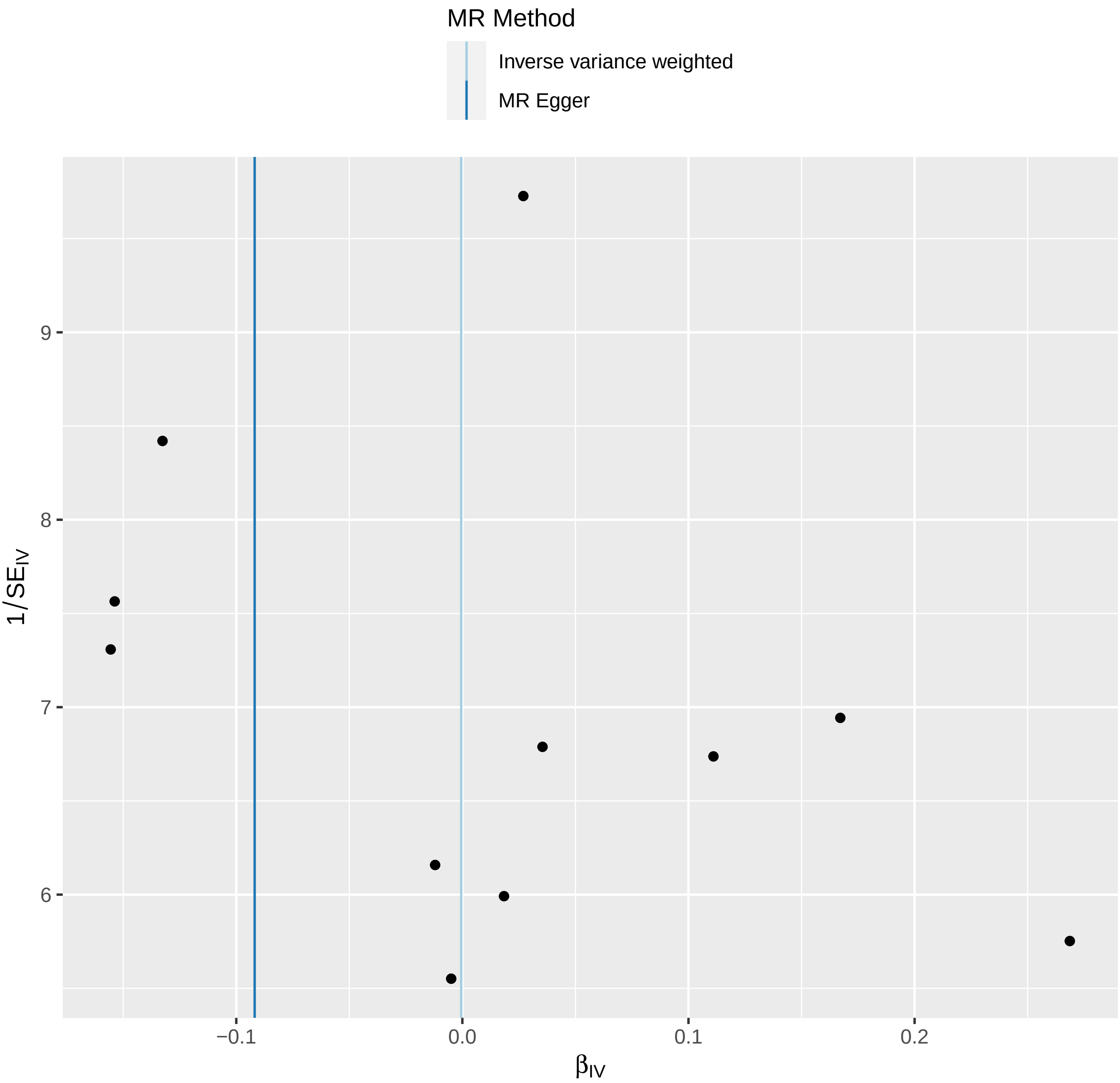

Batch 389 : Gut microbiota abundance (genus Holdemania id.2157) on Obesity due to excess calories

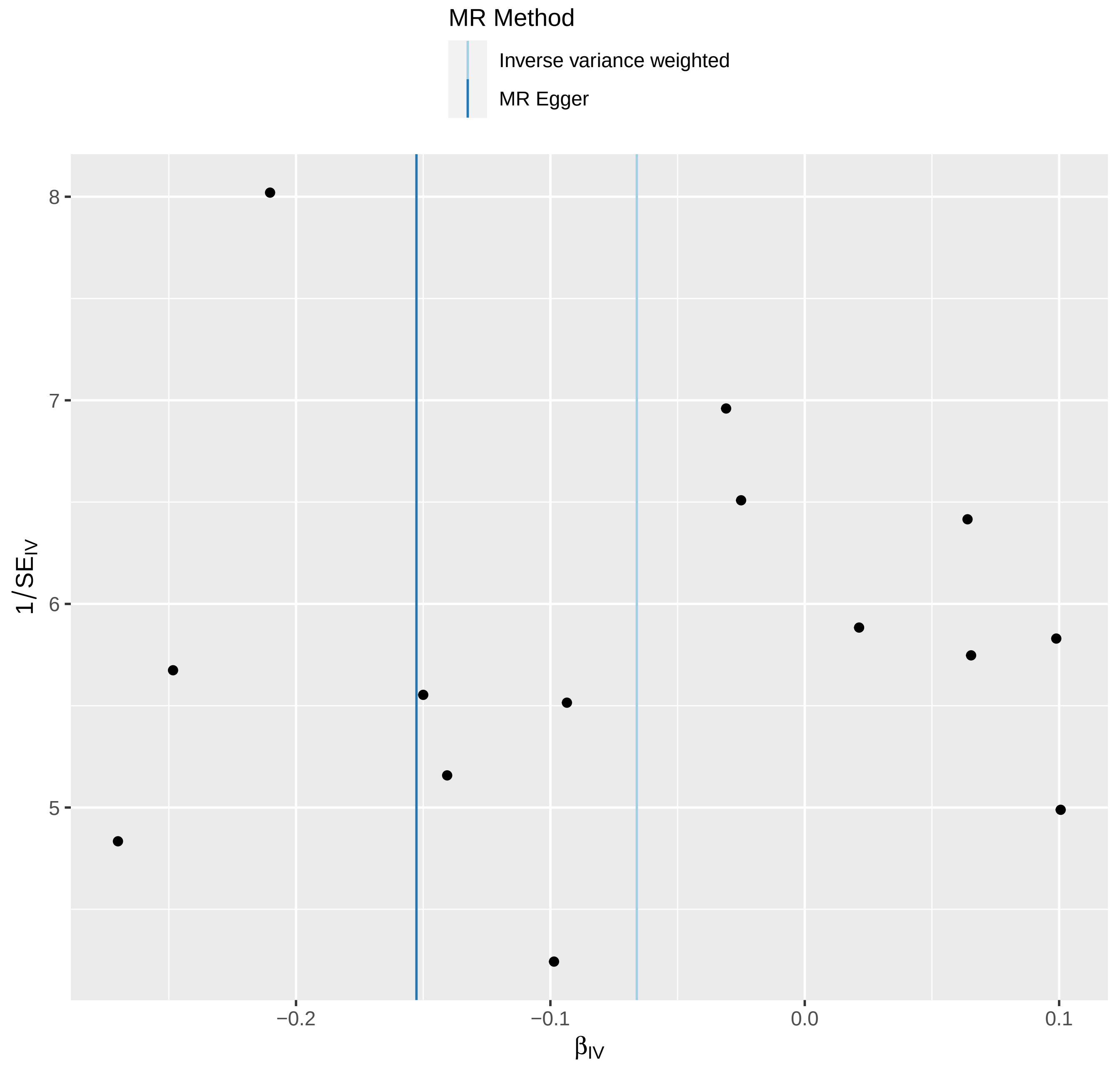

Batch 390 : Gut microbiota abundance (genus Howardella id.2000) on Obesity due to excess calories

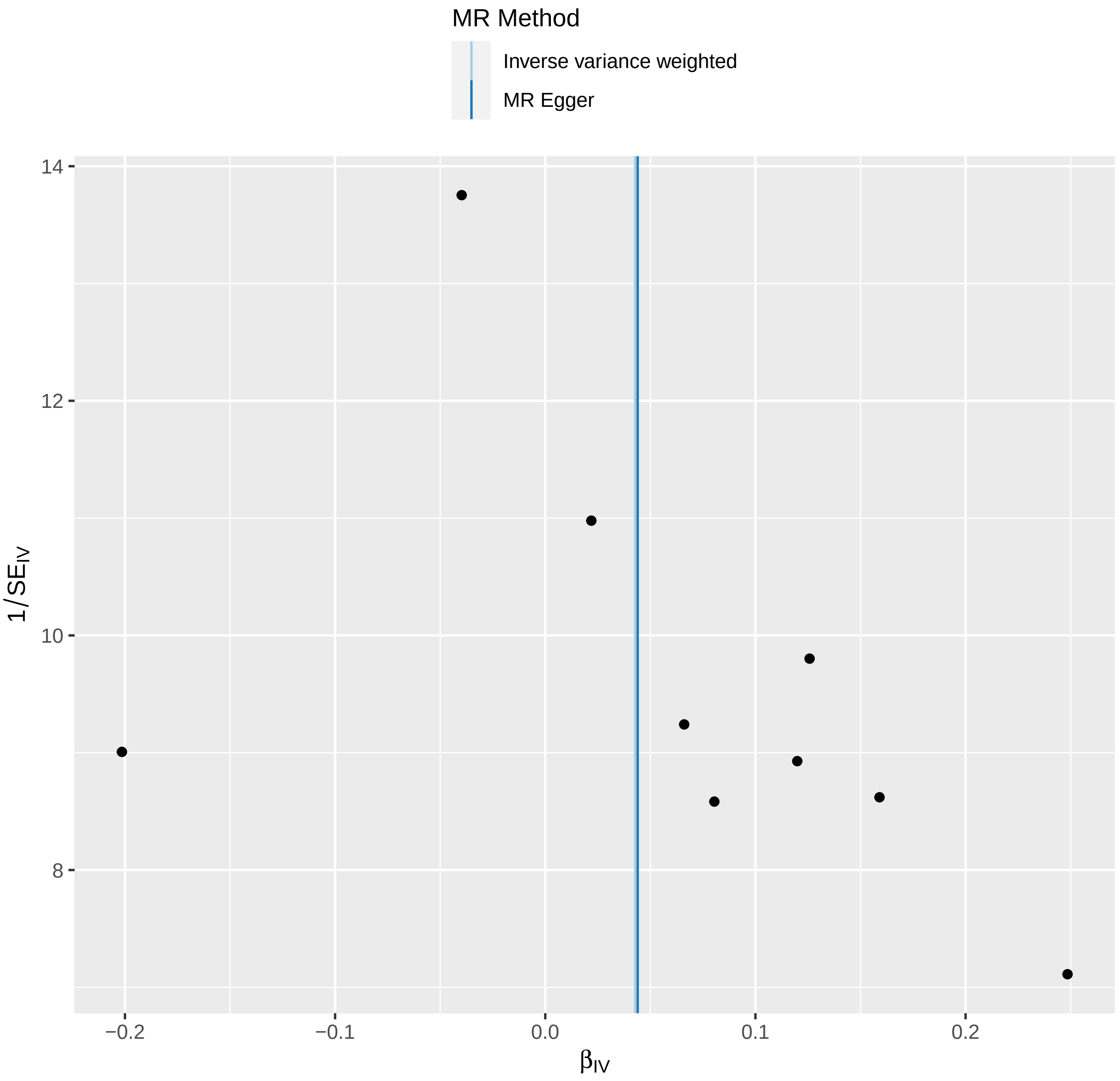

Batch 391 : Gut microbiota abundance (genus Hungatella id.11306) on Obesity due to excess calories

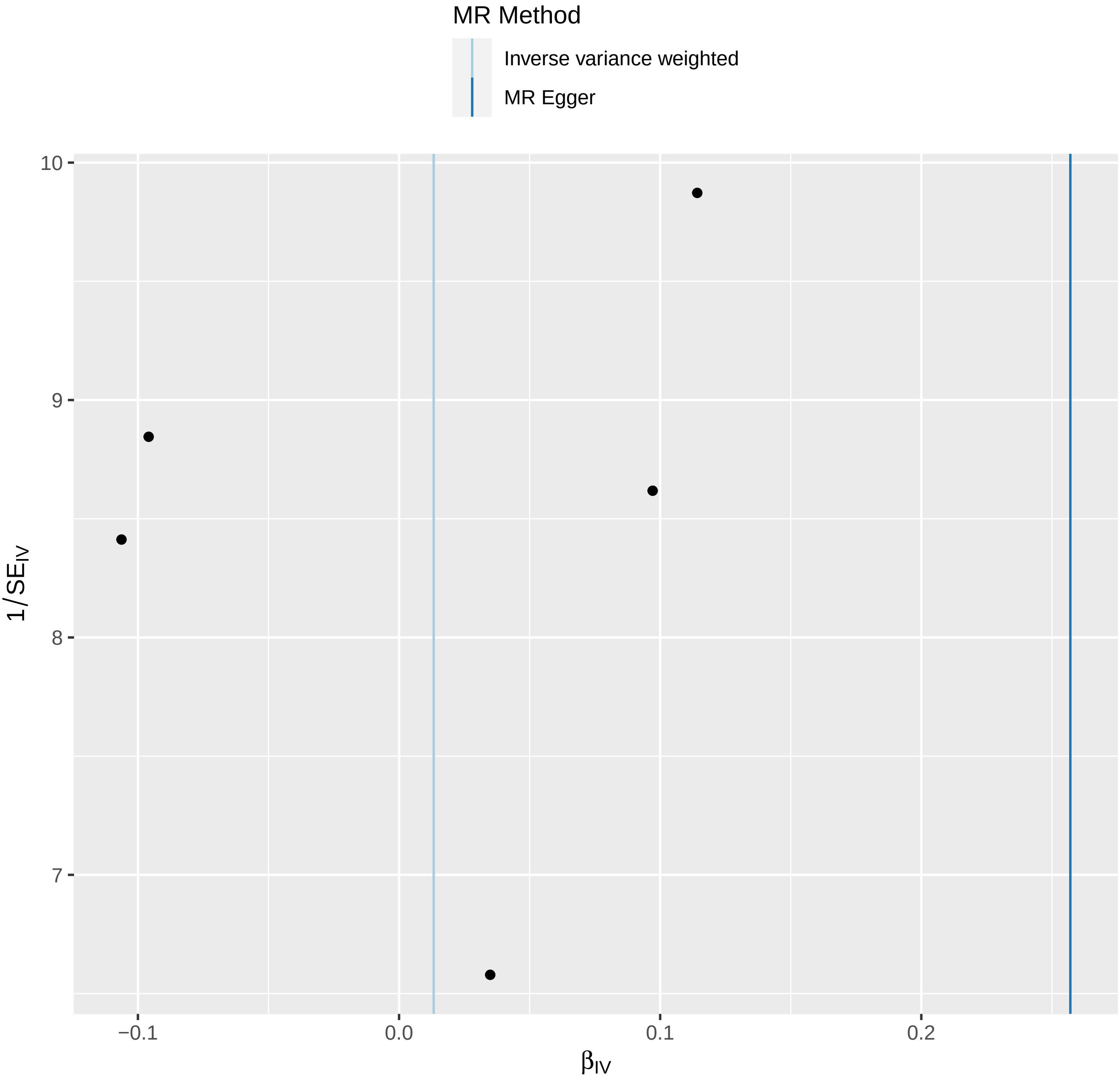

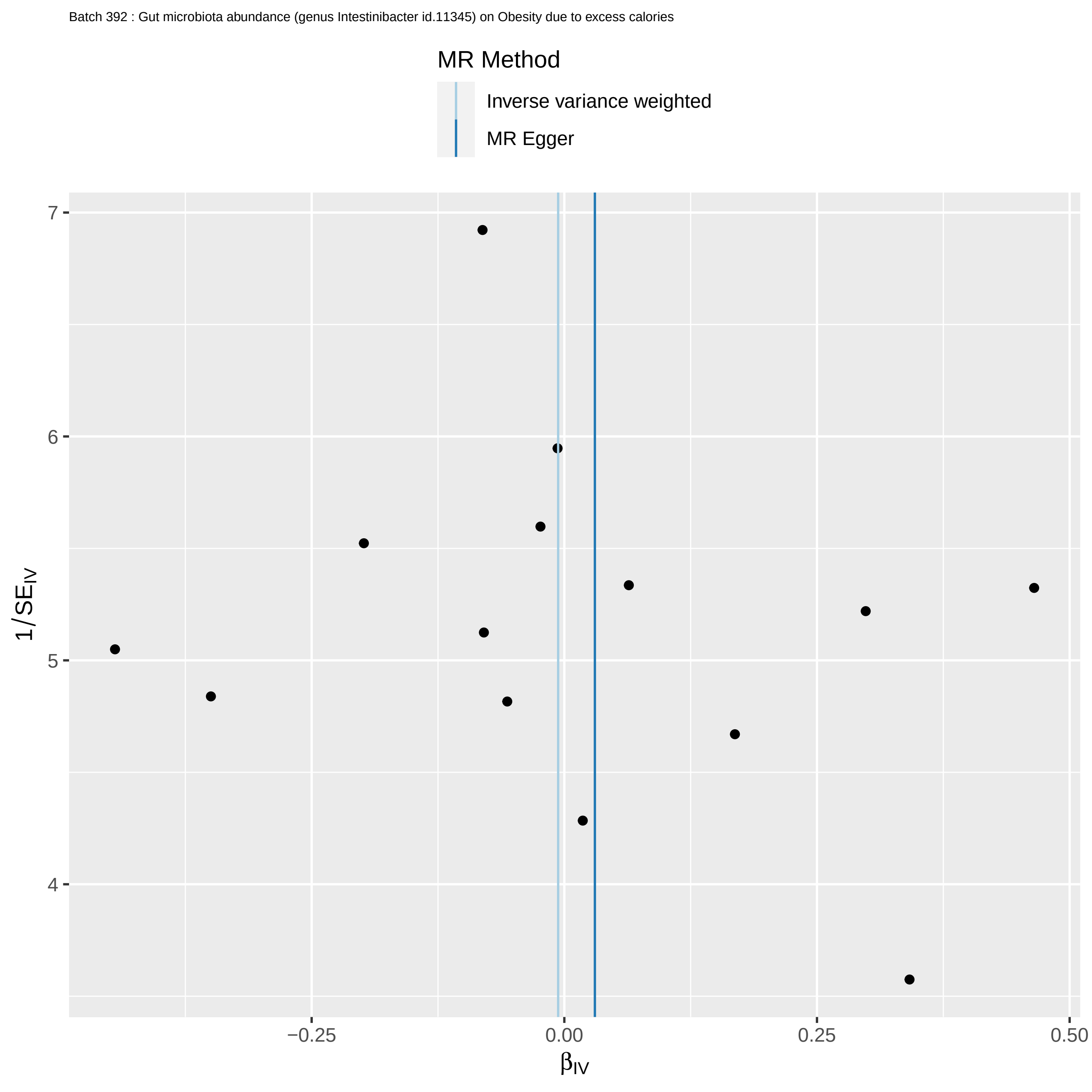

Batch 392 : Gut microbiota abundance (genus Intestinibacter id.11345) on Obesity due to excess calories
MR Method
Inverse variance weighted
MR Egger
1/SE_IV
7
6
5
4
−0.25
0.00
0.25
0.50
β_IV

Batch 393 : Gut microbiota abundance (genus Intestinimonas id.2062) on Obesity due to excess calories

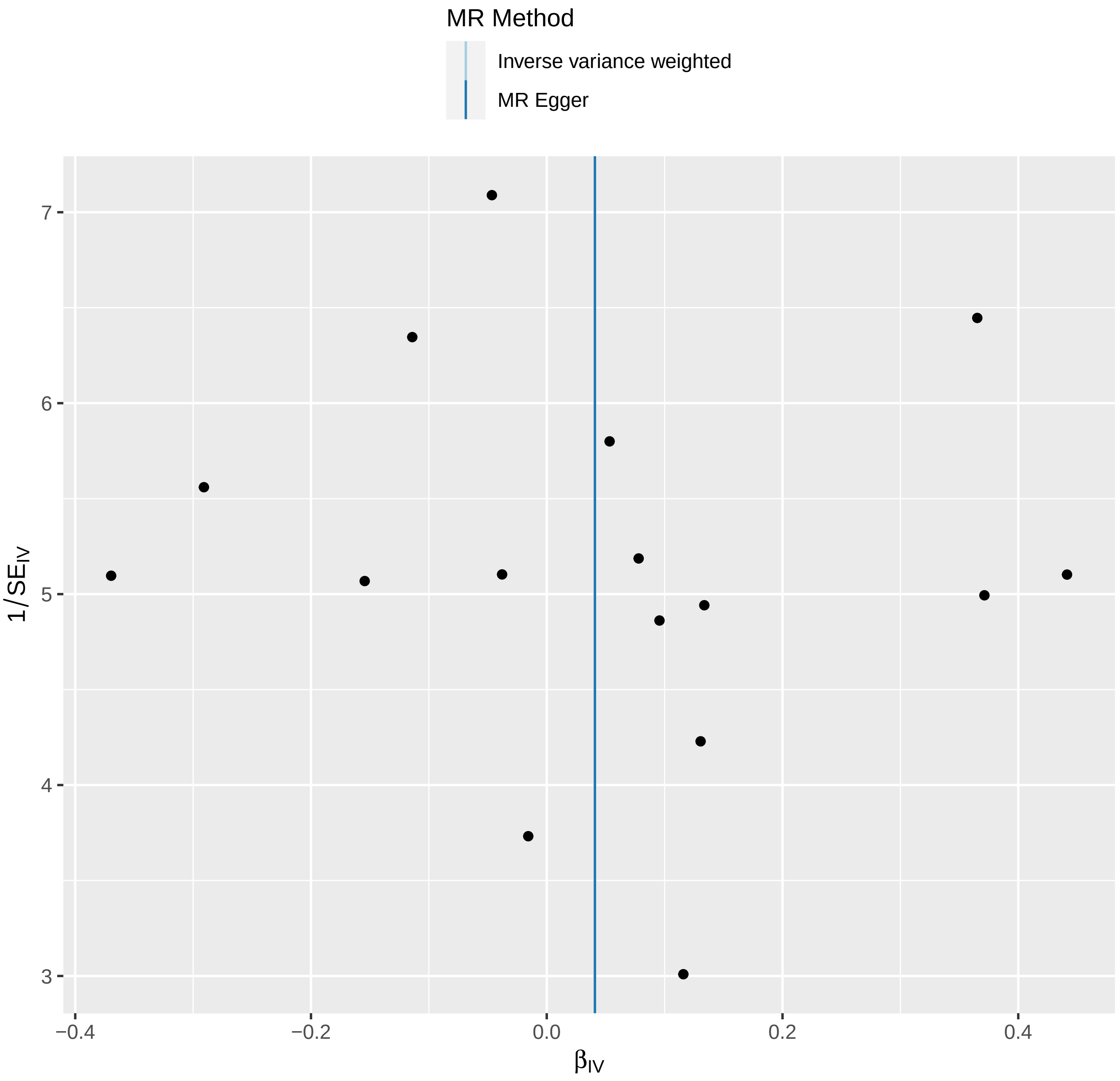

Batch 394 : Gut microbiota abundance (genus Lachnoclostridium id.11308) on Obesity due to excess calories

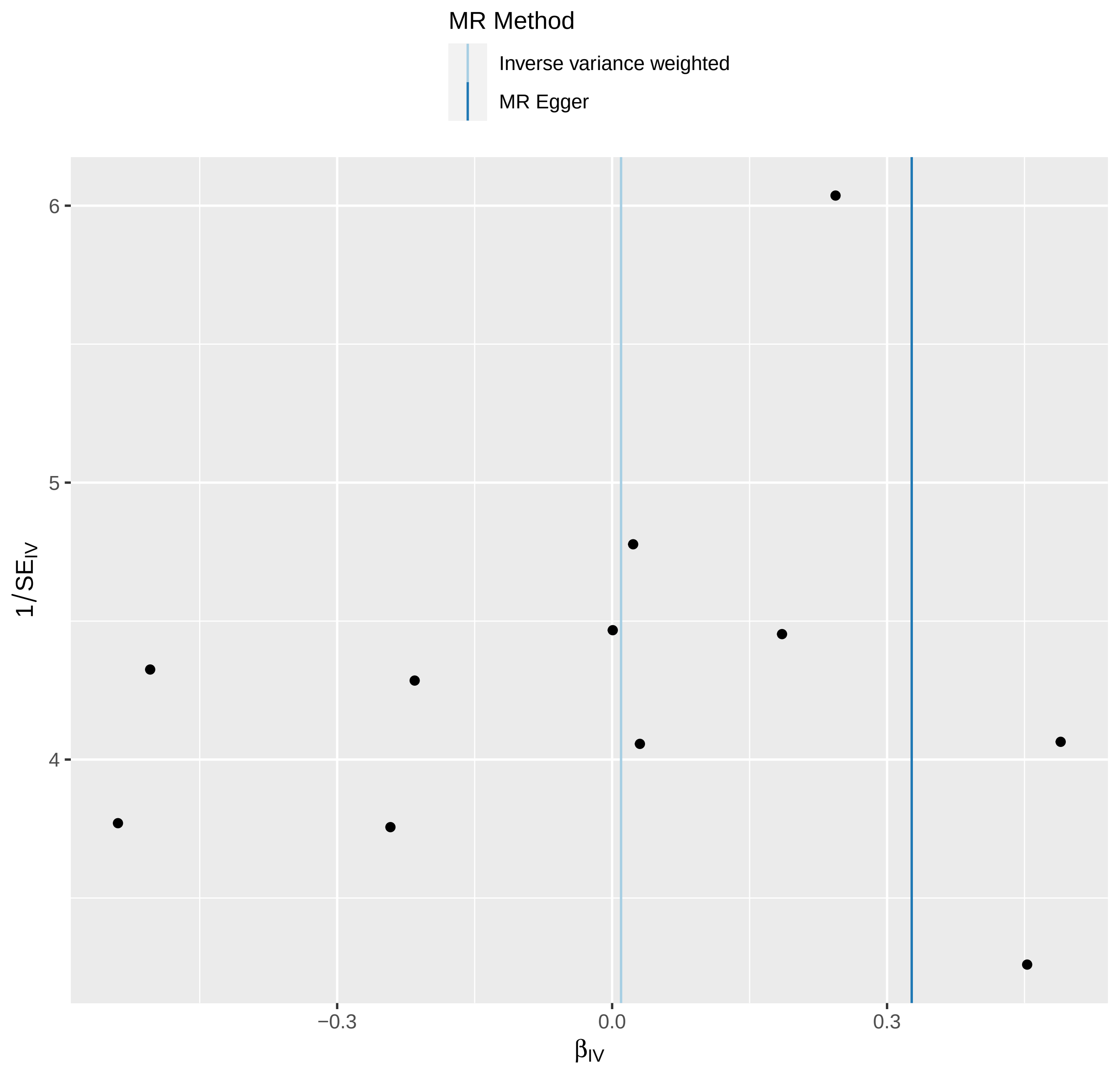

Batch 395 : Gut microbiota abundance (genus Lachnospiraceae FCS020 group id.11314) on Obesity due to excess calories

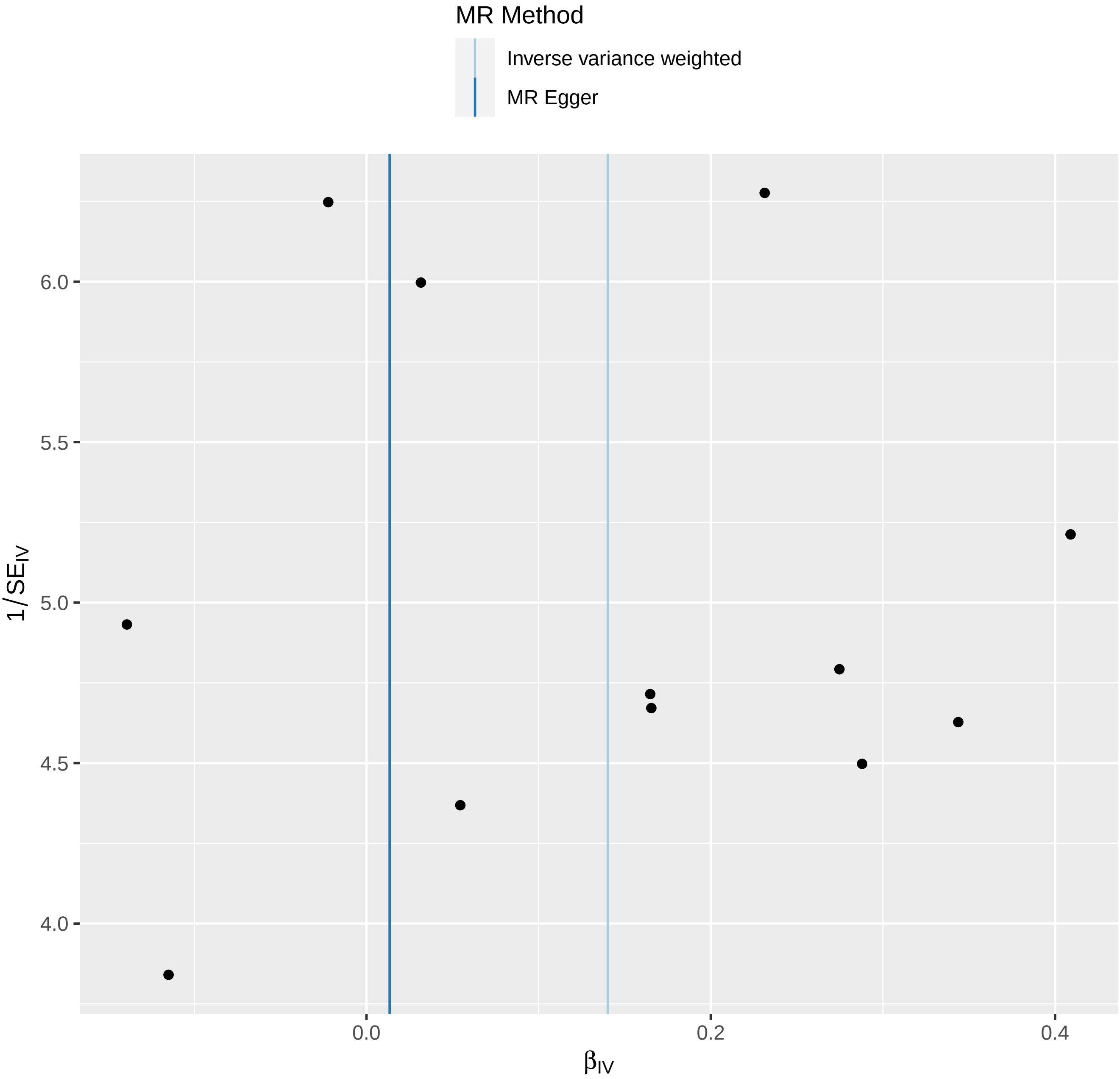

Batch 396 : Gut microbiota abundance (genus Lachnospiraceae NC2004 group id.11316) on Obesity due to excess calories

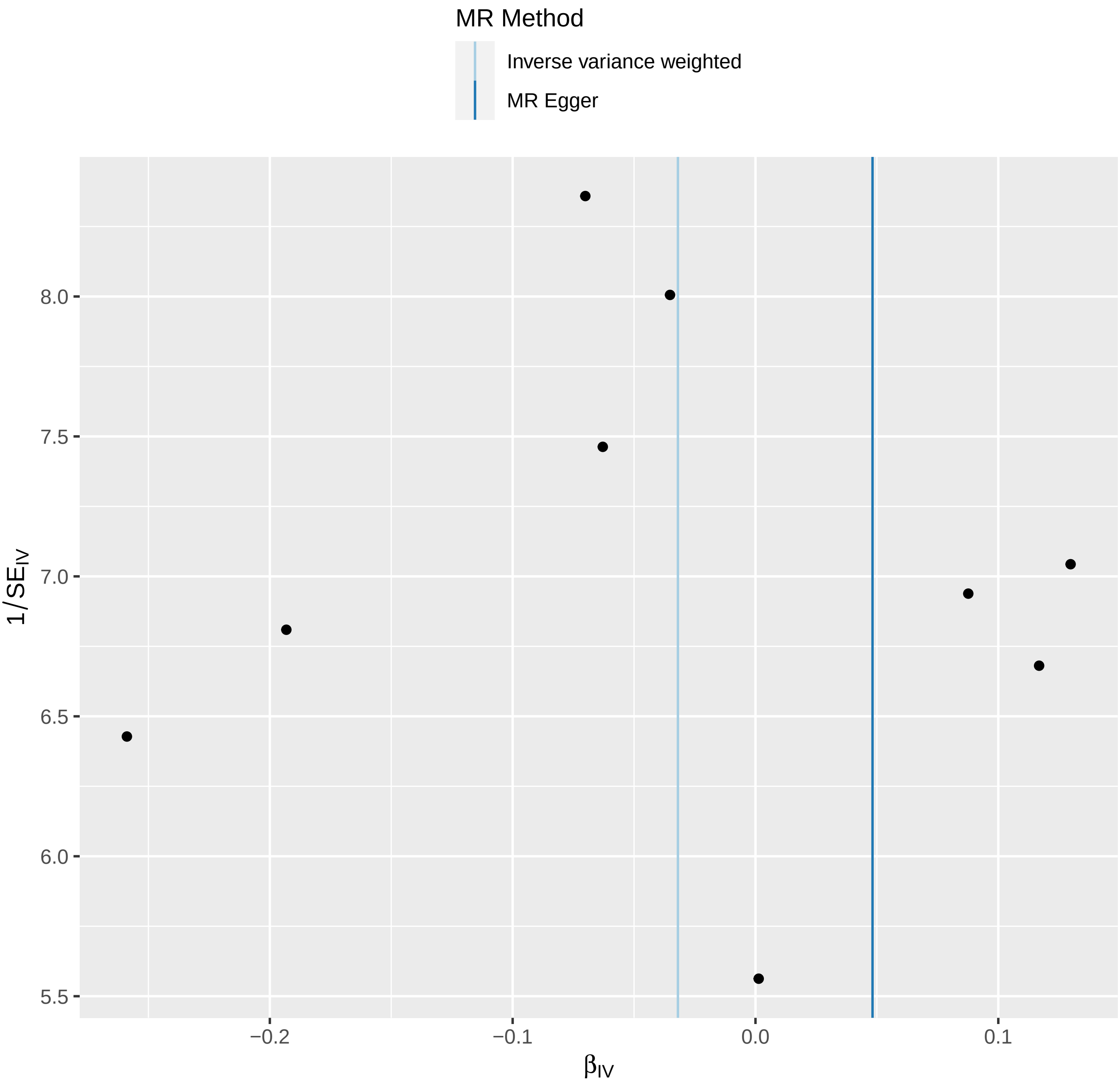

Batch 397 : Gut microbiota abundance (genus Lachnospiraceae ND3007 group id.11317) on Obesity due to excess calories

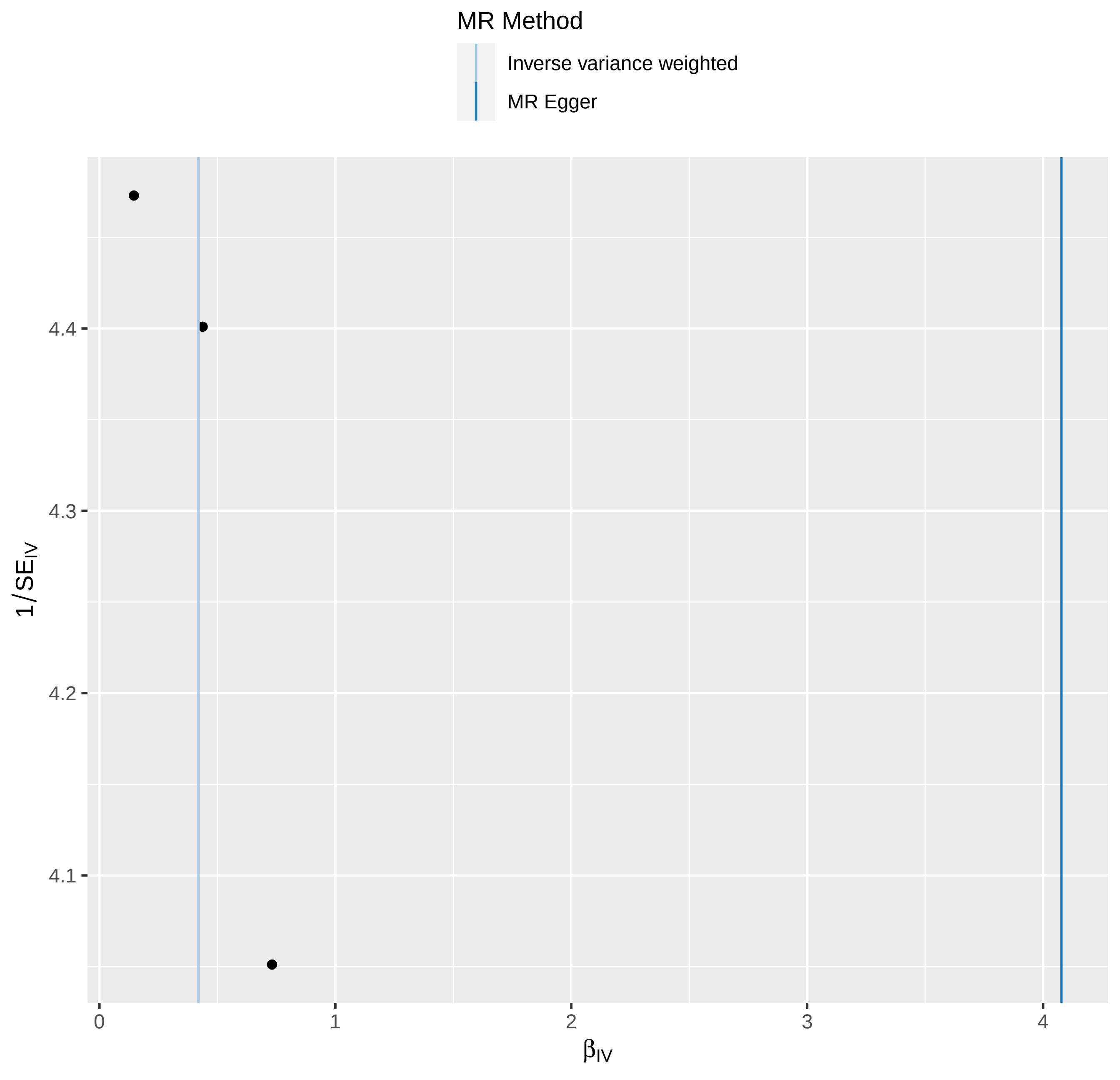

Batch 398 : Gut microbiota abundance (genus Lachnospiraceae NK4A136 group id.11319) on Obesity due to excess calories

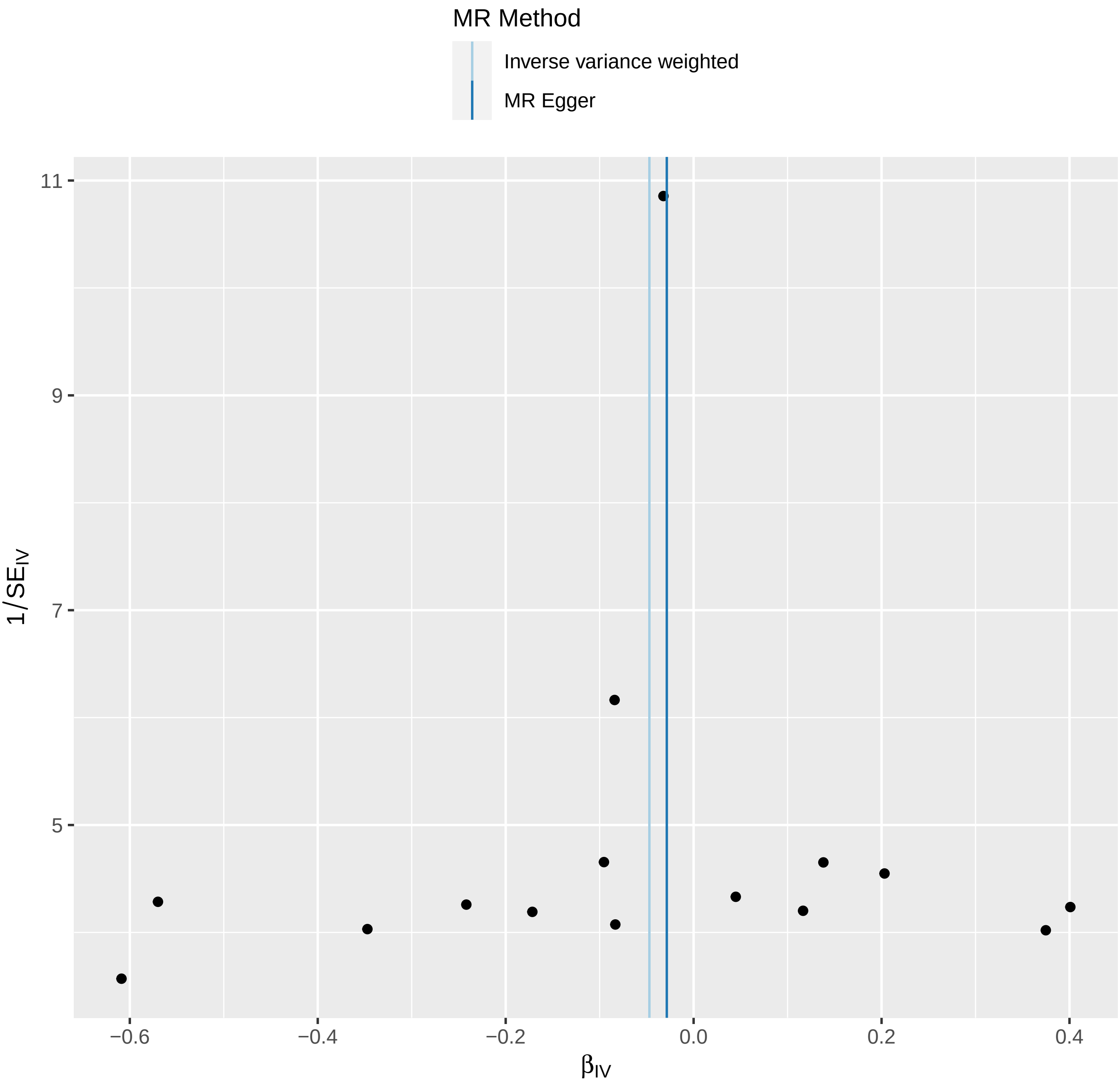

Batch 399 : Gut microbiota abundance (genus Lachnospiraceae UCG001 id.11321) on Obesity due to excess calories

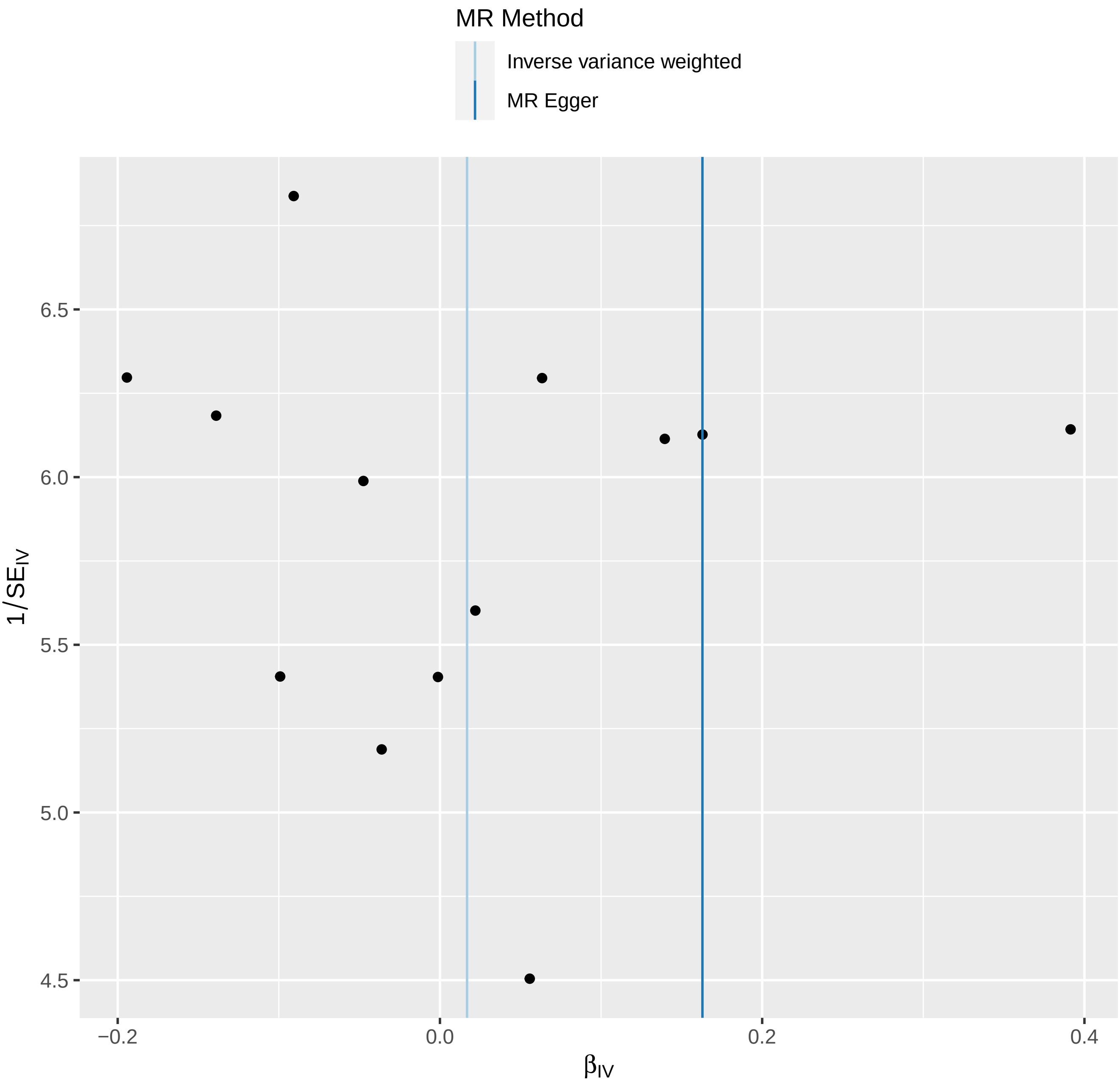

Batch 400 : Gut microbiota abundance (genus Lachnospiraceae UCG004 id.11324) on Obesity due to excess calories

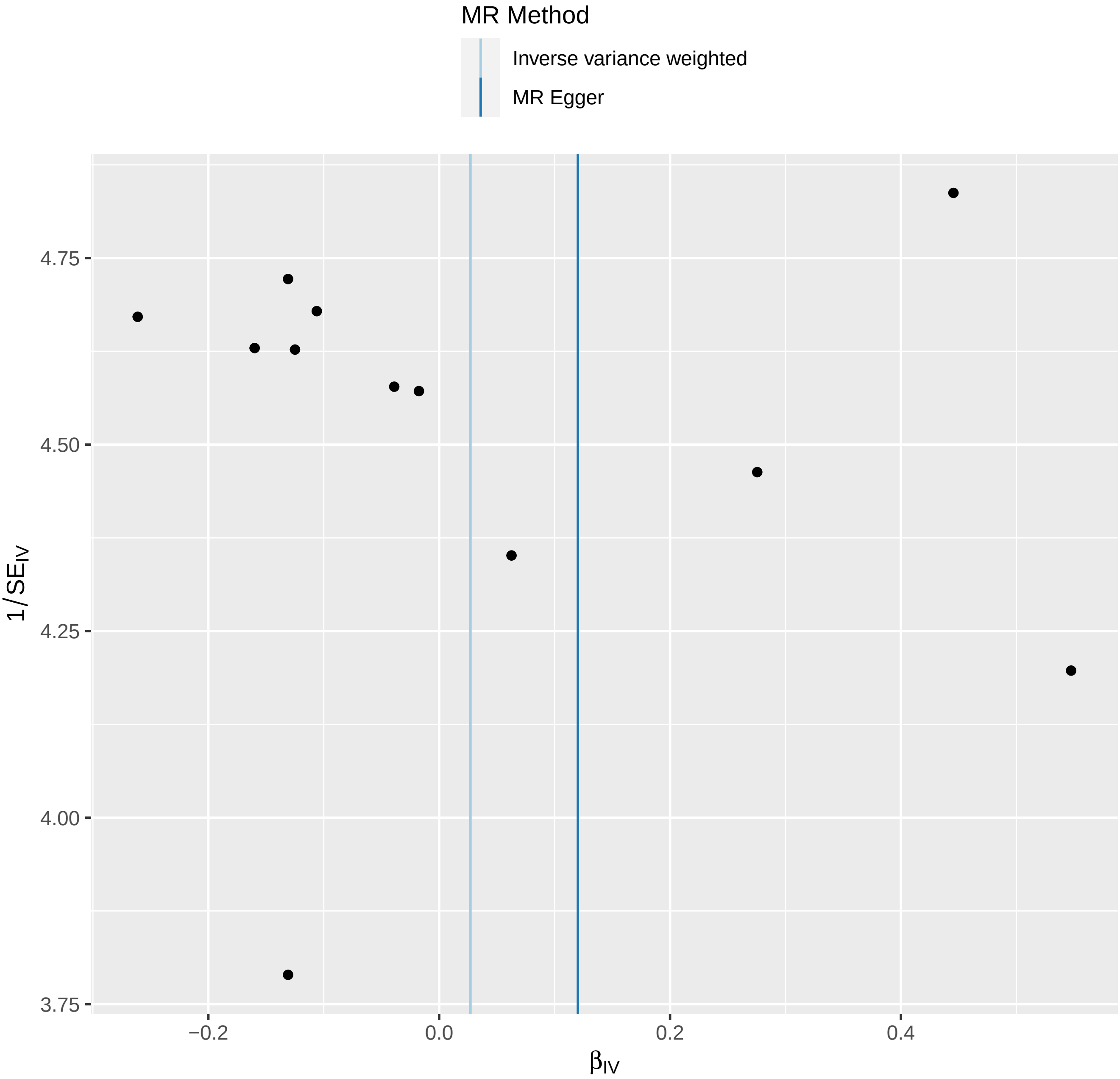

Batch 401 : Gut microbiota abundance (genus Lachnospiraceae UCG008 id.11328) on Obesity due to excess calories

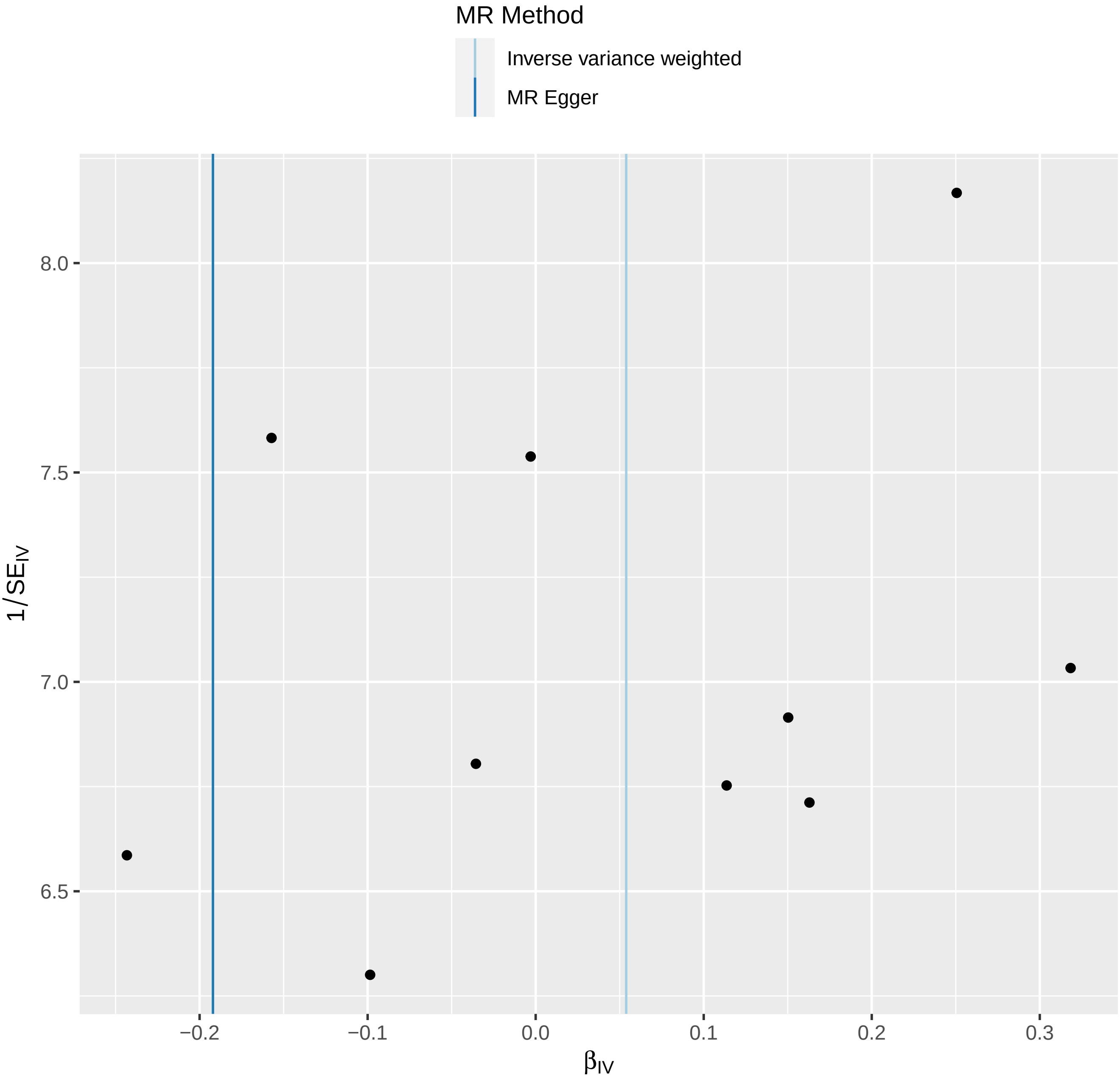

Batch 402 : Gut microbiota abundance (genus Lachnospiraceae UCG010 id.11330) on Obesity due to excess calories

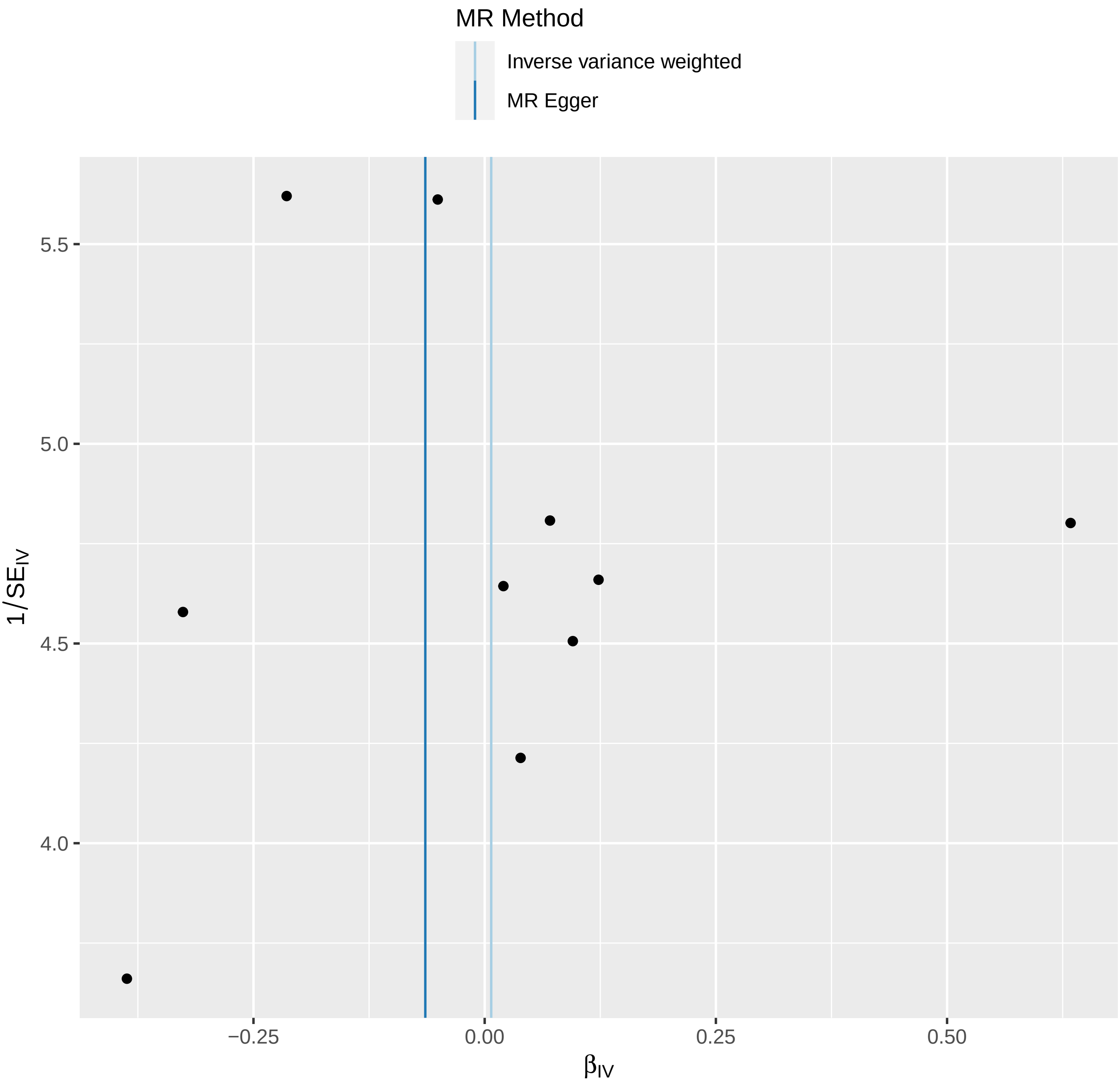

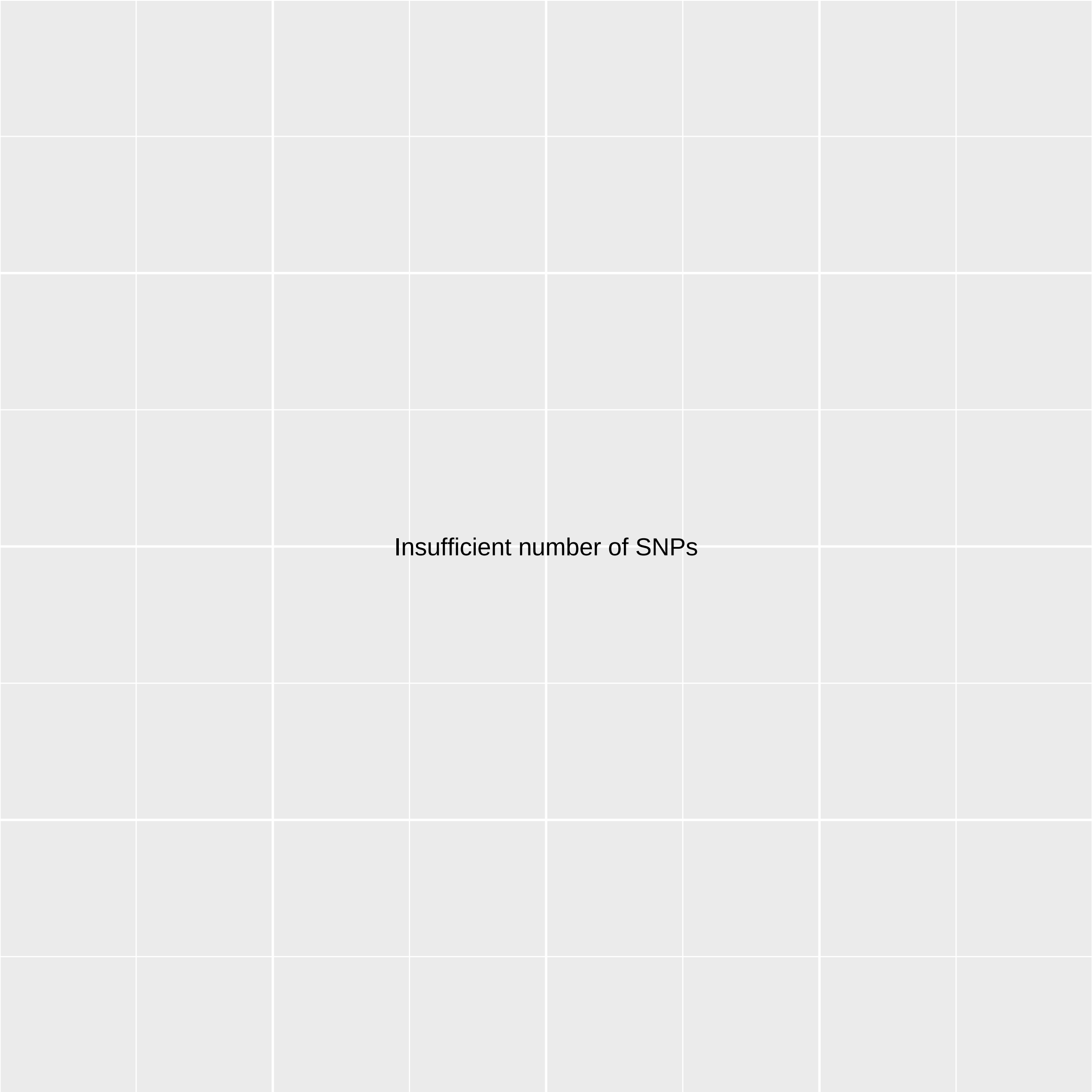
Insufficient number of SNPs

Batch 404 : Gut microbiota abundance (genus Lactobacillus id.1837) on Obesity due to excess calories

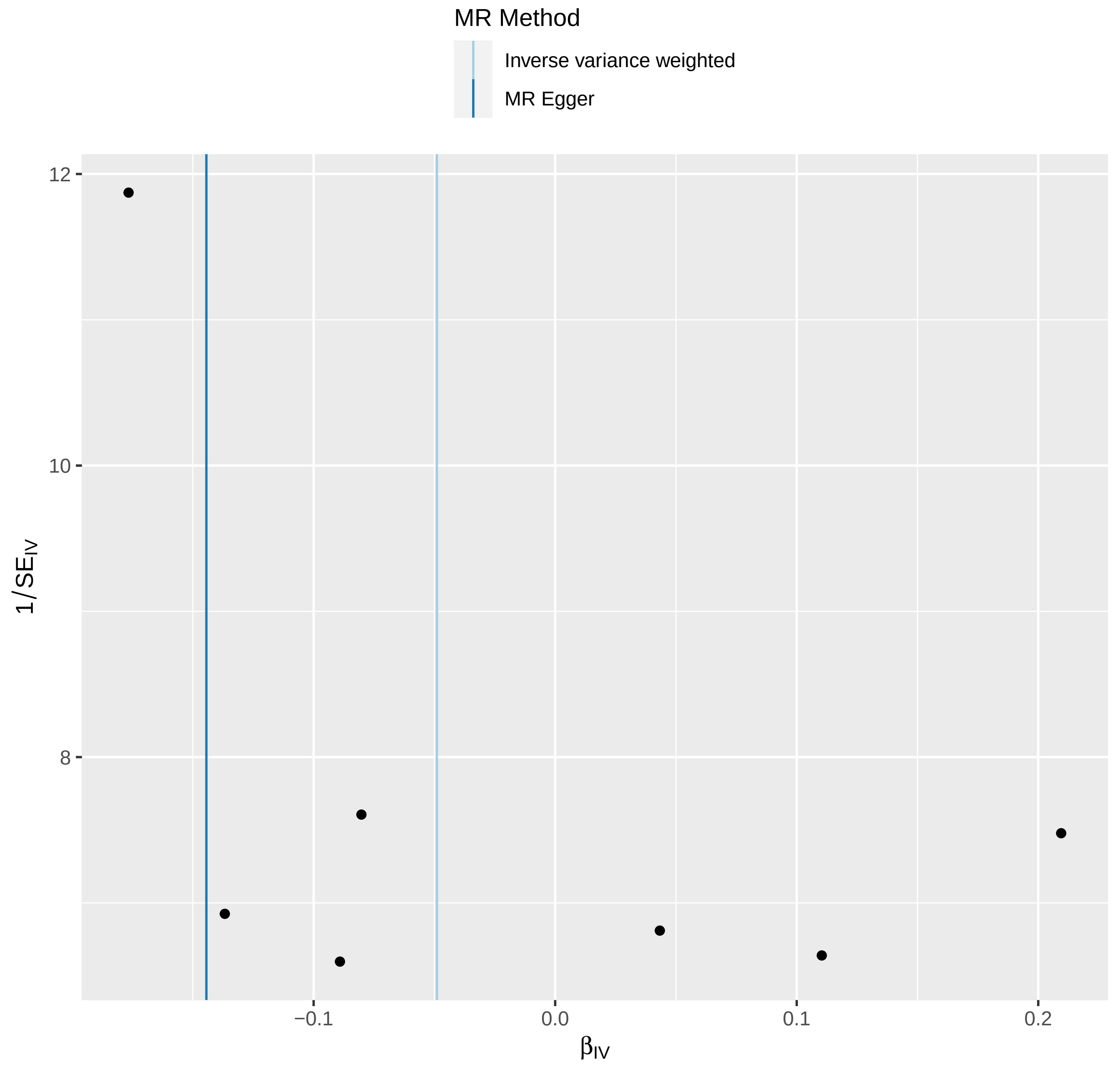

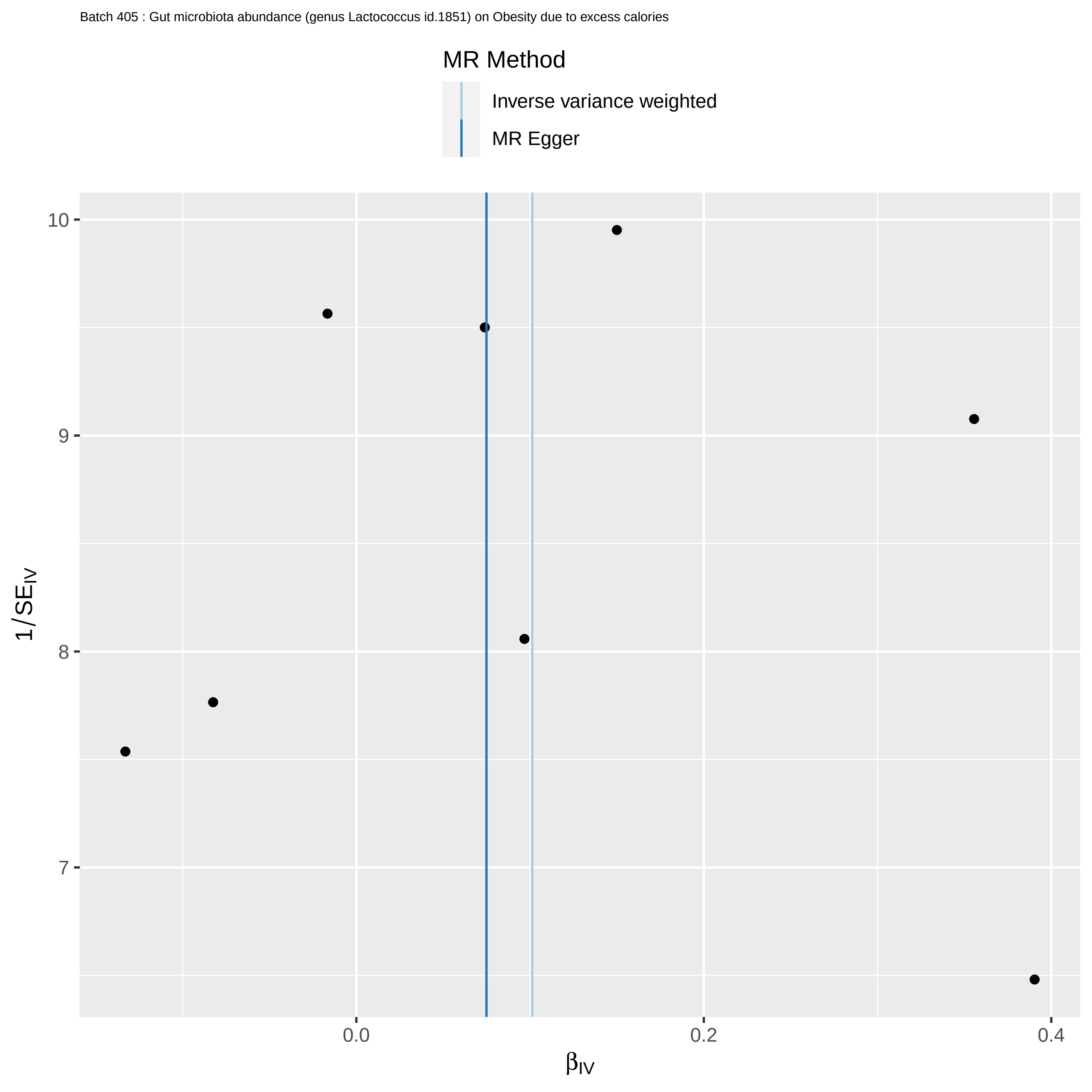
Batch 405 : Gut microbiota abundance (genus Lactococcus id.1851) on Obesity due to excess calories
MR Method
Inverse variance weighted
MR Egger
10
9
8
7
0.0
0.2
0.4
$1/SE_{IV}$
$\beta_{IV}$

Batch 406 : Gut microbiota abundance (genus Marvinbryantia id.2005) on Obesity due to excess calories

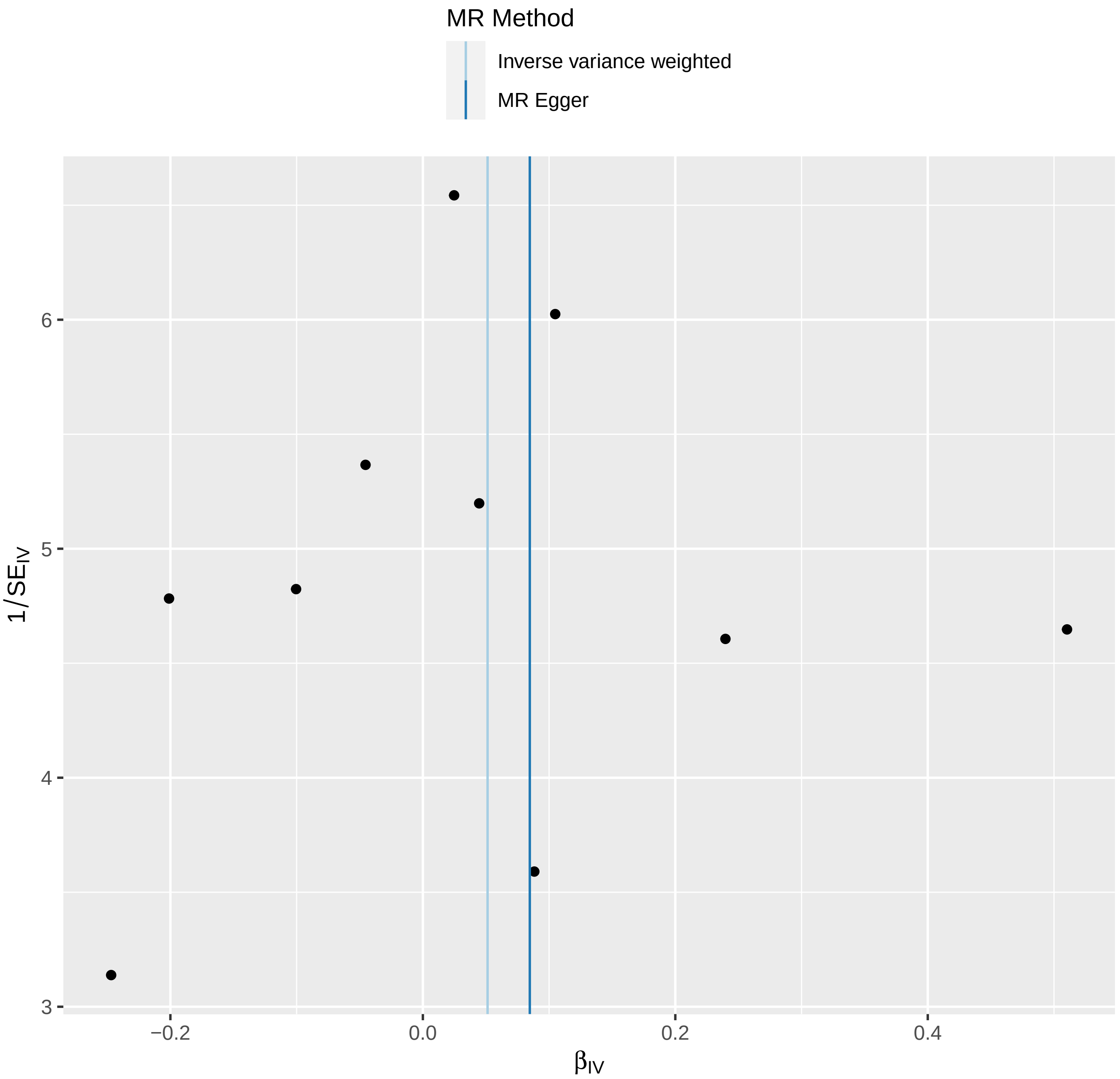

Batch 407 : Gut microbiota abundance (genus Methanobrevibacter id.123) on Obesity due to excess calories

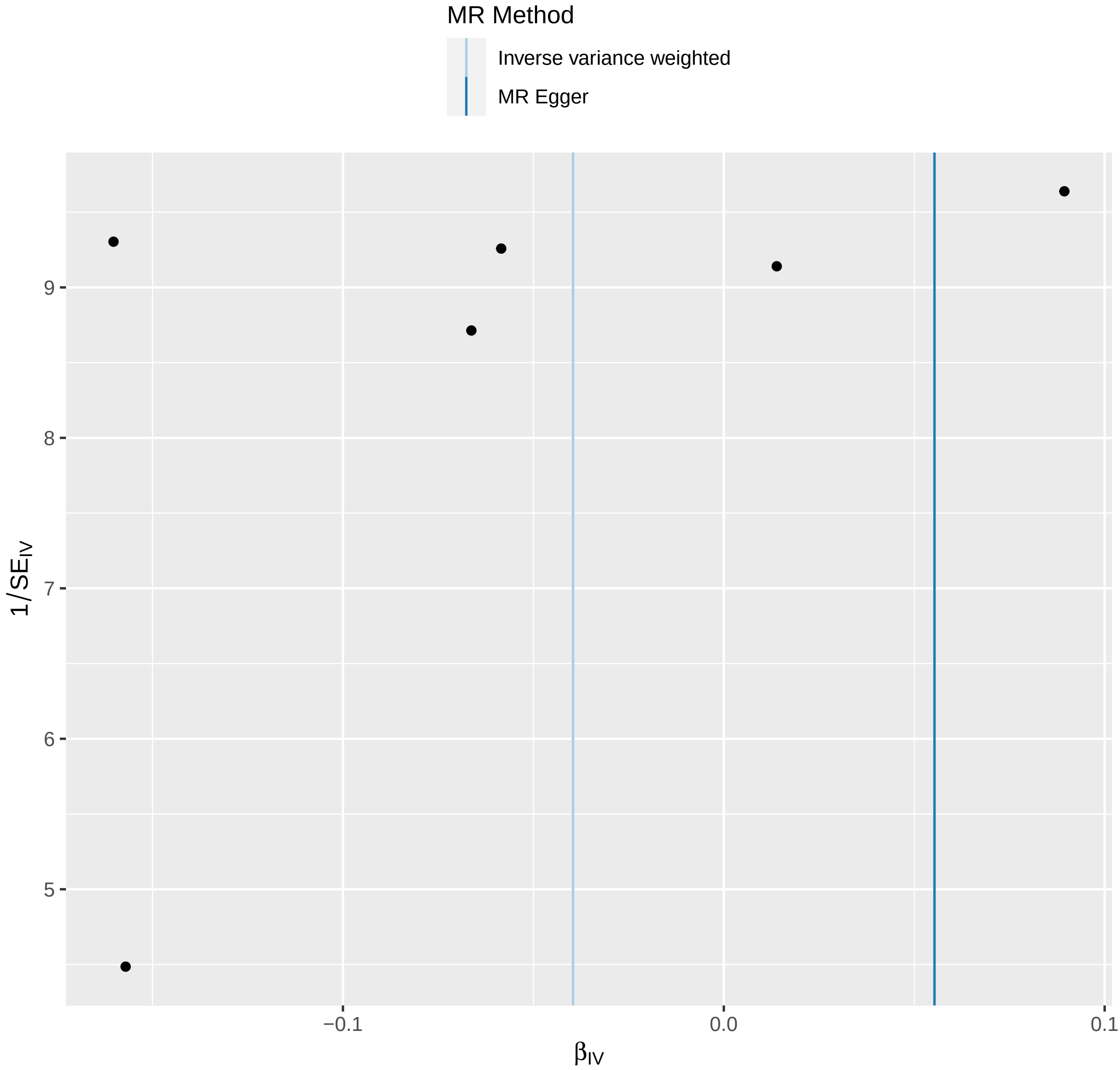

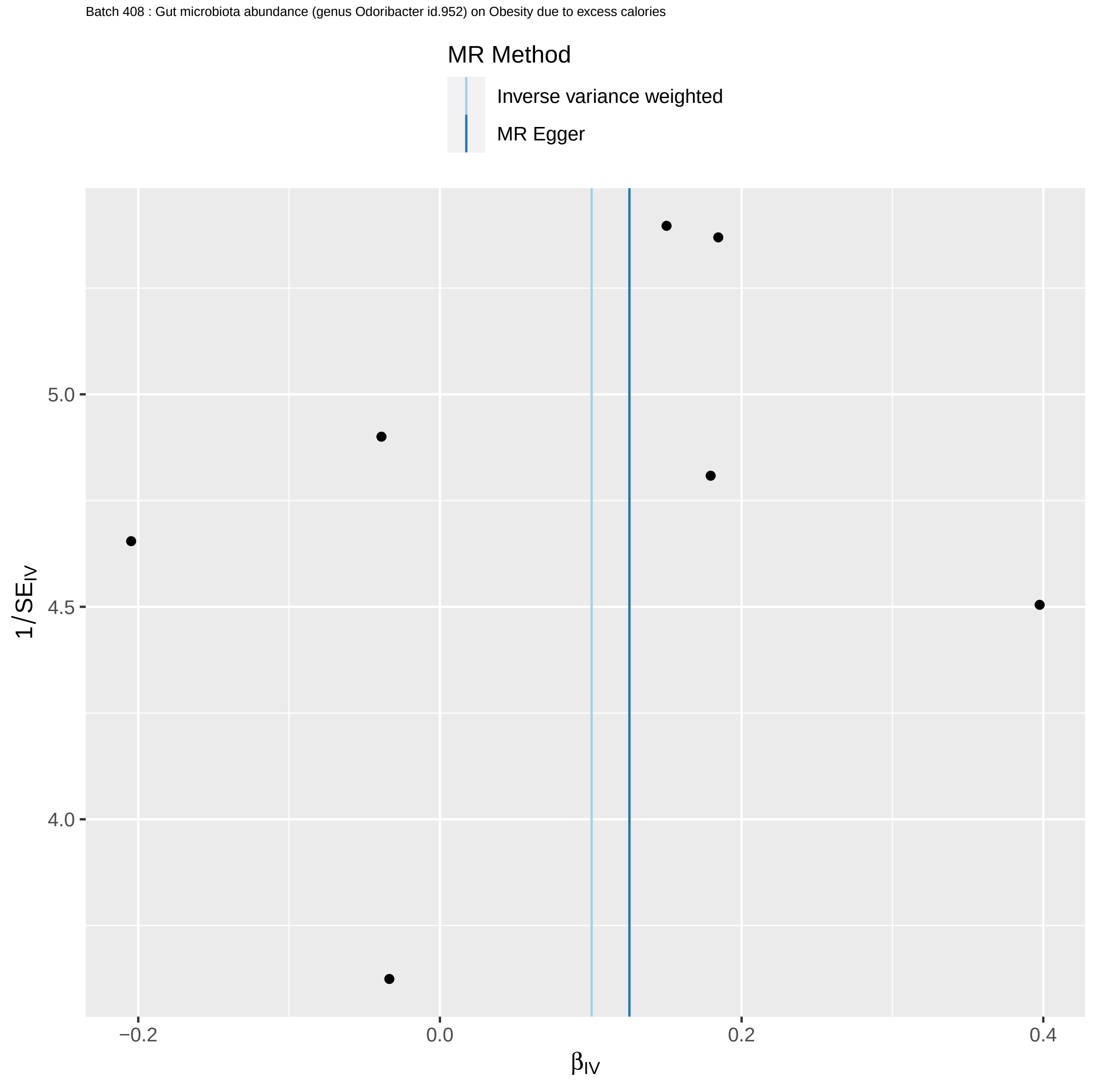

Batch 408 : Gut microbiota abundance (genus Odoribacter id.952) on Obesity due to excess calories
MR Method
Inverse variance weighted
MR Egger
5.0
4.5
4.0
1/SE_IV
−0.2
0.0
0.2
0.4
β_IV

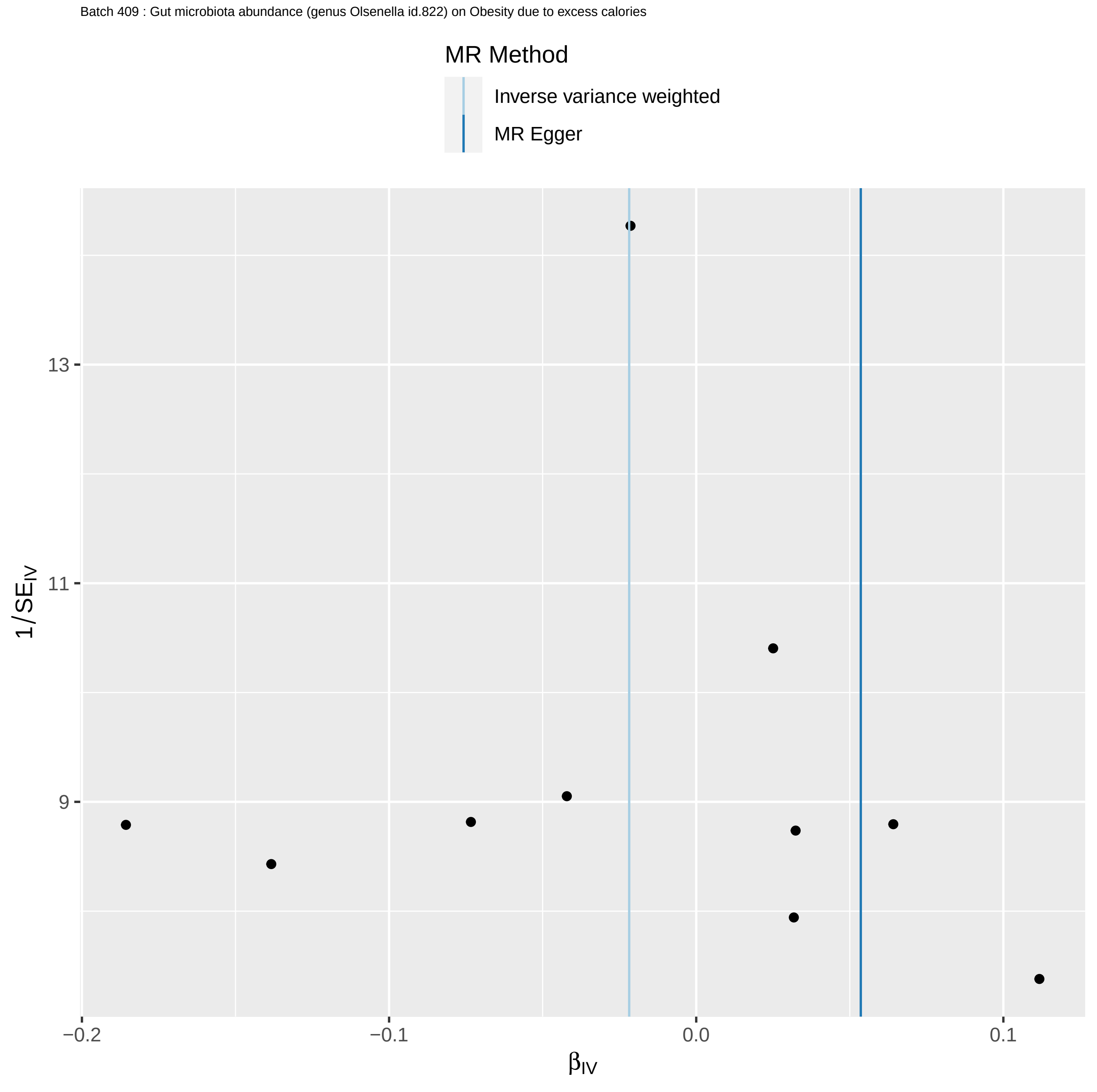
Batch 409 : Gut microbiota abundance (genus Olsenella id.822) on Obesity due to excess calories
MR Method
Inverse variance weighted
MR Egger
13
11
9
1/SE$_{IV}$
−0.2
−0.1
0.0
0.1
β$_{IV}$

Batch 410 : Gut microbiota abundance (genus Oscillibacter id.2063) on Obesity due to excess calories

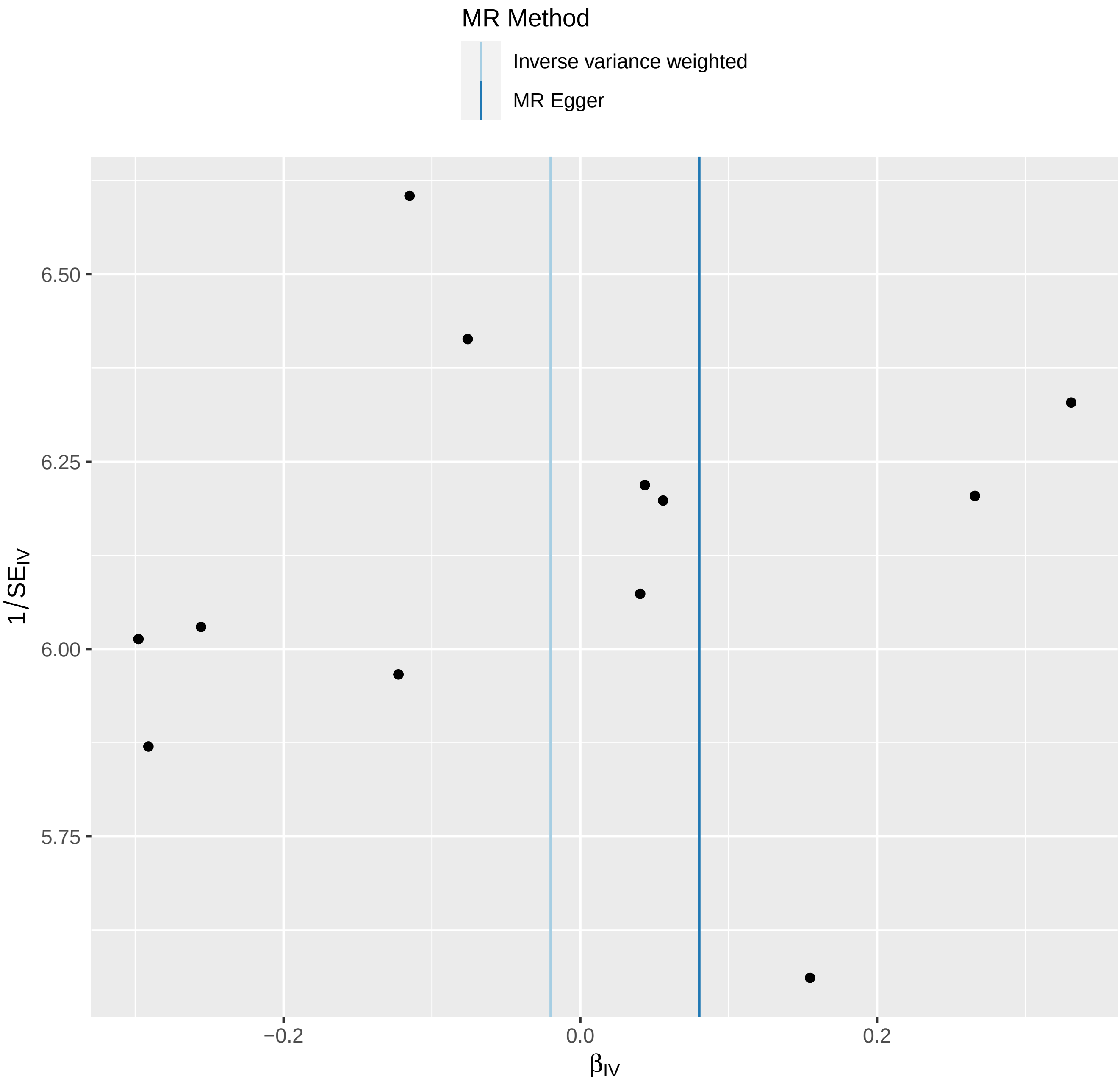

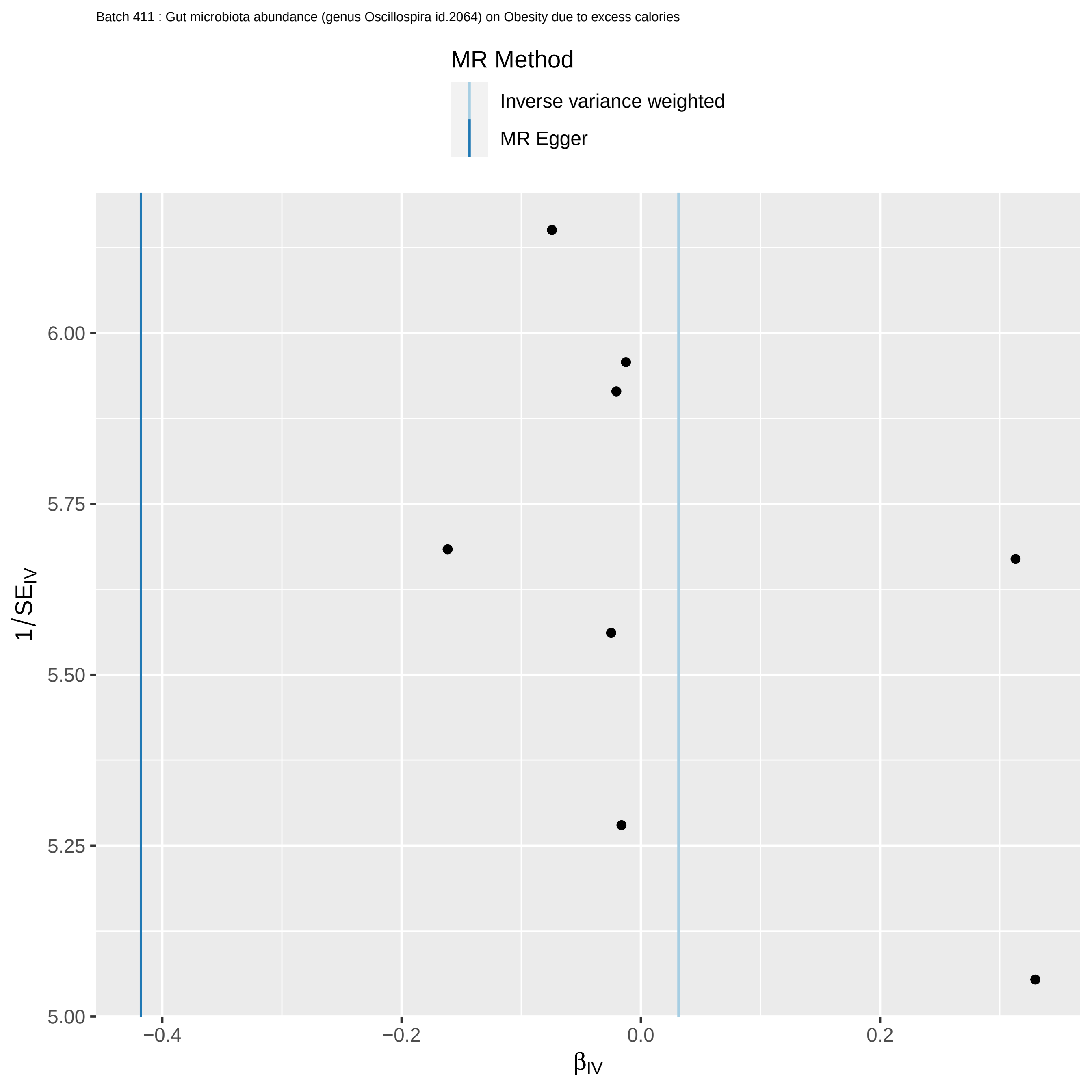
Batch 411 : Gut microbiota abundance (genus Oscillospira id.2064) on Obesity due to excess calories
MR Method
Inverse variance weighted
MR Egger
6.00
5.75
5.50
5.25
5.00
1/SE$_{IV}$
−0.4
−0.2
0.0
0.2
β$_{IV}$

Batch 412 : Gut microbiota abundance (genus Oxalobacter id.2978) on Obesity due to excess calories

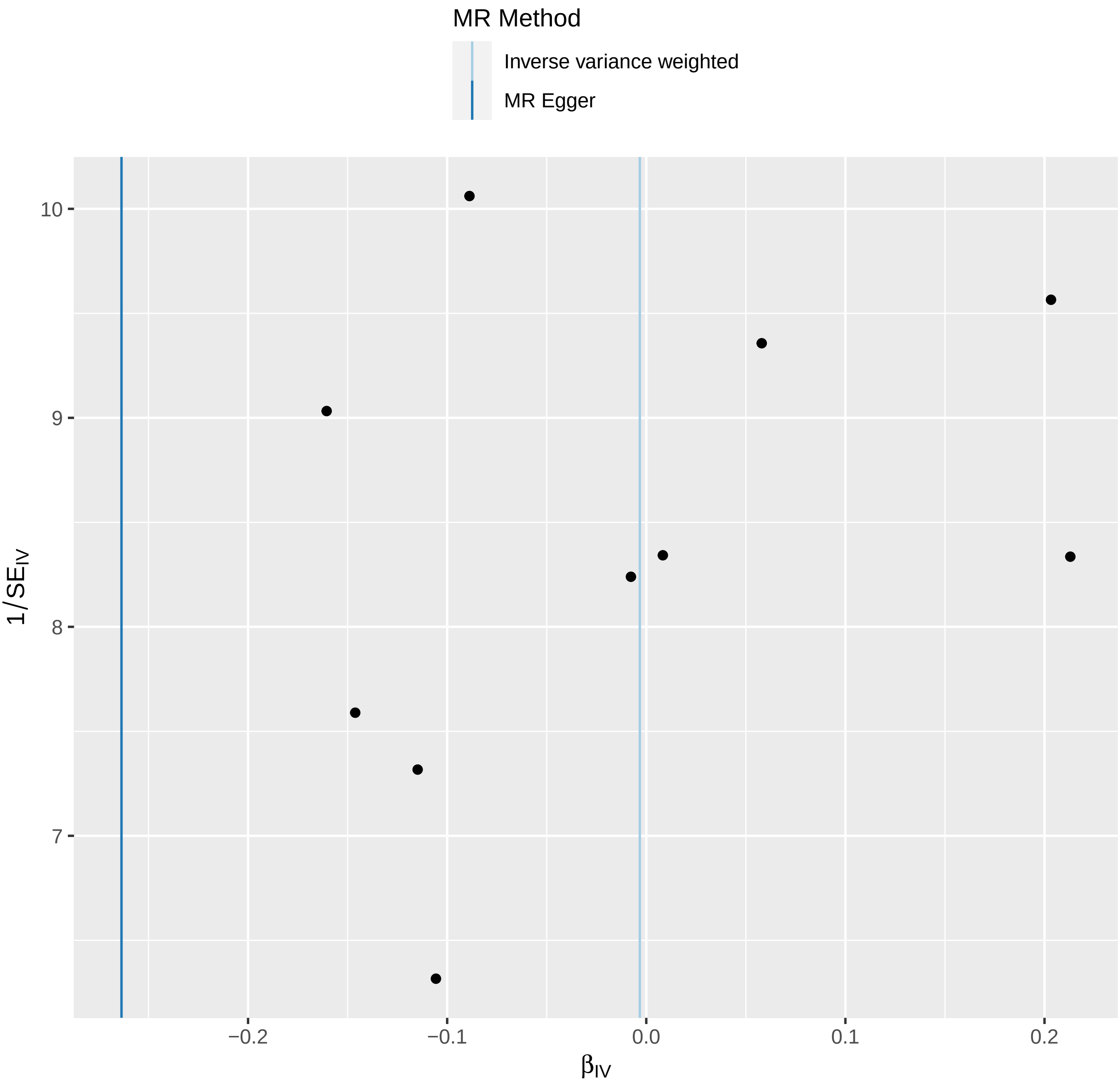

Batch 413 : Gut microbiota abundance (genus Parabacteroides id.954) on Obesity due to excess calories

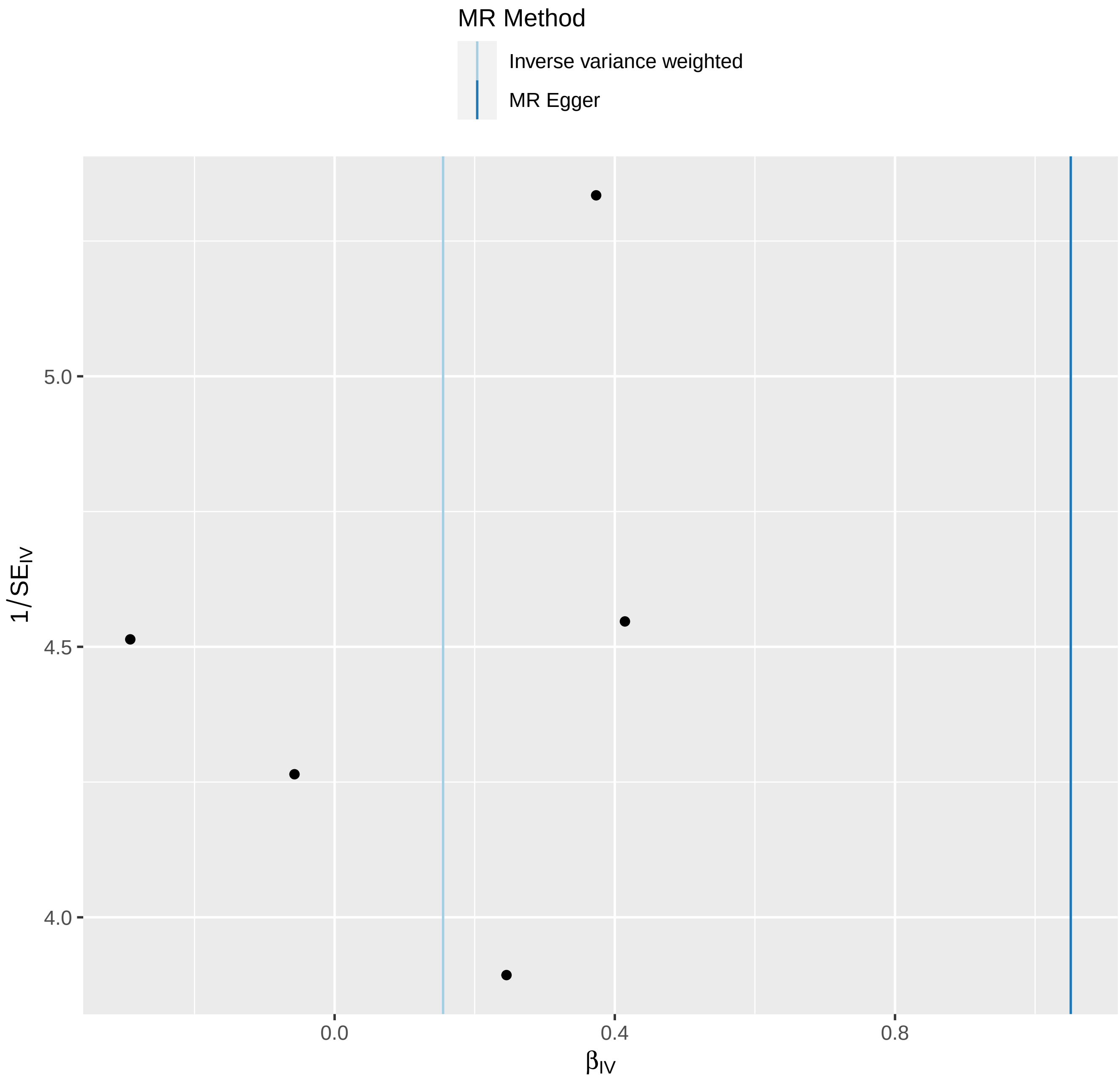

Batch 414 : Gut microbiota abundance (genus Paraprevotella id.962) on Obesity due to excess calories

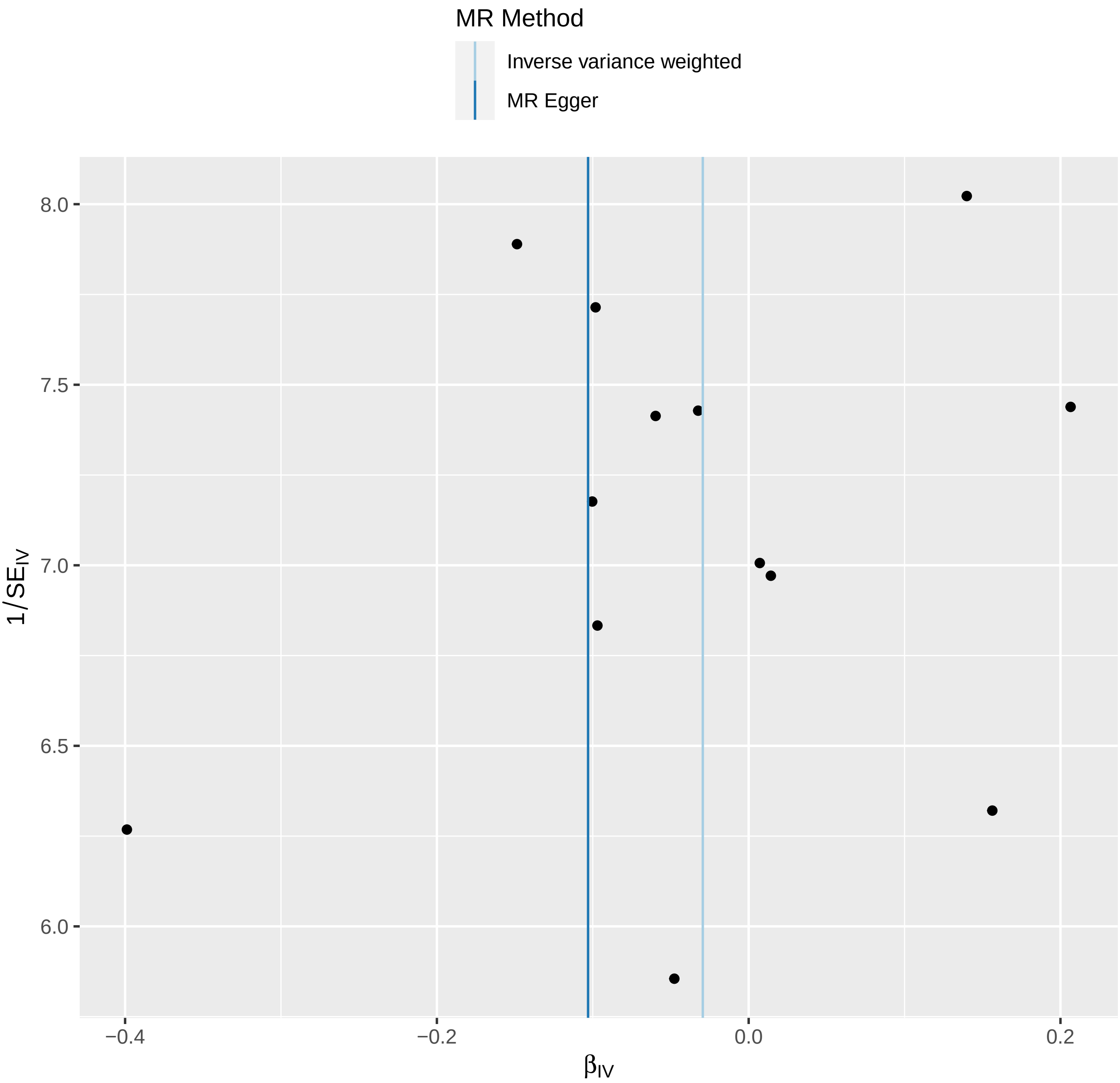

Batch 415 : Gut microbiota abundance (genus Parasutterella id.2892) on Obesity due to excess calories

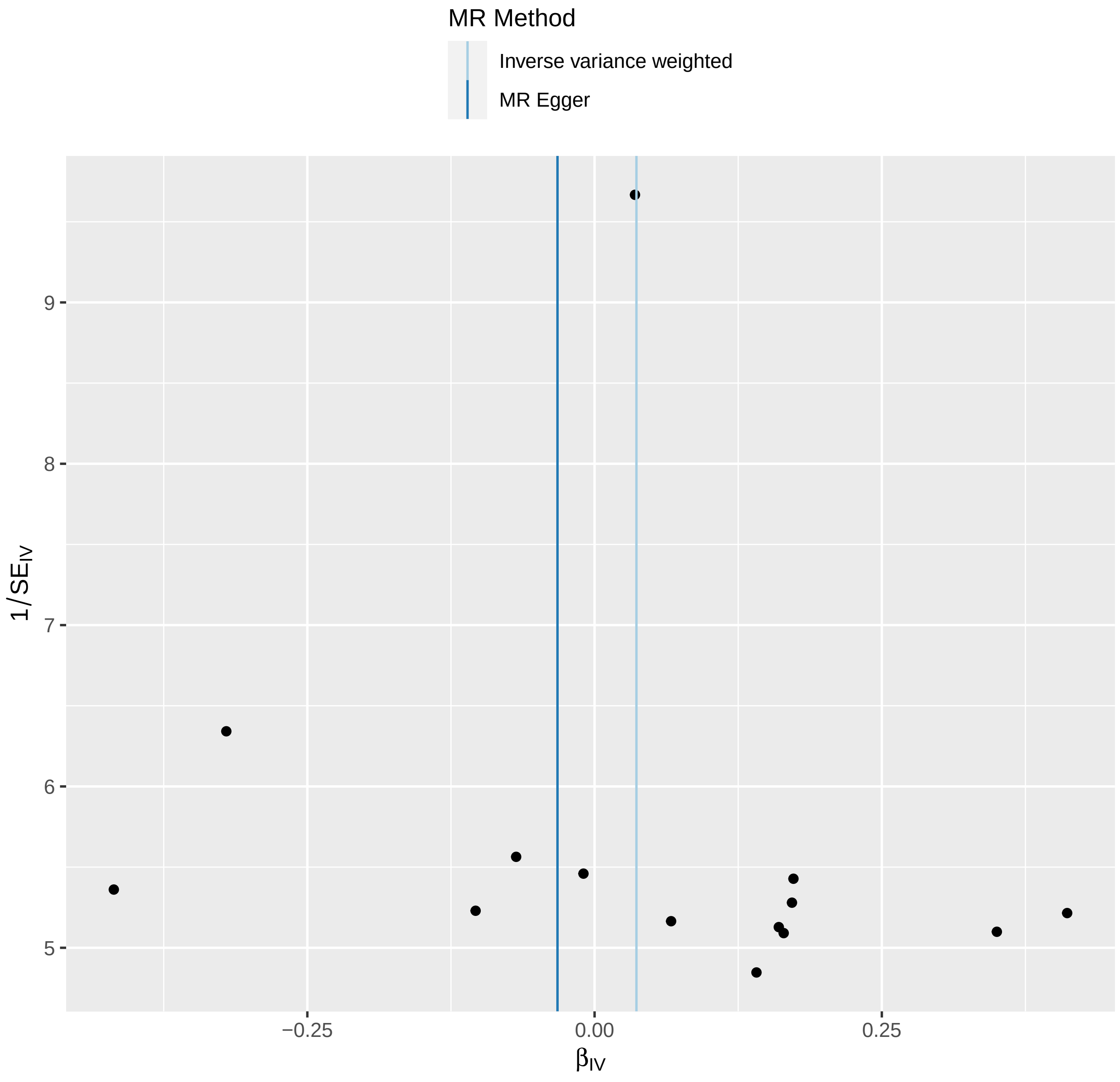

Batch 416 : Gut microbiota abundance (genus Peptococcus id.2037) on Obesity due to excess calories

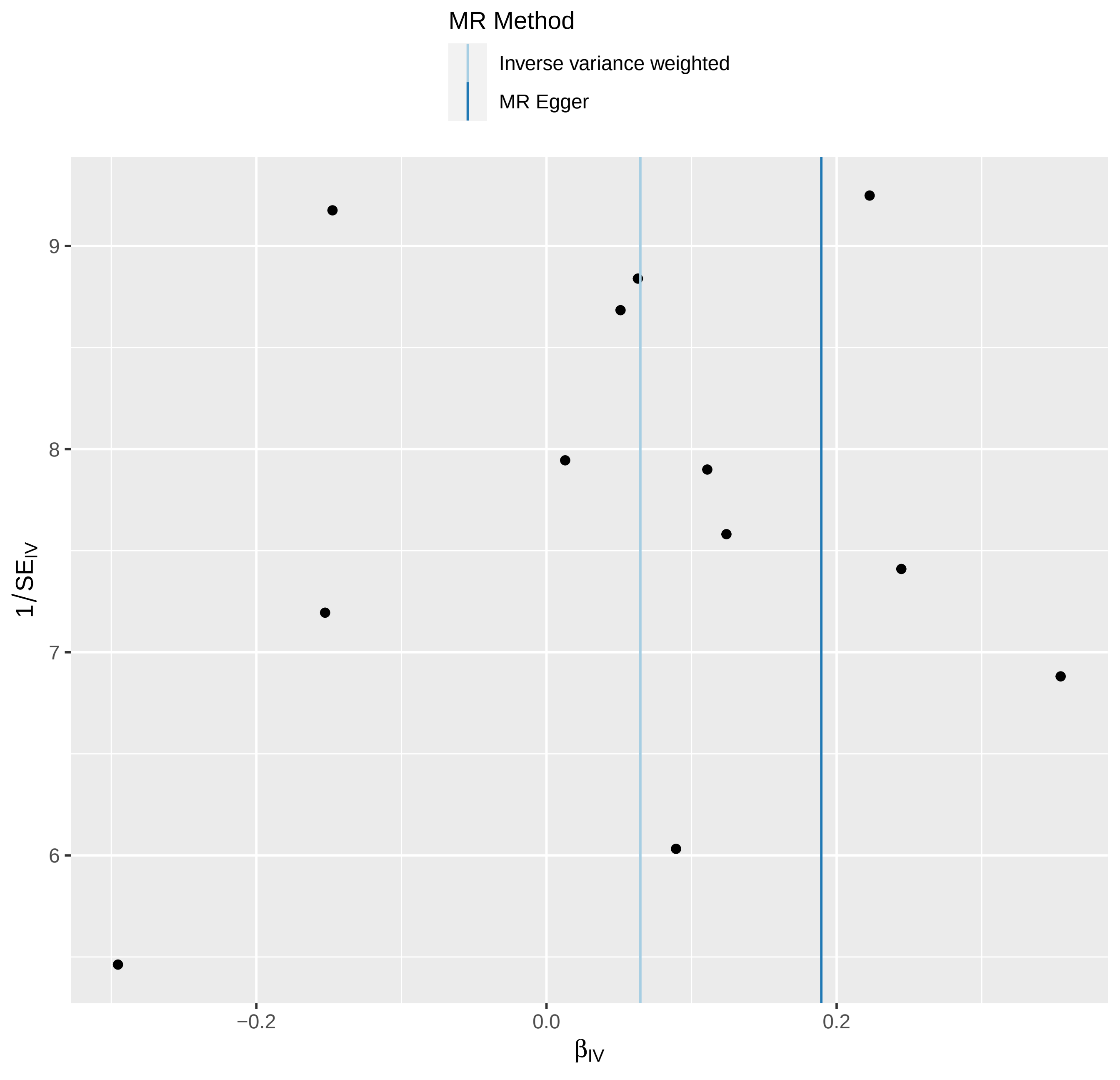

Batch 417 : Gut microbiota abundance (genus Phascolarctobacterium id.2168) on Obesity due to excess calories

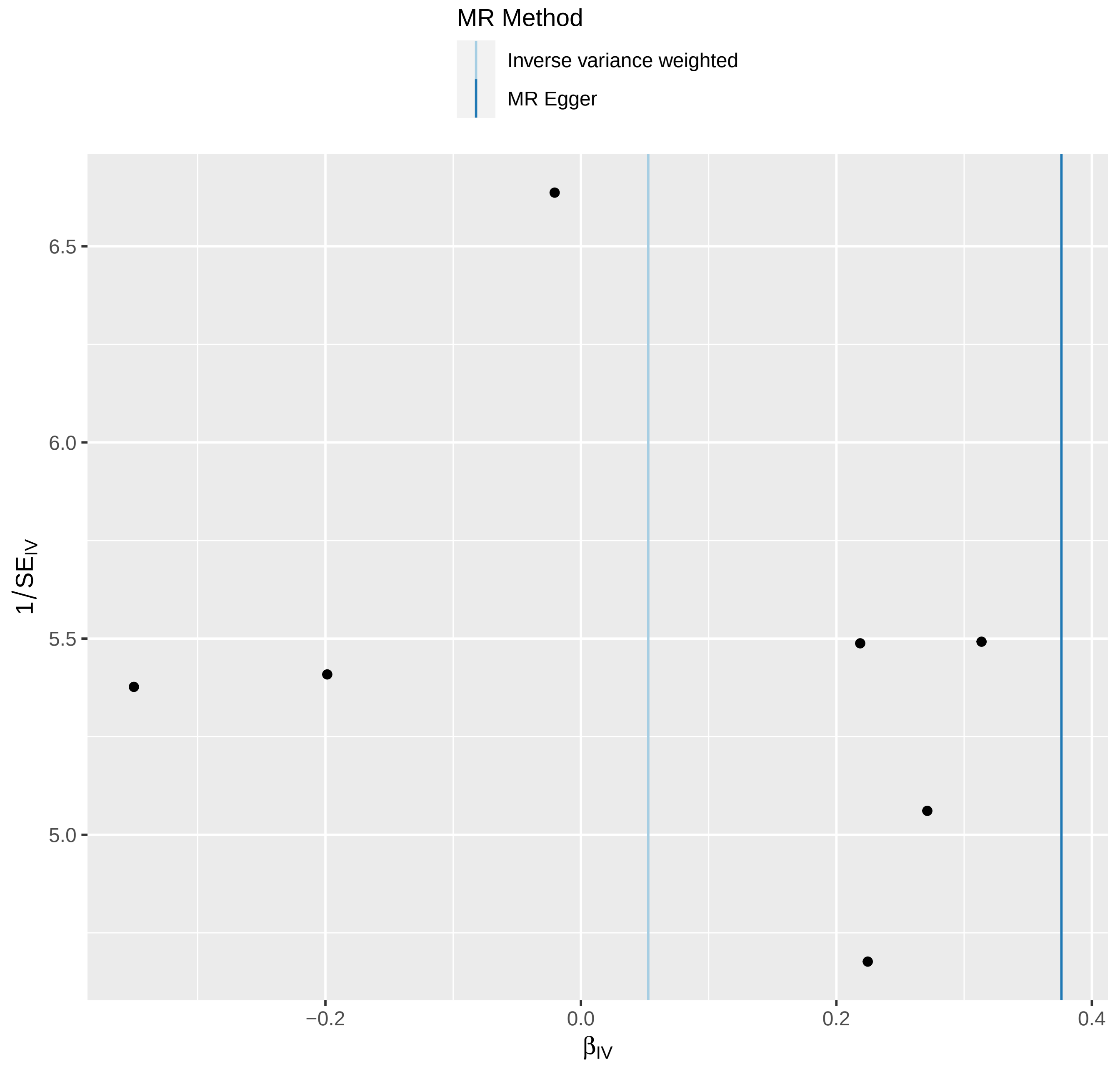

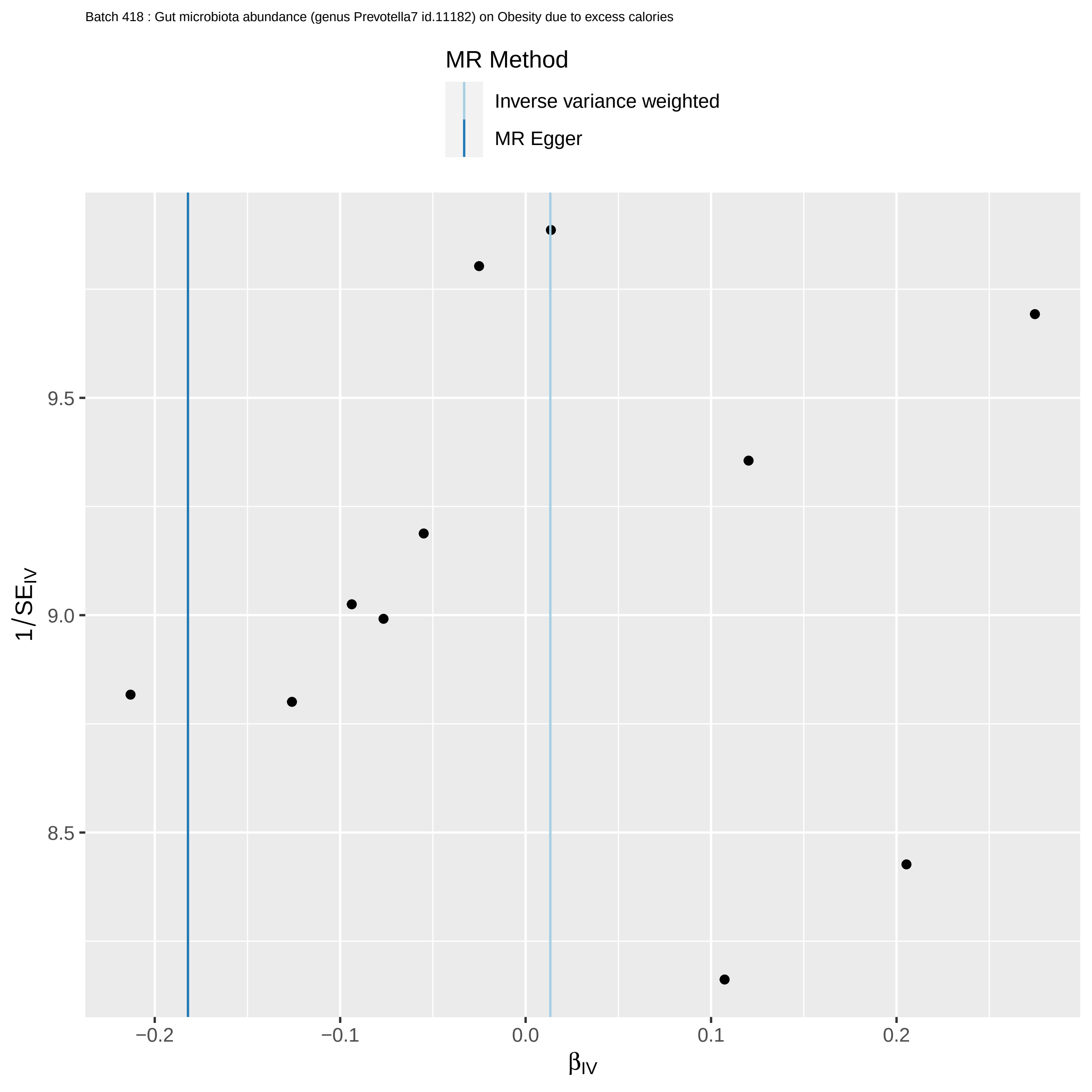
Batch 418 : Gut microbiota abundance (genus Prevotella7 id.11182) on Obesity due to excess calories
MR Method
Inverse variance weighted
MR Egger
9.5
9.0
8.5
1/SE_IV
−0.2
−0.1
0.0
0.1
0.2
β_IV

Batch 419 : Gut microbiota abundance (genus Prevotella9 id.11183) on Obesity due to excess calories

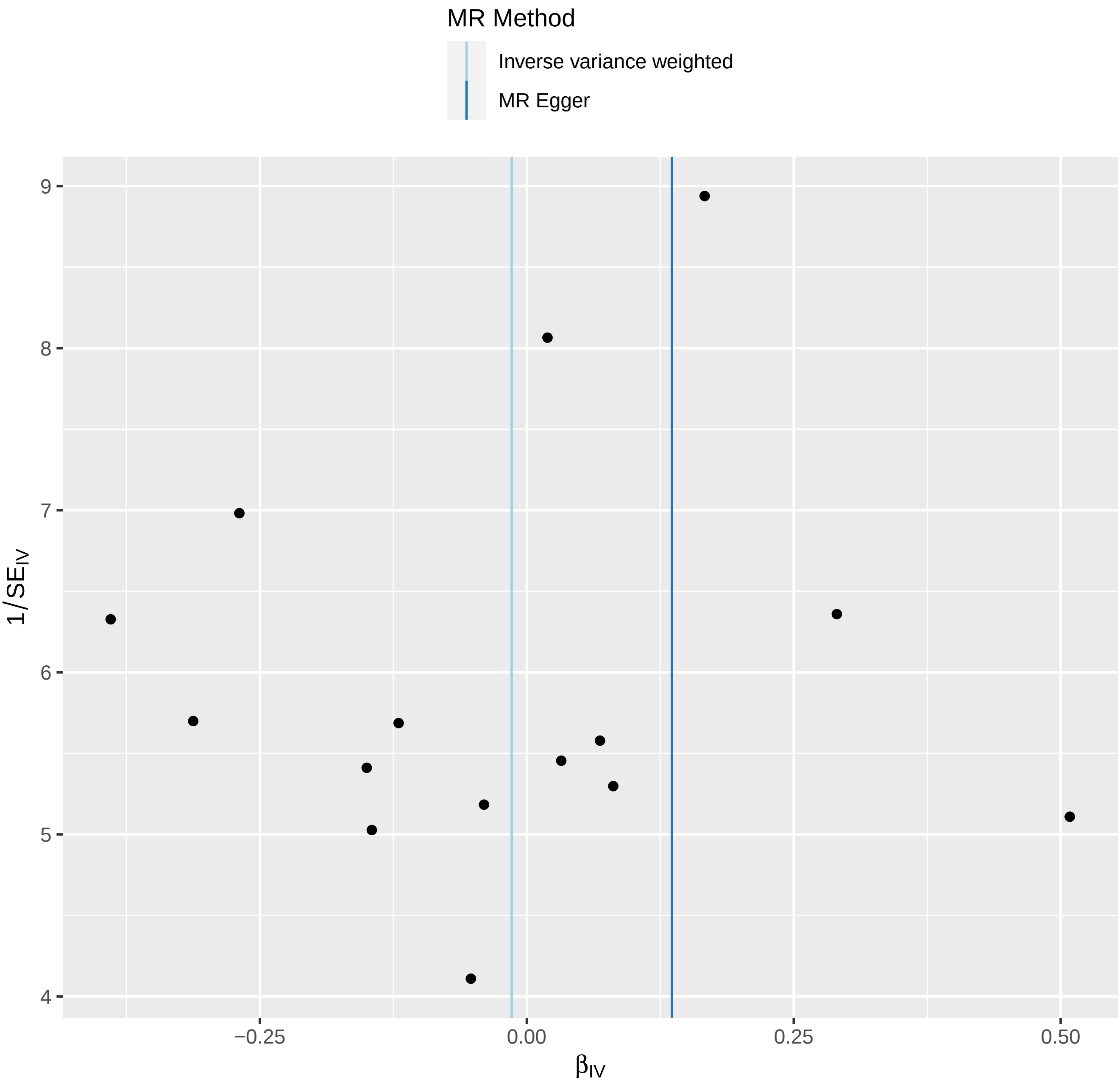

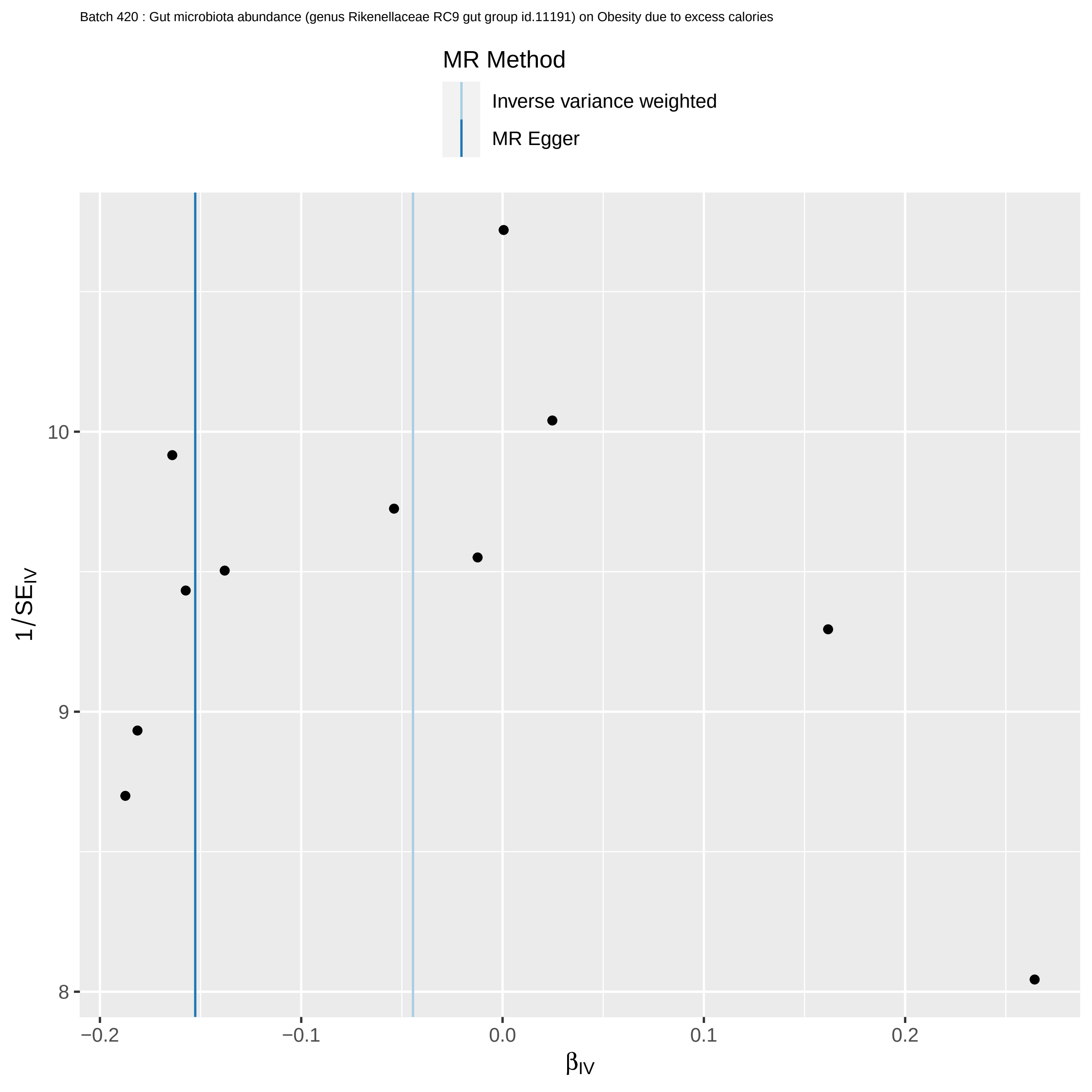

Batch 420 : Gut microbiota abundance (genus Rikenellaceae RC9 gut group id.11191) on Obesity due to excess calories
MR Method
Inverse variance weighted
MR Egger
10
9
8
$1/SE_{IV}$
−0.2
−0.1
0.0
0.1
0.2
$\beta_{IV}$

Batch 421 : Gut microbiota abundance (genus Romboutsia id.11347) on Obesity due to excess calories

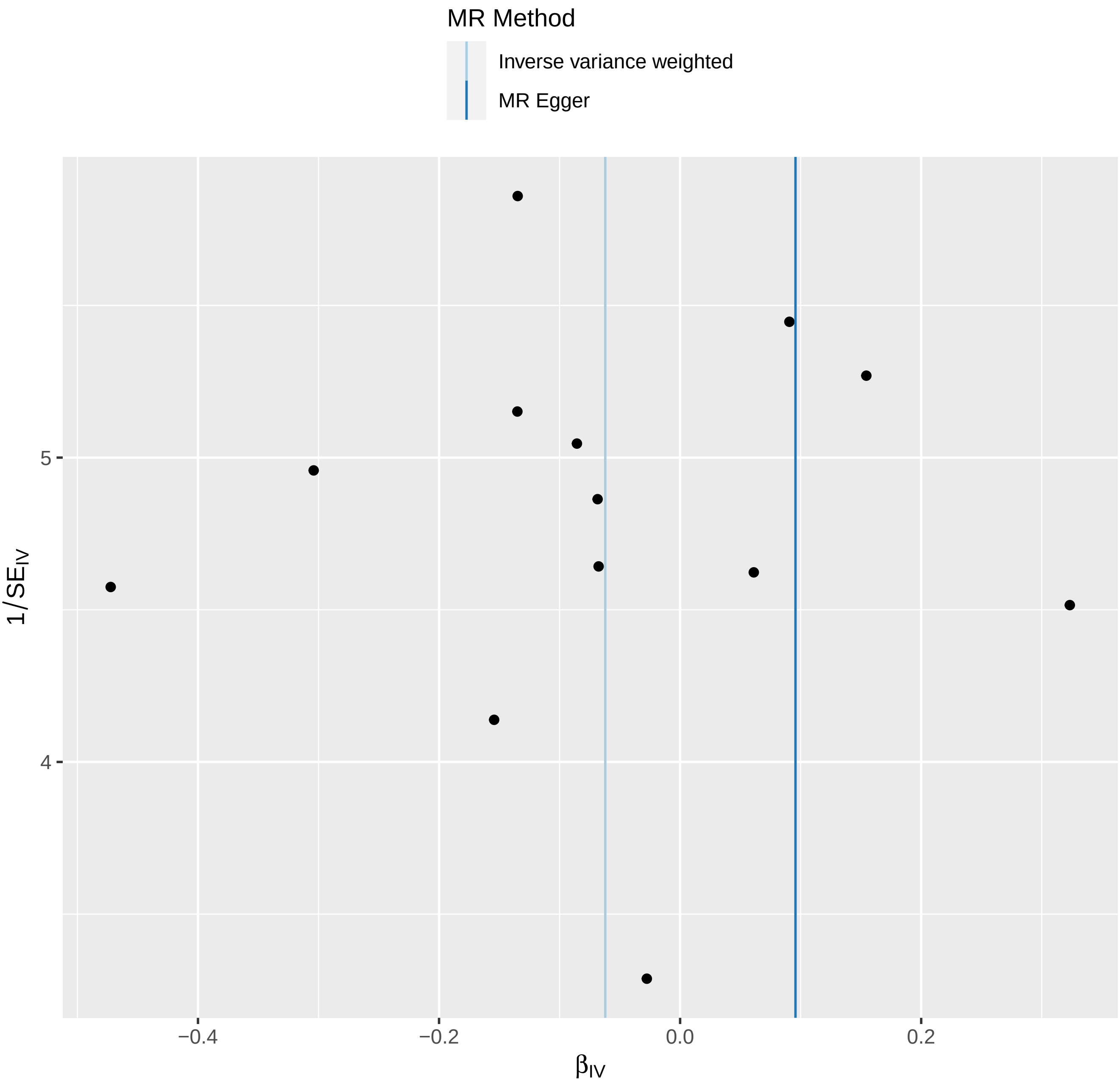

Batch 422 : Gut microbiota abundance (genus Roseburia id.2012) on Obesity due to excess calories

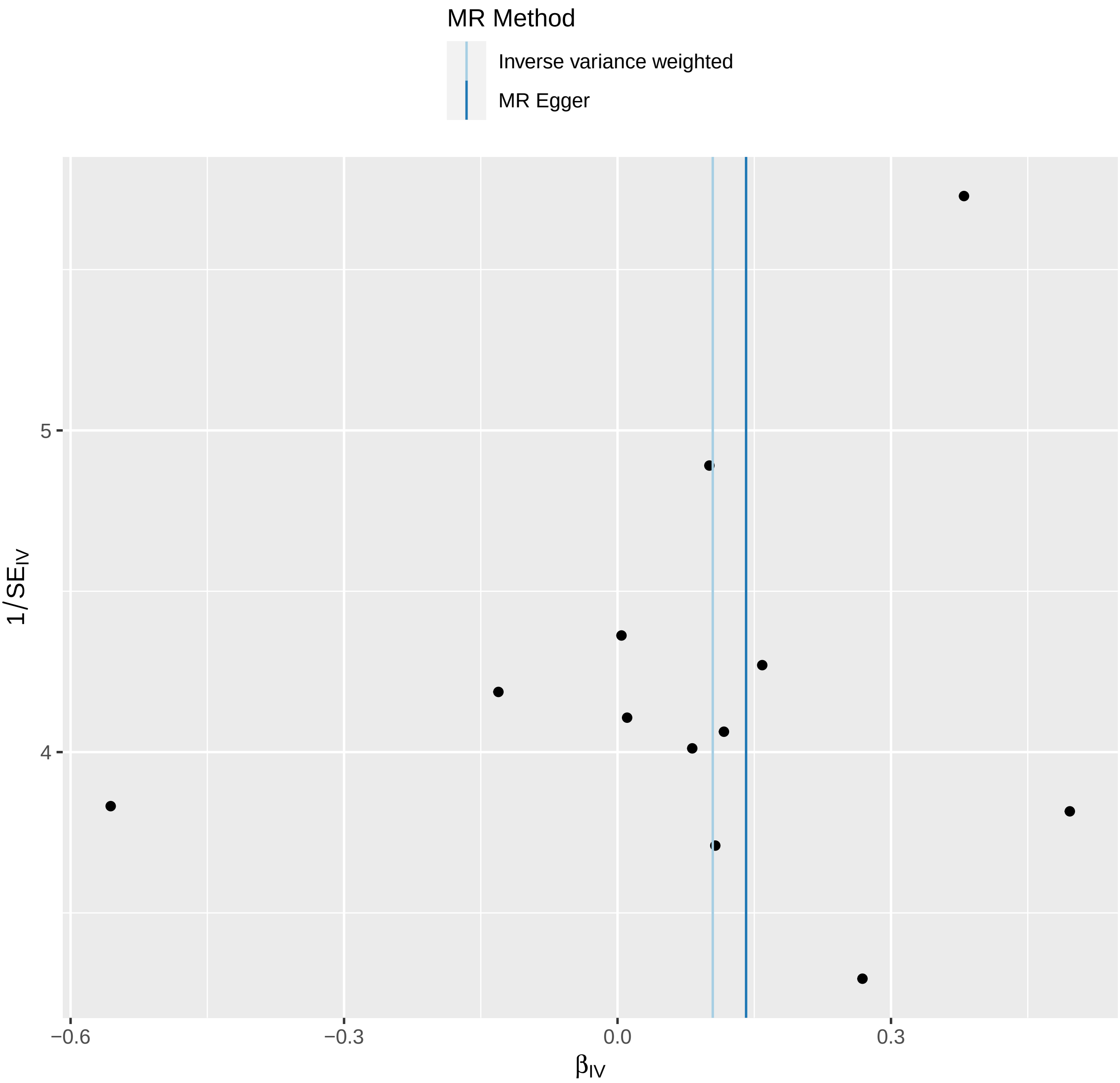

Batch 423 : Gut microbiota abundance (genus Ruminiclostridium5 id.11355) on Obesity due to excess calories

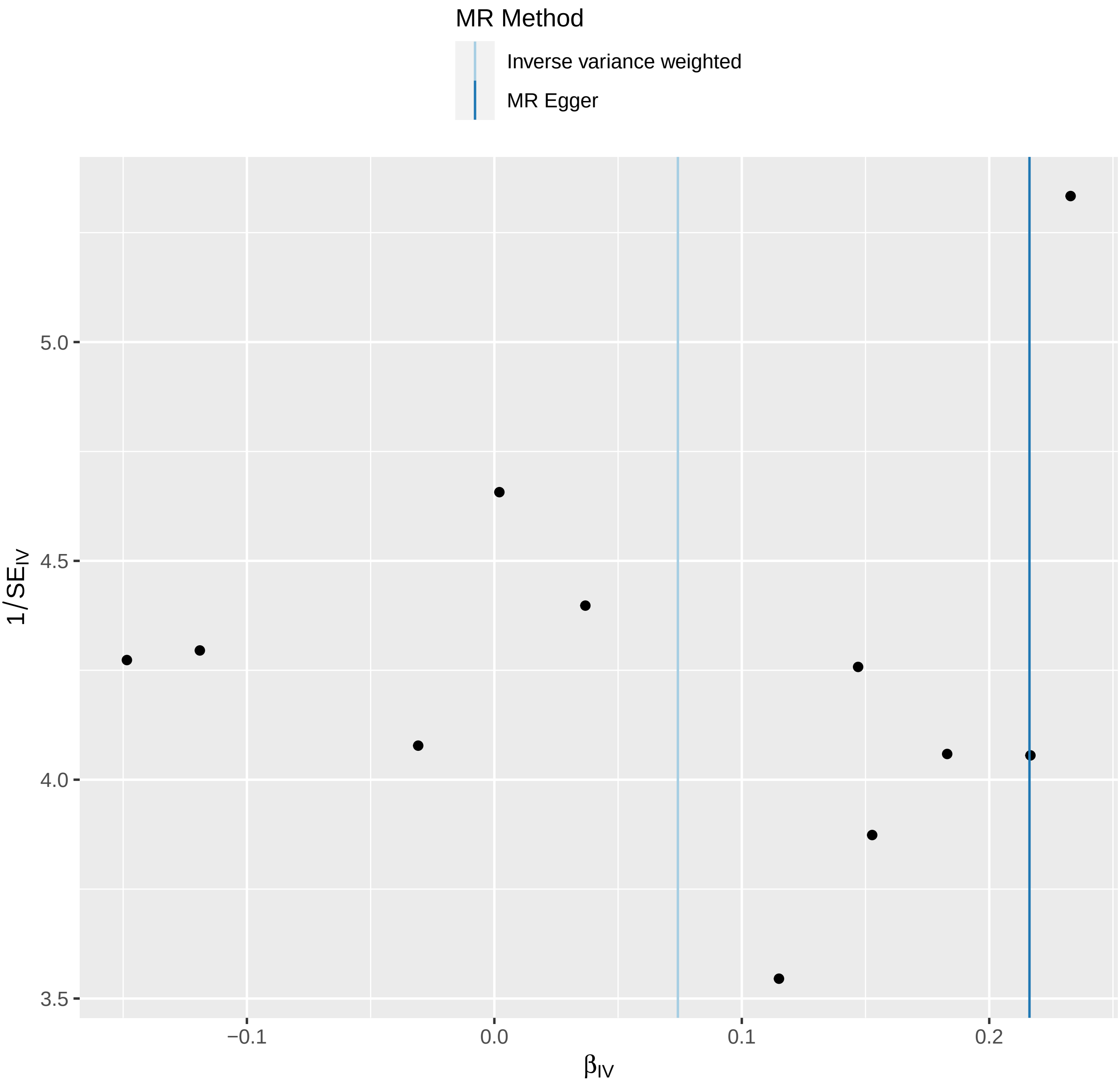

Batch 424 : Gut microbiota abundance (genus Ruminiclostridium6 id.11356) on Obesity due to excess calories

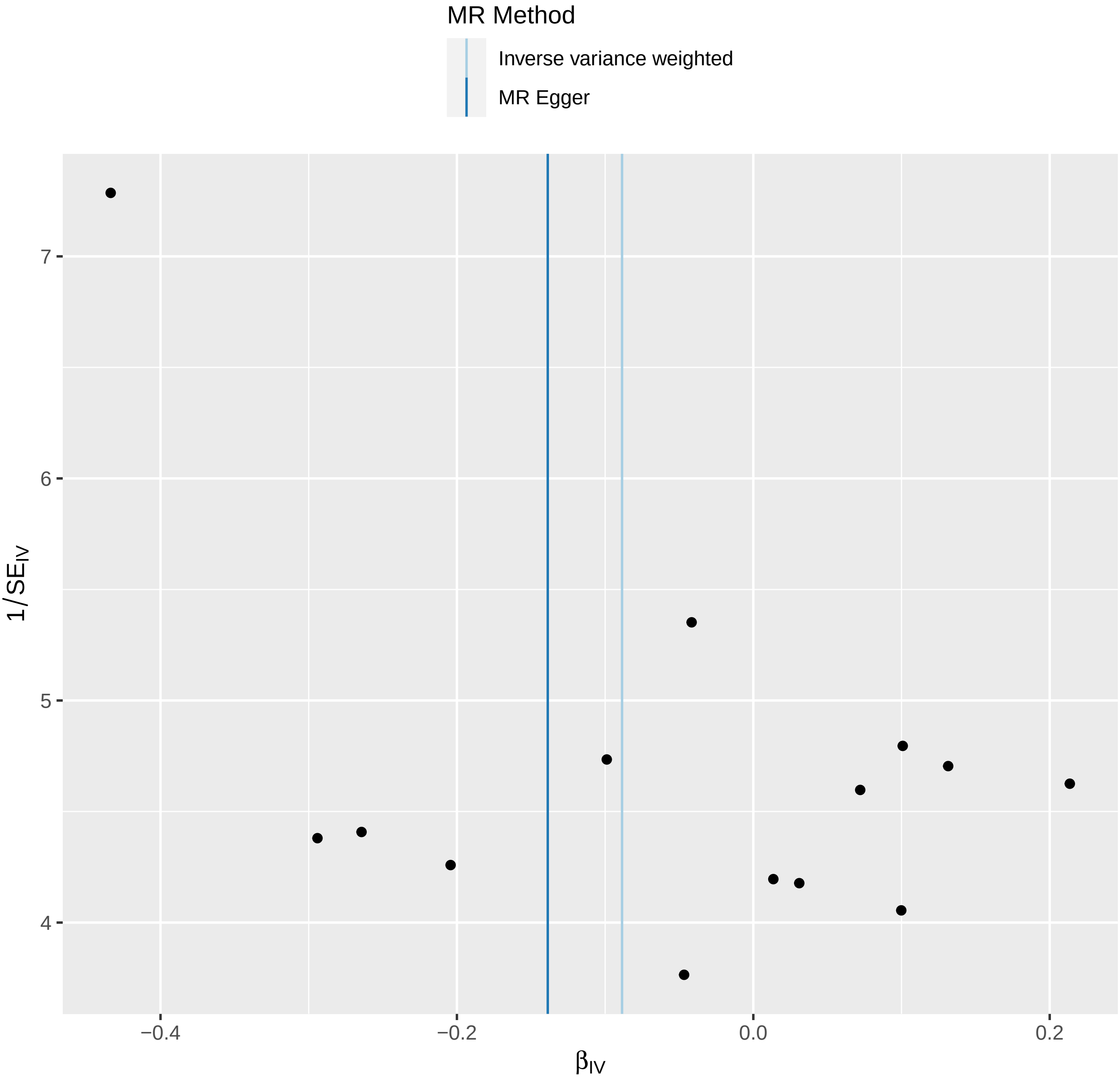

Batch 425 : Gut microbiota abundance (genus Ruminiclostridium9 id.11357) on Obesity due to excess calories

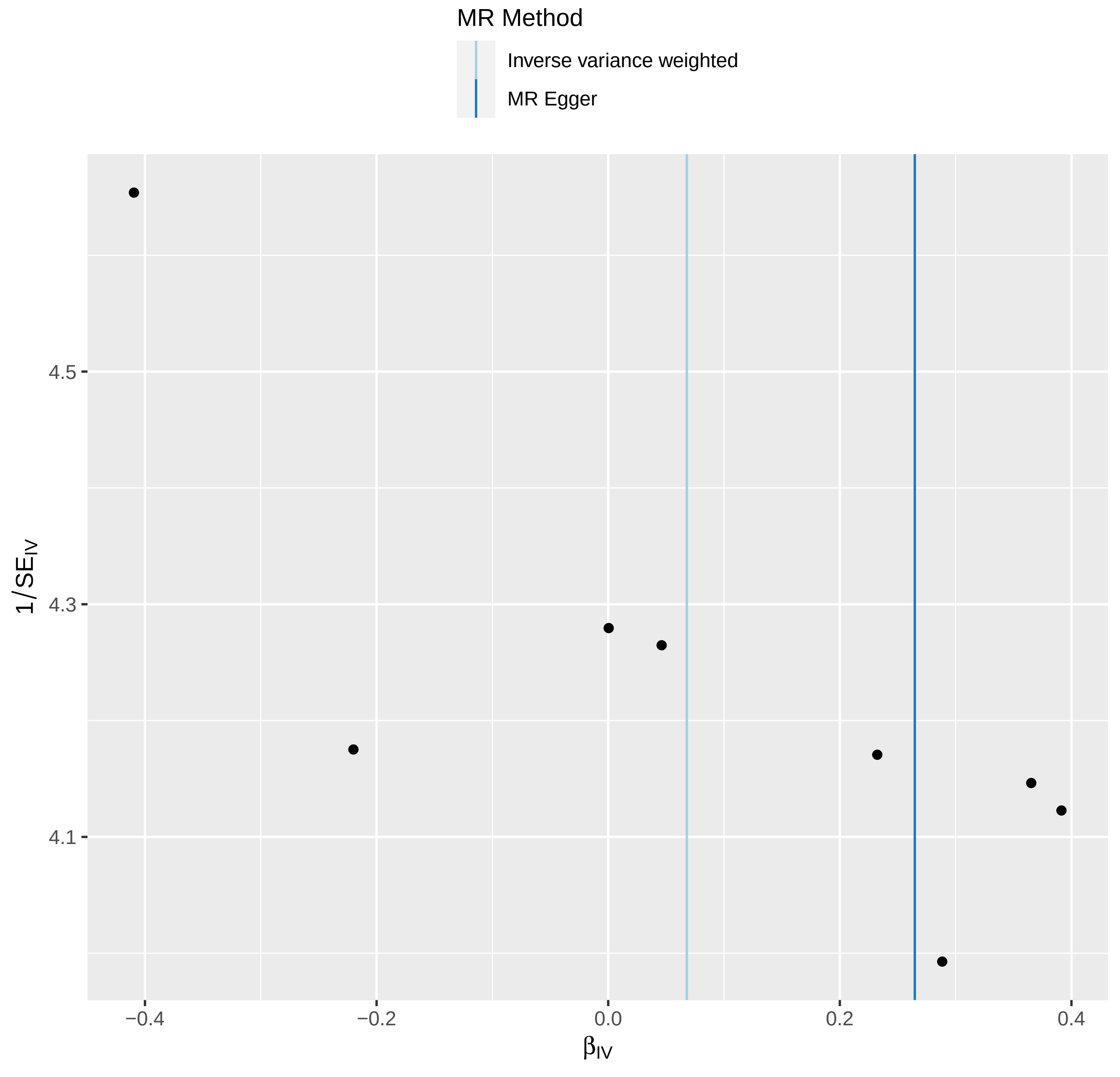

Batch 426 : Gut microbiota abundance (genus Ruminococcaceae NK4A214 group id.11358) on Obesity due to excess calories

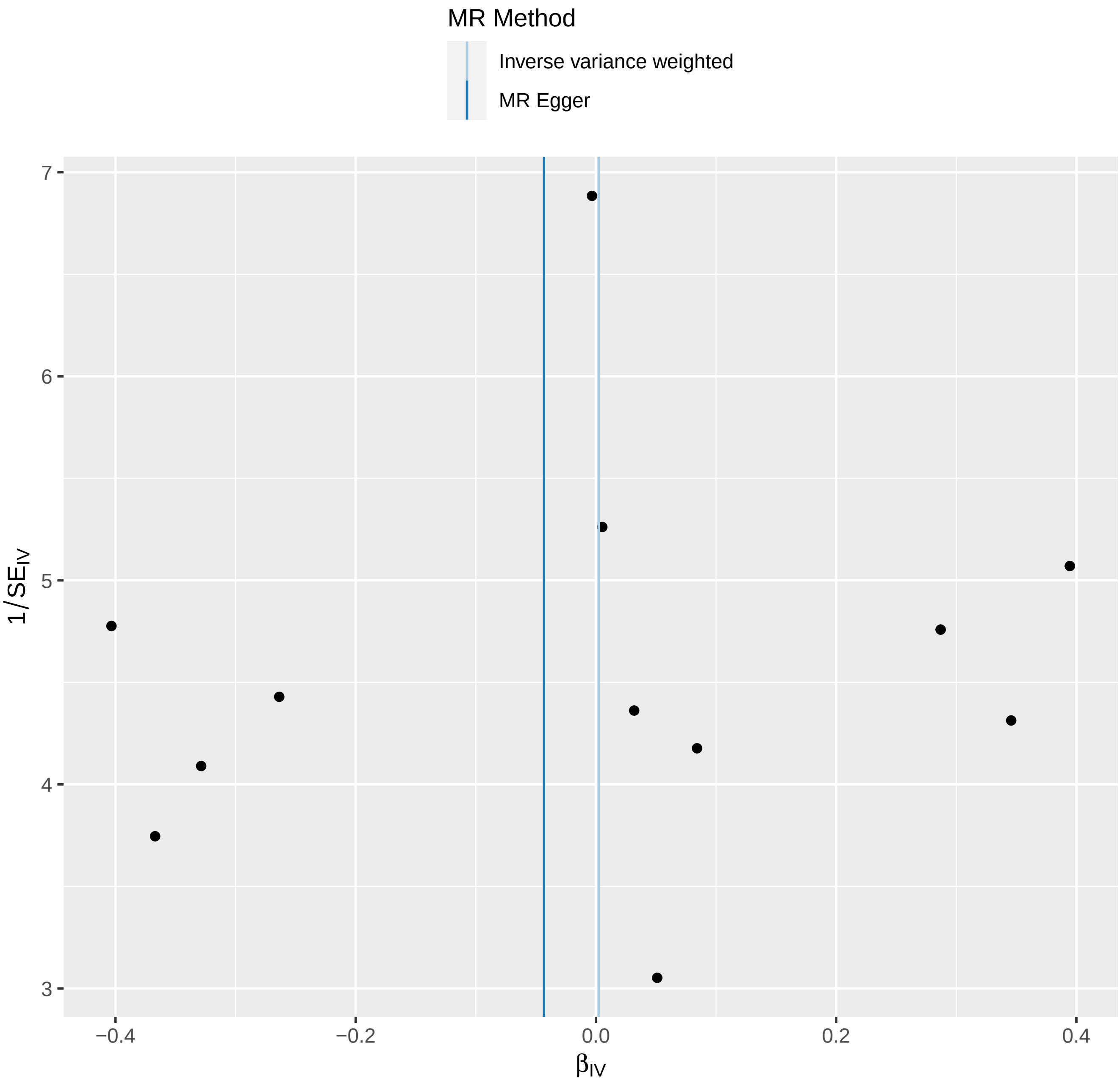

Batch 427 : Gut microbiota abundance (genus Ruminococcaceae UCG002 id.11360) on Obesity due to excess calories

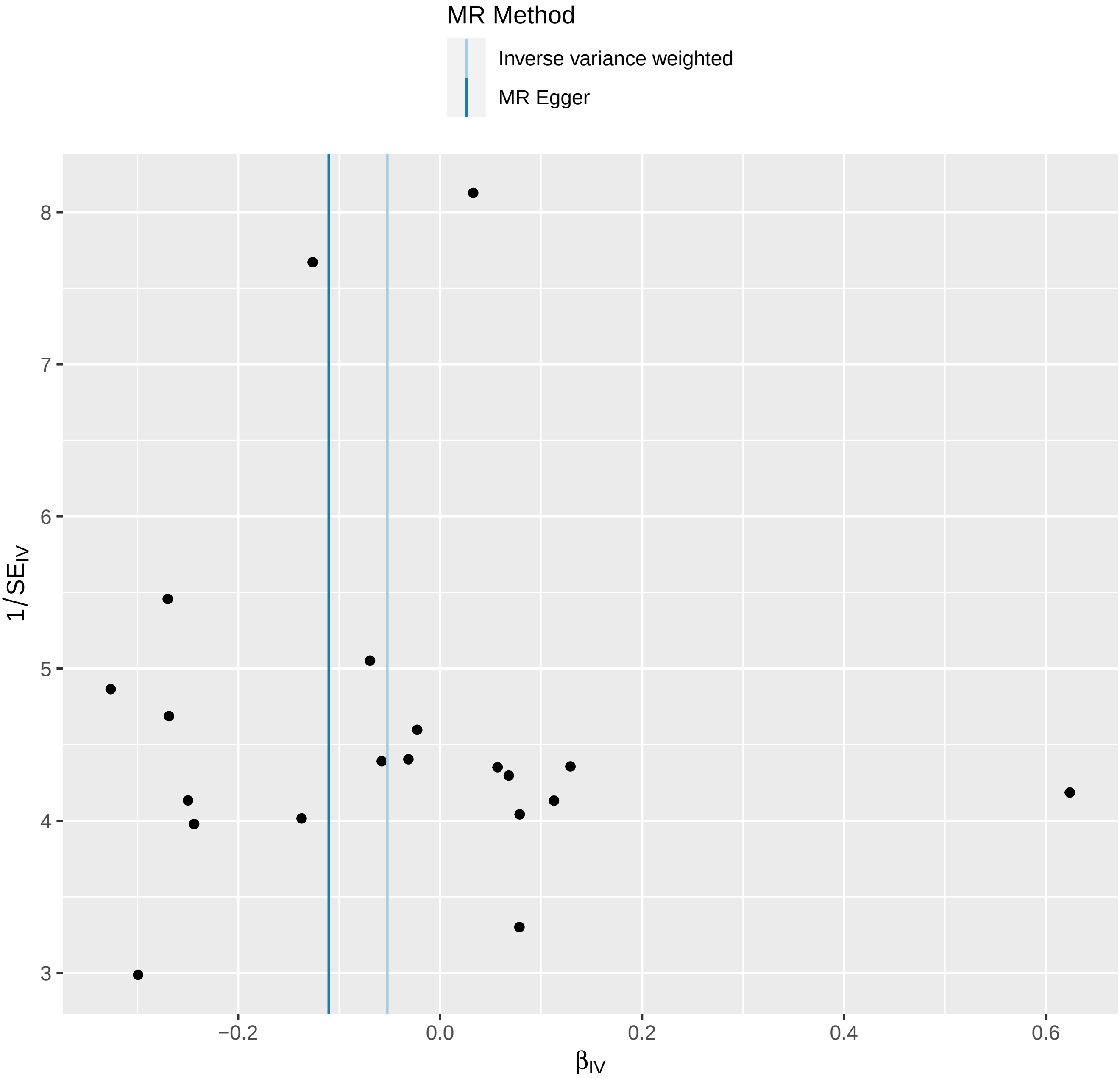

Batch 428 : Gut microbiota abundance (genus Ruminococcaceae UCG003 id.11361) on Obesity due to excess calories

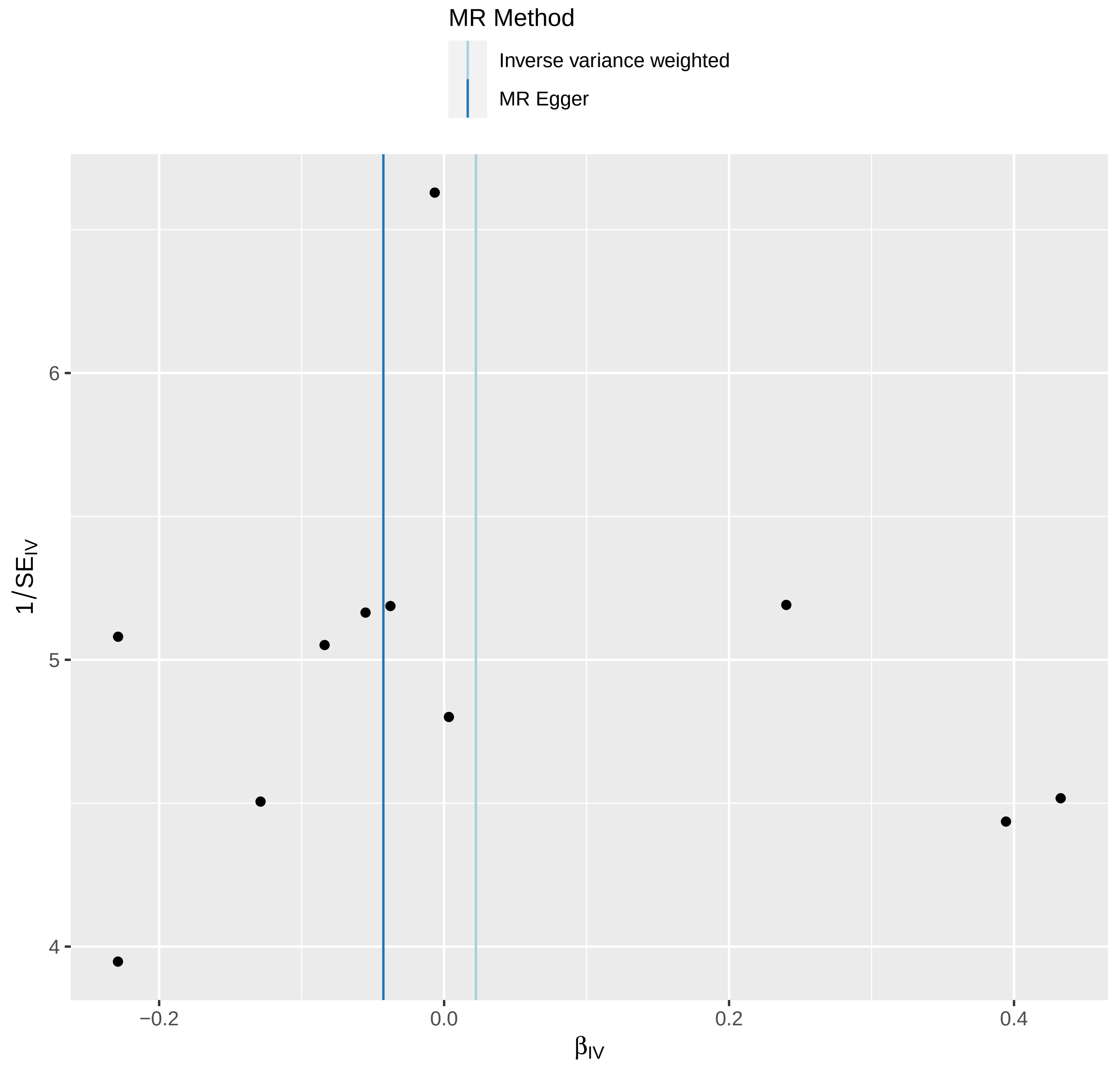

Batch 429 : Gut microbiota abundance (genus Ruminococcaceae UCG004 id.11362) on Obesity due to excess calories

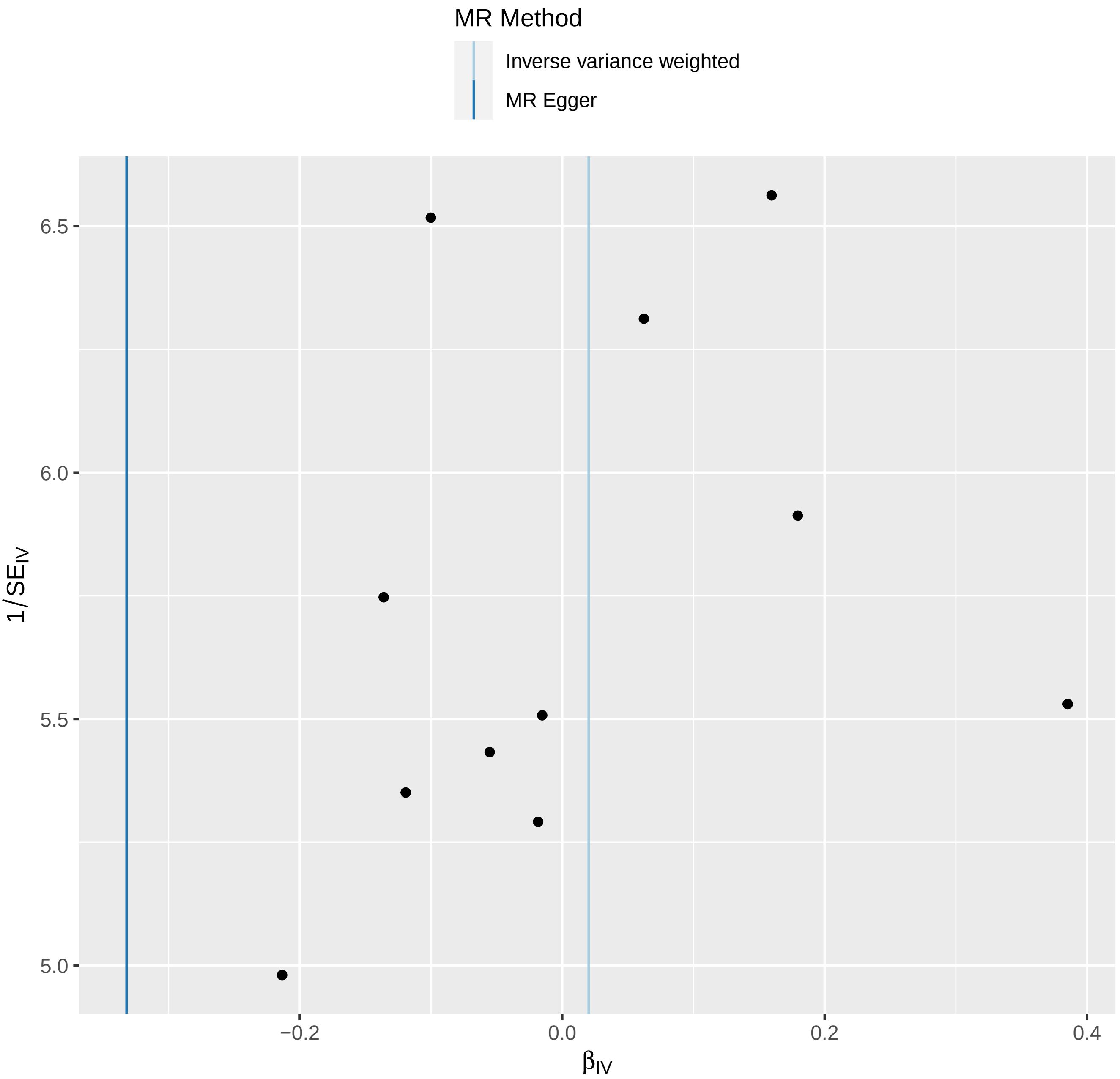

Batch 430 : Gut microbiota abundance (genus Ruminococcaceae UCG005 id.11363) on Obesity due to excess calories

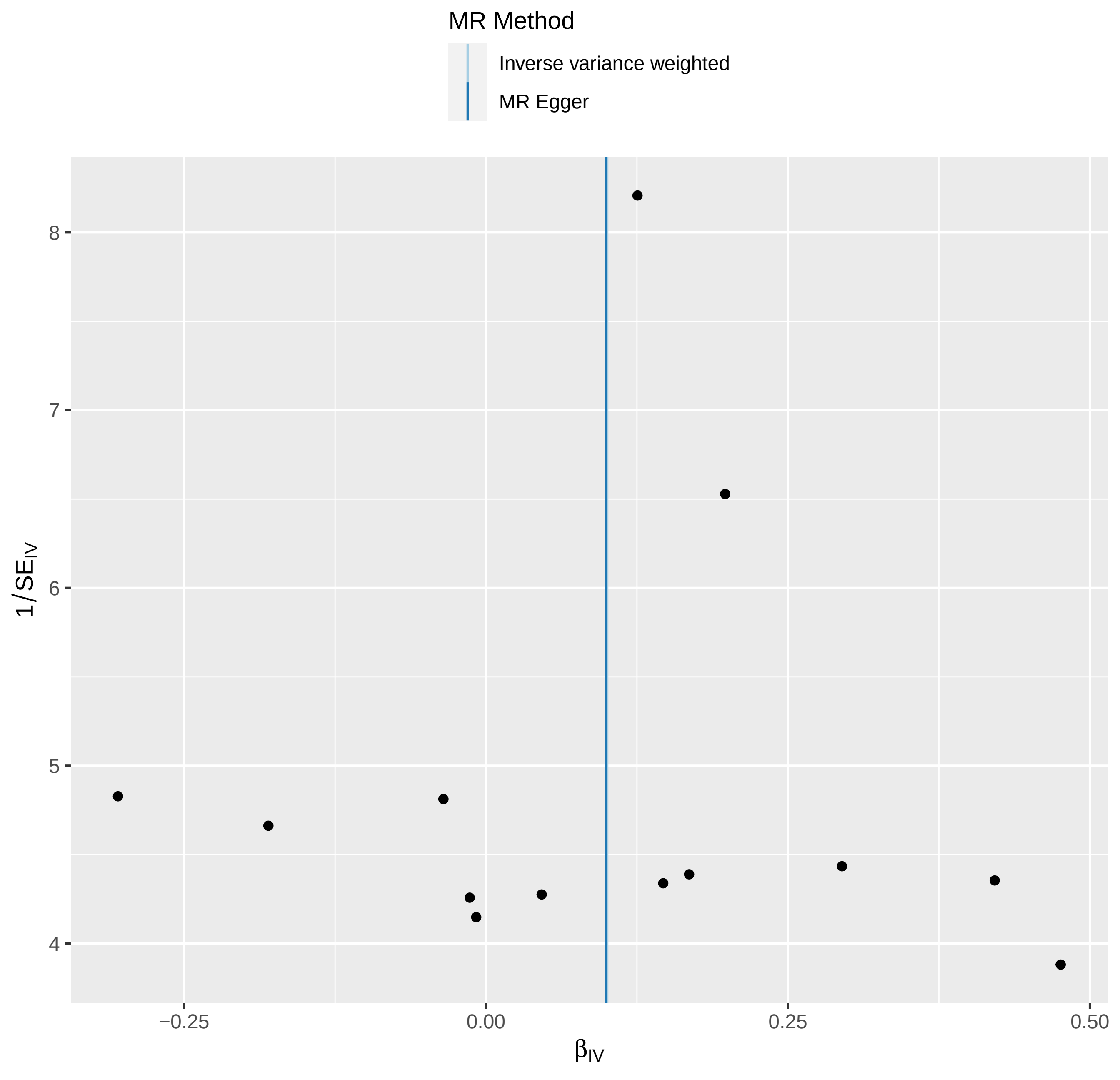

Batch 431 : Gut microbiota abundance (genus Ruminococcaceae UCG009 id.11366) on Obesity due to excess calories

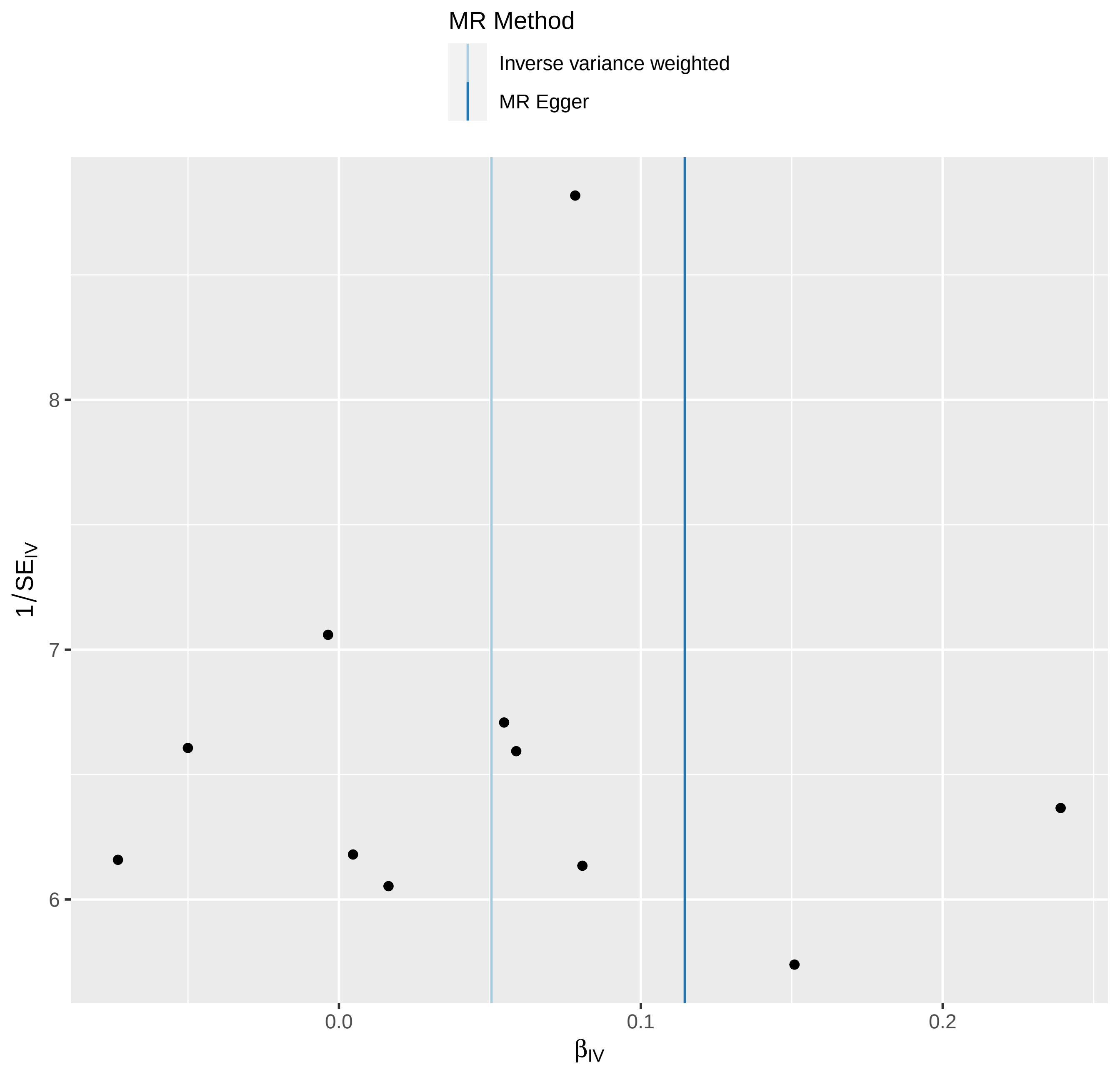

Batch 432 : Gut microbiota abundance (genus Ruminococcaceae UCG010 id.11367) on Obesity due to excess calories

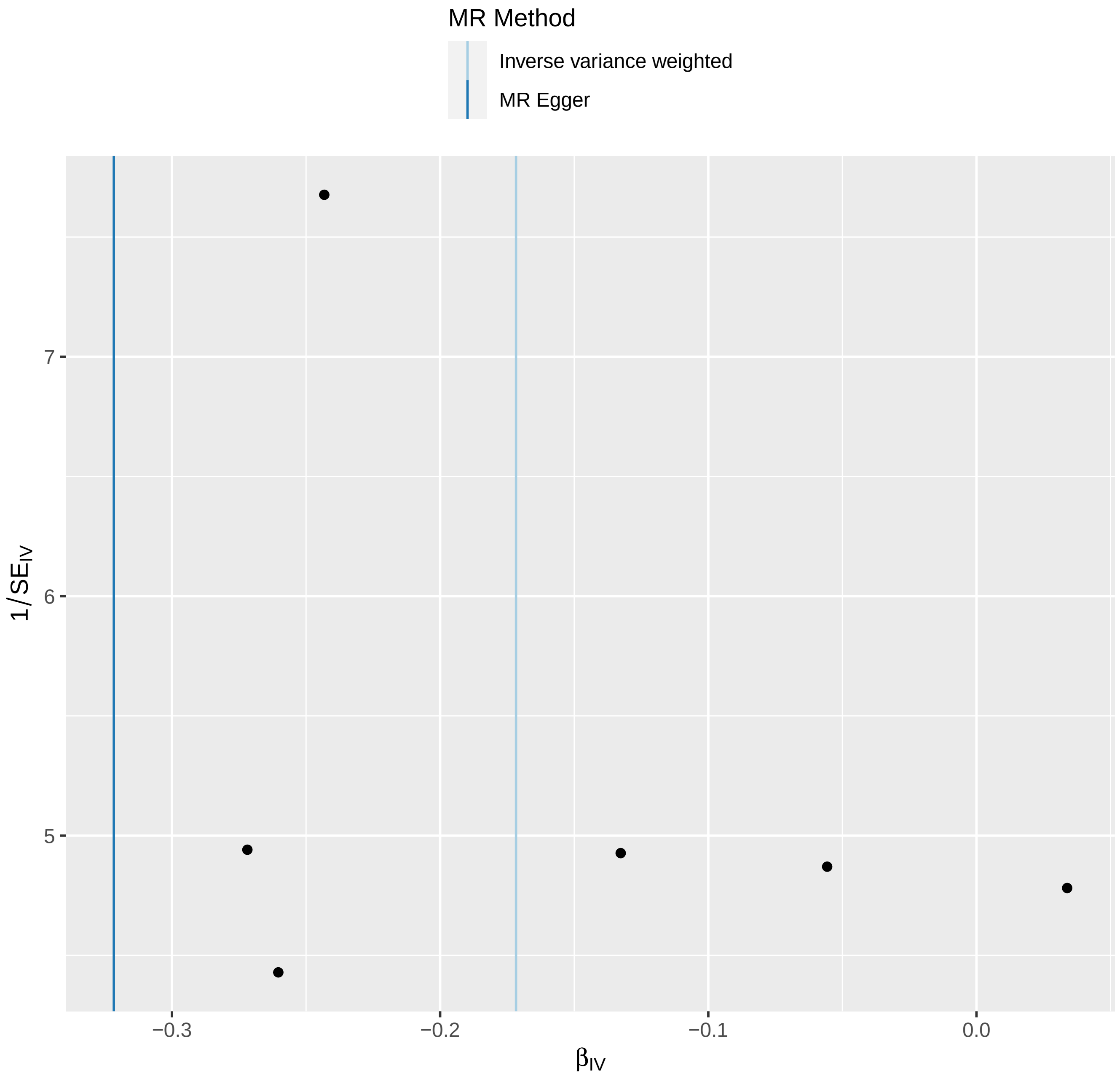

Batch 433 : Gut microbiota abundance (genus Ruminococcaceae UCG011 id.11368) on Obesity due to excess calories

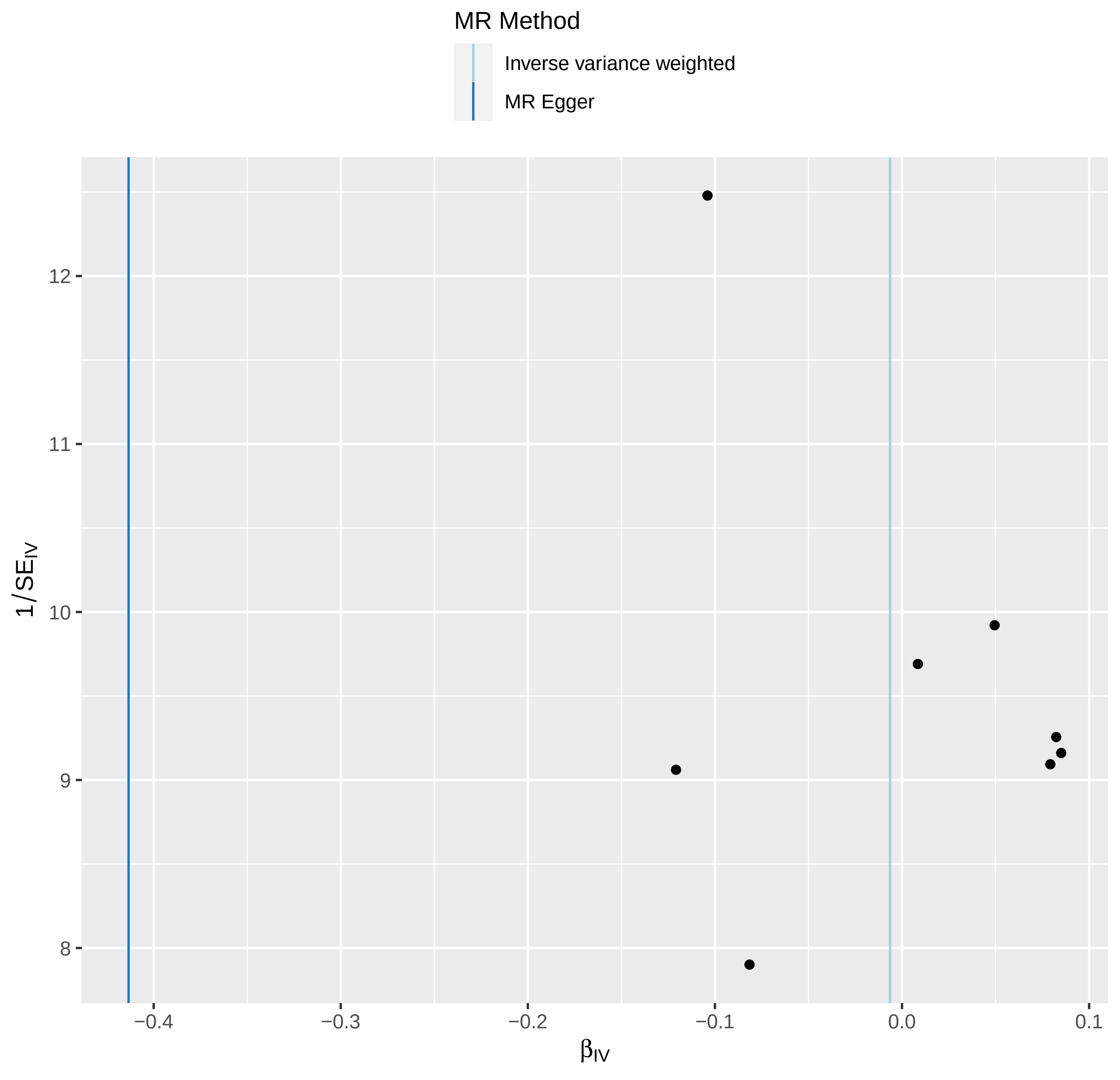

Batch 434 : Gut microbiota abundance (genus Ruminococcaceae UCG013 id.11370) on Obesity due to excess calories

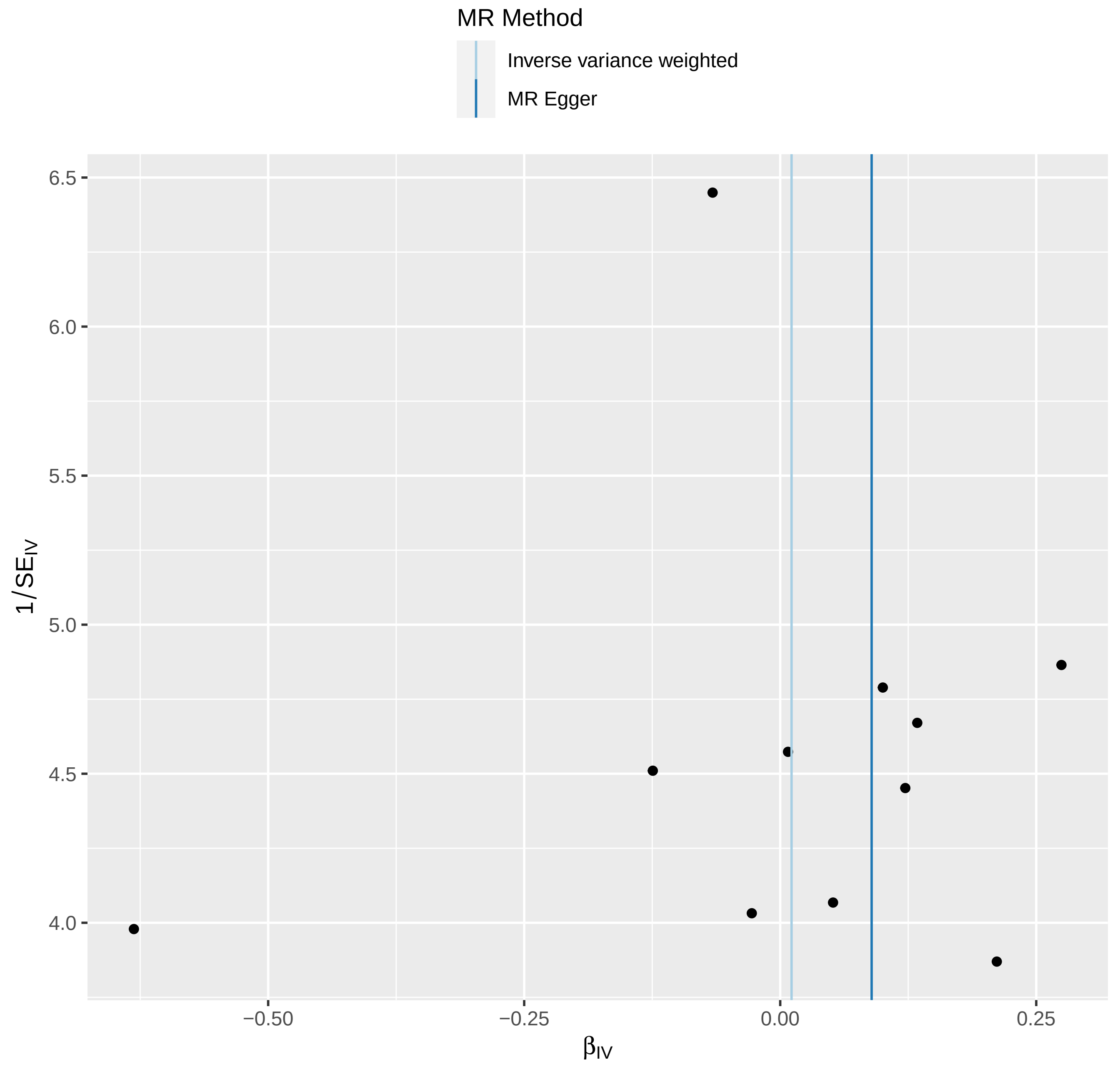

Batch 435 : Gut microbiota abundance (genus Ruminococcaceae UCG014 id.11371) on Obesity due to excess calories

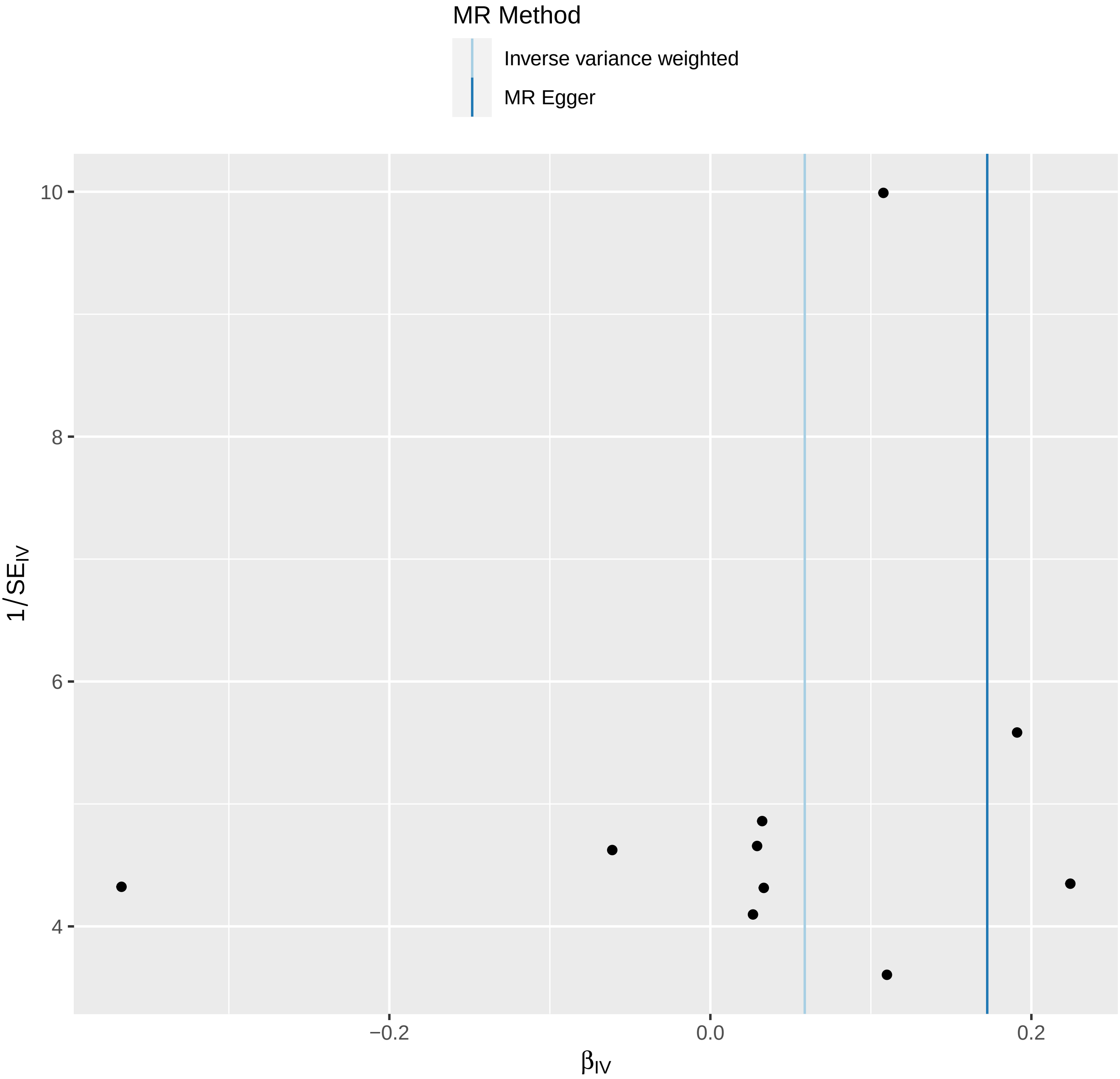

Batch 436 : Gut microbiota abundance (genus Ruminococcus1 id.11373) on Obesity due to excess calories

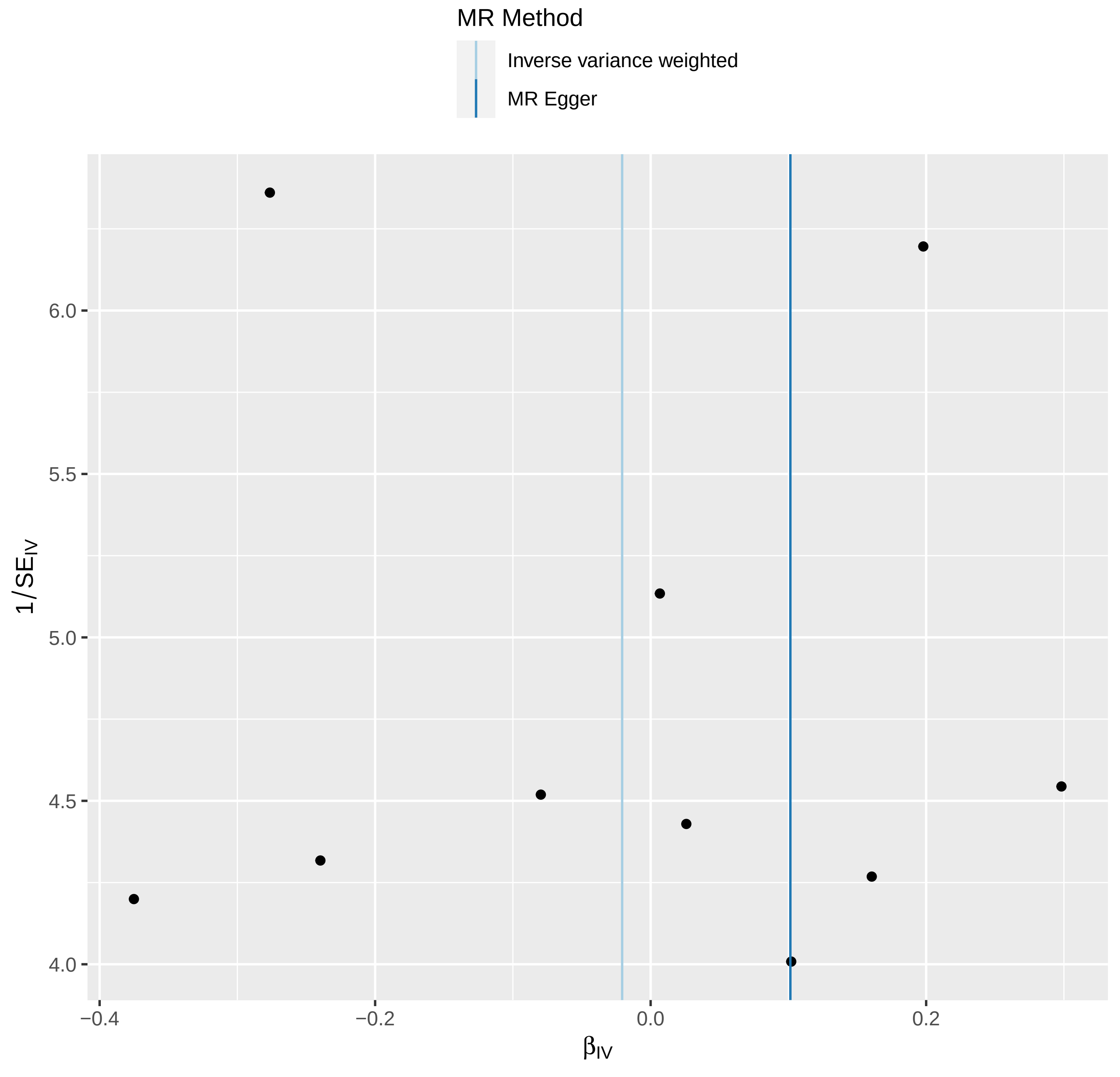

Batch 437 : Gut microbiota abundance (genus Ruminococcus2 id.11374) on Obesity due to excess calories

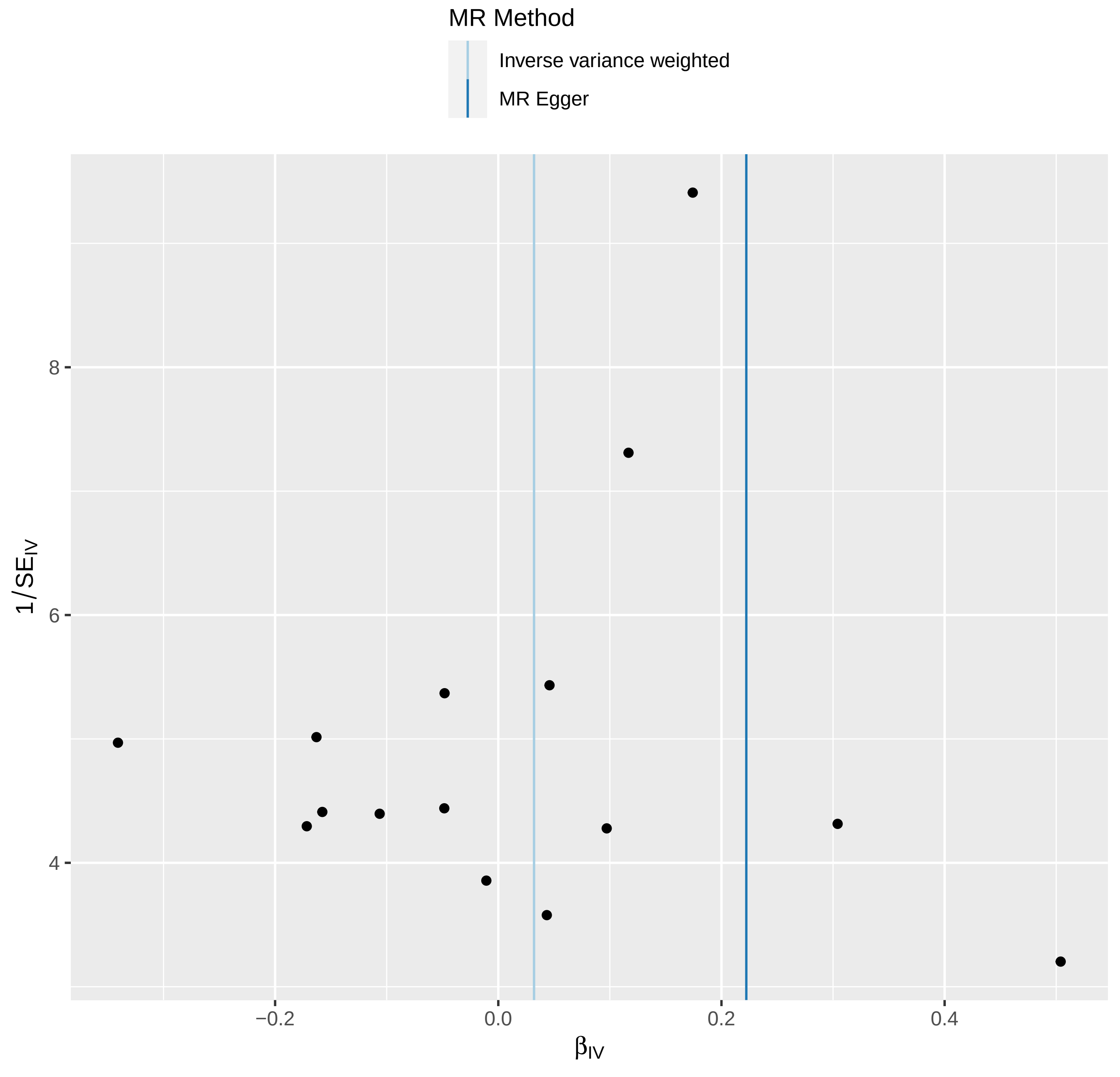

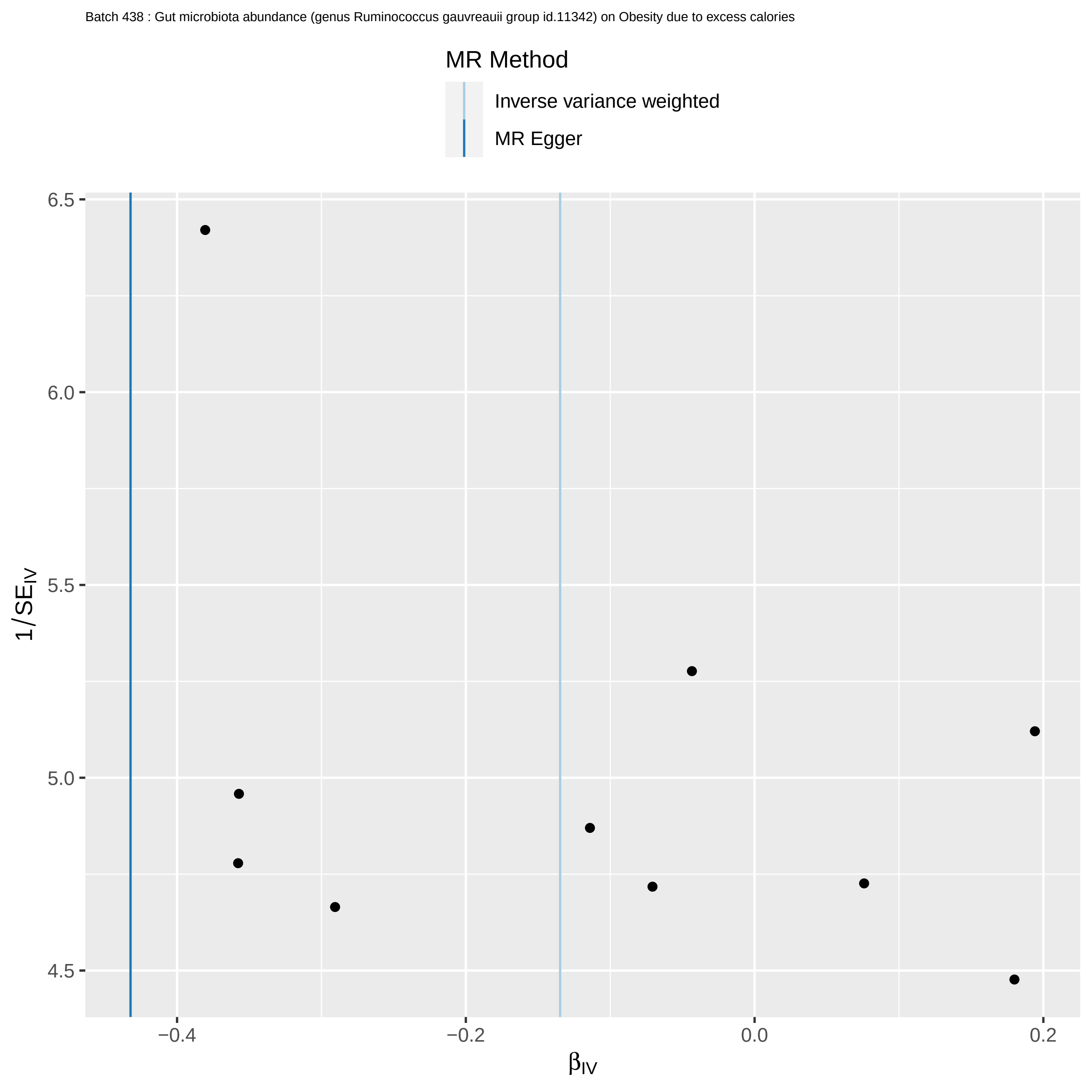

Batch 438 : Gut microbiota abundance (genus Ruminococcus gauvreauii group id.11342) on Obesity due to excess calories
MR Method
Inverse variance weighted
MR Egger
6.5
6.0
5.5
5.0
4.5
1/SE_IV
−0.4
−0.2
0.0
0.2
β_IV

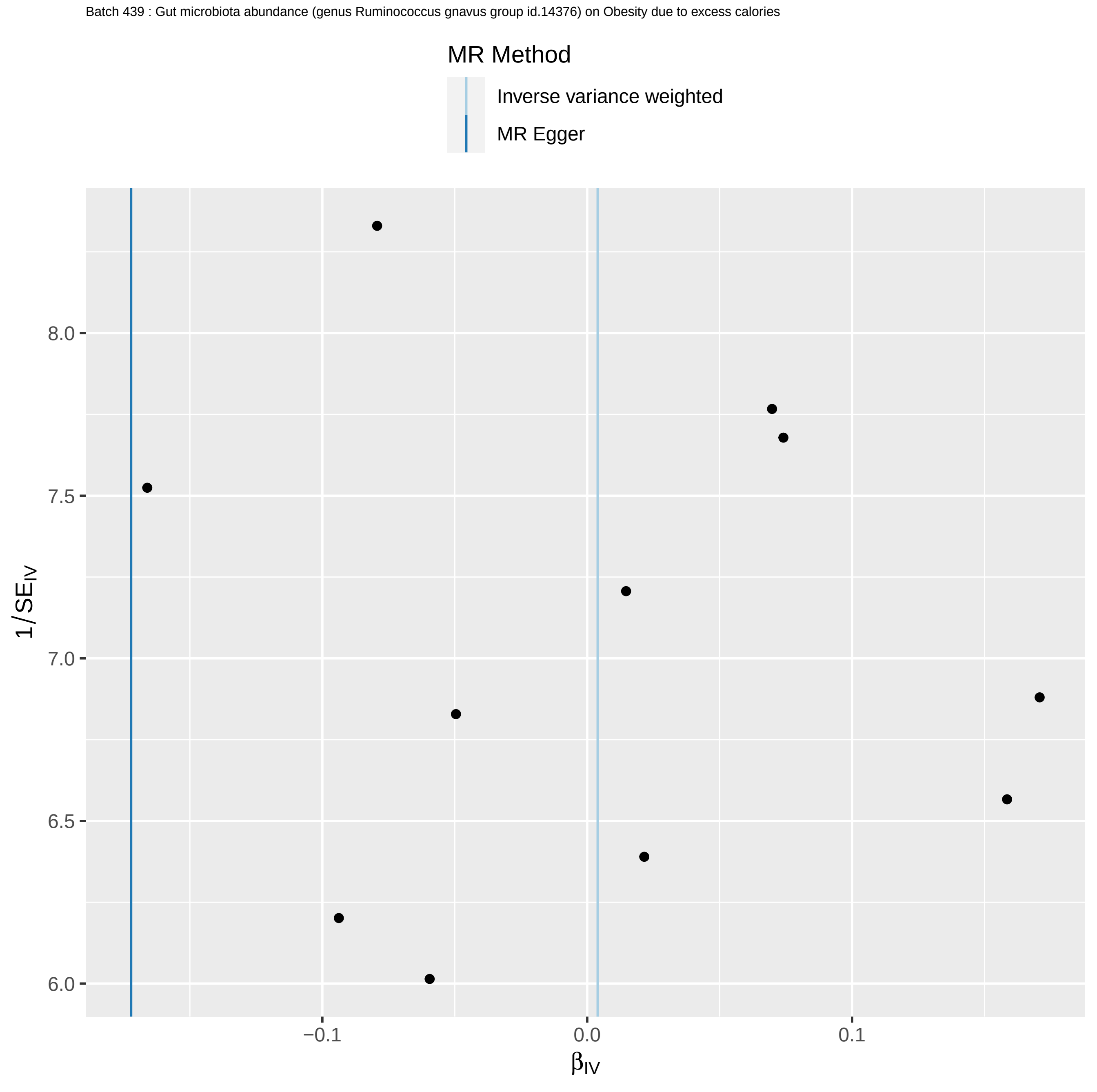

Batch 439 : Gut microbiota abundance (genus Ruminococcus gnavus group id.14376) on Obesity due to excess calories
MR Method
Inverse variance weighted
MR Egger
1/SE_IV
8.0
7.5
7.0
6.5
6.0
−0.1
0.0
0.1
β_IV

Batch 440 : Gut microbiota abundance (genus Ruminococcus torques group id.14377) on Obesity due to excess calories

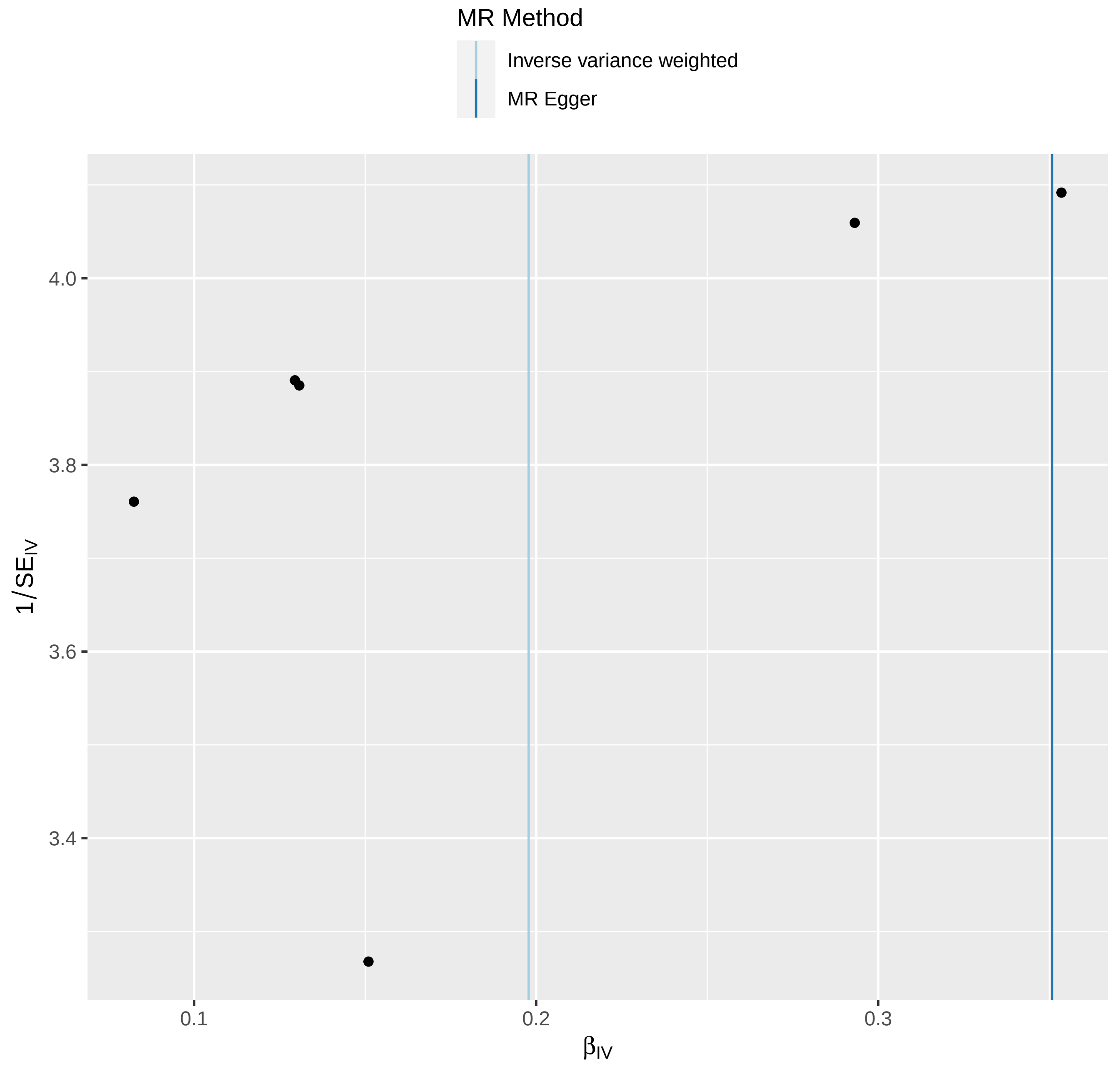

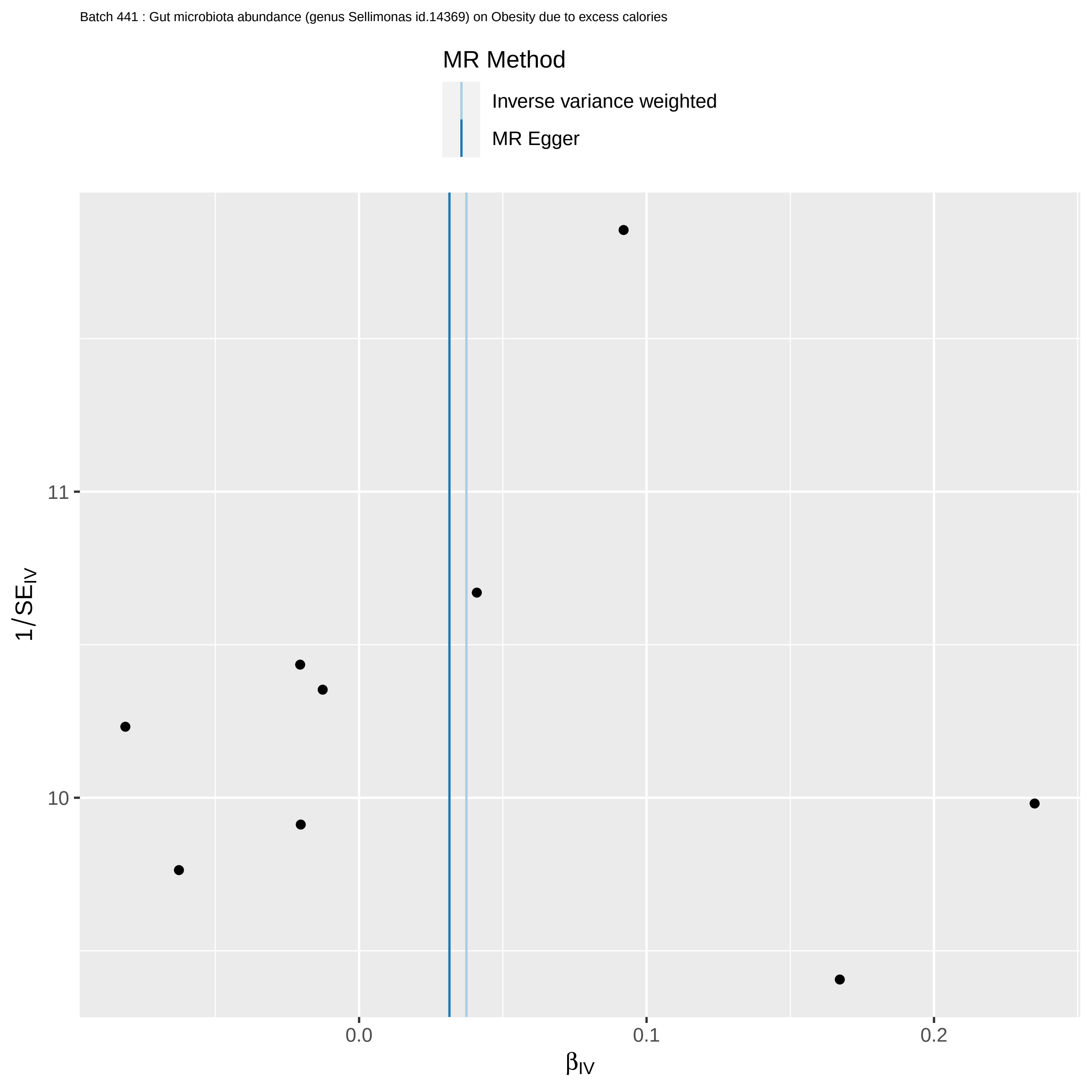
Batch 441 : Gut microbiota abundance (genus Sellimonas id.14369) on Obesity due to excess calories
MR Method
Inverse variance weighted
MR Egger
11
10
1/SE_IV
0.0
0.1
0.2
β_IV

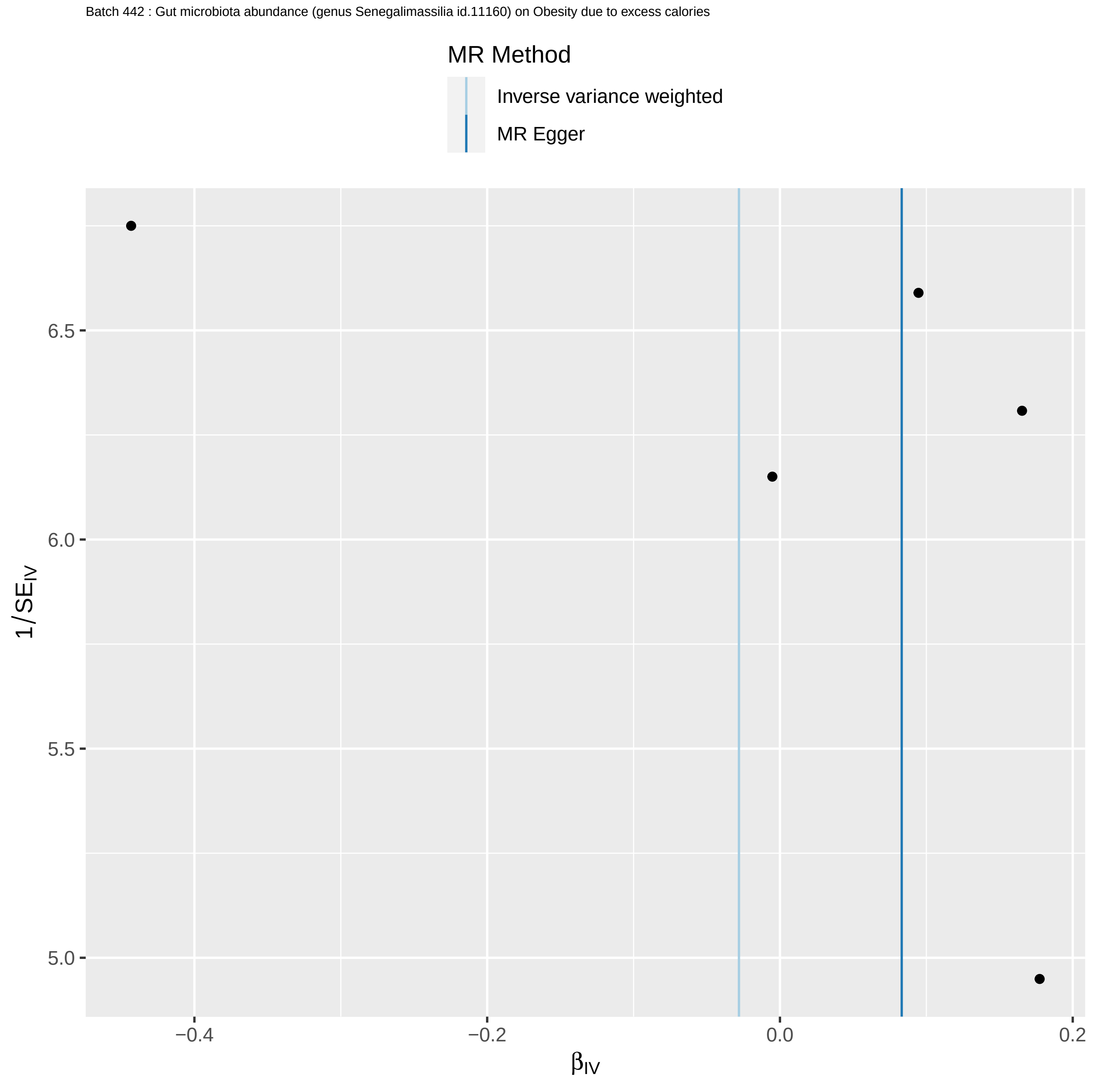
Batch 442 : Gut microbiota abundance (genus Senegalimassilia id.11160) on Obesity due to excess calories
MR Method
Inverse variance weighted
MR Egger
6.5
6.0
5.5
5.0
1/SE_IV
−0.4
−0.2
0.0
0.2
β_IV

Batch 443 : Gut microbiota abundance (genus Slackia id.825) on Obesity due to excess calories

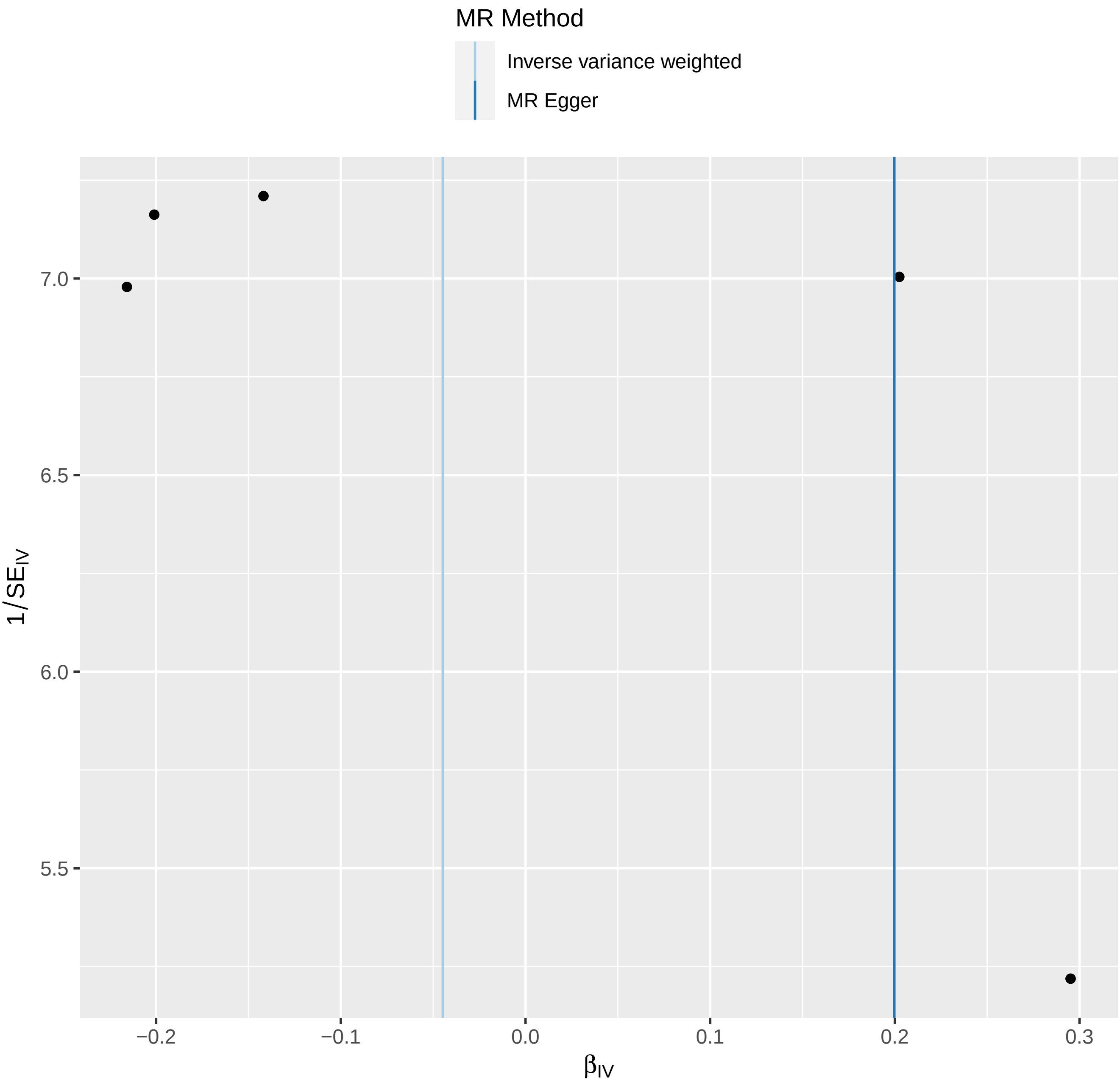

Batch 444 : Gut microbiota abundance (genus Streptococcus id.1853) on Obesity due to excess calories

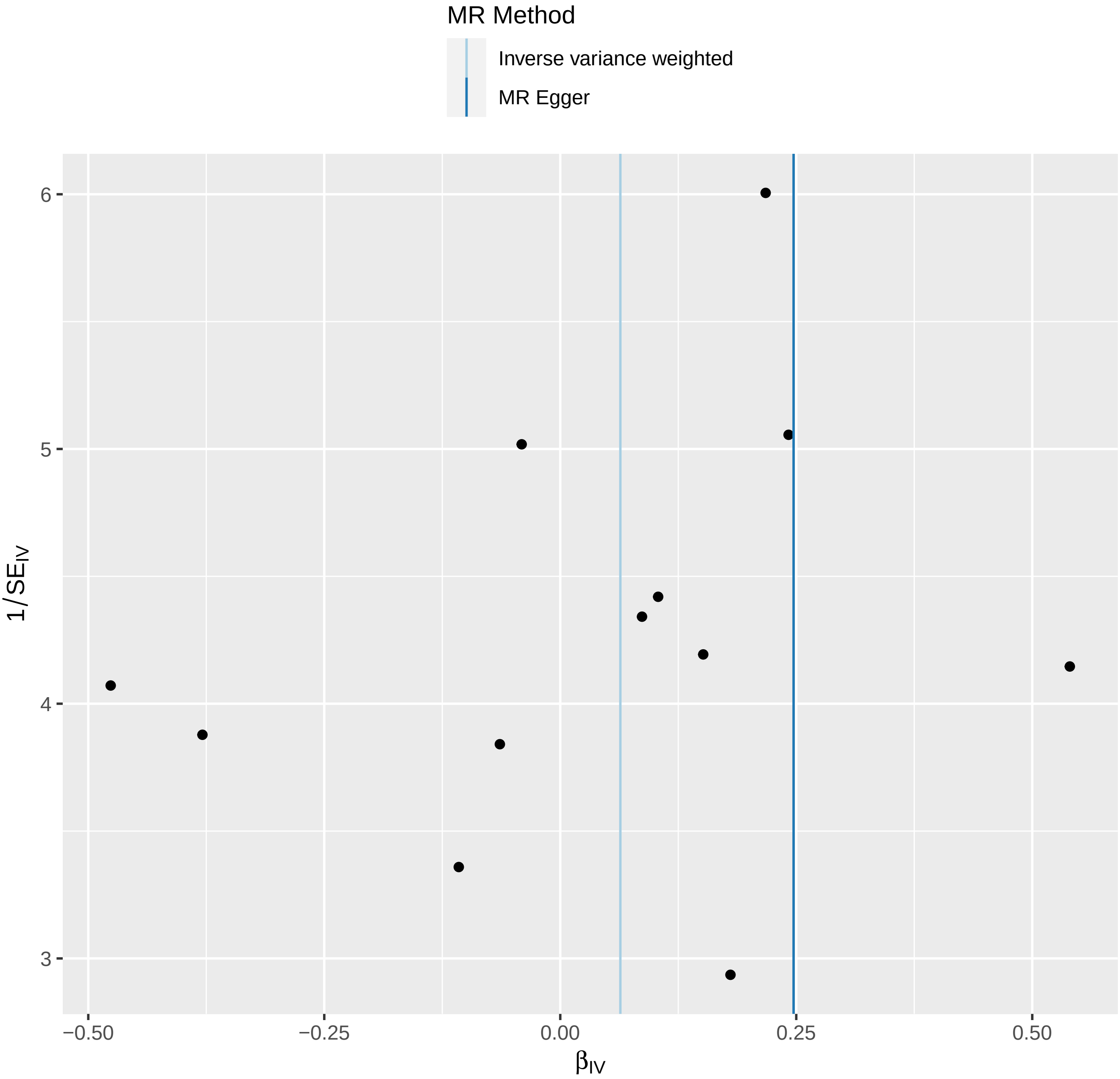

Batch 445 : Gut microbiota abundance (genus Subdoligranulum id.2070) on Obesity due to excess calories

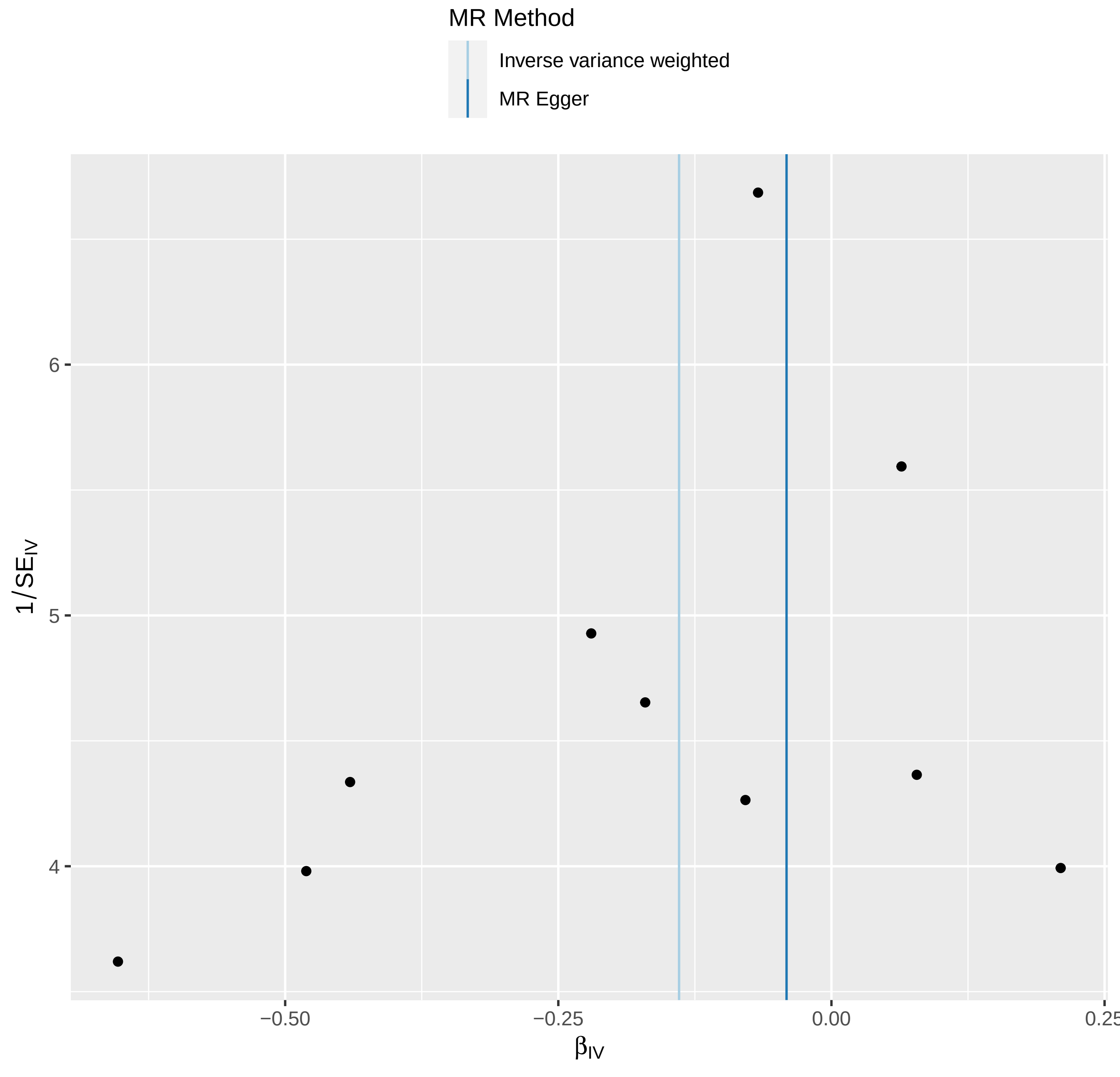

Batch 446 : Gut microbiota abundance (genus Sutterella id.2896) on Obesity due to excess calories

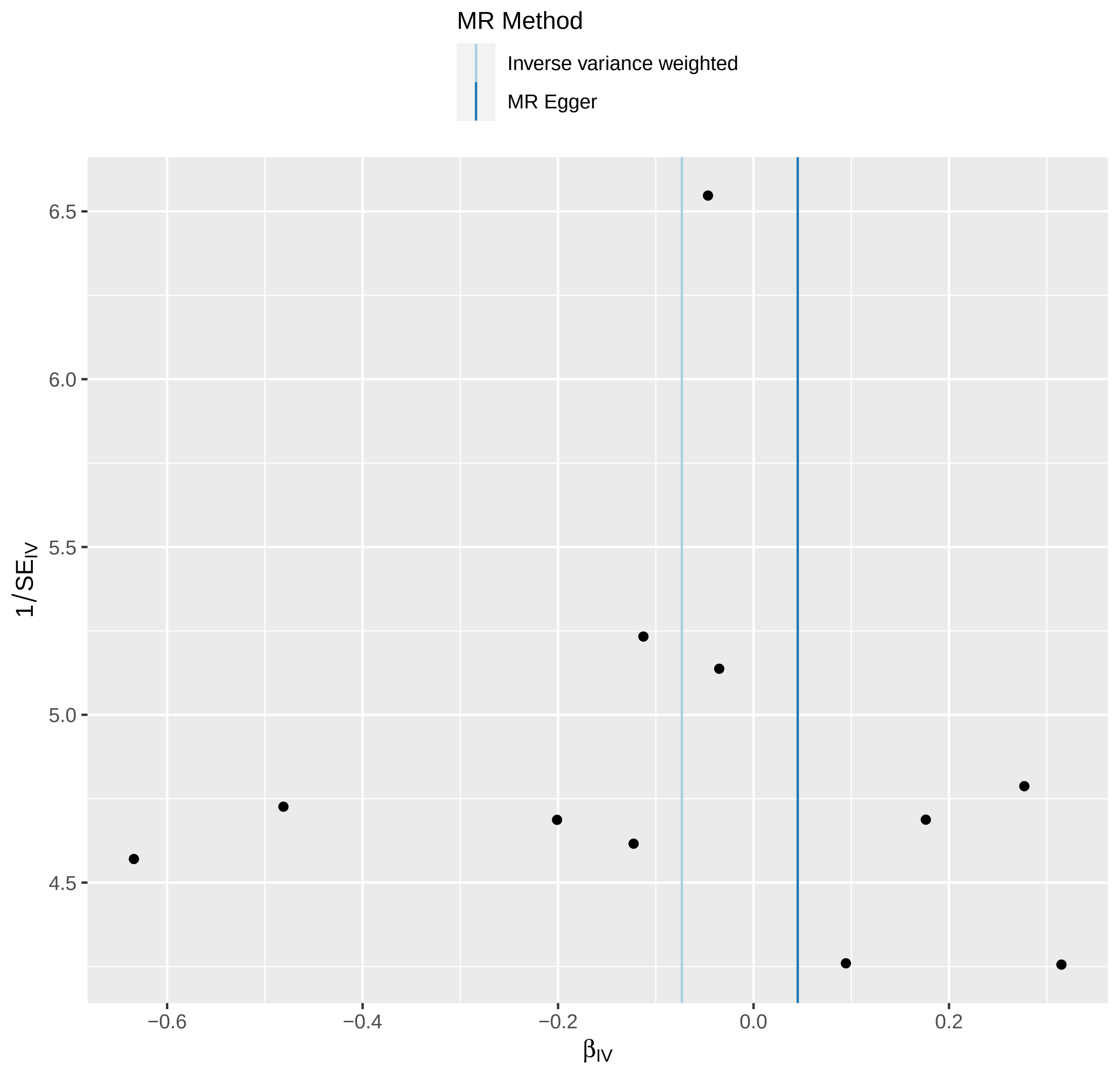

Batch 447 : Gut microbiota abundance (genus Terrisporobacter id.11348) on Obesity due to excess calories

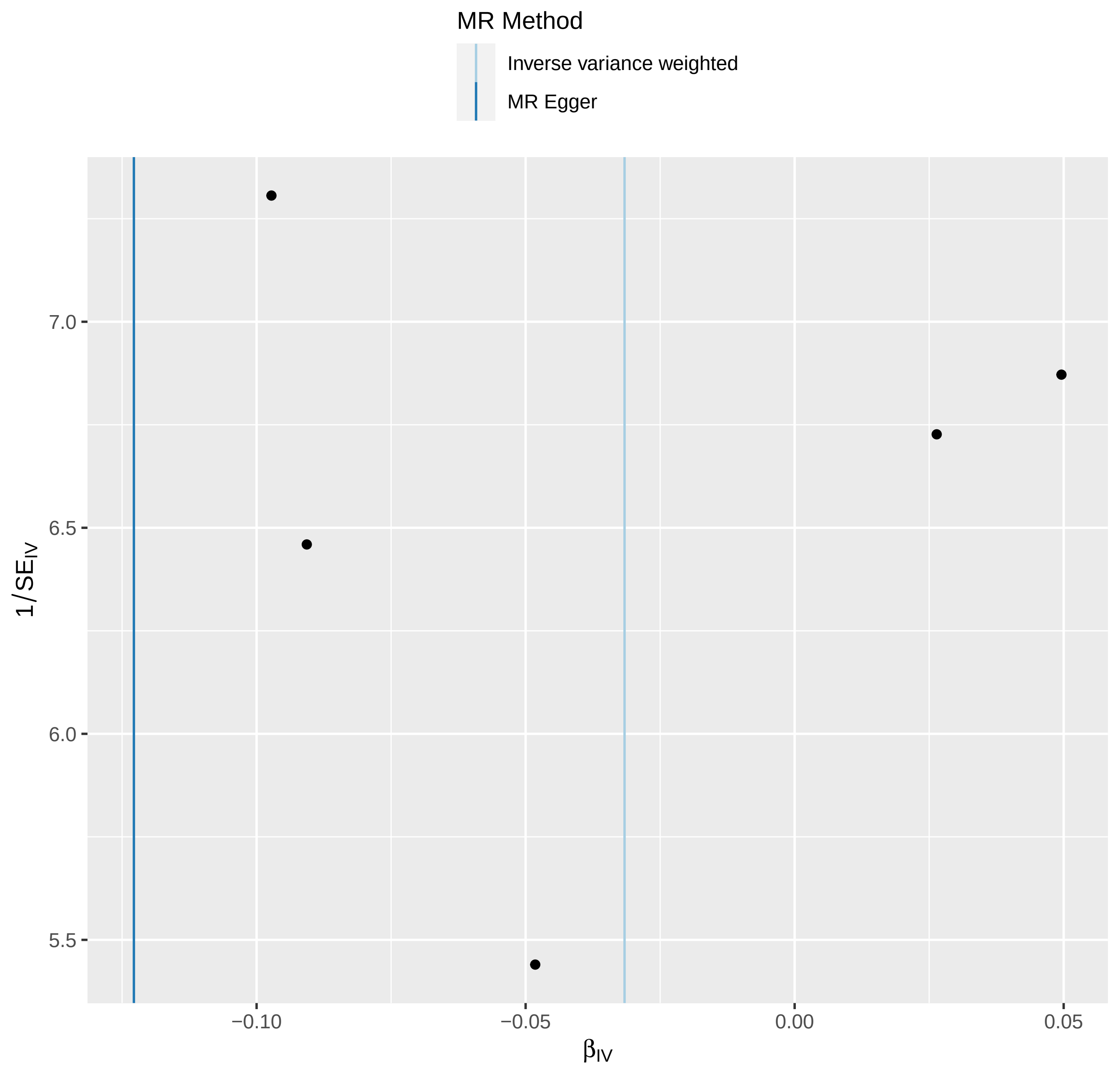

Batch 448 : Gut microbiota abundance (genus Turicibacter id.2162) on Obesity due to excess calories

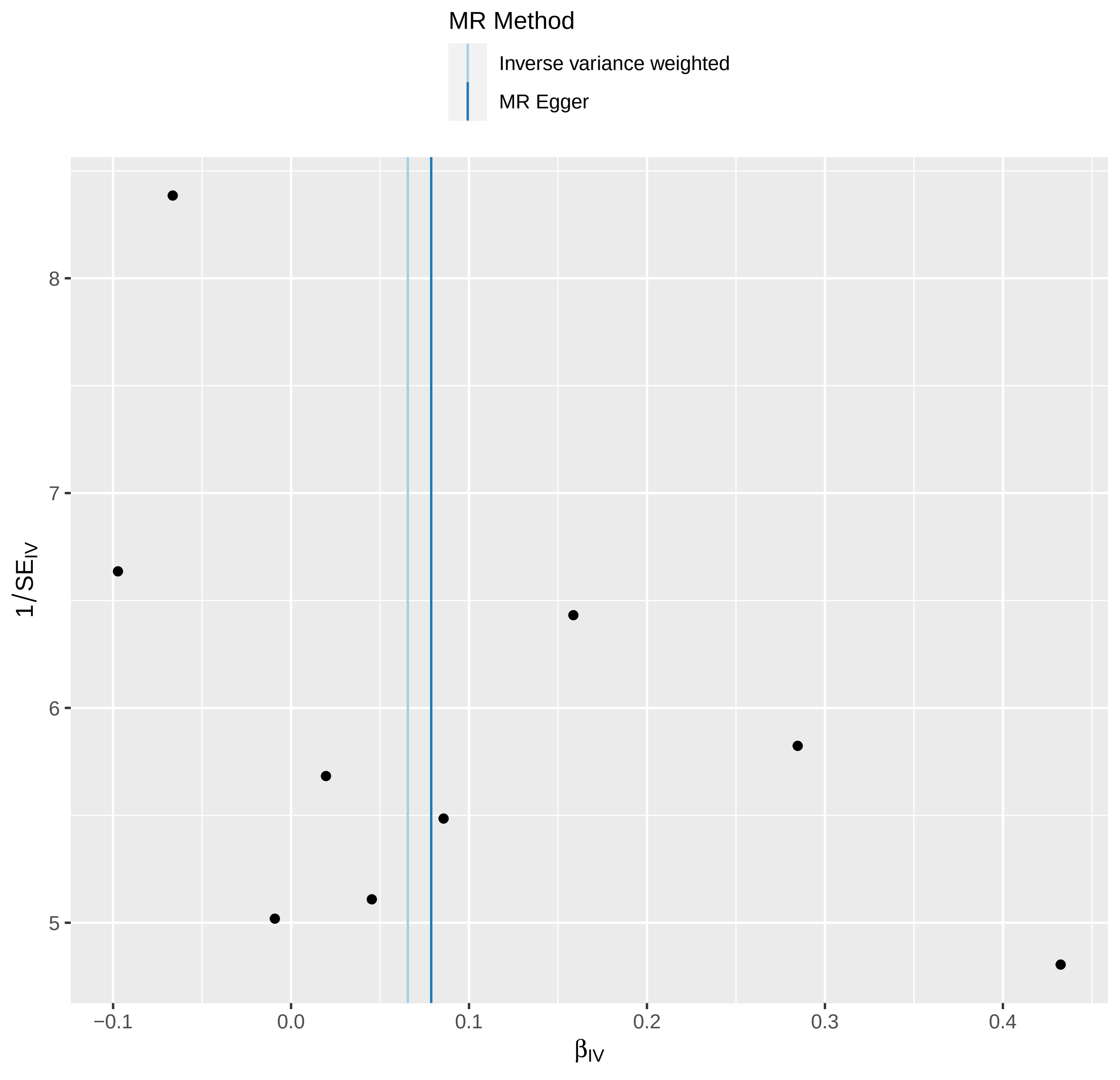

Batch 449 : Gut microbiota abundance (genus Tyzzerella3 id.11335) on Obesity due to excess calories

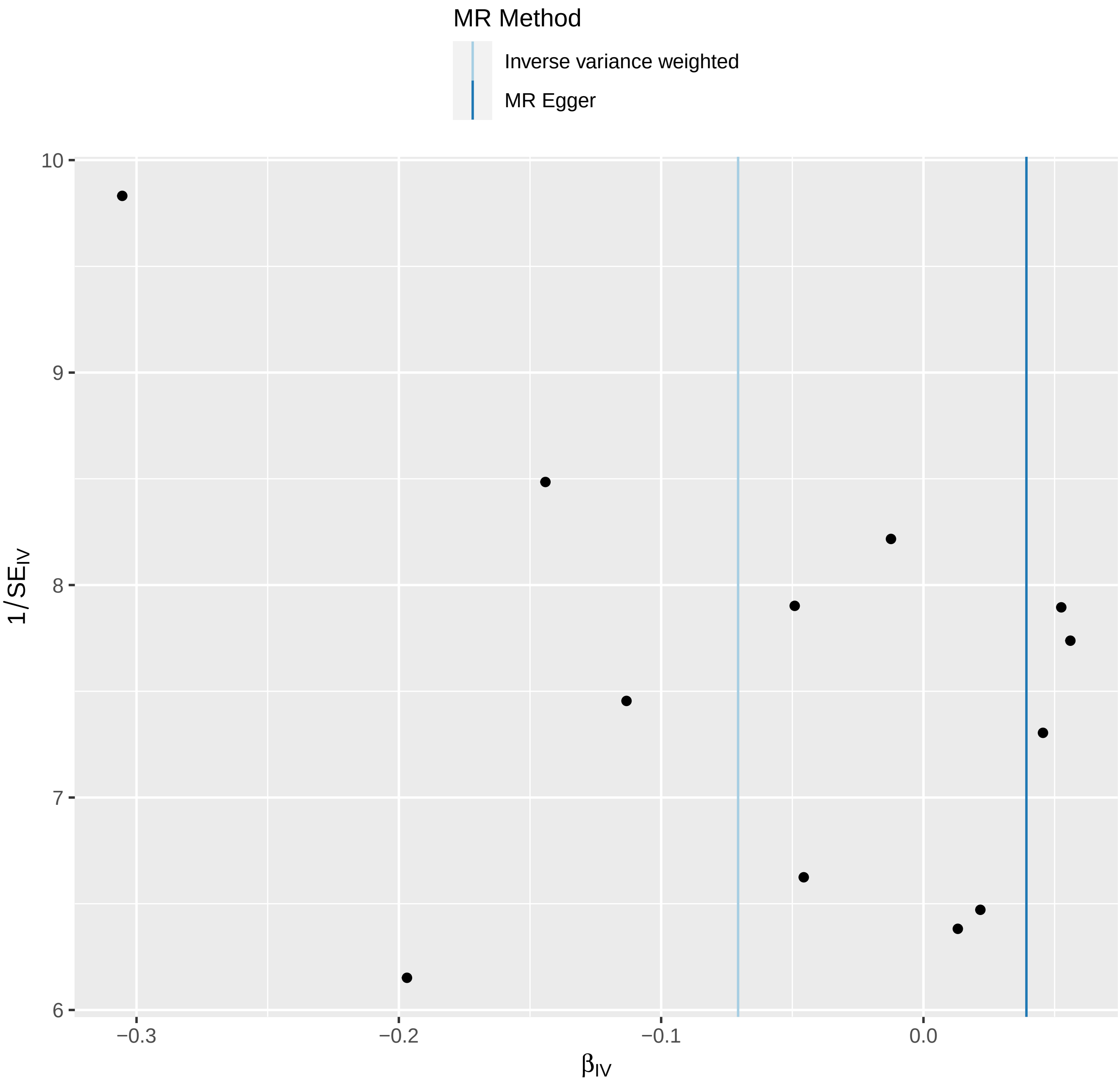

Batch 450 : Gut microbiota abundance (genus Veillonella id.2198) on Obesity due to excess calories

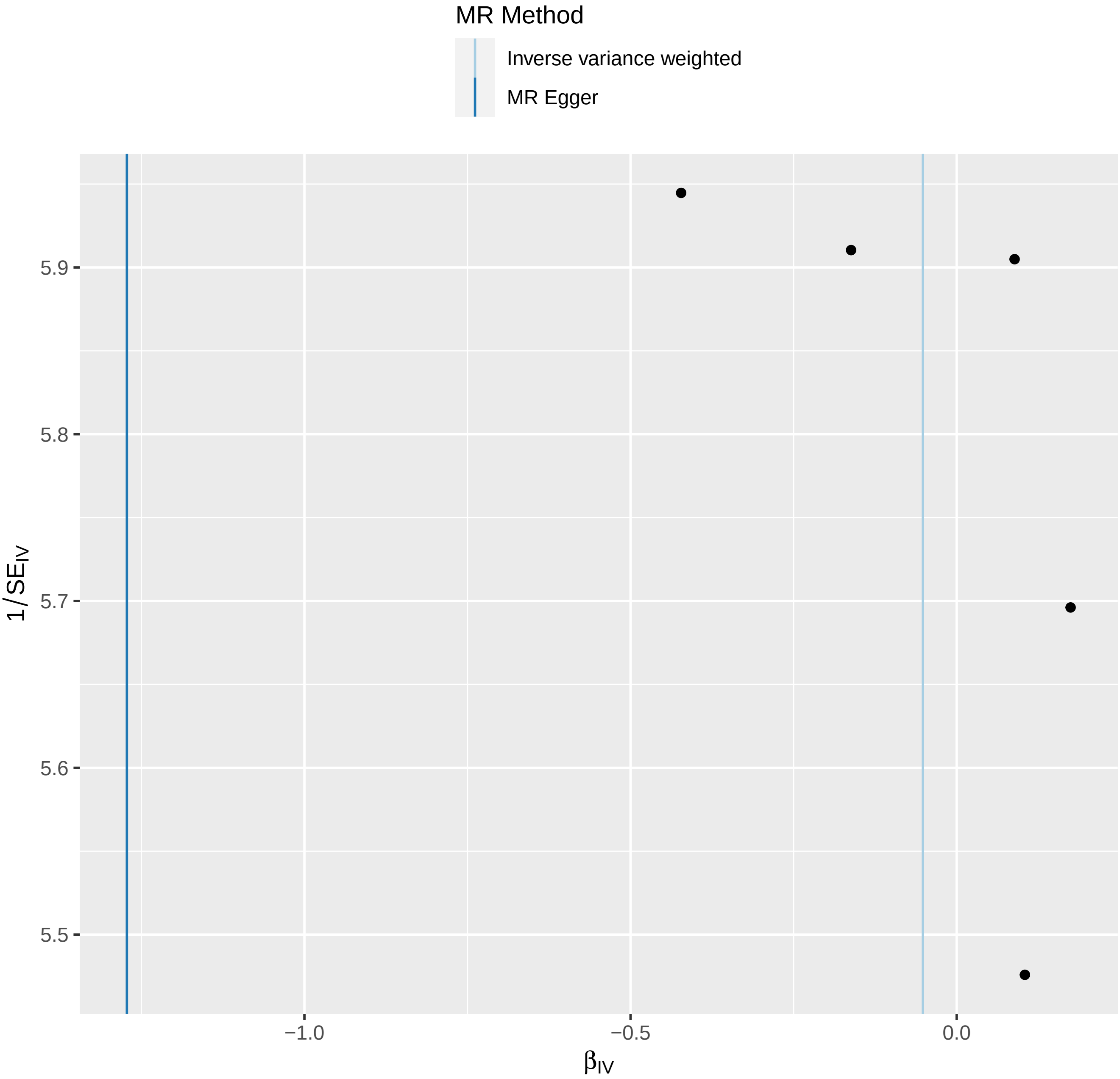

Batch 451 : Gut microbiota abundance (family Acidaminococcaceae id.2166) on Drug−induced obesity

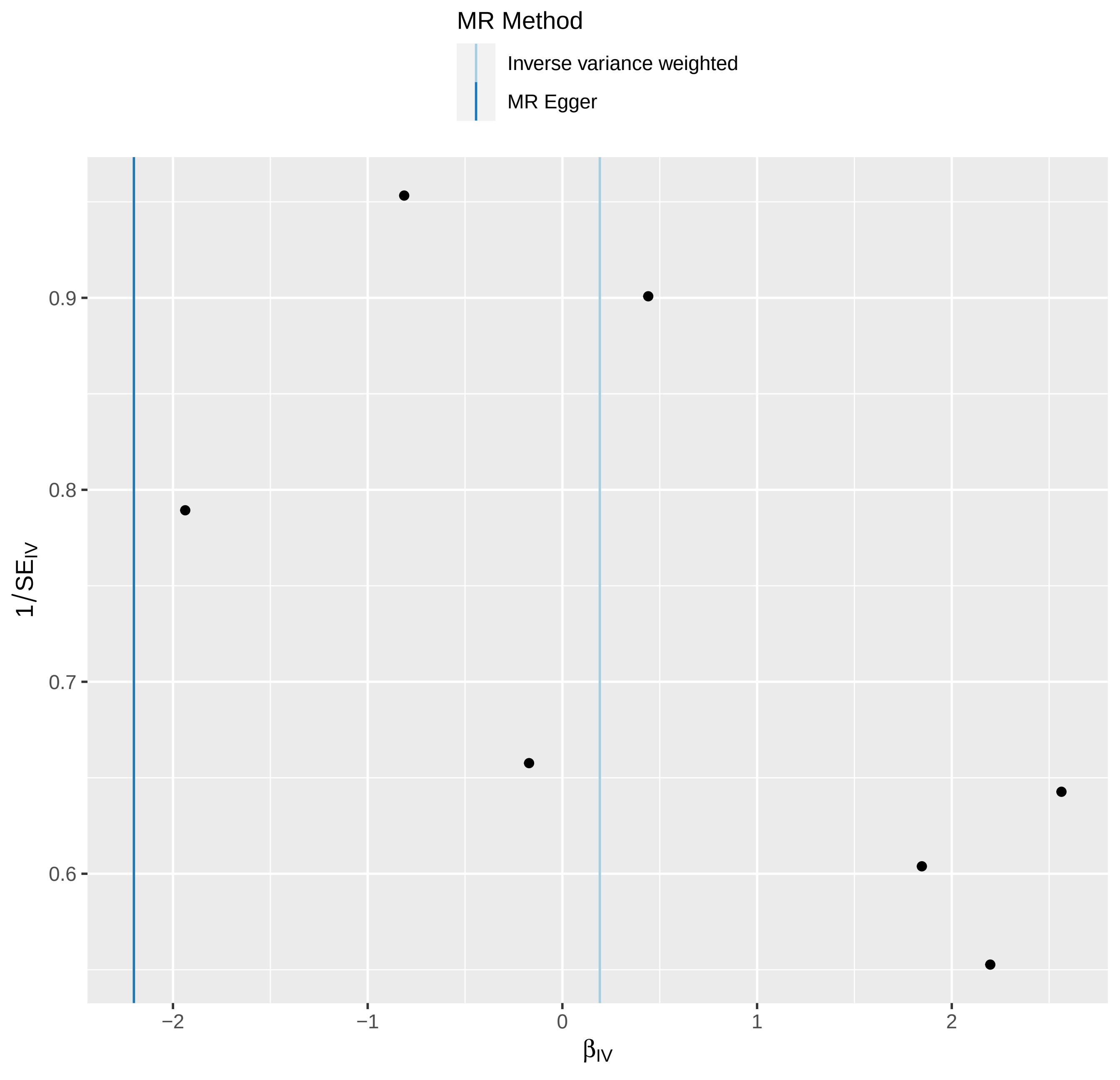

Batch 452 : Gut microbiota abundance (family Actinomycetaceae id.421) on Drug−induced obesity

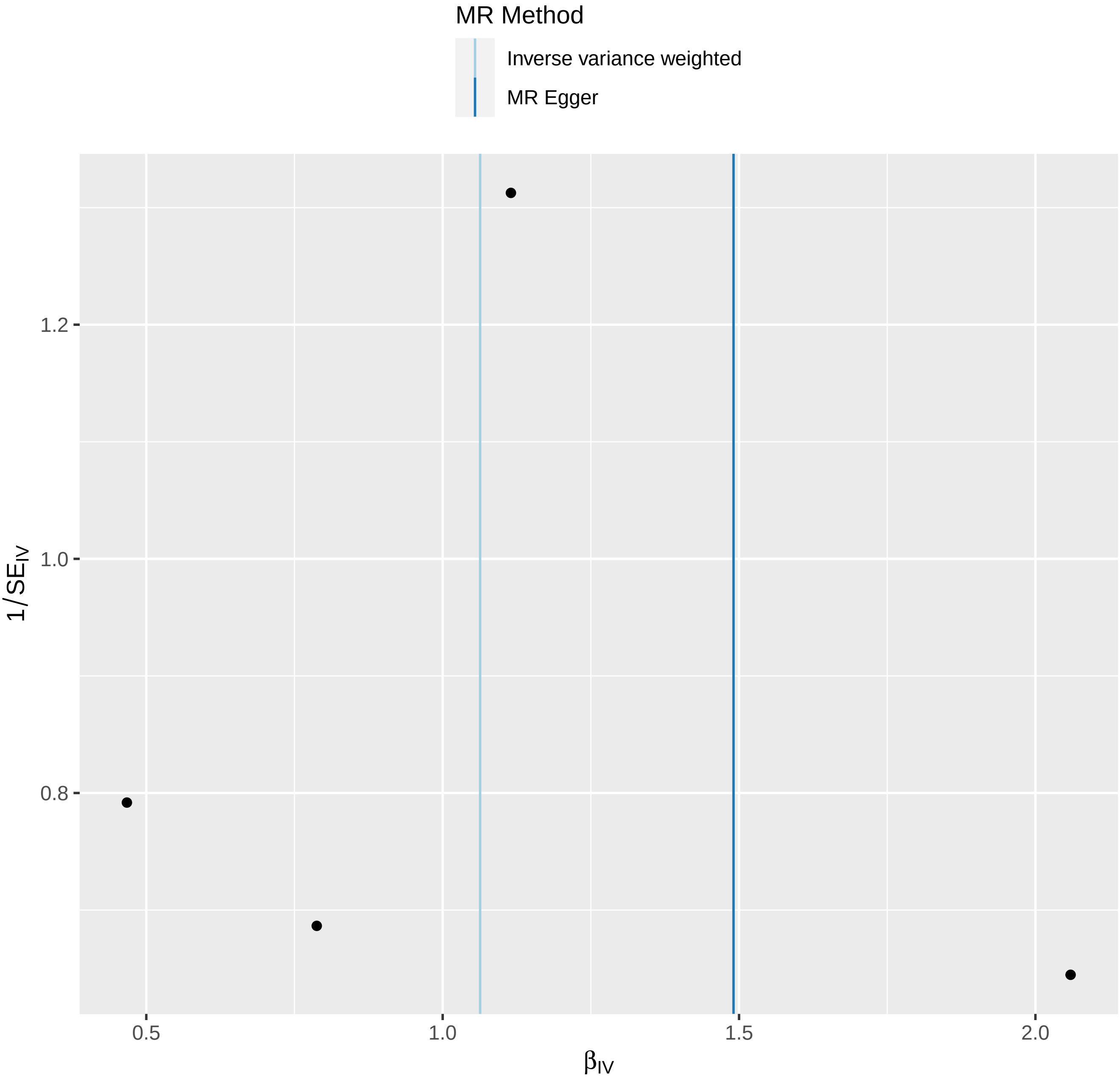

Batch 453 : Gut microbiota abundance (family Alcaligenaceae id.2875) on Drug−induced obesity

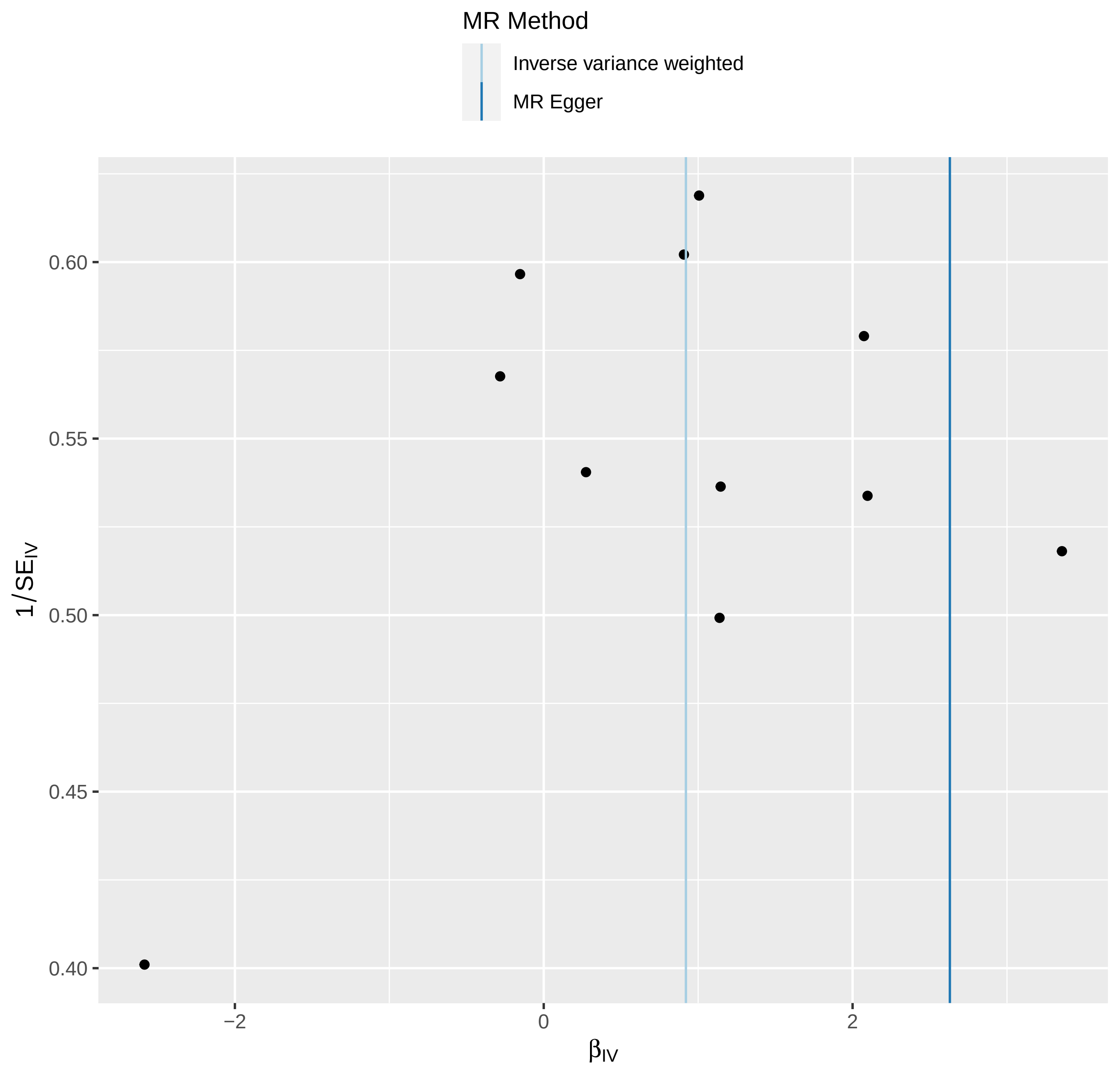

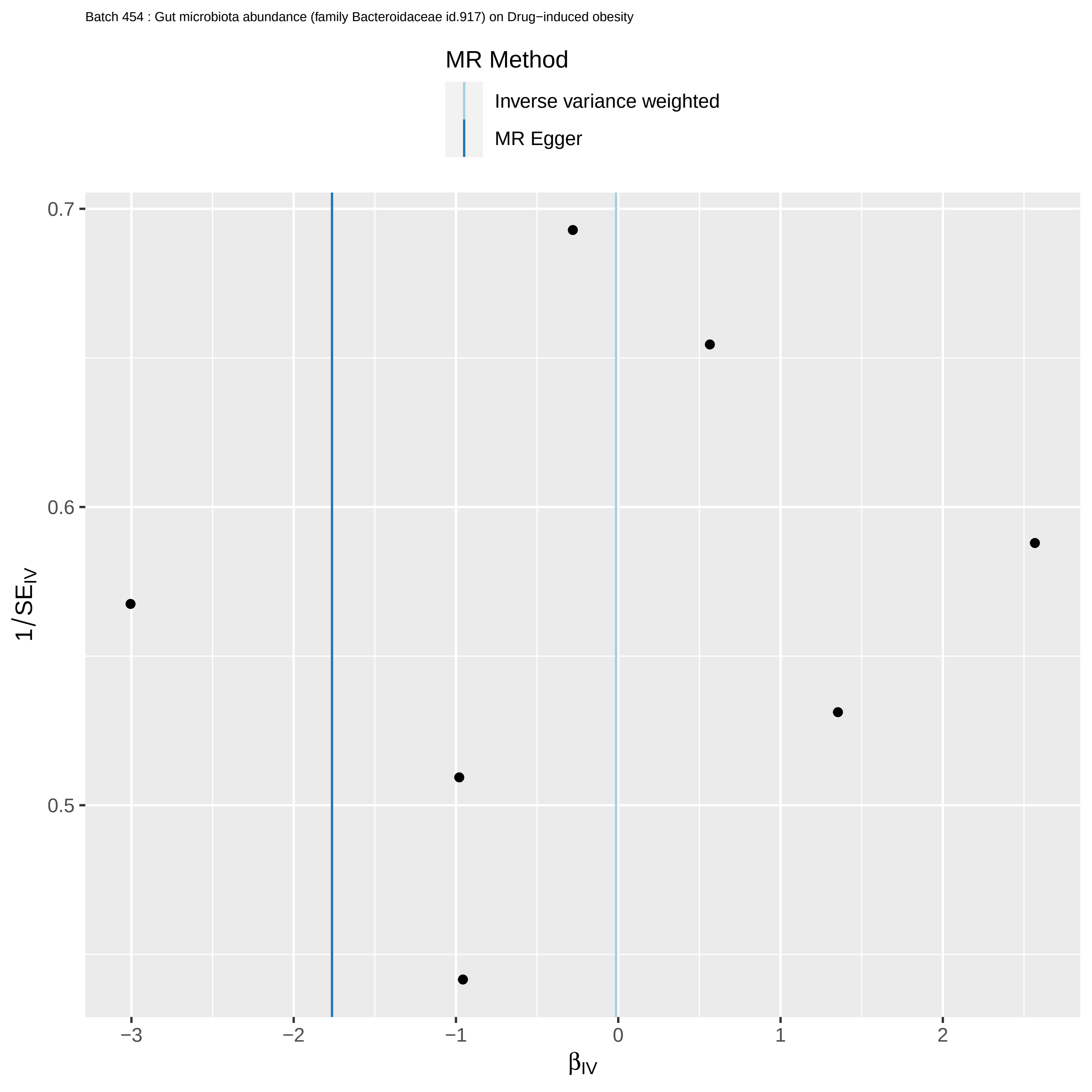

Batch 454 : Gut microbiota abundance (family Bacteroidaceae id.917) on Drug−induced obesity
MR Method
Inverse variance weighted
MR Egger
0.7
0.6
0.5
1/SE_IV
−3
−2
−1
0
1
2
β_IV

Batch 455 : Gut microbiota abundance (family Bacteroidales S24 7group id.11173) on Drug−induced obesity

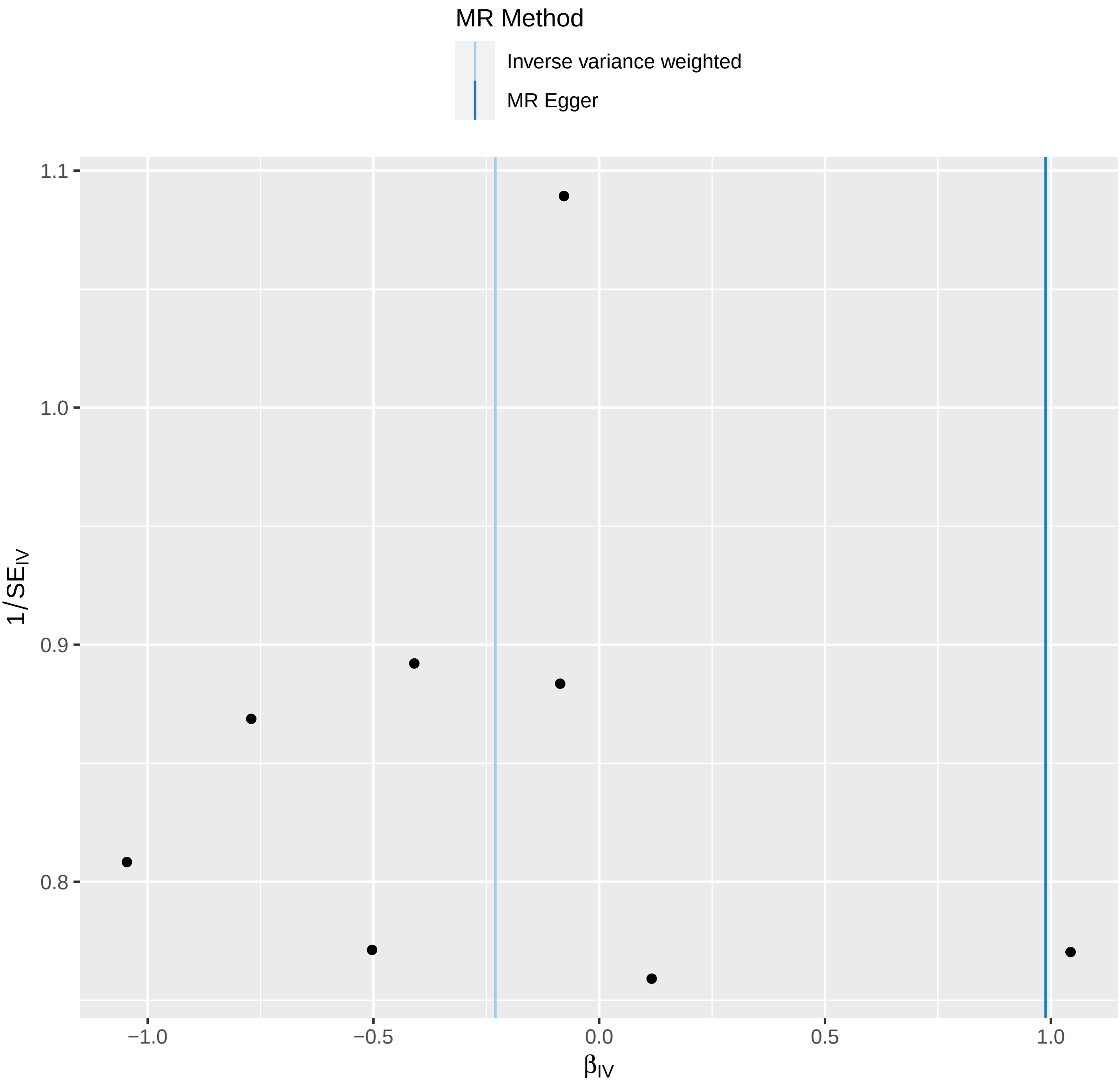

Batch 456 : Gut microbiota abundance (family Bifidobacteriaceae id.433) on Drug−induced obesity

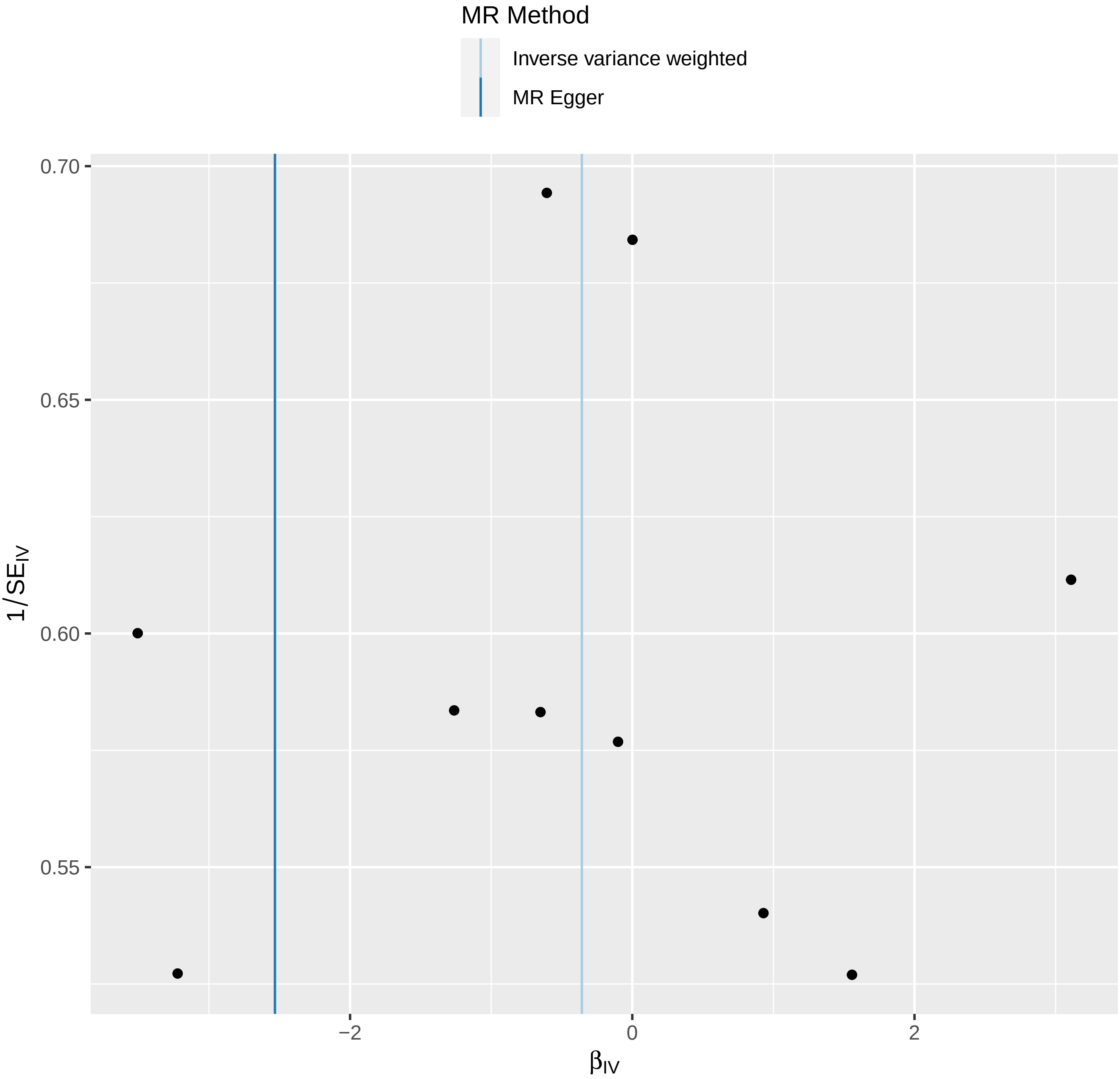

Batch 458 : Gut microbiota abundance (family Clostridiaceae1 id.1869) on Drug−induced obesity

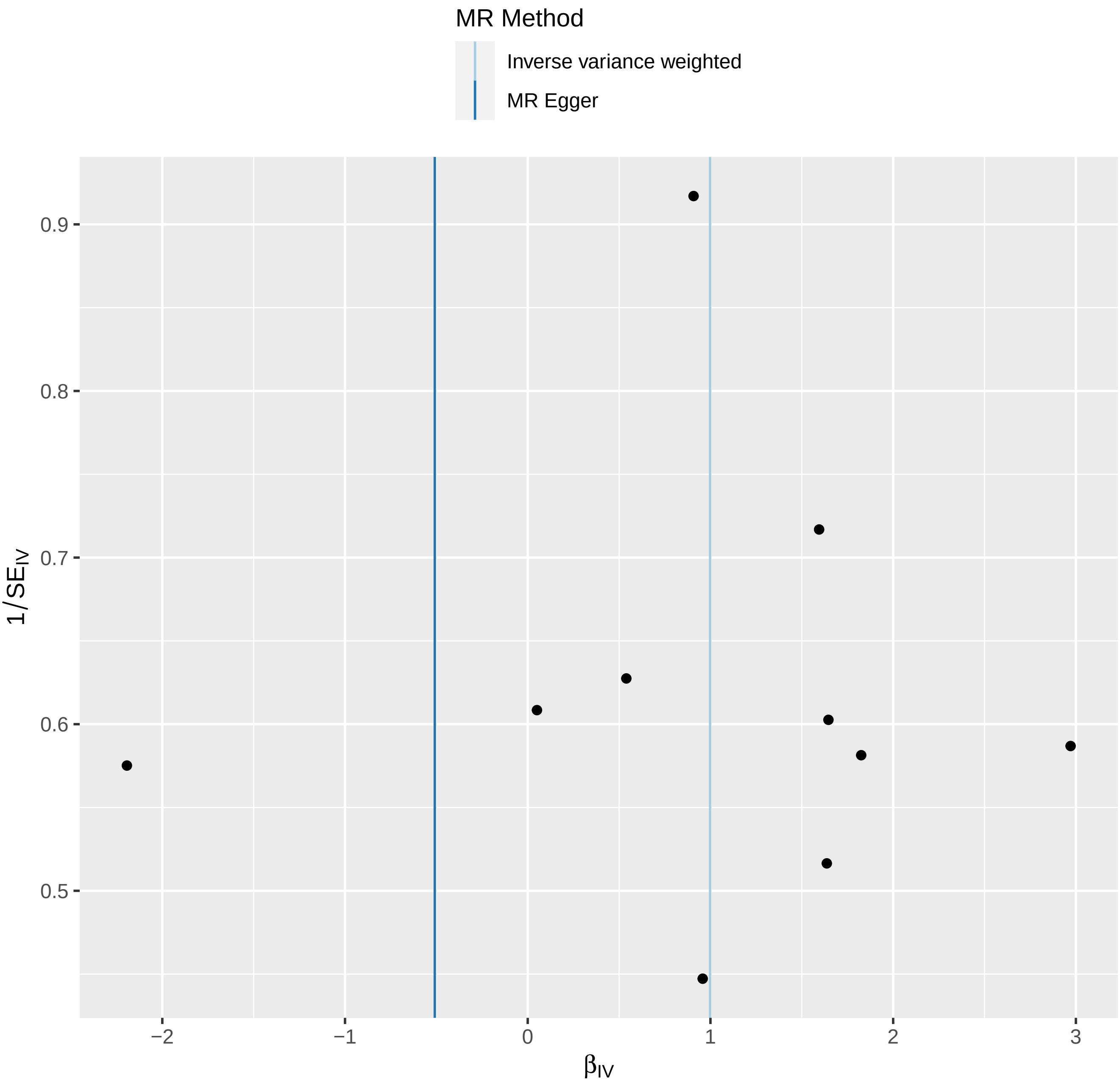

Batch 459 : Gut microbiota abundance (family Clostridiales vadin BB60 group id.11286) on Drug−induced obesity

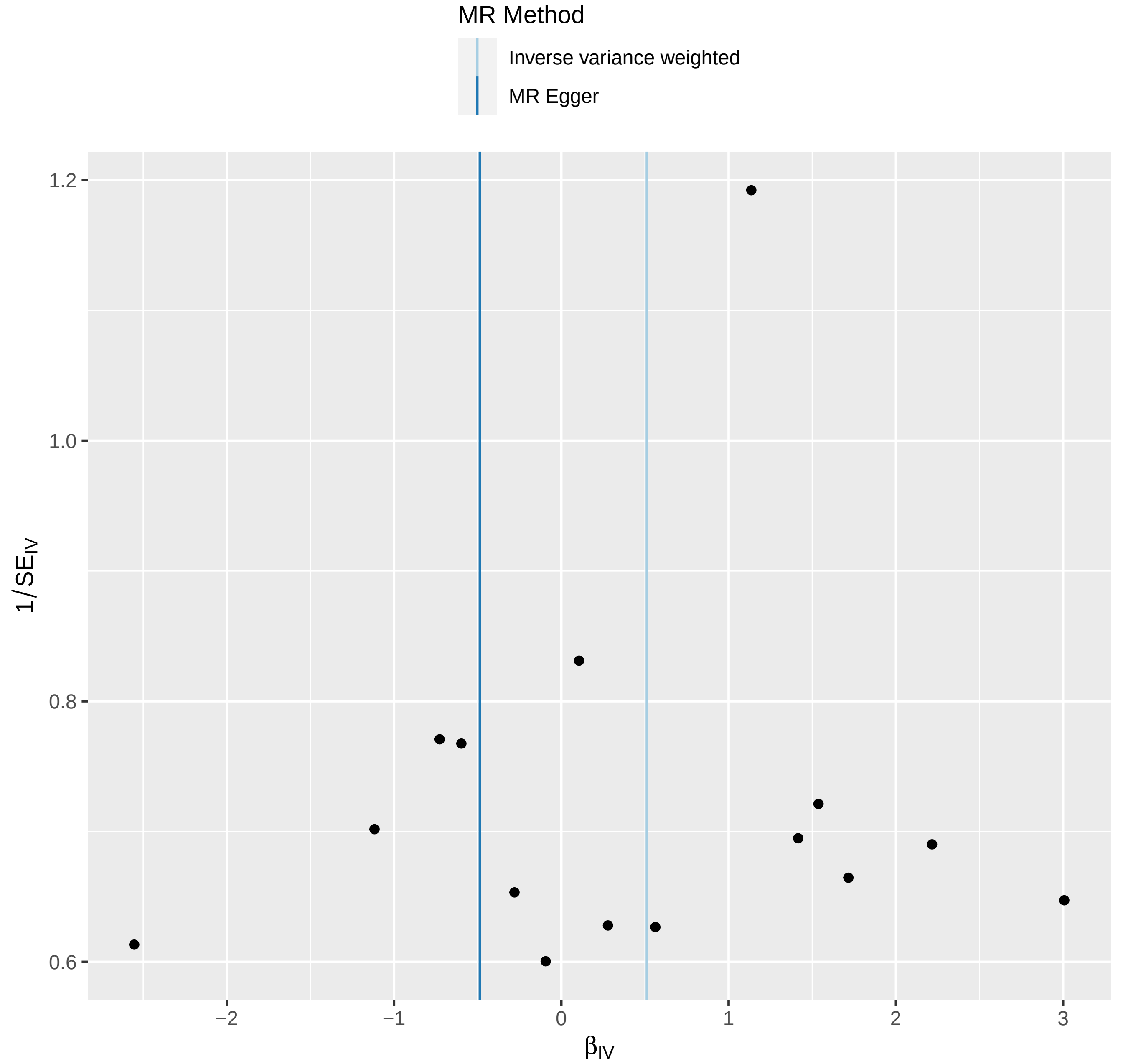

Batch 460 : Gut microbiota abundance (family Coriobacteriaceae id.811) on Drug−induced obesity

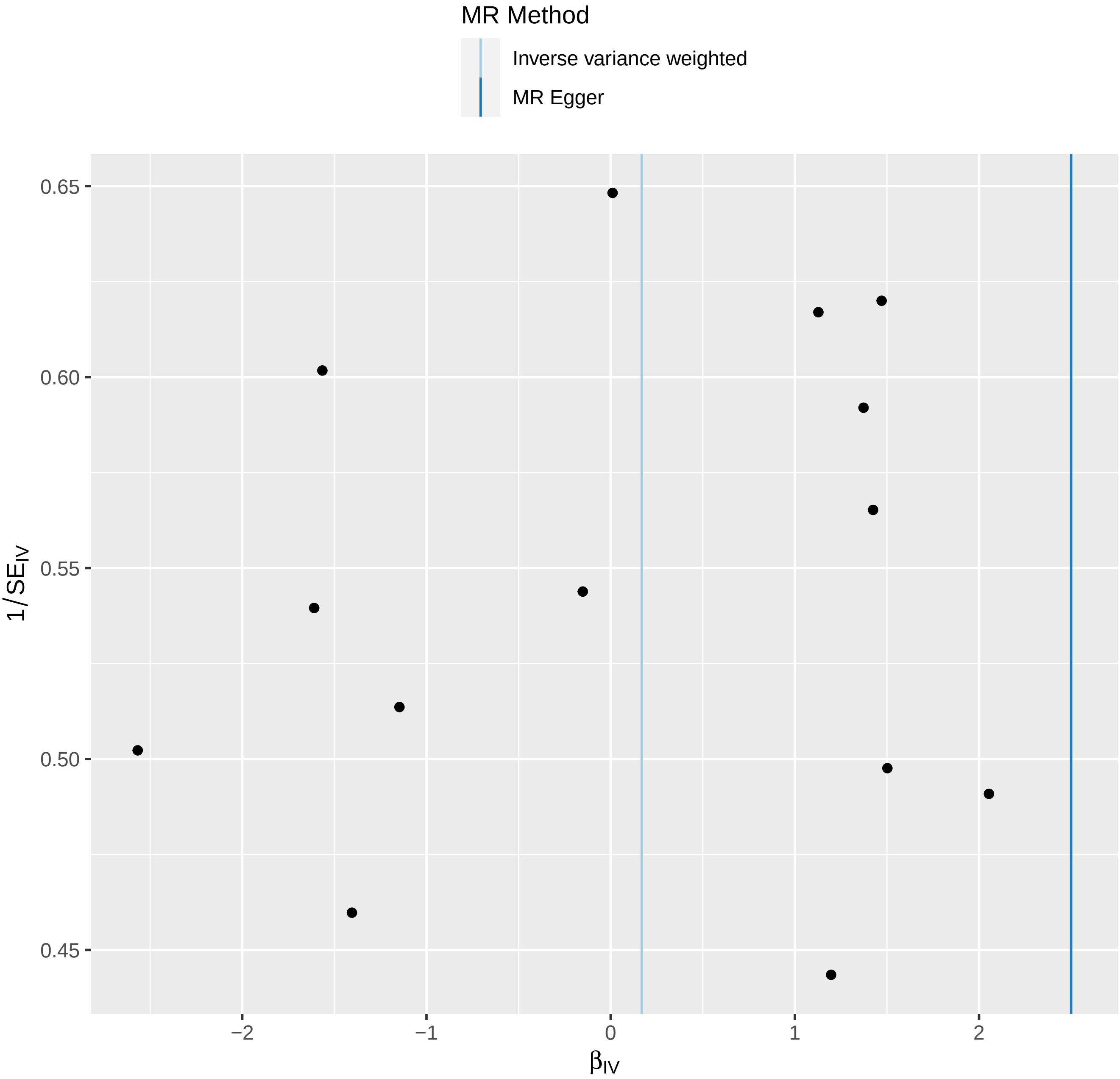

Batch 461 : Gut microbiota abundance (family Defluviitaleaceae id.1924) on Drug−induced obesity

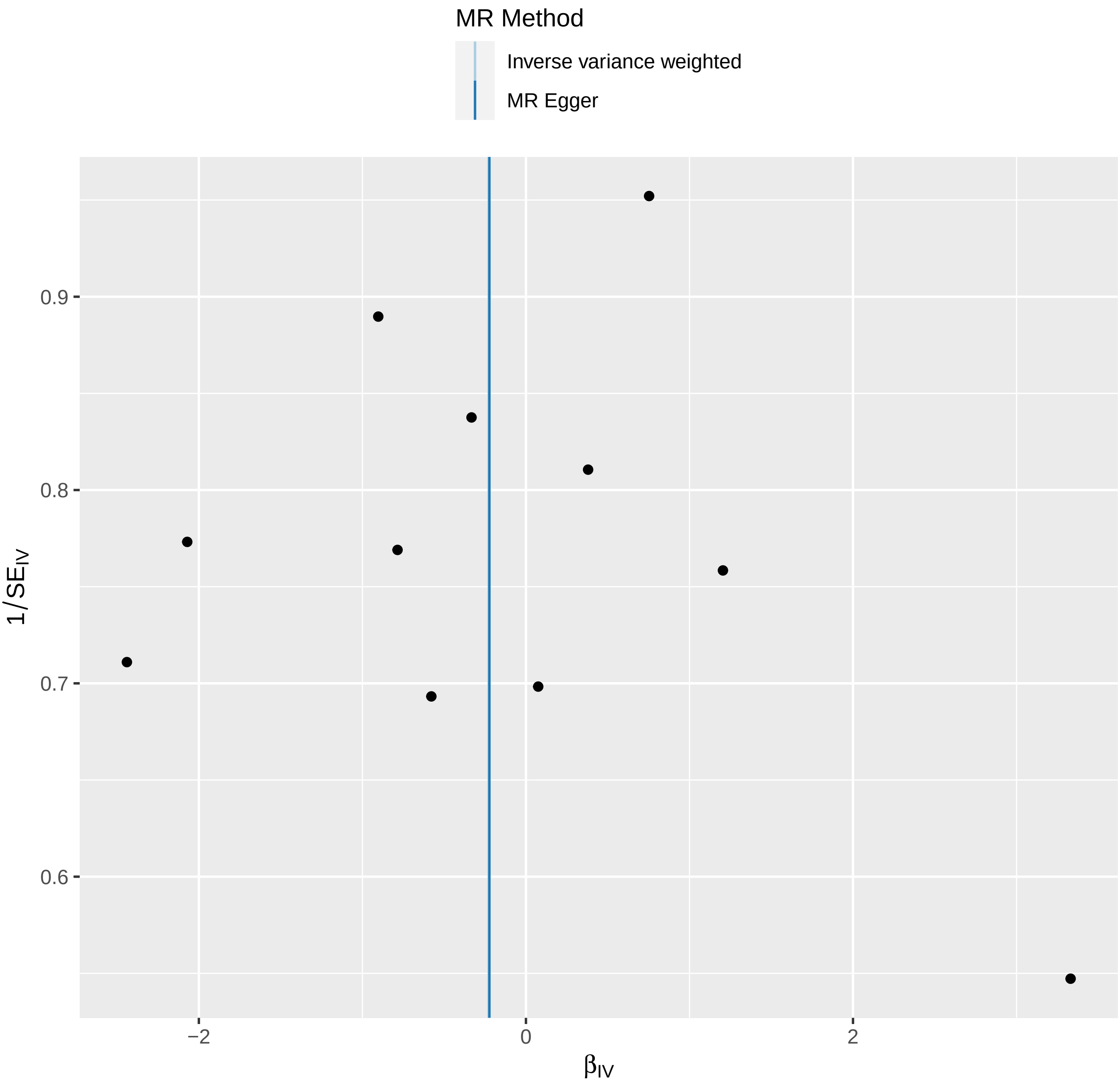

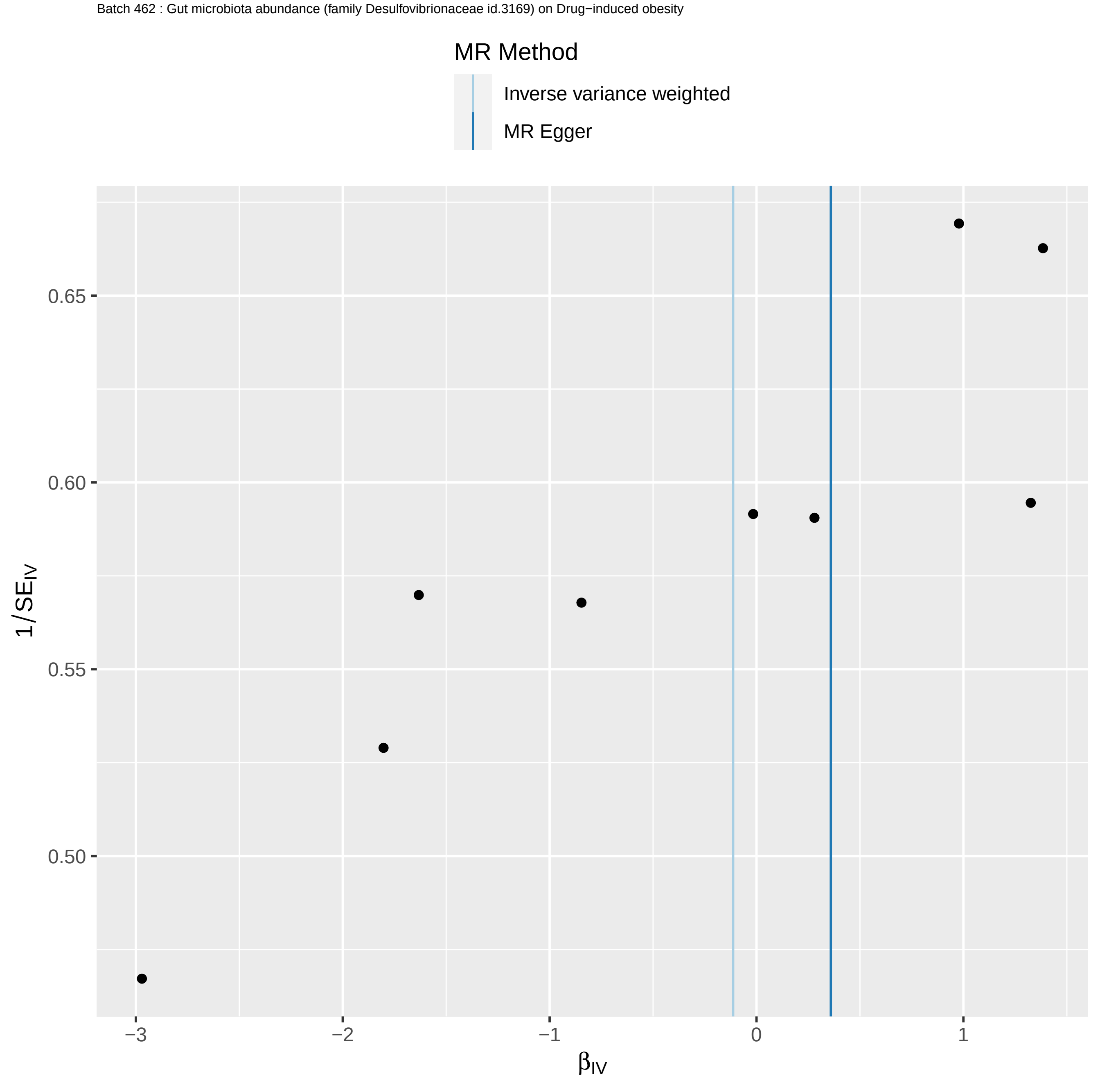

Batch 462 : Gut microbiota abundance (family Desulfovibrionaceae id.3169) on Drug−induced obesity
MR Method
Inverse variance weighted
MR Egger
0.65
0.60
0.55
0.50
1/SE$_{IV}$
−3
−2
−1
0
1
β$_{IV}$

Batch 463 : Gut microbiota abundance (family Enterobacteriaceae id.3469) on Drug−induced obesity

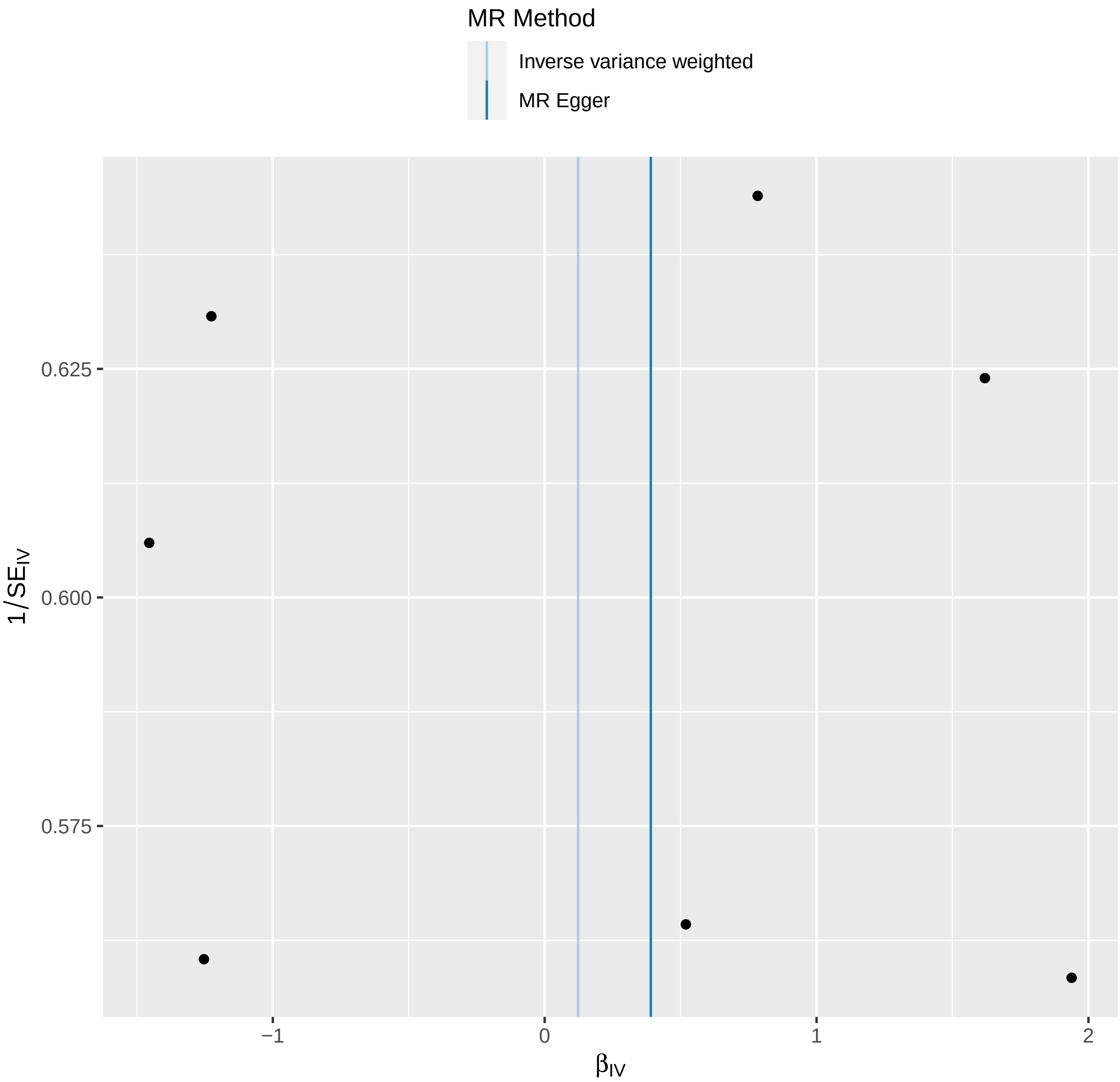

Batch 464 : Gut microbiota abundance (family Erysipelotrichaceae id.2149) on Drug−induced obesity

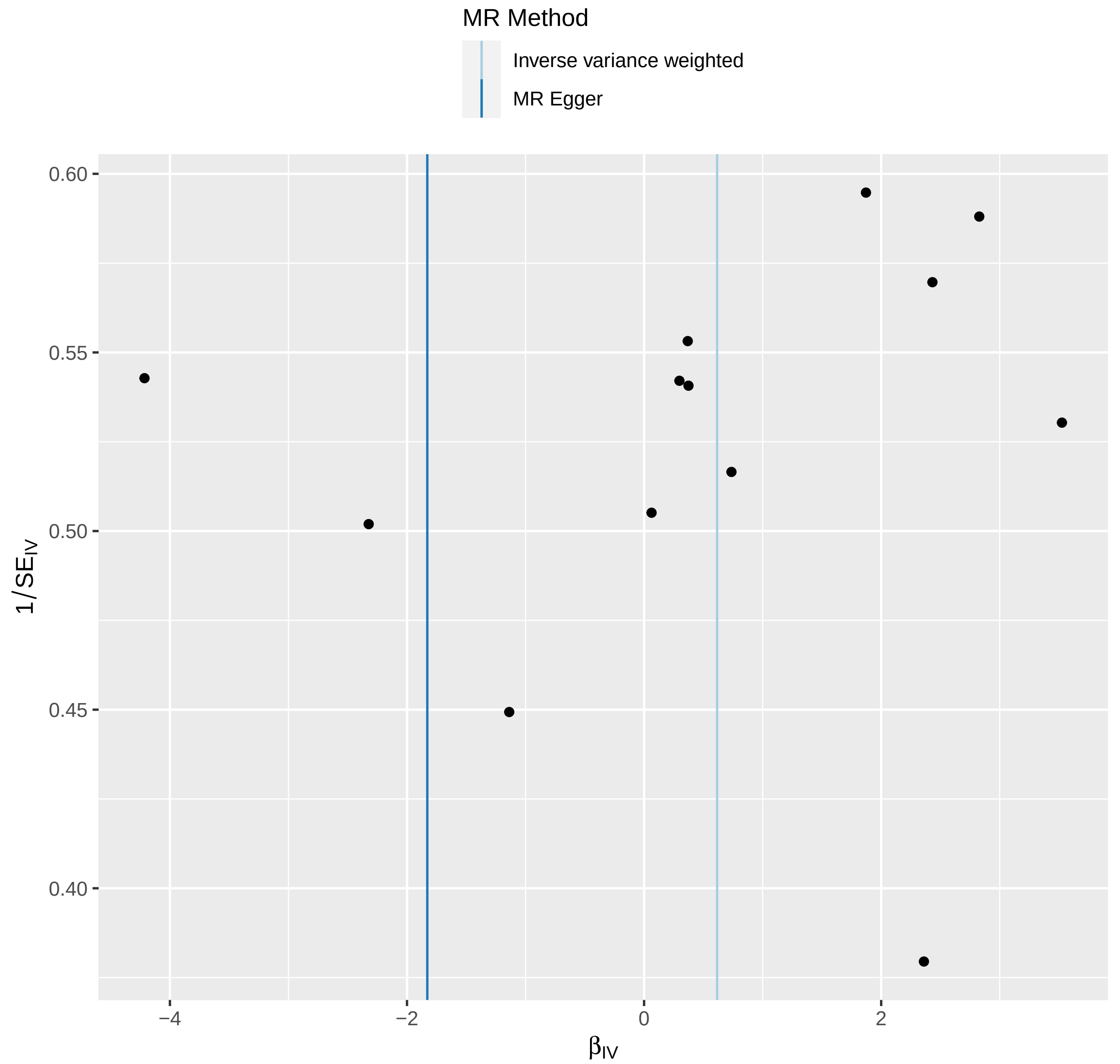

Batch 465 : Gut microbiota abundance (family Family XI id.1936) on Drug−induced obesity

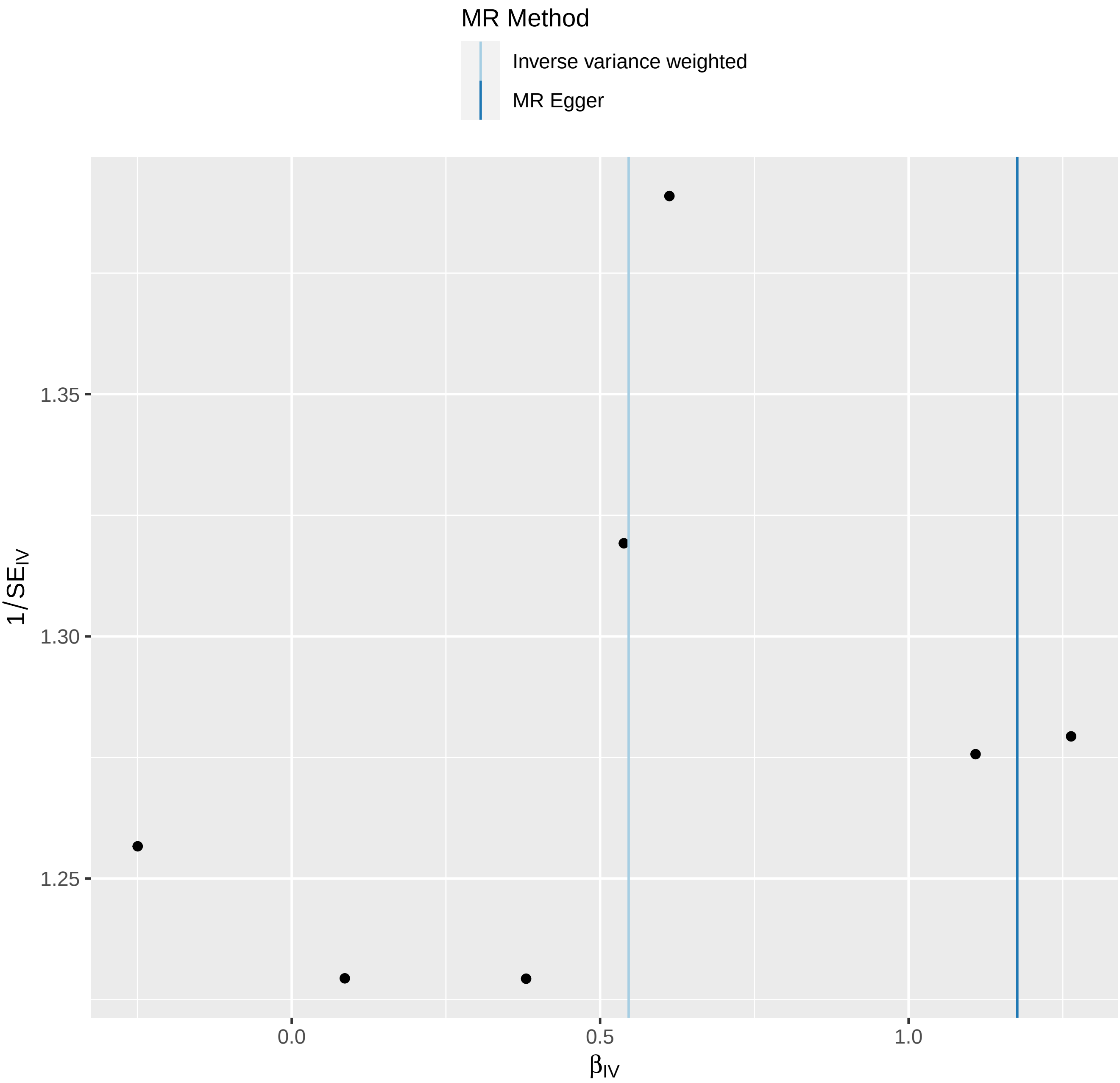

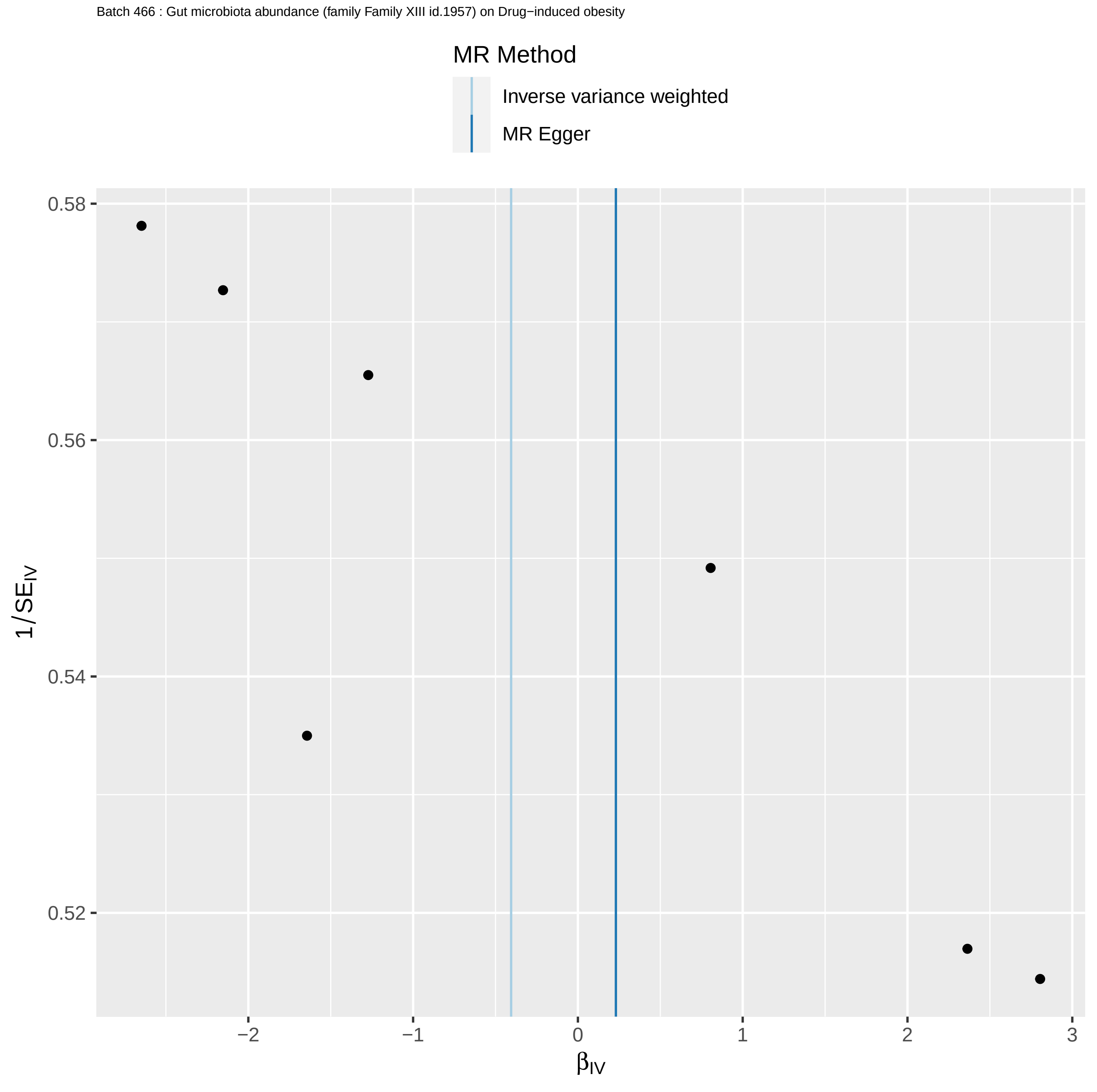
Batch 466 : Gut microbiota abundance (family Family XIII id.1957) on Drug−induced obesity
MR Method
Inverse variance weighted
MR Egger
0.58
0.56
0.54
0.52
1/SE_IV
−2
−1
0
1
2
3
β_IV

Batch 467 : Gut microbiota abundance (family Lachnospiraceae id.1987) on Drug−induced obesity

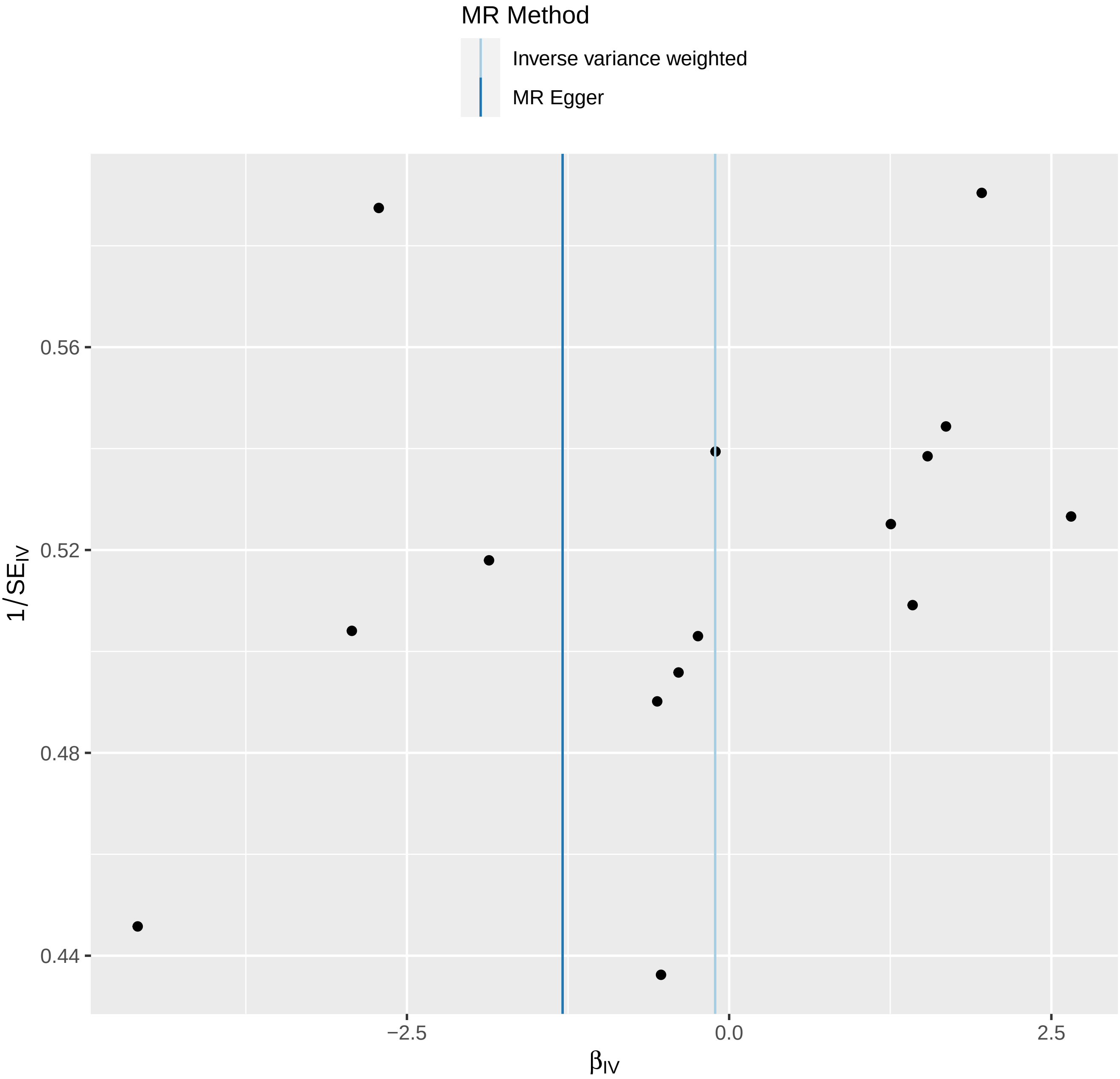

Batch 468 : Gut microbiota abundance (family Lactobacillaceae id.1836) on Drug−induced obesity

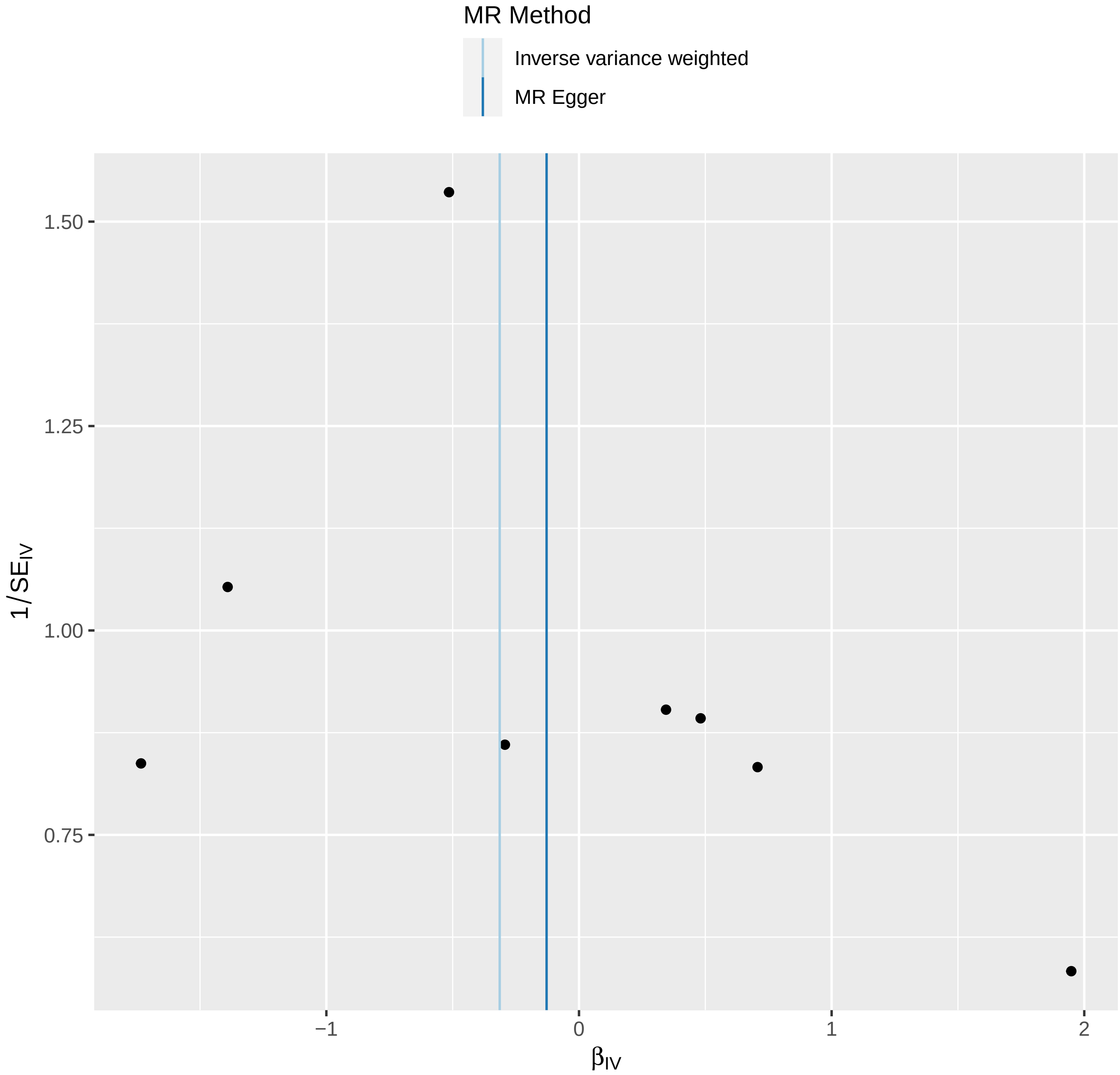

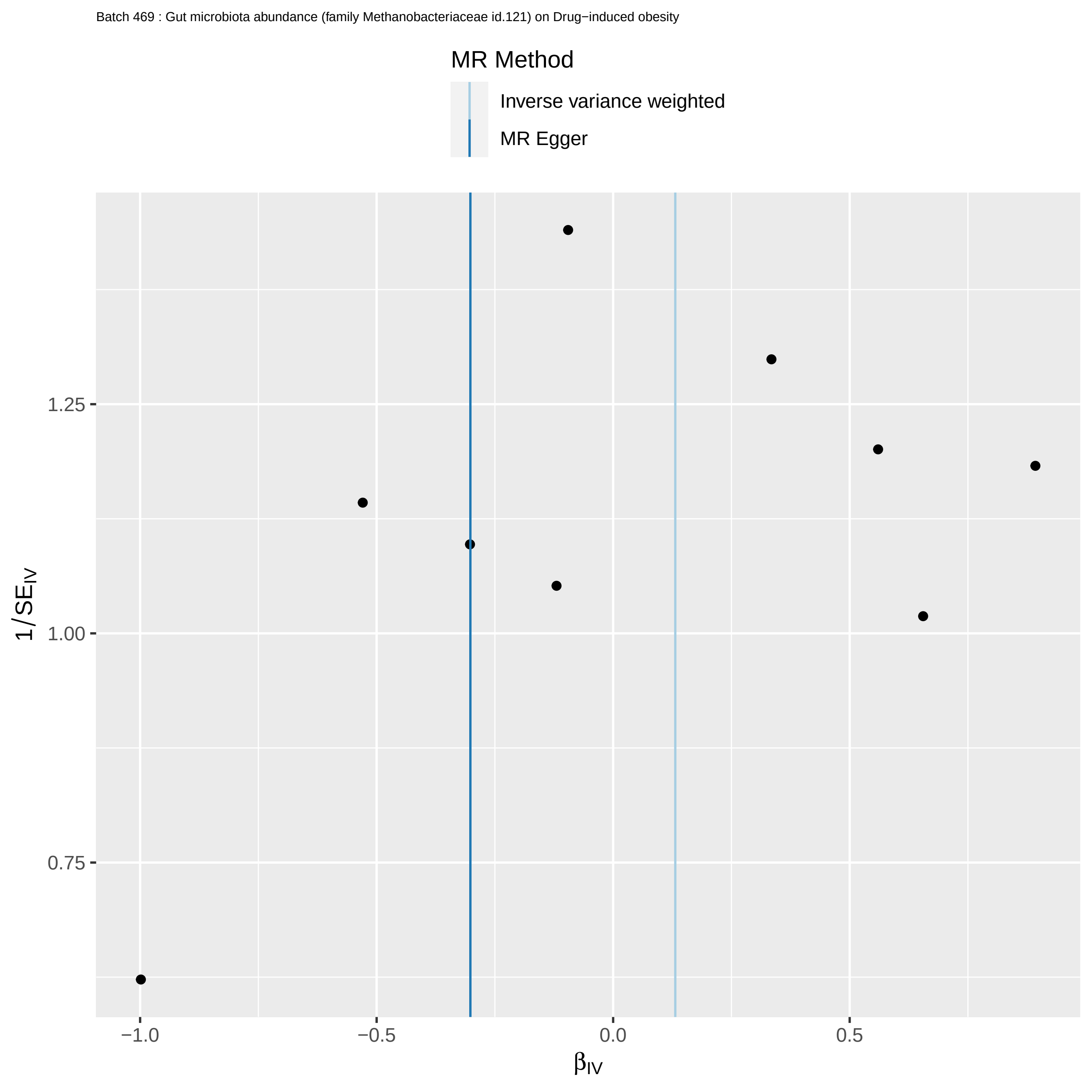

Batch 469 : Gut microbiota abundance (family Methanobacteriaceae id.121) on Drug−induced obesity
MR Method
Inverse variance weighted
MR Egger
1.25
1.00
0.75
1/SE_IV
−1.0
−0.5
0.0
0.5
β_IV

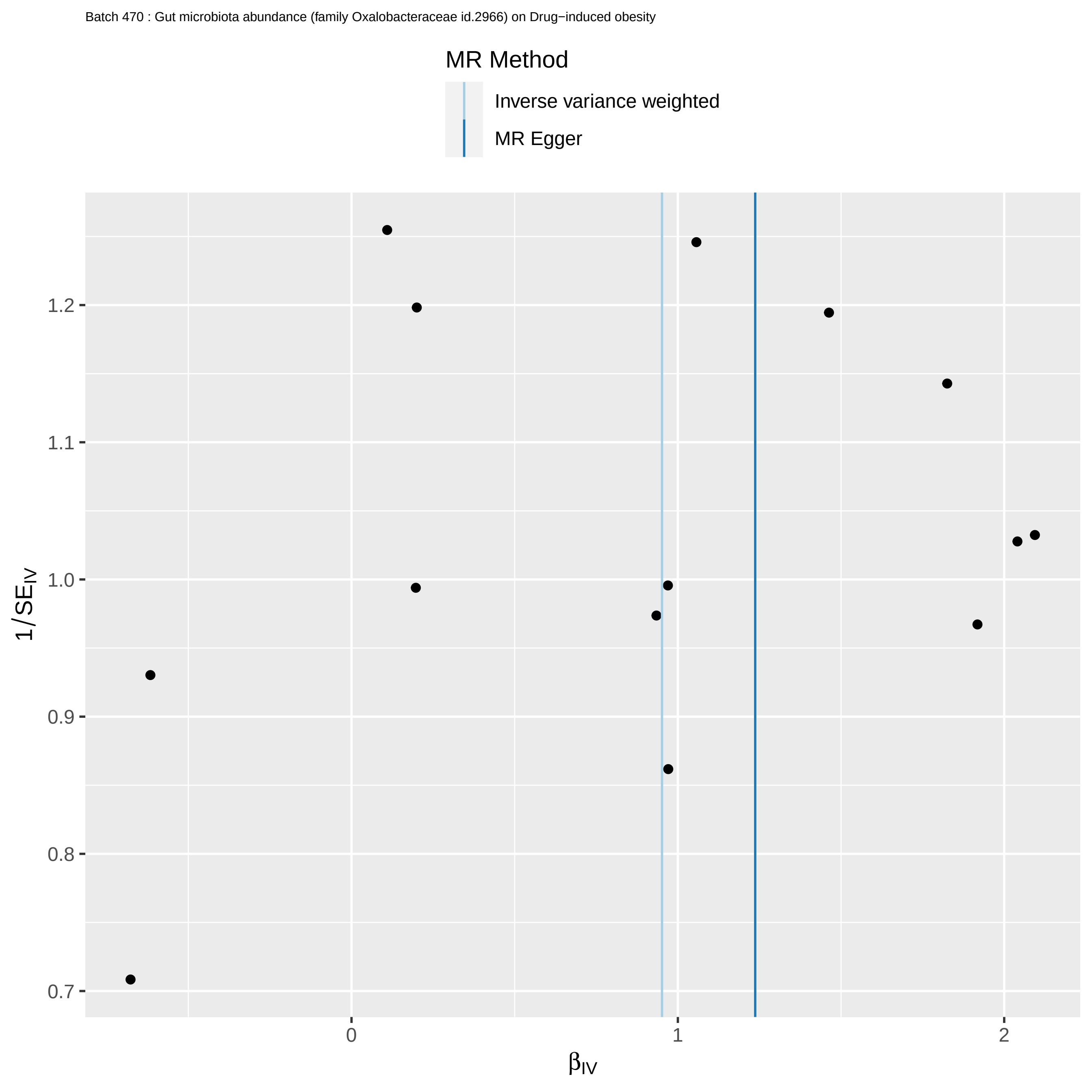
Batch 470 : Gut microbiota abundance (family Oxalobacteraceae id.2966) on Drug−induced obesity
MR Method
Inverse variance weighted
MR Egger
1.2
1.1
1.0
0.9
0.8
0.7
1/SE$_{IV}$
0
1
2
$\beta_{IV}$

Batch 471 : Gut microbiota abundance (family Pasteurellaceae id.3689) on Drug−induced obesity

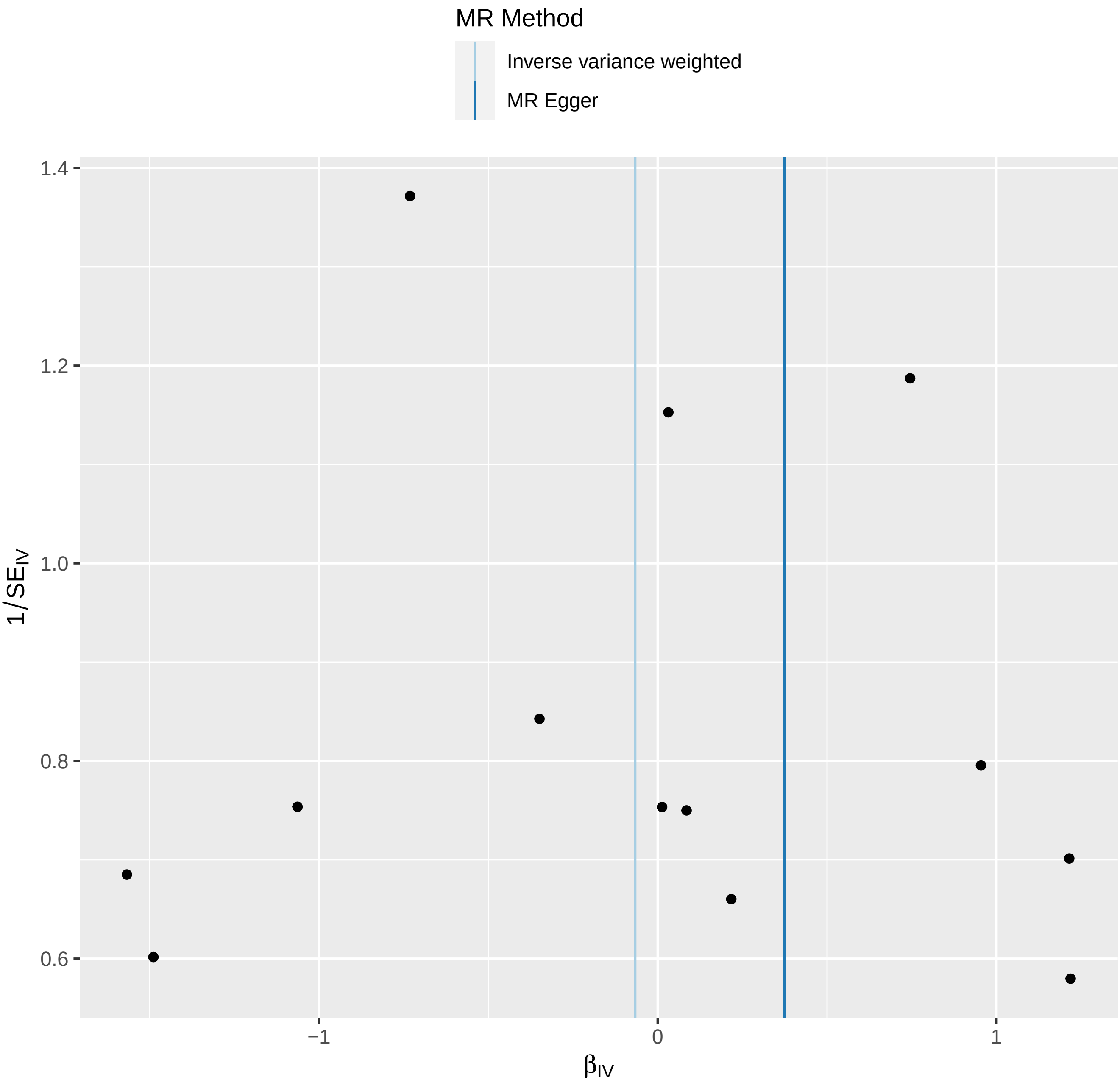

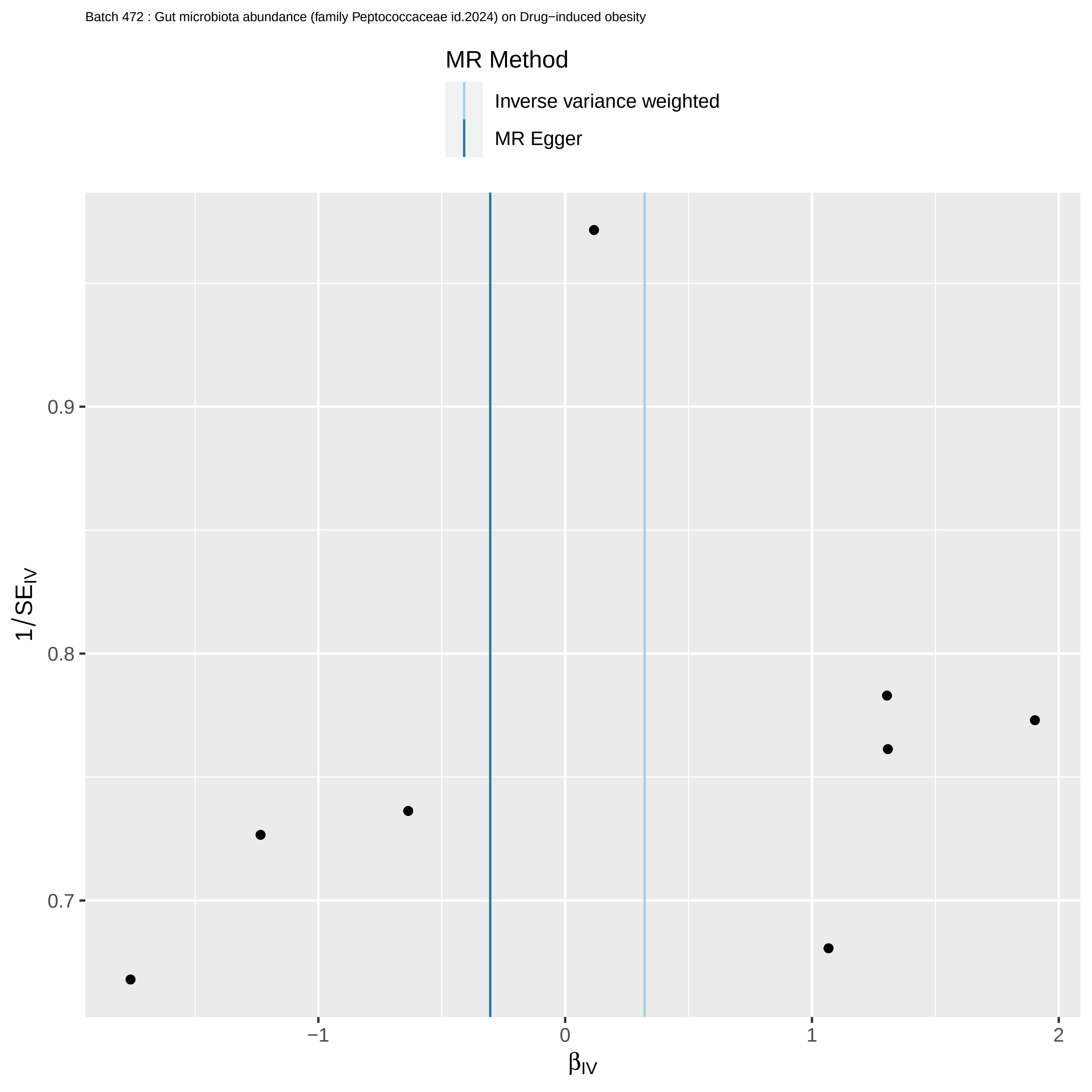

Batch 472 : Gut microbiota abundance (family Peptococcaceae id.2024) on Drug−induced obesity
MR Method
Inverse variance weighted
MR Egger
0.9
0.8
0.7
1/SE_IV
−1
0
1
2
β_IV

Batch 473 : Gut microbiota abundance (family Peptostreptococcaceae id.2042) on Drug−induced obesity

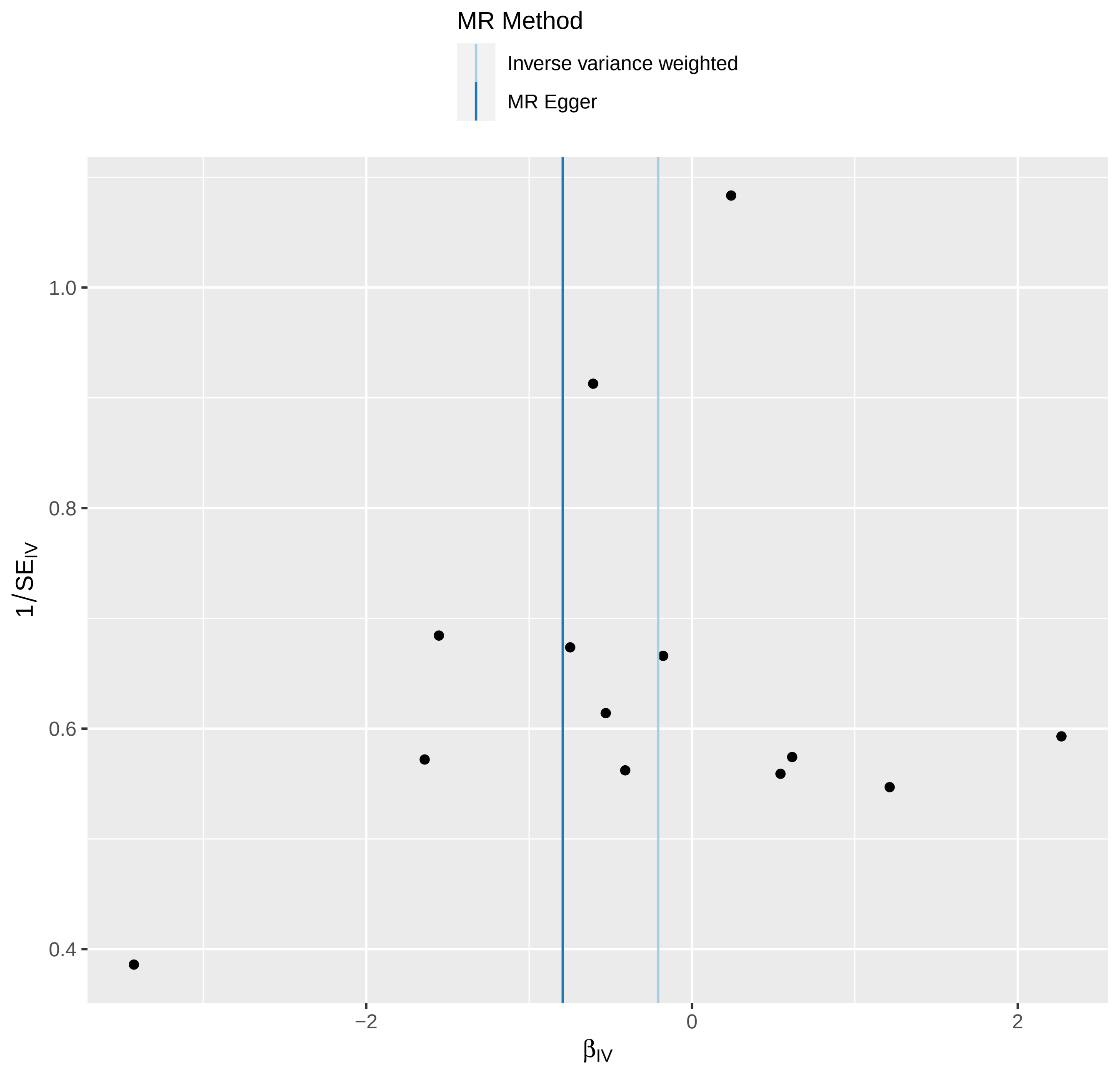

Batch 474 : Gut microbiota abundance (family Porphyromonadaceae id.943) on Drug−induced obesity

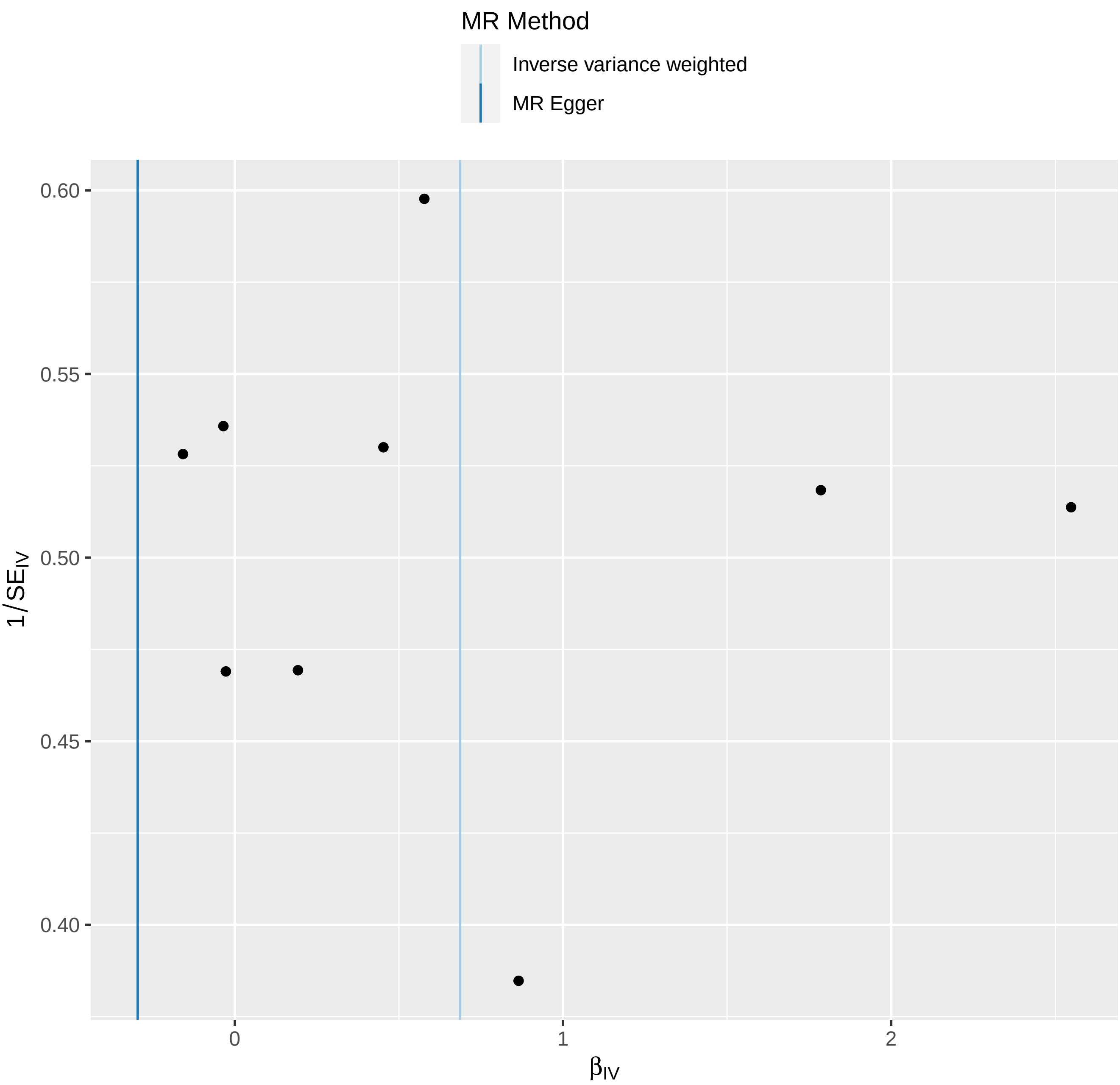

Batch 475 : Gut microbiota abundance (family Prevotellaceae id.960) on Drug−induced obesity

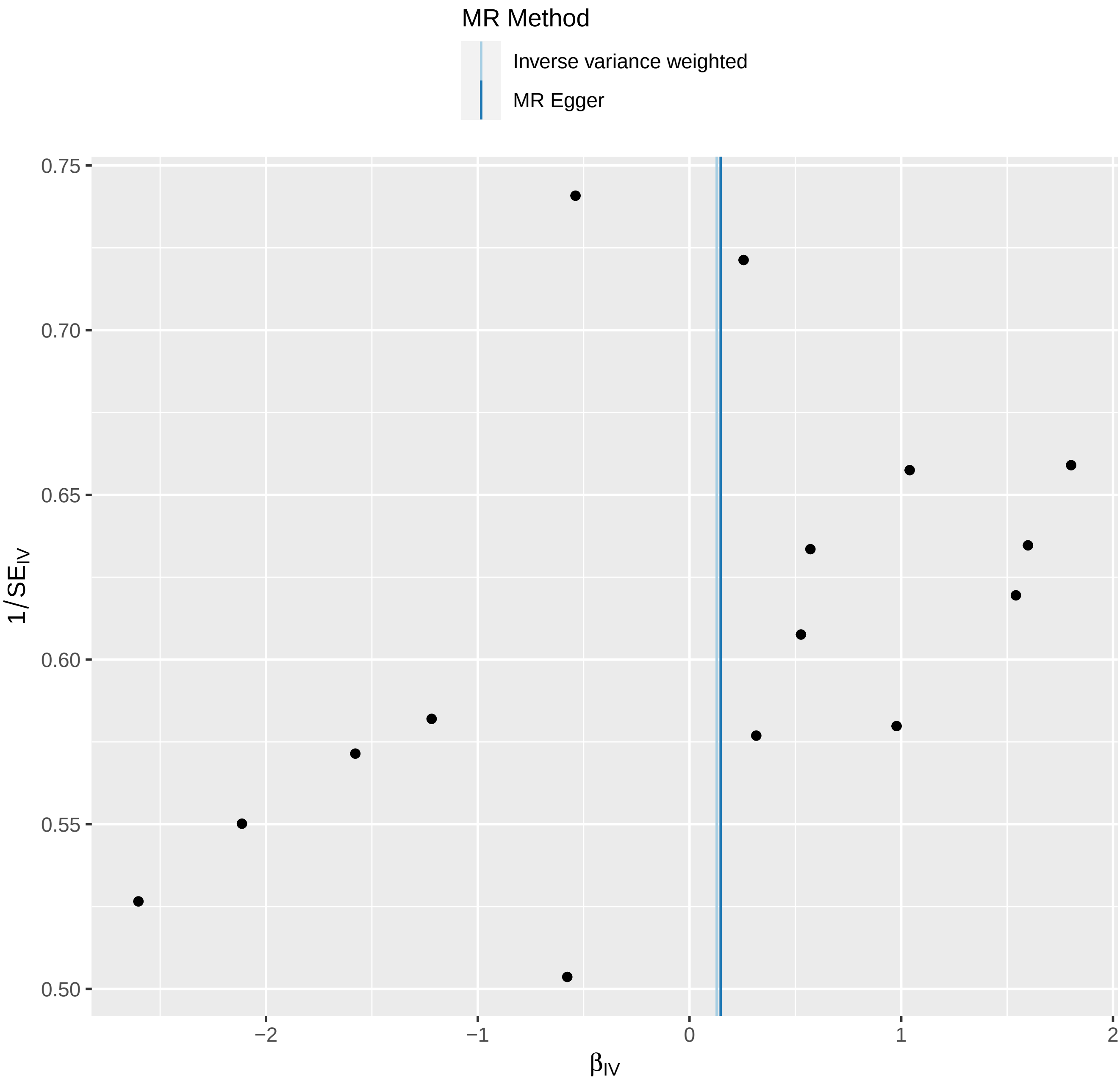

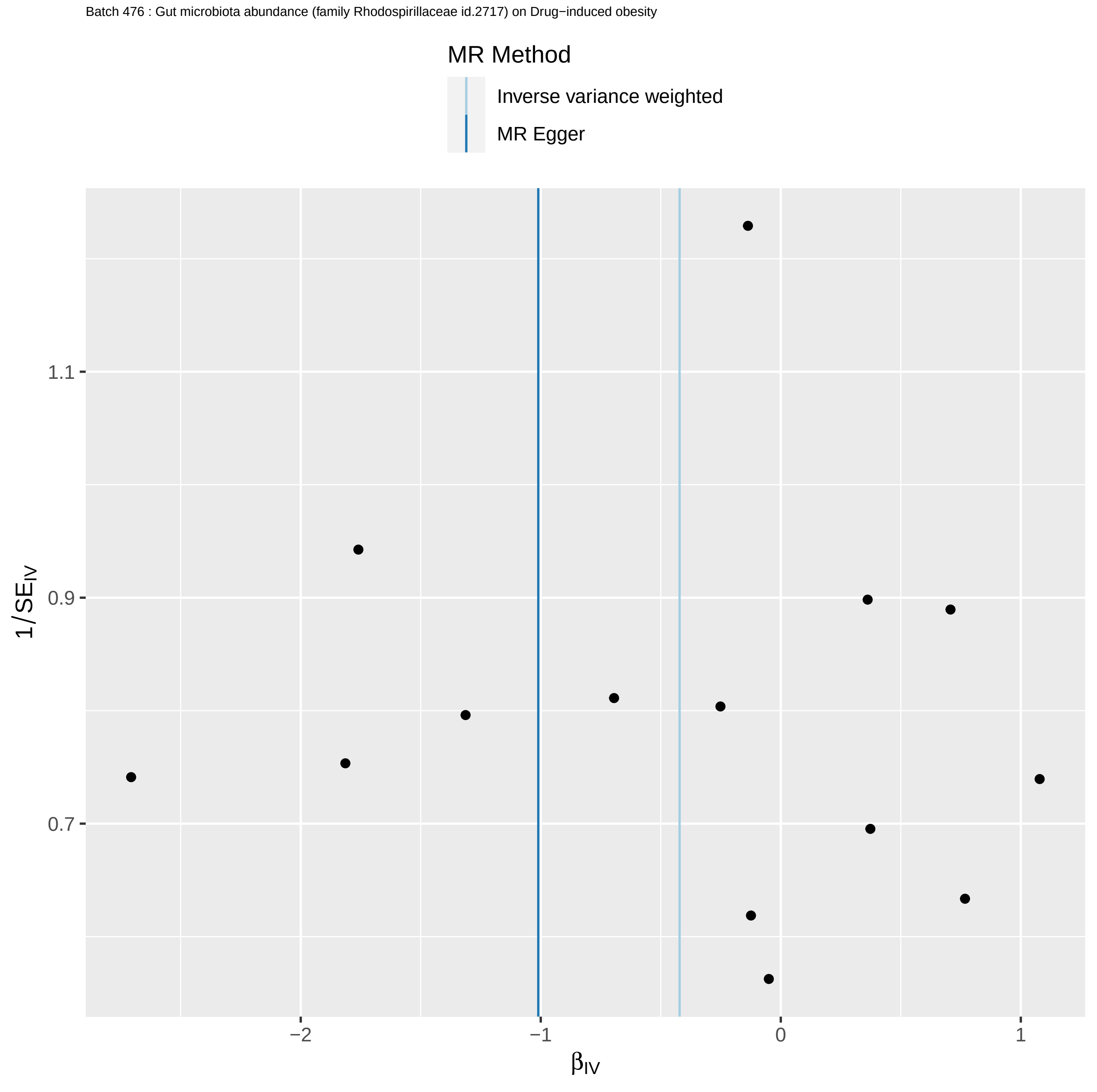

Batch 476 : Gut microbiota abundance (family Rhodospirillaceae id.2717) on Drug−induced obesity
MR Method
Inverse variance weighted
MR Egger
1.1
0.9
0.7
1/SE$_{IV}$
−2
−1
0
1
$\beta_{IV}$

Batch 477 : Gut microbiota abundance (family Rikenellaceae id.967) on Drug−induced obesity

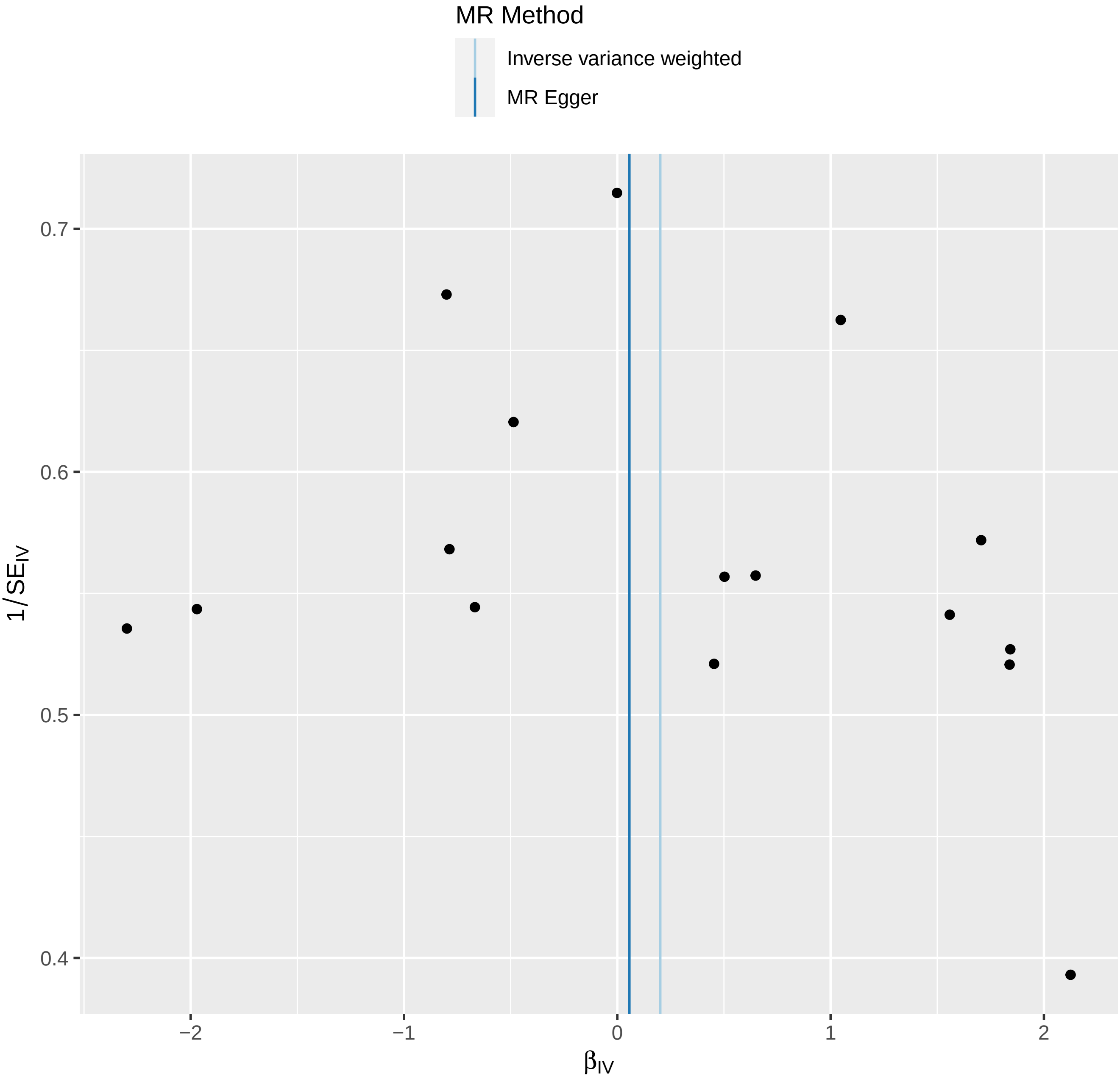

Batch 478 : Gut microbiota abundance (family Ruminococcaceae id.2050) on Drug−induced obesity

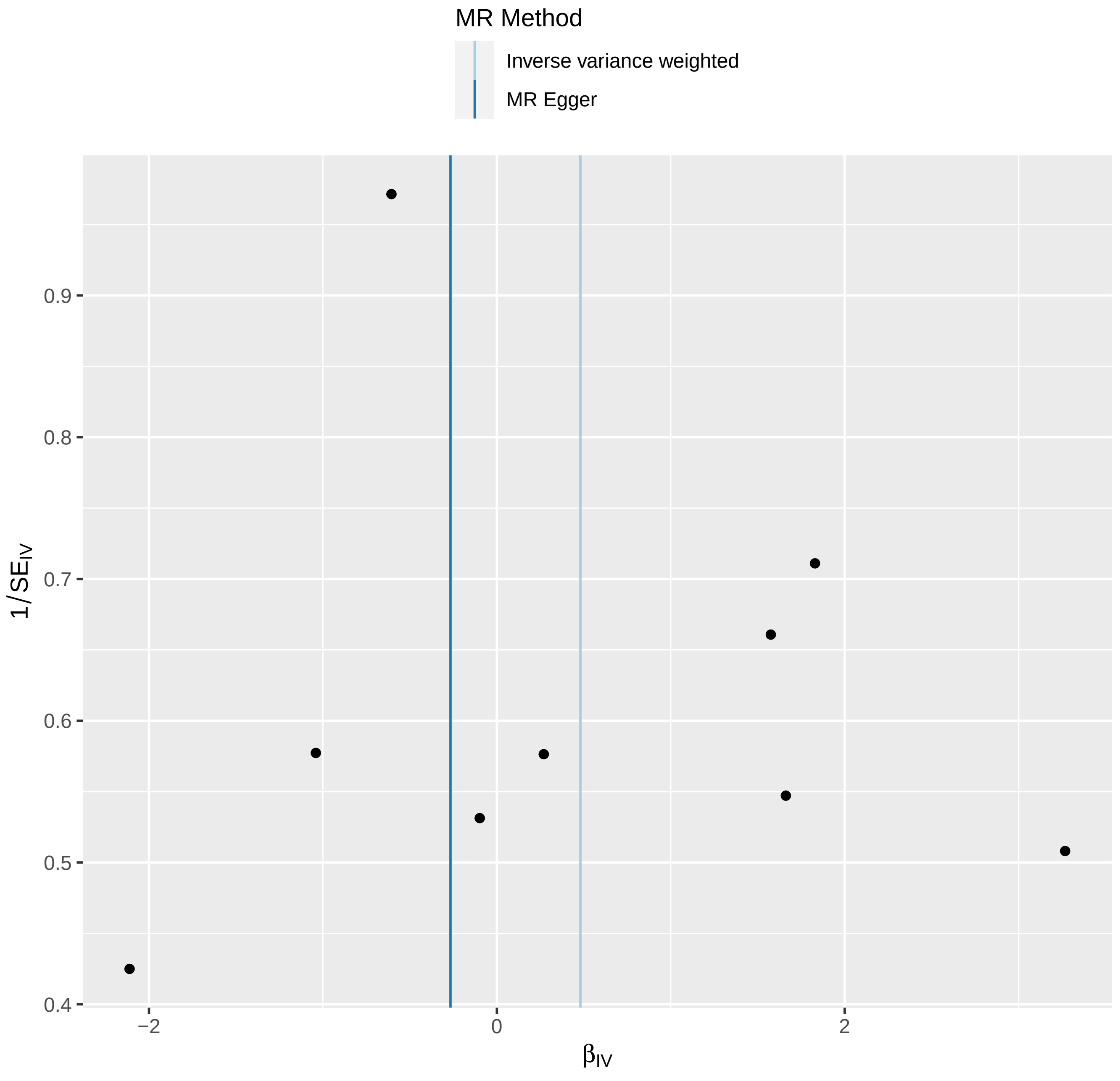

Batch 479 : Gut microbiota abundance (family Streptococcaceae id.1850) on Drug−induced obesity

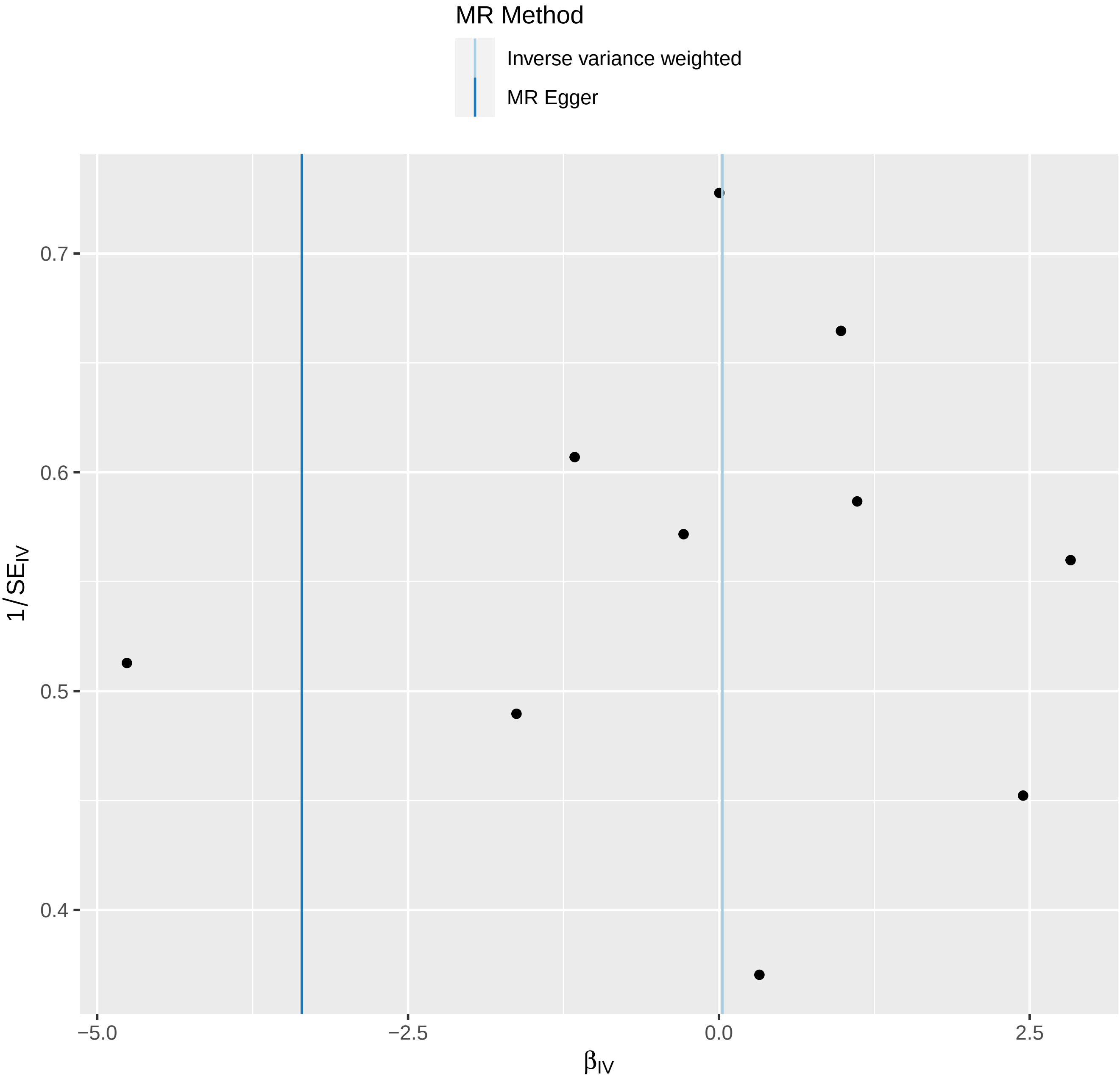

Batch 480 : Gut microbiota abundance (family Veillonellaceae id.2172) on Drug−induced obesity

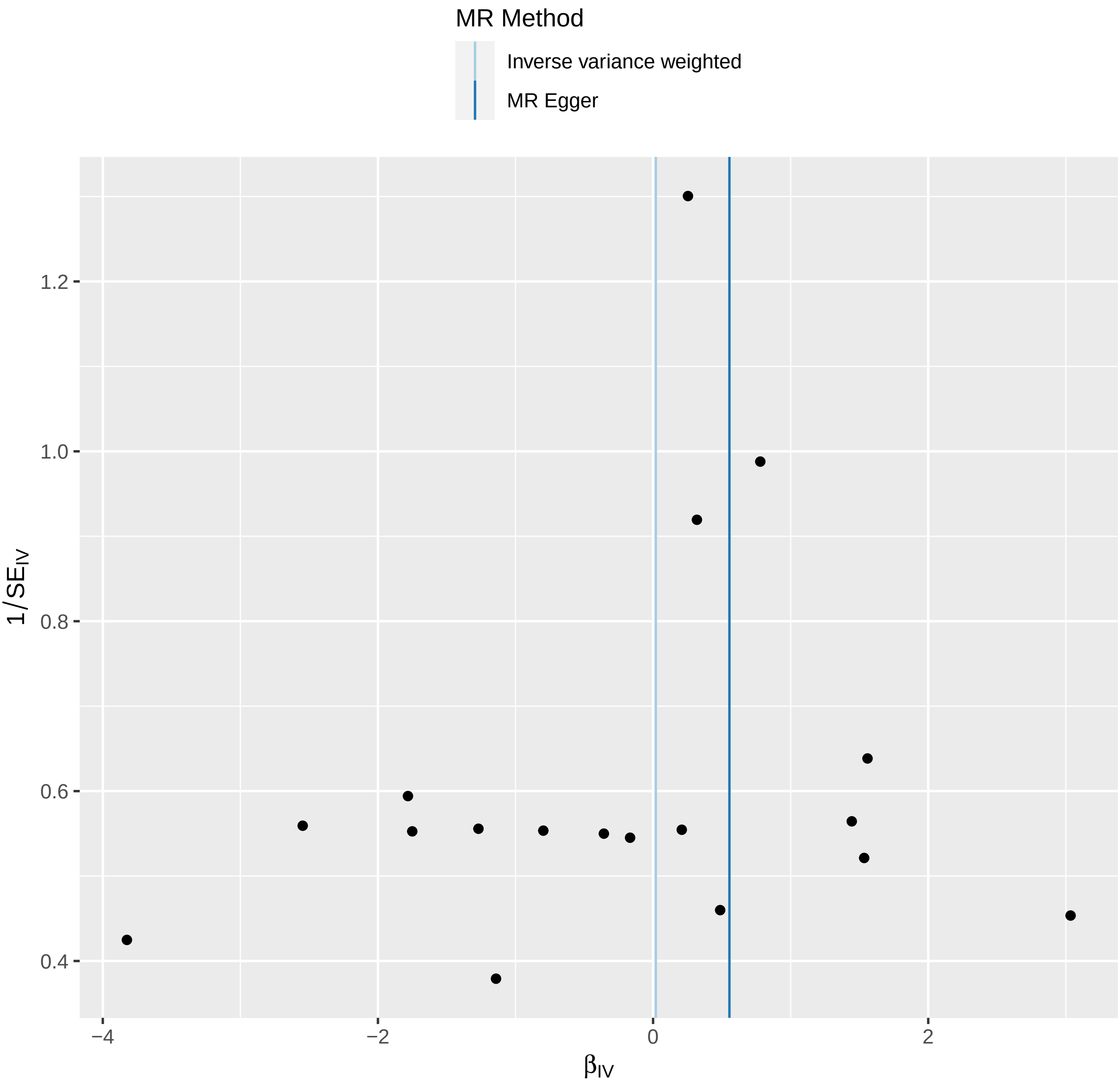

Batch 481 : Gut microbiota abundance (family Verrucomicrobiaceae id.4036) on Drug−induced obesity

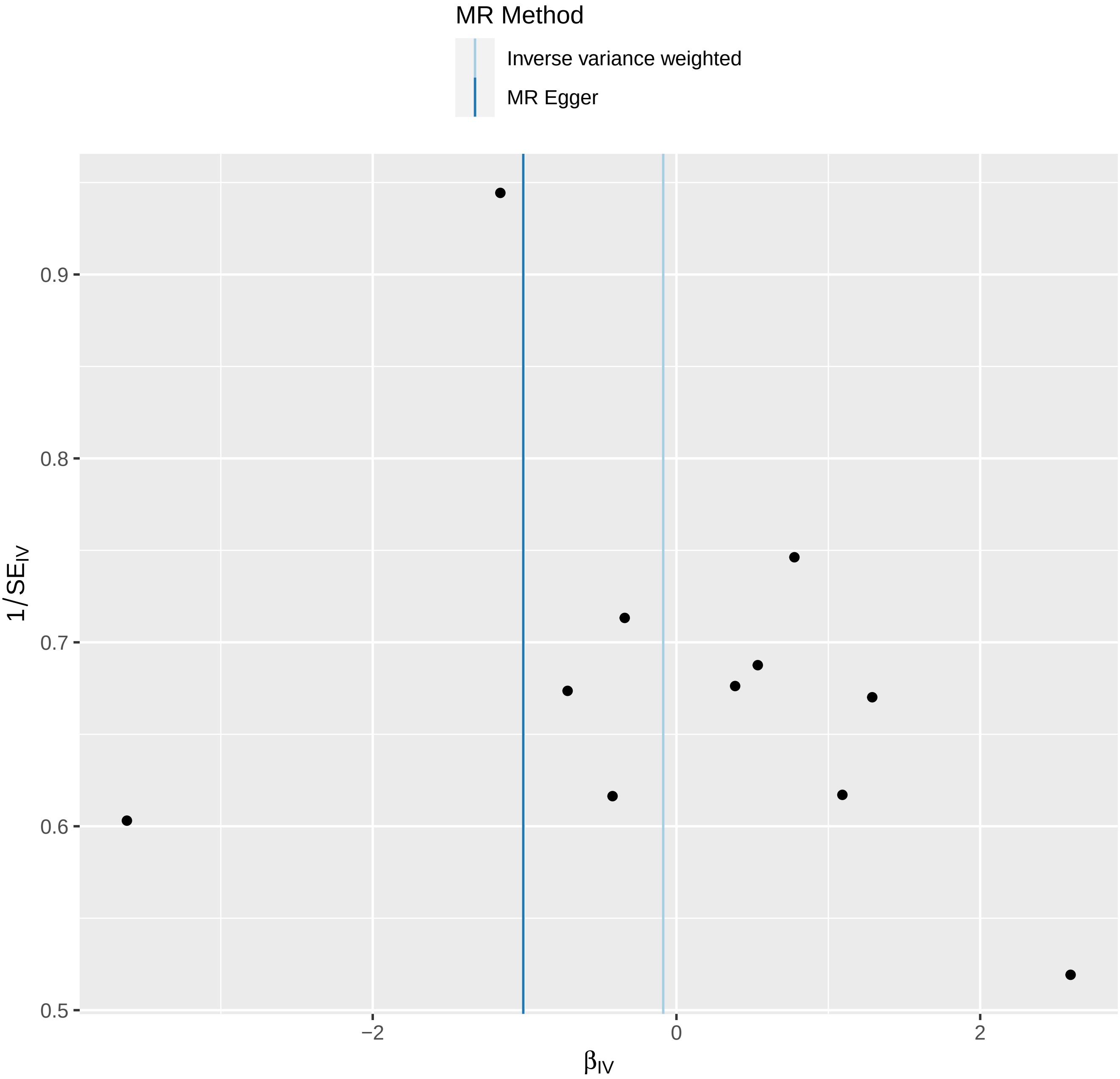

Batch 482 : Gut microbiota abundance (family Victivallaceae id.2255) on Drug−induced obesity

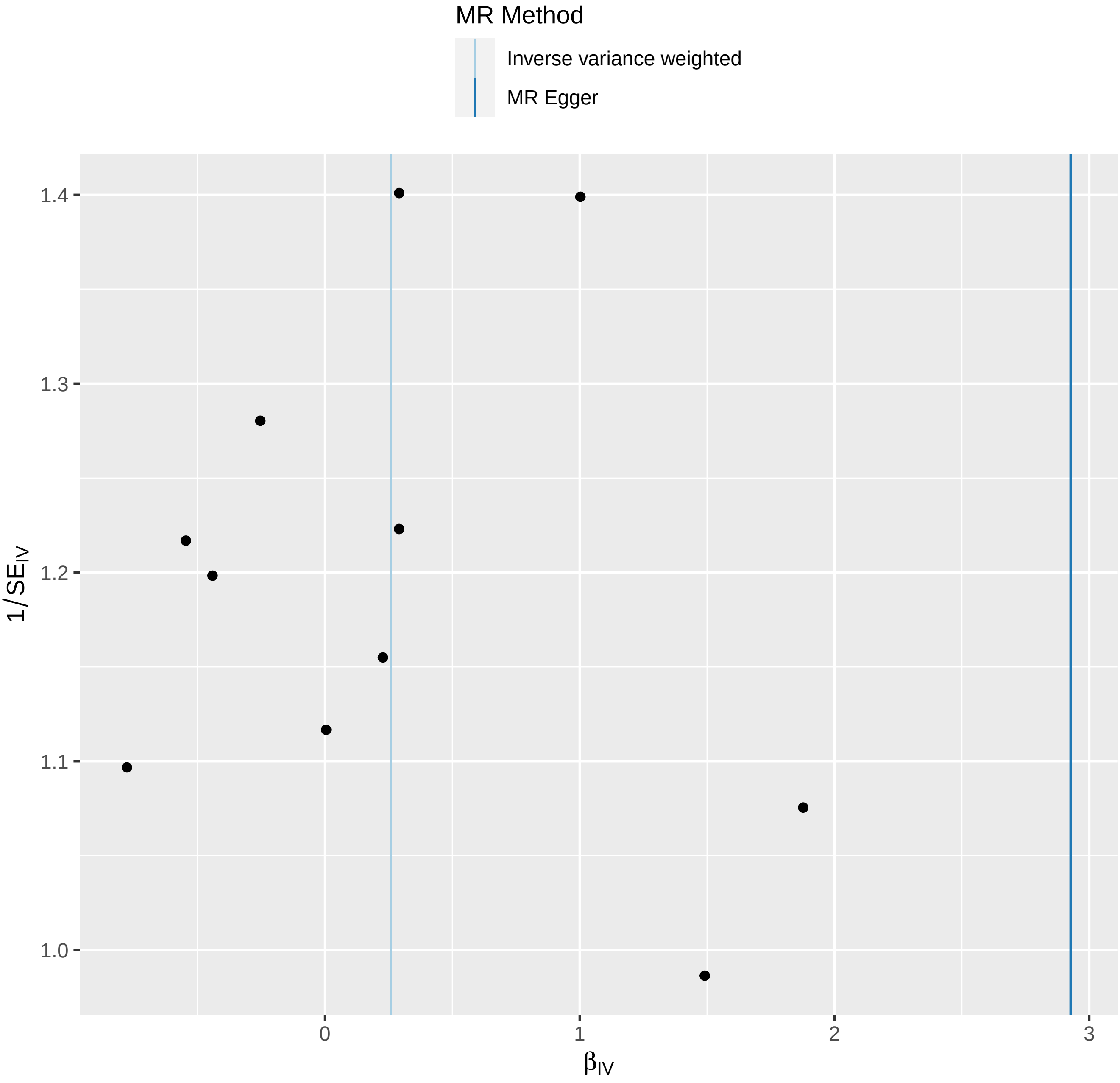

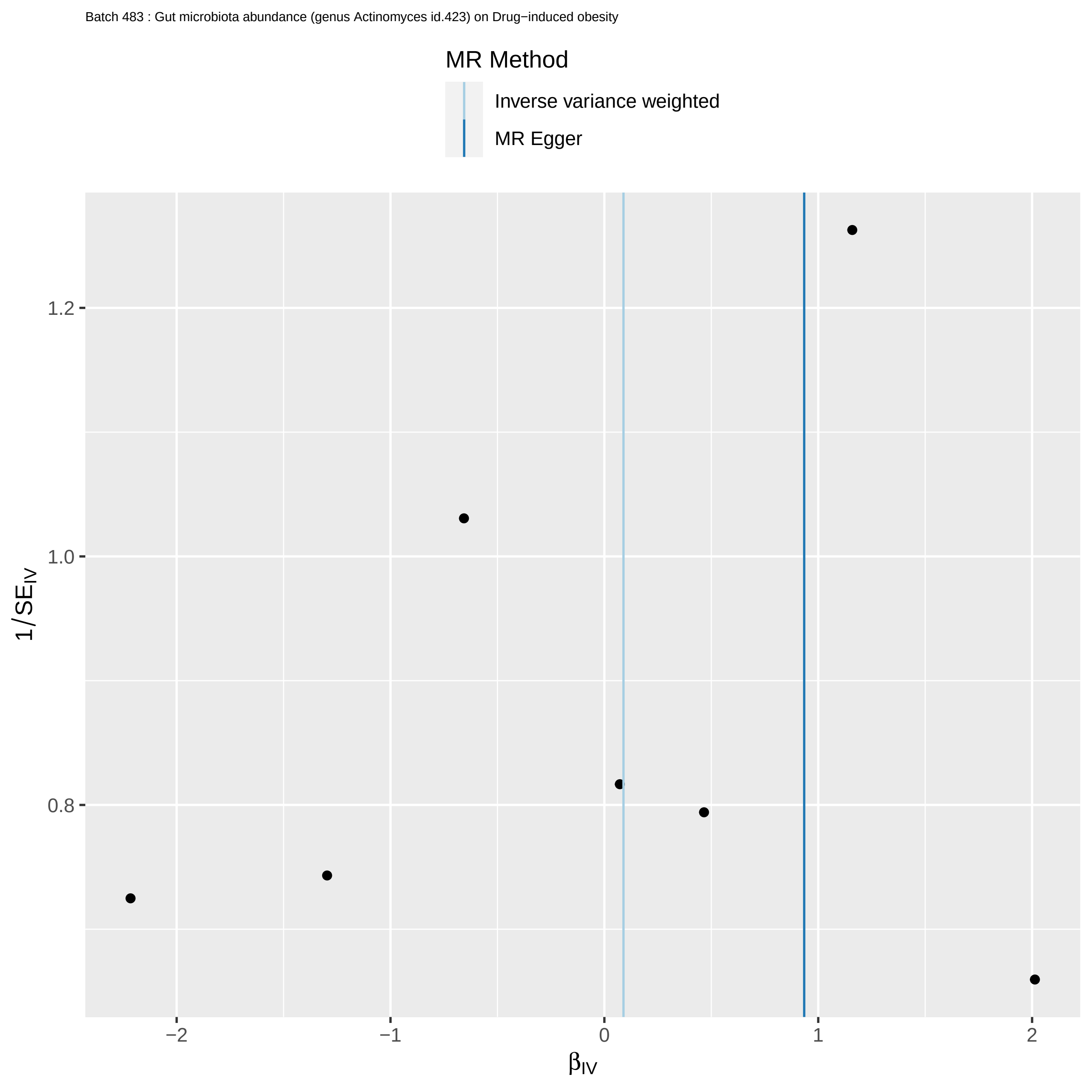
Batch 483 : Gut microbiota abundance (genus Actinomyces id.423) on Drug−induced obesity
MR Method
Inverse variance weighted
MR Egger
1.2
1.0
0.8
1/SE_IV
−2
−1
0
1
2
β_IV

Batch 484 : Gut microbiota abundance (genus Adlercreutzia id.812) on Drug−induced obesity

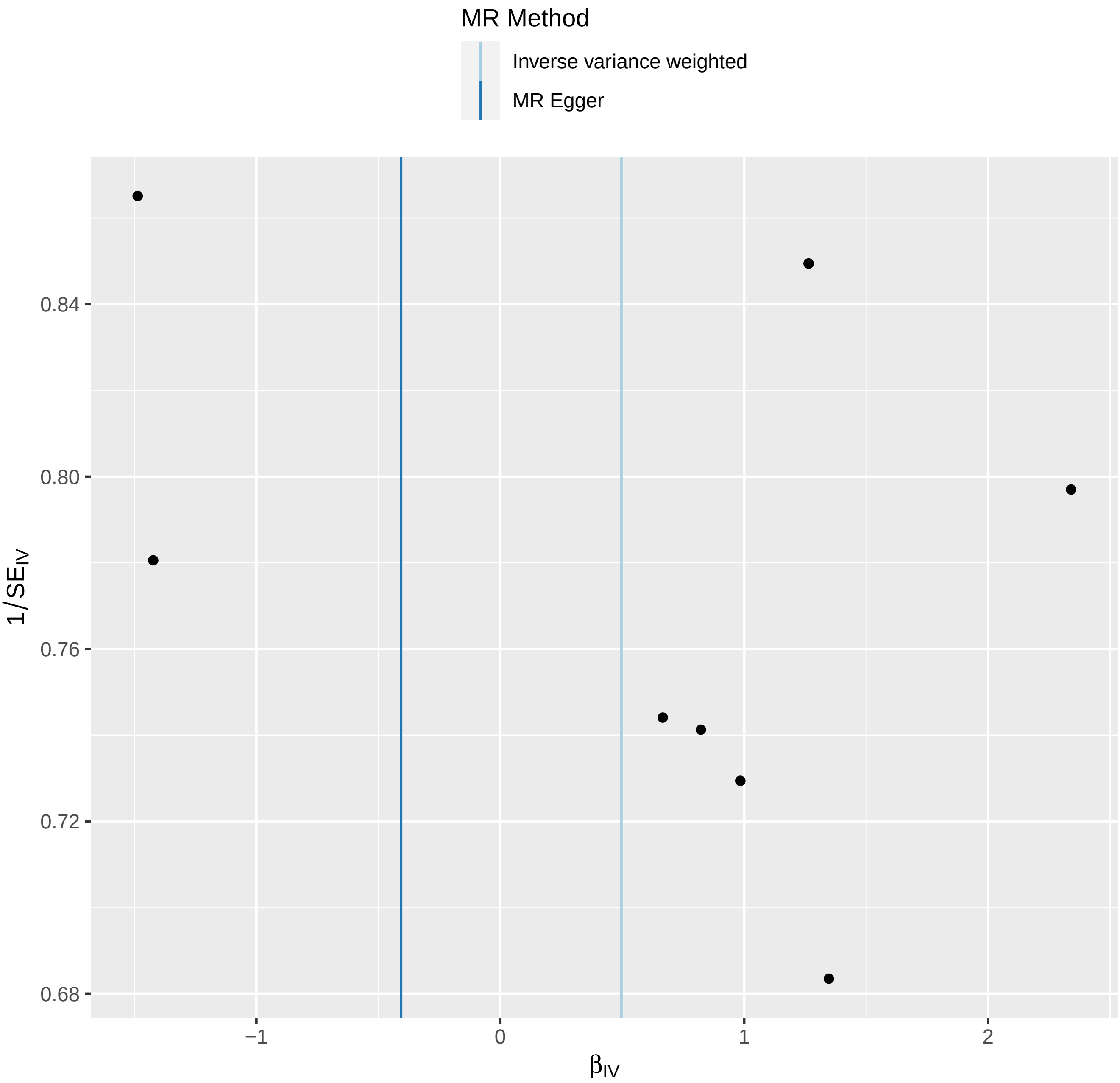

Batch 485 : Gut microbiota abundance (genus Akkermansia id.4037) on Drug−induced obesity

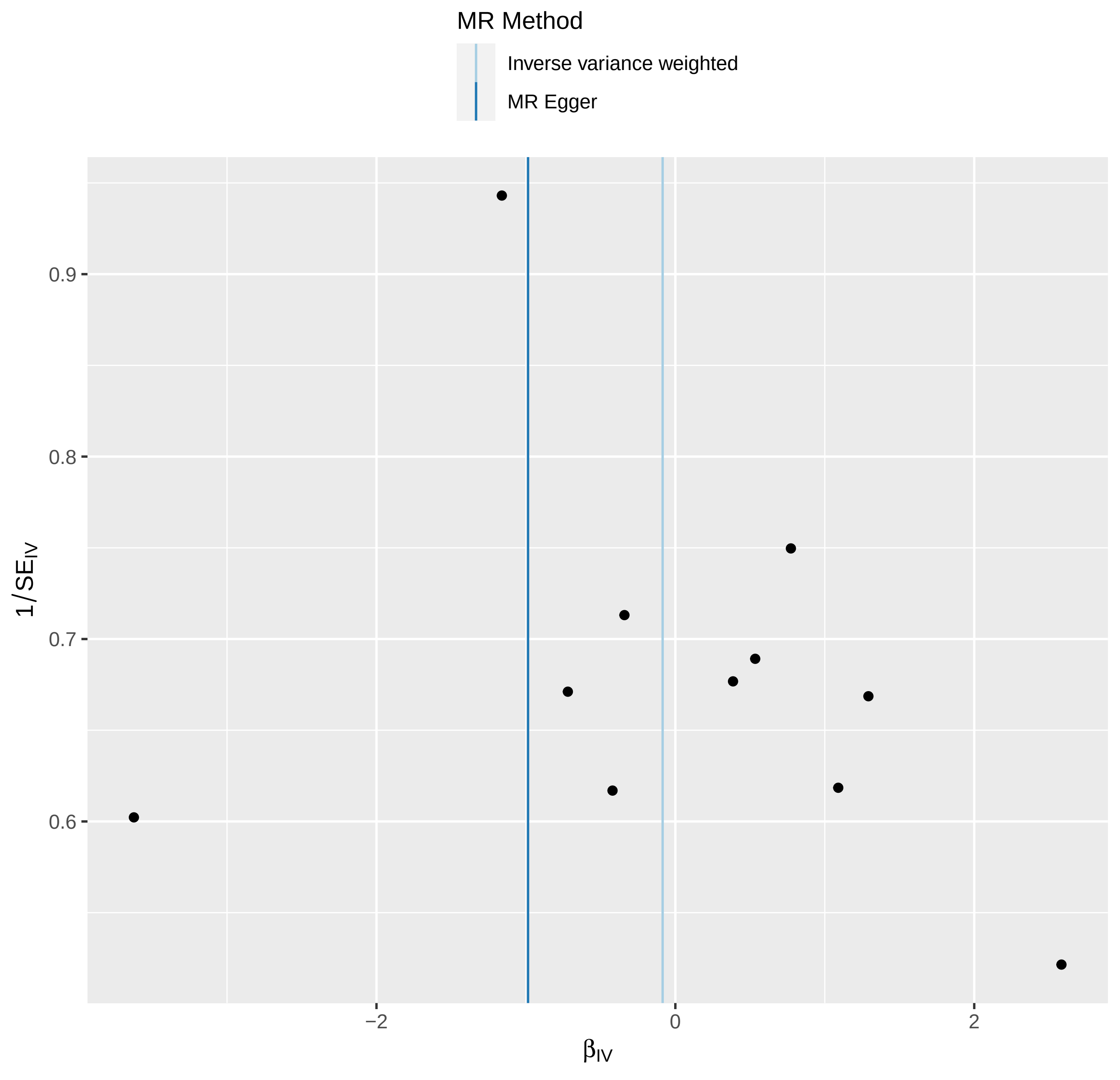

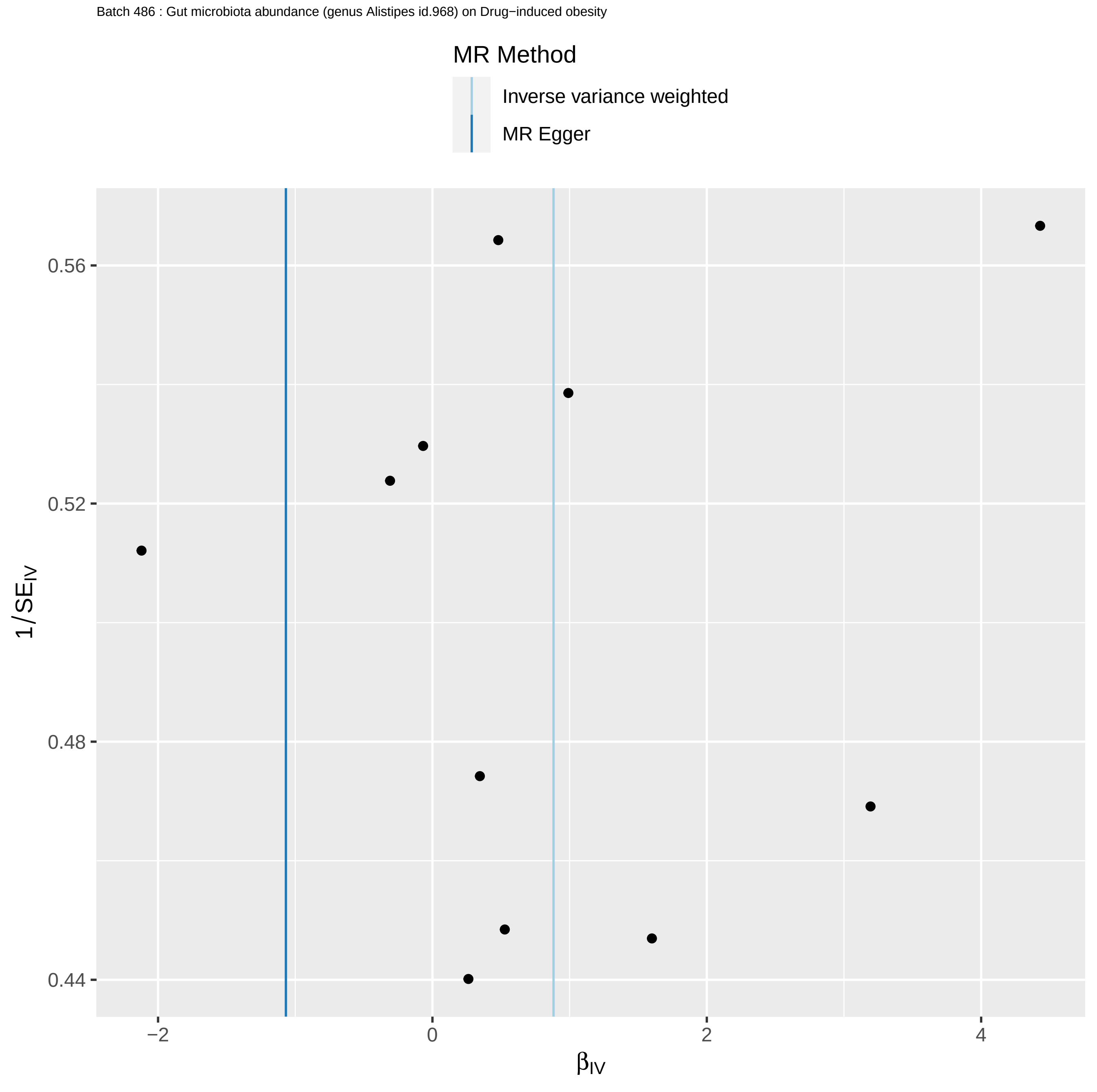

Batch 486 : Gut microbiota abundance (genus Alistipes id.968) on Drug−induced obesity
MR Method
Inverse variance weighted
MR Egger
0.56
0.52
0.48
0.44
1/SE_IV
−2
0
2
4
β_IV

Batch 487 : Gut microbiota abundance (genus Allisonella id.2174) on Drug−induced obesity

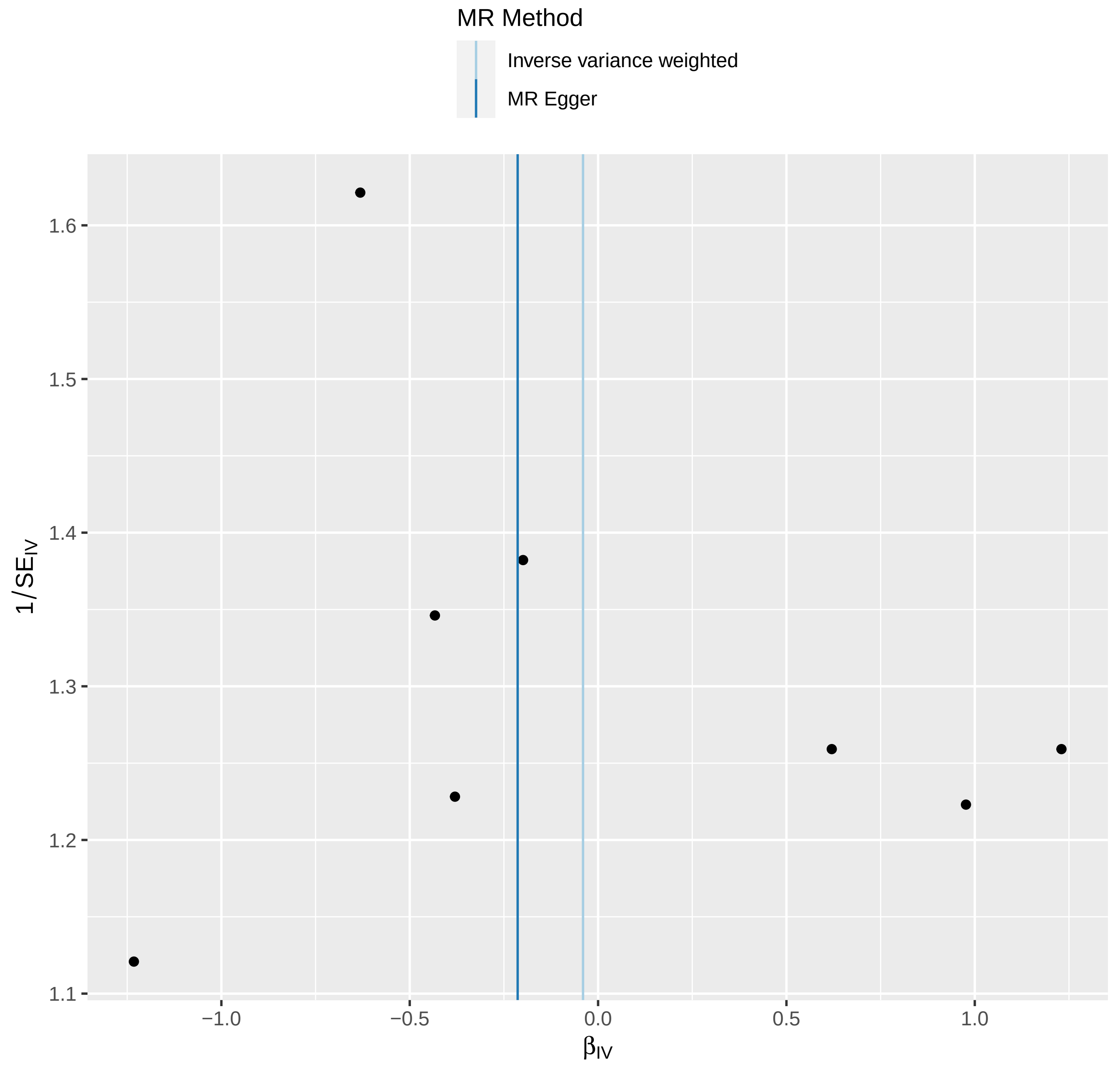

Batch 488 : Gut microbiota abundance (genus Alloprevotella id.961) on Drug−induced obesity

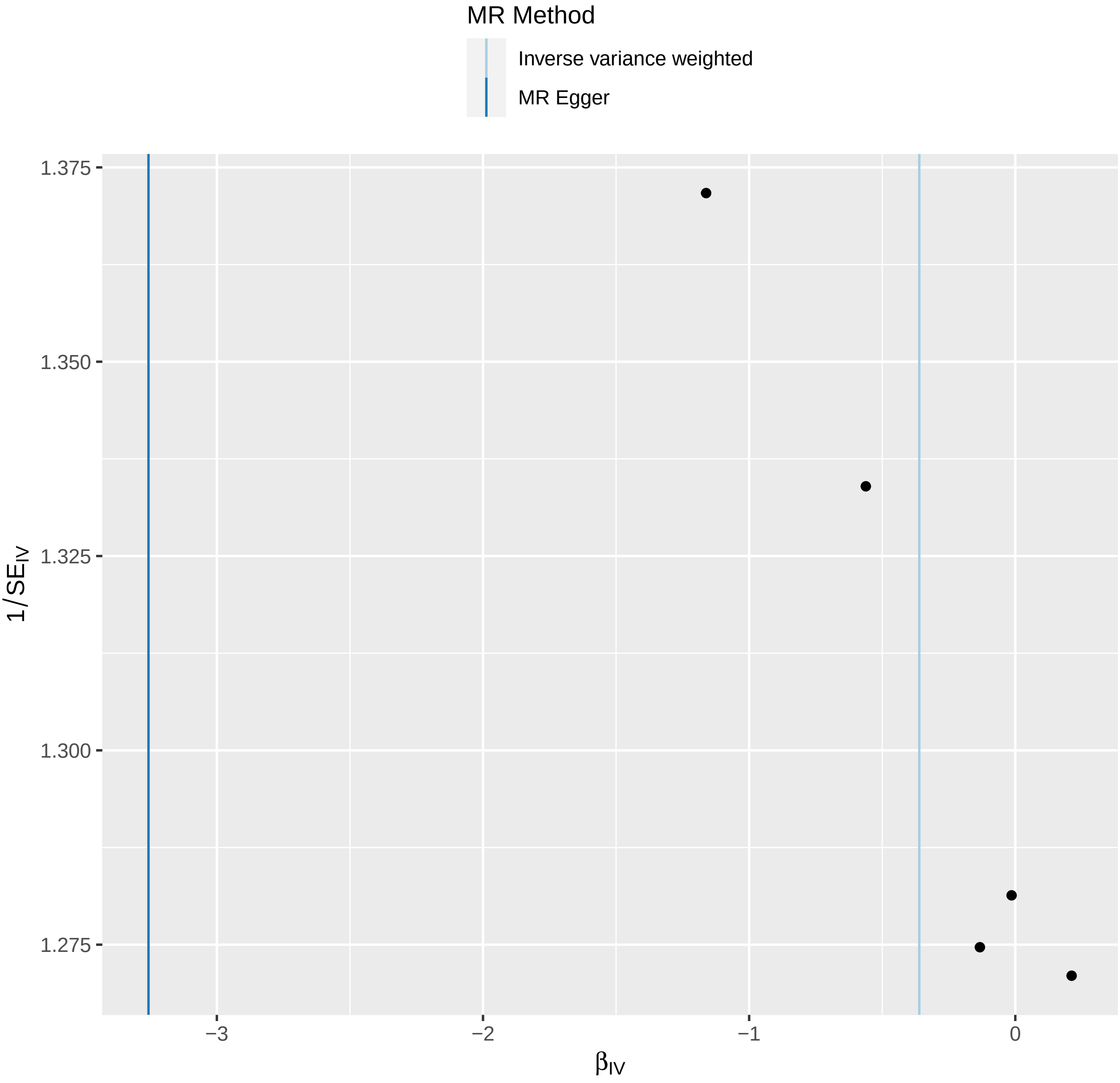

Batch 489 : Gut microbiota abundance (genus Anaerofilum id.2053) on Drug−induced obesity

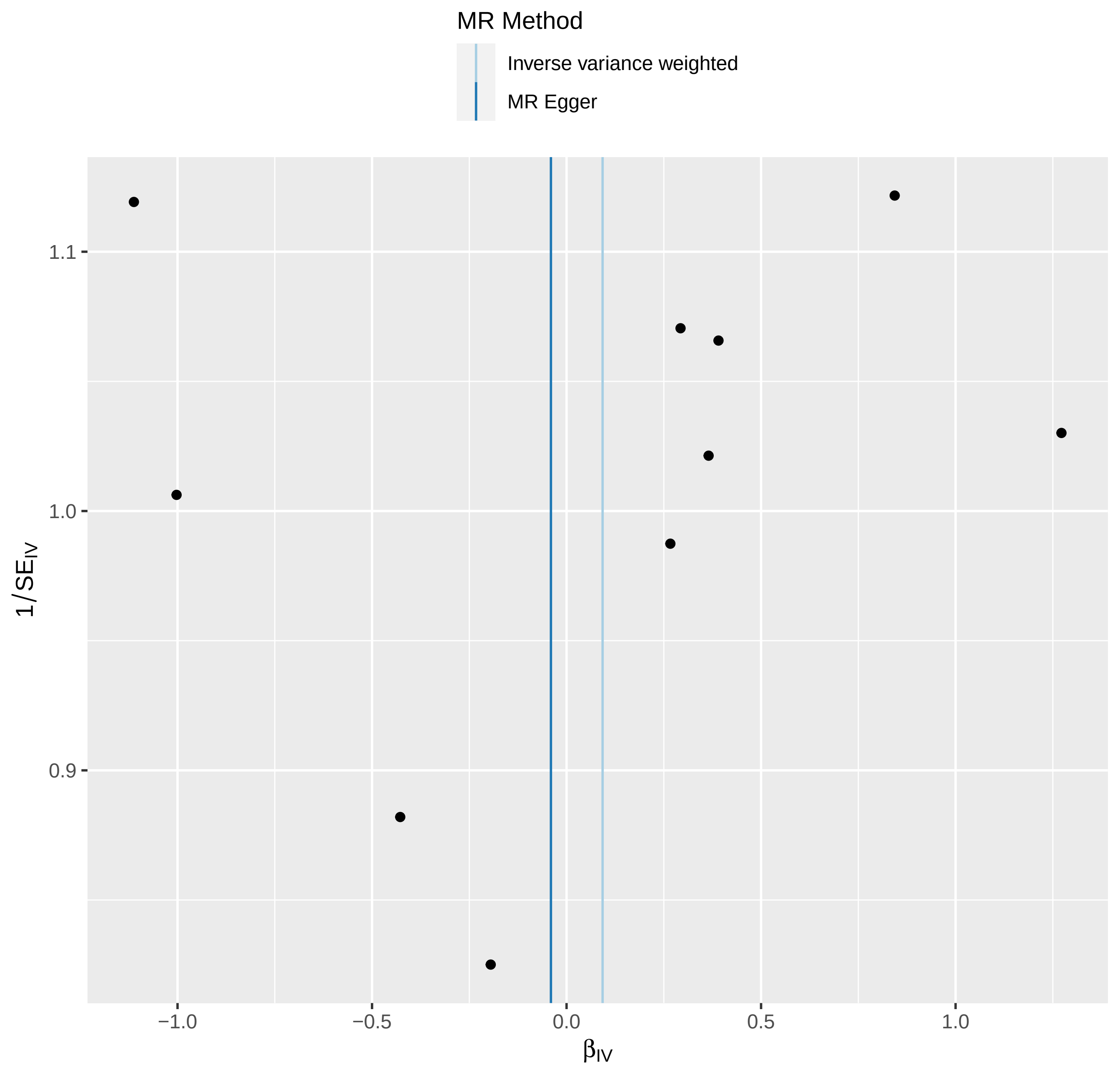

Batch 490 : Gut microbiota abundance (genus Anaerostipes id.1991) on Drug−induced obesity

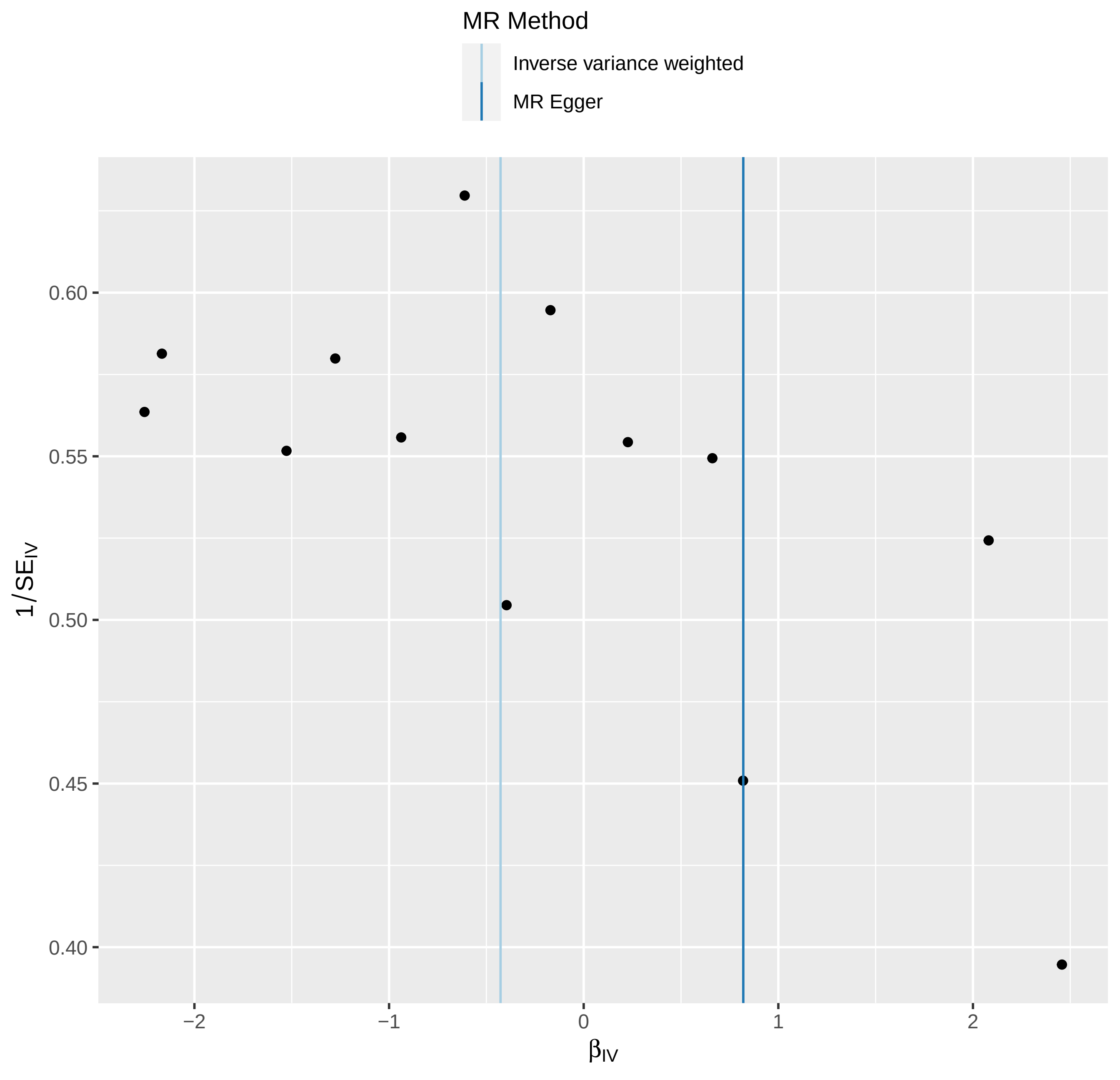

Batch 491 : Gut microbiota abundance (genus Anaerotruncus id.2054) on Drug−induced obesity

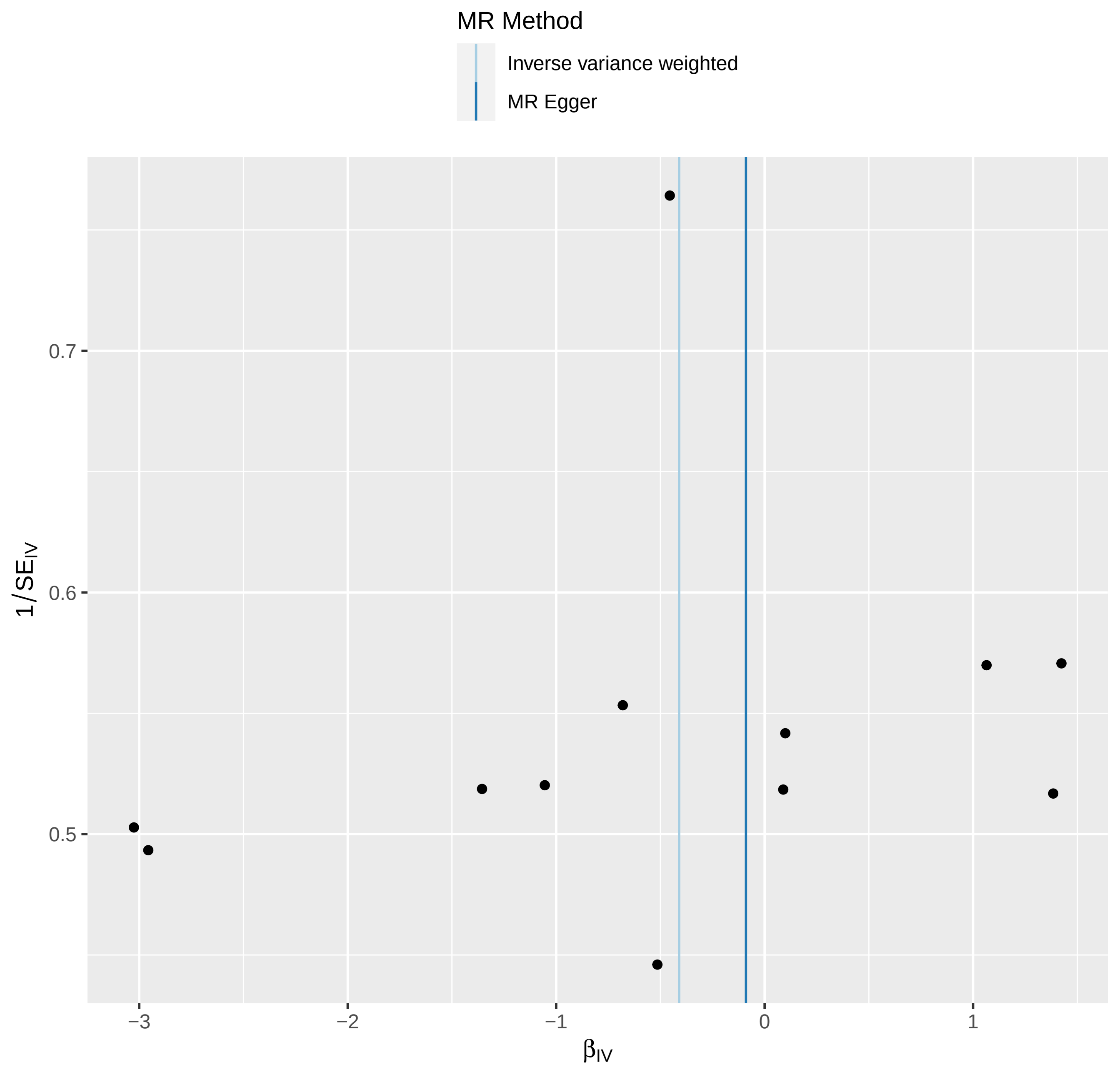

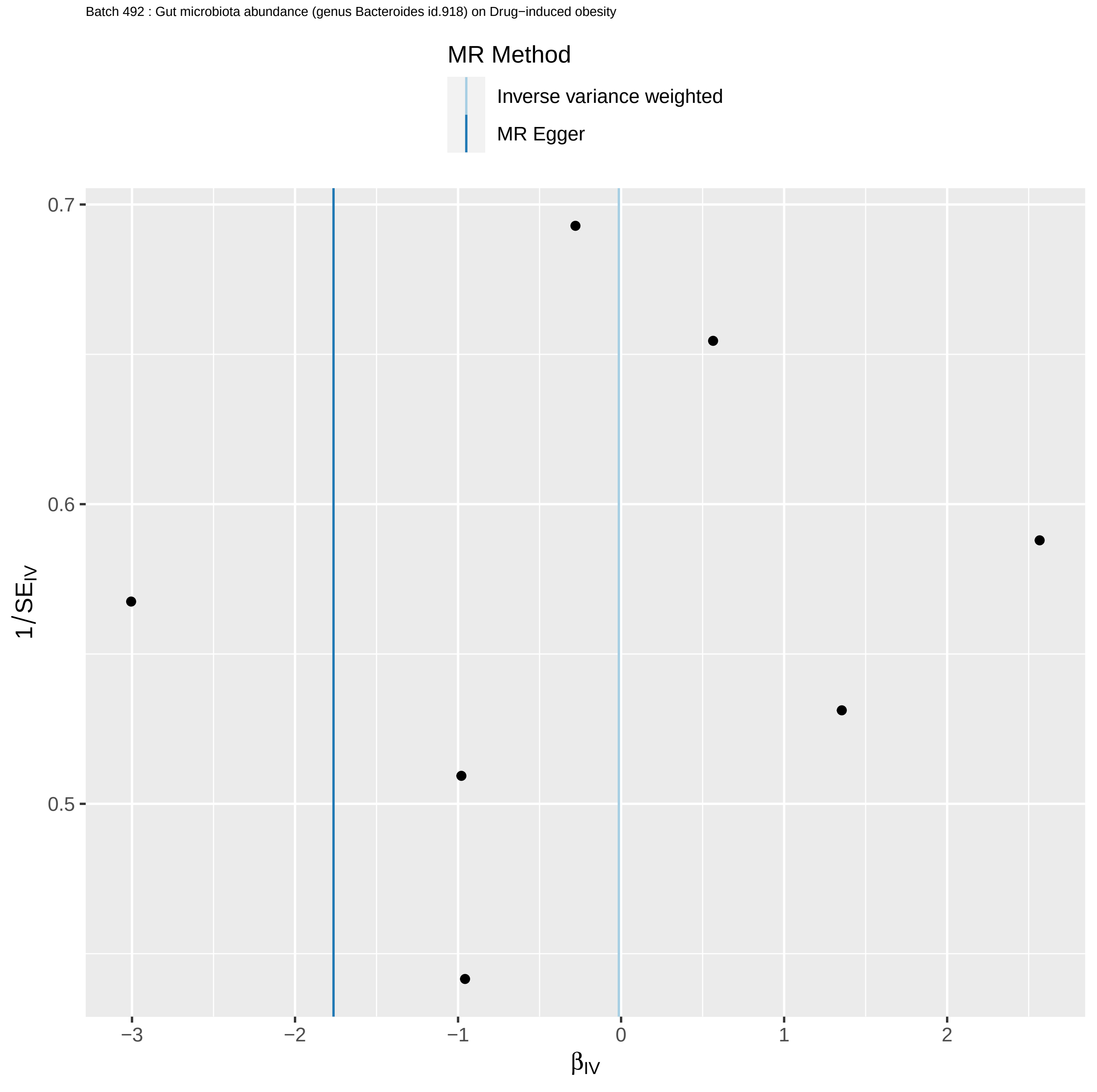

Batch 492 : Gut microbiota abundance (genus Bacteroides id.918) on Drug−induced obesity
MR Method
Inverse variance weighted
MR Egger
0.7
0.6
0.5
1/SE$_{IV}$
−3
−2
−1
0
1
2
$\beta_{IV}$

Batch 493 : Gut microbiota abundance (genus Barnesiella id.944) on Drug−induced obesity

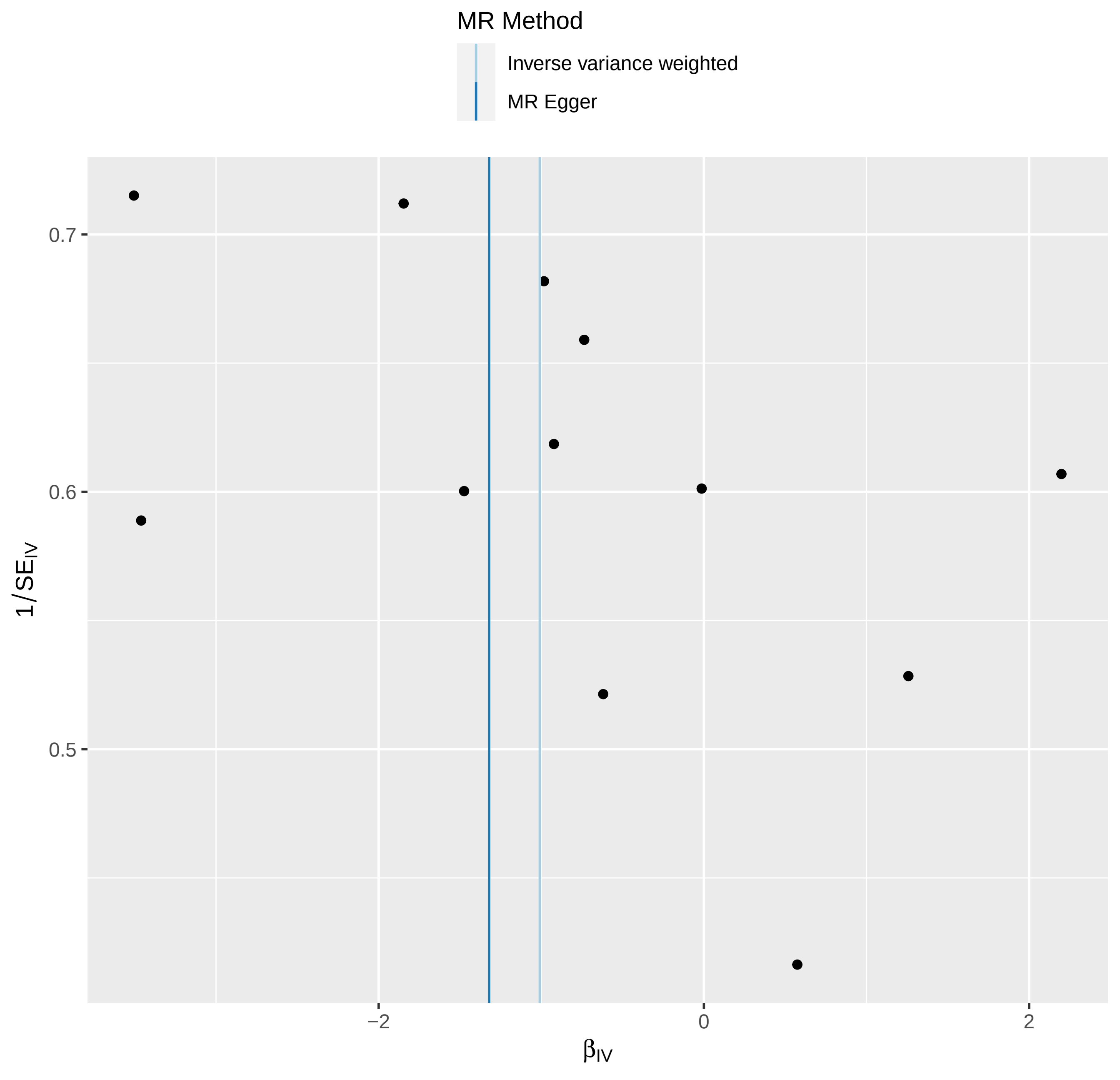

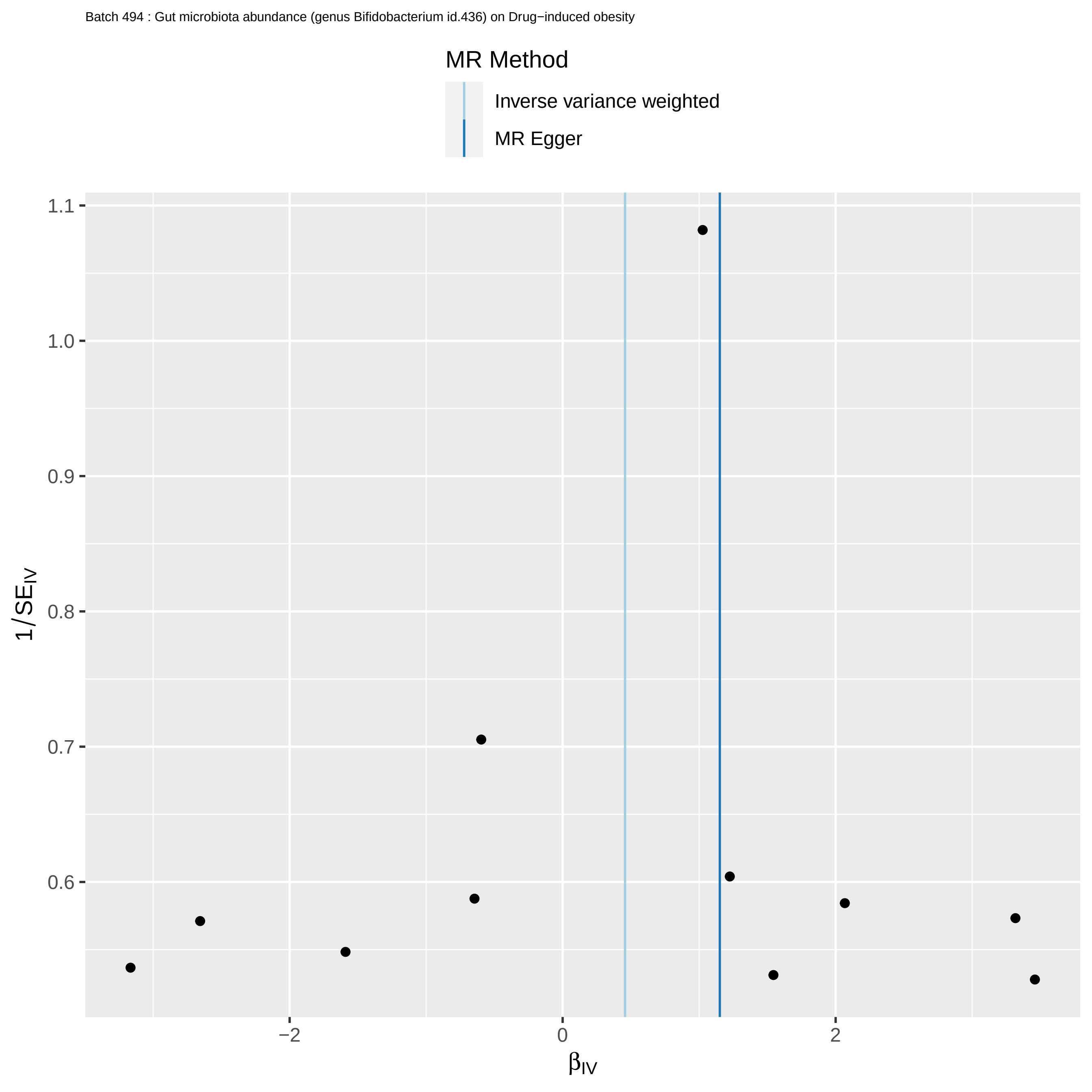

Batch 494 : Gut microbiota abundance (genus Bifidobacterium id.436) on Drug−induced obesity
MR Method
Inverse variance weighted
MR Egger
1.1
1.0
0.9
0.8
0.7
0.6
1/SE$_{IV}$
−2
0
2
β$_{IV}$

Batch 495 : Gut microbiota abundance (genus Bilophila id.3170) on Drug−induced obesity

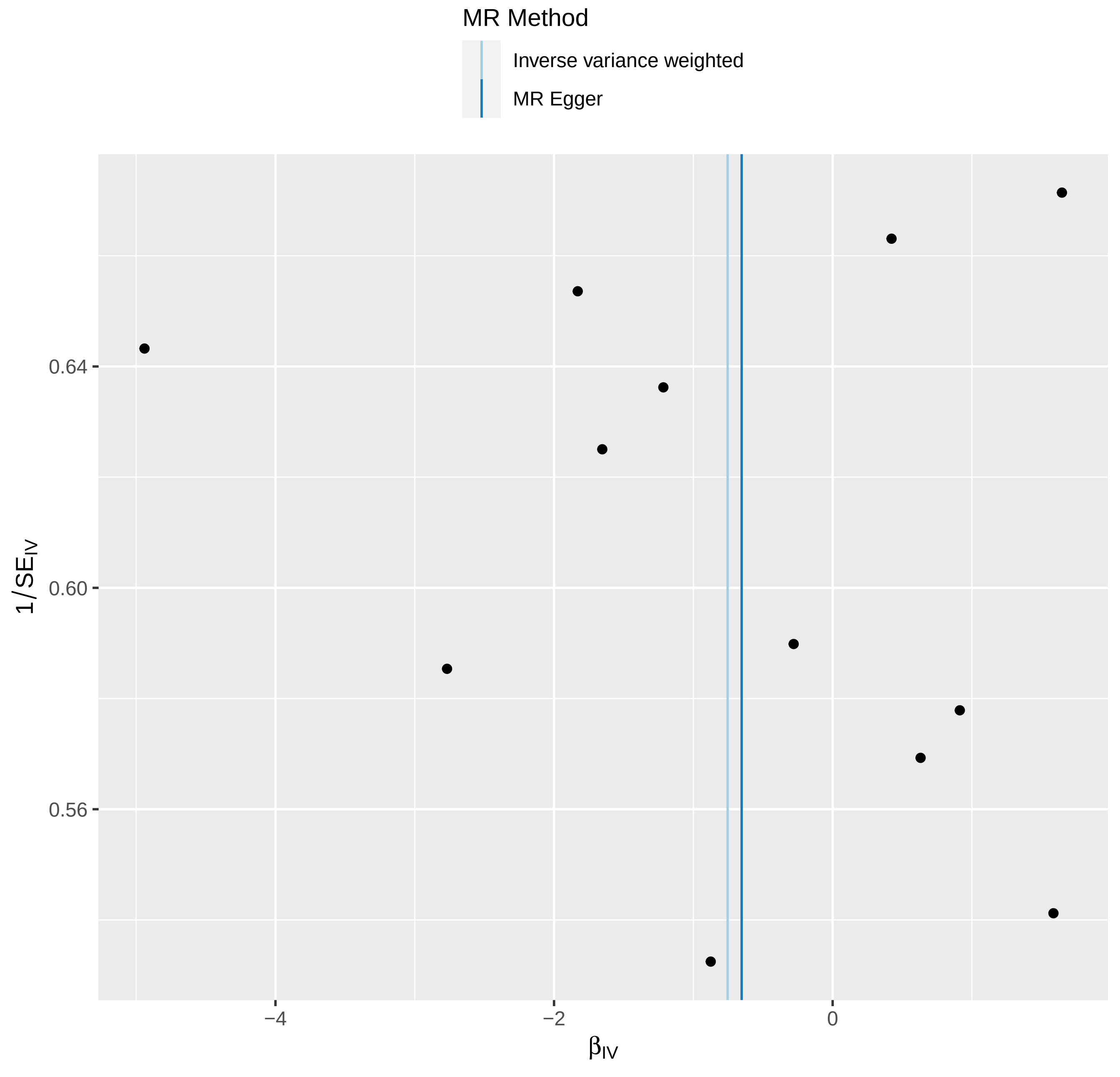

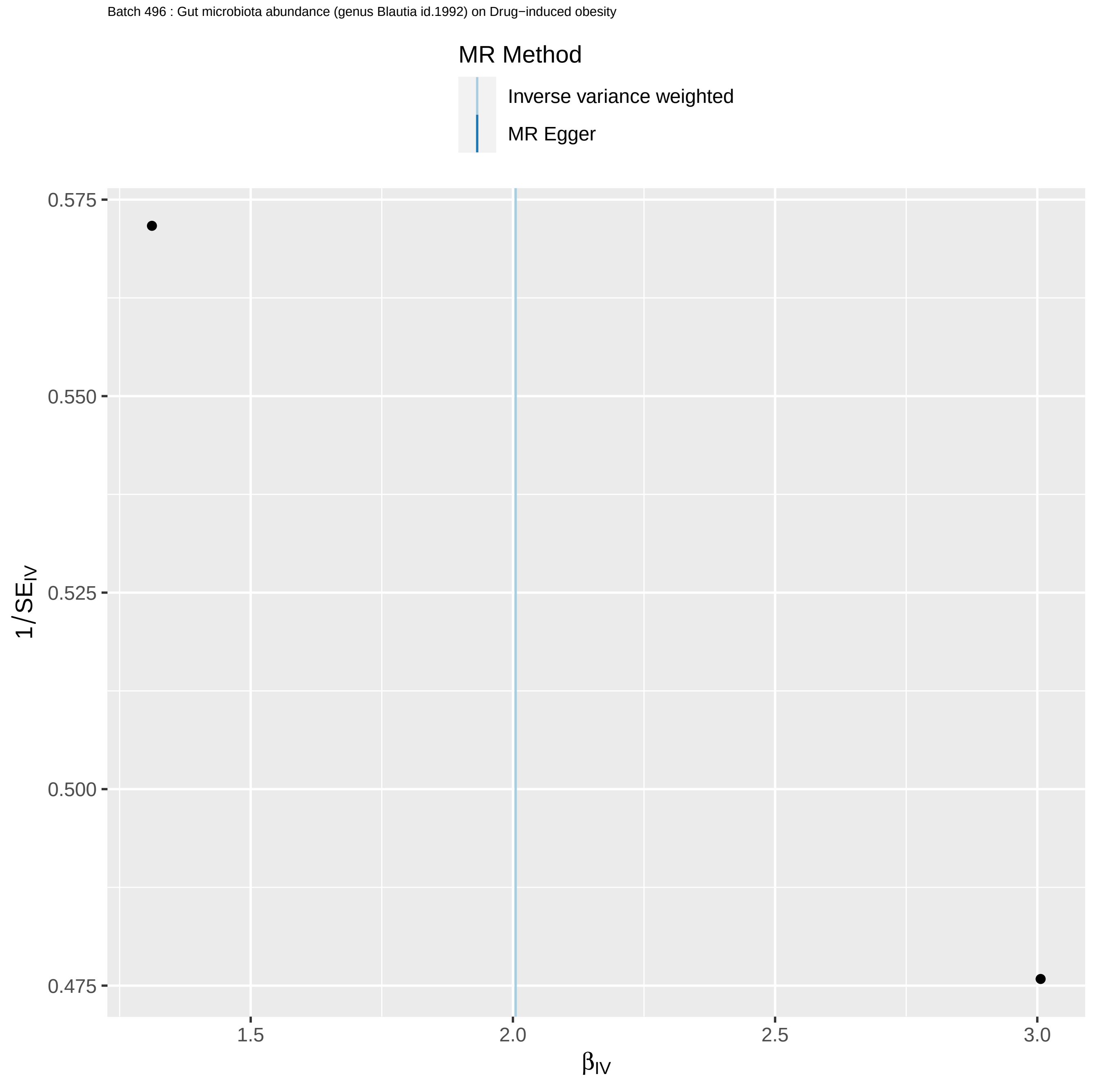

Batch 496 : Gut microbiota abundance (genus Blautia id.1992) on Drug−induced obesity
MR Method
Inverse variance weighted
MR Egger
0.575
0.550
0.525
0.500
0.475
1/SE_IV
1.5
2.0
2.5
3.0
β_IV

Batch 497 : Gut microbiota abundance (genus Butyricicoccus id.2055) on Drug−induced obesity

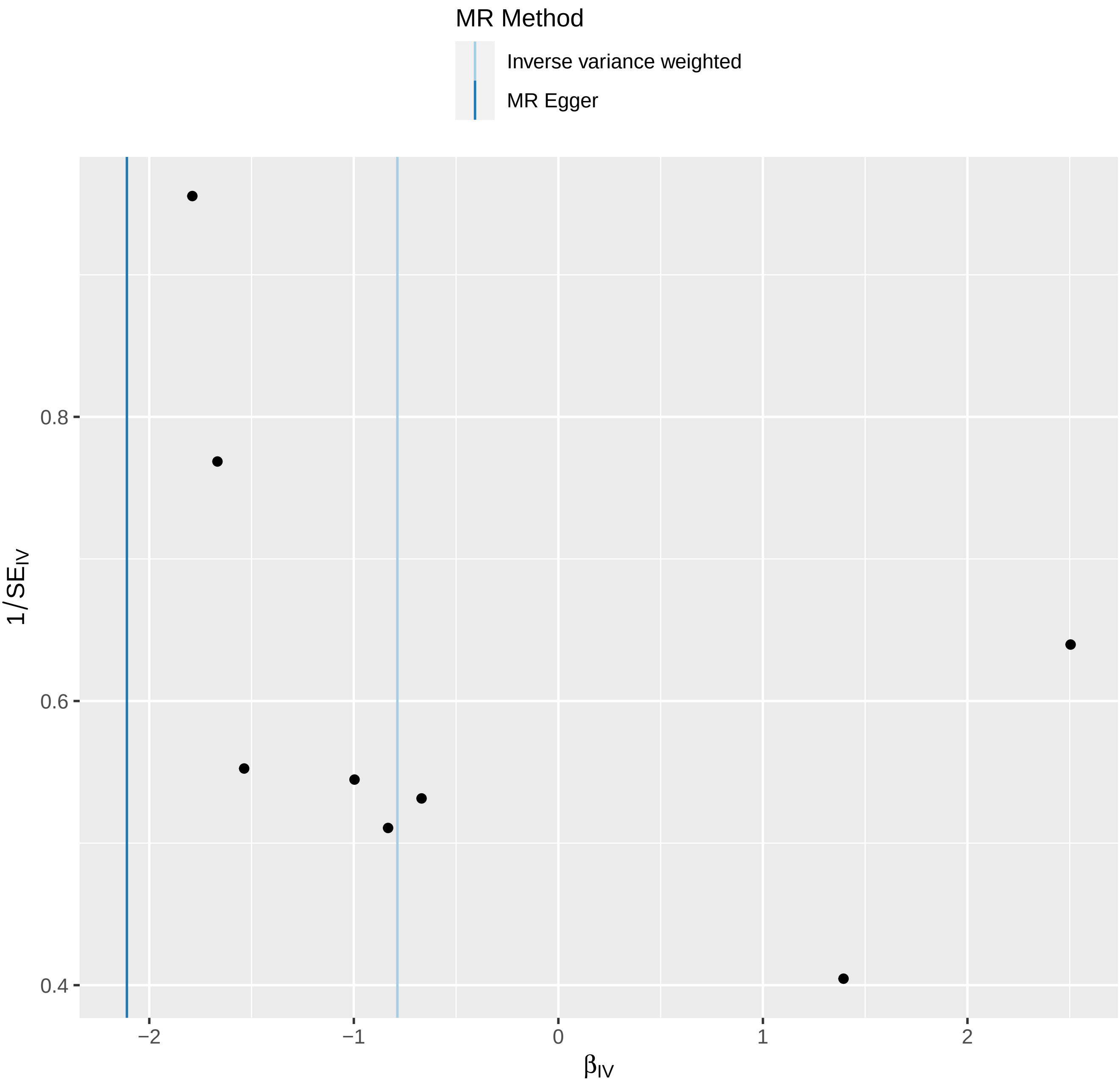

Batch 498 : Gut microbiota abundance (genus Butyricimonas id.945) on Drug−induced obesity

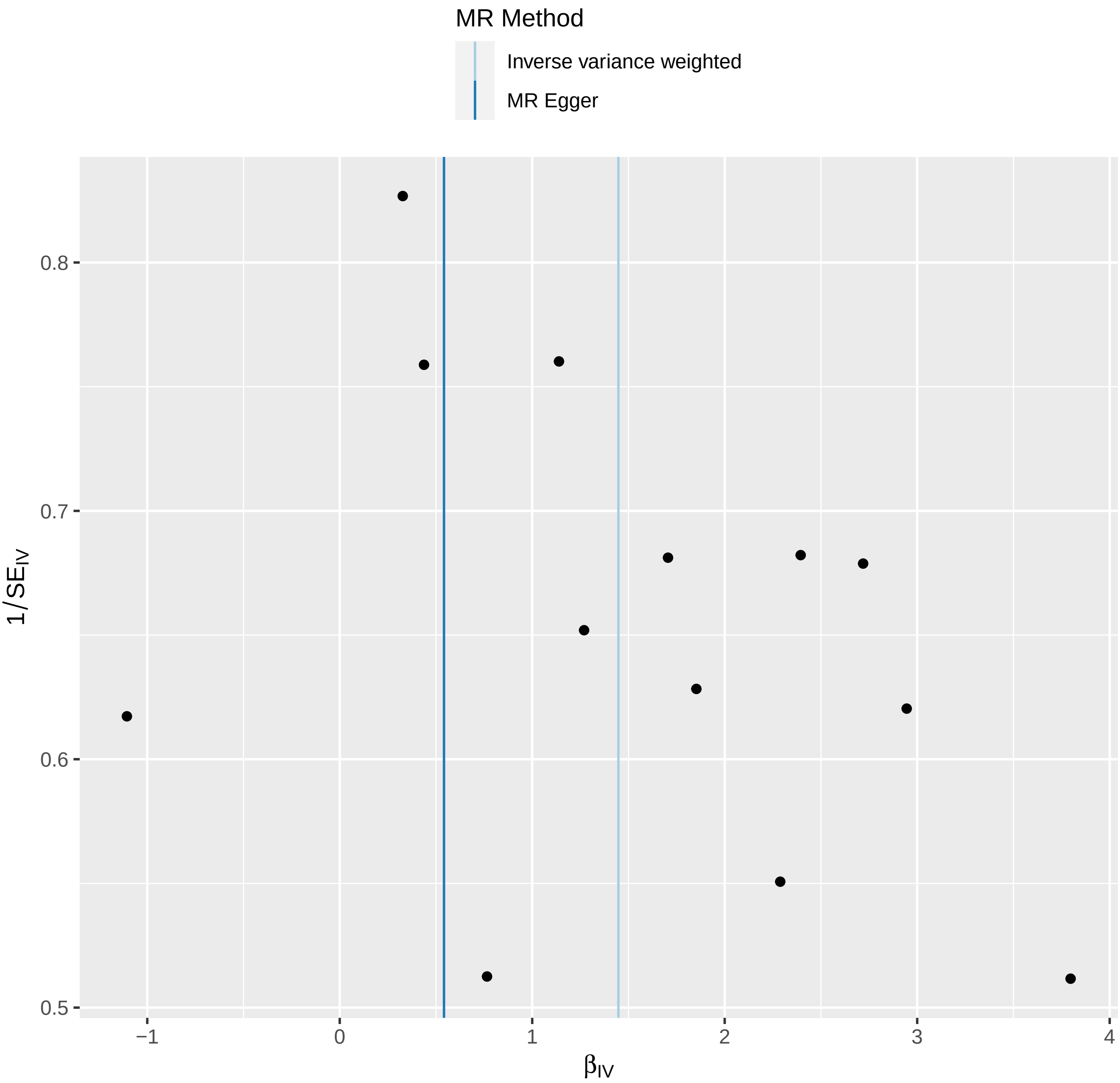

Batch 499 : Gut microbiota abundance (genus Butyrivibrio id.1993) on Drug−induced obesity

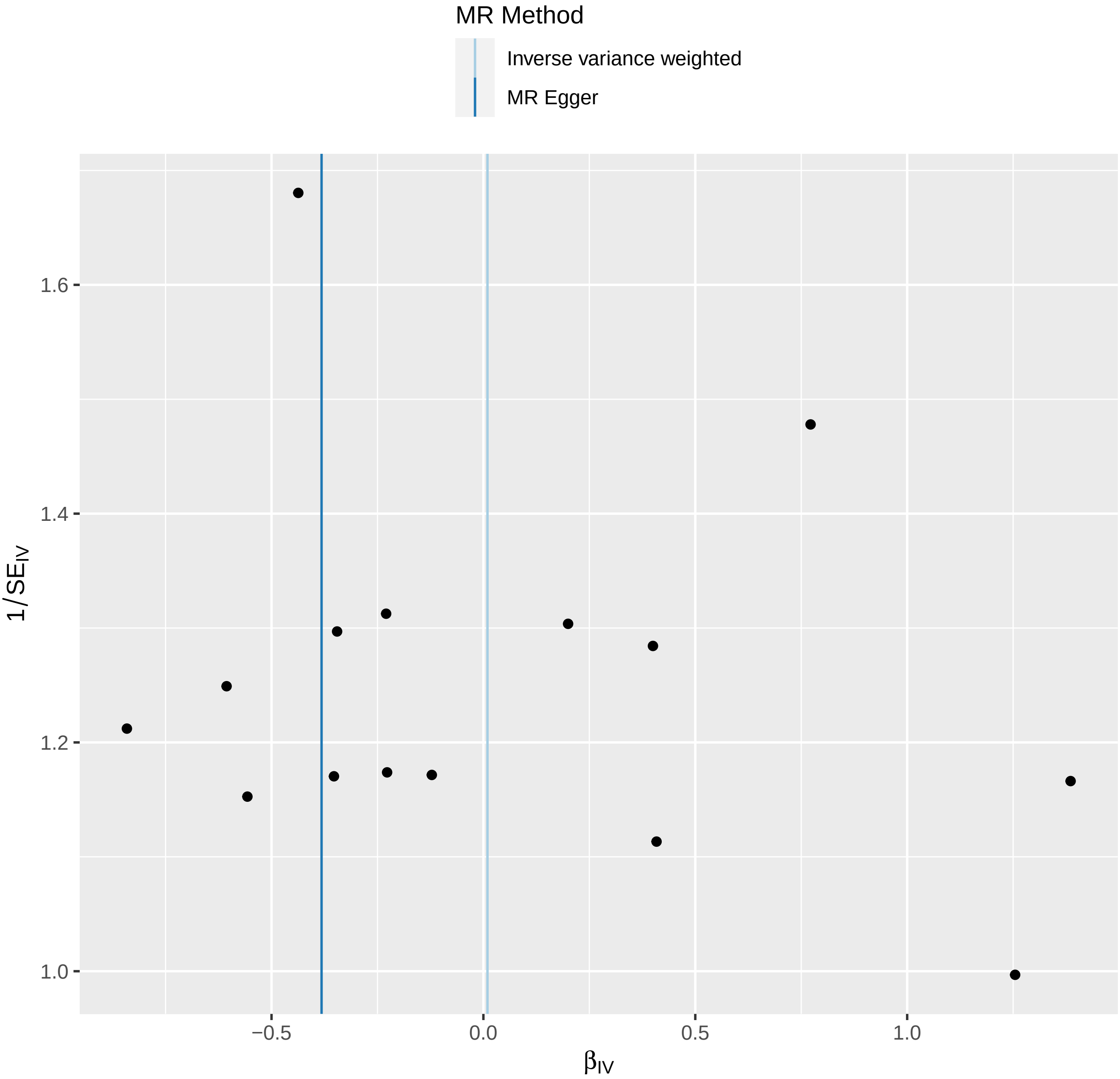

Batch 500 : Gut microbiota abundance (genus Candidatus Soleaferrea id.11350) on Drug−induced obesity

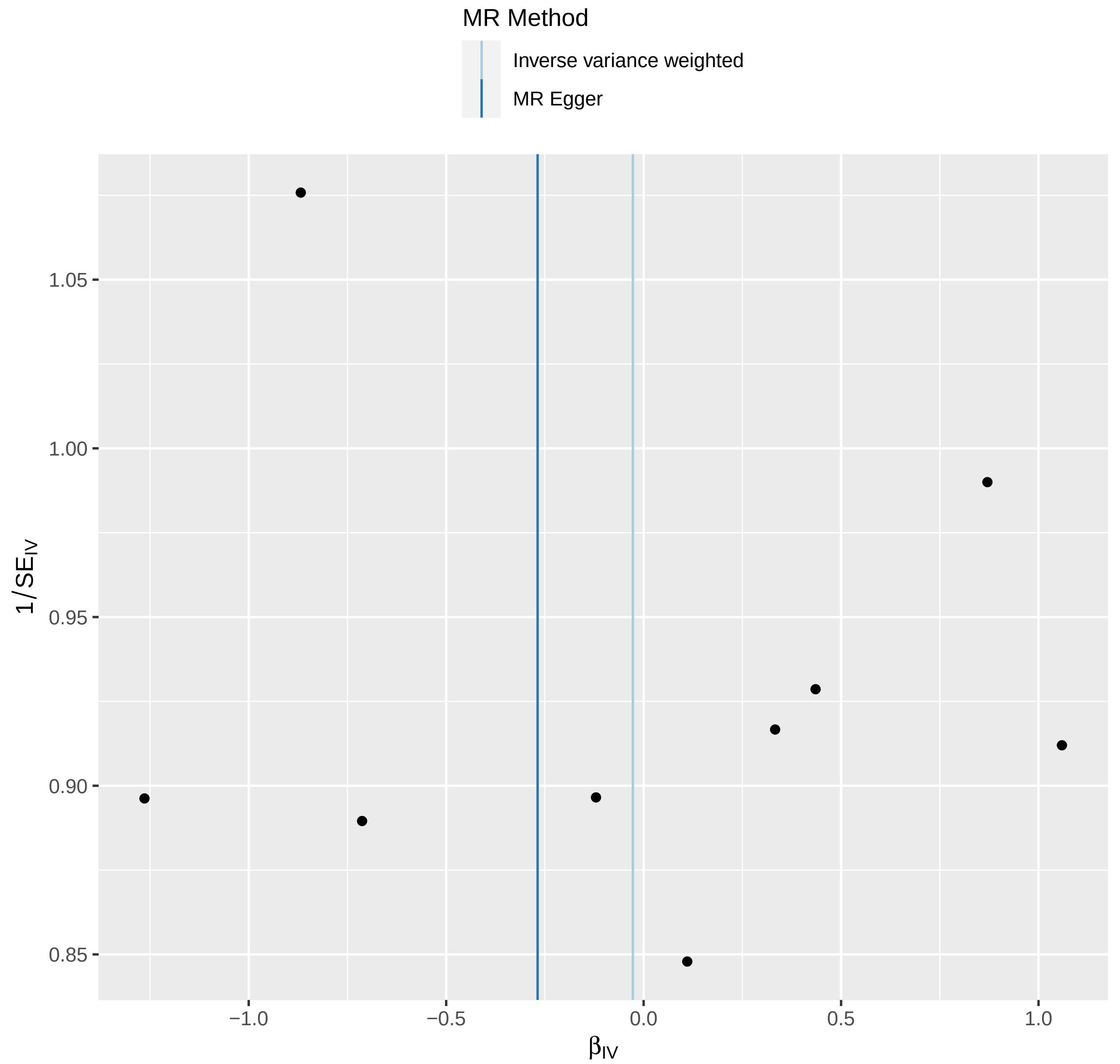

Batch 501 : Gut microbiota abundance (genus Catenibacterium id.2153) on Drug−induced obesity

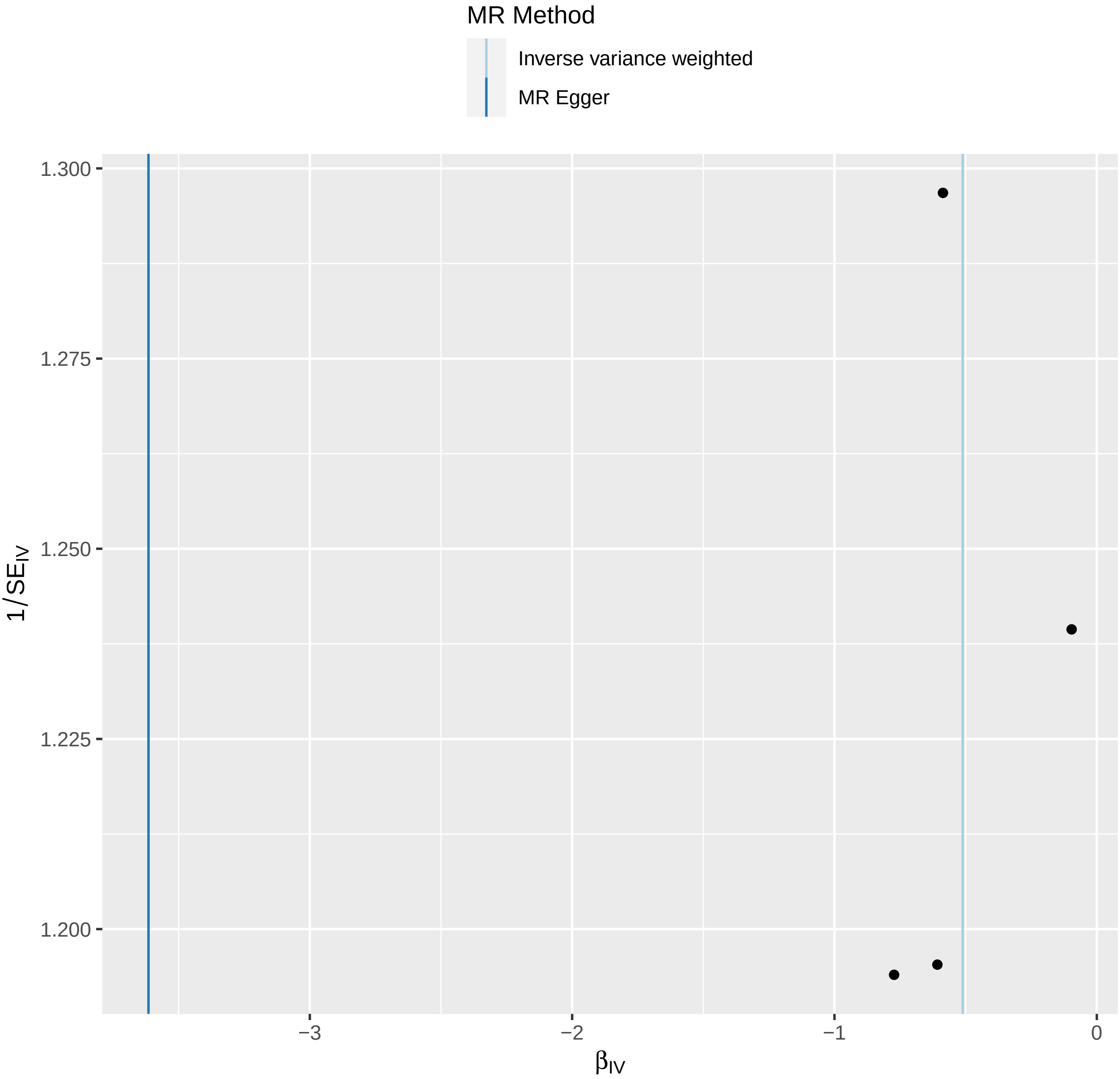

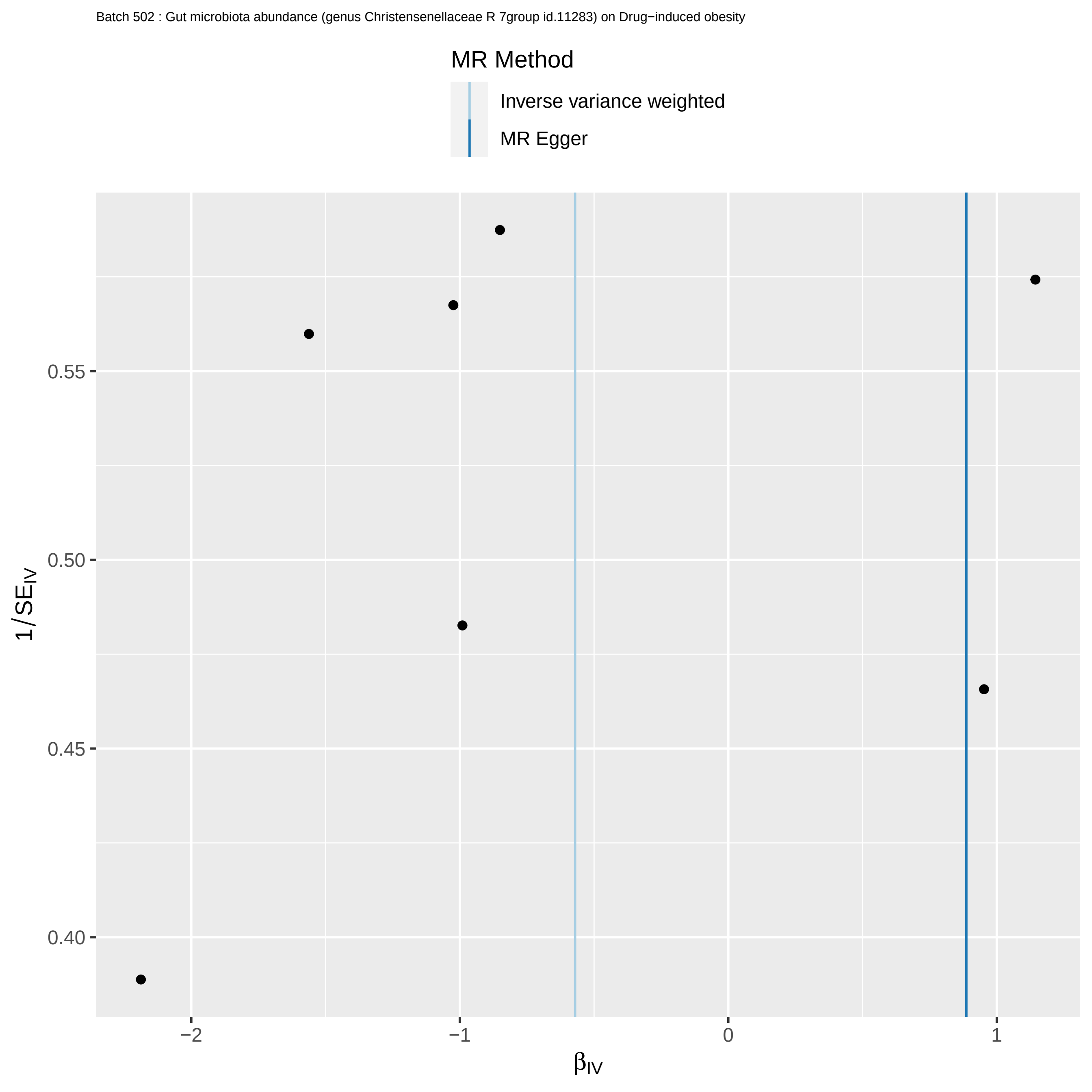

Batch 502 : Gut microbiota abundance (genus Christensenellaceae R 7group id.11283) on Drug−induced obesity
MR Method
Inverse variance weighted
MR Egger
0.55
0.50
0.45
0.40
1/SE_IV
−2
−1
0
1
β_IV

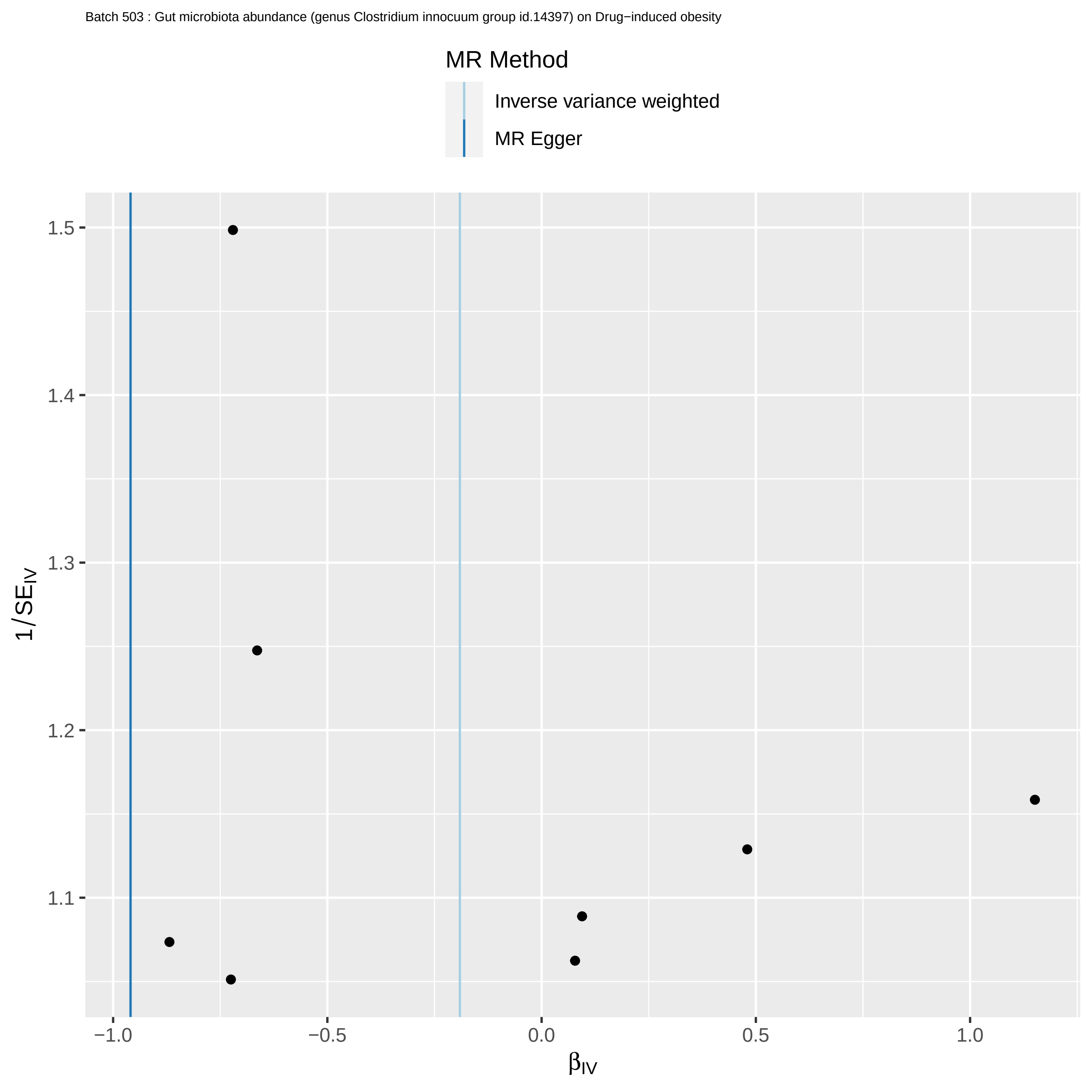

Batch 503 : Gut microbiota abundance (genus Clostridium innocuum group id.14397) on Drug−induced obesity
MR Method
Inverse variance weighted
MR Egger
1.5
1.4
1.3
1.2
1.1
1/SE$_{IV}$
−1.0
−0.5
0.0
0.5
1.0
β$_{IV}$

Batch 504 : Gut microbiota abundance (genus Clostridium sensustricto1 id.1873) on Drug−induced obesity

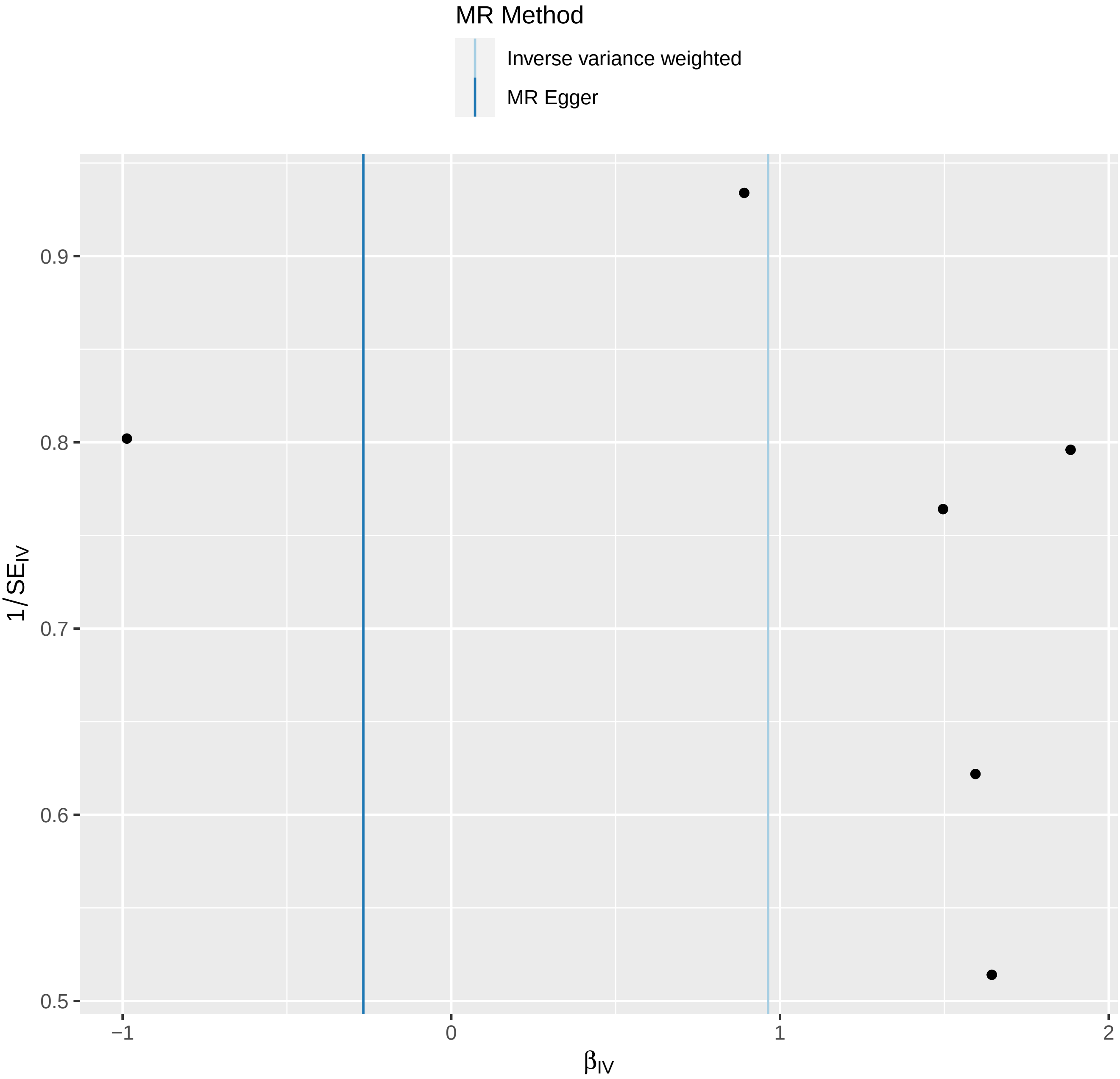

Batch 505 : Gut microbiota abundance (genus Collinsella id.815) on Drug−induced obesity

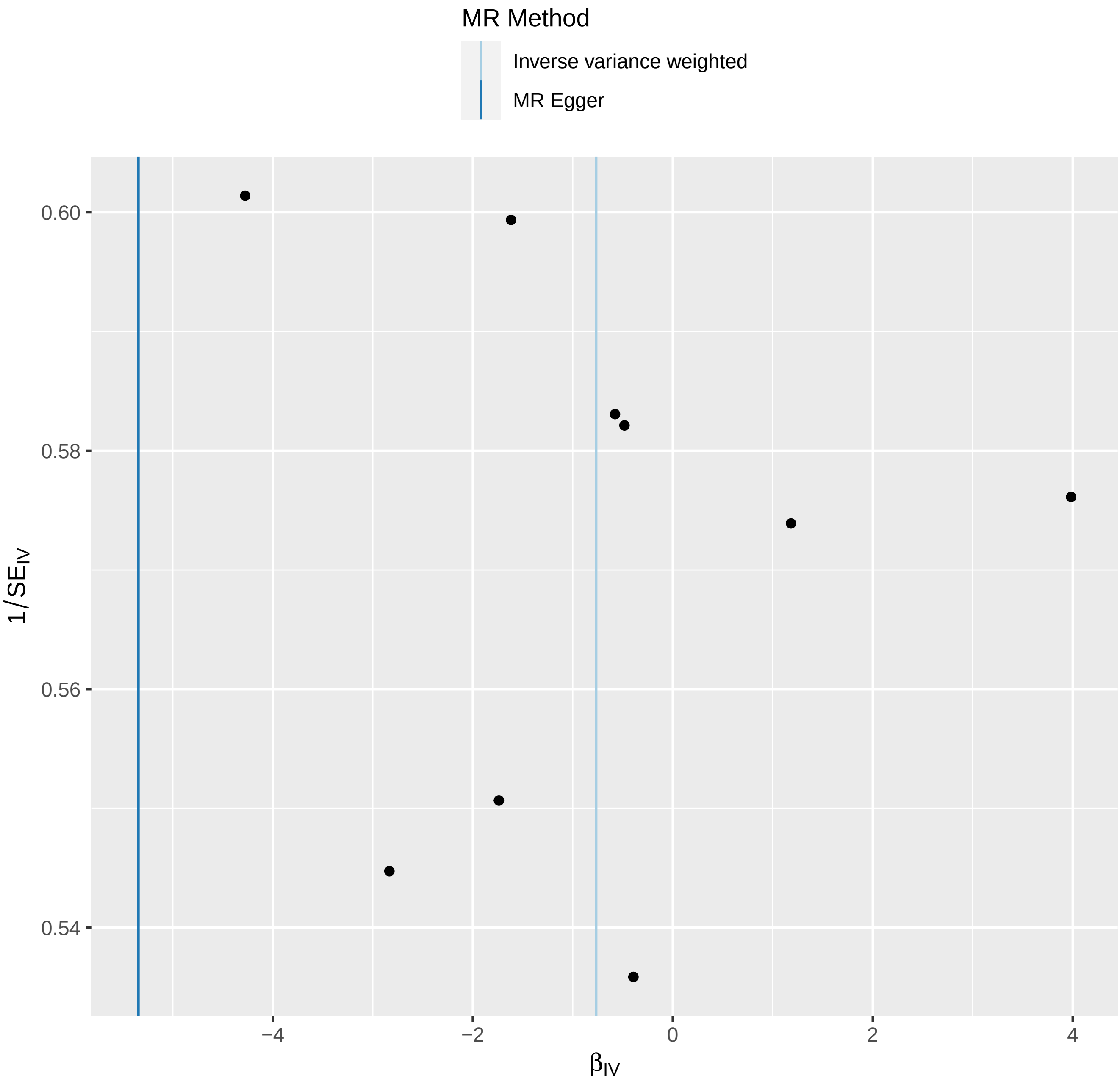

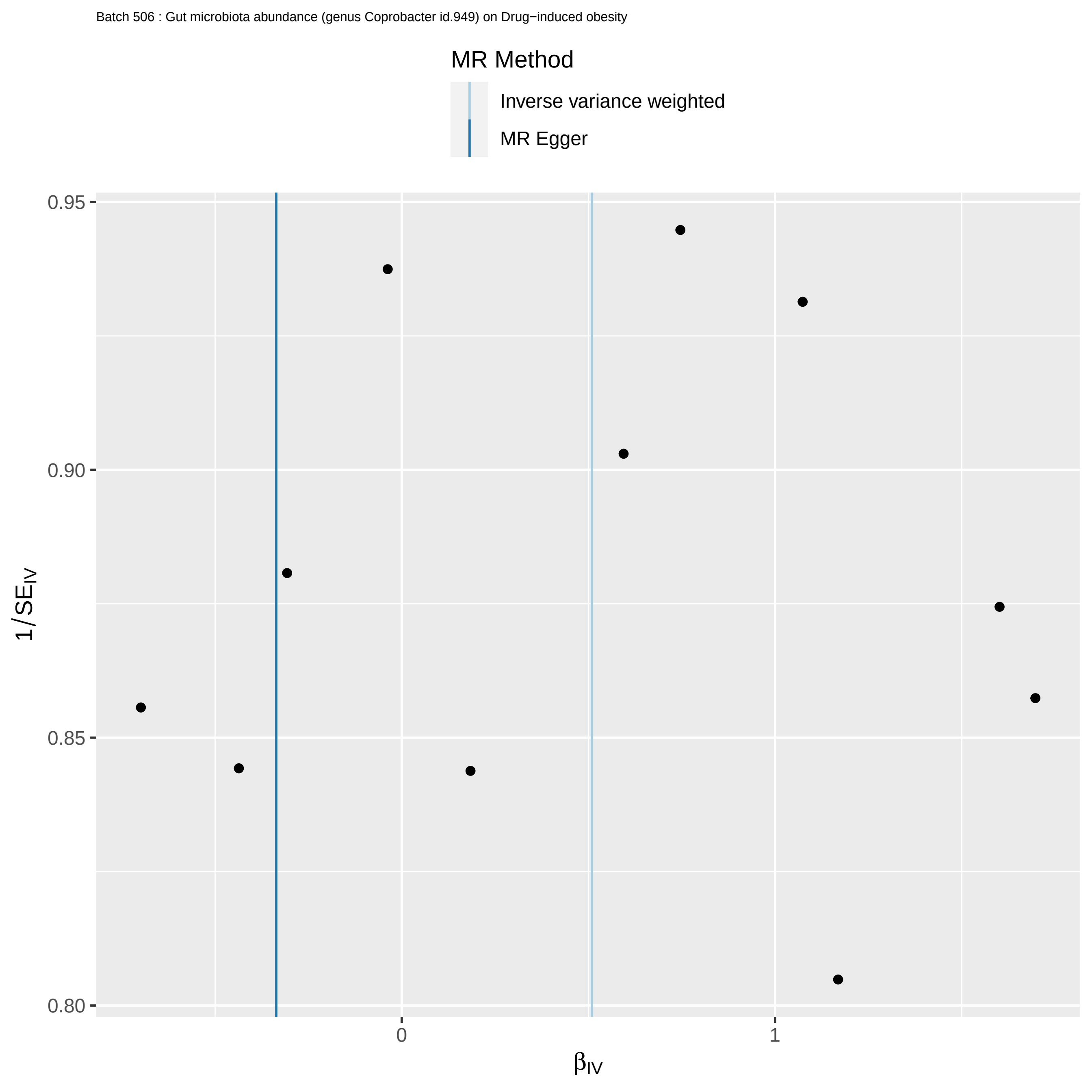

Batch 506 : Gut microbiota abundance (genus Coprobacter id.949) on Drug−induced obesity
MR Method
Inverse variance weighted
MR Egger
0.95
0.90
0.85
0.80
1/SE_IV
0
1
β_IV

Batch 507 : Gut microbiota abundance (genus Coprococcus1 id.11301) on Drug−induced obesity

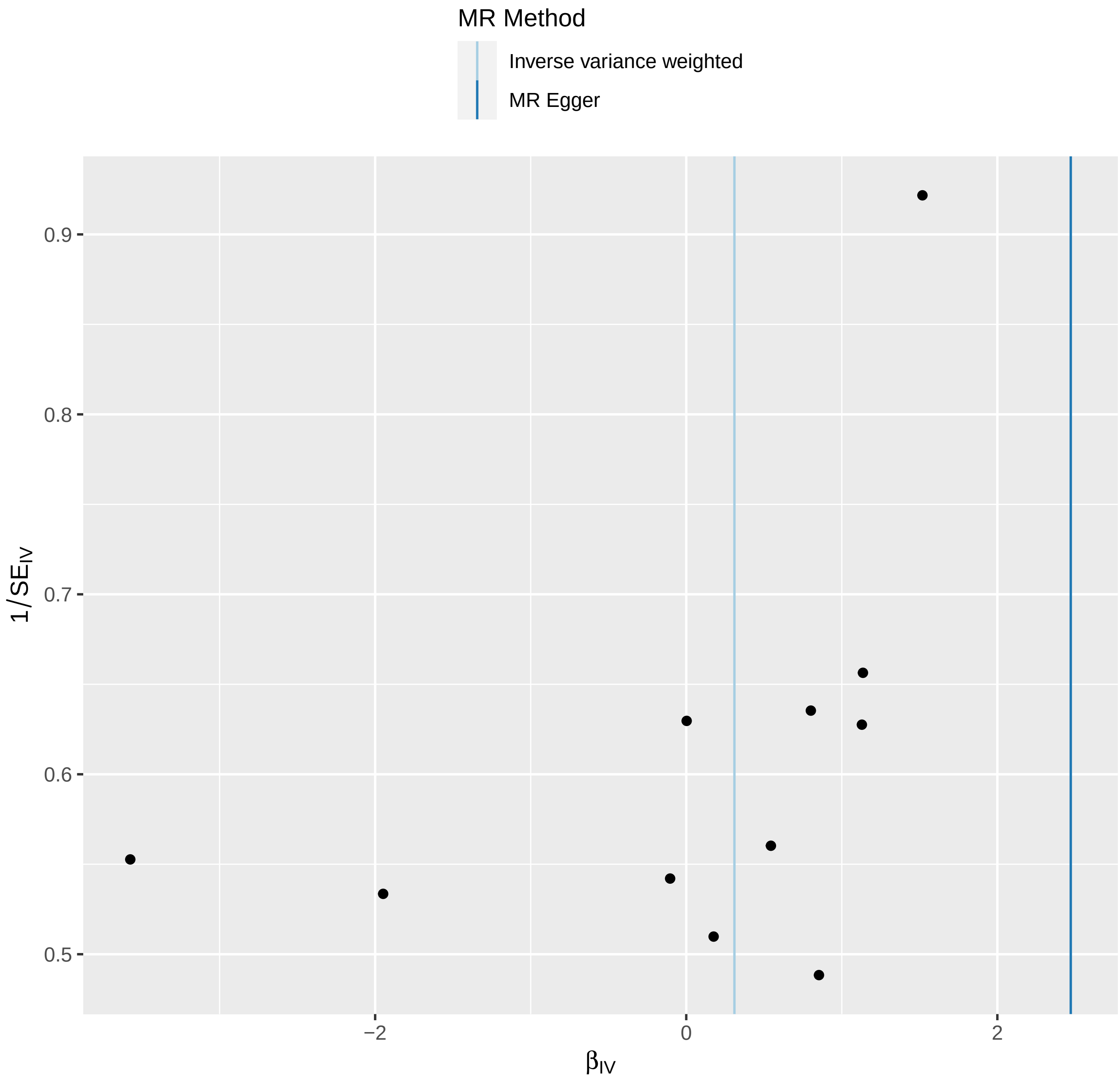

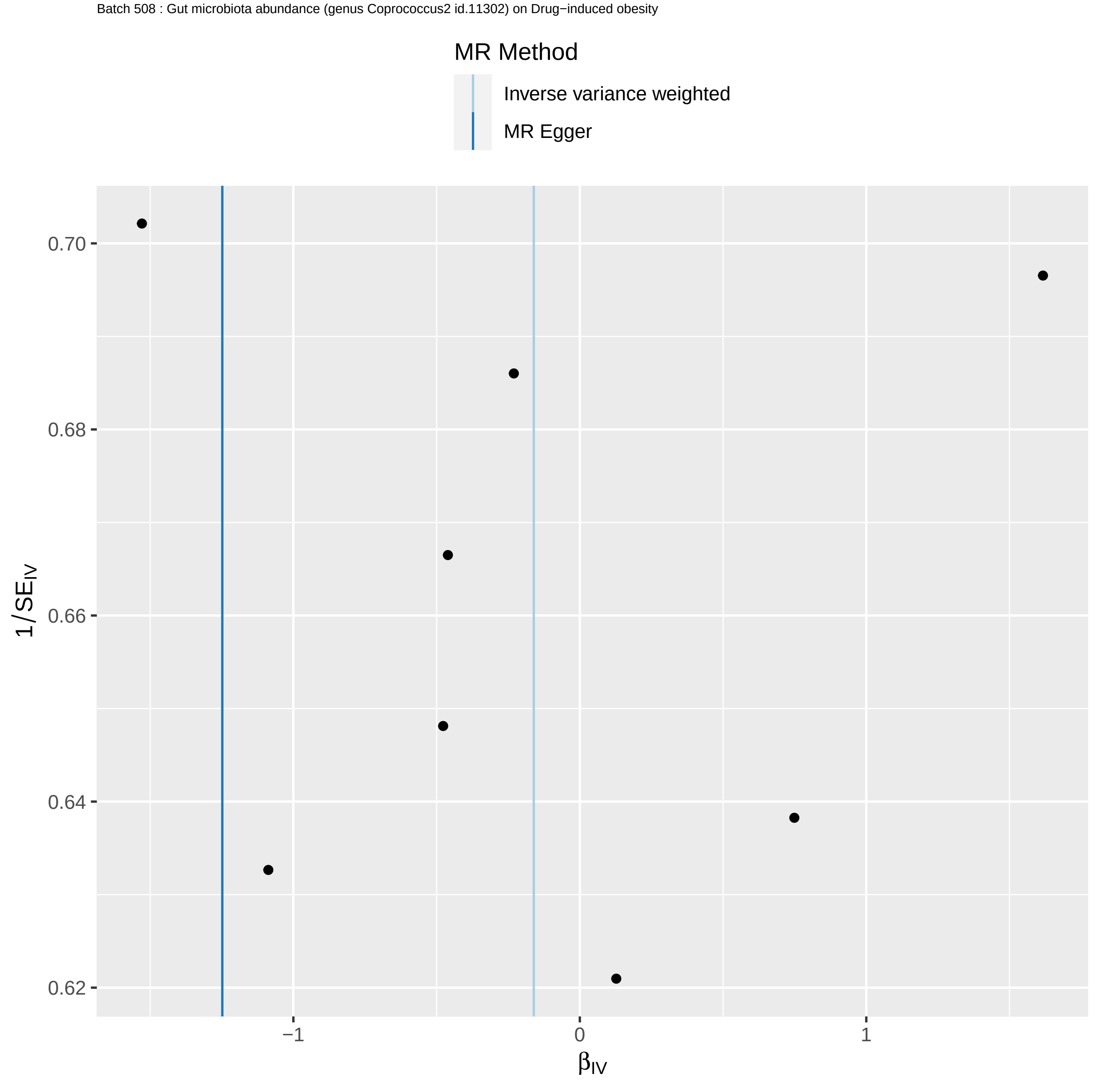
Batch 508 : Gut microbiota abundance (genus Coprococcus2 id.11302) on Drug−induced obesity
MR Method
Inverse variance weighted
MR Egger
0.70
0.68
0.66
0.64
0.62
1/SE_IV
−1
0
1
β_IV

Batch 509 : Gut microbiota abundance (genus Coprococcus3 id.11303) on Drug−induced obesity

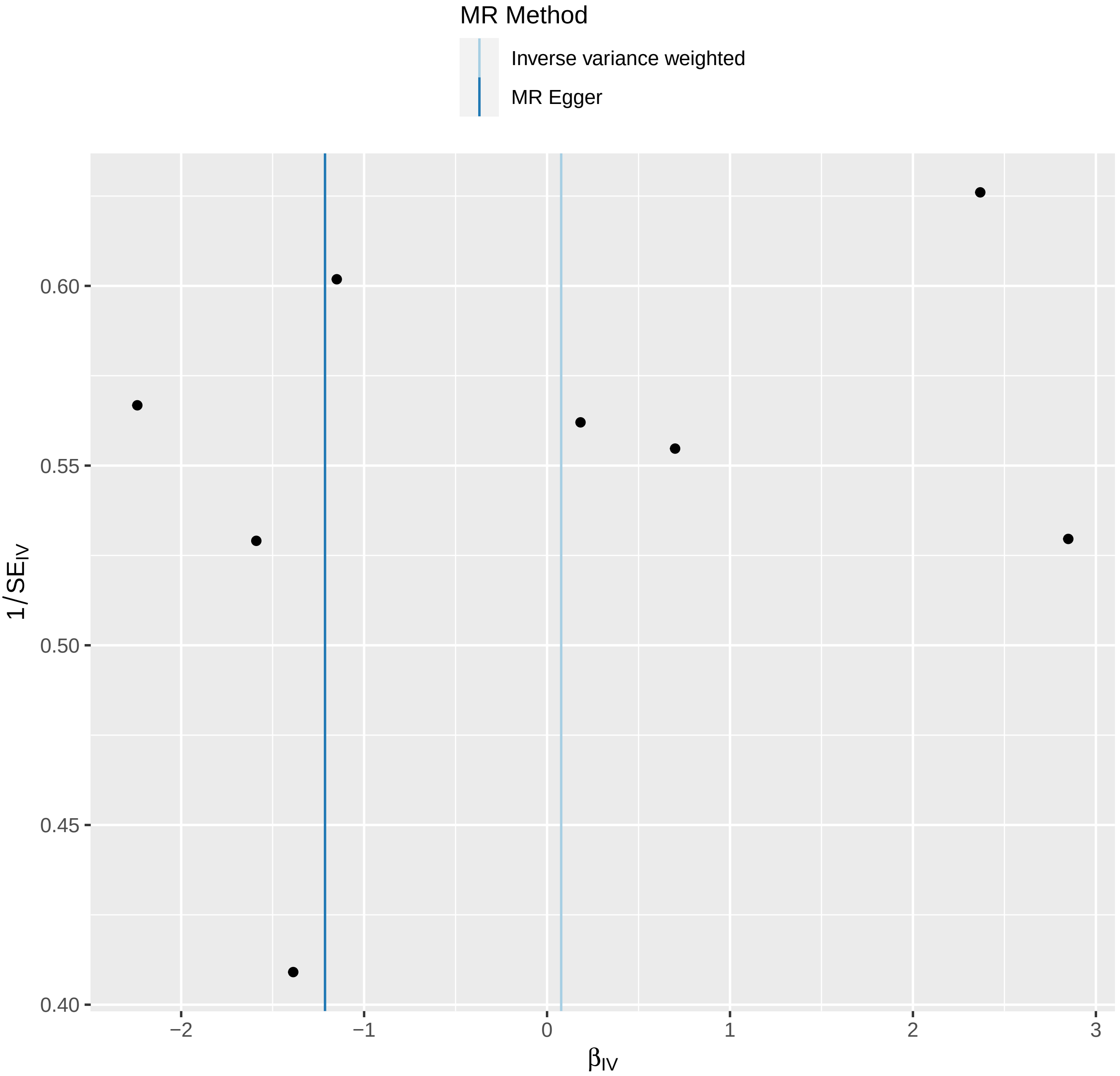

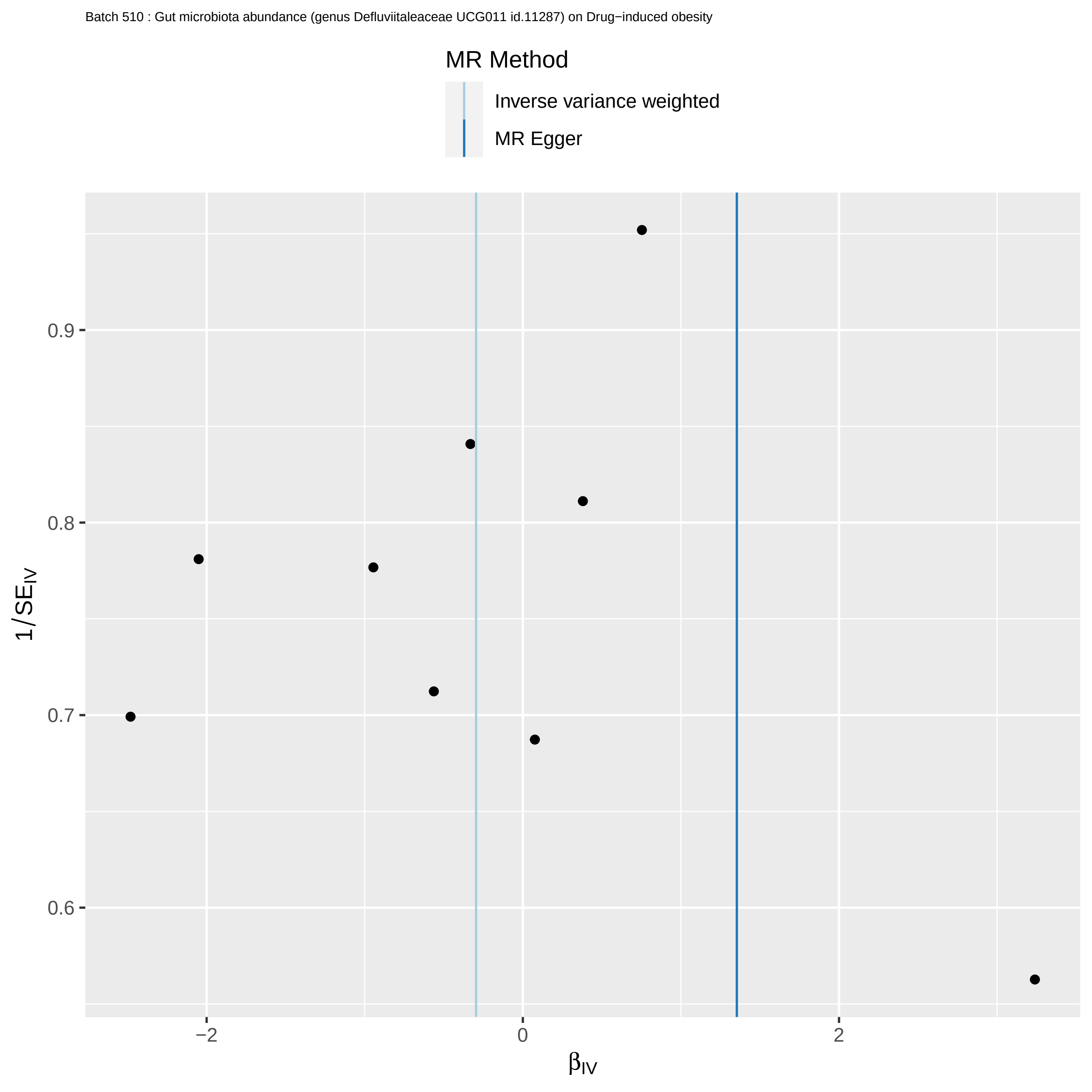
Batch 510 : Gut microbiota abundance (genus Defluviitaleaceae UCG011 id.11287) on Drug−induced obesity
MR Method
Inverse variance weighted
MR Egger
0.9
0.8
0.7
0.6
1/SE_IV
−2
0
2
β_IV

Batch 511 : Gut microbiota abundance (genus Desulfovibrio id.3173) on Drug−induced obesity

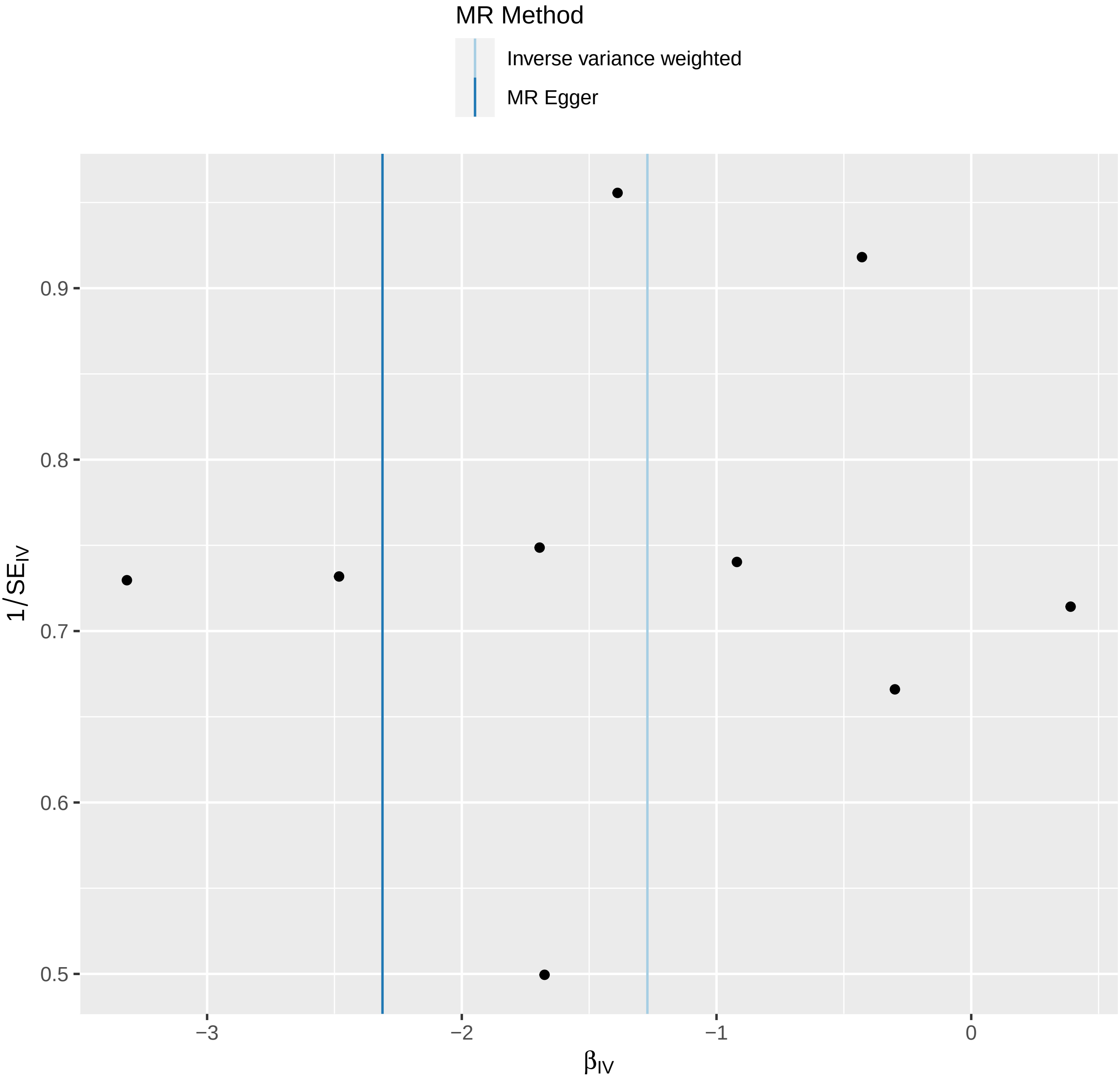

Batch 512 : Gut microbiota abundance (genus Dialister id.2183) on Drug−induced obesity

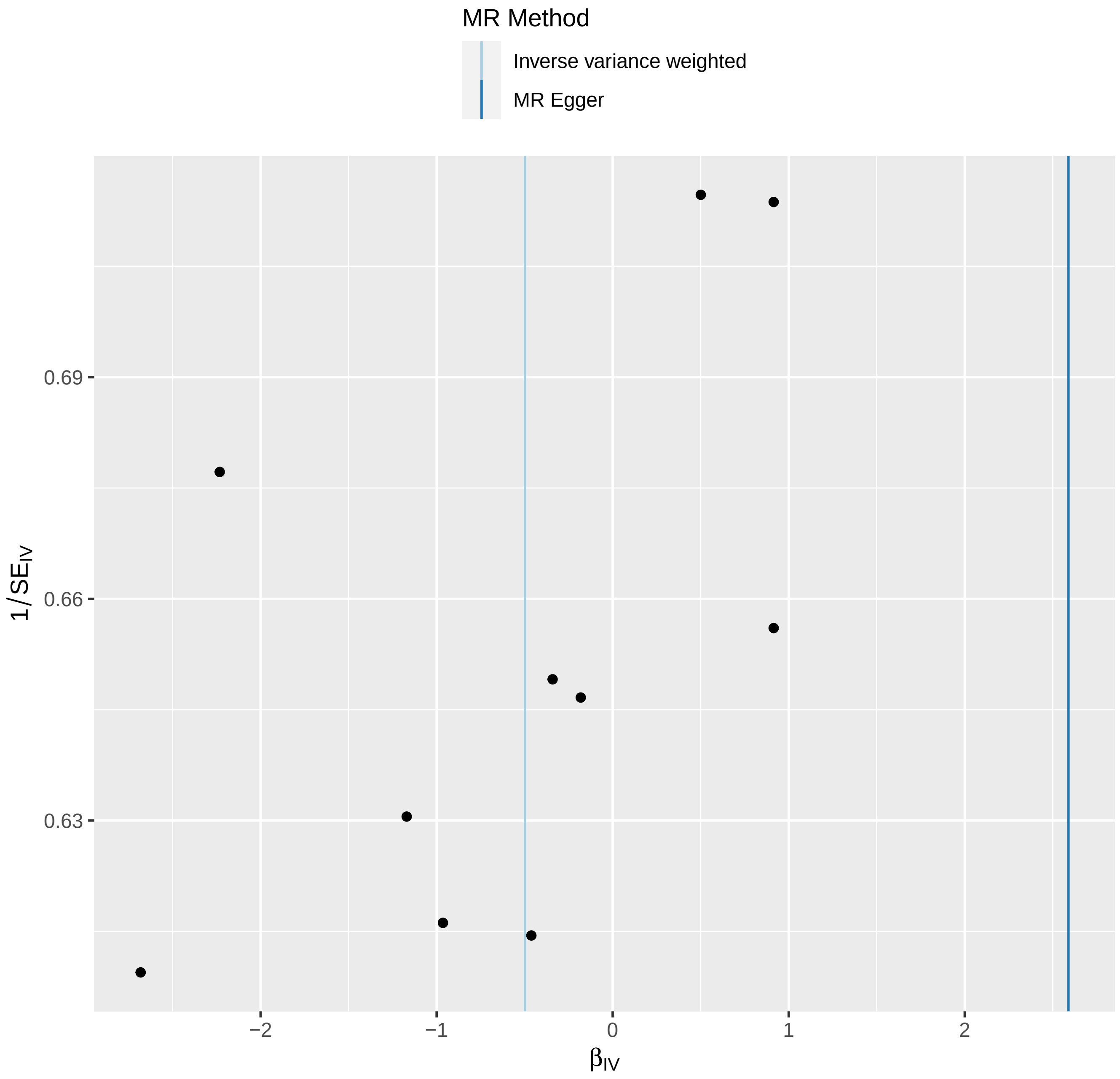

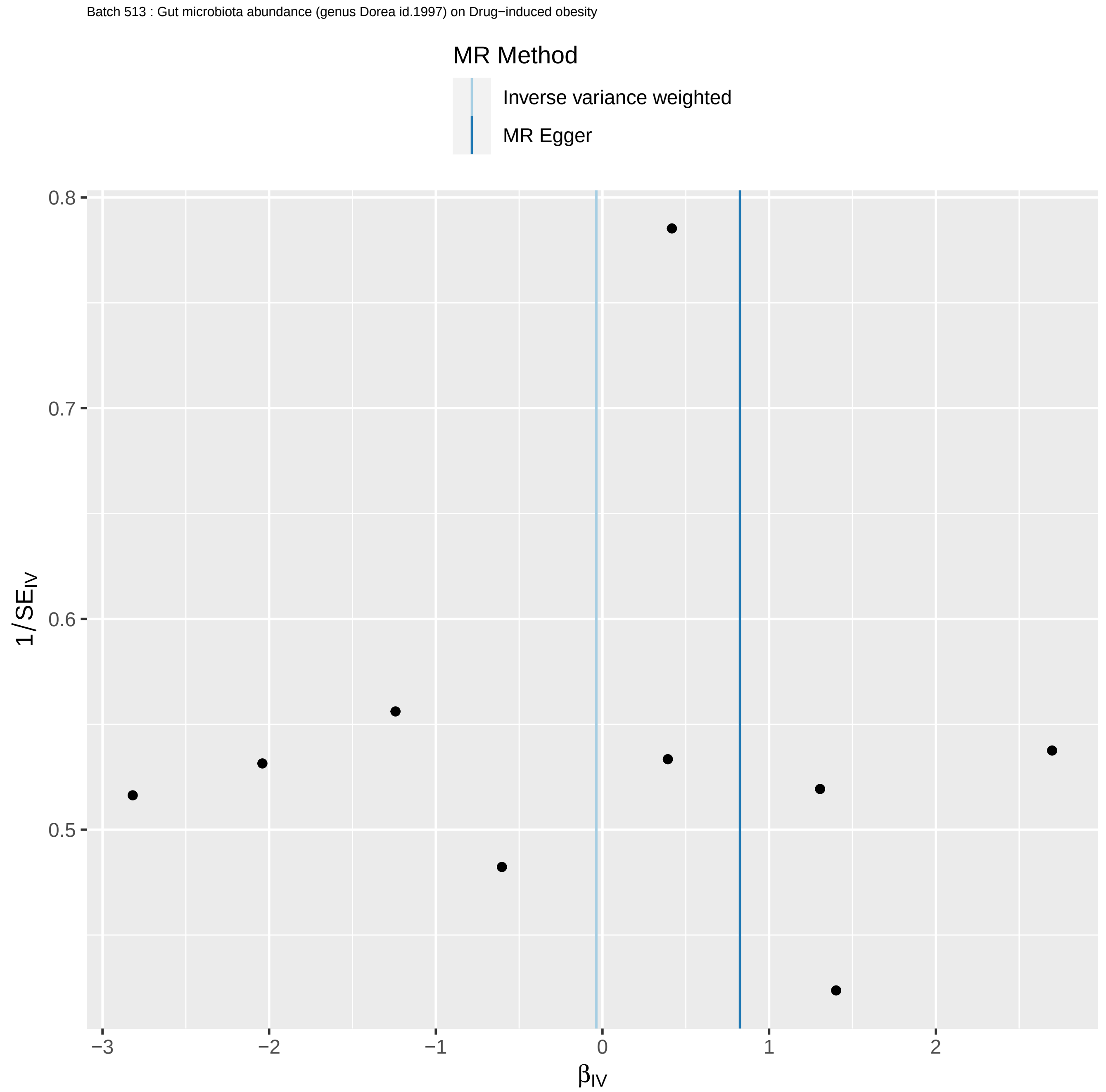

Batch 513 : Gut microbiota abundance (genus Dorea id.1997) on Drug−induced obesity
MR Method
Inverse variance weighted
MR Egger
0.8
0.7
0.6
0.5
1/SE_IV
−3
−2
−1
0
1
2
β_IV

Batch 514 : Gut microbiota abundance (genus Eggerthella id.819) on Drug−induced obesity

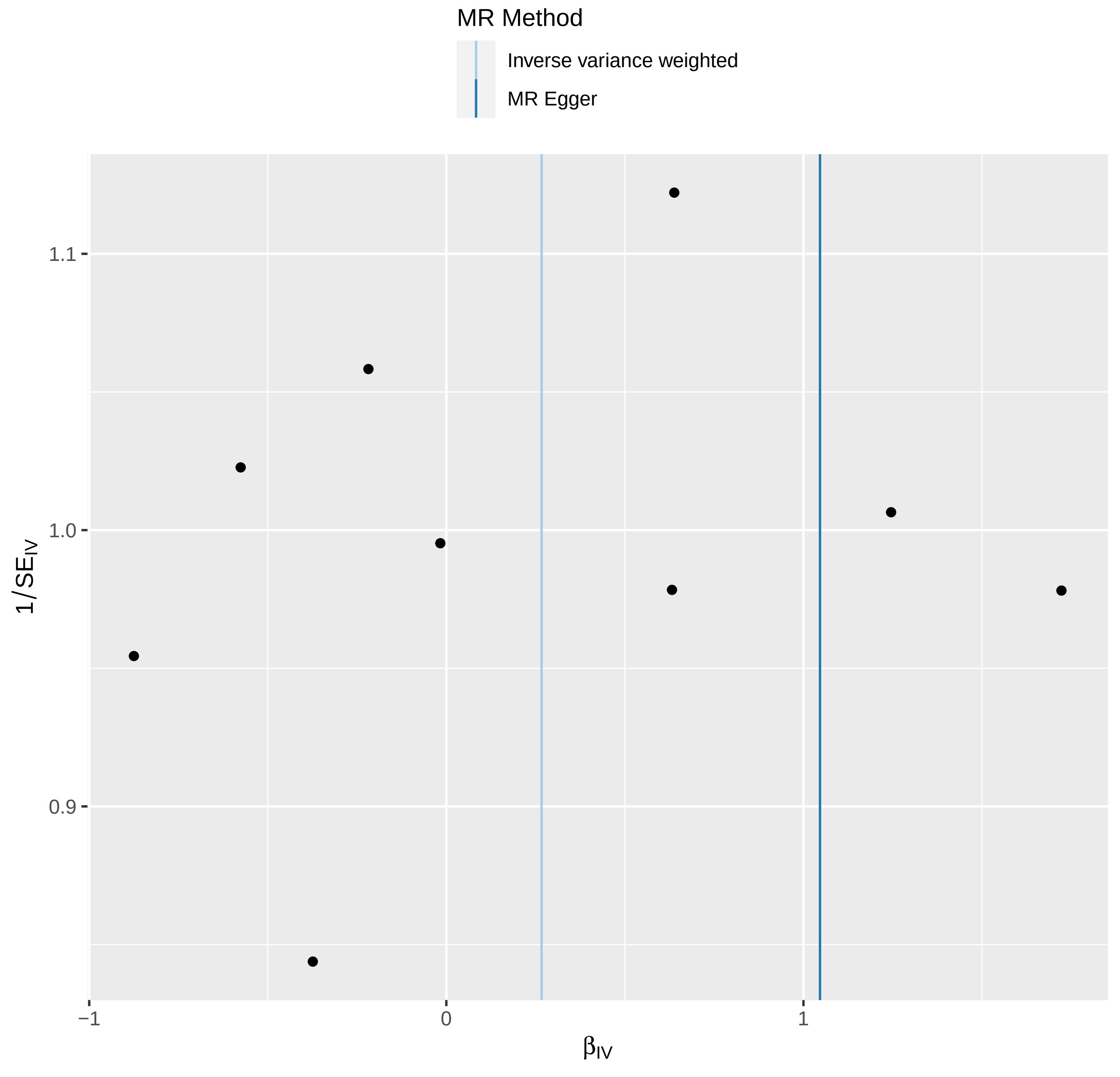

Batch 515 : Gut microbiota abundance (genus Eisenbergiella id.11304) on Drug−induced obesity

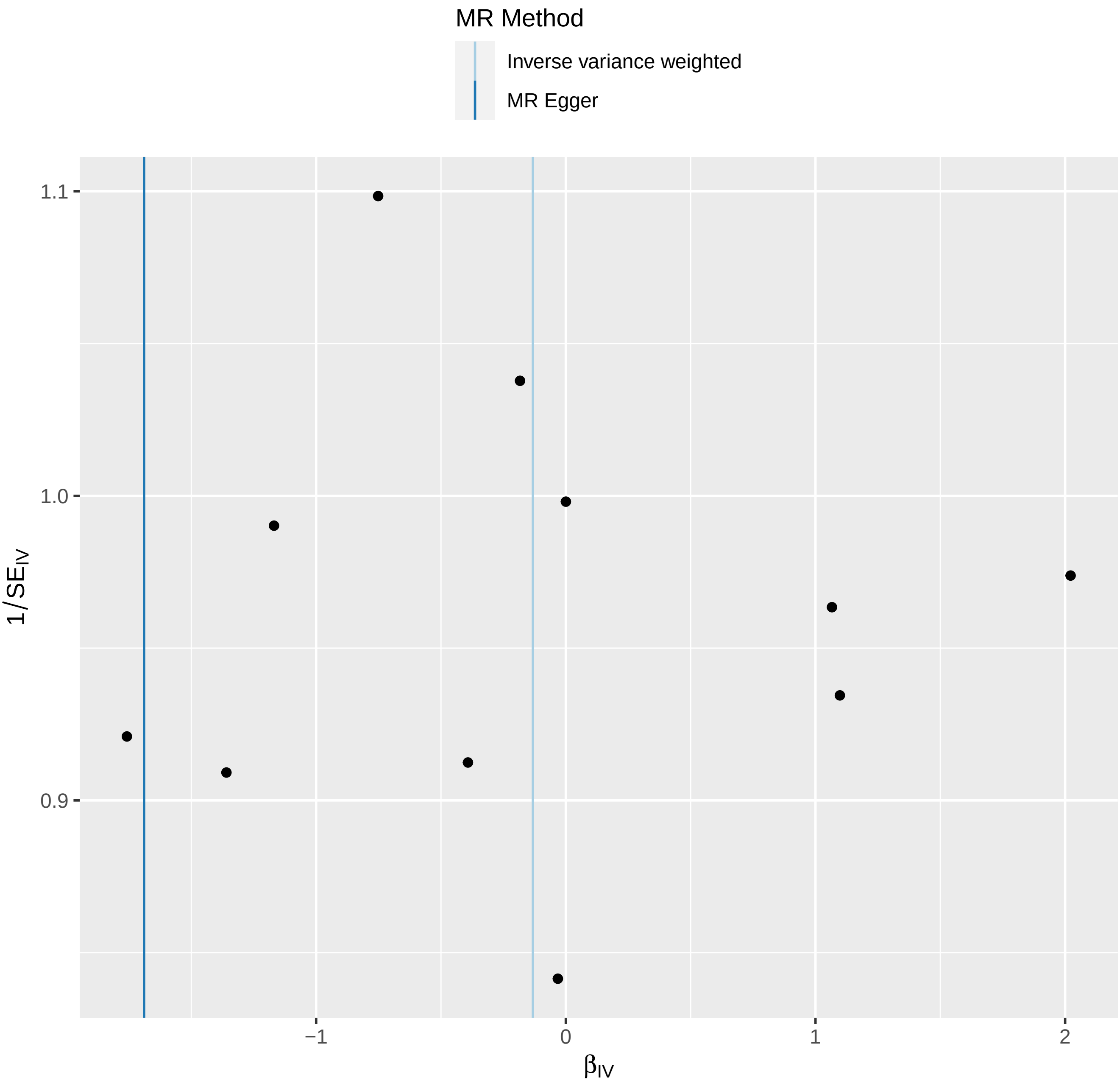

Batch 516 : Gut microbiota abundance (genus Enterorhabdus id.820) on Drug−induced obesity

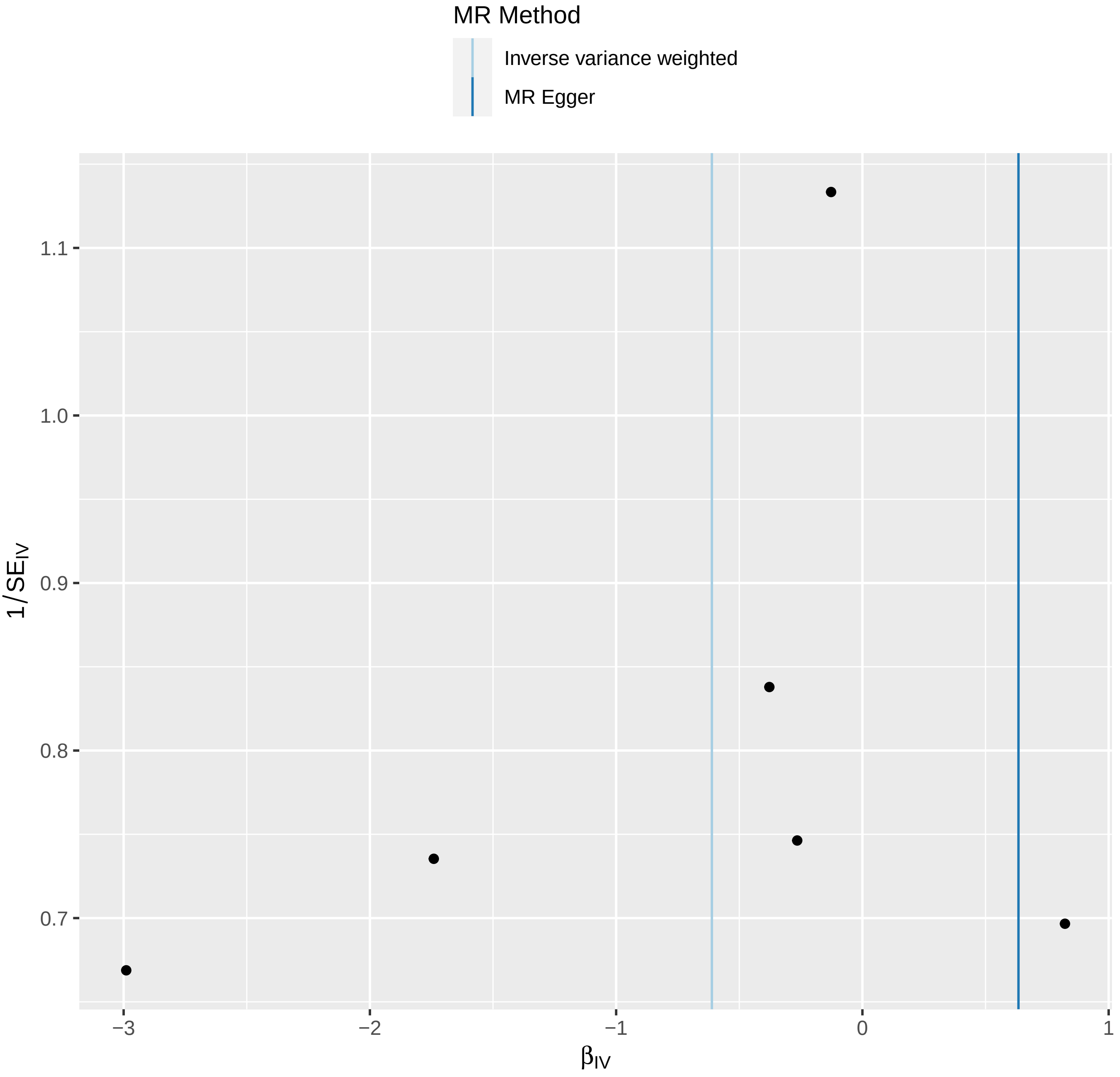

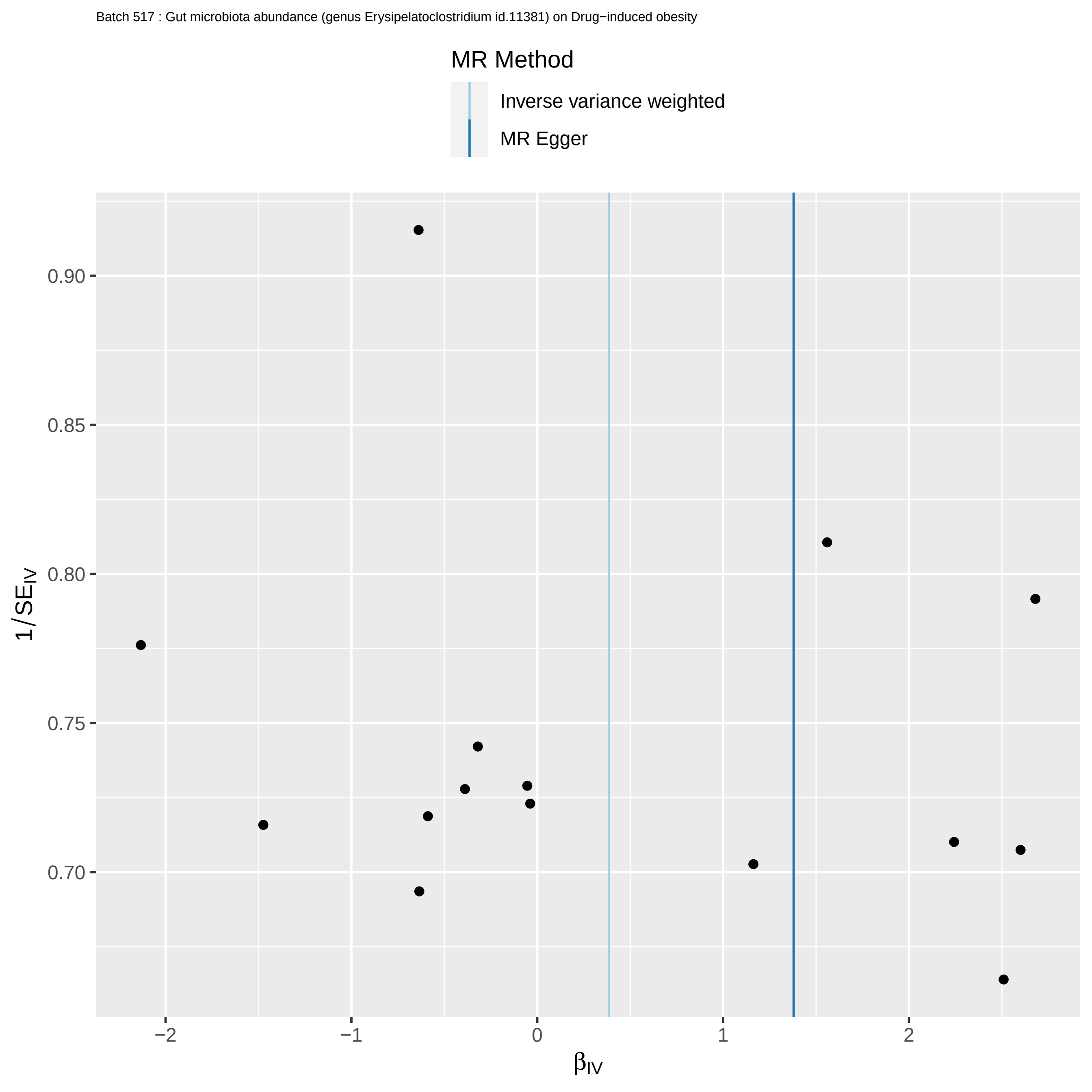

Batch 517 : Gut microbiota abundance (genus Erysipelatoclostridium id.11381) on Drug−induced obesity
MR Method
Inverse variance weighted
MR Egger
0.90
0.85
0.80
0.75
0.70
1/SE_IV
−2
−1
0
1
2
β_IV

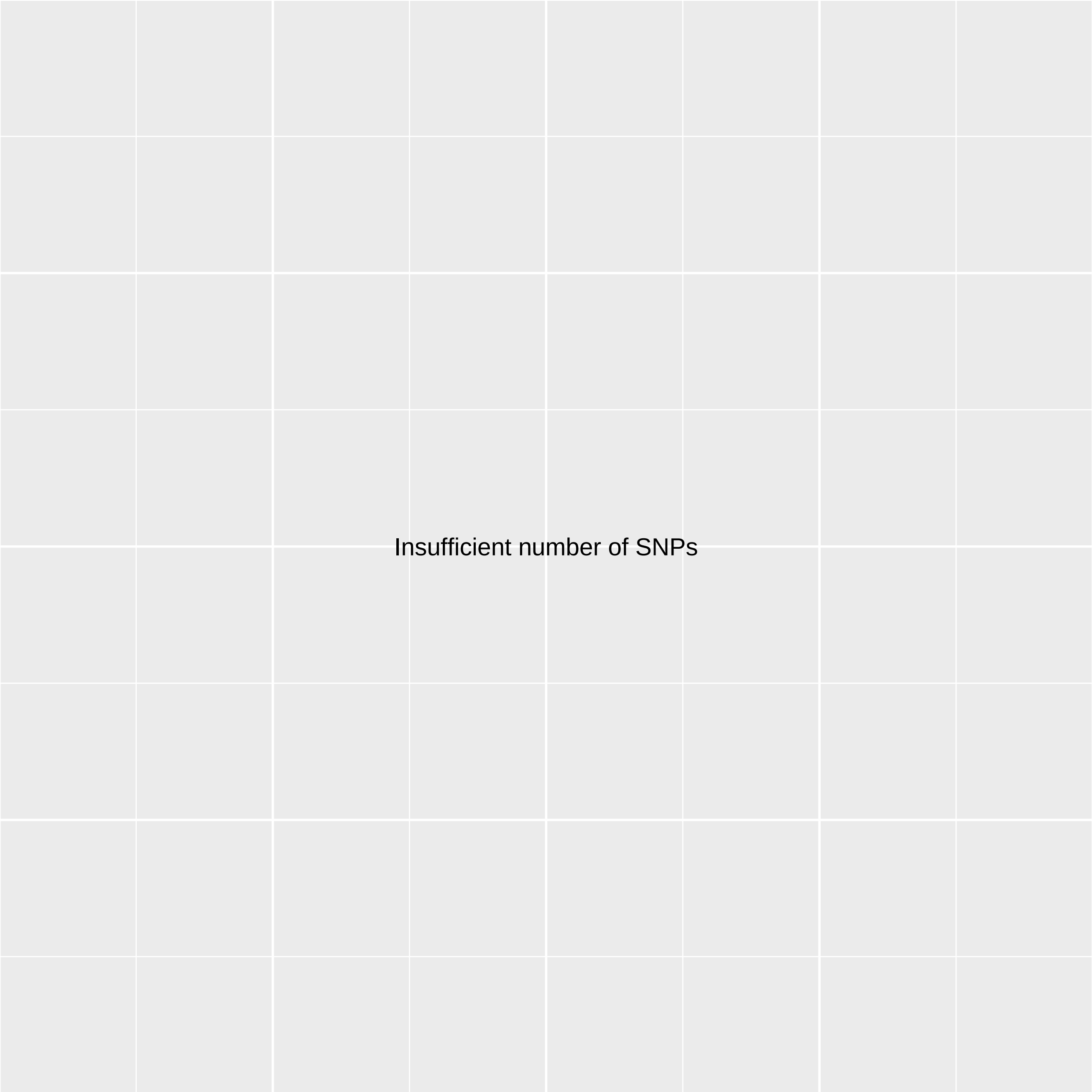
Insufficient number of SNPs

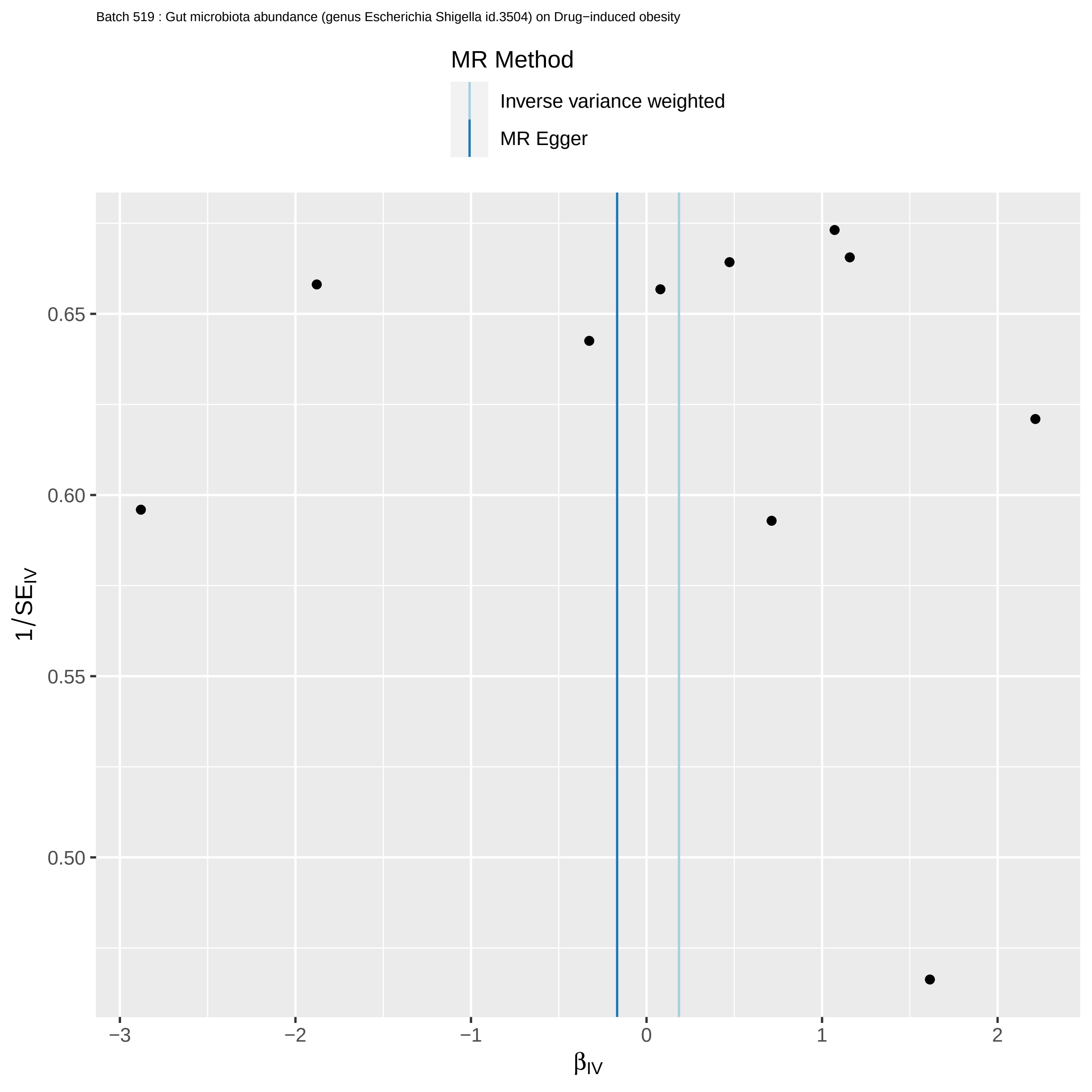

Batch 519 : Gut microbiota abundance (genus Escherichia Shigella id.3504) on Drug−induced obesity
MR Method
Inverse variance weighted
MR Egger
0.65
0.60
0.55
0.50
1/SE_IV
−3
−2
−1
0
1
2
β_IV

Batch 520 : Gut microbiota abundance (genus Eubacterium brachy group id.11296) on Drug−induced obesity

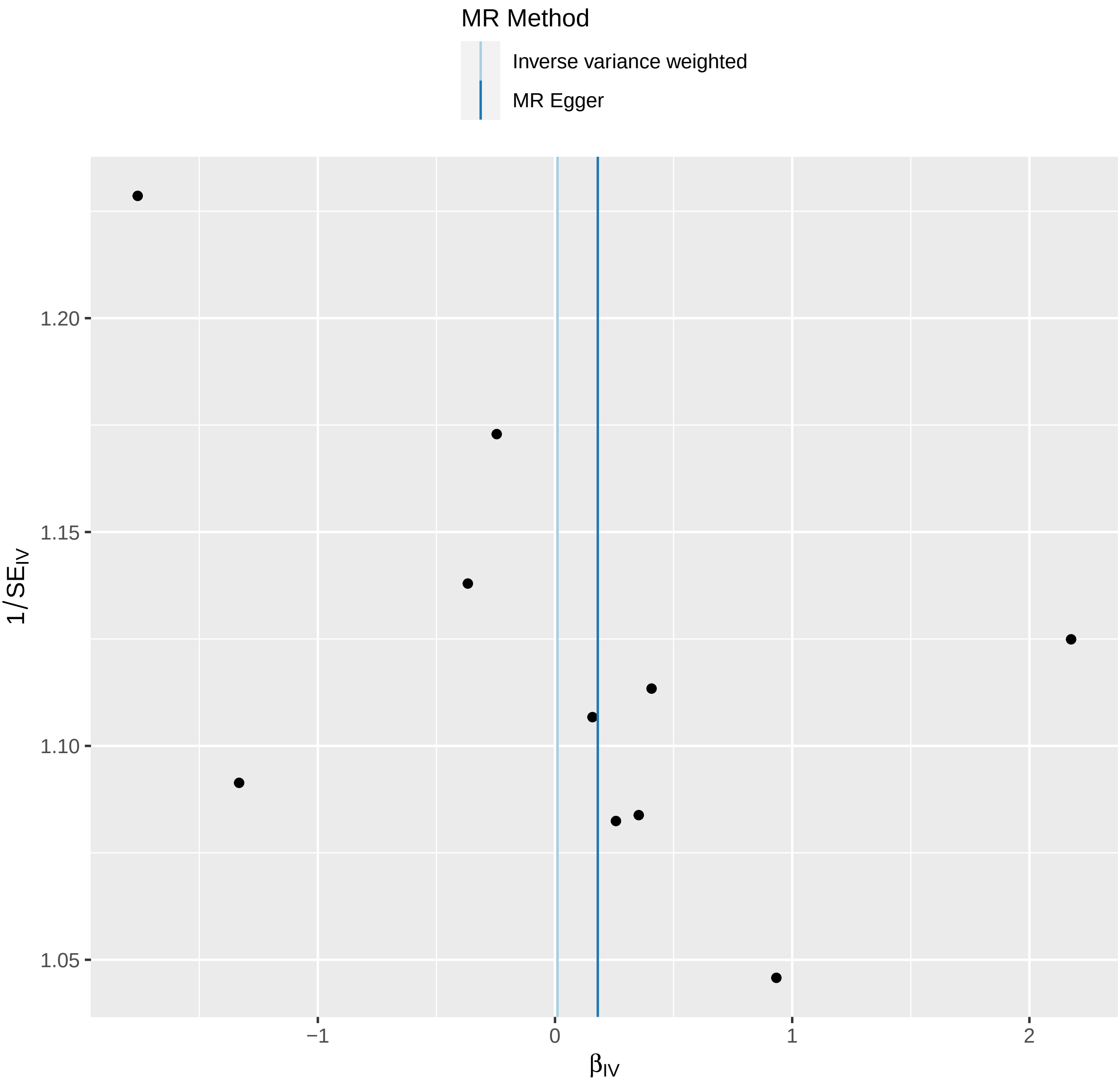

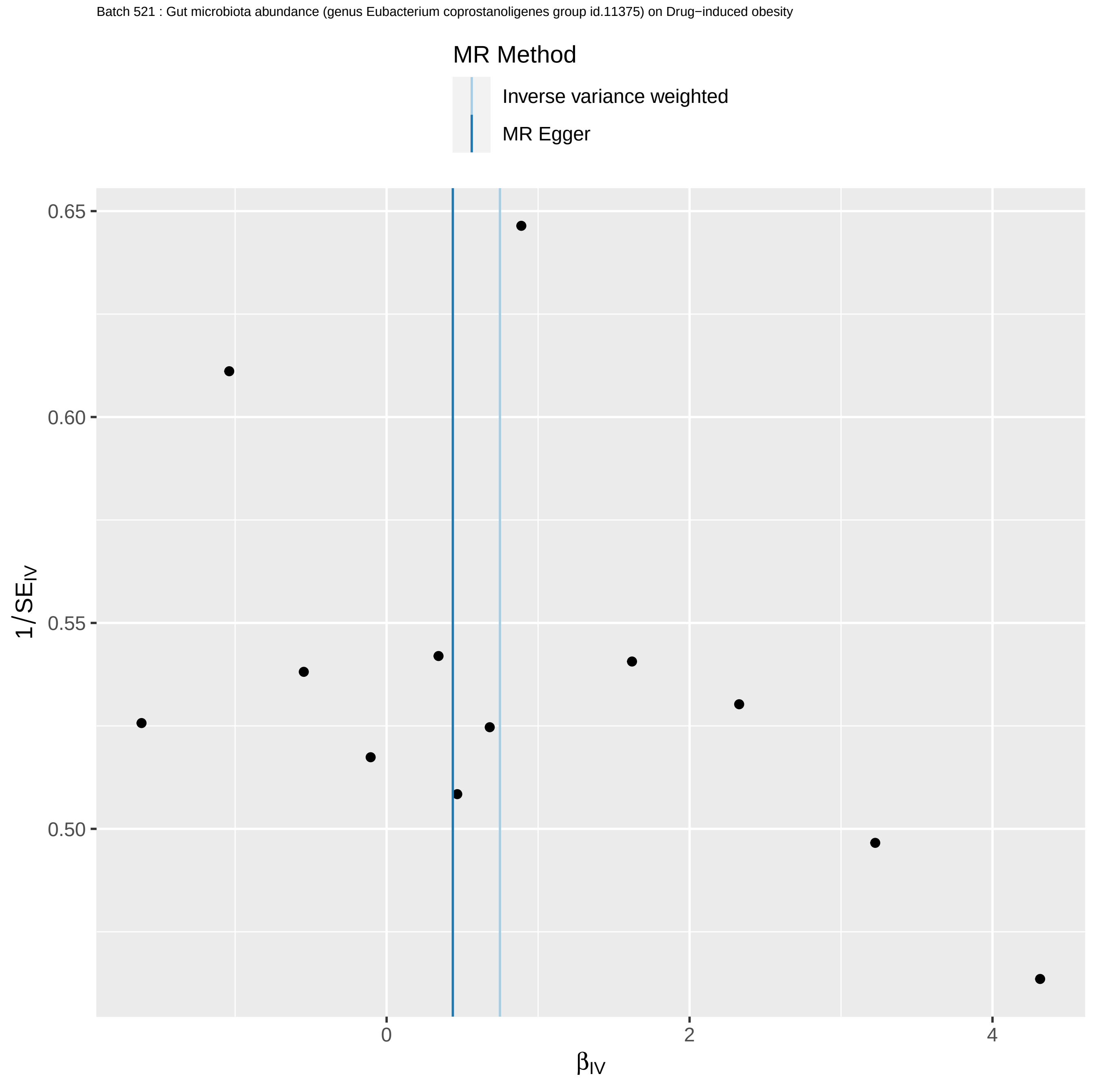

Batch 521 : Gut microbiota abundance (genus Eubacterium coprostanoligenes group id.11375) on Drug−induced obesity
MR Method
Inverse variance weighted
MR Egger
0.65
0.60
0.55
0.50
1/SE_IV
0
2
4
β_IV

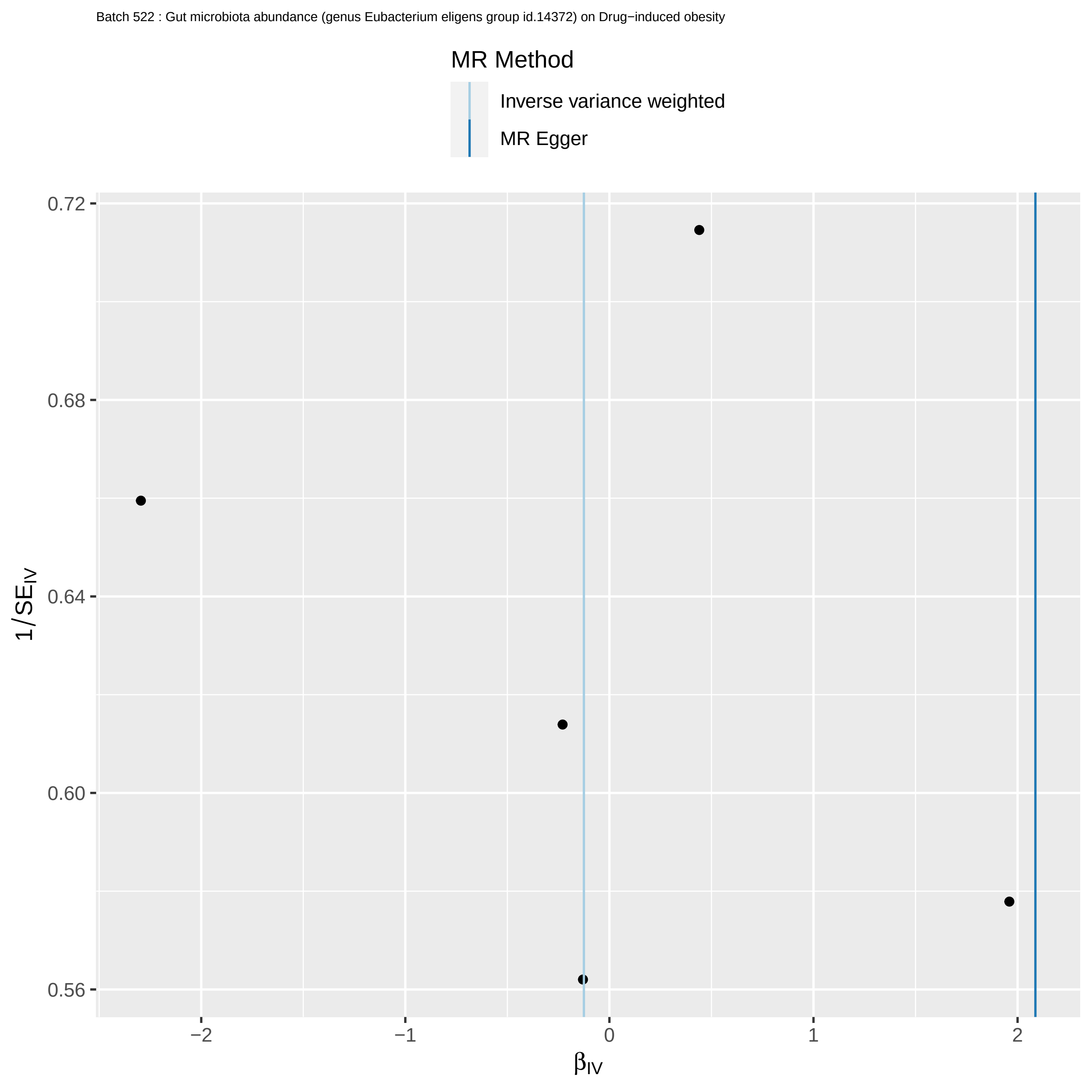
Batch 522 : Gut microbiota abundance (genus Eubacterium eligens group id.14372) on Drug−induced obesity
MR Method
Inverse variance weighted
MR Egger
0.72
0.68
0.64
0.60
0.56
$1/SE_{IV}$
−2
−1
0
1
2
$\beta_{IV}$

Batch 523 : Gut microbiota abundance (genus Eubacterium fissicatena group id.14373) on Drug−induced obesity

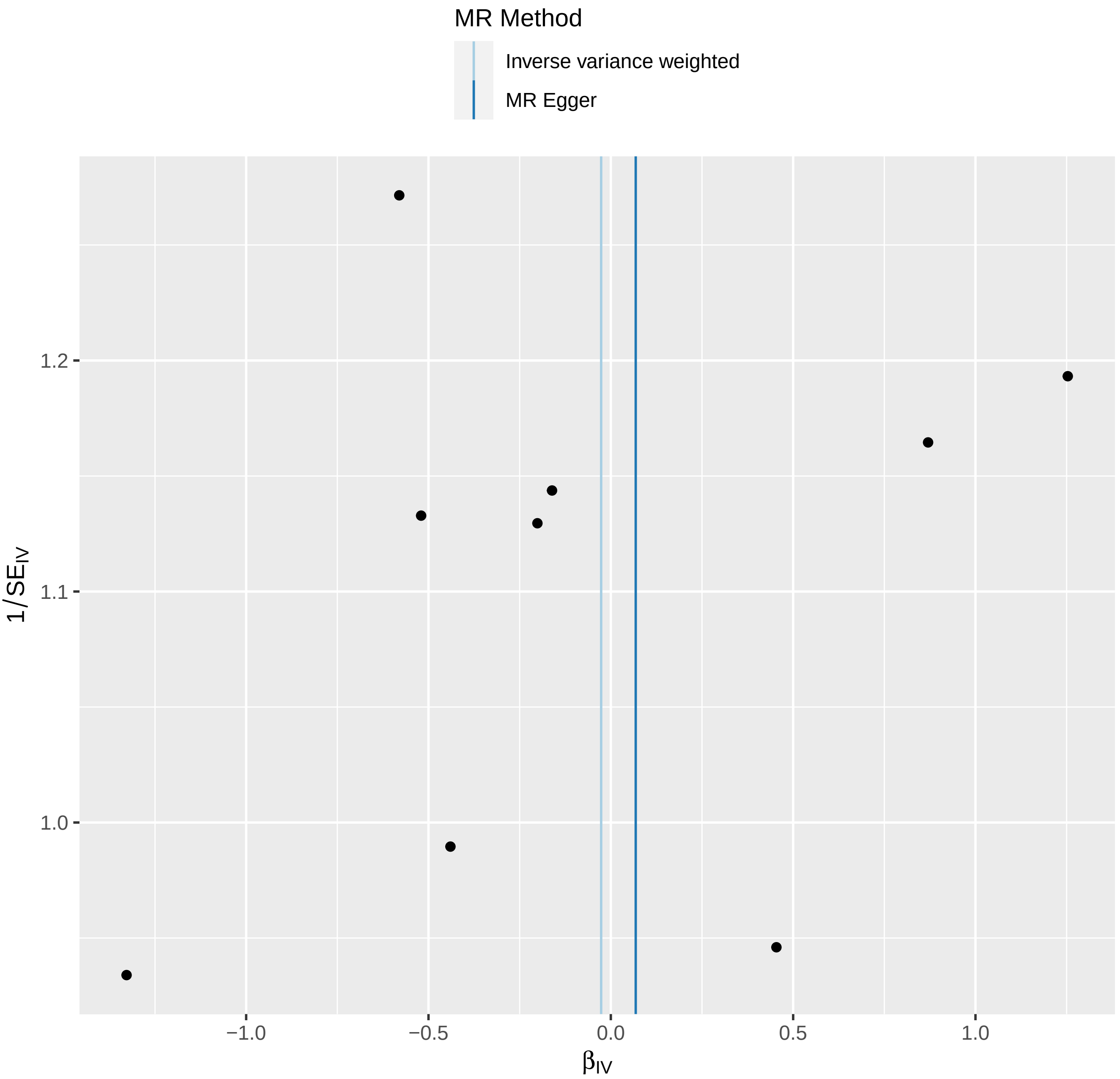

Batch 524 : Gut microbiota abundance (genus Eubacterium hallii group id.11338) on Drug−induced obesity

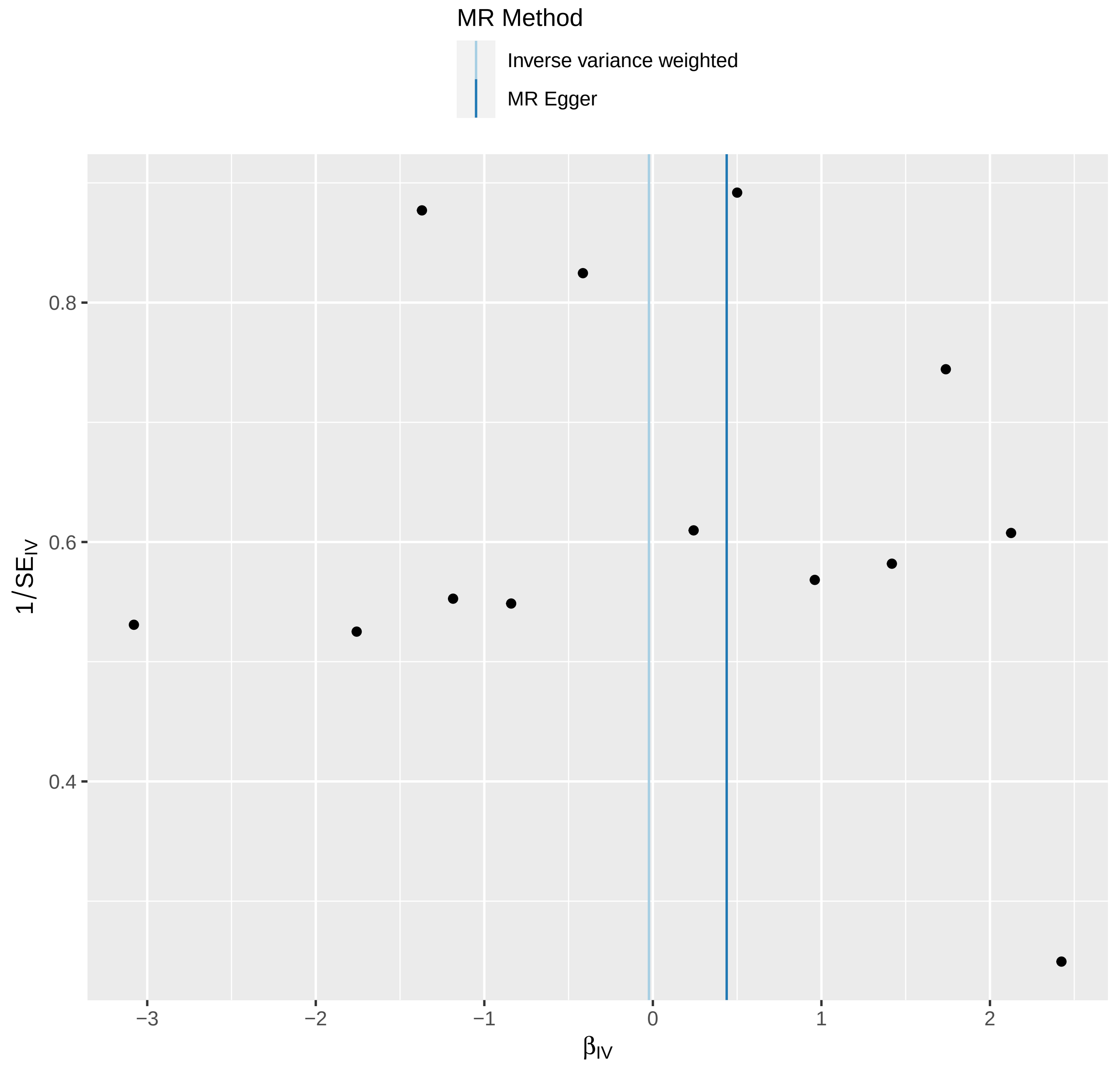

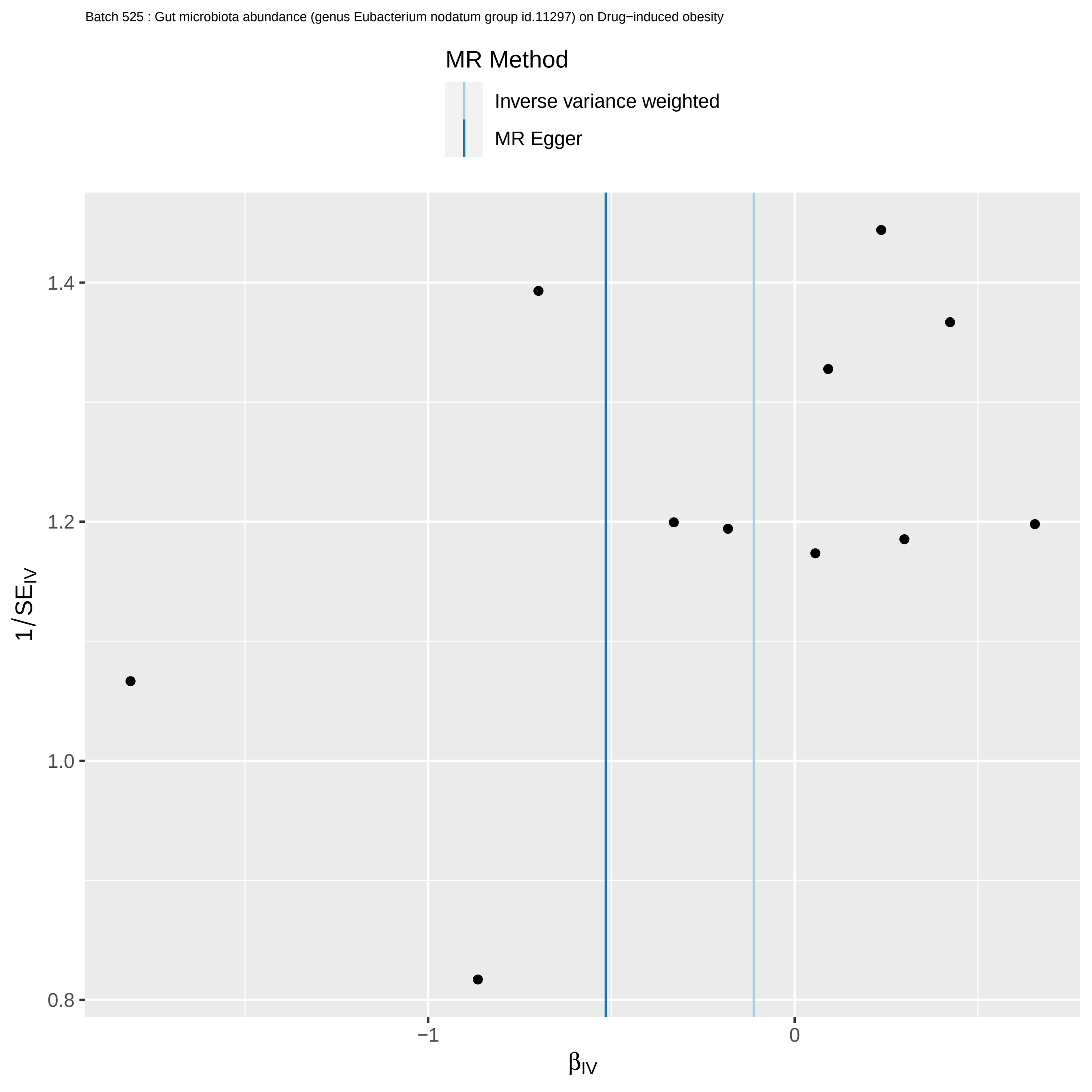
Batch 525 : Gut microbiota abundance (genus Eubacterium nodatum group id.11297) on Drug−induced obesity
MR Method
Inverse variance weighted
MR Egger
1.4
1.2
1.0
0.8
1/SE_IV
−1
0
β_IV

Batch 526 : Gut microbiota abundance (genus Eubacterium oxidoreducens group id.11339) on Drug−induced obesity

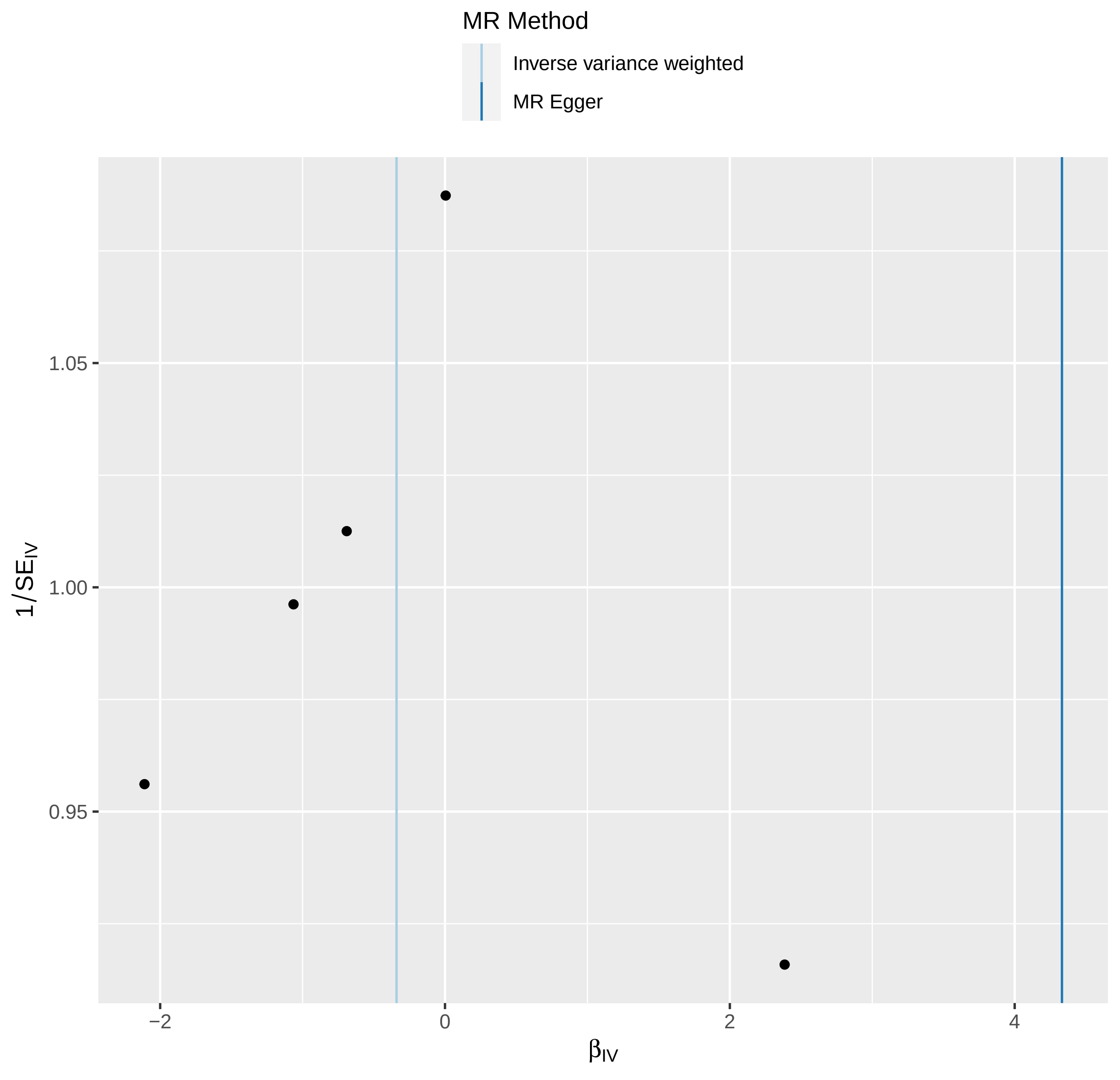

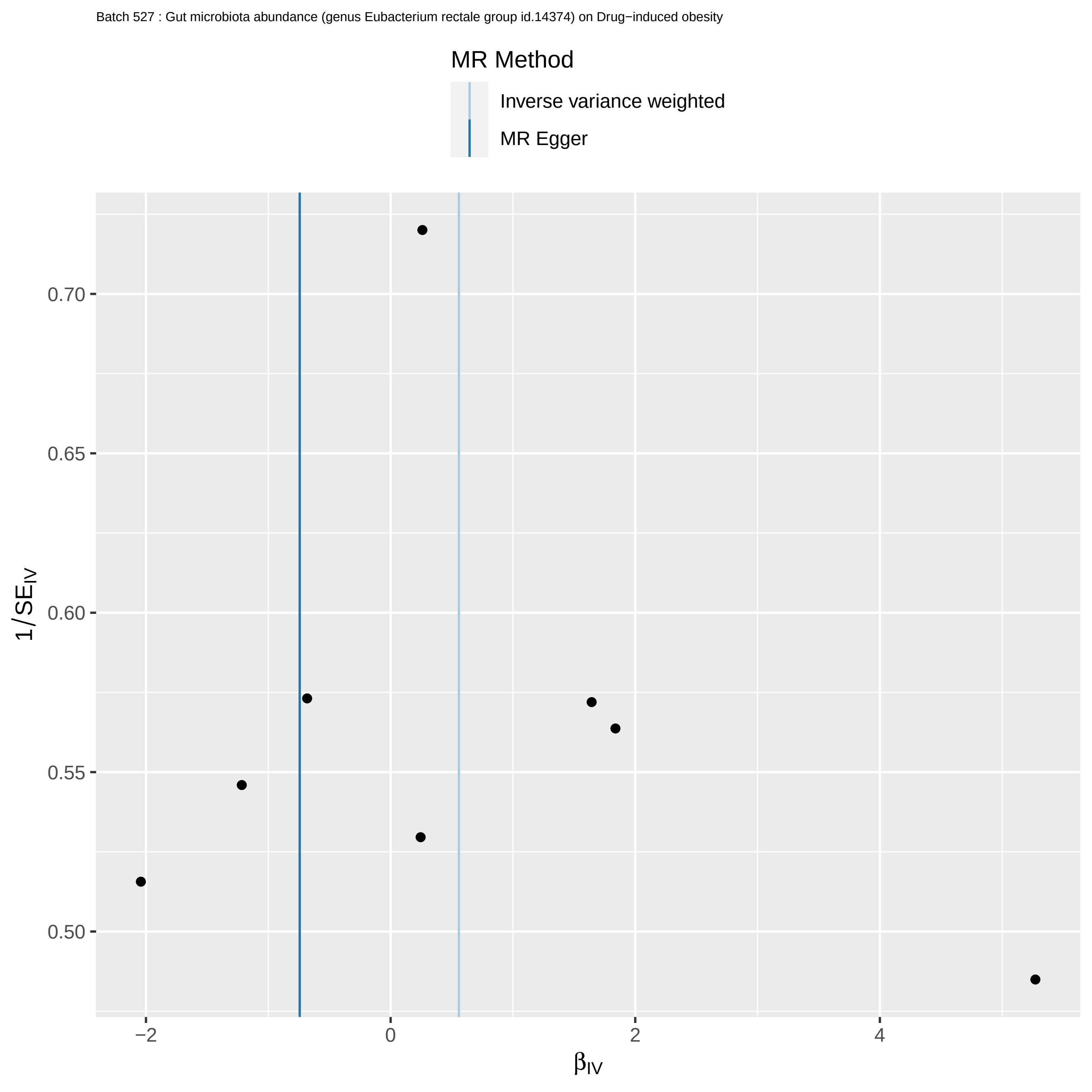

Batch 527 : Gut microbiota abundance (genus Eubacterium rectale group id.14374) on Drug−induced obesity
MR Method
Inverse variance weighted
MR Egger
0.70
0.65
0.60
0.55
0.50
1/SE_IV
−2
0
2
4
β_IV

Batch 528 : Gut microbiota abundance (genus Eubacterium ruminantium group id.11340) on Drug−induced obesity

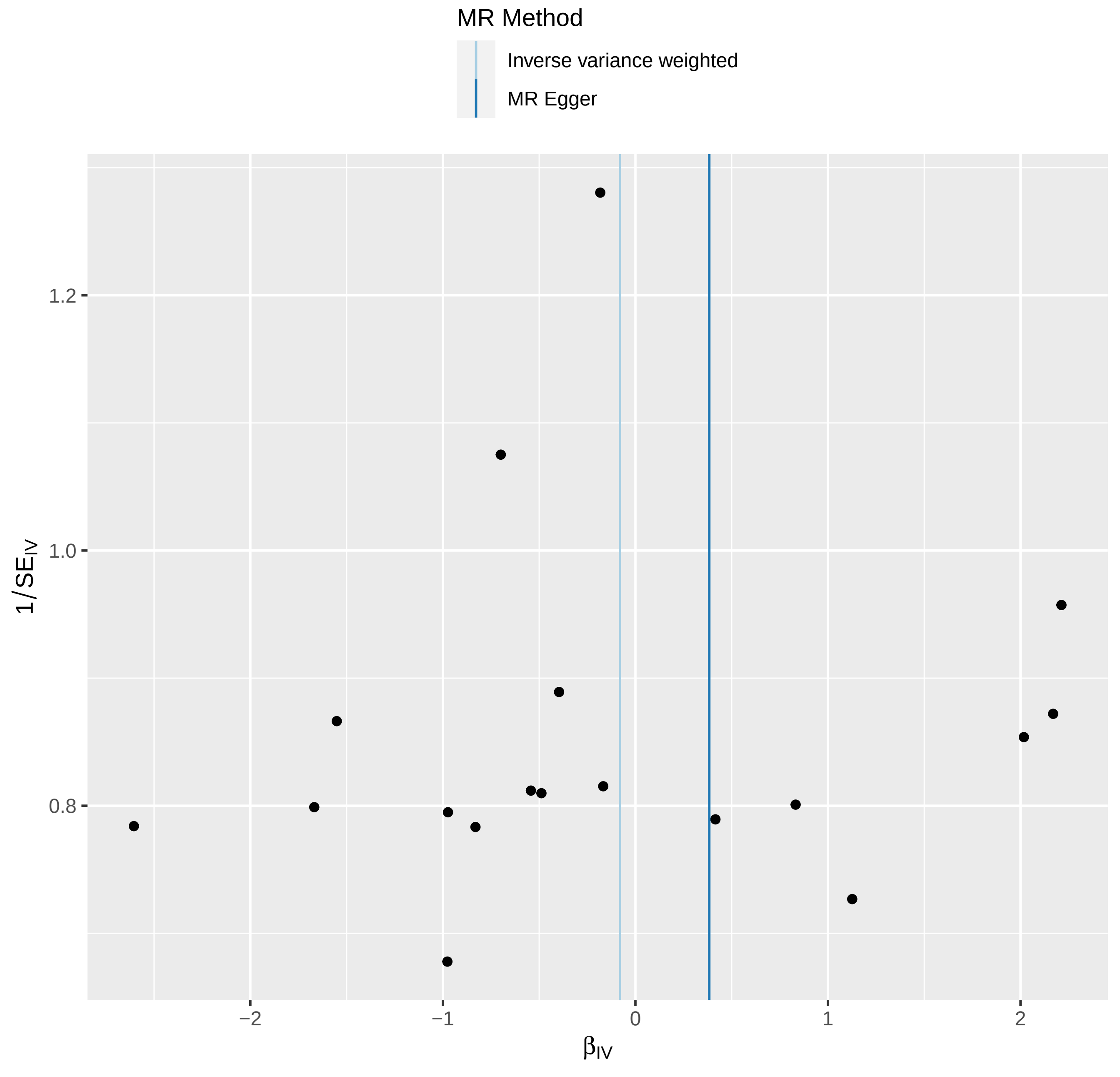

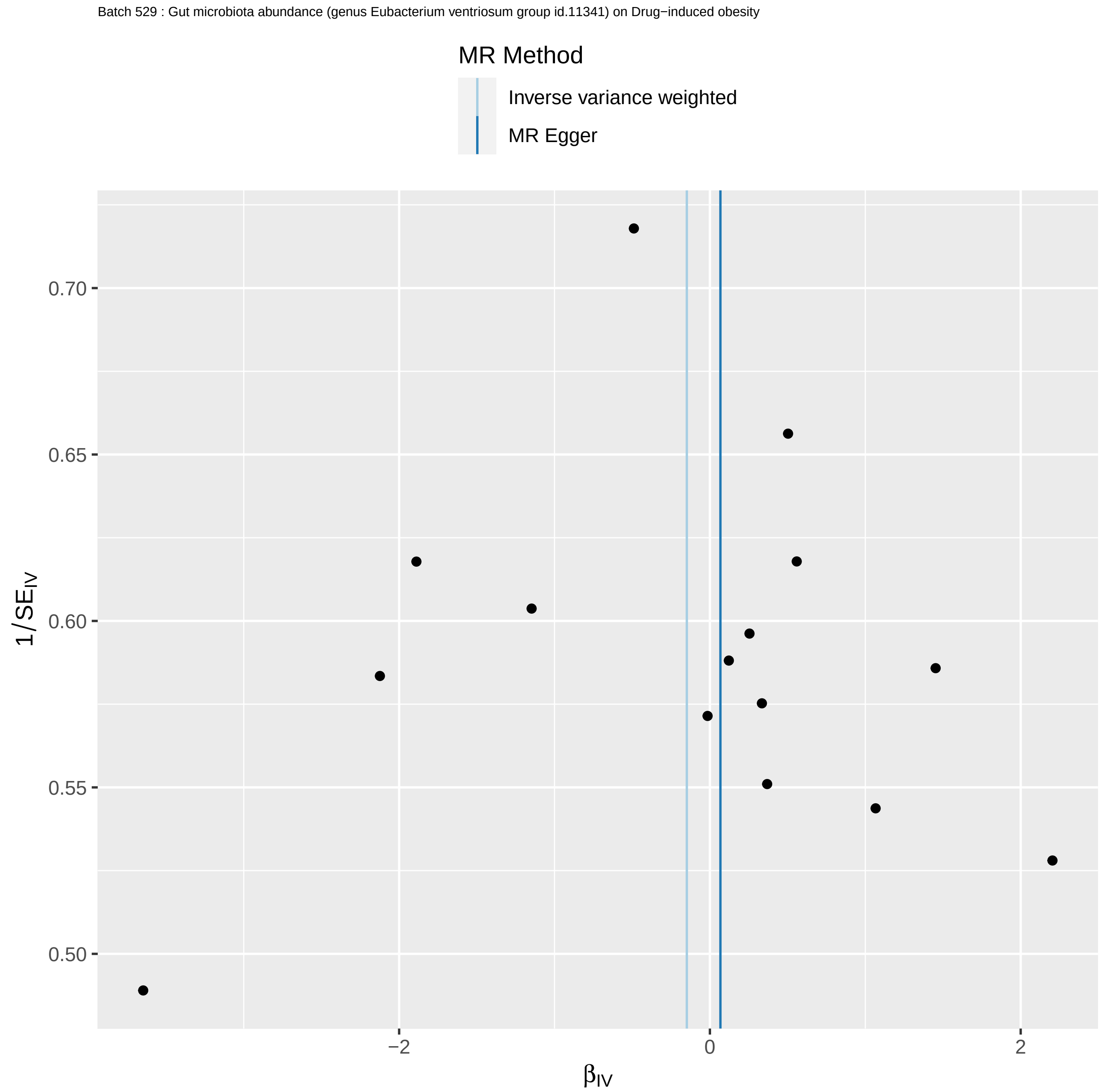
Batch 529 : Gut microbiota abundance (genus Eubacterium ventriosum group id.11341) on Drug−induced obesity
MR Method
Inverse variance weighted
MR Egger
0.70
0.65
0.60
0.55
0.50
1/SE_IV
−2
0
2
β_IV

Batch 530 : Gut microbiota abundance (genus Eubacterium xylanophilum group id.14375) on Drug−induced obesity

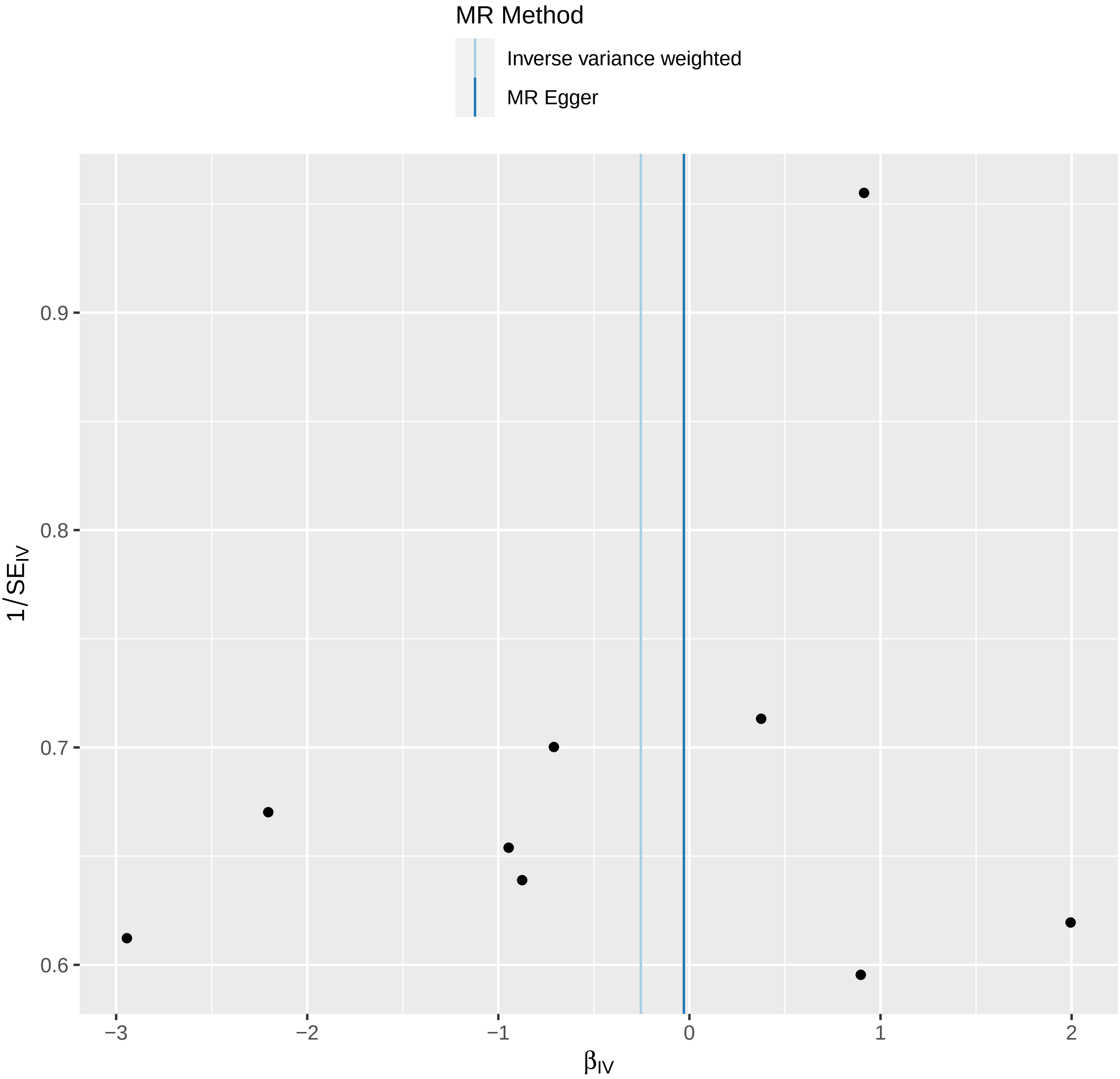

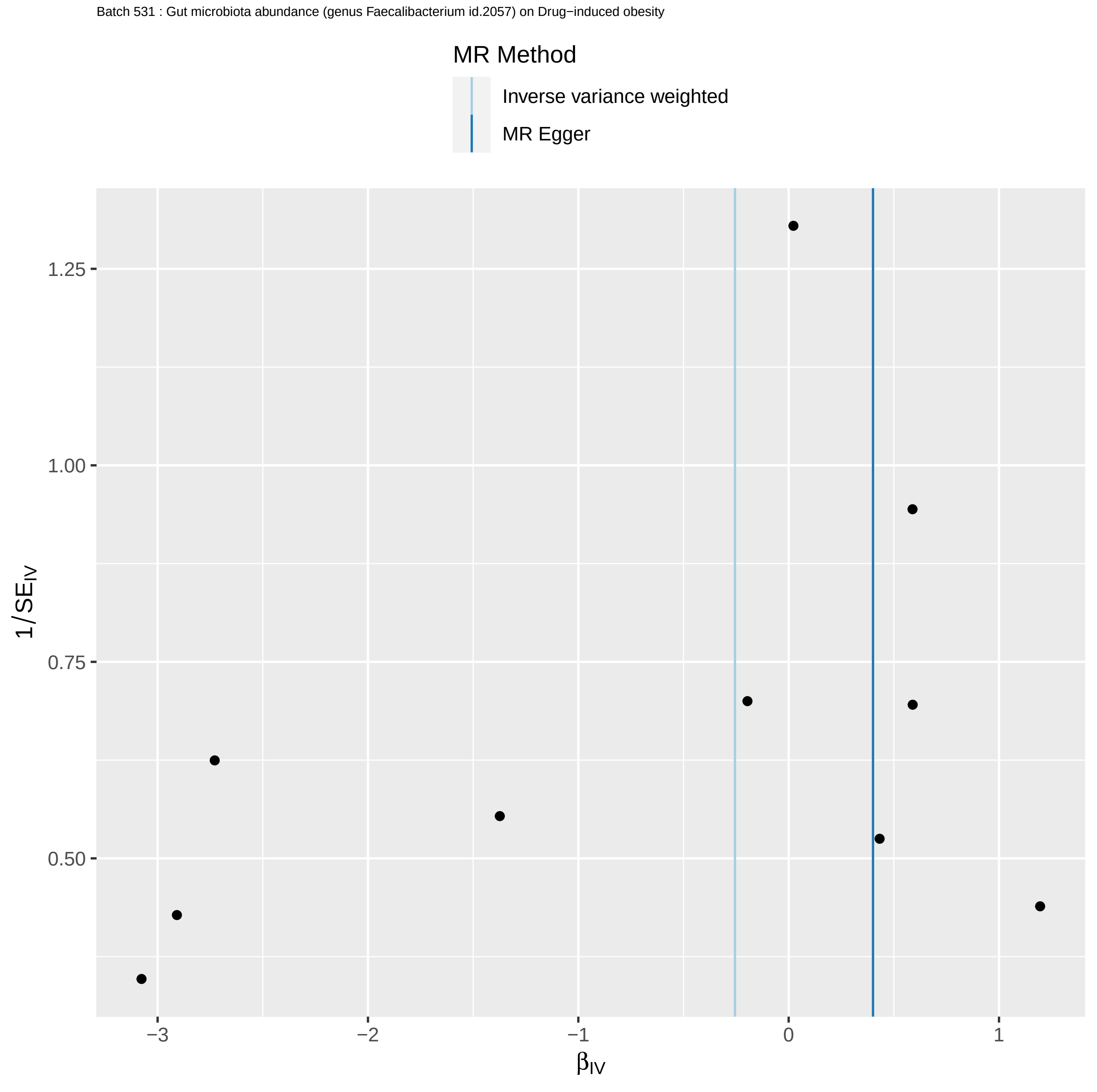

Batch 531 : Gut microbiota abundance (genus Faecalibacterium id.2057) on Drug−induced obesity
MR Method
Inverse variance weighted
MR Egger
1.25
1.00
0.75
0.50
1/SE_IV
−3
−2
−1
0
1
β_IV

Batch 532 : Gut microbiota abundance (genus Family XIII AD3011 group id.11293) on Drug−induced obesity

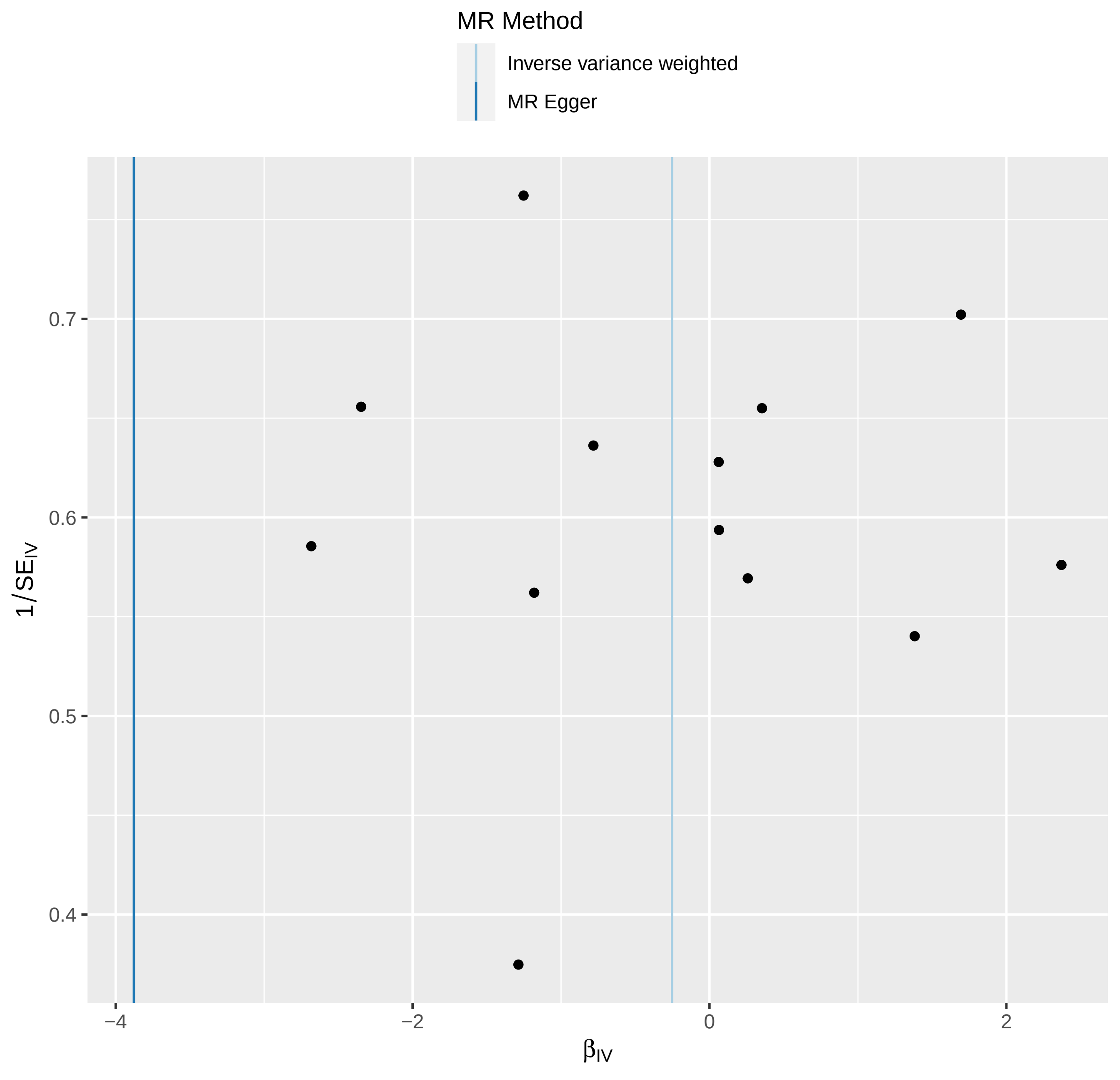

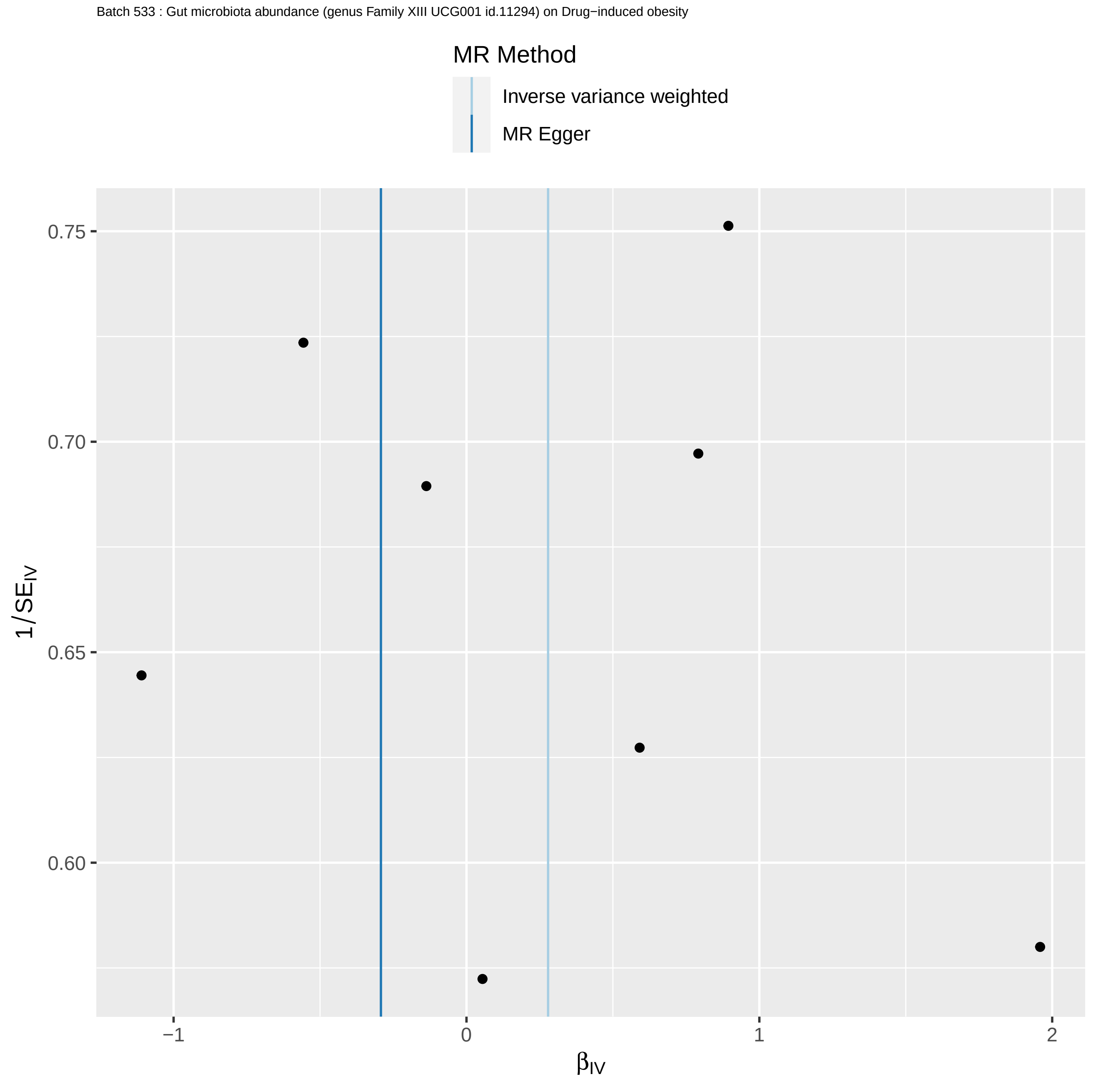

Batch 533 : Gut microbiota abundance (genus Family XIII UCG001 id.11294) on Drug−induced obesity
MR Method
Inverse variance weighted
MR Egger
0.75
0.70
0.65
0.60
1/SE$_{IV}$
−1
0
1
2
$\beta_{IV}$

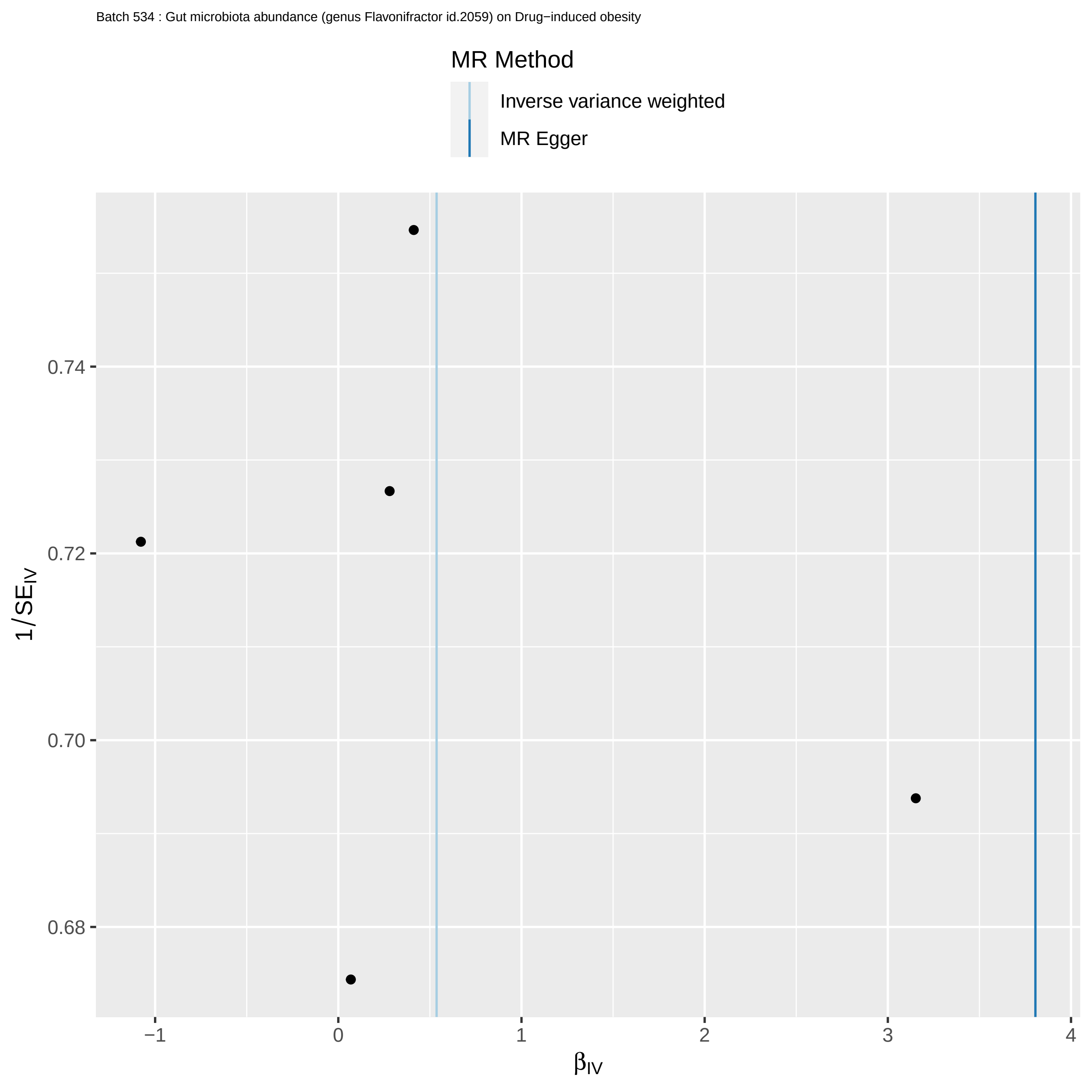
Batch 534 : Gut microbiota abundance (genus Flavonifractor id.2059) on Drug−induced obesity
MR Method
Inverse variance weighted
MR Egger
0.74
0.72
0.70
0.68
1/SE$_{IV}$
−1
0
1
2
3
4
$\beta_{IV}$

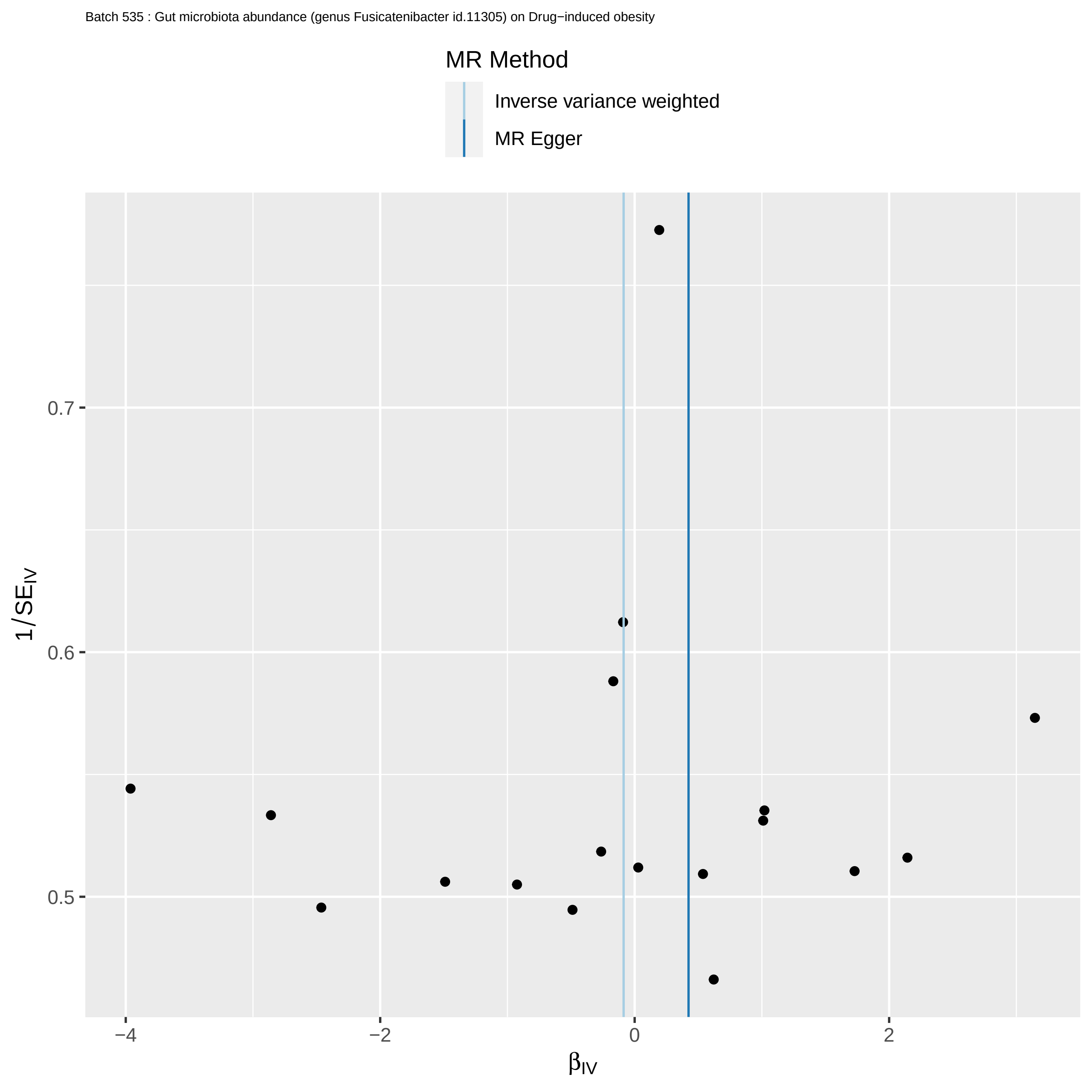
Batch 535 : Gut microbiota abundance (genus Fusicatenibacter id.11305) on Drug−induced obesity
MR Method
Inverse variance weighted
MR Egger
0.7
0.6
0.5
1/SE_IV
−4
−2
0
2
β_IV

Batch 536 : Gut microbiota abundance (genus Gordonibacter id.821) on Drug−induced obesity

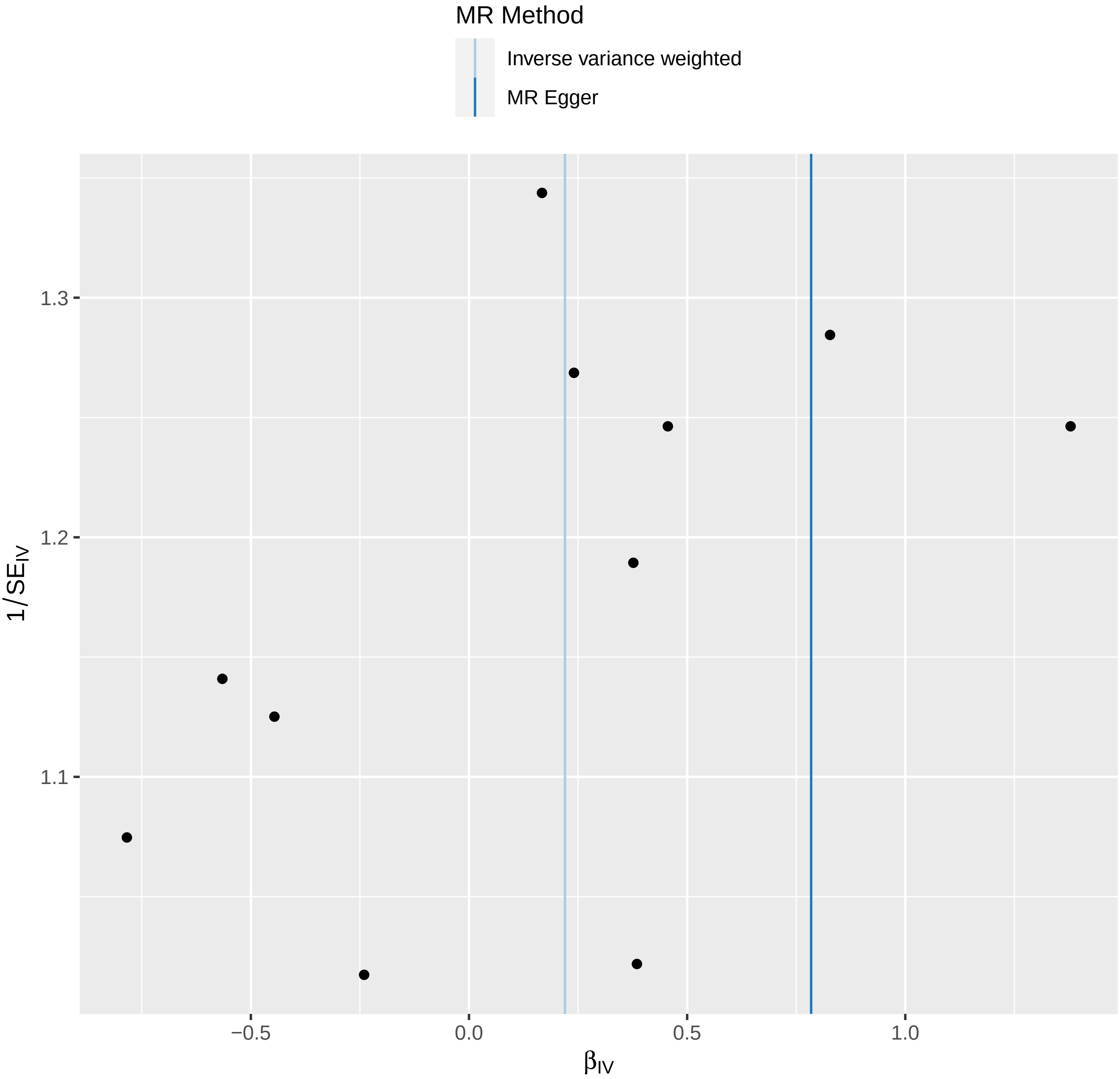

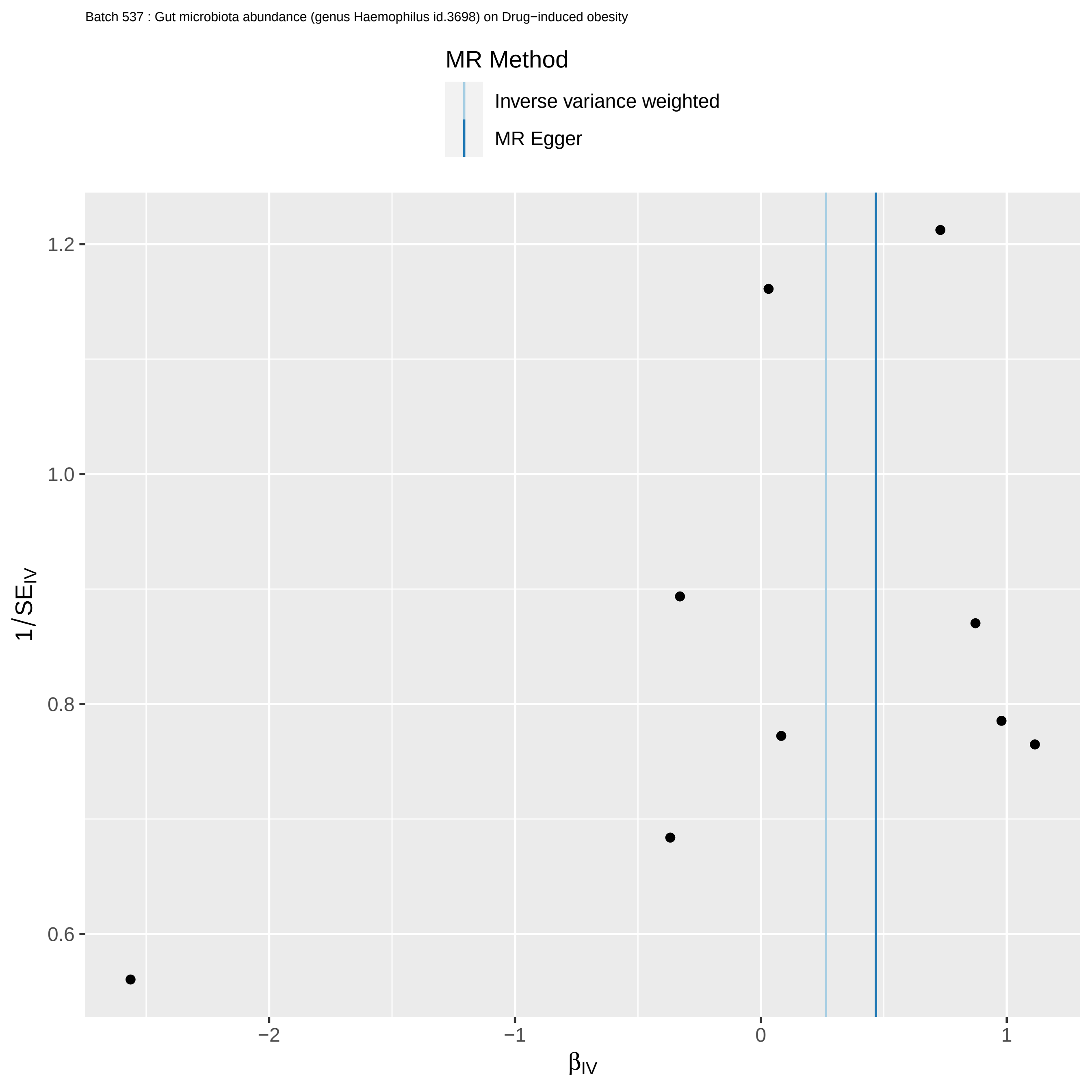
Batch 537 : Gut microbiota abundance (genus Haemophilus id.3698) on Drug−induced obesity
MR Method
Inverse variance weighted
MR Egger
1.2
1.0
0.8
0.6
1/SE_IV
−2
−1
0
1
β_IV

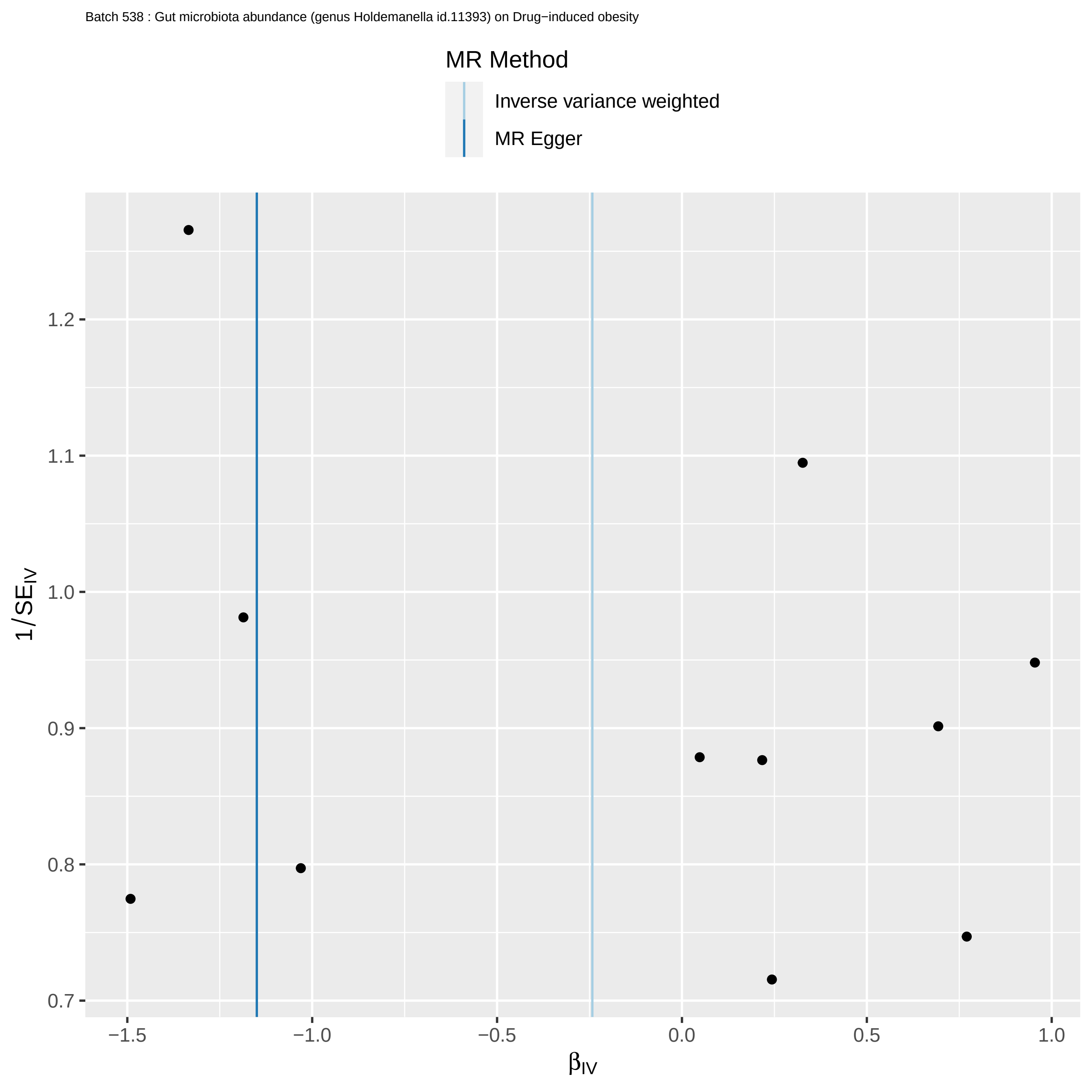
Batch 538 : Gut microbiota abundance (genus Holdemanella id.11393) on Drug−induced obesity
MR Method
Inverse variance weighted
MR Egger
1/SE_IV
1.2
1.1
1.0
0.9
0.8
0.7
−1.5
−1.0
−0.5
0.0
0.5
1.0
β_IV

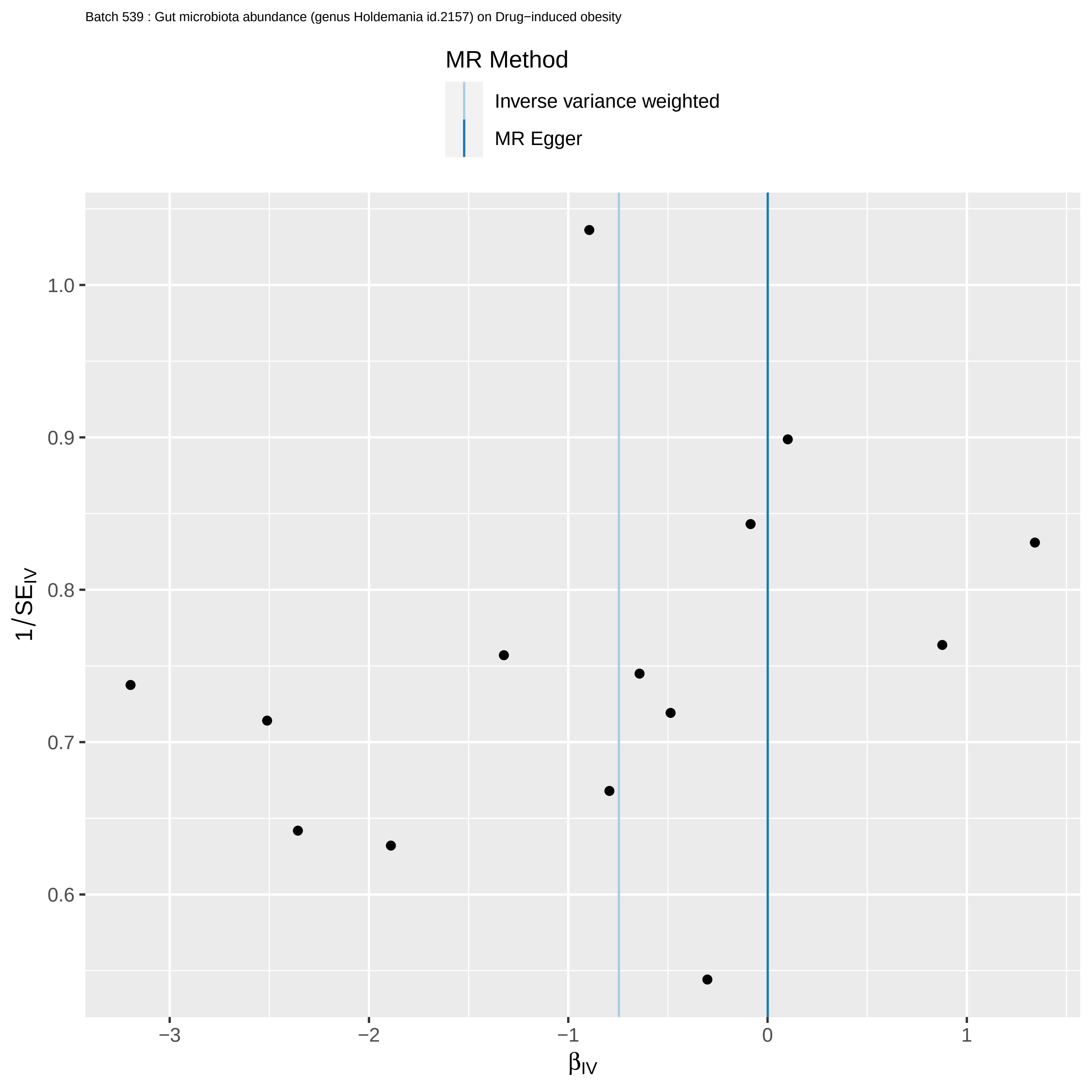
Batch 539 : Gut microbiota abundance (genus Holdemania id.2157) on Drug−induced obesity
MR Method
Inverse variance weighted
MR Egger
1.0
0.9
0.8
0.7
0.6
1/SE$_{IV}$
−3
−2
−1
0
1
β$_{IV}$

Batch 540 : Gut microbiota abundance (genus Howardella id.2000) on Drug−induced obesity

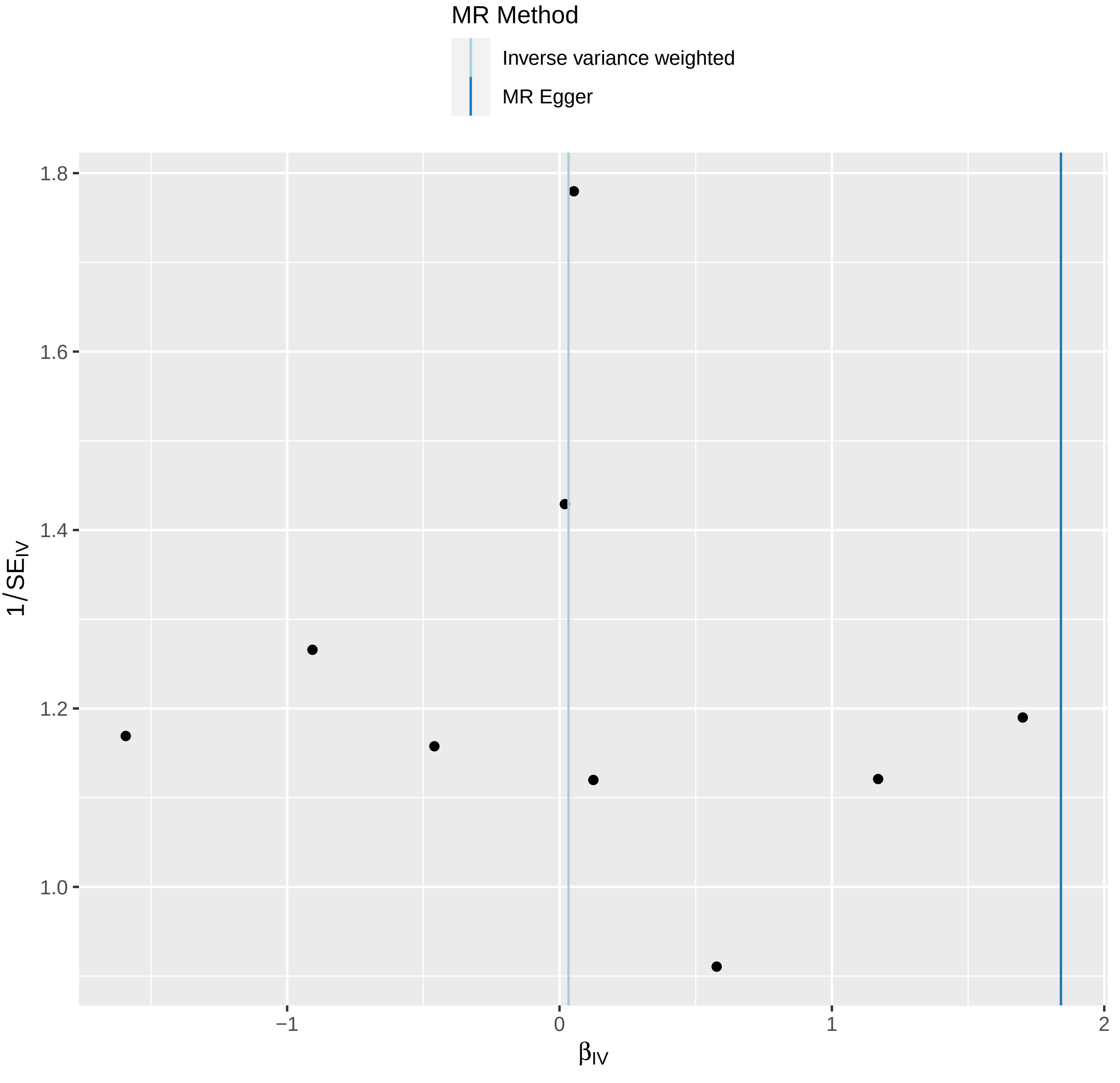

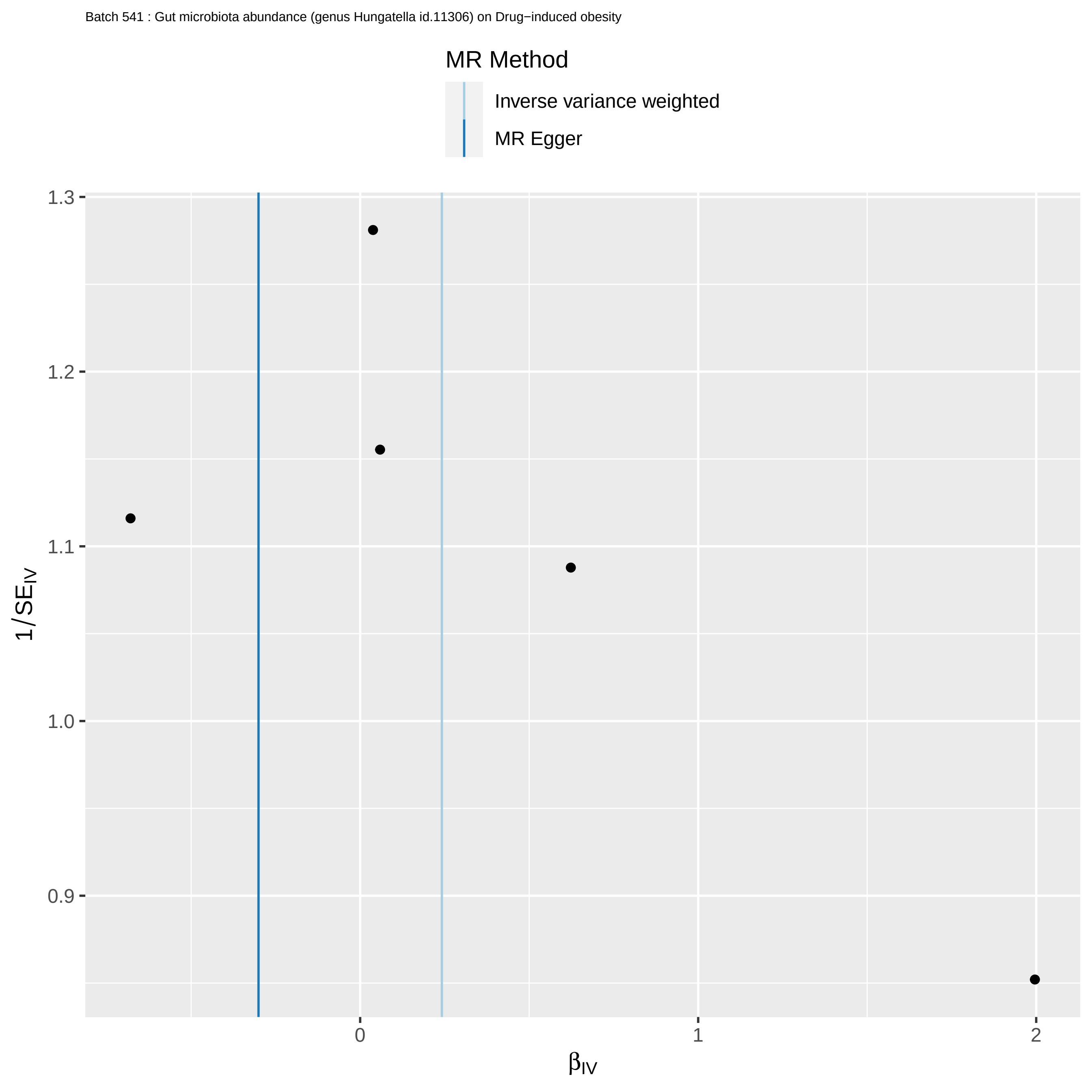
Batch 541 : Gut microbiota abundance (genus Hungatella id.11306) on Drug−induced obesity
MR Method
Inverse variance weighted
MR Egger
1.3
1.2
1.1
1.0
0.9
1/SE_IV
0
1
2
β_IV

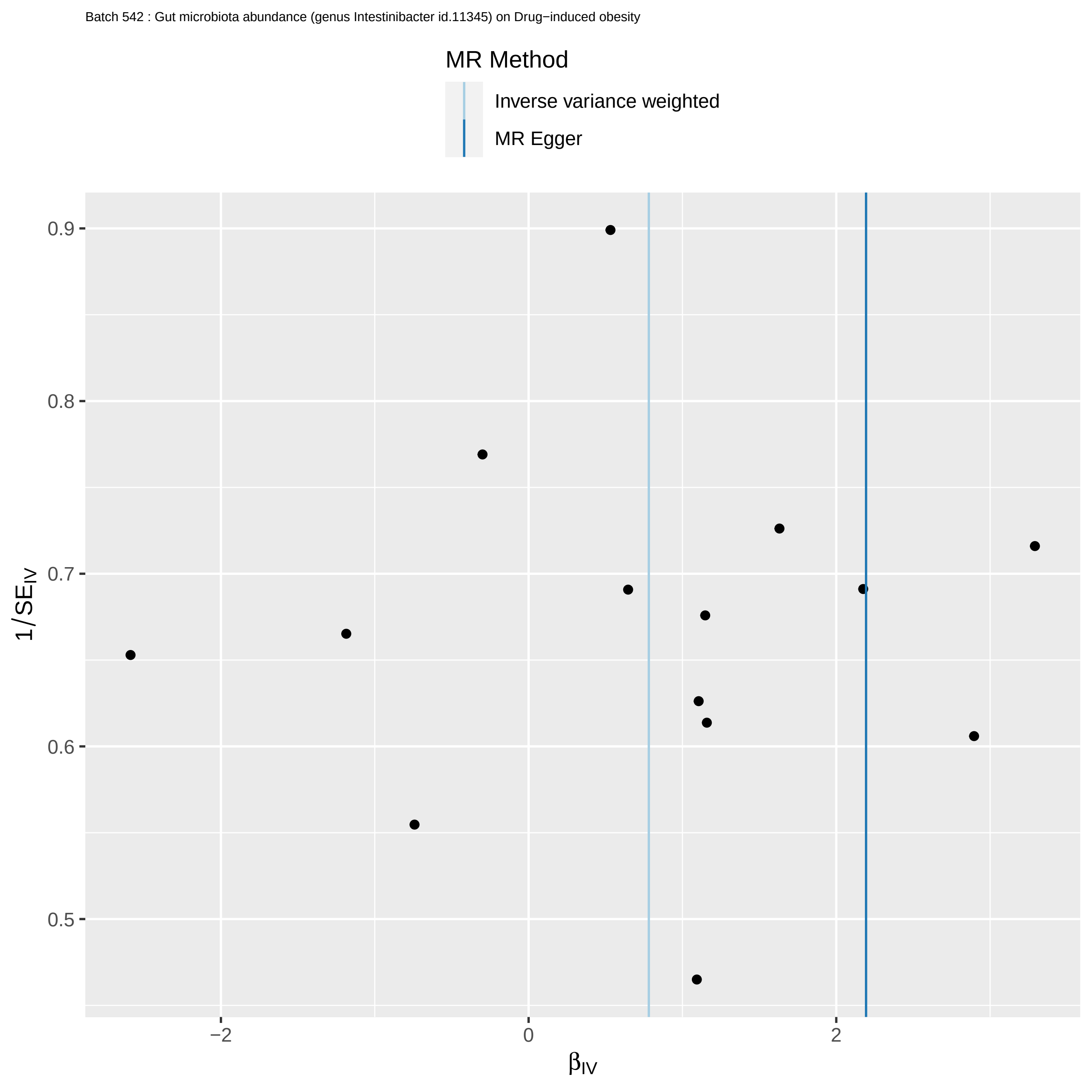

Batch 542 : Gut microbiota abundance (genus Intestinibacter id.11345) on Drug−induced obesity
MR Method
Inverse variance weighted
MR Egger
0.9
0.8
0.7
0.6
0.5
$1/SE_{IV}$
−2
0
2
$\beta_{IV}$

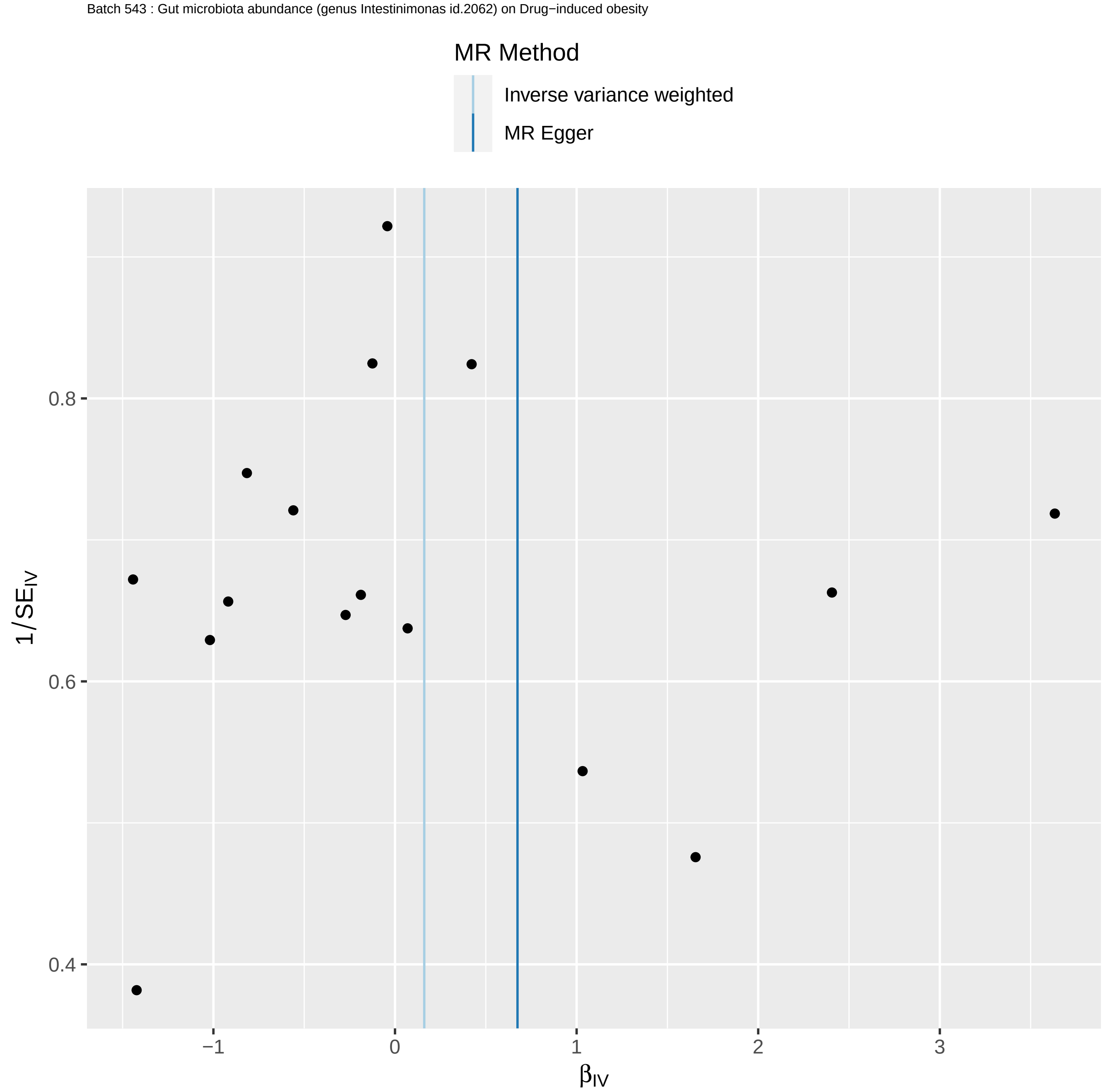

Batch 543 : Gut microbiota abundance (genus Intestinimonas id.2062) on Drug−induced obesity
MR Method
Inverse variance weighted
MR Egger
0.8
0.6
0.4
1/SE_IV
−1
0
1
2
3
β_IV

Batch 544 : Gut microbiota abundance (genus Lachnoclostridium id.11308) on Drug−induced obesity

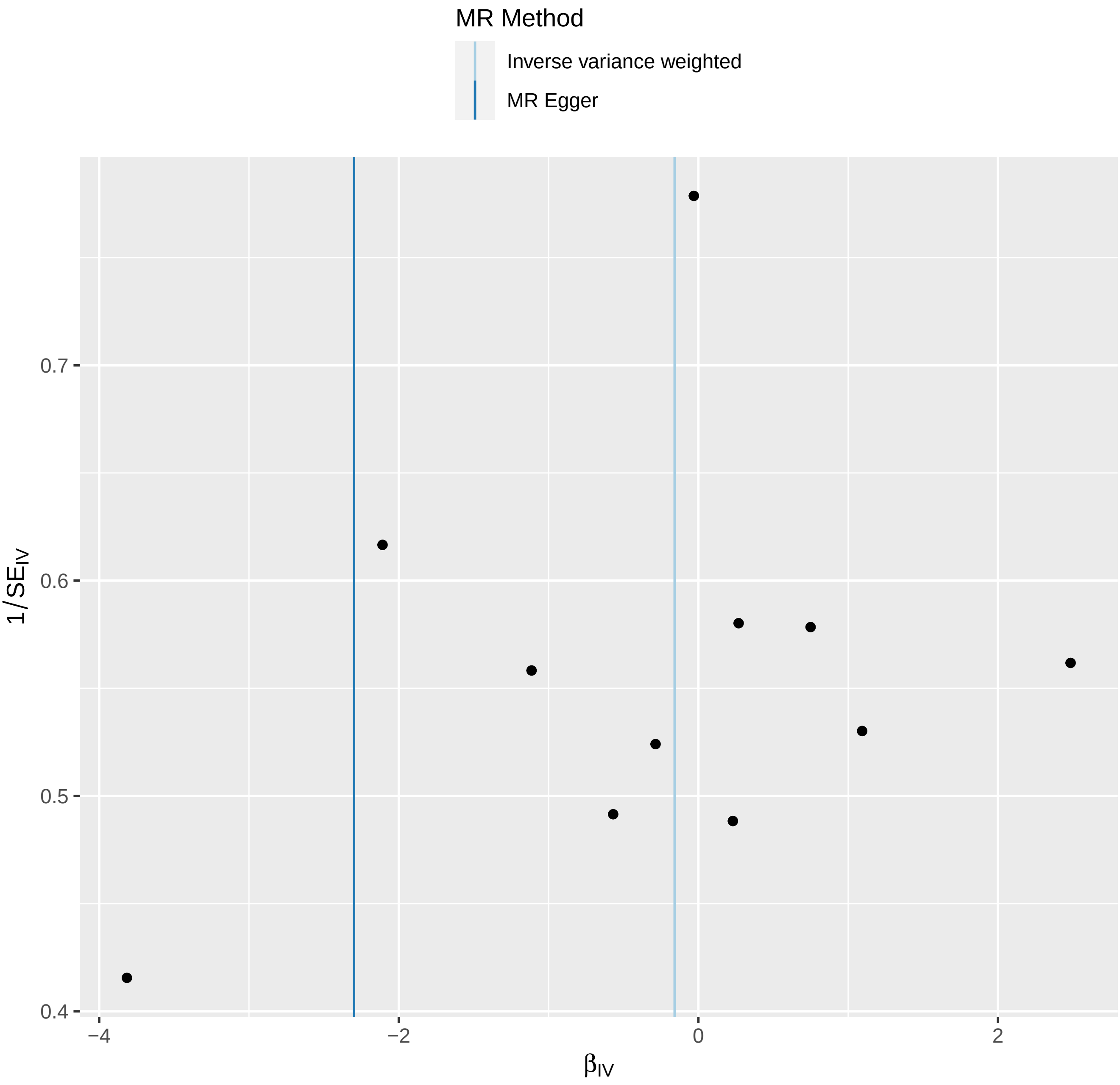

Batch 545 : Gut microbiota abundance (genus Lachnospiraceae FCS020 group id.11314) on Drug−induced obesity

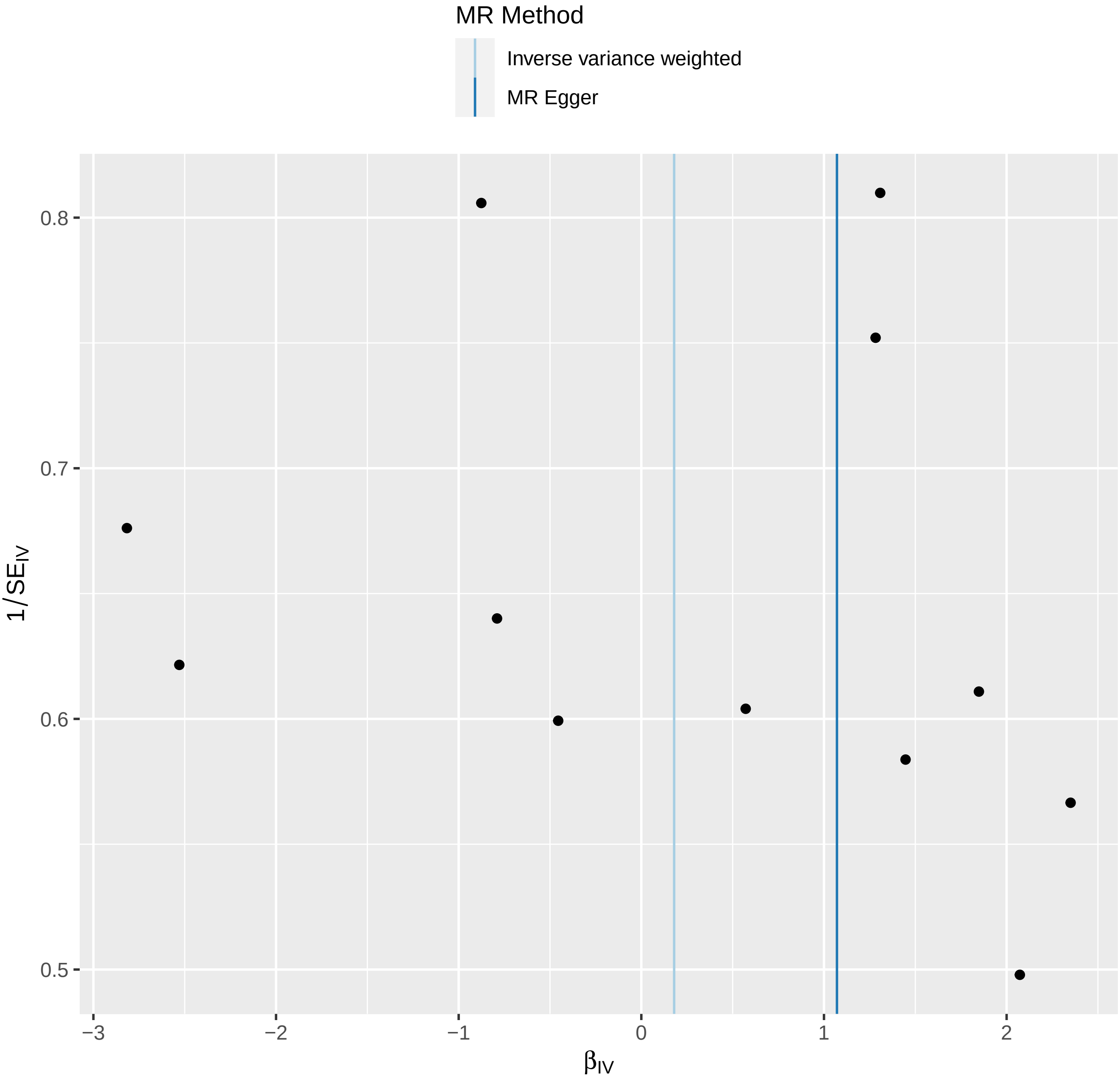

Batch 546 : Gut microbiota abundance (genus Lachnospiraceae NC2004 group id.11316) on Drug−induced obesity

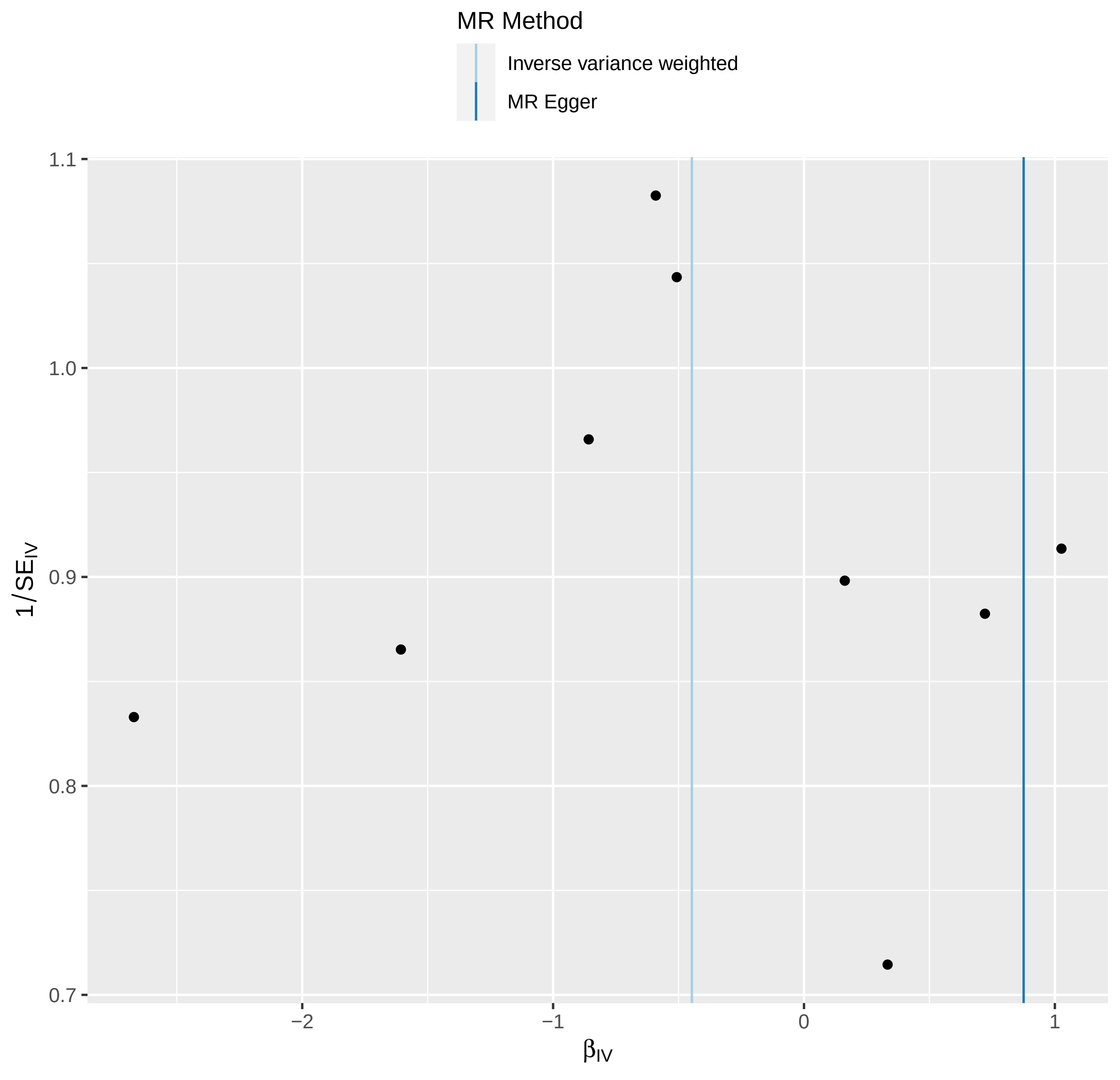

Batch 547 : Gut microbiota abundance (genus Lachnospiraceae ND3007 group id.11317) on Drug−induced obesity

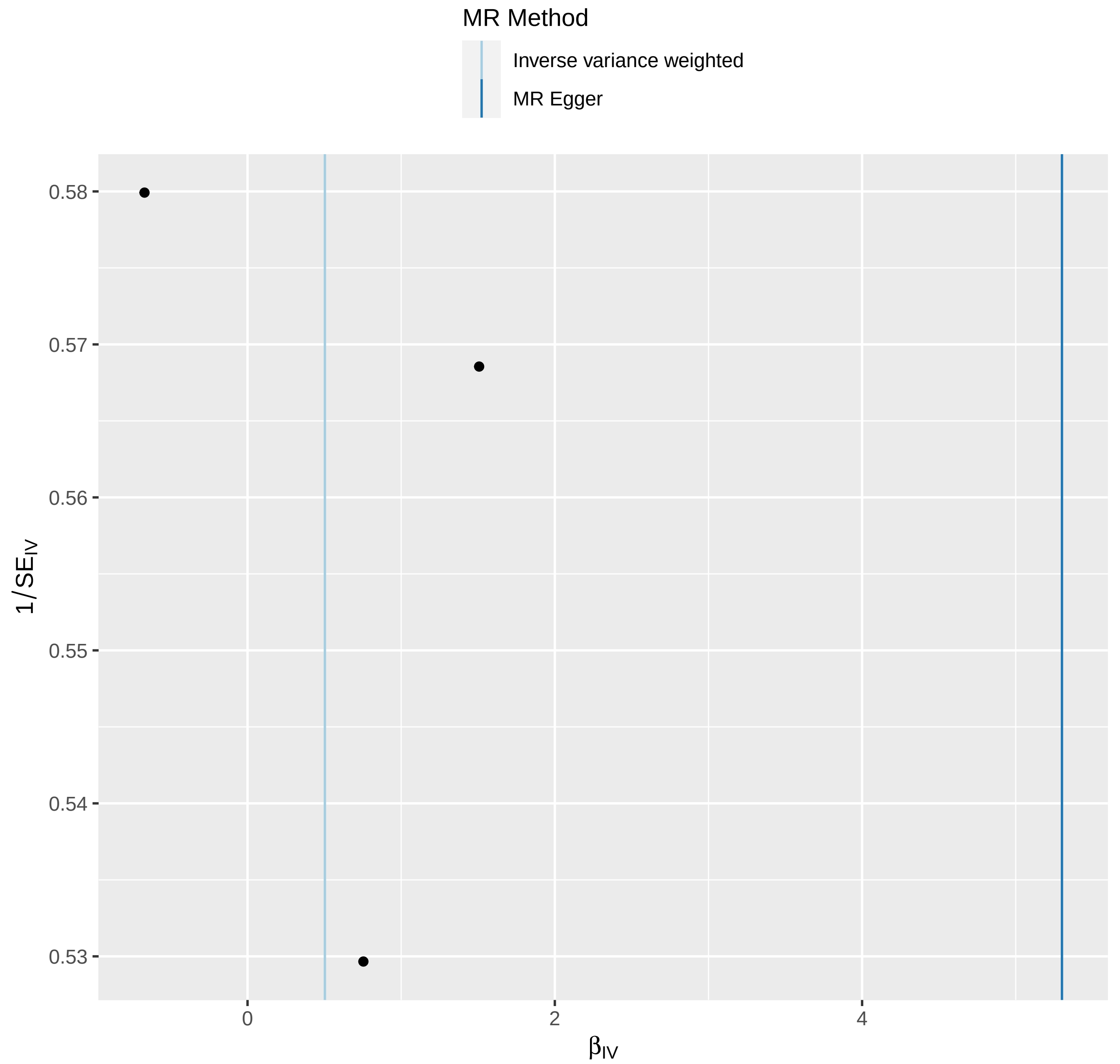

Batch 548 : Gut microbiota abundance (genus Lachnospiraceae NK4A136 group id.11319) on Drug−induced obesity

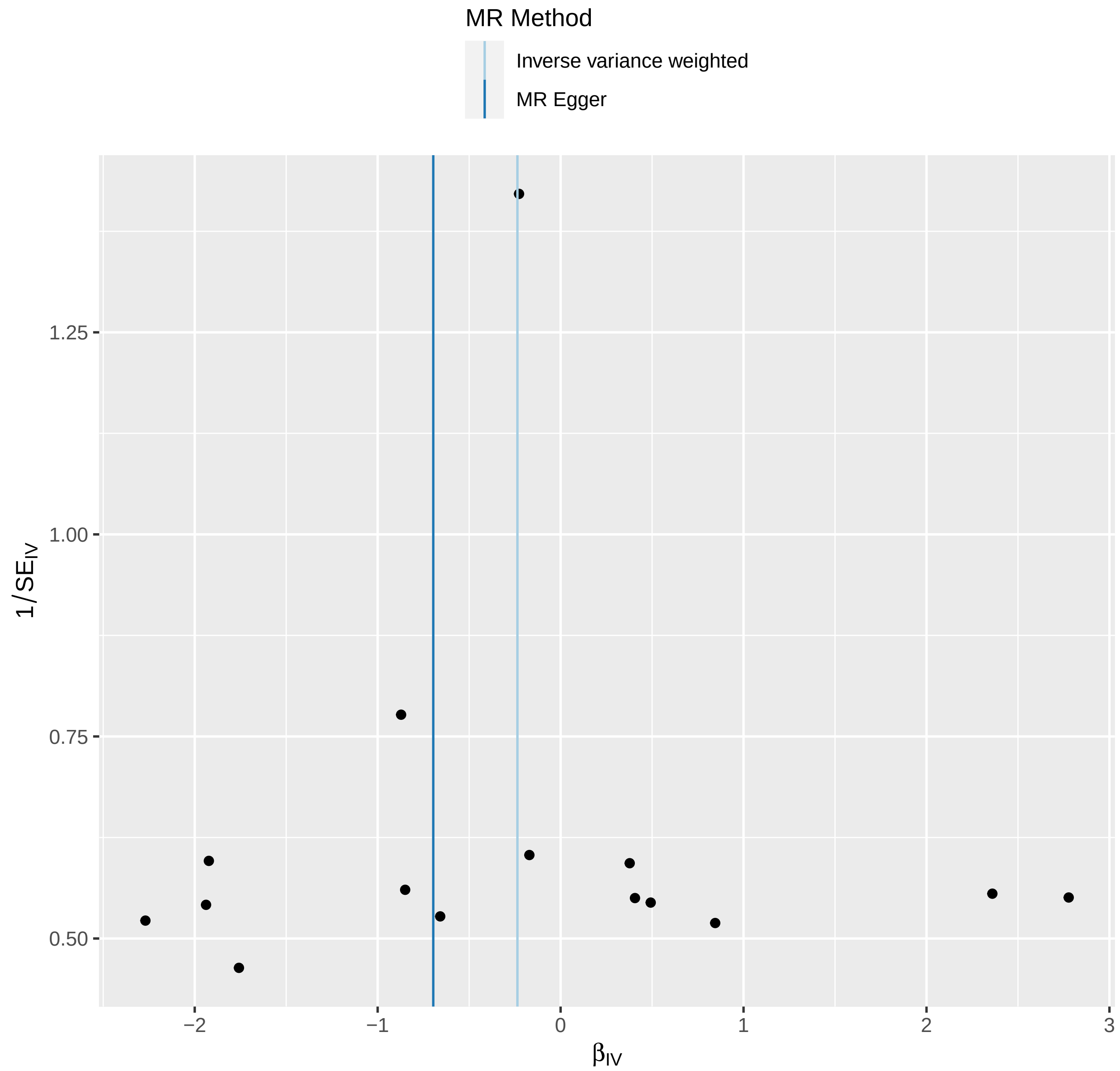

Batch 549 : Gut microbiota abundance (genus Lachnospiraceae UCG001 id.11321) on Drug−induced obesity

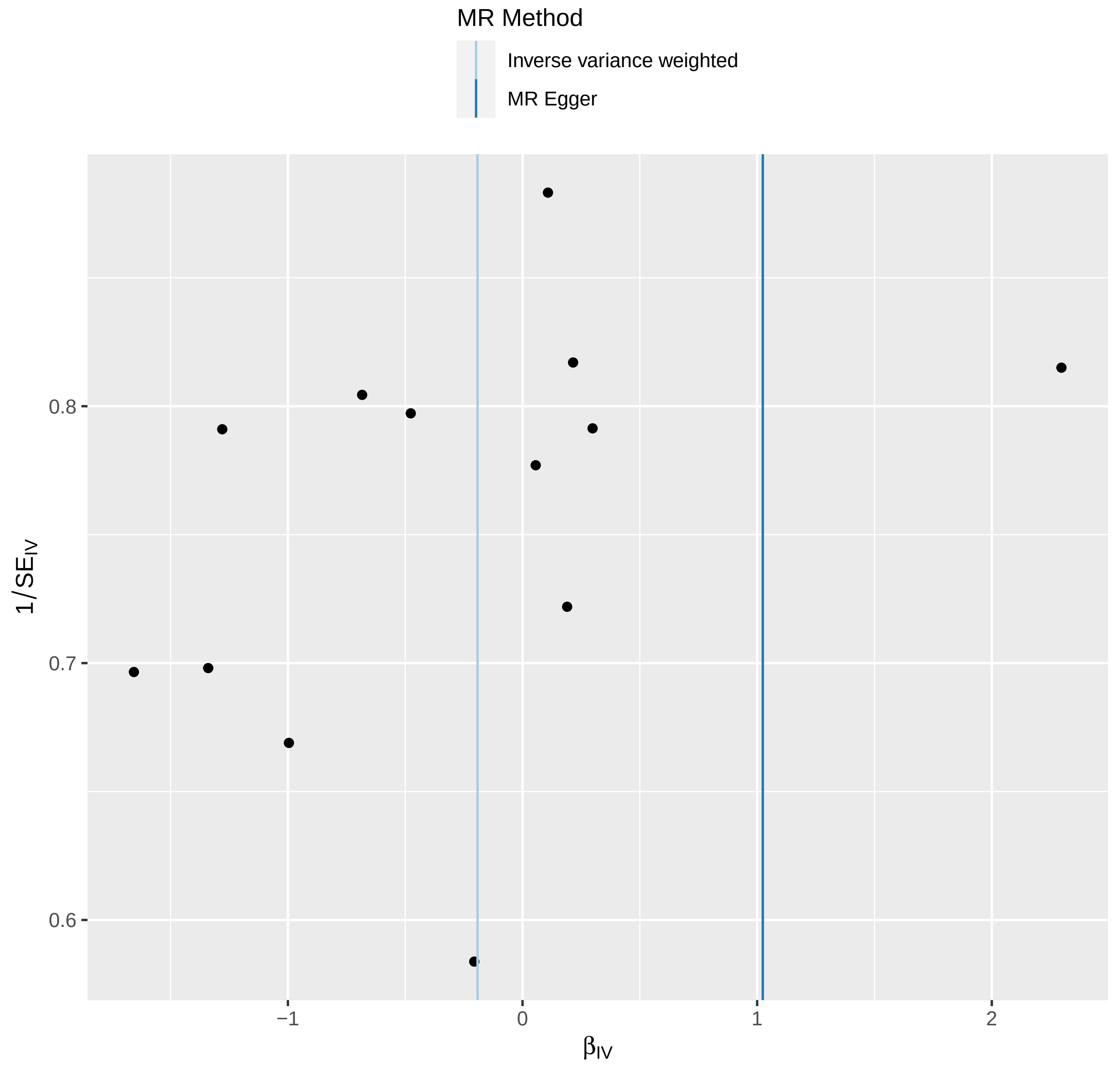

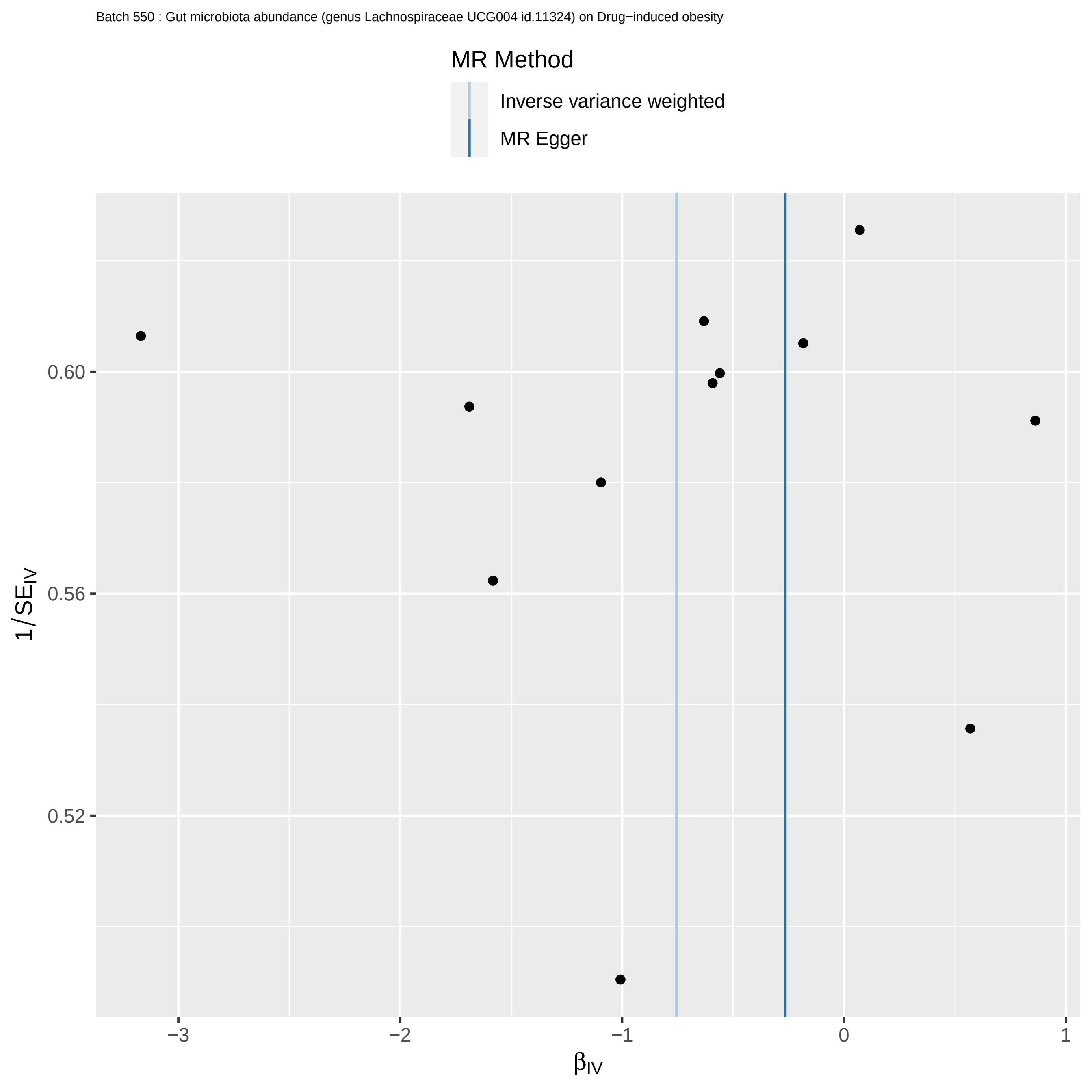

Batch 550 : Gut microbiota abundance (genus Lachnospiraceae UCG004 id.11324) on Drug−induced obesity
MR Method
Inverse variance weighted
MR Egger
0.60
0.56
0.52
−3
−2
−1
0
1
$1/SE_{IV}$
$\beta_{IV}$

Batch 551 : Gut microbiota abundance (genus Lachnospiraceae UCG008 id.11328) on Drug−induced obesity

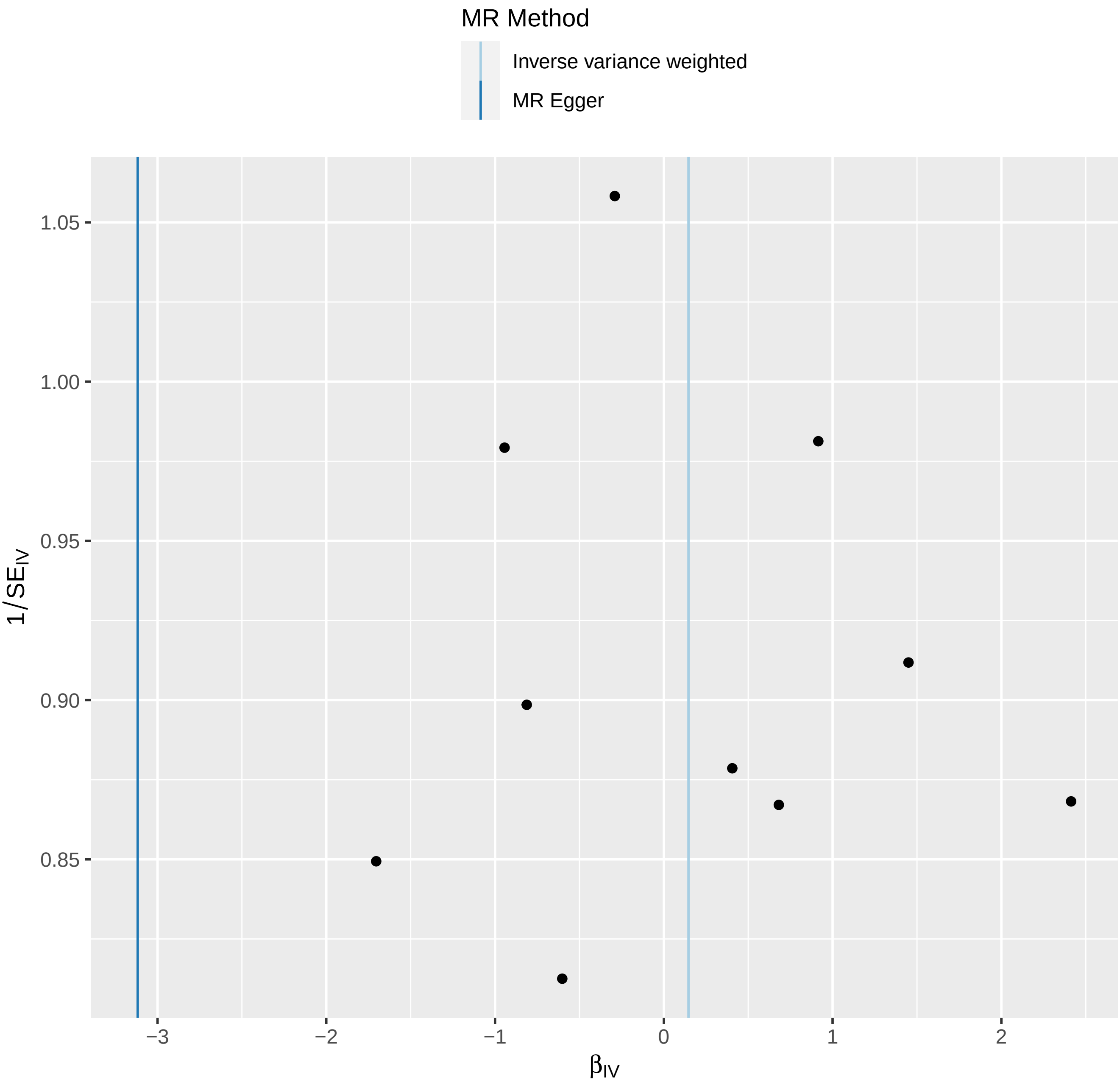

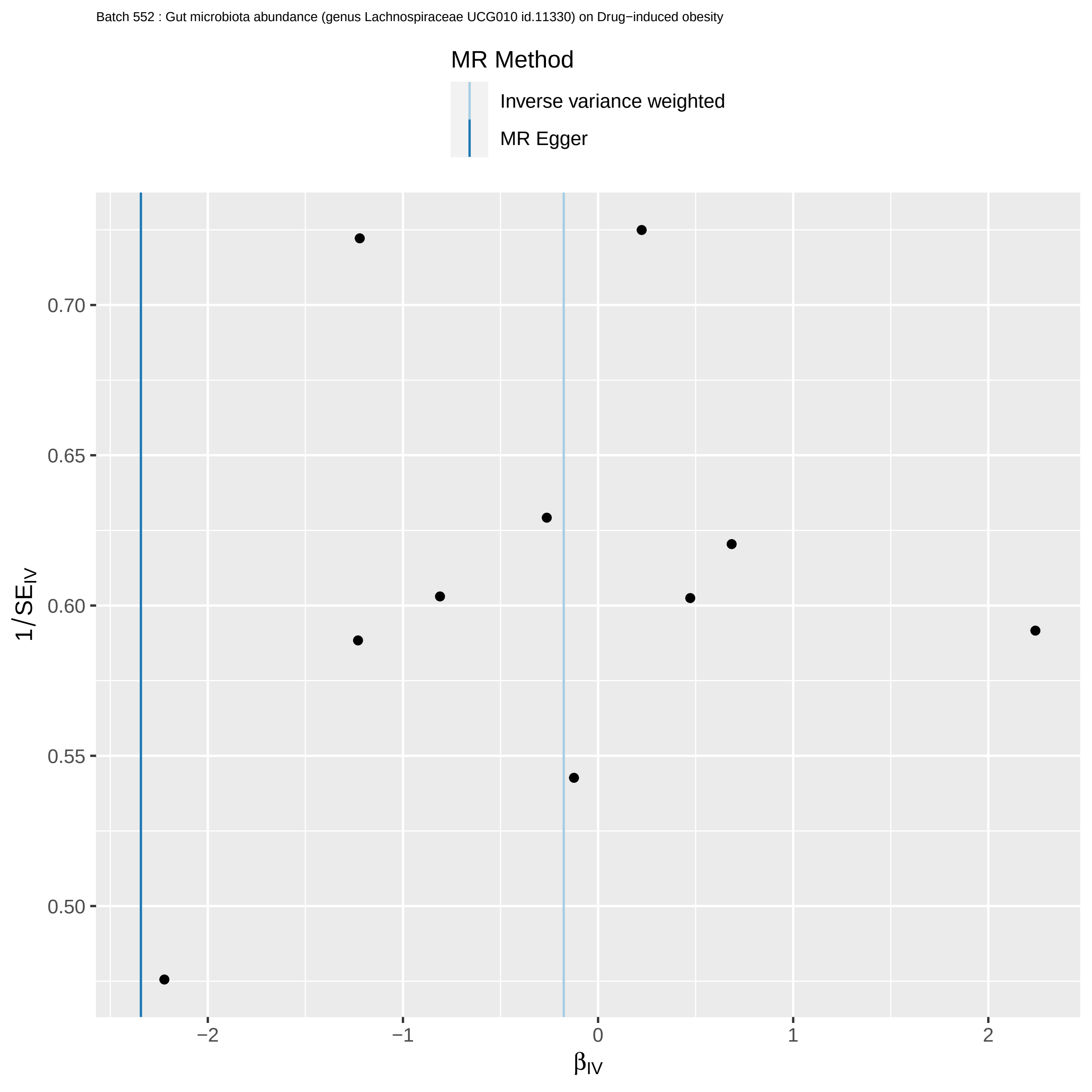

Batch 552 : Gut microbiota abundance (genus Lachnospiraceae UCG010 id.11330) on Drug−induced obesity
MR Method
Inverse variance weighted
MR Egger
0.70
0.65
0.60
0.55
0.50
1/SE_IV
−2
−1
0
1
2
β_IV

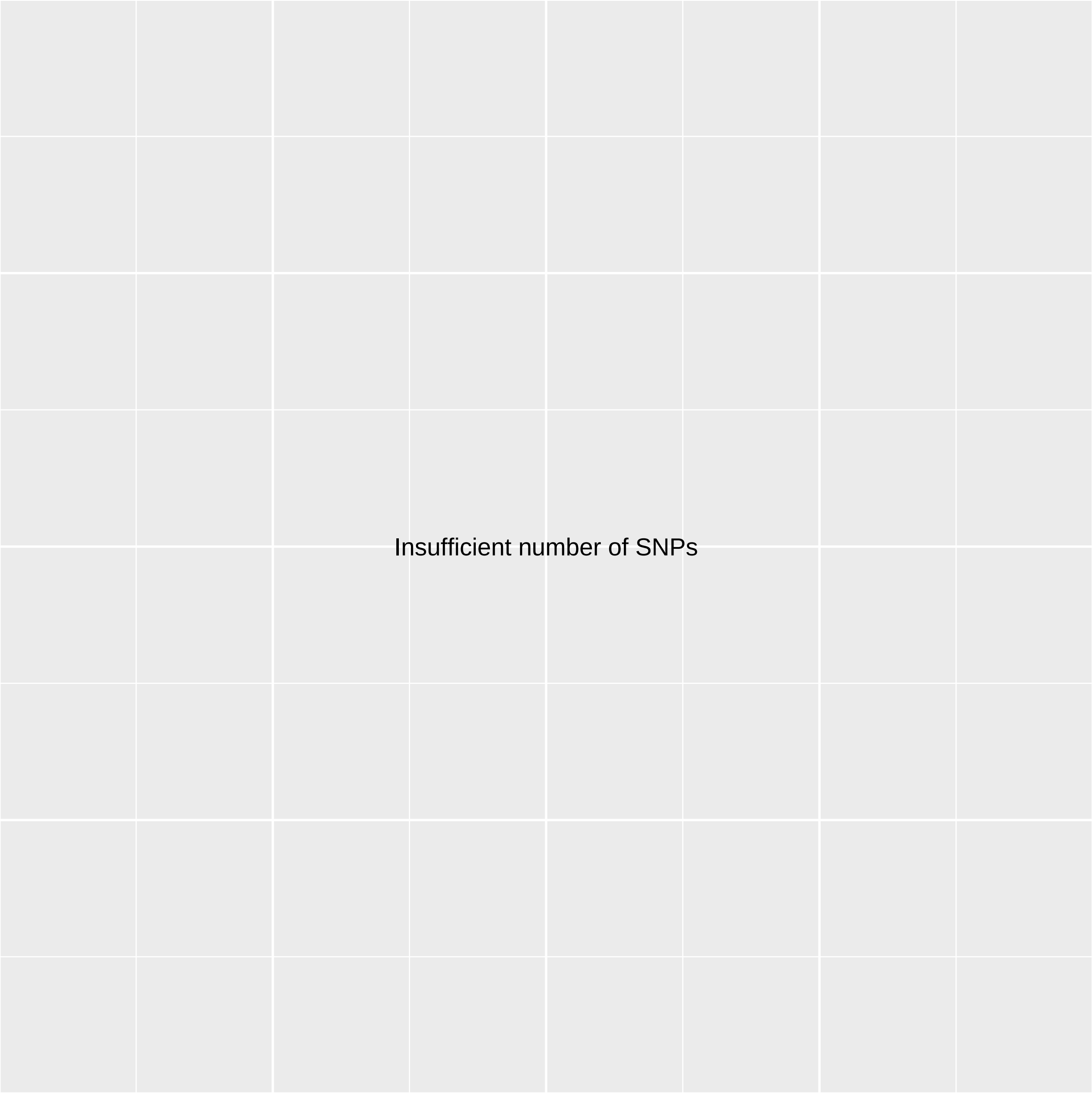
Insufficient number of SNPs

Batch 554 : Gut microbiota abundance (genus Lactobacillus id.1837) on Drug−induced obesity

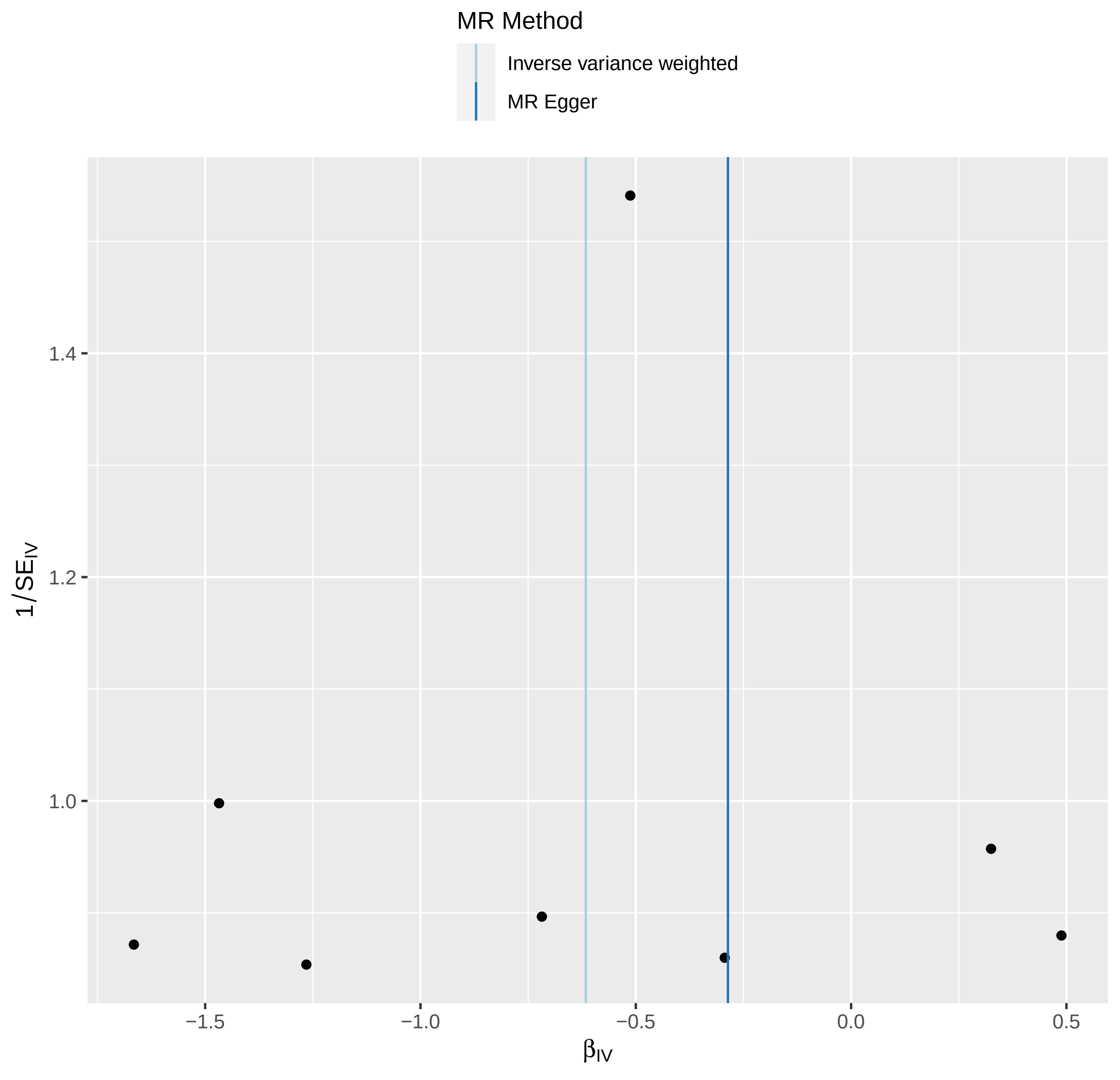

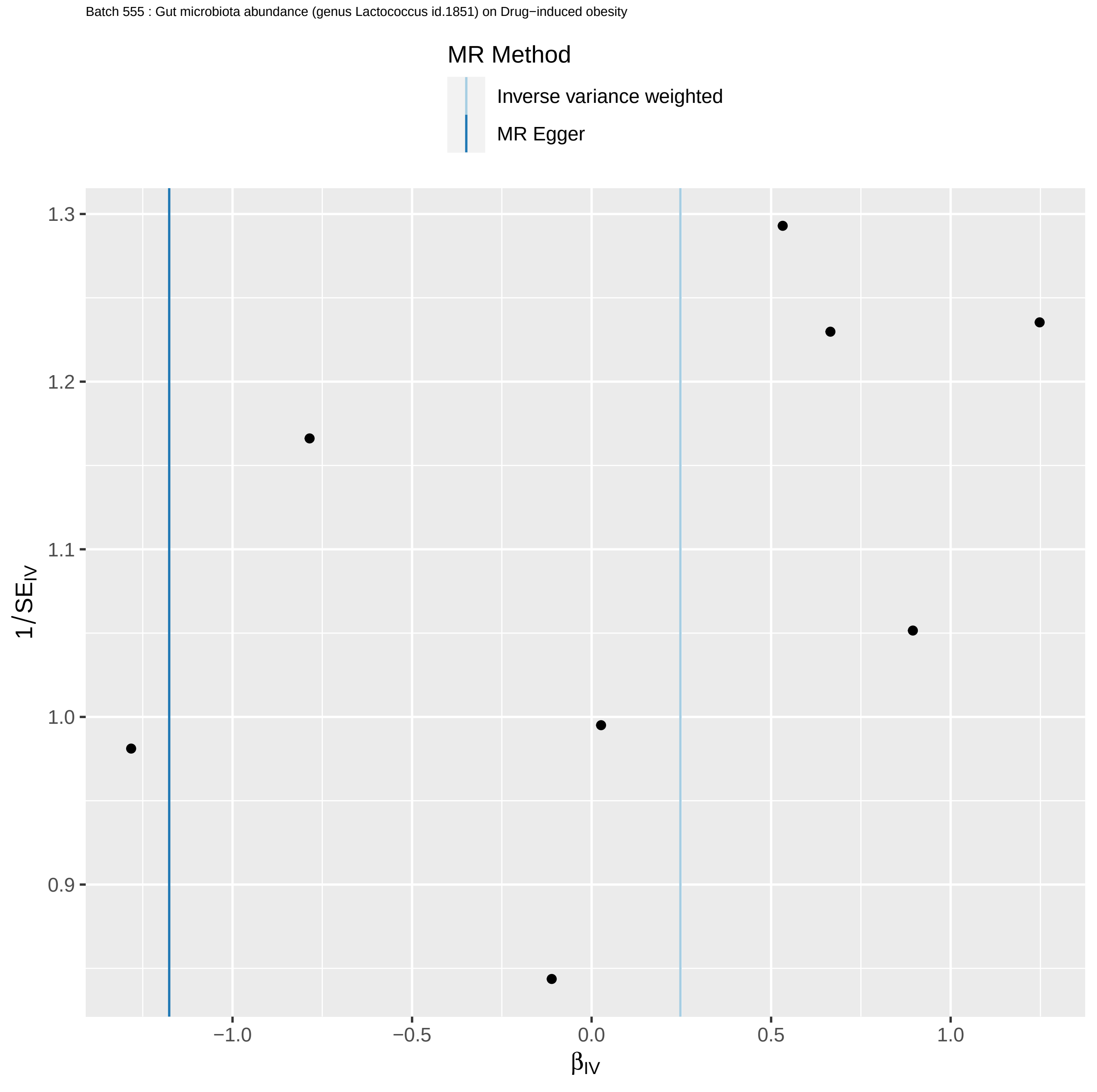

Batch 555 : Gut microbiota abundance (genus Lactococcus id.1851) on Drug−induced obesity
MR Method
Inverse variance weighted
MR Egger
1.3
1.2
1.1
1.0
0.9
1/SE_IV
−1.0
−0.5
0.0
0.5
1.0
β_IV

Batch 556 : Gut microbiota abundance (genus Marvinbryantia id.2005) on Drug−induced obesity

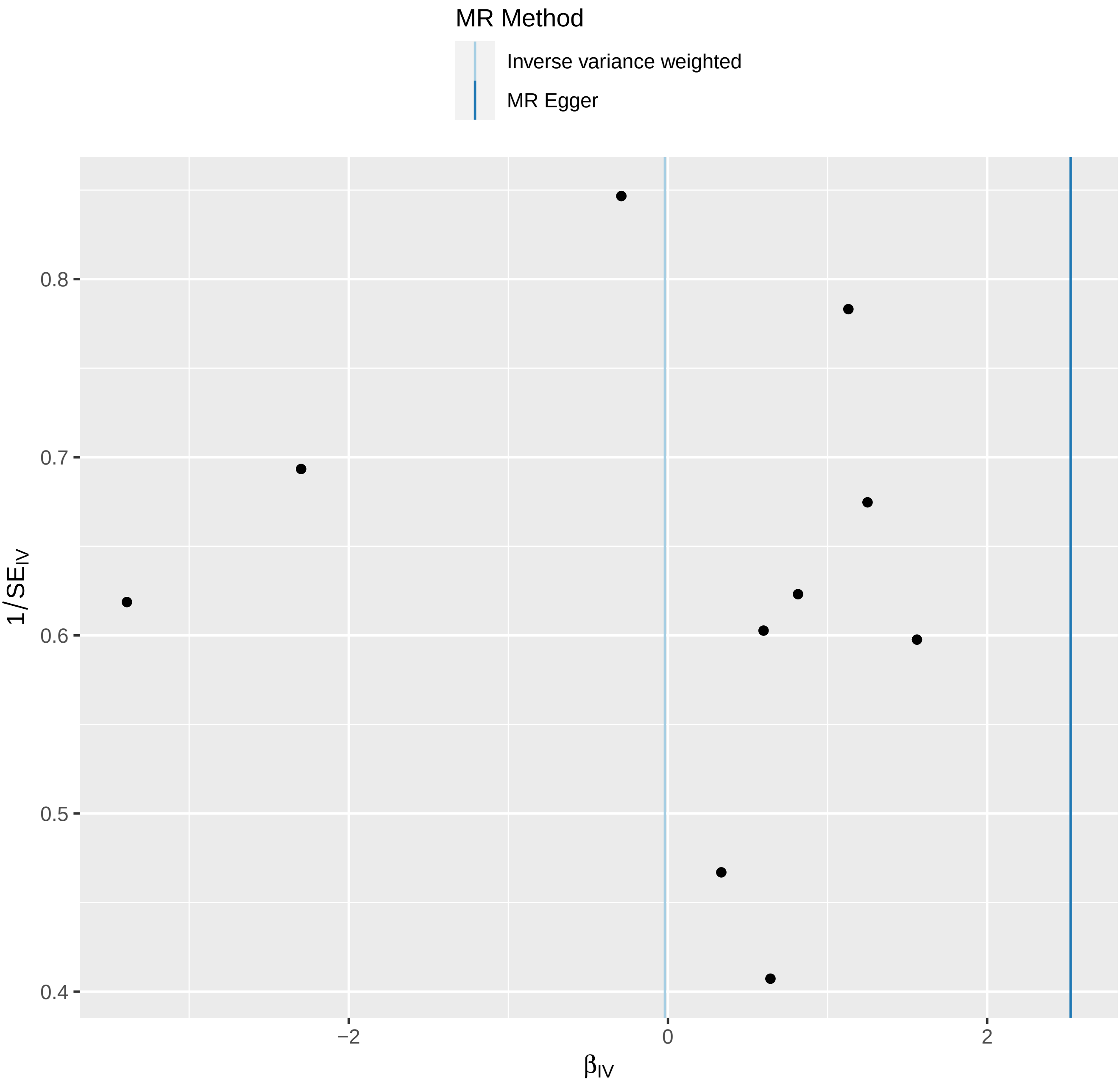

Batch 557 : Gut microbiota abundance (genus Methanobrevibacter id.123) on Drug−induced obesity

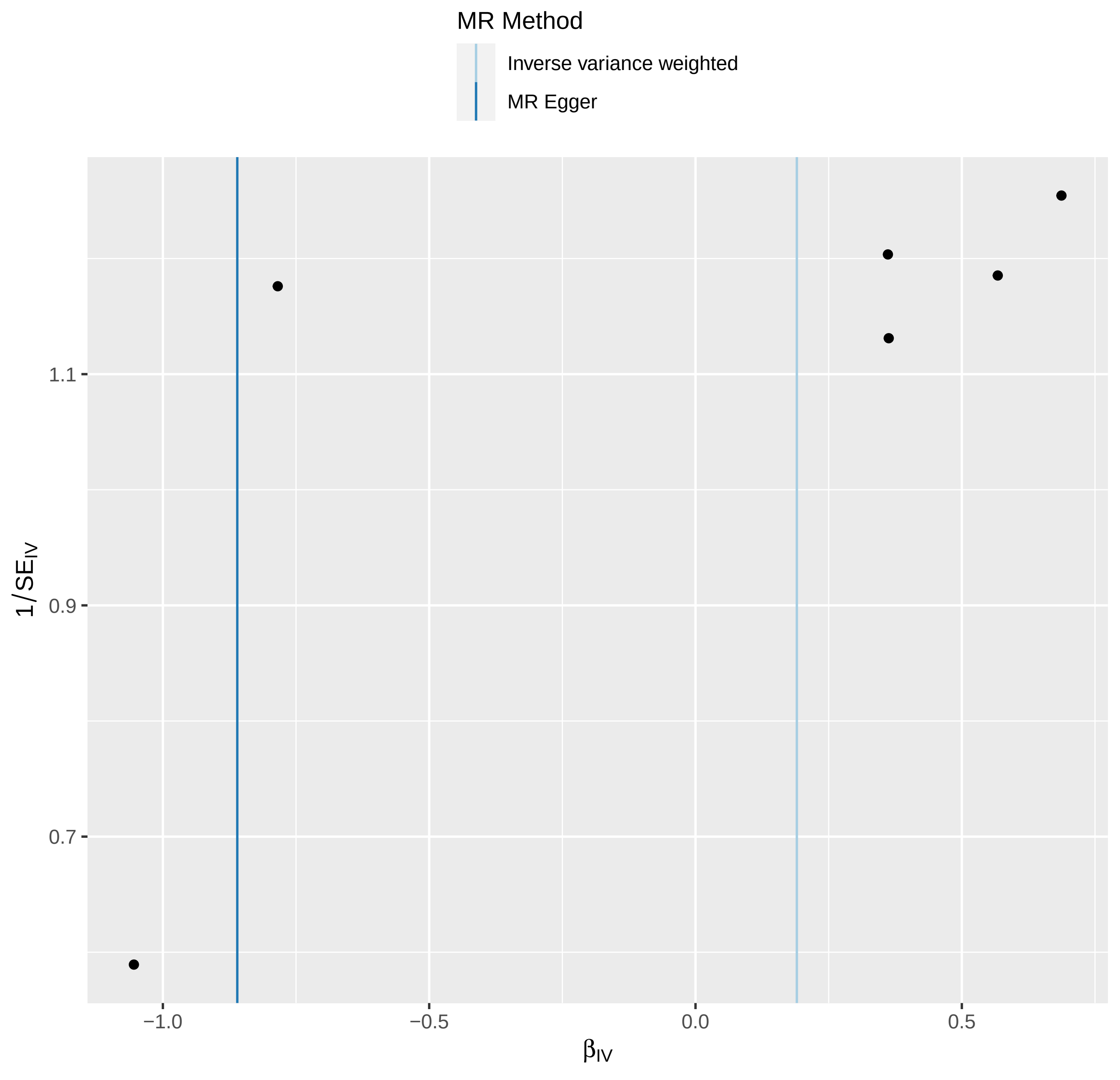

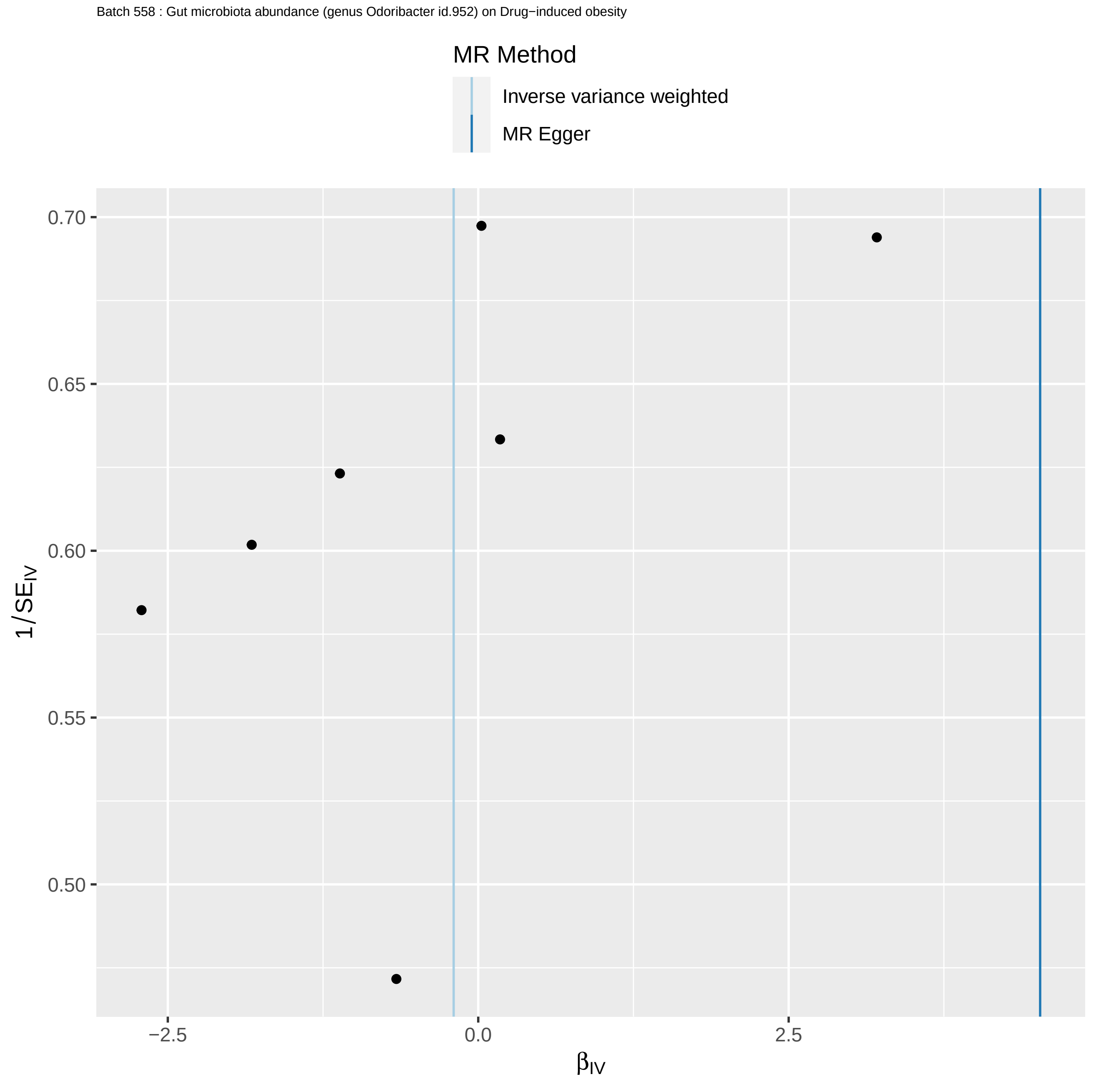
Batch 558 : Gut microbiota abundance (genus Odoribacter id.952) on Drug−induced obesity
MR Method
Inverse variance weighted
MR Egger
0.70
0.65
0.60
0.55
0.50
1/SE_IV
−2.5
0.0
2.5
β_IV

Batch 559 : Gut microbiota abundance (genus Olsenella id.822) on Drug−induced obesity

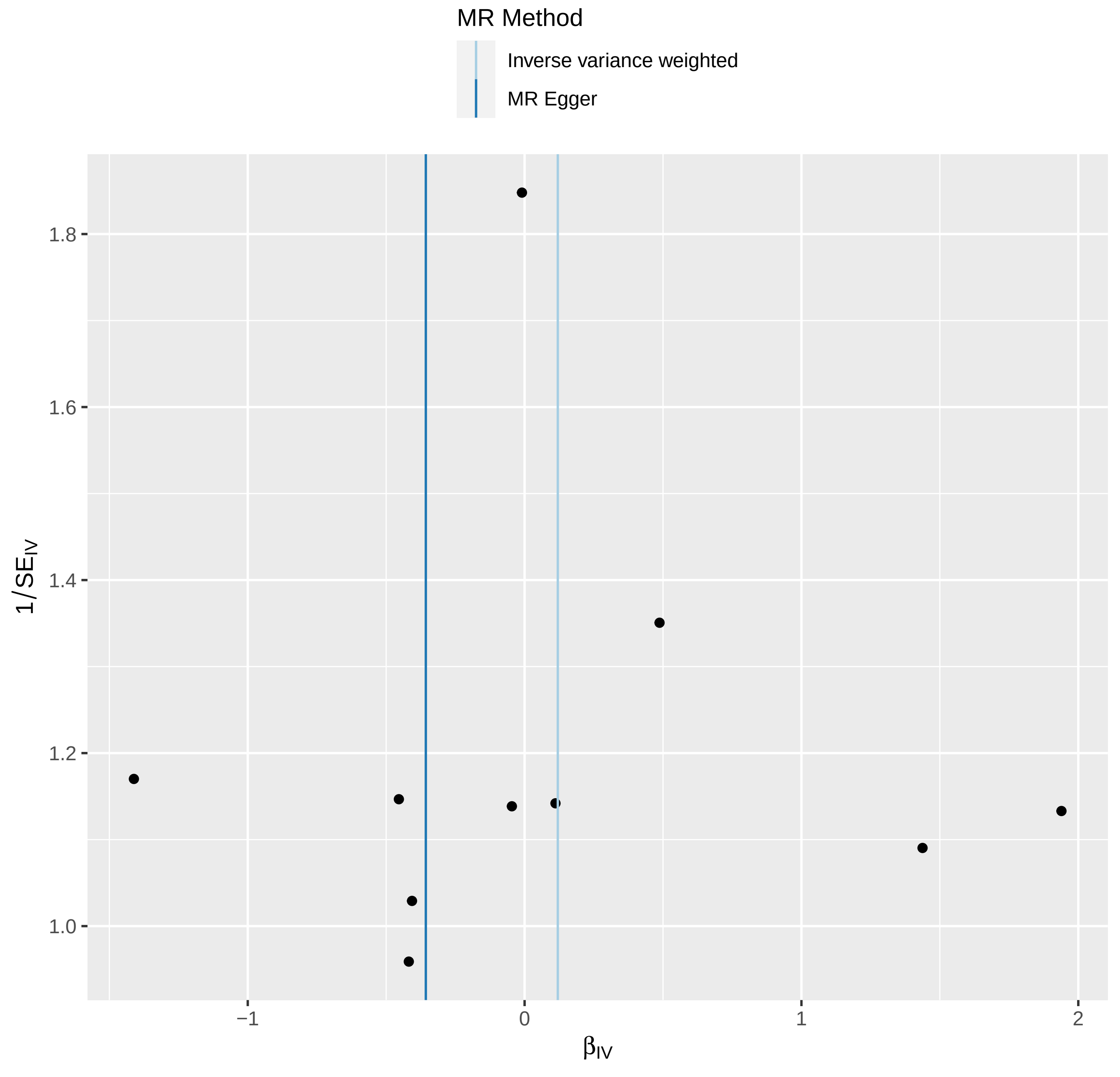

Batch 560 : Gut microbiota abundance (genus Oscillibacter id.2063) on Drug−induced obesity

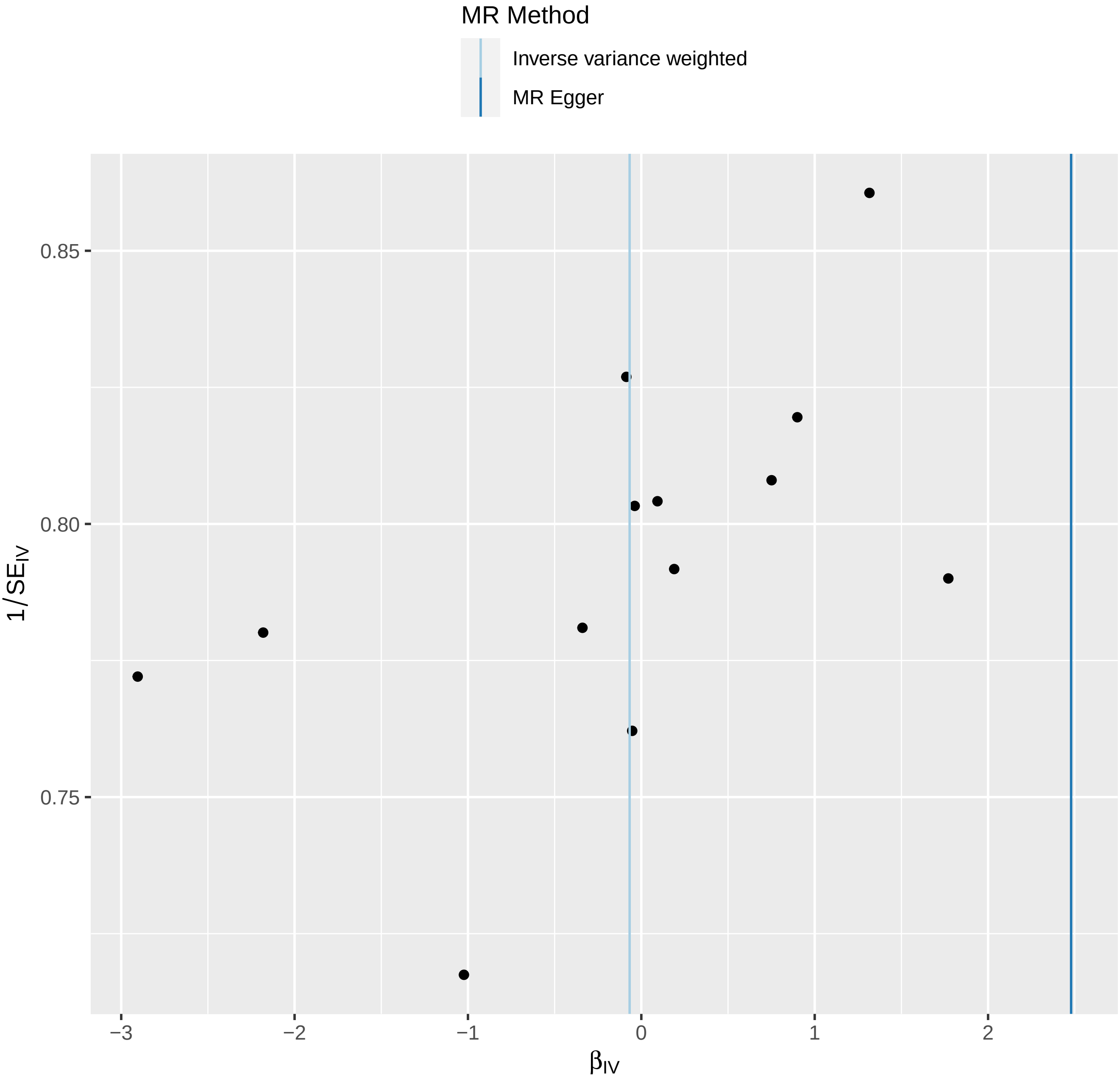

Batch 561 : Gut microbiota abundance (genus Oscillospira id.2064) on Drug−induced obesity

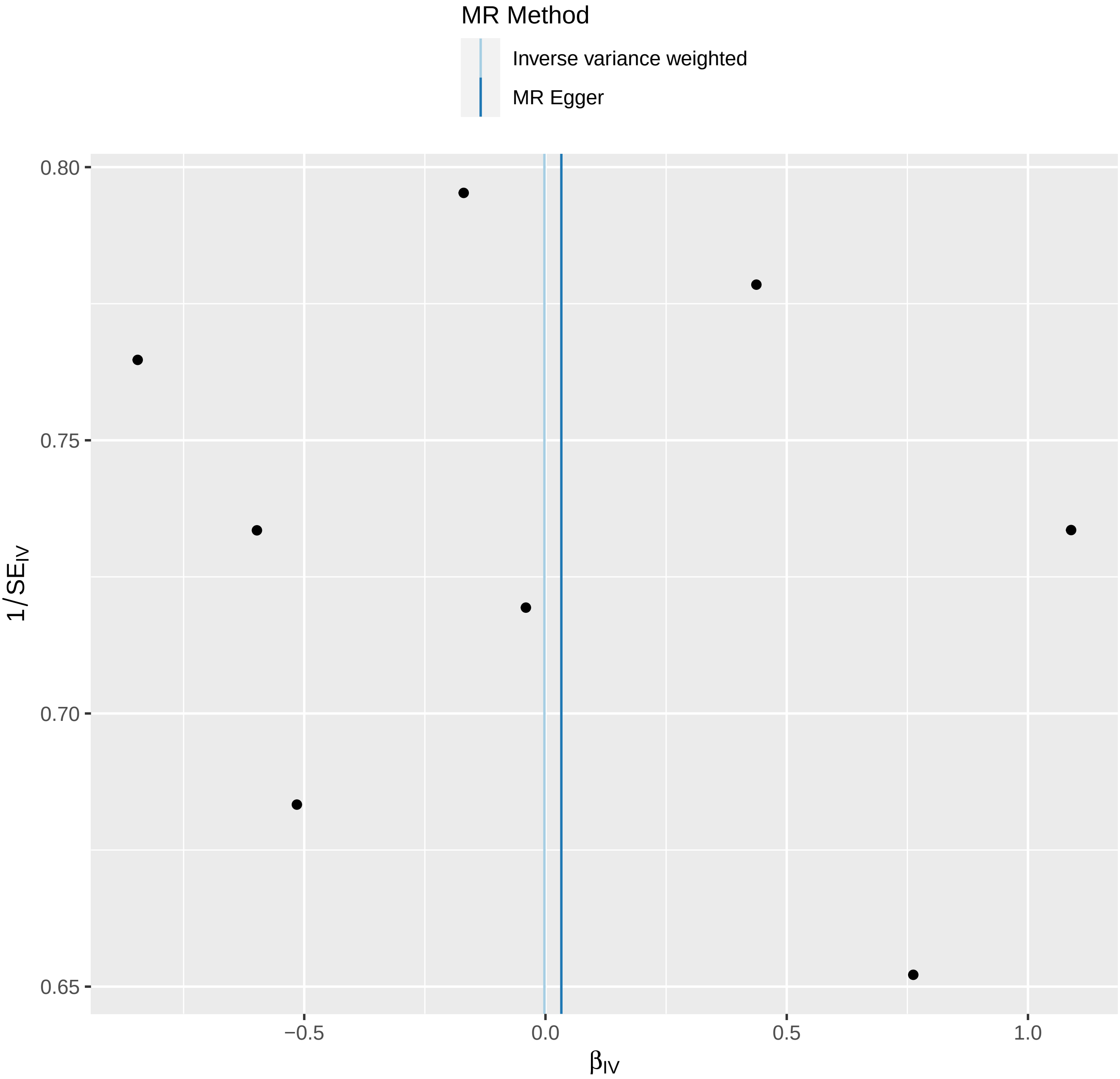

Batch 562 : Gut microbiota abundance (genus Oxalobacter id.2978) on Drug−induced obesity

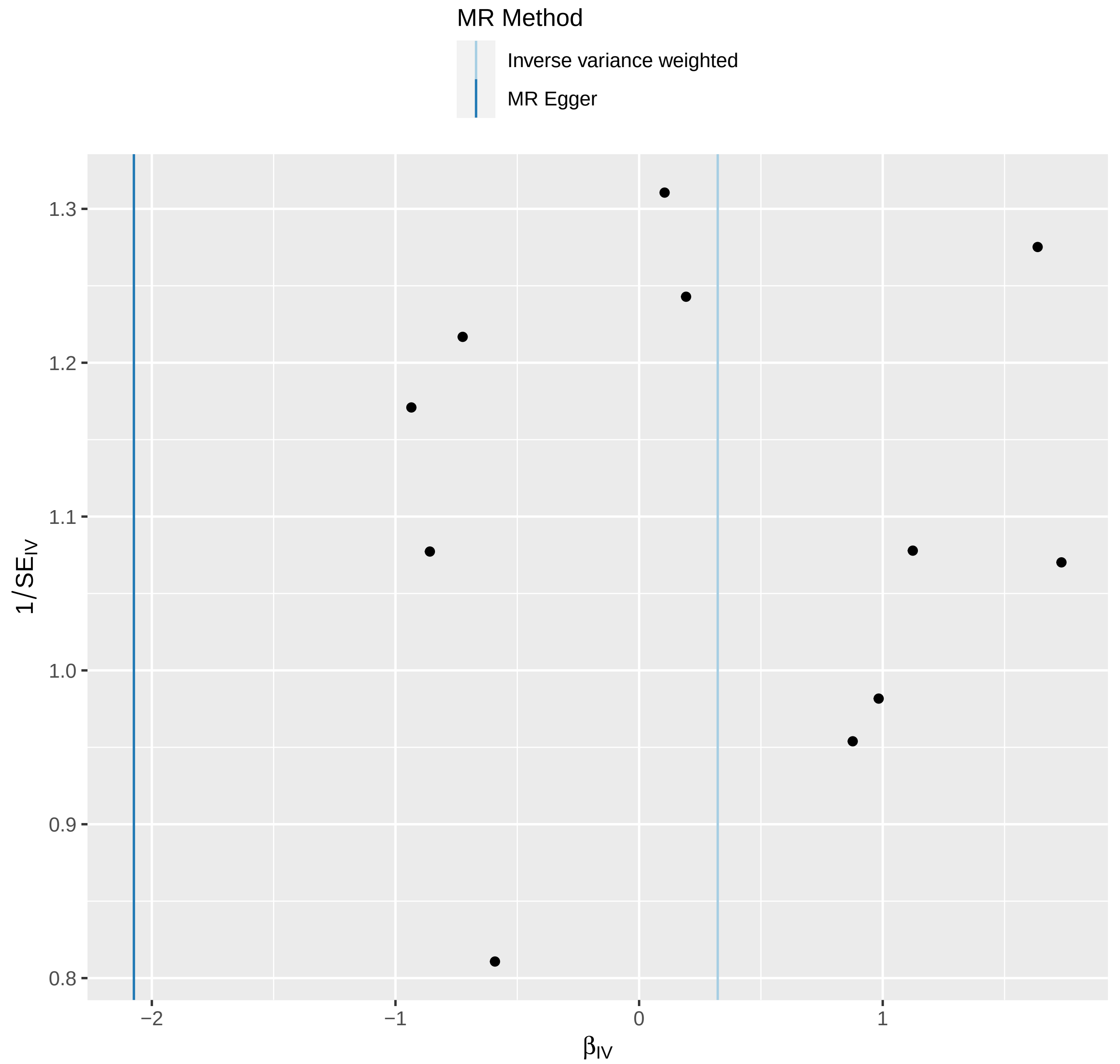

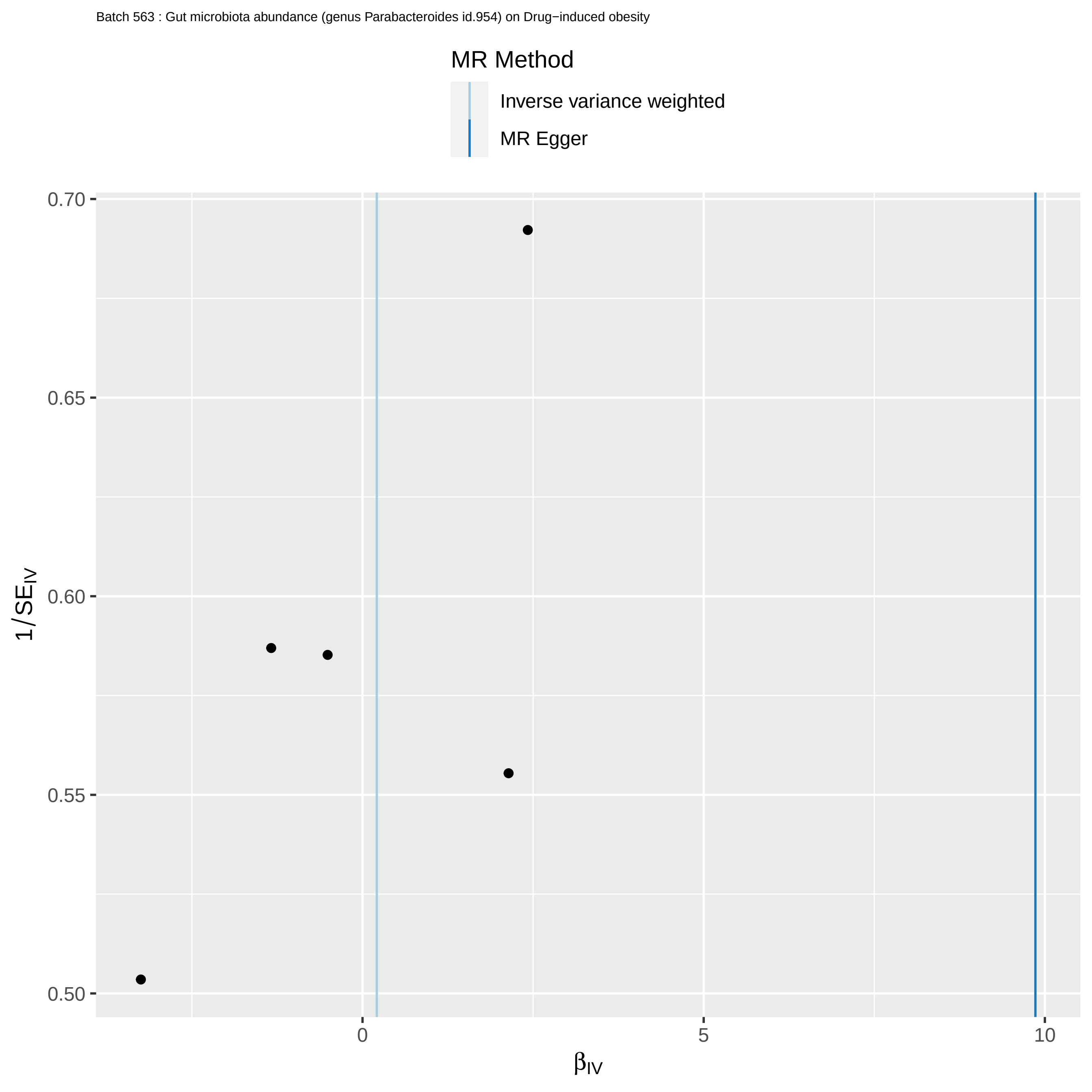

Batch 563 : Gut microbiota abundance (genus Parabacteroides id.954) on Drug−induced obesity
MR Method
Inverse variance weighted
MR Egger
0.70
0.65
0.60
0.55
0.50
1/SE_IV
0
5
10
β_IV

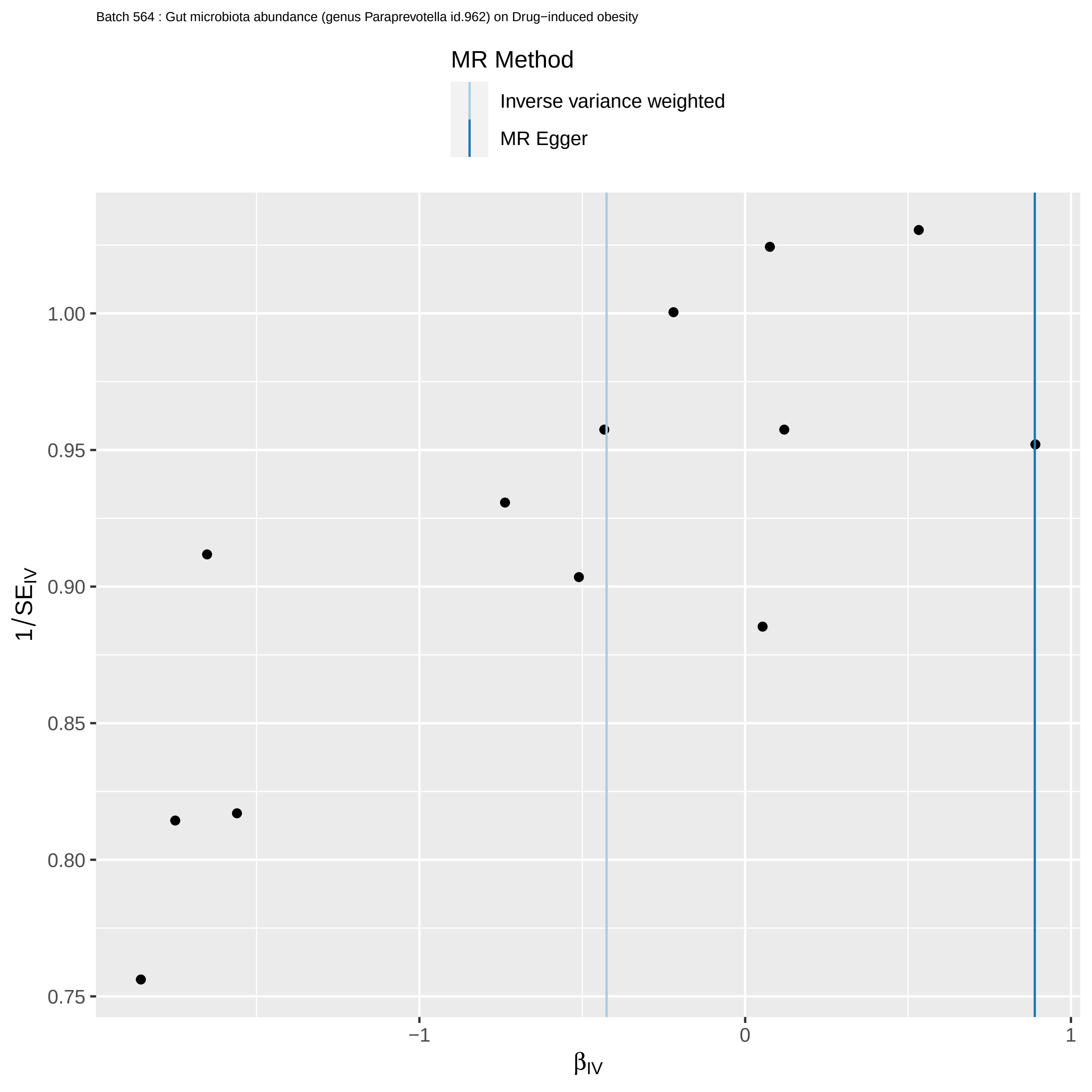

Batch 564 : Gut microbiota abundance (genus Paraprevotella id.962) on Drug−induced obesity
MR Method
Inverse variance weighted
MR Egger
1.00
0.95
0.90
0.85
0.80
0.75
−1
0
1
$1/SE_{IV}$
$\beta_{IV}$

Batch 565 : Gut microbiota abundance (genus Parasutterella id.2892) on Drug−induced obesity

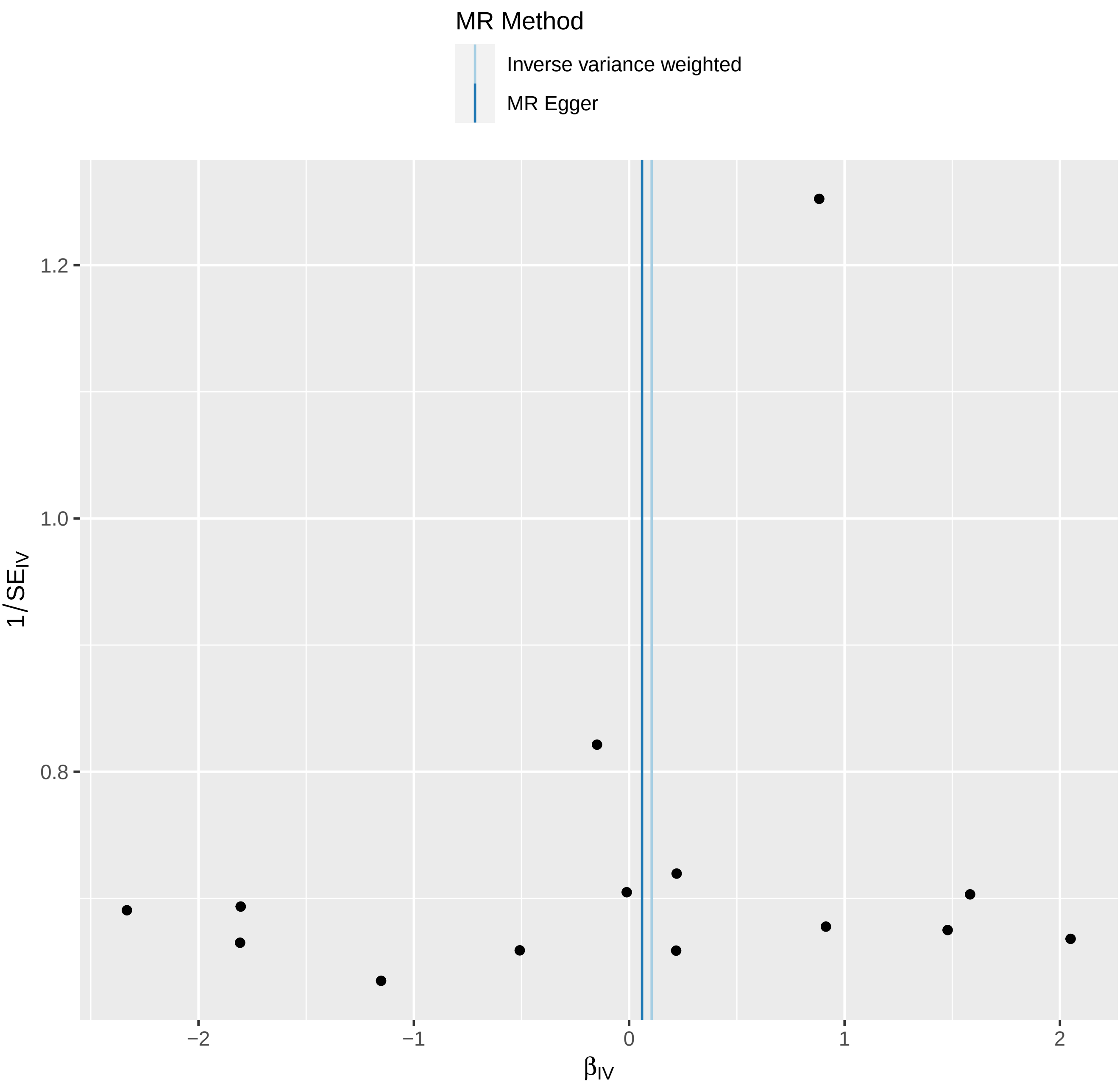

Batch 566 : Gut microbiota abundance (genus Peptococcus id.2037) on Drug−induced obesity

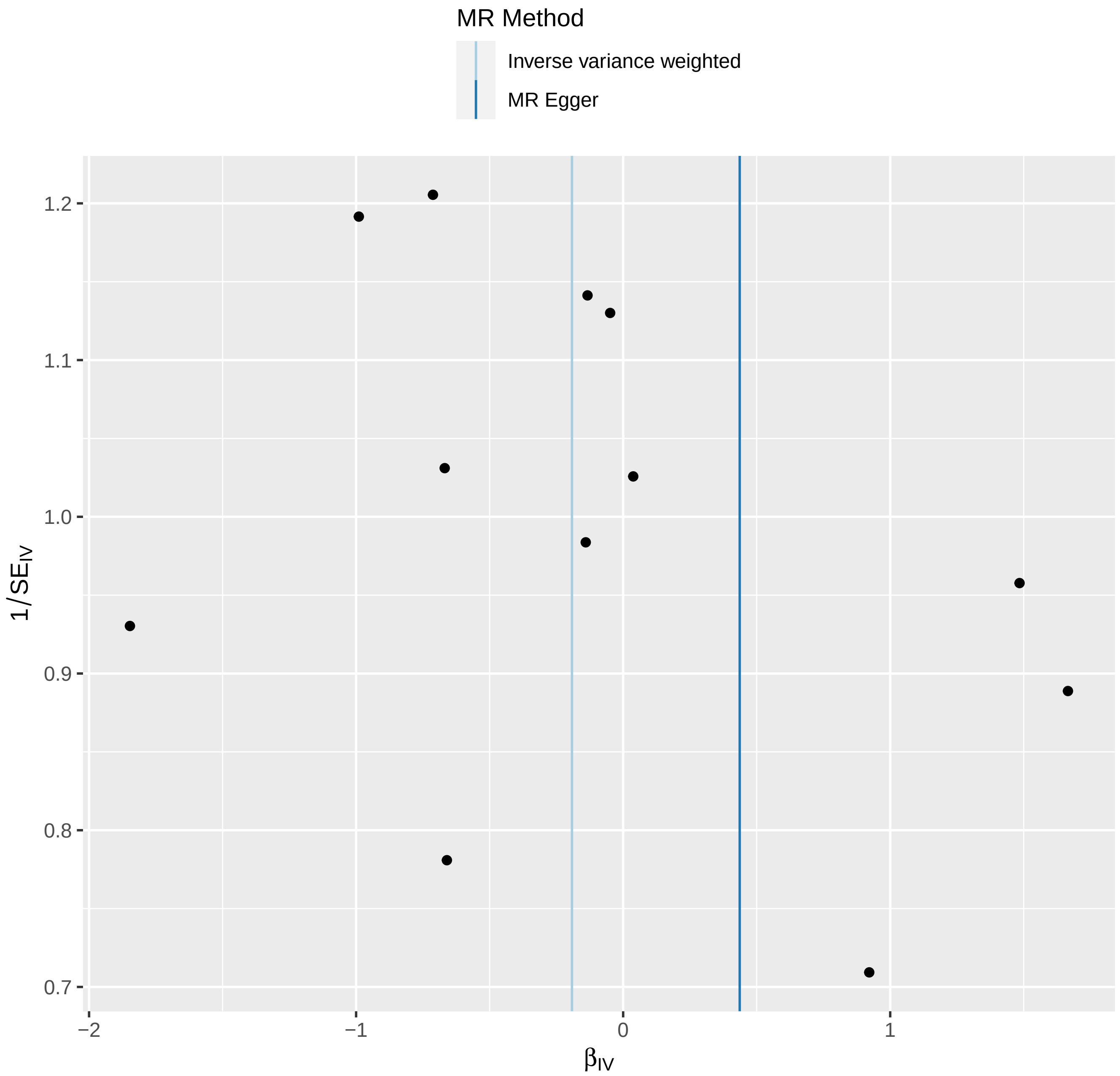

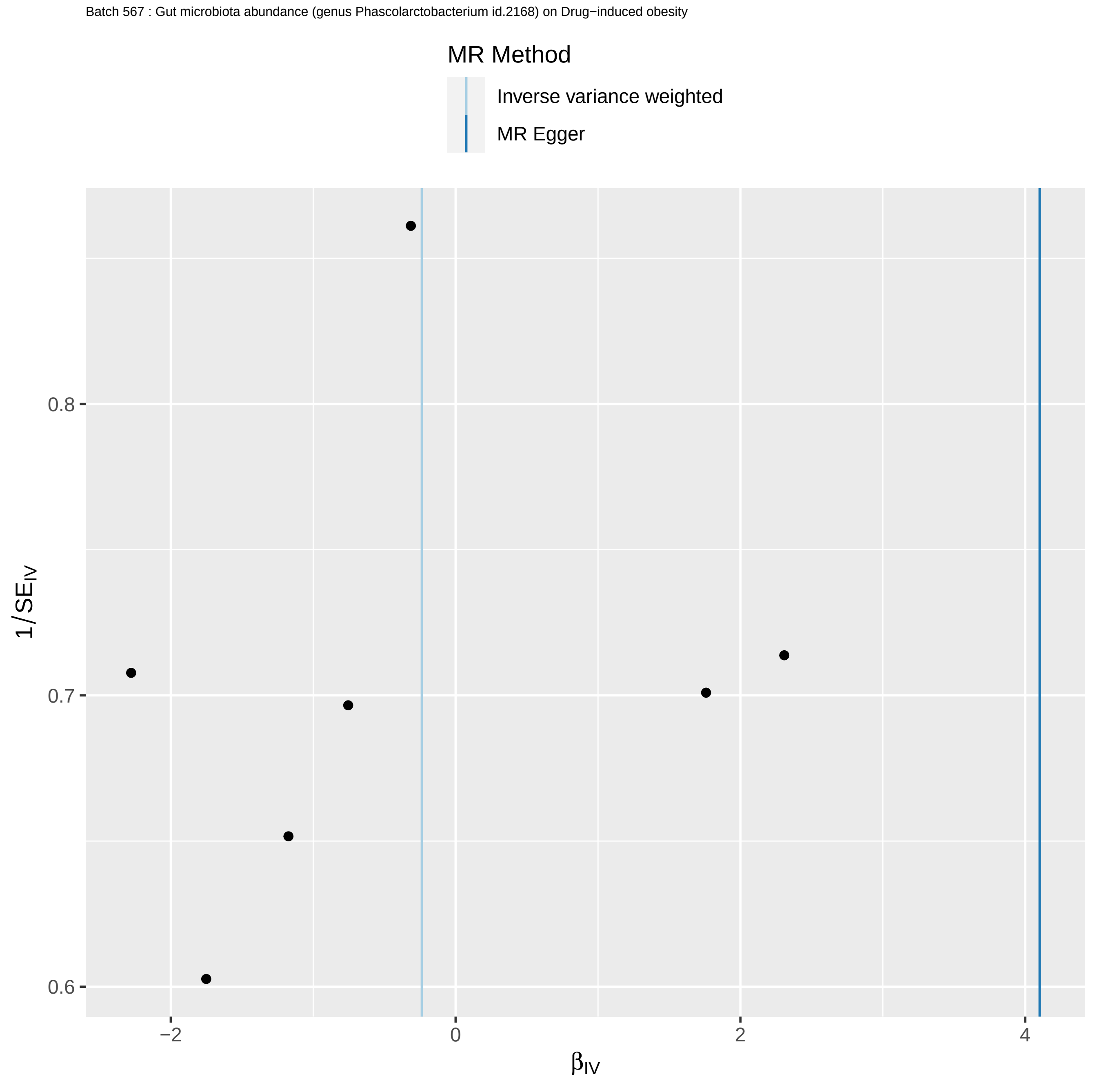

Batch 567 : Gut microbiota abundance (genus Phascolarctobacterium id.2168) on Drug−induced obesity
MR Method
Inverse variance weighted
MR Egger
0.8
0.7
0.6
1/SE_IV
−2
0
2
4
β_IV

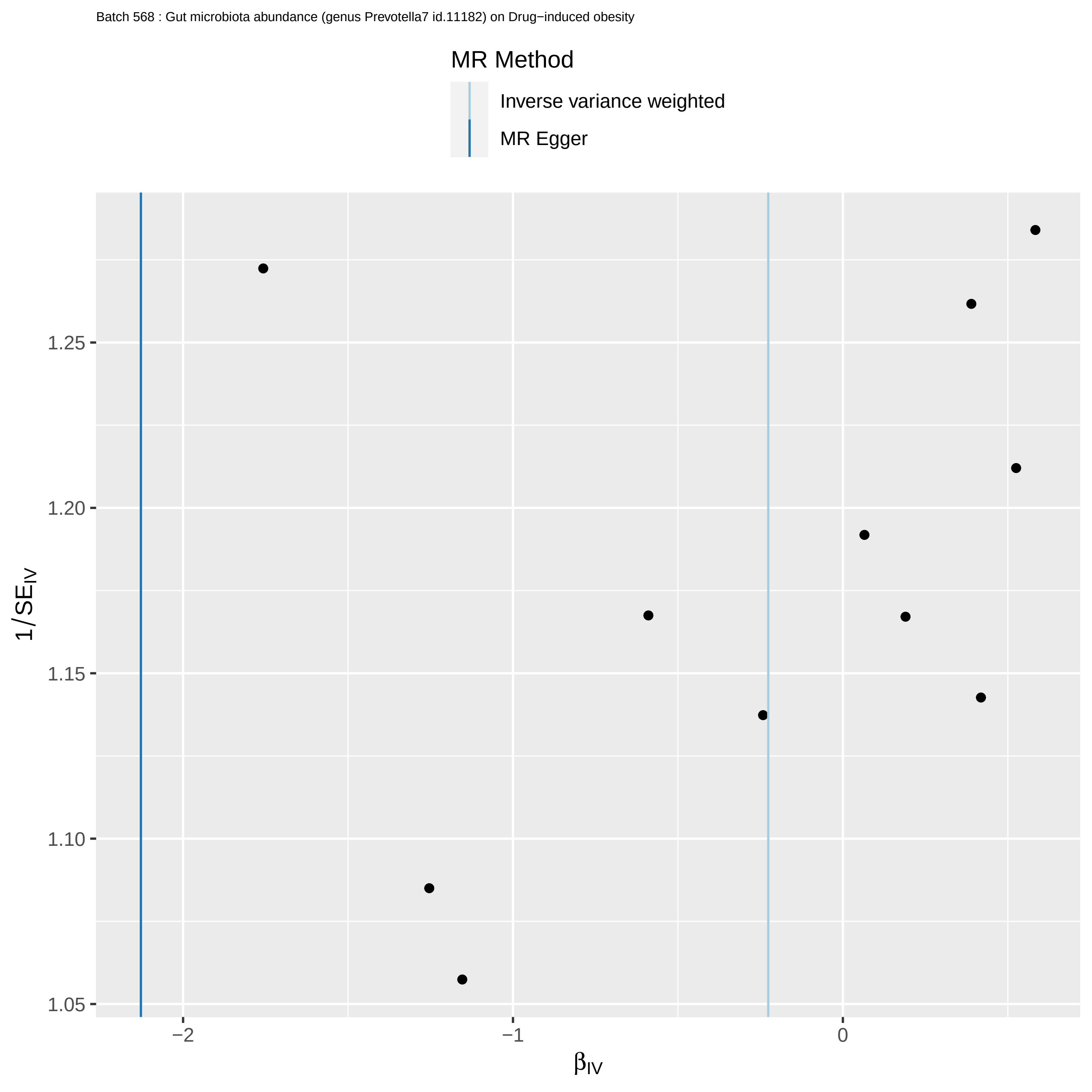
Batch 568 : Gut microbiota abundance (genus Prevotella7 id.11182) on Drug−induced obesity
MR Method
Inverse variance weighted
MR Egger
1.25
1.20
1.15
1.10
1.05
1/SE_IV
−2
−1
0
β_IV

Batch 569 : Gut microbiota abundance (genus Prevotella9 id.11183) on Drug−induced obesity

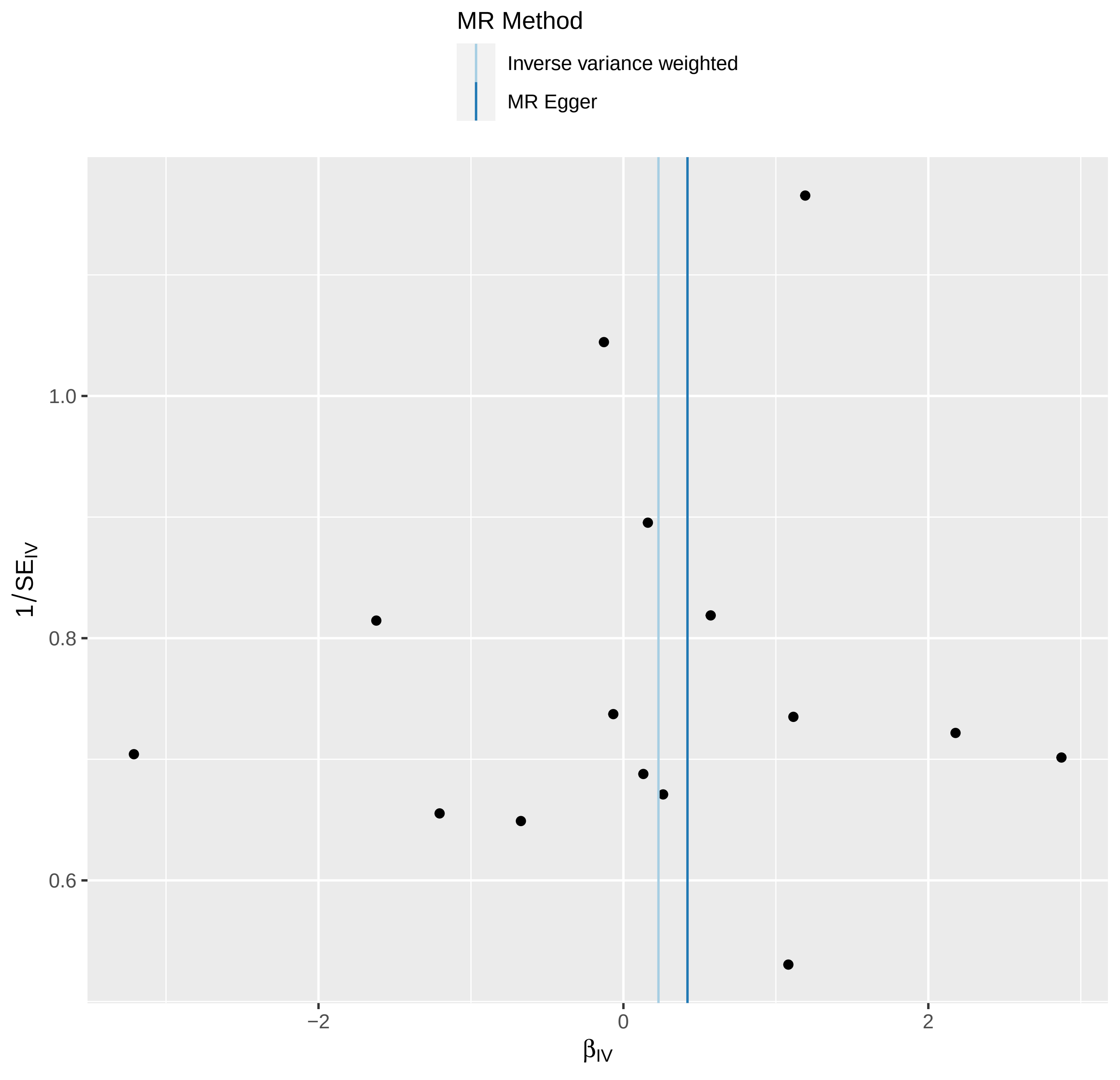

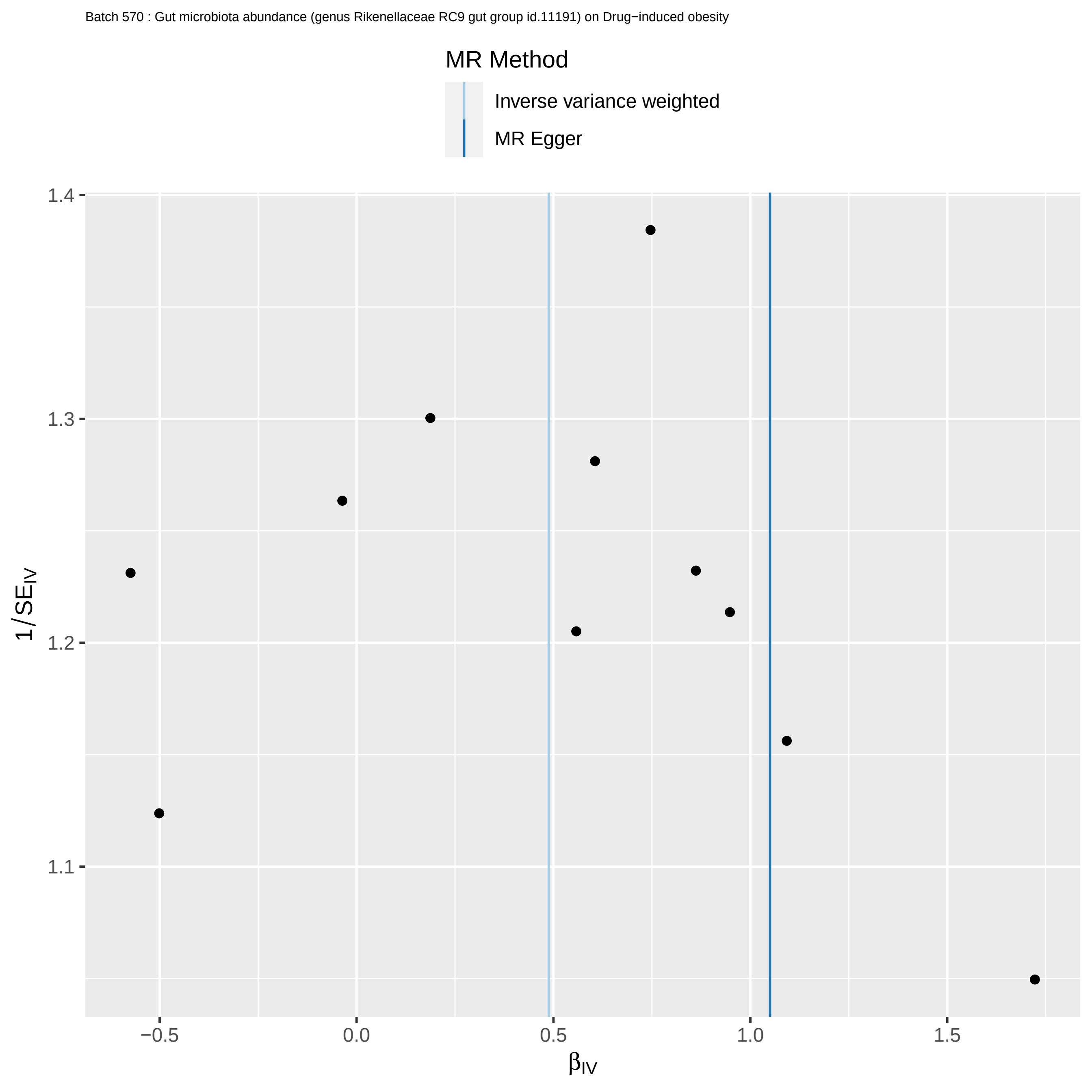

Batch 570 : Gut microbiota abundance (genus Rikenellaceae RC9 gut group id.11191) on Drug−induced obesity
MR Method
Inverse variance weighted
MR Egger
1.4
1.3
1.2
1.1
1/SE$_{IV}$
−0.5
0.0
0.5
1.0
1.5
β$_{IV}$

Batch 571 : Gut microbiota abundance (genus Romboutsia id.11347) on Drug−induced obesity

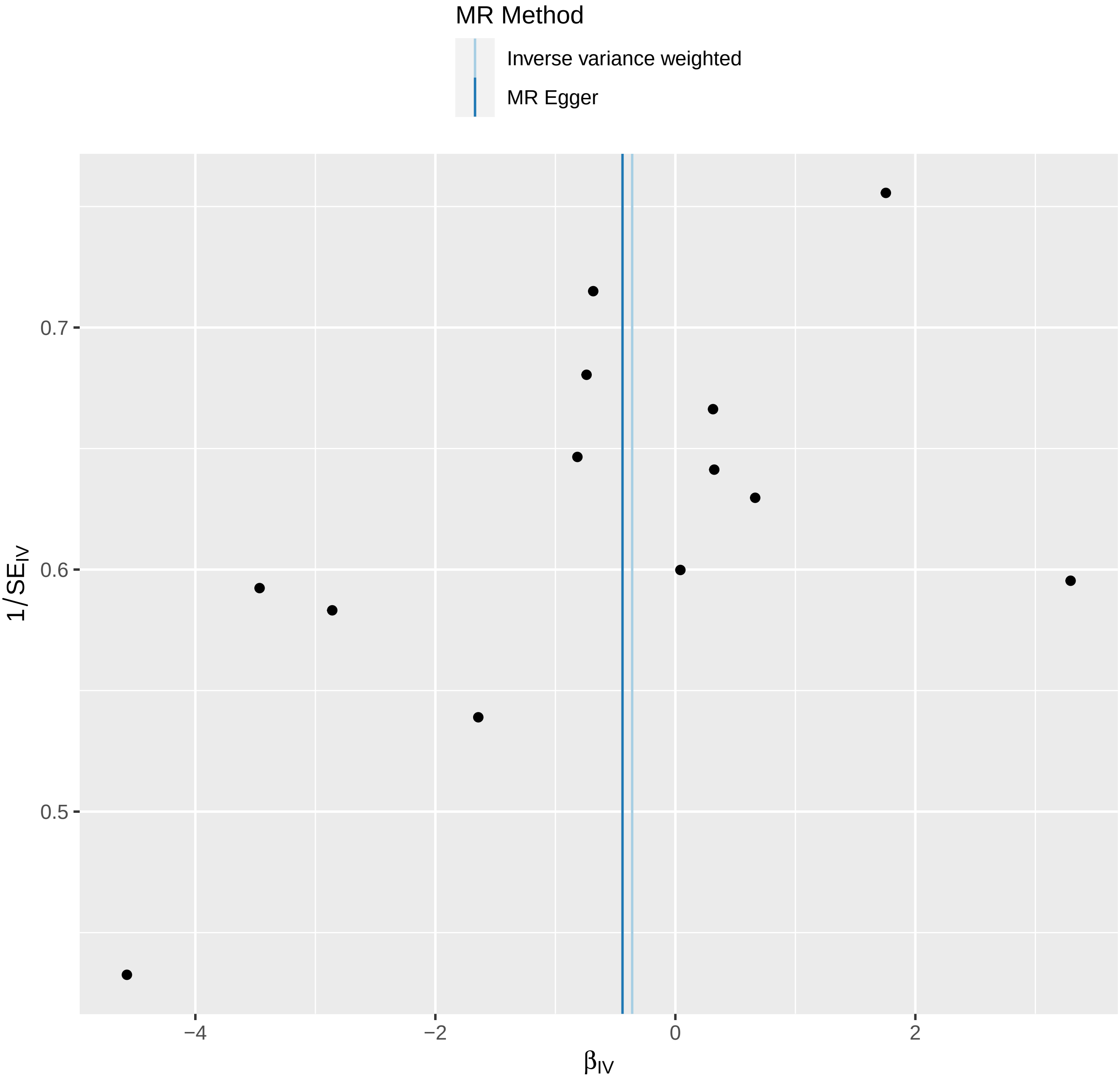

Batch 572 : Gut microbiota abundance (genus Roseburia id.2012) on Drug−induced obesity

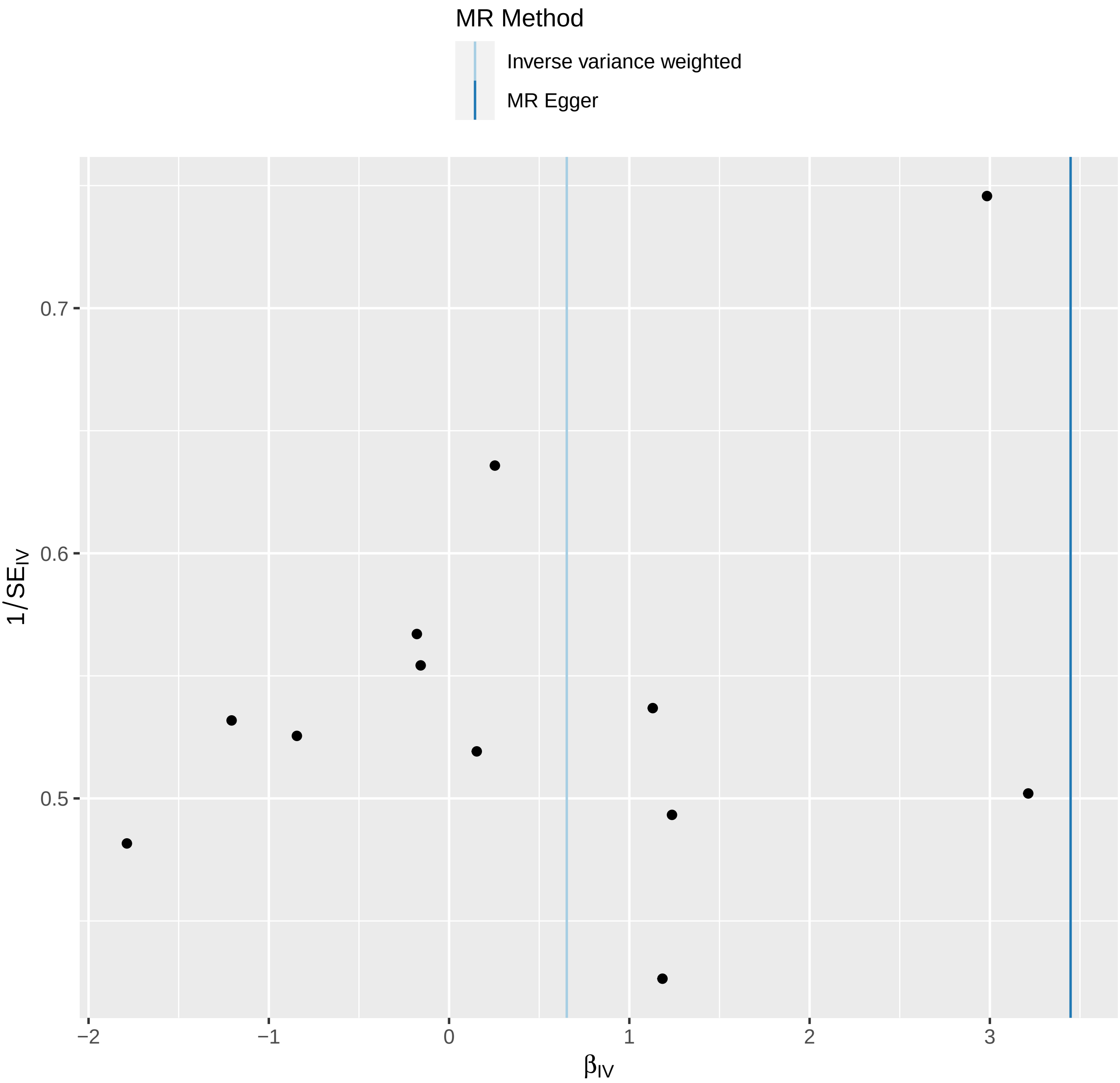

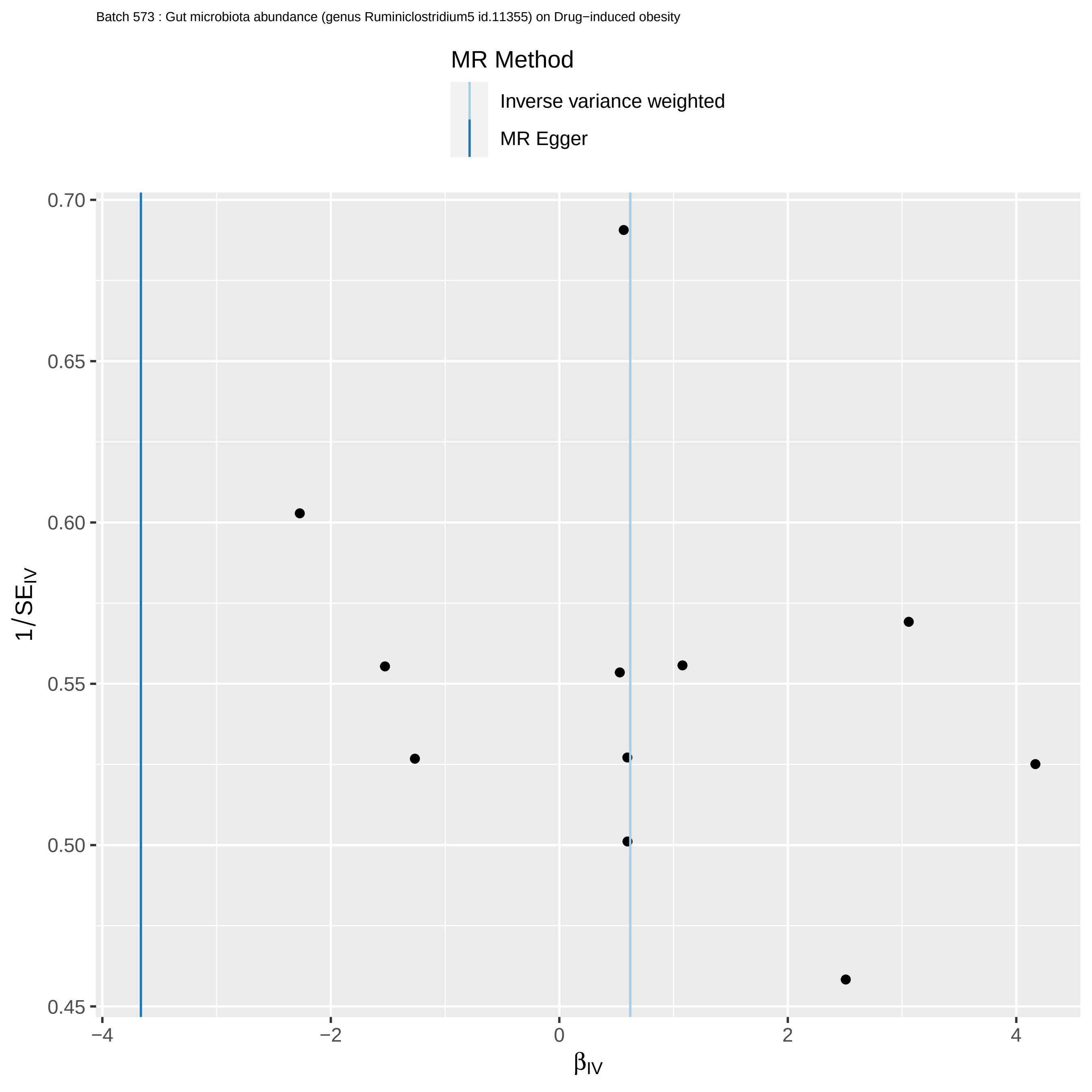

Batch 573 : Gut microbiota abundance (genus Ruminiclostridium5 id.11355) on Drug−induced obesity
MR Method
Inverse variance weighted
MR Egger
0.70
0.65
0.60
0.55
0.50
0.45
1/SE_IV
−4
−2
0
2
4
β_IV

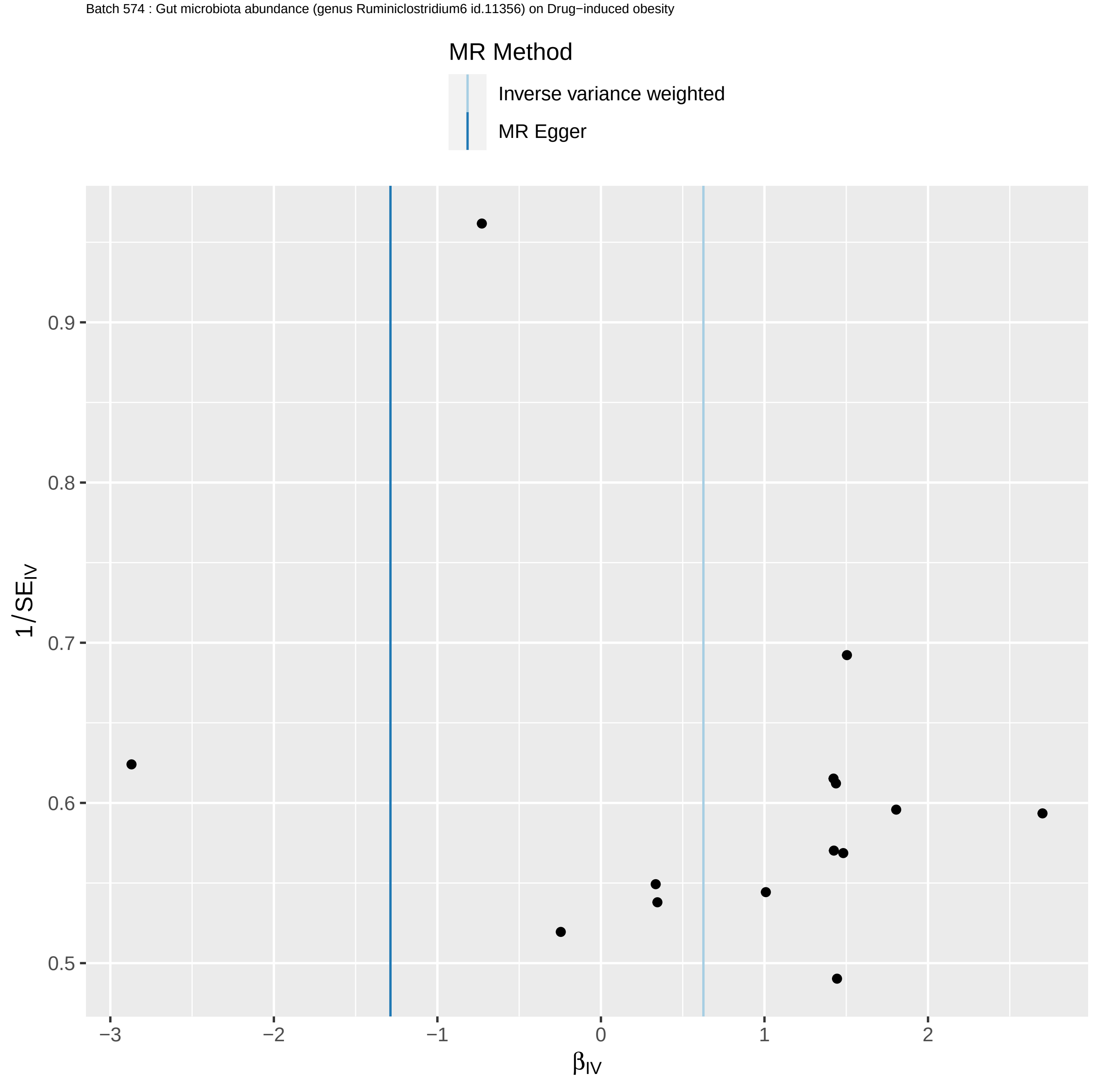
Batch 574 : Gut microbiota abundance (genus Ruminiclostridium6 id.11356) on Drug−induced obesity
MR Method
Inverse variance weighted
MR Egger
0.9
0.8
0.7
0.6
0.5
−3
−2
−1
0
1
2
1/SE_IV
β_IV

Batch 575 : Gut microbiota abundance (genus Ruminiclostridium9 id.11357) on Drug−induced obesity

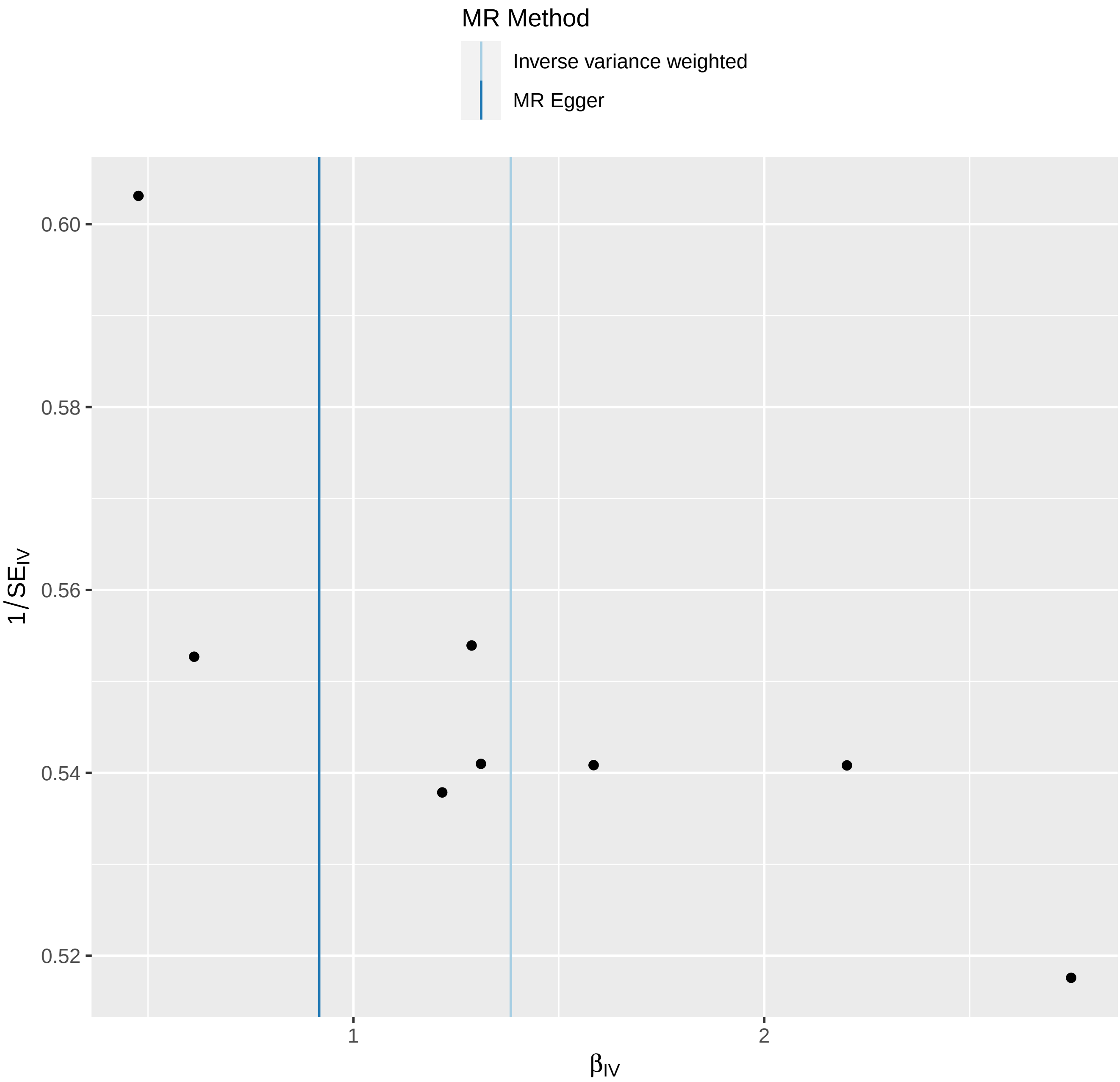

Batch 576 : Gut microbiota abundance (genus Ruminococcaceae NK4A214 group id.11358) on Drug−induced obesity

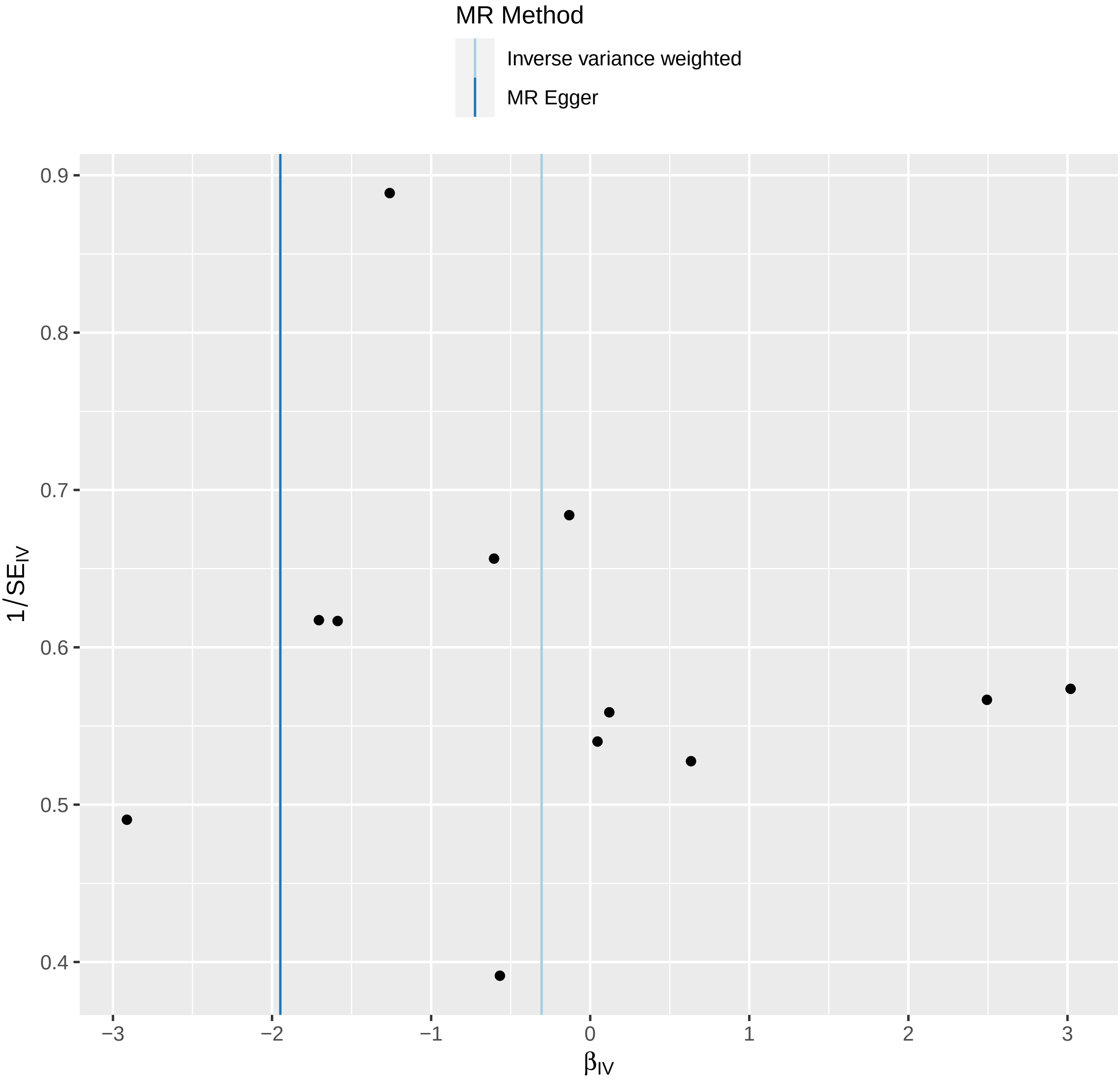

Batch 577 : Gut microbiota abundance (genus Ruminococcaceae UCG002 id.11360) on Drug−induced obesity

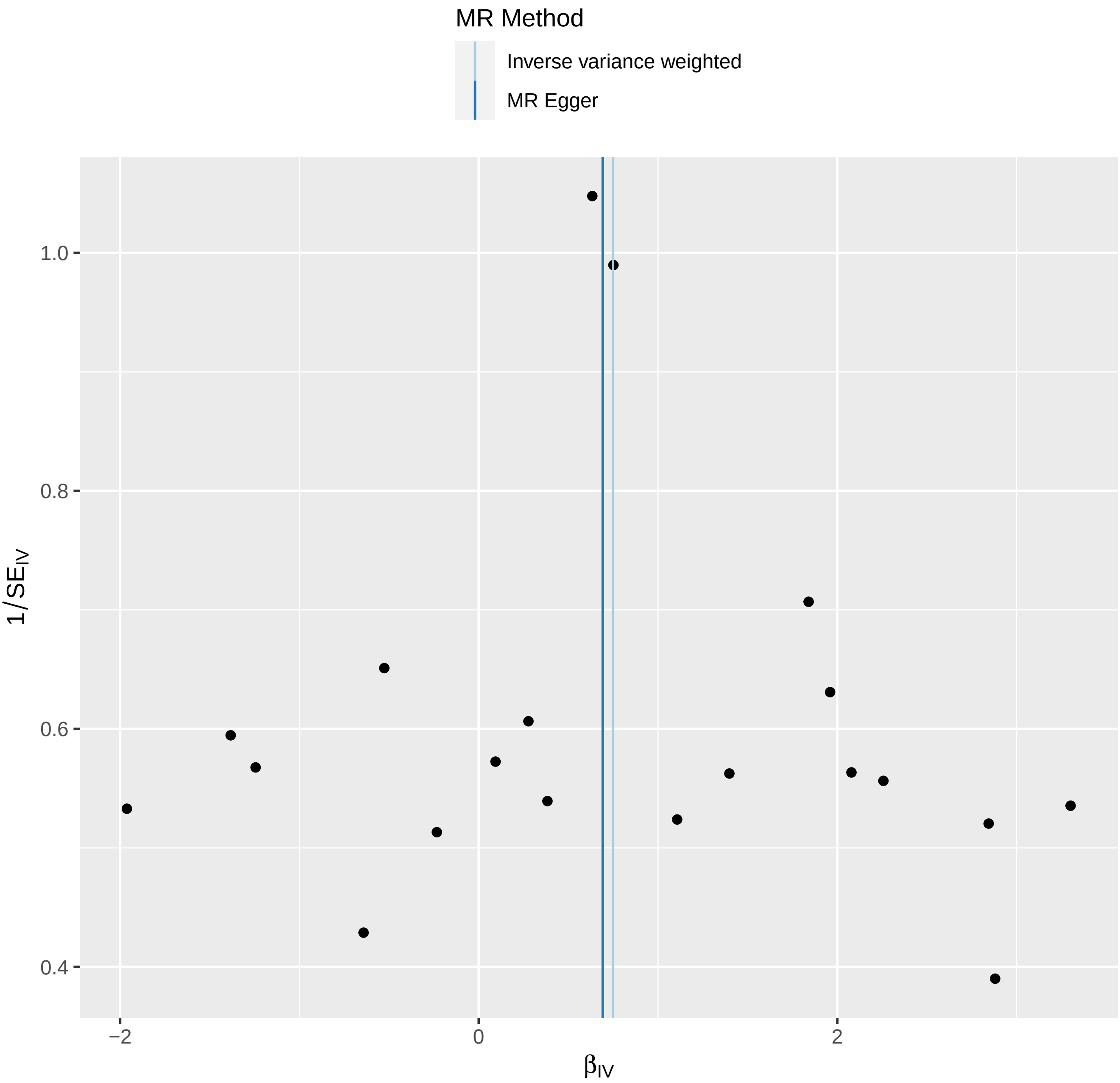

Batch 578 : Gut microbiota abundance (genus Ruminococcaceae UCG003 id.11361) on Drug−induced obesity

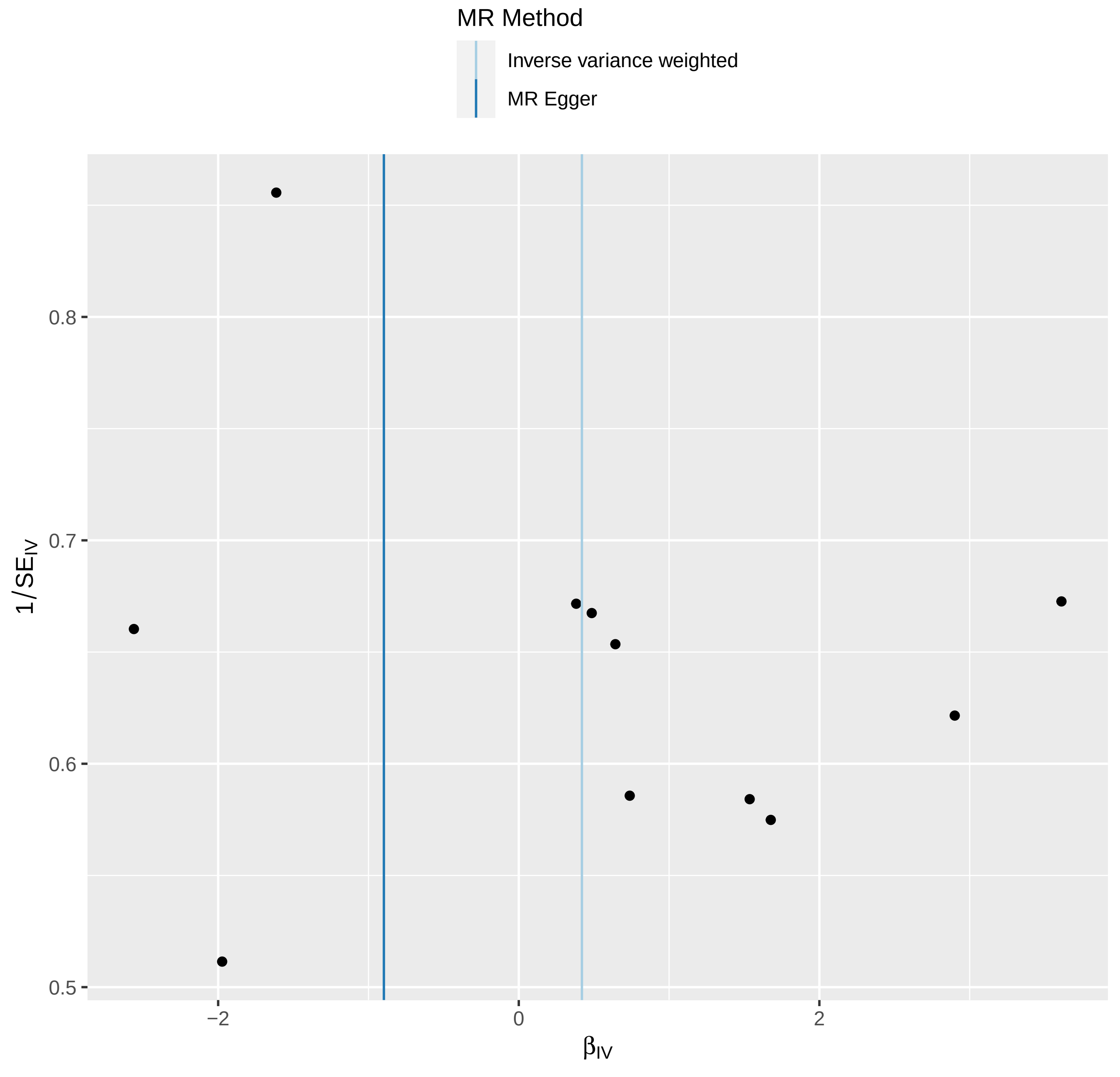

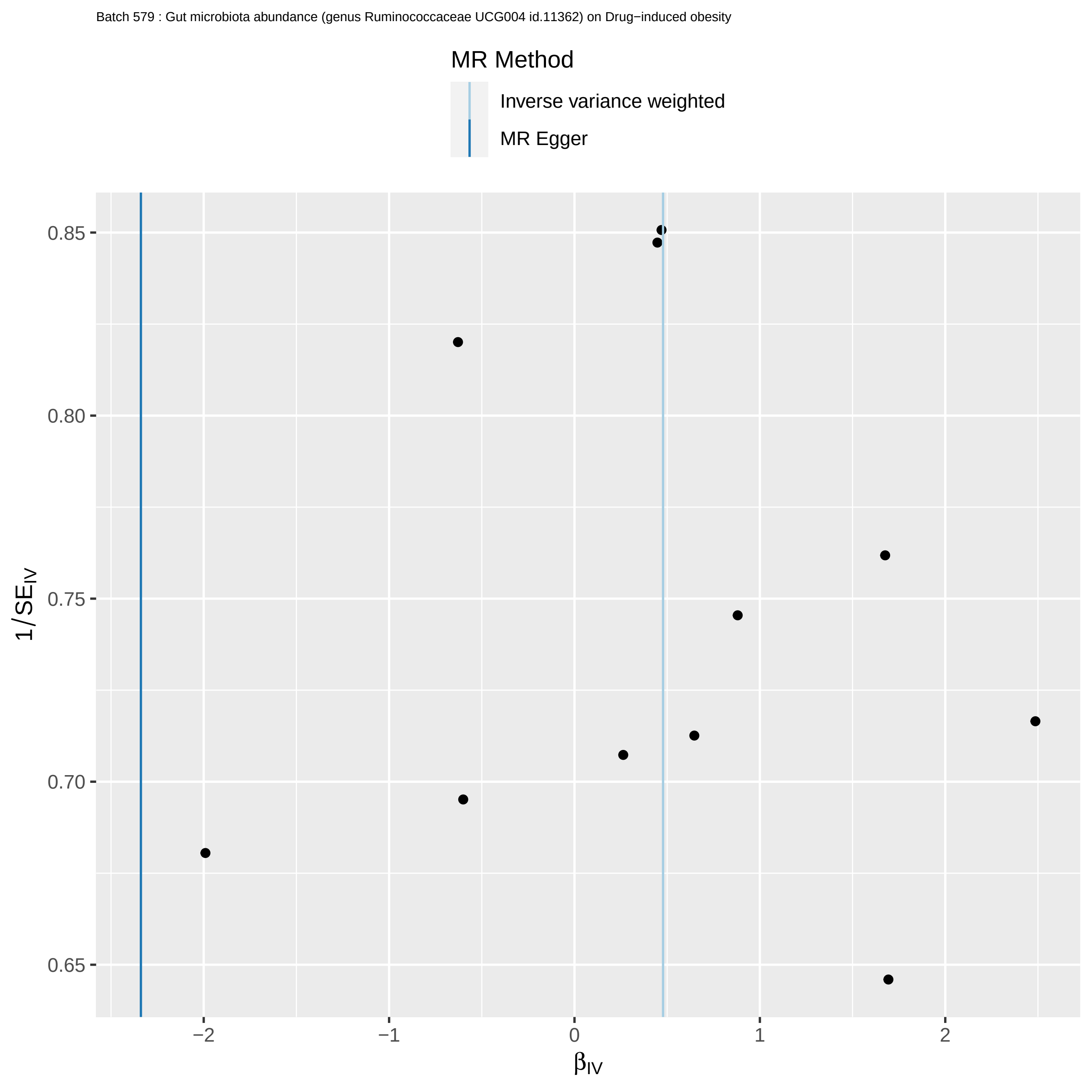
Batch 579 : Gut microbiota abundance (genus Ruminococcaceae UCG004 id.11362) on Drug−induced obesity
MR Method
Inverse variance weighted
MR Egger
0.85
0.80
0.75
0.70
0.65
1/SE_IV
−2
−1
0
1
2
β_IV

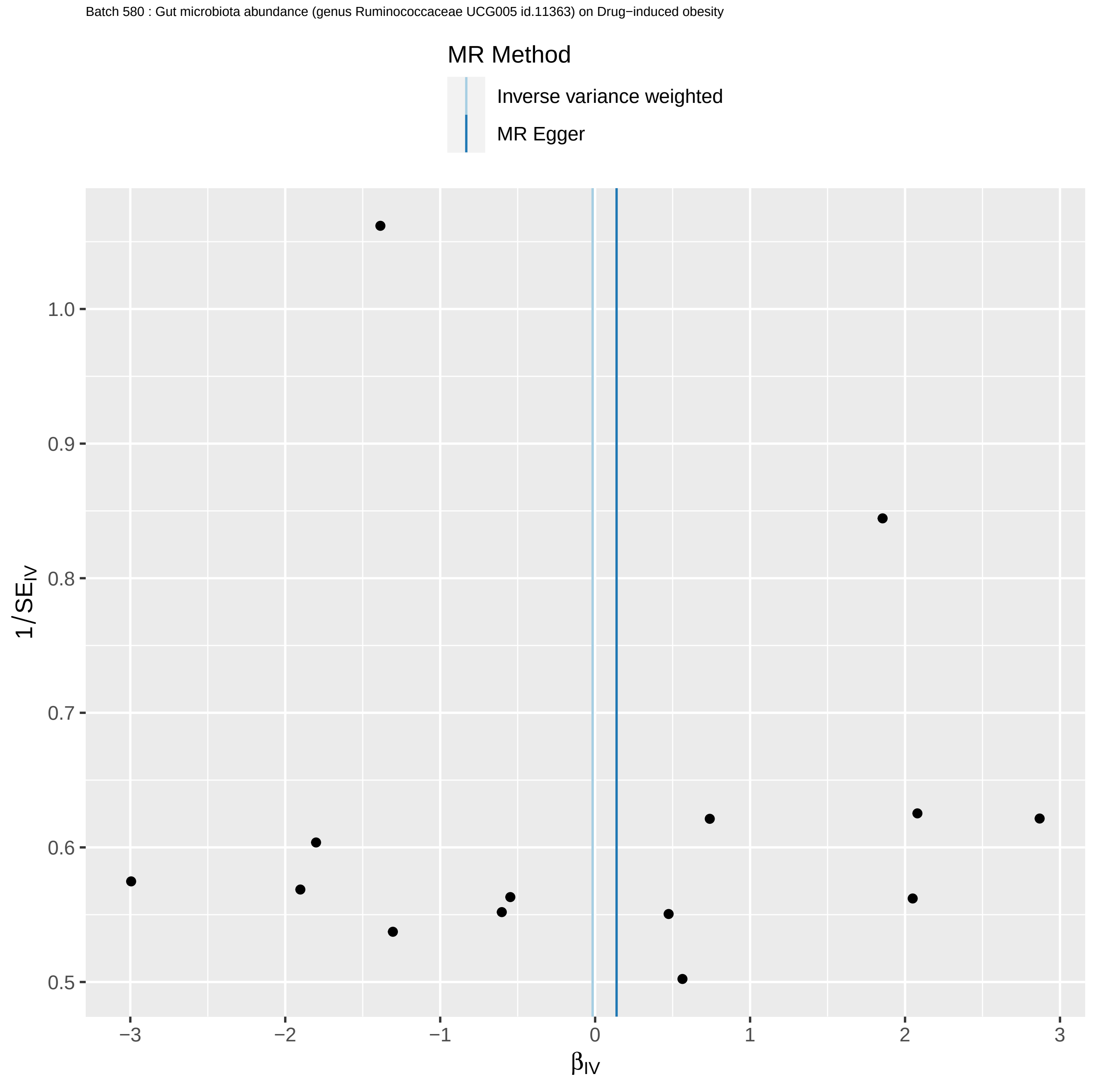
Batch 580 : Gut microbiota abundance (genus Ruminococcaceae UCG005 id.11363) on Drug−induced obesity
MR Method
Inverse variance weighted
MR Egger
1.0
0.9
0.8
0.7
0.6
0.5
1/SE_IV
−3
−2
−1
0
1
2
3
β_IV

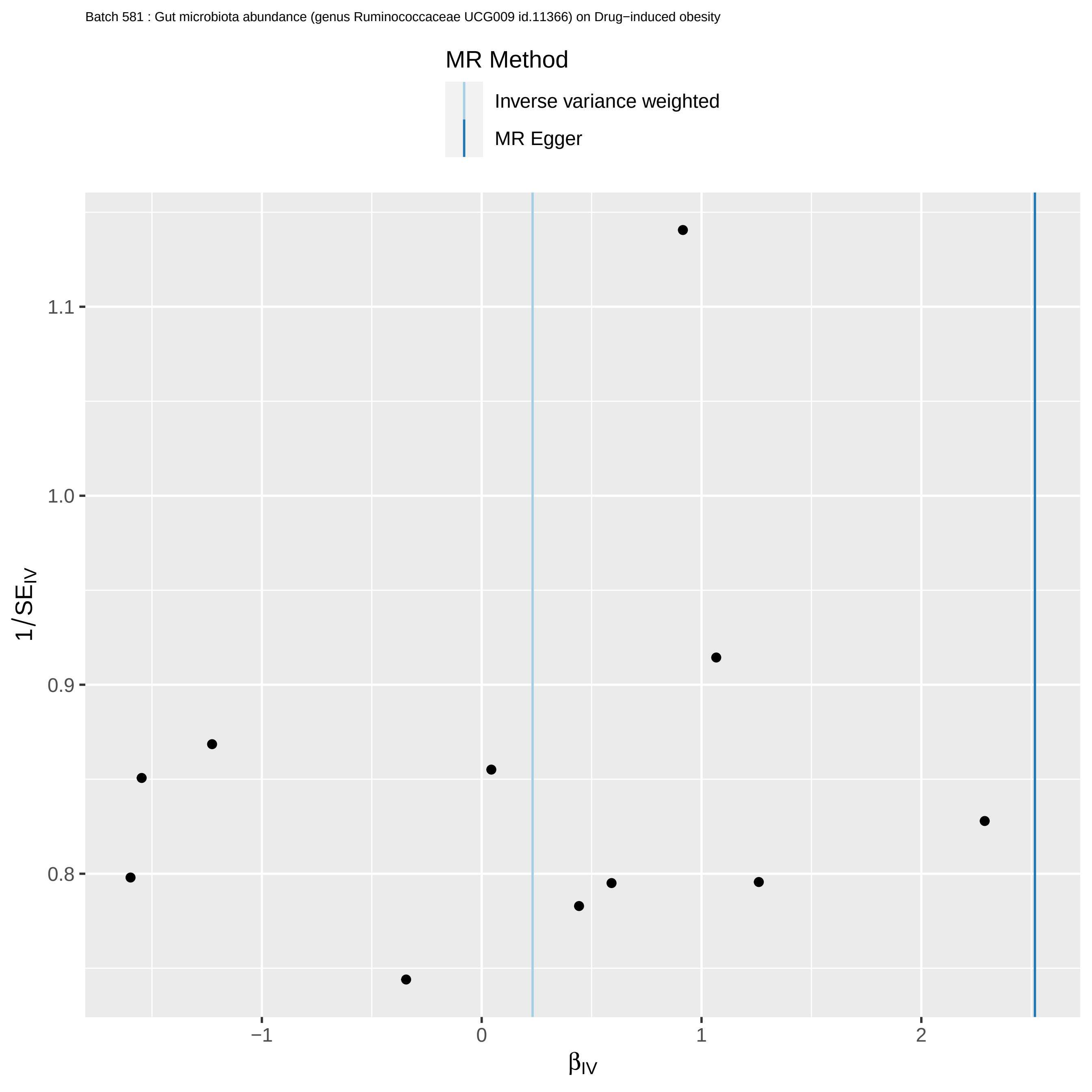

Batch 581 : Gut microbiota abundance (genus Ruminococcaceae UCG009 id.11366) on Drug−induced obesity
MR Method
Inverse variance weighted
MR Egger
1.1
1.0
0.9
0.8
−1
0
1
2
$1/SE_{IV}$
$\beta_{IV}$

Batch 582 : Gut microbiota abundance (genus Ruminococcaceae UCG010 id.11367) on Drug−induced obesity

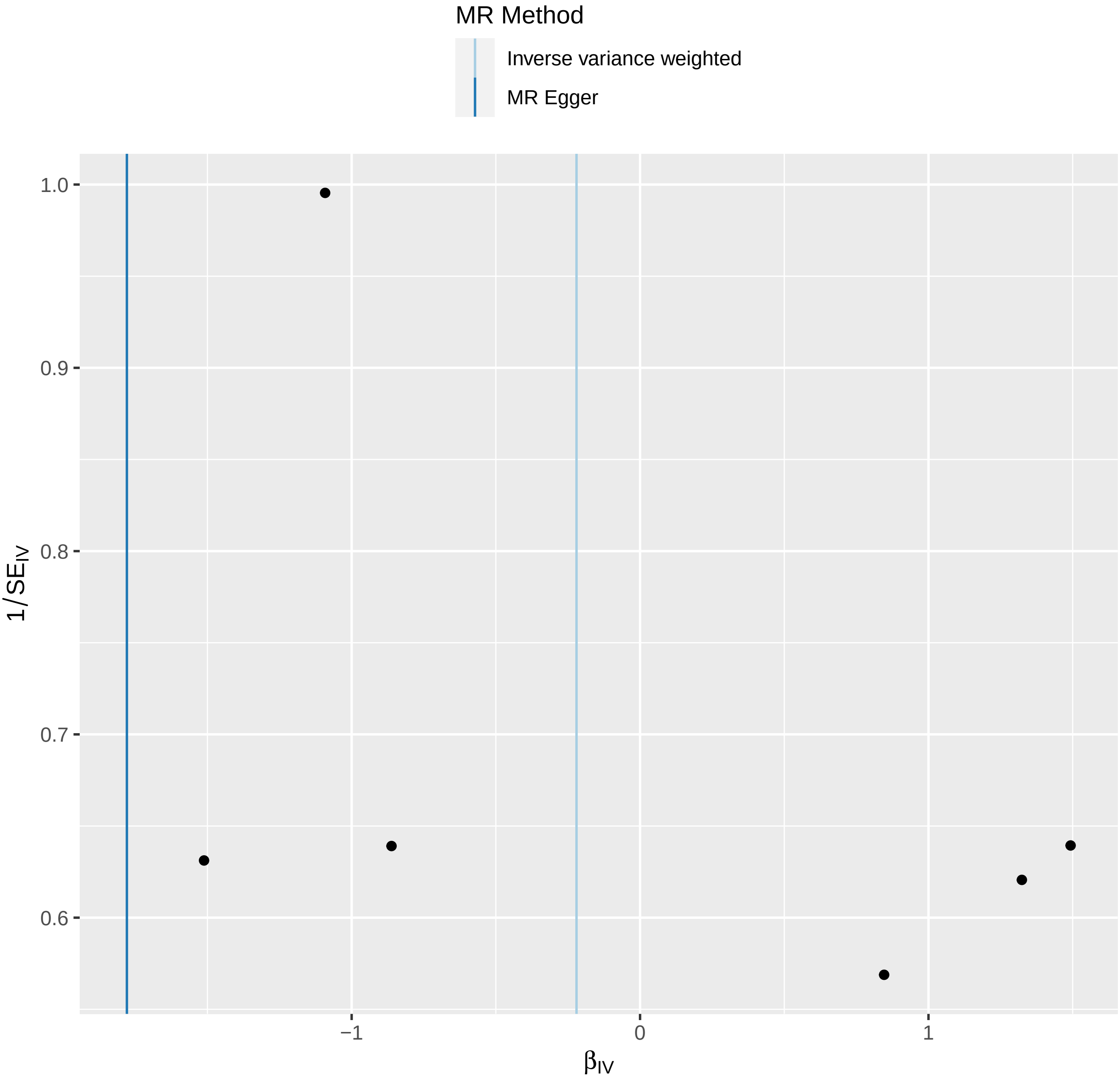

Batch 583 : Gut microbiota abundance (genus Ruminococcaceae UCG011 id.11368) on Drug−induced obesity

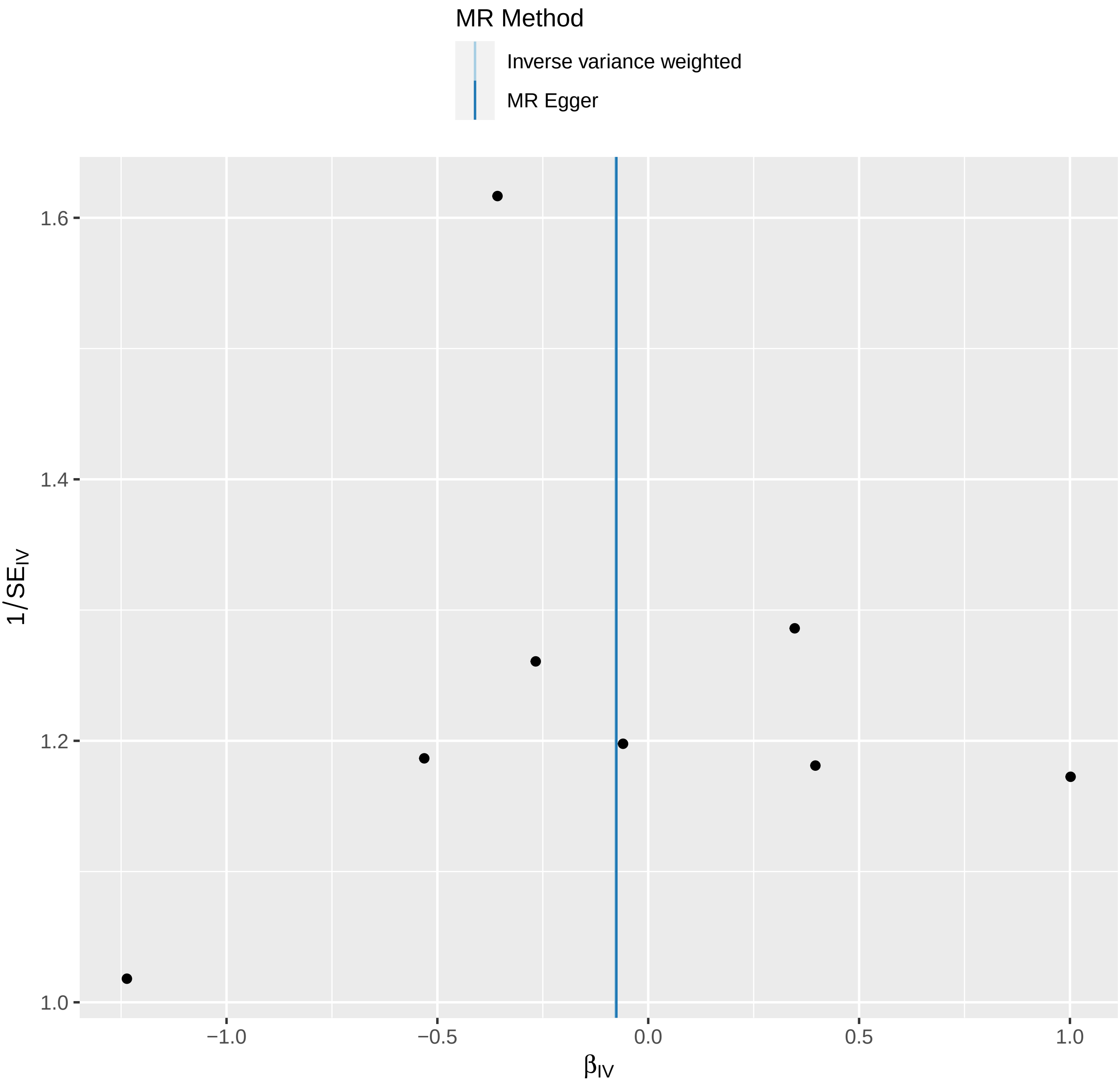

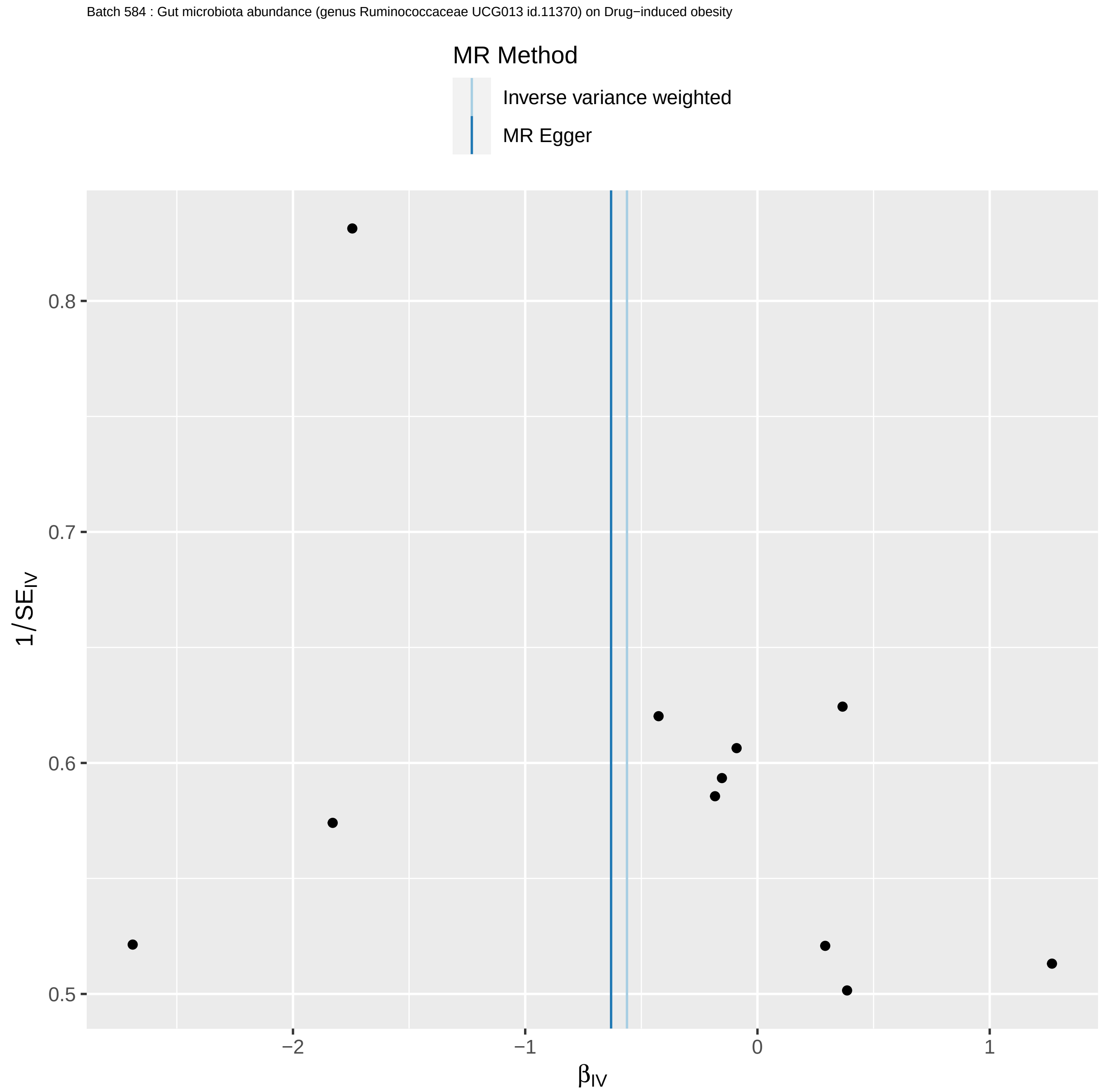

Batch 584 : Gut microbiota abundance (genus Ruminococcaceae UCG013 id.11370) on Drug−induced obesity
MR Method
Inverse variance weighted
MR Egger
0.8
0.7
0.6
0.5
1/SE_IV
−2
−1
0
1
β_IV

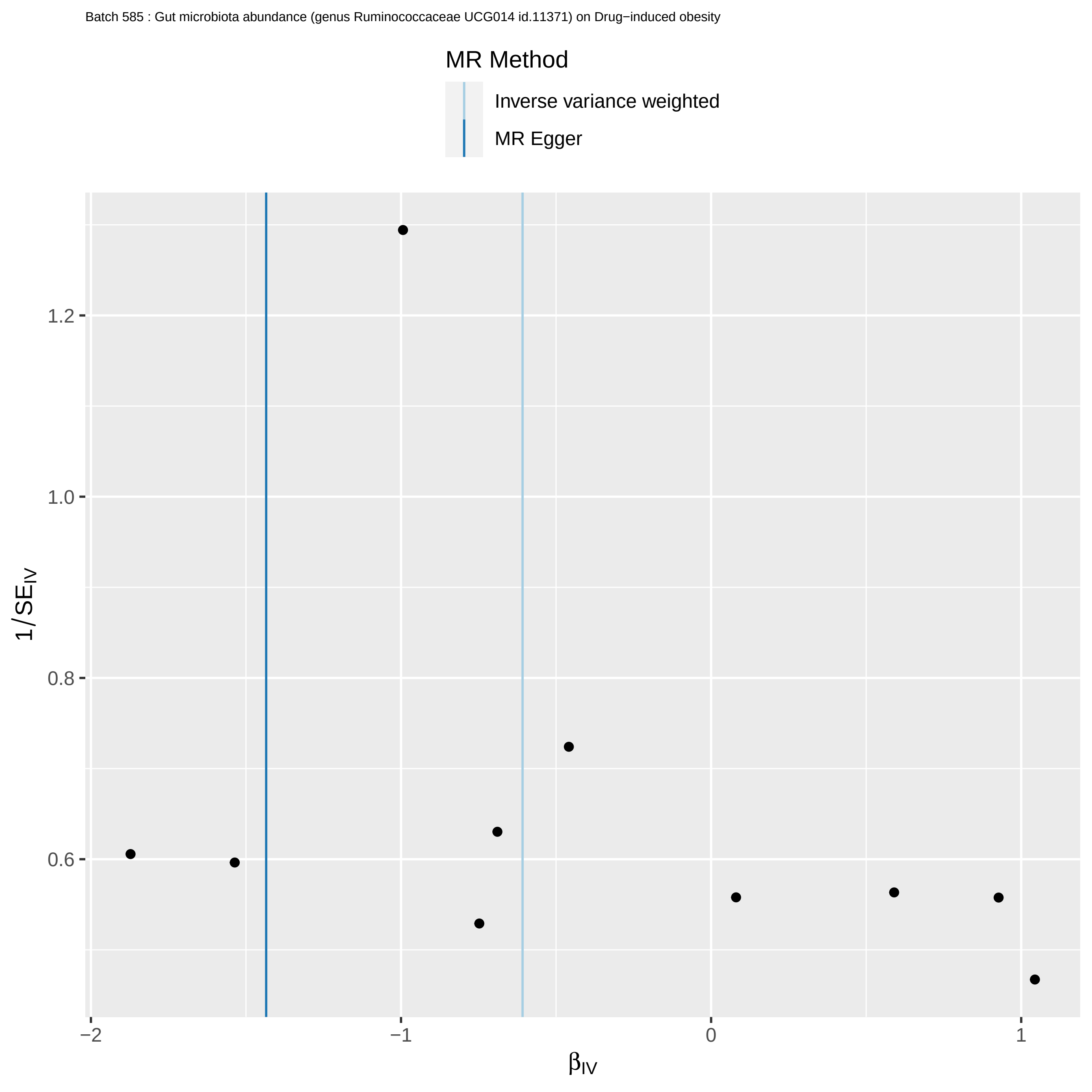
Batch 585 : Gut microbiota abundance (genus Ruminococcaceae UCG014 id.11371) on Drug−induced obesity
MR Method
Inverse variance weighted
MR Egger
1.2
1.0
0.8
0.6
−2
−1
0
1
$1/SE_{IV}$
$\beta_{IV}$

Batch 586 : Gut microbiota abundance (genus Ruminococcus1 id.11373) on Drug−induced obesity

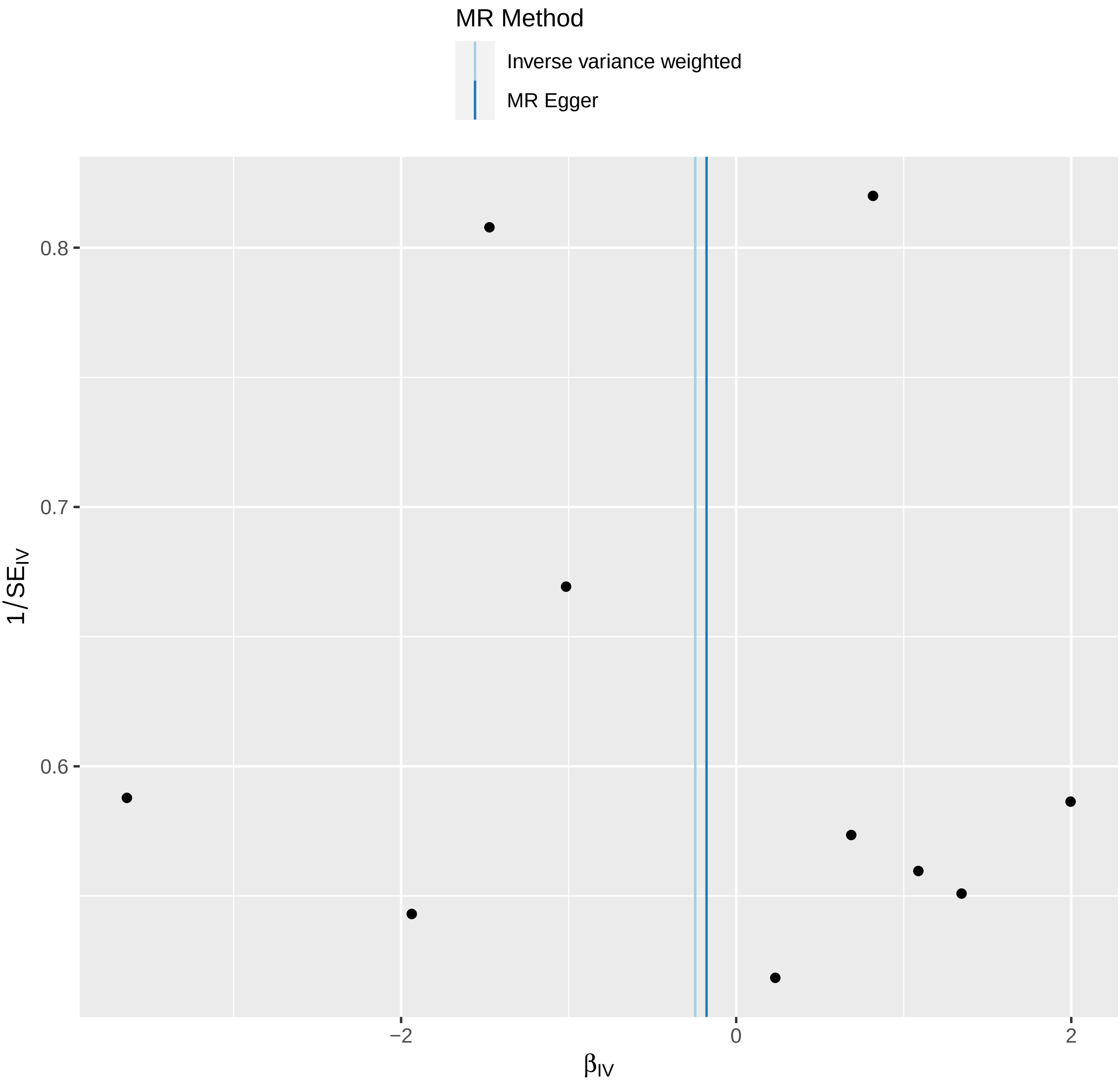

Batch 587 : Gut microbiota abundance (genus Ruminococcus2 id.11374) on Drug−induced obesity

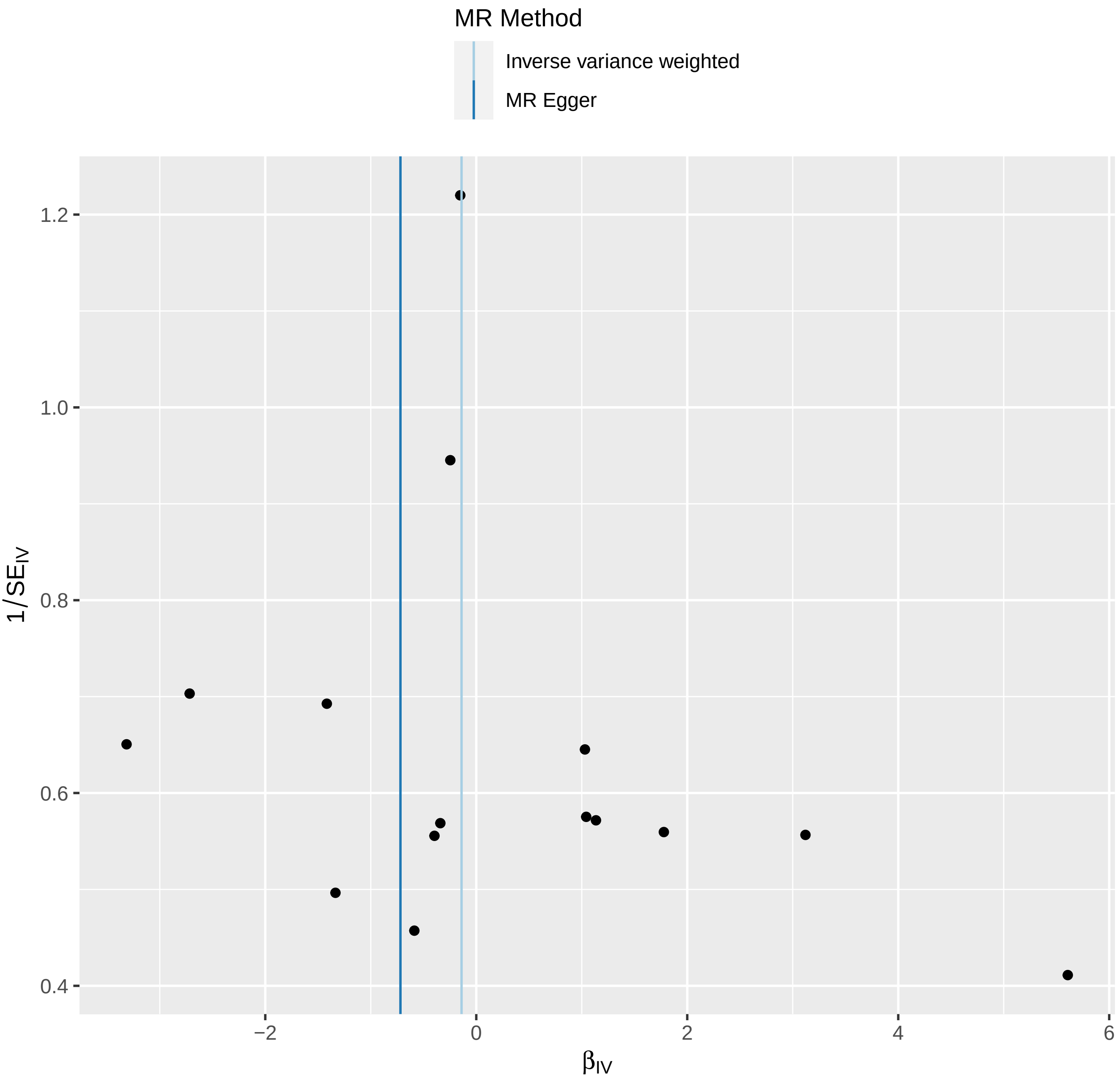

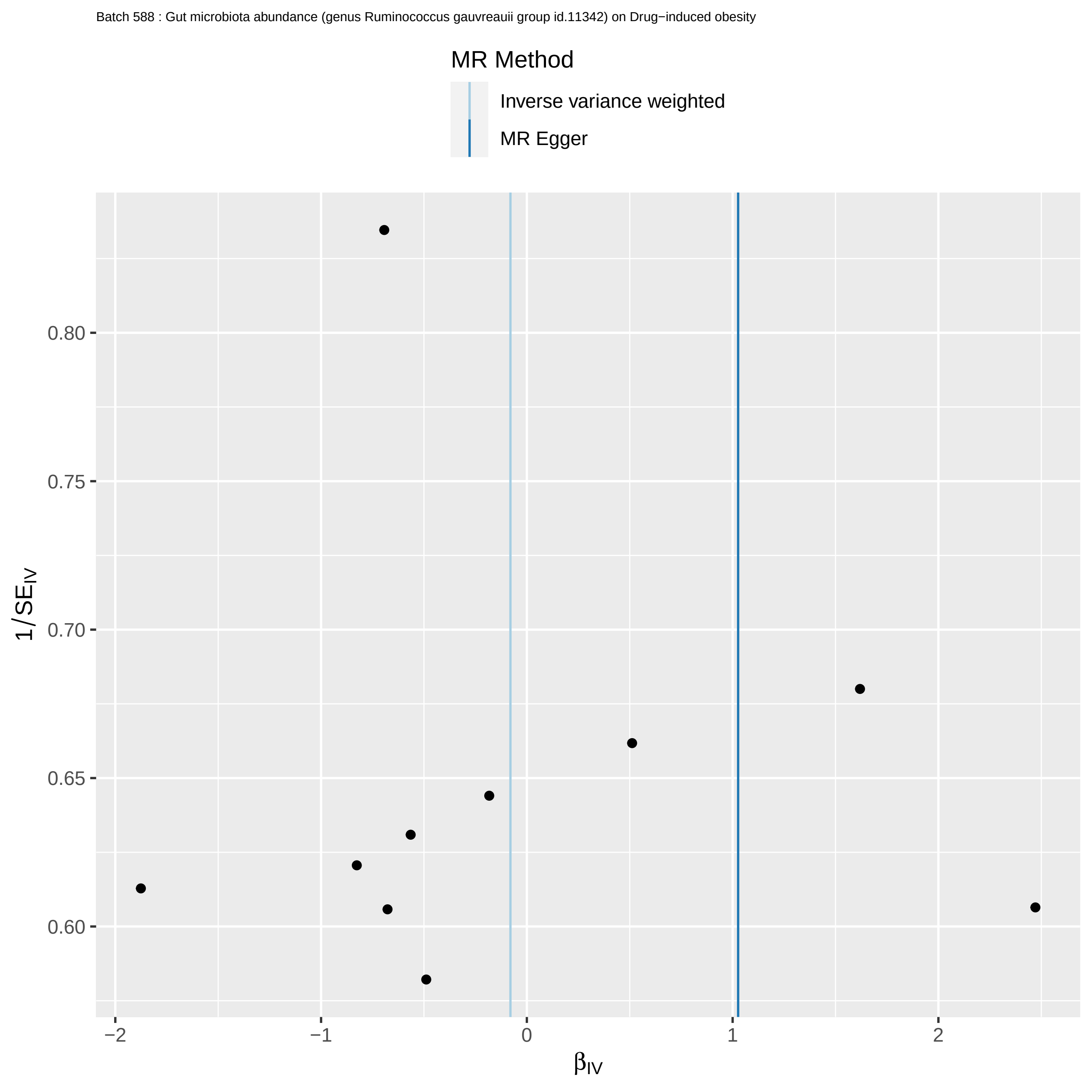

Batch 588 : Gut microbiota abundance (genus Ruminococcus gauvreauii group id.11342) on Drug−induced obesity
MR Method
Inverse variance weighted
MR Egger
0.80
0.75
0.70
0.65
0.60
1/SE_IV
−2
−1
0
1
2
β_IV

Batch 589 : Gut microbiota abundance (genus Ruminococcus gnavus group id.14376) on Drug−induced obesity

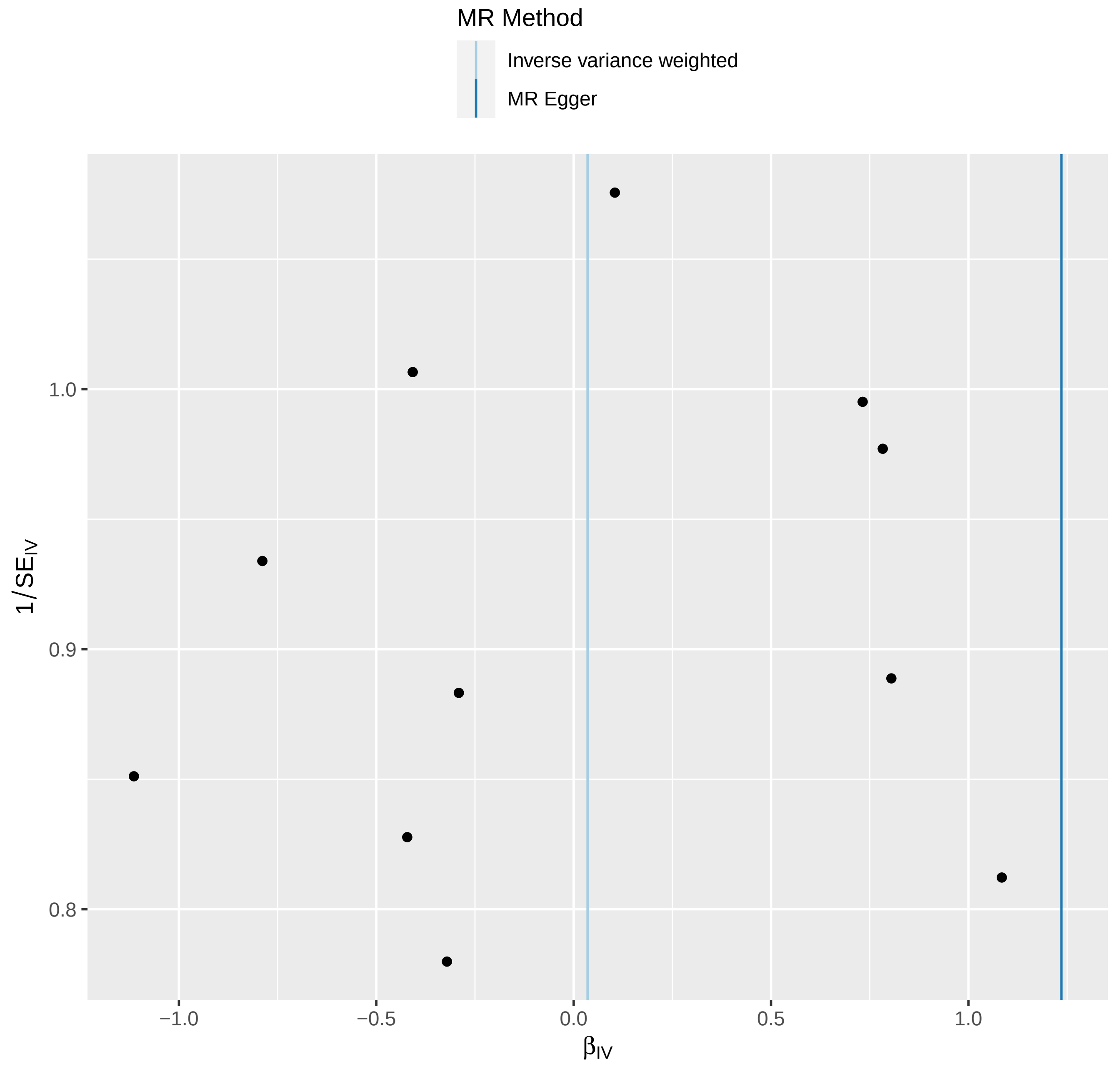

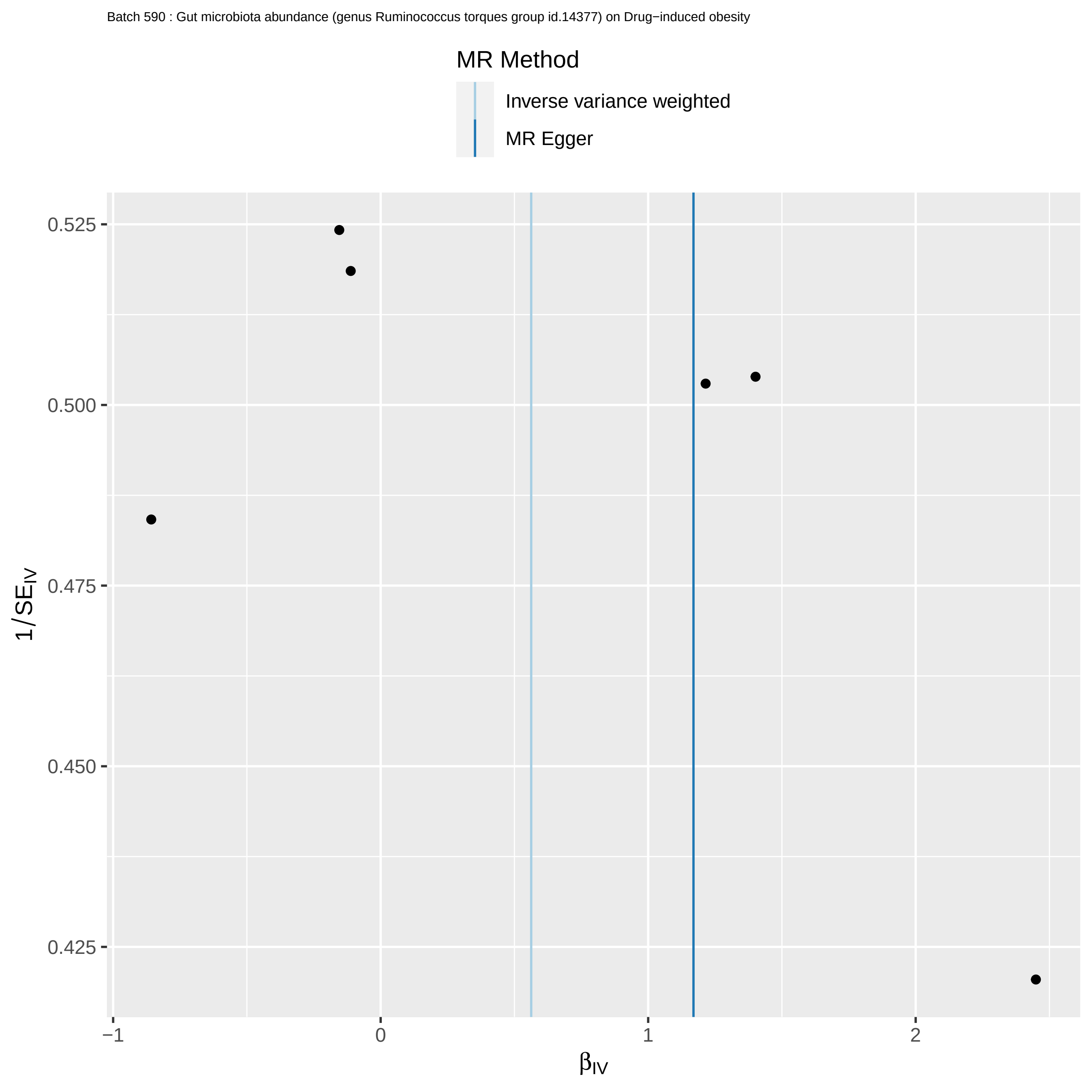

Batch 590 : Gut microbiota abundance (genus Ruminococcus torques group id.14377) on Drug−induced obesity
MR Method
Inverse variance weighted
MR Egger
0.525
0.500
0.475
0.450
0.425
1/SE_IV
−1
0
1
2
β_IV

Batch 591 : Gut microbiota abundance (genus Sellimonas id.14369) on Drug−induced obesity

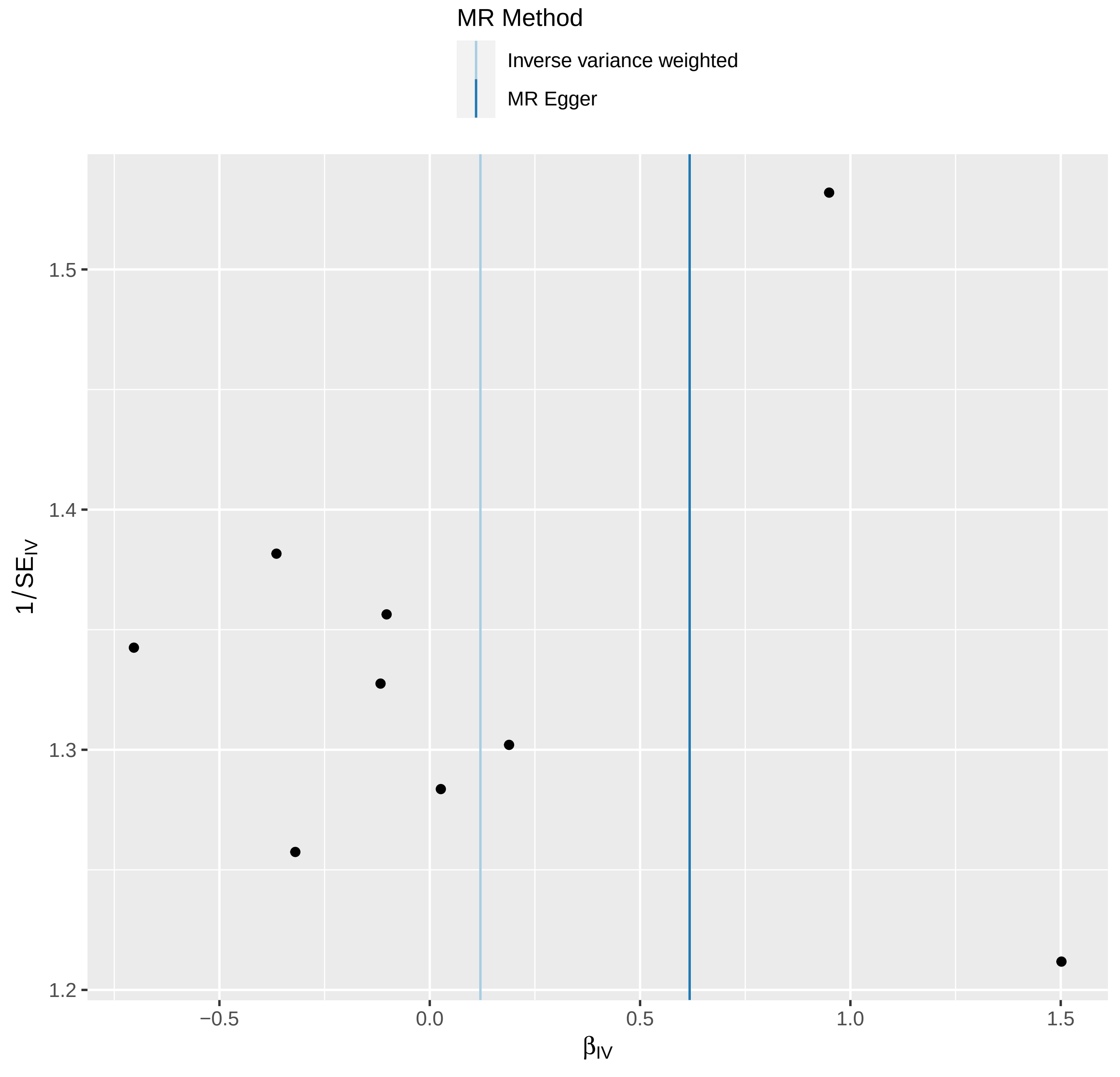

Batch 592 : Gut microbiota abundance (genus Senegalimassilia id.11160) on Drug−induced obesity

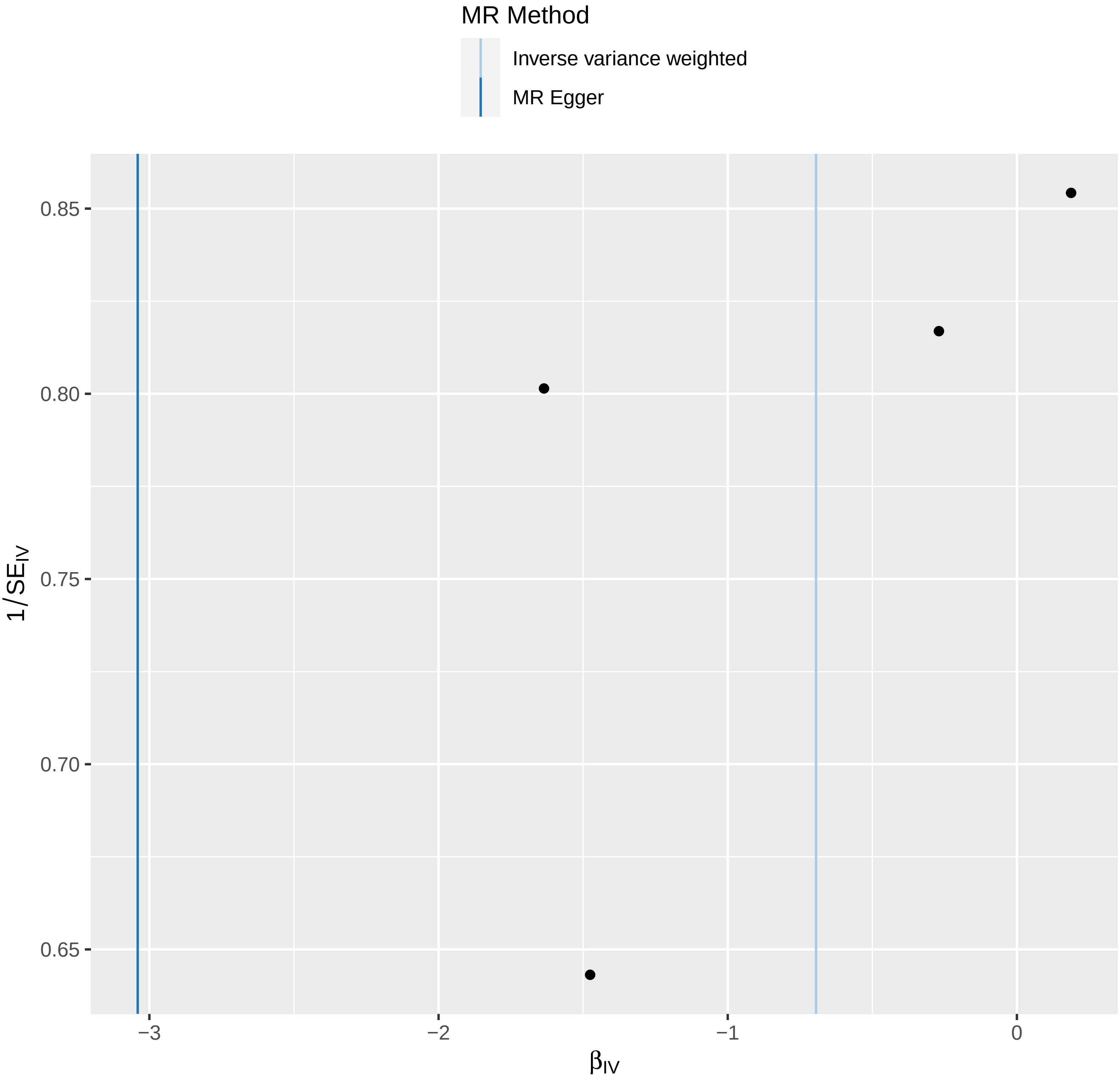

Batch 593 : Gut microbiota abundance (genus Slackia id.825) on Drug−induced obesity

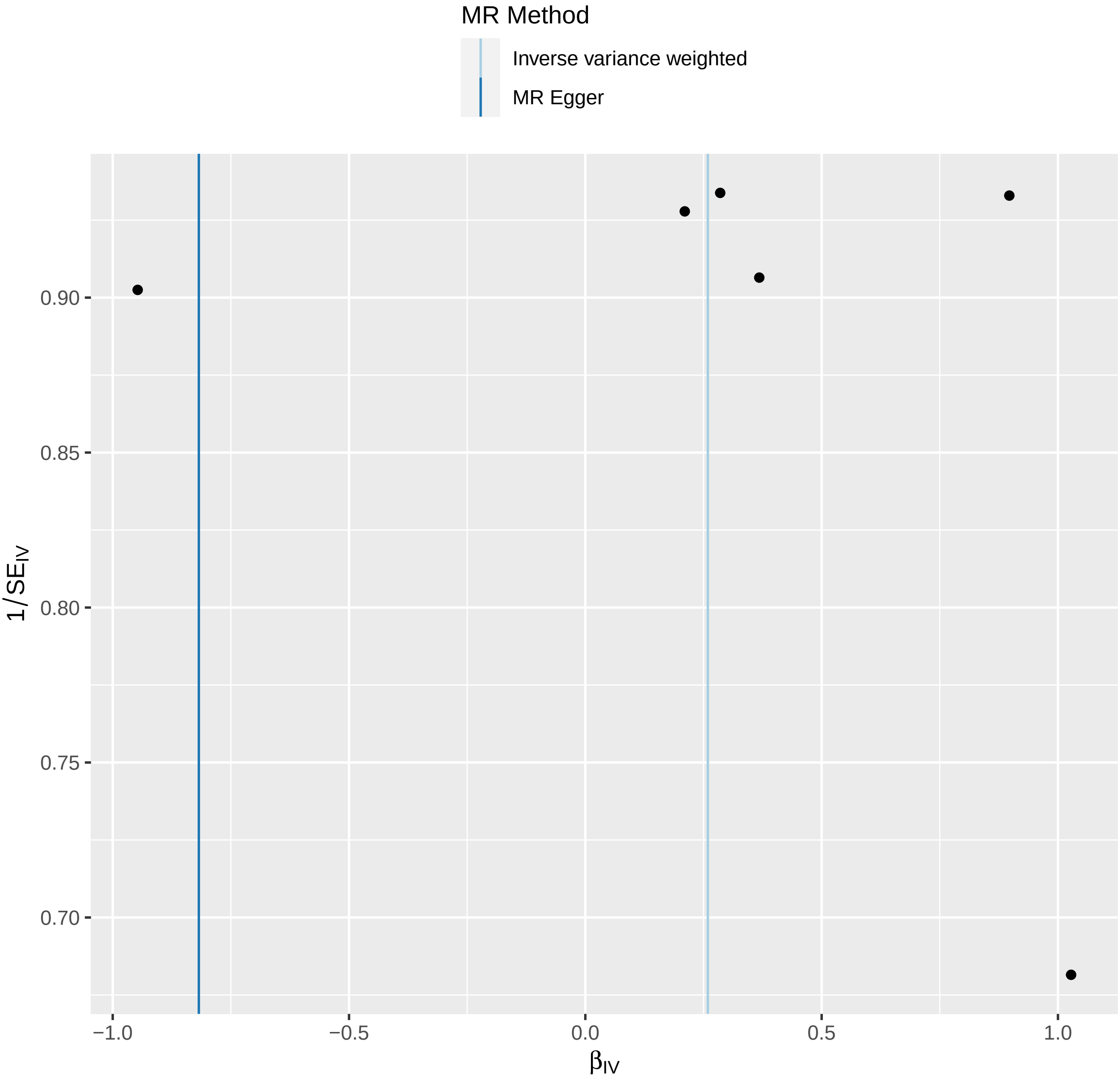

Batch 594 : Gut microbiota abundance (genus Streptococcus id.1853) on Drug−induced obesity

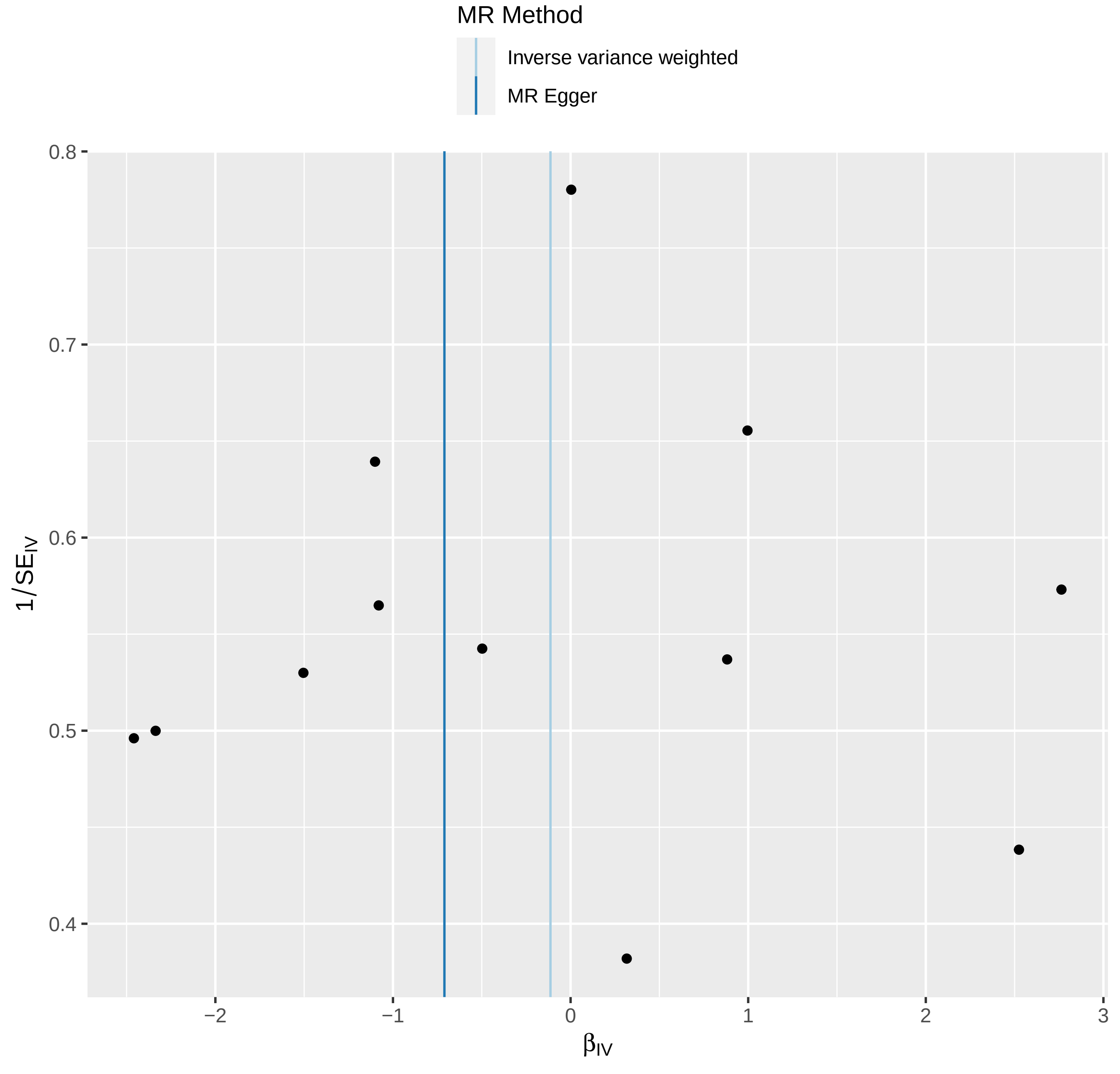

Batch 595 : Gut microbiota abundance (genus Subdoligranulum id.2070) on Drug−induced obesity

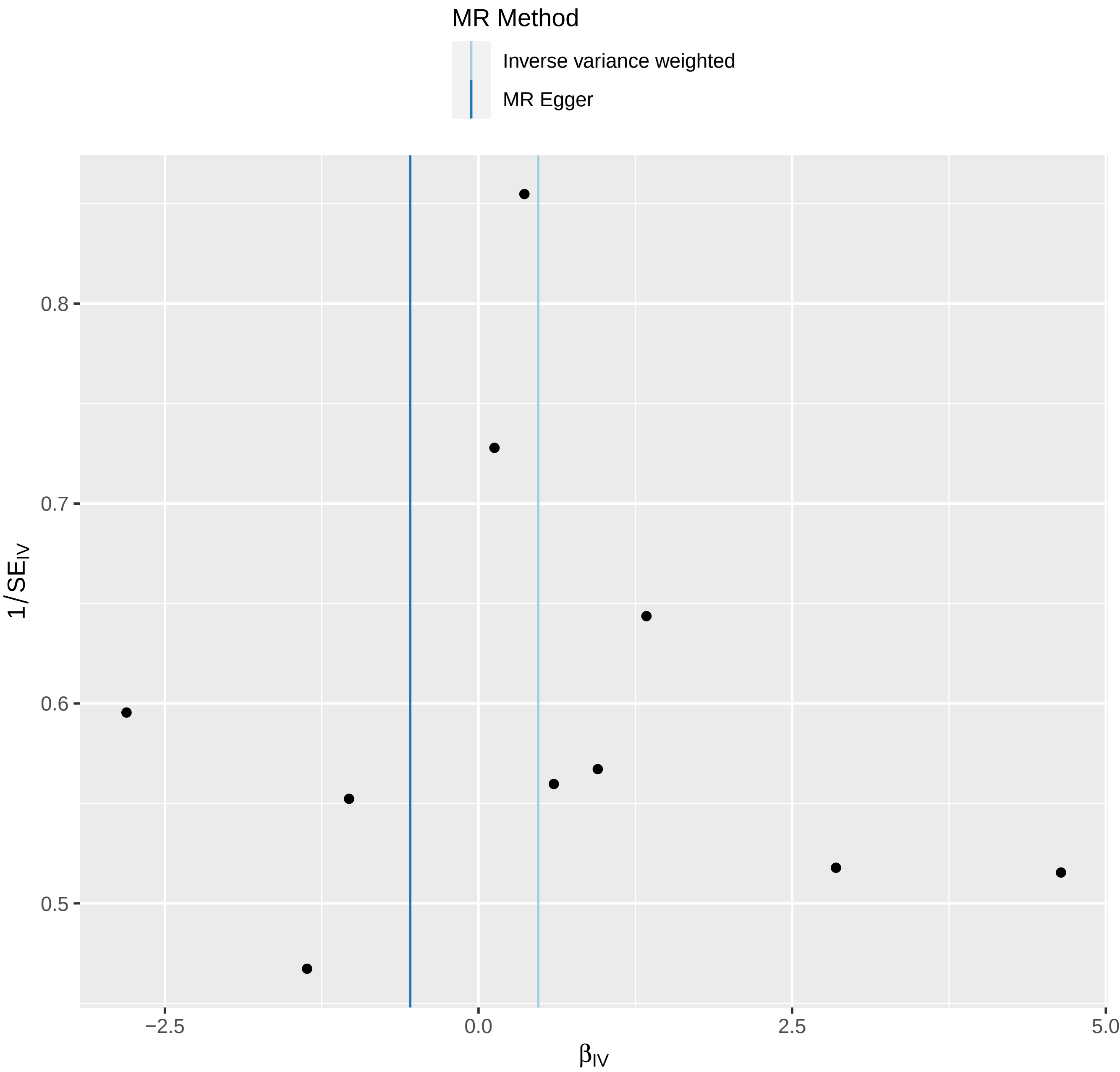

Batch 596 : Gut microbiota abundance (genus Sutterella id.2896) on Drug−induced obesity

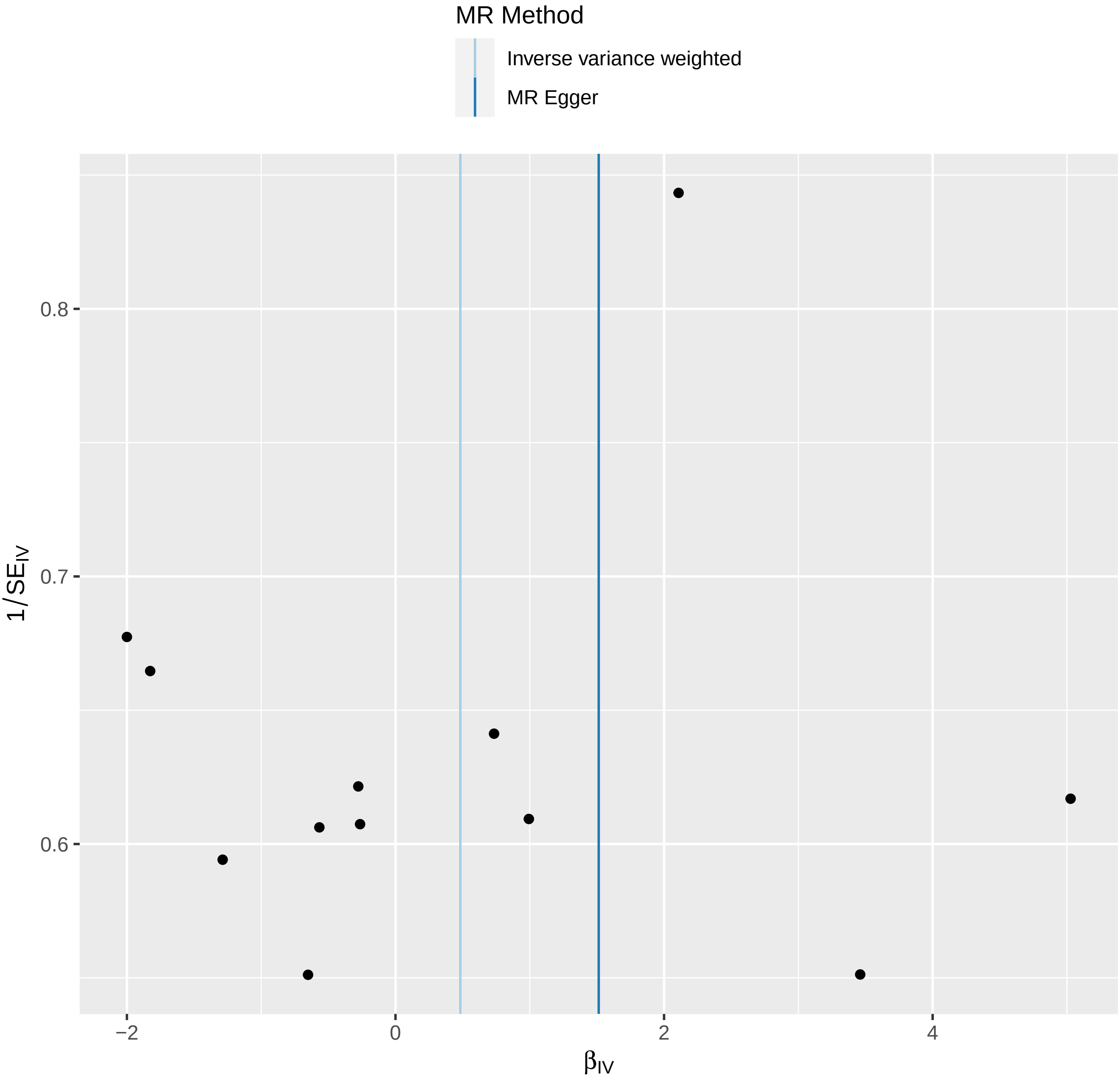

Batch 597 : Gut microbiota abundance (genus Terrisporobacter id.11348) on Drug−induced obesity

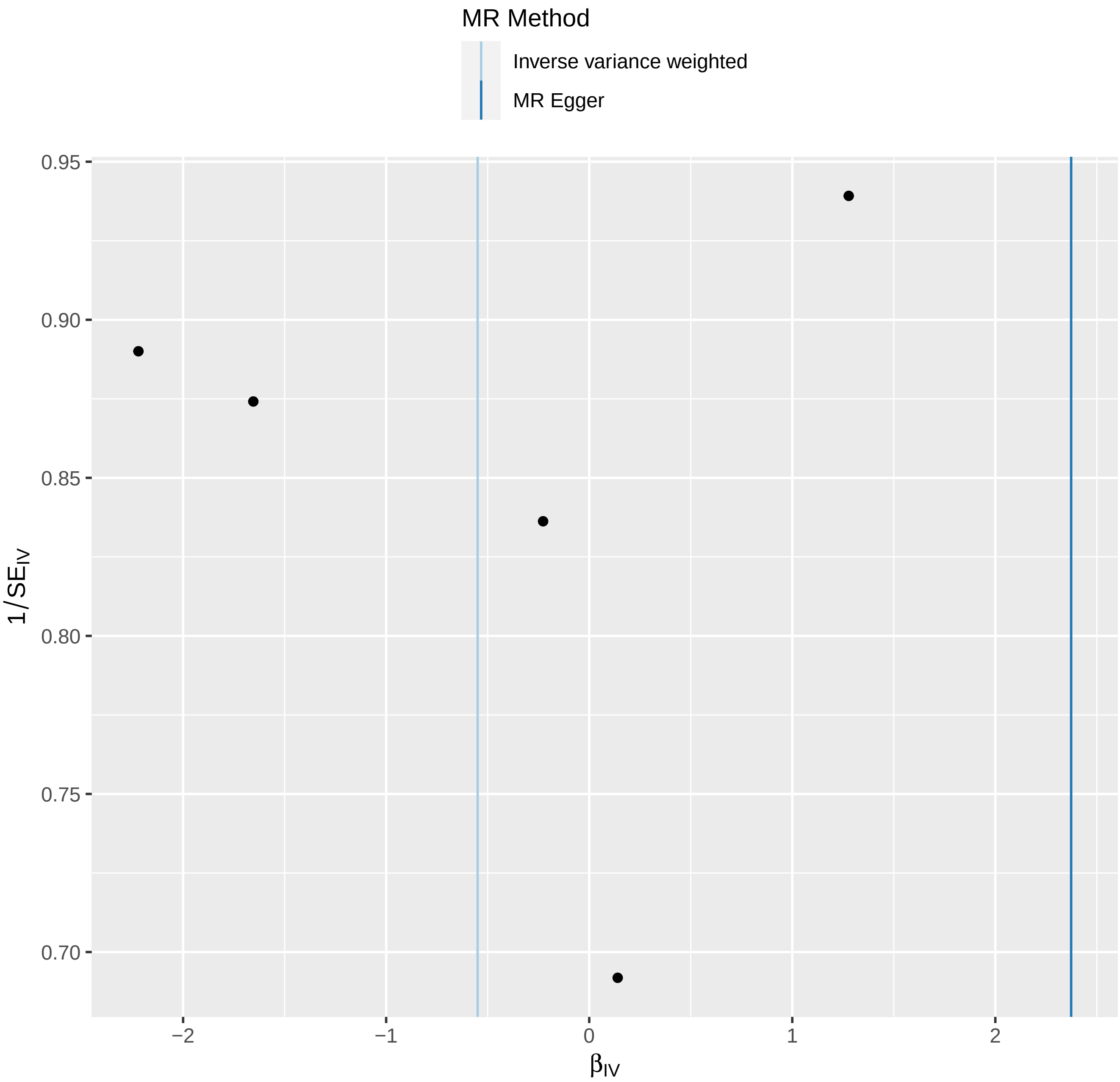

Batch 598 : Gut microbiota abundance (genus Turicibacter id.2162) on Drug−induced obesity

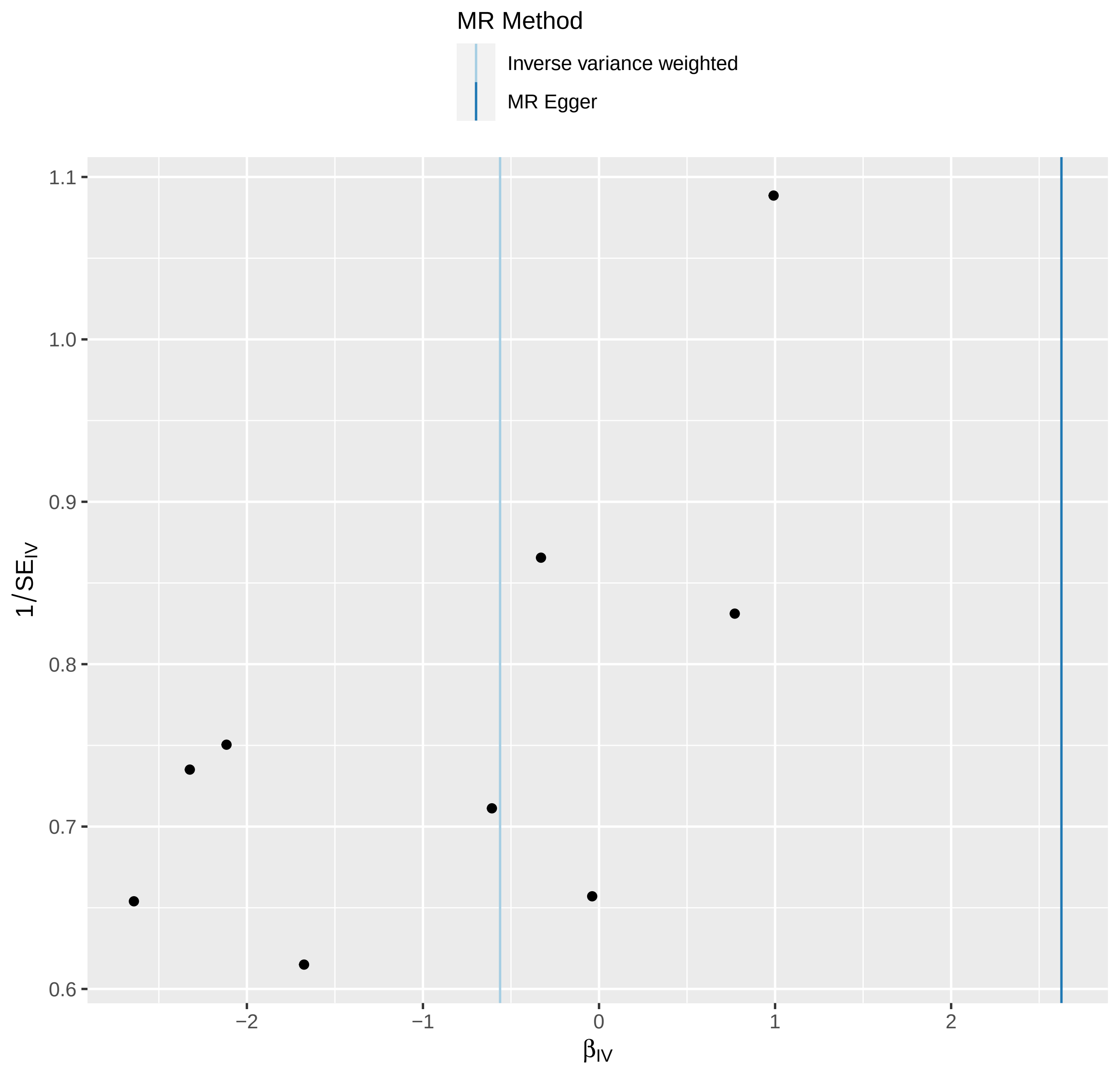

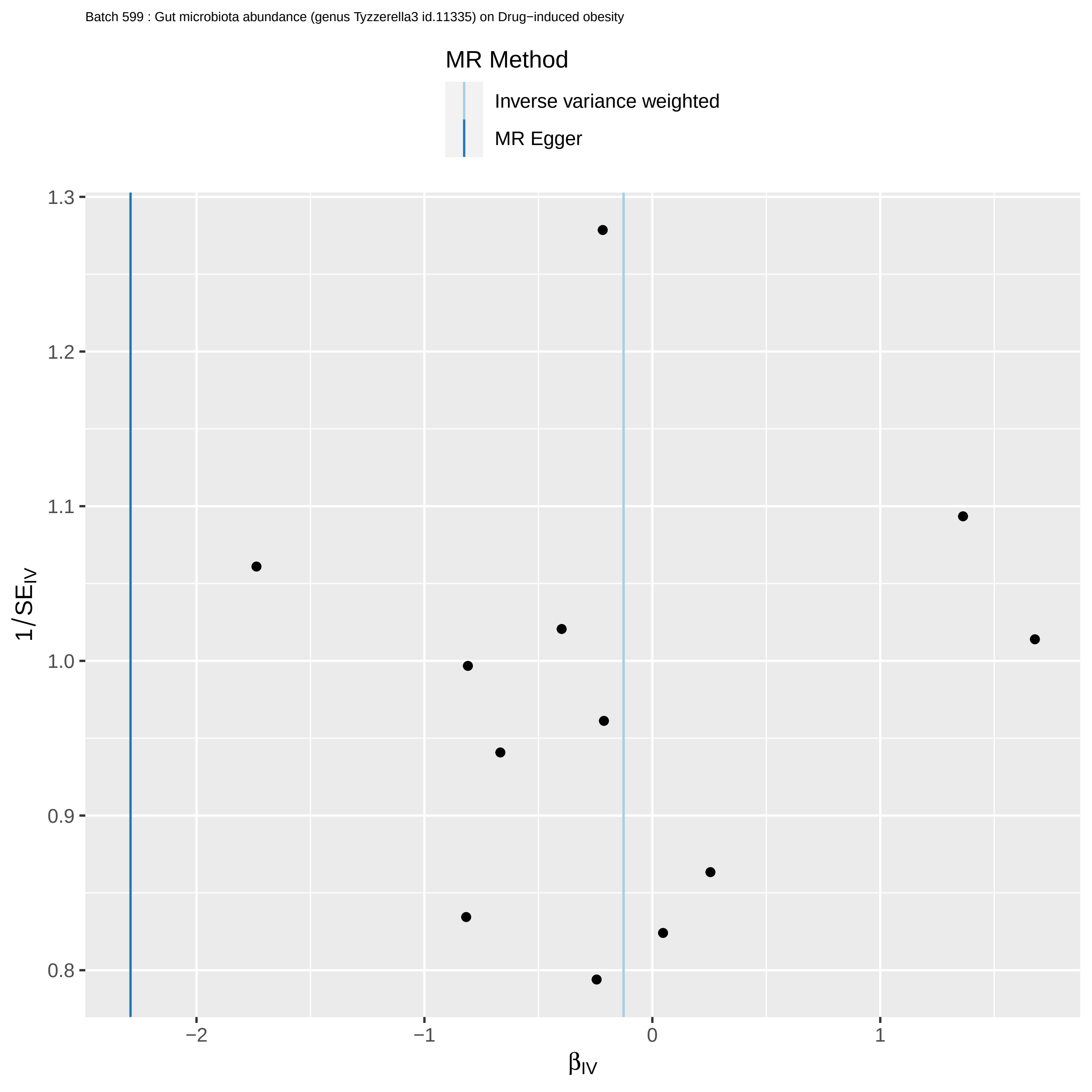

Batch 599 : Gut microbiota abundance (genus Tyzzerella3 id.11335) on Drug−induced obesity
MR Method
Inverse variance weighted
MR Egger
1.3
1.2
1.1
1.0
0.9
0.8
$1/SE_{IV}$
−2
−1
0
1
$\beta_{IV}$

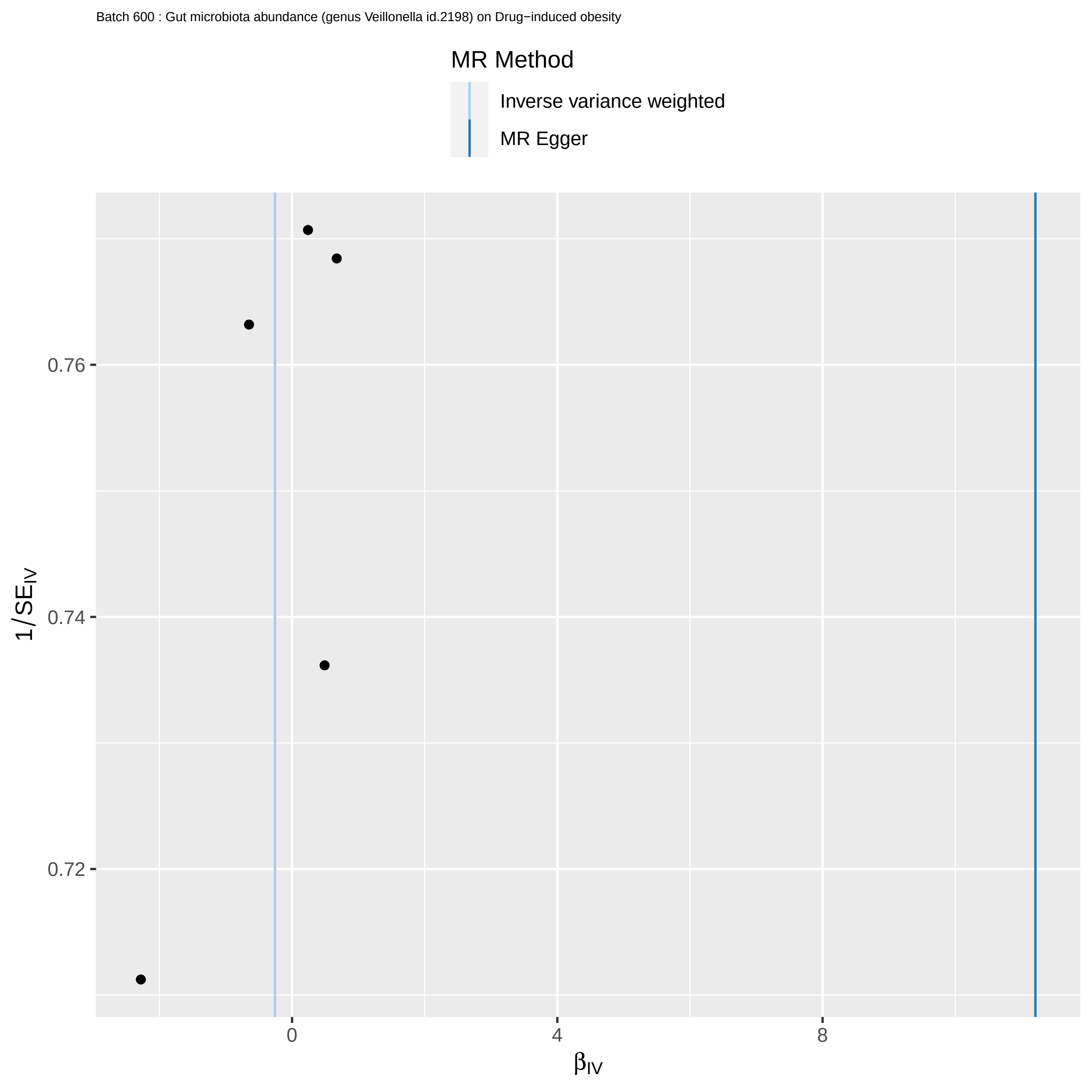
Batch 600 : Gut microbiota abundance (genus Veillonella id.2198) on Drug−induced obesity
MR Method
Inverse variance weighted
MR Egger
0.76
0.74
0.72
1/SE$_{IV}$
0
4
8
$\beta_{IV}$

Batch 601 : Gut microbiota abundance (family Acidaminococcaceae id.2166) on Extreme obesity with alveolar hypoventilation

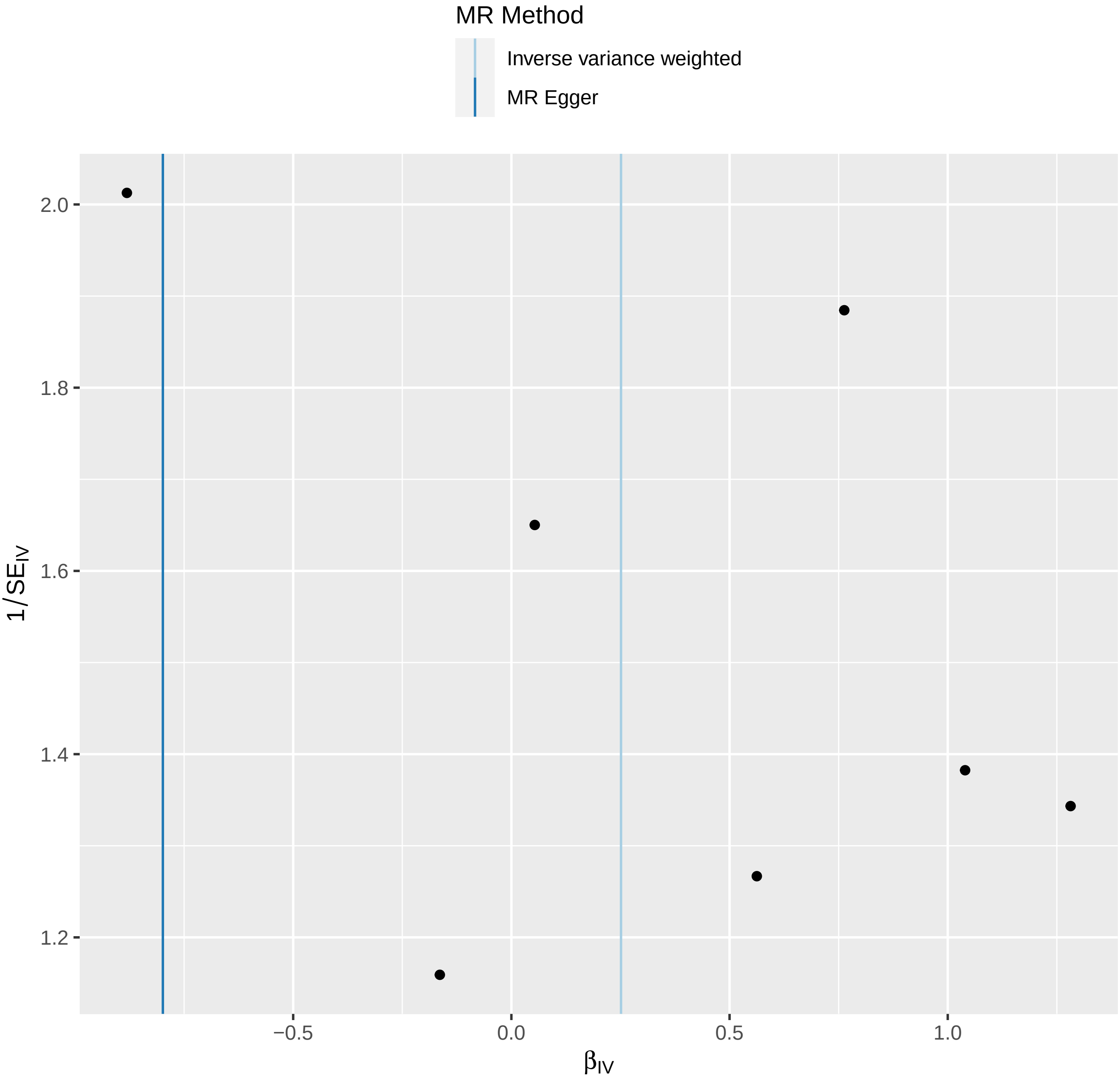

Batch 602 : Gut microbiota abundance (family Actinomycetaceae id.421) on Extreme obesity with alveolar hypoventilation

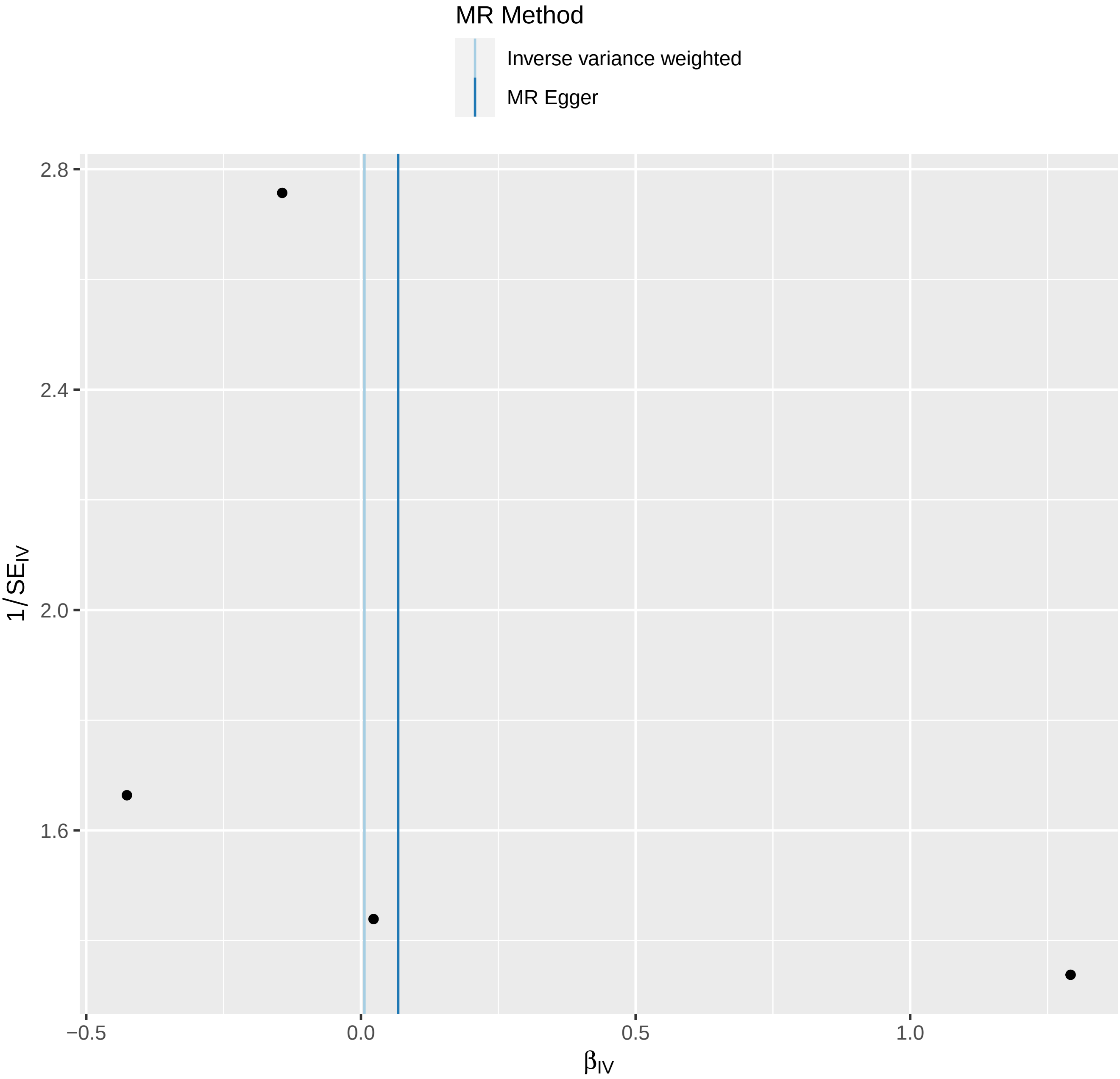

Batch 603 : Gut microbiota abundance (family Alcaligenaceae id.2875) on Extreme obesity with alveolar hypoventilation

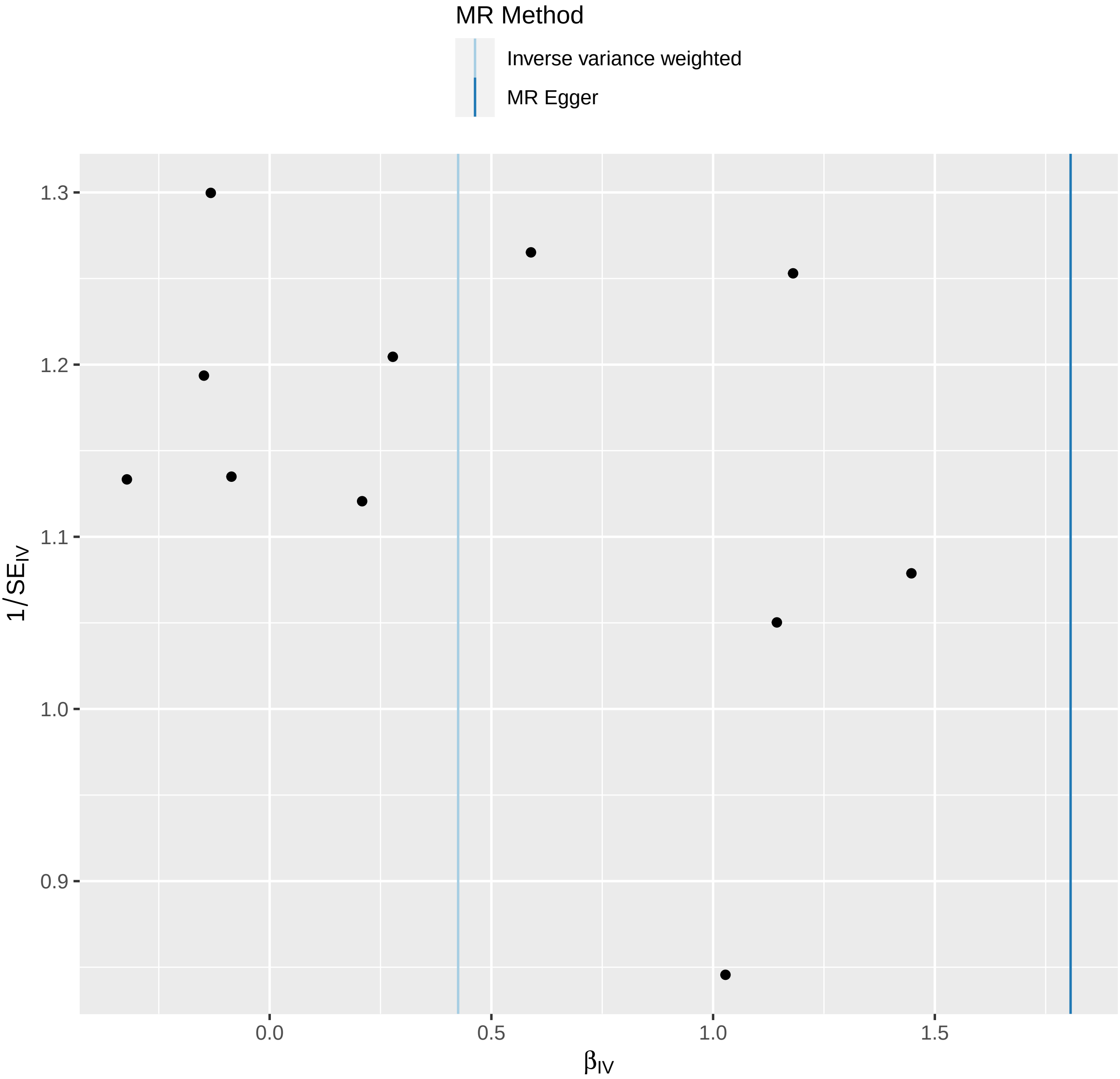

Batch 604 : Gut microbiota abundance (family Bacteroidaceae id.917) on Extreme obesity with alveolar hypoventilation

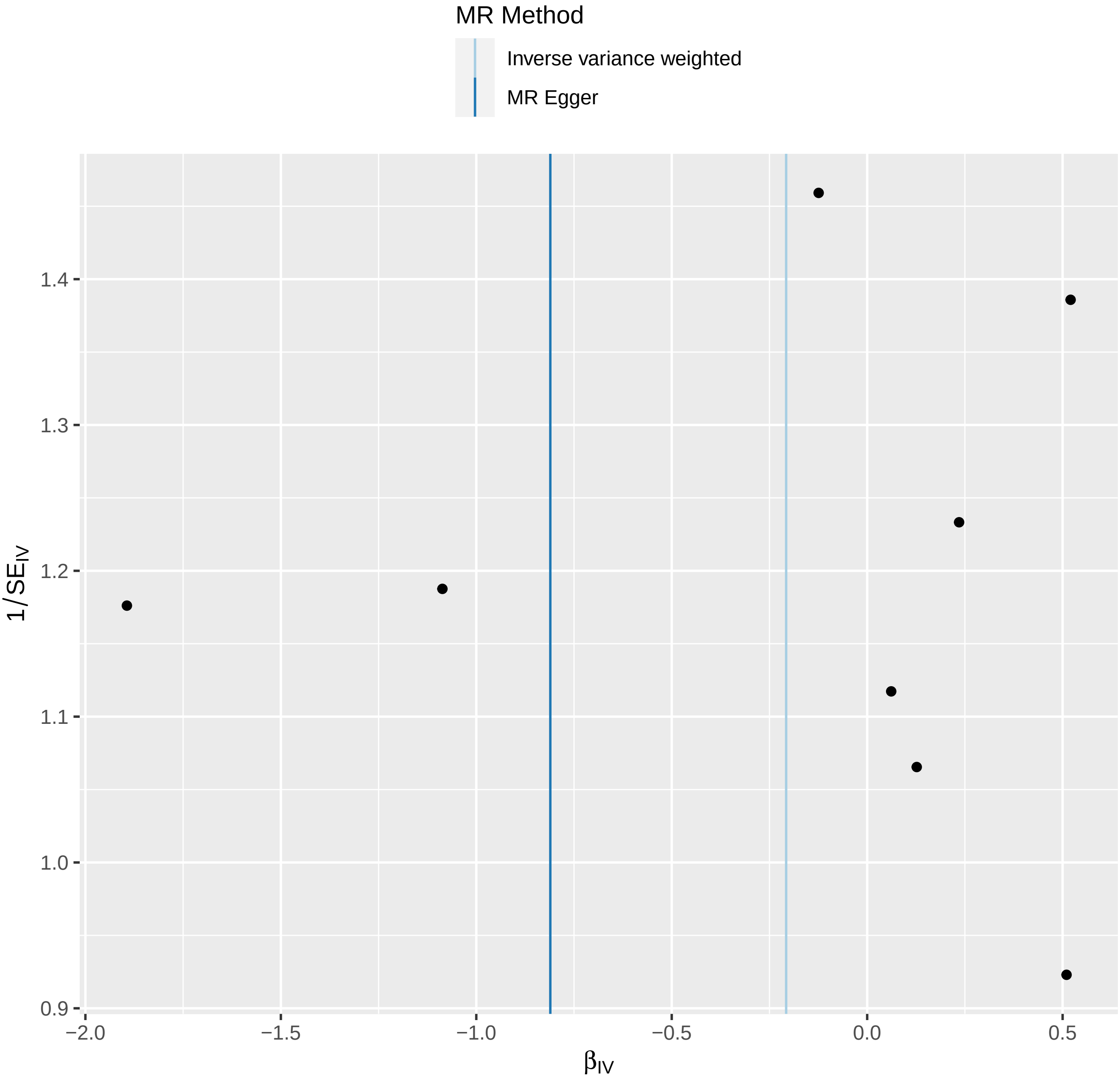

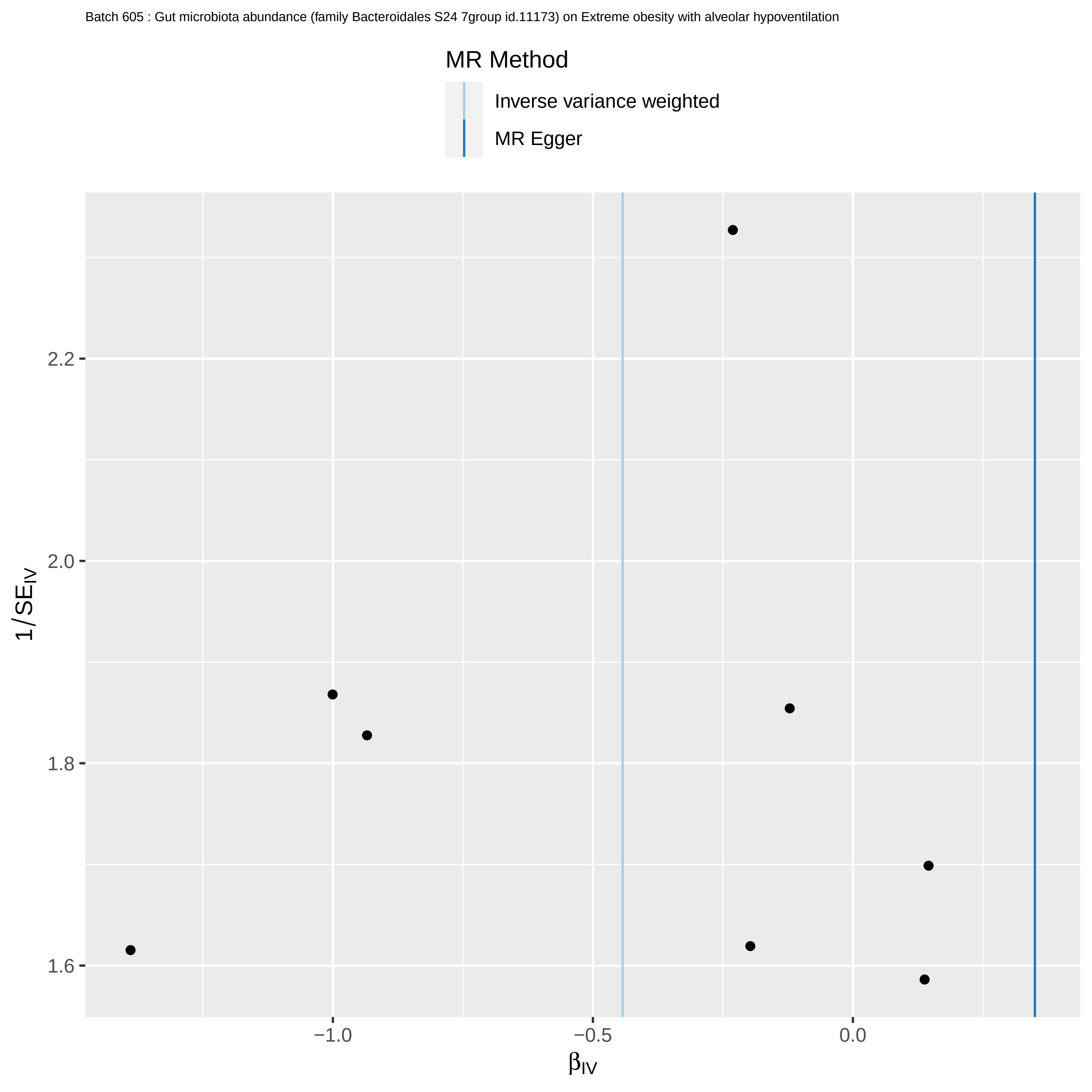

Batch 605 : Gut microbiota abundance (family Bacteroidales S24 7group id.11173) on Extreme obesity with alveolar hypoventilation
MR Method
Inverse variance weighted
MR Egger
2.2
2.0
1.8
1.6
1/SE_IV
−1.0
−0.5
0.0
β_IV

Batch 606 : Gut microbiota abundance (family Bifidobacteriaceae id.433) on Extreme obesity with alveolar hypoventilation

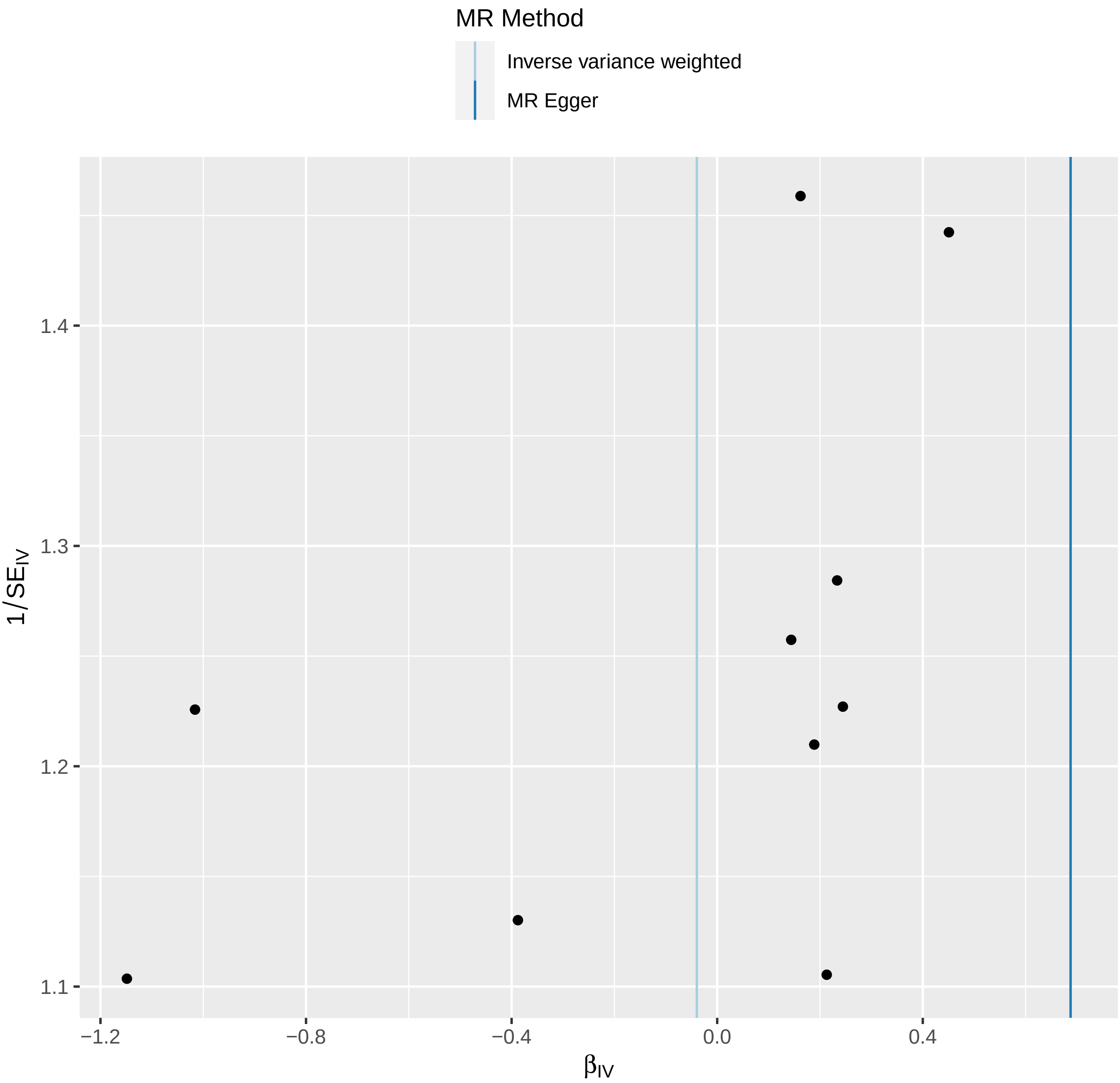

Batch 608 : Gut microbiota abundance (family Clostridiaceae1 id.1869) on Extreme obesity with alveolar hypoventilation

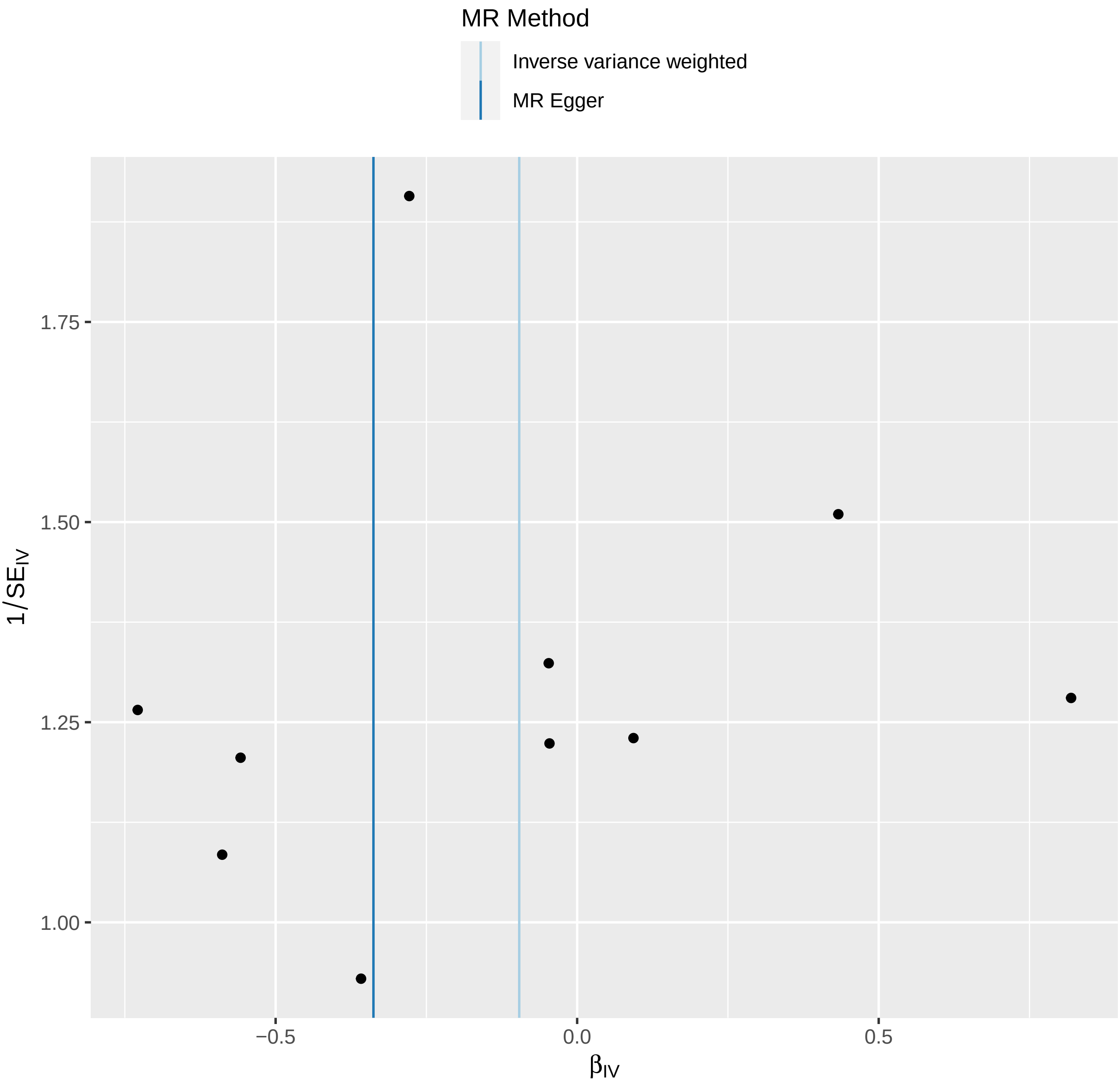

Batch 609 : Gut microbiota abundance (family Clostridiales vadin BB60 group id.11286) on Extreme obesity with alveolar hypoventilation

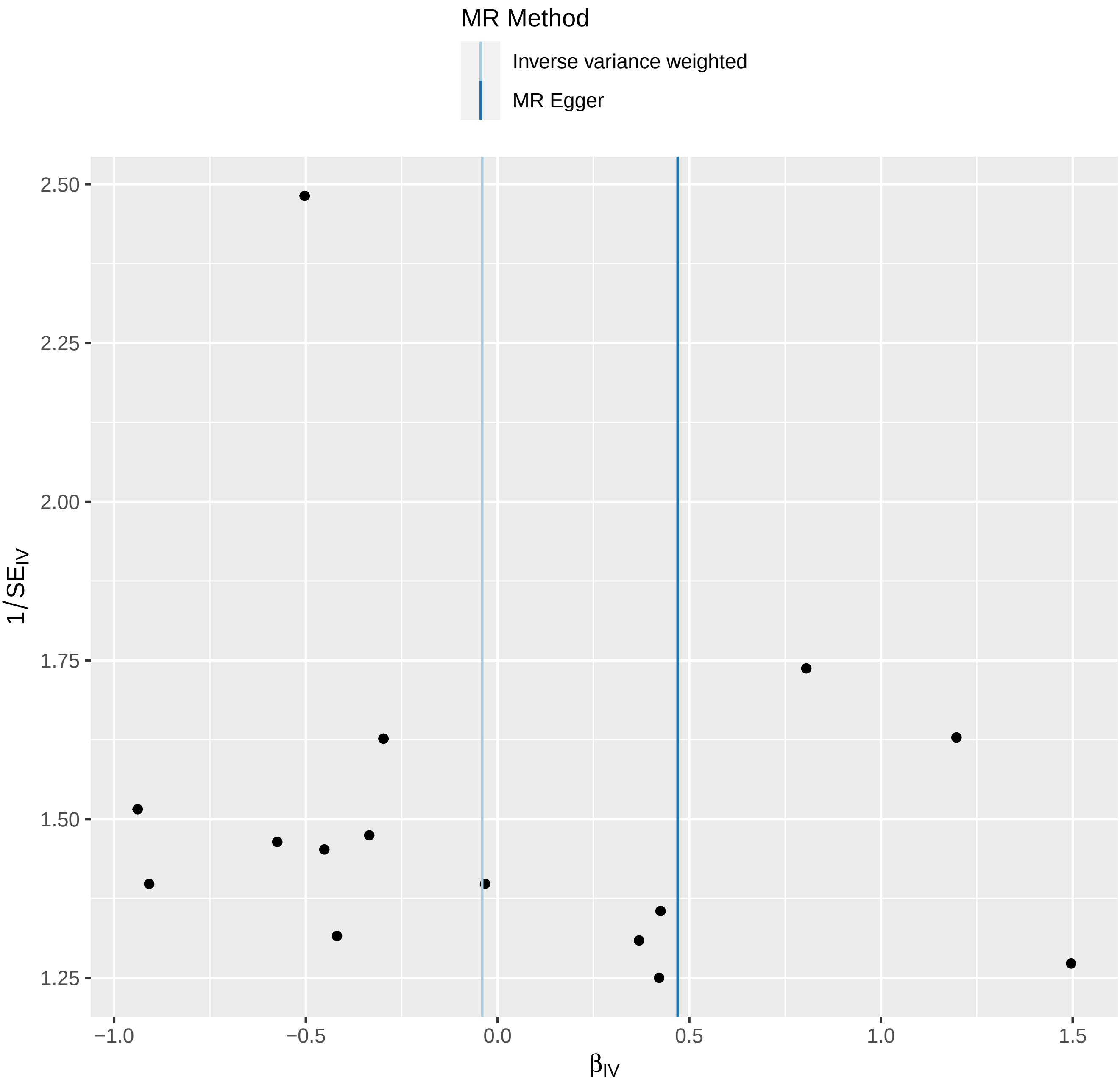

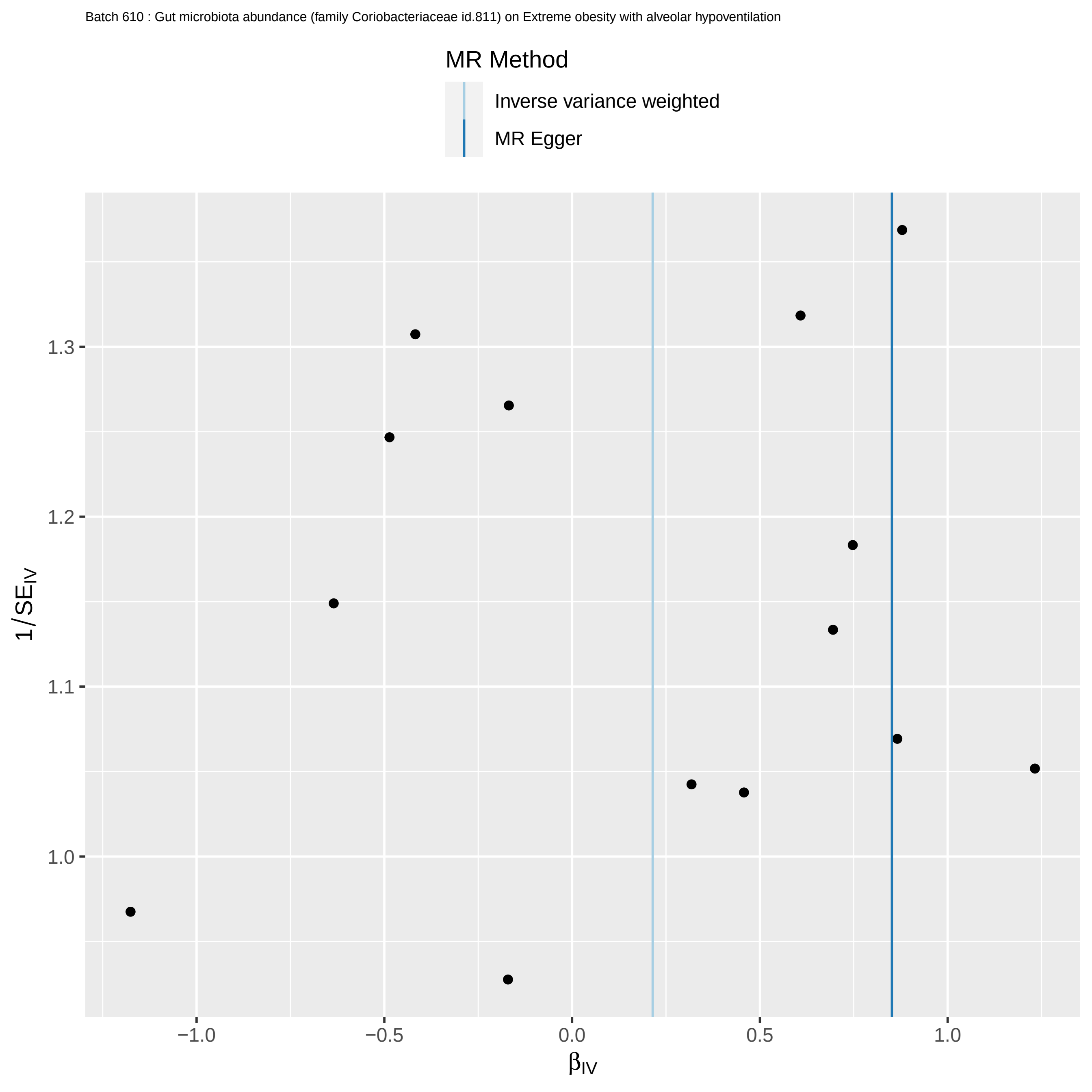

Batch 610 : Gut microbiota abundance (family Coriobacteriaceae id.811) on Extreme obesity with alveolar hypoventilation
MR Method
Inverse variance weighted
MR Egger
1/SE_IV
1.3
1.2
1.1
1.0
−1.0
−0.5
0.0
0.5
1.0
β_IV

Batch 611 : Gut microbiota abundance (family Defluviitaleaceae id.1924) on Extreme obesity with alveolar hypoventilation

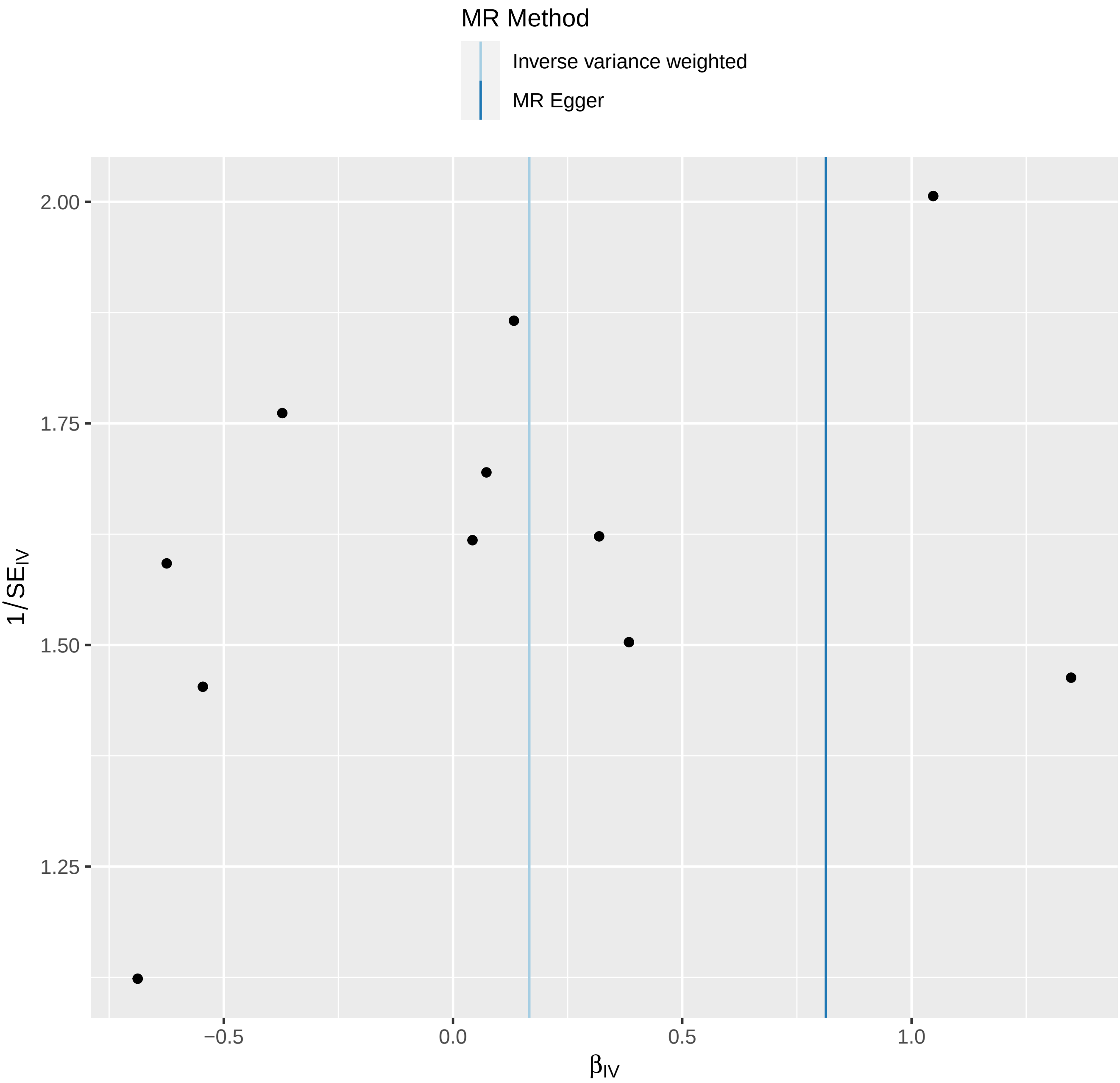

Batch 612 : Gut microbiota abundance (family Desulfovibrionaceae id.3169) on Extreme obesity with alveolar hypoventilation

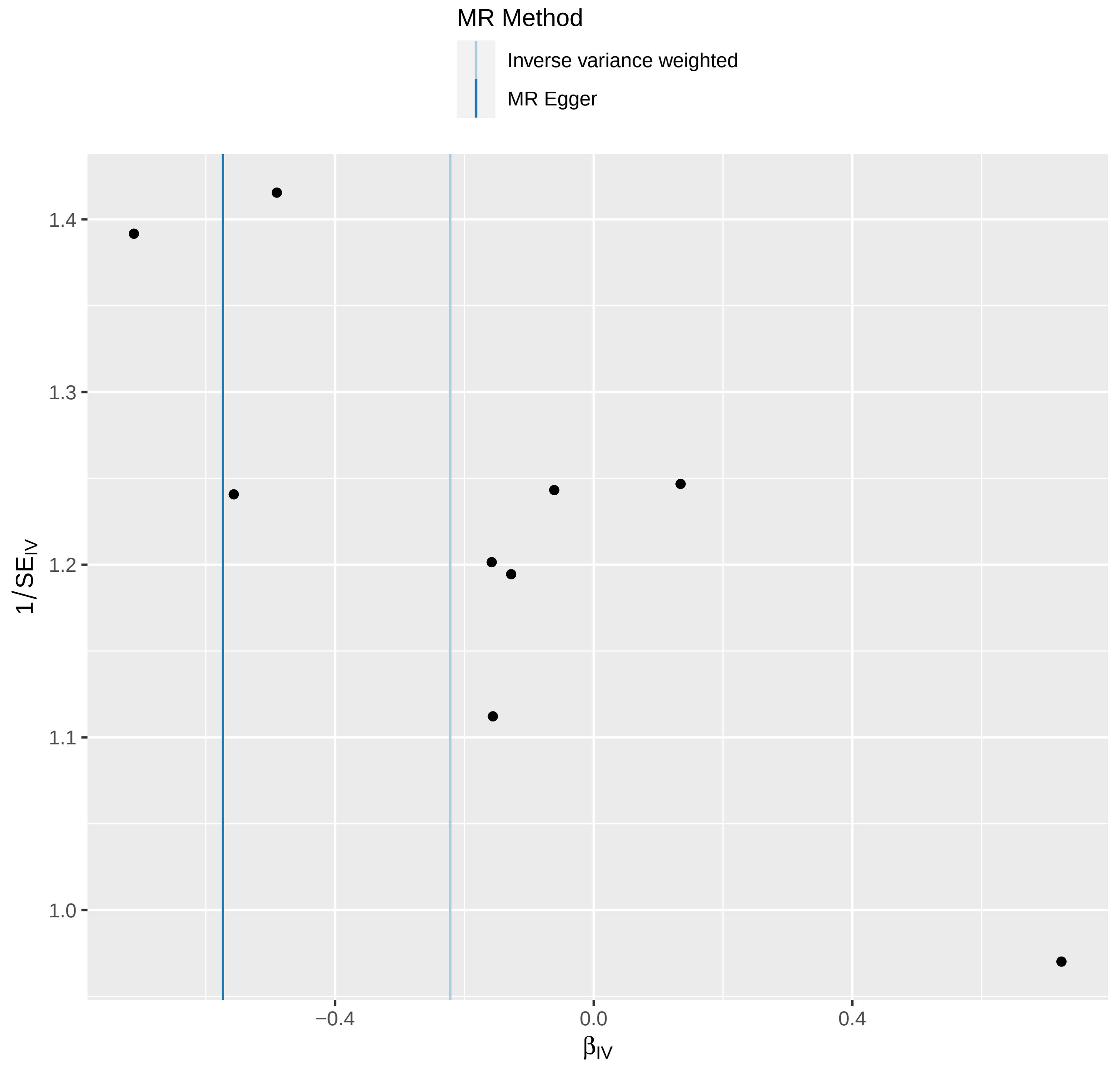

Batch 613 : Gut microbiota abundance (family Enterobacteriaceae id.3469) on Extreme obesity with alveolar hypoventilation

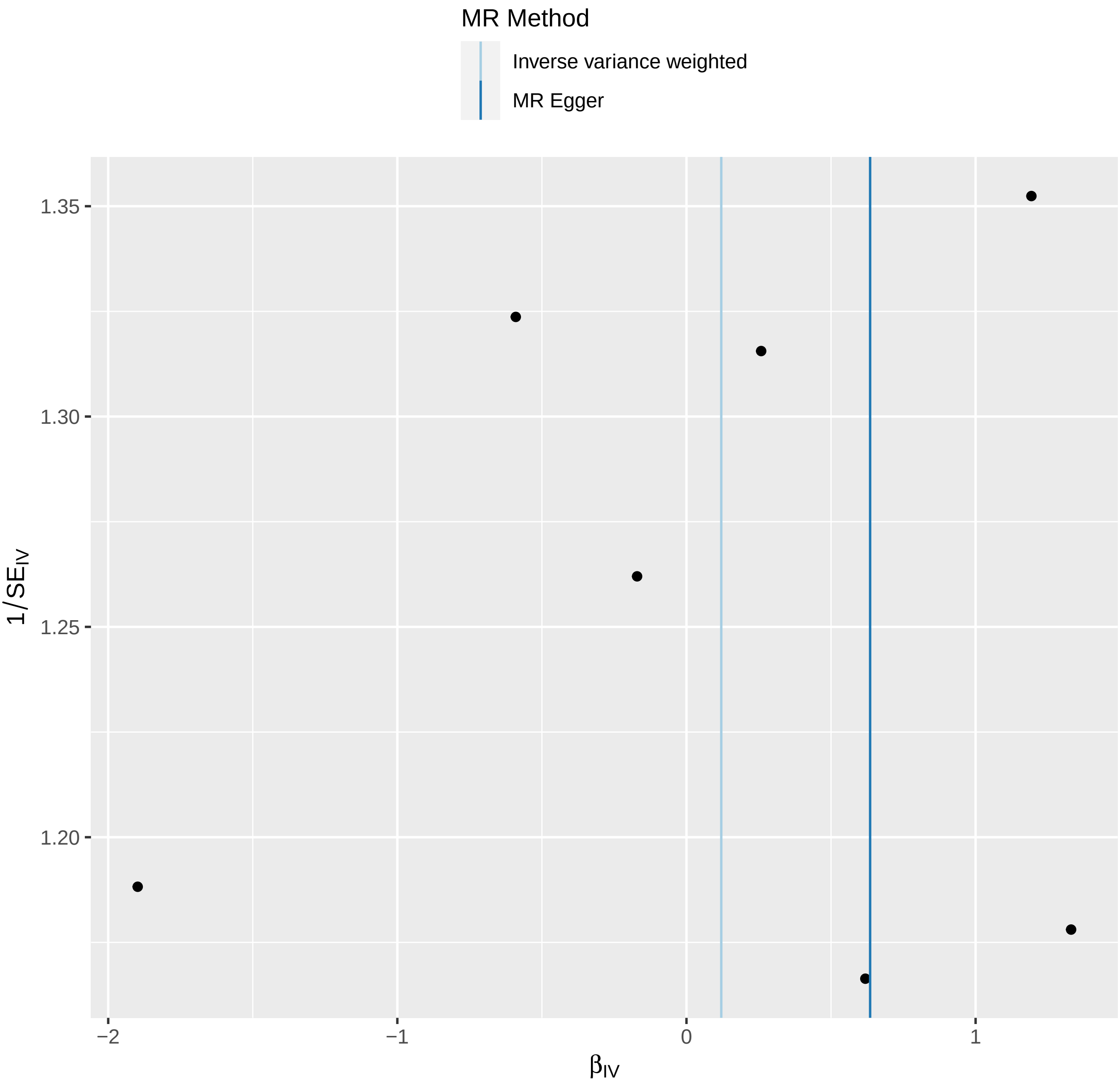

Batch 614 : Gut microbiota abundance (family Erysipelotrichaceae id.2149) on Extreme obesity with alveolar hypoventilation

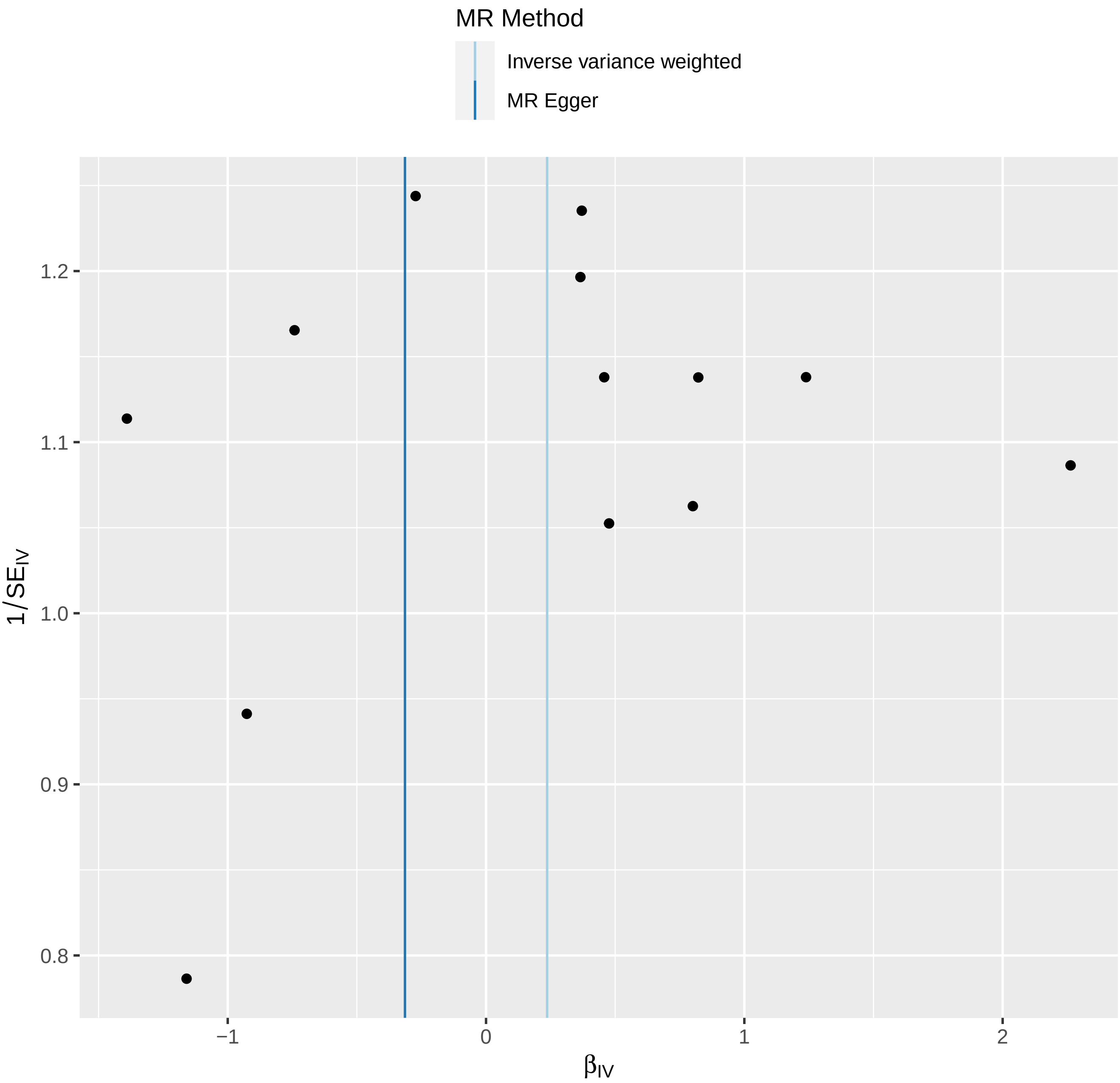

Batch 615 : Gut microbiota abundance (family Family XI id.1936) on Extreme obesity with alveolar hypoventilation

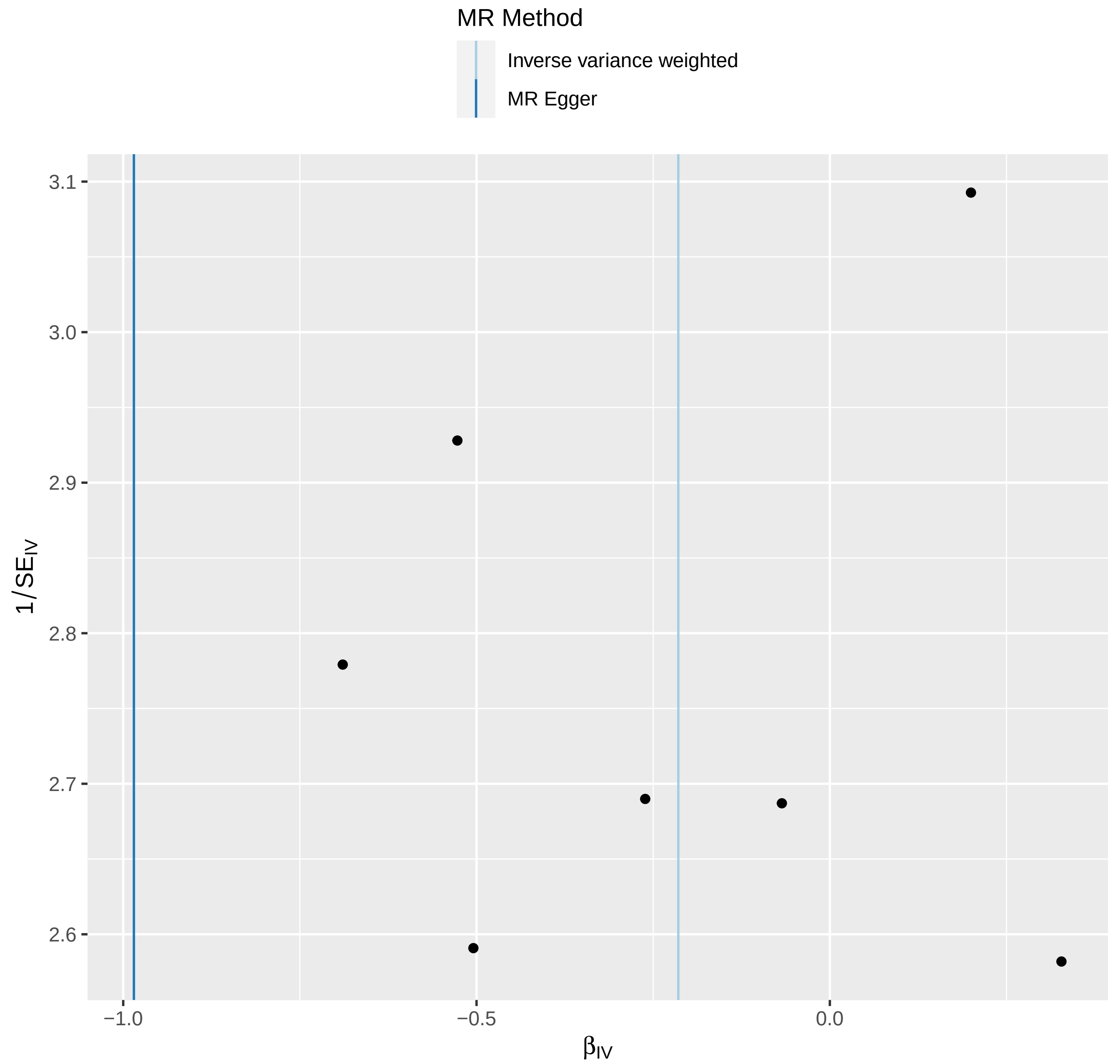

Batch 616 : Gut microbiota abundance (family Family XIII id.1957) on Extreme obesity with alveolar hypoventilation

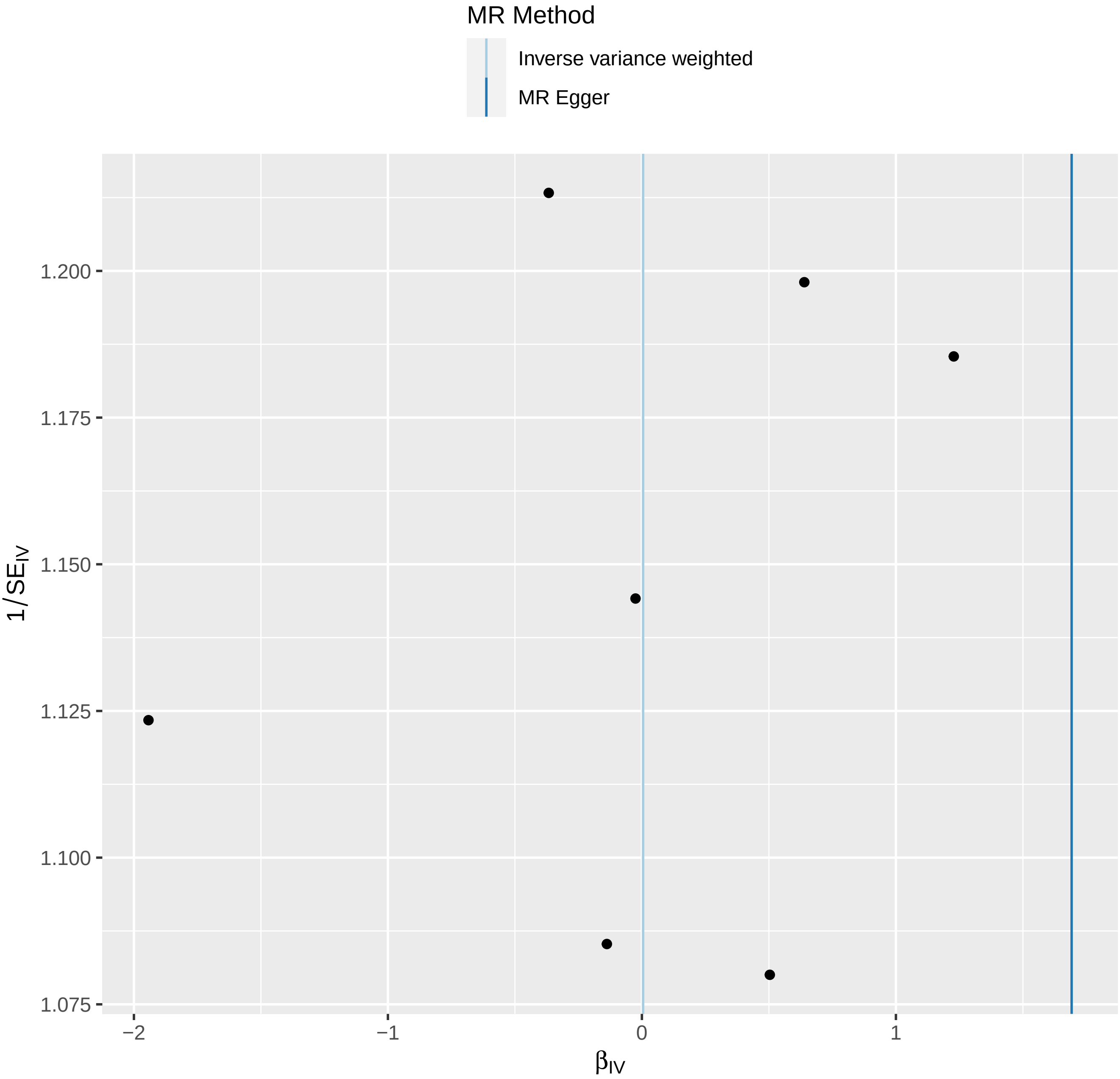

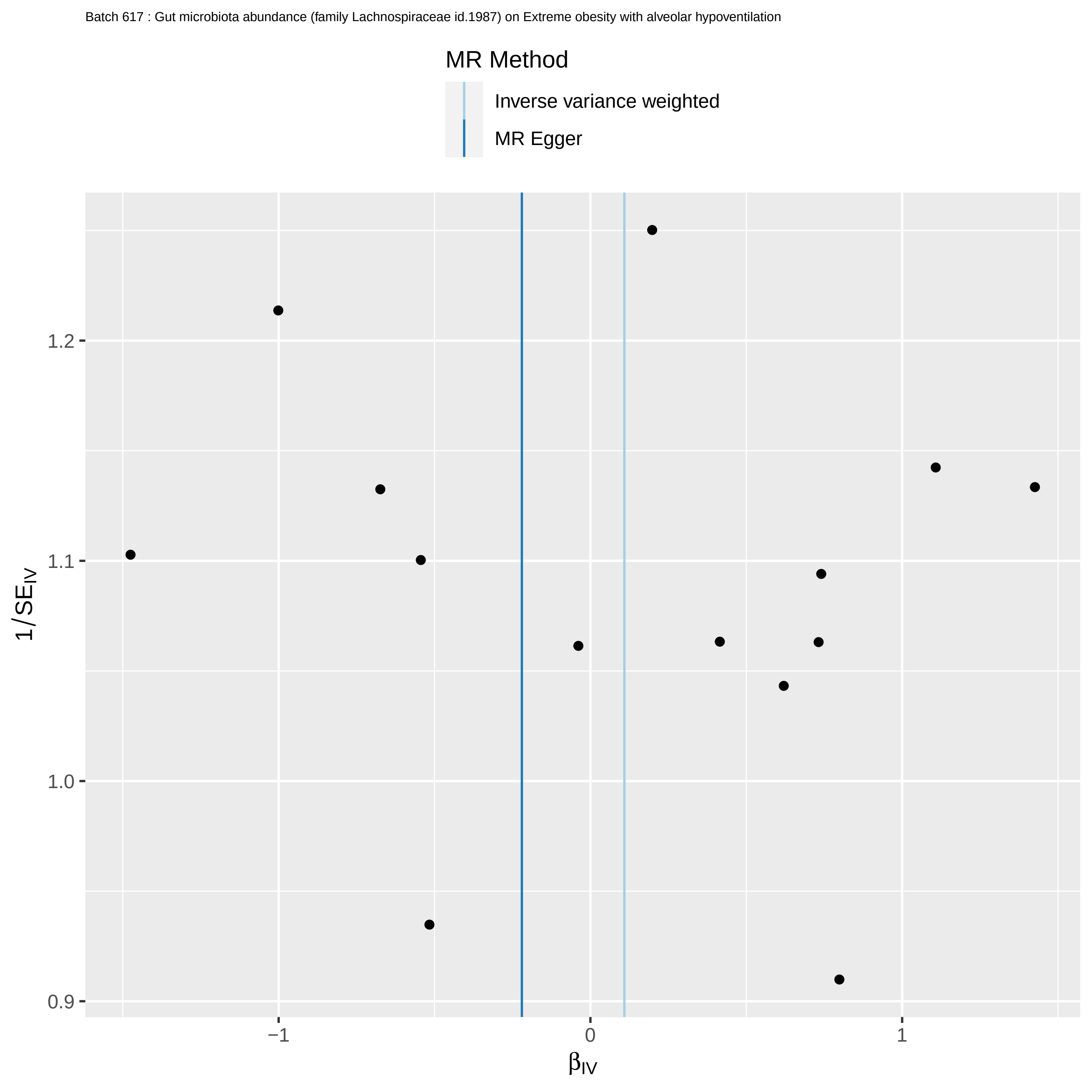

Batch 617 : Gut microbiota abundance (family Lachnospiraceae id.1987) on Extreme obesity with alveolar hypoventilation
MR Method
Inverse variance weighted
MR Egger
1.2
1.1
1.0
0.9
1/SE_IV
−1
0
1
β_IV

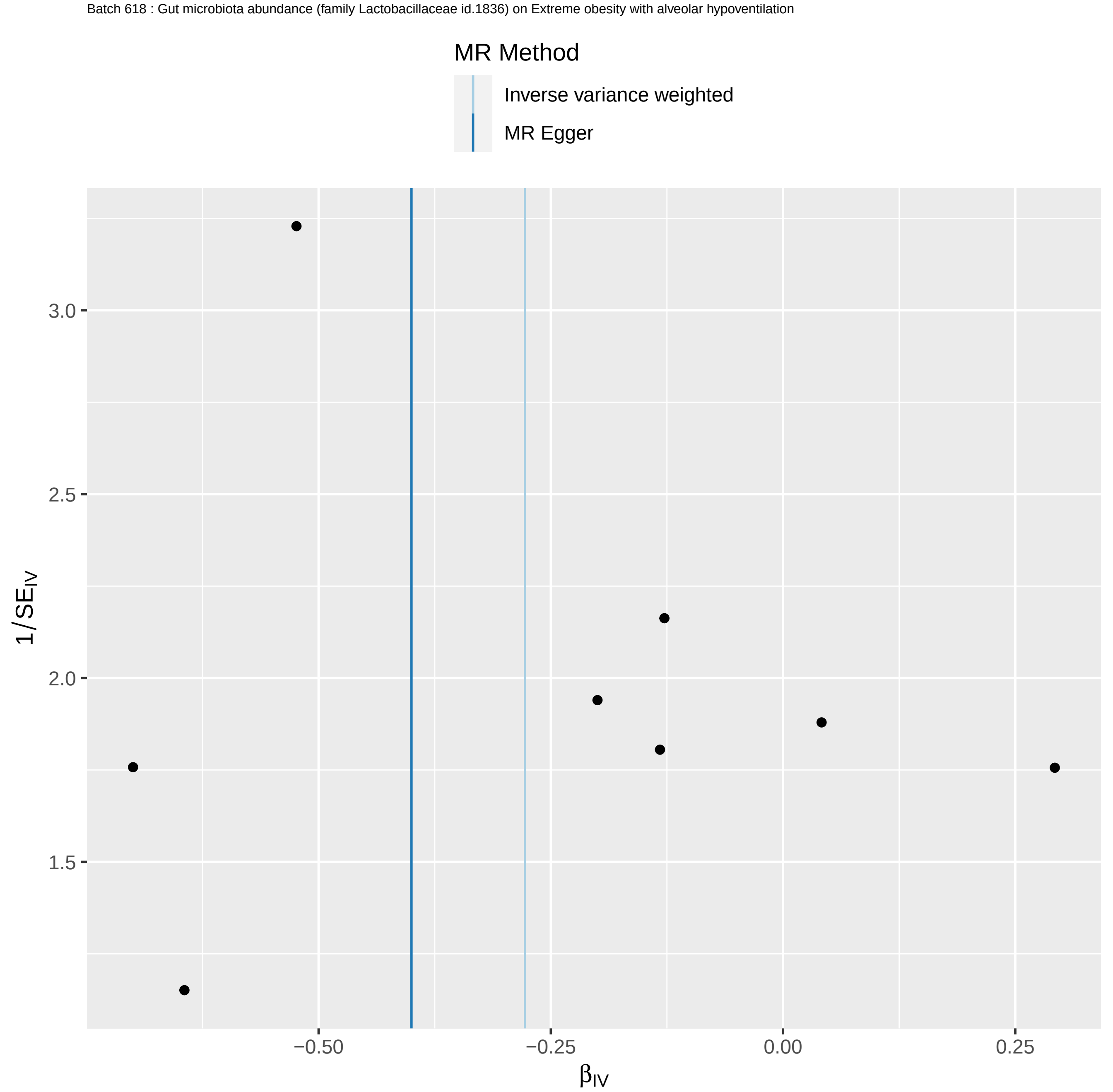
Batch 618 : Gut microbiota abundance (family Lactobacillaceae id.1836) on Extreme obesity with alveolar hypoventilation
MR Method
Inverse variance weighted
MR Egger
3.0
2.5
2.0
1.5
1/SE_IV
−0.50
−0.25
0.00
0.25
β_IV

Batch 619 : Gut microbiota abundance (family Methanobacteriaceae id.121) on Extreme obesity with alveolar hypoventilation

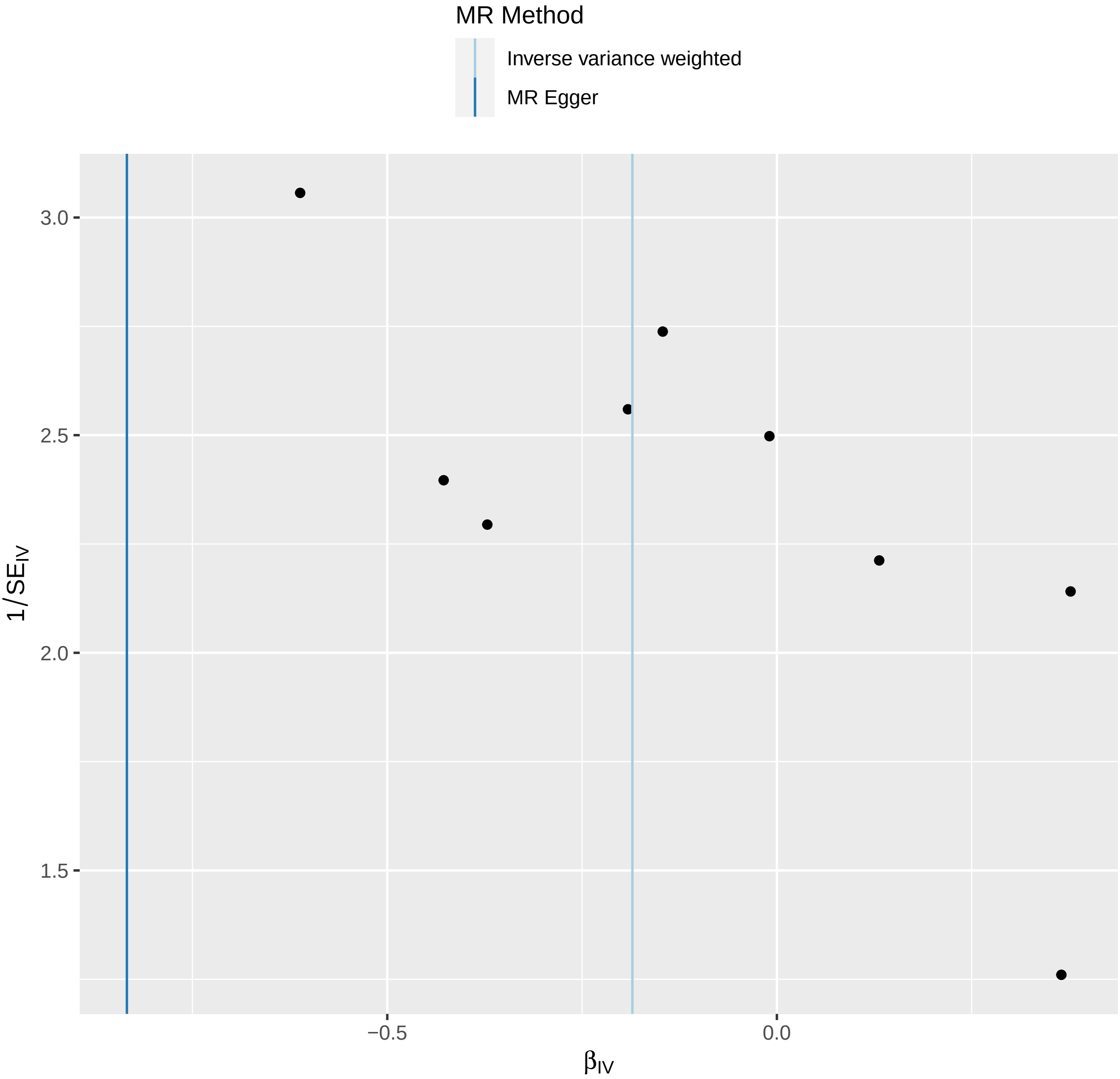

Batch 620 : Gut microbiota abundance (family Oxalobacteraceae id.2966) on Extreme obesity with alveolar hypoventilation

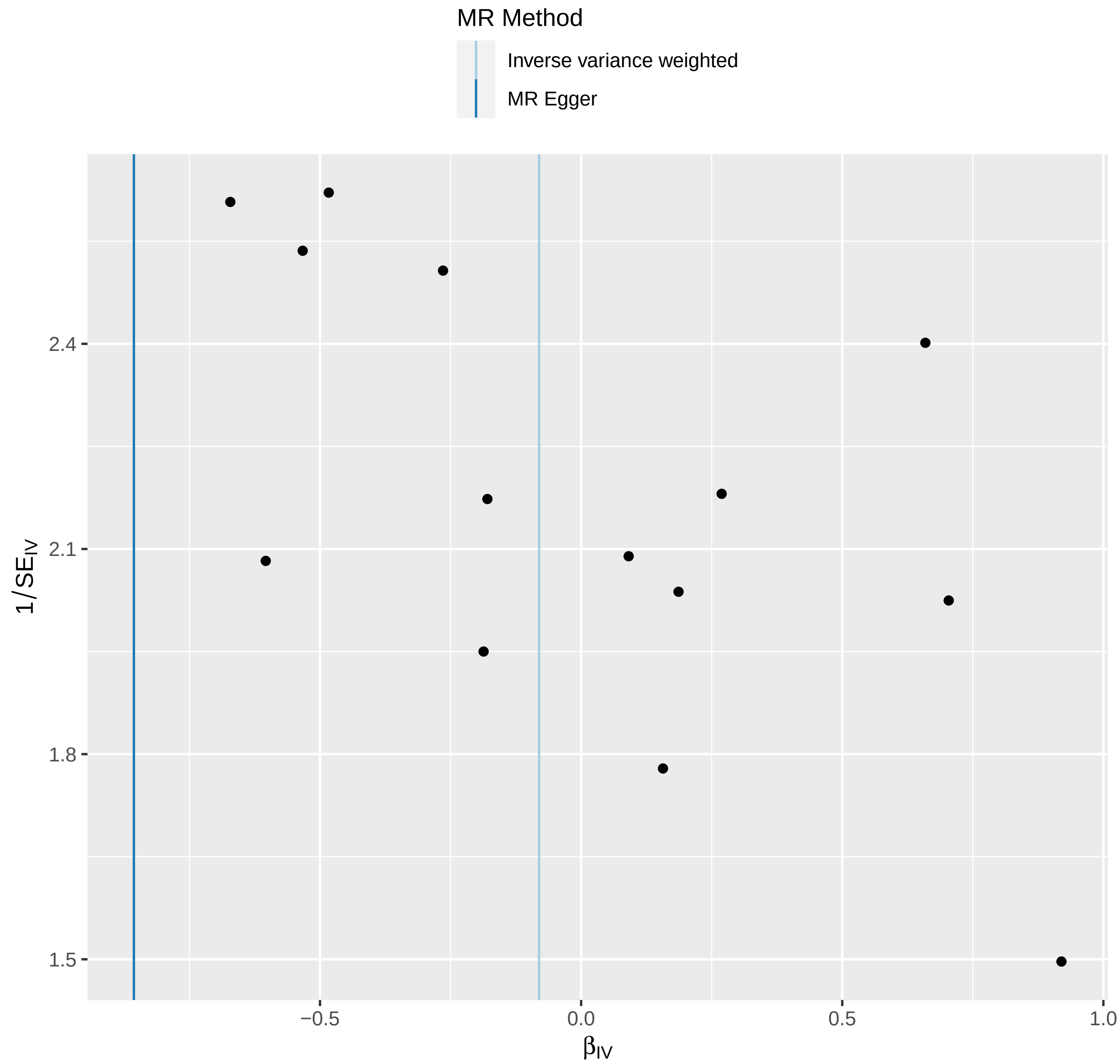

Batch 621 : Gut microbiota abundance (family Pasteurellaceae id.3689) on Extreme obesity with alveolar hypoventilation

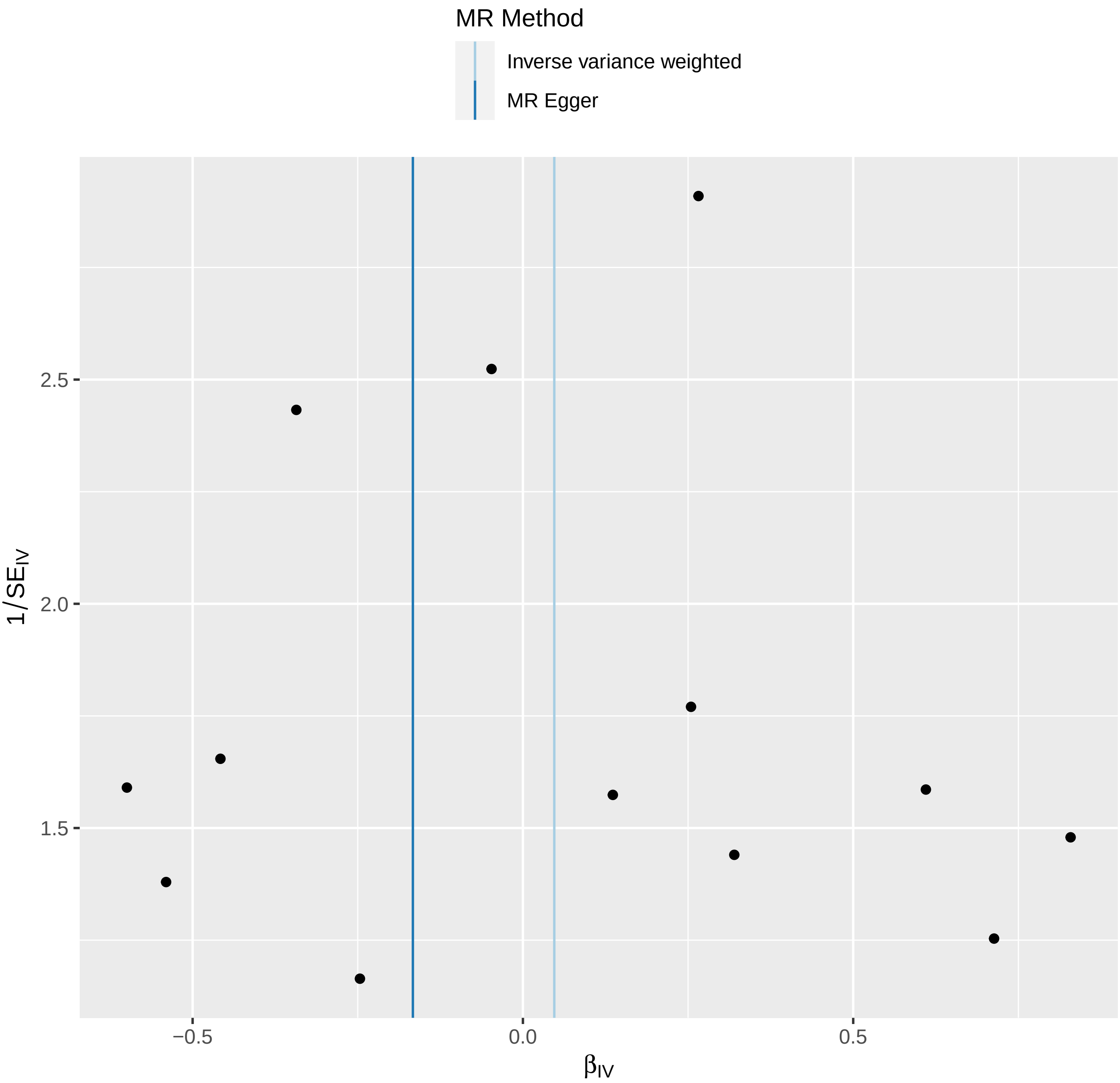

Batch 622 : Gut microbiota abundance (family Peptococcaceae id.2024) on Extreme obesity with alveolar hypoventilation

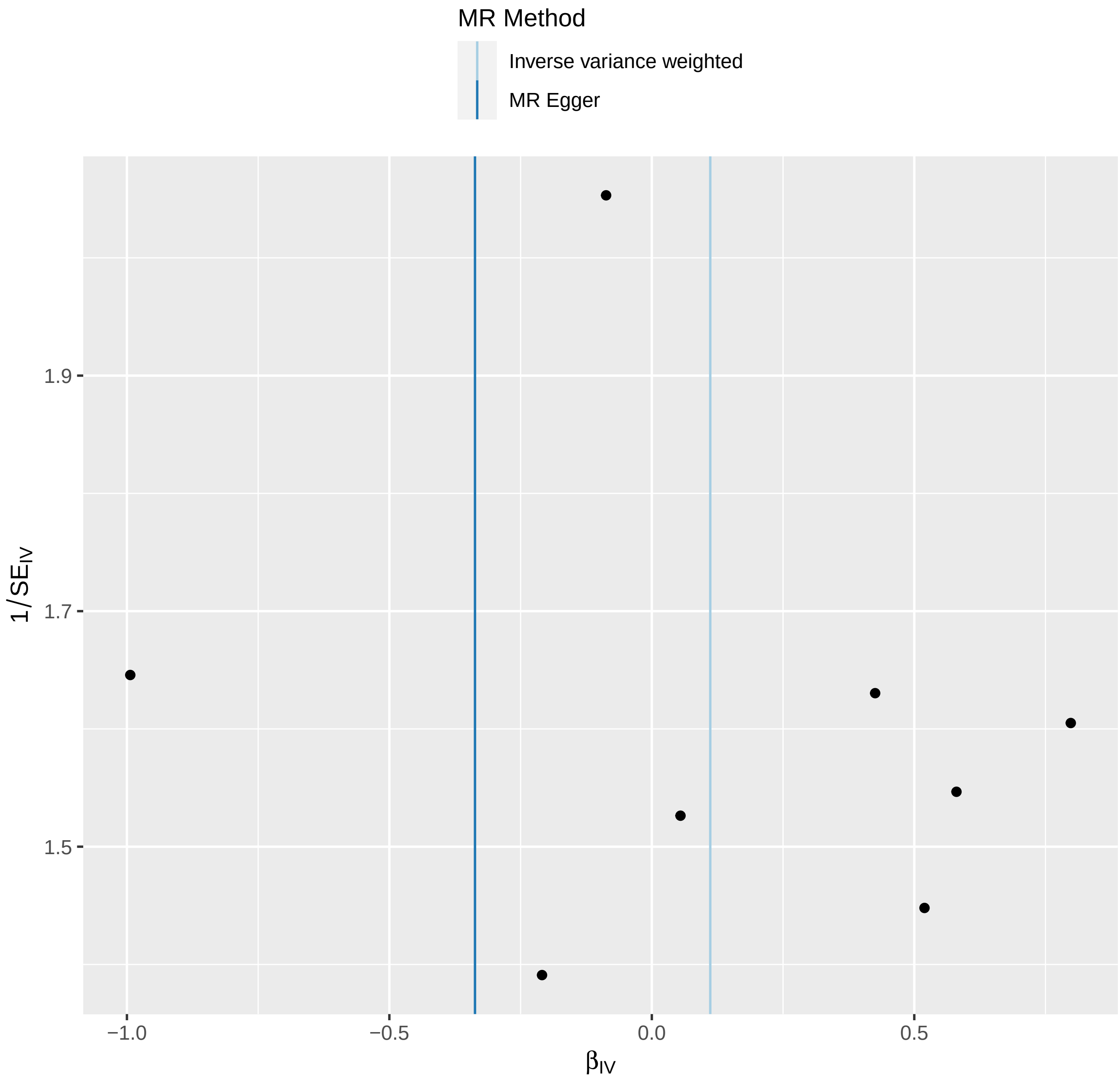

Batch 623 : Gut microbiota abundance (family Peptostreptococcaceae id.2042) on Extreme obesity with alveolar hypoventilation

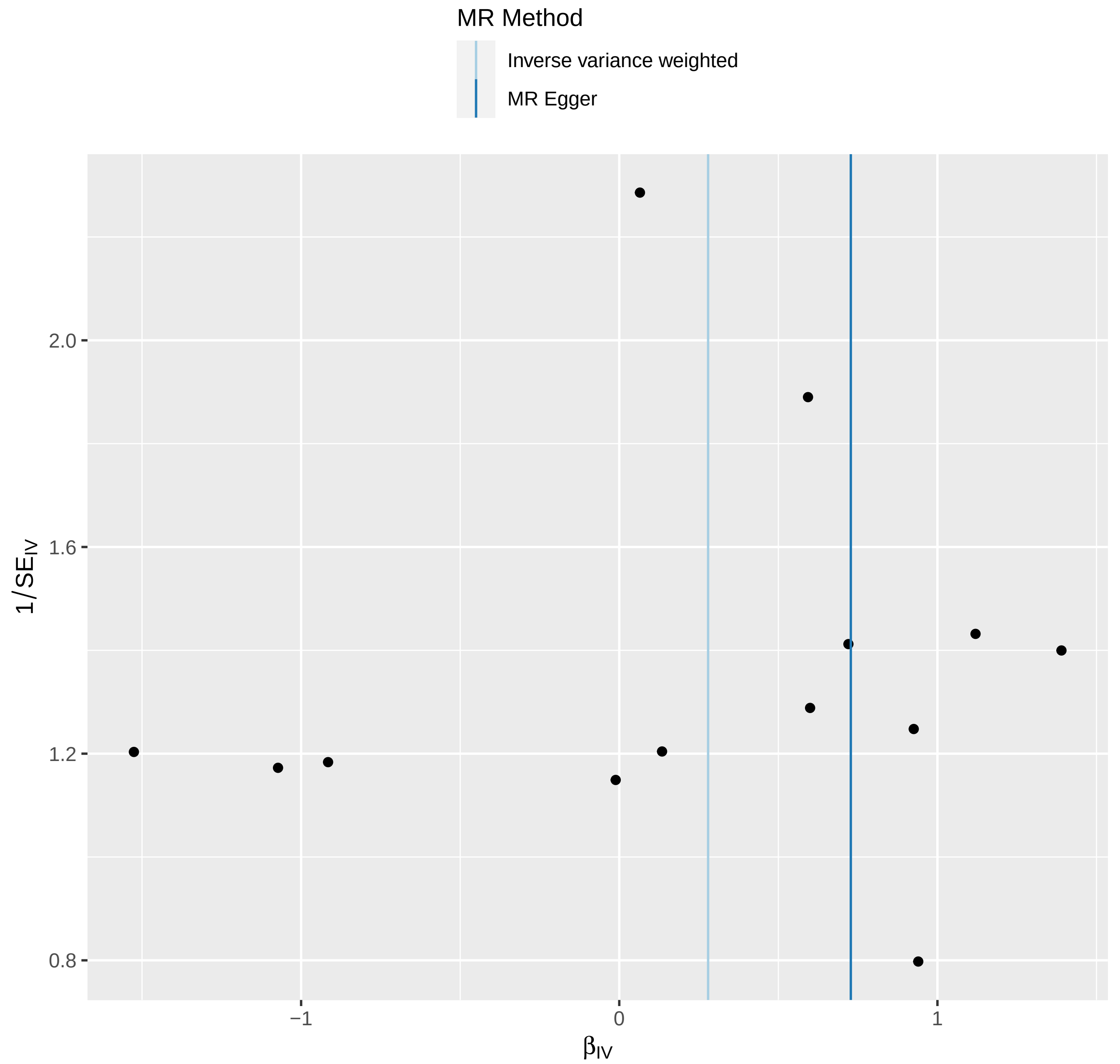

Batch 624 : Gut microbiota abundance (family Porphyromonadaceae id.943) on Extreme obesity with alveolar hypoventilation

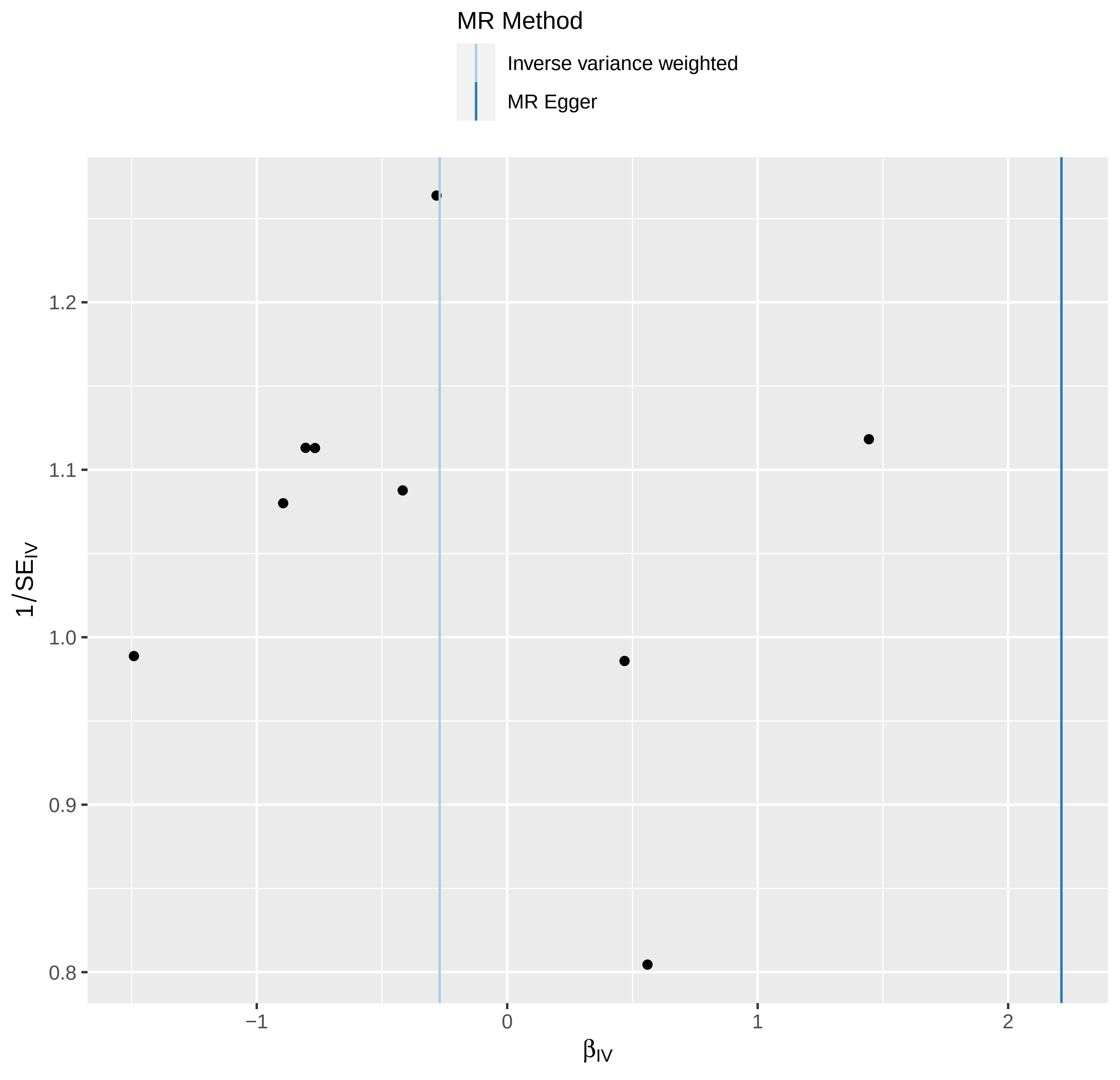

Batch 625 : Gut microbiota abundance (family Prevotellaceae id.960) on Extreme obesity with alveolar hypoventilation

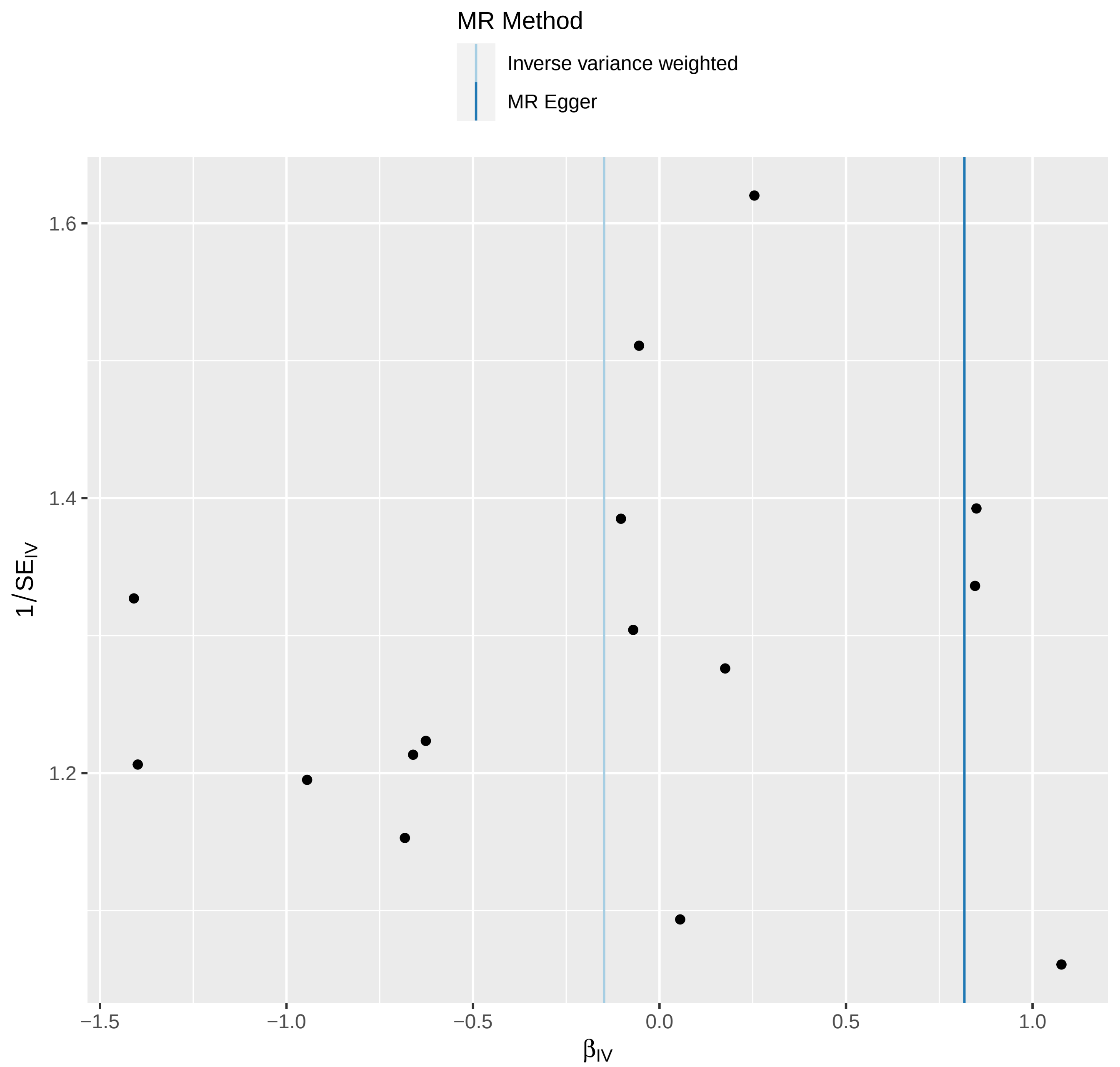

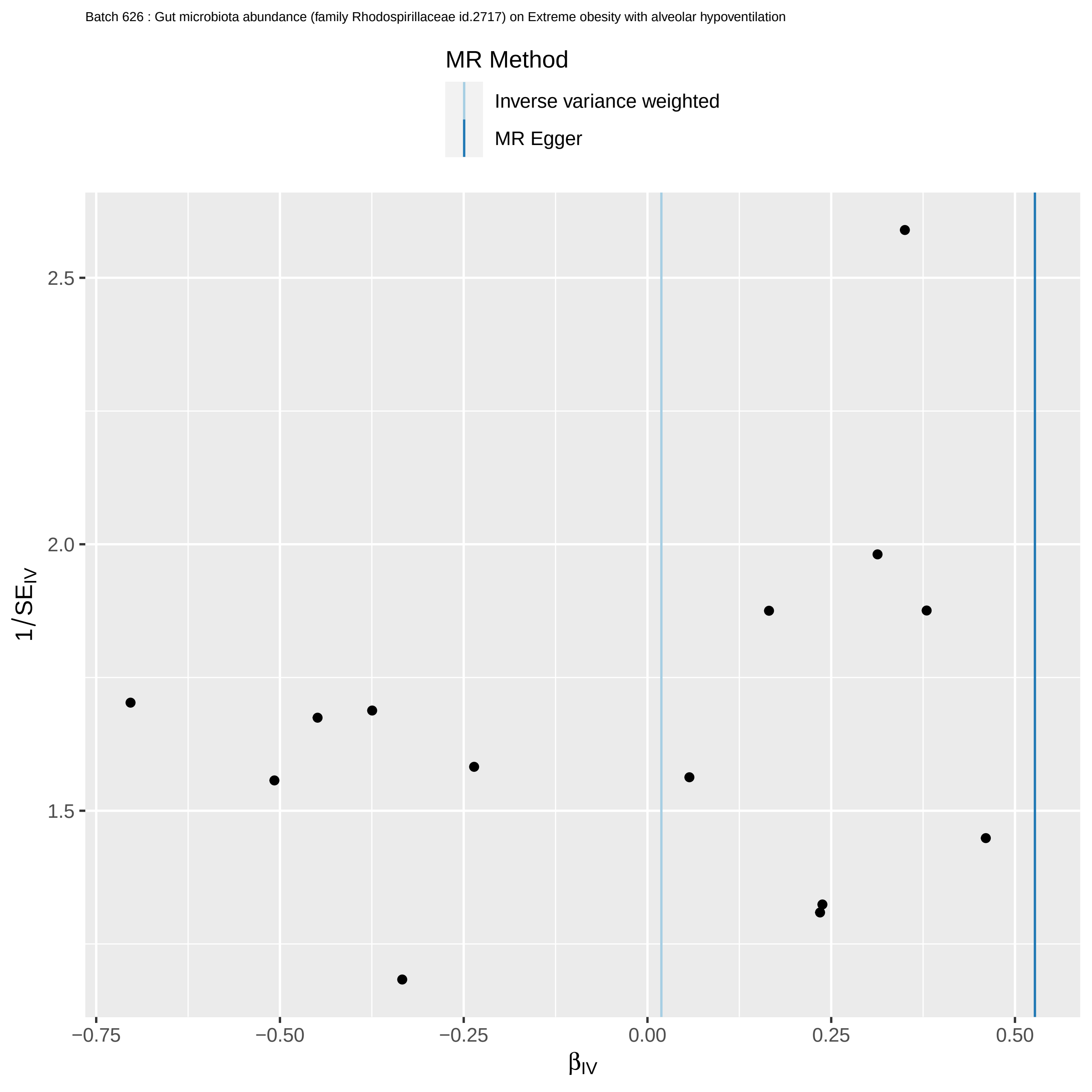
Batch 626 : Gut microbiota abundance (family Rhodospirillaceae id.2717) on Extreme obesity with alveolar hypoventilation
MR Method
Inverse variance weighted
MR Egger
2.5
2.0
1.5
$1/SE_{IV}$
−0.75
−0.50
−0.25
0.00
0.25
0.50
$\beta_{IV}$

Batch 627 : Gut microbiota abundance (family Rikenellaceae id.967) on Extreme obesity with alveolar hypoventilation

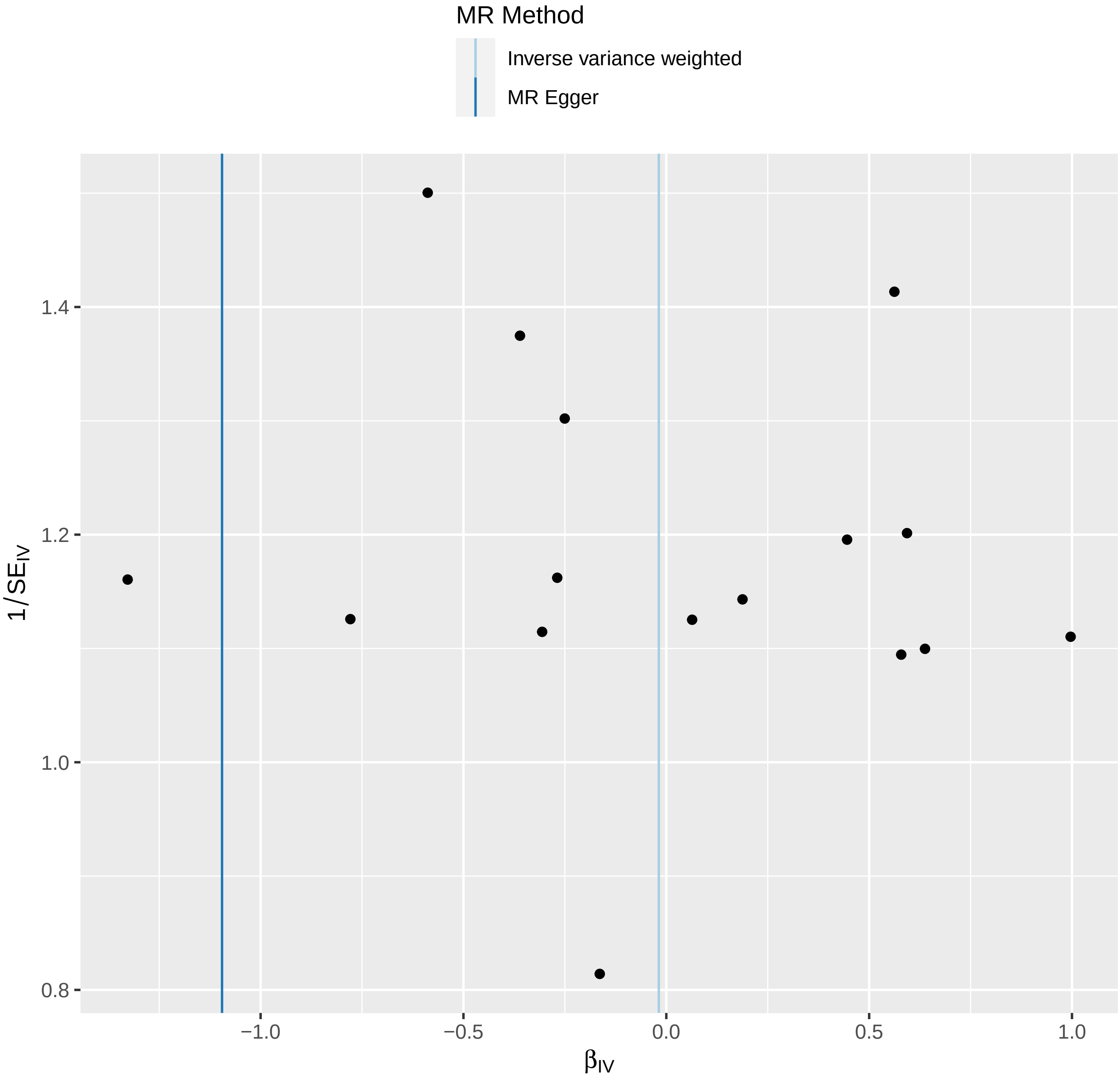

Batch 628 : Gut microbiota abundance (family Ruminococcaceae id.2050) on Extreme obesity with alveolar hypoventilation

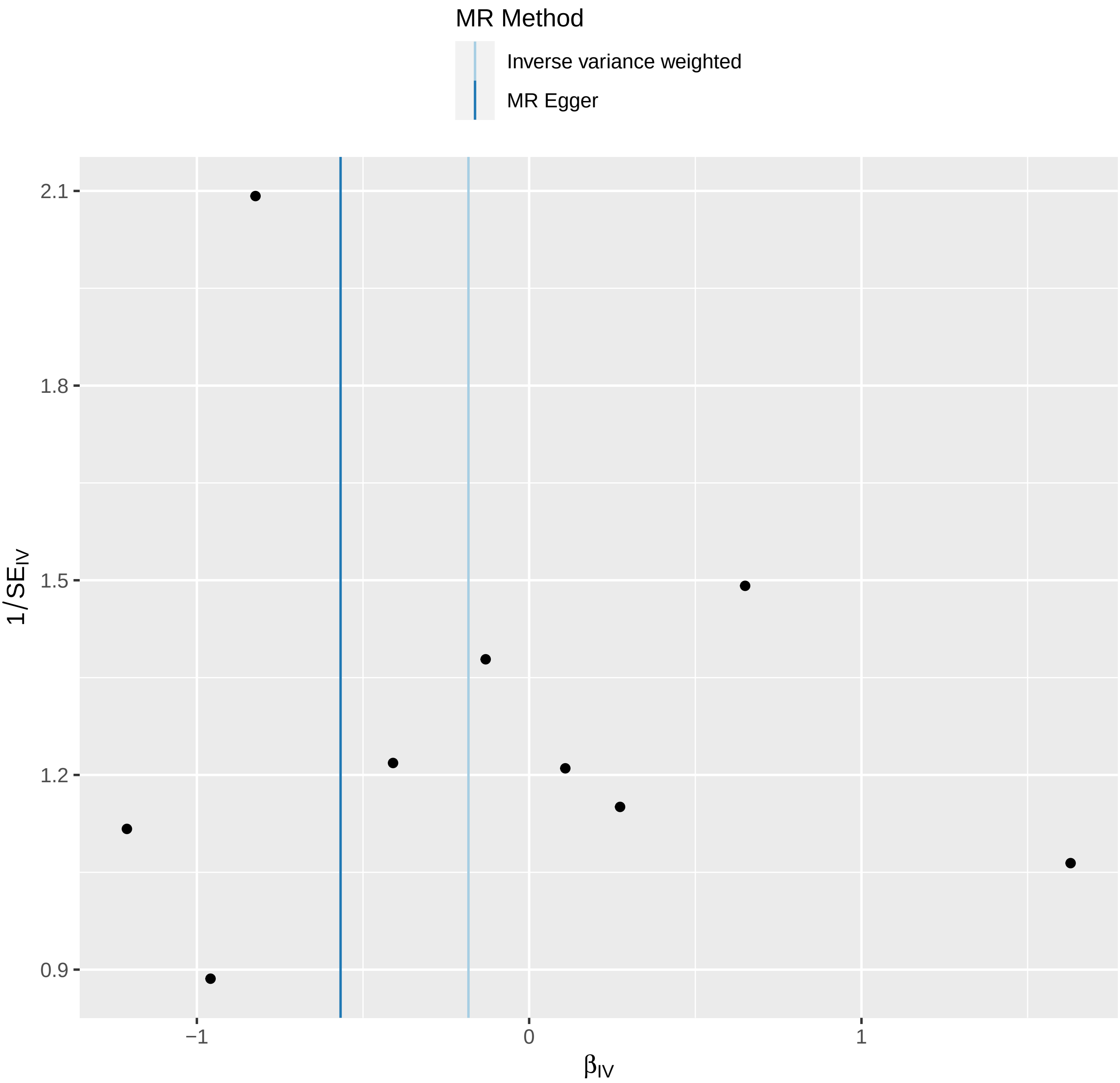

Batch 629 : Gut microbiota abundance (family Streptococcaceae id.1850) on Extreme obesity with alveolar hypoventilation

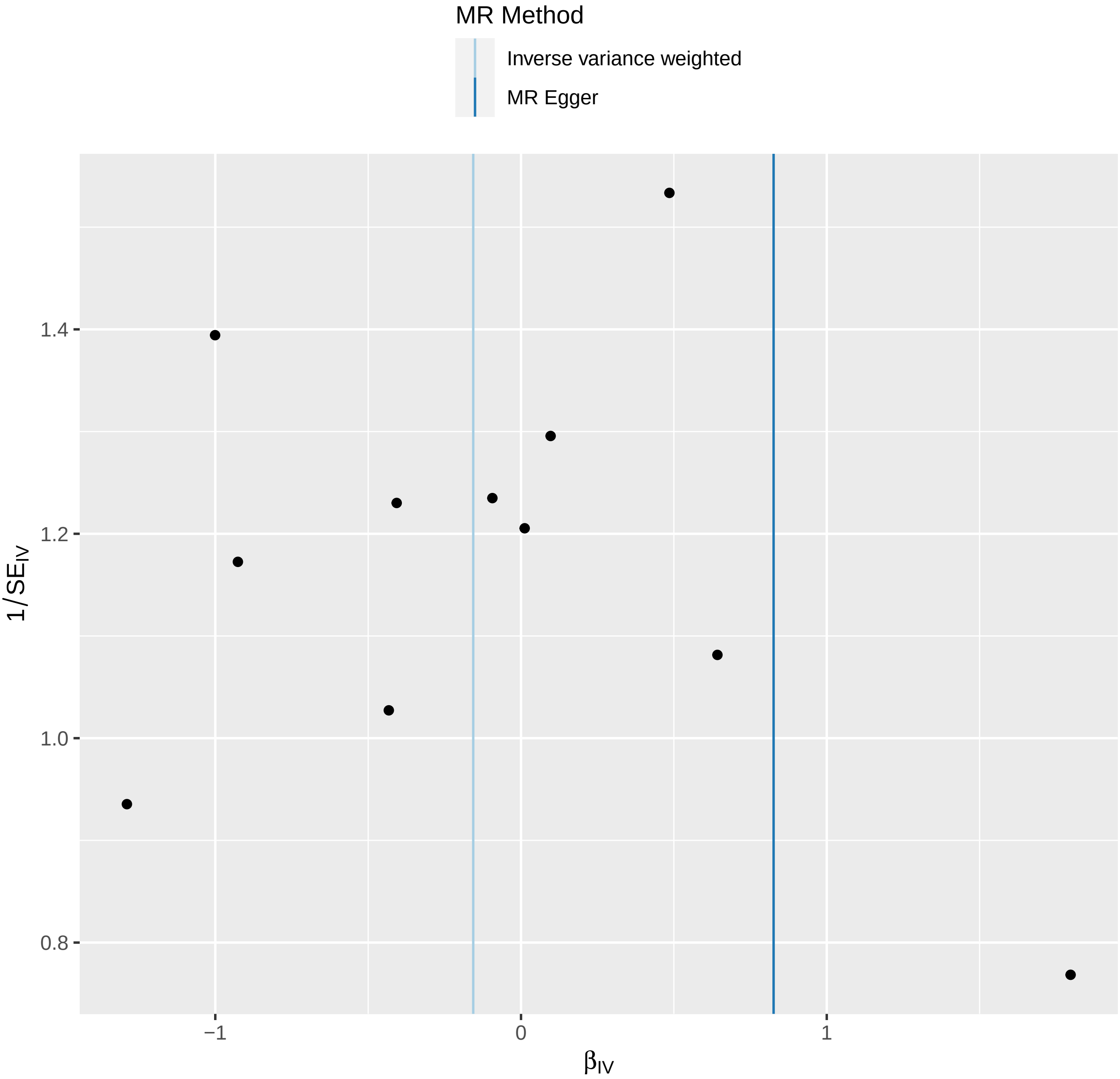

Batch 630 : Gut microbiota abundance (family Veillonellaceae id.2172) on Extreme obesity with alveolar hypoventilation

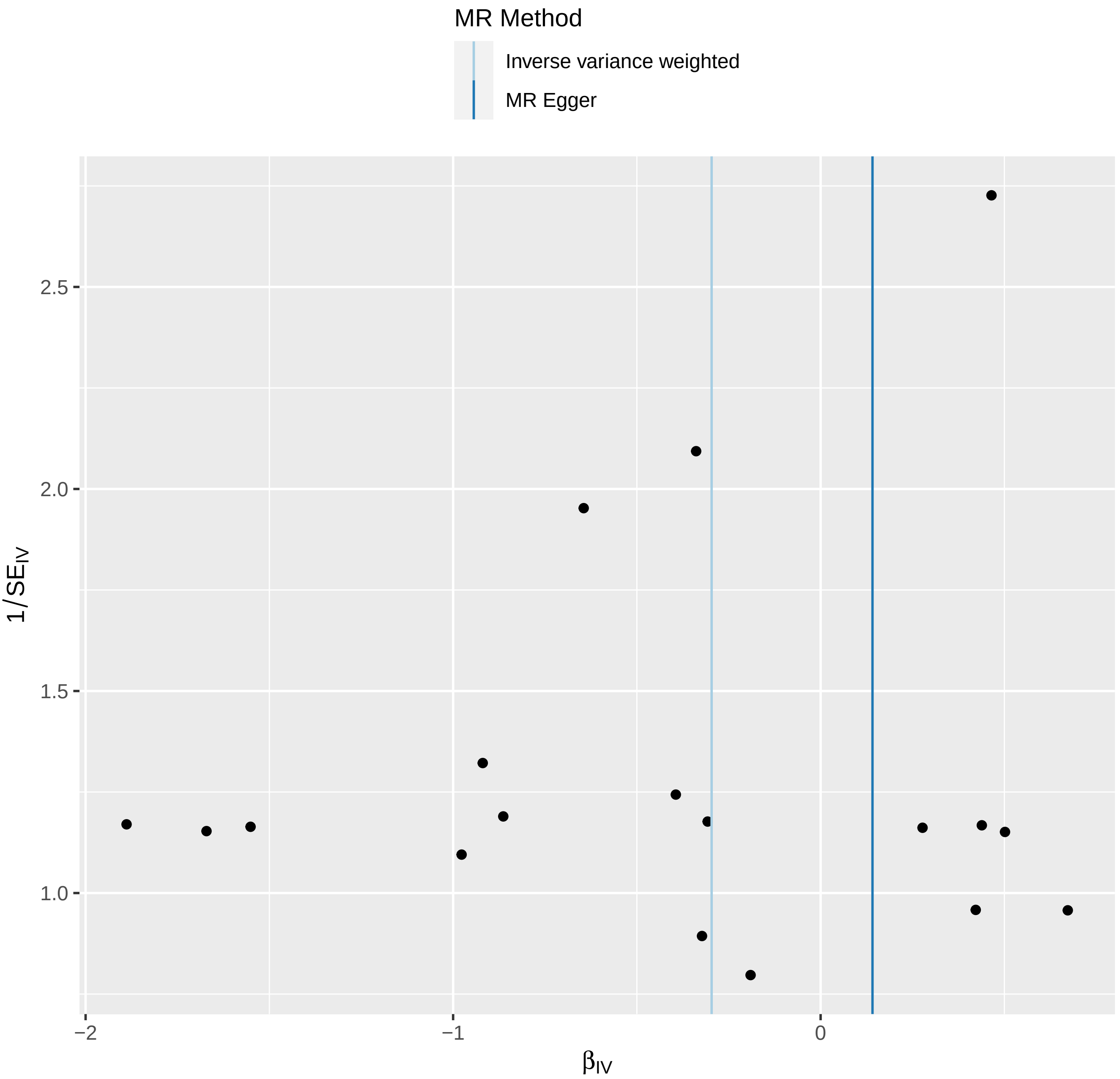

Batch 631 : Gut microbiota abundance (family Verrucomicrobiaceae id.4036) on Extreme obesity with alveolar hypoventilation

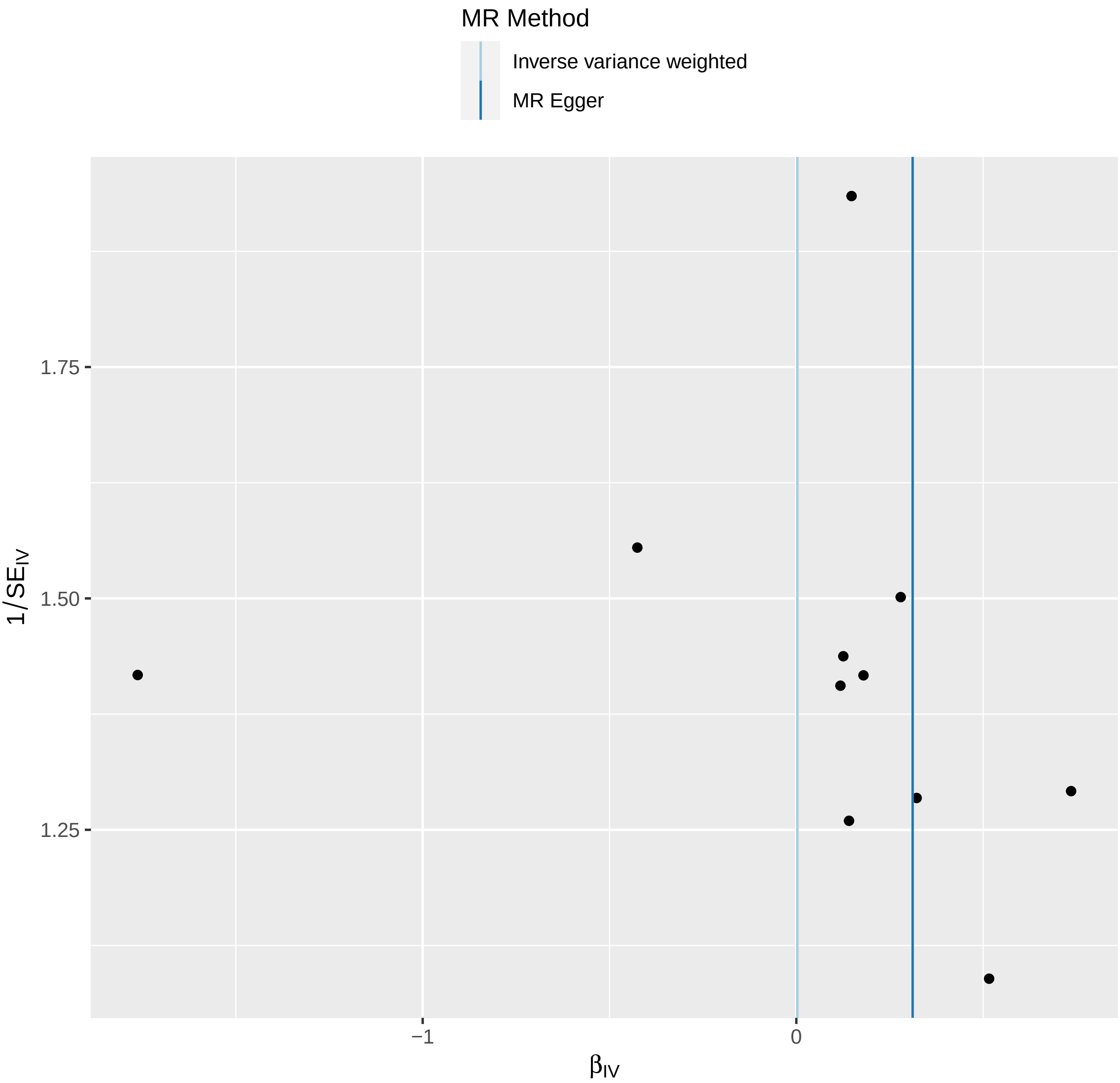

Batch 632 : Gut microbiota abundance (family Victivallaceae id.2255) on Extreme obesity with alveolar hypoventilation

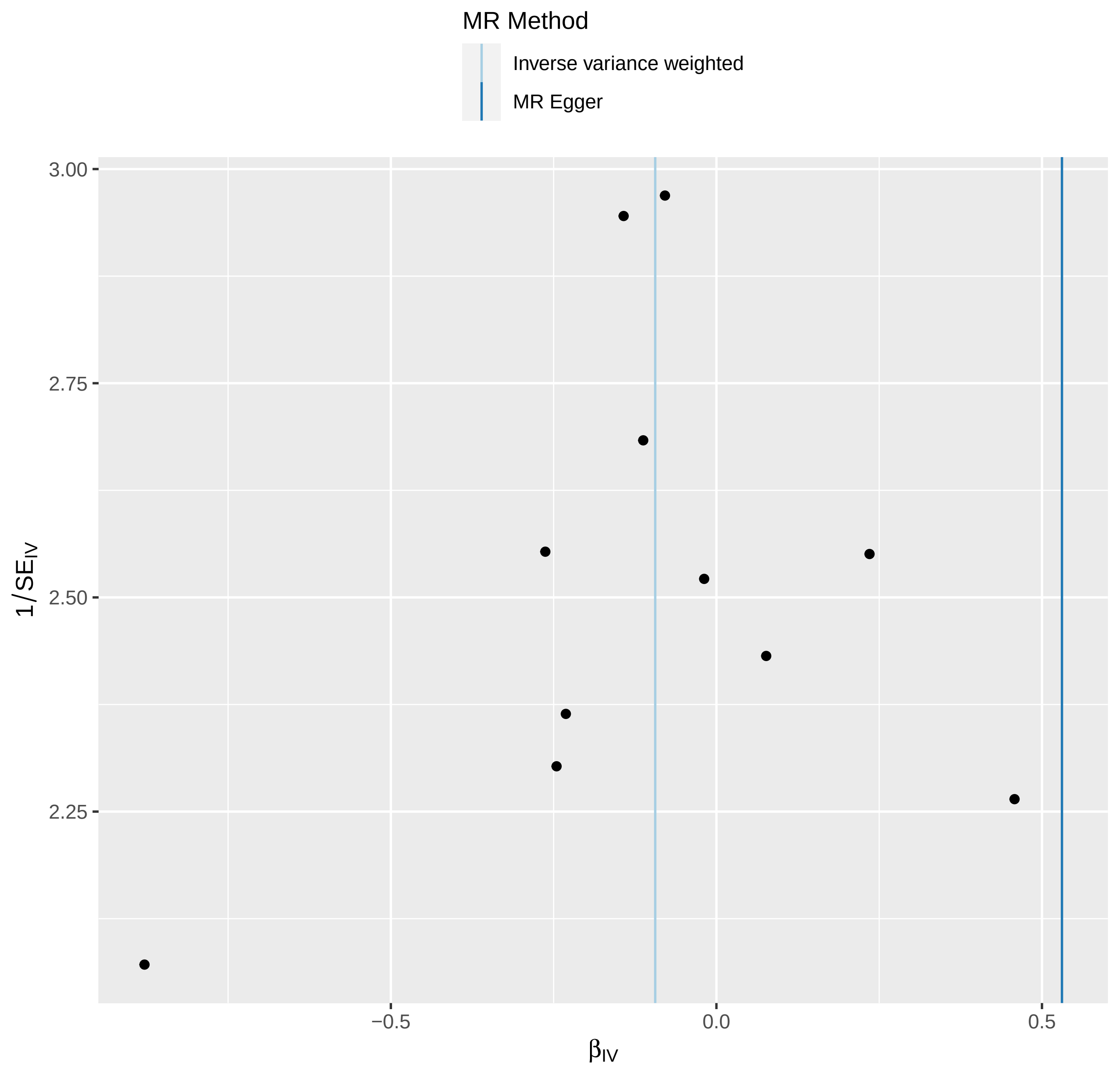

Batch 633 : Gut microbiota abundance (genus Actinomyces id.423) on Extreme obesity with alveolar hypoventilation

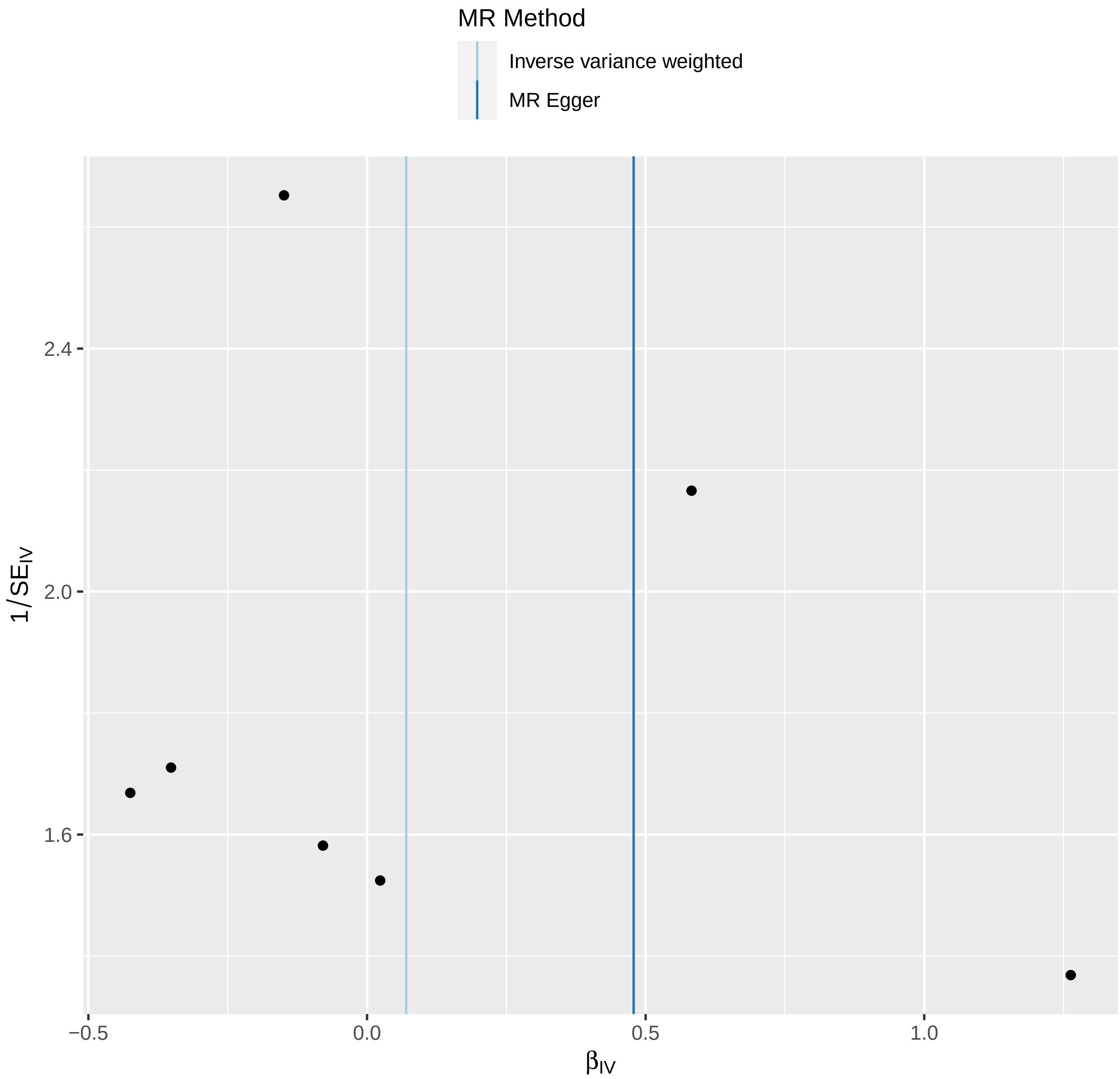

Batch 634 : Gut microbiota abundance (genus Adlercreutzia id.812) on Extreme obesity with alveolar hypoventilation

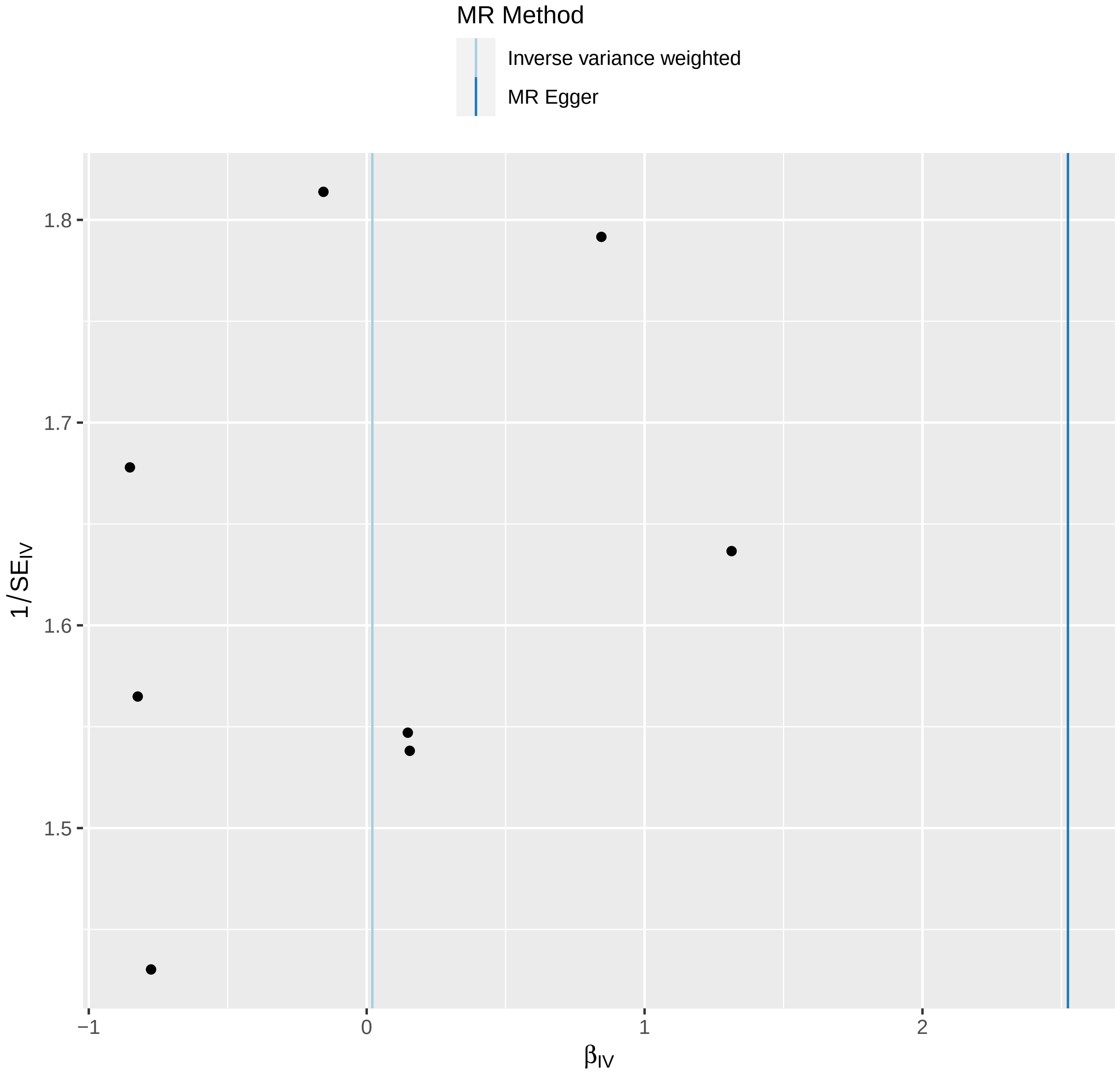

Batch 635 : Gut microbiota abundance (genus Akkermansia id.4037) on Extreme obesity with alveolar hypoventilation

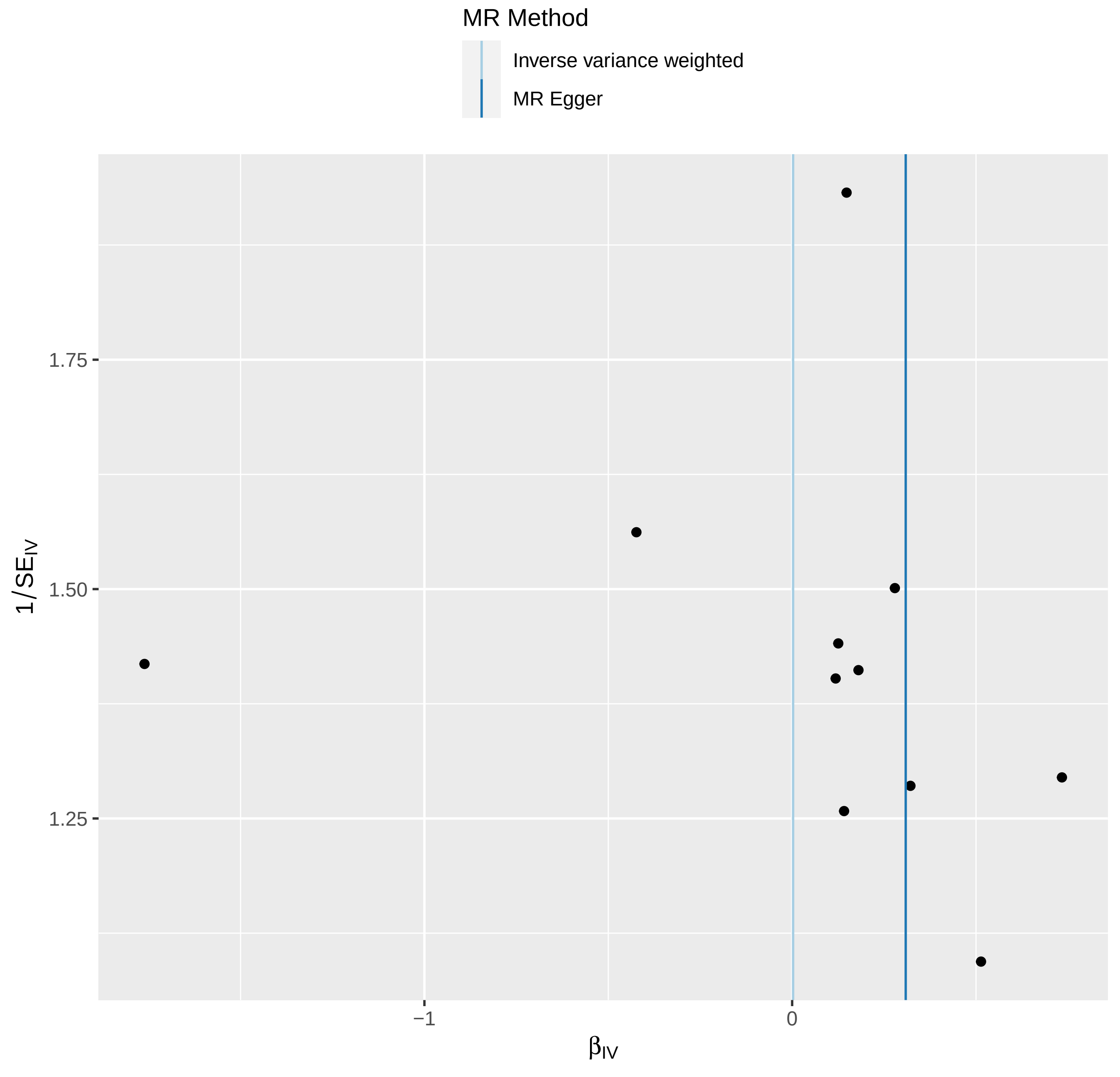

Batch 636 : Gut microbiota abundance (genus Alistipes id.968) on Extreme obesity with alveolar hypoventilation

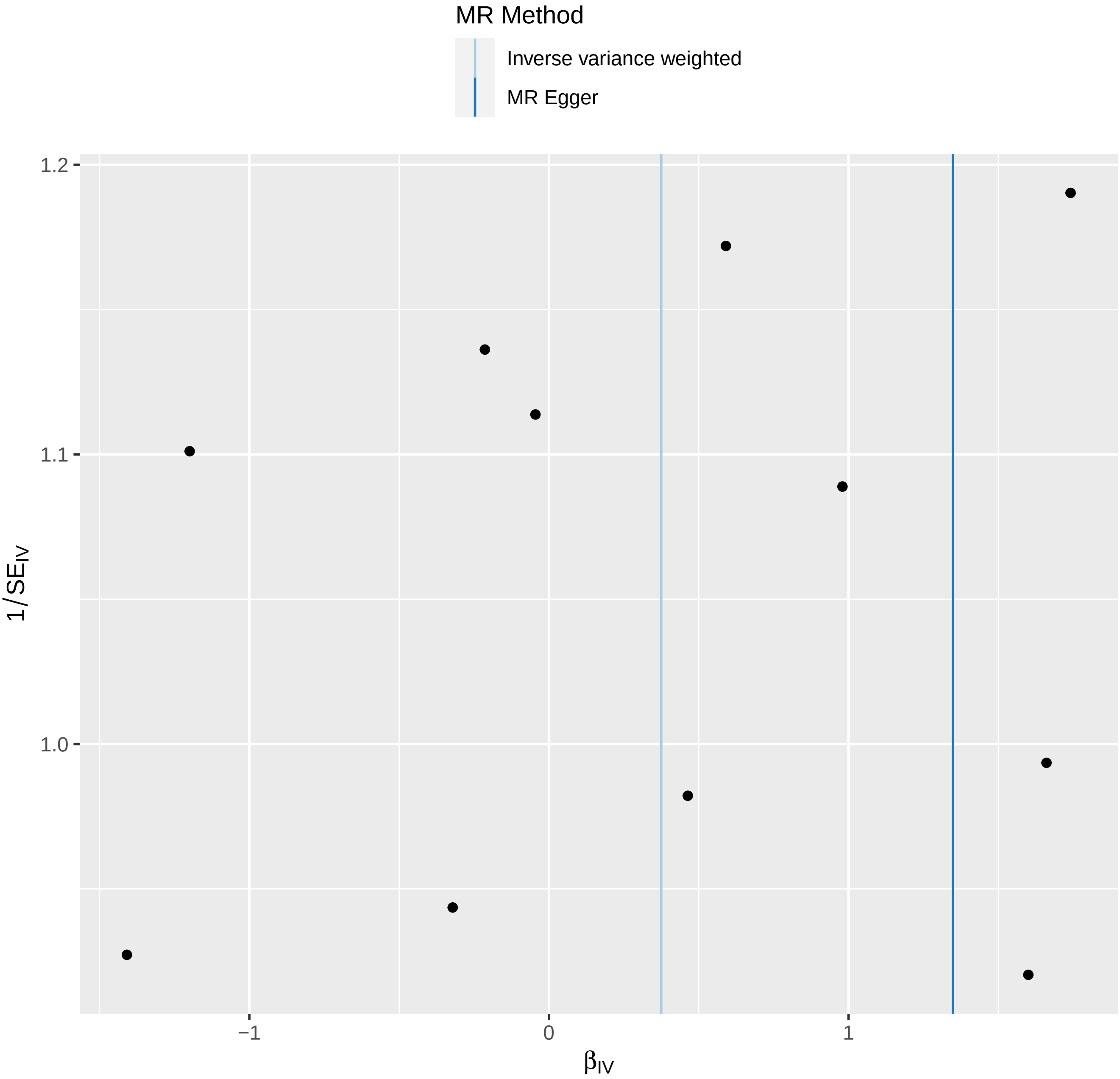

Batch 637 : Gut microbiota abundance (genus Allisonella id.2174) on Extreme obesity with alveolar hypoventilation

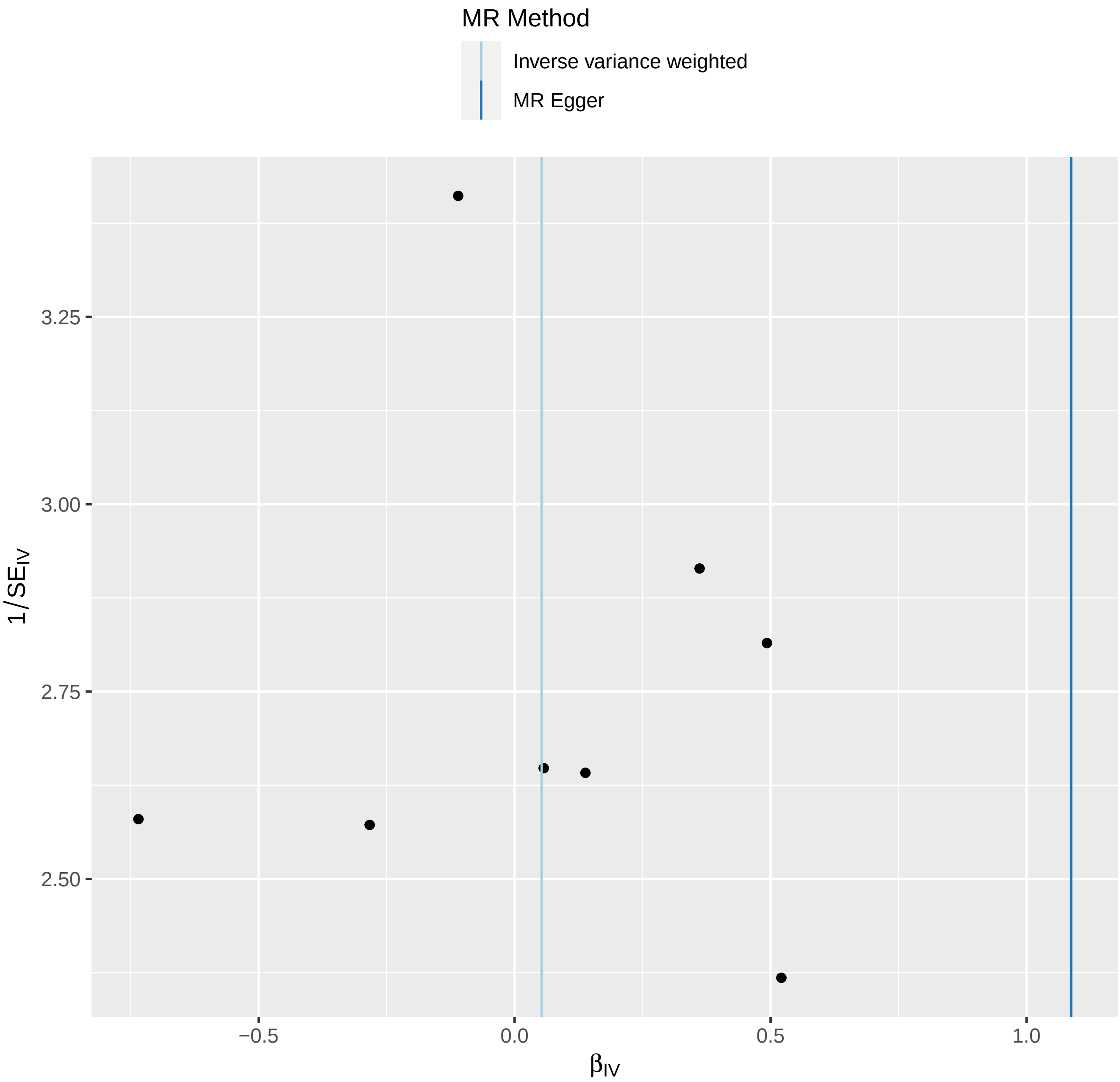

Batch 638 : Gut microbiota abundance (genus Alloprevotella id.961) on Extreme obesity with alveolar hypoventilation

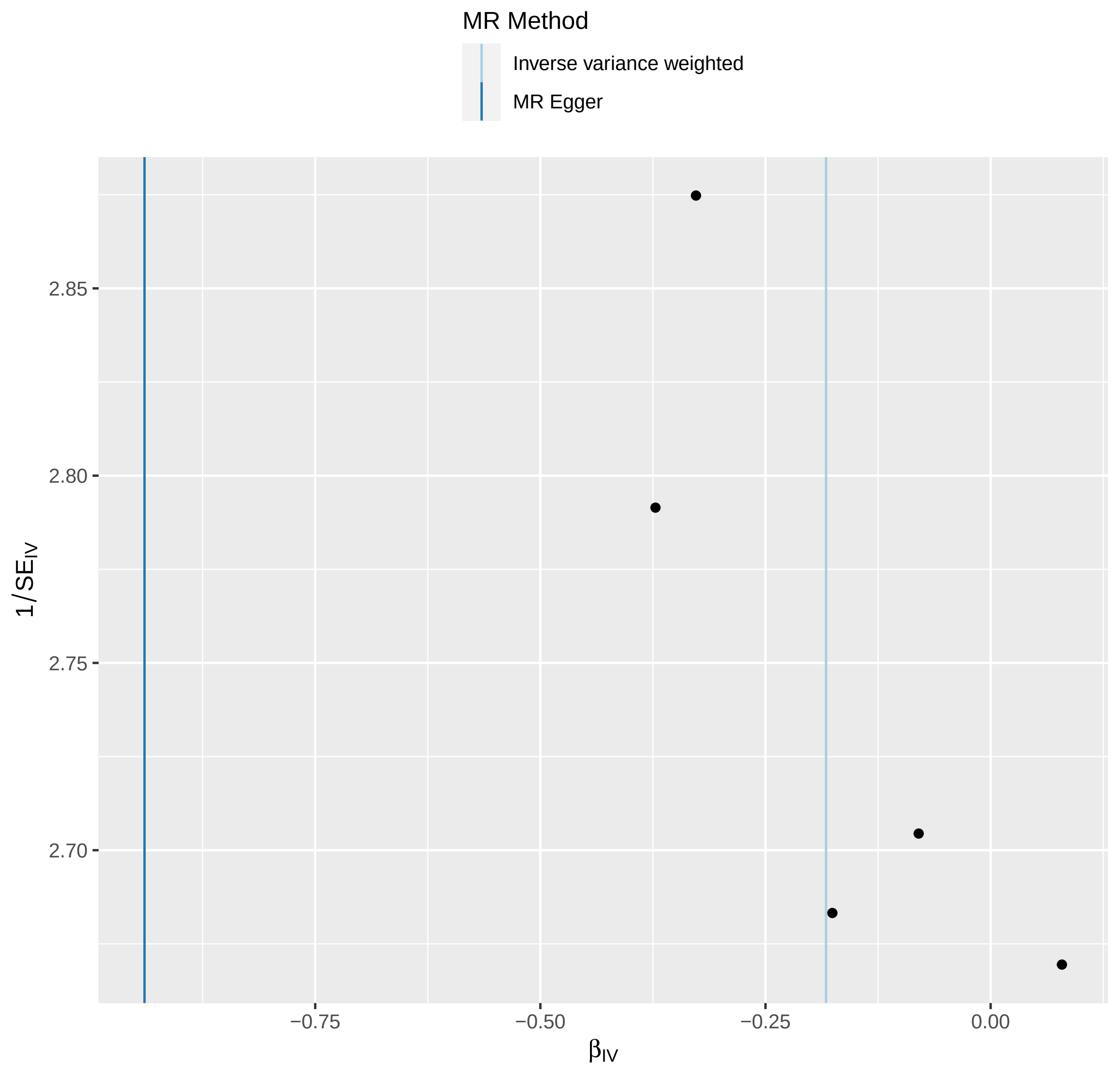

Batch 639 : Gut microbiota abundance (genus Anaerofilum id.2053) on Extreme obesity with alveolar hypoventilation

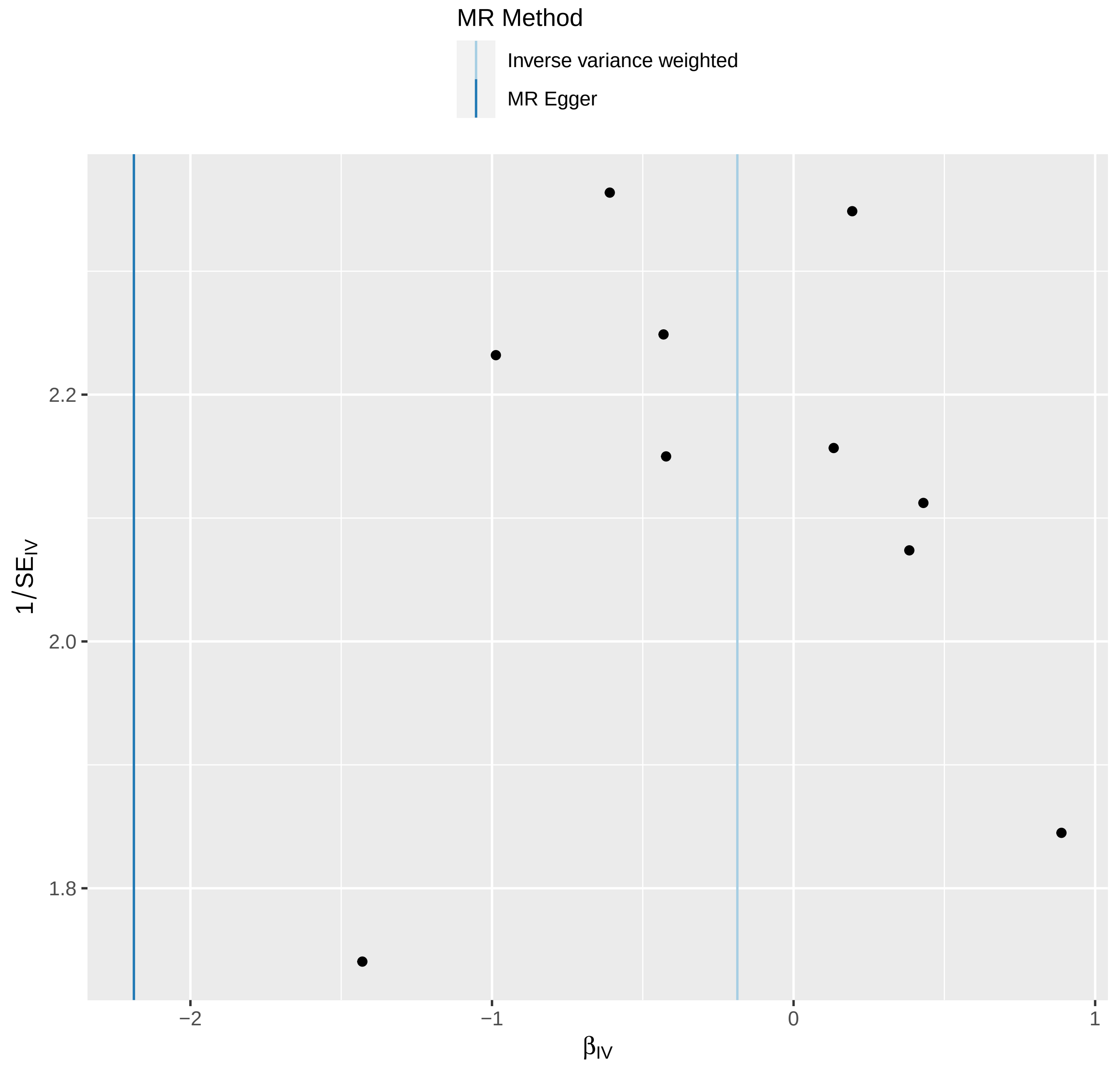

Batch 640 : Gut microbiota abundance (genus Anaerostipes id.1991) on Extreme obesity with alveolar hypoventilation

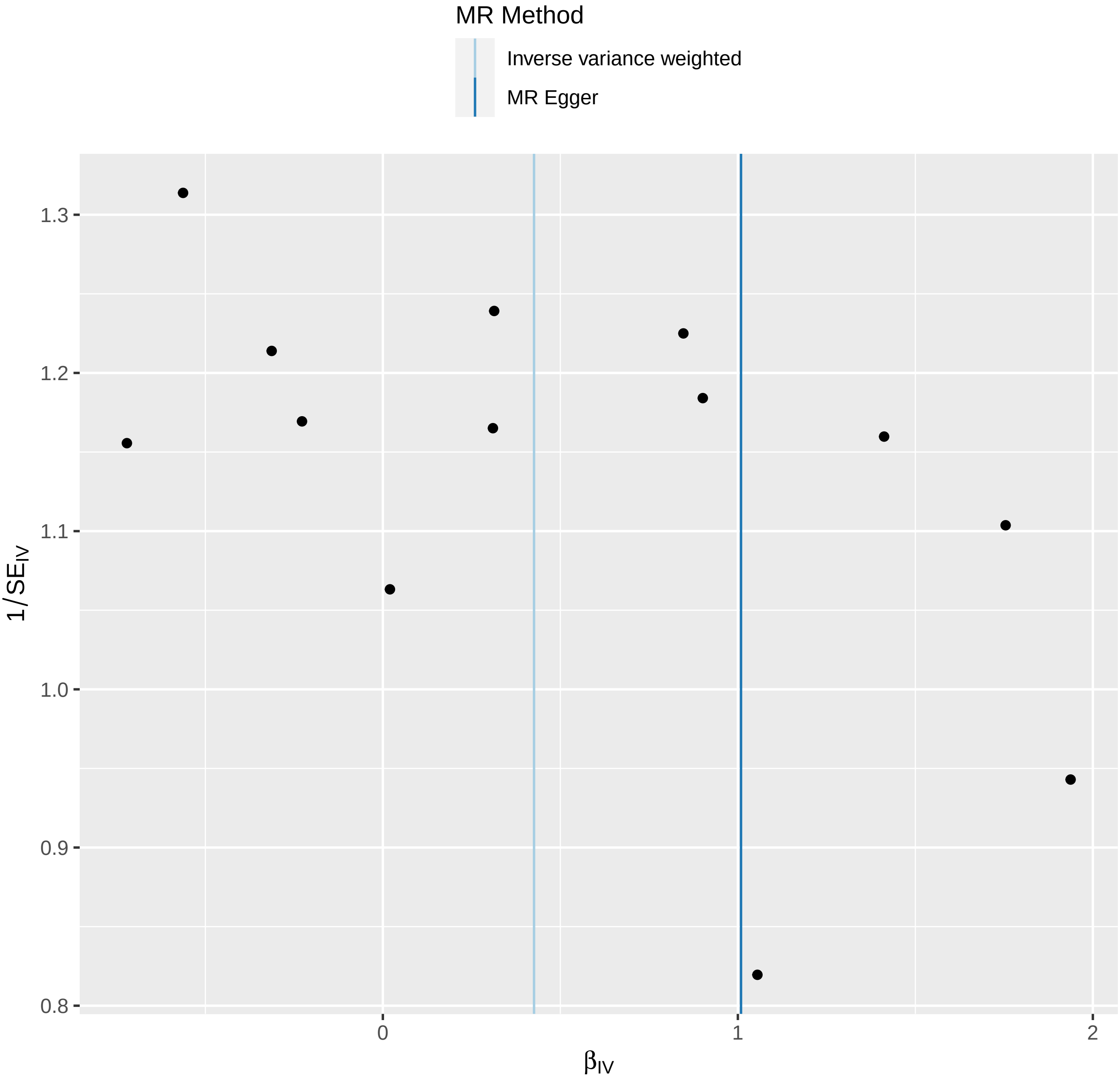

Batch 641 : Gut microbiota abundance (genus Anaerotruncus id.2054) on Extreme obesity with alveolar hypoventilation

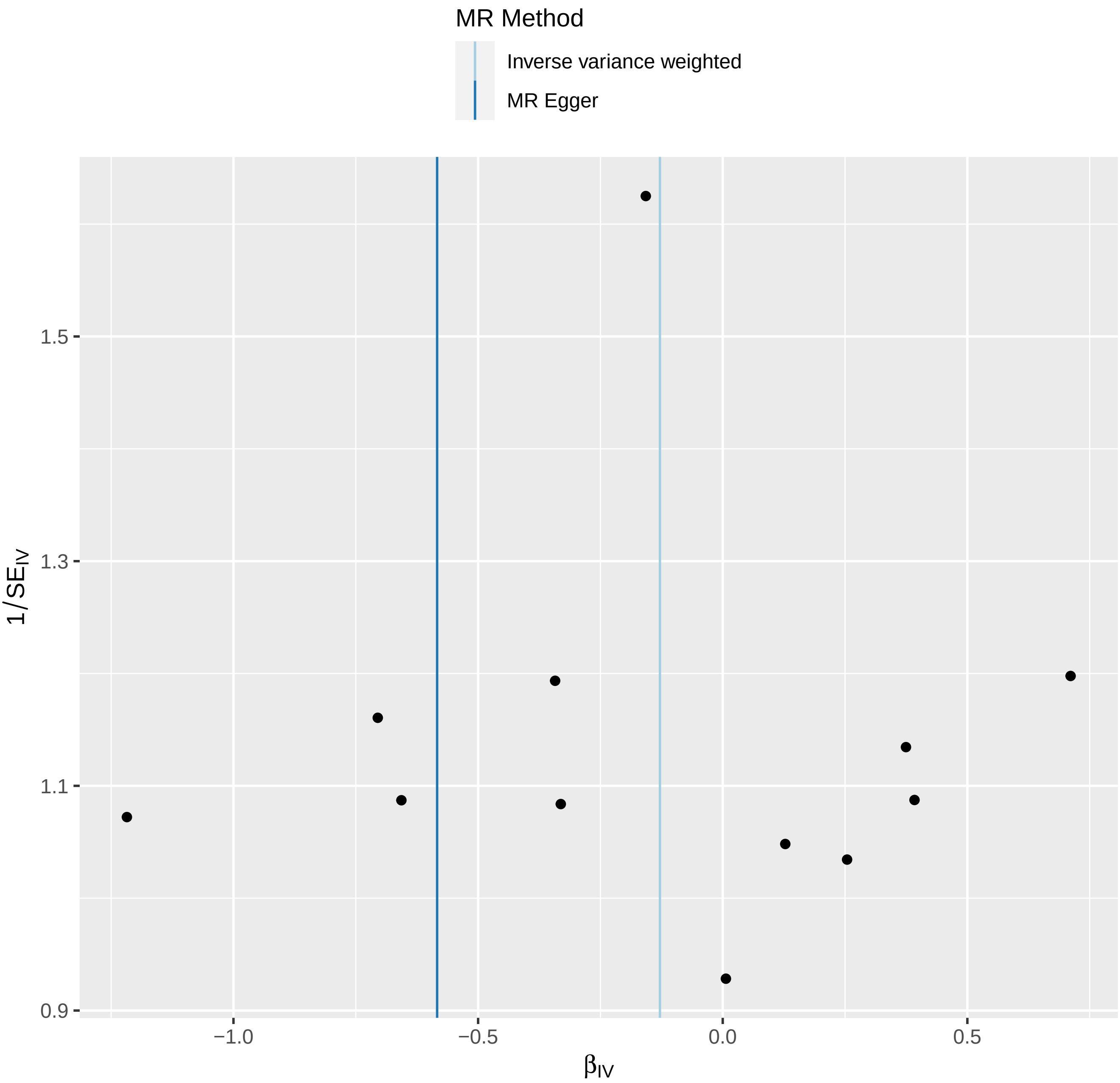

Batch 642 : Gut microbiota abundance (genus Bacteroides id.918) on Extreme obesity with alveolar hypoventilation

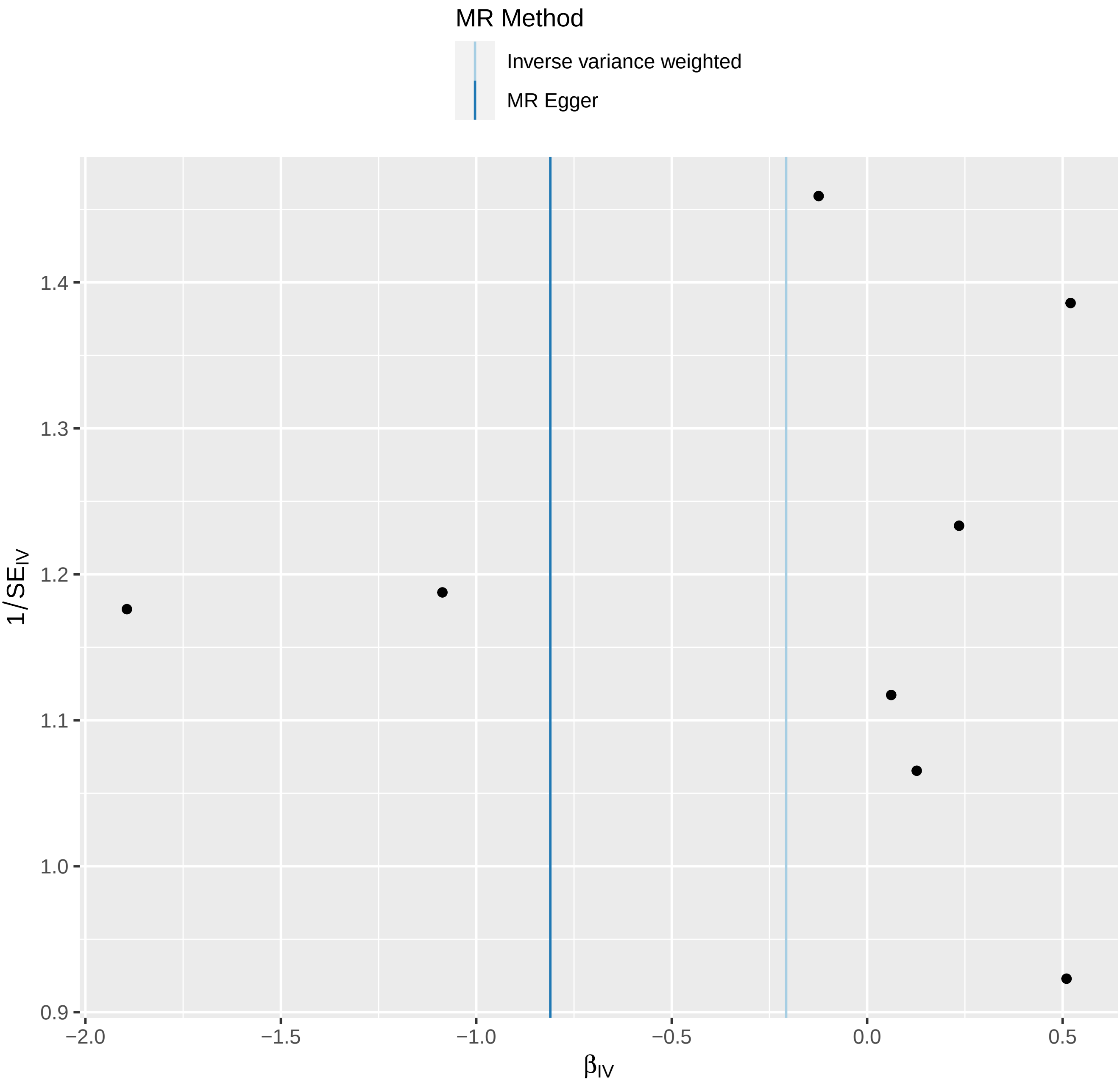

Batch 643 : Gut microbiota abundance (genus Barnesiella id.944) on Extreme obesity with alveolar hypoventilation

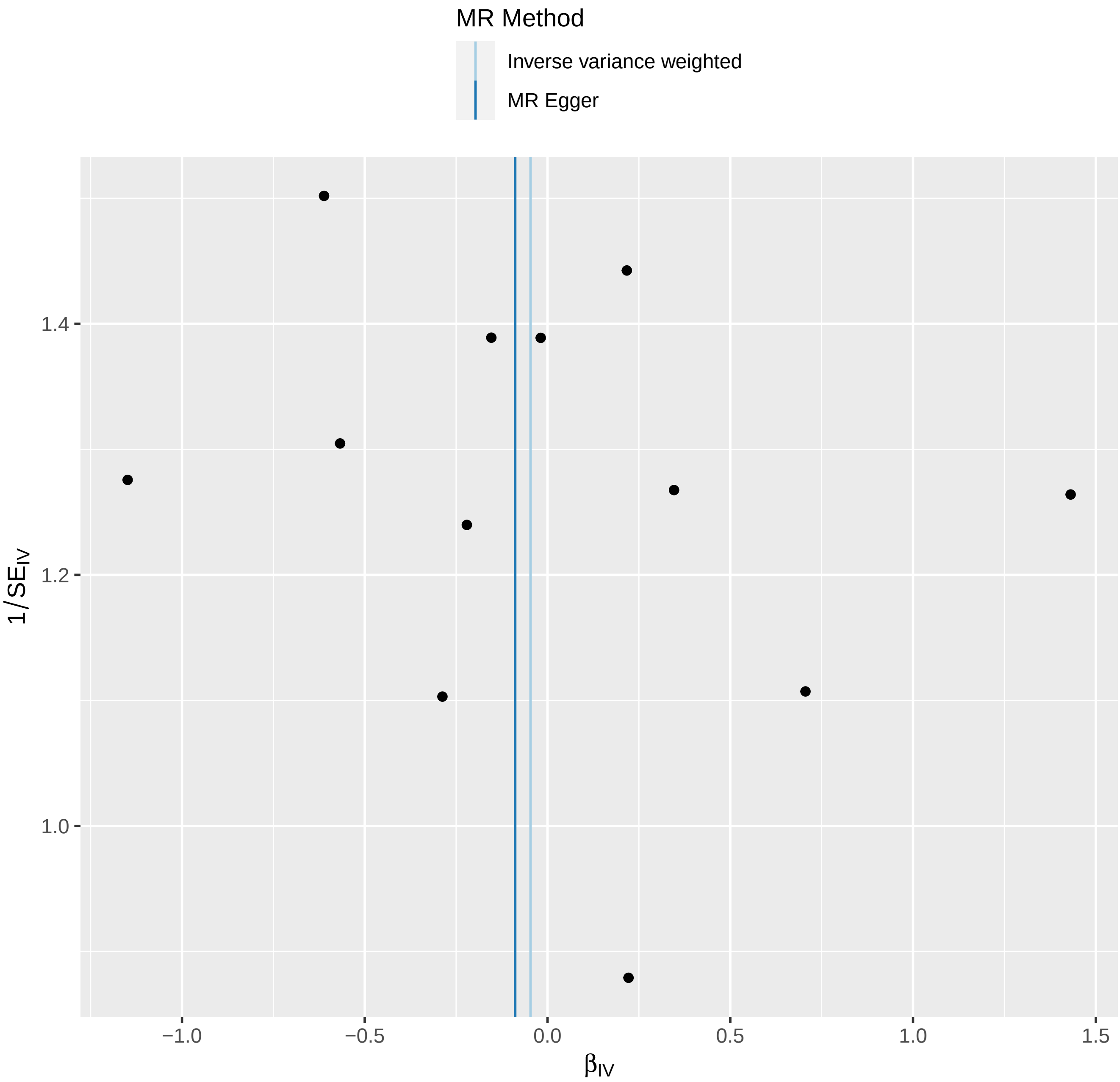

Batch 644 : Gut microbiota abundance (genus Bifidobacterium id.436) on Extreme obesity with alveolar hypoventilation

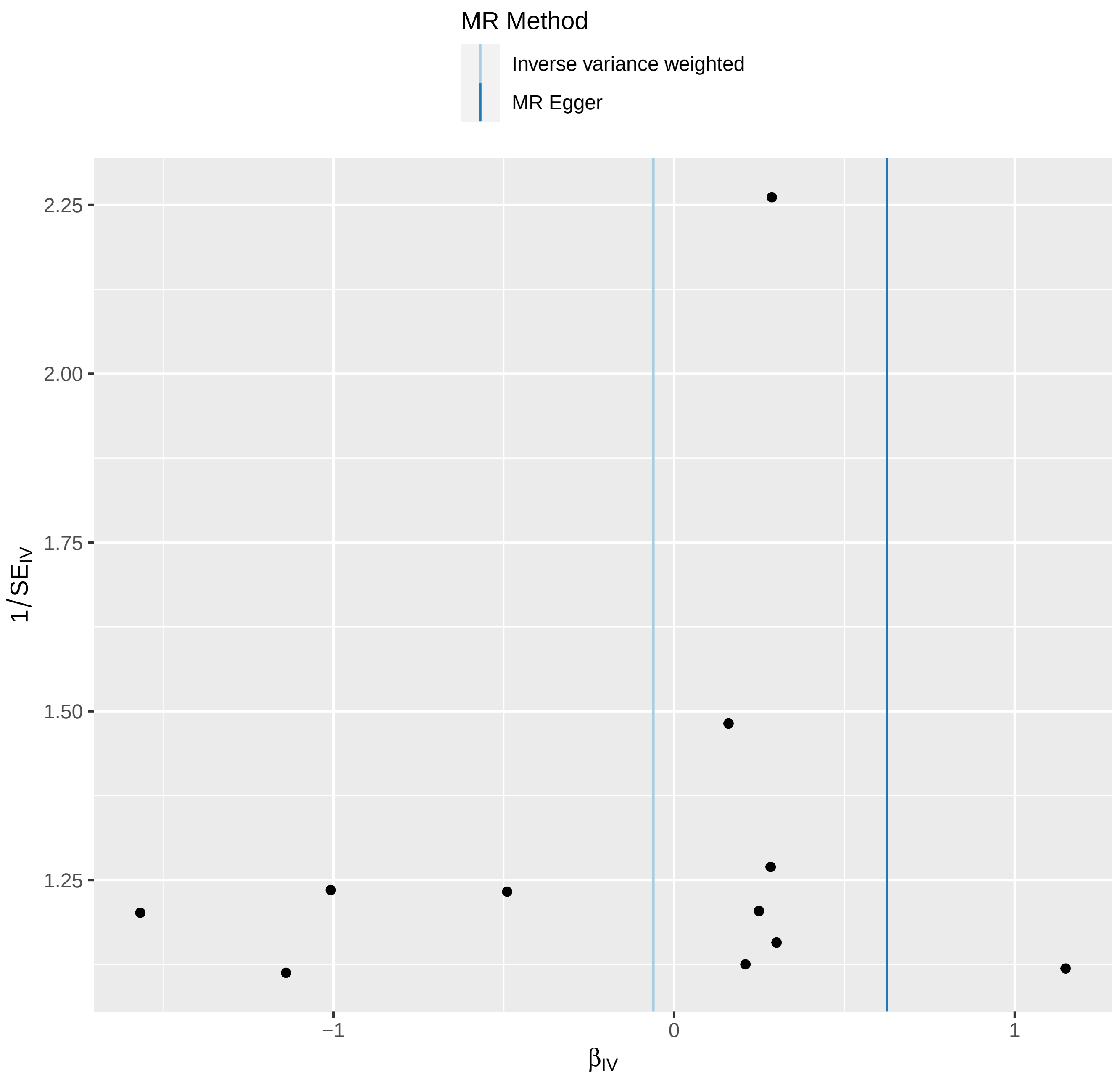

Batch 645 : Gut microbiota abundance (genus Bilophila id.3170) on Extreme obesity with alveolar hypoventilation

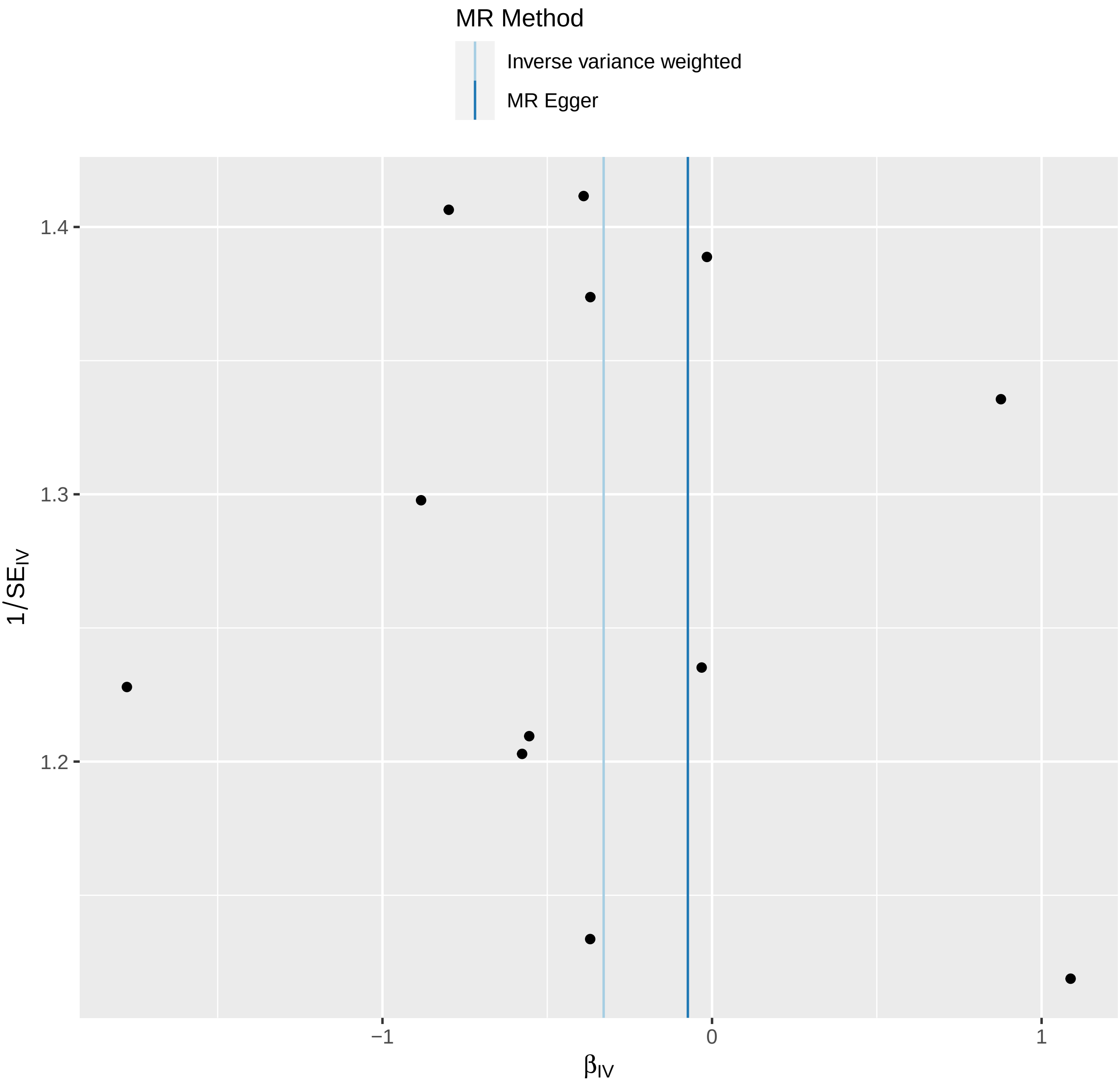

Batch 646 : Gut microbiota abundance (genus Blautia id.1992) on Extreme obesity with alveolar hypoventilation

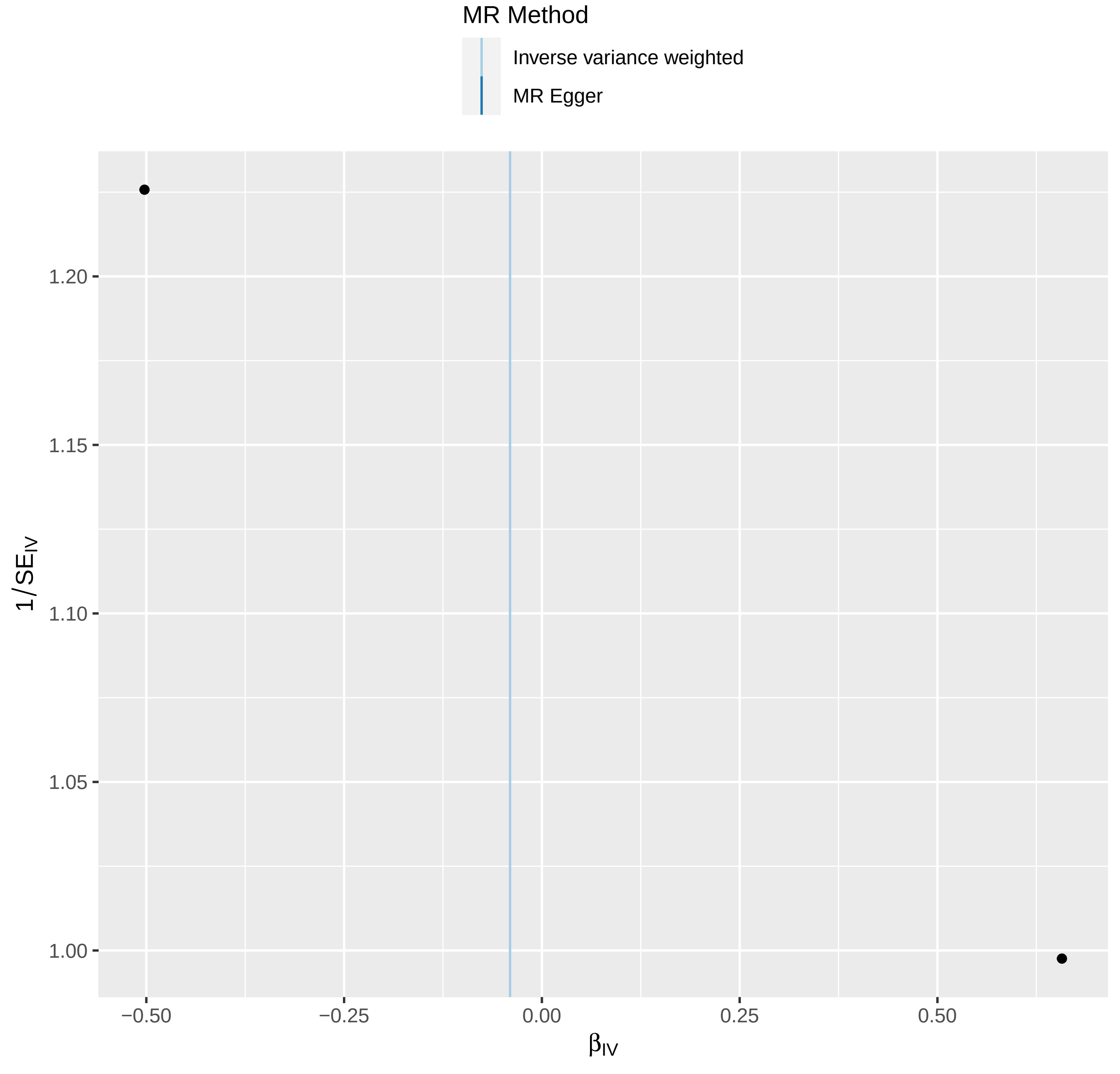

Batch 647 : Gut microbiota abundance (genus Butyricicoccus id.2055) on Extreme obesity with alveolar hypoventilation

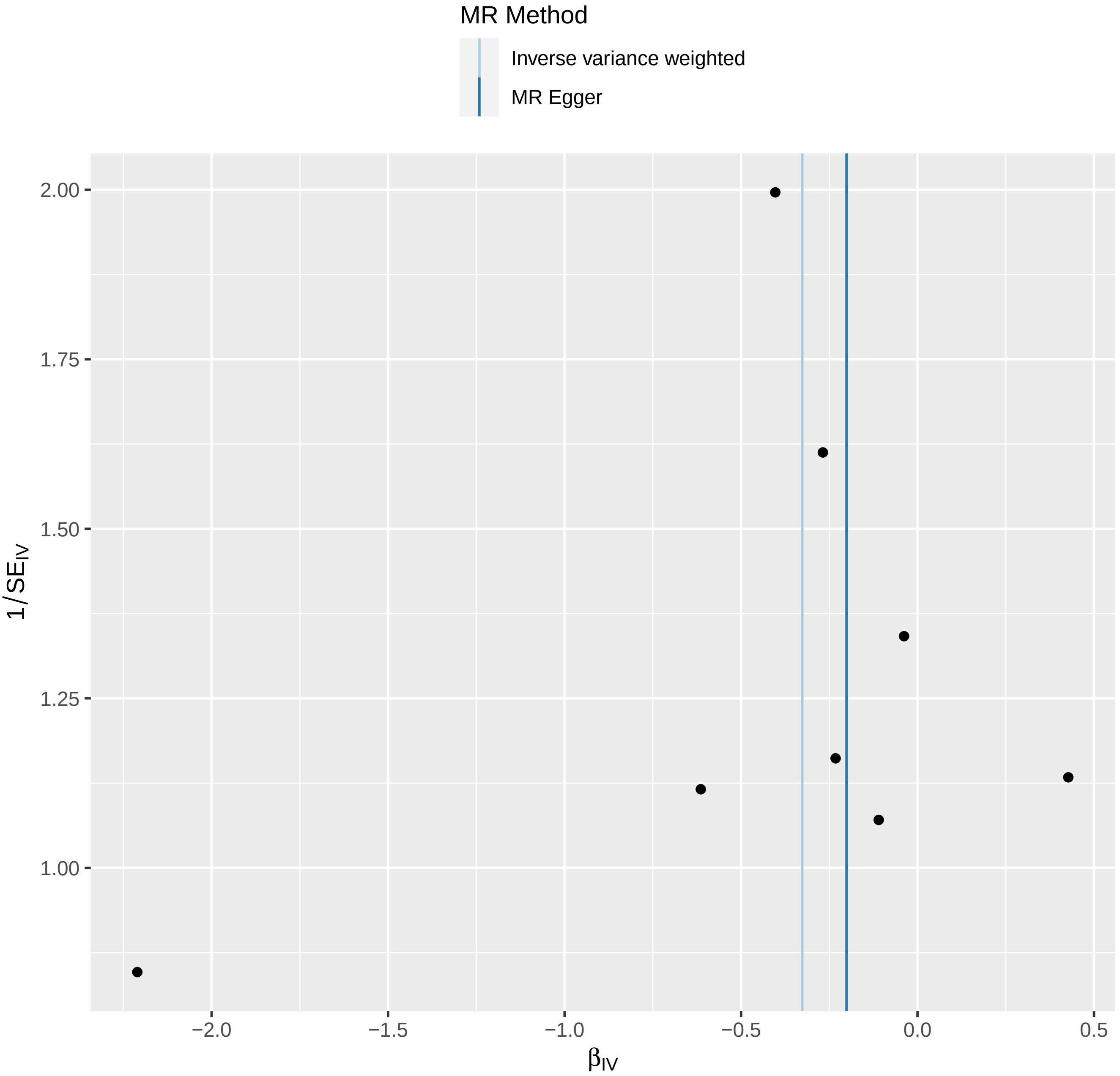

Batch 648 : Gut microbiota abundance (genus Butyricimonas id.945) on Extreme obesity with alveolar hypoventilation

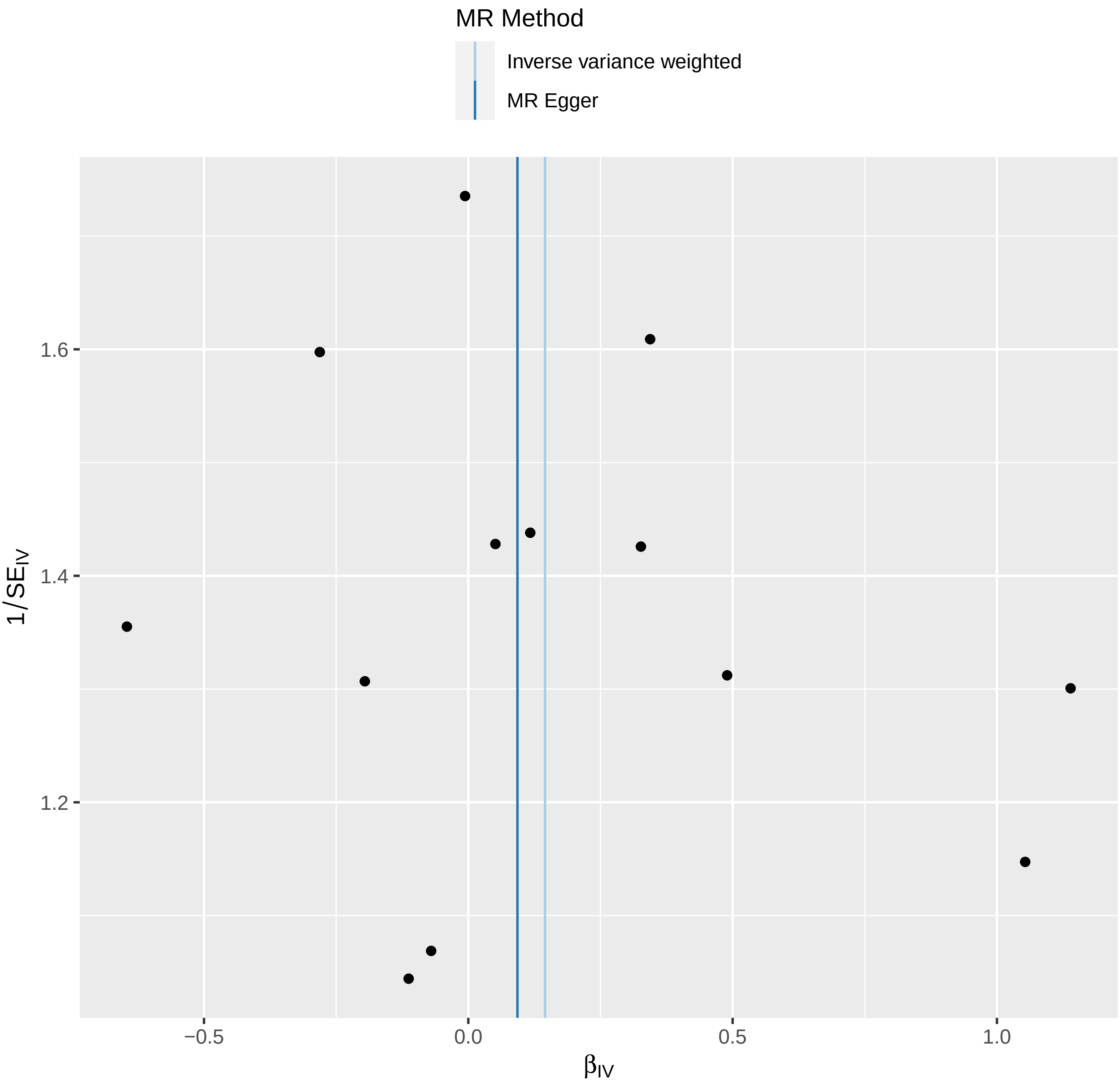

Batch 649 : Gut microbiota abundance (genus Butyrivibrio id.1993) on Extreme obesity with alveolar hypoventilation

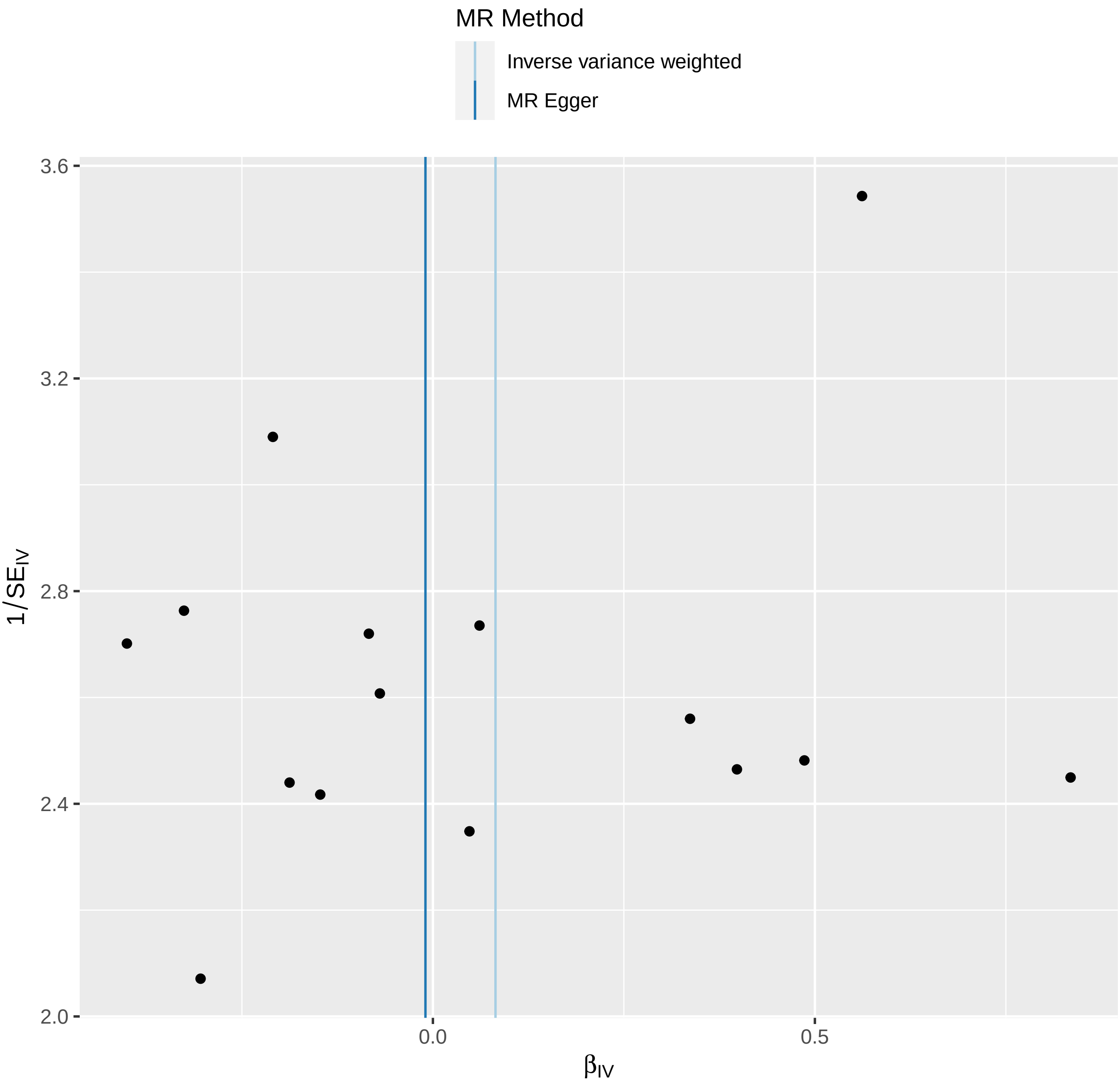

Batch 650 : Gut microbiota abundance (genus Candidatus Soleaferrea id.11350) on Extreme obesity with alveolar hypoventilation

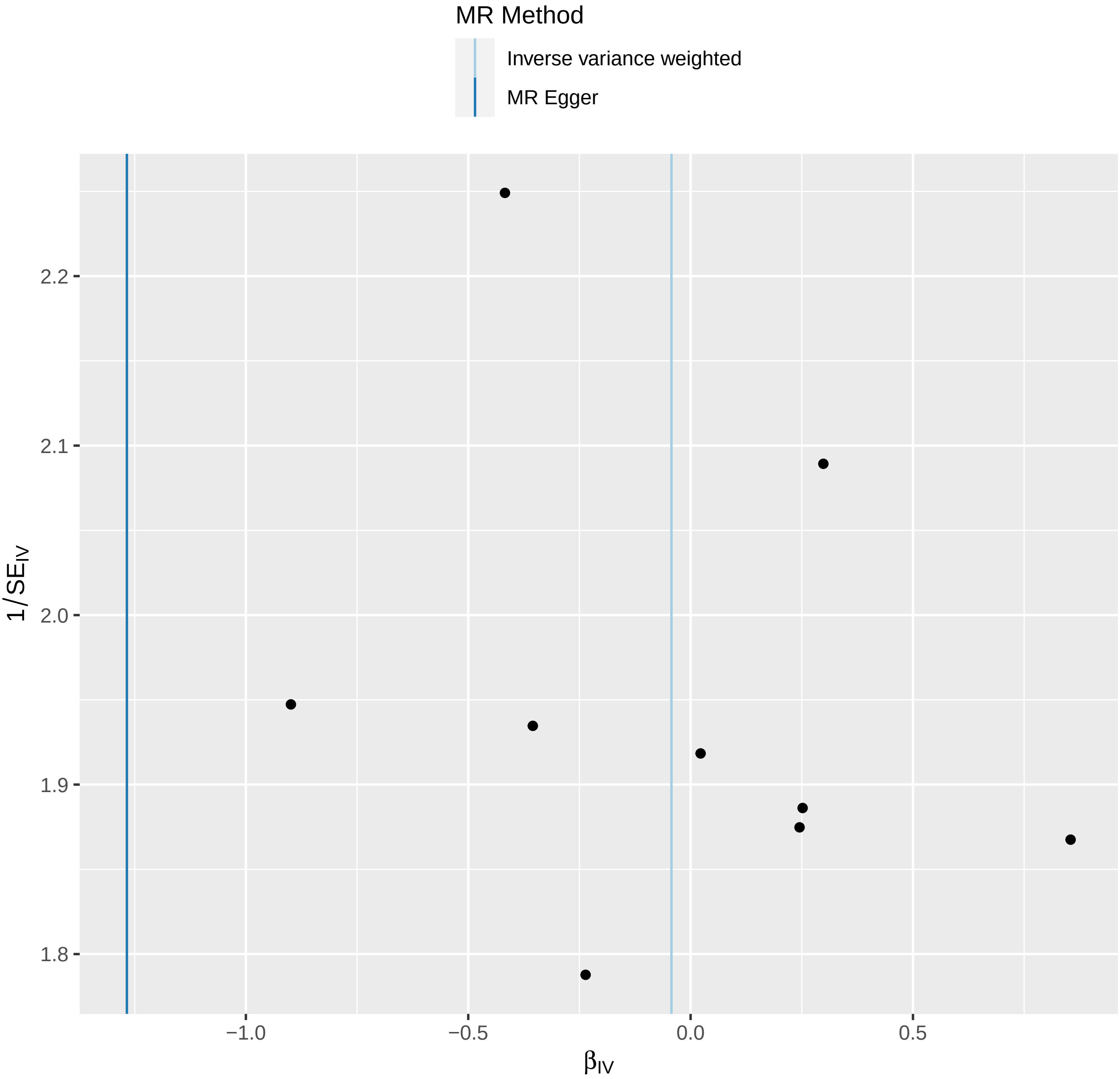

Batch 651 : Gut microbiota abundance (genus Catenibacterium id.2153) on Extreme obesity with alveolar hypoventilation

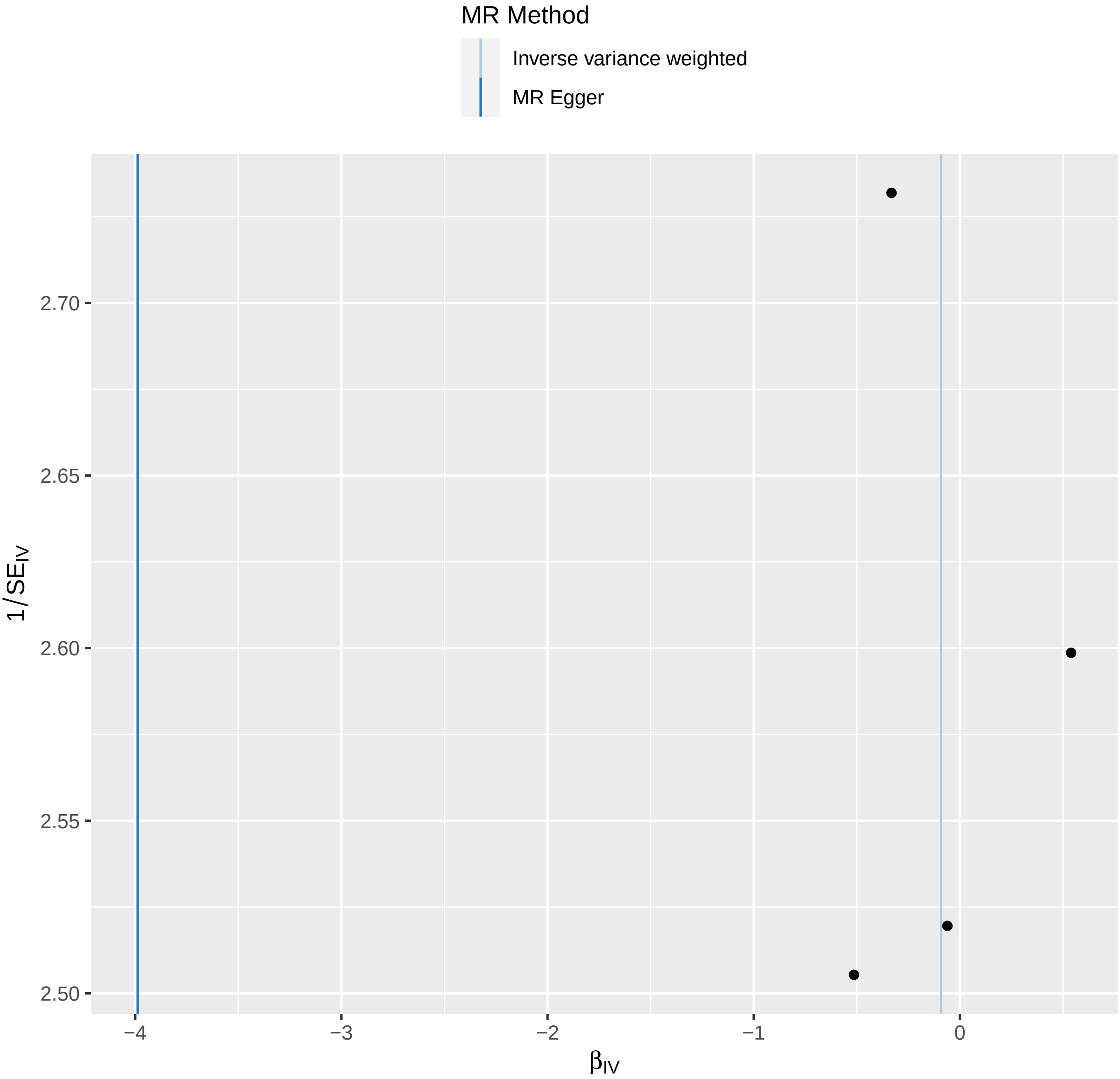

Batch 652 : Gut microbiota abundance (genus Christensenellaceae R 7group id.11283) on Extreme obesity with alveolar hypoventilation

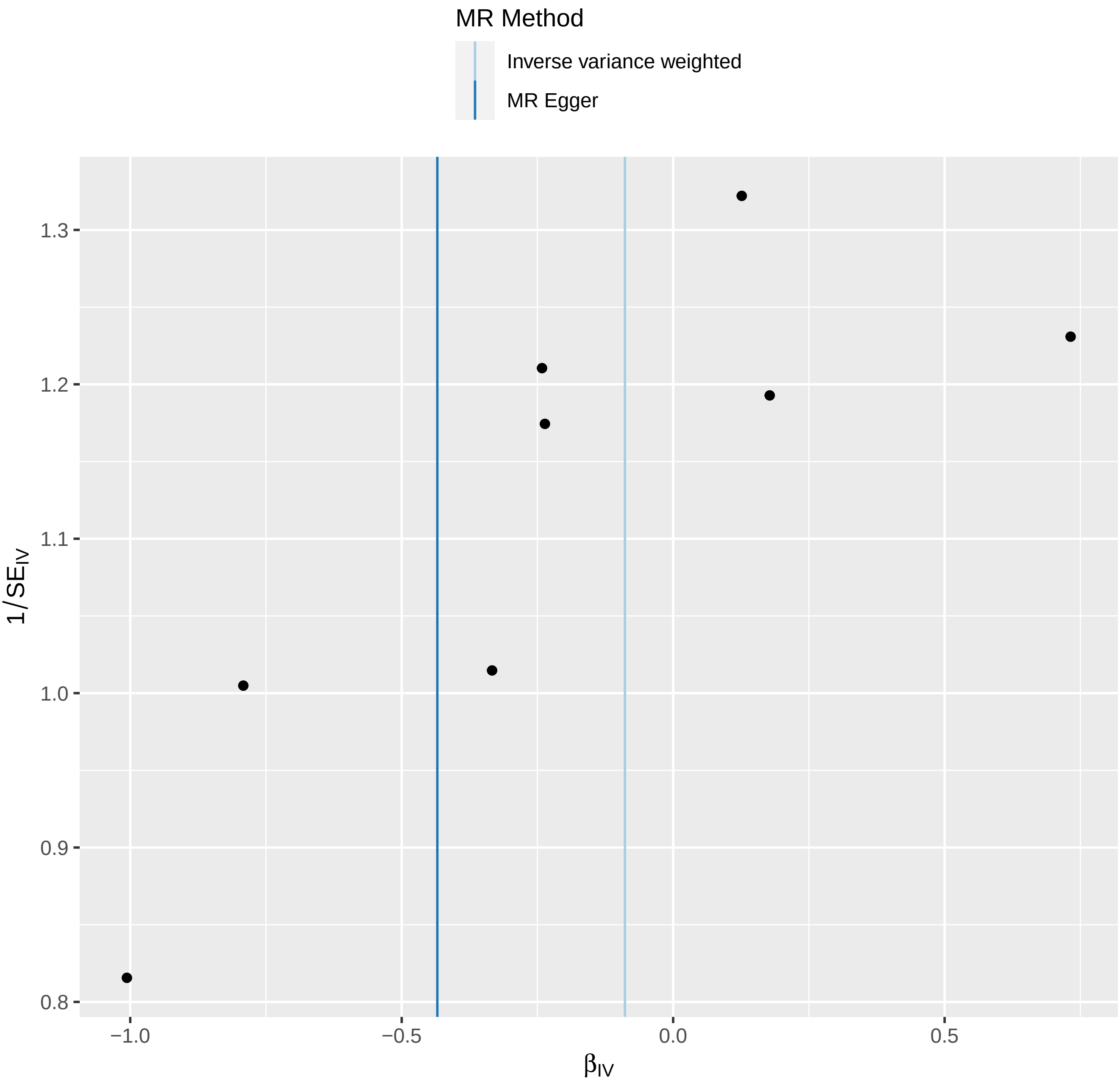

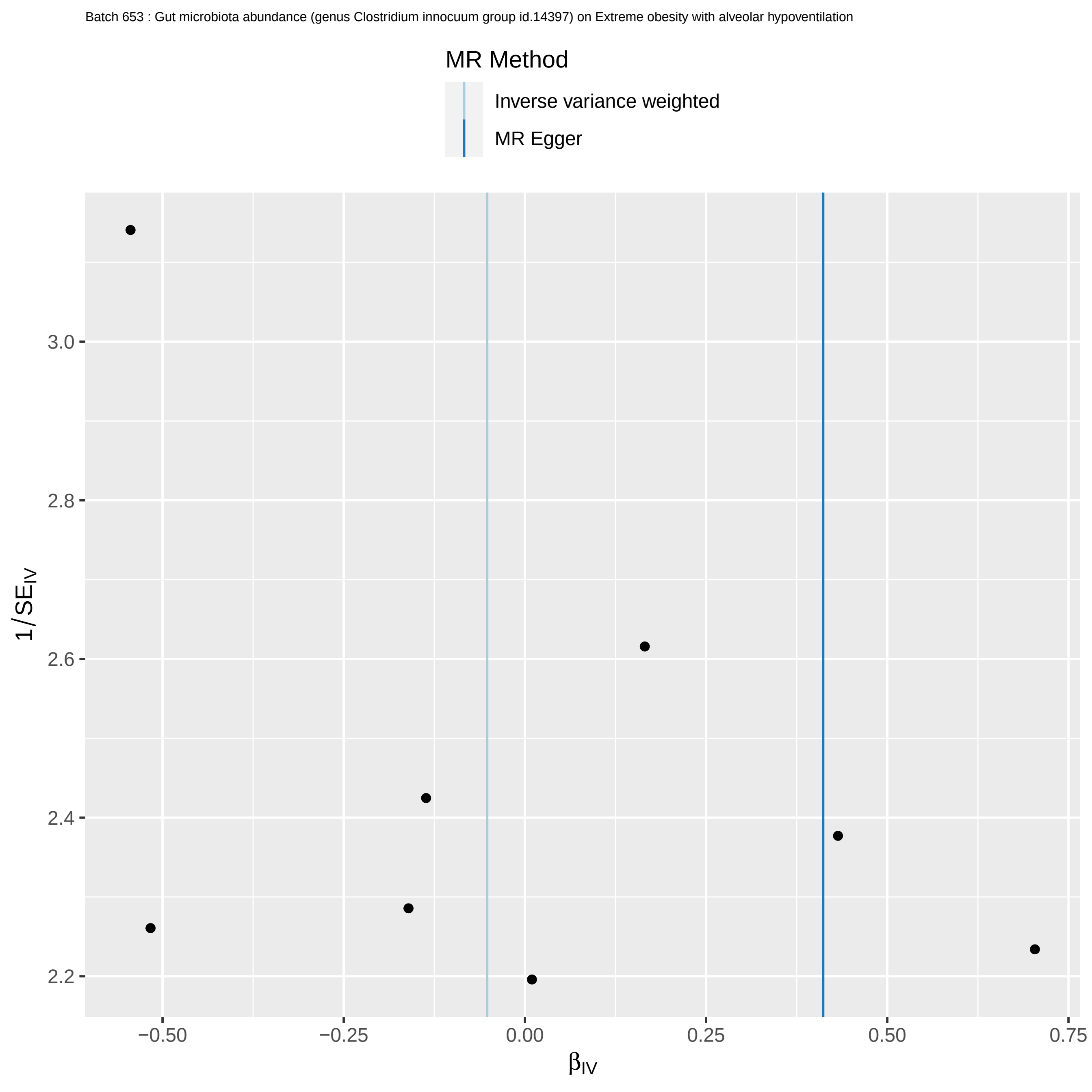

Batch 653 : Gut microbiota abundance (genus Clostridium innocuum group id.14397) on Extreme obesity with alveolar hypoventilation
MR Method
Inverse variance weighted
MR Egger
1/SE_IV
2.2
2.4
2.6
2.8
3.0
−0.50
−0.25
0.00
0.25
0.50
0.75
β_IV

Batch 654 : Gut microbiota abundance (genus Clostridium sensustricto1 id.1873) on Extreme obesity with alveolar hypoventilation

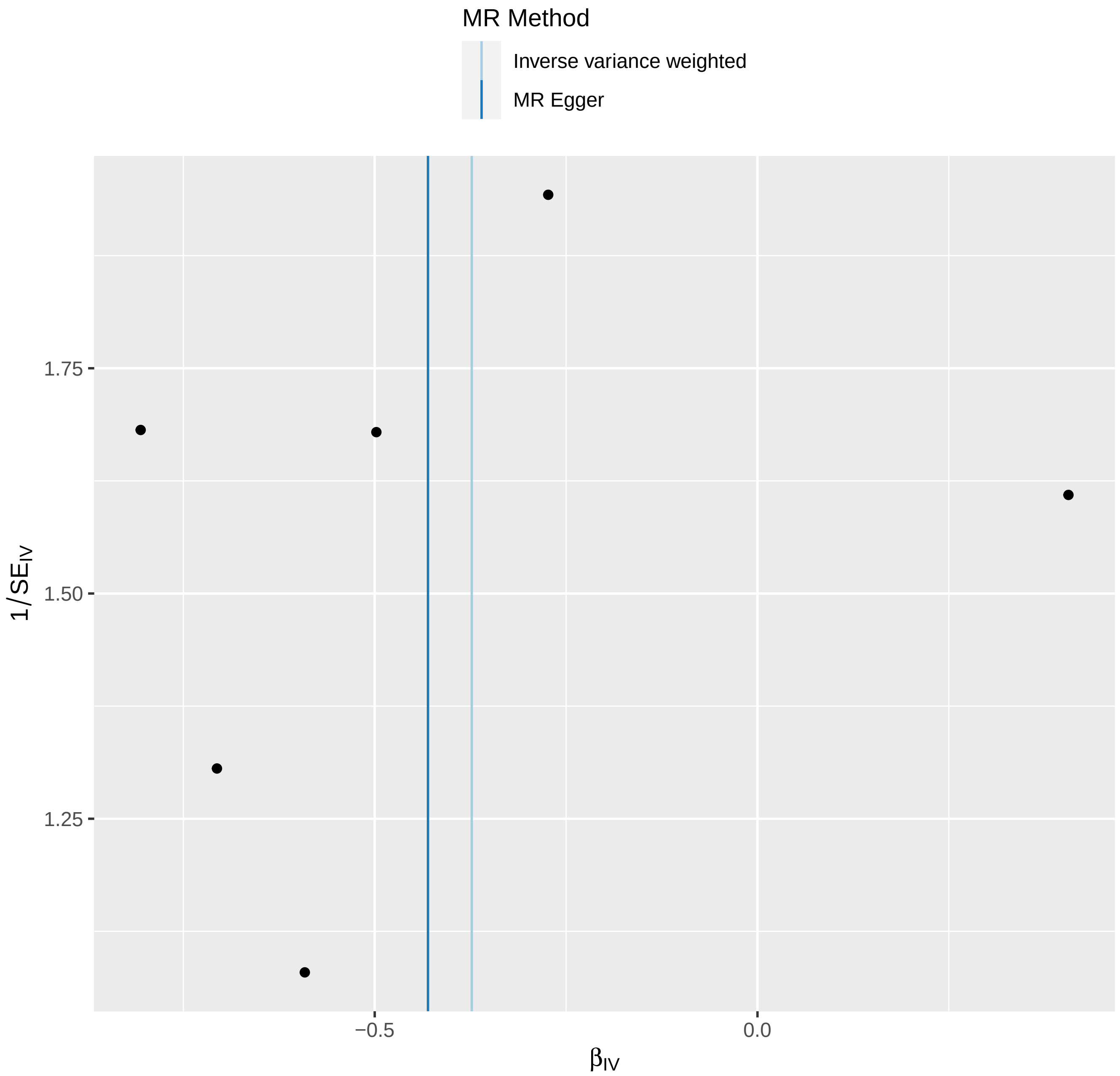

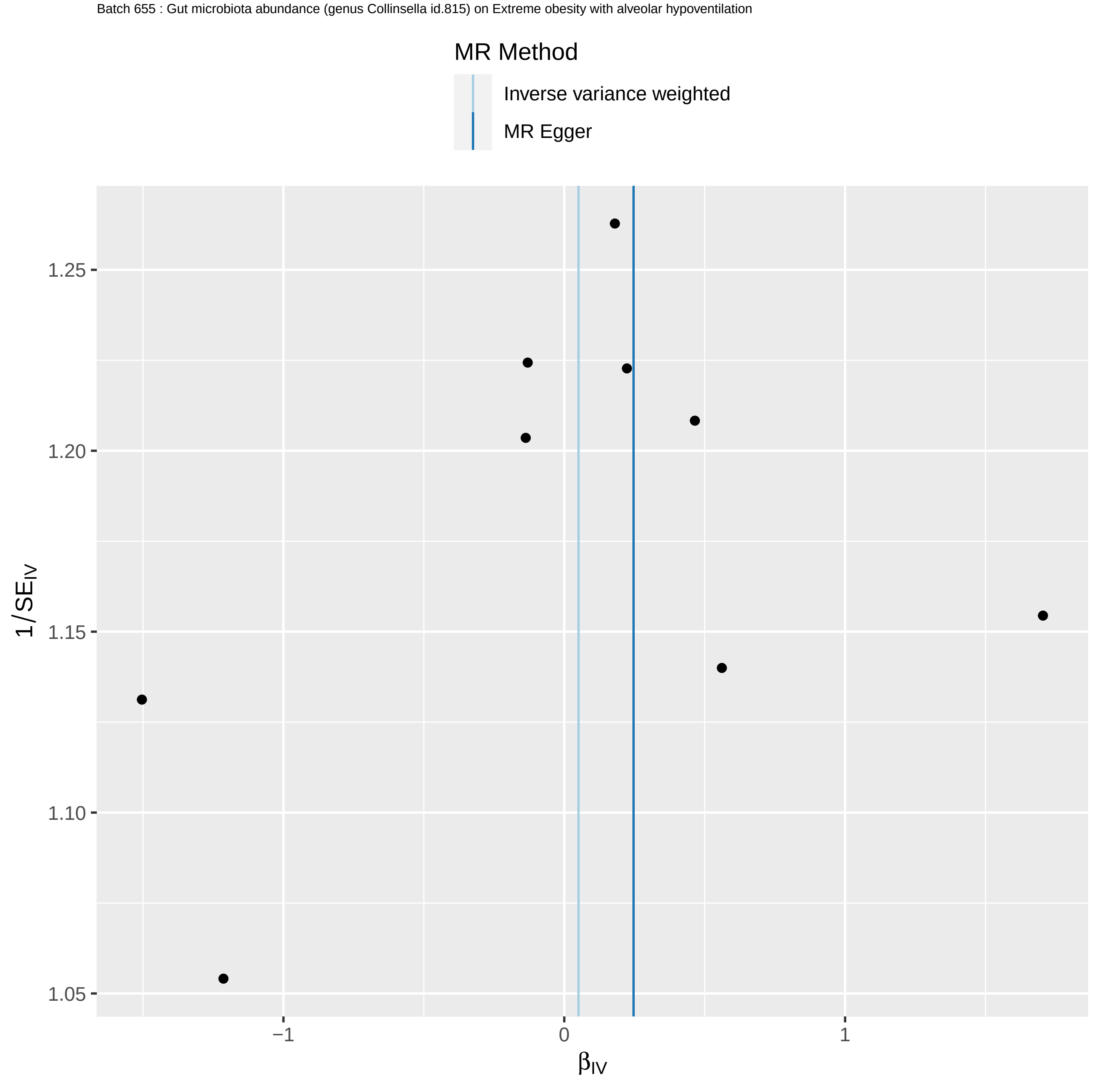
Batch 655 : Gut microbiota abundance (genus Collinsella id.815) on Extreme obesity with alveolar hypoventilation
MR Method
Inverse variance weighted
MR Egger
1.25
1.20
1.15
1.10
1.05
1/SE_IV
−1
0
1
β_IV

Batch 656 : Gut microbiota abundance (genus Coprobacter id.949) on Extreme obesity with alveolar hypoventilation

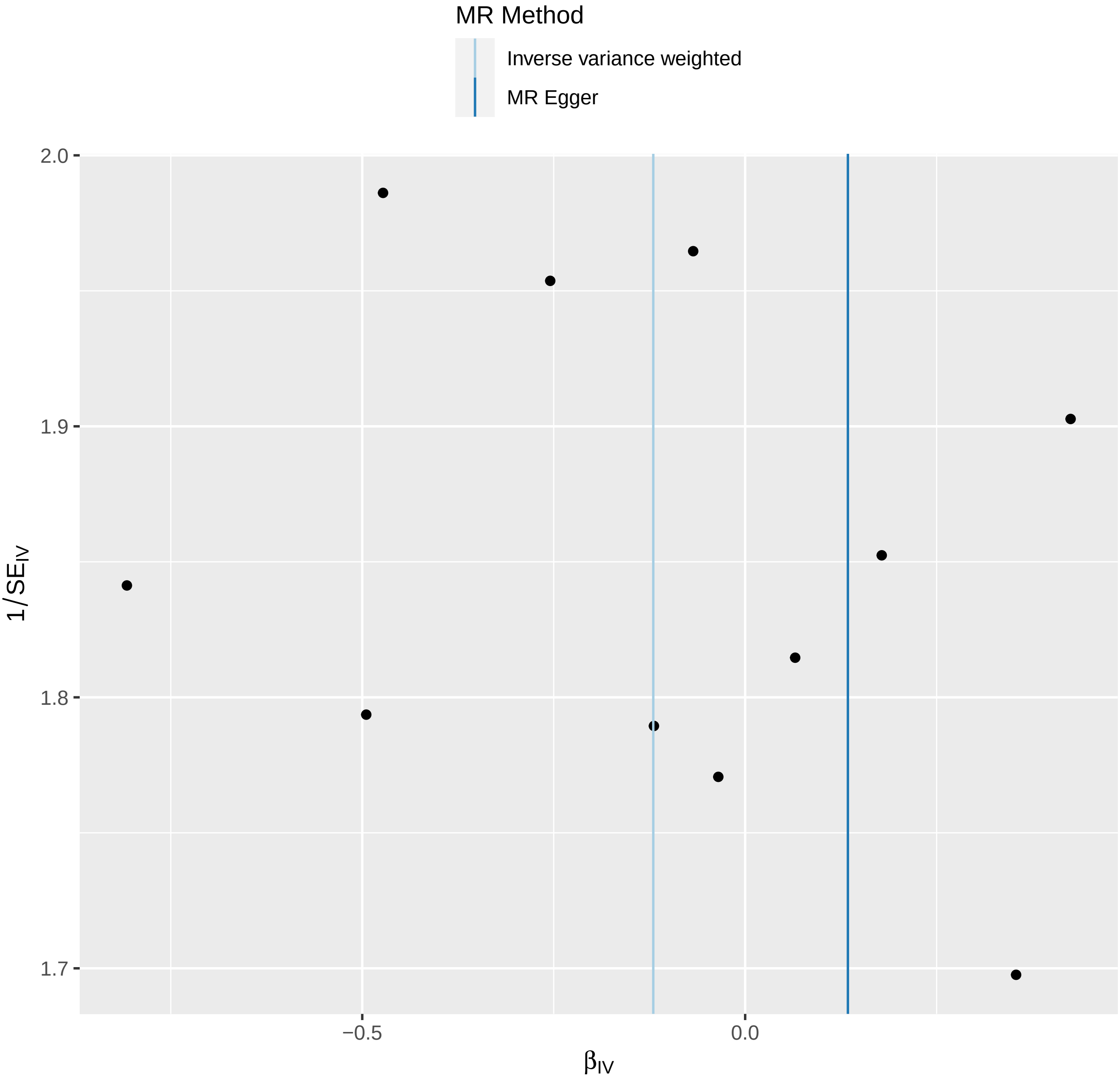

Batch 657 : Gut microbiota abundance (genus Coprococcus1 id.11301) on Extreme obesity with alveolar hypoventilation

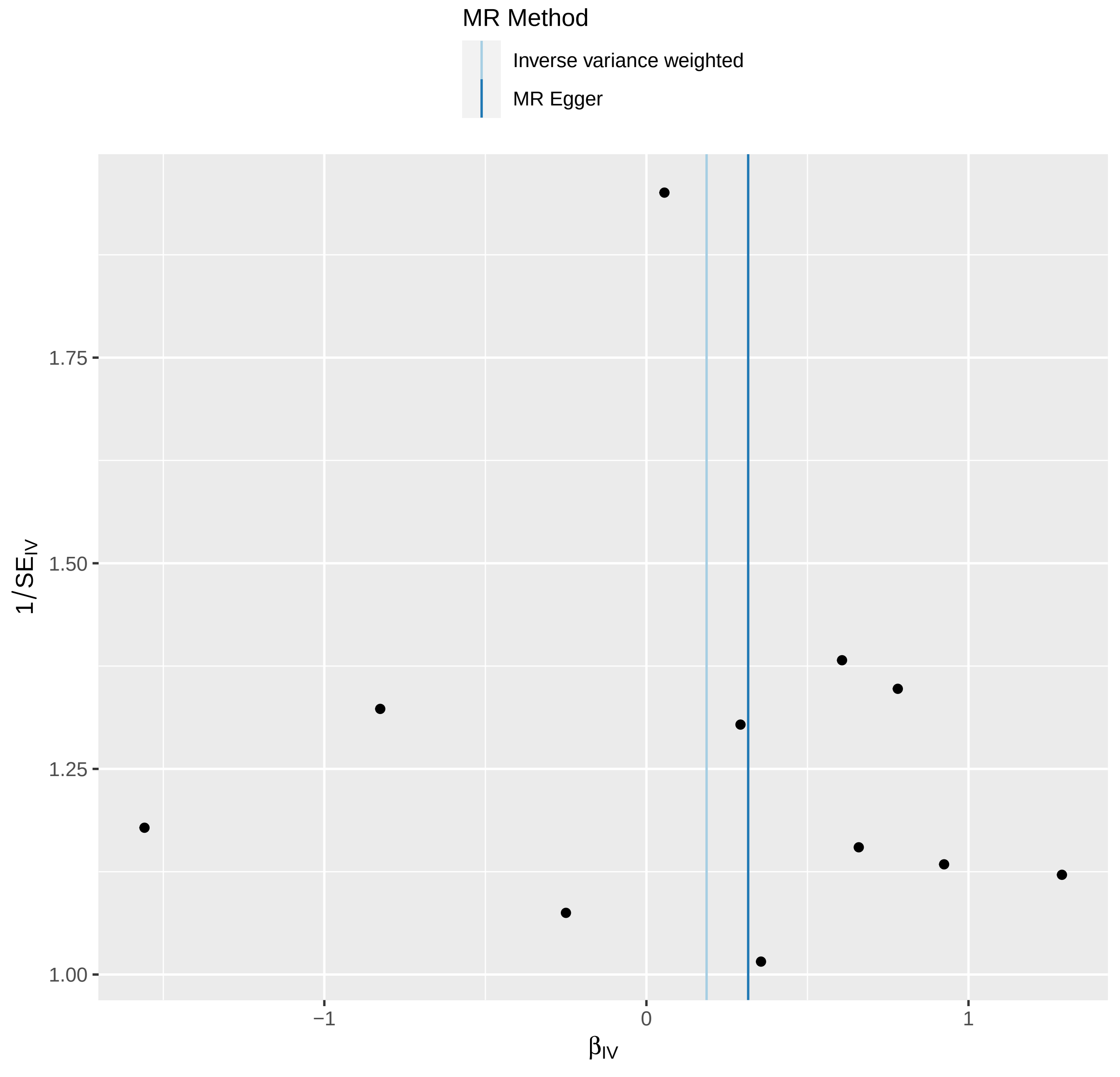

Batch 658 : Gut microbiota abundance (genus Coprococcus2 id.11302) on Extreme obesity with alveolar hypoventilation

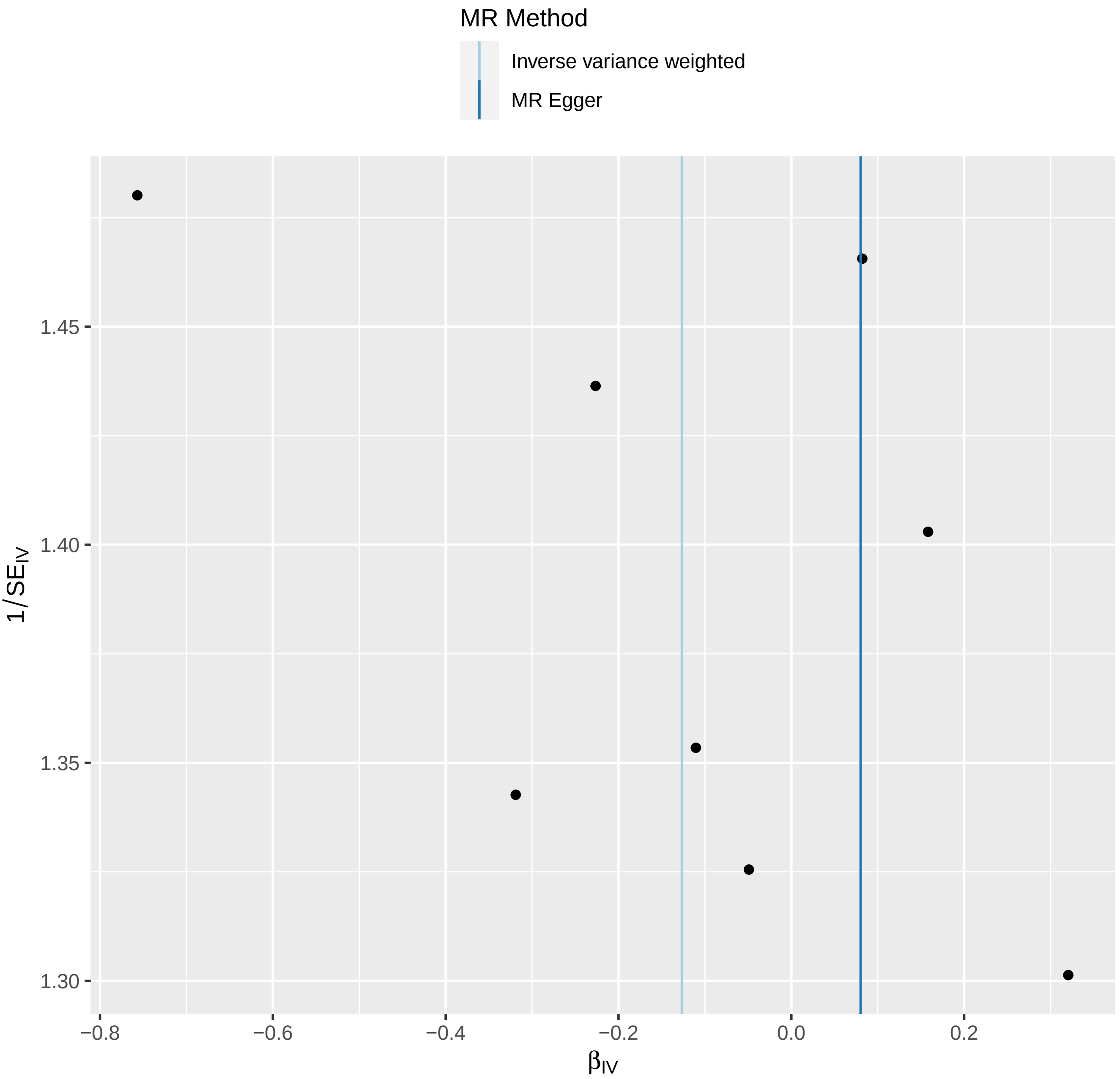

Batch 659 : Gut microbiota abundance (genus Coprococcus3 id.11303) on Extreme obesity with alveolar hypoventilation

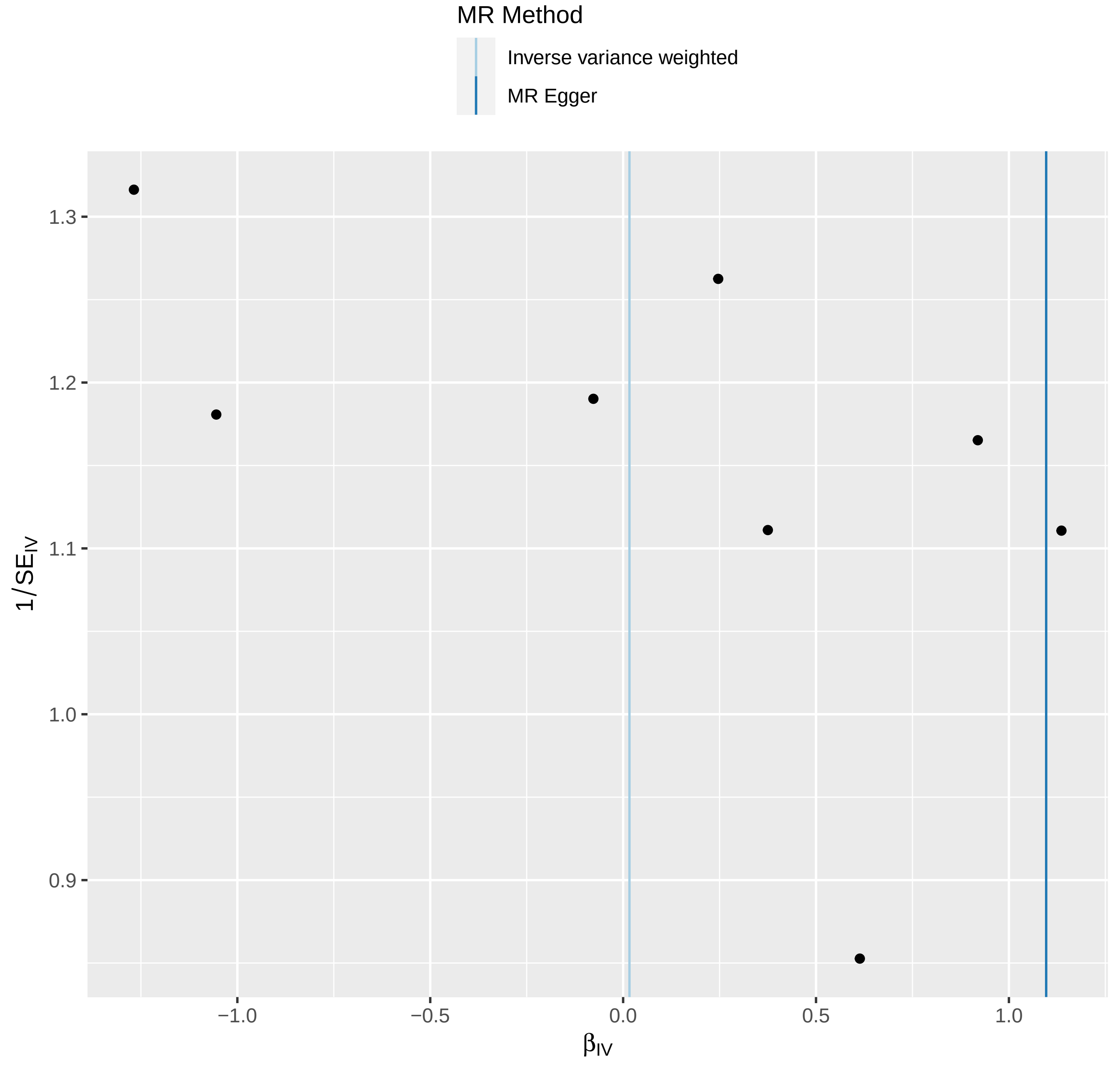

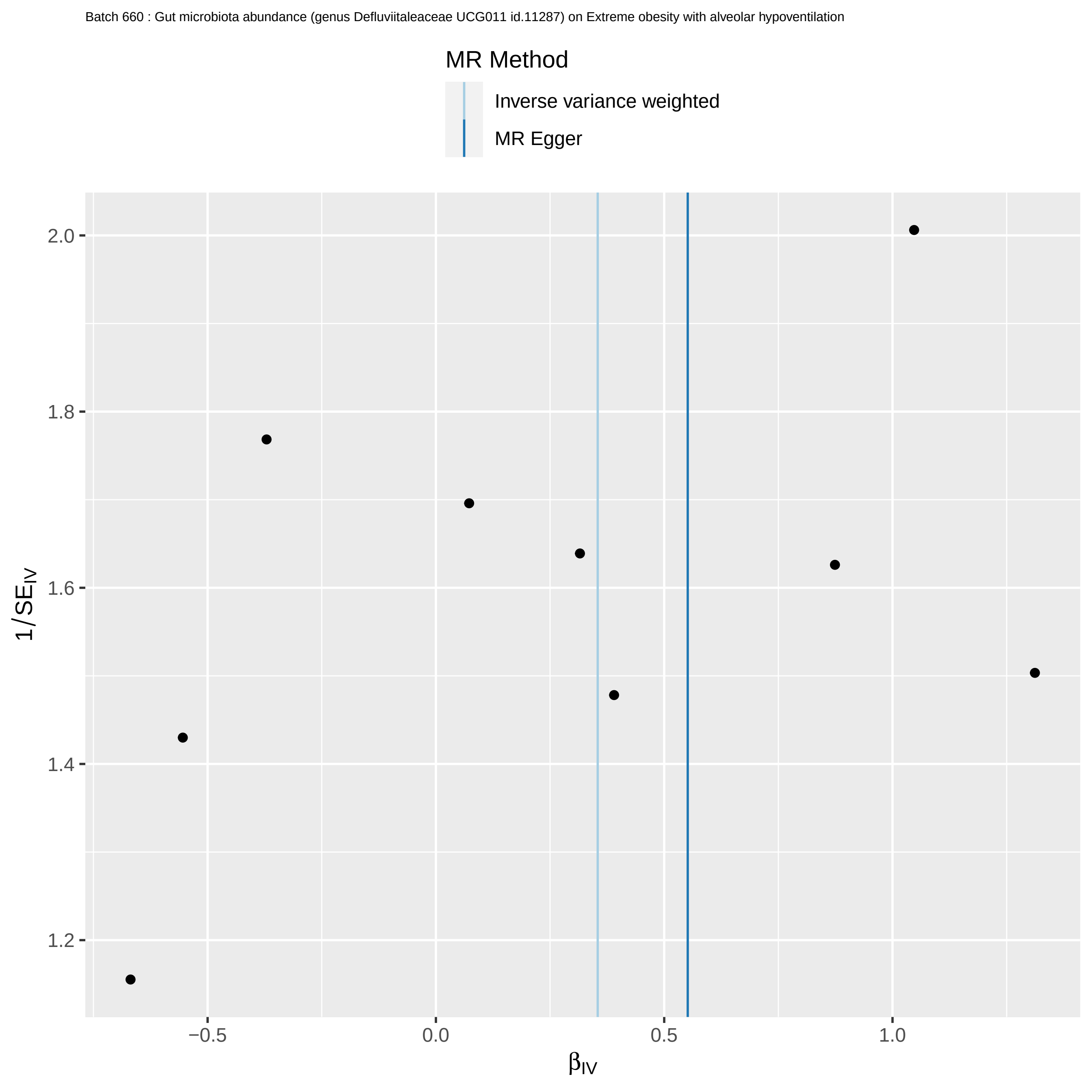
Batch 660 : Gut microbiota abundance (genus Defluviitaleaceae UCG011 id.11287) on Extreme obesity with alveolar hypoventilation
MR Method
Inverse variance weighted
MR Egger
2.0
1.8
1.6
1.4
1.2
1/SE$_{IV}$
−0.5
0.0
0.5
1.0
β$_{IV}$

Batch 661 : Gut microbiota abundance (genus Desulfovibrio id.3173) on Extreme obesity with alveolar hypoventilation

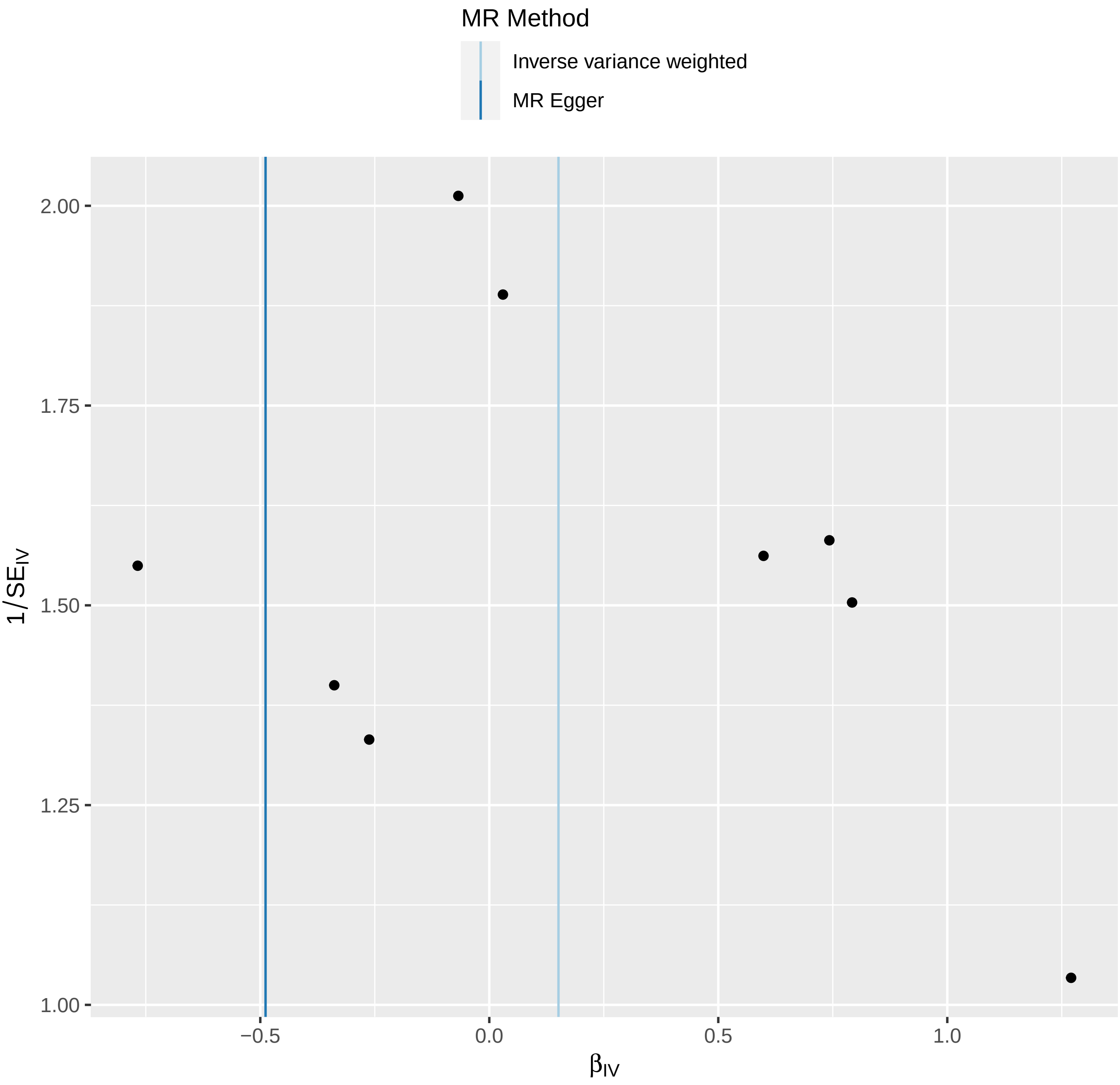

Batch 662 : Gut microbiota abundance (genus Dialister id.2183) on Extreme obesity with alveolar hypoventilation

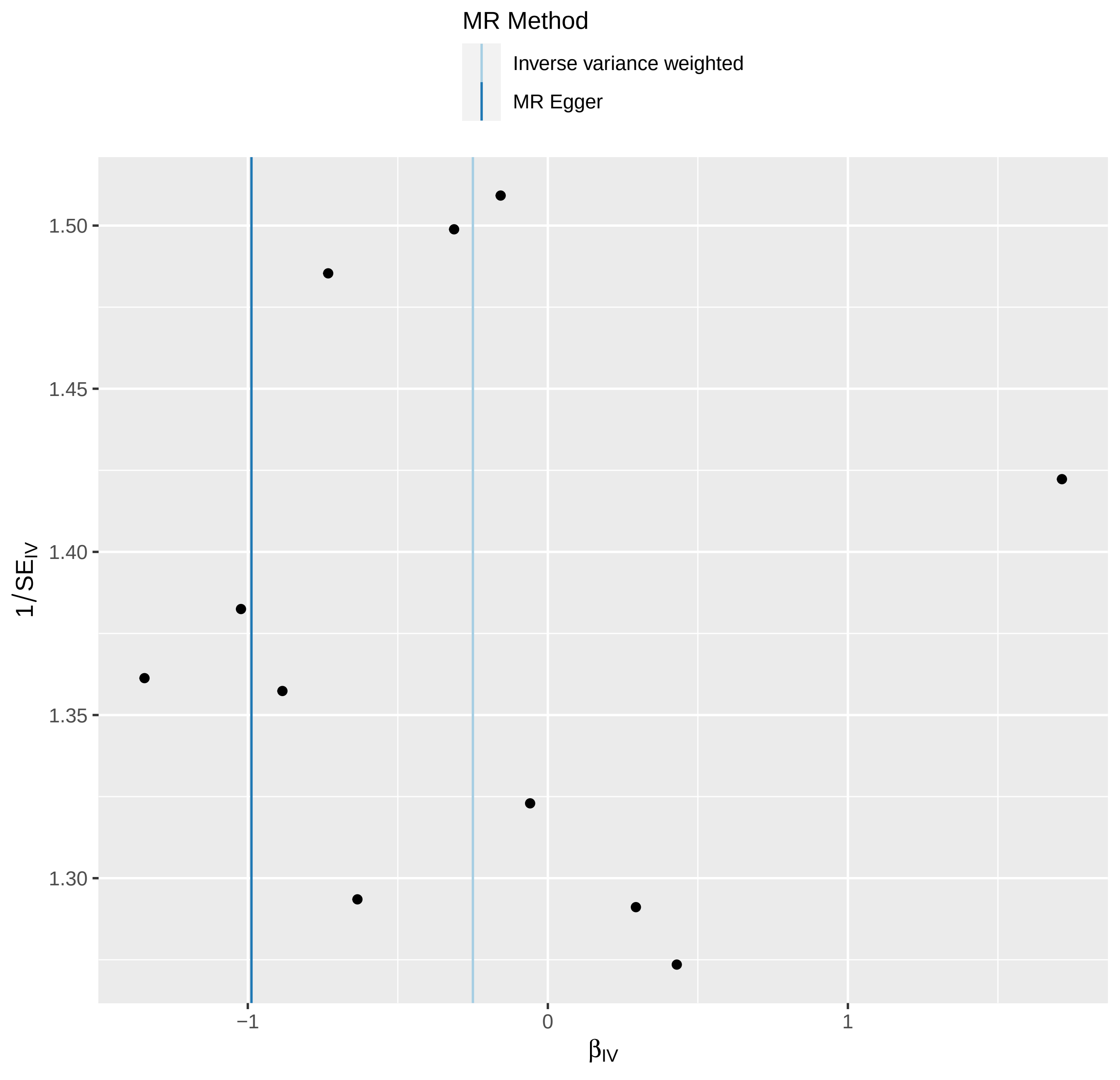

Batch 663 : Gut microbiota abundance (genus Dorea id.1997) on Extreme obesity with alveolar hypoventilation

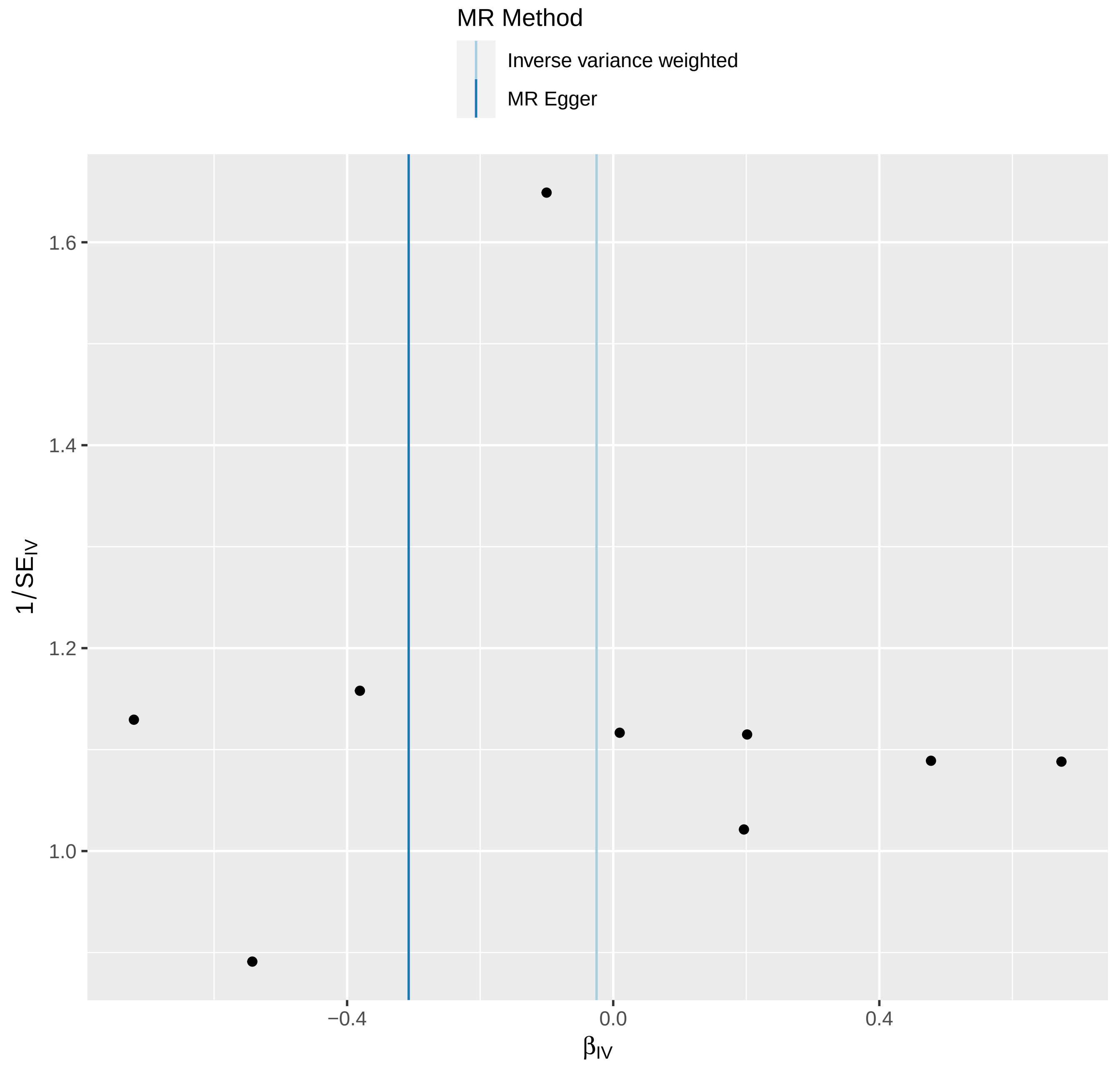

Batch 664 : Gut microbiota abundance (genus Eggerthella id.819) on Extreme obesity with alveolar hypoventilation

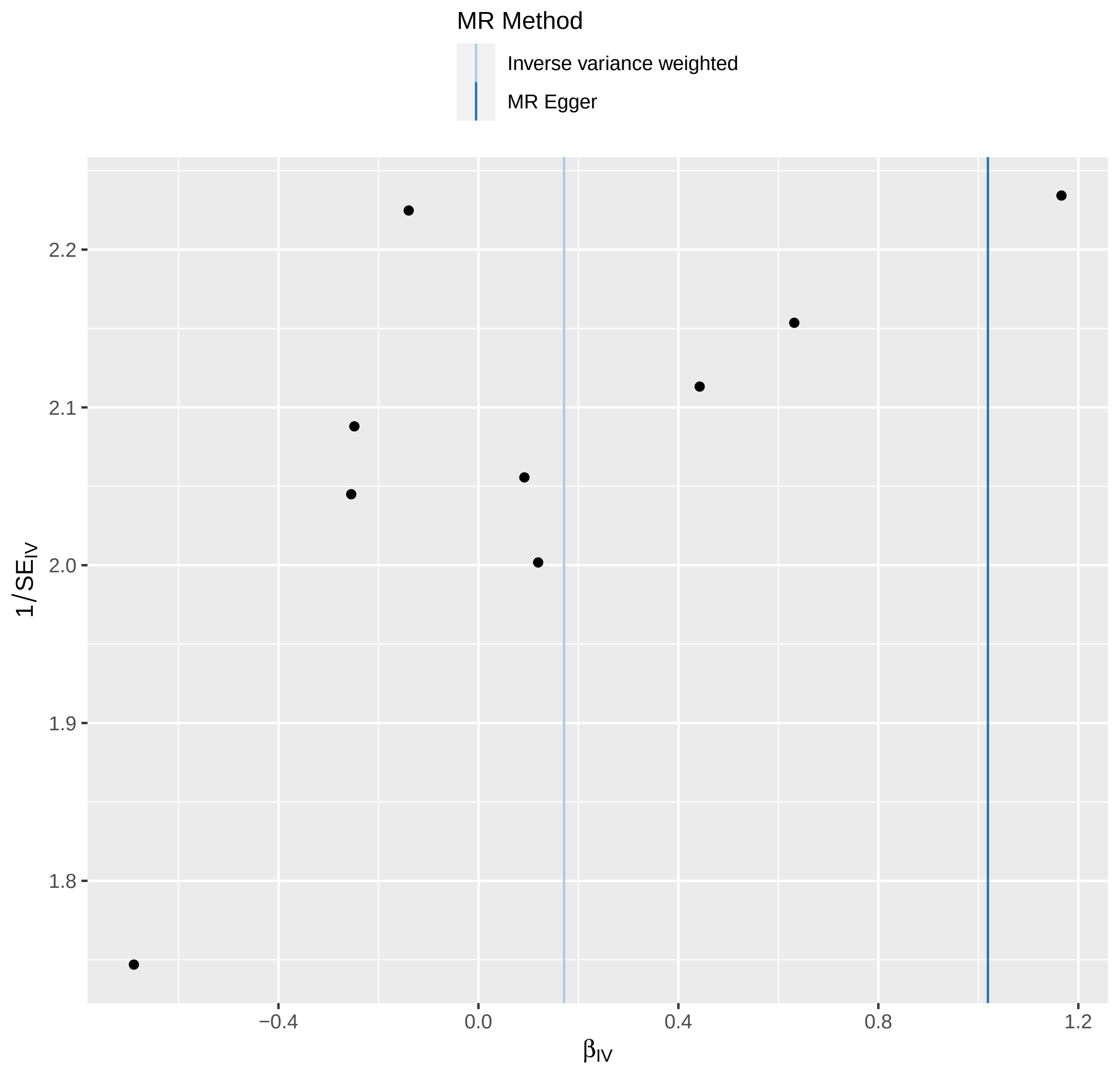

Batch 665 : Gut microbiota abundance (genus Eisenbergiella id.11304) on Extreme obesity with alveolar hypoventilation

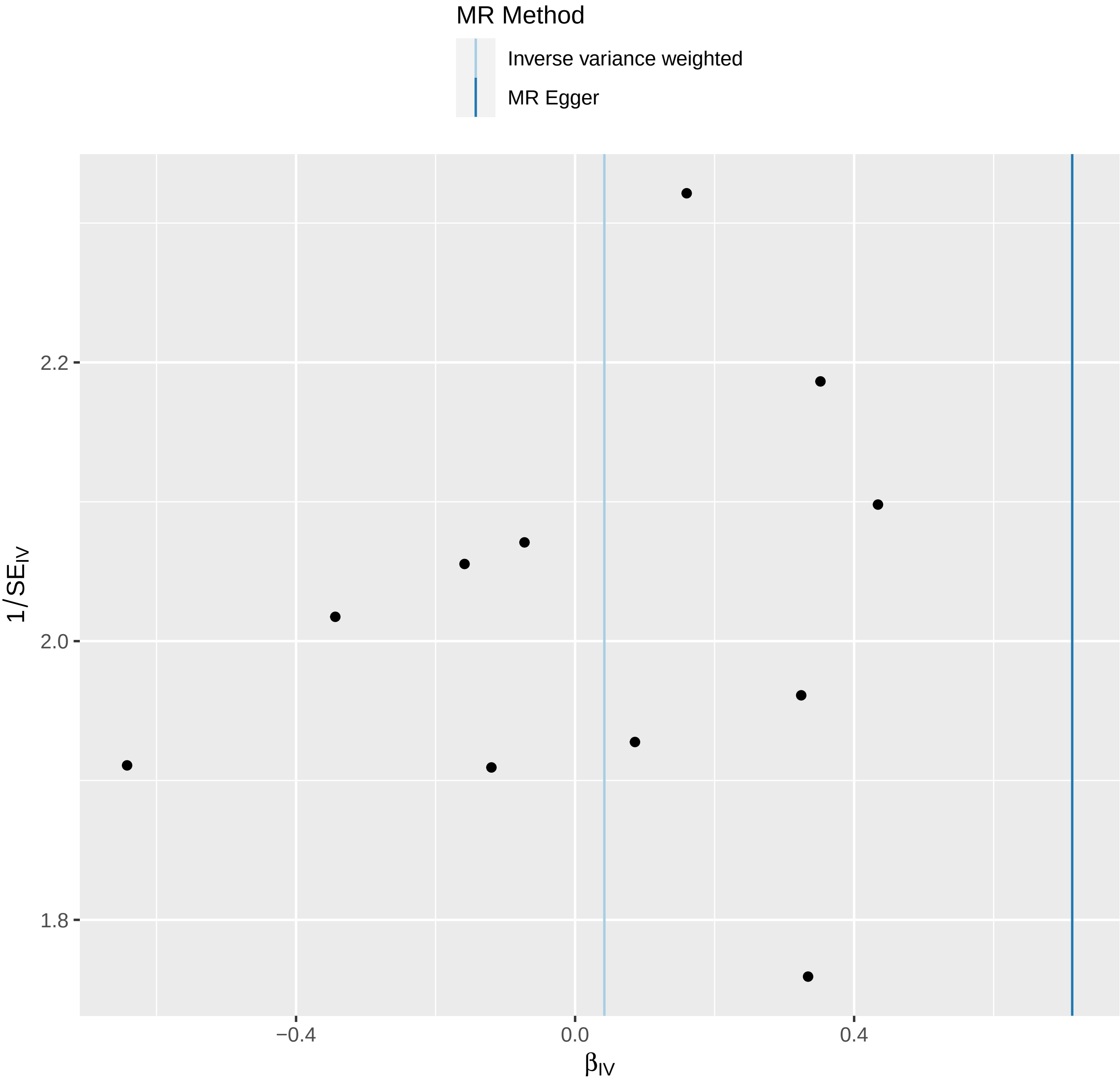

Batch 666 : Gut microbiota abundance (genus Enterorhabdus id.820) on Extreme obesity with alveolar hypoventilation

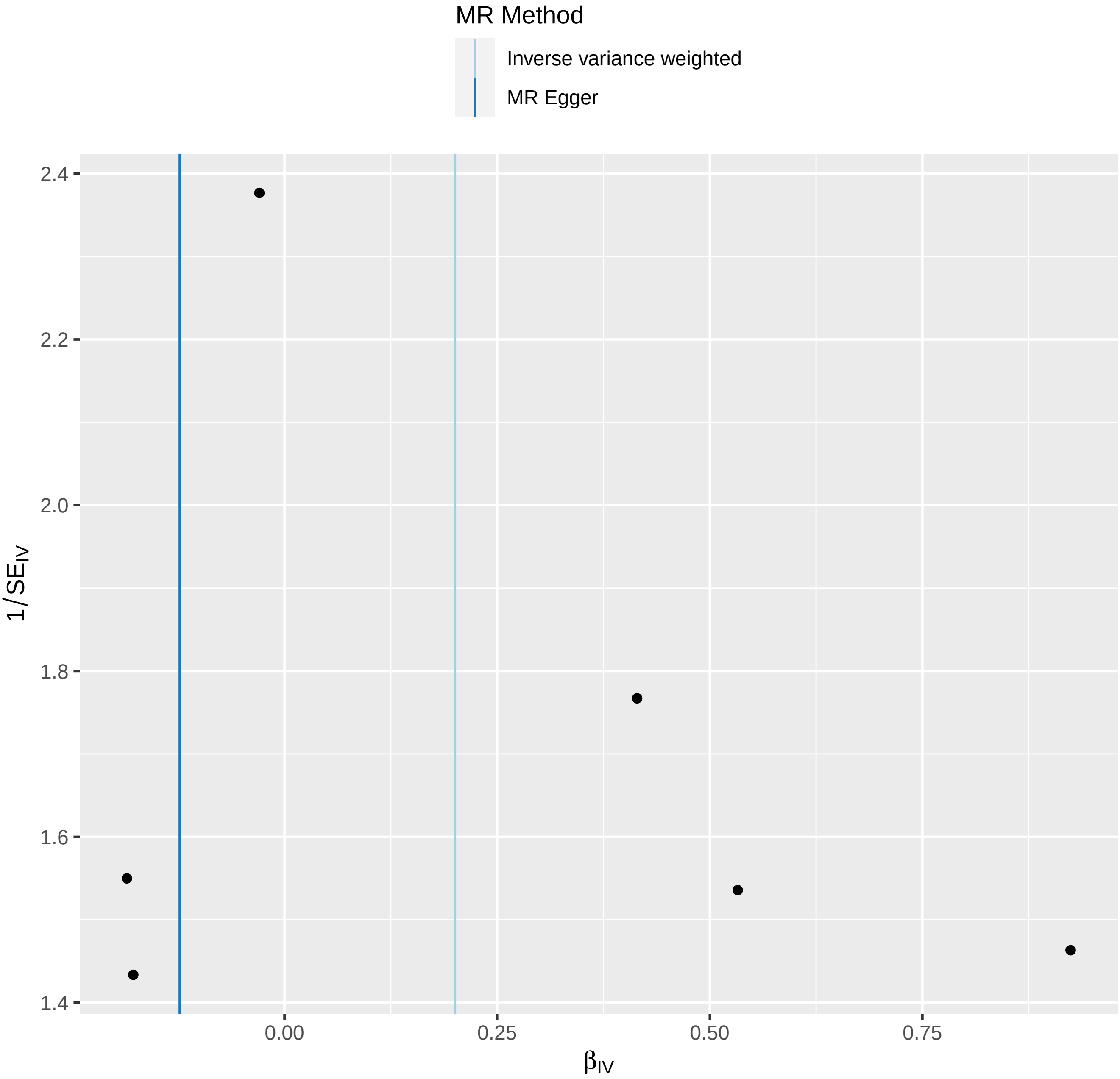

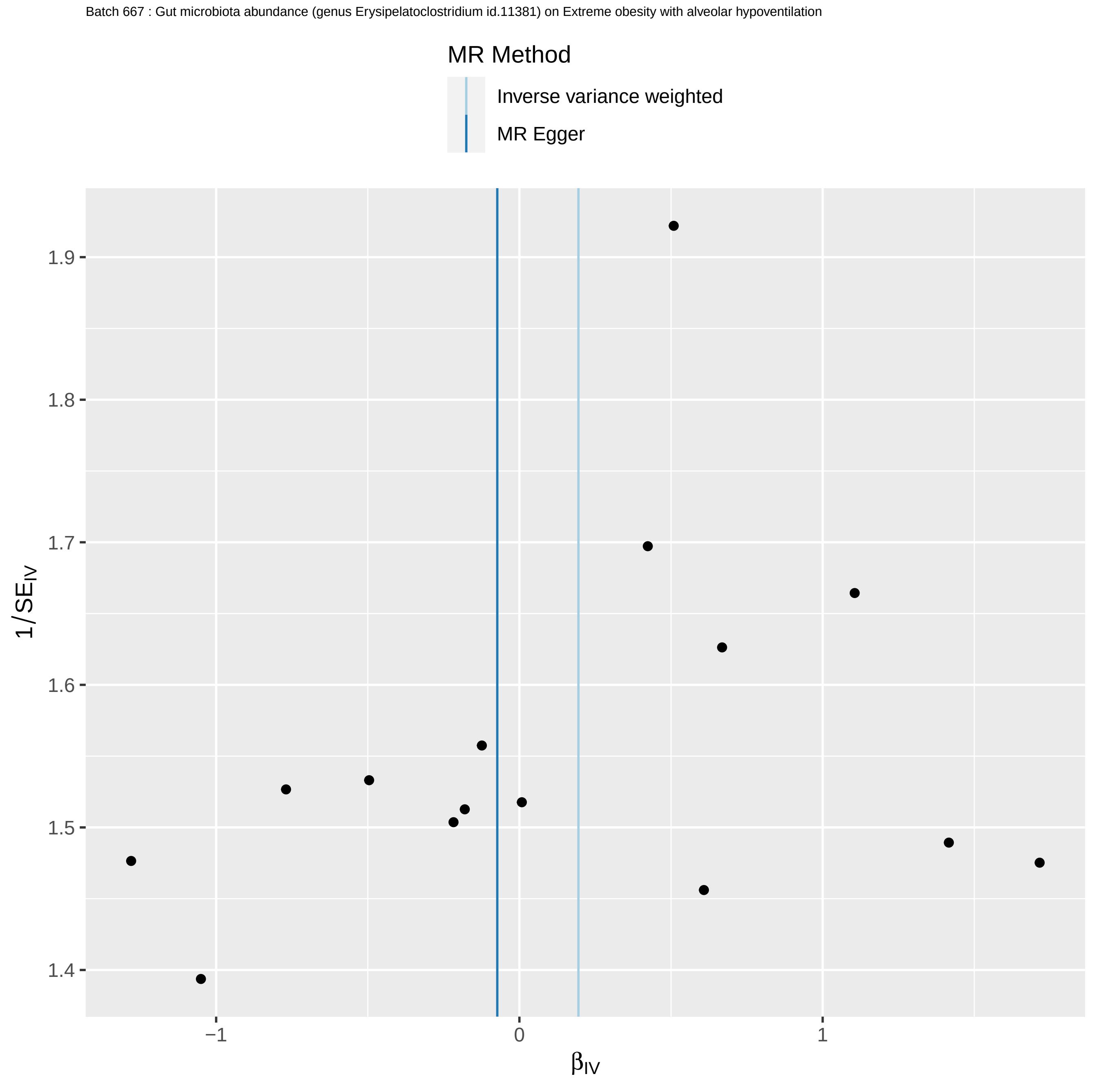
Batch 667 : Gut microbiota abundance (genus Erysipelatoclostridium id.11381) on Extreme obesity with alveolar hypoventilation
MR Method
Inverse variance weighted
MR Egger
1.9
1.8
1.7
1.6
1.5
1.4
1/SE_IV
−1
0
1
β_IV

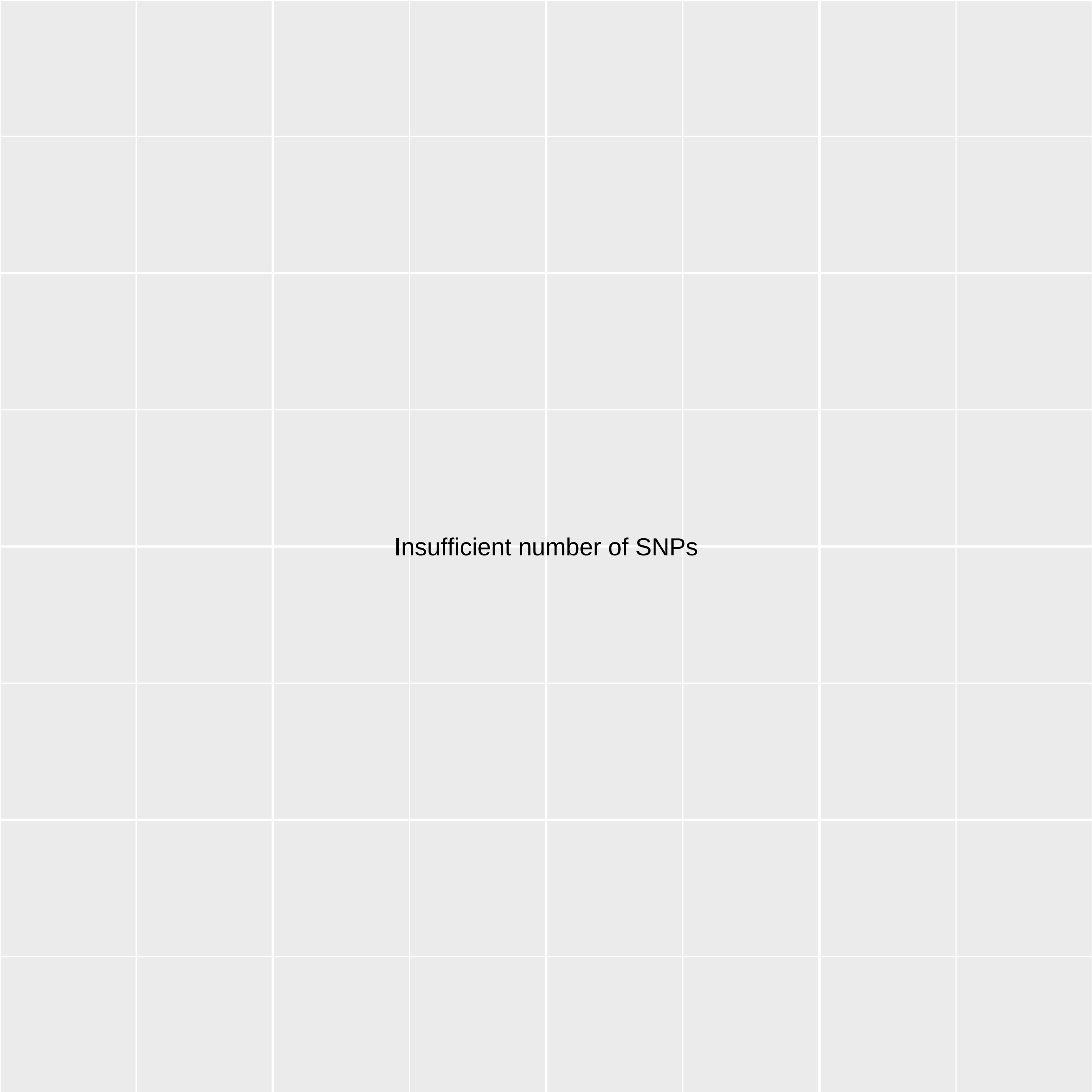
Insufficient number of SNPs

Batch 669 : Gut microbiota abundance (genus Escherichia Shigella id.3504) on Extreme obesity with alveolar hypoventilation

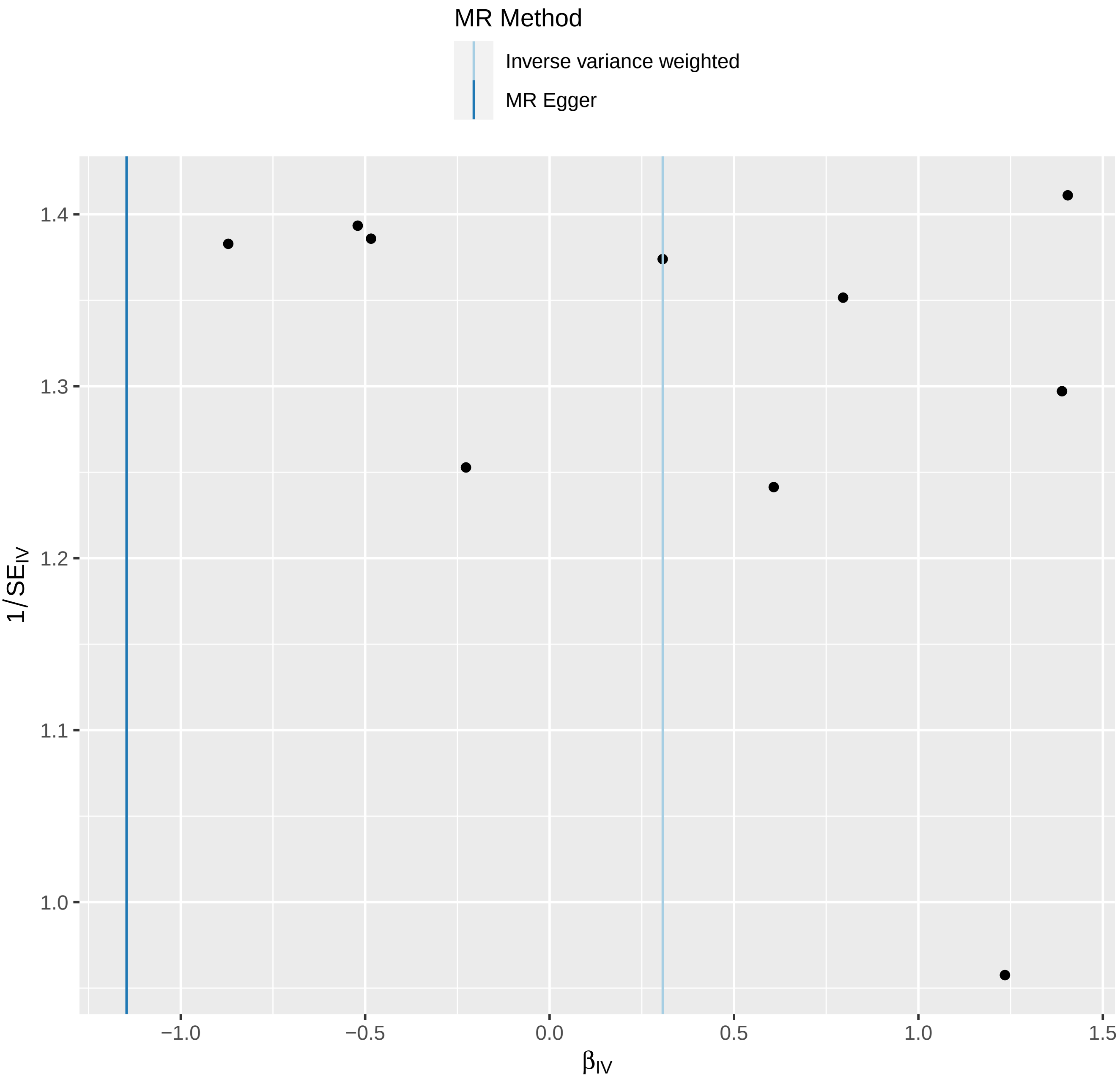

Batch 670 : Gut microbiota abundance (genus Eubacterium brachy group id.11296) on Extreme obesity with alveolar hypoventilation

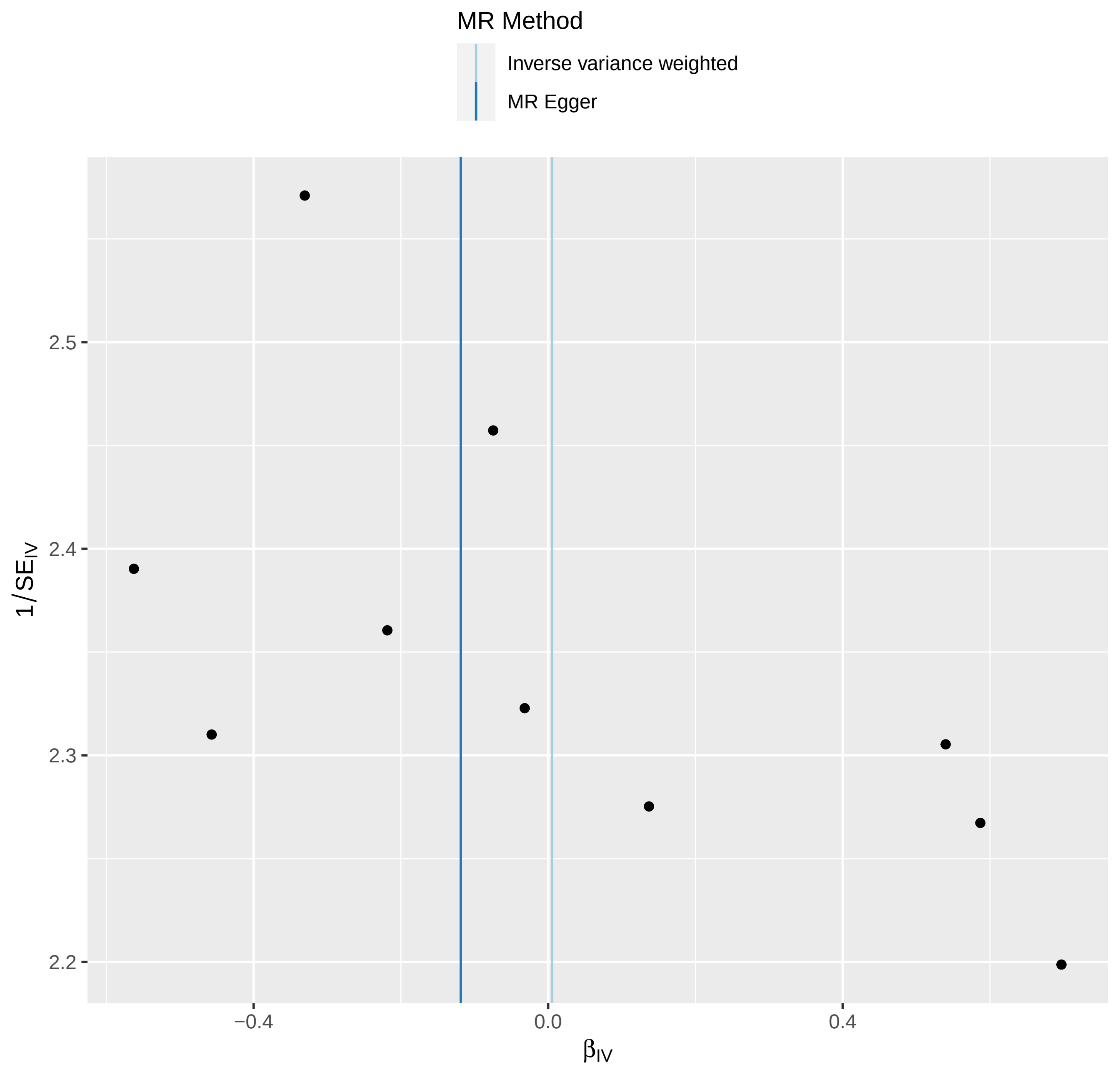

Batch 671 : Gut microbiota abundance (genus Eubacterium coprostanoligenes group id.11375) on Extreme obesity with alveolar hypoventilation

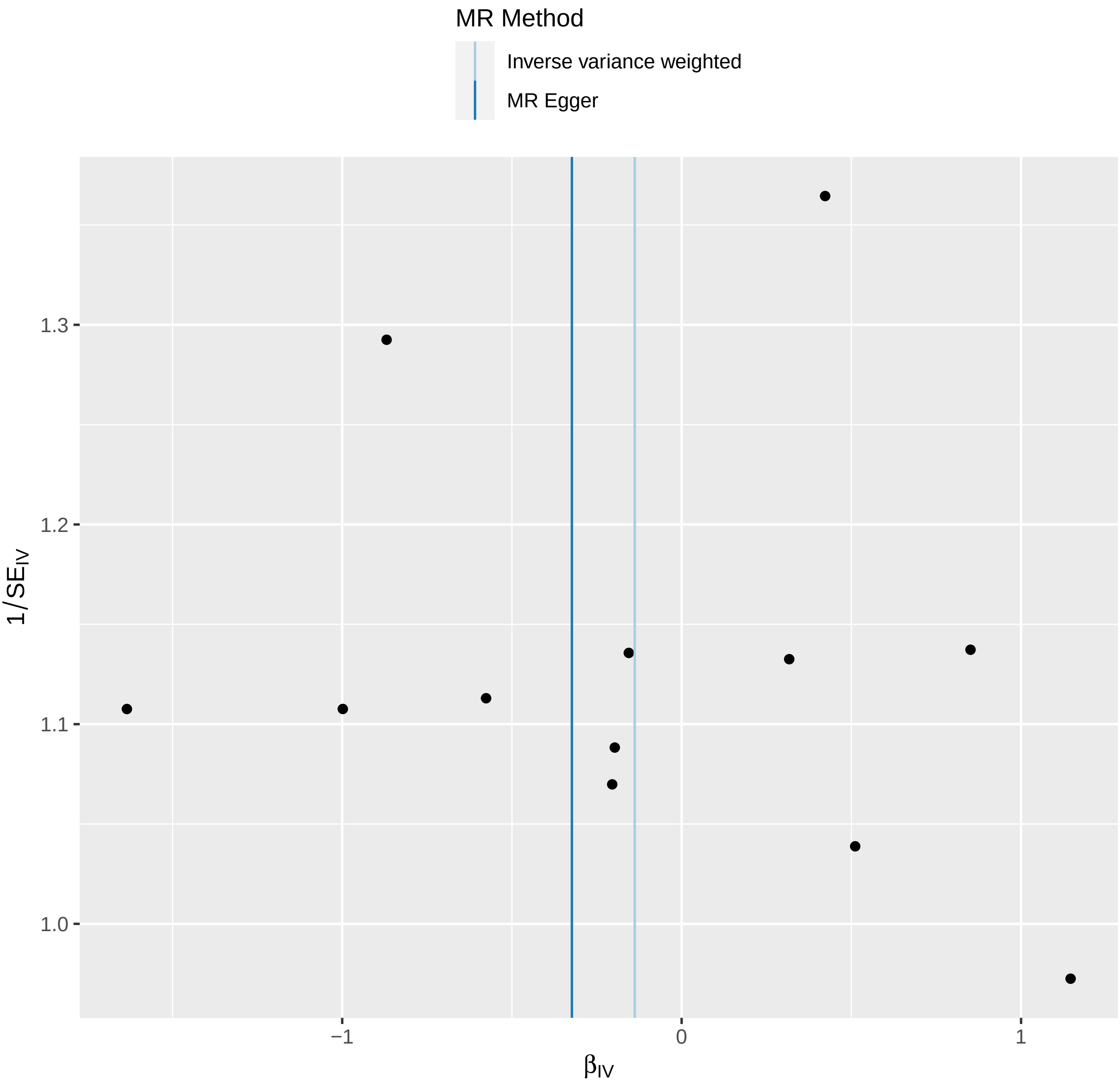

Batch 672 : Gut microbiota abundance (genus Eubacterium eligens group id.14372) on Extreme obesity with alveolar hypoventilation

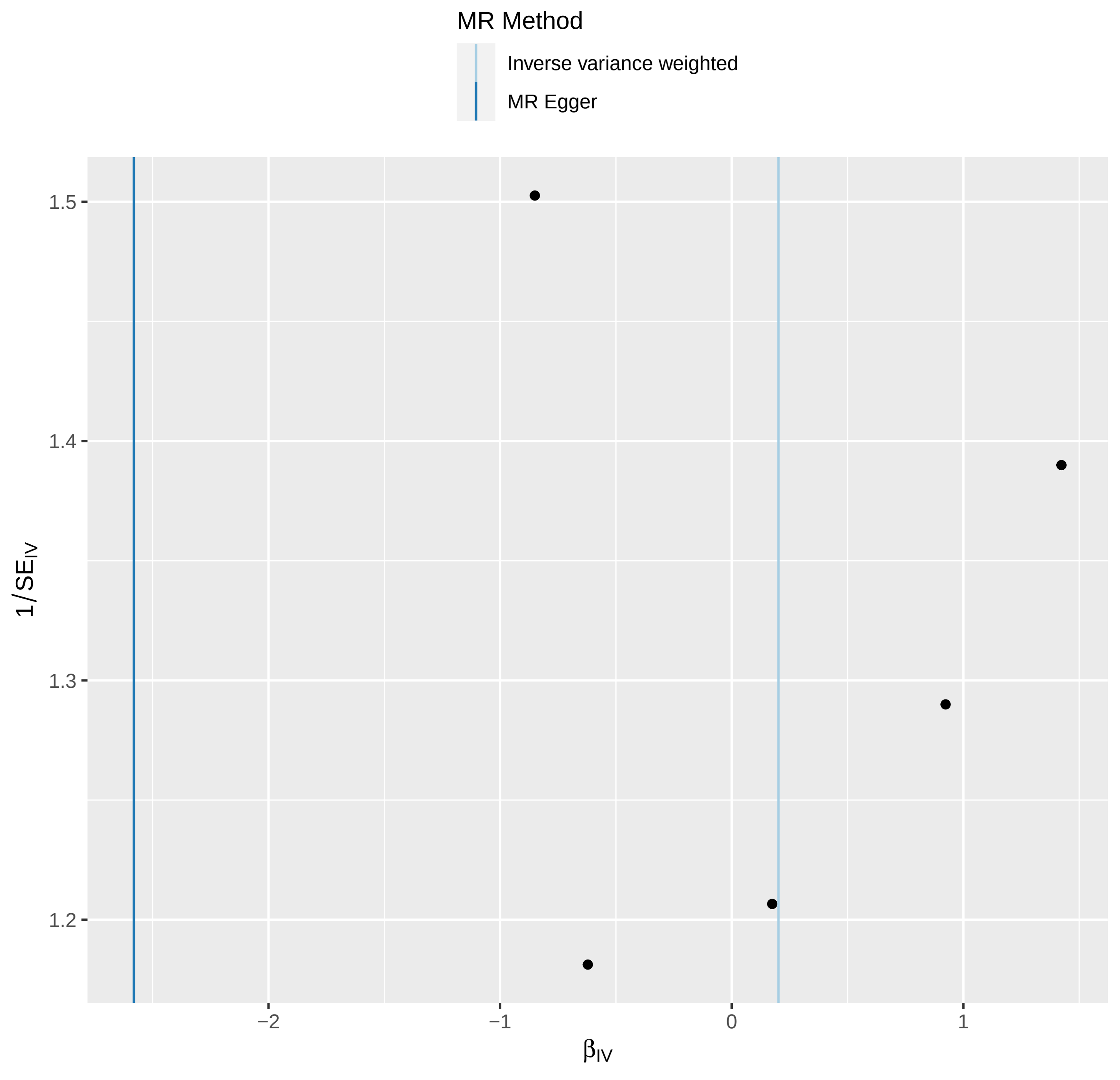

Batch 673 : Gut microbiota abundance (genus Eubacterium fissicatena group id.14373) on Extreme obesity with alveolar hypoventilation

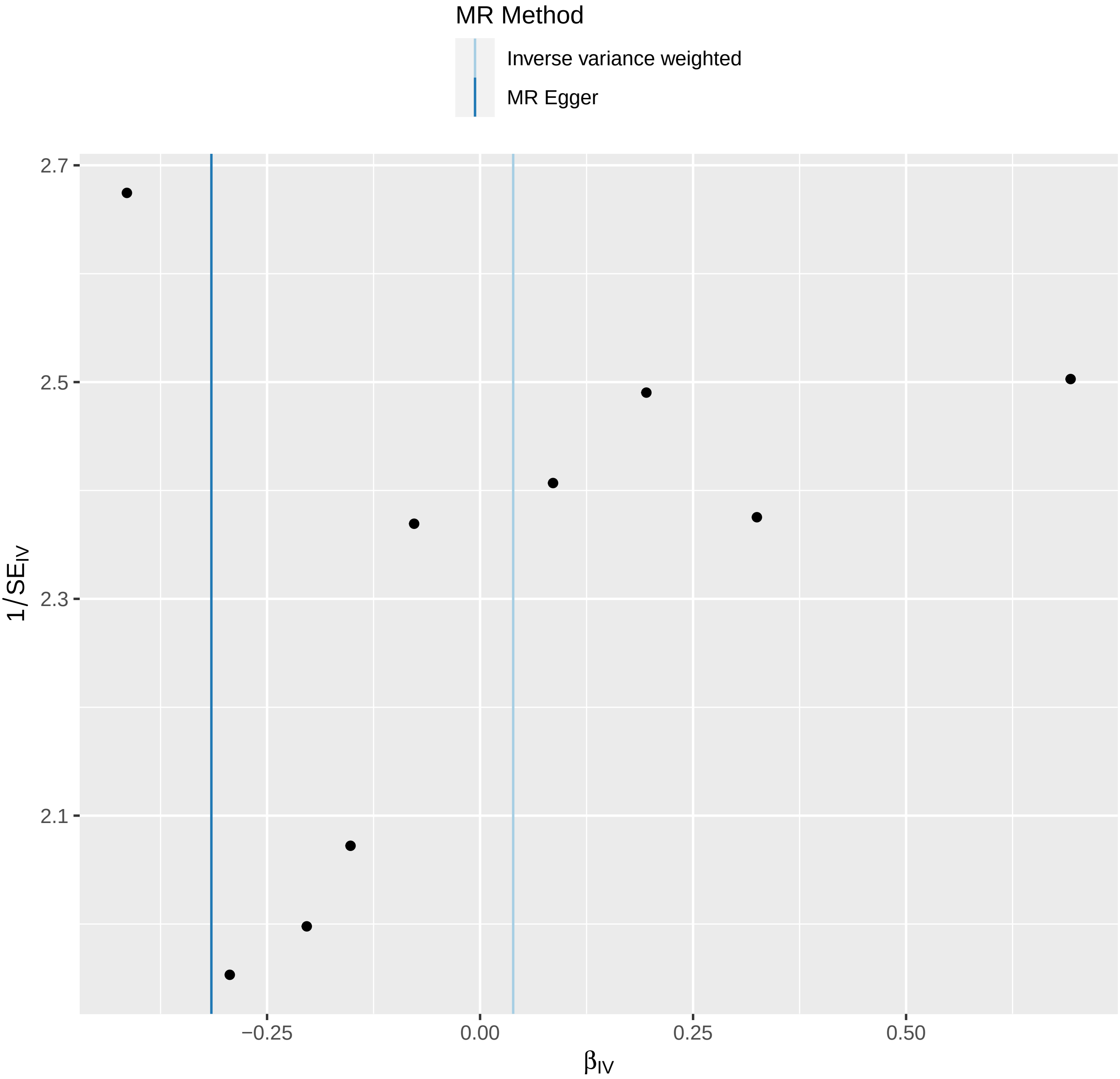

Batch 674 : Gut microbiota abundance (genus Eubacterium hallii group id.11338) on Extreme obesity with alveolar hypoventilation

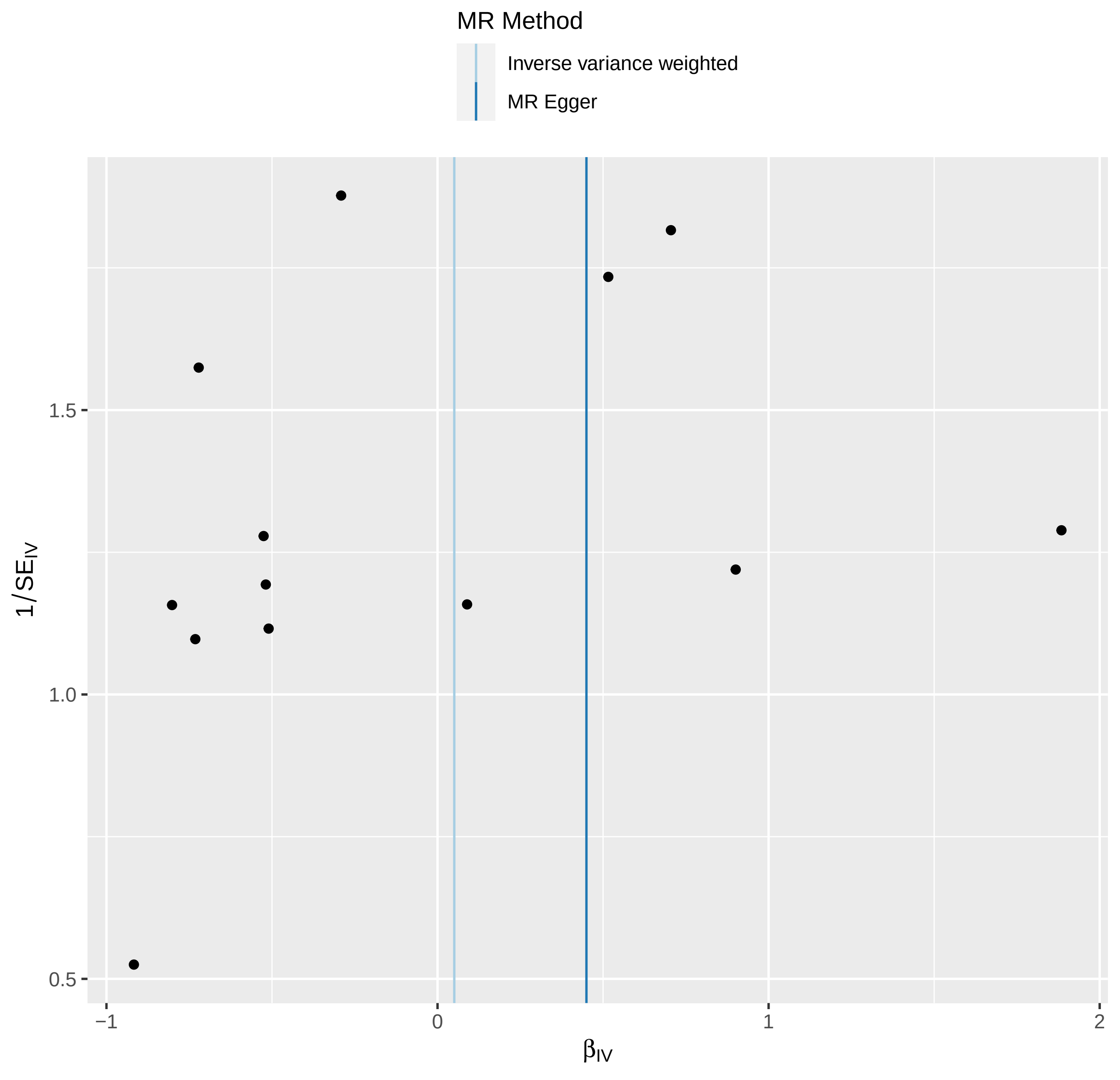

Batch 675 : Gut microbiota abundance (genus Eubacterium nodatum group id.11297) on Extreme obesity with alveolar hypoventilation

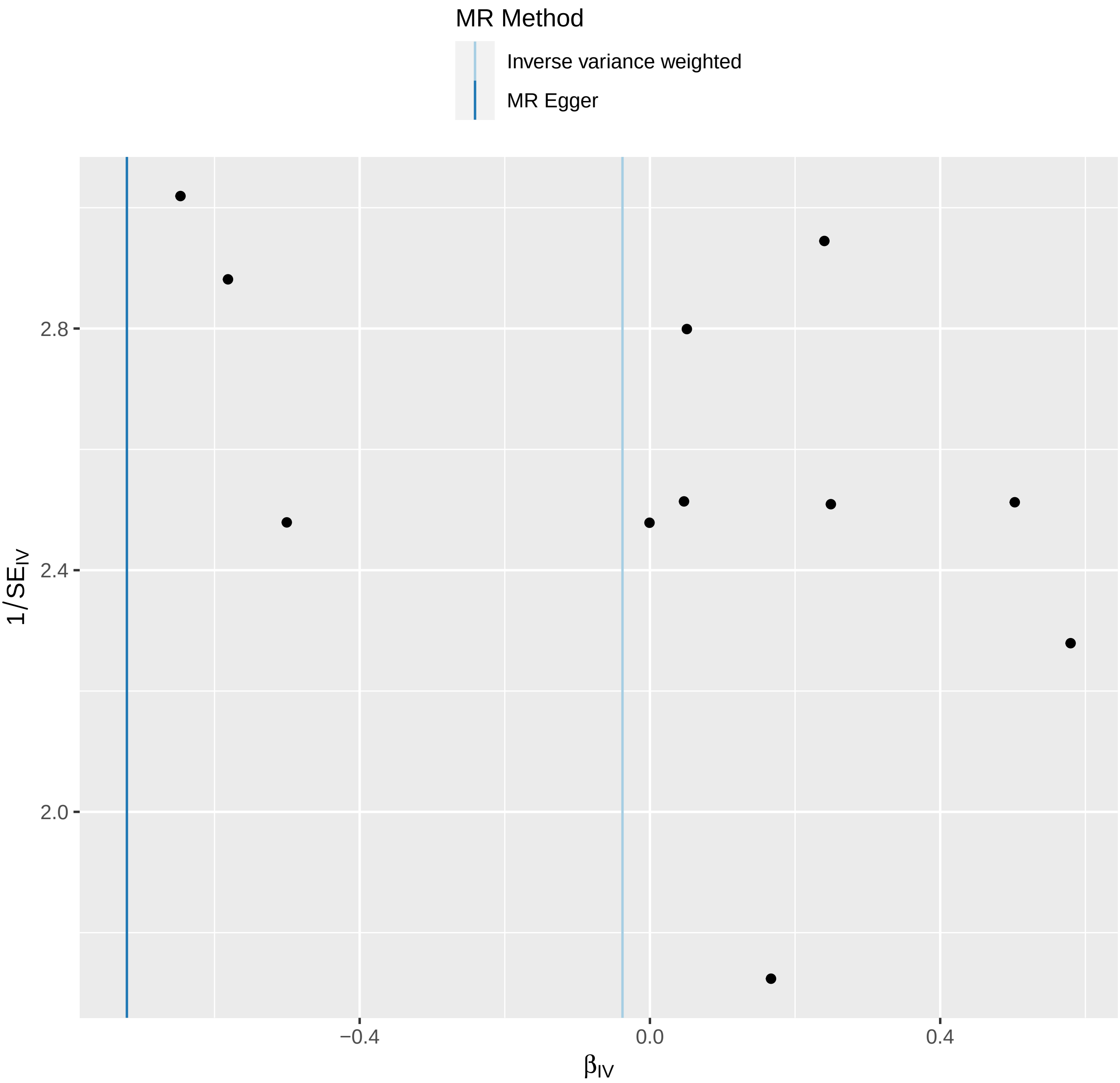

Batch 676 : Gut microbiota abundance (genus Eubacterium oxidoreducens group id.11339) on Extreme obesity with alveolar hypoventilation

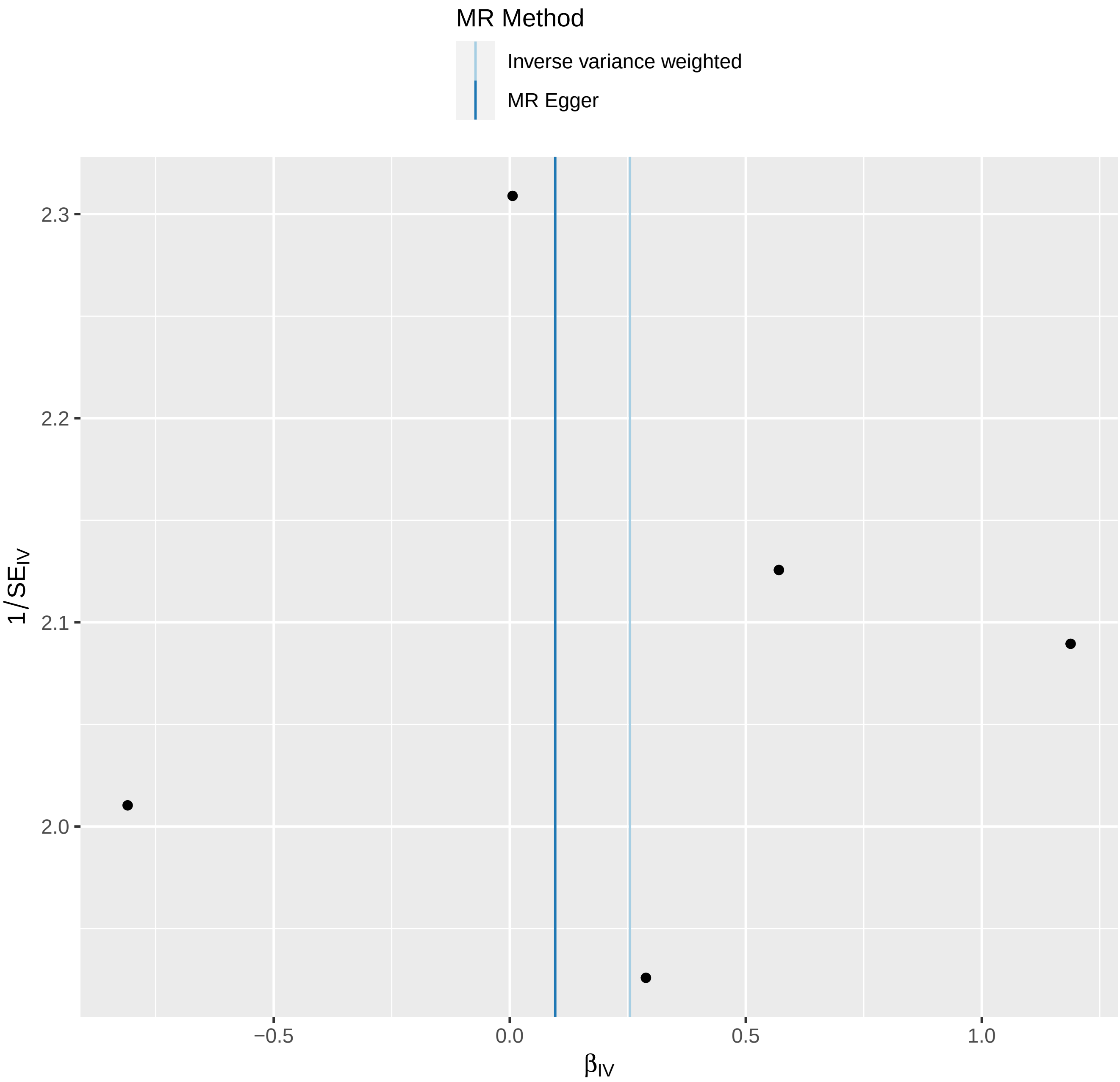

Batch 677 : Gut microbiota abundance (genus Eubacterium rectale group id.14374) on Extreme obesity with alveolar hypoventilation

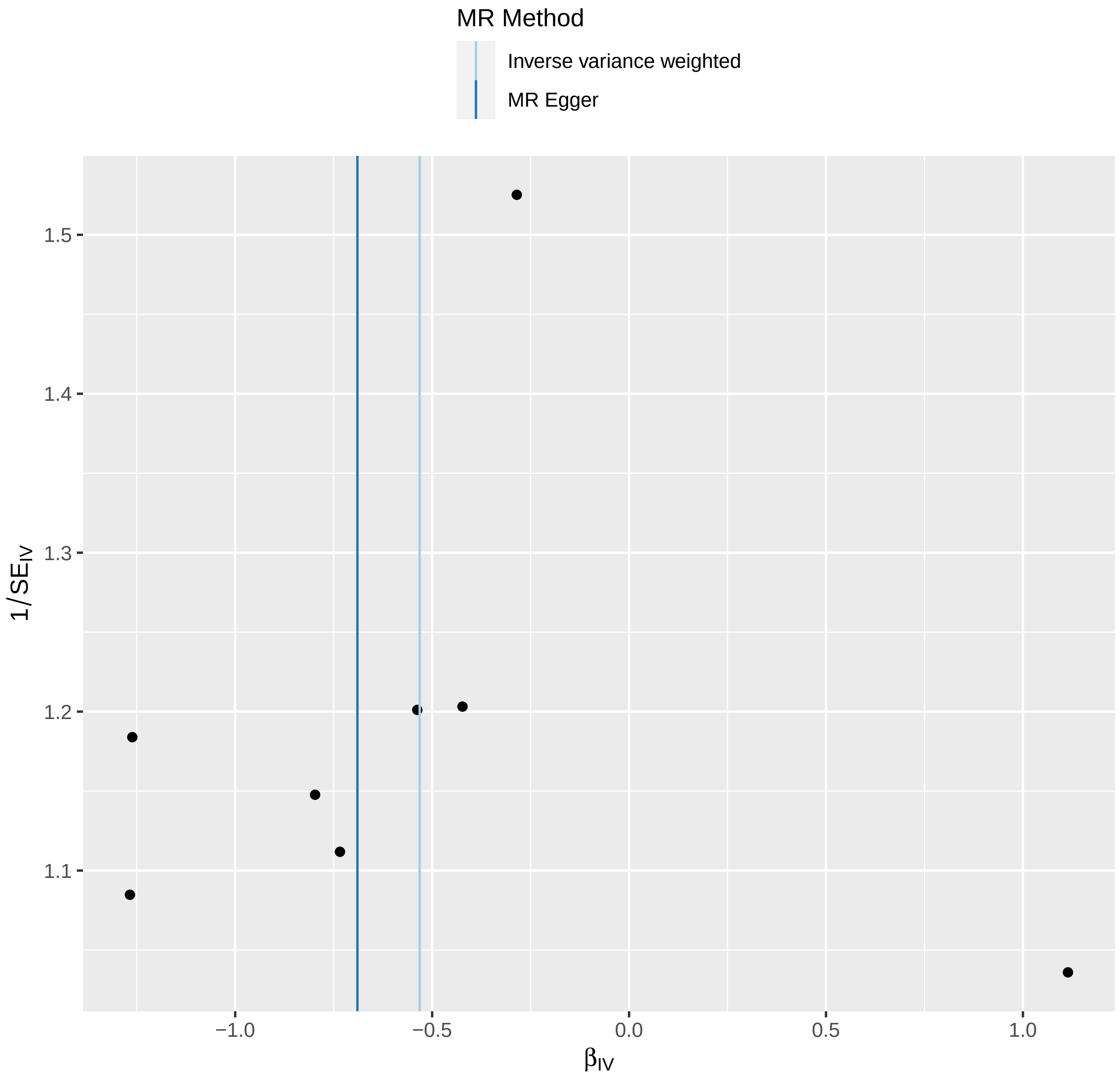

Batch 678 : Gut microbiota abundance (genus Eubacterium ruminantium group id.11340) on Extreme obesity with alveolar hypoventilation

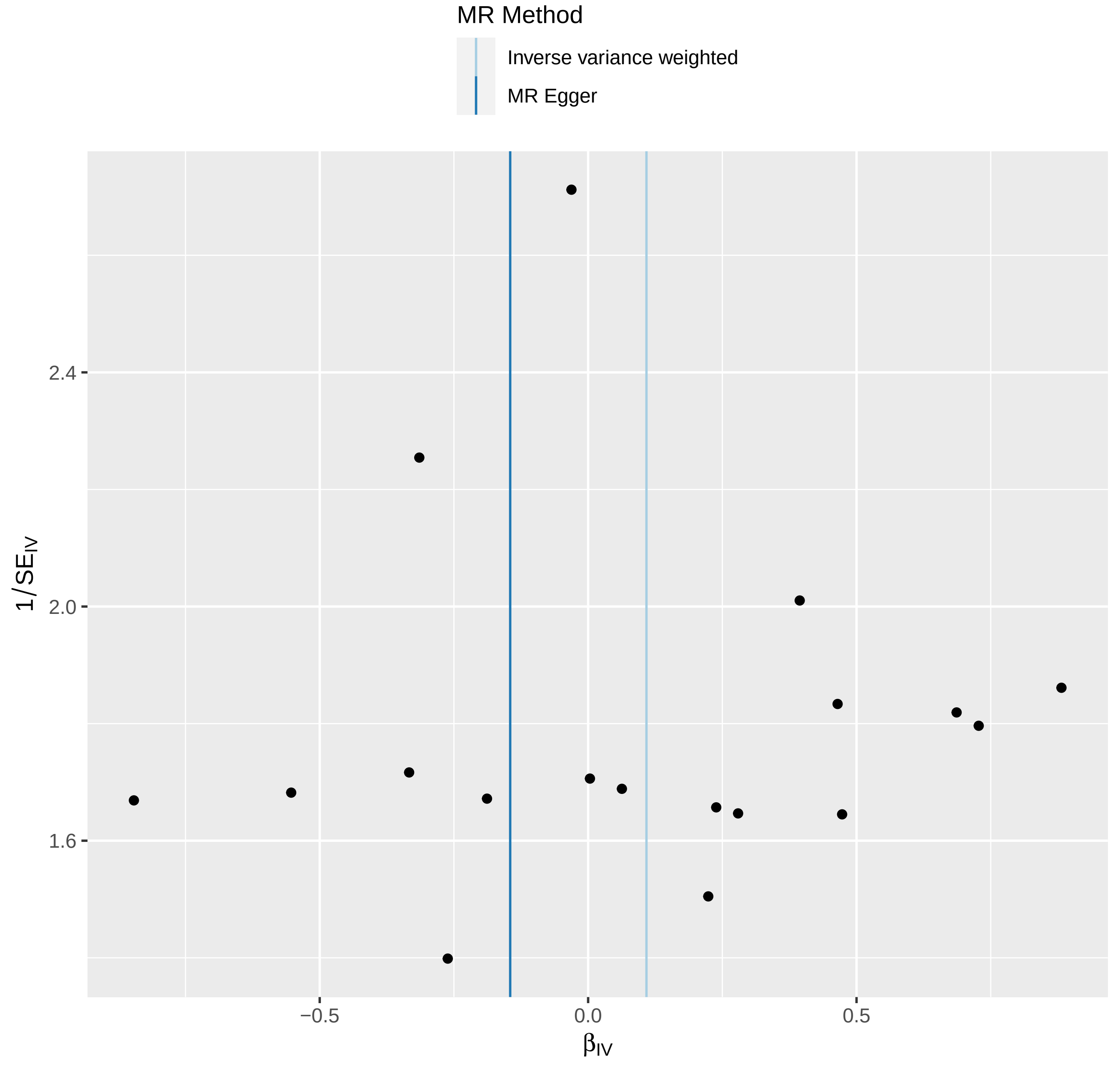

Batch 679 : Gut microbiota abundance (genus Eubacterium ventriosum group id.11341) on Extreme obesity with alveolar hypoventilation

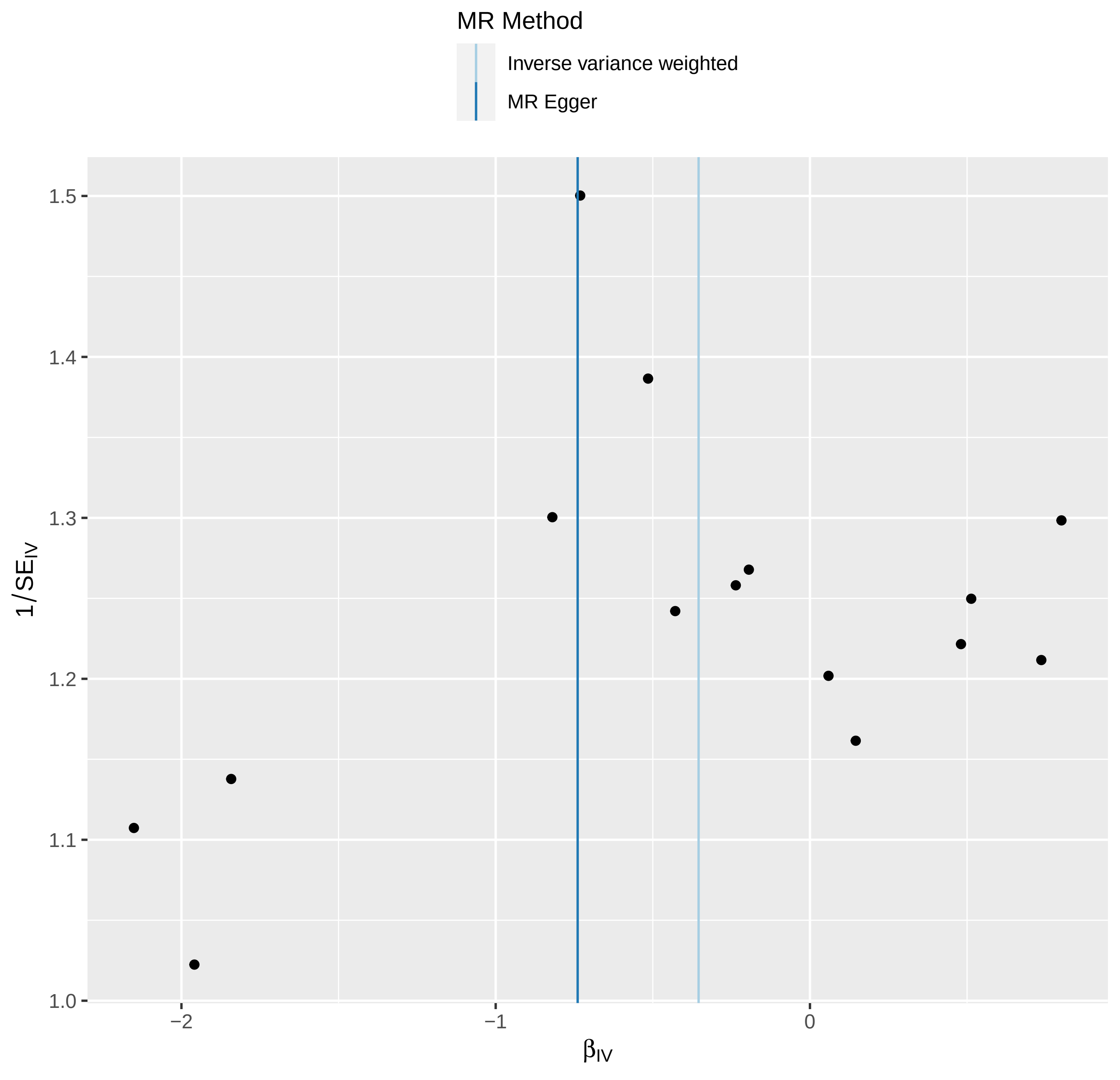

Batch 680 : Gut microbiota abundance (genus Eubacterium xylanophilum group id.14375) on Extreme obesity with alveolar hypoventilation

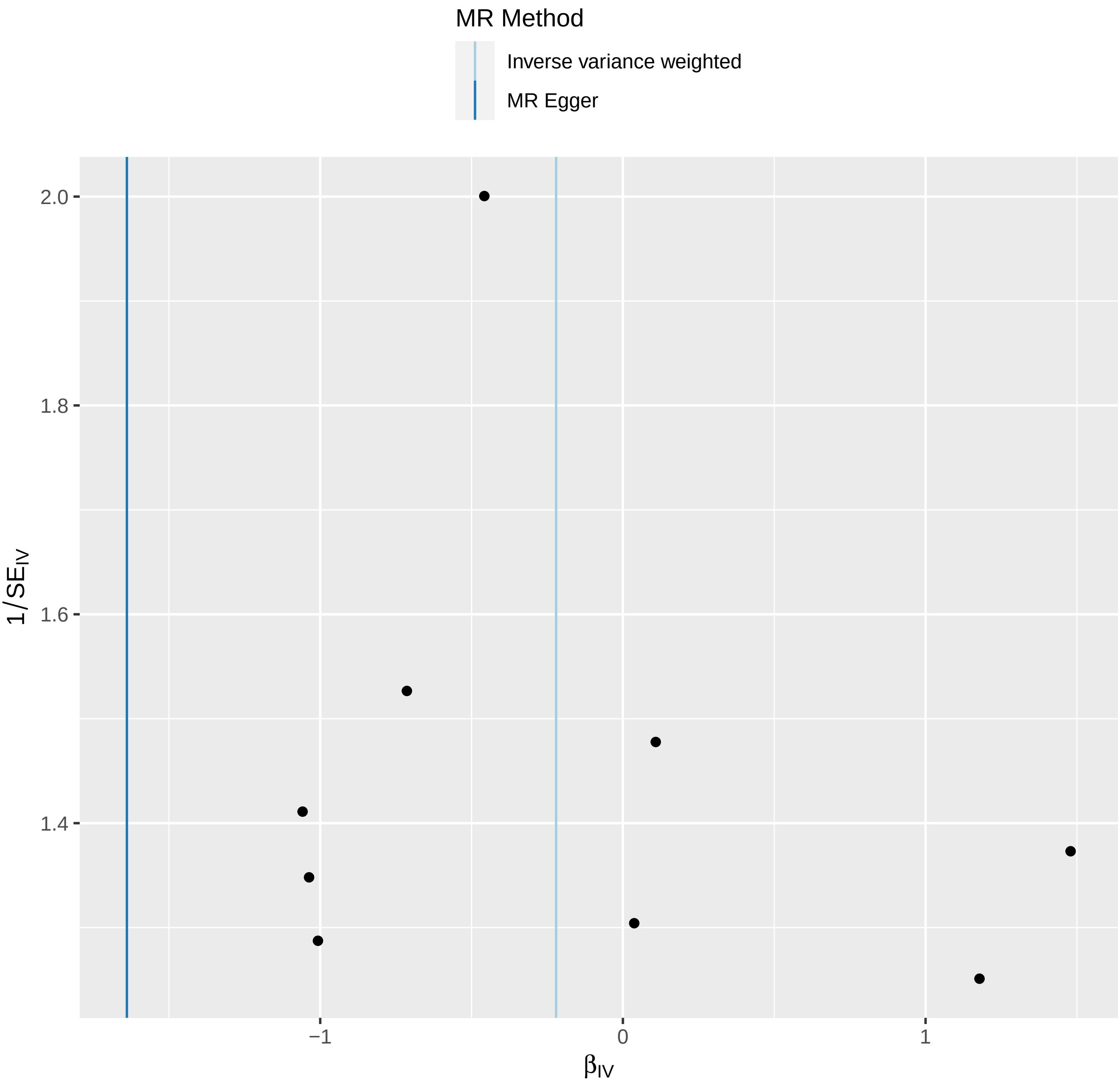

Batch 681 : Gut microbiota abundance (genus Faecalibacterium id.2057) on Extreme obesity with alveolar hypoventilation

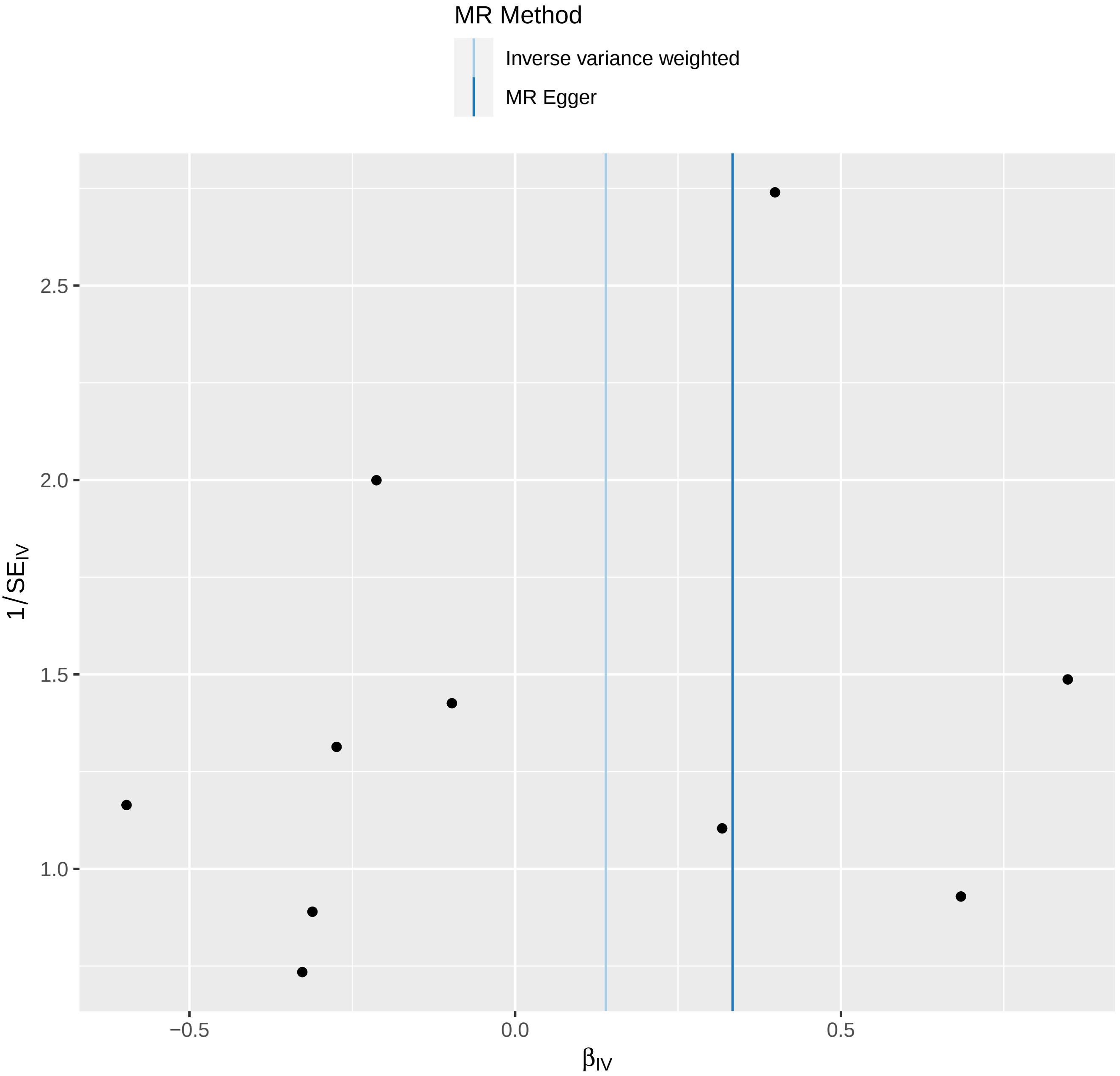

Batch 682 : Gut microbiota abundance (genus Family XIII AD3011 group id.11293) on Extreme obesity with alveolar hypoventilation

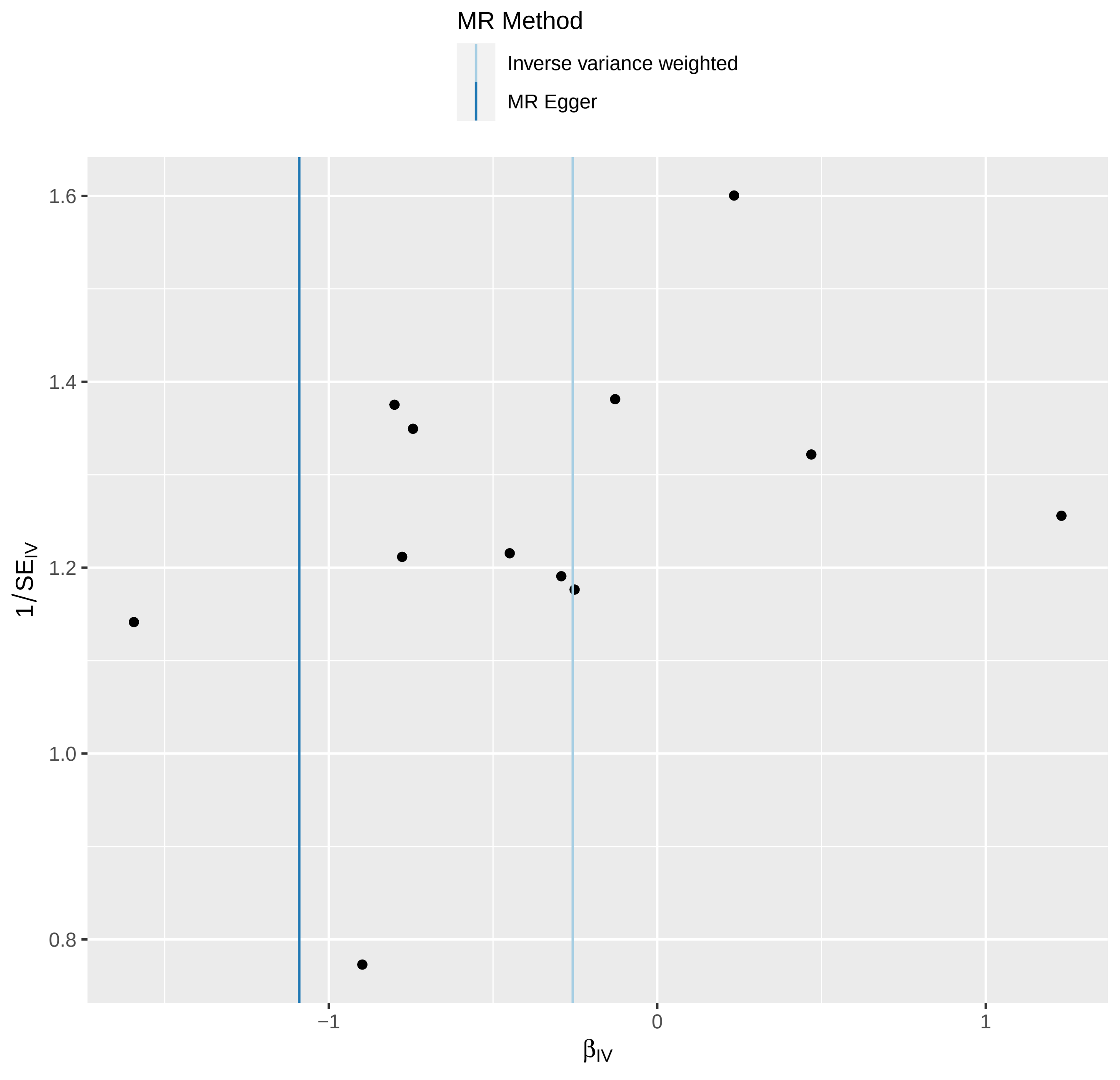

Batch 683 : Gut microbiota abundance (genus Family XIII UCG001 id.11294) on Extreme obesity with alveolar hypoventilation

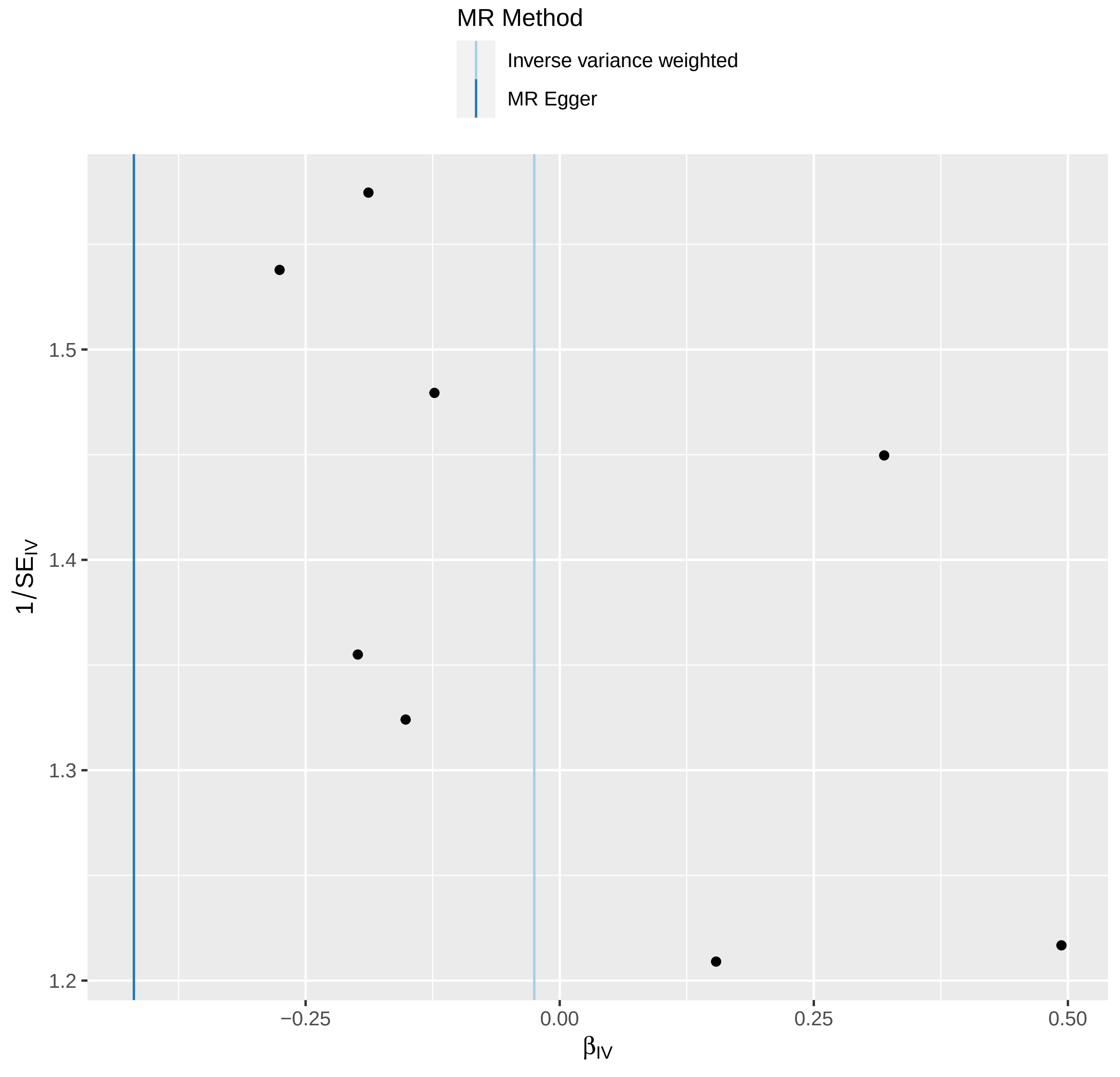

Batch 684 : Gut microbiota abundance (genus Flavonifractor id.2059) on Extreme obesity with alveolar hypoventilation

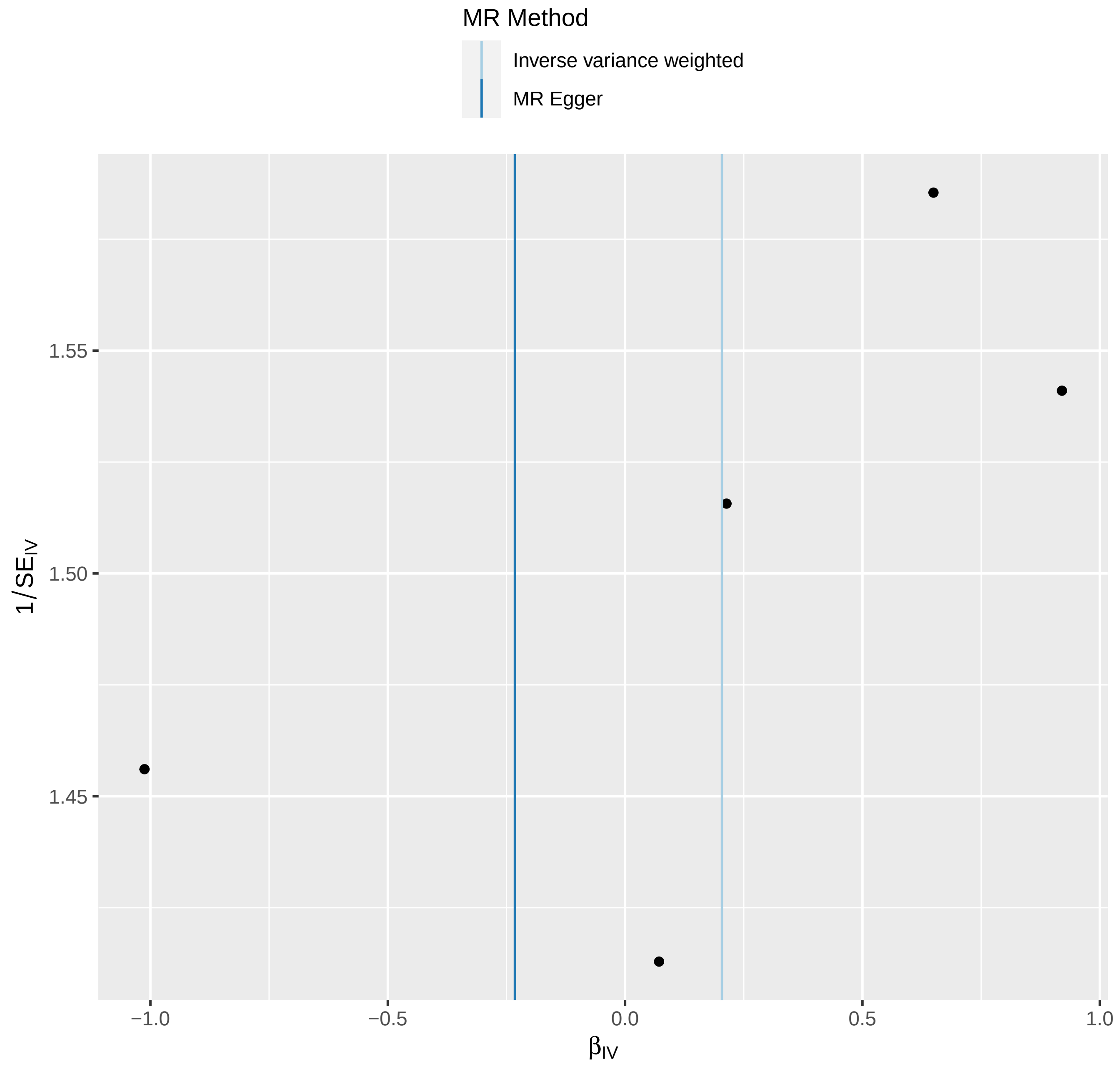

Batch 685 : Gut microbiota abundance (genus Fusicatenibacter id.11305) on Extreme obesity with alveolar hypoventilation

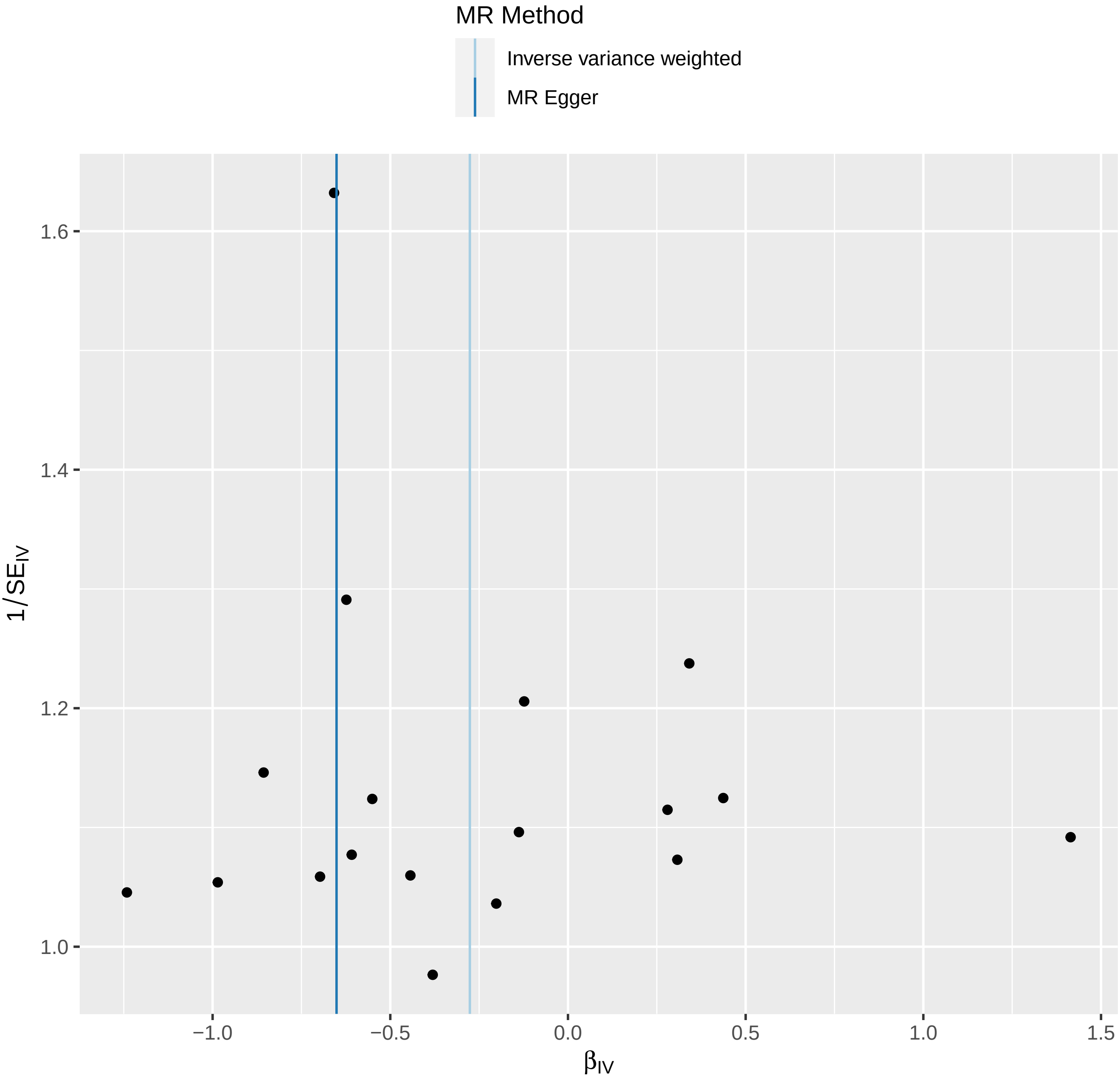

Batch 686 : Gut microbiota abundance (genus Gordonibacter id.821) on Extreme obesity with alveolar hypoventilation

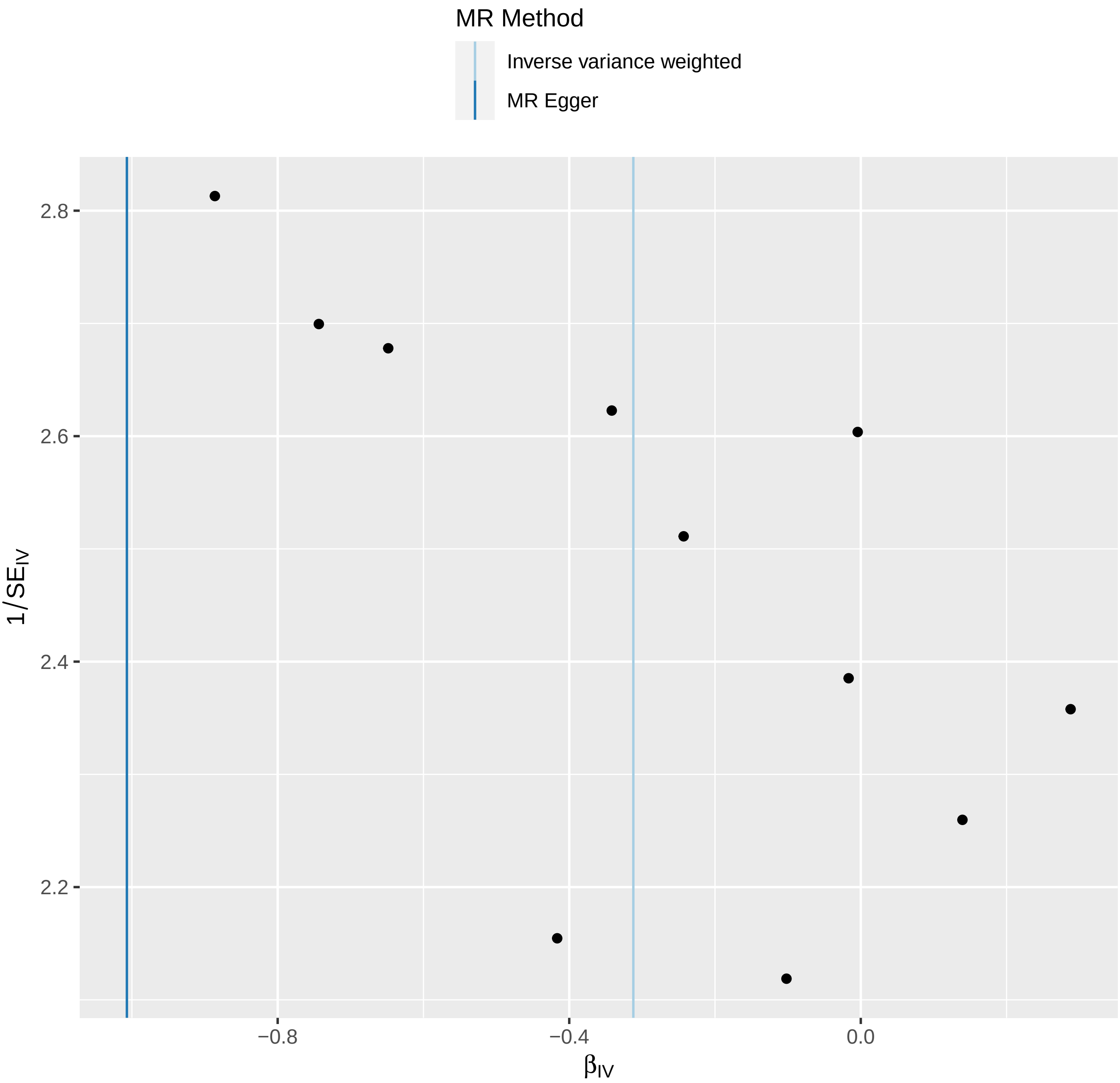

Batch 687 : Gut microbiota abundance (genus Haemophilus id.3698) on Extreme obesity with alveolar hypoventilation

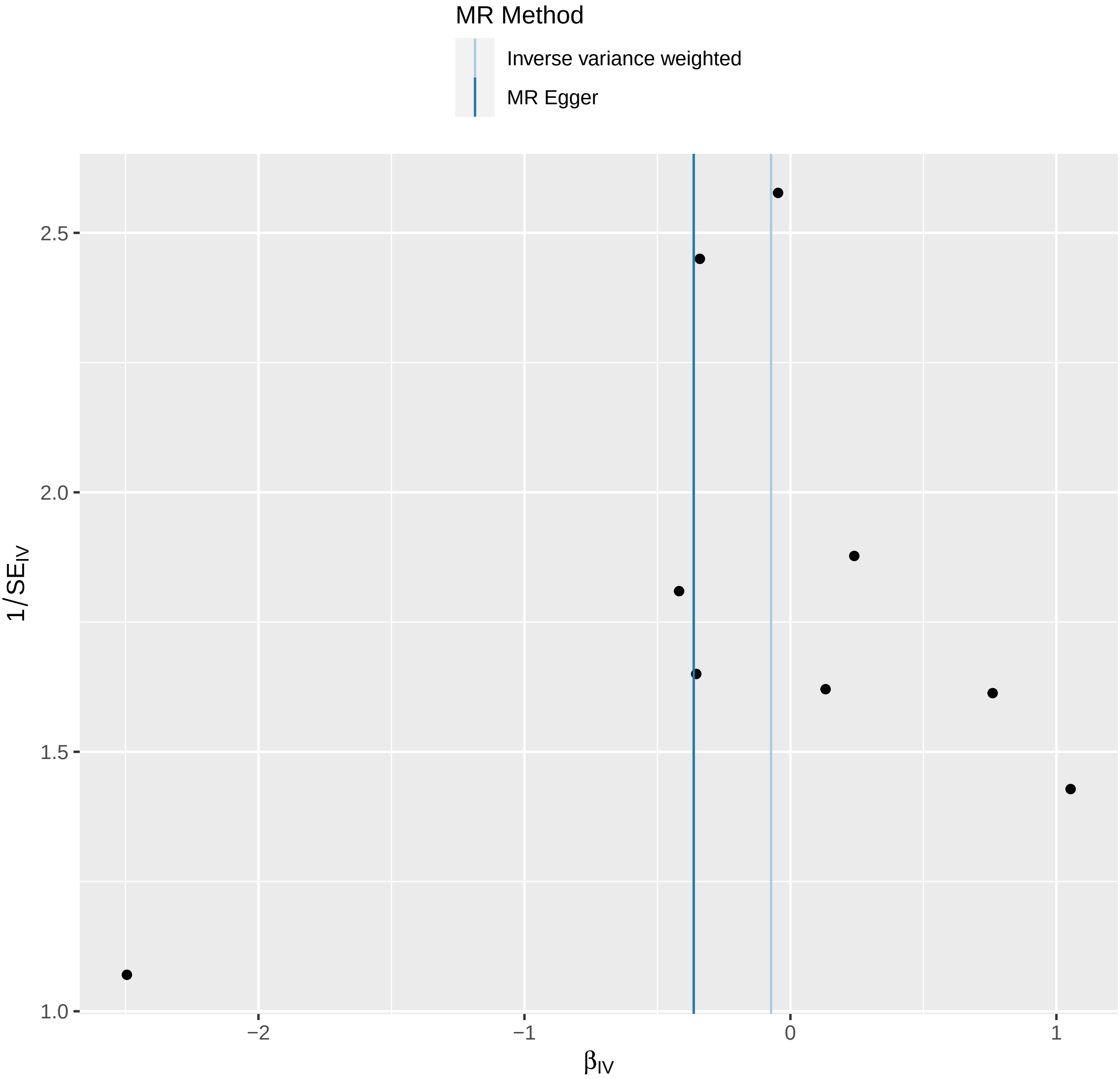

Batch 688 : Gut microbiota abundance (genus Holdemanella id.11393) on Extreme obesity with alveolar hypoventilation

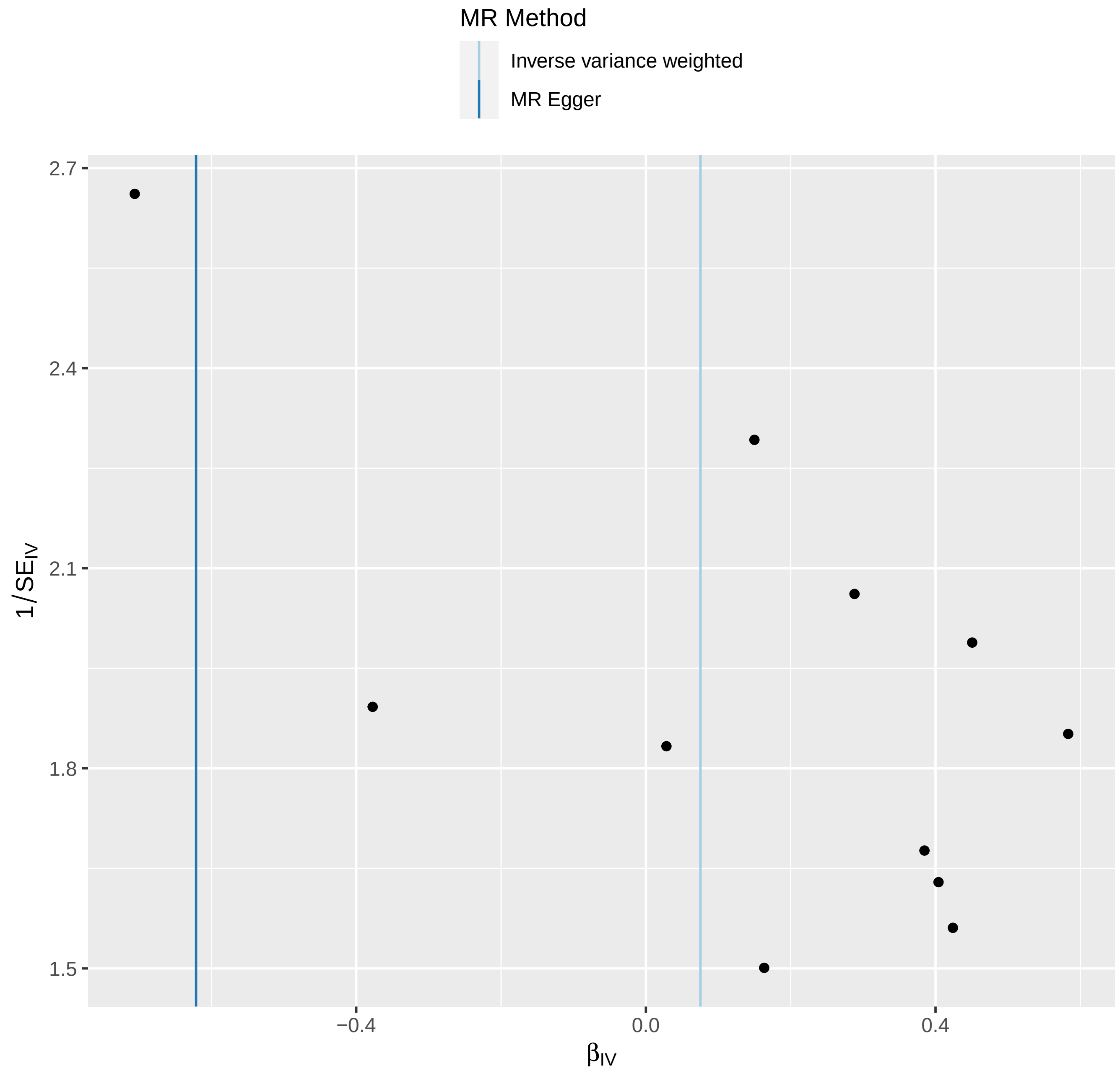

Batch 689 : Gut microbiota abundance (genus Holdemania id.2157) on Extreme obesity with alveolar hypoventilation

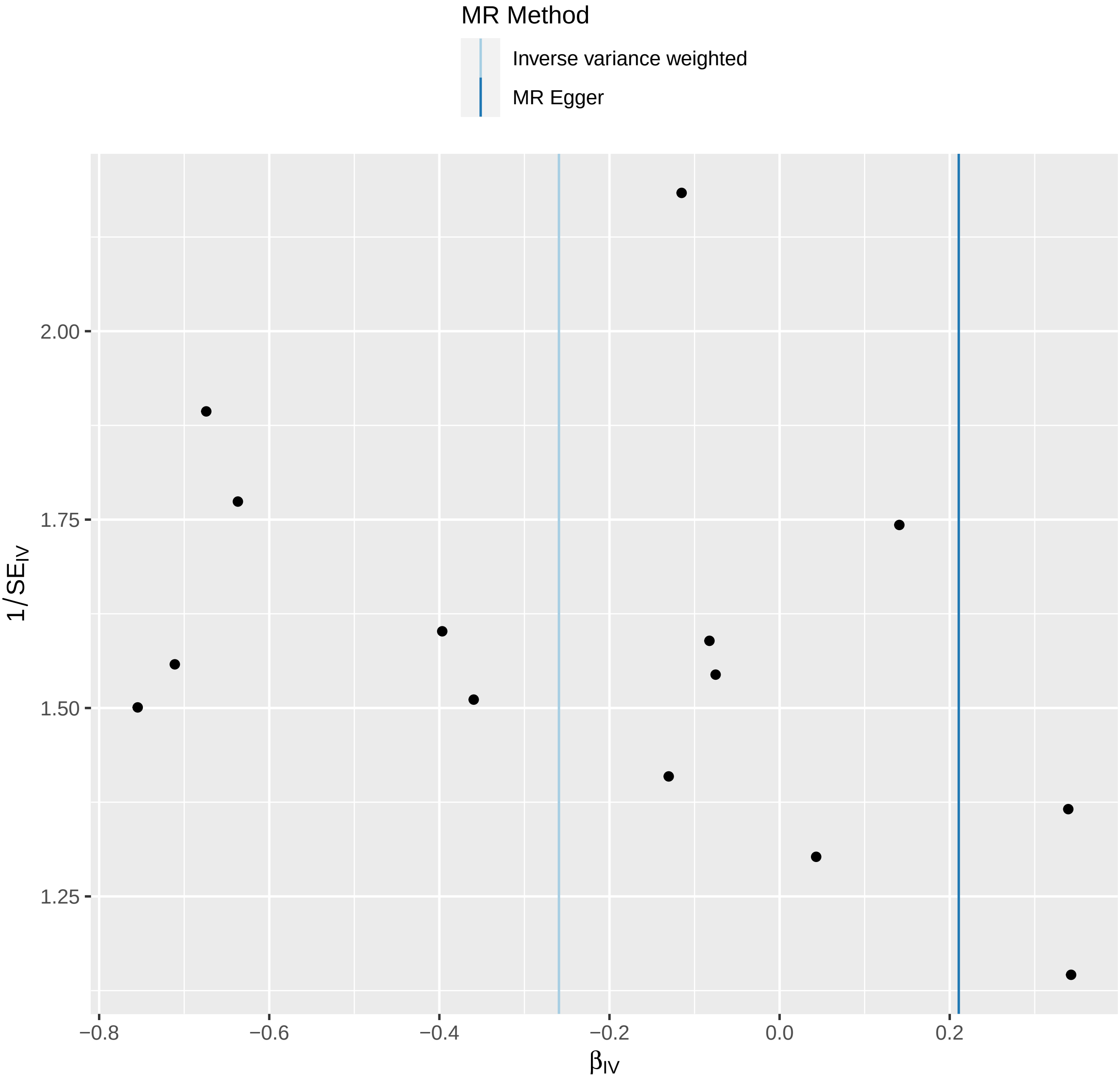

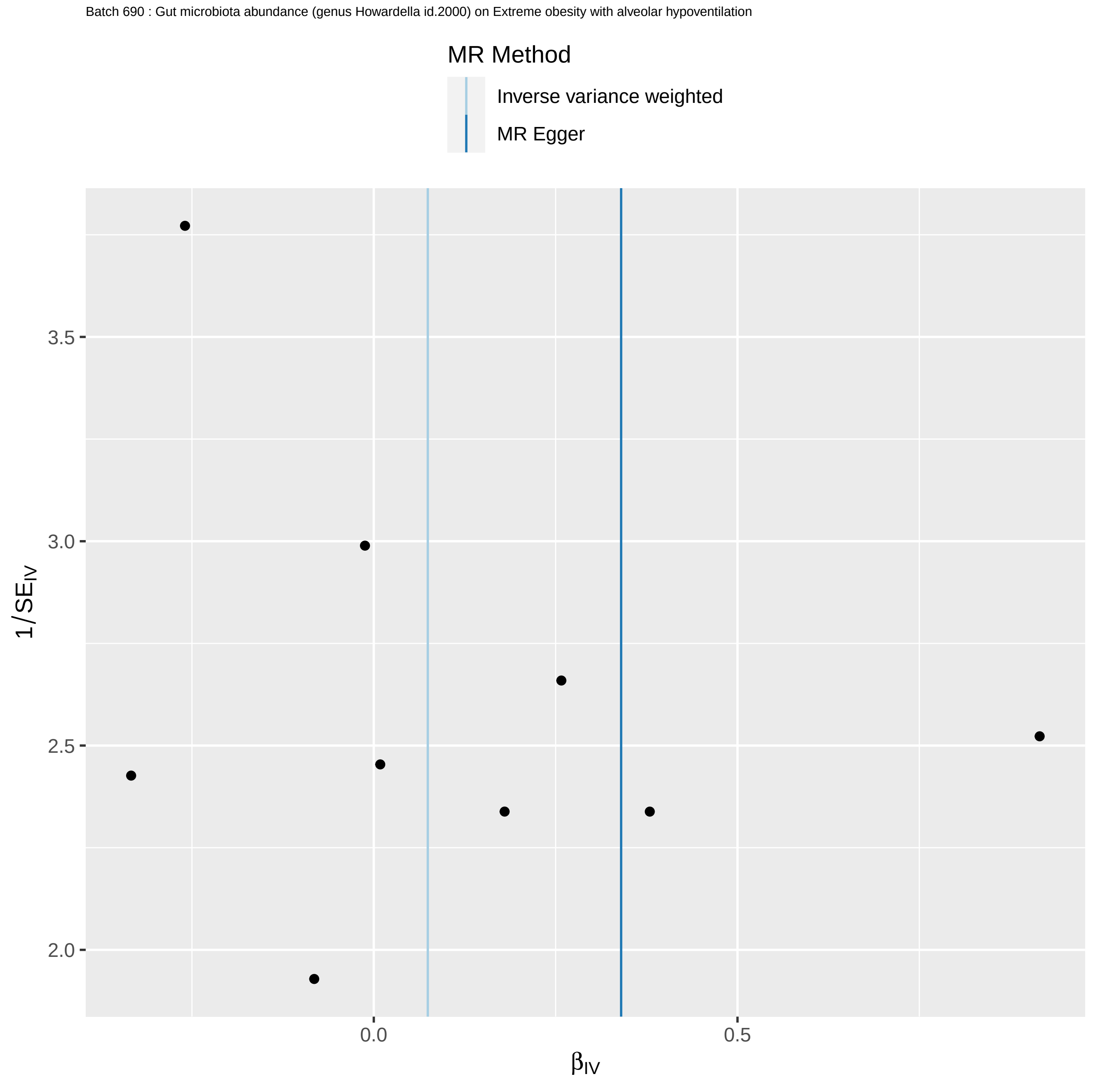
Batch 690 : Gut microbiota abundance (genus Howardella id.2000) on Extreme obesity with alveolar hypoventilation
MR Method
Inverse variance weighted
MR Egger
3.5
3.0
2.5
2.0
1/SE$_{IV}$
0.0
0.5
$\beta_{IV}$

Batch 691 : Gut microbiota abundance (genus Hungatella id.11306) on Extreme obesity with alveolar hypoventilation

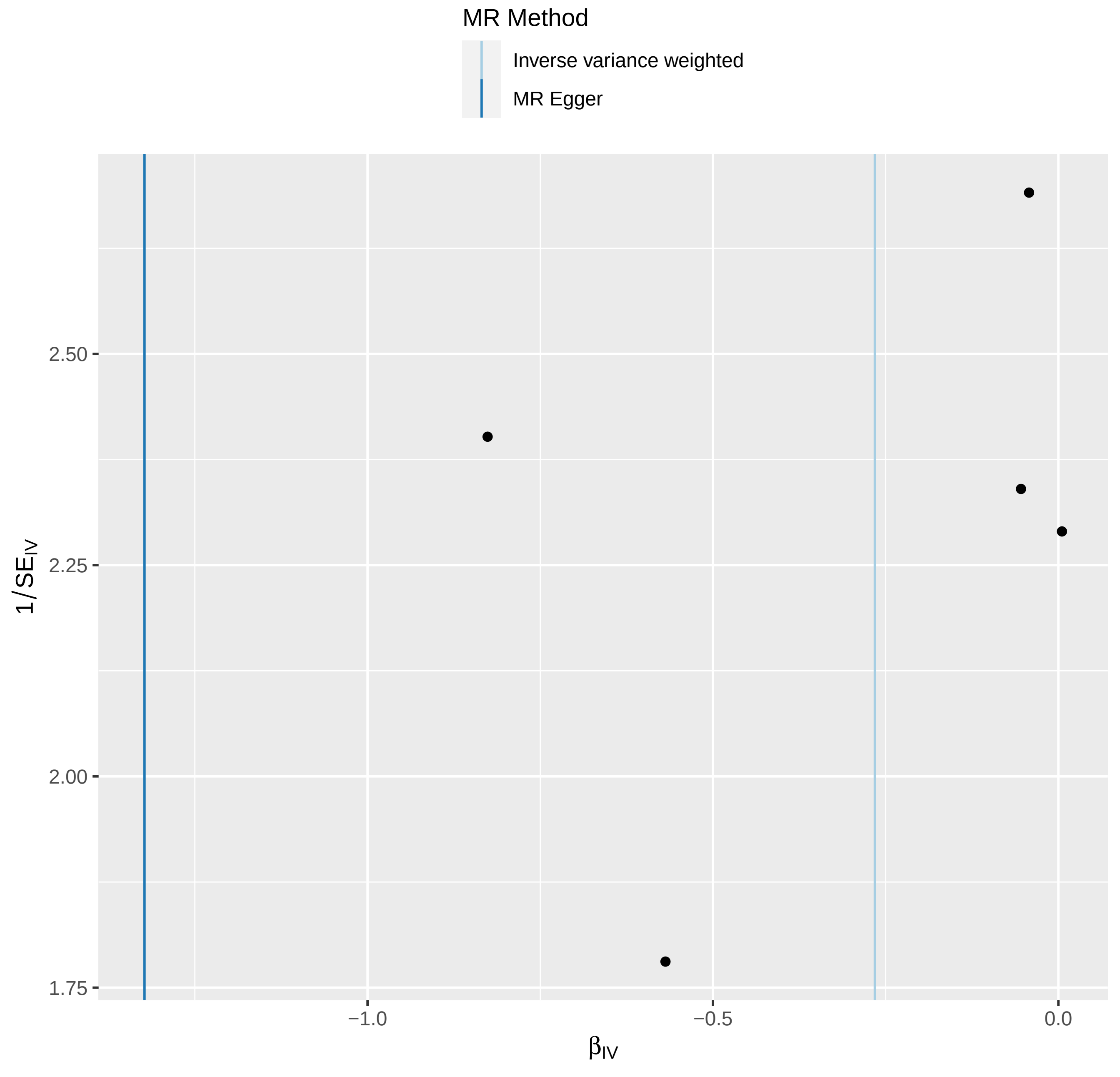

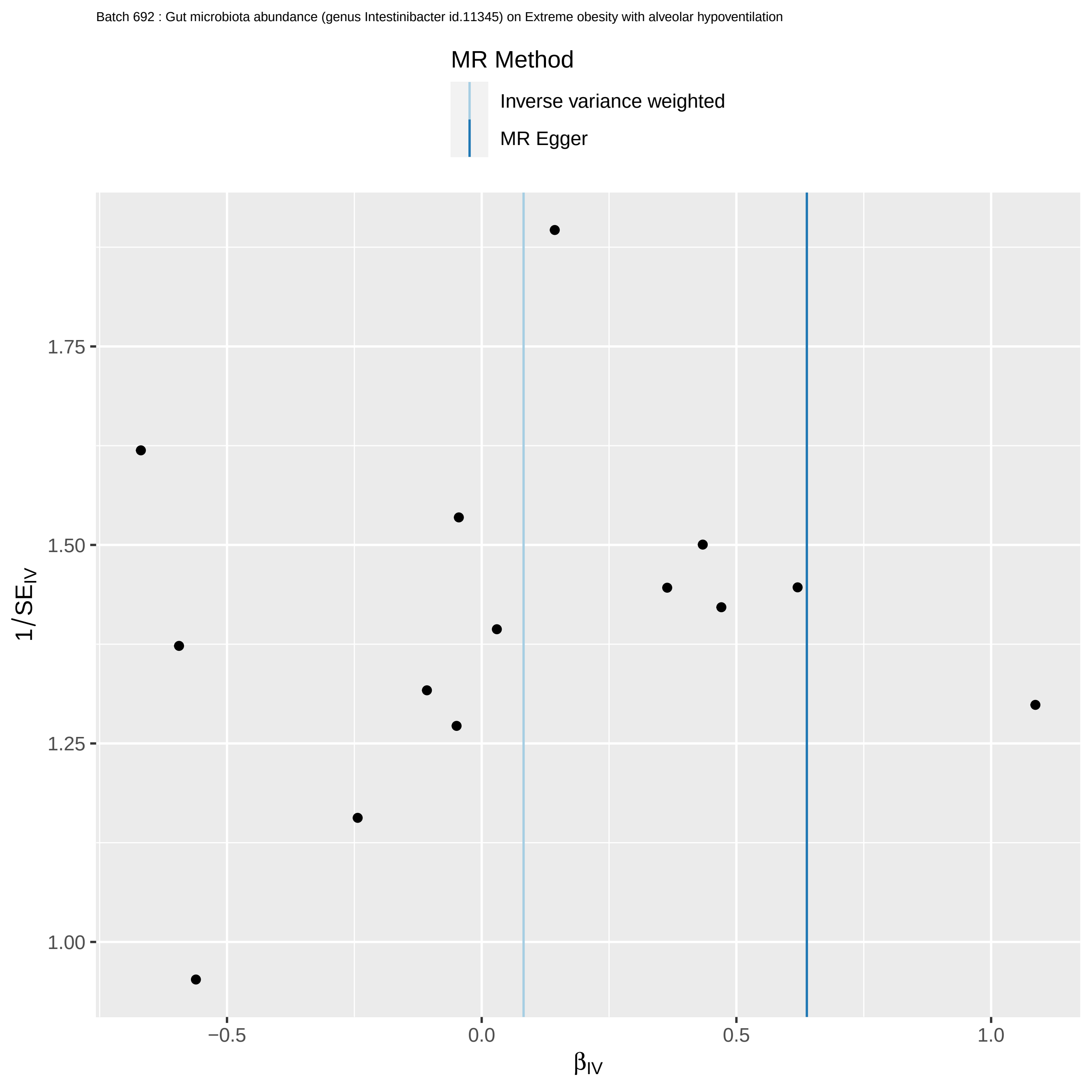

Batch 692 : Gut microbiota abundance (genus Intestinibacter id.11345) on Extreme obesity with alveolar hypoventilation
MR Method
Inverse variance weighted
MR Egger
1.75
1.50
1.25
1.00
1/SE_IV
−0.5
0.0
0.5
1.0
β_IV

Batch 693 : Gut microbiota abundance (genus Intestinimonas id.2062) on Extreme obesity with alveolar hypoventilation

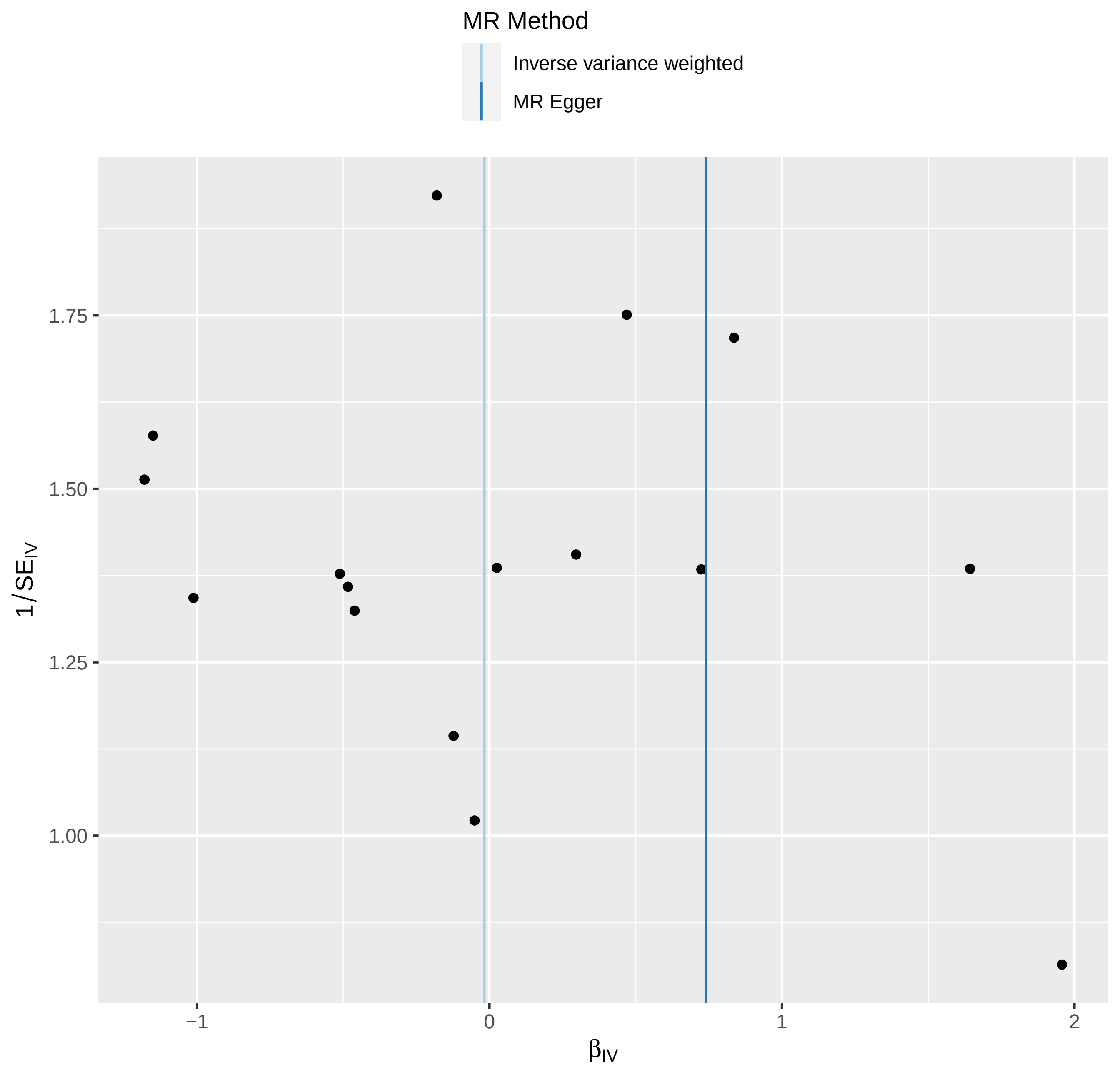

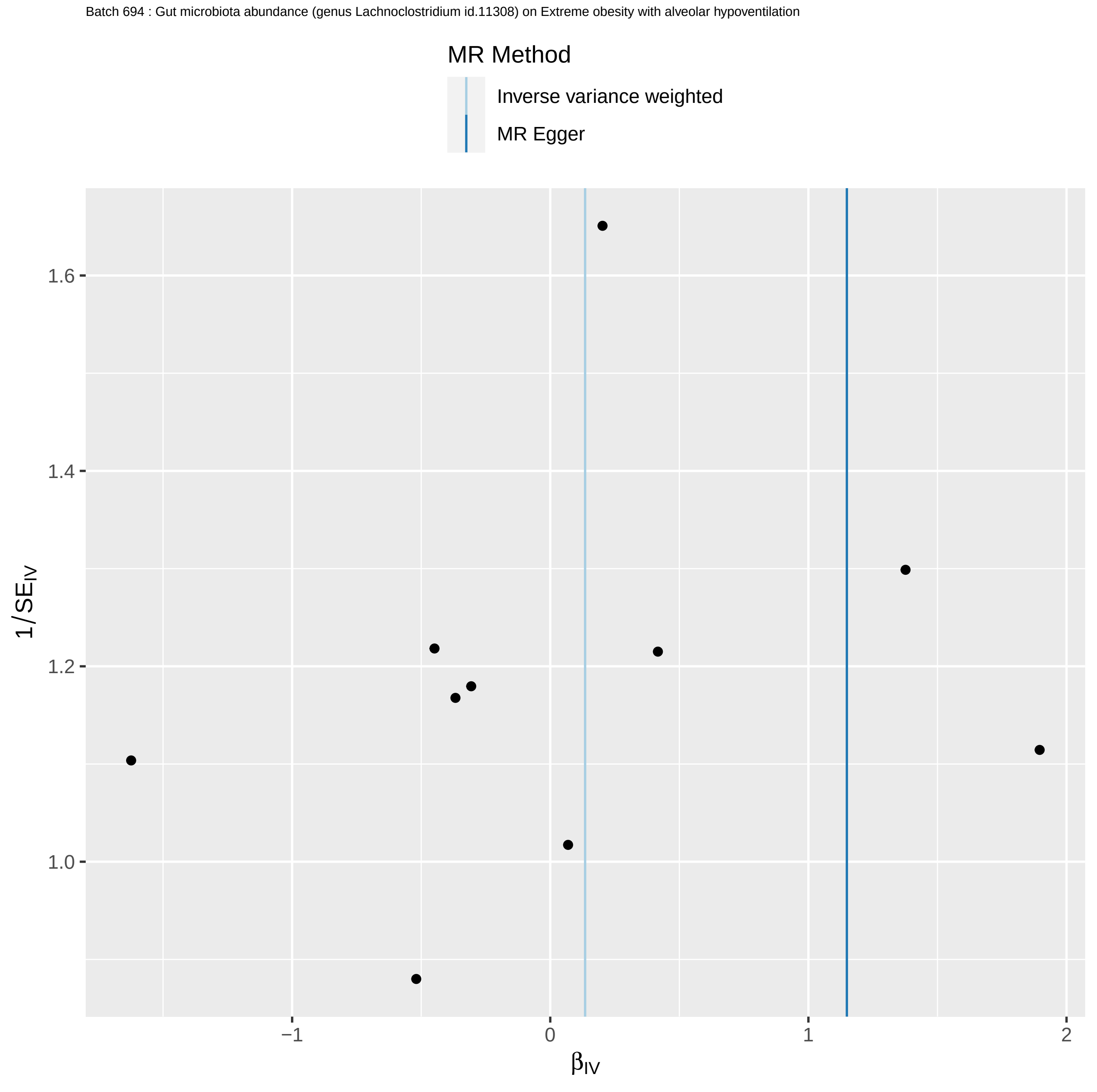
Batch 694 : Gut microbiota abundance (genus Lachnoclostridium id.11308) on Extreme obesity with alveolar hypoventilation
MR Method
Inverse variance weighted
MR Egger
1/SE_IV
1.6
1.4
1.2
1.0
−1
0
1
2
β_IV

Batch 695 : Gut microbiota abundance (genus Lachnospiraceae FCS020 group id.11314) on Extreme obesity with alveolar hypoventilation

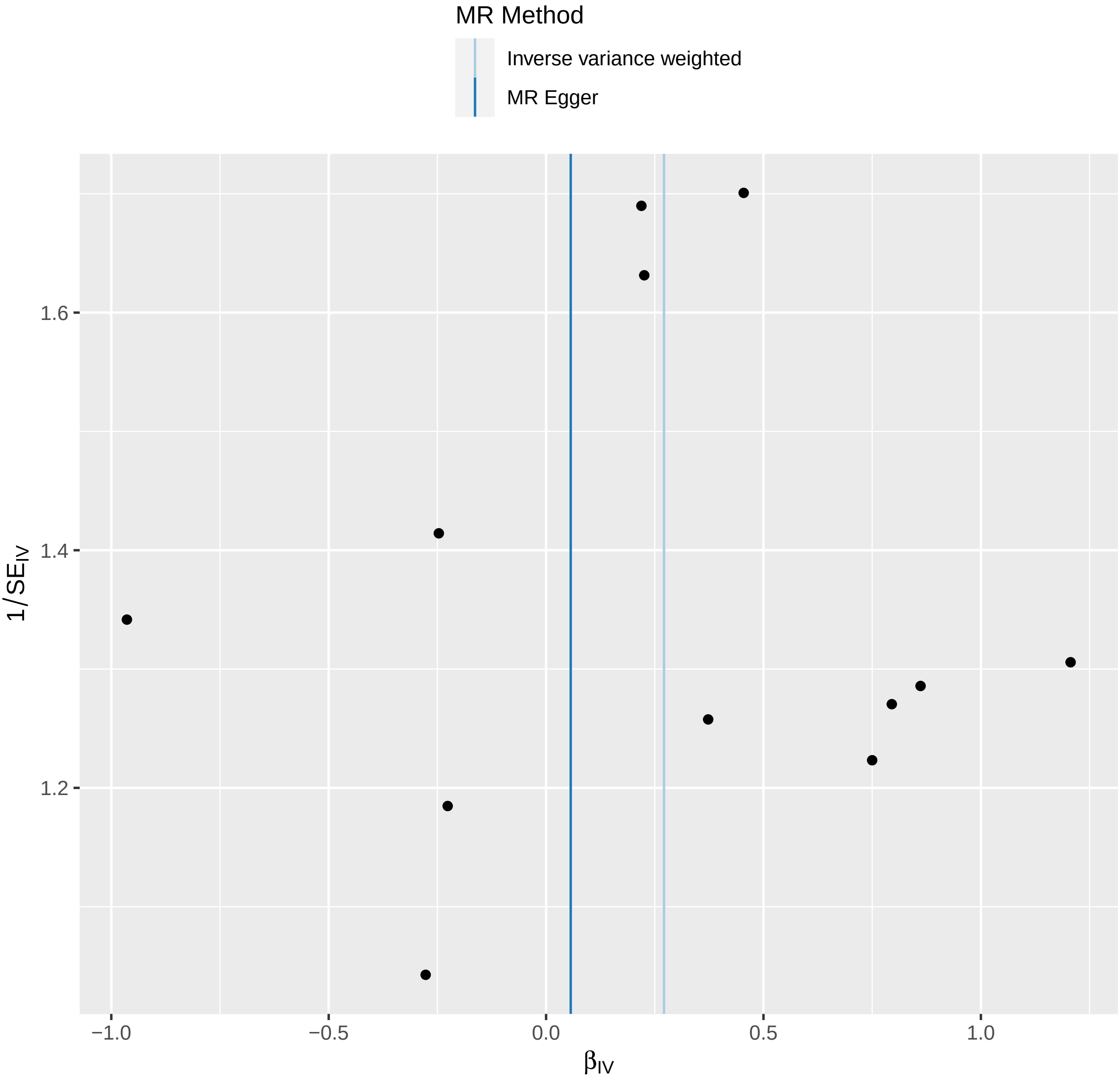

Batch 696 : Gut microbiota abundance (genus Lachnospiraceae NC2004 group id.11316) on Extreme obesity with alveolar hypoventilation

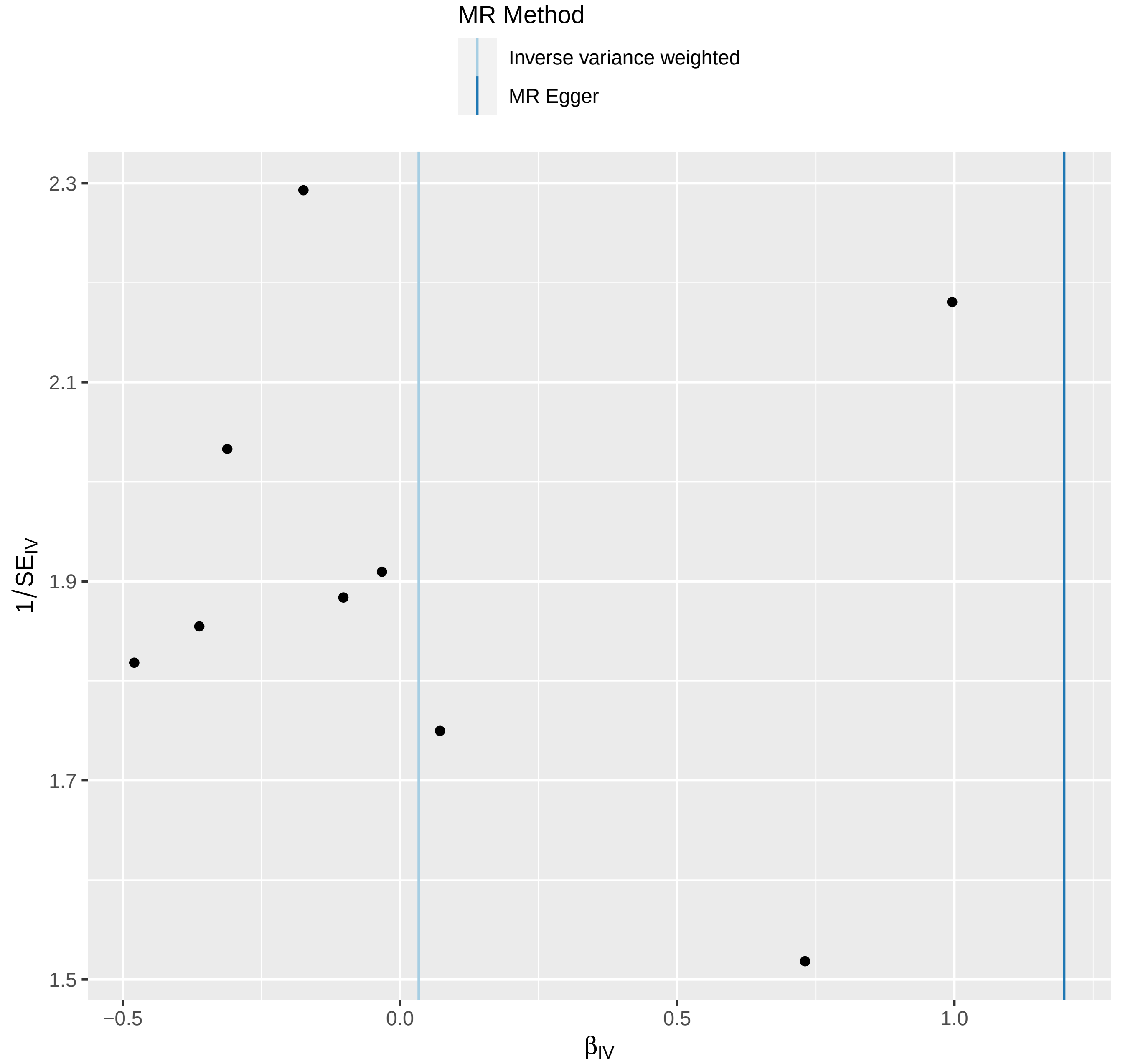

Batch 697 : Gut microbiota abundance (genus Lachnospiraceae ND3007 group id.11317) on Extreme obesity with alveolar hypoventilation

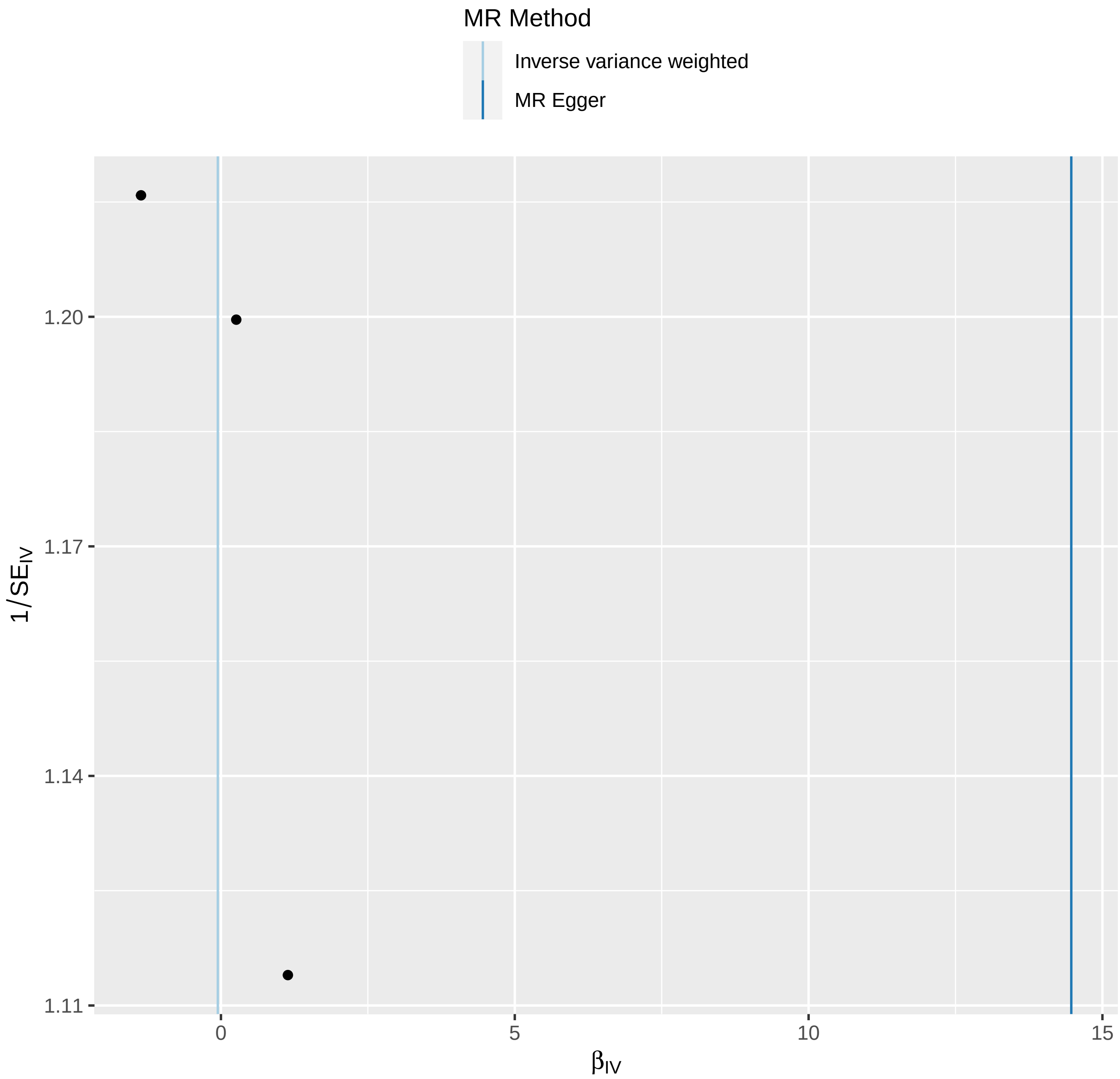

Batch 698 : Gut microbiota abundance (genus Lachnospiraceae NK4A136 group id.11319) on Extreme obesity with alveolar hypoventilation

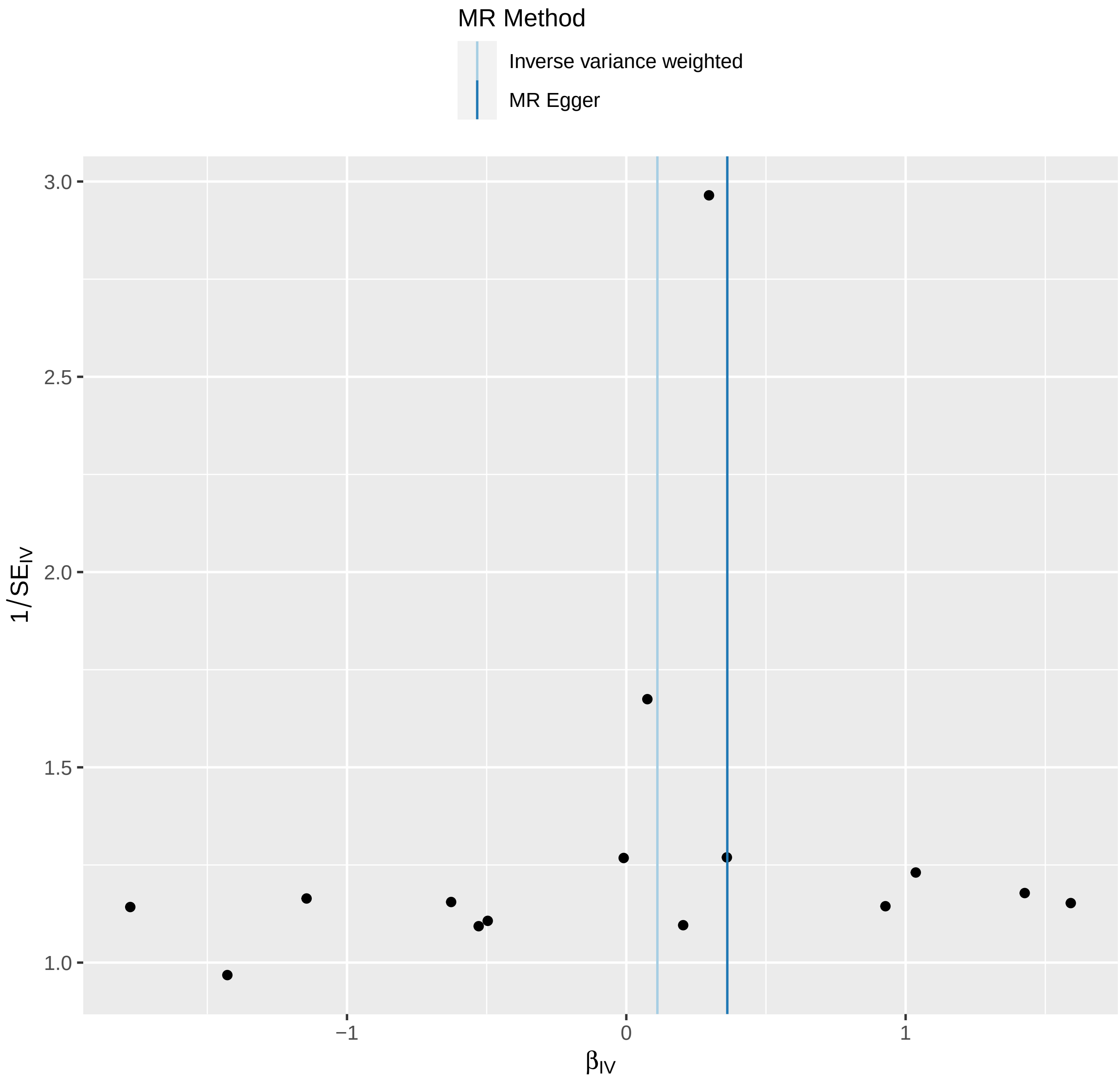

Batch 699 : Gut microbiota abundance (genus Lachnospiraceae UCG001 id.11321) on Extreme obesity with alveolar hypoventilation

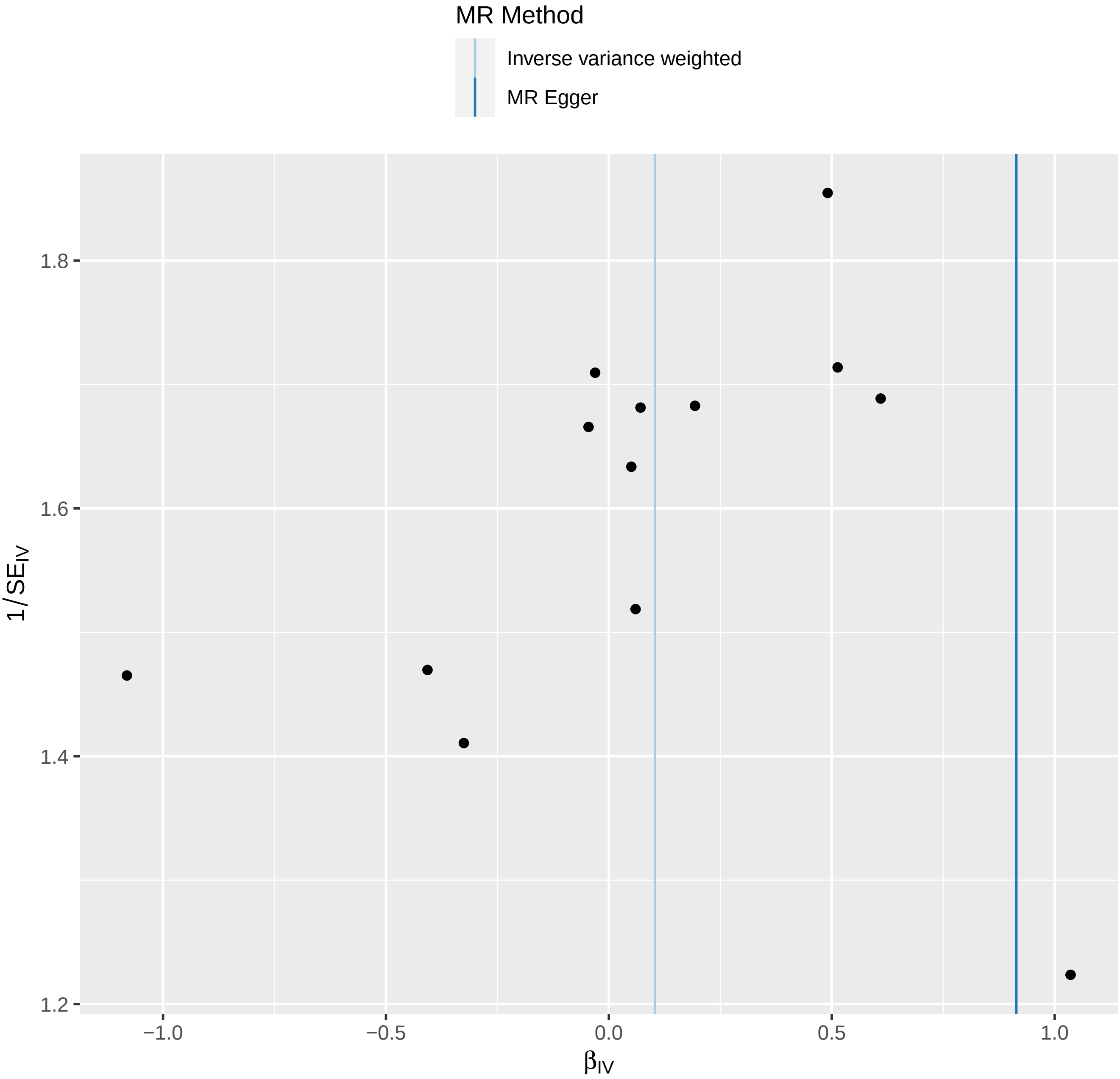

Batch 700 : Gut microbiota abundance (genus Lachnospiraceae UCG004 id.11324) on Extreme obesity with alveolar hypoventilation

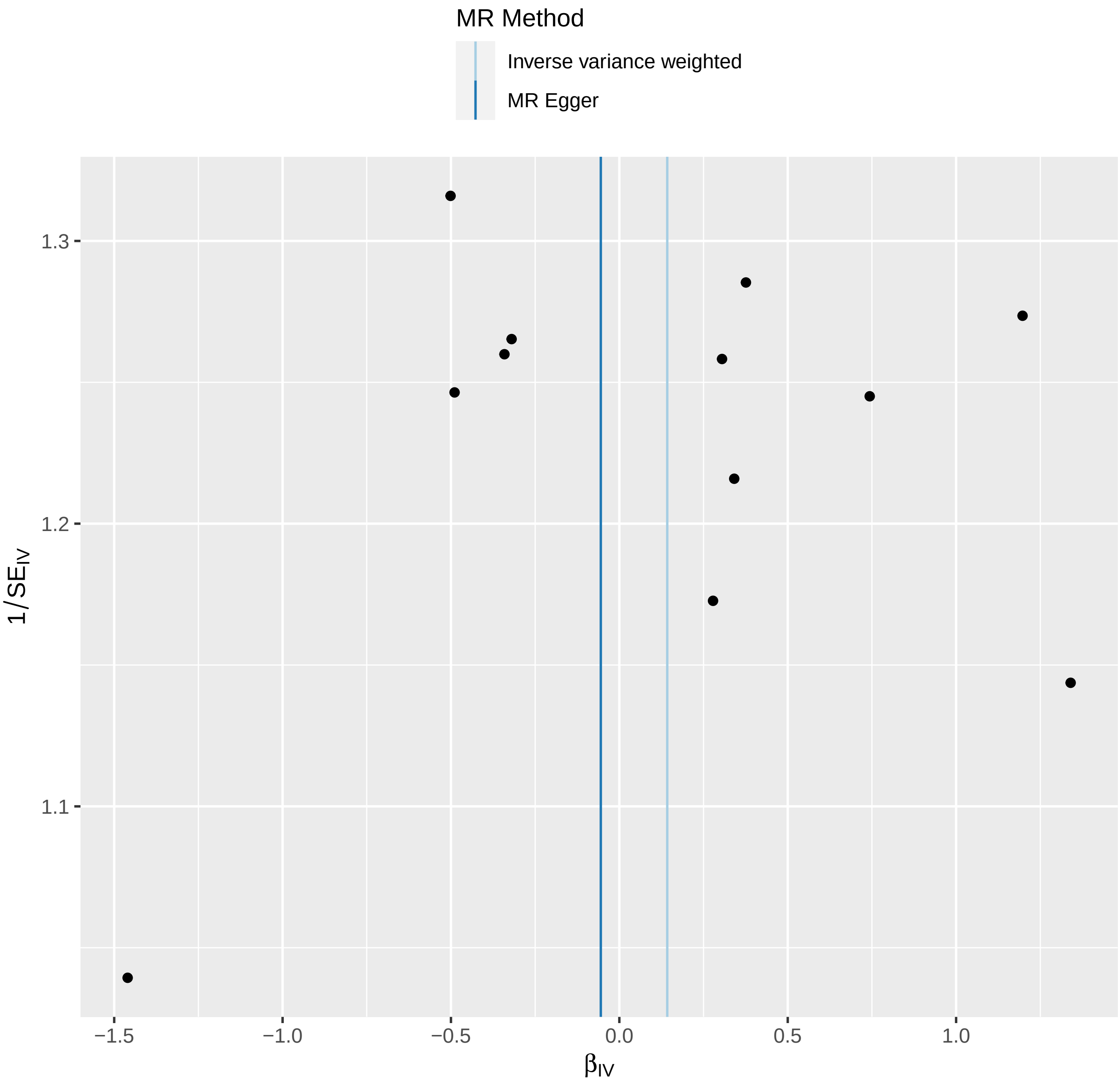

Batch 701 : Gut microbiota abundance (genus Lachnospiraceae UCG008 id.11328) on Extreme obesity with alveolar hypoventilation

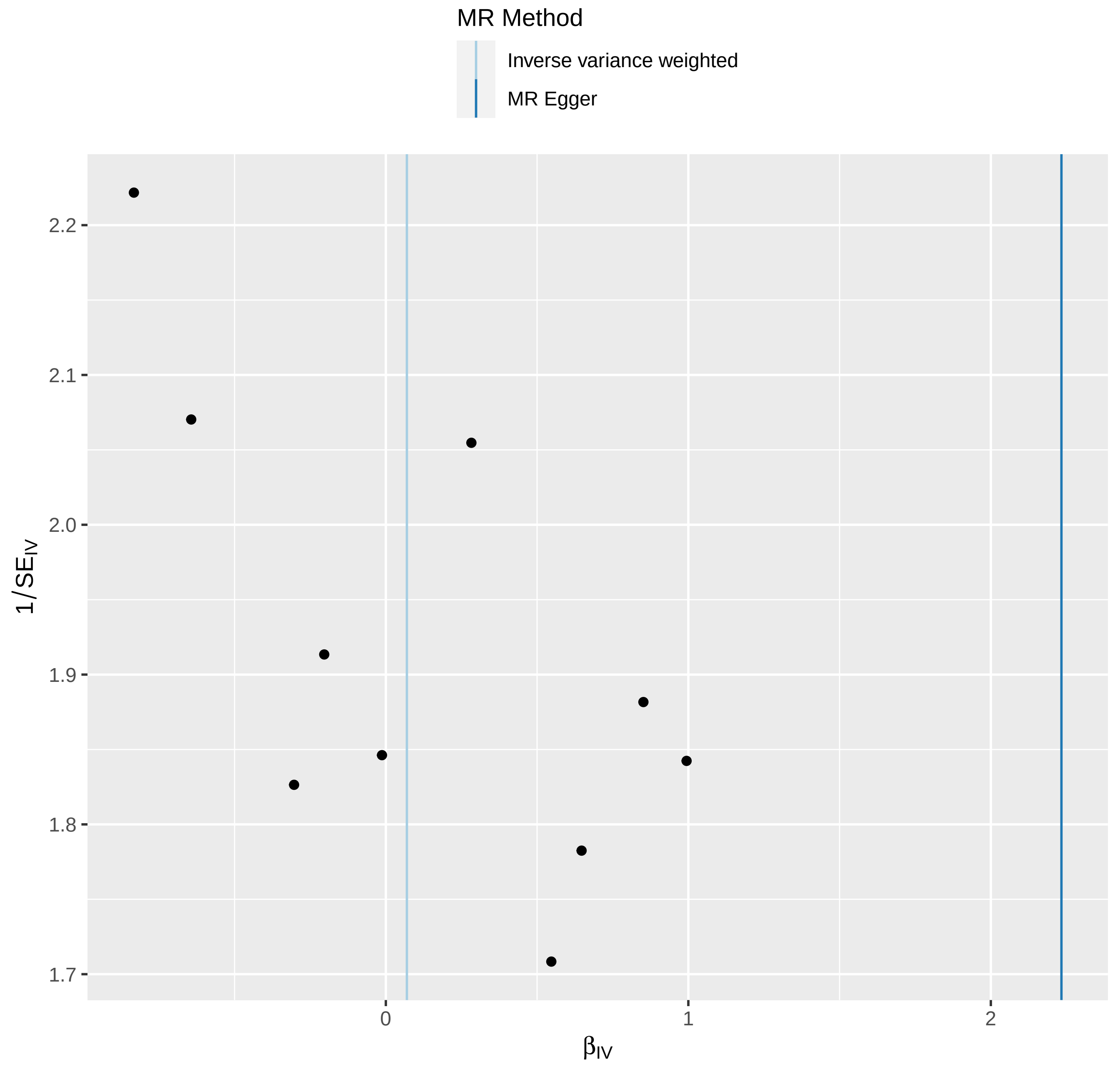

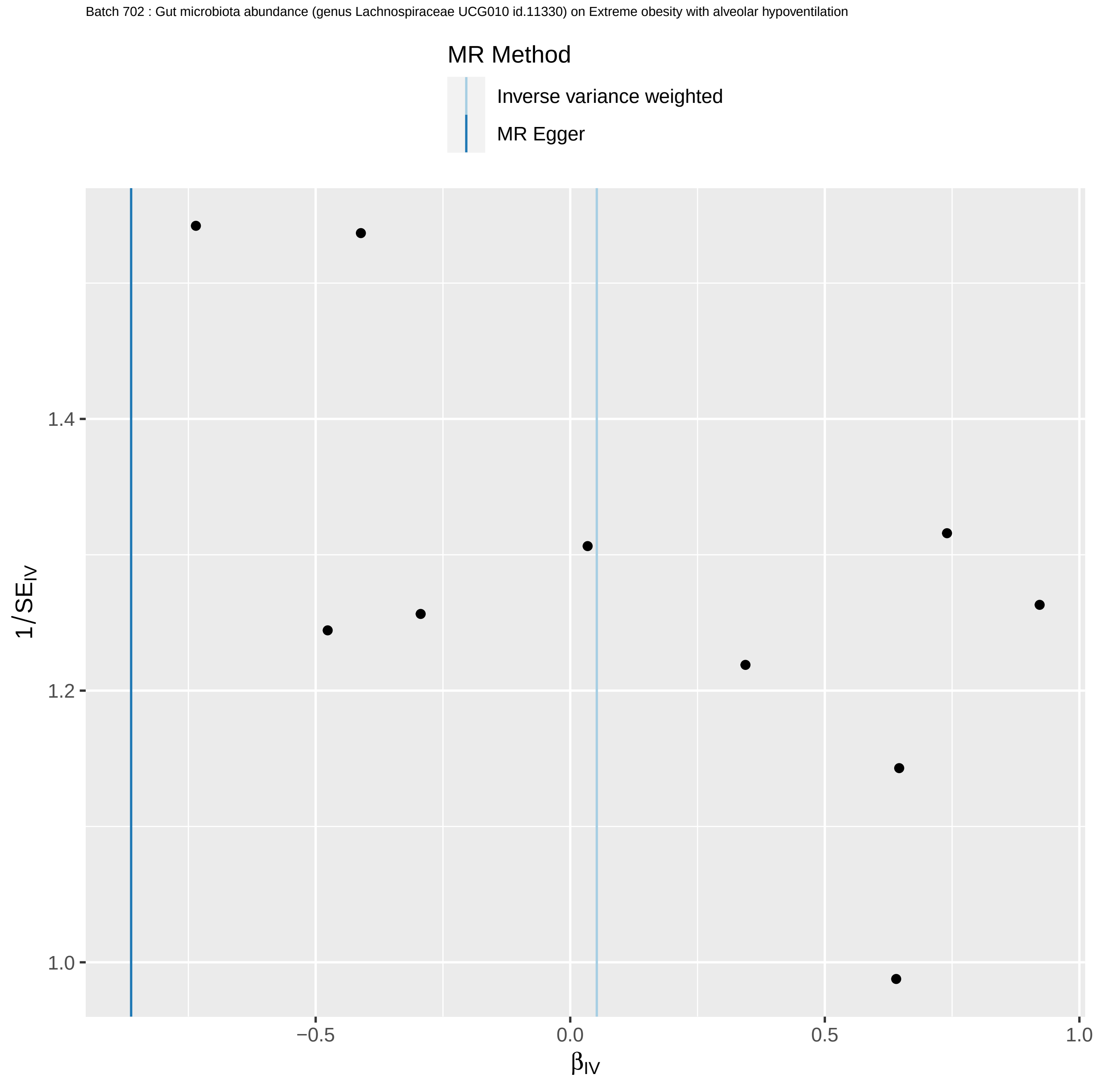

Batch 702 : Gut microbiota abundance (genus Lachnospiraceae UCG010 id.11330) on Extreme obesity with alveolar hypoventilation
MR Method
Inverse variance weighted
MR Egger
1.4
1.2
1.0
1/SE$_{IV}$
−0.5
0.0
0.5
1.0
$\beta_{IV}$

Batch 704 : Gut microbiota abundance (genus Lactobacillus id.1837) on Extreme obesity with alveolar hypoventilation

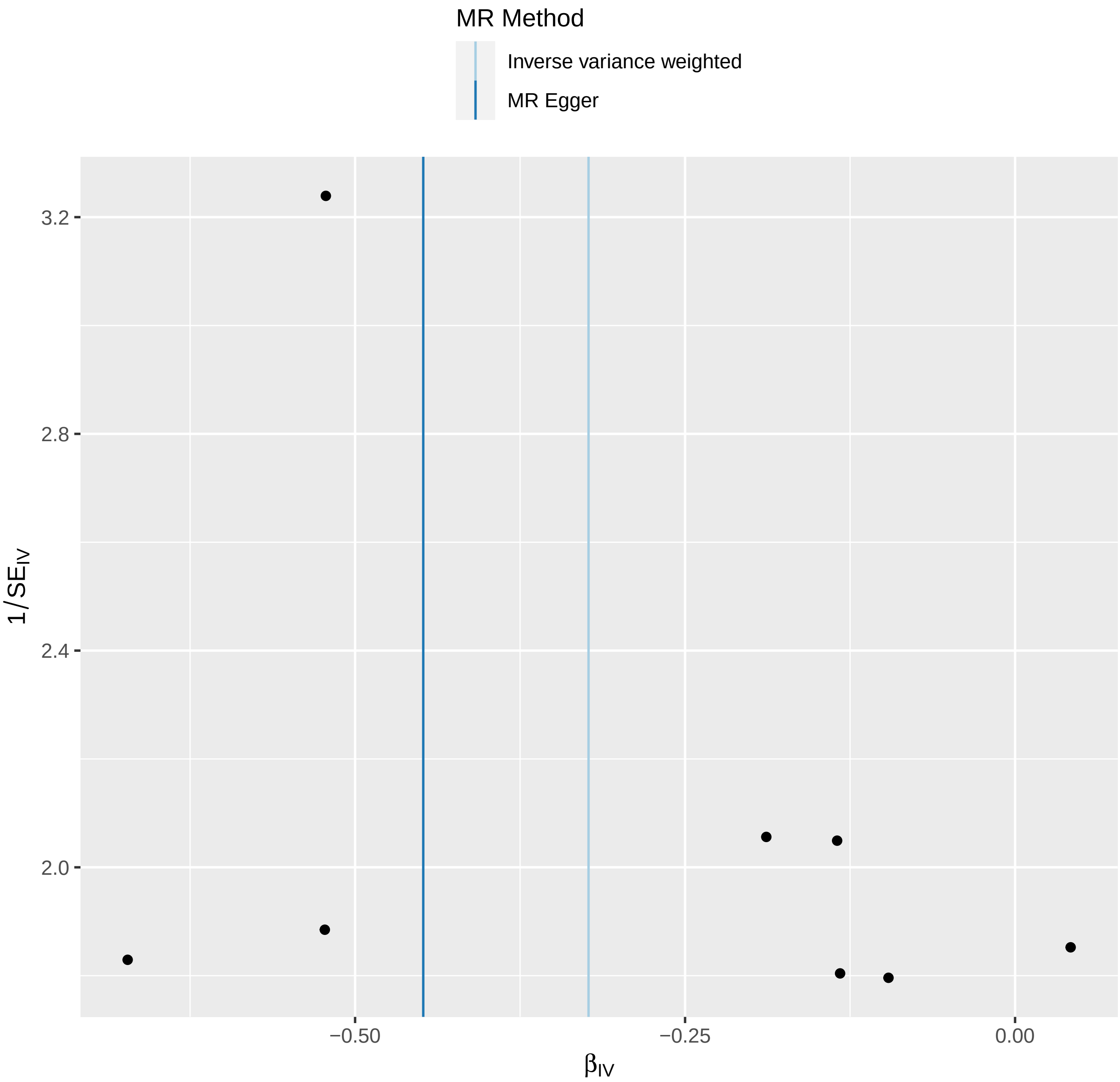

Batch 705 : Gut microbiota abundance (genus Lactococcus id.1851) on Extreme obesity with alveolar hypoventilation

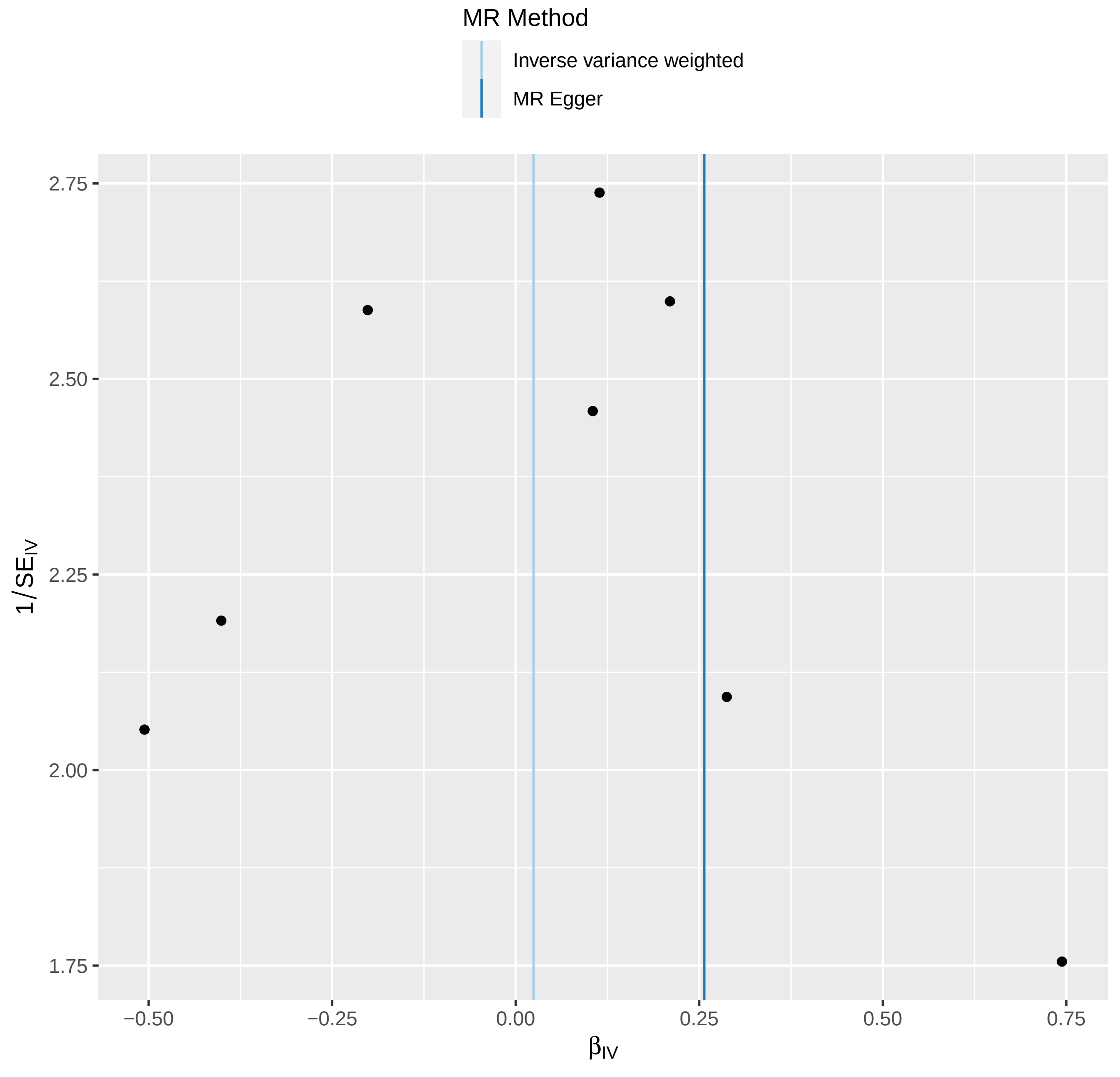

Batch 706 : Gut microbiota abundance (genus Marvinbryantia id.2005) on Extreme obesity with alveolar hypoventilation

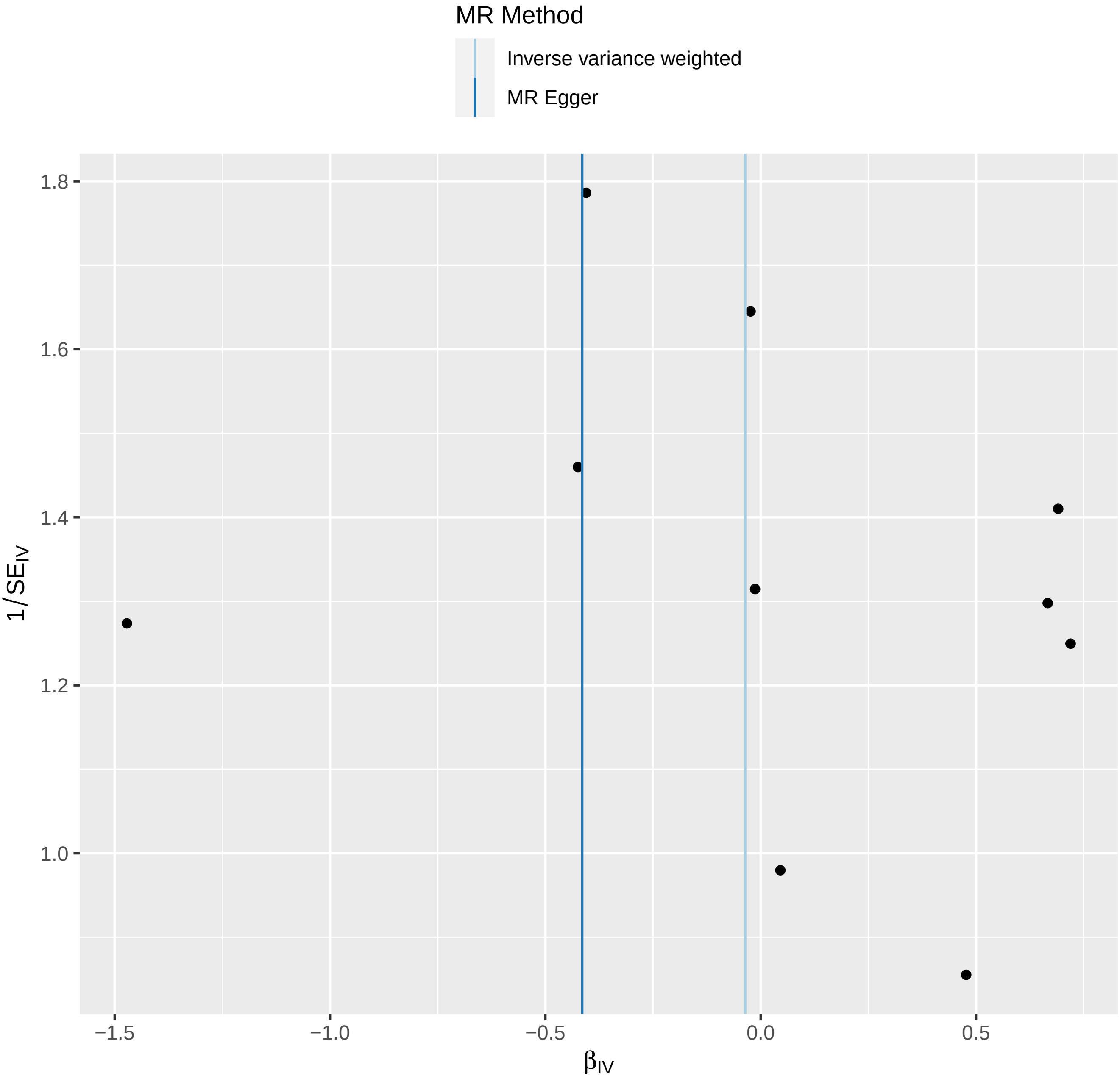

Batch 707 : Gut microbiota abundance (genus Methanobrevibacter id.123) on Extreme obesity with alveolar hypoventilation

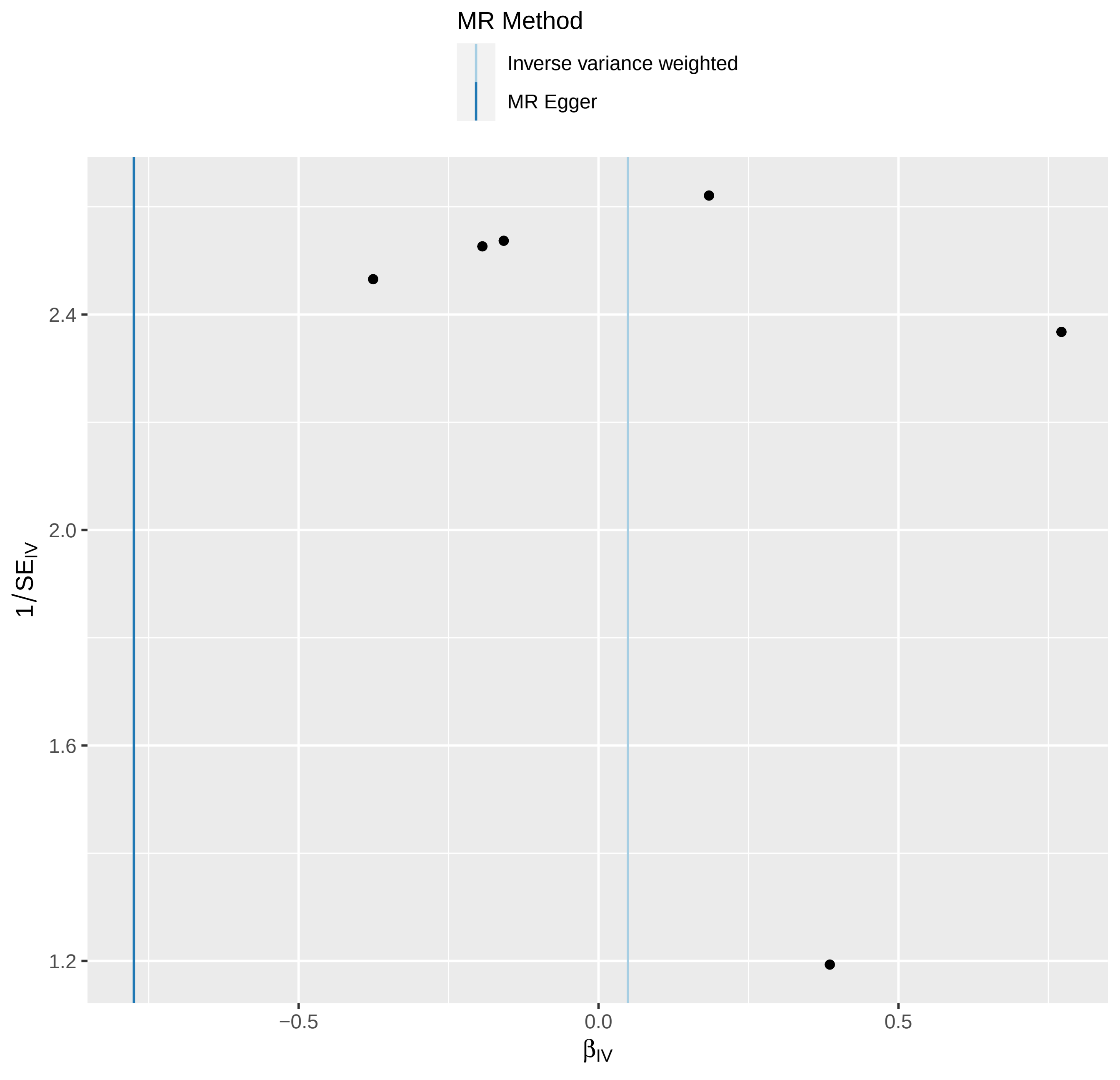

Batch 708 : Gut microbiota abundance (genus Odoribacter id.952) on Extreme obesity with alveolar hypoventilation

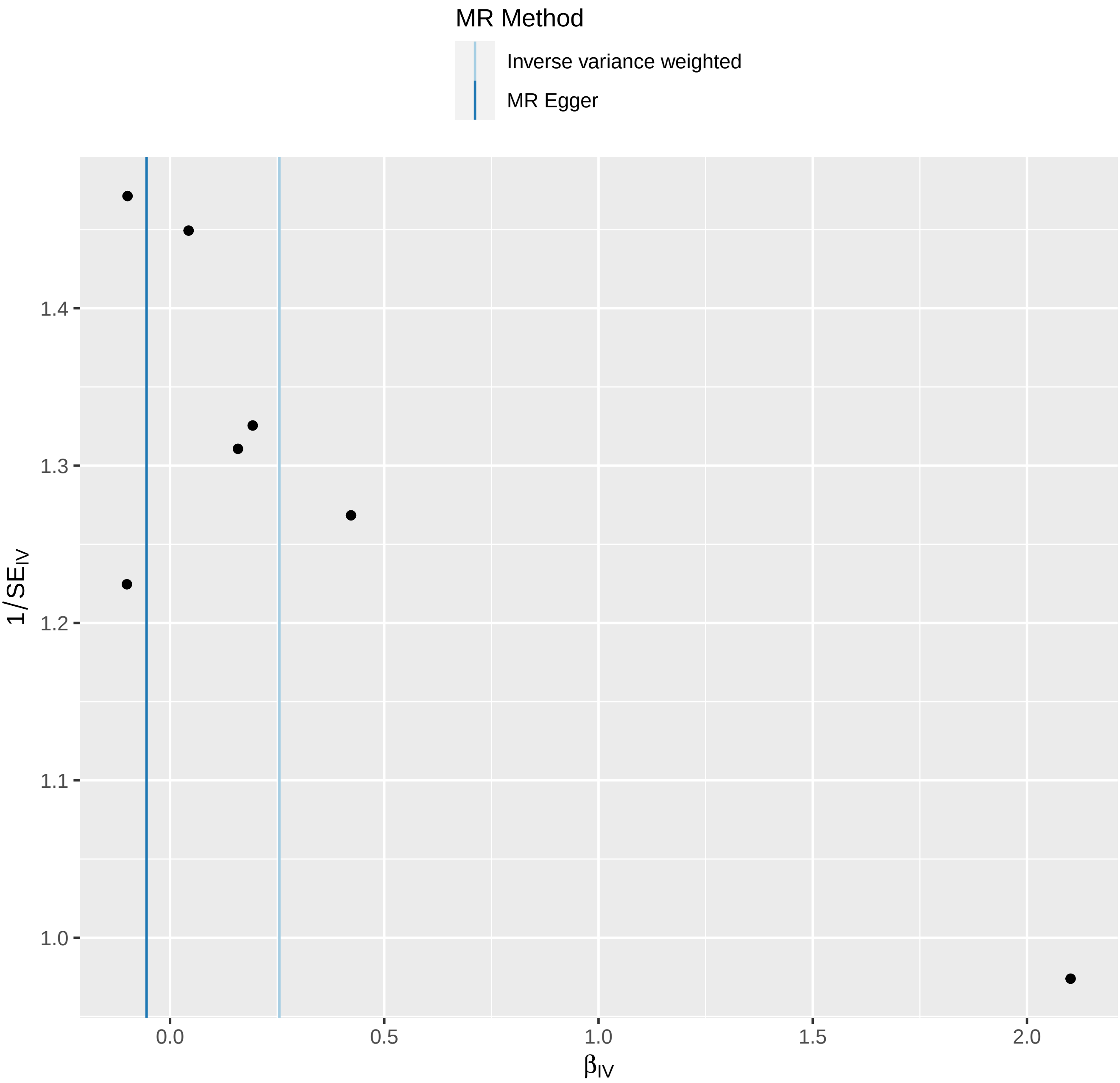

Batch 709 : Gut microbiota abundance (genus Olsenella id.822) on Extreme obesity with alveolar hypoventilation

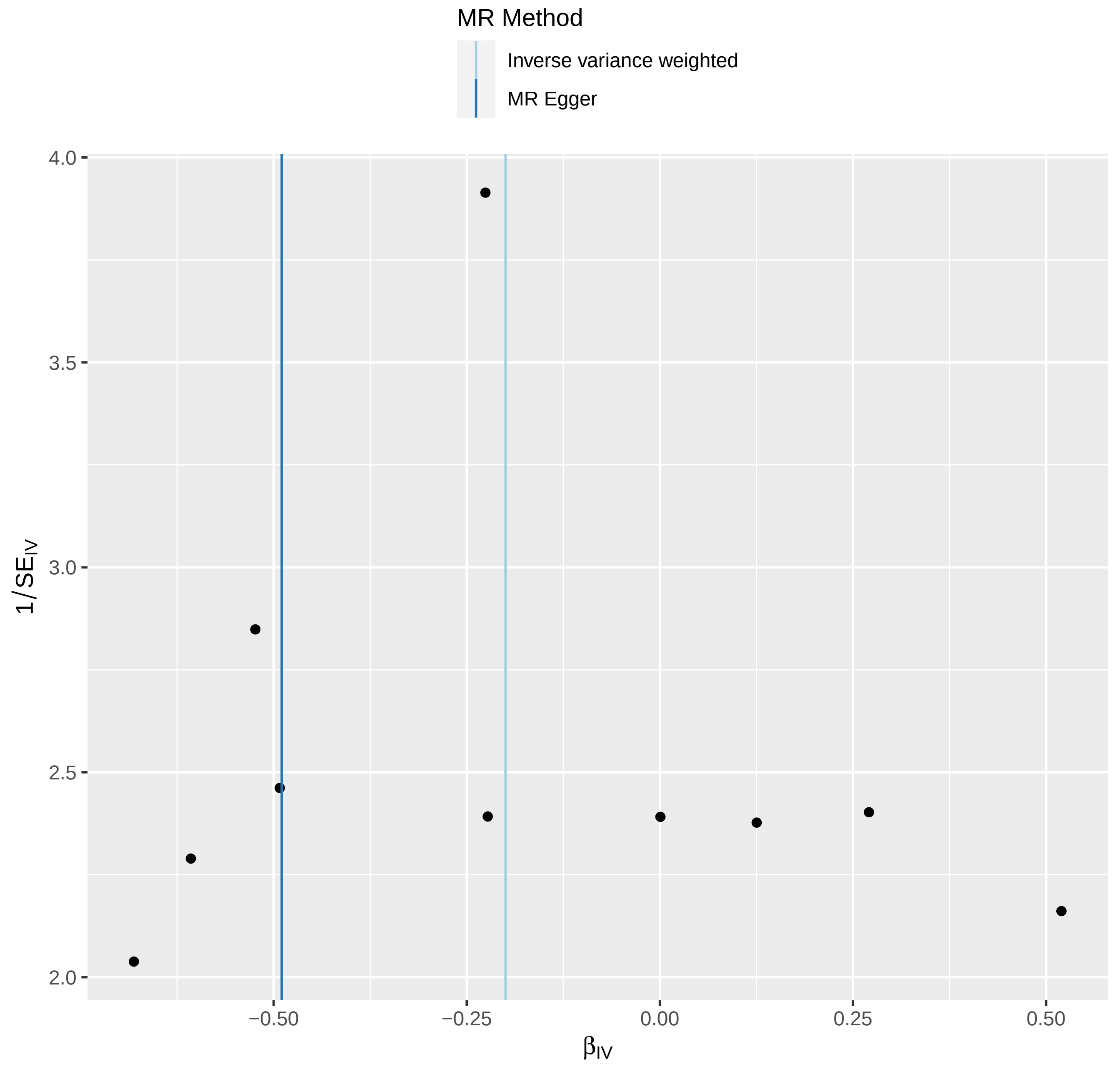

Batch 710 : Gut microbiota abundance (genus Oscillibacter id.2063) on Extreme obesity with alveolar hypoventilation

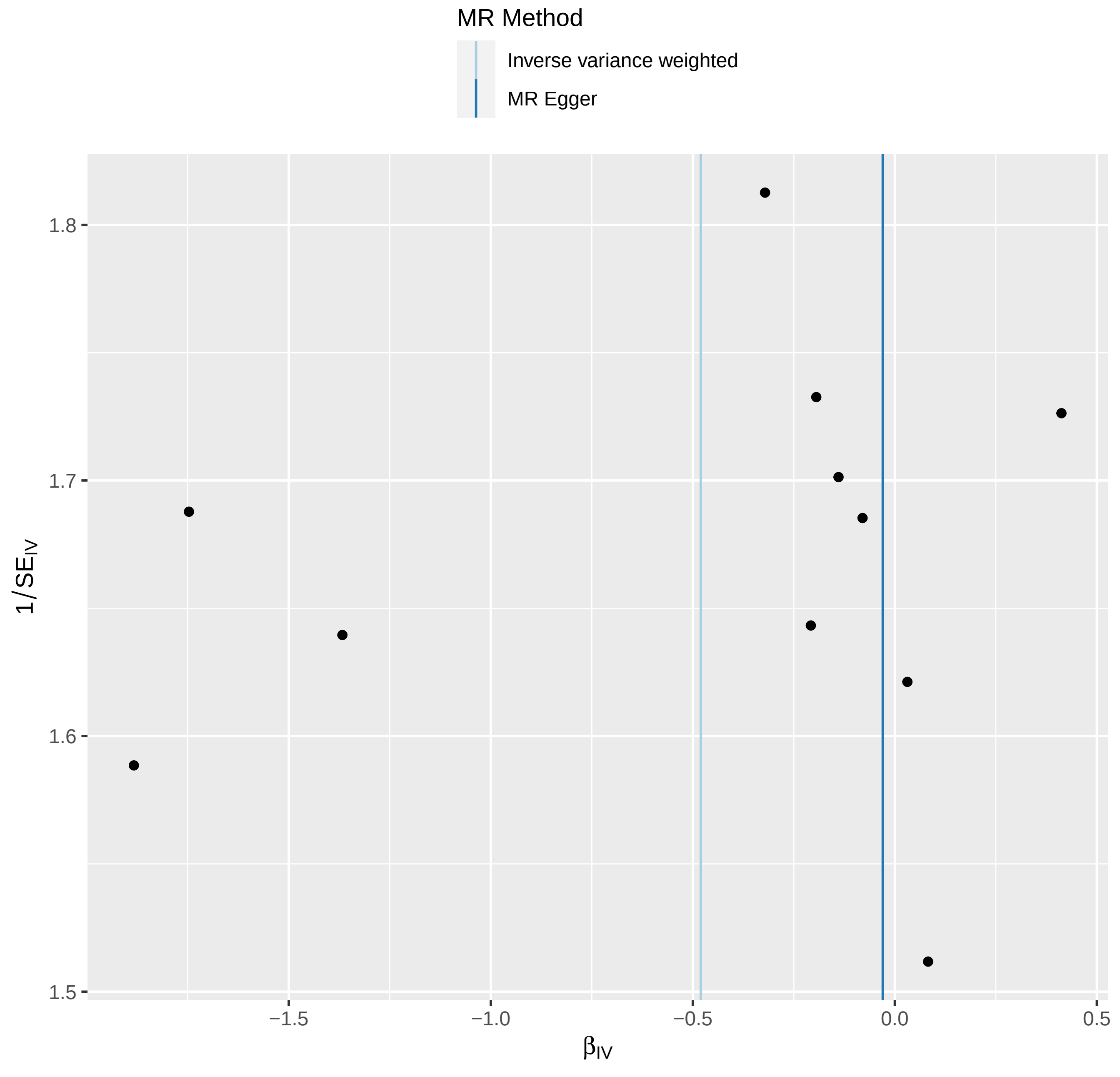

Batch 711 : Gut microbiota abundance (genus Oscillospira id.2064) on Extreme obesity with alveolar hypoventilation

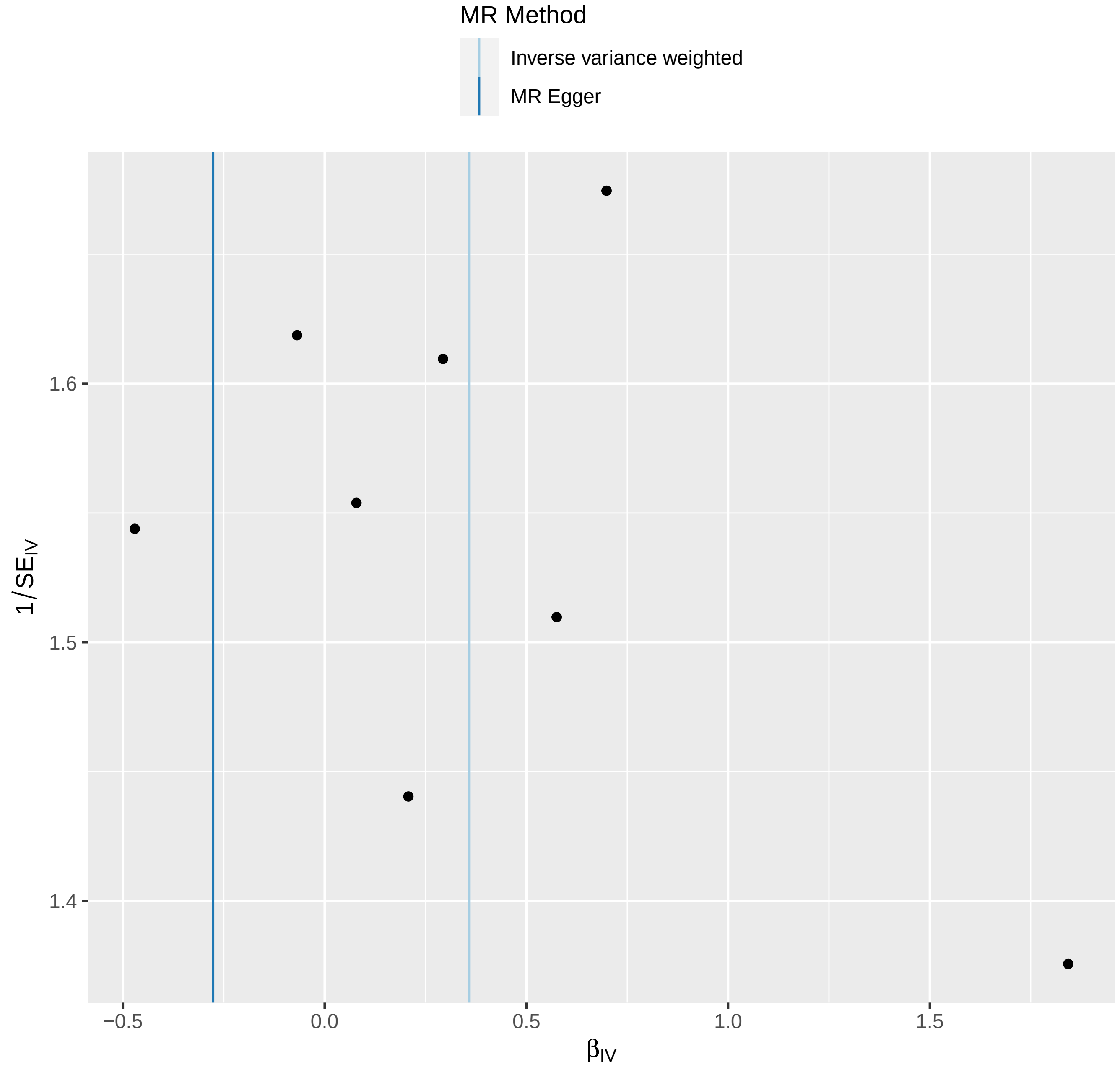

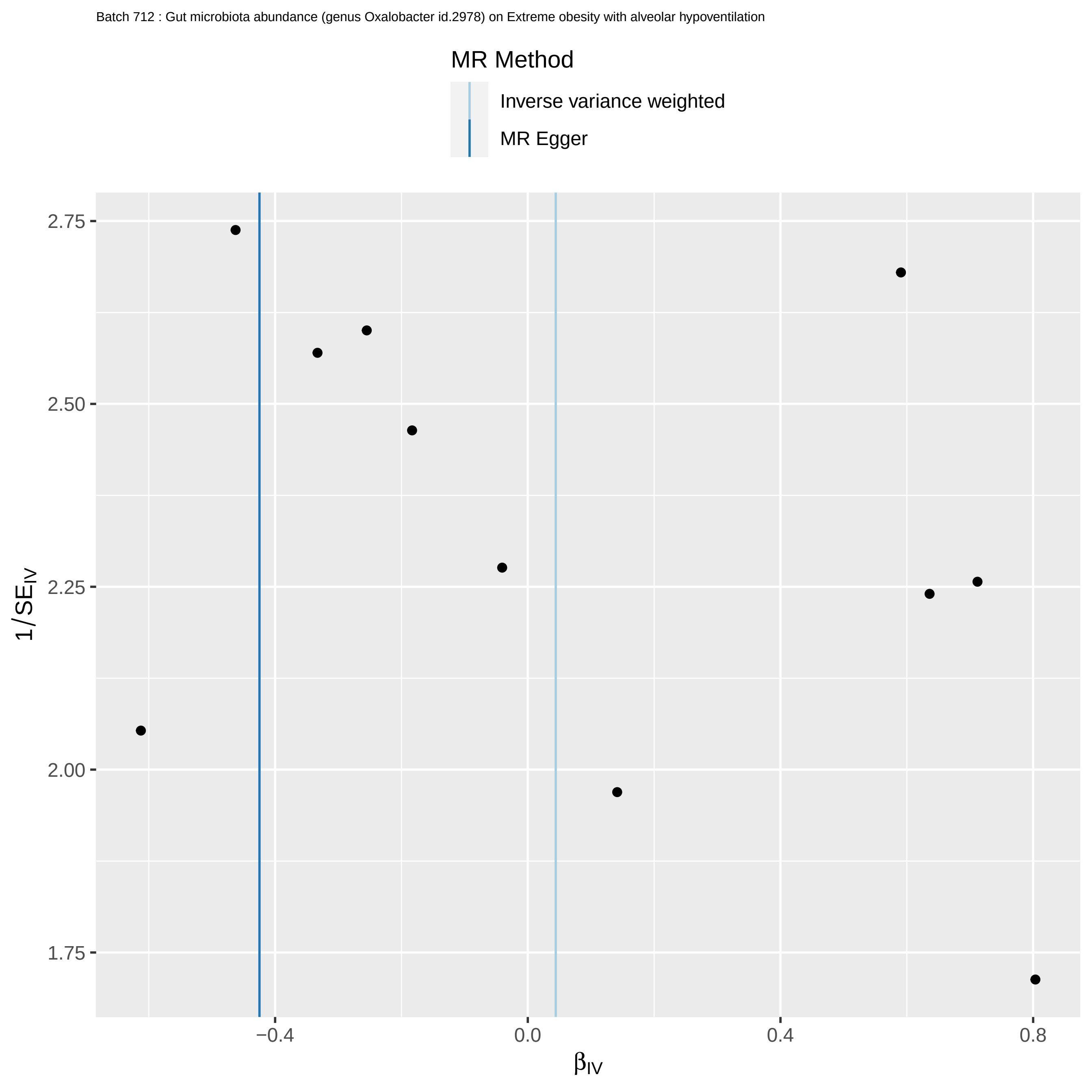

Batch 712 : Gut microbiota abundance (genus Oxalobacter id.2978) on Extreme obesity with alveolar hypoventilation
MR Method
Inverse variance weighted
MR Egger
2.75
2.50
2.25
2.00
1.75
1/SE_IV
−0.4
0.0
0.4
0.8
β_IV

Batch 713 : Gut microbiota abundance (genus Parabacteroides id.954) on Extreme obesity with alveolar hypoventilation

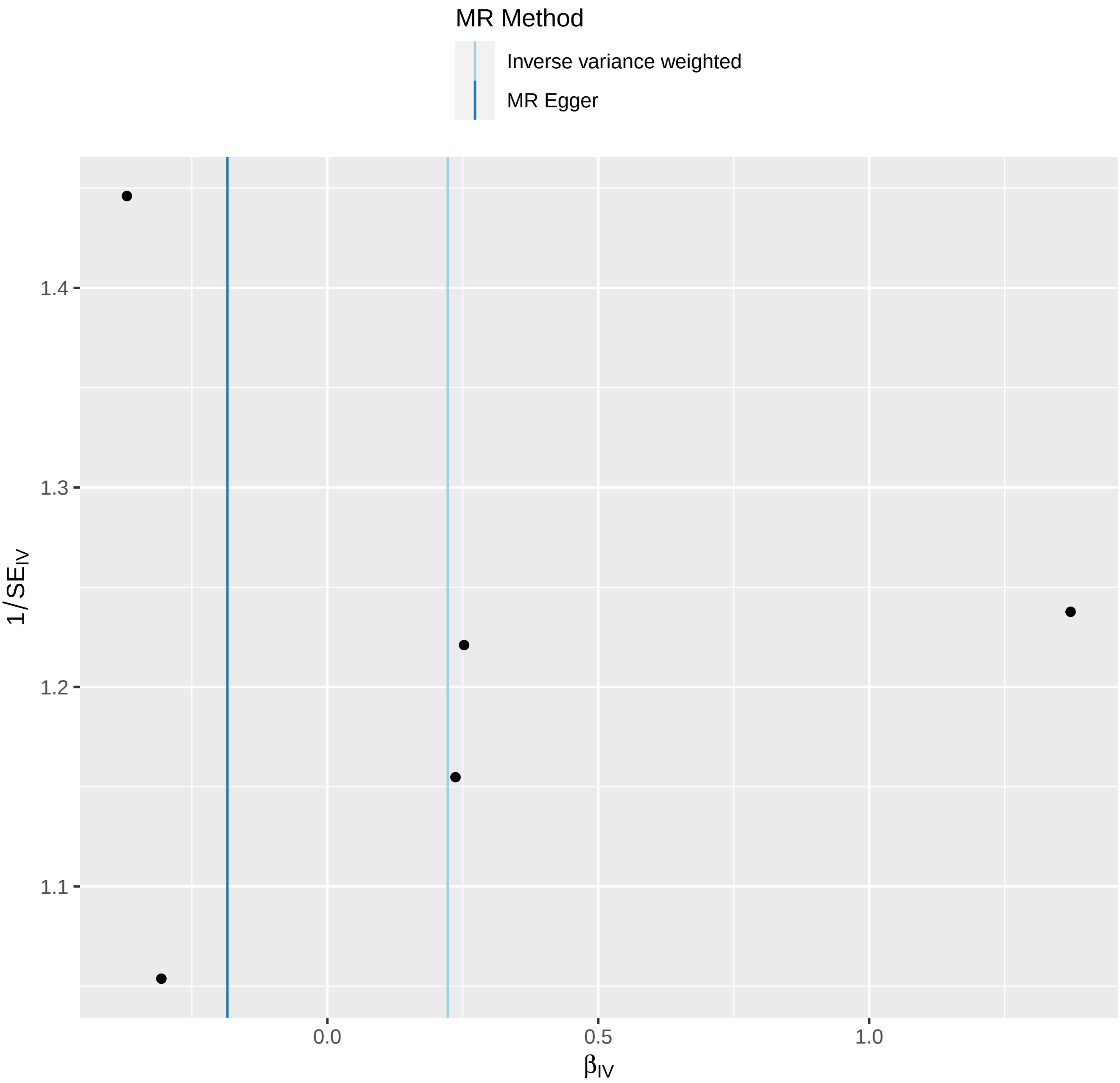

Batch 714 : Gut microbiota abundance (genus Paraprevotella id.962) on Extreme obesity with alveolar hypoventilation

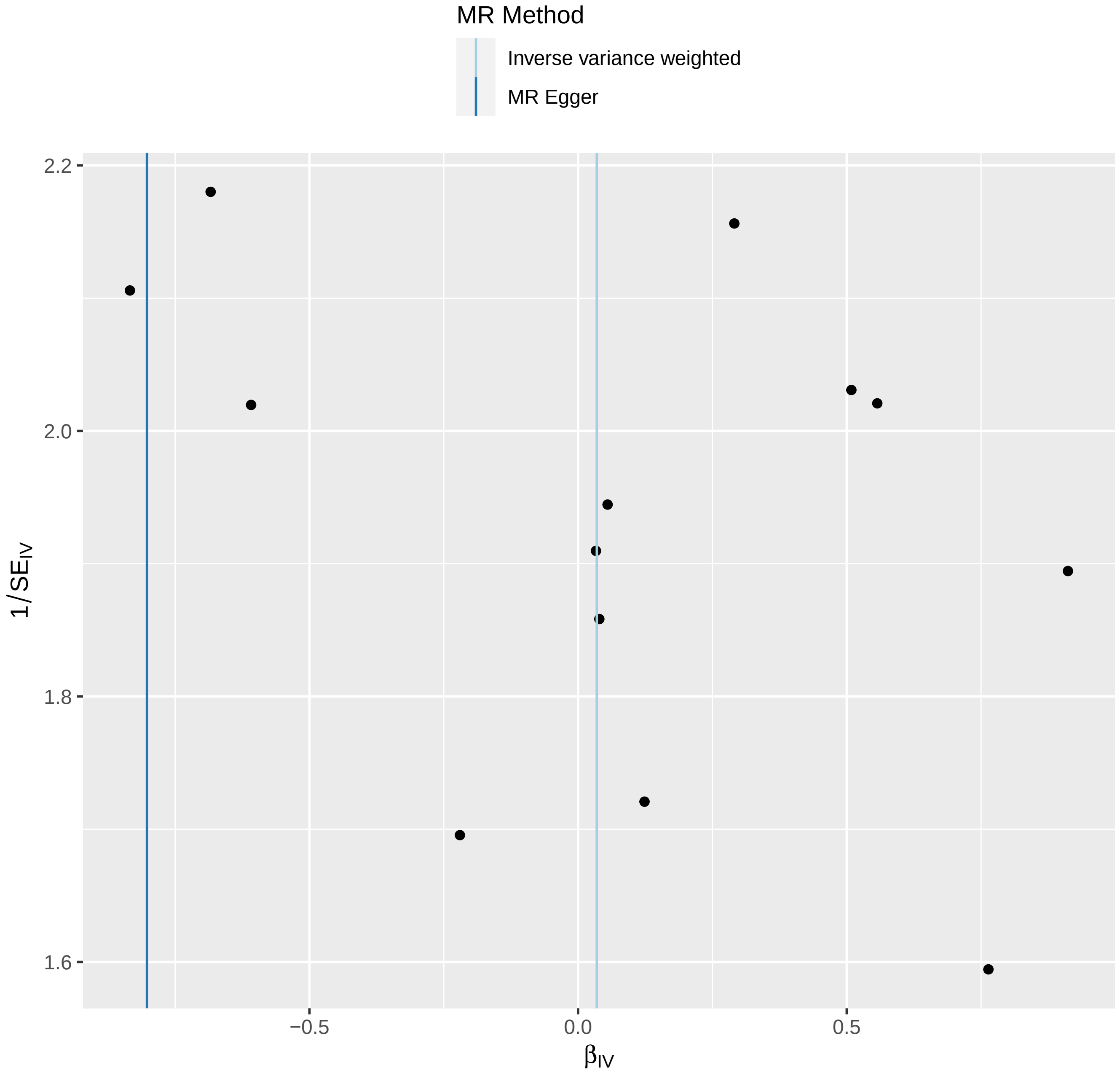

Batch 715 : Gut microbiota abundance (genus Parasutterella id.2892) on Extreme obesity with alveolar hypoventilation

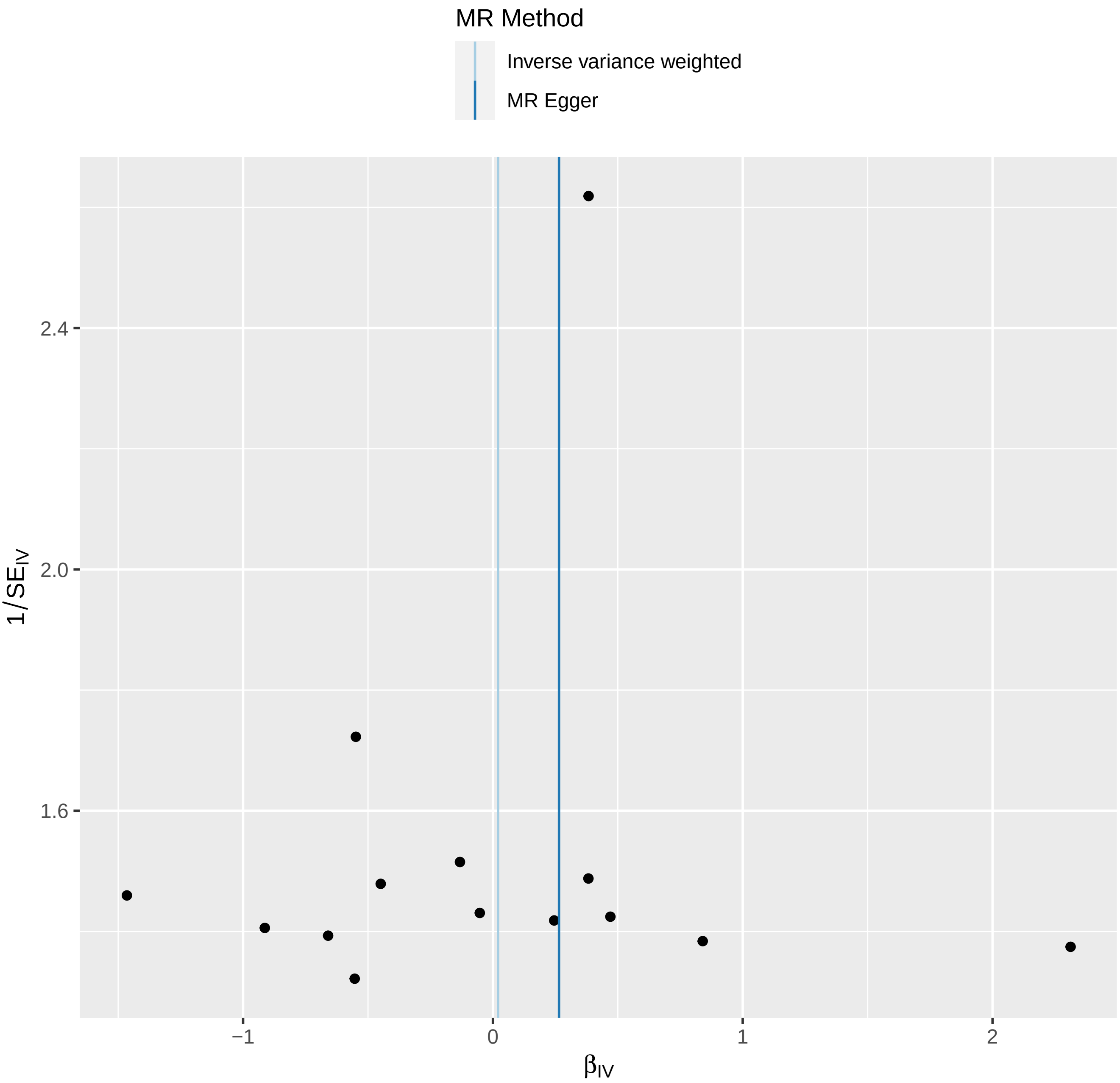

Batch 716 : Gut microbiota abundance (genus Peptococcus id.2037) on Extreme obesity with alveolar hypoventilation

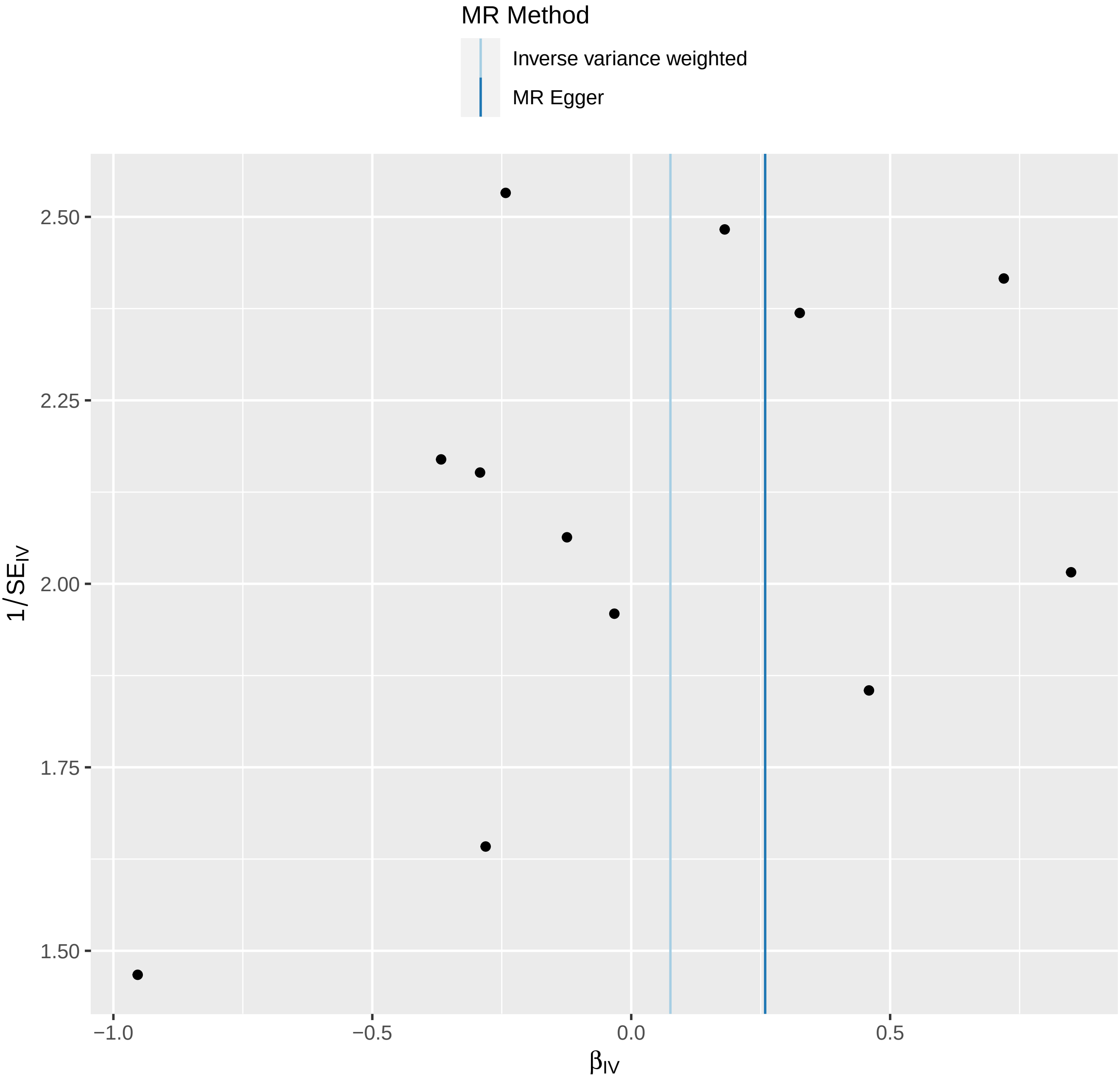

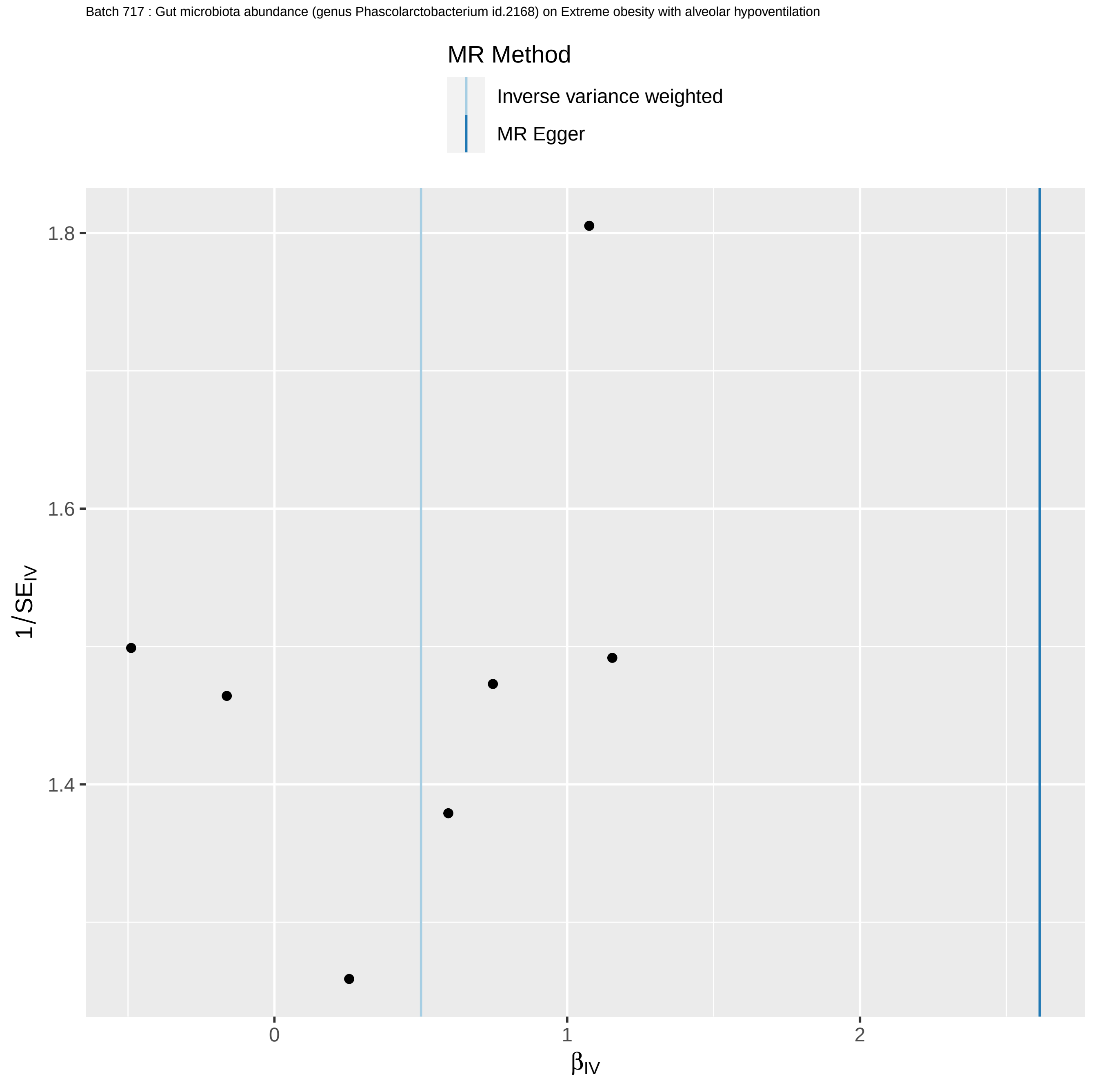

Batch 717 : Gut microbiota abundance (genus Phascolarctobacterium id.2168) on Extreme obesity with alveolar hypoventilation
MR Method
Inverse variance weighted
MR Egger
1.8
1.6
1.4
1/SE_IV
0
1
2
β_IV

Batch 718 : Gut microbiota abundance (genus Prevotella7 id.11182) on Extreme obesity with alveolar hypoventilation

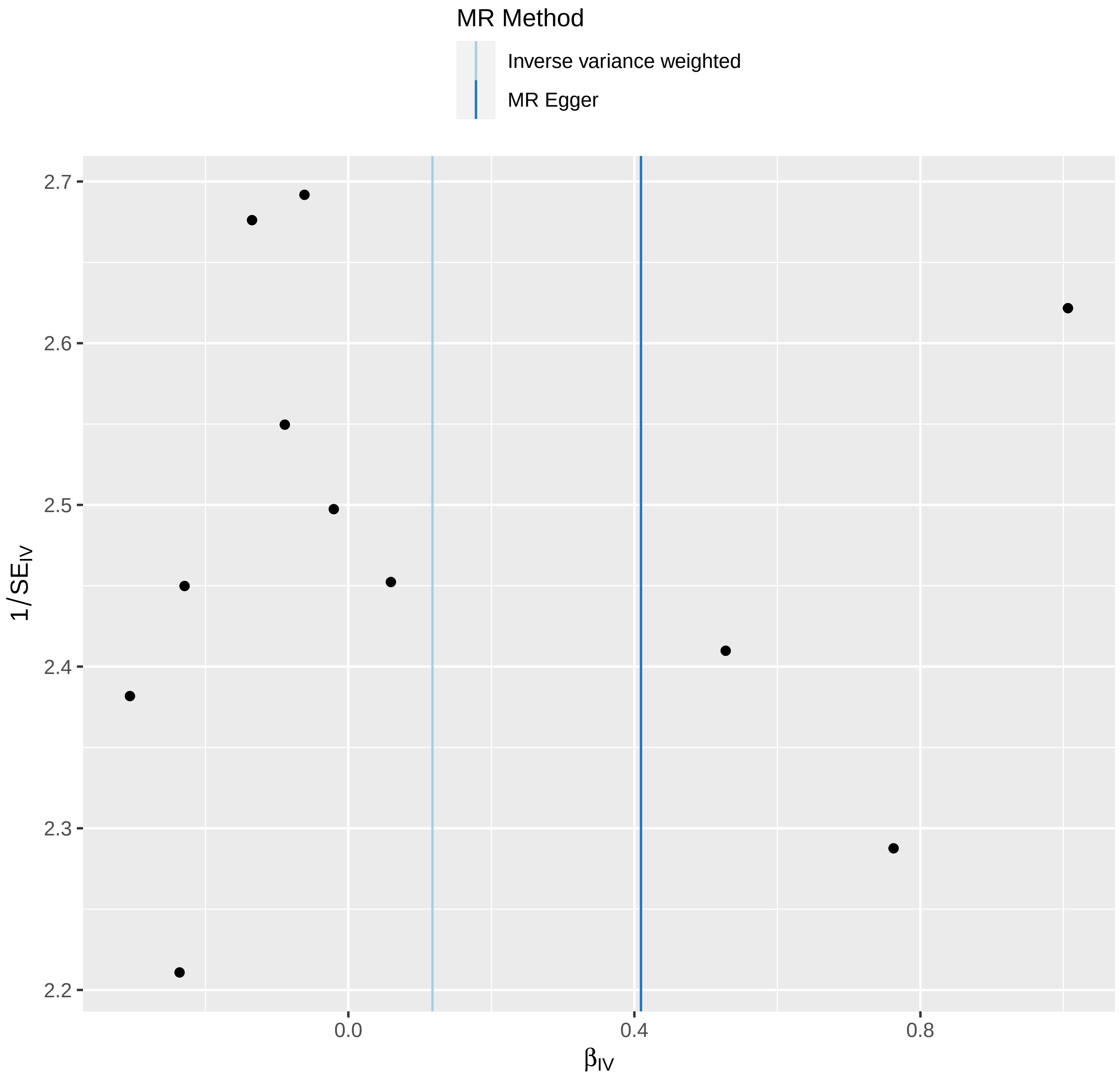

Batch 719 : Gut microbiota abundance (genus Prevotella9 id.11183) on Extreme obesity with alveolar hypoventilation

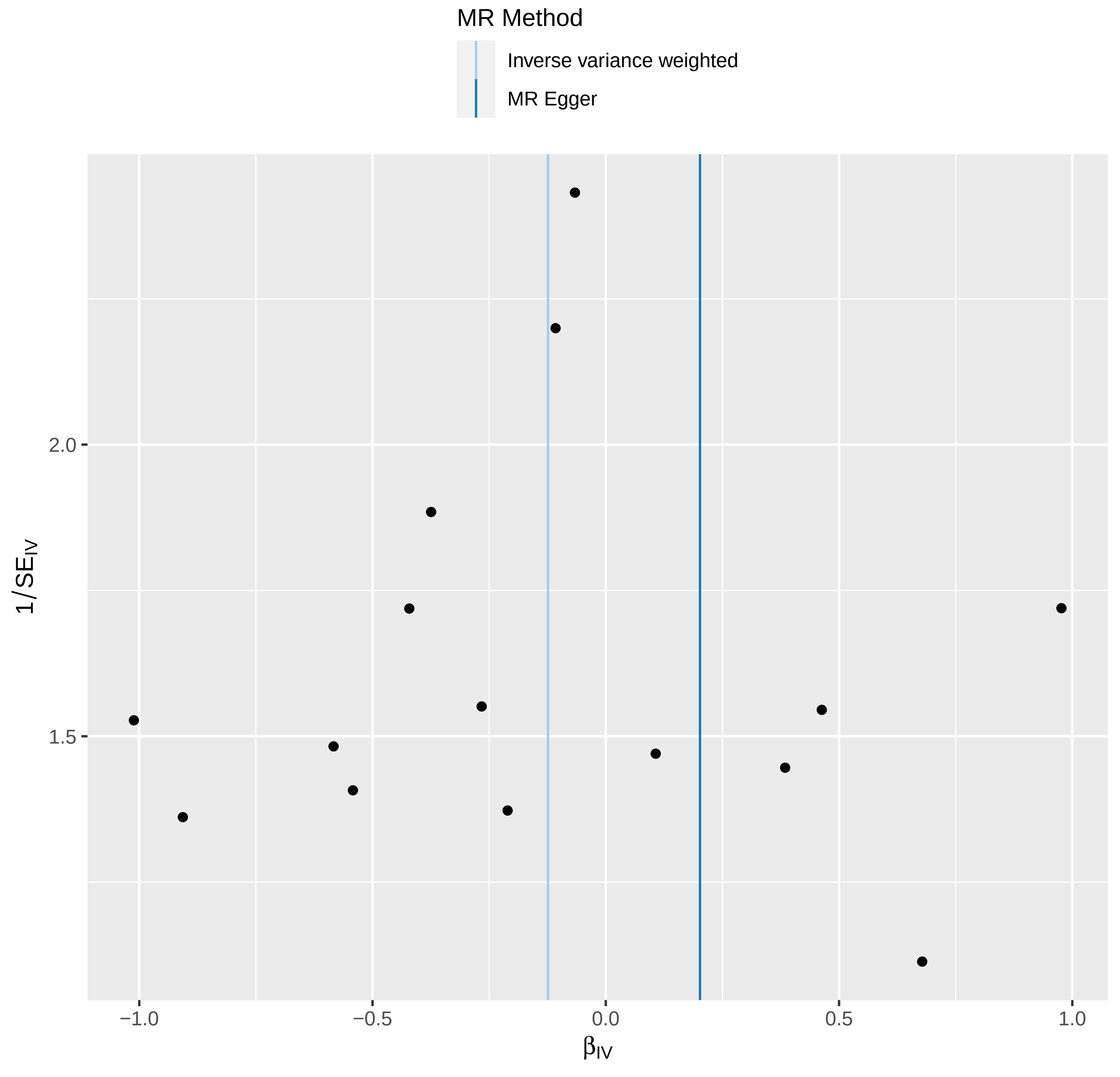

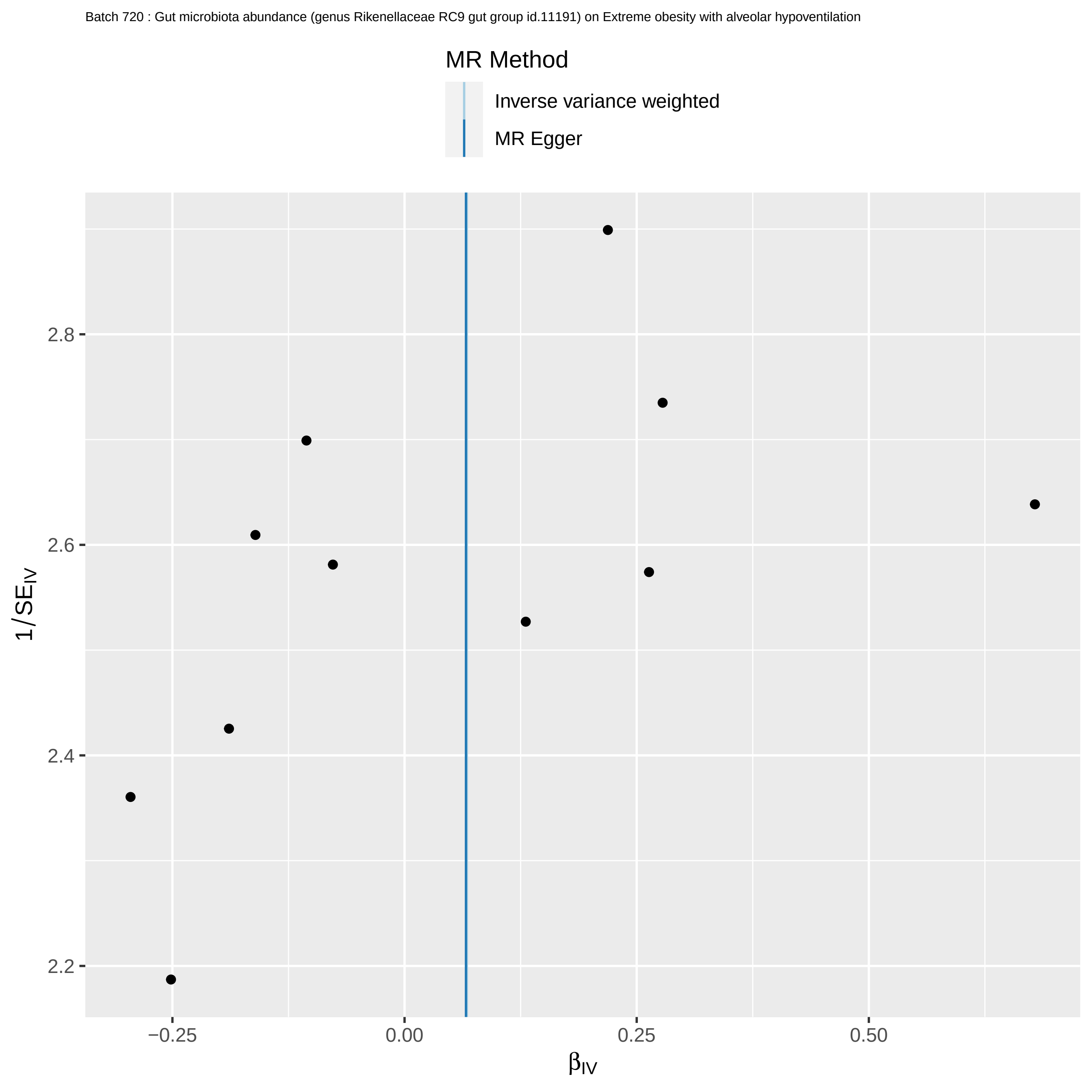

Batch 720 : Gut microbiota abundance (genus Rikenellaceae RC9 gut group id.11191) on Extreme obesity with alveolar hypoventilation
MR Method
Inverse variance weighted
MR Egger
2.8
2.6
2.4
2.2
1/SE$_{IV}$
−0.25
0.00
0.25
0.50
β$_{IV}$

Batch 721 : Gut microbiota abundance (genus Romboutsia id.11347) on Extreme obesity with alveolar hypoventilation

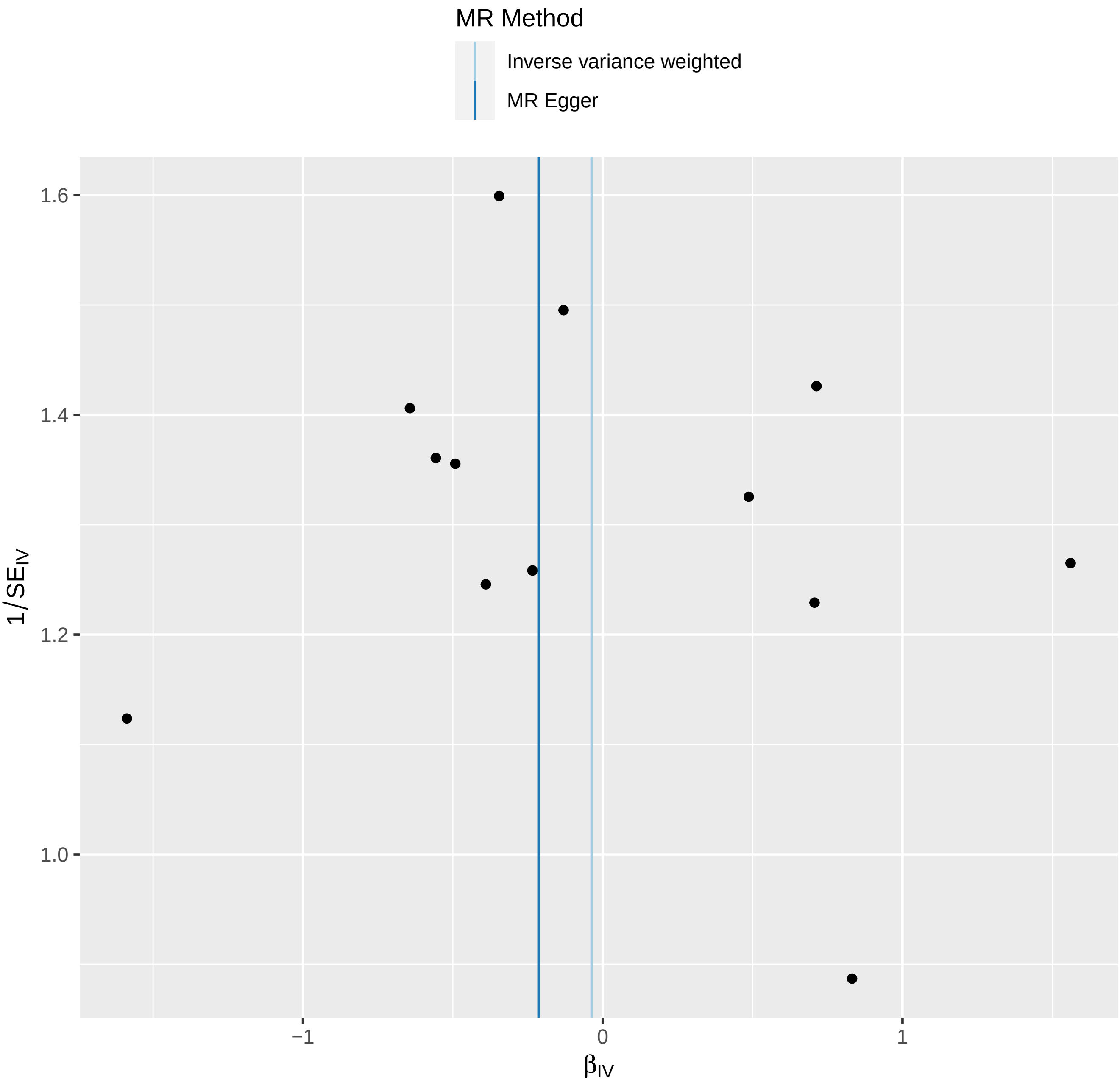

Batch 722 : Gut microbiota abundance (genus Roseburia id.2012) on Extreme obesity with alveolar hypoventilation

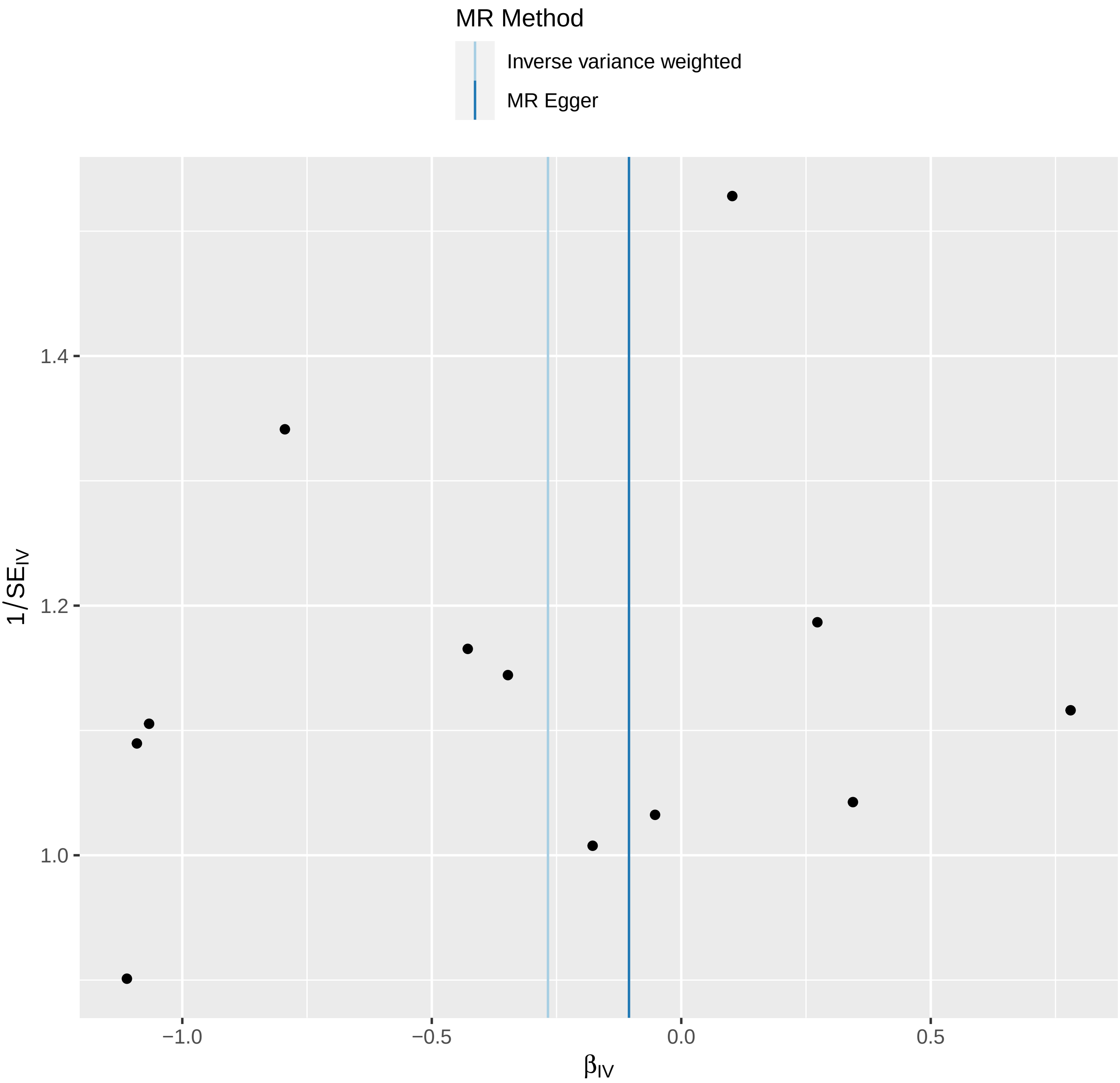

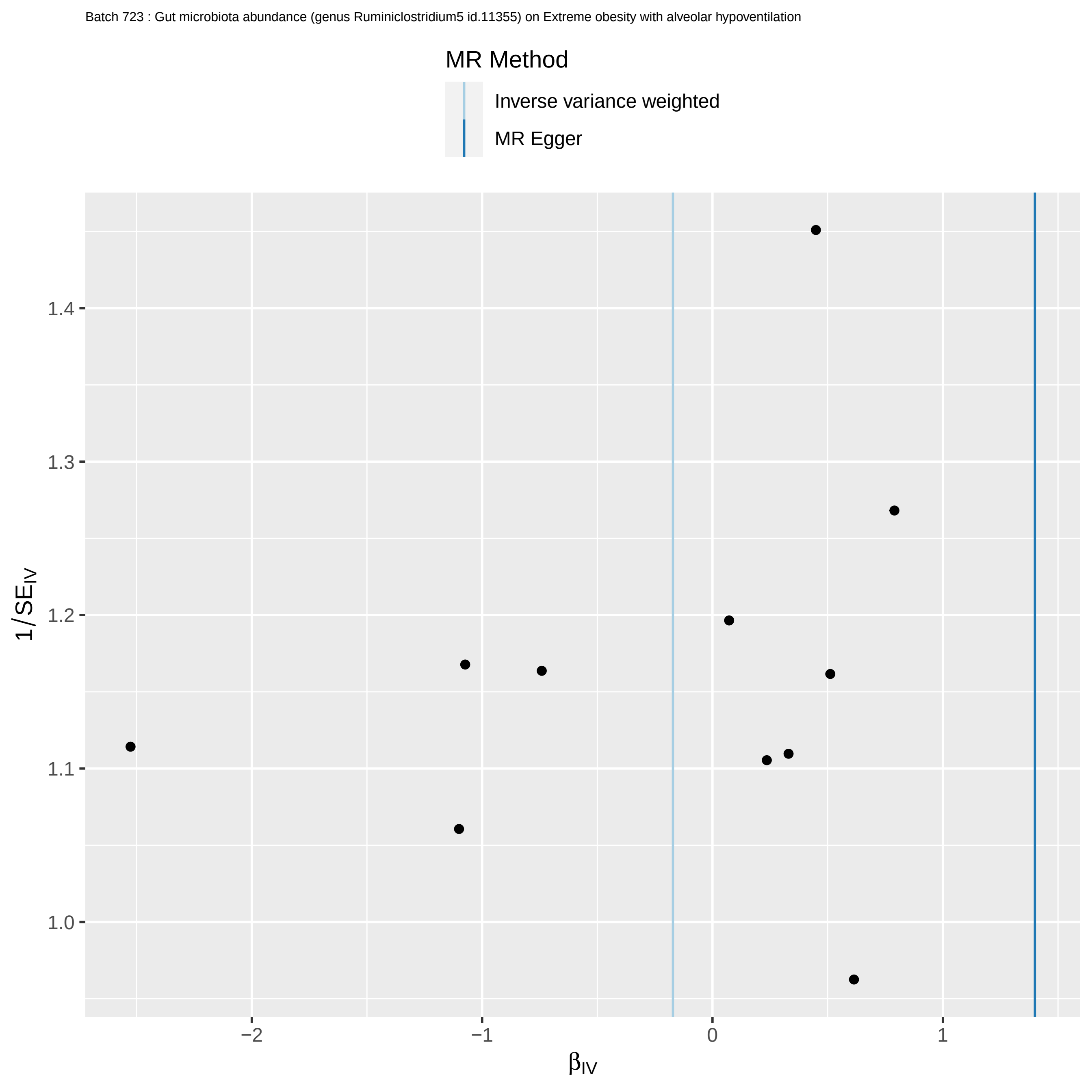
Batch 723 : Gut microbiota abundance (genus Ruminiclostridium5 id.11355) on Extreme obesity with alveolar hypoventilation
MR Method
Inverse variance weighted
MR Egger
1.4
1.3
1.2
1.1
1.0
1/SE$_{IV}$
−2
−1
0
1
β$_{IV}$

Batch 724 : Gut microbiota abundance (genus Ruminiclostridium6 id.11356) on Extreme obesity with alveolar hypoventilation

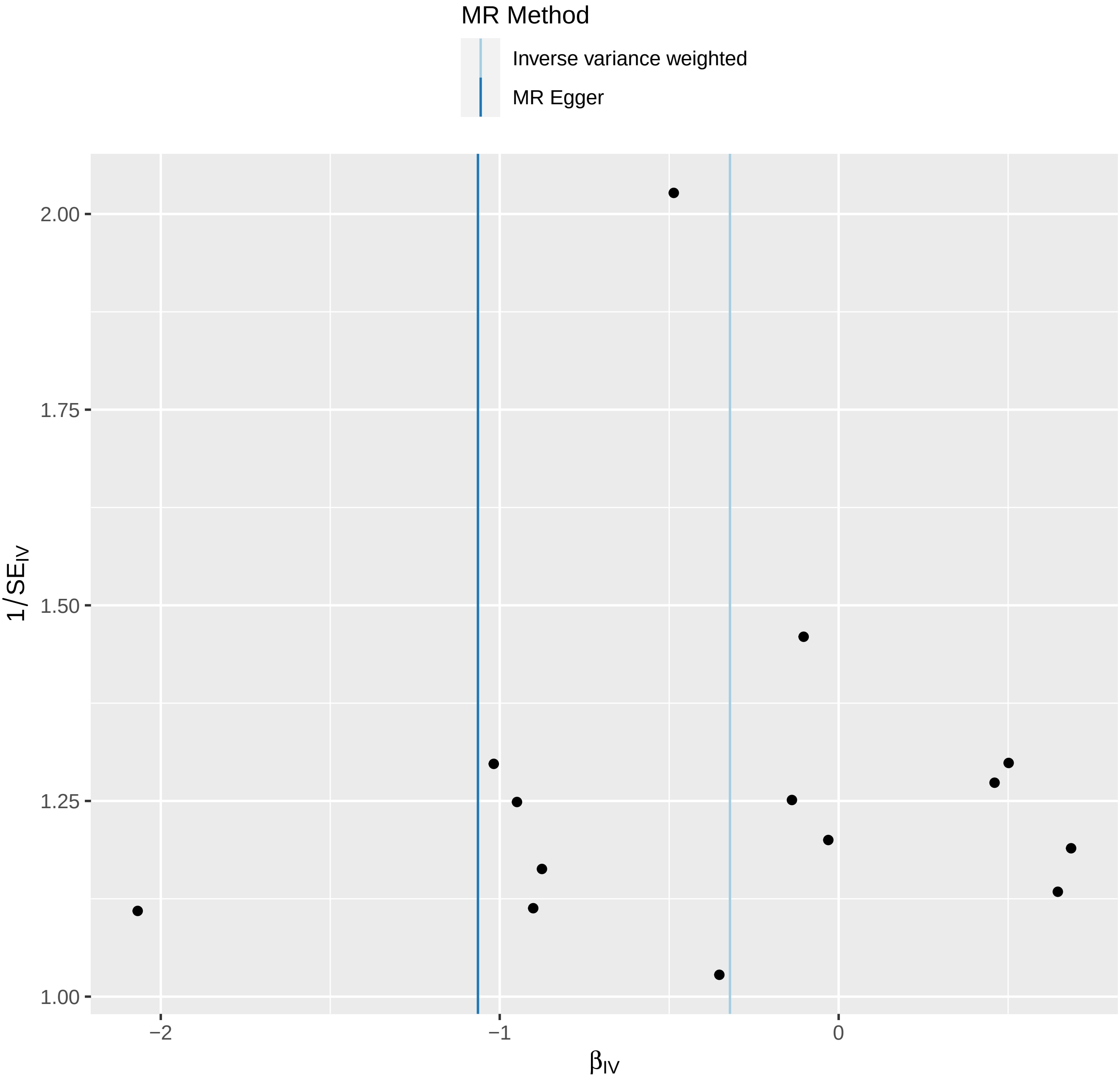

Batch 725 : Gut microbiota abundance (genus Ruminiclostridium9 id.11357) on Extreme obesity with alveolar hypoventilation

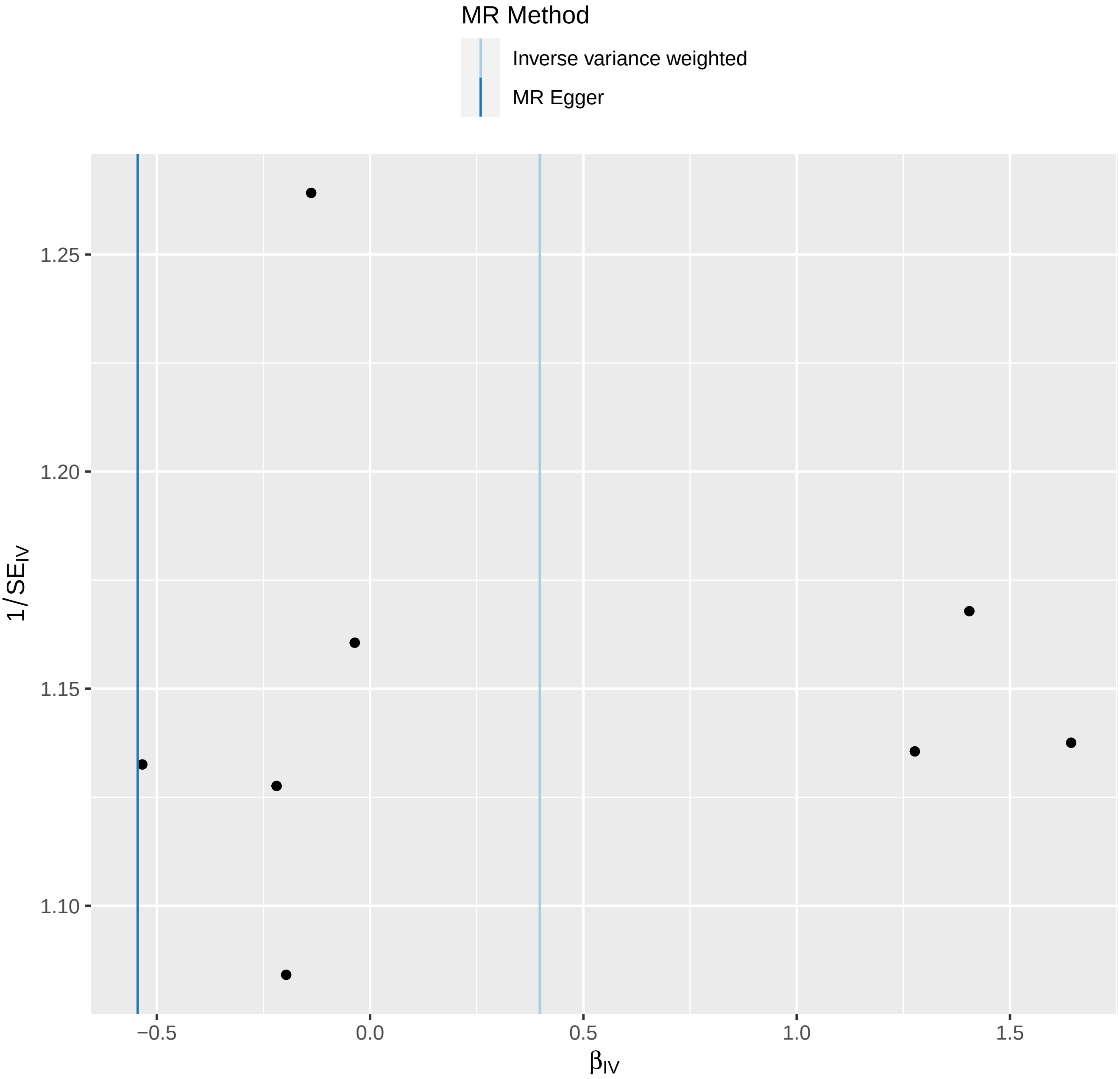

Batch 726 : Gut microbiota abundance (genus Ruminococcaceae NK4A214 group id.11358) on Extreme obesity with alveolar hypoventilation

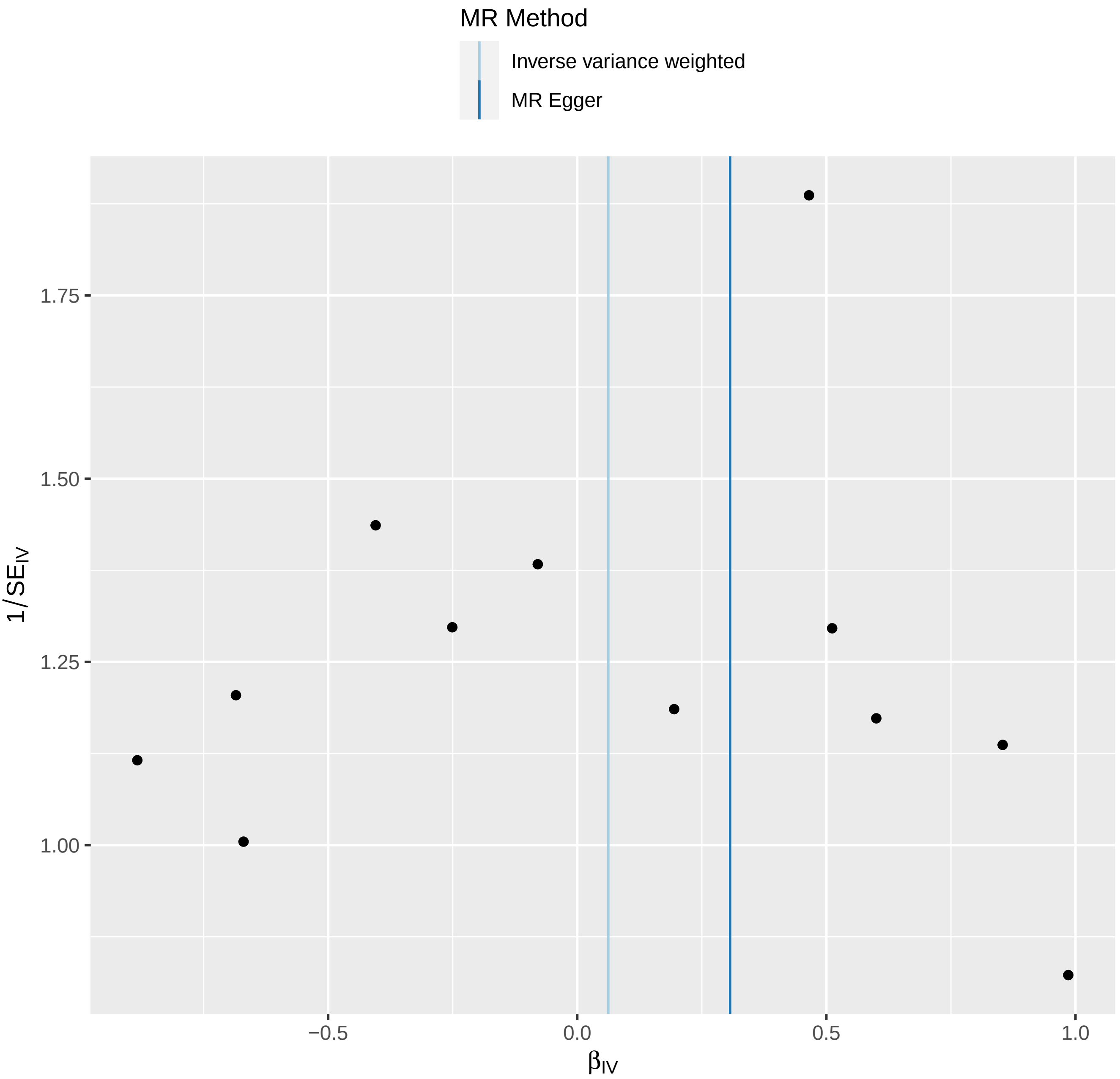

Batch 727 : Gut microbiota abundance (genus Ruminococcaceae UCG002 id.11360) on Extreme obesity with alveolar hypoventilation

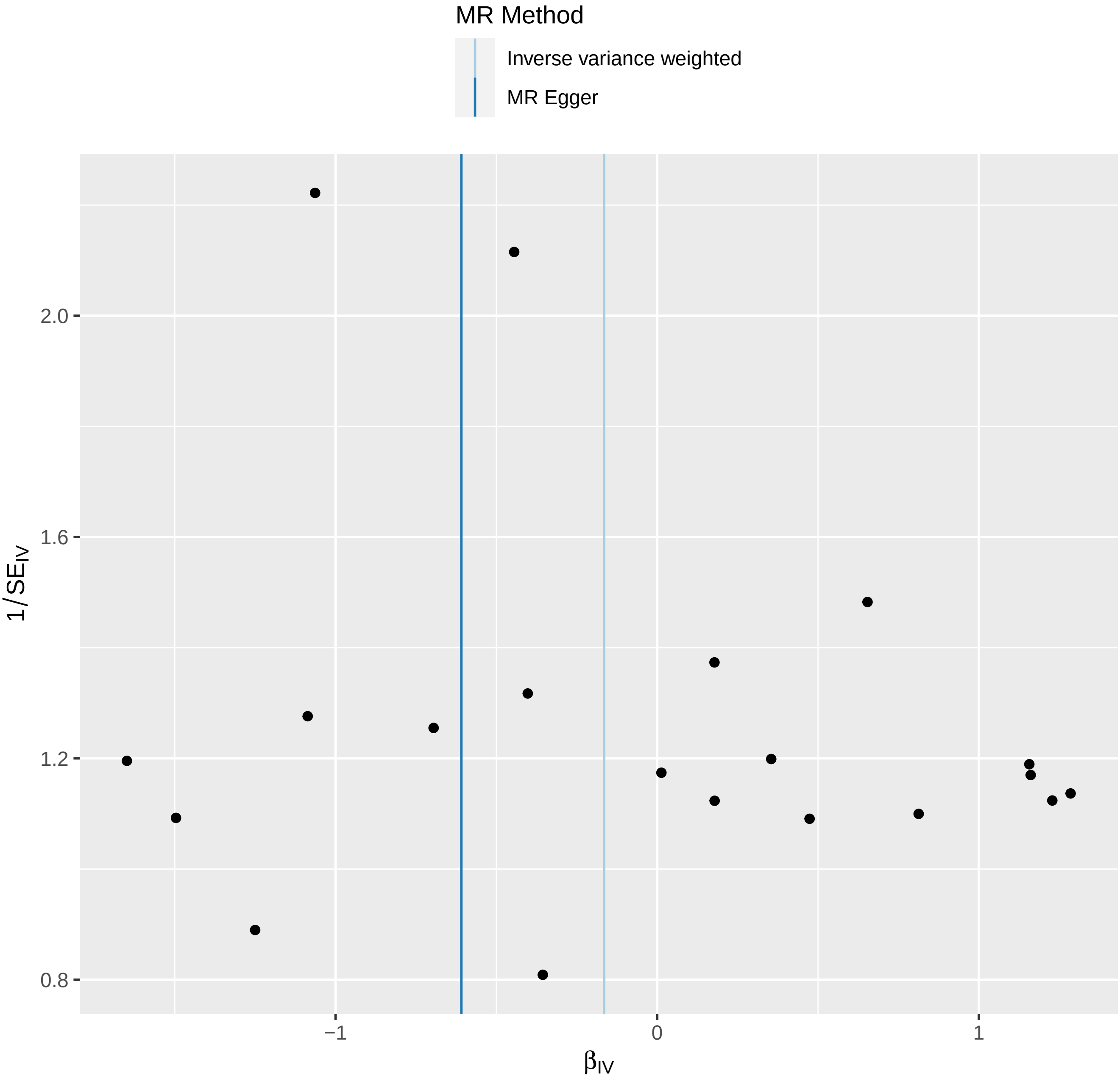

Batch 728 : Gut microbiota abundance (genus Ruminococcaceae UCG003 id.11361) on Extreme obesity with alveolar hypoventilation

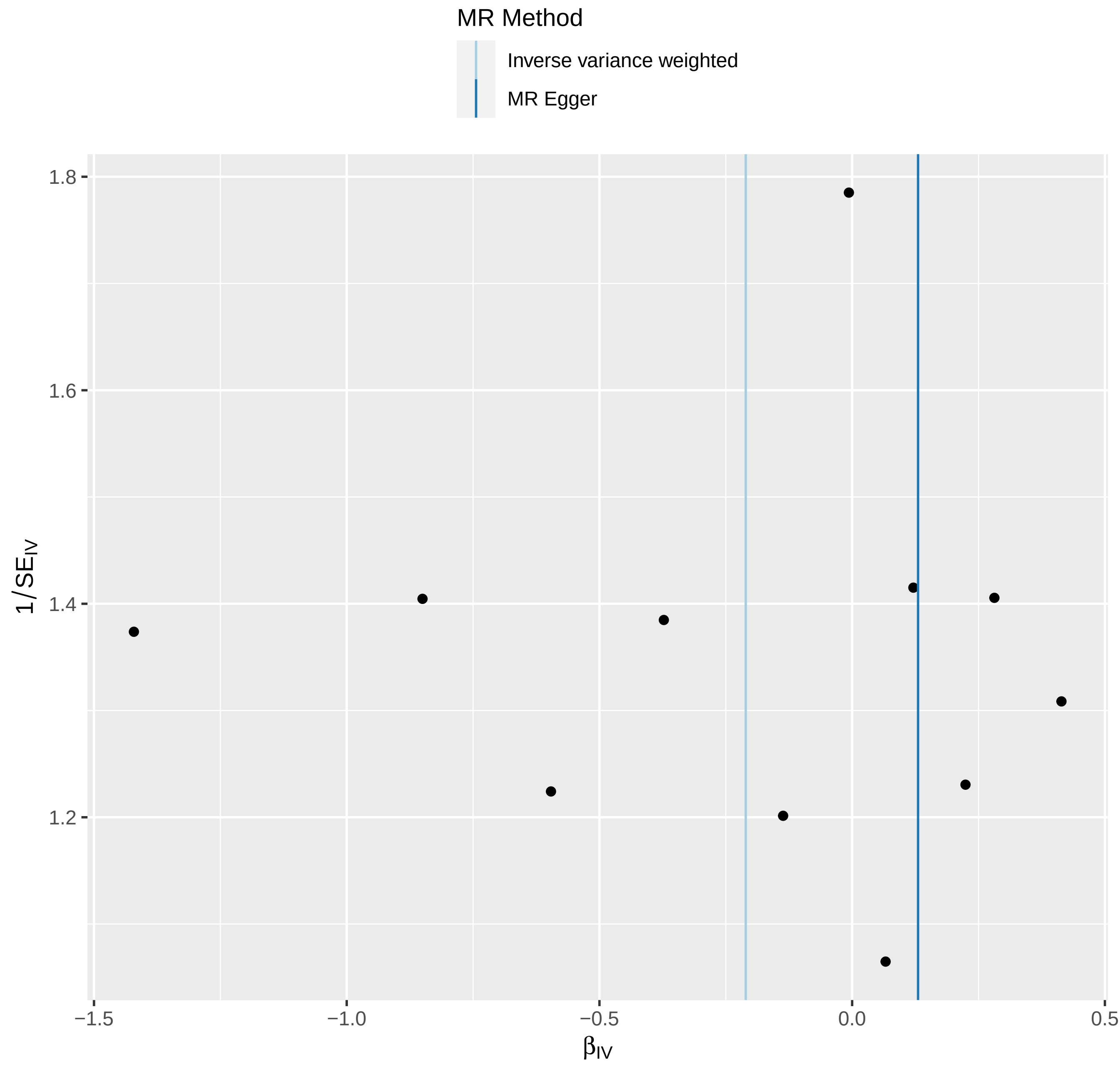

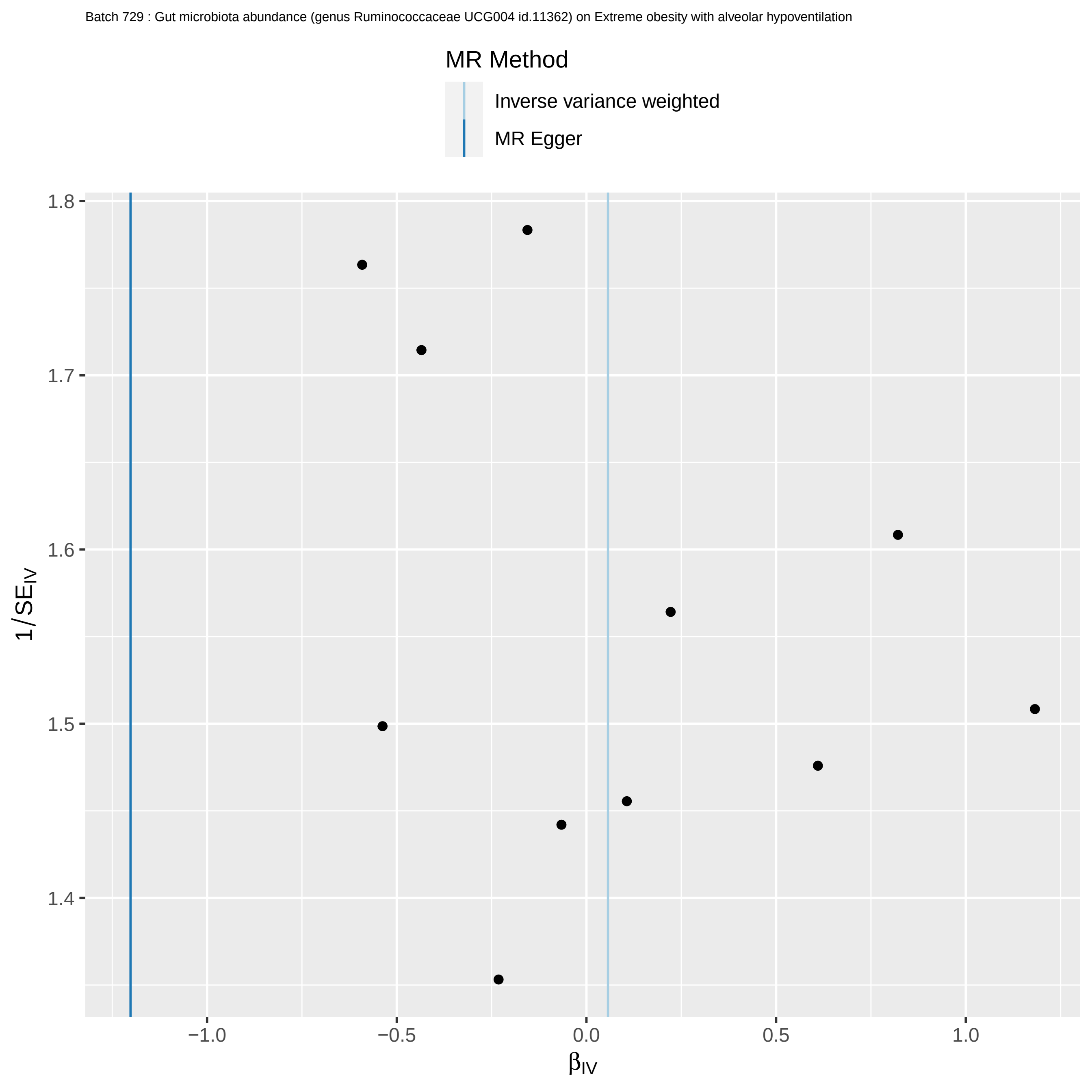

Batch 729 : Gut microbiota abundance (genus Ruminococcaceae UCG004 id.11362) on Extreme obesity with alveolar hypoventilation
MR Method
Inverse variance weighted
MR Egger
1.8
1.7
1.6
1.5
1.4
1/SEIV
−1.0
−0.5
0.0
0.5
1.0
βIV

Batch 730 : Gut microbiota abundance (genus Ruminococcaceae UCG005 id.11363) on Extreme obesity with alveolar hypoventilation

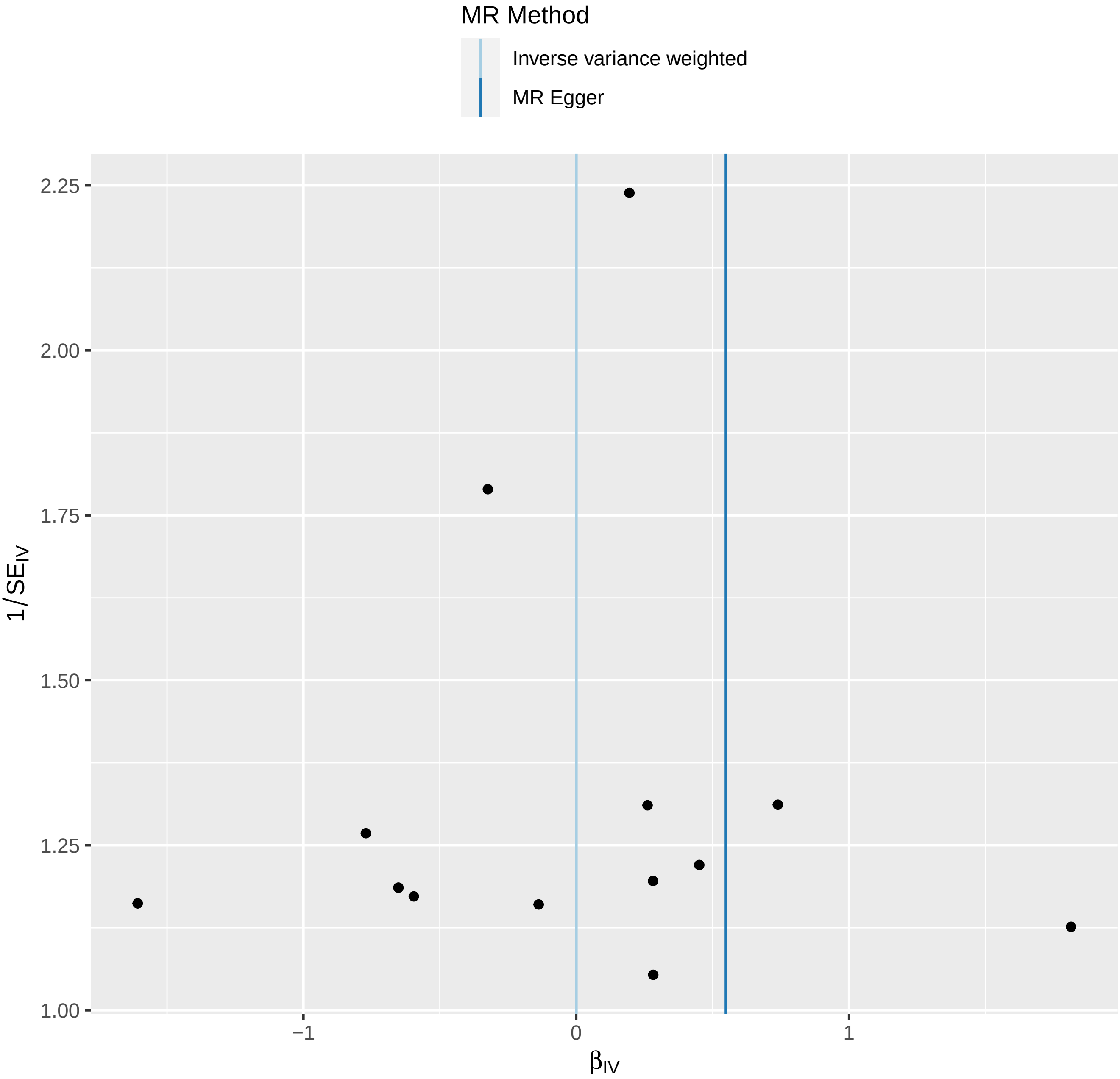

Batch 731 : Gut microbiota abundance (genus Ruminococcaceae UCG009 id.11366) on Extreme obesity with alveolar hypoventilation

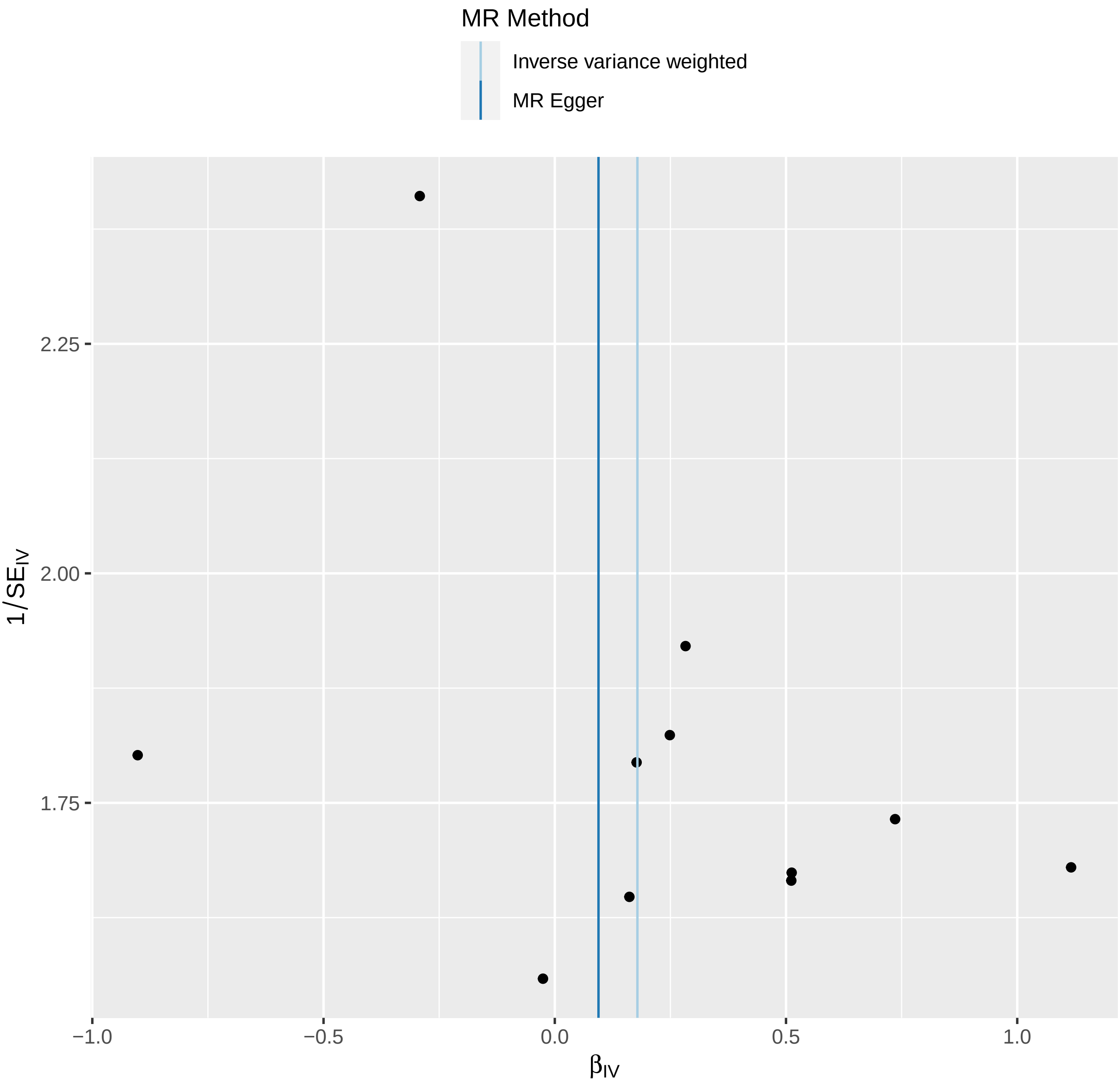

Batch 732 : Gut microbiota abundance (genus Ruminococcaceae UCG010 id.11367) on Extreme obesity with alveolar hypoventilation

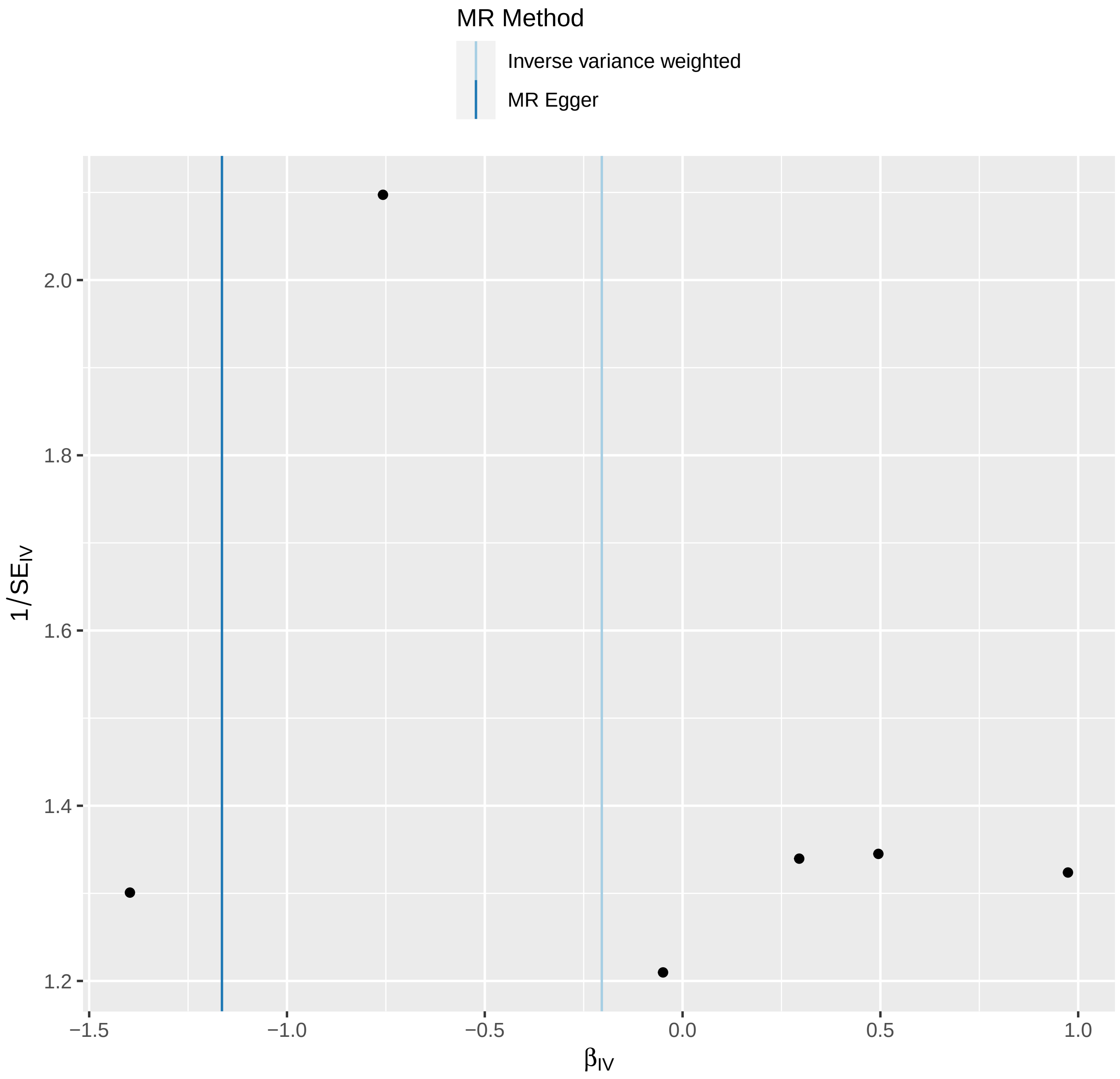

Batch 733 : Gut microbiota abundance (genus Ruminococcaceae UCG011 id.11368) on Extreme obesity with alveolar hypoventilation

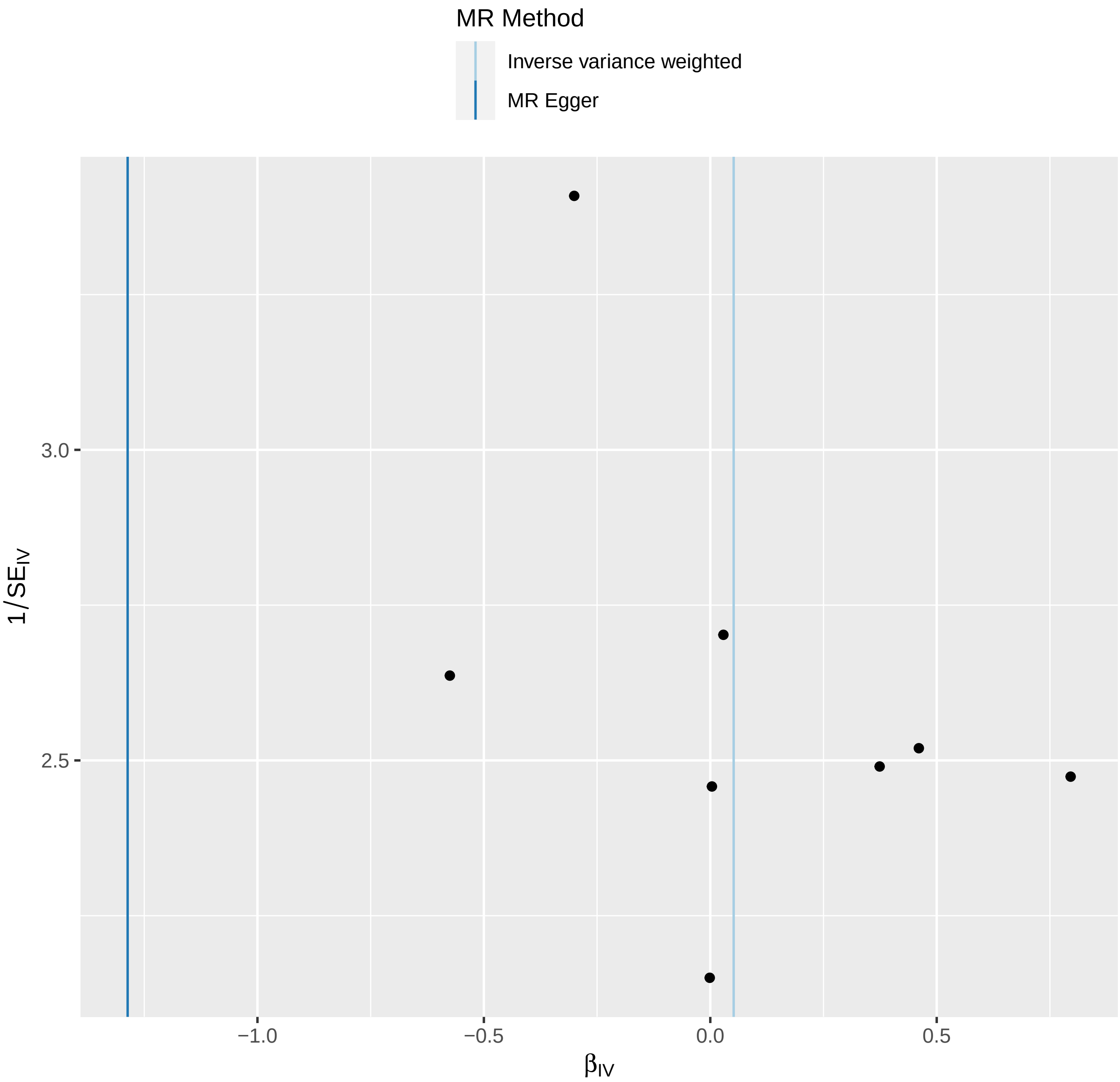

Batch 734 : Gut microbiota abundance (genus Ruminococcaceae UCG013 id.11370) on Extreme obesity with alveolar hypoventilation

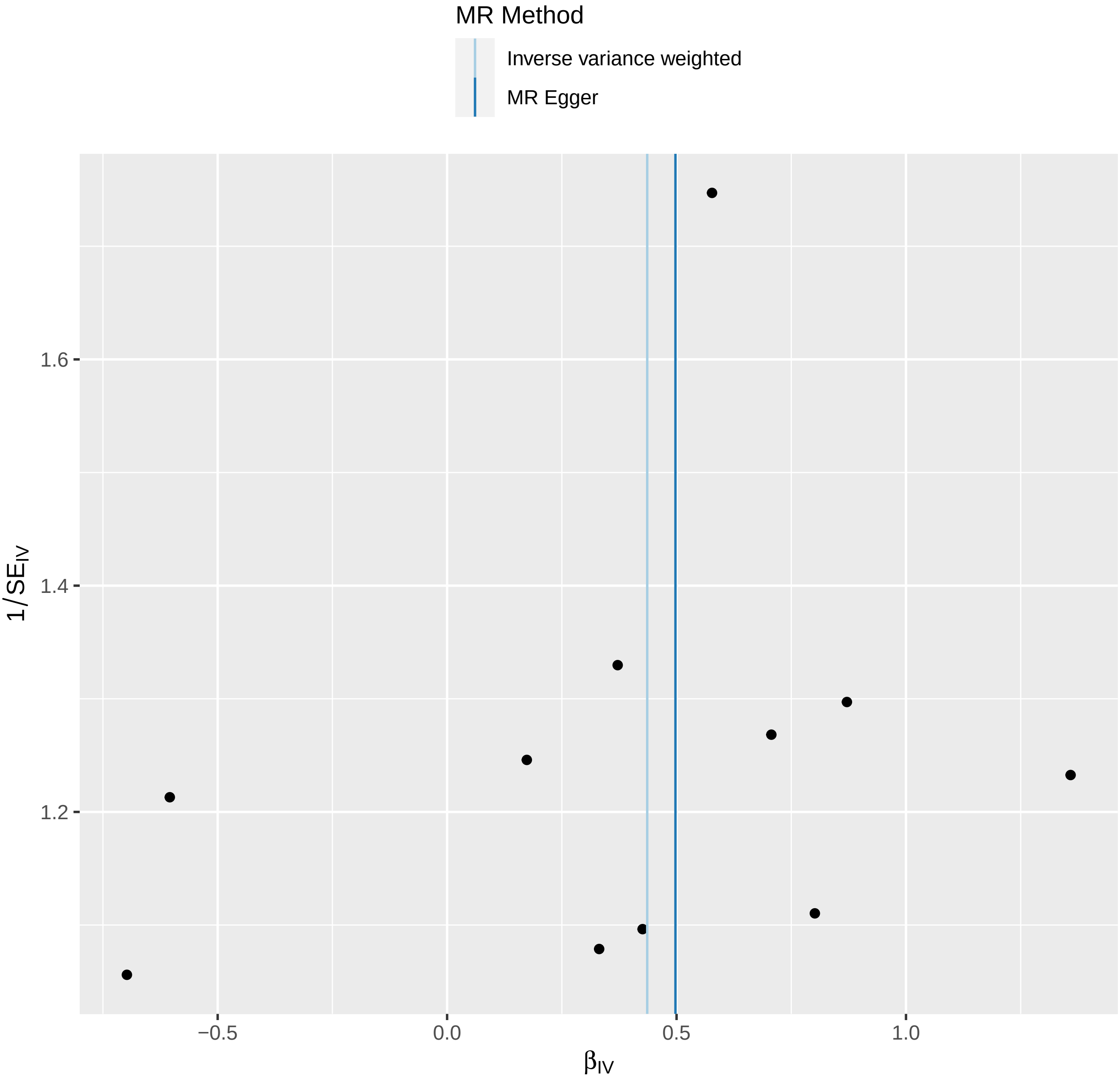

Batch 735 : Gut microbiota abundance (genus Ruminococcaceae UCG014 id.11371) on Extreme obesity with alveolar hypoventilation

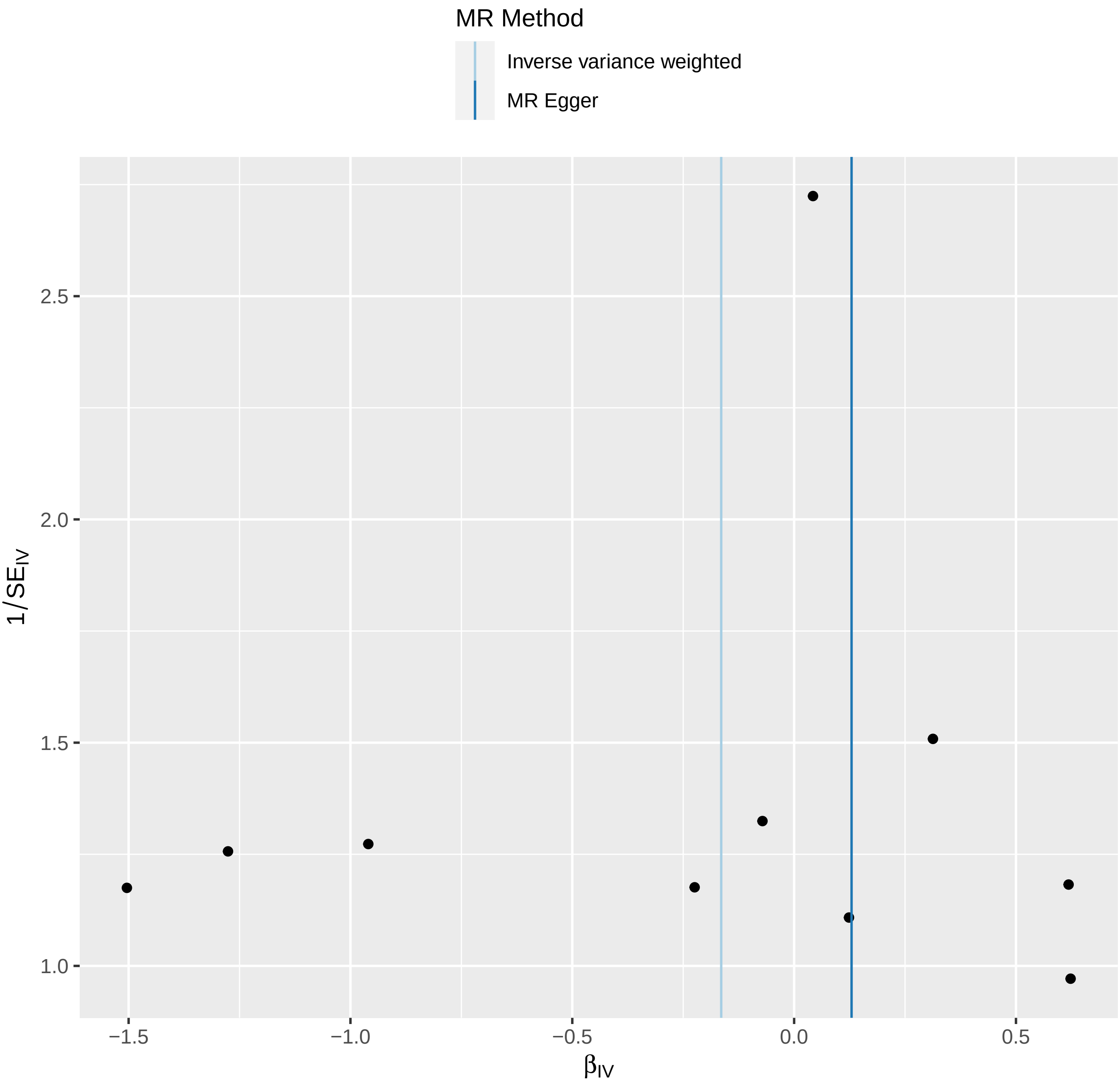

Batch 736 : Gut microbiota abundance (genus Ruminococcus1 id.11373) on Extreme obesity with alveolar hypoventilation

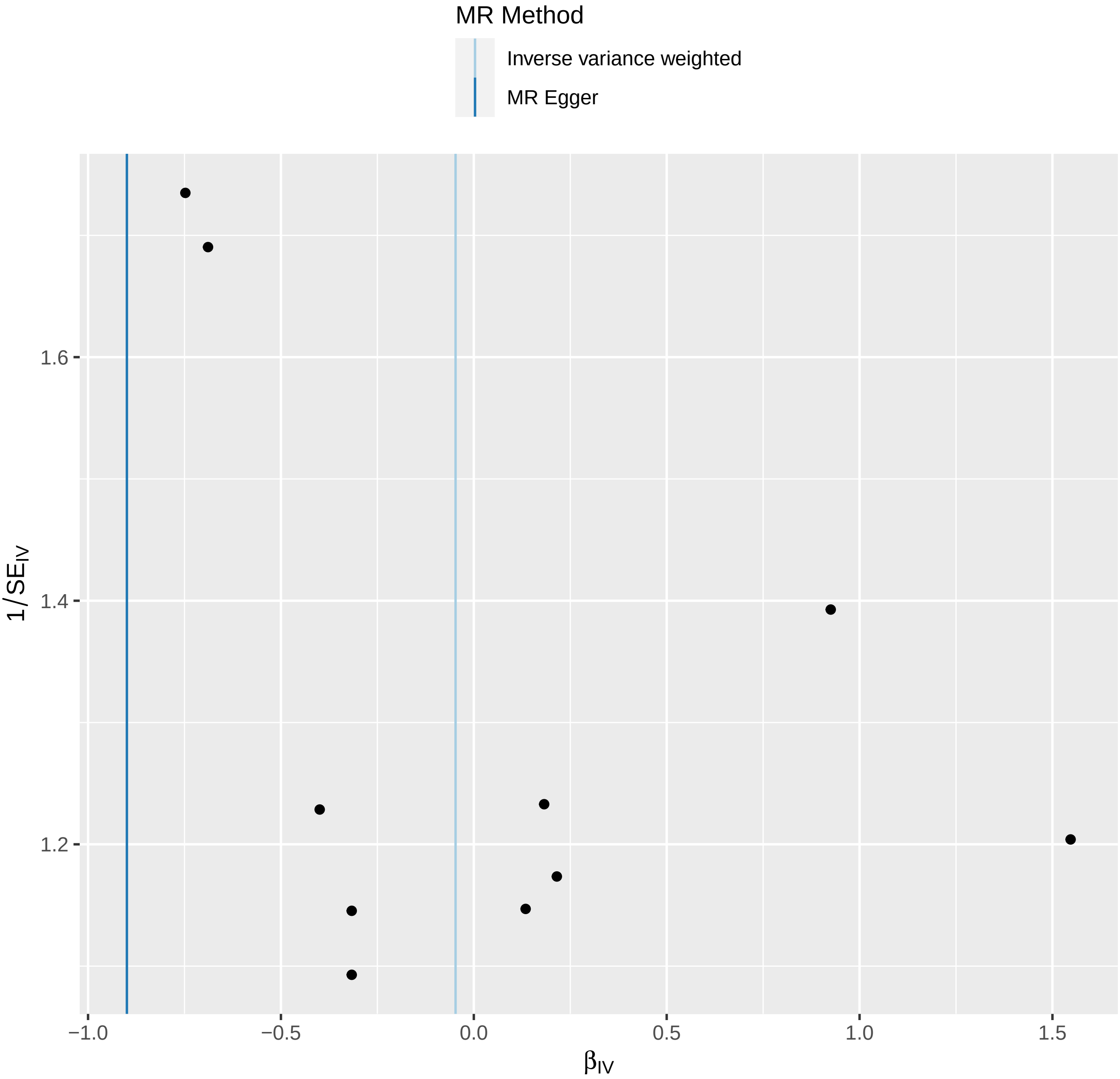

Batch 737 : Gut microbiota abundance (genus Ruminococcus2 id.11374) on Extreme obesity with alveolar hypoventilation

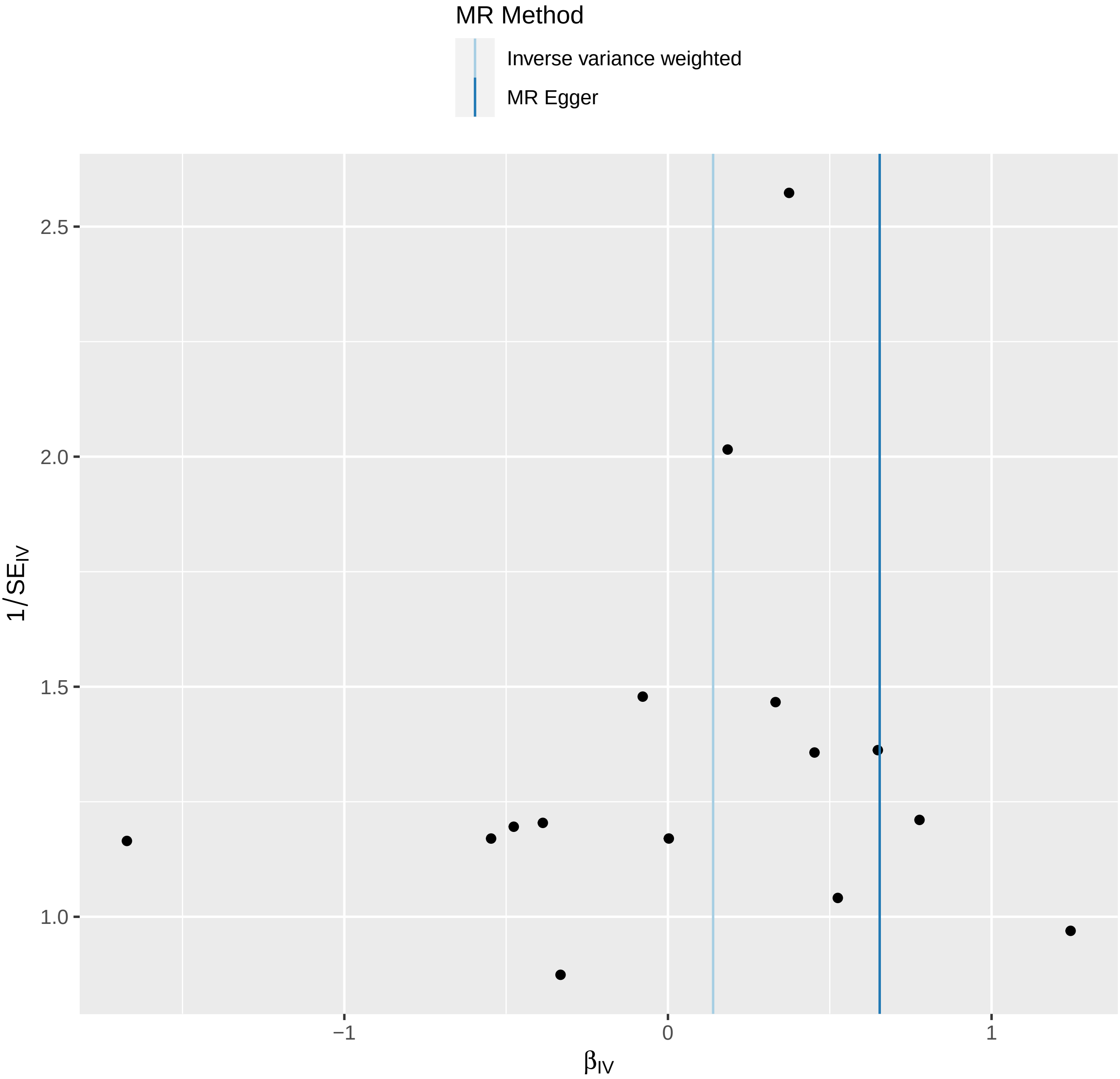

Batch 738 : Gut microbiota abundance (genus Ruminococcus gauvreauii group id.11342) on Extreme obesity with alveolar hypoventilation

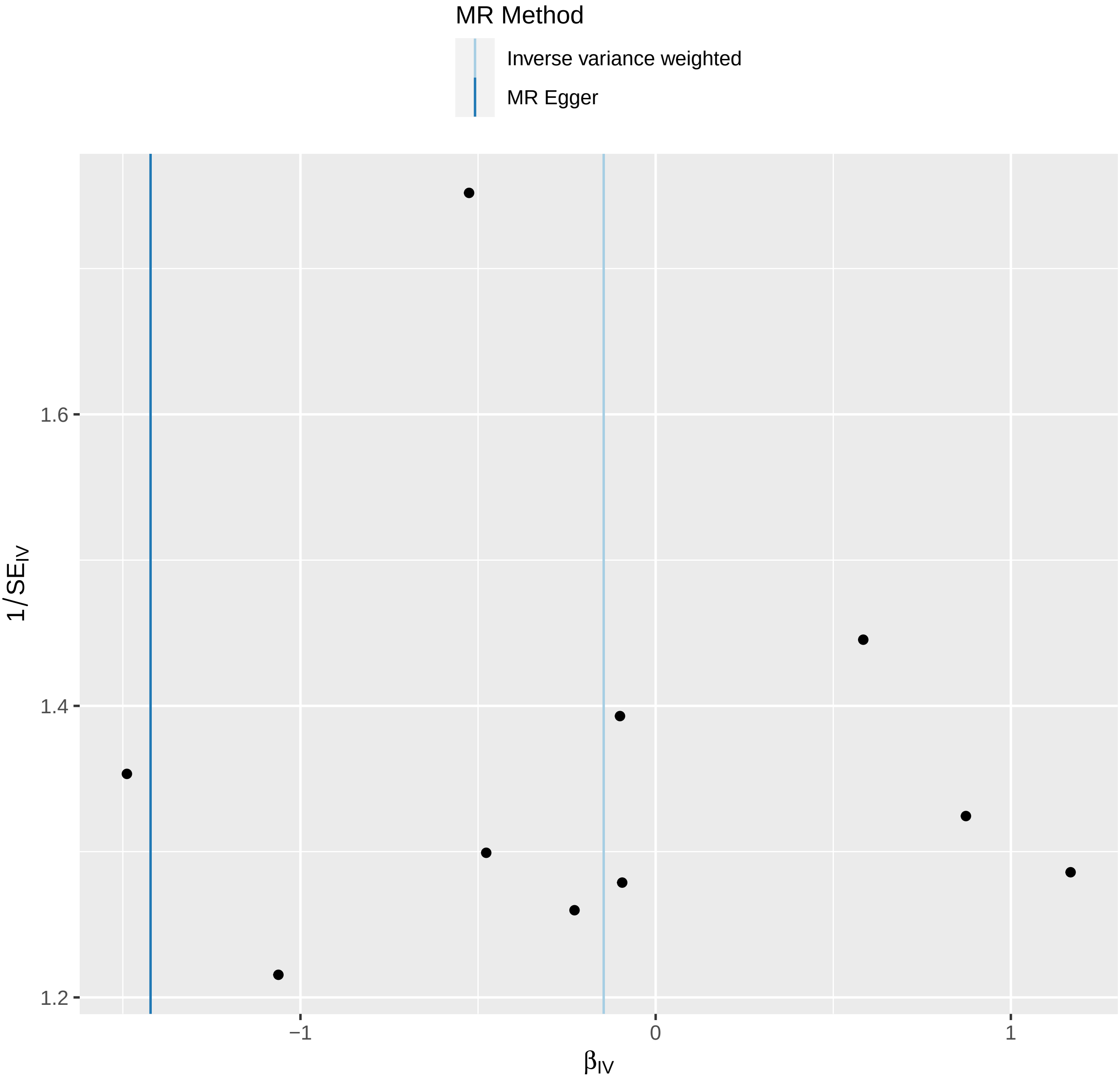

Batch 739 : Gut microbiota abundance (genus Ruminococcus gnavus group id.14376) on Extreme obesity with alveolar hypoventilation

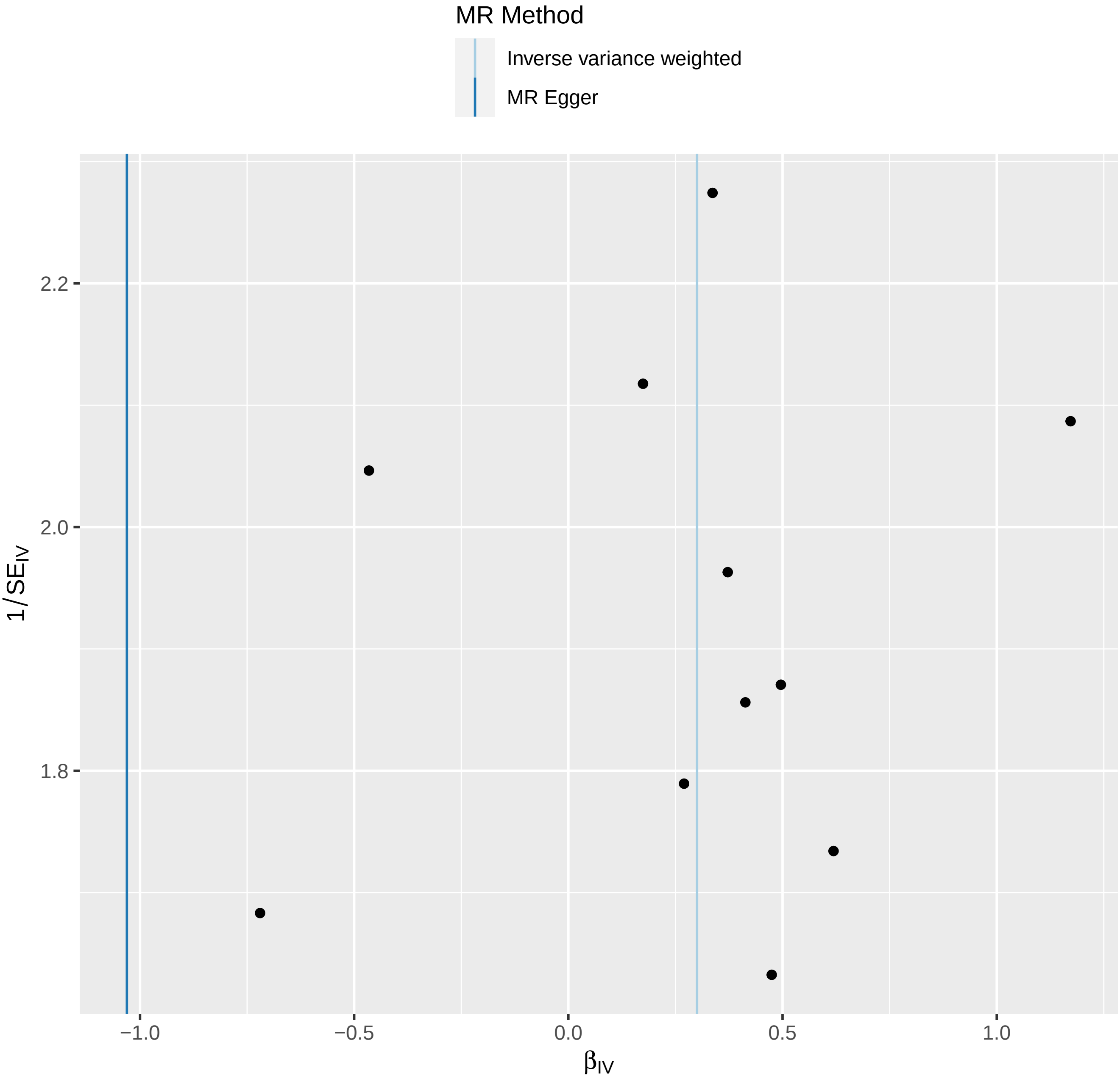

Batch 740 : Gut microbiota abundance (genus Ruminococcus torques group id.14377) on Extreme obesity with alveolar hypoventilation

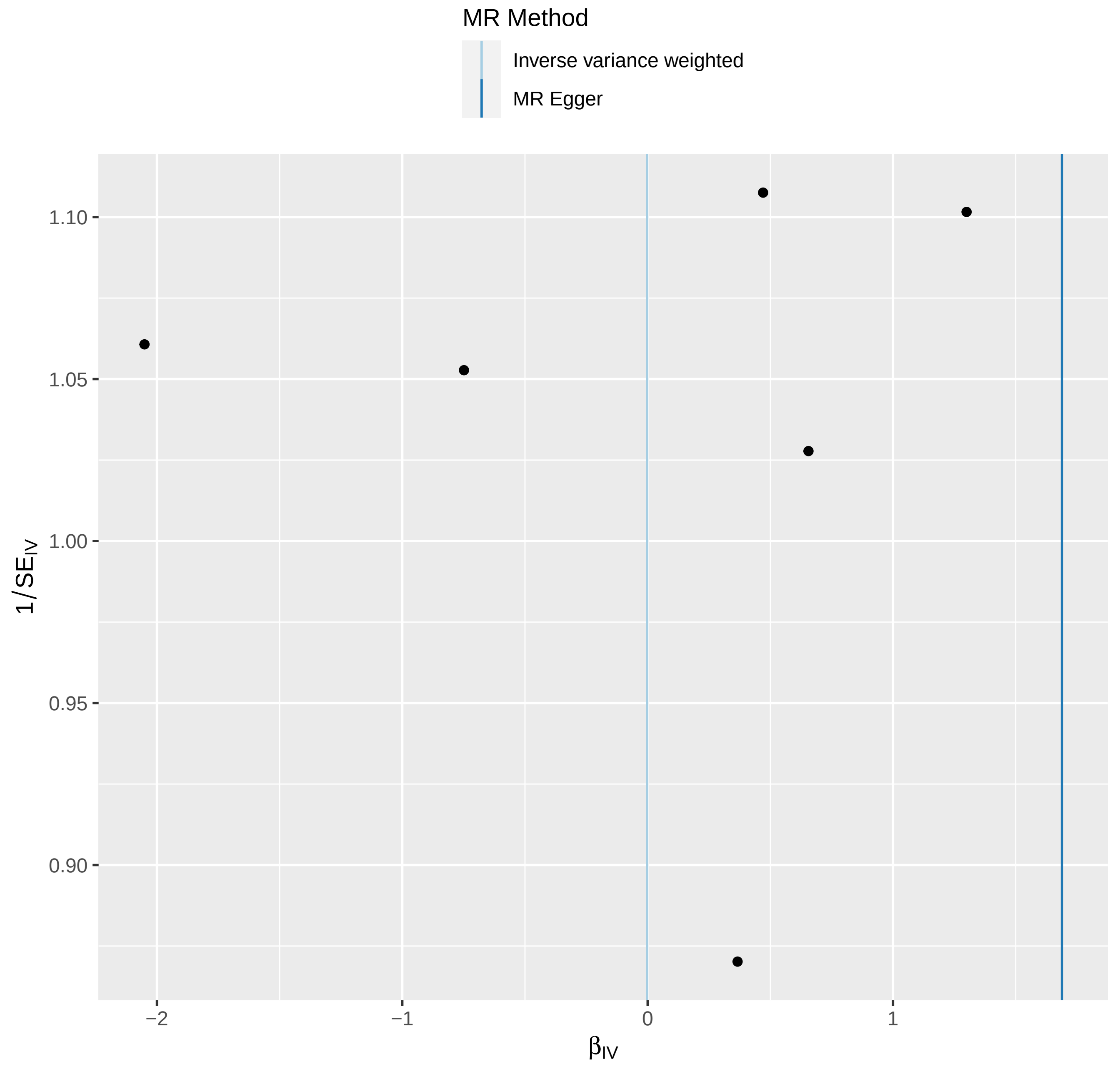

Batch 741 : Gut microbiota abundance (genus Sellimonas id.14369) on Extreme obesity with alveolar hypoventilation

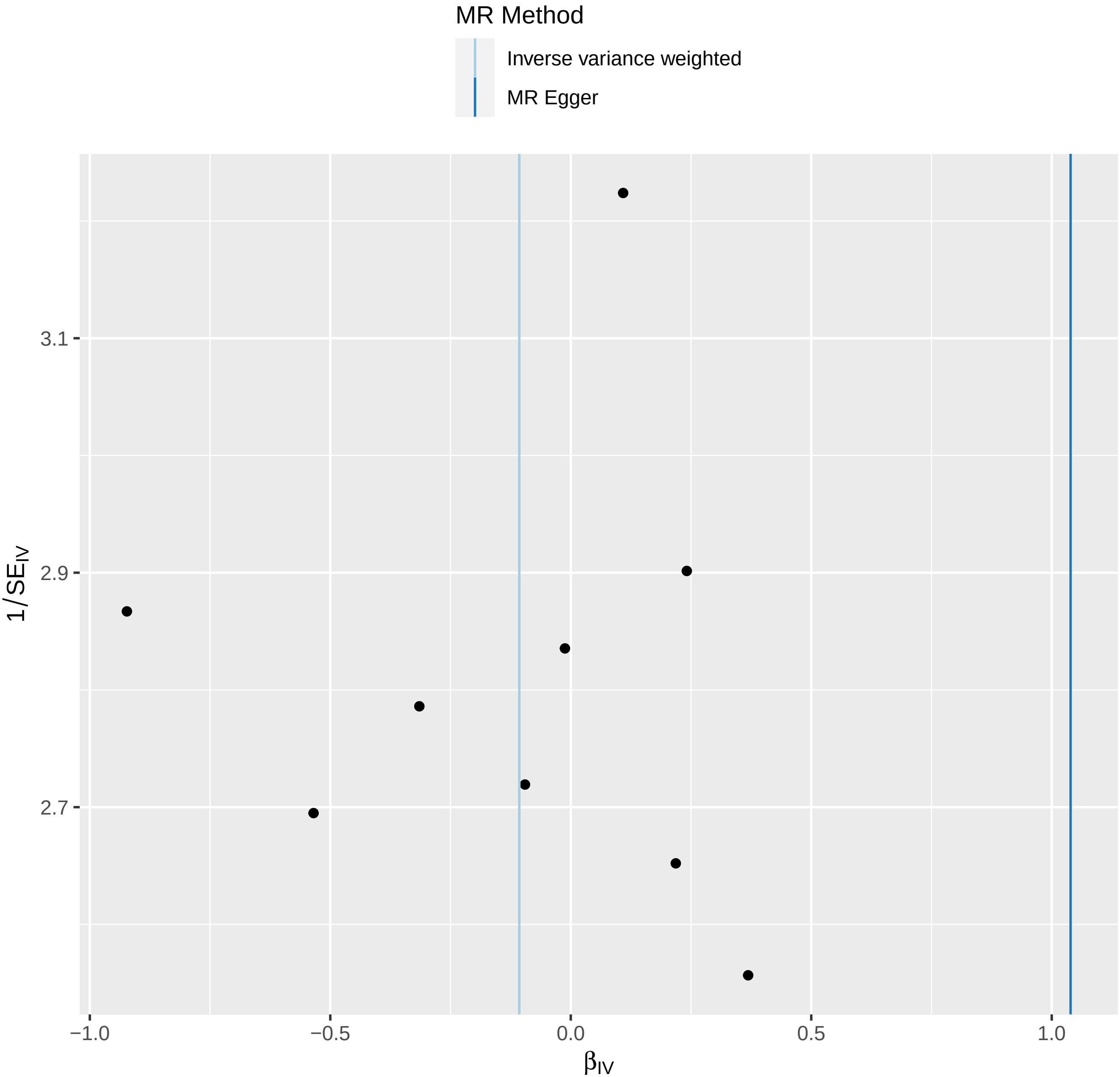

Batch 742 : Gut microbiota abundance (genus Senegalimassilia id.11160) on Extreme obesity with alveolar hypoventilation

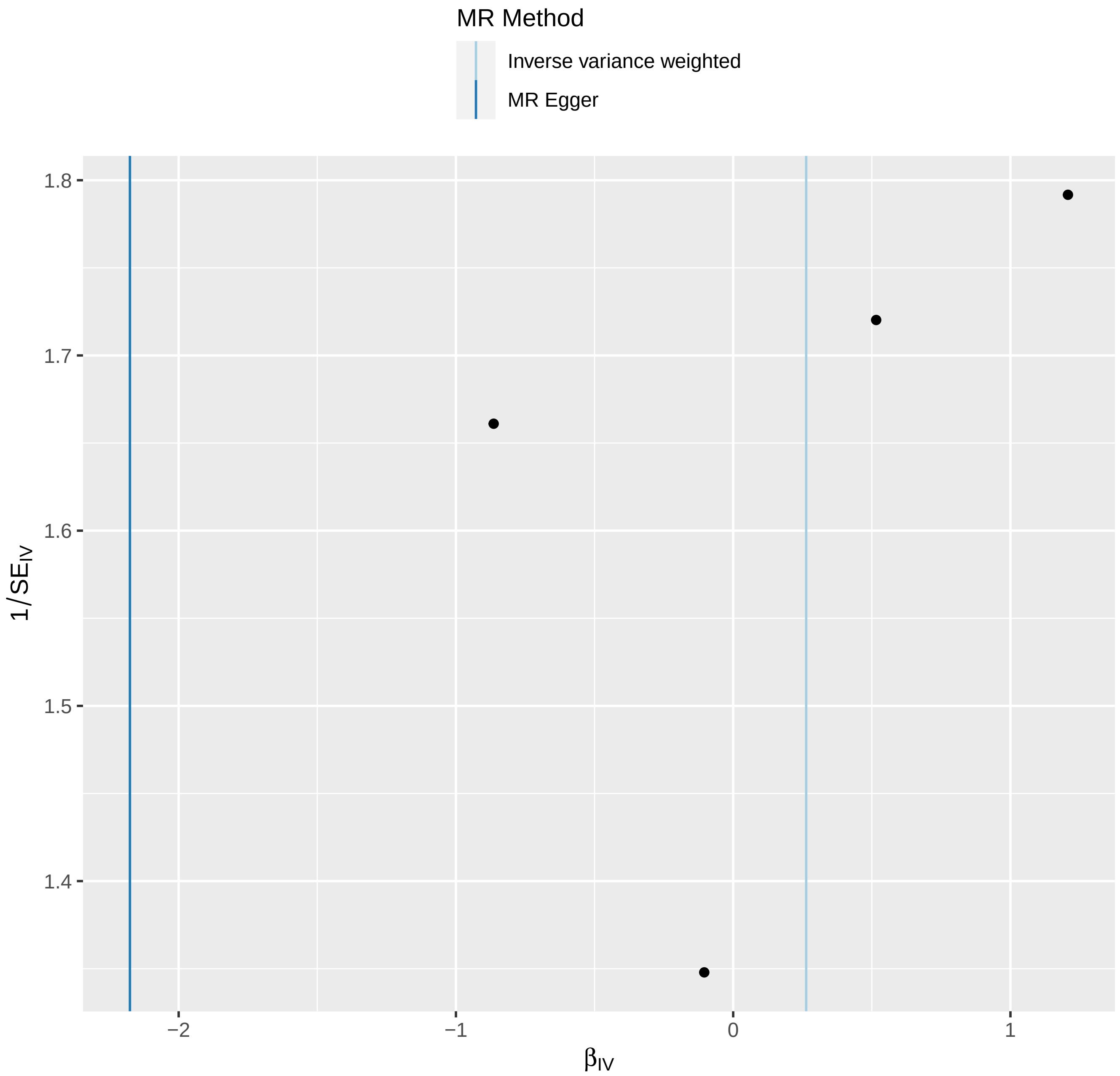

Batch 743 : Gut microbiota abundance (genus Slackia id.825) on Extreme obesity with alveolar hypoventilation

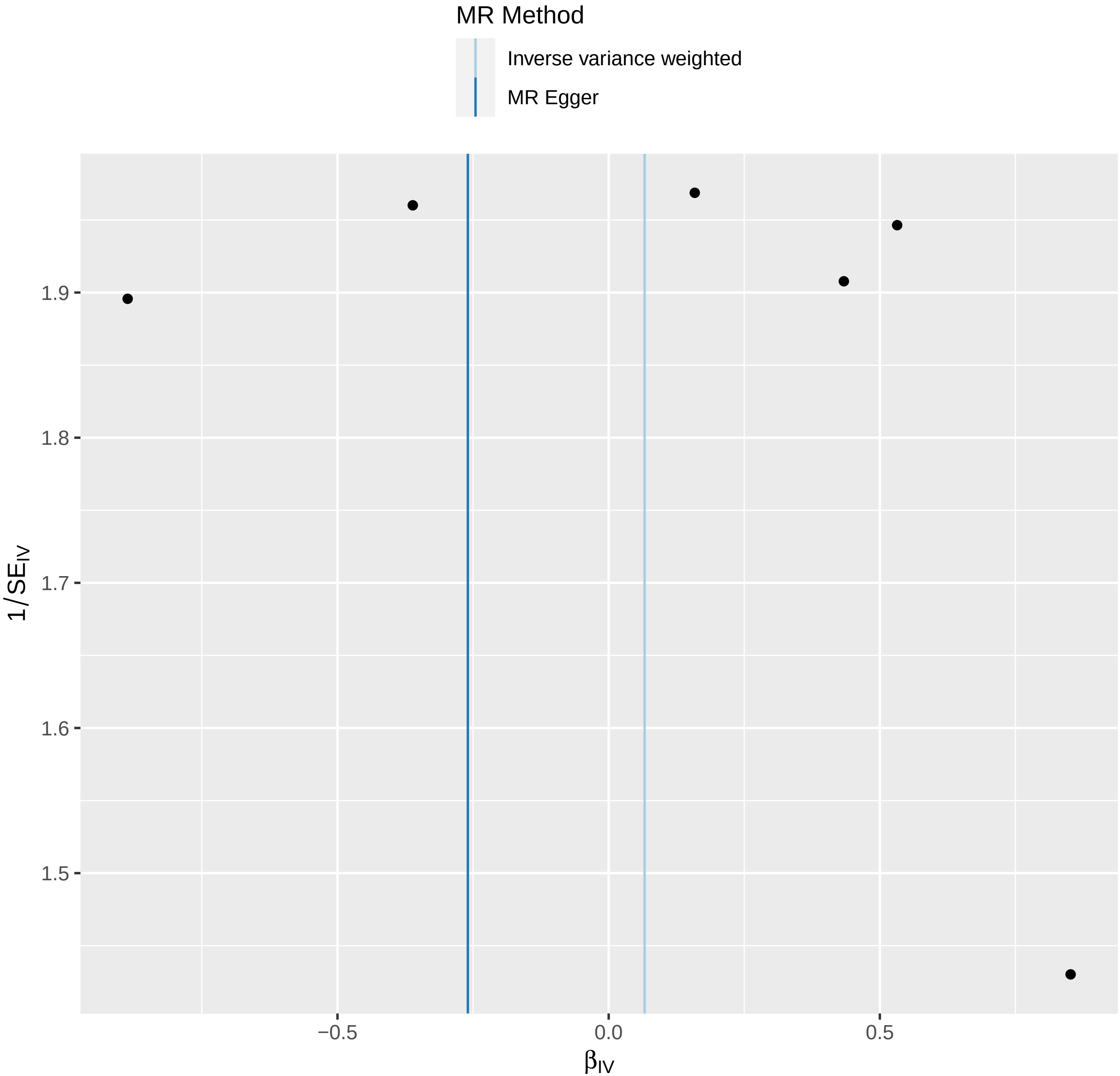

Batch 744 : Gut microbiota abundance (genus Streptococcus id.1853) on Extreme obesity with alveolar hypoventilation

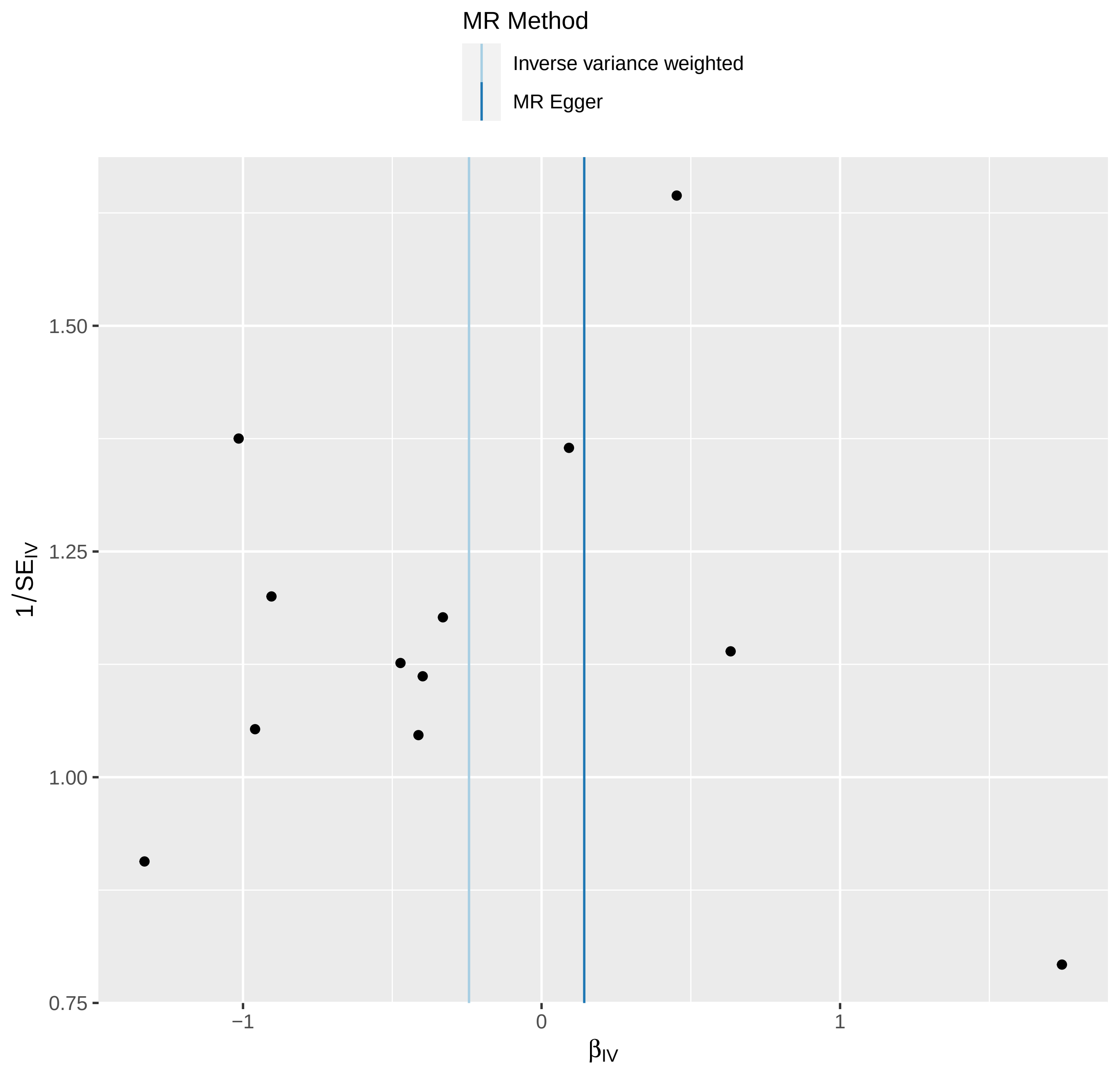

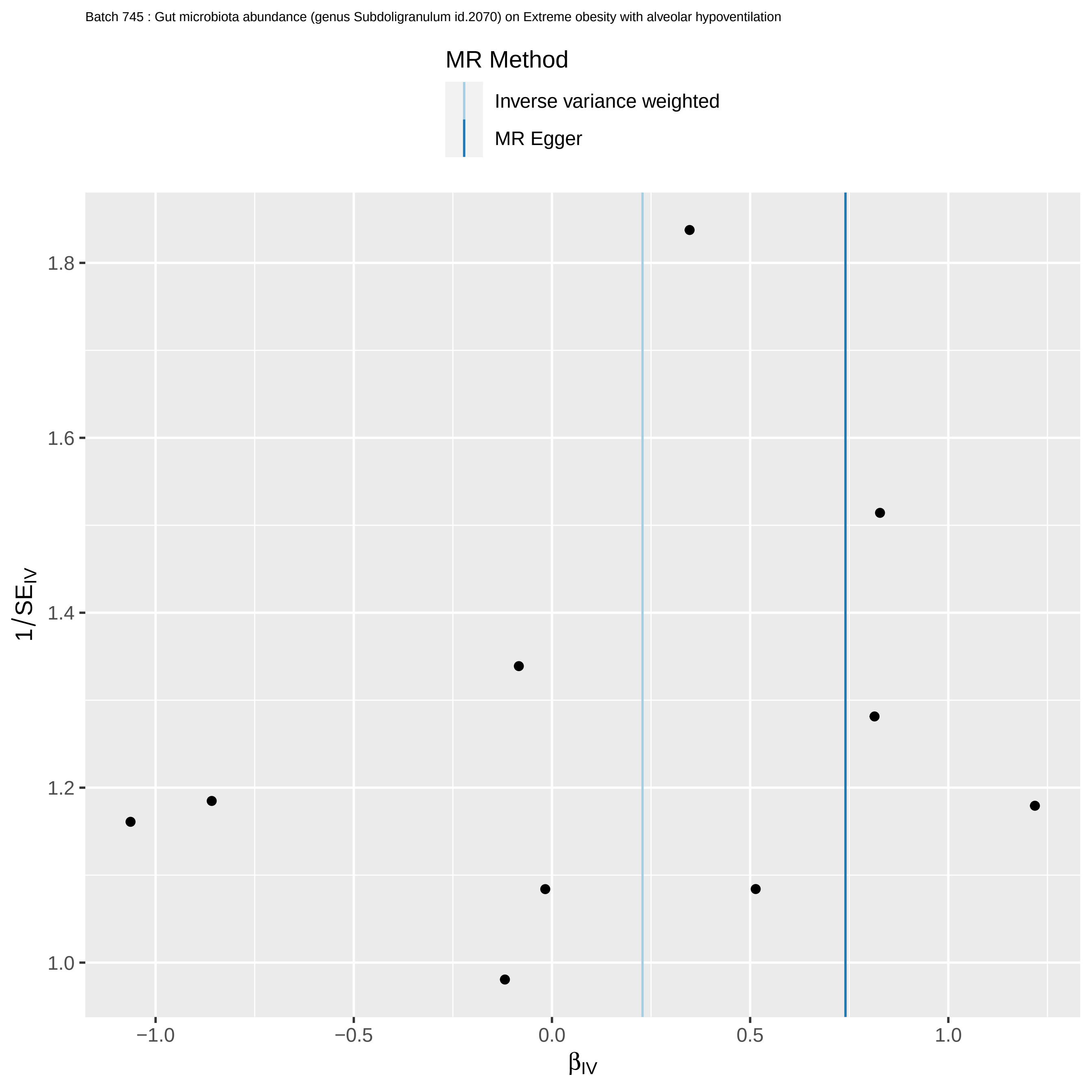

Batch 745 : Gut microbiota abundance (genus Subdoligranulum id.2070) on Extreme obesity with alveolar hypoventilation
MR Method
Inverse variance weighted
MR Egger
1.8
1.6
1.4
1.2
1.0
1/SE_IV
−1.0
−0.5
0.0
0.5
1.0
β_IV

Batch 746 : Gut microbiota abundance (genus Sutterella id.2896) on Extreme obesity with alveolar hypoventilation

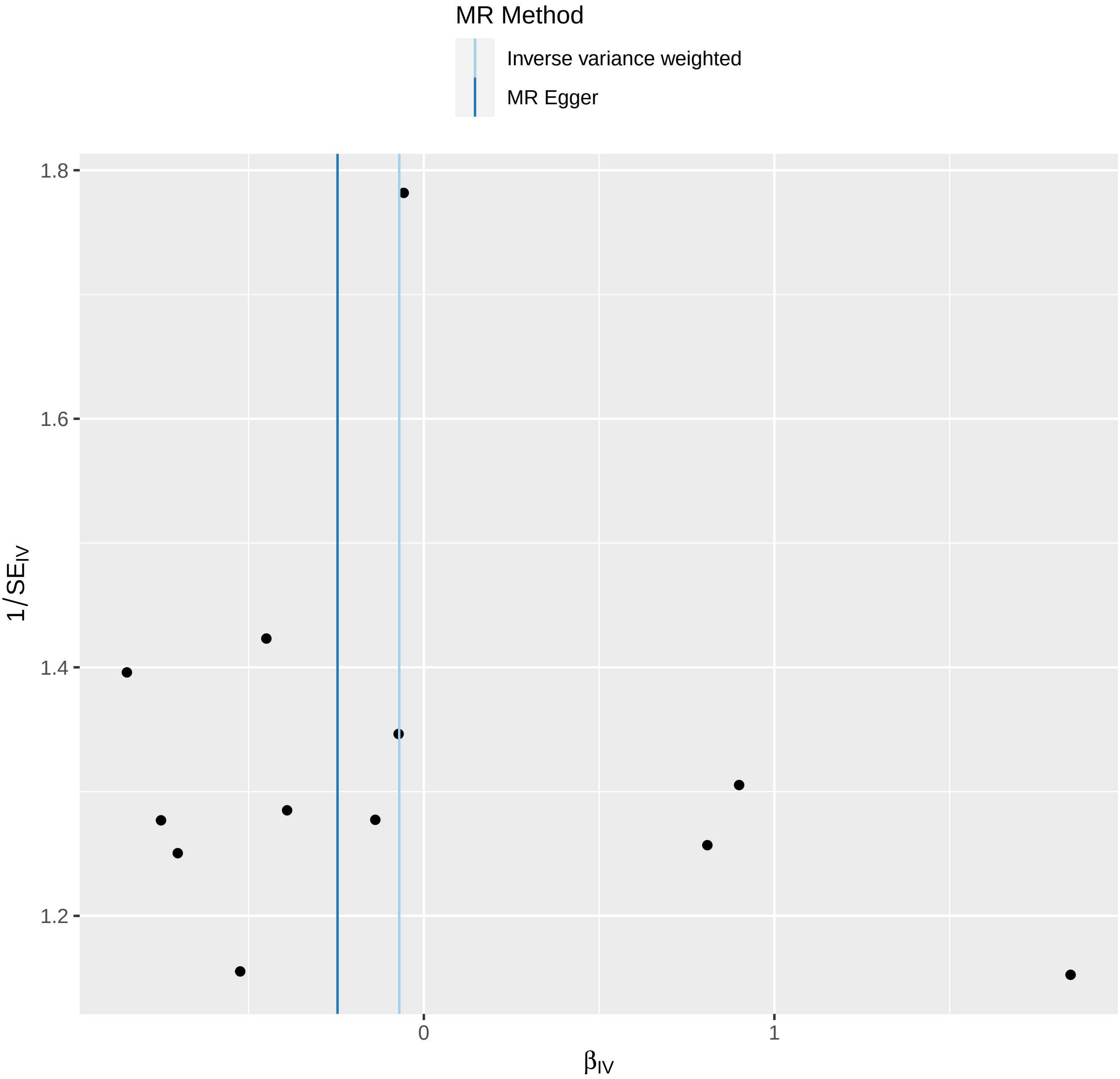

Batch 747 : Gut microbiota abundance (genus Terrisporobacter id.11348) on Extreme obesity with alveolar hypoventilation

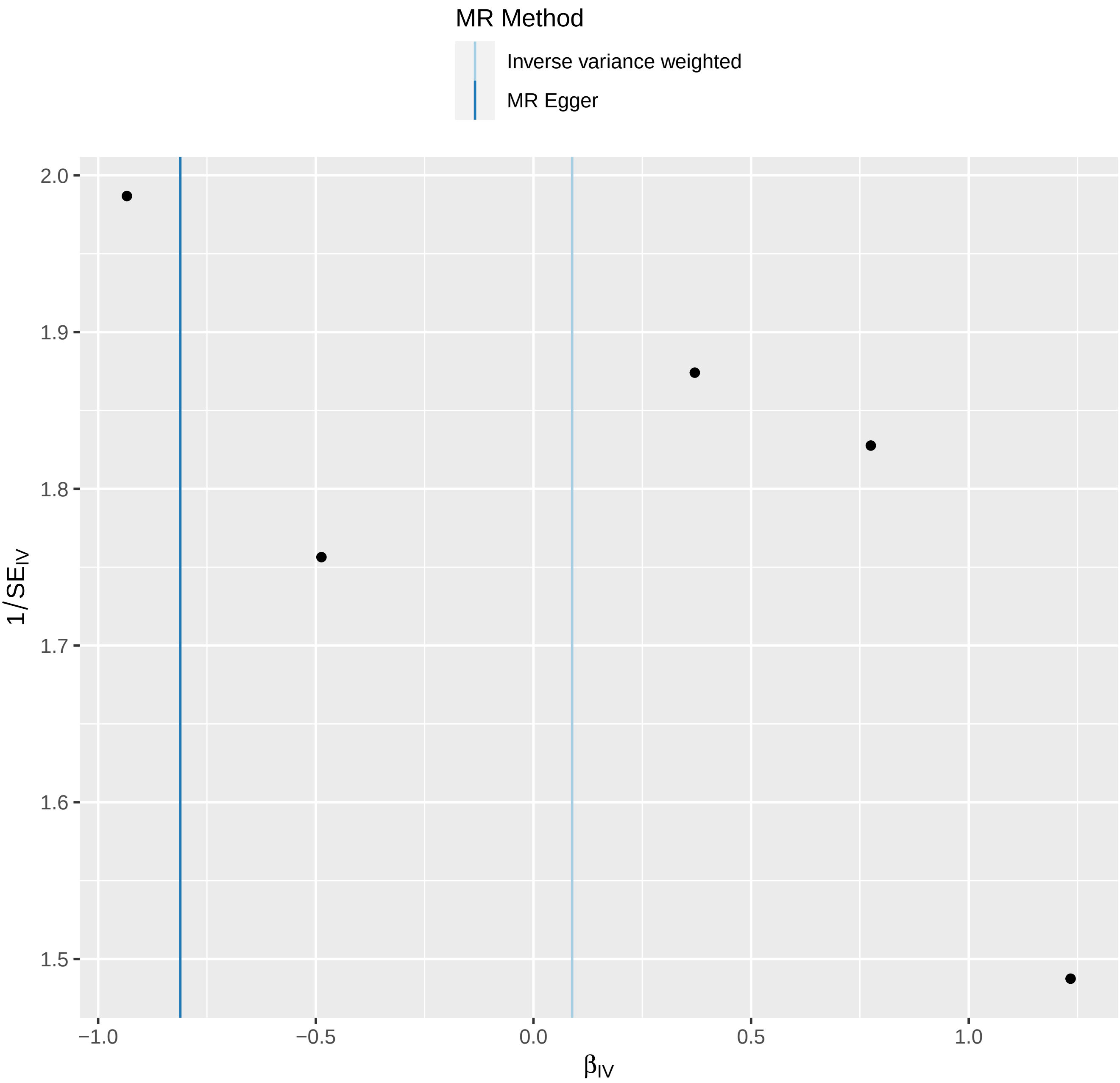

Batch 748 : Gut microbiota abundance (genus Turicibacter id.2162) on Extreme obesity with alveolar hypoventilation

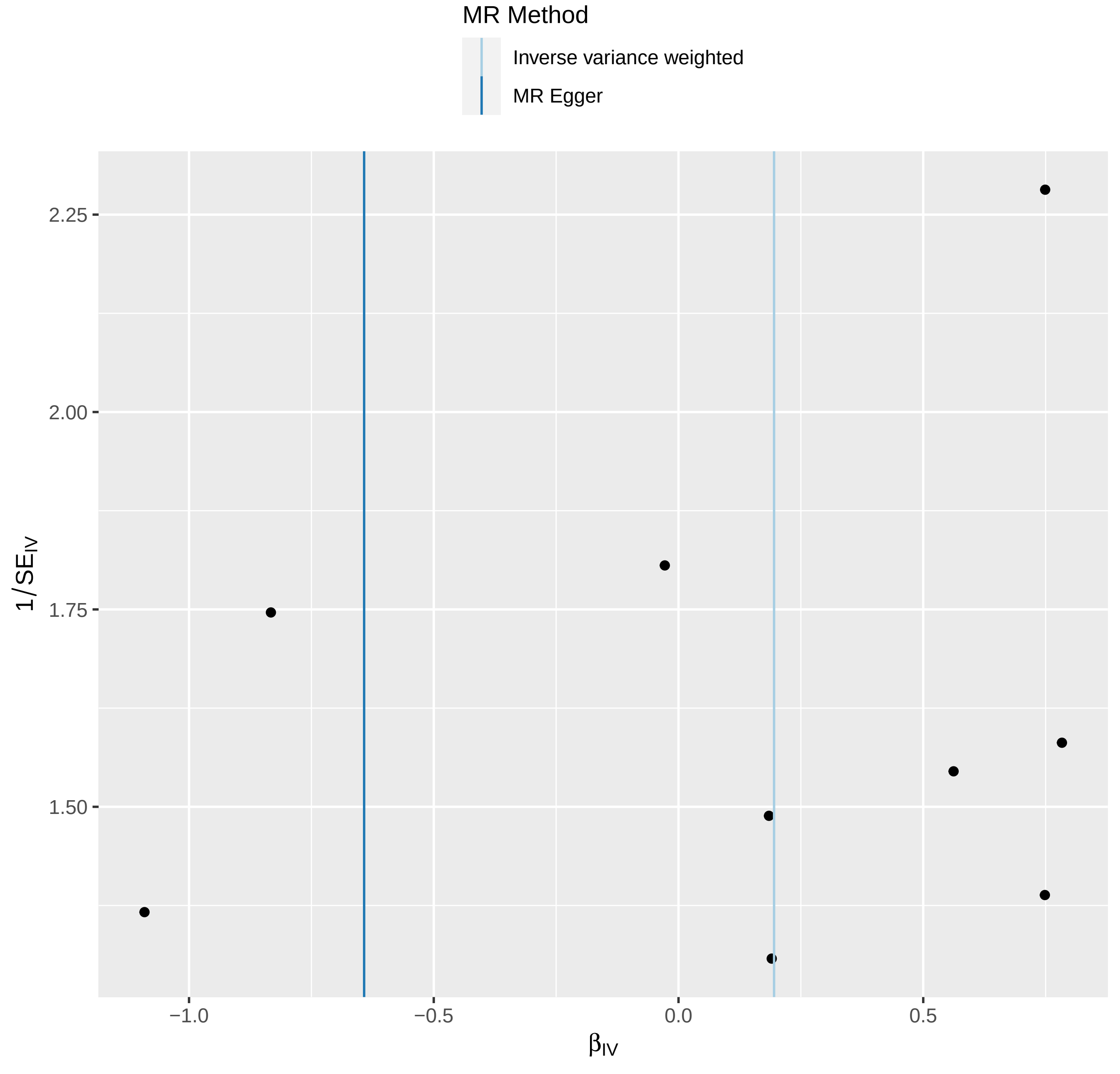

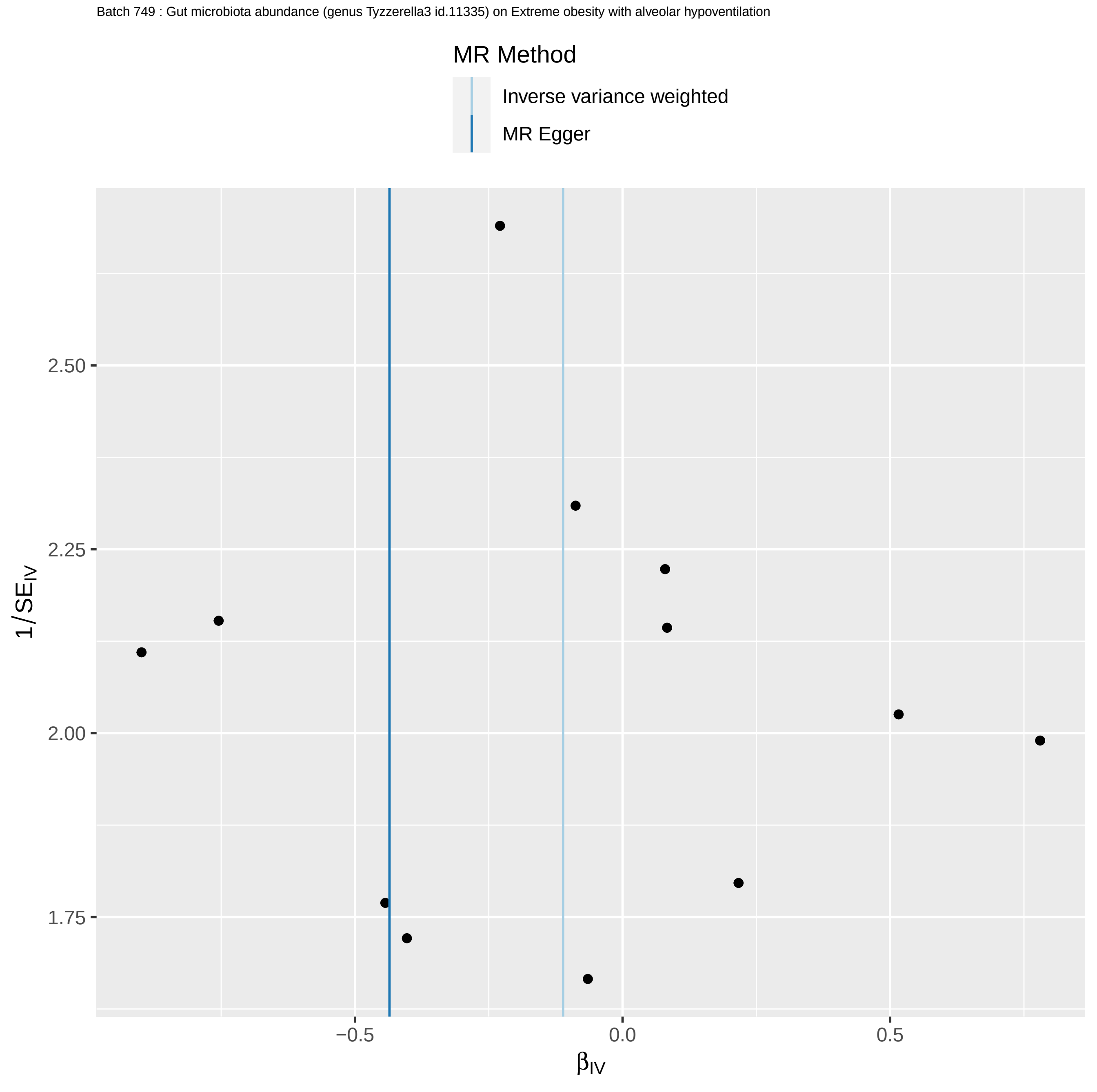

Batch 749 : Gut microbiota abundance (genus Tyzzerella3 id.11335) on Extreme obesity with alveolar hypoventilation
MR Method
Inverse variance weighted
MR Egger
2.50
2.25
2.00
1.75
1/SE_IV
−0.5
0.0
0.5
β_IV

Batch 750 : Gut microbiota abundance (genus Veillonella id.2198) on Extreme obesity with alveolar hypoventilation

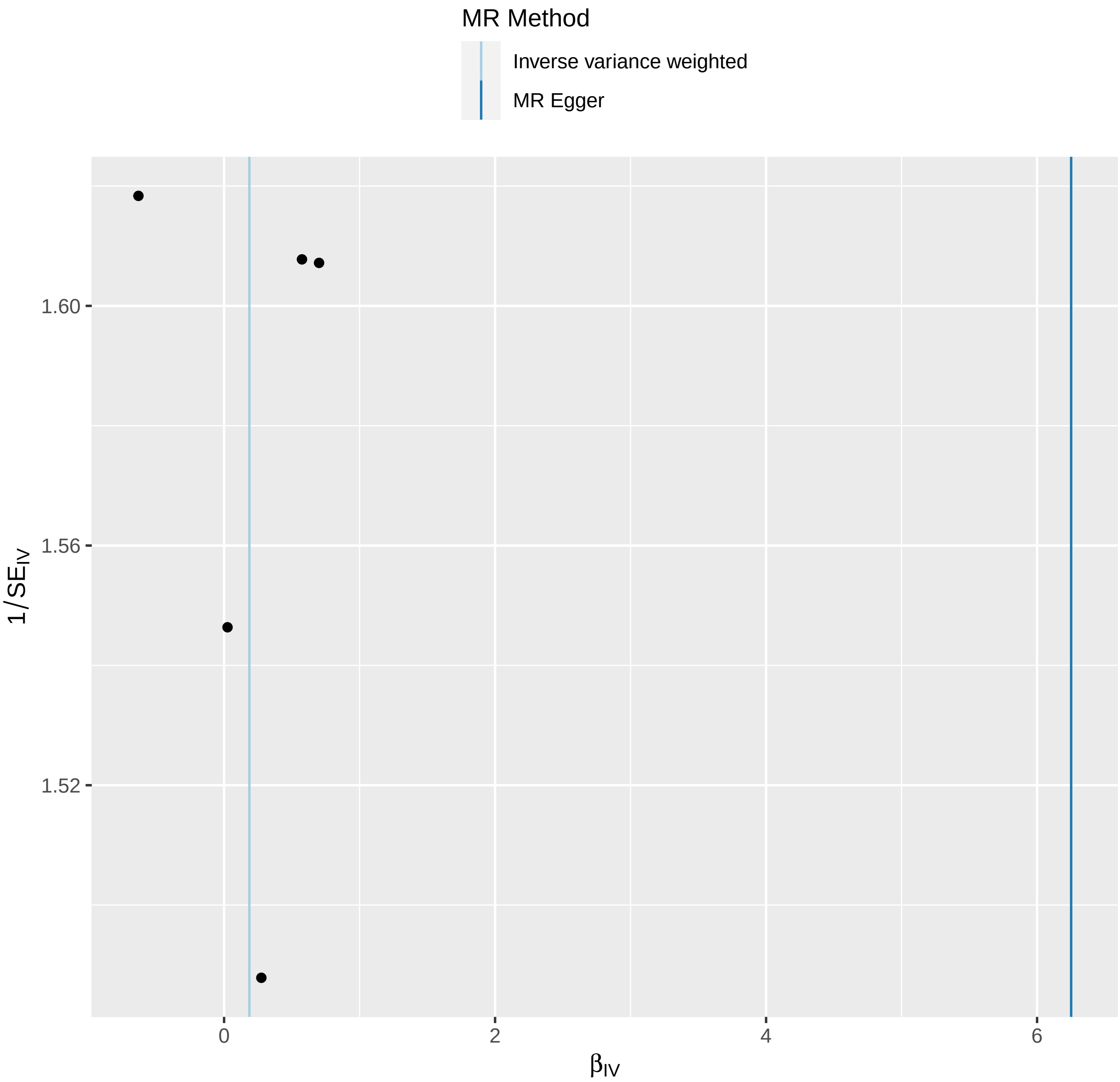

Supplement: Supplementary file 4 [file DataSheet_4.pdf]
